# Exploration of key factors in Gingival Crevicular fluids from patients undergoing Periodontally Accelerated Osteogenic Orthodontics (PAOO) using proteome analysis

**DOI:** 10.1186/s12903-023-03606-7

**Published:** 2023-11-27

**Authors:** Jiaqi Wu, Li Xu, Cuiying Li, Xiujing Wang, Jiuhui Jiang

**Affiliations:** 1 First Clinical Division, Peking University School and Hospital of Stomatology, 100081 Beijing, PR China; 2 Department of Periodontology, Peking University School and Hospital of Stomatology, 100081 Beijing, PR China; 3 Central Laboratory, Peking University School and Hospital of Stomatology, 100081 Beijing, PR China; 4 Department of Orthodontics, Peking University School and Hospital of Stomatology, 100081 Beijing, PR China; 5 National Engineering Research Center of Oral Biomaterials and Digital Medical Devices, Beijing Laboratory of Biomedical Materials & Beijing Key Laboratory of Digital Stomatology, Peking University School and Hospital of Stomatology, 100081 Beijing, PR China

**Keywords:** Gingival crevicular fluid (GCF), Corticotomy, Periodontally Accelerated Osteogenic Orthodontics (PAOO)

## Abstract

**Background:**

The aims of this study are to explore protein changes in gingival crevicular fluid at different time points after PAOO by proteomics method and to select significant bone metabolization-related biomarkers.

**Methods:**

This study included 10 adult patients experiencing PAOO. After orthodontic alignment and leveling, the maxillary anterior teeth were treated with PAOO, which is classified as the experimental area. The traditional orthodontic treatment was performed in the mandibular dentition as the control. Gingival crevicular fluid samples were collected at the following time points: the day before the PAOO (T1) and at 1 week, 2 weeks, 1 month, 2 months and 6 months after PAOO (T2, T3, T4, T5 and T6, respectively). The label-free quantitative proteomic assay was used to evaluate the gingival crevicular fluid in PAOO and control areas at time point T1, T2, and T4. Bioinformatics analysis was carried out to categorize proteins based on biological processes, cellular component and molecular function, which is in compliance with gene ontology (GO) standards. The changes of proteins were confirmed by ELISA.

**Results:**

A total of 134 proteins were selected by keywords (Osteoblast markers, Osteoclast markers, Osteoclastogenesis regulating genes and inflammatory marker). 33 of them were statistically different between groups, and 12 were related to bone metabolism. 5 proteins selected by label-free quantitative proteomics were KLF10, SYT7, APOA1, FBN1 and NOTCH1. KLF10 decreased after PAOO, hitting a trough at T4, and then leveled off. SYT7 increased after PAOO, reaching a peak at T3, and then stabilized until T6. APOA1 ascended to a peak at T4 after PAOO, and then remained stable until T6. The FBN1 rose after PAOO, reaching a peak at T4, and then went down slowly. NOTCH1 ascended rapidly in the first two weeks after PAOO and continued its slow growth trend.

**Conclusion:**

In this study, protein changes in gingival crevicular fluid were detected by proteomics method, and significant bone metabolization-related proteins were selected. It is speculated that APOA1, FBN1, NOTCH1, SYT7 and KLF10 played key roles in regulating bone metabolic balance and in reversible osteopenia after PAOO, which might be involved in the accelerated tooth movement.

**Trial registration:**

This study was registered in the Chinese Clinical Trial Registry (Clinical trial registration number: ChiCTR-ONRC-13,004,129) (26/04/2013).

**Supplementary Information:**

The online version contains supplementary material available at 10.1186/s12903-023-03606-7.

## Background

Tooth movement speed in patients receiving conventional orthodontic treatment is normally 0.8 ~ 1.2 mm per month and the period of comprehensive orthodontic treatment is usually 12 ~ 36 months. Orthodontic treatment period is related to the type and severity of malocclusion, the selection of treatment design and technique, the experience of orthodontists and the compliance of patient. Shortening the orthodontic treatment is of great clinical significance [1].

Periodontally accelerated osteogenic orthodontics (PAOO) proposed by Wilcko in 2001 is a clinical procedure that combines selective alveolar corticotomy, particulate bone grafting, and orthodontic forces. PAOO could result in less cortical resistance during tooth movement, shorter treatment time, increased post-treatment stability, and decreased apical root resorption [2]. Clinical studies have confirmed that the PAOO efficiently accelerates orthodontic movement. However, the internal mechanism remains unclear and further studies are needed [3, 4]. In order to clarify the mechanism of accelerated tooth movement, many scholars have conducted a series of studies and proposed hypotheses. Wilcko et al. believed that PAOO is theoretically based on the bone healing pattern known as the Regional Acceleratory Phenomenon (RAP) that induces the healing of alveolar bone after local trauma [5, 6]. It is believed that corticotomy, an intentional surgical injury to the bone, started a cascade of physiologic events, stimulating osteoclast activity and leading to increased bone turnover with concomitant demineralization and new bone formation at the site of the bone injury [3]. The RAP comprises an acute inflammatory phase and intense osteoclastic and decreased osteoblastic bone formation, which manifests as transient local osteopenia. Lee et al. found the alveolar bone mineral density in the corticotomy site decreased in rats with Micro-CT detection, thus confirming the presence of RAP [7].

Although previous studies have confirmed the uniqueness of bone metabolism in accelerated teeth movement, the specific cellular mechanism such as the signaling pathway of PAOO has not been elucidated. At present, most of the fundamental researches on PAOO concentrate on animal experiments or in vitro experiments, while there are few reports on the mechanism of PAOO in human [8, 9]. Therefore, this study aims to explore the internal mechanism of accelerated tooth movement via PAOO through label-free quantitative proteomic assay and bioinformatics analysis of gingival crevicular fluid, so as to investigate the inherent biological laws of accelerated tooth movement in the human body.

## Materials and methods

This study is a prospective cohort study. PKUSSIRB-202,054,018 (09/03/2020) was reviewed and approved by the Biomedical Ethics Committee of Peking University Hospital of Stomatology. This study was registered in the Chinese Clinical Trial Registry (Clinical trial registration number: ChiCTR-ONRC-13,004,129) (26/04/2013). The study was conducted in full accordance with Declaration of Helsinki.

### Participants

The inclusion criteria for all patients were as follows: (1) 18 to 40 years of age; (2) Class III relationships, ANB < − 4° or Wits < − 6 mm; (3) With combined surgical and orthodontic treatment; (4) Bilateral extraction of maxillary first premolars and erupted third molars; (5) Mild crowding in the maxillary arch (≤ 4 mm) and (6) Without severe or progressive periodontitis and with a probing depth of less than 4 mm.

The exclusion criteria were as follows: (1) Patients with active periodontal disease; (2) Patients with additional teeth loss except tooth extraction for orthodontic treatment; (3) Patients with congenital cleft lip and palate; (4) Patients with acute inflammation in the oral and maxillofacial region; (5) Patients with tumor or systemic severe diseases and (6) Pregnant and lactating patients.

### Gingival crevicular fluid (GCF) collection and processing

Ten adult orthodontic patients were selected in this study. After orthodontic alignment and leveling, the maxillary anterior teeth were treated with periodontally accelerated osteogenic orthodontics (PAOO), which is classified as the experimental area, while traditional orthodontic treatment performed for mandibular dentition, is classified as the control area.

The collection, storage, and processing of all samples were conducted according to a standardized protocol. EP tubes with 35# standard paper points were weighed by the electronic balance (Hundred thousandth). Absorbent paper points were used to collect GCF by inserting the paper points into the mesial and distal buccal gingival sulcus of maxillary anterior teeth (Stop for 30s when mild resistance was detected). The selected site of experimental group is from left upper canine to right upper one, while the selected site of the control group is from left lower canine to right lower one. EP tubes with 35# paper points were weighed again after collection of GCF and then stored at − 80℃ until the time of assay. Gingival crevicular fluid (GCF) was collected at six time points:

T1: the day before the PAOO; the day after orthodontic alignment and leveling.

T2: 1 week after the PAOO; the day postoperative review was scheduled.

T3: 2 weeks after the PAOO; the day of first application of orthodontic force after PAOO.

T4: 1 month after the PAOO; the day of second application of orthodontic force after PAOO.

T5: 2 months after the PAOO;

T6: 6 months after the PAOO.

The protein differences in gingival crevicular fluid between PAOO and the control areas of the same patient were compared. The bone metabolism related proteins in gingival crevicular fluid in the PAOO accelerated tooth movement were selected.

### Label-free quantitative proteomic screening

#### Extraction and enzymolysis of proteins

The microTOF-Q II mass spectrometry was used to evaluate the gingival crevicular fluid in PAOO area and the control area at time points T1, T2, and T4 in the 10 PAOO patients. Briefly, all test paper samples were transferred to a 1.5-mL screw capped tube and the 100 µL of lysis buffer (7 M urea, 2 M thiourea) were added into each sample and then ultrasonication was performed to extract total proteins. Proteins were precipitated with trichloroacetic acid (TCA) for 30 min on ice and centrifuged at 40,000×g for 30 min. Protein concentration was determined using the Qubit fluorescent protein quantification kit (Invitrogen) according to the manufacturer’s instructions.

Following the addition of 100 mmol/L DTT(DL-Dithiothreitol) to a final concentration of 10 mmol/L, the protein fractions were mixed at 56℃ for 60 min, and then diluted 10x with 250 mmol/L IAM(Iodoacetamide) and maintained in dark for 60 min. Finally, the samples were digested with trypsin (substrate to enzyme mass at a mass ratio of 50:1 at 37℃ for 12 h [10].

#### LC-MS/MS analysis

Peptides were acidified with formic acid (FA) (10%, v/v) and desalted byre-versed phase extraction using C18 ZipTip pipette tips and re-suspended in 0.1% FA for high performance liquid chromatography-tandem mass spectrometry (HPLC-MS/MS) analysis. Pressure was applied to a fused silica capillary column packed with 3-µm dionex C18 material (RP; Phenomenex) to load digested peptide combinations. The RP sections with 100Å were each 15 cm long, and the column was washed with buffer A (water, 0.1% formic acid) and buffer B (Acetonitrile, 0.1% formic acid). After desalting, a 5-mm, 300-µm C18 capture tip was placed in line with an Agilent 1100 quaternary HPLC(High Performance Liquid Chromatography) and analyzed using a 12-step separation.

Firstly, a 45-min gradient to 40% buffer B was applied after a 5-min gradient from 0 to 2% buffer B. The buffer B then flowed by a gradient of 40–80% for 3 min and then 80% for 10 min. An approximately 100 µg tryptic peptide mixture was loaded onto the columns after a 2-min buffer B gradient from 80 to 2%, and it was separated using a linear gradient at a flow rate of 0.5 µL/min. After full scanning, 10 debris maps (MS2 scan, HCD) were collected. MS1 has a resolution of 70,000 at M/Z 200, while MS2 has a resolution of 17,500 at M/Z 200. Peptides were electrosprayed into a micrOTOF-Q II mass spectrometer (BRUKER Scientific) with the application of a distal 180 °C source temperature as they were eluted from the micro-capillary column. The mass spectrometer was operated in the MS/MS (auto) mode. Survey MS scans were acquired in the TOF-Q II with the resolution set to a value of 20,000. Each survey scan (50 ~ 2,500) was followed by five data-dependent tandem mass (MS/MS) scans at 2HZ normalized scan speed [11]. The following were the main parameters: 6 for the main search ppm, 2 for the missed cleavage, 20 for the MS/MS tolerance ppm, True set as de-isotopic, trypsin as digestion enzyme, carbamidomethyl as the fixed modification, Oxidation (M) and Acetyl (Protein N-term) as the variable modification.

#### Bioinformatics analysis

Tandem mass spectra were searched against mascot 2.1 (Local Host) human protein database(refseq_human_20140103.fasta). The search results were then filtered using a cutoff of 1% for peptide false identification rate. Peptides with Z score < 4 or Delta-Mass > 5 ppm were rejected. Furthermore, the minimum number of peptides to identify a protein was set to 1. Throughout the analysis, the default parameters for the Quantitative software Profile Analysis 2.0 software were applied.

Bioinformatics analysis was performed to categorize proteins based on biological processes, cellular component and molecular function using annotations in Protein Analysis Through Evolutionary Relationships (PANTHER) database v 6.1 (www.pantherdb.org), which is in compliance with gene ontology (GO) standards. Signaling pathway analysis were performed with the aid of tools on the Kyoto Encyclopedia of Genes and Genome (KEGG) database (http://www.genome.jp/kegg/pathway.html) independently [11–14].

#### Validation by ELISA

ELISA was performed to confirm expression changes of the differentially expressed proteins. Five proteins selected by label-free quantitative proteomics were KLF10, SYT7, APOA1, FBN1 and NOTCH1. Human Krueppel-like factor 10 (KLF10) ELISA Kit (Jiangsu Jingmei Biotechnology Co., Ltd.; the detection limit was 10.0 pg/mL), Human Synaptotagmin 7(SYT7) ELISA Kit (Jiangsu Jingmei Biotechnology Co., Ltd. China; the detection limit was 10 pg/ml), Human apoprotein A1 (APOA1) ELISA Kit (Jiangsu Jingmei Biotechnology Co., Ltd. China; the detection limit was 0.1 µg/mL); Human Fibrillin-1 (FBN1) ELISA Kit (Jiangsu Jingmei Biotechnology Co., Ltd. China; the detection limit was 1.0 pg/mL) and Human NOTCH1 ELISA Kit (Jiangsu Jingmei Biotechnology Co., Ltd. China; the detection limit was 1.0 pg/mL) were used according to the manufacturers’ instructions to measure the concentrations of gingival crevicular fluid proteins in different groups.

### Statistical analysis

#### Statistical analysis of omics data

Fold-change (FC) was calculated by the mean of each group. Selection criteria of different expressed proteins were set as follows: |log(FC)|>0.58.

The intensity data of these regions were used for GraphPad analysis. The biological process was analyzed with Gene Ontology database (http://geneontology.org/). Signaling pathway analysis was performed using the Kyoto Encyclopedia of Genes and Genome (KEGG) database (www.genome.jp/kegg/pathway.html).

#### Statistical analysis of ELISA

The ELISA results were statistically analyzed with RStudio. Paired sample Wilcoxon sign test was used to test these five factors (KLF10, SYT7, APOA1, FBN1, NOTCH1) in the maxillary PAOO area and mandibular control area at six time points. Wilcoxon sign test was used to analyze the trend of five factors related to bone metabolism in gingival crevicular fluid over time with pairwise comparative analysis performed at the same time.

## Result

### Label-free quantitative proteomic analysis

The microTOF-Q II mass spectrometry was used to detect the gingival crevicular fluid of PAOO area and the control area at T1, T2 and T4 in these 10 patients. The detected differential proteins are shown in Table 1.

**Table 1:** The number of differentially expressed proteins in the gingival crevicular fluid (GCF) of PAOO area and the control area at time points T1, T2 and T4

Group	Number of up-regulated differential proteins(>2)		Number of down-regulated differential proteins(≤ 0.5)	
PAOO area T1 vs. T2 vs. T4	T1/T2	T1/T4	T1/T2	T1/T4
	76	45	80	71
The control area T1 vs. T2 vs. T4	T1/T2	T1/T4	T1/T2	T1/T4
	55	39	61	63
Whole T1 vs. T2 vs. T4	T1/T2	T1/T4	T1/T2	T1/T4
	127	70	120	104

Original statistical data in Table 1 could be seen in the folder titled “PAOO area.xls, The control area.xls and Whole.xls” in the supplementary materials

The total number of proteins identified were 2337. 134 proteins were selected by keywords (Osteoblast markers, Osteoclast markers, Osteoclastogenesis regulating genes and inflammatory marker). 33 of them were statistically different between groups, and 12 were related to bone metabolism (Table 2).

**Table 2:** Twelve proteins related to bone metabolism

Gene	GI number	Protein	Median (T1up: T4up)	Median (T2up: T1up)	Median (T4down: T1down)	Median (T2down: T1down)	Median (T2:T1)	Median (T4:T1)
APOA1	4,557,321	apolipoprotein A-I preproprotein	1.32	1.01	1.63	0.86	0.83	0.91
FBN1	281,485,550	fibrillin-1 precursor	0	0	0.87	0.57	0.43	0.41
FBN2	66,346,695	fibrillin-2 precursor	1.09	1.03	1.53	3.53	1.75	0.96
C3	115,298,678	complement C3 precursor	0	0	0	0	0.43	0.57
TRAF6	4,759,254	TNF receptor-associated factor 6			0	0	0.51	0
NOTCH1	148,833,508	neurogenic locus notch homolog protein 1 preproprotein	2.27	0.88	0	0	0.72	0.57
ABCA3	116,734,710	ATP-binding cassette sub-family A member 3	0.87	1.16			2.08	1
ERCC2	195,947,407	TFIIH basal transcription factor complex helicase XPD subunit isoform 2	0	0			0.56	0.99
HDAC1	13,128,860	histone deacetylase 1	0	0.38	0.69	1.02		
KLF10	73,760,403	Krueppel-like factor 10 isoform b	0.33	4.55			1.19	1.08
SYT7	530,397,573	PREDICTED: synaptotagmin-7 isoform X3	1.55	1.74			1.43	0
DCHS1	16,933,557	protocadherin-16 precursor					1.69	0

up: the PAOO area; down: the control area; T1: the day before the PAOO; T2: 1 week after the PAOO; T4: 1 month after the PAOO

Please see supplementary materials for the descriptions of protein function, which shows in the “Supplementary Table 4.txt”

### Statistical analysis of ELISA results

#### KLF10

**Fig. 1:**
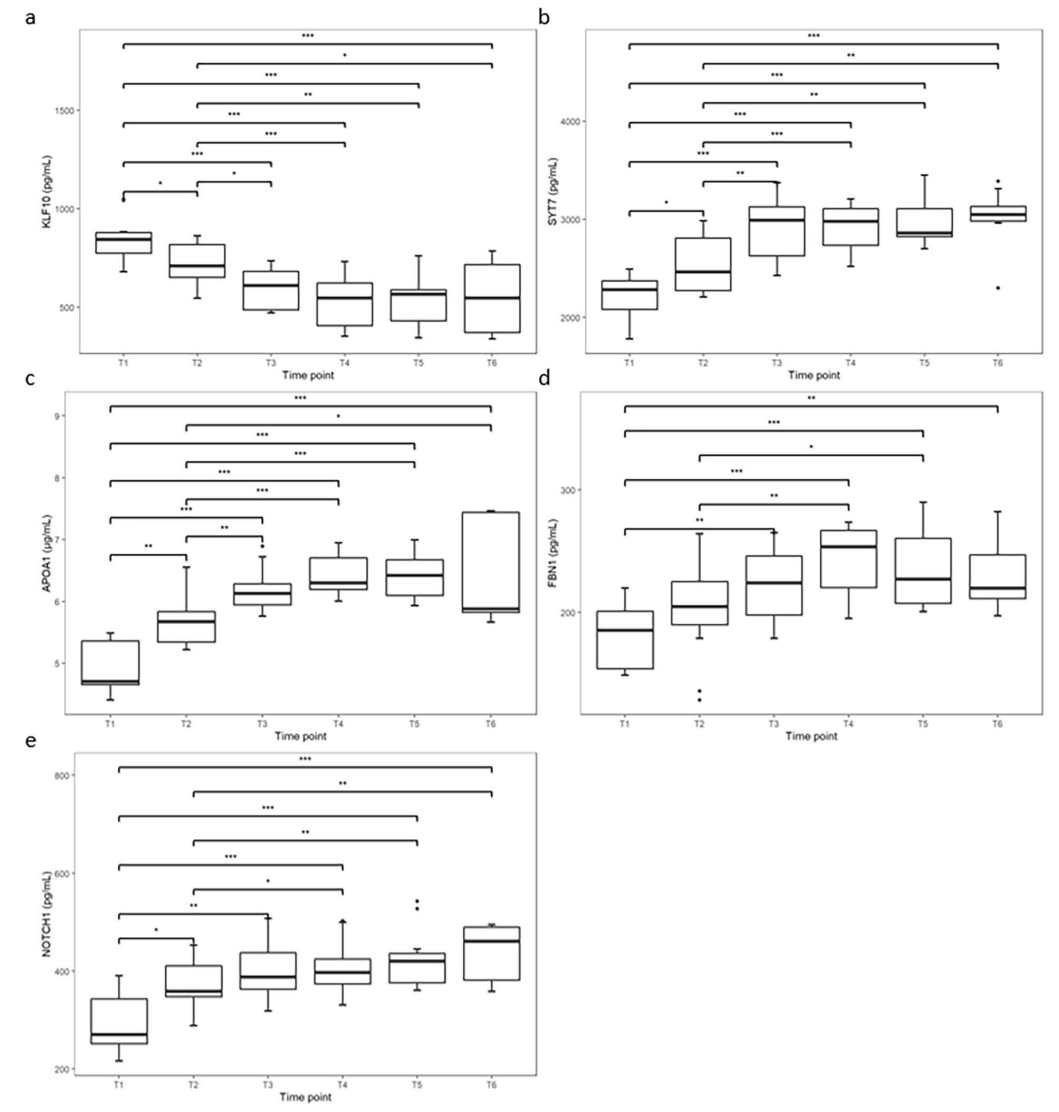
Comparisons of five biomarkers in GCF from patients undergoing PAOO at six time points. The ELISA results were statistically analyzed with RStudio. Wilcoxon sign test was used to analyze the trend of five factors related to bone metabolism in gingival crevicular fluid over time with pairwise comparative analysis performed at the same time

KLF10 decreased after PAOO, hitting a trough at 1 month after PAOO (T4), and then leveled off. A statistically significant difference was found between preoperative (T1) results and postoperative (T2 ~ T6) results (P<0.05). Statistical differences between other groups are shown in Fig. 1a. The ELISA results showed that there was a significant difference in KLF10 between the Maxillary PAOO area and mandibular control area at 2 months after PAOO (T5) (P < 0.05), but no significant difference were found between the two areas at other time points (Table 3).

**Table 3:** Comparisons of five biomarkers at six time points in the PAOO and the control areas

		T1		T2		T3		T4		T5		T6	
Biomarkers		mean ± sd	p	mean ± sd	p	mean ± sd	p	mean ± sd	p	mean ± sd	p	mean ± sd	p
KLF10	PAOO area	679.7 ± 136.87		717.9 ± 110.26		599.2 ± 104.96		530.6 ± 130.78		523.8 ± 134.56		548.3 ± 175.78	
	Control area	683.2 ± 132.56	0.786	711.4 ± 109.65	0.398	598.3 ± 109.88	0.734	530.6 ± 132.56	0.888	538.9 ± 133.11	0.028*	553.0 ± 174.64	0.599
SYT7	PAOO area	2233 ± 276.6		2556 ± 299.9		2904 ± 273.9		2932 ± 222.9		2962 ± 240.8		2964 ± 346.4	
	Control area	2171 ± 247.8	0.068	2519 ± 284.9	0.345	2872 ± 357.4	0.446	2932 ± 233.9	1.000	2983 ± 275.6	0.248	2992 ± 370.8	0.461
APOA1	PAOO area	4.916 ± 0.4684		5.693 ± 0.4203		6.173 ± 0.3096		6.380 ± 0.3315		6.404 ± 0.3766		6.381 ± 0.8202	
	Control area	4.941 ± 0.4124	0.686	5.700 ± 0.4453	0.680	6.185 ± 0.3535	0.674	6.466 ± 0.3403	0.012*	6.429 ± 0.3631	0.498	6.344 ± 0.8615	0.500
FBN1	PAOO area	179.7 ± 22.81		202.4 ± 38.81		224.6 ± 29.13		242.1 ± 27.78		237.9 ± 31.30		231.8 ± 29.21	
	Control area	180.0 ± 31.17	0.893	203.5 ± 41.59	0.612	220.6 ± 33.10	0.310	243.4 ± 29.18	0.779	232.9 ± 33.18	0.149	228.1 ± 31.10	0.249
NOTCH1	PAOO area	294.6 ± 72.09		363.3 ± 50.12		359.4 ± 57.38		413.4 ± 41.21		424.6 ± 52.70		442.3 ± 54.24	
	Control area	292.6 ± 67.77	1.000	275.1 ± 51.07	0.093	406.9 ± 49.88	0.249	400.4 ± 55.51	0.161	421.7 ± 61.75	0.753	441.1 ± 59.59	0.715

(*p < 0.05)

PAOO area: the maxillary anterior teeth were treated with periodontally accelerated osteogenic orthodontics (PAOO); Control area: the mandibular anterior teeth treated with traditional orthodontic treatment; The measurement unit of APOA1 is ug/ml and the measurement unit of KLF10, SYT7, FBN1 and NOTCH1 is pg/ml

#### SYT7

SYT7 increased after PAOO, reaching a peak at 2 weeks after PAOO (T3), and then remained stable until 6 months after PAOO (T6). A statistically significant difference was found between preoperative (T1) results and postoperative (T2 ~ T6) results (P<0.05). Statistical differences between other groups are shown in Fig. 1b. The ELISA results showed that there were no significant difference in SYT7 between the Maxillary PAOO area and the mandibular control area (Table 3).

#### APOA1

The statistical analysis of macroscopic ELISA results was shown in Fig. 1. APOA1 ascended to a peak at 1 month (T4) after PAOO, and then stabilized until 6 months after PAOO (T6). A statistically significant difference was found between preoperative results (T1) and postoperative results (T2 ~ T6) (*P*<0.05). Statistical differences between other groups are shown in Fig. 1c. The ELISA results showed that there was a significant difference in APOA1 between the Maxillary PAOO area and the mandibular control area at one month after PAOO (T4), but no significant difference were found between the two areas at other time points (Table 3).

#### FBN1

FBN1 rose after PAOO, reaching a peak at 1 month after PAOO (T4), and then went down slowly. A statistically significant difference was found between preoperative results (T1) and postoperative (T2 ~ T6) results(P<0.01). Statistical differences between other groups are shown in Fig. 1d. The ELISA results showed that there was no significant difference in FBN1 between the Maxillary PAOO area and the mandibular control area (Table 3).

#### NOTCH1

NOTCH1 ascended rapidly in the first two weeks after PAOO and then continued its slow growth trend. A statistically significant difference was found between preoperative (T1) results and postoperative (T2 ~ T6) results (P<0.05). Statistical differences between other groups are shown in Fig. 1e. The ELISA results showed that there was no significant difference in NOTCH1 between the Maxillary PAOO area and the mandibular control area (Table 3).

## Discussion

It has been documented that transient osteoporosis occurs in damaged bone tissue, and the content of calcium in bone will reduce, resulting in decreased bone mineral density in a certain area around the root. Thus, alveolar bone undergoes biological changes from osteoclastogenesis to osteogenesis, during which alveolar bone goes from mineralization to re-mineralization, providing a time window and theoretical feasibility for accelerating tooth movement [9]. Indeed, the underlying biological process of PAOO is similar to the healing of bone injury. After bone injury, macrophages and monocytes in the altered local microenvironment secrete some growth factors and cytokines. Impaired the lacunocanalicular network, including changed fluid flow, would activate intracellular molecular signaling pathways within osteocytes, actuating the secretion of mediators that could regulate bone remodeling, so as to contribute to the regional acceleration of bone remodeling (RAP). Kumar et al. analyzed ALP and AST in human saliva and crevicular fluid, and found that the enzyme activities of ALP and AST increased after corticotomy and reached a peak at the 6th week, and then decreased at the 3rd and 6th months [10]. Fernandes LSDMCP et al. have shown that GCF during the movement of canines were quantified in a multiplex immunoassay, and differences on biomarkers expression occurred at specific time points (p < 0.05), but a distinct pattern was not observed [11].

In this study, label-free quantitative proteomic assay was employed to detect changes in key proteins in gingival crevicular fluid of PAOO patients at six time points. 12 proteins related to bone metabolism with significant changes were selected. Among them, APOA1 and C3 were also reported in our previous research on the saliva of PAOO patients with the MALDI-TOF MS [15]. The peptides 3052.02 Da and 2775.33 Da detected with MALDI-TOF MS in the saliva of PAOO patients were identified as apolipoprotein A-I precursor (APOA1). APOA1 increased rapidly at one week after PAOO and then decreased. The peptides 1864.17 Da and 2027.72 Da were identified as complement 3 (C3). C3 decreased rapidly at 1 week after PAOO. Compared with our previous studies, this study detected gingival crevicular fluid in the PAOO area and the control area of the same patient separately, and mouth-split control partition was carried out. The label-free proteomics, in a certain range, is more accurate in protein analysis [16, 17].

Based on the results of mass spectrometry, ELISA was further used to verify the trend of bone metabolic factors over time. In this study, twelve bone metabolism-related proteins after PAOO were selected by label-free mass spectrometry, and then the changes of five bone metabolism-related proteins over time after PAOO were detected by ELISA.

KLF10 is known to be vital in bone biology by regulating the expression and activity of Runx2 and Osterix (crucial osteogenic transcription factors) [18, 19]. Additionally, Jong Min Lee et al. found that primary ossification was critically delayed in KLF10 KO mice [20]. KLF10 decreased after PAOO, hitting a trough at 1 month after PAOO (T4), and then leveled off. The above results indicated that the reduction of KLF10 after PAOO weakened the osteogenesis of the PAOO area and reduced the alveolar bone mineral density in the operation area. Therefore, it reduced the resistance of orthodontic tooth movement, and accelerated tooth movement, which occurred within 1 month after PAOO. Haibo Zhao et al. [21] reported SYT7 plays a similar role in osteoblasts and osteoclasts. SYT7 performs a function in bone remodeling and homeostasis by regulating secretory pathways in osteoclasts and osteoblasts. SYT7 increased after PAOO, reaching a peak at 2 weeks after PAOO (T3), and then leveled off until 6 months after PAOO (T6). According to the above results, it could be concluded that SYT7 is a key to the balance between osteogenesis and osteoclastogenesis after PAOO.

In this study, APOA1 and FBN1 ascended, reaching a peak at 1 month after PAOO (T4), and then stabilized until 6 months after PAOO(T6). NOTCH1 increased rapidly in the first two weeks after PAOO(T3) and then continued its slow growth trend. Harry Blair et al. reported that APOA1 deficiency weakened the capacity of MSC to differentiate toward osteoblasts but promoted adipogenesis. The results confirmed that APOA1 was critical in regulating bone remodeling and maintaining bone quality [22]. Harikiran Nistala et al. reported that FBN1 could regulate bone formation by osteoblast differentiation through modulation of endogenous TGFβ and BMP signals. In vivo and in vitro experiments suggested that the fibrillin acted as a negative regulator in the bone resorption [23]. Sekine C. et al. reported that the activation of Notch1/Jag1 could inhibit osteoclast differentiation. NOTCH1 inhibits osteoclastogenesis through the RBPJ pathway and by enhancing OPG levels and Wnt signaling in osteoblasts and osteocytes [24]. It has been documented that transient osteoporosis occurs in damaged bone tissue, and the content of calcium in bone will decrease, resulting in decreased bone mineral density in a certain area around the root. Thus, the alveolar bone undergoes biological changes from osteoclastogenesis to osteogenesis, during which the alveolar bone goes from demineralization to re-mineralization, which could be viewed as a kind of reversible osteopenia, making the acceleration of tooth movement possible [3, 8]. In our study, it could be concluded that increased APOA1, FBN1 and NOTCH1 in PAOO could promote the balance between osteogenesis and osteoclastogenesis, favoring bone formation. These factors exert similar local effects on alveolar bone, which might prevent excessive decrease of alveolar bone mineral density and thus limit the decrease of bone mineral density around the tooth root to a certain range. Therefore, it is speculated that after PAOO, in addition to the increase of cytokines promoting osteoclastic effect, the antagonistic cytokines play roles. These factors negatively regulate the growth of osteoclasts to avoid excessive reduction of local alveolar bone mineral density and excessive loss of local bone tissue, which lays the foundation for the subsequent re-mineralization of alveolar bone. Baloul et al. established an orthodontic rat model of selective corticotomy and found that accelerated tooth movement occurred in the early stage after corticotomy [25]. This study also supports the findings of the previous in vivo studies that the changes of bone metabolic factors are most significant in the early postoperative period.

The ELISA results showed that no significant difference was found between the Maxillary PAOO area and the mandibular control area at time points T1-T6, except for a significant difference in APOA1 at 1 month after PAOO (T4) and in KLF10 at 2 months after PAOO (T5) (P < 0.05) between the two areas. According to a previous study, cytokine level in GCF is not differentiated by type of movement when force is applied to tooth, because GCF presents free circulation in the gingival sulcus [9, 26]. It is inferred that corticotomy affects the oral microenvironment and that gingival crevicular fluid circulates in the body; therefore, there is little difference in bone metabolic factors between surgical area and non-surgical area in the same patient at the same time.

## Conclusions

In this study, protein changes in gingival crevicular fluid at different time points after PAOO were detected by proteomics method, and significant bone metabolization-related proteins were selected. It is speculated that APOA1, FBN1, NOTCH1, SYT7 and KLF10 play key roles in regulating bone metabolic balance and in reversible osteopenia after PAOO, which might be involved in the accelerated tooth movement. Intrinsic mechanism of related bone metabolic factors was further investigated, so as to summarize the changes of the effect of PAOO on alveolar bone with time, in hope of providing theoretical support for accelerating orthodontic tooth movement.

### Electronic supplementary material

Below is the link to the electronic supplementary material.

Supplementary Material 1

Supplementary Material 2

Supplementary Material 3

Supplementary Material 4

Supplementary Material 5

Supplementary Material 6

Supplementary Material 7

Supplementary Material 8

Supplementary Material 9

Supplementary Material 10

## Data Availability

The datasets used and/or analyzed during the current study available from the corresponding author on reasonable request.

## Abbreviations

PAOO: Periodontally accelerated osteogenic orthodontics
RAP: Regional acceleratory phenomenon
GCF: Gingival crevicular fluid
