# Supplementary material for: Exploration of key factors in Gingival Crevicular fluids from patients undergoing Periodontally Accelerated Osteogenic Orthodontics (PAOO) using proteome analysis
Source: BMC Oral Health. 2023 Nov 27;23:934. doi: 10.1186/s12903-023-03606-7 (PMC10683118; doi:10.1186/s12903-023-03606-7)
Supplement: Supplementary file 1 — Supplementary Material 1 [file 12903_2023_3606_MOESM1_ESM.pdf]

Detailed Protein Report

Project Info

Name: teeth-151208 **Date:** Dec 8, 2015
Note:

Sample Info & Protocols

Name: ID-Q **Date:** Dec 8, 2015
Fraction:
Volume: **Concentration:**
Note:
Buffer: **Organism:**

Search Result Info

Search Result	Location	Search Engine	Database
ZT_human_PE_2015-12-08 22:06:22	/teeth-151208/ID-Q/"Combined MS/MS spectra"	Mascot	refseq_human

Protein 1: serum albumin preproprotein [Homo sapiens]

Accession: gi|4502027
Database: refseq_human(refseq_human_20140103.fasta)
Database Date: 2015-11-30
Modification(s): Carbamidomethyl, Oxidation
Score: 1095.1
MW [kDa]: 69.3
pI: 5.9
Sequence Coverage [%]: 40.7
No. of unique Peptides: 23

Quantitation

QD:QU **Median:** 0.89 **CV:** 66.04 % **No. of Peptides:** 12

```
        10         20         30         40         50         60         70         80
MKWVTFISLL FLFSSAYSRG VFRRDAHKSE VAHRFKDLGE ENFKALVLIA FAQYLQQCPF EDHVKLVNEV TEFAKTCVAD
        90        100        110        120        130        140        150        160
ESAENCDKSL HTLFGDKLCT VATLRETYGE MADCCAKQEP ERNECFLQHK DDNPNLPRLV RPEVDVMCTA FHDNEETFLK
       170        180        190        200        210        220        230        240
KYLYEIARRH PYFYAPELLF FAKRYKAAFT ECCQAADKAA CLLPKLDELR DEGKASSAKQ RLKCASLQKF GERAFKAWAV
       250        260        270        280        290        300        310        320
ARLSQRFPKA EFAEVSKLVT DLTKVHTECC HGDLLECADD RADLAKYICE NQDSISSKLK ECCEKPLLEK SHCIAEVEND
       330        340        350        360        370        380        390        400
EMPADLPSLA ADFVESKDVC KNYAEAKDVF LGMFLYEYAR RHPDYSVVLL LRLAKTYETT LEKCCAAADP HECYAKVFDE
       410        420        430        440        450        460        470        480
FKPLVEEPQN LIKQNCELFE QLGEYKFQNA LLVRYTKKVP QVSTPTLVEV SRNLGKVGSK CCKHPEAKRM PCAEDYLSVV
       490        500        510        520        530        540        550        560
LNQLCVLHEK TPVSDRVTKC CTESLVNRRP CFSALEVDET YVPKEFNAET FTFHADICTL SEKERQIKKQ TALVELVKHK
       570        580        590        600        610
PKATKEQLKA VMDDFAAFVE KCCKADDKET CFAEEGKKLV AASQAALGL
```

Cmpd.	No. of Cmpds.	m/z meas.	Δ m/z [ppm]	z	Rt [min]	Score	P	Range	Sequence	Modification	Ratios	
298	12	613.8004	-9.46	2	32.4	71.1	1	35-44	R.FKDLGEENFK.A		QD:QU	0.75
673	12	575.2020	-189.65	2	37.0	62.9	0	66-75	K.LVNEVTEFAK.T			
529	10	509.2667	-10.16	2	36.6	49.5	0	89-97	K.SLHTLFGDK.L			
299	2	467.2609	-4.27	2	33.7	52.0	0	98-105	K.LCTVATLR.E	Carbamidomethyl: 2		
265	2	879.3978	-18.24	3	33.3	25.7	2	118-138	K.QEPERNECFLQHKDDNPNLPR.L	Carbamidomethyl: 8	QD:QU	1.75
279	1	666.3028	-17.84	3	33.5	40.0	1	123-138	R.NECFLQHKDDNPNLPR.L	Carbamidomethyl: 3	QD:QU	0.64
350	2	528.2959	-3.73	2	34.4	39.6	1	161-168	K.KYLYEIAR.R		QD:QU	2.39
532	5	464.1198	-281.27	2	35.3	30.2	0	162-168	K.YLYEIAR.R		QD:QU	1.13
1590	1	977.4188	-47.42	2	50.1	11.8	1	187-205	K.AAFTECCQAADKAACLLPK.L			
21	2	537.7799	9.21	2	29.9	42.9	1	206-214	K.LDELRDEGK.A		QD:QU	0.46

Detailed Protein Report

Cmpd.	No. of Cmpds.	m/z meas.	Δ m/z [ppm]	z	Rt [min]	Score	P	Range	Sequence	Modification	Ratios
235	3	696.2743	-13.94	3	31.7	57.7	0	265-281	K.VHTECCHGDLLECADDR.A	Carbamidomethyl: 5, 6, 13	QD:QU 0.34
45	2	722.3225	-3.01	2	30.4	77.4	0	287-298	K.YICENQDSISSK.L	Carbamidomethyl: 3	QD:QU 0.70
145	1	516.2727	4.30	3	31.8	19.6	1	299-310	K.LKECCEKPLLEK.S	Carbamidomethyl: 4, 5	QD:QU 1.32
2284	1	820.3190	-92.46	2	59.0	33.1	0	348-360	K.DVFLGMFLYEYAR.R	Oxidation: 6	
2041	21	1022.9463	-102.62	2	55.9	37.1	0	397-413	K.VFDEFKPLVEEPQNLIK.Q		
1582	24	829.3539	-31.44	2	48.3	77.6	0	414-426	K.QNCELFEQLGEYK.F	Carbamidomethyl: 3	QD:QU 0.49
742	9	480.7783	-13.77	2	39.3	53.1	0	427-434	K.FQNALLVR.Y		
890	4	820.4506	-26.72	2	41.2	33.9	1	438-452	K.KVPQVSTPTLVEVSR.N		
77	2	569.7395	-23.11	2	30.9	44.1	0	500-508	K.CCTESLVNR.R	Carbamidomethyl: 1, 2	
1753	4	637.6399	-13.90	3	50.5	67.0	0	509-524	R.RPCFSALEVDETYVPK.E	Carbamidomethyl: 3	
628	1	564.7514	-179.84	2	37.9	25.5	1	549-558	K.KQTALVELVK.H		QD:QU 2.11
1207	5	500.6668	-276.87	2	43.6	45.1	0	550-558	K.QTALVELVK.H		QD:QU 0.77
2104	47	671.7654	-82.73	2	56.7	98.0	0	570-581	K.AVMDDFAAFVEK.C		

Detailed Protein Report

Protein 2: keratin, type I cytoskeletal 13 isoform a [Homo sapiens]

Accession: gi|131412225
Database: refseq_human(refseq_human_20140103.fasta)
Database Date: 2015-11-30
Modification(s): Carbamidomethyl

Score: 635.2
MW [kDa]: 49.5
pI: 4.8
Sequence Coverage [%]: 39.7
No. of unique Peptides: 16

Quantitation

QD:QU **Median:** 0.54 **CV:** 50.97 % **No. of Peptides:** 9

10	20	30	40	50	60	70	80
MSLRLQSSSA	SYGGGFGGGS	CQLGGGRGVS	TCSTRFVSGG	SAGGYGGGVS	CGFGGGAGSG	FGGGYGGGLG	GGYGGGLGGG
**90**	**100**	**110**	**120**	**130**	**140**	**150**	**160**
FGGGFAGGFV	DFGACDGGLL	TGNEKITMQN	LNDRLASYLE	KVRALEEANA	DLEVKIRDWH	LKQSPASPER	DYSPYYKTIE
**170**	**180**	**190**	**200**	**210**	**220**	**230**	**240**
ELRDKILTAT	IENNRVILEI	DNARLAADDF	RLKYENELAL	RQSVEADING	LRRVLDELTL	SKTDLEMQIE	SLNEELAYMK
**250**	**260**	**270**	**280**	**290**	**300**	**310**	**320**
KNHEEEMKEF	SNQVVGQVNV	EMDATPGIDL	TRVLAEMREQ	YEAMAERNRR	DAEEWFHAKS	AELNKEVSTN	TAMIQTSKTE
**330**	**340**	**350**	**360**	**370**	**380**	**390**	**400**
ITELRRTLQG	LEIELQSQLS	MKAGLENTVA	ETECRYALQL	QQIQGLISSI	EAQLSELRSE	MECQNQEYKM	LLDIKTRLEQ
**410**	**420**	**430**	**440**	**450**	**460**		
EIATYRSLLE	GQDAKMIGFP	SSAGSVSPRS	TSVTTTSSAS	VTTTSNASGR	RTSDVRRP		

Cmpd.	No. of Cmpds.	m/z meas.	Δ m/z [ppm]	z	Rt [min]	Score	P	Range	Sequence	Modification	Ratios	
337	2	1073.9554	-24.90	2	34.2	51.4	0	5-27	R. LQSSSASYGGGFGGGSCQLGGG G	Carbamidomethyl: 17		
323	3	651.3296	-4.86	2	34.0	78.4	0	124-135	R.ALEEANADLEVK.I			
264	4	572.8011	-32.35	2	33.3	62.0	0	166-175	K.ILTATIENNR.V			
667	3	521.7769	-40.77	2	36.9	58.8	0	176-184	R.VILEIDNAR.L		QD:QU	0.46
518	2	624.8405	-16.71	2	36.5	46.5	1	192-201	R.LKYENELALR.Q		QD:QU	0.57
471	2	601.1764	-225.82	2	34.5	42.6	0	202-212	R.QSVEADINGLR.R		QD:QU	0.37
322	1	679.3584	-6.47	2	34.0	14.0	1	202-213	R.QSVEADINGLRR.V		QD:QU	0.24
686	1	587.3207	-42.26	2	37.1	41.5	1	213-222	R.RVLDELTLSK.T			
727	3	509.2783	-32.74	2	39.1	53.0	0	214-222	R.VLDELTLSK.T			
434	3	566.7484	-14.34	2	35.4	31.5	0	291-299	R.DAEEWFHAK.S		QD:QU	0.49
173	1	705.3260	-34.92	2	30.9	13.5	0	306-318	K.EVSTNTAMIQTSK.T		QD:QU	0.73
2903	1	909.4716	-17.49	2	65.4	28.3	0	327-342	R.TLQGLEIELQSQLSMK.A			
178	2	725.3355	-0.10	2	32.2	38.5	0	343-355	K.AGLENTVAETECR.Y	Carbamidomethyl: 12	QD:QU	0.54
370	2	690.3450	-32.66	2	34.6	18.0	1	396-406	K.TRLEQEIATYR.S		QD:QU	0.55
942	3	696.8484	-3.18	2	41.8	28.0	0	416-429	K.MIGFPSSAGSVSPR.S			
28	2	1001.9840	4.13	2	30.1	29.0	0	430-450	R.STSVTTTSSASVTTTSNASGR. R		QD:QU	1.59

Detailed Protein Report

Protein 3: hemoglobin subunit beta [Homo sapiens]

Accession: gi|4504349
Database: refseq_human(refseq_human_20140103.fasta)
Database Date: 2015-11-30
Modification(s): Carbamidomethyl

Score: 578.7
MW [kDa]: 16.0
pI: 6.9
Sequence Coverage [%]: 84.4
No. of unique Peptides: 12

Quantitation

QD:QU **Median:** 2.34 **CV:** 188.92 % **No. of Peptides:** 4

10 20 30 40 50 60 70 80
MVHLTPEEKS AVTALWGKVN VDEVGGEALG RLLVVYPWTQ RFFESFGDLS TPDAVMGNPK VKAHGKKVLG AFSDGLAHLD
90 100 110 120 130 140 150
NLKGTFATLS ELHCDKLHVD PENFRLLGNV LVCVLAHHFG KEFTPPVQAA YQKVVAGVAN ALAHKYH

Cmpd.	No. of Cmpds.	m/z meas.	Δ m/z [ppm]	z	Rt [min]	Score	P	Range	Sequence	Modification	Ratios	
72	1	476.7493	-19.46	2	30.8	23.9	0	2-9	M.VHLTPEEK.S			
849	5	466.6498	-243.95	2	40.7	45.6	0	10-18	K.SAVTALWGK.V			
496	6	657.8496	20.65	2	36.2	81.3	0	19-31	K.VNVDEVGGEALGR.L		QD:QU	0.35
2380	9	637.8625	-6.20	2	58.5	48.9	0	32-41	R.LLVVYPWTQR.F			
2107	11	1029.8517	-122.16	2	56.7	64.9	0	42-60	R.FFESFGDLSTPDAVMGNPK.V			
2794	3	599.9758	-40.56	3	66.1	37.1	1	67-83	K.KVLGAFSDGLAHLDNLK.G			
2791	13	835.4449	-4.99	2	66.1	74.0	0	68-83	K.VLGAFSDGLAHLDNLK.G			
680	1	739.8715	27.96	2	38.5	19.5	0	84-96	K.GTFATLSELHCDK.L	Carbamidomethyl: 11	QD:QU	6.23
340	6	563.7849	-1.35	2	34.3	45.2	0	97-105	K.LHVDPENFR.L		QD:QU	1.81
532	3	689.8492	-6.46	2	36.6	44.0	0	122-133	K.EFTPPVQAAYQK.V		QD:QU	7.65
307	5	575.3418	2.06	2	33.8	76.7	0	134-145	K.VVAGVANALAHK.Y			
430	1	725.3587	-59.21	2	35.4	17.7	1	134-147	K.VVAGVANALAHKYH.-			

Detailed Protein Report

Protein 4: keratin, type II cytoskeletal 6C [Homo sapiens]

Accession: gi|155969697
Database: refseq_human(refseq_human_20140103.fasta)
Database Date: 2015-11-30
Modification(s): Carbamidomethyl

Score: 429.5
MW [kDa]: 60.0
pI: 8.9
Sequence Coverage [%]: 20.6
No. of unique Peptides: 9

Quantitation

QD:QU **Median:** 1.26 **CV:** 81.26 % **No. of Peptides:** 5

10	20	30	40	50	60	70	80
MASTSTTIRS	HSSSRRGFSA	NSARLPGVSR	SGFSSISVSR	SRGSGGLGGA	CGGAGFGSRS	LYGLGGSKRI	SIGGGSCAIS
**90**	**100**	**110**	**120**	**130**	**140**	**150**	**160**
GGYGSRAGGS	YGFGGAGSGF	GFGGGAGIGF	GLGGGAGLAG	GFGGPGFPVC	PPGGIQEVTV	NQSLLTPLNL	QIDPAIQRVR
**170**	**180**	**190**	**200**	**210**	**220**	**230**	**240**
AEEREQIKTL	NNKFASFIDK	VRFLEQQNKV	LDTKWTLLQE	QGTKTVRQNL	EPLFEQYINN	LRRQLDSIVG	ERGRLDSELR
**250**	**260**	**270**	**280**	**290**	**300**	**310**	**320**
NMQDLVEDLK	NKYEDEINKR	TAAENEFVTL	KKDVDAAYMN	KVELQAKADT	LTDEINFLRA	LYDAELSQMQ	THISDTSVVL
**330**	**340**	**350**	**360**	**370**	**380**	**390**	**400**
SMDNNRNLDL	DSIIAEVKAQ	YEEIAQRSRA	EAESWYQTKY	EELQVTAGRH	GDDLRNTKQE	IAEINRMIQR	LRSEIDHVKK
**410**	**420**	**430**	**440**	**450**	**460**	**470**	**480**
QCASLQAAIA	DAEQRGEMAL	KDAKNKLEGL	EDALQKAKQD	LARLLKEYQE	LMNVKLALDV	EIATYRKLLE	GEECRLNGEG
**490**	**500**	**510**	**520**	**530**	**540**	**550**	**560**
VGQVNVSVVQ	STISSGYGGA	SGVGSGLGLG	GGSSYSYGSG	LGIGGGFSSS	SGRAIGGGLS	SVGGGSSTIK	YTTTSSSSRK
**570**							
SYKH							

Cmpd.	No. of Cmpds.	m/z meas.	Δ m/z [ppm]	z	Rt [min]	Score	P	Range	Sequence	Modification	Ratios
152	2	712.8348	20.14	2	31.9	14.7	0	43-59	R.GSGGLGGACGGAGFGSR.S	Carbamidomethyl: 9	
470	1	799.8881	6.12	2	35.9	18.2	0	70-86	R.ISIGGGSCAISGGYGSR.A	Carbamidomethyl: 8	QD:QU 2.01
910	6	602.3097	-20.53	2	39.9	44.2	0	195-204	K.WTLLQEQGTK.T		QD:QU 1.07
2105	4	945.9050	-88.89	2	56.7	16.4	0	208-222	R.QNLEPLFEQYINNLR.R		
328	2	675.8743	11.03	2	34.1	67.2	1	261-272	R.TAAENEFVTLKK.D		QD:QU 1.16
1975	5	704.2826	-108.99	2	55.1	74.7	0	288-299	K.ADTLTDEINFLR.A		
2868	16	665.3564	-15.34	2	65.0	82.6	0	327-338	R.NLDLDSIIAEVK.A		QD:QU 3.28
231	4	583.2587	-63.96	2	31.6	63.1	0	360-369	K.YEELQVTAGR.H		QD:QU 0.39
2191	9	632.2117	-219.91	2	56.0	48.4	0	456-466	K.LALDVEIATYR.K		

Detailed Protein Report

Protein 5: hemoglobin subunit delta [Homo sapiens]

Accession: gi|4504351
Database: refseq_human(refseq_human_20140103.fasta)
Database Date: 2015-11-30

Score: 366.3
MW [kDa]: 16.0
pI: 9.1
Sequence Coverage [%]: 48.3
No. of unique Peptides: 1

10	20	30	40	50	60	70	80
MVHLTPEEKT	AVNALWGKVN	VDAVGGEALG	RLLVVYPWTQ	RFFESFGDLS	SPDAVMGNPK	VKAHGKKVLG	AFSDGLAHLD
90	100	110	120	130	140	150	
NLKGTFSQLS	ELHCDKLHVD	PENFRLLGNV	LVCVLARNFG	KEFTPQMQAA	YQKVVAGVAN	ALAHKYH	

Cmpd.	No. of Cmpds.	m/z meas.	Δ m/z [ppm]	z	Rt [min]	Score	P	Range	Sequence	Modification
467	1	628.8213	-19.13	2	35.8	42.8	0	19-31	K.VNVDAVGGEALGR.L	

Detailed Protein Report

Protein 6: keratin, type I cytoskeletal 15 [Homo sapiens]

Accession: gi|24430190
Database: refseq_human(refseq_human_20140103.fasta)
Database Date: 2015-11-30

Score: 329.4
MW [kDa]: 49.2
pI: 4.6
Sequence Coverage [%]: 16.0
No. of unique Peptides: 2

Quantitation

QD:QU	**Median:**	0.73	**CV:**	0.00 %	**No. of Peptides:**	1

10	20	30	40	50	60	70	80
MTTTFLQTSS	STFGGGSTRG	GSLLAGGGGF	GGGSLSGGGG	SRSISASSAR	FVSSGSGGGY	GGGMRVCGFG	GGAGSVFGGG
**90**	**100**	**110**	**120**	**130**	**140**	**150**	**160**
FGGGVGGGFG	GGFGGGDGGL	LSGNEKITMQ	NLNDRLASYL	DKVRALEEAN	ADLEVKIHDW	YQKQTPTSPE	CDYSQYFKTI
**170**	**180**	**190**	**200**	**210**	**220**	**230**	**240**
EELRDKIMAT	TIDNSRVILE	IDNARLAADD	FRLKYENELA	LRQGVEADIN	GLRRVLDELT	LARTDLEMQI	EGLNEELAYL
**250**	**260**	**270**	**280**	**290**	**300**	**310**	**320**
KKNHEEEMKE	FSSQLAGQVN	VEMDAAPGVD	LTRVLAEMRE	QYEAMAEKNR	RDVEAWFFSK	TEELNKEVAS	NTEMIQTSKT
**330**	**340**	**350**	**360**	**370**	**380**	**390**	**400**
EITDLRRTMQ	ELEIELQSQL	SMKAGLENSL	AETECRYATQ	LQQIQGLIGG	LEAQLSELRC	EMEAQNQEYK	MLLDIKTRLE
**410**	**420**	**430**	**440**	**450**	**460**		
QEIATYRSLL	EGQDAKMAGI	GIREASSGGG	GSSSNFHINV	EESVDGQVVS	SHKREI		

Cmpd.	No. of Cmpds.	m/z meas.	Δ m/z [ppm]	z	Rt [min]	Score	P	Range	Sequence	Modification	Ratios
296	1	532.8004	-15.38	2	33.7	58.7	1	116-124	R.LASYLDKVR.A		
1065	2	515.1664	-260.45	2	41.8	44.4	0	215-223	R.VLDELTLAR.T		QD:QU 0.73

Detailed Protein Report

Protein 7: hemoglobin subunit alpha [Homo sapiens]

**Accession:**	gi|4504345	**Score:**	326.5
**Database:**	refseq_human(refseq_human_20140103.fasta)	**MW [kDa]:**	15.2
**Database Date:**	2015-11-30	**pI:**	9.4
		**Sequence Coverage [%]:**	50.7
		**No. of unique Peptides:**	6

Quantitation

QD:QU **Median:** 2.08 **CV:** 25.63 % **No. of Peptides:** 2

Alias proteins:

**Accession**	**Name**	**Description**
gi|4504347	refseq_human (refseq_human_20140103.fasta)	hemoglobin subunit alpha [Homo sapiens]

10	20	30	40	50	60	70	80
MVLSPADKTN	VKAAWGKVGA	HAGEYGAEAL	ERMFLSFPTT	KTYFPHFDLS	HGSAQVKGHG	KKVADALTNA	VAHVDDMPNA
**90**	**100**	**110**	**120**	**130**	**140**	**150**	
LSALSDLHAH	KLRVDPVNFK	LLSHCLLVTL	AAHLPAEFTP	AVHASLDKFL	ASVSTVLTSK	YR	

Cmpd.	No. of Cmpds.	m/z meas.	Δ m/z [ppm]	z	Rt [min]	Score	P	Range	Sequence	Modification	Ratios
68	1	586.3403	4.44	2	30.8	24.9	1	2-12	M.VLSPADKTNVK.A		
184	6	765.3829	15.83	2	32.3	76.4	0	18-32	K.VGAHAGEYGAEALER.M		QD:QU 2.68
1886	25	536.2096	-132.70	2	53.9	49.3	0	33-41	R.MFLSFPTTK.T		
1189	11	917.4299	-21.44	2	45.0	51.1	0	42-57	K.TYFPHFDLSHGSAQVK.G		QD:QU 1.62
592	4	544.3095	-13.04	2	37.4	51.7	1	92-100	K.LRVDPVNFK.L		
1619	7	626.8457	-24.38	2	50.5	73.2	0	129-140	K.FLASVSTVLTSK.Y		

Detailed Protein Report

Protein 8: serotransferrin precursor [Homo sapiens]

Accession: gi|4557871
Database: refseq_human(refseq_human_20140103.fasta)
Database Date: 2015-11-30
Modification(s): Carbamidomethyl

Score: 315.8
MW [kDa]: 77.0
pI: 7.0
Sequence Coverage [%]: 17.9
No. of unique Peptides: 10

Quantitation

QD:QU **Median:** 0.72 **CV:** 41.99 % **No. of Peptides:** 7

10	20	30	40	50	60	70	80
MRLAVGALLV	CAVLGLCLAV	PDKTVRWCAV	SEHEATKCQS	FRDHMKSVIP	SDGPSVACVK	KASYLDCIRA	IAANEADAVT
90	100	110	120	130	140	150	160
LDAGLVYDAY	LAPNNLKPVV	AEFYGSKEDP	QTFYYAVAVV	KKDSGFQMNQ	LRGKKSCHTG	LGRSAGWNIP	IGLLYCDLPE
170	180	190	200	210	220	230	240
PRKPLEKAVA	NFFSGSCAPC	ADGTDFPQLC	QLCPGCGCST	LNQYFGYSGA	FKCLKDGAGD	VAFVKHSTIF	ENLANKADRD
250	260	270	280	290	300	310	320
QYELLCLDNT	RKPVDEYKDC	HLAQVPSHTV	VARSMGGKED	LIWELLNQAQ	EHFGKDKSKE	FQLFSSPHGK	DLLFKDSAHG
330	340	350	360	370	380	390	400
FLKVPPRMDA	KMYLGYEYVT	AIRNLREGTC	PEAPTDECKP	VKWCALSHHE	RLKCDEWSVN	SVGKIECVSA	ETTEDCIAKI
410	420	430	440	450	460	470	480
MNGEADAMSL	DGGFVYIAGK	CGLVPVLAEN	YNKSDNCEDT	PEAGYFAVAV	VKKSASDLTW	DNLKGKKSCH	TAVGRTAGWN
490	500	510	520	530	540	550	560
IPMGLLYNKI	NHCRFDEFFS	EGCAPGSKKD	SSLCKLCMGS	GLNLCEPNNK	EGYYGYTGAF	RCLVEKGDVA	FVKHQTVPQN
570	580	590	600	610	620	630	640
TGGKNPDPWA	KNLNEKDYEL	LCLDGTRKPV	EEYANCHLAR	APNHAVVTRK	DKEACVHKIL	RQQQHLFGSN	VTDCSGNFCL
650	660	670	680	690	700		
FRSETKDLLF	RDDTVCLAKL	HDRNTYEKYL	GEEYVKAVGN	LRKCSTSSLL	EACTFRRP		

Cmpd.	No. of Cmpds.	m/z meas.	Δ m/z [ppm]	z	Rt [min]	Score	P	Range	Sequence	Modification	Ratios	
610	3	708.3500	-19.16	2	36.2	66.2	0	47-60	K.SVIPSDGPSVACVK.K	Carbamidomethyl: 12	QD:QU	0.99
583	1	637.3237	-10.51	2	37.3	31.2	0	226-236	K.HSTIFENLANK.A		QD:QU	0.51
302	1	850.4388	3.54	3	33.8	10.5	1	252-273	R. KPVDEYKDCHLAQVPSHTVVAR. S	Carbamidomethyl: 9		
498	1	746.3968	18.25	2	36.2	42.7	1	298-310	K.SKEFQLFSSPHGK.D			
1032	3	638.7248	-148.43	2	41.4	30.2	0	300-310	K.EFQLFSSPHGK.D			
303	1	761.3763	5.77	2	33.8	37.9	1	372-384	R.LKCDEWSVNSVGK.I	Carbamidomethyl: 3	QD:QU	0.82
255	1	863.4074	23.48	2	33.2	14.5	0	385-399	K.IECVSAETTEDCIAK.I	Carbamidomethyl: 3, 12	QD:QU	0.87
954	1	789.3334	1.18	2	40.4	38.9	0	495-508	R.FDEFFSEGCAPGSK.K	Carbamidomethyl: 9	QD:QU	0.80
840	1	853.8816	-5.85	2	40.5	22.5	0	516-530	K.LCMGSGLNLCEPNNK.E	Carbamidomethyl: 2, 10	QD:QU	1.11
330	1	500.6281	-249.21	2	32.8	21.2	0	669-676	K.YLGEEYVK.A		QD:QU	0.32

Detailed Protein Report

Protein 9: keratin, type I cytoskeletal 14 [Homo sapiens]

**Accession:**	gi|15431310	**Score:**	291.3
**Database:**	refseq_human(refseq_human_20140103.fasta)	**MW [kDa]:**	51.6
**Database Date:**	2015-11-30	**pI:**	4.9
		**Sequence Coverage [%]:**	15.5
		**No. of unique Peptides:**	4

Quantitation

QD:QU **Median:** 1.31 **CV:** 22.43 % **No. of Peptides:** 3

10	20	30	40	50	60	70	80
MTTCSRQFTS	SSSMKGSCGI	GGGIGGGSSR	ISSVLAGGSC	RAPSTYGGGL	SVSSSRFSSG	GAYGLGGGYG	GGFSSSSSSF
**90**	**100**	**110**	**120**	**130**	**140**	**150**	**160**
GSGFGGGYGG	GLGAGLGGGF	GGGFAGGDGL	LVGSEKVTMQ	NLNDRLASYL	DKVRALEEAN	ADLEVKIRDW	YQRQRPAEIK
**170**	**180**	**190**	**200**	**210**	**220**	**230**	**240**
DYSPYFKTIE	DLRNKILTAT	VDNANVLLQI	DNARLAADDF	RTKYETELNL	RMSVEADING	LRRVLDELTL	ARADLEMQIE
**250**	**260**	**270**	**280**	**290**	**300**	**310**	**320**
SLKEELAYLK	KNHEEEMNAL	RGQVGGDVNV	EMDAAPGVDL	SRILNEMRDQ	YEKMAEKNRK	DAEEWFFTKT	EELNREVATN
**330**	**340**	**350**	**360**	**370**	**380**	**390**	**400**
SELVQSGKSE	ISELRRTMQN	LEIELQSQLS	MKASLENSLE	ETKGRYCMQL	AQIQEMIGSV	EEQLAQLRCE	MEQQNQEYKI
**410**	**420**	**430**	**440**	**450**	**460**	**470**	**480**
LLDVKTRLEQ	EIATYRRLLE	GEDAHLSSSQ	FSSGSQSSRD	VTSSSRQIRT	KVMDVHDGKV	VSTHEQVLRT	KN

Cmpd.	No. of Cmpds.	m/z meas.	Δ m/z [ppm]	z	Rt [min]	Score	P	Range	Sequence	Modification	Ratios
282	1	633.8578	31.13	2	33.5	20.5	1	202-211	R.TKYETELNLR.M		QD:QU 1.09
639	1	680.8203	-49.04	2	36.6	15.5	1	212-223	R.MSVEADINGLRR.V		QD:QU 1.79
1899	3	586.6929	-125.55	2	52.2	33.2	0	301-309	K.DAEEWFFTK.T		QD:QU 1.17
246	1	512.5147	-194.60	3	33.1	22.6	2	406-417	K.TRLEQEIATYRR.L		

Detailed Protein Report

Protein 10: keratin, type II cytoskeletal 5 [Homo sapiens]

**Accession:**	gi|119395754	**Score:**	260.0
**Database:**	refseq_human(refseq_human_20140103.fasta)	**MW [kDa]:**	62.3
**Database Date:**	2015-11-30	**pI:**	8.6
		**Sequence Coverage [%]:**	12.7
		**No. of unique Peptides:**	2

Quantitation

QD:QU **Median:** 0.60 **CV:** 66.96 % **No. of Peptides:** 2

10	20	30	40	50	60	70	80
MSRQSSVSFR	SGGSRSFSTA	SAITPSVSRT	SFTSVSRSGG	GGGGGFGRVS	LAGACGVGGY	GSRSLYNLGG	SKRISISTSG
**90**	**100**	**110**	**120**	**130**	**140**	**150**	**160**
GSFRNRFGAG	AGGGYGFGGG	AGSGFGFGGG	AGGGFGLGGG	AGFGGGFGGP	GFPVCPPGGI	QEVTVNQSLL	TPLNLQIDPS
**170**	**180**	**190**	**200**	**210**	**220**	**230**	**240**
IQRVRTEERE	QIKTLNNKFA	SFIDKVRFLE	QQNKVLDTKW	TLLQEQGTKT	VRQNLEPLFE	QYINNLRRQL	DSIVGERGRL
**250**	**260**	**270**	**280**	**290**	**300**	**310**	**320**
DSELRNMQDL	VEDFKNKYED	EINKRTTAEN	EFVMLKKDVD	AAYMNKVELE	AKVDALMDEI	NFMKMFFDAE	LSQMQTHVSD
**330**	**340**	**350**	**360**	**370**	**380**	**390**	**400**
TSVVLSMDNN	RNLDLDSIIA	EVKAQYEEIA	NRSRTEAESW	YQTKYEELQQ	TAGRHGDDLR	NTKHEISEMN	RMIQRLRAEI
**410**	**420**	**430**	**440**	**450**	**460**	**470**	**480**
DNVKKQCANL	QNAIADAEQR	GELALKDARN	KLAELEEALQ	KAKQDMARLL	REYQELMNTK	LALDVEIATY	RKLLEGEECR
**490**	**500**	**510**	**520**	**530**	**540**	**550**	**560**
LSGEGVGPVN	ISVVTSSVSS	GYGSGSGYGG	GLGGGLGGGL	GGGLAGGSSG	SYYSSSSGGV	GLGGGLSVGG	SGFSASSGRG
**570**	**580**	**590**	**600**				
LGVGFGSGGG	SSSSVKFVST	TSSSRKSFKS					

Cmpd.	No. of Cmpds.	m/z meas.	Δ m/z [ppm]	z	Rt [min]	Score	P	Range	Sequence	Modification	Ratios	
804	1	572.1668	-261.37	2	38.5	48.3	0	432-441	K.LAELEEALQK.A		QD:QU	0.33
477	2	720.3474	-17.32	2	34.6	20.1	0	560-576	R.GLGVGFGSGGGSSSSVK.F		QD:QU	1.10

Detailed Protein Report

Protein 11: apolipoprotein A-I preproprotein [Homo sapiens]

**Accession:**	gi|4557321	**Score:**	250.5
**Database:**	refseq_human(refseq_human_20140103.fasta)	**MW [kDa]:**	30.8
**Database Date:**	2015-11-30	**pI:**	5.5
		**Sequence Coverage [%]:**	29.2
		**No. of unique Peptides:**	8

Quantitation

QD:QU **Median:** 0.62 **CV:** 31.48 % **No. of Peptides:** 4

Alias proteins:

Accession	Name	Description
gi|530398069	refseq_human (refseq_human_20140103.fasta)	PREDICTED: apolipoprotein A-I isoform X2 [Homo sapiens]
gi|530398067	refseq_human (refseq_human_20140103.fasta)	PREDICTED: apolipoprotein A-I isoform X1 [Homo sapiens]

```
        10         20         30         40         50         60         70         80
MKAAVLTLAV LFLTGSQARH FWQQDEPPQS PWDRVKDLAT VYVDVLKDSG RDYVSQFEGS ALGKQLNLKL LDNWDSVTST
        90        100        110        120        130        140        150        160
FSKLREQLGP VTQEFWDNLE KETEGLRQEM SKDLEEVKAK VQPYLDDFQK KWQEEMELYR QKVEPLRAEL QEGARQKLHE
       170        180        190        200        210        220        230        240
LQEKLSPLGE EMRDRARAHV DALRTHLAPY SDELRQRLAA RLEALKENGG ARLAEYHAKA TEHLSTLSEK AKPALEDLRQ
       250        260        270
GLLPVLESFK VSFLSALEEY TKKLNTQ
```

Cmpd.	No. of Cmpds.	m/z meas.	Δ m/z [ppm]	z	Rt [min]	Score	P	Range	Sequence	Modification	Ratios	
2106	1	731.8566	-99.42	2	56.7	19.8	1	35-47	R.VKDLATVYVDVLK.D			
916	1	700.9155	110.19	2	41.5	24.5	0	52-64	R.DYVSQFEGSALGK.Q			
1831	7	806.7884	-133.73	2	53.2	64.1	0	70-83	K.LLDNWDSVTSTFSK.L			
681	1	626.6736	-224.11	2	37.1	28.6	0	121-130	K.VQPYLDDFQK.K		QD:QU	0.47
451	2	690.8634	2.76	2	35.6	14.6	1	121-131	K.VQPYLDDFQKK.W		QD:QU	1.04
860	2	642.2972	11.37	2	39.2	28.3	0	132-140	K.WQEEMELYR.Q		QD:QU	0.58
1475	3	896.3833	-111.83	1	48.6	11.9	0	158-164	K.LHELQEK.L			
283	3	651.3276	-0.37	2	33.5	58.6	0	185-195	R.THLAPYSDELR.Q		QD:QU	0.53

Detailed Protein Report

Protein 12: keratin, type II cytoskeletal 75 [Homo sapiens]

Accession: gi|153791158
Database: refseq_human(refseq_human_20140103.fasta)
Database Date: 2015-11-30

Score: 247.1
MW [kDa]: 59.5
pI: 8.7
Sequence Coverage [%]: 8.2
No. of unique Peptides: 1

10	20	30	40	50	60	70	80
MSRQSSITFQ	SGSRRGFSTT	SAITPAAGRS	RFSSVSVARS	AAGSGGLGRI	SSAGASFGSR	SLYNLGGAKR	VSINGCGSSC
90	100	110	120	130	140	150	160
RSGFGGRASN	RFGVNSGFGY	GGGVGGGFSG	PSFPVCPPGG	IQEVTVNQSL	LTPLHLQIDP	TIQRVRAEER	EQIKTLNNKF
170	180	190	200	210	220	230	240
ASFIDKVRFL	EQQNKVLETK	WALLQEQGSR	TVRQNLEPLF	DSYTSELRRQ	LESITTERGR	LEAELRNMQD	VVEDFKVRYE
250	260	270	280	290	300	310	320
DEINKRTAAE	NEFVALKKDV	DAAYMNKVEL	EAKVKSLPEE	INFIHSVFDA	ELSQLQTQVG	DTSVVLSMDN	NRNLDLDSII
330	340	350	360	370	380	390	400
AEVKAQYEDI	ANRSRAEAES	WYQTKYEELQ	VTAGRHGDDL	RNTKQEISEM	NRMIQRLRAE	IDSVKKQCSS	LQTAIADAEQ
410	420	430	440	450	460	470	480
RGELALKDAR	AKLVDLEEAL	QKAKQDMARL	LREYQELMNI	KLALDVEIAT	YRKLLEGEEC	RLSGEGVSPV	NISVVTSTLS
490	500	510	520	530	540	550	560
SGYGSGSSIG	GGNLGLGGGS	GYSFTTSGGH	SLGAGLGGSG	FSATSNRGLG	GSGSSVKFVS	TTSSSQKSYT	H

Cmpd.	No. of Cmpds.	m/z meas.	Δ m/z [ppm]	z	Rt [min]	Score	P	Range	Sequence	Modification
566	2	678.9555	96.02	2	35.7	53.0	1	413-424	K.LVDLEEALQKAK.Q	

Detailed Protein Report

Protein 13: PREDICTED: actin, cytoplasmic 2 isoform X1 [Homo sapiens]

Accession: gi|578831328
Database: refseq_human(refseq_human_20140103.fasta)
Database Date: 2015-11-30

Score: 225.8
MW [kDa]: 51.2
pI: 6.9
Sequence Coverage [%]: 19.2
No. of unique Peptides: 6

Quantitation

QD:QU **Median:** 0.99 **CV:** 65.74 % **No. of Peptides:** 3

10	20	30	40	50	60	70	80
MDMFWGRARR	RAARARRFRL	NNGGGGRGRS	QSPLPALALC	SSAAPPSRFS	AGERPAPGPE	LDVAGLRPPT	PAGPASSCRG
90	100	110	120	130	140	150	160
LVAPSPGTKG	GVAMEEEIAA	LVIDNGSGMC	KAGFAGDDAP	RAVFPSIVGR	PRHQGVMVGM	GQKDSYVGDE	AQSKRGILTL
170	180	190	200	210	220	230	240
KYPIEHGIVT	NWDDMEKIWH	HTFYNELRVA	PEEHPVLLTE	APLNPKANRE	KMTQIMFETF	NTPAMYVAIQ	AVLSLYASGR
250	260	270	280	290	300	310	320
TTGIVMDSGD	GVTHTVPIYE	GYALPHAILR	LDLAGRDLTD	YLMKILTERG	YSFTTTAERE	IVRDIKEKLC	YVALDFEQEM
330	340	350	360	370	380	390	400
ATAASSSSLE	KSYELPDGQV	ITIGNERFRC	PEALFQPSFL	GMESCGIHET	TFNSIMKCDV	DIRKDLYANT	VLSGGTTMYP
410	420	430	440	450	460	470	
GIADRMQKEI	TALAPSTMKI	KIIAPPERKY	SVWIGGSILA	SLSTFQQMWI	SKQEYDESGP	SIVHRKCF	

Cmpd.	No. of Cmpds.	m/z meas.	Δ m/z [ppm]	z	Rt [min]	Score	P	Range	Sequence	Modification	Ratios
659	5	599.7070	-249.11	2	36.8	51.4	0	122-132	R.AVFPSIVGRPR.H		
2136	4	651.9448	-125.01	3	55.3	28.9	0	189-206	R.VAPEEHPVLLTEAPLNPK.A		QD:QU 0.93
1678	5	895.8924	-63.80	2	49.5	48.9	0	332-347	K.SYELPDGQVITIGNER.F		
2043	1	1107.8969	-127.87	2	54.1	33.7	0	385-405	K.DLYANTVLSGGTTMYPGIADR.M		
563	1	581.2929	-34.36	2	37.0	51.0	0	409-419	K.EITALAPSTMK.I		QD:QU 2.13
139	1	758.8664	15.04	2	31.7	11.9	0	453-465	K.QEYDESGPSIVHR.K		QD:QU 0.49

Detailed Protein Report

Protein 14: titin isoform IC [Homo sapiens]

**Accession:**	gi|388998877	**Score:**	217.6
**Database:**	refseq_human(refseq_human_20140103.fasta)	**MW [kDa]:**	3992.1
**Database Date:**	2015-11-30	**pI:**	6.0
**Modification(s):**	Carbamidomethyl, Oxidation	**Sequence Coverage [%]:**	0.6
		**No. of unique Peptides:**	14

Quantitation

QD:QU	**Median:**	0.44	**CV:**	19.37 %	**No. of Peptides:**	2	

Detailed Protein Report

10	20	30	40	50	60	70	80
MTTQAPTFTQ	PLQSVVVLEG	STATFEAHIS	GFPVPEVSWF	RDGQVISTST	LPGVQISFSD	GRAKLTIPAV	TKANSGRYSL
90	100	110	120	130	140	150	160
KATNGSGQAT	STAELLVKAE	TAPPNFVQRL	QSMTVRQGSQ	VRLQVRVTGI	PTPVVKFYRD	GAEIQSSLDF	QISQEGDLYS
170	180	190	200	210	220	230	240
LLIAEAYPED	SGTYSVNATN	SVGRATSTAE	LLVQGEEEVP	AKKTKTIVST	AQISESRQTR	IEKKIEAHFD	ARSIATVEMV
250	260	270	280	290	300	310	320
IDGAAGQQLP	HKTPPRIPPK	PKSRSPTPPS	IAAKAQLARQ	QSPSPIRHSP	SPVRHVRAPT	PSPVRSVSPA	ARISTSPIRS
330	340	350	360	370	380	390	400
VRSPLLMRKT	QASTVATGPE	VPPPWKQEGY	VASSSEAEMR	ETTLTTSTQI	RTEERWEGRY	GVQEQVTISG	AAGAAASVSA
410	420	430	440	450	460	470	480
SASYAAEAVA	TGAKEVKQDA	DKSAAVATVV	AAVDMARVRE	PVISAVEQTA	QRTTTTAVHI	QPAQEQVRKE	AEKTAVTKVV
490	500	510	520	530	540	550	560
VAADKAKEQE	LKSRTKEVIT	TKQEQMHVTH	EQIRKETEKT	FVPKVVISAA	KAKEQETRIS	EEITKKQKQV	TQEAIRQETE
570	580	590	600	610	620	630	640
ITAASMVVVA	TAKSTKLETV	PGAQEETTTQ	QDQMHLSYEK	IMKETRKTVV	PKVIVATPKV	KEQDLVSRGR	EGITTKREQV
650	660	670	680	690	700	710	720
QITQEKMRKE	AEKTALSTIA	VATAKAKEQE	TILRTRETMA	TRQEQIQVTH	GKVDVGKKAE	AVATVVAAVD	QARVREPREP
730	740	750	760	770	780	790	800
GHLEESYAQQ	TTLEYGYKER	ISAAKVAEPP	QRPASEPHVV	PKAVKPRVIQ	APSETHIKTT	DQKGMHISSQ	IKKTTDLTTE
810	820	830	840	850	860	870	880
RLVHVDKRPR	TASPHFTVSK	ISVPKTEHGY	EASIAGSAIA	TLQKELSATS	SAQKITKSVK	APTVKPSETR	VRAEPTPLPQ
890	900	910	920	930	940	950	960
FPFADTPDTY	KSEAGVEVKK	EVGVSITGTT	VREERFEVLH	GREAKVTETA	RVPAPVEIPV	TPPTLVSGLK	NVTVIEGESV
970	980	990	1000	1010	1020	1030	1040
TLECHISGYP	SPTVTWYRED	YQIESSIDFQ	ITFQSGIARL	MIREAFAEDS	GRFTCSAVNE	AGTVSTSCYL	AVQVSEEFEK
1050	1060	1070	1080	1090	1100	1110	1120
ETTAVTEKFT	TEEKRFVESR	DVVMTDTSLT	EEQAGPGEPA	APYFITKPVV	QKLVEGGSVV	FGCQVGGNPK	PHVYWKKSGV
1130	1140	1150	1160	1170	1180	1190	1200
PLTTGYRYKV	SYNKQTGECK	LVISMTFADD	AGEYTIVVRN	KHGETSASAS	LLEEADYELL	MKSQQEMLYQ	TQVTAFVQEP
1210	1220	1230	1240	1250	1260	1270	1280
KVGETAPGFV	YSEYEKEYEK	EQALIRKKMA	KDTVVVRTYV	EDQEFHISSF	EERLIKEIEY	RIIKTTLEEL	LEEDGEEKMA
1290	1300	1310	1320	1330	1340	1350	1360
VDISESEAVE	SGFDSRIKNY	RILEGMGVTF	HCKMSGYPLP	KIAWYKDGKR	IKHGERYQMD	FLQDGRASLR	IPVVLPEDEG
1370	1380	1390	1400	1410	1420	1430	1440
IYTAFASNIK	GNAICSGKLY	VEPAAPLGAP	TYIPTLEPVS	RIRSLSPRSV	SRSPIRMSPA	RMSPARMSPA	RMSPARMSPG
1450	1460	1470	1480	1490	1500	1510	1520
RRLEETDESQ	LERLYKPVFV	LKPVSFKCLE	GQTARFDLKV	VGRPMPETFW	FHDGQQIVND	YTHKVVIKED	GTQSLIIVPA
1530	1540	1550	1560	1570	1580	1590	1600
TPSDSGEWTV	VAQNRAGRSS	ISVILTVEAV	EHQVKPMFVE	KLKNVNIKEG	SRLEMKVRAT	GNPNPDIVWL	KNSDIIVPHK
1610	1620	1630	1640	1650	1660	1670	1680
YPKIRIEGTK	GEAALKIDST	VSQDSAWYTA	TAINKAGRDT	TRCKVNVEVE	FAEPEPERKL	IIPRGTYRAK	EIAAPELEPL
1690	1700	1710	1720	1730	1740	1750	1760
HLRYGQEQWE	EGDLYDKEKQ	QKPFFKKKLT	SLRLKRFGPA	HFECRLTPIG	DPTMVVEWLH	DGKPLEAANR	LRMINEFGYC
1770	1780	1790	1800	1810	1820	1830	1840
SLDYGVAYSR	DSGIITCRAT	NKYGTDHTSA	TLIVKDEKSL	VEESQLPEGR	KGLQRIEELE	RMAHEGALTG	VTTDQKEKQK
1850	1860	1870	1880	1890	1900	1910	1920
PDIVLYPEPV	RVLEGETARF	RCRVTGYPQP	KVNWYLNGQL	IRKSKRFRVR	YDGIHYLDIV	DCKSYDTGEV	KVTAENPEGV
1930	1940	1950	1960	1970	1980	1990	2000
IEHKVKLEIQ	QREDFRSVLR	RAPEPRPEFH	VHEPGKLQFE	VQKVDRPVDT	TETKEVVKLK	RAERITHEKV	PEESEELRSK
2010	2020	2030	2040	2050	2060	2070	2080
FKRRTEEGYY	EAITAVELKS	RKKDESYEEL	LRKTKDELLH	WTKELTEEEK	KALAEEGKIT	IPTFKPDKIE	LSPSMEAPKI
2090	2100	2110	2120	2130	2140	2150	2160
FERIQSQTVG	QGSDAHFRVR	VVGKPDPECE	WYKNGVKIER	SDRIYWYWPE	DNVCELVIRD	VTAEDSASIM	VKAINIAGET
2170	2180	2190	2200	2210	2220	2230	2240

Detailed Protein Report

Cmpd.	No. of Cmpds.	m/z meas.	Δ m/z [ppm]	z	Rt [min]	Score	P	Range	Sequence	Modification	Ratios	
1676	1	839.3937	-10.73	2	49.5	10.3	2	1417-1431	R.MSPARMSPARMSPAR.M	Oxidation: 6, 11		
2713	1	698.3756	114.21	2	62.8	14.9	0	8326-8337	K.VDHSDVGEYSCK.A	Carbamidomethyl: 11		
967	1	752.8772	-83.84	2	40.6	12.6	2	10852-10864	K.VPEEPKKPVPEKK.V			
2926	1	713.8984	-28.68	2	65.7	11.1	1	11452-11464	K.VEPPPPPKVPEIK.K			
1106	9	555.2011	-153.95	2	43.9	16.9	0	13889-13899	K.GTAIFACDIAK.D			
1925	1	661.3041	-146.74	2	54.4	13.5	1	15198-15209	R.AAAHLTVIEKLR.I		QD:QU	0.53
707	3	481.1631	-198.64	2	38.8	13.1	0	15703-15710	K.TWVLATDR.A		QD:QU	0.36
1842	1	740.3191	-71.84	2	53.4	15.5	2	16189-16201	K.GYIVERCPRGSDK.W			
2499	1	813.3822	-3.46	2	61.8	12.9	0	19641-19653	R.VPDLLEGCQYEFR.V	Carbamidomethyl: 8		
2869	1	710.3695	-29.92	2	65.0	10.9	0	30145-30156	R.IGHNVHLELPYK.G			
69	1	731.7932	-168.95	2	30.8	10.2	1	31168-31179	K.QLTFTVERLVEK.T			
560	1	607.8375	45.47	2	37.0	10.1	1	32279-32289	R.AQNEKGVSEPR.E			
2686	1	937.4503	-21.14	2	62.5	12.9	0	33057-33072	K.QFTIGGLLEATEYEFR.V			
658	1	677.6643	-16.40	3	38.2	11.8	2	33663-33680	K.DAGFYVVCAKNRFGIDQK.T			

BRUKER

Detailed Protein Report

Protein 15: lactotransferrin isoform 1 precursor [Homo sapiens]

**Accession:**	gi|54607120	**Score:**	206.6
**Database:**	refseq_human(refseq_human_20140103.fasta)	**MW [kDa]:**	78.1
**Database Date:**	2015-11-30	**pI:**	9.6
**Modification(s):**	Carbamidomethyl	**Sequence Coverage [%]:**	12.7
		**No. of unique Peptides:**	7

Quantitation

QD:QU **Median:** 1.20 **CV:** 40.11 % **No. of Peptides:** 3

10	20	30	40	50	60	70	80
MKLVFLVLLF	LGALGLCLAG	RRRSVQWCAV	SQPEATKCFQ	WQRNMRKVRG	PPVSCIKRDS	PIQCIQAIAE	NRADAVTLDG
90	100	110	120	130	140	150	160
GFIYEAGLAP	YKLRPVAAEV	YGTERQPRTH	YYAVAVVKKG	GSFQLNELQG	LKSCHTGLRR	TAGWNVPIGT	LRPFLNWTGP
170	180	190	200	210	220	230	240
PEPIEAAVAR	FFSASCVPGA	DKGQFPNLCR	LCAGTGENKC	AFSSQEPYFS	YSGAFKCLRD	GAGDVAFIRE	STVFEDLSDE
250	260	270	280	290	300	310	320
AERDEYELLC	PDNTRKPVDK	FKDCHLARVP	SHAVVARSVN	GKEDAIWNLL	RQAQEKFGKD	KSPKFQLFGS	PSGQKDLLFK
330	340	350	360	370	380	390	400
DSAIGFSRVP	PRIDSGLYLG	SGYFTAIQNL	RKSEEEVAAR	RARVVWCAVG	EQELRKCNQW	SGLSEGSVTC	SSASTTEDCI
410	420	430	440	450	460	470	480
ALVLKGEADA	MSLDGGYVYT	AGKCGLVPVL	AENYKSQQSS	DPDPNCVDRP	VEGYLAVAVV	RRSDTSLTWN	SVKGKKSCHT
490	500	510	520	530	540	550	560
AVDRTAGWNI	PMGLLFNQTG	SCKFDEYFSQ	SCAPGSDPRS	NLCALCIGDE	QGENKCVPNS	NERYYGYTGA	FRCLAENAGD
570	580	590	600	610	620	630	640
VAFVKDVTVL	QNTDGNNNEA	WAKDLKLADF	ALLCLDGKRK	PVTEARSCHL	AMAPNHAVVS	RMDKVERLKQ	VLLHQQAKFG
650	660	670	680	690	700	710	720
RNGSDCPDKF	CLFQSETKNL	LFNDNTECLA	RLHGKTTYEK	YLGPQYVAGI	TNLKKCSTSP	LLEACEFLRK	

Cmpd.	No. of Cmpds.	m/z meas.	Δ m/z [ppm]	z	Rt [min]	Score	P	Range	Sequence	Modification	Ratios
1629	2	695.8567	-18.94	2	48.9	36.9	0	120-132	K.GGSFQLNELQGLK.S		
739	1	787.3768	-43.94	2	39.2	13.3	1	364-376	R.VVWCAVGEQELRK.C	Carbamidomethyl: 4	
1575	3	681.8469	-16.13	2	49.9	23.1	0	424-435	K.CGLVPVLAENYK.S	Carbamidomethyl: 1	
458	1	619.2938	-20.74	2	35.7	45.8	0	463-473	R.SDTSLTWNSVK.G		QD:QU 1.81
593	1	904.3991	-2.21	2	37.4	38.9	0	520-535	R.SNLCALCIGDEQGENK.C	Carbamidomethyl: 4, 7	QD:QU 1.33
2950	4	668.3368	-23.49	2	66.0	27.5	0	587-598	K.LADFALLCLDGK.R	Carbamidomethyl: 8	
1229	1	790.3991	23.83	2	45.5	20.9	0	659-671	K.NLLFNDNTECLAR.L	Carbamidomethyl: 10	QD:QU 0.71

Detailed Protein Report

Protein 16: keratin, type II cytoskeletal 2 epidermal [Homo sapiens]

Accession: gi|47132620
Database: refseq_human(refseq_human_20140103.fasta)
Database Date: 2015-11-30

Score: 201.3
MW [kDa]: 65.4
pI: 8.9
Sequence Coverage [%]: 5.5
No. of unique Peptides: 1

Quantitation

QD:QU **Median:** 1.22 **CV:** 0.00 % **No. of Peptides:** 1

10	20	30	40	50	60	70	80
MSCQISCKSR	GRGGGGGGFR	GFSSGSAVVS	GGSRRSTSSF	SCLSRHGGGG	GGFGGGGFGS	RSLVGLGGTK	SISISVAGGG
90	100	110	120	130	140	150	160
GGFGAAGGFG	GRGGGFGGGS	SFGGGSGFSG	GGFGGGGFGG	GRFGGFGGPG	GVGGLGGPGG	FGPGGYPGGI	HEVSVNQSLL
170	180	190	200	210	220	230	240
QPLNVKVDPE	IQNVKAQERE	QIKTLNNKFA	SFIDKVRFLE	QQNQVLQTKW	ELLQQMNVGT	RPINLEPIFQ	GYIDSLKRYL
250	260	270	280	290	300	310	320
DGLTAERTSQ	NSELNNMQDL	VEDYKKKYED	EINKRTAAEN	DFVTLKKDVD	NAYMIKVELQ	SKVDLLNQEI	EFLKVLYDAE
330	340	350	360	370	380	390	400
ISQIHQSVTD	TNVILSMDNS	RNLDLDSIIA	EVKAQYEEIA	QRSKEEAEAL	YHSKYEELQV	TVGRHGDSLK	EIKIEISELN
410	420	430	440	450	460	470	480
RVIQRLQGEI	AHVKKQCKNV	QDAIADAEQR	GEHALKDARN	KLNDLEEALQ	QAKEDLARLL	RDYQELMNVK	LALDVEIATY
490	500	510	520	530	540	550	560
RKLLEGEECR	MSGDLSSNVT	VSVTSSTISS	NVASKAAFGG	SGGRGSSSGG	GYSSGSSSYG	SGGRQSGSRG	GSGGGGSISG
570	580	590	600	610	620	630	640
GGYGSGGGSG	GRYGSGGGSK	GGSISGGGYG	SGGGKHSSGG	GSRGGSSSGG	GYGSGGGGSS	SVKGSSGEAF	GSSVTFSFR

Cmpd.	No. of Cmpds.	m/z meas.	Δ m/z [ppm]	z	Rt [min]	Score	P	Range	Sequence	Modification	Ratios
412	1	738.3807	-21.14	2	35.1	70.4	0	198-209	R.FLEQQNQVLQTK.W		QD:QU 1.22

Detailed Protein Report

Protein 17: actin, aortic smooth muscle [Homo sapiens]

**Accession:**	gi|4501883	**Score:**	199.2
**Database:**	refseq_human(refseq_human_20140103.fasta)	**MW [kDa]:**	42.0
**Database Date:**	2015-11-30	**pI:**	5.1
**Modification(s):**	Carbamidomethyl	**Sequence Coverage [%]:**	23.3
		**No. of unique Peptides:**	3

Quantitation

QD:QU **Median:** 0.25 **CV:** 0.00 % **No. of Peptides:** 1

Alias proteins:

Accession	Name	Description
gi|213688375	refseq_huma (refseq_human_20140103.fasta)	actin, aortic smooth muscle [Homo sapiens]

```
        10         20         30         40         50         60         70         80
MCEEEDSTAL VCDNGSGLCK AGFAGDDAPR AVFPSIVGRP RHQGVMVGMG QKDSYVGDEA QSKRGILTLK YPIEHGIITN
        90        100        110        120        130        140        150        160
WDDMEKIWHH SFYNELRVAP EEHPTLLTEA PLNPKANREK MTQIMFETFN VPAMYVAIQA VLSLYASGRT TGIVLDSGDG
       170        180        190        200        210        220        230        240
VTHNVPIYEG YALPHAIMRL DLAGRDLTDY LMKILTERGY SFVTTAEREI VRDIKEKLCY VALDFENEMA TAASSSSLEK
       250        260        270        280        290        300        310        320
SYELPDGQVI TIGNERFRCP ETLFQPSFIG MESAGIHETT YNSIMKCDID IRKDLYANNV LSGGTTMYPG IADRMQKEIT
       330        340        350        360        370        380
ALAPSTMKIK IIAPPERKYS VWIGGSILAS LSTFQQMWIS KQEYDEAGPS IVHRKCF
```

Cmpd.	No. of Cmpds.	m/z meas.	Δ m/z [ppm]	z	Rt [min]	Score	P	Range	Sequence	Modification	Ratios
2365	1	1015.4376	26.15	2	60.0	17.3	0	2-20	M.CEEEDSTALVCDNGSGLCK.A	Carbamidomethyl: 11	
2088	2	652.6205	-100.50	3	54.7	15.4	0	98-115	R.VAPEEHPTLLTEAPLNPK.A		
267	1	750.8467	-14.36	2	32.1	15.2	0	362-374	K.QEYDEAGPSIVHR.K		QD:QU 0.25

Detailed Protein Report

Protein 18: annexin A1 [Homo sapiens]

**Accession:**	gi|4502101	**Score:**	185.4
**Database:**	refseq_human(refseq_human_20140103.fasta)	**MW [kDa]:**	38.7
**Database Date:**	2015-11-30	**pI:**	6.7
		**Sequence Coverage [%]:**	18.2
		**No. of unique Peptides:**	4

Quantitation

QD:QU **Median:** 1.18 **CV:** 49.61 % **No. of Peptides:** 3

10	20	30	40	50	60	70	80
MAMVSEFLKQ	AWFIENEEQE	YVQTVKSSKG	GPGSAVSPYP	TFNPSSDVAA	LHKAIMVKGV	DEATIIDILT	KRNNAQRQQI
**90**	**100**	**110**	**120**	**130**	**140**	**150**	**160**
KAAYLQETGK	PLDETLKKAL	TGHLEEVVLA	LLKTPAQFDA	DELRAAMKGL	GTDEDTLIEI	LASRTNKEIR	DINRVYREEL
**170**	**180**	**190**	**200**	**210**	**220**	**230**	**240**
KRDLAKDITS	DTSGDFRNAL	LSLAKGDRSE	DFGVNEDLAD	SDARALYEAG	ERRKGTDVNV	FNTILTTRSY	PQLRRVFQKY
**250**	**260**	**270**	**280**	**290**	**300**	**310**	**320**
TKYSKHDMNK	VLDLELKGDI	EKCLTAIVKC	ATSKPAFFAE	KLHQAMKGVG	TRHKALIRIM	VSRSEIDMND	IKAFYQKMYG
**330**	**340**	**350**					
ISLCQAILDE	TKGDYEKILV	ALCGGN					

Cmpd.	No. of Cmpds.	m/z meas.	Δ m/z [ppm]	z	Rt [min]	Score	P	Range	Sequence	Modification	Ratios	
1385	1	786.0874	38.51	3	47.5	12.5	0	30-53	K. GGPGSAVSPYPTFNPSSDVAALH A			
537	1	635.6517	-48.42	3	35.3	22.1	1	82-98	K.AAYLQETGKPLDETLKK.A		QD:QU	1.49
422	1	631.7779	-41.67	2	35.3	82.8	0	114-124	K.TPAQFDADELR.A		QD:QU	0.61
174	1	607.2652	-8.28	2	30.9	68.2	0	167-177	K.DITSDTSGDFR.N		QD:QU	1.80

Detailed Protein Report

Protein 19: keratin, type II cytoskeletal 79 [Homo sapiens]

**Accession:**	gi|32567786	**Score:**	141.6
**Database:**	refseq_human(refseq_human_20140103.fasta)	**MW [kDa]:**	57.8
**Database Date:**	2015-11-30	**pI:**	6.9
**Modification(s):**	Oxidation	**Sequence Coverage [%]:**	6.7
		**No. of unique Peptides:**	1

10	20	30	40	50	60	70	80
MRSSVSRQTY	STKGGFSSNS	ASGGSGSQAR	TSFSSVTVSR	SSGSGGGAHC	GPGTGGFGSR	SLYNLGGHKS	ISVSVAGGAL
**90**	**100**	**110**	**120**	**130**	**140**	**150**	**160**
LGRALGGFGF	GSRAFMGQGA	GRQTFGPACP	PGGIQEVTVN	QSLLTPLHVE	IDPEIQRVRT	QEREQIKTLN	NKFASFIDKV
**170**	**180**	**190**	**200**	**210**	**220**	**230**	**240**
RFLEQQNKVL	ETKWALLQEQ	GQNLGVTRNN	LEPLFEAYLG	SMRSTLDRLQ	SERGRLDSEL	RNVQDLVEDF	KNKYEDEINK
**250**	**260**	**270**	**280**	**290**	**300**	**310**	**320**
HTAAENEFVV	LKKDVDAAYM	GRMDLHGKVG	TLTQEIDFLQ	QLYEMELSQV	QTHVSNTNVV	LSMDNNRNLD	LDSIIAEVKA
**330**	**340**	**350**	**360**	**370**	**380**	**390**	**400**
QYELIAQRSR	AEAEAWYQTK	YEELQVTAGK	HGDNLRDTKN	EIAELTRTIQ	RLQGEADAAK	KQCQQLQTAI	AEAEQRGELA
**410**	**420**	**430**	**440**	**450**	**460**	**470**	**480**
LKDAQKKLGD	LDVALHQAKE	DLTRLLRDYQ	ELMNVKLALD	VEIATYRKLL	ESEESRMSGE	CPSAVSISVT	GNSTTVCGGG
**490**	**500**	**510**	**520**	**530**	**540**		
AASFGGGISL	GGSGGATKGG	FSTNVGYSTV	KGGPVSAGTS	ILRKTTTVKT	SSQRY		

Cmpd.	No. of Cmpds.	m/z meas.	Δ m/z [ppm]	z	Rt [min]	Score	P	Range	Sequence	Modification
140	1	773.9092	30.36	2	31.7	10.7	2	1-13	-.MRSSVSRQTYSTK.G	Oxidation: 1

Detailed Protein Report

Protein 20: alpha-2-macroglobulin precursor [Homo sapiens]

Accession: gi|66932947
Database: refseq_human(refseq_human_20140103.fasta)
Database Date: 2015-11-30
Modification(s): Carbamidomethyl

Score: 141.3
MW [kDa]: 163.2
pI: 6.0
Sequence Coverage [%]: 5.4
No. of unique Peptides: 6

Quantitation

QD:QU **Median:** 0.93 **CV:** 0.00 % **No. of Peptides:** 1

10	20	30	40	50	60	70	80
MGKNKLLHPS	LVLLLLVLLP	TDASVSGKPQ	YMVLVPSLLH	TETTEKGCVL	LSYLNETVTV	SASLESVRGN	RSLFTDLEAE
90	100	110	120	130	140	150	160
NDVLHCVAFA	VPKSSSNEEV	MFLTVQVKGP	TQEFKKRTTV	MVKNEDSLVF	VQTDKSIYKP	GQTVKFRVVS	MDENFHPLNE
170	180	190	200	210	220	230	240
LIPLVYIQDP	KGNRIAQWQS	FQLEGGLKQF	SFPLSSEPFQ	GSYKVVVQKK	SGGRTEHPFT	VEEFVLPKFE	VQVTVPKIIT
250	260	270	280	290	300	310	320
ILEEEMNVSV	CGLYTYGKPV	PGHVTVSICR	KYSDASDCHG	EDSQAFCEKF	SGQLNSHGCF	YQQVKTKVFQ	LKRKEYEMKL
330	340	350	360	370	380	390	400
HTEAQIQEEG	TVVELTGRQS	SEITRTITKL	SFVKVDSHFR	QGIPFFGQVR	LVDGKGVPIP	NKVIFIRGNE	ANYYSNATTD
410	420	430	440	450	460	470	480
EHGLVQFSIN	TTNVMGTSLT	VRVNYKDRSP	CYGYQWVSEE	HEEAHHTAYL	VFSPSKSFVH	LEPMSHELPC	GHTQTVQAHY
490	500	510	520	530	540	550	560
ILNGGTLLGL	KKLSFYYLIM	AKGGIVRTGT	HGLLVKQEDM	KGHFSISIPV	KSDIAPVARL	LIYAVLPTGD	VIGDSAKYDV
570	580	590	600	610	620	630	640
ENCLANKVDL	SFSPSQSLPA	SHAHLRVTAA	PQSVCALRAV	DQSVLLMKPD	AELSASSVYN	LLPEKDLTGF	PGPLNDQDDE
650	660	670	680	690	700	710	720
DCINRHNVYI	NGITYTPVSS	TNEKDMYSFL	EDMGLKAFTN	SKIRKPKMCP	QLQQYEMHGP	EGLRVGFYES	DVMGRGHARL
730	740	750	760	770	780	790	800
VHVEEPHTET	VRKYFPETWI	WDLVVVNSAG	VAEVGVTVPD	TITEWKAGAF	CLSEDAGLGI	SSTASLRAFQ	PFFVELTMPY
810	820	830	840	850	860	870	880
SVIRGEAFTL	KATVLNYLPK	CIRVSVQLEA	SPAFLAVPVE	KEQAPHCICA	NGRQTVSWAV	TPKSLGNVNF	TVSAEALESQ
890	900	910	920	930	940	950	960
ELCGTEVPSV	PEHGRKDTVI	KPLLVEPEGL	EKETTFNSLL	CPSGGEVSEE	LSLKLPPNVV	EESARASVSV	LGDILGSAMQ
970	980	990	1000	1010	1020	1030	1040
NTQNLLQMPY	GCGEQNMVLF	APNIYVLDYL	NETQQLTPEI	KSKAIGYLNT	GYQRQLNYKH	YDGSYSTFGE	RYGRNQGNTW
1050	1060	1070	1080	1090	1100	1110	1120
LTAFVLKTFA	QARAYIFIDE	AHITQALIWL	SQRQKDNGCF	RSSGSLLNNA	IKGGVEDEVT	LSAYITIALL	EIPLTVTHPV
1130	1140	1150	1160	1170	1180	1190	1200
VRNALFCLES	AWKTAQEGDH	GSHVYTKALL	AYAFALAGNQ	DKRKEVLKSL	NEEAVKKDNS	VHWERPQKPK	APVGHFYEPQ
1210	1220	1230	1240	1250	1260	1270	1280
APSAEVEMTS	YVLLAYLTAQ	PAPTSEDLTS	ATNIVKWITK	QQNAQGGFSS	TQDTVVALHA	LSKYGAATFT	RTGKAAQVTI
1290	1300	1310	1320	1330	1340	1350	1360
QSSGTFSSKF	QVDNNNRLLL	QQVSLPELPG	EYSMKVTGEG	CVYLQTSLKY	NILPEKEEFP	FALGVQTLPQ	TCDEPKAHTS
1370	1380	1390	1400	1410	1420	1430	1440
FQISLSVSYT	GSRSASNMAI	VDVKMVSGFI	PLKPTVKMLE	RSNHVSRTEV	SSNHVLIYLD	KVSNQTLSLF	FTVLQDVPVR
1450	1460	1470	1480				
DLKPAIVKVY	DYYETDEFAI	AEYNAPCSKD	LGNA				

Cmpd.	No. of Cmpds.	m/z meas.	Δ m/z [ppm]	z	Rt [min]	Score	P	Range	Sequence	Modification	Ratios
743	1	697.8370	-9.33	2	39.3	19.9	0	124-135	K.NEDSLVFVQTDK.S		
2151	2	802.7866	-172.67	2	55.5	12.9	0	175-188	R.IAQWQSFQLEGGLK.Q		
276	1	636.8299	-16.03	2	33.4	30.1	0	587-598	R.VTAAPQSVCALR.A	Carbamidomethyl: 9	
501	1	558.7829	-41.39	2	36.2	34.5	0	854-863	R.QTVSWAVTPK.S		QD:QU 0.93
2729	1	783.4212	1.90	2	65.2	28.1	0	1148-1162	K.ALLAYAFALAGNQDK.R		
2649	1	973.5082	-44.93	2	64.0	15.9	1	1385-	K.MVSGFIPLKPTVKMLER.S		

Detailed Protein Report

Cmpd.	No. of Cmpds.	m/z meas.	Δ m/z [ppm]	z	Rt [min]	Score	P	Range	Sequence	Modification	Ratios
								1401			

Detailed Protein Report

Protein 21: alpha-1-antitrypsin precursor [Homo sapiens]

**Accession:**	gi|50363217	**Score:**	136.1
**Database:**	refseq_human(refseq_human_20140103.fasta)	**MW [kDa]:**	46.7
**Database Date:**	2015-11-30	**pI:**	5.3
		**Sequence Coverage [%]:**	10.5
		**No. of unique Peptides:**	3

Quantitation

QD:QU **Median:** 0.69 **CV:** 7.16 % **No. of Peptides:** 2

Alias proteins:

Accession	Name	Description
gi|189163542	refseq_human (refseq_human_20140103.fasta)	alpha-1-antitrypsin precursor [Homo sapiens]
gi|189163540	refseq_human (refseq_human_20140103.fasta)	alpha-1-antitrypsin precursor [Homo sapiens]
gi|189163538	refseq_human (refseq_human_20140103.fasta)	alpha-1-antitrypsin precursor [Homo sapiens]
gi|189163536	refseq_human (refseq_human_20140103.fasta)	alpha-1-antitrypsin precursor [Homo sapiens]
gi|189163534	refseq_human (refseq_human_20140103.fasta)	alpha-1-antitrypsin precursor [Homo sapiens]
gi|189163532	refseq_human (refseq_human_20140103.fasta)	alpha-1-antitrypsin precursor [Homo sapiens]
gi|189163530	refseq_human (refseq_human_20140103.fasta)	alpha-1-antitrypsin precursor [Homo sapiens]
gi|189163528	refseq_human (refseq_human_20140103.fasta)	alpha-1-antitrypsin precursor [Homo sapiens]
gi|50363221	refseq_human (refseq_human_20140103.fasta)	alpha-1-antitrypsin precursor [Homo sapiens]
gi|50363219	refseq_human (refseq_human_20140103.fasta)	alpha-1-antitrypsin precursor [Homo sapiens]

```
        10         20         30         40         50         60         70         80
MPSSVSWGIL LLAGLCCLVP VSLAEDPQGD AAQKTDTSHH DQDHPTFNKI TPNLAEFAFS LYRQLAHQSN STNIFFSPVS
        90        100        110        120        130        140        150        160
IATAFAMLSL GTKADTHDEI LEGLNFNLTE IPEAQIHEGF QELLRTLNQP DSQLQLTTGN GLFLSEGLKL VDKFLEDVKK
       170        180        190        200        210        220        230        240
LYHSEAFTVN FGDTEEAKKQ INDYVEKGTQ GKIVDLVKEL DRDTVFALVN YIFFKGKWER PFEVKDTEEE DFHVDQVTTV
       250        260        270        280        290        300        310        320
KVPMMKRLGM FNIQHCKKLS SWVLLMKYLG NATAIFFLPD EGKLQHLENE LTHDIITKFL ENEDRRSASL HLPKLSITGT
       330        340        350        360        370        380        390        400
YDLKSVLGQL GITKVFSNGA DLSGVTEEAP LKLSKAVHKA VLTIDEKGTE AAGAMFLEAI PMSIPPEVKF NKPFVFLMIE
       410        420
QNTKSPLFMG KVVNPTQK
```

Cmpd.	No. of Cmpds.	m/z meas.	Δ m/z [ppm]	z	Rt [min]	Score	P	Range	Sequence	Modification	Ratios	
2910	3	858.7649	-23.64	3	65.6	35.3	0	126-149	R.TLNQPDSQLQLTTGNGLFLSEGLKL			
904	5	555.7989	-12.32	2	39.8	70.1	0	315-324	K.LSITGTYDLK.S		QD:QU	0.64
1288	2	508.1666	-283.94	2	44.6	30.8	0	325-334	K.SVLGQLGITK.V		QD:QU	0.74

Detailed Protein Report

Protein 22: keratin, type II cytoskeletal 4 [Homo sapiens]

Accession: gi|331999954
Database: refseq_human(refseq_human_20140103.fasta)
Database Date: 2015-11-30

Score: 127.3
MW [kDa]: 56.1
pI: 6.2
Sequence Coverage [%]: 9.2
No. of unique Peptides: 5

Quantitation

QD:QU **Median:** 0.40 **CV:** 31.96 % **No. of Peptides:** 4

10	20	30	40	50	60	70	80
MIARQQCVRG	GPRGFSCGSA	IVGGGKRGAF	SSVSMSGGAG	RCSSGGFGSR	SLYNLRGNKS	ISMSVAGSRQ	GACFGGAGGF
**90**	**100**	**110**	**120**	**130**	**140**	**150**	**160**
GTGGFGGGFG	GSFSGKGGPG	FPVCPAGGIQ	EVTINQSLLT	PLHVEIDPEI	QKVRTEEREQ	IKLLNNKFAS	FIDKVQFLEQ
**170**	**180**	**190**	**200**	**210**	**220**	**230**	**240**
QNKVLETKWN	LLQQQTTTTS	SKNLEPLFET	YLSVLRKQLD	TLGNDKGRLQ	SELKTMQDSV	EDFKTKYEEE	INKRTAAEND
**250**	**260**	**270**	**280**	**290**	**300**	**310**	**320**
FVVLKKDVDA	AYLNKVELEA	KVDSLNDEIN	FLKVLYDAEL	SQMQTHVSDT	SVVLSMDNNR	NLDLDSIIAE	VRAQYEEIAQ
**330**	**340**	**350**	**360**	**370**	**380**	**390**	**400**
RSKAEAEALY	QTKVQQLQIS	VDQHGDNLKN	TKSEIAELNR	MIQRLRAEIE	NIKKQCQTLQ	VSVADAEQRG	ENALKDAHSK
**410**	**420**	**430**	**440**	**450**	**460**	**470**	**480**
RVELEAALQQ	AKEELARMLR	EYQELMSVKL	ALDIEIATYR	KLLEGEEYRM	SGECQSAVSI	SVVSGSTSTG	GISGGLGSGS
**490**	**500**	**510**	**520**	**530**			
GFGLSSGFGS	GSGSGFGFGG	SVSGSSSSKI	ISTTTLNKRR				

Cmpd.	No. of Cmpds.	m/z meas.	Δ m/z [ppm]	z	Rt [min]	Score	P	Range	Sequence	Modification	Ratios	
950	1	603.8017	-33.37	2	40.4	11.5	0	235-245	R.TAAENDFVVLK.K		QD:QU	0.46
530	1	667.8552	-21.26	2	35.2	56.2	1	235-246	R.TAAENDFVVLKK.D		QD:QU	0.57
2080	1	703.8136	-68.38	2	54.6	13.6	0	262-273	K.VDSLNDEINFLK.V			
906	1	679.3419	-40.90	2	39.8	17.2	0	301-312	R.NLDLDSIIAEVR.A		QD:QU	0.24
554	1	678.3667	-28.00	2	35.5	28.7	1	401-412	K.RVELEAALQQAK.E		QD:QU	0.41

Detailed Protein Report

Protein 23: keratin, type I cytoskeletal 10 [Homo sapiens]

**Accession:**	gi|195972866	**Score:**	126.7
**Database:**	refseq_human(refseq_human_20140103.fasta)	**MW [kDa]:**	58.8
**Database Date:**	2015-11-30	**pI:**	5.0
		**Sequence Coverage [%]:**	6.0
		**No. of unique Peptides:**	1

10	20	30	40	50	60	70	80
MSVRYSSSKH	YSSSRSGGGG	GGGGCGGGGG	VSSLRISSSK	GSLGGGFSSG	GFSGGSFSRG	SSGGGCFGGS	SGGYGGLGGF
90	100	110	120	130	140	150	160
GGGSFRGSYG	SSSFGGSYGG	SFGGGSFGGG	SFGGGSFGGG	GFGGGGFGGG	FGGGFGGDGG	LLSGNEKVTM	QNLNDRLASY
170	180	190	200	210	220	230	240
LDKVRALEES	NYELEGKIKE	WYEKHGNSHQ	GEPRDYSKYY	KTIDDLKNQI	LNLTTDNANI	LLQIDNARLA	ADDFRLKYEN
250	260	270	280	290	300	310	320
EVALRQSVEA	DINGLRRVLD	ELTLTKADLE	MQIESLTEEL	AYLKKNHEEE	MKDLRNVSTG	DVNVEMNAAP	GVDLTQLLNN
330	340	350	360	370	380	390	400
MRSQYEQLAE	QNRKDAEAWF	NEKSKELTTE	IDNNIEQISS	YKSEITELRR	NVQALEIELQ	SQLALKQSLE	ASLAETEGRY
410	420	430	440	450	460	470	480
CVQLSQIQAQ	ISALEEQLQQ	IRAETECQNT	EYQQLLDIKI	RLENEIQTYR	SLLEGEGSSG	GGGRGGGSFG	GGYGGGSSGG
490	500	510	520	530	540	550	560
GSSGGGHGGG	HGGSSGGGYG	GGSSGGGSSG	GGYGGGSSSG	GHGGSSSGGY	GGGSSGGGGG	GYGGGSSGGG	SSSGGGYGGG
570	580	590					
SSSGGHKSSS	SGSVGESSSK	GPRY					

Cmpd.	No. of Cmpds.	m/z meas.	Δ m/z [ppm]	z	Rt [min]	Score	P	Range	Sequence	Modification
348	1	806.4041	46.58	2	34.3	11.3	1	185-198	K.HGNSHQGEPRDYSK.Y	

Detailed Protein Report

Protein 24: POTE ankyrin domain family member E [Homo sapiens]

Accession: gi|134133226
Database: refseq_human(refseq_human_20140103.fasta)
Database Date: 2015-11-30

Score: 123.0
MW [kDa]: 121.3
pI: 5.8
Sequence Coverage [%]: 4.7
No. of unique Peptides: 1

10	20	30	40	50	60	70	80
MVVEVDSMPA	ASSVKKPFGL	RSKMGKWCCR	CFPCYRESGK	SNVGTSGDHD	DSAMKTLRSK	MGKWCHHCFP	CCRGSGKSNV
**90**	**100**	**110**	**120**	**130**	**140**	**150**	**160**
GASGDHDDSA	MKTLRNKMGK	WCCHCFPCCR	GSGKSKVGAW	GDYDDSAFME	PRYHVRGEDL	DKLHRAAWWG	KVPRKDLIVM
**170**	**180**	**190**	**200**	**210**	**220**	**230**	**240**
LRDTDVNKKD	KQKRTALHLA	SANGNSEVVK	LLLDRRCQLN	VLDNKKRTAL	IKAVQCQEDE	CALMLLEHGT	DPNIPDEYGN
**250**	**260**	**270**	**280**	**290**	**300**	**310**	**320**
TTLHYAIYNE	DKLMAKALLL	YGADIESKNK	HGLTPLLLGV	HEQKQQVVKF	LIKKKANLNA	LDRYGRTALI	LAVCCGSASI
**330**	**340**	**350**	**360**	**370**	**380**	**390**	**400**
VSLLLEQNID	VSSQDLSGQT	AREYAVSSHH	HVICQLLSDY	KEKQMLKISS	ENSNPEQELK	LTSEEESQRF	KGSENSQPEK
**410**	**420**	**430**	**440**	**450**	**460**	**470**	**480**
MSQELEINKD	GDREVEEEMK	KHESNNVGLL	ENLTNGVTAG	NGDNGLIPQR	KSRTPENQQF	PDNESEEYHR	ICELLSDYKE
**490**	**500**	**510**	**520**	**530**	**540**	**550**	**560**
KQMPKYSSEN	SNPEQDLKLT	SEEESQRLKG	SENGQPEKRS	QEPEINKDGD	RELENFMAIE	EMKKHGSTHV	GFPENLTNGA
**570**	**580**	**590**	**600**	**610**	**620**	**630**	**640**
TAGNGDDGLI	PPRKSRTPES	QQFPDTENEE	YHSDEQNDTQ	KQFCEEQNTG	ILHDEILIHE	EKQIEVVEKM	NSELSLSCKK
**650**	**660**	**670**	**680**	**690**	**700**	**710**	**720**
EKDVLHENST	LREEIAMLRL	ELDTMKHQSQ	LREKKYLEDI	ESVKKKNDNL	LKALQLNELT	MDDDTAVLVI	DNGSGMCKAG
**730**	**740**	**750**	**760**	**770**	**780**	**790**	**800**
FAGDDAPRAV	FPSIVGRPRQ	QGMMGGMHQK	ESYVGKEAQS	KRGILTLKYP	MEHGIITNWD	DMEKIWHHTF	YNELRVAPEE
**810**	**820**	**830**	**840**	**850**	**860**	**870**	**880**
HPILLTEAPL	NPKANREKMT	QIMFETFNTP	AMYVAIQAVP	SLYTSGRTTG	IVMDSGDGVT	HTVPIYEGNA	LPHATLRLDL
**890**	**900**	**910**	**920**	**930**	**940**	**950**	**960**
AGRELPDYLM	KILTERGYRF	TTMAEREIVR	DIKEKLCYVA	LDFEQEMATA	ASSSSLEKSY	ELPDGQVITI	GNERFRCPEA
**970**	**980**	**990**	**1000**	**1010**	**1020**	**1030**	**1040**
LFQPCFLGME	SCGIHETTFN	SIMKSDVDIR	KDLYTNTVLS	GGTTMYPGMA	HRMQKEIAAL	APSMMKIRII	APPKRKYSVW
**1050**	**1060**	**1070**	**1080**				
VGGSILASLS	TFQQMWISKQ	EYDESGPSIV	HRKCF				

Cmpd.	No. of Cmpds.	m/z meas.	Δ m/z [ppm]	z	Rt [min]	Score	P	Range	Sequence	Modification
1070	1	670.5538	205.94	2	43.5	10.8	2	152-162	K.VPRKDLIVMLR.D	

Detailed Protein Report

Protein 25: **beta-actin-like protein 2 [Homo sapiens]**

**Accession:**	gi|63055057	**Score:**	122.9
**Database:**	refseq_human(refseq_human_20140103.fasta)	**MW [kDa]:**	42.0
**Database Date:**	2015-11-30	**pI:**	5.3
**Modification(s):**	Oxidation	**Sequence Coverage [%]:**	14.6
		**No. of unique Peptides:**	1

10	20	30	40	50	60	70	80
MTDNELSALV	VDNGSGMCKA	GFGGDDAPRA	VFPSMIGRPR	HQGVMVGMGQ	KDCYVGDEAQ	SKRGVLTLKY	PIEHGVVTNW
**90**	**100**	**110**	**120**	**130**	**140**	**150**	**160**
DDMEKIWYHT	FYNELRVAPD	EHPILLTEAP	LNPKINREKM	TQIMFEAFNT	PAMYVAIQAV	LSLYASGRTT	GIVMDSGDGV
**170**	**180**	**190**	**200**	**210**	**220**	**230**	**240**
THIVPIYEGY	ALPHAILRLD	LAGRDLTDYL	MKILTERGYN	FTTTAEREIV	RDVKEKLCYV	ALDFEQEMVR	AAASSSPERS
**250**	**260**	**270**	**280**	**290**	**300**	**310**	**320**
YELPDGQVIT	IGNERFRCPE	AIFQPSFLGI	ESSGIHETTF	NSIMKCDVDI	RKDLYANTVL	SGGSTMYPGI	ADRMQKEIIT
**330**	**340**	**350**	**360**	**370**	**380**		
LAPSTMKIKI	IAPPERKYSV	WIGGSILASL	STFQQMWISK	QEYDEAGPPI	VHRKCF		

Cmpd.	No. of Cmpds.	m/z meas.	Δ m/z [ppm]	z	Rt [min]	Score	P	Range	Sequence	Modification
2065	1	1108.9201	-97.52	2	54.4	45.1	0	293-313	K.DLYANTVLSGGSTMYPGIADR.M	Oxidation: 14

Detailed Protein Report

Protein 26: keratin, type II cytoskeletal 1 [Homo sapiens]

**Accession:**	gi|119395750	**Score:**	122.1
**Database:**	refseq_human(refseq_human_20140103.fasta)	**MW [kDa]:**	66.0
**Database Date:**	2015-11-30	**pI:**	8.8
		**Sequence Coverage [%]:**	5.6
		**No. of unique Peptides:**	1

10	20	30	40	50	60	70	80
MSRQFSSRSG	YRSGGGFSSG	SAGIINYQRR	TTSSSTRRSG	GGGGRFSSCG	GGGGSFGAGG	GFGSRSLVNL	GGSKSISISV
**90**	**100**	**110**	**120**	**130**	**140**	**150**	**160**
ARGGGRGSGF	GGGYGGGGFG	GGGFGGGGFG	GGGIGGGGFG	GFGSGGGGFG	GGGFGGGGYG	GGYGPVCPPG	GIQEVTINQS
**170**	**180**	**190**	**200**	**210**	**220**	**230**	**240**
LLQPLNVEID	PEIQKVKSRE	REQIKSLNNQ	FASFIDKVRF	LEQQNQVLQT	KWELLQQVDT	STRTHNLEPY	FESFINNLRR
**250**	**260**	**270**	**280**	**290**	**300**	**310**	**320**
RVDQLKSDQS	RLDSELKNMQ	DMVEDYRNKY	EDEINKRTNA	ENEFVTIKKD	VDGAYMTKVD	LQAKLDNLQQ	EIDFLTALYQ
**330**	**340**	**350**	**360**	**370**	**380**	**390**	**400**
AELSQMQTQI	SETNVILSMD	NNRSLDLDSI	IAEVKAQYED	IAQKSKAEAE	SLYQSKYEEL	QITAGRHGDS	VRNSKIEISE
**410**	**420**	**430**	**440**	**450**	**460**	**470**	**480**
LNRVIQRLRS	EIDNVKKQIS	NLQQSISDAE	QRGENALKDA	KNKLNDLEDA	LQQAKEDLAR	LLRDYQELMN	TKLALDLEIA
**490**	**500**	**510**	**520**	**530**	**540**	**550**	**560**
TYRTLLEGEE	SRMSGECAPN	VSVSVSTSHT	TISGGGSRGG	GGGGYGSGGS	SYGSGGGSYG	SGGGGGGGRG	SYGSGGSSYG
**570**	**580**	**590**	**600**	**610**	**620**	**630**	**640**
SGGGSYGSGG	GGGGHGSYGS	GSSSGGYRGG	SGGGGGGSSG	GRGSGGGSSG	GSIGGRGSSS	GGVKSSGGSS	SVKFVSTTYS
**650**							
GVTR							

Cmpd.	No. of Cmpds.	m/z meas.	Δ m/z [ppm]	z	Rt [min]	Score	P	Range	Sequence	Modification
1554	1	692.3258	-33.18	2	49.6	24.5	0	186-197	K.SLNNQFASFIDK.V	

Detailed Protein Report

Protein 27: alpha-enolase isoform 1 [Homo sapiens]

**Accession:**	gi|4503571	**Score:**	111.2
**Database:**	refseq_human(refseq_human_20140103.fasta)	**MW [kDa]:**	47.1
**Database Date:**	2015-11-30	**pI:**	7.7
		**Sequence Coverage [%]:**	9.9
		**No. of unique Peptides:**	3

10	20	30	40	50	60	70	80
MSILKIHARE	IFDSRGNPTV	EVDLFTSKGL	FRAAVPSGAS	TGIYEALELR	DNDKTRYMGK	GVSKAVEHIN	KTIAPALVSK
**90**	**100**	**110**	**120**	**130**	**140**	**150**	**160**
KLNVTEQEKI	DKLMIEMDGT	ENKSKFGANA	ILGVSLAVCK	AGAVEKGVPL	YRHIADLAGN	SEVILPVPAF	NVINGGSHAG
**170**	**180**	**190**	**200**	**210**	**220**	**230**	**240**
NKLAMQEFMI	LPVGAANFRE	AMRIGAEVYH	NLKNVIKEKY	GKDATNVGDE	GGFAPNILEN	KEGLELLKTA	IGKAGYTDKV
**250**	**260**	**270**	**280**	**290**	**300**	**310**	**320**
VIGMDVAASE	FFRSGKYDLD	FKSPDDPSRY	ISPDQLADLY	KSFIKDYPVV	SIEDPFDQDD	WGAWQKFTAS	AGIQVVGDDL
**330**	**340**	**350**	**360**	**370**	**380**	**390**	**400**
TVTNPKRIAK	AVNEKSCNCL	LLKVNQIGSV	TESLQACKLA	QANGWGVMVS	HRSGETEDTF	IADLVVGLCT	GQIKTGAPCR
**410**	**420**	**430**	**440**				
SERLAKYNQL	LRIEEELGSK	AKFAGRNFRN	PLAK				

Cmpd.	No. of Cmpds.	m/z meas.	Δ m/z [ppm]	z	Rt [min]	Score	P	Range	Sequence	Modification
1541	1	703.8713	13.64	2	47.8	10.1	0	16-28	R.GNPTVEVDLFTSK.G	
2401	1	902.9549	-22.96	2	58.7	34.1	0	33-50	R.AAVPSGASTGIYEALELR.D	
1618	6	713.3563	-14.47	2	50.5	67.0	0	270-281	R.YISPDQLADLYK.S	

Detailed Protein Report

Protein 28: neutrophil defensin 1 preproprotein [Homo sapiens]

Accession: gi|4758146
Database: refseq_human(refseq_human_20140103.fasta)
Database Date: 2015-11-30
Modification(s): Carbamidomethyl

Score: 108.8
MW [kDa]: 10.2
pI: 7.6
Sequence Coverage [%]: 19.1
No. of unique Peptides: 2

Quantitation

QD:QU **Median:** 0.95 **CV:** 0.00 % **No. of Peptides:** 1

Alias proteins:

Accession	Name	Description
gi|124248516	refseq_huma (refseq_human_20140103.fasta)	neutrophil defensin 1 precursor [Homo sapiens]

```
         10         20         30         40         50         60         70         80
MRTLAILAAI LLVALQAQAE PLQARADEVA AAPEQIAADI PEVVVSLAWD ESLAPKHPGS RKNMACYCRI PACIAGERRY
         90        100
GTCIYQGRLW AFCC
```

Cmpd.	No. of Cmpds.	m/z meas.	Δ m/z [ppm]	z	Rt [min]	Score	P	Range	Sequence	Modification	Ratios
147	2	493.7604	4.77	2	31.8	52.9	0	70-78	R.IPACIAGER.R	Carbamidomethyl: 4	QD:QU 0.95
108	2	559.2630	8.21	2	31.3	55.9	0	80-88	R.YGTCIYQGR.L	Carbamidomethyl: 4	

Detailed Protein Report

Protein 29: haptoglobin isoform 2 preproprotein [Homo sapiens]

**Accession:**	gi|186910296	**Score:**	99.5
**Database:**	refseq_human(refseq_human_20140103.fasta)	**MW [kDa]:**	38.4
**Database Date:**	2015-11-30	**pI:**	6.1
		**Sequence Coverage [%]:**	10.7
		**No. of unique Peptides:**	3

Quantitation

QD:QU **Median:** 0.85 **CV:** 23.78 % **No. of Peptides:** 2

10	20	30	40	50	60	70	80
MSALGAVIAL	LLWGQLFAVD	SGNDVTDIAD	DGCPKPPEIA	HGYVEHSVRY	QCKNYYKLRT	EGDGVYTLNN	EKQWINKAVG
**90**	**100**	**110**	**120**	**130**	**140**	**150**	**160**
DKLPECEAVC	GKPKNPANPV	QRILGGHLDA	KGSFPWQAKM	VSHHNLTTGA	TLINEQWLLT	TAKNLFLNHS	ENATAKDIAP
**170**	**180**	**190**	**200**	**210**	**220**	**230**	**240**
TLTLYVGKKQ	LVEIEKVVLH	PNYSQVDIGL	IKLKQKVSVN	ERVMPICLPS	KDYAEVGRVG	YVSGWGRNAN	FKFTDHLKYV
**250**	**260**	**270**	**280**	**290**	**300**	**310**	**320**
MLPVADQDQC	IRHYEGSTVP	EKKTPKSPVG	VQPILNEHTF	CAGMSKYQED	TCYGDAGSAF	AVHDLEEDTW	YATGILSFDK
**330**	**340**	**350**					
SCAVAEYGVY	VKVTSIQDWV	QKTIAEN					

Cmpd.	No. of Cmpds.	m/z meas.	Δ m/z [ppm]	z	Rt [min]	Score	P	Range	Sequence	Modification	Ratios
239	1	854.8952	-39.14	2	31.7	13.9	1	58-72	K.LRTEGDGVYTLNNEK.Q		QD:QU 0.67
1734	1	645.7555	-175.50	2	52.0	37.8	0	157-168	K.DIAPTLTLYVGK.K		
741	3	602.2951	-44.79	2	39.2	47.7	0	333-342	K.VTSIQDWVQK.T		QD:QU 1.07

Detailed Protein Report

Protein 30: cadherin EGF LAG seven-pass G-type receptor 3 precursor [Homo sapiens]

**Accession:**	gi|145309304	**Score:**	92.8
**Database:**	refseq_human(refseq_human_20140103.fasta)	**MW [kDa]:**	358.0
**Database Date:**	2015-11-30	**pI:**	6.2
**Modification(s):**	Carbamidomethyl, Oxidation	**Sequence Coverage [%]:**	2.9
		**No. of unique Peptides:**	6

Quantitation

QD:QU	**Median:**	7.67	**CV:**	0.00 %	**No. of Peptides:**	1	

Detailed Protein Report

10	20	30	40	50	60	70	80
MMARRPPWRG	LGGRSTPILL	LLLLSLFPLS	QEELGGGGHQ	GWDPGLAATT	GPRAHIGGGA	LALCPESSGV	REDGGPGLGV
90	100	110	120	130	140	150	160
REPIFVGLRG	RRQSARNSRG	PPEQPNEELG	IEHGVQPLGS	RERETGQGPG	SVLYWRPEVS	SCGRTGPLQR	GSLSPGALSS
170	180	190	200	210	220	230	240
GVPGSGNSSP	LPSDFLIRHH	GPKPVSSQRN	AGTGSRKRVG	TARCCGELWA	TGSKGQGERA	TTSGAERTAP	RRNCLPGASG
250	260	270	280	290	300	310	320
SGPELDSAPR	TARTAPASGS	APRESRTAPE	PAPKRMRSRG	LFRCRFLPQR	PGPRPPGLPA	RPEARKVTSA	NRARFRRAAN
330	340	350	360	370	380	390	400
RHPQFPQYNY	QTLVPENEAA	GTAVLRVVAQ	DPDAGEAGRL	VYSLAALMNS	RSLELFSIDP	QSGLIRTAAA	LDRESMERHY
410	420	430	440	450	460	470	480
LRVTAQDHGS	PRLSATTMVA	VTVADRNDHS	PVFEQAQYRE	TLRENVEEGY	PILQLRATDG	DAPPNANLRY	RFVGPPAARA
490	500	510	520	530	540	550	560
AAAAAFEIDP	RSGLISTSGR	VDREHMESYE	LVVEASDQGQ	EPGPRSATVR	VHITVLDEND	NAPQFSEKRY	VAQVREDVRP
570	580	590	600	610	620	630	640
HTVVLRVTAT	DRDKDANGLV	HYNIISGNSR	GHFAIDSLTG	EIQVVAPLDF	EAEREYALRI	RAQDAGRPPL	SNNTGLASIQ
650	660	670	680	690	700	710	720
VVDINDHIPI	FVSTPFQVSV	LENAPLGHSV	IHIQAVDADH	GENARLEYSL	TGVAPDTPFV	INSATGWVSV	SGPLDRESVE
730	740	750	760	770	780	790	800
HYFFGVEARD	HGSPPLSASA	SVTVTVLDVN	DNRPEFTMKE	YHLRLNEDAA	VGTSVVSVTA	VDRDANSAIS	YQITGGNTRN
810	820	830	840	850	860	870	880
RFAISTQGGV	GLVTLALPLD	YKQERYFKLV	LTASDRALHD	HCYVHINITD	ANTHRPVFQS	AHYSVSVNED	RPMGSTIVVI
890	900	910	920	930	940	950	960
SASDDDVGEN	ARITYLLEDN	LPQFRIDADS	GAITLQAPLD	YEDQVTYTLA	ITARDNGIPQ	KADTTYVEVM	VNDVNDNAPQ
970	980	990	1000	1010	1020	1030	1040
FVASHYTGLV	SEDAPPFTSV	LQISATDRDA	HANGRVQYTF	QNGEDGDGDF	TIEPTSGIVR	TVRRLDREAV	SVYELTAYAV
1050	1060	1070	1080	1090	1100	1110	1120
DRGVPPLRTP	VSIQVMVQDV	NDNAPVFPAE	EFEVRVKENS	IVGSVVAQIT	AVDPDEGPNA	HIMYQIVEGN	IPELFQMDIF
1130	1140	1150	1160	1170	1180	1190	1200
SGELTALIDL	DYEARQEYVI	VVQATSAPLV	SRATVHVRLV	DQNDNSPVLN	NFQILFNNYV	SNRSDTFPSG	IIGRIPAYDP
1210	1220	1230	1240	1250	1260	1270	1280
DVSDHLFYSF	ERGNELQLLV	VNQTSGELRL	SRKLDNNRPL	VASMLVTVTD	GLHSVTAQCV	LRVVIITEEL	LANSLTVRLE
1290	1300	1310	1320	1330	1340	1350	1360
NMWQERFLSP	LLGRFLEGVA	AVLATPAEDV	FIFNIQNDTD	VGGTVLNVSF	SALAPRGAGA	GAAGPWFSSE	ELQEQLYVRR
1370	1380	1390	1400	1410	1420	1430	1440
AALAARSLLD	VLPFDDNVCL	REPCENYMKC	VSVLRFDSSA	PFLASASTLF	RPIQPIAGLR	CRCPPGFTGD	FCETELDLCY
1450	1460	1470	1480	1490	1500	1510	1520
SNPCRNGGAC	ARREGGYTCV	CRPRFTGEDC	ELDTEAGRCV	PGVCRNGGTC	TDAPNGGFRC	QCPAGGAFEG	PRCEVAARSF
1530	1540	1550	1560	1570	1580	1590	1600
PPSSFVMFRG	LRQRFHLTLS	LSFATVQQSG	LLFYNGRLNE	KHDFLALELV	AGQVRLTYST	GESNTVVSPT	VPGGLSDGQW
1610	1620	1630	1640	1650	1660	1670	1680
HTVHLRYYNK	PRTDALGGAQ	GPSKDKVAVL	SVDDCDVAVA	LQFGAEIGNY	SCAAAGVQTS	SKKSLDLTGP	LLLGGVPNLP
1690	1700	1710	1720	1730	1740	1750	1760
ENFPVSHKDF	IGCMRDLHID	GRRVDMAAFV	ANNGTMAGCQ	AKLHFCDSGP	CKNSGFCSER	WGSFSCDCPV	GFGGKDCQLT
1770	1780	1790	1800	1810	1820	1830	1840
MAHPHHFRGN	GTLSWNFGSD	MAVSVPWYLG	LAFRTRATQG	VLMQVQAGPH	STLLCQLDRG	LLSVTVTRGS	GRASHLLLDQ
1850	1860	1870	1880	1890	1900	1910	1920
VTVSDGRWHD	LRLELQEEPG	GRRGHHVLMV	SLDFSLFQDT	MAVGSELQGL	KVKQLHVGGL	PPGSAEEAPQ	GLVGCIQGVW
1930	1940	1950	1960	1970	1980	1990	2000
LGSTPSGSPA	LLPPSHRVNA	EPGCVVTNAC	ASGPCPPHAD	CRDLWQTFSC	TCQPGYYGPG	CVDACLLNPC	QNQGSCRHLP
2010	2020	2030	2040	2050	2060	2070	2080
GAPHGYTCDC	VGGYFGHHCE	HRMDQQCPRG	WWGSPTCGPC	NCDVHKGFDP	NCNKTNGQCH	CKEFHYRPRG	SDSCLPCDCY
2090	2100	2110	2120	2130	2140	2150	2160
PVGSTSRSCA	PHSGQCPCRP	GALGRQCNSC	DSPFAEVTAS	GCRVLYDACP	KSLRSGVWWP	QTKFGVLATV	PCPRGALGAA
2170	2180	2190	2200	2210	2220	2230	2240

Detailed Protein Report

Cmpd.	No. of Cmpds.	m/z meas.	Δ m/z [ppm]	z	Rt [min]	Score	P	Range	Sequence	Modification	Ratios
1647	5	942.4685	14.29	2	49.1	22.8	1	232-250	R.RNCLPGASGSGPELDSAPR.T		
2719	1	964.4996	-2.09	2	62.9	13.4	2	1519-1534	R.SFPPSSFVMFRGLRQR.F	Oxidation: 9	
2229	1	956.4132	-125.14	2	56.5	12.5	1	2144-2162	K.FGVLATVPCPRGALGAAVR.L	Carbamidomethyl: 9	
1065	3	470.5947	-263.79	2	43.4	12.8	0	2331-2338	R.MEHPSSPR.G		QD:QU 7.67
2129	1	698.8200	-38.23	2	55.2	13.9	2	2331-2342	R.MEHPSSPRGARR.Y	Oxidation: 1	
118	1	1069.1641	-33.12	3	30.2	17.4	1	3083-3111	R. ERLEEAPAPVLRPLSRPGSQECM L	Carbamidomethyl: 22; Oxidation: 23	

Detailed Protein Report

Protein 31: fibrinogen beta chain isoform 2 preproprotein [Homo sapiens]

**Accession:**	gi|296080754	**Score:**	84.7
**Database:**	refseq_human(refseq_human_20140103.fasta)	**MW [kDa]:**	49.9
**Database Date:**	2015-11-30	**pI:**	9.0
**Modification(s):**	Carbamidomethyl	**Sequence Coverage [%]:**	9.3
		**No. of unique Peptides:**	3

Quantitation

QD:QU **Median:** 1.17 **CV:** 0.00 % **No. of Peptides:** 1

10	20	30	40	50	60	70	80
MKRMVSWSFH	KLKTMKHLLL	LLLCVFLVKS	QGVNDNEEGF	FSARGHRPLD	KKREEALLQQ	ERPIRNSVDE	LNNNVEAVSQ
**90**	**100**	**110**	**120**	**130**	**140**	**150**	**160**
TSSSSFQYMY	LLKDLWQKRQ	KQVKDNENVV	NEYSSELEKH	QLYIDETVNS	NIPTNLRVLR	SILENLRSKI	QKLESDVSAQ
**170**	**180**	**190**	**200**	**210**	**220**	**230**	**240**
MEYCRTPCTV	SCNIPVVSGK	ECEEIIRKGG	ETSEMYLIQP	DSSVKPYRVY	CDMNTENGGW	TVIQNRQDGS	VDFGRKWDPY
**250**	**260**	**270**	**280**	**290**	**300**	**310**	**320**
KQGFGNVATN	TDGKNYCGLP	GEYWLGNDKI	SQLTRMGPTE	LLIEMEDWKG	DKVKAHYGGF	TVQNEANKYQ	ISVNKYRGTA
**330**	**340**	**350**	**360**	**370**	**380**	**390**	**400**
GNALMDGASQ	LMGENRTMTI	HNGMFFSTYD	RDNDGWLTSD	PRKQCSKEDG	GGWWYNRCHA	ANPNGRYYWG	GQYTWDMAKH
**410**	**420**	**430**	**440**				
GTDDGVVWMN	WKGSWYSMRK	MSMKIRPFFP	QQ				

Cmpd.	No. of Cmpds.	m/z meas.	Δ m/z [ppm]	z	Rt [min]	Score	P	Range	Sequence	Modification	Ratios
590	2	809.8948	-6.44	2	37.4	25.6	0	166-180	R.TPCTVSCNIPVVSGK.E	Carbamidomethyl: 3, 7	QD:QU 1.17
1961	3	893.2648	-149.91	2	53.0	20.5	0	255-269	K.NYCGLPGEYWLGNDK.I	Carbamidomethyl: 3	
656	1	620.1188	-231.67	2	36.8	38.6	0	368-377	K.EDGGGWWYNR.C		

Detailed Protein Report

Protein 32: immunoglobulin lambda-like polypeptide 5 isoform 1 [Homo sapiens]

Accession: gi|295986608
Database: refseq_human(refseq_human_20140103.fasta)
Database Date: 2015-11-30
Modification(s): Carbamidomethyl

Score: 83.7
MW [kDa]: 23.0
pI: 10.0
Sequence Coverage [%]: 23.8
No. of unique Peptides: 3

10	20	30	40	50	60	70	80
MRPKTGQVGC	ETPEELGPGP	RQRWPLLLLG	LAMVAHGLLR	PMVAPQSGDP	DPGASVGSSR	SSLRSLWGRL	LLQPSPQRAD
**90**	**100**	**110**	**120**	**130**	**140**	**150**	**160**
PRCWPRGFWS	EPQSLCYVFG	TGTKVTVLGQ	PKANPTVTLF	PPSSEELQAN	KATLVCLISD	FYPGAVTVAW	KADGSPVKAG
**170**	**180**	**190**	**200**	**210**	**220**		
VETTKPSKQS	NNKYAASSYL	SLTPEQWKSH	RSYSCQVTHE	GSTVEKTVAP	TECS		

Cmpd.	No. of Cmpds.	m/z meas.	Δ m/z [ppm]	z	Rt [min]	Score	P	Range	Sequence	Modification
2042	1	1045.4503	-102.49	2	55.9	14.4	2	70-86	R.LLLQPSPQRADPRCWPR.G	Carbamidomethyl: 14
1891	3	1021.8855	-134.81	2	52.1	22.0	0	113-131	K.ANPTVTLFPPSSEELQANK.A	
2200	8	872.4511	20.68	2	56.1	47.3	0	174-188	K.YAASSYLSLTPEQWK.S	

Detailed Protein Report

Protein 33: PREDICTED: plectin isoform X8 [Homo sapiens]

**Accession:**	gi|530389157	**Score:**	83.6
**Database:**	refseq_human(refseq_human_20140103.fasta)	**MW [kDa]:**	513.4
**Database Date:**	2015-11-30	**pI:**	5.5
**Modification(s):**	Carbamidomethyl	**Sequence Coverage [%]:**	1.8
		**No. of unique Peptides:**	3

10	20	30	40	50	60	70	80
MDRYSMEELI	QLGQDERDRV	QKKTFTKWVN	KHLIKAQRHI	SDLYEDLRDG	HNLISLLEVL	SGDSLPREKG	RMRFHKLQNV
90	100	110	120	130	140	150	160
QIALDYLRHR	QVKLVNIRND	DIADGNPKLT	LGLIWTIILH	FQISDIQVSG	QSEDMTAKEK	LLLWSQRMVE	GYQGLRCDNF
170	180	190	200	210	220	230	240
TSSWRDGRLF	NAIIHRHKPL	LIDMNKVYRQ	TNLENLDQAF	SVAERDLGVT	RLLDPEDVDV	PQPDEKSIIT	YVSSLYDAMP
250	260	270	280	290	300	310	320
RVPDVQDGVR	ANELQLRWQE	YRELVLLLLQ	WMRHHTAAFE	ERRFPSSFEE	IEILWSQFLK	FKEMELPAKE	ADKNRSKGIY
330	340	350	360	370	380	390	400
QSLEGAVQAG	QLKVPPGYHP	LDVEKEWGKL	HVAILEREKQ	LRSEFERLEC	LQRIVTKLQM	EAGLCEEQLN	QADALLQSDV
410	420	430	440	450	460	470	480
RLLAAGKVPQ	RAGEVERDLD	KADSMIRLLF	NDVQTLKDGR	HPQGEQMYRR	VYRLHERLVA	IRTEYNLRLK	AGVAAPATQV
490	500	510	520	530	540	550	560
AQVTLQSVQR	RPELEDSTLR	YLQDLLAWVE	ENQHRVDGAE	WGVDLPSVEA	QLGSHRGLHQ	SIEEFRAKIE	RARSDEGQLS
570	580	590	600	610	620	630	640
PATRGAYRDC	LGRLDLQYAK	LLNSSKARLR	SLESLHSFVA	AATKELMWLN	EKEEEEVGFD	WSDRNTNMTA	KKESYSALMR
650	660	670	680	690	700	710	720
ELELKEKKIK	ELQNAGDRLL	REDHPARPTV	ESFQAALQTQ	WSWMLQLCCC	IEAHLKENAA	YFQFFSDVRE	AEGQLQKLQE
730	740	750	760	770	780	790	800
ALRRKYSCDR	SATVTRLEDL	LQDAQDEKEQ	LNEYKGHLSG	LAKRAKAVVQ	LKPRHPAHPM	RGRLPLLAVC	DYKQVEVTVH
810	820	830	840	850	860	870	880
KGDECQLVGP	AQPSHWKVLS	SSGSEAAVPS	VCFLVPPPNQ	EAQEAVTRLE	AQHQALVTLW	HQLHVDMKSL	LAWQSLRRDV
890	900	910	920	930	940	950	960
QLIRSWSLAT	FRTLKPEEQR	QALHSLELHY	QAFLRDSQDA	GGFGPEDRLM	AEREYGSCSH	HYQQLLQSLE	QGAQEESRCQ
970	980	990	1000	1010	1020	1030	1040
RCISELKDIR	LQLEACETRT	VHRLRLPLDK	EPARECAQRI	AEQQKAQAEV	EGLGKGVARL	SAEAEKVLAL	PEPSPAAPTL
1050	1060	1070	1080	1090	1100	1110	1120
RSELELTLGK	LEQVRSLSAI	YLEKLKTISL	VIRGTQGAEE	VLRAHEEQLK	EAQAVPATLP	ELEATKASLK	KLRAQAEAQQ
1130	1140	1150	1160	1170	1180	1190	1200
PTFDALRDEL	RGAQEVGERL	QQRHGERDVE	VERWRERVAQ	LLERWQAVLA	QTDVRQRELE	QLGRQLRYYR	ESADPLGAWL
1210	1220	1230	1240	1250	1260	1270	1280
QDARRRQEQI	QAMPLADSQA	VREQLRQEQA	LLEEIERHGE	KVEECQRFAK	QYINAIKDYE	LQLVTYKAQL	EPVASPAKKP
1290	1300	1310	1320	1330	1340	1350	1360
KVQSGSESVI	QEYVDLRTHY	SELTTLTSQY	IKFISETLRR	MEEEERLAEQ	QRAEERERLA	EVEAALEKQR	QLAEAHAQAK
1370	1380	1390	1400	1410	1420	1430	1440
AQAEREAKEL	QQRMQEEVVR	REEAAVDAQQ	QKRSIQEELQ	QLRQSSEAEI	QAKARQAEAA	ERSRLRIEEE	IRVVRLQLEA
1450	1460	1470	1480	1490	1500	1510	1520
TERQRGGAEG	ELQALRARAE	EAEAQKRQAQ	EEAERLRRQV	QDESQRKRQA	EVELASRVKA	EAEAAREKQR	ALQALEELRL
1530	1540	1550	1560	1570	1580	1590	1600
QAEEAERRLR	QAEVERARQV	QVALETAQRS	AEAELQSKRA	SFAEKTAQLE	RSLQEEHVAV	AQLREEAERR	AQQQAEAERA
1610	1620	1630	1640	1650	1660	1670	1680
REEAERELER	WQLKANEALR	LRLQAEEVAQ	QKSLAQAEAE	KQKEEAEREA	RRRGKAEEQA	VRQRELAEQE	LEKQRQLAEG
1690	1700	1710	1720	1730	1740	1750	1760
TAQQRLAAEQ	ELIRLRAETE	QGEQQRQLLE	EELARLQREA	AAATQKRQEL	EAELAKVRAE	MEVLLASKAR	AEEESRSTSE
1770	1780	1790	1800	1810	1820	1830	1840
KSKQRLEAEA	GRFRELAEEA	ARLRALAEEA	KRQRQLAEED	AARQRAEAER	VLAEKLAAIG	EATRLKTEAE	IALKEKEAEN
1850	1860	1870	1880	1890	1900	1910	1920
ERLRRLAEDE	AFQRRRLEEQ	AAQHKADIEE	RLAQLRKASD	SELERQKGLV	EDTLRQRRQV	EEEILALKAS	FEKAAAGKAE
1930	1940	1950	1960	1970	1980	1990	2000
LELELGRIRS	NAEDTLRSKE	QAELEAARQR	QLAAEEERRR	REAEERVQKS	LAAEEEAARQ	RKAALEEVER	LKAKVEEARR
2010	2020	2030	2040	2050	2060	2070	2080
LRERAEQESA	RQLQLAQEAA	QKRLQAEEKA	HAFAVQQKEQ	ELQQTLQQEQ	SVLDQLRGEA	EAARRAAEEA	EEARVQAERE
2090	2100	2110	2120	2130	2140	2150	2160
AAQSRRQVEE	AERLKQSAEE	QAQARAQAQA	AAEKLRKEAE	QEAARRAQAE	QAALRQKQAA	DAEMEKHKKF	AEQTLRQKAQ
2170	2180	2190	2200	2210	2220	2230	2240

Detailed Protein Report

Cmpd.	No. of Cmpds.	m/z meas.	Δ m/z [ppm]	z	Rt [min]	Score	P	Range	Sequence	Modification
2471	1	588.1664	-172.67	2	61.4	13.5	0	1697-1706	R.AETEQGEQQR.Q	
1806	1	582.2479	-40.78	2	52.9	11.0	0	2352-2361	R.QLEMSAEAER.L	
1932	1	1021.8810	-139.60	2	52.6	12.9	2	3841-3858	R.CRRDDGTGQLLLPLSDAR.K	Carbamidomethyl: 1

Detailed Protein Report

Protein 34: coiled-coil domain-containing protein 168 [Homo sapiens]

**Accession:**	gi|226246554	**Score:**	81.8
**Database:**	refseq_human(refseq_human_20140103.fasta)	**MW [kDa]:**	801.4
**Database Date:**	2015-11-30	**pI:**	9.6
		**Sequence Coverage [%]:**	1.3
		**No. of unique Peptides:**	4

Quantitation

QD:QU	**Median:** 0.93	**CV:** 117.96 %	**No. of Peptides:** 2

Detailed Protein Report

10	20	30	40	50	60	70	80
MSKQYYSFKK	GVGSGLEDNT	FMTLWDFLES	WIIQNDWVAI	FFIILLGIIF	EIILMKACAS	FWKKPTLPEK	GSSDVQETED
90	100	110	120	130	140	150	160
SCPKSRKLAP	ENWSVINSSS	GERVGTFLEK	RITSSLTSEE	KECNFEDRIL	FSREILWSGT	SESEDQVSPS	SESHVPSSNG
170	180	190	200	210	220	230	240
ISSSLPLFYS	EVEETCLSHT	EHPDREYETI	QFSSKKLFSM	MKTNKNKNSG	FSSDLSFSAS	RFTVENEDLD	VAPCPLAHLF
250	260	270	280	290	300	310	320
LSRDQVRLLE	ENVRNQIPSK	PKTKLGSRTT	YQCSRSQESL	NQNQPSVGMV	ISVQAQDSFP	GQNAFQNQGL	YEVQFTSQAQ
330	340	350	360	370	380	390	400
YINHNQESIK	SQPESKASNF	AQPEDVMKKP	FSSSTQDSFQ	SQDLDRNQHF	VEVPSIVEAK	YSVKGLESDE	HLGEDQHCVW
410	420	430	440	450	460	470	480
FIDSNKVKYS	IKGQDTIFKN	AEFLVLTLNP	NLVTEDMPQL	RSVKAQGQQQ	IVSSELNQDS	VYSSVPLLST	IKGQKNRRKT
490	500	510	520	530	540	550	560
PDSKSKLSLN	VPSLKAKKTP	TSQVFQITVC	HTLKNRNELG	CKNNTEKKEL	HERKDISDIA	LHLISVSKLI	LPYVKNYSRK
570	580	590	600	610	620	630	640
QLVKVMPGLI	KCGHFLQKQN	KSPDTEKINY	AGPLEETGIS	DITKKEKEYD	KENKRLKNIS	PKMLPQLEQS	FMVNTVQLKA
650	660	670	680	690	700	710	720
PCLLVETNGK	SKESLKDSIT	QAKGIGITEF	HVLNSKKPFD	LHIPKHKTSL	EEAISKPMQK	LVSSPEMESN	NRMKIQEDLQ
730	740	750	760	770	780	790	800
SSENSHLQLS	NGEELPTSTP	KTQRCFPREN	TQKQKDFLEL	VLELSNVGLL	ISPGSKMHKS	SEELEAIKIQ	VNTESVNLKE
810	820	830	840	850	860	870	880
SKPLILNVTE	DSDLRESEEL	ECNTGSNITN	MHQDKETSDA	FHSATYTTIS	QLPDTETHSI	SKAKADTLRI	IRLSHSASKQ
890	900	910	920	930	940	950	960
EKLPDEKETQ	NAEYIDKSCT	FKKPQQCDRK	EQEKEANSEL	TQGFRFSIHL	KQKPKYVKFQ	MEQISSGSSK	APNKEQEVQP
970	980	990	1000	1010	1020	1030	1040
QTLSTQTILE	NSPCPMMDPF	QVEKVKQSTD	RPTDRESAGD	PKNPLTMPEN	LPVGELLIET	TEYSVPFGGN	LQKTTDSHIA
1050	1060	1070	1080	1090	1100	1110	1120
EEKEDVKRYL	PAVALGSFNN	HLLTLPYFKR	QEIKKKLSET	KSVLSVKYVI	MKVKKPAISL	MPYINICGTS	NHRKKMGGNF
1130	1140	1150	1160	1170	1180	1190	1200
EIIIKQILQD	KIAAGMLLNV	IYPPMSILPN	TRMYSRLNAE	NHSHIKLVQE	ESQIEREEKY	PYFINEGNES	QNTLDAKLQD
1210	1220	1230	1240	1250	1260	1270	1280
EVKGVKETLP	KAVLHDSCNL	GLDAHLEKEI	KTEKEMHQPI	PFTETIIESV	VSPIMELSHA	ENVKSTQKTQ	TDCKCTADSE
1290	1300	1310	1320	1330	1340	1350	1360
TPSPISGKSL	IGDPLNQTRE	SYIPSNGSDT	REMGYCFAEE	KTEIPKDLPA	TSPETFNYCT	PVLSCSKVMK	KRVTFALTTS
1370	1380	1390	1400	1410	1420	1430	1440
TAKPKCVNTK	AVKPSISETV	SVTSHRKKSE	LDFKTKFKKI	NQTKGLVPEC	LNTLCSPMHS	RLQREFCLPA	SQLKQGETAD
1450	1460	1470	1480	1490	1500	1510	1520
KTYTDVFAKN	SISHDREEKL	QDGKEEEHKV	LLEAAPQLSQ	HLGSEAGQMK	EIHLESDPVL	NCLTLELHIN	GQRLQHQTGF
1530	1540	1550	1560	1570	1580	1590	1600
EQTTLETSLQ	MGPLEAEELQ	KANETENDIK	VLGGPKIPPP	KALQALENSD	GLILNAYQKD	NELVKSDEEL	NQPGSTNIQV
1610	1620	1630	1640	1650	1660	1670	1680
QPQTHFTQTI	LKSTSCPTLD	QFPFEKVESH	VRFSPLKSGE	AKVDEIIFYA	REGGISSDSS	HQKEQAGGTE	KKETAIFGSC
1690	1700	1710	1720	1730	1740	1750	1760
MPALSTPKTT	RNLKQFSDMK	TLVNPKCGII	KAKKPSISYM	LNIRAGAGPK	RRKELSCNLT	TKMKELHQGK	KGVDETYAFL
1770	1780	1790	1800	1810	1820	1830	1840
TMTPDINKYS	KVETEKDTLR	EKRLSSTQVK	QDTSPHEDSI	TSRDIKETLL	QDEEQEERKQ	EALLKVIPQH	LQHFMFRSGQ
1850	1860	1870	1880	1890	1900	1910	1920
GKDLDFHKLE	NQGSRKILFV	TKQDVPQQLQ	PAEPIQREET	KKCLQTQNGT	ICTVNSKLLP	LKSEDSVNGE	VLTGAIKRGV
1930	1940	1950	1960	1970	1980	1990	2000
PTDRKCMGEQ	HNSGKGEKAE	FNKDLQATVL	ELQKSPHGGE	AQKANLTDME	SGSSNAMNMN	VQHEREDKNI	QKMLTESVPC
2010	2020	2030	2040	2050	2060	2070	2080
YSQHLRFSTH	QMKDPDPCKS	GSEPKSPEGR	SWNLSHIVQK	TKQETHFRET	VLEPISGYMM	KQSPHMQEGI	KCMEGLKTSF
2090	2100	2110	2120	2130	2140	2150	2160
PKTGKSKIGS	IPRDTPWDEN	PRRKWDSSIS	EKTAWNQKNL	QTVLKPLDFS	SLMSSEYESR	SYTLEFIGKK	SMSPKCVTLK
2170	2180	2190	2200	2210	2220	2230	2240

Detailed Protein Report

Cmpd.	No. of Cmpds.	m/z meas.	Δ m/z [ppm]	z	Rt [min]	Score	P	Range	Sequence	Modification	Ratios	
2367	1	805.3600	-33.80	2	60.0	10.6	0	1883-1897	K.CLQTQNGTICTVNSK.L			
270	1	642.3049	-15.58	2	33.4	11.3	0	2241-2251	R.QNLDGHITEEK.E		QD:QU	0.37
2371	3	974.2361	79.07	3	60.1	12.7	2	5658-5682	R.DEDIYFTGFGTIRSGKRPEWLFTGK			
2107	1	698.3422	-40.67	2	54.9	13.3	1	6680-6691	K.KQSPHENSSLIR.K		QD:QU	2.37

Detailed Protein Report

Protein 35: **PREDICTED: dynein heavy chain 3, axonemal isoform X1 [Homo sapiens]**

**Accession:**	gi|578828592	**Score:**	81.5
**Database:**	refseq_human(refseq_human_20140103.fasta)	**MW [kDa]:**	466.0
**Database Date:**	2015-11-30	**pI:**	6.0
**Modification(s):**	Oxidation	**Sequence Coverage [%]:**	2.1
		**No. of unique Peptides:**	4

Detailed Protein Report

10	20	30	40	50	60	70	80
MGDMDCSSQK	IAKSDSIHHM	SHSQGQPELP	PLPASANEEP	SGLYQTVMSH	SFYPPLMQRT	SWTLAAPFKE	QHHHRGPSDS
90	100	110	120	130	140	150	160
IANNYSLMAQ	DLKLKDLLKV	YQPATISVPR	DRTGQGLPSS	GNRSSSEPMR	KKTKFSSRNK	EDSTRIKLAF	KTSIFSPMKK
170	180	190	200	210	220	230	240
EVKTSLTFPG	SRPMSPEQQL	DVMLQQEMEM	ESKEKKPSES	DLERYYYYLT	NGIRKDMIAP	EEGEVMVRIS	KLISNTLLTS
250	260	270	280	290	300	310	320
PFLEPLMVVL	VQEKENDYYC	SLMKSIVDYI	LMDPMERKRL	FIESIPRLFP	QRVIRAPVPW	HSVYRSAKKW	NEEHLHTVNP
330	340	350	360	370	380	390	400
MMLRLKELWF	AEFRDLRFVR	TAEILAGKLP	LQPQEFWDVI	QKHCLEAHQT	LLNKWIPTCA	QLFTSRKEHW	IHFAPKSNYD
410	420	430	440	450	460	470	480
SSRNIEEYFA	SVASFMSLQL	RELVIKSLED	LVSLFMIHKD	GNDFKEPYQE	MKFFIPQLIM	IKLEVSEPII	VFNPSFDGCW
490	500	510	520	530	540	550	560
ELIRDSFLEI	IKNSNGIPKV	ESVLFPELKG	YNLLLGTVNA	EEKLVSDFLI	QTFKVFQKNQ	VGPCKYLNVY	KKYVDLLDNT
570	580	590	600	610	620	630	640
AEQNIAAFLK	ENHDIDDFVT	KINAIKKRRN	EIASMNITVP	LAMFCLDATA	LNHDLCERAQ	NLKDHLIQFQ	VDVNRDTNTS
650	660	670	680	690	700	710	720
ICNQYSHIAD	KVSEVPANTK	ELVSLIEFLK	KSSAVTVFKL	RRQLRDASER	LEFLMDYADL	PYEDIKLNST	LFLWPDQIED
730	740	750	760	770	780	790	800
IFDNSRNLLL	HKRDQAEMDL	IKRCSEFELR	LEGYHRELES	FRKREVMTTE	EMKHNVEKLN	ELSKNLNRAF	AEFELINKEE
810	820	830	840	850	860	870	880
ELLEKEKSTY	PLLQAMLKNK	VPYEQLWSTA	YEFSIKSEEW	MNGPLFLLNA	EQIAEEIGNM	WRTTYKLIKT	LSDVPAPRRL
890	900	910	920	930	940	950	960
AENVKIKIDK	FKQYIPILSI	SCNPGMKDRH	WQQISEIVGY	EIKPTETTCL	SNMLEFGFGK	FVEKLEPIGA	AASKEYSLEK
970	980	990	1000	1010	1020	1030	1040
NLDRMKLDWV	NVTFSFVKYR	DTDTNILCAI	DDIQMLLDDH	VIKTQTMCGS	PFIKPIEAEC	RKWEEKLIRI	QDNLDAWLKC
1050	1060	1070	1080	1090	1100	1110	1120
QATWLYLEPI	FSSEDIIAQM	PEEGRKFGIV	DSYWKSLMSQ	AVKDNRILVA	ADQPRMAEKL	QEANFLLEDI	QKGLNDYLEK
1130	1140	1150	1160	1170	1180	1190	1200
KRLFFPRFFF	LSNDELLEIL	SETKDPLRVQ	PHLKKCFEGI	AKLEFTDNLE	IVGMISSEKE	TVPFIQKIYP	ANAKGMVEKW
1210	1220	1230	1240	1250	1260	1270	1280
LQQVEQMMLA	SMREVIGLGI	EAYVKVPRNH	WVLQWPGQVV	ICVSSIFWTQ	EVSQALAENT	LLDFLKKSND	QIAQIVQLVR
1290	1300	1310	1320	1330	1340	1350	1360
GKLSSGARLT	LGALTVIDVH	ARDVVAKLSE	DRVSDLNDFQ	WISQLRYYWV	AKDVQVQIIT	TEALYGYEYL	GNSPRLVITP
1370	1380	1390	1400	1410	1420	1430	1440
LTDRCYRTLM	GALKLNLGGA	PEGPAGTGKT	ETTKDLAKAL	AKQCVVFNCS	DGLDYKAMGK	FFKGLAQAGA	WACFDEFNRI
1450	1460	1470	1480	1490	1500	1510	1520
EVEVLSVVAQ	QILSIQQAII	RKLKTFIFEG	TELSLNPTCA	VFITMNPGYA	GRAELPDNLK	ALFRTVAMMV	PDYALIGEIS
1530	1540	1550	1560	1570	1580	1590	1600
LYSMGFLDSR	SLAQKIVATY	RLCSEQLSSQ	HHYDYGMRAV	KSVLTAAGNL	KLKYPEENES	VLLLRALLDV	NLAKFLAQDV
1610	1620	1630	1640	1650	1660	1670	1680
PLFQGIISDL	FPGVVLPKPD	YEVFLKVLND	NIKKMKLQPV	PWFIGKIIQI	YEMMLVRHGY	MIVGDPMGGK	TSAYKVLAAA
1690	1700	1710	1720	1730	1740	1750	1760
LGDLHAANQM	EEFAVEYKII	NPKAITMGQL	YGCFDQVSHE	WMDGVLANAF	REQASSLSDD	RKWIIFDGPV	DAIWIENMNT
1770	1780	1790	1800	1810	1820	1830	1840
VLDDNKKLCL	MSGEIIQMNS	KMSLIFEPAD	LEQASPATVS	RCGMIYMEPH	QLGWKPLKDS	YMDTLPSSLT	KEHKELVNDM
1850	1860	1870	1880	1890	1900	1910	1920
FMWLVQPCLE	FGRLHCKFVV	QTSPIHLAFS	MMRLYSSLLD	EIRAVEEEEM	ELGEGLSSQQ	IFLWLQGLFL	FSLVWTVAGT
1930	1940	1950	1960	1970	1980	1990	2000
INADSRKKFD	VFFRNLIMGM	DDNHPRPKSV	KLTKNNIFPE	RGSIYDFYFI	KQASGHWETW	TQYITKEEEK	VPAGAKVSEL
2010	2020	2030	2040	2050	2060	2070	2080
IIPTMETARQ	SFFLKTYLDH	EIPMLFVGPT	GTGKSAITNN	FLLHLPKNTY	LPNCINFSAR	TSANQTQDII	MSKLDRRRKG
2090	2100	2110	2120	2130	2140	2150	2160
LFGPPIGKKA	VVFVDDLNMP	AKEVYGAQPP	IELLRQWIDH	GYWFDKKDTT	RLDIVDMLLV	TAMGPPGGGR	NDITGRFTRH
2170	2180	2190	2200	2210	2220	2230	2240

Detailed Protein Report

Cmpd.	No. of Cmpds.	m/z meas.	Δ m/z [ppm]	z	Rt [min]	Score	P	Range	Sequence	Modification
565	1	607.8337	-71.29	2	35.7	10.9	1	1562-1573	K.SVLTAAGNLKLK.Y	
315	1	1201.1883	61.60	2	32.6	16.3	1	2128-2150	K.DTTRLDIVDMLLVTAMGPPGGGR.N	Oxidation: 10
1563	1	949.5033	8.02	2	49.7	12.9	2	2269-2283	R.LIDKEDRQVFFNMVK.E	Oxidation: 13
1965	2	572.7269	-161.97	2	54.9	12.8	1	2924-2934	K.LISGLGGEKDR.W	

Detailed Protein Report

Protein 36:	PREDICTED: protein AHNAK2 isoform X1 [Homo sapiens]		
**Accession:**	gi|530403029	**Score:**	80.2
**Database:**	refseq_human(refseq_human_20140103.fasta)	**MW [kDa]:**	605.3
**Database Date:**	2015-11-30	**pI:**	5.1
**Modification(s):**	Oxidation	**Sequence Coverage [%]:**	1.7
		**No. of unique Peptides:**	6

Quantitation

QD:QU	**Median:** 1.08	**CV:** 50.25 %	**No. of Peptides:** 2

Detailed Protein Report

10	20	30	40	50	60	70	80
MSRPEAVQEA	TEVTLKTEVE	AGASGYSVTG	GGDQGIFVKQ	VLKDSSAAKL	FNLREGDQLL	STTVFFENIK	YEDALKILQY
90	100	110	120	130	140	150	160
SEPYKVQFKI	RRQLPAPQDE	EWASSDAQHG	PQGKEKEDTD	VADGCRETPT	KTLEGDGDQE	RLISKPRVGR	GRQSQRERLS
170	180	190	200	210	220	230	240
WPKFQSIKSK	RGPGPQRSHS	SSEAYEPRDA	HDVSPTSTDT	EAQLTVERQE	QKAGPGSQRR	RKFLNLRFRT	GSGQGPSSTG
250	260	270	280	290	300	310	320
QPGRGFQSGV	GRAGVLEELG	PWGDSLEETG	AATGSRREER	AEQDREVMPA	QSMPLPTELG	DPRLCEGTPQ	EGGLRAARLH
330	340	350	360	370	380	390	400
GKTLEGQAQE	TAVAQRKPRA	QPTPGMSREG	EGEGLQSLEI	GIARLSLRDT	TEGGTQIGPP	EIRVRVHDLK	TPKFAFSTEK
410	420	430	440	450	460	470	480
EPERERRLST	PQRGKRQDAS	SKAGTGLKGE	EVEGAGWMPG	REPTTHAEAQ	GDEGDGEEGL	QRTRITEEQD	KGREDTEGQI
490	500	510	520	530	540	550	560
RMPKFKIPSL	GWSPSKHTKT	GREKATEDTE	QGREGEATAT	ADRREQRRTE	EGLKDKEDSD	SMTNTTKIQL	IHDEKRLKKE
570	580	590	600	610	620	630	640
QILTEKEVAT	KDSKFKMPKF	KMPLFGASAP	GKSMEASVDV	SAPKVEADVS	LLSMQGDLKT	TDLSVQTPSA	DLEVQDGQVD
650	660	670	680	690	700	710	720
VKLPEGPLPE	GASLKGHLPK	VQRPSLKMPK	VDLKGPKLDL	KGPKAEVTAP	DVKMSLSSME	VDVQAPRAKL	DGARLEGDLS
730	740	750	760	770	780	790	800
LADKEVTAKD	SKFKMPKFKM	PSFGVSAPGK	SMEDSVDVSA	PKVEADVSLS	SMQGDLKATD	LSIQPPSADL	EVQAGQVDVK
810	820	830	840	850	860	870	880
LPEGPVPEGA	GPKVHLPKVE	MPSFKMPKVD	LKGPQIDVKG	PKLDLKGPKA	EVTAPDGEVS	LPSMEVDVQA	QKAKLDGAWL
890	900	910	920	930	940	950	960
EGDLSLADKD	VTAKDSKFKM	PKFKMPSFGV	SAPGKSIKAL	VDVSAPKVEA	DLSLPSMQGD	LKTTDLSIQP	ASTDLKVQAD
970	980	990	1000	1010	1020	1030	1040
QVDVKLPEGH	LPEGAGLKGH	LPKVEMPSFK	MPKVALKGPQ	VDVKGPKLDL	KSPKAEVTAP	DVEVSLPSVE	VDVEAPGAKL
1050	1060	1070	1080	1090	1100	1110	1120
DSARLEGELS	LADKDVTAKD	SRFKMPKFKM	PSFGASAPGK	SIEASVDVSA	PKVEADVSLP	SMQGDLKTTD	LSIQPPSADL
1130	1140	1150	1160	1170	1180	1190	1200
EVHAGQVDVK	LLEGHVPEGA	GFKGHLPKVQ	MPSLKMPKVD	LKGPQVEVRG	PKLDLKGHKA	EVTAHEVAVS	LPSVEVDMQA
1210	1220	1230	1240	1250	1260	1270	1280
PGAKLDGAQL	DGDLSLADKD	VTAKDSKFKM	PKFKMPSFGV	SAPGKSIEAS	VDLSAPKVEA	DMSLPSMQGD	LKTTDLSIQP
1290	1300	1310	1320	1330	1340	1350	1360
PSTDLELQAG	QLDVKLPEGP	VPEGAGLKGH	LPKLQMPSFK	VPKVDLKGPE	IDIKGPKLDL	KDPKVEVTAP	DVEVSLPSVE
1370	1380	1390	1400	1410	1420	1430	1440
VDVEAPGAKL	DGGRLEEDMS	LADKDLTTKD	SKFKMPKFKM	PSFGVSAPGK	SIEASVDVSA	PKVEADVSLP	SMQGDLKATD
1450	1460	1470	1480	1490	1500	1510	1520
LSIQPPSADL	EVQAGQVDVK	LPEGPVSEGA	GLKGHLPKVQ	MPSFKMPKVD	LKGPQIDVKG	PKLDLKGPKV	EVTAPDVKMS
1530	1540	1550	1560	1570	1580	1590	1600
LSSMEVDVQA	PRAKLDGAQL	EGDLSLADKA	VTAKDSKFKM	PKFKMPSFGV	SAPGKSIEAS	VDVSEPKVEA	DVSLPSMQGD
1610	1620	1630	1640	1650	1660	1670	1680
LKTTDLSIQS	PSADLEVQAG	QVNVKLPEGP	LPEGAGFKGH	LPKVQMPSLK	MPKVALKGPQ	MDVKGPKLDL	KGPKAEVMAP
1690	1700	1710	1720	1730	1740	1750	1760
DVEVSLPSVE	VDVEAPGAKL	DSVRLEGDLS	LADKDVTAKD	SKFKMPKFKM	PSFGVSAPGK	SIEASVDVSA	PKVEAEVSLP
1770	1780	1790	1800	1810	1820	1830	1840
SMQGDLKTTD	LCIPLPSADL	VVQAGQVDMK	LPEGQVPEGA	GLKGHLPKVD	MPSFKMPKVD	LKGPQTDVKG	AKLDLKGPKA
1850	1860	1870	1880	1890	1900	1910	1920
EVTAPDVEVS	LPSMEVDVQA	QKAKLDGARL	EGDLSLADKD	MTAKDSKFKM	PKFKMPSFGV	SAPGRSIEAS	VDVPAPKVEA
1930	1940	1950	1960	1970	1980	1990	2000
DVSLPSMQGD	LKTTDLSIQP	PSADLKVQTG	QVDVKLPEGH	VPEGAGLKGH	LPKVEMPSLK	MPKVDLKGPQ	VDIKGPKLDL
2010	2020	2030	2040	2050	2060	2070	2080
KDPKVEMRVP	DVEVSLPSME	VDVQAPRAKL	DSAHLQGDLT	LANKDLTTKD	SKFKMPKFKM	PSFGVSAPGK	SIEASVDVSP
2090	2100	2110	2120	2130	2140	2150	2160
PKVEADMSLP	SMQGDLKTTD	LSIQPLSADV	KVQAGQVDVK	LLEGPVPEEV	GLKGHLPKLQ	MPSFKVPKVD	LKGPEIDIKG
2170	2180	2190	2200	2210	2220	2230	2240

Detailed Protein Report

Cmpd.	No. of Cmpds.	m/z meas.	Δ m/z [ppm]	z	Rt [min]	Score	P	Range	Sequence	Modification	Ratios	
2046	1	596.7306	-219.53	2	56.0	16.0	2	384-393	R.VRVHDLKTPK.F		QD:QU	1.73
267	1	862.8828	-39.08	2	33.3	11.9	1	1375-1389	R.LEEDMSLADKDLTTK.D	Oxidation: 5		
738	1	769.3703	-105.01	2	39.2	11.2	2	3308-3321	K.SPQVDIKGPKLDLK.V		QD:QU	0.67
1307	2	926.0984	-40.13	3	46.5	19.2	1	3391-3417	R. SIEASLDVSAPKVEADVSLSSMQG A			
2190	3	648.2929	-93.26	2	56.0	10.4	1	5599-5610	R.FPKLGFSSSPTK.K			
1329	1	1023.0254	-12.68	2	46.8	11.6	1	5662-5679	R.VMVTSAARTELILPEQDR.K	Oxidation: 2		

Detailed Protein Report

Protein 37: PREDICTED: dynein heavy chain 5, axonemal isoform X1 [Homo sapiens]

**Accession:**	gi|530378724	**Score:**	75.3
**Database:**	refseq_human(refseq_human_20140103.fasta)	**MW [kDa]:**	526.8
**Database Date:**	2015-11-30	**pI:**	5.7
**Modification(s):**	Carbamidomethyl, Oxidation	**Sequence Coverage [%]:**	1.7
		**No. of unique Peptides:**	4

Quantitation

QD:QU	**Median:** 0.50	**CV:** 0.00 %	**No. of Peptides:** 1

Detailed Protein Report

10	20	30	40	50	60	70	80
MDKLQRLKGE	KEAKRALLDA	RHNYLFAIVA	SCLDLNKTEV	EDAILEGNQI	ERIDQLFAVG	GLRHLMFYYQ	DVEEAETGQL
90	100	110	120	130	140	150	160
GSLGGVNLVS	GKIKKPKVFV	TEGNDVALTG	VCVFFIRTDP	SKAITPDNIH	QEVSFNMLDA	ADGGLLNSVR	RLLSDIFIPA
170	180	190	200	210	220	230	240
LRATSHGWGE	LEGLQDAANI	RQEFLSSLEG	FVNVLSGAQE	SLKEKVNLRK	CDILELKTLK	EPTDYLTLAN	NPETLGKIED
250	260	270	280	290	300	310	320
CMKVWIKQTE	QVLAENNQLL	KEADDVGPRA	ELEHWKKRLS	KFNYLLEQLK	SPDVKAVLAV	LAAAKSKLLK	TWREMDIRIT
330	340	350	360	370	380	390	400
DATNEAKDNV	KYLYTLEKCC	DPLYSSDPLS	MMDAIPTLIN	AIKMIYSISH	YYNTSEKITS	LFVKVTNQII	SACKAYITNN
410	420	430	440	450	460	470	480
GTASIWNQPQ	DVVEEKILSA	IKLKQEYQLC	FHKTKQKLKQ	NPNAKQFDFS	EMYIFGKFET	FHRRLAKIID	IFTTLKTYSV
490	500	510	520	530	540	550	560
LQDSTIEGLE	DMATKYQGIV	ATIKKKEYNF	LDQRKMDFDQ	DYEEFCKQTN	DLHNELRKFM	DVTFAKIQNT	NQALRMLKKF
570	580	590	600	610	620	630	640
ERLNIPNLGI	DDKYQLILEN	YGADIDMISK	LYTKQKYDPP	LARNQPPIAG	KILWARQLFH	RIQQPMQLFQ	QHPAVLSTAE
650	660	670	680	690	700	710	720
AKPIIRSYNR	MAKVLLEFEV	LFHRAWLRQI	EEIHVGLEAS	LLVKAPGTGE	LFVNFDPQIL	ILFRETECMA	QMGLEVSPLA
730	740	750	760	770	780	790	800
TSLFQKRDRY	KRNFSNMKMM	LAEYQRVKSK	IPAAIEQLIV	PHLAKVDEAL	QPGLAALTWT	SLNIEAYLEN	TFAKIKDLEL
810	820	830	840	850	860	870	880
LLDRVNDLIE	FRIDAILEEM	SSTPLCQLPQ	EEPLTCEEFL	QMTKDLCVNG	AQILHFKSSL	VEEAVNELVN	MLLDVEVLSE
890	900	910	920	930	940	950	960
EESEKISNEN	SVNYKNESSA	KREEGNFDTL	TSSINARANA	LLLTTVTRKK	KETEMLGEEA	RELLSHFNHQ	NMDALLKVTR
970	980	990	1000	1010	1020	1030	1040
NTLEAIRKRI	HSSHTINFRD	SNSASNMKQN	SLPIFRASVT	LAIPNIVMAP	ALEDVQQTLN	KAVECIISVP	KGVRQWSSEL
1050	1060	1070	1080	1090	1100	1110	1120
LSKKKIQERK	MAALQSNEDS	DSDVEMGENE	LQDTLEIASV	NLPIPVQTKN	YYKNVSENKE	IVKLVSVLST	IINSTKKEVI
1130	1140	1150	1160	1170	1180	1190	1200
TSMDCFKRYN	HIWQKGKEEA	IKTFITQSPL	LSEFESQILY	FQNLEQEINA	EPEYVCVGSI	ALYTADLKFA	LTAETKAWMV
1210	1220	1230	1240	1250	1260	1270	1280
VIGRHCNKKY	RSEMENIFML	IEEFNKKLNR	PIKDLDDIRI	AMAALKEIRE	EQISIDFQVG	PIEESYALLN	RYGLLIAREE
1290	1300	1310	1320	1330	1340	1350	1360
IDKVDTLHYA	WEKLLARAGE	VQNKLVSLQP	SFKKELISAV	EVFLQDCHQF	YLDYDLNGPM	ASGLKPQEAS	DRLIMFQNQF
1370	1380	1390	1400	1410	1420	1430	1440
DNIYRKYITY	TGGEELFGLP	ATQYPQLLEI	KKQLNLLQKI	YTLYNSVIET	VNSYYDILWS	EVNIEKINNE	LLEFQNRCRK
1450	1460	1470	1480	1490	1500	1510	1520
LPRALKDWQA	FLDLKKIIDD	FSECCPLLEY	MASKAMMERH	WERITTLTGH	SLDVGNESFK	LRNIMEAPLL	KYKEEIEDIC
1530	1540	1550	1560	1570	1580	1590	1600
ISAVKERDIE	QKLKQVINEW	DNKTFTFGSF	KTRGELLLRG	DSTSEIIANM	EDSLMLLGSL	LSNRYNMPFK	AQIQKWVQYL
1610	1620	1630	1640	1650	1660	1670	1680
SNSTDIIESW	MTVQNLWIYL	EAVFVGGDIA	KQLPKEAKRF	SNIDKSWVKI	MTRAHEVPSV	VQCCVGDETL	GQLLPHLLDQ
1690	1700	1710	1720	1730	1740	1750	1760
LEICQKSLTG	YLEKKRLCFP	RFFFVSDPAL	LEILGQASDS	HTIQAHLLNV	FDNIKSVKFH	EKIYDRILSI	SSQEGETIEL
1770	1780	1790	1800	1810	1820	1830	1840
DKPVMAEGNV	EVWLNSLLEE	SQSSLHLVIR	QAAANIQETG	FQLTEFLSSF	PAQVGLLGIQ	MIWTRDSEEA	LRNAKFDKKI
1850	1860	1870	1880	1890	1900	1910	1920
MQKTNQAFLE	LLNTLIDVTT	RDLSSTERVK	YETLITIHVH	QRDIFDDLCH	MHIKSPMDFE	WLKQCRFYFN	EDSDKMMIHI
1930	1940	1950	1960	1970	1980	1990	2000
TDVAFIYQNE	FLGCTDRLVI	TPLTDRCYIT	LAQALGMSMG	GAPAGPAGTG	KTETTKDMGR	CLGKYVVVFN	CSDQMDFRGL
2010	2020	2030	2040	2050	2060	2070	2080
GRIFKGLAQS	GSWGCFDEFN	RIDLPVLSVA	AQQISIILTC	KKEHKKSFIF	TDGDNVTMNP	EFGLFLTMNP	GYAGRQELPE
2090	2100	2110	2120	2130	2140	2150	2160
NLKINFRSVA	MMVPDRQIII	RVKLASCGFI	DNVVLARKFF	TLYKLCEEQL	SKQVHYDFGL	RNILSVLRTL	GAAKRANPMD
2170	2180	2190	2200	2210	2220	2230	2240

Detailed Protein Report

Cmpd.	No. of Cmpds.	m/z meas.	Δ m/z [ppm]	z	Rt [min]	Score	P	Range	Sequence	Modification	Ratios
1349	1	991.5669	90.75	3	47.0	28.2	1	1947-1976	R.CYITLAQALGMSMGGAPAGPAGTD	Carbamidomethyl: 1; Oxidation: 11, 13	
2802	1	849.0671	-21.12	3	66.2	12.2	2	1999-2021	R.GLGRIFKGLAQSGSWGCFDEFNRI		
2750	1	905.4797	-0.39	2	65.5	16.2	2	3993-4008	R.TIAQARKYIVDSMGEK.Y		
592	1	607.1554	-159.80	2	36.0	18.7	0	4415-4424	R.DALDCMFDAR.I	Carbamidomethyl: 5	QD:QU 0.50

Detailed Protein Report

Protein 38: heat shock protein beta-1 [Homo sapiens]

Accession: gi|4504517
Database: refseq_human(refseq_human_20140103.fasta)
Database Date: 2015-11-30

Score: 74.0
MW [kDa]: 22.8
pI: 6.0
Sequence Coverage [%]: 21.5
No. of unique Peptides: 3

10	20	30	40	50	60	70	80
MTERRVPFSL	LRGPSWDPFR	DWYPHSRLFD	QAFGLPRLPE	EWSQWLGGSS	WPGYVRPLPP	AAIESPAVAA	PAYSRALSRQ
**90**	**100**	**110**	**120**	**130**	**140**	**150**	**160**
LSSGVSEIRH	TADRWRVSLD	VNHFAPDELT	VKTKDGVVEI	TGKHEERQDE	HGYISRCFTR	KYTLPPGVDP	TQVSSSLSPE
**170**	**180**	**190**	**200**	**210**			
GTLTVEAPMP	KLATQSNEIT	IPVTFESRAQ	LGGPEAAKSD	ETAAK			

Cmpd.	No. of Cmpds.	m/z meas.	Δ m/z [ppm]	z	Rt [min]	Score	P	Range	Sequence	Modification
1770	3	582.2260	-151.18	2	52.4	43.9	0	28-37	R.LFDQAFGLPR.L	
2031	2	953.3861	-118.85	2	53.9	17.5	0	172-188	K.LATQSNEITIPVTFESR.A	
2630	2	822.4504	42.56	2	63.7	12.6	1	189-205	R.AQLGGPEAAKSDETAAK.-	

Detailed Protein Report

Protein 39: tenascin-X isoform 1 precursor [Homo sapiens]

**Accession:**	gi|188528648	**Score:**	68.8
**Database:**	refseq_human(refseq_human_20140103.fasta)	**MW [kDa]:**	457.9
**Database Date:**	2015-11-30	**pI:**	4.9
**Modification(s):**	Carbamidomethyl	**Sequence Coverage [%]:**	1.9
		**No. of unique Peptides:**	5

Detailed Protein Report

10	20	30	40	50	60	70	80
MMPAQYALTS	SLVLLVLLST	ARAGPFSSRS	NVTLPAPRPP	PQPGGHTVGA	GVGSPSSQLY	EHTVEGGEKQ	VVFTHRINLP
90	100	110	120	130	140	150	160
PSTGCGCPPG	TEPPVLASEV	QALRVRLEIL	EELVKGLKEQ	CTGGCCPASA	QAGTGQTDVR	TLCSLHGVFD	LSRCTCSCEP
170	180	190	200	210	220	230	240
GWGGPTCSDP	TDAEIPPSSP	PSASGSCPDD	CNDQGRCVRG	RCVCFPGYTG	PSCGWPSCPG	DCQGRGRCVQ	GVCVCRAGFS
250	260	270	280	290	300	310	320
GPDCSQRSCP	RGCSQRGRCE	GGRCVCDPGY	TGDDCGMRSC	PRGCSQRGRC	ENGRCVCNPG	YTGEDCGVRS	CPRGCSQRGR
330	340	350	360	370	380	390	400
CKDGRCVCDP	GYTGEDCGTR	SCPWDCGEGG	RCVDGRCVCW	PGYTGEDCST	RTCPRDCRGR	GRCEDGECIC	DTGYSGDDCG
410	420	430	440	450	460	470	480
VRSCPGDCNQ	RGRCEDGRCV	CWPGYTGTDC	GSRACPRDCR	GRGRCENGVC	VCNAGYSGED	CGVRSCPGDC	RGRGRCESGR
490	500	510	520	530	540	550	560
CMCWPGYTGR	DCGTRACPGD	CRGRGRCVDG	RCVCNPGFTG	EDCGSRRCPG	DCRGHGLCED	GVCVCDAGYS	GEDCSTRSCP
570	580	590	600	610	620	630	640
GGCRGRGQCL	DGRCVCEDGY	SGEDCGVRQC	PNDCSQHGVC	QDGVCICWEG	YVSEDCSIRT	CPSNCHGRGR	CEEGRCLCDP
650	660	670	680	690	700	710	720
GYTGPTCATR	MCPADCRGRG	RCVQGVCLCH	VGYGGEDCGQ	EEPPASACPG	GCGPRELCRA	GQCVCVEGFR	GPDCAIQTCP
730	740	750	760	770	780	790	800
GDCRGRGECH	DGSCVCKDGY	AGEDCGEEVP	TIEGMRMHLL	EETTVRTEWT	PAPGPVDAYE	IQFIPTTEGA	SPPFTARVPS
810	820	830	840	850	860	870	880
SASAYDQRGL	APGQEYQVTV	RALRGTSWGL	PASKTITTMI	DGPQDLRVVA	VTPTTLELGW	LRPQAEVDRF	VVSYVSAGNQ
890	900	910	920	930	940	950	960
RVRLEVPPEA	DGTLLTDLMP	GVEYVVTVTA	ERGRAVSYPA	SVRANTGSSP	LGLLGTTDEP	PPSGPSTTQG	AQAPLLQQRP
970	980	990	1000	1010	1020	1030	1040
QELGELRVLG	RDETGRLRVV	WTAQPDTFAY	FQLRMRVPEG	PGAHEEVLPG	DVRQALVPPP	PPGTPYELSL	HGVPPGGKPS
1050	1060	1070	1080	1090	1100	1110	1120
DPIIYQGIMD	KDEEKPGKSS	GPPRLGELTV	TDRTSDSLLL	RWTVPEGEFD	SFVIQYKDRD	GQPQVVPVEG	PQRSAVITSL
1130	1140	1150	1160	1170	1180	1190	1200
DPGRKYKFVL	YGFVGKKRHG	PLVAEAKILP	QSDPSPGTPP	HLGNLWVTDP	TPDSLHLSWT	VPEGQFDTFM	VQYRDRDGRP
1210	1220	1230	1240	1250	1260	1270	1280
QVVPVEGPER	SFVVSSLDPD	HKYRFTLFGI	ANKKRYGPLT	ADGTTAPERK	EEPPRPEFLE	QPLLGELTVT	GVTPDSLRLS
1290	1300	1310	1320	1330	1340	1350	1360
WTVAQGPFDS	FMVQYKDAQG	QPQAVPVAGD	ENEVTVPGLD	PDRKYKMNLY	GLRGRQRVGP	ESVVAKTAPQ	EDVDETPSPT
1370	1380	1390	1400	1410	1420	1430	1440
ELGTEAPESP	EEPLLGELTV	TGSSPDSLSL	FWTVPQGSFD	SFTVQYKDRD	GRPRAVRVGG	KESEVTVGGL	EPGHKYKMHL
1450	1460	1470	1480	1490	1500	1510	1520
YGLHEGQRVG	PVSAVGVTAP	QQEETPPATE	SPLEPRLGEL	TVTDVTPNSV	GLSWTVPEGQ	FDSFIVQYKD	KDGQPQVVPV
1530	1540	1550	1560	1570	1580	1590	1600
AADQREVTVY	NLEPERKYKM	NMYGLHDGQR	MGPLSVVIVT	APLPPAPATE	ASKPPLEPRL	GELTVTDITP	DSVGLSWTVP
1610	1620	1630	1640	1650	1660	1670	1680
EGEFDSFVVQ	YKDRDGQPQV	VPVAADQREV	TIPDLEPSRK	YKFLLFGIQD	GKRRSPVSVE	AKTVARGDAS	PGAPPRLGEL
1690	1700	1710	1720	1730	1740	1750	1760
WVTDPTPDSL	RLSWTVPEGQ	FDSFVVQFKD	KDGPQVVPVE	GHERSVTVTP	LDAGRKYRFL	LYGLLGKKRH	GPLTADGTTE
1770	1780	1790	1800	1810	1820	1830	1840
ARSAMDDTGT	KRPPKPRLGE	ELQVTTVTQN	SVGLSWTVPE	GQFDSFVVQY	KDRDGQPQVV	PVEGSLREVS	VPGLDPAHRY
1850	1860	1870	1880	1890	1900	1910	1920
KLLLYGLHHG	KRVGPISAVA	ITAGREETET	ETTAPTPPAP	EPHLGELTVE	EATSHTLHLS	WMVTEGEFDS	FEIQYTDRDG
1930	1940	1950	1960	1970	1980	1990	2000
QLQMVRIGGD	RNDITLSGLE	SDHRYLVTLY	GFSDGKHVGP	VHVEALTVPE	EEKPSEPPTA	TPEPPIKPRL	GELTVTDATP
2010	2020	2030	2040	2050	2060	2070	2080
DSLSLSWTVP	EGQFDHFLVQ	YRNGDGQPKA	VRVPGHEEGV	TISGLEPDHK	YKMNLYGFHG	GQRMGPVSVV	GVTAAEEETP
2090	2100	2110	2120	2130	2140	2150	2160
SPTEPSMEAP	EPAEEPLLGE	LTVTGSSPDS	LSLSWTVPQG	RFDSFTVQYK	DRDGRPQVVR	VGGEESEVTV	GGLEPGRKYK
2170	2180	2190	2200	2210	2220	2230	2240

Detailed Protein Report

Cmpd.	No. of Cmpds.	m/z meas.	Δ m/z [ppm]	z	Rt [min]	Score	P	Range	Sequence	Modification
2748	1	1085.5225	-113.03	1	65.5	14.6	0	107-115	R.LEILEELVK.G	
15	1	1103.5160	91.05	2	29.8	14.6	1	264-282	R.CVCDPGYTGDDCGMRSCPR.G	Carbamidomethyl: 1, 3, 12
29	1	1103.5152	82.27	2	30.1	10.9	0	445-464	R.CENGVCVCNAGYSGEDCGVR.S	Carbamidomethyl: 6, 8, 17
2799	1	732.8911	134.58	2	64.1	13.0	1	725-737	R.GRGECHDGSCVCK.D	Carbamidomethyl: 10, 12
2882	1	981.9684	-29.71	2	65.2	15.6	1	2349-2366	R.VPGHEDRVTISGLEPDNK.Y	

Detailed Protein Report

Protein 40: zymogen granule protein 16 homolog B precursor [Homo sapiens]

Accession: gi|94536866
Database: refseq_human(refseq_human_20140103.fasta)
Database Date: 2015-11-30

Score: 68.1
MW [kDa]: 22.7
pI: 7.6
Sequence Coverage [%]: 14.4
No. of unique Peptides: 2

Quantitation

QD:QU **Median:** 0.36 **CV:** 0.00 % **No. of Peptides:** 1

10	20	30	40	50	60	70	80
MGAQGAQESI	KAMWRVPGTT	RRPVTGESPG	MHRPEAMLLL	LTLALLGGPT	WAGKMYGPGG	GKYFSTTEDY	DHEITGLRVS
**90**	**100**	**110**	**120**	**130**	**140**	**150**	**160**
VGLLLVKSVQ	VKLGDSWDVK	LGALGGNTQE	VTLQPGEYIT	KVFVAFQAFL	RGMVMYTSKD	RYFYFGKLDG	QISSAYPSQE
**170**	**180**	**190**	**200**	**210**			
GQVLVGIYGQ	YQLLGIKSIG	FEWNYPLEEP	TTEPPVNLTY	SANSPVGR			

Cmpd.	No. of Cmpds.	m/z meas.	Δ m/z [ppm]	z	Rt [min]	Score	P	Range	Sequence	Modification	Ratios
2510	2	464.1779	-296.31	2	60.1	35.2	0	79-87	R.VSVGLLLVK.S		
1743	6	1095.0646	-10.46	2	50.3	32.9	0	101-121	K.LGALGGNTQEVTLQPGEYITK.V		QD:QU 0.36

Detailed Protein Report

Protein 41: uncharacterized protein C4orf21 [Homo sapiens]

**Accession:**	gi|254826809	**Score:**	67.1
**Database:**	refseq_human(refseq_human_20140103.fasta)	**MW [kDa]:**	236.5
**Database Date:**	2015-11-30	**pI:**	5.8
**Modification(s):**	Oxidation	**Sequence Coverage [%]:**	3.3
		**No. of unique Peptides:**	4

Quantitation

QD:QU	**Median:** 2.59	**CV:** 0.00 %	**No. of Peptides:** 1

Alias proteins:

Accession	Name	Description
gi|530377937	refseq_human (refseq_human_20140103.fasta)	PREDICTED: uncharacterized protein C4orf21 isoform X1 [Homo sapiens]

Detailed Protein Report

10	20	30	40	50	60	70	80
MESQEFIVLY	THQKMKKSKV	WQDGILKITH	LGNKAILYDD	KGACLESLFL	KCLEVKPGDD	LESDRYLITV	EEVKVAGAIG
90	100	110	120	130	140	150	160
IVKQNVNKEA	PELNSRTFIS	SGRSLGCQPS	GLKRKFTGFQ	GPRQVPKKMV	IMESGESAAS	HEAKKTGPTI	FSPFCSMPPL
170	180	190	200	210	220	230	240
FPTVGKKDVN	NILADPENIV	TYKNRERNAM	DFSSVFSPSF	QINPEVLCEE	NYFCSPVNSG	NKLSDSLLTN	EPVKRDSLAS
250	260	270	280	290	300	310	320
HYSGVSQNIR	SKAQILALLK	SESSSSCEEL	NSEMTEHFPQ	KQPQGSLKIA	TKPKYLIQQE	ECAEMKSTEN	LYYQHQSENT
330	340	350	360	370	380	390	400
MRNKSRWAMY	LSSQSSPIHS	STVDGNDTER	KPKAQEDDVN	SNLKDLSLQK	IIQFVETYAE	ERKKYNVDQS	VGNNDPSWNQ
410	420	430	440	450	460	470	480
EVKLEIPSFN	ESSSLQVTCS	SAENDGILSE	SDIQEDNKIP	FNQNDKGCIK	GSVLIKENAQ	EVNTCGTLEK	EYEQSESSLP
490	500	510	520	530	540	550	560
ELKHLQIESS	NNSRISDDIT	DMISESKMDN	ESLNSIHESL	SNVTQPFLEV	TFNLNNFETS	DTEEESQESN	KISQDSESWV
570	580	590	600	610	620	630	640
KDILVNDGNS	CFQKRSENTN	CEEIEGEHLP	FLTSVSDKPT	VTFPVKETLP	SQFCDKTYVG	FDMGICKTEN	TGKEIEEYSD
650	660	670	680	690	700	710	720
TLSNFESFKW	TDAVYGDNKE	DANKPIQEVR	INYDFALPPN	KSKGINMNLH	IPHIQNQIAE	NSNLFSEDAQ	PQPFILGSDL
730	740	750	760	770	780	790	800
DKNDEHVLPS	TSSSDNSVQL	LNTNQNHYEC	IALDKSNTHI	SNSLFYPLGK	KHLISKDTEA	HISEPEDLGK	IRSPPPDHVE
810	820	830	840	850	860	870	880
VETAREGKQY	WNPRNSSELS	GLVNTISILK	SLCEHSTALD	SLEILKKKNT	VFQQGTQQTY	EPDSPPEVRK	PFITVVSPKS
890	900	910	920	930	940	950	960
PHLHKDSQQI	LKEDEVELSE	PLQSVQFSSS	GSKEETAFQA	VIPKQIERKT	CDPKPVEFQG	HQVKGSATSG	VMVRGHSSQL
970	980	990	1000	1010	1020	1030	1040
GCSQFPDSTE	YENFMTETPE	LPSTCMQIDF	LQVTSPEENI	STLSPVSTFS	LNSRDEDFMV	EFSETSLKAR	TLPDDLHFLN
1050	1060	1070	1080	1090	1100	1110	1120
LEGMKKSRSL	ENENLQRLSL	LSRTQVPLIT	LPRTDGPPDL	DSHSYMINSN	TYESSGSPML	NLCEKSAVLS	FSIEPEDQNE
1130	1140	1150	1160	1170	1180	1190	1200
TFFSEESREV	NPGDVSLNNI	STQSKWLKYQ	NTSQCNVATP	NRVDKRITDG	FFAEAVSGMH	FRDTSERQSD	AVNESSLDSV
1210	1220	1230	1240	1250	1260	1270	1280
HLQMIKGMLY	QQRQDFSSQD	SVSRKKVLSL	NLKQTSKTEE	IKNVLGGSTC	YNYSVKDLQE	ISGSELCFPS	GQKIKSAYLP
1290	1300	1310	1320	1330	1340	1350	1360
QRQIHIPAVF	QSPAHYKQTF	TSCLIEHLNI	LLFGLAQNLQ	KALSKVDISF	YTSLKGEKLK	NAENNVPSCH	HSQPAKLVMV
1370	1380	1390	1400	1410	1420	1430	1440
KKEGPNKGRL	FYTCDGPKAD	RCKFFKWLED	VTPGYSTQEG	ARPGMVLSDI	KSIGLYLRSQ	KIPLYEECQL	LVRKGFDFQR
1450	1460	1470	1480	1490	1500	1510	1520
KQYGKLKKFT	TVNPEFYNEP	KTKLYLKLSR	KERSSAYSKN	DLWVVSKTLD	FELDTFIACS	AFFGPSSINE	IEILPLKGYF
1530	1540	1550	1560	1570	1580	1590	1600
PSNWPTNMVV	HALLVCNAST	ELTTLKNIQD	YFNPATLPLT	QYLLTTSSPT	IVSNKRVSKR	KFIPPAFTNV	STKFELLSLG
1610	1620	1630	1640	1650	1660	1670	1680
ATLKLASELI	QVHKLNKDQA	TALIQIAQMM	ASHESIEEVK	ELQTHTFPIT	IIHGVFGAGK	SYLLAVVILF	FVQLFEKSEA
1690	1700	1710	1720	1730	1740	1750	1760
PTIGNARPWK	LLISSSTNVA	VDRVLLGLLS	LGFENFIRVG	SVRKIAKPIL	PYSLHAGSEN	ESEQLKELHA	LMKEDLTPTE
1770	1780	1790	1800	1810	1820	1830	1840
RVYVRKSIEQ	HKLGTNRTLL	KQVRVVGVTC	AACPFPCMND	LKFPVVVLDE	CSQITEPASL	LPIARFECEK	LILVGDPKQL
1850	1860	1870	1880	1890	1900	1910	1920
PPTIQGSDAA	HENGLEQTLF	DRLCLMGHKP	ILLRTQYRCH	PAISAIANDL	FYKGALMNGV	TEIERSPLLE	WLPTLCFYNV
1930	1940	1950	1960	1970	1980	1990	2000
KGLEQIERDN	SFHNVAEATF	TLKLIQSLIA	SGIAGSMIGV	ITLYKSQMYK	LCHLLSAVDF	HHPDIKTVQV	STVDAFQGAE
2010	2020	2030	2040	2050	2060	2070	2080
KEIIILSCVR	TRQVGFIDSE	KRMNVALTRG	KRHLLIVGNL	ACLRKNQLWG	RVIQHCEGRE	DGLQHANQYE	PQLNHLLKDY
2090	2100	2110					
FEKQVEEKQK	KKSEKEKSKD	KSHS					

Detailed Protein Report

Cmpd.	No. of Cmpds.	m/z meas.	Δ m/z [ppm]	z	Rt [min]	Score	P	Range	Sequence	Modification	Ratios
1998	1	902.8620	-77.52	2	55.4	13.3	1	128-144	K.KMVIMESGESAASHEAK.K		
2735	1	973.5207	5.77	2	63.2	10.7	1	167-183	K.KDVNNILADPENIVTYK.N		QD:QU 2.59
2930	6	698.3835	-51.56	2	65.8	21.0	1	282-294	K.QPQGSLKIATKPK.Y		
2757	1	849.0563	-13.52	3	65.6	22.0	2	1166-1187	K.RITDGFFAEAVSGMHFRDTSER.Q	Oxidation: 14	

Detailed Protein Report

Protein 42: fanconi-associated nuclease 1 isoform a [Homo sapiens]

**Accession:**	gi|226246523	**Score:**	65.8
**Database:**	refseq_human(refseq_human_20140103.fasta)	**MW [kDa]:**	114.2
**Database Date:**	2015-11-30	**pI:**	7.4
**Modification(s):**	Carbamidomethyl, Oxidation	**Sequence Coverage [%]:**	7.2
		**No. of unique Peptides:**	4

Quantitation

QD:QU	**Median:** 0.58	**CV:** 0.00 %	**No. of Peptides:** 1

Alias proteins:

Accession	Name	Description
gi|578840975	refseq_human (refseq_human_20140103.fasta)	PREDICTED: fanconi-associated nuclease 1 isoform X4 [Homo sapiens]
gi|578840973	refseq_human (refseq_human_20140103.fasta)	PREDICTED: fanconi-associated nuclease 1 isoform X3 [Homo sapiens]
gi|578840971	refseq_human (refseq_human_20140103.fasta)	PREDICTED: fanconi-associated nuclease 1 isoform X2 [Homo sapiens]
gi|578840969	refseq_human (refseq_human_20140103.fasta)	PREDICTED: fanconi-associated nuclease 1 isoform X1 [Homo sapiens]
gi|530405539	refseq_human (refseq_human_20140103.fasta)	PREDICTED: fanconi-associated nuclease 1 isoform X4 [Homo sapiens]
gi|530405537	refseq_human (refseq_human_20140103.fasta)	PREDICTED: fanconi-associated nuclease 1 isoform X3 [Homo sapiens]
gi|530405535	refseq_human (refseq_human_20140103.fasta)	PREDICTED: fanconi-associated nuclease 1 isoform X2 [Homo sapiens]
gi|530405533	refseq_human (refseq_human_20140103.fasta)	PREDICTED: fanconi-associated nuclease 1 isoform X1 [Homo sapiens]

10	20	30	40	50	60	70	80
MMSEGKPPDK	KRPRRSLSIS	KNKKKASNSI	ISCFNNAPPA	KLACPVCSKM	VPRYDLNRHL	DEMCANNDFV	QVDPGQVGLI
**90**	**100**	**110**	**120**	**130**	**140**	**150**	**160**
NSNVSMVDLT	SVTLEDVTPK	KSPPPKTNLT	PGQSDSAKRE	VKQKISPYFK	SNDVVCKNQD	ELRNRSVKVI	CLGSLASKLS
**170**	**180**	**190**	**200**	**210**	**220**	**230**	**240**
RKYVKAKKSI	DKDEEFAGSS	PQSSKSTVVK	SLIDNSSEIE	DEDQILENSS	QKENVFKCDS	LKEECIPEHM	VRGSKIMEAE
**250**	**260**	**270**	**280**	**290**	**300**	**310**	**320**
SQKATRECEK	SALTPGFSDN	AIMLFSPDFT	LRNTLKSTSE	DSLVKQECIK	EVVEKREACH	CEEVKMTVAS	EAKIQLSDSE
**330**	**340**	**350**	**360**	**370**	**380**	**390**	**400**
AKSHSSADDA	SAWSNIQEAP	LQDDSCLNND	IPHSIPLEQG	SSCNGPGQTT	GHPYYLRSFL	VVLKTVLENE	DDMLLFDEQE
**410**	**420**	**430**	**440**	**450**	**460**	**470**	**480**
KGIVTKFYQL	SATGQKLYVR	LFQRKLSWIK	MTKLEYEEIA	LDLTPVIEEL	TNAGFLQTES	ELQELSEVLE	LLSAPELKSL
**490**	**500**	**510**	**520**	**530**	**540**	**550**	**560**
AKTFHLVNPN	GQKQQLVDAF	LKLAKQRSVC	TWGKNKPGIG	AVILKRAKAL	AGQSVRICKG	PRAVFSRILL	LFSLTDSMED
**570**	**580**	**590**	**600**	**610**	**620**	**630**	**640**
EDAACGGQGQ	LSTVLLVNLG	RMEFPSYTIN	RKTHIFQDRD	DLIRYAAATH	MLSDISSAMA	NGNWEEAKEL	AQCAKRDWNR
**650**	**660**	**670**	**680**	**690**	**700**	**710**	**720**
LKNHPSLRCH	EDLPLFLRCF	TVGWIYTRIL	SRFVEILQRL	HMYEEAVREL	ESLLSQRIYC	PDSRGRWWDR	LALNLHQHLK
**730**	**740**	**750**	**760**	**770**	**780**	**790**	**800**
RLEPTIKCIT	EGLADPEVRT	GHRLSLYQRA	VRLRESPSCK	KFKHLFQQLP	EMAVQDVKHV	TITGRLCPQR	GMCKSVFVME
**810**	**820**	**830**	**840**	**850**	**860**	**870**	**880**
AGEAADPTTV	LCSVEELALA	HYRRSGFDQG	IHGEGSTFST	LYGLLLWDII	FMDGIPDVFR	NACQAFPLDL	CTDSFFTSRR
**890**	**900**	**910**	**920**	**930**	**940**	**950**	**960**
PALEARLQLI	HDAPEESLRA	WVAATWHEQE	GRVASLVSWD	RFTSLQQAQD	LVSCLGGPVL	SGVCRHLAAD	FRHCRGGLPD
**970**	**980**	**990**	**1000**	**1010**	**1020**		
LVVWNSQSRH	FKLVEVKGPN	DRLSHKQMIW	LAELQKLGAE	VEVCHVVAVG	AKSQSLS		

Detailed Protein Report

Cmpd.	No. of Cmpds.	m/z meas.	Δ m/z [ppm]	z	Rt [min]	Score	P	Range	Sequence	Modification	Ratios
443	1	874.1657	67.87	3	34.2	13.4	2	25-49	K. KASNSIISCFNNAPPAKLACPVCSH M	Carbamidomethyl: 20	
691	1	765.8997	57.54	2	38.6	18.5	1	223-235	K.EECIPEHMVRGSK.I	Oxidation: 8	QD:QU 0.58
56	3	768.3135	-107.30	2	30.6	16.5	2	233-246	R.GSKIMEAESQKATR.E		
97	1	826.6883	-79.67	3	31.1	17.4	0	922-945	R. FTSLQQAQDLVSCLGGPVLSGVC H		

Detailed Protein Report

Protein 43: PREDICTED: dystonin isoform X7 [Homo sapiens]

**Accession:**	gi|530382430	**Score:**	64.6
**Database:**	refseq_human(refseq_human_20140103.fasta)	**MW [kDa]:**	875.9
**Database Date:**	2015-11-30	**pI:**	5.0
**Modification(s):**	Oxidation	**Sequence Coverage [%]:**	0.9
		**No. of unique Peptides:**	4

Detailed Protein Report

10	20	30	40	50	60	70	80
MGNVCGCVRA	EKEEQYVDPA	KTPLNPEKYS	PGRKYFRRKP	IKKTGGDKES	VGANNENEGK	KKSSSQPSKE	QPAPLSRGLV
90	100	110	120	130	140	150	160
QQESVTLNSA	LGDGIQQKKT	EVVADSVKQK	LLPSAVSSWS	DCVNTSPAKD	SETEVKVSEL	DERISEKDST	PYCAKRKKHL
170	180	190	200	210	220	230	240
DDVNTSEITF	QEKTDVFSFR	KAASLSSIPS	GIERSLEKSG	FPEDPPKSYS	SIQEKQNTER	FCPHATQHFQ	FKKKRCHSLY
250	260	270	280	290	300	310	320
TSMSSVSKDT	DGNEISDIHV	TGESEDMSAK	ERLLLWTQQA	TEGYAGIRCE	NFTTCWRDGK	LFNAIIHKYR	PDLIDMNTVA
330	340	350	360	370	380	390	400
VQSNLANLEH	AFYVAEKIGV	IRLLDPEDVD	VSSPDEKSVI	TYVSSLYDAF	PKVPEGGEGI	GANDVEVKWI	EYQNMVNYLI
410	420	430	440	450	460	470	480
QWIRHHVTTM	SERTFPNNPV	ELKALYNQYL	QFKETEIPPK	ETEKSKIKRL	YKLLEIWIEF	GRIKLLQGYH	PNDIEKEWGK
490	500	510	520	530	540	550	560
LIIAMLEREK	ALRPEVERLE	MLQQIANRVQ	RDSVICEDKL	ILAGNALQSD	SKRLESGVQF	QNEAEIAGYI	LECENLLRQH
570	580	590	600	610	620	630	640
VIDVQILIDG	KYYQADQLVQ	RVAKLRDEIM	ALRNECSSVY	SKGRILTTEQ	TKLMISGITQ	SLNSGFAQTL	HPSLTSGLTQ
650	660	670	680	690	700	710	720
SLTPSLTSSS	MTSGLSSGMT	SRLTPSVTPA	YTPGFPSGLV	PNFSSGVEPN	SLQTLKLMQI	RKPLLKSSLL	DQNLTEEEIN
730	740	750	760	770	780	790	800
MKFVQDLLNW	VDEMQVQLDR	TEWGSDLPSV	ESHLENHKNV	HRAIEEFESS	LKEAKISEIQ	MTAPLKLTYA	EKLHRLESQY
810	820	830	840	850	860	870	880
AKLLNTSRNQ	ERHLDTLHNF	VSRATNELIW	LNEKEEEEVA	YDWSERNTNI	ARKKDYHAEL	MRELDQKEEN	IKSVQEIAEQ
890	900	910	920	930	940	950	960
LLLENHPARL	TIEAYRAAMQ	TQWSWILQLC	QCVEQHIKEN	TAYFEFFNDA	KEATDYLRNL	KDAIQRKYSC	DRSSSIHKLE
970	980	990	1000	1010	1020	1030	1040
DLVQESMEEK	EELLQYKSTI	ANLMGKAKTI	IQLKPRNSDC	PLKTSIPIKA	ICDYRQIEIT	IYKDDECVLA	NNSHRAKWKV
1050	1060	1070	1080	1090	1100	1110	1120
ISPTGNEAMV	PSVCFTVPPP	NKEAVDLANR	IEQQYQNVLT	LWHESHINMK	SVVSWHYLIN	EIDRIRASNV	ASIKTMLPGE
1130	1140	1150	1160	1170	1180	1190	1200
HQQVLSNLQS	RFEDFLEDSQ	ESQVFSGSDI	TQLEKEVNVC	KQYYQELLKS	AEREEQEESV	YNLYISEVRN	IRLRLENCED
1210	1220	1230	1240	1250	1260	1270	1280
RLIRQIRTPL	ERDDLHESVF	RITEQEKLKK	ELERLKDDLG	TITNKCEEFF	SQAAASSSVP	TLRSELNVVL	QNMNQVYSMS
1290	1300	1310	1320	1330	1340	1350	1360
STYIDKLKTV	NLVLKNTQAA	EALVKLYETK	LCEEEAVIAD	KNNIENLIST	LKQWRSEVDE	KRQVFHALED	ELQKAKAISD
1370	1380	1390	1400	1410	1420	1430	1440
EMFKTYKERD	LDFDWHKEKA	DQLVERWQNV	HVQIDNRLRD	LEGIGKSLKY	YRDTYHPLDD	WIQQVETTQR	KIQENQPENS
1450	1460	1470	1480	1490	1500	1510	1520
KTLATQLNQQ	KMLVSEIEMK	QSKMDECQKY	AEQYSATVKD	YELQTMTYRA	MVDSQQKSPV	KRRRMQSSAD	LIIQEFMDLR
1530	1540	1550	1560	1570	1580	1590	1600
TRYTALVTLM	TQYIKFAGDS	LKRLEEEEKS	LEEEKKEHVE	KAKELQKWVS	NISKTLKDAE	KAGKPPFSKQ	KISSEEISTK
1610	1620	1630	1640	1650	1660	1670	1680
KEQLSEALQT	IQLFLAKHGD	KMTDEERNEL	EKQVKTLQES	YNLLFSESLK	QLQESQTSGD	VKVEEKLDKV	IAGTIDQTTG
1690	1700	1710	1720	1730	1740	1750	1760
EVLSVFQAVL	RGLIDYDTGI	RLLETQLMIS	GLISPELRKC	FDLKDAKSHG	LIDEQILCQL	KELSKAKEII	SAASPTTIPV
1770	1780	1790	1800	1810	1820	1830	1840
LDALAQSMIT	ESMAIKVLEI	LLSTGSLVIP	ATGEQLTLQK	AFQQNLVSSA	LFSKVLERQN	MCKDLIDPCT	SEKVSLIDMV
1850	1860	1870	1880	1890	1900	1910	1920
QRSTLQENTG	MWLLPVRPQE	GGRITLKCGR	NISILRAAHE	GLIDRETMFR	LLSAQLLSGG	LINSNSGQRM	TVEEAVREGV
1930	1940	1950	1960	1970	1980	1990	2000
IDRDTASSIL	TYQVQTGGII	QSNPAKRLTV	DEAVQCDLIT	SSSALLVLEA	QRGYVGLIWP	HSGEIFPTSS	SLQQELITNE
2010	2020	2030	2040	2050	2060	2070	2080
LAYKILNGRQ	KIAALYIPES	SQVIGLDAAK	QLGIIDNNTA	SILKNITLPD	KMPDLGDLEA	CKNARRWLSF	CKFQPSTVHD
2090	2100	2110	2120	2130	2140	2150	2160
YRQEEDVFDG	EEPVTTQTSE	ETKKLFLSYL	MINSYMDANT	GQRLLLYDGD	LDEAVGMLLE	GCHAEFDGNT	AIKECLDVLS
2170	2180	2190	2200	2210	2220	2230	2240

Detailed Protein Report

Cmpd.	No. of Cmpds.	m/z meas.	Δ m/z [ppm]	z	Rt [min]	Score	P	Range	Sequence	Modification
1902	1	805.7986	-138.23	2	54.1	11.0	1	3613-3626	R.TKQIMLAIDSEMSK.L	Oxidation: 12
779	1	1105.2011	129.15	2	38.2	12.7	2	4246-4264	K.TLDDIVGRYEDLSKSVNER.N	
2575	1	588.6984	-194.01	2	60.9	10.5	0	6269-6278	K.LLDVMELAEK.F	Oxidation: 5
1793	3	781.2979	-163.01	2	52.8	12.6	2	6886-6899	R.SLKEKTSLADDNLK.L	

Detailed Protein Report

Protein 44: myosin-6 [Homo sapiens]

**Accession:**	gi|156104908	**Score:**	63.9
**Database:**	refseq_human(refseq_human_20140103.fasta)	**MW [kDa]:**	223.6
**Database Date:**	2015-11-30	**pI:**	5.5
**Modification(s):**	Carbamidomethyl	**Sequence Coverage [%]:**	2.7
		**No. of unique Peptides:**	3

Detailed Protein Report

10	20	30	40	50	60	70	80
MTDAQMADFG	AAAQYLRKSE	KERLEAQTRP	FDIRTECFVP	DDKEEFVKAK	ILSREGGKVI	AETENGKTVT	VKEDQVLQQN
90	100	110	120	130	140	150	160
PPKFDKIEDM	AMLTFLHEPA	VLFNLKERYA	AWMIYTYSGL	FCVTVNPYKW	LPVYNAEVVA	AYRGKKRSEA	PPHIFSISDN
170	180	190	200	210	220	230	240
AYQYMLTDRE	NQSILITGES	GAGKTVNTKR	VIQYFASIAA	IGDRGKKDNA	NANKGTLEDQ	IIQANPALEA	FGNAKTVRND
250	260	270	280	290	300	310	320
NSSRFGKFIR	IHFGATGKLA	SADIETYLLE	KSRVIFQLKA	ERNYHIFYQI	LSNKKPELLD	MLLVTNNPYD	YAFVSQGEVS
330	340	350	360	370	380	390	400
VASIDDSEEL	MATDSAFDVL	GFTSEEKAGV	YKLTGAIMHY	GNMKFKQKQR	EEQAEPDGTE	DADKSAYLMG	LNSADLLKGL
410	420	430	440	450	460	470	480
CHPRVKVGNE	YVTKGQSVQQ	VYYSIGALAK	AVYEKMFNWM	VTRINATLET	KQPRQYFIGV	LDIAGFEIFD	FNSFEQLCIN
490	500	510	520	530	540	550	560
FTNEKLQQFF	NHHMFVLEQE	EYKKEGIEWT	FIDFGMDLQA	CIDLIEKPMG	IMSILEEECM	FPKATDMTFK	AKLYDNHLGK
570	580	590	600	610	620	630	640
SNNFQKPRNI	KGKQEAHFSL	IHYAGTVDYN	ILGWLEKNKD	PLNETVVALY	QKSSLKLMAT	LFSSYATADT	GDSGKSKGGK
650	660	670	680	690	700	710	720
KKGSSFQTVS	ALHRENLNKL	MTNLRTTHPH	FVRCIIPNER	KAPGVMDNPL	VMHQLRCNGV	LEGIRICRKG	FPNRILYGDF
730	740	750	760	770	780	790	800
RQRYRILNPV	AIPEGQFIDS	RKGTEKLLSS	LDIDHNQYKF	GHTKVFFKAG	LLGLLEEMRD	ERLSRIITRM	QAQARGQLMR
810	820	830	840	850	860	870	880
IEFKKIVERR	DALLVIQWNI	RAFMGVKNWP	WMKLYFKIKP	LLKSAETEKE	MATMKEEFGR	IKETLEKSEA	RRKELEEKMV
890	900	910	920	930	940	950	960
SLLQEKNDLQ	LQVQAEQDNL	NDAEERCDQL	IKNKIQLEAK	VKEMNERLED	EEEMNAELTA	KKRKLEDECS	ELKKDIDDLE
970	980	990	1000	1010	1020	1030	1040
LTLAKVEKEK	HATENKVKNL	TEEMAGLDEI	IAKLTKEKKA	LQEAHQQALD	DLQVEEDKVN	SLSKSKVKLE	QQVDDLEGSL
1050	1060	1070	1080	1090	1100	1110	1120
EQEKKVRMDL	ERAKRKLEGD	LKLTQESIMD	LENDKLQLEE	KLKKKEFDIN	QQNSKIEDEQ	VLALQLQKKL	KENQARIEEL
1130	1140	1150	1160	1170	1180	1190	1200
EEELEAERTA	RAKVEKLRSD	LSRELEEISE	RLEEAGGATS	VQIEMNKKRE	AEFQKMRRDL	EEATLQHEAT	AAALRKKHAD
1210	1220	1230	1240	1250	1260	1270	1280
SVAELGEQID	NLQRVKQKLE	KEKSEFKLEL	DDVTSNMEQI	IKAKANLEKV	SRTLEDQANE	YRVKLEEAQR	SLNDFTTQRA
1290	1300	1310	1320	1330	1340	1350	1360
KLQTENGELA	RQLEEKEALI	SQLTRGKLSY	TQQMEDLKRQ	LEEEGKAKNA	LAHALQSARH	DCDLLREQYE	EETEAKAELQ
1370	1380	1390	1400	1410	1420	1430	1440
RVLSKANSEV	AQWRTKYETD	AIQRTEELEE	AKKKLAQRLQ	DAEEAVEAVN	AKCSSLEKTK	HRLQNEIEDL	MVDVERSNAA
1450	1460	1470	1480	1490	1500	1510	1520
AAALDKKQRN	FDKILAEWKQ	KYEESQSELE	SSQKEARSLS	TELFKLKNAY	EESLEHLETF	KRENKNLQEE	ISDLTEQLGE
1530	1540	1550	1560	1570	1580	1590	1600
GGKNVHELEK	VRKQLEVEKL	ELQSALEEAE	ASLEHEEGKI	LRAQLEFNQI	KAEIERKLAE	KDEEMEQAKR	NHQRVVDSLQ
1610	1620	1630	1640	1650	1660	1670	1680
TSLDAETRSR	NEVLRVKKKM	EGDLNEMEIQ	LSHANRMAAE	AQKQVKSLQS	LLKDTQIQLD	DAVRANDDLK	ENIAIVERRN
1690	1700	1710	1720	1730	1740	1750	1760
NLLQAELEEL	RAVVEQTERS	RKLAEQELIE	TSERVQLLHS	QNTSLINQKK	KMESDLTQLQ	SEVEEAVQEC	RNAEEKAKKA
1770	1780	1790	1800	1810	1820	1830	1840
ITDAAMMAEE	LKKEQDTSAH	LERMKKNMEQ	TIKDLQHRLD	EAEQIALKGG	KKQLQKLEAR	VRELEGELEA	EQKRNAESVK
1850	1860	1870	1880	1890	1900	1910	1920
GMRKSERRIK	ELTYQTEEDK	KNLLRLQDLV	DKLQLKVKAY	KRQAEEAEEQ	ANTNLSKFRK	VQHELDEAEE	RADIAESQVN
1930	1940						
KLRAKSRDIG	AKQKMHDEE						

Cmpd.	No. of Cmpds.	m/z meas.	Δ m/z [ppm]	z	Rt [min]	Score	P	Range	Sequence	Modification
2955	1	953.4514	-13.99	2	66.1	12.0	2	928-943	R.LEDEEEMNAELTAKKR.K	
202	1	561.6237	-229.75	2	31.3	12.3	0	945-953	K.LEDECSELK.K	Carbamidomethyl: 5
1892	1	628.2831	-7.98	2	54.0	13.1	0	1851-	K.ELTYQTEEDK.K	

Detailed Protein Report

Cmpd.	No. of Cmpds.	m/z meas.	Δ m/z [ppm]	z	Rt [min]	Score	P	Range	Sequence	Modification
								1860		

Detailed Protein Report

Protein 45: **14-3-3 protein sigma [Homo sapiens]**

**Accession:**	gi|5454052	**Score:**	62.7
**Database:**	refseq_human(refseq_human_20140103.fasta)	**MW [kDa]:**	27.8
**Database Date:**	2015-11-30	**pI:**	4.5
		**Sequence Coverage [%]:**	11.3
		**No. of unique Peptides:**	2

10	20	30	40	50	60	70	80
MERASLIQKA	KLAEQAERYE	DMAAFMKGAV	EKGEELSCEE	RNLLSVAYKN	VVGGQRAAWR	VLSSIEQKSN	EEGSEEKGPE
**90**	**100**	**110**	**120**	**130**	**140**	**150**	**160**
VREYREKVET	ELQGVCDTVL	GLLDSHLIKE	AGDAESRVFY	LKMKGDYYRY	LAEVATGDDK	KRIIDSARSA	YQEAMDISKK
**170**	**180**	**190**	**200**	**210**	**220**	**230**	**240**
EMPPTNPIRL	GLALNFSVFH	YEIANSPEEA	ISLAKTTFDE	AMADLHTLSE	DSYKDSTLIM	QLLRDNLTLW	TADNAGEEGG
**250**							
EAPQEPQS							

Cmpd.	No. of Cmpds.	m/z meas.	Δ m/z [ppm]	z	Rt [min]	Score	P	Range	Sequence	Modification
2035	2	1051.3758	-123.72	2	55.8	15.4	2	10-27	K.AKLAEQAERYEDMAAFMK.G	
2725	2	595.3333	-1.39	2	65.2	47.3	0	215-224	K.DSTLIMQLLR.D	

Detailed Protein Report

Protein 46: pyruvate kinase PKM isoform a [Homo sapiens]

**Accession:**	gi|33286418	**Score:**	61.9
**Database:**	refseq_human(refseq_human_20140103.fasta)	**MW [kDa]:**	57.9
**Database Date:**	2015-11-30	**pI:**	9.0
		**Sequence Coverage [%]:**	5.1
		**No. of unique Peptides:**	2

Quantitation

QD:QU **Median:** 1.21 **CV:** 15.59 % **No. of Peptides:** 2

Alias proteins:

Accession	Name	Description
gi|530405979	refseq_human (refseq_human_20140103.fasta)	PREDICTED: pyruvate kinase PKM isoform X3 [Homo sapiens]

```
        10         20         30         40         50         60         70         80
MSKPHSEAGT AFIQTQQLHA AMADTFLEHM CRLDIDSPPI TARNTGIICT IGPASRSVET LKEMIKSGMN VARLNFSHGT
        90        100        110        120        130        140        150        160
HEYHAETIKN VRTATESFAS DPILYRPVAV ALDTKGPEIR TGLIKGSGTA EVELKKGATL KITLDNAYME KCDENILWLD
       170        180        190        200        210        220        230        240
YKNICKVVEV GSKIYVDDGL ISLQVKQKGA DFLVTEVENG GSLGSKKGVN LPGAAVDLPA VSEKDIQDLK FGVEQDVDMV
       250        260        270        280        290        300        310        320
FASFIRKASD VHEVRKVLGE KGKNIKIISK IENHEGVRRF DEILEASDGI MVARGDLGIE IPAEKVFLAQ KMMIGRCNRA
       330        340        350        360        370        380        390        400
GKPVICATQM LESMIKKPRP TRAEGSDVAN AVLDGADCIM LSGETAKGDY PLEAVRMQHL IAREAEAAIY HLQLFEELRR
       410        420        430        440        450        460        470        480
LAPITSDPTE ATAVGAVEAS FKCCSGAIIV LTKSGRSAHQ VARYRPRAPI IAVTRNPQTA RQAHLYRGIF PVLCKDPVQE
       490        500        510        520        530        540
AWAEDVDLRV NFAMNVGKAR GFFKKGDVVI VLTGWRPGSG FTNTMRVVPV P
```

Cmpd.	No. of Cmpds.	m/z meas.	Δ m/z [ppm]	z	Rt [min]	Score	P	Range	Sequence	Modification	Ratios	
859	2	599.3167	-17.70	2	39.2	51.2	0	33-43	R.LDIDSPPITAR.N		QD:QU	1.41
183	1	628.6439	7.16	3	32.3	10.8	0	74-89	R.LNFSHGTHEYHAETIK.N		QD:QU	1.04

Detailed Protein Report

Protein 47: glyceraldehyde-3-phosphate dehydrogenase isoform 2 [Homo sapiens]

**Accession:**	gi|378404908	**Score:**	60.9
**Database:**	refseq_human(refseq_human_20140103.fasta)	**MW [kDa]:**	31.5
**Database Date:**	2015-11-30	**pI:**	7.9
**Modification(s):**	Carbamidomethyl	**Sequence Coverage [%]:**	10.6
		**No. of unique Peptides:**	2

Quantitation

QD:QU **Median:** 3.46 **CV:** 0.00 % **No. of Peptides:** 1

10	20	30	40	50	60	70	80
MVYMFQYDST	HGKFHGTVKA	ENGKLVINGN	PITIFQERDP	SKIKWGDAGA	EYVVESTGVF	TTMEKAGAHL	QGGAKRVIIS
**90**	**100**	**110**	**120**	**130**	**140**	**150**	**160**
APSADAPMFV	MGVNHEKYDN	SLKIISNASC	TTNCLAPLAK	VIHDNFGIVE	GLMTTVHAIT	ATQKTVDGPS	GKLWRDGRGA
**170**	**180**	**190**	**200**	**210**	**220**	**230**	**240**
LQNIIPASTG	AAKAVGKVIP	ELNGKLTGMA	FRVPTANVSV	VDLTCRLEKP	AKYDDIKKVV	KQASEGPLKG	ILGYTEHQVV
**250**	**260**	**270**	**280**	**290**	**300**		
SSDFNSDTHS	STFDAGAGIA	LNDHFVKLIS	WYDNEFGYSN	RVVDLMAHMA	SKE		

Cmpd.	No. of Cmpds.	m/z meas.	Δ m/z [ppm]	z	Rt [min]	Score	P	Range	Sequence	Modification	Ratios
772	2	917.4517	-12.88	2	39.6	40.3	0	104-120	K.IISNASCTTNCLAPLAK.V	Carbamidomethyl: 7, 11	QD:QU 3.46
1648	3	882.4064	1.76	2	50.9	20.6	0	268-281	K.LISWYDNEFGYSNR.V		

Detailed Protein Report

Protein 48: PREDICTED: dedicator of cytokinesis protein 11 isoform X2 [Homo sapiens]

**Accession:**	gi|530422336	**Score:**	60.8
**Database:**	refseq_human(refseq_human_20140103.fasta)	**MW [kDa]:**	235.7
**Database Date:**	2015-11-30	**pI:**	8.1
**Modification(s):**	Oxidation	**Sequence Coverage [%]:**	1.7
		**No. of unique Peptides:**	4

BRUKER

Detailed Protein Report

10	20	30	40	50	60	70	80
MFKEKAKVVE	PLDYENVIAQ	RKTQIYSDPL	RDLLMFPMED	ISISVIGRQR	RTVQSTVPED	AEKRAQSLFV	KECIKTYSTD
90	100	110	120	130	140	150	160
WHVVNYKYED	FSGDFRMLPC	KSLRPEKIPN	HVFEIDEDCE	KDEDSSSLCS	QKGGVIKQGW	LHKANVNSTI	TVTMKVFKRR
170	180	190	200	210	220	230	240
YFYLTQLPDG	SYILNSYKDE	KNSKESKGCI	YLDACIDVVQ	CPKMRRHAFE	LKMLDKYSHY	LAAETEQEME	EWLITLKKII
250	260	270	280	290	300	310	320
QINTDSLVQE	KKETVETAQD	DETSSQGKAE	NIMASLERSM	HPELMKYGRE	TEQLNKLSRG	DGRQNLFSFD	SEVQRLDFSG
330	340	350	360	370	380	390	400
IEPDIKPFEE	KCNKRFLVNC	HDLTFNILGQ	IGDNAKGPPT	NVEPFFINLA	LFDVKNNCKI	SADFHVDLNP	PSVREMLWGS
410	420	430	440	450	460	470	480
STQLASDGSP	KGSSPESYIH	GIAESQLRYI	QQGIFSVTNP	HPEIFLVARI	EKVLQGNITH	CAEPYIKNSD	PVKTAQKVHR
490	500	510	520	530	540	550	560
TAKQVCSRLG	QYRMPFAWAA	RPIFKDTQGS	LDLDGRFSPL	YKQDSSKLSS	EDILKLLSEY	KKPEKTKLQI	IPGQLNITVE
570	580	590	600	610	620	630	640
CVPVDLSNCI	TSSYVPLKPF	EKNCQNITVE	VEEFVPEMTK	YCYPFTIYKN	HLYVYPLQLK	YDSQKTFAKA	RNIAVCVEFR
650	660	670	680	690	700	710	720
DSDESDASAL	KCIYGKPAGS	VFTTNAYAVV	SHHNQNPEFY	DEIKIELPIH	LHQKHHLLFT	FYHVSCEINT	KGTTKKQDTV
730	740	750	760	770	780	790	800
ETPVGFAWVP	LLKDGRIITF	EQQLPVSANL	PPGYLNLNDA	ESRRQCNVDI	KWVDGAKPLL	KIKSHLESTI	YTQDLHVHKF
810	820	830	840	850	860	870	880
FHHCQLIQSG	SKEVPGELIK	YLKCLHAMEI	QVMIQFLPVI	LMQLFRVLTN	MTHEDDVPIN	CTMVLLHIVS	KCHEEGLDSY
890	900	910	920	930	940	950	960
LRSFIKYSFR	PEKPSAPQAQ	LIHETLATTM	IAILKQSADF	LSINKLLKYS	WFFFEIIAKS	MATYLLEENK	IKLPRGQRFP
970	980	990	1000	1010	1020	1030	1040
ETYHHVLHSL	LLAIIPHVTI	RYAEIPDESR	NVNYSLASFL	KRCLTLMDRG	FIFNLINDYI	SGFSPKDPKV	LAEYKFEFLQ
1050	1060	1070	1080	1090	1100	1110	1120
TICNHEHYIP	LNLPMAFAKP	KLQRVQDFFS	FAVDRLTSVD	SNLEYSLSDE	YCKHHFLVGL	LLRETSIALQ	DNYEIRYTAI
1130	1140	1150	1160	1170	1180	1190	1200
SVIKNLLIKH	AFDTRYQHKN	QQAKIAQLYL	PFVGLLLENI	QRLAGRDTLY	SCAAMPNSAS	RDEFPCGFTS	PANRGSLSTD
1210	1220	1230	1240	1250	1260	1270	1280
KDTAYGSFQN	GHGIKREDSR	GSLIPEGATG	FPDQGNTGEN	TRQSSTRSSV	SQYNRLDQYE	IRSLLMCYLY	IVKMISEDTL
1290	1300	1310	1320	1330	1340	1350	1360
LTYWNKVSPQ	ELINILILLE	VCLFHFRYMG	KRNIARVHDA	WLSKHFGIDR	KSQTMPALRN	RSGVMQARLQ	HLSSLESSFT
1370	1380	1390	1400	1410	1420	1430	1440
LNHSSTTTEA	DIFHQALLEG	NTATEVSLTV	LDTISFFTQC	FKTQLLNNDG	HNPLMKKVFD	IHLAFLKNGQ	SEVSLKHVFA
1450	1460	1470	1480	1490	1500	1510	1520
SLRAFISKFP	SAFFKGRVNM	CAAFCYEVLK	CCTSKISSTR	NEASALLYLL	MRNNFEYTKR	KTFLRTHLQI	IIAVSQLIAD
1530	1540	1550	1560	1570	1580	1590	1600
VALSGGSRFQ	ESLFIINNFA	NSDRPMKATA	FPAEVKDLTK	RIRTVLMATA	QMKEHEKDPE	MLIDLQYSLA	KSYASTPELR
1610	1620	1630	1640	1650	1660	1670	1680
KTWLDSMAKI	HVKNGDFSEA	AMCYVHVAAL	VAEFLHRKKL	FPNGCSAFKK	ITPNIDEEGA	MKEDAGMMDV	HYSEEVLLEL
1690	1700	1710	1720	1730	1740	1750	1760
LEQCVDGLWK	AERYEIISEI	SKLIVPIYEK	RREFEKLTQV	YRTLHGAYTK	ILEVMHTKKR	LLGTFFRVAF	YGQSFFEEED
1770	1780	1790	1800	1810	1820	1830	1840
GKEYIYKEPK	LTGLSEISLR	LVKLYGEKFG	TENVKIIQDS	DKVNAKELDP	KYAHIQVTYV	KPYFDDKELT	ERKTEFERNH
1850	1860	1870	1880	1890	1900	1910	1920
NISRFVFEAP	YTLSGKKQGC	IEEQCKRRTI	LTTSNSFPYV	KKRIPINCEQ	QINLKPIDVA	TDEIKDKTAE	LQKLCSSTDV
1930	1940	1950	1960	1970	1980	1990	2000
DMIQLQLKLQ	GCVSVQVNAG	PLAYARAFLN	DSQASKYPPK	KVSELKDMFR	KFIQACSIAL	ELNERLIKED	QVEYHEGLKS
2010	2020	2030	2040	2050	2060		
NFRDMVKELS	DIIHEQILQE	DTMHSPWMSN	TLHVFCAISG	TSSDRGYGSP	RYAEV		

Cmpd.	No. of	m/z meas.	Δ m/z	z	Rt	Score	P	Range	Sequence	Modification

Detailed Protein Report

	Cmpds.		[ppm]		[min]					
180	1	1043.9250	-79.30	2	32.2	13.4	1	269-286	K.AENIMASLERSMHPELMK.Y	
2146	2	682.9130	127.21	2	57.2	18.0	1	279-289	R.SMHPELMKYGR.E	Oxidation: 7
1995	1	682.7325	-137.16	2	53.4	18.8	1	279-289	R.SMHPELMKYGR.E	Oxidation: 2
2045	1	731.3221	-60.47	2	56.0	10.6	1	1650-1662	K.KITPNIDEEGAMK.E	Oxidation: 12

Detailed Protein Report

Protein 49: hemoglobin subunit epsilon [Homo sapiens]

**Accession:**	gi|4885393	**Score:**	60.7
**Database:**	refseq_human(refseq_human_20140103.fasta)	**MW [kDa]:**	16.2
**Database Date:**	2015-11-30	**pI:**	9.4
		**Sequence Coverage [%]:**	15.0
		**No. of unique Peptides:**	1

```
        10         20         30         40         50         60         70         80
MVHFTAEEKA AVTSLWSKMN VEEAGGEALG RLLVVYPWTQ RFFDSFGNLS SPSAILGNPK VKAHGKKVLT SFGDAIKNMD
        90        100        110        120        130        140        150
NLKPAFAKLS ELHCDKLHVD PENFKLLGNV MVIILATHFG KEFTPEVQAA WQKLVSAVAI ALAHKYH
```

Cmpd.	No. of Cmpds.	m/z meas.	Δ m/z [ppm]	z	Rt [min]	Score	P	Range	Sequence	Modification
2153	1	596.7262	-248.11	2	57.3	11.8	0	134-145	K.LVSAVAIALAHK.Y	

Detailed Protein Report

Protein 50: apolipoprotein A-II preproprotein [Homo sapiens]

**Accession:**	gi|4502149	**Score:**	60.5
**Database:**	refseq_human(refseq_human_20140103.fasta)	**MW [kDa]:**	11.2
**Database Date:**	2015-11-30	**pI:**	7.1
		**Sequence Coverage [%]:**	10.0
		**No. of unique Peptides:**	1

```
        10         20         30         40         50         60         70         80
MKLLAATVLL LTICSLEGAL VRRQAKEPCV ESLVSQYFQT VTDYGKDLME KVKSPELQAE AKSYFEKSKE QLTPLIKKAG
        90        100        110
TELVNFLSYF VELGTQPATQ
```

Cmpd.	No. of Cmpds.	m/z meas.	Δ m/z [ppm]	z	Rt [min]	Score	P	Range	Sequence	Modification
457	3	578.8364	-24.29	2	35.7	60.5	1	68-77	K.SKEQLTPLIK.K	

Detailed Protein Report

Protein 51: DNA-dependent protein kinase catalytic subunit isoform 1 [Homo sapiens]

**Accession:**	gi|13654237	**Score:**	59.5
**Database:**	refseq_human(refseq_human_20140103.fasta)	**MW [kDa]:**	468.8
**Database Date:**	2015-11-30	**pI:**	6.8
**Modification(s):**	Carbamidomethyl, Oxidation	**Sequence Coverage [%]:**	1.3
		**No. of unique Peptides:**	4

Quantitation

QD:QU	**Median:** 9.31	**CV:** 0.00 %	**No. of Peptides:** 1

Detailed Protein Report

10	20	30	40	50	60	70	80
MAGSGAGVRC	SLLRLQETLS	AADRCGAALA	GHQLIRGLGQ	ECVLSSSPAV	LALQTSLVFS	RDFGLLVFVR	KSLNSIEFRE
90	100	110	120	130	140	150	160
CREEILKFLC	IFLEKMGQKI	APYSVEIKNT	CTSVYTKDRA	AKCKIPALDL	LIKLLQTFRS	SRLMDEFKIG	ELFSKFYGEL
170	180	190	200	210	220	230	240
ALKKKIPDTV	LEKVYELLGL	LGEVHPSEMI	NNAENLFRAF	LGELKTQMTS	AVREPKLPVL	AGCLKGLSSL	LCNFTKSMEE
250	260	270	280	290	300	310	320
DPQTSREIFN	FVLKAIRPQI	DLKRYAVPSA	GLRLFALHAS	QFSTCLLDNY	VSLFEVLLKW	CAHTNVELKK	AALSALESFL
330	340	350	360	370	380	390	400
KQVSNMVAKN	AEMHKNKLQY	FMEQFYGIIR	NVDSNNKELS	IAIRGYGLFA	GPCKVINAKD	VDFMYVELIQ	RCKQMFLTQT
410	420	430	440	450	460	470	480
DTGDDRVYQM	PSFLQSVASV	LLYLDTVPEV	YTPVLEHLVV	MQIDSFPQYS	PKMQLVCCRA	IVKVFLALAA	KGPVLRNCIS
490	500	510	520	530	540	550	560
TVVHQGLIRI	CSKPVVLPKG	PESESEDHRA	SGEVRTGKWK	VPTYKDYVDL	FRHLLSSDQM	MDSILADEAF	FSVNSSSESL
570	580	590	600	610	620	630	640
NHLLYDEFVK	SVLKIVEKLD	LTLEIQTVGE	QENGDEAPGV	WMIPTSDPAA	NLHPAKPKDF	SAFINLVEFC	REILPEKQAE
650	660	670	680	690	700	710	720
FFEPWVYSFS	YELILQSTRL	PLISGFYKLL	SITVRNAKKI	KYFEGVSPKS	LKHSPEDPEK	YSCFALFVKF	GKEVAVKMKQ
730	740	750	760	770	780	790	800
YKDELLASCL	TFLLSLPHNI	IELDVRAYVP	ALQMAFKLGL	SYTPLAEVGL	NALEEWSIYI	DRHVMQPYYK	DILPCLDGYL
810	820	830	840	850	860	870	880
KTSALSDETK	NNWEVSALSR	AAQKGFNKVV	LKHLKKTKNL	SSNEAISLEE	IRIRVVQMLG	SLGGQINKNL	LTVTSSDEMM
890	900	910	920	930	940	950	960
KSYVAWDREK	RLSFAVPFRE	MKPVIFLDVF	LPRVTELALT	ASDRQTKVAA	CELLHSMVMF	MLGKATQMPE	GGQGAPPMYQ
970	980	990	1000	1010	1020	1030	1040
LYKRTFPVLL	RLACDVDQVT	RQLYEPLVMQ	LIHWFTNNKK	FESQDTVALL	EAILDGIVDP	VDSTLRDFCG	RCIREFLKWS
1050	1060	1070	1080	1090	1100	1110	1120
IKQITPQQQE	KSPVNTKSLF	KRLYSLALHP	NAFKRLGASL	AFNNIYREFR	EEESLVEQFV	FEALVIYMES	LALAHADEKS
1130	1140	1150	1160	1170	1180	1190	1200
LGTIQQCCDA	IDHLCRIIEK	KHVSLNKAKK	RRLPRGFPPS	ASLCLLDLVK	WLLAHCGRPQ	TECRHKSIEL	FYKFVPLLPG
1210	1220	1230	1240	1250	1260	1270	1280
NRSPNLWLKD	VLKEEGVSFL	INTFEGGGCG	QPSGILAQPT	LLYLRGPFSL	QATLCWLDLL	LAALECYNTF	IGERTVGALQ
1290	1300	1310	1320	1330	1340	1350	1360
VLGTEAQSSL	LKAVAFFLES	IAMHDIIAAE	KCFGTGAAGN	RTSPQEGERY	NYSKCTVVVR	IMEFTTTLLN	TSPEGWKLLK
1370	1380	1390	1400	1410	1420	1430	1440
KDLCNTHLMR	VLVQTLCEPA	SIGFNIGDVQ	VMAHLPDVCV	NLMKALKMSP	YKDILETHLR	EKITAQSIEE	LCAVNLYGPD
1450	1460	1470	1480	1490	1500	1510	1520
AQVDRSRLAA	VVSACKQLHR	AGLLHNILPS	QSTDLHHSVG	TELLSLVYKG	IAPGDERQCL	PSLDLSCKQL	ASGLLELAFA
1530	1540	1550	1560	1570	1580	1590	1600
FGGLCERLVS	LLLNPAVLST	ASLGSSQGSV	IHFSHGEYFY	SLFSETINTE	LLKNLDLAVL	ELMQSSVDNT	KMVSAVLNGM
1610	1620	1630	1640	1650	1660	1670	1680
LDQSFRERAN	QKHQGLKLAT	TILQHWKKCD	SWWAKDSPLE	TKMAVLALLA	KILQIDSSVS	FNTSHGSFPE	VFTTYISLLA
1690	1700	1710	1720	1730	1740	1750	1760
DTKLDLHLKG	QAVTLLPFFT	SLTGGSLEEL	RRVLEQLIVA	HFPMQSREFP	PGTPRFNNYV	DCMKKFLDAL	ELSQSPMLLE
1770	1780	1790	1800	1810	1820	1830	1840
LMTEVLCREQ	QHVMEELFQS	SFRRIARRGS	CVTQVGLLES	VYEMFRKDDP	RLSFTRQSFV	DRSLLTLLWH	CSLDALREFF
1850	1860	1870	1880	1890	1900	1910	1920
STIVVDAIDV	LKSRFTKLNE	STFDTQITKK	MGYYKILDVM	YSRLPKDDVH	AKESKINQVF	HGSCITEGNE	LTKTLIKLCY
1930	1940	1950	1960	1970	1980	1990	2000
DAFTENMAGE	NQLLERRRLY	HCAAYNCAIS	VICCVFNELK	FYQGFLFSEK	PEKNLLIFEN	LIDLKRRYNF	PVEVEVPMER
2010	2020	2030	2040	2050	2060	2070	2080
KKKYIEIRKE	AREAANGDSD	GPSYMSSLSY	LADSTLSEEM	SQFDFSTGVQ	SYSYSSQDPR	PATGRFRRRE	QRDPTVHDDV
2090	2100	2110	2120	2130	2140	2150	2160
LELEMDELNR	HECMAPLTAL	VKHMHRSLGP	PQGEEDSVPR	DLPSWMKFLH	GKLGNPIVPL	NIRLFLAKLV	INTEEVFRPY
2170	2180	2190	2200	2210	2220	2230	2240

Detailed Protein Report

Cmpd.	No. of Cmpds.	m/z meas.	Δ m/z [ppm]	z	Rt [min]	Score	P	Range	Sequence	Modification	Ratios
1135	2	473.1745	-251.80	2	44.3	15.1	0	1643-1651	K.MAVLALLAK.I	Oxidation: 1	
935	1	512.1405	-273.82	2	41.8	10.3	0	2434-2441	K.VCLDIIYK.M	Carbamidomethyl: 2	QD:QU 9.31
272	1	898.1750	46.75	3	33.4	12.1	2	3030-3050	K.IWSEPFYQETYLPYMIRSKLK.L		
2232	1	937.4206	-14.54	2	56.5	22.1	1	3682-3696	K.ECSPWMSDFKVEFLR.N		

Detailed Protein Report

Protein 52: myosin-9 [Homo sapiens]

**Accession:**	gi|12667788	**Score:**	59.2
**Database:**	refseq_human(refseq_human_20140103.fasta)	**MW [kDa]:**	226.4
**Database Date:**	2015-11-30	**pI:**	5.4
**Modification(s):**	Oxidation	**Sequence Coverage [%]:**	2.6
		**No. of unique Peptides:**	3

Detailed Protein Report

10	20	30	40	50	60	70	80
MAQQAADKYL	YVDKNFINNP	LAQADWAAKK	LVWVPSDKSG	FEPASLKEEV	GEEAIVELVE	NGKKVKVNKD	DIQKMNPPKF
90	100	110	120	130	140	150	160
SKVEDMAELT	CLNEASVLHN	LKERYYSGLI	YTYSGLFCVV	INPYKNLPIY	SEEIVEMYKG	KKRHEMPPHI	YAITDTAYRS
170	180	190	200	210	220	230	240
MMQDREDQSI	LCTGESGAGK	TENTKKVIQY	LAYVASSHKS	KKDQGELERQ	LLQANPILEA	FGNAKTVKND	NSSRFGKFIR
250	260	270	280	290	300	310	320
INFDVNGYIV	GANIETYLLE	KSRAIRQAKE	ERTFHIFYYL	LSGAGEHLKT	DLLLEPYNKY	RFLSNGHVTI	PGQQDKDMFQ
330	340	350	360	370	380	390	400
ETMEAMRIMG	IPEEEQMGLL	RVISGVLQLG	NIVFKKERNT	DQASMPDNTA	AQKVSHLLGI	NVTDFTRGIL	TPRIKVGRDY
410	420	430	440	450	460	470	480
VQKAQTKEQA	DFAIEALAKA	TYERMFRWLV	LRINKALDKT	KRQGASFIGI	LDIAGFEIFD	LNSFEQLCIN	YTNEKLQQLF
490	500	510	520	530	540	550	560
NHTMFILEQE	EYQREGIEWN	FIDFGLDLQP	CIDLIEKPAG	PPGILALLDE	ECWFPKATDK	SFVEKVMQEQ	GTHPKFQKPK
570	580	590	600	610	620	630	640
QLKDKADFCI	IHYAGKVDYK	ADEWLMKNMD	PLNDNIATLL	HQSSDKFVSE	LWKDVDRIIG	LDQVAGMSET	ALPGAFKTRK
650	660	670	680	690	700	710	720
GMFRTVGQLY	KEQLAKLMAT	LRNTNPNFVR	CIIPNHEKKA	GKLDPHLVLD	QLRCNGVLEG	IRICRQGFPN	RVVFQEFRQR
730	740	750	760	770	780	790	800
YEILTPNSIP	KGFMDGKQAC	VLMIKALELD	SNLYRIGQSK	VFFRAGVLAH	LEEERDLKIT	DVIIGFQACC	RGYLARKAFA
810	820	830	840	850	860	870	880
KRQQQLTAMK	VLQRNCAAYL	KLRNWQWWRL	FTKVKPLLQV	SRQEEEMMAK	EEELVKVREK	QLAAENRLTE	METLQSQLMA
890	900	910	920	930	940	950	960
EKLQLQEQLQ	AETELCAEAE	ELRARLTAKK	QELEEICHDL	EARVEEEEER	CQHLQAEKKK	MQQNIQELEE	QLEEEESARQ
970	980	990	1000	1010	1020	1030	1040
KLQLEKVTTE	AKLKKLEEEQ	IILEDQNCKL	AKEKKLLEDR	IAEFTTNLTE	EEEKSKSLAK	LKNKHEAMIT	DLEERLRREE
1050	1060	1070	1080	1090	1100	1110	1120
KQRQELEKTR	RKLEGDSTDL	SDQIAELQAQ	IAELKMQLAK	KEEELQAALA	RVEEEAAQKN	MALKKIRELE	SQISELQEDL
1130	1140	1150	1160	1170	1180	1190	1200
ESERASRNKA	EKQKRDLGEE	LEALKTELED	TLDSTAAQQE	LRSKREQEVN	ILKKTLEEEA	KTHEAQIQEM	RQKHSQAVEE
1210	1220	1230	1240	1250	1260	1270	1280
LAEQLEQTKR	VKANLEKAKQ	TLENERGELA	NEVKVLLQGK	GDSEHKRKKV	EAQLQELQVK	FNEGERVRTE	LADKVTKLQV
1290	1300	1310	1320	1330	1340	1350	1360
ELDNVTGLLS	QSDSKSSKLT	KDFSALESQL	QDTQELLQEE	NRQKLSLSTK	LKQVEDEKNS	FREQLEEEEE	AKHNLEKQIA
1370	1380	1390	1400	1410	1420	1430	1440
TLHAQVADMK	KKMEDSVGCL	ETAEEVKRKL	QKDLEGLSQR	HEEKVAAYDK	LEKTKTRLQQ	ELDDLLVDLD	HQRQSACNLE
1450	1460	1470	1480	1490	1500	1510	1520
KKQKKFDQLL	AEEKTISAKY	AEERDRAEAE	AREKETKALS	LARALEEAME	QKAELERLNK	QFRTEMEDLM	SSKDDVGKSV
1530	1540	1550	1560	1570	1580	1590	1600
HELEKSKRAL	EQQVEEMKTQ	LEELEDELQA	TEDAKLRLEV	NLQAMKAQFE	RDLQGRDEQS	EEKKKQLVRQ	VREMEAELED
1610	1620	1630	1640	1650	1660	1670	1680
ERKQRSMAVA	ARKKLEMDLK	DLEAHIDSAN	KNRDEAIKQL	RKLQAQMKDC	MRELDDTRAS	REEILAQAKE	NEKKLKSMEA
1690	1700	1710	1720	1730	1740	1750	1760
EMIQLQEELA	AAERAKRQAQ	QERDELADEI	ANSSGKGALA	LEEKRRLEAR	IAQLEEELEE	EQGNTELIND	RLKKANLQID
1770	1780	1790	1800	1810	1820	1830	1840
QINTDLNLER	SHAQKNENAR	QQLERQNKEL	KVKLQEMEGT	VKSKYKASIT	ALEAKIAQLE	EQLDNETKER	QAACKQVRRT
1850	1860	1870	1880	1890	1900	1910	1920
EKKLKDVLLQ	VDDERRNAEQ	YKDQADKAST	RLKQLKRQLE	EAEEEAQRAN	ASRRKLQREL	EDATETADAM	NREVSSLKNK
1930	1940	1950	1960	1970			
LRRGDLPFVV	PRRMARKGAG	DGSDEEVDGK	ADGAEAKPAE				

Cmpd.	No. of Cmpds.	m/z meas.	Δ m/z [ppm]	z	Rt [min]	Score	P	Range	Sequence	Modification
2655	1	822.4643	30.49	2	64.2	12.9	2	1213-1226	K.ANLEKAKQTLENER.G	
187	1	849.4195	-61.73	2	32.3	21.9	2	1358-	K.QIATLHAQVADMKKK.M	Oxidation: 12

Detailed Protein Report

Cmpd.	No. of Cmpds.	m/z meas.	Δ m/z [ppm]	z	Rt [min]	Score	P	Range	Sequence	Modification
								1372		
2426	1	625.6164	-235.71	2	60.8	13.5	0	1593-1602	R.EMEAELEDER.K	

Detailed Protein Report

Protein 53: 14-3-3 protein eta [Homo sapiens]

Accession: gi|4507951
Database: refseq_human(refseq_human_20140103.fasta)
Database Date: 2015-11-30
Modification(s): Oxidation

Score: 59.1
MW [kDa]: 28.2
pI: 4.6
Sequence Coverage [%]: 11.4
No. of unique Peptides: 1

10	20	30	40	50	60	70	80
MGDREQLLQR	ARLAEQAERY	DDMASAMKAV	TELNEPLSNE	DRNLLSVAYK	NVVGARRSSW	RVISSIEQKT	MADGNEKKLE
**90**	**100**	**110**	**120**	**130**	**140**	**150**	**160**
KVKAYREKIE	KELETVCNDV	LSLLDKFLIK	NCNDFQYESK	VFYLKMKGDY	YRYLAEVASG	EKKNSVVEAS	EAAYKEAFEI
**170**	**180**	**190**	**200**	**210**	**220**	**230**	**240**
SKEQMQPTHP	IRLGLALNFS	VFYYEIQNAP	EQACLLAKQA	FDDAIAELDT	LNEDSYKDST	LIMQLLRDNL	TLWTSDQQDE
**250**							
EAGEGN							

Cmpd.	No. of Cmpds.	m/z meas.	Δ m/z [ppm]	z	Rt [min]	Score	P	Range	Sequence	Modification
2722	1	1044.5326	52.39	2	65.2	11.7	2	11-28	R.ARLAEQAERYDDMASAMK.A	Oxidation: 13, 17

Detailed Protein Report

Protein 54: dynein heavy chain 1, axonemal [Homo sapiens]

**Accession:**	gi|197927452	**Score:**	58.6
**Database:**	refseq_human(refseq_human_20140103.fasta)	**MW [kDa]:**	487.2
**Database Date:**	2015-11-30	**pI:**	5.5
**Modification(s):**	Carbamidomethyl, Oxidation	**Sequence Coverage [%]:**	1.5
		**No. of unique Peptides:**	4

Alias proteins:

Accession	Name	Description
gi|578805960	refseq_human (refseq_human_20140103.fasta)	PREDICTED: dynein heavy chain 1, axonemal isoform X1 [Homo sapiens]

Detailed Protein Report

10	20	30	40	50	60	70	80
MEQPNSKGYS	LGRTPQGPEC	SSAPAVQVGT	HRGLEYNPGK	ILPGSDYGLG	NPPALDPKLP	HLPLPPAPPT	LSDLGQPRKS
90	100	110	120	130	140	150	160
PLTGTDKKYP	LMKQRGFYSD	ILSPGTLDQL	GEVCRGPRMS	QNLLRQADLD	KFTPRVGSFE	VPEDFQERME	QQCIGSTTRL
170	180	190	200	210	220	230	240
LAQTDFPLQA	YEPKMQVPFQ	VLPGQHPRKI	EIERRKQQYL	SLDIEQLLFS	QGIDSNKLMP	RHLDHQHPQT	IEQGHDPIFP
250	260	270	280	290	300	310	320
IYLPLKVFDN	EDFDCRTPRE	WINMGLEPGS	LDRKPVPGKA	LLPTDDFLGH	EDPKSQKLKY	KWCEVGVLDY	DEEKKLYLVH
330	340	350	360	370	380	390	400
KTDEKGLVRD	EMGRPILNAG	VTTEGRPPLQ	VCQYWVPRIQ	LLFCAEDPCM	FAQRVVQANA	LRKNTEALLL	YNLYVDCMPS
410	420	430	440	450	460	470	480
DGQHVISEQS	LSKIKQWALS	TPRMRKGPSV	LEHLSSLARE	VSLDYERSMN	KINFDHVVSS	KPETFSYVTL	PKKEEEQVPE
490	500	510	520	530	540	550	560
RGLVSVPKYH	FWEQKEDFTF	VSLLTRPEVI	TALSKVRAEC	NKVTAMSLFH	SSLSKYSHLE	EFEQIQSQTF	SQVQMFLKDS
570	580	590	600	610	620	630	640
WISSLKVAMR	SSLRDMSKGW	YNLYETNWEV	YLMSKLRKLM	ELVKYMLQDT	LRFLVQDSLA	SFSQFISDTC	CSVLNCTDDM
650	660	670	680	690	700	710	720
VWGDDLINSP	YRPRKNPLFI	MDLVLDSSGV	HYSTPLEQFE	ASLLNLFDKG	ILATHAVPQL	EKLVMEDIFI	SGDPLLESVG
730	740	750	760	770	780	790	800
LHEPLVEELR	ATIASAVSKA	MIPLQAYAKE	YRKYLELNNN	DIASFLKTYQ	TQGLLAQEVR	EVVLTHLREK	EILDSSLPSS
810	820	830	840	850	860	870	880
IIIGPFYINT	DNVKQSLSKK	RKALATSVLD	ILAKNLHKEV	DSICEEFRSI	SRKIYEKPNS	IEELAELREW	MKGIPERLVG
890	900	910	920	930	940	950	960
LEERIVKVMD	DYQVMDEFLY	NLSSDDFNDK	WIASNWPSKI	LGQIELVQQQ	HVEDEEKFRK	IQIMDQNNFQ	EKLEGLQLVV
970	980	990	1000	1010	1020	1030	1040
AGFSIHVEIS	RAHEIANEVR	RVKKQLKDCQ	QLAMLYNNRE	RIFSLPITNY	DKLSRMVKEF	QPYLDLWTTA	SDWLRWSESW
1050	1060	1070	1080	1090	1100	1110	1120
MNDPLSAIDA	EQLEKNVVEA	FKTMHKCVKQ	FKDMPACQEV	ALDIRARIEE	FKPYIPLIQG	LRNPGMRIRH	WETLSNQINI
1130	1140	1150	1160	1170	1180	1190	1200
NVRPKANLTF	ARCLEMNLQD	HIESISKVAE	VAGKEYAIEQ	ALDKMEKEWS	TILFNVLPYK	ATDTYILKSP	DEASQLLDDH
1210	1220	1230	1240	1250	1260	1270	1280
IVMTQNMSFS	PYKKPFEQRI	NSWENKLKLT	QEVLEEWLNC	QRSWLYLEPI	FSSEDINQQL	PVESKRYQTM	ERIWKKIMKN
1290	1300	1310	1320	1330	1340	1350	1360
AYENREVINV	CSDLRMLDSL	RDCNKILDLV	QKGLSEYLET	KRSAFPRFYF	LSDDELLEIL	SQTKDPTAVQ	PHLRKCFENI
1370	1380	1390	1400	1410	1420	1430	1440
ARLLFQEDLE	ITHMYSAEGE	EVQLCFSIYP	SSNVEDWLRE	VERSMKASVH	DIIEKAIRAY	PTMPRTQWVL	NWPGQVTIAG
1450	1460	1470	1480	1490	1500	1510	1520
CQTYWTMEVA	EALEAGNLRS	QLFPQLCQQL	SDLVALVRGK	LSRMQRAVLS	ALIVIEVHAK	DVVSKLIQEN	VVSVNDFQWI
1530	1540	1550	1560	1570	1580	1590	1600
SQLRYYWTNN	DLYIRAVNAE	FIYGYEYLGN	SGRLVITPLT	DRCYLTLTGA	LHLKFGGAPA	GPAGTGKTET	TKDLGKALAI
1610	1620	1630	1640	1650	1660	1670	1680
QTVVFNCSDQ	LDFMAMGKFF	KGLASAGAWA	CFDEFNRIDI	EVLSVVAQQI	TTIQKAQQQR	VERFMFEGVE	IPLVPSCAVF
1690	1700	1710	1720	1730	1740	1750	1760
ITMNPGYAGR	TELPDNLKAL	FRPVAMMVPD	YAMITEISLY	SFGFNEASVL	AKKITTTFKL	SSEQLSSQDH	YDFGMRAVKT
1770	1780	1790	1800	1810	1820	1830	1840
VISAAGNLKR	ENPSMNEELI	CLRAIRDVNV	PKFLQEDLKL	FSGIVSDLFP	TIKEEDTDYG	ILDEAIREAC	RNSNLKDVEG
1850	1860	1870	1880	1890	1900	1910	1920
FLTKCIQLYE	TTVVRHGLML	VGPTGSGKST	CYRVLAAAMT	SLKGQPSISG	GMYEAVNYYV	LNPKSITMGQ	LYGEFDLLTH
1930	1940	1950	1960	1970	1980	1990	2000
EWTDGIFSSF	IRAGAITSDT	NKKWYMFDGP	VDAIWIENMN	TVLDDNKKLC	LSSGEIIKLT	EAMTMMFEVQ	DLAVASPATV
2010	2020	2030	2040	2050	2060	2070	2080
SRCGMVYLEP	SILGLMPFIE	CWLRKLPPLL	KPYEEHFKAL	FVSFLEESIS	FVRSSVKEVI	ASTNCNLTMS	LLKLLDCFFK
2090	2100	2110	2120	2130	2140	2150	2160
PFLPREGLKK	IPSEKLSRIV	ELIEPWFIFS	LIWSVGATGD	SSGRTSFSHW	LRLKMENEQL	TLLFPEEGLV	FDYRLEDAGI
2170	2180	2190	2200	2210	2220	2230	2240

Detailed Protein Report

Cmpd.	No. of Cmpds.	m/z meas.	Δ m/z [ppm]	z	Rt [min]	Score	P	Range	Sequence	Modification
1951	1	918.3840	-119.21	2	54.8	12.2	2	559-574	K.DSWISSLKVAMRSSLR.D	
2181	1	990.9291	-65.49	2	55.9	12.3	2	1828-1844	R.EACRNSNLKDVEGFLTK.C	Carbamidomethyl: 3
318	1	715.6717	-189.89	2	32.7	11.8	0	2496-2506	R.CMEQWEVTFNK.V	Oxidation: 2
972	1	730.6935	389.04	1	40.6	12.0	0	3422-3428	K.AAEIQAK.V	

Detailed Protein Report

Protein 55: nesprin-2 isoform 1 [Homo sapiens]

**Accession:**	gi|118918403	**Score:**	58.1
**Database:**	refseq_human(refseq_human_20140103.fasta)	**MW [kDa]:**	795.9
**Database Date:**	2015-11-30	**pI:**	5.1
		**Sequence Coverage [%]:**	1.1
		**No. of unique Peptides:**	2

Detailed Protein Report

10	20	30	40	50	60	70	80
MASSPELPTE	DEQGSWGIDD	LHISLQAEQE	DTQKKAFTCW	INSQLARHTS	PSVISDLFTD	IKKGHVLLDL	LEVLSGQQLP
90	100	110	120	130	140	150	160
RDKGSNTFQC	RINIEHALTF	LRNRSIKLIN	IHVTDIIDGN	PSIILGLIWT	IILHFHIEKL	AQTLSCNYNQ	PSLDDVSVVD
170	180	190	200	210	220	230	240
SSPASSPPAK	KCSKVQARWQ	MSARKALLLW	AQEQCATYES	VNVTDFKSSW	RNGMAFLAII	HALRPDLIDM	KSVKHRSNKD
250	260	270	280	290	300	310	320
NLREAFRIAE	QELKIPRLLE	PEDVDVVDPD	EKSIMTYVAQ	FLQYSKDAPG	TGEEAQGKVK	DAMGWLTLQK	EKLQKLLKDS
330	340	350	360	370	380	390	400
ENDTYFKKYN	SLLSFMESFN	EEKKSFLDVL	SIKRDLDELD	KDHLQLREAW	DGLDHQINAW	KIKLNYALPP	PLHQTEAWLQ
410	420	430	440	450	460	470	480
EVEELMDEDL	SASQDHSQAV	TLIQEKMTLF	KSLMDRFEHH	SNILLTFENK	DENHLPLVPP	NKLEEMKRRI	NNILEKKFIL
490	500	510	520	530	540	550	560
LLEFHYYKCL	VLGLVDEVKS	KLDIWNIKYG	SRESVELLLE	DWHKFIEEKE	FLARLDTSFQ	KCGEIYKNLA	GECQNINKQY
570	580	590	600	610	620	630	640
MMVKSDVCMY	RKNIYNVKST	LQKVLACWAT	YVENLRLLRA	CFEETKKEEI	KEVPFETLAQ	WNLEHATLNE	AGNFLVEVSN
650	660	670	680	690	700	710	720
DVVGSSISKE	LRRLNKRWRK	LVSKTQLEMN	LPLMIKKQDQ	PTFDNSGNIL	SKEEKATVEF	STDMSVELPE	NYNQNIKAGE
730	740	750	760	770	780	790	800
KHEKENEEFT	GQLKVAKDVE	KLIGQVEIWE	AEAKSVLDQD	DVDTSMEESL	KHLIAKGSMF	DELMARSEDM	LQMDIQNISS
810	820	830	840	850	860	870	880
QESFQHVLTT	GLQAKIQEAK	EKVQINVVKL	IAALKNLTDV	SPDLDIRLKM	EESQKELESY	MMRAQQLLGQ	RESPGELISK
890	900	910	920	930	940	950	960
HKEALIISNT	KSLAKYLKAV	EELKNNVTED	IKMSLEEKSR	DVCAKWESLH	HELSLYVQQL	KIDIEKGKLS	DNILKLEKQI
970	980	990	1000	1010	1020	1030	1040
NKEKKLIRRG	RTKGLIKEHE	ACFSEEGCLY	QLNHHMEVLR	ELCEELPSQK	SQQEVKRLLK	DYEQKIERLL	KCASEIHMTL
1050	1060	1070	1080	1090	1100	1110	1120
QPTAGGTSKN	EGTITTSENR	GGDPHSEAPF	AKSDNQPSTE	KAMEPTMKFS	LASVLRPLQE	ESIMEKDYSA	SINSLLERYD
1130	1140	1150	1160	1170	1180	1190	1200
TYRDILEHHL	QNNKFRITSD	FSSEEDRSSS	CLQAKLTDLQ	VIKNETDARW	KEFEIISLKL	ENHVNDIKKP	FVIKERDTLK
1210	1220	1230	1240	1250	1260	1270	1280
ERERELQMTL	NTRMESLETA	LRLVLPVEKA	SLLLCGSDLP	LHKMAIQGFH	LIDADRIYQH	LRNIQDSIAK	QIEICNRLEE
1290	1300	1310	1320	1330	1340	1350	1360
PGNFVLKELH	PFDLHAMQNI	ILKYKTQFEG	MNHRVQRSED	TLKALEDFLA	SLRTAKLSAE	PVTDLSASDT	QVAQENTLTV
1370	1380	1390	1400	1410	1420	1430	1440
KNKEGEIHLM	KDKAKHLDKC	LKMLDMSFKD	AERGDDTSCE	NLLDAFSIKL	SETHGYGVQE	EFTEENKLLE	ACIFKNNELL
1450	1460	1470	1480	1490	1500	1510	1520
KNIQDVQSQI	SKIGLKDPTV	PAVKHRKKSL	IRLDKVLDEY	EEEKRHLQEM	ANSLPHFKDG	REKTVNQQCQ	NTVVLWENTK
1530	1540	1550	1560	1570	1580	1590	1600
ALVTECLEQC	GRVLELLKQY	QNFKSILTTL	IQKEESVISL	QASYMGKENL	KKRIAEIEIV	KEEFNEHLEV	VDKINQVCKN
1610	1620	1630	1640	1650	1660	1670	1680
LQFYLNKMKT	FEEPPFEKEA	NIIVDRWLDI	NEKTEDYYEN	LGRALALWDK	LFNLKNVIDE	WTEKALQKME	LHQLTEEDRE
1690	1700	1710	1720	1730	1740	1750	1760
RLKEELQVHE	QKTSEFSRRV	AEIQFLLQSS	EIPLELQVME	SSILNKMEHV	QKCLTGESNC	HALSGSTAEL	REDLDQAKTQ
1770	1780	1790	1800	1810	1820	1830	1840
IGMTESLLKA	LSPSDSLEIF	TKLEEIQQQI	LQQKHSMILL	ENQIGCLTPE	LSELKKQYES	VSDLFNTKKS	VLQDHFSKLL
1850	1860	1870	1880	1890	1900	1910	1920
NDQCKNFNDW	FSNIKVNLKE	CFESSETKKS	VEQKLQKLSD	FLTLEGRNSK	IKQVDSVLKH	VKKHLPKAHV	KELISWLVGQ
1930	1940	1950	1960	1970	1980	1990	2000
EFELEKMESI	CQARAKELED	SLQQLLRLQD	DHRNLRKWLT	NQEEKWKGME	EPGEKTELFC	QALARKREQF	ESVAQLNNSL
2010	2020	2030	2040	2050	2060	2070	2080
KEYGFTEEEE	IIMEATCLMD	RYQTLLRQLS	EIEEEDKLLP	TEDQSFNDLA	HDVIHWIKEI	KESLMVLNSS	EGKMPLEERI
2090	2100	2110	2120	2130	2140	2150	2160
QKIKEIILLK	PEGDARIETI	MKQAESSEAP	LVQKTLTDIS	NQWDNTLHLA	STYLSHQEKL	LLEGEKYLQS	KEDLRLMLIE
2170	2180	2190	2200	2210	2220	2230	2240

Detailed Protein Report

Cmpd.	No. of Cmpds.	m/z meas.	Δ m/z [ppm]	z	Rt [min]	Score	P	Range	Sequence	Modification
289	1	715.8764	45.86	2	32.3	10.0	0	1817-1828	K.QYESVSDLFNTK.K	
2908	1	922.9773	-1.53	2	65.5	11.3	1	3576-3590	K.ELVQTEIQERHSFTK.E	

Detailed Protein Report

Protein 56: PREDICTED: nesprin-1 isoform X19 [Homo sapiens]

**Accession:**	gi|578812604	**Score:**	57.2
**Database:**	refseq_human(refseq_human_20140103.fasta)	**MW [kDa]:**	1002.8
**Database Date:**	2015-11-30	**pI:**	5.3
		**Sequence Coverage [%]:**	0.8
		**No. of unique Peptides:**	4

Detailed Protein Report

10	20	30	40	50	60	70	80
MATSRGASRC	PRDIANVMQR	LQDEQEIVQK	RTFTKWINSH	LAKRKPPMVV	DDLFEDMKDG	VKLLALLEVL	SGQKLPCEQG
90	100	110	120	130	140	150	160
RRMKRIHAVA	NIGTALKFLE	GRKSMHRGSP	IKLVNINSTD	IADGRPSIVL	GLMWTIILYF	QIEELTSNLP	QLQSLSSSAS
170	180	190	200	210	220	230	240
SVDSIVSSET	PSPPSKRKVT	TKIQGNAKKA	LLKWVQYTAG	KQTGIEVKDF	GKSWRSGVAF	HSVIHAIRPE	LVDLETVKGR
250	260	270	280	290	300	310	320
SNRENLEDAF	TIAETELGIP	RLLDPEDVDV	DKPDEKSIMT	YVAQFLKHYP	DIHNASTDGQ	EDDEILPGFP	SFANSVQNFK
330	340	350	360	370	380	390	400
REDRVIFKEM	KVWIEQFERD	LTRAQMVESN	LQDKYQSFKH	FRVQYEMKRK	QIEHLIQPLH	RDGKLSLDQA	LVKQSWDRVT
410	420	430	440	450	460	470	480
SRLFDWHIQL	DKSLPAPLGT	IGAWLYRAEV	ALREEITVQQ	VHEETANTIQ	RKLEQHKDLL	QNTDAHKRAF	HEIYRTRSVN
490	500	510	520	530	540	550	560
GIPVPPDQLE	DMAERFHFVS	STSELHLMKM	EFLELKYRLL	SLLVLAESKL	KSWIIKYGRR	ESVEQLLQNY	VSFIENSKFF
570	580	590	600	610	620	630	640
EQYEVTYQIL	KQTAEMYVKA	DGSVEEAENV	MKFMNETTAQ	WRNLSVEVRS	VRSMLEEVIS	NWDRYGNTVA	SLQAWLEDAE
650	660	670	680	690	700	710	720
KMLNQSENAK	KDFFRNLPHW	IQQHTAMNDA	GNFLIETCDE	MVSRDLKQQL	LLLNGRWREL	FMEVKQYAQA	DEMDRMKKEY
730	740	750	760	770	780	790	800
TDCVVTLSAF	ATEAHKKLSE	PLEVSFMNVK	LLIQDLEDIE	QRVPVMDAQY	KIITKTAHLI	TKESPQEEGK	EMFATMSKLK
810	820	830	840	850	860	870	880
EQLTKVKECY	SPLLYESQQL	LIPLEELEKQ	MTSFYDSLGK	INEIITVLER	EAQSSALFKQ	KHQELLACQE	NCKKTLTLIE
890	900	910	920	930	940	950	960
KGSQSVQKFV	TLSNVLKHFD	QTRLQRQIAD	IHVAFQSMVK	KTGDWKKHVE	TNSRLMKKFE	ESRAELEKVL	RIAQEGLEEK
970	980	990	1000	1010	1020	1030	1040
GDPEELLRRH	TEFFSQLDQR	VLNAFLKACD	ELTDILPEQE	QQGLQEAVRK	LHKQWKDLQG	EAPYHLLHLK	IDVEKNRFLA
1050	1060	1070	1080	1090	1100	1110	1120
SVEECRTELD	RETKLMPQEG	SEKIIKEHRV	FFSDKGPHHL	CEKRLQLIEE	LCVKLPVRDP	VRDTPGTCHV	TLKELRAAID
1130	1140	1150	1160	1170	1180	1190	1200
STYRKLMEDP	DKWKDYTSRF	SEFSSWISTN	ETQLKGIKGE	AIDTANHGEV	KRAVEEIRNG	VTKRGETLSW	LKSRLKVLTE
1210	1220	1230	1240	1250	1260	1270	1280
VSSENEAQKQ	GDELAKLSSS	FKALVTLLSE	VEKMLSNFGD	CVQYKEIVKN	SLEELISGSK	EVQEQAEKIL	DTENLFEAQQ
1290	1300	1310	1320	1330	1340	1350	1360
LLLHHQQKTK	RISAKKRDVQ	QQIAQAQQGE	GGLPDRGHEE	LRKLESTLDG	LERSRERQER	RIQVTLRKWE	RFETNKETVV
1370	1380	1390	1400	1410	1420	1430	1440
RYLFQTGSSH	ERFLSFSSLE	SLSSELEQTK	EFSKRTESIA	VQAENLVKEA	SEIPLGPQNK	QLLQQQAKSI	KEQVKKLEDT
1450	1460	1470	1480	1490	1500	1510	1520
LEEDIKTMEM	VKTKWDHFGS	NFETLSVWIT	EKEKELNALE	TSSSAMDMQI	SQIKVTIQEI	ESKLSSIVGL	EEEAQSFAQF
1530	1540	1550	1560	1570	1580	1590	1600
VTTGESARIK	AKLTQIRRYG	EELREHAQCL	EGTILGHLSQ	QQKFEENLRK	IQQSVSEFED	KLAVPIKICS	SATETYKVLQ
1610	1620	1630	1640	1650	1660	1670	1680
EHMDLCQALE	SLSSAITAFS	ASARKVVNRD	SCVQEAAALQ	QQYEDILRRA	KERQTALENL	LAHWQRLEKE	LSSFLTWLER
1690	1700	1710	1720	1730	1740	1750	1760
GEAKASSPEM	DISADRVKVE	GELQLIQALQ	NEVVSQASFY	SKLLQLKESL	FSVASKDDVK	MMKLHLEQLD	ERWRDLPQII
1770	1780	1790	1800	1810	1820	1830	1840
NKRINFLQSV	VAEHQQFDEL	LLSFSVWIKL	FLSELQTTSE	ISIMDHQVAL	TRHKDHAAEV	ESKKGELQSL	QGHLAKLGSL
1850	1860	1870	1880	1890	1900	1910	1920
GRAEDLHLLQ	GKAEDCFQLF	EEASQVVERR	QLALSHLAEF	LQSHASLSGI	LRQLRQTVEA	TNSMNKNESD	LIEKDLNDAL
1930	1940	1950	1960	1970	1980	1990	2000
QNAKALESAA	VSLDGILSKA	QYHLKIGSSE	QRTSCRATAD	QLCGEVERIQ	NLLGTKQSEA	DALAVLKKAF	QDQKEELLKS
2010	2020	2030	2040	2050	2060	2070	2080
IEDIEERTDK	ERLKEPTRQA	LQQRLRVFNQ	LEDELNSHEH	ELCWLKDKAK	QIAQKDVAFA	PEVDREINRL	EVTWDDTKRL
2090	2100	2110	2120	2130	2140	2150	2160
IHENQGQCCG	LIDLMREYQN	LKSAVSKVLE	NASSVIVTRT	TIKDQEDLKW	AFSKHETAKN	KMNYKQKDLD	NFTSKGKHLL
2170	2180	2190	2200	2210	2220	2230	2240

Detailed Protein Report

Cmpd.	No. of Cmpds.	m/z meas.	Δ m/z [ppm]	z	Rt [min]	Score	P	Range	Sequence	Modification
1807	1	945.8341	-138.33	2	51.1	10.6	2	1681-1698	R.GEAKASSPEMDISADRVK.V	
742	1	524.3579	134.01	2	37.8	10.5	1	2443-2452	R.TGDSKVLEAK.L	
280	1	607.8593	-49.80	2	33.5	10.5	1	3327-3337	R.TLKLEALLSVK.Q	
2878	1	913.4666	-41.53	2	65.2	14.8	1	7089-7104	K.EVEKIEQNGLALIQNK.K	

Detailed Protein Report

Protein 57: fatty acid-binding protein, epidermal [Homo sapiens]

**Accession:**	gi|4557581	**Score:**	57.2
**Database:**	refseq_human(refseq_human_20140103.fasta)	**MW [kDa]:**	15.2
**Database Date:**	2015-11-30	**pI:**	7.5
**Modification(s):**	Carbamidomethyl	**Sequence Coverage [%]:**	10.4
		**No. of unique Peptides:**	1

Quantitation

QD:QU **Median:** 2.02 **CV:** 0.00 % **No. of Peptides:** 1

```
         10         20         30         40         50         60         70         80
MATVQQLEGR WRLVDSKGFD EYMKELGVGI ALRKMGAMAK PDCIITCDGK NLTIKTESTL KTTQFSCTLG EKFEETTADG
         90        100        110        120        130        140
RKTQTVCNFT DGALVQHQEW DGKESTITRK LKDGKLVVEC VMNNVTCTRI YEKVE
```

Cmpd.	No. of Cmpds.	m/z meas.	Δ m/z [ppm]	z	Rt [min]	Score	P	Range	Sequence	Modification	Ratios	
1139	2	847.8676	-43.89	2	44.4	57.2	0	116-129	K.LVVECVMNNVTCTR.I	Carbamidomethyl: 5, 12	QD:QU	2.02

Detailed Protein Report

Protein 58: PREDICTED: intersectin-1 isoform X1 [Homo sapiens]

Accession: gi|530419020
Database: refseq_human(refseq_human_20140103.fasta)
Database Date: 2015-11-30
Modification(s): Oxidation

Score: 57.2
MW [kDa]: 194.7
pI: 8.5
Sequence Coverage [%]: 3.1
No. of unique Peptides: 3

10	20	30	40	50	60	70	80
MAQFPTPFGG	SLDIWAITVE	ERAKHDQQFH	SLKPISGFIT	GDQARNFFFQ	SGLPQPVLAQ	IWALADMNND	GRMDQVEFSI
90	100	110	120	130	140	150	160
AMKLIKLKLQ	GYQLPSALPP	VMKQQPVAIS	SAPAFGMGGI	ASMPPLTAVA	PVPMGSIPVV	GMSPTLVSSV	PTAAVPPLAN
170	180	190	200	210	220	230	240
GAPPVIQPLP	AFAHPAATLP	KSSSFSRSGP	GSQLNTKLQK	AQSFDVASVP	PVAEWAVPQS	SRLKYRQLFN	SHDKTMSGHL
250	260	270	280	290	300	310	320
TGPQARTILM	QSSLPQAQLA	SIWNLSDIDQ	DGKLTAEEFI	LAMHLIDVAM	SGQPLPPVLP	PEYIPPSFRR	VRSGSGISVI
330	340	350	360	370	380	390	400
SSTSVDQRLP	EEPVLEDEQQ	QLEKKLPVTF	EDKKRENFER	GNLELEKRRQ	ALLEQQRKEQ	ERLAQLERAE	QERKERERQE
410	420	430	440	450	460	470	480
QERKRQLELE	KQLEKQRELE	RQREEERRKE	IERREAAKRE	LERQRQLEWE	RNRRQELLNQ	RNKEQEDIVV	LKAKKKTLEF
490	500	510	520	530	540	550	560
ELEALNDKKH	QLEGKLQDIR	CRLTTQRQEI	ESTNKSRELR	IAEITHLQQQ	LQESQQMLGR	LIPEKQILND	QLKQVQQNSL
570	580	590	600	610	620	630	640
HRDSLVTLKR	ALEAKELARQ	HLRDQLDEVE	KETRSKLQEI	DIFNNQLKEL	REIHNKQQLQ	KQKSMEAERL	KQKEQERKII
650	660	670	680	690	700	710	720
ELEKQKEEAQ	RRAQERDKQW	LEHVQQEDEH	QRPRKLHEEE	KLKREESVKK	KDGEEKGKQE	AQDKLGRLFH	QHQEPAKPAV
730	740	750	760	770	780	790	800
QAPWSTAEKG	PLTISAQENV	KVVYYRALYP	FESRSHDEIT	IQPGDIVMVD	ESQTGEPGWL	GGELKGKTGW	FPANYAEKIP
810	820	830	840	850	860	870	880
ENEVPAPVKP	VTDSTSAPAP	KLALRETPAP	LAVTSSEPST	TPNNWADFSS	TWPTSTNEKP	ETDNWDAWAA	QPSLTVPSAG
890	900	910	920	930	940	950	960
QLRQRSAFTP	ATATGSSPSP	VLGQGEKVEG	LQAQALYPWR	AKKDNHLNFN	KNDVITVLEQ	QDMWWFGEVQ	GQKGWFPKSY
970	980	990	1000	1010	1020	1030	1040
VKLISGPIRK	STSMDSGSSE	SPASLKRVAS	PAAKPVVSGE	EFIAMYTYES	SEQGDLTFQQ	GDVILVTKKD	GDWWTGTVGD
1050	1060	1070	1080	1090	1100	1110	1120
KAGVFPSNYV	RLKDSEGSGT	AGKTGSLGKK	PEIAQVIASY	TATGPEQLTL	APGQLILIRK	KNPGGWWEGE	LQARGKKRQI
1130	1140	1150	1160	1170	1180	1190	1200
GWFPANYVKL	LSPGTSKITP	TEPPKSTALA	AVCQVIGMYD	YTAQNDDELA	FNKGQIINVL	NKEDPDWWKG	EVNGQVGLFP
1210	1220	1230	1240	1250	1260	1270	1280
SNYVKLTTDM	DPSQQWCSDL	HLLDMLTPTE	RKRQGYIHEL	IVTEENYVND	LQLVTEIFQK	PLMESELLTE	KEVAMIFVNW
1290	1300	1310	1320	1330	1340	1350	1360
KELIMCNIKL	LKALRVRKKM	SGEKMPVKMI	GDILSAQLPH	MQPYIRFCSR	QLNGAALIQQ	KTDEAPDFKE	FVKRLAMDPR
1370	1380	1390	1400	1410	1420	1430	1440
CKGMPLSSFI	LKPMQRVTRY	PLIIKNILEN	TPENHPDHSH	LKHALEKAEE	LCSQVNEGVR	EKENSDRLEW	IQAHVQCEGL
1450	1460	1470	1480	1490	1500	1510	1520
SEQLVFNSVT	NCLGPRKFLH	SGKLYKAKSN	KELYGFLFND	FLLLTQITKP	LGSSGTDKVF	SPKSNLQYKM	YKTPIFLNEV
1530	1540	1550	1560	1570	1580	1590	1600
LVKLPTDPSG	DEPIFHISHI	DRVYTLRAES	INERTAWVQK	IKAASELYIE	TEKKKREKAY	LVRSQRATGI	GRLMVNVVEG
1610	1620	1630	1640	1650	1660	1670	1680
IELKPCRSHG	KSNPYCEVTM	GSQCHITKTI	QDTLNPKWNS	NCQFFIRDLE	QEVLCITVFE	RDQFSPDDFL	GRTEIRVADI
1690	1700	1710	1720				
KKDQGSKGPV	TKCLLLHEVP	TGEIVVRLDL	QLFDEP				

Cmpd.	No. of Cmpds.	m/z meas.	Δ m/z [ppm]	z	Rt [min]	Score	P	Range	Sequence	Modification
85	1	827.0046	76.69	2	31.0	15.0	1	73-86	R.MDQVEFSIAMKLIK.L	
2747	1	1045.5347	10.58	2	65.5	12.9	0	886-907	R.SAFTPATATGSSPSPVLGQGEK.V	

Detailed Protein Report

Cmpd.	No. of Cmpds.	m/z meas.	Δ m/z [ppm]	z	Rt [min]	Score	P	Range	Sequence	Modification
2785	1	874.5334	88.10	1	63.9	14.2	1	1354-1360	K.RLAMDPR.C	Oxidation: 4

Detailed Protein Report

Protein 59: **treslin [Homo sapiens]**

**Accession:**	gi|118421085	**Score:**	57.0
**Database:**	refseq_human(refseq_human_20140103.fasta)	**MW [kDa]:**	210.7
**Database Date:**	2015-11-30	**pI:**	9.8
		**Sequence Coverage [%]:**	2.1
		**No. of unique Peptides:**	4

10	20	30	40	50	60	70	80
MACCHKVMLL	LDTAGGAARH	SRVRRAALRL	LTYLSCRFGL	ARVHWAFKFF	DSQGARSRPS	RVSDFRELGS	RSWEDFEEEL
90	100	110	120	130	140	150	160
EARLEDRAHL	PGPAPRATHT	HGALMETLLD	YQWDRPEITS	PTKPILRSSG	RRLLDVESEA	KEAEAALGGL	VNAVFLLAPC
170	180	190	200	210	220	230	240
PHSQRELLQF	VSGCEAQAQR	LPPTPKQVME	KLLPKRVREV	MVARKITFYW	VDTTEWSKLW	ESPDHLGYWT	VCELLHHGGG
250	260	270	280	290	300	310	320
TVLPSESFSW	DFAQAGEMLL	RSGIKLSSEP	HLSPWISMLP	TDATLNRLLY	NSPEYEASFP	RMEGMLFLPV	EAGKEIQETW
330	340	350	360	370	380	390	400
TVTLEPLAMH	QRHFQKPVRI	FLKGSVAQWS	LPTSSTLGTD	SWMLGSPEES	TATQRLLFQQ	LVSRLTAEEL	HLVADVDPGE
410	420	430	440	450	460	470	480
GRPPITGVIS	PLSASAMILT	VCRTKEAEFQ	RHVLQTAVAD	SPRDTASLFS	DVVDSILNQT	HDSLADTASA	ASPVPEWAQQ
490	500	510	520	530	540	550	560
ELGHTTPWSP	AVVEKWFPFC	NISGASSDLM	ESFGLLQAAS	ANKEESSKTE	GELIHCLAEL	YQRKSREEST	IAHQEDSKKK
570	580	590	600	610	620	630	640
RGVPRTPVRQ	KMNTMCRSLK	MLNVARLNVK	AQKLHPDGSP	DVAGEKGIQK	IPSGRTVDKL	EDRGRTLRSS	KPKDFKTEEE
650	660	670	680	690	700	710	720
LLSYIRENYQ	KTVATGEIML	YACARNMIST	VKMFLKSKGT	KELEVNCLNQ	VKSSLLKTSK	SLRQNLGKKL	DKEDKVRECQ
730	740	750	760	770	780	790	800
LQVFLRLEMC	LQCPSINEST	DDMEQVVEEV	TDLLRMVCLT	EDSAYLAEFL	EEILRLYIDS	IPKTLGNLYN	SLGFVIPQKL
810	820	830	840	850	860	870	880
AGVLPTDFFS	DDSMTQENKS	PLLSVPFLSS	ARRSVSGSPE	SDELQELRTR	SAKKRRKNAL	IRHKSIAEVS	QNLRQIEIPK
890	900	910	920	930	940	950	960
VSKRATKKEN	SHPAPQQPSQ	PVKDTVQEVT	KVRRNLFNQE	LLSPSKRSLK	RGLPRSHSVS	AVDGLEDKLD	NFKKNKGYHK
970	980	990	1000	1010	1020	1030	1040
LLTKSVAETP	VHKQISKRLL	HRQIKGRSSD	PGPDIGVVEE	SPEKGDEISL	RRSPRIKQLS	FSRTHSASFY	SVSQPKSRSV
1050	1060	1070	1080	1090	1100	1110	1120
QRVHSFQQDK	SDQRENSPVQ	SIRSPKSLLF	GAMSEMISPS	EKGSARMKKR	SRNTLDSEVP	AAYQTPKKSH	QKSLSFSKTT
1130	1140	1150	1160	1170	1180	1190	1200
PRRISHTPQT	PLYTPERLQK	SPAKMTPTKQ	AAFKESLKDS	SSPGHDSPLD	SKITPQKRHT	QAGEGTSLET	KTPRTPKRQG
1210	1220	1230	1240	1250	1260	1270	1280
TQPPGFLPNC	TWPHSVNSSP	ESPSCPAPPT	SSTAQPRREC	LTPIRDPLRT	PPRAAAFMGT	PQNQTHQQPH	VLRAARAEEP
1290	1300	1310	1320	1330	1340	1350	1360
AQKLKDKAIK	TPKRPGNSTV	TSSPPVTPKK	LFTSPLCDVS	KKSPFRKSKI	ECPSPGELDQ	KEPQMSPSVA	ASLSCPVPST
1370	1380	1390	1400	1410	1420	1430	1440
PPELSQRATL	DTVPPPPPSK	VGKRCRKTSD	PRRSIVECQP	DASATPGVGT	ADSPAAPTDS	RDDQKGLSLS	PQSPPERRGY
1450	1460	1470	1480	1490	1500	1510	1520
PGPGLRSDWH	ASSPLLITSD	TEHVTLLSEA	EHHGIGDLKS	NVLSVEEGEG	LRTADAEKSS	LSHPGIPPSP	PSCGPGSPLM
1530	1540	1550	1560	1570	1580	1590	1600
PSRDVHCTTD	GRQCQASAQL	DNLPASAWHS	TDSASPQTYE	VELEMQASGL	PKLRIKKIDP	SSSLEAEPLS	KEESSLGEES
1610	1620	1630	1640	1650	1660	1670	1680
FLPALSMPRA	SRSLSKPEPT	YVSPPCPRLS	HSTPGKSRGQ	TYICQACTPT	HGPSSTPSPF	QTDGVPWTPS	PKHSGKTTPD
1690	1700	1710	1720	1730	1740	1750	1760
IIKDWPRRKR	AVGCGAGSSS	GRGEVGADLP	GSLSLLESEG	KDHGLELSIH	RTPILEDFEL	EGVCQLPDQS	PPRNSMPKAE
1770	1780	1790	1800	1810	1820	1830	1840
EASSWGQFGL	SSRKRVLLAK	EEADRGAKRI	CDLREDSEVS	KSKEGSPSWS	AWQLPSTGDE	EVFVSGSTPP	PSCAVRSCLS
1850	1860	1870	1880	1890	1900	1910	1920
ASALQALTQS	PLLFQGKTPS	SQSKDPRDED	VDVLPSTVED	SPFSRAFSRR	RPISRTYTRK	KLMGTWLEDL	

Detailed Protein Report

Cmpd.	No. of Cmpds.	m/z meas.	Δ m/z [ppm]	z	Rt [min]	Score	P	Range	Sequence	Modification
1123	3	473.1123	-310.61	2	44.2	19.5	1	1005-1012	K.GDEISLRR.S	
2455	1	669.2124	-220.96	2	61.2	12.2	1	1311-1322	K.LFTSPLCDVSKK.S	
2584	1	658.2319	-124.03	2	61.0	10.7	0	1330-1341	K.IECPSPGELDQK.E	
1623	2	516.2455	-91.18	2	50.5	14.5	2	1786-1794	R.GAKRICDLR.E	

Detailed Protein Report

Protein 60: **PREDICTED: keratin, type I cytoskeletal 24 isoform X1 [Homo sapiens]**

Accession: gi|578830527
Database: refseq_human(refseq_human_20140103.fasta)
Database Date: 2015-11-30
Modification(s): Oxidation

Score: 55.5
MW [kDa]: 46.1
pI: 4.7
Sequence Coverage [%]: 4.9
No. of unique Peptides: 1

10	20	30	40	50	60	70	80
MSCSSRASSS	RAGGSSSARV	SAGGSSFSSG	SRCGLGGSSA	QGFRGGASSC	SLSGGSSDKV	RALEEANTDL	ENKIKEWYDK
**90**	**100**	**110**	**120**	**130**	**140**	**150**	**160**
YGPGSGDGGS	GRDYSKYYSI	IEDLRNQIIA	ATVENAGIIL	HIDNARLAAD	DFRLKYENEL	CLRQSVEADI	NGLRKVLDDL
**170**	**180**	**190**	**200**	**210**	**220**	**230**	**240**
TMTRSDLEMQ	IESFTEELAY	LRKNHEEEMK	NMQGSSGGEV	TVEMNAAPGT	DLTKLLNDMR	AQYEELAEQN	RREAEERFNK
**250**	**260**	**270**	**280**	**290**	**300**	**310**	**320**
QSASLQAQIS	TDAGAATSAK	NEITELKRTL	QALEIELQSQ	LAMKSSLEGT	LADTEAGYVA	QLSEIQTQIS	ALEEEICQIW
**330**	**340**	**350**	**360**	**370**	**380**	**390**	**400**
GETKCQNAEY	KQLLDIKTRL	EVEIETYRRL	LDGEGGGSSF	AEFGGRNSGS	VNMGSRDLVS	GDSRSGSCSG	QGRDSSKTRV
**410**	**420**	**430**					
TKTIVEELVD	GKVVSSQVSS	ISEVKVK					

Cmpd.	No. of Cmpds.	m/z meas.	Δ m/z [ppm]	z	Rt [min]	Score	P	Range	Sequence	Modification
587	1	512.7149	-24.47	2	35.9	12.9	0	367-376	R.NSGSVNMGSR.D	Oxidation: 7

Detailed Protein Report

Protein 61: DNA-directed RNA polymerase I subunit RPA2 isoform 4 [Homo sapiens]

Accession: gi|544346215
Database: refseq_human(refseq_human_20140103.fasta)
Database Date: 2015-11-30
Modification(s): Oxidation

Score: 55.3
MW [kDa]: 107.8
pI: 8.3
Sequence Coverage [%]: 3.8
No. of unique Peptides: 2

10	20	30	40	50	60	70	80
MDPGSRWRNL	PSGPSLKHLT	DPSYGIPREQ	QKAALQELTR	AHVESFNYAV	HEGLGLAVQA	IPPFEFAFKD	ERISFTILDA
90	100	110	120	130	140	150	160
VISPPTVPKG	TICKEANVYP	AECRGRRSTY	RGKLTADINW	AVNGISKGII	KQFLGYVPIM	VKSKLCNLRN	LPPQALIEHH
170	180	190	200	210	220	230	240
EEAEEMGGYF	IINGIEKVIR	MLIMPRRNFP	IAMIRPKWKT	RGPGYTQYGV	SMHCVREEHS	AVNMNLHYLE	NGTVMLNFIY
250	260	270	280	290	300	310	320
RKELFFLPLG	FALKALVSFS	DYQIFQELIK	GKEDDSFLRN	SVSQMLRIVM	EEGCSTQKQV	LNYLGECFRV	KLNVPDWYPN
330	340	350	360	370	380	390	400
EQAAEFLFNQ	CICIHLKSNT	EKFYMLCLMT	RKLFALAKGE	CMEDNPDSLV	NQEVLTPGQL	FLMFLKGSLP	LMELPTDHTV
410	420	430	440	450	460	470	480
LREKRIPPWM	EVVLIPMTGK	PSLYPGLFLF	TTPCRLVRPV	QNLALGKEEL	IGTMEQIFMN	VAIFEDEVFA	GVTTHQELFP
490	500	510	520	530	540	550	560
HSLLSVIANF	IPFSDHNQSP	RNMYQCQMGK	QTMGFPLLTY	QDRSDNKLYR	LQTPQSPLVR	PSMYDYYDMD	NYPIGTNAIV
570	580	590	600	610	620	630	640
AVISYTGYDM	EDAMIVNKAS	WERGFAHGSV	YKSEFIDLSE	KIKQGDSSLV	FGIKPGDPRV	LQKLDDDGLP	FIGAKLQYGD
650	660	670	680	690	700	710	720
PYYSYLNLNT	GESFVMYYKS	KENCVVDNIK	VCSNDTGSGK	FKCVCITMRV	PRNPTIGDKF	ASRHGQKGIL	SRLWPAEDMP
730	740	750	760	770	780	790	800
FTESGMVPDI	LFNPHGFPSR	MTIGMLIESM	AGKSAALHGL	CHDATPFIFS	EENSALEYFG	EMLKAAGYNF	YGTERLYSGI
810	820	830	840	850	860	870	880
SGLELEADIF	IGVVYYQRLR	HMVSDKFQVR	TTGARDRVTN	QPIGGRNVQG	GIRFGEMERD	ALLAHGTSFL	LHDRLFNCSD
890	900	910	920	930	940	950	960
RSVAHVCVKC	GSLLSPLLEK	PPPSWSAMRN	RKYNCTLCSR	SDTIDTVSVP	YVFRYFVAEL	AAMNIKVKLD	VV

Cmpd.	No. of Cmpds.	m/z meas.	Δ m/z [ppm]	z	Rt [min]	Score	P	Range	Sequence	Modification
354	1	1026.5219	-20.89	2	34.4	35.4	1	387-404	K.GSLPLMELPTDHTVLREK.R	Oxidation: 6
2962	1	1019.9724	-91.71	2	66.2	19.9	2	935-952	R.YFVAELAAMNIKVKLDVV.-	Oxidation: 9

Detailed Protein Report

Protein 62: **PREDICTED: striated muscle preferentially expressed protein kinase isoform X14 [Homo sapiens]**

**Accession:**	gi|578803490	**Score:**	55.2
**Database:**	refseq_human(refseq_human_20140103.fasta)	**MW [kDa]:**	283.5
**Database Date:**	2015-11-30	**pI:**	9.4
		**Sequence Coverage [%]:**	1.8
		**No. of unique Peptides:**	2

Quantitation

QD:QU	**Median:** 3.82	**CV:** 0.00 %	**No. of Peptides:** 1

Detailed Protein Report

10	20	30	40	50	60	70	80
MQKARGTRGE	DAGTRAPPSP	GVPPKRAKVG	AGGGAPVAVA	GAPVFLRPLK	NAAVCAGSDV	RLRVVVSGTP	QPSLRWFRDG
90	100	110	120	130	140	150	160
QLLPAPAPEP	SCLWLRRCGA	QDAGVYSCMA	QNERGRASCE	AVLTVLEVGD	SETAEDDISD	VQGTQRLELR	DDGAFSTPTG
170	180	190	200	210	220	230	240
GSDTLVGTSL	DTPPTSVTGT	SEEQVSWWGS	GQTVLEQEAG	SGGGTRRLPG	SPRQAQATGA	GPRHLGVEPL	VRASRANLVG
250	260	270	280	290	300	310	320
ASWGSEDSLS	VASDLYGSAF	SLYRGRALSI	HVSVPQSGLR	REEPDLQPQL	ASEAPRRPAQ	PPPSKSALLP	PPSPRVGKRS
330	340	350	360	370	380	390	400
PPGPPAQPAA	TPTSPHRRTQ	EPVLPEDTTT	EEKRGKKSKS	SGPSLAGTAE	SRPQTPLSEA	SGRLSALGRS	PRLVRAGSRI
410	420	430	440	450	460	470	480
LDKLQFFEER	RRSLERSDSP	PAPLRPWVPL	RKARSLEQPK	SERGAPWGTP	GASQEELRAP	GSVAERRRLF	QQKAASLDER
490	500	510	520	530	540	550	560
TRQRSPASDL	ELRFAQELGR	IRRSTSREEL	VRSHESLRAT	LQRAPSPREP	GEPPLFSRPS	TPKTSRAVSP	AAAQPPSPSS
570	580	590	600	610	620	630	640
AEKPGDEPGR	PRSRGPAGRT	EPGEGPQQEV	RRRDQFPLTR	SRAIQECRSP	VPPPAADPPE	ARTKAPPGRK	REPPAQAVRF
650	660	670	680	690	700	710	720
LPWATPGLEG	AAVPQTLEKN	RAGPEAEKRL	RRGPEEDGPW	GPWDRRGARS	QGKGRRARPT	SPELESSDDS	YVSAGEEPLE
730	740	750	760	770	780	790	800
APVFEIPLQN	VVVAPGADVL	LKCIITANPP	PQVSWHKDGS	ALRSEGRLLL	RAEGERHTLL	LREARAADAG	SYMATATNEL
810	820	830	840	850	860	870	880
GQATCAASLT	VRPGGSTSPF	SSPITSDEEY	LSPPEEFPEP	GETWPRTPTM	KPSPSQNRRS	SDTGSKAPPT	FKVSLMDQSV
890	900	910	920	930	940	950	960
REGQDVIMSI	RVQGEPKPVV	SWLRNRQPVR	PDQRRFAEEA	EGGLCRLRIL	AAERGDAGFY	TCKAVNEYGA	RQCEARLEVR
970	980	990	1000	1010	1020	1030	1040
AHPESRSLAV	LAPLQDVDVG	AGEMALFECL	VAGPTDVEVD	WLCRGRLLQP	ALLKCKMHFD	GRKCKLLLTS	VHEDDSGVYT
1050	1060	1070	1080	1090	1100	1110	1120
CKLSTAKDEL	TCSARLTVRP	SLAPLFTRLL	EDVEVLEGRA	ARFDCKISGT	PPPVVTWTHF	GCPMEESENL	RLRQDGGLHS
1130	1140	1150	1160	1170	1180	1190	1200
LHIAHVGSED	EGLYAVSAVN	THGQAHCSAQ	LYVEEPRTAA	SGPSSKLEKM	PSIPEEPEQG	ELERLSIPDF	LRPLQDLEVG
1210	1220	1230	1240	1250	1260	1270	1280
LAKEAMLECQ	VTGLPYPTIS	WFHNGHRIQS	SDDRRMTQYR	DVHRLVFPAV	GPQHAGVYKS	VIANKLGKAA	CYAHLYVTDV
1290	1300	1310	1320	1330	1340	1350	1360
VPGPPDGAPQ	VVAVTGRMVT	LTWNPPRSLD	MAIDPDSLTY	TVQHQVLGSD	QWTALVTGLR	EPGWAATGLR	KGVQHIFRVL
1370	1380	1390	1400	1410	1420	1430	1440
STTVKSSSKP	SPPSEPVQLL	EHGPTLEEAP	AMLDKPDIVY	VVEGQPASVT	VTFNHVEAQV	VWRSCRGALL	EARAGVYELS
1450	1460	1470	1480	1490	1500	1510	1520
QPDDDQYCLR	ICRVSRRDMG	ALTCTARNRH	GTQTCSVTLE	LAEAPRFESI	MEDVEVGAGE	TARFAVVVEG	KPLPDIMWYK
1530	1540	1550	1560	1570	1580	1590	1600
DEVLLTESSH	VSFVYEENEC	SLVVLSTGAQ	DGGVYTCTAQ	NLAGEVSCKA	ELAVHSAQTA	MEVEGVGEDE	DHRGRRLSDF
1610	1620	1630	1640	1650	1660	1670	1680
YDIHQEIGRG	AFSYLRRIVE	RSSGLEFAAK	FIPSQAKPKA	SARREARLLA	RLQHDCVLYF	HEAFERRRGL	VIVTELCTEE
1690	1700	1710	1720	1730	1740	1750	1760
LLERIARKPT	VCESEIRAYM	RQVLEGIHYL	HQSHVLHLDV	KPENLLVWDG	AAGEQQVRIC	DFGNAQELTP	GEPQYCQYGT
1770	1780	1790	1800	1810	1820	1830	1840
PEFVAPEIVN	QSPVSGVTDI	WPVGVVAFLC	LTGISPFVGE	NDRTTLMNIR	NYNVAFEETT	FLSLSREARG	FLIKVLVQDR
1850	1860	1870	1880	1890	1900	1910	1920
LRPTAEETLE	HPWFKTQAKG	AEVSTDHLKL	FLSRRRWQRS	QISYKCHLVL	RPIPELLRAP	PERVWVTMPR	RPPPSGGLSS
1930	1940	1950	1960	1970	1980	1990	2000
SSDSEEEELE	ELPSVPRPLQ	PEFSGSRVSL	TDIPTEDEAL	GTPETGAATP	MDWQEQGRAP	SQDQEAPSPE	ALPSPGQEPA
2010	2020	2030	2040	2050	2060	2070	2080
AGASPRRGEL	RRGSSAESAL	PRAGPRELGR	GLHKAASVEL	PQRRSPSPGA	TRLARGGLGE	GEYAQRLQAL	RQRLLRGGPE
2090	2100	2110	2120	2130	2140	2150	2160
DGKVSGLRGP	LLESLGGRAR	DPRMARAASS	EAAPHHQPPL	ENRGLQKSSS	FSQGEAEPRG	RHRRAGAPLE	IPVARLGARR
2170	2180	2190	2200	2210	2220	2230	2240

Detailed Protein Report

Cmpd.	No. of Cmpds.	m/z meas.	Δ m/z [ppm]	z	Rt [min]	Score	P	Range	Sequence	Modification	Ratios
146	1	531.9457	-37.14	3	31.8	25.5	2	2089-2103	R.GPLLESLGGRARDPR.M		QD:QU 3.82
257	2	765.3459	-79.23	2	33.2	16.1	0	2578-2592	R.SESVPSLGLEAAIEK.L		

Detailed Protein Report

Protein 63: protein S100-A11 [Homo sapiens]

**Accession:**	gi|5032057	**Score:**	54.9
**Database:**	refseq_human(refseq_human_20140103.fasta)	**MW [kDa]:**	11.7
**Database Date:**	2015-11-30	**pI:**	7.5
**Modification(s):**	Carbamidomethyl	**Sequence Coverage [%]:**	10.5
		**No. of unique Peptides:**	1

```
        10         20         30         40         50         60         70         80
MAKISSPTET ERCIESLIAV FQKYAGKDGY NYTLSKTEFL SFMNTELAAF TKNQKDPGVL DRMMKKLDTN SDGQLDFSEF
        90        100        110
LNLIGGLAMA CHDSFLKAVP SQKRT
```

Cmpd.	No. of Cmpds.	m/z meas.	Δ m/z [ppm]	z	Rt [min]	Score	P	Range	Sequence	Modification
2752	3	654.3389	-24.71	2	65.5	54.9	0	13-23	R.CIESLIAVFQK.Y	Carbamidomethyl: 1

Detailed Protein Report

Protein 64: PREDICTED: ryanodine receptor 1 isoform X3 [Homo sapiens]

**Accession:**	gi|578834746	**Score:**	54.2
**Database:**	refseq_human(refseq_human_20140103.fasta)	**MW [kDa]:**	563.6
**Database Date:**	2015-11-30	**pI:**	5.1
**Modification(s):**	Carbamidomethyl, Oxidation	**Sequence Coverage [%]:**	1.1
		**No. of unique Peptides:**	3

Detailed Protein Report

10	20	30	40	50	60	70	80
MGDAEGEDEV	QFLRTDDEVV	LQCSATVLKE	QLKLCLAAEG	FGNRLCFLEP	TSNAQNVPPD	LAICCFVLEQ	SLSVRALQEM
90	100	110	120	130	140	150	160
LANTVEAGVE	SSQGGGHRTL	LYGHAILLRH	AHSRMYLSCL	TTSRSMTDKL	AFDVGLQEDA	TGEACWWTMH	PASKQRSEGE
170	180	190	200	210	220	230	240
KVRVGDDIIL	VSVSSERYLH	LSTASGELQV	DASFMQTLWN	MNPICSRCEE	GFVTGGHVLR	LFHGHMDECL	TISPADSDDQ
250	260	270	280	290	300	310	320
RRLVYYEGGA	VCTHARSLWR	LEPLRISWSG	SHLRWGQPLR	VRHVTTGQYL	ALTEDQGLVV	VDASKAHTKA	TSFCFRISKE
330	340	350	360	370	380	390	400
KLDVAPKRDV	EGMGPPEIKY	GESLCFVQHV	ASGLWLTYAA	PDPKALRLGV	LKKKAMLHQE	GHMDDALSLT	RCQQEESQAA
410	420	430	440	450	460	470	480
RMIHSTNGLY	NQFIKSLDSF	SGKPRGSGPP	AGTALPIEGV	ILSLQDLIIY	FEPPSEDLQH	EEKQSKLRSL	RNRQSLFQEE
490	500	510	520	530	540	550	560
GMLSMVLNCI	DRLNVYTTAA	HFAEFAGEEA	AESWKEIVNL	LYELLASLIR	GNRSNCALFS	TNLDWLVSKL	DRLEASSGIL
570	580	590	600	610	620	630	640
EVLYCVLIES	PEVLNIIQEN	HIKSIISLLD	KHGRNHKVLD	VLCSLCVCNG	VAVRSNQDLI	TENLLPGREL	LLQTNLINYV
650	660	670	680	690	700	710	720
TSIRPNIFVG	RAEGTTQYSK	WYFEVMVDEV	TPFLTAQATH	LRVGWALTEG	YTPYPGAGEG	WGGNGVGDDL	YSYGFDGLHL
730	740	750	760	770	780	790	800
WTGHVARPVT	SPGQHLLAPE	DVISCCLDLS	VPSISFRING	CPVQGVFESF	NLDGLFFPVV	SFSAGVKVRF	LLGGRHGEFK
810	820	830	840	850	860	870	880
FLPPPGYAPC	HEAVLPRERL	HLEPIKEYRR	EGPRGPHLVG	PSRCLSHTDF	VPCPVDTVQI	VLPPHLERIR	EKLAENIHEL
890	900	910	920	930	940	950	960
WALTRIEQGW	TYGPVRDDNK	RLHPCLVDFH	SLPEPERNYN	LQMSGETLKT	LLALGCHVGM	ADEKAEDNLK	KTKLPKTYMM
970	980	990	1000	1010	1020	1030	1040
SNGYKPAPLD	LSHVRLTPAQ	TTLVDRLAEN	GHNVWARDRV	GQGWSYSAVQ	DIPARRNPRL	VPYRLLDEAT	KRSNRDSLCQ
1050	1060	1070	1080	1090	1100	1110	1120
AVRTLLGYGY	NIEPPDQEPS	QVENQSRCDR	VRIFRAEKSY	TVQSGRWYFE	FEAVTTGEMR	VGWARPELRP	DVELGADELA
1130	1140	1150	1160	1170	1180	1190	1200
YVFNGHRGQR	WHLGSEPFGR	PWQPGDVVGC	MIDLTENTII	FTLNGEVLMS	DSGSETAFRE	IEIGDGFLPV	CSLGPGQVGH
1210	1220	1230	1240	1250	1260	1270	1280
LNLGQDVSSL	RFFAICGLQE	GFEPFAINMQ	RPVTTWFSKG	LPQFEPVPLE	HPHYEVSRVD	GTVDTPPCLR	LTHRTWGSQN
1290	1300	1310	1320	1330	1340	1350	1360
SLVEMLFLRL	SLPVQFHQHF	RCTAGATPLA	PPGLQPPAED	EARAAEPDPD	YENLRRSAGG	WSEAENGKEG	TAKEGAPGGT
1370	1380	1390	1400	1410	1420	1430	1440
PQAGGEAQPA	RAENEKDATT	EKNKKRGFLF	KAKKVAMMTQ	PPATPTLPRL	PHDVVPADNR	DDPEIILNTT	TYYYSVRVFA
1450	1460	1470	1480	1490	1500	1510	1520
GQEPSCVWAG	WVTPDYHQHD	MSFDLSKVRV	VTVTMGDEQG	NVHSSLKCSN	CYMVWGGDFV	SPGQQGRISH	TDLVIGCLVD
1530	1540	1550	1560	1570	1580	1590	1600
LATGLMTFTA	NGKESNTFFQ	VEPNTKLFPA	VFVLPTHQNV	IQFELGKQKN	IMPLSAAMFQ	SERKNPAPQC	PPRLEMQMLM
1610	1620	1630	1640	1650	1660	1670	1680
PVSWSRMPNH	FLQVETRRAG	ERLGWAVQCQ	EPLTMMALHI	PEENRCMDIL	ELSERLDLQR	FHSHTLRLYR	AVCALGNNRV
1690	1700	1710	1720	1730	1740	1750	1760
AHALCSHVDQ	AQLLHALEDA	HLPGPLRAGY	YDLLISIHLE	SACRSRRSML	SEYIVPLTPE	TRAITLFPPG	RSTENGHPRH
1770	1780	1790	1800	1810	1820	1830	1840
GLPGVGVTTS	LRPPHHFSPP	CFVAALPAAG	AAEAPARLSP	AIPLEALRDK	ALRMLGEAVR	DGGQHARDPV	GGSVEFQFVP
1850	1860	1870	1880	1890	1900	1910	1920
VLKLVSTLLV	MGIFGDEDVK	QILKMIEPEV	FTEEEEEEDE	EEEGEEEDEE	EKEEDEEETA	QEKEDEEKEE	EEAAEGEKEE
1930	1940	1950	1960	1970	1980	1990	2000
GLEEGLLQMK	LPESVKLQMC	HLLEYFCDQE	LQHRVESLAA	FAERYVDKLQ	ANQRSRYGLL	IKAFSMTAAE	TARRTREFRS
2010	2020	2030	2040	2050	2060	2070	2080
PPQEQINMLL	QFKDGTDEED	CPLPEEIRQD	LLDFHQDLLA	HCGIQLDGEE	EEPEEETTLG	SRLMSLLEKV	RLVKKKEEKP
2090	2100	2110	2120	2130	2140	2150	2160
EEERSAEESK	PRSLQELVSH	MVVRWAQEDF	VQSPELVRAM	FSLLHRQYDG	LGELLRALPR	AYTISPSSVE	DTMSLLECLG
2170	2180	2190	2200	2210	2220	2230	2240

Detailed Protein Report

Cmpd.	No. of Cmpds.	m/z meas.	Δ m/z [ppm]	z	Rt [min]	Score	P	Range	Sequence	Modification
2169	2	714.6084	-134.05	3	57.5	14.8	1	595-614	R.NHKVLDVLCSLCVCNGVAVR.S	
1512	1	727.4065	108.16	2	49.1	11.1	0	2601-2612	R.DVIEDCLMSLCR.Y	Carbamidomethyl: 11
2682	1	705.8622	37.53	2	64.6	12.1	2	3478-3490	K.MAKSGGSDQERTK.K	Oxidation: 1

Detailed Protein Report

Protein 65: **glutathione S-transferase P [Homo sapiens]**

Accession: gi|4504183
Database: refseq_human(refseq_human_20140103.fasta)
Database Date: 2015-11-30

Score: 53.5
MW [kDa]: 23.3
pI: 5.3
Sequence Coverage [%]: 7.6
No. of unique Peptides: 1

10	20	30	40	50	60	70	80
MPPYTVVYFP	VRGRCAALRM	LLADQGQSWK	EEVVTVETWQ	EGSLKASCLY	GQLPKFQDGD	LTLYQSNTIL	RHLGRTLGLY
90	100	110	120	130	140	150	160
GKDQQEAALV	DMVNDGVEDL	RCKYISLIYT	NYEAGKDDYV	KALPGQLKPF	ETLLSQNQGG	KTFIVGDQIS	FADYNLLDLL
170	180	190	200	210	220		
LIHEVLAPGC	LDAFPLLSAY	VGRLSARPKL	KAFLASPEYV	NLPINGNGKQ			

Cmpd.	No. of Cmpds.	m/z meas.	Δ m/z [ppm]	z	Rt [min]	Score	P	Range	Sequence	Modification
1649	2	942.4739	-4.87	2	50.9	53.5	0	56-71	K.FQDGDLTLYQSNTILR.H	

Detailed Protein Report

Protein 66: coagulation factor VIII isoform a precursor [Homo sapiens]

**Accession:**	gi|4503647	**Score:**	52.7
**Database:**	refseq_human(refseq_human_20140103.fasta)	**MW [kDa]:**	266.8
**Database Date:**	2015-11-30	**pI:**	7.0
**Modification(s):**	Carbamidomethyl, Oxidation	**Sequence Coverage [%]:**	2.3
		**No. of unique Peptides:**	3

Detailed Protein Report

10	20	30	40	50	60	70	80
MQIELSTCFF	LCLLRFCFSA	TRRYYLGAVE	LSWDYMQSDL	GELPVDARFP	PRVPKSFPFN	TSVVYKKTLF	VEFTDHLFNI
90	100	110	120	130	140	150	160
AKPRPPWMGL	LGPTIQAEVY	DTVVITLKNM	ASHPVSLHAV	GVSYWKASEG	AEYDDQTSQR	EKEDDKVFPG	GSHTYVWQVL
170	180	190	200	210	220	230	240
KENGPMASDP	LCLTYSYLSH	VDLVKDLNSG	LIGALLVCRE	GSLAKEKTQT	LHKFILLFAV	FDEGKSWHSE	TKNSLMQDRD
250	260	270	280	290	300	310	320
AASARAWPKM	HTVNGYVNRS	LPGLIGCHRK	SVYWHVIGMG	TTPEVHSIFL	EGHTFLVRNH	RQASLEISPI	TFLTAQTLLM
330	340	350	360	370	380	390	400
DLGQFLLFCH	ISSHQHDGME	AYVKVDSCPE	EPQLRMKNNE	EAEDYDDDLT	DSEMDVVRFD	DDNSPSFIQI	RSVAKKHPKT
410	420	430	440	450	460	470	480
WVHYIAAEEE	DWDYAPLVLA	PDDRSYKSQY	LNNGPQRIGR	KYKKVRFMAY	TDETFKTREA	IQHESGILGP	LLYGEVGDTL
490	500	510	520	530	540	550	560
LIIFKNQASR	PYNIYPHGIT	DVRPLYSRRL	PKGVKHLKDF	PILPGEIFKY	KWTVTVEDGP	TKSDPRCLTR	YYSSFVNMER
570	580	590	600	610	620	630	640
DLASGLIGPL	LICYKESVDQ	RGNQIMSDKR	NVILFSVFDE	NRSWYLTENI	QRFLPNPAGV	QLEDPEFQAS	NIMHSINGYV
650	660	670	680	690	700	710	720
FDSLQLSVCL	HEVAYWYILS	IGAQTDFLSV	FFSGYTFKHK	MVYEDTLTLF	PFSGETVFMS	MENPGLWILG	CHNSDFRNRG
730	740	750	760	770	780	790	800
MTALLKVSSC	DKNTGDYYED	SYEDISAYLL	SKNNAIEPRS	FSQNSRHPST	RQKQFNATTI	PENDIEKTDP	WFAHRTPMPK
810	820	830	840	850	860	870	880
IQNVSSSDLL	MLLRQSPTPH	GLSLSDLQEA	KYETFSDDPS	PGAIDSNNSL	SEMTHFRPQL	HHSGDMVFTP	ESGLQLRLNE
890	900	910	920	930	940	950	960
KLGTTAATEL	KKLDFKVSST	SNNLISTIPS	DNLAAGTDNT	SSLGPPSMPV	HYDSQLDTTL	FGKKSSPLTE	SGGPLSLSEE
970	980	990	1000	1010	1020	1030	1040
NNDSKLLESG	LMNSQESSWG	KNVSSTESGR	LFKGKRAHGP	ALLTKDNALF	KVSISLLKTN	KTSNNSATNR	KTHIDGPSLL
1050	1060	1070	1080	1090	1100	1110	1120
IENSPSVWQN	ILESDTEFKK	VTPLIHDRML	MDKNATALRL	NHMSNKTTSS	KNMEMVQQKK	EGPIPPDAQN	PDMSFFKMLF
1130	1140	1150	1160	1170	1180	1190	1200
LPESARWIQR	THGKNSLNSG	QGPSPKQLVS	LGPEKSVEGQ	NFLSEKNKVV	VGKGEFTKDV	GLKEMVFPSS	RNLFLTNLDN
1210	1220	1230	1240	1250	1260	1270	1280
LHENNTHNQE	KKIQEEIEKK	ETLIQENVVL	PQIHTVTGTK	NFMKNLFLLS	TRQNVEGSYD	GAYAPVLQDF	RSLNDSTNRT
1290	1300	1310	1320	1330	1340	1350	1360
KKHTAHFSKK	GEEENLEGLG	NQTKQIVEKY	ACTTRISPNT	SQQNFVTQRS	KRALKQFRLP	LEETELEKRI	IVDDTSTQWS
1370	1380	1390	1400	1410	1420	1430	1440
KNMKHLTPST	LTQIDYNEKE	KGAITQSPLS	DCLTRSHSIP	QANRSPLPIA	KVSSFPSIRP	IYLTRVLFQD	NSSHLPAASY
1450	1460	1470	1480	1490	1500	1510	1520
RKKDSGVQES	SHFLQGAKKN	NLSLAILTLE	MTGDQREVGS	LGTSATNSVT	YKKVENTVLP	KPDLPKTSGK	VELLPKVHIY
1530	1540	1550	1560	1570	1580	1590	1600
QKDLFPTETS	NGSPGHLDLV	EGSLLQGTEG	AIKWNEANRP	GKVPFLRVAT	ESSAKTPSKL	LDPLAWDNHY	GTQIPKEEWK
1610	1620	1630	1640	1650	1660	1670	1680
SQEKSPEKTA	FKKKDTILSL	NACESNHAIA	AINEGQNKPE	IEVTWAKQGR	TERLCSQNPP	VLKRHQREIT	RTTLQSDQEE
1690	1700	1710	1720	1730	1740	1750	1760
IDYDDTISVE	MKKEDFDIYD	EDENQSPRSF	QKKTRHYFIA	AVERLWDYGM	SSSPHVLRNR	AQSGSVPQFK	KVVFQEFTDG
1770	1780	1790	1800	1810	1820	1830	1840
SFTQPLYRGE	LNEHLGLLGP	YIRAEVEDNI	MVTFRNQASR	PYSFYSSLIS	YEEDQRQGAE	PRKNFVKPNE	TKTYFWKVQH
1850	1860	1870	1880	1890	1900	1910	1920
HMAPTKDEFD	CKAWAYFSDV	DLEKDVHSGL	IGPLLVCHTN	TLNPAHGRQV	TVQEFALFFT	IFDETKSWYF	TENMERNCRA
1930	1940	1950	1960	1970	1980	1990	2000
PCNIQMEDPT	FKENYRFHAI	NGYIMDTLPG	LVMAQDQRIR	WYLLSMGSNE	NIHSIHFSGH	VFTVRKKEEY	KMALYNLYPG
2010	2020	2030	2040	2050	2060	2070	2080
VFETVEMLPS	KAGIWRVECL	IGEHLHAGMS	TLFLVYSNKC	QTPLGMASGH	IRDFQITASG	QYGQWAPKLA	RLHYSGSINA
2090	2100	2110	2120	2130	2140	2150	2160
WSTKEPFSWI	KVDLLAPMII	HGIKTQGARQ	KFSSLYISQF	IIMYSLDGKK	WQTYRGNSTG	TLMVFFGNVD	SSGIKHNIFN
2170	2180	2190	2200	2210	2220	2230	2240

Detailed Protein Report

Cmpd.	No. of Cmpds.	m/z meas.	Δ m/z [ppm]	z	Rt [min]	Score	P	Range	Sequence	Modification
2170	1	937.3499	-130.54	2	55.7	11.5	0	1-15	-.MQIELSTCFFLCLLR.F	Carbamidomethyl: 12
2966	1	953.4397	1.72	2	66.3	11.8	0	1101-1117	K.EGPIPPDAQNPDMSFFK.M	Oxidation: 13
843	1	653.3686	-17.24	2	40.6	10.7	2	1167-1178	K.NKVVVGKGEFTK.D	

Detailed Protein Report

Protein 67: **PREDICTED: centriolin isoform X3 [Homo sapiens]**

**Accession:**	gi|578817016	**Score:**	52.7
**Database:**	refseq_human(refseq_human_20140103.fasta)	**MW [kDa]:**	258.5
**Database Date:**	2015-11-30	**pI:**	5.3
**Modification(s):**	Carbamidomethyl, Oxidation	**Sequence Coverage [%]:**	2.3
		**No. of unique Peptides:**	4

Detailed Protein Report

10	20	30	40	50	60	70	80
MKKGSQQKIF	SKAKIPSSSH	SPIPSSMSNM	RSRSLSPLIG	SETLPFHSGG	QWCEQVEIAD	ENNMLLDYQD	HKGADSHAGV
90	100	110	120	130	140	150	160
RYITEALIKK	LTKQDNLALI	KSLNLSLSKD	GGKKFKYIEN	LEKCVKLEVL	NLSYNLIGKI	EKLDKLLKLR	ELNLSYNKIS
170	180	190	200	210	220	230	240
KIEGIENMCN	LQKLNLAGNE	IEHIPVWLGK	KLKSLRVLNL	KGNKISSLQD	ISKLKPLQDL	ISLILVENPV	VTLPHYLQFT
250	260	270	280	290	300	310	320
IFHLRSLESL	EGQPVTTQDR	QEAFERFSLE	EVERLERDLE	KKMIETEELK	SKQTRFLEEI	KNQDKLNKSL	KEEAMLQKQS
330	340	350	360	370	380	390	400
CEELKSDLNT	KNELLKQKTI	ELTRACQKQY	ELEQELAFYK	IDAKFEPLNY	YPSEYAEIDK	APDESPYIGK	SRYKRNMFAT
410	420	430	440	450	460	470	480
ESYIIDSAQA	VQIKKMEPDE	QLRNDHMNLR	GHTPLDTQLE	DKEKKISAAQ	TRLSELHDEI	EKAEQQILRA	TEEFKQLEEA
490	500	510	520	530	540	550	560
IQLKKISEAG	KDLLYKQLSG	RLQLVNKLRQ	EALDLELQME	KQKQEIAGKQ	KEIKDLQIAI	DSLDSKDPKH	SHMKAQKSGK
570	580	590	600	610	620	630	640
EQQLDIMNKQ	YQQLESRLDE	ILSRIAKETE	EIKDLEEQLT	EGQIAANEAL	KKDLEGVISG	LQEYLGTIKG	QATQAQNECR
650	660	670	680	690	700	710	720
KLRDEKETLL	QRLTEVEQER	DQLEIVAMDA	ENMRKELAEL	ESALQEQHEV	NASLQQTQGD	LSAYEAELEA	RLNLRDAEAN
730	740	750	760	770	780	790	800
QLKEELEKVT	RLTQLEQSAL	QAELEKERQA	LKNALGKAQF	SEEKEQENSE	LHAKLKHLQD	DNNLLKQQLK	DFQNHLNHVV
810	820	830	840	850	860	870	880
DGLVRPEEVA	ARVDELRRKL	KLGTGEMNIH	SPSDVLGKSL	ADLQKQFSEI	LARSKWERDE	AQVRERKLQE	EMALQQEKLA
890	900	910	920	930	940	950	960
TGQEEFRQAC	ERALEARMNF	DKRQHEARIQ	QMENEIHYLQ	ENLKSMEEIQ	GLTDLQLQEA	DEEKERILAQ	LRELEKKKKL
970	980	990	1000	1010	1020	1030	1040
EDAKSQEQVF	GLDKELKKLK	KAVATSDKLA	TAELTIAKDQ	LKSLHGTVMK	INQERAEELQ	EAERFSRKAA	QAARDLTRAE
1050	1060	1070	1080	1090	1100	1110	1120
AEIELLQNLL	RQKGEQFRLE	MEKTGVGTGA	NSQVLEIEKL	NETMERQRTE	IARLQNVLDL	TGSDNKGGFE	NVLEEIAELR
1130	1140	1150	1160	1170	1180	1190	1200
REVSYQNDYI	SSMADPFKRR	GYWYFMPPPP	SSKVSSHSSQ	ATKDSGVGLK	YSASTPVRKP	RPGQQDGKEG	SQPPPASGYW
1210	1220	1230	1240	1250	1260	1270	1280
VYSPIRSGLH	KLFPSRDADS	GGDSQEESEL	DDQEEPPFVP	PPGYMMYTVL	PDGSPVPQGM	ALYAPPPPLP	NNSRPLTPGT
1290	1300	1310	1320	1330	1340	1350	1360
VVYGPPPAGA	PMVYGPPPPN	FSIPFIPMGV	LHCNVPEHHN	LENEVSRLED	IMQHLKSKKR	EERWMRASKR	QSEKEMEELH
1370	1380	1390	1400	1410	1420	1430	1440
HNIDDLLQEK	KSLECEVEEL	HRTVQKRQQQ	KDFIDGNVES	LMTELEIEKS	LKHHEDIVDE	IECIEKTLLK	RRSELREADR
1450	1460	1470	1480	1490	1500	1510	1520
LLAEAESELS	CTKEKTKNAV	EKFTDAKRSL	LQTESDAEEL	ERRAQETAVN	LVKADQQLRS	LQADAKDLEQ	HKIKQEEILK
1530	1540	1550	1560	1570	1580	1590	1600
EINKIVAAKD	SDFQCLSKKK	EKLTEELQKL	QKDIEMAERN	EDHHLQVLKE	SEVLLQAKRA	ELEKLKSQVT	SQQQEMAVLD
1610	1620	1630	1640	1650	1660	1670	1680
RQLGHKKEEL	HLLQGSMVQA	KADLQEALRL	GETEVTEKCN	HIREVKSLLE	ELSFQKGELN	VQISERKTQL	TLIKQEIEKE
1690	1700	1710	1720	1730	1740	1750	1760
EENLQVVLRQ	MSKHKTELKN	ILDMLQLENH	ELQGLKLQHD	QRVSELEKTQ	VAVLEEKLEL	ENLQQISQQQ	KGEIEWQKQL
1770	1780	1790	1800	1810	1820	1830	1840
LERDKREIER	MTAESRALQS	CVECLSKEKE	DLQEKCDIWE	KKLAQTKRVL	AAAEENSKME	QSNLEKLELN	VRKLQQELDQ
1850	1860	1870	1880	1890	1900	1910	1920
LNRDKLSLHN	DISAMQQQLQ	EKREAVNSLQ	EELANVQDHL	NLAKQDLLHT	TKHQDVLLSE	QTRLQKDISE	WANRFEDCQK
1930	1940	1950	1960	1970	1980	1990	2000
EEETKQQQLQ	VLQNEIEENK	LKLVQQEMMF	QRLQKERESE	ESKLETSKVT	LKEQQHQLEK	ELTDQKSKLD	QVLSKVLAAE
2010	2020	2030	2040	2050	2060	2070	2080
ERVRTLQEEE	RWCESLEKTL	SQTKRQLSER	EQQLVEKSGE	LLALQKEADS	MRADFSLLRN	QFLTERKKAE	KQVASLKEAL
2090	2100	2110	2120	2130	2140	2150	2160
KIQRSQLEKN	LLFLPELPAD	LEAILERNEN	LEGELESLKE	NLPFTMNEGP	FEEKLNFSQV	HIMDEHWRGE	ALREKLRHRE
2170	2180	2190	2200	2210	2220	2230	2240

Detailed Protein Report

Cmpd.	No. of Cmpds.	m/z meas.	Δ m/z [ppm]	z	Rt [min]	Score	P	Range	Sequence	Modification
951	1	677.3400	12.76	3	40.4	10.0	1	13-31	K.AKIPSSSHSPIPSSMSNMR.S	Oxidation: 18
1818	1	582.2582	-133.26	1	53.1	16.6	0	1602-1606	R.QLGHK.K	
1797	1	638.2204	-128.45	3	51.0	10.7	1	1771-1787	R.MTAESRALQSCVECLSK.E	Carbamidomethyl: 14
36	1	721.8221	18.43	2	30.3	15.4	1	1915-1925	R.FEDCQKEEETK.Q	Carbamidomethyl: 4

Detailed Protein Report

Protein 68: PREDICTED: rho GTPase-activating protein 26 isoform X6 [Homo sapiens]

**Accession:**	gi|530380462	**Score:**	51.9
**Database:**	refseq_human(refseq_human_20140103.fasta)	**MW [kDa]:**	69.9
**Database Date:**	2015-11-30	**pI:**	9.8
		**Sequence Coverage [%]:**	9.0
		**No. of unique Peptides:**	2

10	20	30	40	50	60	70	80
MKRETRRSAF	CVQGWSGGDP	ARVVLWGSAG	GRGRARHAGV	PAVGPECRAR	RSQRADPIVS	TAPAPLGAEL	LSLECPTVRM
90	100	110	120	130	140	150	160
ERALPRGRCL	PLGKDLSSAK	RKFADSLNEF	KFQCIGDAET	DDEMCIARSL	QEFATVLRNL	EDERIRMIEN	ASEVLITPLE
170	180	190	200	210	220	230	240
KFRKEQIGAA	KEAKKKYDKE	TEKYCGILEK	HLNLSSKKKE	SQLQEADSQV	DLVRQHFYEV	SLEYVFKVQE	VQERKMFEFV
250	260	270	280	290	300	310	320
EPLLAFLQGL	FTFYHHGYEL	AKDFGDFKTQ	LTISIQNTRN	RFEGTRSEVE	SLMKKMKENP	LEHKTISPYT	MEGYLYVQEK
330	340	350	360	370	380	390	400
RHFGTSWVKH	YCTYQRDSKQ	ITMVPFDQKS	GGKGGEDESV	ILKSCTRRKT	DSIEKRFCFD	VEAVDRPGVI	TMQALSEEDR
410	420	430	440	450	460	470	480
RLWMEAMDGR	EPVYNSNKDS	QSEGTAQLDS	IGFSIIRKCI	HAVETRGINE	QGLYRIVGVN	SRVQKLLSVL	MDPKTASETE
490	500	510	520	530	540	550	560
TDICAEWEIK	TITSALKTYL	RMLPGPLMMY	QFQRSFIKAA	KLENQESRVS	EIHSLVHRLP	EKNRQMLQLL	MNHLAKLIVP
570	580	590	600	610	620		
GTLKVTGSVD	MGKSSISLLF	KEMKLLKVRK	QQMLERMWRN	RNDFTLLVGV			

Cmpd.	No. of Cmpds.	m/z meas.	Δ m/z [ppm]	z	Rt [min]	Score	P	Range	Sequence	Modification
2004	1	710.2849	-115.96	2	55.4	11.7	1	35-48	R.ARHAGVPAVGPECR.A	
47	2	768.2750	-160.02	2	30.4	15.4	1	340-353	K.QITMVPFDQKSGGK.G	

Detailed Protein Report

Protein 69: **PREDICTED: protein KIAA1199 isoform X3 [Homo sapiens]**

**Accession:**	gi|578827243	**Score:**	51.9
**Database:**	refseq_human(refseq_human_20140103.fasta)	**MW [kDa]:**	90.7
**Database Date:**	2015-11-30	**pI:**	9.6
		**Sequence Coverage [%]:**	4.2
		**No. of unique Peptides:**	1

10	20	30	40	50	60	70	80
MYSHGNLEIP	WSLPVLITPC	TRQKSSRCFP	ADPAPPTRSK	WQIKDVVGYN	SLGHCFFTED	GPEERNTFDH	CLGLLVKSGT
**90**	**100**	**110**	**120**	**130**	**140**	**150**	**160**
LLPSDRDSKM	CKMITEDSYP	GYIPKPRQDC	NAVSTFWMAN	PNNNLINCAA	AGSEETGFWF	IFHHVPTGPS	VGMYSPGYSE
**170**	**180**	**190**	**200**	**210**	**220**	**230**	**240**
HIPLGKFYNN	RAHSNYRAGM	IIDNGVKTTE	ASAKDKRPFL	SIISARYSPH	QDADPLKPRE	PAIIRHFIAY	KNQDHGAWLR
**250**	**260**	**270**	**280**	**290**	**300**	**310**	**320**
GGDVWLDSCR	FADNGIGLTL	ASGGTFPYDD	GSKQEIKNSL	FVGESGNVGT	EMMDNRIWGP	GGLDHSGRTL	PIGQNFPIRG
**330**	**340**	**350**	**360**	**370**	**380**	**390**	**400**
IQLYDGPINI	QNCTFRKFVA	LEGRHTSALA	FRLNNAWQSC	PHNNVTGIAF	EDVPITSRVF	FGEPGPWFNQ	LDMDGDKTSV
**410**	**420**	**430**	**440**	**450**	**460**	**470**	**480**
FHDVDGSVSE	YPGSYLTKND	NWLVRHPDCI	NVPDWRGAIC	SGCYAQMYIQ	AYKTSNLRMK	IIKNDFPSHP	LYLEGALTRS
**490**	**500**	**510**	**520**	**530**	**540**	**550**	**560**
THYQQYQPVV	TLQKGYTIHW	DQTAPAELAI	WLINFNKGDW	IRVGLCYPRG	TTFSILSDVH	NRLLKQTSKT	GVFVRTLQMD
**570**	**580**	**590**	**600**	**610**	**620**	**630**	**640**
KVEQSYPGRS	HYYWDEDSGL	LFLKLKAQNE	REKFAFCSMK	GCERIKIKAL	IPKNAGVSDC	TATAYPKFTE	RAVVDVPMPK
**650**	**660**	**670**	**680**	**690**	**700**	**710**	**720**
KLFGSQLKTK	DHFLEVKMES	SKQHFFHLWN	DFAYIEVDGK	KYPSSEDGIQ	VVVIDGNQGR	VVSHTSFRNS	ILQGIPWQLF
**730**	**740**	**750**	**760**	**770**	**780**	**790**	**800**
NYVATIPDNS	IVLMASKGRY	VSRGPWTRVL	EKLGADRGLK	LKEQMAFVGF	KGSFRPIWVT	LDTEDHKAKI	FQVVPIPVVK
**810**							
KKKL							

Cmpd.	No. of Cmpds.	m/z meas.	Δ m/z [ppm]	z	Rt [min]	Score	P	Range	Sequence	Modification
737	1	936.9549	11.85	2	39.2	10.8	0	570-584	R.SHYYWDEDSGLLFLK.L	

Detailed Protein Report

Protein 70: myosin-7B [Homo sapiens]

**Accession:**	gi|114842389	**Score:**	51.6
**Database:**	refseq_human(refseq_human_20140103.fasta)	**MW [kDa]:**	225.7
**Database Date:**	2015-11-30	**pI:**	5.8
**Modification(s):**	Oxidation	**Sequence Coverage [%]:**	2.6
		**No. of unique Peptides:**	3

Quantitation

QD:QU	**Median:** 1.05	**CV:** 0.00 %	**No. of Peptides:** 1

Detailed Protein Report

10	20	30	40	50	60	70	80
MSGNKRGSRA	SCPHRGAECL	LPWAALNLQG	FQLLLLHPSA	TAMMDVSELG	ESARYLRQGY	QEMTKVHTIP	WDGKKRVWVP
90	100	110	120	130	140	150	160
DEQDAYVEAE	VKSEATGGRV	TVETKDQKVL	MVREAELQPM	NPPRFDLLED	MAMMTHLNEA	SVLHNLRQRY	ARWMIYTYSG
170	180	190	200	210	220	230	240
LFCVTINPYK	WLPVYTASVV	AAYKGKRRSD	SPPHIYAVAD	NAYNDMLRNR	DNQSMLITGE	SGAGKTVNTK	RVIQYFAIVA
250	260	270	280	290	300	310	320
ALGDGPGKKA	QFLATKTGGT	LEDQIIEANP	AMEAFGNAKT	LRNDNSSRFG	KFIRIHFGPS	GKLASADIDS	YLLEKSRVIF
330	340	350	360	370	380	390	400
QLPGERSYHV	YYQILSGRKP	ELQDMLLLSM	NPYDYHFCSQ	GVITVDNMND	GEELIATDHA	MDILGFSVDE	KCACYKIVGA
410	420	430	440	450	460	470	480
LLHFGNMKFK	QKQREEQAEA	DGTESADKAA	YLMGVSSGDL	LKGLLHPRVR	VGNEYVTKGQ	SVEQVVFAVG	ALAKATYDRL
490	500	510	520	530	540	550	560
FRWLVSRINQ	TLDTKLPRQF	FIGVLDIAGF	EIFEFNSFEQ	LCINFTNEKL	QQFFNQHMFV	LEQEEYKREG	IDWVFIDFGL
570	580	590	600	610	620	630	640
DLQPCIDLIE	KPLGILSILE	EECMFPKASD	ASFRAKLYDN	HAGKSPNFQQ	PRPDKKRKYQ	AHFEVVHYAG	VVPYSIVGWL
650	660	670	680	690	700	710	720
EKNKDPLNET	VVPIFQKSQN	RLLATLYENY	AGSCSTEPPK	SGVKEKRKKA	ASFQTVSQLH	KENLNKLMTN	LRATQPHFVR
730	740	750	760	770	780	790	800
CIVPNENKTP	GVMDAFLVLH	QLRCNGVLEG	IRICRQGFPN	RLLYTDFRQR	YRILNPSAIP	DDTFMDSRKA	TEKLLGSLDL
810	820	830	840	850	860	870	880
DHTQYQFGHT	KVFFKAGLLG	VLEELRDQRL	AKVLTLLQAR	SRGRLMRLEY	QRLLGGRDAL	FTIQWNIRAF	NAVKNWSWMK
890	900	910	920	930	940	950	960
LFFKMKPLLR	SAQAEEELAA	LRAELRGLRG	ALAAAEAKRQ	ELEETHVSIT	QEKNDLALQL	QAEQDNLADA	EERCHLLIKS
970	980	990	1000	1010	1020	1030	1040
KVQLEGKVKE	LSERLEDEEE	VNADLAARRR	KLEDECTELK	KDIDDLKLTL	AKAEKEKQAT	ENKVKNLTEE	MAALDESVAR
1050	1060	1070	1080	1090	1100	1110	1120
LTKEKKALQE	AHQQALGDLQ	AEEDRVSALT	KAKLRLEQQV	EDLECSLEQE	KKLRMDTERA	KRKLEGDLKL	TQESVADAAQ
1130	1140	1150	1160	1170	1180	1190	1200
DKQQLEEKLK	KKDSELSQLS	LRVEDEQLLG	AQMQKKIKEL	QARAEELEEE	LEAERAARAR	VEKQRAEAAR	ELEELSERLE
1210	1220	1230	1240	1250	1260	1270	1280
EAGGASAGQR	EGCRKREAEL	GRLRRELEEA	ALRHEATVAA	LRRKQAEGAA	ELGEQVDSLQ	RVRQKLEKEK	SELRMEVDDL
1290	1300	1310	1320	1330	1340	1350	1360
AANVETLTRA	KASAEKLCRT	YEDQLSEAKI	KVEELQRQLA	DASTQRGRLQ	TESGELSRLL	EEKECLISQL	SRGKALAAQS
1370	1380	1390	1400	1410	1420	1430	1440
LEELRRQLEE	ESKAKSALAH	AVQALRHDCD	LLREQHEEEA	EAQAELQRLL	SKANAEVAQW	RSKYEADAIQ	RTEELEEAKK
1450	1460	1470	1480	1490	1500	1510	1520
KLALRLQEAE	EGVEAANAKC	SSLEKAKLRL	QTESEDVTLE	LERATSAAAA	LDKKQRHLER	ALEERRRQEE	EMQRELEAAQ
1530	1540	1550	1560	1570	1580	1590	1600
RESRGLGTEL	FRLRHGHEEA	LEALETLKRE	NKNLQEEISD	LTDQVSLSGK	SIQELEKTKK	ALEGEKSEIQ	AALEEAEGAL
1610	1620	1630	1640	1650	1660	1670	1680
ELEETKTLRI	QLELSQVKAE	VDRKLAEKDE	ECANLRRNHQ	RAVESLQASL	DAETRARNEA	LRLKKKMEGD	LNDLELQLGH
1690	1700	1710	1720	1730	1740	1750	1760
ATRQATEAQA	ATRLMQAQLK	EEQAGRDEEQ	RLAAELHEQA	QALERRASLL	AAELEELRAA	LEQGERSRRL	AEQELLEATE
1770	1780	1790	1800	1810	1820	1830	1840
RLNLLHSQNT	GLLNQKKKLE	ADLAQLSGEV	EEAAQERREA	EEKAKKAITD	AAMMAEELKK	EQDTSAHLER	MKKTLEQTVR
1850	1860	1870	1880	1890	1900	1910	1920
ELQARLEEAE	QAALRGGKKQ	VQKLEAKVRE	LEAELDAEQK	KHAEALKGVR	KHERRVKELA	YQAEEDRKNL	ARMQDLVDKL
1930	1940	1950	1960	1970	1980	1990	
QSKVKSYKRQ	FEEAEQQANT	NLAKYRKAQH	ELDDAEERAD	MAETQANKLR	ARTRDALGPK	HKE	

Cmpd.	No. of Cmpds.	m/z meas.	Δ m/z [ppm]	z	Rt [min]	Score	P	Range	Sequence	Modification	Ratios
987	1	574.1653	-236.77	2	40.8	12.3	0	1133-1142	K.DSELSQLSLR.V		QD:QU 1.05
2175	1	1031.4134	-120.21	2	55.8	11.8	2	1515-	R.ELEAAQRESRGLGTELFR.L		

Detailed Protein Report

Cmpd.	No. of Cmpds.	m/z meas.	Δ m/z [ppm]	z	Rt [min]	Score	P	Range	Sequence	Modification	Ratios
								1532			
2480	3	625.3103	-63.96	2	61.5	13.4	2	1831-1840	R.MKKTLEQTVR.E	Oxidation: 1	

Detailed Protein Report

Protein 71: lysine-specific demethylase 3A [Homo sapiens]

**Accession:**	gi|20357522	**Score:**	51.2
**Database:**	refseq_human(refseq_human_20140103.fasta)	**MW [kDa]:**	147.2
**Database Date:**	2015-11-30	**pI:**	9.4
**Modification(s):**	Oxidation	**Sequence Coverage [%]:**	2.5
		**No. of unique Peptides:**	2

Alias proteins:

Accession	Name	Description
gi|226442779	refseq_human (refseq_human_20140103.fasta)	lysine-specific demethylase 3A [Homo sapiens]

10	20	30	40	50	60	70	80
MVLTLGESWP	VLVGRRFLSL	SAADGSDGSH	DSWDVERVAE	WPWLSGTIRA	VSHTDVTKKD	LKVCVEFDGE	SWRKRRWIEV
90	100	110	120	130	140	150	160
YSLLRRAFLV	EHNLVLAERK	SPEISERIVQ	WPAITYKPLL	DKAGLGSITS	VRFLGDQQRV	FLSKDLLKPI	QDVNSLRLSL
170	180	190	200	210	220	230	240
TDNQIVSKEF	QALIVKHLDE	SHLLKGDKNL	VGSEVKIYSL	DPSTQWFSAT	VINGNPASKT	LQVNCEEIPA	LKIVDPSLIH
250	260	270	280	290	300	310	320
VEVVHDNLVT	CGNSARIGAV	KRKSSENNGT	LVSKQAKSCS	EASPSMCPVQ	SVPTTVFKEI	LLGCTAATPP	SKDPRQQSTP
330	340	350	360	370	380	390	400
QAANSPPNLG	AKIPQGCHKQ	SLPEEISSCL	NTKSEALRTK	PDVCKAGLLS	KSSQIGTGDL	KILTEPKGSC	TQPKTNTDQE
410	420	430	440	450	460	470	480
NRLESVPQAL	TGLPKECLPT	KASSKAELEI	ANPPELQKHL	EHAPSPSDVS	NAPEVKAGVN	SDSPNNCSGK	KVEPSALACR
490	500	510	520	530	540	550	560
SQNLKESSVK	VDNESCCSRS	NNKIQNAPSR	KSVLTDPAKL	KKLQQSGEAF	VQDDSCVNIV	AQLPKCRECR	LDSLRKDKEQ
570	580	590	600	610	620	630	640
QKDSPVFCRF	FHFRRLQFNK	HGVLRVEGFL	TPNKYDNEAI	GLWLPLTKNV	VGIDLDTAKY	ILANIGDHFC	QMVISEKEAM
650	660	670	680	690	700	710	720
STIEPHRQVA	WKRAVKGVRE	MCDVCDTTIF	NLHWVCPRCG	FGVCVDCYRM	KRKNCQQGAA	YKTFSWLKCV	KSQIHEPENL
730	740	750	760	770	780	790	800
MPTQIIPGKA	LYDVGDIVHS	VRAKWGIKAN	CPCSNRQFKL	FSKPASKEDL	KQTSLAGEKP	TLGAVLQQNP	SVLEPAAVGG
810	820	830	840	850	860	870	880
EAASKPAGSM	KPACPASTSP	LNWLADLTSG	NVNKENKEKQ	PTMPILKNEI	KCLPPLPPLS	KSSTVLHTFN	STILTPVSNN
890	900	910	920	930	940	950	960
NSGFLRNLLN	SSTGKTENGL	KNTPKILDDI	FASLVQNKTT	SDLSKRPQGL	TIKPSILGFD	TPHYWLCDNR	LLCLQDPNNK
970	980	990	1000	1010	1020	1030	1040
SNWNVFRECW	KQGQPVMVSG	VHHKLNSELW	KPESFRKEFG	EQEVDLVNCR	TNEIITGATV	GDFWDGFEDV	PNRLKNEKEP
1050	1060	1070	1080	1090	1100	1110	1120
MVLKLKDWPP	GEDFRDMMPS	RFDDLMANIP	LPEYTRRDGK	LNLASRLPNY	FVRPDLGPKM	YNAYGLITPE	DRKYGTTNLH
1130	1140	1150	1160	1170	1180	1190	1200
LDVSDAANVM	VYVGIPKGQC	EQEEEVLKTI	QDGDSDELTI	KRFIEGKEKP	GALWHIYAAK	DTEKIREFLK	KVSEEQGQEN
1210	1220	1230	1240	1250	1260	1270	1280
PADHDPIHDQ	SWYLDRSLRK	RLHQEYGVQG	WAIVQFLGDV	VFIPAGAPHQ	VHNLYSCIKV	AEDFVSPEHV	KHCFWLTQEF
1290	1300	1310	1320	1330			
RYLSQTHTNH	EDKLQVKNVI	YHAVKDAVAM	LKASESSFGK	P			

Cmpd.	No. of Cmpds.	m/z meas.	Δ m/z [ppm]	z	Rt [min]	Score	P	Range	Sequence	Modification
62	1	675.3854	139.53	2	29.5	11.1	0	457-470	K.AGVNSDSPNNCSGK.K	
755	1	472.1298	-292.09	2	39.4	27.4	0	840-847	K.QPTMPILK.N	Oxidation: 4

Detailed Protein Report

Protein 72: biorientation of chromosomes in cell division protein 1-like 1 [Homo sapiens]

**Accession:**	gi|87299628	**Score:**	51.1
**Database:**	refseq_human(refseq_human_20140103.fasta)	**MW [kDa]:**	330.3
**Database Date:**	2015-11-30	**pI:**	4.9
**Modification(s):**	Oxidation	**Sequence Coverage [%]:**	0.9
		**No. of unique Peptides:**	1

Detailed Protein Report

10	20	30	40	50	60	70	80
MATNPQPQPP	PPAPPPPPPQ	PQPQPPPPPP	GPGAGPGAGG	AGGAGAGAGD	PQLVAMIVNH	LKSQGLFDQF	RRDCLADVDT
90	100	110	120	130	140	150	160
KPAYQNLRQR	VDNFVANHLA	THTWSPHLNK	NQLRNNIRQQ	VLKSGMLESG	IDRIISQVVD	PKINHTFRPQ	VEKAVHEFLA
170	180	190	200	210	220	230	240
TLNHKEEGSG	NTAPDDEKPD	TSLITQGVPT	PGPSANVAND	AMSILETITS	LNQEASAARA	STETSNAKTS	ERASKKLPSQ
250	260	270	280	290	300	310	320
PTTDTSTDKE	RTSEDMADKE	KSTADSGGEG	LETAPKSEEF	SDLPCPVEEI	KNYTKEHNNL	ILLNKDVQQE	SSEQKNKSTD
330	340	350	360	370	380	390	400
KGEKKPDSNE	KGERKKEKKE	KTEKKFDHSK	KSEDTQKVKD	EKQAKEKEVE	SLKLPSEKNS	NKAKTVEGTK	EDFSLIDSDV
410	420	430	440	450	460	470	480
DGLTDITVSS	VHTSDLSSFE	EDTEEEVVTS	DSMEEGEITS	DDEEKNKQNK	TKTQTSDSSE	GKTKSVRHAY	VHKPYLYSKY
490	500	510	520	530	540	550	560
YSDSDDELTV	EQRRQSIAKE	KEERLLRRQI	NREKLEEKRK	QKAEKTKSSK	TKGQGRSSVD	LEESSTKSLE	PKAARIKEVL
570	580	590	600	610	620	630	640
KERKVLEKKV	ALSKKRKKDS	RNVEENSKKK	QQYEEDSKET	LKTSEHCEKE	KISSSKELKH	VHAKSEPSKP	ARRLSESLHV
650	660	670	680	690	700	710	720
VDENKNESKL	EREHKRRTST	PVIMEGVQEE	TDTRDVKRQV	ERSEICTEEP	QKQKSTLKNE	KHLKKDDSET	PHLKSLLKKE
730	740	750	760	770	780	790	800
VKSSKEKPER	EKTPSEDKLS	VKHKYKGDCM	HKTGDETELH	SSEKGLKVEE	NIQKQSQQTK	LSSDDKTERK	SKHRNERKLS
810	820	830	840	850	860	870	880
VLGKDGKPVS	EYIIKTDENV	RKENNKKERR	LSAEKTKAEH	KSRRSSDSKI	QKDSLGSKQH	GITLQRRSES	YSEDKCDMDS
890	900	910	920	930	940	950	960
TNMDSNLKPE	EVVHKEKRRT	KSLLEEKLVL	KSKSKTQGKQ	VKVVETELQE	GATKQATTPK	PDKEKNTEEN	DSEKQRKSKV
970	980	990	1000	1010	1020	1030	1040
EDKPFEETGV	EPVLETASSS	AHSTQKDSSH	RAKLPLAKEK	YKSDKDSTST	RLERKLSDGH	KSRSLKHSSK	DIKKKDENKS
1050	1060	1070	1080	1090	1100	1110	1120
DDKDGKEVDS	SHEKARGNSS	LMEKKLSRRL	CENRRGSLSQ	EMAKGEEKLA	ANTLSTPSGS	SLQRPKKSGD	MTLIPEQEPM
1130	1140	1150	1160	1170	1180	1190	1200
EIDSEPGVEN	VFEVSKTQDN	RNNNSQQDID	SENMKQKTSA	TVQKDELRTC	TADSKATAPA	YKPGRGTGVN	SNSEKHADHR
1210	1220	1230	1240	1250	1260	1270	1280
STLTKKMHIQ	SAVSKMNPGE	KEPIHRGTTE	VNIDSETVHR	MLLSAPSEND	RVQKNLKNTA	AEEHVAQGDA	TLEHSTNLDS
1290	1300	1310	1320	1330	1340	1350	1360
SPSLSSVTVV	PLRESYDPDV	IPLFDKRTVL	EGSTASTSPA	DHSALPNQSL	TVRESEVLKT	SDSKEGGEGF	TVDTPAKASI
1370	1380	1390	1400	1410	1420	1430	1440
TSKRHIPEAH	QATLLDGKQG	KVIMPLGSKL	TGVIVENENI	TKEGGLVDMA	KKENDLNAEP	NLKQTIKATV	ENGKKDGIAV
1450	1460	1470	1480	1490	1500	1510	1520
DHVVGLNTEK	YAETVKLKHK	RSPGKVKDIS	IDVERRNENS	EVDTSAGSGS	APSVLHQRNG	QTEDVATGPR	RAEKTSVATS
1530	1540	1550	1560	1570	1580	1590	1600
TEGKDKDVTL	SPVKAGPATT	TSSETRQSEV	ALPCTSIEAD	EGLIIGTHSR	NNPLHVGAEA	SECTVFAAAE	EGGAVVTEGF
1610	1620	1630	1640	1650	1660	1670	1680
AESETFLTST	KEGESGECAV	AESEDRAADL	LAVHAVKIEA	NVNSVVTEEK	DDAVTSAGSE	EKCDGSLSRD	SEIVEGTITF
1690	1700	1710	1720	1730	1740	1750	1760
ISEVESDGAV	TSAGTEIRAG	SISSEEVDGS	QGNMMRMGPK	KETEGTVTCT	GAEGRSDNFV	ICSVTGAGPR	EERMVTGAGV
1770	1780	1790	1800	1810	1820	1830	1840
VLGDNDAPPG	TSASQEGDGS	VNDGTEGESA	VTSTGITEDG	EGPASCTGSE	DSSEGFAISS	ESEENGESAM	DSTVAKEGTN
1850	1860	1870	1880	1890	1900	1910	1920
VPLVAAGPCD	DEGIVTSTGA	KEEDEEGEDV	VTSTGRGNEI	GHASTCTGLG	EESEGVLICE	SAEGDSQIGT	VVEHVEAEAG
1930	1940	1950	1960	1970	1980	1990	2000
AAIMNANENN	VDSMSGTEKG	SKDTDICSSA	KGIVESSVTS	AVSGKDEVTP	VPGGCEGPMT	SAASDQSDSQ	LEKVEDTTIS
2010	2020	2030	2040	2050	2060	2070	2080
TGLVGGSYDV	LVSGEVPECE	VAHTSPSEKE	DEDIITSVEN	EECDGLMATT	ASGDITNQNS	LAGGKNQGKV	LIISTSTTND
2090	2100	2110	2120	2130	2140	2150	2160
YTPQVSAITD	VEGGLSDALR	TEENMEGTRV	TTEEFEAPMP	SAVSGDDSQL	TASRSEEKDE	CAMISTSIGE	EFELPISSAT
2170	2180	2190	2200	2210	2220	2230	2240
TIKCAESLQP	VAAAVEERAT	GPVLISTADF	EGPMPSAPPE	AESPLASTSK	EEKDECALIS	TSIAEECEAS	VSGVVVESEN
2250	2260	2270	2280	2290	2300	2310	2320
ERAGTVMEEK	DGSGIISTSS	VEDCEGPVSS	AVPQEEGDPS	VTPAEEMGDT	AMISTSTSEG	CEAVMIGAVL	QDEDRLTITR
2330	2340	2350	2360	2370	2380	2390	2400

Detailed Protein Report

Cmpd.	No. of Cmpds.	m/z meas.	Δ m/z [ppm]	z	Rt [min]	Score	P	Range	Sequence	Modification
1228	1	954.9820	37.30	2	45.5	23.8	0	658-674	R.TSTPVIMEGVQEETDTR.D	Oxidation: 7

Detailed Protein Report

Protein 73: triosephosphate isomerase isoform 3 [Homo sapiens]

Accession: gi|384475517
Database: refseq_human(refseq_human_20140103.fasta)
Database Date: 2015-11-30
Modification(s): Carbamidomethyl

Score: 50.7
MW [kDa]: 17.9
pI: 5.3
Sequence Coverage [%]: 18.0
No. of unique Peptides: 2

Quantitation

QD:QU **Median:** 1.54 **CV:** 0.00 % **No. of Peptides:** 1

```
        10         20         30         40         50         60         70         80
MIKDCGATWV VLGHSERRHV FGESDELIGQ KVAHALAEGL GVIACIGEKL DEREAGITEK VVFEQTKVIA DNVKDWSKVV
        90        100        110        120        130        140        150        160
LAYEPVWAIG TGKTATPQQA QEVHEKLRGW LKSNVSDAVA QSTRIIYGGS VTGATCKELA SQPDVDGFLV GGASLKPEFV
       170
DIINAKQ
```

Cmpd.	No. of Cmpds.	m/z meas.	Δ m/z [ppm]	z	Rt [min]	Score	P	Range	Sequence	Modification	Ratios
2923	1	951.4595	-12.75	2	65.7	13.3	1	1-17	-.MIKDCGATWVVLGHSER.R		QD:QU 1.54
219	1	663.8152	-36.93	2	32.7	37.4	0	125-137	R.IIYGGSVTGATCK.E	Carbamidomethyl: 12	

Detailed Protein Report

Protein 74: **dynein heavy chain 9, axonemal isoform 2 [Homo sapiens]**

**Accession:**	gi|114155133	**Score:**	49.5
**Database:**	refseq_human(refseq_human_20140103.fasta)	**MW [kDa]:**	511.6
**Database Date:**	2015-11-30	**pI:**	5.6
**Modification(s):**	Oxidation	**Sequence Coverage [%]:**	1.0
		**No. of unique Peptides:**	3

Detailed Protein Report

10	20	30	40	50	60	70	80
MRLAEERAAL	AAENADGEPG	ADRRLRLLGT	YVAMSLRPAA	GAWERCAGSA	EAEQLLQAFL	GRDAAEGPRP	LLVVRPGPRG
90	100	110	120	130	140	150	160
LAIRPGLEVG	PESGLAGAKA	LFFLRTGPEP	PGPDSFRGAV	VCGDLPAAPL	EHLAALFSEV	VLPVLANEKN	RLNWPHMICE
170	180	190	200	210	220	230	240
DVRRHAHSLQ	CDLSVILEQV	KGKTLLPLPA	GSEKMEFADS	KSETVLDSID	KSVIYAIESA	VIKWSYQVQV	VLKRESSQPL
250	260	270	280	290	300	310	320
LQGENPTPKV	ELEFWKSRYE	DLKYIYNQLR	TITVRGMAKL	LDKLQSSYFP	AFKAMYRDVV	AALAEAQDIH	VHLIPLQRHL
330	340	350	360	370	380	390	400
EALENAEFPE	VKPQLRPLLH	VVCLIWATCK	SYRSPGRLTV	LLQEICNLLI	QQASNYLSPE	DLLRSEVEES	QRKLQVVSDT
410	420	430	440	450	460	470	480
LSFFKQEFQD	RRENLHTYFK	ENQEVKEWDF	QSSLVFVRLD	GFLGQLHVVE	GLLKTALDFH	KLGKVEFSGV	RGNALSQQVQ
490	500	510	520	530	540	550	560
QMHEEFQEMY	RLLSGSSSDC	LYLQSTDFEN	DVSEFNQKVE	DLDRRLGTIF	IQAFDDAPGL	EHAFKLLDIA	GNLLERPLVA
570	580	590	600	610	620	630	640
RDTSDKYLVL	IQMFNKDLDA	VRMIYSQHVQ	EEAELGFSPV	HKNMPTVAGG	LRWAQELRQR	IQGPFSNFGR	ITHPCMESAE
650	660	670	680	690	700	710	720
GKRMQQKYED	MLSLLEKYET	RLYEDWCRTV	SEKSQYNLSQ	PLLKRDPETK	EITINFNPQL	ISVLKEMSYL	EPREMKHMPE
730	740	750	760	770	780	790	800
TAAAMFSSRD	FYRQLVANLE	LMANWYNKVM	KTLLEVEFPL	VEEELQNIDL	RLRAAEETLN	WKTEGICDYV	TEITSSIHDL
810	820	830	840	850	860	870	880
EQRIQKTKDN	VEEIQNIMKT	WVTPIFKTKD	GKRESLLSLD	DRHDRMEKYY	NLIKESGLKI	HALVQENLGL	FSADPTSNIW
890	900	910	920	930	940	950	960
KTYVNSIDNL	LLNGFFLAIE	CSLKYLLENT	ECKAGLTPIF	EAQLSLAIPE	LVFYPSLESG	VKGGFCDIVE	GLITSIFRIP
970	980	990	1000	1010	1020	1030	1040
SLVPRLSPQN	GSPHYQVDLD	GIPDLANMRR	TLMERVQRMM	GLCCGYQSTF	SQYSYLYVED	RKEVLGQFLL	YGHILTPEEI
1050	1060	1070	1080	1090	1100	1110	1120
EDHVEDGIPE	NPPLLSQFKV	QIDSYETLYE	EVCRLEPIKV	FDGWMKIDIR	PFKASLLNII	KRWSLLFKQH	LVDHVTHSLA
1130	1140	1150	1160	1170	1180	1190	1200
NLDAFIKKSE	SGLLKKVEKG	DFQGLVEIMG	HLMAVKERQS	NTDEMFEPLK	QTIELLKTYE	QELPETVFKQ	LEELPEKWNN
1210	1220	1230	1240	1250	1260	1270	1280
IKKVAITVKQ	QVAPLQANEV	TLLRQRCTAF	DAEQQQFWEQ	FHKEAPFRFD	SIHPHQMLDA	RHIEIQQMES	TMASISESAS
1290	1300	1310	1320	1330	1340	1350	1360
LFEVNVPDYK	QLRQCRKEVC	QLKELWDTIG	MVTSSIHAWE	TTPWRNINVE	AMELECKQFA	RHIRNLDKEV	RAWDAFTGLE
1370	1380	1390	1400	1410	1420	1430	1440
STVWNTLSSL	RAVAELQNPA	IRERHWRQLM	QATGVSFTMD	QDTTLAHLLQ	LQLHHYEDEV	RGIVDKAAKE	MGMEKTLKEL
1450	1460	1470	1480	1490	1500	1510	1520
QTTWAGMEFQ	YEPHPRTNVP	LLCSDEDLIE	VLEDNQVQLQ	NLVMSKYVAF	FLEEVSGWQK	KLSTVDAVIS	IWFEVQRTWT
1530	1540	1550	1560	1570	1580	1590	1600
HLESIFTGSE	DIRAQLPQDS	KRFEGIDIDF	KELAYDAQKI	PNVVQTTNKP	GLYEKLEDIQ	GRLCLCEKAL	AEYLDTKRLA
1610	1620	1630	1640	1650	1660	1670	1680
FPRFYFLSSS	DLLDILSNGT	APQQVQRHLS	KLFDNMAKMR	FQLDASGEPT	KTSLGMYSKE	EEYVAFSEPC	DCSGQVEIWL
1690	1700	1710	1720	1730	1740	1750	1760
NHVLGHMKAT	VRHEMTEGVT	AYEEKPREQW	LFDHPAQVAL	TCTQIWWTTE	VGMAFARLEE	GYESAMKDYY	KKQVAQLKTL
1770	1780	1790	1800	1810	1820	1830	1840
ITMLIGQLSK	GDRQKIMTIC	TIDVHARDVV	AKMIAQKVDN	AQAFLWLSQL	RHRWDDEVKH	CFANICDAQF	LYSYEYLGNT
1850	1860	1870	1880	1890	1900	1910	1920
PRLVITPLTD	RCYITLTQSL	HLTMSGAPAG	PAGTGKTETT	KDLGRALGIL	VYVFNCSEQM	DYKSCGNIYK	GLAQTGAWGC
1930	1940	1950	1960	1970	1980	1990	2000
FDEFNRISVE	VLSVVAVQVK	SIQDAIRDKK	QWFSFLGEEI	SLNPSVGIFI	TMNPGYAGRT	ELPENLKSLF	RPCAMVVPDF
2010	2020	2030	2040	2050	2060	2070	2080
ELICEIMLVA	EGFIEAQSLA	RKFITLYQLC	KELLSKQDHY	DWGLRAIKSV	LVVAGSLKRG	DPDRPEDQVL	MRSLRDFNIP
2090	2100	2110	2120	2130	2140	2150	2160
KIVTDDMPIF	MGLIGDLFPA	LDVPRRRDPN	FEALVRKAIV	DLKLQAEDNF	VLKVVQLEEL	LAVRHSVFVV	GGAGTGKSQV
2170	2180	2190	2200	2210	2220	2230	2240

Detailed Protein Report

Cmpd.	No. of Cmpds.	m/z meas.	Δ m/z [ppm]	z	Rt [min]	Score	P	Range	Sequence	Modification
106	1	1068.1367	75.32	2	30.1	13.8	2	182-201	K.GKTLLPLPAGSEKMEFADSK.S	Oxidation: 14
1912	2	634.8166	-103.44	2	54.3	17.9	0	2134-2144	K.VVQLEELLAVR.H	
1679	5	648.2912	-180.05	2	51.3	17.9	0	4475-4486	K.WVLAGVALLLQI.-	

Detailed Protein Report

Protein 75: pogo transposable element with ZNF domain isoform 3 [Homo sapiens]

Accession: gi|302699211
Database: refseq_human(refseq_human_20140103.fasta)
Database Date: 2015-11-30
Modification(s): Carbamidomethyl, Oxidation

Score: 49.3
MW [kDa]: 145.2
pI: 6.8
Sequence Coverage [%]: 2.8
No. of unique Peptides: 4

Quantitation

QD:QU **Median:** 0.31 **CV:** 0.00 % **No. of Peptides:** 1

10	20	30	40	50	60	70	80
MADTDLFMEC	EEEELEPWQK	ISDVIEDSVV	EDYNSVDKTT	TVSVSQQPVS	APVPIAAHAS	VAGHLSTSTT	VSSSGAQNSD
90	100	110	120	130	140	150	160
STKKTLVTLI	ANNNAPGTQF	VKPTVGVPQV	FSQMTPVRPG	STMPVRPTTN	TFTTVIPATL	TIRSTVPQSQ	SQQTKSTPST
170	180	190	200	210	220	230	240
STTPTATQPT	SLGQLAVQSP	GQSNQTTNPK	LAPSFPSPPA	VSIASFVTVK	RPGVTGENSN	EVAKLVNTLN	TIPSLGQSPG
250	260	270	280	290	300	310	320
PVVVSNNSSA	HGSQRTSGPE	SSMKVTSSIP	VFDLQDGGRK	ICPRCNAQFR	VTEALRGHMC	YCCPEMVEYQ	KKGKSLDSEP
330	340	350	360	370	380	390	400
SVPSAAKPPS	PEKTAPVAST	PSSTPIPALS	PPTKVPEPNE	NVGDAVQTKL	IMLVDDFYYG	RDGGKVAQLT	NFPKVATSFR
410	420	430	440	450	460	470	480
CPHCTKRLKN	NIRFMNHMKH	HVELDQQNGE	VDGHTICQHC	YRQFSTPFQL	QCHLENVHSP	YESTTKCKIC	EWAFESEPLF
490	500	510	520	530	540	550	560
LQHMKDTHKP	GEMPYVCQVC	QYRSSLYSEV	DVHFRMIHED	TRHLLCPYCL	KVFKNGNAFQ	QHYMRHQKRN	VYHCNKCRLQ
570	580	590	600	610	620	630	640
FLFAKDKIEH	KLQHHKTFRK	PKQLEGLKPG	TKVTIRASRG	QPRTVPVSSN	DTPPSALQEA	APLTSSMDPL	PVFLYPPVQR
650	660	670	680	690	700	710	720
SIQKRAVRKM	SVMGRQTCLE	CSFEIPDFPN	HFPTYVHCSL	CRYSTCCSRA	YANHMINNHV	PRKSPKYLAL	FKNSVSGIKL
730	740	750	760	770	780	790	800
ACTSCTFVTS	VGDAMAKHLV	FNPSHRSSSI	LPRGLTWIAH	SRHGQTRDRV	HDRNVKNMYP	PPSFPTNKAA	TVKSAGATPA
810	820	830	840	850	860	870	880
EPEELLTPLA	PALPSPASTA	TPPPTPTHPQ	ALALPPLATE	GAECLNVDDQ	DEGSPVTQEP	ELASGGGGSG	GVGKKEQLSV
890	900	910	920	930	940	950	960
KKLRVVLFAL	CCNTEQAAEH	FRNPQRRIRR	WLRRFQASQG	ENLEGKYLSF	EAEEKLAEWV	LTQREQQLPV	NEETLFQKAT
970	980	990	1000	1010	1020	1030	1040
KIGRSLEGGF	KISYEWAVRF	MLRHHLTPHA	RRAVAHTLPK	DVAENAGLFI	DFVQRQIHNQ	DLPLSMIVAI	DEISLFLDTE
1050	1060	1070	1080	1090	1100	1110	1120
VLSSDDRKEN	ALQTVGTGEP	WCDVVLAILA	DGTVLPTLVF	YRGQMDQPAN	MPDSILLEAK	ESGYSDDEIM	ELWSTRVWQK
1130	1140	1150	1160	1170	1180	1190	1200
HTACQRSKGM	LVMDCHRTHL	SEEVLAMLSA	SSTLPAVVPA	GCSSKIQPLD	VCIKRTVKNF	LHKKWKEQAR	EMADTACDSD
1210	1220	1230	1240	1250	1260	1270	1280
VLLQLVLVWL	GEVLGVIGDC	PELVQRSFLV	ASVLPGPDGN	INSPTRNADM	QEELIASLEE	QLKLSGEHSE	SSTPRPRSSP
1290	1300	1310	1320				
EETIEPESLH	QLFEGESETE	SFYGFEEADL	DLMEI				

Cmpd.	No. of Cmpds.	m/z meas.	Δ m/z [ppm]	z	Rt [min]	Score	P	Range	Sequence	Modification	Ratios
343	1	939.2438	-175.61	1	34.3	13.8	0	256-264	R.TSGPESSMK.V	Oxidation: 8	
240	1	732.8262	-63.03	2	31.7	14.2	1	285-296	R.CNAQFRVTEALR.G	Carbamidomethyl: 1	QD:QU 0.31
2117	1	990.8950	2.39	2	55.0	10.3	1	297-312	R.GHMCYCCPEMVEYQKK.G	Oxidation: 3, 10	
2186	1	1031.8206	-95.59	2	55.9	11.0	1	297-312	R.GHMCYCCPEMVEYQKK.G	Carbamidomethyl: 4, 6	

Detailed Protein Report

Protein 76: **SWI/SNF-related matrix-associated actin-dependent regulator of chromatin subfamily A member 5 [Homo sapiens]**

**Accession:**	gi|325651836	**Score:**	49.0
**Database:**	refseq_human(refseq_human_20140103.fasta)	**MW [kDa]:**	121.8
**Database Date:**	2015-11-30	**pI:**	9.0
**Modification(s):**	Oxidation	**Sequence Coverage [%]:**	3.3
		**No. of unique Peptides:**	2

10	20	30	40	50	60	70	80
MSSAAEPPPP	PPPESAPSKP	AASIASGGSN	SSNKGGPEGV	AAQAVASAAS	AGPADAEMEE	IFDDASPGKQ	KEIQEPDPTY
**90**	**100**	**110**	**120**	**130**	**140**	**150**	**160**
EEKMQTDRAN	RFEYLLKQTE	LFAHFIQPAA	QKTPTSPLKM	KPGRPRIKKD	EKQNLLSVGD	YRHRRTEQEE	DEELLTESSK
**170**	**180**	**190**	**200**	**210**	**220**	**230**	**240**
ATNVCTRFED	SPSYVKWGKL	RDYQVRGLNW	LISLYENGIN	GILADEMGLG	KTLQTISLLG	YMKHYRNIPG	PHMVLVPKST
**250**	**260**	**270**	**280**	**290**	**300**	**310**	**320**
LHNWMSEFKR	WVPTLRSVCL	IGDKEQRAAF	VRDVLLPGEW	DVCVTSYEML	IKEKSVFKKF	NWRYLVIDEA	HRIKNEKSKL
**330**	**340**	**350**	**360**	**370**	**380**	**390**	**400**
SEIVREFKTT	NRLLLTGTPL	QNNLHELWSL	LNFLLPDVFN	SADDFDSWFD	TNNCLGDQKL	VERLHMVLRP	FLLRRIKADV
**410**	**420**	**430**	**440**	**450**	**460**	**470**	**480**
EKSLPPKKEV	KIYVGLSKMQ	REWYTRILMK	DIDILNSAGK	MDKMRLLNIL	MQLRKCCNHP	YLFDGAEPGP	PYTTDMHLVT
**490**	**500**	**510**	**520**	**530**	**540**	**550**	**560**
NSGKMVVLDK	LLPKLKEQGS	RVLIFSQMTR	VLDILEDYCM	WRNYEYCRLD	GQTPHDERQD	SINAYNEPNS	TKFVFMLSTR
**570**	**580**	**590**	**600**	**610**	**620**	**630**	**640**
AGGLGINLAT	ADVVILYDSD	WNPQVDLQAM	DRAHRIGQTK	TVRVFRFITD	NTVEERIVER	AEMKLRLDSI	VIQQGRLVDQ
**650**	**660**	**670**	**680**	**690**	**700**	**710**	**720**
NLNKIGKDEM	LQMIRHGATH	VFASKESEIT	DEDIDGILER	GAKKTAEMNE	KLSKMGESSL	RNFTMDTESS	VYNFEGEDYR
**730**	**740**	**750**	**760**	**770**	**780**	**790**	**800**
EKQKIAFTEW	IEPPKRERKA	NYAVDAYFRE	ALRVSEPKAP	KAPRPPKQPN	VQDFQFFPPR	LFELLEKEIL	FYRKTIGYKV
**810**	**820**	**830**	**840**	**850**	**860**	**870**	**880**
PRNPELPNAA	QAQKEEQLKI	DEAESLNDEE	LEEKEKLLTQ	GFTNWNKRDF	NQFIKANEKW	GRDDIENIAR	EVEGKTPEEV
**890**	**900**	**910**	**920**	**930**	**940**	**950**	**960**
IEYSAVFWER	CNELQDIEKI	MAQIERGEAR	IQRRISIKKA	LDTKIGRYKA	PFHQLRISYG	TNKGKNYTEE	EDRFLICMLH
**970**	**980**	**990**	**1000**	**1010**	**1020**	**1030**	**1040**
KLGFDKENVY	DELRQCIRNS	PQFRFDWFLK	SRTAMELQRR	CNTLITLIER	ENMELEEKEK	AEKKKRGPKP	STQKRKMDGA
**1050**	**1060**						
PDGRGRKKKL	KL						

Cmpd.	No. of Cmpds.	m/z meas.	Δ m/z [ppm]	z	Rt [min]	Score	P	Range	Sequence	Modification
2920	2	710.3768	22.23	2	65.7	14.6	1	431-443	K.DIDILNSAGKMDK.M	
971	1	706.9594	14.52	2	42.2	20.7	2	485-496	K.MVVLDKLLPKLK.E	Oxidation: 1

Detailed Protein Report

Protein 77: keratin, type II cuticular Hb4 [Homo sapiens]

**Accession:**	gi|15431316	**Score:**	48.4
**Database:**	refseq_human(refseq_human_20140103.fasta)	**MW [kDa]:**	64.8
**Database Date:**	2015-11-30	**pI:**	9.0
**Modification(s):**	Carbamidomethyl, Oxidation	**Sequence Coverage [%]:**	5.8
		**No. of unique Peptides:**	2

Quantitation

QD:QU **Median:** 1.02 **CV:** 61.49 % **No. of Peptides:** 2

10	20	30	40	50	60	70	80
MSCRSYRVSS	GHRVGNFSSC	SAMTPQNLNR	FRANSVSCWS	GPGFRGLGSF	GSRSVITFGS	YSPRIAAVGS	RPIHCGVRFG
**90**	**100**	**110**	**120**	**130**	**140**	**150**	**160**
AGCGMGFGDG	RGVGLGPRAD	SCVGLGFGAG	SGIGYGFGGP	GFGYRVGGVG	VPAAPSITAV	TVNKSLLTPL	NLEIDPNAQR
**170**	**180**	**190**	**200**	**210**	**220**	**230**	**240**
VKKDEKEQIK	TLNNKFASFI	DKVRFLEQQN	KLLETKWSFL	QEQKCIRSNL	EPLFESYITN	LRRQLEVLVS	DQARLQAERN
**250**	**260**	**270**	**280**	**290**	**300**	**310**	**320**
HLQDVLEGFK	KKYEEEVVCR	ANAENEFVAL	KKDVDAAFMN	KSDLEANVDT	LTQEIDFLKT	LYMEEIQLLQ	SHISETSVIV
**330**	**340**	**350**	**360**	**370**	**380**	**390**	**400**
KMDNSRDLNL	DGIIAEVKAQ	YEEVARRSRA	DAEAWYQTKY	EEMQVTAGQH	CDNLRNIRNE	INELTRLIQR	LKAEIEHAKA
**410**	**420**	**430**	**440**	**450**	**460**	**470**	**480**
QRAKLEAAVA	EAEQQGEATL	SDAKCKLADL	ECALQQAKQD	MARQLCEYQE	LMNAKLGLDI	EIATYRRLLE	GEESRLCEGV
**490**	**500**	**510**	**520**	**530**	**540**	**550**	**560**
GPVNISVSSS	RGGLVCGPEP	LVAGSTLSRG	GVTFSGSSSV	CATSGVLASC	GPSLGGARVA	PATGDLLSTG	TRSGSMLISE
**570**	**580**	**590**	**600**	**610**			
ACVPSVPCPL	PTQGGFSSCS	GGRSSSVRFV	STTTSCRTKY				

Cmpd.	No. of Cmpds.	m/z meas.	Δ m/z [ppm]	z	Rt [min]	Score	P	Range	Sequence	Modification	Ratios	
535	2	607.3148	1.02	2	36.7	15.6	0	54-64	R.SVITFGSYSPR.I		QD:QU	1.80
816	1	652.6427	-181.17	2	38.7	11.9	0	79-91	R.FGAGCGMGFGDGR.G	Carbamidomethyl: 5; Oxidation: 7	QD:QU	0.58

Detailed Protein Report

Protein 78: PREDICTED: sp110 nuclear body protein isoform X3 [Homo sapiens]

**Accession:**	gi|530370295	**Score:**	48.3
**Database:**	refseq_human(refseq_human_20140103.fasta)	**MW [kDa]:**	58.5
**Database Date:**	2015-11-30	**pI:**	10.2
**Modification(s):**	Oxidation	**Sequence Coverage [%]:**	13.5
		**No. of unique Peptides:**	3

10	20	30	40	50	60	70	80
MNAEEDSEEM	PSLLTSTVQV	ASDNLIPQIR	DKEDPQEMPH	SPLGSMPEIR	DNSPEPNDPE	EPQEVSSTPS	DKKGKKRKRC
**90**	**100**	**110**	**120**	**130**	**140**	**150**	**160**
IWSTPKRRHK	KKSLPGGTAS	SRHGIQKKLK	RVDQVPQKKD	DSTCNSTVET	RAQKARTECA	RKSRSEEIID	GTSEMNEGKR
**170**	**180**	**190**	**200**	**210**	**220**	**230**	**240**
SQKTPSTPRR	VTQGAASPGH	GIQEKLQVVD	KVTQRKDDST	WNSEVMMRVQ	KARTKCARKS	RLKEKKKEKD	ICSSSKRRFQ
**250**	**260**	**270**	**280**	**290**	**300**	**310**	**320**
KNIHRRGKPK	SDTVDFHCSK	LPVTCGEAKG	ILYKKKMKHG	SSVKCIRNED	GTWLTPNEFE	VEGKGRNAKN	WKRNIRCEGM
**330**	**340**	**350**	**360**	**370**	**380**	**390**	**400**
TLGELLKRKN	SDECEVCCQG	GQLLCCGTCP	RVFHEDCHIP	PVEAKRMLWS	CTFCRMKRSS	GSQQCHHVSK	TLERQMQPQD
**410**	**420**	**430**	**440**	**450**	**460**	**470**	**480**
QLKCEFLLLK	AYCHPQSSFF	TGIPFNIRDY	GEPFQEAMWL	DLVKERLITE	MYTVAWFVRD	MRLMFRNHKT	FYKASDFGQV
**490**	**500**	**510**	**520**				
GLDLEAEFEK	DLKDVLGFHE	ANDGGFWTLP					

Cmpd.	No. of Cmpds.	m/z meas.	Δ m/z [ppm]	z	Rt [min]	Score	P	Range	Sequence	Modification
220	1	978.1311	-3.65	3	32.7	10.8	2	279-304	K.HGSSVKCIRNEDGTWLTPNEFEVEGK.G	
1829	1	781.3008	-84.17	2	53.2	11.5	2	366-377	K.RMLWSCTFCRMK.R	
2818	1	989.4652	-45.70	2	64.4	15.5	1	395-410	R.QMQPQDQLKCEFLLLK.A	Oxidation: 2

Detailed Protein Report

Protein 79: PREDICTED: ubiquitin carboxyl-terminal hydrolase 24 isoform X2 [Homo sapiens]

**Accession:**	gi|530362260	**Score:**	48.2
**Database:**	refseq_human(refseq_human_20140103.fasta)	**MW [kDa]:**	228.6
**Database Date:**	2015-11-30	**pI:**	6.0
**Modification(s):**	Carbamidomethyl, Oxidation	**Sequence Coverage [%]:**	2.2
		**No. of unique Peptides:**	2

Quantitation

QD:QU	**Median:** 0.45	**CV:** 0.00 %	**No. of Peptides:** 1

Detailed Protein Report

10	20	30	40	50	60	70	80
MDSRQQGGLT	GDYVSLPGYT	ETKQRSSQLN	NPQFVWVVPA	LRQLHEITRS	FIKQTYQKQD	KSIIQDLKKN	FEIVKLVTGS
90	100	110	120	130	140	150	160
LIACHRLAAA	VAGPGGLSGS	TLVDGRYTYR	EYLEAHLKFL	AFFLQEATLY	LGWNRAKEIW	ECLVTGQDVC	ELDREMCFEW
170	180	190	200	210	220	230	240
FTKGQHDLES	DVQQQLFKEK	ILKLESYEIT	MNGFNLFKTF	FENVNLCDHR	LKRQGAQLYV	EKLELIGMDF	IWKIAMESPD
250	260	270	280	290	300	310	320
EEIANEAIQL	IINYSYINLN	PRLKKDSVSL	HKKFIADCYT	RLEAASSALG	GPTLTHAVTR	ATKMLTATAM	PTVATSVQSP
330	340	350	360	370	380	390	400
YRSTKLVIIE	RLLLLAERYV	ITIEDFYSVP	RTILPHGASF	HGHLLTLNVT	YESTKDTFTV	EAHSNETIGS	VRWKIAKQLC
410	420	430	440	450	460	470	480
SPVDNIQIFT	NDSLLTVNKD	QKLLHQLGFS	DEQILTVKTS	GSGTPSGSSA	DSSTSSSSSS	SGVFSSSYAM	EQEKSLPGVV
490	500	510	520	530	540	550	560
MALVCNVFDM	LYQLANLEEP	RITLRVRKLL	LLIPTDPAIQ	EALDQLDSLG	RKKTLLSESS	SQSSKSPSLS	SKQQHQPSAS
570	580	590	600	610	620	630	640
SILESLFRSF	APGMSTFRVL	YNLEVLSSKL	MPTADDDMAR	SCAKSFCENF	LKAGGLSLVV	NVMQRDSIPS	EVDYETRQGV
650	660	670	680	690	700	710	720
YSICLQLARF	LLVGQTMPTL	LDEDLTKDGI	EALSSRPFRN	VSRQTSRQMS	LCGTPEKSSY	RQLSVSDRSS	IRVEEIIPAA
730	740	750	760	770	780	790	800
RVAIQTMEVS	DFTSTVACFM	RLSWAAAAGR	LDLVGSSQPI	KESNSLCPAG	IRNRLSSSGS	NCSSGSEGEP	VALHAGICVR
810	820	830	840	850	860	870	880
QQSVSTKDSL	IAGEALSLLV	TCLQLRSQQL	ASFYNLPCVA	DFIIDILLGS	PSAEIRRVAC	DQLYTLSQTD	TSAHPDVQKP
890	900	910	920	930	940	950	960
NQFLLGVILT	AQLPLWSPTS	IMRGVNQRLL	SQCMEYFDLR	CQLLDDLTTS	EMEQLRISPA	TMLEDEITWL	DNFEPNRTAE
970	980	990	1000	1010	1020	1030	1040
CETSEADNIL	LAGHLRLIKT	LLSLCGAEKE	MLGSSLIKPL	LDDFLFRASR	IILNSHSPAG	SAAISQQDFH	PKCSTANSRL
1050	1060	1070	1080	1090	1100	1110	1120
AAYEVLVMLA	DSSPSNLQII	IKELLSMHHQ	PDPALTKEFD	YLPPVDSRSS	SGFVGLRNGG	ATCYMNAVFQ	QLYMQPGLPE
1130	1140	1150	1160	1170	1180	1190	1200
SLLSVDDDTD	NPDDSVFYQV	QSLFGHLMES	KLQYYVPENF	WKIFKMWNKE	LYVREQQDAY	EFFTSLIDQM	DEYLKKMGRD
1210	1220	1230	1240	1250	1260	1270	1280
QIFKNTFQGI	YSDQKICKDC	PHRYEREEAF	MALNLGVTSC	QSLEISLDQF	VRGEVLEGSN	AYYCEKCKEK	RITVKRTCIK
1290	1300	1310	1320	1330	1340	1350	1360
SLPSVLVIHL	MRFGFDWESG	RSIKYDEQIR	FPWMLNMEPY	TVSGMARQDS	SSEVGENGRS	VDQGGGGSPR	KKVALTENYE
1370	1380	1390	1400	1410	1420	1430	1440
LVGVIVHSGQ	AHAGHYYSFI	KDRRGCGKGK	WYKFNDTVIE	EFDLNDETLE	YECFGGEYRP	KVYDQTNPYT	DVRRRYWNAY
1450	1460	1470	1480	1490	1500	1510	1520
MLFYQRVSDQ	NSPVLPKKSR	VSVVRQEAED	LSLSAPSSPE	ISPQSSPRPH	RPNNDRLSIL	TKLVKKGEKK	GLFVEKMPAR
1530	1540	1550	1560	1570	1580	1590	1600
IYQMVRDENL	KFMKNRDVYS	SDYFSFVLSL	ASLNATKLKH	PYYPCMAKVS	LQLAIQFLFQ	TYLRTKKKLR	VDTEEWIATI
1610	1620	1630	1640	1650	1660	1670	1680
EALLSKSFDA	CQWLVEYFIS	SEGRELIKIF	LLECNVREVR	VAVATILEKT	LDSALFYQDK	LKSLHQLLEV	LLALLDKDVP
1690	1700	1710	1720	1730	1740	1750	1760
ENCKNCAQYF	FLFNTFVQKQ	GIRAGDLLLR	HSALRHMISF	LLGASRQNNQ	IRRWSSAQAR	EFGNLHNTVA	LLVLHSDVSS
1770	1780	1790	1800	1810	1820	1830	1840
QRNVAPGIFK	QRPPISIAPS	SPLLPLHEEV	EALLFMSEGK	PYLLEVMFAL	RELTGSLLAL	IEMVVYCCFC	NEHFSFTMLH
1850	1860	1870	1880	1890	1900	1910	1920
FIKNQLETAP	PHELKNTFQL	LHEILVIEDP	IQVERVKFVF	ETENGLLALM	HHSNHVDSSR	CYQCVKFLVT	LAQKCPAAKE
1930	1940	1950	1960	1970	1980	1990	2000
YFKENSHHWS	WAVQWLQKKM	SEHYWTPQSN	VSNETSTGKT	FQRTISAQDT	LAYATALLNE	KEQSGSSNGS	ESSPANENGD
2010	2020	2030					
RHLQQGSESP	MMIGELRSDL	DDVDP					

Cmpd.	No. of Cmpds.	m/z meas.	Δ m/z [ppm]	z	Rt [min]	Score	P	Range	Sequence	Modification	Ratios
226	1	849.2201	-174.43	2	31.6	10.1	1	590-604	K.LMPTADDDMARSCAK.S	Carbamidomethyl: 13; Oxidation: 2	QD:QU 0.45
2168	1	841.4140	51.38	2	57.5	11.5	1	590-604	K.LMPTADDDMARSCAK.S	Carbamidomethyl: 13	

Detailed Protein Report

Protein 80: PREDICTED: obscurin isoform X19 [Homo sapiens]

**Accession:**	gi|530366567	**Score:**	48.1
**Database:**	refseq_human(refseq_human_20140103.fasta)	**MW [kDa]:**	945.1
**Database Date:**	2015-11-30	**pI:**	5.6
**Modification(s):**	Carbamidomethyl	**Sequence Coverage [%]:**	0.5
		**No. of unique Peptides:**	3

Quantitation

QD:QU	**Median:** 1.75	**CV:** 0.00 %	**No. of Peptides:** 1

10	20	30	40	50	60	70	80
MDQPQFSGAP	RFLTRPKAFV	VSVGKDATLS	CQIVGNPTPQ	VSWEKDQQPV	AAGARFRLAQ	DGDLYRLTIL	DLALGDSGQY
90	100	110	120	130	140	150	160
VCRARNAIGE	AFAAVGLQVD	AEAACAEQAP	HFLLRPTSIR	VREGSEATFR	CRVGGSPRPA	VSWSKDGRRL	GEPDGPRVRV
170	180	190	200	210	220	230	240
EELGEASALR	IRAARPRDGG	TYEVRAENPL	GAASAAAALV	VDSDAADTAS	RPGTSTAALL	AHLQRRREAM	RAEGAPASPP
250	260	270	280	290	300	310	320
STGTRTCTVT	EGKHARLSCY	VTGEPKPETV	WKKDGQLVTE	GRRHVVYEDA	QENFVLKILF	CKQSDRGLYT	CTASNLVGQT
330	340	350	360	370	380	390	400
YSSVLVVVRE	PAVPFKKRLQ	DLEVREKESA	TFLCEVPQPS	TEAAWFKEET	RLWASAKYGI	EEEGTERRLT	VRNVSADDDA
410	420	430	440	450	460	470	480
VYICETPEGS	RTVAELAVQG	NLLRKLPRKT	AVRVGDTAMF	CVELAVPVGP	VHWLRNQEEV	VAGGRVAISA	EGTRHTLTIS
490	500	510	520	530	540	550	560
QCCLEDVGQV	AFMAGDCQTS	TQFCVSAPRK	PPLQPPVDPV	VKARMESSVI	LSWSPPPHGE	RPVTIDGYLV	EKKKLGTYTW
570	580	590	600	610	620	630	640
IRCHEAEWVA	TPELTVADVA	EEGNFQFRVS	ALNSFGQSPY	LEFPGTVHLA	PKLAVRTPLK	AVQAVEGGEV	TFSVDLTVAS
650	660	670	680	690	700	710	720
AGEWFLDGQA	LKASSVYEIH	CDRTRHTLTI	REVPASLHGA	QLKFVANGIE	SSIRMEVRAA	PGLTANKPPA	AAAREVLARL
730	740	750	760	770	780	790	800
HEEAQLLAEL	SDQAAAVTWL	KDGRTLSPGP	KYEVQASAGR	RVLLVRDVAR	DDAGLYECVS	RGGRIAYQLS	VQGLARFLHK
810	820	830	840	850	860	870	880
DMAGSCVDAV	AGGPAQFECE	TSEAHVHVHW	YKDGMELGHS	GERFLQEDVG	TRHRLVAATV	TRQDEGTYSC	RVGEDSVDFR
890	900	910	920	930	940	950	960
LRVSEPKAVF	AKEQPACREV	QAEVGASATL	SCEVAQDQME	VTWYKDGKKL	SSSSKVHVEA	VGCMRRLVVQ	QVGQADSGEY
970	980	990	1000	1010	1020	1030	1040
SCEARGQRVS	FRLDVAEPKV	VFAKEQLARR	KLQAEAGASA	TLSCEVAQAQ	TEVTWYKDGK	KLSSSSKVCM	EATGCTRRLV
1050	1060	1070	1080	1090	1100	1110	1120
VQQAGQADAG	EYSCEAGGQR	LSFHLDVKEP	KVVFAKDQVA	HSEVQAEAGA	SATLSCEVAQ	AQTEVMWYKD	GKKLSSSLKV
1130	1140	1150	1160	1170	1180	1190	1200
HVEAKGCRRR	LVVQQAGKTD	AGDYSCEARG	QRVSFRLHIT	EPKMMFAKEQ	SVHNEVQAEA	GASAMLSCEV	AQAQTEVTWY
1210	1220	1230	1240	1250	1260	1270	1280
KDGKKLSSSS	KVGMEVKGCT	RRLVLPQAGK	ADAGEYSCEA	GGQRVSFHLH	ITEPKGVFAK	EQSVHNEVQA	EAGTTAMLSC
1290	1300	1310	1320	1330	1340	1350	1360
EVAQPQTEVT	WYKDGKKLSS	SSKVRMEVKG	CTRRLVVQQV	GKADAGEYSC	EAGGQRVSFQ	LHITEPKAVF	AKEQLVHNEV
1370	1380	1390	1400	1410	1420	1430	1440
RTEAGASATL	SCEVAQAQTE	VTWYKDGKKL	SSSSKVRIEA	AGCMRQLVVQ	QAGQADAGEY	TCEAGGQRLS	FHLDVSEPKA
1450	1460	1470	1480	1490	1500	1510	1520
VFAKEQLAHR	KVQAEAGAIA	TLSCEVAQAQ	TEVTWYKDGK	KLSSSSKVRM	EAVGCTRRLV	VQQACQADTG	EYSCEAGGQR
1530	1540	1550	1560	1570	1580	1590	1600
LSFSLDVAEP	KVVFAKEQPV	HREVQAQAGA	STTLSCEVAQ	AQTEVMWYKD	GKKLSFSSKV	RMEAVGCTRR	LVVQQAGQAV
1610	1620	1630	1640	1650	1660	1670	1680
AGEYSCEAGS	QRLSFHLHVA	EPKAVFAKEQ	PASREVQAEA	GTSATLSCEV	AQAQTEVTWY	KDGKKLSSSS	KVRMEAVGCT
1690	1700	1710	1720	1730	1740	1750	1760
RRLVVQEAGQ	ADAGEYSCKA	GDQRLSFHLH	VAEPKVVFAK	EQPAHREVQA	EAGASATLSC	EVAQAQTEVT	WYKDGKKLSS
1770	1780	1790	1800	1810	1820	1830	1840
SSKVRVEAVG	CTRRLVVQQA	GQAEAGEYSC	EAGGQQLSFR	LQVAEPKAVF	AKEQVVFAKD	QPVHREVQAE	AGTSTMLSCE
1850	1860	1870	1880	1890	1900	1910	1920
VAQAQTEVMW	YKDGKKLSSS	SKMRVEAVGC	TRRLVVQEAG	QADAGEYSCE	AGGQRLSFHL	HVAEPKVVFA	KEQPACREVQ
1930	1940	1950	1960	1970	1980	1990	2000
AEAGASATLS	CEVAQGQMEV	TWYKDGKKLS	SSSKVHMEAS	GYTRRLVVQQ	AGQADAGEYS	CEAGGQRLSF	RLHVAELEPQ
2010	2020	2030	2040	2050	2060	2070	2080
ISERPCRREP	LVVKEHEDII	LTATLATPSA	ATVTWLKDGV	EIRRSKRHET	ASQGDTHTLT	VHGAQVLDSA	IYSCRVGAEG
2090	2100	2110	2120	2130	2140	2150	2160
QDFPVQVEEV	AAKFCRLLEP	VCGELGGTVT	LACELSPACA	EVVWRCGNTQ	LRVGKRFQMV	AEGPVRSLTV	LGLRAEDAGE
2170	2180	2190	2200	2210	2220	2230	2240

Detailed Protein Report

Cmpd.	No. of Cmpds.	m/z meas.	Δ m/z [ppm]	z	Rt [min]	Score	P	Range	Sequence	Modification	Ratios
961	1	512.1292	-217.77	2	42.1	10.5	0	872-880	R.VGEDSVDFR.L		QD:QU 1.75
2890	1	922.9785	45.19	2	65.3	10.6	2	1021-1037	K.KLSSSSKVCMEATGCTR.R	Carbamidomethyl: 15	
2727	1	846.9286	-83.14	2	65.2	13.0	2	8400-8415	K.ASGRALAAKIIPYHPK.D		

Detailed Protein Report

Protein 81: PREDICTED: dmX-like protein 2 isoform X3 [Homo sapiens]

**Accession:**	gi|530405585	**Score:**	47.9
**Database:**	refseq_human(refseq_human_20140103.fasta)	**MW [kDa]:**	291.5
**Database Date:**	2015-11-30	**pI:**	5.9
**Modification(s):**	Carbamidomethyl, Oxidation	**Sequence Coverage [%]:**	1.8
		**No. of unique Peptides:**	2

Detailed Protein Report

10	20	30	40	50	60	70	80
MHLHQVLTGA	VNPGDNCYSV	GSVGDVPFTA	YGSGCDIVIL	ANDFECVQII	PGAKHGNIQV	SCVECSNQQG	RIAASYGNAV
90	100	110	120	130	140	150	160
CIFEPLGINS	HKRNCQLKCQ	WLKTGQFFLS	SVTYNLAWDP	QDNRLLTATD	SIQLWAPPGD	DILEEEEEID	NTVPPVLNDW
170	180	190	200	210	220	230	240
KCVWQCKTSV	SVHLMEWSPD	GEYFATAGKD	DCLLKVWYPM	TGWKSSIIPQ	DHHEVKRRQS	STQFSFVYLA	HPRAVTGFSW
250	260	270	280	290	300	310	320
RKTSKYMPRG	SVCNVLLTSC	HDGVCRLWAE	TLLPEDCLLG	EQICETTTSS	IASSLSHAGR	HKDRIQHALE	TIHHLKNLRK
330	340	350	360	370	380	390	400
GQRRSSVLVT	HAELMPDQTA	MHEVQRHISH	HANALCHFHI	AASINPATDI	PNVLVGTAFN	VDDGNGGFVV	HWLNNKEFHF
410	420	430	440	450	460	470	480
TSSTEVFMHQ	LRKLSDKQVD	HENDDADRED	EEHSQEDRER	GLHMKLDHDL	SLDRESEAGT	GSSEHEDGER	EGSPRTYSRL
490	500	510	520	530	540	550	560
SVPMPLPTVL	LDRKIETLLT	EWNKNPDMLF	TIHPVDGTFL	VWHVKYLDEY	NPGIFRQVQV	SFSSRIPVAF	PSGDASSLSK
570	580	590	600	610	620	630	640
NIMMYACINA	TKDSHHTLLH	QEGMSVGSPH	GSQPHSRSHS	THMNILAPTV	MMISKHIDGS	LNQWAVTFAD	KSAFTTVLTV
650	660	670	680	690	700	710	720
SHKFRYCGHR	FHLNDLACHS	VLPLLLTSSH	HNALLTPELD	CQWDSDNKLS	RLMDPVKHIK	GSSKQPLRNA	ATRTFHDPNA
730	740	750	760	770	780	790	800
IYSELILWRV	DPIGPLSYTG	GVSELARINS	LHTSAFSNVA	WLPTLIPSYC	LGTYCNSASA	CFVASDGKNL	RLYQAVVDAR
810	820	830	840	850	860	870	880
KLLDELSDPE	SSKLIGEVFN	IVSQQSTARP	GCIIELDAIT	NQCGSNTQLL	HVFQEDFIIG	YKPHKEDMEK	KETEIFFQPS
890	900	910	920	930	940	950	960
QGYRPPPFSE	KFFLVVIEKD	SNNNSILHMW	HLHLKSVQAC	LAKASEGASS	ESLLSVPGQK	NVDSSPETSP	SVSPMPHSSS
970	980	990	1000	1010	1020	1030	1040
IANLQTASKL	ILSSRLVYSQ	PLDLPESVEV	IRATPSAGHL	SSSSIYPVCL	APYLVVTTCS	DNKVRFWKCC	MEANPECNKS
1050	1060	1070	1080	1090	1100	1110	1120
DEKEIYHWKR	WPLMNDEGED	NSSTVSIVGR	PVAVSCSYTG	RLAVAYKQPI	HHNGFVSKEF	SMHVCIFECE	STGGSEWVLE
1130	1140	1150	1160	1170	1180	1190	1200
QTIHLDDLVK	VGSVLDSRVS	VDSNLFVYSK	SDALLSKDRY	LIPNIKHLVH	LDWVSKEDGS	HILTVGVGAN	IFMYGRLSGI
1210	1220	1230	1240	1250	1260	1270	1280
VTEQTNSKDG	VAVITLPLGG	SIKQGVKSRW	VLLRSIDLVS	SVDGTPSLPV	SLSWVRDGIL	VVGMDCEMHV	YAQWKHAVKF
1290	1300	1310	1320	1330	1340	1350	1360
GDTEADSSNA	EEAAMQDHST	FKSNMLARKS	VVEGTAISDD	VFCSPTVIQD	GGLFEAAHVL	SPTLPQYHPT	QLLELMDLGK
1370	1380	1390	1400	1410	1420	1430	1440
VRRAKAILSH	LVKCIAGEVA	IVRDPDAGEG	TKRHLSRTIS	VSGSTAKETV	TVGKDGTRDY	TEIDSIPPLP	LYALLAADQD
1450	1460	1470	1480	1490	1500	1510	1520
TSYRISEEST	KIPQSYEDQT	VSQPEDQYSE	LFQIQDIPTD	DIDLEPEKRE	NKSKVINLSQ	YGPAYFGQEH	ARVLSSHLMH
1530	1540	1550	1560	1570	1580	1590	1600
SSLPGLTRLE	QMFLVALADT	VATTSTELDE	SRDKSCSGRD	TLDECGLRYL	LAMRLHTCLL	TSLPPLYRVQ	LLHQGVSTCH
1610	1620	1630	1640	1650	1660	1670	1680
FAWAFHSEAE	EELINMIPAI	QRGDPQWSEL	RAMGIGWWVR	NINTLRRCIE	KVAKASFQRN	NDALDAALFY	LSMKKKAVVW
1690	1700	1710	1720	1730	1740	1750	1760
GLFRSQHDEK	MTTFFSHNFN	EDRWRKAALK	NAFSLLGKQR	FEQSAAFFLL	AGSLKDAIEV	CLEKMEDIQL	AMVIARLYES
1770	1780	1790	1800	1810	1820	1830	1840
EFETSSTYIS	ILNQKILGCQ	KDGSGFSCKR	LHPDPFLRSL	AYWVMKDYTR	ALDTLLEQTP	KEDDEHQVII	KSCNPVAFSF
1850	1860	1870	1880	1890	1900	1910	1920
YNYLRTHPLL	IRRNLASPEG	TLATLGLKTE	KNFVDKINLI	ERKLFFTTAN	AHFKVGCPVL	ALEVLSKIPK	VTKTSALSAK
1930	1940	1950	1960	1970	1980	1990	2000
KDQPDFISHR	MDDVPSHSKA	LSDGNGSSGI	EWSNVTSSQY	DWSQPIVKVD	EEPLNLDWGE	DHDSALDEEE	DDAVGLVMKS
2010	2020	2030	2040	2050	2060	2070	2080
TDAREKDKQS	DQKASDPNML	LTPQEEDDPE	GDTEVDVIAE	QLKFRACLKI	LMTELRTLAT	GYEVDGGKLR	FQLYNWLEKE
2090	2100	2110	2120	2130	2140	2150	2160
IAALHEICNH	ESVIKEYSSK	TYSKVESDLL	DQEEMVDKPD	IGSYERHQIE	RRRLQAKREH	AERRKSWLQK	NQDLLRVFLS
2170	2180	2190	2200	2210	2220	2230	2240

Detailed Protein Report

Cmpd.	No. of Cmpds.	m/z meas.	Δ m/z [ppm]	z	Rt [min]	Score	P	Range	Sequence	Modification
1518	1	816.3814	-111.76	2	49.2	16.9	2	2557-2569	R.SFKRHLLDIFSLR.K	
1839	1	780.2904	-91.58	2	51.5	14.1	1	2574-2588	R.VSCSSHRGVPGGMGR.K	Carbamidomethyl: 3; Oxidation: 13

Detailed Protein Report

Protein 82: A-kinase anchor protein 9 isoform 3 [Homo sapiens]

**Accession:**	gi|22538393	**Score:**	47.7
**Database:**	refseq_human(refseq_human_20140103.fasta)	**MW [kDa]:**	451.8
**Database Date:**	2015-11-30	**pI:**	4.8
		**Sequence Coverage [%]:**	1.1
		**No. of unique Peptides:**	2

Detailed Protein Report

10	20	30	40	50	60	70	80
MEDEERQKKL	EAGKAKLAQF	RQRKAQSDGQ	SPSKKQKKKR	KTSSSKHDVS	AHHDLNIDQS	QCNEMYINSS	QRVESTVIPE
90	100	110	120	130	140	150	160
STIMRTLHSG	EITSHEQGFS	VELESEISTT	ADDCSSEVNG	CSFVMRTGKP	TNLLREEEFG	VDDSYSEQGA	QDSPTHLEMM
170	180	190	200	210	220	230	240
ESELAGKQHE	IEELNRELEE	MRVTYGTEGL	QQLQEFEAAI	KQRDGIITQL	TANLQQARRE	KDETMREFLE	LTEQSQKLQI
250	260	270	280	290	300	310	320
QFQQLQASET	LRNSTHSSTA	ADLLQAKQQI	LTHQQQLEEQ	DHLLEDYQKK	KEDFTMQISF	LQEKIKVYEM	EQDKKVENSN
330	340	350	360	370	380	390	400
KEEIQEKETI	IEELNTKIIE	EEKKTLELKD	KLTTADKLLG	ELQEQIVQKN	QEIKNMKLEL	TNSKQKERQS	SEEIKQLMGT
410	420	430	440	450	460	470	480
VEELQKRNHK	DSQFETDIVQ	RMEQETQRKL	EQLRAELDEM	YGQQIVQMKQ	ELIRQHMAQM	EEMKTRHKGE	MENALRSYSN
490	500	510	520	530	540	550	560
ITVNEDQIKL	MNVAINELNI	KLQDTNSQKE	KLKEELGLIL	EEKCALQRQL	EDLVEELSFS	REQIQRARQT	IAEQESKLNE
570	580	590	600	610	620	630	640
AHKSLSTVED	LKAEIVSASE	SRKELELKHE	AEVTNYKIKL	EMLEKEKNAV	LDRMAESQEA	ELERLRTQLL	FSHEEELSKL
650	660	670	680	690	700	710	720
KEDLEIEHRI	NIEKLKDNLG	IHYKQQIDGL	QNEMSQKIET	MQFEKDNLIT	KQNQLILEIS	KLKDLQQSLV	NSKSEEMTLQ
730	740	750	760	770	780	790	800
INELQKEIEI	LRQEEKEKGT	LEQEVQELQL	KTELLEKQMK	EKENDLQEKF	AQLEAENSIL	KDEKKTLEDM	LKIHTPVSQE
810	820	830	840	850	860	870	880
ERLIFLDSIK	SKSKDSVWEK	EIEILIEENE	DLKQQCIQLN	EEIEKQRNTF	SFAEKNFEVN	YQELQEEYAC	LLKVKDDLED
890	900	910	920	930	940	950	960
SKNKQELEYK	SKLKALNEEL	HLQRINPTTV	KMKSSVFDED	KTFVAETLEM	GEVVEKDTTE	LMEKLEVTKR	EKLELSQRLS
970	980	990	1000	1010	1020	1030	1040
DLSEQLKQKH	GEISFLNEEV	KSLKQKEKQV	SLRCRELEII	INHNRAENVQ	SCDTQVSSLL	DGVVTMTSRG	AEGSVSKVNK
1050	1060	1070	1080	1090	1100	1110	1120
SFGEESKIMV	EDKVSFENMT	VGEESKQEQL	ILDHLPSVTK	ESSLRATQPS	ENDKLQKELN	VLKSEQNDLR	LQMEAQRICL
1130	1140	1150	1160	1170	1180	1190	1200
SLVYSTHVDQ	VREYMENEKD	KALCSLKEEL	IFAQEEKIKE	LQKIHQLELQ	TMKTQETGDE	GKPLHLLIGK	LQKAVSEECS
1210	1220	1230	1240	1250	1260	1270	1280
YFLQTLCSVL	GEYYTPALKC	EVNAEDKENS	GDYISENEDP	ELQDYRYEVQ	DFQENMHTLL	NKVTEEYNKL	LVLQTRLSKI
1290	1300	1310	1320	1330	1340	1350	1360
WGQQTDGMKL	EFGEENLPKE	ETEFLSIHSQ	MTNLEDIDVN	HKSKLSSLQD	LEKTKLEEQV	QELESLISSL	QQQLKETEQN
1370	1380	1390	1400	1410	1420	1430	1440
YEAEIHCLQK	RLQAVSESTV	PPSLPVDSVV	ITESDAQRTM	YPGSCVKKNI	DGTIEFSGEF	GVKEETNIVK	LLEKQYQEQL
1450	1460	1470	1480	1490	1500	1510	1520
EEEVAKVIVS	MSIAFAQQTE	LSRISGGKEN	TASSKQAHAV	CQQEQHYFNE	MKLSQDQIGF	QTFETVDVKF	KEEFKPLSKE
1530	1540	1550	1560	1570	1580	1590	1600
LGEHGKEILL	SNSDPHDIPE	SKDCVLTISE	EMFSKDKTFI	VRQSIHDEIS	VSSMDASRQL	MLNEEQLEDM	RQELVRQYQE
1610	1620	1630	1640	1650	1660	1670	1680
HQQATELLRQ	AHMRQMERQR	EDQEQLQEEI	KRLNRQLAQR	SSIDNENLVS	ERERVLLEEL	EALKQLSLAG	REKLCCELRN
1690	1700	1710	1720	1730	1740	1750	1760
SSTQTQNGNE	NQGEVEEQTF	KEKELDRKPE	DVPPEILSNE	RYALQKANNR	LLKILLEVVK	TTAAVEETIG	RHVLGILDRS
1770	1780	1790	1800	1810	1820	1830	1840
SKSQSSASLI	WRSEAEASVK	SCVHEEHTRV	TDESIPSYSG	SDMPRNDINM	WSKVTEEGTE	LSQRLVRSGF	AGTEIDPENE
1850	1860	1870	1880	1890	1900	1910	1920
ELMLNISSRL	QAAVEKLLEA	ISETSSQLEH	AKVTQTELMR	ESFRQKQEAT	ESLKCQEELR	ERLHEESRAR	EQLAVELSKA
1930	1940	1950	1960	1970	1980	1990	2000
EGVIDGYADE	KTLFERQIQE	KTDIIDRLEQ	ELLCASNRLQ	ELEAEQQQIQ	EERELLSRQK	EAMKAEAGPV	EQQLLQETEK
2010	2020	2030	2040	2050	2060	2070	2080
LMKEKLEVQC	QAEKVRDDLQ	KQVKALEIDV	EEQVSRFIEL	EQEKNTELMD	LRQQNQALEK	QLEKMRKFLD	EQAIDREHER
2090	2100	2110	2120	2130	2140	2150	2160
DVFQQEIQKL	EQQLKVVPRF	QPISEHQTRE	VEQLANHLKE	KTDKCSELLL	SKEQLQRDIQ	ERNEEIEKLE	FRVRELEQAL
2170	2180	2190	2200	2210	2220	2230	2240

Detailed Protein Report

Cmpd.	No. of Cmpds.	m/z meas.	Δ m/z [ppm]	z	Rt [min]	Score	P	Range	Sequence	Modification
562	1	884.4457	-18.56	2	37.0	21.3	0	3081-3096	R.SLLSEIQALHAQMNGR.K	
49	1	819.8727	-87.95	2	29.3	12.5	1	3777-3790	R.WHRVTGSVSININR.D	

Detailed Protein Report

Protein 83: PREDICTED: kinesin-like protein KIF23 isoform X8 [Homo sapiens]

**Accession:**	gi|578827640	**Score:**	47.1
**Database:**	refseq_human(refseq_human_20140103.fasta)	**MW [kDa]:**	56.1
**Database Date:**	2015-11-30	**pI:**	9.9
		**Sequence Coverage [%]:**	14.0
		**No. of unique Peptides:**	3

10	20	30	40	50	60	70	80
MSFPPLPSCE	ILDINDEQTL	PRLIEALEKR	HNLRQMMIDE	FNKQSNAFKA	LLQEFDNAVL	SKENHMQGKL	NEKEKMISGQ
**90**	**100**	**110**	**120**	**130**	**140**	**150**	**160**
KLEIERLEKK	NKTLEYKIEI	LEKTTTIYEE	DKRNLQQELE	TQNQKLQRQF	SDKRRLEARL	QGMVTETTMK	WEKECERRVA
**170**	**180**	**190**	**200**	**210**	**220**	**230**	**240**
AKQLEMQNKL	WVKDEKLKQL	KAIVTEPKTE	KPERPSRERD	REKVTQRSVS	PSPVPLSSNY	IAQISNGQQL	MSQPQLHRRS
**250**	**260**	**270**	**280**	**290**	**300**	**310**	**320**
NSCSSISVAS	CISEWEQKIP	TYNTPLKVTS	IARRRQQEPG	QSKTCIVSDR	RRGMYWTEGR	EVVPTFRNEI	EIEEDHCGRL
**330**	**340**	**350**	**360**	**370**	**380**	**390**	**400**
LFQPDQNAPP	IRLRHRRSRS	AGDRWVDHKP	ASNMQTETVM	QPHVPHAITV	SVANEKALAK	CEKYMLTHQE	LASDGEIETK
**410**	**420**	**430**	**440**	**450**	**460**	**470**	**480**
LIKGDIYKTR	GGGQSVQFTD	IETLKQESPN	GSRKRRSSTV	APAQPDGAES	EWTDVETRCS	VAVEMRAGSQ	LGPGYQHHAQ
**490**							
PKRKKP							

Cmpd.	No. of Cmpds.	m/z meas.	Δ m/z [ppm]	z	Rt [min]	Score	P	Range	Sequence	Modification
1489	2	1023.0105	58.00	2	48.8	13.4	0	240-258	R.SNSCSSISVASCISEWEQK.I	
1786	1	916.3119	-158.99	2	52.7	11.0	2	275-290	R.RQQEPGQSKTCIVSDR.R	
243	1	838.3874	-37.26	2	31.8	11.3	0	467-482	R.AGSQLGPGYQHHAQPK.R	

Detailed Protein Report

Protein 84: semaphorin-5B isoform 3 precursor [Homo sapiens]

Accession: gi|373432662
Database: refseq_human(refseq_human_20140103.fasta)
Database Date: 2015-11-30
Modification(s): Carbamidomethyl

Score: 47.0
MW [kDa]: 116.0
pI: 9.0
Sequence Coverage [%]: 4.0
No. of unique Peptides: 1

10	20	30	40	50	60	70	80
MVLAGPLAVS	LLLPSLTLLV	SHLSSSQDVS	SEPSSEQQLC	ALSKHPTVAF	EDLQPWVSNF	TYPGARDFSQ	LALDPSGNQL
90	100	110	120	130	140	150	160
IVGARNYLFR	LSLANVSLLQ	ATEWASSEDT	RRSCQSKGKT	EEECQNYVRV	LIVAGRKVFM	CGTNAFSPMC	TSRQVGNLSR
170	180	190	200	210	220	230	240
TIEKINGVAR	CPYDPRHNST	AVISSQGELY	AATVIDFSGR	DPAIYRSLGS	GPPLRTAQYN	SKWLNEPNFV	AAYDIGLFAY
250	260	270	280	290	300	310	320
FFLRENAVEH	DCGRTVYSRV	ARVCKNDVGG	RFLLEDTWTT	FMKARLNCSR	PGEVPFYYNE	LQSAFHLPEQ	DLIYGVFTTN
330	340	350	360	370	380	390	400
VNSIAASAVC	AFNLSAISQA	FNGPFRYQEN	PRAAWLPIAN	PIPNFQCGTL	PETGPNENLT	ERSLQDAQRL	FLMSEAVQPV
410	420	430	440	450	460	470	480
TPEPCVTQDS	VRFSHLVVDL	VQAKDTLYHV	LYIGTESGTI	LKALSTASRS	LHGCYLEELH	VLPPGRREPL	RSLRILHSAR
490	500	510	520	530	540	550	560
ALFVGLRDGV	LRVPLERCAA	YRSQGACLGA	RDPYCGWDGK	QQRCSTLEDS	SNMSLWTQNI	TACPVRNVTR	DGGFGPWSPW
570	580	590	600	610	620	630	640
QPCEHLDGDN	SGSCLCRARS	CDSPRPRCGG	LDCLGPAIHI	ANCSRNGAWT	PWSSWALCST	SCGIGFQVRQ	RSCSNPAPRH
650	660	670	680	690	700	710	720
GGRICVGKSR	EERFCNENTP	CPVPIFWASW	GSWSKCSSNC	GGGMQSRRRA	CENGNSCLGC	GVEFKTCNPE	GCPEVRRNTP
730	740	750	760	770	780	790	800
WTPWLPVNVT	QGGARQEQRF	RFTCRAPLAD	PHGLQFGRRR	TETRTCPADG	SGSCDTDALV	EVLLRSGSTS	PHTVSGGWAA
810	820	830	840	850	860	870	880
WGPWSSCSRD	CELGFRVRKR	TCTNPEPRNG	GLPCVGDAAE	YQDCNPQACP	GEDICLGLHT	EEALCATQAC	PEGWSPWSEW
890	900	910	920	930	940	950	960
SKCTDDGAQS	RSRHCEELLP	GSSACAGNSS	QSRPCPYSEI	PVILPASSME	EATDCAGFNL	IHLVATGISC	FLGSGLLTLA
970	980	990	1000	1010	1020	1030	1040
VYLSCQHCQR	QSQESTLVHP	ATPNHLHYKG	GGTPKNEKYT	PMEFKTLNKN	NLIPDDRANF	YPLQQTNVYT	TTYYPSPLNK
1050	1060						
HSFRPEASPG	QRCFPNS						

Cmpd.	No. of Cmpds.	m/z meas.	Δ m/z [ppm]	z	Rt [min]	Score	P	Range	Sequence	Modification
2242	2	682.6902	75.49	3	58.4	22.5	2	113-129	R.SCQSKGKTEEECQNYVR.V	Carbamidomethyl: 2

Detailed Protein Report

Protein 85: probable helicase senataxin [Homo sapiens]

**Accession:**	gi|113722133	**Score:**	47.0
**Database:**	refseq_human(refseq_human_20140103.fasta)	**MW [kDa]:**	302.7
**Database Date:**	2015-11-30	**pI:**	6.9
**Modification(s):**	Carbamidomethyl, Oxidation	**Sequence Coverage [%]:**	1.8
		**No. of unique Peptides:**	3

Detailed Protein Report

10	20	30	40	50	60	70	80
MSTCCWCTPG	GASTIDFLKR	YASNTPSGEF	QTADEDLCYC	LECVAEYHKA	RDELPFLHEV	LWELETLRLI	NHFEKSMKAE
90	100	110	120	130	140	150	160
IGDDDELYIV	DNNGEMPLFD	ITGQDFENKL	RVPLLEILKY	PYLLLHERVN	ELCVEALCRM	EQANCSFQVF	DKHPGIYLFL
170	180	190	200	210	220	230	240
VHPNEMVRRW	AILTARNLGK	VDRDDYYDLQ	EVLLCLFKVI	ELGLLESPDI	YTSSVLEKGK	LILLPSHMYD	TTNYKSYWLG
250	260	270	280	290	300	310	320
ICMLLTILEE	QAMDSLLLGS	DKQNDFMQSI	LHTMEREADD	DSVDPFWPAL	HCFMVILDRL	GSKVWGQLMD	PIVAFQTIIN
330	340	350	360	370	380	390	400
NASYNREIRH	IRNSSVRTKL	EPESYLDDMV	TCSQIVYNYN	PEKTKKDSGW	RTAICPDYCP	NMYEEMETLA	SVLQSDIGQD
410	420	430	440	450	460	470	480
MRVHNSTFLW	FIPFVQSLMD	LKDLGVAYIA	QVVNHLYSEV	KEVLNQTDAV	CDKVTEFFLL	ILVSVIELHR	NKKCLHLLWV
490	500	510	520	530	540	550	560
SSQQWVEAVV	KCAKLPTTAF	TRSSEKSSGN	CSKGTAMISS	LSLHSMPSNS	VQLAYVQLIR	SLLKEGYQLG	QQSLCKRFWD
570	580	590	600	610	620	630	640
KLNLFLRGNL	SLGWQLTSQE	THELQSCLKQ	IIRNIKFKAP	PCNTFVDLTS	ACKISPASYN	KEESEQMGKT	SRKDMHCLEA
650	660	670	680	690	700	710	720
SSPTFSKEPM	KVQDSVLIKA	DNTIEGDNNE	QNYIKDVKLE	DHLLAGSCLK	QSSKNIFTER	AEDQIKISTR	KQKSVKEISS
730	740	750	760	770	780	790	800
YTPKDCTSRN	GPERGCDRGI	IVSTRLLTDS	STDALEKVST	SNEDFSLKDD	ALAKTSKRKT	KVQKDEICAK	LSHVIKKQHR
810	820	830	840	850	860	870	880
KSTLVDNTIN	LDENLTVSNI	ESFYSRKDTG	VQKGDGFIHN	LSLDPSGVLD	DKNGEQKSQN	NVLPKEKQLK	NEELVIFSFH
890	900	910	920	930	940	950	960
ENNCKIQEFH	VDGKELIPFT	EMTNASEKKS	SPFKDLMTVP	ESRDEEMSNS	TSVIYSNLTR	EQAPDISPKS	DTLTDSQIDR
970	980	990	1000	1010	1020	1030	1040
DLHKLSLLAQ	ASVITFPSDS	PQNSSQLQRK	VKEDKRCFTA	NQNNVGDTSR	GQVIIISDSD	DDDDERILSL	EKLTKQDKIC
1050	1060	1070	1080	1090	1100	1110	1120
LEREHPEQHV	STVNSKEEKN	PVKEEKTETL	FQFEESDSQC	FEFESSSEVF	SVWQDHPDDN	NSVQDGEKKC	LAPIANTTNG
1130	1140	1150	1160	1170	1180	1190	1200
QGCTDYVSEV	VKKGAEGIEE	HTRPRSISVE	EFCEIEVKKP	KRKRSEKPMA	EDPVRPSSSV	RNEGQSDTNK	RDLVGNDFKS
1210	1220	1230	1240	1250	1260	1270	1280
IDRRTSTPNS	RIQRATTVSQ	KKSSKLCTCT	EPIRKVPVSK	TPKKTHSDAK	KGQNRSSNYL	SCRTTPAIVP	PKKFRQCPEP
1290	1300	1310	1320	1330	1340	1350	1360
TSTAEKLGLK	KGPRKAYELS	QRSLDYVAQL	RDHGKTVGVV	DTRKKTKLIS	PQNLSVRNNK	KLLTSQELQM	QRQIRPKSQK
1370	1380	1390	1400	1410	1420	1430	1440
NRRRLSDCES	TDVKRAGSHT	AQNSDIFVPE	SDRSDYNCTG	GTEVLANSNR	KQLIKCMPSE	PETIKAKHGS	PATDDACPLN
1450	1460	1470	1480	1490	1500	1510	1520
QCDSVVLNGT	VPTNEVIVST	SEDPLGGGDP	TARHIEMAAL	KEGEPDSSSD	AEEDNLFLTQ	NDPEDMDLCS	QMENDNYKLI
1530	1540	1550	1560	1570	1580	1590	1600
ELIHGKDTVE	VEEDSVSRPQ	LESLSGTKCK	YKDCLETTKN	QGEYCPKHSE	VKAADEDVFR	KPGLPPPASK	PLRPTTKIFS
1610	1620	1630	1640	1650	1660	1670	1680
SKSTSRIAGL	SKSLETSSAL	SPSLKNKSKG	IQSILKVPQP	VPLIAQKPVG	EMKNSCNVLH	PQSPNNSNRQ	GCKVPFGESK
1690	1700	1710	1720	1730	1740	1750	1760
YFPSSSPVNI	LLSSQSVSDT	FVKEVLKWKY	EMFLNFGQCG	PPASLCQSIS	RPVPVRFHNY	GDYFNVFFPL	MVLNTFETVA
1770	1780	1790	1800	1810	1820	1830	1840
QEWLNSPNRE	NFYQLQVRKF	PADYIKYWEF	AVYLEECELA	KQLYPKENDL	VFLAPERINE	EKKDTERNDI	QDLHEYHSGY
1850	1860	1870	1880	1890	1900	1910	1920
VHKFRRTSVM	RNGKTECYLS	IQTQENFPAN	LNELVNCIVI	SSLVTTQRKL	KAMSLLGSRN	QLARAVLNPN	PMDFCTKDLL
1930	1940	1950	1960	1970	1980	1990	2000
TTTSERIIAY	LRDFNEDQKK	AIETAYAMVK	HSPSVAKICL	IHGPPGTGKS	KTIVGLLYRL	LTENQRKGHS	DENSNAKIKQ
2010	2020	2030	2040	2050	2060	2070	2080
NRVLVCAPSN	AAVDELMKKI	ILEFKEKCKD	KKNPLGNCGD	INLVRLGPEK	SINSEVLKFS	LDSQVNHRMK	KELPSHVQAM
2090	2100	2110	2120	2130	2140	2150	2160
HKRKEFLDYQ	LDELSRQRAL	CRGGREIQRQ	ELDENISKVS	KERQELASKI	KEVQGRPQKT	QSIIILESHI	ICCTLSTSGG
2170	2180	2190	2200	2210	2220	2230	2240

Detailed Protein Report

Cmpd.	No. of Cmpds.	m/z meas.	Δ m/z [ppm]	z	Rt [min]	Score	P	Range	Sequence	Modification
896	1	576.9688	-1.76	3	41.3	12.0	1	679-694	K.LEDHLLAGSCLKQSSK.N	
2700	1	856.9282	-25.41	2	64.9	12.2	2	853-867	K.NGEQKSQNNVLPKEK.Q	
2158	1	682.2802	-2.49	3	57.4	22.7	1	2220-2235	K.AQEYGYDQSMMARFCR.L	Carbamidomethyl: 15; Oxidation: 10, 11

Detailed Protein Report

Protein 86: PREDICTED: protein PRR14L isoform X4 [Homo sapiens]

**Accession:**	gi|578837142	**Score:**	46.5
**Database:**	refseq_human(refseq_human_20140103.fasta)	**MW [kDa]:**	219.9
**Database Date:**	2015-11-30	**pI:**	6.2
**Modification(s):**	Carbamidomethyl	**Sequence Coverage [%]:**	2.1
		**No. of unique Peptides:**	2

Quantitation

QD:QU	**Median:** 0.99	**CV:** 0.00 %	**No. of Peptides:** 1

Detailed Protein Report

10	20	30	40	50	60	70	80
MQSSKELSHV	DLPEDFLRSK	EGNVQITAET	LLKSAEVQGM	KVNGTKTDNN	EGHKNGNVSK	DLSAGCGEFQ	EVDKIMTSDE
90	100	110	120	130	140	150	160
VSETSTLVTP	EPLTFVDPVL	TEATPKEKEC	EELKSCPWLS	LPGNSAISNV	DNGKEELCKP	NLVCEADDNH	QQLHGHHNEQ
170	180	190	200	210	220	230	240
PSSTHDSPTA	TSPLKENSEV	SCFTSDLSGP	ESRTISLENC	GFEGGGLLKR	SAEKTDSSYF	YRGDDQGKNL	ASREENEERL
250	260	270	280	290	300	310	320
LIPRSERGGP	FLFNAREPEK	EISGRCSGEK	EPVVSPKENI	HNNCIQDSLH	TGNSSSLMPN	SFTEATEVML	NKNDLKITVH
330	340	350	360	370	380	390	400
VQGNLTNPED	HKETFTNMSH	PGGHSEESSF	SSLMQIEEAG	QTTPVEPNIL	SKSFYTKDCN	SLVSIQRNLE	GNTQLNEASC
410	420	430	440	450	460	470	480
NDFLFERKSI	VSLMPEDQIS	PVSEVLKPKQ	GTALLLPSPE	FDYRPESEKV	IQTSHDDIPL	LDEQSIACEM	NELSCTNELV
490	500	510	520	530	540	550	560
VNKVESECVL	NQQVSLNSQE	HANLPTDSLL	HLNKEMPLAT	GRDAHQSHHP	PLEGRADVIA	DIQTIPIQTK	IKDISPPGNQ
570	580	590	600	610	620	630	640
TCGASSNCPT	LNIKPVSLER	KKEMADSGTK	ALHSRLRSNK	REAAGFPQVV	SVIECHSVQS	QDISSCHRVR	KNVSQENMCS
650	660	670	680	690	700	710	720
ASAAFKSSKI	SLQVDNSLIT	KYENAFQHRD	HCCQGTGHSV	EKSSCKVSYT	SQERELDGKE	TNGSLPGDKI	RNKMVAGLLN
730	740	750	760	770	780	790	800
SGISNKTIHT	SSSIKLSEEG	LEGKEQDVSK	ETVFCKYNIS	DHAIQELNQT	VNIPGPEKVL	DQSPTVMFSS	FKNVKSVETL
810	820	830	840	850	860	870	880
DQKADEVLDC	QSNQNRPDEC	KSEGQSAKEM	LSSDQRETVT	EPHGEVNHNQ	KDLLVSSGSN	NSLPCGSPKK	CNLKGAFVKM
890	900	910	920	930	940	950	960
SGCDESTEGM	VDIVYTDCSN	KLAEGVLDVK	ASNLLDCGAR	QEKLAFQEDS	RSTLSRRELD	AAHTGTTGQD	SDFPVTAAST
970	980	990	1000	1010	1020	1030	1040
VDFLKIKKSC	EENVCRSLKD	CEMEKCPDSC	AHEMESVADH	EPNKRILGRV	NLSLNDSHYG	QQDKGTSLRE	TQEMTEGSRL
1050	1060	1070	1080	1090	1100	1110	1120
EPNSEFGKES	TFGISSKESM	SCHDESSVSL	RSLKSIEIMP	SQENSETNVN	SEETDLKNLC	KPKDGEMLCE	NVKDCTVLPE
1130	1140	1150	1160	1170	1180	1190	1200
MKEIVSRDWS	NSSDRDSVCT	CVEKNACKAC	HPHENSSDRH	LPLTVKTDIK	VKGEETEEHQ	RGRLGYLTVG	EQSEELVTRE
1210	1220	1230	1240	1250	1260	1270	1280
TGDGDPVSNI	SQTHFKCRGI	LNHAEKQQSP	EVLDYMLQKE	EKYIRQQKAH	TISQQCISSS	LLLDDAQNQN	QPKADKDEST
1290	1300	1310	1320	1330	1340	1350	1360
MINEITLAKL	AKDSIVAQTQ	KLEDQKEERL	HHPLRKDTES	CTSPCLLGAP	RKAQDPSSAG	CDQIHGAFAK	KGVLPLKKQP
1370	1380	1390	1400	1410	1420	1430	1440
HRTCKKVSYQ	EQIIVGRKIG	KIRSSAFLKS	SSNPIPTKAH	RLLSLCTLSA	PTRLEPETAP	TKSLVSHIPK	QMSTPCHPLR
1450	1460	1470	1480	1490	1500	1510	1520
SLNFRKTTKE	SALLNKLSIL	ASKLAPAMKT	QKLRYRRCSS	ELLPMAKSYK	RLRYKRLLDG	FSSSTEQLNP	YLAASGWDKR
1530	1540	1550	1560	1570	1580	1590	1600
PNSKPMALYS	LESIKMTFID	LSNKMPSLLF	GSEIFPVSFH	VKSSSSDCTT	ESSRTFPEHC	APARLALGEA	LQCPSQPPKW
1610	1620	1630	1640	1650	1660	1670	1680
TFSFFLSHGC	PGMATFREDT	GVHSQTHTQA	PPQPPAPLQD	YGGTAIVQTR	ADCSVLGLHT	LLALCSPGCY	RIWTKKRSFS
1690	1700	1710	1720	1730	1740	1750	1760
SHMPTMQRLF	MTQFTQGLKG	LRSPASIADK	VFCSLPYSVG	RVLSIWSQHG	PSVCSFEISS	LHSPHCKRQP	SLGTTSSHTM
1770	1780	1790	1800	1810	1820	1830	1840
LPYVPLPGME	ATYNTSGSQT	RLEPPFPALV	PKSCLVAESA	VSKLLLSASE	FQVRGLDELD	GVKAACPCPQ	SSPPEQKEAE
1850	1860	1870	1880	1890	1900	1910	1920
PEKRPKKVSQ	IRIRKTIPRP	DPNLTPMGLP	RPKRLKKKEF	SLEEIYTNKN	YKSPPANRCL	ETIFEEPKER	NGTLISISQQ
1930	1940	1950	1960	1970	1980	1990	
KRKRVLEFQD	FTVPRKRRAR	GKVKVAGSFT	RAQKAAVQSR	ELDALLIQKL	MELETFFAKE	EEQEQSSGC	

Cmpd.	No. of Cmpds.	m/z meas.	Δ m/z [ppm]	z	Rt [min]	Score	P	Range	Sequence	Modification	Ratios
1682	1	917.4150	-55.34	2	49.6	13.4	2	245-260	R.SERGGPFLFNAREPEK.E		
1702	2	867.8891	-25.32	2	49.8	17.0	1	1316-1331	R.KDTESCTSPCLLGAPR.K	Carbamidomethyl: 10	QD:QU 0.99

Detailed Protein Report

Protein 87: putative RNA polymerase II subunit B1 CTD phosphatase RPAP2 [Homo sapiens]

**Accession:**	gi|226246608	**Score:**	46.2
**Database:**	refseq_human(refseq_human_20140103.fasta)	**MW [kDa]:**	69.5
**Database Date:**	2015-11-30	**pI:**	8.8
		**Sequence Coverage [%]:**	6.4
		**No. of unique Peptides:**	2

10	20	30	40	50	60	70	80
MADFAGPSSA	GRKAGAPRCS	RKAAGTKQTS	TLKQEDASKR	KAELEAAVRK	KIEFERKALH	IVEQLLEENI	TEEFLMECGR
90	100	110	120	130	140	150	160
FITPAHYSDV	VDERSIVKLC	GYPLCQKKLG	IVPKQKYKIS	TKTNKVYDIT	ERKSFCSNFC	YQASKFFEAQ	IPKTPVWVRE
170	180	190	200	210	220	230	240
EERHPDFQLL	KEEQSGHSGE	EVQLCSKAIK	TSDIDNPSHF	EKQYESSSSS	THSDSSSDNE	QDFVSSILPG	NRPNSTNIRP
250	260	270	280	290	300	310	320
QLHQKSIMKK	KAGHKANSKH	KDKEQTVVDV	TEQLGDCKLD	SQEKDATCEL	PLQKVNTQSS	SNSTLPERLK	ASENSESEYS
330	340	350	360	370	380	390	400
RSEITLVGIS	KKSAEHFKRK	FAKSNQVSRS	VSSSVQVCPE	VGKRNLLKVL	KETLIEWKTE	ETLRFLYGQN	YASVCLKPEA
410	420	430	440	450	460	470	480
SLVKEELDED	DIISDPDSHF	PAWRESQNSL	DESLPFRGSG	TAIKPLPSYE	NLKKETEKLN	LRIREFYRGR	YVLGEETTKS
490	500	510	520	530	540	550	560
QDSEEHDSTF	PLIDSSSQNQ	IRKRIVLEKL	SKVLPGLLVP	LQITLGDIYT	QLKNLVRTFR	LTNRNIIHKP	AEWTLIAMVL
570	580	590	600	610	620		
LSLLTPILGI	QKHSQEGMVF	TRFLDTLLEE	LHLKNEDLES	LTIIFRTSCL	PE		

Cmpd.	No. of Cmpds.	m/z meas.	Δ m/z [ppm]	z	Rt [min]	Score	P	Range	Sequence	Modification
1581	1	583.8284	105.83	2	48.3	11.8	0	1-12	-.MADFAGPSSAGR.K	
1728	1	725.3276	-76.17	2	50.1	11.1	0	595-606	K.NEDLESLTIIFR.T	

Detailed Protein Report

Protein 88: TATA element modulatory factor [Homo sapiens]

**Accession:**	gi|110347443	**Score:**	46.1
**Database:**	refseq_human(refseq_human_20140103.fasta)	**MW [kDa]:**	122.8
**Database Date:**	2015-11-30	**pI:**	4.7
**Modification(s):**	Oxidation	**Sequence Coverage [%]:**	4.8
		**No. of unique Peptides:**	3

10	20	30	40	50	60	70	80
MSWFNASQLS	SFAKQALSQA	QKSIDRVLDI	QEEEPSIWAE	TIPYGEPGIS	SPVSGGWDTS	TWGLKSNTEP	QSPPIASPKA
90	100	110	120	130	140	150	160
ITKPVRRTVV	DESENFFSAF	LSPTDVQTIQ	KSPVVSKPPA	KSQRPEEEVK	SSLHESLHIG	QSRTPETTES	QVKDSSLCVS
170	180	190	200	210	220	230	240
GETLAAGTSS	PKTEGKHEET	VNKESDMKVP	TVSLKVSESV	IDVKTTMESI	SNTSTQSLTA	ETKDIALEPK	EQKHEDRQSN
250	260	270	280	290	300	310	320
TPSPPVSTFS	SGTSTTSDIE	VLDHESVISE	SSASSRQETT	DSKSSLHLMQ	TSFQLLSASA	CPEYNRLDDF	QKLTESCCSS
330	340	350	360	370	380	390	400
DAFERIDSFS	VQSLDSRSVS	EINSDDELSG	KGYALVPIIV	NSSTPKSKTV	ESAEGKSEEV	NETLVIPTEE	AEMEESGRSA
410	420	430	440	450	460	470	480
TPVNCEQPDI	LVSSTPINEG	QTVLDKVAEQ	CEPAESQPEA	LSEKEDVCKT	VEFLNEKLEK	REAQLLSLSK	EKALLEEAFD
490	500	510	520	530	540	550	560
NLKDEMFRVK	EESSSISSLK	DEFTQRIAEA	EKKVQLACKE	RDAAKKEIKN	IKEELATRLN	SSETADLLKE	KDEQIRGLME
570	580	590	600	610	620	630	640
EGEKLSKQQL	HNSNIIKKLR	AKDKENENMV	AKLNKKVKEL	EEELQHLKQV	LDGKEEVEKQ	HRENIKKLNS	MVERQEKDLG
650	660	670	680	690	700	710	720
RLQVDMDELE	EKNRSIQAAL	DSAYKELTDL	HKANAAKDSE	AQEAALSREM	KAKEELSAAL	EKAQEEARQQ	QETLAIQVGD
730	740	750	760	770	780	790	800
LRLALQRTEQ	AAARKEDYLR	HEIGELQQRL	QEAENRNQEL	SQSVSSTTRP	LLRQIENLQA	TLGSQTSSWE	KLEKNLSDRL
810	820	830	840	850	860	870	880
GESQTLLAAA	VERERAATEE	LLANKIQMSS	MESQNSLLRQ	ENSRFQAQLE	SEKNRLCKLE	DENNRYQVEL	ENLKDEYVRT
890	900	910	920	930	940	950	960
LEETRKEKTL	LNSQLEMERM	KVEQERKKAI	FTQETIKEKE	RKPFSVSSTP	TMSRSSSISG	VDMAGLQTSF	LSQDESHDHS
970	980	990	1000	1010	1020	1030	1040
FGPMPISANG	SNLYDAVRMG	AGSSIIENLQ	SQLKLREGEI	THLQLEIGNL	EKTRSIMAEE	LVKLTNQNDE	LEEKVKEIPK
1050	1060	1070	1080	1090	1100		
LRTQLRDLDQ	RYNTILQMYG	EKAEEAEELR	LDLEDVKNMY	KTQIDELLRQ	SLS		

Cmpd.	No. of Cmpds.	m/z meas.	Δ m/z [ppm]	z	Rt [min]	Score	P	Range	Sequence	Modification
1573	1	1023.5251	43.32	2	49.9	12.3	0	205-223	K.TTMESISNTSTQSLTAETK.D	Oxidation: 3
2457	1	647.2076	-217.62	2	61.2	10.6	1	489-500	R.VKEESSSISSLK.D	
1116	1	555.1899	-177.69	2	42.4	11.2	0	741-749	R.HEIGELQQR.L	

Detailed Protein Report

Protein 89: voltage-dependent R-type calcium channel subunit alpha-1E isoform 2 [Homo sapiens]

**Accession:**	gi|329663531	**Score:**	45.8
**Database:**	refseq_human(refseq_human_20140103.fasta)	**MW [kDa]:**	254.4
**Database Date:**	2015-11-30	**pI:**	9.3
**Modification(s):**	Carbamidomethyl, Oxidation	**Sequence Coverage [%]:**	2.5
		**No. of unique Peptides:**	4

Quantitation

QD:QU	**Median:** 0.88	**CV:** 0.00 %	**No. of Peptides:** 1

Detailed Protein Report

10	20	30	40	50	60	70	80
MARFGEAVVA	RPGSGDGDSD	QSRNRQGTPV	PASGQAAAYK	QTKAQRARTM	ALYNPIPVRQ	NCFTVNRSLF	IFGEDNIVRK
90	100	110	120	130	140	150	160
YAKKLIDWPP	FEYMILATII	ANCIVLALEQ	HLPEDDKTPM	SRRLEKTEPY	FIGIFCFEAG	IKIVALGFIF	HKGSYLRNGW
170	180	190	200	210	220	230	240
NVMDFIVVLS	GILATAGTHF	NTHVDLRTLR	AVRVLRPLKL	VSGIPSLQIV	LKSIMKAMVP	LLQIGLLLFF	AILMFAIIGL
250	260	270	280	290	300	310	320
EFYSGKLHRA	CFMNNSGILE	GFDPPHPCGV	QGCPAGYECK	DWIGPNDGIT	QFDNILFAVL	TVFQCITMEG	WTTVLYNTND
330	340	350	360	370	380	390	400
ALGATWNWLY	FIPLIIIGSF	FVLNLVLGVL	SGEFAKERER	VENRRAFMKL	RRQQQIEREL	NGYRAWIDKA	EEVMLAEENK
410	420	430	440	450	460	470	480
NAGTSALEVL	RRATIKRSRT	EAMTRDSSDE	HCVDISSVGT	PLARASIKSA	KVDGVSYFRH	KERLLRISIR	HMVKSQVFYW
490	500	510	520	530	540	550	560
IVLSLVALNT	ACVAIVHHNQ	PQWLTHLLYY	AEFLFLGLFL	LEMSLKMYGM	GPRLYFHSSF	NCFDFGVTVG	SIFEVVWAIF
570	580	590	600	610	620	630	640
RPGTSFGISV	LRALRLLRIF	KITKYWASLR	NLVVSLMSSM	KSIISLLFLL	FLFIVVFALL	GMQLFGGRFN	FNDGTPSANF
650	660	670	680	690	700	710	720
DTFPAAIMTV	FQILTGEDWN	EVMYNGIRSQ	GGVSSGMWSA	IYFIVLTLFG	NYTLLNVFLA	IAVDNLANAQ	ELTKDEQEEE
730	740	750	760	770	780	790	800
EAFNQKHALQ	KAKEVSPMSA	PNMPSIERER	RRRHHMSVWE	QRTSQLRKHM	QMSSQEALNR	EEAPTMNPLN	PLNPLSSLNP
810	820	830	840	850	860	870	880
LNAHPSLYRR	PRAIEGLALG	LALEKFEEER	ISRGGSLKGD	GGDRSSALDN	QRTPLSLGQR	EPPWLARPCH	GNCDPTQQEA
890	900	910	920	930	940	950	960
GGGEAVVTFE	DRARHRQSQR	RSRHRRVRTE	GKESSSASRS	RSASQERSLD	EAMPTEGEKD	HELRGNHGAK	EPTIQEERAQ
970	980	990	1000	1010	1020	1030	1040
DLRRTNSLMV	SRGSGLAGGL	DEADTPLVLP	HPELEVGKHV	VLTEQEPEGS	SEQALLGNVQ	LDMGRVISQS	EPDLSCITAN
1050	1060	1070	1080	1090	1100	1110	1120
TDKATTESTS	VTVAIPDVDP	LVDSTVVHIS	NKTDGEASPL	KEAEIREDEE	EVEKKKQKKE	KRETGKAMVP	HSSMFIFSTT
1130	1140	1150	1160	1170	1180	1190	1200
NPIRRACHYI	VNLRYFEMCI	LLVIAASSIA	LAAEDPVLTN	SERNKVLRYF	DYVFTGVFTF	EMVIKMIDQG	LILQDGSYFR
1210	1220	1230	1240	1250	1260	1270	1280
DLWNILDFVV	VVGALVAFAL	ANALGTNKGR	DIKTIKSLRV	LRVLRPLKTI	KRLPKLKAVF	DCVVTSLKNV	FNILIVYKLF
1290	1300	1310	1320	1330	1340	1350	1360
MFIFAVIAVQ	LFKGKFFYCT	DSSKDTEKEC	IGNYVDHEKN	KMEVKGREWK	RHEFHYDNII	WALLTLFTVS	TGEGWPQVLQ
1370	1380	1390	1400	1410	1420	1430	1440
HSVDVTEEDR	GPSRSNRMEM	SIFYVVYFVV	FPFFFVNIFV	ALIIITFQEQ	GDKMMEECSL	EKNERACIDF	AISAKPLTRY
1450	1460	1470	1480	1490	1500	1510	1520
MPQNRHTFQY	RVWHFVVSPS	FEYTIMAMIA	LNTVVLMMKY	YSAPCTYELA	LKYLNIAFTM	VFSLECVLKV	IAFGFLNYFR
1530	1540	1550	1560	1570	1580	1590	1600
DTWNIFDFIT	VIGSITEIIL	TDSKLVNTSG	FNMSFLKLFR	AARLIKLLRQ	GYTIRILLWT	FVQSFKALPY	VCLLIAMLFF
1610	1620	1630	1640	1650	1660	1670	1680
IYAIIGMQVF	GNIKLDEESH	INRHNNFRSF	FGSLMLLFRS	ATGEAWQEIM	LSCLGEKGCE	PDTTAPSGQN	ENERCGTDLA
1690	1700	1710	1720	1730	1740	1750	1760
YVYFVSFIFF	CSFLMLNLFV	AVIMDNFEYL	TRDSSILGPH	HLDEFVRVWA	EYDRAACGRI	HYTEMYEMLT	LMSPPLGLGK
1770	1780	1790	1800	1810	1820	1830	1840
RCPSKVAYKR	LVLMNMPVAE	DMTVHFTSTL	MALIRTALDI	KIAKGGADRQ	QLDSELQKET	LAIWPHLSQK	MLDLLVPMPK
1850	1860	1870	1880	1890	1900	1910	1920
ASDLTVGKIY	AAMMIMDYYK	QSKVKKQRQQ	LEEQKNAPMF	QRMEPSSLPQ	EIIANAKALP	YLQQDPVSGL	SGRSGYPSMS
1930	1940	1950	1960	1970	1980	1990	2000
PLSPQDIFQL	ACMDPADDGQ	FQERQSLVVT	DPSSMRRSFS	TIRDKRSNSS	WLEEFSMERS	SENTYKSRRR	SYHSSLRLSA
2010	2020	2030	2040	2050	2060	2070	2080
HRLNSDSGHK	SDTHRSGGRE	RGRSKERKHL	LSPDVSRCNS	EERGTQADWE	SPERRQSRSP	SEGRSQTPNR	QGTGSLSESS
2090	2100	2110	2120	2130	2140	2150	2160
IPSVSDTSTP	RRSRRQLPPV	PPKPRPLLSY	SSLIRHAGSI	SPPADGSEEG	SPLTSQALES	NNACLTESSN	SPHPQQSQHA
2170	2180	2190	2200	2210	2220	2230	2240

Detailed Protein Report

Cmpd.	No. of Cmpds.	m/z meas.	Δ m/z [ppm]	z	Rt [min]	Score	P	Range	Sequence	Modification	Ratios
2939	1	649.3454	56.93	3	65.9	11.0	1	734-750	K.EVSPMSAPNMPSIERER.R	Oxidation: 10	
27	1	814.2746	-93.66	2	30.0	10.4	1	1296-1308	K.FFYCTDSSKDTEK.E	Carbamidomethyl: 4	
118	1	753.3697	-54.70	2	31.4	13.4	0	1426-1439	R.ACIDFAISAKPLTR.Y		QD:QU 0.88
1976	1	698.7954	-42.34	2	53.2	11.0	1	1728-1739	R.VWAEYDRAACGR.I		

Detailed Protein Report

Protein 90: **C3 and PZP-like alpha-2-macroglobulin domain-containing protein 8 [Homo sapiens]**

**Accession:**	gi|118600977	**Score:**	45.8
**Database:**	refseq_human(refseq_human_20140103.fasta)	**MW [kDa]:**	211.2
**Database Date:**	2015-11-30	**pI:**	6.2
		**Sequence Coverage [%]:**	2.6
		**No. of unique Peptides:**	2

Detailed Protein Report

10	20	30	40	50	60	70	80
MAPGALRGGC	EQRAPGGGAR	TAALGPWPWP	SPGPAPSPGA	APPRSRKMSG	ALLWPLLPLL	LLLLSARDGV	RAAQPQAPGY
90	100	110	120	130	140	150	160
LIAAPSVFRA	GVEEVISVTI	FNSPREVTVQ	AQLVAQGEPV	VQSQGAILDK	GTIKLKVPTG	LRGQALLKVW	GRGWQAEEGP
170	180	190	200	210	220	230	240
LFHNQTSVTV	DGRGASVFIQ	TDKPVYRPQH	RVLISIFTVS	PNLRPVNEKL	EAYILDPRGS	RMIEWRHLKP	FCCGITNMSF
250	260	270	280	290	300	310	320
PLSDQPVLGE	WFIFVEMQGH	AYNKSFEVQK	YVLPKFELLI	DPPRYIQDLD	ACETGTVRAR	YTFGKPVAGA	LMINMTVNGV
330	340	350	360	370	380	390	400
GYYSHEVGRP	VLRTTKILGS	RDFDICVRDM	IPADVPEHFR	GRVSIWAMVT	SVDGSQQVAF	DDSTPVQRQL	VDIRYSKDTR
410	420	430	440	450	460	470	480
KQFKPGLAYV	GKVELSYPDG	SPAEGVTVQI	KAELTPKDNI	YTSEVVSQRG	LVGFEIPSIP	TSAQHVWLET	KVMALNGKPV
490	500	510	520	530	540	550	560
GAQYLPSYLS	LGSWYSPSQC	YLQLQPPSHP	LQVGEEAYFS	VKSTCPCNFT	LYYEVAARGN	IVLSGQQPAH	TTQQRSKRAA
570	580	590	600	610	620	630	640
PALEKPIRLT	HLSETEPPPA	PEAEVDVCVT	SLHLAVTPSM	VPLGRLLVFY	VRENGEGVAD	SLQFAVETFF	ENQVSVTYSA
650	660	670	680	690	700	710	720
NETQPGEVVD	LRIRAARGSC	VCVAAVDKSV	YLLRSGFRLT	PAQVFQELED	YDVSDSFGVS	REDGPFWWAG	LTAQRRRRSS
730	740	750	760	770	780	790	800
VFPWPWGITK	DSGFAFTETG	LVVMTDRVSL	NHRQDGGLYT	DEAVPAFQPH	TGSLVAVAPS	RHPPRTEKRK	RTFFPETWIW
810	820	830	840	850	860	870	880
HCLNISDPSG	EGTLSVKVPD	SITSWVGEAV	ALSTSQGLGI	AEPSLLKTFK	PFFVDFMLPA	LIIRGEQVKI	PLSVYNYMGT
890	900	910	920	930	940	950	960
CAEVYMKLSV	PKGIQFVGHP	GKRHVTKKMC	VAPGEAEPIW	VVLSFSDLGL	NNITAKALAY	GDTNCCRDGR	SSKHPEENHA
970	980	990	1000	1010	1020	1030	1040
DRRVPIGVDH	VRRSVMVEAE	GVPRAYTYSA	FFCPSERVHI	STPNKYEFQY	VQRPLRLTRF	DVAVRAHNDA	RVALSSGPQD
1050	1060	1070	1080	1090	1100	1110	1120
TAGMIEIVLG	GHQNTRSWIS	TSKMGEPVAS	AHTAKILSWD	EFRTFWISWR	GGLIQVGHGP	EPSNESVIVA	WTLPRPPEVQ
1130	1140	1150	1160	1170	1180	1190	1200
FIGFSTGWGS	MGEFRIWRKM	EVDESYSEAF	TLGVPHGAIP	GSERATASII	GDVMGPTLNH	LNNLLRLPFG	CGEQNMIHFA
1210	1220	1230	1240	1250	1260	1270	1280
PNVFVLKYLQ	KTQQLSPEVE	RETTDYLVQG	YQRQLTYKRQ	DGSYSAFGER	DASGSMWLTA	FVLKSFAQAR	SFIFVDPREL
1290	1300	1310	1320	1330	1340	1350	1360
AAAKSWIIQQ	QQADGSFLAV	GRVLNKDIQG	GIHGTVPLTA	YVVVALLETG	TASEEERGST	DKARHFLESA	APLAMDPYSC
1370	1380	1390	1400	1410	1420	1430	1440
ALTTYALTLL	RSPAAPEALR	KLRSLAIMRD	GVTHWSLSNS	WDVDKGTFLS	FSDRVSQSVV	SAEVEMTAYA	LLTYTLLGDV
1450	1460	1470	1480	1490	1500	1510	1520
AAALPVVKWL	SQQRNALGGF	SSTQDTCVAL	QALAEYAILS	YAGGINLTVS	LASTNLDYQE	TFELHRTNQK	VLQTAAIPSL
1530	1540	1550	1560	1570	1580	1590	1600
PTGLFVSAKG	DGCCLMQIDV	TYNVPDPVAK	PAFQLLVSLQ	EPEAQGRPPP	MPASAAEGSR	GDWPPADDDD	PAADQHHQEY
1610	1620	1630	1640	1650	1660	1670	1680
KVMLEVCTRW	LHAGSSNMAV	LEVPLLSGFR	ADIESLEQLL	LDKHMGMKRY	EVAGRRVLFY	FDEIPSRCLT	CVRFRALREC
1690	1700	1710	1720	1730	1740	1750	1760
VVGRTSALPV	SVYDYYEPAF	EATRFYNVST	HSPLARELCA	GPACNEVERA	PARGPGWFPG	ESGPAVAPEE	GAAIARCGCD
1770	1780	1790	1800	1810	1820	1830	1840
HDCGAQGNPV	CGSDGVVYAS	ACRLREAACR	QAAPLEPAPP	SCCALEQRLP	ASSSSTYGDD	LASVAPGPLQ	QDVKLNGAGL
1850	1860	1870	1880	1890	1900	1910	1920
EVEDSDPEPE	GEAEDRVTAG	PRPPVSSGNL	ESSTQSASPF	HRWGQTPAPQ	RHSGRVVGAH	RPGLLSPVFV	YSPAFQSGGE
1930	1940						
EGLWMSNTCT	LR						

Cmpd.	No. of Cmpds.	m/z meas.	Δ m/z [ppm]	z	Rt [min]	Score	P	Range	Sequence	Modification
2723	1	973.5045	12.24	2	65.2	13.4	2	702-717	R.EDGPFWWAGLTAQRRR.R	
968	1	820.0912	-24.82	3	40.6	16.4	1	1285-1306	K.SWIIQQQQADGSFLAVGRVLNK.D	

Detailed Protein Report

Protein 91: leucine-, glutamate- and lysine-rich protein 1 isoform 1 [Homo sapiens]

**Accession:**	gi|262263420	**Score:**	45.7
**Database:**	refseq_human(refseq_human_20140103.fasta)	**MW [kDa]:**	81.2
**Database Date:**	2015-11-30	**pI:**	8.7
**Modification(s):**	Oxidation	**Sequence Coverage [%]:**	5.8
		**No. of unique Peptides:**	2

Alias proteins:

Accession	Name	Description
gi|530374591	refseq_human (refseq_human_20140103.fasta)	PREDICTED: leucine-, glutamate- and lysine-rich protein 1 isoform X1 [Homo sapiens]

10	20	30	40	50	60	70	80
MDHHIPMHAL	PEEIQKMLPE	EKVCKYCGVS	YLILHEFKAM	EEKVKAMEKE	MKFYQGSVDR	EKRLQEKLHS	LSQELEQYKI
**90**	**100**	**110**	**120**	**130**	**140**	**150**	**160**
DNKSKTERIY	DVGMQLKSQQ	NEFQKVKKQL	SHLQDELKIK	YRQSYIFSQR	LSEYKYFWNK	TLSLLTFTKR	ELTSIKNEVY
**170**	**180**	**190**	**200**	**210**	**220**	**230**	**240**
DNYQNWTSLK	GAVFLQIKSI	SETALTEIDI	LNKSLTVSQR	NKVCLEKEMK	NLKLLSDAAI	LRSQQIRTSR	QQEVNLQTRC
**250**	**260**	**270**	**280**	**290**	**300**	**310**	**320**
YDLQKEVLDL	QCLVEALGLK	LQKAVTEMDN	YKEMLMNKSN	EADDCQRELK	KLKFESIISE	SQHTMLLKEK	EDSLMTCQQI
**330**	**340**	**350**	**360**	**370**	**380**	**390**	**400**
YKALQEELTV	KEKQEEDIKR	RINLAENELE	ITKTLLNQTR	EEVLTLKNER	ELMLISHQKS	IEQLQETLRQ	KLLSDDNWKE
**410**	**420**	**430**	**440**	**450**	**460**	**470**	**480**
KIEAELAKER	AQHLVEFEEQ	ALLFKEETKL	QLDIEKEKHQ	DVIQKYKKEQ	EELQMKISDL	ITGATRDLRQ	EVTTLKEKLH
**490**	**500**	**510**	**520**	**530**	**540**	**550**	**560**
KSHIRYTEES	NSKEKEIENL	KNLVAEFESR	LKKEIDSNDS	VSENLRKEME	QKSDELKRVM	LAQTQLIEQF	NQSQEENTFL
**570**	**580**	**590**	**600**	**610**	**620**	**630**	**640**
QETVRRECEE	RFELTEALSQ	AREQLLELSK	LRGSLPFSPC	SLSKGSLTSP	AAAVSNHGER	SLARLNSEKG	IQIPNLRGVS
**650**	**660**	**670**	**680**	**690**	**700**		
KPTTFPTSDK	PKRVRSGVPI	LPQPHPPRGG	ASSANETRQR	LAAILRRRRS	QQ		

Cmpd.	No. of Cmpds.	m/z meas.	Δ m/z [ppm]	z	Rt [min]	Score	P	Range	Sequence	Modification
1884	1	734.7526	-169.10	2	53.9	10.2	1	86-97	K.TERIYDVGMQLK.S	Oxidation: 9
538	1	501.6964	-160.45	2	36.7	11.2	1	494-501	K.EKEIENLK.N	

Detailed Protein Report

Protein 92: PREDICTED: ceruloplasmin isoform X4 [Homo sapiens]

**Accession:**	gi|578807068	**Score:**	45.6
**Database:**	refseq_human(refseq_human_20140103.fasta)	**MW [kDa]:**	103.1
**Database Date:**	2015-11-30	**pI:**	5.4
**Modification(s):**	Carbamidomethyl	**Sequence Coverage [%]:**	1.6
		**No. of unique Peptides:**	1

10	20	30	40	50	60	70	80
MKILILGIFL	FLCSTPAWAK	EKHYYIGIIE	TTWDYASDHG	EKKLISVDTE	HSNIYLQNGP	DRIGRLYKKA	LYLQYTDETF
**90**	**100**	**110**	**120**	**130**	**140**	**150**	**160**
RTTIEKPVWL	GFLGPIIKAE	TGDKVYVHLK	NLASRPYTFH	SHGITYYKEH	EGAIYPDNTT	DFQRADDKVY	PGEQYTYMLL
**170**	**180**	**190**	**200**	**210**	**220**	**230**	**240**
ATEEQSPGEG	DGNCVTRIYH	SHIDAPKDIA	SGLIGPLIIC	KKDSLDKEKE	KHIDREFVVM	FSVVDENFSW	YLEDNIKTYC
**250**	**260**	**270**	**280**	**290**	**300**	**310**	**320**
SEPEKVDKDN	EDFQESNRMY	SVNGYTFGSL	PGLSMCAEDR	VKWYLFGMGN	EVDVHAAFFH	GQALTNKNYR	IDTINLFPAT
**330**	**340**	**350**	**360**	**370**	**380**	**390**	**400**
LFDAYMVAQN	PGEWMLSCQN	LNHLKAGLQA	FFQVQECNKS	SSKDNIRGKH	VRHYYIAAEE	IIWNYAPSGI	DIFTKENLTA
**410**	**420**	**430**	**440**	**450**	**460**	**470**	**480**
PGSDSAVFFE	QGTTRIGGSY	KKLVYREYTD	ASFTNRKERG	PEEEHLGILG	PVIWAEVGDT	IRVTFHNKGA	YPLSIEPIGV
**490**	**500**	**510**	**520**	**530**	**540**	**550**	**560**
RFNKNNEGTY	YSPNYNPQSR	SVPPSASHVA	PTETFTYEWT	VPKEVGPTNA	DPVCLAKMYY	SAVEPTKDIF	TGLIGPMKIC
**570**	**580**	**590**	**600**	**610**	**620**	**630**	**640**
KKGSLHANGR	QKDVDKEFYL	FPTVFDENES	LLLEDNIRMF	TTAPDQVDKE	DEDFQESNKM	HSMNGFMYGN	QPGLTMCKGD
**650**	**660**	**670**	**680**	**690**	**700**	**710**	**720**
SVVWYLFSAG	NEADVHGIYF	SGNTYLWRGE	RRDTANLFPQ	TSLTLHMWPD	TEGTFNVECL	TTDHYTGGMK	QKYTVNQCRR
**730**	**740**	**750**	**760**	**770**	**780**	**790**	**800**
QSEDSTFYLG	ERTYYIAAVE	VEWDYSPQRE	WEKELHHLQE	QNVSNAFLDK	GEFYIGSKYK	KVVYRQYTDS	TFRVPVERKA
**810**	**820**	**830**	**840**	**850**	**860**	**870**	**880**
EEEHLGILGP	QLHADVGDKV	KIIFKNMATR	PYSIHAHGVQ	TESSTVTPTL	PGETLTYVWK	IPERSGAGTE	DSACIPWAYY
**890**	**900**	**910**					
STVDQVKDLY	CKEGEKKWSR	L					

Cmpd.	No. of Cmpds.	m/z meas.	Δ m/z [ppm]	z	Rt [min]	Score	P	Range	Sequence	Modification
2714	3	735.4243	1.19	2	65.0	45.6	0	188-201	K.DIASGLIGPLIICK.K	Carbamidomethyl: 13

Detailed Protein Report

Protein 93: death-inducer obliterator 1 isoform c [Homo sapiens]

**Accession:**	gi|301129165	**Score:**	45.2
**Database:**	refseq_human(refseq_human_20140103.fasta)	**MW [kDa]:**	243.7
**Database Date:**	2015-11-30	**pI:**	8.9
		**Sequence Coverage [%]:**	1.8
		**No. of unique Peptides:**	2

Quantitation

QD:QU	**Median:** 1.22	**CV:** 0.00 %	**No. of Peptides:** 1

Alias proteins:

Accession	Name	Description
gi|301129170	refseq_human (refseq_human_20140103.fasta)	death-inducer obliterator 1 isoform c [Homo sapiens]

Detailed Protein Report

10	20	30	40	50	60	70	80
MDDKGDPSNE	EAPKAIKPTS	KEFRKTWGFR	RTTIAKREGA	GDAEADPLEP	PPPQQQLGLS	LRRSGRQPKR	TERVEQFLTI
90	100	110	120	130	140	150	160
ARRRGRRSMP	VSLEDSGEPT	SCPATDAETA	SEGSVESASE	TRSGPQSAST	AVKERPASSE	KVKGGDDHDD	TSDSDSDGLT
170	180	190	200	210	220	230	240
LKELQNRLRR	KREQEPTERP	LKGIQSRLRK	KRREEGPAET	VGSEASDTVE	GVLPSKQEPE	NDQGVVSQAG	KDDRESKLEG
250	260	270	280	290	300	310	320
KAAQDIKDEE	PGDLGRPKPE	CEGYDPNALY	CICRQPHNNR	FMICCDRCEE	WFHGDCVGIS	EARGRLLERN	GEDYICPNCT
330	340	350	360	370	380	390	400
ILQVQDETHS	ETADQQEAKW	RPGDADGTDC	TSIGTIEQKS	SEDQGIKGRI	EKAANPSGKK	KLKIFQPVIE	APGASKCIGP
410	420	430	440	450	460	470	480
GCCHVAQPDS	VYCSNDCILK	HAAATMKFLS	SGKEQKPKPK	EKMKMKPEKP	SLPKCGAQAG	IKISSVHKRP	APEKKETTVK
490	500	510	520	530	540	550	560
KAVVVPARSE	ALGKEAACES	STPSWASDHN	YNAVKPEKTA	APSPSLLYKS	TKEDRRSEEK	AAAMAASKKT	APPGSAVGKQ
570	580	590	600	610	620	630	640
PAPRNLVPKK	SSFANVAAAT	PAIKKPPSGF	KGTIPKRPWL	SATPSSGASA	ARQAGPAPAA	ATAASKKFPG	SAALVGAVRK
650	660	670	680	690	700	710	720
PVVPSVPMAS	PAPGRLGAMS	AAPSQPNSQI	RQNIRRSLKE	ILWKRVNDSD	DLIMTENEVG	KIALHIEKEM	FNLFQVTDNR
730	740	750	760	770	780	790	800
YKSKYRSIMF	NLKDPKNQGL	FHRVLREEIS	LAKLVRLKPE	ELVSKELSTW	KERPARSVME	SRTKLHNESK	KTAPRQEAIP
810	820	830	840	850	860	870	880
DLEDSPPVSD	SEEQQESARA	VPEKSTAPLL	DVFSSMLKDT	TSQHRAHLFD	LNCKICTGQV	PSAEDEPAPK	KQKLSASVKK
890	900	910	920	930	940	950	960
EDLKSKHDSS	APDPAPDSAD	EVMPEAVPEV	ASEPGLESAS	HPNVDRTYFP	GPPGDGHPEP	SPLEDLSPCP	ASCGSGVVTT
970	980	990	1000	1010	1020	1030	1040
VTVSGRDPRT	APSSSCTAVA	SAASRPDSTH	MVEARQDVPK	PVLTSVMVPK	SILAKPSSSP	DPRYLSVPPS	PNISTSESRS
1050	1060	1070	1080	1090	1100	1110	1120
PPEGDTTLFL	SRLSTIWKGF	INMQSVAKFV	TKAYPVSGCF	DYLSEDLPDT	IHIGGRIAPK	TVWDYVGKLK	SSVSKELCLI
1130	1140	1150	1160	1170	1180	1190	1200
RFHPATEEEE	VAYISLYSYF	SSRGRFGVVA	NNNRHVKDLY	LIPLSAQDPV	PSKLLPFEGP	GLESPRPNII	LGLVICQKIK
1210	1220	1230	1240	1250	1260	1270	1280
RPANSGELDK	MDEKRTRLQP	EEADVPAYPK	VATVPQSEKK	PSKYPLCSAD	AAVSTTPPGS	PPPPPPLPEP	PVLKVLSSLK
1290	1300	1310	1320	1330	1340	1350	1360
PAAPSPATAA	TTAAAASTAA	SSTASSASKT	ASPLEHILQT	LFGKKKSFDP	SAREPPGSTA	GLPQEPKTTA	EDGVPAPPLL
1370	1380	1390	1400	1410	1420	1430	1440
DPIVQQFGQF	SKDKALEEEE	DDRPYDPEEE	YDPERAFDTQ	LVERGRRHEV	ERAPEAAAAE	REEVAYDPED	ETILEEAKVT
1450	1460	1470	1480	1490	1500	1510	1520
VDDLPNRMCA	DVRRNSVERP	AEPVAGAATP	SLVEQQKMLE	ELNKQIEEQK	RQLEEQEEAL	RQQRAAVGVS	MAHFSVSDAL
1530	1540	1550	1560	1570	1580	1590	1600
MSPPPKSSLP	KAELFQQEQQ	SADKPASLPP	ASQASNHRDP	RQARRLATET	GEGEGEPLSR	LSARGAQGAL	PERDASRGGL
1610	1620	1630	1640	1650	1660	1670	1680
VGQAPMPVPE	EKEPASSPWA	SGEKPPAGSE	QDGWKAEPGE	GTRPATVGDS	SARPARRVLL	PTPPCGALQP	GFPLQHDGER
1690	1700	1710	1720	1730	1740	1750	1760
DPFTCPGFAS	QDKALGSAQY	EDPRNLHSAG	RSSSPAGETE	GDREPQARPG	EGTAPLPPPG	QKVGGSQPPF	QGQREPGPHA
1770	1780	1790	1800	1810	1820	1830	1840
LGMSGLHGPN	FPGPRGPAPP	FPEENIASND	GPRGPPPARF	GAQKGPIPSL	FSGQHGPPPY	GDSRGPSPSY	LGGPRGVAPS
1850	1860	1870	1880	1890	1900	1910	1920
QFEERKDPHG	EKREFQDAPY	NEVTGAPAQF	EGTEQAPFLG	SRGGAPFQFG	GQRRPLLSQL	KGPRGGPPPS	QFGGQRGPPP
1930	1940	1950	1960	1970	1980	1990	2000
GHFVGPRGPH	PSQFETARGP	HPNQFEGPRG	QAPNFMPGPR	GIQPQQFEDQ	RVHSPPRFTN	QRAPAPLQFG	GLRGSAPFSE
2010	2020	2030	2040	2050	2060	2070	2080
KNEQTPSRFH	FQGQAPQVMK	PGPRPLLELP	SHPPQHRKDR	WEEAGPPSAL	SSSAPGQGPE	ADGQWASADF	REGKGHEYRN
2090	2100	2110	2120	2130	2140	2150	2160
QTFEGRQRER	FDVGPKEKPL	EEPDAQGRAS	EDRRRERERG	RNWSRERDWD	RPREWDRHRD	KDSSRDWDRN	RERSANRDRE
2170	2180	2190	2200	2210	2220	2230	2240

Detailed Protein Report

Cmpd.	No. of Cmpds.	m/z meas.	Δ m/z [ppm]	z	Rt [min]	Score	P	Range	Sequence	Modification	Ratios
411	1	701.3611	72.76	2	35.1	11.7	1	2-14	M.DDKGDPSNEEAPK.A		QD:QU 1.22
116	5	768.2858	-124.81	2	30.2	17.6	1	1408-1421	R.HEVERAPEAAAAER.E		

Detailed Protein Report

Protein 94: ADAMTS-like protein 1 isoform 4 precursor [Homo sapiens]

Accession: gi|154275767
Database: refseq_human(refseq_human_20140103.fasta)
Database Date: 2015-11-30
Modification(s): Carbamidomethyl

Score: 45.0
MW [kDa]: 193.3
pI: 9.5
Sequence Coverage [%]: 2.4
No. of unique Peptides: 1

10	20	30	40	50	60	70	80
MECCRRATPG	TLLLFLAFLL	LSSRTARSEE	DRDGLWDAWG	PWSECSRTCG	GGASYSLRRC	LSSKSCEGRN	IRYRTCSNVD
90	100	110	120	130	140	150	160
CPPEAGDFRA	QQCSAHNDVK	HHGQFYEWLP	VSNDPDNPCS	LKCQAKGTTL	VVELAPKVLD	GTRCYTESLD	MCISGLCQIV
170	180	190	200	210	220	230	240
GCDHQLGSTV	KEDNCGVCNG	DGSTCRLVRG	QYKSQLSATK	SDDTVVAIPY	GSRHIRLVLK	GPDHLYLETK	TLQGTKGENS
250	260	270	280	290	300	310	320
LSSTGTFLVD	NSSVDFQKFP	DKEILRMAGP	LTADFIVKIR	NSGSADSTVQ	FIFYQPIIHR	WRETDFFPCS	ATCGGGYQLT
330	340	350	360	370	380	390	400
SAECYDLRSN	RVVADQYCHY	YPENIKPKPK	LQECNLDPCP	ASDGYKQIMP	YDLYHPLPRW	EATPWTACSS	SCGGGIQSRA
410	420	430	440	450	460	470	480
VSCVEEDIQG	HVTSVEEWKC	MYTPKMPIAQ	PCNIFDCPKW	LAQEWSPCTV	TCGQGLRYRV	VLCIDHRGMH	TGGCSPKTKP
490	500	510	520	530	540	550	560
HIKEECIVPT	PCYKPKEKLP	VEAKLPWFKQ	AQELEEGAAV	SEEPSFIPEA	WSACTVTCGV	GTQVRIVRCQ	VLLSFSQSVA
570	580	590	600	610	620	630	640
DLPIDECEGP	KPASQRACYA	GPCSGEIPEF	NPDETDGLFG	GLQDFDELYD	WEYEGFTKCS	ESCGGGVQEA	VVSCLNKQTR
650	660	670	680	690	700	710	720
EPAEENLCVT	SRRPPQLLKS	CNLDPCPARW	EIGKWSPCSL	TCGVGLQTRD	VFCSHLLSRE	MNETVILADE	LCRQPKPSTV
730	740	750	760	770	780	790	800
QACNRFNCPP	AWYPAQWQPC	SRTCGGGVQK	REVLCKQRMA	DGSFLELPET	FCSASKPACQ	QACKKDDCPS	EWLLSDWTEC
810	820	830	840	850	860	870	880
STSCGEGTQT	RSAICRKMLK	TGLSTVVNST	LCPPLPFSSS	IRPCMLATCA	RPGRPSTKHS	PHIAAARKVY	IQTRRQRKLH
890	900	910	920	930	940	950	960
FVVGGFAYLL	PKTAVVLRCP	ARRVRKPLIT	WEKDGQHLIS	STHVTVAPFG	YLKIHRLKPS	DAGVYTCSAG	PAREHFVIKL
970	980	990	1000	1010	1020	1030	1040
IGGNRKLVAR	PLSPRSEEEV	LAGRKGGPKE	ALQTHKHQNG	IFSNGSKAEK	RGLAANPGSR	YDDLVSRLLE	QGGWPGELLA
1050	1060	1070	1080	1090	1100	1110	1120
SWEAQDSAER	NTTSEEDPGA	EQVLLHLPFT	MVTEQRRLDD	ILGNLSQQPE	ELRDLYSKHL	VAQLAQEIFR	SHLEHQDTLL
1130	1140	1150	1160	1170	1180	1190	1200
KPSERRTSPV	TLSPHKHVSG	FSSSLRTSST	GDAGGGSRRP	HRKPTILRKI	SAAQQLSASE	VVTHLGQTVA	LASGTLSVLL
1210	1220	1230	1240	1250	1260	1270	1280
HCEAIGHPRP	TISWARNGEE	VQFSDRILLQ	PDDSLQILAP	VEADVGFYTC	NATNALGYDS	VSIAVTLAGK	PLVKTSRMTV
1290	1300	1310	1320	1330	1340	1350	1360
INTEKPAVTV	DIGSTIKTVQ	GVNVTINCQV	AGVPEAEVTW	FRNKSKLGSP	HHLHEGSLLL	TNVSSSDQGL	YSCRAANLHG
1370	1380	1390	1400	1410	1420	1430	1440
ELTESTQLLI	LDPPQVPTQL	EDIRALLAAT	GPNLPSVLTS	PLGTQLVLDP	GNSALLGCPI	KGHPVPNITW	FHGGQPIVTA
1450	1460	1470	1480	1490	1500	1510	1520
TGLTHHILAA	GQILQVANLS	GGSQGEFSCL	AQNEAGVLMQ	KASLVIQDYW	WSVDRLATCS	ASCGNRGVQQ	PRLRCLLNST
1530	1540	1550	1560	1570	1580	1590	1600
EVNPAHCAGK	VRPAVQPIAC	NRRDCPSRWM	VTSWSACTRS	CGGGVQTRRV	TCQKLKASGI	STPVSNDMCT	QVAKRPVDTQ
1610	1620	1630	1640	1650	1660	1670	1680
ACNQQLCVEW	AFSSWGQCNG	PCIGPHLAVQ	HRQVFCQTRD	GITLPSEQCS	ALPRPVSTQN	CWSEACSVHW	RVSLWTLCTA
1690	1700	1710	1720	1730	1740	1750	1760
TCGNYGFQSR	RVECVHARTN	KAVPEHLCSW	GPRPANWQRC	NITPCENMEC	RDTTRYCEKV	KQLKLCQLSQ	FKSRCCGTCG
1770							
KA							

Cmpd.	No. of	m/z meas.	Δ m/z	z	Rt	Score	P	Range	Sequence	Modification

Detailed Protein Report

	Cmpds.		[ppm]		[min]					
1695	1	671.2184	-97.93	3	51.5	14.6	1	172-189	K.EDNCGVCNGDGSTCRLVR.G	Carbamidomethyl: 4, 7

Detailed Protein Report

Protein 95: nuclear receptor coactivator 4 isoform 3 [Homo sapiens]

**Accession:**	gi|14149617	**Score:**	44.9
**Database:**	refseq_human(refseq_human_20140103.fasta)	**MW [kDa]:**	69.7
**Database Date:**	2015-11-30	**pI:**	5.7
**Modification(s):**	Carbamidomethyl	**Sequence Coverage [%]:**	7.0
		**No. of unique Peptides:**	2

Alias proteins:

Accession	Name	Description
gi|223890287	refseq_human (refseq_human_20140103.fasta)	nuclear receptor coactivator 4 isoform 3 [Homo sapiens]
gi|223890284	refseq_human (refseq_human_20140103.fasta)	nuclear receptor coactivator 4 isoform 3 [Homo sapiens]

```
        10         20         30         40         50         60         70         80
MNTFQDQSGS SSNREPLLRC SDARRDLELA IGGVLRAEQQ IKDNLREVKA QIHSCISRHL ECLRSREVWL YEQVDLIYQL
        90        100        110        120        130        140        150        160
KEETLQQQAQ QLYSLLGQFN CLTHQLECTQ NKDLANQVSV CLERLGSLTL KPEDSTVLLF EADTITLRQT ITTFGSLKTI
       170        180        190        200        210        220        230        240
QIPEHLMAHA SSANIGPFLE KRGCISMPEQ KSASGIVAVP FSEWLLGSKP ASGYQAPYIP STDPQDWLTQ KQTLENSQTS
       250        260        270        280        290        300        310        320
SRACNFFNNV GGNLKGLENW LLKSEKSSYQ KCNSHSTTSS FSIEMEKVGD QELPDQDEMD LSDWLVTPQE SHKLRKPENG
       330        340        350        360        370        380        390        400
SRETSEKFKL LFQSYNVNDW LVKTDSCTNC QGNQPKGVEI ENLGNLKCLN DHLEAKKPLS TPSMVTEDWL VQNHQDPCKV
       410        420        430        440        450        460        470        480
EEVCRANEPC TSFAECVCDE NCEKEALYKW LLKKEGKDKN GMPVEPKPEP EKHKDSLNMW LCPRKEVIEQ TKAPKAMTPS
       490        500        510        520        530        540        550        560
RIADSFQVIK NSPLSEWLIR PPYKEGSPKE VPGTEDRAGK QKFKSPMNTS WCSFNTADWV LPGKKMGNLS QLSSGEDKWL
       570        580        590        600        610        620
LRKKAQEVLL NSPLQEEHNF PPDHYGLPAV CDLFACMQLK VDKEKWLYRT PLQM
```

Cmpd.	No. of Cmpds.	m/z meas.	Δ m/z [ppm]	z	Rt [min]	Score	P	Range	Sequence	Modification
2959	2	698.3575	103.79	2	66.2	17.3	0	344-356	K.TDSCTNCQGNQPK.G	
14	2	1103.5194	96.25	2	29.8	16.4	0	406-424	R.ANEPCTSFAECVCDENCEK.E	Carbamidomethyl: 5, 17

Detailed Protein Report

Protein 96: **PREDICTED: beta-enolase isoform X1 [Homo sapiens]**

**Accession:**	gi|530410063	**Score:**	44.7
**Database:**	refseq_human(refseq_human_20140103.fasta)	**MW [kDa]:**	48.0
**Database Date:**	2015-11-30	**pI:**	9.1
**Modification(s):**	Oxidation	**Sequence Coverage [%]:**	7.2
		**No. of unique Peptides:**	1

10	20	30	40	50	60	70	80
MAVMRTLRAM	AMQKIFAREI	LDSRGNPTVE	VDLHTAKGRF	RAAVPSGAST	GIYEALELRD	GDKGRYLGKG	VLKAVENINN
**90**	**100**	**110**	**120**	**130**	**140**	**150**	**160**
TLGPALLQKK	LSVVDQEKVD	KFMIELDGTE	NKSKFGANAI	LGVSLAVCKA	GAAEKGVPLY	RHIADLAGNP	DLILPVPAFN
**170**	**180**	**190**	**200**	**210**	**220**	**230**	**240**
VINGGSHAGN	KLAMQEFMIL	PVGASSFKEA	MRIGAEVYHH	LKGVIKAKYG	KDATNVGDEG	GFAPNILENN	EALELLKTAI
**250**	**260**	**270**	**280**	**290**	**300**	**310**	**320**
QAAGYPDKVV	IGMDVAASEF	YRNGKYDLDF	KSPDDPARHI	TGEKLGELYK	SFIKNYPVVS	IEDPFDQDDW	ATWTSFLSGV
**330**	**340**	**350**	**360**	**370**	**380**	**390**	**400**
NIQIVGDDLT	VTNPKRIAQA	VEKKACNCLL	LKVNQIGSVT	ESIQACKLAQ	SNGWGVMVSH	RSGETEDTFI	ADLVVGLCTG
**410**	**420**	**430**	**440**	**450**			
QIKTGAPCRS	ERLAKYNQLM	RIEEALGDKA	IFAGRKFRNP	KAK			

Cmpd.	No. of Cmpds.	m/z meas.	Δ m/z [ppm]	z	Rt [min]	Score	P	Range	Sequence	Modification
2482	1	835.4239	2.22	2	61.6	10.7	2	1-14	-.MAVMRTLRAMAMQK.I	Oxidation: 4, 10

Detailed Protein Report

Protein 97: **constitutive coactivator of peroxisome proliferator-activated receptor gamma isoform a [Homo sapiens]**

Accession: gi|556503397
Database: refseq_human(refseq_human_20140103.fasta)
Database Date: 2015-11-30
Modification(s): Carbamidomethyl, Oxidation

Score: 44.6
MW [kDa]: 105.1
pI: 5.4
Sequence Coverage [%]: 6.8
No. of unique Peptides: 2

Quantitation

QD:QU **Median:** 0.38 **CV:** 0.00 % **No. of Peptides:** 1

10	20	30	40	50	60	70	80
MDRQMILSRS	SVMGVRGLQG	FVGSTCPHIC	TVVNFKELAE	HHRSKYPGCT	PTIVVDAMCC	LRYWYTPESW	ICGGQWREYF
**90**	**100**	**110**	**120**	**130**	**140**	**150**	**160**
SALRDFVKTF	TAAGIKLIFF	FDGMVEQDKR	DEWVKRRLKN	NREISRIFHY	IKSHKEQPGR	NMFFIPSGLA	VFTRFALKTL
**170**	**180**	**190**	**200**	**210**	**220**	**230**	**240**
GQETLCSLQE	ADYEVASYGL	QHNCLGILGE	DTDYLIYDTC	PYFSISELCL	ESLDTVMLCR	EKLCESLGLC	VADLPLLACL
**250**	**260**	**270**	**280**	**290**	**300**	**310**	**320**
LGNDIIPEGM	FESFRYKCLS	SYTSVKENFD	KKGNIILAVS	DHISKVLYLY	QGEKKLEEIL	PLGPNKALFY	KGMASYLLPG
**330**	**340**	**350**	**360**	**370**	**380**	**390**	**400**
QKSPWFFQKP	KGVITLDKQV	ISTSSDAESR	EEVPMCSDAE	SRQEVPMCTG	PESRREVPVY	TDSEPRQEVP	MCSDPEPRQE
**410**	**420**	**430**	**440**	**450**	**460**	**470**	**480**
VPTCTGPESR	REVPMCSDPE	PRQEVPMCTG	PEARQEVPMY	TDSEPRQEVP	MYTDSEPRQE	VPMYTGSEPR	QEVPMYTGPE
**490**	**500**	**510**	**520**	**530**	**540**	**550**	**560**
SRQEVPMYTG	PESRQEVLIR	TDPESRQEIM	CTGHESKQEV	PICTDPISKQ	EDSMCTHAEI	NQKLPVATDF	EFKLEALMCT
**570**	**580**	**590**	**600**	**610**	**620**	**630**	**640**
NPEIKQEDPT	NVGPEVKQQV	TMVSDTEILK	VARTHHVQAE	SYLVYNIMSS	GEIECSNTLE	DELDQALPSQ	AFIYRPIRQR
**650**	**660**	**670**	**680**	**690**	**700**	**710**	**720**
VYSLLLEDCQ	DVTSTCLAVK	EWFVYPGNPL	RHPDLVRPLQ	MTIPGGTPSL	KILWLNQEPE	IQVRRLDTLL	ACFNLSSSRE
**730**	**740**	**750**	**760**	**770**	**780**	**790**	**800**
ELQAVESPFQ	ALCCLLIYLF	VQVDTLCLED	LHAFIAQALC	LQGKSTSQLV	NLQPDYINPR	AVQLGSLLVR	GLTTLVLVNS
**810**	**820**	**830**	**840**	**850**	**860**	**870**	**880**
ACGFPWKTSD	FMPWNVFDGK	LFHQKYLQSE	KGYAVEVLLE	QNRSRLTKFH	NLKAVVCKAC	MKENRRITGR	AHWGSHHAGR
**890**	**900**	**910**	**920**	**930**			
WGRQGSSYHR	TGSGYSRSSQ	GQPWRDQGPG	SRQYEHDQWR	RY			

Cmpd.	No. of Cmpds.	m/z meas.	Δ m/z [ppm]	z	Rt [min]	Score	P	Range	Sequence	Modification	Ratios
1639	3	839.3743	-22.36	2	49.0	11.4	1	258-271	K.CLSSYTSVKENFDK.K	Carbamidomethyl: 1	
1242	1	666.6715	-165.15	2	45.6	11.1	0	412-422	R.EVPMCSDPEPR.Q	Carbamidomethyl: 5; Oxidation: 4	QD:QU 0.38

Detailed Protein Report

Protein 98: **PREDICTED: lipoxygenase homology domain-containing protein 1 isoform X1 [Homo sapiens]**

**Accession:**	gi|578832310	**Score:**	44.5
**Database:**	refseq_human(refseq_human_20140103.fasta)	**MW [kDa]:**	220.7
**Database Date:**	2015-11-30	**pI:**	5.2
**Modification(s):**	Carbamidomethyl	**Sequence Coverage [%]:**	2.0
		**No. of unique Peptides:**	3

Detailed Protein Report

10	20	30	40	50	60	70	80
MMQLTLNTLF	PVVSTPAITY	IVTVFTGDVR	GAGTKSKIYL	VMYGARGNKN	SGKIFLEGGV	FDRGRTDIFH	IELAVLLSPL
90	100	110	120	130	140	150	160
SRVSVGHGNV	GVNRGWFCEK	VVILCPFTGI	QQTFPCSNWL	DEKKADGLIE	RQLYEMVSLR	KKRLKKFPWS	LWVWTTDLKK
170	180	190	200	210	220	230	240
AGTNSPIFIQ	IYGQKGRTDE	ILLNPNNKWF	KPGIIEKFRI	ELPDLGRFYK	IRVWHDKRSS	GSGWHLERMT	LMNTLNKDKY
250	260	270	280	290	300	310	320
NFNCNRWLDA	NEDDNEIVRE	MTAEGPTVRR	IMGMARYHVT	VCTGELEGAG	TDANVYLCLF	GDVGDTGERL	LYNCRNNTDL
330	340	350	360	370	380	390	400
FEKGNADEFT	IESVTMRNVR	RVRIRHDGKG	SGSGWYLDRV	LVREEGQPES	DNVEFPCLRW	LDKDKDDGQL	VRELLPSDSS
410	420	430	440	450	460	470	480
ATLKNFRYHI	SLKTGDVSGA	STDSRVYIKL	YGDKSDTIKQ	VLLVSDNNLK	DYFERGRVDE	FTLETLNIGN	INRLVIGHDS
490	500	510	520	530	540	550	560
TGMHASWFLG	SVQIRVPRQG	KQYTFPANRW	LDKNQADGRL	EVELYPSEVV	EIQKLVHYEV	EIWTGDVGGA	GTSARVYMQI
570	580	590	600	610	620	630	640
YGEKGKTEVL	FLSSRSKVFE	RASKDTFQLE	AADVGEVYKL	RLGHTGEGFG	PSWFVDTVWL	RHLVVREVDL	TPEEEARKKK
650	660	670	680	690	700	710	720
EKDKLRQLLK	KERLKAKLQR	KKKKRKGSDE	EDEGEEEESS	SSEESSSEEE	EMEEEEEEEE	FGPGMQEVIE	QHKFEAHRWL
730	740	750	760	770	780	790	800
ARGKEDNELV	VELVPAGKPG	PERNTYEVQV	VTGNVPKAGT	DANVYLTIYG	EEYGDTGERP	LKKSDKSNKF	EQGQTDTFTI
810	820	830	840	850	860	870	880
YAIDLGALTK	IRIRHDNTGN	RAGWFLDRID	ITDMNNEITY	YFPCQRWLAV	EEDDGQLSRE	LLPVDESYVL	PQSEEGRGGG
890	900	910	920	930	940	950	960
DNNPLDNLAL	EQKDKSTTFS	VTIKTGVKKN	AGTDANVFIT	LFGTQDDTGM	TLLKSSKTNS	DKFERDSIEI	FTVETLDLGD
970	980	990	1000	1010	1020	1030	1040
LWKVRLGHDN	TGKAPGWFVD	WVEVDAPSLG	KCMTFPCGRW	LAKNEDDGSI	IRDLFHAELQ	TRLYTPFVPY	EITLYTSDVF
1050	1060	1070	1080	1090	1100	1110	1120
AAGTDANIFI	IIYGCDAVCT	QQKYLCTNKR	EQKQFFERKS	ASRFIVELED	VGEIIEKIRI	GHNNTGMNPG	WHCSHVDIRR
1130	1140	1150	1160	1170	1180	1190	1200
LLPDKDGAET	LTFPCDRWLA	TSEDDKKTIR	ELVPYDIFTE	KYMKDGSLRQ	VYKEVEEPLD	IVLYSVQIFT	GNIPGAGTDA
1210	1220	1230	1240	1250	1260	1270	1280
KVYITIYGDL	GDTGERYLGK	SENRTNKFER	GTADTFIIEA	ADLGVIYKIK	LRHDNSKWCA	DWYVEKVEIW	NDTNEDEFLF
1290	1300	1310	1320	1330	1340	1350	1360
LCGRWLSLKK	EDGRLERLFY	EKEYTGDRSS	NCSSPADFWE	IALSSKMADV	DISTVTGPMA	DYVQEGPIIP	YYVSVTTGKH
1370	1380	1390	1400	1410	1420	1430	1440
KDAATDSRAF	IFLIGEDDER	SKRIWLDYPR	GKRGFSRGSV	EEFYVAGLDV	GIIKKIEVLY	EMTVWTGDVV	GGGTDSNIFM
1450	1460	1470	1480	1490	1500	1510	1520
TLYGINGSTE	EMQLDKKKAR	FEREQNDTFI	MEILDIAPFT	KMRIRIDGLG	SRPEWFLERI	LLKNMNTGDL	TMFYYGDWLS
1530	1540	1550	1560	1570	1580	1590	1600
QRKGKKTLVC	EMCAVIDEEE	MMEWTSYTVA	VKTSDILGAG	TDANVFIIIF	GENGDSGTLA	LKQSANWNKF	ERNNTDTFNF
1610	1620	1630	1640	1650	1660	1670	1680
PDMLSLGHLC	KLRVWHDNKG	IFPGWHLSYV	DVKDNSRDET	FHFQCDCWLS	KSEGDGQTVR	DFACANNKIC	DELEETTYEI
1690	1700	1710	1720	1730	1740	1750	1760
VIETGNGGET	RENVWLILEG	RKNRSKEFLM	ENSSRQRAFR	KGTTDTFEFD	SIYLGDIASL	CVGHLAREDR	FIPKRELAWH
1770	1780	1790	1800	1810	1820	1830	1840
VKTITITEME	YGNVYFFNCD	CLIPLKRKRK	YFKVFEVTKT	TESFASKVQS	LVPVKYEVIV	TTGYEPGAGT	DANVFVTIFG
1850	1860	1870	1880	1890	1900	1910	1920
ANGDTGKREL	KQKMRNLFER	GSTDRFFLET	LELGELRKVR	LEHDSSGYCS	GWLVEKVEVT	NTSTGVATIF	NCGRWLDKKR
1930	1940						
GDGLTWRDLF	PSV						

Cmpd.	No. of Cmpds.	m/z meas.	Δ m/z [ppm]	z	Rt [min]	Score	P	Range	Sequence	Modification
2434	1	1045.2535	-261.87	1	60.9	17.6	1	384-392	K.DKDDGQLVR.E	
2071	1	1052.4367	-86.97	2	56.3	14.8	2	1120-1137	R.RLLPDKDGAETLTFPCDR.W	Carbamidomethyl: 16

Detailed Protein Report

Cmpd.	No. of Cmpds.	m/z meas.	Δ m/z [ppm]	z	Rt [min]	Score	P	Range	Sequence	Modification
1581	1	698.7063	-184.90	2	50.0	12.1	1	1707-1717	K.EFLMENSSRQR.A	

Detailed Protein Report

Protein 99: transthyretin precursor [Homo sapiens]

Accession: gi|4507725
Database: refseq_human(refseq_human_20140103.fasta)
Database Date: 2015-11-30

Score: 44.4
MW [kDa]: 15.9
pI: 5.4
Sequence Coverage [%]: 9.5
No. of unique Peptides: 2

Quantitation

QD:QU **Median:** 0.59 **CV:** 12.51 % **No. of Peptides:** 2

```
        10         20         30         40         50         60         70         80
MASHRLLLLC LAGLVFVSEA GPTGTGESKC PLMVKVLDAV RGSPAINVAV HVFRKAADDT WEPFASGKTS ESGELHGLTT
        90        100        110        120        130        140        150
EEEFVEGIYK VEIDTKSYWK ALGISPFHEH AEVVFTANDS GPRRYTIAAL LSPYSYSTTA VVTNPKE
```

Cmpd.	No. of Cmpds.	m/z meas.	Δ m/z [ppm]	z	Rt [min]	Score	P	Range	Sequence	Modification	Ratios	
594	1	761.8596	-3.46	2	37.4	15.4	1	55-68	R.KAADDTWEPFASGK.T		QD:QU	0.66
810	2	697.8221	10.50	2	40.1	29.0	0	56-68	K.AADDTWEPFASGK.T		QD:QU	0.52

Detailed Protein Report

Protein 100: protein inscuteable homolog isoform d [Homo sapiens]

**Accession:**	gi|507417931	**Score:**	44.1
**Database:**	refseq_human(refseq_human_20140103.fasta)	**MW [kDa]:**	57.2
**Database Date:**	2015-11-30	**pI:**	5.4
**Modification(s):**	Carbamidomethyl, Oxidation	**Sequence Coverage [%]:**	9.6
		**No. of unique Peptides:**	3

10	20	30	40	50	60	70	80
MMALPGGRHL	DSVTLPGQRL	HLMQVDSVQR	WMEDLKLMTE	CECMCVLQAK	PISLEEDAQG	DLILAGGPGP	GDPLQLLLKR
**90**	**100**	**110**	**120**	**130**	**140**	**150**	**160**
GWVISTELRR	IGQKLAQDRW	ARVHSMSVRL	TCHARSMVSE	YSAVSRNSLK	EMGEIEKLLM	EKCSELSAVT	ERCLQVENEH
**170**	**180**	**190**	**200**	**210**	**220**	**230**	**240**
VLKSMKACVS	ETLSMLGQHF	GQLLELALTR	EVQALVRKID	ASDNIYTTES	TTGNLFSLTQ	EGAPLCRIIA	KEGGVVALFK
**250**	**260**	**270**	**280**	**290**	**300**	**310**	**320**
VCRQDSFRCL	YPQALRTLAS	ICCVEEGVHQ	LEKVDGVLCL	ADILTDNSHS	EATRAEAAAV	VAQVTSPHLP	VTQHLSSFLE
**330**	**340**	**350**	**360**	**370**	**380**	**390**	**400**
SMEEIVTALV	KLCQEASSGE	VFLLASAALA	NITFFDTMAC	EMLLQLNAIR	VLLEACSDKQ	RVDTPYTRDQ	IVTILANMSV
**410**	**420**	**430**	**440**	**450**	**460**	**470**	**480**
LEQCASDIIQ	ENGVQLIMGM	LSEKPRSGTP	AEVAACERVQ	QKAAVTLARL	SRDPDVAREA	VRLSCMSRLI	ELCRSPSERN
**490**	**500**	**510**	**520**	**530**			
SSDAVLVACL	AMGIQLQVKK	TNISALLLWF	LFWHIQGCSA				

Cmpd.	No. of Cmpds.	m/z meas.	Δ m/z [ppm]	z	Rt [min]	Score	P	Range	Sequence	Modification
2221	1	953.0764	69.85	2	58.2	17.7	1	2-19	M.MALPGGRHLDSVTLPGQR.L	
561	1	592.8217	-63.15	2	35.6	12.3	1	439-449	R.VQQKAAVTLAR.L	
2976	1	754.4009	-4.94	3	66.4	14.1	1	480-500	R.NSSDAVLVACLAMGIQLQVKK.T	Carbamidomethyl: 10; Oxidation: 13

Detailed Protein Report

Protein 101: neuralized-like protein 4 isoform 2 [Homo sapiens]

**Accession:**	gi|53829368	**Score:**	43.7
**Database:**	refseq_human(refseq_human_20140103.fasta)	**MW [kDa]:**	166.6
**Database Date:**	2015-11-30	**pI:**	5.5
		**Sequence Coverage [%]:**	2.0
		**No. of unique Peptides:**	3

10	20	30	40	50	60	70	80
MAAGSGGSGG	SGGGPGPGPG	GGGGPSGSGS	GPGSNGGLGS	GGELHPRTGR	LVSLSACGRT	ARRQQPGQEF	NHGLVLSREP
90	100	110	120	130	140	150	160
LRDGRVFTVR	IDRKVNSWSG	SIEIGVTALD	PSVLDFPSSA	TGLKGGSWVV	SGCSVLRDGR	SVLEEYGQDL	DQLGEGDRVG
170	180	190	200	210	220	230	240
VERTVAGELR	LWVNGRDCGV	AATGLPPRVW	AVVDLYGKCT	QITVLPPEPG	FSPPTPIPTP	PLEPLAPTED	SALAEQGTSA
250	260	270	280	290	300	310	320
DEAFMVSPAQ	ARPETFPNSL	ESHNDFANME	LSEVVSNTIL	SAYNGGLLNV	NLSSPPAGEG	LGSSGAATSP	ILTSNDALLF
330	340	350	360	370	380	390	400
HEKCGTLIKL	SNNNKTAERR	RPLDEFNNGV	VMTNRPLRDN	EMFEIRIDKL	VDKWSGSIEI	GVTTHNPNSL	EYPATMTNLQ
410	420	430	440	450	460	470	480
SGTIMMSGCG	ILTNGKGTRR	EYCEFSLDEL	QEGDHIGLTR	KSNSALHFFI	NGIDQGVATP	LTPPVVYGVV	DLYGMAVKVT
490	500	510	520	530	540	550	560
IVHNNNHSDR	LRRNNAILRA	LSPEGALRRA	APAAQAEPER	LLFHPNCGQK	AAITHEGRTA	LRPHATDDFN	HGVVLSSRAL
570	580	590	600	610	620	630	640
RDGEVFQVRI	DKMVDKWAGS	IEIGVTTHNP	AYLQLPSTMT	NLRSGTWMMT	GNGVMHNGTT	ILDEYGHNLD	RLKAGDTVGV
650	660	670	680	690	700	710	720
VRREDGTLHF	FVNGMTQGPA	AWNVPPGVYA	VVDLYGQAAQ	ATIVDDVEVA	PVPEPLPEGN	NQVSPSSPSS	GAGGSDLRFH
730	740	750	760	770	780	790	800
QLHGSNAVIT	NGGRTALRHN	CRSEFNDAIV	ISNRALRDGE	LFEIVIQKMV	DRWSGSIEAG	VTAIRPEDLE	FPNTMTDIDY
810	820	830	840	850	860	870	880
DTWMLSGTAI	MQDGNTMRNN	YGCDLDALGT	GARIGMMRTA	KGDLHYFING	QDQGAACSGL	PPEVYAVVDL	YGQCVQVSIT
890	900	910	920	930	940	950	960
NATGPMDNSL	ATSNTATEKS	FPLHSPVAGV	AHRFHSTCGK	NVTLEEDGTR	AVRAAGYAHG	LVFSTKELRA	EEVFEVKVEE
970	980	990	1000	1010	1020	1030	1040
LDEKWAGSLR	LGLTTLAPGE	MGPGAGGGGP	GLPPSLPELR	TKTTWMVSSC	EVRRDGQLQR	MNYGRNLERL	GVGSRVGVRR
1050	1060	1070	1080	1090	1100	1110	1120
GADDTMHILV	DGEDMGPAAT	GIAKNVWAVL	DLYGPVRGVS	IVSSTRLEES	EGTQPPSPSS	DTGSEGEEDD	EGEEHGLGGQ
1130	1140	1150	1160	1170	1180	1190	1200
NEVGIIPTTL	EFLENHGKNI	LLSNGNRTAT	RVASYNQGIV	VINQPLVPQL	LVQVRIDFLN	RQWTSSLVLG	VITCAPERLN
1210	1220	1230	1240	1250	1260	1270	1280
FPASACALKR	AAWLLRGRGV	FHNGLKICEK	FGPNLDTCPE	GTILGLRLDS	SGGLHLHVNG	VDQGVAVPDV	PQPCHALVDL
1290	1300	1310	1320	1330	1340	1350	1360
YGQCEQVTIV	NPEPGAASGK	SAGTQGDMEK	ADMVDGIKES	VCWGPPPAAS	PLKSCEYHAL	CSRFQELLLL	PEDYFMPPPK
1370	1380	1390	1400	1410	1420	1430	1440
RSLCYCESCR	KLRGDEAHRR	RGEPPREYAL	PFGWCRFNLR	VNPRLEAGTL	TKKWHMAYHG	SNVAAVRRVL	DRGELGAGTA
1450	1460	1470	1480	1490	1500	1510	1520
SILSCRPLKG	EPGVGFEEPG	ENCAPPREEQ	PPPVLLSPSL	QYAGAETLAS	KVQFRDPKSQ	RTHQAQVAFQ	VCVRPGSYTP
1530	1540	1550	1560	1570			
GPPSAALGEP	PDPHFSPAEL	EWVTKEKGAT	LLCALLVRVE				

Cmpd.	No. of Cmpds.	m/z meas.	Δ m/z [ppm]	z	Rt [min]	Score	P	Range	Sequence	Modification
512	1	457.1269	-288.36	2	35.0	10.5	0	500-508	R.ALSPEGALR.R	
1492	1	555.2127	-156.80	2	47.2	20.0	2	1372-1380	K.LRGDEAHRR.R	
2678	1	693.8667	-63.13	2	64.6	13.3	1	1546-1558	K.EKGATLLCALLVR.V	

Detailed Protein Report

Protein 102: protein furry homolog-like [Homo sapiens]

**Accession:**	gi|119874201	**Score:**	43.5
**Database:**	refseq_human(refseq_human_20140103.fasta)	**MW [kDa]:**	339.4
**Database Date:**	2015-11-30	**pI:**	5.3
		**Sequence Coverage [%]:**	1.4
		**No. of unique Peptides:**	2

Detailed Protein Report

10	20	30	40	50	60	70	80
MSNITIDPDV	KPGEYVIKSL	FAEFAVQAEK	KIEVVMAEPL	EKLLSRSLQR	GEDLQFDQLI	SSMSSVAEHC	LPSLLRTLFD
90	100	110	120	130	140	150	160
WYRRQNGTED	ESYEYRPRSS	TKSKGDEQQR	ERDYLLERRD	LAVDFIFCLV	LVEVLKQIPV	HPVPDPLVHE	VLNLAFKHFK
170	180	190	200	210	220	230	240
HKEGYSGTNT	GNVHIIADLY	AEVIGVLAQS	KFQAVRKKFV	TELKELRQKE	QSPHVVQSVI	SLIMGMKFFR	VKMYPVEDFE
250	260	270	280	290	300	310	320
ASFQFMQECA	QYFLEVKDKD	IKHALAGLFV	EILIPVAAAV	KNEVNVPCLK	NFVEMLYQTT	FELSSRKKHS	LALYPLITCL
330	340	350	360	370	380	390	400
LCVSQKQFFL	NNWHIFLQNC	LSHLKNKDPK	MSRVALESLY	RLLWVYVIRI	KCESNTVTQS	RLMSIVSALF	PKGSRSVVPR
410	420	430	440	450	460	470	480
DTPLNIFVKI	IQFIAQERLD	FAMKEIIFDL	LSVGKSTKTF	TINPERMNIG	LRVFLVIADS	LQQKDGEPPM	PTTGVILPSG
490	500	510	520	530	540	550	560
NTLRVKKIFL	NKTLTDEEAK	VIGMSVYYPQ	VRKALDSILR	HLDKEVGRPM	CMTSVQMSNK	EPEDMITGER	KPKIDLFRTC
570	580	590	600	610	620	630	640
IAAIPRLIPD	GMSRTDLIEL	LARLTIHMDE	ELRALAFNTL	QALMLDFPDW	REDVLSGFVY	FIVREVTDVH	PTLLDNAVKM
650	660	670	680	690	700	710	720
LVQLINQWKQ	AAQMHNKNQD	TQHGVANGAS	HPPPLERSPY	SNVFHVVEGF	ALVILCSSRP	ATRRLAVSVL	REIRALFALL
730	740	750	760	770	780	790	800
EIPKGDDELA	IDVMDRLSPS	ILESFIHLTG	ADQTTLLYCP	SSIDLQTLAE	WNSSPISHQF	DVISPSHIWI	FAHVTQGQDP
810	820	830	840	850	860	870	880
WIISLSSFLK	QENLPKHCST	AVSYAWMFAY	TRLQLLSPQV	DINSPINAKK	VNTTTSSDSY	IGLWRNYLIL	CCSAATSSSS
890	900	910	920	930	940	950	960
TSAGSVRCSP	PETLASTPDS	GYSIDSKIIG	IPSPSSLFKH	IVPMMRSESM	EITESLVLGL	GRTNPGAFRE	LIEELHPIIK
970	980	990	1000	1010	1020	1030	1040
EALERRPENM	KRRRRRDILR	VQLVRIFELL	ADAGVISHSA	SGGLDNETHF	LNNTLLEYVD	LTRQLLEAEN	EKDSDTLKDI
1050	1060	1070	1080	1090	1100	1110	1120
RCHFSALVAN	IIQNVPVHQR	RSIFPQQSLR	HSLFMLFSHW	AGPFSIMFTP	LDRYSDRNMQ	INRHQYCALK	AMSAVLCCGP
1130	1140	1150	1160	1170	1180	1190	1200
VADNVGLSSD	GYLYKWLDNI	LDSLDKKVHQ	LGCEAVTLLL	ELNPDQSNLM	YWAVDRCYTG	SGRVAAGCFK	AIANVFQNRD
1210	1220	1230	1240	1250	1260	1270	1280
YQCDTVMLLN	LILFKAADSS	RSIYEVAMQL	LQILEPKMFR	YAHKLEVQRT	DGVLSQLSPL	PHLYSVSYYQ	LSEELARAYP
1290	1300	1310	1320	1330	1340	1350	1360
ELTLAIFSEI	SQRIQTAHPA	GRQVMLHYLL	PWMNNIELVD	LKPLPTARRH	DEDEDDSLKD	RELMVTSRRW	LRGEGWGSPQ
1370	1380	1390	1400	1410	1420	1430	1440
ATAMVLNNLM	YMTAKYGDEL	AWSEVENVWT	TLADGWPKNL	KIILHFLISI	CGVNSEPSLL	PYVKKVIVYL	GRDKTMQLLE
1450	1460	1470	1480	1490	1500	1510	1520
ELVSELQLTD	PVSSGVTHMD	NPPYYRITSS	YKIPSVTSGT	TSSSNTMVAP	TDGNPDNKPI	KENIEESYVH	LDIYSGLNSH
1530	1540	1550	1560	1570	1580	1590	1600
LNRQHHRLES	RYSSSSGGSY	EEEKSDSMPL	YSNWRLKVME	HNQGEPLPFP	PAGGCWSPLV	DYVPETSSPG	LPLHRCNIAV
1610	1620	1630	1640	1650	1660	1670	1680
ILLTDLIIDH	SVKVEWGSYL	HLLLHAIFIG	FDHCHPEVYE	HCKRLLLHLL	IVMGPNSNIR	TVASVLLRNK	EFNEPRVLTV
1690	1700	1710	1720	1730	1740	1750	1760
KQVAHLDYNF	TAGINDFIPD	YQPSPMTDSG	LSSSSTSSSI	SLGNNSAAIS	HLHTTILNEV	DISVEQDGKV	KTLMEFITSR
1770	1780	1790	1800	1810	1820	1830	1840
KRGPLWNHED	VSAKNPSIKS	AEQLTTFLKH	VVSVFKQSSS	EGIHLEHHLS	EVALQTALSC	SSRHYAGRSF	QIFRALKQPL
1850	1860	1870	1880	1890	1900	1910	1920
TATTLSDVLS	RLVETVGDPG	EDAQGFVIEL	LLTLESAIDT	LAETMKHYDL	LSALSQTSYH	DPIMGNKYAA	NRKSTGQLNL
1930	1940	1950	1960	1970	1980	1990	2000
STSPINSSSY	LGYNSNARSN	SLRLSLIGDR	RGDRRRSNTL	DIMDGRINHS	SSLARTRSLS	SLREKGMYDV	QSTTEPTNLM
2010	2020	2030	2040	2050	2060	2070	2080
ATIFWIAASL	LESDYEYEYL	LALRLLNKLL	IHLPLDKSES	REKIENVQSK	LKWTNFPGLQ	QLFLKGFTSA	STQEMTVHLL
2090	2100	2110	2120	2130	2140	2150	2160
SKLISVSKHT	LVDPSQLSGF	PLNILCLLPH	LIQHFDSPTQ	FCKETASRIA	KVCAEEKCPT	LVNLAHMMSL	YSTHTYSRDC
2170	2180	2190	2200	2210	2220	2230	2240

Detailed Protein Report

Cmpd.	No. of Cmpds.	m/z meas.	Δ m/z [ppm]	z	Rt [min]	Score	P	Range	Sequence	Modification
2945	2	753.3939	-46.69	2	66.0	18.1	1	382-395	R.LMSIVSALFPKGSR.S	
2730	1	725.3873	9.20	3	65.2	13.7	2	541-558	K.EPEDMITGERKPKIDLFR.T	

Detailed Protein Report

Protein 103: rho GTPase-activating protein 11A isoform 3 [Homo sapiens]

**Accession:**	gi|557128992	**Score:**	43.5
**Database:**	refseq_human(refseq_human_20140103.fasta)	**MW [kDa]:**	92.6
**Database Date:**	2015-11-30	**pI:**	9.9
		**Sequence Coverage [%]:**	7.4
		**No. of unique Peptides:**	3

Alias proteins:

Accession	Name	Description
gi|578827655	refseq_human (refseq_human_20140103.fasta)	PREDICTED: rho GTPase-activating protein 11A isoform X3 [Homo sapiens]
gi|557129007	refseq_human (refseq_human_20140103.fasta)	rho GTPase-activating protein 11A isoform 3 [Homo sapiens]

```
        10         20         30         40         50         60         70         80
MDSSNLAVIF APNLLQTSEG HEKMSSNTEK KLRLQAAVVQ TLIDYASDIG RVPDFILEKI PAMLGIDGLC ATPSLEGFEE
        90        100        110        120        130        140        150        160
GEYETPGEYK RKRRQSVGDF VSGALNKFKP NRTPSITPQE ERIAQLSESP VILTPNAKRT LPVDSSHGFS SKKRKSIKHN
       170        180        190        200        210        220        230        240
FNFELLPSNL FNSSSTPVSV HIDTSSEGSS QSSLSPVLIG GNHLITAGVP RRSKRIAGKK VCRVESGKAG CFSPKISHKE
       250        260        270        280        290        300        310        320
KVRRSLRLKF NLGKNGREVN GCSGVNRYES VGWRLANQQS LKNRIESVKT GLLFSPDVDE KLPKKGSEKI SKSEETLLTP
       330        340        350        360        370        380        390        400
ERLVGTNYRM SWTGPNNSSF QEVDANEASS MVENLEVENS LEPDIMVEKS PATSCELTPS NLNNKHNSNI TSSPLSGDEN
       410        420        430        440        450        460        470        480
NMTKETLVKV QKAFSESGSN LHALMNQRQS SVTNVGKVKL TEPSYLEDSP EENLFETNDL TIVESKEKYE HHTGKGEKCF
       490        500        510        520        530        540        550        560
SERDFSPLQT QTFNRETTIK CYSTQMKMEH EKDIHSNMPK DYLSKQEFSS DEEIKKQQSP KDKLNNKLKE NENMMEGNLP
       570        580        590        600        610        620        630        640
KCAAHSKDEA RSSFSQQSTC VVTNLSKPRP MRIAKQQSLE TCEKTVSESS QMTEHRKVSD HIQWFNKLSL NEPNRIKVKS
       650        660        670        680        690        700        710        720
PLKFQRTPVR QSVRRINSLL EYSRQPTGHK LASLGDTASP LVKSVSCDGA LSSCIESASK DSSVSCIKSG PKEQKSMSCE
       730        740        750        760        770        780        790        800
ESNIGAISKS SMELPSKSFL KMRKHPDSVN ASLRSTTVYK QKILSDGQVK VPLDDLTNHD IVKPVVNNNM GISSGINNRV
       810        820        830        840
LRRPSERGRA WYKGSPKHPI GKTQLLPTSK PVDL
```

Cmpd.	No. of Cmpds.	m/z meas.	Δ m/z [ppm]	z	Rt [min]	Score	P	Range	Sequence	Modification
1727	3	682.5948	-72.91	3	51.9	11.1	0	386-404	K.HNSNITSSPLSGDENNMTK.E	
2613	2	822.4595	113.47	2	63.4	14.2	0	684-700	K.SVSCDGALSSCIESASK.D	
2804	1	698.3677	40.53	4	64.2	18.2	2	716-741	K.SMSCEESNIGAISKSSMELPSKSFLK.M	

Detailed Protein Report

Protein 104: ATP-dependent RNA helicase DDX42 [Homo sapiens]

**Accession:**	gi|45446743	**Score:**	43.2
**Database:**	refseq_human(refseq_human_20140103.fasta)	**MW [kDa]:**	102.9
**Database Date:**	2015-11-30	**pI:**	6.6
		**Sequence Coverage [%]:**	5.1
		**No. of unique Peptides:**	3

Alias proteins:

Accession	Name	Description
gi|578830314	refseq_human (refseq_human_20140103.fasta)	PREDICTED: ATP-dependent RNA helicase DDX42 isoform X1 [Homo sapiens]
gi|45446747	refseq_human (refseq_human_20140103.fasta)	ATP-dependent RNA helicase DDX42 [Homo sapiens]

10	20	30	40	50	60	70	80
MNWNKGGPGT	KRGFGFGGFA	ISAGKKEEPK	LPQQSHSAFG	ATSSSSGFGK	SAPPQLPSFY	KIGSKRANFD	EENAYFEDEE
90	100	110	120	130	140	150	160
EDSSNVDLPY	IPAENSPTRQ	QFHSKPVDSD	SDDDPLEAFM	AEVEDQAARD	MKRLEEKDKE	RKNVKGIRDD	IEEEDDQEAY
170	180	190	200	210	220	230	240
FRYMAENPTA	GVVQEEEEDN	LEYDSDGNPI	APTKKIIDPL	PPIDHSEIDY	PPFEKNFYNE	HEEITNLTPQ	QLIDLRHKLN
250	260	270	280	290	300	310	320
LRVSGAAPPR	PGSSFAHFGF	DEQLMHQIRK	SEYTQPTPIQ	CQGVPVALSG	RDMIGIAKTG	SGKTAAFIWP	MLIHIMDQKE
330	340	350	360	370	380	390	400
LEPGDGPIAV	IVCPTRELCQ	QIHAECKRFG	KAYNLRSVAV	YGGGSMWEQA	KALQEGAEIV	VCTPGRLIDH	VKKKATNLQR
410	420	430	440	450	460	470	480
VSYLVFDEAD	RMFDMGFEYQ	VRSIASHVRP	DRQTLLFSAT	FRKKIEKLAR	DILIDPIRVV	QGDIGEANED	VTQIVEILHS
490	500	510	520	530	540	550	560
GPSKWNWLTR	RLVEFTSSGS	VLLFVTKKAN	AEELANNLKQ	EGHNLGLLHG	DMDQSERNKV	ISDFKKKDIP	VLVATDVAAR
570	580	590	600	610	620	630	640
GLDIPSIKTV	INYDVARDID	THTHRIGRTG	RAGEKGVAYT	LLTPKDSNFA	GDLVRNLEGA	NQHVSKELLD	LAMQNAWFRK
650	660	670	680	690	700	710	720
SRFKGGKGKK	LNIGGGGLGY	RERPGLGSEN	MDRGNNNVMS	NYEAYKPSTG	AMGDRLTAMK	AAFQSQYKSH	FVAASLSNQK
730	740	750	760	770	780	790	800
AGSSAAGASG	WTSAGSLNSV	PTNSAQQGHN	SPDSPVTSAA	KGIPGFGNTG	NISGAPVTYP	SAGAQGVNNT	ASGNNSREGT
810	820	830	840	850	860	870	880
GGSNGKRERY	TENRGSSRHS	HGETGNRHSD	SPRHGDGGRH	GDGYRHPESS	SRHTDGHRHG	ENRHGGSAGR	HGENRGANDG
890	900	910	920	930	940		
RNGESRKEAF	NRESKMEPKM	EPKVDSSKMD	KVDSKTDKTA	DGFAVPEPPK	RKKSRWDS		

Cmpd.	No. of Cmpds.	m/z meas.	Δ m/z [ppm]	z	Rt [min]	Score	P	Range	Sequence	Modification
1875	1	990.8378	-137.62	2	51.9	12.0	0	31-50	K.LPQQSHSAFGATSSSSGFGK.S	
2705	1	558.6699	147.14	3	64.9	15.6	1	819-833	R.HSHGETGNRHSDSPR.H	
2100	1	753.3769	8.39	2	56.6	15.7	2	896-908	K.MEPKMEPKVDSSK.M	

Detailed Protein Report

Protein 105: Golgi apparatus protein 1 isoform 3 precursor [Homo sapiens]

**Accession:**	gi|224586817	**Score:**	43.1
**Database:**	refseq_human(refseq_human_20140103.fasta)	**MW [kDa]:**	134.5
**Database Date:**	2015-11-30	**pI:**	6.5
**Modification(s):**	Carbamidomethyl, Oxidation	**Sequence Coverage [%]:**	2.5
		**No. of unique Peptides:**	1

10	20	30	40	50	60	70	80
MAACGRVRRM	FRLSAALHLL	LLFAAGAEKL	PGQGVHSQGQ	GPGANFVSFV	GQAGGGGPAG	QQLPQLPQSS	QLQQQQQQQQ
90	100	110	120	130	140	150	160
QQQQPQPPQP	PFPAGGPPAR	RGGAGAGGGW	KLAEEESCRE	DVTRVCPKHT	WSNNLAVLEC	LQDVREPENE	ISSDCNHLLW
170	180	190	200	210	220	230	240
NYKLNLTTDP	KFESVAREVC	KSTITEIKEC	ADEPVGKGYM	VSCLVDHRGN	ITEYQCHQYI	TKMTAIIFSD	YRLICGFMDD
250	260	270	280	290	300	310	320
CKNDINILKC	GSIRLGEKDA	HSQGEVVSCL	EKGLVKEAEE	REPKIQVSEL	CKKAILRVAE	LSSDDFHLDR	HLYFACRDDR
330	340	350	360	370	380	390	400
ERFCENTQAG	EGRVYKCLFN	HKFEESMSEK	CREALTTRQK	LIAQDYKVSY	SLAKSCKSDL	KKYRCNVENL	PRSREARLSY
410	420	430	440	450	460	470	480
LLMCLESAVH	RGRQVSSECQ	GEMLDYRRML	MEDFSLSPEI	ILSCRGEIEH	HCSGLHRKGR	TLHCLMKVVR	GEKGNLGMNC
490	500	510	520	530	540	550	560
QQALQTLIQE	TDPGADYRID	RALNEACESV	IQTACKHIRS	GDPMILSCLM	EHLYTEKMVE	DCEHRLLELQ	YFISRDWKLD
570	580	590	600	610	620	630	640
PVLYRKCQGD	ASRLCHTHGW	NETSEFMPQG	AVFSCLYRHA	YRTEEQGRRL	SRECRAEVQR	ILHQRAMDVK	LDPALQDKCL
650	660	670	680	690	700	710	720
IDLGKWCSEK	TETGQELECL	QDHLDDLVVE	CRDIVGNLTE	LESEDIQIEA	LLMRACEPII	QNFCHDVADN	QIDSGDLMEC
730	740	750	760	770	780	790	800
LIQNKHQKDM	NEKCAIGVTH	FQLVQMKDFR	FSYKFKMACK	EDVLKLCPNI	KKKVDVVICL	STTVRNDTLQ	EAKEHRVSLK
810	820	830	840	850	860	870	880
CRRQLRVEEL	EMTEDIRLEP	DLYEACKSDI	KNFCSAVQYG	NAQIIECLKE	NKKQLSTRCH	QKVFKLQETE	MMDPELDYTL
890	900	910	920	930	940	950	960
MRVCKQMIKR	FCPEADSKTM	LQCLKQNKNS	ELMDPKCKQM	ITKRQITQNT	DYRLNPMLRK	ACKADIPKFC	HGILTKAKDD
970	980	990	1000	1010	1020	1030	1040
SELEGQVISC	LKLRYADQRL	SSDCEDQIRI	IIQESALDYR	LDPQLQLHCS	DEISSLCAEE	AAAQEQTGQV	EECLKVNLLK
1050	1060	1070	1080	1090	1100	1110	1120
IKTELCKKEV	LNMLKESKAD	IFVDPVLHTA	CALDIKHHCA	AITPGRGRQM	SCLMEALEDK	RVRLQPECKK	RLNDRIEMWS
1130	1140	1150	1160	1170	1180		
YAAKVAPADG	FSDLAMQVMT	SPSKNYILSV	ISGSICILFL	IGLMCGRITK	RVTRELKDR		

Cmpd.	No. of Cmpds.	m/z meas.	Δ m/z [ppm]	z	Rt [min]	Score	P	Range	Sequence	Modification
484	1	546.2461	55.39	2	34.7	15.7	0	538-545	K.MVEDCEHR.L	Carbamidomethyl: 5; Oxidation: 1

Detailed Protein Report

Protein 106: **PREDICTED: zinc finger protein 175 isoform X3 [Homo sapiens]**

**Accession:**	gi|530417172	**Score:**	42.7
**Database:**	refseq_human(refseq_human_20140103.fasta)	**MW [kDa]:**	69.5
**Database Date:**	2015-11-30	**pI:**	10.1
**Modification(s):**	Carbamidomethyl	**Sequence Coverage [%]:**	3.5
		**No. of unique Peptides:**	2

Quantitation

QD:QU **Median:** 1.39 **CV:** 0.00 % **No. of Peptides:** 1

10	20	30	40	50	60	70	80
MPADVNLSQK	PQVLGPEKQD	GSCEASVSFE	DVTVDFSREE	WQQLDPAQRC	LYRDVMLELY	SHLFAVGYHI	PNPEVIFRML
**90**	**100**	**110**	**120**	**130**	**140**	**150**	**160**
KEKEPRVEEA	EVSHQRCQES	LKLNLEVNGQ	NESNDTEQLD	DVVGSGQLFS	HSSSDACSKN	IHTGETFCKG	NQCRKVCGHK
**170**	**180**	**190**	**200**	**210**	**220**	**230**	**240**
QSLKQHQIHT	QKKPDGCSEC	GGSFTQKSHL	FAQQRIHSVG	NLHECGKCGK	AFMPQLKLSV	YLTDHTGDIP	CICKECGKVF
**250**	**260**	**270**	**280**	**290**	**300**	**310**	**320**
IQRSELLTHQ	KTHTRKKPYK	CHDCGKAFFQ	MLSLFRHQRT	HSREKLYECS	ECGKGFSQNS	TLIIHQKIHT	GERQYACSEC
**330**	**340**	**350**	**360**	**370**	**380**	**390**	**400**
GKAFTQKSTL	SLHQRIHSGQ	KSYVCIECGQ	AFIQKAHLIV	HQRSHTGEKP	YQCHNCGKSF	ISKSQLDIHH	RIHTGEKPYE
**410**	**420**	**430**	**440**	**450**	**460**	**470**	**480**
CSDCGKTFTQ	KSHLNIHQKI	HTGERHHVCS	ECGKAFNQKS	ILSMHQRIHT	GEKPYKCSEC	GKAFTSKSQF	KEHQRIHTGE
**490**	**500**	**510**	**520**	**530**	**540**	**550**	**560**
KPYVCTECGK	AFNGRSNFHK	HQITHTRERP	FVCYKCGKAF	VQKSELITHQ	RTHMGEKPYE	CLDCGKSFSK	KPQLKVHQRI
**570**	**580**	**590**	**600**	**610**			
HTGERPYVCS	ECGKAFNNRS	NFNKHQTTHT	RDKSYKCSYS	VKGFTKQ			

Cmpd.	No. of Cmpds.	m/z meas.	Δ m/z [ppm]	z	Rt [min]	Score	P	Range	Sequence	Modification	Ratios
22	1	1205.0462	-16.45	2	30.0	19.7	1	218-238	K.LSVYLTDHTGDIPCICKECGK.V	Carbamidomethyl: 14, 16	QD:QU 1.39
6	2	1205.0350	-25.71	2	29.5	22.9	1	218-238	K.LSVYLTDHTGDIPCICKECGK.V	Carbamidomethyl: 14, 19	

Detailed Protein Report

Protein 107: PREDICTED: histone-lysine N-methyltransferase 2A isoform X1 [Homo sapiens]

**Accession:**	gi|578822195	**Score:**	42.6
**Database:**	refseq_human(refseq_human_20140103.fasta)	**MW [kDa]:**	323.9
**Database Date:**	2015-11-30	**pI:**	9.2
		**Sequence Coverage [%]:**	1.5
		**No. of unique Peptides:**	1

Detailed Protein Report

10	20	30	40	50	60	70	80
MLAQADKLPM	TDKRVASLLK	KAKAQLCKIE	KSKSLKQTDQ	PKAQGQESDS	SETSVRGPRI	KHVCRRAAVA	LGRKRAVFPD
90	100	110	120	130	140	150	160
DMPTLSALPW	EEREKILSSM	GNDDKSSIAG	SEDAEPLAPP	IKPIKPVTRN	KAPQEPPVKK	GRRSRRCGQC	PGCQVPEDCG
170	180	190	200	210	220	230	240
VCTNCLDKPK	FGGRNIKKQC	CKMRKCQNLQ	WMPSKAYLQK	QAKAVKKKEK	KSKTSEKKDS	KESSVVKNVV	DSSQKPTPSA
250	260	270	280	290	300	310	320
REDPAPKKSS	SEPPPRKPVE	EKSEEGNVSA	PGPESKQATT	PASRKSSKQV	SQPALVIPPQ	PPTTGPPRKE	VPKTTPSEPK
330	340	350	360	370	380	390	400
KKQPPPPESG	PEQSKQKKVA	PRPSIPVKQK	PKEKEKPPPV	NKQENAGTLN	ILSTLSNGNS	SKQKIPADGV	HRIRVDFKED
410	420	430	440	450	460	470	480
CEAENVWEMG	GLGILTSVPI	TPRVVCFLCA	SSGHVEFVYC	QVCCEPFHKF	CLEENERPLE	DQLENWCCRR	CKFCHVCGRQ
490	500	510	520	530	540	550	560
HQATKQLLEC	NKCRNSYHPE	CLGPNYPTKP	TKKKKVWICT	KCVRCKSCGS	TTPGKGWDAQ	WSHDFSLCHD	CAKLFAKGNF
570	580	590	600	610	620	630	640
CPLCDKCYDD	DDYESKMMQC	GKCDRWVHSK	CENLSGTEDE	MYEILSNLPE	SVAYTCVNCT	ERHPAEWRLA	LEKELQISLK
650	660	670	680	690	700	710	720
QVLTALLNSR	TTSHLLRYRQ	AAKPPDLNPE	TEESIPSRSS	PEGPDPPVLT	EVSKQDDQQP	LDLEGVKRKM	DQGNYTSVLE
730	740	750	760	770	780	790	800
FSDDIVKIIQ	AAINSDGGQP	EIKKANSMVK	SFFIRQMERV	FPWFSVKKSR	FWEPNKVSSN	SGMLPNAVLP	PSLDHNYAQW
810	820	830	840	850	860	870	880
QEREENSHTE	QPPLMKKIIP	APKPKGPGEP	DSPTPLHPPT	PPILSTDRSR	EDSPELNPPP	GIEDNRQCAL	CLTYGDDSAN
890	900	910	920	930	940	950	960
DAGRLLYIGQ	NEWTHVNCAL	WSAEVFEDDD	GSLKNVHMAV	IRGKQLRCEF	CQKPGATVGC	CLTSCTSNYH	FMCSRAKNCV
970	980	990	1000	1010	1020	1030	1040
FLDDKKVYCQ	RHRDLIKGEV	VPENGFEVFR	RVFVDFEGIS	LRRKFLNGLE	PENIHMMIGS	MTIDCLGILN	DLSDCEDKLF
1050	1060	1070	1080	1090	1100	1110	1120
PIGYQCSRVY	WSTTDARKRC	VYTCKIVECR	PPVVEPDINS	TVEHDENRTI	AHSPTSFTES	SSKESQNTAE	IISPPSPDRP
1130	1140	1150	1160	1170	1180	1190	1200
PHSQTSGSCY	YHVISKVPRI	RTPSYSPTQR	SPGCRPLPSA	GSPTPTTHEI	VTVGDPLLSS	GLRSIGSRRH	STSSLSPQRS
1210	1220	1230	1240	1250	1260	1270	1280
KLRIMSPMRT	GNTYSRNNVS	SVSTTGTATD	LESSAKVVDH	VLGPLNSSTS	LGQNTSTSSN	LQRTVVTVGN	KNSHLDGSSS
1290	1300	1310	1320	1330	1340	1350	1360
SEMKQSSASD	LVSKSSSLKG	EKTKVLSSKS	SEGSAHNVAY	PGIPKLAPQV	HNTTSRELNV	SKIGSFAEPS	SVSFSSKEAL
1370	1380	1390	1400	1410	1420	1430	1440
SFPHLHLRGQ	RNDRDQHTDS	TQSANSSPDE	DTEVKTLKLS	GMSNRSSIIN	EHMGSSSRDR	RQKGKKSCKE	TFKEKHSSKS
1450	1460	1470	1480	1490	1500	1510	1520
FLEPGQVTTG	EEGNLKPEFM	DEVLTPEYMG	QRPCNNVSSD	KIGDKGLSMP	GVPKAPPMQV	EGSAKELQAP	RKRTVKVTLT
1530	1540	1550	1560	1570	1580	1590	1600
PLKMENESQS	KNALKESSPA	SPLQIESTSP	TEPISASENP	GDGPVAQPSP	NNTSCQDSQS	NNYQNLPVQD	RNLMLPDGPK
1610	1620	1630	1640	1650	1660	1670	1680
PQEDGSFKRR	YPRRSARARS	NMFFGLTPLY	GVRSYGEEDI	PFYSSSTGKK	RGKRSAEGQV	DGADDLSTSD	EDDLYYYNFT
1690	1700	1710	1720	1730	1740	1750	1760
RTVISSGGEE	RLASHNLFRE	EEQCDLPKIS	QLDGVDDGTE	SDTSVTATTR	KSSQIPKRNG	KENGTENLKI	DRPEDAGEKE
1770	1780	1790	1800	1810	1820	1830	1840
HVTKSSVGHK	NEPKMDNCHS	VSRVKTQGQD	SLEAQLSSLE	SSRRVHTSTP	SDKNLLDTYN	TELLKSDSDN	NNSDDCGNIL
1850	1860	1870	1880	1890	1900	1910	1920
PSDIMDFVLK	NTPSMQALGE	SPESSSSELL	NLGEGLGLDS	NREKDMGLFE	VFSQQLPTTE	PVDSSVSSSI	SAEEQFELPL
1930	1940	1950	1960	1970	1980	1990	2000
ELPSDLSVLT	TRSPTVPSQN	PSRLAVISDS	GEKRVTITEK	SVASSESDPA	LLSPGVDPTP	EGHMTPDHFI	QGHMDADHIS
2010	2020	2030	2040	2050	2060	2070	2080
SPPCGSVEQG	HGNNQDLTRN	SSTPGLQVPV	SPTVPIQNQK	YVPNSTDSPG	PSQISNAAVQ	TTPPHLKPAT	EKLIVVNQNM
2090	2100	2110	2120	2130	2140	2150	2160
QPLYVLQTLP	NGVTQKIQLT	SSVSSTPSVM	ETNTSVLGPM	GGGLTLTTGL	NPSLPTSQSL	FPSASKGLLP	MSHHQHLHSF
2170	2180	2190	2200	2210	2220	2230	2240

Detailed Protein Report

Cmpd.	No. of Cmpds.	m/z meas.	Δ m/z [ppm]	z	Rt [min]	Score	P	Range	Sequence	Modification
908	1	541.3264	90.29	3	41.4	12.3	2	242-256	R.EDPAPKKSSSEPPPR.K	

Detailed Protein Report

Protein 108: dynein heavy chain 7, axonemal [Homo sapiens]

**Accession:**	gi|151301127	**Score:**	42.5
**Database:**	refseq_human(refseq_human_20140103.fasta)	**MW [kDa]:**	460.9
**Database Date:**	2015-11-30	**pI:**	5.6
		**Sequence Coverage [%]:**	1.0
		**No. of unique Peptides:**	1

Detailed Protein Report

10	20	30	40	50	60	70	80
MSSEQDKSAS	KEKSKKPVRF	LPQLSMEKLA	SKEKFKAPAR	ALPQLSMVST	KPHWQQAAPS	FHLSVKQDDE	SPEPFSVKNE
90	100	110	120	130	140	150	160
QSHAEYMERF	GKKGKLPHQV	DDSYVGPSTS	KSKGKSPHKE	RENFRSTLVN	VIMQQDADLD	SAVPDGSTIP	KPTASAIEKD
170	180	190	200	210	220	230	240
ILRYYYYIHH	GIDTDHVAPM	EDSWLEHVLD	LVPQHLKVFT	DSIVTLSDEM	REDYLLSVRK	SIVDFVLKDP	REKGDDKKTD
250	260	270	280	290	300	310	320
ELPAHRAEME	ILPKPWRKSF	LAASSYIRDH	LNAMNPTMLA	VLDLWHTNFK	KLRLVDIKEF	HNCQDALELS	SFQNIIMRHM
330	340	350	360	370	380	390	400
DSAKETLLKM	WFPEVQNIYY	QGNKKKQLPT	GDSSAKLESF	FNCAAALMTL	QLQDLTLVSM	QDFTDLIAQP	PDSVRAFEHP
410	420	430	440	450	460	470	480
GFIMRLILDN	DTIKFEPELS	DYIDIFLNVY	DVMIKAVSFV	PRVETKLYSK	WESKSKPTTL	KPIILNEIVD	AHKEKIKEVI
490	500	510	520	530	540	550	560
MKESVAPTEH	LRLYDKYDFL	ITRKAERDVD	NFLAENHSYE	KIIDEICKYQ	KLIEEIQYTS	IKTIRLGMFE	MHCEELIRAL
570	580	590	600	610	620	630	640
VKRADIICGK	LLAKMFRDHQ	EVNTRLCDEF	ERIAEKALST	PPNTAELMEM	KAYIQKVEVT	DMIELEQRLV	DSKNCLAFLI
650	660	670	680	690	700	710	720
EYVNFSPADM	RLNNSVFQWY	GRMGEIFEEH	RKIIKEKIEQ	YQEGLKLRCE	RFVEELESYA	KQSEEFYSFG	DLQDVQRYLK
730	740	750	760	770	780	790	800
KAQILNGKLD	LAADKIEQFN	AEEEAFGWLP	SVYPQRKKIQ	DGLNPYLRLY	ETAVEFSSNY	RAWTEGPYHK	VNPDQVEADI
810	820	830	840	850	860	870	880
GNYWRGLYKL	EKTFHDSPYA	LAMTKKVRSK	VEDFKQHIPL	IQVICNPGLR	PRHWEAMSAI	VGYPLQPSDD	STVSSFLDMN
890	900	910	920	930	940	950	960
LEPYIDRFEG	ISEAASKEYS	LEKAMEKMIT	EWDAVEFVIH	SYRETGTFIL	ASVDEIQMLL	DDHIIKTQTM	RGSPFIKPYE
970	980	990	1000	1010	1020	1030	1040
KQMREWEGKL	LLLQEILDEW	LKVQATWLYL	EPIFSSPDIM	SQMPEEGRRF	TAVDKTWRDI	MRSVMQDKHV	LTVVTIDRML
1050	1060	1070	1080	1090	1100	1110	1120
ERLKKSNELL	ELILKGLNEY	LEKKRLFFPR	FFFLSNDELL	EILSETKDPT	RVQPHLKKCF	EGIAKVEFTE	TLDITHMKSS
1130	1140	1150	1160	1170	1180	1190	1200
EGEVVELIEI	ISTAKARGQV	EKWLVELERV	MINSIHKVTG	DATFAYTKYE	RINWVRDWPG	QTVLCVSQIF	WTKEVQTAIP
1210	1220	1230	1240	1250	1260	1270	1280
MGIKALEQYL	KTCNRQIDDI	VTLVRGKLSM	QNRVTLGALV	VLDVHARDVL	SSLVKKNISD	DSDFEWLSQL	RYYWQENHLE
1290	1300	1310	1320	1330	1340	1350	1360
TKMINAGLRY	GYEYLGNSPR	LVITPLTDRC	YRTLFGALHL	HLGGAPEGPA	GTGKTETTKD	LAKAVAKQCV	VFNCSDGLDY
1370	1380	1390	1400	1410	1420	1430	1440
LALGKFFKGL	LSCGAWACFD	EFNRIDLEVL	SVVAQQILTI	QRGINAGADI	LMFEGTELKL	DPTCAVFITM	NPGYAGRSEL
1450	1460	1470	1480	1490	1500	1510	1520
PDNLKALFRT	VAMMVPDYAM	IAEIVLYSCG	FVTARPLSVK	IVATYRLCSE	QLSSQHHYDY	GMRAVKSVLT	AAGNLKLKYP
1530	1540	1550	1560	1570	1580	1590	1600
NENEEILLLR	SIIDVNLPKF	LSHDLPLFEG	ITSDLFPGVK	LPKPDYNDLL	AAIKDNCASM	NLQMTAFFSE	KILQVYEMMI
1610	1620	1630	1640	1650	1660	1670	1680
VRHGFMIVGE	PFGGKTSAYR	VLAGALNDIC	EKGLMEENKV	QITVLNPKSV	TMGQLYGQFD	SVSHEWSDGV	LAVSFRAFAS
1690	1700	1710	1720	1730	1740	1750	1760
SVTPDRKWLI	FDGPVDAVWI	ENMNTVLDDN	KKLCLMSGEI	IQMSPQMNLI	FEPMDLEVAS	PATVSRCGMI	YMEPHMLGWR
1770	1780	1790	1800	1810	1820	1830	1840
PLMLSWVNLL	PASVSVIQKE	FIMGLFDRMV	PVSVEFIRKH	TKELSPTSDT	NLVRSLMNLI	DCFMDDFADE	VKLKERNDRE
1850	1860	1870	1880	1890	1900	1910	1920
TYSLLEGIFL	FSLIWSVGAS	CTDDDRLKFN	KILRELMESP	ISDRTRNTFK	LQSGTEQTSS	KALTVPFPEK	GTIYDYQFVT
1930	1940	1950	1960	1970	1980	1990	2000
EGIGKWEPWI	KKLKEAPPIP	KDVMFNEIIV	PTLDTIRYSA	LMELLTTHQK	PSIFVGPTGT	GKSVYITNFL	LNQLNKEIYK
2010	2020	2030	2040	2050	2060	2070	2080
PLLINFSAQT	TAAQTQNIVM	SKLDKRRKGV	FGPPLGKRMV	VFVDDVNMPA	REVYGAQPPI	ELLRQWLDHW	NWYDLKDCSM
2090	2100	2110	2120	2130	2140	2150	2160
IKLVDIQIMC	AMGPPGGGRN	PVTPRYMRHF	NIITINEFSD	KSMYTIFSRI	LTWHLEICYK	FPDEFLDLTT	QIVNGTMTLY
2170	2180	2190	2200	2210	2220	2230	2240

Detailed Protein Report

Cmpd.	No. of Cmpds.	m/z meas.	Δ m/z [ppm]	z	Rt [min]	Score	P	Range	Sequence	Modification
2726	1	862.4253	-75.80	2	65.2	11.4	2	436-450	K.AVSFVPRVETKLYSK.W	

Detailed Protein Report

Protein 109: **testis-expressed sequence 10 protein isoform 2 [Homo sapiens]**

**Accession:**	gi|239787844	**Score:**	42.4
**Database:**	refseq_human(refseq_human_20140103.fasta)	**MW [kDa]:**	103.8
**Database Date:**	2015-11-30	**pI:**	10.1
		**Sequence Coverage [%]:**	4.4
		**No. of unique Peptides:**	1

10	20	30	40	50	60	70	80
MSRMTKKRKR	QHDFQKVKLK	VGKKKPKLQN	ATPTNFKTKT	IHLPEQLKED	GTLPTNNRKL	NIKDLLSQMH	HYNAGVKQSA
**90**	**100**	**110**	**120**	**130**	**140**	**150**	**160**
LLGLKDLLSQ	YPFIIDAHLS	NILSEVTAVF	TDKDANVRLA	AVQLLQFLAP	KIRAEQISPF	FPLVSAHLSS	AMTHITEGIQ
**170**	**180**	**190**	**200**	**210**	**220**	**230**	**240**
EDSLKVLDIL	LEQYPALITG	RSSILLKNFV	ELISHQQLSK	GLINRDRSQS	WILSVNPNRR	LTSQQWRLKV	LVRLSKFLQA
**250**	**260**	**270**	**280**	**290**	**300**	**310**	**320**
LADGSSRLRE	SEGLQEQKEN	PHATSNSIFI	NWKEHANDQQ	HIQVYENGGS	QPNVSSQFRL	RYLVGGLSGV	DEGLSSTENL
**330**	**340**	**350**	**360**	**370**	**380**	**390**	**400**
KGFIEIIIPL	LIECWVEAVP	PQLATPVGNG	IEREPLQVMQ	QVLNIISLLW	KLSKQQDETH	KLESWLRKNY	LIDFKHHFMS
**410**	**420**	**430**	**440**	**450**	**460**	**470**	**480**
RFPYVLKEIT	KHKRKEPNKS	IKHCTVLSNN	IDHLLLNLTL	SDIMVSLANA	STLQKDCSWI	EMIRKFVTET	LEDGSRLNSK
**490**	**500**	**510**	**520**	**530**	**540**	**550**	**560**
QLNRLLGVSW	RLMQIQPNRE	DTETLIKAVY	TLYQQRGLIL	PVRTLLLKFF	SKIYQTEELR	SCRFRYRSKV	LSRWLAGLPL
**570**	**580**	**590**	**600**	**610**	**620**	**630**	**640**
QLAHLGSRNP	ELSTQLIDII	HTAAARANKE	LLKSLQATAL	RIYDPQEGAV	VVLPADSQQR	LVQLVYFLPS	LPADLLSRLS
**650**	**660**	**670**	**680**	**690**	**700**	**710**	**720**
RCCIMGRLSS	SLAAMLIGIL	HMRSSFSGWK	YSAKDWLMSD	VDYFSFLFST	LTGFSKEELT	WLQSLRGVPH	VIQTQLSPVL
**730**	**740**	**750**	**760**	**770**	**780**	**790**	**800**
LYLTDLDQFL	HHWDVTEAVF	HSLLVIPARS	QNFDILQSAI	SKHLVGLTVI	PDSTAGCVFG	VICKLLDHTC	VVSETLLPFL
**810**	**820**	**830**	**840**	**850**	**860**	**870**	**880**
ASCCYSLLYF	LLTIEKGEAE	HLRKRLMLQS	LRVNRVGPEE	LPVVGQLLRL	LLQHAPLRTH	MLTNAILVQQ	IIKNITTLKS
**890**	**900**	**910**	**920**				
GSVQEQWLTD	LHYCFNVYIT	GHPQGPSALA	TVY				

Cmpd.	No. of Cmpds.	m/z meas.	Δ m/z [ppm]	z	Rt [min]	Score	P	Range	Sequence	Modification
2188	2	945.6234	75.93	2	57.8	12.1	1	508-523	K.AVYTLYQQRGLILPVR.T	

Detailed Protein Report

Protein 110: PREDICTED: uncharacterized protein KIAA1551 isoform X1 [Homo sapiens]

**Accession:**	gi|530399223	**Score:**	42.3
**Database:**	refseq_human(refseq_human_20140103.fasta)	**MW [kDa]:**	194.7
**Database Date:**	2015-11-30	**pI:**	9.7
		**Sequence Coverage [%]:**	2.1
		**No. of unique Peptides:**	2

10	20	30	40	50	60	70	80
MNWNEKPKSA	TLPPLYPKSQ	PPFLHQSLIN	QITTTSQSSF	SYPGSNQEAC	MYPGNSNPIS	QPLLNIQNYP	QQISVSDMHN
90	100	110	120	130	140	150	160
GTVVASHTSV	ERITYANVNG	PKQLTHNLQM	SSGVTQNVWL	NSPMRNPVHS	HIGATVSHQT	DFGANVPNMP	ALQSQLITSD
170	180	190	200	210	220	230	240
TYSMQMQMIP	SNSTRLPVAY	QGNQGLNQSF	SEQQVDWTQQ	CISKGLTYPD	YRPPPKLYRY	SPQSFLPDST	IQKQNFIPHT
250	260	270	280	290	300	310	320
SLQVKNSQLL	NSVLTLPSRQ	TSAVPSQQYA	TQTDKRPPPP	PYNCRYGSQP	LQSTQHITKH	LSMEVPQSRE	MLSSEIRTSF
330	340	350	360	370	380	390	400
QQQWQNPNEN	VSTIGNFTNL	KVNTNSKQPF	NSPIRSSVDG	VQTLAQTNEE	KIMDSCNPTS	NQVLDTSVAK	EKLVRDIKTL
410	420	430	440	450	460	470	480
VEIKQKFSEL	ARKIKINKDL	LMAAGCIKMT	NTSYSEPAQN	SKLSLKQTAK	IQSGPQITPV	MPENAERQTP	TVVESAETNK
490	500	510	520	530	540	550	560
TQCMLNSDIQ	EVNCRRFNQV	DSVLPNPVYS	EKRPMPDSSH	DVKVLTSKTS	AVEMTQAVLN	TQLSSENVTK	VEQNSPAVCE
570	580	590	600	610	620	630	640
TISVPKSMST	EEYKSKIQNE	NMLLLALLSQ	ARKTQKTVLK	DANQTIQDSK	PDSCEMNPNT	QMTGNQLNLK	NMETPSTSNV
650	660	670	680	690	700	710	720
SGRVLDNSFC	SGQESSTKGM	PAKSDSSCSM	EVLATCLSLW	KKQPSDTAKE	KECDKLRTNT	TAVGISKPAN	IHVKSPCSVV
730	740	750	760	770	780	790	800
GNSNSQNKIS	NPSQQTALSM	VMHNYESSGI	NITKGTELQI	AVVSPLVLSE	VKTLSVKGIT	PAVLPETVYP	VIKEGSVCSL
810	820	830	840	850	860	870	880
QNQLAENAKA	TAALKVDVSG	PVASTATSTK	IFPLTQKEKQ	NESTNGNSEV	TPNVNQGKHN	KLESAIHSPM	NDQQISQESR
890	900	910	920	930	940	950	960
NSTVVSSDTL	QIDNICSLVE	GDTSYNSQIA	KIFSSLPLKM	VEPQKPSLPN	QQGIGSREPE	KQLDNTTENK	DFGFQKDKPV
970	980	990	1000	1010	1020	1030	1040
QCTDVSHKIC	DQSKSEPPLE	SSFNNLETNR	VILEKSSLEH	ATEKSTANDT	CSSAAIQEDI	YPQEIDASSN	YTPQDPARNE
1050	1060	1070	1080	1090	1100	1110	1120
IHSDKAPVLY	LHDQLSELLK	EFPYGIEAVN	TREGSVGQQT	TYQTSEDQTA	DKTSSDSKDP	ADQIQITILS	SEQMKEIFPE
1130	1140	1150	1160	1170	1180	1190	1200
QDDQPYVVDK	LAEPQKEEPI	TEVVSQCDLQ	APAAGQSRDS	VILDSEKDDI	HCCALGWLSM	VYEGVPQCQC	NSIKNSSSEE
1210	1220	1230	1240	1250	1260	1270	1280
EKQKEQCSPL	DTNSCKQGER	TSDRDVTVVQ	FKSLVNNPKT	PPDGKSHFPE	LQDDSRKDTP	KTKHKSLPRT	EQELVAGQFS
1290	1300	1310	1320	1330	1340	1350	1360
SKCDKLNPLQ	NHKRKKLRFH	EVTFHSSNKM	TASYEQASQE	TRQKKHVTQN	SRPLKTKTAF	LPNKDVYKKH	SSLGQSLSPE
1370	1380	1390	1400	1410	1420	1430	1440
KIKLKLKSVS	FKQKRKLDQG	NVLDMEVKKK	KHDKQEQKGS	VGATFKLGDS	LSNPNERAIV	KEKMVSNTKS	VDTKASSSKF
1450	1460	1470	1480	1490	1500	1510	1520
SRILTPKEYL	QRQKHKEALS	NKASKKICVK	NVPCDSEHMR	PSKLAVQVES	CGKSNEKHSS	GVQTSKESLN	GLTSHGKNLK
1530	1540	1550	1560	1570	1580	1590	1600
IHHSQESKTY	NILRNVKEKV	GGKQPDKIWI	DKTKLDKLTN	ISNEAQFSQM	PPQVKDQKKL	YLNRVGFKCT	ERESISLTKL
1610	1620	1630	1640	1650	1660	1670	1680
ESSPRKLHKD	KRQENKHKTF	LPVKGNTEKS	NMLEFKLCPD	ILLKNTNSVE	ERKDVKPHPR	KEQAPLQVSG	IKSTKEDWLK
1690	1700	1710	1720	1730	1740	1750	
FVATKKRTQK	DSQERDNVNS	RLSKRSFSAD	GFEMLQNPVK	DSKEMFQTYK	QMYLEKRSRS	LGSSPVK	

Cmpd.	No. of Cmpds.	m/z meas.	Δ m/z [ppm]	z	Rt [min]	Score	P	Range	Sequence	Modification
2776	1	710.3755	7.84	2	63.8	11.0	0	816-830	K.VDVSGPVASTATSTK.I	
258	1	585.2801	-84.98	2	32.0	19.7	0	1662-	K.EQAPLQVSGIK.S	

Detailed Protein Report

Cmpd.	No. of Cmpds.	m/z meas.	Δ m/z [ppm]	z	Rt [min]	Score	P	Range	Sequence	Modification
								1672		

Detailed Protein Report

Protein 111: dynein heavy chain 17, axonemal [Homo sapiens]

**Accession:**	gi|256542310	**Score:**	42.2
**Database:**	refseq_human(refseq_human_20140103.fasta)	**MW [kDa]:**	509.0
**Database Date:**	2015-11-30	**pI:**	5.4
		**Sequence Coverage [%]:**	1.0
		**No. of unique Peptides:**	1

Detailed Protein Report

10	20	30	40	50	60	70	80
MTMAPDVRLE	YLEEVASIVL	KFKPDKWSKL	IGAEENVALF	TEFFEKPDVQ	VLVLTLNAAG	MIIPCLGFPQ	SLKSKGVYFI
90	100	110	120	130	140	150	160
KTKSENINKD	NYRARLLYGD	ISPTPVDQLI	AVVEEVLSSL	LNQSENMAGW	PQVVSEDIVK	QVHRLKNEMF	VMSGKIKGKT
170	180	190	200	210	220	230	240
LLPIPEHLGS	LDGTLESMER	IPSSLDNLLL	HAIETTIIDW	SHQIRDVLSK	DSAQALLDGL	HPLPQVEFEF	WDTRLLNLKC
250	260	270	280	290	300	310	320
IHEQLNRPKV	NKIVEILEKA	KSCYWPALQN	VYTNVTEGLK	EANDIVLYLK	PLRILLEEME	QADFTMLPTF	IAKVLDTICF
330	340	350	360	370	380	390	400
IWATSEYYNT	PARIIVILQE	FCNQIIEMTR	TFLSPEEVLK	GLQGEIEEVL	SGISLAVNVL	KELYQTYDFC	CVNMKLFFKD
410	420	430	440	450	460	470	480
KEPVPWEFPS	SLAFSRINSF	FQRIQTIEEL	YKTAIEFLKL	EKIELGGVRG	NLLGSLVTRI	YDEVFELVKV	FADCKYDPLD
490	500	510	520	530	540	550	560
PGDSNFDRDY	ADFEIKIQDL	DRRLATIFCQ	GFDDCSCIKS	SAKLLYMCGG	LMERPLILAE	VAPRYSVMLE	LFDAELDNAK
570	580	590	600	610	620	630	640
ILYDAQMAAS	EEGNIPLIHK	NMPPVAGQLK	WSLELQERLE	VSMKHLKHVE	HPVMSGAEAK	LTYQKYDEMM	ELLRCHREKI
650	660	670	680	690	700	710	720
YQQWVAGVDQ	DCHFNLGQPL	ILRDAASNLI	HVNFSKALVA	VLREVKYLNF	QQQKEIPDSA	ESLFSENETF	RKFVGNLELI
730	740	750	760	770	780	790	800
VGWYNEIKTI	VKAVEFLLIK	SELEAIDVKL	LSAETTLFWN	GEGVFQYIQE	VREILHNLQN	RMQKAKQNIE	GISQAMKDWS
810	820	830	840	850	860	870	880
ANPLFERKDN	KKEALLDLDG	RIANLNKRYA	AVRDAGVKIQ	AMVAENAELF	RADTLSLPWK	DYVIYIDDMV	LDEFDQFIRK
890	900	910	920	930	940	950	960
SLSFLMDNMV	IDESIAPLFE	IRMELDEDGL	TFNPTLEVGS	DRGFLALIEG	LVNDIYNVAR	LIPRLAKDRM	NYKMDLEDNT
970	980	990	1000	1010	1020	1030	1040
DLIEMREEVS	SLVINAMKEA	EEYQDSFERY	SYLWTDNLQE	FMKNFLIYGC	AVTAEDLDTW	TDDTIPKTPP	TLAQFQEQID
1050	1060	1070	1080	1090	1100	1110	1120
SYEKLYEEVS	KCENTKVFHG	WLQCDCRPFK	QALLSTIRRW	GFMFKRHLSN	HVTNSLADLE	AFMKVARMGL	TKPLKEGDYD
1130	1140	1150	1160	1170	1180	1190	1200
GLVEVMGHLM	KVKERQAATD	NMFEPLKQTI	ELLKTYGEEM	PEEIHLKLQE	LPEHWANTKK	LAIQVKLTVA	PLQANEVSIL
1210	1220	1230	1240	1250	1260	1270	1280
RRKCQQFELK	QHEFRERFRR	EAPFSFSDPN	PYKSLNKQQK	SISAMEGIME	ALSKSGGLFE	VPVPDYKQLK	ACHREVRLLK
1290	1300	1310	1320	1330	1340	1350	1360
ELWDMVVVVN	TSIEDWKTTK	WKDINVEQMD	IDCKKFAKDM	RSLDKEMKTW	DAFVGLDNTV	KNVITSLRAV	SELQNPAIRE
1370	1380	1390	1400	1410	1420	1430	1440
RHWQQLMQAT	QVKFKMSEET	TLADLLQLNL	HSYEDEVRNI	VDKAVKESGM	EKVLKALDST	WSMMEFQHEP	HPRTGTMMLK
1450	1460	1470	1480	1490	1500	1510	1520
SSEVLVETLE	DNQVQLQNLM	MSKYLAHFLK	EVTSWQQKLS	TADSVISIWF	EVQRTWSHLE	SIFIGSEDIR	TQLPGDSQRF
1530	1540	1550	1560	1570	1580	1590	1600
DDINQEFKAL	MEDAVKTPNV	VEATSKPGLY	NKLEALKKSL	AICEKALAEY	LETKRLAFPR	FYFVSSADLL	DILSNGNDPV
1610	1620	1630	1640	1650	1660	1670	1680
EVSRHLSKLF	DSLCKLKFRL	DASDKPLKVG	LGMYSKEDEY	MVFDQECDLS	GQVEVWLNRV	LDRMCSTLRH	EIPEAVVTYE
1690	1700	1710	1720	1730	1740	1750	1760
EKPREQWILD	YPAQVALTCT	QIWWTTEVGL	AFARLEEGYE	NAIRDYNKKQ	ISQLNVLITL	LMGNLNAGDR	MKIMTICTID
1770	1780	1790	1800	1810	1820	1830	1840
VHARDVVAKM	IVAKVESSQA	FTWQAQLRHR	WDEEKRHCFA	NICDAQIQYS	YEYLGNTPRL	VITPLTDRCY	ITLTQSLHLI
1850	1860	1870	1880	1890	1900	1910	1920
MGGAPAGPAG	TGKTETTKDL	GRALGTMVYV	FNCSEQMDYK	SCGNIYKGLA	QTGAWGCFDE	FNRISVEVLS	VIAVQVKCVQ
1930	1940	1950	1960	1970	1980	1990	2000
DAIRAKKKAF	NFLGEIIGLI	PTVGIFITMN	PGYAGRAELP	ENLKALFRPC	AMVVPDFELI	CEIMLMAEGF	LEARLLARKF
2010	2020	2030	2040	2050	2060	2070	2080
ITLYTLCKEL	LSKQDHYDWG	LRAIKSVLVV	AGSLKRGDPS	RAEDQVLMRA	LRDFNIPKIV	TDDLPVFMGL	IGDLFPALDV
2090	2100	2110	2120	2130	2140	2150	2160
PRKRDLNFEK	IIKQSIVELK	LQAEDSFVLK	VVQLEELLQV	RHSVFIVGNA	GSGKSQVLKS	LNKTYQNLKR	KPVAVDLDPK
2170	2180	2190	2200	2210	2220	2230	2240

Detailed Protein Report

Cmpd.	No. of Cmpds.	m/z meas.	Δ m/z [ppm]	z	Rt [min]	Score	P	Range	Sequence	Modification
637	1	635.8011	-148.78	2	36.5	10.8	2	4247-4257	R.RSLKELNLGLK.G	

Detailed Protein Report

Protein 112: **PREDICTED: LOW QUALITY PROTEIN: UPF0627 protein ENSP00000364708 [Homo sapiens]**

**Accession:**	gi|410171120	**Score:**	42.1
**Database:**	refseq_human(refseq_human_20140103.fasta)	**MW [kDa]:**	18.2
**Database Date:**	2015-11-30	**pI:**	9.4
**Modification(s):**	Carbamidomethyl	**Sequence Coverage [%]:**	8.9
		**No. of unique Peptides:**	3

```
        10         20         30         40         50         60         70         80
MGCSKGLWKE RPSAHTSECF STTACPVAFI LLVWNSQSPA GLQSFCTGRH PSLSVRAQRA GPGASREEGT XWTECVGQER
        90        100        110        120        130        140        150        160
RLIHSGSSEN ESQEDEGADL IPYTGLKADN RRKSSTWANE VEDRRPQSTP ALNLTPSHPH PPRPLITFLR SVIGIQILPG
       170
LVAAGGTVA
```

Cmpd.	No. of Cmpds.	m/z meas.	Δ m/z [ppm]	z	Rt [min]	Score	P	Range	Sequence	Modification
2122	1	937.3818	-36.45	2	55.1	13.6	1	0-0	.EEGTHWTECVGQERR.	Carbamidomethyl: 9
1988	1	918.3938	-29.29	2	55.2	12.1	1	67-81	R.EEGTVWTECVGQERR.L	Carbamidomethyl: 9
1963	2	918.3504	-82.67	2	54.9	16.4	2	67-81	R.EEGTRWTECVGQERR.L	

Detailed Protein Report

Protein 113: dynein heavy chain 11, axonemal [Homo sapiens]

**Accession:**	gi|459642365	**Score:**	42.0
**Database:**	refseq_human(refseq_human_20140103.fasta)	**MW [kDa]:**	520.0
**Database Date:**	2015-11-30	**pI:**	6.0
**Modification(s):**	Carbamidomethyl	**Sequence Coverage [%]:**	0.8
		**No. of unique Peptides:**	1

Detailed Protein Report

10	20	30	40	50	60	70	80
MAAQVAAREA	RDFREAPTLR	LTSGAGLEAV	GAVELEEEEE	NEEEAAARRA	RSFAQDARVR	FLGGRLAMML	GFTEEKWSQY
90	100	110	120	130	140	150	160
LESEDNRQVL	GEFLESTSPA	CLVFSFAASG	RLAASQEIPR	DANHKLVFIS	KKITESIGVN	DFSQVVLFGE	LPALSLGHVS
170	180	190	200	210	220	230	240
AFLDEILVPV	LSNKNNHKSW	SCFTSQDMEY	HIEVMKKKMY	IFRGKMSRRT	LLPIPTVAGK	MDLDQNCSEN	KPPSNERIIL
250	260	270	280	290	300	310	320
HAIESVVIEW	SHQIQEIIER	DSVQRLLNGL	HLSPQAELDF	WMMRRENLSC	IYDQLQAPVV	LKMVKILTTK	QSSYFPTLKD
330	340	350	360	370	380	390	400
IFLAVENALL	EAQDVELYLR	PLRRHIQCLQ	ETEFPQTRIL	IAPLFHTICL	IWSHSKFYNT	PARVIVLLQE	FCNLFINQAT
410	420	430	440	450	460	470	480
AYLSPEDLLR	GEIEESLEKV	QVAVNILKTF	KNSFFNYRKK	LASYFMGRKL	RPWDFQSHLV	FCRFDKFLDR	LIKIEDIFAT
490	500	510	520	530	540	550	560
TLEFEKLERL	EFGGTKGAIL	NGQVHEMSEE	LMELCKLFKQ	STYDPSDCTN	MEFESDYVAF	KSKTLEFDRR	LGTIICEAFF
570	580	590	600	610	620	630	640
NCNGLEAAFK	LLTIFGNFLE	KPVVMEIFSL	HYSTLVHMFN	TELDVCKQLY	NEHMKQIECG	HVVLNKNMPF	TSGNMKWAQQ
650	660	670	680	690	700	710	720
VLQRLQMFWS	NFASLRYLFL	GNPDHALVYQ	KYVEMTTLLD	QFESRIYNEW	KSNVDEICEF	NLNQPLVKFS	AINGLLCVNF
730	740	750	760	770	780	790	800
DPKLVAVLRE	VKYLLMLKKQ	DIPDSALAIF	KKRNTILKYI	GNLDLLVQGY	NKLKQTLLEV	EYPLIEDELR	AIDEQLTAAT
810	820	830	840	850	860	870	880
TWLTWQDDCW	GYIERVRAAT	SELEHRVERT	QKNVKVIQQT	MRGWARCVLP	PRREHRREAA	FTLEDKGDLF	TKKYKLIQGD
890	900	910	920	930	940	950	960
GCKIHNLVEE	NRKLFKANPS	LDTWKIYVEF	IDDIVVEGFF	QAIMHDLDFF	LKNTEKQLKP	APFFQAQMIL	LPPEIVFKPS
970	980	990	1000	1010	1020	1030	1040
LDREAGDGFY	DLVEEMLCNS	FRMSAQMNRI	ATHLEIKNYQ	NDMDNMLGLA	EVRQEIMNRV	VNVINKVLDF	RNTLETHTYL
1050	1060	1070	1080	1090	1100	1110	1120
WVDDRAEFMK	HFLLYGHAVS	SDEMDAHANE	EIPEQPPTLE	QFKEQIDIYE	ALYVQMSKFE	DFRVFDSWFK	VDMKPFKVSL
1130	1140	1150	1160	1170	1180	1190	1200
LTIIKKWSWM	FQEHLLRFVI	DSLNELQEFI	KETDSGLQRE	LNEGDHDGLV	DIMVHLLAVR	SRQRATDELF	EPLKETITLL
1210	1220	1230	1240	1250	1260	1270	1280
ESYGQKMPEQ	VYIQLEELPE	RWETTKKIAA	TVRHEVSPLH	NAEVTLIRKK	CILFDAKQAE	FRERFRHYAP	LGFNAENPYT
1290	1300	1310	1320	1330	1340	1350	1360
ALDKANEELE	ALEEEMLQMQ	ESTRLFEVAL	PEYKQMKQCR	KEIKLLKGLW	DVIIYVRRSI	DNWTKTQWRQ	IHVEQMDVEL
1370	1380	1390	1400	1410	1420	1430	1440
RRFAKEIWSL	NKEVRVWDAY	TGLEGTVKDM	TASLRAITEL	QSPALRDRHW	HQLMKAIGVK	FLINEATTLA	DLLALRLHRV
1450	1460	1470	1480	1490	1500	1510	1520
EDDVRRIVDK	AVKELGTEKV	ITEISQTWAT	MKFSYEVHYR	TGIPLLKSDE	QLFETLEHNQ	VQLQTLLQSK	YVEYFIEQVL
1530	1540	1550	1560	1570	1580	1590	1600
SWQNKLNIAD	LVIFTWMEVQ	RTWSHLESIF	VCSEDIRIQL	VKDARRFDGV	DAEFKELMFK	TAKVENVLEA	TCRPNLYEKL
1610	1620	1630	1640	1650	1660	1670	1680
KDLQSRLSLC	EKALAEYLET	KRIAFPRFYF	VSSADLLDIL	SKGAQPKQVT	CHLAKLFDSI	ADLQFEDNQD	VSAHRAVGMY
1690	1700	1710	1720	1730	1740	1750	1760
SKEKEYVPFQ	AECECVGHVE	TWLLQLEQTM	QETVRHSITE	AIVAYEEKPR	ELWIFDFPAQ	VALTSSQIWW	TTDVGIAFSR
1770	1780	1790	1800	1810	1820	1830	1840
LEEGYETALK	DFHKKQISQL	NTLITLLLGE	LPPGDRQKIM	TICTIDVHAR	DVVAKLISQK	VVSPQAFTWL	SQLRHRWEDT
1850	1860	1870	1880	1890	1900	1910	1920
QKHCFVNICD	AQFQYFYEYL	GNSPRLVITP	LTDRCYITLT	QSLHLTMSGA	PAGPAGTGKT	ETTKDLGRAL	GMMVYVFNCS
1930	1940	1950	1960	1970	1980	1990	2000
EQMDYKSIGN	IYKGLVQTGA	WGCFDEFNRI	SVEVLSVVAV	QVKMIHDAIR	NRKKRFVFLG	EAITLKPSVG	IFITMNPGYA
2010	2020	2030	2040	2050	2060	2070	2080
GRTELPENLK	ALFRPCAMVA	PDIELICEIL	LVAEGFVDAR	ALARKFITLY	TLCKELLSKQ	DHYDWGLRAI	KSVLVVAGSL
2090	2100	2110	2120	2130	2140	2150	2160
KRGDKNRPED	QVLMRALRDF	NMPKIVTDDI	PVFLGLVGDL	FPALDVPRRR	KLHFEQMVRQ	STLELRLQPE	ESFILKVVQL
2170	2180	2190	2200	2210	2220	2230	2240

Detailed Protein Report

Cmpd.	No. of Cmpds.	m/z meas.	Δ m/z [ppm]	z	Rt [min]	Score	P	Range	Sequence	Modification
2496	1	822.4302	-68.99	2	61.8	12.6	1	2977-2990	R.LQLKIILCFSPVGR.T	Carbamidomethyl: 8

Detailed Protein Report

Protein 114: PREDICTED: zinc finger protein 665 isoform X5 [Homo sapiens]

Accession: gi|578834915
Database: refseq_human(refseq_human_20140103.fasta)
Database Date: 2015-11-30
Modification(s): Carbamidomethyl, Oxidation

Score: 41.8
MW [kDa]: 76.3
pI: 10.3
Sequence Coverage [%]: 3.3
No. of unique Peptides: 2

Quantitation

QD:QU **Median:** 1.17 **CV:** 0.00 % **No. of Peptides:** 1

10	20	30	40	50	60	70	80
MVCPCSGLLL	AVGGMPPRCQ	GRTLEEEEEE	GKGVRDGSSS	DISCKCVNTD	LPPKGKNNMG	EAFYTVKLER	LESCDTVGLS
90	100	110	120	130	140	150	160
FQEVQKNTYD	FECQWKDDEG	NYKTVLMLQK	ENLPGRRAQR	DRRAAGNRHI	ENQLGVSFQS	HLPELQQFQH	EGKIYEYNQV
170	180	190	200	210	220	230	240
EKSPNNRGKH	YKCDECGKVF	SQNSRLTSHK	RIHTGEKPYQ	CNKCGKAFTV	RSNLTIHQVI	HTGEKPYKCN	ECGKVFSQPS
250	260	270	280	290	300	310	320
NLAGHQRIHT	GEKPYKCNEC	GKAFRAHSKL	TTHQVIHTGE	KPYKCKECGK	CFTQNSHLAS	HRRIHTGEKP	YKCNECGKAF
330	340	350	360	370	380	390	400
SVRSSLTTHQ	TIHTGEKPYK	CNECGKVFRH	NSYLAKHRRI	HTGEKPYKCN	ECGKAFSMHS	NLTKHQIIHT	GEKPFKCNEC
410	420	430	440	450	460	470	480
VKVFTQYSHL	ANHRRIHTGE	KPYRCDECGK	AFSVRSSLTT	HQAIHTGEKP	YKCNDCGKVF	TQNSHLASHR	GIHSGEKPYK
490	500	510	520	530	540	550	560
CDECGKAFSQ	TSQLARHWRV	HTGEKPYKCN	ECGKAFSVHS	SLTIHQTIHT	GQKPYKCNDC	GKVFRHNSYL	AIHQRIHTGE
570	580	590	600	610	620	630	640
KPYKCNECGK	AFSVHSNLAT	HQVIHTGEKP	YKCNECGKVF	TQNSHLANHR	RIHTGEKPYR	CNECGKAFSV	RSTLTTHMAV
650	660	670	680				
HTGDKPYKCN	QCGKVFTQNS	NLAKHRRIHS	G				

Cmpd.	No. of Cmpds.	m/z meas.	Δ m/z [ppm]	z	Rt [min]	Score	P	Range	Sequence	Modification	Ratios
2877	5	937.4629	1.55	2	65.1	10.5	0	1-18	-.MVCPCSGLLLAVGGMPPR.C	Carbamidomethyl: 5; Oxidation: 15	QD:QU 1.17
2957	4	937.4644	3.23	2	66.1	16.2	0	1-18	-.MVCPCSGLLLAVGGMPPR.C	Carbamidomethyl: 3; Oxidation: 15	

Detailed Protein Report

Protein 115: PREDICTED: peroxidasin-like protein isoform X2 [Homo sapiens]

**Accession:**	gi|530388290	**Score:**	41.8
**Database:**	refseq_human(refseq_human_20140103.fasta)	**MW [kDa]:**	95.0
**Database Date:**	2015-11-30	**pI:**	9.5
**Modification(s):**	Carbamidomethyl	**Sequence Coverage [%]:**	2.5
		**No. of unique Peptides:**	2

10	20	30	40	50	60	70	80
MCGSEFFWPC	CDQHVSYSHG	KPHTSSDLLA	QFHYPRDPLI	VEMARAGEIF	EHTLQLIRER	VKQGLTVDLE	GKEFRYNDLV
**90**	**100**	**110**	**120**	**130**	**140**	**150**	**160**
SPRSLSLIAN	LSGCTARRPL	PNCSNRCFHA	KYRAHDGTCN	NLQQPTWGAA	LTAFARLLQP	AYRDGIRAPR	GLGLPVGSRQ
**170**	**180**	**190**	**200**	**210**	**220**	**230**	**240**
PLPPPRLVAT	VWARAAAVTP	DHSYTRMLMH	WGWFLEHDLD	HTVPALSTAR	FSDGRPCSSV	CTNDPPCFPM	NTRHADPRGT
**250**	**260**	**270**	**280**	**290**	**300**	**310**	**320**
HAPCMLFARS	SPACASGRPS	ATVDSVYARE	QINQQTAYID	GSNVYGSSER	ESQALRDPSV	PRGLLKTGFP	WPPSGKPLLP
**330**	**340**	**350**	**360**	**370**	**380**	**390**	**400**
FSTGPPTECA	RQEQESPCFL	AGDHRANEHL	ALAAMHTLWF	REHNRMATEL	SALNPHWEGN	TVYQEARKIV	GAELQHITYS
**410**	**420**	**430**	**440**	**450**	**460**	**470**	**480**
HWLPKVLGDP	GTRMLRGYRG	YNPNVNAGII	NSFATAAFRF	GHTLINPILY	RLNATLGEIS	EGHLPFHKAL	FSPSRIIKEG
**490**	**500**	**510**	**520**	**530**	**540**	**550**	**560**
GIDPVLRGLF	GVAAKWRAPS	YLLSPELTQR	LFSAAYSAAV	DSAATIIQRG	RDHGIPPYVD	FRVFCNLTSV	KNFEDLQNEI
**570**	**580**	**590**	**600**	**610**	**620**	**630**	**640**
KDSEIRQKLR	KLYGSPGDID	LWPALMVEDL	IPGTRVGPTL	MCLFVTQFQR	LRDGDRFWYE	NPGVFTPAQL	TQLKQASLSR
**650**	**660**	**670**	**680**	**690**	**700**	**710**	**720**
VLCDNGDSIQ	QVQADVFVKA	EYPQDYLNCS	EIPKVDLRVW	QDCCADCRSR	GQFRAVTQES	QKKRSAQYSY	PVDKDMELSH
**730**	**740**	**750**	**760**	**770**	**780**	**790**	**800**
LRSRQQDKIY	VGEDARNVTV	LAKTKFSQDF	STFAAEIQET	ITALREQINK	LEARLRQAGC	TDVRGVPRKA	EERWMKEDCT
**810**	**820**	**830**	**840**	**850**			
HCICESGQVT	CVVEICPPAP	CPSPELVKGT	CCPVCRDRGM	PSDSPEKR			

Cmpd.	No. of Cmpds.	m/z meas.	Δ m/z [ppm]	z	Rt [min]	Score	P	Range	Sequence	Modification
1881	1	628.2724	57.53	2	53.9	12.9	0	679-688	R.VWQDCCADCR.S	Carbamidomethyl: 5
1949	1	656.8239	117.11	2	54.8	14.5	0	679-688	R.VWQDCCADCR.S	Carbamidomethyl: 5, 9

Detailed Protein Report

Protein 116: PREDICTED: polyamine-modulated factor 1-binding protein 1 isoform X3 [Homo sapiens]

**Accession:**	gi|578829261	**Score:**	41.6
**Database:**	refseq_human(refseq_human_20140103.fasta)	**MW [kDa]:**	118.6
**Database Date:**	2015-11-30	**pI:**	5.9
**Modification(s):**	Carbamidomethyl	**Sequence Coverage [%]:**	3.5
		**No. of unique Peptides:**	2

Quantitation

QD:QU	**Median:** 0.42	**CV:** 0.00 %	**No. of Peptides:** 1

Alias proteins:

Accession	Name	Description
gi|578829263	refseq_human (refseq_human_20140103.fasta)	PREDICTED: polyamine-modulated factor 1-binding protein 1 isoform X4 [Homo sapiens]

10	20	30	40	50	60	70	80
MKDEAGERDR	EVSSLNSKLL	SLQLDIKNLH	DVCKRQRKTL	QDNQLCMEEA	MNSSHDKKQA	QALAFEESEV	EFGSSKQCHL
90	100	110	120	130	140	150	160
RQLQQLKKKL	LVLQQELEFH	TEELQTSYYS	LRQYQSILEK	QTSDLVLLHH	HCKLKEDEVI	LYEEEMGNHN	ENTGEKLHLA
170	180	190	200	210	220	230	240
QEQLALAGDK	IASLERSLNL	YRDKYQSSLS	NIELLECQVK	MLQGELGGIM	GQEPENKGDH	SKVRIYTSPC	MIQEHQETQK
250	260	270	280	290	300	310	320
RLSEVWQKVS	QQDDLIQELR	NKLACSNALV	LEREKALIKL	QADFASCTAT	HRYPPSSSEE	CEDIKKILKH	LQEQKDSQCL
330	340	350	360	370	380	390	400
HVEEYQNLVK	DLRVELEAVS	EQKRNIMKDM	MKLELDLHGL	REETSAHIER	KDKDITILQC	RLQELQLEFT	ETQKLTLKKD
410	420	430	440	450	460	470	480
KFLQEKDEML	QELEKKLTQV	QNSLLKKEKE	LEKQQCMATE	LEMTVKEAKQ	DKSKEAECKA	LQAEVQKLKN	SLEEAKQQER
490	500	510	520	530	540	550	560
LAAQQAAQCK	EEAALAGCHL	EDTQRKLQKG	LLLDKQKADT	IQELQRELQM	LQKESSMAEK	EQTSNRKRVE	ELSLELSEAL
570	580	590	600	610	620	630	640
RKLENSDKEK	RQLQKTVAEQ	DMKMNDMLDR	IKHQHREQGS	IKCKLEEDLQ	EATKLLEDKR	EQLKKSKEHE	KLMEGELEAL
650	660	670	680	690	700	710	720
RQEFKKKDKT	LKENSRKLEE	ENENLRAELQ	CCSTQLESSL	NKYNTSQQVI	QDLNKEIALQ	KESLMSLQAQ	LDKALQKEKH
730	740	750	760	770	780	790	800
YLQTTITKEA	YDALSRKSAA	CQDDLTQALE	KLNHVTSETK	SLQQSLTQTQ	EKKAQLEEEI	IAYEERMKKL	NTELRKLRGF
810	820	830	840	850	860	870	880
HQESELEVHA	FDKKLEEMSC	QVLQWQKQHQ	NDLKMLAAKE	EQLREFQEEM	AALKENLLED	DKEPCCLPQW	SVPKDTCRLY
890	900	910	920	930	940	950	960
RGNDQIMTNL	EQWAKQQKVA	NEKLGNQLRE	QVKYIAKLSG	EKDHLHSVMV	HLQQENKKLK	KEIEEKKMKA	ENTRLCTKAL
970	980	990	1000	1010	1020		
GPSRTESTQR	EKVCGTLGWK	GLPQDMGQRM	DLTKYIGMPH	CPGTSAICQK	NKCDFFL		

Cmpd.	No. of Cmpds.	m/z meas.	Δ m/z [ppm]	z	Rt [min]	Score	P	Range	Sequence	Modification	Ratios	
293	1	477.5768	-43.77	3	32.4	14.6	1	261-273	R.NKLACSNALVLER.E		QD:QU	0.42
1491	1	472.2634	90.28	2	48.8	14.3	1	1011-1017	K.NKCDFFL.-	Carbamidomethyl: 3		

Detailed Protein Report

Protein 117: DC-STAMP domain-containing protein 1 isoform 2 [Homo sapiens]

Accession: gi|219521926
Database: refseq_human(refseq_human_20140103.fasta)
Database Date: 2015-11-30
Modification(s): Carbamidomethyl, Oxidation

Score: 41.5
MW [kDa]: 77.7
pI: 10.3
Sequence Coverage [%]: 10.0
No. of unique Peptides: 3

10	20	30	40	50	60	70	80
MDIKHHQNGT	RGQRRKQPHT	TVQRLLTWGL	PVSCSWFLWR	QPGEFPVTAL	LLGAGAGGLL	AIGLGAMGWG	TSPHIRCASL
90	100	110	120	130	140	150	160
LLVPKMLGKE	GRLFVLGYAL	AAIYVGPVAN	LRHNLNNVIA	SLGCTVELQI	NNTRAAWRIS	TAPLRAMFKD	LLSSKELLRA
170	180	190	200	210	220	230	240
ETRNISATFE	DLDAQVNSET	GYTPEDTMDS	GETAQGREAR	QAPASRLHLS	TQKMYELKTK	LRCSYVVNQA	ILSCRRWFDR
250	260	270	280	290	300	310	320
KHEQCMKHIW	VPLLTHLLCL	PMKFKFFCGI	AKVMEVWCRN	RIPVEGNFGQ	TYDSLNQSIR	GLDGEFSANI	DFKEEKQAGV
330	340	350	360	370	380	390	400
LGLNTSWERV	STEVRDYVYR	QEARLEWALG	LLHVLLSCTF	LLVLHASFSY	MDSYNHDIRF	DNIYISTYFC	QIDDRRKKLG
410	420	430	440	450	460	470	480
KRTLLPLRKA	EEKTVIFPCK	PTIQASEMSN	VVRELLETLP	ILLLLVVLCG	LDWALYSIFD	TIRHHSFLQY	SFRSSHKLEV
490	500	510	520	530	540	550	560
KVGGDSMLAR	LLRKTIGALN	TSSETVMESN	NMPCLPQPVG	LDARAYWRAA	VPIGLLVCLC	LLQAFGYRLR	RVIAAFYFPK
570	580	590	600	610	620	630	640
REKKRILFLY	NDLLKKRAAF	TKLRRAAILR	RERQQKAPRH	PLADILHRGC	PLLRRWLCRR	CVVCQAPETP	ESYVCRTLDC
650	660	670	680	690			
EAVYCWSCWD	DMRQRCPVCT	PREELSSSAF	SDSNDDTAYA	G			

Cmpd.	No. of Cmpds.	m/z meas.	Δ m/z [ppm]	z	Rt [min]	Score	P	Range	Sequence	Modification
2414	1	964.4799	-71.72	3	60.6	15.7	2	86-112	K.MLGKEGRLFVLGYALAAIYVGPVANLR.H	
748	1	1010.1872	-33.67	3	39.3	12.9	2	248-272	K.HIWVPLLTHLLCLPMKFKFFCGIAK.V	Carbamidomethyl: 21; Oxidation: 15
2267	1	920.9067	1.92	2	58.8	12.9	0	621-636	R.CVVCQAPETPESYVCR.T	Carbamidomethyl: 1

Detailed Protein Report

Protein 118: PREDICTED: chromodomain-helicase-DNA-binding protein 9 isoform X9 [Homo sapiens]

**Accession:**	gi|530424378	**Score:**	41.5
**Database:**	refseq_human(refseq_human_20140103.fasta)	**MW [kDa]:**	269.5
**Database Date:**	2015-11-30	**pI:**	8.8
		**Sequence Coverage [%]:**	1.6
		**No. of unique Peptides:**	3

Quantitation

QD:QU	**Median:** 0.76	**CV:** 0.00 %	**No. of Peptides:** 1

Detailed Protein Report

10	20	30	40	50	60	70	80
MSEKKQRKKV	ESESKQEKAN	RIISEAIAKA	KERGERNIPR	VMSPENFPTA	SVEGKEEKKG	RRMKSKPKDK	DSKKTKTCSK
90	100	110	120	130	140	150	160
LKEKTKIGKL	IITLGKKQKR	KNESSDEISD	AEQMPQHTLK	DQDSQKRRSN	RQIKRKKYAE	DIEGKQSEEE	VKGSMKIKKN
170	180	190	200	210	220	230	240
SAPLPGEQPL	QLFVENPSEE	DAAIVDKILS	SRTVKKEISP	GVMIDTEEFF	VKYKNYSYLH	CEWATEEQLL	KDKRIQQKIK
250	260	270	280	290	300	310	320
RFKLRQAQRA	HFFADMEEEP	FNPDYVEVDR	VLEVSFCEDK	DTGEPVIYYL	VKWCSLPYED	STWELKEDVD	LAKIEEFEQL
330	340	350	360	370	380	390	400
QASRPDTRRL	DRPPSNIWKK	IDQSRDYKNG	NQLREYQLEG	LNWLLFNWYN	RRNCILADEM	GLGKTIQSIT	FLYEILLTGI
410	420	430	440	450	460	470	480
RGPFLIIAPL	STIANWEREF	RTWTDINVVV	YHGSLISRQM	IQQYEMYFRD	SQGRIIRGAY	RFQAIITTFE	MILGGCGELN
490	500	510	520	530	540	550	560
AIEWRCVIID	EAHRLKNKNC	KLLEGLKLMN	LEHKVLLTGT	PLQNTVEELF	SLLHFLEPLR	FPSESTFMQE	FGDLKTEEQV
570	580	590	600	610	620	630	640
QKLQAILKPM	MLRRLKEDVE	KKLAPKEETI	IEVELTNIQK	KYYRAILEKN	FSFLSKGAGQ	TNVPNLVNTM	MELRKCCNHP
650	660	670	680	690	700	710	720
YLIKGAEEKI	LGEFRDTYNP	AASDFHLQAM	IQSAGKLVLI	DKLLPKMKAG	GHKVLIFSQM	VRCLDILEDY	LIHKRYLYER
730	740	750	760	770	780	790	800
IDGRVRGNLR	QAAIDRFSKP	DSDRFVFLLC	TRAGGLGINL	TAADTCIIFD	SDWNPQNDLQ	AQARCHRIGQ	NKAVKVYRLV
810	820	830	840	850	860	870	880
TRNSYEREMF	DRASLKLGLD	KAVLQSMSGR	ESNVGGIQQL	SKKEIEDLLR	RGAYGAIMEE	EDEGSKFCEE	DIDQILLRRT
890	900	910	920	930	940	950	960
KTITIESEGR	GSTFAKASFV	ASGNRTDISL	DDPNFWQKWA	KKAEIDIEAI	SGRNSLVIDT	PRIRKQTRPF	SATKDELAEL
970	980	990	1000	1010	1020	1030	1040
SEAESEGDEK	PKLRRPCDRS	NGYGRTECFR	VEKNLLVYGW	GRWREILSHG	RFKRQLNEHD	VEIICRALLA	YCLVHYRGDE
1050	1060	1070	1080	1090	1100	1110	1120
KIKGFIWDLI	TPTEDGQTRE	LQNHLGLSAP	VPRGRKGKKV	KTQTSSFDIQ	KAEWLRKYNP	EQLLQDEGYK	KHIKHHCNKV
1130	1140	1150	1160	1170	1180	1190	1200
LLRVRMLYYL	KQEVIGNECQ	KVFDGVDASD	IDVWVPEPDH	SEVPAEWWDF	DADKSLLIGV	FKHGYEKYNT	IRADPALCFL
1210	1220	1230	1240	1250	1260	1270	1280
ERVGKPDEKA	VAAEQRANDY	MDGDVEDPEY	KPAPAIFKDD	IEDDVSSPGD	LVIADGDGQL	MEGDKVYWPT	QSALTTRLRR
1290	1300	1310	1320	1330	1340	1350	1360
LITAYQRTNK	NRQIQQIQPT	FSVPTSVMQP	IYEEATLNPK	MAAKIERQQR	WTRREEADFY	RVVSTFGVVF	DPDRGQFDWT
1370	1380	1390	1400	1410	1420	1430	1440
KFRAMARLHK	KTDDSLEKYL	YAFMSMCRRV	CRLPSKEELV	DPNIFIQPIT	EERASRTLYR	IELLRKVREQ	ALRHPQLFER
1450	1460	1470	1480	1490	1500	1510	1520
LKLCHPNPDL	PVWWECGPHD	RDLLIGAAKH	GVSRTDYHIL	RDPELSFMAA	QRNYSQSKMA	HSRTSTPLLQ	QYQVALSASP
1530	1540	1550	1560	1570	1580	1590	1600
LTSLPRLLDA	KGIILEEMKV	KSENLKEEPQ	SSEEESMSSV	ETRTLIKSEP	VSPKNGVLPQ	ATGDQKSGGK	CETDRRMVAA
1610	1620	1630	1640	1650	1660	1670	1680
RTEPLTPNPA	SKKPRVHKRG	SESSSDSDSD	SERSSCSSRS	SSSSSSSSCS	HSRSGSSSSS	SSSCSSASSS	SSSSTSSSSS
1690	1700	1710	1720	1730	1740	1750	1760
SSSSSSEESD	SDEEEAQKRA	ESTTHMKAYD	EESVASLSTT	QDETQDSFQM	NNGTPESAYI	LQGGYMLAAS	YWPKDRVMIN
1770	1780	1790	1800	1810	1820	1830	1840
RLDSICQTVL	KGKWPSARRS	YDANTVASFY	TTKLLDSPGA	ATEYSDPSVP	TPPGAGVKEE	HDQSTQMSKV	KKHVREKEFT
1850	1860	1870	1880	1890	1900	1910	1920
VKIKDEGGLK	LTFQKQGLAQ	KRPFDGEDGA	LGQQQYLTRL	RELQSASETS	LVNFPKSIPV	SGTSIQPTLG	ANGVILDNQP
1930	1940	1950	1960	1970	1980	1990	2000
IVKKRRGRRK	NVEGVDIFFF	NRNKPPNHVS	LGLTSSQIST	GINPALSYTQ	PQGIPDTESP	VPVINLKDGT	RLAGDDAPKR
2010	2020	2030	2040	2050	2060	2070	2080
KDLEKWLKEH	PGYVEDLGAF	IPRMQLHEGR	PKQKRHRCRN	PNKLDVNSLT	GEERVQLINR	RNARKVGGAF	APPLKDLCRF
2090	2100	2110	2120	2130	2140	2150	2160
LKENSEYGVA	PEWGDVVKQS	GFLPESMYER	ILTGPVVREE	VSRRGRRPKS	GIAKATAAAA	AASATSVSGN	PLLANGLLPG
2170	2180	2190	2200	2210	2220	2230	2240

Detailed Protein Report

Cmpd.	No. of Cmpds.	m/z meas.	Δ m/z [ppm]	z	Rt [min]	Score	P	Range	Sequence	Modification	Ratios
2753	2	937.4586	-76.18	2	63.4	14.1	2	813-830	R.ASLKLGLDKAVLQSMSGR.E		
2019	1	719.3122	-83.44	2	55.6	13.7	0	1342-1354	R.VVSTFGVVFDPDR.G		
2569	1	449.6413	-314.31	2	60.8	13.6	1	2055-2061	R.VQLINRR.N		QD:QU 0.76

Detailed Protein Report

Protein 119: ATPase family AAA domain-containing protein 2 [Homo sapiens]

**Accession:**	gi|24497618	**Score:**	41.4
**Database:**	refseq_human(refseq_human_20140103.fasta)	**MW [kDa]:**	158.5
**Database Date:**	2015-11-30	**pI:**	5.9
		**Sequence Coverage [%]:**	2.8
		**No. of unique Peptides:**	2

Quantitation

QD:QU **Median:** 10.05 **CV:** 0.00 % **No. of Peptides:** 1

```
        10         20         30         40         50         60         70         80
MVVLRSSLEL HNHSAASATG SLDLSSDFLS LEHIGRRRLR SAGAAQKKPA ATTAKAGDGS SVKEVETYHR TRALRSLRKD
        90        100        110        120        130        140        150        160
AQNSSDSSFE KNVEITEQLA NGRHFTRQLA RQQADKKKEE HREDKVIPVT RSLRARNIVQ STEHLHEDNG DVEVRRSCRI
       170        180        190        200        210        220        230        240
RSRYSGVNQS MLFDKLITNT AEAVLQKMDD MKKMRRQRMR ELEDLGVFNE TEESNLNMYT RGKQKDIQRT DEETTDNQEG
       250        260        270        280        290        300        310        320
SVESSEEGED QEHEDDGEDE DDEDDDDDDD DDDDDDDEDD EDEEDGEEEN QKRYYLRQRK ATVYYQAPLE KPRHQRKPNI
       330        340        350        360        370        380        390        400
FYSGPASPAR PRYRLSSAGP RSPYCKRMNR RRHAIHSSDS TSSSSSEDEQ HFERRRKRSR NRAINRCLPL NFRKDELKGI
       410        420        430        440        450        460        470        480
YKDRMKIGAS LADVDPMQLD SSVRFDSVGG LSNHIAALKE MVVFPLLYPE VFEKFKIQPP RGCLFYGPPG TGKTLVARAL
       490        500        510        520        530        540        550        560
ANECSQGDKR VAFFMRKGAD CLSKWVGESE RQLRLLFDQA YQMRPSIIFF DEIDGLAPVR SSRQDQIHSS IVSTLLALMD
       570        580        590        600        610        620        630        640
GLDSRGEIVV IGATNRLDSI DPALRRPGRF DREFLFSLPD KEARKEILKI HTRDWNPKPL DTFLEELAEN CVGYCGADIK
       650        660        670        680        690        700        710        720
SICAEAALCA LRRRYPQIYT TSEKLQLDLS SINISAKDFE VAMQKMIPAS QRAVTSPGQA LSTVVKPLLQ NTVDKILEAL
       730        740        750        760        770        780        790        800
QRVFPHAEFR TNKTLDSDIS CPLLESDLAY SDDDVPSVYE NGLSQKSSHK AKDNFNFLHL NRNACYQPMS FRPRILIVGE
       810        820        830        840        850        860        870        880
PGFGQGSHLA PAVIHALEKF TVYTLDIPVL FGVSTTSPEE TCAQVIREAK RTAPSIVYVP HIHVWWEIVG PTLKATFTTL
       890        900        910        920        930        940        950        960
LQNIPSFAPV LLLATSDKPH SALPEEVQEL FIRDYGEIFN VQLPDKEERT KFFEDLILKQ AAKPPISKKK AVLQALEVLP
       970        980        990       1000       1010       1020       1030       1040
VAPPPEPRSL TAEEVKRLEE QEEDTFRELR IFLRNVTHRL AIDKRFRVFT KPVDPDEVPD YVTVIKQPMD LSSVISKIDL
      1050       1060       1070       1080       1090       1100       1110       1120
HKYLTVKDYL RDIDLICSNA LEYNPDRDPG DRLIRHRACA LRDTAYAIIK EELDEDFEQL CEEIQESRKK RGCSSSKYAP
      1130       1140       1150       1160       1170       1180       1190       1200
SYYHVMPKQN STLVGDKRSD PEQNEKLKTP STPVACSTPA QLKRKIRKKS NWYLGTIKKR RKISQAKDDS QNAIDHKIES
      1210       1220       1230       1240       1250       1260       1270       1280
DTEETQDTSV DHNETGNTGE SSVEENEKQQ NASESKLELR NNSNTCNIEN ELEDSRKTTA CTELRDKIAC NGDASSSQII
      1290       1300       1310       1320       1330       1340       1350       1360
HISDENEGKE MCVLRMTRAR RSQVEQQQLI TVEKALAILS QPTPSLVVDH ERLKNLLKTV VKKSQNYNIF QLENLYAVIS
      1370       1380       1390       1400
QCIYRHRKDH DKTSLIQKME QEVENFSCSR
```

Cmpd.	No. of Cmpds.	m/z meas.	Δ m/z [ppm]	z	Rt [min]	Score	P	Range	Sequence	Modification	Ratios
2101	1	1057.6924	66.12	1	54.8	10.3	2	38-47	R.RLRSAGAAQK.K		
2087	7	937.3467	-131.35	2	54.7	19.5	0	407-424	K.IGASLADVDPMQLDSSVR.F		QD:QU 10.05

Detailed Protein Report

Protein 120: PREDICTED: keratin, type I cytoskeletal 26 isoform X2 [Homo sapiens]

Accession: gi|578830847
Database: refseq_human(refseq_human_20140103.fasta)
Database Date: 2015-11-30

Score: 41.4
MW [kDa]: 51.6
pI: 4.7
Sequence Coverage [%]: 8.4
No. of unique Peptides: 3

Quantitation

QD:QU **Median:** 0.81 **CV:** 0.00 % **No. of Peptides:** 1

```
        10         20         30         40         50         60         70         80
MSFRLSGGSR RICSRTGSGR LSGGGTGFVA GNVCVGSGAR SSFSCTLEGI SSGGSFCNSG GGLGSGACAG FLGNEHSLLS
        90        100        110        120        130        140        150        160
GNEKVTMQNL NDRLASYLDH VHALEEANAD LEQKIKGWYE KCEPGSSREH DHDYSRYFSV IEDLKRQIIS ATICNASIVL
       170        180        190        200        210        220        230        240
QNDNARLTAD DFRLKYENEL ALHHSVEADT SGLRRVLDEL TLCTTDLEIQ CETLSEELTY LKKSHEEEME VLQYTAGGNV
       250        260        270        280        290        300        310        320
NVEMNATPGV DLTVLLNNMR AEYEDLAEQN RKDAEAWFNE RSATLQQQIS DHEGAATAAR NELTELKRNL QTLEIELQSL
       330        340        350        360        370        380        390        400
MAVKHSYECS LAETEGNYCN QLQQIQDQIG VMEEQLQQIR TETEGQKLEY EQLLDVKIFL EKEIDIYCNL LDGEERKSKS
       410        420        430        440        450        460        470
TCYKSKGYRP VNSGNQAKEE TIVKTVVEEL DQIGNLLSLR VHSVEEKSSK ISNITVEQRV PSKAP
```

Cmpd.	No. of Cmpds.	m/z meas.	Δ m/z [ppm]	z	Rt [min]	Score	P	Range	Sequence	Modification	Ratios
559	2	625.3768	63.05	2	35.6	11.4	0	368-377	K.LEYEQLLDVK.I		QD:QU 0.81
1697	1	905.3389	-109.42	2	49.8	10.8	1	383-397	K.EIDIYCNLLDGEERK.S		
2812	1	753.3845	-13.35	2	64.3	19.2	1	405-418	K.SKGYRPVNSGNQAK.E		

Detailed Protein Report

Protein 121: hexokinase-1 isoform HKI-R [Homo sapiens]

**Accession:**	gi|15991827	**Score:**	41.3
**Database:**	refseq_human(refseq_human_20140103.fasta)	**MW [kDa]:**	102.1
**Database Date:**	2015-11-30	**pI:**	6.2
**Modification(s):**	Carbamidomethyl	**Sequence Coverage [%]:**	4.3
		**No. of unique Peptides:**	2

10	20	30	40	50	60	70	80
MDCEHSLSLP	CRGAEAWEIG	IDKYLYAMRL	SDETLIDIMT	RFRKEMKNGL	SRDFNPTATV	KMLPTFVRSI	PDGSEKGDFI
**90**	**100**	**110**	**120**	**130**	**140**	**150**	**160**
ALDLGGSSFR	ILRVQVNHEK	NQNVHMESEV	YDTPENIVHG	SGSQLFDHVA	ECLGDFMEKR	KIKDKKLPVG	FTFSFPCQQS
**170**	**180**	**190**	**200**	**210**	**220**	**230**	**240**
KIDEAILITW	TKRFKASGVE	GADVVKLLNK	AIKKRGDYDA	NIVAVVNDTV	GTMMTCGYDD	QHCEVGLIIG	TGTNACYMEE
**250**	**260**	**270**	**280**	**290**	**300**	**310**	**320**
LRHIDLVEGD	EGRMCINTEW	GAFGDDGSLE	DIRTEFDREI	DRGSLNPGKQ	LFEKMVSGMY	LGELVRLILV	KMAKEGLLFE
**330**	**340**	**350**	**360**	**370**	**380**	**390**	**400**
GRITPELLTR	GKFNTSDVSA	IEKNKEGLHN	AKEILTRLGV	EPSDDDCVSV	QHVCTIVSFR	SANLVAATLG	AILNRLRDNK
**410**	**420**	**430**	**440**	**450**	**460**	**470**	**480**
GTPRLRTTVG	VDGSLYKTHP	QYSRRFHKTL	RRLVPDSDVR	FLLSESGSGK	GAAMVTAVAY	RLAEQHRQIE	ETLAHFHLTK
**490**	**500**	**510**	**520**	**530**	**540**	**550**	**560**
DMLLEVKKRM	RAEMELGLRK	QTHNNAVVKM	LPSFVRRTPD	GTENGDFLAL	DLGGTNFRVL	LVKIRSGKKR	TVEMHNKIYA
**570**	**580**	**590**	**600**	**610**	**620**	**630**	**640**
IPIEIMQGTG	EELFDHIVSC	ISDFLDYMGI	KGPRMPLGFT	FSFPCQQTSL	DAGILITWTK	GFKATDCVGH	DVVTLLRDAI
**650**	**660**	**670**	**680**	**690**	**700**	**710**	**720**
KRREEFDLDV	VAVVNDTVGT	MMTCAYEEPT	CEVGLIVGTG	SNACYMEEMK	NVEMVEGDQG	QMCINMEWGA	FGDNGCLDDI
**730**	**740**	**750**	**760**	**770**	**780**	**790**	**800**
RTHYDRLVDE	YSLNAGKQRY	EKMISGMYLG	EIVRNILIDF	TKKGFLFRGQ	ISETLKTRGI	FETKFLSQIE	SDRLALLQVR
**810**	**820**	**830**	**840**	**850**	**860**	**870**	**880**
AILQQLGLNS	TCDDSILVKT	VCGVVSRRAA	QLCGAGMAAV	VDKIRENRGL	DRLNVTVGVD	GTLYKLHPHF	SRIMHQTVKE
**890**	**900**	**910**	**920**				
LSPKCNVSFL	LSEDGSGKGA	ALITAVGVRL	RTEASS				

Cmpd.	No. of Cmpds.	m/z meas.	Δ m/z [ppm]	z	Rt [min]	Score	P	Range	Sequence	Modification
2596	1	658.6717	-179.19	2	61.1	11.3	0	2-12	M.DCEHSLSLPCR.G	Carbamidomethyl: 10
2975	1	953.4552	-46.39	2	66.4	14.7	1	433-450	R.LVPDSDVRFLLSESGSGK.G	

Detailed Protein Report

Protein 122: cystatin-S precursor [Homo sapiens]

Accession: gi|4503109
Database: refseq_human(refseq_human_20140103.fasta)
Database Date: 2015-11-30

Score: 41.3
MW [kDa]: 16.2
pI: 4.8
Sequence Coverage [%]: 7.8
No. of unique Peptides: 1

10	20	30	40	50	60	70	80
MARPLCTLLL	LMATLAGALA	SSSKEENRII	PGGIYDADLN	DEWVQRALHF	AISEYNKATE	DEYYRRPLQV	LRAREQTFGG
**90**	**100**	**110**	**120**	**130**	**140**	**150**	
VNYFFDVEVG	RTICTKSQPN	LDTCAFHEQP	ELQKKQLCSF	EIYEVPWEDR	MSLVNSRCQE	A	

Cmpd.	No. of Cmpds.	m/z meas.	Δ m/z [ppm]	z	Rt [min]	Score	P	Range	Sequence	Modification
887	1	646.8226	-19.68	2	39.6	41.3	0	47-57	R.ALHFAISEYNK.A	

Detailed Protein Report

Protein 123: **PREDICTED: polyribonucleotide nucleotidyltransferase 1, mitochondrial isoform X1 [Homo sapiens]**

**Accession:**	gi|530368250	**Score:**	41.2
**Database:**	refseq_human(refseq_human_20140103.fasta)	**MW [kDa]:**	77.3
**Database Date:**	2015-11-30	**pI:**	6.2
		**Sequence Coverage [%]:**	3.8
		**No. of unique Peptides:**	2

Quantitation

QD:QU **Median:** 0.59 **CV:** 0.00 % **No. of Peptides:** 1

10	20	30	40	50	60	70	80
MVTAVSKTKP	SPSQFMPLVV	DYRQKAAAAG	RIPTNYLRRE	IGTSDKEILT	SRIIDRSIRP	LFPAGYFYDT	QVLCNLLAVD
90	100	110	120	130	140	150	160
GVNEPDVLAI	NGASVALSLS	DIPWNGPVGA	VRIGIIDGEY	VVNPTRKEMS	SSTLNLVVAG	APKSQIVMLE	ASAENILQQD
170	180	190	200	210	220	230	240
FCHAIKVGVK	YTQQIIQGIQ	QLVKETGVTK	RTPQKLFTPS	PEIVKYTHKL	AMERLYAVFT	DYEHDKVSRD	EAVNKIRLDT
250	260	270	280	290	300	310	320
EEQLKEKFPE	ADPYEIIESF	NVVAKEVFRS	IVLNEYKRCD	GRDLTSLRNV	SCEVDMFKTL	HGSALFQRGQ	TQVLCTVTFD
330	340	350	360	370	380	390	400
SLESGIKSDQ	VITAINGIKD	KNFMLHYEFP	PYATNEIGKV	TGLNRRELGH	GALAEKALYP	VIPRDFPFTI	RVTSEVLESN
410	420	430	440	450	460	470	480
GSSSMASACG	GSLALMDSGV	PISSAVAGVA	IGLVTKTDPE	KGEIEDYRLL	TDILGIEDYN	GDMDFKIAGT	NKGITALQAD
490	500	510	520	530	540	550	560
IKLPGIPIKI	VMEAIQQASV	AKKEILQIMN	KTISKPRASR	KENGPVVETV	QVPLSKRAKF	VGPGGYNLKK	LQAETGVTIS
570	580	590	600	610	620	630	640
QVDEETFSVF	APTPSAMHEA	RDFITEICKD	DQEQQLEFGA	VYTATITEIR	DTGVMVKLYP	NMTAVLLHNT	QLDQRKIKHP
650	660	670	680	690	700	710	
TALGLEVGQE	IQVKYFGRDP	ADGRMRLSRK	VLQSPATTVV	RTLNDRSSIV	MGEPISQSSS	NSQ	

Cmpd.	No. of Cmpds.	m/z meas.	Δ m/z [ppm]	z	Rt [min]	Score	P	Range	Sequence	Modification	Ratios
2395	4	973.4016	-133.24	2	60.4	12.8	2	40-56	R.EIGTSDKEILTSRIIDR.S		QD:QU 0.59
80	1	586.3280	116.43	2	30.9	28.4	0	289-298	R.NVSCEVDMFK.T		

Detailed Protein Report

Protein 124: PREDICTED: ankyrin repeat domain-containing protein 11 isoform X4 [Homo sapiens]

**Accession:**	gi|578828959	**Score:**	41.2
**Database:**	refseq_human(refseq_human_20140103.fasta)	**MW [kDa]:**	286.8
**Database Date:**	2015-11-30	**pI:**	6.4
		**Sequence Coverage [%]:**	2.0
		**No. of unique Peptides:**	2

Quantitation

QD:QU	**Median:** 0.61	**CV:** 0.00 %	**No. of Peptides:** 1

Detailed Protein Report

10	20	30	40	50	60	70	80
MGLSGIRAGY	PLSERQQVAL	LMQMTAEESA	NSPVDTTPKH	PSQSTVCQKG	TPNSASKTKD	KVNKRNERGE	TRLHRAAIRG
90	100	110	120	130	140	150	160
DARRIKELIS	EGADVNVKDF	AGWTALHEAC	NRGYYDVAKQ	LLAAGAEVNT	KGLDDDTPLH	DAANNGHYKV	VKLLLRYGGN
170	180	190	200	210	220	230	240
PQQSNRKGET	PLKVANSPTM	VNLLLGKGTY	TSSEESSTES	SEEEDAPSFA	PSSSVDGNNT	DSEFEKGLKH	KAKNPEPQKA
250	260	270	280	290	300	310	320
TAPVKDEYEF	DEDDEQDRVP	PVDDKHLLKK	DYRKETKSNS	FISIPKMEVK	SYTKNNTIAP	KKASHRILSD	TSDEEDASVT
330	340	350	360	370	380	390	400
VGTGEKLRLS	AHTILPGSKT	REPSNAKQQK	EKNKVKKKRK	KETKGREVRF	GKRSDKFCSS	ESESESSESG	EDDRDSLGSS
410	420	430	440	450	460	470	480
GCLKGSPLVL	KDPSLFSSLS	ASSTSSHGSS	AAQKQNPSHT	DQHTKHWRTD	NWKTISSPAW	SEVSSLSDST	RTRLTSESDY
490	500	510	520	530	540	550	560
SSEGSSVESL	KPVRKRQEHR	KRASLSEKKS	PFLSSAEGAV	PKLDKEGKVV	KKHKTKHKHK	NKEKGQCSIS	QELKLKSFTY
570	580	590	600	610	620	630	640
EYEDSKQKSD	KAILLENDLS	TENKLKVLKH	DRDHFKKEEK	LSKMKLEEKE	WLFKDEKSLK	RIKDTNKDIS	RSFREEKDRS
650	660	670	680	690	700	710	720
NKAEKERSLK	EKSPKEEKLR	LYKEERKKKS	KDRPSKLEKK	NDLKEDKISK	EKEKIFKEDK	EKLKKEKVYR	EDSAFDEYCN
730	740	750	760	770	780	790	800
KNQFLENEDT	KFSLSDDQRD	RWFSDLSDSS	FDFKGEDSWD	SPVTDYRDMK	SDSVAKLILE	TVKEDSKERR	RDSRAREKRD
810	820	830	840	850	860	870	880
YREPFFRKKD	RDYLDKNSEK	RKEQTEKHKS	VPGYLSEKDK	KRRESAEAGR	DRKDALESCK	ERRDGRAKPE	EAHREELKEC
890	900	910	920	930	940	950	960
GCESGFKDKS	DGDFGKGLEP	WERHHPAREK	EKKDGPDKER	KEKTKPERYK	EKSSDKDKSE	KSILEKCQKD	KEFDKCFKEK
970	980	990	1000	1010	1020	1030	1040
KDTKEKHKDT	HGKDKERKAS	LDQGKEKKEK	AFPGIISEDF	SEKKDDKKGK	EKSWYIADIF	TDESEDDRDS	CMGSGFKMGE
1050	1060	1070	1080	1090	1100	1110	1120
ASDLPRTDGL	QEKEEGREAY	ASDRHRKSSD	KQHPERQKDK	EPRDRRKDRG	AADAGRDKKE	KVFEKHKEKK	DKESTEKYKD
1130	1140	1150	1160	1170	1180	1190	1200
RKDRASVDST	QDKKNKQKLP	EKAEKKHAAE	DKAKSKHKEK	SDKEHSKERK	SSRSADAEKS	LLEKLEEEAL	HEYREDSNDK
1210	1220	1230	1240	1250	1260	1270	1280
ISEVSSDSFT	DRGQEPGLTA	FLEVSFTEPP	GDDKPRESAC	LPEKLKEKER	HRHSSSSSKK	SHDRERAKKE	KAEKKEKGED
1290	1300	1310	1320	1330	1340	1350	1360
YKEGGSRKDS	GQYEKDFLEA	DAYGVSYNMK	ADIEDELDKT	IELFSTEKKD	KNDSEREPSK	KIEKELKPYG	SSAINILKEK
1370	1380	1390	1400	1410	1420	1430	1440
KKREKHREKW	RDEKERHRDR	HADGLLRHHR	DELLRHHRDE	QKPATRDKDS	PPRVLKDKSR	DEGPRLGDAK	LKEKFKDGAE
1450	1460	1470	1480	1490	1500	1510	1520
KEKGDPVKMS	NGNDKVAPSK	DPGKKDARPR	EKLLGDGDLM	MTSFERMLSQ	KDLEIEERHK	RHKERMKQME	KLRHRSGDPK
1530	1540	1550	1560	1570	1580	1590	1600
LKEKAKPADD	GRKKGLDIPA	KKPPGLDPPF	KDKKLKESTP	IPPAAENKLH	PASGADSKDW	LAGPHMKEVL	PASPRPDQSR
1610	1620	1630	1640	1650	1660	1670	1680
PTGVPTPTSV	LSCPSYEEVM	HTPRTPSCSA	DDYADLVFDC	ADSQHSTPVP	TAPTSACSPS	FFDRFSVASS	GLSENASQAP
1690	1700	1710	1720	1730	1740	1750	1760
ARPLSTNLYR	SVSVDIRRTP	EEEFSVGDKL	FRQQSVPAAS	SYDSPMPPSM	EDRAPLPPVP	AEKFACLSPG	YYSPDYGLPS
1770	1780	1790	1800	1810	1820	1830	1840
PKVDALHCPP	AAVVTVTPSP	EGVFSSLQAK	PSPSPRAELL	VPSLEGALPP	DLDTSEDQQA	TAAIIPPEPS	YLEPLDEGPF
1850	1860	1870	1880	1890	1900	1910	1920
SAVITEEPVE	WAHPSEQALA	SSLIGGTSEN	PVSWPVGSDL	LLKSPQRFPE	SPKRFCPADP	LHSAAPGPFS	ASEAPYPAPP
1930	1940	1950	1960	1970	1980	1990	2000
ASPAPYALPV	AEPGLEDVKD	GVDAVPAAIS	TSEAAPYAPP	SGLESFFSNC	KSLPEAPLDV	APEPACVAAV	AQVEALGPLE
2010	2020	2030	2040	2050	2060	2070	2080
NSFLDGSRGL	SHLGQVEPVP	WADAFAGPED	DLDLGPFSLP	ELPLQTKDAA	DGEAEPVEES	LAPPEEMPPG	APGVINGGDV
2090	2100	2110	2120	2130	2140	2150	2160
STVVAEEPPA	LPPDQASTRL	PAELEPEPSG	EPKLDVALEA	AVEAETVPEE	RARGDPDSSV	EPAPVPPEQR	PLGSGDQGAE
2170	2180	2190	2200	2210	2220	2230	2240

Detailed Protein Report

Cmpd.	No. of Cmpds.	m/z meas.	Δ m/z [ppm]	z	Rt [min]	Score	P	Range	Sequence	Modification	Ratios
793	1	1105.5255	-96.76	2	39.9	11.9	2	167-187	R.KGETPLKVANSPTMVNLLLGK.G		
1383	2	637.4051	150.05	3	45.8	14.2	0	454-471	K.TISSPAWSEVSSLSDSTR.T		QD:QU 0.61

Detailed Protein Report

Protein 125: filaggrin [Homo sapiens]

**Accession:**	gi|60097902	**Score:**	40.9
**Database:**	refseq_human(refseq_human_20140103.fasta)	**MW [kDa]:**	434.9
**Database Date:**	2015-11-30	**pI:**	9.6
		**Sequence Coverage [%]:**	1.1
		**No. of unique Peptides:**	1

Detailed Protein Report

10	20	30	40	50	60	70	80
MSTLLENIFA	IINLFKQYSK	KDKNTDTLSK	KELKELLEKE	FRQILKNPDD	PDMVDVFMDH	LDIDHNKKID	FTEFLLMVFK
90	100	110	120	130	140	150	160
LAQAYYESTR	KENLPISGHK	HRKHSHHDKH	EDNKQEENKE	NRKRPSSLER	RNNRKGNKGR	SKSPRETGGK	RHESSSEKKE
170	180	190	200	210	220	230	240
RKGYSPTHRE	EEYGKNHHNS	SKKEKNKTEN	TRLGDNRKRL	SERLEEKEDN	EEGVYDYENT	GRMTQKWIQS	GHIATYYTIQ
250	260	270	280	290	300	310	320
DEAYDTTDSL	LEENKIYERS	RSSDGKSSSQ	VNRSRHENTS	QVPLQESRTR	KRRGSRVSQD	RDSEGHSEDS	ERHSGSASRN
330	340	350	360	370	380	390	400
HHGSAWEQSR	DGSRHPRSHD	EDRASHGHSA	DSSRQSGTRH	AETSSRGQTA	SSHEQARSSP	GERHGSGHQQ	SADSSRHSAT
410	420	430	440	450	460	470	480
GRGQASSAVS	DRGHRGSSGS	QASDSEGHSE	NSDTQSVSGH	GKAGLRQQSH	QESTRGRSGE	RSGRSGSSLY	QVSTHEQPDS
490	500	510	520	530	540	550	560
AHGRTGTSTG	GRQGSHHEQA	RDSSRHSASQ	EGQDTIRGHP	GSSRGGRQGS	HHEQSVNRSG	HSGSHHSHTT	SQGRSDASHG
570	580	590	600	610	620	630	640
QSGSRSASRQ	TRNEEQSGDG	TRHSGSRHHE	ASSQADSSRH	SQVGQGQSSG	PRTSRNQGSS	VSQDSDSQGH	SEDSERWSGS
650	660	670	680	690	700	710	720
ASRNHHGSAQ	EQSRDGSRHP	RSHHEDRAGH	GHSADSSRKS	GTRHTQNSSS	GQAASSHEQA	RSSAGERHGS	RHQLQSADSS
730	740	750	760	770	780	790	800
RHSGTGHGQA	SSAVRDSGHR	GSSGSQATDS	EGHSEDSDTQ	SVSGHGQAGH	HQQSHQESAR	DRSGERSRRS	GSFLYQVSTH
810	820	830	840	850	860	870	880
KQSESSHGWT	GPSTGVRQGS	HHEQARDNSR	HSASQDGQDT	IRGHPGSSRR	GRQGSHHEQS	VDRSGHSGSH	HSHTTSQGRS
890	900	910	920	930	940	950	960
DASRGQSGSR	SASRTTRNEE	QSRDGSRHSG	SRHHEASSHA	DISRHSQAGQ	GQSEGSRTSR	RQGSSVSQDS	DSEGHSEDSE
970	980	990	1000	1010	1020	1030	1040
RWSGSASRNH	RGSAQEQSRH	GSRHPRSHHE	DRAGHGHSAD	SSRQSGTPHA	ETSSGGQAAS	SHEQARSSPG	ERHGSRHQQS
1050	1060	1070	1080	1090	1100	1110	1120
ADSSRHSGIP	RRQASSAVRD	SGHWGSSGSQ	ASDSEGHSEE	SDTQSVSGHG	QDGPHQQSHQ	ESARDWSGGR	SGRSGSFIYQ
1130	1140	1150	1160	1170	1180	1190	1200
VSTHEQSESA	HGRTRTSTGR	RQGSHHEQAR	DSSRHSASQE	GQDTIRAHPG	SRRGGRQGSH	HEQSVDRSGH	SGSHHSHTTS
1210	1220	1230	1240	1250	1260	1270	1280
QGRSDASHGQ	SGSRSASRQT	RKDKQSGDGS	RHSGSRHHEA	ASWADSSRHS	QVGQEQSSGS	RTSRHQGSSV	SQDSDSERHS
1290	1300	1310	1320	1330	1340	1350	1360
DDSERLSGSA	SRNHHGSSRE	QSRDGSRHPG	FHQEDRASHG	HSADSSRQSG	THHTESSSHG	QAVSSHEQAR	SSPGERHGSR
1370	1380	1390	1400	1410	1420	1430	1440
HQQSADSSRH	SGIGHRQASS	AVRDSGHRGS	SGSQVTNSEG	HSEDSDTQSV	SAHGQAGPHQ	QSHKESARGQ	SGESSGRSRS
1450	1460	1470	1480	1490	1500	1510	1520
FLYQVSSHEQ	SESTHGQTAP	STGGRQGSRH	EQARNSSRHS	ASQDGQDTIR	GHPGSSRGGR	QGSYHEQSVD	RSGHSGYHHS
1530	1540	1550	1560	1570	1580	1590	1600
HTTPQGRSDA	SHGQSGPRSA	SRQTRNEEQS	GDGSRHSGSR	HHEPSTRAGS	SRHSQVGQGE	SAGSKTSRRQ	GSSVSQDRDS
1610	1620	1630	1640	1650	1660	1670	1680
EGHSEDSERR	SESASRNHYG	SAREQSRHGS	RNPRSHQEDR	ASHGHSAESS	RQSGTRHAET	SSGGQAASSQ	EQARSSPGER
1690	1700	1710	1720	1730	1740	1750	1760
HGSRHQQSAD	SSTDSGTGRR	QDSSVVGDSG	NRGSSGSQAS	DSEGHSEESD	TQSVSAHGQA	GPHQQSHQES	TRGQSGERSG
1770	1780	1790	1800	1810	1820	1830	1840
RSGSFLYQVS	THEQSESAHG	RTGPSTGGRQ	RSRHEQARDS	SRHSASQEGQ	DTIRGHPGSS	RGGRQGSHYE	QSVDSSGHSG
1850	1860	1870	1880	1890	1900	1910	1920
SHHSHTTSQE	RSDVSRGQSG	SRSVSRQTRN	EKQSGDGSRH	SGSRHHEASS	RADSSRHSQV	GQGQSSGPRT	SRNQGSSVSQ
1930	1940	1950	1960	1970	1980	1990	2000
DSDSQGHSED	SERWSGSASR	NHLGSAWEQS	RDGSRHPGSH	HEDRAGHGHS	ADSSRQSGTR	HTESSSRGQA	ASSHEQARSS
2010	2020	2030	2040	2050	2060	2070	2080
AGERHGSHHQ	LQSADSSRHS	GIGHGQASSA	VRDSGHRGYS	GSQASDSEGH	SEDSDTQSVS	AQGKAGPHQQ	SHKESARGQS
2090	2100	2110	2120	2130	2140	2150	2160
GESSGRSGSF	LYQVSTHEQS	ESTHGQSAPS	TGGRQGSHYD	QAQDSSRHSA	SQEGQDTIRG	HPGPSRGGRQ	GSHQEQSVDR
2170	2180	2190	2200	2210	2220	2230	2240
SGHSGSHHSH	TTSQGRSDAS	RGQSGSRSAS	RKTYDKEQSG	DGSRHSGSHH	HEASSWADSS	RHSLVGQGQS	SGPRTSRPRG
2250	2260	2270	2280	2290	2300	2310	2320
SSVSQDSDSE	GHSEDSERRS	GSASRNHHGS	AQEQSRDGSR	HPRSHHEDRA	GHGHSAESSR	QSGTHHAENS	SGGQAASSHE
2330	2340	2350	2360	2370	2380	2390	2400

Detailed Protein Report

Cmpd.	No. of Cmpds.	m/z meas.	Δ m/z [ppm]	z	Rt [min]	Score	P	Range	Sequence	Modification
371	1	938.4376	1.41	2	34.6	15.1	2	3797-3814	R.SDASHGQSGSRSASRETR.N	

Detailed Protein Report

Protein 126: zinc finger protein 638 isoform 2 [Homo sapiens]

**Accession:**	gi|357933604	**Score:**	40.9
**Database:**	refseq_human(refseq_human_20140103.fasta)	**MW [kDa]:**	218.1
**Database Date:**	2015-11-30	**pI:**	6.0
		**Sequence Coverage [%]:**	2.0
		**No. of unique Peptides:**	1

Detailed Protein Report

10	20	30	40	50	60	70	80
MSRPRFNPRG	DFPLQRPRAP	NPSGMRPPGP	FMRPGSMGLP	RFYPAGRARG	IPHRFAGHES	YQNMGPQRMN	VQVTQHRTDP
90	100	110	120	130	140	150	160
RLTKEKLDFH	EAQQKKGKPH	GSRWDDEPHI	SASVAVKQSS	VTQVTEQSPK	VQSRYTKESA	SSILASFGLS	NEDLEELSRY
170	180	190	200	210	220	230	240
PDEQLTPENM	PLILRDIRMR	KMGRRLPNLP	SQSRNKETLG	SEAVSSNVID	YGHASKYGYT	EDPLEVRIYD	PEIPTDEVEN
250	260	270	280	290	300	310	320
EFQSQQNISA	SVPNPNVICN	SMFPVEDVFR	QMDFPGESSN	NRSFFSVESG	TKMSGLHISG	GQSVLEPIKS	VNQSINQTVS
330	340	350	360	370	380	390	400
QTMSQSLIPP	SMNQQPFSSE	LISSVSQQER	IPHEPVINSS	NVHVGSRGSK	KNYQSQADIP	IRSPFGIVKA	SWLPKFSHAD
410	420	430	440	450	460	470	480
AQKMKRLPTP	SMMNDYYAAS	PRIFPHLCSL	CNVECSHLKD	WIQHQNTSTH	IESCRQLRQQ	YPDWNPEILP	SRRNEGNRKE
490	500	510	520	530	540	550	560
NETPRRRSHS	PSPRRSRRSS	SSHRFRRSRS	PMHYMYRPRS	RSPRICHRFI	SRYRSRSRSR	SPYRIRNPFR	GSPKCFRSVS
570	580	590	600	610	620	630	640
PERMSRRSVR	SSDRKKALED	VVQRSGHGTE	FNKQKHLEAA	DKGHSPAQKP	KTSSGTKPSV	KPTSATKSDS	NLGGHSIRCK
650	660	670	680	690	700	710	720
SKNLEDDTLS	ECKQVSDKAV	SLQRKLRKEQ	SLHYGSVLLI	TELPEDGCTE	EDVRKLFQPF	GKVNDVLIVP	YRKEAYLEME
730	740	750	760	770	780	790	800
FKEAITAIMK	YIETTPLTIK	GKSVKICVPG	KKKAQNKEVK	KKTLESKKVS	ASTLKRDADA	SKAVEIVTST	SAAKTGQAKA
810	820	830	840	850	860	870	880
SVAKVNKSTG	KSASSVKSVV	TVAVKGNKAS	IKTAKSGGKK	SLEAKKTGNV	KNKDSNKPVT	IPENSEIKTS	IEVKATENCA
890	900	910	920	930	940	950	960
KEAISDAALE	ATENEPLNKE	TEEMCVMLVS	NLPNKGYSVE	EVYDLAKPFG	GLKDILILSS	HKKAYIEINR	KAAESMVKFY
970	980	990	1000	1010	1020	1030	1040
TCFPVLMDGN	QLSISMAPEN	MNIKDEEAIF	ITLVKENDPE	ANIDTIYDRF	VHLDNLPEDG	LQCVLCVGLQ	FGKVDHHVFI
1050	1060	1070	1080	1090	1100	1110	1120
SNRNKAILQL	DSPESAQSMY	SFLKQNPQNI	GDHMLTCSLS	PKIDLPEVQI	EHDPELEKES	PGLKNSPIDE	SEVQTATDSP
1130	1140	1150	1160	1170	1180	1190	1200
SVKPNELEEE	STPSIQTETL	VQQEEPCEEE	AEKATCDSDF	AVETLELETQ	GEEVKEEIPL	VASASVSIEQ	FTENAEECAL
1210	1220	1230	1240	1250	1260	1270	1280
NQQMFNSDLE	KKGAEIINPK	TALLPSDSVF	AEERNLKGIL	EESPSEAEDF	ISGITQTMVE	AVAEVEKNET	VSEILPSTCI
1290	1300	1310	1320	1330	1340	1350	1360
VTLVPGIPTG	DEKTVDKKNI	SEKKGNMDEK	EEEKEFNTKET	RMDLQIGTEK	AEKNEGRMDA	EKVEKMAAMK	EKPAENTLFK
1370	1380	1390	1400	1410	1420	1430	1440
AYPNKGVGQA	NKPDETSKTS	ILAVSDVSSS	KPSIKAVIVS	SPKAKATVSK	TENQKSFPKS	VPRDQINAEK	KLSAKEFGLL
1450	1460	1470	1480	1490	1500	1510	1520
KPTSARSGLA	ESSSKFKPTQ	SSLTRGGSGR	ISALQGKLSK	LDYRDITKQS	QETEARPSIM	KRDDSNNKTL	AEQNTKNPKS
1530	1540	1550	1560	1570	1580	1590	1600
TTGRSSKSKE	EPLFPFNLDE	FVTVDEVIEE	VNPSQAKQNP	LKGKRKETLK	NVPFSELNLK	KKKGKTSTPR	GVEGELSFVT
1610	1620	1630	1640	1650	1660	1670	1680
LDEIGEEEDA	AAHLAQALVT	VDEVIDEEEL	NMEEMVKNSN	SLFTLDELID	QDDCISHSEP	KDVTVLSVAE	EQDLLKQERL
1690	1700	1710	1720	1730	1740	1750	1760
VTVDEIGEVE	ELPLNESADI	TFATLNTKGN	EGDTVRDSIG	FISSQVPEDP	STLVTVDEIQ	DDSSDLHLVT	LDEVTEEDED
1770	1780	1790	1800	1810	1820	1830	1840
SLADFNNLKE	ELNFVTVDEV	GEEEDGDNDL	KVELAQSKND	HPTDKKGNRK	KRAVDTKKTK	LESLSQVGPV	NENVMEEDLK
1850	1860	1870	1880	1890	1900	1910	1920
TMIERHLTAK	TPTKRVRIGK	TLPSEKAVVT	EPAKGEEAFQ	MSEVDEESGL	KDSEPERKRK	KTEDSSSGKS	VASDVPEGEK
1930	1940	1950	1960				
AMTNHCKSTR	HKQNTEKFMA	KQRKEKEQNE	AEERSSR				

Cmpd.	No. of Cmpds.	m/z meas.	Δ m/z [ppm]	z	Rt [min]	Score	P	Range	Sequence	Modification
503	1	658.4149	37.36	2	34.9	13.0	1	703-713	K.VNDVLIVPYRK.E	

Detailed Protein Report

Protein 127: zinc finger protein DZIP1 isoform 1 [Homo sapiens]

**Accession:**	gi|7662436	**Score:**	40.8
**Database:**	refseq_human(refseq_human_20140103.fasta)	**MW [kDa]:**	96.6
**Database Date:**	2015-11-30	**pI:**	5.8
**Modification(s):**	Oxidation	**Sequence Coverage [%]:**	3.9
		**No. of unique Peptides:**	2

10	20	30	40	50	60	70	80
MQAEAADWFS	SMPFQKHVYY	PLASGPEGPD	VAVAAAAAGA	ASMACAPPSA	ASGPLPFFQF	RPRLESVDWR	RLSAIDVDKV
**90**	**100**	**110**	**120**	**130**	**140**	**150**	**160**
AGAVDVLTLQ	ENIMNITFCK	LEDEKCPHCQ	SGVDPVLLKL	IRLAQFTIEY	LLHSQEFLTS	QLHTLEERLR	LSHCDGEQSK
**170**	**180**	**190**	**200**	**210**	**220**	**230**	**240**
KLLTKQAGEI	KTLKEECKRR	KKMISTQQLM	IEAKANYYQC	HFCDKAFMNQ	AFLQSHIQRR	HTEENSHFEY	QKNAQIEKLR
**250**	**260**	**270**	**280**	**290**	**300**	**310**	**320**
SEIVVLKEEL	QLTRSELEAA	HHASAVRFSK	EYEMQKTKEE	DFLKLFDRWK	EEEKEKLVDE	MEKVKEMFMK	EFKELTSKNS
**330**	**340**	**350**	**360**	**370**	**380**	**390**	**400**
ALEYQLSEIQ	KSNMQIKSNI	GTLKDAHEFK	EDRSPYPQDF	HNVMQLLDSQ	ESKWTARVQA	IHQEHKKEKG	RLLSHIEKLR
**410**	**420**	**430**	**440**	**450**	**460**	**470**	**480**
TSMIDDLNAS	NVFYKKRIEE	LGQRLQEQNE	LIITQRQQIK	DFTCNPLNSI	SEPKVNAPAL	HTLETKSSLP	MVHEQAFSSH
**490**	**500**	**510**	**520**	**530**	**540**	**550**	**560**
ILEPIEELSE	EEKGRENEQK	LNNNKMHLRK	ALKSNSSLTK	GLRTMVEQNL	MEKLETLGIN	ADIRGISSDQ	LHRVLKSVES
**570**	**580**	**590**	**600**	**610**	**620**	**630**	**640**
ERHKQEREIP	NFHQIREFLE	HQVSCKIEEK	ALLSSDQCSV	SQMDTLSTGE	VPKMIQLPSK	NRQLIRQKAV	STDRTSVPKI
**650**	**660**	**670**	**680**	**690**	**700**	**710**	**720**
KKNVMEDPFP	RKSSTITTPP	FSSEEEQEDD	DLIRAYASPG	PLPVPPPQNK	GSFGKNTVKS	DADGTEGSEI	EDTDDSPKPA
**730**	**740**	**750**	**760**	**770**	**780**	**790**	**800**
GVAVKTPTEK	VEKMFPHRKN	VNKPVGGTNV	PEMFIKKEEL	QELKCADVED	EDWDISSLEE	EISLGKKSGK	EQKEPPPAKN
**810**	**820**	**830**	**840**	**850**			
EPHFAHVLNA	WGAFNPKGPK	GEGLQENESS	TLKSSLVTVT	DWSDTSDV			

Cmpd.	No. of Cmpds.	m/z meas.	Δ m/z [ppm]	z	Rt [min]	Score	P	Range	Sequence	Modification
1895	3	704.7670	-135.29	2	52.2	17.1	0	183-194	K.MISTQQLMIEAK.A	Oxidation: 8
2012	1	616.7504	-93.75	2	55.5	10.3	1	643-652	K.NVMEDPFPRK.S	

Detailed Protein Report

Protein 128: centromere protein F [Homo sapiens]

**Accession:**	gi|55770834	**Score:**	40.5
**Database:**	refseq_human(refseq_human_20140103.fasta)	**MW [kDa]:**	357.3
**Database Date:**	2015-11-30	**pI:**	4.9
		**Sequence Coverage [%]:**	1.4
		**No. of unique Peptides:**	3

Detailed Protein Report

10	20	30	40	50	60	70	80
MSWALEEWKE	GLPTRALQKI	QELEGQLDKL	KKEKQQRQFQ	LDSLEAALQK	QKQKVENEKT	EGTNLKRENQ	RLMEICESLE
90	100	110	120	130	140	150	160
KTKQKISHEL	QVKESQVNFQ	EGQLNSGKKQ	IEKLEQELKR	CKSELERSQQ	AAQSADVSLN	PCNTPQKIFT	TPLTPSQYYS
170	180	190	200	210	220	230	240
GSKYEDLKEK	YNKEVEERKR	LEAEVKALQA	KKASQTLPQA	TMNHRDIARH	QASSSVFSWQ	QEKTPSHLSS	NSQRTPIRRD
250	260	270	280	290	300	310	320
FSASYFSGEQ	EVTPSRSTLQ	IGKRDANSSF	FDNSSSPHLL	DQLKAQNQEL	RNKINELELR	LQGHEKEMKG	QVNKFQELQL
330	340	350	360	370	380	390	400
QLEKAKVELI	EKEKVLNKCR	DELVRTTAQY	DQASTKYTAL	EQKLKKLTED	LSCQRQNAES	ARCSLEQKIK	EKEKEFQEEL
410	420	430	440	450	460	470	480
SRQQRSFQTL	DQECIQMKAR	LTQELQQAKN	MHNVLQAELD	KLTSVKQQLE	NNLEEFKQKL	CRAEQAFQAS	QIKENELRRS
490	500	510	520	530	540	550	560
MEEMKKENNL	LKSHSEQKAR	EVCHLEAELK	NIKQCLNQSQ	NFAEEMKAKN	TSQETMLRDL	QEKINQQENS	LTLEKLKLAV
570	580	590	600	610	620	630	640
ADLEKQRDCS	QDLLKKREHH	IEQLNDKLSK	TEKESKALLS	ALELKKKEYE	ELKEEKTLFS	CWKSENEKLL	TQMESEKENL
650	660	670	680	690	700	710	720
QSKINHLETC	LKTQQIKSHE	YNERVRTLEM	DRENLSVEIR	NLHNVLDSKS	VEVETQKLAY	MELQQKAEFS	DQKHQKEIEN
730	740	750	760	770	780	790	800
MCLKTSQLTG	QVEDLEHKLQ	LLSNEIMDKD	RCYQDLHAEY	ESLRDLLKSK	DASLVTNEDH	QRSLLAFDQQ	PAMHHSFANI
810	820	830	840	850	860	870	880
IGEQGSMPSE	RSECRLEADQ	SPKNSAILQN	RVDSLEFSLE	SQKQMNSDLQ	KQCEELVQIK	GEIEENLMKA	EQMHQSFVAE
890	900	910	920	930	940	950	960
TSQRISKLQE	DTSAHQNVVA	ETLSALENKE	KELQLLNDKV	ETEQAEIQEL	KKSNHLLEDS	LKELQLLSET	LSLEKKEMSS
970	980	990	1000	1010	1020	1030	1040
IISLNKREIE	ELTQENGTLK	EINASLNQEK	MNLIQKSESF	ANYIDEREKS	ISELSDQYKQ	EKLILLQRCE	ETGNAYEDLS
1050	1060	1070	1080	1090	1100	1110	1120
QKYKAAQEKN	SKLECLLNEC	TSLCENRKNE	LEQLKEAFAK	EHQEFLTKLA	FAEERNQNLM	LELETVQQAL	RSEMTDNQNN
1130	1140	1150	1160	1170	1180	1190	1200
SKSEAGGLKQ	EIMTLKEEQN	KMQKEVNDLL	QENEQLMKVM	KTKHECQNLE	SEPIRNSVKE	RESERNQCNF	KPQMDLEVKE
1210	1220	1230	1240	1250	1260	1270	1280
ISLDSYNAQL	VQLEAMLRNK	ELKLQESEKE	KECLQHELQT	IRGDLETSNL	QDMQSQEISG	LKDCEIDAEE	KYISGPHELS
1290	1300	1310	1320	1330	1340	1350	1360
TSQNDNAHLQ	CSLQTTMNKL	NELEKICEIL	QAEKYELVTE	LNDSRSECIT	ATRKMAEEVG	KLLNEVKILN	DDSGLLHGEL
1370	1380	1390	1400	1410	1420	1430	1440
VEDIPGGEFG	EQPNEQHPVS	LAPLDESNSY	EHLTLSDKEV	QMHFAELQEK	FLSLQSEHKI	LHDQHCQMSS	KMSELQTYVD
1450	1460	1470	1480	1490	1500	1510	1520
SLKAENLVLS	TNLRNFQGDL	VKEMQLGLEE	GLVPSLSSSC	VPDSSSLSSL	GDSSFYRALL	EQTGDMSLLS	NLEGAVSANQ
1530	1540	1550	1560	1570	1580	1590	1600
CSVDEVFCSS	LQEENLTRKE	TPSAPAKGVE	ELESLCEVYR	QSLEKLEEKM	ESQGIMKNKE	IQELEQLLSS	ERQELDCLRK
1610	1620	1630	1640	1650	1660	1670	1680
QYLSENEQWQ	QKLTSVTLEM	ESKLAAEKKQ	TEQLSLELEV	ARLQLQGLDL	SSRSLLGIDT	EDAIQGRNES	CDISKEHTSE
1690	1700	1710	1720	1730	1740	1750	1760
TTERTPKHDV	HQICDKDAQQ	DLNLDIEKIT	ETGAVKPTGE	CSGEQSPDTN	YEPPGEDKTQ	GSSECISELS	FSGPNALVPM
1770	1780	1790	1800	1810	1820	1830	1840
DFLGNQEDIH	NLQLRVKETS	NENLRLLHVI	EDRDRKVESL	LNEMKELDSK	LHLQEVQLMT	KIEACIELEK	IVGELKKENS
1850	1860	1870	1880	1890	1900	1910	1920
DLSEKLEYFS	CDHQELLQRV	ETSEGLNSDL	EMHADKSSRE	DIGDNVAKVN	DSWKERFLDV	ENELSRIRSE	KASIEHEALY
1930	1940	1950	1960	1970	1980	1990	2000
LEADLEVVQT	EKLCLEKDNE	NKQKVIVCLE	EELSVVTSER	NQLRGELDTM	SKKTTALDQL	SEKMKEKTQE	LESHQSECLH
2010	2020	2030	2040	2050	2060	2070	2080
CIQVAEAEVK	EKTELLQTLS	SDVSELLKDK	THLQEKLQSL	EKDSQALSLT	KCELENQIAQ	LNKEKELLVK	ESESLQARLS
2090	2100	2110	2120	2130	2140	2150	2160
ESDYEKLNVS	KALEAALVEK	GEFALRLSST	QEEVHQLRRG	IEKLRVRIEA	DEKKQLHIAE	KLKERERERND	SLKDKVENLE
2170	2180	2190	2200	2210	2220	2230	2240

Detailed Protein Report

Cmpd.	No. of Cmpds.	m/z meas.	Δ m/z [ppm]	z	Rt [min]	Score	P	Range	Sequence	Modification
1209	1	573.1437	-261.24	2	43.6	14.6	0	981-990	K.EINASLNQEK.M	
1276	1	917.4679	30.80	2	46.1	11.7	0	2267-2283	K.ELNEAVAALCGDQEIMK.A	
2721	2	974.5198	32.44	2	63.0	14.2	1	2466-2482	K.AKEQNLSSQVECLELEK.A	

Detailed Protein Report

Protein 129: **PREDICTED: basement membrane-specific heparan sulfate proteoglycan core protein isoform X10 [Homo sapiens]**

**Accession:**	gi|578799003	**Score:**	40.5
**Database:**	refseq_human(refseq_human_20140103.fasta)	**MW [kDa]:**	433.5
**Database Date:**	2015-11-30	**pI:**	6.1
**Modification(s):**	Oxidation	**Sequence Coverage [%]:**	1.5
		**No. of unique Peptides:**	2

Detailed Protein Report

10	20	30	40	50	60	70	80
MGWRAAGALL	LALLLHGRLL	AVTHGLRAYD	GLSLPEDIET	VTASQMRWTH	SYLSDDEDML	ADSISGDDLG	SGDLGSGDFQ
90	100	110	120	130	140	150	160
MVYFRALVNF	TRSIEYSPQL	EDAGSREFRE	VSEAVVDTLE	SEYLKIPGDQ	VVSVVFIKEL	DGWVFVELDV	GSEGNADGAQ
170	180	190	200	210	220	230	240
IQEMLLRVIS	SGSVASYVTS	PQGFQFRRLG	TAQTPPPPPA	AVAVAVTPVP	QFPRACTEAE	FACHSYNECV	ALEYRCDRRP
250	260	270	280	290	300	310	320
DCRDMSDELN	CEEPVLGISP	TFSLLVETTS	LPPRPETTIM	RQPPVTHAPQ	PLLPGSVRPL	PCGPQEAACR	NGHCIPRDYL
330	340	350	360	370	380	390	400
CDGQEDCEDG	SDELDCGPPP	PCEPNEFPCG	NGHCALKLWR	CDGDFDCEDR	TDEANCPTKR	PEEVCGPTQF	RCVSTNMCIP
410	420	430	440	450	460	470	480
ASFHCDEESD	CPDRSDEFGC	MPPQVVTPPR	ESIQASRGQT	VTFTCVAIGV	PTPIINWRLN	WGHIPSHPRV	TVTSEGGRGT
490	500	510	520	530	540	550	560
LIIRDVKESD	QGAYTCEAMN	ARGMVFGIPD	GVLELVPQRA	GPCPDGHFYL	EHSAACLPCF	CFGITSVCQS	TRRFRDQIRL
570	580	590	600	610	620	630	640
RFDQPDDFKG	VNVTMPAQPG	TPPLSSTQLQ	IDPSLHEFQL	VDLSRRFLVH	DSFWALPEQF	LGNKVDSYGG	SLRYNVRYEL
650	660	670	680	690	700	710	720
ARGMLEPVQR	PDVVLMGAGY	RLLSRGHTPT	QPGALNQRQV	QFSEEHWVHE	SGRPVQRAEL	LQVLQSLEAV	LIQTVYNTKM
730	740	750	760	770	780	790	800
ASVGLSDIAM	DTTVTHATSH	GRAHSVEECR	CPIGYSGLSC	ESCDAHFTRV	PGGPYLGTCS	GCNCNGHASS	CDPVYGHCLN
810	820	830	840	850	860	870	880
CQHNTEGPQC	NKCKAGFFGD	AMKATATSCR	PCPCPYIDAS	RRFSDTCFLD	TDGQATCDAC	APGYTGRRCE	SCAPGYEGNP
890	900	910	920	930	940	950	960
IQPGGKCRPV	NQEIVRCDER	GSMGTSGEAC	RCKNNVVGRL	CNECADGSFH	LSTRNPDGCL	KCFCMGVSRH	CTSSSWSRAQ
970	980	990	1000	1010	1020	1030	1040
LHGASEEPGH	FSLTNAASTH	TTNEGIFSPT	PGELGFSSFH	RLLSGPYFWS	LPSRFLGDKV	TSYGGELRFT	VTQRSQPGST
1050	1060	1070	1080	1090	1100	1110	1120
PLHGQPLVVL	QGNNIILEHH	VAQEPSPGQP	STFIVPFREQ	AWQRPDGQPA	TREHLLMALA	GIDTLLIRAS	YAQQPAESRV
1130	1140	1150	1160	1170	1180	1190	1200
SGISMDVAVP	EETGQDPALE	VEQCSCPPGY	RGPSCQDCDT	GYTRTPSGLY	LGTCERCSCH	GHSEACEPET	GACQGCQHHT
1210	1220	1230	1240	1250	1260	1270	1280
EGPRCEQCQP	GYYGDAQRGT	PQDCQLCPCY	GDPAAGQAAH	TCFLDTDGHP	TCDACSPGHS	GRHCERCAPG	YYGNPSQGQP
1290	1300	1310	1320	1330	1340	1350	1360
CQRDSQVPGP	IGCNCDPQGS	VSSQCDAAGQ	CQCKAQVEGL	TCSHCRPHHF	HLSASNPDGC	LPCFCMGITQ	QCASSAYTRH
1370	1380	1390	1400	1410	1420	1430	1440
LISTHFAPGD	FQGFALVNPQ	RNSRLTGEFT	VEPVPEGAQL	SFGNFAQLGH	ESFYWQLPET	YQGDKGEAYF	ARMRRAHQRT
1450	1460	1470	1480	1490	1500	1510	1520
LPMALAPGLW	EVLARLLPPF	HQNRTSLSEE	QLRAAVTAGR	IPEPPEGRDW	AQRASQVDEA	QRRMDAEIWQ	LLSSFAAQPQ
1530	1540	1550	1560	1570	1580	1590	1600
PPPQGLSPHP	QPAAALRAAP	PPSSSSSSSS	SSASLSFSPG	SQFSLSYEGF	SLLPGSLYYW	QLPRAFLGDK	VAAYGGKLRY
1610	1620	1630	1640	1650	1660	1670	1680
TLSYTAGPQG	SPLSDPDVQI	TGNNIMLVAS	QPALQGPERR	SYEIMFREEF	WRRPDGQPAT	REHLLMALAD	LDELLIRATF
1690	1700	1710	1720	1730	1740	1750	1760
SSVPLAASIS	AVSLEVAQPG	PSNRPRALEV	EECRCPPGYI	GLSCQDCAPG	YTRTGSGLYL	GHCELCECNG	HSDLCHPETG
1770	1780	1790	1800	1810	1820	1830	1840
ACSQCQHNAA	GEFCELCAPG	YYGDATAGTP	EDCQPCACPL	TNPENMFSRT	CESLGAGGYR	CTACEPGYTG	QYCEQCGPGY
1850	1860	1870	1880	1890	1900	1910	1920
VGNPSVQGGQ	CLPETNQAPL	VVEVHPARSI	VPQGGSHSLR	CQVSGSPPHY	FYWSREDGRP	VPSGTQQRHQ	GSELHFPSVQ
1930	1940	1950	1960	1970	1980	1990	2000
PSDAGVYICT	CRNLHQSNTS	RAELLVTEAP	SKPITVTVEE	QRSQSVRPGA	DVTFICTAKS	KSPAYTLVWT	RLHNGKLPTR
2010	2020	2030	2040	2050	2060	2070	2080
AMDFNGILTI	RNVQLSDAGT	YVCTGSNMFA	MDQGTATLHV	QASGTLSAPV	VSIHPPQLTV	QPGQLAEFRC	SATGSPTPTL
2090	2100	2110	2120	2130	2140	2150	2160
EWTGGPGGQL	PAKAQIHGGI	LRLPAVEPTD	QAQYLCRAHS	SAGQQVARAV	LHVHGGGGPR	VQVSPERTQV	HAGRTVRLYC
2170	2180	2190	2200	2210	2220	2230	2240
RAAGVPSATI	TWRKEGGSLP	PQARSERTDI	ATLLIPAITT	ADAGFYLCVA	TSPAGTAQAR	IQVVVLSASD	ASPPPVKIES
2250	2260	2270	2280	2290	2300	2310	2320
SSPSVTEGQT	LDLNCVVAGS	AHAQVTWYRR	GGSLPPHTQV	HGSRLRLPQV	SPADSGEYVC	RVENGSGPKE	ASITVSVLHG
2330	2340	2350	2360	2370	2380	2390	2400

Detailed Protein Report

Cmpd.	No. of Cmpds.	m/z meas.	Δ m/z [ppm]	z	Rt [min]	Score	P	Range	Sequence	Modification
2083	1	1052.3950	-135.43	2	56.4	13.6	0	643-661	R.GMLEPVQRPDVVLMGAGYR.L	Oxidation: 2
1714	1	1011.6020	66.80	3	51.7	12.9	1	3926-3953	R.FDAGSGMATIRHPTPLALGHFHTVTLLR.S	Oxidation: 7

Detailed Protein Report

Protein 130: **PREDICTED: zinc finger protein 737 isoform X3 [Homo sapiens]**

**Accession:**	gi|530414417	**Score:**	40.4
**Database:**	refseq_human(refseq_human_20140103.fasta)	**MW [kDa]:**	60.4
**Database Date:**	2015-11-30	**pI:**	10.4
**Modification(s):**	Carbamidomethyl	**Sequence Coverage [%]:**	6.5
		**No. of unique Peptides:**	1

10	20	30	40	50	60	70	80
MGPLQFRDVA	IEFSLEEWHC	LDTAQRNLYR	NVMLENYRNL	VFLGIVVSKP	DLITCLEQGK	KPLTMKKHEM	VANPSVTCSH
90	100	110	120	130	140	150	160
FARDLWPEQS	IKDSFQKVTL	RRYENYGHDN	LQFKKGCESV	DECKVHKRGY	NGLNQYLTTT	QSKIFQCDKY	VKVIHKFSNS
170	180	190	200	210	220	230	240
NRHKIRHTGK	KPFKCIECGK	AFNQSSTLTT	HKKIHTGEKP	FKCEECGKAF	NWSSHLTTHK	RIHTGEKRYK	CEDCGKAFSR
250	260	270	280	290	300	310	320
FSYLTAHKII	HSGEKPYKCE	ECGKAFKRSS	ILTAHKIIHS	GEKPYKCEEC	GKAFKHPSVL	TTHKRIHTGE	KPYKCEECGR
330	340	350	360	370	380	390	400
AFKYFSSLTT	HKIIHSGEKP	YKCEECGKAF	NWSSHLTTHK	RIHTGEKPYK	CEECGEAFKY	SSSLTTHKII	HTGQQPFKCE
410	420	430	440	450	460	470	480
ECGKAFKCFS	ILTTHKRIHT	GEKPYKCEEC	GKAFNSSSHL	TAHKRIHTGE	KPYKCERCGK	AFKRSFILTR	HKRIHTGEKP
490	500	510	520	530			
YKCEECGKGF	KCPSTLTTHK	RSGCPTRKLL	LSSVSTLNNS	EWK			

Cmpd.	No. of Cmpds.	m/z meas.	Δ m/z [ppm]	z	Rt [min]	Score	P	Range	Sequence	Modification
2919	1	981.9597	-7.66	2	65.7	10.8	2	305-320	K.RIHTGEKPYKCEECGR.A	Carbamidomethyl: 11

Detailed Protein Report

Protein 131: **rapamycin-insensitive companion of mTOR isoform 3 [Homo sapiens]**

**Accession:**	gi|550544215	**Score:**	40.3
**Database:**	refseq_human(refseq_human_20140103.fasta)	**MW [kDa]:**	155.8
**Database Date:**	2015-11-30	**pI:**	6.8
		**Sequence Coverage [%]:**	1.9
		**No. of unique Peptides:**	2

10	20	30	40	50	60	70	80
MGIIATFRSW	AGIINLCKPG	NSGIQSLIGV	LCIPNMEIRR	GLLEVLYDIF	RLPLPVVTEE	FIEALLSVDP	GRFQDSWRLS
90	100	110	120	130	140	150	160
DGFVAAEAKT	ILPHRARSRP	DLMDNYLALI	LSAFIRNGLL	EGLVEVITNS	DDHISVRATI	LLGELLHMAN	TILPHSHSHH
170	180	190	200	210	220	230	240
LHCLPTLMNM	AASFDIPKEK	RLRASAALNC	LKRFHEMKKR	GPKPYSLHLD	HIIQKAIATH	QKRDQYLRVQ	KDIFILKDTE
250	260	270	280	290	300	310	320
EALLINLRDS	QVLQHKENLE	WNWNLIGTIL	KWPNVNLRNY	KDEQLHRFVR	RLLYFYKPSS	KLYANLDLDF	AKAKQLTVVG
330	340	350	360	370	380	390	400
CQFTEFLLES	EEDGQGYLED	LVKDIVQWLN	ASSGMKPERS	LQNNGLLTTL	SQHYFLFIGT	LSCHPHGVKM	LEKCSVFQCL
410	420	430	440	450	460	470	480
LNLCSLKNQD	HLLKLTVSSL	DYSRDGLARV	ILSKILTAAT	DACRLYATKH	LRVLLRANVE	FFNNWGIELL	VTQLHDKNKT
490	500	510	520	530	540	550	560
ISSEALDILD	EACEDKANLH	ALIQMKPALS	HLGDKGLLLL	LRFLSIPKGF	SYLNERGYVA	KQLEKWHREY	NSKYVDLIEE
570	580	590	600	610	620	630	640
QLNEALTTYR	KPVDGDNYVR	RSNQRLQRPH	VYLPIHLYGQ	LVHHKTGCHL	LEVQNIITEL	CRNVRTPDLD	KWEEIKKLKA
650	660	670	680	690	700	710	720
SLWALGNIGS	SNWGLNLLQE	ENVIPDILKL	AKQCEVLSIR	GTCVYVLGLI	AKTKQGCDIL	KCHNWDAVRH	SRKHLWPVVP
730	740	750	760	770	780	790	800
DDVEQLCNEL	SSIPSTLSLN	SESTSSRHNS	ESESVPSSMF	ILEDDRFGSS	STSTFFLDIN	EDTEPTFYDR	SGPIKDKNSF
810	820	830	840	850	860	870	880
PFFASSKLVK	NRILNSLTLP	NKKHRSSSDP	KGGKLSSESK	TSNRRIRTLT	EPSVDFNHSD	DFTPISTVQK	TLQLETSFMG
890	900	910	920	930	940	950	960
NKHIEDTGST	PSIGENDLKF	TKNFGTENHR	ENTSRERLVV	ESSTSSHMKI	RSQSFNTDTT	TSGISSMSSS	PSRETVGVDA
970	980	990	1000	1010	1020	1030	1040
TTMDTDCGSM	STVVSTKTIK	TSHYLTPQSN	HLSLSKSNSV	SLVPPGSSHT	LPRRAQSLKA	PSIATIKSLA	DCNFSYTSSR
1050	1060	1070	1080	1090	1100	1110	1120
DAFGYATLKR	LQQQRMHPSL	SHSEALASPA	KDVLFTDTIT	MKANSFESRL	TPSRFMKALS	YASLDKEDLL	SPINQNTLQR
1130	1140	1150	1160	1170	1180	1190	1200
SSSVRSMVSS	ATYGGSDDYI	GLALPVDIND	IFQVKDIPYF	QTKNIPPHDD	RGARAFAHDA	GESTEDTGLQ	EHTDDNCLYC
1210	1220	1230	1240	1250	1260	1270	1280
VCIEILGFQP	SNQLSAICSH	SDFQDIPYSD	WCEQTIHNPL	EVVPSKFSGI	SGCSDGVSQE	GSASSTKSTE	LLLGVKTIPD
1290	1300	1310	1320	1330	1340	1350	1360
DTPMCRILLR	KEVLRLVINL	SSSVSTKCHE	TGLLTIKEKY	PQTFDDICLY	SEVSHLLSHC	TFRLPCRRFI	QELFQDVQFL
1370	1380	1390					
QMHEEAEAVL	ATPPKQPIVD	TSAES					

Cmpd.	No. of Cmpds.	m/z meas.	Δ m/z [ppm]	z	Rt [min]	Score	P	Range	Sequence	Modification
2622	1	658.3808	-6.32	2	61.5	16.9	2	182-193	R.LRASAALNCLKR.F	
2751	1	805.9143	-22.12	2	65.5	23.4	1	435-449	K.ILTAATDACRLYATK.H	

Detailed Protein Report

Protein 132: myosin-2 [Homo sapiens]

**Accession:**	gi|153791586	**Score:**	40.2
**Database:**	refseq_human(refseq_human_20140103.fasta)	**MW [kDa]:**	222.9
**Database Date:**	2015-11-30	**pI:**	5.5
**Modification(s):**	Oxidation	**Sequence Coverage [%]:**	2.1
		**No. of unique Peptides:**	2

Alias proteins:

Accession	Name	Description
gi|153792663	refseq_human (refseq_human_20140103.fasta)	myosin-2 [Homo sapiens]

Detailed Protein Report

10	20	30	40	50	60	70	80
MSSDSELAVF	GEAAPFLRKS	ERERIEAQNR	PFDAKTSVFV	AEPKESFVKG	TIQSREGGKV	TVKTEGGATL	TVKDDQVFPM
90	100	110	120	130	140	150	160
NPPKYDKIED	MAMMTHLHEP	AVLYNLKERY	AAWMIYTYSG	LFCVTVNPYK	WLPVYKPEVV	TAYRGKKRQE	APPHIFSISD
170	180	190	200	210	220	230	240
NAYQFMLTDR	ENQSILITGE	SGAGKTVNTK	RVIQYFATIA	VTGEKKKEEI	TSGKIQGTLE	DQIISANPLL	EAFGNAKTVR
250	260	270	280	290	300	310	320
NDNSSRFGKF	IRIHFGTTGK	LASADIETYL	LEKSRVVFQL	KAERSYHIFY	QITSNKKPEL	IEMLLITTNP	YDYPFVSQGE
330	340	350	360	370	380	390	400
ISVASIDDQE	ELMATDSAID	ILGFTNEEKV	SIYKLTGAVM	HYGNLKFKQK	QREEQAEPDG	TEVADKAAYL	QSLNSADLLK
410	420	430	440	450	460	470	480
ALCYPRVKVG	NEYVTKGQTV	EQVSNAVGAL	AKAVYEKMFL	WMVARINQQL	DTKQPRQYFI	GVLDIAGFEI	FDFNSLEQLC
490	500	510	520	530	540	550	560
INFTNEKLQQ	FFNHHMFVLE	QEEYKKEGIE	WTFIDFGMDL	AACIELIEKP	MGIFSILEEE	CMFPKATDTS	FKNKLYDQHL
570	580	590	600	610	620	630	640
GKSANFQKPK	VVKGKAEAHF	ALIHYAGVVD	YNITGWLEKN	KDPLNETVVG	LYQKSAMKTL	AQLFSGAQTA	EGEGAGGGAK
650	660	670	680	690	700	710	720
KGGKKKGSSF	QTVSALFREN	LNKLMTNLRS	THPHFVRCII	PNETKTPGAM	EHELVLHQLR	CNGVLEGIRI	CRKGFPSRIL
730	740	750	760	770	780	790	800
YADFKQRYKV	LNASAIPEGQ	FIDSKKASEK	LLASIDIDHT	QYKFGHTKVF	FKAGLLGLLE	EMRDDKLAQL	ITRTQARCRG
810	820	830	840	850	860	870	880
FLARVEYQRM	VERREAIFCI	QYNIRSFMNV	KHWPWMKLFF	KIKPLLKSAE	TEKEMATMKE	EFQKIKDELA	KSEAKRKELE
890	900	910	920	930	940	950	960
EKMVTLLKEK	NDLQLQVQAE	AEGLADAEER	CDQLIKTKIQ	LEAKIKEVTE	RAEDEEEINA	ELTAKKRKLE	DECSELKKDI
970	980	990	1000	1010	1020	1030	1040
DDLELTLAKV	EKEKHATENK	VKNLTEEMAG	LDETIAKLTK	EKKALQEAHQ	QTLDDLQAEE	DKVNTLTKAK	IKLEQQVDDL
1050	1060	1070	1080	1090	1100	1110	1120
EGSLEQEKKL	RMDLERAKRK	LEGDLKLAQE	SIMDIENEKQ	QLDEKLKKKE	FEISNLQSKI	EDEQALGIQL	QKKIKELQAR
1130	1140	1150	1160	1170	1180	1190	1200
IEELEEEIEA	ERASRAKAEK	QRSDLSRELE	EISERLEEAG	GATSAQIEMN	KKREAEFQKM	RRDLEEATLQ	HEATAATLRK
1210	1220	1230	1240	1250	1260	1270	1280
KHADSVAELG	EQIDNLQRVK	QKLEKEKSEM	KMEIDDLASN	VETVSKAKGN	LEKMCRTLED	QLSELKSKEE	EQQRLINDLT
1290	1300	1310	1320	1330	1340	1350	1360
AQRGRLQTES	GEFSRQLDEK	EALVSQLSRG	KQAFTQQIEE	LKRQLEEEIK	AKNALAHALQ	SSRHDCDLLR	EQYEEEQESK
1370	1380	1390	1400	1410	1420	1430	1440
AELQRALSKA	NTEVAQWRTK	YETDAIQRTE	ELEEAKKKLA	QRLQAAEEHV	EAVNAKCASL	EKTKQRLQNE	VEDLMLDVER
1450	1460	1470	1480	1490	1500	1510	1520
TNAACAALDK	KQRNFDKILA	EWKQKCEETH	AELEASQKEA	RSLGTELFKI	KNAYEESLDQ	LETLKRENKN	LQQEISDLTE
1530	1540	1550	1560	1570	1580	1590	1600
QIAEGGKRIH	ELEKIKKQVE	QEKCELQAAL	EEAEASLEHE	EGKILRIQLE	LNQVKSEVDR	KIAEKDEEID	QLKRNHIRIV
1610	1620	1630	1640	1650	1660	1670	1680
ESMQSTLDAE	IRSRNDAIRL	KKKMEGDLNE	MEIQLNHANR	MAAEALRNYR	NTQGILKDTQ	IHLDDALRSQ	EDLKEQLAMV
1690	1700	1710	1720	1730	1740	1750	1760
ERRANLLQAE	IEELRATLEQ	TERSRKIAEQ	ELLDASERVQ	LLHTQNTSLI	NTKKKLETDI	SQMQGEMEDI	LQEARNAEEK
1770	1780	1790	1800	1810	1820	1830	1840
AKKAITDAAM	MAEELKKEQD	TSAHLERMKK	NMEQTVKDLQ	LRLDEAEQLA	LKGGKKQIQK	LEARVRELEG	EVESEQKRNA
1850	1860	1870	1880	1890	1900	1910	1920
EAVKGLRKHE	RRVKELTYQT	EEDRKNILRL	QDLVDKLQAK	VKSYKRQAEE	AEEQSNTNLA	KFRKLQHELE	EAEERADIAE
1930	1940	1950					
SQVNKLRVKS	REVHTKVISE	E					

Cmpd.	No. of Cmpds.	m/z meas.	Δ m/z [ppm]	z	Rt [min]	Score	P	Range	Sequence	Modification
2807	1	714.6776	11.25	3	66.4	12.9	1	1228-1246	K.SEMKMEIDDLASNVETVSK.A	Oxidation: 3
1958	1	745.7963	-141.38	2	54.9	15.0	1	1312-	K.QAFTQQIEELKR.Q	

Detailed Protein Report

Cmpd.	No. of Cmpds.	m/z meas.	Δ m/z [ppm]	z	Rt [min]	Score	P	Range	Sequence	Modification
								1323		

Detailed Protein Report

Protein 133: myosin-4 [Homo sapiens]

**Accession:**	gi|110611903	**Score:**	39.8
**Database:**	refseq_human(refseq_human_20140103.fasta)	**MW [kDa]:**	222.9
**Database Date:**	2015-11-30	**pI:**	5.6
		**Sequence Coverage [%]:**	2.0
		**No. of unique Peptides:**	1

Detailed Protein Report

10	20	30	40	50	60	70	80
MSSDSEMAIF	GEAAPFLRKS	EKERIEAQNK	PFDAKTSVFV	VDPKESYVKA	IVQSREGGKV	TAKTEAGATV	TVKEDQVFSM
90	100	110	120	130	140	150	160
NPPKYDKIED	MAMMTHLHEP	AVLYNLKERY	AAWMIYTYSG	LFCVTVNPYK	WLPVYNPEVV	TAYRGKKRQE	APPHIFSISD
170	180	190	200	210	220	230	240
NAYQFMLTDR	ENQSILITGE	SGAGKTVNTK	RVIQYFATIA	VTGEKKKEEP	ASGKMQGTLE	DQIISANPLL	EAFGNAKTVR
250	260	270	280	290	300	310	320
NDNSSRFGKF	IRIHFGATGK	LASADIETYL	LEKSRVTFQL	KAERSYHIFY	QILSNKKPEL	IEMLLITTNP	YDFAFVSQGE
330	340	350	360	370	380	390	400
ITVPSIDDQE	ELMATDSAVD	ILGFTADEKV	AIYKLTGAVM	HYGNMKFKQK	QREEQAEPDG	TEVADKAAYL	TSLNSADLLK
410	420	430	440	450	460	470	480
SLCYPRVKVG	NEFVTKGQTV	QQVYNAVGAL	AKAIYEKMFL	WMVTRINQQL	DTKQPRQYFI	GVLDIAGFEI	FDFNSLEQLC
490	500	510	520	530	540	550	560
INFTNEKLQQ	FFNHHMFVLE	QEEYKKEGIE	WEFIDFGMDL	AACIELIEKP	MGIFSILEEE	CMFPKATDTS	FKNKLYEQHL
570	580	590	600	610	620	630	640
GKSNNFQKPK	PAKGKPEAHF	SLVHYAGTVD	YNIAGWLDKN	KDPLNETVVG	LYQKSAMKTL	AFLFSGAQTA	EAEGGGGKKG
650	660	670	680	690	700	710	720
GKKKGSSFQT	VSALFRENLN	KLMTNLRSTH	PHFVRCIIPN	ETKTPGAMEH	ELVLHQLRCN	GVLEGIRICR	KGFPSRILYA
730	740	750	760	770	780	790	800
DFKQRYKVLN	ASAIPEGQFI	DSKKASEKLL	GSIEIDHTQY	KFGHTKVFFK	AGLLGTLEEM	RDEKLAQLIT	RTQAICRGFL
810	820	830	840	850	860	870	880
MRVEFRKMME	RRESIFCIQY	NIRAFMNVKH	WPWMKLYFKI	KPLLKSAETE	KEMANMKEEF	EKTKEELAKT	EAKRKELEEK
890	900	910	920	930	940	950	960
MVTLMQEKND	LQLQVQAEAD	ALADAEERCD	QLIKTKIQLE	AKIKEVTERA	EDEEEINAEL	TAKKRKLEDE	CSELKKDIDD
970	980	990	1000	1010	1020	1030	1040
LELTLAKVEK	EKHATENKVK	NLTEEMAGLD	ETIAKLTKEK	KALQEAHQQT	LDDLQMEEDK	VNTLTKAKTK	LEQQVDDLEG
1050	1060	1070	1080	1090	1100	1110	1120
SLEQEKKLCM	DLERAKRKLE	GDLKLAQEST	MDTENDKQQL	NEKLKKKEFE	MSNLQGKIED	EQALAIQLQK	KIKELQARIE
1130	1140	1150	1160	1170	1180	1190	1200
ELEEEIEAER	ASRAKAEKQR	SDLSRELEEI	SERLEEAGGA	TSAQIEMNKK	REAEFQKMRR	DLEESTLQHE	ATAAALRKKH
1210	1220	1230	1240	1250	1260	1270	1280
ADSVAELGEQ	IDSLQRVKQK	LEKEKSELKM	EINDLASNME	TVSKAKANFE	KMCRTLEDQL	SEIKTKEEEQ	QRLINELSAQ
1290	1300	1310	1320	1330	1340	1350	1360
KARLHTESGE	FSRQLDEKDA	MVSQLSRGKQ	AFTQQIEELK	RQLEEETKAK	STLAHALQSA	RHDCDLLREQ	YEEEQEAKAE
1370	1380	1390	1400	1410	1420	1430	1440
LQRGMSKANS	EVAQWRTKYE	TDAIQRTEEL	EEAKKKLAQR	LQDAEEHVEA	VNSKCASLEK	TKQRLQNEVE	DLMIDVERSN
1450	1460	1470	1480	1490	1500	1510	1520
AACIALDKKQ	RNFDKVLAEW	KQKYEETQAE	LEASQKESRS	LSTELFKVKN	AYEESLDHLE	TLKRENKNLQ	QEISDLTEQI
1530	1540	1550	1560	1570	1580	1590	1600
AEGGKHIHEL	EKVKKQLDHE	KSELQTSLEE	AEASLEHEEG	KILRIQLELN	QVKSEIDRKI	AEKDEELDQL	KRNHLRVVES
1610	1620	1630	1640	1650	1660	1670	1680
MQSTLDAEIR	SRNDALRIKK	KMEGDLNEME	IQLNHANRQA	AEALRNLRNT	QGILKDTQLH	LDDAIRGQDD	LKEQLAMVER
1690	1700	1710	1720	1730	1740	1750	1760
RANLMQAEVE	ELRASLERTE	RGRKMAEQEL	LDASERVQLL	HTQNTSLINT	KKKLETDISQ	IQGEMEDIVQ	EARNAEEKAK
1770	1780	1790	1800	1810	1820	1830	1840
KAITDAAMMA	EELKKEQDTS	AHLERMKKNM	EQTVKDLQLR	LDEAEQLALK	GGKKQIQKLE	ARVRELESEV	ESEQKHNVEA
1850	1860	1870	1880	1890	1900	1910	1920
VKGLRKHERR	VKELTYQTEE	DRKNILRLQD	LVDKLQTKVK	AYKRQAEEAE	EQSNVNLAKF	RKLQHELEEA	KERADIAESQ
1930	1940						
VNKLRVKSRE	VHTKVISEE						

Cmpd.	No. of Cmpds.	m/z meas.	Δ m/z [ppm]	z	Rt [min]	Score	P	Range	Sequence	Modification
1578	1	873.9352	-55.16	2	48.2	12.5	2	56-73	R.EGGKVTAKTEAGATVTVK.E	

Detailed Protein Report

Protein 134: vacuolar protein sorting-associated protein 13A isoform D [Homo sapiens]

**Accession:**	gi|66346676	**Score:**	39.6
**Database:**	refseq_human(refseq_human_20140103.fasta)	**MW [kDa]:**	347.5
**Database Date:**	2015-11-30	**pI:**	5.8
**Modification(s):**	Carbamidomethyl	**Sequence Coverage [%]:**	1.4
		**No. of unique Peptides:**	2

Quantitation

QD:QU	**Median:** 0.62	**CV:** 0.00 %	**No. of Peptides:** 1

Detailed Protein Report

10	20	30	40	50	60	70	80
MVFESVVVDV	LNRFLGDYVV	DLDTSQLSLG	IWKGAVALKN	LQIKENALSQ	LDVPFKVKVG	HIGNLKLIIP	WKNLYTQPVE
90	100	110	120	130	140	150	160
AVLEEIYLLI	VPSSRIKYDP	LKEEKQLMEA	KQQELKRIEE	AKQKVVDQEQ	HLPEKQDTFA	EKLVTQIIKN	LQVKISSIHI
170	180	190	200	210	220	230	240
RYEDDITNRD	KPLSFGISLQ	NLSMQTTDQY	WVPCLHDETE	KLVRKLIRLD	NLFAYWNVKS	QMFYLSDYDN	SLDDLKNGIV
250	260	270	280	290	300	310	320
NENIVPEGYD	FVFRPISANA	KLVMNRRSDF	DFSAPKINLE	IELHNIAIEF	NKPQYFSIME	LLESVDMMAQ	NLPYRKFKPD
330	340	350	360	370	380	390	400
VPLHHHAREW	WAYAIHGVLE	VNVCPRLWMW	SWKHIRKHRQ	KVKQYKELYK	KKLTSKKPPG	ELLVSLEELE	KTLDVFNITI
410	420	430	440	450	460	470	480
ARQTAEVEVK	KAGYKIYKEG	VKDPEDNKGW	FSWLWSWSEQ	NTNEQQPDVQ	PETLEEMLTP	EEKALLYEAI	GYSETAVDPT
490	500	510	520	530	540	550	560
LLKTFEALKF	FVHLKSMSIV	LRENHQKPEL	VDIVIEEFST	LIVQRPGAQA	IKFETKIDSF	HITGLPDNSE	KPRLLSSLDD
570	580	590	600	610	620	630	640
AMSLFQITFE	INPLDETVSQ	RCIIEAEPLE	IIYDARTVNS	IVEFFRPPKE	VHLAQLTAAT	LTKLEEFRSK	TATGLLYIIE
650	660	670	680	690	700	710	720
TQKVLDLKIN	LKASYIIVPQ	DGIFSPTSNL	LLLDLGHLKV	TSKSRSELPD	VKQGEANLKE	IMDRAYDSFD	IQLTSVQLLY
730	740	750	760	770	780	790	800
SRVGDNWREA	RKLSVSTQHI	LVPMHFNLEL	SKAMVFMDVR	MPKFKIYGKL	PLISLRISDK	KLQGIMELIE	SIPKPEPVTE
810	820	830	840	850	860	870	880
VSAPVKSFQI	QTSTSLGTSQ	ISQKIIPLLE	LPSVSEDDSE	EEFFDAPCSP	LEEPLQFPTG	VKSIRTRKLQ	KQDCSVNMTT
890	900	910	920	930	940	950	960
FKIRFEVPKV	LIEFYHLVGD	CELSVVEILV	LGLGAEIEIR	TYDLKANAFL	KEFCLKCPEY	LDENKKPVYL	VTTLDNTMED
970	980	990	1000	1010	1020	1030	1040
LLTLEYVKAE	KNVPDLKSTY	NNVLQLIKVN	FSSLDIHLHT	EALLNTINYL	HNILPQSEEK	SAPVSTTETE	DKGDVIKKLA
1050	1060	1070	1080	1090	1100	1110	1120
LKLSTNEDII	TLQILAELSC	LQIFIQDQKC	NISEIKIEGL	DSEMIMRPSE	TEINAKLRNI	IVLDSDITAI	YKKAVYITGK
1130	1140	1150	1160	1170	1180	1190	1200
EVFSFKMVSY	MDATAGSAYT	DMNVVDIQVN	LIVGCIEVVF	VTKFLYSILA	FIDNFQAAKQ	ALAEATVQAA	GMAATGVKEL
1210	1220	1230	1240	1250	1260	1270	1280
AQRSSRMALD	INIKAPVVVI	PQSPVSENVF	VADFGLITMT	NTFHMITESQ	SSPPPVIDLI	TIKLSEMRLY	RSRFINDAYQ
1290	1300	1310	1320	1330	1340	1350	1360
EVLDLLLPLN	LEVVVERNLC	WEWYQEVPCF	NVNAQLKPME	FILSQEDITT	IFKTLHGNIW	YEKDGSASPA	VTKDQYSATS
1370	1380	1390	1400	1410	1420	1430	1440
GVTTNASHHS	GGATVVTAAV	VEVHSRALLV	KTTLNISFKT	DDLTMVLYSP	GPKQASFTDV	RDPSLKLAEF	KLENIISTLK
1450	1460	1470	1480	1490	1500	1510	1520
MYTDGSTFSS	FSLKNCILDD	KRPHVKKATP	RMIGLTVGFD	KKDMMDIKYR	KVRDGCVTDA	VFQEMYICAS	VEFLQTVANV
1530	1540	1550	1560	1570	1580	1590	1600
FLEAYTTGTA	VETSVQTWTA	KEEVPTQESV	KWEINVIIKN	PEIVFVADMT	KNDAPALVIT	TQCEICYKGN	LENSTMTAAI
1610	1620	1630	1640	1650	1660	1670	1680
KDLQVRACPF	LPVKRKGKIT	TVLQPCDLFY	QTTQKGTDPQ	VIDMSVKSLT	LKVSPVIINT	MITITSALYT	TKETIPEETA
1690	1700	1710	1720	1730	1740	1750	1760
SSTAHLWEKK	DTKTLKMWFL	EESNETEKIA	PTTELVPKGE	MIKMNIDSIF	IVLEAGIGHR	TVPMLLAKSR	FSGEGKNWSS
1770	1780	1790	1800	1810	1820	1830	1840
LINLHCQLEL	EVHYYNEMFG	VWEPLLEPLE	IDQTEDFRPW	NLGIKMKKKA	KMAIVESDPE	EENYKVPEYK	TVISFHSKDQ
1850	1860	1870	1880	1890	1900	1910	1920
LNITLSKCGL	VMLNNLVKAF	TEAATGSSAD	FVKDLAPFMI	LNSLGLTISV	SPSDSFSVLN	IPMAKSYVLK	NGESLSMDYI
1930	1940	1950	1960	1970	1980	1990	2000
RTKDNDHFNA	MTSLSSKLFF	ILLTPVNHST	ADKIPLTKVG	RRLYTVRHRE	SGVERSIVCQ	IDTVEGSKKV	TIRSPVQIRN
2010	2020	2030	2040	2050	2060	2070	2080
HFSVPLSVYE	GDTLLGTASP	ENEFNIPLGS	YRSFIFLKPE	DENYQMCEGI	DFEEIIKNDG	ALLKKKCRSK	NPSKESFLIN
2090	2100	2110	2120	2130	2140	2150	2160
IVPEKDNLTS	LSVYSEDGWD	LPYIMHLWPP	ILLRNLLPYK	IAYYIEGIEN	SVFTLSEGHS	AQICTAQLGK	ARLHLKLLDY
2170	2180	2190	2200	2210	2220	2230	2240

Detailed Protein Report

Cmpd.	No. of Cmpds.	m/z meas.	Δ m/z [ppm]	z	Rt [min]	Score	P	Range	Sequence	Modification	Ratios
731	1	698.3535	13.36	3	39.1	12.3	1	1970-1988	R.ESGVERSIVCQIDTVEGSK.K	Carbamidomethyl: 10	
1997	2	648.7895	-184.44	2	53.5	13.3	2	1989-1999	K.KVTIRSPVQIR.N		QD:QU 0.62

Detailed Protein Report

Protein 135: ubiquitin carboxyl-terminal hydrolase 6 [Homo sapiens]

**Accession:**	gi|109638751	**Score:**	39.5
**Database:**	refseq_human(refseq_human_20140103.fasta)	**MW [kDa]:**	158.6
**Database Date:**	2015-11-30	**pI:**	8.8
		**Sequence Coverage [%]:**	3.8
		**No. of unique Peptides:**	3

Quantitation

QD:QU	**Median:** 0.83	**CV:** 0.00 %	**No. of Peptides:** 1

Alias proteins:

Accession	Name	Description
gi|530410741	refseq_human (refseq_human_20140103.fasta)	PREDICTED: ubiquitin carboxyl-terminal hydrolase 6 isoform X1 [Homo sapiens]

10	20	30	40	50	60	70	80
MDMVENADSL	QAQERKDILM	KYDKGHRAGL	PEDKGPEPVG	INSSIDRFGI	LHETELPPVT	AREAKKIRRE	MTRTSKWMEM
**90**	**100**	**110**	**120**	**130**	**140**	**150**	**160**
LGEWETYKHS	SKLIDRVYKG	IPMNIRGPVW	SVLLNIQEIK	LKNPGRYQIM	KERGKRSSEH	IHHIDLDVRT	TLRNHVFFRD
**170**	**180**	**190**	**200**	**210**	**220**	**230**	**240**
RYGAKQRELF	YILLAYSEYN	PEVGYCRDLS	HITALFLLYL	PEEDAFWALV	QLLASERHSL	PGFHSPNGGT	VQGLQDQQEH
**250**	**260**	**270**	**280**	**290**	**300**	**310**	**320**
VVPKSQPKTM	WHQDKEGLCG	QCASLGCLLR	NLIDGISLGL	TLRLWDVYLV	EGEQVLMPIT	SIALKVQQKR	LMKTSRCGLW
**330**	**340**	**350**	**360**	**370**	**380**	**390**	**400**
ARLRNQFFDT	WAMNDDTVLK	HLRASTKKLT	RKQGDLPPPA	KREQGSLAPR	PVPASRGGKT	LCKGYRQAPP	GPPAQFQRPI
**410**	**420**	**430**	**440**	**450**	**460**	**470**	**480**
CSASPPWASR	FSTPCPGGAV	REDTYPVGTQ	GVPSLALAQG	GPQGSWRFLE	WKSMPRLPTD	LDIGGPWFPH	YDFEWSCWVR
**490**	**500**	**510**	**520**	**530**	**540**	**550**	**560**
AISQEDQLAT	CWQAEHCGEV	HNKDMSWPEE	MSFTANSSKI	DRQKVPTEKG	ATGLSNLGNT	CFMNSSIQCV	SNTQPLTQYF
**570**	**580**	**590**	**600**	**610**	**620**	**630**	**640**
ISGRHLYELN	RTNPIGMKGH	MAKCYGDLVQ	ELWSGTQKSV	APLKLRRTIA	KYAPKFDGFQ	QQDSQELLAF	LLDGLHEDLN
**650**	**660**	**670**	**680**	**690**	**700**	**710**	**720**
RVHEKPYVEL	KDSDGRPDWE	VAAEAWDNHL	RRNRSIIVDL	FHGQLRSQVK	CKTCGHISVR	FDPFNFLSLP	LPMDSYMDLE
**730**	**740**	**750**	**760**	**770**	**780**	**790**	**800**
ITVIKLDGTT	PVRYGLRLNM	DEKYTGLKKQ	LRDLCGLNSE	QILLAEVHDS	NIKNFPQDNQ	KVQLSVSGFL	CAFEIPVPSS
**810**	**820**	**830**	**840**	**850**	**860**	**870**	**880**
PISASSPTQI	DFSSSPSTNG	MFTLTTNGDL	PKPIFIPNGM	PNTVVPCGTE	KNFTNGMVNG	HMPSLPDSPF	TGYIIAVHRK
**890**	**900**	**910**	**920**	**930**	**940**	**950**	**960**
MMRTELYFLS	PQENRPSLFG	MPLIVPCTVH	TRKKDLYDAV	WIQVSWLARP	LPPQEASIHA	QDRDNCMGYQ	YPFTLRVVQK
**970**	**980**	**990**	**1000**	**1010**	**1020**	**1030**	**1040**
DGNSCAWCPQ	YRFCRGCKID	CGEDRAFIGN	AYIAVDWHPT	ALHLRYQTSQ	ERVVDKHESV	EQSRRAQAEP	INLDSCLRAF
**1050**	**1060**	**1070**	**1080**	**1090**	**1100**	**1110**	**1120**
TSEEELGESE	MYYCSKCKTH	CLATKKLDLW	RLPPFLIIHL	KRFQFVNDQW	IKSQKIVRFL	RESFDPSAFL	VPRDPALCQH
**1130**	**1140**	**1150**	**1160**	**1170**	**1180**	**1190**	**1200**
KPLTPQGDEL	SKPRILAREV	KKVDAQSSAG	KEDMLLSKSP	SSLSANISSS	PKGSPSSSRK	SGTSCPSSKN	SSPNSSPRTL
**1210**	**1220**	**1230**	**1240**	**1250**	**1260**	**1270**	**1280**
GRSKGRLRLP	QIGSKNKPSS	SKKNLDASKE	NGAGQICELA	DALSRGHMRG	GSQPELVTPQ	DHEVALANGF	LYEHEACGNG
**1290**	**1300**	**1310**	**1320**	**1330**	**1340**	**1350**	**1360**
CGDGYSNGQL	GNHSEEDSTD	DQREDTHIKP	IYNLYAISCH	SGILSGGHYI	TYAKNPNCKW	YCYNDSSCEE	LHPDEIDTDS
**1370**	**1380**	**1390**	**1400**	**1410**			
AYILFYEQQG	IDYAQFLPKI	DGKKMADTSS	TDEDSESDYE	KYSMLQ			

Cmpd.	No. of Cmpds.	m/z meas.	Δ m/z [ppm]	z	Rt [min]	Score	P	Range	Sequence	Modification	Ratios
2707	4	673.3583	-36.63	2	65.0	13.5	2	89-99	K.HSSKLIDRVYK.G		
2932	1	965.1226	-27.88	3	65.8	10.3	0	218-244	R. HSLPGFHSPNGGTVQGLQDQQEI S		

Detailed Protein Report

Cmpd.	No. of Cmpds.	m/z meas.	Δ m/z [ppm]	z	Rt [min]	Score	P	Range	Sequence	Modification	Ratios	
598	1	823.8884	-6.53	2	37.5	15.8	0	1230-1245	K.ENGAGQICELADALSR.G		QD:QU	0.83

Detailed Protein Report

Protein 136: PREDICTED: neuroblastoma breakpoint family member 4 isoform X2 [Homo sapiens]

**Accession:**	gi|578798443	**Score:**	39.4
**Database:**	refseq_human(refseq_human_20140103.fasta)	**MW [kDa]:**	70.3
**Database Date:**	2015-11-30	**pI:**	4.9
		**Sequence Coverage [%]:**	7.2
		**No. of unique Peptides:**	2

Quantitation

QD:QU **Median:** 0.99 **CV:** 0.00 % **No. of Peptides:** 1

10	20	30	40	50	60	70	80
MTPPPSNSVI	ATADRYLVRW	LLPPSLLSES	ELQAVPSALS	SGIPVPVFAV	KDSKVLCRVH	QLFLNFPGST	SSATNVSMVV
**90**	**100**	**110**	**120**	**130**	**140**	**150**	**160**
SADPLSSERA	EMNILEINQE	LRSQLAESNQ	QFRDLKEKFL	ITQATAYSLA	NQLKKYKCEE	YKDIIDSVLR	DELQSMEKLA
**170**	**180**	**190**	**200**	**210**	**220**	**230**	**240**
EKLRQAEELR	QYKALVHSQA	KELTQLREKL	REGRDASRWL	NKHLKTLLTP	DDPDKSQGQD	LREQLAEGHR	LAEHLVHKLS
**250**	**260**	**270**	**280**	**290**	**300**	**310**	**320**
PENDEDEDED	EDDKDEEVEK	VQESPAPRHH	DKSNSYRHRE	VSFLALDEQK	VCSAQDVARD	YSNPKWDETS	LGFLEKQSDL
**330**	**340**	**350**	**360**	**370**	**380**	**390**	**400**
EEVKGQETVA	PRLSRGPLRV	DKHEIPQESL	DGCCLTPSIL	PDLTPSYHPY	WSTLYSFEDK	QVSLALVDKI	KKDQEEIEDQ
**410**	**420**	**430**	**440**	**450**	**460**	**470**	**480**
SPPCPRLSQE	LPEVKEQEVP	EDSVNEVYLT	PSVHHDVSDC	HQPYSSTLSS	LEDQLACSAL	DVASPTEAAC	PQGTWSGDLS
**490**	**500**	**510**	**520**	**530**	**540**	**550**	**560**
HHQSEVQVSQ	AQLEPSTLVP	SCLRLQLDQG	FHCGNGLAQR	GLSSTTCSFS	ANADSGNQWP	FQELVLEPSL	GMKNPPQLED
**570**	**580**	**590**	**600**	**610**	**620**	**630**	
DALEGSASNT	QGRQVTGRIR	ASLVLILKTI	RRRLPFSKWR	LAFRFAGPHA	ESAEIPNTAG	RTQRMAG	

Cmpd.	No. of Cmpds.	m/z meas.	Δ m/z [ppm]	z	Rt [min]	Score	P	Range	Sequence	Modification	Ratios
2547	3	1044.5458	-3.00	2	62.5	15.8	1	1-19	-.MTPPPSNSVIATADRYLVR.W		
573	2	666.3169	-41.31	2	37.1	12.9	2	192-202	R.EGRDASRWLNK.H		QD:QU 0.99

Detailed Protein Report

Protein 137: beta/gamma crystallin domain-containing protein 3 [Homo sapiens]

**Accession:**	gi|390979647	**Score:**	39.0
**Database:**	refseq_human(refseq_human_20140103.fasta)	**MW [kDa]:**	330.4
**Database Date:**	2015-11-30	**pI:**	5.0
		**Sequence Coverage [%]:**	1.2
		**No. of unique Peptides:**	1

BRUKER

Detailed Protein Report

10	20	30	40	50	60	70	80
MSSGRRRGSA	PWHSFSRFFA	PRSPSRDKEE	EEEERPGTSP	PPAPGRSAAS	VENEPMSTSQ	KKENVLSSEA	VKIRQSEDKR
90	100	110	120	130	140	150	160
NHAEKPVTLP	VQEDPKKAYD	LSSSTSDTKI	GESDRQPKES	FFQFLGNLFN	ISGKSSLGEA	KQSSFKDDQD	KTEKDLQNPS
170	180	190	200	210	220	230	240
DHHEDGIKRE	REIFSGSLRT	QTHPTEEQDS	NSSELSDAFS	LDTTQDSDQE	TTNLLKQIDG	KPEKPSVTYA	TYRGPRHIGK
250	260	270	280	290	300	310	320
YLKQQTGLAT	VNTLDRENES	SDSSTNRHID	PGSEIEAGVL	PLLLSASTDS	SMKGNLLEGP	LEDSDCSKTS	FNKENSLTNN
330	340	350	360	370	380	390	400
PELQNIASSN	NLLNKNAWGS	IERNRSSPSS	VTNSSYDGES	DSQHHLSCEP	VSQTNRNLVC	SALLTGSNHR	KVPCSPDFQR
410	420	430	440	450	460	470	480
VTTTENTIKE	NSTVMSNRTL	VQREELVEPQ	GPAISDFSCS	KSDGSDTTEQ	ESTNLPSPNK	SIRHEHLQLP	ESECSDKQTI
490	500	510	520	530	540	550	560
DSSSKQAATH	TNIIALQRHA	VTDTEFVNEG	KRLSAQDSQK	NVAVREIRRE	TESASAGESI	ASSHVKAPED	KIESLPKDTD
570	580	590	600	610	620	630	640
QYFETKAKKL	DFRSHDKIPH	IRMNKKDLAS	LNYISESAVV	ASLGNENAPE	LKFELNRSHI	SETPLDSESP	QQAEVSPDAK
650	660	670	680	690	700	710	720
TSLSLDCKKL	NFSISPPTFV	SGVGMLSKLD	IPDLMNEGSP	VPIETGNVNI	VGISYQPRKC	KEENVKNHVE	AAGRKSPPPS
730	740	750	760	770	780	790	800
FCLEYTSAIF	EFKEVLSNSE	KCQVLPGSEA	SGPHLTGLEL	LSFDSGNLSK	DCSSILSQDP	NRVELVSSNT	KANMSIIEKS
810	820	830	840	850	860	870	880
DSLSLEAKTA	NIVSKAEIDG	QNNVLVESHS	GRGKTISLSK	VSLSKVEPRN	ISQDKMSSFP	LKITHVPEKP	ILSELTFLEV
890	900	910	920	930	940	950	960
EQGKRFQSIN	HNEIGEKCSD	AGLKENCQAE	LSPAASKYED	KPEPEVDALG	SPPALLKSNI	SWILPPIHDE	KISRQMAQNC
970	980	990	1000	1010	1020	1030	1040
EAHTCVFHQS	LDICGTKKIS	GHSEMAELSL	TNISPKFQET	GSMKVNSPFL	DSDSSLEKNS	SASEDSSFLK	VPSVLKLEKK
1050	1060	1070	1080	1090	1100	1110	1120
SSSYRKKENI	HFLNGGIDSV	SSSSSYPEEV	SMIVNSHKPQ	NNLDSIQVTK	DLTHEGTSVT	NLLYPTTSYL	EFETSVSIGT
1130	1140	1150	1160	1170	1180	1190	1200
EVTPFQEHFG	IYTGKISIDF	PTAAQFDNLV	EAETGAVAGP	AASVNSSGQQ	CSEASAEHIE	ARRRAHDQLL	DLKSSLLKKA
1210	1220	1230	1240	1250	1260	1270	1280
DTLIGEIFNS	VREELKFKHT	VSTCQEHIAI	EGIMNLGTLK	EDISEKNPSE	VTLTEIQQTE	GLEEQGMENM	SEVKEKPCVS
1290	1300	1310	1320	1330	1340	1350	1360
PTVGEKNLLV	DPNSMNVSCL	LEDKARELVN	EIIYVAQEKL	RNDTFEDTED	TWDSELQANT	SKILNSDSVK	PHDVVREFLV
1370	1380	1390	1400	1410	1420	1430	1440
SEQPVNQSTQ	ISENKVLNEF	FSLSNLASGT	ESIKGGEIVL	YQKSLFSGNG	SGLSDSINLQ	ESDTVLLAED	MSHKRLDDRV
1450	1460	1470	1480	1490	1500	1510	1520
KTHLFRSEDC	NETMEIENVD	NNKTETEDRR	TLVLNFKWPP	LVNDDIHAPG	TSKSSLSDSL	VCISEKNLPG	HSKNTPLAMS
1530	1540	1550	1560	1570	1580	1590	1600
DVGKVHKKDN	EINIGKIELI	PSMLETGKTN	KKDAELNILK	YEAVPPMIEM	GRIHKMDAEL	NVTKTEPKAN	VFKMGEVYQM
1610	1620	1630	1640	1650	1660	1670	1680
DAESCIEKTE	GSAVILGMEK	AYKMKDTEGD	IGKIEVIPMM	PEVKNIHQKD	AEGDIVKTEM	TPVTVDMENI	YQTHAEGDIG
1690	1700	1710	1720	1730	1740	1750	1760
KTGTIALSEV	ENIHQKGGEG	ISEKAEVIPV	TLAMENTYQK	DAEGDIGKAE	VMPVRLEMEN	TYPKDTERDG	GKTEVMPLAL
1770	1780	1790	1800	1810	1820	1830	1840
EVVNTYQKNA	KGFTGNTEGS	VLKMEATYRK	TAEEVIKNTE	IVPCVLKVKE	AHETAPAPLE	MEKACKRDVK	ETIGATVSTP
1850	1860	1870	1880	1890	1900	1910	1920
SVIEMEKISP	EDRGENIGKH	KVLPAVVDIE	KIHGTGLELT	TKQGEAMLPA	FESKTPQEYA	EGSVEETKEE	PTEIKEGLIA
1930	1940	1950	1960	1970	1980	1990	2000
HENRLPTYFR	GYESPTLSKD	YEGYPAPAMP	DFQPGDTTVR	LDKRMSLTAI	YDKRRETDYS	DKGYNLAFVS	QDEQENSSFT
2010	2020	2030	2040	2050	2060	2070	2080
ILYEEPLQEE	DKYASAEARQ	TQSVLFHDTS	ADSMPVLACE	RSESRTDLVH	HFEKGTKLGE	TFDSDSSEMF	LSVEAKRYKI
2090	2100	2110	2120	2130	2140	2150	2160
YPLALSPIYE	DDSSQEDILS	SEVSPGHHGP	RKSRDSENQS	SSVLSLLQSV	SERLKMNFDE	DDREAADEEE	EEEEAAVLHK
2170	2180	2190	2200	2210	2220	2230	2240

Detailed Protein Report

Cmpd.	No. of Cmpds.	m/z meas.	Δ m/z [ppm]	z	Rt [min]	Score	P	Range	Sequence	Modification
2896	1	1012.4872	-151.04	1	65.4	13.7	1	1031-1039	K.VPSVLKLEK.K	

Detailed Protein Report

Protein 138: PREDICTED: sushi, nidogen and EGF-like domain-containing protein 1 isoform X4 [Homo sapiens]

**Accession:**	gi|578804038	**Score:**	39.0
**Database:**	refseq_human(refseq_human_20140103.fasta)	**MW [kDa]:**	142.7
**Database Date:**	2015-11-30	**pI:**	6.4
**Modification(s):**	Carbamidomethyl	**Sequence Coverage [%]:**	4.4
		**No. of unique Peptides:**	2

10	20	30	40	50	60	70	80
MRHGVAWALL	VAAALGLGAR	GVRGAVALAD	FYPFGAERGD	AVTPKQDDGG	SGLRPLSVPF	PFFGAEHSGL	YVNNNGIISF
90	100	110	120	130	140	150	160
LKEVSQFTPV	AFPIAKDRCV	VAAFWADVDN	RRAGDVYYRE	ATDPAMLRRA	TEDVRHYFPE	LLDFNATWVF	VATWYRVTFF
170	180	190	200	210	220	230	240
GGSSSSPVNT	FQTVLITDGK	LSFTIFNYES	IVWTTGTHAS	SGGNATGLGG	IAAQAGFNAG	DGQRYFSIPG	SRTADMAEVE
250	260	270	280	290	300	310	320
TTTNVGVPGR	WAFRIDDAQV	RVGGCGHTTS	VCLALRPCLN	GGKCIDDCVT	GNPSYTCSCL	SGFTGRRCHL	DVNECASQPC
330	340	350	360	370	380	390	400
QNGGTCTHGI	NSFRCQCPAG	FGGPTCETAQ	SPCDTKECQH	GGQCQVENGS	AVCVCQAGYT	GAACEMDVDD	CSPDPCLNGG
410	420	430	440	450	460	470	480
SCVDLVGNYT	CLCAEPFKGL	RCETGDHPVP	DACLSAPCHN	GGTCVDADQG	YVCECPEGFM	GLDCRERVPD	DCECRNGGRC
490	500	510	520	530	540	550	560
LGANTTLCQC	PLGFFGLLCE	FEITAMPCNM	NTQCPDGGYC	MEHGGSYLCV	CHTDHNASHS	LPSPCDSDPC	FNGGSCDAHD
570	580	590	600	610	620	630	640
DSYTCECPRG	FHGKHCEKAR	PHLCSSGPCR	NGGTCKEAGG	EYHCSCPYRF	TGRHCEIGKP	DSCASGPCHN	GGTCFHYIGK
650	660	670	680	690	700	710	720
YKCDCPPGFS	GRHCEIAPSP	CFRSPCVNGG	TCEDRDTDFF	CHCQAGYMGR	RCQAEVDCGP	PEEVKHATLR	FNGTRLGAVA
730	740	750	760	770	780	790	800
LYACDRGYSL	SAPSRIRVCQ	PHGVWSEPPQ	CLEIDECRSQ	PCLHGGSCQD	RVAGYLCLCS	TGYEGAHCEL	ERDECRAHPC
810	820	830	840	850	860	870	880
RNGGSCRNLP	GAYVCRCPAG	FVGVHCETEV	DACDSSPCQH	GGRCESGGGA	YLCVCPESFF	GYHCETVSDP	CFSSPCGGRG
890	900	910	920	930	940	950	960
YCLASNGSHS	CTCKVGYTGE	DCAKELFPPT	ALKMERVEES	GVSISWNPPN	GPAARQMLDG	YAVTYVSSDG	SYRRTDFVDR
970	980	990	1000	1010	1020	1030	1040
TRSSHQLQAL	AAGRAYNISV	FSVKRNSNNK	NDISRPAVLL	ARTRPRPVEG	FEVTNVTAST	ISVQWALHRI	RHATVSGVRV
1050	1060	1070	1080	1090	1100	1110	1120
SIRHPEALRD	QATDVDRSVD	RFTFRALLPG	KRYTIQLTTL	SGLRGEEHPT	ESLATAPTHV	WTRPLPPANL	TAARVTATSA
1130	1140	1150	1160	1170	1180	1190	1200
HVVWDAPTPG	SLLEAYVINV	TTSQSTKSRY	VPNGKLASYT	VRDLLPGRRY	QLSVIAVQST	ELGPQHSEPA	HLYIITSPRD
1210	1220	1230	1240	1250	1260	1270	1280
GADRRWHQGG	HHPRVLKNRP	PPARLPELRL	LNDHSAPETP	TQPPRFSELV	DGRGRVSARF	GGSPSKAATV	RSQPTASAQL
1290	1300	1310	1320	1330			
ENMEEAPKRV	SLALQLPEHG	SKDIGSLDLL	LAPVSTRRTG	LCIRRRE			

Cmpd.	No. of Cmpds.	m/z meas.	Δ m/z [ppm]	z	Rt [min]	Score	P	Range	Sequence	Modification
1017	1	788.6695	-45.57	3	41.2	10.3	2	570-590	R.GFHGKHCEKARPHLCSSGPCR.N	Carbamidomethyl: 15
2079	1	1044.9332	12.94	2	56.4	14.4	1	591-609	R.NGGTCKEAGGEYHCSCPYR.F	Carbamidomethyl: 5

Detailed Protein Report

Protein 139: PREDICTED: nebulin-related-anchoring protein isoform X4 [Homo sapiens]

Accession: gi|530393765
Database: refseq_human(refseq_human_20140103.fasta)
Database Date: 2015-11-30
Modification(s): Oxidation

Score: 39.0
MW [kDa]: 184.9
pI: 9.7
Sequence Coverage [%]: 2.6
No. of unique Peptides: 2

10	20	30	40	50	60	70	80
MNVQPCSRCG	YGVYPAEKIS	CIDQIWHKAC	FHCEVCKMML	SVNNFVSHQK	KPYCHAHNPK	NNTFTSVYHT	PLNLNVRTFP
90	100	110	120	130	140	150	160
EAISGIHDQE	DGEQCKSVFH	WDMKSKDKEG	APNRQPLANE	RAYWTGYGEG	NAWCPGALPD	PEIVRMVEAR	KSLGEEYTED
170	180	190	200	210	220	230	240
YEQPRGKGSF	PAMITPAYQR	AKKANQLASQ	VEYKRGHDER	ISRFSTVVDT	PELLRSKAGA	QLQSDVRYTE	DYEQQRGKGS
250	260	270	280	290	300	310	320
FPAMITPAYQ	IAKRANELAS	DVRYHQQYQK	EMRGMAGPAI	GAEGILTREC	ADQYGQGYPE	EYEEHRGKGS	FPAMITPAYQ
330	340	350	360	370	380	390	400
NAKKAHELAS	DIKYRQDFNK	MKGAAHYHSL	PAQDNLVLKQ	AQSVNKLVSE	VEYKKDLESS	RGHSINYCET	PQFRNVSKIS
410	420	430	440	450	460	470	480
KFTSDNKYKE	NYQNHMRGRY	EGVGMDRRTL	HAMKVGSLAS	NVAYKADYKH	DIVDYNYPAT	LTPSYQTAMK	LVPLKDANYR
490	500	510	520	530	540	550	560
QSIDKLKYSS	VTDTPQIVQA	KINAQQLSHV	NYRADYEKNK	LNYTLPQDVP	QLVKAKTNAK	LFSEVKYKEG	WEKTKGKGFE
570	580	590	600	610	620	630	640
MKLDAMSLLA	AKASGELASN	IKYKEEYEKT	KGKAMGTADS	RLLHSLQIAK	MSSEVEYKKG	FEESKTRFHL	PMDMVNIRHA
650	660	670	680	690	700	710	720
KKAQTLASDL	DYRKKLHEYT	VLPEDMKTQW	AKKAYGLQSE	LQYKADLAWM	KGVGWLTEGS	LNLEQAKKAG	QLVSEKNYRQ
730	740	750	760	770	780	790	800
RVDELKFTSV	TDSSQMEHAK	KSQELQSGVA	YKAGNEQSVH	QYTISKDEPL	FLQARANAAN	LSEKLYKSSW	ENQKAKGFEL
810	820	830	840	850	860	870	880
RLDSLTFLAA	KAKRDLASEV	KYKEDYERSR	GKLIGAKDVQ	GDSQMSHSLQ	MSKLQSELEY	KKGFEDTKSQ	CHVSLDMVHL
890	900	910	920	930	940	950	960
VHARKAQHLA	TDVGYKTAEH	HFTALPTDMK	VEWAKKAYGL	QSDNQYRADV	KWMKGMGWVA	TGSLNVEQAK	KAGELISEKK
970	980	990	1000	1010	1020	1030	1040
YRQHPDALKF	TSIKDTPEMV	QARISYTQAV	DRLYREQGEN	IKHHYTPTAD	LPEVLLAKLN	AMNISETRYK	ESWSKLRDGG
1050	1060	1070	1080	1090	1100	1110	1120
YKLRLDALPF	QAAKASGEII	SDYKYKEAFE	KMKGQMLGSR	SLEDDISLAH	SVYATSLQSD	VNYKKGFEHS	KAQFHLPLDM
1130	1140	1150	1160	1170	1180	1190	1200
AALVHAKKAQ	TLASNQDYKH	PLPQYTSLAE	DLRLSCAKKA	HKLQSENLYR	SDLNFMRGVA	CVIPGTLEIE	GRKKASELIS
1210	1220	1230	1240	1250	1260	1270	1280
ESKYRQHPHS	FKYTAVTDTP	NLLHAKFSNQ	ITNERLYKAA	GEDARHEYTM	TLGLPEFIRA	KTNAANLSDA	RYKESWRNLR
1290	1300	1310	1320	1330	1340	1350	1360
AQGYKLTIEA	LPFQAARASG	DIASDLRYKS	DLIGMKGIGW	LALRSPQMES	AKKAGELISE	TKYRKKPDSI	KFTTVVDSPD
1370	1380	1390	1400	1410	1420	1430	1440
LVHAKNSYMH	CNERMYRSGD	AESLHRYTLI	PDHPDFTRAR	LNALHLSDKV	YRNSWEQTRA	GSYDFRLDAI	PFQTARASRE
1450	1460	1470	1480	1490	1500	1510	1520
IASDFRYKEA	FLRDRGLQIG	YRSVDDDPRM	KHFLNVGRLQ	SDNEYKKDFA	KSRSQFHSST	DQPGLLQAKR	SQQLASDVHY
1530	1540	1550	1560	1570	1580	1590	1600
RQPLPQPTCD	PEQLGLRHAQ	KAHQLQSDVK	YKSDLNLTRG	VGWTPPGSYK	VEMARRAAEL	ANARGLGLQG	AYQRGAEAVE
1610	1620	1630					
AGDHQSGEVN	PDATEILHVK	KKALLL					

Cmpd.	No. of Cmpds.	m/z meas.	Δ m/z [ppm]	z	Rt [min]	Score	P	Range	Sequence	Modification
16	1	537.2110	-171.55	2	29.8	13.1	0	1045-1054	R.LDALPFQAAK.A	
2951	1	823.4152	-52.46	2	66.1	14.2	1	1310-1324	K.SDLIGMKGIGWLALR.S	Oxidation: 6

Detailed Protein Report

Protein 140: low-density lipoprotein receptor-related protein 2 precursor [Homo sapiens]

**Accession:**	gi|126012573	**Score:**	38.8
**Database:**	refseq_human(refseq_human_20140103.fasta)	**MW [kDa]:**	521.6
**Database Date:**	2015-11-30	**pI:**	4.8
**Modification(s):**	Carbamidomethyl	**Sequence Coverage [%]:**	1.2
		**No. of unique Peptides:**	3

Detailed Protein Report

10	20	30	40	50	60	70	80
MDRGPAAVAC	TLLLALVACL	APASGQECDS	AHFRCGSGHC	IPADWRCDGT	KDCSDDADEI	GCAVVTCQQG	YFKCQSEGQC
90	100	110	120	130	140	150	160
IPNSWVCDQD	QDCDDGSDER	QDCSQSTCSS	HQITCSNGQC	IPSEYRCDHV	RDCPDGADEN	DCQYPTCEQL	TCDNGACYNT
170	180	190	200	210	220	230	240
SQKCDWKVDC	RDSSDEINCT	EICLHNEFSC	GNGECIPRAY	VCDHDNDCQD	GSDEHACNYP	TCGGYQFTCP	SGRCIYQNWV
250	260	270	280	290	300	310	320
CDGEDDCKDN	GDEDGCESGP	HDVHKCSPRE	WSCPESGRCI	SIYKVCDGIL	DCPGREDENN	TSTGKYCSMT	LCSALNCQYQ
330	340	350	360	370	380	390	400
CHETPYGGAC	FCPPGYIINH	NDSRTCVEFD	DCQIWGICDQ	KCESRPGRHL	CHCEEGYILE	RGQYCKANDS	FGEASIIFSN
410	420	430	440	450	460	470	480
GRDLLIGDIH	GRSFRILVES	QNRGVAVGVA	FHYHLQRVFW	TDTVQNKVFS	VDINGLNIQE	VLNVSVETPE	NLAVDWVNNK
490	500	510	520	530	540	550	560
IYLVETKVNR	IDMVNLDGSY	RVTLITENLG	HPRGIAVDPT	VGYLFFSDWE	SLSGEPKLER	AFMDGSNRKD	LVKTKLGWPA
570	580	590	600	610	620	630	640
GVTLDMISKR	VYWVDSRFDY	IETVTYDGIQ	RKTVVHGGSL	IPHPFGVSLF	EGQVFFTDWT	KMAVLKANKF	TETNPQVYYQ
650	660	670	680	690	700	710	720
ASLRPYGVTV	YHSLRQPYAT	NPCKDNNGGC	EQVCVLSHRT	DNDGLGFRCK	CTFGFQLDTD	ERHCIAVQNF	LIFSSQVAIR
730	740	750	760	770	780	790	800
GIPFTLSTQE	DVMVPVSGNP	SFFVGIDFDA	QDSTIFFSDM	SKHMIFKQKI	DGTGREILAA	NRVENVESLA	FDWISKNLYW
810	820	830	840	850	860	870	880
TDSHYKSISV	MRLADKTRRT	VVQYLNNPRS	VVVHPFAGYL	FFTDWFRPAK	IMRAWSDGSH	LLPVINTTLG	WPNGLAIDWA
890	900	910	920	930	940	950	960
ASRLYWVDAY	FDKIEHSTFD	GLDRRRLGHI	EQMTHPFGLA	IFGEHLFFTD	WRLGAIIRVR	KADGGEMTVI	RSGIAYILHL
970	980	990	1000	1010	1020	1030	1040
KSYDVNIQTG	SNACNQPTHP	NGDCSHFCFP	VPNFQRVCGC	PYGMRLASNH	LTCEGDPTNE	PPTEQCGLFS	FPCKNGRCVP
1050	1060	1070	1080	1090	1100	1110	1120
NYYLCDGVDD	CHDNSDEQLC	GTLNNTCSSS	AFTCGHGECI	PAHWRCDKRN	DCVDGSDEHN	CPTHAPASCL	DTQYTCDNHQ
1130	1140	1150	1160	1170	1180	1190	1200
CISKNWVCDT	DNDCGDGSDE	KNCNSTETCQ	PSQFNCPNHR	CIDLSFVCDG	DKDCVDGSDE	VGCVLNCTAS	QFKCASGDKC
1210	1220	1230	1240	1250	1260	1270	1280
IGVTNRCDGV	FDCSDNSDEA	GCPTRPPGMC	HSDEFQCQED	GICIPNFWEC	DGHPDCLYGS	DEHNACVPKT	CPSSYFHCDN
1290	1300	1310	1320	1330	1340	1350	1360
GNCIHRAWLC	DRDNDCGDMS	DEKDCPTQPF	RCPSWQWQCL	GHNICVNLSV	VCDGIFDCPN	GTDESPLCNG	NSCSDFNGGC
1370	1380	1390	1400	1410	1420	1430	1440
THECVQEPFG	AKCLCPLGFL	LANDSKTCED	IDECDILGSC	SQHCYNMRGS	FRCSCDTGYM	LESDGRTCKV	TASESLLLLV
1450	1460	1470	1480	1490	1500	1510	1520
ASQNKIIADS	VTSQVHNIYS	LVENGSYIVA	VDFDSISGRI	FWSDATQGKT	WSAFQNGTDR	RVVFDSSIIL	TETIAIDWVG
1530	1540	1550	1560	1570	1580	1590	1600
RNLYWTDYAL	ETIEVSKIDG	SHRTVLISKN	LTNPRGLALD	PRMNEHLLFW	SDWGHHPRIE	RASMDGSMRT	VIVQDKIFWP
1610	1620	1630	1640	1650	1660	1670	1680
CGLTIDYPNR	LLYFMDSYLD	YMDFCDYNGH	HRRQVIASDL	IIRHPYALTL	FEDSVYWTDR	ATRRVMRANK	WHGGNQSVVM
1690	1700	1710	1720	1730	1740	1750	1760
YNIQWPLGIV	AVHPSKQPNS	VNPCAFSRCS	HLCLLSSQGP	HFYSCVCPSG	WSLSPDLLNC	LRDDQPFLIT	VRQHIIFGIS
1770	1780	1790	1800	1810	1820	1830	1840
LNPEVKSNDA	MVPIAGIQNG	LDVEFDDAEQ	YIYWVENPGE	IHRVKTDGTN	RTVFASISMV	GPSMNLALDW	ISRNLYSTNP
1850	1860	1870	1880	1890	1900	1910	1920
RTQSIEVLTL	HGDIRYRKTL	IANDGTALGV	GFPIGITVDP	ARGKLYWSDQ	GTDSGVPAKI	ASANMDGTSV	KTLFTGNLEH
1930	1940	1950	1960	1970	1980	1990	2000
LECVTLDIEE	QKLYWAVTGR	GVIERGNVDG	TDRMILVHQL	SHPWGIAVHD	SFLYYTDEQY	EVIERVDKAT	GANKIVLRDN
2010	2020	2030	2040	2050	2060	2070	2080
VPNLRGLQVY	HRRNAAESSN	GCSNNMNACQ	QICLPVPGGL	FSCACATGFK	LNPDNRSCSP	YNSFIVVSML	SAIRGFSLEL
2090	2100	2110	2120	2130	2140	2150	2160
SDHSETMVPV	AGQGRNALHV	DVDVSSGFIY	WCDFSSSVAS	DNAIRRIKPD	GSSLMNIVTH	GIGENGVRGI	AVDWVAGNLY
2170	2180	2190	2200	2210	2220	2230	2240

Detailed Protein Report

Cmpd.	No. of Cmpds.	m/z meas.	Δ m/z [ppm]	z	Rt [min]	Score	P	Range	Sequence	Modification
573	1	656.8903	-140.87	3	35.8	15.5	1	279-295	R.CISIYKVCDGILDCPGR.E	Carbamidomethyl: 1, 8
1012	1	974.7041	-35.28	3	41.2	13.1	1	1173-1199	K.DCVDGSDEVGCVLNCTASQFKCASGDK.C	Carbamidomethyl: 11, 15, 22
936	1	647.6555	-174.87	2	41.8	10.3	0	3696-3706	K.WAVCNGVDDCR.D	Carbamidomethyl: 4

Detailed Protein Report

Protein 141: **PREDICTED: uveal autoantigen with coiled-coil domains and ankyrin repeats isoform X3 [Homo sapiens]**

**Accession:**	gi|530406156	**Score:**	38.6
**Database:**	refseq_human(refseq_human_20140103.fasta)	**MW [kDa]:**	160.2
**Database Date:**	2015-11-30	**pI:**	6.8
**Modification(s):**	Carbamidomethyl	**Sequence Coverage [%]:**	1.6
		**No. of unique Peptides:**	2

10	20	30	40	50	60	70	80
MMNCWFSCTP	KNRHAADWNK	YDDRLMKAAE	RGDVEKVTSI	LAKKGVNPGK	LDVEGRSVFH	VVTSKGNLEC	LNAILIHGVD
90	100	110	120	130	140	150	160
ITTSDTAGRN	ALHLAAKYGH	ALCLQKLLQY	NCPTEHADLQ	GRTALHDAAM	ADCPSSIQLL	CDHGASVNAK	DVDGRTPLVL
170	180	190	200	210	220	230	240
ATQMSRPTIC	QLLIDRGADV	NSRDKQNRTA	LMLGCEYGCR	DAVEVLIKNG	ADISLLDALG	HDSSYYARIG	DNLDILTLLK
250	260	270	280	290	300	310	320
TASENTNKGR	ELWKKGPSLQ	QRNLTHMQDE	VNVKSHQREH	QNIQDLEIEN	EDLKERLRKI	QQEQRILLDK	VNGLQLQLNE
330	340	350	360	370	380	390	400
REKLKSLLAA	KEKQHEESLR	TIEALKNRFK	YFESDHLGSG	SHFSNRKEDM	LLKQGQMYMA	DSQCTSPGIP	AHMQSRSMLR
410	420	430	440	450	460	470	480
PLELSLPSQT	SYSENEILKK	ELEAMRTFCE	SAKQDRLKLQ	NELAHKVAEC	KALALECERV	KEDSDEQIKQ	LEDALKDVQK
490	500	510	520	530	540	550	560
RMYESEGKVK	QMQTHFLALK	EHLTSEAASG	NHRLTEELKD	QLKDLKVKYE	GASAEVGKLR	NQIKQNEMIV	EEFKRDEGKL
570	580	590	600	610	620	630	640
IEENKRLQKE	LSMCEMEREK	KGRKVTEMEG	QAKELSAKLA	LSIPAEKFEN	MKSSLSNEVN	EKAKKLVEME	REHEKSLSEI
650	660	670	680	690	700	710	720
RQLKRELENV	KAKLAQHVKP	EEHEQVKSRL	EQKSGELGKK	ITELTLKNQT	LQKEIEKVYL	DNKLLKEQAH	NLTIEMKNHY
730	740	750	760	770	780	790	800
VPLKVSEDMK	KSHDAIIDDL	NRKLLDVTQK	YTEKKLEMEK	LLLENDSLSK	DVSRLETVFV	PPEKHEKEII	ALKSNIVELK
810	820	830	840	850	860	870	880
KQLSELKKKC	GEDQEKIHAL	TSENTNLKKM	MSNQYVPVKT	HEEVKMTLND	TLAKTNRELL	DVKKKFEDIN	QEFVKIKDKN
890	900	910	920	930	940	950	960
EILKRNLENT	QNQIKAEYIS	LAEHEAKMSS	LSQSMRKVQD	SNAEILANYR	KGQEEIVTLH	AEIKAQKKEL	DTIQECIKVK
970	980	990	1000	1010	1020	1030	1040
YAPIVSFEEC	ERKFKATEKE	LKDQLSEQTQ	KYSVSEEEVK	KNKQENDKLK	KEIFTLQKDL	RDKTVLIEKS	HEMERALSRK
1050	1060	1070	1080	1090	1100	1110	1120
TDELNKQLKD	LSQKYTEVKN	VKEKLVEENA	KQTSEILAVQ	NLLQKQHVPL	EQVEALKKSL	NGTIENLKEE	LKSMQRCYEK
1130	1140	1150	1160	1170	1180	1190	1200
EQQTVTKLHQ	LLENQKNSSV	PLAEHLQIKE	AFEKEVGIIK	ASLREKEEES	QNKMEEVSKL	QSEVQNTKQA	LKKLETREVV
1210	1220	1230	1240	1250	1260	1270	1280
DLSKYKATKS	DLETQISSLN	EKLANLNRKY	EEVCEEVLHA	KKKEISAKDE	KELLHFSIEQ	EIKDQKERCD	KSLTTITELQ
1290	1300	1310	1320	1330	1340	1350	1360
RRIQESAKQI	EAKDNKITEL	LNDVERLKQA	LNGLSQLTYT	SGNPTKRQSQ	LIDTLQHQVK	SLEQQLADAD	RQHQEVIAIY
1370	1380	1390	1400				
RTHLLSAAQG	HMDEDVQEAL	LQIIQMRQGL	VC				

Cmpd.	No. of Cmpds.	m/z meas.	Δ m/z [ppm]	z	Rt [min]	Score	P	Range	Sequence	Modification
281	2	477.4617	-175.78	3	32.2	22.4	0	189-200	R.TALMLGCEYGCR.D	Carbamidomethyl: 7, 11
1858	2	621.2993	26.87	2	53.6	16.2	1	427-436	R.TFCESAKQDR.L	Carbamidomethyl: 3

Detailed Protein Report

Protein 142: fibrillin-2 precursor [Homo sapiens]

**Accession:**	gi|66346695	**Score:**	38.5
**Database:**	refseq_human(refseq_human_20140103.fasta)	**MW [kDa]:**	314.6
**Database Date:**	2015-11-30	**pI:**	4.6
		**Sequence Coverage [%]:**	1.2
		**No. of unique Peptides:**	2

Quantitation

QD:QU	**Median:**	0.70	**CV:**	18.73 %	**No. of Peptides:**	2

Detailed Protein Report

10	20	30	40	50	60	70	80
MGRRRRLCLQ	LYFLWLGCVV	LWAQGTAGQP	QPPPPKPPRP	QPPPQQVRSA	TAGSEGGFLA	PEYREEGAAV	ASRVRRRGQQ
90	100	110	120	130	140	150	160
DVLRGPNVCG	SRFHSYCCPG	WKTLPGGNQC	IVPICRNSCG	DGFCSRPNMC	TCSSGQISST	CGSKSIQQCS	VRCMNGGTCA
170	180	190	200	210	220	230	240
DDHCQCQKGY	IGTYCGQPVC	ENGCQNGGRC	IGPNRCACVY	GFTGPQCERD	YRTGPCFTQV	NNQMCQGQLT	GIVCTKTLCC
250	260	270	280	290	300	310	320
ATIGRAWGHP	CEMCPAQPQP	CRRGFIPNIR	TGACQDVDEC	QAIPGICQGG	NCINTVGSFE	CRCPAGHKQS	ETTQKCEDID
330	340	350	360	370	380	390	400
ECSIIPGICE	TGECSNTVGS	YFCVCPRGYV	TSTDGSRCID	QRTGMCFSGL	VNGRCAQELP	GRMTKMQCCC	EPGRCWGIGT
410	420	430	440	450	460	470	480
IPEACPVRGS	EEYRRLCMDG	LPMGGIPGSA	GSRPGGTGGN	GFAPSGNGNG	YGPGGTGFIP	IPGGNGFSPG	VGGAGVGAGG
490	500	510	520	530	540	550	560
QGPIITGLTI	LNQTIDICKH	HANLCLNGRC	IPTVSSYRCE	CNMGYKQDAN	GDCIDVDECT	SNPCTNGDCV	NTPGSYYCKC
570	580	590	600	610	620	630	640
HAGFQRTPTK	QACIDIDECI	QNGVLCKNGR	CVNTDGSFQC	ICNAGFELTT	DGKNCVDHDE	CTTTNMCLNG	MCINEDGSFK
650	660	670	680	690	700	710	720
CICKPGFVLA	PNGRYCTDVD	ECQTPGICMN	GHCINSEGSF	RCDCPPGLAV	GMDGRVCVDT	HMRSTCYGGI	KKGVCVRPFP
730	740	750	760	770	780	790	800
GAVTKSECCC	ANPDYGFGEP	CQPCPAKNSA	EFHGLCSSGV	GITVDGRDIN	ECALDPDICA	NGICENLRGS	YRCNCNSGYE
810	820	830	840	850	860	870	880
PDASGRNCID	IDECLVNRLL	CDNGLCRNTP	GSYSCTCPPG	YVFRTETETC	EDINECESNP	CVNGACRNNL	GSFNCECSPG
890	900	910	920	930	940	950	960
SKLSSTGLIC	IDSLKGTCWL	NIQDSRCEVN	INGATLKSEC	CATLGAAWGS	PCERCELDTA	CPRGLARIKG	VTCEDVNECE
970	980	990	1000	1010	1020	1030	1040
VFPGVCPNGR	CVNSKGSFHC	ECPEGLTLDG	TGRVCLDIRM	EQCYLKWDED	ECIHPVPGKF	RMDACCCAVG	AAWGTECEEC
1050	1060	1070	1080	1090	1100	1110	1120
PKPGTKEYET	LCPRGAGFAN	RGDVLTGRPF	YKDINECKAF	PGMCTYGKCR	NTIGSFKCRC	NSGFALDMEE	RNCTDIDECR
1130	1140	1150	1160	1170	1180	1190	1200
ISPDLCGSGI	CVNTPGSFEC	ECFEGYESGF	MMMKNCMDID	ECERNPLLCR	GGTCVNTEGS	FQCDCPLGHE	LSPSREDCVD
1210	1220	1230	1240	1250	1260	1270	1280
INECSLSDNL	CRNGKCVNMI	GTYQCSCNPG	YQATPDRQGC	TDIDECMIMN	GGCDTQCTNS	EGSYECSCSE	GYALMPDGRS
1290	1300	1310	1320	1330	1340	1350	1360
CADIDECENN	PDICDGGQCT	NIPGEYRCLC	YDGFMASMDM	KTCIDVNECD	LNSNICMFGE	CENTKGSFIC	HCQLGYSVKK
1370	1380	1390	1400	1410	1420	1430	1440
GTTGCTDVDE	CEIGAHNCDM	HASCLNIPGS	FKCSCREGWI	GNGIKCIDLD	ECSNGTHQCS	INAQCVNTPG	SYRCACSEGF
1450	1460	1470	1480	1490	1500	1510	1520
TGDGFTCSDV	DECAENINLC	ENGQCLNVPG	AYRCECEMGF	TPASDSRSCQ	DIDECSFQNI	CVFGTCNNLP	GMFHCICDDG
1530	1540	1550	1560	1570	1580	1590	1600
YELDRTGGNC	TDIDECADPI	NCVNGLCVNT	PGRYECNCPP	DFQLNPTGVG	CVDNRVGNCY	LKFGPRGDGS	LSCNTEIGVG
1610	1620	1630	1640	1650	1660	1670	1680
VSRSSCCCSL	GKAWGNPCET	CPPVNSTEYY	TLCPGGEGFR	PNPITIILED	IDECQELPGL	CQGGNCINTF	GSFQCECPQG
1690	1700	1710	1720	1730	1740	1750	1760
YYLSEDTRIC	EDIDECFAHP	GVCGPGTCYN	TLGNYTCICP	PEYMQVNGGH	NCMDMRKSFC	YRSYNGTTCE	NELPFNVTKR
1770	1780	1790	1800	1810	1820	1830	1840
MCCCTYNVGK	AWNKPCEPCP	TPGTADFKTI	CGNIPGFTFD	IHTGKAVDID	ECKEIPGICA	NGVCINQIGS	FRCECPTGFS
1850	1860	1870	1880	1890	1900	1910	1920
YNDLLLVCED	IDECSNGDNL	CQRNADCINS	PGSYRCECAA	GFKLSPNGAC	VDRNECLEIP	NVCSHGLCVD	LQGSYQCICH
1930	1940	1950	1960	1970	1980	1990	2000
NGFKASQDQT	MCMDVDECER	HPCGNGTCKN	TVGSYNCLCY	PGFELTHNND	CLDIDECSSF	FGQVCRNGRC	FNEIGSFKCL
2010	2020	2030	2040	2050	2060	2070	2080
CNEGYELTPD	GKNCIDTNEC	VALPGSCSPG	TCQNLEGSFR	CICPPGYEVK	SENCIDINEC	DEDPNICLFG	SCTNTPGGFQ
2090	2100	2110	2120	2130	2140	2150	2160
CLCPPGFVLS	DNGRRCFDTR	QSFCFTNFEN	GKCSVPKAFN	TTKAKCCCSK	MPGEGWGDPC	ELCPKDDEVA	FQDLCPYGHG
2170	2180	2190	2200	2210	2220	2230	2240

Detailed Protein Report

Cmpd.	No. of Cmpds.	m/z meas.	Δ m/z [ppm]	z	Rt [min]	Score	P	Range	Sequence	Modification	Ratios	
472	1	516.1120	-268.37	2	34.5	12.2	0	1884-1893	K.LSPNGACVDR.N		QD:QU	0.84
388	2	642.3201	56.28	2	34.8	15.4	1	2529-2539	K.TCKDLDECQTK.Q		QD:QU	0.58

Detailed Protein Report

Protein 143: **PREDICTED: immunoglobulin-like domain-containing receptor 2 isoform X6 [Homo sapiens]**

**Accession:**	gi|578800910	**Score:**	38.4
**Database:**	refseq_human(refseq_human_20140103.fasta)	**MW [kDa]:**	65.5
**Database Date:**	2015-11-30	**pI:**	9.4
**Modification(s):**	Carbamidomethyl	**Sequence Coverage [%]:**	4.3
		**No. of unique Peptides:**	3

10	20	30	40	50	60	70	80
MDRVLLRWIS	LFWLTAMVEG	LQVTVPDKKK	VAMLFQPTVL	RCHFSTSSHQ	PAVVQWKFKS	YCQDRMGESL	GMSSTRAQSL
**90**	**100**	**110**	**120**	**130**	**140**	**150**	**160**
SKRNLEWDPY	LDCLDSRRTV	RVVASKQGST	VTLGDFYRGR	EITIVHDADL	QIGKLMWGDS	GLYYCIITTP	DDLEGKNEDS
**170**	**180**	**190**	**200**	**210**	**220**	**230**	**240**
VELLVLGRTG	LLADLLPSFA	VEIMPEWVFV	GLVLLGVFLF	FVLVGICWCQ	CCPHSCCCYV	RCPCCPDSCC	CPQAFRKGYR
**250**	**260**	**270**	**280**	**290**	**300**	**310**	**320**
IQADKERDSM	KVLYYVEKEL	AQFDPARRMR	GRYNNTISEL	SSLHEEDSNF	RQSFHQMRSK	QFPVSGDLES	NPDYWSGVMG
**330**	**340**	**350**	**360**	**370**	**380**	**390**	**400**
GSSGASRGPS	AMEYNKEDRE	SFRHSQPRSK	SEMLSRKNFA	TGVPAVSMDE	LAAFADSYGQ	RPRRADGNSH	EARGGSRFER
**410**	**420**	**430**	**440**	**450**	**460**	**470**	**480**
SESRAHSGFY	QDDSLEEYYG	QRSRSREPLT	DADRGWAFSP	ARRRPAEDAH	LPRLVSRTPG	TAPKYDHSYL	GSARERQARP
**490**	**500**	**510**	**520**	**530**	**540**	**550**	**560**
EGASRGGSLE	TPSKRSAQLG	PRSASYYAWS	PPGTYKAGSS	QDDQEDASDD	ALPPYSELEL	TRGPSYRGRD	LPYHSNSEKK
**570**	**580**	**590**					
RKKEPAKKTN	DFPTRMSLVV						

Cmpd.	No. of Cmpds.	m/z meas.	Δ m/z [ppm]	z	Rt [min]	Score	P	Range	Sequence	Modification
1141	2	902.1920	-134.75	2	42.8	15.4	0	222-236	R.CPCCPDSCCCPQAFR.K	Carbamidomethyl: 3, 4, 8
813	1	625.4938	-120.64	3	38.7	11.2	1	222-237	R.CPCCPDSCCCPQAFRK.G	Carbamidomethyl: 8, 10
1315	1	956.5835	173.01	1	46.6	11.9	0	385-393	R.ADGNSHEAR.G	

Detailed Protein Report

Protein 144: coiled-coil domain-containing protein 33 isoform 1 [Homo sapiens]

**Accession:**	gi|117168273	**Score:**	38.2
**Database:**	refseq_human(refseq_human_20140103.fasta)	**MW [kDa]:**	84.9
**Database Date:**	2015-11-30	**pI:**	6.8
		**Sequence Coverage [%]:**	5.8
		**No. of unique Peptides:**	1

10	20	30	40	50	60	70	80
MGLKNKKNTE	DPEEPLIASQ	STEPEIGHLS	PSKKETIMVT	LHGATNLPAC	KDGSEPWPYV	VVKSTSEEKN	NQSSKAVTSV
**90**	**100**	**110**	**120**	**130**	**140**	**150**	**160**
TSEPTRAPIW	GDTVNVEIQA	EDAGQEDVIL	KVVDNRKKQE	LLSYKIPIKY	LRVFHPYHFE	LVKPTESGKA	DEATAKTQLY
**170**	**180**	**190**	**200**	**210**	**220**	**230**	**240**
ATVVRKSSFI	PRYIGCNHMA	LEIFLRGVNE	PLANNPNPIV	VIARVVPNYK	EFKVSQANRD	LASVGLPITP	LSFPIPSMMN
**250**	**260**	**270**	**280**	**290**	**300**	**310**	**320**
FDVPRVSQNG	CPQLSKPGGP	PEQPLWNQSF	LFQGRDGATS	FSEDTALVLE	YYSSTSMKGS	QPWTLNQPLG	ISVLPLKSRL
**330**	**340**	**350**	**360**	**370**	**380**	**390**	**400**
YQKMLTGKGL	DGLHVERLPI	MDTSLKTIND	EAPTVALSFQ	LLSSERPENF	LTPNNSKALP	TLDPKILDKK	LRTIQESWSK
**410**	**420**	**430**	**440**	**450**	**460**	**470**	**480**
DTVSSTMDLS	TSTPREAEEE	PLVPEMSHDT	EMNNYRRAMQ	KMAEDILSLR	RQASILEGEN	RILRSRLAQQ	EEEEGQGKAS
**490**	**500**	**510**	**520**	**530**	**540**	**550**	**560**
EAQNTVSMKQ	KLLLSELDMK	KLRDRVQHLQ	NELIRKNDRE	KELLLLYQAQ	QPQAALLKQY	QGKLQKMKAL	EETVRHQEKV
**570**	**580**	**590**	**600**	**610**	**620**	**630**	**640**
IEKMERVLED	RLQDRSKPPP	LNRQQGKPYT	GFPMLSASGL	PLGSMGENLP	VELYSVLLAE	NAKLRTELDK	NRHQQAPIIL
**650**	**660**	**670**	**680**	**690**	**700**	**710**	**720**
QQQALPDLLS	GTSDKFNLLA	KLEHAQSRIL	SLESQLEDSA	RRWGREKQDL	ATRLQEQEKG	FRHPSNSIII	EQPSALTHSM
**730**	**740**	**750**	**760**				
DLKQPSELEP	LLPSSDSKLN	KPLSPQKETA	NSQQT				

Cmpd.	No. of Cmpds.	m/z meas.	Δ m/z [ppm]	z	Rt [min]	Score	P	Range	Sequence	Modification
1293	1	679.7520	127.71	3	46.3	12.9	1	52-69	K.DGSEPWPYVVVKSTSEEK.N	

Detailed Protein Report

Protein 145: ubiquitin carboxyl-terminal hydrolase 34 [Homo sapiens]

**Accession:**	gi|110347427	**Score:**	38.2
**Database:**	refseq_human(refseq_human_20140103.fasta)	**MW [kDa]:**	404.0
**Database Date:**	2015-11-30	**pI:**	5.4
		**Sequence Coverage [%]:**	0.7
		**No. of unique Peptides:**	1

Detailed Protein Report

10	20	30	40	50	60	70	80
MCENCADLVE	VLNEISDVEG	GDGLQLRKEH	TLKIFTYINS	WTQRQCLCCF	KEYKHLEIFN	QVVCALINLV	IAQVQVLRDQ
90	100	110	120	130	140	150	160
LCKHCTTINI	DSTWQDESNQ	AEEPLNIDRE	CNEGSTERQK	SIEKKSNSTR	ICNLTEEESS	KSSDPFSLWS	TDEKEKLLLC
170	180	190	200	210	220	230	240
VAKIFQIQFP	LYTAYKHNTH	PTIEDISTQE	SNILGAFCDM	NDVEVPLHLL	RYVCLFCGKN	GLSLMKDCFE	YGTPETLPFL
250	260	270	280	290	300	310	320
IAHAFITVVS	NIRIWLHIPA	VMQHIIPFRT	YVIRYLCKLS	DQELRQSAAR	NMADLMWSTV	KEPLDTTLCF	DKESLDLAFK
330	340	350	360	370	380	390	400
YFMSPTLTMR	LAGLSQITNQ	LHTFNDVCNN	ESLVSDTETS	IAKELADWLI	SNNVVEHIFG	PNLHIEIIKQ	CQVILNFLAA
410	420	430	440	450	460	470	480
EGRLSTQHID	CIWAAAQLKH	CSRYIHDLFP	SLIKNLDPVP	LRHLLNLVSA	LEPSVHTEQT	LYLASMLIKA	LWNNALAAKA
490	500	510	520	530	540	550	560
QLSKQSSFAS	LLNTNIPIGN	KKEEEELRRT	APSPWSPAAS	PQSSDNSDTH	QSGGSDIEMD	EQLINRTKHV	QQRLSDTEES
570	580	590	600	610	620	630	640
MQGSSDETAN	SGEDGSSGPG	SSSGHSDGSS	NEVNSSHASQ	SAGSPGSEVQ	SEDIADIEAL	KEEDEDDDHG	HNPPKSSCGT
650	660	670	680	690	700	710	720
DLRNRKLESQ	AGICLGDSQG	MSERNGTSSG	TGKDLVFNTE	SLPSVDNRMR	MLDACSHSED	PEHDISGEMN	ATHIAQGSQE
730	740	750	760	770	780	790	800
SCITRTGDFL	GETIGNELFN	CRQFIGPQHH	HHHHHHHHHH	DGHMVDDMLS	ADDVSCSSSQ	VSAKSEKNMA	DFDGEESGCE
810	820	830	840	850	860	870	880
EELVQINSHA	ELTSHLQQHL	PNLASIYHEH	LSQGPVVHKH	QFNSNAVTDI	NLDNVCKKGN	TLLWDIVQDE	DAVNLSEGLI
890	900	910	920	930	940	950	960
NEAEKLLCSL	VCWFTDRQIR	MRFIEGCLEN	LGNNRSVVIS	LRLLPKLFGT	FQQFGSSYDT	HWITMWAEKE	LNMMKLFFDN
970	980	990	1000	1010	1020	1030	1040
LVYYIQTVRE	GRQKHALYSH	SAEVQVRLQF	LTCVFSTLGS	PDHFRLSLEQ	VDILWHCLVE	DSECYDDALH	WFLNQVRSKD
1050	1060	1070	1080	1090	1100	1110	1120
QHAMGMETYK	HLFLEKMPQL	KPETISMTGL	NLFQHLCNLA	RLATSAYDGC	SNSELCGMDQ	FWGIALRAQS	GDVSRAAIQY
1130	1140	1150	1160	1170	1180	1190	1200
INSYYINGKT	GLEKEQEFIS	KCMESLMIAS	SSLEQESHSS	LMVIERGLLM	LKTHLEAFRR	RFAYHLRQWQ	IEGTGISSHL
1210	1220	1230	1240	1250	1260	1270	1280
KALSDKQSLP	LRVVCQPAGL	PDKMTIEMYP	SDQVADLRAE	VTHWYENLQK	EQINQQAQLQ	EFGQSNRKGE	FPGGLMGPVR
1290	1300	1310	1320	1330	1340	1350	1360
MISSGHELTT	DYDEKALHEL	GFKDMQMVFV	SLGAPRRERK	GEGVQLPASC	LPPPQKDNIP	MLLLLQEPHL	TTLFDLLEML
1370	1380	1390	1400	1410	1420	1430	1440
ASFKPPSGKV	AVDDSESLRC	EELHLHAENL	SRRVWELLML	LPTCPNMLMA	FQNISDEQSN	DGFNWKELLK	IKSAHKLLYA
1450	1460	1470	1480	1490	1500	1510	1520
LEIIEALGKP	NRRIRRESTG	SYSDLYPDSD	DSSEDQVENS	KNSWSCKFVA	AGGLQQLLEI	FNSGILEPKE	QESWTVWQLD
1530	1540	1550	1560	1570	1580	1590	1600
CLACLLKLIC	QFAVDPSDLD	LAYHDVFAWS	GIAESHRKRT	WPGKSRKAAG	DHAKGLHIPR	LTEVFLVLVQ	GTSLIQRLMS
1610	1620	1630	1640	1650	1660	1670	1680
VAYTYDNLAP	RVLKAQSDHR	SRHEVSHYSM	WLLVSWAHCC	SLVKSSLADS	DHLQDWLKKL	TLLIPETAVR	HESCSGLYKL
1690	1700	1710	1720	1730	1740	1750	1760
SLSGLDGGDS	INRSFLLLAA	STLLKFLPDA	QALKPIRIDD	YEEEPILKPG	CKEYFWLLCK	LVDNIHIKDA	SQTTLLDLDA
1770	1780	1790	1800	1810	1820	1830	1840
LARHLADCIR	SREILDHQDG	NVEDDGLTGL	LRLATSVVKH	KPPFKFSREG	QEFLRDIFNL	LFLLPSLKDR	QQPKCKSHSS
1850	1860	1870	1880	1890	1900	1910	1920
RAAAYDLLVE	MVKGSVENYR	LIHNWVMAQH	MQSHAPYKWD	YWPHEDVRAE	CRFVGLTNLG	ATCYLASTIQ	QLYMIPEARQ
1930	1940	1950	1960	1970	1980	1990	2000
AVFTAKYSED	MKHKTTLLEL	QKMFTYLMES	ECKAYNPRPF	CKTYTMDKQP	LNTGEQKDMT	EFFTDLITKI	EEMSPELKNT
2010	2020	2030	2040	2050	2060	2070	2080
VKSLFGGVIT	NNVVSLDCEH	VSQTAEEFYT	VRCQVADMKN	IYESLDEVTI	KDTLEGDNMY	TCSHCGKKVR	AEKRACFKKL
2090	2100	2110	2120	2130	2140	2150	2160
PRILSFNTMR	YTFNMVTMMK	EKVNTHFSFP	LRLDMTPYTE	DFLMGKSERK	EGFKEVSDHS	KDSESYEYDL	IGVTVHTGTA
2170	2180	2190	2200	2210	2220	2230	2240

Detailed Protein Report

Cmpd.	No. of Cmpds.	m/z meas.	Δ m/z [ppm]	z	Rt [min]	Score	P	Range	Sequence	Modification
597	2	642.8360	18.78	2	36.1	18.4	0	2940-2950	R.SCWTTLISAFR.I	

Detailed Protein Report

Protein 146: GTP-binding nuclear protein Ran [Homo sapiens]

**Accession:**	gi|5453555	**Score:**	38.1
**Database:**	refseq_human(refseq_human_20140103.fasta)	**MW [kDa]:**	24.4
**Database Date:**	2015-11-30	**pI:**	7.8
		**Sequence Coverage [%]:**	13.4
		**No. of unique Peptides:**	1

10	20	30	40	50	60	70	80
MAAQGEPQVQ	FKLVLVGDGG	TGKTTFVKRH	LTGEFEKKYV	ATLGVEVHPL	VFHTNRGPIK	FNVWDTAGQE	KFGGLRDGYY
**90**	**100**	**110**	**120**	**130**	**140**	**150**	**160**
IQAQCAIIMF	DVTSRVTYKN	VPNWHRDLVR	VCENIPIVLC	GNKVDIKDRK	VKAKSIVFHR	KKNLQYYDIS	AKSNYNFEKP
**170**	**180**	**190**	**200**	**210**	**220**		
FLWLARKLIG	DPNLEFVAMP	ALAPPEVVMD	PALAAQYEHD	LEVAQTTALP	DEDDDL		

Cmpd.	No. of Cmpds.	m/z meas.	Δ m/z [ppm]	z	Rt [min]	Score	P	Range	Sequence	Modification
2729	1	671.8236	-44.80	2	63.1	17.4	1	142-152	K.KNLQYYDISAK.S	

Detailed Protein Report

Protein 147: PREDICTED: zinc finger protein 195 isoform X2 [Homo sapiens]

**Accession:**	gi|578820760	**Score:**	38.0
**Database:**	refseq_human(refseq_human_20140103.fasta)	**MW [kDa]:**	56.6
**Database Date:**	2015-11-30	**pI:**	9.7
		**Sequence Coverage [%]:**	5.7
		**No. of unique Peptides:**	1

Quantitation

QD:QU **Median:** 0.47 **CV:** 0.00 % **No. of Peptides:** 1

10	20	30	40	50	60	70	80
MNEAGNPAMS	SHFTQDLLPE	QGIQDAFPKR	ILRGYGNCGL	DNLYLRKDWE	SLDECKLQKD	YNGLNQCSST	THSKIFQYNK
90	100	110	120	130	140	150	160
YVKIFDNFSN	LHRRNISNTG	EKPFKCQECG	KSFQMLSFLT	EHQKIHTGKK	FQKCGECGKT	FIQCSHFTEP	ENIDTGEKPY
170	180	190	200	210	220	230	240
KCQECNNVIK	TCSVLTKNRI	YAGGEHYRCE	EFGKVFNQCS	HLTEHEHGTE	EKPCKYEECS	SVFISCSSLS	NQQMILAGEK
250	260	270	280	290	300	310	320
LSKCETWYKG	FNHSPNPSKH	QRNEIGGKPF	KCEECDSIFK	WFSDLTKHKR	IHTGEKPYKC	DECGKAYTQS	SHLSEHRRIH
330	340	350	360	370	380	390	400
TGEKPYQCEE	CGKVFRTCSS	LSNHKRTHSE	EKPYTCEECG	NIFKQLSDLT	KHKKTHTGEK	PYKCDECGKN	FTQSSNLIVH
410	420	430	440	450	460	470	480
KRIHTGEKPY	KCEECGRVFM	WFSDITKHKK	THTGEKPYKC	DECGKNFTQS	SNLIVHKRIH	TGEKPYKCEK	CGKAFTQFSH
490							
LTVHESIHT							

Cmpd.	No. of Cmpds.	m/z meas.	Δ m/z [ppm]	z	Rt [min]	Score	P	Range	Sequence	Modification	Ratios	
1481	1	727.3860	-18.04	2	48.7	13.2	2	372-383	K.HKKTHTGEKPYK.C		QD:QU	0.47

Detailed Protein Report

Protein 148: PREDICTED: E3 ubiquitin-protein ligase HECTD1 isoform X2 [Homo sapiens]

**Accession:**	gi|578825693	**Score:**	37.8
**Database:**	refseq_human(refseq_human_20140103.fasta)	**MW [kDa]:**	284.3
**Database Date:**	2015-11-30	**pI:**	5.1
**Modification(s):**	Carbamidomethyl, Oxidation	**Sequence Coverage [%]:**	0.8
		**No. of unique Peptides:**	3

Detailed Protein Report

10	20	30	40	50	60	70	80
MADVDPDTLL	EWLQMGQGDE	RDMQLIALEQ	LCMLLLMSDN	VDRCFETCPP	RTFLPALCKI	FLDESAPDNV	LEVTARAITY
90	100	110	120	130	140	150	160
YLDVSAECTR	RIVGVDGAIK	ALCNRLVVVE	LNNRTSRDLA	EQCVKVLELI	CTRESGAVFE	AGGLNCVLTF	IRDSGHLVHK
170	180	190	200	210	220	230	240
DTLHSAMAVV	SRLCGKMEPQ	DSSLEICVES	LSSLLKHEDH	QVSDGALRCF	ASLADRFTRR	GVDPAPLAKH	GLTEELLSRM
250	260	270	280	290	300	310	320
AAAGGTVSGP	SSACKPGRST	TGAPSTTADS	KLSNQVSTIV	SLLSTLCRGS	PVVTHDLLRS	ELPDSIESAL	QGDERCVLDT
330	340	350	360	370	380	390	400
MRLVDLLLVL	LFEGRKALPK	SSAGSTGRIP	GLRRLDSSGE	RSHRQLIDCI	RSKDTDALID	AIDTGAFEVN	FMDDVGQTLL
410	420	430	440	450	460	470	480
NWASAFGTQE	MVEFLCERGA	DVNRGQRSSS	LHYAACFGRP	QVAKTLLRHG	ANPDLRDEDG	KTPLDKARER	GHSEVVAILQ
490	500	510	520	530	540	550	560
SPGDWMCPVN	KGDDKKKKDT	NKDEEECNEP	KGDPEMAPIY	LKRLLPVFAQ	TFQQTMLPSI	RKASLALIRK	MIHFCSEALL
570	580	590	600	610	620	630	640
KEVCDSDVGH	NLPTILVEIT	ATVLDQEDDD	DGHLLALQII	RDLVDKGGDI	FLDQLARLGV	ISKVSTLAGP	SSDDENEEES
650	660	670	680	690	700	710	720
KPEKEDEPQE	DAKELQQGKP	YHWRDWSIIR	GRDCLYIWSD	AAALELSNGS	NGWFRFILDG	KLATMYSSGS	PEGGSDSSES
730	740	750	760	770	780	790	800
RSEFLEKLQR	ARGQVKPSTS	SQPILSAPGP	TKLTVGNWSL	TCLKEGEIAI	HNSDGQQATI	LKEDLPGFVF	ESNRGTKHSF
810	820	830	840	850	860	870	880
TAETSLGSEF	VTGWTGKRGR	KLKSKLEKTK	QKVRTMARDL	YDDHFKAVES	MPRGVVVTLR	NIATQLESSW	ELHTNRQCIE
890	900	910	920	930	940	950	960
SENTWRDLMK	TALENLIVLL	KDENTISPYE	MCSSGLVQAL	LTVLNNSMDL	DMKQDCSQLV	ERINVFKTAF	SENEDDESRP
970	980	990	1000	1010	1020	1030	1040
AVALIRKLIA	VLESIERLPL	HLYDTPGSTY	NLQILTRRLR	FRLERAPGET	ALIDRTGRML	KMEPLATVES	LEQYLLKMVA
1050	1060	1070	1080	1090	1100	1110	1120
KQWYDFDRSS	FVFVRKLREG	QNFIFRHQHD	FDENGIIYWI	GTNAKTAYEW	VNPAAYGLVV	VTSSEGRNLP	YGRLEDILSR
1130	1140	1150	1160	1170	1180	1190	1200
DNSALNCHSN	DDKNAWFAID	LGLWVIPSAY	TLRHARGYGR	SALRNWVFQV	SKDGQNWTSL	YTHVDDCSLN	EPGSTATWPL
1210	1220	1230	1240	1250	1260	1270	1280
DPPKDEKQGW	RHVRIKQMGK	NASGQTHYLS	LSGFELYGTV	NGVCEDQLGK	AAKEAEANLR	RQRRLVRSQV	LKYMVPGARV
1290	1300	1310	1320	1330	1340	1350	1360
IRGLDWKWRD	QDGSPQGEGT	VTGELHNGTT	QSWSSLVKNN	CPDKTSAAAG	SSSRKGSSSS	VCSVASSSDI	SLGSTKTERR
1370	1380	1390	1400	1410	1420	1430	1440
SEIVMEHSIV	SGADVHEPIV	VLSSAENVPQ	TEVGSSSSAS	TSTLTAETGS	ENAERKLGPD	SSVRTPGESS	AISMGIVSVS
1450	1460	1470	1480	1490	1500	1510	1520
SPDVSSVSEL	TNKEAASQRP	LSSSASNRLS	VSSLLAAGAP	MSSSASVPNL	SSRETSSLES	FVRRVANIAR	TNATNNMNLS
1530	1540	1550	1560	1570	1580	1590	1600
RSSSDNNTNT	LGRNVMSTAT	SPLMGAQSFP	NLTTPGTTST	VTMSTSSVTS	SSNVATATTV	LSVGQSLSNT	LTTSLTSTSS
1610	1620	1630	1640	1650	1660	1670	1680
ESDTGQEAEY	SLYDFLDSCR	ASTLLAELDD	DEDLPEPDEE	DDENEDDNQE	DQEYEEVMIL	RRPSLQRRAG	SRSDVTHHAV
1690	1700	1710	1720	1730	1740	1750	1760
TSQLPQVPAG	AGSRPIGEQE	EEEYETKGGR	RRTWDDDYVL	KRQFSALVPA	FDPRPGRTNV	QQTTDLEIPP	PGTPHSELLE
1770	1780	1790	1800	1810	1820	1830	1840
EVECTPSPRL	ALTLKVTGLG	TTREVELPLT	NFRSTIFYYV	QKLLQLSCNG	NVKSDKLRRI	WEPTYTIMYR	EMKDSDKEKE
1850	1860	1870	1880	1890	1900	1910	1920
NGKMGCWSIE	HVEQYLGTDE	LPKNDLITYL	QKNADAAFLR	HWKLTGTNKS	IRKNRNCSQL	IAAYKDFCEH	GTKSGLNQGA
1930	1940	1950	1960	1970	1980	1990	2000
ISTLQSSDIL	NLTKEQPQAK	AGNGQNSCGV	EDVLQLLRIL	YIVASDPYSR	ISQEDGDEQP	QFTFPPDEFT	SKKITTKILQ
2010	2020	2030	2040	2050	2060	2070	2080
QIEEPLALAS	GALPDWCEQL	TSKCPFLIPF	ETRQLYFTCT	AFGASRAIVW	LQNRREATVE	RTRTTSSVRR	DDPGEFRVGR
2090	2100	2110	2120	2130	2140	2150	2160
LKHERVKVPR	GESLMEWAEN	VMQIHADRKS	VLEVEFLGEE	GTGLGPTLEF	YALVAAEFQR	TDLGAWLCDD	NFPDDESRHV
2170	2180	2190	2200	2210	2220	2230	2240

Detailed Protein Report

Cmpd.	No. of Cmpds.	m/z meas.	Δ m/z [ppm]	z	Rt [min]	Score	P	Range	Sequence	Modification
751	1	765.8890	60.31	2	37.9	11.7	1	2226-2238	K.LMCMGDIKSNMSK.L	Carbamidomethyl: 3; Oxidation: 2
2795	1	729.3498	20.88	2	66.1	14.8	1	2226-2238	K.LMCMGDIKSNMSK.L	
1556	1	502.7809	-70.23	2	49.7	11.3	2	2310-2317	R.ARFLKEIK.D	

Detailed Protein Report

Protein 149: PREDICTED: E3 ubiquitin-protein ligase UBR4 isoform X11 [Homo sapiens]

**Accession:**	gi|578798772	**Score:**	37.7
**Database:**	refseq_human(refseq_human_20140103.fasta)	**MW [kDa]:**	574.7
**Database Date:**	2015-11-30	**pI:**	5.6
**Modification(s):**	Carbamidomethyl, Oxidation	**Sequence Coverage [%]:**	1.0
		**No. of unique Peptides:**	3

Detailed Protein Report

10	20	30	40	50	60	70	80
MATSGGEEAA	AAAPAPGTPA	TGADTTPGWE	VAVRPLLSAS	YSAFEMKELP	QLVASVIESE	SEILHHEKQY	EPFYSSFVAL
90	100	110	120	130	140	150	160
STHYITTVCS	LIPRNQLQSV	AAACKVLIEF	SLLRLENPDE	ACAVSQKHLI	LLIKGLCTGC	SRLDRTEIIT	FTAMMKSAKL
170	180	190	200	210	220	230	240
PQTVKTLSDV	EDQKELASPV	SPELRQKEVQ	MNFLNQLTSV	FNPRTVASQP	ISTQTLVEGE	NDEQSSTDQA	SAIKTKNVFI
250	260	270	280	290	300	310	320
AQNVASLQEL	GGSEKLLRVC	LNLPYFLRYI	NRFQDAVLAN	SFFIMPATVA	DATAVRNGFH	SLVIDVTMAL	DTLSLPVLEP
330	340	350	360	370	380	390	400
LNPSRLQDVT	VLSLSCLYAG	VSVATCMAIL	HVGSAQQVRT	GSTSSKEDDY	ESDAATIVQK	CLEIYDMIGQ	AISSSRRAGG
410	420	430	440	450	460	470	480
EHYQNFQLLG	AWCLLNSLFL	ILNLSPTALA	DKGKEKDPLA	ALRVRDILSR	TKEGVGSPKL	GPGKGHQGFG	VLSVILANHA
490	500	510	520	530	540	550	560
IKLLTSLFQD	LQVEALHKGW	ETDGPPAALS	IMAQSTSIQR	IQRLIDSVPL	MNLLLTLLST	SYRKACVLQR	QRKGSMSSDA
570	580	590	600	610	620	630	640
SASTDSNTYY	EDDFSSTEED	SSQDDDSEPI	LGQWFEETIS	PSKEKAAPPP	PPPPPPLESS	PRVKSPSKQA	PGEKGNILAS
650	660	670	680	690	700	710	720
RKDPELFLGL	ASNILNFITS	SMLNSRNNFI	RNYLSVSLSE	HHMATLASII	KEVDKDGLKG	SSDEEFAAAL	YHFNHSLVTS
730	740	750	760	770	780	790	800
DLQSPNLQNT	LLQQLGVAPF	SEGPWPLYIH	PQSLSVLSRL	LLIWQHKASA	QGDPDVPECL	KVWDRFLSTM	KQNALQGVVP
810	820	830	840	850	860	870	880
SETEDLNVEH	LQMLLLIFHN	FTETGRRAIL	SLFVQIIQEL	SVNMDAQMRF	VPLILARLLL	IFDYLLHQYS	KAPVYLFEQV
890	900	910	920	930	940	950	960
QHNLLSPPFG	WASGSQDSNS	RRATTPLYHG	FKEVEENWSK	HFSSDAVPHP	RFYCVLSPEA	SEDDLNRLDS	VACDVLFSKL
970	980	990	1000	1010	1020	1030	1040
VKYDELYAAL	TALLAAGSQL	DTVRRKENKN	VTALEACALQ	YYFLILWRIL	GILPPSKTYI	NQLSMNSPEM	SECDILHTLR
1050	1060	1070	1080	1090	1100	1110	1120
WSSRLRISSY	VNWIKDHLIK	QGMKAEHASS	LLELASTTKC	SSVKYDVEIV	EEYFARQISS	FCSIDCTTIL	QLHEIPSLQS
1130	1140	1150	1160	1170	1180	1190	1200
IYTLDAAISK	VQVSLDEHFS	KMAAETDPHK	SSEITKNLLP	ATLQLIDTYA	SFTRAYLLQN	FNEEGTTEKP	SKEKLQGFAA
1210	1220	1230	1240	1250	1260	1270	1280
VLAIGSSRCK	ANTLGPTLVQ	NLPSSVQTVC	ESWNNINTNE	FPNIGSWRNA	FANDTIPSES	YISAVQAAHL	GTLCSQSLPL
1290	1300	1310	1320	1330	1340	1350	1360
AASLKHTLLS	LVRLTGDLIV	WSDEMNPPQV	IRTLLPLLLE	SSTESVAEIS	SNSLERILGP	AESDEFLARV	YEKLITGCYN
1370	1380	1390	1400	1410	1420	1430	1440
ILANHADPNS	GLDESILEEC	LQYLEKQLES	SQARKAMEEF	FSDSGELVQI	MMATANENLS	AKFCNRVLKF	FTKLFQLTEK
1450	1460	1470	1480	1490	1500	1510	1520
SPNPSLLHLC	GSLAQLACVE	PVRLQAWLTR	MTTSPPKDSD	QLDVIQENRQ	LLQLLTTYIV	RENSQVGEGV	CAVLLGTLTP
1530	1540	1550	1560	1570	1580	1590	1600
MATEMLANGD	GTGFPELMVV	MATLASAGQG	AGHLQLHNAA	VDWLSRCKKY	LSQKNVVEKL	NANVMHGKHV	MILECTCHIM
1610	1620	1630	1640	1650	1660	1670	1680
SYLADVTNAL	SQSNGQGPSH	LSVDGEERAI	EVDSDWVEEL	AVEEEDSQAE	DSDEDSLCNK	LCTFTITQKE	FMNQHWYHCH
1690	1700	1710	1720	1730	1740	1750	1760
TCKMVDGVGV	CTVCAKVCHK	DHEISYAKYG	SFFCDCGAKE	DGSCLALVKR	TPSSGMSSTM	KESAFQSEPR	ISESLVRHAS
1770	1780	1790	1800	1810	1820	1830	1840
TSSPADKAKV	TISDGKVADE	EKPKKSSLCR	TVEGCREELQ	NQANFSFAPL	VLDMLNFLMD	AIQTNFQQAS	AVGSSSRAQQ
1850	1860	1870	1880	1890	1900	1910	1920
ALSELHTVEK	AVEMTDQLMV	PTLGSQEGAF	ENVRMNYSGD	QGQTIRQLIS	AHVLRRVAMC	VLSSPHGRRQ	HLAVSHEKGK
1930	1940	1950	1960	1970	1980	1990	2000
ITVLQLSALL	KQADSSKRKL	TLTRLASAPV	PFTVLSLTGN	PCKEDYLAVC	GLKDCHVLTF	SSSGSVSDHL	VLHPQLATGN
2010	2020	2030	2040	2050	2060	2070	2080
FIIKAVWLPG	SQTELAIVTA	DFVKIYDLCV	DALSPTFYFL	LPSSKIRDVT	FLFNEEGKNI	IVIMSSAGYI	YTQLMEEASS
2090	2100	2110	2120	2130	2140	2150	2160
AQQGPFYVTN	VLEINHEDLK	QDSNSQVAGG	GVSVYYSHVL	QMLFFSYCQG	KSFAATISRT	TLEVLQLFPI	NIKSSNGGSK
2170	2180	2190	2200	2210	2220	2230	2240

Detailed Protein Report

Cmpd.	No. of Cmpds.	m/z meas.	Δ m/z [ppm]	z	Rt [min]	Score	P	Range	Sequence	Modification
1928	1	917.3279	-102.08	2	54.5	15.2	1	2608-2623	K.LPQMETGMDEGKEPQK.Q	Oxidation: 4
1934	1	745.7866	-161.90	2	54.6	10.5	1	3240-3252	R.DLHTLDSHVRGIK.K	
375	1	878.7841	3.73	3	34.7	12.0	2	4668-4690	K.VFLDCFCKIAAGIKNNSNGHQLK.D	Carbamidomethyl: 5, 7

Detailed Protein Report

Protein 150: histone H4 [Homo sapiens]

**Accession:**	gi|4504301	**Score:**	37.5
**Database:**	refseq_human(refseq_human_20140103.fasta)	**MW [kDa]:**	11.4
**Database Date:**	2015-11-30	**pI:**	11.8
		**Sequence Coverage [%]:**	7.8
		**No. of unique Peptides:**	1

Quantitation

QD:QU	**Median:**	1.78	**CV:**	0.00 %	**No. of Peptides:**	1

Alias proteins:

Accession	Name	Description
gi|77539758	refseq_huma (refseq_human_20140103.fasta)	histone H4 [Homo sapiens]
gi|28173560	refseq_huma (refseq_human_20140103.fasta)	histone H4 [Homo sapiens]
gi|11415030	refseq_huma (refseq_human_20140103.fasta)	histone H4 [Homo sapiens]
gi|4504323	refseq_huma (refseq_human_20140103.fasta)	histone H4 [Homo sapiens]
gi|4504321	refseq_huma (refseq_human_20140103.fasta)	histone H4 [Homo sapiens]
gi|4504317	refseq_huma (refseq_human_20140103.fasta)	histone H4 [Homo sapiens]
gi|4504315	refseq_huma (refseq_human_20140103.fasta)	histone H4 [Homo sapiens]
gi|4504313	refseq_huma (refseq_human_20140103.fasta)	histone H4 [Homo sapiens]
gi|4504311	refseq_huma (refseq_human_20140103.fasta)	histone H4 [Homo sapiens]
gi|4504309	refseq_huma (refseq_human_20140103.fasta)	histone H4 [Homo sapiens]
gi|4504307	refseq_huma (refseq_human_20140103.fasta)	histone H4 [Homo sapiens]
gi|4504305	refseq_huma (refseq_human_20140103.fasta)	histone H4 [Homo sapiens]
gi|4504303	refseq_huma (refseq_human_20140103.fasta)	histone H4 [Homo sapiens]

```
        10         20         30         40         50         60         70         80
MSGRGKGGKG LGKGGAKRHR KVLRDNIQGI TKPAIRRLAR RGGVKRISGL IYEETRGVLK VFLENVIRDA VTYTEHAKRK
        90        100        110
TVTAMDVVYA LKRQGRTLYG FGG
```

Cmpd.	No. of Cmpds.	m/z meas.	Δ m/z [ppm]	z	Rt [min]	Score	P	Range	Sequence	Modification	Ratios	
1451	8	495.2222	-141.96	2	48.3	37.5	0	61-68	K.VFLENVIR.D		QD:QU	1.78

Detailed Protein Report

Protein 151: integrin beta-4 isoform 3 precursor [Homo sapiens]

**Accession:**	gi|54607033	**Score:**	37.5
**Database:**	refseq_human(refseq_human_20140103.fasta)	**MW [kDa]:**	194.9
**Database Date:**	2015-11-30	**pI:**	5.7
		**Sequence Coverage [%]:**	1.1
		**No. of unique Peptides:**	1

Alias proteins:

Accession	Name	Description
gi|530412114	refseq_human (refseq_human_20140103.fasta)	PREDICTED: integrin beta-4 isoform X4 [Homo sapiens]

Detailed Protein Report

10	20	30	40	50	60	70	80
MAGPRPSPWA	RLLLAALISV	SLSGTLANRC	KKAPVKSCTE	CVRVDKDCAY	CTDEMFRDRR	CNTQAELLAA	GCQRESIVVM
90	100	110	120	130	140	150	160
ESSFQITEET	QIDTTLRRSQ	MSPQGLRVRL	RPGEERHFEL	EVFEPLESPV	DLYILMDFSN	SMSDDLDNLK	KMGQNLARVL
170	180	190	200	210	220	230	240
SQLTSDYTIG	FGKFVDKVSV	PQTDMRPEKL	KEPWPNSDPP	FSFKNVISLT	EDVDEFRNKL	QGERISGNLD	APEGGFDAIL
250	260	270	280	290	300	310	320
QTAVCTRDIG	WRPDSTHLLV	FSTESAFHYE	ADGANVLAGI	MSRNDERCHL	DTTGTYTQYR	TQDYPSVPTL	VRLLAKHNII
330	340	350	360	370	380	390	400
PIFAVTNYSY	SYYEKLHTYF	PVSSLGVLQE	DSSNIVELLE	EAFNRIRSNL	DIRALDSPRG	LRTEVTSKMF	QKTRTGSFHI
410	420	430	440	450	460	470	480
RRGEVGIYQV	QLRALEHVDG	THVCQLPEDQ	KGNIHLKPSF	SDGLKMDAGI	ICDVCTCELQ	KEVRSARCSF	NGDFVCGQCV
490	500	510	520	530	540	550	560
CSEGWSGQTC	NCSTGSLSDI	QPCLREGEDK	PCSGRGECQC	GHCVCYGEGR	YEGQFCEYDN	FQCPRTSGFL	CNDRGRCSMG
570	580	590	600	610	620	630	640
QCVCEPGWTG	PSCDCPLSNA	TCIDSNGGIC	NGRGHCECGR	CHCHQQSLYT	DTICEINYSA	IHPGLCEDLR	SCVQCQAWGT
650	660	670	680	690	700	710	720
GEKKGRTCEE	CNFKVKMVDE	LKRAEEVVVR	CSFRDEDDDC	TYSYTMEGDG	APGPNSTVLV	HKKKDCPPGS	FWWLIPLLLL
730	740	750	760	770	780	790	800
LLPLLALLLL	LCWKYCACCK	ACLALLPCCN	RGHMVGFKED	HYMLRENLMA	SDHLDTPMLR	SGNLKGRDVV	RWKVTNNMQR
810	820	830	840	850	860	870	880
PGFATHAASI	NPTELVPYGL	SLRLARLCTE	NLLKPDTREC	AQLRQEVEEN	LNEVYRQISG	VHKLQQTKFR	QQPNAGKKQD
890	900	910	920	930	940	950	960
HTIVDTVLMA	PRSAKPALLK	LTEKQVEQRA	FHDLKVAPGY	YTLTADQDAR	GMVEFQEGVE	LVDVRVPLFI	RPEDDDEKQL
970	980	990	1000	1010	1020	1030	1040
LVEAIDVPAG	TATLGRRLVN	ITIIKEQARD	VVSFEQPEFS	VSRGDQVARI	PVIRRVLDGG	KSQVSYRTQD	GTAQGNRDYI
1050	1060	1070	1080	1090	1100	1110	1120
PVEGELLFQP	GEAWKELQVK	LLELQEVDSL	LRGRQVRRFH	VQLSNPKFGA	HLGQPHSTTI	IIRDPDELDR	SFTSQMLSSQ
1130	1140	1150	1160	1170	1180	1190	1200
PPPHGDLGAP	QNPNAKAAGS	RKIHFNWLPP	SGKPMGYRVK	YWIQGDSESE	AHLLDSKVPS	VELTNLYPYC	DYEMKVCAYG
1210	1220	1230	1240	1250	1260	1270	1280
AQGEGPYSSL	VSCRTHQEVP	SEPGRLAFNV	VSSTVTQLSW	AEPAETNGEI	TAYEVCYGLV	NDDNRPIGPM	KKVLVDNPKN
1290	1300	1310	1320	1330	1340	1350	1360
RMLLIENLRE	SQPYRYTVKA	RNGAGWGPER	EAIINLATQP	KRPMSIPIIP	DIPIVDAQSG	EDYDSFLMYS	DDVLRSPSGS
1370	1380	1390	1400	1410	1420	1430	1440
QRPSVSDDTE	HLVNGRMDFA	FPGSTNSLHR	MTTTSAAAYG	THLSPHVPHR	VLSTSSTLTR	DYNSLTRSEH	SHSTTLPRDY
1450	1460	1470	1480	1490	1500	1510	1520
STLTSVSSHD	SRLTAGVPDT	PTRLVFSALG	PTSLRVSWQE	PRCERPLQGY	SVEYQLLNGG	ELHRLNIPNP	AQTSVVVEDL
1530	1540	1550	1560	1570	1580	1590	1600
LPNHSYVFRV	RAQSQEGWGR	EREGVITIES	QVHPQSPLCP	LPGSAFTLST	PSAPGPLVFT	ALSPDSLQLS	WERPRRPNGD
1610	1620	1630	1640	1650	1660	1670	1680
IVGYLVTCEM	AQGGGPATAF	RVDGDSPESR	LTVPGLSENV	PYKFKVQART	TEGFGPEREG	IITIESQDGG	PFPQLGSRAG
1690	1700	1710	1720	1730	1740	1750	1760
LFQHPLQSEY	SSITTTHTSA	TEPFLVDGLT	LGAQHLEAGG	SLTRHVTQEF	VSRTLTTSGT	LSTHMDQQFF	QT

Cmpd.	No. of Cmpds.	m/z meas.	Δ m/z [ppm]	z	Rt [min]	Score	P	Range	Sequence	Modification
522	1	678.9701	154.68	2	36.5	13.0	1	368-379	R.SNLDIRALDSPR.G	

Detailed Protein Report

Protein 152: **eukaryotic peptide chain release factor GTP-binding subunit ERF3A isoform 3 [Homo sapiens]**

**Accession:**	gi|194018522	**Score:**	37.4
**Database:**	refseq_human(refseq_human_20140103.fasta)	**MW [kDa]:**	55.7
**Database Date:**	2015-11-30	**pI:**	5.3
		**Sequence Coverage [%]:**	1.6
		**No. of unique Peptides:**	1

10	20	30	40	50	60	70	80
MELSEPIVEN	GETEMSPEES	WEHKEEISEA	EPGGGSLGDG	RPPEESAHEM	MEEEEEIPKP	KSVVAPPGAP	KKEHVNVVFI
**90**	**100**	**110**	**120**	**130**	**140**	**150**	**160**
GHVDAGKSTI	GGQIMYLTGM	VDKRTLEKYE	REAKEKNRET	WYLSWALDTN	QEERDKGKTV	EVGRAYFETE	KKHFTILDAP
**170**	**180**	**190**	**200**	**210**	**220**	**230**	**240**
GHKSFVPNMI	GGASQADLAV	LVISARKGEF	ETGFEKGGQT	REHAMLAKTA	GVKHLIVLIN	KMDDPTVNWS	NERYEECKEK
**250**	**260**	**270**	**280**	**290**	**300**	**310**	**320**
LVPFLKKVGF	NPKKDIHFMP	CSGLTGANLK	EQSDFCPWYI	GLPFIPYLDN	LPNFNRSVDG	PIRLPIVDKY	KDMGTVVLGK
**330**	**340**	**350**	**360**	**370**	**380**	**390**	**400**
LESGSICKGQ	QLVMMPNKHN	VEVLGILSDD	VETDTVAPGE	NLKIRLKGIE	EEEILPGFIL	CDPNNLCHSG	RTFDAQIVII
**410**	**420**	**430**	**440**	**450**	**460**	**470**	**480**
EHKSIICPGY	NAVLHIHTCI	EEVEITALIC	LVDKKSGEKS	KTRPRFVKQD	QVCIARLRTA	GTICLETFKD	FPQMGRFTLR
**490**	**500**						
DEGKTIAIGK	VLKLVPEKD						

Cmpd.	No. of Cmpds.	m/z meas.	Δ m/z [ppm]	z	Rt [min]	Score	P	Range	Sequence	Modification
668	1	488.2507	-95.56	2	36.9	37.4	1	304-311	R.LPIVDKYK.D	

Detailed Protein Report

Protein 153: PREDICTED: HEAT repeat-containing protein 5B isoform X3 [Homo sapiens]

**Accession:**	gi|578802960	**Score:**	37.3
**Database:**	refseq_human(refseq_human_20140103.fasta)	**MW [kDa]:**	222.9
**Database Date:**	2015-11-30	**pI:**	6.8
**Modification(s):**	Oxidation	**Sequence Coverage [%]:**	1.8
		**No. of unique Peptides:**	2

Detailed Protein Report

10	20	30	40	50	60	70	80
MELAHSLLLN	EEALAQITEA	KRPVFIFEWL	RFLDKVLVAA	NKTDVKEKQK	KLVEQLTGLI	SSSPGPPTRK	LLAKNLAALY
90	100	110	120	130	140	150	160
SIGDTFTVFQ	TLDKCNDIIR	NKDDTAAYLP	TKLAAVACVG	AFYEKMGRML	GSAFPETSQG	RSEILMSLQK	VLSGLGGAAA
170	180	190	200	210	220	230	240
SSHRDIYKNA	RSLLTDRSMA	VRCAVAKCLL	ELQNEAVFMW	TAELENIATL	CFKALENSNY	GVRVAVSKLL	GTVMATALMP
250	260	270	280	290	300	310	320
KQATVMRQNV	KRATFDEVLE	LMATGFLRGG	SGFLKSGGEM	LKVGGSVNRE	VRVGVTQAYV	VFVTTLGGQW	LERSFATFLS
330	340	350	360	370	380	390	400
HVLDLVSHPR	ATQTHVEAVY	SRRCVSFILR	ATVGSLLGEK	AQIAAAKEIC	QAIGKQMKAV	EAVVNDTSGE	NKSGAADIAA
410	420	430	440	450	460	470	480
SQHVMVCALQ	ELGSLVQSLN	ATASPLIQEA	SIGLLEIVTS	VLLHPSMAAR	LAAAWCLRCV	AVALPFQLTP	FLDRCAERLN
490	500	510	520	530	540	550	560
NLKTSPEAVS	GYSFAMAALL	GGVHQCPLGI	PHAKGKMVVS	IAEDLLRTAA	QNSRLSLQRT	QAGWLLLGAL	MTLGPSVVRY
570	580	590	600	610	620	630	640
HLPKMLLLWR	NVFPRSLKEL	EAEKARGDSF	TWQVTLEGRA	GALCAMRSFV	AHCPELLTED	VIRKLMTPIE	CAMTMMSHIP
650	660	670	680	690	700	710	720
SVMKAHGAHL	KASAAMVRLR	LYDILALLPP	KTYEGSFNAL	LRELVAEFTL	TDNSANTTTS	LLRSLCHYDD	SVLLGSWLQE
730	740	750	760	770	780	790	800
TDHKSIEDQL	QPNSASGSGA	LEHDPSSIYL	RIPAGEAVPG	PLPLGVSVID	ASVALFGVVF	PHVSYKHRLQ	MLDHFAECVK
810	820	830	840	850	860	870	880
QAKGVRQQAV	QLNIFTAVLS	ALKGLAENKS	TLGPEEVRKS	ALTLVMGPLD	NPNPILRCAA	GEALGRMAQV	VGEATFIARM
890	900	910	920	930	940	950	960
AQYSFDKLKS	ARDVVSRTGH	SLALGCLHRY	VGGIGSGQHL	KTSVSILLAL	AQDGTSPEVQ	TWSLHSLALI	VDSSGPMYRG
970	980	990	1000	1010	1020	1030	1040
YVEPTLSLVL	TLLLTVPPSH	TEVHQCLGRC	LGAIITTVGP	ELQGNGATTS	TIRSSCLVGC	AITQDHSDSL	VQAAAISCLQ
1050	1060	1070	1080	1090	1100	1110	1120
QLHMFAPRHV	NLSSLVPSLC	VHLCSSHLLL	RRAAVACLRQ	LAQREAAEVC	EYAMSLAKNT	GDKESSSANV	SPFAPGVSSR
1130	1140	1150	1160	1170	1180	1190	1200
TDIHCRHQGV	NITETGLEGL	LFGMLDRETD	RKLCSDIHDT	LGHMLSSLAV	EKLSHWLMLC	KDVLAASSDM	STATLLSSGK
1210	1220	1230	1240	1250	1260	1270	1280
DEEAEKKDEM	DDDTMFTTLG	EEDKSKPFVA	PRWATRVFAA	DCLCRIINLC	ENADQAHFDL	ALARSAKLRN	PTNDLLVLHL
1290	1300	1310	1320	1330	1340	1350	1360
SDLIRMAFMA	ATDHSNQLRM	AGLQALEDII	KKFASVPEPE	FPGHVILEQY	QANVGAALRP	AFSQDTPSDI	IAKACQVCST
1370	1380	1390	1400	1410	1420	1430	1440
WIGSGVVSDL	NDLRRVHNLL	VSSLDKVQAG	KGSSSQLYRE	SATTMEKLAV	LKAWAEVYVV	AMNIKKEAES	KPKRAIKNTD
1450	1460	1470	1480	1490	1500	1510	1520
DDDDDCGTID	ELPPDSLITL	VQPELPTLSR	LWLAALKDYA	LLTLPAEFSS	QLPPDGGAFY	TPETIDTARL	HYRNSWAPIL
1530	1540	1550	1560	1570	1580	1590	1600
HAVALWLNST	GFTCSESTEA	AAISGLQKRS	TSVNLNQASG	AVGSAKSLPE	INKDRMHLIL	GVSIQFLCSP	RPEEPIEHVT
1610	1620	1630	1640	1650	1660	1670	1680
ACLQALHTLL	DSPYARVHIA	EDQLIGVELL	SVLHRLLLTW	NPSSVQLLVT	GVVQQIVRAA	QDYLQEKRNT	LNEDDMEKEA
1690	1700	1710	1720	1730	1740	1750	1760
CTVLGEGGDS	GGLIPGKSLV	FATMELLMFI	LVRHMPHLST	KVSDSPSHIA	TKTRLSEESA	RLVAATVTIL	SDLPSLCSPA
1770	1780	1790	1800	1810	1820	1830	1840
GCMTILPTIL	FLIARILKDT	AIKSADNQVP	PPVSAALQGI	KSIVTLSMAK	TEAGVQKQWT	ALIRSTLACI	LEYSQPEDSV
1850	1860	1870	1880	1890	1900	1910	1920
PTPDEVSMLT	AIALFLWSAS	NEIIGVQSLQ	NGCMNRFKNA	LNSCDPWVQA	KCYQLLLSVF	QHSNRALSTP	YIHSLAPIVV
1930	1940	1950	1960	1970	1980	1990	2000
EKLKAVERNR	PASNIELLAV	QEGIKVLETL	VALGEEQNRV	QLLALLVPTL	ISYLLDENSF	ASASSASKDL	HEFALQNLMH
2010	2020	2030	2040	2050	2060		
IGPLYPHAFK	TVMGAAPELK	VRLETAVRAS	QASKAKAAAR	QPAPAIHSAP	TIKLKTSFF		

Cmpd.	No. of Cmpds.	m/z meas.	Δ m/z [ppm]	z	Rt [min]	Score	P	Range	Sequence	Modification

Detailed Protein Report

Cmpd.	No. of Cmpds.	m/z meas.	Δ m/z [ppm]	z	Rt [min]	Score	P	Range	Sequence	Modification
284	1	635.8126	-19.46	2	32.3	12.1	0	672-682	K.TYEGSFNALLR.E	
1278	1	644.3427	-23.92	2	46.1	14.2	1	2011-2022	K.TVMGAAPELKVR.L	Oxidation: 3

Detailed Protein Report

Protein 154: sortilin isoform 2 [Homo sapiens]

Accession: gi|327315343
Database: refseq_human(refseq_human_20140103.fasta)
Database Date: 2015-11-30
Modification(s): Carbamidomethyl

Score: 37.3
MW [kDa]: 77.3
pI: 5.1
Sequence Coverage [%]: 3.9
No. of unique Peptides: 1

10	20	30	40	50	60	70	80
MTFGQSKLYR	SEDYGKNFKD	ITDLINNTFI	RTEFGMAIGP	ENSGKVVLTA	EVSGGSRGGR	IFRSSDFAKN	FVQTDLPFHP
**90**	**100**	**110**	**120**	**130**	**140**	**150**	**160**
LTQMMYSPQN	SDYLLALSTE	NGLWVSKNFG	GKWEEIHKAV	CLAKWGSDNT	IFFTTYANGS	CTDLGALELW	RTSDLGKSFK
**170**	**180**	**190**	**200**	**210**	**220**	**230**	**240**
TIGVKIYSFG	LGGRFLFASV	MADKDTTRRI	HVSTDQGDTW	SMAQLPSVGQ	EQFYSILAAN	DDMVFMHVDE	PGDTGFGTIF
**250**	**260**	**270**	**280**	**290**	**300**	**310**	**320**
TSDDRGIVYS	KSLDRHLYTT	TGGETDFTNV	TSLRGVYITS	VLSEDNSIQT	MITFDQGGRW	THLRKPENSE	CDATAKNKNE
**330**	**340**	**350**	**360**	**370**	**380**	**390**	**400**
CSLHIHASYS	ISQKLNVPMA	PLSEPNAVGI	VIAHGSVGDA	ISVMVPDVYI	SDDGGYSWTK	MLEGPHYYTI	LDSGGIIVAI
**410**	**420**	**430**	**440**	**450**	**460**	**470**	**480**
EHSSRPINVI	KFSTDEGQCW	QTYTFTRDPI	YFTGLASEPG	ARSMNISIWG	FTESFLTSQW	VSYTIDFKDI	LERNCEEKDY
**490**	**500**	**510**	**520**	**530**	**540**	**550**	**560**
TIWLAHSTDP	EDYEDGCILG	YKEQFLRLRK	SSVCQNGRDY	VVTKQPSICL	CSLEDFLCDF	GYYRPENDSK	CVEQPELKGH
**570**	**580**	**590**	**600**	**610**	**620**	**630**	**640**
DLEFCLYGRE	EHLTTNGYRK	IPGDKCQGGV	NPVREVKDLK	KKCTSNFLSP	EKQNSKSNSV	PIILAIVGLM	LVTVVAGVLI
**650**	**660**	**670**	**680**	**690**	**700**		
VKKYVCGGRF	LVHRYSVLQQ	HAEANGVDGV	DALDTASHTN	KSGYHDDSDE	DLLE		

Cmpd.	No. of Cmpds.	m/z meas.	Δ m/z [ppm]	z	Rt [min]	Score	P	Range	Sequence	Modification
1682	1	1058.4833	-30.52	2	51.3	12.7	1	317-334	K.NKNECSLHIHASYSISQK.L	Carbamidomethyl: 5

Detailed Protein Report

Protein 155: protein SOGA1 isoform 1 [Homo sapiens]

**Accession:**	gi|257467639	**Score:**	37.3
**Database:**	refseq_human(refseq_human_20140103.fasta)	**MW [kDa]:**	183.7
**Database Date:**	2015-11-30	**pI:**	6.1
**Modification(s):**	Carbamidomethyl	**Sequence Coverage [%]:**	2.4
		**No. of unique Peptides:**	2

10	20	30	40	50	60	70	80
MEAPAAEPPV	RGCGPQPAPA	PAPAPERKKS	HRAPSPARPK	DVAGWSLAKG	RRGPGPGSAV	ACSAAFSSRP	DKKGRAVAPG
90	100	110	120	130	140	150	160
ARGAGVRVAG	VRTGVRAKGR	PRSGAGPRPP	PPPPSLTDSS	SEVSDCASEE	ARLLGLELAL	SSDAESAAGG	PAGVRTGQPA
170	180	190	200	210	220	230	240
QPAPSAQQPP	RPPASPDEPS	VAASSVGSSR	LPLSASLAFS	DLTEEMLDCG	PSGLVRELEE	LRSENDYLKD	EIEELRAEML
250	260	270	280	290	300	310	320
EMRDVYMEED	VYQLQELRQQ	LDQASKTCRI	LQYRLRKAER	RSLRAAQTGQ	VDGELIRGLE	QDVKVSKDIS	MRLHKELEVV
330	340	350	360	370	380	390	400
EKKRARLEEE	NEELRQRLIE	TELAKQVLQT	ELERPREHSL	KKRGTRSLGK	ADKKTLVQED	SADLKCQLHF	AKEESALMCK
410	420	430	440	450	460	470	480
KLTKLAKEND	SMKEELLKYR	SLYGDLDSAL	SAEELADAPH	SRETELKVHL	KLVEEEANLL	SRRIVELEVE	NRGLRAEMDD
490	500	510	520	530	540	550	560
MKDHGGGCGG	PEARLAFSAL	GGGECGESLA	ELRRHLQFVE	EEAELLRRSS	AELEDQNKLL	LNELAKFRSE	HELDVALSED
570	580	590	600	610	620	630	640
SCSVLSEPSQ	EELAAAKLQI	GELSGKVKKL	QYENRVLLSN	LQRCDLASCQ	STRPMLETDA	EAGDSAQCVP	APLGETHESH
650	660	670	680	690	700	710	720
AVRLCRAREA	EVLPGLREQA	ALVSKAIDVL	VADANGFTAG	LRLCLDNECA	DFRLHEAPDN	SEGPRDTKLI	HAILVRLSVL
730	740	750	760	770	780	790	800
QQELNAFTRK	ADAVLGCSVK	EQQESFSSLP	PLGSQGLSKE	ILLAKDLGSD	FQPPDFRDLP	EWEPRIREAF	RTGDLDSKPD
810	820	830	840	850	860	870	880
PSRSFRPYRA	EDNDSYASEI	KELQLVLAEA	HDSLRGLQEQ	LSQERQLRKE	EADNFNQKMV	QLKEDQQRAL	LRREFELQSL
890	900	910	920	930	940	950	960
SLQRRLEQKF	WSQEKNMLVQ	ESQQFKHNFL	LLFMKLRWFL	KRWRQGKVLP	SEGDDFLEVN	SMKELYLLME	EEEINAQHSD
970	980	990	1000	1010	1020	1030	1040
NKACTGDSWT	QNTPNEYIKT	LADMKVTLKE	LCWLLRDERR	GLTELQQQFA	KAKATWETER	AELKGHTSQM	ELKTGKGAGE
1050	1060	1070	1080	1090	1100	1110	1120
RAGPDWKAAL	QREREEQQHL	LAESYSAVME	LTRQLQISER	NWSQEKLQLV	ERLQGEKQQV	EQQVKELQNR	LSQLQKAADP
1130	1140	1150	1160	1170	1180	1190	1200
WVLKHSELEK	QDNSWKETRS	EKIHDKEAVS	EVELGGNGLK	RTKSVSSMSE	FESLLDCSPY	LAGGDARGKK	LPNNPAFGFV
1210	1220	1230	1240	1250	1260	1270	1280
SSEPGDPEKD	TKEKPGLSSR	DCNHLGALAC	QDPPGRQMQR	SYTAPDKTGI	RVYYSPPVAR	RLGVPVVHDK	EGKIIIEPGF
1290	1300	1310	1320	1330	1340	1350	1360
LFTTAKPKES	AEADGLAESS	YGRWLCNFSR	QRLDGGSAGS	PSAAGPGFPA	ALHDFEMSGN	MSDDMKEITN	CVRQAMRSGS
1370	1380	1390	1400	1410	1420	1430	1440
LERKVKSTSS	QTVGLASVGT	QTIRTVSVGL	QTDPPRSSLH	GKAWSPRSSS	LVSVRSKQIS	SSLDKVHSRI	ERPCCSPKYG
1450	1460	1470	1480	1490	1500	1510	1520
SPKLQRRSVS	KLDSSKDRSL	WNLHQGKQNG	SAWARSTTTR	DSPVLRNIND	GLSSLFSVVE	HSGSTESVWK	LGMSETRAKP
1530	1540	1550	1560	1570	1580	1590	1600
EPPKYGIVQE	FFRNVCGRAP	SPTSSAGEEG	TKKPEPLSPA	SYHQPEGVAR	ILNKKAAKLG	SSEEVRLTML	PQVGKDGVLR
1610	1620	1630	1640	1650	1660	1670	
DGDGAVVLPN	EDAVCDCSTQ	SLTSCFARSS	RSAIRHSPSK	CRLHPSESSW	GGEERALPPS	E	

Cmpd.	No. of Cmpds.	m/z meas.	Δ m/z [ppm]	z	Rt [min]	Score	P	Range	Sequence	Modification
63	1	1097.5505	-104.35	1	30.7	13.5	2	93-102	R.TGVRAKGRPR.S	
2190	1	701.1048	122.16	3	57.8	13.2	2	1426-1443	K.VHSRIERPCCSPKYGSPK.L	Carbamidomethyl: 9

Detailed Protein Report

Protein 156: PREDICTED: regulator of G-protein signaling 3 isoform X2 [Homo sapiens]

Accession: gi|578817733
Database: refseq_human(refseq_human_20140103.fasta)
Database Date: 2015-11-30
Modification(s): Carbamidomethyl

Score: 37.3
MW [kDa]: 120.1
pI: 5.9
Sequence Coverage [%]: 3.5
No. of unique Peptides: 2

10	20	30	40	50	60	70	80
MERSLHRVSL	GSRRAHPDLS	FYLTTFGQLR	LSIDAQDRVL	LLHIIEGKGL	ISKQPGTCDP	YVKISLIPED	SRLRHQKTQT
90	100	110	120	130	140	150	160
VPDCRDPAFH	EHFFFPVQEE	DDQKRLLVTV	WNRASQSRQS	GLIGCMSFGV	KSLLTPDKEI	SGWYYLLGEH	LGRTKHLKVA
170	180	190	200	210	220	230	240
RRRLRPLRDP	LLRMPGGGDT	ENGKKLKITI	PRGKDGFGFT	ICCDSPVRVQ	AVDSGGPAER	AGLQQLDTVL	QLNERPVEHW
250	260	270	280	290	300	310	320
KCVELAHEIR	SCPSEIILLV	WRMVPQVKPG	PDGGVLRRAS	CKSTHDLQSP	PNKREKNCTH	GVQARPEQRH	SCHLVCDSSD
330	340	350	360	370	380	390	400
GLLLGGWERY	TEVAKRGGQH	TLPALSRATA	PTDPNYIILA	PLNPGSQLLR	PVYQEDTIPE	ESGSPSKGKS	YTGLGKKSRL
410	420	430	440	450	460	470	480
MKTVQTMKGH	GNYQNCPVVR	PHATHSSYGT	YVTLAPKVLV	FPVFVQPLDL	CNPARTLLLS	EELLLYEGRN	KAAEVTLFAY
490	500	510	520	530	540	550	560
SDLLLFTKED	EPGRCDVLRN	PLYLQSVKLQ	EGSSEDLKFC	VLYLAEKAEC	LFTLEAHSQE	QKKRVCWCLS	ENIAKQQQLA
570	580	590	600	610	620	630	640
ASPPDSKMFE	TEADEKREMA	LEEGKGPGAE	DSPPSKEPSP	GQELPPGQDL	PPNKDSPSGQ	EPAPSQEPLS	SKDSATSEGS
650	660	670	680	690	700	710	720
PPGPDAPPSK	DVPPCQEPPP	AQDLSPCQDL	PAGQEPLPHQ	DPLLTKDLPA	IQESPTRDLP	PCQDLPPSQV	SLPAKALTED
730	740	750	760	770	780	790	800
TMSSGDLLAA	TGDPPAAPRP	AFVIPEVRLD	STYSQKAGAE	QGCSGDEEDA	EEAEEVEEGE	EGEEDEDEDT	SDDNYGERSE
810	820	830	840	850	860	870	880
AKRSSMIETG	QGAEGGLSLR	VQNSLRRRTH	SEGSLLQEPR	GPCFASDTTL	HCSDGEGAAS	TWGMPSPSTL	KKELGRNGGS
890	900	910	920	930	940	950	960
MHHLSLFFTG	HRKMSGADTV	GDDDEASRKR	KSKNLAKDMK	NKLGIFRRRN	ESPGAPPAGK	ADKMMKSFKP	TSEEALKWGE
970	980	990	1000	1010	1020	1030	1040
SLEKLLVHKY	GLAVFQAFLR	TEFSEENLEF	WLACEDFKKV	KSQSKMASKA	KKIFAEYIAI	QACKEVNLDS	YTREHTKDNL
1050	1060	1070	1080	1090			
QSVTRGCFDL	AQKRIFGLME	KDSYPRFLRS	DLYLDLINQK	KMSPPL			

Cmpd.	No. of Cmpds.	m/z meas.	Δ m/z [ppm]	z	Rt [min]	Score	P	Range	Sequence	Modification
7	4	819.8312	-118.35	2	29.5	14.6	2	73-85	R.LRHQKTQTVPDCR.D	Carbamidomethyl: 12
512	1	593.1679	-216.84	2	36.4	10.4	0	209-220	R.VQAVDSGGPAER.A	

Detailed Protein Report

Protein 157: histone-lysine N-methyltransferase 2C [Homo sapiens]

**Accession:**	gi|91718902	**Score:**	37.2
**Database:**	refseq_human(refseq_human_20140103.fasta)	**MW [kDa]:**	541.0
**Database Date:**	2015-11-30	**pI:**	6.1
		**Sequence Coverage [%]:**	0.9
		**No. of unique Peptides:**	1

10	20	30	40	50	60	70	80
MSSEEDKSVE	QPQPPPPPPE	EPGAPAPSPA	AADKRPRGRP	RKDGASPFQR	ARKKPRSRGK	TAVEDEDSMD	GLETTETETI
90	100	110	120	130	140	150	160
VETEIKEQSA	EEDAEAEVDN	SKQLIPTLQR	SVSEESANSL	VSVGVEAKIS	EQLCAFCYCG	EKSSLGQGDL	KQFRITPGFI
170	180	190	200	210	220	230	240
LPWRNQPSNK	KDIDDNSNGT	YEKMQNSAPR	KQRGQRKERS	PQQNIVSCVS	VSTQTASDDQ	AGKLWDELSL	VGLPDAIDIQ
250	260	270	280	290	300	310	320
ALFDSTGTCW	AHHRCVEWSL	GVCQMEEPLL	VNVDKAVVSG	STERCAFCKH	LGATIKCCEE	KCTQMYHYPC	AAGAGTFQDF
330	340	350	360	370	380	390	400
SHIFLLCPEH	IDQAPERSKE	DANCAVCDSP	GDLLDQFFCT	TCGQHYHGMC	LDIAVTPLKR	AGWQCPECKV	CQNCKQSGED
410	420	430	440	450	460	470	480
SKMLVCDTCD	KGYHTFCLQP	VMKSVPTNGW	KCKNCRICIE	CGTRSSSQWH	HNCLICDNCY	QQQDNLCPFC	GKCYHPELQK
490	500	510	520	530	540	550	560
DMLHCNMCKR	WVHLECDKPT	DHELDTQLKE	EYICMYCKHL	GAEMDRLQPG	EEVEIAELTT	DYNNEMEVEG	PEDQMVFSEQ
570	580	590	600	610	620	630	640
AANKDVNGQE	STPGIVPDAV	QVHTEEQQKS	HPSESLDTDS	LLIAVSSQHT	VNTELEKQIS	NEVDSEDLKM	SSEVKHICGE
650	660	670	680	690	700	710	720
DQIEDKMEVT	ENIEVVTHQI	TVQQEQLQLL	EEPETVVSRE	ESRPPKLVME	SVTLPLETLV	SPHEESISLC	PEEQLVIERL
730	740	750	760	770	780	790	800
QGEKEQKENS	ELSTGLMDSE	MTPTIEGCVK	DVSYQGGKSI	KLSSETESSF	SSSADISKAD	VSSSPTPSSD	LPSHDMLHNY
810	820	830	840	850	860	870	880
PSALSSSAGN	IMPTTYISVT	PKIGMGKPAI	TKRKFSPGRP	RSKQGAWSTH	NTVSPPSWSP	DISEGREIFK	PRQLPGSAIW
890	900	910	920	930	940	950	960
SIKVGRGSGF	PGKRRPRGAG	LSGRGGRGRS	KLKSGIGAVV	LPGVSTADIS	SNKDDEENSM	HNTVVLFSSS	DKFTLNQDMC
970	980	990	1000	1010	1020	1030	1040
VVCGSFGQGA	EGRLLACSQC	GQCYHPYCVS	IKITKVVLSK	GWRCLECTVC	EACGKATDPG	RLLLCDDCDI	SYHTYCLDPP
1050	1060	1070	1080	1090	1100	1110	1120
LQTVPKGGWK	CKWCVWCRHC	GATSAGLRCE	WQNNYTQCAP	CASLSSCPVC	YRNYREEDLI	LQCRQCDRWM	HAVCQNLNTE
1130	1140	1150	1160	1170	1180	1190	1200
EEVENVADIG	FDCSMCRPYM	PASNVPSSDC	CESSLVAQIV	TKVKELDPPK	TYTQDGVCLT	ESGMTQLQSL	TVTVPRRKRS
1210	1220	1230	1240	1250	1260	1270	1280
KPKLKLKIIN	QNSVAVLQTP	PDIQSEHSRD	GEMDDSREGE	LMDCDGKSES	SPEREAVDDE	TKGVEGTDGV	KKRKRKPYRP
1290	1300	1310	1320	1330	1340	1350	1360
GIGGFMVRQR	SRTGQGKTKR	SVIRKDSSGS	ISEQLPCRDD	GWSEQLPDTL	VDESVSVTES	TEKIKKRYRK	RKNKLEETFP
1370	1380	1390	1400	1410	1420	1430	1440
AYLQEAFFGK	DLLDTSRQSK	ISLDNLSEDG	AQLLYKTNMN	TGFLDPSLDP	LLSSSSAPTK	SGTHGPADDP	LADISEVLNT
1450	1460	1470	1480	1490	1500	1510	1520
DDDILGIISD	DLAKSVDHSD	IGPVTDDPSS	LPQPNVNQSS	RPLSEEQLDG	ILSPELDKMV	TDGAILGKLY	KIPELGGKDV
1530	1540	1550	1560	1570	1580	1590	1600
EDLFTAVLSP	ANTQPTPLPQ	PPPPTQLLPI	HNQDAFSRMP	LMNGLIGSSP	HLPHNSLPPG	SGLGTFSAIA	QSSYPDARDK
1610	1620	1630	1640	1650	1660	1670	1680
NSAFNPMASD	PNNSWTSSAP	TVEGENDTMS	NAQRSTLKWE	KEEALGEMAT	VAPVLYTNIN	FPNLKEEFPD	WTTRVKQIAK
1690	1700	1710	1720	1730	1740	1750	1760
LWRKASSQER	APYVQKARDN	RAALRINKVQ	MSNDSMKRQQ	QQDSIDPSSR	IDSELFKDPL	KQRESEHEQE	WKFRQQMRQK
1770	1780	1790	1800	1810	1820	1830	1840
SKQQAKIEAT	QKLEQVKNEQ	QQQQQQFGS	QHLLVQSGSD	TPSSGIQSPL	TPQPGNGNMS	PAQSFHKELF	TKQPPSTPTS
1850	1860	1870	1880	1890	1900	1910	1920
TSSDDVFVKP	QAPPPPPAPS	RIPIQDSLSQ	AQTSQPPSPQ	VFSPGSSNSR	PPSPMDPYAK	MVGTPRPPPV	GHSFSRRNSA
1930	1940	1950	1960	1970	1980	1990	2000
APVENCTPLS	SVSRPLQMNE	TTANRPSPVR	DLCSSSTTNN	DPYAKPPDTP	RPVMTDQFPK	SLGLSRSPVV	SEQTAKGPIA
2010	2020	2030	2040	2050	2060	2070	2080
AGTSDHFTKP	SPRADVFQRQ	RIPDSYARPL	LTPAPLDSGP	GPFKTPMQPP	PSSQDPYGSV	SQASRRLSVD	PYERPALTPR
2090	2100	2110	2120	2130	2140	2150	2160
PIDNFSHNQS	NDPYSQPPLT	PHPAVNESFA	HPSRAFSQPG	TISRPTSQDP	YSQPPGTPRP	VVDSYSQSSG	TARSNTDPYS
2170	2180	2190	2200	2210	2220	2230	2240

Detailed Protein Report

Cmpd.	No. of Cmpds.	m/z meas.	Δ m/z [ppm]	z	Rt [min]	Score	P	Range	Sequence	Modification
132	1	846.0298	-52.12	3	31.6	13.7	1	4823-4845	R.GVYMFRMDNDHVIDATLTGGPAR.Y	

Detailed Protein Report

Protein 158: protein Jade-2 [Homo sapiens]

Accession: gi|40556370
Database: refseq_human(refseq_human_20140103.fasta)
Database Date: 2015-11-30
Modification(s): Oxidation

Score: 37.2
MW [kDa]: 87.4
pI: 5.0
Sequence Coverage [%]: 6.1
No. of unique Peptides: 2

Quantitation

QD:QU **Median:** 0.42 **CV:** 0.00 % **No. of Peptides:** 1

10	20	30	40	50	60	70	80
MEEKRRKYSI	SSDNSDTTDS	HATSTSASRC	SKLPSSTKSG	WPRQNEKKPS	EVFRTDLITA	MKIPDSYQLS	PDDYYILADP
**90**	**100**	**110**	**120**	**130**	**140**	**150**	**160**
WRQEWEKGVQ	VPAGAEAIPE	PVVRILPPLE	GPPAQASPSS	TMLGEGSQPD	WPGGSRYDLD	EIDAYWLELI	NSELKEMERP
**170**	**180**	**190**	**200**	**210**	**220**	**230**	**240**
ELDELTLERV	LEELETLCHQ	NMARAIETQE	GLGIEYDEDV	VCDVCRSPEG	EDGNEMVFCD	KCNVCVHQAC	YGILKVPTGS
**250**	**260**	**270**	**280**	**290**	**300**	**310**	**320**
WLCRTCALGV	QPKCLLCPKR	GGALKPTRSG	TKWVHVSCAL	WIPEVSIGCP	EKMEPITKIS	HIPASRWALS	CSLCKECTGT
**330**	**340**	**350**	**360**	**370**	**380**	**390**	**400**
CIQCSMPSCV	TAFHVTCAFD	HGLEMRTILA	DNDEVKFKSF	CQEHSDGGPR	NEPTSEPTEP	SQAGEDLEKV	TLRKQRLQQL
**410**	**420**	**430**	**440**	**450**	**460**	**470**	**480**
EEDFYELVEP	AEVAERLDLA	EALVDFIYQY	WKLKRKANAN	QPLLTPKTDE	VDNLAQQEQD	VLYRRLKLFT	HLRQDLERVR
**490**	**500**	**510**	**520**	**530**	**540**	**550**	**560**
NLCYMVTRRE	RTKHAICKLQ	EQIFHLQMKL	IEQDLCRGLS	TSFPIDGTFF	NSWLAQSVQI	TAENMAMSEW	PLNNGHREDP
**570**	**580**	**590**	**600**	**610**	**620**	**630**	**640**
APGLLSEELL	QDEETLLSFM	RDPSLRPGDP	ARKARGRTRL	PAKKKPPPPP	PQDGPGSRTT	PDKAPKKTWG	QDAGSGKGGQ
**650**	**660**	**670**	**680**	**690**	**700**	**710**	**720**
GPPTRKPPRR	TSSHLPSSPA	AGDCPILATP	ESPPPLAPET	PDEAASVAAD	SDVQVPGPAA	SPKPLGRLRP	PRESKVTRRL
**730**	**740**	**750**	**760**	**770**	**780**	**790**	**800**
PGARPDAGMG	PPSAVAERPK	VSLHFDTETD	GYFSDGEMSD	SDVEAEDGGV	QRGPREAGAE	EVVRMGVLAS	

Cmpd.	No. of Cmpds.	m/z meas.	Δ m/z [ppm]	z	Rt [min]	Score	P	Range	Sequence	Modification	Ratios
2789	1	863.0890	-25.83	3	66.0	12.9	2	293-315	K.MEPITKISHIPASRWALSCSLCK.E	Oxidation: 1	
256	1	715.8400	-46.05	2	33.2	12.5	2	624-637	K.APKKTWGQDAGSGK.G		QD:QU 0.42

Detailed Protein Report

Protein 159: tripartite motif-containing protein 65 isoform 2 [Homo sapiens]

**Accession:**	gi|371122551	**Score:**	37.0
**Database:**	refseq_human(refseq_human_20140103.fasta)	**MW [kDa]:**	54.9
**Database Date:**	2015-11-30	**pI:**	6.0
**Modification(s):**	Carbamidomethyl	**Sequence Coverage [%]:**	8.1
		**No. of unique Peptides:**	3

10	20	30	40	50	60	70	80
MAAQLLEEKL	TCAICLGLYQ	DPVTLPCGHN	FCGACIRDWW	DRCGKACPEC	REPFPDGAEL	RRNVALSGVL	EVVRAGPARD
**90**	**100**	**110**	**120**	**130**	**140**	**150**	**160**
PGPDPGPGPD	PAARCPRHGR	PLELFCRTEG	RCVCSVCTVR	ECRLHERALL	DAERLKREAQ	LRASLEVTQQ	QATQAEGQLL
**170**	**180**	**190**	**200**	**210**	**220**	**230**	**240**
ELRKQSSQIQ	NSACILASWV	SGKFSSLLQA	LEIQHTTALR	SIEVAKTQAL	AQARDEEQRL	RVHLEAVARH	GCRIRELLEQ
**250**	**260**	**270**	**280**	**290**	**300**	**310**	**320**
VDEQTFLQES	QLLQPPGPLG	PLTPLQWDED	QQLGDLKQLL	SRLCGLLLEE	GSHPGAPAKP	VDLAPVDYRN	LTFDPVSANR
**330**	**340**	**350**	**360**	**370**	**380**	**390**	**400**
HFYLSRQDQQ	VKHCRQSRGP	GGPGSFELWQ	VQCAQSFQAG	HHYWEVRASD	HSVTLGVSYP	QLPRCRLGPH	TDNIGRGPCS
**410**	**420**	**430**	**440**	**450**	**460**	**470**	**480**
WGLCVQEDSL	QAWHNGEAQR	LPGVSGRLLG	MDLDLASGCL	TFYSLEPQTQ	PLYTFHALFN	QPLTPVFWLL	EGRTLTLCHQ
**490**	**500**						
PGAVFPLGPQ	EEVLS						

Cmpd.	No. of Cmpds.	m/z meas.	Δ m/z [ppm]	z	Rt [min]	Score	P	Range	Sequence	Modification
2836	11	973.5061	53.53	2	64.6	14.4	2	46-62	K.ACPECREPFPDGAELRR.N	
347	1	764.8773	64.49	2	34.3	12.1	1	112-123	R.CVCSVCTVRECR.L	Carbamidomethyl: 1, 3, 6
40	1	661.7546	-162.80	2	30.3	10.4	1	124-134	R.LHERALLDAER.L	

Detailed Protein Report

Protein 160: fibroblast growth factor receptor substrate 2 [Homo sapiens]

**Accession:**	gi|110347408	**Score:**	37.0
**Database:**	refseq_human(refseq_human_20140103.fasta)	**MW [kDa]:**	57.0
**Database Date:**	2015-11-30	**pI:**	5.6
**Modification(s):**	Carbamidomethyl	**Sequence Coverage [%]:**	8.3
		**No. of unique Peptides:**	3

Alias proteins:

Accession	Name	Description
gi|507834075	refseq_human (refseq_human_20140103.fasta)	fibroblast growth factor receptor substrate 2 [Homo sapiens]
gi|507834073	refseq_human (refseq_human_20140103.fasta)	fibroblast growth factor receptor substrate 2 [Homo sapiens]
gi|507834071	refseq_human (refseq_human_20140103.fasta)	fibroblast growth factor receptor substrate 2 [Homo sapiens]
gi|507834069	refseq_human (refseq_human_20140103.fasta)	fibroblast growth factor receptor substrate 2 [Homo sapiens]
gi|507834066	refseq_human (refseq_human_20140103.fasta)	fibroblast growth factor receptor substrate 2 [Homo sapiens]
gi|507834061	refseq_human (refseq_human_20140103.fasta)	fibroblast growth factor receptor substrate 2 [Homo sapiens]
gi|110347414	refseq_human (refseq_human_20140103.fasta)	fibroblast growth factor receptor substrate 2 [Homo sapiens]

```
        10         20         30         40         50         60         70         80
MGSCCSCPDK DTVPDNHRNK FKVINVDDDG NELGSGIMEL TDTELILYTR KRDSVKWHYL CLRRYGYDSN LFSFESGRRC
        90        100        110        120        130        140        150        160
QTGQGIFAFK CARAEELFNM LQEIMQNNSI NVVEEPVVER NNHQTELEVP RTPRTPTTPG FAAQNLPNGY PRYPSFGDAS
       170        180        190        200        210        220        230        240
SHPSSRHPSV GSARLPSVGE ESTHPLLVAE EQVHTYVNTT GVQEERKNRT SVHVPLEARV SNAESSTPKE EPSSIEDRDP
       250        260        270        280        290        300        310        320
QILLEPEGVK FVLGPTPVQK QLMEKEKLEQ LGRDQVSGSG ANNTEWDTGY DSDERRDAPS VNKLVYENIN GLSIPSASGV
       330        340        350        360        370        380        390        400
RRGRLTSTST SDTQNINNSA QRRTALLNYE NLPSLPPVWE ARKLSRDEDD NLGPKTPSLN GYHNNLDPMH NYVNTENVTV
       410        420        430        440        450        460        470        480
PASAHKIEYS RRRDCTPTVF NFDIRRPSLE HRQLNYIQVD LEGGSDSDNP QTPKTPTTPL PQTPTRRTEL YAVIDIERTA
       490        500        510
AMSNLQKALP RDDGTSRKTR HNSTDLPM
```

Cmpd.	No. of Cmpds.	m/z meas.	Δ m/z [ppm]	z	Rt [min]	Score	P	Range	Sequence	Modification
1454	1	450.0854	-157.84	2	48.4	13.2	0	2-10	M.GSCCSCPDK.D	
2420	1	822.3542	-46.73	2	60.7	12.7	1	80-93	R.CQTGQGIFAFKCAR.A	Carbamidomethyl: 1, 12
1513	1	1023.0018	-57.43	2	49.1	11.1	1	304-322	K.LVYENINGLSIPSASGVRR.G	

Detailed Protein Report

Protein 161: PREDICTED: pericentrin isoform X6 [Homo sapiens]

**Accession:**	gi|530419254	**Score:**	36.9
**Database:**	refseq_human(refseq_human_20140103.fasta)	**MW [kDa]:**	355.7
**Database Date:**	2015-11-30	**pI:**	5.2
		**Sequence Coverage [%]:**	0.8
		**No. of unique Peptides:**	1

Detailed Protein Report

10	20	30	40	50	60	70	80
MFTVSDHPPE	QHGMFTVGDH	PPEQRGMFTV	SDHPPEQHGM	FTVSDHPPEQ	RGMFTISDHQ	PEQRGMFTVS	DHTPEQRGIF
90	100	110	120	130	140	150	160
TISDHPAEQR	GMFTKECEQE	CELAITDLES	GREDEAGLHQ	SQAVHGLELE	ALRLSLSNMH	TAQLELTQAN	LQKEKETALT
170	180	190	200	210	220	230	240
ELREMLNSRR	AQELALLQSR	QQHELELLRE	QHAREKEEVV	LRCGQEAAEL	KEKLQSEMEK	NAQIVKTLKE	DWESEKDLCL
250	260	270	280	290	300	310	320
ENLRKELSAK	HQSEMEDLQN	QFQKELAEQR	AELEKIFQDK	NQAERALRNL	ESHHQAAIEK	LREDLQSEHG	RCLEDLEFKF
330	340	350	360	370	380	390	400
KESEKEKQLE	LENLQASYED	LKAQSQEEIR	RLWSQLDSAR	TSRQELSELH	EQLLARTSRV	EDLEQLKQRE	KTQHESELEQ
410	420	430	440	450	460	470	480
LRIYFEKKLR	DAEKTYQEDL	TLLQQRLQGA	REDALLDSVE	VGLSCVGLEE	KPEKGRKDHV	DELEPERHKE	SLPRFQAELE
490	500	510	520	530	540	550	560
ESHRHQLEAL	ESPLCIQHEG	HVSDRCCVET	SALGHEWRLE	PSEGHSQELP	WVHLQGVQDG	DLEADTERAA	RVLGLETEHK
570	580	590	600	610	620	630	640
VQLSLLQTEL	KEEIELLKIE	NRNLYGKLQH	ETRLKDDLEK	VKHNLIEDHQ	KELNNAKQKT	ELMKQEFQRK	ETDWKVMKEE
650	660	670	680	690	700	710	720
LQREAEEKLT	LMLLELREKA	ESEKQTIINK	FELREAEMRQ	LQDQQAAQIL	DLERSLTEQQ	GRLQQLEQDL	TSDDALHCSQ
730	740	750	760	770	780	790	800
CGREPPTAQD	GELAALHVKE	DCALQLMLAR	SRFLEERKEI	TEKFSAEQDA	FLQEAQEQHA	RELQLLQERH	QQQLLSVTAE
810	820	830	840	850	860	870	880
LEARHQAALG	ELTASLESKQ	GALLAARVAE	LQTKHAADLG	ALETRHLSSL	DSLESCYLSE	FQTIREEHRQ	ALELLRADFE
890	900	910	920	930	940	950	960
EQLWKKDSLH	QTILTQELEK	LKRKHEGELQ	SVRDHLRTEV	STELAGTVAH	ELQGVHQGEF	GSEKKTALHE	KEETLRLQSA
970	980	990	1000	1010	1020	1030	1040
QAQPFHQEEK	ESLSLQLQKK	NHQVQQLKDQ	VLSLSHEIEE	CRSELEVLQQ	RRERENREGA	NLLSMLKADV	NLSHSERGAL
1050	1060	1070	1080	1090	1100	1110	1120
QDALRRLLGL	FGETLRAAVT	LRSRIGERVG	LCLDDAGAGL	ALSTAPALEE	TWSDVALPEL	DRTLSECAEM	SSVAEISSHM
1130	1140	1150	1160	1170	1180	1190	1200
RESFLMSPES	VRECEQPIRR	VFQSLSLAVD	GLMEMALDSS	RQLEEARQIH	SRFEKEFSFK	NEETAQVVRK	HQELLECLKE
1210	1220	1230	1240	1250	1260	1270	1280
ESAAKAELAL	ELHKTQGTLE	GFKVETADLK	EVLAGKEDSE	HRLVLELESL	RRQLQQAAQE	QAALREECTR	LWSRGEATAT
1290	1300	1310	1320	1330	1340	1350	1360
DAEAREAALR	KEVEDLTKEQ	SETRKQAEKD	RSALLSQMKI	LESELEEQLS	QHRGCAKQAE	AVTALEQQVA	SLDKHLRNQR
1370	1380	1390	1400	1410	1420	1430	1440
QFMDEQAAER	EHEREEFQQE	IQRLEGQLRQ	AAKPQPWGPR	DSQQAPLDGE	VELLQQKLRE	KLDEFNELAI	QKESADRQVL
1450	1460	1470	1480	1490	1500	1510	1520
MQEEEIKRLE	EMNINIRKKV	AQLQEEVEKQ	KNIVKGLEQD	KEVLKKQQMS	SLLLASTLQS	TLDAGRCPEP	PSGSPPEGPE
1530	1540	1550	1560	1570	1580	1590	1600
IQLEVTQRAL	LRRESEVLDL	KEQLEKMKGD	LESKNEEILH	LNLKLDMQNS	QTAVSLRELE	EENTSLKVIY	TRSSEIEELK
1610	1620	1630	1640	1650	1660	1670	1680
ATIENLQENQ	KRLQKEKAEE	IEQLHEVIEK	LQHELSLMGP	VVHEVSDSQA	GSLQSELLCS	QAGGPRGQAL	QGELEAALEA
1690	1700	1710	1720	1730	1740	1750	1760
KEALSRLLAD	QERRHSQALE	ALQQRLQGAE	EAAELQLAEL	ERNVALREAE	VEDMASRIQE	FEAALKAKEA	TIAERNLEID
1770	1780	1790	1800	1810	1820	1830	1840
ALNQRKAAHS	AELEAVLLAL	ARIRRALEQQ	PLAAGAAPPE	LQWLRAQCAR	LSRQLQVLHQ	RFLRCQVELD	RRQARRATAH
1850	1860	1870	1880	1890	1900	1910	1920
TRVPGAHPQP	RMDGGAKAQV	TGDVEASHDA	ALEPVVPDPQ	GDLQPVLVTL	KDAPLCKQEG	VMSVLTVCQR	QLQSELLLVK
1930	1940	1950	1960	1970	1980	1990	2000
NEMRLSLEDG	GKGKEKVLED	CQLPKVDLVA	QVKQLQEKLN	RLLYSMTFQN	VDAADTKSLW	PMASAHLLES	SWSDDSCDGE
2010	2020	2030	2040	2050	2060	2070	2080
EPDISPHIDT	CDANTATGGV	TDVIKNQAID	ACDANTTPGG	VTDVIKNWDS	LIPDEMPDSP	IQEKSECQDM	SLSSPTSVLG
2090	2100	2110	2120	2130	2140	2150	2160
GSRHQSHTAE	AGPRKSPVGM	LDLSSWSSPE	VLRKDWTLEP	WPSLPVTPHS	GALSLCSADT	SLGDRADTSL	PQTQGPGLLC
2170	2180	2190	2200	2210	2220	2230	2240

Detailed Protein Report

Cmpd.	No. of Cmpds.	m/z meas.	Δ m/z [ppm]	z	Rt [min]	Score	P	Range	Sequence	Modification
2710	2	1044.5270	3.44	2	65.0	25.0	2	1173-1189	R.FEKEFSFKNEETAQVVR.K	

Detailed Protein Report

Protein 162: retinal dehydrogenase 1 [Homo sapiens]

**Accession:**	gi|21361176	**Score:**	36.7
**Database:**	refseq_human(refseq_human_20140103.fasta)	**MW [kDa]:**	54.8
**Database Date:**	2015-11-30	**pI:**	6.3
**Modification(s):**	Carbamidomethyl	**Sequence Coverage [%]:**	6.0
		**No. of unique Peptides:**	1

10	20	30	40	50	60	70	80
MSSSGTPDLP	VLLTDLKIQY	TKIFINNEWH	DSVSGKKFPV	FNPATEEELC	QVEEGDKEDV	DKAVKAARQA	FQIGSPWRTM
**90**	**100**	**110**	**120**	**130**	**140**	**150**	**160**
DASERGRLLY	KLADLIERDR	LLLATMESMN	GGKLYSNAYL	NDLAGCIKTL	RYCAGWADKI	QGRTIPIDGN	FFTYTRHEPI
**170**	**180**	**190**	**200**	**210**	**220**	**230**	**240**
GVCGQIIPWN	FPLVMLIWKI	GPALSCGNTV	VVKPAEQTPL	TALHVASLIK	EAGFPPGVVN	IVPGYGPTAG	AAISSHMDID
**250**	**260**	**270**	**280**	**290**	**300**	**310**	**320**
KVAFTGSTEV	GKLIKEAAGK	SNLKRVTLEL	GGKSPCIVLA	DADLDNAVEF	AHHGVFYHQG	QCCIAASRIF	VEESIYDEFV
**330**	**340**	**350**	**360**	**370**	**380**	**390**	**400**
RRSVERAKKY	ILGNPLTPGV	TQGPQIDKEQ	YDKILDLIES	GKKEGAKLEC	GGGPWGNKGY	FVQPTVFSNV	TDEMRIAKEE
**410**	**420**	**430**	**440**	**450**	**460**	**470**	**480**
IFGPVQQIMK	FKSLDDVIKR	ANNTFYGLSA	GVFTKDIDKA	ITISSALQAG	TVWVNCYGVV	SAQCPFGGFK	MSGNGRELGE
**490**	**500**	**510**					
YGFHEYTEVK	TVTVKISQKN	S					

Cmpd.	No. of Cmpds.	m/z meas.	Δ m/z [ppm]	z	Rt [min]	Score	P	Range	Sequence	Modification
499	1	587.8106	70.58	2	34.9	13.0	0	368-378	K.LECGGGPWGNK.G	Carbamidomethyl: 3

BRUKER

Detailed Protein Report

Protein 163: **PREDICTED: inositol 1,4,5-trisphosphate receptor type 2 isoform X1 [Homo sapiens]**

**Accession:**	gi|578822836	**Score:**	36.6
**Database:**	refseq_human(refseq_human_20140103.fasta)	**MW [kDa]:**	212.9
**Database Date:**	2015-11-30	**pI:**	6.3
		**Sequence Coverage [%]:**	1.8
		**No. of unique Peptides:**	2

10	20	30	40	50	60	70	80
MTEKMSSFLY	IGDIVSLYAE	GSVNGFISTL	GLVDDRCVVH	PEAGDLANPP	KKFRDCLFKV	CPMNRYSAQK	QYWKAKQAKQ
90	100	110	120	130	140	150	160
GNHTEAALLK	KLQHAAELEQ	KQNESENKKL	LGEIVKYSNV	IQLLHIKSNK	YLTVNKRLPA	LLEKNAMRVS	LDAAGNEGSW
170	180	190	200	210	220	230	240
FYIHPFWKLR	SEGDNIVVGD	KVVLMPVNAG	QPLHASNIEL	LDNPGCKEVN	AVNCNTSWKI	TLFMKYSSYR	EDVLKGGDVV
250	260	270	280	290	300	310	320
RLFHAEQEKF	LTCDEYEKKQ	HIFLRTTLRQ	SATSATSSKA	LWEIEVVHHD	PCRGGAGQWN	SLFRFKHLAT	GNYLAAELNP
330	340	350	360	370	380	390	400
DYRDAQNEGK	NVRDGVPPTS	KKKRQAGEKI	MYTLVSVPHG	NDIASLFELD	ATTLQRADCL	VPRNSYVRLR	HLCTNTWVTS
410	420	430	440	450	460	470	480
TSIPIDTDEE	RPVMLKIGTC	QTKEDKEAFA	IVSVPLSEVR	DLDFANDANK	VLATTVKKLE	NGTITQNERR	FVTKLLEDLI
490	500	510	520	530	540	550	560
FFVADVPNNG	QEVLDVVITK	PNRERQKLMR	EQNILAQVFG	ILKAPFKEKA	GEGSMLRLED	LGDQRYAPYK	YMLRLCYRVL
570	580	590	600	610	620	630	640
RHSQQDYRKN	QEYIAKNFCV	MQSQIGYDIL	AEDTITALLH	NNRKLLEKHI	TAKEIETFVS	LLRRNREPRF	LDYLSDLCVS
650	660	670	680	690	700	710	720
NTTAIPVTQE	LICKFMLSPG	NADILIQTKV	VSMQADNPME	SSILSDDIDD	EEVWLYWIDS	NKEPHGKAIR	HLAQEAKEGT
730	740	750	760	770	780	790	800
KADLEVLTYY	RYQLNLFARM	CLDRQYLAIN	QISTQLSVDL	ILRCVSDESL	PFDLRASFCR	LMLHMHVDRD	PQESVVPVRY
810	820	830	840	850	860	870	880
ARLWTEIPTK	ITIHEYDSIT	DSSRNDMKRK	FALTMEFVEE	YLKEVVNQPF	PFGDKEKNKL	TFEVVHLARN	LIYFGFYSFS
890	900	910	920	930	940	950	960
ELLRLTRTLL	AILDIVQAPM	SSYFERLSKF	QDGGNNVMRT	IHGVGEMMTQ	MVLSRGSIFP	MSVPDVPPSI	HPSKQGSPTE
970	980	990	1000	1010	1020	1030	1040
HEDVTVMDTK	LKIIEILQFI	LSVRLDYRIS	YMLSIYKKEF	GEDNDNAETS	ASGSPDTLLP	SAIVPDIDEI	AAQAETMFAG
1050	1060	1070	1080	1090	1100	1110	1120
RKEKNPVQLD	DEGGRTFLRV	LIHLIMHDYP	PLLSGALQLL	FKHFSQRAEV	LQAFKQVQLL	VSNQDVDNYK	QIKADLDQLR
1130	1140	1150	1160	1170	1180	1190	1200
LTVEKSELWV	EKSSNYENGE	IGESQVKGGE	EPIEESNILS	PVQDGTKKPQ	IDSNKSNNYR	IVKEILIRLS	KLCVQNKKCR
1210	1220	1230	1240	1250	1260	1270	1280
NQHQRLLKNM	GAHSVVLDLL	QIPYEKNDEK	MNEVMNLAHT	FLQNFCRGNP	QNQVLLHKHL	NLFLTPGLLE	AETMRHIFMN
1290	1300	1310	1320	1330	1340	1350	1360
NYHLCNEISE	RVVQHFVHCI	ETHGRHVEYL	RFLQTIVKAD	GKYVKKCQDM	VMTELINGGE	DVLIFYNDRA	SFPILLHMMC
1370	1380	1390	1400	1410	1420	1430	1440
SERDRGDESG	PLAYHITLVE	LLAACTEGKN	VYTEIKCNSL	LPLDDIVRVV	THDDCIPEVK	IAYVNFVNHC	YVDTEVEMKE
1450	1460	1470	1480	1490	1500	1510	1520
IYTSNHIWKL	FENFLVDMAR	VCNTTTDRKH	ADIFLEKCVT	ESIMNIVSGF	FNSPFSDNST	SLQTHQPVFI	QLLQSAFRIY
1530	1540	1550	1560	1570	1580	1590	1600
NCTWPNPAQK	ASVESCIRTL	AEVAKNRGIA	IPVDLDSQVN	TLFMKSHSNM	VQRAAMGWRL	SARSGPRFKE	ALGGPAWDYR
1610	1620	1630	1640	1650	1660	1670	1680
NIIEKLQDVV	ASLEHQFSPM	MQAEFSVLVD	VLYSPELLFP	EGSDARIRCG	AFMSKLINHT	KKLMEKEEKL	CIKILQTLRE
1690	1700	1710	1720	1730	1740	1750	1760
MLEKKDSFVE	EGNTLRKILL	NRYFKGDYSI	GVNGHLSGAY	SKTAQVGGSF	SGQDSDKMGI	SMSDIQCLLD	KEGASELVID
1770	1780	1790	1800	1810	1820	1830	1840
VIVNTKNDRI	FSEGIFLGIA	LLEGGNTQTQ	YSFYQQLHEQ	KKSEKFFKVL	YDRMKAAQKE	IRSTVTVNTI	DLGNKKRDDD
1850	1860	1870					
NELMTSGPRM	REFWSWGDPK	TALQD					

Detailed Protein Report

Cmpd.	No. of Cmpds.	m/z meas.	Δ m/z [ppm]	z	Rt [min]	Score	P	Range	Sequence	Modification
725	1	771.2336	-342.03	1	39.0	11.8	0	110-116	K.LLGEIVK.Y	
1690	6	648.2368	-188.91	2	51.4	14.8	2	1199-1208	K.CRNQHQRLLK.N	

Detailed Protein Report

Protein 164: myeloid zinc finger 1 isoform 1 [Homo sapiens]

**Accession:**	gi|37622341	**Score:**	36.5
**Database:**	refseq_human(refseq_human_20140103.fasta)	**MW [kDa]:**	82.0
**Database Date:**	2015-11-30	**pI:**	10.3
**Modification(s):**	Carbamidomethyl	**Sequence Coverage [%]:**	7.2
		**No. of unique Peptides:**	2

Alias proteins:

Accession	Name	Description
gi|578834840	refseq_human (refseq_human_20140103.fasta)	PREDICTED: myeloid zinc finger 1 isoform X5 [Homo sapiens]
gi|578834838	refseq_human (refseq_human_20140103.fasta)	PREDICTED: myeloid zinc finger 1 isoform X4 [Homo sapiens]
gi|37622345	refseq_human (refseq_human_20140103.fasta)	myeloid zinc finger 1 isoform 1 [Homo sapiens]

```
        10         20         30         40         50         60         70         80
MRPAVLGSPD RAPPEDEGPV MVKLEDSEEE GEAALWDPGP EAARLRFRCF RYEEATGPQE ALAQLRELCR QWLRPEVRSK
        90        100        110        120        130        140        150        160
EQMLELLVLE QFLGALPPEI QARVQGQRPG SPEEAAALVD GLRREPGGPR RWVTVQVQGQ EVLSEKMEPS SFQPLPETEP
       170        180        190        200        210        220        230        240
PTPEPGPKTP PRTMQESPLG LQVKEESEVT EDSDFLESGP LAATQESVPT LLPEEAQRCG TVLDQIFPHS KTGPEGPSWR
       250        260        270        280        290        300        310        320
EHPRALWHEE AGGIFSPGFA LQLGSISAGP GSVSPHLHVP WDLGMAGLSG QIQSPSREGG FAHALLLPSD LRSEQDPTDE
       330        340        350        360        370        380        390        400
DPCRGVGPAL ITTRWRSPRG RSRGRPSTGG GVVRGGRCDV CGKVFSQRSN LLRHQKIHTG ERPFVCSECG RSFSRSSHLL
       410        420        430        440        450        460        470        480
RHQLTHTEER PFVCGDCGQG FVRSARLEEH RRVHTGEQPF RCAECGQSFR QRSNLLQHQR IHGDPPGPGA KPPAPPGAPE
       490        500        510        520        530        540        550        560
PPGPFPCSEC RESFARRAVL LEHQAVHTGD KSFGCVECGE RFGRRSVLLQ HRRVHSGERP FACAECGQSF RQRSNLTQHR
       570        580        590        600        610        620        630        640
RIHTGERPFA CAECGKAFRQ RPTLTQHLRV HTGEKPFACP ECGQRFSQRL KLTRHQRTHT GEKPYHCGEC GLGFTQVSRL
       650        660        670        680        690        700        710        720
TEHQRIHTGE RPFACPECGQ SFRQHANLTQ HRRIHTGERP YACPECGKAF RQRPTLTQHL RTHRREKPFA CQDCGRRFHQ
       730        740
STKLIQHQRV HSAE
```

Cmpd.	No. of Cmpds.	m/z meas.	Δ m/z [ppm]	z	Rt [min]	Score	P	Range	Sequence	Modification
254	1	977.7698	-46.68	3	31.9	12.1	2	219-244	R.CGTVLDQIFPHSKTGPEGPSWREHPR.A	
2819	1	937.4520	9.17	2	64.4	12.8	1	673-688	R.RIHTGERPYACPECGK.A	Carbamidomethyl: 11

Detailed Protein Report

Protein 165: DNA-directed RNA polymerase, mitochondrial precursor [Homo sapiens]

Accession: gi|110618253
Database: refseq_human(refseq_human_20140103.fasta)
Database Date: 2015-11-30
Modification(s): Oxidation

Score: 36.5
MW [kDa]: 138.5
pI: 10.1
Sequence Coverage [%]: 2.2
No. of unique Peptides: 1

Quantitation

QD:QU **Median:** 0.69 **CV:** 0.00 % **No. of Peptides:** 1

10	20	30	40	50	60	70	80
MSALCWGRGA	AGLKRALRPC	GRPGLPGKEG	TAGGVCGPRR	SSSASPQEQD	QDRRKDWGHV	ELLEVLQARV	RQLQAESVSE
**90**	**100**	**110**	**120**	**130**	**140**	**150**	**160**
VVVNRVDVAR	LPECGSGDGS	LQPPRKVQMG	AKDATPVPCG	RWAKILEKDK	RTQQMRMQRL	KAKLQMPFQS	GEFKALTRRL
**170**	**180**	**190**	**200**	**210**	**220**	**230**	**240**
QVEPRLLSKQ	MAGCLEDCTR	QAPESPWEEQ	LARLLQEAPG	KLSLDVEQAP	SGQHSQAQLS	GQQQRLLAFF	KCCLLTDQLP
**250**	**260**	**270**	**280**	**290**	**300**	**310**	**320**
LAHHLLVVHH	GQRQKRKLLT	LDMYNAVMLG	WARQGAFKEL	VYVLFMVKDA	GLTPDLLSYA	AALQCMGRQD	QDAGTIERCL
**330**	**340**	**350**	**360**	**370**	**380**	**390**	**400**
EQMSQEGLKL	QALFTAVLLS	EEDRATVLKA	VHKVKPTFSL	PPQLPPPVNT	SKLLRDVYAK	DGRVSYPKLH	LPLKTLQCLF
**410**	**420**	**430**	**440**	**450**	**460**	**470**	**480**
EKQLHMELAS	RVCVVSVEKP	TLPSKEVKHA	RKTLKTLRDQ	WEKALCRALR	ETKNRLEREV	YEGRFSLYPF	LCLLDEREVV
**490**	**500**	**510**	**520**	**530**	**540**	**550**	**560**
RMLLQVLQAL	PAQGESFTTL	ARELSARTFS	RHVVQRQRVS	GQVQALQNHY	RKYLCLLASD	AEVPEPCLPR	QYWEELGAPE
**570**	**580**	**590**	**600**	**610**	**620**	**630**	**640**
ALREQPWPLP	VQMELGKLLA	EMLVQATQMP	CSLDKPHRSS	RLVPVLYHVY	SFRNVQQIGI	LKPHPAYVQL	LEKAAEPTLT
**650**	**660**	**670**	**680**	**690**	**700**	**710**	**720**
FEAVDVPMLC	PPLPWTSPHS	GAFLLSPTKL	MRTVEGATQH	QELLETCPPT	ALHGALDALT	QLGNCAWRVN	GRVLDLVLQL
**730**	**740**	**750**	**760**	**770**	**780**	**790**	**800**
FQAKGCPQLG	VPAPPSEAPQ	PPEAHLPHSA	APARKAELRR	ELAHCQKVAR	EMHSLRAEAL	YRLSLAQHLR	DRVFWLPHNM
**810**	**820**	**830**	**840**	**850**	**860**	**870**	**880**
DFRGRTYPCP	PHFNHLGSDV	ARALLEFAQG	RPLGPHGLDW	LKIHLVNLTG	LKKREPLRKR	LAFAEEVMDD	ILDSADQPLT
**890**	**900**	**910**	**920**	**930**	**940**	**950**	**960**
GRKWWMGAEE	PWQTLACCME	VANAVRASDP	AAYVSHLPVH	QDGSCNGLQH	YAALGRDSVG	AASVNLEPSD	VPQDVYSGVA
**970**	**980**	**990**	**1000**	**1010**	**1020**	**1030**	**1040**
AQVEVFRRQD	AQRGMRVAQV	LEGFITRKVV	KQTVMTVVYG	VTRYGGRLQI	EKRLRELSDF	PQEFVWEASH	YLVRQVFKSL
**1050**	**1060**	**1070**	**1080**	**1090**	**1100**	**1110**	**1120**
QEMFSGTRAI	QHWLTESARL	ISHMGSVVEW	VTPLGVPVIQ	PYRLDSKVKQ	IGGGIQSITY	THNGDISRKP	NTRKQKNGFP
**1130**	**1140**	**1150**	**1160**	**1170**	**1180**	**1190**	**1200**
PNFIHSLDSS	HMMLTALHCY	RKGLTFVSVH	DCYWTHAADV	SVMNQVCREQ	FVRLHSEPIL	QDLSRFLVKR	FCSEPQKILE
**1210**	**1220**	**1230**	**1240**				
ASQLKETLQA	VPKPGAFDLE	QVKRSTYFFS					

Cmpd.	No. of Cmpds.	m/z meas.	Δ m/z [ppm]	z	Rt [min]	Score	P	Range	Sequence	Modification	Ratios	
390	1	685.3636	4.04	2	34.8	10.5	0	992-1003	K.QTVMTVVYGVTR.Y	Oxidation: 4	QD:QU	0.69

Detailed Protein Report

Protein 166: pleckstrin homology domain-containing family G member 4B [Homo sapiens]

**Accession:**	gi|148529025	**Score:**	36.5
**Database:**	refseq_human(refseq_human_20140103.fasta)	**MW [kDa]:**	139.6
**Database Date:**	2015-11-30	**pI:**	6.3
**Modification(s):**	Carbamidomethyl	**Sequence Coverage [%]:**	2.0
		**No. of unique Peptides:**	2

Quantitation

QD:QU **Median:** 1.10 **CV:** 0.00 % **No. of Peptides:** 1

10	20	30	40	50	60	70	80
MEALRNPMPL	GSSEEALGDL	ACSSLTGASR	DLGTGAVASG	TQEETSGPRG	DPQQTPSLEK	ERHTPSRTGP	GAAGRTLPRR
**90**	**100**	**110**	**120**	**130**	**140**	**150**	**160**
SRSWERAPRS	SRGAQAAACH	TSHHSAGSRP	GGHLGGQAVG	TPNCVPVEGP	GCTKEEDVLA	SSACVSTDGG	SLHCHNPSGP
**170**	**180**	**190**	**200**	**210**	**220**	**230**	**240**
SDVPARQPHP	EQEGWPPGTG	DFPSQVPKQV	LDVSQELLQS	GVVTLPGTRD	RHGRAVVQVR	TRSLLWTREH	SSCAELTRLL
**250**	**260**	**270**	**280**	**290**	**300**	**310**	**320**
LYFHSIPRKE	VRDLGLVVLV	DARRSPAAPA	VSQALSGLQN	NTSPIIHSIL	LLVDKESAFR	PDKDAIIQCE	VVSSLKAVHK
**330**	**340**	**350**	**360**	**370**	**380**	**390**	**400**
FVDSCQLTAD	LDGSFPYSHG	DWICFRQRLE	HFAANCEEAI	IFLQNSFCSL	NTHRTPRTAQ	EVAELIDQHE	TMMKLVLEDP
**410**	**420**	**430**	**440**	**450**	**460**	**470**	**480**
LLVSLRLEGG	TVLARLRREE	LGTEDSRDTL	EAATSLYDRV	DEEVHRLVLT	SNNRLQQLEH	LRELASLLEG	NDQQSCQKGL
**490**	**500**	**510**	**520**	**530**	**540**	**550**	**560**
QLAKENPQRT	EEMVQDFRRG	LSAVVSQAEC	REGELARWTR	SSELCETVSS	WMGPLDPEAC	PSSPVAECLR	SCHQEATSVA
**570**	**580**	**590**	**600**	**610**	**620**	**630**	**640**
AEAFPGAGVA	VLKPHALGKP	WASQQDLWLQ	YPQTRLRLEE	ALSEAAPDPS	LPPLAQSPPK	HERAQEAMRR	HQKPPSFPST
**650**	**660**	**670**	**680**	**690**	**700**	**710**	**720**
DSGGGAWEPA	QPLSGLPGRA	LLCGQDGETL	RPGLCALWDP	LSLLRGLPGA	GATTAHLEDS	SACSSEPTQT	LASRPRKHPQ
**730**	**740**	**750**	**760**	**770**	**780**	**790**	**800**
KKMIKKTQSF	EIPQPDSGPR	DSCQPDHTSV	FSKGLEVTST	VATEKKLPLW	QHARSPPVTQ	SRSLSSPSGL	HPAEEDGRQQ
**810**	**820**	**830**	**840**	**850**	**860**	**870**	**880**
VGSSRLRHIM	AEMIATEREY	IRCLGYVIDN	YFPEMERMDL	PQGLRGKHHV	IFGNLEKLHD	FHQQHFLREL	ERCQHCPLAV
**890**	**900**	**910**	**920**	**930**	**940**	**950**	**960**
GRSFLRHEEQ	FGMYVIYSKN	KPQSDALLSS	HGNAFFKDKQ	RELGDKMDLA	SYLLRPVQRV	AKYALLLQDL	LKEASCGLAQ
**970**	**980**	**990**	**1000**	**1010**	**1020**	**1030**	**1040**
GQELGELRAA	EVVVCFQLRH	GNDLLAMDAI	RGCDVNLKEQ	GQLRCRDEFI	VCCGRKKYLR	HVFLFEDLIL	FSKTQKVEGS
**1050**	**1060**	**1070**	**1080**	**1090**	**1100**	**1110**	**1120**
HDVYLYKQSF	KTAEIGMTEN	VGDSGLRFEI	WFRRRRKSQD	TYILQASSAE	VKSAWTDVIG	RILWRQALKS	RELRIQEMAS
**1130**	**1140**	**1150**	**1160**	**1170**	**1180**	**1190**	**1200**
MGIGNQPFMD	VKPRDRTPDC	AVISDRAPKC	AVMSDRVPDS	IVKGTESQMR	GSTAVSSSDH	AAPFKRPHST	ISDSSTSSSS
**1210**	**1220**	**1230**	**1240**	**1250**	**1260**	**1270**	**1280**
SQSSSILGSL	GLLVSSSPAH	PGLWSPAHSP	WSSDIRACVE	EDEPEPELET	GTQAAVCEGA	PAVLLSRTRQ	A

Cmpd.	No. of Cmpds.	m/z meas.	Δ m/z [ppm]	z	Rt [min]	Score	P	Range	Sequence	Modification	Ratios
373	2	627.3450	-10.69	2	34.6	17.1	1	479-489	K.GLQLAKENPQR.T		
2311	4	953.4263	-3.01	2	57.6	19.4	0	823-837	R.CLGYVIDNYFPEMER.M	Carbamidomethyl: 1	QD:QU 1.10

Detailed Protein Report

Protein 167: **PREDICTED: rho guanine nucleotide exchange factor 18 isoform X6 [Homo sapiens]**

**Accession:**	gi|578833130	**Score:**	36.1
**Database:**	refseq_human(refseq_human_20140103.fasta)	**MW [kDa]:**	149.7
**Database Date:**	2015-11-30	**pI:**	6.1
		**Sequence Coverage [%]:**	3.0
		**No. of unique Peptides:**	3

10	20	30	40	50	60	70	80
MEDLSLDLGA	LQGSEYLQDL	GLGAPSHSQP	GETPDSRPTG	EEPGRDSLFS	SLAGSQDLSR	RRSWERSRSC	SESWRRLSLD
**90**	**100**	**110**	**120**	**130**	**140**	**150**	**160**
ASAVDEEPCL	PRTLASLALN	LPGGGLKTWT	QGCLSGGGTP	AESPGKECDS	PKKRGRSRSV	PVSFYEIRSP	EISPGLEVPT
**170**	**180**	**190**	**200**	**210**	**220**	**230**	**240**
PPVQGLEPPV	LECMEKDHVE	PDHVLIVQQV	LQELRQYHGA	RQRACMSASP	GGAHSNLTWF	EFLSESEDGA	GKNEKSDKST
**250**	**260**	**270**	**280**	**290**	**300**	**310**	**320**
SVKRRLSCLR	SRVTRQKEKG	KSPAHLKDKG	QDARERRECV	NGHQLLQGTF	SGPSSCPLCG	KPFLSSASLK	EHPRGTLLSD
**330**	**340**	**350**	**360**	**370**	**380**	**390**	**400**
GSPALSRNVG	MTVSQKGGPQ	PTPSPAGPGT	QLGPITGEMD	EADSAFLKFK	QTADDSLSLT	SPNTESIFVE	DPYTASLRSE
**410**	**420**	**430**	**440**	**450**	**460**	**470**	**480**
IESDGHEFEA	ESWSLAVDAA	YAKKQKREVV	KRQDVLYELM	QTEVHHVRTL	KIMLKVYSRA	LQEELQFSSK	AIGRLFPCAD
**490**	**500**	**510**	**520**	**530**	**540**	**550**	**560**
DLLETHSHFL	ARLKERRQES	LEEGSDRNYV	IQKIGDLLVQ	QFSGENGERM	KEKYGVFCSG	HNEAVSHYKL	LLQQNKKFQN
**570**	**580**	**590**	**600**	**610**	**620**	**630**	**640**
LIKKIGNFSI	VRRLGVQECI	LLVTQRITKY	PVLVERIIQN	TEAGTEDYED	LTQALNLIKD	IISQVDAKVS	ECEKGQRLRE
**650**	**660**	**670**	**680**	**690**	**700**	**710**	**720**
IAGKMDLKSS	SKLKNGLTFR	KEDMLQRQLH	LEGMLCWKTT	SGRLKDILAI	LLTDVLLLLQ	EKDQKYVFAS	VDSKPPVISL
**730**	**740**	**750**	**760**	**770**	**780**	**790**	**800**
QKLIVREVAN	EEKAMFLISA	SLQGPEMYEI	YTSSKEDRNA	WMAHIQRAVE	SCPDEEEGPF	SLPEEERKVV	EARATRLRDF
**810**	**820**	**830**	**840**	**850**	**860**	**870**	**880**
QERLSMKDQL	IAQSLLEKQQ	IYLEMAEMGG	LEDLPQPRGL	FRGGDPSETL	QGELILKSAM	SEIEGIQSLI	CRQLGSANGQ
**890**	**900**	**910**	**920**	**930**	**940**	**950**	**960**
AEDGGSSTGP	PRRAETFAGY	DCTNSPTKNG	SFKKKVSSTD	PRPRDWRGPP	NSPDLKLSDS	DIPGSSEESP	QVVEAPGTES
**970**	**980**	**990**	**1000**	**1010**	**1020**	**1030**	**1040**
DPRLPTVLES	ELVQRIQTLS	QLLLNLQAVI	AHQDSYVETQ	RAAIQEREKQ	FRLQSTRGNL	LLEQERQRNF	EKQREERAAL
**1050**	**1060**	**1070**	**1080**	**1090**	**1100**	**1110**	**1120**
EKLQSQLRHE	QQRWERERQW	QHQELERAGA	RLQEREGEAR	QLRERLEQER	AELERQRQAY	QHDLERLREA	QRAVERERER
**1130**	**1140**	**1150**	**1160**	**1170**	**1180**	**1190**	**1200**
LELLRRLKKQ	NTAPGALPPD	TLAEAQPPSH	PPSFNGEGLE	GPRVSMLPSG	VGPEYAERPE	VARRDSAPTE	NRLAKSDVPI
**1210**	**1220**	**1230**	**1240**	**1250**	**1260**	**1270**	**1280**
QLLSATNQFQ	RQAAVQQQIP	TKLAASTKGG	KDKGGKSRGS	QRWESSASFD	LKQQLLLNKL	MGKDESTSRN	RRSLSPILPG
**1290**	**1300**	**1310**	**1320**	**1330**	**1340**	**1350**	
RHSPAPPPDP	GFPAPSPPPA	DSPSEGFSLK	AGGTALLPGP	PAPSPLPATP	LSAKEDASKE	DVIFF	

Cmpd.	No. of Cmpds.	m/z meas.	Δ m/z [ppm]	z	Rt [min]	Score	P	Range	Sequence	Modification
1663	1	720.3283	-79.69	2	51.1	10.6	1	137-148	R.SRSVPVSFYEIR.S	
2777	1	899.4794	83.49	2	65.9	15.1	0	534-549	K.YGVFCSGHNEAVSHYK.L	
1380	1	683.3455	-90.52	2	45.8	10.4	2	1270-1281	R.NRRSLSPILPGR.H	

Detailed Protein Report

Protein 168: **PREDICTED: neuroblastoma breakpoint family member 6 isoform X2 [Homo sapiens]**

**Accession:**	gi|530363207	**Score:**	36.1
**Database:**	refseq_human(refseq_human_20140103.fasta)	**MW [kDa]:**	80.3
**Database Date:**	2015-11-30	**pI:**	4.8
**Modification(s):**	Oxidation	**Sequence Coverage [%]:**	4.5
		**No. of unique Peptides:**	1

10	20	30	40	50	60	70	80
MTPPPSNSVI	ATADRYLVHW	LLPPSLLSES	ELQAVPSALS	SGIPVPVFAV	KDSKVLCRVH	QLFLNLPGST	SSATNVSMVV
**90**	**100**	**110**	**120**	**130**	**140**	**150**	**160**
SADPLSSERA	EMNILEINQE	LRSQLAESNQ	QFRDLKEKFL	ITQATAYSLA	NQLKKYKCEE	YKDIIDSVLR	DELQSMEKLA
**170**	**180**	**190**	**200**	**210**	**220**	**230**	**240**
EKLRQAEELR	QYKALVHSQA	KELTQLREKL	REGRDASRWL	NKHLKTLLTP	DDPDKSQGQD	LREQLAEGHR	LAEHLVHKLS
**250**	**260**	**270**	**280**	**290**	**300**	**310**	**320**
PENDEDEDED	EDDKDEEVEK	VQESPAPREV	QKTEEKEVPQ	DSLEECAVTC	SNSHNPSNSN	QPHRSTKITF	KEHEVDSALV
**330**	**340**	**350**	**360**	**370**	**380**	**390**	**400**
VESEHPHDEE	EEALNIPPEN	QNDHEEEEGK	APVPPRHHDK	SNSYRHREVS	FLALDEQKVC	SAQDVARDYS	NPKWDETSLG
**410**	**420**	**430**	**440**	**450**	**460**	**470**	**480**
FLEKQSDLEE	VKGQETVAPR	LSRGPLRVDK	HEIPQESLDG	CCLTPSILPD	LTPSYHPYWS	TLYSFEDKQV	SLALVDKIKK
**490**	**500**	**510**	**520**	**530**	**540**	**550**	**560**
DQEEIEDQSP	PCPRLSQELP	EVKEQEVPED	SVNEVYLTPS	VHHDVSDCHQ	PYSSTLSSLE	DQLACSALDV	ASPTEAACPQ
**570**	**580**	**590**	**600**	**610**	**620**	**630**	**640**
GTWSGDLSHH	RSEVQISQAQ	LEPSTLVPSC	LRLQLDQGFH	CGNGLAQRGL	SSTTCSFSAN	ADSGNQWPFQ	ELVLEPSLGM
**650**	**660**	**670**	**680**	**690**	**700**	**710**	**720**
KNPPQLEDDA	LEGSASNTQG	RQVTGRIRAS	LVLILKTIRR	RLPFSKWRLA	FRFAGPHAES	AEIPNTAERM	QRMIG

Cmpd.	No. of Cmpds.	m/z meas.	Δ m/z [ppm]	z	Rt [min]	Score	P	Range	Sequence	Modification
1180	1	384.1589	-56.37	2	44.9	12.5	1	710-715	R.MQRMIG.-	Oxidation: 1, 4

Detailed Protein Report

Protein 169: F-box only protein 41 [Homo sapiens]

**Accession:**	gi|289803020	**Score:**	35.7
**Database:**	refseq_human(refseq_human_20140103.fasta)	**MW [kDa]:**	94.4
**Database Date:**	2015-11-30	**pI:**	9.8
**Modification(s):**	Carbamidomethyl	**Sequence Coverage [%]:**	3.7
		**No. of unique Peptides:**	1

Alias proteins:

Accession	Name	Description
gi|578802734	refseq_human (refseq_human_20140103.fasta)	PREDICTED: F-box only protein 41 isoform X2 [Homo sapiens]

10	20	30	40	50	60	70	80
MASLDLPYRC	PRCGEHKRFR	SLSSLRAHLE	YSHTYETLYI	LSKTNSICDG	AAAAAAAAAA	ASGFPLAPEP	AALLAVPGAR
**90**	**100**	**110**	**120**	**130**	**140**	**150**	**160**
REVFESTSFQ	GKEQAAGPSP	AAPHLLHHHH	HHAPLAHFPG	DLVPASLPCE	ELAEPGLVPA	AAARYALREI	EIPLGELFAR
**170**	**180**	**190**	**200**	**210**	**220**	**230**	**240**
KSVASSACST	PPPGPGPGPC	PGPASASPAS	PSPADVAYEE	GLARLKIRAL	EKLEVDRRLE	RLSEEVEQKI	AGQVGRLQAE
**250**	**260**	**270**	**280**	**290**	**300**	**310**	**320**
LERKAAELET	ARQESARLGR	EKEELEERAS	ELSRQVDVSV	ELLASLKQDL	VHKEQELSRK	QQEVVQIDQF	LKETAAREAS
**330**	**340**	**350**	**360**	**370**	**380**	**390**	**400**
AKLRLQQFIE	ELLERADRAE	RQLQVISSSC	GSTPSASLGR	GGGGGGAGPN	ARGPGRMREH	HVGPAVPNTY	AVSRHGSSPS
**410**	**420**	**430**	**440**	**450**	**460**	**470**	**480**
TGASSRVPAA	SQSSGCYDSD	SLELPRPEEG	APEDSGPGGL	GTRAQAANGG	SERSQPPRSS	GLRRQAIQNW	QRRPRRHSTE
**490**	**500**	**510**	**520**	**530**	**540**	**550**	**560**
GEEGDVSDVG	SRTTESEAEG	PLDAPRPGPA	MAGPLSSCRL	SARPEGGSGR	GRRAERVSPS	RSNEVISPEI	LKMRAALFCI
**570**	**580**	**590**	**600**	**610**	**620**	**630**	**640**
FTYLDTRTLL	HAAEVCRDWR	FVARHPAVWT	RVLLENARVC	SKFLAMLAQW	CTQAHSLTLQ	NLKPRQRGKK	ESKEEYARST
**650**	**660**	**670**	**680**	**690**	**700**	**710**	**720**
RGCLEAGLES	LLKAAGGNLL	ILRISHCPNI	LTDRSLWLAS	CYCRALQAVT	YRSATDPVGH	EVIWALGAGC	REIVSLQVAP
**730**	**740**	**750**	**760**	**770**	**780**	**790**	**800**
LHPCQQPTRF	SNRCLQMIGR	CWPHLRALGV	GGAGCGVQGL	ASLARNCMRL	QVLELDHVSE	ITQEVAAEVC	REGLKGLEML
**810**	**820**	**830**	**840**	**850**	**860**	**870**	**880**
VLTATPVTPK	ALLHFNSICR	NLKSIVVQIG	IADYFKEPSS	PEAQKLFEDM	VTKLQALRRR	PGFSKILHIK	VEGGC

Cmpd.	No. of Cmpds.	m/z meas.	Δ m/z [ppm]	z	Rt [min]	Score	P	Range	Sequence	Modification
2879	2	813.9356	28.83	2	65.2	12.4	1	568-580	R.TLLHAAEVCRDWR.F	Carbamidomethyl: 9

Detailed Protein Report

Protein 170: ral GTPase-activating protein subunit alpha-2 [Homo sapiens]

**Accession:**	gi|118600961	**Score:**	35.6
**Database:**	refseq_human(refseq_human_20140103.fasta)	**MW [kDa]:**	210.6
**Database Date:**	2015-11-30	**pI:**	5.7
**Modification(s):**	Oxidation	**Sequence Coverage [%]:**	2.3
		**No. of unique Peptides:**	1

10	20	30	40	50	60	70	80
MFSRRSHGDV	KKSTQKVLDP	KKDVLTRLKH	LRALLDNVDA	NDLKQFFETN	YSQIYFIFYE	NFIALENSLK	LKGNNKSQRE
90	100	110	120	130	140	150	160
ELDSILFLFE	KILQFLPERI	FFRWHYQSIG	STLKKLLHTG	NSIKIRCEGI	RLFLLWLQAL	QTNCAEEQVL	IFACLVPGFP
170	180	190	200	210	220	230	240
AVMSSRGPCT	LETLINPSPS	VADVKIYPEE	ITPLLPAISG	EKIAEDQTCF	FLQILLKYMV	IQAASLEWKN	KENQDTGFKF
250	260	270	280	290	300	310	320
LFTLFRKYYL	PHLFPSFTKL	TNIYKPVLDI	PHLRPKPVYI	TTTRDNENIY	STKIPYMAAR	VVFIKWIVTF	FLEKKYLTAT
330	340	350	360	370	380	390	400
QNTKNGVDVL	PKIIQTVGGG	AVQERAPELD	GGGPTEQDKS	HSNSSTLSDR	RLSNSSLCSI	EEEHRMVYEM	VQRILLSTRG
410	420	430	440	450	460	470	480
YVNFVNEVFH	QAFLLPSCEI	AVTRKVVQVY	RKWILQDKPV	FMEEPDRKDV	AQEDAEKLGF	SETDSKEASS	ESSGHKRSSS
490	500	510	520	530	540	550	560
WGRTYSFTSA	MSRGCVTEEE	NTNVKAGVQA	LLQVFLTNSA	NIFLLEPCAE	VPVLLKEQVD	ACKAVLIIFR	RMIMELTMNK
570	580	590	600	610	620	630	640
KTWEQMLQIL	LRITEAVMQK	PKDKQIKDLF	AQSLAGLLFR	TLMVAWIRAN	LCVYISRELW	DDFLGVLSSL	TEWEELINEW
650	660	670	680	690	700	710	720
ANIMDSLTAV	LARTVYGVEM	TNLPLDKLSE	QKEKKQRGKG	CVLDPQKGTT	VGRSFSLSWR	SHPDVTEPMR	FRSATTSGAP
730	740	750	760	770	780	790	800
GVEKARNIVR	QKATEVEECQ	QSENAPAAGS	GHLTVGQQQQ	VLRSSSTSDI	PEPLCSDSSQ	GQKAENTQNS	SSSEPQPIQE
810	820	830	840	850	860	870	880
NKGHVKREHE	GITILVRRSS	SPAELDLKDD	LQQTQGKCRE	RQKSESTNSD	TTLGCTNEAE	LSMGPWQTCE	EDPELNTPTD
890	900	910	920	930	940	950	960
VVADADARHW	LQLSPTDASN	LTDSSECLTD	DCSIIAGGSL	TGWHPDSAAV	LWRRVLGILG	DVNNIQSPKI	HARVFCYLYE
970	980	990	1000	1010	1020	1030	1040
LWYKLAKIRD	NLAISLDNQS	SPSPPVLIPP	LRMFASWLFK	AATLPNEYKE	GKLQAYRLIC	AMMTRRQDVL	PNSDFLVHFY
1050	1060	1070	1080	1090	1100	1110	1120
LVMHLGLTSE	DQDILNTIIR	HCPPRFFSLG	FPGFSMLVGD	FITAAARVLS	TDILTAPRSE	AVTVLGSLVC	FPNTYQEIPL
1130	1140	1150	1160	1170	1180	1190	1200
LQSVPEVNEA	ITGTEDVKHY	LINILLKNAT	EEPNEYARCI	AVCSLGVWIC	EELAQCTSHP	QVKEAINVIG	VTLKFPNKIV
1210	1220	1230	1240	1250	1260	1270	1280
AQVACDVLQL	LVSYWEKLQM	FETSLPRKMA	EILVATVAFL	LPSAEYSSVE	TDKKFIVSLL	LCLLDWCMAL	PVSVLLHPVS
1290	1300	1310	1320	1330	1340	1350	1360
TAVLEEQHSA	RAPLLDYIYR	VLHCCVCGSS	TYTQQSHYIL	TLADLSSTDY	DPFLPLANVK	SSEPVQYHSS	AELGNLLTVE
1370	1380	1390	1400	1410	1420	1430	1440
EEKKRRSLEL	IPLTARMVMA	HLVNHLGHYP	LSGGPAILHS	LVSENHDNAH	VEGSELSFEV	FRSPNLQLFV	FNDSTLISYL
1450	1460	1470	1480	1490	1500	1510	1520
QTPTEGPVGG	SPVGSLSDVR	VIVRDISGKY	SWDGKVLYGP	LEGCLAPNGR	NPSFLISSWH	RDTFGPQKDS	SQVEEGDDVL
1530	1540	1550	1560	1570	1580	1590	1600
DKLLENIGHT	SPECLLPSQL	NLNEPSLTPC	GMNYDQEKEI	IEVILRQNAQ	EDEYIQSHNF	DSAMKVTSQG	QPSPVEPRGP
1610	1620	1630	1640	1650	1660	1670	1680
FYFCRLLLDD	LGMNSWDRRK	NFHLLKKNSK	LLRELKNLDS	RQCRETHKIA	VFYIAEGQED	KCSILSNERG	SQAYEDFVAG
1690	1700	1710	1720	1730	1740	1750	1760
LGWEVDLSTH	CGFMGGLQRN	GSTGQTAPYY	ATSTVEVIFH	VSTRMPSDSD	DSLTKKLRHL	GNDEVHIVWS	EHSRDYRRGI
1770	1780	1790	1800	1810	1820	1830	1840
IPTAFGDVSI	IIYPMKNHMF	FIAITKKPEV	PFFGPLFDGA	IVSGKLLPSL	VCATCINASR	AVKCLIPLYQ	SFYEERALYL
1850	1860	1870	1880				
EAIIQNHREV	MTFEDFAAQV	FSPSPSYSLS	GTD				

Cmpd.	No. of Cmpds.	m/z meas.	Δ m/z [ppm]	z	Rt [min]	Score	P	Range	Sequence	Modification

Detailed Protein Report

Cmpd.	No. of Cmpds.	m/z meas.	Δ m/z [ppm]	z	Rt [min]	Score	P	Range	Sequence	Modification
2089	1	1023.9303	-87.12	2	56.5	14.8	1	433-448	K.WILQDKPVFMEEPDRK.D	Oxidation: 10

Detailed Protein Report

**Protein 171:**	**PREDICTED: mediator of RNA polymerase II transcription subunit 13-like isoform X2 [Homo sapiens]**		
**Accession:**	gi|578823479	**Score:**	35.6
**Database:**	refseq_human(refseq_human_20140103.fasta)	**MW [kDa]:**	241.6
**Database Date:**	2015-11-30	**pI:**	5.8
**Modification(s):**	Oxidation	**Sequence Coverage [%]:**	1.4
		**No. of unique Peptides:**	2

Detailed Protein Report

10	20	30	40	50	60	70	80
MLESRKAIAP	SRRKAELTGI	KWRRYNFGGH	GDCGPIISAP	AQDDPILLSF	IRCLQANLLC	VWRRDVKPDC	KELWIFWWGD
90	100	110	120	130	140	150	160
EPNLVGVIHH	ELQVVEEGLW	ENGLSYECRT	LLFKAIHNLL	ERCLMDKNFV	RIGKWFVRPY	EKDEKPVNKS	EHLSCAFTFF
170	180	190	200	210	220	230	240
LHGESNVCTS	VEIAQHQPIY	LINEEHIHMA	QSSPAPFQVL	VSPYGLNGTL	TGQAYKMSDP	ATRKLIEEWQ	YFYPMVLKKK
250	260	270	280	290	300	310	320
EESKEEDELG	YDDDFPVAVE	VIVGGVRMVY	PSAFVLISQN	DIPVPQSVAS	AGGHIAVGQQ	GLGSVKDPSN	CGMPLTPPTS
330	340	350	360	370	380	390	400
PEQAILGESG	GMQSAASHLV	SQDGGMITMH	SPKRSGKIPP	KLHNHMVHRV	WKECILNRTQ	SKRSQMSTPT	LEEEPASNPA
410	420	430	440	450	460	470	480
TWDFVDPTQR	VSCSCSRHKL	LKRCAVGPNR	PPTVSQPGFS	AGPSSSSSLP	PPASSKHKTA	ERQEKGDKLQ	KRPLIPFHHR
490	500	510	520	530	540	550	560
PSVAEELCME	QDTPGQKLGL	AGIDSSLEVS	SSRKYDKQMA	VPSRNTSKQM	NLNPMDSPHS	PISPLPPTLS	PQPRGQETES
570	580	590	600	610	620	630	640
LDPPSVPVNP	ALYGNGLELQ	QLSTLDDRTV	LVGQRLPLMA	EVSETALYCG	IRPSNPESSE	KWWHSYRLPP	SDDAEFRPPE
650	660	670	680	690	700	710	720
LQGERCDAKM	EVNSESTALQ	RLLAQPNKRF	KIWQDKQPQL	QPLHFLDPLP	LSQQPGDSLG	EVNDPYTFED	GDIKYIFTAN
730	740	750	760	770	780	790	800
KKCKQGTEKD	SLKKNKSEDG	FGTKDVTTPG	HSTPVPDGKN	AMSIFSSATK	TDVRQDNAAG	RAGSSSLTQV	TDLAPSLHDL
810	820	830	840	850	860	870	880
DNIFDNSDDD	ELGAVSPALR	SSKMPAVGTE	DRPLGKDGRA	AVPYPPTVAD	LQRMFPTPPS	LEQHPAFSPV	MNYKDGISSE
890	900	910	920	930	940	950	960
TVTALGMMES	PMVSMVSTQL	TEFKMEVEDG	LGSPKPEEIK	DFSYVHKVPS	FQPFVGSSMF	APLKMLPSHC	LLPLKIPDAC
970	980	990	1000	1010	1020	1030	1040
LFRPSWAIPP	KIEQLPMPPA	ATFIRDGYNN	VPSVGSLADP	DYLNTPQMNT	PVTLNSAAPA	SNSGAGVLPS	PATPRFSVPT
1050	1060	1070	1080	1090	1100	1110	1120
PRTPRTPRTP	RGGGTASGQG	SVKYDSTDQG	SPASTPSTTR	PLNSVEPATM	QPIPEAHSLY	VTLILSDSVM	NIFKDRNFDS
1130	1140	1150	1160	1170	1180	1190	1200
CCICACNMNI	KGADVGLYIP	DSSNEDQYRC	TCGFSAIMNR	KLGYNSGLFL	EDELDIFGKN	SDIGQAAERR	LMMCQSTFLP
1210	1220	1230	1240	1250	1260	1270	1280
QVEGTKKPQE	PPISLLLLLQ	NQHTQPFASL	NFLDYISSNN	RQTLPCVSWS	YDRVQADNND	YWTECFNALE	QGRQYVDNPT
1290	1300	1310	1320	1330	1340	1350	1360
GGKVDEALVR	SATVHSWPHS	NVLDISMLSS	QDVVRMLLSL	QPFLQDAIQK	KRTGRTWENI	QHVQGPLTWQ	QFHKMAGRGT
1370	1380	1390	1400	1410	1420	1430	1440
YGSEESPEPL	PIPTLLVGYD	KDFLTISPFS	LPFWERLLLD	PYGGHRDVAY	IVVCPENEAL	LEGAKTFFRD	LSAVYEMCRL
1450	1460	1470	1480	1490	1500	1510	1520
GQHKPICKVL	RDGIMRVGKT	VAQKLTDELV	SEWFNQPWSG	EENDNHSRLK	LYAQVCRHHL	APYLATLQLD	SSLLIPPKYQ
1530	1540	1550	1560	1570	1580	1590	1600
TPPAAAQGQA	TPGNAGPLAP	NGSAAPPAGS	AFNPTSNSSS	TNPAASSSAS	GSSVPPVSSS	ASAPGISQIS	TTSSSGFSGS
1610	1620	1630	1640	1650	1660	1670	1680
VGGQNPSTGG	ISADRTQGNI	GCGGDTDPGQ	SSSQPSQDGQ	ESVTERERIG	IPTEPDSADS	HAHPPAVVIY	MVDPFTYAAE
1690	1700	1710	1720	1730	1740	1750	1760
EDSTSGNFWL	LSLMRCYTEM	LDNLPEHMRN	SFILQIVPCQ	YMLQTMKDEQ	VFYIQYLKSM	AFSVYCQCRR	PLPTQIHIKS
1770	1780	1790	1800	1810	1820	1830	1840
LTGFGPAASI	EMTLKNPERP	SPIQLYSPPF	ILAPIKDKQT	ELGETFGEAS	QKYNVLFVGY	CLSHDQRWLL	ASCTDLHGEL
1850	1860	1870	1880	1890	1900	1910	1920
LETCVVNIAL	PNRSRRSKVS	ARKIGLQKLW	EWCIGIVQMT	SLPWRVVIGR	LGRLGHGELK	DWSILLGECS	LQTISKKLKD
1930	1940	1950	1960	1970	1980	1990	2000
VCRMCGISAA	DSPSILSACL	VAMEPQGSFV	VMPDAVTMGS	VFGRSTALNM	QSSQLNTPQD	ASCTHILVFP	TSSTIQVAPA
2010	2020	2030	2040	2050	2060	2070	2080
NYPNEDGFSP	NNDDMFVDLP	FPDDMDNDIG	ILMTGNLHSS	PNSSPVPSPG	SPSGIGVGSH	FQHSRSQGER	LLSREAPEEL
2090	2100	2110	2120	2130	2140	2150	2160
KQQPLALGYF	VSTAKAENLP	QWFWSSCPQA	QNQCPLFLKA	SLHHHISVAQ	TDELLPARNS	QRVPHPLDSK	TTSDVLRFVL
2170	2180	2190	2200	2210			

Detailed Protein Report

Cmpd.	No. of Cmpds.	m/z meas.	Δ m/z [ppm]	z	Rt [min]	Score	P	Range	Sequence	Modification
2594	2	1044.5480	2.20	2	63.1	15.8	2	115-131	K.AIHNLLERCLMDKNFVR.I	Oxidation: 11
2308	1	892.3159	-67.35	2	57.5	19.8	0	1696-1709	R.CYTEMLDNLPEHMR.N	Oxidation: 5, 13

BRUKER

Detailed Protein Report

Protein 172: PREDICTED: insulin-like growth factor 1 receptor isoform X2 [Homo sapiens]

**Accession:**	gi|530407102	**Score:**	35.6
**Database:**	refseq_human(refseq_human_20140103.fasta)	**MW [kDa]:**	141.1
**Database Date:**	2015-11-30	**pI:**	5.4
**Modification(s):**	Carbamidomethyl, Oxidation	**Sequence Coverage [%]:**	3.7
		**No. of unique Peptides:**	3

10	20	30	40	50	60	70	80
MTNLKDIGLY	NLRNITRGAI	RIEKNADLCY	LSTVDWSLIL	DAVSNNYIVG	NKPPKECGDL	CPGTMEEKPM	CEKTTINNEY
90	100	110	120	130	140	150	160
NYRCWTTNRC	QKMCPSTCGK	RACTENNECC	HPECLGSCSA	PDNDTACVAC	RHYYYAGVCV	PACPPNTYRF	EGWRCVDRDF
170	180	190	200	210	220	230	240
CANILSAESS	DSEGFVIHDG	ECMQECPSGF	IRNGSQSMYC	IPCEGPCPKV	CEEEKKTKTI	DSVTSAQMLQ	GCTIFKGNLL
250	260	270	280	290	300	310	320
INIRRGNNIA	SELENFMGLI	EVVTGYVKIR	HSHALVSLSF	LKNLRLILGE	EQLEGNYSFY	VLDNQNLQQL	WDWDHRNLTI
330	340	350	360	370	380	390	400
KAGKMYFAFN	PKLCVSEIYR	MEEVTGTKGR	QSKGDINTRN	NGERASCESD	VLHFTSTTTS	KNRIIITWHR	YRPPDYRDLI
410	420	430	440	450	460	470	480
SFTVYYKEAP	FKNVTEYDGQ	DACGSNSWNM	VDVDLPPNKD	VEPGILLHGL	KPWTQYAVYV	KAVTLTMVEN	DHIRGAKSEI
490	500	510	520	530	540	550	560
LYIRTNASVP	SIPLDVLSAS	NSSSQLIVKW	NPPSLPNGNL	SYYIVRWQRQ	PQDGYLYRHN	YCSKDKIPIR	KYADGTIDIE
570	580	590	600	610	620	630	640
EVTENPKTEV	CGGEKGPCCA	CPKTEAEKQA	EKEEAEYRKV	FENFLHNSIF	VPRPERKRRD	VMQVANTTMS	SRSRNTTAAD
650	660	670	680	690	700	710	720
TYNITDPEEL	ETEYPFFESR	VDNKERTVIS	NLRPFTLYRI	DIHSCNHEAE	KLGCSASNFV	FARTMPAEGA	DDIPGPVTWE
730	740	750	760	770	780	790	800
PRPENSIFLK	WPEPENPNGL	ILMYEIKYGS	QVEDQRECVS	RQEYRKYGGA	KLNRLNPGNY	TARIQATSLS	GNGSWTDPVF
810	820	830	840	850	860	870	880
FYVQAKTGYE	NFIHLIIALP	VAVLLIVGGL	VIMLYVFHRK	RNNSRLGNGV	LYASVNPEYF	SAADVYVPDE	WEVAREKITM
890	900	910	920	930	940	950	960
SRELGQGSFG	MVYEGVAKGV	VKDEPETRVA	IKTVNEAASM	RERIEFLNEA	SVMKEFNCHH	VVRLLGVVSQ	GQPTLVIMEL
970	980	990	1000	1010	1020	1030	1040
MTRGDLKSYL	RSLRPEMENN	PVLAPPSLSK	MIQMAGEIAD	GMAYLNANKF	VHRDLAARNC	MVAEDFTVKI	GDFGMTRDIY
1050	1060	1070	1080	1090	1100	1110	1120
ETDYYRKGGK	GLLPVRWMSP	ESLKDGVFTT	YSDVWSFGVV	LWEIATLAEQ	PYQGLSNEQV	LRFVMEGGLL	DKPDNCPDML
1130	1140	1150	1160	1170	1180	1190	1200
FELMRMCWQY	NPKMRPSFLE	IISSIKEEME	PGFREVSFYY	SEENKLPEPE	ELDLEPENME	SVPLDPSASS	SSLPLPDRHS
1210	1220	1230	1240	1250			
GHKAENGPGP	GVLVLRASFD	ERQPYAHMNG	GRKNERALPL	PQSSTC			

Cmpd.	No. of Cmpds.	m/z meas.	Δ m/z [ppm]	z	Rt [min]	Score	P	Range	Sequence	Modification
1982	1	1044.8814	-23.69	2	55.2	11.7	0	56-73	K.ECGDLCPGTMEEKPMCEK.T	Carbamidomethyl: 16; Oxidation: 10, 15
1529	1	669.8325	-70.94	2	49.3	13.1	0	271-282	R.HSHALVSLSFLK.N	
2019	1	660.9088	-130.71	3	53.8	10.8	1	325-340	K.MYFAFNPKLCVSEIYR.M	

Detailed Protein Report

Protein 173: **PREDICTED: NADH dehydrogenase [ubiquinone] iron-sulfur protein 2, mitochondrial isoform X2 [Homo sapiens]**

**Accession:**	gi|530364809	**Score:**	35.6
**Database:**	refseq_human(refseq_human_20140103.fasta)	**MW [kDa]:**	41.8
**Database Date:**	2015-11-30	**pI:**	6.1
**Modification(s):**	Oxidation	**Sequence Coverage [%]:**	5.5
		**No. of unique Peptides:**	1

10	20	30	40	50	60	70	80
MELSGEMVRK	CDPHIGLLHR	GTEKLIEYKT	YLQALPYFDR	LDYVSMMCNE	QAYSLAVEKL	LNIRPPPRAQ	WIRVLFGEIT
90	100	110	120	130	140	150	160
RLLNHIMAVT	THALDLGAMT	PFFWLFEERE	KMFEFYERVS	GARMHAAYIR	PGGVHQDLPL	GLMDDIYQFS	KNFSLRLDEL
170	180	190	200	210	220	230	240
EELLTNNRIW	RNRTIDIGVV	TAEEALNYGF	SGVMLRGSGI	QWDLRKTQPY	DVYDQVEFDV	PVGSRGDCYD	RYLCRVEEMR
250	260	270	280	290	300	310	320
QSLRIIAQCL	NKMPPGEIKV	DDAKVSPPKR	AEMKTSMESL	IHHFKLYTEG	YQVPPGATYT	AIEAPKGEFG	VYLVSDGSSR
330	340	350	360	370			
PYRCKIKAPG	FAHLAGLDKM	SKGHMLADVV	AIIGTQDIVF	GEVDR			

Cmpd.	No. of Cmpds.	m/z meas.	Δ m/z [ppm]	z	Rt [min]	Score	P	Range	Sequence	Modification
1905	1	673.3043	-41.33	2	54.2	17.5	0	275-285	K.TSMESLIHHFK.L	Oxidation: 3

Detailed Protein Report

Protein 174: pumilio domain-containing protein KIAA0020 [Homo sapiens]

**Accession:**	gi|109948283	**Score:**	35.0
**Database:**	refseq_human(refseq_human_20140103.fasta)	**MW [kDa]:**	73.5
**Database Date:**	2015-11-30	**pI:**	10.2
**Modification(s):**	Oxidation	**Sequence Coverage [%]:**	5.1
		**No. of unique Peptides:**	2

10	20	30	40	50	60	70	80
MEVKGKKQFT	GKSTKTAQEK	NRFHKNSDSG	SSKTFPTRKV	AKEGGPKVTS	RNFEKSITKL	GKKGVKQFKN	KQQGDKSPKN
**90**	**100**	**110**	**120**	**130**	**140**	**150**	**160**
KFQPANKFNK	KRKFQPDGRS	DESAAKKPKW	DDFKKKKKEL	KQSRQLSDKT	NYDIVVRAKQ	MWEILRRKDC	DKEKRVKLMS
**170**	**180**	**190**	**200**	**210**	**220**	**230**	**240**
DLQKLIQGKI	KTIAFAHDST	RVIQCYIQYG	NEEQRKQAFE	ELRDDLVELS	KAKYSRNIVK	KFLMYGSKPQ	IAEIIRSFKG
**250**	**260**	**270**	**280**	**290**	**300**	**310**	**320**
HVRKMLRHAE	ASAIVEYAYN	DKAILEQRNM	LTEELYGNTF	QLYKSADHRT	LDKVLEVQPE	KLELIMDEMK	QILTPMAQKE
**330**	**340**	**350**	**360**	**370**	**380**	**390**	**400**
AVIKHSLVHK	VFLDFFTYAP	PKLRSEMIEA	IREAVVYLAH	THDGARVAMH	CLWHGTPKDR	KVIVKTMKTY	VEKVANGQYS
**410**	**420**	**430**	**440**	**450**	**460**	**470**	**480**
HLVLLAAFDC	IDDTKLVKQI	IISEIISSLP	SIVNDKYGRK	VLLYLLSPRD	PAHTVREIIE	VLQKGDGNAH	SKKDTEVRRR
**490**	**500**	**510**	**520**	**530**	**540**	**550**	**560**
ELLESISPAL	LSYLQEHAQE	VVLDKSACVL	VSDILGSATG	DVQPTMNAIA	SLAATGLHPG	GKDGELHIAE	HPAGHLVLKW
**570**	**580**	**590**	**600**	**610**	**620**	**630**	**640**
LIEQDKKMKE	NGREGCFAKT	LVEHVGMKNL	KSWASVNRGA	IILSSLLQSC	DLEVANKVKA	ALKSLIPTLE	KTKSTSKGIE
**650**							
ILLEKLST							

Cmpd.	No. of Cmpds.	m/z meas.	Δ m/z [ppm]	z	Rt [min]	Score	P	Range	Sequence	Modification
2240	1	569.1603	-210.52	2	56.7	10.9	0	302-310	K.LELIMDEMK.Q	Oxidation: 8
2534	1	698.3342	-3.44	2	60.4	11.9	0	367-378	R.VAMHCLWHGTPK.D	Oxidation: 3

Detailed Protein Report

Protein 175: TMF-regulated nuclear protein 1 [Homo sapiens]

Accession: gi|61966741
Database: refseq_human(refseq_human_20140103.fasta)
Database Date: 2015-11-30
Modification(s): Carbamidomethyl

Score: 35.0
MW [kDa]: 23.4
pI: 12.2
Sequence Coverage [%]: 14.1
No. of unique Peptides: 2

10	20	30	40	50	60	70	80
MPGCRISACG	PGAQEGTAEQ	RSPPPPRDPM	PSSQPPPPTP	TLTPTPTPGQ	SPPLPDAAGA	SAGAAEDQEL	QRWRQGASGI
90	100	110	120	130	140	150	160
AGLAGPGGGS	GAAAGAGGRA	LELAEARRRL	LEVEGRRRLV	SELESRVLQL	HRVFLAAELR	LAHRAESLSR	LSGGVAQAEL
170	180	190	200	210	220	230	
YLAAHGSRLK	KGPRRGRRGR	PPALLASALG	LGGCVPWGAG	RLRRGHGPEP	DSPFRRSPPR	GPASPQR	

Cmpd.	No. of Cmpds.	m/z meas.	Δ m/z [ppm]	z	Rt [min]	Score	P	Range	Sequence	Modification
1625	2	682.3281	20.05	3	50.6	17.4	1	2-21	M.PGCRISACGPGAQEGTAEQR.S	Carbamidomethyl: 8
1148	1	676.2570	-106.06	2	44.5	17.6	1	205-216	R.GHGPEPDSPFRR.S	

Detailed Protein Report

Protein 176: phenylalanine--tRNA ligase alpha subunit [Homo sapiens]

**Accession:**	gi|4758340	**Score:**	34.9
**Database:**	refseq_human(refseq_human_20140103.fasta)	**MW [kDa]:**	57.5
**Database Date:**	2015-11-30	**pI:**	7.9
**Modification(s):**	Oxidation	**Sequence Coverage [%]:**	5.7
		**No. of unique Peptides:**	1

10	20	30	40	50	60	70	80
MADGQVAELL	LRRLEASDGG	LDSAELAAEL	GMEHQAVVGA	VKSLQALGEV	IEAELRSTKH	WELTAEGEEI	AREGSHEARV
**90**	**100**	**110**	**120**	**130**	**140**	**150**	**160**
FRSIPPEGLA	QSELMRLPSG	KVGFSKAMSN	KWIRVDKSAA	DGPRVFRVVD	SMEDEVQRRL	QLVRGGQAEK	LGEKERSELR
**170**	**180**	**190**	**200**	**210**	**220**	**230**	**240**
KRKLLAEVTL	KTYWVSKGSA	FSTSISKQET	ELSPEMISSG	SWRDRPFKPY	NFLAHGVLPD	SGHLHPLLKV	RSQFRQIFLE
**250**	**260**	**270**	**280**	**290**	**300**	**310**	**320**
MGFTEMPTDN	FIESSFWNFD	ALFQPQQHPA	RDQHDTFFLR	DPAEALQLPM	DYVQRVKRTH	SQGGYGSQGY	KYNWKLDEAR
**330**	**340**	**350**	**360**	**370**	**380**	**390**	**400**
KNLLRTHTTS	ASARALYRLA	QKKPFTPVKY	FSIDRVFRNE	TLDATHLAEF	HQIEGVVADH	GLTLGHLMGV	LREFFTKLGI
**410**	**420**	**430**	**440**	**450**	**460**	**470**	**480**
TQLRFKPAYN	PYTEPSMEVF	SYHQGLKKWV	EVGNSGVFRP	EMLLPMGLPE	NVSVIAWGLS	LERPTMIKYG	INNIRELVGH
**490**	**500**	**510**					
KVNLQMVYDS	PLCRLDAEPR	PPPTQEAA					

Cmpd.	No. of Cmpds.	m/z meas.	Δ m/z [ppm]	z	Rt [min]	Score	P	Range	Sequence	Modification
2536	1	1044.5961	47.08	2	62.3	24.4	2	128-144	R.VVDSMEDEVQRRLQLVR.G	Oxidation: 5

Detailed Protein Report

Protein 177: PREDICTED: zinc finger protein 708 isoform X1 [Homo sapiens]

Accession: gi|578833563
Database: refseq_human(refseq_human_20140103.fasta)
Database Date: 2015-11-30
Modification(s): Carbamidomethyl

Score: 34.7
MW [kDa]: 57.3
pI: 10.4
Sequence Coverage [%]: 4.0
No. of unique Peptides: 2

Quantitation

QD:QU **Median:** 0.30 **CV:** 0.00 % **No. of Peptides:** 1

10	20	30	40	50	60	70	80
MKRHEMAAKP	PAMCSHFAKD	LRPEQYIKNS	FQQVILRRYG	KCGYQKGCKS	VDEHKLHKGG	HKGLNRCVTT	TQSKIVQCDK
**90**	**100**	**110**	**120**	**130**	**140**	**150**	**160**
YVKVFHKYSN	AKRHKIRHTG	KNPFKCKECG	KSFCMLSQLT	QHEIIHTGEK	PYKCEECGKA	FKKSSNLTNH	KIIHTGEKPY
**170**	**180**	**190**	**200**	**210**	**220**	**230**	**240**
KCEECGKAFN	QSSTLTRHKI	IHTGEKLYKC	EECGKAFNRS	SNLTKHKIVH	TGEKPYKCEE	CGKAFKQSSN	LTNHKKIHTG
**250**	**260**	**270**	**280**	**290**	**300**	**310**	**320**
EKPYKCGECG	KAFTLSSHLT	THKRIHTGEK	PYKCEECGKA	FSVFSTLTKH	KIIHTEEKPY	KCEECGKAFN	RSSHLTNHKV
**330**	**340**	**350**	**360**	**370**	**380**	**390**	**400**
IHTGEKPYKC	EECGKAFTKS	STLTYHKVIH	TGKKPYKCEE	CGKAFSIFSI	LTKHKVIHTE	DKPYKCEECG	KTFNYSSNFT
**410**	**420**	**430**	**440**	**450**	**460**	**470**	**480**
NHKKIHTGEK	PYKCEECGKS	FILSSHLTTH	KIIHTGEKPY	KCKECGKAFN	QSSTLMKHKI	IHTGEKPYKC	EECGKAFNQS
**490**	**500**						
PNLTKHKRIH	TKEKPYKCK						

Cmpd.	No. of Cmpds.	m/z meas.	Δ m/z [ppm]	z	Rt [min]	Score	P	Range	Sequence	Modification	Ratios	
328	2	605.6684	-199.06	2	32.8	13.8	2	102-111	K.NPFKCKECGK.S	Carbamidomethyl: 8	QD:QU	0.30
1258	6	586.7739	28.23	2	45.9	20.9	1	330-339	K.CEECGKAFTK.S	Carbamidomethyl: 4		

Detailed Protein Report

Protein 178: **PREDICTED: centlein isoform X2 [Homo sapiens]**

Accession: gi|578816553
Database: refseq_human(refseq_human_20140103.fasta)
Database Date: 2015-11-30

Score: 34.4
MW [kDa]: 155.6
pI: 9.1
Sequence Coverage [%]: 1.9
No. of unique Peptides: 1

10	20	30	40	50	60	70	80
MAARSPPSPH	PSPPARQLGP	RSPRVGRGAE	VHAMRSEASG	FAGAAREVVA	DESDKIWADK	EFVWSLWKRL	QVTNPDLTQV
90	100	110	120	130	140	150	160
VSLVVEREKQ	KSEAKDRKVL	EILQVKDAKI	QEFEQRESVL	KQEINDLVKR	KIAVDEENAF	LRKEFSDLEK	KFKDKSQEIK
170	180	190	200	210	220	230	240
DTKECVQNKE	EQNRLVIKNL	EEENKKLSTR	CTDLLNDLEK	LRKQEAHLRK	EKYSTDAKIK	TFEDNLIEAR	KEVEVSQSKY
250	260	270	280	290	300	310	320
NALSLQLSNK	QTELIQKDMD	ITLVRKELQE	LQNLYKQNST	HTAQQAELIQ	QLQVLNMDTQ	KVLRNQEDVH	TAESISYQKL
330	340	350	360	370	380	390	400
YNELHICFET	TKSNEAMLRQ	SVTNLQDQLL	QKEQENAKLK	EKLQESQGAP	LPLPQESDPD	YSAQVPHRPS	LSSLETLMVS
410	420	430	440	450	460	470	480
QKSEIEYLQE	KLKIANEKLS	ENISANKGFS	RKSIMTSAEG	KHKEPPVKRS	RSLSPKSSFT	DSEELQKLRK	AERKIENLEK
490	500	510	520	530	540	550	560
ALQLKSQEND	ELRDAHEKRK	ERLQMLQTNY	RAVKEQLKQW	EEGSGMTEIR	KIKRADPQQL	RQEDSDAVWN	ELAYFKRENQ
570	580	590	600	610	620	630	640
ELMIQKMNLE	EELDELKVHI	SIDKAAIQEL	NRCVAERREE	QLFRSGEDDE	VKRSTPEKNG	KEMLEQTLQK	VTELENRLKS
650	660	670	680	690	700	710	720
FEKRSRKLKE	GNKKLMKEND	FLKSLLKQQQ	EDTETREKEL	EQIIKGSKDV	EKENTELQVK	ISELETEVTS	LRRQVAEANA
730	740	750	760	770	780	790	800
LRNENEELIN	PMEKSHQSAD	RAKSEMATMK	VRSGRYDCKT	TMTKVKFKAA	KKNCSVGRHH	TVLNHSIKVM	SNVFENLSKD
810	820	830	840	850	860	870	880
GWEDVSESSS	DSEAQTSQTL	GTIIVETSQK	ISPTEDGKDQ	KESDPTEDSQ	TQGKEIVQTY	LNIDGKTPKD	YFHDKNAKKP
890	900	910	920	930	940	950	960
TFQKKNCKMQ	KSSHTAVPTR	VNREKYKNIT	AQKSSSNIIL	LRERIISLQQ	QNSVLQNAKK	TAELSVKEYK	EVNEKLLHQQ
970	980	990	1000	1010	1020	1030	1040
QVSDQRFQTS	RQTIKKLNLD	LAGLRKEKED	LLKKLESSSE	ITSLAEENSQ	VTFPRIQVTS	LSPSRSMDLE	MKQLQYKLKN
1050	1060	1070	1080	1090	1100	1110	1120
ATNELTKQSS	NVKTLKFELL	AKEEHIKEMH	EKISRMERDI	TMKRHLIEDL	KFRQKVNLES	NKSFSEMLQN	LDKKVKTLTE
1130	1140	1150	1160	1170	1180	1190	1200
ECSNKKVSID	SLKQRLNVAV	KEKSQYEQMY	QKSKEELEKK	DLKLTLLVSR	ISETESAMAE	IETAASKQLQ	ELALQSEQVL
1210	1220	1230	1240	1250	1260	1270	1280
EGAQKTLLLA	NEKVEEFTTF	VKALAKELQN	DVHVVRRQIR	ELKKMKKNRD	ACKTSTHKAQ	TLAASILNIS	RSDLEEILDT
1290	1300	1310	1320	1330	1340	1350	
EDQVEIEKTK	IDAENDKEWM	LYIQKLLEGQ	LPFASYLLEA	VLEKINEKKK	LVEGYFTIMK	DIR	

Cmpd.	No. of Cmpds.	m/z meas.	Δ m/z [ppm]	z	Rt [min]	Score	P	Range	Sequence	Modification
848	1	512.1520	-290.70	2	40.7	15.4	1	1084-1091	K.RHLIEDLK.F	

Detailed Protein Report

Protein 179: protein MROH8 isoform 3 [Homo sapiens]

**Accession:**	gi|538260586	**Score:**	34.3
**Database:**	refseq_human(refseq_human_20140103.fasta)	**MW [kDa]:**	64.5
**Database Date:**	2015-11-30	**pI:**	8.0
		**Sequence Coverage [%]:**	4.4
		**No. of unique Peptides:**	1

10	20	30	40	50	60	70	80
MPLSRRGRGS	RLAGSRAATS	NLPRATALSP	KSAGRGALSI	SHAQLLTRRR	HSSEQVPPES	EPRADFRSGK	WLQEPATGDA
**90**	**100**	**110**	**120**	**130**	**140**	**150**	**160**
RDSRQALRAR	MSSKHRICSQ	EEVVIPCAYD	SDSESVDLEL	SNLEIIKKGS	SSIELTDLDI	PDIPGLHCEP	LSHSPRHLTQ
**170**	**180**	**190**	**200**	**210**	**220**	**230**	**240**
QDPLSEAIVE	KLIQSIQKVF	NGELKGELEK	LKFLGDLSSL	SQALPYDETA	KSFIHSHIAD	IVHTLNVLVQ	EERPHSLSSS
**250**	**260**	**270**	**280**	**290**	**300**	**310**	**320**
MRQEVFVTIA	DLSYQDVHLL	LGSEDRAELF	SLTIKSIITL	PSVRTLTQIQ	EIMPNGTCNT	ECLYRQTFQA	FSEMLQSLVV
**330**	**340**	**350**	**360**	**370**	**380**	**390**	**400**
KDPHLENLDT	IIKLPLRFQR	LGHLVALMAL	LCGDPQEKVA	EEAAEGIHSL	LHITLRLKYI	THDKKDQQNL	KRALTKCREF
**410**	**420**	**430**	**440**	**450**	**460**	**470**	**480**
LELHSSAAKC	FYNCPFRIAQ	VFEGFLDSNE	LCQFIMTTFD	TLKTLKHPCI	QRSAGELLLT	LAKNTESQFE	KVPEIMGVIC
**490**	**500**	**510**	**520**	**530**	**540**	**550**	**560**
AQLSIISQPR	VRQQIINTVS	LFISRPKYTD	IVLSFLLCHP	VPYNRHLAEV	WRMLSVELPS	TTWILWRLLR	KLQKCHNEPA
**570**	**580**						
QEKMAYVAVA	VSP						

Cmpd.	No. of Cmpds.	m/z meas.	Δ m/z [ppm]	z	Rt [min]	Score	P	Range	Sequence	Modification
222	1	493.5794	24.80	3	32.8	11.1	0	51-63	R.HSSEQVPPESEPR.A	

Detailed Protein Report

Protein 180: uncharacterized protein LOC284677 [Homo sapiens]

Accession: gi|197116396
Database: refseq_human(refseq_human_20140103.fasta)
Database Date: 2015-11-30
Modification(s): Carbamidomethyl, Oxidation

Score: 34.2
MW [kDa]: 25.1
pI: 10.6
Sequence Coverage [%]: 8.1
No. of unique Peptides: 2

10	20	30	40	50	60	70	80
MGMLAPGPLQ	GRRPRKGHKG	QEDAVAPGCK	ASGRGSRVTH	LLGYPTQNVS	RSLRRKYAPP	PCGGPEDVAL	APCTAAAACE
**90**	**100**	**110**	**120**	**130**	**140**	**150**	**160**
AGPSPVYVKV	KSAEPADCAE	GPVQCKNGLL	VSSPHCEEPC	AHSCAHPGLP	PHLVHKLPLS	YLQTQDTDAA	SRRINAPLAA
**170**	**180**	**190**	**200**	**210**	**220**	**230**	**240**
GWSWLRLWLV	TLASGVDFPQ	VSAWMRALPS	PDCPGLRTTG	EQMQKLLLKE	NKVKTRKSKR	RSGEGSHLTT	SILEQ

Cmpd.	No. of Cmpds.	m/z meas.	Δ m/z [ppm]	z	Rt [min]	Score	P	Range	Sequence	Modification
2167	3	1051.4380	-62.66	2	57.5	23.3	1	187-205	R.ALPSPDCPGLRTTGEQMQK.L	Carbamidomethyl: 7; Oxidation: 17
2182	1	682.2713	-87.72	3	57.7	10.9	1	187-205	R.ALPSPDCPGLRTTGEQMQK.L	Oxidation: 17

Detailed Protein Report

Protein 181: PREDICTED: uncharacterized protein LOC101929108 [Homo sapiens]

Accession: gi|578795301
Database: refseq_human(refseq_human_20140103.fasta)
Database Date: 2015-11-30
Modification(s): Carbamidomethyl, Oxidation

Score: 34.2
MW [kDa]: 23.1
pI: 12.7
Sequence Coverage [%]: 16.9
No. of unique Peptides: 2

10	20	30	40	50	60	70	80
MTKTLSLQAF	PQLRSLLGLG	PFLLLSKGVA	ANQPCPPALL	TPSFSSVPEL	CMGSELWLPP	GVPVLPQFSQ	RSMGPSTPPL
**90**	**100**	**110**	**120**	**130**	**140**	**150**	**160**
AAMHWGLRRV	PGPLPPPWPG	LPAPGRSCAR	FSASRVCVAC	SAQRPPPRSA	PCRTPRCCGR	GGSVAVRAVS	VRPHRLPSAD
**170**	**180**	**190**	**200**	**210**	**220**		
FLRRGPELPA	RSDAAECRNG	LPVCQSGLPR	TPSEATRSSR	GVSVALLKQK	QQLHSPASV		

Cmpd.	No. of Cmpds.	m/z meas.	Δ m/z [ppm]	z	Rt [min]	Score	P	Range	Sequence	Modification
278	1	998.9505	-45.38	2	33.5	21.2	1	72-89	R.SMGPSTPPLAAMHWGLRR.V	Oxidation: 2, 12
2912	1	1012.4649	-62.98	2	65.6	13.0	2	137-155	R.CCGRGGSVAVRAVSVRPHR.L	Carbamidomethyl: 1

Detailed Protein Report

Protein 182: PREDICTED: uncharacterized protein C11orf85 isoform X7 [Homo sapiens]

Accession: gi|578821305
Database: refseq_human(refseq_human_20140103.fasta)
Database Date: 2015-11-30
Modification(s): Carbamidomethyl, Oxidation

Score: 34.1
MW [kDa]: 24.4
pI: 10.6
Sequence Coverage [%]: 14.9
No. of unique Peptides: 2

```
        10         20         30         40         50         60         70         80
MSLKPFTYPF PETRFLHAGP NVYKFKIRYG KSIRGEEIEN KEVITQELEL VLKVAFGSLR MGSFLHCAFM NCEQLWKGGV
        90        100        110        120        130        140        150        160
TGICFLLKVP VEKKAVGAVM RKRKHMDEPS SPSRPGLDRA KIGTSSQGPS KKKPPVETRR NRERKTQQGL QETLASDITD
       170        180        190        200        210        220
VQKQDSEWGH SLPGRIVPPL QHNSPPPKER AATGFFGFLS SLFPFRYFFR KSSHS
```

Cmpd.	No. of Cmpds.	m/z meas.	Δ m/z [ppm]	z	Rt [min]	Score	P	Range	Sequence	Modification
2875	1	922.9828	-58.69	2	65.1	22.3	2	78-94	K.GGVTGICFLLKVPVEKK.A	Carbamidomethyl: 7
930	1	848.9319	50.50	2	41.7	11.9	0	105-119	K.HMDEPSSPSRPGLDR.A	Oxidation: 2

Detailed Protein Report

Protein 183: PREDICTED: ATP-dependent RNA helicase DDX55 isoform X1 [Homo sapiens]

Accession: gi|530401708
Database: refseq_human(refseq_human_20140103.fasta)
Database Date: 2015-11-30
Modification(s): Carbamidomethyl, Oxidation

Score: 34.1
MW [kDa]: 50.9
pI: 9.9
Sequence Coverage [%]: 6.0
No. of unique Peptides: 2

10	20	30	40	50	60	70	80
MEHVTEGSWE	SLPVPLHPQV	LGALRELGFP	YMTPVQSATI	PLFMRNKDVA	AEAVTGSGKT	LAFVIPILEI	LLRREEKLKK
**90**	**100**	**110**	**120**	**130**	**140**	**150**	**160**
SQVGAIIITP	TRELAIQIDE	VLSHFTKHFP	EFSQILWIGG	RNPGEDVERF	KQQGGNIIVA	TPGRLEDMFR	RKAEGLDLAS
**170**	**180**	**190**	**200**	**210**	**220**	**230**	**240**
CVRSLDVLVL	DEADRLLDMG	FEASINTILE	FLPKQRRTGL	FSATQTQEVE	NLVRAGLRNP	VRVSVKEKGV	AASSAQKTPS
**250**	**260**	**270**	**280**	**290**	**300**	**310**	**320**
RLENYYMVCK	ADEKFNQLVH	FLRNHKQEKH	LVFFSTCACV	EYYGKALEVL	VKGVKIMCIH	GKMKYKRNKI	FMEFRKLQSG
**330**	**340**	**350**	**360**	**370**	**380**	**390**	**400**
ILVCTDVMAR	GIDIPEVNWV	LQYDPPSNAS	AFVHRCGRTA	RIGHGGSALV	FLLPMEESYI	NFLAINQKCP	LQEMKPQRNT
**410**	**420**	**430**	**440**	**450**	**460**		
ADLLPKLKSM	ALADRAVFEK	GMKAFVSYVQ	AYAKHECNLI	FRLKALLEVL	PC		

Cmpd.	No. of Cmpds.	m/z meas.	Δ m/z [ppm]	z	Rt [min]	Score	P	Range	Sequence	Modification
408	1	655.8392	-35.43	2	35.1	10.7	0	132-144	K.QQGGNIIVATPGR.L	
2795	1	874.5325	120.22	1	64.0	11.6	0	296-302	K.IMCIHGK.M	Carbamidomethyl: 3; Oxidation: 2

Detailed Protein Report

Protein 184: dynein heavy chain domain-containing protein 1 isoform 1 [Homo sapiens]

**Accession:**	gi|222144249	**Score:**	34.1
**Database:**	refseq_human(refseq_human_20140103.fasta)	**MW [kDa]:**	533.3
**Database Date:**	2015-11-30	**pI:**	6.3
**Modification(s):**	Oxidation	**Sequence Coverage [%]:**	0.5
		**No. of unique Peptides:**	1

Detailed Protein Report

10	20	30	40	50	60	70	80
MVPEERRVGL	SSDETSSDSL	KSWHSICVLD	SKEQPLACQQ	KQRQFVKPVT	ESEQPTVLEL	LLAELRTLFS	AVLQDSSPAA
90	100	110	120	130	140	150	160
WRYLHAVLGL	LPPYRELLVG	HLDLLPFLEQ	LYCWAPWVQT	HLHLDLLGAI	VQAFPPDSSL	LDSASHADCC	PQKRRLHHRP
170	180	190	200	210	220	230	240
PCPACPFVQA	QWSRQQVKEE	LATWLRPLTL	PELQRCLGIV	GAQVALEEAV	WLDGLSLLPL	ALAADIPVRY	ESSDTDNAEV
250	260	270	280	290	300	310	320
EPVGRKETRS	QLDYEVPREK	AFQKSSTGFS	PETSFLDSQV	MTALKMERYL	KKIHFLYLNV	APSRYFRPYS	LMVVPPDKVN
330	340	350	360	370	380	390	400
PEHYIFSPFG	ILHVHPVEGS	ETMTLGTWHH	HCVLWQQLQF	IPFFKYCLLR	KSFTCWKKNV	RLQGLHRLQK	FLENHLLLAV
410	420	430	440	450	460	470	480
PHFGAGLLHI	SRLLQELHSV	SWLPQELDRC	YELLDLQTAL	AEEKHKALRL	LHRCLNLCTS	ILRLVHEDTY	HMQQCLQERV
490	500	510	520	530	540	550	560
QNCDRIRTGQ	GSIYLQRVQH	KQLEQKLKQA	EAWWLQLGKF	ARLVDYMICQ	SLISVLEEQI	TSFVANILQA	PRQKPFLSSQ
570	580	590	600	610	620	630	640
LVFDDHGQLS	HVPCVENMIQ	TLTGGLQSVK	TSALQVVQSA	DLKTSSDSLY	SEEEDEEEDS	KDEFLMPKFQ	GQPSDAVSIF
650	660	670	680	690	700	710	720
CGPNVGLVWP	WKSHPIAGIL	EVRGCRLRGQ	YFPHNYKQLE	EDLDNNPKIQ	QALNIQQVLL	EGVLCKVQEF	CREHHWITGI
730	740	750	760	770	780	790	800
YEFLQSWGPQ	KLEDMRGGPI	KNYVTLVSRL	NVWQARVSSM	PIELLTKGGL	LLLSCHDVQA	EMESKLNSIR	KDILAHVQNE
810	820	830	840	850	860	870	880
CWNLSQQLMT	ELTDFMHIFR	TINSDIHAIA	QCTQKLNEAN	EQYVELEERM	EYVRALHELI	RNHFSLFSAE	NEALDISVRR
890	900	910	920	930	940	950	960
QFGESPIPPC	PPPPQPHLLH	CPLLAPQLLD	MWEAFQFEKS	QASEFLLSKR	HAIMPKLQQL	MAAALAELEG	LLAKALSGPF
970	980	990	1000	1010	1020	1030	1040
MDPTQDQRST	EHQLVSLERQ	FQNTVSDLSE	LHHAYAIFTE	DETPVPLPIC	GTRPIVQQQR	IWHLYRVISE	NISEWKCMAF
1050	1060	1070	1080	1090	1100	1110	1120
AKFSPAMAQE	KTEGWLTEAA	RMSTTLELHS	PVLQHCMRIL	GEFRSYLPLL	TKLGSLHPQS	LNCQCLLRAL	GLGSLQTIEL
1130	1140	1150	1160	1170	1180	1190	1200
LTLGQLLTYP	LLEFADRINQ	VWQNENERIH	AQETIRRLQR	YWEARQLRLL	NFILHVPYEP	PASERSKRQV	LRSPQWEVVD
1210	1220	1230	1240	1250	1260	1270	1280
KDSGTFILSD	YSNLQDSIQE	SLQVLSKILA	IEKSGDLNKI	ALEWVAIMHG	LGALLEVWLT	FQQKWIFLNK	VLHEMKIQFP
1290	1300	1310	1320	1330	1340	1350	1360
NADLNSRFKV	MDDQYRTLMR	ISVADPMVLS	LVVPSAERSP	YFQGQQLQQL	LQAGSVELEG	IIMSLESVLY	GVCAHFPRLF
1370	1380	1390	1400	1410	1420	1430	1440
FLSDSELVAL	LAARLESCEA	QLWVRRCFPH	VHAVSFRSCP	TGEKNTDDWE	SSPNTQTQVE	ALAVLGAGGE	EVKLQGPLPL
1450	1460	1470	1480	1490	1500	1510	1520
HPDLPKWLAS	LEKCLRLALV	HMLQGCVAAR	LARGPSLGEA	LKQLPKQNKL	YLQLYVQHWI	DLVQAFPWQC	VLVAEEVVWR
1530	1540	1550	1560	1570	1580	1590	1600
AEMEEALLEW	GTLAMVSMHM	RKLEVLVNFM	RAQRASQGGQ	SLPSVRQTSL	LSALLVMAVT	HRDIAQLLEQ	HQVSDLTDFH
1610	1620	1630	1640	1650	1660	1670	1680
WVRQLKYHLG	SPHIIPKSPL	QSLKTIASSE	PSLSPAACWI	DVLGRSFLYN	YEYLGPRLGP	LPSLLPERPA	LVLLLALEEV
1690	1700	1710	1720	1730	1740	1750	1760
ACGTVLGPNG	VGKRAIVNSL	AQALGRQLVM	LPCSPQIEAQ	CLSNYLNGAL	QGGAWLLLEK	VHQLPPGLLS	ALGQRLGELH
1770	1780	1790	1800	1810	1820	1830	1840
HLYAPLYQEA	SRNTSTIDPT	QPQLLGSSFF	EKHHVSVRLG	YGCLLVLRAL	SSAVPANLHL	LLRPVALALP	DLRQVAELTL
1850	1860	1870	1880	1890	1900	1910	1920
LGAGMRDAFQ	MATRLSKFFS	LERELVSGPL	PCRLPLLKQI	LEDTIRTLNV	TKEEPKCQKP	RSLAAIEEAA	LLRSPLFSIL
1930	1940	1950	1960	1970	1980	1990	2000
NGLHLHNLRG	LLCALFPSAS	QVLAEPMTYK	LMKPLVVEEL	QQVGLDPSPD	ILGSLEQLSQ	ALSRASGILL	LGPAGSGKTT
2010	2020	2030	2040	2050	2060	2070	2080
CWHSLFKIQN	RLAAMEDTST	QGCQPVEITH	LYPSGLSPQE	FLGWLEGSCW	HHGIFPKVLR	AAGQCNNMGQ	KRQTEESIGI
2090	2100	2110	2120	2130	2140	2150	2160
QHWIICDGAS	NGAWLDSITC	LLSELPQLSL	PSGQQIARPP	GTFLLMEVAD	TTGISPTVVG	CCALVWCGGE	QTWQCILSAL
2170	2180	2190	2200	2210	2220	2230	2240
MASLPYEYRL	QHRTVAELNH	MAEVLVPATL	RFLTCQGVSS	LLQVHGQQAV	CAGVAEVTSM	ARILHSLLDL	HLRLKEEKAP
2250	2260	2270	2280	2290	2300	2310	2320
GPEDLSYSDP	VAQSFRSSKS	SFLNRSQVDS	DDVPDKCREH	LLAVSSFLFA	LIWGFGAHLP	SRFWPIFDTF	IRDSISRLSN
2330	2340	2350	2360	2370	2380	2390	2400

Detailed Protein Report

Cmpd.	No. of Cmpds.	m/z meas.	Δ m/z [ppm]	z	Rt [min]	Score	P	Range	Sequence	Modification
2087	1	753.3822	17.22	2	56.5	20.7	1	2058-2071	K.VLRAAGQCNNMGQK.R	Oxidation: 11

Detailed Protein Report

Protein 185: PREDICTED: putative Polycomb group protein ASXL2 isoform X2 [Homo sapiens]

Accession: gi|578802980
Database: refseq_human(refseq_human_20140103.fasta)
Database Date: 2015-11-30

Score: 34.0
MW [kDa]: 125.4
pI: 8.8
Sequence Coverage [%]: 2.5
No. of unique Peptides: 2

10	20	30	40	50	60	70	80
MKRTKCADID	VETPDSILVN	TNLRALINKH	TFSVLPGDCQ	QRLLLLLPEV	DRQVGPDGLM	KLNGSALNNE	FFTSAAQGWK
90	100	110	120	130	140	150	160
ERLSEGEFTP	EMQVRIRQEI	EKEKKVEPWK	EQFFESYYGQ	SSGLSLEDSK	KLTASPSDPK	VKKTPAEQPK	SMPVSEASLI
170	180	190	200	210	220	230	240
RIVPVVSQSE	CKEEALQMSS	PGRKEECESQ	GEVQPNFSTS	SEPLLSSALN	THELSSILPI	KCPKDEDLLE	QKPVTSAEQE
250	260	270	280	290	300	310	320
SEKNHLTTAS	NYNKSESQES	LVTSPSKPKS	PGVEKPIVKP	TAGAGPQETN	MKEPLATLVD	QSPESLKRKS	SLTQEEAPVS
330	340	350	360	370	380	390	400
WEKRPRVTEN	RQHQQPFQVS	PQPFLNRGDR	IQVRKVPPLK	IPVSRISPMP	FHPSQVSPRA	RFPVSITSPN	RTGARTLADI
410	420	430	440	450	460	470	480
KAKAQLVKAQ	RAAAAAAAAA	AAAASVGGTI	PGPGPGGGQG	PGEGGEGQTA	RGGSPGSDRV	SETGKGPTLE	LAGTGSRGGT
490	500	510	520	530	540	550	560
RELLPCGPET	QPQSETKTTP	SQAQPHSVSG	AQLQQTPPVP	PTPAVSGACT	SVPSPAHIEK	LDNEKLNPTR	ATATVASVSH
570	580	590	600	610	620	630	640
PQGPSSCRQE	KAPSPTGPAL	ISGASPVHCA	ADGTVELKAG	PSKNIPNPSA	SSKTDASVPV	AVTPSPLTSL	LTTATLEKLP
650	660	670	680	690	700	710	720
VPQVSATTAP	AGSAPPSSTL	PAASSLKTPG	TSLNMNGPTL	RPTSSIPANN	PLVTQLLQGK	DVPMEQILPK	PLTKVEMKTV
730	740	750	760	770	780	790	800
PLTAKEERGM	GALIATNTTE	NSTREEVNER	QSHPATQQQL	GKTLQSKQLP	QVPRPLQLFS	AKELRDSSID	THQYHEGLSK
810	820	830	840	850	860	870	880
ATQDQILQTL	IQRVRRQNLL	SVVPPSQFNF	AHSGFQLEDI	STSQRFMLGF	AGRRTSKPAM	AGHYLLNIST	YGRGSESFRR
890	900	910	920	930	940	950	960
THSVNPEDRF	CLSSPTEALK	MGYTDCKNAT	GESSSSKEDD	TDEESTGDEQ	ESVTVKEEPQ	VSQSAGKGDT	SSGPHSRETL
970	980	990	1000	1010	1020	1030	1040
STSDCLASKN	VKAEIPLNEQ	TTLSKENYLF	TRGQTFDEKT	LARDLIQAAQ	KQMAHAVRGK	AIRSSPELFS	STVLPLPADS
1050	1060	1070	1080	1090	1100	1110	1120
PTHQPLLLPP	LQTPKLYGSP	TQIGPSYRGM	INVSTSSDMD	HNSAVPGSQV	SSNVGDVMSF	SVTVTTIPAS	QAMNPSSHGQ
1130	1140	1150	1160	1170	1180		
TIPVQAFSEE	NSIEGTPSKC	YCRLKAMIMC	KGCGAFCHDD	CIGPSKLCVS	CLVVR		

Cmpd.	No. of Cmpds.	m/z meas.	Δ m/z [ppm]	z	Rt [min]	Score	P	Range	Sequence	Modification
326	1	761.7364	-167.46	2	34.1	10.4	0	83-95	R.LSEGEFTPEMQVR.I	
2134	8	937.3539	-101.03	2	55.3	23.7	2	874-889	R.GSESFRRTHSVNPEDR.F	

Detailed Protein Report

Protein 186: PREDICTED: tau-tubulin kinase 2 isoform X5 [Homo sapiens]

**Accession:**	gi|578826647	**Score:**	34.0
**Database:**	refseq_human(refseq_human_20140103.fasta)	**MW [kDa]:**	129.8
**Database Date:**	2015-11-30	**pI:**	6.5
		**Sequence Coverage [%]:**	2.8
		**No. of unique Peptides:**	1

10	20	30	40	50	60	70	80
MLPGKDHVCR	FIGCGRNDRF	NYVVMQLQGR	NLADLRRSQS	RGTFTISTTL	RLGRQILESI	ESIHSVGFLH	RDIKPSNFAM
**90**	**100**	**110**	**120**	**130**	**140**	**150**	**160**
GRFPSTCRKC	YMLDFGLARQ	FTNSCGDVRP	PRAVAGFRGT	VRYASINAHR	NREMGRHDDL	WSLFYMLVEF	VVGQLPWRKI
**170**	**180**	**190**	**200**	**210**	**220**	**230**	**240**
KDKEQVGSIK	ERYDHRLMLK	HLPPEFSIFL	DHISSLDYFT	KPDYQLLTSV	FDNSIKTFGV	IESDPFDWEK	TGNDGSLTTT
**250**	**260**	**270**	**280**	**290**	**300**	**310**	**320**
TTSTTPQLHT	RLTPAAIGIA	NATPIPGDLL	RENTDEVFPD	EQLSDGENGI	PVGVSPDKLP	GSLGHPRPQE	KDVWEEMDAN
**330**	**340**	**350**	**360**	**370**	**380**	**390**	**400**
KNKIKLGICK	AATEEENSHG	QANGLLNAPS	LGSPIRVRSE	ITQPDRDIPL	VRKLRSIHSF	ELEKRLTLEP	KPDTDKFLET
**410**	**420**	**430**	**440**	**450**	**460**	**470**	**480**
CLEKMQKDTS	AGKESILPAL	LHKPCVPAVS	RTDHIWHYDE	EYLPDASKPA	SANTPEQADG	GGSNGFIAVN	LSSCKQEIDS
**490**	**500**	**510**	**520**	**530**	**540**	**550**	**560**
KEWVIVDKEQ	DLQDFRTNEA	VGHKTTGSPS	DEEPEVLQVL	EASPQDEKLQ	LGPWAENDHL	KKETSGVVLA	LSAEGPPTAA
**570**	**580**	**590**	**600**	**610**	**620**	**630**	**640**
SEQYTDRLEL	QPGAASQFIA	ATPTSLMEAQ	AEGPLTAITI	PRPSVASTQS	TSGSFHCGQQ	PEKKDLQPME	PTVELYSPRE
**650**	**660**	**670**	**680**	**690**	**700**	**710**	**720**
NFSGLVVTEG	EPPSGGSRTD	LGLQIDHIGH	DMLPNIRESN	KSQDLGPKEL	PDHNRLVVRE	FENLPGETEE	KSILLESDNE
**730**	**740**	**750**	**760**	**770**	**780**	**790**	**800**
DEKLSRGQHC	IEISSLPGDL	VIVEKDHSAT	TEPLDVTKTQ	TFSVVPNQDK	NNEIMKLLTV	GTSEISSRDI	DPHVEGQIGQ
**810**	**820**	**830**	**840**	**850**	**860**	**870**	**880**
VAEMQKNKIS	KDDDIMSEDL	PGHQGDLSTF	LHQEGKREKI	TPRNGELFHC	VSENEHGAPT	RKDMVRSSFV	TRHSRIPVLA
**890**	**900**	**910**	**920**	**930**	**940**	**950**	**960**
QEIDSTLESS	SPVSAKEKLL	QKKAYQPDLV	KLLVEKRQFK	SFLGDLSSAS	DKLLEEKLAT	VPAPFCEEEV	LTPFSRLTVD
**970**	**980**	**990**	**1000**	**1010**	**1020**	**1030**	**1040**
SHLSRSAEDS	FLSPIISQSR	KSKIPRPVSW	VNTDQVNSST	SSQFFPRPPP	GKPPTRPGVE	ARLRRYKVLG	SSNSDSDLFS
**1050**	**1060**	**1070**	**1080**	**1090**	**1100**	**1110**	**1120**
RLAQILQNGS	QKPRSTTQCK	SPGSPHNPKT	PPKSPVVPRR	SPSASPRSSS	LPRTSSSSPS	RAGRPHHDQR	SSSPHLGRSK
**1130**	**1140**	**1150**	**1160**	**1170**	**1180**		
SPPSHSGSSS	SRRSCQQEHC	KPSKNGLKGS	GSLHHHSAST	KTPQGKSKPA	SKLSR		

Cmpd.	No. of Cmpds.	m/z meas.	Δ m/z [ppm]	z	Rt [min]	Score	P	Range	Sequence	Modification
2024	1	694.3465	21.44	2	55.7	10.8	1	1119-1132	R.SKSPPSHSGSSSSR.R	

Detailed Protein Report

Protein 187: fibrillin-1 precursor [Homo sapiens]

**Accession:**	gi|281485550	**Score:**	34.0
**Database:**	refseq_human(refseq_human_20140103.fasta)	**MW [kDa]:**	312.1
**Database Date:**	2015-11-30	**pI:**	4.7
**Modification(s):**	Carbamidomethyl, Oxidation	**Sequence Coverage [%]:**	1.3
		**No. of unique Peptides:**	2

Quantitation

QD:QU	**Median:** 0.71	**CV:** 0.00 %	**No. of Peptides:** 1

Detailed Protein Report

10	20	30	40	50	60	70	80
MRRGRLLEIA	LGFTVLLASY	TSHGADANLE	AGNVKETRAS	RAKRRGGGGH	DALKGPNVCG	SRYNAYCCPG	WKTLPGGNQC
90	100	110	120	130	140	150	160
IVPICRHSCG	DGFCSRPNMC	TCPSGQIAPS	CGSRSIQHCN	IRCMNGGSCS	DDHCLCQKGY	IGTHCGQPVC	ESGCLNGGRC
170	180	190	200	210	220	230	240
VAPNRCACTY	GFTGPQCERD	YRTGPCFTVI	SNQMCQGQLS	GIVCTKTLCC	ATVGRAWGHP	CEMCPAQPHP	CRRGFIPNIR
250	260	270	280	290	300	310	320
TGACQDVDEC	QAIPGLCQGG	NCINTVGSFE	CKCPAGHKLN	EVSQKCEDID	ECSTIPGICE	GGECTNTVSS	YFCKCPPGFY
330	340	350	360	370	380	390	400
TSPDGTRCID	VRPGYCYTAL	TNGRCSNQLP	QSITKMQCCC	DAGRCWSPGV	TVAPEMCPIR	ATEDFNKLCS	VPMVIPGRPE
410	420	430	440	450	460	470	480
YPPPPLGPIP	PVLPVPPGFP	PGPQIPVPRP	PVEYLYPSRE	PPRVLPVNVT	DYCQLVRYLC	QNGRCIPTPG	SYRCECNKGF
490	500	510	520	530	540	550	560
QLDLRGECID	VDECEKNPCA	GGECINNQGS	YTCQCRAGYQ	STLTRTECRD	IDECLQNGRI	CNNGRCINTD	GSFHCVCNAG
570	580	590	600	610	620	630	640
FHVTRDGKNC	EDMDECSIRN	MCLNGMCINE	DGSFKCICKP	GFQLASDGRY	CKDINECETP	GICMNGRCVN	TDGSYRCECF
650	660	670	680	690	700	710	720
PGLAVGLDGR	VCVDTHMRST	CYGGYKRGQC	IKPLFGAVTK	SECCCASTEY	AFGEPCQPCP	AQNSAEYQAL	CSSGPGMTSA
730	740	750	760	770	780	790	800
GSDINECALD	PDICPNGICE	NLRGTYKCIC	NSGYEVDSTG	KNCVDINECV	LNSLLCDNGQ	CRNTPGSFVC	TCPKGFIYKP
810	820	830	840	850	860	870	880
DLKTCEDIDE	CESSPCINGV	CKNSPGSFIC	ECSSESTLDP	TKTICIETIK	GTCWQTVIDG	RCEININGAT	LKSQCCSSLG
890	900	910	920	930	940	950	960
AAWGSPCTLC	QVDPICGKGY	SRIKGTQCED	IDECEVFPGV	CKNGLCVNTR	GSFKCQCPSG	MTLDATGRIC	LDIRLETCFL
970	980	990	1000	1010	1020	1030	1040
RYEDEECTLP	IAGRHRMDAC	CCSVGAAWGT	EECEECPMRN	TPEYEELCPR	GPGFATKEIT	NGKPFFKDIN	ECKMIPSLCT
1050	1060	1070	1080	1090	1100	1110	1120
HGKCRNTIGS	FKCRCDSGFA	LDSEERNCTD	IDECRISPDL	CGRGQCVNTP	GDFECKCDEG	YESGFMMMKN	CMDIDECQRD
1130	1140	1150	1160	1170	1180	1190	1200
PLLCRGGVCH	NTEGSYRCEC	PPGHQLSPNI	SACIDINECE	LSAHLCPNGR	CVNLIGKYQC	ACNPGYHSTP	DRLFCVDIDE
1210	1220	1230	1240	1250	1260	1270	1280
CSIMNGGCET	FCTNSEGSYE	CSCQPGFALM	PDQRSCTDID	ECEDNPNICD	GGQCTNIPGE	YRCLCYDGFM	ASEDMKTCVD
1290	1300	1310	1320	1330	1340	1350	1360
VNECDLNPNI	CLSGTCENTK	GSFICHCDMG	YSGKKGKTGC	TDINECEIGA	HNCGKHAVCT	NTAGSFKCSC	SPGWIGDGIK
1370	1380	1390	1400	1410	1420	1430	1440
CTDLDECSNG	THMCSQHADC	KNTMGSYRCL	CKEGYTGDGF	TCTDLDECSE	NLNLCGNGQC	LNAPGGYRCE	CDMGFVPSAD
1450	1460	1470	1480	1490	1500	1510	1520
GKACEDIDEC	SLPNICVFGT	CHNLPGLFRC	ECEIGYELDR	SGGNCTDVNE	CLDPTTCISG	NCVNTPGSYI	CDCPPDFELN
1530	1540	1550	1560	1570	1580	1590	1600
PTRVGCVDTR	SGNCYLDIRP	RGDNGDTACS	NEIGVGVSKA	SCCCSLGKAW	GTPCEMCPAV	NTSEYKILCP	GGEGFRPNPI
1610	1620	1630	1640	1650	1660	1670	1680
TVILEDIDEC	QELPGLCQGG	KCINTFGSFQ	CRCPTGYYLN	EDTRVCDDVN	ECETPGICGP	GTCYNTVGNY	TCICPPDYMQ
1690	1700	1710	1720	1730	1740	1750	1760
VNGGNNCMDM	RRSLCYRNYY	ADNQTCDGEL	LFNMTKKMCC	CSYNIGRAWN	KPCEQCPIPS	TDEFATLCGS	QRPGFVIDIY
1770	1780	1790	1800	1810	1820	1830	1840
TGLPVDIDEC	REIPGVCENG	VCINMVGSFR	CECPVGFFYN	DKLLVCEDID	ECQNGPVCQR	NAECINTAGS	YRCDCKPGYR
1850	1860	1870	1880	1890	1900	1910	1920
FTSTGQCNDR	NECQEIPNIC	SHGQCIDTVG	SFYCLCHTGF	KTNDDQTMCL	DINECERDAC	GNGTCRNTIG	SFNCRCNHGF
1930	1940	1950	1960	1970	1980	1990	2000
ILSHNNDCID	VDECASGNGN	LCRNGQCINT	VGSFQCQCNE	GYEVAPDGRT	CVDINECLLE	PRKCAPGTCQ	NLDGSYRCIC
2010	2020	2030	2040	2050	2060	2070	2080
PPGYSLQNEK	CEDIDECVEE	PEICALGTCS	NTEGSFKCLC	PEGFSLSSSG	RRCQDLRMSY	CYAKFEGGKC	SSPKSRNHSK
2090	2100	2110	2120	2130	2140	2150	2160
QECCCALKGE	GWGDPCELCP	TEPDEAFRQI	CPYGSGIIVG	PDDSAVDMDE	CKEPDVCKHG	QCINTDGSYR	CECPFGYILA
2170	2180	2190	2200	2210	2220	2230	2240

Detailed Protein Report

Cmpd.	No. of Cmpds.	m/z meas.	Δ m/z [ppm]	z	Rt [min]	Score	P	Range	Sequence	Modification	Ratios	
1872	1	1057.6895	-79.32	3	53.8	11.0	2	610-636	R. YCKDINECETPGICMNGRCVNTD[illegible] C	Carbamidomethyl: 2, 19; Oxidation: 15		
976	6	635.1635	-177.36	2	40.7	23.1	1	2482-2492	R.SCKDLDECATK.Q	Carbamidomethyl: 8	QD:QU	0.71

Detailed Protein Report

Protein 188: SCO-spondin precursor [Homo sapiens]

**Accession:**	gi|134031945	**Score:**	34.0
**Database:**	refseq_human(refseq_human_20140103.fasta)	**MW [kDa]:**	547.1
**Database Date:**	2015-11-30	**pI:**	5.6
**Modification(s):**	Carbamidomethyl	**Sequence Coverage [%]:**	1.0
		**No. of unique Peptides:**	2

Detailed Protein Report

10	20	30	40	50	60	70	80
MLLPALLFGM	AWALADGRWC	EWTETIRVEE	EVAPRQEDLV	PCASLDHYSR	LGWRLDLPWS	GRSGLTRSPA	PGLCPIYKPP
90	100	110	120	130	140	150	160
ETRPAKWNRT	VRTCCPGWGG	AHCTEALAKA	SPEGHCFAMW	QCQLQAGSAN	ASAGSLEECC	ARPWGQSWWD	GSSQACRSCS
170	180	190	200	210	220	230	240
SRHLPGSASS	PALLQPLAGA	VGQLWSQHQR	PSATCASWSG	FHYRTFDGRH	YHFLGRCTYL	LAGAADSTWA	VHLTPGDRCP
250	260	270	280	290	300	310	320
QPGHCQRVTM	GPEEVLIQAG	NVSVKGQLVP	EGQSWLLHGL	SLQWLGDWLV	LSGGLGVVVR	LDRTGSISIS	VDHELWGQTQ
330	340	350	360	370	380	390	400
GLCGLYNGWP	EDDFMEPGGG	LAMLAATFGN	SWRLPGSESG	CLDAVEVAQG	CDPLGLIDAD	VEPGHLRAEA	QDVCHQLLEG
410	420	430	440	450	460	470	480
PFGQCHAQVS	PAEYHEACLF	AYCAGAMAGS	GQEGRQQAVC	ATFASYVQAC	ARRHIHIRWR	KPGFCERLCP	GGQLYSDCVS
490	500	510	520	530	540	550	560
LCPPSCEAVG	QGEEESCREE	CVSGCECPRG	LFWNGTLCVP	AAHCPCYYCR	QRYVPGDTVR	QLCNPCVCRD	GRWHCAQALC
570	580	590	600	610	620	630	640
PAECAVGGDG	HYLTFDGRSY	SFWGGQGCRY	SLVQDYVKGQ	LLILLEHGAC	DAGSCLHAIS	VSLEDTHIQL	RDSGAVLVNG
650	660	670	680	690	700	710	720
QDVGLPWIGA	EGLSVRRASS	AFLLLRWPGA	QVLWGLSDPV	AYITLDPRHA	HQVQGLCGTF	TQNQQDDFLT	PAGDVETSIA
730	740	750	760	770	780	790	800
AFASKFQVAG	KGRCPSEDSA	LLSPCTTHSQ	RHAFAEAACA	ILHSSVFQEC	HRLVDKEPFY	LRCLAAVCGC	DPGSDCLCPV
810	820	830	840	850	860	870	880
LSAYARRCAQ	EGASPPWRNQ	TLCPVMCPGG	QEYRECAPAC	GQHCGKPEDC	GELGSCVAGC	NCPLGLLWDP	EGQCVPPSLC
890	900	910	920	930	940	950	960
PCQLGARRYA	PGSATMKECN	RCICQERGLW	NCTARHCPSQ	AFCPRELVYA	PGACLLTCDS	PSANHSCPAG	STDGCVCPPG
970	980	990	1000	1010	1020	1030	1040
TVLLDERCVP	PDLCPCRHSG	QWYLPNATIQ	EDCNVCVCRG	RQWHCTGQRR	SGRCQASGAP	HYVTFDGLAF	TYPGACEYLL
1050	1060	1070	1080	1090	1100	1110	1120
VREASGLFTV	SAQNLPCGAS	GLTCTKALAV	RLEGTVVHML	RGRAVTVNGV	SVTPPKVYTG	PGLSLRRAGL	FLLLSTHLGL
1130	1140	1150	1160	1170	1180	1190	1200
TLLWDGGTRV	LVQLSPQFRG	RVAGLCGDFD	GDASNDLRSR	QGVLEPTAEL	AAHSWRLSPL	CPEPGDLPHP	CTMNTHRAGW
1210	1220	1230	1240	1250	1260	1270	1280
ARARCGALLQ	PLFTLCHAEV	PPQQHYEWCL	YDACGCDSGG	DCECLCSAIA	TYADECARHG	HHVRWRSQEL	CSLQCEGGQV
1290	1300	1310	1320	1330	1340	1350	1360
YEACGPTCPP	TCHEQHPEPG	WHCQVVACVE	GCFCPEGTLL	HGGACLEPAS	CPCEWGRNSF	PPGSVLQKDC	GNCTCQEGQW
1370	1380	1390	1400	1410	1420	1430	1440
HCGGDGGHCE	ELVPACAEGE	ALCQENGHCV	PHGWLCDNQD	DCGDGSDEEG	CAAPGCGEGQ	MTCSSGHCLP	LALLCDRQDD
1450	1460	1470	1480	1490	1500	1510	1520
CGDGTDEPSY	PCPQGLLACA	DGRCLPPALL	CDGHPDCLDA	ADEESCLGQV	TCVPGEVSCV	DGTCLGAIQL	CDGVWDCPDG
1530	1540	1550	1560	1570	1580	1590	1600
ADEGPGHCPL	PSLPTPPAST	LPGPSPGSLD	TASSPLASAS	PAPPCGPFEF	RCGSGECTPR	GWRCDQEEDC	ADGSDERGCG
1610	1620	1630	1640	1650	1660	1670	1680
GPCAPHHAPC	ARGPHCVSPE	QLCDGVRQCP	DGSDEGPDAC	GGLPALGGPN	RTGLPCPEYT	CPNGTCIGFQ	LVCDGQPDCG
1690	1700	1710	1720	1730	1740	1750	1760
RPGQVGPSPE	EQGCGAWGPW	SPWGPCSRTC	GPWGQGRSRR	CSPLGLLVLQ	NCPGPEHQSQ	ACFTAACPVD	GEWSTWSPWS
1770	1780	1790	1800	1810	1820	1830	1840
VCSEPCRGTM	TRQRQCHSPQ	NGGRTCAALP	GGLHSTRQTK	PCPQDGCPNA	TCSGELMFQP	CAPCPLTCDD	ISGQVTCPPD
1850	1860	1870	1880	1890	1900	1910	1920
WPCGSPGCWC	PEGQVLGSEG	WCVWPRQCPC	LVDGARYWPG	QRIKADCQLC	ICQDGRPRRC	RLNPDCAVDC	GWSSWSPWAK
1930	1940	1950	1960	1970	1980	1990	2000
CLGPCGSQSI	QWSFRSSNNP	RPSGRGRQCR	GIHRKARRCQ	TEPCEGCEHQ	GQVHRVGERW	HGGPCRVCQC	LHNLTAHCSP
2010	2020	2030	2040	2050	2060	2070	2080
YCPLGSCPQG	WVLVEGTGES	CCHCALPGEN	QTVQPMATPA	AAPAPSPQIR	FPLATYILPP	SGDPCYSPLG	LAGLAEGSLH
2090	2100	2110	2120	2130	2140	2150	2160
ASSQQLEHPT	QAALLGAPTQ	GPSPQGWHAG	GDAYAKWHTR	PHYLQLDLLQ	PRNLTGILVP	ETGSSNAYAS	SFSLQFSSNG
2170	2180	2190	2200	2210	2220	2230	2240

Detailed Protein Report

Cmpd.	No. of Cmpds.	m/z meas.	Δ m/z [ppm]	z	Rt [min]	Score	P	Range	Sequence	Modification
1387	1	927.1161	66.38	3	47.5	19.2	0	2250-2277	R.GGPCDGVLDCEDGSDEEGCVLLPEGTGR.F	
1675	2	1136.5319	16.36	2	49.5	14.8	1	2854-2875	R.GRSCSSLAPGDTTCPGPHSQTR.D	Carbamidomethyl: 4

Detailed Protein Report

Protein 189: **PREDICTED: leucine-rich repeat serine/threonine-protein kinase 2 isoform X1 [Homo sapiens]**

**Accession:**	gi|530399725	**Score:**	33.9
**Database:**	refseq_human(refseq_human_20140103.fasta)	**MW [kDa]:**	285.2
**Database Date:**	2015-11-30	**pI:**	6.3
		**Sequence Coverage [%]:**	1.2
		**No. of unique Peptides:**	1

Quantitation

QD:QU	**Median:** 4.52	**CV:** 0.00 %	**No. of Peptides:** 1

Detailed Protein Report

10	20	30	40	50	60	70	80
MASGSCQGCE	EDEETLKKLI	VRLNNVQEGK	QIETLVQILE	DLLVFTYSER	ASKLFQGKNI	HVPLLIVLDS	YMRVASVQQV
90	100	110	120	130	140	150	160
GWSLLCKLIE	VCPGTMQSLM	GPQDVGNDWE	VLGVHQLILK	MLTVHNASVN	LSVIGLKTLD	LLLTSGKITL	LILDEESDIF
170	180	190	200	210	220	230	240
MLIFDAMHSF	PANDEVQKLG	CKALHVLFER	VSEEQLTEFV	ENKDYMILLS	ALTNFKDEEE	IVLHVLHCLH	SLAIPCNNVE
250	260	270	280	290	300	310	320
VLMSGNVRCY	NIVVEAMKAF	PMSERIQEVS	CCLLHRLTLG	NFFNILVLNE	VHEFVVKAVQ	QYPENAALQI	SALSCLALLT
330	340	350	360	370	380	390	400
ETIFLNQDLE	EKNENQENDD	EGEEDKLFWL	EACYKALTWH	RKNKHVQEAA	CWALNNLLMY	QNSLHEKIGD	EDGHFPAHRE
410	420	430	440	450	460	470	480
VMLSMLMHSS	SKEVFQASAN	ALSTLLEQNV	NFRKILLSKG	IHLNVLELMQ	KHIHSPEVAE	SGCKMLNHLF	EGSNTSLDIM
490	500	510	520	530	540	550	560
AAVVPKILTV	MKRHETSLPV	QLEALRAILH	FIVPGMPEES	REDTEFHHKL	NMVKKQCFKN	DIHKLVLAAL	NRFIGNPGIQ
570	580	590	600	610	620	630	640
KCGLKVISSI	VHFPDALEML	SLEGAMDSVL	HTLQMYPDDQ	EIQCLGLSLI	GYLITKKNVF	IGTGHLLAKI	LVSSLYRFKD
650	660	670	680	690	700	710	720
VAEIQTKGFQ	TILAILKLSA	SFSKLLVHHS	FDLVIFHQMS	SNIMEQKDQQ	FLNLCCKCFA	KVAMDDYLKN	VMLERACDQN
730	740	750	760	770	780	790	800
NSIMVECLLL	LGADANQAKE	GSSLICQVCE	KESSPKLVEL	LLNSGSREQD	VRKALTISIG	KGDSQIISLL	LRRLALDVAN
810	820	830	840	850	860	870	880
NSICLGGFCI	GKVEPSWLGP	LFPDKTSNLR	KQTNIASTLA	RMVIRYQMKS	AVEEGTASGS	DGNFSEDVLS	KFDEWTFIPD
890	900	910	920	930	940	950	960
SSMDSVFAQS	DDLDSEGSEG	SFLVKKKSNS	ISVGEFYRDA	VLQRCSPNLQ	RHSNSLGPIF	DHEDLLKRKR	KILSSDDSLR
970	980	990	1000	1010	1020	1030	1040
SSKLQSHMRH	SDSISSLASE	REYITSLDLS	ANELRDIDAL	SQKCCISVHL	EHLEKLELHQ	NALTSFPQQL	CETLKSLTHL
1050	1060	1070	1080	1090	1100	1110	1120
DLHSNKFTSF	PSYLLKMSCI	ANLDVSRNDI	GPSVVLDPTV	KCPTLKQFNL	SYNQLSFVPE	NLTDVVEKLE	QLILEGNKIS
1130	1140	1150	1160	1170	1180	1190	1200
GICSPLRLKE	LKILNLSKNH	ISSLSENFLE	ACPKVESFSA	RMNFLAAMPF	LPPSMTILKL	SQNKFSCIPE	AILNLPHLRS
1210	1220	1230	1240	1250	1260	1270	1280
LDMSSNDIQY	LPGPAHWKSL	NLRELLFSHN	QISILDLSEK	AYLWSRVEKL	HLSHNKLKEI	PPEIGCLENL	TSLDVSYNLE
1290	1300	1310	1320	1330	1340	1350	1360
LRSFPNEMGK	LSKIWDLPLD	ELHLNFDFKH	IGCKAKDIIR	FLQQRLKKAV	PYNRMKLMIV	GNTGSGKTTL	LQQLMKTKKS
1370	1380	1390	1400	1410	1420	1430	1440
DLGMQSATVG	IDVKDWPIQI	RDKRKRDLVL	NVWDFAGREE	FYSTHPHFMT	QRALYLAVYD	LSKGQAEVDA	MKPWLFNIKA
1450	1460	1470	1480	1490	1500	1510	1520
RASSSPVILV	GTHLDVSDEK	QRKACMSKIT	KELLNKRGFP	AIRDYHFVNA	TEESDALAKL	RKTIINESLN	FKIRDQLVVG
1530	1540	1550	1560	1570	1580	1590	1600
QLIPDCYVEL	EKIILSERKN	VPIEFPVIDR	KRLLQLVREN	QLQLDENELP	HAVHFLNESG	VLLHFQDPAL	QLSDLYFVEP
1610	1620	1630	1640	1650	1660	1670	1680
KWLCKIMAQI	LTVKVEGCPK	HPKGIISRRD	VEKFLSKKRK	FPKNYMSQYF	KLLEKFQIAL	PIGEEYLLVP	SSLSDHRPVI
1690	1700	1710	1720	1730	1740	1750	1760
ELPHCENSEI	IIRLYEMPYF	PMGFWSRLIN	RLLEISPYML	SGRERALRPN	RMYWRQGIYL	NWSPEAYCLV	GSEVLDNHPE
1770	1780	1790	1800	1810	1820	1830	1840
SFLKITVPSC	RKGCILLGQV	VDHIDSLMEE	WFPGLLEIDI	CGEGETLLKK	WALYSFNDGE	EHQKILLDDL	MKKAEEGDLL
1850	1860	1870	1880	1890	1900	1910	1920
VNPDQPRLTI	PISQIAPDLI	LADLPRNIML	NNDELEFEQA	PEFLLGDGSF	GSVYRAAYEG	EEVAVKIFNK	HTSLRLLRQE
1930	1940	1950	1960	1970	1980	1990	2000
LVVLCHLHHP	SLISLLAAGI	RPRMLVMELA	SKGSLDRLLQ	QDKASLTRTL	QHRIALHVAD	GLRYLHSAMI	IYRDLKPHNV
2010	2020	2030	2040	2050	2060	2070	2080
LLFTLYPNAA	IIAKIADYGI	AQYCCRMGIK	TSEGTPGFRA	PEVARGNVIY	NQQADVYSFG	LLLYDILTTG	GRIVEGLKFP
2090	2100	2110	2120	2130	2140	2150	2160
NEFDELEIQG	KLPDPVKEYG	CAPWPMVEKL	IKQCLKENPQ	ERPTSAQVFD	ILNSAELVCL	TRRILLPKNV	IVECMVATHH
2170	2180	2190	2200	2210	2220	2230	2240

Detailed Protein Report

Cmpd.	No. of Cmpds.	m/z meas.	Δ m/z [ppm]	z	Rt [min]	Score	P	Range	Sequence	Modification	Ratios
394	1	591.8037	-59.20	2	34.9	10.0	1	1964-1973	K.ASLTRTLQHR.I		QD:QU 4.52

Detailed Protein Report

Protein 190: collagen alpha-6(IV) chain isoform 5 precursor [Homo sapiens]

**Accession:**	gi|567757604	**Score:**	33.7
**Database:**	refseq_human(refseq_human_20140103.fasta)	**MW [kDa]:**	158.0
**Database Date:**	2015-11-30	**pI:**	10.0
**Modification(s):**	Carbamidomethyl, Oxidation	**Sequence Coverage [%]:**	1.8
		**No. of unique Peptides:**	2

10	20	30	40	50	60	70	80
MHPGLWLLLV	TLCLTEELAA	AGEKSYGKPC	GGQDCSGSCQ	CFPEKGARGR	PGPIGIQGPT	GPQGFTGSTG	LSGLKGERGF
90	100	110	120	130	140	150	160
PGLLGPYGPK	GDKGPMGVPG	FLGINGIPGH	PGQPGPRGPP	GLDGCNGTQG	AVGFPGPDGY	PGLLGPPGLP	GQKGSKGDPV
170	180	190	200	210	220	230	240
LAPGSFKGMK	GDPGLPGLDG	ITGPQGAPGF	PGAVGPAGPP	GLQGPPGPPG	PLGPDGNMGL	GFQGEKGVKG	DVGLPGPAGP
250	260	270	280	290	300	310	320
PPSTGELEFM	GFPKGKKGSK	GEPGPKGFPG	ISGPPGFPGL	GTTGEKGEKG	EKGIPGLPGP	RGPMGSEGVQ	GPPGQQGKKG
330	340	350	360	370	380	390	400
TLGFPGLNGF	QGIEGQKGDI	GLPGPDVFID	IDGAVISGNP	GDPGVPGLPG	LKGDEGIQGL	RGPSGVPGLP	ALSGVPGALG
410	420	430	440	450	460	470	480
PQGFPGLKGD	QGNPGRTTIG	AAGLPGRDGL	PGPPGPPGPP	SPEFETETLH	NKESGFPGLR	GEQGPKGNLG	LKGIKGDSGF
490	500	510	520	530	540	550	560
CACDGGVPNT	GPPGEPGPPG	PWGLIGLPGL	KGARGDRGSG	GAQGPAGAPG	LVGPLGPSGP	KGKKGEPILS	TIQGMPGDRG
570	580	590	600	610	620	630	640
DSGSQGFRGV	IGEPGKDGVP	GLPGLPGLPG	DGGQGFPGEK	GLPGLPGEKG	HPGPPGLPGN	GLPGLPGPRG	LPGDKGKDGL
650	660	670	680	690	700	710	720
PGQQGLPGSK	GITLPCIIPG	SYGPSGFPGT	PGFPGPKGSR	GLPGTPGQPG	SSGSKGEPGS	PGLVHLPELP	GFPGPRGEKG
730	740	750	760	770	780	790	800
LPGFPGLPGK	DGLPGMIGSP	GLPGSKGATG	DIFGAENGAP	GEQGLQGLTG	HKGFLGDSGL	PGLKGVHGKP	GLLGPKGERG
810	820	830	840	850	860	870	880
SPGTPGQVGQ	PGTPGSSGPY	GIKGKSGLPG	APGFPGISGH	PGKKGTRGKK	GPPGSIVKKG	LPGLKGLPGN	PGLVGLKGSP
890	900	910	920	930	940	950	960
GSPGVAGLPA	LSGPKGEKGS	VGFVGFPGIP	GLPGIPGTRG	LKGIPGSTGK	MGPSGRAGTP	GEKGDRGNPG	PVGIPSPRRP
970	980	990	1000	1010	1020	1030	1040
MSNLWLKGDK	GSQGSAGSNG	FPGPRGDKGE	AGRPGPPGLP	GAPGLPGIIK	GVSGKPGPPG	FMGIRGLPGL	KGSSGITGFP
1050	1060	1070	1080	1090	1100	1110	1120
GMPGESGSQG	IRGSPGLPGA	SGLPGLKGDN	GQTVEISGSP	GPKGQPGESG	FKGTKGRDGL	IGNIGFPGNK	GEDGKVGVSG
1130	1140	1150	1160	1170	1180	1190	1200
DVGLPGAPGF	PGVAGMRGEP	GLPGSSGHQG	AIGPLGSPGL	IGPKGPSITG	VPGPAGLPGP	KGEKGYPGIG	IGAPGKPGLR
1210	1220	1230	1240	1250	1260	1270	1280
GQKGDRGFPG	LQGPAGLPGA	PGISLPSLIA	GQPGDPGRPG	LDGERGRPGP	AGPPGPPGPS	SNQGDTGDPG	FPGIPGPKGP
1290	1300	1310	1320	1330	1340	1350	1360
KGDQGIPGFS	GLPGELGLKG	SSGLQGDPGQ	TPTAEAVQVP	PGPLGLPGID	GIPGLTGDPG	AQGPVGLQGS	KGLPGIPGKD
1370	1380	1390	1400	1410	1420	1430	1440
GPSGLPGPPG	ALGDPGLPGL	QGPPGFEGAP	GQQGPFGMPG	MPGQSMRVGY	TLVKHSQSEQ	VPPCPIGMSQ	LWVGYSLLFV
1450	1460	1470	1480	1490	1500	1510	1520
EGQEKAHNQD	LGFAGSCLPR	FSTMPFIYCN	INEVCHYARR	NDKSYWLSTT	APIPMMPVSQ	TQIPQYISRC	SVCEAPSQAI
1530	1540	1550	1560	1570	1580	1590	1600
AVHSQDITIP	QCPLGWRSLW	IGYSFLMHTA	AGAEGGGQSL	VSPGSCLEDF	RATPFIECSG	ARGTCHYFAN	KYSFWLTTVE
1610	1620	1630	1640				
ERQQFGELPV	SETLKAGQLH	TRVSRCQVCM	KSL				

Cmpd.	No. of Cmpds.	m/z meas.	Δ m/z [ppm]	z	Rt [min]	Score	P	Range	Sequence	Modification
2734	2	710.3788	14.25	2	63.1	11.1	1	157-170	K.GDPVLAPGSFKGMK.G	Oxidation: 13
2485	1	821.9375	56.09	2	61.6	11.5	0	1446-1460	K.AHNQDLGFAGSCLPR.F	Carbamidomethyl: 12

Detailed Protein Report

Protein 191: semaphorin-3A precursor [Homo sapiens]

**Accession:**	gi|5174673	**Score:**	33.4
**Database:**	refseq_human(refseq_human_20140103.fasta)	**MW [kDa]:**	88.8
**Database Date:**	2015-11-30	**pI:**	7.2
		**Sequence Coverage [%]:**	2.6
		**No. of unique Peptides:**	2

Quantitation

QD:QU **Median:** 0.59 **CV:** 0.00 % **No. of Peptides:** 1

Alias proteins:

Accession	Name	Description
gi|578813881	refseq_human (refseq_human_20140103.fasta)	PREDICTED: semaphorin-3A isoform X4 [Homo sapiens]
gi|578813879	refseq_human (refseq_human_20140103.fasta)	PREDICTED: semaphorin-3A isoform X3 [Homo sapiens]
gi|530385417	refseq_human (refseq_human_20140103.fasta)	PREDICTED: semaphorin-3A isoform X2 [Homo sapiens]
gi|530385415	refseq_human (refseq_human_20140103.fasta)	PREDICTED: semaphorin-3A isoform X1 [Homo sapiens]

```
        10         20         30         40         50         60         70         80
MGWLTRIVCL FWGVLLTARA NYQNGKNNVP RLKLSYKEML ESNNVITFNG LANSSSYHTF LLDEERSRLY VGAKDHIFSF
        90        100        110        120        130        140        150        160
DLVNIKDFQK IVWPVSYTRR DECKWAGKDI LKECANFIKV LKAYNQTHLY ACGTGAFHPI CTYIEIGHHP EDNIFKLENS
       170        180        190        200        210        220        230        240
HFENGRGKSP YDPKLLTASL LIDGELYSGT AADFMGRDFA IFRTLGHHHP IRTEQHDSRW LNDPKFISAH LISESDNPED
       250        260        270        280        290        300        310        320
DKVYFFFREN AIDGEHSGKA THARIGQICK NDFGGHRSLV NKWTTFLKAR LICSVPGPNG IDTHFDELQD VFLMNFKDPK
       330        340        350        360        370        380        390        400
NPVVYGVFTT SSNIFKGSAV CMYSMSDVRR VFLGPYAHRD GPNYQWVPYQ GRVPYPRPGT CPSKTFGGFD STKDLPDDVI
       410        420        430        440        450        460        470        480
TFARSHPAMY NPVFPMNNRP IVIKTDVNYQ FTQIVVDRVD AEDGQYDVMF IGTDVGTVLK VVSIPKETWY DLEEVLLEEM
       490        500        510        520        530        540        550        560
TVFREPTAIS AMELSTKQQQ LYIGSTAGVA QLPLHRCDIY GKACAECCLA RDPYCAWDGS ACSRYFPTAK RRTRRQDIRN
       570        580        590        600        610        620        630        640
GDPLTHCSDL HHDNHHGHSP EERIIYGVEN SSTFLECSPK SQRALVYWQF QRRNEERKEE IRVDDHIIRT DQGLLLRSLQ
       650        660        670        680        690        700        710        720
QKDSGNYLCH AVEHGFIQTL LKVTLEVIDT EHLEELLHKD DDGDGSKTKE MSNSMTPSQK VWYRDFMQLI NHPNLNTMDE
       730        740        750        760        770        780
FCEQVWKRDR KQRRQRPGHT PGNSNKWKHL QENKKGRNRR THEFERAPRS V
```

Cmpd.	No. of Cmpds.	m/z meas.	Δ m/z [ppm]	z	Rt [min]	Score	P	Range	Sequence	Modification	Ratios
1358	2	436.6061	-206.99	2	47.1	18.6	0	213-219	R.TEQHDSR.W		QD:QU 0.59
2058	1	722.8469	-17.26	2	56.1	14.8	1	265-277	R.IGQICKNDFGGHR.S		

Detailed Protein Report

Protein 192: tenascin-N precursor [Homo sapiens]

**Accession:**	gi|62988324	**Score:**	33.4
**Database:**	refseq_human(refseq_human_20140103.fasta)	**MW [kDa]:**	143.9
**Database Date:**	2015-11-30	**pI:**	5.3
		**Sequence Coverage [%]:**	1.5
		**No. of unique Peptides:**	1

10	20	30	40	50	60	70	80
MSLQEMFRFP	MGLLLGSVLL	VASAPATLEP	PGCSNKEQQV	TVSHTYKIDV	PKSALVQVDA	DPQPLSDDGA	SLLALGEARE
90	100	110	120	130	140	150	160
EQNIIFRHNI	RLQTPQKDCE	LAGSVQDLLA	RVKKLEEEMV	EMKEQCSAQR	CCQGVTDLSR	HCSGHGTFSL	ETCSCHCEEG
170	180	190	200	210	220	230	240
REGPACERLA	CPGACSGHGR	CVDGRCLCHE	PYVGADCGYP	ACPENCSGHG	ECVRGVCQCH	EDFMSEDCSE	KRCPGDCSGH
250	260	270	280	290	300	310	320
GFCDTGECYC	EEGFTGLDCA	QVVTPQGLQL	LKNTEDSLLV	SWEPSSQVDH	YLLSYYPLGK	ELSGKQIQVP	KEQHSYEILG
330	340	350	360	370	380	390	400
LLPGTKYIVT	LRNVKNEVSS	SPQHLLATTD	LAVLGTAWVT	DETENSLDVE	WENPSTEVDY	YKLRYGPMTG	QEVAEVTVPK
410	420	430	440	450	460	470	480
SSDPKSRYDI	TGLHPGTEYK	ITVVPMRGEL	EGKPILLNGR	TEIDSPTNVV	TDRVTEDTAT	VSWDPVQAVI	DKYVVRYTSA
490	500	510	520	530	540	550	560
DGDTKEMAVH	KDESSTVLTG	LKPGEAYKVY	VWAERGNQGS	KKADTNALTE	IDSPANLVTD	RVTENTATIS	WDPVQATIDK
570	580	590	600	610	620	630	640
YVVRYTSADD	QETREVLVGK	EQSSTVLTGL	RPGVEYTVHV	WAQKGDRESK	KADTNAPTDI	DSPKNLVTDR	VTENMATVSW
650	660	670	680	690	700	710	720
DPVQAAIDKY	VVRYTSAGGE	TREVPVGKEQ	SSTVLTGLRP	GMEYMVHVWA	QKGDQESKKA	DTKAQTDIDS	PQNLVTDRVT
730	740	750	760	770	780	790	800
ENMATVSWDP	VRATIDRYVV	RYTSAKDGET	REVPVGKEQS	STVLTGLRPG	VEYTVHVWAQ	KGAQESKKAD	TKAQTDIDSP
810	820	830	840	850	860	870	880
QNLVTDWVTE	NTATVSWDPV	QATIDRYVVH	YTSANGETRE	VPVGKEQSST	VLTGLRPGME	YTVHVWAQKG	NQESKKADTK
890	900	910	920	930	940	950	960
AQTEIDGPKN	LVTDWVTENM	ATVSWDPVQA	TIDKYMVRYT	SADGETREVP	VGKEHSSTVL	TGLRPGMEYM	VHVWAQKGAQ
970	980	990	1000	1010	1020	1030	1040
ESKKADTKAQ	TELDPPRNLR	PSAVTQSGGI	LTWTPPSAQI	HGYILTYQFP	DGTVKEMQLG	REDQRFALQG	LEQGATYPVS
1050	1060	1070	1080	1090	1100	1110	1120
LVAFKGGRRS	RNVSTTLSTV	GARFPHPSDC	SQVQQNSNAA	SGLYTIYLHG	DASRPLQVYC	DMETDGGGWI	VFQRRNTGQL
1130	1140	1150	1160	1170	1180	1190	1200
DFFKRWRSYV	EGFGDPMKEF	WLGLDKLHNL	TTGTPARYEV	RVDLQTANES	AYAIYDFFQV	ASSKERYKLT	VGKYRGTAGD
1210	1220	1230	1240	1250	1260	1270	1280
ALTYHNGWKF	TTFDRDNDIA	LSNCALTHHG	GWWYKNCHLA	NPNGRYGETK	HSEGVNWEPW	KGHEFSIPYV	ELKIRPHGYS
1290	1300						
REPVLGRKKR	TLRGRLRTF						

Cmpd.	No. of Cmpds.	m/z meas.	Δ m/z [ppm]	z	Rt [min]	Score	P	Range	Sequence	Modification
2573	1	590.4386	252.94	1	62.8	11.4	0	516-521	R.GNQGSK.K	

Detailed Protein Report

Protein 193: heat shock 70 kDa protein 1A/1B [Homo sapiens]

Accession: gi|167466173
Database: refseq_human(refseq_human_20140103.fasta)
Database Date: 2015-11-30

Score: 33.3
MW [kDa]: 70.0
pI: 5.4
Sequence Coverage [%]: 3.9
No. of unique Peptides: 2

Quantitation

QD:QU **Median:** 1.11 **CV:** 0.00 % **No. of Peptides:** 1

Alias proteins:

Accession	Name	Description
gi|194248072	refseq_human (refseq_human_20140103.fasta)	heat shock 70 kDa protein 1A/1B [Homo sapiens]

10	20	30	40	50	60	70	80
MAKAAAIGID	LGTTYSCVGV	FQHGKVEIIA	NDQGNRTTPS	YVAFTDTERL	IGDAAKNQVA	LNPQNTVFDA	KRLIGRKFGD
90	100	110	120	130	140	150	160
PVVQSDMKHW	PFQVINDGDK	PKVQVSYKGE	TKAFYPEEIS	SMVLTKMKEI	AEAYLGYPVT	NAVITVPAYF	NDSQRQATKD
170	180	190	200	210	220	230	240
AGVIAGLNVL	RIINEPTAAA	IAYGLDRTGK	GERNVLIFDL	GGGTFDVSIL	TIDDGIFEVK	ATAGDTHLGG	EDFDNRLVNH
250	260	270	280	290	300	310	320
FVEEFKRKHK	KDISQNKRAV	RRLRTACERA	KRTLSSSTQA	SLEIDSLFEG	IDFYTSITRA	RFEELCSDLF	RSTLEPVEKA
330	340	350	360	370	380	390	400
LRDAKLDKAQ	IHDLVLVGGS	TRIPKVQKLL	QDFFNGRDLN	KSINPDEAVA	YGAAVQAAIL	MGDKSENVQD	LLLLDVAPLS
410	420	430	440	450	460	470	480
LGLETAGGVM	TALIKRNSTI	PTKQTQIFTT	YSDNQPGVLI	QVYEGERAMT	KDNNLLGRFE	LSGIPPAPRG	VPQIEVTFDI
490	500	510	520	530	540	550	560
DANGILNVTA	TDKSTGKANK	ITITNDKGRL	SKEEIERMVQ	EAEKYKAEDE	VQRERVSAKN	ALESYAFNMK	SAVEDEGLKG
570	580	590	600	610	620	630	640
KISEADKKKV	LDKCQEVISW	LDANTLAEKD	EFEHKRKELE	QVCNPIISGL	YQGAGGPGPG	GFGAQGPKGG	SGSGPTIEEV
650							
D							

Cmpd.	No. of Cmpds.	m/z meas.	Δ m/z [ppm]	z	Rt [min]	Score	P	Range	Sequence	Modification	Ratios
602	1	744.3677	18.07	2	37.5	10.9	0	37-49	R.TTPSYVAFTDTER.L		QD:QU 1.11
1835	3	599.2683	-138.16	2	53.3	22.4	0	160-171	K.DAGVIAGLNVLR.I		

Detailed Protein Report

Protein 194: **PREDICTED: lysine-specific demethylase 2A isoform X3 [Homo sapiens]**

Accession: gi|578821239
Database: refseq_human(refseq_human_20140103.fasta)
Database Date: 2015-11-30

Score: 33.2
MW [kDa]: 120.2
pI: 9.2
Sequence Coverage [%]: 2.1
No. of unique Peptides: 2

10	20	30	40	50	60	70	80
MVDVMDVNTQ	KGIEMTMAQW	TRYYETPEEE	REKLYNVISL	EFSHTRLENM	VQRPSTVDFI	DWVDNMWPRH	LKESQTESTN
90	100	110	120	130	140	150	160
AILEMQYPKV	QKYCLMSVRG	CYTDFHVDFG	GTSVWYHIHQ	GGKVFWLIPP	TAHNLELYEN	WLLSGKQGDI	FLGDRVSDCQ
170	180	190	200	210	220	230	240
RIELKQGYTF	VIPSGWIHAV	YTPTDTLVFG	GNFLHSFNIP	MQLKIYNIED	RTRVPNKFRY	PFYYEMCWYV	LERYVYCITN
250	260	270	280	290	300	310	320
RSHLTKEFQK	ESLSMDLELN	GLESGNGDEE	AVDREPRRLS	SRRSVLTSPV	ANGVNLDYDG	LGKTCRSLPS	LKKTLAGDSS
330	340	350	360	370	380	390	400
SDCSRGSHNG	QVWDPQCAPR	KDRQVHLTHF	ELEGLRCLVD	KLESLPLHKK	CVPTGIEDED	ALIADVKILL	EELANSDPKL
410	420	430	440	450	460	470	480
ALTGVPIVQW	PKRDKLKFPT	RPKVRVPTIP	ITKPHTMKPA	PRLTPVRPAA	ASPIVSGARR	RRVRCRKCKA	CVQGECGVCH
490	500	510	520	530	540	550	560
YCRDMKKFGG	PGRMKQSCVL	RQCLAPRLPH	SVTCSLCGEV	DQNEETQDFE	KKLMECCICN	EIVHPGCLQM	DGEGLLNEEL
570	580	590	600	610	620	630	640
PNCWECPKCY	QEDSSEKAQK	RKMEESDEEA	VQAKVLRPLR	SCDEPLTPPP	HSPTSMLQLI	HDPVSPRGMV	TRSSPGAGPS
650	660	670	680	690	700	710	720
DHHSASRDER	FKRRQLLRLQ	ATERTMVREK	ENNPSGKKEL	SEVEKAKIRG	SYLTVTLQRP	TKELHGTSIV	PKLQAITASS
730	740	750	760	770	780	790	800
ANLRHSPRVL	VQHCPARTPQ	RGDEEGLGGE	EEEEEEEEEE	DDSAEEGGAA	RLNGRGSWAQ	DGDESWMQRE	VWMSVFRYLS
810	820	830	840	850	860	870	880
RRELCECMRV	CKTWYKWCCD	KRLWTKIDLS	RCKAIVPQAL	SGIIKRQPVS	LDLSWTNISK	KQLTWLVNRL	PGLKDLLLAG
890	900	910	920	930	940	950	960
CSWSAVSALS	TSSCPLLRTL	DLRWAVGIKD	PQIRDLLTPP	ADKPGQDNRS	KLRNMTDFRL	AGLDITDATL	RLIIRHMPLL
970	980	990	1000	1010	1020	1030	1040
SRLDLSHCSH	LTDQSSNLLT	AVGSSTRYSL	TELNMAGCNK	LTDQTLIYLR	RIANVTLIDL	RGCKQITRKA	CEHFISDLSI
1050	1060						
NSLYCLSDEK	LIQKIS						

Cmpd.	No. of Cmpds.	m/z meas.	Δ m/z [ppm]	z	Rt [min]	Score	P	Range	Sequence	Modification
846	1	663.6790	-184.30	2	40.6	10.3	1	313-325	K.KTLAGDSSSDCSR.G	
762	1	599.6213	-221.03	2	39.5	10.5	0	314-325	K.TLAGDSSSDCSR.G	

Detailed Protein Report

Protein 195: PREDICTED: thyroglobulin isoform X9 [Homo sapiens]

**Accession:**	gi|530389281	**Score:**	33.0
**Database:**	refseq_human(refseq_human_20140103.fasta)	**MW [kDa]:**	232.1
**Database Date:**	2015-11-30	**pI:**	5.1
		**Sequence Coverage [%]:**	1.1
		**No. of unique Peptides:**	2

Quantitation

QD:QU	**Median:** 0.78	**CV:** 0.00 %	**No. of Peptides:** 1

BRUKER

Detailed Protein Report

10	20	30	40	50	60	70	80
MALVLEIFTL	LASICWVSAN	IFEYQVDAQP	LRPCELQRET	AFLKQADYVP	QCAEDGSFQT	VQCQNDGRSC	WCVGANGSEV
90	100	110	120	130	140	150	160
LGSRQPGRPV	ACLSFCQLQK	QQILLSGYIN	STDTSYLPQC	QDSGDYAPVQ	CDVQQVQCWC	VDAEGMEVYG	TRQLGRPKRC
170	180	190	200	210	220	230	240
PRSCEIRNRR	LLHGVGDKSP	PQCSAEGEFM	PVQCKFVNTT	DMMIFDLVHS	YNRFPDAFVT	FSSFQRRFPE	VSGYCHCADS
250	260	270	280	290	300	310	320
QGRELAETGL	ELLLDEIYDT	IFAGLDLPST	FTETTLYRIL	QRRFLAVQSV	ISGRFRCPTK	CEVERFTATS	FGHPYVPSCR
330	340	350	360	370	380	390	400
RNGDYQAVQC	QTEGPCWCVD	AQGKEMHGTR	QQGEPPSCAE	GQSCASERQQ	ALSRLYFGTS	GYFSQHDLFS	SPEKRWASPR
410	420	430	440	450	460	470	480
VARFATSCPP	TIKELFVDSG	LLRPMVEGQS	QQFSVSENLL	KEAIRAIFPS	RGLARLALQF	TTNPKRLQQN	LFGGKFLVNV
490	500	510	520	530	540	550	560
GQFNLSGALG	TRGTFNFSQF	FQQLGLASFL	NGGRQEDLAK	PLSVGLDSNS	STGTPEAAKK	DGTMNKPTVG	SFGFEINLQE
570	580	590	600	610	620	630	640
NQNALKFLAS	LLELPEFLLF	LQHAISVPED	VARDLGDVME	TVLSSQTCEQ	TPERLFVPSC	TTEGSYEDVQ	CFSGECWCVN
650	660	670	680	690	700	710	720
SWGKELPGSR	VRGGQPRCPT	DCEKQRARMQ	SLMGSQPAGS	TLFVPACTSE	GHFLPVQCFN	SECYCVDAEG	QAIPGTRSAI
730	740	750	760	770	780	790	800
GKPKKCPTPC	QLQSEQAFLR	TVQALLSNSS	MLPTLSDTYI	PQCSTDGQWR	QVQCNGPPEQ	VFELYQRWEA	QNKGQDLTPA
810	820	830	840	850	860	870	880
KLLVKIMSYR	EAASGNFSLF	IQSLYEAGQQ	DVFPVLSQYP	SLQDVPLAAL	EGKRPQPREN	ILLEPYLFWQ	ILNGQLSQYP
890	900	910	920	930	940	950	960
GSYSDFSTPL	AHFDLRNCWC	VDEAGQELEG	MRSEPSKLPT	CPGSCEEAKL	RVLQFIRETE	EIVSASNSSR	FPLGESFLVA
970	980	990	1000	1010	1020	1030	1040
KGIRLRNEDL	GLPPLFPPRE	AFAEQFLRGS	DYAIRLAAQS	TLSFYQRRRF	SPDDSAGASA	LLRSGPYMPQ	CDAFGSWEPV
1050	1060	1070	1080	1090	1100	1110	1120
QCHAGTGHCW	CVDEKGGFIP	GSLTARSLQI	PQCPTTCEKS	RTSGLLSSWK	QARSQENPSP	KDLFVPACLE	TGEYARLQAS
1130	1140	1150	1160	1170	1180	1190	1200
GAGTWCVDPA	SGEELRPGSS	SSAQCPSLCN	VLKSGVLSRR	VSPGYVPACR	AEDGGFSPVQ	CDQAQGSCWC	VMDSGEEVPG
1210	1220	1230	1240	1250	1260	1270	1280
TRVTGGQPAC	ESPRCPLPFN	ASEVVGGTIL	CETISGPTGS	AMQQCQLLCR	QGSWSVFPPG	PLICSLESGR	WESQLPQPRA
1290	1300	1310	1320	1330	1340	1350	1360
CQRPQLWQTI	QTQGHFQLQL	PPGKMCSADY	ADLLQTFQVF	ILDELTARGF	CQIQVKTFGT	LVSIPVCNNS	SVQVGCLTRE
1370	1380	1390	1400	1410	1420	1430	1440
RLGVNVTWKS	RLEDIPVASL	PDLHDIERAL	VGKDLLGRFT	DLIQSGSFQL	HLDSKTFPAE	TIRFLQGDHF	GTSPRTWFGC
1450	1460	1470	1480	1490	1500	1510	1520
SEGFYQVLTS	EASQDGLGCV	KCPEGSYSQD	EECIPCPVGF	YQEQAGSLAC	VPCPVGRTTI	SAGAFSQTHC	VTDCQRNEAG
1530	1540	1550	1560	1570	1580	1590	1600
LQCDQNGQYR	ASQKDRGSGK	AFCVDGEGRR	LPWWETEAPL	EDSQCLMMQK	FEKVPESKVI	FDANAPVAVR	SKVPDSEFPV
1610	1620	1630	1640	1650	1660	1670	1680
MQCLTDCTED	EACSFFTVST	TEPEISCDFY	AWTSDNVACM	TSDQKRDALG	NSKATSFGSL	RCQVKVRSHG	QDSPAVYLKK
1690	1700	1710	1720	1730	1740	1750	1760
GQGSTTTLQK	RFEPTGFQNM	LSGLYNPIVF	SASGANLTDA	HLFCLLACDR	DLCCDGFVLT	QVQGGAIICG	LLSSPSVLLC
1770	1780	1790	1800	1810	1820	1830	1840
NVKDWMDPSE	AWANATCPGV	TYDQESHQVI	LRLGDQEFIK	SLTPLEGTQD	TFTNFQQVYL	WKDSDMGSRP	ESMGCRKDTV
1850	1860	1870	1880	1890	1900	1910	1920
PRPASPTEAG	LTTELFSPVD	LNQVIVNGNQ	SLSSQKHWLF	KHLFSAQQAN	LWCLSRCVQE	HSFCQLAEIT	ESASLYFTCT
1930	1940	1950	1960	1970	1980	1990	2000
LYPEAQVCDD	IMESNAQGCR	LILPQMPKAL	FRKKVILEDK	VKNFYTRLPF	QKLMGISIRN	KVPMSEKSIS	NGFFECERRC
2010	2020	2030	2040	2050	2060	2070	2080
DADPCCTGFG	FLNVSQLKGG	EVTCLTLNSL	GIQMCSEENG	GAWRILDCGS	PDIEVHTYPF	GWYQKPIAQN	NAPSFCPLVV
2090	2100	2110					
LPSLTEKVFK	VREVRGTQLK	RME					

Detailed Protein Report

Cmpd.	No. of Cmpds.	m/z meas.	Δ m/z [ppm]	z	Rt [min]	Score	P	Range	Sequence	Modification	Ratios
1580	1	502.6699	-218.64	2	50.0	19.9	0	467-475	R.LQQNLFGGK.F		
1861	1	719.7564	-125.05	2	51.8	13.2	2	1537-1550	R.GSGKAFCVDGEGRR.L		QD:QU 0.78

Detailed Protein Report

Protein 196: PREDICTED: rho GTPase-activating protein 36 isoform X1 [Homo sapiens]

**Accession:**	gi|578838609	**Score:**	33.0
**Database:**	refseq_human(refseq_human_20140103.fasta)	**MW [kDa]:**	46.2
**Database Date:**	2015-11-30	**pI:**	9.9
		**Sequence Coverage [%]:**	3.6
		**No. of unique Peptides:**	2

10	20	30	40	50	60	70	80
MQVEEATGQA	AGRRRGNVVR	RVFGRIRRFF	SRRRNEPTLP	REFTRRGRRG	AVSVDSLAEL	EDGALLLQTL	QLSKISFPIG
**90**	**100**	**110**	**120**	**130**	**140**	**150**	**160**
QRLLGSKRKM	SLNPIAKQIP	QVVEACCQFI	EKHGLSAVGI	FTLEYSVQRV	RQLREEFDQG	LDVVLDDNQN	VHDVAALLKE
**170**	**180**	**190**	**200**	**210**	**220**	**230**	**240**
FFRDMKDSLL	PDDLYMSFLL	TATLKPQDQL	SALQLLVYLM	PPCHSDTLER	LLKALHKITE	NCEDSIGIDG	QLVPGNRMTS
**250**	**260**	**270**	**280**	**290**	**300**	**310**	**320**
TNLALVFGSA	LLKKGKFGKR	ESRKTKLGID	HYVASVNVVR	AMIDNWDVLF	QVPPHIQRQV	AKRVWKSSPE	ALDFIRRRNL
**330**	**340**	**350**	**360**	**370**	**380**	**390**	**400**
RKIQSARIKM	EEDALLSDPV	ETSAEARAAV	LAQSKPSDEG	SSEEPAVPSG	TARSHDDEEG	AGNPPIPEQD	RPLLRVPREK
**410**	**420**						
EAKTGVSYFF	P						

Cmpd.	No. of Cmpds.	m/z meas.	Δ m/z [ppm]	z	Rt [min]	Score	P	Range	Sequence	Modification
1422	1	459.2842	44.39	2	47.9	16.9	0	75-82	K.ISFPIGQR.L	
2474	1	822.4025	-186.55	1	61.5	16.1	1	211-217	R.LLKALHK.I	

Detailed Protein Report

Protein 197: zinc finger protein 90 [Homo sapiens]

Accession: gi|209862791
Database: refseq_human(refseq_human_20140103.fasta)
Database Date: 2015-11-30
Modification(s): Carbamidomethyl

Score: 33.0
MW [kDa]: 69.0
pI: 10.3
Sequence Coverage [%]: 4.3
No. of unique Peptides: 2

Quantitation

QD:QU **Median:** 1.73 **CV:** 0.00 % **No. of Peptides:** 1

10	20	30	40	50	60	70	80
MGPLEFRDVA	IEFSLEEWHC	LDTAQQNLYR	DVMLENYRHL	VFLGIVVTKP	DLITCLEQGK	KPFTVKRHEM	IAKSPVMCFH
**90**	**100**	**110**	**120**	**130**	**140**	**150**	**160**
FAQDLCPEQS	LKDSFQKVIV	TRYEKREYGN	LELKKGCESV	DEGKVHKRGY	NGLNQCLTAT	QSKVFQCDTY	VKVSHIFSNS
**170**	**180**	**190**	**200**	**210**	**220**	**230**	**240**
NRHKIRDTGK	KPFKCIECGK	AFNQSSTLAT	HKKIHTGEIT	CKCEECGKAF	NRSSHLTSHK	RIHTGEKRYK	CEDCGKELKY
**250**	**260**	**270**	**280**	**290**	**300**	**310**	**320**
SSTLTAHKRI	HTGEKRYKCE	DCGKELKYSS	TLTAHKRIHT	GEKPYKCDKC	GRAFISSSIL	YVHKISHTEE	KPYKCEECGK
**330**	**340**	**350**	**360**	**370**	**380**	**390**	**400**
AFKLSSILST	HKRIHTGEKP	YKCEECGKAF	RRSLVLRTHK	RIHTGEKPYK	CDKCGKAFIS	SSLLYKHKIS	HSEKKPYKCE
**410**	**420**	**430**	**440**	**450**	**460**	**470**	**480**
ECGKAFKRSS	TLTIHKISHT	EEKPYKCQEC	DKVFKRSSAL	STHKIIHSGE	KPYKCEECGK	AFKRSSNLTT	HKISHTEEKL
**490**	**500**	**510**	**520**	**530**	**540**	**550**	**560**
YKCQECDKAF	KYSSALSTHK	IIHSGENPYK	CEECGKAFKR	SSVLSKHKII	HTGAKPYKCE	ECGKAFKRSS	QLTSHKISHT
**570**	**580**	**590**	**600**	**610**			
GEKPYKCEEC	GKAFNLSSDL	NTHKRIHIGQ	KAYIVKNMAN	L			

Cmpd.	No. of Cmpds.	m/z meas.	Δ m/z [ppm]	z	Rt [min]	Score	P	Range	Sequence	Modification	Ratios
1099	1	917.4452	-8.48	2	43.8	11.6	1	529-544	K.IIHTGAKPYKCEECGK.A	Carbamidomethyl: 11	
1760	1	568.7537	-99.49	2	52.3	21.4	1	592-601	K.AYIVKNMANL.-		QD:QU 1.73

Detailed Protein Report

Protein 198: E3 ubiquitin-protein ligase MARCH7 isoform c [Homo sapiens]

**Accession:**	gi|544346219	**Score:**	33.0
**Database:**	refseq_human(refseq_human_20140103.fasta)	**MW [kDa]:**	73.7
**Database Date:**	2015-11-30	**pI:**	6.0
**Modification(s):**	Oxidation	**Sequence Coverage [%]:**	2.7
		**No. of unique Peptides:**	1

10	20	30	40	50	60	70	80
MIGNYDHLMS	LVTSTSASAS	ASPFQSAWYS	ESEITQGARS	RSQNQQRDHD	SKRPKLSCTN	CTTSAGRNVG	NGLNTLSDSS
**90**	**100**	**110**	**120**	**130**	**140**	**150**	**160**
WRHSQVPRSS	SMVLGSFGTD	LMRERRDLER	RTDSSISNLM	DYSHRSGDFT	TSSYVQDRVP	SYSQGARPKE	NSMSTLQLNT
**170**	**180**	**190**	**200**	**210**	**220**	**230**	**240**
SSTNHQLPSE	HQTILSSRDS	RNSLRSNFSS	RESESSRSNT	QPGFSYSSSR	DEAPIISNSE	RVVSSQRPFQ	ESSDNEGRRT
**250**	**260**	**270**	**280**	**290**	**300**	**310**	**320**
TRRLLSRIAS	SMSSTFFSRR	SSQDSLNTRS	LNSENSYVSP	RILTASQSRS	NVPSASEVPD	NRASEASQGF	RFLRRRWGLS
**330**	**340**	**350**	**360**	**370**	**380**	**390**	**400**
SLSHNHSSES	DSENFNQESE	GRNTGPWLSS	SLRNRCTPLF	SRRRREGRDE	SSRIPTSDTS	SRSHIFRRES	NEVVHLEAQN
**410**	**420**	**430**	**440**	**450**	**460**	**470**	**480**
DPLGAAANRP	QASAASSSAT	TGGSTSDSAQ	GGRNTGISGI	LPGSLFRFAV	PPALGSNLTD	NVMITVDIIP	SGWNSADGKS
**490**	**500**	**510**	**520**	**530**	**540**	**550**	**560**
DKTKSAPSRD	PERLQKIKES	LLLEDSEEEE	GDLCRICQMA	AASSSNLLIE	PCKCTGSLQY	VHQDCMKKWL	QAKINSGSSL
**570**	**580**	**590**	**600**	**610**	**620**	**630**	**640**
EAVTTCELCK	EKLELNLEDF	DIHELHRAHA	NEQAEYEFIS	SGLYLVVLLH	LCEQSFSDMM	GNTNEPSTRV	RFINLARTLQ
**650**	**660**	**670**					
AHMEDLETSE	DDSEEDGDHN	RTFDIA					

Cmpd.	No. of Cmpds.	m/z meas.	Δ m/z [ppm]	z	Rt [min]	Score	P	Range	Sequence	Modification
1613	1	682.3429	11.62	3	50.4	22.6	2	89-106	R.SSSMVLGSFGTDLMRERR.D	Oxidation: 4

Detailed Protein Report

Protein 199: crooked neck-like protein 1 isoform c [Homo sapiens]

**Accession:**	gi|520975425	**Score:**	32.9
**Database:**	refseq_human(refseq_human_20140103.fasta)	**MW [kDa]:**	68.6
**Database Date:**	2015-11-30	**pI:**	5.9
**Modification(s):**	Carbamidomethyl	**Sequence Coverage [%]:**	4.3
		**No. of unique Peptides:**	2

Alias proteins:

Accession	Name	Description
gi|520975430	refseq_human (refseq_human_20140103.fasta)	crooked neck-like protein 1 isoform c [Homo sapiens]

```
        10         20         30         40         50         60         70         80
MEMKNRQVNH ARNIWDRAIT TLPRVNQFWY KYTYMEEMLG NVAGARQVFE RWMEWQPEEQ AWHSYINFEL RYKEVDRART
        90        100        110        120        130        140        150        160
IYERFVLVHP DVKNWIKYAR FEEKHAYFAH ARKVYERAVE FFGDEHMDEH LYVAFAKFEE NQKEFERVRV IYKYALDRIS
       170        180        190        200        210        220        230        240
KQDAQELFKN YTIFEKKFGD RRGIEDIIVS KRRFQYEEEV KANPHNYDAW FDYLRLVESD AEAEAVREVY ERAIANVPPI
       250        260        270        280        290        300        310        320
QEKRHWKRYI YLWINYALYE ELEAKDPERT RQVYQASLEL IPHKKFTFAK MWILYAQFEI RQKNLSLARR ALGTSIGKCP
       330        340        350        360        370        380        390        400
KNKLFKVYIE LELQLREFDR CRKLYEKFLE FGPENCTSWI KFAELETILG DIDRARAIYE LAISQPRLDM PEVLWKSYID
       410        420        430        440        450        460        470        480
FEIEQEETER TRNLYRRLLQ RTQHVKVWIS FAQFELSSGK EGSLTKCRQI YEEANKTMRN CEEKEERLML LESWRSFEEE
       490        500        510        520        530        540        550        560
FGTASDKERV DKLMPEKVKK RRKVQTDDGS DAGWEEYFDY IFPEDAANQP NLKLLAMAKL WKKQQQEKED AEHHPDEDVD
       570
ESES
```

Cmpd.	No. of Cmpds.	m/z meas.	Δ m/z [ppm]	z	Rt [min]	Score	P	Range	Sequence	Modification
2727	1	698.3840	-35.80	2	63.0	10.6	2	148-158	R.VRVIYKYALDR.I	
1986	1	687.3345	-69.29	2	55.2	22.3	2	311-323	R.ALGTSIGKCPKNK.L	Carbamidomethyl: 9

Detailed Protein Report

Protein 200: PREDICTED: diacylglycerol kinase delta isoform X3 [Homo sapiens]

**Accession:**	gi|530371455	**Score:**	32.8
**Database:**	refseq_human(refseq_human_20140103.fasta)	**MW [kDa]:**	104.0
**Database Date:**	2015-11-30	**pI:**	9.2
**Modification(s):**	Carbamidomethyl	**Sequence Coverage [%]:**	2.9
		**No. of unique Peptides:**	2

Quantitation

QD:QU **Median:** 0.45 **CV:** 0.00 % **No. of Peptides:** 1

10	20	30	40	50	60	70	80
MAAAAGAPPP	GPPQPPPPPP	PEESSDSEPE	AEPGSPQKLI	RKVSTSGQIR	QKTIIKEGML	TKQNNSFQRS	KRRYFKLRGR
**90**	**100**	**110**	**120**	**130**	**140**	**150**	**160**
TLYYAKTAKS	IIFDEVDLTD	ASVAESSTKN	VNNSFTVITP	CRKLILCADN	RKEMEDWIAA	LKTVQNREHF	EPTQYSMDHF
**170**	**180**	**190**	**200**	**210**	**220**	**230**	**240**
SGMHNWYACS	HARPTYCNVC	REALSGVTSH	GLSCEVCKFK	AHKRCAVRAT	NNCKWTTLAS	IGKDIIEDAD	GIAMPHQWLE
**250**	**260**	**270**	**280**	**290**	**300**	**310**	**320**
GNLPVSAKCT	VCDKTCGSVL	RLQDWRCLWC	KAMVHTSCKE	SLLTKCPLGL	CKVSVIPPTA	LNSIDSDGFW	KASCPPSCTS
**330**	**340**	**350**	**360**	**370**	**380**	**390**	**400**
PLLVFVNSKS	GDNQGVKFLR	RFKQLLNPAQ	VFDLMNGGPH	LGLRLFQKFD	TFRILVCGGD	GSVGWVLSEI	DSLNLHKQCQ
**410**	**420**	**430**	**440**	**450**	**460**	**470**	**480**
LGVLPLGTGN	DLARVLGWGS	ACDDDTQLPQ	ILEKLERAST	KMLDRWSVMA	YEAKLPRQAS	SSTVTEDFSE	DSEVQQILFY
**490**	**500**	**510**	**520**	**530**	**540**	**550**	**560**
EDSVAAHLSK	ILTSDQHSVV	ISSAKVLCET	VKDFVARVGK	AYEKTTESSE	ESEVMAKKCS	VLKEKLDSLL	KTLDDESQAS
**570**	**580**	**590**	**600**	**610**	**620**	**630**	**640**
SSLPNPPPTI	AEEAEDGDGS	GSICGSTGDR	LVASACPARP	QIFRPREQLM	LRANSLKKAI	RQIIEHTEKA	VDEQNAQTQE
**650**	**660**	**670**	**680**	**690**	**700**	**710**	**720**
QEGFVLGLSE	SEEKMDHRVC	PPLSHSESFG	VPKGRSQRKV	SKSPCEKLIS	KGSLSLGSSA	SLPPQPGSRD	GLPALNTKIL
**730**	**740**	**750**	**760**	**770**	**780**	**790**	**800**
YPNVRAGMSG	SLPGGSVISR	LLINADPFNS	EPETLEYYTE	KCVMNNYFGI	GLDAKISLDF	NNKRDEHPEK	CRSRTKNMMW
**810**	**820**	**830**	**840**	**850**	**860**	**870**	**880**
YGVLGTKELL	HRTYKNLEQK	VLLECDGRPI	PLPSLQGIAV	LNIPSYAGGT	NFWGGTKEDD	TFAAPSFDDK	ILEVVAVFGS
**890**	**900**	**910**	**920**	**930**	**940**	**950**	
MQMAVSRVIR	LQHHRIAQCR	TVKISILGDE	GVPVQVDGEA	WVQPPGYIRI	VHKNRAQTLT	RDRL	

Cmpd.	No. of Cmpds.	m/z meas.	Δ m/z [ppm]	z	Rt [min]	Score	P	Range	Sequence	Modification	Ratios
1745	1	917.3940	-36.10	2	50.3	17.0	0	182-198	R.EALSGVTSHGLSCEVCK.F	Carbamidomethyl: 13, 16	
22	1	657.3901	130.41	2	28.9	15.8	2	785-794	R.DEHPEKCRSR.T	Carbamidomethyl: 7	QD:QU 0.45

Detailed Protein Report

Protein 201: cystatin-B [Homo sapiens]

Accession: gi|4503117
Database: refseq_human(refseq_human_20140103.fasta)
Database Date: 2015-11-30

Score: 32.7
MW [kDa]: 11.1
pI: 7.9
Sequence Coverage [%]: 12.2
No. of unique Peptides: 1

Quantitation

QD:QU **Median:** 1.47 **CV:** 0.00 % **No. of Peptides:** 1

10	20	30	40	50	60	70	80
MMCGAPSATQ	PATAETQHIA	DQVRSQLEEK	ENKKFPVFKA	VSFKSQVVAG	TNYFIKVHVG	DEDFVHLRVF	QSLPHENKPL
90	100						
TLSNYQTNKA	KHDELTYF						

Cmpd.	No. of Cmpds.	m/z meas.	Δ m/z [ppm]	z	Rt [min]	Score	P	Range	Sequence	Modification	Ratios
953	2	663.8439	-18.59	2	40.4	32.7	0	45-56	K.SQVVAGTNYFIK.V		QD:QU 1.47

Detailed Protein Report

Protein 202: coiled-coil domain-containing protein 88B precursor [Homo sapiens]

**Accession:**	gi|262118216	**Score:**	32.7
**Database:**	refseq_human(refseq_human_20140103.fasta)	**MW [kDa]:**	164.7
**Database Date:**	2015-11-30	**pI:**	4.9
		**Sequence Coverage [%]:**	1.9
		**No. of unique Peptides:**	1

10	20	30	40	50	60	70	80
MEGGKGPRLR	DFLSGSLATW	ALGLAGLVGE	AEDSEGEEEE	EEEEPPLWLE	KRFLRLSDGA	LLLRVLGIIA	PSSRGGPRML
90	100	110	120	130	140	150	160
RGLDGPAAWR	VWNLNHLWGR	LRDFYQEELQ	LLILSPPPDL	QTLGFDPLSE	EAVEQLEGVL	RLLLGASVQC	EHRELFIRHI
170	180	190	200	210	220	230	240
QGLSLEVQSE	LAAAIQEVTQ	PGAGVVLALS	GPDPGELAPA	ELEMLSRSLM	GTLSKLARER	DLGAQRLAEL	LLEREPLCLR
250	260	270	280	290	300	310	320
PEAPSRAPAE	GPSHHLALQL	ANAKAQLRRL	RQELEEKAEL	LLDSQAEVQG	LEAEIRRLRQ	EAQALSGQAK	RAELYREEAE
330	340	350	360	370	380	390	400
ALRERAGRLP	RLQEELRRCR	ERLQAAEAYK	SQLEEERVLS	GVLEASKALL	EEQLEAARER	CARLHETQRE	NLLLRTRLGE
410	420	430	440	450	460	470	480
AHAELDSLRH	QVDQLAEENV	ELELELQRSL	EPPPGSPGEA	PLAGAAPSLQ	DEVREAEAGR	LRTLERENRE	LRGLLQVLQG
490	500	510	520	530	540	550	560
QPGGQHPLLE	APREDPVLPV	LEEAPQTPVA	FDHSPQGLVQ	KARDGGPQAL	DLAPPALDSV	LEASAECPQA	PDSDPQEAES
570	580	590	600	610	620	630	640
PLQAAAMDPQ	ASDWSPQESG	SPVETQESPE	KAGRRSSLQS	PASVAPPQGP	GTKIQAPQLL	GGETEGREAP	QGELVPEAWG
650	660	670	680	690	700	710	720
LRQEGPEHKP	GPSEPSSVQL	EEQEGPNQGL	DLATGQAEAR	EHDQRLEGTV	RDPAWQKPQQ	KSEGALEVQV	WEGPIPGESL
730	740	750	760	770	780	790	800
ASGVAEQEAL	REEVAQLRRK	AEALGDELEA	QARKLEAQNT	EAARLSKELA	QARRAEAEAH	REAEAQAWEQ	ARLREAVEAA
810	820	830	840	850	860	870	880
GQELESASQE	REALVEALAA	AGRERRQWER	EGSRLRAQSE	AAEERMQVLE	SEGRQHLEEA	ERERREKEAL	QAELEKAVVR
890	900	910	920	930	940	950	960
GKELGDRLEH	LQRELEQAAL	ERQEFLREKE	SQHQRYQGLE	QRLEAELQAA	ATSKEEALME	LKTRALQLEE	ELFQLRQGPA
970	980	990	1000	1010	1020	1030	1040
GLGPKKRAEP	QLVETQNVRL	IEVERSNAML	VAEKAALQGQ	LQHLEGQLGS	LQGRAQELLL	QSQRAQEHSS	RLQAEKSVLE
1050	1060	1070	1080	1090	1100	1110	1120
IQGQELHRKL	EVLEEEVRAA	RQSQEETRGQ	QQALLRDHKA	LAQLQRRQEA	ELEGLLVRHR	DLKANMRALE	LAHRELQGRH
1130	1140	1150	1160	1170	1180	1190	1200
EQLQAQRASV	EAQEVALLAE	RERLMQDGHR	QRGLEEELRR	LQSEHDRAQM	LLAELSRERG	ELQGERGELR	GRLARLELER
1210	1220	1230	1240	1250	1260	1270	1280
AQLEMQSQQL	RESNQQLDLS	ACRLTTQCEL	LTQLRSAQEE	ENRQLLAEVQ	ALSRENRELL	ERSLESRDHL	HREQREYLDQ
1290	1300	1310	1320	1330	1340	1350	1360
LNALRREKQK	LVEKIMDQYR	VLEPVPLPRT	KKGSWLADKV	KRLMRPRREG	GPPGGLRLGA	DGAGSTESLG	GPPETELPEG
1370	1380	1390	1400	1410	1420	1430	1440
READGTGSPS	PAPMRRAQSS	LCLRDETLAG	GQRRKLSSRF	PVGRSSESFS	PGDTPRQRFR	QRHPGPLGAP	VSHSKGPGVG
1450	1460	1470	1480				
WENSAETLQE	HETDANREGP	EVQEPEKRPL	TPSLSQ				

Cmpd.	No. of Cmpds.	m/z meas.	Δ m/z [ppm]	z	Rt [min]	Score	P	Range	Sequence	Modification
245	1	715.7361	-205.90	2	33.1	13.1	1	755-767	K.LEAQNTEAARLSK.E	

Detailed Protein Report

Protein 203: laminin subunit alpha-2 isoform b precursor [Homo sapiens]

**Accession:**	gi|119466532	**Score:**	32.6
**Database:**	refseq_human(refseq_human_20140103.fasta)	**MW [kDa]:**	343.3
**Database Date:**	2015-11-30	**pI:**	6.0
		**Sequence Coverage [%]:**	0.5
		**No. of unique Peptides:**	1

Quantitation

QD:QU	**Median:** 1.08	**CV:** 0.00 %	**No. of Peptides:** 1

Detailed Protein Report

10	20	30	40	50	60	70	80
MPGAAGVLLL	LLLSGGLGGV	QAQRPQQQRQ	SQAHQQRGLF	PAVLNLASNA	LITTNATCGE	KGPEMYCKLV	EHVPGQPVRN
90	100	110	120	130	140	150	160
PQCRICNQNS	SNPNQRHPIT	NAIDGKNTWW	QSPSIKNGIE	YHYVTITLDL	QQVFQIAYVI	VKAANSPRPG	NWILERSLDD
170	180	190	200	210	220	230	240
VEYKPWQYHA	VTDTECLTLY	NIYPRTGPPS	YAKDDEVICT	SFYSKIHPLE	NGEIHISLIN	GRPSADDPSP	ELLEFTSARY
250	260	270	280	290	300	310	320
IRLRFQRIRT	LNADLMMFAH	KDPREIDPIV	TRRYYYSVKD	ISVGGMCICY	GHARACPLDP	ATNKSRCECE	HNTCGDSCDQ
330	340	350	360	370	380	390	400
CCPGFHQKPW	RAGTFLTKTE	CEACNCHGKA	EECYYDENVA	RRNLSLNIRG	KYIGGGVCIN	CTQNTAGINC	ETCTDGFFRP
410	420	430	440	450	460	470	480
KGVSPNYPRP	CQPCHCDPIG	SLNEVCVKDE	KHARRGLAPG	SCHCKTGFGG	VSCDRCARGY	TGYPDCKACN	CSGLGSKNED
490	500	510	520	530	540	550	560
PCFGPCICKE	NVEGGDCSRC	KSGFFNLQED	NWKGCDECFC	SGVSNRCQSS	YWTYGKIQDM	SGWYLTDLPG	RIRVAPQQDD
570	580	590	600	610	620	630	640
LDSPQQISIS	NAEARQALPH	SYYWSAPAPY	LGNKLPAVGG	QLTFTISYDL	EEEEEDTERV	LQLMIILEGN	DLSISTAQDE
650	660	670	680	690	700	710	720
VYLHPSEEHT	NVLLLKEESF	TIHGTHFPVR	RKEFMTVLAN	LKRVLLQITY	SFGMDAIFRL	SSVNLESAVS	YPTDGSIAAA
730	740	750	760	770	780	790	800
VEVCQCPPGY	TGSSCESCWP	RHRRVNGTIF	GGICEPCQCF	GHAESCDDVT	GECLNCKDHT	GGPYCDKCLP	GFYGEPTKGT
810	820	830	840	850	860	870	880
SEDCQPCACP	LNIPSNNFSP	TCHLDRSLGL	ICDGCPVGYT	GPRCERCAEG	YFGQPSVPGG	SCQPCQCNDN	LDFSIPGSCD
890	900	910	920	930	940	950	960
SLSGSCLICK	PGTTGRYCEL	CADGYFGDAV	DAKNCQPCRC	NAGGSFSEVC	HSQTGQCECR	ANVQGQRCDK	CKAGTFGLQS
970	980	990	1000	1010	1020	1030	1040
ARGCVPCNCN	SFGSKSFDCE	ESGQCWCQPG	VTGKKCDRCA	HGYFNFQEGG	CTACECSHLG	NNCDPKTGRC	ICPPNTIGEK
1050	1060	1070	1080	1090	1100	1110	1120
CSKCAPNTWG	HSITTGCKAC	NCSTVGSLDF	QCNVNTGQCN	CHPKFSGAKC	TECSRGHWNY	PRCNLCDCFL	PGTDATTCDS
1130	1140	1150	1160	1170	1180	1190	1200
ETKKCSCSDQ	TGQCTCKVNV	EGIHCDRCRP	GKFGLDAKNP	LGCSSCYCFG	TTTQCSEAKG	LIRTWVTLKA	EQTILPLVDE
1210	1220	1230	1240	1250	1260	1270	1280
ALQHTTTKGI	VFQHPEIVAH	MDLMREDLHL	EPFYWKLPEQ	FEGKKLMAYG	GKLKYAIYFE	AREETGFSTY	NPQVIIRGGT
1290	1300	1310	1320	1330	1340	1350	1360
PTHARIIVRH	MAAPLIGQLT	RHEIEMTEKE	WKYYGDDPRV	HRTVTREDFL	DILYDIHYIL	IKATYGNFMR	QSRISEISME
1370	1380	1390	1400	1410	1420	1430	1440
VAEQGRGTTM	TPPADLIEKC	DCPLGYSGLS	CEACLPGFYR	LRSQPGGRTP	GPTLGTCVPC	QCNGHSSLCD	PETSICQNCQ
1450	1460	1470	1480	1490	1500	1510	1520
HHTAGDFCER	CALGYYGIVK	GLPNDCQQCA	CPLISSSNNF	SPSCVAEGLD	DYRCTACPRG	YEGQYCERCA	PGYTGSPGNP
1530	1540	1550	1560	1570	1580	1590	1600
GGSCQECECD	PYGSLPVPCD	PVTGFCTCRP	GATGRKCDGC	KHWHAREGWE	CVFCGDECTG	LLLGDLARLE	QMVMSINLTG
1610	1620	1630	1640	1650	1660	1670	1680
PLPAPYKMLY	GLENMTQELK	HLLSPQRAPE	RLIQLAEGNL	NTLVTEMNEL	LTRATKVTAD	GEQTGQDAER	TNTRAKSLGE
1690	1700	1710	1720	1730	1740	1750	1760
FIKELARDAE	AVNEKAIKLN	ETLGTRDEAF	ERNLEGLQKE	IDQMIKELRR	KNLETQKEIA	EDELVAAEAL	LKKVKKLFGE
1770	1780	1790	1800	1810	1820	1830	1840
SRGENEEMEK	DLREKLADYK	NKVDDAWDLL	REATDKIREA	NRLFAVNQKN	MTALEKKKEA	VESGKRQIEN	TLKEGNDILD
1850	1860	1870	1880	1890	1900	1910	1920
EANRLADEIN	SIIDYVEDIQ	TKLPPMSEEL	NDKIDDLSQE	IKDRKLAEKV	SQAESHAAQL	NDSSAVLDGI	LDEAKNISFN
1930	1940	1950	1960	1970	1980	1990	2000
ATAAFKAYSN	IKDYIDEAEK	VAKEAKDLAH	EATKLATGPR	GLLKEDAKGC	LQKSFRILNE	AKKLANDVKE	NEDHLNGLKT
2010	2020	2030	2040	2050	2060	2070	2080
RIENADARNG	DLLRTLNDTL	GKLSAIPNDT	AAKLQAVKDK	ARQANDTAKD	VLAQITELHQ	NLDGLKKNYN	KLADSVAKTN
2090	2100	2110	2120	2130	2140	2150	2160
AVVKDPSKNK	IIADADATVK	NLEQEADRLI	DKLKPIKELE	DNLKKNISEI	KELINQARKQ	ANSIKVSVSS	GGDCIRTYKP
2170	2180	2190	2200	2210	2220	2230	2240

Detailed Protein Report

Cmpd.	No. of Cmpds.	m/z meas.	Δ m/z [ppm]	z	Rt [min]	Score	P	Range	Sequence	Modification	Ratios	
2430	1	531.0471	-454.49	1	60.8	17.8	0	2291-2295	K.ADAVR.V		QD:QU	1.08

Detailed Protein Report

Protein 204: **protein FAM194B [Homo sapiens]**

**Accession:**	gi|210147567	**Score:**	32.5
**Database:**	refseq_human(refseq_human_20140103.fasta)	**MW [kDa]:**	81.6
**Database Date:**	2015-11-30	**pI:**	4.6
		**Sequence Coverage [%]:**	4.3
		**No. of unique Peptides:**	2

10	20	30	40	50	60	70	80
MSAENNQLSG	ASPPHPPTTP	QYSTQNLPSE	KEDTEVELDE	ESLQDESPFS	PEGESLEDKE	YLEEEEDLEE	EEYLGKEEYL
**90**	**100**	**110**	**120**	**130**	**140**	**150**	**160**
KEEEYLGKEE	HLEEEEYLEK	AGYLEEEEYI	EEEEYLGKEG	YLEEEEYLGK	EEHLEEEEYL	GKEGYLEKED	YIEEVDYLGK
**170**	**180**	**190**	**200**	**210**	**220**	**230**	**240**
KAYLEEEEYL	GKKSYLEEEK	ALEKEENLEE	EEALEKEENL	DGKENLYKKY	LKEPKASYSS	QTMLLRDARS	PDAGPSQVTT
**250**	**260**	**270**	**280**	**290**	**300**	**310**	**320**
FLTVPLTFAT	PSPVSESATE	SSELLLTLYR	RSQASQTDWC	YDRTAVKSLK	SKSETEQETT	TKLAPEEHVN	TKVQQKKEEN
**330**	**340**	**350**	**360**	**370**	**380**	**390**	**400**
VLEFASKENF	WDGITDESID	KLEVEDLDEN	FLNSSYQTVF	KTIIKEMAAH	NELEEDFDIP	LTKLLESENR	WKLVIMLKKN
**410**	**420**	**430**	**440**	**450**	**460**	**470**	**480**
YEKFKETILR	IKRRREAQKL	TEMTSFTFHL	MSKPTPEKPE	TEEIQKPQRV	VHHRKKLERD	KEWIQKKTVV	HQGDGKLILY
**490**	**500**	**510**	**520**	**530**	**540**	**550**	**560**
PNKNVYQILF	PDGTGQIHYP	SGNLAMLILY	AKMKKFTYII	LEDSLEGRIR	ALINNSGNAT	FYDENSDIWL	NLSSNLGYYF
**570**	**580**	**590**	**600**	**610**	**620**	**630**	**640**
PKDKRQKAWN	WWNLNIHVHA	PPVQPISLKI	NEYIQVQIRS	QDKIIFCFTY	EQKQICLNLG	TRYKFVIPEV	LSEMKKKTIL
**650**	**660**	**670**	**680**	**690**	**700**		
EAEPGPTAQK	IRVLLGKMNR	LLNYATTPDL	ENFIEAVSIS	LMDNKYLKKM	LSKLWF		

Cmpd.	No. of Cmpds.	m/z meas.	Δ m/z [ppm]	z	Rt [min]	Score	P	Range	Sequence	Modification
2059	1	1051.4459	-42.83	2	56.1	10.5	0	366-383	K.EMAAHNELEEDFDIPLTK.L	
2977	23	710.3760	-33.01	2	66.5	22.0	1	625-636	K.FVIPEVLSEMKK.K	

Detailed Protein Report

Protein 205: probable ATP-dependent RNA helicase DDX10 [Homo sapiens]

Accession: gi|13514831
Database: refseq_human(refseq_human_20140103.fasta)
Database Date: 2015-11-30

Score: 32.3
MW [kDa]: 100.8
pI: 9.3
Sequence Coverage [%]: 4.3
No. of unique Peptides: 1

10	20	30	40	50	60	70	80
MGKTANSPGS	GARPDPVRSF	NRWKKKHSHR	QNKKKQLRKQ	LKKPEWQVER	ESISRLMQNY	EKINVNEITR	FSDFPLSKKT
90	100	110	120	130	140	150	160
LKGLQEAQYR	LVTEIQKQTI	GLALQGKDVL	GAAKTGSGKT	LAFLVPVLEA	LYRLQWTSTD	GLGVLIISPT	RELAYQTFEV
170	180	190	200	210	220	230	240
LRKVGKNHDF	SAGLIIGGKD	LKHEAERINN	INILVCTPGR	LLQHMDETVS	FHATDLQMLV	LDEADRILDM	GFADTMNAVI
250	260	270	280	290	300	310	320
ENLPKKRQTL	LFSATQTKSV	KDLARLSLKN	PEYVWVHEKA	KYSTPATLEQ	NYIVCELQQK	ISVLYSFLRS	HLKKKSIVFF
330	340	350	360	370	380	390	400
SSCKEVQYLY	RVFCRLRPGV	SILALHGRQQ	QMRRMEVYNE	FVRKRAAVLF	ATDIAARGLD	FPAVNWVLQF	DCPEDANTYI
410	420	430	440	450	460	470	480
HRAGRTARYK	EDGEALLILL	PSEKAMVQQL	LQKKVPVKEI	KINPEKLIDV	QKKLESILAQ	DQDLKERAQR	CFVSYVRSVY
490	500	510	520	530	540	550	560
LMKDKEVFDV	SKLPIPEYAL	SLGLAVAPRV	RFLQKMQKQP	TKELVRSQAD	KVIEPRAPSL	TNDEVEEFRA	YFNEKMSILQ
570	580	590	600	610	620	630	640
KGGKRLEGTE	HRQDNDTGNE	EQEEEEDDEE	EMEEKLAKAK	GSQAPSLPNT	SEAQKIKEVP	TQFLDRDEEE	EDADFLKVKR
650	660	670	680	690	700	710	720
HNVFGLDLKD	EKTLQKKEPS	KSSIKKKMTK	VAEAKKVMKR	NFKVNKKITF	TDEGELVQQW	PQMQKSAIKD	AEEDDDTGGI
730	740	750	760	770	780	790	800
NLHKAKERLQ	EEDKFDKEEY	RKKIKAKHRE	KRLKEREARR	EANKRQAKAK	DEEEAFLDWS	DDDDDDDGF	DPSTLPDPDK
810	820	830	840	850	860	870	880
YRSSEDSDSE	DMENKISDTK	KKQGMKKRSN	SEVEDVGPTS	HNRKKARWDT	LEPLDTGLSL	AEDEELVLHL	LRSQS

Cmpd.	No. of Cmpds.	m/z meas.	Δ m/z [ppm]	z	Rt [min]	Score	P	Range	Sequence	Modification
2761	1	713.3401	-111.86	2	65.7	11.2	2	638-649	K.VKRHNVFGLDLK.D	

Detailed Protein Report

Protein 206: **protein MON2 homolog isoform 5 [Homo sapiens]**

Accession: gi|510937041
Database: refseq_human(refseq_human_20140103.fasta)
Database Date: 2015-11-30
Modification(s): Carbamidomethyl

Score: 32.2
MW [kDa]: 182.4
pI: 5.6
Sequence Coverage [%]: 2.1
No. of unique Peptides: 1

10	20	30	40	50	60	70	80
MGCGTKEPKI	TQLCLAAIQR	LMSHEVVSET	AAGNIINMLW	QLMENSLEEL	KLLQTVLVLL	TTNTVVHDEA	LSKAIVLCFR
90	100	110	120	130	140	150	160
LHFTKDNITN	NTAAATVRQV	VTVVFERMVA	EDERHRDIIE	QPVLVQGNSN	RRSVSTLKPC	AKDAYMLFQD	LCQLVNADAP
170	180	190	200	210	220	230	240
YWLVGMTEMT	RTFGLELLES	VLNDFPQVFL	QHQEFSFLLK	ERVCPLVIKL	FSPNIKFRQG	SSTSSSPAPV	EKPYFPICMR
250	260	270	280	290	300	310	320
LLRVVSVLIK	QFYSLLVTEC	EIFLSLLVKF	LDADKPQWLR	AVAVESIHRF	CVQPQLLRSF	CQSYDMKQHS	TKVFRDIVNA
330	340	350	360	370	380	390	400
LGSFIQSLFL	VPPTGNPATS	NQAGNNNLGG	SVSAPANSGM	VGIGGGVTLL	PAFEYRGTWI	PILTITVQGS	AKATYLEMLD
410	420	430	440	450	460	470	480
KVEPPTIPEG	YAMSVAFHCL	LDLVRGITSM	IEGELGELET	ECQTTTEEGS	SPTQSTEQQD	LQSTSDQMDK	EIVSRAVWEE
490	500	510	520	530	540	550	560
MVNACWCGLL	AALSLLLDAS	TDEAATENIL	KAELTMAALC	GRLGLVTSRD	AFITAICKGS	LPPHYALTVL	NTTTAATLSN
570	580	590	600	610	620	630	640
KSYSVQGQSV	MMISPSSESH	QQVVAVGQPL	AVQPQGTVML	TSKNIQCMRT	LLNLAHCHGA	VLGTSWQLVL	ATLQHLVWIL
650	660	670	680	690	700	710	720
GLKPSSGGAL	KPGRAVEGPS	TVLTTAVMTD	LPVISNILSR	LFESSQYLDD	VSLHHLINAL	CSLSLEAMDM	AYGNNKEPSL
730	740	750	760	770	780	790	800
FAVAKLLETG	LVNMHRIEIL	WRPLTGHLLE	VCQHPNSRMR	EWGAEALTSL	IKAGLTFNHD	PPLSQNQRLQ	LLLLNPLKEM
810	820	830	840	850	860	870	880
SNINHPDIRL	KQLECVLQIL	QSQGDSLGPG	WPLVLGVMGA	IRNDQGESLI	RTAFQCLQLV	VTDFLPTMPC	TCLQIVVDVA
890	900	910	920	930	940	950	960
GSFGLHNQEL	NISLTSIGLL	WNISDYFFQR	GETIEKELNK	EEAAQQKQAE	EKGVVLNRPF	HPAPPFDCLW	LCLYAKLGEL
970	980	990	1000	1010	1020	1030	1040
CVDPRPAVRK	SAGQTLFSTI	GAHGTLLQHS	TWHTVIWKVL	FHLLDRVRES	STTADKEKIE	SGGGNILIHH	SRDTAEKQWA
1050	1060	1070	1080	1090	1100	1110	1120
ETWVLTLAGV	ARIFNTRRYL	LQPLGDFSRA	WDVLLDHIQS	AALSKNNEVS	LAALKSFQEI	LQIVSPVRDS	DKPETPPVVN
1130	1140	1150	1160	1170	1180	1190	1200
VPVPVLIGPI	SGMSRPFVRT	DSIGEKLGRY	SSSEPPIVTD	ELEDLNLWWA	AWNTWYRIGS	ESTKPPITFD	KLTFIPSQPF
1210	1220	1230	1240	1250	1260	1270	1280
LTALIQIFPA	LYQHIKTGFN	MDDLQKLGVI	LHSAISVPIS	SDASPFILPS	YTEAVLTSLQ	EAVLTALDVL	QKAICVGPEN
1290	1300	1310	1320	1330	1340	1350	1360
MQIMYPAIFD	QLLAFVEFSC	KPPQYGQLET	KHIANAKYNQ	IQLFAPAEWV	ALNYVPFAER	SLEVVVDLYQ	KTACHKAVVN
1370	1380	1390	1400	1410	1420	1430	1440
EKVLQNIIKT	LRVPLSLKYS	CPSESTWKLA	VSSLLRVLSI	GLPVARQHAS	SGKFDSMWPE	LANTFEDFLF	TKSIPPDNLS
1450	1460	1470	1480	1490	1500	1510	1520
IQEFQRNENI	DVEVVQLISN	EILPYANFIP	KEFVGQIMTM	LNKGSIHSQS	SSFTEAEIDI	RLREEFSKMC	FETLLQFSFS
1530	1540	1550	1560	1570	1580	1590	1600
NKVTTPQEGY	ISRMALSVLL	KRSQDVLHRY	IEDERLSGKC	PLPRQQVTEI	IFVLKAVSTL	IDSLKKTQPE	NVDGNTWAQV
1610	1620	1630	1640	1650			
IALYPTLVEC	ITCSSSEVCS	ALKEALVPFK	DFMQPPASRV	QNGES			

Cmpd.	No. of Cmpds.	m/z meas.	Δ m/z [ppm]	z	Rt [min]	Score	P	Range	Sequence	Modification
2422	1	1044.2014	136.78	2	60.8	16.5	2	2-20	M.GCGTKEPKITQLCLAAIQR.L	Carbamidomethyl: 13

Detailed Protein Report

Protein 207: **PREDICTED: alpha-actinin-4 isoform X1 [Homo sapiens]**

Accession: gi|530417302
Database: refseq_human(refseq_human_20140103.fasta)
Database Date: 2015-11-30

Score: 32.1
MW [kDa]: 104.3
pI: 5.2
Sequence Coverage [%]: 2.4
No. of unique Peptides: 2

10	20	30	40	50	60	70	80
MVDYHAANQS	YQYGPSSAGN	GAGGGGSMGD	YMAQEDDWDR	DLLLDPAWEK	QQRKTFTAWC	NSHLRKAGTQ	IENIDEDFRD
90	100	110	120	130	140	150	160
GLKLMLLLEV	ISGERLPKPE	RGKMRVHKIN	NVNKALDFIA	SKGVKLVSIG	AEEIVDGNAK	MTLGMIWTII	LRFAIQDISV
170	180	190	200	210	220	230	240
EETSAKEGLL	LWCQRKTAPY	KNVNVQNFHI	SWKDGLAFNA	LIHRHRPELI	EYDKLRKDDP	VTNLNNAFEV	AEKYLDIPKM
250	260	270	280	290	300	310	320
LDAEDIVNTA	RPDEKAIMTY	VSSFYHAFSG	AQKAETAANR	ICKVLAVNQE	NEHLMEDYEK	LASDLLEWIR	RTIPWLEDRV
330	340	350	360	370	380	390	400
PQKTIQEMQQ	KLEDFRDYRR	VHKPPKVQEK	CQLEINFNTL	QTKLRLSNRP	AFMPSEGKMV	SDINNGWQHL	EQAEKGYEEW
410	420	430	440	450	460	470	480
LLNEIRRLER	LDHLAEKFRQ	KASIHEAWTD	GKEAMLKHRD	YETATLSDIK	ALIRKHEAFE	SDLAAHQDRV	EQIAAIAQEL
490	500	510	520	530	540	550	560
NELDYYDSHN	VNTRCQKICD	QWDALGSLTH	SRREALEKTE	KQLEAIDQLH	LEYAKRAAPF	NNWMESAMED	LQDMFIVHTI
570	580	590	600	610	620	630	640
EEIEGLISAH	DQFKSTLPDA	DREREAILAI	HKEAQRIAES	NHIKLSGSNP	YTTVTPQIIN	SKWEKVQQLV	PKRDHALLEE
650	660	670	680	690	700	710	720
QSKQQSNEHL	RRQFASQANV	VGPWIQTKME	EIGRISIEMN	GTLEDQLSHL	KQYERSIVDY	KPNLDLLEQQ	HQLIQEALIF
730	740	750	760	770	780	790	800
DNKHTNYTME	HIRVGWEQLL	TTIARTINEV	ENQILTRDAK	GISQEQMQEF	RASFNHFDKK	QTGSMDSDDF	RALLISTGYS
810	820	830	840	850	860	870	880
LGEAEFNRIM	SLVDPNHSGL	VTFQAFIDFM	SRETTDTDTA	DQVIASFKVL	AGDKNFITAE	ELRRELPPDQ	AEYCIARMAP
890	900	910					
YQGPDAVPGA	LDYKSFSTAL	YGESDL					

Cmpd.	No. of Cmpds.	m/z meas.	Δ m/z [ppm]	z	Rt [min]	Score	P	Range	Sequence	Modification
875	1	648.3760	9.03	2	41.0	11.8	2	337-346	R.DYRRVHKPPK.V	
2706	1	693.8970	9.14	2	64.9	20.3	0	734-745	R.VGWEQLLTTIAR.T	

Detailed Protein Report

Protein 208: PREDICTED: centrosomal protein of 95 kDa isoform X10 [Homo sapiens]

**Accession:**	gi|578831608	**Score:**	32.1
**Database:**	refseq_human(refseq_human_20140103.fasta)	**MW [kDa]:**	71.1
**Database Date:**	2015-11-30	**pI:**	10.2
**Modification(s):**	Carbamidomethyl	**Sequence Coverage [%]:**	2.3
		**No. of unique Peptides:**	1

Alias proteins:

Accession	Name	Description
gi|578831610	refseq_human (refseq_human_20140103.fasta)	PREDICTED: centrosomal protein of 95 kDa isoform X11 [Homo sapiens]

10	20	30	40	50	60	70	80
MKICCTLLVF	CPRVGHPLLK	TVSCNGSETL	SVSGIPNARK	LGEPIRAAIP	LHPPYHPSEP	RAPCPIGKEY	LHSSHCSPAV
**90**	**100**	**110**	**120**	**130**	**140**	**150**	**160**
NSTGEHTEFS	GDLDDGLFLI	SKLPKGSKWE	VYPAQVQGPR	TRKPPKGKRN	ENRATASSCN	SPFPQRPRKR	LTEQELHDVS
**170**	**180**	**190**	**200**	**210**	**220**	**230**	**240**
EKLSQRLSEL	DWMLKSALGD	RIKEKTDHKE	ENTGNEEVED	GTEETLSQHS	DGIVEYGPKK	SRPGLSMRRK	PPYRSHSLSP
**250**	**260**	**270**	**280**	**290**	**300**	**310**	**320**
SPVNKHKQFH	LERKRQRKPR	ETDVRQFQAQ	AFTEAFEREL	RRHKVQENIG	PLRIHEKEEE	TEKIYRGEAV	RKGTPECSQP
**330**	**340**	**350**	**360**	**370**	**380**	**390**	**400**
WKIYSRKTTT	QSLRGGLPKP	NKAVPMKVSE	HSLLPLMLEQ	FPFLYVSGPT	LSKMWKQQIA	QVEQLKKEAC	RENRSKKKLQ
**410**	**420**	**430**	**440**	**450**	**460**	**470**	**480**
DEIEEALRRH	DLLTTLVKKE	YEHNKRLQDF	KDCIRRQRLT	QSKIKENRQQ	IVRARKYYDD	YRVQLCAKMM	RMRTREEMIF
**490**	**500**	**510**	**520**	**530**	**540**	**550**	**560**
KKLFEEGLNI	QKQRLRDLRN	YAKEKRDEQR	RRHQDELDSM	ENYYKDQFSL	LAEAISQEHQ	ELKAREKSQA	QVIIKIEAKS
**570**	**580**	**590**	**600**	**610**	**620**		
CTAWQCFFQQ	GTSYIGGWSN	RLYHIAPVCS	GLYSLGLCKY	TDILHNNKII			

Cmpd.	No. of Cmpds.	m/z meas.	Δ m/z [ppm]	z	Rt [min]	Score	P	Range	Sequence	Modification
1748	1	568.7494	-72.31	2	52.2	14.3	1	463-471	R.VQLCAKMMR.M	Carbamidomethyl: 4

Detailed Protein Report

Protein 209: desmoglein-2 preproprotein [Homo sapiens]

**Accession:**	gi|116534898	**Score:**	32.1
**Database:**	refseq_human(refseq_human_20140103.fasta)	**MW [kDa]:**	122.2
**Database Date:**	2015-11-30	**pI:**	5.0
		**Sequence Coverage [%]:**	1.7
		**No. of unique Peptides:**	1

10	20	30	40	50	60	70	80
MARSPGRAYA	LLLLLICFNV	GSGLHLQVLS	TRNENKLLPK	HPHLVRQKRA	WITAPVALRE	GEDLSKKNPI	AKIHSDLAEE
90	100	110	120	130	140	150	160
RGLKITYKYT	GKGITEPPFG	IFVFNKDTGE	LNVTSILDRE	ETPFFLLTGY	ALDARGNNVE	KPLELRIKVL	DINDNEPVFT
170	180	190	200	210	220	230	240
QDVFVGSVEE	LSAAHTLVMK	INATDADEPN	TLNSKISYRI	VSLEPAYPPV	FYLNKDTGEI	YTTSVTLDRE	EHSSYTLTVE
250	260	270	280	290	300	310	320
ARDGNGEVTD	KPVKQAQVQI	RILDVNDNIP	VVENKVLEGM	VEENQVNVEV	TRIKVFDADE	IGSDNWLANF	TFASGNEGGY
330	340	350	360	370	380	390	400
FHIETDAQTN	EGIVTLIKEV	DYEEMKNLDF	SVIVANKAAF	HKSIRSKYKP	TPIPIKVKVK	NVKEGIHFKS	SVISIYVSES
410	420	430	440	450	460	470	480
MDRSSKGQII	GNFQAFDEDT	GLPAHARYVK	LEDRDNWISV	DSVTSEIKLA	KLPDFESRYV	QNGTYTVKIV	AISEDYPRKT
490	500	510	520	530	540	550	560
ITGTVLINVE	DINDNCPTLI	EPVQTICHDA	EYVNVTAEDL	DGHPNSGPFS	FSVIDKPPGM	AEKWKIARQE	STSVLLQQSE
570	580	590	600	610	620	630	640
KKLGRSEIQF	LISDNQGFSC	PEKQVLTLTV	CECLHGSGCR	EAQHDSYVGL	GPAAIALMIL	AFLLLLLVPL	LLLMCHCGKG
650	660	670	680	690	700	710	720
AKGFTPIPGT	IEMLHPWNNE	GAPPEDKVVP	SFLPVDQGGS	LVGRNGVGGM	AKEATMKGSS	SASIVKGQHE	MSEMDGRWEE
730	740	750	760	770	780	790	800
HRSLLSGRAT	QFTGATGAIM	TTETTKTARA	TGASRDMAGA	QAAAVALNEE	FLRNYFTDKA	ASYTEEDENH	TAKDCLLVYS
810	820	830	840	850	860	870	880
QEETESLNAS	IGCCSFIEGE	LDDRFLDDLG	LKFKTLAEVC	LGQKIDINKE	IEQRQKPATE	TSMNTASHSL	CEQTMVNSEN
890	900	910	920	930	940	950	960
TYSSGSSFPV	PKSLQEANAE	KVTQEIVTER	SVSSRQAQKV	ATPLPDPMAS	RNVIATETSY	VTGSTMPPTT	VILGPSQPQS
970	980	990	1000	1010	1020	1030	1040
LIVTERVYAP	ASTLVDQPYA	NEGTVVVTER	VIQPHGGGSN	PLEGTQHLQD	VPYVMVRERE	SFLAPSSGVQ	PTLAMPNIAV
1050	1060	1070	1080	1090	1100	1110	1120
GQNVTVTERV	LAPASTLQSS	YQIPTENSMT	ARNTTVSGAG	VPGPLPDFGL	EESGHSNSTI	TTSSTRVTKH	STVQHSYS

Cmpd.	No. of Cmpds.	m/z meas.	Δ m/z [ppm]	z	Rt [min]	Score	P	Range	Sequence	Modification
1168	1	955.7541	206.12	1	44.8	19.8	1	33-40	R.NENKLLPK.H	

Detailed Protein Report

Protein 210: pregnancy zone protein precursor [Homo sapiens]

Accession: gi|162809334
Database: refseq_human(refseq_human_20140103.fasta)
Database Date: 2015-11-30

Score: 31.9
MW [kDa]: 163.8
pI: 5.9
Sequence Coverage [%]: 2.1
No. of unique Peptides: 1

10	20	30	40	50	60	70	80
MRKDRLLHLC	LVLLLILLSA	SDSNSTEPQY	MVLVPSLLHT	EAPKKGCVLL	SHLNETVTVS	ASLESGRENR	SLFTDLVAEK
90	100	110	120	130	140	150	160
DLFHCVSFTL	PRISASSEVA	FLSIQIKGPT	QDFRKRNTVL	VLNTQSLVFV	QTDKPMYKPG	QTVRFRVVSV	DENFRPRNEL
170	180	190	200	210	220	230	240
IPLIYLENPR	RNRIAQWQSL	KLEAGINQLS	FPLSSEPIQG	SYRVVVQTES	GGRIQHPFTV	EEFVLPKFEV	KVQVPKIISI
250	260	270	280	290	300	310	320
MDEKVNITVC	GEYTYGKPVP	GLATVSLCRK	LSRVLNCDKQ	EVCEEFSQQL	NSNGCITQQV	HTKMLQITNT	GFEMKLRVEA
330	340	350	360	370	380	390	400
RIREEGTDLE	VTANRISEIT	NIVSKLKFVK	VDSHFRQGIP	FFAQVLLVDG	KGVPIPNKLF	FISVNDANYY	SNATTNEQGL
410	420	430	440	450	460	470	480
AQFSINTTSI	SVNKLFVRVF	TVHPNLCFHY	SWVAEDHQGA	QHTANRVFSL	SGSYIHLEPV	AGTLPCGHTE	TITAHYTLNR
490	500	510	520	530	540	550	560
QAMGELSELS	FHYLIMAKGV	IVRSGTHTLP	VESGDMKGSF	ALSFPVESDV	APIARMFIFA	ILPDGEVVGD	SEKFEIENCL
570	580	590	600	610	620	630	640
ANKVDLSFSP	AQSPPASHAH	LQVAAAPQSL	CALRAVDQSV	LLMKPEAELS	VSSVYNLLTV	KDLTNFPDNV	DQQEEEQGHC
650	660	670	680	690	700	710	720
PRPFFIHNGA	IYVPLSSNEA	DIYSFLKGMG	LKVFTNSKIR	KPKSCSVIPS	VSAGAVGQGY	YGAGLGVVER	PYVPQLGTYN
730	740	750	760	770	780	790	800
VIPLNNEQSS	GPVPETVRSY	FPETWIWELV	AVNSSGVAEV	GVTVPDTITE	WKAGAFCLSE	DAGLGISSTA	SLRAFQPFFV
810	820	830	840	850	860	870	880
ELTMPYSVIR	GEVFTLKATV	LNYLPKCIRV	SVQLKASPAF	LASQNTKGEE	SYCICGNERQ	TLSWTVTPKT	LGNVNFSVSA
890	900	910	920	930	940	950	960
EAMQSLELCG	NEVVEVPEIK	RKDTVIKTLL	VEAEGIEQEK	TFSSMTCASG	ANVSEQLSLK	LPSNVVKESA	RASFSVLGDI
970	980	990	1000	1010	1020	1030	1040
LGSAMQNIQN	LLQMPYGCGE	QNMVLFAPNI	YVLNYLNETQ	QLTQEIKAKA	VGYLITGYQR	QLNYKHQDGS	YSTFGERYGR
1050	1060	1070	1080	1090	1100	1110	1120
NQGNTWLTAF	VLKTFAQARS	YIFIDEAHIT	QSLTWLSQMQ	KDNGCFRSSG	SLLNNAIKGG	VEDEATLSAY	VTIALLEIPL
1130	1140	1150	1160	1170	1180	1190	1200
PVTNPIVRNA	LFCLESAWNV	AKEGTHGSHV	YTKALLAYAF	SLLGKQNQNR	EILNSLDKEA	VKEDNLVHWE	RPQRPKAPVG
1210	1220	1230	1240	1250	1260	1270	1280
HLYQTQAPSA	EVEMTSYVLL	AYLTAQPAPT	SGDLTSATNI	VKWIMKQQNA	QGGFSSTQDT	VVALHALSRY	GAATFTRTEK
1290	1300	1310	1320	1330	1340	1350	1360
TAQVTVQDSQ	TFSTNFQVDN	NNLLLLQQIS	LPELPGEYVI	TVTGERCVYL	QTSMKYNILP	EKEDSPFALK	VQTVPQTCDG
1370	1380	1390	1400	1410	1420	1430	1440
HKAHTSFQIS	LTISYTGNRP	ASNMVIVDVK	MVSGFIPLKP	TVKMLERSSS	VSRTEVSNNH	VLIYVEQVTN	QTLSFSFMVL
1450	1460	1470	1480	1490			
QDIPVGDLKP	AIVKVYDYYE	TDESVVAEYI	APCSTDTEHG	NV			

Cmpd.	No. of Cmpds.	m/z meas.	Δ m/z [ppm]	z	Rt [min]	Score	P	Range	Sequence	Modification
705	1	802.0356	159.48	2	38.8	16.0	1	322-335	R.IREEGTDLEVTANR.I	

Detailed Protein Report

Protein 211: oligodendrocyte transcription factor 3 [Homo sapiens]

**Accession:**	gi|28411948	**Score:**	31.9
**Database:**	refseq_human(refseq_human_20140103.fasta)	**MW [kDa]:**	29.3
**Database Date:**	2015-11-30	**pI:**	10.3
**Modification(s):**	Oxidation	**Sequence Coverage [%]:**	11.4
		**No. of unique Peptides:**	2

Quantitation

QD:QU **Median:** 0.84 **CV:** 0.00 % **No. of Peptides:** 1

10	20	30	40	50	60	70	80
MNSDSSSVSS	RASSPDMDEM	YLRDHHHRHH	HHQESRLNSV	SSTQGDMMQK	MPGESLSRAG	AKAAGESSKY	KIKKQLSEQD
**90**	**100**	**110**	**120**	**130**	**140**	**150**	**160**
LQQLRLKING	RERKRMHDLN	LAMDGLREVM	PYAHGPSVRK	LSKIATLLLA	RNYILMLTSS	LEEMKRLVGE	IYGGHHSAFH
**170**	**180**	**190**	**200**	**210**	**220**	**230**	**240**
CGTVGHSAGH	PAHAANSVHP	VHPILGGALS	SGNASSPLSA	ASLPAIGTIR	PPHSLLKAPS	TPPALQLGSG	FQHWAGLPCP
**250**	**260**	**270**	**280**				
CTICQMPPPP	HLSALSTANM	ARLSAESKDL	LK				

Cmpd.	No. of Cmpds.	m/z meas.	Δ m/z [ppm]	z	Rt [min]	Score	P	Range	Sequence	Modification	Ratios
1287	1	611.9489	-43.61	3	46.2	14.1	2	51-69	K.MPGESLSRAGAKAAGESSK.Y		
399	1	701.3413	9.92	2	35.0	17.8	0	96-107	R.MHDLNLAMDGLR.E	Oxidation: 1	QD:QU 0.84

Detailed Protein Report

Protein 212: PREDICTED: myotubularin-related protein 10 isoform X2 [Homo sapiens]

**Accession:**	gi|530406109	**Score:**	31.9
**Database:**	refseq_human(refseq_human_20140103.fasta)	**MW [kDa]:**	64.0
**Database Date:**	2015-11-30	**pI:**	9.3
		**Sequence Coverage [%]:**	6.6
		**No. of unique Peptides:**	2

Alias proteins:

Accession	Name	Description
gi|578840990	refseq_human (refseq_human_20140103.fasta)	PREDICTED: myotubularin-related protein 10 isoform X2 [Homo sapiens]

10	20	30	40	50	60	70	80
MFSLKPPKPT	FRSYLLPPPQ	TDDKINSEPK	IKKLEPVLLP	GEIVVNEVNF	VRKCIATDTS	QYDLWGKLIC	SNFKISFITD
**90**	**100**	**110**	**120**	**130**	**140**	**150**	**160**
DPMPLQKFHY	RNLLLGEHDV	PLTCIEQIVT	VNDHKRKQKV	LGPNQKLKFN	PTELIIYCKD	FRIVRFRFDE	SGPESAKKVC
**170**	**180**	**190**	**200**	**210**	**220**	**230**	**240**
LAIAHYSQPT	DLQLLFAFEY	VGKKYHNSAN	KINGIPSGDG	GGGGGGGNGA	GGGSSQKTPL	FETYSDWDRE	IKRTGASGWR
**250**	**260**	**270**	**280**	**290**	**300**	**310**	**320**
VCSINEGYMI	STCLPEYIVV	PSSLADQDLK	IFSHSFVGRR	MPLWCWSHSN	GSALVRMALI	KDVLQQRKID	QRICNAITKS
**330**	**340**	**350**	**360**	**370**	**380**	**390**	**400**
HPQRSDVYKS	DLDKTLPNIQ	EVQAAFVKLK	QLCVNEPFEE	TEEKWLSSLE	NTRWLEYVRA	FLKHSAELVY	MLESKHLSVV
**410**	**420**	**430**	**440**	**450**	**460**	**470**	**480**
LQEEEGRDLS	CCVASLVQVM	LDPYFRTITG	FQSLIQKEWV	MAGYQFLDRC	NHLKRSEKES	PLFLLFLDAT	WQLLEQYPAA
**490**	**500**	**510**	**520**	**530**	**540**	**550**	**560**
FEFSETYLAV	LYDSTRISLF	GTFLFNSPHQ	RVKQSTKIWR	VLKGTEQRIR	HFPSSSDFVS	GVISSSELLC	IHCCGSLLPS
**570**							
IS							

Cmpd.	No. of Cmpds.	m/z meas.	Δ m/z [ppm]	z	Rt [min]	Score	P	Range	Sequence	Modification
2250	1	752.7461	-190.38	2	56.8	15.6	0	75-87	K.ISFITDDPMPLQK.F	
1260	1	903.4372	-43.14	3	45.9	16.3	2	325-348	R.SDVYKSDLDKTLPNIQEVQAAFVK.L	

Detailed Protein Report

Protein 213: PREDICTED: Bloom syndrome protein isoform X1 [Homo sapiens]

**Accession:**	gi|578827270	**Score:**	31.5
**Database:**	refseq_human(refseq_human_20140103.fasta)	**MW [kDa]:**	85.6
**Database Date:**	2015-11-30	**pI:**	10.0
**Modification(s):**	Carbamidomethyl	**Sequence Coverage [%]:**	5.2
		**No. of unique Peptides:**	2

10	20	30	40	50	60	70	80
MMKIFHKKFG	LHNFRTNQLE	AINAALLGED	CFILMPTGGG	KSLCYQLPAC	VSPGVTVVIS	PLRSLIVDQV	QKLTSLDIPA
90	100	110	120	130	140	150	160
TYLTGDKTDS	EATNIYLQLS	KKDPIIKLLY	VTPEKICASN	RLISTLENLY	ERKLLARFVI	DEAHCVSQWG	HDFRQDYKRM
170	180	190	200	210	220	230	240
NMLRQKFPSV	PVMALTATAN	PRVQKDILTQ	LKILRPQVFS	MSFNRHNLKY	YVLPKKPKKV	AFDCLEWIRK	HHPYDSGIIY
250	260	270	280	290	300	310	320
CLSRRECDTM	ADTLQRDGLA	ALAYHAGLSD	SARDEVQQKW	INQDGCQVIC	ATIAFGMGID	KPDVRFVIHA	SLPKSVEGYY
330	340	350	360	370	380	390	400
QESGRAGRDG	EISHCLLFYT	YHDVTRLKRL	IMMEKDGNHH	TRETHFNNLY	SMVHYCENIT	ECRRIQLLAY	FGENGFNPDF
410	420	430	440	450	460	470	480
CKKHPDVSCD	NCCKTKDYKT	RDVTDDVKSI	VRFVQEHSSS	QGMRNIKHVG	PSGRFTMNML	VDIFLGSKSA	KIQSGIFGKG
490	500	510	520	530	540	550	560
SAYSRHNAER	LFKKLILDKI	LDEDLYINAN	DQAIAYVMLG	NKAQTVLNGN	LKVDFMETEN	SSSVKKQKAL	VAKVSQREEM
570	580	590	600	610	620	630	640
VKKCLGELTE	VCKSLGKVFG	VHYFNIFNTV	TLKKLAESLS	SDPEVLLQID	GVTEDKLEKY	GAEVISVLQK	YSEWTSPAED
650	660	670	680	690	700	710	720
SSPGISLSSS	RGPGRSAAEE	LDEEIPVSSH	YFASKTRNER	KRKKMPASQR	SKRRKTASSG	SKAKGGSATC	RKISSKTKSS
730	740	750	760	770			
SIIGSSSASH	TSQATSGANS	KLGIMAPPKP	INRPFLKPSY	AFS			

Cmpd.	No. of Cmpds.	m/z meas.	Δ m/z [ppm]	z	Rt [min]	Score	P	Range	Sequence	Modification
2797	1	937.4532	-7.25	2	64.1	14.4	1	231-245	K.HHPYDSGIIYCLSRR.E	Carbamidomethyl: 11
1181	1	955.7839	-9.69	3	44.9	17.1	2	656-680	R.SAAEELDEEIPVSSHYFASKTRNER.K	

Detailed Protein Report

Protein 214: eukaryotic initiation factor 4A-III [Homo sapiens]

**Accession:**	gi|7661920	**Score:**	31.3
**Database:**	refseq_human(refseq_human_20140103.fasta)	**MW [kDa]:**	46.8
**Database Date:**	2015-11-30	**pI:**	6.3
**Modification(s):**	Oxidation	**Sequence Coverage [%]:**	5.4
		**No. of unique Peptides:**	1

```
        10         20         30         40         50         60         70         80
MATTATMATS GSARKRLLKE EDMTKVEFET SEEVDVTPTF DTMGLREDLL RGIYAYGFEK PSAIQQRAIK QIIKGRDVIA
        90        100        110        120        130        140        150        160
QSQSGTGKTA TFSISVLQCL DIQVRETQAL ILAPTRELAV QIQKGLLALG DYMNVQCHAC IGGTNVGEDI RKLDYGQHVV
       170        180        190        200        210        220        230        240
AGTPGRVFDM IRRRSLRTRA IKMLVLDEAD EMLNKGFKEQ IYDVYRYLPP ATQVVLISAT LPHEILEMTN KFMTDPIRIL
       250        260        270        280        290        300        310        320
VKRDELTLEG IKQFFVAVER EEWKFDTLCD LYDTLTITQA VIFCNTKRKV DWLTEKMREA NFTVSSMHGD MPQKERESIM
       330        340        350        360        370        380        390        400
KEFRSGASRV LISTDVWARG LDVPQVSLII NYDLPNNREL YIHRIGRSGR YGRKGVAINF VKNDDIRILR DIEQYYSTQI
       410        420
DEMPMNVADL I
```

Cmpd.	No. of Cmpds.	m/z meas.	Δ m/z [ppm]	z	Rt [min]	Score	P	Range	Sequence	Modification
1883	2	621.2961	4.72	2	53.9	17.2	0	2-14	M.ATTATMATSGSAR.K	Oxidation: 6

Detailed Protein Report

Protein 215: PREDICTED: uncharacterized protein LOC100996842 isoform X2 [Homo sapiens]

Accession: gi|530438094
Database: refseq_human(refseq_human_20140103.fasta)
Database Date: 2015-11-30

Score: 31.2
MW [kDa]: 25.7
pI: 12.0
Sequence Coverage [%]: 7.0
No. of unique Peptides: 1

Quantitation

QD:QU	**Median:** 4.73	**CV:** 0.00 %	**No. of Peptides:** 1

Alias proteins:

Accession	Name	Description
gi|578830177	refseq_human (refseq_human_20140103.fasta)	PREDICTED: uncharacterized protein LOC102724164 isoform X2 [Homo sapiens]
gi|578797315	refseq_human (refseq_human_20140103.fasta)	PREDICTED: uncharacterized protein LOC102724164 isoform X2 [Homo sapiens]

10	20	30	40	50	60	70	80
MSAPATAGPG	TVKQRRRWGR	RAQVGEGQHL	NHPWGPDPQE	SPGSRGLARQ	SRDSPPPRWP	EGAPRAGQRL	RRGRVDPAAL
**90**	**100**	**110**	**120**	**130**	**140**	**150**	**160**
LGKGHLGLPG	GGVVLRYRPP	GRQPTVPTRA	SFRCGSALWR	PPSKVYKVTR	MSAGRGGIGA	CQVLRGFNRA	LLLALNMVDR
**170**	**180**	**190**	**200**	**210**	**220**	**230**	**240**
TGHSHCSGAT	GAAFGNEVPQ	PPTHCFGCLL	HLKLYEGQEE	GCGDEWLYRL	ATDGELEQPV	APRGGRKGGT	ADPTPAVPGF
**250**							
GG							

Cmpd.	No. of Cmpds.	m/z meas.	Δ m/z [ppm]	z	Rt [min]	Score	P	Range	Sequence	Modification	Ratios
1852	1	612.7877	-44.99	2	53.5	10.4	1	59-69	R.WPEGAPRAGQR.L		QD:QU 4.73

Detailed Protein Report

Protein 216: PREDICTED: estrogen receptor isoform X1 [Homo sapiens]

Accession: gi|578812488
Database: refseq_human(refseq_human_20140103.fasta)
Database Date: 2015-11-30
Modification(s): Oxidation

Score: 31.0
MW [kDa]: 51.7
pI: 9.5
Sequence Coverage [%]: 7.5
No. of unique Peptides: 1

10	20	30	40	50	60	70	80
MTMTLHTKAS	GMALLHQIQG	NELEPLNRPQ	LKIPLERPLG	EVYLDSSKPA	VYNYPEGAAY	EFNAAAAANA	QVYGQTGLPY
90	100	110	120	130	140	150	160
GPGSEAAAFG	SNGLGGFPPL	NSVSPSPLML	LHPPPQLSPF	LQPHGQQVPY	YLENEPSGYT	VREAGPPAFY	RPNSDNRRQG
170	180	190	200	210	220	230	240
GRERLASTND	KGSMAMESAK	ETRYCAVCND	YASGYHYGVW	SCEGCKAFFK	RSIQGHNDYM	CPATNQCTID	KNRRKSCQAC
250	260	270	280	290	300	310	320
RLRKCYEVGM	MKGGIRKDRR	GGRMLKHKRQ	RDDGEGRGEV	GSAGDMRAAN	LWPSPLMIKR	SKKNSLALSL	TADQMVSALL
330	340	350	360	370	380	390	400
DAEPPILYSE	YDPTRPFSEA	SMMGLLTNLA	DRELVHMINW	AKRVPGFVDL	TLHDQVHLLE	CAWLEILMIG	LVWRSMEHPG
410	420	430	440	450	460	470	
KLLFAPNLLL	DRNQGKCVEG	MVEIFDMLLA	TSSRFRMMNL	QGEEFVCLKS	IILLNSVTKA	WSICTA	

Cmpd.	No. of Cmpds.	m/z meas.	Δ m/z [ppm]	z	Rt [min]	Score	P	Range	Sequence	Modification
2049	1	812.3468	12.24	2	56.0	10.2	1	272-287	R.DDGEGRGEVGSAGDMR.A	Oxidation: 15

Detailed Protein Report

Protein 217: caspase-10 isoform 6 preproprotein [Homo sapiens]

Accession: gi|330864823
Database: refseq_human(refseq_human_20140103.fasta)
Database Date: 2015-11-30
Modification(s): Carbamidomethyl

Score: 30.9
MW [kDa]: 51.8
pI: 5.4
Sequence Coverage [%]: 7.0
No. of unique Peptides: 1

10	20	30	40	50	60	70	80
MKSQGQHWYS	SSDKNCKVSF	REKLLIIDSN	LGVQDVENLK	FLCIGLVPNK	KLEKSSSASD	VFEHLLAEDL	LSEEDPFFLA
**90**	**100**	**110**	**120**	**130**	**140**	**150**	**160**
ELLYIIRQKK	LLQHLNCTKE	EVERLLPTRQ	RVSLFRNLLY	ELSEGIDSEN	LKDMIFLLKD	SLPKTEMTSL	SFLAFLEKQG
**170**	**180**	**190**	**200**	**210**	**220**	**230**	**240**
KIDEDNLTCL	EDLCKTVVPK	LLRNIEKYKR	EKAIQIVTPP	VDKEAESYQG	EEELVSQTDV	KTFLEALPQE	SWQNKHAGSN
**250**	**260**	**270**	**280**	**290**	**300**	**310**	**320**
EILSHVFQWL	GFTVHIHNNV	TKVEMEMVLQ	KQKCNPAHAD	GDCFVFCILT	HGRFGAVYSS	DEALIPIREI	MSHFTALQCP
**330**	**340**	**350**	**360**	**370**	**380**	**390**	**400**
RLAEKPKLFF	IQACQGEEIQ	PSVSIEADAL	NPEQAPTSLQ	DSIPAEADFL	LGLATVPGYV	SFRHVEEGSW	YIQSLCNHLK
**410**	**420**	**430**	**440**	**450**	**460**		
KLVPRHEDIL	SILTAVNDDV	SRRVDKQGTK	KQMPQPAFTL	RKKLVFPVPL	DALSL		

Cmpd.	No. of Cmpds.	m/z meas.	Δ m/z [ppm]	z	Rt [min]	Score	P	Range	Sequence	Modification
2646	1	1145.5422	35.87	2	61.8	11.6	0	274-293	K.CNPAHADGDCFVFCILTHGR.F	Carbamidomethyl: 10, 14

Detailed Protein Report

Protein 218: **LIM/homeobox protein Lhx1 [Homo sapiens]**

**Accession:**	gi|30410785	**Score:**	30.9
**Database:**	refseq_human(refseq_human_20140103.fasta)	**MW [kDa]:**	44.8
**Database Date:**	2015-11-30	**pI:**	9.1
**Modification(s):**	Carbamidomethyl	**Sequence Coverage [%]:**	7.6
		**No. of unique Peptides:**	1

10	20	30	40	50	60	70	80
MVHCAGCKRP	ILDRFLLNVL	DRAWHVKCVQ	CCECKCNLTE	KCFSREGKLY	CKNDFFRCFG	TKCAGCAQGI	SPSDLVRRAR
**90**	**100**	**110**	**120**	**130**	**140**	**150**	**160**
SKVFHLNCFT	CMMCNKQLST	GEELYIIDEN	KFVCKEDYLS	NSSVAKENSL	HSATTGSDPS	LSPDSQDPSQ	DDAKDSESAN
**170**	**180**	**190**	**200**	**210**	**220**	**230**	**240**
VSDKEAGSNE	NDDQNLGAKR	RGPRTTIKAK	QLETLKAAFA	ATPKPTRHIR	EQLAQETGLN	MRVIQVWFQN	RRSKERRMKQ
**250**	**260**	**270**	**280**	**290**	**300**	**310**	**320**
LSALGARRHA	FFRSPRRMRP	LVDRLEPGEL	IPNGPFSFYG	DYQSEYYGPG	GNYDFFPQGP	PSSQAQTPVD	LPFVPSSGPS
**330**	**340**	**350**	**360**	**370**	**380**	**390**	**400**
GTPLGGLEHP	LPGHHPSSEA	QRFTDILAHP	PGDSPSPEPS	LPGPLHSMSA	EVFGPSPPFS	SLSVNGGASY	GNHLSHPPEM
**410**							
NEAAVW							

Cmpd.	No. of Cmpds.	m/z meas.	Δ m/z [ppm]	z	Rt [min]	Score	P	Range	Sequence	Modification
2236	1	916.4377	77.30	2	56.6	12.2	1	28-41	K.CVQCCECKCNLTEK.C	Carbamidomethyl: 4, 5, 7, 9

Detailed Protein Report

Protein 219: pre-mRNA 3' end processing protein WDR33 isoform 1 [Homo sapiens]

**Accession:**	gi|56243590	**Score:**	30.8
**Database:**	refseq_human(refseq_human_20140103.fasta)	**MW [kDa]:**	145.8
**Database Date:**	2015-11-30	**pI:**	9.9
		**Sequence Coverage [%]:**	2.5
		**No. of unique Peptides:**	1

10	20	30	40	50	60	70	80
MATEIGSPPR	FFHMPRFQHQ	APRQLFYKRP	DFAQQQAMQQ	LTFDGKRMRK	AVNRKTIDYN	PSVIKYLENR	IWQRDQRDMR
90	100	110	120	130	140	150	160
AIQPDAGYYN	DLVPPIGMLN	NPMNAVTTKF	VRTSTNKVKC	PVFVVRWTPE	GRRLVTGASS	GEFTLWNGLT	FNFETILQAH
170	180	190	200	210	220	230	240
DSPVRAMTWS	HNDMWMLTAD	HGGYVKYWQS	NMNNVKMFQA	HKEAIREASF	SPTDNKFATC	SDDGTVRIWD	FLRCHEERIL
250	260	270	280	290	300	310	320
RGHGADVKCV	DWHPTKGLVV	SGSKDSQQPI	KFWDPKTGQS	LATLHAHKNT	VMEVKLNLNG	NWLLTASRDH	LCKLFDIRNL
330	340	350	360	370	380	390	400
KEELQVFRGH	KKEATAVAWH	PVHEGLFASG	GSDGSLLFWH	VGVEKEVGGM	EMAHEGMIWS	LAWHPLGHIL	CSGSNDHTSK
410	420	430	440	450	460	470	480
FWTRNRPGDK	MRDRYNLNLL	PGMSEDGVEY	DDLEPNSLAV	IPGMGIPEQL	KLAMEQEQMG	KDESNEIEMT	IPGLDWGMEE
490	500	510	520	530	540	550	560
VMQKDQKKVP	QKKVPYAKPI	PAQFQQAWMQ	NKVPIPAPNE	VLNDRKEDIK	LEEKKKTQAE	IEQEMATLQY	TNPQLLEQLK
570	580	590	600	610	620	630	640
IERLAQKQVE	QIQPPPSSGT	PLLGPQPFPG	QGPMSQIPQG	FQQPHPSQQM	PMNMAQMGPP	GPQGQFRPPG	PQGQMGPQGP
650	660	670	680	690	700	710	720
PLHQGGGGPQ	GFMGPQGPQG	PPQGLPRPQD	MHGPQGMQRH	PGPHGPLGPQ	GPPGPQGSSG	PQGHMGPQGP	PGPQGHIGPQ
730	740	750	760	770	780	790	800
GPPGPQGHLG	PQGPPGTQGM	QGPPGPRGMQ	GPPHPHGIQG	GPGSQGIQGP	VSQGPLMGLN	PRGMQGPPGP	RENQGPAPQG
810	820	830	840	850	860	870	880
MIMGHPPQEM	RGPHPPGGLL	GHGPQEMRGP	QEIRGMQGPP	PQGSMLGPPQ	ELRGPPGSQS	QQGPPQGSLG	PPPQGGMQGP
890	900	910	920	930	940	950	960
PGPQGQQNPA	RGPHPSQGPI	PFQQQKTPLL	GDGPRAPFNQ	EGQSTGPPPL	IPGLGQQGAQ	GRIPPLNPGQ	GPGPNKGDSR
970	980	990	1000	1010	1020	1030	1040
GPPNHHMGPM	SERRHEQSGG	PEHGPERGPF	RGGQDCRGPP	DRRGPHPDFP	DDFSRPDDFH	PDKRFGHRLR	EFEGRGGPLP
1050	1060	1070	1080	1090	1100	1110	1120
QEEKWRRGGP	GPPFPPDHRE	FSEGDGRGAA	RGPPGAWEGR	RPGDERFPRD	PEDPRFRGRR	EESFRRGAPP	RHEGRAPPRG
1130	1140	1150	1160	1170	1180	1190	1200
RDGFPGPEDF	GPEENFDASE	EAARGRDLRG	RGRGTPRGGR	KGLLPTPDEF	PRFEGGRKPD	SWDGNREPGP	GHEHFRDTPR
1210	1220	1230	1240	1250	1260	1270	1280
PDHPPHDGHS	PASRERSSSL	QGMDMASLPP	RKRPWHDGPG	TSEHREMEAP	GGPSEDRGGK	GRGGPGPAQR	VPKSGRSSSL
1290	1300	1310	1320	1330	1340		
DGEHHDGYHR	DEPFGGPPGS	GTPSRGGRSG	SNWGRGSNMN	SGPPRRGASR	GGGRGR		

Cmpd.	No. of Cmpds.	m/z meas.	Δ m/z [ppm]	z	Rt [min]	Score	P	Range	Sequence	Modification
2698	1	937.4703	7.09	2	62.7	13.8	1	1-16	-.MATEIGSPPRFFHMPR.F	

Detailed Protein Report

Protein 220: PREDICTED: arginine/serine-rich protein PNISR isoform X3 [Homo sapiens]

**Accession:**	gi|530383562	**Score:**	30.8
**Database:**	refseq_human(refseq_human_20140103.fasta)	**MW [kDa]:**	99.0
**Database Date:**	2015-11-30	**pI:**	10.4
		**Sequence Coverage [%]:**	4.1
		**No. of unique Peptides:**	1

10	20	30	40	50	60	70	80
MVWLQMTNGC	YYSLLPGETN	RAPSCLVPEE	VEASKALERC	YRNGGDKGVP	ELEWKIKYIL	KEGMWDQGGQ	PWQQWPLNQQ
**90**	**100**	**110**	**120**	**130**	**140**	**150**	**160**
QWMQSFQHQQ	DPSQIDWAAL	AQAWIAQREA	SGQQSMVEQP	PGMMPNGQDM	STMESGPNNH	GNFQGDSNFN	RMWQPEWGMH
**170**	**180**	**190**	**200**	**210**	**220**	**230**	**240**
QQPPHPPPDQ	PWMPPTPGPM	DIVPPSEDSN	SQDSGEFAPD	NRHIFNQNNH	NFGGPPDNFA	VGPVNQFDYQ	HGAAFGPPQG
**250**	**260**	**270**	**280**	**290**	**300**	**310**	**320**
GFHPPYWQPG	PPGPPAPPQN	RRERPSSFRD	RQRSPIALPV	KQEPPQIDAV	KRRTLPAWIR	EGLEKMEREK	QKKLEKERME
**330**	**340**	**350**	**360**	**370**	**380**	**390**	**400**
QQRSQLSKKE	KKATEDAEGG	DGPRLPQRSK	FDSDEEEEDT	ENVEAASSGK	VTRSPSPVPQ	EEHSDPEMTE	EEKEYQMMLL
**410**	**420**	**430**	**440**	**450**	**460**	**470**	**480**
TKMLLTEILL	DVTDEEIYYV	AKDAHRKATK	GGLGGYGSGD	SEDERSDRGS	ESSDTDDEEL	RHRIRQKQEA	FWRKEKEQQL
**490**	**500**	**510**	**520**	**530**	**540**	**550**	**560**
LHDKQMEEEK	QQTERVTKEM	NEFIHKEQNS	LSLLEAREAD	GDVVNEKKRT	PNETTSVLEP	KKEHKEKEKQ	GRSRSGSSSS
**570**	**580**	**590**	**600**	**610**	**620**	**630**	**640**
GSSSSNSRTS	STSSTVSSSS	YSSSSGSSRT	SSRSSSPKRK	KRHSRSRSPT	IKARRSRSRS	YSRRIKIESN	RARVKIRDRR
**650**	**660**	**670**	**680**	**690**	**700**	**710**	**720**
RSNRNSIERE	RRRNRSPSRE	RRRSRSRSRD	RRTNRASRSR	SRDRRKIDDQ	RGNLSGNSHK	HKGEAKEQER	KKERSRSIDK
**730**	**740**	**750**	**760**	**770**	**780**	**790**	**800**
DRKKKDKERE	REQDKRKEKQ	KREEKDFKFS	SQDDRLKRKR	ESERTFSRSG	SISVKIIRHD	SRQDSKKSTT	KDSKKHSGSD
**810**	**820**	**830**	**840**	**850**	**860**		
SSGRSSSESP	GSSKEKKAKK	PKHSRSRSVE	KSQRSGKKAS	RKHKSKSRSR	STTPPRRKR		

Cmpd.	No. of Cmpds.	m/z meas.	Δ m/z [ppm]	z	Rt [min]	Score	P	Range	Sequence	Modification
2313	2	599.8930	-40.29	3	59.4	18.5	1	446-461	R.SDRGSESSDTDDEELR.H	

Detailed Protein Report

Protein 221: **PREDICTED: leucine-rich PPR motif-containing protein, mitochondrial isoform X2 [Homo sapiens]**

Accession: gi|578802653
Database: refseq_human(refseq_human_20140103.fasta)
Database Date: 2015-11-30

Score: 30.7
MW [kDa]: 119.1
pI: 5.9
Sequence Coverage [%]: 2.1
No. of unique Peptides: 1

10	20	30	40	50	60	70	80
MAALLRSARW	LLRAGAAPRL	PLSLRLLPGG	PGRLHAASYL	PAARAGPVAG	GLLSPARLYA	IAAKEKDIQE	ESTFSSRKIS
90	100	110	120	130	140	150	160
NQFDWALMRL	DLSVRRTGRI	PKKLLQKVFN	DTCRSGGLGG	SHALLLLRSC	GSLLPELKLE	ERTEFAHRIW	DTLQKLGAVY
170	180	190	200	210	220	230	240
DVSHYNALLK	VYLQNEYKFS	PTDFLAKMEE	ANIQPNRVTY	QRLIASYCNV	GDIEGASKIL	GFMKTKDLPV	TEAVFSALVT
250	260	270	280	290	300	310	320
GHARAGDMEN	AENILTVMRD	AGIEPGPDTY	LALLNAYAEK	GDIDHVKQTL	EKVEKSELHL	MDRDLLQIIF	SFSKAGYPQY
330	340	350	360	370	380	390	400
VSEILEKVTC	ERRYIPDAMN	LILLLVTEKL	EDVALQILLA	CPVSKEDGPS	VFGSFFLQHC	VTMNTPVEKL	TDYCKKLKEV
410	420	430	440	450	460	470	480
QMHSFPLQFT	LHCALLANKT	DLAKALMKAV	KEEGFPIRPH	YFWPLLVGRR	KEKNVQGIIE	ILKGMQELGV	HPDQETYTDY
490	500	510	520	530	540	550	560
VIPCFDSVNS	ARAILQENGC	LSDSDMFSQA	GLRSEAANGN	LDFVLSFLKS	NTLPISLQSI	RSSLLLGFRR	SMNINLWSEI
570	580	590	600	610	620	630	640
TELLYKDGRY	CQEPRGPTEA	VGYFLYNLID	SMSDSEVQAK	EEHLRQYFHQ	LEKMNVKIPE	NIYRGIRNLL	ESYHVPELIK
650	660	670	680	690	700	710	720
DAHLLVESKN	LDFQKTVQLT	SSELESTLET	LKAENQPIRD	VLKQLILVLC	SEENMQKALE	LKAKYESDMV	TGGYAALINL
730	740	750	760	770	780	790	800
CCRHDKVEDA	LNLKEEFDRL	DSSAVLDTGK	YVGLVRVLAK	HGKLQDAINI	LKEMKEKDVL	IKDTTALSFF	HMLNGAALRG
810	820	830	840	850	860	870	880
EIETVKQLHE	AIVTLGLAEP	STNISFPLVT	VHLEKGDLST	ALEVAIDCYE	KYKVLPRIHD	VLCKLVEKGE	TDLIQKAMDF
890	900	910	920	930	940	950	960
VSQEQGEMVM	LYDLFFAFLQ	TGNYKEAKKI	IETPGIRARS	ARLQWFCDRC	VANNQVETLE	KLVELTQKLF	ECDRDQMYYN
970	980	990	1000	1010	1020	1030	1040
LLKLYKINGD	WQRADAVWNK	IQEENVIPRE	KTLRLLAEIL	REGNQEVPFD	VPELWYEDEK	HSLNSSSAST	TEPDFQKDIL
1050	1060						
IACRLNQKKR	GI						

Cmpd.	No. of Cmpds.	m/z meas.	Δ m/z [ppm]	z	Rt [min]	Score	P	Range	Sequence	Modification
1368	1	428.2118	-70.40	3	47.2	12.4	1	673-683	K.AENQPIRDVLK.Q	

Detailed Protein Report

Protein 222: PREDICTED: protein prune homolog 2 isoform X10 [Homo sapiens]

**Accession:**	gi|530390628	**Score:**	30.6
**Database:**	refseq_human(refseq_human_20140103.fasta)	**MW [kDa]:**	337.3
**Database Date:**	2015-11-30	**pI:**	4.2
		**Sequence Coverage [%]:**	0.7
		**No. of unique Peptides:**	1

Quantitation

QD:QU	**Median:** 0.95	**CV:** 0.00 %	**No. of Peptides:** 1

Detailed Protein Report

10	20	30	40	50	60	70	80
MEEFLQRAKS	KLNRSKRLEK	VHVVIGPKSC	DLDSLISTFT	YAYFLDKVSP	PGVLCLPVLN	IPRTEFNYFT	ETRFILEELN
90	100	110	120	130	140	150	160
ISESFHIFRD	EINLHQLNDE	GKLSITLVGS	SVLASEDKTL	ESAVVKVINP	VEQSDANVEF	RESSSSLVLK	EILQEAPELI
170	180	190	200	210	220	230	240
TEQLAHRLRG	SILFKWMTME	SEKISEKQEE	ILSILEEKFP	NLPPREDIIN	VLQETQFSAQ	GLSIEQTMLK	DLKELSDGEI
250	260	270	280	290	300	310	320
KVAISTVSMN	LENCLFHSNI	TSDLKAFTDK	FGFDVLILFS	SYLSEEQQPR	RQIAVYSENM	ELCSQICCEL	EECQNPCLEL
330	340	350	360	370	380	390	400
EPFDCGCDEI	LVYQQEDPSV	TCDQVVLVVK	EVINRRCPEM	VSNSRTSSTE	AVAGSAPLSQ	GSSGIMELYG	SDIEPQPSSV
410	420	430	440	450	460	470	480
NFIENPPDLN	DSNQAQVDAN	VDLVSPDSGL	ATIRSSRSSK	ESSVFLSDDS	PVGEGAGPHH	TLLPGLDSYS	PIPEGAVAEE
490	500	510	520	530	540	550	560
HAWSGEHGEH	FDLFNFDPAP	MASGQSQQSS	HSADYSPADD	FFPNSDLSEG	QLPAGPEGLD	GMGTNMSNYS	SSSLLSGAGK
570	580	590	600	610	620	630	640
DSLVEHDEEF	VQRQDSPRDN	SERNLSLTDF	VGDESPSPER	LKNTGKRIPP	TPMNSLVESS	PSTEEPASLY	TEDMTQKATD
650	660	670	680	690	700	710	720
TGHMGPPQTH	ARCSSWWGGL	EIDSKNIADA	WSSSEQESVF	QSPESWKEHK	PSSIDRRASD	SVFQPKSLEF	TKSGPWESEF
730	740	750	760	770	780	790	800
GQPELGSNDI	QDKNEESLPF	QNLPMEKSPL	PNTSPQGTNH	LIEDFASLWH	SGRSPTAMPE	PWGNPTDDGE	PAAVAPFPAW
810	820	830	840	850	860	870	880
SAFGKEDHDE	ALKNTWNLHP	TSSKTPSVRD	PNEWAMAKSG	FAFSSSELLD	NSPSEINNEA	APEIWGKKNN	DSRDHIFAPG
890	900	910	920	930	940	950	960
NPSSDLDHTW	TNSKPPKEDQ	NGLVDPKTRG	KVYEKVDSWN	LFEENMKKGG	SDVLVPWEDS	FLSYKCSDYS	ASNLGEDSVP
970	980	990	1000	1010	1020	1030	1040
SPLDTNYSTS	DSYTSPTFAG	DEKETEHKPF	AKEEGFESKD	GNSTAEETDI	PPQSLQQSSR	NRISSGPGNL	DMWASPHTDN
1050	1060	1070	1080	1090	1100	1110	1120
SSEINTTHNL	DENELKTEHT	DGKNISMEDD	VGESSQSSYD	DPSMMQLYNE	TNRQLTLLHS	STNSRQTAPD	SLDLWNRVIL
1130	1140	1150	1160	1170	1180	1190	1200
EDTQSTATIS	DMDNDLDWDD	CSGGAAIPSD	GQTEGYMAEG	SEPETRFTVR	QLEPWGLEYQ	EANQVDWELP	ASDEHTKDSA
1210	1220	1230	1240	1250	1260	1270	1280
PSEHHTLNEK	SGQLIANSIW	DSVMRDKDMS	SFMLPGSSHI	TDSEQRELPP	EIPSHSANVK	DTHSPDAPAA	SGTSESEALI
1290	1300	1310	1320	1330	1340	1350	1360
SHLDKQDTER	ETLQSDAASL	ATRLENPGYF	PHPDPWKGHG	DGQSESEKEA	QGATDRGHLD	EEEVIASGVE	NASGISEKGQ
1370	1380	1390	1400	1410	1420	1430	1440
SDQELSSLVA	SEHQEICIKS	GKISSLAVTF	SPQTEEPEEV	LEYEEGSYNL	DSRDVQTGMS	ADNLQPKDTH	EKHLMSQRNS
1450	1460	1470	1480	1490	1500	1510	1520
GETTETSDGM	NFTKYVSVPE	KDLEKTEECN	FLEPENVGGG	PPHRVPRSLD	FGDVPIDSDV	HVSSTCSEIT	KNLDVKGSEN
1530	1540	1550	1560	1570	1580	1590	1600
SLPGAGSSGN	FDRDTISSEY	THSSASSPEL	NDSSVALSSW	GQQPSSGYQE	ENQGNWSEQN	HQESELITTD	GQVEIVTKVK
1610	1620	1630	1640	1650	1660	1670	1680
DLEKNRINEF	EKSFDRKTPT	FLEIWNDSVD	GDSFSSLSSP	ETGKYSEHSG	THQESNLIAS	YQEKNEHDIS	ATVQPEDARV
1690	1700	1710	1720	1730	1740	1750	1760
ISTSSGSDDD	SVGGEESIEE	EIQVANCHVA	EDESRAWDSL	NESNKFLVTA	DPKSENIYDY	LDSSEPAENE	NKSNPFCDNQ
1770	1780	1790	1800	1810	1820	1830	1840
QSSPDPWTFS	PLTETEMQIT	AVEKEKRSSP	ETGTTGDVAW	QISPKASFPK	NEDNSQLEML	GFSADSTEWW	KASPQEGRLI
1850	1860	1870	1880	1890	1900	1910	1920
ESPFERELSD	SSGVLEINSS	VHQNASPWGV	PVQGDIEPVE	THYTNPFSDN	HQSPFLEGNG	KNSHEQLWNI	QPRQPDPDAD
1930	1940	1950	1960	1970	1980	1990	2000
KFSQLVKLDQ	IKEKDSREQT	FVSAAGDELT	PETPTQEQCQ	DTMLPVCDHP	DTAFTHAEEN	SCVTSNVSTN	EGQETNQWEQ
2010	2020	2030	2040	2050	2060	2070	2080
EKSYLGEMTN	SSIATENFPA	VSSPTQLIMK	PGSEWDGSTP	SEDSRGTFVP	DILHGNFQEG	GQLASAAPDL	WIDAKKPFSL
2090	2100	2110	2120	2130	2140	2150	2160
KADGENPDIL	THCEHDSNSQ	ASDSPDICHD	SEAKQETEKH	LSACMGPEVE	SSELCLTEPE	IDEEPIYEPG	REFVPSNAEL
2170	2180	2190	2200	2210	2220	2230	2240
DSENATVLPP	IGYQADIKGS	SQPASHKGSP	EPSEINGDNS	TGLQVSEKGA	SPDMAPILEP	VDRRIPRIEN	VATSIFVTHQ
2250	2260	2270	2280	2290	2300	2310	2320
EPTPEGDGSW	ISDSFSPESQ	PGARALFDGD	PHLSTENPAL	VPDALLASDT	CLDISEAAFD	HSFSDASGLN	TSTGTIDDMS
2330	2340	2350	2360	2370	2380	2390	2400

Detailed Protein Report

Cmpd.	No. of Cmpds.	m/z meas.	Δ m/z [ppm]	z	Rt [min]	Score	P	Range	Sequence	Modification	Ratios
797	1	534.6313	-269.15	2	40.0	10.3	0	688-696	K.EHKPSSIDR.R		QD:QU 0.95

Detailed Protein Report

Protein 223: PREDICTED: unconventional myosin-XVIIIb isoform X4 [Homo sapiens]

**Accession:**	gi|578837445	**Score:**	30.5
**Database:**	refseq_human(refseq_human_20140103.fasta)	**MW [kDa]:**	272.3
**Database Date:**	2015-11-30	**pI:**	6.5
		**Sequence Coverage [%]:**	1.5
		**No. of unique Peptides:**	1

Detailed Protein Report

10	20	30	40	50	60	70	80
MTSINGEKAQ	ELGSSATPTK	KTVPFKRGVR	RGDVLLMVAK	LDPDSAKPEK	THPHDAPPCK	TSPPATDTGK	EKKGETSRTP
90	100	110	120	130	140	150	160
CGSQASTEIL	APKAEKTRTG	GLGDPGQGTV	ALKKGEEGQS	IVGKGLGTPK	TTELKEAEPQ	GKDRQGTRPQ	AQGPGEGVRP
170	180	190	200	210	220	230	240
GKAEKEGAEP	TNTVEKGNVS	KDVGSEGKHV	RPQIPGRKWG	GFLGRRSKWD	GPQNKKDKEG	VLLSKAEKTG	EPQTQMEKTS
250	260	270	280	290	300	310	320
QVQGELGDDL	RMGEKAGELR	STTGKAGESW	DKKEKMGQPQ	GKSGNAGEAR	SQTEKGCEAP	KEVSTMVESP	AAPGKGGWPG
330	340	350	360	370	380	390	400
SRGQEAEEPC	SRAGDGAGAL	ETELEGPSQP	ALEKDAERPR	IRKENQDGPA	PQEEGKGGQS	RDSDQAPEDR	WYEAEKVWLA
410	420	430	440	450	460	470	480
QKDGFTLATV	LKPDEGTADL	PAGRVRLWID	ADKTITEVDE	EHVHRANPPE	LDQVEDLASL	ISVNESSVLN	TLLQRYKAQL
490	500	510	520	530	540	550	560
LHTCTGPDLI	VLQPRGPSVP	SAGKVPKGRR	DGLPAHIGSM	AQRAYWALLN	QRRDQSIVAL	GWSGAGKTTC	CEQVLEHLVG
570	580	590	600	610	620	630	640
MAGSVDGRVS	VEKIRATFTV	LRAFGSVSMA	HSRSATRFSM	VMSLDFNATG	RITAAQLQTM	LLEKSRVARQ	PEGESNFLVF
650	660	670	680	690	700	710	720
SQMLAGLDLD	LRTELNLHQM	ADSSSFGMGV	WSKPEDKQKA	AAAFAQLQGA	MEMLGISESE	QRAVWRVLAA	IYHLGAAGAC
730	740	750	760	770	780	790	800
KVGRKQFMRF	EWANYAAEAL	GCEYEELNTA	TFKHHLRQII	QQMTFGPSRW	GLEDEETSSG	LKMTGVDCVE	GMASGLYQEL
810	820	830	840	850	860	870	880
FAAVVSLINR	SFSSHHLSMA	SIMVVDSPGF	QNPRHQGKDR	AATFEELCHN	YAHERLQLLF	YQRTFVSTLQ	RYQEEGVPVQ
890	900	910	920	930	940	950	960
FDLPDPSPGT	TVAVVDQNPS	QQVRLPAGGG	AQDARGLFWV	LDEEVHVEGS	SDSVVLERLC	AAFEKKGAGT	EGSSALRTCE
970	980	990	1000	1010	1020	1030	1040
QPLQCEIFHQ	LGWDPVRYDL	TGWLHRAKPN	LSALDAPQVL	HQSKREELRS	LFQARAKLPP	VCRAVAGLEG	TSQQALQRSR
1050	1060	1070	1080	1090	1100	1110	1120
MVRRTFASSL	AAVRRKAPCS	QIKLQMDALT	SMIKRSRLHF	IHCLVPNPVV	ESRSGQESPP	PPQPGRDKPG	AGGPLALDIP
1130	1140	1150	1160	1170	1180	1190	1200
ALRVQLAGFH	ILEALRLHRT	GYADHMGLTR	FRRQFQVLDA	PLLKKLMSTS	EGIDERKAVE	ELLETLDLEK	KAVAVGHSQV
1210	1220	1230	1240	1250	1260	1270	1280
FLKAGVISRL	EKQREKLVSQ	SIVLFQAACK	GFLSRQEFKK	LKIRRLAAQC	IQKNVAVFLA	VKDWPWWQLL	GSLQPLLSAT
1290	1300	1310	1320	1330	1340	1350	1360
IGTEQLRAKE	EELTTLRRKL	EKSEKLRNEL	RQNTDLLESK	IADLTSDLAD	ERFKGDVACQ	VLESERAERL	QAFREVQELK
1370	1380	1390	1400	1410	1420	1430	1440
SKHEQVQKKL	GDVNKQLEEA	QQKIQLNDLE	RNPTGGADEW	QMRFDCAQME	NEFLRKRLQQ	CEERLDSELT	ARKELEQKLG
1450	1460	1470	1480	1490	1500	1510	1520
ELQSAYDGAK	KMAHQLKRKC	HHLTCDLEDT	CVLLENQQSR	NHELEKKQKK	FDLQLAQALG	ESVFEKGLRE	KVTQENTSVR
1530	1540	1550	1560	1570	1580	1590	1600
WELGQLQQQL	KQKEQEASQL	KQQVEMLQDH	KRELLGSPSL	GENCVAGLKE	RLWKLESSAL	EQQKIQSQQE	NTIKQLEQLR
1610	1620	1630	1640	1650	1660	1670	1680
QRFELEIERM	KQMHQKDRED	QEEELEDVRQ	SCQKRLHQLE	MQLEQEYEEK	QMVLHEKQDL	EGLIGTLCDQ	IGHRDFDVEK
1690	1700	1710	1720	1730	1740	1750	1760
RLRRDLRRTH	ALLSDVQLLL	GTMEDGKTSV	SKEELEKVHS	QLEQSEAKCE	EALKTQKVLT	ADLESMHSEL	ENMTRNKSLV
1770	1780	1790	1800	1810	1820	1830	1840
DEQLYRLQFE	KADLLKRIDE	DQDDLNELMQ	KHKDLIAQSA	ADIGQIQELQ	LQLEEAKKEK	HKLQEQLQVA	QMRIEYLEQS
1850	1860	1870	1880	1890	1900	1910	1920
TVDRAIVSRQ	EAVICDLENK	TEFQKVQIKR	FEVLVIRLRD	SLIKMGEELS	QAATSESQQR	ESSQYYQRRL	EELKADMEEL
1930	1940	1950	1960	1970	1980	1990	2000
VQREAEASRR	CMELEKYVEE	LAAVRQTLQT	DLETSIRRIA	DLQAALEEVA	SSDSDTESVQ	TAVDCGSSGR	KEMDNVSILS
2010	2020	2030	2040	2050	2060	2070	2080
SQPEGSLQSW	LSCTLSLATD	TMRTPSRQSA	TSSRILSPRI	NEEAGDTERT	QSALALSRAR	STNVHSKTSG	DKPVSPHFVR
2090	2100	2110	2120	2130	2140	2150	2160
RQKYCHFGDG	EVLAVQRKST	ERLEPASSPL	ASRSTNTSPL	SREKLPSPSA	ALSEFVEGLR	RKRAQRGQGS	TLGLEDWPTL
2170	2180	2190	2200	2210	2220	2230	2240

Detailed Protein Report

Cmpd.	No. of Cmpds.	m/z meas.	Δ m/z [ppm]	z	Rt [min]	Score	P	Range	Sequence	Modification
2500	1	937.1908	150.69	2	60.0	17.9	1	1124-1139	R.VQLAGFHILEALRLHR.T	

Detailed Protein Report

Protein 224: hypoxia up-regulated protein 1 precursor [Homo sapiens]

Accession: gi|5453832
Database: refseq_human(refseq_human_20140103.fasta)
Database Date: 2015-11-30

Score: 30.5
MW [kDa]: 111.3
pI: 5.0
Sequence Coverage [%]: 1.4
No. of unique Peptides: 1

Alias proteins:

Accession	Name	Description
gi|195976805	refseq_human (refseq_human_20140103.fasta)	hypoxia up-regulated protein 1 precursor [Homo sapiens]

10	20	30	40	50	60	70	80
MADKVRRQRP	RRRVCWALVA	VLLADLLALS	DTLAVMSVDL	GSESMKVAIV	KPGVPMEIVL	NKESRRKTPV	IVTLKENERF
90	100	110	120	130	140	150	160
FGDSAASMAI	KNPKATLRYF	QHLLGKQADN	PHVALYQARF	PEHELTFDPQ	RQTVHFQISS	QLQFSPEEVL	GMVLNYSRSL
170	180	190	200	210	220	230	240
AEDFAEQPIK	DAVITVPVFF	NQAERRAVLQ	AARMAGLKVL	QLINDNTATA	LSYGVFRRKD	INTTAQNIMF	YDMGSGSTVC
250	260	270	280	290	300	310	320
TIVTYQMVKT	KEAGMQPQLQ	IRGVGFDRTL	GGLEMELRLR	ERLAGLFNEQ	RKGQRAKDVR	ENPRAMAKLL	REANRLKTVL
330	340	350	360	370	380	390	400
SANADHMAQI	EGLMDDVDFK	AKVTRVEFEE	LCADLFERVP	GPVQQALQSA	EMSLDEIEQV	ILVGGATRVP	RVQEVLLKAV
410	420	430	440	450	460	470	480
GKEELGKNIN	ADEAAAMGAV	YQAAALSKAF	KVKPFVVRDA	VVYPILVEFT	REVEEEPGIH	SLKHNKRVLF	SRMGPYPQRK
490	500	510	520	530	540	550	560
VITFNRYSHD	FNFHINYGDL	GFLGPEDLRV	FGSQNLTTVK	LKGVGDSFKK	YPDYESKGIK	AHFNLDESGV	LSLDRVESVF
570	580	590	600	610	620	630	640
ETLVEDSAEE	ESTLTKLGNT	ISSLFGGGTT	PDAKENGTDT	VQEEEESPAE	GSKDEPGEQV	ELKEEAEAPV	EDGSQPPPPE
650	660	670	680	690	700	710	720
PKGDATPEGE	KATEKENGDK	SEAQKPSEKA	EAGPEGVAPA	PEGEKKQKPA	RKRRMVEEIG	VELVVLDLPD	LPEDKLAQSV
730	740	750	760	770	780	790	800
QKLQDLTLRD	LEKQEREKAA	NSLEAFIFET	QDKLYQPEYQ	EVSTEEQREE	ISGKLSAAST	WLEDEGVGAT	TVMLKEKLAE
810	820	830	840	850	860	870	880
LRKLCQGLFF	RVEERKKWPE	RLSALDNLLN	HSSMFLKGAR	LIPEMDQIFT	EVEMTTLEKV	INETWAWKNA	TLAEQAKLPA
890	900	910	920	930	940	950	960
TEKPVLLSKD	IEAKMMALDR	EVQYLLNKAK	FTKPRPRPKD	KNGTRAEPPL	NASASDQGEK	VIPPAGQTED	AEPISEPEKV
970	980	990	1000				
ETGSEPGDTE	PLELGGPGAE	PEQKEQSTGQ	KRPLKNDEL				

Cmpd.	No. of Cmpds.	m/z meas.	Δ m/z [ppm]	z	Rt [min]	Score	P	Range	Sequence	Modification
419	1	643.8239	-17.73	2	35.2	10.7	2	298-308	K.DVRENPRAMAK.L	

Detailed Protein Report

Protein 225: splicing factor, proline- and glutamine-rich [Homo sapiens]

Accession: gi|4826998
Database: refseq_human(refseq_human_20140103.fasta)
Database Date: 2015-11-30

Score: 30.5
MW [kDa]: 76.1
pI: 9.9
Sequence Coverage [%]: 3.8
No. of unique Peptides: 1

Quantitation

QD:QU **Median:** 0.54 **CV:** 0.00 % **No. of Peptides:** 1

Alias proteins:

Accession	Name	Description
gi|530363144	refseq_human (refseq_human_20140103.fasta)	PREDICTED: splicing factor, proline- and glutamine-rich isoform X3 [Homo sapiens]
gi|530363142	refseq_human (refseq_human_20140103.fasta)	PREDICTED: splicing factor, proline- and glutamine-rich isoform X2 [Homo sapiens]
gi|530363140	refseq_human (refseq_human_20140103.fasta)	PREDICTED: splicing factor, proline- and glutamine-rich isoform X1 [Homo sapiens]

```
        10         20         30         40         50         60         70         80
MSRDRFRSRG GGGGGFHRRG GGGGRGGLHD FRSPPPGMGL NQNRGPMGPG PGQSGPKPPI PPPPPHQQQQ QPPPQQPPPQ
        90        100        110        120        130        140        150        160
QPPPHQPPPH PQPHQQQQPP PPPQDSSKPV VAQGPGPAPG VGSAPPASSS APPATPPTSG APPGSGPGPT PTPPPAVTSA
       170        180        190        200        210        220        230        240
PPGAPPPTPP SSGVPTTPPQ AGGPPPPPAA VPGPGPGPKQ GPGPGGPKGG KMPGGPKPGG GPGLSTPGGH PKPPHRGGGE
       250        260        270        280        290        300        310        320
PRGGRQHHPP YHQQHHQGPP PGGPGGRSEE KISDSEGFKA NLSLLRRPGE KTYTQRCRLF VGNLPADITE DEFKRLFAKY
       330        340        350        360        370        380        390        400
GEPGEVFINK GKGFGFIKLE SRALAEIAKA ELDDTPMRGR QLRVRFATHA AALSVRNLSP YVSNELLEEA FSQFGPIERA
       410        420        430        440        450        460        470        480
VVIVDDRGRS TGKGIVEFAS KPAARKAFER CSEGVFLLTT TPRPVIVEPL EQLDDEDGLP EKLAQKNPMY QKERETPPRF
       490        500        510        520        530        540        550        560
AQHGTFEYEY SQRWKSLDEM EKQQREQVEK NMKDAKDKLE SEMEDAYHEH QANLLRQDLM RRQEELRRME ELHNQEMQKR
       570        580        590        600        610        620        630        640
KEMQLRQEEE RRRREEEMMI RQREMEEQMR RQREESYSRM GYMDPRERDM RMGGGGAMNM GDPYGSGGQK FPPLGGGGGI
       650        660        670        680        690        700        710
GYEANPGVPP ATMSGSMMGS DMRTERFGQG GAGPVGGQGP RGMGPGTPAG YGRGREEYEG PNKKPRF
```

Cmpd.	No. of Cmpds.	m/z meas.	Δ m/z [ppm]	z	Rt [min]	Score	P	Range	Sequence	Modification	Ratios
284	1	671.3405	5.90	2	33.5	10.1	0	667-681	R.FGQGGAGPVGGQGPR.G		QD:QU 0.54

Detailed Protein Report

Protein 226: PREDICTED: synaptojanin-1 isoform X6 [Homo sapiens]

**Accession:**	gi|530419130	**Score:**	30.5
**Database:**	refseq_human(refseq_human_20140103.fasta)	**MW [kDa]:**	139.8
**Database Date:**	2015-11-30	**pI:**	6.8
**Modification(s):**	Carbamidomethyl	**Sequence Coverage [%]:**	2.8
		**No. of unique Peptides:**	2

10	20	30	40	50	60	70	80
MAFSKGFRIY	HKLDPPPFSL	IVETRHKEEC	LMFESGAVAV	LSSAEKEAIK	GTYSKVLDAY	GLLGVLRLNL	GDTMLHYLVL
**90**	**100**	**110**	**120**	**130**	**140**	**150**	**160**
VTGCMSVGKI	QESEVFRVTS	TEFISLRIDS	SDEDRISEVR	KVLNSGNFYF	AWSASGISLD	LSLNAHRSMQ	EQTTDNRFFW
**170**	**180**	**190**	**200**	**210**	**220**	**230**	**240**
NQSLHLHLKH	YGVNCDDWLL	RLMCGGVEIR	TIYAAHKQAK	ACLISRLSCE	RAGTRFNVRG	TNDDGHVANF	VETEQVVYLD
**250**	**260**	**270**	**280**	**290**	**300**	**310**	**320**
DSVSSFIQIR	GSVPLFWEQP	GLQVGSHRVR	MSRGFEANAP	AFDRHFRTLK	NLYGKQIIVN	LLGSKEGEHM	LSKAFQSHLK
**330**	**340**	**350**	**360**	**370**	**380**	**390**	**400**
ASEHAADIQM	VNFDYHQMVK	GGKAEKLHSV	LKPQVQKFLD	YGFFYFNGSE	VQRCQSGTVR	TNCLDCLDRT	NSVQAFLGLE
**410**	**420**	**430**	**440**	**450**	**460**	**470**	**480**
MLAKQLEALG	LAEKPQLVTR	FQEVFRSMWS	VNGDSISKIY	AGTGALEGKA	KAGKLKDGAR	SVTRTIQNNF	FDSSKQEAID
**490**	**500**	**510**	**520**	**530**	**540**	**550**	**560**
VLLLGNTLNS	DLADKARALL	TTGSLRASSK	VLKSMCENFY	KYSKPKKIRV	CVGTWNVNGG	KQFRSIAFKN	QTLTDWLLDA
**570**	**580**	**590**	**600**	**610**	**620**	**630**	**640**
PKLAGIQEFQ	DKRSKPTDIF	AIGFEEMVEL	NAGNIVSAST	TNQKLWAVEL	QKTISRDNKY	VLLASEQLVG	VCLFVFIRPQ
**650**	**660**	**670**	**680**	**690**	**700**	**710**	**720**
HAPFIRDVAV	DTVKTGMGGA	TGNKGAVAIR	MLFHTTSLCF	VCSHFAAGQS	QVKERNEDFI	EIARKLSFPM	GRMLFSHDYV
**730**	**740**	**750**	**760**	**770**	**780**	**790**	**800**
FWCGDFNYRI	DLPNEEVKEL	IRQQNWDSLI	AGDQLINQKN	AGQVFRGFLE	GKVTFAPTYK	YDLFSDDYDT	SEKCRTPAWT
**810**	**820**	**830**	**840**	**850**	**860**	**870**	**880**
DRVLWRRRKW	PFDRSAEDLD	LLNASFQDES	KILYTWTPGT	LLHYGRAELK	TSDHRPVVAL	IDIDIFEVEA	EERQNIYKEV
**890**	**900**	**910**	**920**	**930**	**940**	**950**	**960**
IAVQGPPDGT	VLVSIKSSLP	ENNFFDDALI	DELLQQFASF	GEVILIRFVE	DKMWVTFLEG	SSALNVLSLN	GKELLNRTIT
**970**	**980**	**990**	**1000**	**1010**	**1020**	**1030**	**1040**
IALKSPDWIK	NLEEEMSLEK	ISIALPSSTS	STLLGEDAEV	AADFDMEGDV	DDYSAEVEEL	LPQHLQPSSS	SGLGTSPSSS
**1050**	**1060**	**1070**	**1080**	**1090**	**1100**	**1110**	**1120**
PRTSPCQSPT	ISEGPVPSLP	IRPSRAPSRT	PGPPSAQSSP	IDAQPATPLP	QKDPAQPLEP	KRPPPPRPVA	PPTRPAPPQR
**1130**	**1140**	**1150**	**1160**	**1170**	**1180**	**1190**	**1200**
PPPPSGRSQP	SPQAGLAGPG	PAGYSTARPT	IPPRAGVISA	PQSHARASAG	RLTPESQSKT	SETSKGSTFL	PEPLKPQAAF
**1210**	**1220**	**1230**	**1240**	**1250**	**1260**	**1270**	
PPQSSLPPPA	QRLQEPLVPV	AAPMPQSGPQ	PNLETPPQPP	PRSRSSHSLP	SEASSQPQQE	QPSG	

Cmpd.	No. of Cmpds.	m/z meas.	Δ m/z [ppm]	z	Rt [min]	Score	P	Range	Sequence	Modification
1994	1	1044.9084	-141.16	2	55.3	14.4	2	182-200	R.LMCGGVEIRTIYAAHKQAK.A	
2282	1	614.2542	-33.53	3	59.0	16.0	1	374-389	R.CQSGTVRTNCLDCLDR.T	Carbamidomethyl: 10

Detailed Protein Report

Protein 227: N-alpha-acetyltransferase 10 isoform 2 [Homo sapiens]

**Accession:**	gi|371121601	**Score:**	30.5
**Database:**	refseq_human(refseq_human_20140103.fasta)	**MW [kDa]:**	24.8
**Database Date:**	2015-11-30	**pI:**	5.2
**Modification(s):**	Oxidation	**Sequence Coverage [%]:**	10.5
		**No. of unique Peptides:**	1

10	20	30	40	50	60	70	80
MNIRNARPED	LMNMQHCNLL	CLPENYQMKY	YFYHGLSWPQ	LSYIAEDENG	KIVGYVLAKM	EEDPDDVPHG	HITSLAVKRS
**90**	**100**	**110**	**120**	**130**	**140**	**150**	**160**
HRRLGLAQKL	MDQASRAMIE	NFNAKYVSLH	VRKRISEVEP	KYYADGEDAY	AMKRDLTQMA	DELRRHLELK	EKGRHVVLGA
**170**	**180**	**190**	**200**	**210**	**220**	**230**	
IENKVESKGN	SPPSSGEACR	EEKGLAAEDS	GGDSKDLSEV	SETTESTDVK	DSSEASDSAS		

Cmpd.	No. of Cmpds.	m/z meas.	Δ m/z [ppm]	z	Rt [min]	Score	P	Range	Sequence	Modification
223	1	636.6401	-38.73	3	32.8	18.5	1	97-112	R.AMIENFNAKYVSLHVR.K	Oxidation: 2

Detailed Protein Report

Protein 228: mitogen-activated protein kinase kinase kinase 5 [Homo sapiens]

Accession: gi|5174547
Database: refseq_human(refseq_human_20140103.fasta)
Database Date: 2015-11-30
Modification(s): Carbamidomethyl, Oxidation

Score: 30.4
MW [kDa]: 154.4
pI: 5.4
Sequence Coverage [%]: 2.5
No. of unique Peptides: 2

Quantitation

QD:QU **Median:** 1.82 **CV:** 0.00 % **No. of Peptides:** 1

10	20	30	40	50	60	70	80
MSTEADEGIT	FSVPPFAPSG	FCTIPEGGIC	RRGGAAAVGE	GEEHQLPPPP	PGSFWNVESA	AAPGIGCPAA	TSSSSATRGR
**90**	**100**	**110**	**120**	**130**	**140**	**150**	**160**
GSSVGGGSRR	TTVAYVINEA	SQGQLVVAES	EALQSLREAC	ETVGATLETL	HFGKLDFGET	TVLDRFYNAD	IAVVEMSDAF
**170**	**180**	**190**	**200**	**210**	**220**	**230**	**240**
RQPSLFYHLG	VRESFSMANN	IILYCDTNSD	SLQSLKEIIC	QKNTMCTGNY	TFVPYMITPH	NKVYCCDSSF	MKGLTELMQP
**250**	**260**	**270**	**280**	**290**	**300**	**310**	**320**
NFELLLGPIC	LPLVDRFIQL	LKVAQASSSQ	YFRESILNDI	RKARNLYTGK	ELAAELARIR	QRVDNIEVLT	ADIVINLLLS
**330**	**340**	**350**	**360**	**370**	**380**	**390**	**400**
YRDIQDYDSI	VKLVETLEKL	PTFDLASHHH	VKFHYAFALN	RRNLPGDRAK	ALDIMIPMVQ	SEGQVASDMY	CLVGRIYKDM
**410**	**420**	**430**	**440**	**450**	**460**	**470**	**480**
FLDSNFTDTE	SRDHGASWFK	KAFESEPTLQ	SGINYAVLLL	AAGHQFESSF	ELRKVGVKLS	SLLGKKGNLE	KLQSYWEVGF
**490**	**500**	**510**	**520**	**530**	**540**	**550**	**560**
FLGASVLAND	HMRVIQASEK	LFKLKTPAWY	LKSIVETILI	YKHFVKLTTE	QPVAKQELVD	FWMDFLVEAT	KTDVTVVRFP
**570**	**580**	**590**	**600**	**610**	**620**	**630**	**640**
VLILEPTKIY	QPSYLSINNE	VEEKTISIWH	VLPDDKKGIH	EWNFSASSVR	GVSISKFEER	CCFLYVLHNS	DDFQIYFCTE
**650**	**660**	**670**	**680**	**690**	**700**	**710**	**720**
LHCKKFFEMV	NTITEEKGRS	TEEGDCESDL	LEYDYEYDEN	GDRVVLGKGT	YGIVYAGRDL	SNQVRIAIKE	IPERDSRYSQ
**730**	**740**	**750**	**760**	**770**	**780**	**790**	**800**
PLHEEIALHK	HLKHKNIVQY	LGSFSENGFI	KIFMEQVPGG	SLSALLRSKW	GPLKDNEQTI	GFYTKQILEG	LKYLHDNQIV
**810**	**820**	**830**	**840**	**850**	**860**	**870**	**880**
HRDIKGDNVL	INTYSGVLKI	SDFGTSKRLA	GINPCTETFT	GTLQYMAPEI	IDKGPRGYGK	AADIWSLGCT	IIEMATGKPP
**890**	**900**	**910**	**920**	**930**	**940**	**950**	**960**
FYELGEPQAA	MFKVGMFKVH	PEIPESMSAE	AKAFILKCFE	PDPDKRACAN	DLLVDEFLKV	SSKKKTQPK	LSALSAGSNE
**970**	**980**	**990**	**1000**	**1010**	**1020**	**1030**	**1040**
YLRSISLPVP	VLVEDTSSSS	EYGSVSPDTE	LKVDPFSFKT	RAKSCGERDV	KGIRTLFLGI	PDENFEDHSA	PPSPEEKDSG
**1050**	**1060**	**1070**	**1080**	**1090**	**1100**	**1110**	**1120**
FFMLRKDSER	RATLHRILTE	DQDKIVRNLM	ESLAQGAEEP	KLKWEHITTL	IASLREFVRS	TDRKIIATTL	SKLKLELDFD
**1130**	**1140**	**1150**	**1160**	**1170**	**1180**	**1190**	**1200**
SHGISQVQVV	LFGFQDAVNK	VLRNHNIKPH	WMFALDSIIR	KAVQTAITIL	VPELRPHFSL	ASESDTADQE	DLDVEDDHEE
**1210**	**1220**	**1230**	**1240**	**1250**	**1260**	**1270**	**1280**
QPSNQTVRRP	QAVIEDAVAT	SGVSTLSSTV	SHDSQSAHRS	LNVQLGRMKI	ETNRLLEELV	RKEKELQALL	HRAIEEKDQE
**1290**	**1300**	**1310**	**1320**	**1330**	**1340**	**1350**	**1360**
IKHLKLKSQP	IEIPELPVFH	LNSSGTNTED	SELTDWLRVN	GADEDTISRF	LAEDYTLLDV	LYYVTRDDLK	CLRLRGGMLC
**1370**	**1380**						
TLWKAIIDFR	NKQT						

Cmpd.	No. of Cmpds.	m/z meas.	Δ m/z [ppm]	z	Rt [min]	Score	P	Range	Sequence	Modification	Ratios
2651	1	993.9806	1.15	2	64.1	16.5	0	536-551	K.QELVDFWMDFLVEATK.T	Oxidation: 8	
2925	1	1018.9798	-57.02	2	65.7	13.8	2	927-944	R.ACANDLLVDEFLKVSSKK.K	Carbamidomethyl: 2	QD:QU 1.82

Detailed Protein Report

Protein 229: DNA-directed RNA polymerase I subunit RPA49 isoform 2 [Homo sapiens]

**Accession:**	gi|544346178	**Score:**	30.4
**Database:**	refseq_human(refseq_human_20140103.fasta)	**MW [kDa]:**	39.8
**Database Date:**	2015-11-30	**pI:**	9.6
**Modification(s):**	Carbamidomethyl, Oxidation	**Sequence Coverage [%]:**	6.3
		**No. of unique Peptides:**	1

Quantitation

QD:QU **Median:** 0.52 **CV:** 0.00 % **No. of Peptides:** 1

10	20	30	40	50	60	70	80
MMLNCSTCSH	YFQVFHQESK	IIFWKEPRTC	FLILSLPLPG	ELTWDVSVES	ELALESQTKT	YREKMDSCIE	AFGTTKQKRA
**90**	**100**	**110**	**120**	**130**	**140**	**150**	**160**
LNTRRMNRVG	NESLNRAVAK	AAETIIDTKG	VTALVSDAIH	NDLQDDSLYL	PPCYDDAAKP	EDVYKFEDLL	SPAEYEALQS
**170**	**180**	**190**	**200**	**210**	**220**	**230**	**240**
PSEAFRNVTS	EEILKMIEEN	SHCTFVIEAL	KSLPSDVESR	DRQARCIWFL	DTLIKFRAHR	VVKRKSALGP	GVPHIINTKL
**250**	**260**	**270**	**280**	**290**	**300**	**310**	**320**
LKHFTCLTYN	NGRLRNLISD	SMKAKITAYV	IILALHIHDF	QIDLTVLQRD	LKLSEKRMME	IAKAMRLKIS	KRRVSVAAGS
**330**	**340**	**350**					
EEDHKLGTLS	LPLPPAQTSD	RLAKRRKIT					

Cmpd.	No. of Cmpds.	m/z meas.	Δ m/z [ppm]	z	Rt [min]	Score	P	Range	Sequence	Modification	Ratios
182	1	688.3002	4.63	2	31.0	12.9	0	65-76	K.MDSCIEAFGTTK.Q	Carbamidomethyl: 4; Oxidation: 1	QD:QU 0.52

Detailed Protein Report

Protein 230: **PREDICTED: leukocyte immunoglobulin-like receptor subfamily B member 5 isoform X5 [Homo sapiens]**

Accession: gi|578833860
Database: refseq_human(refseq_human_20140103.fasta)
Database Date: 2015-11-30
Modification(s): Carbamidomethyl, Oxidation

Score: 30.3
MW [kDa]: 49.1
pI: 6.8
Sequence Coverage [%]: 6.2
No. of unique Peptides: 1

10	20	30	40	50	60	70	80
MTLTLSVLIC	LGLSVGPRTC	VQAGTLPKPT	LWAEPASVIA	RGKPVTLWCQ	GPLETEEYRL	DKEGLPWARK	RQNPLEPGAK
**90**	**100**	**110**	**120**	**130**	**140**	**150**	**160**
AKFHIPSTVY	DSAGRYRCYY	ETPAGWSEPS	DPLELVATGF	YAEPTLLALP	SPVVASGGNV	TLQCDTLDGL	LTFVLVEEEQ
**170**	**180**	**190**	**200**	**210**	**220**	**230**	**240**
KLPRTLYSQK	LPKGPSQALF	PVGPVTPSCR	WRFRCYYYYR	KNPQVWSNPS	DLLEILVPGV	SRKPSLLIPQ	GSVVARGGSL
**250**	**260**	**270**	**280**	**290**	**300**	**310**	**320**
TLQCRSDVGY	DIFVLYKEGE	HDLVQGSGQQ	PQAGLSQANF	TLGPVSRSHG	GQYRCYGAHN	LSPRWSAPSD	PLDILIAGLI
**330**	**340**	**350**	**360**	**370**	**380**	**390**	**400**
PDIPALSVQP	GPKVASGENV	TLLCQSWHQI	DTFFLTKEGA	AHPPLCLKSK	YQSYRHQAEF	SMSPVTSAQG	GTYRCYSAIR
**410**	**420**	**430**	**440**	**450**	**460**		
SYPYLLSSPS	YPQELVVSGP	SGDPSLSPTG	STPTPGPEDQ	PLTPTGLDPQ	SGE		

Cmpd.	No. of Cmpds.	m/z meas.	Δ m/z [ppm]	z	Rt [min]	Score	P	Range	Sequence	Modification
2477	1	973.4893	-56.45	2	61.5	15.4	0	1-18	-.MTLTLSVLICLGLSVGPR.T	Carbamidomethyl: 10; Oxidation: 1

Detailed Protein Report

Protein 231: ataxin-2 [Homo sapiens]

Accession: gi|171543895
Database: refseq_human(refseq_human_20140103.fasta)
Database Date: 2015-11-30

Score: 30.2
MW [kDa]: 140.2
pI: 10.2
Sequence Coverage [%]: 2.4
No. of unique Peptides: 2

10	20	30	40	50	60	70	80
MRSAAAAPRS	PAVATESRRF	AAARWPGWRS	LQRPARRSGR	GGGGAAPGPY	PSAAPPPPGP	GPPPSRQSSP	PSASDCFGSN
**90**	**100**	**110**	**120**	**130**	**140**	**150**	**160**
GNGGGAFRPG	SRRLLGLGGP	PRPFVVLLLP	LASPGAPPAA	PTRASPLGAR	ASPPRSGVSL	ARPAPGCPRP	ACEPVYGPLT
**170**	**180**	**190**	**200**	**210**	**220**	**230**	**240**
MSLKPQQQQQ	QQQQQQQQQQ	QQQQQQQQPP	PAAANVRKPG	GSGLLASPAA	APSPSSSSVS	SSSATAPSSV	VAATSGGGRP
**250**	**260**	**270**	**280**	**290**	**300**	**310**	**320**
GLGRGRNSNK	GLPQSTISFD	GIYANMRMVH	ILTSVVGSKC	EVQVKNGGIY	EGVFKTYSPK	CDLVLDAAHE	KSTESSSGPK
**330**	**340**	**350**	**360**	**370**	**380**	**390**	**400**
REEIMESILF	KCSDFVVVQF	KDMDSSYAKR	DAFTDSAISA	KVNGEHKEKD	LEPWDAGELT	ANEELEALEN	DVSNGWDPND
**410**	**420**	**430**	**440**	**450**	**460**	**470**	**480**
MFRYNEENYG	VVSTYDSSLS	SYTVPLERDN	SEEFLKREAR	ANQLAEEIES	SAQYKARVAL	ENDDRSEEEK	YTAVQRNSSE
**490**	**500**	**510**	**520**	**530**	**540**	**550**	**560**
REGHSINTRE	NKYIPPGQRN	REVISWGSGR	QNSPRMGQPG	SGSMPSRSTS	HTSDFNPNSG	SDQRVVNGGV	PWPSPCPSPS
**570**	**580**	**590**	**600**	**610**	**620**	**630**	**640**
SRPPSRYQSG	PNSLPPRAAT	PTRPPSRPPS	RPSRPPSHPS	AHGSPAPVST	MPKRMSSEGP	PRMSPKAQRH	PRNHRVSAGR
**650**	**660**	**670**	**680**	**690**	**700**	**710**	**720**
GSISSGLEFV	SHNPPSEAAT	PPVARTSPSG	GTWSSVVSGV	PRLSPKTHRP	RSPRQNSIGN	TPSGPVLASP	QAGIIPTEAV
**730**	**740**	**750**	**760**	**770**	**780**	**790**	**800**
AMPIPAASPT	PASPASNRAV	TPSSEAKDSR	LQDQRQNSPA	GNKENIKPNE	TSPSFSKAEN	KGISPVVSEH	RKQIDDLKKF
**810**	**820**	**830**	**840**	**850**	**860**	**870**	**880**
KNDFRLQPSS	TSESMDQLLN	KNREGEKSRD	LIKDKIEPSA	KDSFIENSSS	NCTSGSSKPN	SPSISPSILS	NTEHKRGPEV
**890**	**900**	**910**	**920**	**930**	**940**	**950**	**960**
TSQGVQTSSP	ACKQEKDDKE	EKKDAAEQVR	KSTLNPNAKE	FNPRSFSQPK	PSTTPTSPRP	QAQPSPSMVG	HQQPTPVYTQ
**970**	**980**	**990**	**1000**	**1010**	**1020**	**1030**	**1040**
PVCFAPNMMY	PVPVSPGVQP	LYPIPMTPMP	VNQAKTYRAV	PNMPQQRQDQ	HHQSAMMHPA	SAAGPPIAAT	PPAYSTQYVA
**1050**	**1060**	**1070**	**1080**	**1090**	**1100**	**1110**	**1120**
YSPQQFPNQP	LVQHVPHYQS	QHPHVYSPVI	QGNARMMAPP	THAQPGLVSS	SATQYGAHEQ	THAMYACPKL	PYNKETSPSF
**1130**	**1140**	**1150**	**1160**	**1170**	**1180**	**1190**	**1200**
YFAISTGSLA	QQYAHPNATL	HPHTPHPQPS	ATPTGQQQSQ	HGGSHPAPSP	VQHHQHQAAQ	ALHLASPQQQ	SAIYHAGLAP
**1210**	**1220**	**1230**	**1240**	**1250**	**1260**	**1270**	**1280**
TPPSMTPASN	TQSPQNSFPA	AQQTVFTIHP	SHVQPAYTNP	PHMAHVPQAH	VQSGMVPSHP	TAHAPMMLMT	TQPPGGPQAA
**1290**	**1300**	**1310**	**1320**				
LAQSALQPIP	VSTTAHFPYM	THPSVQAHHQ	QQL				

Cmpd.	No. of Cmpds.	m/z meas.	Δ m/z [ppm]	z	Rt [min]	Score	P	Range	Sequence	Modification
67	2	1018.8972	-98.75	2	29.5	14.3	1	438-455	R.EARANQLAEEIESSAQYK.A	
536	1	786.8751	-28.43	2	36.7	16.0	1	502-515	R.EVISWGSGRQNSPR.M	

Detailed Protein Report

Protein 232: PREDICTED: ubl carboxyl-terminal hydrolase 18 isoform X1 [Homo sapiens]

**Accession:**	gi|578836791	**Score:**	30.2
**Database:**	refseq_human(refseq_human_20140103.fasta)	**MW [kDa]:**	34.6
**Database Date:**	2015-11-30	**pI:**	9.4
**Modification(s):**	Oxidation	**Sequence Coverage [%]:**	8.1
		**No. of unique Peptides:**	1

Quantitation

QD:QU **Median:** 1.15 **CV:** 0.00 % **No. of Peptides:** 1

10	20	30	40	50	60	70	80
MNVDFTRILK	RITVPRGADE	QRRSVPFQML	LLLEKMQDSR	QKAVRPLELA	YCLQKCNVPL	FVQHDAAQLY	LKLWNLIKDQ
**90**	**100**	**110**	**120**	**130**	**140**	**150**	**160**
ITDVHLVERL	QALYTIRVKD	SLICVDCAME	SSRNSSMLTL	PLSLFDVDSK	PLKTLEDALH	CFFQPRELSS	KSKCFCENCG
**170**	**180**	**190**	**200**	**210**	**220**	**230**	**240**
KKTRGKQVLK	LTHLPQTLTI	HLMRFSIRNS	QTRKICHSLY	FPQSLDFSQI	LPMKRESCDA	EEQSGGQYEL	FAVIAHVGMA
**250**	**260**	**270**	**280**	**290**	**300**		
DSGHYCVYIR	NAVDGKWFCF	NDSNICLVSW	EDIQCTYGNP	NYHWQETAYL	LVYMKMEC		

Cmpd.	No. of Cmpds.	m/z meas.	Δ m/z [ppm]	z	Rt [min]	Score	P	Range	Sequence	Modification	Ratios	
374	1	795.4149	-53.97	2	34.7	14.0	1	23-35	R.RSVPFQMLLLLEK.M	Oxidation: 7	QD:QU	1.15

Detailed Protein Report

Protein 233: ATP-dependent DNA helicase Q1 [Homo sapiens]

**Accession:**	gi|14591902	**Score:**	30.1
**Database:**	refseq_human(refseq_human_20140103.fasta)	**MW [kDa]:**	73.4
**Database Date:**	2015-11-30	**pI:**	9.0
		**Sequence Coverage [%]:**	3.9
		**No. of unique Peptides:**	1

Alias proteins:

Accession	Name	Description
gi|578823000	refseq_human (refseq_human_20140103.fasta)	PREDICTED: ATP-dependent DNA helicase Q1 isoform X5 [Homo sapiens]
gi|530399347	refseq_human (refseq_human_20140103.fasta)	PREDICTED: ATP-dependent DNA helicase Q1 isoform X4 [Homo sapiens]
gi|530399345	refseq_human (refseq_human_20140103.fasta)	PREDICTED: ATP-dependent DNA helicase Q1 isoform X3 [Homo sapiens]
gi|530399343	refseq_human (refseq_human_20140103.fasta)	PREDICTED: ATP-dependent DNA helicase Q1 isoform X2 [Homo sapiens]
gi|530399341	refseq_human (refseq_human_20140103.fasta)	PREDICTED: ATP-dependent DNA helicase Q1 isoform X1 [Homo sapiens]
gi|14591904	refseq_human (refseq_human_20140103.fasta)	ATP-dependent DNA helicase Q1 [Homo sapiens]

```
        10         20         30         40         50         60         70         80
MASVSALTEE LDSITSELHA VEIQIQELTE RQQELIQKKK VLTKKIKQCL EDSDAGASNE YDSSPAAWNK EDFPWSGKVK
        90        100        110        120        130        140        150        160
DILQNVFKLE KFRPLQLETI NVTMAGKEVF LVMPTGGGKS LCYQLPALCS DGFTLVICPL ISLMEDQLMV LKQLGISATM
       170        180        190        200        210        220        230        240
LNASSSKEHV KWVHAEMVNK NSELKLIYVT PEKIAKSKMF MSRLEKAYEA RRFTRIAVDE VHCCSQWGHD FRPDYKALGI
       250        260        270        280        290        300        310        320
LKRQFPNASL IGLTATATNH VLTDAQKILC IEKCFTFTAS FNRPNLYYEV RQKPSNTEDF IEDIVKLING RYKGQSGIIY
       330        340        350        360        370        380        390        400
CFSQKDSEQV TVSLQNLGIH AGAYHANLEP EDKTTVHRKW SANEIQVVVA TVAFGMGIDK PDVRFVIHHS MSKSMENYYQ
       410        420        430        440        450        460        470        480
ESGRAGRDDM KADCILYYGF GDIFRISSMV VMENVGQQKL YEMVSYCQNI SKCRRVLMAQ HFDEVWNSEA CNKMCDNCCK
       490        500        510        520        530        540        550        560
DSAFERKNIT EYCRDLIKIL KQAEELNEKL TPLKLIDSWM GKGAAKLRVA GVVAPTLPRE DLEKIIAHFL IQQYLKEDYS
       570        580        590        600        610        620        630        640
FTAYATISYL KIGPKANLLN NEAHAITMQV TKSTQNSFRA ESSQTCHSEQ GDKKMEEKNS GNFQKKAANM LQQSGSKNTG
       650
AKKRKIDDA
```

Cmpd.	No. of Cmpds.	m/z meas.	Δ m/z [ppm]	z	Rt [min]	Score	P	Range	Sequence	Modification
2931	1	753.8886	104.41	2	65.8	11.5	0	600-613	R.AESSQTCHSEQGDK.K	

Detailed Protein Report

Protein 234: **structural maintenance of chromosomes protein 4 isoform 2 [Homo sapiens]**

**Accession:**	gi|570700827	**Score:**	30.1
**Database:**	refseq_human(refseq_human_20140103.fasta)	**MW [kDa]:**	144.4
**Database Date:**	2015-11-30	**pI:**	6.7
		**Sequence Coverage [%]:**	1.2
		**No. of unique Peptides:**	1

10	20	30	40	50	60	70	80
MPRKGTQPST	ARRREEGPPP	PSPDGASSDA	EPEPPSGRTE	SPATAAAMTN	EAGAPRLMIT	HIVNQNFKSY	AGEKILGPFH
**90**	**100**	**110**	**120**	**130**	**140**	**150**	**160**
KRFSCIIGPN	GSGKSNVIDS	MLFVFGYRAQ	KIRSKKLSVL	IHNSDEHKDI	QSCTVEVHFQ	KIIDKEGDDY	EVIPNSNFYV
**170**	**180**	**190**	**200**	**210**	**220**	**230**	**240**
SRTACRDNTS	VYHISGKKKT	FKDVGNLLRS	HGIDLDHNRF	LILQGEVEQI	AMMKPKGQTE	HDEGMLEYLE	DIIGCGRLNE
**250**	**260**	**270**	**280**	**290**	**300**	**310**	**320**
PIKVLCRRVE	ILNEHRGEKL	NRVKMVEKEK	DALEGEKNIA	IEFLTLENEI	FRKKNHVCQY	YIYELQKRIA	EMETQKEKIH
**330**	**340**	**350**	**360**	**370**	**380**	**390**	**400**
EDTKEINEKS	NILSNEMKAK	NKDVKDTEKK	LNKITKFIEE	NKEKFTQLDL	EDVQVREKLK	HATSKAKKLE	KQLQKDKEKV
**410**	**420**	**430**	**440**	**450**	**460**	**470**	**480**
EEFKSIPAKS	NNIINETTTR	NNALEKEKEK	EEKKLKEVMD	SLKQETQGLQ	KEKESREKEL	MGFSKSVNEA	RSKMDVAQSE
**490**	**500**	**510**	**520**	**530**	**540**	**550**	**560**
LDIYLSRHNT	AVSQLTKAKE	ALIAASETLK	ERKAAIRDIE	GKLPQTEQEL	KEKEKELQKL	TQEETNFKSL	VHDLFQKVEE
**570**	**580**	**590**	**600**	**610**	**620**	**630**	**640**
AKSSLAMNRS	RGKVLDAIIQ	EKKSGRIPGI	YGRLGDLGAI	DEKYDVAISS	CCHALDYIVV	DSIDIAQECV	NFLKRQNIGV
**650**	**660**	**670**	**680**	**690**	**700**	**710**	**720**
ATFIGLDKMA	VWAKKMTEIQ	TPENTPRLFD	LVKVKDEKIR	QAFYFALRDT	LVADNLDQAT	RVAYQKDRRW	RVVTLQGQII
**730**	**740**	**750**	**760**	**770**	**780**	**790**	**800**
EQSGTMTGGG	SKVMKGRMGS	SLVIEISEEE	VNKMESQLQN	DSKKAMQIQE	QKVQLEERVV	KLRHSEREMR	NTLEKFTASI
**810**	**820**	**830**	**840**	**850**	**860**	**870**	**880**
QRLIEQEEYL	NVQVKELEAN	VLATAPDKKK	QKLLEENVSA	FKTEYDAVAE	KAGKVEAEVK	RLHNTIVEIN	NHKLKAQQDK
**890**	**900**	**910**	**920**	**930**	**940**	**950**	**960**
LDKINKQLDE	CASAITKAQV	AIKTADRNLQ	KAQDSVLRTE	KEIKDTEKEV	DDLTAELKSL	EDKAAEVVKN	TNAAEESLPE
**970**	**980**	**990**	**1000**	**1010**	**1020**	**1030**	**1040**
IQKEHRNLLQ	ELKVIQENEH	ALQKDALSIK	LKLEQIDGHI	AEHNSKIKYW	HKEISKISLH	PIEDNPIEEI	SVLSPEDLEA
**1050**	**1060**	**1070**	**1080**	**1090**	**1100**	**1110**	**1120**
IKNPDSITNQ	IALLEARCHE	MKPNLGAIAE	YKKKEELYLQ	RVAELDKITY	ERDSFRQAYE	DLRKQRLNEF	MAGFYIITNK
**1130**	**1140**	**1150**	**1160**	**1170**	**1180**	**1190**	**1200**
LKENYQMLTL	GGDAELELVD	SLDPFSEGIM	FSVRPPKKSW	KKIFNLSGGE	KTLSSLALVF	ALHHYKPTPL	YFMDEIDAAL
**1210**	**1220**	**1230**	**1240**	**1250**	**1260**	**1270**	
DFKNVSIVAF	YIYEQTKNAQ	FIIISLRNNM	FEISDRLIGI	YKTYNITKSV	AVNPKEIASK	GLC	

Cmpd.	No. of Cmpds.	m/z meas.	Δ m/z [ppm]	z	Rt [min]	Score	P	Range	Sequence	Modification
452	3	862.8266	-126.10	2	34.3	30.1	2	457-471	R.EKELMGFSKSVNEAR.S	

Detailed Protein Report

Protein 235: **PREDICTED: sprouty-related, EVH1 domain-containing protein 2 isoform X2 [Homo sapiens]**

**Accession:**	gi|530367354	**Score:**	30.1
**Database:**	refseq_human(refseq_human_20140103.fasta)	**MW [kDa]:**	25.4
**Database Date:**	2015-11-30	**pI:**	7.8
**Modification(s):**	Carbamidomethyl, Oxidation	**Sequence Coverage [%]:**	14.0
		**No. of unique Peptides:**	2

10	20	30	40	50	60	70	80
MPRPYRQVSF	PDDDEEIVRI	NPREKIWMTG	YEDYRHAPVR	GKYPDPSEDA	DSSYVRFAKG	EVPKHDYNYP	YVDSSDFGLG
**90**	**100**	**110**	**120**	**130**	**140**	**150**	**160**
EDPKGRGGSV	IKTQPSRGKS	RRRKEDGERS	RCVYCRDMFN	HEENRRGHCQ	DAPDSVRTCI	RRVSCMWCAD	SMLYHCMSDP
**170**	**180**	**190**	**200**	**210**	**220**	**230**	
EGDYTDPCSC	DTSDEKFCLR	WMALIALSFL	APCMCCYLPL	RACYHCGVMC	RCCGGKHKAA	A	

Cmpd.	No. of Cmpds.	m/z meas.	Δ m/z [ppm]	z	Rt [min]	Score	P	Range	Sequence	Modification
2553	3	973.5096	122.27	2	62.6	16.7	1	112-125	R.CVYCRDMFNHEENR.R	Carbamidomethyl: 1, 4; Oxidation: 7
1552	3	993.4662	70.96	2	49.6	13.4	2	202-218	R.ACYHCGVMCRCCGGKHK.A	Carbamidomethyl: 2, 5; Oxidation: 8

Detailed Protein Report

Protein 236: cytochrome b-245 heavy chain [Homo sapiens]

**Accession:**	gi|6996021	**Score:**	30.1
**Database:**	refseq_human(refseq_human_20140103.fasta)	**MW [kDa]:**	65.3
**Database Date:**	2015-11-30	**pI:**	9.8
		**Sequence Coverage [%]:**	4.9
		**No. of unique Peptides:**	1

10	20	30	40	50	60	70	80
MGNWAVNEGL	SIFVILVWLG	LNVFLFVWYY	RVYDIPPKFF	YTRKLLGSAL	ALARAPAACL	NFNCMLILLP	VCRNLLSFLR
90	100	110	120	130	140	150	160
GSSACCSTRV	RRQLDRNLTF	HKMVAWMIAL	HSAIHTIAHL	FNVEWCVNAR	VNNSDPYSVA	LSELGDRQNE	SYLNFARKRI
170	180	190	200	210	220	230	240
KNPEGGLYLA	VTLLAGITGV	VITLCLILII	TSSTKTIRRS	YFEVFWYTHH	LFVIFFIGLA	IHGAERIVRG	QTAESLAVHN
250	260	270	280	290	300	310	320
ITVCEQKISE	WGKIKECPIP	QFAGNPPMTW	KWIVGPMFLY	LCERLVRFWR	SQQKVVITKV	VTHPFKTIEL	QMKKKGFKME
330	340	350	360	370	380	390	400
VGQYIFVKCP	KVSKLEWHPF	TLTSAPEEDF	FSIHIRIVGD	WTEGLFNACG	CDKQEFQDAW	KLPKIAVDGP	FGTASEDVFS
410	420	430	440	450	460	470	480
YEVVMLVGAG	IGVTPFASIL	KSVWYKYCNN	ATNLKLKKIY	FYWLCRDTHA	FEWFADLLQL	LESQMQERNN	AGFLSYNIYL
490	500	510	520	530	540	550	560
TGWDESQANH	FAVHHDEEKD	VITGLKQKTL	YGRPNWDNEF	KTIASQHPNT	RIGVFLCGPE	ALAETLSKQS	ISNSESGPRG
570	580						
VHFIFNKENF							

Cmpd.	No. of Cmpds.	m/z meas.	Δ m/z [ppm]	z	Rt [min]	Score	P	Range	Sequence	Modification
906	1	676.2230	-177.87	2	41.4	13.2	1	560-570	R.GVHFIFNKENF.-	

Detailed Protein Report

Protein 237: **PREDICTED: zinc finger CCHC domain-containing protein 10 isoform X4 [Homo sapiens]**

Accession: gi|530380033
Database: refseq_human(refseq_human_20140103.fasta)
Database Date: 2015-11-30

Score: 30.0
MW [kDa]: 13.8
pI: 11.3
Sequence Coverage [%]: 21.9
No. of unique Peptides: 2

Quantitation

QD:QU **Median:** 0.41 **CV:** 13.14 % **No. of Peptides:** 2

```
         10         20         30         40         50         60         70         80
MATPMHRLIA RRQAFDTELQ PVKTFWILIQ PSIVISEANK QHVRCQKCLE FGHWTYECTG KRKYLHRPSR TAELKKALKE
         90        100        110        120
KENRLLLQQR SFFPPRVYQH WRNQCRKKGQ EKKV
```

Cmpd.	No. of Cmpds.	m/z meas.	Δ m/z [ppm]	z	Rt [min]	Score	P	Range	Sequence	Modification	Ratios	
1467	1	1095.0485	53.51	2	48.5	15.5	2	45-62	R.CQKCLEFGHWTYECTGKR.K		QD:QU	0.36
493	1	464.6529	-228.72	2	34.8	14.5	0	64-70	K.YLHRPSR.T		QD:QU	0.46

Detailed Protein Report

Protein 238: uncharacterized protein C5orf55 precursor [Homo sapiens]

**Accession:**	gi|156523054	**Score:**	30.0
**Database:**	refseq_human(refseq_human_20140103.fasta)	**MW [kDa]:**	12.7
**Database Date:**	2015-11-30	**pI:**	12.0
**Modification(s):**	Carbamidomethyl	**Sequence Coverage [%]:**	27.7
		**No. of unique Peptides:**	1

```
        10         20         30         40         50         60         70         80
MPAVFMLASS SALQCGRGVP RFPRTEVGAG HSVNEETKAE KVGNQTSVIP ATSRQAALGT SWTQRRTQPL QERSHWHPRG
        90        100        110        120
NNASGMGGHR MFPGPLRGPA AQVLENECGS LGRAAEGRS
```

Cmpd.	No. of Cmpds.	m/z meas.	Δ m/z [ppm]	z	Rt [min]	Score	P	Range	Sequence	Modification
1854	1	829.2700	-158.89	2	51.7	10.9	0	98-113	R.GPAAQVLENECGSLGR.A	Carbamidomethyl: 11

Detailed Protein Report

Protein 239: PREDICTED: microtubule-associated protein 4 isoform X15 [Homo sapiens]

**Accession:**	gi|530372482	**Score:**	30.0
**Database:**	refseq_human(refseq_human_20140103.fasta)	**MW [kDa]:**	241.1
**Database Date:**	2015-11-30	**pI:**	6.0
		**Sequence Coverage [%]:**	1.7
		**No. of unique Peptides:**	1

10	20	30	40	50	60	70	80
MADLSLADAL	TEPSPDIEGE	IKRDFIATLE	AEAFDDVVGE	TVGKTDYIPL	LDVDEKTGNS	ESKKKPCSET	SQIEDTPSSK
90	100	110	120	130	140	150	160
PTLLANGGHG	VEGSDTTGSP	TEFLEEKMAY	QEYPNSQNWP	EDTNFCFQPE	QVVDPIQTDP	FKMYHDDDLA	DLVFPSSATA
170	180	190	200	210	220	230	240
DTSIFAGQND	PLKDSYVPLE	LAKEIEMASE	ERPPAQALEI	MMGLKTTDMA	PSKETEMALA	KDMALATKTE	VALAKDMESP
250	260	270	280	290	300	310	320
TKLDVTLAKD	MQPSMESDMA	LVKDMELPTE	KEVALVKDVR	WPTETDVSSA	KNVVLPTETE	VAPAKDVTLL	KETERASPIK
330	340	350	360	370	380	390	400
MDLAPSKDMG	PPKENKKETE	RASPIKMDLA	PSKDMGPPKE	NKIVPAKDLV	LLSEIEVAQA	NDIISSTEIS	SAEKVALSSE
410	420	430	440	450	460	470	480
TEVALARDMT	LPPETNVILT	KDKALPLEAE	VAPVKDMAQL	PETEIAPAKD	VAPSTVKEVG	LLKDMSPLSE	TEMALGKDVT
490	500	510	520	530	540	550	560
PPPETEVVLI	KNVCLPPEME	VALTEDQVPA	LKTEAPLAKD	GVLTLANNVT	PAKDVPPLSE	TEATPVPIKD	MEIAQTQKGI
570	580	590	600	610	620	630	640
SEDSHLESLQ	DVGQSAAPTF	MISPETVTGT	GKKCSLPAEE	DSVLEKLGER	KPCNSQPSEL	SSETSANFMY	CGTPPTQAKQ
650	660	670	680	690	700	710	720
VCRPSDRRST	RPKPARVPPE	LLGGSPPWKT	LDHRLGHCSL	SESGWVSGSS	SCGGPGNQRK	SIHVDSLEPQ	RDLGREAWDI
730	740	750	760	770	780	790	800
ESTPIMMKKK	KKKPKQKRYS	QPRAGGPSDD	DNADKPKGHP	FAADTQKSGV	LPSQPTTMGT	EYGLVSGENL	KRECLVNSSA
810	820	830	840	850	860	870	880
ARLVAENFVS	ESLRIPLYPS	EEAPKTAISS	QSKLRVEEES	KSNKSVLQNQ	DKKLLKQHEY	KPQPAPHLKT	PVDKSQSVGP
890	900	910	920	930	940	950	960
LNLKGPLAEV	SAYNVETPLD	IRLKEGCSPF	LDQEVMGVVS	KPTAAKEIPN	LVPTLIASNP	LECNLKEGNN	ESKMTKLQNV
970	980	990	1000	1010	1020	1030	1040
KLKEFPEGAE	EDKELKKEAF	PNERQEISIF	TSEQLQGQVL	VQVPGVENEP	FKRMAGDGKS	RKGRGSSGKM	RTDSGKVKAK
1050	1060	1070	1080	1090	1100	1110	1120
SELPFLLDSQ	KDGRAVLIPS	EPVSKTEGMT	TQDKSEELGL	NSSKQPGTKA	DLTEAVVMGE	PKEMTQPKVA	GTMQALIPLE
1130	1140	1150	1160	1170	1180	1190	1200
SGSGMTQTSG	VSTETGDVVK	DMGVNNQSKE	GRCPWKDHEA	APWISEKPKK	RGNEGKSKKF	KNNYSTQPAR	MERKEEILNP
1210	1220	1230	1240	1250	1260	1270	1280
PFEGKDGDTG	SIPHKSKEIG	FTFPKMHDSS	FSHTPDTPTV	EAVDRKGGNF	QVNFVELGTL	GENKISTVKA	STVTEPPAKV
1290	1300	1310	1320	1330	1340	1350	1360
TDVSCQEQIQ	GAGFVPSVVS	EENKTDAANR	YTAVADKPSK	RSNDGKSKKV	KNSSPEKHIL	ENKIDATKIH	VPMETTGDQG
1370	1380	1390	1400	1410	1420	1430	1440
IEGMAYMDEN	RNITFTCPRT	PSELINKSSP	LEVLESAACE	KLPTPTPQVV	KEGDSFPDTL	AKNGQEIAPA	QISKSLMVDN
1450	1460	1470	1480	1490	1500	1510	1520
YTKDGVPGQE	RPKGPSAVVP	STSTGGVALP	ITTAIETVNI	HGDHSLKNKA	ELADSMKNEA	GIDEGHVIGE	SESVHSGASK
1530	1540	1550	1560	1570	1580	1590	1600
HSVEKVTELA	KGHLLPGVPV	EDQSLPGEAR	ALEGYADRGN	FPAHPVNEEK	ETKEGSVAVQ	IPDLLEDKAQ	KLSFCEDQNA
1610	1620	1630	1640	1650	1660	1670	1680
QDRNSKGSDS	LNKKVDLTLL	SPKSENDKLK	EISLACKITE	LESVSLPTPE	IQSDFLHSKV	EAPPSEVADT	LVIMTASKGV
1690	1700	1710	1720	1730	1740	1750	1760
RLPEPKDKIL	ETPQKMTEKS	ESKTPGEGKK	EDKSRMAEPM	KGYMRPTKSR	GLTPLLPKST	IQEQERHKQL	KSAVCLSSST
1770	1780	1790	1800	1810	1820	1830	1840
VYQQLGMSVY	GIARPEEGRP	VVSGTGNDIT	TPPNKELPPS	PEKKTKPLAT	TQPAKTSTSK	AKTQPTSLPK	QPAPTTIGGL
1850	1860	1870	1880	1890	1900	1910	1920
NKKPMSLASG	LVPAAPPKRP	AVASARPSIL	PSKDVKPKPI	ADAKAPEKRA	SPSKPASAPA	SRSGSKSTQT	VAKTTTAAAV
1930	1940	1950	1960	1970	1980	1990	2000
ASTGPSSRSP	STLLPKKPTA	IKTEGKPAEV	KKMTAKSVPA	DLSRPKSTST	SSMKKTTTLS	GTAPAAGVVP	SRVKATPMPS
2010	2020	2030	2040	2050	2060	2070	2080
RPSTTPFIDK	KPTSAKPSST	TPRLSRLATN	TSAPDLKNVR	SKVGSTENIK	HQPGGGRAKV	EKKTEAAATT	RKPESNAVTK
2090	2100	2110	2120	2130	2140	2150	2160
TAGPIASAQK	QPAGKVQIVS	KKVSYSHIQS	KCGSKDNIKH	VPGGGNVQIQ	NKKVDISKVS	SKCGSKANIK	HKPGGGDVKI
2170	2180	2190	2200	2210	2220	2230	2240

Detailed Protein Report

Cmpd.	No. of Cmpds.	m/z meas.	Δ m/z [ppm]	z	Rt [min]	Score	P	Range	Sequence	Modification
1574	1	949.4919	-30.30	2	49.9	14.9	1	2072-2090	R.KPESNAVTKTAGPIASAQK.Q	

Detailed Protein Report

Protein 240: folate receptor gamma precursor [Homo sapiens]

Accession: gi|112382370
Database: refseq_human(refseq_human_20140103.fasta)
Database Date: 2015-11-30
Modification(s): Carbamidomethyl

Score: 29.8
MW [kDa]: 27.9
pI: 9.2
Sequence Coverage [%]: 11.0
No. of unique Peptides: 2

Quantitation

QD:QU **Median:** 0.07 **CV:** 0.00 % **No. of Peptides:** 1

10	20	30	40	50	60	70	80
MDMAWQMMQL	LLLALVTAAG	SAQPRSARAR	TDLLNVCMNA	KHHKTQPSPE	DELYGQCSPW	KKNACCTAST	SQELHKDTSR
**90**	**100**	**110**	**120**	**130**	**140**	**150**	**160**
LYNFNWDHCG	KMEPTCKRHF	IQDSCLYECS	PNLGPWIRQV	NQSWRKERIL	NVPLCKEDCE	RWWEDCRTSY	TCKSNWHKGW
**170**	**180**	**190**	**200**	**210**	**220**	**230**	**240**
NWTSGINECP	AGALCSTFES	YFPTPAALCE	GLWSHSFKVS	NYSRGSGRCI	QMWFDSAQGN	PNEEVAKFYA	AAMNAGAPSR
**250**							
GIIDS							

Cmpd.	No. of Cmpds.	m/z meas.	Δ m/z [ppm]	z	Rt [min]	Score	P	Range	Sequence	Modification	Ratios
2968	1	937.4525	-14.98	2	66.3	16.9	2	127-141	K.ERILNVPLCKEDCER.W	Carbamidomethyl: 9	QD:QU 0.07
782	1	1105.5604	108.49	2	39.8	12.9	2	137-153	K.EDCERWWEDCRTSYTCK.S		

Detailed Protein Report

Protein 241: **1-phosphatidylinositol 4,5-bisphosphate phosphodiesterase epsilon-1 isoform 2 [Homo sapiens]**

**Accession:**	gi|260166694	**Score:**	29.8
**Database:**	refseq_human(refseq_human_20140103.fasta)	**MW [kDa]:**	223.7
**Database Date:**	2015-11-30	**pI:**	6.2
**Modification(s):**	Carbamidomethyl	**Sequence Coverage [%]:**	1.2
		**No. of unique Peptides:**	1

Quantitation

QD:QU	**Median:** 0.57	**CV:** 0.00 %	**No. of Peptides:** 1

Detailed Protein Report

10	20	30	40	50	60	70	80
MVSEGSAAGR	DFAGMEEVRQ	LHVRFCKGIK	IWHQAWFLCS	LLGREPQERE	AGCQLWLCTL	SAVLKVGWLF	PLSEVPNFTL
90	100	110	120	130	140	150	160
LKDGCGCWRL	KEDQIYNAVR	REETENTVGS	LLHFLTKLPA	SETAHGRISV	GPCLKQCVRD	TVCEYRATLQ	RTSISQYITG
170	180	190	200	210	220	230	240
SLLEATTSLG	ARSGLLSTFG	GSTGRMMLKE	RQPGPSVANS	NALPSSSAGI	SKELIDLQPL	IQFPEEVASI	LMEQEQTIYR
250	260	270	280	290	300	310	320
RVLPVDYLCF	LTRDLGTPEC	QSSLPCLKAS	ISASILTTQN	GEHNALEDLV	MRFNEVSSWV	TWLILTAGSM	EEKREVFSYL
330	340	350	360	370	380	390	400
VHVAKCCWNM	GNYNAVMEFL	AGLRSRKVLK	MWQFMDQSDI	ETMRSLKDAM	AQHESSCEYR	KVVTRALHIP	GCKVVPFCGV
410	420	430	440	450	460	470	480
FLKELCEVLD	GASGLMKLCP	RYNSQEETLE	FVADYSGQDN	FLQRVGQNGL	KNSEKESTVN	SIFQVIRSCN	RSLETDEEDS
490	500	510	520	530	540	550	560
PSEGNSSRKS	SLKDKSRWQF	IIGDLLDSDN	DIFEQSKEYD	SHGSEDSQKA	FDHGTELIPW	YVLSIQADVH	QFLLQGATVI
570	580	590	600	610	620	630	640
HYDQDTHLSA	RCFLQLQPDN	STLTWVKPTT	ASPASSKAKL	GVLNNTAEPG	KFPLLGNAGL	SSLTEGVLDL	FAVKAVYMGH
650	660	670	680	690	700	710	720
PGIDIHTVCV	QNKLGSMFLS	ETGVTLLYGL	QTTDNRLLHF	VAPKHTAKML	FSGLLELTRA	VRKMRKFPDQ	RQQWLRKQYV
730	740	750	760	770	780	790	800
SLYQEDGRYE	GPTLAHAVEL	FGGRRWSARN	PSPGTSAKNA	EKPNMQRNNT	LGISTTKKKK	KILMRGESGE	VTDDEMATRK
810	820	830	840	850	860	870	880
AKMHKECRSR	SGSDPQDINE	QEESEVNAIA	NPPNPLPSRR	AHSLTTAGSP	NLAAGTSSPI	RPVSSPVLSS	SNKSPSSAWS
890	900	910	920	930	940	950	960
SSSWHGRIKG	GMKGFQSFMV	SDSNMSFVEF	VELFKSFSVR	SRKDLKDLFD	VYAVPCNRSG	SESAPLYTNL	TIDENTSDLQ
970	980	990	1000	1010	1020	1030	1040
PDLDLLTRNV	SDLGLFIKSK	QQLSDNQRQI	SDAIAAASIV	TNGTGIESTS	LGIFGVGILQ	LNDFLVNCQG	EHCTYDEILS
1050	1060	1070	1080	1090	1100	1110	1120
IIQKFEPSIS	MCHQGLMSFE	GFARFLMDKE	NFASKNDESQ	ENIKELQLPL	SYYYIESSHN	TYLTGHQLKG	ESSVELYSQV
1130	1140	1150	1160	1170	1180	1190	1200
LLQGCRSVEL	DCWDGDDGMP	IIYHGHTLTT	KIPFKEVVEA	IDRSAFINSD	LPIIISIENH	CSLPQQRKMA	EIFKTVFGEK
1210	1220	1230	1240	1250	1260	1270	1280
LVTKFLFETD	FSDDPMLPSP	DQLRKKVLLK	NKKLKAHQTP	VDILKQKAHQ	LASMQVQAYN	GGNANPRPAN	NEEEEDEEDE
1290	1300	1310	1320	1330	1340	1350	1360
YDYDYESLSD	DNILEDRPEN	KSCNDKLQFE	YNEEIPKRIK	KADNSACNKG	KVYDMELGEE	FYLDQNKKES	RQIAPELSDL
1370	1380	1390	1400	1410	1420	1430	1440
VIYCQAVKFP	GLSTLNASGS	SRGKERKSRK	SIFGNNPGRM	SPGETASFNK	TSGKSSCEGI	RQTWEESSSP	LNPTTSLSAI
1450	1460	1470	1480	1490	1500	1510	1520
IRTPKCYHIS	SLNENAAKRL	CRRYSQKLTQ	HTACQLLRTY	PAATRIDSSN	PNPLMFWLHG	IQLVALNYQT	DDLPLHLNAA
1530	1540	1550	1560	1570	1580	1590	1600
MFEANGGCGY	VLKPPVLWDK	NCPMYQKFSP	LERDLDSMDP	AVYSLTIVSG	QNVCPSNSMG	SPCIEVDVLG	MPLDSCHFRT
1610	1620	1630	1640	1650	1660	1670	1680
KPIHRNTLNP	MWNEQFLFHV	HFEDLVFLRF	AVVENNSSAV	TAQRIIPLKA	LKRGYRHLQL	RNLHNEVLEI	SSLFINSRRM
1690	1700	1710	1720	1730	1740	1750	1760
EENSSGNTMS	ASSMFNTEER	KCLQTHRVTV	HGVPGPEPFT	VFTINGGTKA	KQLLQQILTN	EQDIKPVTTD	YFLMEEKYFI
1770	1780	1790	1800	1810	1820	1830	1840
SKEKNECRKQ	PFQRAIGPEE	EIMQILSSWF	PEEGYMGRIV	LKTQQENLEE	KNIVQDDKEV	ILSSEEESFF	VQVHDVSPEQ
1850	1860	1870	1880	1890	1900	1910	1920
PRTVIKAPRV	STAQDVIQQT	LCKAKYSYSI	LSNPNPSDYV	LLEEVVKDTT	NKKTTTPKSS	QRVLLDQECV	FQAQSKWKGA
1930	1940	1950	1960	1970	1980	1990	2000
GKFILKLKEQ	VQASREDKKK	GISFASELKK	LTKSTKQPRG	LTSPSQLLTS	ESIQTKEEKP	VGGLSSSDTM	DYRQ

Cmpd.	No. of Cmpds.	m/z meas.	Δ m/z [ppm]	z	Rt [min]	Score	P	Range	Sequence	Modification	Ratios	
547	1	624.6856	-218.06	2	35.5	11.3	2	1319-1329	R.IKKADNSACNK.G	Carbamidomethyl: 9	QD:QU	0.57

Detailed Protein Report

Protein 242: PREDICTED: MAX gene-associated protein isoform X16 [Homo sapiens]

**Accession:**	gi|578826753	**Score:**	29.8
**Database:**	refseq_human(refseq_human_20140103.fasta)	**MW [kDa]:**	290.2
**Database Date:**	2015-11-30	**pI:**	8.7
**Modification(s):**	Oxidation	**Sequence Coverage [%]:**	1.1
		**No. of unique Peptides:**	2

Quantitation

QD:QU	**Median:** 0.84	**CV:** 0.00 %	**No. of Peptides:** 1

Detailed Protein Report

10	20	30	40	50	60	70	80
MFKLDTGKMP	VVYLEPCAVT	RSTVKISELP	DNMLSTSRKD	KSSMLAELEY	LPTYIENSNE	TAFCLGKESE	NGLRKHSPDL
90	100	110	120	130	140	150	160
RVVQKYPLLK	EPQWKYPDIS	DSISTERILD	DSKDSVGDSL	SGKEDLGRKR	TTMLKIATAA	KVVNANQNAS	PNVPGKRGRP
170	180	190	200	210	220	230	240
RKLKLCKAGR	PPKNTGKSLI	STKNTPVSPG	STFPDVKPDL	EDVDGVLFVS	FESKEALDIH	AVDGTTEESS	SLQASTTNDS
250	260	270	280	290	300	310	320
GYRARISQLE	KELIEDLKTL	RHKQVIHPGL	QEVGLKLNSV	DPTMSIDLKY	LGVQLPLAPA	TSFPFWNLTG	TNPASPDAGF
330	340	350	360	370	380	390	400
PFVSRTGKTN	DFTKIKGWRG	KFHSASASRN	EGGNSESSLK	NRSAFCSDKL	DEYLENEGKL	METSMGFSSN	APTSPVVYQL
410	420	430	440	450	460	470	480
PTKSTSYVRT	LDSVLKKQST	ISPSTSYSLK	PHSVPPVSRK	AKSQNRQATF	SGRTKSSYKS	ILPYPVSPKQ	KYSHVILGDK
490	500	510	520	530	540	550	560
VTKNSSGIIS	ENQANNFVVP	TLDENIFPKQ	ISLRQAQQQQ	QQQQGSRPPG	LSKSQVKLMD	LEDCALWEGK	PRTYITEERA
570	580	590	600	610	620	630	640
DVSLTTLLTA	QASLKTKPIH	TIIRKRAPPC	NNDFCRLGCV	CSSLALEKRQ	PAHCRRPDCM	FGCTCLKRKV	VLVKGGSKTK
650	660	670	680	690	700	710	720
HFQRKAAHRD	PVFYDTLGEE	AREEEEGIRE	EEEQLKEKKK	RKKLEYTICE	TEPEQPVRHY	PLWVKVEGEV	DPEPVYIPTP
730	740	750	760	770	780	790	800
SVIEPMKPLL	LPQPEVLSPT	VKGKLLTGIK	SPRSYTPKPN	PVIREEDKDP	VYLYFESMMT	CARVRVYERK	KEDQRQPSSS
810	820	830	840	850	860	870	880
SSPSPSFQQQ	TSCHSSPENH	NNAKEPDSEQ	QPLKQLTCDL	EDDSDKLQEK	SWKSSCNEGE	SSSTSYMHQR	SPGGPTKLIE
890	900	910	920	930	940	950	960
IISDCNWEED	RNKILSILSQ	HINSNMPQSL	KVGSFIIELA	SQRKSRGEKN	PPVYSSRVKI	SMPSCQDQDD	MAEKSGSETP
970	980	990	1000	1010	1020	1030	1040
DGPLSPGKME	DISPVQTDAL	DSVRERLHGG	KGLPFYAGLS	PAGKLVAYKR	KPSSSTSGLI	QVNGKSYPQA	KLLLGQMGAL
1050	1060	1070	1080	1090	1100	1110	1120
HPANRLAAYI	TGRLRPSVLD	LSTLSTVISK	VASNAKVAAS	RKPRTLLPST	SNSKMASSSG	TATNRPGKNL	KAFVPAKRPI
1130	1140	1150	1160	1170	1180	1190	1200
AARPSPGGVF	TQFVMSKVGA	LQQKIPGVST	PQTLAGTQKF	SIRPSPVMVV	TPVVSSEPVQ	VCSPVTAAVT	TTTPQVFLEN
1210	1220	1230	1240	1250	1260	1270	1280
TTAVTPMTAI	SDVETKETTY	SSGATTTGVV	EVSETNTSTS	VTSTQSTATV	NLTKTTGITT	PVASVAFPKS	LVASPSTITL
1290	1300	1310	1320	1330	1340	1350	1360
PVASTASTSL	VVVTAAASSS	MVTTPTSSLG	SVPIILSGIN	GSPPVSQRPE	NAAQIPVATP	QVSPNTVKRA	GPRLLLIPVQ
1370	1380	1390	1400	1410	1420	1430	1440
QGSPTLRPVS	NTQLQGHRMV	LQPVRSPSGM	NLFRHPNGQI	VQLLPLHQLR	GSNTQPNLQP	VMFRNPGSVM	GIRLPAPSKP
1450	1460	1470	1480	1490	1500	1510	1520
SETPPSSTSS	SAFSVMNPVI	QAVGSSSAVN	VITQAPSLLS	SGASFVSQAG	TLTLRISPPE	PQSFASKTGS	ETKITYSSGG
1530	1540	1550	1560	1570	1580	1590	1600
QPVGTASLIP	LQSGSFALLQ	LPGQKPVPSS	ILQHVASLQM	KRESQNPDQK	DETNSIKREQ	ETKKVLQSEG	EAVDPEANVI
1610	1620	1630	1640	1650	1660	1670	1680
KQNSGAATSE	ETLNDSLEDR	GDHLDEECLP	EEGCATVKPS	EHSCITGSHT	DQDYKDVNEE	YGARNRKSSK	EKVAVLEVRT
1690	1700	1710	1720	1730	1740	1750	1760
ISEKASNKTV	QNLSKVQHQK	LGDVKVEQQK	GFDNPEENSS	EFPVTFKEES	KFELSGSKVM	EQQSNLQPEA	KEKECGDSLE
1770	1780	1790	1800	1810	1820	1830	1840
KDRERWRKHL	KGPLTRKCVG	ASQECKKEAD	EQLIKETKTC	QENSDVFQQE	QGISDLLGKS	GITEDARVLK	TECDSWSRIS
1850	1860	1870	1880	1890	1900	1910	1920
NPSAFSIVPR	RAAKSSRGNG	HFQGHLLLPG	EQIQPKQEKK	GGRSSADFTV	LDLEEDDEDD	NEKTDDSIDE	IVDVVSDYQS
1930	1940	1950	1960	1970	1980	1990	2000
EEVDDVEKVN	NCVEYIEDDE	EHVDIETVEE	LSEEINVAHL	KTTAAHTQSF	KQPSCTHISA	DEKAAERSRK	APPIPLKLKP
2010	2020	2030	2040	2050	2060	2070	2080
DYWSDKLQKE	AEAFAYYRRT	HTANERRRRG	EMRDLFEKLK	ITLGLLHSSK	VSKSLILTRA	FSEIQGLTDQ	ADKLIGQKNL
2090	2100	2110	2120	2130	2140	2150	2160
LTRKRNILIR	KVSSLSGKTE	EVVLKKLEYI	YAKQQALEAQ	KRKKKMGSDE	FDISPRISKQ	QEGSSASSVD	LGQMFINNRR
2170	2180	2190	2200	2210	2220	2230	2240

Detailed Protein Report

Cmpd.	No. of Cmpds.	m/z meas.	Δ m/z [ppm]	z	Rt [min]	Score	P	Range	Sequence	Modification	Ratios
2438	1	611.7346	-126.77	2	60.9	12.8	0	597-608	R.LGCVCSSLALEK.R		
238	1	897.4097	-91.61	2	31.7	17.0	1	2603-2620	K.VMPCLAPIAAKVGSVGHK.M	Oxidation: 2	QD:QU 0.84

Detailed Protein Report

Protein 243: SH3 and multiple ankyrin repeat domains protein 3 [Homo sapiens]

**Accession:**	gi|380748963	**Score:**	29.7
**Database:**	refseq_human(refseq_human_20140103.fasta)	**MW [kDa]:**	184.6
**Database Date:**	2015-11-30	**pI:**	9.6
		**Sequence Coverage [%]:**	1.5
		**No. of unique Peptides:**	1

10	20	30	40	50	60	70	80
MDGPGASAVV	VRVGIPDLQQ	TKCLRLDPAA	PVWAAKQRVL	CALNHSLQDA	LNYGLFQPPS	RGRAGKFLDE	ERLLQEYPPN
90	100	110	120	130	140	150	160
LDTPLPYLEF	RYKRRVYAQN	LIDDKQFAKL	HTKANLKKFM	DYVQLHSTDK	VARLLDKGLD	PNFHDPDSGE	CPLSLAAQLD
170	180	190	200	210	220	230	240
NATDLLKVLK	NGGAHLDFRT	RDGLTAVHCA	TRQRNAAALT	TLLDLGASPD	YKDSRGLTPL	YHSALGGGDA	LCCELLLHDH
250	260	270	280	290	300	310	320
AQLGITDENG	WQEIHQACRF	GHVQHLEHLL	FYGADMGAQN	ASGNTALHIC	ALYNQESCAR	VLLFRGANRD	VRNYNSQTAF
330	340	350	360	370	380	390	400
QVAIIAGNFE	LAEVIKTHKD	SDVVPFRETP	SYAKRRRLAG	PSGLASPRPL	QRSASDINLK	GEAQPAASPG	PSLRSLPHQL
410	420	430	440	450	460	470	480
LLQRLQEEKD	RDRDADQESN	ISGPLAGRAG	QSKISPSGPG	GPGPAPGPGP	APPAPPAPPP	RGPKRKLYSA	VPGRKFIAVK
490	500	510	520	530	540	550	560
AHSPQGEGEI	PLHRGEAVKV	LSIGEGGFWE	GTVKGRTGWF	PADCVEEVQM	RQHDTRPETR	EDRTKRLFRH	YTVGSYDSLT
570	580	590	600	610	620	630	640
SHSDYVIDDK	VAVLQKRDHE	GFGFVLRGAK	AETPIEEFTP	TPAFPALQYL	ESVDVEGVAW	RAGLRTGDFL	IEVNGVNVVK
650	660	670	680	690	700	710	720
VGHKQVVALI	RQGGNRLVMK	VVSVTRKPEE	DGARRRAPPP	PKRAPSTTLT	LRSKSMTAEL	EELASIRRRK	GEKLDEMLAA
730	740	750	760	770	780	790	800
AAEPTLRPDI	ADADSRAATV	KQRPTSRRIT	PAEISSLFER	QGLPGPEKLP	GSLRKGIPRT	KSVGEDEKLA	SLLEGRFPRS
810	820	830	840	850	860	870	880
TSMQDPVREG	RGIPPPPQTA	PPPPPAPYYF	DSGPPPAFSP	PPPPGRAYDT	VRSSFKPGLE	ARLGAGAAGL	YEPGAALGPL
890	900	910	920	930	940	950	960
PYPERQKRAR	SMIILQDSAP	ESGDAPRPPP	AATPPERPKR	RPRPPGPDSP	YANLGAFSAS	LFAPSKPQRR	KSPLVKQLQV
970	980	990	1000	1010	1020	1030	1040
EDAQERAALA	VGSPGPGGGS	FAREPSPTHR	GPRPGGLDYG	AGDGPGLAFG	GPGPAKDRRL	EERRRSTVFL	SVGAIEGSAP
1050	1060	1070	1080	1090	1100	1110	1120
GADLPSLQPS	RSIDERLLGT	GPTAGRDLLL	PSPVSALKPL	VSGPSLGPSG	STFIHPLTGK	PLDPSSPLAL	ALAARERALA
1130	1140	1150	1160	1170	1180	1190	1200
SQAPSRSPTP	VHSPDADRPG	PLFVDVQARD	PERGSLASPA	FSPRSPAWIP	VPARREAEKV	PREERKSPED	KKSMILSVLD
1210	1220	1230	1240	1250	1260	1270	1280
TSLQRPAGLI	VVHATSNGQE	PSRLGGAEEE	RPGTPELAPA	PMQSAAVAEP	LPSPRAQPPG	GTPADAGPGQ	GSSEEEPELV
1290	1300	1310	1320	1330	1340	1350	1360
FAVNLPPAQL	SSSDEETREE	LARIGLVPPP	EEFANGVLLA	TPLAGPGPSP	TTVPSPASGK	PSSEPPPAPE	SAADSGVEEA
1370	1380	1390	1400	1410	1420	1430	1440
DTRSSSDPHL	ETTSTISTVS	SMSTLSSESG	ELTDTHTSFA	DGHTFLLEKP	PVPPKPKLKS	PLGKGPVTFR	DPLLKQSSDS
1450	1460	1470	1480	1490	1500	1510	1520
ELMAQQHHAA	SAGLASAAGP	ARPRYLFQRR	SKLWGDPVES	RGLPGPEDDK	PTVISELSSR	LQQLNKDTRS	LGEEPVGGLG
1530	1540	1550	1560	1570	1580	1590	1600
SLLDPAKKSP	IAAARLFSSL	GELSSISAQR	SPGGPGGGAS	YSVRPSGRYP	VARRAPSPVK	PASLERVEGL	GAGAGGAGRP
1610	1620	1630	1640	1650	1660	1670	1680
FGLTPPTILK	SSSLSIPHEP	KEVRFVVRSV	SARSRSPSPS	PLPSPASGPG	PGAPGPRRPF	QQKPLQLWSK	FDVGDWLESI
1690	1700	1710	1720	1730	1740		
HLGEHRDRFE	DHEIEGAHLP	ALTKDDFVEL	GVTRVGHRMN	IERALRQLDG	S		

Cmpd.	No. of Cmpds.	m/z meas.	Δ m/z [ppm]	z	Rt [min]	Score	P	Range	Sequence	Modification
498	1	546.2697	-47.83	2	34.8	13.2	0	853-862	R.SSFKPGLEAR.L	

Detailed Protein Report

Protein 244: cGMP-specific 3',5'-cyclic phosphodiesterase isoform 3 [Homo sapiens]

**Accession:**	gi|61744432	**Score:**	29.6
**Database:**	refseq_human(refseq_human_20140103.fasta)	**MW [kDa]:**	93.6
**Database Date:**	2015-11-30	**pI:**	5.7
**Modification(s):**	Carbamidomethyl	**Sequence Coverage [%]:**	4.1
		**No. of unique Peptides:**	1

10	20	30	40	50	60	70	80
MVNAWFAERV	HTIPVCKEGI	RGHTESCSCP	LQQSPRADNS	APGTPTRKIS	ASEFDRPLRP	IVVKDSEGTV	SFLSDSEKKE
**90**	**100**	**110**	**120**	**130**	**140**	**150**	**160**
QMPLTPPRFD	HDEGDQCSRL	LELVKDISSH	LDVTALCHKI	FLHIHGLISA	DRYSLFLVCE	DSSNDKFLIS	RLFDVAEGST
**170**	**180**	**190**	**200**	**210**	**220**	**230**	**240**
LEEVSNNCIR	LEWNKGIVGH	VAALGEPLNI	KDAYEDPRFN	AEVDQITGYK	TQSILCMPIK	NHREEVVGVA	QAINKKSGNG
**250**	**260**	**270**	**280**	**290**	**300**	**310**	**320**
GTFTEKDEKD	FAAYLAFCGI	VLHNAQLYET	SLLENKRNQV	LLDLASLIFE	EQQSLEVILK	KIAATIISFM	QVQKCTIFIV
**330**	**340**	**350**	**360**	**370**	**380**	**390**	**400**
DEDCSDSFSS	VFHMECEELE	KSSDTLTREH	DANKINYMYA	QYVKNTMEPL	NIPDVSKDKR	FPWTTENTGN	VNQQCIRSLL
**410**	**420**	**430**	**440**	**450**	**460**	**470**	**480**
CTPIKNGKKN	KVIGVCQLVN	KMEENTGKVK	PFNRNDEQFL	EAFVIFCGLG	IQNTQMYEAV	ERAMAKQMVT	LEVLSYHASA
**490**	**500**	**510**	**520**	**530**	**540**	**550**	**560**
AEEETRELQS	LAAAVVPSAQ	TLKITDFSFS	DFELSDLETA	LCTIRMFTDL	NLVQNFQMKH	EVLCRWILSV	KKNYRKNVAY
**570**	**580**	**590**	**600**	**610**	**620**	**630**	**640**
HNWRHAFNTA	QCMFAALKAG	KIQNKLTDLE	ILALLIAALS	HDLDHRGVNN	SYIQRSEHPL	AQLYCHSIME	HHHFDQCLMI
**650**	**660**	**670**	**680**	**690**	**700**	**710**	**720**
LNSPGNQILS	GLSIEEYKTT	LKIIKQAILA	TDLALYIKRR	GEFFELIRKN	QFNLEDPHQK	ELFLAMLMTA	CDLSAITKPW
**730**	**740**	**750**	**760**	**770**	**780**	**790**	**800**
PIQQRIAELV	ATEFFDQGDR	ERKELNIEPT	DLMNREKKNK	IPSMQVGFID	AICLQLYEAL	THVSEDCFPL	LDGCRKNRQK
**810**	**820**	**830**					
WQALAEQQEK	MLINGESGQA	KRN					

Cmpd.	No. of Cmpds.	m/z meas.	Δ m/z [ppm]	z	Rt [min]	Score	P	Range	Sequence	Modification
2072	1	1071.4033	-85.02	2	56.3	13.5	1	18-36	K.EGIRGHTESCSCPLQQSPR.A	Carbamidomethyl: 10

Detailed Protein Report

Protein 245: transport and Golgi organization protein 6 homolog [Homo sapiens]

**Accession:**	gi|153791502	**Score:**	29.6
**Database:**	refseq_human(refseq_human_20140103.fasta)	**MW [kDa]:**	120.7
**Database Date:**	2015-11-30	**pI:**	5.7
		**Sequence Coverage [%]:**	3.2
		**No. of unique Peptides:**	1

10	20	30	40	50	60	70	80
MAARQAVGSG	AQETCGLDRI	LEALKLLLSP	GGSGSSSLQV	TKHDVLLATL	KSNLSALEDK	FLKDPQWKNL	KLLRDEIADK
90	100	110	120	130	140	150	160
AEWPQNSVDV	TWSFTSQTLL	LLLCLKETMI	RLAANFNPGK	PNPRTPEVAP	ALSPDALSIS	QQKTVQFVLQ	FVVTLGICPY
170	180	190	200	210	220	230	240
LMPGVGVPLR	YRTEFGAVVQ	DVVCFDAAPD	ATRRLYTSCK	ALLNVAQHTS	LGSLIFCHHF	GDIAAGLCQL	GFCPTKRKLL
250	260	270	280	290	300	310	320
TPAEEVLTEE	ERTLSRGALR	DMLDQVYQPL	AVRELLILQG	GPPQSCTDVK	TQMRCRAPAW	LRRLCGQLLS	ERLMRPNGVQ
330	340	350	360	370	380	390	400
AVVRGILEGA	GAGAAGGSDA	EVTAADWKKC	DLIAKILASC	PQQSLSPENY	YRDICPQVLD	LFHFQDKLTA	RQFQRVATTT
410	420	430	440	450	460	470	480
FITLSRERPH	LAAKYLLQPV	LAPLHRCLNT	AELSESDMVP	GTILVTEEEL	SRCIEDVFKV	YVVGNEPLTV	LMDSLLPVLG
490	500	510	520	530	540	550	560
VLFLLYCFTK	QSVSHIRSLC	QEILLWILGK	LERKKAIASL	KGFAGLDKAV	PSLHSLCQFR	VATQGGIMIT	IKEAISDEDE
570	580	590	600	610	620	630	640
DEALYQKVSS	EQGRVEHLGD	LLSHCQECGL	AGDFFIFCLK	ELTHVASENE	TELKTEPFSS	KSLLELEQHQ	TLLVEGQERK
650	660	670	680	690	700	710	720
LLVLQLMAVL	CERMSEQIFT	NVTQVVDFVA	ATLQRACASL	AHQAESTVES	QTLSMSMGLV	AVMLGGAVQL	KSSDFAVLKQ
730	740	750	760	770	780	790	800
LLPLLEKVSN	TYPDPVIQEL	AVDLRITIST	HGAFATEAVS	MAAQSTLNRK	DLEGKIEEQQ	QTSHERPTDV	AHSHLEQQQS
810	820	830	840	850	860	870	880
HETAPQTGLQ	SNAPIIPQGV	NEPSTTTSQK	SGSVTTEQLQ	EVLLSAYDPQ	IPTRAAALRT	LSHWIEQREA	KALEMQEKLL
890	900	910	920	930	940	950	960
KIFLENLEHE	DTFVYLSAIQ	GVALLSDVYP	EKILPDLLAQ	YDSSKDKHTP	ETRMKVGEVL	MRIVRALGDM	VSKYREPLIH
970	980	990	1000	1010	1020	1030	1040
TFLRGVRDPD	GAHRASSLAN	LGELCQRLDF	LLGSVVHEVT	ACLIAVAKTD	GEVQVRRAAI	HVVVLLLRGL	SQKATEVLSA
1050	1060	1070	1080	1090	1100		
VLKDLYHLLK	HVVCLEPDDV	AKLHAQLALE	ELDDIMKNFL	FPPQKLEKKI	MVLP		

Cmpd.	No. of Cmpds.	m/z meas.	Δ m/z [ppm]	z	Rt [min]	Score	P	Range	Sequence	Modification
1030	1	809.4708	-1.60	3	43.0	11.9	2	1029-1050	R.GLSQKATEVLSAVLKDLYHLLK.H	

Detailed Protein Report

Protein 246: **cell cycle checkpoint control protein RAD9B isoform 1 [Homo sapiens]**

**Accession:**	gi|169234617	**Score:**	29.6
**Database:**	refseq_human(refseq_human_20140103.fasta)	**MW [kDa]:**	48.1
**Database Date:**	2015-11-30	**pI:**	6.5
**Modification(s):**	Carbamidomethyl, Oxidation	**Sequence Coverage [%]:**	7.7
		**No. of unique Peptides:**	2

Quantitation

QD:QU **Median:** 1.00 **CV:** 0.00 % **No. of Peptides:** 1

10	20	30	40	50	60	70	80
MAAMLKCVMS	GSQVKVFGKA	VQALSRISDE	FWLDPSKKGL	ALRCVNSSRS	AYGCVLFSPV	FFQHYQWSAL	VKMSENELDT
**90**	**100**	**110**	**120**	**130**	**140**	**150**	**160**
TLHLKCKLGM	KSILPIFRCL	NSLERNIEKC	RIFTRSDKCK	VVIQFFYRHG	IKRTHNICFQ	ESQPLQVIFD	KNVCTNTLMI
**170**	**180**	**190**	**200**	**210**	**220**	**230**	**240**
QPRLLADAIV	LFTSSQEEVT	LAVTPLNFCL	KSSNEESMDL	SNAVHSEMFV	GSDEFDFFQI	GMDTEITFCF	KELKGILTFS
**250**	**260**	**270**	**280**	**290**	**300**	**310**	**320**
EATHAPISIY	FDFPGKPLAL	SIDDMLVEAN	FILATLADEQ	SRASSPQSLC	LSQKRKRSDL	IEKKAGKNVT	GQALECISKK
**330**	**340**	**350**	**360**	**370**	**380**	**390**	**400**
AAPRRLYPKE	TLTNISALEN	CGSPAMKRVD	GDVSEVSESS	VSNTEEVPGS	LCLRKFSCMF	FGAVSSDQQE	HFNHPFDSLA
**410**	**420**	**430**					
RASDSEEDMN	NVCCRKEFNG	SDAKYFCII					

Cmpd.	No. of Cmpds.	m/z meas.	Δ m/z [ppm]	z	Rt [min]	Score	P	Range	Sequence	Modification	Ratios
2570	8	1044.5429	14.94	2	62.8	15.9	2	1-19	-.MAAMLKCVMSGSQVKVFGK.A	Carbamidomethyl: 7; Oxidation: 4	
421	1	846.3629	-53.88	2	35.2	13.6	2	416-429	R.KEFNGSDAKYFCII.-	Carbamidomethyl: 12	QD:QU 1.00

Detailed Protein Report

Protein 247: **PREDICTED: cadherin EGF LAG seven-pass G-type receptor 2 isoform X2 [Homo sapiens]**

**Accession:**	gi|578798514	**Score:**	29.5
**Database:**	refseq_human(refseq_human_20140103.fasta)	**MW [kDa]:**	316.2
**Database Date:**	2015-11-30	**pI:**	5.0
		**Sequence Coverage [%]:**	0.9
		**No. of unique Peptides:**	1

Detailed Protein Report

10	20	30	40	50	60	70	80
MRSPATGVPL	PTPPPPLLLL	LLLLLPPPLL	GDQVGPCRSL	GSRGRGSSGA	CAPMGWLCPS	SASNLWLYTS	RCRDAGTELT
90	100	110	120	130	140	150	160
GHLVPHHDGL	RVWCPESEAH	IPLPPAPEGC	PWSCRLLGIG	GHLSPQGKLT	LPEEHPCLKA	PRLRCQSCKL	AQAPGLRAGE
170	180	190	200	210	220	230	240
RSPEESLGGR	RKRNVNTAPQ	FQPPSYQATV	PENQPAGTPV	ASLRAIDPDE	GEAGRLEYTM	DALFDSRSNQ	FFSLDPVTGA
250	260	270	280	290	300	310	320
VTTAEELDRE	TKSTHVFRVT	AQDHGMPRRS	ALATLTILVT	DTNDHDPVFE	QQEYKESLRE	NLEVGYEVLT	VRATDGDAPP
330	340	350	360	370	380	390	400
NANILYRLLE	GSGGSPSEVF	EIDPRSGVIR	TRGPVDREEV	ESYQLTVEAS	DQGRDPGPRS	TTAAVFLSVE	DDNDNAPQFS
410	420	430	440	450	460	470	480
EKRYVVQVRE	DVTPGAPVLR	VTASDRDKGS	NAVVHYSIMS	GNARGQFYLD	AQTGALDVVS	PLDYETTKEY	TLRVRAQDGG
490	500	510	520	530	540	550	560
RPPLSNVSGL	VTVQVLDIND	NAPIFVSTPF	QATVLESVPL	GYLVLHVQAI	DADAGDNARL	EYRLAGVGHD	FPFTINNGTG
570	580	590	600	610	620	630	640
WISVAAELDR	EEVDFYSFGV	EARDHGTPAL	TASASVSVTV	LDVNDNNPTF	TQPEYTVRLN	EDAAVGTSVV	TVSAVDRDAH
650	660	670	680	690	700	710	720
SVITYQITSG	NTRNRFSITS	QSGGGLVSLA	LPLDYKLERQ	YVLAVTASDG	TRQDTAQIVV	NVTDANTHRP	VFQSSHYTVN
730	740	750	760	770	780	790	800
VNEDRPAGTT	VVLISATDED	TGENARITYF	MEDSIPQFRI	DADTGAVTTQ	AELDYEDQVS	YTLAITARDN	GIPQKSDTTY
810	820	830	840	850	860	870	880
LEILVNDVND	NAPQFLRDSY	QGSVYEDVPP	FTSVLQISAT	DRDSGLNGRV	FYTFQGGDDG	DGDFIVESTS	GIVRTLRRLD
890	900	910	920	930	940	950	960
RENVAQYVLR	AYAVDKGMPP	ARTPMEVTVT	VLDVNDNPPV	FEQDEFDVFV	EENSPIGLAV	ARVTATDPDE	GTNAQIMYQI
970	980	990	1000	1010	1020	1030	1040
VEGNIPEVFQ	LDIFSGELTA	LVDLDYEDRP	EYVLVIQATS	APLVSRATVH	VRLLDRNDNP	PVLGNFEILF	NNYVTNRSSS
1050	1060	1070	1080	1090	1100	1110	1120
FPGGAIGRVP	AHDPDISDSL	TYSFERGNEL	SLVLLNASTG	ELKLSRALDN	NRPLEAIMSV	LVSDGVHSVT	AQCALRVTII
1130	1140	1150	1160	1170	1180	1190	1200
TDEMLTHSIT	LRLEDMSPER	FLSPLLGLFI	QAVAATLATP	PDHVVVFNVQ	RDTDAPGGHI	LNVSLSVGQP	PGPGGGPPFL
1210	1220	1230	1240	1250	1260	1270	1280
PSEDLQERLY	LNRSLLTAIS	AQRVLPFDDN	ICLREPCENY	MRCVSVLRFD	SSAPFIASSS	VLFRPIHPVG	GLRCRCPPGF
1290	1300	1310	1320	1330	1340	1350	1360
TGDYCETEVD	LCYSRPCGPH	GRCRSREGGY	TCLCRDGYTG	EHCEVSARSG	RCTPGVCKNG	GTCVNLLVGG	FKCDCPSGDF
1370	1380	1390	1400	1410	1420	1430	1440
EKPYCQVTTR	SFPAHSFITF	RGLRQRFHFT	LALSFATKER	DGLLLYNGRF	NEKHDFVALE	VIQEQVQLTF	SAGESTTTVS
1450	1460	1470	1480	1490	1500	1510	1520
PFVPGGVSDG	QWHTVQLKYY	NKPLLGQTGL	PQGPSEQKVA	VVTVDGCDTG	VALRFGSVLG	NYSCAAQGTQ	GGSKKSLDLT
1530	1540	1550	1560	1570	1580	1590	1600
GPLLLGGVPD	LPESFPVRMR	QFVGCMRNLQ	VDSRHIDMAD	FIANNGTVPG	CPAKKNVCDS	NTCHNGGTCV	NQWDAFSCEC
1610	1620	1630	1640	1650	1660	1670	1680
PLGFGGKSCA	QEMANPQHFL	GSSLVAWHGL	SLPISQPWYL	SLMFRTRQAD	GVLLQAITRG	RSTITLQLRE	GHVMLSVEGT
1690	1700	1710	1720	1730	1740	1750	1760
GLQASSLRLE	PGRANDGDWH	HAQLALGASG	GPGHAILSFD	YGQQRAEGNL	GPRLHGLHLS	NITVGGIPGP	AGGVARGFRG
1770	1780	1790	1800	1810	1820	1830	1840
CLQGVRVSDT	PEGVNSLDPS	HGESINVEQG	CSLPDPCDSN	PCPANSYCSN	DWDSYSCSCD	PGYYGDNCTN	VCDLNPCEHQ
1850	1860	1870	1880	1890	1900	1910	1920
SVCTRKPSAP	HGYTCECPPN	YLGPYCETRI	DQPCPRGWWG	HPTCGPCNCD	VSKGFDPDCN	KTSGECHCKE	NHYRPPGSPT
1930	1940	1950	1960	1970	1980	1990	2000
CLLCDCYPTG	SLSRVCDPED	GQCPCKPGVI	GRQCDRCDNP	FAEVTTNGCE	VNYDSCPRAI	EAGIWWPRTR	FGLPAAAPCP
2010	2020	2030	2040	2050	2060	2070	2080
KGSFGTAVRH	CDEHRGWLPP	NLFNCTSITF	SELKGFAERL	QRNESGLDSG	RSQQLALLLR	NATQHTAGYF	GSDVKVAYQL
2090	2100	2110	2120	2130	2140	2150	2160
ATRLLAHEST	QRGFGLSATQ	DVHFTENLLR	VGSALLDTAN	KRHWELIQQT	EGGTAWLLQH	YEAYASALAQ	NMRHTYLSPF
2170	2180	2190	2200	2210	2220	2230	2240
TIVTPNIVIS	VVRLDKGNFA	GAKLPRYEAL	RGEQPPDLET	TVILPESVFR	ETPPVVRPAG	PGEAQEPEEL	ARRQRRHPEL
2250	2260	2270	2280	2290	2300	2310	2320
SQGEAVASVI	IYRTLAGLLP	HNYDPDKRSL	RVPKRPIINT	PVVSISVHDD	EELLPRALDK	PVTVQFRLLE	TEERTKPICV
2330	2340	2350	2360	2370	2380	2390	2400

Detailed Protein Report

Cmpd.	No. of Cmpds.	m/z meas.	Δ m/z [ppm]	z	Rt [min]	Score	P	Range	Sequence	Modification
2164	1	937.1664	148.30	2	57.5	14.0	2	129-144	K.LTLPEEHPCLKAPRLR.C	

Detailed Protein Report

Protein 248: centrosomal protein of 55 kDa [Homo sapiens]

**Accession:**	gi|187608537	**Score:**	29.5
**Database:**	refseq_human(refseq_human_20140103.fasta)	**MW [kDa]:**	54.1
**Database Date:**	2015-11-30	**pI:**	6.6
		**Sequence Coverage [%]:**	2.8
		**No. of unique Peptides:**	1

Alias proteins:

Accession	Name	Description
gi|187608545	refseq_human (refseq_human_20140103.fasta)	centrosomal protein of 55 kDa [Homo sapiens]

10	20	30	40	50	60	70	80
MSSRSTKDLI	KSKWGSKPSN	SKSETTLEKL	KGEIAHLKTS	VDEITSGKGK	LTDKERHRLL	EKIRVLEAEK	EKNAYQLTEK
**90**	**100**	**110**	**120**	**130**	**140**	**150**	**160**
DKEIQRLRDQ	LKARYSTTAL	LEQLEETTRE	GERREQVLKA	LSEEKDVLKQ	QLSAATSRIA	ELESKTNTLR	LSQTVAPNCF
**170**	**180**	**190**	**200**	**210**	**220**	**230**	**240**
NSSINNIHEM	EIQLKDALEK	NQQWLVYDQQ	REVYVKGLLA	KIFELEKKTE	TAAHSLPQQT	KKPESEGYLQ	EEKQKCYNDL
**250**	**260**	**270**	**280**	**290**	**300**	**310**	**320**
LASAKKDLEV	ERQTITQLSF	ELSEFRRKYE	ETQKEVHNLN	QLLYSQRRAD	VQHLEDDRHK	TEKIQKLREE	NDIARGKLEE
**330**	**340**	**350**	**360**	**370**	**380**	**390**	**400**
EKKRSEELLS	QVQFLYTSLL	KQQEEQTRVA	LLEQQMQACT	LDFENEKLDR	QHVQHQLHVI	LKELRKARNQ	ITQLESLKQL
**410**	**420**	**430**	**440**	**450**	**460**	**470**	
HEFAITEPLV	TFQGETENRE	KVAASPKSPT	AALNESLVEC	PKCNIQYPAT	EHRDLLVHVE	YCSK	

Cmpd.	No. of Cmpds.	m/z meas.	Δ m/z [ppm]	z	Rt [min]	Score	P	Range	Sequence	Modification
1005	1	423.6388	-147.47	2	41.1	17.5	0	309-315	R.EENDIAR.G	

Detailed Protein Report

Protein 249: OTU domain-containing protein 7A [Homo sapiens]

**Accession:**	gi|18702331	**Score:**	29.5
**Database:**	refseq_human(refseq_human_20140103.fasta)	**MW [kDa]:**	100.6
**Database Date:**	2015-11-30	**pI:**	9.5
		**Sequence Coverage [%]:**	4.2
		**No. of unique Peptides:**	2

10	20	30	40	50	60	70	80
MVSSVLPNPT	SAECWAALLH	DPMTLDMDAV	LSDFVRSTGA	EPGLARDLLE	GKNWDLTAAL	SDYEQLRQVH	TANLPHVFNE
**90**	**100**	**110**	**120**	**130**	**140**	**150**	**160**
GRGPKQPERE	PQPGHKVERP	CLQRQDDIAQ	EKRLSRGISH	ASSAIVSLAR	SHVASECNNE	QFPLEMPIYT	FQLPDLSVYS
**170**	**180**	**190**	**200**	**210**	**220**	**230**	**240**
EDFRSFIERD	LIEQATMVAL	EQAGRLNWWS	TVCTSCKRLL	PLATTGDGNC	LLHAASLGMW	GFHDRDLVLR	KALYTMMRTG
**250**	**260**	**270**	**280**	**290**	**300**	**310**	**320**
AEREALKRRW	RWQQTQQNKE	EEWEREWTEL	LKLASSEPRT	HFSKNGGTGG	GVDNSEDPVY	ESLEEFHVFV	LAHILRRPIV
**330**	**340**	**350**	**360**	**370**	**380**	**390**	**400**
VVADTMLRDS	GGEAFAPIPF	GGIYLPLEVP	PNRCHCSPLV	LAYDQAHFSA	LVSMEQRDQQ	REQAVIPLTD	SEHKLLPLHF
**410**	**420**	**430**	**440**	**450**	**460**	**470**	**480**
AVDPGKDWEW	GKDDNDNARL	AHLILSLEAK	LNLLHSYMNV	TWIRIPSETR	APLAQPESPT	ASAGEDVQSL	ADSLDSDRDS
**490**	**500**	**510**	**520**	**530**	**540**	**550**	**560**
VCSNSNSNNG	KNGKDKEKEK	QRKEKDKTRA	DSVANKLGSF	SKTLGIKLKK	NMGGLGGLVH	GKMGRANSAN	GKNGDSAERG
**570**	**580**	**590**	**600**	**610**	**620**	**630**	**640**
KEKKAKSRKG	SKEESGASAS	TSPSEKTTPS	PTDKAAGASP	AEKGGGPRGD	AWKYSTDVKL	SLNILRAAMQ	GERKFIFAGL
**650**	**660**	**670**	**680**	**690**	**700**	**710**	**720**
LLTSHRHQFH	EEMIGYYLTS	AQERFSAEQE	QRRRDAATAA	AAAAAAAAAT	AKRPPRRPET	EGVPVPERAS	PGPPTQLVLK
**730**	**740**	**750**	**760**	**770**	**780**	**790**	**800**
LKERPSPGPA	AGRAARAAAG	GTASPGGGAR	RASASGPVPG	RSPPAPARQS	VIHVQASGAR	DEACAPAVGA	LRPCATYPQQ
**810**	**820**	**830**	**840**	**850**	**860**	**870**	**880**
NRSLSSQSYS	PARAAALRTV	NTVESLARAV	PGALPGAAGT	AGAAEHKSQT	YTNGFGALRD	GLEFADADAP	TARSNGECGR
**890**	**900**	**910**	**920**	**930**			
GGPGPVQRRC	QRENCAFYGR	AETEHYCSYC	YREELRRRRE	ARGARP			

Cmpd.	No. of Cmpds.	m/z meas.	Δ m/z [ppm]	z	Rt [min]	Score	P	Range	Sequence	Modification
2690	1	724.8511	-130.11	2	64.7	15.6	1	709-722	R.ASPGPPTQLVLKLK.E	
41	3	1068.4771	-72.42	2	30.4	13.9	2	737-761	R.AAAGGTASPGGGARRASASGPVPGR.S	

Detailed Protein Report

Protein 250: SPOC domain-containing protein 1 isoform 3 [Homo sapiens]

**Accession:**	gi|530354695	**Score:**	29.5
**Database:**	refseq_human(refseq_human_20140103.fasta)	**MW [kDa]:**	76.5
**Database Date:**	2015-11-30	**pI:**	9.2
**Modification(s):**	Oxidation	**Sequence Coverage [%]:**	4.2
		**No. of unique Peptides:**	2

10	20	30	40	50	60	70	80
MEVSSPSPAQ	RLRRKKRPMV	QGPAGCQVFQ	PSPSGGTAGD	PGGLSDPFYP	PRSGSLALGD	PSSDPACSQS	GPMEAEEDSL
**90**	**100**	**110**	**120**	**130**	**140**	**150**	**160**
PEQPEDSAQL	QQEKPSLYIG	VRGTVVRSMQ	EVLWTRLREL	PDPVLSEEVV	EGIAAGIEAA	LWDLTQGTNG	RYKTKYRSLL
**170**	**180**	**190**	**200**	**210**	**220**	**230**	**240**
FNLRDPRNLD	LFLKVVHGDV	TPYDLVRMSS	MQLAPQELAR	WRDQEEKRGL	NIIEQQQKEP	CRLPASKMTH	KGEVEIQRDM
**250**	**260**	**270**	**280**	**290**	**300**	**310**	**320**
DQTLTLEDLV	GPQMFMDCSP	QALPIASEDT	TGQHDHHFLD	PNCHICKDWE	PSNELLGSFE	AAKSCGDNIF	QKALSQTPMP
**330**	**340**	**350**	**360**	**370**	**380**	**390**	**400**
APEMPKTREL	SPTEPQDRVP	PSGLHVPAAP	TKALPCLPPW	EGVLDMFSIK	RFRARAQLVS	GHSCRLVQAL	PTVIRSAGCI
**410**	**420**	**430**	**440**	**450**	**460**	**470**	**480**
PSNIVWDLLA	SICPAKAKDV	CVVRLCPHGA	RDTQNCRLLY	SYLNDRQRHG	LASVEHMGMV	LLPLPAFQPL	PTRLRPLGGP
**490**	**500**	**510**	**520**	**530**	**540**	**550**	**560**
GLEVTHSSLL	LAVLLPKEGL	PDTAGSSPWL	GKVQKMVSFN	SKVEKRYYQP	DDRRPNVPLK	GTPPPGGAWQ	QSQGRGSIAP
**570**	**580**	**590**	**600**	**610**	**620**	**630**	**640**
RGISAWQRPP	RGRGRLWPEP	ENWQHPGRGQ	WPPEPGLRQS	QHPYSVAPAG	HGFGRGQHFH	RDSCPHQALL	RHLESLATMS
**650**	**660**	**670**	**680**	**690**	**700**		
HQLQALLCPQ	TKSSIPRPLQ	RLSSALAAPE	PPGPARDSSL	GPTDEAGSEC	PFPRKA		

Cmpd.	No. of Cmpds.	m/z meas.	Δ m/z [ppm]	z	Rt [min]	Score	P	Range	Sequence	Modification
1959	1	757.2595	-164.75	2	54.9	16.2	2	219-231	K.EPCRLPASKMTHK.G	Oxidation: 10
78	1	877.8658	-95.96	2	30.9	13.3	1	313-328	K.ALSQTPMPAPEMPKTR.E	

Detailed Protein Report

Protein 251: PREDICTED: protein TANC1 isoform X8 [Homo sapiens]

**Accession:**	gi|578805065	**Score:**	29.5
**Database:**	refseq_human(refseq_human_20140103.fasta)	**MW [kDa]:**	183.3
**Database Date:**	2015-11-30	**pI:**	9.8
**Modification(s):**	Oxidation	**Sequence Coverage [%]:**	2.1
		**No. of unique Peptides:**	1

10	20	30	40	50	60	70	80
MTVSYKQVLK	MLKAVLKKSR	EGGKGGKKEA	GSDFGPETSP	VLHLDHSADS	PVSSLPTAED	TYRVSLAKGV	SMSLPSSPLL
90	100	110	120	130	140	150	160
PRQSHLVQSR	VNKKSPGPVR	KPKYVESPRV	PGDAVIMPFR	EVAKPTEPDE	HATSSTKLED	LSYLDGQRNA	PLRTSIRLPW
170	180	190	200	210	220	230	240
HNTAGGRAQE	VKARFAPYKP	QDILLKPLLF	EVPSITTDSV	FVGRDWLFHQ	IEENLRNTEL	AENRGAVVVG	NVGFGKTAII
250	260	270	280	290	300	310	320
SKLVALSCHG	SRMRQIASNS	PGSSPKTSDP	TQDLHFTPLL	SPSSSTSASS	TAKTPLGSIS	AENQRPREDA	VKYLASKVVA
330	340	350	360	370	380	390	400
YHYCQADNTY	TCLVPEFVHS	IAALLCRSHQ	LAAYRDLLIK	EPQLQSMLSL	RSCVQDPVAA	FKRGVLEPLT	NLRNEQKIPE
410	420	430	440	450	460	470	480
EEYIILIDGL	NEAEFHKPDY	GDTLSSFITK	IISKFPAWLK	LIVTVRANFQ	EIISALPFVK	LSLDDFPDNK	DIHSDLHAYV
490	500	510	520	530	540	550	560
QHRVHSSQDI	LSNISLNGKA	DATLIGKVSS	HLVLRSLGSY	LYLKLTLDLF	QRGHLVIKSA	SYKVVPVSLS	ELYLLQCNMK
570	580	590	600	610	620	630	640
FMTQSAFERA	LPILNVALAS	LHPMTDEQIF	QAINAGHIQG	EQGWEDFQQR	MDALSCFLIK	RRDKTRMFCH	PSFREWLVWR
650	660	670	680	690	700	710	720
ADGENTAFLC	EPRNGHALLA	FMFSRQEGKL	NRQQTMELGH	HILKAHIFKG	LSKKTGISSS	HLQALWIGYS	TEGLSAALAS
730	740	750	760	770	780	790	800
LRNLYTPNVK	VSRLLILGGA	NVNYRTEVLN	NAPILCVQSH	LGHEEVVTLL	LEFGACLDGT	SENGMTALCY	AAAAGHMKLV
810	820	830	840	850	860	870	880
CLLTKKGVRV	DHLDKKGQCA	LVHSALRGHG	DILQYLLTCE	WSPGPPQPGT	LRKSHALQQA	LTAAASMGHS	SVVQCLLGME
890	900	910	920	930	940	950	960
KEHEVEVNGT	DTLWGETALT	AAAGRGKLEV	CELLLGHGAA	VSRTNRRGVP	PLFCAARQGH	WQIVRLLLER	GCDVNLSDKQ
970	980	990	1000	1010	1020	1030	1040
GRTPLMVAAC	EGHLSTVEFL	LSKGAALSSL	DKEGLSALSW	ACLKGHRAVV	QYLVEEGAAI	DQTDKNGRTP	LDLAAFYGDA
1050	1060	1070	1080	1090	1100	1110	1120
ETVLYLVEKG	AVIEHVDHSG	MRPLDRAIGC	RNTSVVVALL	RKGAKLGNAA	WAMATSKPDI	LIILLQKLME	EGNVMYKKGK
1130	1140	1150	1160	1170	1180	1190	1200
MKEAAQRYQY	ALRKFPREGF	GEDMRPFNEL	RVSLYLNLSR	CRRKTNDFGM	AEEFASKALE	LKPKSYEAFY	ARARAKRNSR
1210	1220	1230	1240	1250	1260	1270	1280
QFVAALADLQ	EAVKLCPTNQ	EVKRLLARVE	EECKQLQRSQ	QQKQQGPLPA	PLNDSENEED	TPTPGLSDHF	HSEETEEEET
1290	1300	1310	1320	1330	1340	1350	1360
SPQEESVSPT	PRSQPSSSVP	SSYIRNLQEG	LQSKGRPVSP	QSRAGIGKSL	REPVAQPGLL	LQPSKQAQIV	KTSQHLGSGQ
1370	1380	1390	1400	1410	1420	1430	1440
SAVRNGSMKV	QISSQNPPPS	PMPGRIAATP	AGSRTQHLEG	TGTFTTRAGC	GHFGDRLGPS	QNVRLQCGEN	GPAHPLPSKT
1450	1460	1470	1480	1490	1500	1510	1520
KTTERLLSHS	SVAVDAAPPN	QGGLATCSDV	RHPASLTSSG	SSGSPSSSIK	MSSSTSSLTS	SSSFSDGFKV	QGPDTRIKDK
1530	1540	1550	1560	1570	1580	1590	1600
VVTHVQSGTA	EHRPRNTPFM	GIMDKTARFQ	QQSNPPSRSW	HCPAPEGLLT	NTSSAAGLQS	ANTEKPSLMQ	VGGYNNQAKT
1610	1620	1630	1640	1650	1660	1670	1680
CSVSTLSASV	HNGAQVKELE	ESKCQIPVHS	QENRITKTVS	HLYQESISKQ	QPHISNEAHR	SHLTAAKPKR	SFIESNV

Cmpd.	No. of Cmpds.	m/z meas.	Δ m/z [ppm]	z	Rt [min]	Score	P	Range	Sequence	Modification
2803	2	973.5138	96.14	2	64.1	10.3	0	1491-1509	K.MSSSTSSLTSSSSFSDGFK.V	Oxidation: 1

Detailed Protein Report

Protein 252: PREDICTED: alpha-tubulin N-acetyltransferase isoform X9 [Homo sapiens]

**Accession:**	gi|530382649	**Score:**	29.4
**Database:**	refseq_human(refseq_human_20140103.fasta)	**MW [kDa]:**	25.8
**Database Date:**	2015-11-30	**pI:**	9.6
		**Sequence Coverage [%]:**	11.6
		**No. of unique Peptides:**	1

Alias proteins:

Accession	Name	Description
gi|530429736	refseq_human (refseq_human_20140103.fasta)	PREDICTED: alpha-tubulin N-acetyltransferase isoform X9 [Homo sapiens]
gi|530429452	refseq_human (refseq_human_20140103.fasta)	PREDICTED: alpha-tubulin N-acetyltransferase isoform X9 [Homo sapiens]
gi|530429087	refseq_human (refseq_human_20140103.fasta)	PREDICTED: alpha-tubulin N-acetyltransferase isoform X9 [Homo sapiens]
gi|530428827	refseq_human (refseq_human_20140103.fasta)	PREDICTED: alpha-tubulin N-acetyltransferase isoform X9 [Homo sapiens]
gi|530428535	refseq_human (refseq_human_20140103.fasta)	PREDICTED: alpha-tubulin N-acetyltransferase isoform X9 [Homo sapiens]
gi|530428190	refseq_human (refseq_human_20140103.fasta)	PREDICTED: alpha-tubulin N-acetyltransferase isoform X9 [Homo sapiens]

```
        10         20         30         40         50         60         70         80
MEFPFDVDAL FPERITVLDQ HLRPPARRPG TTTPARVDLQ QQIMTIIDEL GKASAKAQNL SAPITSASRM QSNRHVVYIL
        90        100        110        120        130        140        150        160
KDSSARPAGK GAIIGFIKVG YKKLFVLDDR EAHNEVEPLC ILDFYIHESV QRHGHGRELF QYMLQKERVE PHQLAIDRPS
       170        180        190        200        210        220        230
QKLLKFLNKH YNLETTVPQV NNFVIFEGFF AHQHPPARKL PPKRAEGDIK PYSSSDREWG LPQVW
```

Cmpd.	No. of Cmpds.	m/z meas.	Δ m/z [ppm]	z	Rt [min]	Score	P	Range	Sequence	Modification
109	1	712.4379	47.78	3	30.1	12.9	2	82-102	K.DSSARPAGKGAIIGFIKVGYK.K	

Detailed Protein Report

Protein 253: calmodulin-regulated spectrin-associated protein 1 [Homo sapiens]

**Accession:**	gi|186659512	**Score:**	29.4
**Database:**	refseq_human(refseq_human_20140103.fasta)	**MW [kDa]:**	177.9
**Database Date:**	2015-11-30	**pI:**	6.3
		**Sequence Coverage [%]:**	1.9
		**No. of unique Peptides:**	1

10	20	30	40	50	60	70	80
MVDASGRAAA	EGWRKMEAPP	DGAADLVPLD	RYDAARAKIA	ANLQWICAKA	YGRDNIPEDL	RDPFYVDQYE	QEHIKPPVIK
90	100	110	120	130	140	150	160
LLLSSELYCR	VCSLILKGDQ	VAALQGHQSV	IQALSRKGIY	VMESDDTPVT	ESDLSRAPIK	MSAHMAMVDA	LMMAYTVEMI
170	180	190	200	210	220	230	240
SIEKVVASVK	RFSTFSASKE	LPYDLEDAMV	FWINKVNLKM	REITEKEVKL	KQQLLESPAH	QKVRYRREHL	SARQSPYFPL
250	260	270	280	290	300	310	320
LEDLMRDGSD	GAALLAVIHY	YCPEQMKLDD	ICLKEVTSMA	DSLYNIRLLR	EFSNEYLNKC	FYLTLEDMLY	APLVLKPNVM
330	340	350	360	370	380	390	400
VFIAELFWWF	ENVKPDFVQP	RDVQELKDAK	TVLHQKSSRP	PVPISNATKR	SFLGSPAAGT	LAELQPPVQL	PAEGCHRHYL
410	420	430	440	450	460	470	480
HPEEPEYLGK	GTAAFSPSHP	LLPLRQKQQK	SIQGEDIPDQ	RHRSNSLTRV	DGQPRGAAIA	WPEKKTRPAS	QPTPFALHHA
490	500	510	520	530	540	550	560
ASCEVDPSSG	DSISLARSIS	KDSLASNIVN	LTPQNQPHPT	ATKSHGKSLL	SNVSIEDEEE	ELVAIVRADV	VPQQADPEFP
570	580	590	600	610	620	630	640
RASPRALGLT	ANARSPQGQL	DTSESKPDSF	FLEPLMPAVL	KPAKEKQVIT	KEDERGEGRP	RSIVSRRPSE	GPQPLVRRKM
650	660	670	680	690	700	710	720
TGSRDLNRTF	TPIPCSEFPM	GIDPTETGPL	SVETAGEVCG	GPLALGGFDP	FPQGPSTDGF	FLHVGRADED	TEGRLYVSCS
730	740	750	760	770	780	790	800
KSPNSHDSEP	WTLLRQDSDS	DVVDIEEAEH	DFMGEAHPVV	FSRYIGEEES	AKLQEDMKVK	EHEDKDDASG	RSSPCLSTAS
810	820	830	840	850	860	870	880
QMSSVSMASG	SVKMTSFAER	KLQRLNSCET	KSSTSSSQKT	TPDASESCPA	PLTTWRQKRE	QSPSQHGKDP	ASLLASELVQ
890	900	910	920	930	940	950	960
LHMQLEEKRR	AIEAQKKKME	ALSARQRLKL	GKAAFLHVVK	KGKAEAAPPL	RPEHFAKEYS	QHNGEDCGDA	VSKTEDFLVK
970	980	990	1000	1010	1020	1030	1040
EEQREELLHE	PQDVDKESLA	FAQQHKAKDP	VALHELERNK	VISAALLEDT	VGEVVDVNEC	DLSIEKLNET	ISTLQQAILK
1050	1060	1070	1080	1090	1100	1110	1120
ISQQQEQLLM	KSPTVPVPGS	KNNSQDHKVK	APVHFVEPLS	PTGVAGHRKA	PRLGQGRNSR	SGRPAELKVP	KDRPQGSSRS
1130	1140	1150	1160	1170	1180	1190	1200
KTPTPSVETL	PHLRPFPASS	HPRTPTDPGL	DSALEPSGDP	HGKCLFDSYR	LHDESNQRTL	TLSSSKDANI	LSEQMSLKEV
1210	1220	1230	1240	1250	1260	1270	1280
LDASVKEVGS	SSSDVSGKES	VPVEEPLRSR	ASLIEVDLSD	LKAPDEDGEL	VSLDGSADLV	SEGDQKPGVG	FFFKDEQKAE
1290	1300	1310	1320	1330	1340	1350	1360
DELAKKRAAF	LLKQQRKAEE	ARVRKQQLEA	EVELKRDEAR	RKAEEDRVRK	EEEKARRELI	KQEYLRRKQQ	QILEEQGLGK
1370	1380	1390	1400	1410	1420	1430	1440
PKSKPKKPRP	KSVHREESCS	DSGTKCSSTP	DNLSRTQSGS	SLSLASAATT	EPESVHSGGT	PSQRVESMEA	LPILSRNPSR
1450	1460	1470	1480	1490	1500	1510	1520
STDRDWETAS	AASSLASVAE	YTGPKLFKEP	SSKSNKPIIH	NAISHCCLAG	KVNEPHKNSI	LEELEKCDAN	HYIILFRDAG
1530	1540	1550	1560	1570	1580	1590	1600
CQFRALYCYY	PDTEEIYKLT	GTGPKNITKK	MIDKLYKYSS	DRKQFNLIPA	KTMSVSVDAL	TIHNHLWQPK	RPAVPKKAQT
1610							
RK							

Cmpd.	No. of Cmpds.	m/z meas.	Δ m/z [ppm]	z	Rt [min]	Score	P	Range	Sequence	Modification
2677	1	862.3965	-106.85	2	64.6	15.1	1	1348-1362	R.KQQQILEEQGLGKPK.S	

Detailed Protein Report

Protein 254: PREDICTED: zinc finger protein 845 isoform X3 [Homo sapiens]

**Accession:**	gi|578835119	**Score:**	29.4
**Database:**	refseq_human(refseq_human_20140103.fasta)	**MW [kDa]:**	89.9
**Database Date:**	2015-11-30	**pI:**	10.5
**Modification(s):**	Carbamidomethyl, Oxidation	**Sequence Coverage [%]:**	5.2
		**No. of unique Peptides:**	1

10	20	30	40	50	60	70	80
MALSQGLLTF	RDVAIEFSQE	EWKCLDPAQR	TLYRDVMLEN	YRNLVSLDIS	SKCMMKEFSS	TAQGNTEVIH	TGTLQRHERH
**90**	**100**	**110**	**120**	**130**	**140**	**150**	**160**
HIGDFCFQEM	EKDIHDFEFQ	WKEDERNSHE	APMTEIKQLT	GSTNRHDQRH	AGNKPIKDQL	GSSFHSHLPE	LHMFQTEGKI
**170**	**180**	**190**	**200**	**210**	**220**	**230**	**240**
GNQVEKSINS	ASLVSTSQRI	SCRPKTHISK	NYGNNFLNSS	LLTQKQEVHM	REKSFQCNES	GKAFNYSSVL	RKHQIIHLGA
**250**	**260**	**270**	**280**	**290**	**300**	**310**	**320**
KQYKCDVCGK	VFNQKRYLAC	HRRCHTGKKP	YKCNDCGKTF	SQELTLTCHH	RLHTGEKHYK	CSECGKTFSR	NSALVIHKAI
**330**	**340**	**350**	**360**	**370**	**380**	**390**	**400**
HTGEKSYKCN	ECGKTFSQTS	YLVYHRRLHT	GEKPYKCEEC	DKAFSFKSNL	ERHRKIHTGE	KPYKCNECSR	TFSRKSSLTR
**410**	**420**	**430**	**440**	**450**	**460**	**470**	**480**
HRRLHTGEKP	YKCNDCGKTF	SQMSSLVYHR	RLHTGEKPYK	CEECDEAFSF	KSNLQRHRRI	HTGEKPYRCN	ECGKTFSRKS
**490**	**500**	**510**	**520**	**530**	**540**	**550**	**560**
YLTCHRRLHT	GEKPYKCNEC	GKTFGRNSAL	IIHKAIHTGE	KPYKCNECGK	AFSQKSSLTC	HLRLHTGEKP	YKCEECDKVF
**570**	**580**	**590**	**600**	**610**	**620**	**630**	**640**
SRKSSLEKHR	RIHTGEKPYK	CKVCDKAFGR	DSHLAQHTRI	HTGEKPYKCN	ECGKNFRHNS	ALVIHKAIHS	GEKPYKCNEC
**650**	**660**	**670**	**680**	**690**	**700**	**710**	**720**
GKTFRHNSAL	EIHKAIHTGE	KPYKCSECGK	VFNRKANLSR	HHRLHTGEKP	YKCNKCGKVF	NQQAHLACHH	RIHTGEKPYK
**730**	**740**	**750**	**760**	**770**	**780**		
CNECGKTFRH	NSVLVIHKTI	HTGEKPYKCN	ECGKVFNRKA	KLARHHRIHT	GKKH		

Cmpd.	No. of Cmpds.	m/z meas.	Δ m/z [ppm]	z	Rt [min]	Score	P	Range	Sequence	Modification
2753	1	920.1206	31.91	3	65.6	17.8	1	53-76	K.CMMKEFSSTAQGNTEVIHTGTLQR.H	Carbamidomethyl: 1; Oxidation: 2, 3

Detailed Protein Report

Protein 255: **PREDICTED: nuclear factor 1 X-type isoform X4 [Homo sapiens]**

**Accession:**	gi|530414877	**Score:**	29.4
**Database:**	refseq_human(refseq_human_20140103.fasta)	**MW [kDa]:**	54.1
**Database Date:**	2015-11-30	**pI:**	9.7
**Modification(s):**	Oxidation	**Sequence Coverage [%]:**	6.7
		**No. of unique Peptides:**	2

10	20	30	40	50	60	70	80
MDEFHPFIEA	LLPHVRAFSY	TWFNLQARKR	KYFKKHEKRM	SKDEERAVKD	ELLGEKPEIK	QKWASRLLAK	LRKDIRPEFR
**90**	**100**	**110**	**120**	**130**	**140**	**150**	**160**
EDFVLTITGK	KPPCCVLSNP	DQKGKIRRID	CLRQADKVWR	LDLVMVILFK	GIPLESTDGE	RLYKSPQCSN	PGLCVQPHHI
**170**	**180**	**190**	**200**	**210**	**220**	**230**	**240**
GVTIKELDLY	LAYFVHTPES	GQSDSSNQQG	DADIKPLPNG	HLSFQDCFVT	SGVWNVTELV	RVSQTPVATA	SGPNFSLADL
**250**	**260**	**270**	**280**	**290**	**300**	**310**	**320**
ESPSYYNINQ	VTLGRRSITS	PPSTSTTKRP	KSIDDSEMES	PVDDVFYPGT	GRSPAAGSSQ	SSGWPNDVDA	GPASLKKSGK
**330**	**340**	**350**	**360**	**370**	**380**	**390**	**400**
LDFCSALSSQ	GSSPRMAFTH	HPLPVLAGVR	PGSPRATASA	LHFPSTSIIQ	QSSPYFTHPT	IRYHHHHGQD	SLKEFVQFVC
**410**	**420**	**430**	**440**	**450**	**460**	**470**	**480**
SDGSGQATGQ	PNGSGQGKVP	GSFLLPPPPP	VARPVPLPMP	DSKSTSTAPD	GAALTPPSPS	FATTGASSAN	RFVSIGPRDG
**490**	**500**						
NFLNIPQQSQ	SWFL						

Cmpd.	No. of Cmpds.	m/z meas.	Δ m/z [ppm]	z	Rt [min]	Score	P	Range	Sequence	Modification
1084	2	530.7982	-0.72	2	43.7	16.4	1	73-80	R.KDIRPEFR.E	
392	1	874.3959	-106.40	3	34.9	13.0	0	419-443	K.VPGSFLLPPPPPVARPVPLPMPDSK.S	Oxidation: 21

Detailed Protein Report

Protein 256: collagen alpha-1(XXVIII) chain precursor [Homo sapiens]

**Accession:**	gi|154759255	**Score:**	29.4
**Database:**	refseq_human(refseq_human_20140103.fasta)	**MW [kDa]:**	116.6
**Database Date:**	2015-11-30	**pI:**	6.1
		**Sequence Coverage [%]:**	3.4
		**No. of unique Peptides:**	1

10	20	30	40	50	60	70	80
MWNRYFVFYL	LLLSAFTSQT	VSGQRKKGPK	SNLLARKSDV	QGSICFIDIV	FIVDSSESSK	IALFDKQKDF	VDSLSDKIFQ
90	100	110	120	130	140	150	160
LTPGRSLEYD	IKLAALQFSS	SVQIDPPFSS	WKDLQTFKQK	VKSMNLIGQG	TFSYYAISNA	TRLLKREGRK	DGVKVVLLMT
170	180	190	200	210	220	230	240
DGIDHPKNPD	VQSISEDARI	SGISFITIAL	STVVNEAKLR	LISGDSSSEP	TLLLSDPTLV	DKIQDRLDIL	FEKKCERKIC
250	260	270	280	290	300	310	320
ECEKGDPGDP	GPPGTHGNPG	IKGERGPKGN	PGNAQKGEAG	ERGPGGIPGY	KGDKGERGEC	GKPGIKGDKG	SPGPYGPKGP
330	340	350	360	370	380	390	400
RGIQGITGPP	GDPGPKGFQG	NKGEPGPPGP	YGSPGAPGIG	QQGIKGERGQ	EGRPGAPGPI	GVGEPGQPGP	RGPEGVPGER
410	420	430	440	450	460	470	480
GLPGEGFPGP	KGEKGSEGPT	GPQGLQGLSI	KGEKGDIGPV	GPQGPMGIPG	IGSQGEQGIQ	GPIGPPGPQG	PAGQGLPGSK
490	500	510	520	530	540	550	560
GEVGQMGPTG	PRGPVGIGVQ	GPKGEPGSIG	LPGQPGVPGE	DGAAGKKGEA	GLPGARGPEG	PPGKGQPGPK	GDEGKKGSKG
570	580	590	600	610	620	630	640
NQGQRGLPGP	EGPKGEPGIM	GPFGMPGTSI	PGPPGPKGDR	GGPGIPGFKG	EPGLSIRGPK	GVQGPRGPVG	APGLKGDGYP
650	660	670	680	690	700	710	720
GVPGPRGLPG	PPGPMGLRGV	GDTGAKGEPG	VRGPPGPSGP	RGVGTQGPKG	DTGQKGLPGP	PGPPGYGSQG	IKGEQGPQGF
730	740	750	760	770	780	790	800
PGPKGTMGHG	LPGQKGEHGE	RGDVGKKGDK	GEIGEPGSPG	KQGLQGPKGD	LGLTKEEIIK	LITEICGCGP	KCKETPLELV
810	820	830	840	850	860	870	880
FVIDSSESVG	PENFQIIKNF	VKTMADRVAL	DLATARIGII	NYSHKVEKVA	NLKQFSSKDD	FKLAVDNMQY	LGEGTYTATA
890	900	910	920	930	940	950	960
LQAANDMFED	ARPGVKKVAL	VITDGQTDSR	DKEKLTEVVK	NASDTNVEIF	VIGVVKKNDP	NFEIFHKEMN	LIATDPEHVY
970	980	990	1000	1010	1020	1030	1040
QFDDFFTLQD	TLKQKLFQKI	CEDFDSYLVQ	IFGSSSPQPG	FGMSGEELSE	STPEPQKEIS	ESLSVTRDQD	EDDKAPEPTW
1050	1060	1070	1080	1090	1100	1110	1120
ADDLPATTSS	EATTTPRPLL	STPVDGAEDP	RCLEALKPGN	CGEYVVRWYY	DKQVNSCARF	WFSGCNGSGN	RFNSEKECQE
1130							
TCIQG							

Cmpd.	No. of Cmpds.	m/z meas.	Δ m/z [ppm]	z	Rt [min]	Score	P	Range	Sequence	Modification
201	1	714.3696	-12.54	3	32.5	18.4	2	667-689	K.GEPGVRGPPGPSGPRGVGTQGPK.G	

Detailed Protein Report

Protein 257: **PREDICTED: nucleobindin-2 isoform X9 [Homo sapiens]**

**Accession:**	gi|578820568	**Score:**	29.3
**Database:**	refseq_human(refseq_human_20140103.fasta)	**MW [kDa]:**	40.3
**Database Date:**	2015-11-30	**pI:**	4.9
		**Sequence Coverage [%]:**	3.8
		**No. of unique Peptides:**	2

10	20	30	40	50	60	70	80
MRWRTILLQY	CFLLITCLLT	ALEAVPIDID	KTKVQNIHPV	ESAKIEPPDT	GLYYDEYLKQ	VIDVLETDKH	FREKLQKADI
**90**	**100**	**110**	**120**	**130**	**140**	**150**	**160**
EEIKSGRLSK	ELDLVSHHVR	TKLDELKRQE	VGRLRMLIKA	KLDSLQDIGM	DHQALLKQFD	HLNHLNPDKF	ESTDLDMLIK
**170**	**180**	**190**	**200**	**210**	**220**	**230**	**240**
AATSDLEHYD	KTRHEEFKKY	EMMKEHERRE	YLKTLNEEKR	KEEESKFEEM	KKKHENHPKV	NHPGSKDQLK	EVWEETDGLD
**250**	**260**	**270**	**280**	**290**	**300**	**310**	**320**
PNDFDPKTFF	KLHDVNSDGF	LDEQELEALF	TKELEKVYDP	KNEEDDMVEM	EEERLRMREH	VMNEVDTNKD	RLVTLEEFLK
**330**	**340**						
ATEKKEFLEP	DSWESPSI						

Cmpd.	No. of Cmpds.	m/z meas.	Δ m/z [ppm]	z	Rt [min]	Score	P	Range	Sequence	Modification
585	1	537.7782	-18.47	2	37.3	11.4	2	172-179	K.TRHEEFKK.Y	
1116	1	701.1743	-178.79	1	44.1	17.9	0	180-184	K.YEMMK.E	

BRUKER

Detailed Protein Report

Protein 258: PREDICTED: phosphatidylinositol 4-phosphate 3-kinase C2 domain-containing subunit beta isoform X2 [Homo sapiens]

**Accession:**	gi|530364909	**Score:**	29.3
**Database:**	refseq_human(refseq_human_20140103.fasta)	**MW [kDa]:**	181.7
**Database Date:**	2015-11-30	**pI:**	7.2
**Modification(s):**	Carbamidomethyl	**Sequence Coverage [%]:**	2.1
		**No. of unique Peptides:**	1

10	20	30	40	50	60	70	80
MSSTQGNGEH	WKSLESVGIS	RKELAMAEAL	QMEYDALSRL	RHDKEENRAK	QNADPSLISW	DEPGVDFYSK	PAGRRTDLKL
90	100	110	120	130	140	150	160
LRGLSGSDPT	LNYNSLSPQE	GPPNHSTSQG	PQPGSDPWPK	GSLSGDYLYI	FDGSDGGVSS	SPGPGDIEGS	CKKLSPPPLP
170	180	190	200	210	220	230	240
PRASIWDTPP	LPPRKGSPSS	SKISQPSDIN	TFSLVEQLPG	KLLEHRILEE	EEVLGGGGQG	RLLGSVDYDG	INDAITRLNL
250	260	270	280	290	300	310	320
KSTYDAEMLR	DATRGWKEGR	GPLDFSKDTS	GKPVARSKTM	PPQVPPRTYA	SRYGNRKNAT	PGKNRRISAA	PVGSRPHTVA
330	340	350	360	370	380	390	400
NGHELFEVSE	ERDEEVAAFC	HMLDILRSGS	DIQDYFLTGY	VWSAVTPSPE	HLGDEVNLKV	TVLCDRLQEA	LTFTCNCSST
410	420	430	440	450	460	470	480
VDLLIYQTLC	YTHDDLRNVD	VGDFVLKPCG	LEEFLQNKHA	LGSHEYIQYC	RKFDIDIRLQ	LMEQKVVRSD	LARTVNDDQS
490	500	510	520	530	540	550	560
PSTLNYLVHL	QERPVKQTIS	RQALSLLFDT	YHNEVDAFLL	ADGDFPLKAD	RVVQSVKAIC	NALAAVETPE	ITSALNQLPP
570	580	590	600	610	620	630	640
CPSRMQPKIQ	KDPSVLAVRE	NREKVVEALT	AAILDLVELY	CNTFNADFQT	AVPGSRKHDL	VQEACHFARS	LAFTVYATHR
650	660	670	680	690	700	710	720
IPIIWATSYE	DFYLSCSLSH	GGKELCSPLQ	TRRAHFSKYL	FHLIVWDQQI	CFPVQVNRLP	RETLLCATLY	ALPIPPPGSS
730	740	750	760	770	780	790	800
SEANKQRRVP	EALGWVTTPL	FNFRQVLTCG	RKLLGLWPAT	QENPSARWSA	PNFHQPDSVI	LQIDFPTSAF	DIKFTSPPGD
810	820	830	840	850	860	870	880
KFSPRYEFGS	LREEDQRKLK	DIMQKESLYW	LTDADKKRLW	EKRYYCHSEQ	WTHMNHQDAL	GLLHATFPDQ	EVRRMAVQWI
890	900	910	920	930	940	950	960
GSLSDAELLD	YLPQLVQALK	YECYLDSPLV	RFLLKRAVSD	LRVTHYFFWL	LKDGLKDSQF	SIRYQYLLAA	LLCCCGKGLR
970	980	990	1000	1010	1020	1030	1040
EEFNRQCWLV	NALAKLAQQV	REAAPSARQG	ILRTGLEEVK	QFFALNGSCR	LPLSPSLLVK	GIVPRDCSYF	NSNAVPLKLS
1050	1060	1070	1080	1090	1100	1110	1120
FQNVDPLGEN	IRVIFKCGDD	LRQDMLTLQM	IRIMSKIWVQ	EGLDMRMVIF	RCFSTGRGRG	MVEMIPNAET	LRKIQVEHGV
1130	1140	1150	1160	1170	1180	1190	1200
TGSFKDRPLA	DWLQKHNPGE	DEYEKAVENF	IYSCAGCCVA	TYVLGICDRH	NDNIMLKTTG	HMFHIDFGRF	LGHAQMFGNI
1210	1220	1230	1240	1250	1260	1270	1280
KRDRAPFVFT	SDMAYVINGG	DKPSSRFHDF	VDLCCQAYNL	IRKHTHLFLN	LLGLMLSCGI	PELSDLEDLK	YVYDALRPQD
1290	1300	1310	1320	1330	1340	1350	1360
TEANATTYFT	RLIESSLGSV	ATKLNFFIHN	LAQMKFTGSD	DRLTLSFASR	THTLKSSGRI	SDVFLCRHEK	IFHPNKGYIY
1370	1380	1390	1400	1410	1420	1430	1440
VVKVMRENTH	EATYIQRTFE	EFQELHNKLR	LLFPSSHLPS	FPSRFVIGRS	RGEAVAERRR	EELNGYIWHL	IHAPPEVAEC
1450	1460	1470	1480	1490	1500	1510	1520
DLVYTFFHPL	PRDEKAMGTS	PAPKSSDGTW	ARPVGKVGGE	VKLSISYKNN	KLFIMVMHIR	GLQLLQDGND	PDPYVKIYLL
1530	1540	1550	1560	1570	1580	1590	1600
PDPQKTTKRK	TKVARKTCNP	TYNEMLVYDG	IPKGDLQQRE	LQLSVLSEQG	FWENVLLGEV	NIRLRELDLA	QEKTGWFALG
1610							
SRSHGTL							

Cmpd.	No. of Cmpds.	m/z meas.	Δ m/z [ppm]	z	Rt [min]	Score	P	Range	Sequence	Modification
2067	1	714.5942	-120.32	3	56.2	13.5	1	1227-1243	R.FHDFVDLCCQAYNLIRK.H	Carbamidomethyl: 8

Detailed Protein Report

Protein 259: phosphatidylinositol 4-phosphate 5-kinase type-1 alpha isoform 4 [Homo sapiens]

**Accession:**	gi|208431776	**Score:**	29.3
**Database:**	refseq_human(refseq_human_20140103.fasta)	**MW [kDa]:**	56.0
**Database Date:**	2015-11-30	**pI:**	7.8
		**Sequence Coverage [%]:**	5.2
		**No. of unique Peptides:**	2

Quantitation

QD:QU **Median:** 1.88 **CV:** 0.00 % **No. of Peptides:** 1

10	20	30	40	50	60	70	80
MASASSGPSS	SVGFSSFDPA	VPSCTLSSAS	GIKRPMASEV	PYASGMPIKK	IGHRSVDSSG	ETTYKKTTSS	ALKGAIQLGI
**90**	**100**	**110**	**120**	**130**	**140**	**150**	**160**
THTVGSLSTK	PERDVLMQDF	YVVESIFFPS	EGSNLTPAHH	YNDFRFKTYA	PVAFRYFREL	FGIRPDDYLY	SLCSEPLIEL
**170**	**180**	**190**	**200**	**210**	**220**	**230**	**240**
CSSGASGSLF	YVSSDDEFII	KTVQHKEAEF	LQKLLPGYYM	NLNQNPRTLL	PKFYGLYCVQ	AGGKNIRIVV	MNNLLPRSVK
**250**	**260**	**270**	**280**	**290**	**300**	**310**	**320**
MHIKYDLKGS	TYKRRASQKE	REKPLPTFKD	LDFLQDIPDG	LFLDADMYNA	LCKTLQRDCL	VLQSFKIMDY	SLLMSIHNID
**330**	**340**	**350**	**360**	**370**	**380**	**390**	**400**
HAQREPLSSE	TQYSVDTRRP	APQKALYSTA	MESIQGEARR	GGTMETDDHM	GGIPARNSKG	ERLLLYIGII	DILQSYRFVK
**410**	**420**	**430**	**440**	**450**	**460**	**470**	**480**
KLEHSWKALV	HDGDTVSVHR	PGFYAERFQR	FMCNTVFKKI	PCVHLGRPDV	LPQTPPLEEI	SEGSPIPDPS	FSPLVGETLQ
**490**	**500**	**510**					
MLTTSTTLEK	LEVAESEFTH						

Cmpd.	No. of Cmpds.	m/z meas.	Δ m/z [ppm]	z	Rt [min]	Score	P	Range	Sequence	Modification	Ratios
2199	1	933.6289	74.91	2	56.1	14.3	2	225-240	K.NIRIVVMNNLLPRSVK.M		
989	3	581.1819	-159.70	2	42.5	14.9	0	491-500	K.LEVAESEFTH.-		QD:QU 1.88

Detailed Protein Report

Protein 260: leukocyte receptor cluster member 9 [Homo sapiens]

**Accession:**	gi|206725452	**Score:**	29.2
**Database:**	refseq_human(refseq_human_20140103.fasta)	**MW [kDa]:**	53.1
**Database Date:**	2015-11-30	**pI:**	9.5
		**Sequence Coverage [%]:**	8.2
		**No. of unique Peptides:**	2

```
        10         20         30         40         50         60         70         80
MGSRPPCGAT SSARRACQFP APMAAAREPE LPQEAPATEP APPPACRFFL EGRCRFGARC RQPHPGAPAP PGREAQPEAG
        90        100        110        120        130        140        150        160
AKKPPLRTAA DVIQRIRWDP RLDPADFSVG YVDRFLGVRE EPFSAFCWDQ PLAALGPGVL AVPQHRVRFF RFHGRLVWDR
       170        180        190        200        210        220        230        240
ASRTDLVFGS GSAAGRGPTI LDAPNTEGAH GAEGAEWTLA GTGQEAQAAP KRGSTRPLCT GHQEPGVEEP GELEAAQERA
       250        260        270        280        290        300        310        320
LGTAADLGTL APRGRLAGVT EEALKPTAAT RTTLLGGKEA QALGVPGGSA ETTEAEWGPA AWPEDKRARL SVAAPCQPRP
       330        340        350        360        370        380        390        400
THFVALMVTE PGLQAEVTKA QEYLVHVAPH CANFLVPSQN LHLTLALLRL AGAGEEAAAI GALRRALLAP GLNAPPRLSF
       410        420        430        440        450        460        470        480
RKLVLLGPHV LCAPPSPTLE SMAQVLSQRL EAEGLSTLQS PGQLHPHLTV AKVPHGSQVH LPKLEFTLSQ EVGCQPLQTL
       490        500        510
WLCRIGRTGG PFQPLAEIRL E
```

Cmpd.	No. of Cmpds.	m/z meas.	Δ m/z [ppm]	z	Rt [min]	Score	P	Range	Sequence	Modification
1675	1	1011.7711	-47.99	3	51.2	10.4	1	212-239	K.RGSTRPLCTGHQEPGVEEPGELEAAQER.A	
2744	6	673.3579	-72.57	2	65.4	18.8	1	385-397	R.RALLAPGLNAPPR.L	

Detailed Protein Report

Protein 261: putative uncharacterized zinc finger protein 814 [Homo sapiens]

Accession: gi|222418585
Database: refseq_human(refseq_human_20140103.fasta)
Database Date: 2015-11-30
Modification(s): Carbamidomethyl

Score: 29.1
MW [kDa]: 97.3
pI: 10.2
Sequence Coverage [%]: 3.3
No. of unique Peptides: 2

Quantitation

QD:QU **Median:** 0.60 **CV:** 0.00 % **No. of Peptides:** 1

10	20	30	40	50	60	70	80
MAAAATLRLS	AQGTVTFEDV	AVNFTWEEWN	LLSEAQRCLY	RDVTLENLAL	ISSLGCWCGV	EDEAAPSKQS	IYIQRETQVR
**90**	**100**	**110**	**120**	**130**	**140**	**150**	**160**
TPMAGVSPKK	AHPCEMCGPI	LGDILHVADH	QGTHHKQKLH	RCEAWGNKLY	DSGNFHQHQN	EHIGEKPYRG	SVEEALFAKR
**170**	**180**	**190**	**200**	**210**	**220**	**230**	**240**
CKLHVSGESS	VFSESGKDFL	PRSGLLQQEA	SHTGEKSNSK	TECVSPIQCG	GAHYSCGESM	KHFSTKHILS	QHQRLLTREE
**250**	**260**	**270**	**280**	**290**	**300**	**310**	**320**
CYVCCECGKS	FSKYASLSNH	QRVHTEKKHE	CGECGKSFSK	YVSFSNHQRV	HTEKKHECGE	CGKSFSKYVS	FSNHQRVHTG
**330**	**340**	**350**	**360**	**370**	**380**	**390**	**400**
KRPYECGECG	KSFSKYASFS	NHQRVHTEKK	HYECGECGKS	FSKYVSFSNH	QRVHTGKRPY	ECGECGKSFS	KYASFSNHQR
**410**	**420**	**430**	**440**	**450**	**460**	**470**	**480**
VHTDKKHYEC	GECGKSFSQK	SSLIQHQRFH	TGEKPYGCEE	CGKSFSSEGH	LRSHQRVHAG	ERPFKCGECV	KSFSHKRSLV
**490**	**500**	**510**	**520**	**530**	**540**	**550**	**560**
HHQRVHSGER	PYQCGECGKS	FSQKGNLVLH	QRVHTGARPY	ECGECGKSFS	SKGHLRNHQQ	IHTGDRLYEC	GECGKSFSHK
**570**	**580**	**590**	**600**	**610**	**620**	**630**	**640**
GTLILHQRVH	PRERSYGCGE	CGKSFSSIGH	LRSHQRVHTG	ERPYECGECG	KSFSHKRSLV	HHQRMHTGER	PYKCGDCGKS
**650**	**660**	**670**	**680**	**690**	**700**	**710**	**720**
FNEKGHLRNH	QRVHTTERPF	KCGECGKCFS	HKGNLILHQH	GHTGERPYVC	RECGKLFKKK	SHLLVHQRIH	NGEKPYACEA
**730**	**740**	**750**	**760**	**770**	**780**	**790**	**800**
CQKFFRNKYQ	LIAHQRVHTG	ERPYECNDCG	KSFTHSSTFC	VHKRIHTGEK	PYECSECGKS	FAESSSFTKH	KRVHTGEKPY
**810**	**820**	**830**	**840**	**850**	**860**		
ECSECGKSFA	ESSSLTKHKR	VHTGEKPYKC	EKCGKLFNKK	SHLLVHQSSH	WRKAI		

Cmpd.	No. of Cmpds.	m/z meas.	Δ m/z [ppm]	z	Rt [min]	Score	P	Range	Sequence	Modification	Ratios
2009	1	766.2777	-53.66	2	55.5	17.9	1	351-363	K.HYECGECGKSFSK.Y	Carbamidomethyl: 7	
417	1	564.5160	-156.98	3	35.2	11.2	1	653-667	R.VHTTERPFKCGECGK.C		QD:QU 0.60

Detailed Protein Report

Protein 262: PREDICTED: uncharacterized protein C17orf85 isoform X2 [Homo sapiens]

**Accession:**	gi|578829984	**Score:**	29.1
**Database:**	refseq_human(refseq_human_20140103.fasta)	**MW [kDa]:**	57.0
**Database Date:**	2015-11-30	**pI:**	5.6
		**Sequence Coverage [%]:**	4.8
		**No. of unique Peptides:**	1

10	20	30	40	50	60	70	80
MSTQDVFSYF	KEYPPAHIEW	LDDTSCNVVW	LDEMTATRAL	INMSSLPAQD	KIRSRDASED	KSAEKRKKDK	QEDSSDDDEA
**90**	**100**	**110**	**120**	**130**	**140**	**150**	**160**
EEGEVEDENS	SDVELDTLSQ	VEEESLLRND	LRPANKLAKG	NRLFMRFATK	DDKKELGAAR	RSQYYMKYGN	PNYGGMKGIL
**170**	**180**	**190**	**200**	**210**	**220**	**230**	**240**
SNSWKRRYHS	RRIQRDVIKK	RALIGDDVGL	TSYKHRHSGL	VNVPEEPIEE	EEEEEEEEEE	EEEEDQDMDA	DDRVVVEYHE
**250**	**260**	**270**	**280**	**290**	**300**	**310**	**320**
ELPALKQPRE	RSASRRSSAS	SSDSDEMDYD	LELKMISTPS	PKKSMKMTMY	ADEVESQLKN	IRNSMRADSV	SSSNIKNRIG
**330**	**340**	**350**	**360**	**370**	**380**	**390**	**400**
NKLPPEKFAD	VRHLLDEKRQ	HSRPRPPVSS	TKSDIRQRLG	KRPHSPEKAF	SSNPVVRREP	SSDVHSRLGV	PRQDSKGLYA
**410**	**420**	**430**	**440**	**450**	**460**	**470**	**480**
DTREKKSGNL	WTRLGSAPKT	KEKNTKKVDH	RAPGAEEDDS	ELQRAWGALI	KEKEQSRQKK	SRCYQHPFPK	KSQFPGAYWT
**490**	**500**						
SFEGEEEGSG	QLTLPGP						

Cmpd.	No. of Cmpds.	m/z meas.	Δ m/z [ppm]	z	Rt [min]	Score	P	Range	Sequence	Modification
2116	1	945.7998	-143.91	2	56.8	14.7	1	284-299	K.SMKMTMYADEVESQLK.N	

Detailed Protein Report

Protein 263: plakophilin-1 isoform 1a [Homo sapiens]

**Accession:**	gi|53729344	**Score:**	29.1
**Database:**	refseq_human(refseq_human_20140103.fasta)	**MW [kDa]:**	80.4
**Database Date:**	2015-11-30	**pI:**	10.0
**Modification(s):**	Carbamidomethyl	**Sequence Coverage [%]:**	3.3
		**No. of unique Peptides:**	1

10	20	30	40	50	60	70	80
MNHSPLKTAL	AYECFQDQDN	STLALPSDQK	MKTGTSGRQR	VQEQVMMTVK	RQKSKSSQSS	TLSHSNRGSM	YDGLADNYNY
**90**	**100**	**110**	**120**	**130**	**140**	**150**	**160**
GTTSRSSYYS	KFQAGNGSWG	YPIYNGTLKR	EPDNRRFSSY	SQMENWSRHY	PRGSCNTTGA	GSDICFMQKI	KASRSEPDLY
**170**	**180**	**190**	**200**	**210**	**220**	**230**	**240**
CDPRGTLRKG	TLGSKGQKTT	QNRYSFYSTC	SGQKAIKKCP	VRPPSCASKQ	DPVYIPPISC	NKDLSFGHSR	ASSKICSEDI
**250**	**260**	**270**	**280**	**290**	**300**	**310**	**320**
ECSGLTIPKA	VQYLSSQDEK	YQAIGAYYIQ	HTCFQDESAK	QQVYQLGGIC	KLVDLLRSPN	QNVQQAAAGA	LRNLVFRSTT
**330**	**340**	**350**	**360**	**370**	**380**	**390**	**400**
NKLETRRQNG	IREAVSLLRR	TGNAEIQKQL	TGLLWNLSST	DELKEELIAD	ALPVLADRVI	IPFSGWCDGN	SNMSREVVDP
**410**	**420**	**430**	**440**	**450**	**460**	**470**	**480**
EVFFNATGCL	RNLSSADAGR	QTMRNYSGLI	DSLMAYVQNC	VAASRCDDKS	VENCMCVLHN	LSYRLDAEVP	TRYRQLEYNA
**490**	**500**	**510**	**520**	**530**	**540**	**550**	**560**
RNAYTEKSST	GCFSNKSDKM	MNNNYDCPLP	EEETNPKGSG	WLYHSDAIRT	YLNLMGKSKK	DATLEACAGA	LQNLTASKGL
**570**	**580**	**590**	**600**	**610**	**620**	**630**	**640**
MSSGMSQLIG	LKEKGLPQIA	RLLQSGNSDV	VRSGASLLSN	MSRHPLLHRV	MGNQVFPEVT	RLLTSHTGNT	SNSEDILSSA
**650**	**660**	**670**	**680**	**690**	**700**	**710**	**720**
CYTVRNLMAS	QPQLAKQYFS	SSMLNNIINL	CRSSASPKAA	EAARLLLSDM	WSSKELQGVL	RQQGFDRNML	GTLAGANSLR
**730**							
NFTSRF							

Cmpd.	No. of Cmpds.	m/z meas.	Δ m/z [ppm]	z	Rt [min]	Score	P	Range	Sequence	Modification
1592	1	783.4244	89.54	2	50.1	14.5	1	152-164	K.ASRSEPDLYCDPR.G	Carbamidomethyl: 10

Detailed Protein Report

Protein 264: myosin-10 isoform 2 [Homo sapiens]

**Accession:**	gi|367460087	**Score:**	29.1
**Database:**	refseq_human(refseq_human_20140103.fasta)	**MW [kDa]:**	228.9
**Database Date:**	2015-11-30	**pI:**	5.3
		**Sequence Coverage [%]:**	1.5
		**No. of unique Peptides:**	1

Detailed Protein Report

10	20	30	40	50	60	70	80
MAQRTGLEDP	ERYLFVDRAV	IYNPATQADW	TAKKLVWIPS	ERHGFEAASI	KEERGDEVMV	ELAENGKKAM	VNKDDIQKMN
90	100	110	120	130	140	150	160
PPKFSKVEDM	AELTCLNEAS	VLHNLKDRYY	SGLIYTYSGL	FCVVINPYKN	LPIYSENIIE	MYRGKKRHEM	PPHIYAISES
170	180	190	200	210	220	230	240
AYRCMLQDRE	DQSILCTGES	GAGKTENTKK	VIQYLAHVAS	SHKGRKDHNI	PGELERQLLQ	ANPILESFGN	AKTVKNDNSS
250	260	270	280	290	300	310	320
RFGKFIRINF	DVTGYIVGAN	IETYLLEKSR	AVRQAKDERT	FHIFYQLLSG	AGEHLKSDLL	LEGFNNYRFL	SNGYIPIPGQ
330	340	350	360	370	380	390	400
QDKDNFQETM	EAMHIMGFSH	EEILSMLKVV	SSVLQFGNIS	FKKERNTDQA	SMPENTVAQK	LCHLLGMNVM	EFTRAILTPR
410	420	430	440	450	460	470	480
IKVGRDYVQK	AQTKEQADFA	VEALAKATYE	RLFRWLVHRI	NKALDRTKRQ	GASFIGILDI	AGFEIFELNS	FEQLCINYTN
490	500	510	520	530	540	550	560
EKLQQLFNHT	MFILEQEEYQ	REGIEWNFID	FGLDLQPCID	LIERPANPPG	VLALLDEECW	FPKATDKTFV	EKLVQEQGSH
570	580	590	600	610	620	630	640
SKFQKPRQLK	DKADFCIIHY	AGKVDYKADE	WLMKNMDPLN	DNVATLLHQS	SDRFVAELWK	DVDRIVGLDQ	VTGMTETAFG
650	660	670	680	690	700	710	720
SAYKTKKGMF	RTVGQLYKES	LTKLMATLRN	TNPNFVRCII	PNHEKRAGKL	DPHLVLDQLR	CNGVLEGIRI	CRQGFPNRIV
730	740	750	760	770	780	790	800
FQEFRQRYEI	LTPNAIPKGF	MDGKQACERM	IRALELDPNL	YRIGQSKIFF	RAGVLAHLEE	ERDLKITDII	IFFQAVCRGY
810	820	830	840	850	860	870	880
LARKAFAKKQ	QQLSALKVLQ	RNCAAYLKLR	HWQWWRVFTK	VKPLLQVTRQ	EEELQAKDEE	LLKVKEKQTK	VEGELEEMER
890	900	910	920	930	940	950	960
KHQQLLEEKN	ILAEQLQAET	ELFAEAEEMR	ARLAAKKQEL	EEILHDLESR	VEEEEERNQI	LQNEKKKMQA	HIQDLEEQLD
970	980	990	1000	1010	1020	1030	1040
EEEGARQKLQ	LEKVTAEAKI	KKMEEEILLL	EDQNSKFIKE	KKLMEDRIAE	CSSQLAEEEE	KAKNLAKIRN	KQEVMISDLE
1050	1060	1070	1080	1090	1100	1110	1120
ERLKKEEKTR	QELEKAKRKL	DGETTDLQDQ	IAELQAQIDE	LKLQLAKKEE	ELQGALARGD	DETLHKNNAL	KVVRELQAQI
1130	1140	1150	1160	1170	1180	1190	1200
AELQEDFESE	KASRNKAEKQ	KRDLSEELEA	LKTELEDTLD	TTAAQQELRT	KREQEVAELK	KALEEETKNH	EAQIQDMRQR
1210	1220	1230	1240	1250	1260	1270	1280
HATALEELSE	QLEQAKRFKA	NLEKNKQGLE	TDNKELACEV	KVLQQVKAES	EHKRKKLDAQ	VQELHAKVSE	GDRLRVELAE
1290	1300	1310	1320	1330	1340	1350	1360
KASKLQNELD	NVSTLLEEAE	KKGIKFAKDA	ASLESQLQDT	QELLQEETRQ	KLNLSSRIRQ	LEEEKNSLQE	QQEEEEEARK
1370	1380	1390	1400	1410	1420	1430	1440
NLEKQVLALQ	SQLADTKKKV	DDDLGTIESL	EEAKKKLLKD	AEALSQRLEE	KALAYDKLEK	TKNRLQQELD	DLTVDLDHQR
1450	1460	1470	1480	1490	1500	1510	1520
QVASNLEKKQ	KKFDQLLAEE	KSISARYAEE	RDRAEAEARE	KETKALSLAR	ALEEALEAKE	EFERQNKQLR	ADMEDLMSSK
1530	1540	1550	1560	1570	1580	1590	1600
DDVGKNVHEL	EKSKRALEQQ	VEEMRTQLEE	LEDELQATED	AKLRLEVNMQ	AMKAQFERDL	QTRDEQNEEK	KRLLIKQVRE
1610	1620	1630	1640	1650	1660	1670	1680
LEAELEDERK	QRALAVASKK	KMEIDLKDLE	AQIEAANKAR	DEVIKQLRKL	QAQMKDYQRE	LEEARASRDE	IFAQSKESEK
1690	1700	1710	1720	1730	1740	1750	1760
KLKSLEAEIL	QLQEELASSE	RARRHAEQER	DELADEITNS	ASGKSALLDE	KRRLEARIAQ	LEEELEEEQS	NMELLNDRFR
1770	1780	1790	1800	1810	1820	1830	1840
KTTLQVDTLN	AELAAERSAA	QKSDNARQQL	ERQNKELKAK	LQELEGAVKS	KFKATISALE	AKIGQLEEQL	EQEAKERAAA
1850	1860	1870	1880	1890	1900	1910	1920
NKLVRRTEKK	LKEIFMQVED	ERRHADQYKE	QMEKANARMK	QLKRQLEEAE	EEATRANASR	RKLQRELDDA	TEANEGLSRE
1930	1940	1950	1960	1970	1980		
VSTLKNRLRR	GGPISFSSSR	SGRRQLHLEG	ASLELSDDDT	ESKTSDVNET	QPPQSE		

Cmpd.	No. of Cmpds.	m/z meas.	Δ m/z [ppm]	z	Rt [min]	Score	P	Range	Sequence	Modification
2562	1	648.3620	94.94	2	62.7	10.9	0	1853-1862	K.EIFMQVEDER.R	

BRUKER

Detailed Protein Report

Protein 265: hemicentin-1 precursor [Homo sapiens]

**Accession:**	gi|118572606	**Score:**	29.1
**Database:**	refseq_human(refseq_human_20140103.fasta)	**MW [kDa]:**	613.0
**Database Date:**	2015-11-30	**pI:**	6.1
**Modification(s):**	Carbamidomethyl	**Sequence Coverage [%]:**	0.7
		**No. of unique Peptides:**	1

Detailed Protein Report

10	20	30	40	50	60	70	80
MISWEVVHTV	FLFALLYSSL	AQDASPQSEI	RAEEIPEGAS	TLAFVFDVTG	SMYDDLVQVI	EGASKILETS	LKRPKRPLFN
90	100	110	120	130	140	150	160
FALVPFHDPE	IGPVTITTDP	KKFQYELREL	YVQGGGDCPE	MSIGAIKIAL	EISLPGSFIY	VFTDARSKDY	RLTHEVLQLI
170	180	190	200	210	220	230	240
QQKQSQVVFV	LTGDCDDRTH	IGYKVYEEIA	STSSGQVFHL	DKKQVNEVLK	WVEEAVQASK	VHLLSTDHLE	QAVNTWRIPF
250	260	270	280	290	300	310	320
DPSLKEVTVS	LSGPSPMIEI	RNPLGKLIKK	GFGLHELLNI	HNSAKVVNVK	EPEAGMWTVK	TSSSGRHSVR	ITGLSTIDFR
330	340	350	360	370	380	390	400
AGFSRKPTLD	FKKTVSRPVQ	GIPTYVLLNT	SGISTPARID	LLELLSISGS	SLKTIPVKYY	PHRKPYGIWN	ISDFVPPNEA
410	420	430	440	450	460	470	480
FFLKVTGYDK	DDYLFQRVSS	VSFSSIVPDA	PKVTMPEKTP	GYYLQPGQIP	CSVDSLLPFT	LSFVRNGVTL	GVDQYLKESA
490	500	510	520	530	540	550	560
SVNLDIAKVT	LSDEGFYECI	AVSSAGTGRA	QTFFDVSEPP	PVIQVPNNVT	VTPGERAVLT	CLIISAVDYN	LTWQRNDRDV
570	580	590	600	610	620	630	640
RLAEPARIRT	LANLSLELKS	VKFNDAGEYH	CMVSSEGGSS	AASVFLTVQE	PPKVTVMPKN	QSFTGGSEVS	IMCSATGYPK
650	660	670	680	690	700	710	720
PKIAWTVNDM	FIVGSHRYRM	TSDGTLFIKN	AAPKDAGIYG	CLASNSAGTD	KQNSTLRYIE	APKLMVVQSE	LLVALGDITV
730	740	750	760	770	780	790	800
MECKTSGIPP	PQVKWFKGDL	ELRPSTFLII	DPLLGLLKIQ	ETQDLDAGDY	TCVAINEAGR	ATGKITLDVG	SPPVFIQEPA
810	820	830	840	850	860	870	880
DVSMEIGSNV	TLPCYVQGYP	EPTIKWRRLD	NMPIFSRPFS	VSSISQLRTG	ALFILNLWAS	DKGTYICEAE	NQFGKIQSET
890	900	910	920	930	940	950	960
TVTVTGLVAP	LIGISPSVAN	VIEGQQLTLP	CTLLAGNPIP	ERRWIKNSAM	LLQNPYITVR	SDGSLHIERV	QLQDGGEYTC
970	980	990	1000	1010	1020	1030	1040
VASNVAGTNN	KTTSVVVHVL	PTIQHGQQIL	STIEGIPVTL	PCKASGNPKP	SVIWSKKGEL	ISTSSAKFSA	GADGSLYVVS
1050	1060	1070	1080	1090	1100	1110	1120
PGGEESGEYV	CTATNTAGYA	KRKVQLTVYV	RPRVFGDQRG	LSQDKPVEIS	VLAGEEVTLP	CEVKSLPPPI	ITWAKETQLI
1130	1140	1150	1160	1170	1180	1190	1200
SPFSPRHTFL	PSGSMKITET	RTSDSGMYLC	VATNIAGNVT	QAVKLNVHVP	PKIQRGPKHL	KVQVGQRVDI	PCNAQGTPLP
1210	1220	1230	1240	1250	1260	1270	1280
VITWSKGGST	MLVDGEHHVS	NPDGTLSIDQ	ATPSDAGIYT	CVATNIAGTD	ETEITLHVQE	PPTVEDLEPP	YNTTFQERVA
1290	1300	1310	1320	1330	1340	1350	1360
NQRIEFPCPA	KGTPKPTIKW	LHNGRELTGR	EPGISILEDG	TLLVIASVTP	YDNGEYICVA	VNEAGTTERK	YNLKVHVPPV
1370	1380	1390	1400	1410	1420	1430	1440
IKDKEQVTNV	SVLLNQLTNL	FCEVEGTPSP	IIMWYKDNVQ	VTESSTIQTV	NNGKILKLFR	ATPEDAGRYS	CKAINIAGTS
1450	1460	1470	1480	1490	1500	1510	1520
QKYFNIDVLV	PPTIIGTNFP	NEVSVVLNRD	VALECQVKGT	PFPDIHWFKD	GKPLFLGDPN	VELLDRGQVL	HLKNARRNDK
1530	1540	1550	1560	1570	1580	1590	1600
GRYQCTVSNA	AGKQAKDIKL	TIYIPPSIKG	GNVTTDISVL	INSLIKLECE	TRGLPMPAIT	WYKDGQPIMS	SSQALYIDKG
1610	1620	1630	1640	1650	1660	1670	1680
QYLHIPRAQV	SDSATYTCHV	ANVAGTAEKS	FHVDVYVPPM	IEGNLATPLN	KQVVIAHSLT	LECKAAGNPS	PILTWLKDGV
1690	1700	1710	1720	1730	1740	1750	1760
PVKANDNIRI	EAGGKKLEIM	SAQEIDRGQY	ICVATSVAGE	KEIKYEVDVL	VPPAIEGGDE	TSYFIVMVNN	LLELDCHVTG
1770	1780	1790	1800	1810	1820	1830	1840
SPPPTIMWLK	DGQLIDERDG	FKILLNGRKL	VIAQAQVSNT	GLYRCMAANT	AGDHKKEFEV	TVHVPPTIKS	SGLSERVVVK
1850	1860	1870	1880	1890	1900	1910	1920
YKPVALQCIA	NGIPNPSITW	LKDDQPVNTA	QGNLKIQSSG	RVLQIAKTLL	EDAGRYTCVA	TNAAGETQQH	IQLHVHEPPS
1930	1940	1950	1960	1970	1980	1990	2000
LEDAGKMLNE	TVLVSNPVQL	ECKAAGNPVP	VITWYKDNRL	LSGSTSMTFL	NRGQIIDIES	AQISDAGIYK	CVAINSAGAT
2010	2020	2030	2040	2050	2060	2070	2080
ELFYSLQVHV	APSISGSNNM	VAVVVNNPVR	LECEARGIPA	PSLTWLKDGS	PVSSFSNGLQ	VLSGGRILAL	TSAQISDTGR
2090	2100	2110	2120	2130	2140	2150	2160
YTCVAVNAAG	EKQRDIDLRV	YVPPNIMGEE	QNVSVLISQA	VELLCQSDAI	PPPTLTWLKD	GHPLLKKPGL	SISENRSVLK
2170	2180	2190	2200	2210	2220	2230	2240

Detailed Protein Report

Cmpd.	No. of Cmpds.	m/z meas.	Δ m/z [ppm]	z	Rt [min]	Score	P	Range	Sequence	Modification
1025	1	676.6792	37.90	3	41.3	16.6	2	5247-5264	K.NTRGGYKCIDLCPNGMTK.A	Carbamidomethyl: 8

Detailed Protein Report

Protein 266: PREDICTED: notchless protein homolog 1 isoform X5 [Homo sapiens]

Accession: gi|578831085
Database: refseq_human(refseq_human_20140103.fasta)
Database Date: 2015-11-30
Modification(s): Carbamidomethyl

Score: 28.9
MW [kDa]: 42.7
pI: 10.1
Sequence Coverage [%]: 4.1
No. of unique Peptides: 1

10	20	30	40	50	60	70	80
MRAGSCWVPR	STCPWTSPRT	GCSSCATRYW	PRYLASGSGD	TTVRFWDLST	ETPHFTCKGH	RHWVLSISWS	PDGRKLASGC
**90**	**100**	**110**	**120**	**130**	**140**	**150**	**160**
KNGQILLWDP	STGKQVGRTL	AGHSKWITGL	SWEPLHANPE	CRYVASSSKD	GSVRIWDTTA	GRCERILTGH	TQSVTCLRWG
**170**	**180**	**190**	**200**	**210**	**220**	**230**	**240**
GDGLLYSASQ	DRTIKVWRAH	DGVLCRTLQG	HGHWVNTMAL	STDYALRTGA	FEPAEASVNP	QDLQGSLQEL	KERALSRYNL
**250**	**260**	**270**	**280**	**290**	**300**	**310**	**320**
VRGQGPERLV	SGSDDFTLFL	WSPAEDKKPL	TRMTGHQALI	NQVLFSPDSR	IVASASFDKS	IKLWDGRTGK	YLASLRGHVA
**330**	**340**	**350**	**360**	**370**	**380**	**390**	
AVYQIAWSAD	SRLLVSGSSD	STLKVWDVKA	QKLAMDLPGH	ADEVYAVDWS	PDGQRVASGG	KDKCLRM	

Cmpd.	No. of Cmpds.	m/z meas.	Δ m/z [ppm]	z	Rt [min]	Score	P	Range	Sequence	Modification
2764	1	937.4600	-13.04	2	63.6	12.4	1	143-158	R.CERILTGHTQSVTCLR.W	Carbamidomethyl: 1

Detailed Protein Report

Protein 267: rap guanine nucleotide exchange factor 4 isoform e [Homo sapiens]

**Accession:**	gi|544583481	**Score:**	28.9
**Database:**	refseq_human(refseq_human_20140103.fasta)	**MW [kDa]:**	90.9
**Database Date:**	2015-11-30	**pI:**	6.3
**Modification(s):**	Carbamidomethyl, Oxidation	**Sequence Coverage [%]:**	4.7
		**No. of unique Peptides:**	1

10	20	30	40	50	60	70	80
MIRDRKYHLK	TYRQCCVGTE	LVDWMMQQTP	CVHSRTQAVG	MWQVLLEDGV	LNHVDQEHHF	QDKYLFYRFL	DDEHEDAPLP
**90**	**100**	**110**	**120**	**130**	**140**	**150**	**160**
TEEEKKECDE	ELQDTMLLLS	QMGPDAHMRM	ILRKPPGQRT	VDDLEIIYEE	LLHIKALSHL	STTVKRELAG	VLIFESHAKG
**170**	**180**	**190**	**200**	**210**	**220**	**230**	**240**
GTVLFNQGEE	GTSWYIILKG	SVNVVIYGKG	VVCTLHEGDD	FGKLALVNDA	PRAASIVLRE	DNCHFLRVDK	EDFNRILRDV
**250**	**260**	**270**	**280**	**290**	**300**	**310**	**320**
EANTVRLKEH	DQDVLVLEKV	PAGNRASNQG	NSQPQQKYTV	MSGTPEKILE	HFLETIRLEA	TLNEATDSVL	NDFIMMHCVF
**330**	**340**	**350**	**360**	**370**	**380**	**390**	**400**
MPNTQLCPAL	VAHYHAQPSQ	GTEQEKMDYA	LNNKRRVIRL	VLQWAAMYGD	LLQEDDVSMA	FLEEFYVSVS	DDARMIAALK
**410**	**420**	**430**	**440**	**450**	**460**	**470**	**480**
EQLPELEKIV	KQISEDAKAP	QKKHKVLLQQ	FNTGDERAQK	RQPIRGSDEV	LFKVYCMDHT	YTTIRVPVAT	SVKEVISAVA
**490**	**500**	**510**	**520**	**530**	**540**	**550**	**560**
DKLGSGEGLI	IVKMSSGGEK	VVLKPNDVSV	FTTLTINGRL	FACPREQFDS	LTPLPEQEGP	TVGTVGTFEL	MSSKDLAYQM
**570**	**580**	**590**	**600**	**610**	**620**	**630**	**640**
TIYDWELFNC	VHELELIYHT	FGRHNFKKTT	ANLDLFLRRF	NEIQFWVVTE	ICLCSQLSKR	VQLLKKFIKI	AAHCKEYKNL
**650**	**660**	**670**	**680**	**690**	**700**	**710**	**720**
NSFFAIVMGL	SNVAVSRLAL	TWEKLPSKFK	KFYAEFESLM	DPSRNHRAYR	LTVAKLEPPL	IPFMPLLIKD	MTFTHEGNKT
**730**	**740**	**750**	**760**	**770**	**780**	**790**	**800**
FIDNLVNFEK	MRMIANTART	VRYYRSQPFN	PDAAQANKNH	QDVRSYVRQL	NVIDNQRTLS	QMSHRLEPRR	P

Cmpd.	No. of Cmpds.	m/z meas.	Δ m/z [ppm]	z	Rt [min]	Score	P	Range	Sequence	Modification
2090	2	917.8279	107.57	3	54.7	15.4	0	87-109	K.ECDEELQDTMLLLSQMGPDAHMR.M	Carbamidomethyl: 2; Oxidation: 10, 22

Detailed Protein Report

Protein 268: **ras-related protein Rab-44 [Homo sapiens]**

**Accession:**	gi|390635651	**Score:**	28.9
**Database:**	refseq_human(refseq_human_20140103.fasta)	**MW [kDa]:**	110.8
**Database Date:**	2015-11-30	**pI:**	4.9
**Modification(s):**	Carbamidomethyl	**Sequence Coverage [%]:**	5.0
		**No. of unique Peptides:**	2

10	20	30	40	50	60	70	80
METGQRTSRK	VRKLGSNRRR	QTREPADGEG	AAVAPEPESW	SSQAAAELQA	FFQDCGAKER	GFVTREDLAV	AKFSFLGSKE
90	100	110	120	130	140	150	160
ESEMIFDWVD	VERKGHLSLE	EFSSGLKNIF	GSSQSPHRLR	RRKPLPSKRV	SATTSFPALE	EADAEEKEAF	LAFMEQLGTG
170	180	190	200	210	220	230	240
HLLPKQMEIW	QLWGQLRQEE	PQLAGNLAGF	LAKMTSRLQE	AQADKEALEL	TLRKRDSDHH	REVQQLYEEM	EQQIRQEKQQ
250	260	270	280	290	300	310	320
LQAESDSRGL	ALTSQMQDVL	EAKEREVQRL	AEGQRELEAQ	LSHLRSTHQE	AASENQQLQE	AKRDLAGRLE	EVRGQLQVTR
330	340	350	360	370	380	390	400
GRLDAARGRV	SWQVEEKLSF	PGAGEKTPDP	QAASPEEAPL	PGLFGDNDDW	DQLLSNFGSP	PHGALQLCWS	PPPTPRATSG
410	420	430	440	450	460	470	480
PQTPRVVRQI	SISEPQAFLF	GQEPSSDPDG	APRTPPGVTF	SAKDNKGVDP	HEQDIRAEQP	VEPHDPDPNQ	EPGSTPEGRL
490	500	510	520	530	540	550	560
LWGLSGSLVA	PAFKVLIPLE	DGPPPPANSP	PPQAPAGSSK	QIQASDPDDK	GPGSWAPPSG	AQPGAGAGPQ	EPTQTPPTMT
570	580	590	600	610	620	630	640
ERETQPGPSP	TTALTGVGPA	KPPRQRDALQ	QDLHATGSEP	RLGTQRARAL	TLGPAEPFQG	LEFVGPVPTE	RLEQGQAGPA
650	660	670	680	690	700	710	720
VQEGLPEGLR	EAHGQVLGLG	ELSAFPHQEL	EEEPRSEEGK	QEGRGGQDLS	SEQSEQSVEA	HGLETAHSEL	PQQDSLLVSL
730	740	750	760	770	780	790	800
PSATPQAQVE	AEGPTPGKSA	PPRGSPPRGA	QPGAGAGPQE	PTQTPPTMAE	QEAQPRPSLT	TAHAEEQGPP	HSREPRAESR
810	820	830	840	850	860	870	880
LEDPGMDSRE	AGLTPSPGDP	MAGGGPQANP	DYLFHVIFLG	DSNVGKTSFL	HLLHQNSFAT	GLTATVGVDF	RVKTLLVDNK
890	900	910	920	930	940	950	960
CFVLQLWDTA	GQERYHSMTR	QLLRKADGVV	LMYDITSQES	FAHVRYWLDC	LQDAGSDGVV	ILLLGNKMDC	EEERQVSVEA
970	980	990	1000	1010	1020	1030	
GQQLAQELGV	YFGECSAALG	HNILEPVVNL	ARSLRMQEEG	LKDSLVKVAP	KRPPKRFGCC	S	

Cmpd.	No. of Cmpds.	m/z meas.	Δ m/z [ppm]	z	Rt [min]	Score	P	Range	Sequence	Modification
1160	1	1207.8157	-48.25	3	44.6	14.3	0	24-58	R.EPADGEGAAVAPEPESWSSQAAAELQAFFQDCGAK.E	Carbamidomethyl: 32
2821	1	914.4775	-18.24	2	64.4	14.6	1	198-213	R.LQEAQADKEALELTLR.K	

Detailed Protein Report

Protein 269: tripartite motif-containing protein 46 isoform 4 [Homo sapiens]

**Accession:**	gi|375298680	**Score:**	28.9
**Database:**	refseq_human(refseq_human_20140103.fasta)	**MW [kDa]:**	81.8
**Database Date:**	2015-11-30	**pI:**	9.6
**Modification(s):**	Carbamidomethyl, Oxidation	**Sequence Coverage [%]:**	3.8
		**No. of unique Peptides:**	1

```
        10         20         30         40         50         60         70         80
MGAKGNGLTS MKNMEKELLC PVCQEMYKQP LVLPCTHNVC QACAREVLGQ QGYIGHGGDP SSEPTSPAST PSTRSPRLSR
        90        100        110        120        130        140        150        160
RTLPKPDRLD RLLKSGFGTY PGRKRGALHP QVIMFPCPAC QGDVELGERG LAGLFRNLTL ERVVERYRQS VSVGGAILCQ
       170        180        190        200        210        220        230        240
LCKPPPLEAT KGCTECRATF CNECFKLFHP WGTQKAQHEP TLPTLSFRPK GLMCPDHKEE VTHYCKTCQR LVCQLCRVRR
       250        260        270        280        290        300        310        320
THSGHKITPV LSAYQALKDK LTKSLTYILG NQDTVQTQIC ELEEAVRHTE VSGQQAKEEV SQLVRGLGAV LEEKRASLLQ
       330        340        350        360        370        380        390        400
AIEECQQERL ARLSAQIQEH RSLLDGSGLV GYAQEVLKET DQPCFVQAAK QLHNRIARAT EALQTFRPAA SSSFRHCQLD
       410        420        430        440        450        460        470        480
VGREMKLLTE LNFLRVPEAP VIDTQRTFAY DQIFLCWRLP PHSPPAWHYT VEFRRTDVPA QPGPTRWQRR EEVRGTSALL
       490        500        510        520        530        540        550        560
ENPDTGSVYV LRVRGCNKAG YGEYSEDVHL HTPPAPVLHF FLDSRWGASR ERLAISKDQR AVRSVPGLPL LLAADRLLTG
       570        580        590        600        610        620        630        640
CHLSVDVVLG DVAVTQGRSY WACAVDPASY LVKVGVGLES KLQESFQGAP DVISPRYDPD SGHDSGAEDA TVEASPPFAF
       650        660        670        680        690        700        710        720
LTIGMGKILL GSGASSNAGL TGRDGPTAGC TVPLPPRLGI CLDYERGRVS FLDAVSFRGL LECPLDCSGP VCPAFCFIGG
       730        740        750
GAVQLQEPVG TKPERKVTIG GFAKLD
```

Cmpd.	No. of Cmpds.	m/z meas.	Δ m/z [ppm]	z	Rt [min]	Score	P	Range	Sequence	Modification
1590	1	945.6946	-76.21	3	48.4	12.7	2	5-28	K.GNGLTSMKNMEKELLCPVCQEMYK.Q	Carbamidomethyl: 16; Oxidation: 7, 22

Detailed Protein Report

Protein 270: PREDICTED: zinc finger protein 675 isoform X4 [Homo sapiens]

**Accession:**	gi|530414624	**Score:**	28.8
**Database:**	refseq_human(refseq_human_20140103.fasta)	**MW [kDa]:**	57.4
**Database Date:**	2015-11-30	**pI:**	10.2
**Modification(s):**	Carbamidomethyl	**Sequence Coverage [%]:**	3.7
		**No. of unique Peptides:**	1

Alias proteins:

Accession	Name	Description
gi|578833063	refseq_human (refseq_human_20140103.fasta)	PREDICTED: zinc finger protein 675 isoform X6 [Homo sapiens]

10	20	30	40	50	60	70	80
MCSHFAQEFW	PEQNIKDSFE	KVTLRRYEKC	GNDNFQLKGC	KSVDECKLHK	GGYNGLNQCL	PTMQSKMFQC	DKYVKVFNKF
**90**	**100**	**110**	**120**	**130**	**140**	**150**	**160**
SHSDRHKIKH	MENKPFKCKE	CGRSFCMLSH	LTRHERNYTK	VNFCKCEECE	KAVNQSSKLT	KHKRIYTCEK	LYKCQECDRT
**170**	**180**	**190**	**200**	**210**	**220**	**230**	**240**
FNQFSNLTEY	KKDYAREKPY	KCEECGKAFN	QSSHLTTHKI	IHTGEKPYKC	EECGKAFNQF	SNLTTHKKIH	TGEQPYICEE
**250**	**260**	**270**	**280**	**290**	**300**	**310**	**320**
CGKAFTQSST	LTTHKRIHTG	EKPYKCEECG	KAFNRSSKLT	EHKNIHTGEQ	PYKCEECGKA	FNRSSNLTEH	RKIHTEEKPY
**330**	**340**	**350**	**360**	**370**	**380**	**390**	**400**
KCKECGKAFK	HSSALTTHKR	IHTGEKPYKC	EECGKAFNRS	SKLTEHKKLH	TGKKPYKCEE	CGKAFIQSSK	LTEHKKIHSG
**410**	**420**	**430**	**440**	**450**	**460**	**470**	**480**
EIPYKCEECG	KAFKHSSSLT	THKRIHTGEK	PYKCEECGKA	FSRSSKLTEH	KIIHTGEKPY	KCERCDKAFN	QSANLTKHKK
**490**	**500**						
IHTGEKLQNW	NV						

Cmpd.	No. of Cmpds.	m/z meas.	Δ m/z [ppm]	z	Rt [min]	Score	P	Range	Sequence	Modification
2266	1	682.3051	5.92	3	58.8	17.1	2	30-47	K.CGNDNFQLKGCKSVDECK.L	Carbamidomethyl: 1

Detailed Protein Report

Protein 271: 3-hydroxyisobutyryl-CoA hydrolase, mitochondrial isoform 2 precursor [Homo sapiens]

**Accession:**	gi|37594469	**Score:**	28.8
**Database:**	refseq_human(refseq_human_20140103.fasta)	**MW [kDa]:**	38.0
**Database Date:**	2015-11-30	**pI:**	9.5
		**Sequence Coverage [%]:**	11.8
		**No. of unique Peptides:**	1

10	20	30	40	50	60	70	80
MGQREMWRLM	SRFNAFKRTN	TILHHLRMSK	HTDAAEEVLL	EKKGCTGVIT	LNRPKFLNAL	TLNMIRQIYP	QLKKWEQDPE
90	100	110	120	130	140	150	160
TFLIIIKGAG	GKAFCAGGDI	RVISEAEKAK	QKIAPVFFRE	EYMLNNAVGS	CQKPYVALIH	GITMGGGVGL	SVHGQFRVAT
170	180	190	200	210	220	230	240
EKCLFAMPET	AIGLFPDVGG	GYFLPRLQGK	LGYFLALTGF	RLKGRDVYRA	GIATHFVDSE	KLAMLEEDLL	ALKSPSKENI
250	260	270	280	290	300	310	320
ASVLENYHTE	SKIDRDKSFI	LEEHMDKINS	CFSANTVEEI	IENLQQDGSS	FALEQLKVIN	KMSPTSLKIT	LRQLMEGSSK
330	340						
TLQEVLTMEY	RLSQACMF						

Cmpd.	No. of Cmpds.	m/z meas.	Δ m/z [ppm]	z	Rt [min]	Score	P	Range	Sequence	Modification
2405	1	904.4370	-16.87	2	60.5	15.9	1	206-221	R.DVYRAGIATHFVDSEK.L	

Detailed Protein Report

Protein 272: plastin-2 [Homo sapiens]

**Accession:**	gi|167614506	**Score:**	28.7
**Database:**	refseq_human(refseq_human_20140103.fasta)	**MW [kDa]:**	70.2
**Database Date:**	2015-11-30	**pI:**	5.2
		**Sequence Coverage [%]:**	4.1
		**No. of unique Peptides:**	2

Alias proteins:

Accession	Name	Description
gi|530402335	refseq_human (refseq_human_20140103.fasta)	PREDICTED: plastin-2 isoform X2 [Homo sapiens]

10	20	30	40	50	60	70	80
MARGSVSDEE	MMELREAFAK	VDTDGNGYIS	FNELNDLFKA	ACLPLPGYRV	REITENLMAT	GDLDQDGRIS	FDEFIKIFHG
**90**	**100**	**110**	**120**	**130**	**140**	**150**	**160**
LKSTDVAKTF	RKAINKKEGI	CAIGGTSEQS	SVGTQHSYSE	EEKYAFVNWI	NKALENDPDC	RHVIPMNPNT	NDLFNAVGDG
**170**	**180**	**190**	**200**	**210**	**220**	**230**	**240**
IVLCKMINLS	VPDTIDERTI	NKKKLTPFTI	QENLNLALNS	ASAIGCHVVN	IGAEDLKEGK	PYLVLGLLWQ	VIKIGLFADI
**250**	**260**	**270**	**280**	**290**	**300**	**310**	**320**
ELSRNEALIA	LLREGESLED	LMKLSPEELL	LRWANYHLEN	AGCNKIGNFS	TDIKDSKAYY	HLLEQVAPKG	DEEGVPAVVI
**330**	**340**	**350**	**360**	**370**	**380**	**390**	**400**
DMSGLREKDD	IQRAECMLQQ	AERLGCRQFV	TATDVVRGNP	KLNLAFIANL	FNRYPALHKP	ENQDIDWGAL	EGETREERTF
**410**	**420**	**430**	**440**	**450**	**460**	**470**	**480**
RNWMNSLGVN	PRVNHLYSDL	SDALVIFQLY	EKIKVPVDWN	RVNKPPYPKL	GGNMKKLENC	NYAVELGKNQ	AKFSLVGIGG
**490**	**500**	**510**	**520**	**530**	**540**	**550**	**560**
QDLNEGNRTL	TLALIWQLMR	RYTLNILEEI	GGGQKVNDDI	IVNWVNETLR	EAKKSSSISS	FKDPKISTSL	PVLDLIDAIQ
**570**	**580**	**590**	**600**	**610**	**620**	**630**	
PGSINYDLLK	TENLNDDEKL	NNAKYAISMA	RKIGARVYAL	PEDLVEVNPK	MVMTVFACLM	GKGMKRV	

Cmpd.	No. of Cmpds.	m/z meas.	Δ m/z [ppm]	z	Rt [min]	Score	P	Range	Sequence	Modification
2609	1	658.3945	34.02	2	61.3	14.5	1	77-88	K.IFHGLKSTDVAK.T	
2231	1	793.3805	-58.93	2	56.5	14.2	0	597-610	R.VYALPEDLVEVNPK.M	

Detailed Protein Report

Protein 273: collagen alpha-1(IV) chain preproprotein [Homo sapiens]

**Accession:**	gi|148536825	**Score:**	28.7
**Database:**	refseq_human(refseq_human_20140103.fasta)	**MW [kDa]:**	160.5
**Database Date:**	2015-11-30	**pI:**	9.4
		**Sequence Coverage [%]:**	2.3
		**No. of unique Peptides:**	1

10	20	30	40	50	60	70	80
MGPRLSVWLL	LLPAALLLHE	EHSRAAAKGG	CAGSGCGKCD	CHGVKGQKGE	RGLPGLQGVI	GFPGMQGPEG	PQGPPGQKGD
90	100	110	120	130	140	150	160
TGEPGLPGTK	GTRGPPGASG	YPGNPGLPGI	PGQDGPPGPP	GIPGCNGTKG	ERGPLGPPGL	PGFAGNPGPP	GLPGMKGDPG
170	180	190	200	210	220	230	240
EILGHVPGML	LKGERGFPGI	PGTPGPPGLP	GLQGPVGPPG	FTGPPGPPGP	PGPPGEKGQM	GLSFQGPKGD	KGDQGVSGPP
250	260	270	280	290	300	310	320
GVPGQAQVQE	KGDFATKGEK	GQKGEPGFQG	MPGVGEKGEP	GKPGPRGKPG	KDGDKGEKGS	PGFPGEPGYP	GLIGRQGPQG
330	340	350	360	370	380	390	400
EKGEAGPPGP	PGIVIGTGPL	GEKGERGYPG	TPGPRGEPGP	KGFPGLPGQP	GPPGLPVPGQ	AGAPGFPGER	GEKGDRGFPG
410	420	430	440	450	460	470	480
TSLPGPSGRD	GLPGPPGSPG	PPGQPGYTNG	IVECQPGPPG	DQGPPGIPGQ	PGFIGEIGEK	GQKGESCLIC	DIDGYRGPPG
490	500	510	520	530	540	550	560
PQGPPGEIGF	PGQPGAKGDR	GLPGRDGVAG	VPGPQGTPGL	IGQPGAKGEP	GEFYFDLRLK	GDKGDPGFPG	QPGMTGRAGS
570	580	590	600	610	620	630	640
PGRDGHPGLP	GPKGSPGSVG	LKGERGPPGG	VGFPGSRGDT	GPPGPPGYGP	AGPIGDKGQA	GFPGGPGSPG	LPGPKGEPGK
650	660	670	680	690	700	710	720
IVPLPGPPGA	EGLPGSPGFP	GPQGDRGFPG	TPGRPGLPGE	KGAVGQPGIG	FPGPPGPKGV	DGLPGDMGPP	GTPGRPGFNG
730	740	750	760	770	780	790	800
LPGNPGVQGQ	KGEPGVGLPG	LKGLPGLPGI	PGTPGEKGSI	GVPGVPGEHG	AIGPPGLQGI	RGEPGPPGLP	GSVGSPGVPG
810	820	830	840	850	860	870	880
IGPPGARGPP	GGQGPPGLSG	PPGIKGEKGF	PGFPGLDMPG	PKGDKGAQGL	PGITGQSGLP	GLPGQQGAPG	IPGFPGSKGE
890	900	910	920	930	940	950	960
MGVMGTPGQP	GSPGPVGAPG	LPGEKGDHGF	PGSSGPRGDP	GLKGDKGDVG	LPGKPGSMDK	VDMGSMKGQK	GDQGEKGQIG
970	980	990	1000	1010	1020	1030	1040
PIGEKGSRGD	PGTPGVPGKD	GQAGQPGQPG	PKGDPGISGT	PGAPGLPGPK	GSVGGMGLPG	TPGEKGVPGI	PGPQGSPGLP
1050	1060	1070	1080	1090	1100	1110	1120
GDKGAKGEKG	QAGPPGIGIP	GLRGEKGDQG	IAGFPGSPGE	KGEKGSIGIP	GMPGSPGLKG	SPGSVGYPGS	PGLPGEKGDK
1130	1140	1150	1160	1170	1180	1190	1200
GLPGLDGIPG	VKGEAGLPGT	PGPTGPAGQK	GEPGSDGIPG	SAGEKGEPGL	PGRGFPGFPG	AKGDKGSKGE	VGFPGLAGSP
1210	1220	1230	1240	1250	1260	1270	1280
GIPGSKGEQG	FMGPPGPQGQ	PGLPGSPGHA	TEGPKGDRGP	QGQPGLPGLP	GPMGPPGLPG	IDGVKGDKGN	PGWPGAPGVP
1290	1300	1310	1320	1330	1340	1350	1360
GPKGDPGFQG	MPGIGGSPGI	TGSKGDMGPP	GVPGFQGPKG	LPGLQGIKGD	QGDQGVPGAK	GLPGPPGPPG	PYDIIKGEPG
1370	1380	1390	1400	1410	1420	1430	1440
LPGPEGPPGL	KGLQGLPGPK	GQQGVTGLVG	IPGPPGIPGF	DGAPGQKGEM	GPAGPTGPRG	FPGPPGPDGL	PGSMGPPGTP
1450	1460	1470	1480	1490	1500	1510	1520
SVDHGFLVTR	HSQTIDDPQC	PSGTKILYHG	YSLLYVQGNE	RAHGQDLGTA	GSCLRKFSTM	PFLFCNINNV	CNFASRNDYS
1530	1540	1550	1560	1570	1580	1590	1600
YWLSTPEPMP	MSMAPITGEN	IRPFISRCAV	CEAPAMVMAV	HSQTIQIPPC	PSGWSSLWIG	YSFVMHTSAG	AEGSGQALAS
1610	1620	1630	1640	1650	1660	1670	
PGSCLEEFRS	APFIECHGRG	TCNYYANAYS	FWLATIERSE	MFKKPTPSTL	KAGELRTHVS	RCQVCMRRT	

Cmpd.	No. of Cmpds.	m/z meas.	Δ m/z [ppm]	z	Rt [min]	Score	P	Range	Sequence	Modification
177	1	942.3962	-112.61	2	32.2	12.0	1	808-828	R.GPPGGQGPPGLSGPPGIKGEK.G	

Detailed Protein Report

Protein 274: **ATP-sensitive inward rectifier potassium channel 1 isoform a [Homo sapiens]**

**Accession:**	gi|4504837	**Score:**	28.6
**Database:**	refseq_human(refseq_human_20140103.fasta)	**MW [kDa]:**	44.8
**Database Date:**	2015-11-30	**pI:**	9.9
**Modification(s):**	Oxidation	**Sequence Coverage [%]:**	9.0
		**No. of unique Peptides:**	1

10	20	30	40	50	60	70	80
MNASSRNVFD	TLIRVLTESM	FKHLRKWVVT	RFFGHSRQRA	RLVSKDGRCN	IEFGNVEAQS	RFIFFVDIWT	TVLDLKWRYK
**90**	**100**	**110**	**120**	**130**	**140**	**150**	**160**
MTIFITAFLG	SWFFFGLLWY	AVAYIHKDLP	EFHPSANHTP	CVENINGLTS	AFLFSLETQV	TIGYGFRCVT	EQCATAIFLL
**170**	**180**	**190**	**200**	**210**	**220**	**230**	**240**
IFQSILGVII	NSFMCGAILA	KISRPKKRAK	TITFSKNAVI	SKRGGKLCLL	IRVANLRKSL	LIGSHIYGKL	LKTTVTPEGE
**250**	**260**	**270**	**280**	**290**	**300**	**310**	**320**
TIILDQININ	FVVDAGNENL	FFISPLTIYH	VIDHNSPFFH	MAAETLLQQD	FELVVFLDGT	VESTSATCQV	RTSYVPEEVL
**330**	**340**	**350**	**360**	**370**	**380**	**390**	**400**
WGYRFAPIVS	KTKEGKYRVD	FHNFSKTVEV	ETPHCAMCLY	NEKDVRARMK	RGYDNPNFIL	SEVNETDDTK	M

Cmpd.	No. of Cmpds.	m/z meas.	Δ m/z [ppm]	z	Rt [min]	Score	P	Range	Sequence	Modification
2895	1	859.1037	-1.61	3	65.4	17.8	2	1-22	-.MNASSRNVFDTLIRVLTESMFK.H	Oxidation: 20

Detailed Protein Report

Protein 275: calmodulin-like protein 3 [Homo sapiens]

Accession: gi|4885111
Database: refseq_human(refseq_human_20140103.fasta)
Database Date: 2015-11-30

Score: 28.6
MW [kDa]: 16.9
pI: 4.1
Sequence Coverage [%]: 8.1
No. of unique Peptides: 1

Quantitation

QD:QU **Median:** 0.35 **CV:** 0.00 % **No. of Peptides:** 1

10	20	30	40	50	60	70	80
MADQLTEEQV	TEFKEAFSLF	DKDGDGCITT	RELGTVMRSL	GQNPTEAELR	DMMSEIDRDG	NGTVDFPEFL	GMMARKMKDT
**90**	**100**	**110**	**120**	**130**	**140**	**150**	
DNEEEIREAF	RVFDKDGNGF	VSAAELRHVM	TRLGEKLSDE	EVDEMIRAAD	TDGDGQVNYE	EFVRVLVSK	

Cmpd.	No. of Cmpds.	m/z meas.	Δ m/z [ppm]	z	Rt [min]	Score	P	Range	Sequence	Modification	Ratios
245	2	657.8057	-46.21	2	31.8	28.6	0	39-50	R.SLGQNPTEAELR.D		QD:QU 0.35

Detailed Protein Report

Protein 276: **A disintegrin and metalloproteinase with thrombospondin motifs 16 preproprotein [Homo sapiens]**

**Accession:**	gi|110735441	**Score:**	28.6
**Database:**	refseq_human(refseq_human_20140103.fasta)	**MW [kDa]:**	136.1
**Database Date:**	2015-11-30	**pI:**	10.2
**Modification(s):**	Carbamidomethyl	**Sequence Coverage [%]:**	1.9
		**No. of unique Peptides:**	2

10	20	30	40	50	60	70	80
MKPRARGWRG	LAALWMLLAQ	VAEQAPACAM	GPAAAAPGSP	SVPRPPPPAE	RPGWMEKGEY	DLVSAYEVDH	RGDYVSHEIM
**90**	**100**	**110**	**120**	**130**	**140**	**150**	**160**
HHQRRRRAVP	VSEVESLHLR	LKGSRHDFHM	DLRTSSSLVA	PGFIVQTLGK	TGTKSVQTLP	PEDFCFYQGS	LRSHRNSSVA
**170**	**180**	**190**	**200**	**210**	**220**	**230**	**240**
LSTCQGLSGM	IRTEEADYFL	RPLPSHLSWK	LGRAAQGSSP	SHVLYKRSTE	PHAPGASEVL	VTSRTWELAH	QPLHSSDLRL
**250**	**260**	**270**	**280**	**290**	**300**	**310**	**320**
GLPQKQHFCG	RRKKYMPQPP	KEDLFILPDE	YKSCLRHKRS	LLRSHRNEEL	NVETLVVVDK	KMMQNHGHEN	ITTYVLTILN
**330**	**340**	**350**	**360**	**370**	**380**	**390**	**400**
MVSALFKDGT	IGGNINIAIV	GLILLEDEQP	GLVISHHADH	TLSSFCQWQS	GLMGKDGTRH	DHAILLTGLD	ICSWKNEPCD
**410**	**420**	**430**	**440**	**450**	**460**	**470**	**480**
TLGFAPISGM	CSKYRSCTIN	EDTGLGLAFT	IAHESGHNFG	MIHDGEGNMC	KKSEGNIMSP	TLAGRNGVFS	WSPCSRQYLH
**490**	**500**	**510**	**520**	**530**	**540**	**550**	**560**
KFLSTAQAIC	LADQPKPVKE	YKYPEKLPGE	LYDANTQCKW	QFGEKAKLCM	LDFKKDICKA	LWCHRIGRKC	ETKFMPAAEG
**570**	**580**	**590**	**600**	**610**	**620**	**630**	**640**
TICGHDMWCR	GGQCVKYGDE	GPKPTHGHWS	DWSSWSPCSR	TCGGGVSHRS	RLCTNPKPSH	GGKFCEGSTR	TLKLCNSQKC
**650**	**660**	**670**	**680**	**690**	**700**	**710**	**720**
PRDSVDFRAA	QCAEHNSRRF	RGRHYKWKPY	TQVEDQDLCK	LYCIAEGFDF	FFSLSNKVKD	GTPCSEDSRN	VCIDGICERV
**730**	**740**	**750**	**760**	**770**	**780**	**790**	**800**
GCDNVLGSDA	VEDVCGVCNG	NNSACTIHRG	LYTKHHHTNQ	YYHMVTIPSG	ARSIRIYEMN	VSTSYISVRN	ALRRYYLNGH
**810**	**820**	**830**	**840**	**850**	**860**	**870**	**880**
WTVDWPGRYK	FSGTTFDYRR	SYNEPENLIA	TGPTNETLIV	ELLFQGRNPG	VAWEYSMPRL	GTEKQPPAQP	SYTWAIVRSE
**890**	**900**	**910**	**920**	**930**	**940**	**950**	**960**
CSVSCGGGQM	TVREGCYRDL	KFQVNMSFCN	PKTRPVTGLV	PCKVSACPPS	WSVGNWSACS	RTCGGGAQSR	PVQCTRRVHY
**970**	**980**	**990**	**1000**	**1010**	**1020**	**1030**	**1040**
DSEPVPASLC	PQPAPSSRQA	CNSQSCPPAW	SAGPWAECSH	TCGKGWRKRA	VACKSTNPSA	RAQLLPDAVC	TSEPKPRMHE
**1050**	**1060**	**1070**	**1080**	**1090**	**1100**	**1110**	**1120**
ACLLQRCHKP	KKLQWLVSAW	SQCSVTCERG	TQKRFLKCAE	KYVSGKYREL	ASKKCSHLPK	PSLELERACA	PLPCPRHPPF
**1130**	**1140**	**1150**	**1160**	**1170**	**1180**	**1190**	**1200**
AAAGPSRGSW	FASPWSQCTA	SCGGGVQTRS	VQCLAGGRPA	SGCLLHQKPS	ASLACNTHFC	PIAEKKDAFC	KDYFHWCYLV
**1210**	**1220**	**1230**					
PQHGMCSHKF	YGKQCCKTCS	KSNL					

Cmpd.	No. of Cmpds.	m/z meas.	Δ m/z [ppm]	z	Rt [min]	Score	P	Range	Sequence	Modification
1904	1	724.3383	-56.32	2	54.2	12.7	2	631-642	R.TLKLCNSQKCPR.D	Carbamidomethyl: 10
1728	1	636.2813	0.97	2	51.9	15.9	2	1214-1224	K.QCCKTCSKSNL.-	Carbamidomethyl: 2

Detailed Protein Report

Protein 277: **PREDICTED: probable threonine--tRNA ligase 2, cytoplasmic isoform X3 [Homo sapiens]**

**Accession:**	gi|578826598	**Score:**	28.5
**Database:**	refseq_human(refseq_human_20140103.fasta)	**MW [kDa]:**	82.5
**Database Date:**	2015-11-30	**pI:**	5.7
**Modification(s):**	Carbamidomethyl, Oxidation	**Sequence Coverage [%]:**	6.5
		**No. of unique Peptides:**	2

10	20	30	40	50	60	70	80
MAAEALAAEA	VASRLERQEE	DIRWLWSEVE	RLRDEQLNAP	YSCQAEGPCL	TREVAQLRAE	NCDLRHRLCS	LRLCLAEERS
**90**	**100**	**110**	**120**	**130**	**140**	**150**	**160**
RQATLESAEL	EAAQEAGAQP	PPSQSQDKDM	KKKKMKESEA	DSEVKHQPIF	IKERLKLFEI	LKKDHQLLLA	IYGKKGDTSN
**170**	**180**	**190**	**200**	**210**	**220**	**230**	**240**
IITVRVADGQ	TVQGEVWKTT	PYQVAAEISQ	ELAESTVIAK	VNGELWDLDR	PLEGDSSLEL	LTFDNEEAQA	VYWHSSAHIL
**250**	**260**	**270**	**280**	**290**	**300**	**310**	**320**
GEAMELYYGG	HLCYGPPIEN	GFYYDMFIED	RAVSSTELSA	LENICKAIIK	EKQPFERLEV	SKEILLEMFK	YNKFKCRILN
**330**	**340**	**350**	**360**	**370**	**380**	**390**	**400**
EKVNTATTTV	YRCGPLIDLC	KGPHVRHTGK	IKTIKIFKNS	STYWEGNPEM	ETLQRIYGIS	FPDNKMMRDW	EKFQEEAKNR
**410**	**420**	**430**	**440**	**450**	**460**	**470**	**480**
DHRKIGKEQE	LFFFHDLSPG	SCFFLPRGAF	IYNTLTDFIR	EEYHKRDFTE	VLSPNMYNSK	LWEASGHWQH	YSENMFTFEI
**490**	**500**	**510**	**520**	**530**	**540**	**550**	**560**
EKDTFALKPM	NCPGHCLMFA	HRPRSWREMP	IRFADFGVLH	RNELSGTLSG	LTRVRRFQQD	DAHIFCTVEQ	IEEEIKGCLQ
**570**	**580**	**590**	**600**	**610**	**620**	**630**	**640**
FLQSVYSTFG	FSFQLNLSTR	PENFLGEIEM	WNEAEKQLQN	SLMDFGEPWK	MNPGDGAFYG	PKIDIKIKDA	IGRYHQCATI
**650**	**660**	**670**	**680**	**690**	**700**	**710**	**720**
QLDFQLPIRF	NLTYVSKDGD	DKKRPVIIHR	AILGSVERMI	AILSENYGGK	WYPVNFLKKD	LWLTLTWITV	VH

Cmpd.	No. of Cmpds.	m/z meas.	Δ m/z [ppm]	z	Rt [min]	Score	P	Range	Sequence	Modification
2780	1	849.0600	-40.11	3	65.9	10.9	2	1-23	-.MAAEALAAEAVASRLERQEEDIR.W	Oxidation: 1
569	1	689.8555	-24.02	4	37.1	17.6	2	627-649	K.IKDAIGRYHQCATIQLDFQLPIR.F	Carbamidomethyl: 11

Detailed Protein Report

Protein 278: PREDICTED: replication initiator 1 isoform X5 [Homo sapiens]

**Accession:**	gi|578814165	**Score:**	28.4
**Database:**	refseq_human(refseq_human_20140103.fasta)	**MW [kDa]:**	69.8
**Database Date:**	2015-11-30	**pI:**	12.1
		**Sequence Coverage [%]:**	2.7
		**No. of unique Peptides:**	1

Alias proteins:

Accession	Name	Description
gi|578814167	refseq_human (refseq_human_20140103.fasta)	PREDICTED: replication initiator 1 isoform X6 [Homo sapiens]

10	20	30	40	50	60	70	80
MGIGVSLLLQ	FSLTPGGYRS	VGRSRRCSRG	SIPRNIPKRS	WKKPHPQLCS	LQEEEPMLER	RCRGPLAMGL	AQPRLLSGPS
**90**	**100**	**110**	**120**	**130**	**140**	**150**	**160**
QESPQTLGKE	SRGLRQQGTS	VAQSGAQAPG	RAHRCAHCRR	HFPGWVALWL	HTRRCQARLP	LPCPECGRRF	RHAPFLALHR
**170**	**180**	**190**	**200**	**210**	**220**	**230**	**240**
QVHAAATPDL	GFACHLCGQS	FRGWVALVLH	LRAHSAAKRP	IACPKCERRF	WRRKQLRAHL	RRCHPPAPEA	RPFICGNCGR
**250**	**260**	**270**	**280**	**290**	**300**	**310**	**320**
SFAQWDQLVA	HKRVHVAEAL	EEAAAKALGP	RPRGRPAVTA	PRPGGDAVDR	PFQCACCGKR	FRHKPNLIAH	RRVHTGERPH
**330**	**340**	**350**	**360**	**370**	**380**	**390**	**400**
QCPECGKRFT	NKPYLTSHRR	IHTGEKPYPC	KECGRRFRHK	PNLLSHSKIH	KRSEGSAQAA	PGPGSPQLPA	GPQESAAEPT
**410**	**420**	**430**	**440**	**450**	**460**	**470**	**480**
PAVPLKPAQE	PPPGAPPEHP	QDPIEAPPSL	YSCDDCGRSF	RLERFLRAHQ	RQHTGERPFT	CAECGKNFGK	KTHLVAHSRV
**490**	**500**	**510**	**520**	**530**	**540**	**550**	**560**
HSGERPFACE	ECGRRFSQGS	HLAAHRRDHA	PDRPFVCPDC	GKAFRHKPYL	AAHRRIHTGE	KPYVCPDCGK	AFSQKSNLVS
**570**	**580**	**590**	**600**	**610**	**620**	**630**	
HRRIHTGERP	YACPDCDRSF	SQKSNLITHR	KSHIRDGAFC	CAICGQTFDD	EERLLAHQKK	HDV	

Cmpd.	No. of Cmpds.	m/z meas.	Δ m/z [ppm]	z	Rt [min]	Score	P	Range	Sequence	Modification
1480	1	555.1330	-245.75	2	47.0	11.1	2	112-120	R.AHRCAHCRR.H	

Detailed Protein Report

Protein 279: PREDICTED: protein EMSY isoform X10 [Homo sapiens]

**Accession:**	gi|578821618	**Score:**	28.4
**Database:**	refseq_human(refseq_human_20140103.fasta)	**MW [kDa]:**	122.8
**Database Date:**	2015-11-30	**pI:**	10.2
**Modification(s):**	Oxidation	**Sequence Coverage [%]:**	2.3
		**No. of unique Peptides:**	2

10	20	30	40	50	60	70	80
MPVVWPTLLD	LSRDECKRIL	RKLELEAYAG	VISALRAQGD	LTKEKKDLLG	ELSKVLSIST	ERHRAEVRRA	VNDERLTTIA
**90**	**100**	**110**	**120**	**130**	**140**	**150**	**160**
HNMSGPNSSS	EWSIEGRRLV	PLMPRLVPQT	AFTVTANAVA	NAAIQHNASL	PVPAETGSKE	GVSCSDEDEK	PRKRRRTNSS
**170**	**180**	**190**	**200**	**210**	**220**	**230**	**240**
SSSPVVLKEV	PKAVVPVSKT	ITVPVSGSPK	MSNIMQSIAN	SLPPHMSPVK	ITFTKPSTQT	TNTTTQKVII	VTTSPSSTFV
**250**	**260**	**270**	**280**	**290**	**300**	**310**	**320**
PNILSKSHNY	AAVTKLVPTS	VIASTTQKPP	VVITASQSSL	VSNSSSGSSS	STPSPIPNTV	AVTAVVSSTP	SVVMSTVAQG
**330**	**340**	**350**	**360**	**370**	**380**	**390**	**400**
VSTSAIKMAS	TRLPSPKSLV	SAPTQILAQF	PKQHQQSPKQ	QLYQVQQQTQ	QQVAQPSPVS	HQQQPQQSPL	PPGIKPTIQI
**410**	**420**	**430**	**440**	**450**	**460**	**470**	**480**
KQESGVKIIT	QQVQPSKILP	KPVTATLPTS	SNSPIMVVSS	NGAIMTTKLV	TTPTGTQATY	TRPTVSPSIG	RMAATPGAAT
**490**	**500**	**510**	**520**	**530**	**540**	**550**	**560**
YVKTTSGSII	TVVPKSLATL	GGKIISSNIV	SGTTTKITTI	PMTSKPNVIV	VQKTTGKGTT	IQGLPGKNVV	TTLLNAGGEK
**570**	**580**	**590**	**600**	**610**	**620**	**630**	**640**
TIQTVPTGAK	PAILTATRPI	TKMIVTQPKG	IGSTVQPAAK	IIPTKIVYGQ	QGKTQVLIKP	KPVTFQATVV	SEQTRQLVTE
**650**	**660**	**670**	**680**	**690**	**700**	**710**	**720**
TLQQASRVAE	AGNSSIQEGK	EEPQNYTDSS	SSSTESSQSS	QVSHRSQPQQ	PSQPQRTLLQ	HVAQSQTATQ	TSVVVKSIPA
**730**	**740**	**750**	**760**	**770**	**780**	**790**	**800**
SSPGAITHIM	QQALSSHTAF	TKHSEELGTE	EGEVEEMDTL	DPQTGLFYRS	ALTQSQSAKQ	QKLSQPPLEQ	TQLQVKTLQC
**810**	**820**	**830**	**840**	**850**	**860**	**870**	**880**
FQTKQKQTIH	LQADQLQHKL	PQMPQLSIRH	QKLTPLQQEQ	AQPKPDVQHT	QHPMVAKDRQ	LPTLMAQPPQ	TVVQVLAVKT
**890**	**900**	**910**	**920**	**930**	**940**	**950**	**960**
TQQLPKLQQA	PNQPKIYVQP	QTPQSQMSLP	ASSEKQTASQ	VEQPIITQGS	SVTKITFEGR	QPPTVTKITG	GSSVPKLTSP
**970**	**980**	**990**	**1000**	**1010**	**1020**	**1030**	**1040**
VTSISPIQAS	EKTAVSDILK	MSLMEAQIDT	NVEHMIVDPP	KKALATSMLT	GEAGSLPSTH	MVVAGMANST	PQQQKCRESC
**1050**	**1060**	**1070**	**1080**	**1090**	**1100**	**1110**	**1120**
SSPSTVGSSL	TTRKIDPPAV	PATGQFMRIQ	NVGQKKAEES	PAEIIIQAIP	QYAIPCHSSS	NVVVEPSGLL	ELNNFTSQQL
**1130**	**1140**	**1150**	**1160**				
DDEETAMEQD	IDSSTEDGTE	PSPSQSSAER	S				

Cmpd.	No. of Cmpds.	m/z meas.	Δ m/z [ppm]	z	Rt [min]	Score	P	Range	Sequence	Modification
2690	5	698.3734	-42.95	2	62.6	16.9	0	2-13	M.PVVWPTLLDLSR.D	
2683	2	822.4436	13.69	2	64.6	11.5	1	1054-1068	R.KIDPPAVPATGQFMR.I	Oxidation: 14

Detailed Protein Report

Protein 280: NF-kappa-B inhibitor zeta isoform a [Homo sapiens]

Accession: gi|13899229
Database: refseq_human(refseq_human_20140103.fasta)
Database Date: 2015-11-30

Score: 28.4
MW [kDa]: 78.0
pI: 6.2
Sequence Coverage [%]: 4.5
No. of unique Peptides: 1

10	20	30	40	50	60	70	80
MIVDKLLDDS	RGGEGLRDAA	GGCGLMTSPL	NLSYFYGASP	PAAAPGACDA	SCSVLGPSAP	GSPGSDSSDF	SSASSVSSCG
**90**	**100**	**110**	**120**	**130**	**140**	**150**	**160**
AVESRSRGGA	RAERQPVEPH	MGVGRQQRGP	FQGVRVKNSV	KELLLHIRSH	KQKASGQAVD	DFKTQGVNIE	QFRELKNTVS
**170**	**180**	**190**	**200**	**210**	**220**	**230**	**240**
YSGKRKGPDS	LSDGPACKRP	ALLHSQFLTP	PQTPTPGESM	EDVHLNEPKQ	ESSADLLQNI	INIKNECSPV	SLNTVQVSWL
**250**	**260**	**270**	**280**	**290**	**300**	**310**	**320**
NPVVVPQSSP	AEQCQDFHGG	QVFSPPQKCQ	PFQVRGSQQM	IDQASLYQYS	PQNQHVEQQP	HYTHKPTLEY	SPFPIPPQSP
**330**	**340**	**350**	**360**	**370**	**380**	**390**	**400**
AYEPNLFDGP	ESQFCPNQSL	VSLLGDQRES	ENIANPMQTS	SSVQQQNDAH	LHSFSMMPSS	ACEAMVGHEM	ASDSSNTSLP
**410**	**420**	**430**	**440**	**450**	**460**	**470**	**480**
FSNMGNPMNT	TQLGKSLFQW	QVEQEESKLA	NISQDQFLSK	DADGDTFLHI	AVAQGRRALS	YVLARKMNAL	HMLDIKEHNG
**490**	**500**	**510**	**520**	**530**	**540**	**550**	**560**
QSAFQVAVAA	NQHLIVQDLV	NIGAQVNTTD	CWGRTPLHVC	AEKGHSQVLQ	AIQKGAVGSN	QFVDLEATNY	DGLTPLHCAV
**570**	**580**	**590**	**600**	**610**	**620**	**630**	**640**
IAHNAVVHEL	QRNQQPHSPE	VQELLLKNKS	LVDTIKCLIQ	MGAAVEAKDR	KSGRTALHLA	AEEANLELIR	LFLELPSCLS
**650**	**660**	**670**	**680**	**690**	**700**	**710**	**720**
FVNAKAYNGN	TALHVAASLQ	YRLTQLDAVR	LLMRKGADPS	TRNLENEQPV	HLVPDGPVGE	QIRRILKGKS	IQQRAPPY

Cmpd.	No. of Cmpds.	m/z meas.	Δ m/z [ppm]	z	Rt [min]	Score	P	Range	Sequence	Modification
1761	1	823.3000	-143.58	2	52.3	10.0	2	129-143	R.SHKQKASGQAVDDFK.T	

Detailed Protein Report

Protein 281: FERM and PDZ domain-containing protein 1 [Homo sapiens]

Accession: gi|239582741
Database: refseq_human(refseq_human_20140103.fasta)
Database Date: 2015-11-30
Modification(s): Carbamidomethyl, Oxidation

Score: 28.4
MW [kDa]: 173.3
pI: 5.0
Sequence Coverage [%]: 1.5
No. of unique Peptides: 2

10	20	30	40	50	60	70	80
MEELETSLFQ	TRKAHRIEQM	VARWLRRSRD	SSARAKVAAA	DGPARNPTQT	LIPVRHTVKI	DKDTLLQDYG	FHISESLPLT
90	100	110	120	130	140	150	160
VVAVTAGGSA	HGKLFPGDQI	LQMNNEPAED	LSWERAVDIL	REAEDSLSIT	VVRCTSGVPK	SSFLTEEKRA	RLKTNPVKVH
170	180	190	200	210	220	230	240
FAEEVLISGH	SQGNSLLCMP	NVLKLYLENG	QTKAFKFEAN	TTVKDIILTV	KEKLSIRSIE	YFALALEEQY	SISRLHLLHE
250	260	270	280	290	300	310	320
EELIQQVVER	EESHDYRCLF	RVCFVPKDPL	DLLKEDPVAF	EYLYLQSCSD	VLQERFAVEM	KCSSALRLAA	LHIQERIYAC
330	340	350	360	370	380	390	400
AQPQKISLKY	IEKDWGIENF	ISPTLLRNMK	GKDIKKAISF	HMKRNQNLLE	PRQKQLISAA	QLRLNYLQIL	GELKTYGGRI
410	420	430	440	450	460	470	480
FNATLMLQDR	ESYIALLVGA	KYGISQVINS	KLNIMSTLAE	FANISRVELT	EESEKVSVVK	VYLQDVKVLT	LLLESNSAKD
490	500	510	520	530	540	550	560
LACLIAGYYR	LLVDPVTSIF	LWPGNKQQAH	RVSAEEGYES	RACSDSEESS	EVDCVLEPLS	DRRLVKLAPC	RSLIKEEQPP
570	580	590	600	610	620	630	640
GNSPTPEVAR	RGPSTCGASS	TTDSAESEAS	DSANTESRGY	RTSGSSESMD	ALEEDDLDTC	SSSRSTFFHF	GSPGLAESID
650	660	670	680	690	700	710	720
SDSQEERSGI	ETSGFLCLLD	LAQRANPQCQ	KTEFSESAAL	ETFGWAPELS	TVRLDPRLYE	GSHADYYSLC	SSVSPASYLS
730	740	750	760	770	780	790	800
DSSESTASRQ	GGAPPAWGQQ	GWTEAQPSSM	LEPLALHPPL	AFEDGSSDEE	YYDAADKLTP	PGPPSGPRDV	STAEPSATSL
810	820	830	840	850	860	870	880
QNKASTSSPE	NSLPCGPDGR	QPSRRGGVKK	YAKTLRKRRS	FLQTDYTSQV	SFPLVPSASL	ESVDDVCYYD	REPYLALGAP
890	900	910	920	930	940	950	960
SPTVSSLQDM	QGEPGLLETK	ALGLLAPLRE	TKSTNPASRV	MEMEPETMET	KSVIDSRVSS	ISAIRFRIDP	NNKENSGVVP
970	980	990	1000	1010	1020	1030	1040
AASSSASTPH	CSNPGSSGPD	TAQARPSQIL	PLSQDLDGIA	PKEPTIEHGD	SSFSLSSGDP	NPDRACLASN	PGLNNVSQGD
1050	1060	1070	1080	1090	1100	1110	1120
TLELQLEPHV	QLEMGLESFC	TNHIQETAPK	YTEPLLSPRD	EPRSDECGIN	PGEKIASIPT	KEEPQGQLSL	ERDREVTNKN
1130	1140	1150	1160	1170	1180	1190	1200
GTNVFQEESR	KDSGDSPGDV	SNNVSQTLDI	SSPAGKIVTS	LSLDAPVTGT	EQIPPHPPRD	PQGQSREPPG	QGCQAQEQKL
1210	1220	1230	1240	1250	1260	1270	1280
FVELDLDPDF	FLGKQTVSPA	VPPEGIKAEA	PNHVTGQDIA	PRDSPEWVCF	NPEPSLPEPL	PCPQEDPHLE	TSNHCLLSEG
1290	1300	1310	1320	1330	1340	1350	1360
KSDSSSICLS	AEKSFLCFAP	ESHPEVSASL	RVATSLGFAG	MNEMVAPRIG	MDQCSCQFSY	ATCFRGPQPE	TEEEDRDLEA
1370	1380	1390	1400	1410	1420	1430	1440
HPMAPLTSPP	SAGSPVVLPW	RPARAHSCTT	APLSRKSHIW	PEYCSRALRQ	LKATPASTPE	GFIQLMESLL	ELQDILETSW
1450	1460	1470	1480	1490	1500	1510	1520
GVGNKHPPEK	CTWHFTESRS	RLCMGSQKLL	SSCRHVIRMD	QSPEEMQGAV	RDTFQHLVQL	AGLCFQFTDC	SRCSARHREA
1530	1540	1550	1560	1570	1580		
AGNLRDVVYT	YHQFIEAAKS	TCERGYHDLS	VKLLARQCTA	LTAAVFCLTQ	KFRASTAL		

Cmpd.	No. of Cmpds.	m/z meas.	Δ m/z [ppm]	z	Rt [min]	Score	P	Range	Sequence	Modification
2745	1	849.4011	-32.55	1	65.4	14.4	0	357-363	K.AISFHMK.R	Oxidation: 6
2634	1	994.5127	-8.52	2	63.7	13.9	2	1462-1478	R.LCMGSQKLLSSCRHVIR.M	Carbamidomethyl: 2

Detailed Protein Report

Protein 282: PREDICTED: T-complex protein 11-like protein 1 isoform X2 [Homo sapiens]

Accession: gi|578820625
Database: refseq_human(refseq_human_20140103.fasta)
Database Date: 2015-11-30
Modification(s): Oxidation

Score: 28.3
MW [kDa]: 36.6
pI: 6.2
Sequence Coverage [%]: 9.3
No. of unique Peptides: 2

10	20	30	40	50	60	70	80
MMGTLCAPAR	DEEVKKLKDI	KEIVPLFREI	FSVLDLMKVD	MANFAISSIR	PHLMQQSVEY	ERKKFQEILE	RQPNSLDFVT
**90**	**100**	**110**	**120**	**130**	**140**	**150**	**160**
QWLEEASEDL	MTQKYKHALP	VGGMAAGSGD	MPRLSPVAVQ	NYAYLKLLKW	DHLQRPFPET	VLMDQSRFHE	LQLQLEQLTI
**170**	**180**	**190**	**200**	**210**	**220**	**230**	**240**
LGAVLLVTFS	MAAPGISSQA	DFAEKLKMIV	KILLTDMHLP	SFHLKDVLTT	IGEKVCLEVS	SCLSLCGSSP	FTTDKETVLK
**250**	**260**	**270**	**280**	**290**	**300**	**310**	**320**
GQIQAVASPD	DPIRRIMESR	ILTFLETYLA	SGHQKPLPTV	PGGLSPVQRE	LEEVAIKFAR	LVNYNKMVFC	PYYDAILSKI
**330**							
LVRS							

Cmpd.	No. of Cmpds.	m/z meas.	Δ m/z [ppm]	z	Rt [min]	Score	P	Range	Sequence	Modification
780	1	541.6146	-237.37	2	39.7	10.1	0	1-10	-.MMGTLCAPAR.D	Oxidation: 1, 2
117	1	1096.9944	-98.96	2	30.2	18.2	2	236-255	K.ETVLKGQIQAVASPDDPIRR.I	

Detailed Protein Report

Protein 283: sodium/hydrogen exchanger 9B1 isoform 2 [Homo sapiens]

**Accession:**	gi|154937338	**Score:**	28.1
**Database:**	refseq_human(refseq_human_20140103.fasta)	**MW [kDa]:**	52.2
**Database Date:**	2015-11-30	**pI:**	9.5
**Modification(s):**	Carbamidomethyl	**Sequence Coverage [%]:**	4.4
		**No. of unique Peptides:**	2

10	20	30	40	50	60	70	80
MHTTESKNEH	LEDENFQTST	TPQSLIDPNN	TAHEETKTVL	SDTEEIKPQT	KKETYISCPL	RGVLNVIITN	GVILFVIWCM
**90**	**100**	**110**	**120**	**130**	**140**	**150**	**160**
TWSILGSEAL	PGGNLFGLFI	IFYSAIIGGK	ILQLIRIPLV	PPLPPLLGML	LAGFTIRNVP	FINEHVHVPN	TWSSILRSIA
**170**	**180**	**190**	**200**	**210**	**220**	**230**	**240**
LTIILIRAGL	GLDPQALRHL	KVVCFRLAVG	PCLMEASAAA	VFSHFIMKFP	WQWAFLLGFV	LGAVSPAVVV	PYMMVLQENG
**250**	**260**	**270**	**280**	**290**	**300**	**310**	**320**
YGVEEGIPTL	LMAASSMDDI	LAITGFNTCL	SIVFSSGGIL	NNAIASIRNV	CISLLAGIVL	GFFVRYFPSE	DQKKLTLKRG
**330**	**340**	**350**	**360**	**370**	**380**	**390**	**400**
FLVLTMCVSA	VLGSQRIGLH	GSGGLCTLVL	SFIAGTKWSQ	EKMKVQKIIT	TVWDIFQPLL	FGLVGAEVSV	SSLESNIVGI
**410**	**420**	**430**	**440**	**450**	**460**	**470**	**480**
SVATLSLALC	VRILTTYLLM	CFAGFSFKEK	IFIALAWMPK	ATVQINQAIL	LLFLLREEWT	NCKVAKKCEY	TKERQ

Cmpd.	No. of Cmpds.	m/z meas.	Δ m/z [ppm]	z	Rt [min]	Score	P	Range	Sequence	Modification
2206	1	682.1436	141.92	3	58.0	11.6	0	337-357	R.IGLHGSGGLCTLVLSFIAGTK.W	
2202	1	701.1391	121.39	3	57.9	16.6	0	337-357	R.IGLHGSGGLCTLVLSFIAGTK.W	Carbamidomethyl: 10

Detailed Protein Report

Protein 284: PREDICTED: serine/threonine-protein kinase ULK2 isoform X1 [Homo sapiens]

Accession: gi|578830126
Database: refseq_human(refseq_human_20140103.fasta)
Database Date: 2015-11-30
Modification(s): Oxidation

Score: 28.1
MW [kDa]: 110.3
pI: 9.8
Sequence Coverage [%]: 2.1
No. of unique Peptides: 2

Quantitation

QD:QU **Median:** 4.25 **CV:** 0.00 % **No. of Peptides:** 1

10	20	30	40	50	60	70	80
MEVVGDFEYS	KRDLVGHGAF	AVVFRGRHRQ	KTDWEVAIKS	INKKNLSKSQ	ILLGKEIKIL	KELQHENIVA	LYDVQELPNS
90	100	110	120	130	140	150	160
VFLVMEYCNG	GDLADYLQAK	GTLSEDTIRV	FLHQIAAAMR	ILHSKGIIHR	DLKPQNILLS	YANRRKSSVS	GIRIKIADFG
170	180	190	200	210	220	230	240
FARYLHSNMM	AATLCGSPMY	MAPEVIMSQH	YDAKADLWSI	GTVIYQCLVG	KPPFQANSPQ	DLRMFYEKNR	SLMPSIPRET
250	260	270	280	290	300	310	320
SPYLANLLLG	LLQRNQKDRM	DFEAFFSHPF	LEQGPVKKSC	PVPVPMYSGS	VSGSSCGSSP	SCRFASPPSL	PDMQHIQEEN
330	340	350	360	370	380	390	400
LSSPPLGPPN	YLQVSKDSAS	TSSKNSSCDT	DDFVLVPHNI	SSDHSCDMPV	GTAGRRASNE	FLVCGGQCQP	TVSPHSETAP
410	420	430	440	450	460	470	480
IPVPTQIRNY	QRIEQNLTST	ASSGTNVHGS	PRSAVVRRSN	TSPMGFLRPG	SCSPVPADTA	QTVGRRLSTG	SSRPYSPSPL
490	500	510	520	530	540	550	560
VGTIPEQFSQ	CCCGHPQGHD	SRSRNSSGSP	VPQAQSPQSL	LSGARLQSAP	TLTDIYQNKQ	KLRKQHSDPV	CPSHTGAGYS
570	580	590	600	610	620	630	640
YSPQPSRPGS	LGTSPTKHLG	SSPRSSDWFF	KTPLPTIIGS	PTKTTAPFKI	PKTQASSNLL	ALVTRHGPAE	EQSKDGNEPR
650	660	670	680	690	700	710	720
ECAHCLLVQG	SERQRAEQQS	KAVFGRSVST	GKLSDQQGKT	PICRHQGSTD	SLNTERPMDI	GSPPHSAAAP	TCTHMFLRTR
730	740	750	760	770	780	790	800
TTSVGPSNSG	GSLCAMSGRV	CVGSPPGPGF	GSSPPGAEAA	PSLRYVPYGA	SPPSLEGLIT	FEAPELPEET	LMEREHTDTL
810	820	830	840	850	860	870	880
RHLNVMLMFT	ECVLDLTAMR	GGNPELCTSA	VSLYQIQESV	VVDQISQLSK	DWGRVEQLVL	YMKAAQLLAA	SLHLAKAQIK
890	900	910	920	930	940	950	960
SGKLSPSTAV	KQVVKNLNER	YKFCITMCKK	LTEKLNRFFS	DKQRFIDEIN	SVTAEKLIYN	CAVEMVQSAA	LDEMFQQTED
970	980	990	1000	1010			
IVYRYHKAAL	LLEGLSRILQ	DPADIENVHK	YKCSIERRLS	ALCHSTATV			

Cmpd.	No. of Cmpds.	m/z meas.	Δ m/z [ppm]	z	Rt [min]	Score	P	Range	Sequence	Modification	Ratios
2179	1	766.3372	-117.94	2	55.9	10.0	2	32-44	K.TDWEVAIKSINKK.N		QD:QU 4.25
1434	1	458.6103	-303.83	2	48.1	18.1	0	231-238	R.SLMPSIPR.E	Oxidation: 3	

Detailed Protein Report

Protein 285: mitochondrial ornithine transporter 1 [Homo sapiens]

**Accession:**	gi|7657585	**Score:**	28.1
**Database:**	refseq_human(refseq_human_20140103.fasta)	**MW [kDa]:**	32.7
**Database Date:**	2015-11-30	**pI:**	10.1
		**Sequence Coverage [%]:**	11.0
		**No. of unique Peptides:**	1

10	20	30	40	50	60	70	80
MKSNPAIQAA	IDLTAGAAGG	TACVLTGQPF	DTMKVKMQTF	PDLYRGLTDC	CLKTYSQVGF	RGFYKGTSPA	LIANIAENSV
**90**	**100**	**110**	**120**	**130**	**140**	**150**	**160**
LFMCYGFCQQ	VVRKVAGLDK	QAKLSDLQNA	AAGSFASAFA	ALVLCPTELV	KCRLQTMYEM	ETSGKIAKSQ	NTVWSVIKSI
**170**	**180**	**190**	**200**	**210**	**220**	**230**	**240**
LRKDGPLGFY	HGLSSTLLRE	VPGYFFFFGG	YELSRSFFAS	GRSKDELGPV	PLMLSGGVGG	ICLWLAVYPV	DCIKSRIQVL
**250**	**260**	**270**	**280**	**290**	**300**	**310**	
SMSGKQAGFI	RTFINVVKNE	GITALYSGLK	PTMIRAFPAN	GALFLAYEYS	RKLMMNQLEA	Y	

Cmpd.	No. of Cmpds.	m/z meas.	Δ m/z [ppm]	z	Rt [min]	Score	P	Range	Sequence	Modification
1595	1	932.5310	29.48	2	50.2	10.2	0	259-275	K.NEGITALYSGLKPTMIR.A	

Detailed Protein Report

Protein 286: **PREDICTED: huntingtin-interacting protein 1-related protein isoform X2 [Homo sapiens]**

**Accession:**	gi|530401786	**Score:**	28.0
**Database:**	refseq_human(refseq_human_20140103.fasta)	**MW [kDa]:**	118.6
**Database Date:**	2015-11-30	**pI:**	6.2
		**Sequence Coverage [%]:**	2.6
		**No. of unique Peptides:**	1

10	20	30	40	50	60	70	80
MEARFSPINQ	ILPWCRQDLA	ISISKAINTQ	EAPVKEKHAR	RIILGTHHEK	GAFTFWSYAI	GLPLPSSSIL	SWKFCHVLHK
**90**	**100**	**110**	**120**	**130**	**140**	**150**	**160**
VLRDGHPNVL	HDCQRYRSNI	REIGDLWGHL	HDRYGQLVNV	YTKLLLTKIS	FHLKHPQFPA	GLEVTDEVLE	KAAGTDVNNI
**170**	**180**	**190**	**200**	**210**	**220**	**230**	**240**
FQLTVEMFDY	MDCELKLSES	VFRQLNTAIA	VSQMSSGQCR	LAPLIQVIQD	CSHLYHYTVK	LLFKLHSCLP	ADTLQGHRDR
**250**	**260**	**270**	**280**	**290**	**300**	**310**	**320**
FHEQFHSLRN	FFRRASDMLY	FKRLIQIPRL	PEGPPNFLRA	SALAEHIKPV	VVIPEEAPED	EEPENLIEIS	TGPPAGEPVV
**330**	**340**	**350**	**360**	**370**	**380**	**390**	**400**
VADLFDQTFG	PPNGSVKDDR	TSVPRDLQIE	SLKREVEMLR	SELEKIKLEA	QRYIAQLKSQ	VNALEGELEE	QRKQKQKALV
**410**	**420**	**430**	**440**	**450**	**460**	**470**	**480**
DNEQLRHELA	QLRAAQLEGE	RSQGLREEAE	RKASATEARY	NKLKEKHSEL	VHVHAELLRK	NADTAKQLTV	TQQSQEEVAR
**490**	**500**	**510**	**520**	**530**	**540**	**550**	**560**
VKEQLAFQVE	QVKRESELKL	EEKSDQLEKL	KRELEAKAGE	LARAQEALSH	TEQSKSELSS	RLDTLSAEKD	ALSGAVRQRE
**570**	**580**	**590**	**600**	**610**	**620**	**630**	**640**
ADLLAAQSLV	RETEAALSRE	QQRSSQEQGE	LQGRLAERES	QEQGLRQRLL	DEQFAVLRGA	AAEAAGILQD	AVSKLDDPLH
**650**	**660**	**670**	**680**	**690**	**700**	**710**	**720**
LRCTSSPDYL	VSRAQEALDA	VSTLEEGHAQ	YLTSLADASA	LVAALTRFSH	LAADTIINGG	ATSHLAPTDP	ADRLIDTCRE
**730**	**740**	**750**	**760**	**770**	**780**	**790**	**800**
CGARALELMG	QLQDQQALRH	MQASLVRTPL	QGILQLGQEL	KPKSLDVRQE	ELGAVVDKEM	AATSAAIEDA	VRRIEDMMNQ
**810**	**820**	**830**	**840**	**850**	**860**	**870**	**880**
ARHASSGVKL	EVNERILNSC	TDLMKAIRLL	VTTSTSLQKE	IVESGRGAAT	QQEFYAKNSR	WTEGLISASK	AVGWGATQLV
**890**	**900**	**910**	**920**	**930**	**940**	**950**	**960**
EAADKVVLHT	GKYEELIVCS	HEIAASTAQL	VAASKVKANK	HSPHLSRLQE	CSRTVNERAA	NVVASTKSGQ	EQIEDRDTMD
**970**	**980**	**990**	**1000**	**1010**	**1020**	**1030**	**1040**
FSGLSLIKLK	KQEMETQVRV	LELEKTLEAE	RMRLGELRKQ	HYVLAGASGS	PGEEVAIRPS	TAPRSVTTKK	PPLAQKPSVA
**1050**	**1060**	**1070**					
PRQDHQLDKK	DGIYPAQLVN	Y					

Cmpd.	No. of Cmpds.	m/z meas.	Δ m/z [ppm]	z	Rt [min]	Score	P	Range	Sequence	Modification
662	1	593.6995	-211.24	2	38.3	11.3	1	513-523	R.ELEAKAGELAR.A	

Detailed Protein Report

Protein 287: **PREDICTED: otoferlin isoform X1 [Homo sapiens]**

**Accession:**	gi|530368284	**Score:**	28.0
**Database:**	refseq_human(refseq_human_20140103.fasta)	**MW [kDa]:**	225.9
**Database Date:**	2015-11-30	**pI:**	5.3
		**Sequence Coverage [%]:**	1.5
		**No. of unique Peptides:**	1

Detailed Protein Report

10	20	30	40	50	60	70	80
MALLIHLKTV	SELRGRGDRI	AKVTFRGQSF	YSRVLENCED	VADFDETFRW	PVASSIDRNE	MLEIQVFNYS	KVFSNKLIGT
90	100	110	120	130	140	150	160
FRMVLQKVVE	ESHVEVTDTL	IDDNNAIIKT	SLCVEVRYQA	TDGTVGSWDD	GDFLGDESLQ	EEEKDSQETD	GLLPGSRPSS
170	180	190	200	210	220	230	240
RPPGEKSFRS	KGREKTKGGR	DDEHKAGRSV	FSAMKLGKNR	SHKEEPQRPD	EPAVLEMEDL	DHLAIRLGDG	LDPDSVSLAS
250	260	270	280	290	300	310	320
VTALTTNVSN	KRSKPDIKME	PSAGRPMDYQ	VSITVIEARQ	LVGLNMDPVV	CVEVGDDKKY	TSMKESTNCP	YYNEYFVFDF
330	340	350	360	370	380	390	400
HVSPDVMFDK	IIKISVIHSK	NLLRSGTLVG	SFKMDVGTVY	SQPEHQFHHK	WAILSDPDDI	SSGLKGYVKC	DVAVVGKGDN
410	420	430	440	450	460	470	480
IKTPHKANET	DEDDIEGNLL	LPEGVPPERQ	WARFYVKIYR	AEGLPRMNTS	LMANVKKAFI	GENKDLVDPY	VQVFFAGQKG
490	500	510	520	530	540	550	560
KTSVQKSSYE	PLWNEQVVFT	DLFPPLCKRM	KVQIRDSDKV	NDVAIGTHFI	DLRKISNDGD	KGFLPTLGPA	WVNMYGSTRN
570	580	590	600	610	620	630	640
YTLLDEHQDL	NEGLGEGVSF	RARLLLGLAV	EIVDTSNPEL	TSSTEVQVEQ	ATPISESCAG	KMEEFFLFGA	FLEASMIDRR
650	660	670	680	690	700	710	720
NGDKPITFEV	TIGNYGNEVD	GLSRPQRPRP	RKEPGDEEEV	DLIQNASDDE	AGDAGDLASV	SSTPPMRPQV	TDRNYFHLPY
730	740	750	760	770	780	790	800
LERKPCIYIK	SWWPDQRRRL	YNANIMDHIA	DKLEEGLNDI	QEMIKTEKSY	PERRLRGVLE	ELSCGCCRFL	SLADKDQGHS
810	820	830	840	850	860	870	880
SRTRLDRERL	KSCMRELENM	GQQARMLRAQ	VKRHTVRDKL	RLCQNFLQKL	RFLADEPQHS	IPDIFIWMMS	NNKRVAYARV
890	900	910	920	930	940	950	960
PSKDLLFSIV	EEETGKDCAK	VKTLFLKLPG	KRGFGSAGWT	VQAKVELYLW	LGLSKQRKEF	LCGLPCGFQE	VKAAQGLGLH
970	980	990	1000	1010	1020	1030	1040
AFPPVSLVYT	KKQAFQLRAH	MYQARSLFAA	DSSGLSDPFA	RVFFINQSQC	TEVLNETLCP	TWDQMLVFDN	LELYGEAHEL
1050	1060	1070	1080	1090	1100	1110	1120
RDDPPIIVIE	IYDQDSMGKA	DFMGRTFAKP	LVKMADEAYC	PPRFPPQLEY	YQIYRGNATA	GDLLAAFELL	QIGPAGKADL
1130	1140	1150	1160	1170	1180	1190	1200
PPINGPVDVD	RGPIMPVPMG	IRPVLSKYRV	EVLFWGLRDL	KRVNLAQVDR	PRVDIECAGK	GVQSSLIHNY	KKNPNFNTLV
1210	1220	1230	1240	1250	1260	1270	1280
KWFEVDLPEN	ELLHPPLNIR	VVDCRAFGRY	TLVGSHAVSS	LRRFIYRPPD	RSAPSWNTTG	EVVVTMEPEV	PIKKLETMVK
1290	1300	1310	1320	1330	1340	1350	1360
LDATSEAVVK	VDVAEEEKEK	KKKKKGTAEE	PEEEEPDESM	LDWWSKYFAS	IDTMKEQLRQ	QEPSGIDLEE	KEEVDNTEGL
1370	1380	1390	1400	1410	1420	1430	1440
KGSMKGKEKA	RAAKEEKKKK	TQSSGSGQGS	EAPEKKKPKI	DELKVYPKEL	ESEFDNFEDW	LHTFNLLRGK	TGDDEDGSTE
1450	1460	1470	1480	1490	1500	1510	1520
EERIVGRFKG	SLCVYKVPLP	EDVSREAGYD	STYGMFQGIP	SNDPINVLVR	VYVVRATDLH	PADINGKADP	YIAIRLGKTD
1530	1540	1550	1560	1570	1580	1590	1600
IRDKENYISK	QLNPVFGKSF	DIEASFPMES	MLTVAVYDWD	LVGTDDLIGE	TKIDLENRFY	SKHRATCGIA	QTYSTHGYNI
1610	1620	1630	1640	1650	1660	1670	1680
WRDPMKPSQI	LTRLCKDGKV	DGPHFGPPGR	VKVANRVFTG	PSEIEDENGQ	RKPTDEHVAL	LALRHWEDIP	RAGCRLVPEH
1690	1700	1710	1720	1730	1740	1750	1760
VETRPLLNPD	KPGIEQGRLE	LWVDMFPMDM	PAPGTPLDIS	PRKPKKYELR	VIIWNTDEVV	LEDDDFFTGE	KSSDIFVRGW
1770	1780	1790	1800	1810	1820	1830	1840
LKGQQEDKQD	TDVHYHSLTG	EGNFNWRYLF	PFDYLAAEEK	IVISKKESMF	SWDETEYKIP	ARLTLQIWDA	DHFSADDFLG
1850	1860	1870	1880	1890	1900	1910	1920
AIELDLNRFP	RGAKTAKQCT	MEMATGEVDV	PLVSIFKQKR	VKGWWPLLAR	NENDEFELTG	KVEAELHLLT	AEEAEKNPVG
1930	1940	1950	1960	1970	1980	1990	2000
LARNEPDPLE	KPNRPDTAFV	WFLNPLKSIK	YLICTRYKWL	IIKIVLALLG	LLMLGLFLYS	LPGYMVKKLL	GA

Cmpd.	No. of Cmpds.	m/z meas.	Δ m/z [ppm]	z	Rt [min]	Score	P	Range	Sequence	Modification
1211	2	839.3736	-36.62	2	45.3	13.7	2	181-195	R.DDEHKAGRSVFSAMK.L	

Detailed Protein Report

Protein 288: PREDICTED: YLP motif-containing protein 1 isoform X1 [Homo sapiens]

**Accession:**	gi|530404193	**Score:**	28.0
**Database:**	refseq_human(refseq_human_20140103.fasta)	**MW [kDa]:**	231.6
**Database Date:**	2015-11-30	**pI:**	6.0
**Modification(s):**	Oxidation	**Sequence Coverage [%]:**	0.9
		**No. of unique Peptides:**	2

Detailed Protein Report

10	20	30	40	50	60	70	80
MYPNWGRYGG	SSHYPPPPVP	PPPPVALPEA	SPGPGYSSST	TPAAPSSSGF	MSFREQHLAQ	LQQLQQMHQK	QMQCVLQPHH
90	100	110	120	130	140	150	160
LPPPPLPPPP	VMPGGGYGDW	QPPPPPMPPP	PGPALSYQKQ	QQYKHQMLHH	QRDGPPGLVP	MELESPPESP	PVPPGSYMPP
170	180	190	200	210	220	230	240
SQSYMPPPQP	PPSYYPPTSS	QPYLPPAQPS	PSQSPPSQSY	LAPTPSYSSS	SSSSQSYLSH	SQSYLPSSQA	SPSRPSQGHS
250	260	270	280	290	300	310	320
KSQLLAPPPP	SAPPGNKTTV	QQEPLESGAK	NKSTEQQQAA	PEPDPSTMTP	QEQQQYWYRQ	HLLSLQQRTK	VHLPGHKKGP
330	340	350	360	370	380	390	400
VVAKDTPEPV	KEEVTVPATS	QVPESPSSEE	PPLPPPNEEV	PPPLPPEEPQ	SEDPEEDARL	KQLQAAAAHW	QQHQQHRVGF
410	420	430	440	450	460	470	480
QYQGIMQKHT	QLQQILQQYQ	QIIQPPPHIQ	TMSVDMQLRH	YEMQQQQFQH	LYQEWEREFQ	LWEEQLHSYP	HKDQLQEYEK
490	500	510	520	530	540	550	560
QWKTWQGHMK	ATQSYLQEKV	NSFQNMKNQY	MGNMSMPPPF	VPYSQMPPPL	PTMPPPVLPP	SLPPPVMPPA	LPATVPPPGM
570	580	590	600	610	620	630	640
PPPVMPPSLP	TSVPPPGMPP	SLSSAGPPPV	LPPPSLSSAG	PPPVLPPPSL	SSTAPPPVMP	LPPLSSATPP	PGIPPPGVPQ
650	660	670	680	690	700	710	720
GIPPQLTAAP	VPPASSSQSS	QVPEKPRPAL	LPTPVSFGSA	PPTTYHPPLQ	SAGPSEQVNS	KAPLSKSALP	YSSFSSDQGL
730	740	750	760	770	780	790	800
GESSAAPSQP	ITAVKDMPVR	SGGLLPDPPR	SSYLESPRGP	RFDGPRRFED	LGSRCEGPRP	KGPRFEGNRP	DGPRPRYEGH
810	820	830	840	850	860	870	880
PAEGTKSKWG	MIPRGPASQF	YITPSTSLSP	RQSGPQWKGP	KPAFGQQHQQ	QPKSQAEPLS	GNKEPLADTS	SNQQKNFKMQ
890	900	910	920	930	940	950	960
SAAFSIAADV	KDVKAAQSNE	NLSDSQQEPP	KSEVSEGPVE	PSNWDQNVQS	METQIDKAQA	VTQPVPLANK	PVPAQSTFPS
970	980	990	1000	1010	1020	1030	1040
KTGGMEGGTA	VATSSLTADN	DFKPVGIGLP	HSENNQDKGL	PRPDNRDNRL	EGNRGNSSSY	RGPGQSRMED	TRDKGLVNRG
1050	1060	1070	1080	1090	1100	1110	1120
RGQAISRGPG	LVKQEDFRDK	MMGRREDSRE	KMNRGEGSRD	RGLVRPGSSR	EKVPGGLQGS	QDRGAAGSRE	RGPPRRAGSQ
1130	1140	1150	1160	1170	1180	1190	1200
ERGPLRRAGS	RERIPPRRAG	SRERGPPRGP	GSRERGLGRS	DFGRDRGPFR	PEPGDGGEKM	YPYHRDEPPR	APWNHGEERG
1210	1220	1230	1240	1250	1260	1270	1280
HEEFPLDGRN	APMERERLDD	WDRERYWREC	ERDYQDDTLE	LYNREDRFSA	PPSRSHDGDR	RGPWWDDWER	DQDMDEDYNR
1290	1300	1310	1320	1330	1340	1350	1360
EMERDMDRDV	DRISRPMDMY	DRSLDNEWDR	DYGRPLDEQE	SQFRERDIPS	LPPLPPLPPL	PPLDRYRDDR	WREERNREHG
1370	1380	1390	1400	1410	1420	1430	1440
YDRDFRDRGE	LRIREYPERG	DTWREKRDYV	PDRMDWERER	LSDRWYPSDV	DRHSPMAEHM	PSSHHSSEMM	GSDASLDSDQ
1450	1460	1470	1480	1490	1500	1510	1520
GLGGVMVLSQ	RQHEIILKAA	QELKMLREQK	EQLQKMKDFG	SEPQMADHLP	PQESRLQNTS	SRPGMYPPPG	SYRPPPPMGK
1530	1540	1550	1560	1570	1580	1590	1600
PPGSIVRPSA	PPARSSVPVT	RPPVPIPPPP	PPPPLPPPPP	VIKPQTSAVE	QERWDEDSFY	GLWDTNDEQG	LNSEFKSETA
1610	1620	1630	1640	1650	1660	1670	1680
AIPSAPVLPP	PPVHSSIPPP	GPVPMGMPPM	SKPPPVQQTV	DYGHGRDIST	NKVEQIPYGE	RITLRPDPLP	ERSTFETEHA
1690	1700	1710	1720	1730	1740	1750	1760
GQRDRYDRER	DREPYFDRQS	NVIADHRDFK	RDRETHRDRD	RDRGVIDYDR	DRFDRERRPR	DDRAQSYRDK	KDHSSSRRGG
1770	1780	1790	1800	1810	1820	1830	1840
FDRPSYDRKS	DRPVYEGPSM	FGGERRTYPE	ERMPLPAPSL	SHQPPPAPRV	EKKPESKNVD	DILKPPGRES	RPERIVVIMR
1850	1860	1870	1880	1890	1900	1910	1920
GLPGSGKTHV	AKLIRDKEVE	FGGPAPRVLS	LDDYFITEVE	KEEKDPDSGK	KVKKKVMEYE	YEAEMEETYR	TSMFKTFKKT
1930	1940	1950	1960	1970	1980	1990	2000
LDDGFFPFII	LDAINDRVRH	FDQFWSAAKT	KGFEVYLAEM	SADNQTCGKR	NIHGRKLKEI	NKMADHWETA	PRHMMRLDIR
2010	2020	2030	2040	2050	2060	2070	
SLLQDAAIEE	VEMEDFDANI	EEQKEEKKDA	EEEESELGYI	PKSKWEMDTS	EAKLVCHVEE	V	

Cmpd.	No. of Cmpds.	m/z meas.	Δ m/z [ppm]	z	Rt [min]	Score	P	Range	Sequence	Modification

Detailed Protein Report

Cmpd.	No. of Cmpds.	m/z meas.	Δ m/z [ppm]	z	Rt [min]	Score	P	Range	Sequence	Modification
44	1	767.2360	-155.99	2	30.4	11.8	1	1673-1685	R.STFETEHAGQRDR.Y	
2554	2	746.4789	26.21	1	62.6	16.2	0	1835-1840	R.IVVIMR.G	Oxidation: 5

Detailed Protein Report

Protein 289: **PREDICTED: CLIP-associating protein 1 isoform X9 [Homo sapiens]**

**Accession:**	gi|578803955	**Score:**	27.9
**Database:**	refseq_human(refseq_human_20140103.fasta)	**MW [kDa]:**	162.2
**Database Date:**	2015-11-30	**pI:**	9.4
**Modification(s):**	Oxidation	**Sequence Coverage [%]:**	1.4
		**No. of unique Peptides:**	1

10	20	30	40	50	60	70	80
MEPRMESCLA	QVLQKDVGKR	LQVGQELIDY	FSDKQKSADL	EHDQTMLDKL	VDGLATSWVN	SSNYKVVLLG	MDILSALVTR
90	100	110	120	130	140	150	160
LQDRFKAQIG	TVLPSLIDRL	GDAKDSVREQ	DQTLLLKIMD	QAANPQYVWD	RMLGGFKHKN	FRTREGICLC	LIATLNASGA
170	180	190	200	210	220	230	240
QTLTLSKIVP	HICNLLGDPN	SQVRDAAINS	LVEIYRHVGE	RVRADLSKKG	LPQSRLNVIF	TKFDEVQKSG	NMIQSANDKN
250	260	270	280	290	300	310	320
FDDEDSVDGN	RPSSASSTSS	KAPPSSRRNV	GMGTTRRLGS	STLGSKSSAA	KEGAGAVDEE	DFIKAFDDVP	VVQIYSSRDL
330	340	350	360	370	380	390	400
EESINKIREI	LSDDKHDWEQ	RVNALKKIRS	LLLAGAAEYD	NFFQHLRLLD	GAFKLSAKDL	RSQVVREACI	TLGHLSSVLG
410	420	430	440	450	460	470	480
NKFDHGAEAI	MPTIFNLIPN	SAKIMATSGV	VAVRLIIRHT	HIPRLIPVIT	SNCTSKSVAV	RRRCFEFLDL	LLQEWQTHSL
490	500	510	520	530	540	550	560
ERHISVLAET	IKKGIHDADS	EARIEARKCY	WGFHSHFSRE	AEHLYHTLES	SYQKALQSHL	KNSDSIVSLP	QSDRSSSSSQ
570	580	590	600	610	620	630	640
ESLNRPLSAK	RSPTGSTTSR	ASTVSTKSVS	TTGSLQRSRS	DIDVNAAASA	KSKVSSSSGT	TPFSSAAALP	PGSYASLESR
650	660	670	680	690	700	710	720
HMREDMEYIG	LDSGRIRTRR	QSSGSATNVA	STPDNRGRSR	AKVVSQSQPG	SRSSSPGKLL	GSGYGGLTGG	SSRGPPVTPS
730	740	750	760	770	780	790	800
SEKRSKIPRS	QGCSRETSPN	RIGLDRFGLG	QPGRIPGSVN	AMRVLSTSTD	LEAAVADALK	KPVRRRYEPY	GMYSDDDANS
810	820	830	840	850	860	870	880
DASSVCSERS	YGSRNGGIPH	YLRQTEDVAE	VLNHCASSNW	SERKEGLLGL	QNLLKSQRTL	SRVELKRLCE	IFTRMFADPH
890	900	910	920	930	940	950	960
SKRVFSMFLE	TLVDFIIIHK	DDLQDWLFVL	LTQLLKKMGA	DLLGSVQAKV	QKALDVTRDS	FPFDQQFNIL	MRFIVDQTQT
970	980	990	1000	1010	1020	1030	1040
PNLKVKVAIL	KYIESLARQM	DPTDFVNSSE	TRLAVSRIIT	WTTEPKSSDV	RKAAQIVLIS	LFELNTPEFT	MLLGALPKTF
1050	1060	1070	1080	1090	1100	1110	1120
QDGATKLLHN	HLKNSSNTSV	GSPSNTIGRT	PSRHTSSRTS	PLTSPTNCSH	GGLSPSMLDY	DTENLNSEEI	YSSLRGVTEA
1130	1140	1150	1160	1170	1180	1190	1200
IEKFSFRSQE	DLNEPIKRDG	KKECDIVSRD	GGAASPATEG	RGGSEVEGGR	TALDNKTSLL	NTQPPRAFPG	PRARDYNPYP
1210	1220	1230	1240	1250	1260	1270	1280
YSDAINTYDK	TALKEAVFDD	DMEQLRDVPI	DHSDLVADLL	KELSNHNERV	EERKGALLEL	LKITREDSLG	VWEEHFKTIL
1290	1300	1310	1320	1330	1340	1350	1360
LLLLETLGDK	DHSIRALALR	VLREILRNQP	ARFKNYAELT	IMKTLEAHKD	SHKEVVRAAE	EAASTLASSI	HPEQCIKVLC
1370	1380	1390	1400	1410	1420	1430	1440
PIIQTADYPI	NLAAIKMQTK	VVERIAKESL	LQLLVDIIPG	LLQGYDNTES	SVRKASVFCL	VAIYSVIGED	LKPHLAQLTG
1450	1460	1470	1480				
SKMKLLNLYI	KRAQTTNSNS	SSSSDVSTHS					

Cmpd.	No. of Cmpds.	m/z meas.	Δ m/z [ppm]	z	Rt [min]	Score	P	Range	Sequence	Modification
2270	1	614.2104	-96.89	3	58.8	10.1	1	641-655	R.HMREDMEYIGLDSGR.I	Oxidation: 2, 6

Detailed Protein Report

Protein 290: **PREDICTED: peregrin isoform X6 [Homo sapiens]**

**Accession:**	gi|530373123	**Score:**	27.9
**Database:**	refseq_human(refseq_human_20140103.fasta)	**MW [kDa]:**	78.0
**Database Date:**	2015-11-30	**pI:**	6.9
		**Sequence Coverage [%]:**	4.3
		**No. of unique Peptides:**	1

10	20	30	40	50	60	70	80
MGVDFDVKTF	CHNLRATKPP	YECPVETCRK	VYKSYSGIEY	HLYHYDHDNP	PPPQQTPLRK	HKKKGRQSRP	ANKQSPSPSE
**90**	**100**	**110**	**120**	**130**	**140**	**150**	**160**
VSQSPGREVM	SYAQAQRMVE	VDLHGRVHRI	SIFDNLDVVS	EDEEAPEEAP	ENGSNKENTE	TPAATPKSGK	HKNKEKRKDS
**170**	**180**	**190**	**200**	**210**	**220**	**230**	**240**
NHHHHHNVSA	STTPKLPEVV	YRELEQDTPD	APPRPTSYYR	YIEKSAEELD	EEVEYDMDEE	DYIWLDIMNE	RRKTEGVSPI
**250**	**260**	**270**	**280**	**290**	**300**	**310**	**320**
PQEIFEYLMD	RLEKESYFES	HNKGDPNALV	DEDAVCCICN	DGECQNSNVI	LFCDMCNLAV	HQECYGVPYI	PEGQWLCRRC
**330**	**340**	**350**	**360**	**370**	**380**	**390**	**400**
LQSPSRAVDC	ALCPNKGGAF	KQTDDGRWAH	VVCALWIPEV	CFANTVFLEP	IDSIEHIPPA	RWKLTCYICK	QRGSGACIQC
**410**	**420**	**430**	**440**	**450**	**460**	**470**	**480**
HKANCYTAFH	VTCAQQAGLY	MKMEPVRETG	ANGTSFSVRK	TAYCDIHTPP	GSARRLPALS	HSEGEEDEDE	EEDEGKGWSS
**490**	**500**	**510**	**520**	**530**	**540**	**550**	**560**
EKVKKAKAKS	RIKMKKARKI	LAEKRAAAPV	VSVPCIPPHR	LSKITNRLTI	QRKSQFMQRL	HSYWTLKRQS	RNGVPLLRRL
**570**	**580**	**590**	**600**	**610**	**620**	**630**	**640**
QTHLQSQRNC	DQVGRDSEDK	NWALKEQLKS	WQRLRHDLER	ARLLVELIRK	REKLKRETIK	VQQIAMEMQL	TPFLILLRKT
**650**	**660**	**670**	**680**	**690**			
LEQLQEKDTG	NIFSEPVPLS	EGPEWEAVCT	PSPEAGTESA	K			

Cmpd.	No. of Cmpds.	m/z meas.	Δ m/z [ppm]	z	Rt [min]	Score	P	Range	Sequence	Modification
1974	1	757.2741	-191.30	2	55.1	10.7	0	506-520	R.AAAPVVSVPCIPPHR.L	

Detailed Protein Report

Protein 291: trinucleotide repeat-containing gene 18 protein [Homo sapiens]

**Accession:**	gi|169658378	**Score:**	27.9
**Database:**	refseq_human(refseq_human_20140103.fasta)	**MW [kDa]:**	314.3
**Database Date:**	2015-11-30	**pI:**	9.6
		**Sequence Coverage [%]:**	0.8
		**No. of unique Peptides:**	1

Alias proteins:

Accession	Name	Description
gi|578813684	refseq_human (refseq_human_20140103.fasta)	PREDICTED: trinucleotide repeat-containing gene 18 protein isoform X1 [Homo sapiens]

Detailed Protein Report

10	20	30	40	50	60	70	80
MDGRDFGPQR	SVHGPPPPLL	SGLAMDSHRV	GAATAGRLPA	SGLPGPLPPG	KYMAGLNLHP	HPGEAFLGSF	VASGMGPSAS
90	100	110	120	130	140	150	160
SHGSPVPLPS	DLSFRSPTPS	NLPMVQLWAA	HAHEGFSHLP	SGLYPSYLHL	NHLEPPSSGS	PLLSQLGQPS	IFDTQKGQGP
170	180	190	200	210	220	230	240
GGDGFYLPTA	GAPGSLHSHA	PSARTPGGGH	SSGAPAKGSS	SRDGPAKERA	GRGGEPPPLF	GKKDPRARGE	EASGPRGVVD
250	260	270	280	290	300	310	320
LTQEARAEGR	QDRGPPRLAE	RLSPFLAESK	TKNAALQPSV	LTMCNGGAGD	VGLPALVAEA	GRGGAKEAAR	QDEGARLLRR
330	340	350	360	370	380	390	400
TETLLPGPRP	CPSPLPPPPA	PPKGPPAPPA	ATPAGVYTVF	REQGREHRVV	APTFVPSVEA	FDERPGPIQI	ASQARDARAR
410	420	430	440	450	460	470	480
EREAGRPGVL	QAPPGSPRPL	DRPEGLREKN	SVIRSLKRPP	PADAPTVRAT	RASPDPRAYV	PAKELLKPEA	DPRPCERAPR
490	500	510	520	530	540	550	560
GPAGPAAQQA	AKLFGLEPGR	PPPTGPEHKW	KPFELGNFAA	TQMAVLAAQH	HHSRAEEEAA	VVAASSSKKA	YLDPGAVLPR
570	580	590	600	610	620	630	640
SAATCGRPVA	DMHSAAHGSG	EASAMQSLIK	YSGSFARDAV	AVRPGGCGKK	SPFGGLGTMK	PEPAPTSAGA	SRAQARLPHS
650	660	670	680	690	700	710	720
GGPAAGGGRQ	LKRDPERPES	AKAFGREGSG	AQGEAEVRHP	PVGIAVAVAR	QKDSGGSGRL	GPGLVDQERS	LSLSNVKGHG
730	740	750	760	770	780	790	800
RADEDCVDDR	ARHREERLLG	ARLDRDQEKL	LRESKELADL	ARLHPTSCAP	NGLNPNLMVT	GGPALAGSGR	WSADPAAHLA
810	820	830	840	850	860	870	880
THPWLPRSGN	ASMWLAGHPY	GLGPPSLHQG	MAPAFPPGLG	GSLPSAYQFV	RDPQSGQLVV	IPSDHLPHFA	ELMERATVPP
890	900	910	920	930	940	950	960
LWPALYPPGR	SPLHHAQQLQ	LFSQQHFLRQ	QEFLYLQQQA	AQALELQRSA	QLVQERLKAQ	EHRAEMEEKG	SKRGLEAAGK
970	980	990	1000	1010	1020	1030	1040
AGLATAGPGL	LPRKPPGLAA	GPAGTYGKAV	SPPPSPRASP	VAALKAKVIQ	KLEDVSKPPA	YAYPATPSSH	PTSPPPASPP
1050	1060	1070	1080	1090	1100	1110	1120
PTPGITRKEE	APENVVEKKD	LELEKEAPSP	FQALFSDIPP	RYPFQALPPH	YGRPYPFLLQ	PTAAADADGL	APDVPLPADG
1130	1140	1150	1160	1170	1180	1190	1200
PERLALSPED	KPIRLSPSKI	TEPLREGPEE	EPLAEREVKA	EVEDMDEGPT	ELPPLESPLP	LPAAEAMATP	SPAGGCGGGL
1210	1220	1230	1240	1250	1260	1270	1280
LEAQALSATG	QSCAEPSECP	DFVEGPEPRV	DSPGRTEPCT	AALDLGVQLT	PETLVEAKEE	PVEVPVAVPV	VEAVPEEGLA
1290	1300	1310	1320	1330	1340	1350	1360
QVAPSESQPT	LEMSDCDVPA	GEGQCPSLEP	QEAVPVLGST	CFLEEASSDQ	FLPSLEDPLA	GMNALAAAAE	LPQARPLPSP
1370	1380	1390	1400	1410	1420	1430	1440
GAAGAQALEK	LEAAESLVLE	QSFLHGITLL	SEIAELELER	RSQEMGGAER	ALVARPSLES	LLAAGSHMLR	EVLDGPVVDP
1450	1460	1470	1480	1490	1500	1510	1520
LKNLRLPREL	KPNKKYSWMR	KKEERMYAMK	SSLEDMDALE	LDFRMRLAEV	QRQYKEKQRE	LVKLQRRRDS	EDRREEPHRS
1530	1540	1550	1560	1570	1580	1590	1600
LARRGPGRPR	KRTHAPSALS	PPRKRGKSGH	SSGKLSSKSL	LTSDDYELGA	GIRKRHKGSE	EEHDALIGMG	KARGRNQTWD
1610	1620	1630	1640	1650	1660	1670	1680
EHEASSDFIS	QLKIKKKKMA	SDQEQLASKL	DKALSLTKQD	KLKSPFKFSD	SAGGKSKTSG	GCGRYLTPYD	SLLGKNRKAL
1690	1700	1710	1720	1730	1740	1750	1760
AKGLGLSLKS	SREGKHKRAA	KTRKMEVGFK	ARGQPKSAHS	PFASEVSSYS	YNTDSEEDEE	FLKDEWPAQG	PSSSKLTPSL
1770	1780	1790	1800	1810	1820	1830	1840
LCSMVAKNSK	AAGGPKLTKR	GLAAPRTLKP	KPATSRKQPF	CLLLREAEAR	SSFSDSSEES	FDQDESSEEE	DEEEELEEED
1850	1860	1870	1880	1890	1900	1910	1920
EASGGGYRLG	ARERALSPGL	EESGLGLLAR	FAASALPSPT	VGPSLSVVQL	EAKQKARKKE	ERQSLLGTEF	EYTDSESEVK
1930	1940	1950	1960	1970	1980	1990	2000
VRKRSPAGLL	RPKKGLGEPG	PSLAAPTPGA	RGPDPSSPDK	AKLAVEKGRK	ARKLRGPKEP	GFEAGPEASD	DDLWTRRRSE
2010	2020	2030	2040	2050	2060	2070	2080
RIFLHDASAA	APAPVSTAPA	TKTSRCAKGG	PLSPRKDAGR	AKDRKDPRKK	KKGKEAGPGA	GLPPPRAPAL	PSEARAPHAS
2090	2100	2110	2120	2130	2140	2150	2160
SLTAAKRSKA	KAKGKEVKKE	NRGKGGAVSK	LMESMAAEED	FEPNQDSSFS	EDEHLPRGGA	VERPLTPAPR	SCIIDKDELK
2170	2180	2190	2200	2210	2220	2230	2240

Detailed Protein Report

Cmpd.	No. of Cmpds.	m/z meas.	Δ m/z [ppm]	z	Rt [min]	Score	P	Range	Sequence	Modification
1643	1	713.3402	-67.63	2	50.8	15.8	2	2464-2476	K.LDHEGVTSPKSKK.A	

Detailed Protein Report

Protein 292: uncharacterized protein C12orf60 [Homo sapiens]

Accession: gi|157389009
Database: refseq_human(refseq_human_20140103.fasta)
Database Date: 2015-11-30
Modification(s): Oxidation

Score: 27.9
MW [kDa]: 27.6
pI: 8.9
Sequence Coverage [%]: 11.0
No. of unique Peptides: 1

Alias proteins:

Accession	Name	Description
gi|530399044	refseq_human (refseq_human_20140103.fasta)	PREDICTED: uncharacterized protein C12orf60 isoform X2 [Homo sapiens]
gi|530399042	refseq_human (refseq_human_20140103.fasta)	PREDICTED: uncharacterized protein C12orf60 isoform X1 [Homo sapiens]

```
        10         20         30         40         50         60         70         80
MSSESEKDKE RLIQAAKMFF FHVQDLASVI NTLTELFSRS MNTQILLMAV KNNSYIKDFF EQMLKIFKEM QSVVDARHDK
        90        100        110        120        130        140        150        160
IQKESLCSKV AMAMCSVVQK STNVEELHQS AKEVFKSAHT PVIISVLNSS NILGSLESSL SHLMKFPIMN LQLSDFYTED
       170        180        190        200        210        220        230        240
TKEQSDVTTS ERTRSPPGSS KTTMIDTLKK LQDVLKTEDS KNPTKSAADL LEQIVKAMGP ILEILQKAIK TMEMNISVFK
       250
KASDK
```

Cmpd.	No. of Cmpds.	m/z meas.	Δ m/z [ppm]	z	Rt [min]	Score	P	Range	Sequence	Modification
2000	1	918.3556	-135.68	2	55.4	15.0	2	173-189	R.TRSPPGSSKTTMIDTLK.K	Oxidation: 12

Detailed Protein Report

Protein 293: **PREDICTED: protein 4.1 isoform X29 [Homo sapiens]**

Accession: gi|578798602
Database: refseq_human(refseq_human_20140103.fasta)
Database Date: 2015-11-30
Modification(s): Oxidation

Score: 27.8
MW [kDa]: 90.9
pI: 6.1
Sequence Coverage [%]: 3.9
No. of unique Peptides: 2

10	20	30	40	50	60	70	80
MHCKVSLLDD	TVYECVVEKH	AKGQDLLKRV	CEHLNLLEED	YFGLAIWDNA	TSKTWLDSAK	EIKKQVRGVP	WNFTFNVKFY
**90**	**100**	**110**	**120**	**130**	**140**	**150**	**160**
PPDPAQLTED	ITRYYLCLQL	RQDIVAGRLP	CSFATLALLG	SYTIQSELGD	YDPELHGVDY	VSDFKLAPNQ	TKELEEKVME
**170**	**180**	**190**	**200**	**210**	**220**	**230**	**240**
LHKSYRSMTP	AQADLEFLEN	AKKLSMYGVD	LHKAKDLEGV	DIILGVCSSG	LLVYKDKLRI	NRFPWPKVLK	ISYKRSSFFI
**250**	**260**	**270**	**280**	**290**	**300**	**310**	**320**
KIRPGEQEQY	ESTIGFKLPS	YRAAKKLWKV	CVEHHTFFRL	TSTDTIPKSK	FLALGSKFRY	SGRTQAQTRQ	ASALIDRPAP
**330**	**340**	**350**	**360**	**370**	**380**	**390**	**400**
HFERTASKRA	SRSLDGAAAV	DSADRSPRPT	SAPAITQGQV	AEGGVLDASA	KKTVVPKAQK	ETVKAEVKKE	DEPPEQAEPE
**410**	**420**	**430**	**440**	**450**	**460**	**470**	**480**
PTEAWKVEKT	HIEVTVPTSN	GDQTQKLAEK	TEDLIRMRKK	KRERLDGENI	YIRHSNLMLE	DLDKSQEEIK	KHHASISELK
**490**	**500**	**510**	**520**	**530**	**540**	**550**	**560**
KNFMESVPEP	RPSEWDKRLS	THSPFRTLNI	NGQIPTGEGV	KKTSVLPSER	KSINGIRTEE	VAVVTKGPST	NPDSEWEGPK
**570**	**580**	**590**	**600**	**610**	**620**	**630**	**640**
HSVVPSKSQM	TTSSESLQSF	AFGSLSISSK	ETEEKEEGAA	GYLDIKEMPR	GPTGGCIGVE	EQASALKFSV	TPASCQLQPG
**650**	**660**	**670**	**680**	**690**	**700**	**710**	**720**
VKKAESSEEH	VTPGEPPGKQ	NGSFLDFHVG	NQFPTLIRSF	QPPLVKTQTV	TISDNANAVK	SEIPTKDVPI	VHTETKTITY
**730**	**740**	**750**	**760**	**770**	**780**	**790**	**800**
EAAQTDDNSG	DLDPGVLLTA	QTITSETPSS	TTTTQITKTV	KGGISETRIE	KRIVITGDAD	IDHDQVLVQA	IKEAKEQHPD
**810**	**820**						
MSVTKVVVHQ	ETEIADE						

Cmpd.	No. of Cmpds.	m/z meas.	Δ m/z [ppm]	z	Rt [min]	Score	P	Range	Sequence	Modification
1704	1	682.2585	-109.76	3	51.6	12.4	1	454-470	R.HSNLMLEDLDKSQEEIK.K	Oxidation: 5
100	1	565.6045	-3.81	3	31.2	15.5	1	596-610	K.EEGAAGYLDIKEMPR.G	Oxidation: 13

Detailed Protein Report

Protein 294: ubiquitin carboxyl-terminal hydrolase 35 [Homo sapiens]

**Accession:**	gi|148746183	**Score:**	27.8
**Database:**	refseq_human(refseq_human_20140103.fasta)	**MW [kDa]:**	113.3
**Database Date:**	2015-11-30	**pI:**	5.3
**Modification(s):**	Carbamidomethyl	**Sequence Coverage [%]:**	3.2
		**No. of unique Peptides:**	1

10	20	30	40	50	60	70	80
MDKILEAVVT	SSYPVSVKQG	LVRRVLEAAR	QPLEREQCLA	LLALGARLYV	GGAEELPRRV	GCQLLHVAGR	HHPDVFAEFF
**90**	**100**	**110**	**120**	**130**	**140**	**150**	**160**
SARRVLRLLQ	GGAGPPGPRA	LACVQLGLQL	LPEGPAADEV	FALLRREVLR	TVCERPGPAA	CAQVARLLAR	HPRCVPDGPH
**170**	**180**	**190**	**200**	**210**	**220**	**230**	**240**
RLLFCQQLVR	CLGRFRCPAE	GEEGAVEFLE	QAQQVSGLLA	QLWRAQPAAI	LPCLKELFAV	ISCAEEEPPS	SALASVVQHL
**250**	**260**	**270**	**280**	**290**	**300**	**310**	**320**
PLELMDGVVR	NLSNDDSVTD	SQMLTAISRM	IDWVSWPLGK	NIDKWIIALL	KGLAAVKKFS	ILIEVSLTKI	EKVFSKLLYP
**330**	**340**	**350**	**360**	**370**	**380**	**390**	**400**
IVRGAALSVL	KYMLLTFQHS	HEAFHLLLPH	IPPMVASLVK	EDSNSGTSCL	EQLAELVHCM	VFRFPGFPDL	YEPVMEAIKD
**410**	**420**	**430**	**440**	**450**	**460**	**470**	**480**
LHVPNEDRIK	QLLGQDAWTS	QKSELAGFYP	RLMAKSDTGK	IGLINLGNTC	YVNSILQALF	MASDFRHCVL	RLTENNSQPL
**490**	**500**	**510**	**520**	**530**	**540**	**550**	**560**
MTKLQWLFGF	LEHSQRPAIS	PENFLSASWT	PWFSPGTQQD	CSEYLKYLLD	RLHEEEKTGT	RICQKLKQSS	SPSPPEEPPA
**570**	**580**	**590**	**600**	**610**	**620**	**630**	**640**
PSSTSVEKMF	GGKIVTRICC	LCCLNVSSRE	EAFTDLSLAF	PPPERCRRRR	LGSVMRPTED	ITARELPPPT	SAQGPGRVGP
**650**	**660**	**670**	**680**	**690**	**700**	**710**	**720**
RRQRKHCITE	DTPPTSLYIE	GLDSKEAGGQ	SSQEERIERE	EEGKEERTEK	EEVGEEEEST	RGEGEREKEE	EVEEEEEKVE
**730**	**740**	**750**	**760**	**770**	**780**	**790**	**800**
KETEKEAEQE	KEEDSLGAGT	HPDAAIPSGE	RTCGSEGSRS	VLDLVNYFLS	PEKLTAENRY	YCESCASLQD	AEKVVELSQG
**810**	**820**	**830**	**840**	**850**	**860**	**870**	**880**
PCYLILTLLR	FSFDLRTMRR	RKILDDVSIP	LLLRLPLAGG	RGQAYDLCSV	VVHSGVSSES	GHYYCYAREG	AARPAASLGT
**890**	**900**	**910**	**920**	**930**	**940**	**950**	**960**
ADRPEPENQW	YLFNDTRVSF	SSFESVSNVT	SFFPKDTAYV	LFYRQRPREG	PEAELGSSRV	RTEPTLHKDL	MEAISKDNIL
**970**	**980**	**990**	**1000**	**1010**	**1020**		
YLQEQEKEAR	SRAAYISALP	TSPHWGRGFD	EDKDEDEGSP	GGCNPAGGNG	GDFHRLVF		

Cmpd.	No. of Cmpds.	m/z meas.	Δ m/z [ppm]	z	Rt [min]	Score	P	Range	Sequence	Modification
1261	1	1070.0005	-62.40	2	45.9	12.2	1	131-150	R.TVCERPGPAACAQVARLLAR.H	Carbamidomethyl: 3

BRUKER

Detailed Protein Report

Protein 295: PREDICTED: cullin-9 isoform X3 [Homo sapiens]

**Accession:**	gi|578811520	**Score:**	27.8
**Database:**	refseq_human(refseq_human_20140103.fasta)	**MW [kDa]:**	268.2
**Database Date:**	2015-11-30	**pI:**	5.3
**Modification(s):**	Oxidation	**Sequence Coverage [%]:**	1.3
		**No. of unique Peptides:**	2

Detailed Protein Report

10	20	30	40	50	60	70	80
MVGERHAGDL	MVPLGPRLQA	YPEELIRQRP	GHDGHPEYLI	RWSVLKCGEV	GKVGVEEGKA	EHILMWLSAP	EVYANCPGLL
90	100	110	120	130	140	150	160
GERALSKGLQ	HEPAGVSGSF	PRDPGGLDEV	AMGEMEADVQ	ALVRRAARQL	AESGTPSLTA	AVLHTIHVLS	AYASIGPLTG
170	180	190	200	210	220	230	240
VFRETGALDL	LMHMLCNPEP	QIRRSAGKML	QALAAHDAGS	RAHVLLSLSQ	QDGIEQHMDF	DSRYTLLELF	AETTSSEEHC
250	260	270	280	290	300	310	320
MAFEGIHLPQ	IPGKLLFSLV	KRYLCVTSLL	DQLNSSPELG	AGDQSSPCAT	REKSRGQREL	EFSMAVGNLI	SELVRSMGWA
330	340	350	360	370	380	390	400
RNLSEQGMSP	PRPTRSIFQP	YISGPSLLLP	TIVTTPRRQG	WVFRQRSEFS	SRSGYGEYVQ	QTLQPGMRVR	MLDDYEEISA
410	420	430	440	450	460	470	480
GDEGEFRQSN	NGIPPVQTLG	EKALGEISVS	VEMAESLLQV	LSSRFEGSTL	NDLLNSQIYT	KYGLLSNEPS	SSSTSRNHSC
490	500	510	520	530	540	550	560
TPDPEEESKS	EASFSEEETE	SLKAKAEAPK	TEAEPTKTRT	ETPMAQSDSQ	LFNQLLVTEG	MTLPTEMKEA	ASEMARALRG
570	580	590	600	610	620	630	640
PGPRSSLDQH	VAAVVATVQI	SSLDTNLQLS	GLSALSQAVE	EVTERDHPLV	RPDRSLREKL	VKMLVELLTN	QVGEKMVVVQ
650	660	670	680	690	700	710	720
ALRLLYLLMT	KHEWRPLFAR	EGGIYAVLVC	MQEYKTSVLV	QQAGLAALKM	LAVASSSEIP	TFVTGRDSIH	SLFDAQMTRE
730	740	750	760	770	780	790	800
IFASIDSATR	PGSESLLLTV	PAAVILMLNT	EGCSSAARNG	LLLLNLLLCN	HHTLGDQIIT	QELRDTLFRH	SGIAPRTEPM
810	820	830	840	850	860	870	880
PTTRTILMML	LNRYSEPPGS	PERAALETPI	IQGQDGSPEL	LIRSLVGGPS	AELLLDLERV	LCREGSPGGA	VRPLLKRLQQ
890	900	910	920	930	940	950	960
ETQPFLLLLR	TLDAPGPNKT	LLLSVLRVIT	RLLDFPEAMV	LPWHEVLEPC	LNCLSGPSSD	SEIVQELTCF	LHRLASMHKD
970	980	990	1000	1010	1020	1030	1040
YAVVLCCLGA	KEILSKVLDK	HSAQLLLGCE	LRDLVTECEK	YAQLYSNLTS	SILAGCIQMV	LGQIEDHRRT	HQPINIPFFD
1050	1060	1070	1080	1090	1100	1110	1120
VFLRHLCQGS	SVEVKEDKCW	EKVEVSSNPH	RASKLTDHNP	KTYWESNGST	GSHYITLHMH	RGVLVRQLTL	LVASEDSSYM
1130	1140	1150	1160	1170	1180	1190	1200
PARVVVFGGD	STSCIGTELN	TVNVMPSASR	VILLENLNRF	WPIIQIRIKR	CQQGGIDTRV	RGVEVLGPKP	TFWPLFREQL
1210	1220	1230	1240	1250	1260	1270	1280
CRRTCLFYTI	RAQAWSRDIA	EDHRRLLQLC	PRLNRVLRHE	QNFADRFLPD	DEAAQALGKT	CWEALVSPLV	QNITSPDAEG
1290	1300	1310	1320	1330	1340	1350	1360
VSALGWLLDQ	YLEQRETSRN	PLSRAASFAS	RVRRLCHLLV	HVEPPPGPSP	EPSTRPFSKN	SKGRDRSPAP	SPVLPSSSLR
1370	1380	1390	1400	1410	1420	1430	1440
NITQCWLSVV	QEQVSRFLAA	AWRAPDFVPR	YCKLYEHLQR	AGSELFGPRA	AFMLALRSGF	SGALLQQSFL	TAAHMSEQFA
1450	1460	1470	1480	1490	1500	1510	1520
RYIDQQIQGG	LIGGAPGVEM	LGQLQRHLEP	IMVLSGLELA	TTFEHFYQHY	MADRLLSFGS	SWLEGAVLEQ	IGLCFPNRLP
1530	1540	1550	1560	1570	1580	1590	1600
QLMLQSLSTS	EELQRQFHLF	QLQRLDKLFL	EQEDEEEKRL	EEEEEEEEEE	EAEKELFIED	PSPAISILVL	SPRCWPVSPL
1610	1620	1630	1640	1650	1660	1670	1680
CYLYHPRKCL	PTEFCDALDR	FSSFYSQSQN	HPVLDMGPHR	RLQWTWLGRA	ELQFGKQILH	VSTVQMWLLL	KFNQTEEVSV
1690	1700	1710	1720	1730	1740	1750	1760
ETLLKDSDLS	PELLLQALVP	LTSGNGPLTL	HEGQDFPHGG	VLRLHEPGPQ	RSGEALWLIP	PQAYLNVEKD	EGRTLEQKRN
1770	1780	1790	1800	1810	1820	1830	1840
LLSCLLVRIL	KAHGEKGLHI	DQLVCLVLEA	WQKGPNPPGT	LGHTVAGGVA	CTSTDVLSCI	LHLLGQGYVK	RRDDRPQILM
1850	1860	1870	1880	1890	1900	1910	1920
YAAPEPMGPC	RGQADVPFCG	SQSETSKPSP	EAVATLASLQ	LPAGRTMSPQ	EVEGLMKQTV	RQVQETLNLE	PDVAQHLLAH
1930	1940	1950	1960	1970	1980	1990	2000
SHWGAEQLLQ	SYSEDPEPLL	LAAGLCVHQA	QAVPVRPDHC	PVCVSPLGCD	DDLPSLCCMH	YCCKSCWNEY	LTTRIEQNLV
2010	2020	2030	2040	2050	2060	2070	2080
LNCTCPIADC	PAQPTGAFIR	AIVSSPEVIS	KYEKALLRGY	VESCSNLTWC	TNPQGCDRIL	CRQGLGCGTT	CSKCGWASCF
2090	2100	2110	2120	2130	2140	2150	2160
NCSFPEAHYP	ASCGHMSQWV	DDGGYYDGMS	VEAQSKHLAK	LISKRCPSCQ	APIEKNEGCL	HMTCAKCNHG	FCWRCLKSWK
2170	2180	2190	2200	2210	2220	2230	2240

Detailed Protein Report

Cmpd.	No. of Cmpds.	m/z meas.	Δ m/z [ppm]	z	Rt [min]	Score	P	Range	Sequence	Modification
2528	1	938.1784	46.28	3	60.3	13.1	2	164-188	R.ETGALDLLMHMLCNPEPQIRRSAGK.M	Oxidation: 9, 11
949	1	737.3420	-85.80	1	40.4	14.7	0	790-796	R.HSGIAPR.T	

Detailed Protein Report

Protein 296: PREDICTED: sterol regulatory element-binding protein 1 isoform X2 [Homo sapiens]

**Accession:**	gi|578830023	**Score:**	27.8
**Database:**	refseq_human(refseq_human_20140103.fasta)	**MW [kDa]:**	119.1
**Database Date:**	2015-11-30	**pI:**	9.9
		**Sequence Coverage [%]:**	2.0
		**No. of unique Peptides:**	1

Quantitation

QD:QU **Median:** 1.31 **CV:** 0.00 % **No. of Peptides:** 1

```
        10         20         30         40         50         60         70         80
MDCTFEDMLQ LINNQDSDFP GLFDPPYAGS GAGGTDPASP DTSSPGSLSP PPATLSSSLE AFLSGPQAAP SPLSPPQPAP
        90        100        110        120        130        140        150        160
TPLKMYPSMP AFSPGPGIKE ESVPLSILQT PTPQPLPGAL LPQSFPAPAP PQFSSTPVLG YPSPPGGFST GSPPGNTQQP
       170        180        190        200        210        220        230        240
LPGLPLASPP GVPPVSLHTQ VQSVVPQQLL TVTAAPTAAP VTTTVTSQIQ QVPVLLQPHF IKADSLLLTA MKTDGATVKA
       250        260        270        280        290        300        310        320
AGLSPLVSGT TVQTGPLPTL VSGGTILATV PLVVDAEKLP INRLAAGSKA PASAQSRGEK RTAHNAIEKR YRSSINDKII
       330        340        350        360        370        380        390        400
ELKDLVVGTE AKLNKSAVLR KAIDYIRFLQ HSNQKLKQEN LSLRTAVHKS KSLKDLVSAC GSGGNTDVLM EGVKTEVEDT
       410        420        430        440        450        460        470        480
LTPPPSDAGS PFQSSPLSLG SRGSGSGGSG SDSEPDSPVF EDSKAKPEQR PSLHSRGMLD RSRLALCTLV FLCLSCNPLA
       490        500        510        520        530        540        550        560
SLLGARGLPS PSDTTSVYHS PGRNVLGTES RDGPGWAQWL LPPVVWLLNG LLVLVSLVLL FVYGEPVTRP HSGPAVYFWR
       570        580        590        600        610        620        630        640
HRKQADLDLA RGDFAQAAQQ LWLALRALGR PLPTSHLDLA CSLLWNLIRH LLQRLWVGRW LAGRAGGLQQ DCALRVDASA
       650        660        670        680        690        700        710        720
SARDAALVYH KLHQLHTMGK HTGGHLTATN LALSALNLAE CAGDAVSVAT LAEIYVAAAL RVKTSLPRAL HFLTRFFLSS
       730        740        750        760        770        780        790        800
ARQACLAQSG SVPPAMQWLC HPVGHRFFVD GDWSVLSTPW ESLYSLAGNP VDPLAQVTQL FREHLLERAL NCVTQPNPSP
       810        820        830        840        850        860        870        880
GSADGDKEFS DALGYLQLLN SCSDAAGAPA YSFSISSSMA TTTGVDPVAK WWASLTAVVI HWLRRDEEAA ERLCPLVEHL
       890        900        910        920        930        940        950        960
PRVLQESERP LPRAALHSFK AARALLGCAK AESGPASLTI CEKASGYLQD SLATTPASSS IDKAVQLFLC DLLLVVRTSL
       970        980        990       1000       1010       1020       1030       1040
WRQQQPPAPA PAAQGTSSRP QASALELRGF QRDLSSLRRL AQSFRPAMRR VFLHEATARL MAGASPTRTH QLLDRSLRRR
      1050       1060       1070       1080       1090       1100       1110       1120
AGPGGKGGAV AELEPRPTRR EHAEALLLAS CYLPPGFLSA PGQRVGMLAE AARTLEKLGD RRLLHDCQQM LMRLGGGTTV
      1130
TSS
```

Cmpd.	No. of Cmpds.	m/z meas.	Δ m/z [ppm]	z	Rt [min]	Score	P	Range	Sequence	Modification	Ratios
783	1	676.8811	19.88	2	39.8	12.5	0	1047-1059	K.GGAVAELEPRPTR.R		QD:QU 1.31

Detailed Protein Report

Protein 297: **PREDICTED: ubiquitin-protein ligase E3C isoform X3 [Homo sapiens]**

**Accession:**	gi|530387311	**Score:**	27.7
**Database:**	refseq_human(refseq_human_20140103.fasta)	**MW [kDa]:**	116.7
**Database Date:**	2015-11-30	**pI:**	6.2
		**Sequence Coverage [%]:**	1.4
		**No. of unique Peptides:**	1

10	20	30	40	50	60	70	80
MFSFEGDFKT	RPKVSLGGAS	RKEEKASLLH	RTQEERRKRE	EERRRLKNAI	IIQSFIRGYR	DRKQQYSIQR	SAFDRCATLS
90	100	110	120	130	140	150	160
QSGGAFPIAN	GPNLTLLVRQ	LLFFYKQNED	SKRLIWLYQN	LIKHSSLFVK	QLDGSERLTC	LFQIKRLMSL	CCRLLQNCND
170	180	190	200	210	220	230	240
DSLNVALPMR	MLEVFSSENT	YLPVLQDASY	VVSVIEQILH	YMIHNGYYRS	LYLLINSKLP	SSIEYSDLSR	VPIAKILLEN
250	260	270	280	290	300	310	320
VLKPLHFTYN	SCPEGARQQV	FTAFTEEFLA	APFTDQIFHF	IIPALADAQT	VFPYEPFLNA	LLLIESRCSR	KSGGAPWLFY
330	340	350	360	370	380	390	400
FVLTVGENYL	GALSEEGLLV	YLRVLQTFLS	QLPVSPASAS	CHDSASDSEE	ESEEADKPSS	PEDGRLSVSY	ITEECLKKLD
410	420	430	440	450	460	470	480
TKQQTNTLLN	LVWRDSASEE	VFTTMASVCH	TLMVQHRMMV	PKVRLLYSLA	FNARFLRHLW	FLISSMSTRM	ITGSMVPLLQ
490	500	510	520	530	540	550	560
VISRGSPMSF	EDSSRIIPLF	YLFSSLFSHS	LISIHDNEFF	GDPIEVVGQR	QSSMMPFTLE	ELIMLSRCLR	DACLGIIKLA
570	580	590	600	610	620	630	640
YPETKPEVRE	EYITAFQSIG	VTTSSEMQQC	IQMEQKRWIQ	LFKVITNLVK	MLKSRDTRRN	FCPPNHWLSE	QEDIKADKIF
650	660	670	680	690	700	710	720
QRLIYADKQE	VQGDGPFLDG	INVTIRRNYI	YEDAYDKLSP	ENEPDLKKRI	RVHLLNAHGL	DEAGIDGGGI	FREFLNELLK
730	740	750	760	770	780	790	800
SGFNPNQGFF	KTTNEGLLYP	NPAAQMLVGD	SFARHYYFLG	RMLGKALYEN	MLVELPFAGF	FLSKLLGTSA	DVDIHHLASL
810	820	830	840	850	860	870	880
DPEVYKNLLF	LKSYEDDVEE	LGLNFTVVNN	DLGEAQVVEL	KFGGKDIPVT	SANRIAYIHL	VADYRLNRQI	RQHCLAFRQG
890	900	910	920	930	940	950	960
LANVVSLEWL	RMFDQQEIQV	LISGAQVPIS	LEDLKSFTNY	SGGYSADHPV	IKVFWRVVEG	FTDEEKRKLL	KFVTSCSRPP
970	980	990	1000	1010	1020	1030	
LLGFKELYPA	FCIHNGGSDL	ERLPTASTCM	NLLKLPEFYD	ETLLRSKLLY	AIECAAGFEL	S	

Cmpd.	No. of Cmpds.	m/z meas.	Δ m/z [ppm]	z	Rt [min]	Score	P	Range	Sequence	Modification
2489	4	817.4835	32.82	1	61.7	16.0	0	124-130	K.HSSLFVK.Q	

Detailed Protein Report

Protein 298: PREDICTED: protein FAM65C isoform X2 [Homo sapiens]

**Accession:**	gi|578835791	**Score:**	27.7
**Database:**	refseq_human(refseq_human_20140103.fasta)	**MW [kDa]:**	105.1
**Database Date:**	2015-11-30	**pI:**	7.0
**Modification(s):**	Carbamidomethyl	**Sequence Coverage [%]:**	1.0
		**No. of unique Peptides:**	1

Quantitation

QD:QU **Median:** 0.99 **CV:** 0.00 % **No. of Peptides:** 1

10	20	30	40	50	60	70	80
MVTTMSVRLR	FLSPGDTGAV	GVVGRSASFA	GFSSAQSRRI	ANSVRSRMPA	KSSKMYGTLR	KGSVCADPKP	QQVKKIFEAL
90	100	110	120	130	140	150	160
KRGLKEYLCV	QQAELDHLSG	RHKDTRRNSR	LAFYYDLDKQ	TRCVERHIRK	MEFHISKVDE	LYEDYCIQCR	LRDGASSMQR
170	180	190	200	210	220	230	240
AFARCPPSRA	ARESLQELGR	SLHECAEDMW	LIEGALEVHL	GEFHIRMKGL	VGYARLCPGD	HYEVLMRLGR	QRWKLKGRIE
250	260	270	280	290	300	310	320
SDDSQTWDEE	EKAFIPTLHE	NLDIKVTELR	GLGSLAVGAV	TCDIADFFTT	RPQVIVVDIT	ELGTIKLQLE	VQWNPFDTES
330	340	350	360	370	380	390	400
FLVSPSPTGK	FSMGSRKGSL	YNWTPPSTPS	FRERYYLSVL	QQPTQQALLL	GGPRATSILS	YLSDSDLRGP	SLRSQSQELP
410	420	430	440	450	460	470	480
EMDSFSSEDP	RDTETSTSAS	TSDVGFLPLT	FGPHASIEEE	AREDPLPPGL	LPEMAHLSGG	PFAEQPGWRN	LGGESPSLPQ
490	500	510	520	530	540	550	560
GSLFHSGTAS	SSQNGHEEGA	TGDREDGPGV	ALEGPLQEVL	ELLRPTDSTQ	PQLRELEYQV	LGFRDRLKPC	RARQEHTSAE
570	580	590	600	610	620	630	640
SLMECILESF	AFLNADFALD	ELSLFGGSQG	LRKDRPLPPP	SSLKASSREL	TAGAPELDVL	LMVHLQVCKA	LLQKLASPNL
650	660	670	680	690	700	710	720
SRLVQECLLE	EVAQQKHVLE	TLSVLDFEKV	GKATSIEEII	PQASRTKGCL	KLWRGCTGPG	RVLSCPATTL	LNQLKKTFQH
730	740	750	760	770	780	790	800
RVRGKYPGQL	EIACRRLLEQ	VVSCGGLLPG	AGLPEEQIIT	WFQFHSYLQR	QSVSDLEKHF	TQLTKEVTLI	EELHCAGQAK
810	820	830	840	850	860	870	880
VVRKLQGKRL	GQLQPLPQTL	RAWALLQLDG	TPRVCRAASA	RLAGAVRNRS	FREKALLFYT	NALAENDARL	QQAACLALKH
890	900	910	920	930	940	950	
LKGIESIDQT	ASLCQSDLEA	VRAAARETTL	SFGEKGRLAF	EKMDKLCSEQ	REVFCQEADV	EITIF	

Cmpd.	No. of Cmpds.	m/z meas.	Δ m/z [ppm]	z	Rt [min]	Score	P	Range	Sequence	Modification	Ratios	
1153	2	583.8250	97.26	2	44.6	14.3	1	923-931	K.MDKLCSEQR.E	Carbamidomethyl: 5	QD:QU	0.99

Detailed Protein Report

Protein 299: prokineticin-2 isoform b precursor [Homo sapiens]

**Accession:**	gi|17530787	**Score:**	27.6
**Database:**	refseq_human(refseq_human_20140103.fasta)	**MW [kDa]:**	11.7
**Database Date:**	2015-11-30	**pI:**	12.3
**Modification(s):**	Carbamidomethyl, Oxidation	**Sequence Coverage [%]:**	15.7
		**No. of unique Peptides:**	2

```
        10         20         30         40         50         60         70         80
MRSLCCAPLL LLLLLPPLLL TPRAGDAAVI TGACDKDSQC GGGMCCAVSI WVKSIRICTP MGKLGDSCHP LTRKVPFFGR
        90        100        110
RMHHTCPCLP GLACLRTSFN RFICLAQK
```

Cmpd.	No. of Cmpds.	m/z meas.	Δ m/z [ppm]	z	Rt [min]	Score	P	Range	Sequence	Modification
2732	3	937.4633	79.63	2	63.1	12.6	0	37-53	K.DSQCGGGMCCAVSIWVK.S	Carbamidomethyl: 4, 10; Oxidation: 8
2648	2	937.4596	75.76	2	61.9	15.1	0	37-53	K.DSQCGGGMCCAVSIWVK.S	Carbamidomethyl: 4, 9; Oxidation: 8

Detailed Protein Report

Protein 300: PREDICTED: protein angel homolog 2 isoform X1 [Homo sapiens]

**Accession:**	gi|530366645	**Score:**	27.6
**Database:**	refseq_human(refseq_human_20140103.fasta)	**MW [kDa]:**	59.7
**Database Date:**	2015-11-30	**pI:**	7.3
**Modification(s):**	Oxidation	**Sequence Coverage [%]:**	5.2
		**No. of unique Peptides:**	1

Quantitation

QD:QU	**Median:** 1.13	**CV:** 0.00 %	**No. of Peptides:** 1

Alias proteins:

Accession	Name	Description
gi|530366649	refseq_human (refseq_human_20140103.fasta)	PREDICTED: protein angel homolog 2 isoform X3 [Homo sapiens]
gi|530366647	refseq_human (refseq_human_20140103.fasta)	PREDICTED: protein angel homolog 2 isoform X2 [Homo sapiens]

```
        10         20         30         40         50         60         70         80
MFPHHSRSLG RDWTTPWENL QRCCWNRHIS SCMRWPGHYS RAPYPYFSSR HFSLNWRPPC LFESRTQFQY CNWRPDNLSQ
        90        100        110        120        130        140        150        160
TSLIHLSSYV MNAEGDEPSS KRRKHQGVIK RNWEYICSHD KEKTKILGDK NVDPKCEDSE NKFDFSVMSY NILSQDLLED
       170        180        190        200        210        220        230        240
NSHLYRHCRR PVLHWSFRFP NILKEIKHFD ADVLCLQEVQ EDHYGAEIRP SLESLGYHCE YKMRTGRKPD GCAICFKHSK
       250        260        270        280        290        300        310        320
FSLLSVNPVE FFRPDISLLD RDNVGLVLLL QPKIPYAACP AICVANTHLL YNPRRGDIKL TQLAMLLAEI SSVAHQKDGS
       330        340        350        360        370        380        390        400
FCPIVMCGDF NSVPGSPLYS FIKEGKLNYE GLPIGKVSGQ EQSSRGQRIL SIPIWPPNLG ISQNCVYEVQ QVPKVEKTDS
       410        420        430        440        450        460        470        480
DLTQTQLKQT EVLVTAEKLS SNLQHHFSLS SVYSHYFPDT GIPEVTTCHS RSAITVDYIF YSAEKEDVAG HPGAEVALVG
       490        500        510        520        530
GLKLLARLSL LTEQDLWTVN GLPNENNSSD HLPLLAKFRL EL
```

Cmpd.	No. of Cmpds.	m/z meas.	Δ m/z [ppm]	z	Rt [min]	Score	P	Range	Sequence	Modification	Ratios
910	1	866.9745	93.61	2	41.4	12.5	1	28-41	R.HISSCMRWPGHYSR.A	Oxidation: 6	QD:QU 1.13

Detailed Protein Report

Protein 301: **EGF domain-specific O-linked N-acetylglucosamine transferase isoform a precursor [Homo sapiens]**

**Accession:**	gi|522838254	**Score:**	27.6
**Database:**	refseq_human(refseq_human_20140103.fasta)	**MW [kDa]:**	62.0
**Database Date:**	2015-11-30	**pI:**	6.6
**Modification(s):**	Carbamidomethyl	**Sequence Coverage [%]:**	4.7
		**No. of unique Peptides:**	1

Alias proteins:

Accession	Name	Description
gi|578805996	refseq_human (refseq_human_20140103.fasta)	PREDICTED: EGF domain-specific O-linked N-acetylglucosamine transferase isoform X3 [Homo sapiens]
gi|530373685	refseq_human (refseq_human_20140103.fasta)	PREDICTED: EGF domain-specific O-linked N-acetylglucosamine transferase isoform X2 [Homo sapiens]
gi|530373683	refseq_human (refseq_human_20140103.fasta)	PREDICTED: EGF domain-specific O-linked N-acetylglucosamine transferase isoform X1 [Homo sapiens]

10	20	30	40	50	60	70	80
MLMLFVFGVL	LHEVSLSGQN	EAPPNTHSIP	GEPLYNYASI	RLPEEHIPFF	LHNNRHIATV	CRKDSLCPYK	KHLEKLKYCW
**90**	**100**	**110**	**120**	**130**	**140**	**150**	**160**
GYEKSCKPEF	RFGYPVCSYV	DMGWTDTLES	AEDIFWKQAD	FGYARERLEE	MHVLCQPKET	SDSSLVCSRY	LQYCRATNLY
**170**	**180**	**190**	**200**	**210**	**220**	**230**	**240**
LDLRNIKRNH	DRFKEDFFQS	GEIGGHCKLD	IRTLTSEGQR	KSPLQSWFAE	LQSYTQLNFR	PIEDAKCDIV	IEKPTYFMKL
**250**	**260**	**270**	**280**	**290**	**300**	**310**	**320**
DAGVNMYHHF	CDFINLYITQ	HVNNSFSTDV	YIVMWDTSSY	GYGDLFSDTW	NAFTDYDVIH	LKTYDSKRVC	FKEAVFSLLP
**330**	**340**	**350**	**360**	**370**	**380**	**390**	**400**
RMRYGLFYNT	PLISGCQNTG	LFRAFAQHVL	HRLNITQEGP	KDGKIRVTIL	ARSTEYRKIL	NQNELVNALK	TVSTFEVQIV
**410**	**420**	**430**	**440**	**450**	**460**	**470**	**480**
DYKYRELGFL	DQLRITHNTD	IFIGMHGAGL	THLLFLPDWA	AVFELYNCED	ERCYLDLARL	RGVHYITWRR	QNKVFPQDKG
**490**	**500**	**510**	**520**	**530**			
HHPTLGEHPK	FTNYSFDVEE	FMYLVLQAAD	HVLQHPKWPF	KKKHDEL			

Cmpd.	No. of Cmpds.	m/z meas.	Δ m/z [ppm]	z	Rt [min]	Score	P	Range	Sequence	Modification
674	1	698.6888	27.74	3	38.4	13.0	2	76-91	K.LKYCWGYEKSCKPEFR.F	Carbamidomethyl: 11

Detailed Protein Report

Protein 302: SPRY domain-containing SOCS box protein 2 [Homo sapiens]

Accession: gi|14249178
Database: refseq_human(refseq_human_20140103.fasta)
Database Date: 2015-11-30
Modification(s): Oxidation

Score: 27.6
MW [kDa]: 28.6
pI: 9.2
Sequence Coverage [%]: 9.9
No. of unique Peptides: 2

Quantitation

QD:QU **Median:** 2.18 **CV:** 483.35 % **No. of Peptides:** 2

Alias proteins:

Accession	Name	Description
gi|226423903	refseq_human (refseq_human_20140103.fasta)	SPRY domain-containing SOCS box protein 2 [Homo sapiens]

10	20	30	40	50	60	70	80
MGQTALAGGS	SSTPTPQALY	PDLSCPEGLE	ELLSAPPPDL	GAQRRHGWNP	KDCSENIEVK	EGGLYFERRP	VAQSTDGARG
**90**	**100**	**110**	**120**	**130**	**140**	**150**	**160**
KRGYSRGLHA	WEISWPLEQR	GTHAVVGVAT	ALAPLQTDHY	AALLGSNSES	WGWDIGRGKL	YHQSKGPGAP	QYPAGTQGEQ
**170**	**180**	**190**	**200**	**210**	**220**	**230**	**240**
LEVPERLLVV	LDMEEGTLGY	AIGGTYLGPA	FRGLKGRTLY	PAVSAVWGQC	QVRIRYLGER	RAEPHSLLHL	SRLCVRHNLG
**250**	**260**	**270**					
DTRLGQVSAL	PLPPAMKRYL	LYQ					

Cmpd.	No. of Cmpds.	m/z meas.	Δ m/z [ppm]	z	Rt [min]	Score	P	Range	Sequence	Modification	Ratios	
290	2	642.3243	-17.35	2	32.3	15.8	1	233-243	R.LCVRHNLGDTR.L		QD:QU	0.37
2133	2	797.3986	-78.05	2	57.0	11.7	1	244-258	R.LGQVSALPLPPAMKR.Y	Oxidation: 13	QD:QU	13.04

Detailed Protein Report

Protein 303: PREDICTED: nucleoprotein TPR isoform X1 [Homo sapiens]

**Accession:**	gi|530365345	**Score:**	27.5
**Database:**	refseq_human(refseq_human_20140103.fasta)	**MW [kDa]:**	274.8
**Database Date:**	2015-11-30	**pI:**	4.9
		**Sequence Coverage [%]:**	0.8
		**No. of unique Peptides:**	1

Detailed Protein Report

10	20	30	40	50	60	70	80
MPSSGSVSRS	RQRGRSMASR	GFDGSRRGSL	RRAGAREPPS	EVIEAAAQAS	GSCWSSPFFS	ASTPSAAATG	VPGPTDMAAV
90	100	110	120	130	140	150	160
LQQVLERTEL	NKLPKSVQNK	LEKFLADQQS	EIDGLKGRHE	KFKVESEQQY	FEIEKRLSHS	QERLVNETRE	CQSLRLELEK
170	180	190	200	210	220	230	240
LNNQLKALTE	KNKELEIAQD	RNIAIQSQFT	RTKEELEAEK	RDLIRTNERL	SQELEYLTED	VKRLNEKLKE	SNTTKGELQL
250	260	270	280	290	300	310	320
KLDELQASDV	SVKYREKRLE	QEKELLHSQN	TWLNTELKTK	TDELLALGRE	KGNEILELKC	NLENKKEEVS	RLEEQMNGLK
330	340	350	360	370	380	390	400
TSNEHLQKHV	EDLLTKLKEA	KEQQASMEEK	FHNELNAHIK	LSNLYKSAAD	DSEAKSNELT	RAVEELHKLL	KEAGEANKAI
410	420	430	440	450	460	470	480
QDHLLEVEQS	KDQMEKEMLE	KIGRLEKELE	NANDLLSATK	RKGAILSEEE	LAAMSPTAAA	VAKIVKPGMK	LTELYNAYVE
490	500	510	520	530	540	550	560
TQDQLLLEKL	ENKRINKYLD	EIVKEVEAKA	PILKRQREEY	ERAQKAVASL	SVKLEQAMKE	IQRLQEDTDK	ANKQSSVLER
570	580	590	600	610	620	630	640
DNRRMEIQVK	DLSQQIRVLL	MELEEARGNH	VIRDEEVSSA	DISSSSEVIS	QHLVSYRNIE	ELQQQNQRLL	VALRELGETR
650	660	670	680	690	700	710	720
EREEQETTSS	KITELQLKLE	SALTELEQLR	KSRQHQMQLV	DSIVRQRDMY	RILLSQTTGV	AIPLHASSLD	DVSLASTPKR
730	740	750	760	770	780	790	800
PSTSQTVSTP	APVPVIESTE	AIEAKAALKQ	LQEIFENYKK	EKAENEKIQN	EQLEKLQEQV	TDLRSQNTKI	STQLDFASKR
810	820	830	840	850	860	870	880
YEMLQDNVEG	YRREITSLHE	RNQKLTATTQ	KQEQIINTMT	QDLRGANEKL	AVAEVRAENL	KKEKEMLKLS	EVRLSQQRES
890	900	910	920	930	940	950	960
LLAEQRGQNL	LLTNLQTIQG	ILERSETETK	QRLSSQIEKL	EHEISHLKKK	LENEVEQRHT	LTRNLDVQLL	DTKRQLDTET
970	980	990	1000	1010	1020	1030	1040
NLHLNTKELL	KNAQKEIATL	KQHLSNMEVQ	VASQSSQRTG	KGQPSNKEDV	DDLVSQLRQT	EEQVNDLKER	LKTSTSNVEQ
1050	1060	1070	1080	1090	1100	1110	1120
YQAMVTSLEE	SLNKEKQVTE	EVRKNIEVRL	KESAEFQTQL	EKKLMEVEKE	KQELQDDKRR	AIESMEQQLS	ELKKTLSSVQ
1130	1140	1150	1160	1170	1180	1190	1200
NEVQEALQRA	STALSNEQQA	RRDCQEQAKI	AVEAQNKYER	ELMLHAADVE	ALQAAKEQVS	KMASVRQHLE	ETTQKAESQL
1210	1220	1230	1240	1250	1260	1270	1280
LECKASWEER	ERMLKDEVSK	CVCRCEDLEK	QNRLLHDQIE	KLSDKVVASV	KEGVQGPLNV	SLSEEGKSQE	QILEILRFIR
1290	1300	1310	1320	1330	1340	1350	1360
REKEIAETRF	EVAQVESLRY	RQRVELLERE	LQELQDSLNA	EREKVQVTAK	TMAQHEELMK	KTETMNVVME	TNKMLREEKE
1370	1380	1390	1400	1410	1420	1430	1440
RLEQDLQQMQ	AKVRKLELDI	LPLQEANAEL	SEKSGMLQAE	KKLLEEDVKR	WKARNQHLVS	QQKDPDTEEY	RKLLSEKEVH
1450	1460	1470	1480	1490	1500	1510	1520
TKRIQQLTEE	IGRLKAEIAR	SNASLTNNQN	LIQSLKEDLN	KVRTEKETIQ	KDLDAKIIDI	QEKVKTITQV	KKIGRRYKTQ
1530	1540	1550	1560	1570	1580	1590	1600
YEELKAQQDK	VMETSAQSSG	DHQEQHVSVQ	EMQELKETLN	QAETKSKSLE	SQVENLQKTL	SEKETEARNL	QEQTVQLQSE
1610	1620	1630	1640	1650	1660	1670	1680
LSRLRQDLQD	RTTQEEQLRQ	QITEKEEKTR	KAIVAAKSKI	AHLAGVKDQL	TKENEELKQR	NGALDQQKDE	LDVRITALKS
1690	1700	1710	1720	1730	1740	1750	1760
QYEGRISRLE	RELREHQERH	LEQRDEPQEP	SNKVPEQQRQ	ITLKTTPASG	ERGIASTSDP	PTANIKPTPV	VSTPSKVTAA
1770	1780	1790	1800	1810	1820	1830	1840
AMAGNKSTPR	ASIRPMVTPA	TVTNPTTTPT	ATVMPTTQVE	SQEAMQSEGP	VEHVPVFGST	SGSVRSTSPN	VQPSISQPIL
1850	1860	1870	1880	1890	1900	1910	1920
TVQQQTQATA	FVQPTQQSHP	QIEPANQELS	SNIVEVVQSS	PVERPSTSTA	VFGTVSATPS	SSLPKRTREE	EEDSTIEASD
1930	1940	1950	1960	1970	1980	1990	2000
QVSDDTVEMP	LPKKLKSVTP	VGTEEEVMAE	ESTDGEVETQ	VYNQDSQDSI	GEGVTQGDYT	PMEDSEETSQ	SLQIDLGPLQ
2010	2020	2030	2040	2050	2060	2070	2080
SDQQTTTSSQ	DGQGKGDDVI	VIDSDDEEED	DDENDGEHED	YEEDEEDDDD	DEDDTGMGDE	GEDSNEGTGS	ADGNDGYEAD
2090	2100	2110	2120	2130	2140	2150	2160
DAEGGDGTDP	GTETEESMGG	GEGNHRAADS	QNSGEGNTGA	AESSFSQEVS	REQQPSSASE	RQAPRAPQSP	RRPPHPLPPR
2170	2180	2190	2200	2210	2220	2230	2240
LTIHAPPQEL	GPPVQRIQMT	RRQSVGRGLQ	LTPGIGGMQQ	HFFDDEDRTV	PSTPTLVVPH	RTDGFAEAIH	SPQVAGVPRF
2250	2260	2270	2280	2290	2300	2310	2320
RFGPPEDMPQ	TSSSHSDLGQ	LASQGGLGMY	ETPLFLAHEE	ESGGRSVPTT	PLQVAAPVTV	FTESTTSDAS	EHASQSVPMV
2330	2340	2350	2360	2370	2380	2390	2400

Detailed Protein Report

Cmpd.	No. of Cmpds.	m/z meas.	Δ m/z [ppm]	z	Rt [min]	Score	P	Range	Sequence	Modification
1647	1	524.8144	75.03	2	50.8	12.8	2	12-20	R.QRGRSMASR.G	

Detailed Protein Report

Protein 304: zinc finger protein 622 [Homo sapiens]

Accession: gi|15529978
Database: refseq_human(refseq_human_20140103.fasta)
Database Date: 2015-11-30
Modification(s): Oxidation

Score: 27.5
MW [kDa]: 54.2
pI: 5.8
Sequence Coverage [%]: 5.5
No. of unique Peptides: 2

10	20	30	40	50	60	70	80
MATYTCITCR	VAFRDADMQR	AHYKTDWHRY	NLRRKVASMA	PVTAEGFQER	VRAQRAVAEE	ESKGSATYCT	VCSKKFASFN
90	100	110	120	130	140	150	160
AYENHLKSRR	HVELEKKAVQ	AVNRKVEMMN	EKNLEKGLGV	DSVDKDAMNA	AIQQAIKAQP	SMSPKKAPPA	PAKEARNVVA
170	180	190	200	210	220	230	240
VGTGGRGTHD	RDPSEKPPRL	QWFEQQAKKL	AKQQEEDSEE	EEEDLDGDDW	EDIDSDEELE	CEDTEAMDDV	VEQDAEEEEA
250	260	270	280	290	300	310	320
EEGPPLGAIP	ITDCLFCSHH	SSSLMKNVAH	MTKDHSFFIP	DIEYLSDIKG	LIKYLGEKVG	VGKICLWCNE	KGKSFYSTEA
330	340	350	360	370	380	390	400
VQAHMNDKSH	CKLFTDGDAA	LEFADFYDFR	SSYPDHKEGE	DPNKAEELPS	EKNLEYDDET	MELILPSGAR	VGHRSLMRYY
410	420	430	440	450	460	470	480
KQRFGLSRAV	AVAKNRKAVG	RVLQQYRALG	WTGSTGAALM	RERDMQYVQR	MKSKWMLKTG	MKNNATKQMH	FRVQVRF

Cmpd.	No. of Cmpds.	m/z meas.	Δ m/z [ppm]	z	Rt [min]	Score	P	Range	Sequence	Modification
2687	2	973.5040	65.98	2	64.7	14.9	2	15-29	R.DADMQRAHYKTDWHR.Y	Oxidation: 4
1757	1	560.2452	1.92	2	52.3	12.6	0	64-74	K.GSATYCTVCSK.K	

Detailed Protein Report

Protein 305: retinoblastoma-associated protein [Homo sapiens]

**Accession:**	gi|108773787	**Score:**	27.5
**Database:**	refseq_human(refseq_human_20140103.fasta)	**MW [kDa]:**	106.1
**Database Date:**	2015-11-30	**pI:**	8.9
**Modification(s):**	Carbamidomethyl, Oxidation	**Sequence Coverage [%]:**	2.0
		**No. of unique Peptides:**	1

10	20	30	40	50	60	70	80
MPPKTPRKTA	ATAAAAAAEP	PAPPPPPPPE	EDPEQDSGPE	DLPLVRLEFE	ETEEPDFTAL	CQKLKIPDHV	RERAWLTWEK
**90**	**100**	**110**	**120**	**130**	**140**	**150**	**160**
VSSVDGVLGG	YIQKKKELWG	ICIFIAAVDL	DEMSFTFTEL	QKNIEISVHK	FFNLLKEIDT	STKVDNAMSR	LLKKYDVLFA
**170**	**180**	**190**	**200**	**210**	**220**	**230**	**240**
LFSKLERTCE	LIYLTQPSSS	ISTEINSALV	LKVSWITFLL	AKGEVLQMED	DLVISFQLML	CVLDYFIKLS	PPMLLKEPYK
**250**	**260**	**270**	**280**	**290**	**300**	**310**	**320**
TAVIPINGSP	RTPRRGQNRS	ARIAKQLEND	TRIIEVLCKE	HECNIDEVKN	VYFKNFIPFM	NSLGLVTSNG	LPEVENLSKR
**330**	**340**	**350**	**360**	**370**	**380**	**390**	**400**
YEEIYLKNKD	LDARLFLDHD	KTLQTDSIDS	FETQRTPRKS	NLDEEVNVIP	PHTPVRTVMN	TIQQLMMILN	SASDQPSENL
**410**	**420**	**430**	**440**	**450**	**460**	**470**	**480**
ISYFNNCTVN	PKESILKRVK	DIGYIFKEKF	AKAVGQGCVE	IGSQRYKLGV	RLYYRVMESM	LKSEEERLSI	QNFSKLLNDN
**490**	**500**	**510**	**520**	**530**	**540**	**550**	**560**
IFHMSLLACA	LEVVMATYSR	STSQNLDSGT	DLSFPWILNV	LNLKAFDFYK	VIESFIKAEG	NLTREMIKHL	ERCEHRIMES
**570**	**580**	**590**	**600**	**610**	**620**	**630**	**640**
LAWLSDSPLF	DLIKQSKDRE	GPTDHLESAC	PLNLPLQNNH	TAADMYLSPV	RSPKKKGSTT	RVNSTANAET	QATSAFQTQK
**650**	**660**	**670**	**680**	**690**	**700**	**710**	**720**
PLKSTSLSLF	YKKVYRLAYL	RLNTLCERLL	SEHPELEHII	WTLFQHTLQN	EYELMRDRHL	DQIMMCSMYG	ICKVKNIDLK
**730**	**740**	**750**	**760**	**770**	**780**	**790**	**800**
FKIIVTAYKD	LPHAVQETFK	RVLIKEEEYD	SIIVFYNSVF	MQRLKTNILQ	YASTRPPTLS	PIPHIPRSPY	KFPSSPLRIP
**810**	**820**	**830**	**840**	**850**	**860**	**870**	**880**
GGNIYISPLK	SPYKISEGLP	TPTKMTPRSR	ILVSIGESFG	TSEKFQKINQ	MVCNSDRVLK	RSAEGSNPPK	PLKKLRFDIE
**890**	**900**	**910**	**920**	**930**			
GSDEADGSKH	LPGESKFQQK	LAEMTSTRTR	MQKQKMNDSM	DTSNKEEK			

Cmpd.	No. of Cmpds.	m/z meas.	Δ m/z [ppm]	z	Rt [min]	Score	P	Range	Sequence	Modification
2018	1	1044.9422	-30.44	2	55.6	16.6	1	699-715	R.HLDQIMMCSMYGICKVK.N	Carbamidomethyl: 14; Oxidation: 6, 10

Detailed Protein Report

Protein 306: **PREDICTED: delta and Notch-like epidermal growth factor-related receptor isoform X1 [Homo sapiens]**

**Accession:**	gi|530371157	**Score:**	27.5
**Database:**	refseq_human(refseq_human_20140103.fasta)	**MW [kDa]:**	73.8
**Database Date:**	2015-11-30	**pI:**	5.0
**Modification(s):**	Carbamidomethyl	**Sequence Coverage [%]:**	6.9
		**No. of unique Peptides:**	2

10	20	30	40	50	60	70	80
MQPRRAQAPG	AQLLPALALL	LLLLGAGPRG	SSLANPVPAA	PLSAPGPCAA	QPCRNGGVCT	SRPEPDPQHP	APAGEPGYSC
**90**	**100**	**110**	**120**	**130**	**140**	**150**	**160**
TCPAGISGAN	CQLVADPCAS	NPCHHGNCSS	SSSSSSDGYL	CICNEGYEGP	NCEQALPSLP	ATGWTESMAP	RQLQPVPATQ
**170**	**180**	**190**	**200**	**210**	**220**	**230**	**240**
EPDKILPRSQ	ATVTLPTWQP	KTGQKVVEMK	WDQVEVIPDI	ACGNASSNSS	AGGRLVSFEV	PQNTSVKIRQ	DATASLILLW
**250**	**260**	**270**	**280**	**290**	**300**	**310**	**320**
KVTATGFQQC	SLIDGRSVTP	LQASGGLVLL	EEMLALGNNH	FIGFVNDSVT	KSIVALRLTL	VVKVSTCVPG	ESHANDLECS
**330**	**340**	**350**	**360**	**370**	**380**	**390**	**400**
GKGKCTTKPS	EATFSCTCEE	QYVGTFCEEY	DACQRKPCQN	NASCIDANEK	QDGSNFTCVC	LPGYTGELCQ	SKIDYCILDP
**410**	**420**	**430**	**440**	**450**	**460**	**470**	**480**
CRNGATCISS	LSGFTCQCPE	GYFGSACEEK	VDPCASSPCQ	NNGTCYVDGV	HFTCNCSPGF	TGPTCAQLID	FCALSPCAHG
**490**	**500**	**510**	**520**	**530**	**540**	**550**	**560**
TCRSVGTSYK	CLCDPGTHCE	LYKDPCANVS	CLNGATCDSD	GLNGTCICAP	GFTGEECDID	INECDSNPCH	HGGSCLDQPN
**570**	**580**	**590**	**600**	**610**	**620**	**630**	**640**
GYNCHCPHGW	VGANCEIHLQ	WKSGHMAESL	TNMPRHSLYI	IIGALCVAFI	LMLIILIVGI	CRISRIEYQG	SSRPAYEEFY
**650**	**660**	**670**	**680**	**690**	**700**		
NCRSIDSEFS	NAIASIRHAR	FGKKSRPAMY	DVSPIAYEDY	SPDDKPLVTL	IKTKDL		

Cmpd.	No. of Cmpds.	m/z meas.	Δ m/z [ppm]	z	Rt [min]	Score	P	Range	Sequence	Modification
2308	1	1002.6780	75.60	3	59.3	12.4	2	1-29	-.MQPRRAQAPGAQLLPALALLLLLLGAGPR.G	
1231	1	1023.8326	-111.10	2	45.5	15.1	0	304-322	K.VSTCVPGESHANDLECSGK.G	Carbamidomethyl: 4, 16

Detailed Protein Report

Protein 307: protein dopey-2 [Homo sapiens]

**Accession:**	gi|45827701	**Score:**	27.4
**Database:**	refseq_human(refseq_human_20140103.fasta)	**MW [kDa]:**	258.1
**Database Date:**	2015-11-30	**pI:**	5.9
		**Sequence Coverage [%]:**	1.0
		**No. of unique Peptides:**	1

Alias proteins:

Accession	Name	Description
gi|578836735	refseq_human (refseq_human_20140103.fasta)	PREDICTED: protein dopey-2 isoform X1 [Homo sapiens]

Detailed Protein Report

10	20	30	40	50	60	70	80
MDPEEQELLN	DYRYRSYSSV	IEKALRNFES	SSEWADLISS	LGKLNKALQS	NLRYSLLPRR	LLISKRLAQC	LHPALPSGVH
90	100	110	120	130	140	150	160
LKALETYEII	FKIVGTKWLA	KDLFLYSCGL	FPLLAHAAVS	VRPVLLTLYE	KYFLPLQKLL	LPSLQAFIVG	LLPGLEEGSE
170	180	190	200	210	220	230	240
ISDRTDALLL	RLSLVVGKEV	FYTALWGSVL	ASPSIRLPAS	VFVVGHINRD	APGREQKYML	GTNHQLTVKS	LRASLLDSNV
250	260	270	280	290	300	310	320
LVQRNNLEIV	LFFFPFYTCL	DSNERAIPLL	RSDIVRILSA	ATQTLLRRDM	SLNRRLYAWL	LGSDIKGNTV	VPESEISNSY
330	340	350	360	370	380	390	400
EDQSSYFFEK	YSKDLLVEGL	AEILHQKFID	ADVEERHHAY	LKPFRVLISL	LDKPEIGPQV	VGNLFLEVIR	AFYSYCRDAL
410	420	430	440	450	460	470	480
GSDLKLSYTQ	SGNSLISAIK	ENRNASEIVK	TVNLLITSLS	TDFLWDYMTR	CFEECFRPVK	QRYSVRNSVS	PPPTVSELCA
490	500	510	520	530	540	550	560
LLVFLLDVIP	LELYSEVQTQ	YLPQVLGCLV	QPLAEDMEAL	SLPELTHALK	TCFKVLSKVQ	MPPSYLDTES	TSGTSSPVKG
570	580	590	600	610	620	630	640
ENGKIILETK	AVIPGDEDAS	FPPLKSEDSG	IGLSASSPEL	SEHLRVPRVS	LERDDVWKKG	GSMQRTFLCI	QELIANFASK
650	660	670	680	690	700	710	720
NIFGVQLTAS	GEESKSEEPA	GKRDRDGTQS	LAANDSSRKN	SWEPKPITVP	QFKQMLSDLF	TARGSPFKTK	SSESPSSSPS
730	740	750	760	770	780	790	800
SPARKNGGEW	DVEKVVIDLG	GSREERREAF	AAACHLLLDC	ATFPVYLSEE	ETEQLCATLF	QLPGAGDSSF	PSWLKSLMTI
810	820	830	840	850	860	870	880
CCCVTDCYLQ	NVAISTLLEV	INHSQSLALV	IEDKMKRYKS	SGHNPFFGKL	QMVTVPPIAP	GILKVIAEKT	DFYQRVARVL
890	900	910	920	930	940	950	960
WNQLNKETRE	HHVTCVELFY	RLHCLAPTAN	ICEDIICHAL	LDPDKGTRLE	ALFRFSVIWH	LTREIQGSRV	TSHNRSFDRS
970	980	990	1000	1010	1020	1030	1040
LFVVLDSLAC	TDGAIGAAAQ	GWLVRALSLG	DVARILEPVL	LLLLQPKTQR	TSIHCLKQEN	SADDLHRWFN	RKKTSFREAC
1050	1060	1070	1080	1090	1100	1110	1120
AVPEPQESGS	EEHLPLSQFT	TVDREAIWAE	VEKEPEKYPL	RGELSEEELP	YYVELPDRTA	HGAPDSSEHT	ESADTSSCHT
1130	1140	1150	1160	1170	1180	1190	1200
DSENTSSFSS	PSHDLQELSN	EENCCAPIPM	GGRAYPKRSA	LLAAFQSESF	KAGAKLSLVR	VDSDKTQASE	SFSSDEEADL
1210	1220	1230	1240	1250	1260	1270	1280
ELQALTTSRL	LKQQRERQEA	VEALFKHILL	YLQPYDSRRV	LYAFSVLEAV	LKTNPKEFIE	AVSRTSMDTS	STAHLNLISN
1290	1300	1310	1320	1330	1340	1350	1360
LLARHQEALI	GQSFYGKLQT	QVPNVCPHSL	LLELLTYLCL	SFLRSYYPCY	LKVSHRDILG	NRDVQVKSVE	VLIRIMMQLV
1370	1380	1390	1400	1410	1420	1430	1440
SVAKSSEGKN	VEFIHSLLQR	CKVQEFVLLS	LSASMYTSQK	RYGLATAHHG	RALPEDSLFE	ESLINLGQDQ	IWSEHPLQIE
1450	1460	1470	1480	1490	1500	1510	1520
LLKLLQVLIV	LEHHLGRAHE	EAENQPDLSR	EWQRALNFQQ	AISALQYVQP	HPLTSQGLLV	SAVVRGLQPA	YGYGMHPAWV
1530	1540	1550	1560	1570	1580	1590	1600
SLVTHSLPYF	GKSLGWTVTP	FVVQICKNLD	DLVKQYESES	VKLSVSTTSK	RENISPDYPL	TLLEGLTTIS	HFCLLEQANQ
1610	1620	1630	1640	1650	1660	1670	1680
NKKTMAAGDP	ANLRNARNAI	LEELPRTVNT	MALLWNVLRK	EETQKRPVDL	LGATKGSSSV	YFKTTKTIRQ	KILDFLNPLT
1690	1700	1710	1720	1730	1740	1750	1760
AHLGVQLTAA	VAAVWSRKKA	QRHSKMKIIP	TASASQLTLV	DLVCALSTLQ	TDTLLHLVKE	VVKRPPQVKG	GDEKSPLVDI
1770	1780	1790	1800	1810	1820	1830	1840
PVLQFCYAFL	QRLPVPALQE	NFSSLLGVLK	ESVQLNLAPP	GYFLLLSMLN	DFVTRTPNLE	NKKDQKDLQE	ITQKILEAVG
1850	1860	1870	1880	1890	1900	1910	1920
NIAGSSLEQT	SWLSRNLEVK	AQPQASLEES	DAEEDLYDAA	AASAMVSSSA	PSVYSVQALS	LLAEVLASLL	DMVYRSDEKE
1930	1940	1950	1960	1970	1980	1990	2000
KAVPLISRLL	YYVFPYLRNH	SAYNAPSFRA	GAQLLSSLSG	YAYTKRAWRK	EVLELFLDPA	FFQMDTSCVH	WKSIIDHLLT
2010	2020	2030	2040	2050	2060	2070	2080
HEKTMFKDLM	NMQSSSLKLF	SSFEQKAMLL	KRQAFAVFSG	ELDQYHLYLP	LIQERLTDNL	RVGQTSIVAA	QMFLFFRVLL
2090	2100	2110	2120	2130	2140	2150	2160
LRISPQHLTS	LWPIMVSELI	QTFTQLEEDL	KDEDESLRST	NKVNRTKVSV	PDANGPSVGE	IPQSELILYL	SACKFLDTAL
2170	2180	2190	2200	2210	2220	2230	2240
SFPPDKMPLF	QIYRWAFIPE	VDTEGPAFLS	DVEENHQECK	PHTVRILELL	KLKFGEISSS	DEITMKSEFP	LLRQHSVSSI
2250	2260	2270	2280	2290	2300		
RQLMPFFMTL	NGAFKTQRQL	PADSPGTPFL	DFPVTDSPRI	LKQLEECIEY	DFLEHPEC		

Detailed Protein Report

Cmpd.	No. of Cmpds.	m/z meas.	Δ m/z [ppm]	z	Rt [min]	Score	P	Range	Sequence	Modification
1889	1	673.7777	-150.29	2	54.0	16.1	0	1929-1938	R.LLYYVFPYLR.N	

Detailed Protein Report

Protein 308: phosphoglycerate kinase 1 [Homo sapiens]

Accession: gi|4505763
Database: refseq_human(refseq_human_20140103.fasta)
Database Date: 2015-11-30
Modification(s): Carbamidomethyl

Score: 27.4
MW [kDa]: 44.6
pI: 9.2
Sequence Coverage [%]: 4.1
No. of unique Peptides: 1

Quantitation

QD:QU **Median:** 1.26 **CV:** 0.00 % **No. of Peptides:** 1

10	20	30	40	50	60	70	80
MSLSNKLTLD	KLDVKGKRVV	MRVDFNVPMK	NNQITNNQRI	KAAVPSIKFC	LDNGAKSVVL	MSHLGRPDGV	PMPDKYSLEP
90	100	110	120	130	140	150	160
VAVELKSLLG	KDVLFLKDCV	GPEVEKACAN	PAAGSVILLE	NLRFHVEEEG	KGKDASGNKV	KAEPAKIEAF	RASLSKLGDV
170	180	190	200	210	220	230	240
YVNDAFGTAH	RAHSSMVGVN	LPQKAGGFLM	KKELNYFAKA	LESPERPFLA	ILGGAKVADK	IQLINNMLDK	VNEMIIGGGM
250	260	270	280	290	300	310	320
AFTFLKVLNN	MEIGTSLFDE	EGAKIVKDLM	SKAEKNGVKI	TLPVDFVTAD	KFDENAKTGQ	ATVASGIPAG	WMGLDCGPES
330	340	350	360	370	380	390	400
SKKYAEAVTR	AKQIVWNGPV	GVFEWEAFAR	GTKALMDEVV	KATSRGCITI	IGGGDTATCC	AKWNTEDKVS	HVSTGGGASL
410	420						
ELLEGKVLPG	VDALSNI						

Cmpd.	No. of Cmpds.	m/z meas.	Δ m/z [ppm]	z	Rt [min]	Score	P	Range	Sequence	Modification	Ratios	
2285	1	884.8843	-99.45	2	59.0	27.4	0	107-123	K.ACANPAAGSVILLENLR.F	Carbamidomethyl: 2	QD:QU	1.26

Detailed Protein Report

Protein 309: chromodomain-helicase-DNA-binding protein 2 isoform 1 [Homo sapiens]

**Accession:**	gi|118421089	**Score:**	27.3
**Database:**	refseq_human(refseq_human_20140103.fasta)	**MW [kDa]:**	211.2
**Database Date:**	2015-11-30	**pI:**	8.8
**Modification(s):**	Oxidation	**Sequence Coverage [%]:**	1.7
		**No. of unique Peptides:**	2

10	20	30	40	50	60	70	80
MMRNKDKSQE	EDSSLHSNAS	SHSASEEASG	SDSGSQSESE	QGSDPGSGHG	SESNSSSESS	ESQSESESES	AGSKSQPVLP
90	100	110	120	130	140	150	160
EAKEKPASKK	ERIADVKKMW	EEYPDVYGVR	RSNRSRQEPS	RFNIKEEASS	GSESGSPKRR	GQRQLKKQEK	WKQEPSEDEQ
170	180	190	200	210	220	230	240
EQGTSAESEP	EQKKVKARRP	VPRRTVPKPR	VKKQPKTQRG	KRKKQDSSDE	DDDDDEAPKR	QTRRRAAKNV	SYKEDDDFET
250	260	270	280	290	300	310	320
DSDDLIEMTG	EGVDEQQDNS	ETIEKVLDSR	LGKKGATGAS	TTVYAIEANG	DPSGDFDTEK	DEGEIQYLIK	WKGWSYIHST
330	340	350	360	370	380	390	400
WESEESLQQQ	KVKGLKKLEN	FKKKEDEIKQ	WLGKVSPEDV	EYFNCQQELA	SELNKQYQIV	ERVIAVKTSK	STLGQTDFPA
410	420	430	440	450	460	470	480
HSRKPAPSNE	PEYLCKWMGL	PYSECSWEDE	ALIGKKFQNC	IDSFHSRNNS	KTIPTRECKA	LKQRPRFVAL	KKQPAYLGGE
490	500	510	520	530	540	550	560
NLELRDYQLE	GLNWLAHSWC	KNNSVILADE	MGLGKTIQTI	SFLSYLFHQH	QLYGPFLIVV	PLSTLTSWQR	EFEIWAPEIN
570	580	590	600	610	620	630	640
VVVYIGDLMS	RNTIREYEWI	HSQTKRLKFN	ALITTYEILL	KDKTVLGSIN	WAFLGVDEAH	RLKNDDSLLY	KTLIDFKSNH
650	660	670	680	690	700	710	720
RLLITGTPLQ	NSLKELWSLL	HFIMPEKFEF	WEDFEEDHGK	GRENGYQSLH	KVLEPFLLRR	VKKDVEKSLP	AKVEQILRVE
730	740	750	760	770	780	790	800
MSALQKQYYK	WILTRNYKAL	AKGTRGSTSG	FLNIVMELKK	CCNHCYLIKP	PEENERENGQ	EILLSLIRSS	GKLILLDKLL
810	820	830	840	850	860	870	880
TRLRERGNRV	LIFSQMVRML	DILAEYLTIK	HYPFQRLDGS	IKGEIRKQAL	DHFNADGSED	FCFLLSTRAG	GLGINLASAD
890	900	910	920	930	940	950	960
TVVIFDSDWN	PQNDLQAQAR	AHRIGQKKQV	NIYRLVTKGT	VEEEIIERAK	KKMVLDHLVI	QRMDTTGRTI	LENNSGRSNS
970	980	990	1000	1010	1020	1030	1040
NPFNKEELTA	ILKFGAEDLF	KELEGEESEP	QEMDIDEILR	LAETRENEVS	TSATDELLSQ	FKVANFATME	DEEELEERPH
1050	1060	1070	1080	1090	1100	1110	1120
KDWDEIIPEE	QRKKVEEEER	QKELEEIYML	PRIRSSTKKA	QTNDSDSDTE	SKRQAQRSSA	SESETEDSDD	DKKPKRRGRP
1130	1140	1150	1160	1170	1180	1190	1200
RSVRKDLVEG	FTDAEIRRFI	KAYKKFGLPL	ERLECIARDA	ELVDKSVADL	KRLGELIHNS	CVSAMQEYEE	QLKENASEGK
1210	1220	1230	1240	1250	1260	1270	1280
GPGKRRGPTI	KISGVQVNVK	SIIQHEEEFE	MLHKSIPVDP	EEKKKYCLTC	RVKAAHFDVE	WGVEDDSRLL	LGIYEHGYGN
1290	1300	1310	1320	1330	1340	1350	1360
WELIKTDPEL	KLTDKILPVE	TDKKPQGKQL	QTRADYLLKL	LRKGLEKKGA	VTGGEEAKLK	KRKPRVKKEN	KVPRLKEEHG
1370	1380	1390	1400	1410	1420	1430	1440
IELSSPRHSD	NPSEEGEVKD	DGLEKSPMKK	KQKKKENKEN	KEKQMSSRKD	KEGDKERKKS	KDKKEKPKSG	DAKSSSKSKR
1450	1460	1470	1480	1490	1500	1510	1520
SQGPVHITAG	SEPVPIGEDE	DDDLDQETFS	ICKERMRPVK	KALKQLDKPD	KGLNVQEQLE	HTRNCLLKIG	DRIAECLKAY
1530	1540	1550	1560	1570	1580	1590	1600
SDQEHIKLWR	RNLWIFVSKF	TEFDARKLHK	LYKMAHKKRS	QEEEEQKKKD	DVTGGKKPFR	PEASGSSRDS	LISQSHTSHN
1610	1620	1630	1640	1650	1660	1670	1680
LHPQKPHLPA	SHGPQMHGHP	RDNYNHPNKR	HFSNADRGDW	QRERKFNYGG	GNNNPPWGSD	RHHQYEQHWY	KDHHYGDRRH
1690	1700	1710	1720	1730	1740	1750	1760
MDAHRSGSYR	PNNMSRKRPY	DQYSSDRDHR	GHRDYYDRHH	HDSKRRRSDE	FRPQNYHQQD	FRRMSDHRPA	MGYHGQGPSD
1770	1780	1790	1800	1810	1820	1830	
HYRSFHTDKL	GEYKQPLPPL	HPAVSDPRSP	PSQKSPHDSK	SPLDHRSPLE	RSLEQKNNPD	YNWNVRKT	

Cmpd.	No. of	m/z meas.	Δ m/z	z	Rt	Score	P	Range	Sequence	Modification

Detailed Protein Report

	Cmpds.		[ppm]		[min]					
1777	2	1022.8922	-139.78	2	52.6	14.7	2	932-948	K.KMVLDHLVIQRMDTTGR.T	Oxidation: 2, 12
2192	1	885.2814	-167.85	2	57.8	12.6	0	1221-1234	K.SIIQHEEEFEMLHK.S	

Detailed Protein Report

Protein 310: **pyrroline-5-carboxylate reductase 2 isoform 2 [Homo sapiens]**

**Accession:**	gi|410991931	**Score:**	27.3
**Database:**	refseq_human(refseq_human_20140103.fasta)	**MW [kDa]:**	25.9
**Database Date:**	2015-11-30	**pI:**	10.2
		**Sequence Coverage [%]:**	8.5
		**No. of unique Peptides:**	1

```
        10         20         30         40         50         60         70         80
MSVGFIGAGQ LAYALARGFT AAGILSAHKI IASSPEMNLP TVSALRKMGV NLTRSNKETV KHSDVLFLAV KPHIIPFILD
        90        100        110        120        130        140        150        160
EIGADVQARH IVVSCAAGVT ISSVEKAFMA LDALADGGVK MGLPRRLAIQ LGAQALLGAA KMLLDSEQHP CQLKDNVCSP
       170        180        190        200        210        220        230        240
GGATIHALHF LESGGFRSLL INAVEASCIR TRELQSMADQ EKISPAALKK TLLDRVKLES PTVSTLTPSS PGKLLTRSLA
       250
LGGKKD
```

Cmpd.	No. of Cmpds.	m/z meas.	Δ m/z [ppm]	z	Rt [min]	Score	P	Range	Sequence	Modification
325	1	1074.4954	-137.82	2	34.1	27.3	2	121-141	K.MGLPRRLAIQLGAQALLGAAK.M	

Detailed Protein Report

Protein 311: insulin receptor-related protein precursor [Homo sapiens]

**Accession:**	gi|31657140	**Score:**	27.3
**Database:**	refseq_human(refseq_human_20140103.fasta)	**MW [kDa]:**	143.6
**Database Date:**	2015-11-30	**pI:**	6.0
**Modification(s):**	Carbamidomethyl	**Sequence Coverage [%]:**	2.2
		**No. of unique Peptides:**	1

Quantitation

QD:QU **Median:** 0.15 **CV:** 0.00 % **No. of Peptides:** 1

10	20	30	40	50	60	70	80
MAVPSLWPWG	ACLPVIFLSL	GFGLDTVEVC	PSLDIRSEVA	ELRQLENCSV	VEGHLQILLM	FTATGEDFRG	LSFPRLTQVT
90	100	110	120	130	140	150	160
DYLLLFRVYG	LESLRDLFPN	LAVIRGTRLF	LGYALVIFEM	PHLRDVALPA	LGAVLRGAVR	VEKNQELCHL	STIDWGLLQP
170	180	190	200	210	220	230	240
APGANHIVGN	KLGEECADVC	PGVLGAAGEP	CAKTTFSGHT	DYRCWTSSHC	QRVCPCPHGM	ACTARGECCH	TECLGGCSQP
250	260	270	280	290	300	310	320
EDPRACVACR	HLYFQGACLW	ACPPGTYQYE	SWRCVTAERC	ASLHSVPGRA	STFGIHQGSC	LAQCPSGFTR	NSSSIFCHKC
330	340	350	360	370	380	390	400
EGLCPKECKV	GTKTIDSIQA	AQDLVGCTHV	EGSLILNLRQ	GYNLEPQLQH	SLGLVETITG	FLKIKHSFAL	VSLGFFKNLK
410	420	430	440	450	460	470	480
LIRGDAMVDG	NYTLYVLDNQ	NLQQLGSWVA	AGLTIPVGKI	YFAFNPRLCL	EHIYRLEEVT	GTRGRQNKAE	INPRTNGDRA
490	500	510	520	530	540	550	560
ACQTRTLRFV	SNVTEADRIL	LRWERYEPLE	ARDLLSFIVY	YKESPFQNAT	EHVGPDACGT	QSWNLLDVEL	PLSRTQEPGV
570	580	590	600	610	620	630	640
TLASLKPWTQ	YAVFVRAITL	TTEEDSPHQG	AQSPIVYLRT	LPAAPTVPQD	VISTSNSSSH	LLVRWKPPTQ	RNGNLTYYLV
650	660	670	680	690	700	710	720
LWQRLAEDGD	LYLNDYCHRG	LRLPTSNNDP	RFDGEDGDPE	AEMESDCCPC	QHPPPGQVLP	PLEAQEASFQ	KKFENFLHNA
730	740	750	760	770	780	790	800
ITIPISPWKV	TSINKSPQRD	SGRHRRAAGP	LRLGGNSSDF	EIQEDKVPRE	RAVLSGLRHF	TEYRIDIHAC	NHAAHTVGCS
810	820	830	840	850	860	870	880
AATFVFARTM	PHREADGIPG	KVAWEASSKN	SVLLRWLEPP	DPNGLILKYE	IKYRRLGEEA	TVLCVSRLRY	AKFGGVHLAL
890	900	910	920	930	940	950	960
LPPGNYSARV	RATSLAGNGS	WTDSVAFYIL	GPEEEDAGGL	HVLLTATPVG	LTLLIVLAAL	GFFYGKKRNR	TLYASVNPEY
970	980	990	1000	1010	1020	1030	1040
FSASDMYVPD	EWEVPREQIS	IIRELGQGSF	GMVYEGLARG	LEAGEESTPV	ALKTVNELAS	PRECIEFLKE	ASVMKAFKCH
1050	1060	1070	1080	1090	1100	1110	1120
HVVRLLGVVS	QGQPTLVIME	LMTRGDLKSH	LRSLRPEAEN	NPGLPQPALG	EMIQMAGEIA	DGMAYLAANK	FVHRDLAARN
1130	1140	1150	1160	1170	1180	1190	1200
CMVSQDFTVK	IGDFGMTRDV	YETDYYRKGG	KGLLPVRWMA	PESLKDGIFT	THSDVWSFGV	VLWEIVTLAE	QPYQGLSNEQ
1210	1220	1230	1240	1250	1260	1270	1280
VLKFVMDGGV	LEELEGCPLQ	LQELMSRCWQ	PNPRLRPSFT	HILDSIQEEL	RPSFRLLSFY	YSPECRGARG	SLPTTDAEPD
1290	1300						
SSPTPRDCSP	QNGGPGH						

Cmpd.	No. of Cmpds.	m/z meas.	Δ m/z [ppm]	z	Rt [min]	Score	P	Range	Sequence	Modification	Ratios	
608	1	730.3714	88.53	2	37.6	15.3	0	213-225	R.VCPCPHGMACTAR.G	Carbamidomethyl: 2, 4	QD:QU	0.15

Detailed Protein Report

Protein 312: fibronectin isoform 6 preproprotein [Homo sapiens]

**Accession:**	gi|47132549	**Score:**	27.1
**Database:**	refseq_human(refseq_human_20140103.fasta)	**MW [kDa]:**	239.5
**Database Date:**	2015-11-30	**pI:**	5.5
		**Sequence Coverage [%]:**	1.1
		**No. of unique Peptides:**	1

Detailed Protein Report

10	20	30	40	50	60	70	80
MLRGPGPGLL	LLAVQCLGTA	VPSTGASKSK	RQAQQMVQPQ	SPVAVSQSKP	GCYDNGKHYQ	INQQWERTYL	GNALVCTCYG
90	100	110	120	130	140	150	160
GSRGFNCESK	PEAEETCFDK	YTGNTYRVGD	TYERPKDSMI	WDCTCIGAGR	GRISCTIANR	CHEGGQSYKI	GDTWRRPHET
170	180	190	200	210	220	230	240
GGYMLECVCL	GNGKGEWTCK	PIAEKCFDHA	AGTSYVVGET	WEKPYQGWMM	VDCTCLGEGS	GRITCTSRNR	CNDQDTRTSY
250	260	270	280	290	300	310	320
RIGDTWSKKD	NRGNLLQCIC	TGNGRGEWKC	ERHTSVQTTS	SGSGPFTDVR	AAVYQPQPHP	QPPPYGHCVT	DSGVVYSVGM
330	340	350	360	370	380	390	400
QWLKTQGNKQ	MLCTCLGNGV	SCQETAVTQT	YGGNSNGEPC	VLPFTYNGRT	FYSCTTEGRQ	DGHLWCSTTS	NYEQDQKYSF
410	420	430	440	450	460	470	480
CTDHTVLVQT	RGGNSNGALC	HFPFLYNNHN	YTDCTSEGRR	DNMKWCGTTQ	NYDADQKFGF	CPMAAHEEIC	TTNEGVMYRI
490	500	510	520	530	540	550	560
GDQWDKQHDM	GHMMRCTCVG	NGRGEWTCIA	YSQLRDQCIV	DDITYNVNDT	FHKRHEEGHM	LNCTCFGQGR	GRWKCDPVDQ
570	580	590	600	610	620	630	640
CQDSETGTFY	QIGDSWEKYV	HGVRYQCYCY	GRGIGEWHCQ	PLQTYPSSSG	PVEVFITETP	SQPNSHPIQW	NAPQPSHISK
650	660	670	680	690	700	710	720
YILRWRPKNS	VGRWKEATIP	GHLNSYTIKG	LKPGVVYEGQ	LISIQQYGHQ	EVTRFDFTTT	STSTPVTSNT	VTGETTPFSP
730	740	750	760	770	780	790	800
LVATSESVTE	ITASSFVVSW	VSASDTVSGF	RVEYELSEEG	DEPQYLDLPS	TATSVNIPDL	LPGRKYIVNV	YQISEDGEQS
810	820	830	840	850	860	870	880
LILSTSQTTA	PDAPPDPTVD	QVDDTSIVVR	WSRPQAPITG	YRIVYSPSVE	GSSTELNLPE	TANSVTLSDL	QPGVQYNITI
890	900	910	920	930	940	950	960
YAVEENQEST	PVVIQQETTG	TPRSDTVPSP	RDLQFVEVTD	VKVTIMWTPP	ESAVTGYRVD	VIPVNLPGEH	GQRLPISRNT
970	980	990	1000	1010	1020	1030	1040
FAEVTGLSPG	VTYYFKVFAV	SHGRESKPLT	AQQTTKLDAP	TNLQFVNETD	STVLVRWTPP	RAQITGYRLT	VGLTRRGQPR
1050	1060	1070	1080	1090	1100	1110	1120
QYNVGPSVSK	YPLRNLQPAS	EYTVSLVAIK	GNQESPKATG	VFTTLQPGSS	IPPYNTEVTE	TTIVITWTPA	PRIGFKLGVR
1130	1140	1150	1160	1170	1180	1190	1200
PSQGGEAPRE	VTSDSGSIVV	SGLTPGVEYV	YTIQVLRDGQ	ERDAPIVNKV	VTPLSPPTNL	HLEANPDTGV	LTVSWERSTT
1210	1220	1230	1240	1250	1260	1270	1280
PDITGYRITT	TPTNGQQGNS	LEEVVHADQS	SCTFDNLSPG	LEYNVSVYTV	KDDKESVPIS	DTIIPAVPPP	TDLRFTNIGP
1290	1300	1310	1320	1330	1340	1350	1360
DTMRVTWAPP	PSIDLTNFLV	RYSPVKNEED	VAELSISPSD	NAVVLTNLLP	GTEYVVSVSS	VYEQHESTPL	RGRQKTGLDS
1370	1380	1390	1400	1410	1420	1430	1440
PTGIDFSDIT	ANSFTVHWIA	PRATITGYRI	RHHPEHFSGR	PREDRVPHSR	NSITLTNLTP	GTEYVVSIVA	LNGREESPLL
1450	1460	1470	1480	1490	1500	1510	1520
IGQQSTVSDV	PRDLEVVAAT	PTSLLISWDA	PAVTVRYYRI	TYGETGGNSP	VQEFTVPGSK	STATISGLKP	GVDYTITVYA
1530	1540	1550	1560	1570	1580	1590	1600
VTGRGDSPAS	SKPISINYRT	EIDKPSQMQV	TDVQDNSISV	KWLPSSSPVT	GYRVTTTPKN	GPGPTKTKTA	GPDQTEMTIE
1610	1620	1630	1640	1650	1660	1670	1680
GLQPTVEYVV	SVYAQNPSGE	SQPLVQTAVT	TIPAPTDLKF	TQVTPTSLSA	QWTPPNVQLT	GYRVRVTPKE	KTGPMKEINL
1690	1700	1710	1720	1730	1740	1750	1760
APDSSSVVVS	GLMVATKYEV	SVYALKDTLT	SRPAQGVVTT	LENVSPPRRA	RVTDATETTI	TISWRTKTET	ITGFQVDAVP
1770	1780	1790	1800	1810	1820	1830	1840
ANGQTPIQRT	IKPDVRSYTI	TGLQPGTDYK	IYLYTLNDNA	RSSPVVIDAS	TAIDAPSNLR	FLATTPNSLL	VSWQPPRARI
1850	1860	1870	1880	1890	1900	1910	1920
TGYIIKYEKP	GSPPREVVPR	PRPGVTEATI	TGLEPGTEYT	IYVIALKNNQ	KSEPLIGRKK	TGQEALSQTT	ISWAPFQDTS
1930	1940	1950	1960	1970	1980	1990	2000
EYIISCHPVG	TDEEPLQFRV	PGTSTSATLT	GLTRGATYNI	IVEALKDQQR	HKVREEVVTV	GNSVNEGLNQ	PTDDSCFDPY
2010	2020	2030	2040	2050	2060	2070	2080
TVSHYAVGDE	WERMSESGFK	LLCQCLGFGS	GHFRCDSSRW	CHDNGVNYKI	GEKWDRQGEN	GQMMSCTCLG	NGKGEFKCDP
2090	2100	2110	2120	2130	2140	2150	2160
HEATCYDDGK	TYHVGEQWQK	EYLGAICSCT	CFGGQRGWRC	DNCRRPGGEP	SPEGTTGQSY	NQYSQRYHQR	TNTNVNCPIE
2170	2180						
CFMPLDVQAD	REDSRE						

Cmpd.	No. of	m/z meas.	Δ m/z	z	Rt	Score	P	Range	Sequence	Modification

Detailed Protein Report

	Cmpds.		[ppm]		[min]					
2765	1	904.4689	14.28	2	65.7	16.9	0	923-938	K.VTIMWTPPESAVTGYR.V	

Detailed Protein Report

Protein 313: sterol 26-hydroxylase, mitochondrial precursor [Homo sapiens]

**Accession:**	gi|4503211	**Score:**	27.1
**Database:**	refseq_human(refseq_human_20140103.fasta)	**MW [kDa]:**	60.2
**Database Date:**	2015-11-30	**pI:**	9.7
		**Sequence Coverage [%]:**	5.3
		**No. of unique Peptides:**	1

10	20	30	40	50	60	70	80
MAALGCARLR	WALRGAGRGL	CPHGARAKAA	IPAALPSDKA	TGAPGAGPGV	RRRQRSLEEI	PRLGQLRFFF	QLFVQGYALQ
**90**	**100**	**110**	**120**	**130**	**140**	**150**	**160**
LHQLQVLYKA	KYGPMWMSYL	GPQMHVNLAS	APLLEQVMRQ	EGKYPVRNDM	ELWKEHRDQH	DLTYGPFTTE	GHHWYQLRQA
**170**	**180**	**190**	**200**	**210**	**220**	**230**	**240**
LNQRLLKPAE	AALYTDAFNE	VIDDFMTRLD	QLRAESASGN	QVSDMAQLFY	YFALEAICYI	LFEKRIGCLQ	RSIPEDTVTF
**250**	**260**	**270**	**280**	**290**	**300**	**310**	**320**
VRSIGLMFQN	SLYATFLPKW	TRPVLPFWKR	YLDGWNAIFS	FGKKLIDEKL	EDMEAQLQAA	GPDGIQVSGY	LHFLLASGQL
**330**	**340**	**350**	**360**	**370**	**380**	**390**	**400**
SPREAMGSLP	ELLMAGVDTT	SNTLTWALYH	LSKDPEIQEA	LHEEVVGVVP	AGQVPQHKDF	AHMPLLKAVL	KETLRLYPVV
**410**	**420**	**430**	**440**	**450**	**460**	**470**	**480**
PTNSRIIEKE	IEVDGFLFPK	NTQFVFCHYV	VSRDPTAFSE	PESFQPHRWL	RNSQPATPRI	QHPFGSVPFG	YGVRACLGRR
**490**	**500**	**510**	**520**	**530**	**540**		
IAELEMQLLL	ARLIQKYKVV	LAPETGELKS	VARIVLVPNK	KVGLQFLQRQ	C		

Cmpd.	No. of Cmpds.	m/z meas.	Δ m/z [ppm]	z	Rt [min]	Score	P	Range	Sequence	Modification
2189	1	937.1073	154.72	2	57.8	12.8	2	124-137	K.YPVRNDMELWKEHR.D	

Detailed Protein Report

Protein 314: syntaxin-binding protein 5 isoform b [Homo sapiens]

**Accession:**	gi|189217915	**Score:**	27.1
**Database:**	refseq_human(refseq_human_20140103.fasta)	**MW [kDa]:**	127.5
**Database Date:**	2015-11-30	**pI:**	7.1
		**Sequence Coverage [%]:**	2.2
		**No. of unique Peptides:**	2

10	20	30	40	50	60	70	80
MRKFNIRKVL	DGLTAGSSSA	SQQQQQQHPP	GNREPEIQET	LQSEHFQLCK	TVRHGFPYQP	SALAFDPVQK	ILAVGTQTGA
90	100	110	120	130	140	150	160
LRLFGRPGVE	CYCQHDSGAA	VIQLQFLINE	GALVSALADD	TLHLWNLRQK	RPAILHSLKF	CRERVTFCHL	PFQSKWLYVG
170	180	190	200	210	220	230	240
TERGNIHIVN	VESFTLSGYV	IMWNKAIELS	SKSHPGPVVH	ISDNPMDEGK	LLIGFESGTV	VLWDLKSKKA	DYRYTYDEAI
250	260	270	280	290	300	310	320
HSVAWHHEGK	QFICSHSDGT	LTIWNVRSPA	KPVQTITPHG	KQLKDGKKPE	PCKPILKVEF	KTTRSGEPFI	ILSGGLSYDT
330	340	350	360	370	380	390	400
VGRRPCLTVM	HGKSTAVLEM	DYSIVDFLTL	CETPYPNDFQ	EPYAVVVLLE	KDLVLIDLAQ	NGYPIFENPY	PLSIHESPVT
410	420	430	440	450	460	470	480
CCEYFADCPV	DLIPALYSVG	ARQKRQGYSK	KEWPINGGNW	GLGAQSYPEI	IITGHADGSV	KFWDASAITL	QVLYKLKTSK
490	500	510	520	530	540	550	560
VFEKSRNKDD	RPNTDIVDED	PYAIQIISWC	PESRMLCIAG	VSAHVIIYRF	SKQEVITEVI	PMLEVRLLYE	INDVETPEGE
570	580	590	600	610	620	630	640
QPPPLPTPVG	GSNPQPIPPQ	SHPSTSSSSS	DGLRDNVPCL	KVKNSPLKQS	PGYQTELVIQ	LVWVGGEPPQ	QITSLAVNSS
650	660	670	680	690	700	710	720
YGLVVFGNCN	GIAMVDYLQK	AVLLNLGTIE	LYGSNDPYRR	EPRSPRKSRQ	PSGAGLCDIS	EGTVVPEDRC	KSPTSGSSSP
730	740	750	760	770	780	790	800
HNSDDEQKMN	NFIEKVKTKS	RKFSKMVAND	IAKMSRKLSL	PTDLKPDLDV	KDNSFSRSRS	SSVTSIDKES	REAISALHFC
810	820	830	840	850	860	870	880
ETFTRKTDSS	PSPCLWVGTT	LGTVLVIALN	LPPGGEQRLL	QPVIVSPSGT	ILRLKGAILR	MAFLDTTGCL	IPPAYEPWRE
890	900	910	920	930	940	950	960
HNVPEEKDEK	EKLKKRRPVS	VSPSSSQEIS	ENQYAVICSE	KQAKVISLPT	QNCAYKQNIT	ETSFVLRGDI	VALSNSICLA
970	980	990	1000	1010	1020	1030	1040
CFCANGHIMT	FSLPSLRPLL	DVYYLPLTNM	RIARTFCFTN	NGQALYLVSP	TEIQRLTYSQ	ETCENLQEML	GELFTPVETP
1050	1060	1070	1080	1090	1100	1110	1120
EAPNRGFFKG	LFGGGAQSLD	REELFGESSS	GKASRSLAQH	IPGPGGIEGV	KGAASGVVGE	LARARLALDE	RGQKLGDLEE
1130	1140	1150	1160				
RTAAMLSSAE	SFSKHAHEIM	LKYKDKKWYQ	F				

Cmpd.	No. of Cmpds.	m/z meas.	Δ m/z [ppm]	z	Rt [min]	Score	P	Range	Sequence	Modification
1487	1	676.3270	-69.10	2	48.8	11.5	2	742-753	R.KFSKMVANDIAK.M	
1944	1	665.3207	-0.95	2	54.7	15.6	0	1122-1134	R.TAAMLSSAESFSK.H	

Detailed Protein Report

Protein 315: PREDICTED: serine/threonine-protein kinase WNK2 isoform X12 [Homo sapiens]

**Accession:**	gi|578817784	**Score:**	27.0
**Database:**	refseq_human(refseq_human_20140103.fasta)	**MW [kDa]:**	227.1
**Database Date:**	2015-11-30	**pI:**	5.4
		**Sequence Coverage [%]:**	1.7
		**No. of unique Peptides:**	1

Quantitation

QD:QU	**Median:** 2.32	**CV:** 0.00 %	**No. of Peptides:** 1

Detailed Protein Report

10	20	30	40	50	60	70	80
MDGDGGRRDV	PGTLMEPGRG	AGPAGMAEPR	AKAARPGPQR	FLRRSVVESD	QEEPPGLEAA	EAPGPQPPQP	LQRRVLLLCK
90	100	110	120	130	140	150	160
TRRLIAERAR	GRPAAPAPAA	LVAQPGAPGA	PADAGPEPVG	TQEPGPDPIA	AAVETAPAPD	GGPREEAAAT	VRKEDEGAAE
170	180	190	200	210	220	230	240
AKPEPGRTRR	DEPEEEEDDE	DDLKAVATSL	DGRFLKFDIE	LGRGSFKTVY	KGLDTETWVE	VAWCELQDRK	LTKLERQRFK
250	260	270	280	290	300	310	320
EEAEMLKGLQ	HPNIVRFYDF	WESSAKGKRC	IVLVTELMTS	GTLKTYLKRF	KVMKPKVLRS	WCRQILKGLL	FLHTRTPPII
330	340	350	360	370	380	390	400
HRDLKCDNIF	ITGPTGSVKI	GDLGLATLKR	ASFAKSVIGT	PEFMAPEMYE	EHYDESVDVY	AFGMCMLEMA	TSEYPYSECQ
410	420	430	440	450	460	470	480
NAAQIYRKVT	CGIKPASFEK	VHDPEIKEII	GECICKNKEE	RYEIKDLLSH	AFFAEDTGVR	VELAEEDHGR	KSTIALRLWV
490	500	510	520	530	540	550	560
EDPKKLKGKP	KDNGAIEFTF	DLEKETPDEV	AQEMIESGFF	HESDVKIVAK	SIRDRVALIQ	WRRERIWPAL	QPKEQQDVGS
570	580	590	600	610	620	630	640
PDKARGPPVP	LQVQVTYHAQ	AGQPGPPEPE	EPEADQHLLP	PTLPTSATSL	ASDSTFDSGQ	GSTVYSDSQS	SQQSVMLGSL
650	660	670	680	690	700	710	720
ADAAPSPAQC	VCSPPVSEGP	VLPQSLPSLG	AYQQPTAAPG	LPVGSVPAPA	CPPSLQQHFP	DPAMSFAPVL	PPPSTPMPTG
730	740	750	760	770	780	790	800
PGQPAPPGQQ	PPPLAQPTPL	PQVLAPQPVV	PLQPVPPHLP	PYLAPASQVG	APAQLKPLQM	PQAPLQPLAQ	VPPQMPPIPV
810	820	830	840	850	860	870	880
VPPITPLAGI	DGLPPALPDL	PTATVPPVPP	PQYFSPAVIL	PSLAAPLPPA	SPALPLQAVK	LPHPPGAPLA	MPCRTIVPNA
890	900	910	920	930	940	950	960
PATIPLLAVA	PPGVAALSIH	SAVAQLPGQP	VYPAAFPQMA	PTDVPPSPHH	TVQNMRATPP	QPALPPQPTL	PPQPVLPPQP
970	980	990	1000	1010	1020	1030	1040
TLPPQPVLPP	QPTRPPQPVL	PPQPMLPPQP	VLPPQPALPV	RPEPLQPHLP	EQAAPAATPG	SQILLGHPAP	YAVDVAAQVP
1050	1060	1070	1080	1090	1100	1110	1120
TVPVPPAAVL	SPPLPEVLLP	AAPELLPQFP	SSLATVSASV	QSVPTQTATL	LPPANPPLPG	GPGIASPCPT	VQLTVEPVQE
1130	1140	1150	1160	1170	1180	1190	1200
EQASQDKPPG	LPQSCESYGG	SDVTSGKELS	DSCEGAFGGG	RLEGRAARKH	HRRSTRARSR	QERASRPRLT	ILNVCNTGDK
1210	1220	1230	1240	1250	1260	1270	1280
MVECQLETHN	HKMVTFKFDL	DGDAPDEIAT	YMVEHDFILQ	AERETFIEQM	KDVMDKAEDM	LSEDTDADRG	SDPGTSPPHL
1290	1300	1310	1320	1330	1340	1350	1360
STCGLGTGEE	SRQSQANAPV	YQQNVLHTGK	RWFIICPVAE	HPAPEAPESS	PPLPLSSLPP	EASQDSAPYK	DQLSSKEQPS
1370	1380	1390	1400	1410	1420	1430	1440
FLASQQLLSQ	AGPSNPPGAP	PAPLAPSSPP	VTALPQDGAA	PATSTMPEPA	SGTASQAGGP	GTPQGLTSEL	ETSQPLAETH
1450	1460	1470	1480	1490	1500	1510	1520
EAPLAVQPLV	VGLAPCTPAP	EAASTRDASA	PREPLPPPAP	EPSPHSGTPQ	PALGQPAPLL	PAAVGAVSLA	TSQLPSPPLG
1530	1540	1550	1560	1570	1580	1590	1600
PTVPPQPPSA	LESDGEGPPP	RVGFVDSTIK	SLDEKLRTLL	YQEHVPTSSA	SAGTPVEVGD	RDFTLEPLRG	DQPRSEVCGG
1610	1620	1630	1640	1650	1660	1670	1680
DLALPPVPKE	AVSGRVQLPQ	PLVEKSELAP	TRGAVMEQGT	SSSMTAESSP	RSMLGYDRDG	RQVASDSHVV	PSVPQDVPAF
1690	1700	1710	1720	1730	1740	1750	1760
VRPARVEPTD	RDGGEAGESS	AEPPPSDMGT	VGGQASHPQT	LGARALGSPR	KRPEQQDVSS	PAKTVGRFSV	VSTQDEWTLA
1770	1780	1790	1800	1810	1820	1830	1840
SPHSLRYSAP	PDVYLDEAPS	SPDVKLAVRR	AQTASSIEVG	VGEPVSSDSG	DEGPRARPPV	QKQASLPVSG	SVAGDFVKKA
1850	1860	1870	1880	1890	1900	1910	1920
TAFLQRPSRA	GSLGPETPSR	VGMKVPTISV	TSFHSQSSYI	SSDNDSELED	ADIKKELQSL	REKHLKEISE	LQSQQKQEIE
1930	1940	1950	1960	1970	1980	1990	2000
ALYRRLGKPL	PPNVGFFHTA	PPTGRRRKTS	KSKLKAGKLL	NPLVRQLKVV	ASSTGSSTSS	LAPGPEPGPQ	PALHVQAQVN
2010	2020	2030	2040	2050	2060	2070	2080
NSNNKKGTFT	DDLHKLVDEW	TSKTVGAAQL	KPTLNQLKQT	QKLQDMEAQA	GWAAPGEARA	MTAPRAGVGM	PRLPPAPGPL
2090	2100	2110	2120	2130	2140	2150	
STTVIPGAAP	TLSVPTPDGA	LGTARRNQVW	FGLRVPPTAC	CGHSTQPRGG	QRVGSKTASF	AASDPVRS	

Detailed Protein Report

Cmpd.	No. of Cmpds.	m/z meas.	Δ m/z [ppm]	z	Rt [min]	Score	P	Range	Sequence	Modification	Ratios
1286	1	945.9319	-64.24	2	46.2	11.9	2	2129-2147	R.GGQRVGSKTASFAASDPVR.S		QD:QU 2.32

Detailed Protein Report

Protein 316: **PREDICTED: cyclin-dependent kinase-like 1 isoform X1 [Homo sapiens]**

**Accession:**	gi|530404810	**Score:**	27.0
**Database:**	refseq_human(refseq_human_20140103.fasta)	**MW [kDa]:**	108.9
**Database Date:**	2015-11-30	**pI:**	9.8
		**Sequence Coverage [%]:**	3.1
		**No. of unique Peptides:**	1

10	20	30	40	50	60	70	80
MELSTDTGWN	QKSNFPPCDS	RVSKEEAQLP	FPTPSSYGAD	SSNQTYAHKS	QSEREQHKEV	RAVLNHCSGL	DGGEESGLCG
90	100	110	120	130	140	150	160
TSTRIDAGTG	NGTDDKVTDQ	HRHRRCLQGT	KGNNRGSEVW	GLLLQGNVDR	SGGAPSAGVL	LRRRGYSCAL	HGLRKFANLA
170	180	190	200	210	220	230	240
GLLSRQQDSA	RGVSHHSRLK	IHFKKIYSSM	MEKYEKIGKI	GEGSYGVVFK	CRNRDTGQIV	AIKKFLESED	DPVIKKIALR
250	260	270	280	290	300	310	320
EIRMLKAPSP	YAAEPSLCGM	KMVRRGKKEF	LPAVAEKVDA	PSGVGGQGQD	SVTVGSLGRR	STYGRKQEKQ	VRQREGIIYC
330	340	350	360	370	380	390	400
YVAVLLRIYY	FDQGCVAREE	EQFQELVFGP	FCHIGSYFTG	HRTNVRPYIL	LLSRPSPFKT	AAGTYEAGLV	ILECSYFLAE
410	420	430	440	450	460	470	480
QEPYCPTQAL	QQPHPIIGPW	ALEGGGVESK	EDRHPPPKEA	PASCEGFLRS	AVPKQAYTPF	KTSPDKRLSD	CVATPPWAPP
490	500	510	520	530	540	550	560
TPLIISSGVL	VAICSMIDPV	PEFHSEGLLA	KATSGSAGIL	VWIFLCNDAF	IYGKYILRSG	VLWVRGLPGF	KSEAAALCHK
570	580	590	600	610	620	630	640
CYSSADPKRE	QQQDLLQRVK	EQSFHSVNGA	QARHKGSPSP	HQTQEPSWPH	PVDPTPGHRW	SCLPVPCRAP	ALLSPWVVDG
650	660	670	680	690	700	710	720
TGCCGAGGGS	DRGGSAAQEP	TQLKHPNLVN	LLEVFRRKRR	LHLVFEYCDH	TVLHELDRYQ	RGICNIFVCT	GRRLGEHTEA
730	740	750	760	770	780	790	800
LSKKKKKGGG	GPFLKLRAAS	CRITLFKNVG	CGLETTGDLS	LNSGGGAASR	GVAAALRALV	CGTELTSSDS	PQRCIHRDVK
810	820	830	840	850	860	870	880
PENILITKHS	VIKLCDFGFA	RLLTGPSDYY	TDYVATRWYR	SPELLVGDTQ	YGPPVDVWAI	GCVFAELLSG	VPLWPGKSDV
890	900	910	920	930	940	950	960
DQLYLIRKTL	GDLIPRHQQV	FSTNQYFSGV	KIPDPEDMSL	CLSVTLTEGG	LLASGAVKRS	QMGSSVSQAT	SWPHPDIVAE
970	980	990	1000				
TAELDDIAMA	RQTPVMLRFN	RQKEQEKYLS	YGA				

Cmpd.	No. of Cmpds.	m/z meas.	Δ m/z [ppm]	z	Rt [min]	Score	P	Range	Sequence	Modification
2221	1	1032.3950	-114.27	2	56.4	16.5	2	247-265	K.APSPYAAEPSLCGMKMVRR.G	

Detailed Protein Report

Protein 317: **PREDICTED: transmembrane channel-like protein 8 isoform X4 [Homo sapiens]**

Accession: gi|578830481
Database: refseq_human(refseq_human_20140103.fasta)
Database Date: 2015-11-30
Modification(s): Oxidation

Score: 26.9
MW [kDa]: 45.7
pI: 10.7
Sequence Coverage [%]: 2.2
No. of unique Peptides: 1

Quantitation

QD:QU **Median:** 0.73 **CV:** 0.00 % **No. of Peptides:** 1

10	20	30	40	50	60	70	80
MLLPRSVSSE	RAPGVPEPEE	LWEAEMERLR	GSGTPVRGLP	YAMMDKRLIW	QLREPAGVQT	LRWQRWQRRR	QTVERRLREA
90	100	110	120	130	140	150	160
AQRLARGLGL	WEGALYEIGG	LFGTGIRSYF	TFLRFLLLLN	LLSLLLTASF	VLLPLVWLRP	PDPGPTLNLT	LQCPGSRQSP
170	180	190	200	210	220	230	240
PGVLRFHNQL	WHVLTGRAFT	NTYLFYGAYR	VGPESSSVYS	IRLAYLLSPL	ACLLLCFCGT	LRRMVKGLPQ	KTLLGQGYQA
250	260	270	280	290	300	310	320
PLSAKVFSSW	DFCIRVQEAA	TIKKHEISNE	FKVELEEGRR	FQLMQQQTRA	QTACRLLSYL	RVNVLNGLLV	VGAISAIFWA
330	340	350	360	370	380	390	400
TKYSQDNKEV	SGVPVSAAPV	PAPWGHRPGQ	LPGSPAVHIS	GPAGELPSQH	GGQPHSDLVR	GAEAGQLGDV	LRLPGSDHTV
410							
HWQRQEQL							

Cmpd.	No. of Cmpds.	m/z meas.	Δ m/z [ppm]	z	Rt [min]	Score	P	Range	Sequence	Modification	Ratios
1031	1	598.1512	-245.53	2	43.0	26.9	0	281-289	R.FQLMQQQTR.A	Oxidation: 4	QD:QU 0.73

Detailed Protein Report

Protein 318: **E3 ubiquitin-protein ligase TRIP12 isoform c [Homo sapiens]**

**Accession:**	gi|10863903	**Score:**	26.9
**Database:**	refseq_human(refseq_human_20140103.fasta)	**MW [kDa]:**	220.3
**Database Date:**	2015-11-30	**pI:**	9.6
		**Sequence Coverage [%]:**	1.7
		**No. of unique Peptides:**	2

Detailed Protein Report

10	20	30	40	50	60	70	80
MSNRPNNNPG	GSLRRSQRNT	AGAQPQDDSI	GGRSCSSSSA	VIVPQPEDPD	RANTSERQKT	GQVPKKDNSR	GVKRSASPDY
90	100	110	120	130	140	150	160
NRTNSPSSAK	KPKALQHTES	PSETNKPHSK	SKKRHLDQEQ	QLKSAQSPST	SKAHTRKSGA	TGGSRSQKRK	RTESSCVKSG
170	180	190	200	210	220	230	240
SGSESTGAEE	RSAKPTKLAS	KSATSAKAGC	STITDSSSAA	STSSSSSAVA	SASSTVPPGA	RVKQGKDQNK	ARRSRSASSP
250	260	270	280	290	300	310	320
SPRRSSREKE	QSKTGGSSKF	DWAARFSPKV	SLPKTKLSLP	GSSKSETSKP	GPSGLQAKLA	SLRKSTKKRS	ESPPAELPSL
330	340	350	360	370	380	390	400
RRSTRQKTTG	SCASTSRRGS	GLGKRGAAEA	RRQEKMADPE	SNQEAVNSSA	ARTDEAPQGA	AGAVGMTTSG	ESESDDSEMG
410	420	430	440	450	460	470	480
RLQALLEARG	LPPHLFGPLG	PRMSQLFHRT	IGSGASSKAQ	QLLQGLQASD	ESQQLQAVIE	MCQLLVMGNE	ETLGGFPVKS
490	500	510	520	530	540	550	560
VVPALITLLQ	MEHNFDIMNH	ACRALTYMME	ALPRSSAVVV	DAIPVFLEKL	QVIQCIDVAE	QALTALEMLS	RRHSKAILQA
570	580	590	600	610	620	630	640
GGLADCLLYL	EFFSINAQRN	ALAIAANCCQ	SITPDEFHFV	ADSLPLLTQR	LTHQDKKSVE	STCLCFARLV	DNFQHEENLL
650	660	670	680	690	700	710	720
QQVASKDLLT	NVQQLLVVTP	PILSSGMFIM	VVRMFSLMCS	NCPTLAVQLM	KQNIAETLHF	LLCGASNGSC	QEQIDLVPRS
730	740	750	760	770	780	790	800
PQELYELTSL	ICELMPCLPK	EGIFAVDTML	KKGNAQNTDG	AIWQWRDDRG	LWHPYNRIDS	RIIEQINEDT	GTARAIQRKP
810	820	830	840	850	860	870	880
NPLANSNTSG	YSESKKDDAR	AQLMKEDPEL	AKSFIKTLFG	VLYEVYSSSA	GPAVRHKCLR	AILRIIYFAD	AELLKDVLKN
890	900	910	920	930	940	950	960
HAVSSHIASM	LSSQDLKIVV	GALQMAEILM	QKLPDIFSVY	FRREGVMHQV	KHLAESESLL	TSPPKACTNG	SGSMGSTTSV
970	980	990	1000	1010	1020	1030	1040
SSGTATAATH	AAADLGSPSL	QHSRDDSLDL	SPQGRLSDVL	KRKRLPKRGP	RRPKYSPPRD	DDKVDNQAKS	PTTTQSPKSS
1050	1060	1070	1080	1090	1100	1110	1120
FLASLNPKTW	GRLSTQSNSN	NIEPARTAGG	SGLARAASKD	TISNNREKIK	GWIKEQAHKF	VERYFSSENM	DGSNPALNVL
1130	1140	1150	1160	1170	1180	1190	1200
QRLCAATEQL	NLQVDGGAEC	LVEIRSIVSE	SDVSSFEIQH	SGFVKQLLLY	LTSKSEKDAV	SREIRLKRFL	HVFFSSPLPG
1210	1220	1230	1240	1250	1260	1270	1280
EEPIGRVEPV	GNAPLLALVH	KMNNCLSQME	QFPVKVHDFP	SGNGTGGSFS	LNRGSQALKF	FNTHQLKCQL	QRHPDCANVK
1290	1300	1310	1320	1330	1340	1350	1360
QWKGGPVKID	PLALVQAIER	YLVVRGYGRV	REDDEDSDDD	GSDEEIDESL	AAQFLNSGNV	RHRLQFYIGE	HLLPYNMTVY
1370	1380	1390	1400	1410	1420	1430	1440
QAVRQFSIQA	EDERESTDDE	SNPLGRAGIW	TKTHTIWYKP	VREDEESNKD	CVGGKRGRAQ	TAPTKTSPRN	AKKHDELWHD
1450	1460	1470	1480	1490	1500	1510	1520
GVCPSVSNPL	EVYLIPTPPE	NITFEDPSLD	VILLLRVLHA	ISRYWYYLYD	NAMCKEIIPT	SEFINSKLTA	KANRQLQDPL
1530	1540	1550	1560	1570	1580	1590	1600
VIMTGNIPTW	LTELGKTCPF	FFPFDTRQML	FYVTAFDRDR	AMQRLLDTNP	EINQSDSQDS	RVAPRLDRKK	RTVNREELLK
1610	1620	1630	1640	1650	1660	1670	1680
QAESVMQDLG	SSRAMLEIQY	ENEVGTGLGP	TLEFYALVSQ	ELQRADLGLW	RGEEVTLSNP	KGSQEGTKYI	QNLQGLFALP
1690	1700	1710	1720	1730	1740	1750	1760
FGRTAKPAHI	AKVKMKFRFL	GKLMAKAIMD	FRLVDLPLGL	PFYKWMLRQE	TSLTSHDLFD	IDPVVARSVY	HLEDIVRQKK
1770	1780	1790	1800	1810	1820	1830	1840
RLEQDKSQTK	ESLQYALETL	TMNGCSVEDL	GLDFTLPGFP	NIELKKGGKD	IPVTIHNLEE	YLRLVIFWAL	NEGVSRQFDS
1850	1860	1870	1880	1890	1900	1910	1920
FRDGFESVFP	LSHLQYFYPE	ELDQLLCGSK	ADTWDAKTLM	ECCRPDHGYT	HDSRAVKFLF	EILSSFDNEQ	QRLFLQFVTG
1930	1940	1950	1960	1970	1980	1990	2000
SPRLPVGGFR	SLNPPLTIVR	KTFESTENPD	DFLPSVMTCV	NYLKLPDYSS	IEIMREKLLI	AAREGQQSFH	LS

Cmpd.	No. of Cmpds.	m/z meas.	Δ m/z [ppm]	z	Rt [min]	Score	P	Range	Sequence	Modification
1915	1	643.3072	-50.38	2	54.3	12.5	2	133-145	K.AHTRKSGATGGSR.S	
2359	1	974.1497	149.86	2	60.0	14.5	2	1067-1086	R.TAGGSGLARAASKDTISNNR.E	

Detailed Protein Report

Protein 319: **PREDICTED: MAM domain-containing glycosylphosphatidylinositol anchor protein 1 isoform X2 [Homo sapiens]**

**Accession:**	gi|578811587	**Score:**	26.9
**Database:**	refseq_human(refseq_human_20140103.fasta)	**MW [kDa]:**	59.5
**Database Date:**	2015-11-30	**pI:**	9.6
**Modification(s):**	Carbamidomethyl	**Sequence Coverage [%]:**	7.8
		**No. of unique Peptides:**	1

10	20	30	40	50	60	70	80
MEVTCLLLLA	LIPFHCRGQG	VYAPAQAQIV	HAGQACVVKE	DNISERVYTI	REGDTLMLQC	LVTGHPRPQV	RWTKTAGSAS
**90**	**100**	**110**	**120**	**130**	**140**	**150**	**160**
DKFQETSVFN	ETLRIERIAR	TQGGRYYCKA	ENGVGVPAIK	SIRVDVQYLD	EPMLTVHQTV	SDVRGNFYQE	KTVFLRCTVN
**170**	**180**	**190**	**200**	**210**	**220**	**230**	**240**
SNPPARFIWK	RGSDTLSHSQ	DNGVDIYEPL	YTQGETKVLK	LKNLRPQDYA	SYTCQVSVRN	VCGIPDKAIT	FRLTNTTAPP
**250**	**260**	**270**	**280**	**290**	**300**	**310**	**320**
ALKLSVNETL	VVNPGENVTV	QCLLTGGDPL	PQLQWSHGPG	PLPLGALAQG	GTLSIPSVQA	RDSGYYNCTA	TNNVGNPAKK
**330**	**340**	**350**	**360**	**370**	**380**	**390**	**400**
TVNLLVRSMK	NATFQITPDV	IKESENIQLG	QDLKLSCHVD	AVPQEKVTYQ	WFKNGKPARM	SKRLLVTRND	PELPAVTSSL
**410**	**420**	**430**	**440**	**450**	**460**	**470**	**480**
ELIDLHFSDY	GTYLCMASFP	GAPVPDLSVE	VNISSETVPP	TISVPKGRAV	VTVREGSPAE	LQCEVRGKPR	PPVLWSRVDK
**490**	**500**	**510**	**520**	**530**	**540**		
EAALLPSGLP	LEETPDGKLR	LERVSRDMSG	TYRCQTARYN	GFNVRPREAQ	VQLNVQWTR		

Cmpd.	No. of Cmpds.	m/z meas.	Δ m/z [ppm]	z	Rt [min]	Score	P	Range	Sequence	Modification
1451	1	652.4568	150.96	3	46.7	16.5	0	2-17	M.EVTCLLLLALIPFHCR.G	Carbamidomethyl: 4, 15

Detailed Protein Report

Protein 320: **PREDICTED: probable E3 ubiquitin-protein ligase HECTD2 isoform X2 [Homo sapiens]**

**Accession:**	gi|530393122	**Score:**	26.9
**Database:**	refseq_human(refseq_human_20140103.fasta)	**MW [kDa]:**	76.8
**Database Date:**	2015-11-30	**pI:**	8.4
		**Sequence Coverage [%]:**	4.8
		**No. of unique Peptides:**	1

```
        10         20         30         40         50         60         70         80
MDASSSEMKA PVLPEPILPI QPKTVKDFQE DVEKVKSSGD WKAVHDFYLT TFDSFPELNA AFKKDATASF NTIEDSGINA
        90        100        110        120        130        140        150        160
KFVNAVYDTL LNTPQDVQKT VLKGIINSLL REWKGPRTKD DLRAYFILLQ NPQFNNTSTY VIYAHLLRQI ATLVEADHHF
       170        180        190        200        210        220        230        240
LVHWFKKLSQ KRFKQLVERL LQFISLRLFP AKPEEFPPIT KCSWWIPSAA KVLALLNTAN NLVHPPLIPY TDFYNSTLDH
       250        260        270        280        290        300        310        320
IDLMEEYHTW QNFGNSHRFS FCQYPFVISV AAKKIIIQRD SEQQMINIAR QSLVDKVSRR QRPDMNILFL NMKVRRTHLV
       330        340        350        360        370        380        390        400
SDSLDELTRK RADLKKKLKV TFVGEAGLDM GGLTKEWFLL LIRQIFHPDY GMFTYHKDSH CHWFSSFKCD NYSEFRLVGI
       410        420        430        440        450        460        470        480
LMGLAVYNSI TLDIRFPPCC YKKLLSPPII PSDQNIPVGI CNVTVDDLCQ IMPELAHGLS ELLSHEGNVE EDFYSTFQVF
       490        500        510        520        530        540        550        560
QEEFGIIKSY NLKPGGDKIS VTNQNRKEYV QLYTDFLLNK SIYKQFAAFY YGFHSVCASN ALMLLRPEEV EILVCGSPDL
       570        580        590        600        610        620        630        640
DMHALQRSTQ YDGYAKTDLT IKYFWDVVLG FPLDLQKKLL HFTTGSDRVP VGGMADLNFK ISKNETSTNC LPVAHTCFNQ
       650        660        670
LCLPPYKSKK DLKQKLIIGI SNSEGFGLE
```

Cmpd.	No. of Cmpds.	m/z meas.	Δ m/z [ppm]	z	Rt [min]	Score	P	Range	Sequence	Modification
2703	1	724.8604	-34.28	2	64.9	13.1	0	656-669	K.LIIGISNSEGFGLE.-	

Detailed Protein Report

Protein 321: ERC protein 2 [Homo sapiens]

**Accession:**	gi|34577114	**Score:**	26.9
**Database:**	refseq_human(refseq_human_20140103.fasta)	**MW [kDa]:**	110.5
**Database Date:**	2015-11-30	**pI:**	6.5
		**Sequence Coverage [%]:**	2.7
		**No. of unique Peptides:**	1

10	20	30	40	50	60	70	80
MYGSARTITN	LEGSPSRSPR	LPRSPRLGHR	RTSSGGGGGT	GKTLSMENIQ	SLNAAYATSG	PMYLSDHEGV	ASTTYPKGTM
90	100	110	120	130	140	150	160
TLGRATNRAV	YGGRVTAMGS	SPNIASAGLS	HTDVLSYTDQ	HGGLTGSSHH	HHHQVPSMLR	QVRDSTMLDL	QAQLKELQRE
170	180	190	200	210	220	230	240
NDLLRKELDI	KDSKLGSSMN	SIKTFWSPEL	KKERVLRKEE	AARMSVLKEQ	MRVSHEENQH	LQLTIQALQD	ELRTQRDLNH
250	260	270	280	290	300	310	320
LLQQESGNRG	AEHFTIELTE	ENFRRLQAEH	DRQAKELFLL	RKTLEEMELR	IETQKQTLNA	RDESIKKLLE	MLQSKGLPSK
330	340	350	360	370	380	390	400
SLEDDNERTR	RMAEAESQVS	HLEVILDQKE	KENIHLREEL	HRRSQLQPEP	AKTKALQTVI	EMKDTKIASL	ERNIRDLEDE
410	420	430	440	450	460	470	480
IQMLKANGVL	NTEDREEEIK	QIEVYKSHSK	FMKTKIDQLK	QELSKKESEL	LALQTKLETL	SNQNSDCKQH	IEVLKESLTA
490	500	510	520	530	540	550	560
KEQRAAILQT	EVDALRLRLE	EKESFLNKKT	KQLQDLTEEK	GTLAGEIRDM	KDMLEVKERK	INVLQKKIEN	LQEQLRDKDK
570	580	590	600	610	620	630	640
QLTNLKDRVK	SLQTDSSNTD	TALATLEEAL	SEKERIIERL	KEQRERDDRE	RLEEIESFRK	ENKDLKEKVN	ALQAELTEKE
650	660	670	680	690	700	710	720
SSLIDLKEHA	SSLASAGLKR	DSKLKSLEIA	IEQKKEECSK	LEAQLKKAHN	IEDDSRMNPE	FADQIKQLDK	EASYYRDECG
730	740	750	760	770	780	790	800
KAQAEVDRLL	EILKEVENEK	NDKDKKIAEL	ESLTLRHMKD	QNKKVANLKH	NQQLEKKKNA	QLLEEVRRRE	DSMADNSQHL
810	820	830	840	850	860	870	880
QIEELMNALE	KTRQELDATK	ARLASTQQSL	AEKEAHLANL	RIERRKQLEE	ILEMKQEALL	AAISEKDANI	ALLELSASKK
890	900	910	920	930	940	950	960
KKTQEEVMAL	KREKDRLVHQ	LKQQTQNRMK	LMADNYDDDH	HHYHHHHHHH	HHRSPGRSQH	SNHRPSPDQD	DEEGIWA

Cmpd.	No. of Cmpds.	m/z meas.	Δ m/z [ppm]	z	Rt [min]	Score	P	Range	Sequence	Modification
790	1	572.7630	-120.83	2	38.4	14.6	0	747-756	K.IAELESLTLR.H	

Detailed Protein Report

Protein 322: pleckstrin homology domain-containing family G member 1 [Homo sapiens]

**Accession:**	gi|71274148	**Score:**	26.8
**Database:**	refseq_human(refseq_human_20140103.fasta)	**MW [kDa]:**	155.3
**Database Date:**	2015-11-30	**pI:**	5.8
**Modification(s):**	Oxidation	**Sequence Coverage [%]:**	2.7
		**No. of unique Peptides:**	1

Alias proteins:

Accession	Name	Description
gi|578812862	refseq_human (refseq_human_20140103.fasta)	PREDICTED: pleckstrin homology domain-containing family G member 1 isoform X7 [Homo sapiens]
gi|578812859	refseq_human (refseq_human_20140103.fasta)	PREDICTED: pleckstrin homology domain-containing family G member 1 isoform X6 [Homo sapiens]
gi|530383900	refseq_human (refseq_human_20140103.fasta)	PREDICTED: pleckstrin homology domain-containing family G member 1 isoform X4 [Homo sapiens]
gi|530383898	refseq_human (refseq_human_20140103.fasta)	PREDICTED: pleckstrin homology domain-containing family G member 1 isoform X3 [Homo sapiens]
gi|530383896	refseq_human (refseq_human_20140103.fasta)	PREDICTED: pleckstrin homology domain-containing family G member 1 isoform X2 [Homo sapiens]

10	20	30	40	50	60	70	80
MELSDSDRPV	SFGSTSSSAS	SRDSHGSFGS	RMTLVSNSHM	GLFNQDKEVG	AIKLELIPAR	PFSSSELQRD	NPATGQQNAD
90	100	110	120	130	140	150	160
EGSERPPRAQ	WRVDSNGAPK	TIADSATSPK	LLYVDRVVQE	ILETERTYVQ	DLKSIVEDYL	DCIRDQTKLP	LGTEERSALF
170	180	190	200	210	220	230	240
GNIQDIYHFN	SELLQDLENC	ENDPVAIAEC	FVSKSEEFHI	YTQYCTNYPR	SVAVLTECMR	NKILAKFFRE	RQETLKHSLP
250	260	270	280	290	300	310	320
LGSYLLKPVQ	RILKYHLLLH	EIENHLDKDT	EGYDVVLDAI	DTMQRVAWHI	NDMKRKHEHA	VRLQEIQSLL	TNWKGPDLTS
330	340	350	360	370	380	390	400
YGELVLEGTF	RIQRAKNERT	LFLFDKLLLI	TKKRDDTFTY	KAHILCGNLM	LVEVIPKEPL	SFSVFHYKNP	KLQHTVQAKS
410	420	430	440	450	460	470	480
QQDKRLWVLH	LKRLILENHA	AKIPAKAKQA	ILEMDAIHHP	GFCYSPEGGT	KALFGSKEGS	APYRLRRKSE	PSSRSHKVLK
490	500	510	520	530	540	550	560
TSETAQDIQK	VSREEGSPQL	SSARPSPAQR	NSQPSSSTMI	SVLRAGGALR	NIWTDHQIRQ	ALFPSRRSPQ	ENEDDEDDYQ
570	580	590	600	610	620	630	640
MFVPSFSSSD	LNSTRLCEDS	TSSRPCSWHM	GQMESTETSS	SGHRIVRRAS	SAGESNTCPP	EIGTSDRTRE	LQNSPKTEGQ
650	660	670	680	690	700	710	720
EEMTPFGSSI	ELTIDDIDHV	YDNISYEDLK	LMVAKREEAE	STPSKSARDS	VRPKSTPELA	FTKRQAGHSK	GSLYAQTDGT
730	740	750	760	770	780	790	800
LSGGEASSQS	THELQAVEEN	IYDTIGLPDP	PSLGFKCSSL	KRAKRSTFLG	LEADFVCCDS	LRPFVSQDSL	QLSEDEAPYH
810	820	830	840	850	860	870	880
QATPDHGYLS	LLYDSPSGNL	SMPHKPVSDK	LSEEVDEIWN	DLENYIKKNE	DKARDRLLAA	FPVSKDDVPD	RLHAESTPEL
890	900	910	920	930	940	950	960
SRDVGRSVST	LSLPESQALL	TPVKSRAGRA	SRANCPFEED	LISKEGSFMS	LNRLSLASEM	PLMDNPYDLA	NSGLSQTDPE
970	980	990	1000	1010	1020	1030	1040
NPDLGMEATD	KTKSRVFMMA	RQYSQKIKKA	NQLLKVKSLE	LEQPPASQHQ	KSMHKDLAAI	LEEKKQGGPA	IGARIAEYSQ
1050	1060	1070	1080	1090	1100	1110	1120
LYDQIVFRES	PLKIQKDGWA	SPQESSLLRS	VSPSQVHHGS	GDWLLHSTYS	NGELADFCLP	PEQDLRSRYP	TFEINTKSTP
1130	1140	1150	1160	1170	1180	1190	1200
RQLSAACSVP	SLQTSDPLPG	SVQRCSVVVS	QPNKENWCQD	HLYNSLGRKG	ISAKSQPYHR	SQSSSSVLIN	KSMDSINYPS
1210	1220	1230	1240	1250	1260	1270	1280
DVGKQQLLSL	HRSSRCESHQ	DLLPDIADSH	QQGTEKLSDL	TLQDSQKVVV	VNRNLPLNAQ	IATQNYFSNF	KETDGDEDDY
1290	1300	1310	1320	1330	1340	1350	1360
VEIKSEEDES	ELELSHNRRR	KSDSKFVDAD	FSDNVCSGNT	LHSLNSPRTP	KKPVNSKLGL	SPYLTPYNDS	DKLNDYLWRG
1370	1380	1390					
PSPNQQNIVQ	SLREKFQCLS	SSSFA					

Cmpd.	No. of	m/z meas.	Δ m/z	z	Rt	Score	P	Range	Sequence	Modification

Detailed Protein Report

	Cmpds.		[ppm]		[min]					
2063	1	697.5833	-140.16	3	56.2	11.8	1	998-1015	K.SLELEQPPASQHQKSMHK.D	Oxidation: 16

Detailed Protein Report

Protein 323: PREDICTED: collagen alpha-2(I) chain-like [Homo sapiens]

**Accession:**	gi|578803403	**Score:**	26.8
**Database:**	refseq_human(refseq_human_20140103.fasta)	**MW [kDa]:**	69.1
**Database Date:**	2015-11-30	**pI:**	12.4
		**Sequence Coverage [%]:**	3.6
		**No. of unique Peptides:**	1

10	20	30	40	50	60	70	80
MKNRRPGTPS	HRDSWVPFRP	TPHTPYTLWR	CALARAILSQ	CMYFLAGFLT	ENREGGELGD	VRVWSKSLPP	TTSCTSRRED
**90**	**100**	**110**	**120**	**130**	**140**	**150**	**160**
ELSAPLQAAG	AASPPRWLSG	SGLRESGRRA	RRLPTFPKHN	PGKFRLREGD	GAVGRGGDEN	RGRRFQPGSS	SHTQRLPLTA
**170**	**180**	**190**	**200**	**210**	**220**	**230**	**240**
RGGGVNGGIP	TASRGRRLLG	EAAPRRSRRS	GGARSPESHI	RAGSESGRPP	IRLGGAGQHR	AVRSAAAGQG	LRLSAAPPPG
**250**	**260**	**270**	**280**	**290**	**300**	**310**	**320**
RRRPPSALPA	SPLSAAARSP	PASPRSPAVS	SPSVVCVCAR	ARAHGSVWGN	RKFCRLGEQY	GKSEPRRQRW	RAGEAARGRS
**330**	**340**	**350**	**360**	**370**	**380**	**390**	**400**
GGRVSRGAGT	PRGTPGCGGA	GLDARAGGRG	WPWMGPAGPR	ISRGPCGQHC	LLEPGMGEWR	GPCCPRGLGT	LPLPTPTRAA
**410**	**420**	**430**	**440**	**450**	**460**	**470**	**480**
AGRSSLRSSG	PSVTLSRDAV	IHGGGGGGRG	RDKHGSGEVL	NSVLLSCLSL	GTFSPLGSVL	DFAGCENLLT	SESSVEPRLG
**490**	**500**	**510**	**520**	**530**	**540**	**550**	**560**
LWSAYFLQSD	CSCLRWSLSV	PLWGTSALIS	ALTAAVRAAG	KWGAPSGGRV	AVSLLISSVT	FKLSLEATAA	AGHVRRALGC
**570**	**580**	**590**	**600**	**610**	**620**	**630**	**640**
WGKISSAGSR	DRAGGRRGTA	PRPRLGTWEG	RAGALGPGAR	PSWDTREDGG	GEEVGSAAGE	LGIMQMGKEN	LRPRPSAKSS
**650**	**660**	**670**					
LGICRYCGHV	MEGSGKYGLI	F					

Cmpd.	No. of Cmpds.	m/z meas.	Δ m/z [ppm]	z	Rt [min]	Score	P	Range	Sequence	Modification
195	1	572.8054	9.14	2	32.4	16.7	2	312-323	R.AGEAARGRSGGR.V	

Detailed Protein Report

Protein 324: sialate O-acetylesterase isoform 2 [Homo sapiens]

**Accession:**	gi|321267600	**Score:**	26.8
**Database:**	refseq_human(refseq_human_20140103.fasta)	**MW [kDa]:**	54.5
**Database Date:**	2015-11-30	**pI:**	7.1
**Modification(s):**	Carbamidomethyl	**Sequence Coverage [%]:**	3.5
		**No. of unique Peptides:**	1

10	20	30	40	50	60	70	80
MVLQKEPAGA	VIWGFGTPGA	TVTVTLRQGQ	ETIMKKVTSV	KAHSDTWMVV	LDPMKPGGPF	EVMAQQTLEK	INFTLRVHDV
90	100	110	120	130	140	150	160
LFGDVWLCSG	QSNMQMTVLQ	IFNATRELSN	TAAYQSVRIL	SVSPIQAEQE	LEDLVAVDLQ	WSKPTSENLG	HGYFKYMSAV
170	180	190	200	210	220	230	240
CWLFGRHLYD	TLQYPIGLIA	SSWGGTPIEA	WSSGRSLKAC	GVPKQGSIPY	DSVTGPSKHS	VLWNAMIHPL	CNMTLKGVVW
250	260	270	280	290	300	310	320
YQGESNINYN	TDLYNCTFPA	LIEDWRETFH	RGSQGQTERF	FPFGLVQLSS	DLSKKSSDDG	FPQIRWHQTA	DFGYVPNPKM
330	340	350	360	370	380	390	400
PNTFMAVAMD	LCDRDSPFGS	IHPRDKQTVA	YRLHLGARAL	AYGEKNLTFE	GPLPEKIELL	AHKGLLNLTY	YQQIQVQKKD
410	420	430	440	450	460	470	480
NKIFEISCCS	DHRCKWLPAS	MNTVSTQSLT	LAIDSCHGTV	VALRYAWTTW	PCEYKQCPLY	HPSSALPAPP	FIAFITDQGP
490							
GHQSNVAK							

Cmpd.	No. of Cmpds.	m/z meas.	Δ m/z [ppm]	z	Rt [min]	Score	P	Range	Sequence	Modification
693	1	1035.0409	84.42	2	38.6	14.9	2	400-415	K.DNKIFEISCCSDHRCK.W	Carbamidomethyl: 9, 10, 15

Detailed Protein Report

Protein 325: tyrosine-protein kinase SYK isoform 2 [Homo sapiens]

**Accession:**	gi|205277435	**Score:**	26.7
**Database:**	refseq_human(refseq_human_20140103.fasta)	**MW [kDa]:**	69.5
**Database Date:**	2015-11-30	**pI:**	7.8
		**Sequence Coverage [%]:**	4.9
		**No. of unique Peptides:**	2

Alias proteins:

Accession	Name	Description
gi|293332611	refseq_human (refseq_human_20140103.fasta)	tyrosine-protein kinase SYK isoform 2 [Homo sapiens]

10	20	30	40	50	60	70	80
MASSGMADSA	NHLPFFFGNI	TREEAEDYLV	QGGMSDGLYL	LRQSRNYLGG	FALSVAHGRK	AHHYTIEREL	NGTYAIAGGR
**90**	**100**	**110**	**120**	**130**	**140**	**150**	**160**
THASPADLCH	YHSQESDGLV	CLLKKPFNRP	QGVQPKTGPF	EDLKENLIRE	YVKQTWNLQG	QALEQAIISQ	KPQLEKLIAT
**170**	**180**	**190**	**200**	**210**	**220**	**230**	**240**
TAHEKMPWFH	GKISREESEQ	IVLIGSKTNG	KFLIRARDNN	GSYALCLLHE	GKVLHYRIDK	DKTGKLSIPE	GKKFDTLWQL
**250**	**260**	**270**	**280**	**290**	**300**	**310**	**320**
VEHYSYKADG	LLRVLTVPCQ	KIGTQGNVNF	GGRPQLPGSH	PASSPAQGNR	QESTVSFNPY	EPELAPWAAD	KGPQREALPM
**330**	**340**	**350**	**360**	**370**	**380**	**390**	**400**
DTEVYESPYA	DPEEIRPKEV	YLDRKLLTLE	DKELGSGNFG	TVKKGYYQMK	KVVKTVAVKI	LKNEANDPAL	KDELLAEANV
**410**	**420**	**430**	**440**	**450**	**460**	**470**	**480**
MQQLDNPYIV	RMIGICEAES	WMLVMEMAEL	GPLNKYLQQN	RHVKDKNIIE	LVHQVSMGMK	YLEESNFVHR	DLAARNVLLV
**490**	**500**	**510**	**520**	**530**	**540**	**550**	**560**
TQHYAKISDF	GLSKALRADE	NYYKAQTHGK	WPVKWYAPEC	INYYKFSSKS	DVWSFGVLMW	EAFSYGQKPY	RGMKGSEVTA
**570**	**580**	**590**	**600**	**610**	**620**		
MLEKGERMGC	PAGCPREMYD	LMNLCWTYDV	ENRPGFAAVE	LRLRNYYYDV	VN		

Cmpd.	No. of Cmpds.	m/z meas.	Δ m/z [ppm]	z	Rt [min]	Score	P	Range	Sequence	Modification
2468	2	1044.5140	-52.13	2	61.4	15.4	2	173-191	K.ISREESEQIVLIGSKTNGK.F	
1940	2	643.2750	-142.67	2	54.6	11.3	0	476-486	R.NVLLVTQHYAK.I	

Detailed Protein Report

Protein 326: cullin-associated NEDD8-dissociated protein 2 isoform 2 [Homo sapiens]

Accession: gi|112420977
Database: refseq_human(refseq_human_20140103.fasta)
Database Date: 2015-11-30
Modification(s): Carbamidomethyl, Oxidation

Score: 26.7
MW [kDa]: 122.6
pI: 5.5
Sequence Coverage [%]: 3.3
No. of unique Peptides: 2

10	20	30	40	50	60	70	80
MSTAAFHISS	LLEKMTSSDK	DFRFMATSDL	MSELQKDSIQ	LDEDSERKVV	KMLLRLLEDK	NGEVQNLAVK	WLGVPLGAFH
90	100	110	120	130	140	150	160
ASLLHCLLPQ	LSSPRLAVRK	RAVGALGHLA	AACSTDLFVE	LADHLLDRLP	GPRVPTSPTA	IRTLIQCLGS	VGRQAGHRLG
170	180	190	200	210	220	230	240
AHLDRLVPLV	EDFCNLDDDE	LRESCLQAFE	AFLRKCPKEM	GPHVPNVTSL	CLQYIKHDPN	YNYDSDEDEE	QMETEDSEFS
250	260	270	280	290	300	310	320
EQESEDEYSD	DDDMSWKVRR	AAAKCIAALI	SSRPDLLPDF	HCTLAPVLIR	RFKEREENVK	ADVFTAYIVL	LRQTQPPKGW
330	340	350	360	370	380	390	400
LEAMEEPTQT	GSNLHMLRGQ	VPLVVKALQR	QLKDRSVRAR	QGCFSLLTEL	AGVLPGSLAE	HMPVLVSGII	FSLADRSSSS
410	420	430	440	450	460	470	480
TIRMDALAFL	QGLLGTEPAE	AFHPHLPILL	PPVMACVADS	FYKIAAEALV	VLQELVRALW	PLHRPRMLDP	EPYVGEMSAV
490	500	510	520	530	540	550	560
TLARLRATDL	DQEVKERAIS	CMGHLVGHLG	DRLGDDLEPT	LLLLLDRLRN	EITRLPAIKA	LTLVAVSPLQ	LDLQPILAEA
570	580	590	600	610	620	630	640
LHILASFLRK	NQRALRLATL	AALDALAQSQ	GLSLPPSAVQ	AVLAELPALV	NESDMHVAQL	AVDFLATVTQ	AQPASLVEVS
650	660	670	680	690	700	710	720
GPVLSELLRL	LRSPLLPAGV	LAAAEGFLQA	LVGTRPPCVD	YAKLISLLTA	PVYEQAVDGG	PGLHKQVFHS	LARCVAALSA
730	740	750	760	770	780	790	800
ACPQEAASTA	SRLVCDARSP	HSSTGVKVLA	FLSLAEVGQV	AGPGHQRELK	AVLLEALGSP	SEDVRAAASY	ALGRVGAGSL
810	820	830	840	850	860	870	880
PDFLPFLLEQ	IEAEPRRQYL	LLHSLREALG	AAQPDSLKPY	AEDIWALLFQ	RCEGAEEGTR	GVVAECIGKL	VLVNPSFLLP
890	900	910	920	930	940	950	960
RLRKQLAAGR	PHTRSTVITA	VKFLISDQPH	PIDPLLKSFI	AVHNKPSLVR	DLLDDILPLL	YQETKIRRDL	IREVEMGPFK
970	980	990	1000	1010	1020	1030	1040
HTVDDGLDVR	KAAFECMYSL	LESCLGQLDI	CEFLNHVEDG	LKDHYDIRML	TFIMVARLAT	LCPAPVLQRV	DRLIEPLRAT
1050	1060	1070	1080	1090	1100	1110	1120
CTAKVKAGSV	KQEFEKQDEL	KRSAMRAVAA	LLTIPEVGKS	PIMADFSSQI	RSNPELAALF	ESIQKDSASA	PSTDSMELS

Cmpd.	No. of Cmpds.	m/z meas.	Δ m/z [ppm]	z	Rt [min]	Score	P	Range	Sequence	Modification
1132	1	701.3400	-16.93	3	44.3	12.1	0	199-216	K.EMGPHVPNVTSLCLQYIK.H	Carbamidomethyl: 13; Oxidation: 2
2247	1	1017.5903	-9.72	2	58.5	14.5	2	884-902	R.KQLAAGRPHTRSTVITAVK.F	

Detailed Protein Report

Protein 327: PREDICTED: sperm flagellar protein 2 isoform X5 [Homo sapiens]

**Accession:**	gi|578810001	**Score:**	26.7
**Database:**	refseq_human(refseq_human_20140103.fasta)	**MW [kDa]:**	176.7
**Database Date:**	2015-11-30	**pI:**	5.7
		**Sequence Coverage [%]:**	1.6
		**No. of unique Peptides:**	2

Quantitation

QD:QU **Median:** 2.59 **CV:** 0.00 % **No. of Peptides:** 1

```
        10         20         30         40         50         60         70         80
MSEILCQWLN KELKVSRTVS PKSFAKAFSS GYLLGEVLHK FELQDDFSEF LDSRVSSAKL NNFSRLEPTL NLLGVQFDQN
        90        100        110        120        130        140        150        160
VAHGIITEKP GVATKLLYQL YIALQKKKKS GLTGVEMQTM QRLTNLRLQN MKSDTFQERL RHMIPRQTDF NLMRITYRFQ
       170        180        190        200        210        220        230        240
EKYKHVKEDL AHLHFEKLER FQKLKEEQRC FDIEKQYLNR RRQNEIMAKI QAAIIQIPKP ASNRTLKALE AQKMMKKKKE
       250        260        270        280        290        300        310        320
AEDVADEIKK FEALIKKDLQ AKESASKTSL DTAGQTTTDL LNTYSDDEYI KKIQKRLEED AFAREQREKR RRKLLMDQLI
       330        340        350        360        370        380        390        400
AHEAQEEAYR EEQLINRLMR QSQQERRIAV QLMHVRHEKE VLWQNRIFRE KQHEERRLKD FQDALDREAA LAKQAKIDFE
       410        420        430        440        450        460        470        480
EQFLKEKRFH DQIAVERAQA RYEKHYSVCA EILDQIVDLS TKVADYRMLT NNLIPYKLMH DWKELFFNAK PIYEQASVKT
       490        500        510        520        530        540        550        560
LPANPSREQL TELEKRDLLD TNDYEEYKNM VGEWALPEEM VDNLPPSNNC ILGHILHRLA EKSLPPRAES TTPELPSFAV
       570        580        590        600        610        620        630        640
KGCLLGKTLS GKTTILRSLQ KDFPIQILSI DTLVQEAIQA FHDNEKVSEV LPIQKNDEED ALPVLQEEIK ESQDPQHVFS
       650        660        670        680        690        700        710        720
AGPVSDEVLP ETEGETMLSA NADKTPKAEE VKSSDSFLKL TTRAQLGAKS EQLLKKGKSI PDVLLVDIIV NAINEIPVNQ
       730        740        750        760        770        780        790        800
DCILDGFPMT LNQAQLLEEA LTGCNRNLTE VERKKAQKST LAIDPATSKE IPLPSPAFDF VILLDVSDTS SMSRMNDIIA
       810        820        830        840        850        860        870        880
EELSYKTAHE DISQRVAAEN QDKDGDQNLR DQIQHRIIGF LDNWPLLEQW FSEPENILIK INAEIDKESL CEKVKEILTT
       890        900        910        920        930        940        950        960
EIAKKKNKVE KKLEEKEAEK KAAASLAELP LPTPPPAPPP EPEKEKEIHQ SHVASKTPTA KGKPQSEAPH GKQESLQEGK
       970        980        990       1000       1010       1020       1030       1040
GKKGETALKR KGSPKGKSSG GKVPVKKSPA DSTDTSPVAI VPQPPKPGSE EWVYVNEPVP EEMPLFLVPY WELIENSYIN
      1050       1060       1070       1080       1090       1100       1110       1120
TIKTVLRHLR EDQHTVLAYL YEIRTSFQEF LKRPDHKQDF VAQWQADFNS LPDDLWDDEE TKAELHQRVN DLRDRLWDIC
      1130       1140       1150       1160       1170       1180       1190       1200
DARKEEAEQE RLDIINESWL QDTLGMTMNH FFSLMQAELN RFQDTKRLLQ DYYWGMESKI PVEDNKRFTR IPLVQLDSKD
      1210       1220       1230       1240       1250       1260       1270       1280
NSESQLRIPL VPRISISLET VTPKPKTKSV LKGKMDNSLE NVESNFEADE KLVMDTWQQA SLAVSHMVAA EIHQRLMEEE
      1290       1300       1310       1320       1330       1340       1350       1360
KENQPADPKE KSPQMGANKK VKKEPPKKKQ EDKKPKGKSP PMAEATPVIV TTEEIAEIKR KNELRVKIKE EHLAALQFEE
      1370       1380       1390       1400       1410       1420       1430       1440
IATQFRLELI KTKALALLED LVTKVVDVYK LMEKWLGERY LNEMASTEKL TDVARYHIET STKIQNELYL SQEDFFINGN
      1450       1460       1470       1480       1490       1500       1510       1520
IKVFPDPPPS IRPPPVEKEE DGTLTIEQLD SLRDQFLDMA PKECSENCFQ RPSPSTCDAF SNRRQEVASW ITGSPPELSQ
      1530       1540
GISCNCSATA LSRQRQK
```

Cmpd.	No. of Cmpds.	m/z meas.	Δ m/z [ppm]	z	Rt [min]	Score	P	Range	Sequence	Modification	Ratios
2407	2	973.4149	-118.32	2	60.6	12.1	2	2-17	M.SEILCQWLNKELKVSR.T		QD:QU 2.59
1569	1	480.6396	-230.14	2	48.1	14.6	1	1292-1300	K.SPQMGANKK.V		

Detailed Protein Report

Protein 328: zinc finger protein 570 [Homo sapiens]

**Accession:**	gi|21389599	**Score:**	26.5
**Database:**	refseq_human(refseq_human_20140103.fasta)	**MW [kDa]:**	62.3
**Database Date:**	2015-11-30	**pI:**	9.7
		**Sequence Coverage [%]:**	7.5
		**No. of unique Peptides:**	2

10	20	30	40	50	60	70	80
MAVGLLKAMY	QELVTFRDVA	VDFSQEEWDC	LDSSQRHLYS	NVMLENYRIL	VSLGLCFSKP	SVILLLEQGK	APWMVKRELT
**90**	**100**	**110**	**120**	**130**	**140**	**150**	**160**
KGLCSGWEPI	CETEELTPKQ	DFYEEHQSQK	IIETLTSYNL	EYSSLREEWK	CEGYFERQPG	NQKACFKEEI	ITHEEPLFDE
**170**	**180**	**190**	**200**	**210**	**220**	**230**	**240**
REQEYKSWGS	FHQNPLLCTQ	KIIPKEEKVH	KHDTQKRSFK	KNLMAIKPKS	VCAEKKLLKC	NDCEKVFSQS	SSLTLHQRIH
**250**	**260**	**270**	**280**	**290**	**300**	**310**	**320**
TGEKPYKCIE	CGKAFSQRSN	LVQHQRIHTG	EKPYECKECR	KAFSQNAHLV	QHLRVHTGEK	PYECKVCRKA	FSQFAYLAQH
**330**	**340**	**350**	**360**	**370**	**380**	**390**	**400**
QRVHTGEKPY	ECIECGKAFS	NRSSIAQHQR	VHTGEKPYEC	NVCGKAFSLR	AYLTVHQRIH	TGERPYECKE	CGKAFSQNSH
**410**	**420**	**430**	**440**	**450**	**460**	**470**	**480**
LAQHQRIHTG	EKPYKCQECR	KAFSQIAYLA	QHQRVHTGEK	PYECIECGKA	FSNDSSLTQH	QRVHTGEKPY	ECTVCGKAFS
**490**	**500**	**510**	**520**	**530**	**540**		
YCGSLAQHQR	IHTGERPYEC	KECKKTFRQH	AHLAHHQRIH	IGESLSPPNP	VNHQVL		

Cmpd.	No. of Cmpds.	m/z meas.	Δ m/z [ppm]	z	Rt [min]	Score	P	Range	Sequence	Modification
1383	1	1179.1487	-41.33	2	47.4	15.2	0	49-70	R.ILVSLGLCFSKPSVILLLEQGK.A	
17	1	1103.4962	-28.67	2	29.9	11.4	1	144-161	K.ACFKEEIITHEEPLFDER.E	

Detailed Protein Report

Protein 329: small proline-rich protein 3 [Homo sapiens]

Accession: gi|4885607
Database: refseq_human(refseq_human_20140103.fasta)
Database Date: 2015-11-30

Score: 26.5
MW [kDa]: 18.1
pI: 9.9
Sequence Coverage [%]: 18.3
No. of unique Peptides: 1

Quantitation

QD:QU **Median:** 0.84 **CV:** 0.00 % **No. of Peptides:** 1

Alias proteins:

Accession	Name	Description
gi|148229144	r e f s e q _ h u m a (refseq_human_20140103.fasta)	small proline-rich protein 3 [Homo sapiens]

10	20	30	40	50	60	70	80
MSSYQQKQTF	TPPPQLQQQQ	VKQPSQPPPQ	EIFVPTTKEP	CHSKVPQPGN	TKIPEPGCTK	VPEPGCTKVP	EPGCTKVPEP
**90**	**100**	**110**	**120**	**130**	**140**	**150**	**160**
GCTKVPEPGC	TKVPEPGCTK	VPEPGYTKVP	EPGSIKVPDQ	GFIKFPEPGA	IKVPEQGYTK	VPVPGYTKLP	EPCPSTVTPG
**170**							
PAQQKTKQK							

Cmpd.	No. of Cmpds.	m/z meas.	Δ m/z [ppm]	z	Rt [min]	Score	P	Range	Sequence	Modification	Ratios	
828	1	897.4861	12.13	2	40.4	15.1	0	23-38	K.QPSQPPPQEIFVPTTK.E		QD:QU	0.84

Detailed Protein Report

Protein 330: protein phosphatase 1A isoform 3 [Homo sapiens]

Accession: gi|193211600
Database: refseq_human(refseq_human_20140103.fasta)
Database Date: 2015-11-30
Modification(s): Oxidation

Score: 26.5
MW [kDa]: 51.3
pI: 6.8
Sequence Coverage [%]: 8.1
No. of unique Peptides: 2

10	20	30	40	50	60	70	80
MFCSGRKWVA	EATICTKLMK	REKRRMGKRR	AKKAKREEKK	KGGERRRNEK	RGNQMKRMCE	RKKYETDLED	QDIMGAFLDK
**90**	**100**	**110**	**120**	**130**	**140**	**150**	**160**
PKMEKHNAQG	QGNGLRYGLS	SMQGWRVEME	DAHTAVIGLP	SGLESWSFFA	VYDGHAGSQV	AKYCCEHLLD	HITNNQDFKG
**170**	**180**	**190**	**200**	**210**	**220**	**230**	**240**
SAGAPSVENV	KNGIRTGFLE	IDEHMRVMSE	KKHGADRSGS	TAVGVLISPQ	HTYFINCGDS	RGLLCRNRKV	HFFTQDHKPS
**250**	**260**	**270**	**280**	**290**	**300**	**310**	**320**
NPLEKERIQN	AGGSVMIQRV	NGSLAVSRAL	GDFDYKCVHG	KGPTEQLVSP	EPEVHDIERS	EEDDQFIILA	CDGIWDVMGN
**330**	**340**	**350**	**360**	**370**	**380**	**390**	**400**
EELCDFVRSR	LEVTDDLEKV	CNEVVDTCLY	KGSRDNMSVI	LICFPNAPKV	SPEAVKKEAE	LDKYLECRVE	EIIKKQGEGV
**410**	**420**	**430**	**440**	**450**	**460**		
PDLVHVMRTL	ASENIPSLPP	GGELASKRNV	IEAVYNRLNP	YKNDDTDSTS	TDDMW		

Cmpd.	No. of Cmpds.	m/z meas.	Δ m/z [ppm]	z	Rt [min]	Score	P	Range	Sequence	Modification
2669	1	974.0887	123.98	2	62.2	10.6	2	1-17	-.MFCSGRKWVAEATICTK.L	Oxidation: 1
2937	1	1018.9845	-66.59	2	65.9	15.9	1	409-428	R.TLASENIPSLPPGGELASKR.N	

Detailed Protein Report

Protein 331: **zinc finger RNA-binding protein [Homo sapiens]**

**Accession:**	gi|34101286	**Score:**	26.5
**Database:**	refseq_human(refseq_human_20140103.fasta)	**MW [kDa]:**	116.9
**Database Date:**	2015-11-30	**pI:**	9.8
		**Sequence Coverage [%]:**	3.4
		**No. of unique Peptides:**	2

Quantitation

QD:QU **Median:** 0.96 **CV:** 0.00 % **No. of Peptides:** 1

10	20	30	40	50	60	70	80
MIPICPVVSF	TYVPSRLGED	AKMATGNYFG	FTHSGAAAAA	AAAQYSQQPA	SGVAYSHPTT	VASYTVHQAP	VAAHTVTAAY
90	100	110	120	130	140	150	160
APAAATVAVA	RPAPVAVAAA	ATAAAYGGYP	TAHTATDYGY	TQRQQEAPPP	PPPATTQNYQ	DSYSYVRSTA	PAVAYDSKQY
170	180	190	200	210	220	230	240
YQQPTATAAA	VAAAAQPQPS	VAETYYQTAP	KAGYSQGATQ	YTQAQQTRQV	TAIKPATPSP	ATTTFSIYPV	SSTVQPVAAA
250	260	270	280	290	300	310	320
ATVVPSYTQS	ATYSTTAVTY	SGTSYSGYEA	AVYSAASSYY	QQQQQQQKQA	AAAAAAAAAT	AAWTGTTFTK	KAPFQNKQLK
330	340	350	360	370	380	390	400
PKQPPKPPQI	HYCDVCKISC	AGPQTYKEHL	EGQKHKKKEA	ALKASQNTSS	SNSSTRGTQN	QLRCELCDVS	CTGADAYAAH
410	420	430	440	450	460	470	480
IRGAKHQKVV	KLHTKLGKPI	PSTEPNVVSQ	ATSSTAVSAS	KPTASPSSIA	ANNCTVNTSS	VATSSMKGLT	TTGNSSLNST
490	500	510	520	530	540	550	560
SNTKVSAVPT	NMAAKKTSTP	KINFVGGNKL	QSTGNKAEDI	KGTECVKSTP	VTSAVQIPEV	KQDTVSEPVT	PASLAALQSD
570	580	590	600	610	620	630	640
VQPVGHDYVE	EVRNDEGKVI	RFHCKLCECS	FNDPNAKEMH	LKGRRHRLQY	KKKVNPDLQV	EVKPSIRARK	IQEEKMRKQM
650	660	670	680	690	700	710	720
QKEEYWRRRE	EEERWRMEMR	RYEEDMYWRR	MEEEQHHWDD	RRRMPDGGYP	HGPPGPLGLL	GVRPGMPPQP	QGPAPLRRPD
730	740	750	760	770	780	790	800
SSDDRYVMTK	HATIYPTEEE	LQAVQKIVSI	TERALKLVSD	SLSEHEKNKN	KEGDDKKEGG	KDRALKGVLR	VGVLAKGLLL
810	820	830	840	850	860	870	880
RGDRNVNLVL	LCSEKPSKTL	LSRIAENLPK	QLAVISPEKY	DIKCAVSEAA	IILNSCVEPK	MQVTITLTSP	IIREENMREG
890	900	910	920	930	940	950	960
DVTSGMVKDP	PDVLDRQKCL	DALAALRHAK	WFQARANGLQ	SCVIIIRILR	DLCQRVPTWS	DFPSWAMELL	VEKAISSASS
970	980	990	1000	1010	1020	1030	1040
PQSPGDALRR	VFECISSGII	LKGSPGLLDP	CEKDPFDTLA	TMTDQQREDI	TSSAQFALRL	LAFRQIHKVL	GMDPLPQMSQ
1050	1060	1070	1080				
RFNIHNNRKR	RRDSDGVDGF	EAEGKKDKKD	YDNF				

Cmpd.	No. of Cmpds.	m/z meas.	Δ m/z [ppm]	z	Rt [min]	Score	P	Range	Sequence	Modification	Ratios	
1045	1	839.3856	-82.99	2	41.6	11.3	0	2-16	M.IPICPVVSFTYVPSR.L		QD:QU	0.96
103	1	1240.0318	-79.59	2	31.2	15.2	1	726-746	R.YVMTKHATIYPTEEELQAVQK.I			

Detailed Protein Report

Protein 332: **origin recognition complex subunit 1 isoform 2 [Homo sapiens]**

**Accession:**	gi|299890795	**Score:**	26.4
**Database:**	refseq_human(refseq_human_20140103.fasta)	**MW [kDa]:**	96.7
**Database Date:**	2015-11-30	**pI:**	10.1
		**Sequence Coverage [%]:**	2.5
		**No. of unique Peptides:**	1

10	20	30	40	50	60	70	80
MAHYPTRLKT	RKTYSWVGRP	LLDRKLHYQT	YREMCVKTEG	CSTEIHIQIG	QFVLIEGDDD	ENPYVAKLLE	LFEDDSDPPP
**90**	**100**	**110**	**120**	**130**	**140**	**150**	**160**
KKRARVQWFV	RFCEVPACKR	HLLGRKPGAQ	EIFWYDYPAC	DSNINAETII	GLVRVIPLAP	KDVVPTNLKN	EKTLFVKLSW
**170**	**180**	**190**	**200**	**210**	**220**	**230**	**240**
NEKKFRPLSS	ELFAELNKPQ	ESAAKCQKPV	RAKSKSAESP	SWTPAEHVAK	RIESRHSASK	SRQTPTHPLT	PRARKRLELG
**250**	**260**	**270**	**280**	**290**	**300**	**310**	**320**
NLGNPQMSQQ	TSCASLDSPG	RIKRKVAFSE	ITSPSKRSQP	DKLQTLSPAL	KAPEKTRETG	LSYTEDDKKA	SPEHRIILRT
**330**	**340**	**350**	**360**	**370**	**380**	**390**	**400**
RIAASKTIDI	REERTLTPIS	GGQRSSVVPS	VILKPENIKK	RDAKEAKAQN	EATSTPHRIR	RKSSVLTMNR	IRQQLRFLGN
**410**	**420**	**430**	**440**	**450**	**460**	**470**	**480**
SKSDQEEKEI	LPAAEISDSS	SDEEEASTPP	LPRRAPRTVS	RNLRSSLKSS	LHTLTKLKPR	TPRCAAPQIR	SRSLAAQEPA
**490**	**500**	**510**	**520**	**530**	**540**	**550**	**560**
SVLEEARLRL	HVSAVPESLP	CREQEFQDIY	NFVESKLLDH	TGGCMYISGV	PGTGKTATVH	EVIRCLQQAA	QANDVPPFQY
**570**	**580**	**590**	**600**	**610**	**620**	**630**	**640**
IEVNGMKLTE	PHQVYVQILQ	KLTGQKATAN	HAAELLAKQF	CTRGSPQETT	VLLVDELDLL	WTHKQDIMYN	LFDWPTHKEA
**650**	**660**	**670**	**680**	**690**	**700**	**710**	**720**
RLVVLAIANT	MDLPERIMMN	RVSSRLGLTR	MCFQPYTYSQ	LQQILRSRLK	HLKAFEDDAI	QLVARKVAAL	SGDARRCLDI
**730**	**740**	**750**	**760**	**770**	**780**	**790**	**800**
CRRATEICEF	SQQKPDSPGL	VTIAHSMEAV	DEMFSSSYIT	AIKNSSVLEQ	SFLRAILAEF	RRSGLEEATF	QQIYSQHVAL
**810**	**820**	**830**	**840**	**850**	**860**		
CRMEGLPYPT	MSETMAVCSH	LGSCRLLLVE	PSRNDLLLRV	RLNVSQDDVL	YALKDE		

Cmpd.	No. of Cmpds.	m/z meas.	Δ m/z [ppm]	z	Rt [min]	Score	P	Range	Sequence	Modification
1039	1	516.1512	-244.92	2	41.5	10.2	1	327-334	K.TIDIREER.T	

Detailed Protein Report

Protein 333: PREDICTED: PH and SEC7 domain-containing protein 3 isoform X3 [Homo sapiens]

**Accession:**	gi|578815261	**Score:**	26.4
**Database:**	refseq_human(refseq_human_20140103.fasta)	**MW [kDa]:**	112.4
**Database Date:**	2015-11-30	**pI:**	5.6
		**Sequence Coverage [%]:**	2.7
		**No. of unique Peptides:**	2

Quantitation

QD:QU **Median:** 0.45 **CV:** 0.00 % **No. of Peptides:** 1

10	20	30	40	50	60	70	80
MGNCWSYSNL	CDHGGSTLLP	PNVTNEFPEY	GTMEEGGEGL	RASLEFDGEA	LPCHPQEQQG	VQPLTGCHSG	LDSVTEGPKD
90	100	110	120	130	140	150	160
VREAPSQSHL	KEQSLQPIDS	LISALKATEA	RIISGTLQAT	KVLDQDAVSS	FSVQQVEKEL	DTASRKTQRV	NKTLPAGQKN
170	180	190	200	210	220	230	240
LPEIPLSAEV	TTEESFYLSI	QKDLTALLTG	DTQAEISQIM	NNGRKGAVCV	QEPSCPLASL	GSSAVTCHSA	GSVGFLKEQR
250	260	270	280	290	300	310	320
SALGREHPGG	CDRSSSMGRP	GRVKHVEFQG	VEILWTGGDK	RETQHPIDFE	TSLQRTASPD	SKESSKVPRH	LISSAGLCNS
330	340	350	360	370	380	390	400
SSLTENVWDE	SWKAPSERPG	TSSGTFSPVR	LDESGEDEVF	LQENKQHLEK	TPKPERDRER	ISEQEEHVKG	EDEDILGPGY
410	420	430	440	450	460	470	480
TEDSTDVYSS	QFETILDNTS	LYYSAESLET	LYSEPDSYFS	FEMPLTPMIQ	QRIKEGGQFL	ERTSGGGHQD	ILSVSADGGI
490	500	510	520	530	540	550	560
VMGYSSGVTN	GLNDASDSIY	TKGTPEIAFW	GSNAGVKTTR	LEAHSEMGST	EILEKETPEN	LSNGTSSNVE	AAKRLAKRLY
570	580	590	600	610	620	630	640
QLDRFKRSDV	AKHLGKNNEF	SKLVAEEYLK	FFDFTGMTLD	QSLRYFFKAF	SLVGETQERE	RVLIHFSNRY	FYCNPDTIAS
650	660	670	680	690	700	710	720
QDGVHCLTCA	IMLLNTDLHG	HNIGKKMTCQ	EFIANLQGVN	EGVDFSKDLL	KALYNSIKNE	KLEWAVDDEE	KKKSPSESTE
730	740	750	760	770	780	790	800
EKANGTHPKT	ISRIGSTTNP	FLDIPHDPNA	AVYKSGFLAR	KIHADMDGKK	TPRGKRGWKT	FYAVLKGTVL	YLQKDEYKPE
810	820	830	840	850	860	870	880
KALSEEDLKN	AVSVHHALAS	KATDYEKKPN	VFKLKTADWR	VLLFQTQSPE	EMQGWINKIN	CVAAVFSAPP	FPAAIGSQKK
890	900	910	920	930	940	950	960
FSRPLLPATT	TKLSQEEQLK	SHESKLKQIT	TELAEHRSYP	PDKKVKAKDV	DEYKLKDHYL	EFEKTRYEMY	VSILKEGGKE
970	980	990	1000	1010	1020		
LLSNDESEAA	GLKKSHSSPS	LNPDTSPITA	KVKRNVSERK	DHRPETPSIK	QKVT		

Cmpd.	No. of Cmpds.	m/z meas.	Δ m/z [ppm]	z	Rt [min]	Score	P	Range	Sequence	Modification	Ratios
909	1	578.3188	46.40	3	41.4	13.5	0	334-350	K.APSERPGTSSGTFSPVR.L		QD:QU 0.45
249	1	561.2240	-81.38	2	31.8	12.9	1	713-722	K.KSPSESTEEK.A		

Detailed Protein Report

Protein 334: **PREDICTED: poly [ADP-ribose] polymerase 14 isoform X1 [Homo sapiens]**

**Accession:**	gi|530374806	**Score:**	26.4
**Database:**	refseq_human(refseq_human_20140103.fasta)	**MW [kDa]:**	187.2
**Database Date:**	2015-11-30	**pI:**	8.1
		**Sequence Coverage [%]:**	1.2
		**No. of unique Peptides:**	2

10	20	30	40	50	60	70	80
MEDIPEECEN	ISSLVAFENL	KANVTDIMLI	LLVENISGLS	NDDFQVEIIR	DFDVAVVTFQ	KHIDTIRFVD	DCTKHHSIKQ
**90**	**100**	**110**	**120**	**130**	**140**	**150**	**160**
LQLSPRLLEV	TNTIRVENLP	PGADDYSLKL	FFENPYNGGG	RVANVEYFPE	ESSALIEFFD	RKVLDTIMAT	KLDFNKMPLS
**170**	**180**	**190**	**200**	**210**	**220**	**230**	**240**
VFPYYASLGT	ALYGKEKPLI	KLPAPFEESL	DLPLWKFLQK	KNHLIEEIND	EMRRCHCELT	WSQLSGKVTI	RPAATLVNEG
**250**	**260**	**270**	**280**	**290**	**300**	**310**	**320**
RPRIKTWQAD	TSTTLSSIRS	KYKVNPIKVD	PTMWDTIKND	VKDDRILIEF	DTLKEMVILA	GKSEDVQSIE	VQVRELIEST
**330**	**340**	**350**	**360**	**370**	**380**	**390**	**400**
TQKIKREEQS	LKEKMIISPG	RYFLLCHSSL	LDHLLTECPE	IEICYDRVTQ	HLCLKGPSAD	VYKAKCEIQE	KVYTMAQKNI
**410**	**420**	**430**	**440**	**450**	**460**	**470**	**480**
QVSPEIFQFL	QQVNWKEFSK	CLFIAQKILA	LYELEGTTVL	LTSCSSEALL	EAEKQMLSAL	NYKRIEVENK	EVLHGKKWKG
**490**	**500**	**510**	**520**	**530**	**540**	**550**	**560**
LTHNLLKKQN	SSPNTVIINE	LTSETTAEVI	ITGCVKEVNE	TYKLLFNFVE	QNMKIERLVE	VKPSLVIDYL	KTEKKLFWPK
**570**	**580**	**590**	**600**	**610**	**620**	**630**	**640**
IKKVNVQVSF	NPENKQKGIL	LTGSKTEVLK	AVDIVKQVWD	SVCVKSVHTD	KPGAKQFFQD	KARFYQSEIK	RLFGCYIELQ
**650**	**660**	**670**	**680**	**690**	**700**	**710**	**720**
ENEVMKEGGS	PAGQKCFSRT	VLAPGVVLIV	QQGDLARLPV	DVVVNASNED	LKHYGGLAAA	LSKAAGPELQ	ADCDQIVKRE
**730**	**740**	**750**	**760**	**770**	**780**	**790**	**800**
GRLLPGNATI	SKAGKLPYHH	VIHAVGPRWS	GYEAPRCVYL	LRRAVQLSLC	LAEKYKYRSI	AIPAISSGVF	GFPLGRCVET
**810**	**820**	**830**	**840**	**850**	**860**	**870**	**880**
IVSAIKENFQ	FKKDGHCLKE	IYLVDVSEKT	VEAFAEAVKT	VFKATLPDTA	APPGLPPAAA	GPGKTSWEKG	SLVSPGGLQM
**890**	**900**	**910**	**920**	**930**	**940**	**950**	**960**
LLVKEGVQNA	KTDVVVNSVP	LDLVLSRGPL	SKSLLEKAGP	ELQEELDTVG	QGVAVSMGTV	LKTSSWNLDC	RYVLHVVAPE
**970**	**980**	**990**	**1000**	**1010**	**1020**	**1030**	**1040**
WRNGSTSSLK	IMEDIIRECM	EITESLSLKS	IAFPAIGTGN	LGFPKNIFAE	LIISEVFKFS	SKNQLKTLQE	VHFLLHPSDH
**1050**	**1060**	**1070**	**1080**	**1090**	**1100**	**1110**	**1120**
ENIQAFSDEF	ARRANGNLVS	DKIPKAKDTQ	GFYGTVSSPD	SGVYEMKIGS	IIFQVASGDI	TKEEADVIVN	STSNSFNLKA
**1130**	**1140**	**1150**	**1160**	**1170**	**1180**	**1190**	**1200**
GVSKAILECA	GQNVERECSQ	QAQQRKNDYI	ITGGGFLRCK	NIIHVIGGND	VKSSVSSVLQ	ECEKKNYSSI	CLPAIGTGNA
**1210**	**1220**	**1230**	**1240**	**1250**	**1260**	**1270**	**1280**
KQHPDKVAEA	IIDAIEDFVQ	KGSAQSVKKV	KVVIFLPQVL	DVFYANMKKR	EGTQLSSQQS	VMSKLASFLG	FSKQSPQKKN
**1290**	**1300**	**1310**	**1320**	**1330**	**1340**	**1350**	**1360**
HLVLEKKTES	ATFRVCGENV	TCVEYAISWL	QDLIEKEQCP	YTSEDECIKD	FDEKEYQELN	ELQKKLNINI	SLDHKRPLIK
**1370**	**1380**	**1390**	**1400**	**1410**	**1420**	**1430**	**1440**
VLGISRDVMQ	ARDEIEAMIK	RVRLAKEQES	RADCISEFIE	WQYNDNNTSH	CFNKMTNLKL	EDARREKKKT	VDVKINHRHY
**1450**	**1460**	**1470**	**1480**	**1490**	**1500**	**1510**	**1520**
TVNLNTYTAT	DTKGHSLSVQ	RLTKSKVDIP	AHWSDMKQQN	FCVVELLPSD	PEYNTVASKF	NQTCSHFRIE	KIERIQNPDL
**1530**	**1540**	**1550**	**1560**	**1570**	**1580**	**1590**	**1600**
WNSYQAKKKT	MDAKNGQTMN	EKQLFHGTDA	GSVPHVNRNG	FNRSYAGKNA	VAYGKGTYFA	VNANYSANDT	YSRPDANGRK
**1610**	**1620**	**1630**	**1640**	**1650**	**1660**	**1670**	
HVYYVRVLTG	IYTHGNHSLI	VPPSKNPQNP	TDLYDTVTDN	VHHPSLFVAF	YDYQAYPEYL	ITFRK	

Cmpd.	No. of Cmpds.	m/z meas.	Δ m/z [ppm]	z	Rt [min]	Score	P	Range	Sequence	Modification
554	1	476.1400	-265.76	2	36.9	12.8	1	1019-1026	K.FSSKNQLK.T	
2539	1	648.3564	69.94	2	62.4	13.6	0	1173-1184	K.SSVSSVLQECEK.K	

Detailed Protein Report

Protein 335: sodium-dependent phosphate transport protein 2B isoform a [Homo sapiens]

**Accession:**	gi|110611906	**Score:**	26.3
**Database:**	refseq_human(refseq_human_20140103.fasta)	**MW [kDa]:**	75.7
**Database Date:**	2015-11-30	**pI:**	9.8
		**Sequence Coverage [%]:**	2.9
		**No. of unique Peptides:**	1

10	20	30	40	50	60	70	80
MAPWPELGDA	QPNPDKYLEG	AAGQQPTAPD	KSKETNKTDN	TEAPVTKIEL	LPSYSTATLI	DEPTEVDDPW	NLPTLQDSGI
**90**	**100**	**110**	**120**	**130**	**140**	**150**	**160**
KWSERDTKGK	ILCFFQGIGR	LILLLGFLYF	FVCSLDILSS	AFQLVGGKMA	GQFFSNSSIM	SNPLLGLVIG	VLVTVLVQSS
**170**	**180**	**190**	**200**	**210**	**220**	**230**	**240**
STSTSIVVSM	VSSSLLTVRA	AIPIIMGANI	GTSITNTIVA	LMQVGDRSEF	RRAFAGATVH	DFFNWLSVLV	LLPVEVATHY
**250**	**260**	**270**	**280**	**290**	**300**	**310**	**320**
LEIITQLIVE	SFHFKNGEDA	PDLLKVITKP	FTKLIVQLDK	KVISQIAMND	EKAKNKSLVK	IWCKTFTNKT	QINVTVPSTA
**330**	**340**	**350**	**360**	**370**	**380**	**390**	**400**
NCTSPSLCWT	DGIQNWTMKN	VTYKENIAKC	QHIFVNFHLP	DLAVGTILLI	LSLLVLCGCL	IMIVKILGSV	LKGQVATVIK
**410**	**420**	**430**	**440**	**450**	**460**	**470**	**480**
KTINTDFPFP	FAWLTGYLAI	LVGAGMTFIV	QSSSVFTSAL	TPLIGIGVIT	IERAYPLTLG	SNIGTTTTAI	LAALASPGNA
**490**	**500**	**510**	**520**	**530**	**540**	**550**	**560**
LRSSLQIALC	HFFFNISGIL	LWYPIPFTRL	PIRMAKGLGN	ISAKYRWFAV	FYLIIFFFLI	PLTVFGLSLA	GWRVLVGVGV
**570**	**580**	**590**	**600**	**610**	**620**	**630**	**640**
PVVFIIILVL	CLRLLQSRCP	RVLPKKLQNW	NFLPLWMRSL	KPWDAVVSKF	TGCFQMRCCC	CCRVCCRACC	LLCGCPKCCR
**650**	**660**	**670**	**680**	**690**	**700**		
CSKCCEDLEE	AQEGQDVPVK	APETFDNITI	SREAQGEVPA	SDSKTECTAL			

Cmpd.	No. of Cmpds.	m/z meas.	Δ m/z [ppm]	z	Rt [min]	Score	P	Range	Sequence	Modification
1700	1	538.2445	-41.77	2	51.5	12.2	0	38-47	K.TDNTEAPVTK.I	

Detailed Protein Report

Protein 336: PREDICTED: dynein intermediate chain 1, axonemal isoform X3 [Homo sapiens]

**Accession:**	gi|578816466	**Score:**	26.3
**Database:**	refseq_human(refseq_human_20140103.fasta)	**MW [kDa]:**	76.4
**Database Date:**	2015-11-30	**pI:**	5.9
		**Sequence Coverage [%]:**	3.9
		**No. of unique Peptides:**	1

Quantitation

QD:QU **Median:** 0.67 **CV:** 0.00 % **No. of Peptides:** 1

10	20	30	40	50	60	70	80
MIPASAKAPH	KQPHKQSISI	GRGTRKRDED	SGTEVGEGTD	EWAQSKATVR	PPDQLELTDA	ELKEEFTRIL	TANNPHAPQN
**90**	**100**	**110**	**120**	**130**	**140**	**150**	**160**
IVRYSFKKQW	SLGFIPKLKR	KIPVNYWGQD	EGEISNETEG	TYKPIGFVNQ	LAVHYTQVGN	LIPKDSDEGR	RQHYRDELVA
**170**	**180**	**190**	**200**	**210**	**220**	**230**	**240**
VSYQGSQESV	KVISETGNLE	EDEEPKELET	EPGSQTDVPA	AGAAEKVTEE	ELMTPKQPKE	RKLTNQFNFS	ERASQTYNNP
**250**	**260**	**270**	**280**	**290**	**300**	**310**	**320**
VRDRECQTEP	PPRTNFSATA	NQWEIYDAYV	EELEKQEKTK	EKEKAKTPVA	KKSGKMAMRK	LTSMESQTDD	LIKLSQAAKI
**330**	**340**	**350**	**360**	**370**	**380**	**390**	**400**
MERMVNQNTY	DDIAQDFKYY	DDAADEYRDQ	VGTLLPLWKF	QNDKAKRLSV	TALCWNPKYR	DLFAVGYGSY	DFMKQSRGML
**410**	**420**	**430**	**440**	**450**	**460**	**470**	**480**
LLYSLKNPSF	PEYMFSSNSG	VMCLDIHVDH	PYLVAVGHYD	GNVAIYNLKK	PHSQPSFCSS	AKSGKHSDPV	WQVKWQKDDM
**490**	**500**	**510**	**520**	**530**	**540**	**550**	**560**
DQNLNFFSVS	SDGRIVSWTL	VKRKLVHIDV	IKLKVEGSTT	EVPEGLQLHP	VGCGTAFDFH	KEIDYMFLVG	TEEGKIYKCS
**570**	**580**	**590**	**600**	**610**	**620**	**630**	**640**
KSYSSQFLDT	YDAHNMSVDT	VSWNPYHTKV	FMSCSSDWTV	KIWDHTIKTP	MFIYDLNSAV	GDVAWAPYSS	TVFAAVTTDG
**650**	**660**	**670**	**680**				
KEKKGQEVQK	GPAVEIAKLD	KLLNLVREVK	IKT				

Cmpd.	No. of Cmpds.	m/z meas.	Δ m/z [ppm]	z	Rt [min]	Score	P	Range	Sequence	Modification	Ratios
255	1	838.3679	-55.82	2	31.9	12.9	1	450-465	K.KPHSQPSFCSSAKSGK.H		QD:QU 0.67

Detailed Protein Report

Protein 337: SPATS2-like protein isoform b [Homo sapiens]

**Accession:**	gi|154426312	**Score:**	26.2
**Database:**	refseq_human(refseq_human_20140103.fasta)	**MW [kDa]:**	54.1
**Database Date:**	2015-11-30	**pI:**	10.3
		**Sequence Coverage [%]:**	2.9
		**No. of unique Peptides:**	1

Alias proteins:

Accession	Name	Description
gi|530370155	refseq_human (refseq_human_20140103.fasta)	PREDICTED: SPATS2-like protein isoform X7 [Homo sapiens]
gi|530370153	refseq_human (refseq_human_20140103.fasta)	PREDICTED: SPATS2-like protein isoform X6 [Homo sapiens]

10	20	30	40	50	60	70	80
MAELNTHVNV	KEKIYAVRSV	VPNKSNNEIV	LVLQQFDFNV	DKAVQAFVDG	SAIQVLKEWN	MTGKKKNNKR	KRSKSKQHQG
**90**	**100**	**110**	**120**	**130**	**140**	**150**	**160**
NKDAKDKVER	PEAGPLQPQP	PQIQNGPMNG	CEKDSSSTDS	ANEKPALIPR	EKKISILEEP	SKALRGVTGP	NIEKSVKDLQ
**170**	**180**	**190**	**200**	**210**	**220**	**230**	**240**
RCTVSLTRYR	VMIKEEVDSS	VKKIKAAFAE	LHNCIIDKEV	SLMAEMDKVK	EEAMEILTAR	QKKAEELKRL	TDLASQMAEM
**250**	**260**	**270**	**280**	**290**	**300**	**310**	**320**
QLAELRAEIK	HFVSERKYDE	ELGKAARFSC	DIEQLKAQIM	LCGEITHPKN	NYSSRTPCSS	LLPLLNAHAA	TSGKQSNFSR
**330**	**340**	**350**	**360**	**370**	**380**	**390**	**400**
KSSTHNKPSE	GKAANPKMVS	SLPSTADPSH	QTMPANKQNG	SSNQRRRFNP	QYHNNRLNGP	AKSQGSGNEA	EPLGKGNSRH
**410**	**420**	**430**	**440**	**450**	**460**	**470**	**480**
EHRRQPHNGF	RPKNKGGAKN	QEASLGMKTP	EAPAHSEKPR	RRQHAADTSE	ARPFRGSVGR	VSQCNLCPTR	IEVSTDAAVL
**490**							
SVPAVTLVA							

Cmpd.	No. of Cmpds.	m/z meas.	Δ m/z [ppm]	z	Rt [min]	Score	P	Range	Sequence	Modification
1896	1	621.2840	-141.50	1	54.1	16.2	0	14-18	K.IYAVR.S	

Detailed Protein Report

Protein 338: integrator complex subunit 1 [Homo sapiens]

**Accession:**	gi|160948599	**Score:**	26.2
**Database:**	refseq_human(refseq_human_20140103.fasta)	**MW [kDa]:**	244.1
**Database Date:**	2015-11-30	**pI:**	5.7
**Modification(s):**	Carbamidomethyl	**Sequence Coverage [%]:**	1.7
		**No. of unique Peptides:**	2

Detailed Protein Report

10	20	30	40	50	60	70	80
MNRAKPTTVR	RPSAAAKPSG	HPPPGDFIAL	GSKGQANESK	TASTLLKPAP	SGLPSERKRD	AAAALSSASA	LTGLTKRPKL
90	100	110	120	130	140	150	160
SSTPPLSALG	RLAEAAVAEK	RAISPSIKEP	SVVPIEVLPT	VLLDEIEAAE	LEGNDDRIEG	VLCGAVKQLK	VTRAKPDSTL
170	180	190	200	210	220	230	240
YLSLMYLAKI	KPNIFATEGV	IEALCSLLRR	DASINFKAKG	NSLVSVLACN	LLMAAYEEDE	NWPEIFVKVY	IEDSLGERIW
250	260	270	280	290	300	310	320
VDSPHCKTFV	DNIQTAFNTR	MPPRSVLLQG	EAGRVAGDLG	AGSSPHPSLT	EEEDSQTELL	IAEEKLSPEQ	EGQLMPRYEE
330	340	350	360	370	380	390	400
LAESVEEYVL	DMLRDQLNRR	QPIDNVSRNL	LRLLTSTCGY	KEVRLLAVQK	LEMWLQNPKL	TRPAQDLLMS	VCMNCNTHGS
410	420	430	440	450	460	470	480
EDMDVISHLI	KIRLKPKVLL	NHFMLCIREL	LSAHKDNLGT	TIKLVIFNEL	SSARNPNNMQ	VLYTALQHSS	ELAPKFLAMV
490	500	510	520	530	540	550	560
FQDLLTNKDD	YLRASRALLR	EIIKQTKHEI	NFQAFCLGLM	QERKEPQYLE	MEFKERFVVH	ITDVLAVSMM	LGITAQVKEA
570	580	590	600	610	620	630	640
GIAWDKGEKR	NLEVLRSFQN	QIAAIQRDAV	WWLHTVVPSI	SKLAPKDYVH	CLHKVLFTEQ	PETYYKWDNW	PPESDRNFFL
650	660	670	680	690	700	710	720
RLCSEVPILE	DTLMRILVIG	LSRELPLGPA	DAMELADHLV	KRAAAVQADD	VEVLKVGRTQ	LIDAVLNLCT	YHHPENIQLP
730	740	750	760	770	780	790	800
PGYQPPNLAI	STLYWKAWPL	LLVVAAFNPE	NIGLAAWEEY	PTLKMLMEMV	MTNNYSYPPC	TLTDEETRTE	MLNRELQTAQ
810	820	830	840	850	860	870	880
REKQEILAFE	GHLAAASTKQ	TITESSSLLL	SQLTSLDPQG	PPRRPPPHIL	DQVKSLNQSL	RLGHLLCRSR	NPDFLLHIIQ
890	900	910	920	930	940	950	960
RQASSQSMPW	LADLVQSSEG	SLDVLPVQCL	CEFLLHDAVD	DAASGEEDDE	GESKEQKAKK	RQRQQKQRQL	LGRLQDLLLG
970	980	990	1000	1010	1020	1030	1040
PKADEQTTCE	VLDYFLRRLG	SSQVASRVLA	MKGLSLVLSE	GSLRDGEEKE	PPMEEDVGDT	DVLQGYQWLL	RDLPRLPLFD
1050	1060	1070	1080	1090	1100	1110	1120
SVRSTTALAL	QQAIHMETDP	QTISAYLIYL	SQHTPVEEQA	QHSDLALDVA	RLVVERSTIM	SHLFSKLSPS	AASDAVLSAL
1130	1140	1150	1160	1170	1180	1190	1200
LSIFSRYVRR	MRQSKEGEEV	YSWSESQDQV	FLRWSSGETA	TMHILVVHAM	VILLTLGPPR	ADDSEFQALL	DIWFPEEKPL
1210	1220	1230	1240	1250	1260	1270	1280
PTAFLVDTSE	EALLLPDWLK	LRMIRSEVLR	LVDAALQDLE	PQQLLLFVQS	FGIPVSSMSK	LLQFLDQAVA	HDPQTLEQNI
1290	1300	1310	1320	1330	1340	1350	1360
MDKNYMAHLV	EVQHERGASG	GQTFHSLLTA	SLPPRRDSTE	APKPKSSPEQ	PIGQGRIRVG	TQLRVLGPED	DLAGMFLQIF
1370	1380	1390	1400	1410	1420	1430	1440
PLSPDPRWQS	SSPRPVALAL	QQALGQELAR	VVQGSPEVPG	ITVRVLQALA	TLLSSPHGGA	LVMSMHRSHF	LACPLLRQLC
1450	1460	1470	1480	1490	1500	1510	1520
QYQRCVPQDT	GFSSLFLKVL	LQMLQWLDSP	GVEGGPLRAQ	LRMLASQASA	GRRLSDVRGG	LLRLAEALAF	RQDLEVVSST
1530	1540	1550	1560	1570	1580	1590	1600
VRAVIATLRS	GEQCSVEPDL	ISKVLQGLIE	VRSPHLEELL	TAFFSATADA	ASPFPACKPV	VVVSSLLLQE	EEPLAGGKPG
1610	1620	1630	1640	1650	1660	1670	1680
ADGGSLEAVR	LGPSSGLLVD	WLEMLDPEVV	SSCPDLQLRL	LFSRRKGKGQ	AQVPSFRPYL	LTLFTHQSSW	PTLHQCIRVL
1690	1700	1710	1720	1730	1740	1750	1760
LGKSREQRFD	PSASLDFLWA	CIHVPRIWQG	RDQRTPQKRR	EELVLRVQGP	ELISLVELIL	AEAETRSQDG	DTAACSLIQA
1770	1780	1790	1800	1810	1820	1830	1840
RLPLLLSCCC	GDDESVRKVT	EHLSGCIQQW	GDSVLGRRCR	DLLLQLYLQR	PELRVPVPEV	LLHSEGAASS	SVCKLDGLIH
1850	1860	1870	1880	1890	1900	1910	1920
RFITLLADTS	DSRALENRGA	DASMACRKLA	VAHPLLLLRH	LPMIAALLHG	RTHLNFQEFR	QQNHLSCFLH	VLGLLELLQP
1930	1940	1950	1960	1970	1980	1990	2000
HVFRSEHQGA	LWDCLLSFIR	LLLNYRKSSR	HLAAFINKFV	QFIHKYITYN	APAAISFLQK	HADPLHDLSF	DNSDLVMLKS
2010	2020	2030	2040	2050	2060	2070	2080
LLAGLSLPSR	DDRTDRGLDE	EGEEESSAGS	LPLVSVSLFT	PLTAAEMAPY	MKRLSRGQTV	EDLLEVLSDI	DEMSRRRPEI
2090	2100	2110	2120	2130	2140	2150	2160
LSFFSTNLQR	LMSSAEECCR	NLAFSLALRS	MQNSPSIAAA	FLPTFMYCLG	SQDFEVVQTA	LRNLPEYALL	CQEHAAVLLH
2170	2180	2190	2200				

Detailed Protein Report

Cmpd.	No. of Cmpds.	m/z meas.	Δ m/z [ppm]	z	Rt [min]	Score	P	Range	Sequence	Modification
1586	1	948.9589	-43.25	2	50.1	10.1	0	1297-1315	R.GASGGQTFHSLLTASLPPR.R	
464	1	724.3818	41.56	3	35.8	16.2	1	2091-2109	R.LMSSAEECCRNLAFSLALR.S	Carbamidomethyl: 8

Detailed Protein Report

Protein 339: **5'-nucleotidase isoform 2 preproprotein [Homo sapiens]**

**Accession:**	gi|325651886	**Score:**	26.1
**Database:**	refseq_human(refseq_human_20140103.fasta)	**MW [kDa]:**	57.9
**Database Date:**	2015-11-30	**pI:**	6.6
**Modification(s):**	Carbamidomethyl, Oxidation	**Sequence Coverage [%]:**	3.2
		**No. of unique Peptides:**	1

10	20	30	40	50	60	70	80
MCPRAARAPA	TLLLALGAVL	WPAAGAWELT	ILHTNDVHSR	LEQTSEDSSK	CVNASRCMGG	VARLFTKVQQ	IRRAEPNVLL
**90**	**100**	**110**	**120**	**130**	**140**	**150**	**160**
LDAGDQYQGT	IWFTVYKGAE	VAHFMNALRY	DAMALGNHEF	DNGVEGLIEP	LLKEAKFPIL	SANIKAKGPL	ASQISGLYLP
**170**	**180**	**190**	**200**	**210**	**220**	**230**	**240**
YKVLPVGDEV	VGIVGYTSKE	TPFLSNPGTN	LVFEDEITAL	QPEVDKLKTL	NVNKIIALGH	SGFEMDKLIA	QKVRGVDVVV
**250**	**260**	**270**	**280**	**290**	**300**	**310**	**320**
GGHSNTFLYT	GNPPSKEVPA	GKYPFIVTSD	DGRKVPVVQA	YAFGKYLGYL	KIEFDERGNV	ISSHGNPILL	NSSIPEDPSI
**330**	**340**	**350**	**360**	**370**	**380**	**390**	**400**
KADINKWRIK	LDNYSTQELG	KTIVYLDGSS	QSCRFRECNM	GNLICDAMIN	NNLRHTDEMF	WNHVSMCILN	GGGIRSPIDE
**410**	**420**	**430**	**440**	**450**	**460**	**470**	**480**
RNNGIHVVYD	LSRKPGDRVV	KLDVLCTKCR	VPSYDPLKMD	EVYKVILPNF	LANGGDGFQM	IKDELLRHDS	GDQDINVVST
**490**	**500**	**510**	**520**	**530**			
YISKMKVIYP	AVEGRIKFST	GSHCHGSFSL	IFLSLWAVIF	VLYQ			

Cmpd.	No. of Cmpds.	m/z meas.	Δ m/z [ppm]	z	Rt [min]	Score	P	Range	Sequence	Modification
1850	1	727.3153	-15.31	2	51.6	13.1	1	51-63	K.CVNASRCMGGVAR.L	Carbamidomethyl: 1, 7; Oxidation: 8

Detailed Protein Report

Protein 340: PREDICTED: sodium/myo-inositol cotransporter 2 isoform X3 [Homo sapiens]

**Accession:**	gi|530407547	**Score:**	26.1
**Database:**	refseq_human(refseq_human_20140103.fasta)	**MW [kDa]:**	56.6
**Database Date:**	2015-11-30	**pI:**	6.3
**Modification(s):**	Carbamidomethyl	**Sequence Coverage [%]:**	6.6
		**No. of unique Peptides:**	2

Quantitation

QD:QU **Median:** 0.73 **CV:** 0.00 % **No. of Peptides:** 1

10	20	30	40	50	60	70	80
MYAGAIFIQQ	SLHLDLYLAI	VGLLAITAVY	TVAGGLAAVI	YTDALQTLIM	LIGALTLMGY	SFAAVGGMEG	LKEKYFLALA
90	100	110	120	130	140	150	160
SNRSENSSCG	LPREDAFHIF	RDPLTSDLPW	PGVLFGMSIP	SLWYWCTDQV	IVQRTLAAKN	LSHAKGGALM	AAYLKVLPLF
170	180	190	200	210	220	230	240
IMVFPGMVSR	ILFPDQVACA	DPEICQKICS	NPSGCSDIAY	PKLVLELLPT	GLRGLMMAVM	VAALMSSLTS	IFNSASTIFT
250	260	270	280	290	300	310	320
MDLWNHLRPR	ASEKELMIVG	RVFVLLLVLV	SILWIPVVQA	SQGGQLFIYI	QSISSYLQPP	VAVVFIMGCF	WKRTNEKGAF
330	340	350	360	370	380	390	400
WGLISGLLLG	LVRLVLDFIY	VQPRCDQPDE	RPVLVKSIHY	LYFSMILSTV	TLITVSTVSW	FTEPPSKEMV	SHLTWFTRHD
410	420	430	440	450	460	470	480
PVVQKEQAPP	AAPLSLTLSQ	NGMPEASSSS	SVQFEMVQEN	TSKTHSCDMT	PKQSKVVKAI	LWLCGIQEKG	KEELPARAEA
490	500	510	520				
IIVSLEENPL	VKTLLDVNLI	FCVSCAIFIW	GYFA				

Cmpd.	No. of Cmpds.	m/z meas.	Δ m/z [ppm]	z	Rt [min]	Score	P	Range	Sequence	Modification	Ratios
2244	1	714.6704	-20.12	3	58.5	12.0	1	75-93	K.YFLALASNRSENSSCGLPR.E	Carbamidomethyl: 15	
816	1	812.9497	-14.41	2	40.2	14.1	0	478-492	R.AEAIIVSLEENPLVK.T		QD:QU 0.73

Detailed Protein Report

Protein 341: **PREDICTED: coiled-coil domain-containing protein 136 isoform X4 [Homo sapiens]**

**Accession:**	gi|530386309	**Score:**	26.0
**Database:**	refseq_human(refseq_human_20140103.fasta)	**MW [kDa]:**	131.9
**Database Date:**	2015-11-30	**pI:**	4.5
		**Sequence Coverage [%]:**	1.8
		**No. of unique Peptides:**	1

10	20	30	40	50	60	70	80
MEAGAGAGAG	AAGWSCPGPG	PTVTTLGSYE	ASEGCERKKG	QRWGSLERRG	MQAMEGEVLL	PALYEEEEEE	EEEEEEVEEE
90	100	110	120	130	140	150	160
EEQVQKGGSV	GSLSVNKHRG	LSLTETELEE	LRAQVLQLVA	ELEETRELAG	QHEDDSLELQ	GLLEDERLAS	AQQAEVFTKQ
170	180	190	200	210	220	230	240
IQQLQGELRS	LREEISLLEH	EKESELKEIE	QELHLAQAEI	QSLRQAAEDS	ATEHESDIAS	LQEDLCRMQN	ELEDMERIRG
250	260	270	280	290	300	310	320
DYEMEIASLR	AEMEMKSSEP	SGSLGLSDYS	GLQEELQELR	ERYHFLNEEY	RALQESNSSL	TGQLADLESE	RTQRATERWL
330	340	350	360	370	380	390	400
QSQTLSMTSA	ESQTSEMDFL	EPDPEMQLLR	QQLRDAEEQM	HGMKNKCQEL	CCELEELQHH	RQVSEEEQRR	LQRELKCAQN
410	420	430	440	450	460	470	480
EVLRFQTSHS	VTQNEELKSR	LCTLQKKYDT	SQDEQNELLK	MQLQLQTELR	QLKVMKSTLV	ENQSEKELLC	RLQKLHLQHQ
490	500	510	520	530	540	550	560
NVTCEKEKLL	ERQQQLQEEL	QCHEAELQHL	RDTVASFKES	NEKDTETHAQ	LQEMKQLYQA	SKDELERQKH	MYDQLEQDLL
570	580	590	600	610	620	630	640
LCQLELKELK	ASHPIPEDKG	KCANKSQELL	TKLEDLCELQ	LLYQGMQEEQ	KKLIQNQDCV	LKEQLEIHEE	LRRFKESHFQ
650	660	670	680	690	700	710	720
EVLENPDDSK	LAKSSKCNRN	KQSKLLMEQM	QALQVMYDAG	QAKQELLQQE	QGRLLEERKR	LQADLQLCLE	EMQLLQVQSP
730	740	750	760	770	780	790	800
SIKMSLESYG	KSYGSMVPSN	ENCRKTYDTT	VDDNESYYKS	YTSTQTSSKS	FLKSYDSSTS	ASEAYGKSYC	TTSNSSITYK
810	820	830	840	850	860	870	880
KSYGSTSSSD	TCQKSFVSSC	TDEEPAEPED	MERFEEMVVK	VLIKLQAVQA	MYQISQEEHS	QLQEQMEKLL	AKQKDLKEEL
890	900	910	920	930	940	950	960
DACEREFKEC	MECLEKPMAP	QNDKNEIKEL	QTKLRELQLQ	YQASMDEQGR	LLVVQEQLEG	QLQCCQEELR	QLREKRPSVV
970	980	990	1000	1010	1020	1030	1040
KEARGKNANK	NMNKNANGVK	MKKVTKPCSD	TSESDLETRK	SLEVVLYYKA	SQRKLDGLAK	EEEKKEEMEE	EKKQVKEEAK
1050	1060	1070	1080	1090	1100	1110	1120
EQCGDELVAE	PADPEEAKST	EDQEENEEDK	EEEEKEEDSE	EEEDDADSSL	ESPEENNPLR	LSESKKNMFG	LWKPMVFLAI
1130	1140	1150					
AAVALYVLPN	MRQQESEFCL	ME					

Cmpd.	No. of Cmpds.	m/z meas.	Δ m/z [ppm]	z	Rt [min]	Score	P	Range	Sequence	Modification
488	1	686.3221	-115.45	2	36.1	10.5	2	905-915	K.NEIKELQTKLR.E	

Detailed Protein Report

Protein 342: PREDICTED: ubiquitin carboxyl-terminal hydrolase 31 isoform X1 [Homo sapiens]

**Accession:**	gi|530408620	**Score:**	26.0
**Database:**	refseq_human(refseq_human_20140103.fasta)	**MW [kDa]:**	119.3
**Database Date:**	2015-11-30	**pI:**	10.2
		**Sequence Coverage [%]:**	2.2
		**No. of unique Peptides:**	1

Alias proteins:

Accession	Name	Description
gi|578828616	refseq_human (refseq_human_20140103.fasta)	PREDICTED: ubiquitin carboxyl-terminal hydrolase 31 isoform X2 [Homo sapiens]

10	20	30	40	50	60	70	80
MMPEGPSFPV	CSTFVQELFQ	AQYRSSLTCP	HCQKQSNTFD	PFLCISLPIP	LPHTRPLYVT	VVYQGKCSHC	MRIGVAVPLS
90	100	110	120	130	140	150	160
GTVARLREAV	SMETKIPTDQ	IVLTEMYYDG	FHRSFCDTDD	LETVHESDCI	FAFETPEIFR	PEGILSQRGI	HLNNNLNHLK
170	180	190	200	210	220	230	240
FGLDYHRLSS	PTQTAAKQGK	MDSPTSRAGS	DKIVLLVCNR	ACTGQQGKRF	GLPFVLHLEK	TIAWDLLQKE	ILEKMKYFLR
250	260	270	280	290	300	310	320
PTVCIQVCPF	SLRVVSVVGI	TYLLPQEEQP	LCHPIVERAL	KSCGPGGTAH	VKLVVEWDKE	TRDFLFVNTE	DEYIPDAESV
330	340	350	360	370	380	390	400
RLQRERHHQP	QTCTLSQCFQ	LYTKEERLAP	DDAWRCPHCK	QLQQGSITLS	LWTLPDVLII	HLKRFRQEGD	RRMKLQNMVK
410	420	430	440	450	460	470	480
FPLTGLDMTP	HVVKRSQSSW	SLPSHWSPWR	RPYGLGRDPE	DYIYDLYAVC	NHHGTMQGGH	YTAYCKNSVD	GLWYCFDDSD
490	500	510	520	530	540	550	560
VQQLSEDEVC	TQTAYILFYQ	RRTAIPSWSA	NSSVAGSTSS	SLCEHWVSRL	PGSKPASVTS	AASSRRTSLA	SLSESVEMTG
570	580	590	600	610	620	630	640
ERSEDDGGFS	TRPFVRSVQR	QSLSSRSSVT	SPLAVNENCM	RPSWSLSAKL	QMRSNSPSRF	SGDSPIHSSA	STLEKIGEAA
650	660	670	680	690	700	710	720
DDKVSISCFG	SLRNLSSSYQ	EPSDSHSRRE	HKAVGRAPLA	VMEGVFKDES	DTRRLNSSVV	DTQSKHSAQG	DRLPPLSGPF
730	740	750	760	770	780	790	800
DNNNQIAYVD	QSDSVDSSPV	KEVKAPSHPG	SLAKKPESTT	KRSPSSKGTS	EPEKSLRKGR	PALASQESSL	SSTSPSSPLP
810	820	830	840	850	860	870	880
VKVSLKPSRS	RSKADSSSRG	SGRHSSPAPA	QPKKESSPKS	QDSVSSPSPQ	KQKSASALTY	TASSTSAKKA	SGPATRSPFP
890	900	910	920	930	940	950	960
PGKSRTSDHS	LSREGSRQSL	GSDRASATST	SKPNSPRVSQ	ARAGEGRGAG	KHVRSSSMAS	LRSPSTSIKS	GLKRDSKSED
970	980	990	1000	1010	1020	1030	1040
KGLSFFKSAL	RQKETRRSTD	LGKTALLSKK	AGGSSVKSVC	KNTGDDEAER	GHQPPASQQP	NANTTGKEQL	VTKDPASAKH
1050	1060	1070	1080	1090			
SLLSARKSKS	SQLDSGVPSS	PGGRQSAEKS	SKKLSSSMQT	SARPSQKPQ			

Cmpd.	No. of Cmpds.	m/z meas.	Δ m/z [ppm]	z	Rt [min]	Score	P	Range	Sequence	Modification
2244	1	792.8941	-36.04	2	56.7	11.2	2	958-971	K.SEDKGLSFFKSALR.Q	

Detailed Protein Report

Protein 343: zinc finger protein 512 isoform b [Homo sapiens]

Accession: gi|404501451
Database: refseq_human(refseq_human_20140103.fasta)
Database Date: 2015-11-30

Score: 26.0
MW [kDa]: 61.3
pI: 10.5
Sequence Coverage [%]: 3.7
No. of unique Peptides: 1

10	20	30	40	50	60	70	80
MSSRLGAVPA	TSGPTTFKQQ	RSTRIVGAKN	RTQCSIKDNS	FQYTIPHDDS	LSGSSSASSC	EPVSDFPASF	RKSTYWMKMR
90	100	110	120	130	140	150	160
RIKPAATSHV	EGSGGVSAKG	KRKPRQEEDE	DYREFPQKKH	KLYGSLEEQW	YLEIVDKGSV	SCPTCQAVGR	KTIEGLKKHM
170	180	190	200	210	220	230	240
ENCKQEMFTC	HHCGKQLRSL	AGMKYHVMAN	HNSLPILKAG	DEIDEPSERE	RLRTVLKRLG	KLRCMRESCS	SSFTSIMGYL
250	260	270	280	290	300	310	320
YHVRKCGKGA	AELEKMTLKC	HHCGKPYRSK	AGLAYHLRSE	HGPISFFPES	GQPECLKEMN	LESKSGGRVQ	RRSAKIAVYH
330	340	350	360	370	380	390	400
LQELASAELA	KEWPKRKVLQ	DLVPDDRKLK	YTRPGLPTFS	QEVLHKWKTD	IKKYHRIQCP	NQGCEAVYSS	VSGLKAHLGS
410	420	430	440	450	460	470	480
CTLGNFVAGK	YKCLLCQKEF	VSESGVKYHI	NSVHAEDWFV	VNPTTTKSFE	KLMKIKQRQQ	EEEKRRQQHR	SRRSLRRRQQ
490	500	510	520	530	540		
PGIELPETEL	SLRVGKDQRR	NNEELVVSAS	CKEPEQEPVP	AQFQKVKPPK	TNHKRGRK		

Cmpd.	No. of Cmpds.	m/z meas.	Δ m/z [ppm]	z	Rt [min]	Score	P	Range	Sequence	Modification
1367	1	388.0811	-337.50	2	45.6	13.6	1	19-24	K.QQRSTR.I	

Detailed Protein Report

Protein 344: protocadherin gamma-B5 isoform 1 precursor [Homo sapiens]

Accession: gi|11128031
Database: refseq_human(refseq_human_20140103.fasta)
Database Date: 2015-11-30
Modification(s): Carbamidomethyl

Score: 26.0
MW [kDa]: 99.8
pI: 4.8
Sequence Coverage [%]: 3.4
No. of unique Peptides: 1

Quantitation

QD:QU **Median:** 0.08 **CV:** 0.00 % **No. of Peptides:** 1

10	20	30	40	50	60	70	80
MGSGAGELGR	AERLPVLFLF	LLSLFCPALC	EQIRYRIPEE	MPKGSVVGNL	ATDLGFSVQE	LPTRKLRVSS	EKPYFTVSAE
**90**	**100**	**110**	**120**	**130**	**140**	**150**	**160**
SGELLVSSRL	DREEICGKKP	ACALEFEAVA	ENPLNFYHVN	VEIEDINDHT	PKFTQNSFEL	QISESAQPGT	RFILEVAEDA
**170**	**180**	**190**	**200**	**210**	**220**	**230**	**240**
DIGLNSLQKY	KLSLNPSFSL	IIKEKQDGSK	YPELALEKTL	DREQQSYHRL	VLTALDGGHP	PLSGTTELRI	QVTDANDNPP
**250**	**260**	**270**	**280**	**290**	**300**	**310**	**320**
VFNRDVYRVS	LRENVPPGTT	VLQVSATDQD	EGINSEITYS	FYRTGQIFSL	NSKSGEITTQ	KKLDFEETKE	YSMVVEGRDG
**330**	**340**	**350**	**360**	**370**	**380**	**390**	**400**
GGLVAQCTVE	INIQDENDNS	PEVTFHSLLE	MILENAVPGT	LIALIKIHDQ	DSGENGEVNC	QLQGEVPFKI	ISSSKNSYKL
**410**	**420**	**430**	**440**	**450**	**460**	**470**	**480**
VTDGTLDREQ	TPEYNVTITA	TDRGKPPLSS	SISVILHIRD	VNDNAPVFHQ	ASYLVSVPEN	NPPGASIAQV	CASDLDLGLN
**490**	**500**	**510**	**520**	**530**	**540**	**550**	**560**
GQVSYSIMAS	DLEPLALASY	VSMSAQSGVV	FAQRAFDYEQ	LRTFELTLQA	RDQGSPALSA	NVSLRVLVGD	RNDNAPRVLY
**570**	**580**	**590**	**600**	**610**	**620**	**630**	**640**
PALGPDGSAL	FDMVPRAAEP	GYLVTKVVAV	DADSGHNAWL	SYHVLQASEP	GLFSLGLRTG	EVRTARALGD	RDAARQRLLV
**650**	**660**	**670**	**680**	**690**	**700**	**710**	**720**
AVRDGGQPPL	SATATLHLVF	ADSLQEVLPD	ITDRPVPSDP	QAELQFYLVV	ALALISVLFL	LAVILAVALR	LRRSSSPAAW
**730**	**740**	**750**	**760**	**770**	**780**	**790**	**800**
SCFQPGLCVK	SGPVVPPNYS	QGTLPYSYNL	CVAHTGKTEF	NFLKCSEQLS	SGQDILCGDS	SGALFPLCNS	SESTSHPELQ
**810**	**820**	**830**	**840**	**850**	**860**	**870**	**880**
APPNTDWRFS	QAQRPGTSGS	QNGDDTGTWP	NNQFDTEMLQ	AMILASASEA	ADGSSTLGGG	AGTMGLSARY	GPQFTLQHVP
**890**	**900**	**910**	**920**	**930**			
DYRQNVYIPG	SNATLTNAAG	KRDGKAPAGG	NGNKKKSGKK	EKK			

Cmpd.	No. of Cmpds.	m/z meas.	Δ m/z [ppm]	z	Rt [min]	Score	P	Range	Sequence	Modification	Ratios	
1752	1	990.9543	-20.48	2	50.4	10.1	1	713-730	R.RSSSPAAWSCFQPGLCVK.S	Carbamidomethyl: 16	QD:QU	0.08

Detailed Protein Report

Protein 345: **PREDICTED: deleted in lung and esophageal cancer protein 1 isoform X6 [Homo sapiens]**

Accession: gi|578806835
Database: refseq_human(refseq_human_20140103.fasta)
Database Date: 2015-11-30
Modification(s): Carbamidomethyl

Score: 26.0
MW [kDa]: 160.3
pI: 6.1
Sequence Coverage [%]: 2.1
No. of unique Peptides: 1

10	20	30	40	50	60	70	80
METRSSKTRR	SLASRTNECQ	GTMWAPTSPP	AGSSSPSQPT	WKSSLYSSLA	YSEAFHYSFA	ARPRRLTQLA	LAQRPEPQLL
**90**	**100**	**110**	**120**	**130**	**140**	**150**	**160**
RLRPSSLRTQ	DISHLLTGVF	RNLYSAEVIG	DEVSASLIKA	RGSENERHEE	FVDQLQQIRE	LYKQRLDEFE	MLERHITQAQ
**170**	**180**	**190**	**200**	**210**	**220**	**230**	**240**
ARAIAENERV	MSQAGVQDLE	SLVRLPPVKS	VSRWCIDSEL	LRKHHLISPE	DYYTDTVPFH	SAPKGISLPG	CSKLTFSCEK
**250**	**260**	**270**	**280**	**290**	**300**	**310**	**320**
RSVQKKELNK	KLEDSCRKKL	AEFEDELDHT	VDSLTWNLTP	KAKERTREPL	KKASQPRNKN	WMNHLRVPQR	ELDRLLLARM
**330**	**340**	**350**	**360**	**370**	**380**	**390**	**400**
ESRNHFLKNP	RFFPPNTRYG	GKSLVFPPKK	PAPIGEFQST	EPEQSCADTP	VFLAKPPIGF	FTDYEIGPVY	EMVIALQNTT
**410**	**420**	**430**	**440**	**450**	**460**	**470**	**480**
TTSRYLRVLP	PSTPYFALGL	GMFPGKGGMV	APGMTCQYIV	QFFPDCLGDF	DDFILVETQS	AHTLLIPLQA	RRPPPVLTLS
**490**	**500**	**510**	**520**	**530**	**540**	**550**	**560**
PVLDCGYCLI	GGVKMTRFIC	KNVGFSVGRF	CIMPKTSWPP	LSFKAIATVG	FVEQPPFGIL	PSVFELAPGH	AILVEVLFSP
**570**	**580**	**590**	**600**	**610**	**620**	**630**	**640**
KSLGKAEQTF	IIMCDNCQIK	ELVTIGIGQL	IALDLIYISG	EKSQPDPGEL	TDLTAQHFIR	FEPENLRSTA	RKQLIIRNAT
**650**	**660**	**670**	**680**	**690**	**700**	**710**	**720**
HVELAFYWQI	MKPNLQPLMP	GETFSMDSIK	CYPDKETAFS	IMPRKGVLSP	HTDHEFILSF	SPHELRDFHS	VLQMVLEEVP
**730**	**740**	**750**	**760**	**770**	**780**	**790**	**800**
EPVSSEAESL	GHSSYSVDDV	IVLEIEVKGS	VEPFQVLLEP	YALIIPGENY	IGINVKKAFK	MWNNSKSPIR	YLWGKISDCH
**810**	**820**	**830**	**840**	**850**	**860**	**870**	**880**
IIEVEPGTGV	IEPSEVGDFE	LNFTGGVPGP	TSQDLLCEIE	DSPSPVVLHI	EAVFKGPALI	INVSALQFGL	LRLGQKATNS
**890**	**900**	**910**	**920**	**930**	**940**	**950**	**960**
IQIRNVSQLP	ATWRMKESPV	SLQERPEDVS	PFDIEPSSGQ	LHSLGECRVD	ITLEALHCQH	LETVLELEVE	NGAWSYLPVY
**970**	**980**	**990**	**1000**	**1010**	**1020**	**1030**	**1040**
AEVQKPHVYL	QSSQVEVRNL	YLGVPTKTTI	TLINGTLLPT	QFHWGKLLGH	QAEFCMVTVS	PKHGLLGPSE	ECQLKLELTA
**1050**	**1060**	**1070**	**1080**	**1090**	**1100**	**1110**	**1120**
HTQEELTHLA	LPCHVSGMKK	PLVLGISGKP	QGLQVAITIS	KESSDCSVFS	TEQWPGHPKE	LRLDFGSAVP	LRTRVTRQLI
**1130**	**1140**	**1150**	**1160**	**1170**	**1180**	**1190**	**1200**
LTNRSPIRTR	FSLKFEYFGS	PQNSLSKKTS	LPNMPPALLK	TVRMQEHLAK	REQLDFMESM	LSHGKGAAFF	PHFSQGMLGP
**1210**	**1220**	**1230**	**1240**	**1250**	**1260**	**1270**	**1280**
YQQLCIDITG	CANMWGEYWD	NLICTVGDLL	PEVIPVHMAA	VGCPISSLRT	TSYTIDQAQK	EPAMRFGTQV	SGGDTVTRTL
**1290**	**1300**	**1310**	**1320**	**1330**	**1340**	**1350**	**1360**
RLNNSSPCDI	RLDWETYVPE	DKEDRLVELL	VFYGPPFPLR	DQAGNELVCP	DTPEGGCLLW	SPGPSSSSEF	SHETDSSVEG
**1370**	**1380**	**1390**	**1400**	**1410**	**1420**	**1430**	**1440**
SSSASNRVAQ	KLISVILQAH	EGVPSGHLYC	ISPKQVVVPA	GGSSTIYISF	TPMVLSPEIL	HKVECTGYAL	GFMSLDSK

Cmpd.	No. of Cmpds.	m/z meas.	Δ m/z [ppm]	z	Rt [min]	Score	P	Range	Sequence	Modification
34	1	628.3634	65.98	3	29.1	13.9	2	225-241	K.GISLPGCSKLTFSCEKR.S	Carbamidomethyl: 14

Detailed Protein Report

Protein 346: **PREDICTED: conserved oligomeric Golgi complex subunit 4 isoform X1 [Homo sapiens]**

**Accession:**	gi|578828932	**Score:**	26.0
**Database:**	refseq_human(refseq_human_20140103.fasta)	**MW [kDa]:**	73.7
**Database Date:**	2015-11-30	**pI:**	5.1
		**Sequence Coverage [%]:**	4.3
		**No. of unique Peptides:**	1

10	20	30	40	50	60	70	80
MDGVQTALRS	EDYEQAAAHT	HRYLCLDKSV	IELSRQGKEG	SMIDANLKLL	QEAEQRLKAI	VAEKFAIATK	EGDLPQVERF
**90**	**100**	**110**	**120**	**130**	**140**	**150**	**160**
FKIFPLLGLH	EEGLRKFSEY	LCKQVASKAE	ENLLMVLGTD	MSDRRAAVIF	ADTLTLLFEG	IARIVETHQP	IVETYYGPGR
**170**	**180**	**190**	**200**	**210**	**220**	**230**	**240**
LYTLIKYLQV	ECDRQVEKVV	DKFIKQRDYH	QQFRHVQNNL	MRNSTTEKIE	PRELDPILTE	VTLMNARSEL	YLRFLKKRIS
**250**	**260**	**270**	**280**	**290**	**300**	**310**	**320**
SDFEVGDSMA	SEEVKQEHQK	CLDKLLNNCL	LSCTMQELIG	LYVTMEEYFM	RETVNKAVAL	DTYEKGQLTS	SMVDDVFYIV
**330**	**340**	**350**	**360**	**370**	**380**	**390**	**400**
KKCIGRALSS	SSIDCLCAMI	NLATTELESD	FRDVLCNKLR	MGFPATTFQD	IQRGVTSAVN	IMHSSLQQGK	FDTKGIESTD
**410**	**420**	**430**	**440**	**450**	**460**	**470**	**480**
EAKMSFLVTL	NNVEVCSENI	STLKKTLESD	CTKLFSQGIG	GEQAQAKFDS	CLSDLAAVSN	KFRDLLQEGL	TELNSTAIKP
**490**	**500**	**510**	**520**	**530**	**540**	**550**	**560**
QVQPWINSFF	SVSHNIEEEE	FNDYEANDPW	VQQFILNLEQ	QMAEFKASLS	PVIYDSLTGL	MTSLVAVELE	KVVLKSTFNR
**570**	**580**	**590**	**600**	**610**	**620**	**630**	**640**
LGGLQFDKEL	RSLIAYLTTV	TTWTIRDKFA	RLSQMATILN	LERVTEILDY	WGPNSGPLTW	RLTPAEVRQV	LALRIDFRSE
**650**							
DIKRLRL							

Cmpd.	No. of Cmpds.	m/z meas.	Δ m/z [ppm]	z	Rt [min]	Score	P	Range	Sequence	Modification
1637	1	993.4172	-42.79	2	50.7	12.5	1	238-255	K.RISSDFEVGDSMASEEVK.Q	

Detailed Protein Report

Protein 347: **PREDICTED: ankyrin repeat domain-containing protein 18A isoform X1 [Homo sapiens]**

**Accession:**	gi|578816446	**Score:**	26.0
**Database:**	refseq_human(refseq_human_20140103.fasta)	**MW [kDa]:**	124.6
**Database Date:**	2015-11-30	**pI:**	9.3
**Modification(s):**	Oxidation	**Sequence Coverage [%]:**	3.1
		**No. of unique Peptides:**	1

10	20	30	40	50	60	70	80
MRKLFSFGRR	LGQALLSSMD	QEYAGPGYDI	RDWELRKIHR	AAIKGDAAEV	ERCLTRRFRD	LDARDRKDRT	VLHLACAHGR
90	100	110	120	130	140	150	160
VQVVTLLLHR	RCQIDICDRL	NRTPLMKAVH	SQEEACAIVL	LECGANPNIE	DIYGNTALHY	AVYNKGTSLA	ERLLSHHANI
170	180	190	200	210	220	230	240
EALNKEGNTP	LLFAINSRRQ	HMVEFLLKNQ	ANIHAVDNFK	RTALILAVQH	NLSSIVTLLL	QQNIRISSQD	MFGQTAEDYA
250	260	270	280	290	300	310	320
LCSDLRSIRQ	QILEHKNKML	KNHLRNDNQE	AAAMKNESFK	TQGASSKETA	AMKPANLKKR	KERAKAEHNL	KVASEEKQER
330	340	350	360	370	380	390	400
LQRSENKQPQ	DSQSYGKKKD	AMYGNFMLKK	DIAMLKEELY	AIKNDSLRKE	KKYIQEIKSI	TEINANFEKS	VRLNEKMITK
410	420	430	440	450	460	470	480
TVARYSQQLN	DLKAENARLN	SELEKEKHNK	ERLEAEVESL	HSSLATAINE	YNEIVERKDL	ELVLWRADDV	SRHEKMGSNI
490	500	510	520	530	540	550	560
SQLTDKNELL	TEQVHKARVK	FNTLKGKLRE	TRDALREKTL	ALGSVQLDLR	QAQHRIKEMK	QMHPNGEAKE	SQSIGKQNSL
570	580	590	600	610	620	630	640
EERIRQQELE	NLLLERQLED	ARKEGDNKEI	VINIHRDCLE	NGKEDLLEER	NKELMKEYNY	LKEKLLQCEK	EKAEREVIVR
650	660	670	680	690	700	710	720
EFQEELVDHL	KTFSISESPL	EGTSHCHINL	NETWTSKKKL	FQVEIQPEEK	HEEFRKLFEL	ISLLNYTADQ	IRKKNRELEE
730	740	750	760	770	780	790	800
EATGYKKCLE	MTINMLNAFA	NEDFSCHGDL	NTDQLKMDIL	FKKLKQKFND	LVAEKEAVSS	ECVNLAKDNE	VLHQELLSMR
810	820	830	840	850	860	870	880
NVQEKCEKLE	KDKKMLEEEV	LNLKTHMEKD	MVELGKLQEY	KSELDERAVQ	EIEKLEEIHL	QKQAEYEKQL	EQLNKDNTAS
890	900	910	920	930	940	950	960
LKKKELTLKD	VECKFSKMKT	AYEEVTTELE	EFKEAFAGAV	KANNSMSKKL	MKSDKKIAVI	STKLFTEKQR	MKYFLSTLPT
970	980	990	1000	1010	1020	1030	1040
RPEPELPCVE	NLNSIELNRK	YIPKTAIRIP	TSNPQTSNNC	KNFLTEMELD	RVEQIITGTK	KSFAMLSTCS	RLLSFVESTA
1050	1060	1070	1080				
PRKHRRALPI	MRSLVPNRRT	TVASTEVKGT	SQHS				

Cmpd.	No. of Cmpds.	m/z meas.	Δ m/z [ppm]	z	Rt [min]	Score	P	Range	Sequence	Modification
702	1	733.4640	131.96	3	38.8	12.2	1	788-805	K.DNEVLHQELLSMRNVQEK.C	Oxidation: 12

Detailed Protein Report

Protein 348: catenin alpha-1 [Homo sapiens]

**Accession:**	gi|55770844	**Score:**	25.9
**Database:**	refseq_human(refseq_human_20140103.fasta)	**MW [kDa]:**	100.0
**Database Date:**	2015-11-30	**pI:**	5.9
**Modification(s):**	Carbamidomethyl, Oxidation	**Sequence Coverage [%]:**	2.9
		**No. of unique Peptides:**	2

Alias proteins:

Accession	Name	Description
gi|578810148	refseq_human (refseq_human_20140103.fasta)	PREDICTED: catenin alpha-1 isoform X4 [Homo sapiens]

10	20	30	40	50	60	70	80
MTAVHAGNIN	FKWDPKSLEI	RTLAVERLLE	PLVTQVTTLV	NTNSKGPSNK	KRGRSKKAHV	LAASVEQATE	NFLEKGDKIA
**90**	**100**	**110**	**120**	**130**	**140**	**150**	**160**
KESQFLKEEL	VAAVEDVRKQ	GDLMKAAAGE	FADDPCSSVK	RGNMVRAARA	LLSAVTRLLI	LADMADVYKL	LVQLKVVEDG
**170**	**180**	**190**	**200**	**210**	**220**	**230**	**240**
ILKLRNAGNE	QDLGIQYKAL	KPEVDKLNIM	AAKRQQELKD	VGHRDQMAAA	RGILQKNVPI	LYTASQACLQ	HPDVAAYKAN
**250**	**260**	**270**	**280**	**290**	**300**	**310**	**320**
RDLIYKQLQQ	AVTGISNAAQ	ATASDDASQH	QGGGGGELAY	ALNNFDKQII	VDPLSFSEER	FRPSLEERLE	SIISGAALMA
**330**	**340**	**350**	**360**	**370**	**380**	**390**	**400**
DSSCTRDDRR	ERIVAECNAV	RQALQDLLSE	YMGNAGRKER	SDALNSAIDK	MTKKTRDLRR	QLRKAVMDHV	SDSFLETNVP
**410**	**420**	**430**	**440**	**450**	**460**	**470**	**480**
LLVLIEAAKN	GNEKEVKEYA	QVFREHANKL	IEVANLACSI	SNNEEGVKLV	RMSASQLEAL	CPQVINAALA	LAAKPQSKLA
**490**	**500**	**510**	**520**	**530**	**540**	**550**	**560**
QENMDLFKEQ	WEKQVRVLTD	AVDDITSIDD	FLAVSENHIL	EDVNKCVIAL	QEKDVDGLDR	TAGAIRGRAA	RVIHVVTSEM
**570**	**580**	**590**	**600**	**610**	**620**	**630**	**640**
DNYEPGVYTE	KVLEATKLLS	NTVMPRFTEQ	VEAAVEALSS	DPAQPMDENE	FIDASRLVYD	GIRDIRKAVL	MIRTPEELDD
**650**	**660**	**670**	**680**	**690**	**700**	**710**	**720**
SDFETEDFDV	RSRTSVQTED	DQLIAGQSAR	AIMAQLPQEQ	KAKIAEQVAS	FQEEKSKLDA	EVSKWDDSGN	DIIVLAKQMC
**730**	**740**	**750**	**760**	**770**	**780**	**790**	**800**
MIMMEMTDFT	RGKGPLKNTS	DVISAAKKIA	EAGSRMDKLG	RTIADHCPDS	ACKQDLLAYL	QRIALYCHQL	NICSKVKAEV
**810**	**820**	**830**	**840**	**850**	**860**	**870**	**880**
QNLGGELVVS	GVDSAMSLIQ	AAKNLMNAVV	QTVKASYVAS	TKYQKSQGMA	SLNLPAVSWK	MKAPEKKPLV	KREKQDETQT
**890**	**900**	**910**					
KIKRASQKKH	VNPVQALSEF	KAMDSI					

Cmpd.	No. of Cmpds.	m/z meas.	Δ m/z [ppm]	z	Rt [min]	Score	P	Range	Sequence	Modification
2291	1	682.7592	-174.39	2	59.1	12.9	0	138-149	R.LLILADMADVYK.L	
2280	1	920.8411	-26.04	2	58.9	13.1	0	718-731	K.QMCMIMMEMTDFTR.G	Carbamidomethyl: 3; Oxidation: 2

Detailed Protein Report

Protein 349: centrosomal protein of 162 kDa isoform b [Homo sapiens]

**Accession:**	gi|555289992	**Score:**	25.9
**Database:**	refseq_human(refseq_human_20140103.fasta)	**MW [kDa]:**	153.0
**Database Date:**	2015-11-30	**pI:**	5.3
**Modification(s):**	Oxidation	**Sequence Coverage [%]:**	1.5
		**No. of unique Peptides:**	2

Quantitation

QD:QU	**Median:** 5.44	**CV:** 0.00 %	**No. of Peptides:** 1

Alias proteins:

Accession	Name	Description
gi|578812505	refseq_human (refseq_human_20140103.fasta)	PREDICTED: centrosomal protein of 162 kDa isoform X7 [Homo sapiens]

```
        10         20         30         40         50         60         70         80
MEIEEESAEK IQFLKSSGTS LLSTDSLETN ELVVSELNHS SLGVGLDTLE EQEEKEQFFA RLEKGLTSSI DYSRLNKELD
        90        100        110        120        130        140        150        160
SNDSTHFKAL HSNQANAELT DDEHENESKH EELAENYSDD FEDEYVGAPL TTKDEEMPSK ENSKSEKISV PKQEEEKTGM
       170        180        190        200        210        220        230        240
LANVVLLDSL DSVAEVNLDE QDKITPKPRC LPEMTENEMT GTGVSYGQSS SDVEALHQAY CHIAHSLGDE DKQKIESNTV
       250        260        270        280        290        300        310        320
EDIKSSVKGH PQENEENSKN ISTMESDLPT VEELMKPIRI DSFGISGFDL QPVSSEKVAE RKETEFFSSL PLKMNPNILS
       330        340        350        360        370        380        390        400
QDSQHVNLFF DKNDENVILQ KTTNESMENS CPQVTEVTAT EEHVDKMYLN ILRKKITVNS SSLSQDDKIN KTYRSQLSSE
       410        420        430        440        450        460        470        480
EEGAVMGKQV PYKKARSAPP LLKRKPQSGL YASVRSSGYG KPSSPLKMFS TLEKKTSEDI IKSKNLRSIS TSNQPRKKEI
       490        500        510        520        530        540        550        560
LSGTKLIKPA ALDKPAHKTE SCLSTRKKSE NPTETDSCIQ FQTDSLGYCG ENKEKKLLMF KRVQEAEDKW RGAQALIEQI
       570        580        590        600        610        620        630        640
KATFSEKEKE LENKLEELKK QQEKELFKLN QDNYILQAKL SSFEETNKKQ RWLHFGEAAD PVTGEKLKQI QKEIQEQETL
       650        660        670        680        690        700        710        720
LQGYQQENER LYNQVKDLQE QNKKNEERMF KENQSLFSEV ASLKEQMHKS RFLSQVVEDS EPTRNQNFTD LLAELRMAQK
       730        740        750        760        770        780        790        800
EKDSLLEDIK RLKQDKQALE VDFEKMKKER DQAKDQIAYV TGEKLYEIKI LEETHKQEIS RLQKRLQWYA ENQELLDKDA
       810        820        830        840        850        860        870        880
LRLREANEEI EKLKLEIEKL KAESGNPSIR QKIRLKDKAA DAKKIQDLER QVKEMEGILK RRYPNSLPAL ILAASAAGDT
       890        900        910        920        930        940        950        960
VDKNTVEFME KRIKKLEADL EGKDEDAKKS LRTMEQQFQK MKIQYEQRLE QQEQLLACKL NQHDSPRIKA LEKELDDIKE
       970        980        990       1000       1010       1020       1030       1040
AHQITVRNLE AEIDVLKHQN AELDVKKNDK DDEDFQSIEF QVEQAHAKAK LVRLNEELAA KKREIQDLSK TVERLQKDRR
      1050       1060       1070       1080       1090       1100       1110       1120
MMLSNQNSKG REEMSAKRAK KDVLHSSKGN ANSFPGTLDS KLYQPHTFTD SHVSEVLQEN YRLKNELEGL ISEKNELKMK
      1130       1140       1150       1160       1170       1180       1190       1200
SEAVMNQFEN SMRRVKEDTA AHIASLKASH QREIEKLLCQ NAVENSSSKV AELNRKIATQ EVLIRHFQSQ VNELQSKQES
      1210       1220       1230       1240       1250       1260       1270       1280
LVVSEVREEI LQKEITKLLE ELREAKENHT PEMKHFVGLE KKIKQMEMRH AQREQELQQI IQQTHQVVET EQNKEVEKWK
      1290       1300       1310       1320       1330
RLAQLKNREL EKFRTELDSI LDVLRELHRQ GVVVPVAFAD EMNAPEY
```

Cmpd.	No. of Cmpds.	m/z meas.	Δ m/z [ppm]	z	Rt [min]	Score	P	Range	Sequence	Modification	Ratios
377	1	657.6657	-223.40	2	34.7	10.5	1	913-922	R.TMEQQFQKMK.I	Oxidation: 2	QD:QU 5.44
1864	1	612.7893	-8.93	2	53.7	15.4	1	1040-1049	R.RMMLSNQNSK.G	Oxidation: 2	

Detailed Protein Report

Protein 350: serine/threonine-protein kinase WNK3 isoform 2 [Homo sapiens]

**Accession:**	gi|50845416	**Score:**	25.9
**Database:**	refseq_human(refseq_human_20140103.fasta)	**MW [kDa]:**	191.7
**Database Date:**	2015-11-30	**pI:**	5.4
		**Sequence Coverage [%]:**	2.6
		**No. of unique Peptides:**	1

Alias proteins:

Accession	Name	Description
gi|578838231	refseq_human (refseq_human_20140103.fasta)	PREDICTED: serine/threonine-protein kinase WNK3 isoform X2 [Homo sapiens]
gi|578838229	refseq_human (refseq_human_20140103.fasta)	PREDICTED: serine/threonine-protein kinase WNK3 isoform X1 [Homo sapiens]

Detailed Protein Report

10	20	30	40	50	60	70	80
MATDSGDPAS	TEDSEKPDGI	SFENRVPQVA	ATLTVEARLK	EKNSTFSASG	ETVERKRFFR	KSVEMTEDDK	VAESSPKDER
90	100	110	120	130	140	150	160
IKAAMNIPRV	DKLPSNVLRG	GQEVKYEQCS	KSTSEISKDC	FKEKNEKEME	EEAEMKAVAT	SPSGRFLKFD	IELGRGAFKT
170	180	190	200	210	220	230	240
VYKGLDTETW	VEVAWCELQD	RKLTKAEQQR	FKEEAEMLKG	LQHPNIVRFY	DSWESILKGK	KCIVLVTELM	TSGTLKTYLK
250	260	270	280	290	300	310	320
RFKVMKPKVL	RSWCRQILKG	LQFLHTRTPP	IIHRDLKCDN	IFITGPTGSV	KIGDLGLATL	MRTSFAKSVI	GTPEFMAPEM
330	340	350	360	370	380	390	400
YEEHYDESVD	VYAFGMCMLE	MATSEYPYSE	CQNAAQIYRK	VTSGIKPASF	NKVTDPEVKE	IIEGCIRQNK	SERLSIRDLL
410	420	430	440	450	460	470	480
NHAFFAEDTG	LRVELAEEDD	CSNSSLALRL	WVEDPKKLKG	KHKDNEAIEF	SFNLETDTPE	EVAYEMVKSG	FFHESDSKAV
490	500	510	520	530	540	550	560
AKSIRDRVTP	IKKTREKKPA	GCLEERRDSQ	CKSMGNVFPQ	PQNTTLPLAP	AQQTGAECEE	TEVDQHVRQQ	LLQRKPQQHC
570	580	590	600	610	620	630	640
SSVTGDNLSE	AGAASVIHSD	TSSQPSVAYS	SNQTMGSQMV	SNIPQAEVNV	PGQIYSSQQL	VGHYQQVSGL	QKHSKLTQPQ
650	660	670	680	690	700	710	720
ILPLVQGQST	VLPVHVLGPT	VVSQPQVSPL	TVQKVPQIKP	VSQPVGAEQQ	AALLKPDLVR	SLNQDVATTK	ENVSSPDNPS
730	740	750	760	770	780	790	800
GNGKQDRIKQ	RRASCPRPEK	GTKFQLTVLQ	VSTSGDNMVE	CQLETHNNKM	VTFKFDVDGD	APEDIADYMV	EDNFVLESEK
810	820	830	840	850	860	870	880
EKFVEELRAI	VGQAQEILHV	HFATERATGV	DSITVDSNSS	QTGSSEQVQI	NSTSTQTSNE	SAPQSSPVGR	WRFCINQTIR
890	900	910	920	930	940	950	960
NRETQSPPSL	QHSMSAVPGR	HPLPSPKNTS	NKEISRDTLL	TIENNPCHRA	LFTSKSEHKD	VVDGKISECA	SVETKQPAIL
970	980	990	1000	1010	1020	1030	1040
YQVEDNRQIM	APVTNSSSYS	TTSVRAVPAE	CEGLTKQASI	FIPVYPCHQT	ASQADALMSH	PGESTQTSGN	SLTTLAFDQK
1050	1060	1070	1080	1090	1100	1110	1120
PQTLSVQQPA	MDAEFISQEG	ETTVNTEASS	PKTVIPTQTP	GLEPTTLQPT	TVLESDGERP	PKLEFADNRI	KTLDEKLRNL
1130	1140	1150	1160	1170	1180	1190	1200
LYQEHSISSI	YPESQKDTQS	IDSPFSSSAE	DTLSCPVTEV	IAISHCGIKD	SPVQSPNFQQ	TGSKLLSNVA	ASQPANISVF
1210	1220	1230	1240	1250	1260	1270	1280
KRDLNVITSV	PSELCLHEMS	SDASLPGDPE	AYPAAVSSGG	AIHLQTGVET	EEMRSAIAPD	PIPLTRESTA	DTRALNRCKA
1290	1300	1310	1320	1330	1340	1350	1360
MSGSFQRGRF	QVITIPQQQS	AKMTSFGIEH	ISVFSETNHS	SEEAFIKTAK	SQLVEIEPAT	QNPKTSFSYE	KLQALQETCK
1370	1380	1390	1400	1410	1420	1430	1440
ENKGVPKQGD	NFLSFSAACE	TDVSSVTPEK	EFEETSATGS	SMQSGSELLL	KEREILTAGK	QPSSDSEFSA	SLAGSGKSVA
1450	1460	1470	1480	1490	1500	1510	1520
KTGPESNQCL	PHHEEQAYAQ	TQSSLFYSPS	SPMSSDDESE	IEDEDLKVEL	QRLREKHIQE	VVNLQTQQNK	ELQELYERLR
1530	1540	1550	1560	1570	1580	1590	1600
SIKDSKTQST	EIPLPPASPR	RPRSFKSKLR	SRPQSLTHVD	NGIVATDPLC	VESNAASCQQ	SPASKKGMFT	DDLHKLVDDW
1610	1620	1630	1640	1650	1660	1670	1680
TKEAVGNSLI	KPSLNQLKQS	QHKLETENWN	KVSENTPSTM	GYTSTWISSL	SQIRGAVPTS	LPQGLSLPSF	PGPLSSYGMP
1690	1700	1710	1720	1730	1740	1750	
HVCQYNAVAG	AGYPVQWVGI	SGTTQQSVVI	PAQSGGPFQP	GMNMQAFPTS	SVQNPATIPP	GPK	

Cmpd.	No. of Cmpds.	m/z meas.	Δ m/z [ppm]	z	Rt [min]	Score	P	Range	Sequence	Modification
1472	1	918.1282	-10.85	3	46.9	10.8	2	1414-1441	R.EILTAGKQPSSDSEFSASLAGSGKSVAK.T	

Detailed Protein Report

Protein 351: teneurin-1 isoform 3 [Homo sapiens]

**Accession:**	gi|110347400	**Score:**	25.9
**Database:**	refseq_human(refseq_human_20140103.fasta)	**MW [kDa]:**	304.8
**Database Date:**	2015-11-30	**pI:**	6.0
		**Sequence Coverage [%]:**	1.4
		**No. of unique Peptides:**	2

Detailed Protein Report

10	20	30	40	50	60	70	80
MEQTDCKPYQ	PLPKVKHEMD	LAYTSSSDES	EDGRKPRQSY	NSRETLHEYN	QELRMNYNSQ	SRKRKEVEKS	TQEMEFCETS
90	100	110	120	130	140	150	160
HTLCSGYQTD	MHSVSRHGYQ	LEMGSDVDTE	TEGAASPDHA	LRMWIRGMKS	EHSSCLSSRA	NSALSLTDTD	HERKSDGENG
170	180	190	200	210	220	230	240
FKFSPVCCDM	EAQAGSTQDV	QSSPHNQFTF	RPLPPPPPPP	HACTCARKPP	PAADSLQRRS	MTTRSQPSPA	APAPPTSTQD
250	260	270	280	290	300	310	320
SVHLHNSWVL	NSNIPLETRH	FLFKHGSGSS	AIFSAASQNY	PLTSNTVYSP	PPRPLPRSTF	SRPAFTFNKP	YRCCNWKCTA
330	340	350	360	370	380	390	400
LSATAITVTL	ALLLAYVIAV	HLFGLTWQLQ	PVEGELYANG	VSKGNRGTES	MDTTYSPIGG	KVSDKSEKKV	FQKGRAIDTG
410	420	430	440	450	460	470	480
EVDIGAQVMQ	TIPPGLFWRF	QITIHHPIYL	KFNISLAKDS	LLGIYGRRNI	PPTHTQFDFV	KLMDGKQLVK	QDSKGSDDTQ
490	500	510	520	530	540	550	560
HSPRNLILTS	LQETGFIEYM	DQGPWYLAFY	NDGKKMEQVF	VLTTAIEIMD	DCSTNCNGNG	ECISGHCHCF	PGFLGPDCAR
570	580	590	600	610	620	630	640
DSCPVLCGGN	GEYEKGHCVC	RHGWKGPECD	VPEEQCIDPT	CFGHGTCIMG	VCICVPGYKG	EICEEEDCLD	PMCSNHGICV
650	660	670	680	690	700	710	720
KGECHCSTGW	GGVNCETPLP	VCQEQCSGHG	TFLLDAGVCS	CDPKWTGSDC	STELCTMECG	SHGVCSRGIC	QCEEGWVGPT
730	740	750	760	770	780	790	800
CEERSCHSHC	TEHGQCKDGK	CECSPGWEGD	HCTIAHYLDA	VRDGCPGLCF	GNGRCTLDQN	GWHCVCQVGW	SGTGCNVVME
810	820	830	840	850	860	870	880
MLCGDNLDND	GDGLTDCVDP	DCCQQSNCYI	SPLCQGSPDP	LDLIQQSQTL	FSQHTSRLFY	DRIKFLIGKD	STHVIPPEVS
890	900	910	920	930	940	950	960
FDSRRACVIR	GQVVAIDGTP	LVGVNVSFLH	HSDYGFTISR	QDGSFDLVAI	GGISVILIFD	RSPFLPEKRT	LWLPWNQFIV
970	980	990	1000	1010	1020	1030	1040
VEKVTMQRVV	SDPPSCDISN	FISPNPIVLP	SPLTSFGGSC	PERGTIVPEL	QVVQEEIPIP	SSFVRLSYLS	SRTPGYKTLL
1050	1060	1070	1080	1090	1100	1110	1120
RILLTHSTIP	VGMIKVHLTV	AVEGRLTQKW	FPAAINLVYT	FAWNKTDIYG	QKVWGLAEAL	VSVGYEYETC	PDFILWEQRT
1130	1140	1150	1160	1170	1180	1190	1200
VVLQGFEMDA	SNLGGWSLNK	HHILNPQSGI	IHKGNGENMF	ISQQPPVIST	IMGNGHQRSV	ACTNCNGPAH	NNKLFAPVAL
1210	1220	1230	1240	1250	1260	1270	1280
ASGPDGSVYV	GDFNFVRRIF	PSGNSVSILE	LSTSPAHKYY	LAMDPVSESL	YLSDTNTRKV	YKLKSLVETK	DLSKNFEVVA
1290	1300	1310	1320	1330	1340	1350	1360
GTGDQCLPFD	QSHCGDGGRA	SEASLNSPRG	ITVDRHGFIY	FVDGTMIRKI	DENAVITTVI	GSNGLTSTQP	LSCDSGMDIT
1370	1380	1390	1400	1410	1420	1430	1440
QVRLEWPTDL	AVNPMDNSLY	VLDNNIVLQI	SENRRVRIIA	GRPIHCQVPG	IDHFLVSKVA	IHSTLESARA	ISVSHSGLLF
1450	1460	1470	1480	1490	1500	1510	1520
IAETDERKVN	RIQQVTTNGE	IYIIAGAPTD	CDCKIDPNCD	CFSGDGGYAK	DAKMKAPSSL	AVSPDGTLYV	ADLGNVRIRT
1530	1540	1550	1560	1570	1580	1590	1600
ISRNQAHLND	MNIYEIASPA	DQELYQFTVN	GTHLHTLNLI	TRDYVYNFTY	NSEGDLGAIT	SSNGNSVHIR	RDAGGMPLWL
1610	1620	1630	1640	1650	1660	1670	1680
VVPGGQVYWL	TISSNGVLKR	VSAQGYNLAL	MTYPGNTGLL	ATKSNENGWT	TVYEYDPEGH	LTNATFPTGE	VSSFHSDLEK
1690	1700	1710	1720	1730	1740	1750	1760
LTKVELDTSN	RENVLMSTNL	TATSTIYILK	QENTQSTYRV	NPDGSLRVTF	ASGMEIGLSS	EPHILAGAVN	PTLGKCNISL
1770	1780	1790	1800	1810	1820	1830	1840
PGEHNANLIE	WRQRKEQNKG	NVSAFERRLR	AHNRNLLSID	FDHITRTGKI	YDDHRKFTLR	ILYDQTGRPI	LWSPVSRYNE
1850	1860	1870	1880	1890	1900	1910	1920
VNITYSPSGL	VTFIQRGTWN	EKMEYDQSGK	IISRTWADGK	IWSYTYLEKS	VMLLLHSQRR	YIFEYDQPDC	LLSVTMPSMV
1930	1940	1950	1960	1970	1980	1990	2000
RHSLQTMLSV	GYYRNIYTPP	DSSTSFIQDY	SRDGRLLQTL	HLGTGRRVLY	KYTKQARLSE	VLYDTTQVTL	TYEESSGVIK
2010	2020	2030	2040	2050	2060	2070	2080
TIHLMHDGFI	CTIRYRQTGP	LIGRQIFRFS	EEGLVNARFD	YSYNNFRVTS	MQAVINETPL	PIDLYRYVDV	SGRTEQFGKF
2090	2100	2110	2120	2130	2140	2150	2160
SVINYDLNQV	ITTTVMKHTK	IFSANGQVIE	VQYEILKAIA	YWMTIQYDNV	GRMVICDIRV	GVDANITRYF	YEYDADGQLQ
2170	2180	2190	2200	2210	2220	2230	2240

Detailed Protein Report

Cmpd.	No. of Cmpds.	m/z meas.	Δ m/z [ppm]	z	Rt [min]	Score	P	Range	Sequence	Modification
286	1	879.7298	-17.42	3	32.3	12.4	2	15-37	K.VKHEMDLAYTSSSDESEDGRKPR.Q	
2523	1	856.5611	167.23	2	62.1	13.5	0	2222-2236	R.GNDIFEYNSNGLLQK.A	

Detailed Protein Report

Protein 352: PREDICTED: chondroadherin-like protein isoform X2 [Homo sapiens]

**Accession:**	gi|530419599	**Score:**	25.9
**Database:**	refseq_human(refseq_human_20140103.fasta)	**MW [kDa]:**	81.7
**Database Date:**	2015-11-30	**pI:**	10.9
**Modification(s):**	Carbamidomethyl	**Sequence Coverage [%]:**	4.4
		**No. of unique Peptides:**	2

Quantitation

QD:QU **Median:** 0.73 **CV:** 0.00 % **No. of Peptides:** 1

10	20	30	40	50	60	70	80
MEGPRSSTHV	PLVLPLLVLL	LLAPARQAAA	QRCPQACICD	NSRRHVACRY	QNLTEVPDAI	PELTQRLDLQ	GNLLKVIPAA
**90**	**100**	**110**	**120**	**130**	**140**	**150**	**160**
AFQGVPHLTH	LDLRHCEVEL	VAEGAFRGLG	RLLLLNLASN	HLRELPQEAL	DGLGSLRRLE	LEGNALEELR	PGTFGALGAL
**170**	**180**	**190**	**200**	**210**	**220**	**230**	**240**
ATLNLAHNAL	VYLPAMAFQG	LLRVRWLRLS	HNALSVLAPE	ALAGLPALRR	LSLHHNELQA	LPGPVLSQAR	GLARLELGHN
**250**	**260**	**270**	**280**	**290**	**300**	**310**	**320**
PLTYAGEEDG	LALPGLRELL	LDGGALQALG	PRAFAHCPRL	HTLDLRGNQL	DTLPPLQGPG	QLRRLRLQGN	PLWCGCQARP
**330**	**340**	**350**	**360**	**370**	**380**	**390**	**400**
LLEWLARARV	RSDGACQGPR	RLRGEALDAL	RPWDLRCPGD	AAQEEEELEE	RAVAGPRAPP	RGPPRGPGEE	RAVAPCPRAC
**410**	**420**	**430**	**440**	**450**	**460**	**470**	**480**
VCVPESRHSS	CEGCGLQAVP	RGFPSDTQLL	DLRRNHFPSV	PRAAFPGLGH	LVSLHLQHCG	IAELEAGALA	GLGRLIYLYL
**490**	**500**	**510**	**520**	**530**	**540**	**550**	**560**
SDNQLAGLSA	AALEGAPRLG	YLYLERNRFL	QVPGAALRAL	PSLFSLHLQD	NAVDRLAPGD	LGRTRALRWV	YLSGNRITEV
**570**	**580**	**590**	**600**	**610**	**620**	**630**	**640**
SLGALGPARE	LEKLHLDRNQ	LREVPTGALE	GLPALLELQL	SGNPLRALRD	GAFQPVGRSL	QHLFLNSSGL	EQICPGAFSG
**650**	**660**	**670**	**680**	**690**	**700**	**710**	**720**
LGPGLQSLHL	QKNQLRALPA	LPSLSQLELI	DLSSNPFHCD	CQLLPLHRWL	TGLNLRVGAT	CATPPNARGQ	RVKAAAAVFE
**730**	**740**	**750**	**760**				
DCPGWAARKA	KRTPASRPSA	RRTPIKGRQC	GADKGTL				

Cmpd.	No. of Cmpds.	m/z meas.	Δ m/z [ppm]	z	Rt [min]	Score	P	Range	Sequence	Modification	Ratios
173	1	679.8611	32.65	2	32.1	14.2	2	330-341	R.VRSDGACQGPRR.L	Carbamidomethyl: 7	QD:QU 0.73
2204	1	1130.1280	10.71	2	58.0	11.7	1	549-569	R.WVYLSGNRITEVSLGALGPAR.E		

Detailed Protein Report

Protein 353: interferon-induced guanylate-binding protein 2 [Homo sapiens]

**Accession:**	gi|38327558	**Score:**	25.8
**Database:**	refseq_human(refseq_human_20140103.fasta)	**MW [kDa]:**	67.2
**Database Date:**	2015-11-30	**pI:**	5.4
**Modification(s):**	Oxidation	**Sequence Coverage [%]:**	5.2
		**No. of unique Peptides:**	1

10	20	30	40	50	60	70	80
MAPEINLPGP	MSLIDNTKGQ	LVVNPEALKI	LSAITQPVVV	VAIVGLYRTG	KSYLMNKLAG	KKNGFSLGST	VKSHTKGIWM
**90**	**100**	**110**	**120**	**130**	**140**	**150**	**160**
WCVPHPKKPE	HTLVLLDTEG	LGDIEKGDNE	NDSWIFALAI	LLSSTFVYNS	MGTINQQAMD	QLHYVTELTD	RIKANSSPGN
**170**	**180**	**190**	**200**	**210**	**220**	**230**	**240**
NSVDDSADFV	SFFPAFVWTL	RDFTLELEVD	GEPITADDYL	ELSLKLRKGT	DKKSKSFNDP	RLCIRKFFPK	RKCFVFDWPA
**250**	**260**	**270**	**280**	**290**	**300**	**310**	**320**
PKKYLAHLEQ	LKEEELNPDF	IEQVAEFCSY	ILSHSNVKTL	SGGIPVNGPR	LESLVLTYVN	AISSGDLPCM	ENAVLALAQI
**330**	**340**	**350**	**360**	**370**	**380**	**390**	**400**
ENSAAVEKAI	AHYEQQMGQK	VQLPTETLQE	LLDLHRDSER	EAIEVFMKNS	FKDVDQMFQR	KLGAQLEARR	DDFCKQNSKA
**410**	**420**	**430**	**440**	**450**	**460**	**470**	**480**
SSDCCMALLQ	DIFGPLEEDV	KQGTFSKPGG	YRLFTQKLQE	LKNKYYQVPR	KGIQAKEVLK	KYLESKEDVA	DALLQTDQSL
**490**	**500**	**510**	**520**	**530**	**540**	**550**	**560**
SEKEKAIEVE	RIKAESAEAA	KKMLEEIQKK	NEEMMEQKEK	SYQEHVKQLT	EKMERDRAQL	MAEQEKTLAL	KLQEQERLLK
**570**	**580**	**590**	**600**				
EGFENESKRL	QKDIWDIQMR	SKSLEPICNI	L				

Cmpd.	No. of Cmpds.	m/z meas.	Δ m/z [ppm]	z	Rt [min]	Score	P	Range	Sequence	Modification
2870	1	1027.5482	62.66	2	65.0	11.1	2	503-518	K.MLEEIQKKNEEMMEQK.E	Oxidation: 12

Detailed Protein Report

Protein 354: zinc finger protein 865 [Homo sapiens]

Accession: gi|307219238
Database: refseq_human(refseq_human_20140103.fasta)
Database Date: 2015-11-30

Score: 25.8
MW [kDa]: 111.0
pI: 10.6
Sequence Coverage [%]: 2.7
No. of unique Peptides: 2

10	20	30	40	50	60	70	80
MEANPAGSGA	GGGGSSGIGG	EDGVHFQSYP	FDFLEFLNHQ	RFEPMELYGE	HAKAVAALPC	APGPPPQPPP	QPPPPQYDYP
**90**	**100**	**110**	**120**	**130**	**140**	**150**	**160**
PQSTFKPKAE	VPSSSSSSSS	SSSSSSSSSS	SSSSSSSQAK	KPDPPLPPAF	GAPPPPLFDA	AFPTPQWGIV	DLSGHQHLFG
**170**	**180**	**190**	**200**	**210**	**220**	**230**	**240**
NLKRGGPASG	PGVTPGLGAP	AGAPGPLPAP	SQTPPGPPAA	AACDPTKDDK	GYFRRLKYLM	ERRFPCGVCQ	KSFKQSSHLV
**250**	**260**	**270**	**280**	**290**	**300**	**310**	**320**
QHMLVHSGER	PYECGVCGRT	YNHVSSLIRH	RRCHKDVPPA	AGGPPQPGPH	LPPLGLPAPA	ASAATAAAPS	TVSSGPPATP
**330**	**340**	**350**	**360**	**370**	**380**	**390**	**400**
VAPAPSADGS	AAPAGVGVPP	PATGGGDGPF	ACPLCWKVFK	KPSHLHQHQI	IHTGEKPFSC	SVCSKSFNRR	ESLKRHVKTH
**410**	**420**	**430**	**440**	**450**	**460**	**470**	**480**
SADLLRLPCG	ICGKAFRDAS	YLLKHQAAHA	GAGAGGPRPV	YPCDLCGKSY	SAPQSLLRHK	AAHAPPAAAA	EAPKDGAASA
**490**	**500**	**510**	**520**	**530**	**540**	**550**	**560**
PQPPPTFPPG	PYLLPPDPPT	TDSEKAAAAA	AAVVYGAVPV	PLLGAHPLLL	GGAGTSGAGG	SGASVPGKTF	CCGICGRGFG
**570**	**580**	**590**	**600**	**610**	**620**	**630**	**640**
RRETLKRHER	IHTGEKPHQC	PVCGKRFRES	FHLSKHHVVH	TRERPYKCEL	CGKVFGYPQS	LTRHRQVHRL	QLPCALAGAA
**650**	**660**	**670**	**680**	**690**	**700**	**710**	**720**
GLPSTQGTPG	ACGPGASGTS	AGPTDGLSYA	CSDCGEHFPD	LFHVMSHKEV	HMAEKPYGCD	ACGKTFGFIE	NLMWHKLVHQ
**730**	**740**	**750**	**760**	**770**	**780**	**790**	**800**
AAPERLLPPA	PGGLQPPDGS	SGTDAASVLD	NGLAGEVGAA	VAALAGVSGG	EDAGGAAVAG	AGGGASSGPE	RFSCATCGQS
**810**	**820**	**830**	**840**	**850**	**860**	**870**	**880**
FKHFLGLVTH	KYVHLVRRTL	GCGLCGQSFA	GAYDLLLHRR	SHRQKRGFRC	PVCGKRFWEA	ALLMRHQRCH	TEQRPYRCGV
**890**	**900**	**910**	**920**	**930**	**940**	**950**	**960**
CGRGFLRSWY	LRQHRVVHTG	ERAFKCGVCA	KRFAQSSSLA	EHRRLHAVAR	PQRCSACGKT	FRYRSNLLEH	QRLHLGERAY
**970**	**980**	**990**	**1000**	**1010**	**1020**	**1030**	**1040**
RCEHCGKGFF	YLSSVLRHQR	AHEPPRPELR	CPACLKAFKD	PGYFRKHLAA	HQGGRPFRCS	SCGEGFANTY	GLKKHRLAHK
**1050**	**1060**						
AENLGGPGAG	AGTLAGKDA						

Cmpd.	No. of Cmpds.	m/z meas.	Δ m/z [ppm]	z	Rt [min]	Score	P	Range	Sequence	Modification
13	1	819.8462	-127.06	2	29.7	15.8	2	260-272	R.TYNHVSSLIRHRR.C	
2279	1	869.2738	-114.47	2	58.9	10.1	0	689-704	K.EVHMAEKPYGCDACGK.T	

Detailed Protein Report

Protein 355: centrosomal protein of 290 kDa [Homo sapiens]

**Accession:**	gi|109255234	**Score:**	25.8
**Database:**	refseq_human(refseq_human_20140103.fasta)	**MW [kDa]:**	290.2
**Database Date:**	2015-11-30	**pI:**	5.7
		**Sequence Coverage [%]:**	0.9
		**No. of unique Peptides:**	2

Detailed Protein Report

10	20	30	40	50	60	70	80
MPPNINWKEI	MKVDPDDLPR	QEELADNLLI	SLSKVEVNEL	KSEKQENVIH	LFRITQSLMK	MKAQEVELAL	EEVEKAGEEQ
90	100	110	120	130	140	150	160
AKFENQLKTK	VMKLENELEM	AQQSAGGRDT	RFLRNEICQL	EKQLEQKDRE	LEDMEKELEK	EKKVNEQLAL	RNEEAENENS
170	180	190	200	210	220	230	240
KLRRENKRLK	KKNEQLCQDI	IDYQKQIDSQ	KETLLSRRGE	DSDYRSQLSK	KNYELIQYLD	EIQTLTEANE	KIEVQNQEMR
250	260	270	280	290	300	310	320
KNLEESVQEM	EKMTDEYNRM	KAIVHQTDNV	IDQLKKENDH	YQLQVQELTD	LLKSKNEEDD	PIMVAVNAKV	EEWKLILSSK
330	340	350	360	370	380	390	400
DDEIIEYQQM	LHNLREKLKN	AQLDADKSNV	MALQQGIQER	DSQIKMLTEQ	VEQYTKEMEK	NTCIIEDLKN	ELQRNKGAST
410	420	430	440	450	460	470	480
LSQQTHMKIQ	STLDILKEKT	KEAERTAELA	EADAREKDKE	LVEALKRLKD	YESGVYGLED	AVVEIKNCKN	QIKIRDREIE
490	500	510	520	530	540	550	560
ILTKEINKLE	LKISDFLDEN	EALRERVGLE	PKTMIDLTEF	RNSKHLKQQQ	YRAENQILLK	EIESLEEERL	DLKKKIRQMA
570	580	590	600	610	620	630	640
QERGKRSATS	GLTTEDLNLT	ENISQGDRIS	ERKLDLLSLK	NMSEAQSKNE	FLSRELIEKE	RDLERSRTVI	AKFQNKLKEL
650	660	670	680	690	700	710	720
VEENKQLEEG	MKEILQAIKE	MQKDPDVKGG	ETSLIIPSLE	RLVNAIESKN	AEGIFDASLH	LKAQVDQLTG	RNEELRQELR
730	740	750	760	770	780	790	800
ESRKEAINYS	QQLAKANLKI	DHLEKETSLL	RQSEGSNVVF	KGIDLPDGIA	PSSASIINSQ	NEYLIHLLQE	LENKEKKLKN
810	820	830	840	850	860	870	880
LEDSLEDYNR	KFAVIRHQQS	LLYKEYLSEK	ETWKTESKTI	KEEKRKLEDQ	VQQDAIKVKE	YNNLLNALQM	DSDEMKKILA
890	900	910	920	930	940	950	960
ENSRKITVLQ	VNEKSLIRQY	TTLVELERQL	RKENEKQKNE	LLSMEAEVCE	KIGCLQRFKE	MAIFKIAALQ	KVVDNSVSLS
970	980	990	1000	1010	1020	1030	1040
ELELANKQYN	ELTAKYRDIL	QKDNMLVQRT	SNLEHLECEN	ISLKEQVESI	NKELEITKEK	LHTIEQAWEQ	ETKLGNESSM
1050	1060	1070	1080	1090	1100	1110	1120
DKAKKSITNS	DIVSISKKIT	MLEMKELNER	QRAEHCQKMY	EHLRTSLKQM	EERNFELETK	FAELTKINLD	AQKVEQMLRD
1130	1140	1150	1160	1170	1180	1190	1200
ELADSVSKAV	SDADRQRILE	LEKNEMELKV	EVSKLREISD	IARRQVEILN	AQQQSRDKEV	ESLRMQLLDY	QAQSDEKSLI
1210	1220	1230	1240	1250	1260	1270	1280
AKLHQHNVSL	QLSEATALGK	LESITSKLQK	MEAYNLRLEQ	KLDEKEQALY	YARLEGRNRA	KHLRQTIQSL	RRQFSGALPL
1290	1300	1310	1320	1330	1340	1350	1360
AQQEKFSKTM	IQLQNDKLKI	MQEMKNSQQE	HRNMENKTLE	MELKLKGLEE	LISTLKDTKG	AQKVINWHMK	IEELRLQELK
1370	1380	1390	1400	1410	1420	1430	1440
LNRELVKDKE	EIKYLNNIIS	EYERTISSLE	EEIVQQNKFH	EERQMAWDQR	EVDLERQLDI	FDRQQNEILN	AAQKFEEATG
1450	1460	1470	1480	1490	1500	1510	1520
SIPDPSLPLP	NQLEIALRKI	KENIRIILET	RATCKSLEEK	LKEKESALRL	AEQNILSRDK	VINELRLRLP	ATAEREKLIA
1530	1540	1550	1560	1570	1580	1590	1600
ELGRKEMEPK	SHHTLKIAHQ	TIANMQARLN	QKEEVLKKYQ	RLLEKAREEQ	REIVKKHEED	LHILHHRLEL	QADSSLNKFK
1610	1620	1630	1640	1650	1660	1670	1680
QTAWDLMKQS	PTPVPTNKHF	IRLAEMEQTV	AEQDDSLSSL	LVKLKKVSQD	LERQREITEL	KVKEFENIKL	QLQENHEDEV
1690	1700	1710	1720	1730	1740	1750	1760
KKVKAEVEDL	KYLLDQSQKE	SQCLKSELQA	QKEANSRAPT	TTMRNLVERL	KSQLALKEKQ	QKALSRALLE	LRAEMTAAAE
1770	1780	1790	1800	1810	1820	1830	1840
ERIISATSQK	EAHLNVQQIV	DRHTRELKTQ	VEDLNENLLK	LKEALKTSKN	RENSLTDNLN	DLNNELQKKQ	KAYNKILREK
1850	1860	1870	1880	1890	1900	1910	1920
EEIDQENDEL	KRQIKRLTSG	LQGKPLTDNK	QSLIEELQRK	VKKLENQLEG	KVEEVDLKPM	KEKNAKEELI	RWEEGKKWQA
1930	1940	1950	1960	1970	1980	1990	2000
KIEGIRNKLK	EKEGEVFTLT	KQLNTLKDLF	AKADKEKLTL	QRKLKTTGMT	VDQVLGIRAL	ESEKELEELK	KRNLDLENDI
2010	2020	2030	2040	2050	2060	2070	2080
LYMRAHQALP	RDSVVEDLHL	QNRYLQEKLH	ALEKQFSKDT	YSKPSISGIE	SDDHCQREQE	LQKENLKLSS	ENIELKFQLE
2090	2100	2110	2120	2130	2140	2150	2160
QANKDLPRLK	NQVRDLKEMC	EFLKKEKAEV	QRKLGHVRGS	GRSGKTIPEL	EKTIGLMKKV	VEKVQRENEQ	LKKASGILTS
2170	2180	2190	2200	2210	2220	2230	2240

Detailed Protein Report

Cmpd.	No. of Cmpds.	m/z meas.	Δ m/z [ppm]	z	Rt [min]	Score	P	Range	Sequence	Modification
2146	2	937.4291	-49.48	2	55.4	15.4	2	115-129	R.NEICQLEKQLEQKDR.E	
1648	1	858.4049	-102.70	1	49.1	10.3	0	2438-2444	K.NILLEEK.V	

Detailed Protein Report

Protein 356: zinc finger protein 534 isoform 1 [Homo sapiens]

**Accession:**	gi|221139777	**Score:**	25.8
**Database:**	refseq_human(refseq_human_20140103.fasta)	**MW [kDa]:**	75.7
**Database Date:**	2015-11-30	**pI:**	10.5
**Modification(s):**	Carbamidomethyl	**Sequence Coverage [%]:**	3.6
		**No. of unique Peptides:**	1

10	20	30	40	50	60	70	80
MALTQGQLSF	SDVAIEFSQE	EWKCLDPGQK	ALYRDVMLEN	YRNLVSLGIC	LPDLSVTSML	EQKRDPWTLQ	SEVKIINNPD
**90**	**100**	**110**	**120**	**130**	**140**	**150**	**160**
GRECIKGVNT	EKSSKLGSSA	GNKSLKNQHG	LTLQLHLTEW	QPFQAVRNIY	GCKHVEKSIS	DNSSVSPVQI	SFFSVKTHIF
**170**	**180**	**190**	**200**	**210**	**220**	**230**	**240**
NNYRNDFLFS	TLLPQEQKVH	IREKPYGCNE	HGKVFRVSSS	LTNRQVIHIA	DKTYKCSDCG	EIFSSNSNFA	QHQRIHTGEK
**250**	**260**	**270**	**280**	**290**	**300**	**310**	**320**
PYKYNECGKV	FNQNSHLAQH	QKIHTGQKPY	NNKECGKVFS	HHAYLAQHRK	IHTGEKPYKC	SECGKAFSVC	SSLTAHLVIH
**330**	**340**	**350**	**360**	**370**	**380**	**390**	**400**
TGEKPYDCKE	CGKVFRHKSS	LTTHQTVHTG	ERPYKCNECG	KGFSRIAFLA	RHRKVHTGEK	PYKCNECGKV	FIGNSRLARH
**410**	**420**	**430**	**440**	**450**	**460**	**470**	**480**
RKIHTGGRRY	KCNECGKAFR	TCSDLTAHLL	IHTGEKPYEC	IDCGKVFRHK	SSLTYHCRIH	TGEKPYKCNE	CGKVFSQNSN
**490**	**500**	**510**	**520**	**530**	**540**	**550**	**560**
LQRHRKIHTG	EKLYKCNECG	KVFRQNSHLA	QHRDIHTGEK	PYSCNECGKV	FRRNSHLVRH	RNVHTGEKPY	SCNECGKVFS
**570**	**580**	**590**	**600**	**610**	**620**	**630**	**640**
RNSHLARHRN	IHTGEKPHSC	NECGKVFSRN	SHLARHRKIH	TGEKLYKCNE	CSKVFSRNSR	LAQHRNIHTG	VKPYSCNECG
**650**	**660**	**670**					
KVFSKNSILV	QHCSIHTREK	P					

Cmpd.	No. of Cmpds.	m/z meas.	Δ m/z [ppm]	z	Rt [min]	Score	P	Range	Sequence	Modification
2242	1	961.4221	-46.22	2	56.7	10.2	2	290-305	R.KIHTGEKPYKCSECGK.A	Carbamidomethyl: 11, 14

Detailed Protein Report

Protein 357: fibrous sheath-interacting protein 2 [Homo sapiens]

**Accession:**	gi|297206791	**Score:**	25.8
**Database:**	refseq_human(refseq_human_20140103.fasta)	**MW [kDa]:**	789.4
**Database Date:**	2015-11-30	**pI:**	6.2
**Modification(s):**	Carbamidomethyl, Oxidation	**Sequence Coverage [%]:**	0.7
		**No. of unique Peptides:**	2

10	20	30	40	50	60	70	80
MKFQLWSSQI	RPLGPIFPAL	RALYALARAA	PQEATALLGP	LPLEAWNAGG	GAEVVGLWPF	LVRSGQRDNG	VLEKESGAGE
90	100	110	120	130	140	150	160
EGAEGAVPAM	ELYLGACSKP	AKVAVTKTVA	SVLAADTQQC	RDGVHKTHFA	GVGPAQLLDL	PLGVKLPVIP	GSNAVFYTTN
170	180	190	200	210	220	230	240
FGEKLFRPSY	GFNLTDPYCR	LLENQYKSLH	DPHLKAYYKR	KDILKRLKKG	GYITSNNKVV	CTLRELNKYR	QYLTSLKLDF
250	260	270	280	290	300	310	320
ERNYIKEQRI	LAKQLHNIPE	NNQIPQHCDV	AQVQNWLLKE	GTESIKDQER	LMRHRYLDMI	SRKLEQLERT	AEEQRLFLMD
330	340	350	360	370	380	390	400
REERRQREHT	RRKLTLRRKI	EEEWKTKEML	LLTRMAEDVK	REERIEEQQH	RNREESDRKK	QDLLEKKMAY	HLQKMQDTGF
410	420	430	440	450	460	470	480
NGEDIGKNTF	KYRGQDGTHA	SPKNKKKTSE	DIMLVYPAGD	QNTYKETHGH	TANAAHQRQN	SSNNFTKKNS	ASVVYQADVQ
490	500	510	520	530	540	550	560
DNGINQKRDG	MVSKNSSIFD	DRGGINISGQ	GSIISAQVSP	TRNFSRVSQA	FLDPSKEEKE	TNADWDGRPT	KRSSYLCESG
570	580	590	600	610	620	630	640
PQAHATDPGI	FSSPVYTNMQ	QNLLQNCLQE	KVTSEELNII	IQNVMTWVVA	TVTSILYPAI	TKYEKRLQNN	TYPVSDDSIL
650	660	670	680	690	700	710	720
SSDSSSFCST	CSEDFTYRSY	TSATTKTFQA	EPCAFVVDTS	VRRPTTPIKP	PPAHVEKTVV	GKTCHIKGQS	IISKHKYNKT
730	740	750	760	770	780	790	800
NLLYSYPKLR	SCKSDSHLLA	SFETGTKKSK	DATTETDSLG	SSLHCDKTAK	AMDEMKNLKN	VFVNFKCYLK	GETEVILESI
810	820	830	840	850	860	870	880
LREIMSDLTQ	AIPSLSSVTA	EVFVEQCERE	KEILLSNAHI	PSVASEIVEN	MLEKLESAVE	KKCVEMFSQD	LSVDIKPSLA
890	900	910	920	930	940	950	960
ASDELLTSSN	GKPLKNSMPH	TLDPMCDIAE	DMVHAILEKL	MTLVSFKQNE	FLHLKDTNKL	SCQQHKTDPI	CMFLQRAGKN
970	980	990	1000	1010	1020	1030	1040
KSSLESDEAS	LIVNEEVQNL	ISNIFSQSSL	VAYIEEAINA	ILGYIQTELN	NERIIASEET	VVLLQLLEDI	LFQLHQEPVN
1050	1060	1070	1080	1090	1100	1110	1120
ESFQKSRQPR	ISSPSDTKEK	YRLTGTRLSN	SPRSGRPFPP	INVPGMVLYS	DDENEEIDNI	VKNVLDSTFK	DEKVKSQEQI
1130	1140	1150	1160	1170	1180	1190	1200
PNHWFTKGNT	CFECKRNIKP	PTKPGSRSKA	AFHDWELKTE	PPSTNHEDIL	KKKLSSNKDI	STFSQDQKHQ	IEKASENIVT
1210	1220	1230	1240	1250	1260	1270	1280
SILKEMLKDI	SSVPFGHLDS	KTGSEASVLV	SEKPQGLSHQ	EWIDQMFSVS	EISTVAQEIT	DSVLNILHKA	SNYISNTTKS
1290	1300	1310	1320	1330	1340	1350	1360
SISSSVHQIS	LHNSDTEHIV	KEAPNKYPLK	TWFDSEKKMK	YLSLFDVDPE	KPPWLKSGKS	EPKPVDDIND	KIIRTIFKRL
1370	1380	1390	1400	1410	1420	1430	1440
KSFICPKLHM	GFKSSLRSQL	SKYTAKIVNI	VLCAIQNELE	LHKENLNLRE	IDHTKSLTDK	GFFANTDKKL	ESLVTSIDDD
1450	1460	1470	1480	1490	1500	1510	1520
ILASPLLTCI	YDMLLSSENA	HQRSISLSSR	KPKSATDSVD	VQSILPNRQD	KKSFHKYLAT	PCTHHSVNGG	NHIKENAKLQ
1530	1540	1550	1560	1570	1580	1590	1600
VLERIGETLH	EMLSKLLGTH	LHSQLSCSQQ	SREMTNKNQK	MAAALQSNIQ	LISKAILDYI	LAKLCGVDMD	TSFASCGLKA
1610	1620	1630	1640	1650	1660	1670	1680
ISESLDIDNP	SFASIIEKMA	KSTKIISSIV	SRRVQEDNKE	ETKSKAKPVA	PVSSKTPSTK	EMHPNKLKAV	ASDILNMVFA
1690	1700	1710	1720	1730	1740	1750	1760
KLEGFANGHL	EILGAINDGN	KKSNKIGWEY	ESTNISRDTH	EASFLSALYM	HAKKVSSAIL	KVIQTELNVT	SSDLKTSVEN
1770	1780	1790	1800	1810	1820	1830	1840
PPPETQILKY	VVKLILDAVS	SDMFNEMESE	GGGIETYRYR	PTYGSLPGGA	ESDSFLEDDA	YTAKKIIDER	SPQREEVKTR
1850	1860	1870	1880	1890	1900	1910	1920
SLKQWALEKT	LNKIEVKLKE	PHISPIAPII	RNILNEIFQS	TLINQLNVLS	LSHSNFNGMP	HNVDEPTPQT	SVQFMDKMMD
1930	1940	1950	1960	1970	1980	1990	2000
PLLSEADITI	VTDNIVRTVF	HKLYSAAMTE	RNVRENRYKT	ITFSANVSSH	EHTYKGKSSV	TALDENPCTF	QSRFSVADKE
2010	2020	2030	2040	2050	2060	2070	2080
TKVNLAEDIV	QAILTNLETF	ATSKVKSLFY	SQVNFTVPVA	LPIQQDHSTL	SKALSAKDSY	SDEQFSCCSV	DHTKSGKTNL
2090	2100	2110	2120	2130	2140	2150	2160
CQLSLSKLNT	YALQVARRNL	QGIKQELDKE	RENPFLTHDI	GISESIASQI	VNALLDIISR	KGKCDKNSSD	KEIDLDQQKG
2170	2180	2190	2200	2210	2220	2230	2240
VIEKLLNETK	YRKVLQLQIQ	DTIEGILCDI	YEKTLFQNNL	SFATPTLKCS	IADKHSEENS	EMFMEGANKI	IPKLSVPKSD
2250	2260	2270	2280	2290	2300	2310	2320
VILISNDIVN	IVLHNLSSAA	TLVINAKNPT	SARLPLTFCD	TFPKIDCQQP	LKGSKTERKT	ERFSYSRNQK	SAYADDNQIT
2330	2340	2350	2360	2370	2380	2390	2400

Detailed Protein Report

Cmpd.	No. of Cmpds.	m/z meas.	Δ m/z [ppm]	z	Rt [min]	Score	P	Range	Sequence	Modification
2211	1	933.6966	-60.77	3	56.3	14.8	0	896-919	K.NSMPHTLDPMCDIAEDMVHAILEK.L	Carbamidomethyl: 11; Oxidation: 3, 10
59	1	841.4513	24.83	3	30.6	11.0	2	5590-5613	K.SGMINLTSGLATGVTNKKEVDENK.V	Oxidation: 3

Detailed Protein Report

Protein 358: short transient receptor potential channel 4 isoform zeta [Homo sapiens]

**Accession:**	gi|209863032	**Score:**	25.8
**Database:**	refseq_human(refseq_human_20140103.fasta)	**MW [kDa]:**	92.2
**Database Date:**	2015-11-30	**pI:**	9.3
		**Sequence Coverage [%]:**	2.5
		**No. of unique Peptides:**	1

10	20	30	40	50	60	70	80
MAQFYYKRNV	NAPYRDRIPL	RIVRAESELS	PSEKAYLNAV	EKGDYASVKK	SLEEAEIYFK	ININCIDPLG	RTALLIAIEN
**90**	**100**	**110**	**120**	**130**	**140**	**150**	**160**
ENLELIELLL	SFNVYVGDAL	LHAIRKEVVG	AVELLLNHKK	PSGEKQFVAQ	PNCQQLLASR	WYDEFPGWRR	RHWAVKMVTC
**170**	**180**	**190**	**200**	**210**	**220**	**230**	**240**
FIIGLLFPVF	SVCYLIAPKS	PLGLFIRKPF	IKFICHTASY	LTFLFLLLLA	SQHIDRSDLN	RQGPPPTIVE	WMILPWVLGF
**250**	**260**	**270**	**280**	**290**	**300**	**310**	**320**
IWGEIKQMWD	GGLQDYIHDW	WNLMDFVMNS	LYLATISLKI	VAFVKYSALN	PRESWDMWHP	TLVAEALFAI	ANIFSSLRLI
**330**	**340**	**350**	**360**	**370**	**380**	**390**	**400**
SLFTANSHLG	PLQISLGRML	LDILKFLFIY	CLVLLAFANG	LNQLYFYYEE	TKGLTCKGIR	CEKQNNAFST	LFETLQSLFW
**410**	**420**	**430**	**440**	**450**	**460**	**470**	**480**
SIFGLINLYV	TNVKAQHEFT	EFVGATMFGT	YNVISLVVLL	NMLIAMMNNS	YQLIADHADI	EWKFARTKLW	MSYFEEGGTL
**490**	**500**	**510**	**520**	**530**	**540**	**550**	**560**
PTPFNVIPSP	KSLWYLIKWI	WTHLCKKKMR	RKPESFGTIG	RRAADNLRRH	HQYQEVMRNL	VKRYVAAMIR	DAKTEEGLTE
**570**	**580**	**590**	**600**	**610**	**620**	**630**	**640**
ENFKELKQDI	SSFRFEVLGL	LRGSKLSTIQ	SANASKESSN	SADSDEKSDS	EGNSKDKKKN	FSLFDLTTLI	HPRSAAIASE
**650**	**660**	**670**	**680**	**690**	**700**	**710**	**720**
RHNISNGSAL	VVQEPPREKQ	RKVNFVTDIK	NFGLFHRRSK	QNAAEQNANQ	IFSVSEEVAR	QQAAGPLERN	IQLESRGLAS
**730**	**740**	**750**	**760**	**770**	**780**	**790**	**800**
RGDLSIPGLS	EQCVLVDHRE	RNTDTLGLQV	GKRVCPFKSE	KVVVEDTVPI	IPKEKHAKEE	DSSIDYDLNL	PDTVTHEDYV
**810**							
TTRL							

Cmpd.	No. of Cmpds.	m/z meas.	Δ m/z [ppm]	z	Rt [min]	Score	P	Range	Sequence	Modification
547	1	607.8337	8.15	2	36.8	10.5	1	499-507	K.WIWTHLCKK.K	

Detailed Protein Report

Protein 359: complement component C7 precursor [Homo sapiens]

**Accession:**	gi|45580688	**Score:**	25.8
**Database:**	refseq_human(refseq_human_20140103.fasta)	**MW [kDa]:**	93.5
**Database Date:**	2015-11-30	**pI:**	6.1
**Modification(s):**	Carbamidomethyl, Oxidation	**Sequence Coverage [%]:**	4.4
		**No. of unique Peptides:**	2

10	20	30	40	50	60	70	80
MKVISLFILV	GFIGEFQSFS	SASSPVNCQW	DFYAPWSECN	GCTKTQTRRR	SVAVYGQYGG	QPCVGNAFET	QSCEPTRGCP
90	100	110	120	130	140	150	160
TEEGCGERFR	CFSGQCISKS	LVCNGDSDCD	EDSADEDRCE	DSERRPSCDI	DKPPPNIELT	GNGYNELTGQ	FRNRVINTKS
170	180	190	200	210	220	230	240
FGGQCRKVFS	GDGKDFYRLS	GNVLSYTFQV	KINNDFNYEF	YNSTWSYVKH	TSTEHTSSSR	KRSFFRSSSS	SSRSYTSHTN
250	260	270	280	290	300	310	320
EIHKGKSYQL	LVVENTVEVA	QFINNNPEFL	QLAEPFWKEL	SHLPSLYDYS	AYRRLIDQYG	THYLQSGSLG	GEYRVLFYVD
330	340	350	360	370	380	390	400
SEKLKQNDFN	SVEEKKCKSS	GWHFVVKFSS	HGCKELENAL	KAASGTQNNV	LRGEPFIRGG	GAGFISGLSY	LELDNPAGNK
410	420	430	440	450	460	470	480
RRYSAWAESV	TNLPQVIKQK	LTPLYELVKE	VPCASVKKLY	LKWALEEYLD	EFDPCHCRPC	QNGGLATVEG	THCLCHCKPY
490	500	510	520	530	540	550	560
TFGAACEQGV	LVGNQAGGVD	GGWSCWSSWS	PCVQGKKTRS	RECNNPPPSG	GGRSCVGETT	ESTQCEDEEL	EHLRLLEPHC
570	580	590	600	610	620	630	640
FPLSLVPTEF	CPSPPALKDG	FVQDEGTMFP	VGKNVVYTCN	EGYSLIGNPV	ARCGEDLRWL	VGEMHCQKIA	CVLPVLMDGI
650	660	670	680	690	700	710	720
QSHPQKPFYT	VGEKVTVSCS	GGMSLEGPSA	FLCGSSLKWS	PEMKNARCVQ	KENPLTQAVP	KCQRWEKLQN	SRCVCKMPYE
730	740	750	760	770	780	790	800
CGPSLDVCAQ	DERSKRILPL	TVCKMHVLHC	QGRNYTLTGR	DSCTLPASAE	KACGACPLWG	KCDAESSKCV	CREASECEEE
810	820	830	840	850			
GFSICVEVNG	KEQTMSECEA	GALRCRGQSI	SVTSIRPCAA	ETQ			

Cmpd.	No. of Cmpds.	m/z meas.	Δ m/z [ppm]	z	Rt [min]	Score	P	Range	Sequence	Modification
1397	1	786.4380	113.40	3	47.6	11.7	2	717-736	K.MPYECGPSLDVCAQDERSKR.I	Carbamidomethyl: 5; Oxidation: 1
2693	1	559.3108	17.19	4	64.8	14.1	2	736-753	K.RILPLTVCKMHVLHCQGR.N	Carbamidomethyl: 8, 15; Oxidation: 10

Detailed Protein Report

Protein 360: squamous cell carcinoma antigen recognized by T-cells 3 [Homo sapiens]

**Accession:**	gi|7661952	**Score:**	25.8
**Database:**	refseq_human(refseq_human_20140103.fasta)	**MW [kDa]:**	109.9
**Database Date:**	2015-11-30	**pI:**	5.3
		**Sequence Coverage [%]:**	2.7
		**No. of unique Peptides:**	1

10	20	30	40	50	60	70	80
MATAAETSAS	EPEAESKAGP	KADGEEDEVK	AARTRRKVLS	RAVAAATYKT	MGPAWDQQEE	GVSESDGDEY	AMASSAESSP
**90**	**100**	**110**	**120**	**130**	**140**	**150**	**160**
GEYEWEYDEE	EEKNQLEIER	LEEQLSINVY	DYNCHVDLIR	LLRLEGELTK	VRMARQKMSE	IFPLTEELWL	EWLHDEISMA
**170**	**180**	**190**	**200**	**210**	**220**	**230**	**240**
QDGLDREHVY	DLFEKAVKDY	ICPNIWLEYG	QYSVGGIGQK	GGLEKVRSVF	ERALSSVGLH	MTKGLALWEA	YREFESAIVE
**250**	**260**	**270**	**280**	**290**	**300**	**310**	**320**
AARLEKVHSL	FRRQLAIPLY	DMEATFAEYE	EWSEDPIPES	VIQNYNKALQ	QLEKYKPYEE	ALLQAEAPRL	AEYQAYIDFE
**330**	**340**	**350**	**360**	**370**	**380**	**390**	**400**
MKIGDPARIQ	LIFERALVEN	CLVPDLWIRY	SQYLDRQLKV	KDLVLSVHNR	AIRNCPWTVA	LWSRYLLAME	RHGVDHQVIS
**410**	**420**	**430**	**440**	**450**	**460**	**470**	**480**
VTFEKALNAG	FIQATDYVEI	WQAYLDYLRR	RVDFKQDSSK	ELEELRAAFT	RALEYLKQEV	EERFNESGDP	SCVIMQNWAR
**490**	**500**	**510**	**520**	**530**	**540**	**550**	**560**
IEARLCNNMQ	KARELWDSIM	TRGNAKYANM	WLEYYNLERA	HGDTQHCRKA	LHRAVQCTSD	YPEHVCEVLL	TMERTEGSLE
**570**	**580**	**590**	**600**	**610**	**620**	**630**	**640**
DWDIAVQKTE	TRLARVNEQR	MKAAEKEAAL	VQQEEEKAEQ	RKRARAEKKA	LKKKKKIRGP	EKRGADEDDE	KEWGDDEEEQ
**650**	**660**	**670**	**680**	**690**	**700**	**710**	**720**
PSKRRRVENS	IPAAGETQNV	EVAAGPAGKC	AAVDVEPPSK	QKEKAASLKR	DMPKVLHDSS	KDSITVFVSN	LPYSMQEPDT
**730**	**740**	**750**	**760**	**770**	**780**	**790**	**800**
KLRPLFEACG	EVVQIRPIFS	NRGDFRGYCY	VEFKEEKSAL	QALEMDRKSV	EGRPMFVSPC	VDKSKNPDFK	VFRYSTSLEK
**810**	**820**	**830**	**840**	**850**	**860**	**870**	**880**
HKLFISGLPF	SCTKEELEEI	CKAHGTVKDL	RLVTNRAGKP	KGLAYVEYEN	ESQASQAVMK	MDGMTIKENI	IKVAISNPPQ
**890**	**900**	**910**	**920**	**930**	**940**	**950**	**960**
RKVPEKPETR	KAPGGPMLLP	QTYGARGKGR	TQLSLLPRAL	QRPSAAAPQA	ENGPAAAPAV	AAPAATEAPK	MSNADFAKLF
**970**							
LRK							

Cmpd.	No. of Cmpds.	m/z meas.	Δ m/z [ppm]	z	Rt [min]	Score	P	Range	Sequence	Modification
1898	1	686.2375	-164.62	2	52.2	12.0	1	670-682	K.CAAVDVEPPSKQK.E	

Detailed Protein Report

Protein 361: PREDICTED: centromere protein T isoform X3 [Homo sapiens]

Accession: gi|578829227
Database: refseq_human(refseq_human_20140103.fasta)
Database Date: 2015-11-30
Modification(s): Carbamidomethyl, Oxidation

Score: 25.7
MW [kDa]: 34.4
pI: 5.0
Sequence Coverage [%]: 4.7
No. of unique Peptides: 1

10	20	30	40	50	60	70	80
MSWCCLWLCL	SSVGRTGSAG	PSLPFSELCS	LGLLRLRPVF	SPLHSGPGKP	AQFLAGEAEE	VNAFALGFLS	TSSGVSGEDE
90	100	110	120	130	140	150	160
VEPLHDGVEE	AEKKMEEEGV	SVSEMEATGA	QGPSRVEEAE	GHTEVTEAEG	SQGTAEADGP	GASSGDEDAS	GRAASPESAS
170	180	190	200	210	220	230	240
STPESLQARR	HHQFLEPAPA	PGAAVLSSEP	AEPLLVRHPP	RPRTTGPRPR	QDPHKAGLSH	YVKLFSFYAK	MPMERKALEM
250	260	270	280	290	300	310	320
VEKCLDKYFQ	HLCDDLEVFA	AHAGRKTVKP	EDLELLMRRQ	GLVTDQVSLH	VLVERHLPLE	YRQLLIPCAY	SGNSVFPAQ

Cmpd.	No. of Cmpds.	m/z meas.	Δ m/z [ppm]	z	Rt [min]	Score	P	Range	Sequence	Modification
2474	1	965.7758	-154.68	2	59.6	14.1	0	1-15	-.MSWCCLWLCLSSVGR.T	Carbamidomethyl: 4, 5, 9; Oxidation: 1

Detailed Protein Report

Protein 362: PREDICTED: ALS2 C-terminal-like protein isoform X7 [Homo sapiens]

**Accession:**	gi|578805946	**Score:**	25.7
**Database:**	refseq_human(refseq_human_20140103.fasta)	**MW [kDa]:**	58.0
**Database Date:**	2015-11-30	**pI:**	10.0
		**Sequence Coverage [%]:**	5.2
		**No. of unique Peptides:**	1

10	20	30	40	50	60	70	80
MCNPEEAALL	RLEEVFSATL	AHVNSLVLQP	LLPAAPDPSD	PWGRECLRLL	QQLHKSSQQL	WEVTEESLHS	LQERLRYPDS
**90**	**100**	**110**	**120**	**130**	**140**	**150**	**160**
TGLESLLLLR	GADRVLQAHI	EYIESYTSCM	VVQAFQKAAK	RRSEYWRGQR	KALRQLLSGV	SSEGSVGASL	GQALHQPLAH
**170**	**180**	**190**	**200**	**210**	**220**	**230**	**240**
HVQQYVLLLL	SLGDTIGEHH	PTRELVVNAV	TLFGNLQSFM	KQELDQAVAT	QALWHTLRGR	LRDVLCTPAH	RLLQDSQDVP
**250**	**260**	**270**	**280**	**290**	**300**	**310**	**320**
VTVAPLRAER	VLLFDDALVL	LQGHNVHTFD	LKLVWVDPGQ	DGCTFHLLTP	EEEFSFCAKD	SQGQAVWQWK	VTWAVHQALH
**330**	**340**	**350**	**360**	**370**	**380**	**390**	**400**
GKKDFPVLGA	GLEPSQPPDC	RCAEYTFQAE	GRLCQATYEG	EWCRGRPHGK	GTLKWPDGRN	HVGNFCQGLE	HGFGIRLLPQ
**410**	**420**	**430**	**440**	**450**	**460**	**470**	**480**
ASEDKFDCYK	CHWREGSMCG	YGICTAPTRC	TRATSRRACG	TDLGSLRVVR	RPPSPSGTRA	TGRGARGAAM	ALRRMVTEVS
**490**	**500**	**510**	**520**	**530**			
ATLACGRLVS	ATAQGSWSPR	QVSATRAPSR	RTRRCVSLVN	DK			

Cmpd.	No. of Cmpds.	m/z meas.	Δ m/z [ppm]	z	Rt [min]	Score	P	Range	Sequence	Modification
1521	1	828.8811	-23.41	2	49.2	13.7	1	397-410	R.LLPQASEDKFDCYK.C	

Detailed Protein Report

Protein 363: mucin-17 precursor [Homo sapiens]

**Accession:**	gi|91982772	**Score:**	25.7
**Database:**	refseq_human(refseq_human_20140103.fasta)	**MW [kDa]:**	451.5
**Database Date:**	2015-11-30	**pI:**	3.9
**Modification(s):**	Carbamidomethyl	**Sequence Coverage [%]:**	0.6
		**No. of unique Peptides:**	2

Quantitation

QD:QU	**Median:**	0.75	**CV:**	0.00 %	**No. of Peptides:**	1	

Detailed Protein Report

10	20	30	40	50	60	70	80
MPRPGTMALC	LLTLVLSLLP	PQAAAEQDLS	VNRAVWDGGG	CISQGDVLNR	QCQQLSQHVR	TGSAANTATG	TTSTNVVEPR
90	100	110	120	130	140	150	160
MYLSCSTNPE	MTSIESSVTS	DTPGVSSTRM	TPTESRTTSE	STSDSTTLFP	SSTEDTSSPT	TPEGTDVPMS	TPSEESISST
170	180	190	200	210	220	230	240
MAFVSTAPLP	SFEAYTSLTY	KVDMSTPLTT	STQASSSPTT	PESTTIPKST	NSEGSTPLTS	MPASTMKVAS	SEAITLLTTP
250	260	270	280	290	300	310	320
VEISTPVTIS	AQASSSPTTA	EGPSLSNSAP	SGGSTPLTRM	PLSVMLVVSS	EASTLSTTPA	ATNIPVITST	EASSSPTTAE
330	340	350	360	370	380	390	400
GTSIPTSTYT	EGSTPLTSTP	ASTMPVATSE	MSTLSITPVD	TSTLVTTSTE	PSSLPTTAEA	TSMLTSTLSE	GSTPLTNMPV
410	420	430	440	450	460	470	480
STILVASSEA	STTSTIPVDS	KTFVTTASEA	SSSPTTAEDT	SIATSTPSEG	STPLTSMPVS	TTPVASSEAS	NLSTTPVDSK
490	500	510	520	530	540	550	560
TQVTTSTEAS	SSPPTAEVNS	MPTSTPSEGS	TPLTSMSVST	MPVASSEAST	LSTTPVDTST	PVTTSSEASS	SSTTPEGTSI
570	580	590	600	610	620	630	640
PTSTPSEGST	PLTNMPVSTR	LVVSSEASTT	STTPADSNTF	VTTSSEASSS	STTAEGTSMP	TSTYSERGTT	ITSMSVSTTL
650	660	670	680	690	700	710	720
VASSEASTLS	TTPVDSNTPV	TTSTEATSSS	TTAEGTSMPT	STYTEGSTPL	TSMPVNTTLV	ASSEASTLST	TPVDTSTPVT
730	740	750	760	770	780	790	800
TSTEASSSPT	TADGASMPTS	TPSEGSTPLT	SMPVSKTLLT	SSEASTLSTT	PLDTSTHITT	STEASCSPTT	TEGTSMPIST
810	820	830	840	850	860	870	880
PSEGSPLLTS	IPVSITPVTS	PEASTLSTTP	VDSNSPVTTS	TEVSSSPTPA	EGTSMPTSTY	SEGRTPLTSM	PVSTTLVATS
890	900	910	920	930	940	950	960
AISTLSTTPV	DTSTPVTNST	EARSSPTTSE	GTSMPTSTPG	EGSTPLTSMP	DSTTPVVSSE	ARTLSATPVD	TSTPVTTSTE
970	980	990	1000	1010	1020	1030	1040
ATSSPTTAEG	TSIPTSTPSE	GTTPLTSTPV	SHTLVANSEA	STLSTTPVDS	NTPLTTSTEA	SSPPPTAEGT	SMPTSTPSEG
1050	1060	1070	1080	1090	1100	1110	1120
STPLTRMPVS	TTMVASSETS	TLSTTPADTS	TPVTTYSQAS	SSSTTADGTS	MPTSTYSEGS	TPLTSVPVST	RLVVSSEAST
1130	1140	1150	1160	1170	1180	1190	1200
LSTTPVDTSI	PVTTSTEASS	SPTTAEGTSI	PTSPPSEGTT	PLASMPVSTT	LVVSSEANTL	STTPVDSKTQ	VATSTEASSP
1210	1220	1230	1240	1250	1260	1270	1280
PPTAEVTSMP	TSTPGERSTP	LTSMPVRHTP	VASSEASTLS	TSPVDTSTPV	TTSAETSSSP	TTAEGTSLPT	STTSEGSTLL
1290	1300	1310	1320	1330	1340	1350	1360
TSIPVSTTLV	TSPEASTLLT	TPVDTKGPVV	TSNEVSSSPT	PAEGTSMPTS	TYSEGRTPLT	SIPVNTTLVA	SSAISILSTT
1370	1380	1390	1400	1410	1420	1430	1440
PVDNSTPVTT	STEACSSPTT	SEGTSMPNSN	PSEGTTPLTS	IPVSTTPVVS	SEASTLSATP	VDTSTPGTTS	AEATSSPTTA
1450	1460	1470	1480	1490	1500	1510	1520
EGISIPTSTP	SEGKTPLKSI	PVSNTPVANS	EASTLSTTPV	DSNSPVVTST	AVSSSPTPAE	GTSIAISTPS	EGSTALTSIP
1530	1540	1550	1560	1570	1580	1590	1600
VSTTTVASSE	INSLSTTPAV	TSTPVTTYSQ	ASSSPTTADG	TSMQTSTYSE	GSTPLTSLPV	STMLVVSSEA	NTLSTTPIDS
1610	1620	1630	1640	1650	1660	1670	1680
KTQVTASTEA	SSSTTAEGSS	MTISTPSEGS	PLLTSIPVST	TPVASPEAST	LSTTPVDSNS	PVITSTEVSS	SPTPAEGTSM
1690	1700	1710	1720	1730	1740	1750	1760
PTSTYTEGRT	PLTSITVRTT	PVASSAISTL	STTPVDNSTP	VTTSTEARSS	PTTSEGTSMP	NSTPSEGTTP	LTSIPVSTTP
1770	1780	1790	1800	1810	1820	1830	1840
VLSSEASTLS	ATPIDTSTPV	TTSTEATSSP	TTAEGTSIPT	STLSEGMTPL	TSTPVSHTLV	ANSEASTLST	TPVDSNSPVV
1850	1860	1870	1880	1890	1900	1910	1920
TSTAVSSSPT	PAEGTSIATS	TPSEGSTALT	SIPVSTTTVA	SSETNTLSTT	PAVTSTPVTT	YAQVSSSPTT	ADGSSMPTST
1930	1940	1950	1960	1970	1980	1990	2000
PREGRPPLTS	IPVSTTTVAS	SEINTLSTTL	ADTRTPVTTY	SQASSSPTTA	DGTSMPTPAY	SEGSTPLTSM	PLSTTLVVSS
2010	2020	2030	2040	2050	2060	2070	2080
EASTLSTTPV	DTSTPATTST	EGSSSPTTAG	GTSIQTSTPS	ERTTPLAGMP	VSTTLVVSSE	GNTLSTTPVD	SKTQVTNSTE
2090	2100	2110	2120	2130	2140	2150	2160
ASSSATAEGS	SMTISAPSEG	SPLLTSIPLS	TTPVASPEAS	TLSTTPVDSN	SPVITSTEVS	SSPIPTEGTS	MQTSTYSDRR
2170	2180	2190	2200	2210	2220	2230	2240

Detailed Protein Report

Cmpd.	No. of Cmpds.	m/z meas.	Δ m/z [ppm]	z	Rt [min]	Score	P	Range	Sequence	Modification	Ratios
193	1	613.7845	-35.89	2	32.4	14.1	0	51-60	R.QCQQLSQHVR.T		QD:QU 0.75
490	1	636.2255	-63.89	3	36.1	11.6	1	4132-4148	R.TTTCFGDGCQNTASRCK.N	Carbamidomethyl: 9, 16	

Detailed Protein Report

Protein 364: **PREDICTED: transmembrane protein 255B isoform X13 [Homo sapiens]**

Accession: gi|578825377
Database: refseq_human(refseq_human_20140103.fasta)
Database Date: 2015-11-30
Modification(s): Carbamidomethyl

Score: 25.6
MW [kDa]: 17.8
pI: 10.7
Sequence Coverage [%]: 10.4
No. of unique Peptides: 1

Quantitation

QD:QU **Median:** 1.06 **CV:** 0.00 % **No. of Peptides:** 1

10	20	30	40	50	60	70	80
MGLRGRKNLT	SSLACLPEGL	SRRKKTSLWF	VGSLLLVSVL	IVTVGLAATT	RTENVTVGGY	YPGIILGFGS	FLGIIGINLV
**90**	**100**	**110**	**120**	**130**	**140**	**150**	**160**
ENRRQMLVAA	IVFISFGVVA	AFCCAIVDGV	FAAQHIEPRP	LTTGRCQFYS	SGVGYLYDVY	QTEVTCHSLD	GKCQLKRRAL
**170**							
ARLL							

Cmpd.	No. of Cmpds.	m/z meas.	Δ m/z [ppm]	z	Rt [min]	Score	P	Range	Sequence	Modification	Ratios
2276	1	873.4565	-8.97	2	58.9	13.3	1	7-22	R.KNLTSSLACLPEGLSR.R	Carbamidomethyl: 9	QD:QU 1.06

Detailed Protein Report

Protein 365: polypeptide N-acetylgalactosaminyltransferase 9 isoform A [Homo sapiens]

Accession: gi|169790926
Database: refseq_human(refseq_human_20140103.fasta)
Database Date: 2015-11-30

Score: 25.6
MW [kDa]: 68.3
pI: 9.4
Sequence Coverage [%]: 3.8
No. of unique Peptides: 1

Quantitation

QD:QU **Median:** 0.37 **CV:** 0.00 % **No. of Peptides:** 1

10	20	30	40	50	60	70	80
MAVARKIRTL	LTVNILVFVG	IVLFSVYCRL	QGRSQELVRI	VSGDRRVRSR	HAKVGTLGDR	EAILQRLDHL	EEVVYNQLNG
90	100	110	120	130	140	150	160
LAKPIGLVEG	PGGLGQGGLA	ATLRDDGQEA	EGKYEEYGYN	AQLSDRISLD	RSIPDYRPRK	CRQMSYAQDL	PQVSVVFIFV
170	180	190	200	210	220	230	240
NEALSVILRS	VHSVVNHTPS	QLLKEVILVD	DNSDNVELKF	NLDQYVNKRY	PGLVKIVRNS	RREGLIRARL	QGWKAATAPV
250	260	270	280	290	300	310	320
VGFFDAHVEF	NTGWAEPALS	RIREDRRRIV	LPAIDNIKYS	TFEVQQYANA	AHGYNWGLWC	MYIIPPQDWL	DRGDESAPIR
330	340	350	360	370	380	390	400
TPAMIGCSFV	VDREYFGDIG	LLDPGMEVYG	GENVELGMRV	WQCGGSMEVL	PCSRVAHIER	TRKPYNNDID	YYAKRNALRA
410	420	430	440	450	460	470	480
AEVWMDDFKS	HVYMAWNIPM	SNPGVDFGDV	SERLALRQRL	KCRSFKWYLE	NVYPEMRVYN	NTLTYGEVRN	SKASAYCLDQ
490	500	510	520	530	540	550	560
GAEDGDRAIL	YPCHGMSSQL	VRYSADGLLQ	LGPLGSTAFL	PDSKCLVDDG	TGRMPTLKKC	EDVARPTQRL	WDFTQSGPIV
570	580	590	600	610			
SRATGRCLEV	EMSKDANFGL	RLVVQRCSGQ	KWMIRNWIKH	ARH			

Cmpd.	No. of Cmpds.	m/z meas.	Δ m/z [ppm]	z	Rt [min]	Score	P	Range	Sequence	Modification	Ratios
393	1	548.2880	-98.86	2	34.9	10.8	0	269-278	R.IVLPAIDNIK.Y		QD:QU 0.37

Detailed Protein Report

Protein 366: PREDICTED: alpha-tectorin isoform X1 [Homo sapiens]

**Accession:**	gi|530398338	**Score:**	25.6
**Database:**	refseq_human(refseq_human_20140103.fasta)	**MW [kDa]:**	238.8
**Database Date:**	2015-11-30	**pI:**	5.1
**Modification(s):**	Carbamidomethyl	**Sequence Coverage [%]:**	1.3
		**No. of unique Peptides:**	1

Quantitation

QD:QU	**Median:** 0.90	**CV:** 0.00 %	**No. of Peptides:** 1

Detailed Protein Report

10	20	30	40	50	60	70	80
MNYSSFLRIW	VSFIFALVQH	QAQPRELMYP	FWQNDTKTPK	VDDGSSSEIK	LAIPVFFFGV	PYRTVYVNNN	GVVSFNVLVS
90	100	110	120	130	140	150	160
QFTPESFPLT	DGRAFVAPFW	ADVHNGIRGE	IYYRETMEPA	ILKRATKDIR	KYFKDMATFS	ATWVFIVTWE	EVTFYGGSST
170	180	190	200	210	220	230	240
TPVNTFQAVL	VSDGSYTFTL	FNYYEINWTT	GTASGGDPLT	GLGGVMAQAG	FNGGNLTNFF	SLPGSRTPEI	VNIQETTNVN
250	260	270	280	290	300	310	320
VPGRWAFKVD	GKEIDPANGC	TSRGQFLRRG	EVFWDDLNCT	VKCRCLDFNN	EIYCQEASCS	PYEVCEPKGK	FFYCSAVETS
330	340	350	360	370	380	390	400
TCVVFGEPHY	HTFDGFLFHF	QGSCAYLLAR	QCLQTSSLPF	FSVEAKNEHR	RGSAVSWVKE	LSVEVNGYKI	LIPKGSYGRV
410	420	430	440	450	460	470	480
KVNDLVTSLP	VTLDLGTVKI	YQSGISTAVE	TDFGLLVTFD	GQHYASISVP	GSYINSTCGL	CGNYNKNPLD	DFLRPDGRPA
490	500	510	520	530	540	550	560
MSVLDLGESW	RVYHADWKCD	SGCVDNCTQC	DAATEALYFG	SDYCGFLNKT	DGPLWECGTV	VDPTAFVHSC	VYDLCSVRDN
570	580	590	600	610	620	630	640
GTLLCQAIQA	YALVCQALGI	PIGDWRTQTG	CVSTVQCPSF	SHYSVCTSSC	PDTCSDLTAS	RNCATPCTEG	CECNQGFVLS
650	660	670	680	690	700	710	720
TSQCVPLHKC	GCDFDGHYYT	MGEFFWATAN	CTVQCLCEEG	GDVYCFNKTC	GSGEVCAVED	GYQGCFPKRE	TVCLLSQNQV
730	740	750	760	770	780	790	800
LHTFDGASYA	FPSEFSYTLL	KTCPERPEYL	EIDINKKKPD	AGPAWLRGLR	ILVADQEVKI	GGIGASEVKL	NGQEVELPFF
810	820	830	840	850	860	870	880
HPSGKLEIYR	NKNSTTVESK	GVVTVQYSDI	GLLYIRLSTT	YFNCTGGLCG	FYNANASDEF	CLPNGKCTDN	LAVFLESWTT
890	900	910	920	930	940	950	960
FEEICNGECG	DLLKACNNDS	ELLKFYRSRS	RCGIINDPSN	SSFLECHGVV	NVTAYYRTCL	FRLCQSGGNE	SELCDSVARY
970	980	990	1000	1010	1020	1030	1040
ASACKNADVE	VGPWRTYDFC	PLECPENSHF	EECITCTETC	ETLTLGPICV	DSCSEGCQCD	EGYALLGSQC	VTRSECGCNF
1050	1060	1070	1080	1090	1100	1110	1120
EGHQLATNET	FWVDLDCQIF	CYCSGTDNRV	HCETIPCKDD	EYCMEEGGLY	YCQARTDASC	IVSGYGHYLT	FDGFPFDFQT
1130	1140	1150	1160	1170	1180	1190	1200
SCPLILCTTG	SRPSSDSFPK	FVVTAKNEDR	DPSLALWVKQ	VDVTVFGYSI	VIHRAYKHTV	LVNSERLYLP	LKLGQGKINI
1210	1220	1230	1240	1250	1260	1270	1280
FSFGFHVVVE	TDFGLKVVYD	WKTFLSITVP	RSMQNSTYGL	CGRYNGNPDD	DLEMPMGLLA	SSVNEFGQSW	VKRDTFCQVG
1290	1300	1310	1320	1330	1340	1350	1360
CGDRCPSCAK	VEGFSKVQQL	CSLIPNQNAA	FSKCHSKVNP	TFFYKNCLFD	SCIDGGAVQT	ACSWLQNYAS	TCQTQGITVT
1370	1380	1390	1400	1410	1420	1430	1440
GWRNYTSCTV	TCPPNSHYES	CVSVCQPRCA	AIRLKSDCSH	YCVEGCHCDA	GYVLNGKSCI	LPHSCGCYSD	GKYYEPKQLF
1450	1460	1470	1480	1490	1500	1510	1520
WNSDCTRRCR	CFRRNVIQCD	PRQCKSDEEC	ALRNGVRGCF	STKTSYCLAA	GGGVFRTFDG	AFLRFPANCA	FVLSTICQKL
1530	1540	1550	1560	1570	1580	1590	1600
PDISFQLIIN	FDKWSAPNLT	IISPVYFYIN	EEQILINDRN	TVKVNGTQVN	VPFITGLATK	IYSSEGFLVI	DTSPDIQIYY
1610	1620	1630	1640	1650	1660	1670	1680
NGFNVIKISI	SERLQNKVCG	LCGNFNGDLT	DDYVTLRGKP	VVSSVVLAQS	WKTNGMQKSC	NELQFSQYAA	MCDNVHIQKM
1690	1700	1710	1720	1730	1740	1750	1760
QGDGYCLKLT	DMKGFFQPCY	GLLDPLPFYE	SCYLDGCYSH	KKFQLCGSLA	AYGEACRSFG	ILSTEWIEKE	NCSGVVEDPC
1770	1780	1790	1800	1810	1820	1830	1840
VGADCPNRTC	ELGNGRELCG	CIEPPPYGNN	SHDIIDAEVT	CKAAQMEVSI	SKCKLFQLGF	EREGVRINDR	QCTGIEGEDF
1850	1860	1870	1880	1890	1900	1910	1920
ISFQINNTKG	NCGNIVQSNG	THIMYKNTLW	IESANNTGNI	ITRDRTINVE	FSCAYELDIK	ISLDSVVKPM	LSVINLTVPT
1930	1940	1950	1960	1970	1980	1990	2000
QEGSFITKMA	LYKNASYKHP	YRQGEVVLTT	RDVLYVGVFV	VGADATHLIL	TLNKCYATPT	RDSNDKLRYF	IIEGGCQNLK
2010	2020	2030	2040	2050	2060	2070	2080
DNTIGIEENA	VSLTCRFHVT	VFKFIGDYDE	VHLHCAVSLC	DSEKYSCKIT	CPHNSRIATD	YTKEPKEQII	SVGPIRRKRL
2090	2100	2110	2120	2130	2140	2150	2160
DWCEDNGGCE	QICTSRVDGP	LCSCVTGTLQ	EDGKSCRASN	SSMELQVWTL	LLIMIQISLW	HFVYKSGTTS	

Detailed Protein Report

Cmpd.	No. of Cmpds.	m/z meas.	Δ m/z [ppm]	z	Rt [min]	Score	P	Range	Sequence	Modification	Ratios	
132	1	673.3206	73.69	3	30.4	10.7	1	1274-1290	R.DTFCQVGCGDRCPSCAK.V	Carbamidomethyl: 4, 8, 12, 15	QD:QU	0.90

Detailed Protein Report

Protein 367: dynein heavy chain 10, axonemal [Homo sapiens]

**Accession:**	gi|198442844	**Score:**	25.5
**Database:**	refseq_human(refseq_human_20140103.fasta)	**MW [kDa]:**	514.5
**Database Date:**	2015-11-30	**pI:**	5.6
**Modification(s):**	Oxidation	**Sequence Coverage [%]:**	0.4
		**No. of unique Peptides:**	1

Detailed Protein Report

10	20	30	40	50	60	70	80
MVPEEVEVEI	DEIPVLSEEG	EEEEETYSQK	VESVDKVRAK	RVSLRTESLG	QPLNREDEEM	DKEISEKLPS	KRTAKHIMEK
90	100	110	120	130	140	150	160
MHLHMLCTPL	PEEFLDQNVV	FFLRNTKEAI	SEATDMKEAM	EIMPETLEYG	IINANVLHFL	KNIICQVFLP	ALSFNQHRTS
170	180	190	200	210	220	230	240
TTVGVTSGEV	SNSSEHESDL	PPMPGEAVEY	HSIQLIRDEF	LMNVQKFASN	IQRTMQQLEG	EIKLEMPIIS	VEGEVSDLAA
250	260	270	280	290	300	310	320
DPETVDILEQ	CVINWLNQIS	TAVEAQLKKT	PQGKGPLAEI	EFWRERNATL	SALHEQTKLP	IVRKVLDVIK	ESDSMLVANL
330	340	350	360	370	380	390	400
QPVFTELFKF	HTEASDNVRF	LSTVERYFKN	ITHGSGFHVV	LDTIPAMMSA	LRMVWIISRH	YNKDERMIPL	MERIAWEIAE
410	420	430	440	450	460	470	480
RVCRVVNLRT	LFKENRASAQ	SKTLEARNTL	RLWKKAYFDT	RAKIEASGRE	DRWEFDRKRL	FERTDYMATI	CQDLSDVLQI
490	500	510	520	530	540	550	560
LEEFYNIFGP	ELKAVTGDPK	RIDDVLCRVD	GLVTPMENLT	FDPFSIKSSQ	FWKYVMDEFK	IEVLIDIINK	IFVQNLENPP
570	580	590	600	610	620	630	640
LYKNHPPVAG	AIYWERSLFF	RIKHTILRFQ	EVQEILDSDR	GQEVKQKYLE	VGRTMKEYED	RKYEQWMEVT	EQVLPALMKK
650	660	670	680	690	700	710	720
SLLTKSSIAT	EEPSTLERGA	VFAINFSPAL	REIINETKYL	EQLGFTVPEL	ARNVALQEDK	FLRYTAGIQR	MLDHYHMLIG
730	740	750	760	770	780	790	800
TLNDAESVLL	KDHSQELLRV	FRSGYKRLNW	NSLGIGDYIT	GCKQAIGKFE	SLVHQIHKNA	DDISSRLTLI	EAINLFKYPA
810	820	830	840	850	860	870	880
AKSEEELPGV	KEFFEHIERE	RASDVDHMVR	WYLAIGPLLT	KVEGLVVHTN	TGKAPKLASY	YKYWEKKIYE	VLTKLILKNL
890	900	910	920	930	940	950	960
QSFNSLILGN	VPLFHTETIL	TAPEIILHPN	TNEIDKMCFH	CVRNCVEITK	HFVRWMNGSC	IECPPQKGEE	EEVVIINFYN
970	980	990	1000	1010	1020	1030	1040
DISLNPQIIE	QAVMIPQNVH	RILINLMKYL	QKWKRYRPLW	KLDKAIVMEK	FAAKKPPCVA	YDEKLQFYSK	IAYEVMRHPL
1050	1060	1070	1080	1090	1100	1110	1120
IKDEHCIRLQ	LRHLANTVQE	NAKSWVISLG	KLLNESAKEE	LYNLHEEMEH	LAKNLRKIPN	TLEDLKFVLA	TIAEIRSKSL
1130	1140	1150	1160	1170	1180	1190	1200
VMELRYRDVQ	ERYRTMAMYN	LFPPDAEKEL	VDKIESIWSN	LFNDSVNVEH	ALGDIKRTFT	ELTRGEIMNY	RVQIEEFAKR
1210	1220	1230	1240	1250	1260	1270	1280
FYSEGPGSVG	DDLDKGVELL	GVYERELARH	EKSRQELANA	EKLFDLPITM	YPELLKVQKE	MSGLRMIYEL	YEGLKVAKEE
1290	1300	1310	1320	1330	1340	1350	1360
WSQTLWINLN	VQILQEGIEG	FLRALRKLPR	PVRGLSVTYY	LEAKMKAFKD	SIPLLLDLKN	EALRDRHWKE	LMEKTSVFFE
1370	1380	1390	1400	1410	1420	1430	1440
MTETFTLENM	FAMELHKHTD	VLNEIVTAAI	KEVAIEKAVK	EILDTWENMK	FTVVKYCKGT	QERGYILGSV	DEIIQSLDDN
1450	1460	1470	1480	1490	1500	1510	1520
TFNLQSISGS	RFVGPFLQTV	HKWEKTLSLI	GEVIEIWMLV	QRKWMYLESI	FIGGDIRSQL	PEEAKKFDNI	DKVFKRIMGE
1530	1540	1550	1560	1570	1580	1590	1600
TLKDPVIKRC	CEAPNRLSDL	QNVSEGLEKC	QKSLNDYLDS	KRNAFPRFFF	ISDDELLSIL	GSSDPLCVQE	HMIKMYDNIA
1610	1620	1630	1640	1650	1660	1670	1680
SLRFNDGDSG	EKLVSAMISA	EGEVMEFRKI	LRAEGRVEDW	MTAVLNEMRR	TNRLITKEAI	FRYCEDRSRV	DWMLLYQGMV
1690	1700	1710	1720	1730	1740	1750	1760
VLAASQVWWT	WEVEDVFHKA	QKGEKQAMKN	YGRKMHRQID	ELVTRITMPL	SKNDRKKYNT	VLIIDVHARD	IVDSFIRGSI
1770	1780	1790	1800	1810	1820	1830	1840
LEAREFDWES	QLRFYWDREP	DELNIRQCTG	TFGYGYEYMG	LNGRLVITPL	TDRIYLTLTQ	ALSMYLGGAP	AGPAGTGKTE
1850	1860	1870	1880	1890	1900	1910	1920
TTKDLAKALG	LLCVVTNCGE	GMDYRAVGKI	FSGLAQCGAW	GCFDEFNRID	ASVLSVISSQ	IQTIRNALIH	QLTTFQFEGQ
1930	1940	1950	1960	1970	1980	1990	2000
EISLDSRMGI	FITMNPGYAG	RTELPESVKA	LFRPVVVIVP	DLQQICEIML	FSEGFLEAKT	LAKKMTVLYK	LAREQLSKQY
2010	2020	2030	2040	2050	2060	2070	2080
HYDFGLRALK	SVLVMAGELK	RGSSDLREDV	VLMRALRDMN	LPKFVFEDVP	LFLGLISDLF	PGLDCPRVRY	PDFNDAVEQV
2090	2100	2110	2120	2130	2140	2150	2160
LEENGYAVLP	IQVDKVVQMF	ETMLTRHTTM	VVGPTRGGKS	VVINTLCQAQ	TKLGLTTKLY	ILNPKAVSVI	ELYGILDPTT
2170	2180	2190	2200	2210	2220	2230	2240

Detailed Protein Report

Cmpd.	No. of Cmpds.	m/z meas.	Δ m/z [ppm]	z	Rt [min]	Score	P	Range	Sequence	Modification
2459	1	1045.2266	-235.83	1	61.2	14.2	0	822-830	R.ASDVDHMVR.W	Oxidation: 7

BRUKER

Detailed Protein Report

Protein 368: PREDICTED: centrosomal protein of 192 kDa isoform X12 [Homo sapiens]

Accession: gi|578832148
Database: refseq_human(refseq_human_20140103.fasta)
Database Date: 2015-11-30
Modification(s): Carbamidomethyl

Score: 25.5
MW [kDa]: 154.9
pI: 4.5
Sequence Coverage [%]: 2.4
No. of unique Peptides: 2

Quantitation

QD:QU **Median:** 0.88 **CV:** 0.00 % **No. of Peptides:** 1

10	20	30	40	50	60	70	80
MEDFRGIAEE	SFPSFLTNSL	FGNSGILENV	TLSSNLGLPV	AVSTLARDRS	STDNRYPDIQ	ASYLVEGRFS	VPSGSSPGSQ
90	100	110	120	130	140	150	160
SDAEPRERLQ	LSFQDDDSIS	RKKSYVESQR	LSNALSKQSA	LQMETAGPEE	EPAGATESLQ	GQDLFNRASP	LEQAQDSPID
170	180	190	200	210	220	230	240
FHLQSWMNNK	EPKIVVLDAG	KHFEDKTLKS	DLSHTSLLEN	EKLILPTSLE	DSSDDDIDDE	MFYDDHLEAY	FEQLAIPGMI
250	260	270	280	290	300	310	320
YEDLEGPEPP	EKGFKLPTNG	LRQANENGSL	NCKFQSENNS	SLISLDSHSS	ETTHKESEES	QVICLPGTSN	SIGTGDSRRY
330	340	350	360	370	380	390	400
TDGMLPFSSG	TWGTEKEIEN	LKGIVPDLNS	ECASKDVLVK	TLRAIDVKLN	SDNFHDANAN	RGGFDLTDPV	KQGAECPHQN
410	420	430	440	450	460	470	480
KTVLHMDGCL	DTETPTVSIQ	ENVDVASLKP	ISDSGINFTD	AIWSPTCERR	TCECHESIEK	NKDKTDLPQS	VVYQNEEGRW
490	500	510	520	530	540	550	560
VTDLAYYTSF	NSKQNLNVSL	SDEMNEDFRS	GSEAFDLIAQ	DEEEFNKEHQ	FIQEENIDAH	NTSVALGDTS	WGATINYSLL
570	580	590	600	610	620	630	640
RKSRSTSDLD	KDDASYLRLS	LGEFFAQRSE	ALGCLGGGNN	VKRPSFGYFI	RSPEKREPIA	LIRKSDVSRG	NLEKEMAHLN
650	660	670	680	690	700	710	720
HDLYSGDLNE	QSQAQLSEGS	ITLQVEAVES	TSQVDENDVT	LTADKGKTED	TFFMSNKPQR	YKDKLPDSGD	SMLRISTIAS
730	740	750	760	770	780	790	800
AIAEASVNTD	PSQLAAMIKA	LSNKTRDKTF	QEDEKQKDYS	HVRHFLPNDL	EKSNGSNALD	MEKYLKKTEV	SRYESALENF
810	820	830	840	850	860	870	880
SRASMSDTWD	LSLPKEQTTQ	DIHPVDLSAT	SVSVRAPEEN	TAAIVYVENG	ESENQESFRT	INSSNSVTNR	ENNSAVVDVK
890	900	910	920	930	940	950	960
TCSIDNKLQD	VGNDEKATSI	STPSDSYSSV	RNPRITSLCL	LKDCEEIRDN	RENQRQNECV	SEISNSEKHV	TFENHRIVSP
970	980	990	1000	1010	1020	1030	1040
KNSDLKNTSP	EHGGRGSEDE	QESFRPSTSP	LSHSSPSEIS	GTSSSGCALE	SFGSAAQQQQ	PPCEQELSPL	VCSPAGVSRL
1050	1060	1070	1080	1090	1100	1110	1120
TYVSEPESSY	PTTATDDALE	DRKSDITSEL	STTIIQGSPA	ALEERAMEKL	REKVPFQNRG	KGTLSSIIQN	NSDTRKATET
1130	1140	1150	1160	1170	1180	1190	1200
TSLSSKPEYV	KPDFRWSKDP	SSKSGNLLET	SEVGWTSNPE	ELDPIRLALL	GKSGLSCQVG	SATSHPVSCQ	EPIDEDQRIS
1210	1220	1230	1240	1250	1260	1270	1280
PKDKSTAGRE	FSGQVSHQTT	SENQCTPIPS	STVHSSVADM	QNMPAAVHAL	LTQPSLSAAP	FAQRYLGTLP	STGSTTLPQC
1290	1300	1310	1320	1330	1340	1350	1360
HAGNATVCGF	SGGLPYPAVA	GEPVQNSVAV	GICLGSNIGS	GWMGTSSLCN	PYSNTLNQNL	LSTTKPFPVP	SVGTNCGIEP
1370	1380	1390	1400	1410	1420		
WDSGVTSGLG	SVRVPEELKL	PHACCVGIAS	QTLLSVLNPT	DRWLQVSIGV	LSISVNGEK		

Cmpd.	No. of Cmpds.	m/z meas.	Δ m/z [ppm]	z	Rt [min]	Score	P	Range	Sequence	Modification	Ratios
1517	1	993.4897	-5.03	2	49.2	13.0	1	256-273	K.LPTNGLRQANENGSLNCK.F	Carbamidomethyl: 17	QD:QU 0.88
2156	2	955.8889	-44.17	2	55.6	12.5	0	494-509	K.QNLNVSLSDEMNEDFR.S		

Detailed Protein Report

Protein 369: zinc finger protein 471 [Homo sapiens]

Accession: gi|150170667
Database: refseq_human(refseq_human_20140103.fasta)
Database Date: 2015-11-30

Score: 25.4
MW [kDa]: 73.0
pI: 9.9
Sequence Coverage [%]: 4.6
No. of unique Peptides: 1

Quantitation

QD:QU **Median:** 0.29 **CV:** 0.00 % **No. of Peptides:** 1

10	20	30	40	50	60	70	80
MNVEVVKVMP	QDLVTFKDVA	IDFSQEEWQW	MNPAQKRLYR	SMMLENYQSL	VSLGLCISKP	YVISLLEQGR	EPWEMTSEMT
**90**	**100**	**110**	**120**	**130**	**140**	**150**	**160**
RSPFSDWESI	YVTQELPLKQ	FMYDDACMEG	ITSYGLECST	FEENWKWEDL	FEKQMGSHEM	FSKKEIITHK	ETITKETEFK
**170**	**180**	**190**	**200**	**210**	**220**	**230**	**240**
YTKFGKCIHL	ENIEESIYNH	TSDKKSFSKN	SMVIKHKKVY	VGKKLFKCNE	CDKTFTHSSS	LTVHFRIHTG	EKPYACEECG
**250**	**260**	**270**	**280**	**290**	**300**	**310**	**320**
KAFKQRQHLA	QHHRTHTGEK	LFECKECRKA	FKQSEHLIQH	QRIHTGEKPY	KCKECRKAFR	QPAHLAQHQR	IHTGEKPYEC
**330**	**340**	**350**	**360**	**370**	**380**	**390**	**400**
KECGKAFSDG	SSFARHQRCH	TGKRPYECIE	CGKAFRYNTS	FIRHWRSYHT	GEKPFNCIDC	GKAFSVHIGL	ILHRRIHTGE
**410**	**420**	**430**	**440**	**450**	**460**	**470**	**480**
KPYKCGVCGK	TFSSGSSRTV	HQRIHTGEKP	YECDICGKDF	SHHASLTQHQ	RVHSGEKPYE	CKECGKAFRQ	NVHLVSHLRI
**490**	**500**	**510**	**520**	**530**	**540**	**550**	**560**
HTGEKPYECK	ECGKAFRISS	QLATHQRIHT	GEKPYECIEC	GNAFKQRSHL	AQHQKTHTGE	KPYECNECGK	AFSQTSNLTQ
**570**	**580**	**590**	**600**	**610**	**620**	**630**	
HQRIHTGEKP	YKCTECGKAF	SDSSSCAQHQ	RLHTGQRPYQ	CFECGKAFRR	KLSLICHQRS	HTGEEP	

Cmpd.	No. of Cmpds.	m/z meas.	Δ m/z [ppm]	z	Rt [min]	Score	P	Range	Sequence	Modification	Ratios
520	1	690.4558	150.35	2	36.5	14.8	1	357-366	R.YNTSFIRHWR.S		QD:QU 0.29

Detailed Protein Report

Protein 370: ubiquitin-like-conjugating enzyme ATG10 [Homo sapiens]

**Accession:**	gi|18594496	**Score:**	25.4
**Database:**	refseq_human(refseq_human_20140103.fasta)	**MW [kDa]:**	25.3
**Database Date:**	2015-11-30	**pI:**	5.3
**Modification(s):**	Carbamidomethyl, Oxidation	**Sequence Coverage [%]:**	6.8
		**No. of unique Peptides:**	2

Quantitation

QD:QU **Median:** 2.86 **CV:** 0.00 % **No. of Peptides:** 1

Alias proteins:

Accession	Name	Description
gi|530379522	refseq_human (refseq_human_20140103.fasta)	PREDICTED: ubiquitin-like-conjugating enzyme ATG10 isoform X2 [Homo sapiens]
gi|530379520	refseq_human (refseq_human_20140103.fasta)	PREDICTED: ubiquitin-like-conjugating enzyme ATG10 isoform X1 [Homo sapiens]
gi|196162717	refseq_human (refseq_human_20140103.fasta)	ubiquitin-like-conjugating enzyme ATG10 [Homo sapiens]

```
        10         20         30         40         50         60         70         80
MEEDEFIGEK TFQRYCAEFI KHSQQIGDSW EWRPSKDCSD GYMCKIHFQI KNGSVMSHLG ASTHGQTCLP MEEAFELPLD
        90        100        110        120        130        140        150        160
DCEVIETAAA SEVIKYEYHV LYSCSYQVPV LYFRASFLDG RPLTLKDIWE GVHECYKMRL LQGPWDTITQ QEHPILGQPF
       170        180        190        200        210        220        230
FVLHPCKTNE FMTPVLKNSQ KINKNVNYIT SWLSIVGPVV GLNLPLSYAK ATSQDERNVP
```

Cmpd.	No. of Cmpds.	m/z meas.	Δ m/z [ppm]	z	Rt [min]	Score	P	Range	Sequence	Modification	Ratios
1635	1	576.3013	186.62	2	49.0	10.1	0	37-45	K.DCSDGYMCK.I	Carbamidomethyl: 2, 8; Oxidation: 7	
2921	2	922.9870	83.40	2	65.7	15.3	1	37-51	K.DCSDGYMCKIHFQIK.N	Carbamidomethyl: 2	QD:QU 2.86

Detailed Protein Report

Protein 371: PREDICTED: cysteine-rich with EGF-like domain protein 1 isoform X2 [Homo sapiens]

**Accession:**	gi|578806578	**Score:**	25.4
**Database:**	refseq_human(refseq_human_20140103.fasta)	**MW [kDa]:**	42.9
**Database Date:**	2015-11-30	**pI:**	6.2
**Modification(s):**	Carbamidomethyl	**Sequence Coverage [%]:**	7.1
		**No. of unique Peptides:**	2

```
        10         20         30         40         50         60         70         80
MAPWPPKGLV PAMLWGLSLF LNLPGPIWLQ PSPPPQSSPP PQPHPCHTCR GLVDSFNKGL ERTIRDNFGG GNTAWEEENL
        90        100        110        120        130        140        150        160
SKYKDSETRL VEVLEGVCSK SDFECHRLLE LSEELVESWW FHKQQEAPDL FQWLCSDSLK LCCPAGTFGP SCLPCPGGTE
       170        180        190        200        210        220        230        240
RPCGGYGQCE GEGTRGGSGH CDCQAGYGGE ACGQCGLGYF EAERNASHLV CSACFGPCAR CSGPEESNCL QCKKGWALHH
       250        260        270        280        290        300        310        320
LKCVDCAKAC LGCMGAGPGR CKKCSPGYQQ VGSKCLDVDE CETEVCPGEN KQCENTEGGY RCICAEGYKQ MEGICVKEQI
       330        340        350        360        370        380        390        400
PGAFPILTDL TPETTRRWKL GSHPHSTYVK MKMQRDEATF PGLYGKQVAK LGSQSRQSDR GTRLIHSQQA SSQR
```

Cmpd.	No. of Cmpds.	m/z meas.	Δ m/z [ppm]	z	Rt [min]	Score	P	Range	Sequence	Modification
2830	1	913.4702	92.84	2	64.5	10.8	1	243-260	K.CVDCAKACLGCMGAGPGR.C	Carbamidomethyl: 4, 8
2495	1	607.3216	119.88	2	61.8	14.6	0	292-301	K.QCENTEGGYR.C	Carbamidomethyl: 2

Detailed Protein Report

Protein 372: zinc finger protein 670 isoform 2 [Homo sapiens]

**Accession:**	gi|323635444	**Score:**	25.4
**Database:**	refseq_human(refseq_human_20140103.fasta)	**MW [kDa]:**	44.5
**Database Date:**	2015-11-30	**pI:**	9.8
		**Sequence Coverage [%]:**	5.2
		**No. of unique Peptides:**	1

10	20	30	40	50	60	70	80
MDSVSFEDVA	VAFTQEEWAL	LDPSQKNLYR	DVMQEIFRNL	ASVGNKSEDQ	NIQDDFKNPG	RNLSHVVERL	FEIKEGSQYG
**90**	**100**	**110**	**120**	**130**	**140**	**150**	**160**
ETFSQDSNLN	LNKKVSTGVK	PCECSVCGKV	FICHSALHRH	ILSHIGNKLF	ECEECPEKLY	HCKQCGKAFI	SLTSVDRHMV
**170**	**180**	**190**	**200**	**210**	**220**	**230**	**240**
THTSNGPYKG	PVYEKPFDFP	SVFQMPQSTY	TGEKTYKCKH	CDKAFNYSSY	LREHERTHTG	EKPYACKKCG	KSFTFSSSLR
**250**	**260**	**270**	**280**	**290**	**300**	**310**	**320**
QHERSHTGEK	PYECKECGKA	FSRSTYLGIH	ERTHTGEKPY	ECIKCGKAFR	CSRVLRVHER	THSGEKPYEC	KQCGKAFKYS
**330**	**340**	**350**	**360**	**370**	**380**	**390**	
SNLCEHERTH	TGVKPYGCKE	CGKSFTSSSA	LRSHERTHTG	EKPYECKKCG	KAFSCSSSLR	KHERAYMW	

Cmpd.	No. of Cmpds.	m/z meas.	Δ m/z [ppm]	z	Rt [min]	Score	P	Range	Sequence	Modification
1723	2	1108.8589	233.76	1	51.9	12.9	0	148-157	K.AFISLTSVDR.H	

Detailed Protein Report

Protein 373: **PREDICTED: pleckstrin homology domain-containing family G member 3 isoform X5 [Homo sapiens]**

**Accession:**	gi|578825697	**Score:**	25.4
**Database:**	refseq_human(refseq_human_20140103.fasta)	**MW [kDa]:**	148.9
**Database Date:**	2015-11-30	**pI:**	5.3
		**Sequence Coverage [%]:**	2.1
		**No. of unique Peptides:**	2

Quantitation

QD:QU **Median:** 1.18 **CV:** 0.00 % **No. of Peptides:** 1

10	20	30	40	50	60	70	80
MNLPGVPSPG	SNARMPVSTS	LHQDGSQERP	VSLTSTTSSS	GSSCDSRSAM	EEPSSSEAPA	KNGAGSLRSR	HLPNSNNNSS
90	100	110	120	130	140	150	160
SWLNVKGPLS	PFNSRAAAGP	AHHKLSYLGR	VVREIVETER	MYVQDLRSIV	EDYLLKIIDT	PGLLKPEQVS	ALFGNIENIY
170	180	190	200	210	220	230	240
ALNSQLLRDL	DSCNSDPVAV	ASCFVERSQE	FDIYTQYCNN	YPNSVAALTE	CMRDKQQAKF	FRDRQELLQH	SLPLGSYLLK
250	260	270	280	290	300	310	320
PVQRILKYHL	LLQEIAKHFD	EEEDGFEVVE	DAIDTMTCVA	WYINDMKRRH	EHAVRLQEIQ	SLLINWKGPD	LTTYGELVLE
330	340	350	360	370	380	390	400
GTFRVHRVRN	ERTFFLFDKT	LLITKKRGDH	FVYKGNIPCS	SLMLIESTRD	SLCFTVTHYK	HSKQQYSIQA	KTVEEKRNWT
410	420	430	440	450	460	470	480
HHIKRLILEN	HHATIPQKAK	EAILEMDSYY	PNRYRCSPER	LKKAWSSQDE	VSTNVRQGRR	QSEPTKHLLR	QLNEKARAAG
490	500	510	520	530	540	550	560
MKHAGSAGTL	LDFGQPSRTR	GLQPEAEGAT	QEEEEEEEEV	VEEEEEEEEE	EQAFQVSLED	LTGHEGNEKG	AGPEPPGSEE
570	580	590	600	610	620	630	640
EEEEQEESLA	VAEQVADFAS	SLLAALHCWH	YRANALLFSR	GAMGKGRRES	ESSRSSRRPS	GRSPTSTEKR	MSFESISSLP
650	660	670	680	690	700	710	720
EVEPDPEAGS	EQEVFSAVEG	PSAEETPSDT	ESPEVLETQL	DAHQGLLGMD	PPGDMVDFVA	AESTEDLKAL	SSEEEEEMGG
730	740	750	760	770	780	790	800
AAQEPESLLP	PSVLDQASVI	AERFVSSFSR	RSSVAQEDSK	SSGFGSPRLV	SRSSSVLSLE	GSEKGLARHG	SATDSLSCQL
810	820	830	840	850	860	870	880
SPEVDISVGV	ATEDSPSVNG	MEPPSPGCPV	EPDRSSCKKK	ESALSTRDRL	LLDKIKSYYE	NAEHHDAGFS	VRRRESLSYI
890	900	910	920	930	940	950	960
PKGLVRNSIS	RFNSLPRPDP	EPVPPVGSKR	QVGSRPTSWA	LFELPGPSQA	VKGDPPPISD	AEFRPSSEIV	KIWEGMESSG
970	980	990	1000	1010	1020	1030	1040
GSPGKGPGQG	QANGFDLHEP	LFILEEHELG	AITEESATAS	PESSSPTEGR	SPAHLARELK	ELVKELSSST	QGELVAPLHP
1050	1060	1070	1080	1090	1100	1110	1120
RIVQLSHVMD	SHVSERVKNK	VYQLARQYSL	RIKSNKPVMA	RPPLQWEKVA	PERDGKSPTV	PCLQEEAGEP	LGGKGKRKPV
1130	1140	1150	1160	1170	1180	1190	1200
LSLFDYEQLM	AQEHSPPKPS	SAGEMSPQRF	FFNPSAVSQR	TTSPGGRPSA	RSPLSPTETF	SWPDVRELCS	KYASRDEARR
1210	1220	1230	1240	1250	1260	1270	1280
AGGGRPRGPP	VNRSHSVPEN	MVEPPLSGRV	GRCRSLSTKR	GRGGGEAAQS	PGPLPQSKPD	GGETLYVTAD	LTLEDNRRVI
1290	1300	1310	1320	1330	1340	1350	1360
VMEKGPLPSP	TAGLEESSGQ	GPSSPVALLG	QVQDFQQSAE	CQPKEEGSRD	PADPSQQGRV	RNLREKFQAL	NSVG

Cmpd.	No. of Cmpds.	m/z meas.	Δ m/z [ppm]	z	Rt [min]	Score	P	Range	Sequence	Modification	Ratios
2000	1	698.7973	-80.70	2	53.5	15.3	0	1-14	-.MNLPGVPSPGSNAR.M		QD:QU 1.18
2130	1	789.2959	-119.98	2	57.0	10.1	2	609-622	R.ESESSRSSRRPSGR.S		

Detailed Protein Report

Protein 374: serine/threonine-protein kinase 36 isoform 2 [Homo sapiens]

Accession: gi|343183344
Database: refseq_human(refseq_human_20140103.fasta)
Database Date: 2015-11-30

Score: 25.3
MW [kDa]: 141.6
pI: 5.5
Sequence Coverage [%]: 2.8
No. of unique Peptides: 1

10	20	30	40	50	60	70	80
MEKYHVLEMI	GEGSFGRVYK	GRRKYSAQVV	ALKFIPKLGR	SEKELRNLQR	EIEIMRGLRH	PNIVHMLDSF	ETDKEVVVVT
**90**	**100**	**110**	**120**	**130**	**140**	**150**	**160**
DYAEGELFQI	LEDDGKLPED	QVQAIAAQLV	SALYYLHSHR	ILHRDMKPQN	ILLAKGGGIK	LCDFGFARAM	STNTMVLTSI
**170**	**180**	**190**	**200**	**210**	**220**	**230**	**240**
KGTPLYMSPE	LVEERPYDHT	ADLWSVGCIL	YELAVGTPPF	YATSIFQLVS	LILKDPVRWP	STISPCFKNF	LQGLLTKDPR
**250**	**260**	**270**	**280**	**290**	**300**	**310**	**320**
QRLSWPDLLY	HPFIAGHVTI	ITEPAGPDLG	TPFTSRLPPE	LQVLKDEQAH	RLAPKGNQSR	ILTQAYKRMA	EEAMQKKHQN
**330**	**340**	**350**	**360**	**370**	**380**	**390**	**400**
TGPALEQEDK	TSKVAPGTAP	LPRLGATPQE	SSLLAGILAS	ELKSSWAKSG	TGEVPSAPRE	NRTTPDCERA	FPEERPEVLG
**410**	**420**	**430**	**440**	**450**	**460**	**470**	**480**
QRSTDVVDLE	NEEPDSDNEW	QHLLETTEPV	PIQLKAPLTL	LCNPDFCQRI	QSQLHEAGGQ	ILKGILEGAS	HILPAFRVLS
**490**	**500**	**510**	**520**	**530**	**540**	**550**	**560**
SLLSSCSDSV	ALYSFCREAG	LPGLLLSLLR	HSQESNSLQQ	QSWYGTFLQD	LMAVIQAYFA	CTFNLERSQT	SDSLQVFQEA
**570**	**580**	**590**	**600**	**610**	**620**	**630**	**640**
ANLFLDLLGK	LLAQPDDSEQ	TLRRDSLMCF	TVLCEAMDGN	SRAISKAFYS	SLLTTQQVVL	DGLLHGLTVP	QLPVHTPQGA
**650**	**660**	**670**	**680**	**690**	**700**	**710**	**720**
PQVSQPLREQ	SEDIPGAISS	ALAAICTAPV	GLPDCWDAKE	QVCWHLANQL	TEDSSQLRPS	LISGLQHPIL	CLHLLKVLYS
**730**	**740**	**750**	**760**	**770**	**780**	**790**	**800**
CCLVSEGLCR	LLGQEPLALE	SLFMLIQGKV	KVVDWEESTE	VTLYFLSLLV	FRLQNLPCGM	EKLGSDVATL	FTHSHVVSLV
**810**	**820**	**830**	**840**	**850**	**860**	**870**	**880**
SAAACLLGQL	GQQGVTFDLQ	PMEWMAAATH	ALSAPAELLT	EQGKASLIRD	MSSSEMWTVL	WHRFSMVLRL	PEEASAQEGE
**890**	**900**	**910**	**920**	**930**	**940**	**950**	**960**
LSLSSPPSPE	PDWTLISPQG	MAALLSLAMA	TFTQEPQLCL	SCLSQHGSIL	MSILKHLLCP	SFLNQLRQAP	HGSEFLPVVV
**970**	**980**	**990**	**1000**	**1010**	**1020**	**1030**	**1040**
LSVCQLLCFP	FALDMDADLL	IGVLADLRDS	EVAAHLLQVC	CYHLPLMQVE	LPISLLTRLA	LMDPTSLNQF	VNTVSASPRT
**1050**	**1060**	**1070**	**1080**	**1090**	**1100**	**1110**	**1120**
IVSFLSVALL	SDQPLLTSDL	LSLLAHTARV	LSPSHLSFIQ	ELLAGSDESY	RPLRSLLGHP	ENSVRAHTYR	LLGHLLQHSM
**1130**	**1140**	**1150**	**1160**	**1170**	**1180**	**1190**	**1200**
ALRGALQSQS	GLLSLLLLGL	GDKDPVVRCS	ASFAVGNAAY	QAGPLGPALA	AAVPSMTQLL	GDPQAGIRRN	VASALGNLGP
**1210**	**1220**	**1230**	**1240**	**1250**	**1260**	**1270**	**1280**
EGLGEELLQC	EVPQRLLEMA	CGDPQPNVKE	AALIALRSLQ	QEPGIHQVLV	SLGASEKLSL	LSLGNQSLPH	SSPRPASAKH
**1290**	**1300**						
CRKLIHLLRP	AHSM						

Cmpd.	No. of Cmpds.	m/z meas.	Δ m/z [ppm]	z	Rt [min]	Score	P	Range	Sequence	Modification
259	1	898.4195	-41.72	3	33.2	13.6	0	1190-1215	R.NVASALGNLGPEGLGEELLQCEVPQR.L	

Detailed Protein Report

Protein 375: PREDICTED: PR domain zinc finger protein 16 isoform X3 [Homo sapiens]

**Accession:**	gi|530360370	**Score:**	25.3
**Database:**	refseq_human(refseq_human_20140103.fasta)	**MW [kDa]:**	129.7
**Database Date:**	2015-11-30	**pI:**	5.9
		**Sequence Coverage [%]:**	1.9
		**No. of unique Peptides:**	1

10	20	30	40	50	60	70	80
MRSKARARKL	AKSDGDVVNN	MYEPNRDLLA	SHSAEDEAED	SAMSPIPVGP	PSPFPTSEDF	TPKEGSPYEA	PVYIPEDIPI
**90**	**100**	**110**	**120**	**130**	**140**	**150**	**160**
PADFELRESS	IPGAGLGVWA	KRKMEAGERL	GPCVVVPRAA	AKETDFGWEQ	ILTDVEVSPQ	EGCITKQISE	DLGSEKFCVD
**170**	**180**	**190**	**200**	**210**	**220**	**230**	**240**
ANQAGAGSWL	KYIRVACSCD	DQNLTMCQIS	EQIYYKVIKD	IEPGEELLVH	VKEGVYPLGT	VPPGLDEEPT	FRCDECDELF
**250**	**260**	**270**	**280**	**290**	**300**	**310**	**320**
QSKLDLRRHK	KYTCGSVGAA	LYEGLAEELK	PEGLGGGSGQ	AHECKDCERM	FPNKYSLEQH	MVIHTEEREY	KCDQCPKAFN
**330**	**340**	**350**	**360**	**370**	**380**	**390**	**400**
WKSNLIRHQM	SHDSGKRFEC	ENCVKVFTDP	SNLQRHIRSQ	HVGARAHACP	DCGKTFATSS	GLKQHKHIHS	TVKPFICEVC
**410**	**420**	**430**	**440**	**450**	**460**	**470**	**480**
HKSYTQFSNL	CRHKRMHADC	RTQIKCKDCG	QMFSTTSSLN	KHRRFCEGKN	HYTPGGIFAP	GLPLTPSPMM	DKAKPSPSLN
**490**	**500**	**510**	**520**	**530**	**540**	**550**	**560**
HASLGFNEYF	PSRPHPGSLP	FSTAPPTFPA	LTPGFPGIFP	PSLYPRPPLL	PPTSLLKSPL	NHTQDAKLPS	PLGNPALPLV
**570**	**580**	**590**	**600**	**610**	**620**	**630**	**640**
SAVSNSSQGT	TAAAGPEEKF	ESRLEDSCVE	KLKTRSSDMS	DGSDFEDVNT	TTGTDLDTTT	GTGSDLDSDV	DSDPDKDKGK
**650**	**660**	**670**	**680**	**690**	**700**	**710**	**720**
GKSAEGQPKF	GGGLAPPGAP	NSVAEVPVFY	SQHSFFPPPD	EQLLTATGAA	GDSIKAIASI	AEKYFGPGFM	GMQEKKLGSL
**730**	**740**	**750**	**760**	**770**	**780**	**790**	**800**
PYHSAFPFQF	LPNFPHSLYP	FTDRALAHNL	LVKAEPKSPR	DALKVGGPSA	ECPFDLTTKP	KDVKPILPMP	KGPSAPASGE
**810**	**820**	**830**	**840**	**850**	**860**	**870**	**880**
EQPLDLSIGS	RARASQNGGG	REPRKNHVYG	ERKLGAGEGL	PQVCPARMPQ	QPPLHYAKPS	PFFMDPIYSR	VEKRKVTDPV
**890**	**900**	**910**	**920**	**930**	**940**	**950**	**960**
GALKEKYLRP	SPLLFHPQMS	AIETMTEKLE	SFAAMKADSG	SSLQPLPHHP	FNFRSPPPTL	SDPILRKGKE	RYTCRYCGKI
**970**	**980**	**990**	**1000**	**1010**	**1020**	**1030**	**1040**
FPRSANLTRH	LRTHTGEQPY	RCKYCDRSFS	ISSNLQRHVR	NIHNKEKPFK	CHLCNRCFGQ	QTNLDRHLKK	HEHENAPVSQ
**1050**	**1060**	**1070**	**1080**	**1090**	**1100**	**1110**	**1120**
HPGVLTNHLG	TSASSPTSES	DNHALLDEKE	DSYFSEIRNF	IANSEMNQAS	TRTEKRADMQ	IVDGSAQCPG	LASEKQEDVE
**1130**	**1140**	**1150**	**1160**	**1170**	**1180**		
EEDDDDLEED	DEDSLAGKSQ	DDTVSPAPEP	QAAYEDEEDE	EPAASLAVGF	DHTRRHMQ		

Cmpd.	No. of Cmpds.	m/z meas.	Δ m/z [ppm]	z	Rt [min]	Score	P	Range	Sequence	Modification
2972	1	646.8226	-64.07	2	66.4	11.8	0	935-946	R.SPPPTLSDPILR.K	

Detailed Protein Report

Protein 376: brain mitochondrial carrier protein 1 isoform 2 precursor [Homo sapiens]

Accession: gi|532524956
Database: refseq_human(refseq_human_20140103.fasta)
Database Date: 2015-11-30

Score: 25.3
MW [kDa]: 35.9
pI: 10.2
Sequence Coverage [%]: 8.4
No. of unique Peptides: 1

10	20	30	40	50	60	70	80
MGIFPGIILI	FLRVKFATAA	VIHQKSTTVS	HEMSGLNWKP	FVYGGLASIV	AEFGTFPVDL	TKTRLQVQGQ	SIDARFKEIK
**90**	**100**	**110**	**120**	**130**	**140**	**150**	**160**
YRGMFHALFR	ICKEEGVLAL	YSGIAPALLR	QASYGTIKIG	IYQSLKRLFV	ERLEDETLLI	NMICGVVSGV	ISSTIANPTD
**170**	**180**	**190**	**200**	**210**	**220**	**230**	**240**
VLKIRMQAQG	SLFQGSMIGS	FIDIYQQEGT	RGLWRGVVPT	AQRAAIVVGV	ELPVYDITKK	HLILSGMMGD	TILTHFVSSF
**250**	**260**	**270**	**280**	**290**	**300**	**310**	**320**
TCGLAGALAS	NPVDVVRTRM	MNQRAIVGHV	DLYKGTVDGI	LKMWKHEGFF	ALYKGFWPNW	LRLGPWNIIF	FITYEQLKRL
**330**							
QI							

Cmpd.	No. of Cmpds.	m/z meas.	Δ m/z [ppm]	z	Rt [min]	Score	P	Range	Sequence	Modification
1961	1	656.7950	-145.20	2	54.9	11.0	1	14-25	R.VKFATAAVIHQK.S	

Detailed Protein Report

Protein 377: PREDICTED: disks large homolog 5 isoform X3 [Homo sapiens]

**Accession:**	gi|578819987	**Score:**	25.2
**Database:**	refseq_human(refseq_human_20140103.fasta)	**MW [kDa]:**	176.9
**Database Date:**	2015-11-30	**pI:**	6.5
		**Sequence Coverage [%]:**	1.6
		**No. of unique Peptides:**	2

Alias proteins:

Accession	Name	Description
gi|578839810	refseq_human (refseq_human_20140103.fasta)	PREDICTED: disks large homolog 5 isoform X3 [Homo sapiens]

10	20	30	40	50	60	70	80
MEPQRRELLA	QCQQSLAQAM	TEVEAVLGLL	EAAGALSPGE	RRQLDEEAGG	AKAELLLKLL	LAKERDHFQD	LRAALEKTQP
90	100	110	120	130	140	150	160
HLLPILYLNG	VVGPPQPAEG	AGSTYSVLST	MPSDSESSSS	LSSVGTTGKA	PSPPPLLTDQ	QVNEKVENLS	IQLRLMTRER
170	180	190	200	210	220	230	240
NELRKRLAFA	THGTAFDKRP	YHRLNPDYER	LKIQCVRAMS	DLQSLQNQHT	NALKRCEEVA	KETDFYHTLH	SRLLSDQTRL
250	260	270	280	290	300	310	320
KDDVDMLRRE	NGQLLRERNL	LQQSWEDMKR	LHEEDQKEIG	DLRAQQQQVL	KHNGSSEILN	KLYDTAMDKL	EVVKKDYDAL
330	340	350	360	370	380	390	400
RKRYSEKVAI	HNADLSRLEQ	LGEENQRLLK	QTEMLTQQRD	TAIQLQHQCA	LSLRRFEAIH	HELNKATAQN	KDLQWEMELL
410	420	430	440	450	460	470	480
QSELTELRTT	QVKTAKESEK	YREERDAVYS	EYKLIMSERD	QVISELDKLQ	TEVELAESKL	KSSTSEKKAA	NEEMEALRQI
490	500	510	520	530	540	550	560
KDTVTMDAGR	ANKEVEILRK	QCKALCQELK	EALQEADVAK	CRRDWAFQER	DKIVAERDSI	RTLCDNLRRE	RDRAVSELAE
570	580	590	600	610	620	630	640
ALRSLDDTRK	QKNDVSRELK	ELKEQMESQL	EKEARFRQLM	AHSSHDSAID	TDSMEWETEV	VEFERETEDI	DLKALGFDMA
650	660	670	680	690	700	710	720
EGVNEPCFPG	DCGIFVTKVD	KGSIADGRLR	VNDWLLRIND	VDLINKDKKQ	AIKALLNGEG	AINMVVRRRK	SLGGKVVTPL
730	740	750	760	770	780	790	800
HINLSGQKDS	GISLENGVYA	AAVLPGSPAA	KEGSLAVGDR	IVAINGIALD	NKSLNECESL	LRSCQDSLTL	SLLKEQKCVP
810	820	830	840	850	860	870	880
ASGELSPELQ	EWAPYSPGHS	SRHSNPPLYP	SRPSVGTVPR	SLTPSTTVSS	ILRNPIYTVR	SHRVGPCSSP	PAARDAGPQG
890	900	910	920	930	940	950	960
LHPSVQHQGR	LSLDLSHRTC	SDYSEMRATH	GSNSLPSSAR	LGSSSNLQFK	AERIKIPSTP	RYPRSVVGSE	RGSVSHSECS
970	980	990	1000	1010	1020	1030	1040
TPPQSPLNID	TLSSCSQSQT	SASTLPRIAV	NPASLGERRK	DRPYVEEPRH	VKVQKGSEPL	GISIVSGEKG	GIYVSKVTVG
1050	1060	1070	1080	1090	1100	1110	1120
SIAHQAGLEY	GDQLLEFNGI	NLRSATEQQA	RLIIGQQCDT	ITILAQYNPH	VHQLSSHSRS	SSHLDPAGTH	STLQGSGTTT
1130	1140	1150	1160	1170	1180	1190	1200
PEHPSVIDPL	MEQDEGPSTP	PAKQSSSRIA	GDANKKTLEP	RVVFIKKSQL	ELGVHLCGGN	LHGVFVAEVE	DDSPAKGPDG
1210	1220	1230	1240	1250	1260	1270	1280
LVPGDLILEY	GSLDVRNKTV	EEVYVEMLKP	RDGVRLKVQY	RPEEFTKAKG	LPGDSFYIRA	LYDRLADVEQ	ELSFKKDDIL
1290	1300	1310	1320	1330	1340	1350	1360
YVDDTLPQGT	FGSWMAWQLD	ENAQKIQRGQ	IPSKYVMDQE	FSRRLSMSEV	KDDNSATKTL	SAAARRSFFR	RKHKHKRSGS
1370	1380	1390	1400	1410	1420	1430	1440
KDGKDLLALD	AFSSDSIPLF	EDSVSLAYQR	VQKVDCTALR	PVLILGPLLD	VVKEMLVNEA	PGKFCRCPLE	VMKASQQAIE
1450	1460	1470	1480	1490	1500	1510	1520
RGVKDCLFVD	YKRRSGHFDV	TTVASIKEIT	EKNRHCLLDI	APHAIERLHH	MHIYPIVIFI	HYKSAKHIKE	QRDPIYLRDK
1530	1540	1550	1560	1570	1580		
VTQRHSKEQF	EAAQKLEQEY	SRYFTGVIQG	GALSSICTQI	LAMVNQEQNK	VLWIPACPL		

Cmpd.	No. of Cmpds.	m/z meas.	Δ m/z [ppm]	z	Rt [min]	Score	P	Range	Sequence	Modification
2115	1	916.8144	-155.80	2	55.0	10.3	2	257-270	R.ERNLLQQSWEDMKR.L	
1673	1	662.2991	-86.48	2	49.4	14.9	1	1248-	K.AKGLPGDSFYIR.A	

Detailed Protein Report

Cmpd.	No. of Cmpds.	m/z meas.	Δ m/z [ppm]	z	Rt [min]	Score	P	Range	Sequence	Modification
								1259		

Detailed Protein Report

Protein 378: **PREDICTED: extracellular matrix protein FRAS1 isoform X4 [Homo sapiens]**

**Accession:**	gi|578809385	**Score:**	25.1
**Database:**	refseq_human(refseq_human_20140103.fasta)	**MW [kDa]:**	430.3
**Database Date:**	2015-11-30	**pI:**	5.3
**Modification(s):**	Carbamidomethyl	**Sequence Coverage [%]:**	0.6
		**No. of unique Peptides:**	1

Detailed Protein Report

10	20	30	40	50	60	70	80
MGVLKVWLGL	ALALAEFAVL	PHHSEGACVY	QDSLLADATI	WKPDSCQSCR	CHGDIVICKP	AVCRNPQCAF	EKGEVLQIAA
90	100	110	120	130	140	150	160
NQCCPECVLR	TPGSCHHEKK	IHEHGTEWAS	SPCSVCSCNH	GEVRCTPQPC	PPLSCGHQEL	AFIPEGSCCP	VCVGLGKPCS
170	180	190	200	210	220	230	240
YEGHVFQDGE	DWRLSRCAKC	LCRNGVAQCF	TAQCQPLFCN	QDETVVRVPG	KCCPQCSARS	CSAAGQVYEH	GEQWSENACT
250	260	270	280	290	300	310	320
TCICDRGEVR	CHKQACLPLR	CGKGQSRARR	HGQCCEECVS	PAGSCSYDGV	VRYQDEMWKG	SACEFCMCDH	GQVTCQTGEC
330	340	350	360	370	380	390	400
AKVECARDEE	LIHLDGKCCP	ECISRNGYCV	YEETGEFMSS	NASEVKRIPE	GEKWEDGPCK	VCECRGAQVT	CYEPSCPPCP
410	420	430	440	450	460	470	480
VGTLALEVKG	QCCPDCTSVH	CHPDCLTCSQ	SPDHCDLCQD	PTKLLQNGWC	VHSCGLGFYQ	AGSLCLACQP	QCSTCTSGLE
490	500	510	520	530	540	550	560
CSSCQPPLLM	RHGQCVPTCG	DGFYQDRHSC	AVCHESCAGC	WGPTEKHCLA	CRDPLHVLRD	GGCESSCGKG	FYNRQGTCSA
570	580	590	600	610	620	630	640
CDQSCDSCGP	SSPRCLTCTE	KTVLHDGKCM	SECPGGYYAD	ATGRCKVCHN	SCASCSGPTP	SHCTACSPPK	ALRQGHCLPR
650	660	670	680	690	700	710	720
CGEGFYSDHG	VCKACHSSCL	ACMGPAPSHC	TGCKKPEEGL	QVEQLSDVGI	PSGECLAQCR	AHFYLESTGI	CEACHQSCFR
730	740	750	760	770	780	790	800
CAGKSPHNCT	DCGPSHVLLD	GQCLSQCPDG	YFHQEGSCTE	CHPTCRQCHG	PLESDCISCY	PHISLTNGNC	RTSCREEQFL
810	820	830	840	850	860	870	880
NLVGYCADCH	HLCQHCAADL	HNTGSICLRC	QNAHYLLLGD	HCVPDCPSGY	YAERGACKKC	HSSCRTCQGR	GPFSCSSCDT
890	900	910	920	930	940	950	960
NLVLSHTGTC	STTCFPGHYL	DDNHVCQPCN	THCGSCDSQA	SCTSCRDPNK	VLLFGECQYE	SCAPQYYLDF	STNTCKECDW
970	980	990	1000	1010	1020	1030	1040
SCSACSGPLK	TDCLQCMDGY	VLQDGACVEQ	CLSSFYQDSG	LCKNCDSYCL	QCQGPHECTR	CKGPFLLLEA	QCVQECGKGY
1050	1060	1070	1080	1090	1100	1110	1120
FADHAKHKCT	ACPQGCLQCS	HRDRCHLCDH	GFFLKSGLCV	YNCVPGFSVH	TSNETCSGKI	HTPSLHVNGS	LILPIGSIKP
1130	1140	1150	1160	1170	1180	1190	1200
LDFSLLNVQD	QEGRVEDLLF	HVVSTPTNGQ	LVLSRNGKEV	QLDKAGRFSW	KDVNEKKVRF	VHSKEKLRKG	YLFLKISDQQ
1210	1220	1230	1240	1250	1260	1270	1280
FFSEPQLINI	QAFSTQAPYV	LRNEVLHISR	GERATITTQM	LDIRDDDNPQ	DVVIEIIDPP	LHGQLLQTLQ	SPATPIYQFQ
1290	1300	1310	1320	1330	1340	1350	1360
LDELSRGLLH	YAHDGSDSTS	DVAVLQANDG	HSFHNILFQV	KTVPQNDRGL	QLVANSMVWV	PEGGMLQITN	RILQAEAPGA
1370	1380	1390	1400	1410	1420	1430	1440
SAEEIIYKIT	QDYPQFGEVV	LLVNMPADSP	ADEGQHLPDG	RTATPTSTFT	QQDINEGIVW	YRHSGAPAQS	DSFRFEVSSA
1450	1460	1470	1480	1490	1500	1510	1520
SNAQTRLESH	MFNIAILPQT	PEAPKVSLEA	SLHMTAREDG	LTVIQPHSLS	FINSEKPSGK	IVYNITLPLH	PNQGIIEHRD
1530	1540	1550	1560	1570	1580	1590	1600
HPHSPIRYFT	QEDINQGKVM	YRPPPAAPHL	QELMAFSFAG	LPESVKFHFT	VSDGEHTSPE	MVLTIHLLPS	DQQLPVFQVT
1610	1620	1630	1640	1650	1660	1670	1680
APRLAVSPGG	STSVGLQVVV	RDAETAPKEL	FFELRRPPQH	GVLLKHTAEF	RRPMATGDTF	TYEDVEKNAL	QYIHDGSSTR
1690	1700	1710	1720	1730	1740	1750	1760
EDSMEISVTD	GLTVTMLEVR	VEVSLSEDRG	PRLAAGSSLS	ITVASKSTAI	ITRSHLAYVD	DSSPDPEIWI	QLNYLPSYGT
1770	1780	1790	1800	1810	1820	1830	1840
LLRISGSEVE	ELSEVSNFTM	EDINNKKIRY	SAVFETDGHL	VTDSFYFSVS	DMDHNHLDNQ	IFTIMITPAE	NPPPVIAFAD
1850	1860	1870	1880	1890	1900	1910	1920
LITVDEGGRA	PLSFHHFFAT	DDDDNLQRDA	IIKLSALPKY	GCIENTGTGH	VLWRQTASEP	LENGRVLVQG	STFTYQDILA
1930	1940	1950	1960	1970	1980	1990	2000
GLVGYVPSVP	GMVVDEFQFS	LTDGLHVDTG	RMKIYTELPA	SDTPHLAINQ	GLQLSAGSVA	RITEQHLKVT	DIDSDDHQVM
2010	2020	2030	2040	2050	2060	2070	2080
YIMKEDPGAG	RLQMMKHGNL	EQISIKGPIR	SFTQADISQG	HVEYSHGTGE	PGGSFAFKFD	VVDGEGNRLI	DKSFSISILE
2090	2100	2110	2120	2130	2140	2150	2160
DKSPPVITTN	KGLVLDENSV	KKITTLQLSA	TDQDSGPTEL	IYRITRQPQL	GHLEHAASPG	IQISSFTQAD	LTSRNVQYVH
2170	2180	2190	2200	2210	2220	2230	2240
SSEAEKHSDA	FSFTLSDGVS	EVTQTFHITL	HPVDDSLPVV	QNLGMRVQEG	MRKTITEFEL	KAVDADTEAE	SVTFTIVQPP
2250	2260	2270	2280	2290	2300	2310	2320
RHGTIERTSN	GQHFHLTSTF	TMKDIYQNRV	SYSHDGSNSL	KDRFTFTVSD	GTNPFFIIEE	GGKEIMTAAP	QPFRVDILPV
2330	2340	2350	2360	2370	2380	2390	2400

Detailed Protein Report

Cmpd.	No. of Cmpds.	m/z meas.	Δ m/z [ppm]	z	Rt [min]	Score	P	Range	Sequence	Modification
2707	1	710.3638	64.25	2	62.8	10.1	1	208-219	R.VPGKCCPQCSAR.S	Carbamidomethyl: 5, 6, 9

Detailed Protein Report

Protein 379: **putative polypeptide N-acetylgalactosaminyltransferase-like protein 3 [Homo sapiens]**

**Accession:**	gi|22538495	**Score:**	25.1
**Database:**	refseq_human(refseq_human_20140103.fasta)	**MW [kDa]:**	67.7
**Database Date:**	2015-11-30	**pI:**	9.8
**Modification(s):**	Carbamidomethyl	**Sequence Coverage [%]:**	5.9
		**No. of unique Peptides:**	2

10	20	30	40	50	60	70	80
MASLRRVKVL	LVLNLIAVAG	FVLFLAKCRP	IAVRSGDAFH	EIRPRAEVAN	LSAHSASPIQ	DAVLKRLSLL	EDIVYRQLNG
**90**	**100**	**110**	**120**	**130**	**140**	**150**	**160**
LSKSLGLIEG	YGGRGKGGLP	ATLSPAEEEK	AKGPHEKYGY	NSYLSEKISL	DRSIPDYRPT	KCKELKYSKD	LPQISIIFIF
**170**	**180**	**190**	**200**	**210**	**220**	**230**	**240**
VNEALSVILR	SVHSAVNHTP	THLLKEIILV	DDNSDEEELK	VPLEEYVHKR	YPGLVKVVRN	QKREGLIRAR	IEGWKVATGQ
**250**	**260**	**270**	**280**	**290**	**300**	**310**	**320**
VTGFFDAHVE	FTAGWAEPVL	SRIQENRKRV	ILPSIDNIKQ	DNFEVQRYEN	SAHGYSWELW	CMYISPPKDW	WDAGDPSLPI
**330**	**340**	**350**	**360**	**370**	**380**	**390**	**400**
RTPAMIGCSF	VVNRKFFGEI	GLLDPGMDVY	GGENIELGIK	VWLCGGSMEV	LPCSRVAHIE	RKKKPYNSNI	GFYTKRNALR
**410**	**420**	**430**	**440**	**450**	**460**	**470**	**480**
VAEVWMDDYK	SHVYIAWNLP	LENPGIDIGD	VSERRALRKS	LKCKNFQWYL	DHVYPEMRRY	NNTVAYGELR	NNKAKDVCLD
**490**	**500**	**510**	**520**	**530**	**540**	**550**	**560**
QGPLENHTAI	LYPCHGWGPQ	LARYTKEGFL	HLGALGTTTL	LPDTRCLVDN	SKSRLPQLLD	CDKVKSSLYK	RWNFIQNGAI
**570**	**580**	**590**	**600**				
MNKGTGRCLE	VENRGLAGID	LILRSCTGQR	WTIKNSIK				

Cmpd.	No. of Cmpds.	m/z meas.	Δ m/z [ppm]	z	Rt [min]	Score	P	Range	Sequence	Modification
33	1	1069.0298	-31.46	2	30.2	13.7	1	28-45	K.CRPIAVRSGDAFHEIRPR.A	Carbamidomethyl: 1
2906	1	981.9571	-51.41	2	65.5	11.4	2	551-567	K.RWNFIQNGAIMNKGTGR.C	

Detailed Protein Report

Protein 380: **rRNA 2'-O-methyltransferase fibrillarin [Homo sapiens]**

**Accession:**	gi|12056465	**Score:**	25.1
**Database:**	refseq_human(refseq_human_20140103.fasta)	**MW [kDa]:**	33.8
**Database Date:**	2015-11-30	**pI:**	10.7
		**Sequence Coverage [%]:**	10.3
		**No. of unique Peptides:**	1

10	20	30	40	50	60	70	80
MKPGFSPRGG	GFGGRGGFGD	RGGRGGRGGF	GGGRGRGGGF	RGRGRGGGGG	GGGGGGGGRG	GGGFHSGGNR	GRGRGGKRGN
**90**	**100**	**110**	**120**	**130**	**140**	**150**	**160**
QSGKNVMVEP	HRHEGVFICR	GKEDALVTKN	LVPGESVYGE	KRVSISEGDD	KIEYRAWNPF	RSKLAAAILG	GVDQIHIKPG
**170**	**180**	**190**	**200**	**210**	**220**	**230**	**240**
AKVLYLGAAS	GTTVSHVSDI	VGPDGLVYAV	EFSHRSGRDL	INLAKKRTNI	IPVIEDARHP	HKYRMLIAMV	DVIFADVAQP
**250**	**260**	**270**	**280**	**290**	**300**	**310**	**320**
DQTRIVALNA	HTFLRNGGHF	VISIKANCID	STASAEAVFA	SEVKKMQQEN	MKPQEQLTLE	PYERDHAVVV	GVYRPPPKVK
**330**							
N							

Cmpd.	No. of Cmpds.	m/z meas.	Δ m/z [ppm]	z	Rt [min]	Score	P	Range	Sequence	Modification
2667	1	1057.0851	99.43	2	62.2	13.7	2	46-72	R.GGGGGGGGGGGGGRGGGGFHSGGNRGR.G	

Detailed Protein Report

Protein 381: pleckstrin homology domain-containing family A member 4 isoform 2 [Homo sapiens]

Accession: gi|238859653
Database: refseq_human(refseq_human_20140103.fasta)
Database Date: 2015-11-30

Score: 25.1
MW [kDa]: 64.2
pI: 10.7
Sequence Coverage [%]: 4.5
No. of unique Peptides: 2

Quantitation

QD:QU **Median:** 0.40 **CV:** 37.08 % **No. of Peptides:** 2

10	20	30	40	50	60	70	80
MEGSRPRSSL	SLASSASTIS	SLSSLSPKKP	TRAVNKIHAF	GKRGNALRRD	PNLPVHIRGW	LHKQDSSGLR	LWKRRWFVLS
**90**	**100**	**110**	**120**	**130**	**140**	**150**	**160**
GHCLFYYKDS	REESVLGSVL	LPSYNIRPDG	PGAPRGRRFT	FTAEHPGMRT	YVLAADTLED	LRGWLRALGR	ASRAEGDDYG
**170**	**180**	**190**	**200**	**210**	**220**	**230**	**240**
QPRSPARPQP	GEGPGGPGGP	PEVSRGEEGR	ISESPEVTRL	SRGRGRPRLL	TPSPTTDLHS	GLQMRRARSP	DLFTPLSRPP
**250**	**260**	**270**	**280**	**290**	**300**	**310**	**320**
SPLSLPRPRS	APARRPPAPS	GDTAPPARPH	TPLSRIDVRP	PLDWGPQRQT	LSRPPTPRRG	PPSEAGGGKP	PRSPQHWSQE
**330**	**340**	**350**	**360**	**370**	**380**	**390**	**400**
PRTQPGPPLE	STFHQSLETD	TLLTKLCGQD	RLLRRLQEEI	DQKQEEKEQL	EAALELTRQQ	LGQATREAGA	PGRAWGRQRL
**410**	**420**	**430**	**440**	**450**	**460**	**470**	**480**
LQDRLVSVRA	TLCHLTQERE	RVWDTYSGLE	QELGTLRETL	EYLLHLGSPQ	DRVSAQQQLW	MVEDTLAGLG	GPQKPPPHTE
**490**	**500**	**510**	**520**	**530**	**540**	**550**	**560**
PDSPSPVLQG	EESSERESLP	ESLELSSPRS	PETDWGRPPG	GDKDLASPHL	GLGSPRVSRA	SSPEGRHLPS	PQLGTKSKEH
**570**	**580**	**590**					
HPLLADFRRS	PGAGSQPLPS	PGY					

Cmpd.	No. of Cmpds.	m/z meas.	Δ m/z [ppm]	z	Rt [min]	Score	P	Range	Sequence	Modification	Ratios
526	1	711.1747	-209.60	2	35.2	11.2	1	151-163	R.ASRAEGDDYGQPR.S		QD:QU 0.28
489	2	603.8256	17.71	2	36.1	13.9	0	300-312	R.GPPSEAGGGKPPR.S		QD:QU 0.58

Detailed Protein Report

Protein 382: growth/differentiation factor 2 precursor [Homo sapiens]

**Accession:**	gi|7705308	**Score:**	25.1
**Database:**	refseq_human(refseq_human_20140103.fasta)	**MW [kDa]:**	47.3
**Database Date:**	2015-11-30	**pI:**	6.0
		**Sequence Coverage [%]:**	5.6
		**No. of unique Peptides:**	1

10	20	30	40	50	60	70	80
MCPGALWVAL	PLLSLLAGSL	QGKPLQSWGR	GSAGGNAHSP	LGVPGGGLPE	HTFNLKMFLE	NVKVDFLRSL	NLSGVPSQDK
**90**	**100**	**110**	**120**	**130**	**140**	**150**	**160**
TRVEPPQYMI	DLYNRYTSDK	STTPASNIVR	SFSMEDAISI	TATEDFPFQK	HILLFNISIP	RHEQITRAEL	RLYVSCQNHV
**170**	**180**	**190**	**200**	**210**	**220**	**230**	**240**
DPSHDLKGSV	VIYDVLDGTD	AWDSATETKT	FLVSQDIQDE	GWETLEVSSA	VKRWVRSDST	KSKNKLEVTV	ESHRKGCDTL
**250**	**260**	**270**	**280**	**290**	**300**	**310**	**320**
DISVPPGSRN	LPFFVVFSND	HSSGTKETRL	ELREMISHEQ	ESVLKKLSKD	GSTEAGESSH	EEDTDGHVAA	GSTLARRKRS
**330**	**340**	**350**	**360**	**370**	**380**	**390**	**400**
AGAGSHCQKT	SLRVNFEDIG	WDSWIIAPKE	YEAYECKGGC	FFPLADDVTP	TKHAIVQTLV	HLKFPTKVGK	ACCVPTKLSP
**410**	**420**	**430**					
ISVLYKDDMG	VPTLKYHYEG	MSVAECGCR					

Cmpd.	No. of Cmpds.	m/z meas.	Δ m/z [ppm]	z	Rt [min]	Score	P	Range	Sequence	Modification
1810	1	599.2960	-61.59	2	53.0	13.6	1	226-235	K.LEVTVESHRK.G	

Detailed Protein Report

Protein 383: sterile alpha motif domain-containing protein 9 [Homo sapiens]

**Accession:**	gi|38201706	**Score:**	25.1
**Database:**	refseq_human(refseq_human_20140103.fasta)	**MW [kDa]:**	184.2
**Database Date:**	2015-11-30	**pI:**	8.7
**Modification(s):**	Carbamidomethyl	**Sequence Coverage [%]:**	1.8
		**No. of unique Peptides:**	2

Alias proteins:

Accession	Name	Description
gi|300863105	refseq_human (refseq_human_20140103.fasta)	sterile alpha motif domain-containing protein 9 [Homo sapiens]

10	20	30	40	50	60	70	80
MAKQLNLPEN	TDDWTKEDVN	QWLESHKIDQ	KHREILTEQD	VNGAVLKWLK	KEHLVDMGIT	HGPAIQIEEL	FKELRKTAIE
90	100	110	120	130	140	150	160
DSIQTSKMGK	PSKNAPKDQT	VSQKERRETS	KQKQKGKENP	DMANPSAMST	TAKGSKSLKV	ELIEDKIDYT	KERQPSIDLT
170	180	190	200	210	220	230	240
CVSYPFDEFS	NPYRYKLDFS	LQPETGPGNL	IDPIHEFKAF	TNTATATEED	VKMKFSNEVF	RFASACMNSR	TNGTIHFGVK
250	260	270	280	290	300	310	320
DKPHGKIVGI	KVTNDTKEAL	INHFNLMINK	YFEDHQVQQA	KKCIREPRFV	EVLLPNSTLS	DRFVIEVDII	PQFSECQYDY
330	340	350	360	370	380	390	400
FQIKMQNYNN	KIWEQSKKFS	LFVRDGTSSK	DITKNKVDFR	AFKADFKTLA	ESRKAAEEKF	RAKTNKKERE	GPKLVKLLTG
410	420	430	440	450	460	470	480
NQDLLDNSYY	EQYILVTNKC	HPDQTKHLDF	LKEIKWFAVL	EFDPESNING	VVKAYKESRV	ANLHFPSVYV	EQKTTPNETI
490	500	510	520	530	540	550	560
STLNLYHQPS	WIFCNGRLDL	DSEKYKPFDP	SSWQRERASD	VRKLISFLTH	EDIMPRGKFL	VVFLLLSSVD	DPRDPLIETF
570	580	590	600	610	620	630	640
CAFYQDLKGM	ENILCICVHP	HIFQGWKDLL	EARLIKHQDE	ISSQCISALS	LEEINGTILK	LKSVTQSSKR	LLPSIGLSTV
650	660	670	680	690	700	710	720
LLKKEEDIMT	ALEIICENEC	EGTLLEKDKN	KFLEFKASKE	EDFYRGGKVS	WWNFYFSSES	YSSPFVKRDK	YERLEAMIQN
730	740	750	760	770	780	790	800
CADSSKPTST	KIIHLYHHPG	CGGTTLAMHI	LWELRKKFRC	AVLKNKTVDF	SEIGEQVTSL	ITYGAMNRQE	YVPVLLLVDD
810	820	830	840	850	860	870	880
FEEQDNVYLL	QYSIQTAIAK	KYIRYEKPLV	IILNCMRSQN	PEKSARIPDS	IAVIQQLSPK	EQRAFELKLK	EIKEQHKNFE
890	900	910	920	930	940	950	960
DFYSFMIMKT	NFNKEYIENV	VRNILKGQNI	FTKEAKLFSF	LALLNSYVPD	TTISLSQCEK	FLGIGNKKAF	WGTEKFEDKM
970	980	990	1000	1010	1020	1030	1040
GTYSTILIKT	EVIECGNYCG	VRIIHSLIAE	FSLEELKKSY	HLNKSQIMLD	MLTENLFFDT	GMGKSKFLQD	MHTLLLTRHR
1050	1060	1070	1080	1090	1100	1110	1120
DEHEGETGNW	FSPFIEALHK	DEGNEAVEAV	LLESIHRFNP	NAFICQALAR	HFYIKKKDFG	NALNWAKQAK	IIEPDNSYIS
1130	1140	1150	1160	1170	1180	1190	1200
DTLGQVYKSK	IRWWIEENGG	NGNISVDDLI	ALLDLAEHAS	SAFKESQQQS	EDREYEVKER	LYPKSKRRYD	TYNIAGYQGE
1210	1220	1230	1240	1250	1260	1270	1280
IEVGLYTIQI	LQLIPFFDNK	NELSKRYMVN	FVSGSSDIPG	DPNNEYKLAL	KNYIPYLTKL	KFSLKKSFDF	FDEYFVLLKP
1290	1300	1310	1320	1330	1340	1350	1360
RNNIKQNEEA	KTRRKVAGYF	KKYVDIFCLL	EESQNNTGLG	SKFSEPLQVE	RCRRNLVALK	ADKFSGLLEY	LIKSQEDAIS
1370	1380	1390	1400	1410	1420	1430	1440
TMKCIVNEYT	FLLEQCTVKI	QSKEKLNFIL	ANIILSCIQP	TSRLVKPVEK	LKDQLREVLQ	PIGLTYQFSE	PYFLASLLFW
1450	1460	1470	1480	1490	1500	1510	1520
PENQQLDQHS	EQMKEYAQAL	KNSFKGQYKH	MHRTKQPIAY	FFLGKGKRLE	RLVHKGKIDQ	CFKKTPDINS	LWQSGDVWKE
1530	1540	1550	1560	1570	1580	1590	
EKVQELLLRL	QGRAENNCLY	IEYGINEKIT	IPITPAFLGQ	LRSGRSIEKV	SFYLGFSIGG	PLAYDIEIV	

Cmpd.	No. of Cmpds.	m/z meas.	Δ m/z [ppm]	z	Rt [min]	Score	P	Range	Sequence	Modification
1785	1	660.9025	-122.86	3	50.8	11.7	0	714-731	R.LEAMIQNCADSSKPTSTK.I	Carbamidomethyl: 8
382	1	591.8148	-46.01	2	34.8	13.4	0	1344-	K.FSGLLEYLIK.S	

Detailed Protein Report

Cmpd.	No. of Cmpds.	m/z meas.	Δ m/z [ppm]	z	Rt [min]	Score	P	Range	Sequence	Modification
								1353		

Detailed Protein Report

Protein 384: **PREDICTED: probable E3 ubiquitin-protein ligase HERC1 isoform X6 [Homo sapiens]**

**Accession:**	gi|530406610	**Score:**	25.0
**Database:**	refseq_human(refseq_human_20140103.fasta)	**MW [kDa]:**	524.9
**Database Date:**	2015-11-30	**pI:**	5.7
		**Sequence Coverage [%]:**	1.0
		**No. of unique Peptides:**	1

Detailed Protein Report

10	20	30	40	50	60	70	80
MATMIPPVKL	KWLEHLNSSW	ITEDSESIAT	REGVAVLYSK	LVSNKEVVPL	PQQVLCLKGP	QLPDFERESL	SSDEQDHYLD
90	100	110	120	130	140	150	160
ALLSSQLALA	KMVCSDSPFA	GALRKRLLVL	QRVFYALSNK	YHDKGKVKQQ	QHSPESSSGS	ADVHSVSERP	RSSTDALIEM
170	180	190	200	210	220	230	240
GVRTGLSLLF	ALLRQSWMMP	VSGPGLSLCN	DVIHTAIEVV	SSLPPLSLAN	ESKIPPMGLD	CLSQVTTFLK	GVTIPNSGAD
250	260	270	280	290	300	310	320
TLGRRLASEL	LLGLAAQRGS	LRYLLEWIEM	ALGASAVVHT	MEKGKLLSSQ	EGMISFDCFM	TILMQMRRSL	GSSADRSQWR
330	340	350	360	370	380	390	400
EPTRTSDGLC	SLYEAALCLF	EEIEAGQYCT	FVISTDGSVR	ACGKGSYGRL	GLGDSNNQST	LKKLTFEPHR	SIKKVSSSKG
410	420	430	440	450	460	470	480
SDGHTLAFTT	EGEVFSWGDG	DYGKLGHGNS	STQKYPKLIQ	GPLQGKVVVC	VSAGYRHSAA	VTEDGELYTW	GEGDFGRLGH
490	500	510	520	530	540	550	560
GDSNSRNIPT	LVKDISNVGE	VSCGSSHTIA	LSKDGRTVWS	FGGGDNGKLG	HGDTNRVYKP	KVIEALQGMF	IRKVCAGSQS
570	580	590	600	610	620	630	640
SLALTSTGQV	YAWGCGACLG	CGSSEATALR	PKLIEELAAT	RIVDVSIGDS	HCLALSHDNE	VYAWGNNSMG	QCGQGNSTGP
650	660	670	680	690	700	710	720
ITKPKKVSGL	DGIAIQQISA	GTSHSLAWTA	LPRDRQVVAW	HRPYCVDLEE	STFSHLRSFL	ERYCDKINSE	IPPLPFPSSR
730	740	750	760	770	780	790	800
EHHSFLKLCL	KLLSNHLALA	LAGGVATSIL	GRQAGPLRNL	LFRLMDSTVP	DEIQEVVIET	LSVGATMLLP	PLRERMELLH
810	820	830	840	850	860	870	880
SLLPQGPDRW	ESLSKGQRMQ	LDIILTSLQD	HTHVASLLGY	SSPSDAADLS	SVCTGYGNLS	DQPYGTQSCH	PDTHLAEILM
890	900	910	920	930	940	950	960
KTLLRNLGFY	TDQAFGELEK	NSDKFLLGTS	SSENSQPAHL	HELLCSLQKQ	LLAFCHINNI	SENSSSVALL	HKHLQLLLPH
970	980	990	1000	1010	1020	1030	1040
ATDIYSRSAN	LLKESPWNGS	VGEKLRDVIY	VSAAGSMLCQ	IVNSLLLLPV	SVARPLLSYL	LDLLPPLDCL	NRLLPAADLL
1050	1060	1070	1080	1090	1100	1110	1120
EDQELQWPLH	GGPELIDPAG	LPLPQPAQSW	VWLVDLERTI	ALLIGRCLGG	MLQGSPVSPE	EQDTAYWMKT	PLFSDGVEMD
1130	1140	1150	1160	1170	1180	1190	1200
TPQLDKCMSC	LLEVALSGNE	EQKPFDYKLR	PEIAVYVDLA	LGCSKEPARS	LWISMQDYAV	SKDWDSATLS	NESLLDTVSR
1210	1220	1230	1240	1250	1260	1270	1280
FVLAALLKHT	NLLSQACGES	RYQPGKHLSE	VYRCVYKVRS	RLLACKNLEL	IQTRSSSRDR	WISENQDSAD	VDPQEHSFTR
1290	1300	1310	1320	1330	1340	1350	1360
TIDEEAEMEE	QAERDREEGH	PEPEDEEEER	EHEVMTAGKI	FQCFLSAREV	ARSRDRDRMN	SGAGSGARAD	DPPPQSQQER
1370	1380	1390	1400	1410	1420	1430	1440
RVSTDLPEGQ	DVYTAACNSV	IHRCALLILG	VSPVIDELQK	RREEGQLQQP	STSASEGGGL	MTRSESLTAE	SRLVHTSPNY
1450	1460	1470	1480	1490	1500	1510	1520
RLIKSRSESD	LSQPESDEEG	YALSGRRNVD	LDLAASHRKR	GPMHSQLESL	SDSWARLKHS	RDWLCNSSYS	FESDFDLTKS
1530	1540	1550	1560	1570	1580	1590	1600
LGVHTLIENV	VSFVSGDVGN	APGFKEPEES	MSTSPQASII	AMEQQQLRAE	LRLEALHQIL	VLLSGMEEKG	SISLAGSRLS
1610	1620	1630	1640	1650	1660	1670	1680
SGFQSSTLLT	SVRLQFLAGC	FGLGTVGHTG	GKGESGRLHH	YQDGIRAAKR	NIQIEIQVAV	HKIYQQLSAT	LERALQANKH
1690	1700	1710	1720	1730	1740	1750	1760
HIEAQQRLLL	VTVFALSVHY	QPVDVSLAIS	TGLLNVLSQL	CGTDTMLGQP	LQLLPKTGVS	QLSTALKVAS	TRLLQILAIT
1770	1780	1790	1800	1810	1820	1830	1840
TGTYADKLSP	KVVQSLLDLL	CSQLKNLLSQ	TGVLHMASFG	EGEQEDGEEE	EKKVDSSGET	EKKDFRAALR	KQHAAELHLG
1850	1860	1870	1880	1890	1900	1910	1920
DFLVFLRRVV	SSKAIQSKMA	SPKWTEVLLN	IASQKCSSGI	PLVGNLRTRL	LALHVLEAVL	PACESGVEDD	QMAQIVERLF
1930	1940	1950	1960	1970	1980	1990	2000
SLLSDCMWET	PIAQAKHAIQ	IKEKEQEIKL	QKQGELEEED	ENLPIQEVSF	DPEKAQCCLV	ENGQILTHGS	GGKGYGLAST
2010	2020	2030	2040	2050	2060	2070	2080
GVTSGCYQWK	FYIVKENRGN	EGTCVGVSRW	PVHDFNHRTT	SDMWLYRAYS	GNLYHNGEQT	LTLSSFTQGD	FITCVLDMEA
2090	2100	2110	2120	2130	2140	2150	2160
RTISFGKNGE	EPKLAFEDVD	AAELYPCVMF	YSSNPGEKVK	ICDMQMRGTP	RDLLPGDPIC	SPVAAVLAEA	TIQLIRILHR
2170	2180	2190	2200	2210	2220	2230	2240

Detailed Protein Report

Cmpd.	No. of Cmpds.	m/z meas.	Δ m/z [ppm]	z	Rt [min]	Score	P	Range	Sequence	Modification
2832	1	923.8157	-4.48	3	64.6	12.0	1	487-513	R.NIPTLVKDISNVGEVSCGSSHTIALSK.D	

Detailed Protein Report

Protein 385: protein LAP2 isoform 7 [Homo sapiens]

Accession: gi|55770895
Database: refseq_human(refseq_human_20140103.fasta)
Database Date: 2015-11-30

Score: 25.0
MW [kDa]: 146.0
pI: 5.0
Sequence Coverage [%]: 1.3
No. of unique Peptides: 1

10	20	30	40	50	60	70	80
MTTKRSLFVR	LVPCRCLRGE	EETVTTLDYS	HCSLEQVPKE	IFTFEKTLEE	LYLDANQIEE	LPKQLFNCQS	LHKLSLPDND
**90**	**100**	**110**	**120**	**130**	**140**	**150**	**160**
LTTLPASIAN	LINLRELDVS	KNGIQEFPEN	IKNCKVLTIV	EASVNPISKL	PDGFSQLLNL	TQLYLNDAFL	EFLPANFGRL
**170**	**180**	**190**	**200**	**210**	**220**	**230**	**240**
TKLQILELRE	NQLKMLPKTM	NRLTQLERLD	LGSNEFTEVP	EVLEQLSGLK	EFWMDANRLT	FIPGFIGSLK	QLTYLDVSKN
**250**	**260**	**270**	**280**	**290**	**300**	**310**	**320**
NIEMVEEGIS	TCENLQDLLL	SSNSLQQLPE	TIGSLKNITT	LKIDENQLMY	LPDSIGGLIS	VEELDCSFNE	VEALPSSIGQ
**330**	**340**	**350**	**360**	**370**	**380**	**390**	**400**
LTNLRTFAAD	HNYLQQLPPE	IGSWKNITVL	FLHSNKLETL	PEEMGDMQKL	KVINLSDNRL	KNLPFSFTKL	QQLTAMWLSD
**410**	**420**	**430**	**440**	**450**	**460**	**470**	**480**
NQSKPLIPLQ	KETDSETQKM	VLTNYMFPQQ	PRTEDVMFIS	DNESFNPSLW	EEQRKQRAQV	AFECDEDKDE	REAPPREGNL
**490**	**500**	**510**	**520**	**530**	**540**	**550**	**560**
KRYPTPYPDE	LKNMVKTVQT	IVHRLKDEET	NEDSGRDLKP	HEDQQDINKD	VGVKTSESTT	TVKSKVDERE	KYMIGNSVQK
**570**	**580**	**590**	**600**	**610**	**620**	**630**	**640**
ISEPEAEISP	GSLPVTANMK	ASENLKHIVN	HDDVFEESEE	LSSDEEMKMA	EMRPPLIETS	INQPKVVALS	NNKKDDTKET
**650**	**660**	**670**	**680**	**690**	**700**	**710**	**720**
DSLSDEVTHN	SNQNNSNCSS	PSRMSDSVSL	NTDSSQDTSL	CSPVKQTHID	INSKIRQEDE	NFNSLLQNGD	ILNSSTEEKF
**730**	**740**	**750**	**760**	**770**	**780**	**790**	**800**
KAHDKKDFNL	PEYDLNVEER	LVLIEKSVDS	TATADDTHKL	DHINMNLNKL	ITNDTFQPEI	MERSKTQDIV	LGTSFLSINS
**810**	**820**	**830**	**840**	**850**	**860**	**870**	**880**
KEETEHLENG	NKYPNLESVN	KVNGHSEETS	QSPNRTEPHD	SDCSVDLGIS	KSTEDLSPQK	SGPVGSVVKS	HSITNMEIGG
**890**	**900**	**910**	**920**	**930**	**940**	**950**	**960**
LKIYDILSDN	GPQQPSTTVK	ITSAVDGKNI	VRSKSATLLY	DQPLQVFTGS	SSSSDLISGT	KAIFKFDSNH	NPEEPNIIRG
**970**	**980**	**990**	**1000**	**1010**	**1020**	**1030**	**1040**
PTSGPQSAPQ	IYGPPQYNIQ	YSSSAAVKDT	LWHSKQNPQI	DHASFPPQLL	PRSESTENQS	YAKHSANMNF	SNHNNVRANT
**1050**	**1060**	**1070**	**1080**	**1090**	**1100**	**1110**	**1120**
AYHLHQRLGP	ARHGEMWAIS	PNDRLIPAVT	RSTIQRQSSV	SSTASVNLGD	PGSTRRAQIP	EGDYLSYREF	HSAGRTPPMM
**1130**	**1140**	**1150**	**1160**	**1170**	**1180**	**1190**	**1200**
PGSQRPLSAR	TYSIDGPNAS	RPQSARPSIN	EIPERTMSVS	DFNYSRTSPS	KRPNARVGSE	HSLLDPPGKS	KVPRDWREQV
**1210**	**1220**	**1230**	**1240**	**1250**	**1260**	**1270**	**1280**
LRHIEAKKLE	KIRVRVEKDP	ELGFSISGGV	GGRGNPFRPD	DDGIFVTRVQ	PEGPASKLLQ	PGDKIIQANG	YSFINIEHGQ
**1290**	**1300**	**1310**					
AVSLLKTFQN	TVELIIVREV	SS					

Cmpd.	No. of Cmpds.	m/z meas.	Δ m/z [ppm]	z	Rt [min]	Score	P	Range	Sequence	Modification
2597	1	913.4719	-133.82	1	63.1	13.2	2	1209-1215	K.LEKIRVR.V	

Detailed Protein Report

Protein 386: **vitamin D3 receptor isoform VDRA [Homo sapiens]**

**Accession:**	gi|4507883	**Score:**	25.0
**Database:**	refseq_human(refseq_human_20140103.fasta)	**MW [kDa]:**	48.3
**Database Date:**	2015-11-30	**pI:**	6.1
**Modification(s):**	Carbamidomethyl, Oxidation	**Sequence Coverage [%]:**	2.6
		**No. of unique Peptides:**	2

Alias proteins:

Accession	Name	Description
gi|578824200	refseq_human (refseq_human_20140103.fasta)	PREDICTED: vitamin D3 receptor isoform X1 [Homo sapiens]
gi|63054845	refseq_human (refseq_human_20140103.fasta)	vitamin D3 receptor isoform VDRA [Homo sapiens]

10	20	30	40	50	60	70	80
MEAMAASTSL	PDPGDFDRNV	PRICGVCGDR	ATGFHFNAMT	CEGCKGFFRR	SMKRKALFTC	PFNGDCRITK	DNRRHCQACR
**90**	**100**	**110**	**120**	**130**	**140**	**150**	**160**
LKRCVDIGMM	KEFILTDEEV	QRKREMILKR	KEEEALKDSL	RPKLSEEQQR	IIAILLDAHH	KTYDPTYSDF	CQFRPPVRVN
**170**	**180**	**190**	**200**	**210**	**220**	**230**	**240**
DGGGSHPSRP	NSRHTPSFSG	DSSSSCSDHC	ITSSDMMDSS	SFSNLDLSEE	DSDDPSVTLE	LSQLSMLPHL	ADLVSYSIQK
**250**	**260**	**270**	**280**	**290**	**300**	**310**	**320**
VIGFAKMIPG	FRDLTSEDQI	VLLKSSAIEV	IMLRSNESFT	MDDMSWTCGN	QDYKYRVSDV	TKAGHSLELI	EPLIKFQVGL
**330**	**340**	**350**	**360**	**370**	**380**	**390**	**400**
KKLNLHEEEH	VLLMAICIVS	PDRPGVQDAA	LIEAIQDRLS	NTLQTYIRCR	HPPPGSHLLY	AKMIQKLADL	RSLNEEHSKQ
**410**	**420**	**430**					
YRCLSFQPEC	SMKLTPLVLE	VFGNEIS					

Cmpd.	No. of Cmpds.	m/z meas.	Δ m/z [ppm]	z	Rt [min]	Score	P	Range	Sequence	Modification
2656	1	673.3675	125.73	2	64.2	12.0	0	403-413	R.CLSFQPECSMK.L	Carbamidomethyl: 1; Oxidation: 10
2332	3	665.1572	-192.83	2	59.6	12.9	0	403-413	R.CLSFQPECSMK.L	Carbamidomethyl: 1

Detailed Protein Report

Protein 387: SKI family transcriptional corepressor 1 [Homo sapiens]

Accession: gi|384407369
Database: refseq_human(refseq_human_20140103.fasta)
Database Date: 2015-11-30

Score: 25.0
MW [kDa]: 93.7
pI: 8.5
Sequence Coverage [%]: 4.1
No. of unique Peptides: 2

10	20	30	40	50	60	70	80
MIPKSLVQAA	ETQRGLCEPG	GKGREGRSGG	KGETRPRGVG	TQEGKGTIPG	SPREKRGERG	EGDPALLPAE	DLWRLPGSKD
90	100	110	120	130	140	150	160
RLRSSLLPPP	GPPSSDSGPG	PPSSHSGKTA	QGPRTLNWAR	KQSARTSSNL	CAWSLAMATK	MAEIPSSPYE	PGQRGMKDTQ
170	180	190	200	210	220	230	240
RGDAPQRGSP	EPRILQLARV	GNVYLKEESS	RSGRLEAKKW	IPTKFLENLS	VPHVDLGGNQ	LWSGGMEALT	TQLGPGREGS
250	260	270	280	290	300	310	320
SSPNSKQELQ	PYSGSSALKP	NQVGETSLYG	VPIVSLVIDG	QERLCLAQIS	NTLLKNYSYN	EIHNRRVALG	ITCVQCTPVQ
330	340	350	360	370	380	390	400
LEILRRAGAM	PISSRRCGMI	TKREAERLCK	SFLGEHKPPK	LPENFAFDVV	HECAWGSRGS	FIPARYNSSR	AKCIKCGYCS
410	420	430	440	450	460	470	480
MYFSPNKFIF	HSHRTPDAKY	TQPDAANFNS	WRRHLKLSDK	SATDELSHAW	EDRGLGLATG	ASGPAGPGGP	GGGAGVRSYP
490	500	510	520	530	540	550	560
VIPVPSKGFG	LLQKLPPPLF	PHPYGFPTAF	GLCPKKDDPV	LGAGEPKGGS	YVSAFRPVVK	DTESIAKLYG	SAREAYGAGP
570	580	590	600	610	620	630	640
ARGPGPGAGS	GGYVSPDFLS	EGSSSYNSAS	PDVDTADEPE	VDVESNRFPD	DEDAQEETEP	SAPSAGGGPD	AGRPAFGDLA
650	660	670	680	690	700	710	720
AEDLVRRPER	SPPSGGGGYE	LREPCGPLGG	PAPAKVFAPE	RDEHVKSAAV	ALGPAASYVC	TPEAHEPDKE	DNHSPADDLE
730	740	750	760	770	780	790	800
TRKSYPDQRS	ISQPSPANTD	RGEDGLTLDV	TGTHLVEKDI	ENLAREELQK	LLLEQMELRK	KLEREFQSLK	DNFQDQMKRE
810	820	830	840	850	860	870	
LAYREEMVQQ	LQIVRDTLCN	ELDQERKARY	AIQQKLKEAH	DALHHFSCKM	LTPRHCTGNC	SFKPPLLP	

Cmpd.	No. of Cmpds.	m/z meas.	Δ m/z [ppm]	z	Rt [min]	Score	P	Range	Sequence	Modification
2243	1	945.9550	-39.77	2	58.5	11.7	0	454-477	R.GLGLATGASGPAGPGGPGGGAGVR.S	
2760	1	729.3797	-5.05	2	65.6	13.2	1	759-770	K.DIENLAREELQK.L	

Detailed Protein Report

Protein 388: **PREDICTED: tetratricopeptide repeat protein 13 isoform X7 [Homo sapiens]**

Accession: gi|578802328
Database: refseq_human(refseq_human_20140103.fasta)
Database Date: 2015-11-30

Score: 25.0
MW [kDa]: 57.4
pI: 9.3
Sequence Coverage [%]: 4.4
No. of unique Peptides: 1

10	20	30	40	50	60	70	80
MMLYHHGSLQ	EALKNFKRCL	QLEPYNEVCQ	YMKGLSHVAM	GQFYEGIKAQ	TKVMLNDPLP	GQKASPEYLK	VKYLREYSRY
**90**	**100**	**110**	**120**	**130**	**140**	**150**	**160**
LHAHLDTPLT	EYNIDVDLPG	SFKDHWAKNL	PFLIEDYEEQ	PGLQPHIKDV	LHQNFESYKP	EVQELICVAD	RLGSLMQYET
**170**	**180**	**190**	**200**	**210**	**220**	**230**	**240**
PGFLPNKRIH	RAMGLAALEV	MQAVQRTWTN	SKVRMNGKTR	LMQWRDMFDI	AVKWRRIADP	DQPVLWLDQM	PARSLSRGFN
**250**	**260**	**270**	**280**	**290**	**300**	**310**	**320**
NHINLIRGQV	INMRYLEYFE	KILHFIKDRI	LVYHGANNPK	GLLEVREALE	KVHKVEDLLP	IMKQFNTKTK	DGFTVNTKVP
**330**	**340**	**350**	**360**	**370**	**380**	**390**	**400**
SLKDQGKEYD	GFTITITGDK	VGNILFSVET	QTTEERTQLY	HAEIDALYKD	LTAKGKVLIL	SSEFGEADAV	CNLILSLVYY
**410**	**420**	**430**	**440**	**450**	**460**	**470**	**480**
FYNLMPLSRG	SSVIAYSVIV	GALMASGKEV	AGKIPKGKLV	DFEAMTAPGS	EAFSKVAKSW	MNLKSISPSY	KTLPSVSETF
**490**	**500**	**510**					
PTLRSMIEVL	NTDSSPRCLK	KL					

Cmpd.	No. of Cmpds.	m/z meas.	Δ m/z [ppm]	z	Rt [min]	Score	P	Range	Sequence	Modification
1705	2	820.8385	-139.02	2	49.9	10.6	1	234-247	R.SLSRGFNNHINLIR.G	

Detailed Protein Report

Protein 389: **PREDICTED: coiled-coil domain-containing protein 38 isoform X1 [Homo sapiens]**

**Accession:**	gi|578823285	**Score:**	25.0
**Database:**	refseq_human(refseq_human_20140103.fasta)	**MW [kDa]:**	46.1
**Database Date:**	2015-11-30	**pI:**	6.0
		**Sequence Coverage [%]:**	6.8
		**No. of unique Peptides:**	1

10	20	30	40	50	60	70	80
MNLLMTRETG	FCSRAAQETI	NKLQMTAELK	KASMEVQAVK	SEIAKTEFLL	REYMKYGFFL	LQMSPKHWQI	QQALKRAQAS
**90**	**100**	**110**	**120**	**130**	**140**	**150**	**160**
KSKANIILPK	ILAKLSLHSS	NKEGILEESG	RTAVLSEDAS	QGRDSQGKPS	RSLTRTPEKK	KSNLAESFGS	EDSLEFLLDD
**170**	**180**	**190**	**200**	**210**	**220**	**230**	**240**
EMDVDLEPAL	YFKEPEELLQ	VLRELEEQNL	TLFQYSQDVD	ENLEEVNKRE	KVIQDKTNSN	IEFLLEQEKM	LKANCVREEE
**250**	**260**	**270**	**280**	**290**	**300**	**310**	**320**
KAAELQLKSK	LFSFGEFNSD	AQEILIDSLS	KKITQVYKVC	IGDAEDDGLN	PIQKLVKVES	RLVELCDLIE	SIPKENVEAI
**330**	**340**	**350**	**360**	**370**	**380**	**390**	**400**
ERMKQKEWRQ	KFRDEKMKEK	QRHQQERLKA	ALEKAVAQPK	KKLGRRLVFH	SKPPSGNKQQ	LPLVNETKTK	SQEEEYFFT

Cmpd.	No. of Cmpds.	m/z meas.	Δ m/z [ppm]	z	Rt [min]	Score	P	Range	Sequence	Modification
353	1	676.4443	133.31	3	33.1	11.6	1	279-297	K.VCIGDAEDDGLNPIQKLVK.V	

Detailed Protein Report

Protein 390: **PREDICTED: KN motif and ankyrin repeat domain-containing protein 3 isoform X2 [Homo sapiens]**

**Accession:**	gi|530427478	**Score:**	25.0
**Database:**	refseq_human(refseq_human_20140103.fasta)	**MW [kDa]:**	91.0
**Database Date:**	2015-11-30	**pI:**	4.9
**Modification(s):**	Carbamidomethyl	**Sequence Coverage [%]:**	5.8
		**No. of unique Peptides:**	1

10	20	30	40	50	60	70	80
MCGGRWLGGG	ELLGPVCLDS	KAGPRGSSQR	DSAQPHLHPF	CRVPLTAAAG	NMAKFALNQN	LPDLGGPRLC	PVPAAGGARS
90	100	110	120	130	140	150	160
PSSPYSVETP	YGFHLDLDFL	KYIEELERGP	AARRAPGPPT	SRRPRAPRPG	LAGARSPGAW	TSSESLASDD	GGAPGILSQG
170	180	190	200	210	220	230	240
APSGLLMQPL	SPRAPVRNPR	VEHTLRETSR	RLELAQTHER	APSPGRGVPR	SPRGSGRSSP	APNLAPASPG	PAQLQLVREQ
250	260	270	280	290	300	310	320
MAAALRRLRE	LEDQARTLPE	LQEQVRALRA	EKARLLAGRA	QPEPDGEAET	RPDKLAQLRR	LTERLATSER	GGRARASPRA
330	340	350	360	370	380	390	400
DSPDGLAAGR	SEGALQVLDG	EVGSLDGTPQ	TREVAAEAVP	ETREAGAQAV	PETREAGVEA	APETVEADAW	VTEALLGLPA
410	420	430	440	450	460	470	480
AAERELELLR	ASLEHQRGVS	ELLRGRLREL	EEAREAAEEA	AAGARAQLRE	ATTQTPWSCA	EKAAQTESPA	EAPSLTQESS
490	500	510	520	530	540	550	560
PGSMDGDRAV	APAGILKSIM	KKRDGTPGAQ	PSSGPKSLQF	VGVLNGEYES	SSSEDASDSD	GDSENGGAEP	PGSSSGSGDD
570	580	590	600	610	620	630	640
SGGGSDSGTP	GPPSGGDIRD	PEPEAEAEPQ	QVAQGRCELS	PRLREACVAL	QRQLSRPRGV	ASDGGAVRLV	AQEWFRVSSQ
650	660	670	680	690	700	710	720
RRSQAEPVAR	MLEGVRRLGP	ELLAHVVNLA	DGNGNTALHY	SVSHGNLAIA	SLLLDTGACE	VNRQNRAGYS	ALMLAALTSV
730	740	750	760	770	780	790	800
RQEEEDMAVV	QRLFCMGDVN	AKASQTGQTA	LMLAISHGRQ	DMVATLLACG	ADVNAQDADG	ATALMCASEY	GRLDTVRLLL
810	820	830	840	850	860	870	880
TQPGCDPAIL	DNEGTSALAI	ALEAEQDEVA	ALLHAHLSSG	QPDTQSESPP	GSQTATPGEG	ECGDNGENPQ	VQ

Cmpd.	No. of Cmpds.	m/z meas.	Δ m/z [ppm]	z	Rt [min]	Score	P	Range	Sequence	Modification
428	1	1293.7593	91.80	2	34.0	12.9	2	1-25	-.MCGGRWLGGGELLGPVCLDSKAGPR.G	Carbamidomethyl: 17

Detailed Protein Report

Protein 391: PREDICTED: E3 ubiquitin-protein ligase UBR5 isoform X5 [Homo sapiens]

**Accession:**	gi|530389110	**Score:**	24.9
**Database:**	refseq_human(refseq_human_20140103.fasta)	**MW [kDa]:**	308.4
**Database Date:**	2015-11-30	**pI:**	5.5
		**Sequence Coverage [%]:**	1.1
		**No. of unique Peptides:**	1

Quantitation

QD:QU	**Median:** 0.96	**CV:** 0.00 %	**No. of Peptides:** 1

10	20	30	40	50	60	70	80
MTSIHFVVHP	LPGTEDQLND	RLREVSEKLN	KYNLNSHPPL	NVLEQATIKQ	CVVGPNHAAF	LLEDGRVCRI	GFSVQPDRLE
90	100	110	120	130	140	150	160
LGKPDNNDGS	KLNSNSGAGR	TSRPGRTSDS	PWFLSGSETL	GRLAGNTLGS	RWSSGVGGSG	GGSSGRSSAG	ARDSRRQTRV
170	180	190	200	210	220	230	240
IRTGRDRGSG	LLGSQPQPVI	PASVIPEELI	SQAQVVLQGK	SRSVIIRELQ	RTNLDVNLAV	NNLLSRDDED	GDDGDDTASE
250	260	270	280	290	300	310	320
SYLPGEDLMS	LLDADIHSAH	PSVIIDADAM	FSEDISYFGY	PSFRRSSLSR	LGSSRERDSE	LLRERESVLR	LRERRWLDGA
330	340	350	360	370	380	390	400
SFDNERGSTS	KEGEPNLDKK	NTPVQSPVSL	GEDLQWWPDK	DGTKFICIGA	LYSELLAVSS	KGELYQWKWS	ESEPYRNAQN
410	420	430	440	450	460	470	480
PSLHHPRATF	LGLTNEKIVL	LSANSIRATV	ATENNKVATW	VDETLSSVAS	KLEHTAQTYS	ELQGERIVSL	HCCALYTCAQ
490	500	510	520	530	540	550	560
LENSLYWWGV	VPFSQRKKML	EKARAKNKKP	KSSAGISSMP	NITVGTQVCL	RNNPLYHAGA	VAFSISAGIP	KVGVLMESVW
570	580	590	600	610	620	630	640
NMNDSCRFQL	RSPESLKNME	KASKTTEAKP	ESKQEPVKTE	MGPPPSPAST	CSDASSIASS	ASMPYKRRRS	TPAPKEEEKV
650	660	670	680	690	700	710	720
NEEQWSLREV	VFVEDVKNVP	VGKVLKVDGA	YVAVKFPGTS	SNTNCQNSSG	PDADPSSLLQ	DCRLLRIDEL	QVVKTGGTPK
730	740	750	760	770	780	790	800
VPDCFQRTPK	KLCIPEKTEI	LAVNVDSKGV	HAVLKTGNWV	RYCIFDLATG	KAEQENNFPT	SSIAFLGQNE	RNVAIFTAGQ
810	820	830	840	850	860	870	880
ESPIILRDGN	GTIYPMAKDC	MGGIRDPDWL	DLPPISSLGM	GVHSLINLPA	NSTIKKKAAV	IIMAVEKQTL	MQHILRCDYE
890	900	910	920	930	940	950	960
ACRQYLMNLE	QAVVLEQNLQ	MLQTFISHRC	DGNRNILHAC	VSVCFPTSNK	ETKEEEEAER	SERNTFAERL	SAVEAIANAI
970	980	990	1000	1010	1020	1030	1040
SVVSSNGPGN	RAGSSSSRSL	RLREMMRRSL	RAAGLGRHEA	GASSSDHQDP	VSPPIAPPSW	VPDPPAMDPD	GDIDFILAPA
1050	1060	1070	1080	1090	1100	1110	1120
VGSLTTAATG	TGQGPSTSTI	PGPSTEPSVV	ESKDRKANAH	FILKLLCDSV	VLQPYLRELL	SAKDARGMTP	FMSAVSGRAY
1130	1140	1150	1160	1170	1180	1190	1200
PAAITILETA	QKIAKAEISS	SEKEEDVFMG	MVCPSGTNPD	DSPLYVLCCN	DTCSFTWTGA	EHINQDIFEC	RTCGLLESLC
1210	1220	1230	1240	1250	1260	1270	1280
CCTECARVCH	KGHDCKLKRT	SPTAYCDCWE	KCKCKTLIAG	QKSARLDLLY	RLLTATNLVT	LPNSRGEHLL	LFLVQTVARQ
1290	1300	1310	1320	1330	1340	1350	1360
TVEHCQYRPP	RIREDRNRKT	ASPEDSDMPD	HDLEPPRFAQ	LALERVLQDW	NALKSMIMFG	SQENKDPLSA	SSRIGHLLPE
1370	1380	1390	1400	1410	1420	1430	1440
EQVYLNQQSG	TIRLDCFTHC	LIVKCTADIL	LLDTLLGTLV	KELQNKYTPG	RREEAIAVTM	RFLRSVARVF	VILSVEMASS
1450	1460	1470	1480	1490	1500	1510	1520
KKKNNFIPQP	IGKCKRVFQA	LLPYAVEELC	NVAESLIVPV	RMGIARPTAP	FTLASTSIDA	MQGSEELFSV	EPLPPRPSSD
1530	1540	1550	1560	1570	1580	1590	1600
QSSSSSQSQS	SYIIRNPQQR	RISQSQPVRG	RDEEQDDIVS	ADVEEVEVVE	GVAGEEDHHD	EQEEHGEENA	EAEGQHDEHD
1610	1620	1630	1640	1650	1660	1670	1680
EDGSDMELDL	LAAAETESDS	ESNHSNQDNA	SGRRSVVTAA	TAGSEAGASS	VPAFFSEDDS	QSNDSSDSDS	SSSQSDDIEQ
1690	1700	1710	1720	1730	1740	1750	1760
ETFMLDEPLE	RTTNSSHANG	AAQAPRSMQW	AVRNTQHQRA	ASTAPSSTST	PAASSAGLIY	IDPSNLRRSG	TISTSAAAAA
1770	1780	1790	1800	1810	1820	1830	1840
AALEASNASS	YLTSASSLAR	AYSIVIRQIS	DLMGLIPKYN	HLVYSQIPAA	VKLTYQDAVN	LQNYVEEKLI	PTWNWMVSIM
1850	1860	1870	1880	1890	1900	1910	1920
DSTEAQLRYG	SALASAGDPG	HPNHPLHASQ	NSARRERMTA	REEASLRTLE	GRRRATLLSA	RQGMMSARGD	FLNYALSLMR
1930	1940	1950	1960	1970	1980	1990	2000
SHNDEHSDVL	PVLDVCSLKH	VAYVFQALIY	WIKAMNQQTT	LDTPQLERKR	TRELLELGID	NEDSEHENDD	DTNQSATLND
2010	2020	2030	2040	2050	2060	2070	2080
KDDDSLPAET	GQNHPFFRRS	DSMTFLGCIP	PNPFEVPLAE	AIPLADQPHL	LQPNARKEDL	FGRPSQGLYS	SSASSGKCLM
2090	2100	2110	2120	2130	2140	2150	2160
EVTVDRNCLE	VLPTKMSYAA	NLKNVMNMQN	RQKKEGEEQP	VLPEETESSK	PGPSAHDLAA	QLKSSLLAEI	GLTESEGPPL
2170	2180	2190	2200	2210	2220	2230	2240

Detailed Protein Report

Cmpd.	No. of Cmpds.	m/z meas.	Δ m/z [ppm]	z	Rt [min]	Score	P	Range	Sequence	Modification	Ratios	
1093	1	647.6752	-180.54	2	42.2	14.8	0	132-146	R.WSSGVGGSGGGSSGR.S		QD:QU	0.96

Detailed Protein Report

Protein 392: dnaJ homolog subfamily C member 14 [Homo sapiens]

**Accession:**	gi|119943096	**Score:**	24.9
**Database:**	refseq_human(refseq_human_20140103.fasta)	**MW [kDa]:**	78.5
**Database Date:**	2015-11-30	**pI:**	9.4
**Modification(s):**	Carbamidomethyl	**Sequence Coverage [%]:**	3.4
		**No. of unique Peptides:**	2

Quantitation

QD:QU **Median:** 0.77 **CV:** 37.11 % **No. of Peptides:** 2

10	20	30	40	50	60	70	80
MAQKHPGERG	LYGAHHSGGA	SLRTLGPSVD	PEIPSFSGLR	DSAGTAPNGT	RCLTEHSGPK	HTQHPNPAHW	LDPSHGPPGG
**90**	**100**	**110**	**120**	**130**	**140**	**150**	**160**
PGPPRDAEDP	DQSETSSEEE	SGVDQELSKE	NETGNQKDGN	SFLSIPSACN	CQGTPGIPEG	PYSEGGNGSS	SNFCHHCTSP
**170**	**180**	**190**	**200**	**210**	**220**	**230**	**240**
ALGEDELEEE	YDDEESLKFP	SDFSRVSSGK	KPPSRRQRHR	FPTKEDTREG	GRRDPRSPGR	HRLGRKRSQA	DKRKGLGLWG
**250**	**260**	**270**	**280**	**290**	**300**	**310**	**320**
AEELCQLGQA	GFWWLIELLV	LVGEYVETCG	HLIYACRQLK	SSDLDLFRVW	MGVWTGRLGG	WAQVMFQFLS	QGFYCGVGLF
**330**	**340**	**350**	**360**	**370**	**380**	**390**	**400**
TRFLKLLGAL	LLLALALFLG	FLQLGWRFLV	GLGDRLGWRD	KATWLFSWLD	SPALQRCLTL	LRDSRPWQRL	VRIVQWGWLE
**410**	**420**	**430**	**440**	**450**	**460**	**470**	**480**
LPWVKQNINR	QGNAPVASGR	YCQPEEEVAR	LLTMAGVPED	ELNPFHVLGV	EATASDVELK	KAYRQLAVMV	HPDKNHHPRA
**490**	**500**	**510**	**520**	**530**	**540**	**550**	**560**
EEAFKVLRAA	WDIVSNAEKR	KEYEMKRMAE	NELSRSVNEF	LSKLQDDLKE	AMNTMMCSRC	QGKHRRFEMD	REPKSARYCA
**570**	**580**	**590**	**600**	**610**	**620**	**630**	**640**
ECNRLHPAEE	GDFWAESSML	GLKITYFALM	DGKVYDITEW	AGCQRVGISP	DTHRVPYHIS	FGSRIPGTRG	RQRATPDAPP
**650**	**660**	**670**	**680**	**690**	**700**	**710**	
ADLQDFLSRI	FQVPPGQMPN	GNFFAAPQPA	PGAAAASKPN	STVPKGEAKP	KRRKKVRRPF	QR	

Cmpd.	No. of Cmpds.	m/z meas.	Δ m/z [ppm]	z	Rt [min]	Score	P	Range	Sequence	Modification	Ratios	
2138	1	661.2888	-106.46	2	55.3	10.6	1	278-288	R.QLKSSDLDLFR.V		QD:QU	0.53
1717	4	820.8378	-35.57	2	50.0	14.3	2	552-564	R.EPKSARYCAECNR.L	Carbamidomethyl: 8, 11	QD:QU	1.10

Detailed Protein Report

Protein 393: krev interaction trapped protein 1 isoform 2 [Homo sapiens]

**Accession:**	gi|61742817	**Score:**	24.8
**Database:**	refseq_human(refseq_human_20140103.fasta)	**MW [kDa]:**	78.6
**Database Date:**	2015-11-30	**pI:**	9.6
		**Sequence Coverage [%]:**	4.5
		**No. of unique Peptides:**	1

Alias proteins:

Accession	Name	Description
gi|578814707	refseq_human (refseq_human_20140103.fasta)	PREDICTED: krev interaction trapped protein 1 isoform X15 [Homo sapiens]

```
        10         20         30         40         50         60         70         80
MGNPENIEDA YVAVIRPKNT ASLNSREYRA KSYEILLHEV PIEGQKKKRK KVLLETKLQG NSEITQGILD YVVETTKPIS
        90        100        110        120        130        140        150        160
PANQGIRGKR VVLMKKFPLD GEKMGREASL FIVPSVVKDN TKYTYTPGCP IFYCLQDIMR VCSESSTHFA TLTARMLIAL
       170        180        190        200        210        220        230        240
DKWLDERHAQ SHFIPALFRP SPLERIKTNV INPAYATESG QTENSLHMGY SALEIKSKML ALEKADTCIY NPLFGSDLQY
       250        260        270        280        290        300        310        320
TNRVDKVVIN PYFGLGAPDY SKIQIPKQEK WQRSMSSVTE DKYGKVEATR ILLEKGKCNP NLLNGQLSSP LHFAAGGGHA
       330        340        350        360        370        380        390        400
EIVQILLNHP ETDRHITDQQ GRSPLNICEE NKQNNWEEAA KLLKEAINKP YEKVRIYRMD GSYRSVELKH GNNTTVQQIM
       410        420        430        440        450        460        470        480
EGMRLSQETQ QYFTIWICSE NLSLQLKPYH KPLQHVRDWP EILAELTNLD PQRETPQLFL RRDVRLPLEV EKQIEDPLAI
       490        500        510        520        530        540        550        560
LILFDEARYN LLKGFYTAPD AKLITLASLL LQIVYGNYES KKHKQGFLNE ENLKSIVPVT KLKSKAPHWT NRILHEYKNL
       570        580        590        600        610        620        630        640
STSEGVSKEM HHLQRMFLQN CWEIPTYGAA FFTGQIFTKA SPSNHKVIPV YVGVNIKGLH LLNMETKALL ISLKYGCFMW
       650        660        670        680        690
QLGDTDTCFQ IHSMENKMSF IVHTKQAGLV VKLLMKLNGQ LMPTERNS
```

Cmpd.	No. of Cmpds.	m/z meas.	Δ m/z [ppm]	z	Rt [min]	Score	P	Range	Sequence	Modification
1509	1	829.3458	-157.64	2	49.1	14.1	1	658-672	K.MSFIVHTKQAGLVVK.L	

Detailed Protein Report

Protein 394: PREDICTED: integrin beta-7 isoform X3 [Homo sapiens]

Accession: gi|578823673
Database: refseq_human(refseq_human_20140103.fasta)
Database Date: 2015-11-30
Modification(s): Carbamidomethyl

Score: 24.8
MW [kDa]: 85.0
pI: 5.7
Sequence Coverage [%]: 3.3
No. of unique Peptides: 2

10	20	30	40	50	60	70	80
MVALPMVLVL	LLVLSRGESE	LDAKIPSTGD	ATEWRNPHLS	MLGSCQPAPS	CQKCILSHPS	CAWCKQLNFT	ASGEAEARRC
90	100	110	120	130	140	150	160
ARREELLARG	CPLEELEEPR	GQQEVLQDQP	LSQGARGEGA	TQLAPQRVRV	TLRPGEPQQL	QVRFLRAEGY	PVDLYYLMDL
170	180	190	200	210	220	230	240
SYSMKDDLER	VRQLGHALLV	RLQEVTHSVR	IGFGSFVDKT	VLPFVSTVPS	KLRHPCPTRL	ERCQSPFSFH	HVLSLTGDAQ
250	260	270	280	290	300	310	320
AFEREVGRQS	VSGNLDSPEG	GFDAILQAAL	CQEQIGWRNV	SRLLVFTSDD	TFHTAGDGKL	GGIFMPSDGH	CHLDSNGLYS
330	340	350	360	370	380	390	400
RSTEFDYPSV	GQVAQALSAA	NIQPIFAVTS	AALPVYQELS	KLIPKSAVGE	LSEDSSNVVQ	LIMDAYNSLS	STVTLEHSSL
410	420	430	440	450	460	470	480
PPGVHISYES	QCEGPEKREG	KAEDRGQCNH	VRINQTVTFW	VSLQATHCLP	EPHLLRLRAL	GFSEELIVEL	HTLCDCNCSD
490	500	510	520	530	540	550	560
TQPQAPHCSD	GQGHLQCGVC	SCAPGRLGRL	CECSVAELSS	PDLESGCRAP	NGTGPLCSGK	GHCQCGRCSC	SGQSSGHLCE
570	580	590	600	610	620	630	640
CDDASCERHE	GILCGGFGRC	QCGVCHCHAN	RTGRACECSG	DMDSCISPEG	GLCSGHGRCK	CNRCQCLDGY	YGALCDQCPG
650	660	670	680	690	700	710	720
CKTPCERHRD	CAECGAFRTG	PLATNCSTAC	AHTNVTLALA	PILDDGWCKE	RTLDNQLFFF	LVEDDARGTV	VLRVRPQEKG
730	740	750	760	770	780	790	
ADHTQAIVLG	CVGGIVAVGL	GLVLAYRLSV	EIYDRREYSR	FEKEQQQLNW	KQLLGFGSRL	AS	

Cmpd.	No. of Cmpds.	m/z meas.	Δ m/z [ppm]	z	Rt [min]	Score	P	Range	Sequence	Modification
2949	1	1019.4212	-21.13	3	66.0	11.9	2	569-594	R.HEGILCGGFGRCQCGVCHCHANRTGR.A	Carbamidomethyl: 6, 12, 14, 17, 19
143	1	722.7732	-4.06	2	30.5	13.0	0	580-591	R.CQCGVCHCHANR.T	Carbamidomethyl: 1, 3

Detailed Protein Report

Protein 395: neuropeptides B/W receptor type 1 [Homo sapiens]

**Accession:**	gi|53828924	**Score:**	24.8
**Database:**	refseq_human(refseq_human_20140103.fasta)	**MW [kDa]:**	36.1
**Database Date:**	2015-11-30	**pI:**	10.1
**Modification(s):**	Carbamidomethyl	**Sequence Coverage [%]:**	8.5
		**No. of unique Peptides:**	1

10	20	30	40	50	60	70	80
MDNASFSEPW	PANASGPDPA	LSCSNASTLA	PLPAPLAVAV	PVVYAVICAV	GLAGNSAVLY	VLLRAPRMKT	VTNLFILNLA
**90**	**100**	**110**	**120**	**130**	**140**	**150**	**160**
IADELFTLVL	PINIADFLLR	QWPFGELMCK	LIVAIDQYNT	FSSLYFLTVM	SADRYLVVLA	TAESRRVAGR	TYSAARAVSL
**170**	**180**	**190**	**200**	**210**	**220**	**230**	**240**
AVWGIVTLVV	LPFAVFARLD	DEQGRRQCVL	VFPQPEAFWW	RASRLYTLVL	GFAIPVSTIC	VLYTTLLCRL	HAMRLDSHAK
**250**	**260**	**270**	**280**	**290**	**300**	**310**	**320**
ALERAKKRVT	FLVVAILAVC	LLCWTPYHLS	TVVALTTDLP	QTPLVIAISY	FITSLSYANS	CLNPFLYAFL	DASFRRNLRQ
**330**							
LITCRAAA							

Cmpd.	No. of Cmpds.	m/z meas.	Δ m/z [ppm]	z	Rt [min]	Score	P	Range	Sequence	Modification
42	1	706.9621	-93.24	3	30.4	10.4	1	186-201	R.RQCVLVFPQPEAFWWR.A	Carbamidomethyl: 3

Detailed Protein Report

Protein 396: PREDICTED: ankyrin repeat domain-containing protein 12 isoform X6 [Homo sapiens]

**Accession:**	gi|578832112	**Score:**	24.8
**Database:**	refseq_human(refseq_human_20140103.fasta)	**MW [kDa]:**	202.9
**Database Date:**	2015-11-30	**pI:**	6.5
**Modification(s):**	Oxidation	**Sequence Coverage [%]:**	1.7
		**No. of unique Peptides:**	1

10	20	30	40	50	60	70	80
MVNVPELLLG	QSPSRNDTKI	INSEEAQSVN	PSSVDENIDS	ETEKDSLICE	SKQILPSKTP	LPSALDEYEF	KDDDDEEINK
90	100	110	120	130	140	150	160
MIDDRHILRK	EQRKENEPEA	EKTHLFAKQE	KAFYPKSFKS	KKQKPSRVLY	SSTESSDEEA	LQNKKISTSC	SVIPETSNSD
170	180	190	200	210	220	230	240
MQTKKEYVVS	GEHKQKGKVK	RKLKNQNKNK	ENQELKQEKE	GKENTRITNL	TVNTGLDCSE	KTREEGNFRK	SFSPKDDTSL
250	260	270	280	290	300	310	320
HLFHISTGKS	PKHSCGLSEK	QSTPLKQEHT	KTCLSPGSSE	MSLQPDLVRY	DNTESEFLPE	SSSVKSCKHK	EKSKHQKDFH
330	340	350	360	370	380	390	400
LEFGEKSNAK	IKDEDHSPTF	ENSDCTLKKM	DKEGKTLKKH	KLKHKEREKE	KHKKEIEGEK	EKYKTKDSAK	ELQRSVEFDR
410	420	430	440	450	460	470	480
EFWKENFFKS	DETEDLFLNM	EHESLTLEKK	SKLEKNIKDD	KSTKEKHVSK	ERNFKEERDK	IKKESEKSFR	EEKIKDLKEE
490	500	510	520	530	540	550	560
RENIPTDKDS	EFTSLGMSAI	EESIGLHLVE	KEIDIEKQEK	HIKESKEKPE	KRSQIKEKDI	EKMERKTFEK	EKKIKHEHKS
570	580	590	600	610	620	630	640
EKDKLDLSEC	VDKIKEKDKL	YSHHTEKCHK	EGEKSKNTAA	IKKTDDREKS	REKMDRKHDK	EKPEKERHLA	ESKEKHLMEK
650	660	670	680	690	700	710	720
KNKQSDNSEY	SKSEKGKNKE	KDRELDKKEK	SRDKESINIT	NSKHIQEEKK	SSIVDGNKAQ	HEKPLSLKEK	TKDEPLKTPD
730	740	750	760	770	780	790	800
GKEKDKKDKD	IDRYKERDKH	KDKIQINSLL	KLKSEADKPK	PKSSPASKDT	RPKEKRLVND	DLMQTSFERM	LSLKDLEIEQ
810	820	830	840	850	860	870	880
WHKKHKEKIK	QKEKERLRNR	NCLELKIKDK	EKTKHTPTES	KNKELTRSKS	SEVTDAYTKE	KQPKDAVSNR	SQSVDTKNVM
890	900	910	920	930	940	950	960
TLGKSSFVSD	NSLNRSPRSE	NEKPGLSSRS	VSMISVASSE	DSCHTTVTTP	RPPVEYDSDF	MLESSESQMS	FSQSPFLSIA
970	980	990	1000	1010	1020	1030	1040
KSPALHEREL	DSLADLPERI	KPPYANRLST	SHLRSSSVED	VKLIISEGRP	TIEVRRCSMP	SVICEHTKQF	QTISEESNQG
1050	1060	1070	1080	1090	1100	1110	1120
SLLTVPGDTS	PSPKPEVFSN	VPERDLSNVS	NIHSSFATSP	TGASNSKYVS	ADRNLIKNTA	PVNTVMDSPV	HLEPSSQVGV
1130	1140	1150	1160	1170	1180	1190	1200
IQNKSWEMPV	DRLETLSTRD	FICPNSNIPD	QESSLQSFCN	SENKVLKENA	DFLSLRQTEL	PGNSCAQDPA	SFMPPQQPCS
1210	1220	1230	1240	1250	1260	1270	1280
FPSQSLSDAE	SISKHMSLSY	VANQEPGILQ	QKNAVQIISS	ALDTDNESTK	DTENTFVLGD	VQKTDAFVPV	YSDSTIQEAS
1290	1300	1310	1320	1330	1340	1350	1360
PNFEKAYTLP	VLPSEKDFNG	SDASTQLNTH	YAFSKLTYKS	SSGHEVENST	TDTQVISHEK	ENKLESLVLT	HLSRCDSDLC
1370	1380	1390	1400	1410	1420	1430	1440
EMNAGMPKGN	LNEQDPKHCP	ESEKCLLSIE	DEESQQSILS	SLENHSQQST	QPEMHKYGQL	VKVELEENAE	DDKTENQIPQ
1450	1460	1470	1480	1490	1500	1510	1520
RMTRNKANTM	ANQSKQILAS	CTLLSEKDSE	SSSPRGRIRL	TEDDDPQIHH	PRKRKVSRVP	QPVQVSPSLL	QAKEKTQQSL
1530	1540	1550	1560	1570	1580	1590	1600
AAIVDSLKLD	EIQPYSSERA	NPYFEYLHIR	KKIEEKRKLL	CSVIPQAPQY	YDEYVTFNGS	YLLDGNPLSK	ICIPTITPPP
1610	1620	1630	1640	1650	1660	1670	1680
SLSDPLKELF	RQQEVVRMKL	RLQHSIEREK	LIVSNEQEVL	RVHYRAARTL	ANQTLPFSAC	TVLLDAEVYN	VPLDSQSDDS
1690	1700	1710	1720	1730	1740	1750	1760
KTSVRDRFNA	RQFMSWLQDV	DDKFDKLKTC	LLMRQQHEAA	ALNAVQRLEW	QLKLQELDPA	TYKSISIYEI	QEFYVPLVDV
1770							
NDDFELTPI							

Cmpd.	No. of Cmpds.	m/z meas.	Δ m/z [ppm]	z	Rt [min]	Score	P	Range	Sequence	Modification
110	1	1083.4576	-80.32	2	31.3	13.6	2	865-884	K.DAVSNRSQSVDTKNVMTLGK.S	Oxidation: 16

Detailed Protein Report

Protein 397: **PREDICTED: plasma membrane calcium-transporting ATPase 2 isoform X9 [Homo sapiens]**

**Accession:**	gi|578806173	**Score:**	24.8
**Database:**	refseq_human(refseq_human_20140103.fasta)	**MW [kDa]:**	129.4
**Database Date:**	2015-11-30	**pI:**	6.0
**Modification(s):**	Oxidation	**Sequence Coverage [%]:**	2.6
		**No. of unique Peptides:**	1

10	20	30	40	50	60	70	80
MGDMTNSDFY	SKNQRNESSH	GGEFGCTMEE	LRSLMELRGT	EAVVKIKETY	GDTEAICRRL	KTSPVEGLPG	TAPDLEKRKQ
**90**	**100**	**110**	**120**	**130**	**140**	**150**	**160**
IFGQNFIPPK	KPKTFLQLVW	EALQDVTLII	LEIAAIISLG	LSFYHPPGEG	NEGCATAQGG	AEDEGEAEAG	WIEGAAILLS
**170**	**180**	**190**	**200**	**210**	**220**	**230**	**240**
VICVVLVTAF	NDWSKEKQFR	GLQSRIEQEQ	KFTVVRAGQV	VQIPVAEIVV	GDIAQVKYGD	LLPADGLFIQ	GNDLKIDESS
**250**	**260**	**270**	**280**	**290**	**300**	**310**	**320**
LTGESDQVRK	SVDKDPMLLS	GTHVMEGSGR	MLVTAVGVNS	QTGIIFTLLG	AGGEEEEKKD	KKGKMQDGNV	DASQSKAKQQ
**330**	**340**	**350**	**360**	**370**	**380**	**390**	**400**
DGAAAMEMQP	LKSAEGGDAD	DRKKASMHKK	EKSVLQGKLT	KLAVQIGKAG	LVMSAITVII	LVLYFTVDTF	VVNKKPWLPE
**410**	**420**	**430**	**440**	**450**	**460**	**470**	**480**
CTPVYVQYFV	KFFIIGVTVL	VVAVPEGLPL	AVTISLAYSV	KKMMKDNNLV	RHLDACETMG	NATAICSDKT	GTLTTNRMTV
**490**	**500**	**510**	**520**	**530**	**540**	**550**	**560**
VQAYVGDVHY	KEIPDPSSIN	TKTMELLINA	IAINSAYTTK	ILPPEKEGAL	PRQVGNKTEC	GLLGFVLDLK	QDYEPVRSQM
**570**	**580**	**590**	**600**	**610**	**620**	**630**	**640**
PEEKLYKVYT	FNSVRKSMST	VIKLPDESFR	MYSKGASEIV	LKKCCKILNG	AGEPRVFRPR	DRDEMVKKVI	EPMACDGLRT
**650**	**660**	**670**	**680**	**690**	**700**	**710**	**720**
ICVAYRDFPS	SPEPDWDNEN	DILNELTCIC	VVGIEDPVRP	EVPEAIRKCQ	RAGITVRMVT	GDNINTARAI	AIKCGIIHPG
**730**	**740**	**750**	**760**	**770**	**780**	**790**	**800**
EDFLCLEGKE	FNRRIRNEKG	EIEQERIDKI	WPKLRVLARS	SPTDKHTLVK	GIIDSTHTEQ	RQVVAVTGDG	TNDGPALKKA
**810**	**820**	**830**	**840**	**850**	**860**	**870**	**880**
DVGFAMGIAG	TDVAKEASDI	ILTDDNFSSI	VKAVMWGRNV	YDSISKFLQF	QLTVNVVAVI	VAFTGACITQ	DSPLKAVQML
**890**	**900**	**910**	**920**	**930**	**940**	**950**	**960**
WVNLIMDTFA	SLALATEPPT	ETLLLRKPYG	RNKPLISRTM	MKNILGHAVY	QLALIFTLLF	VGEKMFQIDS	GRNAPLHSPP
**970**	**980**	**990**	**1000**	**1010**	**1020**	**1030**	**1040**
SEHYTIIFNT	FVMMQLFNEI	NARKIHGERN	VFDGIFRNPI	FCTIVLGTFA	IQIVIVQFGG	KPFSCSPLQL	DQWMWCIFIG
**1050**	**1060**	**1070**	**1080**	**1090**	**1100**	**1110**	**1120**
LGELVWGQVI	ATIPTSRLKF	LKEAGRLTQK	EEIPEEELNE	DVEEIDHAER	ELRRGQILWF	RGLNRIQTQI	EVVNTFKSGA
**1130**	**1140**	**1150**	**1160**	**1170**	**1180**		
SFQGALRRQS	SVTSQSQDVA	NLSSPSRVSL	SNALSSPTSL	PPAAAGHPRR	EGVP		

Cmpd.	No. of Cmpds.	m/z meas.	Δ m/z [ppm]	z	Rt [min]	Score	P	Range	Sequence	Modification
336	1	625.9844	-32.51	3	34.2	10.9	2	568-583	K.VYTFNSVRKSMSTVIK.L	Oxidation: 11

Detailed Protein Report

Protein 398: beta-1,4 N-acetylgalactosaminyltransferase 2 isoform c [Homo sapiens]

**Accession:**	gi|227497758	**Score:**	24.7
**Database:**	refseq_human(refseq_human_20140103.fasta)	**MW [kDa]:**	54.2
**Database Date:**	2015-11-30	**pI:**	8.9
		**Sequence Coverage [%]:**	4.6
		**No. of unique Peptides:**	1

10	20	30	40	50	60	70	80
MFGSMFLQAV	FSSPKPELPS	PAPGVQKLKL	LPEERLRNLF	SYDGIWLFPK	NQCKCEANKE	QGGYNFQDAY	GQSDLPAVKA
**90**	**100**	**110**	**120**	**130**	**140**	**150**	**160**
RRQAEFEHFQ	RREGLPRPLP	LLVQPNLPFG	YPVHGVEVMP	LHTVPIPGLQ	FEGPDAPVYE	VTLTASLGTL	NTLADVPDSV
**170**	**180**	**190**	**200**	**210**	**220**	**230**	**240**
VQGRGQKQLI	ISTSDRKLLK	FILQHVTYTS	TGYQHQKVDI	VSLESRSSVA	KFPVTIRHPV	IPKLYDPGPE	RKLRNLVTIA
**250**	**260**	**270**	**280**	**290**	**300**	**310**	**320**
TKTFLRPHKL	MIMLRSIREY	YPDLTVIVAD	DSQKPLEIKD	NHVEYYTMPF	GKGWFAGRNL	AISQVTTKYV	LWVDDDFLFN
**330**	**340**	**350**	**360**	**370**	**380**	**390**	**400**
EETKIEVLVD	VLEKTELDVV	GGSVLGNVFQ	FKLLLEQSEN	GACLHKRMGF	FQPLDGFPSC	VVTSGVVNFF	LAHTERLQRV
**410**	**420**	**430**	**440**	**450**	**460**	**470**	**480**
GFDPRLQRVA	HSEFFIDGLG	TLLVGSCPEV	IIGHQSRSPV	VDSELAALEK	TYNTYRSNTL	TRVQFKLALH	YFKNHLQCAA
**490**							

Cmpd.	No. of Cmpds.	m/z meas.	Δ m/z [ppm]	z	Rt [min]	Score	P	Range	Sequence	Modification
26	1	537.7695	-77.75	2	30.0	13.9	0	299-308	R.NLAISQVTTK.Y	

Detailed Protein Report

Protein 399: leucine-rich repeat and WD repeat-containing protein KIAA1239 [Homo sapiens]

**Accession:**	gi|222418587	**Score:**	24.7
**Database:**	refseq_human(refseq_human_20140103.fasta)	**MW [kDa]:**	197.3
**Database Date:**	2015-11-30	**pI:**	5.8
		**Sequence Coverage [%]:**	1.3
		**No. of unique Peptides:**	1

10	20	30	40	50	60	70	80
MWPAGAGTKL	PCPRDSALRR	AAFSGNLTAL	PSHLVPAGRS	VRVFISANPE	DTGAERQALR	ENVYPKLREF	CRENYGLEFQ
90	100	110	120	130	140	150	160
VIDLYWGVEE	DEWDSPELQK	TRMKLLENCL	KTSAGPCFVG	LLGEKYGNIR	IPGEVEASEF	EMILDAAIEA	KLETKLLEEW
170	180	190	200	210	220	230	240
YCRDENSVPA	AYYLRPKSEM	LRSNRNAMQP	STNAENEKTW	QEISDEIKKI	FKAAVKLLHE	KGKMKHSQAK	RYLFSAIEDE
250	260	270	280	290	300	310	320
FDFALGKQTP	AFLKKCVCYI	RKIANIERFV	KIPEMGKYMD	ITGTEPRIIR	DPEAQEKLIK	LRDEFIPTIV	ASSNLRVYTS
330	340	350	360	370	380	390	400
VTHCDMKLGY	SQEIENHYIE	GLGKQFYEDM	IDIIQATIQQ	NFDTETDTLY	DEILQHSSLC	KTYASFYEYK	CESLNIVHNY
410	420	430	440	450	460	470	480
ILPSKAGHIN	PLIIYGGPCT	GKTLLLAEVA	KKAYGWLHED	TGPESDPVVI	VRFLGTTDMS	SDLRTLLLSV	CEQLAVNYRC
490	500	510	520	530	540	550	560
LVQSYPKKIH	DLCDLFINLL	NESSLQRPLV	IIFDALEQLS	ENDDARKLWW	LPAHLPRFVR	IVLSTLPNKH	GILQKLRCLI
570	580	590	600	610	620	630	640
HEEDNYIELI	PRDRKMCSQV	LKHQLLRVKR	KVTSGQQIYV	NNALSKCTLP	MFVNLTFREV	RHWRSHKDVD	ESSLSVTVHE
650	660	670	680	690	700	710	720
SIEQLFWSLE	KKCGQKLVSR	ALGYITMAKM	GLSEMELEDV	LALDNSVMSE	LKENTRPSNP	LRVPYLYIAR	LKEGLSGYLI
730	740	750	760	770	780	790	800
ERHVKNVTLL	VWANRHLQLI	AQKLYLQDDN	DLREMHTILA	DYFLGVWSGG	RRKAFCLEDP	YLNGCLDLEN	RSLLEEEKHF
810	820	830	840	850	860	870	880
MEQASFDRQA	PDQPWVFQCN	PLEPDIFFVN	HRKMSELLYH	LTRCGKTDDL	LYGIIMNFSW	LYTMIKIGQF	DKVLSDIELA
890	900	910	920	930	940	950	960
YNYSQEKELK	FLANTLRSIK	NKVTAFPGSL	SAELQQRLLP	VVSSLPKLRH	LLLECDKDGP	KYCSIVPLHS	SMDVTYSPER
970	980	990	1000	1010	1020	1030	1040
LPLSSSHLHV	TEILPTCNPS	TVLTALENGS	ISTWDVETRQ	LLRQITTAQS	VILGMKLTSD	EKYLVVATTN	NTLLIYDNVN
1050	1060	1070	1080	1090	1100	1110	1120
SCLLSEVEIK	GTKHGSSATY	INGFTLSANH	ALAWLEASKD	VTVIDLLYGW	PLYQFHCWYE	VTCVQCSLDG	LYAFCGQYLN
1130	1140	1150	1160	1170	1180	1190	1200
TTTIFHLGSG	EKLCTVTSEF	SGGFVKFLLI	LDTAQEMVMV	DSEGSLSVWN	TEDISSPQLT	DDFDCRREDS	EVVSIELSED
1210	1220	1230	1240	1250	1260	1270	1280
QSAVLICKAL	SIELLDTGLW	KVAEKFRAKH	NERFISAVLS	KNGDCIIATM	ENTSAVFFWR	RDTGQCMASL	QEISGSIVKL
1290	1300	1310	1320	1330	1340	1350	1360
VKSSHHNMLL	SLSTSGVLSI	WDIDIITAMS	NIDKTGKPIQ	SLLLPARGEI	IYSLDGSDCV	HKWNFSSGFI	EAVFKHEGIV
1370	1380	1390	1400	1410	1420	1430	1440
EHCVLTSTGD	IMVTSDDKSS	QYVWHTSSGE	NLFRINGQRI	SQLLITHNDQ	FVVSLCEENA	SRVWRLATGH	RVCNILTTLQ
1450	1460	1470	1480	1490	1500	1510	1520
NAFITSANTF	VVGMTKSKVL	AVSLWTGSIT	KKFCCEDGTT	IVNFKLIPDC	PDIIVFITSA	ETVNIWSLTD	EVICRRVQLP
1530	1540	1550	1560	1570	1580	1590	1600
NNFLKNLEDF	EISPNGKLGI	IARGDENINV	LDLYSGKLRV	VHASGIIWRQ	RLSRDGRYLV	YICFRNGEEE	DENGAIFSLI
1610	1620	1630	1640	1650	1660	1670	1680
VMRLADGKNI	GACSLYKTPT	FLALSQRHLN	IIVGFDDGSI	GIYTVVDRVD	AALKIKIATS	NSRQIFNNAT	HTSRPKCNSY
1690	1700	1710	1720	1730	1740	1750	
CFKISVDCLW	RESTEVFARD	SPITVSDSTE	SNEATPSKKH	NSCYERVCSA	LEARGHSYAP	DN	

Cmpd.	No. of Cmpds.	m/z meas.	Δ m/z [ppm]	z	Rt [min]	Score	P	Range	Sequence	Modification
607	1	766.5212	180.82	2	36.2	10.1	0	1343-1355	K.WNFSSGFIEAVFK.H	

Detailed Protein Report

Protein 400: spectrin beta chain, non-erythrocytic 1 isoform 2 [Homo sapiens]

**Accession:**	gi|112382252	**Score:**	24.7
**Database:**	refseq_human(refseq_human_20140103.fasta)	**MW [kDa]:**	251.2
**Database Date:**	2015-11-30	**pI:**	5.3
		**Sequence Coverage [%]:**	0.9
		**No. of unique Peptides:**	2

Quantitation

QD:QU	**Median:** 0.37	**CV:** 0.00 %	**No. of Peptides:** 1

Detailed Protein Report

10	20	30	40	50	60	70	80
MELQRTSSIS	GPLSPAYTGQ	VPYNYNQLEG	RFKQLQDERE	AVQKKTFTKW	VNSHLARVSC	RITDLYTDLR	DGRMLIKLLE
90	100	110	120	130	140	150	160
VLSGERLPKP	TKGRMRIHCL	ENVDKALQFL	KEQRVHLENM	GSHDIVDGNH	RLTLGLIWTI	ILRFQIQDIS	VETEDNKEKK
170	180	190	200	210	220	230	240
SAKDALLLWC	QMKTAGYPNV	NIHNFTTSWR	DGMAFNALIH	KHRPDLIDFD	KLKKSNAHYN	LQNAFNLAEQ	HLGLTKLLDP
250	260	270	280	290	300	310	320
EDISVDHPDE	KSIITYVVTY	YHYFSKMKAL	AVEGKRIGKV	LDNAIETEKM	IEKYESLASD	LLEWIEQTII	ILNNRKFANS
330	340	350	360	370	380	390	400
LVGVQQQLQA	FNTYRTVEKP	PKFTEKGNLE	VLLFTIQSKM	RANNQKVYMP	REGKLISDIN	KAWERLEKAE	HERELALRNE
410	420	430	440	450	460	470	480
LIRQEKLEQL	ARRFDRKAAM	RETWLSENQR	LVSQDNFGFD	LPAVEAATKK	HEAIETDIAA	YEERVQAVVA	VARELEAENY
490	500	510	520	530	540	550	560
HDIKRITARK	DNVIRLWEYL	LELLRARRQR	LEMNLGLQKI	FQEMLYIMDW	MDEMKVLVLS	QDYGKHLLGV	EDLLQKHTLV
570	580	590	600	610	620	630	640
EADIGIQAER	VRGVNASAQK	FATDGEGYKP	CDPQVIRDRV	AHMEFCYQEL	CQLAAERRAR	LEESRRLWKF	FWEMAEEEGW
650	660	670	680	690	700	710	720
IREKEKILSS	DDYGKDLTSV	MRLLSKHRAF	EDEMSGRSGH	FEQAIKEGED	MIAEEHFGSE	KIRERIIYIR	EQWANLEQLS
730	740	750	760	770	780	790	800
AIRKKRLEEA	SLLHQFQADA	DDIDAWMLDI	LKIVSSSDVG	HDEYSTQSLV	KKHKDVAEEI	ANYRPTLDTL	HEQASALPQE
810	820	830	840	850	860	870	880
HAESPDVRGR	LSGIEERYKE	VAELTRLRKQ	ALQDTLALYK	MFSEADACEL	WIDEKEQWLN	NMQIPEKLED	LEVIQHRFES
890	900	910	920	930	940	950	960
LEPEMNNQAS	RVAVVNQIAR	QLMHSGHPSE	KEIKAQQDKL	NTRWSQFREL	VDRKKDALLS	ALSIQNYHLE	CNETKSWIRE
970	980	990	1000	1010	1020	1030	1040
KTKVIESTQD	LGNDLAGVMA	LQRKLTGMER	DLVAIEAKLS	DLQKEAEKLE	SEHPDQAQAI	LSRLAEISDV	WEEMKTTLKN
1050	1060	1070	1080	1090	1100	1110	1120
REASLGEASK	LQQFLRDLDD	FQSWLSRTQT	AIASEDMPNT	LTEAEKLLTQ	HENIKNEIDN	YEEDYQKMRD	MGEMVTQGQT
1130	1140	1150	1160	1170	1180	1190	1200
DAQYMFLRQR	LQALDTGWNE	LHKMWENRQN	LLSQSHAYQQ	FLRDTKQAEA	FLNNQEYVLA	HTEMPTTLEG	AEAAIKKQED
1210	1220	1230	1240	1250	1260	1270	1280
FMTTMDANEE	KINAVVETGR	RLVSDGNINS	DRIQEKVDSI	DDRHRKNRET	ASELLMRLKD	NRDLQKFLQD	CQELSLWINE
1290	1300	1310	1320	1330	1340	1350	1360
KMLTAQDMSY	DEARNLHSKW	LKHQAFMAEL	ASNKEWLDKI	EKEGMQLISE	KPETEAVVKE	KLTGLHKMWE	VLESTTQTKA
1370	1380	1390	1400	1410	1420	1430	1440
QRLFDANKAE	LFTQSCADLD	KWLHGLESQI	QSDDYGKDLT	SVNILLKKQQ	MLENQMEVRK	KEIEELQSQA	QALSQEGKST
1450	1460	1470	1480	1490	1500	1510	1520
DEVDSKRLTV	QTKFMELLEP	LNERKHNLLA	SKEIHQFNRD	VEDEILWVGE	RMPLATSTDH	GHNLQTVQLL	IKKNQTLQKE
1530	1540	1550	1560	1570	1580	1590	1600
IQGHQPRIDD	IFERSQNIVT	DSSSLSAEAI	RQRLADLKQL	WGLLIEETEK	RHRRLEEAHR	AQQYYFDAAE	AEAWMSEQEL
1610	1620	1630	1640	1650	1660	1670	1680
YMMSEEKAKD	EQSAVSMLKK	HQILEQAVED	YAETVHQLSK	TSRALVADSH	PESERISMRQ	SKVDKLYAGL	KDLAEERRGK
1690	1700	1710	1720	1730	1740	1750	1760
LDERHRLFQL	NREVDDLEQW	IAEREVVAGS	HELGQDYEHV	TMLQERFREF	ARDTGNIGQE	RVDTVNHLAD	ELINSGHSDA
1770	1780	1790	1800	1810	1820	1830	1840
ATIAEWKDGL	NEAWADLLEL	IDTRTQILAA	SYELHKFYHD	AKEIFGRIQD	KHKKLPEELG	RDQNTVETLQ	RMHTTFEHDI
1850	1860	1870	1880	1890	1900	1910	1920
QALGTQVRQL	QEDAARLQAA	YAGDKADDIQ	KRENEVLEAW	KSLLDACESR	RVRLVDTGDK	FRFFSMVRDL	MLWMEDVIRQ
1930	1940	1950	1960	1970	1980	1990	2000
IEAQEKPRDV	SSVELLMNNH	QGIKAEIDAR	NDSFTTCIEL	GKSLLARKHY	ASEEIKEKLL	QLTEKRKEMI	DKWEDRWEWL
2010	2020	2030	2040	2050	2060	2070	2080
RLILEVHQFS	RDASVAEAWL	LGQEPYLSSR	EIGQSVDEVE	KLIKRHEAFE	KSAATWDERF	SALERLTTLE	LLEVRRQQEE
2090	2100	2110	2120	2130	2140	2150	2160
EERKRRPPSP	EPSTKVSEEA	ESQQQWDTSK	GEQVSQNGLP	AEQGSPRVSY	RSQTYQNYKN	FNSRRTASDQ	PWSGL

Detailed Protein Report

Cmpd.	No. of Cmpds.	m/z meas.	Δ m/z [ppm]	z	Rt [min]	Score	P	Range	Sequence	Modification	Ratios
577	3	607.8304	-55.95	2	35.8	12.5	2	820-829	K.EVAELTRLRK.Q		
601	2	548.1743	-250.21	2	36.1	12.2	0	1087-1095	K.LLTQHENIK.N		QD:QU 0.37

Detailed Protein Report

Protein 401: RING finger protein 39 isoform 2 [Homo sapiens]

**Accession:**	gi|297139767	**Score:**	24.6
**Database:**	refseq_human(refseq_human_20140103.fasta)	**MW [kDa]:**	38.2
**Database Date:**	2015-11-30	**pI:**	10.1
		**Sequence Coverage [%]:**	7.6
		**No. of unique Peptides:**	1

10	20	30	40	50	60	70	80
MWWRDLTRLR	LWLKREAIPG	EGRKAAKVNA	GVGEKGIYTA	SSRGGPPSAR	SKAVTVVAEG	AASRSWLSMD	APELGPGLVE
**90**	**100**	**110**	**120**	**130**	**140**	**150**	**160**
RLEQLATCPL	CGGSFEDPVL	LACEHSFCRA	CLARRWGTPP	ATGTEASPTA	CPCCGLPCPR	RSLRSNVRLA	VEVRISRELR
**170**	**180**	**190**	**200**	**210**	**220**	**230**	**240**
EKLAEPGARA	GRRRGGRIPT	MGCLDLPGED	MRKTWRRFEV	PTSKSSNSED	DLPEDYPVVK	KMLHRLTADL	TLDPGTAHRR
**250**	**260**	**270**	**280**	**290**	**300**	**310**	**320**
LLISADRRSV	QLAPPGTPAP	PDGPKRFDQL	PAVLGAQGFG	AGRHCWEVET	ADAASCRDSS	GEDADDEESH	YAVGAAGESV
**330**	**340**	**350**	**360**				
QRKGCAPGPL	GERIFPLFCT	CDPRAPLRIV	PAES				

Cmpd.	No. of Cmpds.	m/z meas.	Δ m/z [ppm]	z	Rt [min]	Score	P	Range	Sequence	Modification
732	1	472.2592	-60.62	2	39.1	11.7	1	241-248	R.LLISADRR.S	

Detailed Protein Report

Protein 402: PREDICTED: ceramide-1-phosphate transfer protein isoform X2 [Homo sapiens]

**Accession:**	gi|530360429	**Score:**	24.6
**Database:**	refseq_human(refseq_human_20140103.fasta)	**MW [kDa]:**	17.2
**Database Date:**	2015-11-30	**pI:**	8.0
**Modification(s):**	Carbamidomethyl	**Sequence Coverage [%]:**	21.2
		**No. of unique Peptides:**	2

```
        10         20         30         40         50         60         70         80
MERLRGGPQS EHYRSLQAMV AHELSNRLVD LERRSHHPES GCRTVLRLHR ALHWLQLFLE GLRTSPEDAR TSALCADSYN
        90        100        110        120        130        140        150        160
ASLAAYHPWV VRRAVTVAFC TLPTREVFLE AMNVGPPEQA VQMLGEALPF IQRVYNVSQK LYAEHSLLDL P
```

Cmpd.	No. of Cmpds.	m/z meas.	Δ m/z [ppm]	z	Rt [min]	Score	P	Range	Sequence	Modification
2793	1	846.4331	4.83	2	66.1	13.0	2	34-47	R.RSHHPESGCRTVLR.L	Carbamidomethyl: 9
2006	1	1044.9286	-117.90	2	55.5	11.6	1	134-151	R.VYNVSQKLYAEHSLLDLP.-	

Detailed Protein Report

Protein 403: 1-phosphatidylinositol 3-phosphate 5-kinase isoform 2 [Homo sapiens]

**Accession:**	gi|121583483	**Score:**	24.6
**Database:**	refseq_human(refseq_human_20140103.fasta)	**MW [kDa]:**	237.0
**Database Date:**	2015-11-30	**pI:**	6.2
		**Sequence Coverage [%]:**	1.3
		**No. of unique Peptides:**	2

Detailed Protein Report

10	20	30	40	50	60	70	80
MATDDKTSPT	LDSANDLPRS	PTSPSHLTHF	KPLTPDQDEP	PFKSAYSSFV	NLFRFNKERA	EGGQGEQQPL	SGSWTSPQLP
90	100	110	120	130	140	150	160
SRTQSVRSPT	PYKKQLNEEL	QRRSSALDTR	RKAEPTFGGH	DPRTAVQLRS	LSTVLKRLKE	IMEGKSQDSD	LKQYWMPDSQ
170	180	190	200	210	220	230	240
CKECYDCSEK	FTTFRRRHHC	RLCGQIFCSR	CCNQEIPGKF	MGYTGDLRAC	TYCRKIALSY	AHSTDSNSIG	EDLNALSDSA
250	260	270	280	290	300	310	320
CSVSVLDPSE	PRTPVGSRKA	SRNIFLEDDL	AWQSLIHPDS	SNTPLSTRLV	SVQEDAGKSP	ARNRSASITN	LSLDRSGSPM
330	340	350	360	370	380	390	400
VPSYETSVSP	QANRTYVRTE	TTEDERKILL	DSVQLKDLWK	KICHHSSGME	FQDHRYWLRT	HPNCIVGKEL	VNWLIRNGHI
410	420	430	440	450	460	470	480
ATRAQAIAIG	QAMVDGRWLD	CVSHHDQLFR	DEYALYRPLQ	STEFSETPSP	DSDSVNSVEG	HSEPSWFKDI	KFDDSDTEQI
490	500	510	520	530	540	550	560
AEEGDDNLAN	SASPSKRTSV	SSFQSTVDSD	SAASISLNVE	LDNVNFHIKK	PSKYPHVPPH	PADQKEYLIS	DTGGQQLSIS
570	580	590	600	610	620	630	640
DAFIKESLFN	RRVEEKSKEL	PFTPLGWHHN	NLELLREENG	EKQAMERLLS	ANHNHMMALL	QQLLHSDSLS	SSWRDIIVSL
650	660	670	680	690	700	710	720
VCQVVQTVRP	DVKNQDDDMD	IRQFVHIKKI	PGGKKFDSVV	VNGFVCTKNI	AHKKMSSCIK	NPKILLLKCS	IEYLYREETK
730	740	750	760	770	780	790	800
FTCIDPIVLQ	EREFLKNYVQ	RIVDVRPTLV	LVEKTVSRIA	QDMLLEHGIT	LVINVKSQVL	ERISRMTQGD	LVMSMDQLLT
810	820	830	840	850	860	870	880
KPHLGTCHKF	YMQIFQLPNE	QTKTLMFFEG	CPQHLGCTIK	LRGGSDYELA	RVKEILIFMI	CVAYHSQLEI	SFLMDEFAMP
890	900	910	920	930	940	950	960
PTLMQNPSFH	SLIEGRGHEG	AVQEQYGGGS	IPWDPDIPPE	SLPCDDSSLL	ELRIVFEKGE	QENKNLPQAV	ASVKHQEHST
970	980	990	1000	1010	1020	1030	1040
TACPAGLPCA	FFAPVPESLL	PLPVDDQQDA	LGSEQPETLQ	QTVVLQDPKS	QIRAFRDPLQ	DDTGLYVTEE	VTSSEDKRKT
1050	1060	1070	1080	1090	1100	1110	1120
YSLAFKQELK	DVILCISPVI	TFREPFLLTE	KGMRCSTRDY	FAEQVYWSPL	LNKEFKEMEN	RRKKQLLRDL	SGLQGMNGSI
1130	1140	1150	1160	1170	1180	1190	1200
QAKSIQVLPS	HELVSTRIAE	HLGDSQSLGR	MLADYRARGG	RIQPKNSDPF	AHSKDASSTS	SGQSGSKNEG	DEERGLILSD
1210	1220	1230	1240	1250	1260	1270	1280
AVWSTKVDCL	NPINHQRLCV	LFSSSSAQSS	NAPSACVSPW	IVTMEFYGKN	DLTLGIFLER	YCFRPSYQCP	SMFCDTPMVH
1290	1300	1310	1320	1330	1340	1350	1360
HIRRFVHGQG	CVQIILKELD	SPVPGYQHTI	LTYSWCRICK	QVTPVVALSN	ESWSMSFAKY	LELRFYGHQY	TRRANAEPCG
1370	1380	1390	1400	1410	1420	1430	1440
HSIHHDYHQY	FSYNQMVASF	SYSPIRLLEV	CVPLPKIFIK	RQAPLKVSLL	QDLKDFFQKV	SQVYVAIDER	LASLKTDTFS
1450	1460	1470	1480	1490	1500	1510	1520
KTREEKMEDI	FAQKEMEEGE	FKNWIEKMQA	RLMSSSVDTP	QQLQSVFESL	IAKKQSLCEV	LQAWNNRLQD	LFQQEKGRKR
1530	1540	1550	1560	1570	1580	1590	1600
PSVPPSPGRL	RQGEESKISA	MDASPRNISP	GLQNGEKEDR	FLTTLSSQSS	TSSTHLQLPT	PPEVMSEQSV	GGPPELDTAS
1610	1620	1630	1640	1650	1660	1670	1680
SSEDVFDGHL	LGSTDSQVKE	KSTMKAIFAN	LLPGNSYNPI	PFPFDPDKHY	LMYEHERVPI	AVCEKEPSSI	IAFALSCKEY
1690	1700	1710	1720	1730	1740	1750	1760
RNALEELSKA	TQWNSAEEGL	PTNSTSDSRP	KSSSPIRLPE	MSGGQTNRTT	ETEPQPTKKA	SGMLSFFRGT	AGKSPDLSSQ
1770	1780	1790	1800	1810	1820	1830	1840
KRETLRGADS	AYYQVGQTGK	EGTENQGVEP	QDEVDGGDTQ	KKQLINPHVE	LQFSDANAKF	YCRLYYAGEF	HKMREVILDS
1850	1860	1870	1880	1890	1900	1910	1920
SEEDFIRSLS	HSSPWQARGG	KSGAAFYATE	DDRFILKQMP	RLEVQSFLDF	APHYFNYITN	AVQQKRPTAL	AKILGVYRIG
1930	1940	1950	1960	1970	1980	1990	2000
YKNSQNNTEK	KLDLLVMENL	FYGRKMAQVF	DLKGSLRNRN	VKTDTGKESC	DVVLLDENLL	KMVRDNPLYI	RSHSKAVLRT
2010	2020	2030	2040	2050	2060	2070	2080
SIHSDSHFLS	SHLIIDYSLL	VGRDDTSNEL	VVGIIDYIRT	FTWDKKLEMV	VKSTGILGGQ	GKMPTVVSPE	LYRTRFCEAM
2090	2100						
DKYFLMVPDH	WTGLGLNC						

Detailed Protein Report

Cmpd.	No. of Cmpds.	m/z meas.	Δ m/z [ppm]	z	Rt [min]	Score	P	Range	Sequence	Modification
596	1	656.3887	96.05	2	36.0	14.0	1	112-123	R.KAEPTFGGHDPR.T	
1652	1	911.3660	-108.75	2	50.9	10.6	0	2024-2039	R.DDTSNELVVGIIDYIR.T	

Detailed Protein Report

Protein 404: PREDICTED: WD repeat-containing protein 59 isoform X2 [Homo sapiens]

**Accession:**	gi|530424315	**Score:**	24.6
**Database:**	refseq_human(refseq_human_20140103.fasta)	**MW [kDa]:**	76.6
**Database Date:**	2015-11-30	**pI:**	9.7
**Modification(s):**	Oxidation	**Sequence Coverage [%]:**	4.5
		**No. of unique Peptides:**	1

10	20	30	40	50	60	70	80
MMMWSWSSSG	GSRRKLCAND	ILDGVDEFIE	SISLLPEPEK	TLHTEDTDHQ	HTASHGEEEA	LKEDPPRNLL	EERKSDQLGL
**90**	**100**	**110**	**120**	**130**	**140**	**150**	**160**
PQTLQQEFSL	INVQIRNVNV	EMDAADRSCT	VSVHCSNHRV	KMLVKFPAQY	PNNAAPSFQF	INPTTITSTM	KAKLLKILKD
**170**	**180**	**190**	**200**	**210**	**220**	**230**	**240**
TALQKVKRGQ	SCLEPCLRQL	VSCLESFVNQ	EDSASSNPFA	LPNSVTPPLP	TFARVTTAYG	SYQDANIPFP	RTSGARFCGA
**250**	**260**	**270**	**280**	**290**	**300**	**310**	**320**
GYLVYFTRPM	TMHRAVSPTE	PTPRSLSALS	AYHTGLIAPM	KIRTEAPGNL	RLYSGSPTRS	EKEQVSISSF	YYKERMSPRS
**330**	**340**	**350**	**360**	**370**	**380**	**390**	**400**
ARRRWSIQAI	NDFPKSRRWK	SKREGSDSGN	RQIKAAGKVI	IQDIACLLPV	HKSLGELYIL	NVNDIQETCQ	KNAASALLVG
**410**	**420**	**430**	**440**	**450**	**460**	**470**	**480**
RKDLVQVWSL	ATVATDLCLG	PKSDPDLETP	WARHPFGRQL	LESLLAHYCR	LRDVQTLAML	CSVFEAQSRP	QGLPNPFGPF
**490**	**500**	**510**	**520**	**530**	**540**	**550**	**560**
PNRSSNLVVS	HSRYPSFTSS	GSCSSMSDPG	LNTGGWNIAG	REAEHLSSPW	GESSPEELRF	GSLTYSDPRE	RERDQHDKNK
**570**	**580**	**590**	**600**	**610**	**620**	**630**	**640**
RLLDPANTQQ	FDDFKKCYGE	ILYRWGLREK	RAEVLKFVSC	PPDPHKGIEF	GVYCSHCRSE	VRGTQCAICK	GFTFQCAICH
**650**	**660**	**670**	**680**	**690**			
VAVRGSSNFC	LTCGHGGHTS	HMMEWFRTQE	VCPTGCGCHC	LLESTF			

Cmpd.	No. of Cmpds.	m/z meas.	Δ m/z [ppm]	z	Rt [min]	Score	P	Range	Sequence	Modification
1549	1	1023.0071	-55.90	2	49.6	10.6	1	265-283	R.SLSALSAYHTGLIAPMKIR.T	Oxidation: 16

Detailed Protein Report

Protein 405: **up-regulator of cell proliferation isoform 2 [Homo sapiens]**

**Accession:**	gi|117968345	**Score:**	24.6
**Database:**	refseq_human(refseq_human_20140103.fasta)	**MW [kDa]:**	100.3
**Database Date:**	2015-11-30	**pI:**	6.2
		**Sequence Coverage [%]:**	3.3
		**No. of unique Peptides:**	2

10	20	30	40	50	60	70	80
MEGDDCEFRY	GDGTNEAQDN	DFPTVERSRL	QEMLSLLGLE	TYQVQKLSLQ	DSLQISFDSM	KNWAPQVPKD	LPWNFLRKLQ
90	100	110	120	130	140	150	160
ALNADARNTT	MVLDVLPDAR	PVEKESQMEE	EIIYWDPADD	LAADIYSFSE	LPTPDTPVNP	LDLLCALLLS	SDSFLQQEIA
170	180	190	200	210	220	230	240
LKMALCQFAL	PLVLPDSENH	YHTFLLWAMR	GIVRTWWSQP	PRGMGSFRED	SVVLSRAPAF	AFVRMDVSSN	SKSQLLNAVL
250	260	270	280	290	300	310	320
SPGHRQWDCF	WHRDLNLGTN	AREISDGLVE	ISWFFPSGRE	DLDIFPEPVA	FLNLRGDIGS	HWLQFKLLTE	ISSAVFILTD
330	340	350	360	370	380	390	400
NISKKEYKLL	YSMKESTTKY	YFILSPYRGK	RNTNLRFLNK	LIPVLKIDHS	HVLVKVSSTD	SDSFVKRIRA	IVGNVLRAPC
410	420	430	440	450	460	470	480
RRVSVEDMAH	AARKLGLKVD	EDCEECQKAK	DRMERITRKI	KDSDAYRRDE	LRLQGDPWRK	AAQVEKEFCQ	LQWAVDPPEK
490	500	510	520	530	540	550	560
HRAELRRRLL	ELRMQQNGHD	PSSGVQEFIS	GISSPSLSEK	QYFLRWMEWG	LARVAQPRLR	QPPETLLTLR	PKHGGTTDVG
570	580	590	600	610	620	630	640
EPLWPEPLGV	EHFLREMGQF	YEAESCLVEA	GRLPAGQRRF	AHFPGLASEL	LLTGLPLELI	DGSTLSMPVR	WVTGLLKELH
650	660	670	680	690	700	710	720
VRLERRSRLV	VLSTVGVPGT	GKSTLLNTMF	GLRFATGKSC	GPRGAFMQLI	TVAEGFSQDL	GCDHILVIDS	GGLIGGALTS
730	740	750	760	770	780	790	800
AGDRFELEAS	LATLLMGLSN	VTVISLAETK	DIPAAILHAF	LRLEKTGHMP	NYQFVYQNLH	DVSVPGPRPR	DKRQLLDPPG
810	820	830	840	850	860	870	880
DLSRAAAQME	KQGDGFRALA	GLAFCDPEKQ	HIWHIPGLWH	GAPPMAAVSL	AYSEAIFELK	RCLLENIRNG	LSNQNKNIQQ
890							
LIELVRRL							

Cmpd.	No. of Cmpds.	m/z meas.	Δ m/z [ppm]	z	Rt [min]	Score	P	Range	Sequence	Modification
1901	12	698.8088	27.42	2	52.3	14.5	1	419-430	K.VDEDCEECQKAK.D	
2800	1	863.4092	-128.64	2	66.2	10.1	2	646-662	R.RSRLVVLSTVGVPGTGK.S	

Detailed Protein Report

Protein 406: PREDICTED: protein Shroom4 isoform X2 [Homo sapiens]

Accession: gi|578838225
Database: refseq_human(refseq_human_20140103.fasta)
Database Date: 2015-11-30
Modification(s): Carbamidomethyl, Oxidation

Score: 24.6
MW [kDa]: 147.5
pI: 6.0
Sequence Coverage [%]: 1.9
No. of unique Peptides: 2

Quantitation

QD:QU **Median:** 0.25 **CV:** 0.00 % **No. of Peptides:** 1

```
        10         20         30         40         50         60         70         80
MESLEQPGQA TYESHLLPID QNMYPNQRDS AYSSFSASSN ASDCALSLRP EEPASTDCIM QGPGPTKAPS GRPNVAETSG
        90        100        110        120        130        140        150        160
GSRRTNGGHL TPSSQMSSRP QEGYQSGPAK AVRGPPQPPV RRDSLQASRA QLLNGEQRRA SEPVVPLPQK EKLSLEPVLP
       170        180        190        200        210        220        230        240
ARNPNRFCCL SGHDQVTSEG HQNCEFSQPP ESSQQGSEHL LMQASTKAVG SPKACDRASS VDSNPLNEAS AELAKASFGR
       250        260        270        280        290        300        310        320
PPHLIGPTGH RHSAPEQLLA SHLQHVHLDT RGSKGMELPP VQDGHQWTLS PLHSSHKGKK SPCPPTGGTH DQSSKERKTR
       330        340        350        360        370        380        390        400
QVDDRSLVLG HQSQSSPPHG EADGHPSEKG FLDPNRTSRA ASELANQQPS ASGSLVQQAT DCSSTTKAAS GTEAGEEGDS
       410        420        430        440        450        460        470        480
EPKECSRMGG RRSGGTRGRS IQNRRKSERF ATNLRNEIQR RKAQLQKSKG PLSQLCDTKE PVEETQEPPE SPPLTASNTS
       490        500        510        520        530        540        550        560
LLSSCKKPPS PRDKLFNKSM MLRARSSECL SQAPESHESR TGLEGRISPG QRPGQSSLGL NTWWKAPDPS SSDPEKAHAH
       570        580        590        600        610        620        630        640
CGVRGGHWRW SPEHNSQPLV AAAMEGPSNP GDNKELKAST AQAGEDAILL PFADRRKFFE ESSKSLSTSH LPGLTTHSNK
       650        660        670        680        690        700        710        720
TFTQRPKPID QNFQPMSSSC RELRRHPMDQ SYHSADQPYH ATDQSYHSMS PLQSETPTYS ECFASKGLEN SMCCKPLHCG
       730        740        750        760        770        780        790        800
DFDYHRTCSY SCSVQGALVH DPCIYCSGEI CPALLKRNMM PNCYNCRCHH HQCIRCSVCY HNPQHSALED SSLAPGNTWK
       810        820        830        840        850        860        870        880
PRKLTVQEFP GDKWNPITGN RKTSQSGREM AHSKTSFSWA TPFHPCLENP ALDLSSYRAI SSLDLLGDFK HALKKSEETS
       890        900        910        920        930        940        950        960
VYEEGSSLAS MPHPLRSRAF SESHISLAPQ STRAWGQHRR ELFSKGDETQ SDLLGARKKA FPPPRPPPPN WEKYRLFRAA
       970        980        990       1000       1010       1020       1030       1040
QQQKQQQQQQ KQQEEEEEEE EEEEEEEEEE EEEAEEEEEE LPPQYFSSET SGSCALNPEE VLEQPQPLSF GHLEGSRQGS
      1050       1060       1070       1080       1090       1100       1110       1120
QSVPAEQESF ALHSSDFLPP IRGHLGSQPE QAQPPCYYGI GGLWRTSGQE ATESAKQEFQ HFSPPSGAPG IPTSYSAYYN
      1130       1140       1150       1160       1170       1180       1190       1200
ISVAKAELLN KLKDQPEMAE IGLGEEEVDH ELAQKKIQLI ESISRKLSVL REAQRGLLED INANSALGEE VEANLKAVCK
      1210       1220       1230       1240       1250       1260       1270       1280
SNEFEKYHLF VGDLDKVVNL LLSLSGRLAR VENALNSIDS EANQEKLVLI EKKQQLTGQL ADAKELKEHV DRREKLVFGM
      1290       1300       1310       1320       1330       1340
VSRYLPQDQL QDYQHFVKMK SALIIEQREL EEKIKLGEEQ LKCLRESLLL GPSNF
```

Cmpd.	No. of Cmpds.	m/z meas.	Δ m/z [ppm]	z	Rt [min]	Score	P	Range	Sequence	Modification	Ratios
43	1	750.8112	-65.69	2	30.4	14.4	1	557-569	K.AHAHCGVRGGHWR.W	Carbamidomethyl: 5	QD:QU 0.25
2564	1	731.7179	-190.01	2	60.7	10.2	2	822-834	R.KTSQSGREMAHSK.T	Oxidation: 9	

Detailed Protein Report

Protein 407: **carboxyl-terminal PDZ ligand of neuronal nitric oxide synthase protein isoform 3 [Homo sapiens]**

Accession: gi|258613965
Database: refseq_human(refseq_human_20140103.fasta)
Database Date: 2015-11-30
Modification(s): Oxidation

Score: 24.6
MW [kDa]: 55.5
pI: 5.9
Sequence Coverage [%]: 6.2
No. of unique Peptides: 1

10	20	30	40	50	60	70	80
MPSKTKYNLV	DDGHDLRIPL	HNEDAFQHGI	CFEAKYVGSL	DVPRPNSRVE	IVAAMRRIRY	EFKAKNIKKK	KVSIMVSVDG
90	100	110	120	130	140	150	160
VKVILKKKKK	KKEWTWDESK	MLVMQDPIYR	IFYVSHDSQD	LKIFSYIARD	GASNIFRCNV	FKSKKKSQAM	RIVRTVGQAF
170	180	190	200	210	220	230	240
EVCHKLSLQH	TQQNADGQED	GESERNSNSS	GDPGRQLTGA	ERASTATAEE	TDIDAVEVPL	PGNDVLEFSR	GVTDLDAVGK
250	260	270	280	290	300	310	320
EGGSHTGSKV	SHPQEPMLTA	SPRMLLPSSS	SKPPGLGTET	PLSTHHQMQL	LQQLLQQQQQ	QTQVAVAQVH	LLKDQLAAEA
330	340	350	360	370	380	390	400
AARLEAQARV	HQLLLQNKDM	LQHISLLVKQ	VQELELKLSG	QNAMGSQDSL	LEITFRSGAL	PVLCDPTTPK	PEDLHSPPLG
410	420	430	440	450	460	470	480
AGLADFAHPA	GSPLGRRDCL	VKLECFRFLP	PEDTPPPAQG	EALLGGLELI	KFRESGIASE	YESNTDESEE	RDSWSQEELP
490	500	510					
RLLNVLQRQE	LGDGLDDEIA	V					

Cmpd.	No. of Cmpds.	m/z meas.	Δ m/z [ppm]	z	Rt [min]	Score	P	Range	Sequence	Modification
1604	1	783.2767	-145.45	2	50.3	12.9	0	250-263	K.VSHPQEPMLTASPR.M	Oxidation: 8

Detailed Protein Report

Protein 408: smoothelin isoform e [Homo sapiens]

Accession: gi|333360860
Database: refseq_human(refseq_human_20140103.fasta)
Database Date: 2015-11-30
Modification(s): Oxidation

Score: 24.5
MW [kDa]: 104.8
pI: 9.9
Sequence Coverage [%]: 2.9
No. of unique Peptides: 1

10	20	30	40	50	60	70	80
MGGMGTPGDL	HAACPSTTRR	DALCLGPEFE	FDLRHSAGCP	GDLGFFPLDP	DTEAGGSGCL	PTWLPLRRLA	RGRLEVTADL
90	100	110	120	130	140	150	160
AERRRIRSAI	RELQRQELER	EEEALASKRF	RAERQDNKEN	WLHSQQREAE	QRAALARLAG	QLESMNDVEE	LTALLRSAGE
170	180	190	200	210	220	230	240
YEERKLIRAA	IRRVRAQEIE	AATLAGRLYS	GRPNSGSRED	SKGLAAHRLE	QCEVPEREEQ	EQQAEVSKPT	PTPEGTSQDV
250	260	270	280	290	300	310	320
TTVTLLLRAP	PGSTSSSPAS	PSSSPTPASP	EPPLEPAEAQ	CLTAEVPGSP	EPPPSPPKTT	SPEPQESPTL	PSTEGQVVNK
330	340	350	360	370	380	390	400
LLSGPKETPA	AQSPTRGPSD	TKRADVAGPR	PCQRSLSVLS	PRQPAQNRES	TPLASGPSSF	QRAGSVRDRV	HKFTSDSPMA
410	420	430	440	450	460	470	480
ARLQDGTPQA	ALSPLTPARL	LGPSLTSTTP	ASSSSGSSSR	GPSDTSSRFS	KEQRGVAQPL	AQLRSCPQEE	GPRGRGLAAR
490	500	510	520	530	540	550	560
PLENRAGGPV	ARSEEPGAPL	PVAVGTAEPG	GSMKTTFTIE	IKDGRGQAST	GRVLLPTGNQ	RAELTLGLRA	PPTLLSTSSG
570	580	590	600	610	620	630	640
GKSTITRVNS	PGTLARLGSV	THVTSFSHAP	PSSRGGCSIK	MEAEPAEPLA	AAVEAANGAE	QTRVNKAPEG	RSPLSAEELM
650	660	670	680	690	700	710	720
TIEDEGVLDK	MLDQSTDFEE	RKLIRAALRE	LRQRKRDQRD	KERERRLQEA	RGRPGEGRGN	TATETTTRHS	QRAADGSAVS
730	740	750	760	770	780	790	800
TVTKTERLVH	SNDGTRTART	TTVESSFVRR	SENGSGSTMM	QTKTFSSSSS	SKKMGSIFDR	EDQASPRAGS	LAALEKRQAE
810	820	830	840	850	860	870	880
KKKELMKAQS	LPKTSASQAR	KAMIEKLEKE	GAAGSPGGPR	AAVQRSTSFG	VPNANSIKQM	LLDWCRAKTR	GYEHVDIQNF
890	900	910	920	930	940	950	960
SSSWSDGMAF	CALVHNFFPE	AFDYGQLSPQ	NRRQNFEVAF	SSAETHADCP	QLLDTEDMVR	LREPDWKCVY	TYIQEFYRCL
970	980						
VQKGLVKTKK	S						

Cmpd.	No. of Cmpds.	m/z meas.	Δ m/z [ppm]	z	Rt [min]	Score	P	Range	Sequence	Modification
2098	2	938.2855	-133.64	2	54.8	13.7	0	1-19	-.MGGMGTPGDLHAACPSTTR.R	Oxidation: 1

Detailed Protein Report

Protein 409: PREDICTED: PDZ domain-containing protein 2 isoform X8 [Homo sapiens]

**Accession:**	gi|578809880	**Score:**	24.5
**Database:**	refseq_human(refseq_human_20140103.fasta)	**MW [kDa]:**	289.4
**Database Date:**	2015-11-30	**pI:**	7.4
		**Sequence Coverage [%]:**	1.4
		**No. of unique Peptides:**	1

Detailed Protein Report

10	20	30	40	50	60	70	80
MPITQDNAVL	HLPLLYQWLQ	NSLQEGGDGP	EQRLCQAAIQ	KLQEYIQLNF	AVDESTVPPD	HSPPEMEICT	VYLTKELGDT
90	100	110	120	130	140	150	160
ETVGLSFGNI	PVFGDYGEKR	RGGKKRKTHQ	GPVLDVGCIW	VTELRKNSPA	GKSGKVRLRD	EILSLNGQLM	VGVDVSGASY
170	180	190	200	210	220	230	240
LAEQCWNGGF	IYLIMLRRFK	HKAHSTYNGN	SSNSSEPGET	PTLELGDRTA	KKGKRTRKFG	VISRPPANKA	PEESKGSAGC
250	260	270	280	290	300	310	320
EVSSDPSTEL	ENGPDPELGN	GHVFQLENGP	DSLKEVAGPH	LERSEVDRGT	EHRIPKTDAP	LTTSNDKRRF	SKGGKTDFQS
330	340	350	360	370	380	390	400
SDCLAREEVG	RIWKMELLKE	SDGLGIQVSG	GRGSKRSPHA	IVVTQVKEGG	AAHRDGRLSL	GDELLVINGH	LLVGLSHEEA
410	420	430	440	450	460	470	480
VAILRSATGM	VQLVVASKEN	SAEDLLRLTS	KSLPDLTSSV	EDVSSWTDNE	DQEADGEEDE	GTSSSVQRAM	PGTDEPQDVC
490	500	510	520	530	540	550	560
GAEESKGNLE	SPKQGSNKIK	LKSRLSGGVH	RLESVEEYNE	LMVRNGDPRI	RMLEVSRDGR	KHSLPQLLDS	SSASQEYHIV
570	580	590	600	610	620	630	640
KKSTRSLSTT	QVESPWRLIR	PSVISIIGLY	KEKGKGLGFS	IAGGRDCIRG	QMGIFVKTIF	PNGSAAEDGR	LKEGDEILDV
650	660	670	680	690	700	710	720
NGIPIKGLTF	QEAIHTFKQI	RSGLFVLTVR	TKLVSPSLTP	CSTPTHMSRS	ASPNFNTSGG	ASAGGSDEGS	SSSLGRKTPG
730	740	750	760	770	780	790	800
PKDRIVMEVT	LNKEPRVGLG	IGACCLALEN	SPPGIYIHSL	APGSVAKMES	NLSRGDQILE	VNSVNVRHAA	LSKVHAILSK
810	820	830	840	850	860	870	880
CPPGPVRLVI	GRHPNPKVSE	QEMDEVIARS	TYQESKEANS	SPGLGTPLKS	PSLAKKDSLI	SESELSQYFA	HDVPGPLSDF
890	900	910	920	930	940	950	960
MVAGSEDEDH	PGSGCSTSEE	GSLPPSTSTH	KEPGKPRANS	LVTLGSHRAS	GLFHKQVTVA	RQASLPGSPQ	ALRNPLLRQR
970	980	990	1000	1010	1020	1030	1040
KVGCYDANDA	SDEEEFDREG	DCISLPGALP	GPIRPLSEDD	PRRVSISSSK	GMDVHNQEER	PRKTLESLGK	LTTGDACVST
1050	1060	1070	1080	1090	1100	1110	1120
SCELASALSH	LDASHLTENL	PKAASELGQQ	PMTELDSSSD	LISSPGKKGA	AHPDPSKTSV	DTGQVSRPEN	PSQPASPRVT
1130	1140	1150	1160	1170	1180	1190	1200
KCKARSPVRL	PHEGSPSPGE	KAAAPPDYSK	TRSASETSTP	HNTRRVAALR	GAGPGAEGMT	PAGAVLPGDP	LTSQEQRQGA
1210	1220	1230	1240	1250	1260	1270	1280
PGNHSKALEM	TGIHAPESSQ	EPSLLEGADS	VSSRAPQASL	SMLPSTDNTK	EACGHVSGHC	CPGGSRESPV	TDIDSFIKEL
1290	1300	1310	1320	1330	1340	1350	1360
DASAARSPSS	QTGDSGSQEG	SAQGHPPAGA	GGGSSCRAEP	VPGGQTSSPR	RAWAAGAPAY	PQWASQPSVL	DSINPDKHFT
1370	1380	1390	1400	1410	1420	1430	1440
VNKNFLSNYS	RNFSSFHEDS	TSLSGLGDST	EPSLSSMYGD	AEDSSSDPES	LTEAPRASAR	DGWSPPRSRV	SLHKEDPSES
1450	1460	1470	1480	1490	1500	1510	1520
EEEQIEICST	RGCPNPPSSP	AHLPTQAAIC	PASAKVLSLK	YSTPRESVAS	PREKAACLPG	SYTSGPDSSQ	PSSLLEMSSQ
1530	1540	1550	1560	1570	1580	1590	1600
EHETHADIST	SQNHRPSCAE	ETTEVTSASS	AMENSPLSKV	ARHFHSPPII	LSSPNMVNGL	EHDLLDDETL	NQYETSINAA
1610	1620	1630	1640	1650	1660	1670	1680
ASLSSFSVDV	PKNGESVLEN	LHISESQDLD	DLLQKPKMIA	RRPIMAWFKE	INKHNQGTHL	RSKTEKEQPL	MPARSPDSKI
1690	1700	1710	1720	1730	1740	1750	1760
QMVSSSQKKG	VTVPHSPPQP	KTNLENKDLS	KKSPAEMLLT	NGQKAKCGPK	LKRLSLKGKA	KVNSEAPAAN	AVKAGGTDHR
1770	1780	1790	1800	1810	1820	1830	1840
KPLISPQTSH	KTLSKAVSQR	LHVADHEDPD	RNTTAAPRSP	QCVLESKPPL	ATSGPLKPSV	SDTSIRTFVS	PLTSPKPVPE
1850	1860	1870	1880	1890	1900	1910	1920
QGMWSRFHMA	VLSEPDRGCP	TTPKSPKCRA	EGRAPRADSG	PVSPAASRNG	MSVAGNRQSE	PRLASHVAAD	TAQPRPTGEK
1930	1940	1950	1960	1970	1980	1990	2000
GGNIMASDRL	ERTNQLKIVE	ISAEAVSETV	CGNKPAESDR	RGGCLAQGNC	QEKSEIRLYR	QVAESSTSHP	SSLPSHASQA
2010	2020	2030	2040	2050	2060	2070	2080
EQEMSRSFSM	AKLASSSSSL	QTAIRKAEYS	QGKSSLMSDS	RGVPRNSIPG	GPSGEDHLYF	TPRPATRTYS	MPAQFSSHFG
2090	2100	2110	2120	2130	2140	2150	2160
REGHPPHSLG	RSRDSQVPVT	SSVVPEAKAS	RGGLPSLANG	QGIYSVKPLL	DTSRNLPATD	EGDIISVQET	SCLVTDKIKV
2170	2180	2190	2200	2210	2220	2230	2240

Detailed Protein Report

Cmpd.	No. of Cmpds.	m/z meas.	Δ m/z [ppm]	z	Rt [min]	Score	P	Range	Sequence	Modification
892	1	675.8467	18.44	4	41.2	12.0	1	76-100	K.ELGDTETVGLSFGNIPVFGDYGEKR.R	

Detailed Protein Report

Protein 410: zinc finger protein 99 [Homo sapiens]

**Accession:**	gi|339418230	**Score:**	24.5
**Database:**	refseq_human(refseq_human_20140103.fasta)	**MW [kDa]:**	100.7
**Database Date:**	2015-11-30	**pI:**	10.5
		**Sequence Coverage [%]:**	2.9
		**No. of unique Peptides:**	1

10	20	30	40	50	60	70	80
MGSLTFWDVT	IEFALEEWQC	LDMAQQNLYR	NVMLENYRNL	VFLGIAVSKL	DLITCLKQGK	EPWNMKRHEM	VTKPPVISSH
**90**	**100**	**110**	**120**	**130**	**140**	**150**	**160**
FTQDFWPDQS	IKDSFQEIIL	RTYARCGHKN	LRLRKDCESV	NEGKMHEEAY	NKLNQCWTTT	QGKIFQCNKY	VKVFHKYSNS
**170**	**180**	**190**	**200**	**210**	**220**	**230**	**240**
NRYKIRHTKK	KTFKCMKCSK	SFFMLSHLIQ	HKRIHTRENI	YKCEERGKAF	KWFSTLIKHK	IIHTEDKPYK	YKKCGKAFNI
**250**	**260**	**270**	**280**	**290**	**300**	**310**	**320**
SSMFTKCKII	HTGKKPCKCE	ECGKVFNNSS	TLMKHKIIHT	GKKPYKCEEC	GKAFKQSSHL	TRHKAIHTGE	KPYKCEECGK
**330**	**340**	**350**	**360**	**370**	**380**	**390**	**400**
AFNHFSALRK	HQIIHTGKKP	YKCEECGKAF	SQSSTLRKHE	IIHTEEKPYK	YEECGKAFSN	LSALRKHEII	HTGQKPYKCE
**410**	**420**	**430**	**440**	**450**	**460**	**470**	**480**
ECGKAFKWSS	KLTVHKVIHT	AEKPCKCEEC	GKAFKRFSAL	RKHKIIHTGK	QPYKCEECSK	AFSNFSALRK	HEIIHTGEKP
**490**	**500**	**510**	**520**	**530**	**540**	**550**	**560**
YKCEECGKAF	KWSSKLTVHK	VIHMEEKPCK	CEECGKAFKH	FSALRKHKII	HTGKKPYKCE	ECGKAFNNSS	TLMKHKIIHT
**570**	**580**	**590**	**600**	**610**	**620**	**630**	**640**
GKKPYKCEEC	GKAFKQSSHL	TRHKAIHTGE	KPYKCEECGK	AFNHFSALRK	HQIIHTGKKP	YKCEECGKAF	SQSSTLRKHE
**650**	**660**	**670**	**680**	**690**	**700**	**710**	**720**
IIHTGEKPYK	CEECGKAFKW	SSHLTRHKVI	HTEEKPYKCE	ECGKAFNHFS	ALRKHKIIHT	GKKPYKCEEC	GKAFSQSSTL
**730**	**740**	**750**	**760**	**770**	**780**	**790**	**800**
RKHEIIHTGE	KPYKCEECGK	AFKWSSKLTV	HKVIHTAEKP	CKCEECGKAF	KHFSALRKHK	IIHTGKKPYK	CEECGKAFNN
**810**	**820**	**830**	**840**	**850**	**860**	**870**	
SSTLRKHEII	HTGEKSYKCE	ECGKAFQWSS	KLTLHKVIHM	ERNPANVKNV	AKLLNISQPL	ENMR	

Cmpd.	No. of Cmpds.	m/z meas.	Δ m/z [ppm]	z	Rt [min]	Score	P	Range	Sequence	Modification
2579	1	822.4636	21.90	2	62.9	10.6	1	181-193	K.SFFMLSHLIQHKR.I	

Detailed Protein Report

Protein 411: PREDICTED: protein transport protein Sec16A isoform X5 [Homo sapiens]

**Accession:**	gi|530426857	**Score:**	24.4
**Database:**	refseq_human(refseq_human_20140103.fasta)	**MW [kDa]:**	240.9
**Database Date:**	2015-11-30	**pI:**	5.7
		**Sequence Coverage [%]:**	1.0
		**No. of unique Peptides:**	1

Detailed Protein Report

10	20	30	40	50	60	70	80
MGAAGLGLGP	TTCSLTLFKQ	VRNLTPERQM	LAEQIGPGAT	SAGLKLSLHR	LRPCWRGTDI	TPSLTSRRPS	WGQGWLPPRE
90	100	110	120	130	140	150	160
DLSRCWELSL	PRCHRAGTPT	TAPALWKEQQ	RKAAALPCPC	QAGVQLQLRN	SYILLPSVSS	IDLCKGSVMQ	PPPQTVPSGM
170	180	190	200	210	220	230	240
AGPPPAGNPR	SVFWASSPYR	RRANNNAAVA	PTTCPLQPVT	DPFAFSRQAL	QSTPLGSSSK	SSPPVLQGPA	PAGFSQHPGL
250	260	270	280	290	300	310	320
LVPHTHARDS	SQGPCEPLPG	PLTQPRAHAS	PFSGALTPSA	PPGPEMNRSA	EVGPSSEPEV	QTLPYLPHYI	PGVDPETSHG
330	340	350	360	370	380	390	400
GHPHGNMPGL	DRPLSRQNPH	DGVVTPAASP	SLPQPGLQMP	GQWGPVQGGP	QPSGQHRSPC	PEGPVPSGVP	CATSVPHFPT
410	420	430	440	450	460	470	480
PSILHQGPGH	EQHSPLVAPP	AALPSDGRDE	VSHLQSGSHL	ANNSDPESTF	RQNPRIVNHW	ASPELRQNPG	VKNEHRPASA
490	500	510	520	530	540	550	560
LVNPLARGDS	PENRTHHPLG	AGAGSGCAPL	EADSGASGAL	AMFFQGGETE	NEENLSSEKA	GLSGQADFDD	FCSSPGLGRP
570	580	590	600	610	620	630	640
PAPTHVGAGS	LCQALLPGPS	NEAAGDVWGD	TASTGVPDAS	GSQYENVENL	EFVQNQEVLP	SEPLNLDPSS	PSDQFRYGPL
650	660	670	680	690	700	710	720
PGPAVPRHGA	VCHTGAPDAT	LHTVHPDSVS	SSYSSRSHGR	LSGSARPQEL	VGTFIQQEVG	KPEDEASGSF	FKQIDSSPVG
730	740	750	760	770	780	790	800
GETDETTVSQ	NYRGSVSQPS	TPSPPKPTGI	FQTSANSSFE	PVKSHLVGVK	PFEADRANVV	GEVRETCVRQ	KQCRPAAALP
810	820	830	840	850	860	870	880
DASPGNLEQP	PDNMETLCAP	QVCPLPLNST	TEAVHMLPHA	GAPPLDTVYP	APEKRPSART	QGPVKCESPA	TTLWAQSELP
890	900	910	920	930	940	950	960
DFGGNVLLAP	AAPALYVCAK	PQPPVVQPPE	EAMSGQQSRN	PSSAAPVQSR	GGIGASENLE	NPPKMGEEEA	LQSQVTKDAQ
970	980	990	1000	1010	1020	1030	1040
GQPGLERAQQ	ELVPPQQQAS	PPQLPKAMFS	ELSNPESLPA	QGQAQNSAQS	PASLVLVDAG	QQLPPRPPQS	SSVSLVSSGS
1050	1060	1070	1080	1090	1100	1110	1120
GQAAVPSEQP	WPQPVPALAP	GPPPQDLAAY	YYYRPLYDAY	QPQYSLPYPP	EPGAASLYYQ	DVYSLYEPRY	RPYDGAASAY
1130	1140	1150	1160	1170	1180	1190	1200
AQNYRYPEPE	RPSSRASHSS	ERPPPRQGYP	EGYYSSKSGW	SSQSDYYASY	YSSQYDYGDP	GHWDRYHYSA	RVRDPRTYDR
1210	1220	1230	1240	1250	1260	1270	1280
RYWCDAEYDA	YRREHSAFGD	RPEKRDNNWR	YDPRFTGSFD	DDPDPHRDPY	GEEVDRRSVH	SEHSARSLHS	AHSLASRRSS
1290	1300	1310	1320	1330	1340	1350	1360
LSSHSHQSQI	YRSHNVAAGS	YEAPLPPGSF	HGDFAYGTYR	SNFSSGPGFP	EYGYPADTVW	PAMEQVSSRP	TSPEKFSVPH
1370	1380	1390	1400	1410	1420	1430	1440
VCARFGPGGQ	LIKVIPNLPS	EGQPALVEVH	SMEALLQHTS	EQEEMRAFPG	PLAKDDTHKV	DVINFAQNKA	MKCLQNENLI
1450	1460	1470	1480	1490	1500	1510	1520
DKESASLLWN	FIVLLCRQNG	TVVGTDIAEL	LLRDHRTVWL	PGKSPNEANL	IDFTNEAVEQ	VEEEESGEAQ	LSFLTGGPAA
1530	1540	1550	1560	1570	1580	1590	1600
AASSLERETE	RFRELLLYGR	KKDALESAMK	NGLWGHALLL	ASKMDSRTHA	RVMTRFANSL	PINDPLQTVY	QLMSGRMPAA
1610	1620	1630	1640	1650	1660	1670	1680
STCCGDEKWG	DWRPHLAMVL	SNLNNNMDVE	SRTMATMGDT	LASRGLLDAA	HFCYLMAQAG	FGVYTKKTTK	LVLIGSNHSL
1690	1700	1710	1720	1730	1740	1750	1760
PFLKFATNEA	IQRTEAYEYA	QSLGAETCPL	PSFQVFKFIY	SCRLAEMGLA	TQAFHYCEAI	AKSILTQPHL	YSPVLISQLV
1770	1780	1790	1800	1810	1820	1830	1840
QMASQLRLFD	PQLKEKPEEE	SLAAPTWLVH	LQQVERQIKE	GAGVWHQDGA	LPQQCPGTPS	SEMEQLDRPG	LSQPGALGIA
1850	1860	1870	1880	1890	1900	1910	1920
NPLLAVPAPS	PEHSSPSVRL	LPSAPQTLPD	GPLASPARVP	MFPVPLPPGP	LEPGPGCVTP	GPALGFLEPS	GPGLPPGVPP
1930	1940	1950	1960	1970	1980	1990	2000
LQERRHLLQE	ARSPDPGIVP	QEAPVGNSLS	ELSEENFDGK	FANLTPSRTV	PDSEAPPGWD	RADSGPTQPP	LSLSPAPETK
2010	2020	2030	2040	2050	2060	2070	2080
RPGQAAKKET	KEPKKGESWF	FRWLPGKKKT	EAYLPDDKNK	SIVWDEKKNQ	WVNLNEPEEE	KKAPPPPPTS	MPKTVQAAPP
2090	2100	2110	2120	2130	2140	2150	2160
ALPGPPGAPV	NMYSRRAAGT	RARYVDVLNP	SGTQRSEPAL	APADFVAPLA	PLPIPSNLFV	PTPDAEEPQL	PDGTGREGPA
2170	2180	2190	2200	2210	2220	2230	2240

Detailed Protein Report

Cmpd.	No. of Cmpds.	m/z meas.	Δ m/z [ppm]	z	Rt [min]	Score	P	Range	Sequence	Modification
2846	2	607.3426	-19.17	2	64.7	10.1	2	2001-2011	K.RPGQAAKKETK.E	

Detailed Protein Report

Protein 412: putative RNA-binding protein 15B [Homo sapiens]

**Accession:**	gi|54607124	**Score:**	24.4
**Database:**	refseq_human(refseq_human_20140103.fasta)	**MW [kDa]:**	97.1
**Database Date:**	2015-11-30	**pI:**	10.3
		**Sequence Coverage [%]:**	3.0
		**No. of unique Peptides:**	1

10	20	30	40	50	60	70	80
MKRQSERDSS	PSGRGSSSSA	KRPREREREA	EAGGRRAAHK	ASGGAKHPVP	ARARDKPRGS	GSGGGGHRDG	RGTGDANHRA
**90**	**100**	**110**	**120**	**130**	**140**	**150**	**160**
SSGRSSGSGA	GGGGRGGKAS	GDPGASGMSP	RASPLPPPPP	PPGAEPACPG	SSAAAPEYKT	LLISSLSPAL	PAEHLEDRLF
**170**	**180**	**190**	**200**	**210**	**220**	**230**	**240**
HQFKRFGEIS	LRLSHTPELG	RVAYVNFRHP	QDAREARQHA	LARQLLLYDR	PLKVEPVYLR	GGGGSSRRSS	SSSAAASTPP
**250**	**260**	**270**	**280**	**290**	**300**	**310**	**320**
PGPPAPADPL	GYLPLHGGYQ	YKQRSLSPVA	APPLREPRAR	HAAAAFALDA	AAAAAVGLSR	ERALDYYGLY	DDRGRPYGYP
**330**	**340**	**350**	**360**	**370**	**380**	**390**	**400**
AVCEEDLMPE	DDQRATRNLF	IGNLDHSVSE	VELRRAFEKY	GIIEEVVIKR	PARGQGGAYA	FLKFQNLDMA	HRAKVAMSGR
**410**	**420**	**430**	**440**	**450**	**460**	**470**	**480**
VIGRNPIKIG	YGKANPTTRL	WVGGLGPNTS	LAALAREFDR	FGSIRTIDHV	KGDSFAYIQY	ESLDAAQAAC	AKMRGFPLGG
**490**	**500**	**510**	**520**	**530**	**540**	**550**	**560**
PDRRLRVDFA	KAEETRYPQQ	YQPSPLPVHY	ELLTDGYTRH	RNLDADLVRD	RTPPHLLYSD	RDRTFLEGDW	TSPSKSSDRR
**570**	**580**	**590**	**600**	**610**	**620**	**630**	**640**
NSLEGYSRSV	RSRSGERWGA	DGDRGLPKPW	EERRKRRSLS	SDRGRTTHSP	YEERSRTKGS	GQQSERGSDR	TPERSRKENH
**650**	**660**	**670**	**680**	**690**	**700**	**710**	**720**
SSEGTKESSS	NSLSNSRHGA	EERGHHHHHH	EAADSSHGKK	ARDSERNHRT	TEAEPKPLEE	PKHETKKLKN	LSEYAQTLQL
**730**	**740**	**750**	**760**	**770**	**780**	**790**	**800**
GWNGLLVLKN	SCFPTSMHIL	EGDQGVISSL	LKDHTSGSKL	TQLKIAQRLR	LDQPKLDEVT	RRIKQGSPNG	YAVLLATQAT
**810**	**820**	**830**	**840**	**850**	**860**	**870**	**880**
PSGLGTEGMP	TVEPGLQRRL	LRNLVSYLKQ	KQAAGVISLP	VGGSKGRDGT	GMLYAFPPCD	FSQQYLQSAL	RTLGKLEEEH
**890**	**900**						
MVIVIVRDTA							

Cmpd.	No. of Cmpds.	m/z meas.	Δ m/z [ppm]	z	Rt [min]	Score	P	Range	Sequence	Modification
609	1	666.9243	159.05	2	36.2	14.3	1	604-614	R.GRTTHSPYEER.S	

Detailed Protein Report

Protein 413: prolactin-inducible protein precursor [Homo sapiens]

Accession: gi|4505821
Database: refseq_human(refseq_human_20140103.fasta)
Database Date: 2015-11-30
Modification(s): Carbamidomethyl

Score: 24.3
MW [kDa]: 16.6
pI: 9.3
Sequence Coverage [%]: 10.3
No. of unique Peptides: 1

10	20	30	40	50	60	70	80
MRLLQLLFRA	SPATLLLVLC	LQLGANKAQD	NTRKIIIKNF	DIPKSVRPND	EVTAVLAVQT	ELKECMVVKT	YLISSIPLQG
**90**	**100**	**110**	**120**	**130**	**140**	**150**	
AFNYKYTACL	CDDNPKTFYW	DFYTNRTVQI	AAVVDVIREL	GICPDDAAVI	PIKNNRFYTI	EILKVE	

Cmpd.	No. of Cmpds.	m/z meas.	Δ m/z [ppm]	z	Rt [min]	Score	P	Range	Sequence	Modification
2317	1	805.9036	-28.46	2	57.6	24.3	0	119-133	R.ELGICPDDAAVIPIK.N	Carbamidomethyl: 5

Detailed Protein Report

Protein 414: xylosyltransferase 1 precursor [Homo sapiens]

**Accession:**	gi|28269693	**Score:**	24.3
**Database:**	refseq_human(refseq_human_20140103.fasta)	**MW [kDa]:**	107.5
**Database Date:**	2015-11-30	**pI:**	10.0
**Modification(s):**	Oxidation	**Sequence Coverage [%]:**	4.5
		**No. of unique Peptides:**	2

10	20	30	40	50	60	70	80
MVAAPCARRL	ARRSHSALLA	ALTVLLLQTL	VVWNFSSLDS	GAGERRGGAA	VGGGEQPPPA	PAPRRERRDL	PAEPAAARGG
**90**	**100**	**110**	**120**	**130**	**140**	**150**	**160**
GGGGGGGGGG	RGPQARARGG	GPGEPRGQQP	ASRGALPARA	LDPHPSPLIT	LETQDGYFSH	RPKEKVRTDS	NNENSVPKDF
**170**	**180**	**190**	**200**	**210**	**220**	**230**	**240**
ENVDNSNFAP	RTQKQKHQPE	LAKKPPSRQK	ELLKRKLEQQ	EKGKGHTFPG	KGPGEVLPPG	DRAAANSSHG	KDVSRPPHAR
**250**	**260**	**270**	**280**	**290**	**300**	**310**	**320**
KTGGSSPETK	YDQPPKCDIS	GKEAISALSR	AKSKHCRQEI	GETYCRHKLG	LLMPEKVTRF	CPLEGKANKN	VQWDEDSVEY
**330**	**340**	**350**	**360**	**370**	**380**	**390**	**400**
MPANPVRIAF	VLVVHGRASR	QLQRMFKAIY	HKDHFYYIHV	DKRSNYLHRQ	VLQVSRQYSN	VRVTPWRMAT	IWGGASLLST
**410**	**420**	**430**	**440**	**450**	**460**	**470**	**480**
YLQSMRDLLE	MTDWPWDFFI	NLSAADYPIR	TNDQLVAFLS	RYRDMNFLKS	HGRDNARFIR	KQGLDRLFLE	CDAHMWRLGD
**490**	**500**	**510**	**520**	**530**	**540**	**550**	**560**
RRIPEGIAVD	GGSDWFLLNR	RFVEYVTFST	DDLVTKMKQF	YSYTLLPAES	FFHTVLENSP	HCDTMVDNNL	RITNWNRKLG
**570**	**580**	**590**	**600**	**610**	**620**	**630**	**640**
CKCQYKHIVD	WCGCSPNDFK	PQDFHRFQQT	ARPTFFARKF	EAVVNQEIIG	QLDYYLYGNY	PAGTPGLRSY	WENVYDEPDG
**650**	**660**	**670**	**680**	**690**	**700**	**710**	**720**
IHSLSDVTLT	LYHSFARLGL	RRAETSLHTD	GENSCRYYPM	GHPASVHLYF	LADRFQGFLI	KHHATNLAVS	KLETLETWVM
**730**	**740**	**750**	**760**	**770**	**780**	**790**	**800**
PKKVFKIASP	PSDFGRLQFS	EVGTDWDAKE	RLFRNFGGLL	GPMDEPVGMQ	KWGKGPNVTV	TVIWVDPVNV	IAATYDILIE
**810**	**820**	**830**	**840**	**850**	**860**	**870**	**880**
STAEFTHYKP	PLNLPLRPGV	WTVKILHHWV	PVAETKFLVA	PLTFSNRQPI	KPEEALKLHN	GPLRNAYMEQ	SFQSLNPVLS
**890**	**900**	**910**	**920**	**930**	**940**	**950**	**960**
LPINPAQVEQ	ARRNAASTGT	ALEGWLDSLV	GGMWTAMDIC	ATGPTACPVM	QTCSQTAWSS	FSPDPKSELG	AVKPDGRLR

Cmpd.	No. of Cmpds.	m/z meas.	Δ m/z [ppm]	z	Rt [min]	Score	P	Range	Sequence	Modification
87	1	669.7442	115.37	3	29.8	12.8	2	68-91	R.RDLPAEPAAARGGGGGGGGGGGR.G	
2175	1	701.2622	-127.85	3	57.6	11.6	0	388-406	R.MATIWGGASLLSTYLQSMR.D	Oxidation: 18

Detailed Protein Report

Protein 415: PREDICTED: E3 ubiquitin-protein ligase MIB2 isoform X7 [Homo sapiens]

**Accession:**	gi|578798430	**Score:**	24.3
**Database:**	refseq_human(refseq_human_20140103.fasta)	**MW [kDa]:**	111.6
**Database Date:**	2015-11-30	**pI:**	10.4
		**Sequence Coverage [%]:**	1.9
		**No. of unique Peptides:**	2

```
        10         20         30         40         50         60         70         80
MAGALRRGRA LGSRPSGPTV SSRRSPQCPV AQEGLGARSR PRVAPRSLAR CGPSSRLMGW KPSEARGQSQ SFQASGLQPR
        90        100        110        120        130        140        150        160
SLKAARRATG RPDRSRAARP TMDPSAHRSR AAPPNMDPDP QAGVQVGMRV VRGVDWKWGQ QDGGEGGVGT VVELGRHGSP
       170        180        190        200        210        220        230        240
STPDRTVVVQ WDQGTRTNYR AGYQGAHDLL LYDNAQIGVR HPNIICDCCK KHGLRGMRWK CRVCLDYDLC TQCYMHNKHE
       250        260        270        280        290        300        310        320
LAHAFDRYET AHSRPVTLSP RQGLPRIPLR GIFQGAKVVR GPDWEWGSQD GGEGKPGRVV DIRGWDVETG RSVASVTWAD
       330        340        350        360        370        380        390        400
GTTNVYRVGH KGKVDLKCVG EAAGGFYYKD HLPRLGKPAE LQRRVSADSQ PFQHGDKVKC LLDTDVLREM QEGHGGWNPR
       410        420        430        440        450        460        470        480
MAEHHSFWVG DVVRVIGDLD TVKRLQAGHG EWTDDMAPAL GRVGKVVKVF GDGNLRVAVA GQRWTFSPSC LVAYRPEEDA
       490        500        510        520        530        540        550        560
NLDVAERARE NKSSLSVALD KLRAQKSDPE HPGRLVVEVA LGNAARALDL LRRRPEQVDT KNQGRTALQV AAYLGQVELI
       570        580        590        600        610        620        630        640
RLLLQARAGV DLPDDEGNTA LHYAALGNQP EATRVLLSAG CRADAINSTQ STALHVAVQR GFLEVVRALC ERGCDVNLPD
       650        660        670        680        690        700        710        720
AHSDTPLHSA ISAGTGASGI VEVLTEVPNI DVTATNSQGF TLLHHASLKG HALAVRKILA RARQLVDAKK EDGFTALHLA
       730        740        750        760        770        780        790        800
ALNNHREVAQ ILIREGRCDV NVRNRKLQSP LHLAVQQAHV GLVPLLVDAG CSVNAEDEEG DTALHVALQR HQLLPLVADG
       810        820        830        840        850        860        870        880
AGGDPGPLQL LSRLQASGLP GSAELTVGAA VACFLALEGA DVSYTNHRGR SPLDLAAEGR VLKALQGCAQ RFRERQAGGG
       890        900        910        920        930        940        950        960
AAPGPRQTLG TPNTVTNLHV GAAPGPEAAE CLVCSELALL VLFSPCQHRT VCEECARRMK KCIRCQVVVS KKLRPDGSEV
       970        980        990       1000       1010       1020       1030       1040
ASAAPAPGPP RQLVEELQSR YRQMEERITC PICIDSHIRL VFQCGHGACA PCGSALSACP ICRQPIRDRI QIFV
```

Cmpd.	No. of Cmpds.	m/z meas.	Δ m/z [ppm]	z	Rt [min]	Score	P	Range	Sequence	Modification
2600	1	835.4660	80.35	2	63.2	12.9	0	401-414	R.MAEHHSFWVGDVVR.V	
1170	1	713.1709	-414.81	1	43.1	11.4	0	562-567	R.LLLQAR.A	

Detailed Protein Report

Protein 416: DNA replication ATP-dependent helicase/nuclease DNA2 [Homo sapiens]

Accession: gi|320461728
Database: refseq_human(refseq_human_20140103.fasta)
Database Date: 2015-11-30

Score: 24.3
MW [kDa]: 120.3
pI: 8.9
Sequence Coverage [%]: 2.5
No. of unique Peptides: 1

Quantitation

QD:QU **Median:** 2.58 **CV:** 0.00 % **No. of Peptides:** 1

10	20	30	40	50	60	70	80
MEQLNELELL	MEKSFWEEAE	LPAELFQKKV	VASFPRTVLS	TGMDNRYLVL	AVNTVQNKEG	NCEKRLVITA	SQSLENKELC
90	100	110	120	130	140	150	160
ILRNDWCSVP	VEPGDIIHLE	GDCTSDTWII	DKDFGYLILY	PDMLISGTSI	ASSIRCMRRA	VLSETFRSSD	PATRQMLIGT
170	180	190	200	210	220	230	240
VLHEVFQKAI	NNSFAPEKLQ	ELAFQTIQEI	RHLKEMYRLN	LSQDEIKQEV	EDYLPSFCKW	AGDFMHKNTS	TDFPQMQLSL
250	260	270	280	290	300	310	320
PSDNSKDNST	CNIEVVKPMD	IEESIWSPRF	GLKGKIDVTV	GVKIHRGYKT	KYKIMPLELK	TGKESNSIEH	RSQVVLYTLL
330	340	350	360	370	380	390	400
SQERRADPEA	GLLLYLKTGQ	MYPVPANHLD	KRELLKLRNQ	MAFSLFHRIS	KSATRQKTQL	ASLPQIIEEE	KTCKYCSQIG
410	420	430	440	450	460	470	480
NCALYSRAVE	QQMDCSSVPI	VMLPKIEEET	QHLKQTHLEY	FSLWCLMLTL	ESQSKDNKKN	HQNIWLMPAS	EMEKSGSCIG
490	500	510	520	530	540	550	560
NLIRMEHVKI	VCDGQYLHNF	QCKHGAIPVT	NLMAGDRVIV	SGEERSLFAL	SRGYVKEINM	TTVTCLLDRN	LSVLPESTLF
570	580	590	600	610	620	630	640
RLDQEEKNCD	IDTPLGNLSK	LMENTFVSKK	LRDLIIDFRE	PQFISYLSSV	LPHDAKDTVA	CILKGLNKPQ	RQAMKKVLLS
650	660	670	680	690	700	710	720
KDYTLIVGMP	GTGKTTTICT	LVRILYACGF	SVLLTSYTHS	AVDNILLKLA	KFKIGFLRLG	QIQKVHPAIQ	QFTEQEICRS
730	740	750	760	770	780	790	800
KSIKSLALLE	ELYNSQLIVA	TTCMGINHPI	FSRKIFDFCI	VDEASQISQP	ICLGPLFFSR	RFVLVGDHQQ	LPPLVLNREA
810	820	830	840	850	860	870	880
RALGMSESLF	KRLEQNKSAV	VQLTVQYRMN	SKIMSLSNKL	TYEGKLECGS	DKVANAVINL	RHFKDVKLEL	EFYADYSDNP
890	900	910	920	930	940	950	960
WLMGVFEPNN	PVCFLNTDKV	PAPEQVEKGG	VSNVTEAKLI	VFLTSIFVKA	GCSPSDIGII	APYRQQLKII	NDLLARSIGM
970	980	990	1000	1010	1020	1030	1040
VEVNTVDKYQ	GRDKSIVLVS	FVRSNKDGTV	GELLKDWRRL	NVAITRAKHK	LILLGCVPSL	NCYPPLEKLL	NHLNSEKLII
1050	1060	1070					
DLPSREHESL	CHILGDFQRE						

Cmpd.	No. of Cmpds.	m/z meas.	Δ m/z [ppm]	z	Rt [min]	Score	P	Range	Sequence	Modification	Ratios
236	1	862.4518	-58.13	2	32.9	10.3	2	853-867	K.VANAVINLRHFKDVK.L		QD:QU 2.58

Detailed Protein Report

Protein 417: PREDICTED: E3 ubiquitin-protein ligase DTX4 isoform X1 [Homo sapiens]

**Accession:**	gi|578821243	**Score:**	24.2
**Database:**	refseq_human(refseq_human_20140103.fasta)	**MW [kDa]:**	55.2
**Database Date:**	2015-11-30	**pI:**	10.5
		**Sequence Coverage [%]:**	3.9
		**No. of unique Peptides:**	1

Quantitation

QD:QU **Median:** 2.11 **CV:** 0.00 % **No. of Peptides:** 1

```
        10         20         30         40         50         60         70         80
MEVGITIQHA YEKQHPWIDL TSIGFSYVID FNTMGQINRQ TQRQRRVRRR LDLIYPMVTG TLPKAQSWPV SPGPATSPPM
        90        100        110        120        130        140        150        160
SPCSCPQCVL VMSVKAAVVN GSTGPLQLPV TRKNMPPPGV VKLPPLPGSG AKPLDSTGTI RGPLKTAPSQ VIRRQASSMP
       170        180        190        200        210        220        230        240
TGTTMGSPAS PPGPNSKTGR VALATLNRTN LQRLAIAQSR VLIASGVPTV PVKNLNGSSP VNPALAGITG ILMSAAGLPV
       250        260        270        280        290        300        310        320
CLTRPPKLVL HPPPVSKSEI KSIPGVSNTS RKTTKKQAKK GKTPEEVLKK YLQKVRHPPD EDCTICMERL TAPSGYKGPQ
       330        340        350        360        370        380        390        400
PTVKPDLVGK LSRCGHVYHI YCLVAMYNNG NKDGSLQCPT CKTIYGVKTG TQPPGKMEYH LIPHSLPGHP DCKTIRIIYS
       410        420        430        440        450        460        470        480
IPPGIQGPEH PNPGKSFSAR GFPRHCYLPD SEKGRKVLKL LLVAWDRRLI FAIGTSSTTG ESDTVIWNEV HHKTEFGSNL
       490        500        510        520
TGHGYPDANY LDNVLAELAA QGISEDSTAQ EKD
```

Cmpd.	No. of Cmpds.	m/z meas.	Δ m/z [ppm]	z	Rt [min]	Score	P	Range	Sequence	Modification	Ratios
690	1	564.6885	-259.06	2	37.2	10.9	2	280-289	K.KGKTPEEVLK.K		QD:QU 2.11

Detailed Protein Report

Protein 418: **PREDICTED: RAF proto-oncogene serine/threonine-protein kinase isoform X5 [Homo sapiens]**

Accession: gi|530372930
Database: refseq_human(refseq_human_20140103.fasta)
Database Date: 2015-11-30
Modification(s): Carbamidomethyl, Oxidation

Score: 24.2
MW [kDa]: 60.5
pI: 10.0
Sequence Coverage [%]: 6.4
No. of unique Peptides: 2

10	20	30	40	50	60	70	80
MMANSQILLR	QATLSVFSCR	TSKEQCKKAR	LDWNTDAASL	IGEELQVDFL	DHVPLTTHNF	ARKTFLKLAF	CDICQKFLLN
**90**	**100**	**110**	**120**	**130**	**140**	**150**	**160**
GFRCQTCGYK	FHEHCSTKVP	TMCVDWSNIR	QLFSQHRYST	PHAFTFNTSS	PSSEGSLSQR	QRSTSTPNVH	MVSTTLPVDS
**170**	**180**	**190**	**200**	**210**	**220**	**230**	**240**
RMIEDAIRSH	SESASPSALS	SSPNNLSPTG	WSQPKTPVPA	QRERAPVSGT	QEKNKIRPRG	QRDSSYYWEI	EASEVMLSTR
**250**	**260**	**270**	**280**	**290**	**300**	**310**	**320**
IGSGSFGTVY	KGKWHGDVAV	KILKVVDPTP	EQFQAFRNEV	AVLRKTRHVN	ILLFMGYMTK	DNLAIVTQWC	EGSSLYKHLH
**330**	**340**	**350**	**360**	**370**	**380**	**390**	**400**
VQETKFQMFQ	LIDIARQTAQ	GMDYLHAKNI	IHRDMKSNNI	FLHEGLTVKI	GDFGLATVKS	RWSGSQQVEQ	PTGSVLWMAP
**410**	**420**	**430**	**440**	**450**	**460**	**470**	**480**
EVIRMQDNNP	FSFQSDVYSY	GIVLYELMTG	ELPYSHINNR	DQIIFMVGRG	YASPDLSKLY	KNCPKAMKRL	VADCVKKVKE
**490**	**500**	**510**	**520**	**530**	**540**		
ERPLFPQILS	SIELLQHSLP	KINRSASEPS	LHRAAHTEDI	NACTLTTSPR	LPVF		

Cmpd.	No. of Cmpds.	m/z meas.	Δ m/z [ppm]	z	Rt [min]	Score	P	Range	Sequence	Modification
2671	1	822.4677	60.63	2	64.5	10.7	1	64-76	K.TFLKLAFCDICQK.F	Carbamidomethyl: 8, 11
2739	1	806.4174	-6.55	3	65.4	13.5	2	441-461	R.DQIIFMVGRGYASPDLSKLYK.N	Oxidation: 6

Detailed Protein Report

Protein 419: **PREDICTED: pecanex-like protein 3 isoform X4 [Homo sapiens]**

**Accession:**	gi|530396772	**Score:**	24.2
**Database:**	refseq_human(refseq_human_20140103.fasta)	**MW [kDa]:**	115.4
**Database Date:**	2015-11-30	**pI:**	6.0
		**Sequence Coverage [%]:**	2.0
		**No. of unique Peptides:**	1

10	20	30	40	50	60	70	80
MGSQVLQILR	QGVWASLTGG	WFFDPHQSTF	SNCFHLYVWI	FLLIFPFLLY	MVLPPSLMVA	GVYCLVVAVI	FATIKTVNYR
**90**	**100**	**110**	**120**	**130**	**140**	**150**	**160**
LHAMFDQGEI	VEKRSSTMGE	LEEEPAQGDS	NPPRDPGVEM	TVFRKVSSTP	PVRCSSQHSV	FGFNQVSELL	PRMEDSGPLR
**170**	**180**	**190**	**200**	**210**	**220**	**230**	**240**
DIKELVREQG	SNNVIVTSAD	REMLKLSSQE	KLIGDLPQTP	PGAVPDPSLA	STDSSEPSPL	AGDGAPWSGS	SMADTPMSPL
**250**	**260**	**270**	**280**	**290**	**300**	**310**	**320**
LKGSLSQELS	KSFLTLTQPD	RALVRTSSRR	EQRRGAGGYQ	PLDRRGSGEP	TPQKAGSSDS	CFSGTDRETL	SSFKSEKTNS
**330**	**340**	**350**	**360**	**370**	**380**	**390**	**400**
THLDSPPGGP	APEGSDTDPP	SEAELPASPD	AGVPSDDTLR	SFDTVIGAGT	PPGLAEPLLV	VRPKDLALLR	PSKRQPPLRR
**410**	**420**	**430**	**440**	**450**	**460**	**470**	**480**
HSPPGRAPRR	PLLEGGGFFE	DEDTSEGSEL	SPASSLRSQR	RYSTDSSSST	SCYSPESSRG	AAGGPRKRRA	PHGAEEGTAV
**490**	**500**	**510**	**520**	**530**	**540**	**550**	**560**
PPKRPYGTQR	TPSTASAKTH	ARVLSMDGAG	GDVLRPPLAG	CKAELEAQVG	VEQAASEPVV	LPAEARRGPA	ANQPGWRGEL
**570**	**580**	**590**	**600**	**610**	**620**	**630**	**640**
QEEGAVGGAA	EETGRRDRSS	SVRRTQAIRR	RHNAGSNPTP	PASVMGSPPS	SLQEAQRGRA	ASHSRALTLP	SALHFASSLL
**650**	**660**	**670**	**680**	**690**	**700**	**710**	**720**
LTRAGANVHE	ACTFDDTSEG	AVHYFYDESG	VRRSYTFGLA	GGGYENPVGQ	QGEQTANGAW	DRHSHSSSFH	SADVPEATGG
**730**	**740**	**750**	**760**	**770**	**780**	**790**	**800**
LNLLQPRPVV	LQGMQVRRVP	LEIPEFDLLD	QDSLHESQEQ	TLMEEAPPRA	QHSYKYWLLP	GRWTSVRYER	LALLALLDRT
**810**	**820**	**830**	**840**	**850**	**860**	**870**	**880**
RGVLENIFGV	GLSSLVAFLG	YLLLLKGFFT	DIWVFQFCLV	IASCQYSLLK	SVQPDAASPM	HGHNWVIAYS	RPVYFCICCL
**890**	**900**	**910**	**920**	**930**	**940**	**950**	**960**
LIWLLDALGS	AQPFPPVSLY	GLTLFSASFF	FCARDVATVF	TLCFPFVFLL	GLLPQVNTCL	MYLLEQIDMH	GFGGTAATSP
**970**	**980**	**990**	**1000**	**1010**	**1020**	**1030**	**1040**
LTAVFSLSRS	LLAAALLYGF	CLGAIKTPWP	EQHVPVLFSV	FCGLLVALSY	HLSRQSSDPT	VLWSLIRSKL	FPELEERSLE
**1050**	**1060**	**1070**					
TARAEPPDPL	PDKMRQSVLN	VSPGPAA					

Cmpd.	No. of Cmpds.	m/z meas.	Δ m/z [ppm]	z	Rt [min]	Score	P	Range	Sequence	Modification
1593	1	1172.1148	-1.72	2	50.1	24.2	2	161-181	R.DIKELVREQGSNNVIVTSADR.E	

Detailed Protein Report

Protein 420: myosin light chain kinase, smooth muscle isoform 3B [Homo sapiens]

**Accession:**	gi|116008190	**Score:**	24.1
**Database:**	refseq_human(refseq_human_20140103.fasta)	**MW [kDa]:**	197.3
**Database Date:**	2015-11-30	**pI:**	6.1
		**Sequence Coverage [%]:**	1.7
		**No. of unique Peptides:**	2

Quantitation

QD:QU	**Median:** 0.93	**CV:** 0.00 %	**No. of Peptides:** 1

Detailed Protein Report

10	20	30	40	50	60	70	80
MGDVKLVASS	HISKTSLSVD	PSRVDSMPLT	EAPAFILPPR	NLCIKEGATA	KFEGRVRGYP	EPQVTWHRNG	QPITSGGRFL
90	100	110	120	130	140	150	160
LDCGIRGTFS	LVIHAVHEED	RGKYTCEATN	GSGARQVTVE	LTVEGSFAKQ	LGQPVVSKTL	GDRFSAPAVE	TRPSIWGECP
170	180	190	200	210	220	230	240
PKFATKLGRV	VVKEGQMGRF	SCKITGRPQP	QVTWLKGNVP	LQPSARVSVS	EKNGMQVLEI	HGVNQDDVGV	YTCLVVNGSG
250	260	270	280	290	300	310	320
KASMSAELSI	QGLDSANRSF	VRETKATNSD	VRKEVTNVIS	KESKLDSLEA	AAKSKNCSSP	QRGGSPPWAA	NSQPQPPRES
330	340	350	360	370	380	390	400
KLESCKDSPR	TAPQTPVLQK	TSSSITLQAA	RVQPEPRAPG	LGVLSPSGEE	RKRPAPPRPA	TFPTRQPGLG	SQDVVSKAAN
410	420	430	440	450	460	470	480
RRIPMEGQRD	SAFPKFESKP	QSQEVKENQT	VKFRCEGLAV	MEVAPSFSSV	LKDCAVIEGQ	DFVLQCSVRG	TPVPRITWLL
490	500	510	520	530	540	550	560
NGQPIQYARS	TCEAGVAELH	IQDALPEDHG	TYTCLAENAL	GQVSCSAWVT	VHEKKSSRKS	EYLLPVAPSK	PTAPIFLQGL
570	580	590	600	610	620	630	640
SDLKVMDGSQ	VTMTVQVSGN	PPPEVIWLHN	GNEIQESEDF	HFEQRGTQHS	LCIQEVFPED	TGTYTCEAWN	SAGEVRTQAV
650	660	670	680	690	700	710	720
LTVQEPHDGT	QPWFISKPRS	VTASLGQSVL	ISCAIAGDPF	PTVHWLRDGK	ALCKDTGHFE	VLQNEDVFTL	VLKKVQPWHA
730	740	750	760	770	780	790	800
GQYEILLKNR	VGECSCQVSL	MLQNSSARAL	PRGREPASCE	DLCGGGVGAD	GGGSDRYGSL	RPGWPARGQG	WLEEEDGEDV
810	820	830	840	850	860	870	880
RGVLKRRVET	RQHTEEAIRQ	QEVEQLDFRD	LLGKKVSTKT	LSEDDLKEIP	AEQMDFRANL	QRQVKPKTVS	EEERKVHSPQ
890	900	910	920	930	940	950	960
QVDFRSVLAK	KGTSKTPVPE	KVPPPKPATP	DFRSVLGGKK	KLPAENGSSS	AETLNAKAVE	SSKPLSNAQP	SGPLKPVGNA
970	980	990	1000	1010	1020	1030	1040
KPAETLKPMG	NAKPAETLKP	MGNAKPDENL	KSASKEELKK	DVKNDVNCKR	GHAGTTDNEK	RSESQGTAPA	FKQKLQDVHV
1050	1060	1070	1080	1090	1100	1110	1120
AEGKKLLLQC	QVSSDPPATI	IWTLNGKTLK	TTKFIILSQE	GSLCSVSIEK	ALPEDRGLYK	CVAKNDAGQA	ECSCQVTVDD
1130	1140	1150	1160	1170	1180	1190	1200
APASENTKAP	EMKSRRPKSS	LPPVLGTESD	ATVKKKPAPK	TPPKAAMPPQ	IIQFPEDQKV	RAGESVELFG	KVTGTQPITC
1210	1220	1230	1240	1250	1260	1270	1280
TWMKFRKQIQ	ESEHMKVENS	ENGSKLTILA	ARQEHCGCYT	LLVENKLGSR	QAQVNLTVVD	KPDPPAGTPC	ASDIRSSSLT
1290	1300	1310	1320	1330	1340	1350	1360
LSWYGSSYDG	GSAVQSYSIE	IWDSANKTWK	ELATCRSTSF	NVQDLLPDHE	YKFRVRAINV	YGTSEPSQES	ELTTVGEKPE
1370	1380	1390	1400	1410	1420	1430	1440
EPKDEVEVSD	DDEKEPEVDY	RTVTINTEQK	VSDFYDIEER	LGSGKFGQVF	RLVEKKTRKV	WAGKFFKAYS	AKEKENIRQE
1450	1460	1470	1480	1490	1500	1510	1520
ISIMNCLHHP	KLVQCVDAFE	EKANIVMVLE	IVSGGELFER	IIDEDFELTE	RECIKYMRQI	SEGVEYIHKQ	GIVHLDLKPE
1530	1540	1550	1560	1570	1580	1590	1600
NIMCVNKTGT	RIKLIDFGLA	RRLENAGSLK	VLFGTPEFVA	PEVINYEPIG	YATDMWSIGV	ICYILNRLDC	TQCLQHPWLM
1610	1620	1630	1640	1650	1660	1670	1680
KDTKNMEAKK	LSKDRMKKYM	ARRKWQKTGN	AVRAIGRLSS	MAMISGLSGR	KSSTGSPTSP	LNAEKLESEE	DVSQAFLEAV
1690	1700	1710	1720	1730	1740	1750	1760
AEEKPHVKPY	FSKTIRDLEV	VEGSAARFDC	KIEGYPDPEV	VWFKDDQSIR	ESRHFQIDYD	EDGNCSLIIS	DVCGDDDAKY
1770	1780	1790	1800				
TCKAVNSLGE	ATCTAELIVE	TMEEGEGEGE	EEEE				

Cmpd.	No. of Cmpds.	m/z meas.	Δ m/z [ppm]	z	Rt [min]	Score	P	Range	Sequence	Modification	Ratios
678	1	670.8078	-108.06	2	38.5	14.1	1	2-14	M.GDVKLVASSHISK.T		QD:QU 0.93
2643	1	995.0088	-31.93	2	63.9	10.1	1	180-196	R.FSCKITGRPQPQVTWLK.G		

Detailed Protein Report

Protein 421: zinc finger protein 493 isoform 1 [Homo sapiens]

Accession: gi|115511042
Database: refseq_human(refseq_human_20140103.fasta)
Database Date: 2015-11-30
Modification(s): Carbamidomethyl, Oxidation

Score: 24.1
MW [kDa]: 75.3
pI: 10.4
Sequence Coverage [%]: 4.8
No. of unique Peptides: 2

Alias proteins:

Accession	Name	Description
gi|578833199	refseq_human (refseq_human_20140103.fasta)	PREDICTED: zinc finger protein 493 isoform X3 [Homo sapiens]

10	20	30	40	50	60	70	80
MNECNVHKEG	YNELNQYLTT	TQSKIFQCDK	YVKVFHKLLN	SNRHNTKHTG	KKPFKCKKCG	KSFCMLLHLC	QHKRIHIREN
90	100	110	120	130	140	150	160
SYRCEECGKA	FIWFSTLTRH	RRVHTGEKSY	KYECGKSFNQ	DSNLTTHKRI	HTGQKPYKCE	ECGTSFYQFS	YLTRHKLIHT
170	180	190	200	210	220	230	240
REKPYKCEQY	GKTFNQSSTL	TGHKIIHNGE	KPYKCEECGK	AFSIFSTPTK	HKIIHTEEKS	HRCEEYCKAY	KESSHLTTHK
250	260	270	280	290	300	310	320
RIHTGEKPYK	CEECGKAFSI	FSTLTKHKII	HTEEKSHRCE	ECGKAYKESS	HLTTHKRIHT	GEKPYKCEEC	GKTFSVFSIL
330	340	350	360	370	380	390	400
TKHKIIHTEE	KPYKCEECGK	AFKRSSTLTK	HRIIHTEEKP	YKCEECGKAF	NQSSTLSIHK	IIHTGEKPYK	CEECGKAFKR
410	420	430	440	450	460	470	480
SSTLTIHKMI	HTGEKPYKCE	ECGKAFNRSS	HLTTHKRIHT	GHKPYKCKEC	GKSFSVFSTL	TKHKIIHTDK	KPYKCEECGK
490	500	510	520	530	540	550	560
AFNRSSILSI	HKKIHTGEKP	YKCEECGKAF	KRSSHLAGHK	QIHSVQKPYK	CEECGKAFSI	FSTLTKHKII	HTEEKPYKCE
570	580	590	600	610	620	630	640
KCGKTFYRFS	NLNTHKIIHT	GEKPCKCEEC	GKAFNHSSNL	IKHKLIHTGD	KPYKCEACGK	AFRRSSHLSR	HKIIHIGIHT
650							
EETVQK							

Cmpd.	No. of Cmpds.	m/z meas.	Δ m/z [ppm]	z	Rt [min]	Score	P	Range	Sequence	Modification
2085	1	939.2954	-149.37	2	56.4	12.7	1	59-73	K.CGKSFCMLLHLCQHK.R	Carbamidomethyl: 1, 6; Oxidation: 7
1854	1	938.8329	-130.94	2	53.5	11.5	1	521-536	K.QIHSVQKPYKCEECGK.A	

Detailed Protein Report

Protein 422: phosphoserine aminotransferase isoform 2 [Homo sapiens]

**Accession:**	gi|10863955	**Score:**	24.1
**Database:**	refseq_human(refseq_human_20140103.fasta)	**MW [kDa]:**	35.2
**Database Date:**	2015-11-30	**pI:**	6.3
		**Sequence Coverage [%]:**	11.4
		**No. of unique Peptides:**	1

10	20	30	40	50	60	70	80
MDAPRQVVNF	GPGPAKLPHS	VLLEIQKELL	DYKGVGISVL	EMSHRSSDFA	KIINNTENLV	RELLAVPDNY	KVIFLQGGGC
**90**	**100**	**110**	**120**	**130**	**140**	**150**	**160**
GQFSAVPLNL	IGLKAGRCAD	YVVTGAWSAK	AAEEAKKFGT	INIVHPKLGS	YTKIPDPSTW	NLNPDASYVY	YCANETVHGV
**170**	**180**	**190**	**200**	**210**	**220**	**230**	**240**
EFDFIPDVKG	AVLVCDMSSN	FLSKPVDVSK	FGVIFAGAQK	NVGSAGVTVV	IVRDDLLGFA	LRECPSVLEY	KVQAGNSSLY
**250**	**260**	**270**	**280**	**290**	**300**	**310**	**320**
NTPPCFSIYV	MGLVLEWIKN	NGGAAAMEKL	SSIKSQTIYE	IIDNSQGFYV	SVGGIRASLY	NAVTIEDVQK	LAAFMKKFLE
**330**							
MHQL							

Cmpd.	No. of Cmpds.	m/z meas.	Δ m/z [ppm]	z	Rt [min]	Score	P	Range	Sequence	Modification
1320	1	1136.1196	-22.05	2	46.6	12.1	1	201-222	K.NVGSAGVTVVIVRDDLLGFALR.E	

Detailed Protein Report

Protein 423: **titin isoform novex-3 [Homo sapiens]**

**Accession:**	gi|110349721	**Score:**	24.1
**Database:**	refseq_human(refseq_human_20140103.fasta)	**MW [kDa]:**	631.2
**Database Date:**	2015-11-30	**pI:**	5.4
		**Sequence Coverage [%]:**	0.6
		**No. of unique Peptides:**	1

Detailed Protein Report

10	20	30	40	50	60	70	80
MTTQAPTFTQ	PLQSVVVLEG	STATFEAHIS	GFPVPEVSWF	RDGQVISTST	LPGVQISFSD	GRAKLTIPAV	TKANSGRYSL
90	100	110	120	130	140	150	160
KATNGSGQAT	STAELLVKAE	TAPPNFVQRL	QSMTVRQGSQ	VRLQVRVTGI	PTPVVKFYRD	GAEIQSSLDF	QISQEGDLYS
170	180	190	200	210	220	230	240
LLIAEAYPED	SGTYSVNATN	SVGRATSTAE	LLVQGEEEVP	AKKTKTIVST	AQISESRQTR	IEKKIEAHFD	ARSIATVEMV
250	260	270	280	290	300	310	320
IDGAAGQQLP	HKTPPRIPPK	PKSRSPTPPS	IAAKAQLARQ	QSPSPIRHSP	SPVRHVRAPT	PSPVRSVSPA	ARISTSPIRS
330	340	350	360	370	380	390	400
VRSPLLMRKT	QASTVATGPE	VPPPWKQEGY	VASSSEAEMR	ETTLTTSTQI	RTEERWEGRY	GVQEQVTISG	AAGAAASVSA
410	420	430	440	450	460	470	480
SASYAAEAVA	TGAKEVKQDA	DKSAAVATVV	AAVDMARVRE	PVISAVEQTA	QRTTTTAVHI	QPAQEQVRKE	AEKTAVTKVV
490	500	510	520	530	540	550	560
VAADKAKEQE	LKSRTKEVIT	TKQEQMHVTH	EQIRKETEKT	FVPKVVISAA	KAKEQETRIS	EEITKKQKQV	TQEAIRQETE
570	580	590	600	610	620	630	640
ITAASMVVVA	TAKSTKLETV	PGAQEETTTQ	QDQMHLSYEK	IMKETRKTVV	PKVIVATPKV	KEQDLVSRGR	EGITTKREQV
650	660	670	680	690	700	710	720
QITQEKMRKE	AEKTALSTIA	VATAKAKEQE	TILRTRETMA	TRQEQIQVTH	GKVDVGKKAE	AVATVVAAVD	QARVREPREP
730	740	750	760	770	780	790	800
GHLEESYAQQ	TTLEYGYKER	ISAAKVAEPP	QRPASEPHVV	PKAVKPRVIQ	APSETHIKTT	DQKGMHISSQ	IKKTTDLTTE
810	820	830	840	850	860	870	880
RLVHVDKRPR	TASPHFTVSK	ISVPKTEHGY	EASIAGSAIA	TLQKELSATS	SAQKITKSVK	APTVKPSETR	VRAEPTPLPQ
890	900	910	920	930	940	950	960
FPFADTPDTY	KSEAGVEVKK	EVGVSITGTT	VREERFEVLH	GREAKVTETA	RVPAPVEIPV	TPPTLVSGLK	NVTVIEGESV
970	980	990	1000	1010	1020	1030	1040
TLECHISGYP	SPTVTWYRED	YQIESSIDFQ	ITFQSGIARL	MIREAFAEDS	GRFTCSAVNE	AGTVSTSCYL	AVQVSEEFEK
1050	1060	1070	1080	1090	1100	1110	1120
ETTAVTEKFT	TEEKRFVESR	DVVMTDTSLT	EEQAGPGEPA	APYFITKPVV	QKLVEGGSVV	FGCQVGGNPK	PHVYWKKSGV
1130	1140	1150	1160	1170	1180	1190	1200
PLTTGYRYKV	SYNKQTGECK	LVISMTFADD	AGEYTIVVRN	KHGETSASAS	LLEEADYELL	MKSQQEMLYQ	TQVTAFVQEP
1210	1220	1230	1240	1250	1260	1270	1280
KVGETAPGFV	YSEYEKEYEK	EQALIRKKMA	KDTVVVRTYV	EDQEFHISSF	EERLIKEIEY	RIIKTTLEEL	LEEDGEEKMA
1290	1300	1310	1320	1330	1340	1350	1360
VDISESEAVE	SGFDSRIKNY	RILEGMGVTF	HCKMSGYPLP	KIAWYKDGKR	IKHGERYQMD	FLQDGRASLR	IPVVLPEDEG
1370	1380	1390	1400	1410	1420	1430	1440
IYTAFASNIK	GNAICSGKLY	VEPAAPLGAP	TYIPTLEPVS	RIRSLSPRSV	SRSPIRMSPA	RMSPARMSPA	RMSPARMSPG
1450	1460	1470	1480	1490	1500	1510	1520
RRLEETDESQ	LERLYKPVFV	LKPVSFKCLE	GQTARFDLKV	VGRPMPETFW	FHDGQQIVND	YTHKVVIKED	GTQSLIIVPA
1530	1540	1550	1560	1570	1580	1590	1600
TPSDSGEWTV	VAQNRAGRSS	ISVILTVEAV	EHQVKPMFVE	KLKNVNIKEG	SRLEMKVRAT	GNPNPDIVWL	KNSDIIVPHK
1610	1620	1630	1640	1650	1660	1670	1680
YPKIRIEGTK	GEAALKIDST	VSQDSAWYTA	TAINKAGRDT	TRCKVNVEVE	FAEPEPERKL	IIPRGTYRAK	EIAAPELEPL
1690	1700	1710	1720	1730	1740	1750	1760
HLRYGQEQWE	EGDLYDKEKQ	QKPFFKKKLT	SLRLKRFGPA	HFECRLTPIG	DPTMVVEWLH	DGKPLEAANR	LRMINEFGYC
1770	1780	1790	1800	1810	1820	1830	1840
SLDYGVAYSR	DSGIITCRAT	NKYGTDHTSA	TLIVKDEKSL	VEESQLPEGR	KGLQRIEELE	RMAHEGALTG	VTTDQKEKQK
1850	1860	1870	1880	1890	1900	1910	1920
PDIVLYPEPV	RVLEGETARF	RCRVTGYPQP	KVNWYLNGQL	IRKSKRFRVR	YDGIHYLDIV	DCKSYDTGEV	KVTAENPEGV
1930	1940	1950	1960	1970	1980	1990	2000
IEHKVKLEIQ	QREDFRSVLR	RAPEPRPEFH	VHEPGKLQFE	VQKVDRPVDT	TETKEVVKLK	RAERITHEKV	PEESEELRSK
2010	2020	2030	2040	2050	2060	2070	2080
FKRRTEEGYY	EAITAVELKS	RKKDESYEEL	LRKTKDELLH	WTKELTEEEK	KALAEEGKIT	IPTFKPDKIE	LSPSMEAPKI
2090	2100	2110	2120	2130	2140	2150	2160
FERIQSQTVG	QGSDAHFRVR	VVGKPDPECE	WYKNGVKIER	SDRIYWYWPE	DNVCELVIRD	VTAEDSASIM	VKAINIAGET
2170	2180	2190	2200	2210	2220	2230	2240
SSHAFLLVQA	KQLITFTQEL	QDVVAKEKDT	MATFECETSE	PFVKVKWYKD	GMEVHEGDKY	RMHSDRKVHF	LSILTIDTSD
2250	2260	2270	2280	2290	2300	2310	2320
AEDYSCVLVE	DENVKTTAKL	IVEGAVVEFV	KELQDIEVPE	SYSGELECIV	SPENIEGKWY	HNDVELKSNG	KYTITSRRGR
2330	2340	2350	2360	2370	2380	2390	2400

Detailed Protein Report

Cmpd.	No. of Cmpds.	m/z meas.	Δ m/z [ppm]	z	Rt [min]	Score	P	Range	Sequence	Modification
2534	1	921.5697	156.01	2	62.3	13.8	1	4501-4516	R.ESRSSNLNANMYQAEK.M	

Detailed Protein Report

Protein 424: **PREDICTED: centrosomal protein of 164 kDa isoform X10 [Homo sapiens]**

**Accession:**	gi|530397903	**Score:**	24.1
**Database:**	refseq_human(refseq_human_20140103.fasta)	**MW [kDa]:**	151.3
**Database Date:**	2015-11-30	**pI:**	5.2
		**Sequence Coverage [%]:**	1.8
		**No. of unique Peptides:**	1

10	20	30	40	50	60	70	80
MGPAGNPPTG	LAPRLRLQAL	GSSLAPVHVP	LGGLAPLRGL	VDTPPSALRG	SQSVSLGSSV	ESGRQLGELM	LPSQGLKTSA
**90**	**100**	**110**	**120**	**130**	**140**	**150**	**160**
YTKGLLGSIY	EDKTALSLLG	LGEETNEEDE	EESDNQSVHS	SSEPLRNLHL	DIGALGGDFE	YEESLRTSQP	EEKKDVSLDS
**170**	**180**	**190**	**200**	**210**	**220**	**230**	**240**
DAAGPPTPCK	PSSPGADSSL	SSAVGKGRQG	SGARPGLPEK	EENEKSEPKI	CRNLVTPKAD	PTGSEPAKAS	EKEAPEDTVD
**250**	**260**	**270**	**280**	**290**	**300**	**310**	**320**
AGEEGSRREE	AAKEPKKKAS	ALEEGSSDAS	QELEISEHMK	EPQLSDSIAS	DPKSFHGLDF	GFRSRISEHL	LDVDVLSPVL
**330**	**340**	**350**	**360**	**370**	**380**	**390**	**400**
GGACRQAQQP	LGIEDKDDSQ	SSQDELQSKQ	SKGLEERYHR	LSPPLPHEER	AQSPPRSLAT	EEEPPQGPEG	QPEWKEAEEL
**410**	**420**	**430**	**440**	**450**	**460**	**470**	**480**
GEDSAASLSL	QLSLQREQAP	SPPAACEKGK	EQHSQAEELG	PGQEEAEDPE	EKVAVSPTPP	VSPEVRSTEP	VAPPEQLSEA
**490**	**500**	**510**	**520**	**530**	**540**	**550**	**560**
ALKAMEEAVA	QVLEQDQRHL	LESKQEKMQQ	LREKLCQEEE	EEILRLHQQK	EQSLSSLRER	LQKAIEEEEA	RMREEESQRL
**570**	**580**	**590**	**600**	**610**	**620**	**630**	**640**
SWLRAQVQSS	TQADEDQIRA	EQEASLQKLR	EELESQQKAE	RASLEQKNRQ	MLEQLKEEIE	ASEKSEQAAL	NAAKEKALQQ
**650**	**660**	**670**	**680**	**690**	**700**	**710**	**720**
LREQLEGERK	EAVATLEKEH	SAELERLCSS	LEAKHREVVS	SLQKKIQEAQ	QKEEAQLQKC	LGQVEHRVHQ	KSYHVAGYEH
**730**	**740**	**750**	**760**	**770**	**780**	**790**	**800**
ELSSLLREKR	QEVEGEHERR	LDKMKEEHQQ	VMAKAREQYE	AEERKQRAEL	LGHLTGELER	LQRAHERELE	TVRQEQHKRL
**810**	**820**	**830**	**840**	**850**	**860**	**870**	**880**
EDLRRRHREQ	ERKLQDLELD	LETRAKDVKA	RLALLEVQEE	TARREKQQLL	DVQRQVALKS	EEATATHQQL	EEAQKEHTHL
**890**	**900**	**910**	**920**	**930**	**940**	**950**	**960**
LQSNQQLREI	LDELQARKLK	LESQVDLLQA	QSQQLQKHFS	SLEAEAQKKQ	HLLREVTVEE	NNASPHFEPD	LHIEDLRKSL
**970**	**980**	**990**	**1000**	**1010**	**1020**	**1030**	**1040**
GTNQTKEVSS	SLSQSKEDLY	LDSLSSHNVW	HLLSAEGVAL	RSAKEFLVQQ	TRSMRRRQTA	LKAAQQHWRH	ELASAQEVAK
**1050**	**1060**	**1070**	**1080**	**1090**	**1100**	**1110**	**1120**
DPPGIKALED	MRKNLEKETR	HLDEMKSAMR	KGHNLLKKKE	EKLNQLESSL	WEEASDEGTL	GGSPTKKAVT	FDLSDMDSLS
**1130**	**1140**	**1150**	**1160**	**1170**	**1180**	**1190**	**1200**
SESSESFSPP	HREWWRQQRI	DSTPSLTSRK	IHGLSHSLRQ	ISSQLSSVLS	ILDSLNPQSP	PPLLASMPAQ	LPPRDPKSTP
**1210**	**1220**	**1230**	**1240**	**1250**	**1260**	**1270**	**1280**
TPTYYGSLAR	FSALSSATPT	STQWAWDSGQ	GPRLPSSVAQ	TVDDFLLEKW	RKYFPSGIPL	LSNSPTPLES	RLGYMSASEQ
**1290**	**1300**	**1310**	**1320**	**1330**	**1340**	**1350**	**1360**
LRLLQHSHSQ	VPEAGSTTFQ	GIIEANRRWL	ERVKNDPRLP	LFSSTPKPKA	TLSLLQLGLD	EHNRVKVYRF	

Cmpd.	No. of Cmpds.	m/z meas.	Δ m/z [ppm]	z	Rt [min]	Score	P	Range	Sequence	Modification
142	1	797.3752	-80.90	2	31.7	10.3	2	1017-1029	R.RQTALKAAQQHWR.H	

Detailed Protein Report

Protein 425: **PREDICTED: zinc finger CCCH domain-containing protein 3 isoform X7 [Homo sapiens]**

Accession: gi|578815908
Database: refseq_human(refseq_human_20140103.fasta)
Database Date: 2015-11-30

Score: 24.1
MW [kDa]: 107.0
pI: 12.1
Sequence Coverage [%]: 3.1
No. of unique Peptides: 1

10	20	30	40	50	60	70	80
MSIQGTLLSD	VFSSGHTPPW	PAPPRPREKG	RVAMTSWGPG	RPSRTREMEE	KEILRRQIRL	LQGLIDDYKT	LHGNAPAPGT
**90**	**100**	**110**	**120**	**130**	**140**	**150**	**160**
PAASGWQPPT	YHSGRAFSAR	YPRPSRRGYS	SHHGPSWRKK	YSLVNRPPGP	SDPPADHAVR	PLHGARGGQP	PVPQQHVLER
**170**	**180**	**190**	**200**	**210**	**220**	**230**	**240**
QVQLSQGQNV	VIKVKPPSKS	GSASASGAQR	GSLEEFEETP	WSDQRPREGE	GEPPRGQLQP	SRPTRARGTC	SVEDPLLVCQ
**250**	**260**	**270**	**280**	**290**	**300**	**310**	**320**
KEPGKPRMVK	SVGSVGDSPR	EPRRTVSESV	IAVKASFPSS	ALPPRTGVAL	GRKLGSHSVA	SCAPQLLGDR	RVDAGHTDQP
**330**	**340**	**350**	**360**	**370**	**380**	**390**	**400**
VPSGSVGGPA	RPASGPRQAR	EASLVVTCRT	NKFRKNNYKW	VAASSKSPRV	ARRALSPRVA	AENVCKASAG	MANKVEKPQL
**410**	**420**	**430**	**440**	**450**	**460**	**470**	**480**
IADPEPKPRK	PATSSKPGSA	PSKYKWKASS	PSASSSSSFR	WQSEASSKDH	ASQLSPVLSR	SPSGDRPAVG	HSGLKPLSGE
**490**	**500**	**510**	**520**	**530**	**540**	**550**	**560**
TPLSAYKVKS	RTKIIRRRSS	TSLPGDKKSG	TSPAATAKSH	LSLRRRQALR	GKSSPVLKKT	PNKGLVQVTT	HRLCRLPPSR
**570**	**580**	**590**	**600**	**610**	**620**	**630**	**640**
AHLPTKEASS	LHAVRTAPTS	KVIKTRYRIV	KKTPASPLSA	PPFPLSLPSW	RARRLSLSRS	LVLNRLRPVA	SGGGKAQPGS
**650**	**660**	**670**	**680**	**690**	**700**	**710**	**720**
PWWRSKGYRC	IGGVLYKVSA	NKLSKTSGQP	SDAGSRPLLR	TGRLDPAGSC	SRSLASRAVQ	RSLAIIRQAR	QRREKRKEYC
**730**	**740**	**750**	**760**	**770**	**780**	**790**	**800**
MYYNRFGRCN	RGERCPYIHD	PEKVAVCTRF	VRGTCKKTDG	TCPFSHHVSK	EKMPVCSYFL	KGICSNSNCP	YSHVYVSRKA
**810**	**820**	**830**	**840**	**850**	**860**	**870**	**880**
EVCSDFLKGY	CPLGAKCKKK	HTLLCPDFAR	RGACPRGAQC	QLLHRTQKRH	SRRAATSPAP	GPSDATARSR	VSASHGPRKP
**890**	**900**	**910**	**920**	**930**	**940**	**950**	**960**
SASQRPTRQT	PSSAALTAAA	VAAPPHCPGG	SASPSSSKAS	SSSSSSSSPP	ASLDHEAPSL	QEAALAAACS	NRLCKLPSFI
**970**	**980**	**990**	**1000**				
SLQSSPSPGA	QPRVRAPRAP	LTKDSGKPLH	IKPRL				

Cmpd.	No. of Cmpds.	m/z meas.	Δ m/z [ppm]	z	Rt [min]	Score	P	Range	Sequence	Modification
2689	1	701.3840	39.36	2	64.7	10.2	0	32-44	R.VAMTSWGPGRPSR.T	

Detailed Protein Report

Protein 426: potassium channel subfamily K member 2 isoform b [Homo sapiens]

Accession: gi|14589851
Database: refseq_human(refseq_human_20140103.fasta)
Database Date: 2015-11-30

Score: 24.0
MW [kDa]: 45.5
pI: 8.8
Sequence Coverage [%]: 8.3
No. of unique Peptides: 1

10	20	30	40	50	60	70	80
MAAPDLLDPK	SAAQNSKPRL	SFSTKPTVLA	SRVESDTTIN	VMKWKTVSTI	FLVVVLYLII	GATVFKALEQ	PHEISQRTTI
**90**	**100**	**110**	**120**	**130**	**140**	**150**	**160**
VIQKQTFISQ	HSCVNSTELD	ELIQQIVAAI	NAGIIPLGNT	SNQISHWDLG	SSFFFAGTVI	TTIGFGNISP	RTEGGKIFCI
**170**	**180**	**190**	**200**	**210**	**220**	**230**	**240**
IYALLGIPLF	GFLLAGVGDQ	LGTIFGKGIA	KVEDTFIKWN	VSQTKIRIIS	TIIFILFGCV	LFVALPAIIF	KHIEGWSALD
**250**	**260**	**270**	**280**	**290**	**300**	**310**	**320**
AIYFVVITLT	TIGFGDYVAG	GSDIEYLDFY	KPVVWFWILV	GLAYFAAVLS	MIGDWLRVIS	KKTKEEVGEF	RAHAAEWTAN
**330**	**340**	**350**	**360**	**370**	**380**	**390**	**400**
VTAEFKETRR	RLSVEIYDKF	QRATSIKRKL	SAELAGNHNQ	ELTPCRRTLS	VNHLTSERDV	LPPLLKTESI	YLNGLTPHCA
**410**	**420**						
GEEIAVIENI	K						

Cmpd.	No. of Cmpds.	m/z meas.	Δ m/z [ppm]	z	Rt [min]	Score	P	Range	Sequence	Modification
1783	1	823.3074	-115.03	2	52.6	10.7	0	312-326	R.AHAAEWTANVTAEFK.E	

Detailed Protein Report

Protein 427: PREDICTED: focal adhesion kinase 1 isoform X12 [Homo sapiens]

**Accession:**	gi|530389222	**Score:**	24.0
**Database:**	refseq_human(refseq_human_20140103.fasta)	**MW [kDa]:**	108.1
**Database Date:**	2015-11-30	**pI:**	5.7
		**Sequence Coverage [%]:**	3.2
		**No. of unique Peptides:**	2

10	20	30	40	50	60	70	80
MEMLLMSGYE	LRIRYLPKGF	LNQFTEDKPT	LNFFYQQVKS	DYMLEIADQV	DQEIALKLGC	LEIRRSYWEM	RGNALEKKSN
**90**	**100**	**110**	**120**	**130**	**140**	**150**	**160**
YEVLEKDVGL	KRFFPKSLLD	SVKAKTLRKL	IQQTFRQFAN	LNREESILKF	FEILSPVYRF	DKECFKCALG	SSWIISVELA
**170**	**180**	**190**	**200**	**210**	**220**	**230**	**240**
IGPEEGISYL	TDKGCNPTHL	ADFTQVQTIQ	YSNSEDKDRK	GMLQLKIAGA	PEPLTVTAPS	LTIAENMADL	IDGYCRLVNG
**250**	**260**	**270**	**280**	**290**	**300**	**310**	**320**
TSQSFIIRPQ	KEGERALPSI	PKLANSEKQG	MRTHAVSVSD	EISGDETDDY	AEIIDEEDTY	TMPSKSYGID	EARDYEIQRE
**330**	**340**	**350**	**360**	**370**	**380**	**390**	**400**
RIELGRCIGE	GQFGDVHQGI	YMSPENPALA	VAIKTCKNCT	SDSVREKFLQ	EALTMRQFDH	PHIVKLIGVI	TENPVWIIME
**410**	**420**	**430**	**440**	**450**	**460**	**470**	**480**
LCTLGELRSF	LQVRKYSLDL	ASLILYAYQL	STALAYLESK	RFVHRDIAAR	NVLVSSNDCV	KLGDFGLSRY	MEDSTYYKAS
**490**	**500**	**510**	**520**	**530**	**540**	**550**	**560**
KGKLPIKWMA	PESINFRRFT	SASDVWMFGV	CMWEILMHGV	KPFQGVKNND	VIGRIENGER	LPMPPNCPPT	LYSLMTKCWA
**570**	**580**	**590**	**600**	**610**	**620**	**630**	**640**
YDPSRRPRFT	ELKAQLSTIL	EEEKAQQEER	MRMESRRQAT	VSWDSGGSDE	APPKPSRPGY	PSPRSSEGFY	PSPQHMVQTN
**650**	**660**	**670**	**680**	**690**	**700**	**710**	**720**
HYQVSGYPGS	HGITAMAGSI	YPGQASLLDQ	TDSWNHRPQE	IAMWQPNVED	STVLDLRGIG	QVLPTHLMEE	RLIRQQQEME
**730**	**740**	**750**	**760**	**770**	**780**	**790**	**800**
EDQRWLEKEE	RFLKPDVRLS	RGSIDREDGS	LQGPIGNQHI	YQPVGKPDPA	APPKKPPRPG	APGHLGSLAS	LSSPADSYNE
**810**	**820**	**830**	**840**	**850**	**860**	**870**	**880**
GVKPWRLQPQ	EISPPPTANL	DRSNDKVYEN	VTGLVKAVIE	MSSKIQPAPP	EEYVPMVKEV	GLALRTLLAT	VDETIPLLPA
**890**	**900**	**910**	**920**	**930**	**940**	**950**	**960**
STHREIEMAQ	KLLNSDLGEL	INKMKLAQQY	VMTSLQQEYK	KQMLTAAHAL	AVDAKNLLDV	IDQARLKMLG	QTRPH

Cmpd.	No. of Cmpds.	m/z meas.	Δ m/z [ppm]	z	Rt [min]	Score	P	Range	Sequence	Modification
2292	1	920.6271	128.21	2	59.1	13.3	2	256-272	R.ALPSIPKLANSEKQGMR.T	
2062	1	720.2626	-173.22	2	54.3	10.7	0	922-935	K.QMLTAAHALAVDAK.N	

Detailed Protein Report

Protein 428: **PREDICTED: probable phospholipid-transporting ATPase IIB isoform X6 [Homo sapiens]**

**Accession:**	gi|530414190	**Score:**	24.0
**Database:**	refseq_human(refseq_human_20140103.fasta)	**MW [kDa]:**	114.6
**Database Date:**	2015-11-30	**pI:**	7.3
**Modification(s):**	Carbamidomethyl	**Sequence Coverage [%]:**	2.7
		**No. of unique Peptides:**	1

10	20	30	40	50	60	70	80
MADQIPLYPV	RSAAAAAANR	KRAAYYSAAG	PRPGADRHSR	YQLEDESAHL	DEMPLMMSEE	GFENEESDYH	TLPRARIMQR
**90**	**100**	**110**	**120**	**130**	**140**	**150**	**160**
KRGLEWFVCD	GWKFLCTSCC	GWLINICRRK	KELKARTVWL	GCPEKCEEKH	PRNSIKNQKY	NVFTFIPGVL	YEQFKFFLNL
**170**	**180**	**190**	**200**	**210**	**220**	**230**	**240**
YFLVISCSQF	VPALKIGYLY	TYWAPLGFVL	AVTMTREAID	EFRRFQRDKE	VNSQLYSKLT	VRGKVQVKSS	DIQVGDLIIV
**250**	**260**	**270**	**280**	**290**	**300**	**310**	**320**
EKNQRIPSDM	VFLRTSEKAG	SCFIRTDQLD	GETDWKLKVA	VSCTQQLPAL	GDLFSISAYV	YAQKPQMDIH	SFEGTFTRED
**330**	**340**	**350**	**360**	**370**	**380**	**390**	**400**
SDPPIHESLS	IENTLWASTI	VASGTVIGVV	IYTGKETRSV	MNTSNPKNKV	GLLDLELNRL	TKALFLALVA	LSIVMVTLQG
**410**	**420**	**430**	**440**	**450**	**460**	**470**	**480**
FVGPWYRNLF	RFLLLFSYII	PISLRVNLDM	GKAVYGWMMM	KDENIPGTVV	RTSTIPEELG	RLVYLLTDKT	GTLTQNEMIF
**490**	**500**	**510**	**520**	**530**	**540**	**550**	**560**
KRLHLGTVSY	GADTMDEIQS	HVRDSYSQMQ	SQAGGNNTGS	TPLRKAQSSA	PKVRKSVSSR	IHEAVKAIVL	CHNVTPVYES
**570**	**580**	**590**	**600**	**610**	**620**	**630**	**640**
RAGVTEETEF	AEADQDFSDE	NRTYQASSPD	EVALVQWTES	VGLTLVSRDL	TSMQLKTPSG	QVLSFCILQL	FPFTSESKRM
**650**	**660**	**670**	**680**	**690**	**700**	**710**	**720**
GVIVRDESTA	EITFYMKGAD	VAMSPIVQYN	DWLEEECGNM	AREGLRTLVV	AKKALTEEQY	QDFESRYTQA	KLSMHDRSLK
**730**	**740**	**750**	**760**	**770**	**780**	**790**	**800**
VAAVVESLER	EMELLCLTGV	EDQLQADVRP	TLEMLRNAGI	KIWMLTGDKL	ETATCIAKSS	HLVSRTQDIH	IFRQVTSRGE
**810**	**820**	**830**	**840**	**850**	**860**	**870**	**880**
AHLELNAFRR	KHDCALVISG	DSLEVCLKYY	EHEFVELACQ	CPAVVCCRCS	PTQKARIVTL	LQQHTGRRTC	AIGDGGNDVS
**890**	**900**	**910**	**920**	**930**	**940**	**950**	**960**
MIQAADCGIG	IEGKEGKQAS	LAADFSITQF	RHIGRLLMVH	GRNSYKRSAA	LGQFVMHRGL	IISTMQAVFS	SVFYFASVPL
**970**	**980**	**990**	**1000**	**1010**	**1020**		
YQGFLMVGYA	TIYTMFPVFS	LVLDQDVKPE	MAMLYPELYK	DLTKFAKVAQ	TAALCVVV		

Cmpd.	No. of Cmpds.	m/z meas.	Δ m/z [ppm]	z	Rt [min]	Score	P	Range	Sequence	Modification
2606	1	973.0002	44.61	2	63.3	10.2	1	94-109	K.FLCTSCCGWLINICRR.K	Carbamidomethyl: 6

Detailed Protein Report

Protein 429: PREDICTED: coiled-coil domain-containing protein 129 isoform X1 [Homo sapiens]

**Accession:**	gi|530384574	**Score:**	24.0
**Database:**	refseq_human(refseq_human_20140103.fasta)	**MW [kDa]:**	104.8
**Database Date:**	2015-11-30	**pI:**	5.3
		**Sequence Coverage [%]:**	3.2
		**No. of unique Peptides:**	1

10	20	30	40	50	60	70	80
MVQMTVKDYM	RSLHQFSETP	ILSRGTSFNS	CYSTASVPQS	IPEWLEFWEI	DPVEILLDLG	FGADEPDICM	QIPARFLGCG
90	100	110	120	130	140	150	160
SAARGINIRV	FLEAQKQRMD	IENPNLYGRF	RQLEILDHVT	NAFSSLLSDV	SILPNRAEEK	AGGESVQRTS	VSAAKEHRRR
170	180	190	200	210	220	230	240
MGKLLRRASK	QNIRRDCNPE	VSESFKVKDE	VFVPFTKPWD	CGAELAATSI	NHKQNHLSLS	VEHQSLQACD	DLLPYPPHGL
250	260	270	280	290	300	310	320
LSKQWPCSSM	PAKQAPPSCV	SEGSVKGRTQ	KENLFQTNKL	KSLSHLAGKG	PDSFEMEEVQ	SFEEETGNPL	DMTSGTVGAR
330	340	350	360	370	380	390	400
VDRANSCQSD	SSGFLEEPLE	PLPLQMPSLP	NSQSPAENGG	RKPRDQSHSL	VSSQDCQLES	DGPDSKSRAS	MSFSSQEANA
410	420	430	440	450	460	470	480
LEQRASVSVM	EEEFLLEAME	GPPELYIPDM	ACAKTTTRGE	CPRKDSHLWQ	LLPMPHAEYE	VTRPTATSKY	DHPLGFMVTH
490	500	510	520	530	540	550	560
VTEMQDSFVR	PEGAGKVQSH	HNESQRSPGN	DHTQDKFLHV	DSEAPREEES	SGFCPHTNHS	LLVPESSSQC	IPKHSEITPY
570	580	590	600	610	620	630	640
ATDLAQTSEK	LIPHLHKLPG	DPAQVKSRSG	TLGQILPGTE	AEMENLPLNT	GSSRSVMTQM	SSSLVSAAQR	AVALGTGPRG
650	660	670	680	690	700	710	720
TSLECTVCDP	VTATETRLGT	KARQLNDASI	QTSALSNKTL	THGPQPLTKS	VSLDSGFSSI	CPMGTCHAIP	AHCCICCHHH
730	740	750	760	770	780	790	800
PHCHGERQSP	GPEPSVCRHC	LCSLTGHQEA	QFMTTLKALQ	DTTVRELCSC	TVHEMEAMKT	ICQSFREYLE	EIEQHLMGQQ
810	820	830	840	850	860	870	880
ALFSRDMSEE	EREEAEQLQT	LREALRQQVA	ELEFQLGDRA	QQIREGILLQ	LEVLTAEPPE	HYSNLHQYNW	IEESNGQTSC
890	900	910	920	930	940	950	960
SKIHPGMAPR	TVFPPDDGQE	APCSGGTQLA	AFTPPTLENS	TRMSPSSSAW	AKLGPTPLSN	CPVGEKDADV	FL

Cmpd.	No. of Cmpds.	m/z meas.	Δ m/z [ppm]	z	Rt [min]	Score	P	Range	Sequence	Modification
2294	1	841.8204	-110.73	2	59.1	12.2	0	615-630	R.SVMTQMSSSLVSAAQR.A	

Detailed Protein Report

Protein 430: zinc finger homeobox protein 3 isoform B [Homo sapiens]

**Accession:**	gi|258613987	**Score:**	24.0
**Database:**	refseq_human(refseq_human_20140103.fasta)	**MW [kDa]:**	306.5
**Database Date:**	2015-11-30	**pI:**	6.5
**Modification(s):**	Carbamidomethyl	**Sequence Coverage [%]:**	1.5
		**No. of unique Peptides:**	2

BRUKER

Detailed Protein Report

10	20	30	40	50	60	70	80
MRLGGGQLVS	EELMNLGESF	IQTNDPSLKL	FQCAVCNKFT	TDNLDMLGLH	MNVERSLSED	EWKAVMGDSY	QCKLCRYNTQ
90	100	110	120	130	140	150	160
LKANFQLHCK	TDKHVQKYQL	VAHIKEGGKA	NEWRLKCVAI	GNPVHLKCNA	CDYYTNSLEK	LRLHTVNSRH	EASLKLYKHL
170	180	190	200	210	220	230	240
QQHESGVEGE	SCYYHCVLCN	YSTKAKLNLI	QHVRSMKHQR	SESLRKLQRL	QKGLPEEDED	LGQIFTIRRC	PSTDPEEAIE
250	260	270	280	290	300	310	320
DVEGPSETAA	DPEELAKDQE	GGASSSQAEK	ELTDSPATSK	RISFPGSSES	PLSSKRPKTA	EEIKPEQMYQ	CPYCKYSNAD
330	340	350	360	370	380	390	400
VNRLRVHAMT	QHSVQPMLRC	PLCQDMLNNK	IHLQLHLTHL	HSVAPDCVEK	LIMTVTTPEM	VMPSSMFLPA	AVPDRDGNSN
410	420	430	440	450	460	470	480
LEEAGKQPET	SEDLGKNILP	SASTEQSGDL	KPSPADPGSV	REDSGFICWK	KGCNQVFKTS	AALQTHFNEV	HAKRPQLPVS
490	500	510	520	530	540	550	560
DRHVYKYRCN	QCSLAFKTIE	KLQLHSQYHV	IRAATMCCLC	QRSFRTFQAL	KKHLETSHLE	LSEADIQQLY	GGLLANGDLL
570	580	590	600	610	620	630	640
AMGDPTLAED	HTIIVEEDKE	EESDLEDKQS	PTGSDSGSVQ	EDSGSEPKRA	LPFRKGPNFT	MEKFLDPSRP	YKCTVCKESF
650	660	670	680	690	700	710	720
TQKNILLVHY	NSVSHLHKLK	RALQESATGQ	PEPTSSPDNK	PFKCNTCNVA	YSQSSTLEIH	MRSVLHQTKA	RAAKLEAASG
730	740	750	760	770	780	790	800
SSNGTGNSSS	ISLSSSTPSP	VSTSGSNTFT	TSNPSSAGIA	PSSNLLSQVP	TESVGMPPLG	NPIGANIASP	SEPKEANRKK
810	820	830	840	850	860	870	880
LADMIASRQQ	QQQQQQQQQQ	QQQQQQQAQT	LAQAQAQVQA	HLQQELQQQA	ALIQSQLFNP	TLLPHFPMTT	ETLLQLQQQQ
890	900	910	920	930	940	950	960
HLLFPFYIPS	AEFQLNPEVS	LPVTSGALTL	TGTGPGLLED	LKAQVQVPQQ	SHQQILPQQQ	QNQLSIAQSH	SALLQPSQHP
970	980	990	1000	1010	1020	1030	1040
EKKNKLVIKE	KEKESQRERD	SAEGGEGNTG	PKETLPDALK	AKEKKELAPG	GGSEPSMLPP	RIASDARGNA	TKALLENFGF
1050	1060	1070	1080	1090	1100	1110	1120
ELVIQYNENK	QKVQKKNGKT	DQGENLEKLE	CDSCGKLFSN	ILILKSHQEH	VHQNYFPFKQ	LERFAKQYRD	HYDKLYPLRP
1130	1140	1150	1160	1170	1180	1190	1200
QTPEPPPPPP	PPPPPPLPAA	PPQPASTPAI	PASAPPITSP	TIAPAQPSVP	LTQLSMPMEL	PIFSPLMMQT	MPLQTLPAQL
1210	1220	1230	1240	1250	1260	1270	1280
PPQLGPVEPL	PADLAQLYQH	QLNPTLLQQQ	NKRPRTRITD	DQLRVLRQYF	DINNSPSEEQ	IKEMADKSGL	PQKVIKHWFR
1290	1300	1310	1320	1330	1340	1350	1360
NTLFKERQRN	KDSPYNFSNP	PITSLEELKI	DSRPPSPEPP	KQEYWGSKRS	SRTRFTDYQL	RVLQDFFDAN	AYPKDDEFEQ
1370	1380	1390	1400	1410	1420	1430	1440
LSNLLNLPTR	VIVVWFQNAR	QKARKNYENQ	GEGKDGERRE	LTNDRYIRTS	NLNYQCKKCS	LVFQRIFDLI	KHQKKLCYKD
1450	1460	1470	1480	1490	1500	1510	1520
EDEEGQDDSQ	NEDSMDAMEI	LTPTSSSCST	PMPSQAYSAP	APSANNTASS	AFLQLTAEAE	ELATFNSKTE	AGDEKPKLAE
1530	1540	1550	1560	1570	1580	1590	1600
APSAQPNQTQ	EKQGQPKPEL	QQQEQPEQKT	NTPQQKLPQL	VSLPSLPQPP	PQAPPPQCPL	PQSSPSPSQL	SHLPLKPLHT
1610	1620	1630	1640	1650	1660	1670	1680
STPQQLANLP	PQLIPYQCDQ	CKLAFPSFEH	WQEHQQLHFL	SAQNQFIHPQ	FLDRSLDMPF	MLFDPSNPLL	ASQLLSGAIP
1690	1700	1710	1720	1730	1740	1750	1760
QIPASSATSP	STPTSTMNTL	KRKLEEKASA	SPGENDSGTG	GEEPQRDKRL	RTTITPEQLE	ILYQKYLLDS	NPTRKMLDHI
1770	1780	1790	1800	1810	1820	1830	1840
AHEVGLKKRV	VQVWFQNTRA	RERKGQFRAV	GPAQAHRRCP	FCRALFKAKT	ALEAHIRSRH	WHEAKRAGYN	LTLSAMLLDC
1850	1860	1870	1880	1890	1900	1910	1920
DGGLQMKGDI	FDGTSFSHLP	PSSSDGQGVP	LSPVSKTMEL	SPRTLLSPSS	IKVEGIEDFE	SPSMSSVNLN	FDQTKLDNDD
1930	1940	1950	1960	1970	1980	1990	2000
CSSVNTAITD	TTTGDEGNAD	NDSATGIATE	TKSSSAPNEG	LTKAAMMAMS	EYEDRLSSGL	VSPAPSFYSK	EYDNEGTVDY
2010	2020	2030	2040	2050	2060	2070	2080
SETSSLADPC	SPSPGASGSA	GKSGDSGDRP	GQKRFRTQMT	NLQLKVLKSC	FNDYRTPTML	ECEVLGNDIG	LPKRVVQVWF
2090	2100	2110	2120	2130	2140	2150	2160
QNARAKEKKS	KLSMAKHFGI	NQTSYEGPKT	ECTLCGIKYS	ARLSVRDHIF	SQQHISKVKD	TIGSQLDKEK	EYFDPATVRQ
2170	2180	2190	2200	2210	2220	2230	2240
LMAQQELDRI	KKANEVLGLA	AQQQGMFDNT	PLQALNLPTA	YPALQGIPPV	LLPGLNSPSL	PGFTPSNTAL	TSPKPNLMGL
2250	2260	2270	2280	2290	2300	2310	2320
PSTTVPSPGL	PTSGLPNKPS	SASLSSPTPA	QATMAMGPQQ	PPQQQQQQQQ	PQVQQPPPPP	AAQPPPTPQL	PLQQQQQRKD
2330	2340	2350	2360	2370	2380	2390	2400

Detailed Protein Report

Cmpd.	No. of Cmpds.	m/z meas.	Δ m/z [ppm]	z	Rt [min]	Score	P	Range	Sequence	Modification
2629	1	973.4760	-77.59	2	61.6	11.7	1	1730-1745	R.LRTTITPEQLEILYQK.Y	
2712	1	1006.4372	-42.21	3	65.0	12.3	2	2097-2122	K.HFGINQTSYEGPKTECTLCGIKYSAR.L	Carbamidomethyl: 16, 19

Detailed Protein Report

Protein 431: PREDICTED: rotatin isoform X3 [Homo sapiens]

**Accession:**	gi|578832429	**Score:**	24.0
**Database:**	refseq_human(refseq_human_20140103.fasta)	**MW [kDa]:**	229.9
**Database Date:**	2015-11-30	**pI:**	6.1
**Modification(s):**	Carbamidomethyl, Oxidation	**Sequence Coverage [%]:**	2.0
		**No. of unique Peptides:**	2

Detailed Protein Report

10	20	30	40	50	60	70	80
MVLAGLIRKL	GHQLAEIRER	ALKSILCKIE	HNLICYADLI	QERQLFLHLL	EWFNFPSVPM	KEEVLNLLSR	LVKYPPAVQH
90	100	110	120	130	140	150	160
LVDVGAVEFL	SKLRSNVEPN	LQAEIDGILD	GLFLLPSEVP	ALSSASYQTN	QTELSKNPEI	LTGYFPQDKS	NFQQMEVPPR
170	180	190	200	210	220	230	240
PVVNQTVKCL	KFSTFPWLPL	TTTDRHVLSS	NESSLRSSNH	TLIWNTCELL	KDVIMQDFPA	EIFLQRPKIV	QSLLSLLKLA
250	260	270	280	290	300	310	320
FGDGKHRLAL	QSVSCLQQLC	MYLRNRLNFH	RDPGFFSNKH	DTVSQNSSLS	YCHEARGTHH	SQNPSPGSSS	PRPSVVGRTG
330	340	350	360	370	380	390	400
QRPRGDGQDW	DAASSSGSSS	HAHVNSRISV	HSPLDMGHID	LPELETEDTL	ELQFQQLSLP	QFCVSILESA	VPLLRTGSRQ
410	420	430	440	450	460	470	480
VIIRVLELLT	EDMTLIGEAI	STDIWDDSSL	FGIDMKEKLL	LVLGALGETM	CYHKSSISLE	QPEVMLVHHR	MAFISISLFA
490	500	510	520	530	540	550	560
VRLLQTLLPV	EKASEFLSEP	MSTALFLLSL	DMPISLEYPN	IHEAVVAYLE	QLNSENYSIY	KRTAEAVYSI	ECTCNFLSDI
570	580	590	600	610	620	630	640
GKEGEKNLLE	LVELADQALR	SFSYHQHFPL	IKEIISICSK	IWKSAQASPL	LQGESQKVLL	HMLSHPLPRV	KAETYHCCLE
650	660	670	680	690	700	710	720
ITKECLGVHN	VTKPVSSLCN	GIHFLLHPKV	LYEISVFGIQ	EPESEQVNTA	AKAILLYLLQ	GRLMMTALTW	NKFIESLCPV
730	740	750	760	770	780	790	800
IPILQGYADT	EDPLGNCILL	LSKASSDTEE	MLPCTTRLKS	MLRLLLVKKP	SVRSLALKLL	AFHLTSEEGA	DTKRPLIDAR
810	820	830	840	850	860	870	880
VLSRVTDLFI	GKKPIELRLD	DRRELVIKLE	TVEKVYEIFT	SDDVDLVLRK	SAAEQLAVIM	QDIKMHAVVK	KLCLIDKIIE
890	900	910	920	930	940	950	960
YLNECVSQDG	KVVECLVQPC	LTLLRKVLCG	DPVMRVSLSQ	QSSLLTVLFR	VSLIFHEDCS	VVTEVGALFC	LLLFDEVSRM
970	980	990	1000	1010	1020	1030	1040
DMWSVNPSNK	PSLPSVFSLP	VSVFRRYHLP	VHVIGHHAVS	PYSIVLPLSA	DCLALKPVSD	MLRIAWNLSW	YHGSDNLLKQ
1050	1060	1070	1080	1090	1100	1110	1120
MNSETKTQEI	LDALKLSTED	ILTLKITHMA	SGLQDCLHSI	VQAATHREVR	AAVTRMSFYL	LNDRLSLKGC	PGPCGVTLKS
1130	1140	1150	1160	1170	1180	1190	1200
LAWHTALNRF	LQVLPACTED	EKLLIDIIHF	LNKLIKEQRK	NSSLELLNWI	LELLLRHSAN	PLLDLLVLTE	SQAREETDDI
1210	1220	1230	1240	1250	1260	1270	1280
RTAVRQQLQK	ELIALFDTLL	LNFMEVTDRK	CSELLYVFQT	QLALKLLQCL	KVTDAPHFYG	LPSLERTLRG	MANLTAFPGW
1290	1300	1310	1320	1330	1340	1350	1360
SSHSPLTKPL	DICVKYLSGL	LEVITSFYVE	RGGNAMSFMG	KGVTKSTILC	LLHLSHEMMA	QAGSLEWMSL	WFLPLGSHSE
1370	1380	1390	1400	1410	1420	1430	1440
EHIPTQQGLA	WLIPLWVDRD	PEVRFTSLGL	GSALTTLETG	CVALANSCQN	ISGGLWGTVV	NILLDQSECS	MVRREAAFIL
1450	1460	1470	1480	1490	1500	1510	1520
QNLLVIPMPT	EIIKDYTWQG	PCVHDEDSGL	SLIGKPALQA	LLYHCHFYEH	LNQMVKHCYL	GRCMFDLNFS	AFDRNSESND
1530	1540	1550	1560	1570	1580	1590	1600
LNGLDDSFKF	WRAPSRTSQD	RDPSSLSTSE	TTVAPSLGST	EFQPLVQSTT	LLPEASHDQF	VAQGHQESTS	PRPPHDSSLS
1610	1620	1630	1640	1650	1660	1670	1680
APLPKLCVFV	TPSLLSAMCS	LLDNLLTIAP	RDTAKAFRQA	HLIELLCSIA	DATLIQTCVQ	ELRALLPSSP	PAEHTQAQVS
1690	1700	1710	1720	1730	1740	1750	1760
FLLEYLSSLS	RLLQSCLLVE	PDLVIQDELV	KPLITNIIGI	LTICTKDVLD	KELISAFYHT	WTHLFNLLAM	LLRKAGAITL
1770	1780	1790	1800	1810	1820	1830	1840
PFVTVALAKH	WTAAIDMFCT	CAGLSATCPA	LYTASLQFLS	VLLTEEAKGH	LQAKSKTHLC	CSPTVASLLD	DSQENQKSLE
1850	1860	1870	1880	1890	1900	1910	1920
QLSDVILQCY	EGKSSKDILK	RVAANALMSL	LAVSRRAQKH	ALKANLIDNC	MEQMKHINAQ	LNLDSLRPGK	AALKKKEDGV
1930	1940	1950	1960	1970	1980	1990	2000
IKELSIAMQL	LRNCLYQNEE	CKEAALEAHL	VPVLHSLWPW	ILMDDSLMQI	SLQLLCVYTA	NFPNGCSSLC	WSSCGQHPVQ
2010	2020	2030	2040	2050	2060		
ATHRGAVSNS	LMLCILKLAS	QMPLENTTVQ	QMVFMLLSNL	ALSHDCKGVI	QKAKINCRS		

Cmpd.	No. of Cmpds.	m/z meas.	Δ m/z [ppm]	z	Rt [min]	Score	P	Range	Sequence	Modification

Detailed Protein Report

Cmpd.	No. of Cmpds.	m/z meas.	Δ m/z [ppm]	z	Rt [min]	Score	P	Range	Sequence	Modification
10	1	1205.0326	-122.89	2	29.6	12.9	1	71-92	R.LVKYPPAVQHLVDVGAVEFLSK.L	
2647	1	753.6332	-97.01	3	61.9	11.1	2	1877-1895	R.AQKHALKANLIDNCMEQMK.H	Carbamidomethyl: 14; Oxidation: 15

Detailed Protein Report

Protein 432: tubulin polyglutamylase TTLL5 [Homo sapiens]

**Accession:**	gi|50658079	**Score:**	23.9
**Database:**	refseq_human(refseq_human_20140103.fasta)	**MW [kDa]:**	143.5
**Database Date:**	2015-11-30	**pI:**	9.5
		**Sequence Coverage [%]:**	1.5
		**No. of unique Peptides:**	1

10	20	30	40	50	60	70	80
MPIVMARDLE	ETASSSEDEE	VISQEDHPCI	MWTGGCRRIP	VLVFHADAIL	TKDNNIRVIG	ERYHLSYKIV	RTDSRLVRSI
90	100	110	120	130	140	150	160
LTAHGFHEVH	PSSTDYNLMW	TGSHLKPFLL	RTLSEAQKVN	HFPRSYELTR	KDRLYKNIIR	MQHTHGFKAF	HILPQTFLLP
170	180	190	200	210	220	230	240
AEYAEFCNSY	SKDRGPWIVK	PVASSRGRGV	YLINNPNQIS	LEENILVSRY	INNPLLIDDF	KFDVRLYVLV	TSYDPLVIYL
250	260	270	280	290	300	310	320
YEEGLARFAT	VRYDQGAKNI	RNQFMHLTNY	SVNKKSGDYV	SCDDPEVEDY	GNKWSMSAML	RYLKQEGRDT	TALMAHVEDL
330	340	350	360	370	380	390	400
IIKTIISAEL	AIATACKTFV	PHRSSCFELY	GFDVLIDSTL	KPWLLEVNLS	PSLACDAPLD	LKIKASMISD	MFTVVGFVCQ
410	420	430	440	450	460	470	480
DPAQRASTRP	IYPTFESSRR	NPFQKPQRCR	PLSASDAEMK	NLVGSAREKG	PGKLGGSVLG	LSMEEIKVLR	RVKEENDRRG
490	500	510	520	530	540	550	560
GFIRIFPTSE	TWEIYGSYLE	HKTSMNYMLA	TRLFQDRMTA	DGAPELKIES	LNSKAKLHAA	LYERKLLSLE	VRKRRRRSSR
570	580	590	600	610	620	630	640
LRAMRPKYPV	ITQPAEMNVK	TETESEEEEE	VALDNEDEEQ	EASQEESAGF	LRENQAKYTP	SLTALVENTP	KENSMKVREW
650	660	670	680	690	700	710	720
NNKGGHCCKL	ETQELEPKFN	LMQILQDNGN	LSKMQARIAF	SAYLQHVQIR	LMKDSGGQTF	SASWAAKEDE	QMELVVRFLK
730	740	750	760	770	780	790	800
RASNNLQHSL	RMVLPSRRLA	LLERRRILAH	QLGDFIIVYN	KETEQMAEKK	SKKKVEEEEE	DGVNMENFQE	FIRQASEAEL
810	820	830	840	850	860	870	880
EEVLTFYTQK	NKSASVFLGT	HSKISKNNNN	YSDSGAKGDH	PETIMEEVKI	KPPKQQQTTE	IHSDKLSRFT	TSAEKEAKLV
890	900	910	920	930	940	950	960
YSNSSSGPTA	TLQKIPNTHL	SSVTTSDLSP	GPCHHSSLSQ	IPSAIPSMPH	QPTILLNTVS	ASASPCLHPG	AQNIPSPTGL
970	980	990	1000	1010	1020	1030	1040
PRCRSGSHTI	GPFSSFQSAA	HIYSQKLSRP	SSAKAGSCYL	NKHHSGIAKT	QKEGEDASLY	SKRYNQSMVT	AELQRLAEKQ
1050	1060	1070	1080	1090	1100	1110	1120
AARQYSPSSH	INLLTQQVTN	LNLATGIINR	SSASAPPTLR	PIISPSGPTW	STQSDPQAPE	NHSSSPGSRS	LQTGGFAWEG
1130	1140	1150	1160	1170	1180	1190	1200
EVENNVYSQA	TGVVPQHKYH	PTAGSYQLQF	ALQQLEQQKL	QSRQLLDQSR	ARHQAIFGSQ	TLPNSNLWTM	NNGAGCRISS
1210	1220	1230	1240	1250	1260	1270	1280
ATASGQKPTT	LPQKVVPPPS	SCASLVPKPP	PNHEQVLRRA	TSQKASKGSS	AEGQLNGLQS	SLNPAAFVPI	TSSTDPAHTK
1290							
I							

Cmpd.	No. of Cmpds.	m/z meas.	Δ m/z [ppm]	z	Rt [min]	Score	P	Range	Sequence	Modification
2086	1	720.2523	-137.01	2	54.7	11.9	0	1024-1035	R.YNQSMVTAELQR.L	

Detailed Protein Report

Protein 433: peroxisome biogenesis factor 1 isoform 3 [Homo sapiens]

**Accession:**	gi|544186104	**Score:**	23.9
**Database:**	refseq_human(refseq_human_20140103.fasta)	**MW [kDa]:**	119.5
**Database Date:**	2015-11-30	**pI:**	5.7
		**Sequence Coverage [%]:**	2.5
		**No. of unique Peptides:**	1

10	20	30	40	50	60	70	80
MMKELQTKQL	QSNTVGITES	NENESEIPVD	SSSVASLWTM	IGSIFSFQSE	KKQETSWGLT	EINAFKNMQS	KVVPLDNIFR
**90**	**100**	**110**	**120**	**130**	**140**	**150**	**160**
VCKSQPPSIY	NASATSVFHK	HCAIHVFPWD	QEYFDVEPSF	TVTYGKLVKL	LSPKQQQSKT	KQNVLSPEKE	KQMSEPLDQK
**170**	**180**	**190**	**200**	**210**	**220**	**230**	**240**
KIRSDHNEED	EKACVLQVVW	NGLEELNNAI	KYTKNVEVLH	LGKVWIPDDL	RKRLNIEMHA	VVRITPVEVT	PKIPRSLKLQ
**250**	**260**	**270**	**280**	**290**	**300**	**310**	**320**
PRENLPKDIS	EEDIKTVFYS	WLQQSTTTML	PLVISEEEFI	KLETKDGLKE	FSLSIVHSWE	KEKDKNIFLL	SPNLLQKTTI
**330**	**340**	**350**	**360**	**370**	**380**	**390**	**400**
QVLLDPMVKE	ENSEEIDFIL	PFLKLSSLGG	VNSLGVSSLE	HITHSLLGRP	LSRQLMSLVA	GLRNGALLLT	GGKGSGKSTL
**410**	**420**	**430**	**440**	**450**	**460**	**470**	**480**
AKAICKEAFD	KLDAHVERVD	CKALRGKRLE	NIQKTLEVAF	SEAVWMQPSV	VLLDDLDLIA	GLPAVPEHEH	SPDAVQSQRL
**490**	**500**	**510**	**520**	**530**	**540**	**550**	**560**
AHALNDMIKE	FISMGSLVAL	IATSQSQQSL	HPLLVSAQGV	HIFQCVQHIQ	PPNQEQRCEI	LCNVIKNKLD	CDINKFTDLD
**570**	**580**	**590**	**600**	**610**	**620**	**630**	**640**
LQHVAKETGG	FVARDFTVLV	DRAIHSRLSR	QSISTREKLV	LTTLDFQKAL	RGFLPASLRS	VNLHKPRDLG	WDKIGGLHEV
**650**	**660**	**670**	**680**	**690**	**700**	**710**	**720**
RQILMDTIQL	PAKYPELFAN	LPIRQRTGIL	LYGPPGTGKT	LLAGVIARES	RMNFISVKGP	ELLSKYIGAS	EQAVRDIFIR
**730**	**740**	**750**	**760**	**770**	**780**	**790**	**800**
AQAAKPCILF	FDEFESIAPR	RGHDNTGVTD	RVVNQLLTQL	DGVEGLQGVY	VLAATSRPDL	IDPALLRPGR	LDKCVYCPPP
**810**	**820**	**830**	**840**	**850**	**860**	**870**	**880**
DQVSRLEILN	VLSDSLPLAD	DVDLQHVASV	TDSFTGADLK	ALLYNAQLEA	LHGMLLSSGL	QDGSSSSDSD	LSLSSMVFLN
**890**	**900**	**910**	**920**	**930**	**940**	**950**	**960**
HSSGSDDSAG	DGECGLDQSL	VSLEMSEILP	DESKFNMYRL	YFGSSYESEL	GNGTSSDLSS	QCLSAPSSMT	QDLPGVPGKD
**970**	**980**	**990**	**1000**	**1010**	**1020**	**1030**	**1040**
QLFSQPPVLR	TASQEGCQEL	TQEQRDQLRA	DISIIKGRYR	SQSGEDESMN	QPGPIKTRLA	ISQSHLMTAL	GHTRPSISED
**1050**	**1060**	**1070**	**1080**				
DWKNFAELYE	SFQNPKRRKN	QSGTMFRPGQ	KVTLA				

Cmpd.	No. of Cmpds.	m/z meas.	Δ m/z [ppm]	z	Rt [min]	Score	P	Range	Sequence	Modification
1391	1	1207.8676	86.30	1	45.9	12.7	2	233-242	K.IPRSLKLQPR.E	

Detailed Protein Report

Protein 434: short transient receptor potential channel 7 isoform 3 [Homo sapiens]

**Accession:**	gi|262399375	**Score:**	23.9
**Database:**	refseq_human(refseq_human_20140103.fasta)	**MW [kDa]:**	86.2
**Database Date:**	2015-11-30	**pI:**	8.4
		**Sequence Coverage [%]:**	5.1
		**No. of unique Peptides:**	1

10	20	30	40	50	60	70	80
MLRNSTFKNM	QRRHTTLREK	GRRQAIRGPA	YMFNEKGTSL	TPEEERFLDS	AEYGNIPVVR	KMLEESKTLN	FNCVDYMGQN
90	100	110	120	130	140	150	160
ALQLAVGNEH	LEVTELLLKK	ENLARVGDAL	LLAISKGYVR	IVEAILNHPA	FAQGQRLTLS	PLEQELRDDD	FYAYDEDGTR
170	180	190	200	210	220	230	240
FSHDITPIIL	AAHCQEYEIV	HILLLKGARI	ERPHDYFCKC	NECTEKQRKD	SFSHSRSRMN	AYKGLASAAY	LSLSSEDPVL
250	260	270	280	290	300	310	320
TALELSNELA	RLANIETEFK	LGRTLRSPFM	KFVAHAVSFT	IFLGLLVVNA	SDRFEGVKTL	PNETFTDYPK	QIFRVKTTQF
330	340	350	360	370	380	390	400
SWTEMLIMKW	VLGMIWSECK	EIWEEGPREY	VLHLWNLLDF	GMLSIFVASF	TARFMAFLKA	TEAQLYVDQH	VQDDTLHNVS
410	420	430	440	450	460	470	480
LPPEVAYFTY	ARDKWWPSDP	QIISEGLYAI	AVVLSFSRIA	YILPANESFG	PLQISLGRTV	KDIFKFMVIF	IMVFVAFMIG
490	500	510	520	530	540	550	560
MFNLYSYYRG	AKYNPAFTTV	EESFKTLFWS	IFGLSEVISV	VLKYDHKFIE	NIGYVLYGVY	NVTMVVVLLN	MLIAMINNSY
570	580	590	600	610	620	630	640
QEIEEDADVE	WKFARAKLWL	SYFDEGRTLP	APFNLVPSPK	SFYYLIMRIK	MCLIKLCKSK	AKSCENDLEM	GMLNSKFKKT
650	660	670	680	690	700	710	720
RYQAGMRNSE	NLTANNTLSK	PTRYQKIMKR	LIKRYVLKAQ	VDRENDEVNE	GELKEIKQDI	SSLRYELLEE	KSQATGELAD
730	740	750					
LIQQLSEKFG	KNLNKDHLRV	NKGKDI					

Cmpd.	No. of Cmpds.	m/z meas.	Δ m/z [ppm]	z	Rt [min]	Score	P	Range	Sequence	Modification
81	1	1120.1328	21.81	2	30.9	11.6	1	117-136	K.GYVRIVEAILNHPAFAQGQR.L	

Detailed Protein Report

Protein 435: phosphoglycolate phosphatase [Homo sapiens]

**Accession:**	gi|108796653	**Score:**	23.9
**Database:**	refseq_human(refseq_human_20140103.fasta)	**MW [kDa]:**	34.0
**Database Date:**	2015-11-30	**pI:**	5.8
**Modification(s):**	Carbamidomethyl	**Sequence Coverage [%]:**	13.4
		**No. of unique Peptides:**	2

10	20	30	40	50	60	70	80
MAAAEAGGDD	ARCVRLSAER	AQALLADVDT	LLFDCDGVLW	RGETAVPGAP	EALRALRARG	KRLGFITNNS	SKTRAAYAEK
**90**	**100**	**110**	**120**	**130**	**140**	**150**	**160**
LRRLGFGGPA	GPGASLEVFG	TAYCTALYLR	QRLAGAPAPK	AYVLGSPALA	AELEAVGVAS	VGVGPEPLQG	EGPGDWLHAP
**170**	**180**	**190**	**200**	**210**	**220**	**230**	**240**
LEPDVRAVVV	GFDPHFSYMK	LTKALRYLQQ	PGCLLVGTNM	DNRLPLENGR	FIAGTGCLVR	AVEMAAQRQA	DIIGKPSRFI
**250**	**260**	**270**	**280**	**290**	**300**	**310**	**320**
FDCVSQEYGI	NPERTVMVGD	RLDTDILLGA	TCGLKTILTL	TGVSTLGDVK	NNQESDCVSK	KKMVPDFYVD	SIADLLPALQ
**330**							
G							

Cmpd.	No. of Cmpds.	m/z meas.	Δ m/z [ppm]	z	Rt [min]	Score	P	Range	Sequence	Modification
44	1	634.4553	188.58	2	29.2	13.2	0	42-54	R.GETAVPGAPEALR.A	
2202	1	1057.5324	-23.37	3	56.1	10.7	2	81-110	K.LRRLGFGGPAGPGASLEVFGTAYCTALYLR.Q	Carbamidomethyl: 24

Detailed Protein Report

Protein 436: **PREDICTED: probable phospholipid-transporting ATPase IG isoform X3 [Homo sapiens]**

**Accession:**	gi|578838665	**Score:**	23.8
**Database:**	refseq_human(refseq_human_20140103.fasta)	**MW [kDa]:**	126.6
**Database Date:**	2015-11-30	**pI:**	6.4
**Modification(s):**	Carbamidomethyl, Oxidation	**Sequence Coverage [%]:**	2.8
		**No. of unique Peptides:**	2

Quantitation

QD:QU **Median:** 0.67 **CV:** 88.43 % **No. of Peptides:** 2

Alias proteins:

Accession	Name	Description
gi|578838667	refseq_human (refseq_human_20140103.fasta)	PREDICTED: probable phospholipid-transporting ATPase IG isoform X4 [Homo sapiens]

10	20	30	40	50	60	70	80
MFRRSLNRFC	AGEEKRVGTR	TVFVGNHPVS	ETEAYIAQRF	CDNRIVSSKY	TLWNFLPKNL	FEQFRRIANF	YFLIIFLVQV
**90**	**100**	**110**	**120**	**130**	**140**	**150**	**160**
TVDTPTSPVT	SGLPLFFVIT	VTAIKQGYED	CLRHRADNEV	NKSTVYIIEN	AKRVRKESEK	IKVGDVVEVQ	ADETFPCDLI
**170**	**180**	**190**	**200**	**210**	**220**	**230**	**240**
LLSSCTTDGT	CYVTTASLDG	ESNCKTHYAV	RDTIALCTAE	SIDTLRAAIE	CEQPQPDLYK	FVGRINIYSN	SLEAVARSLG
**250**	**260**	**270**	**280**	**290**	**300**	**310**	**320**
PENLLLKGAT	LKNTEKIYGV	AVYTGMETKM	ALNYQGKSQK	RSAVEKSINA	FLIVYLFILL	TKAAVCTTLK	YVWQSTPYND
**330**	**340**	**350**	**360**	**370**	**380**	**390**	**400**
EPWYNQKTQK	ERETLKVLKM	FTDFLSFMVL	FNFIIPVSMY	VTVEMQKFLG	SFFISWDKDF	YDEEINEGAL	VNTSDLNEEL
**410**	**420**	**430**	**440**	**450**	**460**	**470**	**480**
GQVDYVFTDK	TGTLTENSME	FIECCIDGHK	YKGVTQEVDG	LSQTDGTLTY	FDKVDKNREE	LFLRALCLCH	TVEIKTNDAV
**490**	**500**	**510**	**520**	**530**	**540**	**550**	**560**
DGATESAELT	YISSSPDEIA	LVKGAKRYGF	TFLGNRNGYM	RVENQRKEIE	EYELLHTLNF	DAVRRRMSVI	VKTQEGDILL
**570**	**580**	**590**	**600**	**610**	**620**	**630**	**640**
FCKGADSAVF	PRVQNHEIEL	TKVHVERNAM	DGYRTLCVAF	KEIAPDDYER	INRQLIEAKM	ALQDREEKME	KVFDDIETNM
**650**	**660**	**670**	**680**	**690**	**700**	**710**	**720**
NLIGATAVED	KLQDQAAETI	EALHAAGLKV	WVLTGDKMET	AKSTCYACRL	FQTNTELLEL	TTKTIEESER	KEDRLHELLI
**730**	**740**	**750**	**760**	**770**	**780**	**790**	**800**
EYRKKLLHEF	PKSTRSFKKA	WTEHQEYGLI	IDGSTLSLIL	NSSQDSSSNN	YKSIFLQICM	KCTAVLCCRM	APLQKAQIVR
**810**	**820**	**830**	**840**	**850**	**860**	**870**	**880**
MVKNLKGSPI	TLSIGDGAND	VSMILESHVG	IGIKGKEGRQ	AARNSDYSVP	KFKHLKKLLL	AHGHLYYVRI	AHLVQYFFYK
**890**	**900**	**910**	**920**	**930**	**940**	**950**	**960**
NLCFILPQFL	YQFFCGFSQQ	PLYDAAYLTM	YNICFTSLPI	LAYSLLEQHI	NIDTLTSDPR	LYMKISGNAM	LQLGPFLYWT
**970**	**980**	**990**	**1000**	**1010**	**1020**	**1030**	**1040**
FLAAFEGTVF	FFGTYFLFQT	ASLEENGKVY	GNWTFGTIVF	TVLVFTVTLK	LALDTRFWTW	INHFVIWGSL	AFYVFFSFFW
**1050**	**1060**	**1070**	**1080**	**1090**	**1100**	**1110**	
GGIIWPFLKQ	QRMYFVFAQM	LSSVSTWLAI	ILLIFISLFP	EILLIVLKNV	RRRSARVHHL	ISSSA	

Cmpd.	No. of Cmpds.	m/z meas.	Δ m/z [ppm]	z	Rt [min]	Score	P	Range	Sequence	Modification	Ratios	
237	1	898.4036	-13.81	3	33.0	10.9	2	588-610	R.NAMDGYRTLCVAFKEIAPDDYER.I	Oxidation: 3	QD:QU	1.44
223	1	491.6240	-189.17	2	31.5	12.8	0	782-789	K.CTAVLCCR.M	Carbamidomethyl: 1, 6	QD:QU	0.32

Detailed Protein Report

Protein 437: tubulin-specific chaperone E isoform a [Homo sapiens]

**Accession:**	gi|4507375	**Score:**	23.8
**Database:**	refseq_human(refseq_human_20140103.fasta)	**MW [kDa]:**	59.3
**Database Date:**	2015-11-30	**pI:**	6.3
		**Sequence Coverage [%]:**	4.4
		**No. of unique Peptides:**	1

Alias proteins:

Accession	Name	Description
gi|118442828	refseq_human (refseq_human_20140103.fasta)	tubulin-specific chaperone E isoform a [Homo sapiens]

10	20	30	40	50	60	70	80
MSDTLTADVI	GRRVEVNGEH	ATVRFAGVVP	PVAGPWLGVE	WDNPERGKHD	GSHEGTVYFK	CRHPTGGSFI	RPNKVNFGTD
**90**	**100**	**110**	**120**	**130**	**140**	**150**	**160**
FLTAIKNRYV	LEDGPEEDRK	EQIVTIGNKP	VETIGFDSIM	KQQSQLSKLQ	EVSLRNCAVS	CAGEKGGVAE	ACPNIRKVDL
**170**	**180**	**190**	**200**	**210**	**220**	**230**	**240**
SKNLLSSWDE	VIHIADQLRH	LEVLNVSENK	LKFPSGSVLT	GTLSVLKVLV	LNQTGITWAE	VLRCVAGCPG	LEELYLESNN
**250**	**260**	**270**	**280**	**290**	**300**	**310**	**320**
IFISERPTDV	LQTVKLLDLS	SNQLIDENQL	YLIAHLPRLE	QLILSDTGIS	SLHFPDAGIG	CKTSMFPSLK	YLVVNDNQIS
**330**	**340**	**350**	**360**	**370**	**380**	**390**	**400**
QWSFFNELEK	LPSLRALSCL	RNPLTKEDKE	AETARLLIIA	SIGQLKTLNK	CEILPEERRR	AELDYRKAFG	NEWKQAGGHK
**410**	**420**	**430**	**440**	**450**	**460**	**470**	**480**
DPEKNRLSEE	FLTAHPRYQF	LCLKYGAPED	WELKTQQPLM	LKNQLLTLKI	KYPHQLDQKV	LEKQLPGSMT	IQKVKGLLSR
**490**	**500**	**510**	**520**	**530**			
LLKVPVSDLL	LSYESPKKPG	REIELENDLK	SLQFYSVENG	DCLLVRW			

Cmpd.	No. of Cmpds.	m/z meas.	Δ m/z [ppm]	z	Rt [min]	Score	P	Range	Sequence	Modification
486	1	535.7503	-186.51	2	34.7	13.3	1	443-451	K.NQLLTLKIK.Y	

Detailed Protein Report

Protein 438: PREDICTED: ankyrin repeat domain-containing protein SOWAHC-like [Homo sapiens]

Accession: gi|341914886
Database: refseq_human(refseq_human_20140103.fasta)
Database Date: 2015-11-30

Score: 23.7
MW [kDa]: 25.7
pI: 12.2
Sequence Coverage [%]: 7.8
No. of unique Peptides: 1

Quantitation

QD:QU **Median:** 0.78 **CV:** 0.00 % **No. of Peptides:** 1

10	20	30	40	50	60	70	80
MRSAAFTIPS	LKQWSPRCST	GRAELGKGEP	SPCTGRPEQR	GRRKSSLAPP	APTPDAGPQT	SKDLEPPPHG	CQEADRGGSW
**90**	**100**	**110**	**120**	**130**	**140**	**150**	**160**
GATTSRPFRQ	NLSDLGRHSV	LPLKRNLCPG	GSSLGAPPLR	TPQRRVTLAA	QRWLSRPQRR	AVGLGELDQG	AQLAALGLRW
**170**	**180**	**190**	**200**	**210**	**220**	**230**	**240**
GVDSLGGCPR	ASQPAGHSGL	HHTLTCLRRH	LALQAGASGH	AGQLPQRASA	AWEQQGQSYT	ALHLAAMYLG	DGEAASGNIG
**250**							
RRC							

Cmpd.	No. of Cmpds.	m/z meas.	Δ m/z [ppm]	z	Rt [min]	Score	P	Range	Sequence	Modification	Ratios
1819	1	719.7489	-178.16	2	51.3	10.6	0	106-120	R.NLCPGGSSLGAPPLR.T		QD:QU 0.78

Detailed Protein Report

Protein 439: reticulon-4 receptor-like 1 precursor [Homo sapiens]

Accession: gi|30425553
Database: refseq_human(refseq_human_20140103.fasta)
Database Date: 2015-11-30
Modification(s): Carbamidomethyl

Score: 23.7
MW [kDa]: 49.0
pI: 10.3
Sequence Coverage [%]: 6.1
No. of unique Peptides: 1

10	20	30	40	50	60	70	80
MLRKGCCVEL	LLLLVAAELP	LGGGCPRDCV	CYPAPMTVSC	QAHNFAAIPE	GIPVDSERVF	LQNNRIGLLQ	PGHFSPAMVT
**90**	**100**	**110**	**120**	**130**	**140**	**150**	**160**
LWIYSNNITY	IHPSTFEGFV	HLEELDLGDN	RQLRTLAPET	FQGLVKLHAL	YLYKCGLSAL	PAGVFGGLHS	LQYLYLQDNH
**170**	**180**	**190**	**200**	**210**	**220**	**230**	**240**
IEYLQDDIFV	DLVNLSHLFL	HGNKLWSLGP	GTFRGLVNLD	RLLLHENQLQ	WVHHKAFHDL	RRLTTLFLFN	NSLSELQGEC
**250**	**260**	**270**	**280**	**290**	**300**	**310**	**320**
LAPLGALEFL	RLNGNPWDCG	CRARSLWEWL	QRFRGSSSAV	PCVSPGLRHG	QDLKLLRAED	FRNCTGPASP	HQIKSHTLTT
**330**	**340**	**350**	**360**	**370**	**380**	**390**	**400**
TDRAARKEHH	SPHGPTRSKG	HPHGPRPGHR	KPGKNCTNPR	NRNQISKAGA	GKQAPELPDY	APDYQHKFSF	DIMPTARPKR
**410**	**420**	**430**	**440**	**450**			
KGKCARRTPI	RAPSGVQQAS	SASSLGASLL	AWTLGLAVTL	R			

Cmpd.	No. of Cmpds.	m/z meas.	Δ m/z [ppm]	z	Rt [min]	Score	P	Range	Sequence	Modification
2008	1	687.3490	4.76	2	55.5	10.2	0	275-288	R.GSSSAVPCVSPGLR.H	Carbamidomethyl: 8

Detailed Protein Report

Protein 440: PAB-dependent poly(A)-specific ribonuclease subunit 2 isoform 3 [Homo sapiens]

**Accession:**	gi|188536112	**Score:**	23.7
**Database:**	refseq_human(refseq_human_20140103.fasta)	**MW [kDa]:**	134.9
**Database Date:**	2015-11-30	**pI:**	5.6
**Modification(s):**	Oxidation	**Sequence Coverage [%]:**	3.5
		**No. of unique Peptides:**	2

10	20	30	40	50	60	70	80
MNFEGLDPGL	AEYAPAMHSA	LDPVLDAHLN	PSLLQNVELD	PEGVALEALP	VQESVHIMEG	VYSELHSVVA	EVGVPVSVSH
**90**	**100**	**110**	**120**	**130**	**140**	**150**	**160**
FDLHEEMLWV	GSHGGHATSF	FGPALERYSS	FQVNGSDDIR	QIQSLENGIL	FLTKNNLKYM	ARGGLIIFDY	LLDENEDMHS
**170**	**180**	**190**	**200**	**210**	**220**	**230**	**240**
LLLTDSSTLL	VGGLQNHILE	IDLNTVQETQ	KYAVETPGVT	IMRQTNRFFF	CGHTSGKVSL	RDLRTFKVEH	EFDAFSGSLS
**250**	**260**	**270**	**280**	**290**	**300**	**310**	**320**
DFDVHGNLLA	ACGFSSRLTG	LACDRFLKVY	DLRMMRAITP	LQVHVDPAFL	RFIPTYTSRL	AIISQSGQCQ	FCEPTGLANP
**330**	**340**	**350**	**360**	**370**	**380**	**390**	**400**
ADIFHVNPVG	PLLMTFDVSA	SKQALAFGDS	EGCVHLWTDS	PEPSFNPYSR	ETEFALPCLV	DSLPPLDWSQ	DLLPLSLIPV
**410**	**420**	**430**	**440**	**450**	**460**	**470**	**480**
PLTTDTLLSD	WPAANSAPAP	RRAPPVDAEI	LRTMKKVGFI	GYAPNPRTRL	RNQIPYRLKE	SDSEFDSFSQ	VTESPVGREE
**490**	**500**	**510**	**520**	**530**	**540**	**550**	**560**
EPHLHMVSKK	YRKVTIKYSK	LGLEDFDFKH	YNKTLFAGLE	PHIPNAYCNC	MIQVLYFLEP	VRCLIQNHLC	QKEFCLACEL
**570**	**580**	**590**	**600**	**610**	**620**	**630**	**640**
GFLFHMLDLS	RGDPCQGNNF	LRAFRTIPEA	SALGLILADS	DEASGKGNLA	RLIQRWNRFI	LTQLHQDMQE	LEIPQAYRGA
**650**	**660**	**670**	**680**	**690**	**700**	**710**	**720**
GGSSFCSSGD	SVIGQLFSCE	MENCSLCRCG	SETVRASSTL	LFTLSYPDDK	TGKNYDFAQV	LKRSICLDQN	TQAWCDTCEK
**730**	**740**	**750**	**760**	**770**	**780**	**790**	**800**
YQPTIQTRNI	RHLPDILVIN	CEVNSSKEAD	FWRMQAEVAF	KMAVKKHGGE	ISKNKEFALA	DWKELGSPEG	VLVCPSIEEL
**810**	**820**	**830**	**840**	**850**	**860**	**870**	**880**
KNVWLPFSIR	MKMTKNKGLD	VCNWTDGDEM	QWGPARAEEE	HGVYVYDLMA	TVVHILDSRT	GGSLVAHIKV	GETYHQRKEG
**890**	**900**	**910**	**920**	**930**	**940**	**950**	**960**
VTHQQWYLFN	DFLIEPIDKH	EAVQFDMNWK	VPAILYYVKR	NLNSRYNLNI	KNPIEASVLL	AEASLARKQR	KTHTTFIPLM
**970**	**980**	**990**	**1000**	**1010**	**1020**	**1030**	**1040**
LNEMPQIGDL	VGLDAEFVTL	NEEEAELRSD	GTKSTIKPSQ	MSVARITCVR	GQGPNEGIPF	IDDYISTQEQ	VVDYLTQYSG
**1050**	**1060**	**1070**	**1080**	**1090**	**1100**	**1110**	**1120**
IKPGDLDAKI	SSKHLTTLKS	TYLKLRFLID	IGVKFVGHGL	QKDFRVINLM	VPKDQVLDTV	YLFHMPRKRM	ISLRFLAWYF
**1130**	**1140**	**1150**	**1160**	**1170**	**1180**	**1190**	**1200**
LDLKIQGETH	DSIEDARTAL	QLYRKYLELS	KNGTEPESFH	KVLKGLYEKG	RKMDWKVPEP	EGQTSPKNAA	VFSSVLAL

Cmpd.	No. of Cmpds.	m/z meas.	Δ m/z [ppm]	z	Rt [min]	Score	P	Range	Sequence	Modification
1833	1	1010.9916	-90.84	3	53.3	11.9	0	639-668	R.GAGGSSFCSSGDSVIGQLFSCEMENCSLCR.C	
1929	1	650.7900	-100.99	2	54.5	11.7	2	762-773	K.MAVKKHGGEISK.N	Oxidation: 1

Detailed Protein Report

Protein 441: **PREDICTED: type I inositol 3,4-bisphosphate 4-phosphatase isoform X4 [Homo sapiens]**

**Accession:**	gi|578804269	**Score:**	23.6
**Database:**	refseq_human(refseq_human_20140103.fasta)	**MW [kDa]:**	110.5
**Database Date:**	2015-11-30	**pI:**	6.7
**Modification(s):**	Carbamidomethyl, Oxidation	**Sequence Coverage [%]:**	3.6
		**No. of unique Peptides:**	2

Quantitation

QD:QU **Median:** 0.50 **CV:** 0.00 % **No. of Peptides:** 1

10	20	30	40	50	60	70	80
MTAREHSPRH	GARARAMQRA	STIDVAADML	GLSLAGNIQD	PDEPILEFSL	ACSELHTPSL	DRKPNSFVAV	SVTTPPQAFW
**90**	**100**	**110**	**120**	**130**	**140**	**150**	**160**
TKHAQTEIIE	GTNNPIFLSS	IAFFQDSLIN	QMTQVKLSVY	DVKDRSQGTM	YLLGSGTFIV	KDLLQDRHHR	LHLTLRSAES
**170**	**180**	**190**	**200**	**210**	**220**	**230**	**240**
DRVGNITVIG	WQMEEKSDQR	PPVTRSVDTV	NGRMVLPVDE	SLTEALGIRS	KYASLRKDTL	LKSVFGGAIC	RMYRFPTTDG
**250**	**260**	**270**	**280**	**290**	**300**	**310**	**320**
NHLRILEQMA	ESVLSLHVPR	QFVKLLLEED	AARVCELEEL	GELSPCWESL	RRQIVTQYQT	IILTYQENLT	DLHQYRGPSF
**330**	**340**	**350**	**360**	**370**	**380**	**390**	**400**
KASSLKADKK	LEFVPTNLHI	QRMRVQDDGG	SDQNYDIVTI	GAPAAHCQGF	KSGGLRKKLH	KFEETKKHSF	EECCTSSGCQ
**410**	**420**	**430**	**440**	**450**	**460**	**470**	**480**
SIIYIPQDVV	RAKEIIAQIN	TLKTQVSYYA	ERLSRAAKDR	SATGLERTLA	ILADKTRQLV	TVCDCKLLAN	SIHGLNAARP
**490**	**500**	**510**	**520**	**530**	**540**	**550**	**560**
DYIASKASPT	STEEEQVMLR	NDQDTLMARW	TGRNSRSSLQ	VDWHEEEWEK	VWLNVDKSLE	CIIQRVDKLL	QKERLHGEGC
**570**	**580**	**590**	**600**	**610**	**620**	**630**	**640**
EDVFPCAGSC	TSKKGNPDSH	AYWIRPEDPF	CDVPSSPCPS	TMPSTACHPH	LTTREWSEAL	YPLLTTLTDC	VAMMSDKAKK
**650**	**660**	**670**	**680**	**690**	**700**	**710**	**720**
AMVFLLMQDS	APTIATYLSL	QYRRDVVFCQ	TLTALICGFI	IKLRNCLHDD	GFLRQLYTIG	LLAQFESLLS	TYGEELAMLE
**730**	**740**	**750**	**760**	**770**	**780**	**790**	**800**
DMSLGIMDLR	NVTFKVTQAT	SSASADMLPV	ITGNRDGFNV	RVPLPGPLFD	ALPREIQSGM	LLRVQPVLFN	VGINEQQTLA
**810**	**820**	**830**	**840**	**850**	**860**	**870**	**880**
ERFGDTSLQE	VINVESLVRL	NSYFEQFKEV	LPEDCLPRSR	SQTCLPELLR	FLGQNVHARK	NKNVDILWQA	AEICRRLNGV
**890**	**900**	**910**	**920**	**930**	**940**	**950**	**960**
RFTSCKSAKD	RTAMSVTLEQ	CLILQHEHGM	APQVFTQALE	CMRSIGTREV	VTQKNLSGLV	PIRDLRLDPS	LLCSIPLLAL
**970**	**980**	**990**					
SPNLLIVWLF	LSIAYLVTKL	RCK					

Cmpd.	No. of Cmpds.	m/z meas.	Δ m/z [ppm]	z	Rt [min]	Score	P	Range	Sequence	Modification	Ratios
2694	1	963.8006	5.32	3	64.8	12.9	1	615-639	R. EWSEALYPLLTTLTDCVAMMSDK. K	Carbamidomethyl: 16; Oxidation: 19	
675	1	562.6665	-233.07	2	37.0	10.7	1	877-886	R.LNGVRFTSCK.S		QD:QU 0.50

Detailed Protein Report

Protein 442: PREDICTED: allograft inflammatory factor 1 isoform X2 [Homo sapiens]

**Accession:**	gi|530381505	**Score:**	23.6
**Database:**	refseq_human(refseq_human_20140103.fasta)	**MW [kDa]:**	20.9
**Database Date:**	2015-11-30	**pI:**	9.9
		**Sequence Coverage [%]:**	8.6
		**No. of unique Peptides:**	1

Alias proteins:

Accession	Name	Description
gi|530429518	refseq_human (refseq_human_20140103.fasta)	PREDICTED: allograft inflammatory factor 1 isoform X2 [Homo sapiens]
gi|530429177	refseq_human (refseq_human_20140103.fasta)	PREDICTED: allograft inflammatory factor 1 isoform X2 [Homo sapiens]
gi|530428888	refseq_human (refseq_human_20140103.fasta)	PREDICTED: allograft inflammatory factor 1 isoform X2 [Homo sapiens]
gi|530428260	refseq_human (refseq_human_20140103.fasta)	PREDICTED: allograft inflammatory factor 1 isoform X2 [Homo sapiens]
gi|530427928	refseq_human (refseq_human_20140103.fasta)	PREDICTED: allograft inflammatory factor 1 isoform X2 [Homo sapiens]

10	20	30	40	50	60	70	80
MSQTRDLQGG	KAFGLLKAQQ	EERLDEINKQ	FLDDPKYSSD	EDLPSKLEGF	KEKYMEFDLN	GNGDIGEKRV	ICGGRVVCRP
**90**	**100**	**110**	**120**	**130**	**140**	**150**	**160**
KKTEVSPTCS	IPHDLGGGPP	TTVGGRRMGM	RKWERRERVS	PPSPHPHPLP	PDIMSLKRML	EKLGVPKTHL	ELKKLIGEVS
**170**	**180**	**190**					
SGSGETFSYP	DFLRMMLGKR	SAILKM					

Cmpd.	No. of Cmpds.	m/z meas.	Δ m/z [ppm]	z	Rt [min]	Score	P	Range	Sequence	Modification
1677	1	619.9559	5.16	3	51.2	23.6	1	54-69	K.YMEFDLNGNGDIGEKR.V	

Detailed Protein Report

Protein 443: probable ubiquitin carboxyl-terminal hydrolase FAF-Y [Homo sapiens]

**Accession:**	gi|74319833	**Score:**	23.6
**Database:**	refseq_human(refseq_human_20140103.fasta)	**MW [kDa]:**	290.9
**Database Date:**	2015-11-30	**pI:**	5.5
**Modification(s):**	Carbamidomethyl, Oxidation	**Sequence Coverage [%]:**	1.6
		**No. of unique Peptides:**	1

Detailed Protein Report

10	20	30	40	50	60	70	80
MTAITHGSPV	GGNDSQGQVL	DGQSQHLFQQ	NQTSSPDSSN	ENSVATPPPE	EQGQGDAPPQ	HEDEEPAFPH	TELANLDDMI
90	100	110	120	130	140	150	160
NRPRWVVPVL	PKGELEVLLE	AAIDLSVKGL	DVKSEACQRF	FRDGLTISFT	KILMDEAVSG	WKFEIHRCII	NNTHRLVELC
170	180	190	200	210	220	230	240
VAKLSQDWFP	LLELLAMALN	PHCKFHIYNG	TRPCELISSN	AQLPEDELFA	RSSDPRSPKG	WLVDLINKFG	TLNGFQILHD
250	260	270	280	290	300	310	320
RFFNGSALNI	QIIAALIKPF	GQCYEFLSQH	TLKKYFIPVI	EIVPHLLENL	TDEELKKEAK	NEAKNDALSM	IIKSLKNLAS
330	340	350	360	370	380	390	400
RISGQDETIK	NLEIFRLKMI	LRLLQISSFN	GKMNALNEIN	KVISSVSYYT	HRHSNPEEEE	WLTAERMAEW	IQQNNILSIV
410	420	430	440	450	460	470	480
LQDSLHQPQY	VEKLEKILRF	VIKEKALTLQ	DLDNIWAAQA	GKHEAIVKNV	HDLLAKLAWD	FSPGQLDHLF	DCFKASWTNA
490	500	510	520	530	540	550	560
SKKQREKLLE	LIRRLAEDDK	DGVMAHKVLN	LLWNLAQSDD	VPVDIMDLAL	SAHIKILDYS	CSQDRDAQKI	QWIDHFIEEL
570	580	590	600	610	620	630	640
RTNDKWVIPA	LKQIREICSL	FGEASQNLSQ	TQRSPHIFYR	HDLINQLQQN	HALVTLVAEN	LATYMNSIRL	YAGDHEDYDP
650	660	670	680	690	700	710	720
QTVRLGSRYS	HVQEVQERLN	FLRFLLKDGQ	LWLCAPQAKQ	IWKCLAENAV	YLCDREACFK	WYSKLMGDEP	DLDPDINKDF
730	740	750	760	770	780	790	800
FESNVLQLDP	SLLTENGMKC	FERFFKAVNC	RERKLIAKRR	SYMMDDLELI	GLDYLWRVVI	QSSDEIANRA	IDLLKEIYTN
810	820	830	840	850	860	870	880
LGPRLKANQV	VIHEDFIQSC	FDRLKASYDT	LCVFDGDKNS	INCARQEAIR	MVRVLTVIKE	YINECDSDYH	KERMILPMSR
890	900	910	920	930	940	950	960
AFRGKHLSLI	VRFPNQGRQV	DELDIWSHTN	DTIGSVRRCI	VNRIKANVAH	KKIELFVGGE	LIDSEDDRKL	IGQLNLKDKS
970	980	990	1000	1010	1020	1030	1040
LITAKLTQIN	FNMPSSPDSS	SDSSTASPGN	HRNHYNDGPN	LEVESCLPGV	IMSVHPRYIS	FLWQVADLGS	NLNMPPLRDG
1050	1060	1070	1080	1090	1100	1110	1120
ARVLMKLMPP	DRTAVEKLRA	VCLDHAKLGE	GKLSPPLDSL	FFGPSASQVL	YLTEVVYALL	MPAGVPLTDG	SSDFQVHFLK
1130	1140	1150	1160	1170	1180	1190	1200
SGGLPLVLSM	LIRNNFLPNT	DMETRRGAYL	NALKIAKLLL	TAIGYGHVRA	VAEACQPVVD	GTDPITQINQ	VTHDQAVVLQ
1210	1220	1230	1240	1250	1260	1270	1280
SALQSIPNPS	SECVLRNESI	LLAQEISNEA	SRYMPDICVI	RAIQKIIWAS	ACGALGLVFS	PNEEITKIYQ	MTTNGSNKLE
1290	1300	1310	1320	1330	1340	1350	1360
VEDEQVCCEA	LEVMTLCFAL	LPTALDALSK	EKAWQTFIID	LLLHCPSKTV	RQLAQEQFFL	MCTRCCMGHR	PLLFFITLLF
1370	1380	1390	1400	1410	1420	1430	1440
TILGSTAREK	GKYSGDYFTL	LRHLLNYAYN	GNINIPNAEV	LLVSEIDWLK	RIRDNVKNTG	ETGVEEPILE	GHLGVTKELL
1450	1460	1470	1480	1490	1500	1510	1520
AFQTSEKKYH	FGCEKGGANL	IKELIDDFIF	PASKVYLQYL	RSGELPAEQA	IPVCSSPVTI	NAGFELLVAL	AIGCVRNLKQ
1530	1540	1550	1560	1570	1580	1590	1600
IVDCLTEMYY	MGTAITTCEA	LTEWEYLPPV	GPRPPKGFVG	LKNAGATCYM	NSVIQQLYMI	PSIRNSILAI	EGTGSDLHDD
1610	1620	1630	1640	1650	1660	1670	1680
MFGDEKQDSE	SNVDPRDDVF	GYPHQFEDKP	ALSKTEDRKE	YNIGVLRHLQ	VIFGHLAASQ	LQYYVPRGFW	KQFRLWGEPV
1690	1700	1710	1720	1730	1740	1750	1760
NLREQHDALE	FFNSLVDSLD	EALKALGHPA	ILSKVLGGSF	ADQKICQGCP	HRYECEESFT	TLNVDIRNHQ	NLLDSLEQYI
1770	1780	1790	1800	1810	1820	1830	1840
KGDLLEGANA	YHCEKCDKKV	DTVKRLLIKK	LPRVLAIQLK	RFDYDWEREC	AIKFNDYFEF	PRELDMGPYT	VAGVANLERD
1850	1860	1870	1880	1890	1900	1910	1920
NVNSENELIE	QKEQSDNETA	GGTKYRLVGV	LVHSGQASGG	HYYSYIIQRN	GKDDQTDHWY	KFDDGDVTEC	KMDDDEEMKN
1930	1940	1950	1960	1970	1980	1990	2000
QCFGGEYMGE	VFDHMMKRMS	YRRQKRWWNA	YILFYEQMDM	IDEDDEMIRY	ISELTIARPH	QIIMSPAIER	SVRKQNVKFM
2010	2020	2030	2040	2050	2060	2070	2080
HNRLQYSLEY	FQFVKKLLTC	NGVYLNPAPG	QDYLLPEAEE	ITMISIQLAA	RFLFTTGFHT	KKIVRGPASD	WYDALCVLLR
2090	2100	2110	2120	2130	2140	2150	2160
HSKNVRFWFT	HNVLFNVSNR	FSEYLLECPS	AEVRGAFAKL	IVFIAHFSLQ	DGSCPSPFAS	PGPSSQACDN	LSLSDHLLRA
2170	2180	2190	2200	2210	2220	2230	2240
TLNLLRREVS	EHGHHLQQYF	NLFVMYANLG	VAEKTQLLKL	NVPATFMLVS	LDEGPGPPIK	YQYAELGKLY	SVVSQLIRCC
2250	2260	2270	2280	2290	2300	2310	2320
NVSSTMQSSI	NGNPPLPNPF	GDLNLSQPIM	PIQQNVLDIL	FVRTSYVKKI	IEDCSNSEDT	IKLLRFCSWE	NPQFSSTVLS
2330	2340	2350	2360	2370	2380	2390	2400

Detailed Protein Report

Cmpd.	No. of Cmpds.	m/z meas.	Δ m/z [ppm]	z	Rt [min]	Score	P	Range	Sequence	Modification
387	1	1294.0546	-51.53	2	34.8	12.0	0	2391-2413	K.CMVALFSSCPVAYQILQGNGDLK.R	Carbamidomethyl: 1, 9; Oxidation: 2

Detailed Protein Report

Protein 444: fibrinogen alpha chain isoform alpha preproprotein [Homo sapiens]

**Accession:**	gi|11761629	**Score:**	23.6
**Database:**	refseq_human(refseq_human_20140103.fasta)	**MW [kDa]:**	69.7
**Database Date:**	2015-11-30	**pI:**	9.1
		**Sequence Coverage [%]:**	3.7
		**No. of unique Peptides:**	1

10	20	30	40	50	60	70	80
MFSMRIVCLV	LSVVGTAWTA	DSGEGDFLAE	GGGVRGPRVV	ERHQSACKDS	DWPFCSDEDW	NYKCPSGCRM	KGLIDEVNQD
**90**	**100**	**110**	**120**	**130**	**140**	**150**	**160**
FTNRINKLKN	SLFEYQKNNK	DSHSLTTNIM	EILRGDFSSA	NNRDNTYNRV	SEDLRSRIEV	LKRKVIEKVQ	HIQLLQKNVR
**170**	**180**	**190**	**200**	**210**	**220**	**230**	**240**
AQLVDMKRLE	VDIDIKIRSC	RGSCSRALAR	EVDLKDYEDQ	QKQLEQVIAK	DLLPSRDRQH	LPLIKMKPVP	DLVPGNFKSQ
**250**	**260**	**270**	**280**	**290**	**300**	**310**	**320**
LQKVPPEWKA	LTDMPQMRME	LERPGGNEIT	RGGSTSYGTG	SETESPRNPS	SAGSWNSGSS	GPGSTGNRNP	GSSGTGGTAT
**330**	**340**	**350**	**360**	**370**	**380**	**390**	**400**
WKPGSSGPGS	TGSWNSGSSG	TGSTGNQNPG	SPRPGSTGTW	NPGSSERGSA	GHWTSESSVS	GSTGQWHSES	GSFRPDSPGS
**410**	**420**	**430**	**440**	**450**	**460**	**470**	**480**
GNARPNNPDW	GTFEEVSGNV	SPGTRREYHT	EKLVTSKGDK	ELRTGKEKVT	SGSTTTTRRS	CSKTVTKTVI	GPDGHKEVTK
**490**	**500**	**510**	**520**	**530**	**540**	**550**	**560**
EVVTSEDGSD	CPEAMDLGTL	SGIGTLDGFR	HRHPDEAAFF	DTASTGKTFP	GFFSPMLGEF	VSETESRGSE	SGIFTNTKES
**570**	**580**	**590**	**600**	**610**	**620**	**630**	**640**
SSHHPGIAEF	PSRGKSSSYS	KQFTSSTSYN	RGDSTFESKS	YKMADEAGSE	ADHEGTHSTK	RGHAKSRPVR	GIHTSPLGKP
**650**							
SLSP							

Cmpd.	No. of Cmpds.	m/z meas.	Δ m/z [ppm]	z	Rt [min]	Score	P	Range	Sequence	Modification
1392	1	721.3777	-27.06	2	47.6	12.5	0	226-238	K.MKPVPDLVPGNFK.S	

Detailed Protein Report

Protein 445: trifunctional enzyme subunit alpha, mitochondrial precursor [Homo sapiens]

Accession: gi|20127408
Database: refseq_human(refseq_human_20140103.fasta)
Database Date: 2015-11-30
Modification(s): Carbamidomethyl, Oxidation

Score: 23.6
MW [kDa]: 82.9
pI: 9.8
Sequence Coverage [%]: 5.0
No. of unique Peptides: 2

10	20	30	40	50	60	70	80
MVACRAIGIL	SRFSAFRILR	SRGYICRNFT	GSSALLTRTH	INYGVKGDVA	VVRINSPNSK	VNTLSKELHS	EFSEVMNEIW
**90**	**100**	**110**	**120**	**130**	**140**	**150**	**160**
ASDQIRSAVL	ISSKPGCFIA	GADINMLAAC	KTLQEVTQLS	QEAQRIVEKL	EKSTKPIVAA	INGSCLGGGL	EVAISCQYRI
**170**	**180**	**190**	**200**	**210**	**220**	**230**	**240**
ATKDRKTVLG	TPEVLLGALP	GAGGTQRLPK	MVGVPAALDM	MLTGRSIRAD	RAKKMGLVDQ	LVEPLGPGLK	PPEERTIEYL
**250**	**260**	**270**	**280**	**290**	**300**	**310**	**320**
EEVAITFAKG	LADKKISPKR	DKGLVEKLTA	YAMTIPFVRQ	QVYKKVEEKV	RKQTKGLYPA	PLKIIDVVKT	GIEQGSDAGY
**330**	**340**	**350**	**360**	**370**	**380**	**390**	**400**
LCESQKFGEL	VMTKESKALM	GLYHGQVLCK	KNKFGAPQKD	VKHLAILGAG	LMGAGIAQVS	VDKGLKTILK	DATLTALDRG
**410**	**420**	**430**	**440**	**450**	**460**	**470**	**480**
QQQVFKGLND	KVKKKALTSF	ERDSIFSNLT	GQLDYQGFEK	ADMVIEAVFE	DLSLKHRVLK	EVEAVIPDHC	IFASNTSALP
**490**	**500**	**510**	**520**	**530**	**540**	**550**	**560**
ISEIAAVSKR	PEKVIGMHYF	SPVDKMQLLE	IITTEKTSKD	TSASAVAVGL	KQGKVIIVVK	DGPGFYTTRC	LAPMMSEVIR
**570**	**580**	**590**	**600**	**610**	**620**	**630**	**640**
ILQEGVDPKK	LDSLTTSFGF	PVGAATLVDE	VGVDVAKHVA	EDLGKVFGER	FGGGNPELLT	QMVSKGFLGR	KSGKGFYIYQ
**650**	**660**	**670**	**680**	**690**	**700**	**710**	**720**
EGVKRKDLNS	DMDSILASLK	LPPKSEVSSD	EDIQFRLVTR	FVNEAVMCLQ	EGILATPAEG	DIGAVFGLGF	PPCLGGPFRF
**730**	**740**	**750**	**760**	**770**			
VDLYGAQKIV	DRLKKYEAAY	GKQFTPCQLL	ADHANSPNKK	FYQ			

Cmpd.	No. of Cmpds.	m/z meas.	Δ m/z [ppm]	z	Rt [min]	Score	P	Range	Sequence	Modification
1343	2	652.4474	143.56	3	46.9	12.8	2	1-17	-.MVACRAIGILSRFSAFR.I	Carbamidomethyl: 4
1289	1	1136.5599	-53.35	2	46.3	10.8	2	188-208	R.LPKMVGVPAALDMMLTGRSIR.A	Oxidation: 4

Detailed Protein Report

Protein 446: PREDICTED: metabotropic glutamate receptor 4 isoform X1 [Homo sapiens]

**Accession:**	gi|578811622	**Score:**	23.6
**Database:**	refseq_human(refseq_human_20140103.fasta)	**MW [kDa]:**	58.9
**Database Date:**	2015-11-30	**pI:**	9.8
		**Sequence Coverage [%]:**	6.7
		**No. of unique Peptides:**	1

10	20	30	40	50	60	70	80
MTSDRERIGQ	DSAYEQEGKV	QFVIDAVYAM	GHALHAMHRD	LCPGRVGLCP	RMDPVDGTQL	LKYIRNVNFS	GIAGNPVTFN
**90**	**100**	**110**	**120**	**130**	**140**	**150**	**160**
ENGDAPGRYD	IYQYQLRNDS	AEYKVIGSWT	DHLHLRIERM	HWPGSGQQLP	RSICSLPCQP	GERKKTVKGM	PCCWHCEPCT
**170**	**180**	**190**	**200**	**210**	**220**	**230**	**240**
GYQYQVDRYT	CKTCPYDMRP	TENRTGCRPI	PIIKLEWGSP	WAVLPLFLAV	VGIAATLFVV	ITFVRYNDTP	IVKASGRELS
**250**	**260**	**270**	**280**	**290**	**300**	**310**	**320**
YVLLAGIFLC	YATTFLMIAE	PDLGTCSLRR	IFLGLGMSIS	YAALLTKTNR	IYRIFEQGKR	SVSAPRFISP	ASQLAITFSL
**330**	**340**	**350**	**360**	**370**	**380**	**390**	**400**
ISLQLLGICV	WFVVDPSHSV	VDFQDQRTLD	PRFARGVLKC	DISDLSLICL	LGYSMLLMVT	CTVYAIKTRG	VPETFNEAKP
**410**	**420**	**430**	**440**	**450**	**460**	**470**	**480**
IGFTMYTTCI	VWLAFIPIFF	GTSQSADKLY	IQTTTLTVSV	SLSASVSLGM	LYMPKVYIIL	FHPEQNVPKR	KRSLKAVVTA
**490**	**500**	**510**	**520**	**530**			
ATMSNKFTQK	GNFRPNGEAK	SELCENLEAP	ALATKQTYVT	YTNHAI			

Cmpd.	No. of Cmpds.	m/z meas.	Δ m/z [ppm]	z	Rt [min]	Score	P	Range	Sequence	Modification
1390	1	977.5200	-44.32	2	47.5	12.4	1	270-287	R.RIFLGLGMSISYAALLTK.T	

Detailed Protein Report

Protein 447: deoxyribonuclease-2-beta isoform 1 precursor [Homo sapiens]

Accession: gi|194473657
Database: refseq_human(refseq_human_20140103.fasta)
Database Date: 2015-11-30

Score: 23.5
MW [kDa]: 41.7
pI: 10.1
Sequence Coverage [%]: 9.1
No. of unique Peptides: 2

10	20	30	40	50	60	70	80
MKQKMMARLL	RTSFALLFLG	LFGVLGAATI	SCRNEEGKAV	DWFTFYKLPK	RQNKESGETG	LEYLYLDSTT	RSWRKSEQLM
**90**	**100**	**110**	**120**	**130**	**140**	**150**	**160**
NDTKSVLGRT	LQQLYEAYAS	KSNNTAYLIY	NDGVPKPVNY	SRKYGHTKGL	LLWNRVQGFW	LIHSIPQFPP	IPEEGYDYPP
**170**	**180**	**190**	**200**	**210**	**220**	**230**	**240**
TGRRNGQSGI	CITFKYNQYE	AIDSQLLVCN	PNVYSCSIPA	TFHQELIHMP	QLCTRASSSE	IPGRLLTTLQ	SAQGQKFLHF
**250**	**260**	**270**	**280**	**290**	**300**	**310**	**320**
AKSDSFLDDI	FAAWMAQRLK	THLLTETWQR	KRQELPSNCS	LPYHVYNIKA	IKLSRHSYFS	SYQDHAKWCI	SQKGTKNRWT
**330**	**340**	**350**	**360**	**370**			
CIGDLNRSPH	QAFRSGGFIC	TQNWQIYQAF	QGLVLYYESC	K			

Cmpd.	No. of Cmpds.	m/z meas.	Δ m/z [ppm]	z	Rt [min]	Score	P	Range	Sequence	Modification
2252	1	569.2585	-75.58	3	56.8	10.9	2	75-89	R.KSEQLMNDTKSVLGR.T	
2758	1	1086.0280	-8.69	2	65.6	12.6	2	317-334	K.NRWTCIGDLNRSPHQAFR.S	

Detailed Protein Report

Protein 448: dual specificity protein kinase TTK isoform 2 [Homo sapiens]

**Accession:**	gi|262399361	**Score:**	23.5
**Database:**	refseq_human(refseq_human_20140103.fasta)	**MW [kDa]:**	96.9
**Database Date:**	2015-11-30	**pI:**	9.2
**Modification(s):**	Carbamidomethyl	**Sequence Coverage [%]:**	4.1
		**No. of unique Peptides:**	1

10	20	30	40	50	60	70	80
MESEDLSGRE	LTIDSIMNKV	RDIKNKFKNE	DLTDELSLNK	ISADTTDNSG	TVNQIMMAN	NPEDWLSLLL	KLEKNSVPLS
**90**	**100**	**110**	**120**	**130**	**140**	**150**	**160**
DALLNKLIGR	YSQAIEALPP	DKYGQNESFA	RIQVRFAELK	AIQEPDDARD	YFQMARANCK	KFAFVHISFA	QFELSQGNVK
**170**	**180**	**190**	**200**	**210**	**220**	**230**	**240**
KSKQLLQKAV	ERGAVPLEML	EIALRNLNLQ	KKQLLSEEEK	KNLSASTVLT	AQESFSGSLG	HLQNRNNSCD	SRGQTTKARF
**250**	**260**	**270**	**280**	**290**	**300**	**310**	**320**
LYGENMPPQD	AEIGYRNSLR	QTNKTKQSCP	FGRVPVNLLN	SPDCDVKTDD	SVVPCFMKRQ	TSRSECRDLV	VPGSKPSGND
**330**	**340**	**350**	**360**	**370**	**380**	**390**	**400**
SCELRNLKSV	QNSHFKEPLV	SDEKSSELII	TDSITLKNKT	ESSLLAKLEE	TKEYQEPEVP	ESNQKQWQSK	RKSECINQNP
**410**	**420**	**430**	**440**	**450**	**460**	**470**	**480**
AASSNHWQIP	ELARKVNTEK	HTTFEQPVFS	VSKQSPPIST	SKWFDPKSIC	KTPSSNTLDD	YMSCFRTPVV	KNDFPPACQL
**490**	**500**	**510**	**520**	**530**	**540**	**550**	**560**
STPYGQPACF	QQQQHQILAT	PLQNLQVLAS	SSANECISVK	GRIYSILKQI	GSGGSSKVFQ	VLNEKKQIYA	IKYVNLEEAD
**570**	**580**	**590**	**600**	**610**	**620**	**630**	**640**
NQTLDSYRNE	IAYLNKLQQH	SDKIIRLYDY	EITDQYIYMV	MECGNIDLNS	WLKKKKSIDP	WERKSYWKNM	LEAVHTIHQH
**650**	**660**	**670**	**680**	**690**	**700**	**710**	**720**
GIVHSDLKPA	NFLIVDGMLK	LIDFGIANQM	QPDTTSVVKD	SQVGTVNYMP	PEAIKDMSSS	RENGKSKSKI	SPKSDVWSLG
**730**	**740**	**750**	**760**	**770**	**780**	**790**	**800**
CILYYMTYGK	TPFQQIINQI	SKLHAIIDPN	HEIEFPDIPE	KDLQDVLKCC	LKRDPKQRIS	IPELLAHPYV	QIQTHPVNQM
**810**	**820**	**830**	**840**	**850**	**860**		
AKGTTEEMKY	VLGQLVGLNS	PNSILKAAKT	LYEHYSGGES	HNSSSKTFE	KKRGKK		

Cmpd.	No. of Cmpds.	m/z meas.	Δ m/z [ppm]	z	Rt [min]	Score	P	Range	Sequence	Modification
309	1	936.1804	52.20	3	32.6	11.1	2	391-414	K.RKSECINQNPAASSNHWQIPELAR.K	Carbamidomethyl: 5

Detailed Protein Report

Protein 449: lysine-specific demethylase 5B [Homo sapiens]

**Accession:**	gi|57242796	**Score:**	23.5
**Database:**	refseq_human(refseq_human_20140103.fasta)	**MW [kDa]:**	175.5
**Database Date:**	2015-11-30	**pI:**	6.3
		**Sequence Coverage [%]:**	1.4
		**No. of unique Peptides:**	2

Quantitation

QD:QU **Median:** 0.78 **CV:** 0.00 % **No. of Peptides:** 1

10	20	30	40	50	60	70	80
MEAATTLHPG	PRPALPLGGP	GPLGEFLPPP	ECPVFEPSWE	EFADPFAFIH	KIRPIAEQTG	ICKVRPPPDW	QPPFACDVDK
90	100	110	120	130	140	150	160
LHFTPRIQRL	NELEAQTRVK	LNFLDQIAKY	WELQGSTLKI	PHVERKILDL	FQLNKLVAEE	GGFAVVCKDR	KWTKIATKMG
170	180	190	200	210	220	230	240
FAPGKAVGSH	IRGHYERILN	PYNLFLSGDS	LRCLQKPNLT	TDTKDKEYKP	HDIPQRQSVQ	PSETCPPARR	AKRMRAEAMN
250	260	270	280	290	300	310	320
IKIEPEETTE	ARTHNLRRRM	GCPTPKCENE	KEMKSSIKQE	PIERKDYIVE	NEKEKPKSRS	KKATNAVDLY	VCLLCGSGND
330	340	350	360	370	380	390	400
EDRLLLCDGC	DDSYHTFCLI	PPLHDVPKGD	WRCPKCLAQE	CSKPQEAFGF	EQAARDYTLR	TFGEMADAFK	SDYFNMPVHM
410	420	430	440	450	460	470	480
VPTELVEKEF	WRLVSTIEED	VTVEYGADIA	SKEFGSGFPV	RDGKIKLSPE	EEEYLDSGWN	LNNMPVMEQS	VLAHITADIC
490	500	510	520	530	540	550	560
GMKLPWLYVG	MCFSSFCWHI	EDHWSYSINY	LHWGEPKTWY	GVPGYAAEQL	ENVMKKLAPE	LFVSQPDLLH	QLVTIMNPNT
570	580	590	600	610	620	630	640
LMTHEVPVYR	TNQCAGEFVI	TFPRAYHSGF	NQGFNFAEAV	NFCTVDWLPL	GRQCVEHYRL	LHRYCVFSHD	EMICKMASKA
650	660	670	680	690	700	710	720
DVLDVVVAST	VQKDMAIMIE	DEKALRETVR	KLGVIDSERM	DFELLPDDER	QCVKCKTTCF	MSAISCSCKP	GLLVCLHHVK
730	740	750	760	770	780	790	800
ELCSCPPYKY	KLRYRYTLDD	LYPMMNALKL	RAESYNEWAL	NVNEALEAKI	NKKKSLVSFK	ALIEESEMKK	FPDNDLLRHL
810	820	830	840	850	860	870	880
RLVTQDAEKC	ASVAQQLLNG	KRQTRYRSGG	GKSQNQLTVN	ELRQFVTQLY	ALPCVLSQTP	LLKDLLNRVE	DFQQHSQKLL
890	900	910	920	930	940	950	960
SEETPSAAEL	QDLLDVSFEF	DVELPQLAEM	RIRLEQARWL	EEVQQACLDP	SSLTLDDMRR	LIDLGVGLAP	YSAVEKAMAR
970	980	990	1000	1010	1020	1030	1040
LQELLTVSEH	WDDKAKSLLK	ARPRHSLNSL	ATAVKEIEEI	PAYLPNGAAL	KDSVQRARDW	LQDVEGLQAG	GRVPVLDTLI
1050	1060	1070	1080	1090	1100	1110	1120
ELVTRGRSIP	VHLNSLPRLE	TLVAEVQAWK	ECAVNFTLTE	NSPYSLLEVL	CPRCDIGLLG	LKRKQRKLKE	PLPNGKKKST
1130	1140	1150	1160	1170	1180	1190	1200
KLESLSDLER	ALTESKETAS	AMATLGEARL	REMEALQSLR	LANEGKLLSP	LQDVDIKICL	CQKAPAAPMI	QCELCRDAFH
1210	1220	1230	1240	1250	1260	1270	1280
TSCVAVPSIS	QGLRIWLCPH	CRRSEKPPLE	KILPLLASLQ	RIRVRLPEGD	ALRYMIERTV	NWQHRAQQLL	SSGNLKFVQD
1290	1300	1310	1320	1330	1340	1350	1360
RVGSGLLYSR	WQASAGQVSD	TNKVSQPPGT	TSFSLPDDWD	NRTSYLHSPF	STGRSCIPLH	GVSPEVNELL	MEAQLLQVSL
1370	1380	1390	1400	1410	1420	1430	1440
PEIQELYQTL	LAKPSPAQQT	DRSSPVRPSS	EKNDCCRGKR	DGINSLERKL	KRRLEREGLS	SERWERVKKM	RTPKKKKIKL
1450	1460	1470	1480	1490	1500	1510	1520
SHPKDMNNFK	LERERSYELV	RSAETHSLPS	DTSYSEQEDS	EDEDAICPAV	SCLQPEGDEV	DWVQCDGSCN	QWFHQVCVGV
1530	1540	1550					
SPEMAEKEDY	ICVRCTVKDA	PSRK					

Cmpd.	No. of Cmpds.	m/z meas.	Δ m/z [ppm]	z	Rt [min]	Score	P	Range	Sequence	Modification	Ratios
333	1	734.3935	-67.03	2	34.1	13.3	0	1033-1045	R.VPVLDTLIELVTR.G		QD:QU 0.78
1877	1	1059.3256	-215.69	1	53.8	10.2	1	1400-1408	K.RDGINSLER.K		

Detailed Protein Report

Protein 450: protein phosphatase 1 regulatory subunit 29 precursor [Homo sapiens]

Accession: gi|116268101
Database: refseq_human(refseq_human_20140103.fasta)
Database Date: 2015-11-30
Modification(s): Carbamidomethyl

Score: 23.5
MW [kDa]: 89.6
pI: 8.4
Sequence Coverage [%]: 3.2
No. of unique Peptides: 1

Alias proteins:

Accession	Name	Description
gi|578836905	refseq_human (refseq_human_20140103.fasta)	PREDICTED: protein phosphatase 1 regulatory subunit 29 isoform X3 [Homo sapiens]
gi|578836903	refseq_human (refseq_human_20140103.fasta)	PREDICTED: protein phosphatase 1 regulatory subunit 29 isoform X2 [Homo sapiens]
gi|530419503	refseq_human (refseq_human_20140103.fasta)	PREDICTED: protein phosphatase 1 regulatory subunit 29 isoform X1 [Homo sapiens]

```
        10         20         30         40         50         60         70         80
MLRLGLCAAA LLCVCRPGAV RADCWLIEGD KGYVWLAICS QNQPPYETIP QHINSTVHDL RLNENKLKAV LYSSLNRFGN
        90        100        110        120        130        140        150        160
LTDLNLTKNE ISYIEDGAFL GQSSLQVLQL GYNKLSNLTE GMLRGMSRLQ FLFVQHNLIE VVTPTAFSEC PSLISIDLSS
       170        180        190        200        210        220        230        240
NRLSRLDGAT FASLASLMVC ELAGNPFNCE CDLFGFLAWL VVFNNVTKNY DRLQCESPRE FAGYPLLVPR PYHSLNAITV
       250        260        270        280        290        300        310        320
LQAKCRNGSL PARPVSHPTP YSTDAQREPD ENSGFNPDEI LSVEPPASST TDASAGPAIK LHHVTFTSAT LVVIIPHPYS
       330        340        350        360        370        380        390        400
KMYILVQYNN SYFSDVMTLK NKKEIVTLDK LRAHTEYTFC VTSLRNSRRF NHTCLTFTTR DPVPGDLAPS TSTTTHYIMT
       410        420        430        440        450        460        470        480
ILGCLFGMVI VLGAVYYCLR KRRMQEEKQK SVNVKKTILE MRYGADVDAG SIVHAAQKLG EPPVLPVSRM ASIPSMIGEK
       490        500        510        520        530        540        550        560
LPTAKGLEAG LDTPKVATKG NYIEVRTGAG GDGLARPEDD LPDLENGQGS AAEISTIAKE VDKVNQIINN CIDALKLDSA
       570        580        590        600        610        620        630        640
SFLGGGSSSG DPELAFECQS LPAAAAASSA TGPGALERPS FLSPPYKESS HHPLQRQLSA DAAVTRKTCS VSSSGSIKSA
       650        660        670        680        690        700        710        720
KVFSLDVPDH PAATGLAKGD SKYIEKGSPL NSPLDRLPLV PAGSGGGSGG GGGIHHLEVK PAYHCSEHRH SFPALYYEEG
       730        740        750        760        770        780        790        800
ADSLSQRVSF LKPLTRSKRD STYSQLSPRH YYSGYSSSPE YSSESTHKIW ERFRPYKKHH REEVYMAAGH ALRKKVQFAK
       810        820        830
DEDLHDILDY WKGVSAQQKL
```

Cmpd.	No. of Cmpds.	m/z meas.	Δ m/z [ppm]	z	Rt [min]	Score	P	Range	Sequence	Modification
1279	1	955.9599	-14.52	2	46.1	12.2	2	366-380	R.NSRRFNHTCLTFTTR.D	Carbamidomethyl: 9

Detailed Protein Report

Protein 451: **PREDICTED: zinc finger protein 263 isoform X4 [Homo sapiens]**

**Accession:**	gi|578827945	**Score:**	23.3
**Database:**	refseq_human(refseq_human_20140103.fasta)	**MW [kDa]:**	67.8
**Database Date:**	2015-11-30	**pI:**	7.1
		**Sequence Coverage [%]:**	2.7
		**No. of unique Peptides:**	1

10	20	30	40	50	60	70	80
MASGPGSQER	EGLLIVKLEE	DCAWSQELPP	PDPGPSPEAS	HLRFRRFRFQ	EAAGPREALS	RLQELCHGWL	RPEMRTKEQI
**90**	**100**	**110**	**120**	**130**	**140**	**150**	**160**
LELLVLEQFL	TILPQEIQSR	VQELHPESGE	EAVTLVEDMQ	RELGRLRQQL	PESLEDVAMY	ISQEEWGHQD	PSKRALSRDT
**170**	**180**	**190**	**200**	**210**	**220**	**230**	**240**
VQESYENVDS	LESHIPSQEV	PGTQVGQGGK	LWDPSVQSCK	EGLSPRGPAP	GEEKFENLEG	VPSVCSENIH	PQVLLPDQAR
**250**	**260**	**270**	**280**	**290**	**300**	**310**	**320**
GEVPWSPELG	RPHDRSQGDW	APPPEGGMEQ	ALAGASSGRE	LGRPKELQPK	KLHLCPLCGK	NFSNNSNLIR	HQRIHAAERL
**330**	**340**	**350**	**360**	**370**	**380**	**390**	**400**
CMGVDCTEIF	GGNPRFLSLH	RAHLGEEAHK	CLECGKCFSQ	NTHLTRHQRT	HTGEKPYQCN	ICGKCFSCNS	NLHRHQRTHT
**410**	**420**	**430**	**440**	**450**	**460**	**470**	**480**
GEKPYKCPEC	GEIFAHSSNL	LRHQRIHTGE	RPYKCPECGK	SFSRSSHLVI	HERTHERERL	YPFSECGEAV	SDSTPFLTNH
**490**	**500**	**510**	**520**	**530**	**540**	**550**	**560**
GAHKAEKKLF	ECLTCGKSFR	QGMHLTRHQR	THTGEKPYKC	TLCGENFSHR	SNLIRHQRIH	TGEKPYTCHE	CGDSFSHSSN
**570**	**580**	**590**	**600**				
RIRHLRTHTG	ERPYKCSECG	ESFSRSSRLM	SHQRTHTG				

Cmpd.	No. of Cmpds.	m/z meas.	Δ m/z [ppm]	z	Rt [min]	Score	P	Range	Sequence	Modification
1617	1	820.8186	-162.40	2	50.5	10.3	1	2-17	M.ASGPGSQEREGLLIVK.L	

Detailed Protein Report

Protein 452: **PREDICTED: zinc finger and BTB domain-containing protein 38 isoform X2 [Homo sapiens]**

**Accession:**	gi|530374206	**Score:**	23.3
**Database:**	refseq_human(refseq_human_20140103.fasta)	**MW [kDa]:**	134.3
**Database Date:**	2015-11-30	**pI:**	9.3
**Modification(s):**	Carbamidomethyl	**Sequence Coverage [%]:**	2.1
		**No. of unique Peptides:**	1

10	20	30	40	50	60	70	80
MMTVMSLSRD	LKDDFHSDTV	LSILNEQRIR	GILCDVTIIV	EDTKFKAHSN	VLAASSLYFK	NIFWSHTICI	SSHVLELDDL
**90**	**100**	**110**	**120**	**130**	**140**	**150**	**160**
KAEVFTEILN	YIYSSTVVVK	RQETVTDLAA	AGKKLGISFL	EDLTDRNFSN	SPGPYVFCIT	EKGVVKEEKN	EKRHEEPAIT
**170**	**180**	**190**	**200**	**210**	**220**	**230**	**240**
NGPRITNAFS	IIETENSNNM	FSPLDLRASF	KKVSDSMRTA	SLCLERTDVC	HEAEPVRTLA	EHSYAVSSVA	EAYRSQPVRE
**250**	**260**	**270**	**280**	**290**	**300**	**310**	**320**
HDGSSPGNTG	KENCEALAAK	PKTCRKPKTF	SIPQDSDSAT	ENIPPPPVSN	LEVNQERSPQ	PAAVLTRSKS	PNNEGDVHFS
**330**	**340**	**350**	**360**	**370**	**380**	**390**	**400**
REDENQSSDV	PGPPAAEVPP	LVYNCSCCSK	AFDSSTLLSA	HMQLHKPTQE	PLVCKYCNKQ	FTTLNRLDRH	EQICMRSSHM
**410**	**420**	**430**	**440**	**450**	**460**	**470**	**480**
PIPGGNQRFL	ENYPTIGQNG	GSFTGPEPLL	SENRIGEFSS	TGSTLPDTDH	MVKFVNGQML	YSCVVCKRSY	VTLSSLRRHA
**490**	**500**	**510**	**520**	**530**	**540**	**550**	**560**
NVHSWRRTYP	CHYCNKVFAL	AEYRTRHEIW	HTGERRYQCI	FCLETFMTYY	ILKNHQKSFH	AIDHRLSISK	KTANGGLKPS
**570**	**580**	**590**	**600**	**610**	**620**	**630**	**640**
VYPYKLYRLL	PMKCKRAPYK	SYRNSSYENA	RENSQMNESA	PGTYVVQNPH	SSELPTLNFQ	DTVNTLTNSP	AIPLETSACQ
**650**	**660**	**670**	**680**	**690**	**700**	**710**	**720**
DIPTSANVQN	AEGTKWGEEA	LKMDLDNNFY	STEVSVSSTE	NAVSSDLRAG	DVPVLSLSNS	SENAASVISY	SGSAPSVIVH
**730**	**740**	**750**	**760**	**770**	**780**	**790**	**800**
SSQFSSVIMH	SNAIAAMTSS	NHRAFSDPAV	SQSLKDDSKP	EPDKVGRFAS	RPKSIKEKKK	TTSHTRGEIP	EESNYVADPG
**810**	**820**	**830**	**840**	**850**	**860**	**870**	**880**
GSLSKTTNIA	EETSKIETYI	AKPALPGTST	NSNVAPLCQI	TVKIGNEAIV	KRHILGSKLF	YKRGRRPKYQ	MQEEPLPQGN
**890**	**900**	**910**	**920**	**930**	**940**	**950**	**960**
DPEPSGDSPL	GLCQSECMEM	SEVFDDASDQ	DSTDKPWRPY	YNYKPKKKSR	QLKKMRKVNW	RKEHGNRSPS	HKCKYPAELD
**970**	**980**	**990**	**1000**	**1010**	**1020**	**1030**	**1040**
CAVGKAPQDK	PFEEEETKEM	PKLQCELCDG	DKAVGAGNQG	RPHRHLTSRP	YACELCAKQF	QSPSTLKMHM	RCHTGEKPYQ
**1050**	**1060**	**1070**	**1080**	**1090**	**1100**	**1110**	**1120**
CKTCGRCFSV	QGNLQKHERI	HLGLKEFVCQ	YCNKAFTLNE	TLKIHERIHT	GEKRYHCQFC	FQRFLYLSTK	RNHEQRHIRE
**1130**	**1140**	**1150**	**1160**	**1170**	**1180**	**1190**	**1200**
HNGKGYACFQ	CPKICKTAAA	LGMHQKKHLF	KSPSQQEKIG	DVCHENSNPL	ENQHFIGSED	NDQKDNIQTG	VENVVL

Cmpd.	No. of Cmpds.	m/z meas.	Δ m/z [ppm]	z	Rt [min]	Score	P	Range	Sequence	Modification
2863	1	922.9727	-41.66	2	65.0	11.0	1	29-44	R.IRGILCDVTIIVEDTK.F	Carbamidomethyl: 6

Detailed Protein Report

Protein 453: PREDICTED: interferon regulatory factor 2 isoform X2 [Homo sapiens]

Accession: gi|530377663
Database: refseq_human(refseq_human_20140103.fasta)
Database Date: 2015-11-30
Modification(s): Oxidation

Score: 23.3
MW [kDa]: 39.1
pI: 7.8
Sequence Coverage [%]: 6.9
No. of unique Peptides: 1

10	20	30	40	50	60	70	80
MPVERMRMRP	WLEEQINSNT	IPGLKWLNKE	KKIFQIPWMH	AARHGWDVEK	DAPLFRNWAI	HTGKHQPGVD	KPDPKTWKAN
**90**	**100**	**110**	**120**	**130**	**140**	**150**	**160**
FRCAMNSLPD	IEEVKDKSIK	KGNNAFRVYR	MLPLSERPSK	KGKKPKTEKE	DKVKHIKQEP	VESSLGLSNG	VSDLSPEYAV
**170**	**180**	**190**	**200**	**210**	**220**	**230**	**240**
LTSTIKNEVD	STVNIIGQSH	LDSNIENQEI	VTNPPDICQV	VEVTTESDEQ	PVSMSELYPL	QISPVSSYAE	SETTDSVPSD
**250**	**260**	**270**	**280**	**290**	**300**	**310**	**320**
EESAEGRPHW	RKRNIEGKQY	LSNMGTRGSY	LLPGMASFVT	SNKPDLQVTI	KEESNPVPYN	SSWPPFQDLP	LSSSMTPASS
**330**	**340**	**350**					
SSRPDRETRA	SVIKKTSDIT	QARVKSC					

Cmpd.	No. of Cmpds.	m/z meas.	Δ m/z [ppm]	z	Rt [min]	Score	P	Range	Sequence	Modification
513	1	587.1711	-250.83	2	35.0	11.4	0	111-120	R.MLPLSERPSK.K	Oxidation: 1

Detailed Protein Report

Protein 454: protocadherin Fat 1 precursor [Homo sapiens]

**Accession:**	gi|66346693	**Score:**	23.3
**Database:**	refseq_human(refseq_human_20140103.fasta)	**MW [kDa]:**	506.0
**Database Date:**	2015-11-30	**pI:**	4.7
		**Sequence Coverage [%]:**	0.7
		**No. of unique Peptides:**	1

Alias proteins:

Accession	Name	Description
gi|578808907	refseq_human (refseq_human_20140103.fasta)	PREDICTED: protocadherin Fat 1 isoform X3 [Homo sapiens]

Detailed Protein Report

10	20	30	40	50	60	70	80
MGRHLALLLL	LLLLFQHFGD	SDGSQRLEQT	PLQFTHLEYN	VTVQENSAAK	TYVGHPVKMG	VYITHPAWEV	RYKIVSGDSE
90	100	110	120	130	140	150	160
NLFKAEEYIL	GDFCFLRIRT	KGGNTAILNR	EVKDHYTLIV	KALEKNTNVE	ARTKVRVQVL	DTNDLRPLFS	PTSYSVSLPE
170	180	190	200	210	220	230	240
NTAIRTSIAR	VSATDADIGT	NGEFYYSFKD	RTDMFAIHPT	SGVIVLTGRL	DYLETKLYEM	EILAADRGMK	LYGSSGISSM
250	260	270	280	290	300	310	320
AKLTVHIEQA	NECAPVITAV	TLSPSELDRD	PAYAIVTVDD	CDQGANGDIA	SLSIVAGDLL	QQFRTVRSFP	GSKEYKVKAI
330	340	350	360	370	380	390	400
GGIDWDSHPF	GYNLTLQAKD	KGTPPQFSSV	KVIHVTSPQF	KAGPVKFEKD	VYRAEISEFA	PPNTPVVMVK	AIPAYSHLRY
410	420	430	440	450	460	470	480
VFKSTPGKAK	FSLNYNTGLI	SILEPVKRQQ	AAHFELEVTT	SDRKASTKVL	VKVLGANSNP	PEFTQTAYKA	AFDENVPIGT
490	500	510	520	530	540	550	560
TVMSLSAVDP	DEGENGYVTY	SIANLNHVPF	AIDHFTGAVS	TSENLDYELM	PRVYTLRIRA	SDWGLPYRRE	VEVLATITLN
570	580	590	600	610	620	630	640
NLNDNTPLFE	KINCEGTIPR	DLGVGEQITT	VSAIDADELQ	LVQYQIEAGN	ELDFFSLNPN	SGVLSLKRSL	MDGLGAKVSF
650	660	670	680	690	700	710	720
HSLRITATDG	ENFATPLYIN	ITVAASHKLV	NLQCEETGVA	KMLAEKLLQA	NKLHNQGEVE	DIFFDSHSVN	AHIPQFRSTL
730	740	750	760	770	780	790	800
PTGIQVKENQ	PVGSSVIFMN	STDLDTGFNG	KLVYAVSGGN	EDSCFMIDME	TGMLKILSPL	DRETTDKYTL	NITVYDLGIP
810	820	830	840	850	860	870	880
QKAAWRLLHV	VVVDANDNPP	EFLQESYFVE	VSEDKEVHSE	IIQVEATDKD	LGPNGHVTYS	IVTDTDTFSI	DSVTGVVNIA
890	900	910	920	930	940	950	960
RPLDRELQHE	HSLKIEARDQ	AREEPQLFST	VVVKVSLEDV	NDNPPTFIPP	NYRVKVREDL	PEGTVIMWLE	AHDPDLGQSG
970	980	990	1000	1010	1020	1030	1040
QVRYSLLDHG	EGNFDVDKLS	GAVRIVQQLD	FEKKQVYNLT	VRAKDKGKPV	SLSSTCYVEV	EVVDVNENLH	PPVFSSFVEK
1050	1060	1070	1080	1090	1100	1110	1120
GTVKEDAPVG	SLVMTVSAHD	EDARRDGEIR	YSIRDGSGVG	VFKIGEETGV	IETSDRLDRE	STSHYWLTVF	ATDQGVVPLS
1130	1140	1150	1160	1170	1180	1190	1200
SFIEIYIEVE	DVNDNAPQTS	EPVYYPEIME	NSPKDVSVVQ	IEAFDPDSSS	NDKLMYKITS	GNPQGFFSIH	PKTGLITTTS
1210	1220	1230	1240	1250	1260	1270	1280
RKLDREQQDE	HILEVTVTDN	GSPPKSTIAR	VIVKILDEND	NKPQFLQKFY	KIRLPEREKP	DRERNARREP	LYHVIATDKD
1290	1300	1310	1320	1330	1340	1350	1360
EGPNAEISYS	IEDGNEHGKF	FIEPKTGVVS	SKRFSAAGEY	DILSIKAVDN	GRPQKSSTTR	LHIEWISKPK	PSLEPISFEE
1370	1380	1390	1400	1410	1420	1430	1440
SFFTFTVMES	DPVAHMIGVI	SVEPPGIPLW	FDITGGNYDS	HFDVDKGTGT	IIVAKPLDAE	QKSNYNLTVE	ATDGTTTILT
1450	1460	1470	1480	1490	1500	1510	1520
QVFIKVIDTN	DHRPQFSTSK	YEVVIPEDTA	PETEILQISA	VDQDEKNKLI	YTLQSSRDPL	SLKKFRLDPA	TGSLYTSEKL
1530	1540	1550	1560	1570	1580	1590	1600
DHEAVHQHTL	TVMVRDQDVP	VKRNFARIVV	NVSDTNDHAP	WFTASSYKGR	VYESAAVGSV	VLQVTALDKD	KGKNAEVLYS
1610	1620	1630	1640	1650	1660	1670	1680
IESGNIGNSF	MIDPVLGSIK	TAKELDRSNQ	AEYDLMVKAT	DKGSPPMSEI	TSVRIFVTIA	DNASPKFTSK	EYSVELSETV
1690	1700	1710	1720	1730	1740	1750	1760
SIGSFVGMVT	AHSQSSVVYE	IKDGNTGDAF	DINPHSGTII	TQKALDFETL	PIYTLIIQGT	NMAGLSTNTT	VLVHLQDEND
1770	1780	1790	1800	1810	1820	1830	1840
NAPVFMQAEY	TGLISESASI	NSVVLTDRNV	PLVIRAADAD	KDSNALLVYH	IVEPSVHTYF	AIDSSTGAIH	TVLSLDYEET
1850	1860	1870	1880	1890	1900	1910	1920
SIFHFTVQVH	DMGTPRLFAE	YAANVTVHVI	DINDCPPVFA	KPLYEASLLL	PTYKGVKVIT	VNATDADSSA	FSQLIYSITE
1930	1940	1950	1960	1970	1980	1990	2000
GNIGEKFSMD	YKTGALTVQN	TTQLRSRYEL	TVRASDGRFA	GLTSVKINVK	ESKESHLKFT	QDVYSAVVKE	NSTEAETLAV
2010	2020	2030	2040	2050	2060	2070	2080
ITAIGNPINE	PLFYHILNPD	RRFKISRTSG	VLSTTGTPFD	REQQEAFDVV	VEVTEEHKPS	AVAHVVVKVI	VEDQNDNAPV
2090	2100	2110	2120	2130	2140	2150	2160
FVNLPYYAVV	KVDTEVGHVI	RYVTAVDRDS	GRNGEVHYYL	KEHHEHFQIG	PLGEISLKKQ	FELDTLNKEY	LVTVVAKDGG
2170	2180	2190	2200	2210	2220	2230	2240

Detailed Protein Report

Cmpd.	No. of Cmpds.	m/z meas.	Δ m/z [ppm]	z	Rt [min]	Score	P	Range	Sequence	Modification
2147	1	719.8600	-56.51	2	55.4	10.9	1	1300-1312	K.FFIEPKTGVVSSK.R	

Detailed Protein Report

Protein 455: PREDICTED: autophagy-related protein 2 homolog B isoform X1 [Homo sapiens]

**Accession:**	gi|578825925	**Score:**	23.3
**Database:**	refseq_human(refseq_human_20140103.fasta)	**MW [kDa]:**	232.5
**Database Date:**	2015-11-30	**pI:**	5.4
		**Sequence Coverage [%]:**	1.3
		**No. of unique Peptides:**	1

Detailed Protein Report

10	20	30	40	50	60	70	80
MPWPFSESIK	KRACRYLLQR	YLGHFLQEKL	SLEQLSLDLY	QGTGSLAQVP	LDKWCLNEIL	ESADAPLEVT	EGFIQSISLS
90	100	110	120	130	140	150	160
VPWGSLLQDN	CALEVRGLEM	VFRPRPRPAT	GSEPMYWSSF	MTSSMQLAKE	CLSQKLTDEQ	GEGSQPFEGL	EKFAETIETV
170	180	190	200	210	220	230	240
LRRVKVTFID	TVLRIEHVPE	NSKTGTALEI	RIERTVYCDE	TADESSGINV	HQPTAFAHKL	LQLSGVSLFW	DEFSASAKSS
250	260	270	280	290	300	310	320
PVCSTAPVET	EPKLSPSWNP	KIIYEPHPQL	TRNLPEIAPS	DPVQIGRLIG	RLELSLTLKQ	NEVLPGAKLD	VDGQIDSIHL
330	340	350	360	370	380	390	400
LLSPRQVHLL	LDMLAAIAGP	ENSSKIGLAN	KDRKNRPMQQ	EDEYRIQMEL	NRYYLRKDSL	SVGVSSEQSF	YETETARTPS
410	420	430	440	450	460	470	480
SREEVFFSMA	DMDMSHSLSS	LPPLGDPPNM	DLELSLTSTY	TNTPAGSPLS	ATVLQPTWGE	FLDHHKEQPV	RGSTFPSNLV
490	500	510	520	530	540	550	560
HPTPLQKTSL	PSRSVSVDES	RPELIFRLAV	GTFSISVLHI	DPLSPPETSQ	NLNPLTPMAV	AFFTCIEKID	PARFSTEDFK
570	580	590	600	610	620	630	640
SFRAVFAEAC	SHDHLRFIGT	GIKVSYEQRQ	RSASRYFSTD	MSIGQMEFLE	CLFPTDFHSV	PPHYTELLTF	HSKEETGSHS
650	660	670	680	690	700	710	720
PVCLQLHYKH	SENRGPQGNQ	ARLSSVPHKA	ELQIKLNPVC	CELDISIVDR	LNSLLQPQKL	ATVEMMASHM	YTSYNKHISL
730	740	750	760	770	780	790	800
HKAFTEVFLD	DSHSPANCRI	SVQVATPALN	LSVRFPIPDL	RSDQERGPWF	KKSLQKEILY	LAFTDLEFKT	EFIGGSTPEQ
810	820	830	840	850	860	870	880
IKLELTFREL	IGSFQEEKGD	PSIKFFHVSS	GVDGDTTSSD	DFDWPRIVLK	INPPAMHSIL	ERIAAEEEEE	NDGHYQEEEE
890	900	910	920	930	940	950	960
GGAHSLKDVC	DLRRPAPSPF	SSRRVMFENE	QMVMPGDPVE	MTEFQDKAIS	NSHYVLELTL	PNIYVTLPNK	SFYEKLYNRI
970	980	990	1000	1010	1020	1030	1040
FNDLLLWEPT	APSPVETFEN	ISYGIGLSVA	SQLINTFNKD	SFSAFKSAVH	YDEESGSEEE	TLQYFSTVDP	NYRSRRKKKL
1050	1060	1070	1080	1090	1100	1110	1120
DSQNKNSQSF	LSVLLNINHG	LIAVFTDVKQ	DNGDLLENKH	GEFWLEFNSG	SLFCVTKYEG	FDDKHYICLH	SSSFSLYHKG
1130	1140	1150	1160	1170	1180	1190	1200
IVNGVILPTE	TRLPSSTRPH	WLEPTIYSSE	EDGLSKTSSD	GVGGDSLNML	SVAVKILSDK	SESNTKEFLI	AVGLKGATLQ
1210	1220	1230	1240	1250	1260	1270	1280
HRMLPSGLSW	HEQILYFLNI	ADEPVLGYNP	PTSFTTFHVH	LWSCALDYRP	LYLPIRSLLT	VETFSVSSSV	ALDKSSSTLR
1290	1300	1310	1320	1330	1340	1350	1360
IILDEAALHL	SDKCNTVTIN	LSRDYVRVMD	MGLLELTITA	VKSDSDGEQT	EPRFELHCSS	DVVHIRTCSD	SCAALMNLIQ
1370	1380	1390	1400	1410	1420	1430	1440
YIASYGDLQT	PNKADMKPGA	FQRRSKVDSS	GRSSSRGPVL	PEADQQMLRD	LMSDAMEEID	MQQGTSSVKP	QANGVLDEKS
1450	1460	1470	1480	1490	1500	1510	1520
QIQEPCCSDL	FLFPDESGNV	SQESGPTYAS	FSHHFISDAM	TGVPTENDDF	CILFAPKAAM	QEKEEEPVIK	IMVDDAIVIR
1530	1540	1550	1560	1570	1580	1590	1600
DNYFSLPVNK	TDTSKAPLHF	PIPVIRYVVK	EVSLVWHLYG	GKDFGIVPPT	SPAKSYISPH	SSPSHTPTRH	GRNTVCGGKG
1610	1620	1630	1640	1650	1660	1670	1680
RNHDFLMEIQ	LSKVKFQHEV	YPPCKPDCDS	SLSEHPVSRQ	VFIVQDLEIR	DRLATSQMNK	FLYLYCSKEM	PRKAHSNMLT
1690	1700	1710	1720	1730	1740	1750	1760
VKALHVCPES	GRSPQECCLR	VSLMPLRLNI	DQDALFFLKD	FFTSLSAEVE	LQMTPDPEVK	KSPGADVTCS	LPRHLSTSKE
1770	1780	1790	1800	1810	1820	1830	1840
PNLVISFSGP	KQPSQNDSAN	SVEVVNGMEE	KNFSAEEASF	RDQPVFFREF	RFTSEVPIRL	DYHGKHVSMD	QGTLAGILIG
1850	1860	1870	1880	1890	1900	1910	1920
LAQLNCSELK	LKRLSYRHGL	LGVDKLFSYA	ITEWLNDIKK	NQLPGILGGV	GPMHSLVQLV	QGLKDLVWLP	IEQYRKDGRI
1930	1940	1950	1960	1970	1980	1990	2000
VRGFQRGAAS	FGTSTAMAAL	ELTNRMVQTI	QAAAETAYDM	VSPGTLSIEP	KKTKRFPHHR	LAHQPVDLRE	GVAKAYSVVK
2010	2020	2030	2040	2050	2060	2070	2080
EGITDTAQTI	YETAAREHES	RGVTGAVGEV	LRQIPPAVVK	PLIVATEATS	NVLGGMRNQI	RPDVRQDESQ	KWRHGDD

Cmpd.	No. of Cmpds.	m/z meas.	Δ m/z [ppm]	z	Rt [min]	Score	P	Range	Sequence	Modification
1611	1	621.3254	12.46	2	50.4	11.2	2	1590-1601	R.HGRNTVCGGKGR.N	

Detailed Protein Report

Protein 456: low-density lipoprotein receptor-related protein 1B precursor [Homo sapiens]

**Accession:**	gi|93102379	**Score:**	23.3
**Database:**	refseq_human(refseq_human_20140103.fasta)	**MW [kDa]:**	515.2
**Database Date:**	2015-11-30	**pI:**	5.0
**Modification(s):**	Oxidation	**Sequence Coverage [%]:**	0.6
		**No. of unique Peptides:**	1

Detailed Protein Report

10	20	30	40	50	60	70	80
MSEFLLALLT	LSGLLPIARV	LTVGADRDQQ	LCDPGEFLCH	DHVTCVSQSW	LCDGDPDCPD	DSDESLDTCP	EEVEIKCPLN
90	100	110	120	130	140	150	160
HIACLGTNKC	VHLSQLCNGV	LDCPDGYDEG	VHCQELLSNC	QQLNCQYKCT	MVRNSTRCYC	EDGFEITEDG	RSCKDQDECA
170	180	190	200	210	220	230	240
VYGTCSQTCR	NTHGSYTCSC	VEGYLMQPDN	RSCKAKIEPT	DRPPILLIAN	FETIEVFYLN	GSKMATLSSV	NGNEIHTLDF
250	260	270	280	290	300	310	320
IYNEDMICWI	ESRESSNQLK	CIQITKAGGL	TDEWTINILQ	SFHNVQQMAI	DWLTRNLYFV	DHVGDRIFVC	NSNGSVCVTL
330	340	350	360	370	380	390	400
IDLELHNPKA	IAVDPIAGKL	FFTDYGNVAK	VERCDMDGMN	RTRIIDSKTE	QPAALALDLV	NKLVYWVDLY	LDYVGVVDYQ
410	420	430	440	450	460	470	480
GKNRHTVIQG	RQVRHLYGIT	VFEDYLYATN	SDNYNIVRIN	RFNGTDIHSL	IKIENAWGIR	IYQKRTQPTV	RSHACEVDPY
490	500	510	520	530	540	550	560
GMPGGCSHIC	LLSSSYKTRT	CRCRTGFNLG	SDGRSCKRPK	NELFLFYGKG	RPGIVRGMDL	NTKIADEYMI	PIENLVNPRA
570	580	590	600	610	620	630	640
LDFHAETNYI	YFADTTSFLI	GRQKIDGTER	ETILKDDLDN	VEGIAVDWIG	NNLYWTNDGH	RKTINVARLE	KASQSRKTLL
650	660	670	680	690	700	710	720
EGEMSHPRGI	VVDPVNGWMY	WTDWEEDEID	DSVGRIEKAW	MDGFNRQIFV	TSKMLWPNGL	TLDFHTNTLY	WCDAYYDHIE
730	740	750	760	770	780	790	800
KVFLNGTHRK	IVYSGRELNH	PFGLSHHGNY	VFWTDYMNGS	IFQLDLITSE	VTLLRHERPP	LFGLQIYDPR	KQQGDNMCRV
810	820	830	840	850	860	870	880
NNGGCSTLCL	AIPGGRVCAC	ADNQLLDENG	TTCTFNPGEA	LPHICKAGEF	RCKNRHCIQA	RWKCDGDDDC	LDGSDEDSVN
890	900	910	920	930	940	950	960
CFNHSCPDDQ	FKCQNNRCIP	KRWLCDGAND	CGSNEDESNQ	TCTARTCQVD	QFSCGNGRCI	PRAWLCDRED	DCGDQTDEMA
970	980	990	1000	1010	1020	1030	1040
SCEFPTCEPL	TQFVCKSGRC	ISSKWHCDSD	DDCGDGSDEV	GCVHSCFDNQ	FRCSSGRCIP	GHWACDGDND	CGDFSDEAQI
1050	1060	1070	1080	1090	1100	1110	1120
NCTKEEIHSP	AGCNGNEFQC	HPDGNCVPDL	WRCDGEKDCE	DGSDEKGCNG	TIRLCDHKTK	FSCWSTGRCI	NKAWVCDGDI
1130	1140	1150	1160	1170	1180	1190	1200
DCEDQSDEDD	CDSFLCGPPK	HPCANDTSVC	LQPEKLCNGK	KDCPDGSDEG	YLCDECSLNN	GGCSNHCSVV	PGRGIVCSCP
1210	1220	1230	1240	1250	1260	1270	1280
EGLQLNKDNK	TCEIVDYCSN	HLKCSQVCEQ	HKHTVKCSCY	EGWKLDVDGE	SCTSVDPFEA	FIIFSIRHEI	RRIDLHKRDY
1290	1300	1310	1320	1330	1340	1350	1360
SLLVPGLRNT	IALDFHFNQS	LLYWTDVVED	RIYRGKLSES	GGVSAIEVVV	EHGLATPEGL	TVDWIAGNIY	WIDSNLDQIE
1370	1380	1390	1400	1410	1420	1430	1440
VAKLDGSLRT	TLIAGAMEHP	RAIALDPRYG	ILFWTDWDAN	FPRIESASMS	GAGRKTIYKD	MKTGAWPNGL	TVDHFEKRIV
1450	1460	1470	1480	1490	1500	1510	1520
WTDARSDAIY	SALYDGTNMI	EIIRGHEYLS	HPFAVSLYGS	EVYWTDWRTN	TLSKANKWTG	QNVSVIQKTS	AQPFDLQIYH
1530	1540	1550	1560	1570	1580	1590	1600
PSRQPQAPNP	CAANDGKGPC	SHMCLINHNR	SAACACPHLM	KLSSDKKTCY	EMKKFLLYAR	RSEIRGVDID	NPYFNFITAF
1610	1620	1630	1640	1650	1660	1670	1680
TVPDIDDVTV	IDFDASEERL	YWTDIKTQTI	KRAFINGTGL	ETVISRDIQS	IRGLAVDWVS	RNLYWISSEF	DETQINVARL
1690	1700	1710	1720	1730	1740	1750	1760
DGSLKTSIIH	GIDKPQCLAA	HPVRGKLYWT	DGNTINMANM	DGSNSKILFQ	NQKEPVGLSI	DYVENKLYWI	SSGNGTINRC
1770	1780	1790	1800	1810	1820	1830	1840
NLDGGNLEVI	ESMKEELTKA	TALTIMDKKL	WWADQNLAQL	GTCSKRDGRN	PTILRNKTSG	VVHMKVYDKE	AQQGSNSCQL
1850	1860	1870	1880	1890	1900	1910	1920
NNGGCSQLCL	PTSETTRTCM	CTVGYYLQKN	RMSCQGIESF	LMYSVHEGIR	GIPLEPSDKM	DALMPISGTS	FAVGIDFHAE
1930	1940	1950	1960	1970	1980	1990	2000
NDTIYWTDMG	FNKISRAKRD	QTWKEDIITN	GLGRVEGIAV	DWIAGNIYWT	DHGFNLIEVA	RLNGSFRYVI	ISQGLDQPRS
2010	2020	2030	2040	2050	2060	2070	2080
IAVHPEKGLL	FWTEWGQMPC	IGKARLDGSE	KVVLVSMGIA	WPNGISIDYE	ENKLYWCDAR	TDKIERIDLE	TGGNREMVLS
2090	2100	2110	2120	2130	2140	2150	2160
GSNVDMFSVA	VFGAYIYWSD	RAHANGSVRR	GHKNDATETI	TMRTGLGVNL	KEVKIFNRVR	EKGTNVCARD	NGGCKQLCLY
2170	2180	2190	2200	2210	2220	2230	2240

Detailed Protein Report

Cmpd.	No. of Cmpds.	m/z meas.	Δ m/z [ppm]	z	Rt [min]	Score	P	Range	Sequence	Modification
2037	2	977.3860	-129.29	2	54.0	11.5	1	1364-1381	K.LDGSLRTTLIAGAMEHPR.A	Oxidation: 14

Detailed Protein Report

Protein 457: ectoderm-neural cortex protein 1 isoform 2 [Homo sapiens]

**Accession:**	gi|375268727	**Score:**	23.2
**Database:**	refseq_human(refseq_human_20140103.fasta)	**MW [kDa]:**	57.8
**Database Date:**	2015-11-30	**pI:**	5.7
**Modification(s):**	Carbamidomethyl	**Sequence Coverage [%]:**	6.6
		**No. of unique Peptides:**	1

10	20	30	40	50	60	70	80
MFSGGLKESQ	DSEVNFDNSI	HPEVLELLLD	YAYSSRVIIN	EENAESLLEA	GDMLEFQDIR	DACAEFLEKN	LHPTNCLGML
**90**	**100**	**110**	**120**	**130**	**140**	**150**	**160**
LLSDAHQCTK	LYELSWRMCL	SNFQTIRKNE	DFLQLPQDMV	VQLLSSEELE	TEDERLVYES	AINWISYDLK	KRYCYLPELL
**170**	**180**	**190**	**200**	**210**	**220**	**230**	**240**
QTVRLALLPA	IYLMENVAME	ELITKQRKSK	EIVEEAIRCK	LKILQNDGVV	TSLCARPRKT	GHALFLLGGQ	TFMCDKLYLV
**250**	**260**	**270**	**280**	**290**	**300**	**310**	**320**
DQKAKEIIPK	ADIPSPRKEF	SACAIGCKVY	ITGGRGSENG	VSKDVWVYDT	LHEEWSKAAP	MLVARFGHGS	AELKHCLYVV
**330**	**340**	**350**	**360**	**370**	**380**	**390**	**400**
GGHTAATGCL	PASPSVSLKQ	VEHYDPTINK	WTMVAPLREG	VSNAAVVSAK	LKLFAFGGTS	VSHDKLPKVQ	CYDQCENRWT
**410**	**420**	**430**	**440**	**450**	**460**	**470**	**480**
VPATCPQPWR	YTAAAVLGNQ	IFIMGGDTEF	SACSAYKFNS	ETYQWTKVGD	VTAKRMSCHA	VASGNKLYVV	GGYFGIQRCK
**490**	**500**	**510**	**520**				
TLDCYDPTLD	VWNSITTVPY	SLIPTAFVST	WKHLPS				

Cmpd.	No. of Cmpds.	m/z meas.	Δ m/z [ppm]	z	Rt [min]	Score	P	Range	Sequence	Modification
1958	2	948.3829	-82.11	2	53.0	13.0	0	220-236	K.TGHALFLLGGQTFMCDK.L	Carbamidomethyl: 15

Detailed Protein Report

Protein 458: **tripartite motif-containing protein 5 isoform alpha [Homo sapiens]**

**Accession:**	gi|283046694	**Score:**	23.2
**Database:**	refseq_human(refseq_human_20140103.fasta)	**MW [kDa]:**	56.3
**Database Date:**	2015-11-30	**pI:**	5.7
**Modification(s):**	Oxidation	**Sequence Coverage [%]:**	5.9
		**No. of unique Peptides:**	2

Alias proteins:

Accession	Name	Description
gi|530395903	refseq_human (refseq_human_20140103.fasta)	PREDICTED: tripartite motif-containing protein 5 isoform X1 [Homo sapiens]

10	20	30	40	50	60	70	80
MASGILVNVK	EEVTCPICLE	LLTQPLSLDC	GHSFCQACLT	ANHKKSMLDK	GESSCPVCRI	SYQPENIRPN	RHVANIVEKL
**90**	**100**	**110**	**120**	**130**	**140**	**150**	**160**
REVKLSPEGQ	KVDHCARHGE	KLLLFCQEDG	KVICWLCERS	QEHRGHHTFL	TEEVAREYQV	KLQAALEMLR	QKQQEAEELE
**170**	**180**	**190**	**200**	**210**	**220**	**230**	**240**
ADIREEKASW	KTQIQYDKTN	VLADFEQLRD	ILDWEESNEL	QNLEKEEEDI	LKSLTNSETE	MVQQTQSLRE	LISDLEHRLQ
**250**	**260**	**270**	**280**	**290**	**300**	**310**	**320**
GSVMELLQGV	DGVIKRTENV	TLKKPETFPK	NQRRVFRAPD	LKGMLEVFRE	LTDVRRYWVD	VTVAPNNISC	AVISEDKRQV
**330**	**340**	**350**	**360**	**370**	**380**	**390**	**400**
SSPKPQIIYG	ARGTRYQTFV	NFNYCTGILG	SQSITSGKHY	WEVDVSKKTA	WILGVCAGFQ	PDAMCNIEKN	ENYQPKYGYW
**410**	**420**	**430**	**440**	**450**	**460**	**470**	**480**
VIGLEEGVKC	SAFQDSSFHT	PSVPFIVPLS	VIICPDRVGV	FLDYEACTVS	FFNITNHGFL	IYKFSHCSFS	QPVFPYLNPR
**490**	**500**						
KCGVPMTLCS	PSS						

Cmpd.	No. of Cmpds.	m/z meas.	Δ m/z [ppm]	z	Rt [min]	Score	P	Range	Sequence	Modification
1780	1	945.9067	-62.20	2	50.8	10.3	2	85-101	K.LSPEGQKVDHCARHGEK.L	
1800	1	599.2749	39.82	2	52.8	12.9	0	482-493	K.CGVPMTLCSPSS.-	Oxidation: 5

Detailed Protein Report

Protein 459: **GRINL1A combined protein isoform 3 [Homo sapiens]**

**Accession:**	gi|553726985	**Score:**	23.2
**Database:**	refseq_human(refseq_human_20140103.fasta)	**MW [kDa]:**	87.8
**Database Date:**	2015-11-30	**pI:**	5.7
		**Sequence Coverage [%]:**	3.1
		**No. of unique Peptides:**	1

Quantitation

QD:QU **Median:** 0.67 **CV:** 0.00 % **No. of Peptides:** 1

10	20	30	40	50	60	70	80
MLRSTSTVTL	LSGGAARTPG	APSRRANVCR	LRLTVPPESP	VPEQCEKKIE	RKEQLLDLSN	GEPTRKLPQG	VVYGVVRRSD
90	100	110	120	130	140	150	160
QNQQKEMVVY	GWSTSQLKEE	MNYIKDVRAT	LEKVRKRMYG	DYDEMRQKIR	QLTQELSVSH	AQQEYLENHI	QTQSSALDRF
170	180	190	200	210	220	230	240
NAMNSALASD	SIGLQKTLVD	VTLENSNIKD	QIRNLQQTYE	ASMDKLREKQ	RQLEVAQVEN	QLLKMKVESS	QEANAEVMRE
250	260	270	280	290	300	310	320
MTKKLYSQYE	EKLQEEQRKH	SAEKEALLEE	TNSFLKAIEE	ANKKMQAAEI	SLEEKDQRIG	ELDRLIERME	KERHQLQLQL
330	340	350	360	370	380	390	400
LEHETEMSGE	LTDSDKERYQ	QLEEASASLR	ERIRHLDDMV	HCQQKKVKQM	VEEIESLKKK	LQQKQLLILQ	LLEKISFLEG
410	420	430	440	450	460	470	480
ENNELQSRLD	YLTETQAKTE	VETREIGVGC	DLLPRKFICK	LPDKGKKIFD	SFAKLKAAIA	ECEEVRRKSE	LFNPVSLDCK
490	500	510	520	530	540	550	560
LRQKAIAEVD	VGTDKAQNSD	PILDTSSLVP	GCSSVDNIKS	SQTSQNQGLG	RPTLEGDEET	SEVEYTVNKG	PASSNRDRVP
570	580	590	600	610	620	630	640
PSSEASEHHP	RHRVSSQAED	TSSSFDNLFI	DRLQRITIAD	QGEQQSEENA	STKNLTGLSS	GTEKKPHYME	VLEMRAKNPV
650	660	670	680	690	700	710	720
PQLRKFKTNV	LPFRQNDSSS	HCQKSGSPIS	SEERRRDKQ	HLDDITAARL	LPLHHMPTQL	LSIEESLALQ	KQQKQNYEEM
730	740	750	760	770			
QAKLAAQKLA	ERLNIKMRSY	NPEGESSGRY	REVRDEDDDW	SSDEF			

Cmpd.	No. of Cmpds.	m/z meas.	Δ m/z [ppm]	z	Rt [min]	Score	P	Range	Sequence	Modification	Ratios
205	1	854.9273	-8.70	2	32.5	10.1	2	355-368	R.HLDDMVHCQQKKVK.Q		QD:QU 0.67

Detailed Protein Report

Protein 460: **PREDICTED: arf-GAP with GTPase, ANK repeat and PH domain-containing protein 3 isoform X2 [Homo sapiens]**

Accession: gi|530386889
Database: refseq_human(refseq_human_20140103.fasta)
Database Date: 2015-11-30

Score: 23.2
MW [kDa]: 61.7
pI: 11.8
Sequence Coverage [%]: 5.2
No. of unique Peptides: 1

10	20	30	40	50	60	70	80
MERGWPQGDS	CPGERPAACR	RAHSVCDSLD	LHGASAGRAA	AALQAALCAA	SEQPARPRSV	CSGGPEPPPT	GARGLLLGLL
**90**	**100**	**110**	**120**	**130**	**140**	**150**	**160**
RPRLGRRGLA	PSGPPVSPAP	SPASSPAPTR	RSRTRGEPTP	RPRPASMTFL	EVNRLELAAA	EAPGAGLGRA	GSAGFLRGAA
**170**	**180**	**190**	**200**	**210**	**220**	**230**	**240**
LWSSQRWPVL	RGGRGPEGPR	RGLAALRKSF	SFRLRRGQEV	RRSESGLLAR	PPRARTRSDG	DAGSLGAFPS	RRDLLGSDAP
**250**	**260**	**270**	**280**	**290**	**300**	**310**	**320**
RAAPEPGRPR	TAAGLWRLLT	SRFRRREPAP	AAPLWGRRAA	AAPELLRAPS	DSFVNSQEWT	LSRSVPELKV	GIVGNLSSGK
**330**	**340**	**350**	**360**	**370**	**380**	**390**	**400**
SALVHRYLTG	TYVQEESPEG	GRFKKEIVVD	GQSYLLLIRD	EGGPPELQFA	AWVDAVVFVF	SLEDEISFQT	VYNYFLRLCS
**410**	**420**	**430**	**440**	**450**	**460**	**470**	**480**
FRNASEVPMV	LVGTQDAISA	ANPRVIDDSR	ARKLSTDLKR	CTYYETCATY	GLNVERVFQD	VAQKVVALRK	KQQLAIGPCK
**490**	**500**	**510**	**520**	**530**	**540**	**550**	**560**
SLPNSPSHSA	VSAASIPAVH	INQATNGGGS	AFSDYSSSVP	STPSISQREL	RIETIAASST	PTPIRKQSKR	RSNIFTICAT
**570**	**580**						
VSNFSSTKRP	FQLLPN						

Cmpd.	No. of Cmpds.	m/z meas.	Δ m/z [ppm]	z	Rt [min]	Score	P	Range	Sequence	Modification
1530	1	873.6409	147.98	2	47.7	10.1	1	345-359	K.KEIVVDGQSYLLLIR.D	

Detailed Protein Report

Protein 461: **PREDICTED: serine/arginine-rich splicing factor 4 isoform X1 [Homo sapiens]**

**Accession:**	gi|578799577	**Score:**	23.2
**Database:**	refseq_human(refseq_human_20140103.fasta)	**MW [kDa]:**	42.4
**Database Date:**	2015-11-30	**pI:**	12.5
		**Sequence Coverage [%]:**	6.2
		**No. of unique Peptides:**	1

Alias proteins:

Accession	Name	Description
gi|578799579	refseq_human (refseq_human_20140103.fasta)	PREDICTED: serine/arginine-rich splicing factor 4 isoform X2 [Homo sapiens]

10	20	30	40	50	60	70	80
MRQAGEVTYA	DAHKGRKNEG	VIEFVSYSDM	KRALEKLDGT	EVNGRKIRLV	EDKPGSRRRR	SYSRSRSHSR	SRSRSRHSRK
**90**	**100**	**110**	**120**	**130**	**140**	**150**	**160**
SRSRSGSSKS	SHSKSRSRSR	SGSRSRSKSR	SRSQSRSRSK	KEKSRSPSKE	KSRSRSHSAG	KSRSKSKDQA	EEKIQNNDNV
**170**	**180**	**190**	**200**	**210**	**220**	**230**	**240**
GKPKSRSPSR	HKSKSKSRSR	SQERRVEEEK	RGSVSRGRSQ	EKSLRQSRSR	SRSKGGSRSR	SRSRSKSKDK	RKGRKRSREE
**250**	**260**	**270**	**280**	**290**	**300**	**310**	**320**
SRSRSRSRSK	SERSRKRGSK	RDSKAGSSKK	KKKEDTDRSQ	SRSPSRSVSK	EREHAKSESS	QREGRGESEN	AGTNQETRSR
**330**	**340**	**350**	**360**	**370**	**380**		
SRSNSKSKPN	LPSESRSRSK	SASKTRSRSK	SRSRSASRSP	SRSRSRSHSR	S		

Cmpd.	No. of Cmpds.	m/z meas.	Δ m/z [ppm]	z	Rt [min]	Score	P	Range	Sequence	Modification
2024	1	788.7867	-118.83	2	53.8	11.0	1	1-14	-.MRQAGEVTYADAHK.G	

Detailed Protein Report

Protein 462: transcriptional protein SWT1 [Homo sapiens]

Accession: gi|157502207
Database: refseq_human(refseq_human_20140103.fasta)
Database Date: 2015-11-30

Score: 23.1
MW [kDa]: 103.2
pI: 9.7
Sequence Coverage [%]: 3.4
No. of unique Peptides: 2

Alias proteins:

Accession	Name	Description
gi|157502209	refseq_human (refseq_human_20140103.fasta)	transcriptional protein SWT1 [Homo sapiens]

10	20	30	40	50	60	70	80
MSSKESCGKK	ETSQRKDTTT	SSPNFGEKDK	KERKTPASST	SSSSIRSVSS	EKRKLKSDHT	DVLYYNIKRR	QGLKRLSVEI
**90**	**100**	**110**	**120**	**130**	**140**	**150**	**160**
DTLRRRPKIG	SSSQRPIKLK	EASYSNDNQI	ILQSPSSNGT	KKDIHKCVDF	KPKDIKLTNA	GSKLDHGIKS	LSSPKIASDV
**170**	**180**	**190**	**200**	**210**	**220**	**230**	**240**
KPKAEGQASE	NKWSHLLVQR	EKMKELKKGR	NSKFRDNSEK	CVLEKWKRNQ	FSQDYNSNKI	IKEPLGSRRQ	KISFKIPIKS
**250**	**260**	**270**	**280**	**290**	**300**	**310**	**320**
RDTLQKLVEE	NVFNIDSNNS	KTKQEEREYL	ESSQVSLNVT	RQKTEHLLSD	FTYKRTVHEW	KRKHHYDHQE	SNDSHSRENL
**330**	**340**	**350**	**360**	**370**	**380**	**390**	**400**
TQSFEAPCCS	VSSESIQDAD	QEMQIVEELH	AARVGKSVDL	PGELMSMEID	LEDDVHSSSA	NNTSDRKLLI	VIDTNILMNH
**410**	**420**	**430**	**440**	**450**	**460**	**470**	**480**
LKFVRILKTT	EVPGFDKLVL	IIPWVVMQEL	DRMKEGKLLK	RAQHKAIPAV	HFINDSLKNQ	DRKLWGQSIQ	LASQKHYGLS
**490**	**500**	**510**	**520**	**530**	**540**	**550**	**560**
DENNDDRVLK	CCLQHQELFP	CSFVILCTDD	RNLRNKGLIS	GVKSLSKEEL	SAELLHLSLN	TDVCHQPCIP	KQQLKAETTP
**570**	**580**	**590**	**600**	**610**	**620**	**630**	**640**
LKESYKEEST	NSGLSILLES	IVSDLEKSLG	TGLSSILETE	MKIAFGNLWM	EILYLKPPWT	LLHLLQCFKK	HWLAVFGLVM
**650**	**660**	**670**	**680**	**690**	**700**	**710**	**720**
EKNLLLTIES	LYKNLRKANK	AVDFTTVKFL	LQDSRSLLHA	FSTRSNYDGI	LPQTFAQVNN	LLQTFAEVKT	KLKPNSSENT
**730**	**740**	**750**	**760**	**770**	**780**	**790**	**800**
VTKKQEGTSL	KNSHNQEITV	FSSSHLPQPS	RHQEIWSILE	SVWITIYQNS	TDVFQRLGSN	SALTTSNIAS	FEEAFICLQK
**810**	**820**	**830**	**840**	**850**	**860**	**870**	**880**
LMAAVRDILE	GIQRILAPNS	NYQDVETLYN	FLIKYEVNKN	VKFTAQEIYD	CVSQTEYREK	LTIGCRQLVE	MEYTMQQCNA
**890**	**900**	**910**					
SVYMEAKNRG	WCEDMLNYRI						

Cmpd.	No. of Cmpds.	m/z meas.	Δ m/z [ppm]	z	Rt [min]	Score	P	Range	Sequence	Modification
1636	1	682.3058	-115.98	3	50.7	10.7	2	441-458	K.RAQHKAIPAVHFINDSLK.N	
69	1	665.8831	-34.85	2	29.6	12.5	2	515-527	R.NKGLISGVKSLSK.E	

Detailed Protein Report

Protein 463: nuclear receptor corepressor 2 isoform 2 [Homo sapiens]

**Accession:**	gi|331284176	**Score:**	23.0
**Database:**	refseq_human(refseq_human_20140103.fasta)	**MW [kDa]:**	267.9
**Database Date:**	2015-11-30	**pI:**	8.0
**Modification(s):**	Oxidation	**Sequence Coverage [%]:**	1.4
		**No. of unique Peptides:**	1

Quantitation

QD:QU	**Median:** 0.07	**CV:** 0.00 %	**No. of Peptides:** 1

Detailed Protein Report

10	20	30	40	50	60	70	80
MSGSTQPVAQ	TWRATEPRYP	PHSLSYPVQI	ARTHTDVGLL	EYQHHSRDYA	SHLSPGSIIQ	PQRRRPSLLS	EFQPGNERSQ
90	100	110	120	130	140	150	160
ELHLRPESHS	YLPELGKSEM	EFIESKRPRL	ELLPDPLLRP	SPLLATGQPA	GSEDLTKDRS	LTGKLEPVSP	PSPPHTDPEL
170	180	190	200	210	220	230	240
ELVPPRLSKE	ELIQNMDRVD	REITMVEQQI	SKLKKKQQQL	EEEAAKPPEP	EKPVSPPPIE	SKHRSLVQII	YDENRKKAEA
250	260	270	280	290	300	310	320
AHRILEGLGP	QVELPLYNQP	SDTRQYHENI	KINQAMRKKL	ILYFKRRNHA	RKQWEQKFCQ	RYDQLMEAWE	KKVERIENNP
330	340	350	360	370	380	390	400
RRRAKESKVR	EYYEKQFPEI	RKQRELQERM	QRVGQRGSGL	SMSAARSEHE	VSEIIDGLSE	QENLEKQMRQ	LAVIPPMLYD
410	420	430	440	450	460	470	480
ADQQRIKFIN	MNGLMADPMK	VYKDRQVMNM	WSEQEKETFR	EKFMQHPKNF	GLIASFLERK	TVAECVLYYY	LTKKNENYKS
490	500	510	520	530	540	550	560
LVRRSYRRRG	KSQQQQQQQQ	QQQQQQQQQP	MPRSSQEEKD	EKEKEKEAEK	EEEKPEVEND	KEDLLKEKTD	DTSGEDNDEK
570	580	590	600	610	620	630	640
EAVASKGRKT	ANSQGRRKGR	ITRSMANEAN	SEEAITPQQS	AELASMELNE	SSRWTEEEME	TAKKGLLEHG	RNWSAIARMV
650	660	670	680	690	700	710	720
GSKTVSQCKN	FYFNYKKRQN	LDEILQQHKL	KMEKERNARR	KKKKAPAAAS	EEAAFPPVVE	DEEMEASGVS	GNEEEMVEEA
730	740	750	760	770	780	790	800
EATVNNSSDT	ESIPSPHTEA	AKDTGQNGPK	PPATLGADGP	PPGPPTPPPE	DIPAPTEPTP	ASEATGAPTP	PPAPPSPSAP
810	820	830	840	850	860	870	880
PPVVPKEEKE	EETAAAPPVE	EGEEQKPPAA	EELAVDTGKA	EEPVKSECTE	EAEEGPAKGK	DAEAAEATAE	GALKAEKKEG
890	900	910	920	930	940	950	960
GSGRATTAKS	SGAPQDSDSS	ATCSADEVDE	AEGGDKNRLL	SPRPSLLTPT	GDPRANASPQ	KPLDLKQLKQ	RAAAIPPIQV
970	980	990	1000	1010	1020	1030	1040
TKVHEPPRED	AAPTKPAPPA	PPPPQNLQPE	SDAPQQPGSS	PRGKSRSPAP	PADKEAEKPV	FFPAFAAEAQ	KLPGDPPCWT
1050	1060	1070	1080	1090	1100	1110	1120
SGLPFPVPPR	EVIKASPHAP	DPSAFSYAPP	GHPLPLGLHD	TARPVLPRPP	TISNPPPLIS	SAKHPSVLER	QIGAISQGMS
1130	1140	1150	1160	1170	1180	1190	1200
VQLHVPYSEH	AKAPVGPVTM	GLPLPMDPKK	LAPFSGVKQE	QLSPRGQAGP	PESLGVPTAQ	EASVLRGTAL	GSVPGGSITK
1210	1220	1230	1240	1250	1260	1270	1280
GIPSTRVPSD	SAITYRGSIT	HGTPADVLYK	GTITRIIGED	SPSRLDRGRE	DSLPKGHVIY	EGKKGHVLSY	EGGMSVTQCS
1290	1300	1310	1320	1330	1340	1350	1360
KEDGRSSSGP	PHETAAPKRT	YDMMEGRVGR	AISSASIEGL	MGRAIPPERH	SPHHLKEQHH	IRGSITQGIP	RSYVEAQEDY
1370	1380	1390	1400	1410	1420	1430	1440
LRREAKLLKR	EGTPPPPPPS	RDLTEAYKTQ	ALGPLKLKPA	HEGLVATVKE	AGRSIHEIPR	EELRHTPELP	LAPRPLKEGS
1450	1460	1470	1480	1490	1500	1510	1520
ITQGTPLKYD	TGASTTGSKK	HDVRSLIGSP	GRTFPPVHPL	DVMADARALE	RACYEESLKS	RPGTASSSGG	SIARGAPVIV
1530	1540	1550	1560	1570	1580	1590	1600
PELGKPRQSP	LTYEDHGAPF	AGHLPRGSPV	TTREPTPRLQ	EGSLSSSKAS	QDRKLTSTPR	EIAKSPHSTV	PEHHPHPISP
1610	1620	1630	1640	1650	1660	1670	1680
YEHLLRGVSG	VDLYRSHIPL	AFDPTSIPRG	IPLDAAAAYY	LPRHLAPNPT	YPHLYPPYLI	RGYPDTAALE	NRQTIINDYI
1690	1700	1710	1720	1730	1740	1750	1760
TSQQMHHNAA	TAMAQRADML	RGLSPRESSL	ALNYAAGPRG	IIDLSQVPHL	PVLVPPTPGT	PATAMDRLAY	LPTAPQPFSS
1770	1780	1790	1800	1810	1820	1830	1840
RHSSSPLSPG	GPTHLTKPTT	TSSSERERDR	DRERDRDRER	EKSILTSTTT	VEHAPIWRPG	TEQSSGSSGG	GGGSSSRPAS
1850	1860	1870	1880	1890	1900	1910	1920
HSHAHQHSPI	SPRTQDALQQ	RPSVLHNTGM	KGIITAVEPS	TPTVLRSTST	SSPVRPAATF	PPATHCPLGG	TLDGVYPTLM
1930	1940	1950	1960	1970	1980	1990	2000
EPVLLPKEAP	RVARPERPRA	DTGHAFLAKP	PARSGLEPAS	SPSKGSEPRP	LVPPVSGHAT	IARTPAKNLA	PHHASPDPPA
2010	2020	2030	2040	2050	2060	2070	2080
PPASASDPHR	EKTQSKPFSI	QELELRSLGY	HGSSYSPEGV	EPVSPVSSPS	LTHDKGLPKH	LEELDKSHLE	GELRPKQPGP
2090	2100	2110	2120	2130	2140	2150	2160
VKLGGEAAHL	PHLRPLPESQ	PSSSPLLQTA	PGVKGHQRVV	TLAQHISEVI	TQDYTRHHPQ	QLSAPLPAPL	YSFPGASCPV
2170	2180	2190	2200	2210	2220	2230	2240

Detailed Protein Report

Cmpd.	No. of Cmpds.	m/z meas.	Δ m/z [ppm]	z	Rt [min]	Score	P	Range	Sequence	Modification	Ratios	
1282	1	530.6426	-232.27	2	44.5	10.1	1	441-448	R.EKFMQHPK.N	Oxidation: 4	QD:QU	0.07

Detailed Protein Report

Protein 464: **PREDICTED: immunoglobulin-like and fibronectin type III domain-containing protein 1 isoform X3 [Homo sapiens]**

**Accession:**	gi|578801693	**Score:**	23.0
**Database:**	refseq_human(refseq_human_20140103.fasta)	**MW [kDa]:**	355.2
**Database Date:**	2015-11-30	**pI:**	5.6
**Modification(s):**	Oxidation	**Sequence Coverage [%]:**	1.1
		**No. of unique Peptides:**	2

Quantitation

QD:QU	**Median:** 1.75	**CV:** 0.00 %	**No. of Peptides:** 1

BRUKER

Detailed Protein Report

10	20	30	40	50	60	70	80
MAGKLRKSHI	PGVSIWQLVE	EIPEGCSTPD	FEQKPVTSAL	PEGKNAVFRA	VVCGEPRPEV	RWQNSKGDLS	DSSKYKISSS
90	100	110	120	130	140	150	160
PGSKEHVLQI	NKLTGEDTDL	YRCTAVNAYG	EAACSVRLTV	IEVGFRKNRK	RHREPQEDLR	KELMDFRKLL	KKRAPPAPKK
170	180	190	200	210	220	230	240
KMDLEQIWQL	LMTADRKDYE	KICLKYGIVD	YRGMLRRLQE	MKKEQEDKMA	QYINTISSLR	HIRVTKDGNA	KFDLELDLKD
250	260	270	280	290	300	310	320
SQSKIYLYKD	GEMIPYGFNN	QTKHCLRRLG	KRYEFQIQDL	RPEDSGIYQV	KVEDAVVFST	ELEASAIPPR	VVVPLAETHC
330	340	350	360	370	380	390	400
EEQGDAVFEC	TLSSPCPSAA	WHFRHRLLHP	SDKYEVYVSP	DGLTHRLVVR	GARFSDMGPY	SLGTGLYTSS	AWLVVEAGKD
410	420	430	440	450	460	470	480
KDLQSTSADH	KLQRQGAQAS	GAEESGSIES	QGEKSREQGP	RGGSLEGAGP	ASGLQHIASP	DRDGLGRHGY	SLMGDKGTAD
490	500	510	520	530	540	550	560
SAWGPGQEGE	GFPVAEGSRA	TLPRENQSHR	EGGWARSLAE	RPHLQGESSE	SGLGLPEKQQ	QDRGRDSNSD	ECWRKAGGWE
570	580	590	600	610	620	630	640
AGSSRLQAGG	LGSSREGKEH	RGDSGRQLDR	HAPEQLWDAR	LGPGRGKSDL	QGCQSDPVGS	WPRGKQIEIS	QDDSLAEMDR
650	660	670	680	690	700	710	720
GDAPSRERGR	GIVVWGGGTG	LGEAGDSNGA	GGPGTLELTG	GRGSGSKVGM	APESWGSQGG	RDADYGEARG	YWGSGELLEQ
730	740	750	760	770	780	790	800
IPGGKDFQEP	SISGGRKFLL	GDGSPEIKAE	DSLQEADGIC	RGESVVTGSA	YKTGPGGPGD	PRGCEGVLQE	LRGRDGQETA
810	820	830	840	850	860	870	880
WASGEVEYDP	RSFQSSQGWT	AGHRAAGGIG	RIESKGTSPW	DDTPSSLRKT	GAHHGPGVLG	PSGGQEGMGG	IWVAGLTESG
890	900	910	920	930	940	950	960
QGVDARSHWL	SRAPGLGAQG	SGGTLGDKKG	LRGPGSIGSE	PDFWNGSGSS	RVKGPRGETG	YKDGLEGPGR	MESRYEGGLG
970	980	990	1000	1010	1020	1030	1040
YSREISSKSG	AGYSYGSGVP	GEMGSGHGAG	CRVSPRAPAG	VESEEGGGYR	HGSGAPGGVW	SGNEDSGPAG	GGSGRVASLK
1050	1060	1070	1080	1090	1100	1110	1120
NGSGGPDGAP	MNDTRNWASA	CQAGMDPRGG	HHSDGGLGSP	GVTGSAGRGG	LKAPGVVETV	GMGCVEAEPE	SSGRIRPWGQ
1130	1140	1150	1160	1170	1180	1190	1200
TGNYGGFRAS	EALGAFGEGG	YEDGSGGPGA	MGPGSLRAGS	KVGEGDGTRC	PGAKASGAGA	GYRDDTRHPE	SLAPHNGAAS
1210	1220	1230	1240	1250	1260	1270	1280
GSQWAYGAGN	VLGYEDGSEL	PGPQGTGVRT	AYGERSRGLG	PRSTGPGGEA	GFRDGSGGLQ	GMGSADGPGC	RKGIGSSGEM
1290	1300	1310	1320	1330	1340	1350	1360
GSVDKEGYKK	DLGAPENMGS	GSKADYRDGV	GGSGAMGSMD	EAGYRKDLGA	PEGISSGSKA	DYRGGLQDSR	EAGSGSKADY
1370	1380	1390	1400	1410	1420	1430	1440
SGGLKGSREI	GSMDETDNRK	DLGVPEGMGA	GYRAGLRGPG	EMGSLDESGH	RNGIGGYGEM	GSGYREDLGA	PEGMGTGSKA
1450	1460	1470	1480	1490	1500	1510	1520
GYRDGLRGSG	EMRSMDEAGY	RKNLGAPERM	DSGSKAGYRG	GLRGSGEMGL	IEAGYRKDLG	VSEGGGSGSK	AGYRGGLGSG
1530	1540	1550	1560	1570	1580	1590	1600
EMGSVDKAGY	RKDLGASEAI	GSGSKAGFTD	GLGGSEEMGS	VNKAGYRKDL	GAPKGMGSGS	KASFRDGLGG	SGEMGSVNEA
1610	1620	1630	1640	1650	1660	1670	1680
GYRKDLGVPE	GIGSGSKAGF	RDGLGGSEEM	GSVNKAGYRK	DLGAPKGIGS	GSKAGFRDGL	GSSGEMGSMD	EAGYRKNLGA
1690	1700	1710	1720	1730	1740	1750	1760
PEGIGSGSKA	GFRDGLGSSV	EMGSVNEAGY	RKDLGAPEGM	GSGSKAGFRD	GLGGSGEMGS	VNEAGYRKDL	GAPKGIGSGS
1770	1780	1790	1800	1810	1820	1830	1840
KADFRDALGS	SGEMGSMDEA	GYRKDLWAPE	GIGSGSKAGF	RDGLGSSVEM	GSVNEAGYRK	DLGAPEGMGS	GSKAGFRDGL
1850	1860	1870	1880	1890	1900	1910	1920
GGSGEMGSVN	EAGYRKDLGA	PKGIGSGSKA	DFRDALGSSG	EMGSMDEAGY	RKDLWAPEGI	GSGSKAGFRD	GLGSSVEMGS
1930	1940	1950	1960	1970	1980	1990	2000
VNEAGYRKDL	GAPEGMGSGS	KEGFRDGLGG	SEEMGSVNKA	GYRKDLGAPK	GMGSGSKEGF	RDGLGGSEEM	GSMDEAGYRK
2010	2020	2030	2040	2050	2060	2070	2080
DLGAPEGIGS	GSKAGFRDGL	GGSEEMRSMD	EAGYRKDLGA	PERIGSGSKA	GFRDGLGSSV	EMGSVNEAGY	RKDLGAPKGM
2090	2100	2110	2120	2130	2140	2150	2160
GSGSKTGFRD	GLGGSEEMES	MDEAGYRKDL	GAPEGIGSGS	KAGFRDGLGS	STEMGSVNEA	GYRKDLGAPK	GMGSESKAGF
2170	2180	2190	2200	2210	2220	2230	2240

Detailed Protein Report

Cmpd.	No. of Cmpds.	m/z meas.	Δ m/z [ppm]	z	Rt [min]	Score	P	Range	Sequence	Modification	Ratios
548	1	512.1851	-91.29	2	35.5	10.1	0	468-476	R.HGYSLMGDK.G	Oxidation: 6	QD:QU 1.75
765	1	937.7156	-58.56	3	38.1	12.9	1	2411-2439	R. ETRLVDGAGPGVEPGMAGMPGT. D	Oxidation: 16, 19	

Detailed Protein Report

Protein 465: PREDICTED: kinesin-like protein KIF7 isoform X4 [Homo sapiens]

**Accession:**	gi|578826899	**Score:**	23.0
**Database:**	refseq_human(refseq_human_20140103.fasta)	**MW [kDa]:**	146.9
**Database Date:**	2015-11-30	**pI:**	6.1
		**Sequence Coverage [%]:**	2.3
		**No. of unique Peptides:**	1

10	20	30	40	50	60	70	80
MGLEAQRLPG	AEEAPVRVAL	RVRPLLPKEL	LHGHQSCLQV	EPGLGRVTLG	RDRHFGFHVV	LAEDAGQEAV	YQACVQPLLE
90	100	110	120	130	140	150	160
AFFEGFNATV	FAYGQTGSGK	TYTMGEASVA	SLLEDEQGIV	PRAMAEAFKL	IDENDLLDCL	VHVSYLEVYK	EEFRDLLEVG
170	180	190	200	210	220	230	240
TASRDIQLRE	DERGNVVLCG	VKEVDVEGLD	EVLSLLEMGN	AARHTGATHL	NHLSSRSHTV	FTVTLEQRGR	APSRLPRPAP
250	260	270	280	290	300	310	320
GQLLVSKFHF	VDLAGSERVL	KTGSTGERLK	ESIQINSSLL	ALGNVISALG	DPQRRGSHIP	YRDSKITRIL	KDSLGGNAKT
330	340	350	360	370	380	390	400
VMIACVSPSS	SDFDETLNTL	NYASRAQNIR	NRATVNWRPE	AERPPEETAS	GARGPPRHRS	ETRIIHRGRR	APGPATASAA
410	420	430	440	450	460	470	480
AAMRLGAECA	RYRACTDAAY	SLLRELQAEP	GLPGAAARKV	RDWLCAVEGE	RSALSSASGP	DSGIESASVE	DQAAQGAGGR
490	500	510	520	530	540	550	560
KEDEGAQQLL	TLQNQVARLE	EENRDFLAAL	EDAMEQYKLQ	SDRLREQQEE	MVELRLRLEL	VRPGWGGPRL	LNGLPPGSFV
570	580	590	600	610	620	630	640
PRPHTAPLGG	AHAHVLGMVP	PACLPGDEVG	SEQRGEQVTN	GREAGAELLT	EVNRLGSGSS	AASEEEEEEE	EPPRRTLHLR
650	660	670	680	690	700	710	720
RNRISNCSQR	AGARPGSLPE	RKGPELCLEE	LDAAIPGSRA	VGGSKARVQA	RQVPPATASE	WRLAQAQQKI	RELAINIRMK
730	740	750	760	770	780	790	800
EELIGELVRT	GKAAQALNRQ	HSQRIRELEQ	EAEQVRAELS	EGQRQLRELE	GKELQDAGER	SRLQEFRRRV	AAAQSQVQVL
810	820	830	840	850	860	870	880
KEKKQATERL	VSLSAQSEKR	LQELERNVQL	MRQQQGQLQR	RLREETEQKR	RLEAEMSKRQ	HRVKELELKH	EQQQKILKIK
890	900	910	920	930	940	950	960
TEEIAAFQRK	RRSGSNGSVV	SLEQQQKIEE	QKKWLDQEME	KVLQQRRALE	ELGEELHKRE	AILAKKEALM	QEKTGLESKR
970	980	990	1000	1010	1020	1030	1040
LRSSQALNED	IVRVSSRLEH	LEKELSEKSG	QLRQGSAQSQ	QQIRGEIDSL	RQEKDSLLKQ	RLEIDGKLRQ	GSLLSPEEER
1050	1060	1070	1080	1090	1100	1110	1120
TLFQLDEAIE	ALDAAIEYKN	EAITCRQRVL	RASASLLSQC	EMNLMAKLSY	LSSSETRALL	CKYFDKVVTL	REEQHQQQIA
1130	1140	1150	1160	1170	1180	1190	1200
FSELEMQLEE	QQRLVYWLEV	ALERQRLEMD	RQLTLQQKEH	EQNMQLLLQQ	SRDHLGEGLA	DSRRQYEARI	QALEKELGRY
1210	1220	1230	1240	1250	1260	1270	1280
MWINQELKQK	LGGVNAVGHS	RGGEKRSLCS	EGRQAPGNED	ELHLAPELLW	LSPLTEGAPR	TREETRDLVH	APLPLTWKRS
1290	1300	1310	1320				
SLCGDSSTTP	ISGPGSEDLE	EPHAQGLLHT	TCN				

Cmpd.	No. of Cmpds.	m/z meas.	Δ m/z [ppm]	z	Rt [min]	Score	P	Range	Sequence	Modification
2376	1	847.3981	-71.01	2	60.2	11.0	2	1209-1225	K.QKLGGVNAVGHSRGGEK.R	

Detailed Protein Report

Protein 466: PREDICTED: thyroid receptor-interacting protein 11 isoform X2 [Homo sapiens]

**Accession:**	gi|530404931	**Score:**	22.9
**Database:**	refseq_human(refseq_human_20140103.fasta)	**MW [kDa]:**	177.3
**Database Date:**	2015-11-30	**pI:**	5.1
		**Sequence Coverage [%]:**	2.2
		**No. of unique Peptides:**	1

10	20	30	40	50	60	70	80
MSLLKLNNEY	EVIKSTATRD	ISLDSELHDL	RLNLEAKEQE	LNQSISEKET	LIAEIEELDR	QNQEATKHMI	LIKDQLSKQQ
90	100	110	120	130	140	150	160
NEGDSIISKL	KQDLNDEKKR	VHQLEDDKMD	ITKELDVQKE	KLIQSEVALN	DLHLTKQKLE	DKVENLVDQL	NKSQESNVSI
170	180	190	200	210	220	230	240
QKENLELKEH	IRQNEEELSR	IRNELMQSLN	QDSNSNFKDT	LLKEREAEVR	NLKQNLSELE	QLNENLKKVA	FDVKMENEKL
250	260	270	280	290	300	310	320
VLACEDVRHQ	LEECLAGNNQ	LSLEKNTIVE	TLKMEKGEIE	AELCWAKKRL	LEEANKYEKT	IEELSNARNL	NTSALQLEHE
330	340	350	360	370	380	390	400
HLIKLNQKKD	MEIAELKKNI	EQMDTDHKET	KDVLSSSLEE	QKQLTQLINK	KEIFIEKLKE	RSSKLQEELD	KYSQALRKNE
410	420	430	440	450	460	470	480
ILRQTIEEKD	RSLGSMKEEN	NHLQEELERL	REEQSRTAPV	ADPKTLDSVT	ELASEVSQLN	TIKEHLEEEI	KHHQKIIEDQ
490	500	510	520	530	540	550	560
NQSKMQLLQS	LQEQKKEMDE	FRYQHEQMNA	THTQLFLEKD	EEIKSLQKTI	EQIKTQLHEE	RQDIQTDNSD	IFQETKVQSL
570	580	590	600	610	620	630	640
NIENGSEKHD	LSKAETERLV	KGIKERELEI	KLLNEKNISL	TKQIDQLSKD	EVGKLTQIIQ	QKDLEIQALH	ARISSTSHTQ
650	660	670	680	690	700	710	720
DVVYLQQQLQ	AYAMEREKVF	AVLNEKTREN	SHLKTEYHKM	MDIVAAKEAA	LIKLQDENKK	LSTRFESSGQ	DMFRETIQNL
730	740	750	760	770	780	790	800
SRIIREKDIE	IDALSQKCQT	LLAVLQTSST	GNEAGGVNSN	QFEELLQERD	KLKQQVKKME	EWKQQVMTTV	QNMQHESAQL
810	820	830	840	850	860	870	880
QEELHQLQAQ	VLVDSDNNSK	LQVDYTGLIQ	SYEQNETKLK	NFGQELAQVQ	HSIGQLCNTK	DLLLGKLDII	SPQLSSASLL
890	900	910	920	930	940	950	960
TPQSAECLRA	SKSEVLSESS	ELLQQELEEL	RKSLQEKDAT	IRTLQENNHR	LSDSIAATSE	LERKEHEQTD	SEIKQLKEKQ
970	980	990	1000	1010	1020	1030	1040
DVLQKLLKEK	DLLIKAKSDQ	LLSSNENFTN	KVNENELLRQ	AVTNLKERIL	ILEMDIGKLK	GENEKIVETY	RGKETEYQAL
1050	1060	1070	1080	1090	1100	1110	1120
QETNMKFSMM	LREKEFECHS	MKEKALAFEQ	LLKEKEQGKT	GELNQLLNAV	KSMQEKTVVF	QQERDQVMLA	LKQKQMENTA
1130	1140	1150	1160	1170	1180	1190	1200
LQNEVQRLRD	KEFRSNQELE	RLRNHLLESE	DSYTREALAA	EDREAKLRKK	VTVLEEKLVS	SSNAMENASH	QASVQVESLQ
1210	1220	1230	1240	1250	1260	1270	1280
EQLNVVSKQR	DETALQLSVS	QEQVKQYALS	LANLQMVLEH	FQQEEKAMYS	AELEKQKQLI	AEWKKNAENL	EGKVISLQEC
1290	1300	1310	1320	1330	1340	1350	1360
LDEANAALDS	ASRLTEQLDV	KEEQIEELKR	QNELRQEMLD	DVQKKLMSLA	NSSEGKVDKV	LMRNLFIGHF	HTPKNQRHEV
1370	1380	1390	1400	1410	1420	1430	1440
LRLMGSILGV	RREEMEQLFH	DDQGGVTRWM	TGWLGGGSKS	VPNTPLRPNQ	QSVVNSSFSE	LFVKFLETES	HPSIPPPKLS
1450	1460	1470	1480	1490	1500	1510	1520
VHDMKPLDSP	GRRKRDTNAP	ESFKDTAESR	SGRRTDVNPF	LAPRSAAVPL	INPAGLGPGG	PGHLLLKPIS	DVLPTFTPLP
1530	1540						
ALPDNSAGVV	LKDLLKQ						

Cmpd.	No. of Cmpds.	m/z meas.	Δ m/z [ppm]	z	Rt [min]	Score	P	Range	Sequence	Modification
2902	1	836.7384	-37.57	3	65.4	10.0	2	1092-1112	K.SMQEKTVVFQQERDQVMLALK.Q	

Detailed Protein Report

Protein 467: cadherin-6 preproprotein [Homo sapiens]

**Accession:**	gi|4826673	**Score:**	22.9
**Database:**	refseq_human(refseq_human_20140103.fasta)	**MW [kDa]:**	88.3
**Database Date:**	2015-11-30	**pI:**	4.6
		**Sequence Coverage [%]:**	3.0
		**No. of unique Peptides:**	1

10	20	30	40	50	60	70	80
MRTYRYFLLL	FWVGQPYPTL	STPLSKRTSG	FPAKKRALEL	SGNSKNELNR	SKRSWMWNQF	FLLEEYTGSD	YQYVGKLHSD
90	100	110	120	130	140	150	160
QDRGDGSLKY	ILSGDGAGDL	FIINENTGDI	QATKRLDREE	KPVYILRAQA	INRRTGRPVE	PESEFIIKIH	DINDNEPIFT
170	180	190	200	210	220	230	240
KEVYTATVPE	MSDVGTFVVQ	VTATDADDPT	YGNSAKVVYS	ILQGQPYFSV	ESETGIIKTA	LLNMDRENRE	QYQVVIQAKD
250	260	270	280	290	300	310	320
MGGQMGGLSG	TTTVNITLTD	VNDNPPRFPQ	STYQFKTPES	SPPGTPIGRI	KASDADVGEN	AEIEYSITDG	EGLDMFDVIT
330	340	350	360	370	380	390	400
DQETQEGIIT	VKKLLDFEKK	KVYTLKVEAS	NPYVEPRFLY	LGPFKDSATV	RIVVEDVDEP	PVFSKLAYIL	QIREDAQINT
410	420	430	440	450	460	470	480
TIGSVTAQDP	DAARNPVKYS	VDRHTDMDRI	FNIDSGNGSI	FTSKLLDRET	LLWHNITVIA	TEINNPKQSS	RVPLYIKVLD
490	500	510	520	530	540	550	560
VNDNAPEFAE	FYETFVCEKA	KADQLIQTLH	AVDKDDPYSG	HQFSFSLAPE	AASGSNFTIQ	DNKDNTAGIL	TRKNGYNRHE
570	580	590	600	610	620	630	640
MSTYLLPVVI	SDNDYPVQSS	TGTVTVRVCA	CDHHGNMQSC	HAEALIHPTG	LSTGALVAIL	LCIVILLVTV	VLFAALRRQR
650	660	670	680	690	700	710	720
KKEPLIISKE	DIRDNIVSYN	DEGGGEEDTQ	AFDIGTLRNP	EAIEDNKLRR	DIVPEALFLP	RRTPTARDNT	DVRDFINQRL
730	740	750	760	770	780	790	800
KENDTDPTAP	PYDSLATYAY	EGTGSVADSL	SSLESVTTDA	DQDYDYLSDW	GPRFKKLADM	YGGVDSDKDS	

Cmpd.	No. of Cmpds.	m/z meas.	Δ m/z [ppm]	z	Rt [min]	Score	P	Range	Sequence	Modification
2713	2	701.4134	71.40	2	65.0	11.3	2	702-713	R.RTPTARDNTDVR.D	

Detailed Protein Report

Protein 468: PREDICTED: ubiquilin-4 isoform X3 [Homo sapiens]

**Accession:**	gi|530365094	**Score:**	22.9
**Database:**	refseq_human(refseq_human_20140103.fasta)	**MW [kDa]:**	45.6
**Database Date:**	2015-11-30	**pI:**	5.3
**Modification(s):**	Oxidation	**Sequence Coverage [%]:**	7.0
		**No. of unique Peptides:**	2

10	20	30	40	50	60	70	80
MAEPSGAETR	PPIRVTVKTP	KDKEEIVICD	RASVKEFKEE	ISRRFKAQQD	QLVLIFAGKI	LKDGDTLNQH	GIKDGLTVHL
**90**	**100**	**110**	**120**	**130**	**140**	**150**	**160**
VIKTPQKAQD	PAAATASSPS	TPDPASAPST	TPASPATPAQ	PSTSGSASSD	AGSGSRRSSG	GGPSPGAGEG	SPSATASILS
**170**	**180**	**190**	**200**	**210**	**220**	**230**	**240**
GFGGILGLGS	LGLGSANFME	LQQQMQRQLM	SNPEMLSQIM	ENPLVQDMMS	NPDLMRHMIM	ANPQMQQLME	RNPEISHMLN
**250**	**260**	**270**	**280**	**290**	**300**	**310**	**320**
NPELMRQTME	LARNPAMMQE	MMRNQDRALS	NLESIPGGYN	ALRRMYTDIQ	EPMFSAAREQ	FGNNPFSSLA	GNSDSSSSQP
**330**	**340**	**350**	**360**	**370**	**380**	**390**	**400**
LRTENREPLP	NPWSPSPPTS	QAPGSGGEGT	GGSGTSQVHP	TVSNPFGINA	ASLGSGMFNS	PEMQALLQQI	SENPQLMQNV
**410**	**420**	**430**					
ISAPYMRSMM	QTLAQNPDFA	AQAFRIL					

Cmpd.	No. of Cmpds.	m/z meas.	Δ m/z [ppm]	z	Rt [min]	Score	P	Range	Sequence	Modification
2774	2	937.4636	35.15	2	63.8	12.4	0	217-231	R.HMIMANPQMQQLMER.N	Oxidation: 13
1812	1	916.3298	-105.96	2	53.0	10.4	1	284-298	R.RMYTDIQEPMFSAAR.E	Oxidation: 2

Detailed Protein Report

Protein 469: septin-7 isoform 3 [Homo sapiens]

Accession: gi|339639597
Database: refseq_human(refseq_human_20140103.fasta)
Database Date: 2015-11-30

Score: 22.9
MW [kDa]: 46.5
pI: 9.1
Sequence Coverage [%]: 5.7
No. of unique Peptides: 2

Quantitation

QD:QU **Median:** 1.22 **CV:** 0.00 % **No. of Peptides:** 1

10	20	30	40	50	60	70	80
MSVSARSAAA	EERSVNSSTM	GESGLGKSTL	INSLFLTDLY	SPEYPGPSHR	IKKTVQVEQS	KVLIKEGGVQ	LLLTIVDTPG
90	100	110	120	130	140	150	160
FGDAVDNSNC	WQPVIDYIDS	KFEDYLNAES	RVNRRQMPDN	RVQCCLYFIA	PSGHGLKPLD	IEFMKRLHEK	VNIIPLIAKA
170	180	190	200	210	220	230	240
DTLTPEECQQ	FKKQIMKEIQ	EHKIKIYEFP	ETDDEEENKL	VKKIKDRLPL	AVVGSNTIIE	VNGKRVRGRQ	YPWGVAEVEN
250	260	270	280	290	300	310	320
GEHCDFTILR	NMLIRTHMQD	LKDVTNNVHY	ENYRSRKLAA	VTYNGVDNNK	NKGQLTKSPL	AQMEEERREH	VAKMKKMEME
330	340	350	360	370	380	390	400
MEQVFEMKVK	EKVQKLKDSE	AELQRRHEQM	KKNLEAQHKE	LEEKRRQFED	EKANWEAQQR	ILEQQNSSRT	LEKNKKKGKI
410							
F							

Cmpd.	No. of Cmpds.	m/z meas.	Δ m/z [ppm]	z	Rt [min]	Score	P	Range	Sequence	Modification	Ratios
1252	1	918.2353	-276.27	1	45.8	10.9	0	54-61	K.TVQVEQSK.V		
150	1	894.4204	-71.26	2	31.8	11.9	2	381-395	R.ILEQQNSSRTLEKNK.K		QD:QU 1.22

Detailed Protein Report

Protein 470: **PREDICTED: histone-lysine N-methyltransferase EHMT1 isoform X7 [Homo sapiens]**

**Accession:**	gi|530426806	**Score:**	22.8
**Database:**	refseq_human(refseq_human_20140103.fasta)	**MW [kDa]:**	99.7
**Database Date:**	2015-11-30	**pI:**	5.5
**Modification(s):**	Carbamidomethyl	**Sequence Coverage [%]:**	3.5
		**No. of unique Peptides:**	1

10	20	30	40	50	60	70	80
MAAADAEAVP	ARGEPQQDCC	VKTELLGEET	PMAADEGSAE	KQAGEAHMAA	DGETNGSCEN	SDASSHANAA	KHTQDSARVN
**90**	**100**	**110**	**120**	**130**	**140**	**150**	**160**
PQDGTNTLTR	IAENGVSERD	SEAAKQNHVT	ADDFVQTSVI	GSNGYILNKP	ALQAQPLRTT	STLASSLPGH	AAKTLPGGAG
**170**	**180**	**190**	**200**	**210**	**220**	**230**	**240**
KGRTPSAFPQ	TPAAPPATLG	EGSADTEDRK	LPAPGADVKV	HRARKTMPKS	VVGLHAASKD	PREVREARDH	KEPKEEINKN
**250**	**260**	**270**	**280**	**290**	**300**	**310**	**320**
ISDFGRQQLL	PPFPSLHQSL	PQNQCYMATT	KSQTACLPFV	LAAAVSRKKK	RRMGTYSLVP	KKKTKVLKQR	TVIEMFKSIT
**330**	**340**	**350**	**360**	**370**	**380**	**390**	**400**
HSTVGSKGEK	DLGASSLHVN	GESLEMDSDE	DDSEELEEDD	GHGAEQAAAF	PTEDSRTSKE	SMSEADRAQK	MDGESEEEQE
**410**	**420**	**430**	**440**	**450**	**460**	**470**	**480**
SVDTGEEEEG	GDESDLSSES	SIKKKFLKRK	GKTDSPWIKP	ARKRRRRSRK	KPSGALGSES	YKSSAGSAEQ	TAPGDSTGYM
**490**	**500**	**510**	**520**	**530**	**540**	**550**	**560**
EVSLDSLDLR	VKGILSSQAE	GLANGPDVLE	TDGLQEVPLC	SCRMETPKSR	EITTLANNQC	MATESVDHEL	GRCTNSVVKY
**570**	**580**	**590**	**600**	**610**	**620**	**630**	**640**
ELMRPSNKAP	LLVLCEDHRG	RMVKHQCCPG	CGYFCTAGNF	MECQPESSIS	HRFHKDCASR	VNNASYCPHC	GEESSKAKEV
**650**	**660**	**670**	**680**	**690**	**700**	**710**	**720**
TIAKADTTST	VTPVPGQEKG	SALEGRADTT	TGSAAGPPLS	EDDKLQGAAS	HVPEGFDPTG	PAGLGRPTPG	LSQGPGKETL
**730**	**740**	**750**	**760**	**770**	**780**	**790**	**800**
ESALIALDSE	KPKKLRFHPK	QLYFSARQGE	LQKVLLMLVD	GIDPNFKMEH	QNKRSPLHAA	AEAGHVDICH	MLVQAGANID
**810**	**820**	**830**	**840**	**850**	**860**	**870**	**880**
TCSEDQRTPL	MEAAAENNHLE	AVKYLIKAGA	LVDPKDAEGS	TCLHLAAKKG	HYEVVQYLLS	NGQMDVNCQD	DGGWTPMIWA
**890**	**900**	**910**	**920**	**930**			
TEYKHVDLVK	LLLSKGSDIN	IRDNDDGTMK	RGALSRQKEE	KREAEEP			

Cmpd.	No. of Cmpds.	m/z meas.	Δ m/z [ppm]	z	Rt [min]	Score	P	Range	Sequence	Modification
1764	1	990.9536	23.24	2	50.6	10.5	1	621-638	R.VNNASYCPHCGEESSKAK.E	Carbamidomethyl: 7

Detailed Protein Report

Protein 471: otogelin precursor [Homo sapiens]

**Accession:**	gi|471270262	**Score:**	22.8
**Database:**	refseq_human(refseq_human_20140103.fasta)	**MW [kDa]:**	314.6
**Database Date:**	2015-11-30	**pI:**	5.5
**Modification(s):**	Carbamidomethyl	**Sequence Coverage [%]:**	1.1
		**No. of unique Peptides:**	1

Detailed Protein Report

10	20	30	40	50	60	70	80
MGVLASALCW	LLCVWLPWGE	QAAESLRVQR	LGERVVDSGR	SGARGMRNVK	GMRNGPAQTR	VSSSSSHQEA	TLAMGDKATV
90	100	110	120	130	140	150	160
VGGQQAEAPD	SVAMSSWERR	LHRAKCAPSY	LFSCFNGGEC	VHPAFCDCRR	FNATGPRCQM	VYNAGPERDS	ICRAWGQHHV
170	180	190	200	210	220	230	240
ETFDGLYYYL	SGKGSYTLVG	RHEPEGQSFS	IQVHNDPQCG	SSPYTCSRAV	SLFFVGEQEI	HLAKEVTHGG	MRVQLPHVMG
250	260	270	280	290	300	310	320
SARLQQLAGY	VIVRHQSAFT	LAWDGASAVY	IKMSPELLGW	THGLCGNNNA	DPKDDLVTSS	GKLTDDVVEF	VHSWQEQAPN
330	340	350	360	370	380	390	400
QPPGPTTSSL	PRPPCLQQNP	GTMQGVYEQC	EALLRPPFDA	CHAYVSPLPF	TASCTSDLCQ	SMGDVATWCR	ALAEYARACA
410	420	430	440	450	460	470	480
QAGRPLQGWR	TQLRQCTVHC	KEKAFTYNEC	IACCPASCHP	RASCVDSEIA	CVDGCYCPNG	LIFEDGGCVA	PAECPCEFHG
490	500	510	520	530	540	550	560
TLYPPGSVVK	EDCNTCTCTS	GKWECSTAVC	PAECSVTGDI	HFTTFDGRRY	TFPATCQYIL	AKSRSSGTFT	VTLQNAPCGL
570	580	590	600	610	620	630	640
NQDGACVQSV	SVILHQDPRR	QVTLTQAGDV	LLFDQYKIIP	PYTDDAFEIR	RLSSVFLRVR	TNVGVRVLYD	REGLRLYLQV
650	660	670	680	690	700	710	720
DQRWVEDTVG	LCGTFNGNTQ	DDFLSPVGVP	ESTPQLFGNS	WKTLSACSPL	VSGSPLDPCD	VHLQAASYSV	QACSVLTGEM
730	740	750	760	770	780	790	800
FAPCSAFLSP	VPYFEQCRRD	ACRCGQPCLC	ATLAHYAHLC	RRHGLPVDFR	ARLPACALSC	EASKEYSPCV	APCGRTCQDL
810	820	830	840	850	860	870	880
ASPEACGVDG	GDDLSRDECV	EGCACPPDTY	LDTQADLCVP	RNQCSCHFQG	VDYPPGDSDI	PSLGHCHCKD	GVMSCDSRAP
890	900	910	920	930	940	950	960
AAACPAGQVF	VNCSDLHTDL	ELSRERTCEQ	QLLNLSVSAR	GPCLSGCACP	QGLLRHGDAC	FLPEECPCTW	KGKEYFPGDQ
970	980	990	1000	1010	1020	1030	1040
VMSPCHTCVC	QRGSFQCTLH	PCASTCTAYG	DRHYRTFDGL	PFDFVGACKV	HLVKSTSDVS	FSVIVENVNC	YSSGMICRKF
1050	1060	1070	1080	1090	1100	1110	1120
ISINVGNSLI	VFDDDSGNPS	PESFLDDKQE	VHTWRVGFFT	LVHFPQEHIT	LLWDQRTTVH	VQAGPQWQGQ	LAGLCGNFDL
1130	1140	1150	1160	1170	1180	1190	1200
KTINEMRTPE	NLELTNPQEF	GSSWAAVECP	DTLDPRDMCV	LNPLREPFAK	KECSILLSEV	FEICHPVVDV	TWFYSNCLTD
1210	1220	1230	1240	1250	1260	1270	1280
TCGCSQGGDC	ECFCASVSAY	AHQCCQHGVA	VDWRTPRLCP	YDCDFFNKVL	GKGPYQLSSL	AAGGALVGMK	AVGDDIVLVR
1290	1300	1310	1320	1330	1340	1350	1360
TEDVAPADIV	SFLLTAALYK	AKAHDPDVVS	LEAADRPNFF	LHVTANGSLE	LAKWQGRDTF	QQHASFLLHR	GTRQAGLVAL
1370	1380	1390	1400	1410	1420	1430	1440
ESLAKPSSFL	YVSGAVLALR	LYEHTEVFRR	GTLFRLLDAK	PSGAAYPICE	WRYDACASPC	FQTCRDPRAA	SCRDVPRVEG
1450	1460	1470	1480	1490	1500	1510	1520
CVPVCPTPQV	LDEVTQRCVY	LEDCVEPAVW	VPTEALGNET	LPPSQGLPTP	SDEEPQLSQE	SPRTPTHRPA	LTPAAPLTTA
1530	1540	1550	1560	1570	1580	1590	1600
LNPPVTATEE	PVVSPGPTQT	TLQQPLELTA	SQLPAGPTES	PASKGVTASL	LAIPHTPESS	SLPVALQTPT	PGMVSGAMET
1610	1620	1630	1640	1650	1660	1670	1680
TRVTVIFAGS	PNITVSSRSP	PAPRFPLMTK	AVTVRGHGSL	PVRTTPPQPS	LTASPSSRPV	ASPGAISRSP	TSSGSHKAVL
1690	1700	1710	1720	1730	1740	1750	1760
TPAVTKVISR	TGVPQPTQAQ	SASSPSTPLT	VAGTAAEQVP	VSPLATRSLE	IVLSTEKGEA	GHSQPMGSPA	SPQPHPLPSA
1770	1780	1790	1800	1810	1820	1830	1840
PPRPAQHTTM	ATRSPALPPE	TPAAASLSTA	TDGLAATPFM	SLESTRPSQL	LSGLPPDTSL	PLAKVGTSAP	VATPGPKASV
1850	1860	1870	1880	1890	1900	1910	1920
ITTPLQPQAT	TLPAQTLSPV	LPFTPAAMTQ	AHPPTHIAPP	AAGTAPGLLL	GATLPTSGVL	PVAEGTASMV	SVVPRKSTTG
1930	1940	1950	1960	1970	1980	1990	2000
KVAILSKQVS	LPTSMYGSAE	GGPTELTPAT	SHPLTPLVAE	PEGAQAGTAL	PVPTSYALSR	VSARTAPQDS	MLVLLPQLAE
2010	2020	2030	2040	2050	2060	2070	2080
AHGTSAGPHL	AAEPVDEATT	EPSGRSAPAL	SIVEGLAEAL	ATTTEANTST	TCVPIAEQDC	VRHICLEGQL	IRVNQSQHCP
2090	2100	2110	2120	2130	2140	2150	2160
QGAAPPRCGI	LGLAVRVGGD	RCCPLWECAC	RCSIFPDLSF	VTFDGSHVAL	FKEAIYILSQ	SPDEMLTVHV	LDCKSANLGH
2170	2180	2190	2200	2210	2220	2230	2240

Detailed Protein Report

Cmpd.	No. of Cmpds.	m/z meas.	Δ m/z [ppm]	z	Rt [min]	Score	P	Range	Sequence	Modification
2044	1	1135.9200	78.97	3	54.1	22.8	1	921-951	R.GPCLSGCACPQGLLRHGDACFLPEECPCTWK.G	Carbamidomethyl: 7, 9

Detailed Protein Report

Protein 472: round spermatid basic protein 1-like protein [Homo sapiens]

**Accession:**	gi|158534055	**Score:**	22.8
**Database:**	refseq_human(refseq_human_20140103.fasta)	**MW [kDa]:**	94.8
**Database Date:**	2015-11-30	**pI:**	9.6
		**Sequence Coverage [%]:**	1.2
		**No. of unique Peptides:**	1

10	20	30	40	50	60	70	80
MAEPPSPVHC	VAAAAPTATV	SEKEPFGKLQ	LSSRDPPGSL	SAKKVRTEEK	KAPRRVNGEG	GSGGNSRQLQ	PPAAPSPQSY
**90**	**100**	**110**	**120**	**130**	**140**	**150**	**160**
GSPASWSFAP	LSAAPSPSSS	RSSFSFSAGT	AVPSSASASL	SQPVPRKLLV	PPTLLHAQPH	HLLLPAAAAA	ASANAKSRRP
**170**	**180**	**190**	**200**	**210**	**220**	**230**	**240**
KEKREKERRR	HGLGGAREAG	GASREENGEV	KPLPRDKIKD	KIKERDKEKE	REKKKHKVMN	EIKKENGEVK	ILLKSGKEKP
**250**	**260**	**270**	**280**	**290**	**300**	**310**	**320**
KTNIEDLQIK	KVKKKKKKKH	KENEKRKRPK	MYSKSIQTIC	SGLLTDVEDQ	AAKGILNDNI	KDYVGKNLDT	KNYDSKIPEN
**330**	**340**	**350**	**360**	**370**	**380**	**390**	**400**
SEFPFVSLKE	PRVQNNLKRL	DTLEFKQLIH	IEHQPNGGAS	VIHAYSNELS	HLSPMEMERF	AEEFVGLVFS	ENENSAAFYV
**410**	**420**	**430**	**440**	**450**	**460**	**470**	**480**
MGIVHGAATY	LPDFLDYFSF	NFPNSPVKME	ILGKKDIETT	TMSNFHAQVK	RTYSHGTYRA	GPMRQISLVG	AVDEEVGDYF
**490**	**500**	**510**	**520**	**530**	**540**	**550**	**560**
PEFLDMLEES	PFLKCTLPWG	TLSSLKLQSR	KDSDDGPIMW	VRPGEQMIPV	ADMPKSPFKR	KRTTNEIKNL	QYLPRTSEPR
**570**	**580**	**590**	**600**	**610**	**620**	**630**	**640**
EMLFEDRTRA	HADHIGQGFE	RQTTAAVGVL	KAVHCGEWPD	QPRITKDVIC	FHAEDFLEVV	QRMQLDLHEP	PLSQCVQWVD
**650**	**660**	**670**	**680**	**690**	**700**	**710**	**720**
DAKLNQLRRE	GIRYARIQLY	DNDIYFIPRN	VVHQFKTVSA	VCSLAWHIRL	KLYHSEEDTS	QNTATHETGT	SSDSTSSVLG
**730**	**740**	**750**	**760**	**770**	**780**	**790**	**800**
PHTDNMICAV	SKASLDSVFS	DKLHSKYELQ	QIKHEPIASV	RIKEEPVNVN	IPEKTTALNN	MDGKNVKAKL	DHVQFAEFKI
**810**	**820**	**830**	**840**	**850**			
DMDSKFENSN	KDLKEELCPG	NLSLVDTRQH	SSAHSNQDKK	DDDILC			

Cmpd.	No. of Cmpds.	m/z meas.	Δ m/z [ppm]	z	Rt [min]	Score	P	Range	Sequence	Modification
1767	4	627.2880	-110.44	2	50.6	22.8	2	330-339	K.EPRVQNNLKR.L	

Detailed Protein Report

Protein 473: **PREDICTED: arf-GAP with GTPase, ANK repeat and PH domain-containing protein 1 isoform X8 [Homo sapiens]**

**Accession:**	gi|578803598	**Score:**	22.8
**Database:**	refseq_human(refseq_human_20140103.fasta)	**MW [kDa]:**	85.9
**Database Date:**	2015-11-30	**pI:**	9.8
		**Sequence Coverage [%]:**	3.7
		**No. of unique Peptides:**	1

10	20	30	40	50	60	70	80
MDAGGRFKKE	IVVDGQSYLL	LIRDEGGPPE	AQFAMWVDAV	IFVFSLEDEI	SFQTVYHYYS	RMANYRNTSE	IPLVLVGTQD
**90**	**100**	**110**	**120**	**130**	**140**	**150**	**160**
AISSANPRVI	DDARARKLSN	DLKRCTYYET	CATYGLNVER	VFQDVAQKIV	ATRKKQQLSI	GPCKSLPNSP	SHSSVCSAQV
**170**	**180**	**190**	**200**	**210**	**220**	**230**	**240**
SAVHISQTSN	GGGSLSDYSS	SVPSTPSTSQ	KELRIDVPPT	ANTPTPVRKQ	SKRRSNLFTS	RKGSDPDKEK	KGLESRADSI
**250**	**260**	**270**	**280**	**290**	**300**	**310**	**320**
GSGRAIPIKQ	GMLLKRSGKS	LNKEWKKKYV	TLCDNGVLTY	HPSLHDYMQN	VHGKEIDLLR	TTVKVPGKRP	PRATSACAPI
**330**	**340**	**350**	**360**	**370**	**380**	**390**	**400**
SSPKTNGLSK	DMSSLHISPN	SGNVTSASGS	QMASGISLVS	FNSRPDGMHQ	RSYSVSSADQ	WSEATVIANS	AISSDTGLGD
**410**	**420**	**430**	**440**	**450**	**460**	**470**	**480**
SVCSSPSISS	TTSPKLDPPP	SPHANRKKHR	RKKSTSNFKA	DGLSGTAEAK	RKAWKLNRVG	SLRNIYSSST	NTEEQEENFE
**490**	**500**	**510**	**520**	**530**	**540**	**550**	**560**
FIIVSLTGQT	WHFEATTYEE	RDAWVQAIES	QILASLQSCE	SSKNKSRLTS	QSEAMALQSI	RNMRGNSHCV	DCETQNPNWA
**570**	**580**	**590**	**600**	**610**	**620**	**630**	**640**
SLNLGALMCI	ECSGIHRNLG	THLSRVRSLD	LDDWPVELIK	VMSSIGNELA	NSVWEESSQG	RTKPSVDSTR	EEKERWIRAK
**650**	**660**	**670**	**680**	**690**	**700**	**710**	**720**
YEQKLFLAPL	PCTELSLGQH	LLRATADEDL	RTAILLLAHG	SRDEVNETCG	EGDGRTALHL	ACRKGNVVLA	QLLIWYGVDV
**730**	**740**	**750**	**760**	**770**	**780**	**790**	
TARDAHGNTA	LAYARQASSQ	ECIDVLLQYG	CPDERFVLMA	TPNLSRRNNN	RNNSSGRVPT	II	

Cmpd.	No. of Cmpds.	m/z meas.	Δ m/z [ppm]	z	Rt [min]	Score	P	Range	Sequence	Modification
139	1	768.0380	181.88	2	30.5	12.7	0	528-541	R.LTSQSEAMALQSIR.N	

Detailed Protein Report

Protein 474: PREDICTED: LOW QUALITY PROTEIN: hemicentin-2 [Homo sapiens]

**Accession:**	gi|578818143	**Score:**	22.7
**Database:**	refseq_human(refseq_human_20140103.fasta)	**MW [kDa]:**	546.0
**Database Date:**	2015-11-30	**pI:**	5.5
		**Sequence Coverage [%]:**	1.0
		**No. of unique Peptides:**	1

BRUKER

Detailed Protein Report

10	20	30	40	50	60	70	80
MMPGAPLLRL	LTAVSAAVAV	AVAGAPGTVM	PPTTGDATLA	FVFDVTGSMW	DELMQVIDGA	SRILERSLSR	RSQAIANYAL
90	100	110	120	130	140	150	160
VPFHDPDIGP	VTLTADPTVF	QRELRELYVQ	GGGDCPEMSV	GAIKAAVEVA	NPGSFIYVFS	DARAKDYHKK	EELLRLLQLK
170	180	190	200	210	220	230	240
QSQVVFVLTG	DCGDRTHPGY	LAYEEIAATS	SGQVFHLDKQ	QVTEVLKWVE	SAIQASKVHL	LSTDHEEEGE	HTWRLPFDPS
250	260	270	280	290	300	310	320
LKEVTISLSG	PGPEIEVQDP	LGRILQEDEG	LNVLLNIPDS	AKVVAFKPEH	PGLWSIKVYS	SGRHSVRITG	VSNIDFRAGF
330	340	350	360	370	380	390	400
STQPLLDLNH	TLEWPLQGVP	ISLVINSTGL	KAPGRLDSVE	LAQSSGKPLL	TLPTKPLSNG	STHQLWGGPP	FHTPKERFYL
410	420	430	440	450	460	470	480
KVKGKDHEGN	PLLRVSGVSY	SGVAPGAPLV	SMAPRIHGYL	HQPLLVSCSV	HSALPFRLQL	RRGEARLGEE	RHFQESGNSS
490	500	510	520	530	540	550	560
WEILRASKAE	EGTYECTAVS	RAGTGRAKAQ	IVVTDPPPQL	VPAPNVTVSP	GETAVLSCRV	LGEAPYNLTW	VRDWRVLPAS
570	580	590	600	610	620	630	640
TGRVAQLADL	SLEISGIIPT	DGGRYQCVAS	NANGVTRASV	WLLVREAPQV	SIHTSSQHFS	QGVEVKVSCS	ASGYPTPHIS
650	660	670	680	690	700	710	720
WSRESQALQE	DSRIHVDAQG	TLIIQGVAPE	DAGNYSCQAT	NEVGTDQETV	TLYYTDPPSV	SAVNAVVLVA	VGEEAVLVCE
730	740	750	760	770	780	790	800
ASGVPPPRVI	WYRGGLEMIL	APEGSSSGKL	RIPAAQERDA	GTYTCRAVNE	LGDASAEIQL	AVGHAPQLTE	LPRDVTVELG
810	820	830	840	850	860	870	880
RSALLACRAT	GRPPPTVTWR	RGDGQPLGLR	LGAGRGSRSR	QPDSGVLFFE	SVAPEDQAPY	VCEARNVFGK	VQAEARLIVT
890	900	910	920	930	940	950	960
GHAPPQIASS	APTVRVLEGQ	PVSLPCIVLA	GRPLPERHWL	KDGRPLPPGS	RHSIRADGSL	HLDRALQEHA	GRYSCVATNT
970	980	990	1000	1010	1020	1030	1040
AGSQHRDVEL	VVQVPPRIHP	TATHHITNEG	VAASLPCVAS	GVPAPTITWT	KETNALTSRG	PHYNVSKEGT	LLIAQPSAQD
1050	1060	1070	1080	1090	1100	1110	1120
AGAYVCTATN	TVGFSSQEMR	LSVNTKPRIH	MNGSRNADVP	LQVTAKAGEE	VTLDCEAKGS	PPPLVTWTKD	SRPVPPITNR
1130	1140	1150	1160	1170	1180	1190	1200
YGLLPSGSLR	LAQVQVGDSG	HYECTASNPA	GSASHRYVLG	VQVPPQVQPG	PRVLKVLVGE	ALDLNCVAEG	NPEPQLSWSK
1210	1220	1230	1240	1250	1260	1270	1280
DGVVLQGRGP	QGSVHFAAIR	TSDAGRYRCE	ASNSAGVDAW	EVELRVLEPP	HWGADETSGL	LERVAGENAS	LPCPARGTPK
1290	1300	1310	1320	1330	1340	1350	1360
PQVTWRKGPS	SEPLHGQPGV	AVLEEGSLFL	ASVSPADSGD	YECQATNEVG	STSRRAKLVV	YVPPSIREDG	RKANVSGMAG
1370	1380	1390	1400	1410	1420	1430	1440
QSLTLECDAN	GFPVPEIVWL	KDAQLIPKVG	GHRLLDEGQS	LHFPRIQEGD	SGLYSCRAEN	QAGTAQRDFH	LLVLTPPSVL
1450	1460	1470	1480	1490	1500	1510	1520
GAGAAQEVLG	LAGADVELQC	WTSGVPTPQV	EWTKDRQPVL	PGGPHLQVQE	DGQVLRITGS	HVGDEGRYQC	VAFSPAGQQA
1530	1540	1550	1560	1570	1580	1590	1600
RDFQLRVHAP	PTIWGSNETG	EVAVMEDHLV	QLLCEARGVP	TPNITWFKDG	ALLPTSTKVV	YTRGGRQLQL	GRAQSSDAGV
1610	1620	1630	1640	1650	1660	1670	1680
YTCKASNAVG	AAEKATRLDV	YVPPTIEGAG	GRPYVVKAVA	GRPVALECVA	RGHPSPTLSW	HHEGLPVAES	NESRLETDGS
1690	1700	1710	1720	1730	1740	1750	1760
VLRLESPGEA	SSGLYSCVAS	SPAGEAVLQY	SVEVQVPPQL	LVAEGLGQVT	TIVGQPLELP	CQASGSPVPT	IQWLQNGRPA
1770	1780	1790	1800	1810	1820	1830	1840
EELAGVQVAS	QGTTLHIDHV	ELDHSGLFAC	QATNEAGTAG	AEVEVSVHGE	WAPGVLEVPI	GQYHWGXEHH	SSFPAACDPP
1850	1860	1870	1880	1890	1900	1910	1920
VHRGWGAHPK	PPLVEGWCSP	GSLWGEPTGI	PVAYSLPPPL	QIEKVDLRDE	GIYTCAATNL	AGESKREVAL	KVLVPPNIEP
1930	1940	1950	1960	1970	1980	1990	2000
GPVNKAVLEN	ASVTLECLAS	GVPPPDVSWF	KGHQPVSSWM	GVTVSVDGRV	LRIEQAQLSD	AGSYRCVASN	VAGSTELRYG
2010	2020	2030	2040	2050	2060	2070	2080
LRVNVPPRIT	LPPSLPGPVL	VNTPVRLTCN	ATGAPSPTLM	WLKDGNPVSP	AGTPGLQVFP	GGRVLTLASA	RASDSGRYSC
2090	2100	2110	2120	2130	2140	2150	2160
VAVSAVGEDR	QDVVLQVHMP	PSILGEELNV	SVVANESVAL	ECQSHAMPPP	VLSWWKDGRP	LEPRPGVHLS	ADKALLQVDR
2170	2180	2190	2200	2210	2220	2230	2240

Detailed Protein Report

Cmpd.	No. of Cmpds.	m/z meas.	Δ m/z [ppm]	z	Rt [min]	Score	P	Range	Sequence	Modification
61	1	623.8088	95.46	5	29.5	12.6	2	866-895	R.NVFGKVQAEARLIVTGHAPPQIASSAPTVR.V	

Detailed Protein Report

Protein 475: rap1 GTPase-activating protein 1 isoform c [Homo sapiens]

**Accession:**	gi|224809582	**Score:**	22.7
**Database:**	refseq_human(refseq_human_20140103.fasta)	**MW [kDa]:**	73.3
**Database Date:**	2015-11-30	**pI:**	5.5
		**Sequence Coverage [%]:**	4.7
		**No. of unique Peptides:**	1

10	20	30	40	50	60	70	80
MIEKMQGSRM	DEQRCSFPPP	LKTEEDYIPY	PSVHEVLGRE	GPFPLILLPQ	FGGYWIEGTN	HEITSIPETE	PLQSPTTKVK
**90**	**100**	**110**	**120**	**130**	**140**	**150**	**160**
LECNPTARIY	RKHFLGKEHF	NYYSLDAALG	HLVFSLKYDV	IGDQEHLRLL	LRTKCRTYHD	VIPISCLTEF	PNVVQMAKLV
**170**	**180**	**190**	**200**	**210**	**220**	**230**	**240**
CEDVNVDRFY	PVLYPKASRL	IVTFDEHVIS	NNFKFGVIYQ	KLGQTSEEEL	FSTNEESPAF	VEFLEFLGQK	VKLQDFKGFR
**250**	**260**	**270**	**280**	**290**	**300**	**310**	**320**
GGLDVTHGQT	GTESVYCNFR	NKEIMFHVST	KLPYTEGDAQ	QLQRKRHIGN	DIVAVVFQDE	NTPFVPDMIA	SNFLHAYVVV
**330**	**340**	**350**	**360**	**370**	**380**	**390**	**400**
QAEGGGPDGP	LYKVSVTARD	DVPFFGPPLP	DPAVFRKGPE	FQEFLLTKLI	NAEYACYKAE	KFAKLEERTR	AALLETLYEE
**410**	**420**	**430**	**440**	**450**	**460**	**470**	**480**
LHIHSQSMMG	LGGDEDKMEN	GSGGGGFFES	FKRVIRSRSQ	SMDAMGLSNK	KPNTVSTSHS	GSFAPNNPDL	AKAAGISLIV
**490**	**500**	**510**	**520**	**530**	**540**	**550**	**560**
PGKSPTRKKS	GPFGSRRSSA	IGIENIQEVQ	EKRESPPAGQ	KTPDSGHVSQ	EPKSENSSTQ	SSPEMPTTKN	RAETAAQRAE
**570**	**580**	**590**	**600**	**610**	**620**	**630**	**640**
ALKDFSRSSS	SASSFASVVE	ETEGVDGEDT	GLESVSSSGT	PHKRDSFIYS	TWLEDSVSTT	SGGSSPGPSR	SPHPDAGKLG
**650**	**660**	**670**					
DPACPEIKIQ	LEASEQHMPQ	LGC					

Cmpd.	No. of Cmpds.	m/z meas.	Δ m/z [ppm]	z	Rt [min]	Score	P	Range	Sequence	Modification
2007	1	660.8874	-123.62	3	53.6	11.5	1	534-551	K.SENSSTQSSPEMPTTKNR.A	

Detailed Protein Report

Protein 476: PREDICTED: protein aurora borealis isoform X1 [Homo sapiens]

**Accession:**	gi|578825016	**Score:**	22.7
**Database:**	refseq_human(refseq_human_20140103.fasta)	**MW [kDa]:**	55.2
**Database Date:**	2015-11-30	**pI:**	4.6
**Modification(s):**	Oxidation	**Sequence Coverage [%]:**	5.5
		**No. of unique Peptides:**	1

10	20	30	40	50	60	70	80
MGDVKESKMQ	ITPETPGRIP	VLNPFESPSD	YSNLHEQTLA	SPSVFKSTKL	PFFTKDVIVP	SPWTDHEGKQ	LSQCHSSKCT
**90**	**100**	**110**	**120**	**130**	**140**	**150**	**160**
NINSDSPVGK	KLTIHSEKSD	AACQTLLSLP	VDFNLENILG	DYFRADEFAD	QSPGNLSSSS	LRRKLFLDGN	GSISDSLPSA
**170**	**180**	**190**	**200**	**210**	**220**	**230**	**240**
SPGSPHSGVQ	TSLEMFYSID	LSPVKCRSPL	QTPSSGQFSS	SPIQASAKKY	SLGSITSPSP	ISSPTFSPIE	FQIGETPLSE
**250**	**260**	**270**	**280**	**290**	**300**	**310**	**320**
QRKFTVHSPD	ASSGTNSNGI	TNPCIRSPYI	DGCSPIKNWS	PMRLQMYSGG	TQYRTSVIQI	PFTLETQGED	EEDKENIPST
**330**	**340**	**350**	**360**	**370**	**380**	**390**	**400**
DVSSPAMDAA	GIHLRQFSNE	ASTHGTHLVV	TAMSVTQNQS	SASEKELALL	QDVEREKDNN	TVDMVDPIEI	ADETTWIKEP
**410**	**420**	**430**	**440**	**450**	**460**	**470**	**480**
VDNGSLPMTD	FVSGIAFSIE	NSHMCMSPLA	ESSVIPCESS	NIQMDSGYNT	QNCGSNIMDT	VGAESYCKES	DAQTCEVESK
**490**	**500**	**510**					
SQAFNMKQDH	TTQRCWMKTA	SPFQCSSP					

Cmpd.	No. of Cmpds.	m/z meas.	Δ m/z [ppm]	z	Rt [min]	Score	P	Range	Sequence	Modification
1061	1	660.1801	-190.79	2	41.8	10.0	0	284-294	R.LQMYSGGTQYR.T	Oxidation: 3

Detailed Protein Report

Protein 477: E3 ubiquitin-protein ligase listerin [Homo sapiens]

**Accession:**	gi|231573214	**Score:**	22.7
**Database:**	refseq_human(refseq_human_20140103.fasta)	**MW [kDa]:**	205.0
**Database Date:**	2015-11-30	**pI:**	6.1
		**Sequence Coverage [%]:**	1.4
		**No. of unique Peptides:**	1

10	20	30	40	50	60	70	80
MDRVGGGKGP	GGSYYRVSSS	AARSRKLCPG	RVNRGLSAQS	TAATATMGGK	NKQRTKGNLR	PSNSGRAAEL	LAKEQGTVPG
90	100	110	120	130	140	150	160
FIGFGTSQSD	LGYVPAIQGA	EEIDSLVDSD	FRMVLRKLSK	KDVTTKLKAM	QEFGTMCTER	DTETVKGVLP	YWPRIFCKIS
170	180	190	200	210	220	230	240
LDHDRRVREA	TQQAFEKLIL	KVKKQLAPYL	KSLMGYWLMA	QCDTYTPAAF	AAKDAFEAAF	PPSKQPEAIA	FCKDEITSVL
250	260	270	280	290	300	310	320
QDHLIKETPD	TLSDPQTVPE	EEREAKFYRV	VTCSLLALKR	LLCLLPDNEL	DSLEEKFKSL	LSQNKFWKYG	KHSVPQIRSA
330	340	350	360	370	380	390	400
YFELVSALCQ	RIPQLMKEEA	SKVSPSVLLS	IDDSDPIVCP	ALWEAVLYTL	TTIEDCWLHV	NAKKSVFPKL	STVIREGGRG
410	420	430	440	450	460	470	480
LATVIYPYLL	PFISKLPQSI	TNPKLDFFKN	FLTSLVAGLS	TERTKTSSLE	SSAVISAFFE	CLRFIMQQNL	GEEEIEQMLV
490	500	510	520	530	540	550	560
NDQLIPFIDA	VLKDPGLQHG	QLFNHLAETL	SSWEAKADTE	KDEKTAHNLE	NVLIHFWERL	SEICVAKISE	PEADVESVLG
570	580	590	600	610	620	630	640
VSNLLQVLQK	PKSSLKSSKK	KNGKVRFADE	ILESNKENEK	CVSSEGEKIE	GWELTTEPSL	THNSSGLLSP	LRKKPLEDLV
650	660	670	680	690	700	710	720
CKLADISINY	VNERKSEQHL	RFLSTLLDSF	SSSRVFKMLL	GDEKQSIVQA	KPLEIAKLVQ	KNPAVQFLYQ	KLIGWLNEDQ
730	740	750	760	770	780	790	800
RKDFGFLVDI	LYSALRCCDN	DMERKKVLDD	LTKVDLKWNS	LLKIIEKACP	SSDKHALVTP	WLKGDILGEK	LVNLADCLCN
810	820	830	840	850	860	870	880
EDLESRVSSE	SHFSERWTLL	SLVLSQHVKN	DYLIGDVYVE	RIIVRLHETL	FKTKKLSEAE	SSDSSVSFIC	DVAYNYFSSA
890	900	910	920	930	940	950	960
KGCLLMPSSE	DLLLTLFQLC	AQSKEKTHLP	DFLICKLKNT	WLSGVNLLVH	QTDSSYKEST	FLHLSALWLK	NQVQASSLDI
970	980	990	1000	1010	1020	1030	1040
NSLQVLLSAV	DDLLNTLLES	EDSYLMGVYI	GSVMPNDSEW	EKMRQSLPMQ	WLHRPLLEGR	LSLNYECFKT	DFKEQDIKTL
1050	1060	1070	1080	1090	1100	1110	1120
PSHLCTSALL	SKMVLIALRK	ETVLENNELE	KIIAELLYSL	QWCEELDNPP	IFLIGFCEIL	QKMNITYDNL	RVLGNTSGLL
1130	1140	1150	1160	1170	1180	1190	1200
QLLFNRSREH	GTLWSLIIAK	LILSRSISSD	EVKPHYKRKE	SFFPLTEGNL	HTIQSLCPFL	SKEEKKEFSA	QCIPALLGWT
1210	1220	1230	1240	1250	1260	1270	1280
KKDLCSTNGG	FGHLAIFNSC	LQTKSIDDGE	LLHGILKIII	SWKKEHEDIF	LFSCNLSEAS	PEVLGVNIEI	IRFLSLFLKY
1290	1300	1310	1320	1330	1340	1350	1360
CSSPLAESEW	DFIMCSMLAW	LETTSENQAL	YSIPLVQLFA	CVSCDLACDL	SAFFDSTTLD	TIGNLPVNLI	SEWKEFFSQG
1370	1380	1390	1400	1410	1420	1430	1440
IHSLLLPILV	TVTGENKDVS	ETSFQNAMLK	PMCETLTYIS	KEQLLSHKLP	ARLVADQKTN	LPEYLQTLLN	TLAPLLLFRA
1450	1460	1470	1480	1490	1500	1510	1520
RPVQIAVYHM	LYKLMPELPQ	YDQDNLKSYG	DEEEEPALSP	PAALMSLLSI	QEDLLENVLG	CIPVGQIVTI	KPLSEDFCYV
1530	1540	1550	1560	1570	1580	1590	1600
LGYLLTWKLI	LTFFKAASSQ	LRALYSMYLR	KTKSLNKLLY	HLFRLMPENP	TYAETAVEVP	NKDPKTFFTE	ELQLSIRETT
1610	1620	1630	1640	1650	1660	1670	1680
MLPYHIPHLA	CSVYHMTLKD	LPAMVRLWWN	SSEKRVFNIV	DRFTSKYVSS	VLSFQEISSV	QTSTQLFNGM	TVKARATTRE
1690	1700	1710	1720	1730	1740	1750	1760
VMATYTIEDI	VIELIIQLPS	NYPLGSIIVE	SGKRVGVAVQ	QWRNWMLQLS	TYLTHQNGSI	MEGLALWKNN	VDKRFEGVED
1770	1780	1790	1800	1810	1820		
CMICFSVIHG	FNYSLPKKAC	RTCKKKFHSA	CLYKWFTSSN	KSTCPLCRET	FF		

Cmpd.	No. of Cmpds.	m/z meas.	Δ m/z [ppm]	z	Rt [min]	Score	P	Range	Sequence	Modification
48	1	1097.0025	-131.66	2	30.5	11.6	2	1127-1145	R.SREHGTLWSLIIAKLILSR.S	

Detailed Protein Report

Protein 478: **coiled-coil domain-containing protein 171 [Homo sapiens]**

**Accession:**	gi|38348729	**Score:**	22.7
**Database:**	refseq_human(refseq_human_20140103.fasta)	**MW [kDa]:**	152.7
**Database Date:**	2015-11-30	**pI:**	6.4
		**Sequence Coverage [%]:**	1.7
		**No. of unique Peptides:**	1

10	20	30	40	50	60	70	80
MNLNTSSNTG	DTQRLKIASL	DVKQILKNET	ELDITDNLRK	KLHWAKKEKL	EITTKHNAEL	ASYESQIAKL	RSEVEKGEAL
90	100	110	120	130	140	150	160
RQSLEYDLAV	ARKEAGLGRR	AAEERLAEAH	RIQEKLCAQN	SELQAKTNET	EKAFQTSQQK	WKEECRRFEH	DLEERDNMIQ
170	180	190	200	210	220	230	240
NCNREYDLLM	KEKSRLEKTL	QEALEKHQRE	KNEMESHIRE	TALEEFRLQE	EQWEAERREL	QFIVQEQDTA	VQNMHKKVEK
250	260	270	280	290	300	310	320
LETEHMDCSD	LLRRQTSELE	FSTQREERLR	KEFEATTLRV	RKLEENIEAE	RAAHLESKFN	SEIIQLRIRD	LEGALQVEKA
330	340	350	360	370	380	390	400
SQAEAVADLE	IIKNEFKEVE	SAYEREKHNA	QESFAKLNLL	EKEYFSKNKK	LNEDIEEQKK	VIIDLSKRLQ	YNEKSCSELQ
410	420	430	440	450	460	470	480
EELVMAKKHQ	AFLVETCENN	VKELESILDS	FTVSGQWTSG	IHKDKDKPPS	FSVVLERLRR	TLTDYQNKLE	DASNEEKACN
490	500	510	520	530	540	550	560
ELDSTKQKID	SHTKNIKELQ	DKLADVNKEL	SHLHTKCADR	EALISTLKVE	LQNVLHCWEK	EKAQAAQSES	ELQKLSQAFH
570	580	590	600	610	620	630	640
KDAEEKLTFL	HTLYQHLVAG	CVLIKQPEGM	LDKFSWSELC	AVLQENVDAL	IADLNRANEK	IRHLEYICKN	KSDTMRELQQ
650	660	670	680	690	700	710	720
TQEDTFTKVA	EQIKAQESCW	HRQKKELELQ	YSELFLEVQK	RAQKFQEIAE	KNMEKLNHIE	KSHEQLVLEN	SHFKKLLSQT
730	740	750	760	770	780	790	800
QREQMSLLAA	CALMAGALYP	LYSRSCALST	QRDFLQEQVN	TFELFKLEIR	TLAQALSTVE	EKKQEEAKMK	KKTFKGLIRI
810	820	830	840	850	860	870	880
FRKGVIAVLA	ANRLKILGQS	CASLFTWMES	FKEGIGMLVC	TGEPQDKHKF	PKHQKEQLRC	LQALSWLTSS	DLLAAIISSM
890	900	910	920	930	940	950	960
AELQDVIGKA	DPNSRICGHL	LIGAAKNSFA	KLMDKISLVM	ECIPLHSSRS	ITYVEKDSLV	QRLAHGLHKV	NTLALKYGLR
970	980	990	1000	1010	1020	1030	1040
GHVPITKSTA	SLQKQILGFT	QRLHAAEVER	RSLRLEVTEF	KRSVNEMKKE	LDKAQGLQMQ	LNEFKQSKLI	THEKFESACE
1050	1060	1070	1080	1090	1100	1110	1120
ELNNALLREE	QAQMLLNEQA	QQLQELNYKL	ELHSSEEADK	NQTLGEAVKS	LSEAKMELRR	KDQSLRQLNR	HLTQLEQDKR
1130	1140	1150	1160	1170	1180	1190	1200
RLEENIHDAE	SALRMAAKDK	ECVANHMRAV	ENTLHKVRDQ	ISLSWSAASR	NDFTLQLPKL	HLETFAMEGL	KGGPEVVACQ
1210	1220	1230	1240	1250	1260	1270	1280
AMIKSFMDVY	QLASTRIMTL	EKEMTSHRSH	IAALKSELHT	ACLRENASLQ	SIGSRDHSNL	SIPSRAPLPA	DTTGIGDFLP
1290	1300	1310	1320	1330			
LKAELDTTYT	FLKETFINTV	PHALTSSHSS	PVTMSANANR	PTQIGL			

Cmpd.	No. of Cmpds.	m/z meas.	Δ m/z [ppm]	z	Rt [min]	Score	P	Range	Sequence	Modification
151	1	773.9066	22.69	2	31.9	12.1	1	541-554	K.EKAQAAQSESELQK.L	

BRUKER

Detailed Protein Report

Protein 479: CUB and sushi domain-containing protein 3 isoform 3 [Homo sapiens]

**Accession:**	gi|205277354	**Score:**	22.7
**Database:**	refseq_human(refseq_human_20140103.fasta)	**MW [kDa]:**	387.1
**Database Date:**	2015-11-30	**pI:**	5.5
**Modification(s):**	Carbamidomethyl	**Sequence Coverage [%]:**	0.5
		**No. of unique Peptides:**	1

Detailed Protein Report

10	20	30	40	50	60	70	80
MKGIRKGESR	AKESKPWEPG	KRRCAKCGRL	DFILMKKMGI	KSGFTFWNLV	FLLTVSCVKG	FIYTCGGTLK	GLNGTIESPG
90	100	110	120	130	140	150	160
FPYGYPNGAN	CTWVIIAEER	NRIQIVFQSF	ALEEEYDYLS	LYDGHPHPTN	FRTRLTGFHL	PPPVTSTKSV	FSLRLTSDFA
170	180	190	200	210	220	230	240
VSAHGFKVYY	EELQSSSCGN	PGVPPKGVLY	GTRFDVGDKI	RYSCVTGYIL	DGHPQLTCIA	NSVNTASWDF	PVPICRAEDA
250	260	270	280	290	300	310	320
CGGTMRGSSG	IISSPSFPNE	YHNNADCTWT	IVAEPGDTIS	LIFTDFQMEE	KYDYLEIEGS	EPPTIWLSGM	NIPPPIISNK
330	340	350	360	370	380	390	400
NWLRLHFVTD	SNHRYRGFSA	PYQVKKAIDF	KSRGFKLFPG	KDNSNKFSIL	NEGGIKTASN	LCPDPGEPEN	GKRIGSDFSL
410	420	430	440	450	460	470	480
GSTVQFSCDE	DYVLQGAKSI	TCQRIAEVFA	AWSDHRPVCK	VKTCGSNLQG	PSGTFTSPNF	PFQYDSNAQC	VWVITAVNTN
490	500	510	520	530	540	550	560
KVIQINFEEF	DLEIGYDTLT	IGDGGEVGDP	RTVLQVLTGS	FVPDLIVSMS	SQMWLHLQTD	ESVGSVGFKV	NYKEIEKESC
570	580	590	600	610	620	630	640
GDPGTPLYGI	REGDGFSNRD	VLRFECQFGF	ELIGEKSIVC	QENNQWSANI	PICIFPCLSN	FTAPMGTVLS	PDYPEGYGNN
650	660	670	680	690	700	710	720
LNCIWTIISD	PGSRIHLSFN	DFDLESQFDF	LAVKDGDSPE	SPILGTFTGA	EVPSHLTSNS	HILRLEFQAD	HSMSGRGFNI
730	740	750	760	770	780	790	800
TYNTFGHNEC	PDPGIPINAR	RFGDNFQLGS	SISVICEEGF	IKTQGTETIT	CILMDGKVMW	SGLIPKCGAP	CGGHFSAPSG
810	820	830	840	850	860	870	880
VILSPGWPGY	YKDSLNCEWV	IEAEPGHSIK	ITFERFQTEL	NYDVLEVHDG	PNLLSPLLGS	YNGTQVPQFL	FSSSNFIYLL
890	900	910	920	930	940	950	960
FTTDNSRSNN	GFKIHYESVT	VNTYSCLDPG	IPVHGRRYGH	DFSIGSTVSF	SCDSGYRLSH	EEPLLCEKNH	WWSHPLPTCD
970	980	990	1000	1010	1020	1030	1040
ALCGGDVRGP	SGTILSPGYP	EFYPNSLNCT	WTVDVTHGKG	VQFNFHTFHL	EDHHDYLLIT	ENGSFTQPLA	RLTGSDLPPT
1050	1060	1070	1080	1090	1100	1110	1120
INAGLYGNFR	AQLRFISDFS	ISYEGFNITF	SEYNLEPCED	PGIPQYGSRI	GFNFGIGDTL	TFSCSSGYRL	EGTSEIICLG
1130	1140	1150	1160	1170	1180	1190	1200
GGRRVWSAPL	PRCVAECGAS	ATNNEGILLS	PNYPLNYENN	HECIYSIQVQ	AGKGINISAR	TFHLAQGDVL	KIYDGKDKTT
1210	1220	1230	1240	1250	1260	1270	1280
HLLGAFTGAS	MRGLTLSSTS	NQLWLEFNSD	TEGTDEGFQL	VYTSFELSHC	EDPGIPQFGY	KISDQGHFAG	STIIYGCNPG
1290	1300	1310	1320	1330	1340	1350	1360
YTLHGSSLLK	CMTGERRAWD	YPLPSCIAEC	GGRFKGESSG	RILSPGYPFP	YDNNLRCMWM	IEVDPGNIVS	LQFLAFDTEA
1370	1380	1390	1400	1410	1420	1430	1440
SHDILRVWDG	PPENDMLLKE	ISGSLIPEGI	HSTLNIVTIQ	FDTDFYISKS	GFAIQFSSSV	ATACRDPGVP	MNGTRNGDGR
1450	1460	1470	1480	1490	1500	1510	1520
EPGDTVVFQC	DPGYELQGEE	RITCIQVENR	YFWQPSPPVC	IAPCGGNLTG	SSGFILSPNF	PHPYPHSRDC	DWTITVNADY
1530	1540	1550	1560	1570	1580	1590	1600
VISLAFISFS	IEPNYDFLYI	YDGPDSNSPL	IGSFQDSKLP	ERIESSSNTM	HLAFRSDGSV	SYTGFHLEYK	AKLRESCFDP
1610	1620	1630	1640	1650	1660	1670	1680
GNIMNGTRLG	MDYKLGSTVT	YYCDAGYVLQ	GYSTLTCIMG	DDGRPGWNRA	LPSCHAPCGS	RSTGSEGTVL	SPNYPKNYSV
1690	1700	1710	1720	1730	1740	1750	1760
GHNCVYSIAV	PKEFVVFGQF	VFFQTSLHDV	VEVYDGPTQQ	SSLLSSLSGS	HSGESLPLSS	GNQITIRFTS	VGPITAKGFH
1770	1780	1790	1800	1810	1820	1830	1840
FVYQAVPRTS	STQCSSVPEP	RFGRRIGNEF	AVGSSVLFDC	NPGYILHGSI	AIRCETVPNS	LAQWNDSLPT	CIVPCGGILT
1850	1860	1870	1880	1890	1900	1910	1920
KRKGTILSPG	YPEPYDNNLN	CVWKITVPEG	AGIQVQVVSF	ATEHNWDSLD	FYDGGDNNAP	RLGSYSGTTI	PHLLNSTSNN
1930	1940	1950	1960	1970	1980	1990	2000
LYLNFQSDIS	VSAAGFHLEY	TAIGLDSCPE	PQTPSSGIKI	GDRYMVGDVV	SFQCDQGYSL	QGHSHITCMP	GPVRRWNYPI
2010	2020	2030	2040	2050	2060	2070	2080
PICLAQCGGA	MSDFSGVILS	PGFPGNYPSS	LDCTWTINLP	IGFGVHLQFV	NFSTETIHDY	LEVRSGSSET	STVIGRLSGP
2090	2100	2110	2120	2130	2140	2150	2160
QIPSSLFSTT	HETSLYFHSD	YSQNKQGFHI	VYQAYQLQSC	PDPRPFRNGF	VIGNDFTVGQ	TISFECFPGY	TLIGNSALTC
2170	2180	2190	2200	2210	2220	2230	2240

Detailed Protein Report

Cmpd.	No. of Cmpds.	m/z meas.	Δ m/z [ppm]	z	Rt [min]	Score	P	Range	Sequence	Modification
1304	1	661.3930	144.57	1	46.4	11.2	0	2937-2941	R.QPECK.A	Carbamidomethyl: 4

Detailed Protein Report

Protein 480: PREDICTED: fatty-acid amide hydrolase 2 isoform X4 [Homo sapiens]

Accession: gi|530426388
Database: refseq_human(refseq_human_20140103.fasta)
Database Date: 2015-11-30

Score: 22.7
MW [kDa]: 49.2
pI: 9.3
Sequence Coverage [%]: 4.5
No. of unique Peptides: 1

10	20	30	40	50	60	70	80
MINGIVKYRF	EEAMKEAHAV	DQKLAEKQED	EATLENKWPF	LGVPLTVKEA	FQLQGMPNSS	GLMNRRDAIA	KTDATVVALL
**90**	**100**	**110**	**120**	**130**	**140**	**150**	**160**
KGAGAIPLGI	TNCSELCMWY	ESSNKIYGRS	NNPYDLQHIV	GGSSGGEGCT	LAAACSVIGV	GSDIGGSIRM	PAFFNGIFGH
**170**	**180**	**190**	**200**	**210**	**220**	**230**	**240**
KPSPGVVPNK	GQFPLAVGAQ	ELFLCTGPMC	RYAEDLAPML	KVMAGPGIKR	LKLDTKVHLK	DLKFYWMEHD	GGSFLMSKVD
**250**	**260**	**270**	**280**	**290**	**300**	**310**	**320**
QDLIMTQKKV	VVHLETILGA	SVQHVKLKKM	KYSFQLWIAM	MSAKGHDGKE	PVKFVDLLGD	HGKHVSPLWE	LIKWCLGLSV
**330**	**340**	**350**	**360**	**370**	**380**	**390**	**400**
YTIPSIGLAL	LEEKLRYSNE	KYQKFKAVEE	SLRKELVDML	GDDGVFLYPS	HPTVAPKHHV	PLTRPFNFAY	TGVFSALGLP
**410**	**420**	**430**	**440**	**450**			
VTQCPLGLNA	KGLPLGIQVV	AGPFNDHLTL	AVAQYLEKTF	GGWVCPGKF			

Cmpd.	No. of Cmpds.	m/z meas.	Δ m/z [ppm]	z	Rt [min]	Score	P	Range	Sequence	Modification
701	1	1099.6923	17.73	2	38.7	22.7	2	249-268	K.KVVVHLETILGASVQHVKLK.K	

Detailed Protein Report

Protein 481: **PREDICTED: NAD-dependent protein deacylase sirtuin-5, mitochondrial isoform X3 [Homo sapiens]**

Accession: gi|530381706
Database: refseq_human(refseq_human_20140103.fasta)
Database Date: 2015-11-30
Modification(s): Oxidation

Score: 22.7
MW [kDa]: 31.7
pI: 11.1
Sequence Coverage [%]: 9.9
No. of unique Peptides: 1

10	20	30	40	50	60	70	80
MRPLQIVPSR	LISQLYCGLK	PPASTRNQIC	LKMARPSSSM	ADFRKFFAKA	KHIVIISGAG	VSAESGVPTF	RGAGGYWRKW
**90**	**100**	**110**	**120**	**130**	**140**	**150**	**160**
QAQDLATPLA	FAHNPSRVWE	FYHYRREVMG	SKEPNAGHRA	IAECETRLGK	QGRRVVVITQ	NIDELHRKAG	TKNLLEIHGS
**170**	**180**	**190**	**200**	**210**	**220**	**230**	**240**
LFKTRCTSCG	VVAENYKSPI	CPALSGKGAP	EPGTQDASIP	VEKLPRWALP	LWCTQQPCLP	PRWLPGACQW	LNLTRRPPQL
**250**	**260**	**270**	**280**	**290**			
RTDSGFISRD	PVERLFLKPL	PVMKMKLFLK	CPGEERNYSI	SKN			

Cmpd.	No. of Cmpds.	m/z meas.	Δ m/z [ppm]	z	Rt [min]	Score	P	Range	Sequence	Modification
605	1	730.9957	75.86	2	36.2	11.8	1	255-266	R.LFLKPLPVMKMK.L	Oxidation: 9

Detailed Protein Report

Protein 482: **multiple epidermal growth factor-like domains protein 8 isoform 2 precursor [Homo sapiens]**

**Accession:**	gi|145701025	**Score:**	22.7
**Database:**	refseq_human(refseq_human_20140103.fasta)	**MW [kDa]:**	295.7
**Database Date:**	2015-11-30	**pI:**	6.5
**Modification(s):**	Carbamidomethyl, Oxidation	**Sequence Coverage [%]:**	1.6
		**No. of unique Peptides:**	2

Detailed Protein Report

10	20	30	40	50	60	70	80
MALGKVLAMA	LVLALAVLGS	LSPGARAGDC	KGQRQVLREA	PGFVTDGAGN	YSVNGNCEWL	IEAPSPQHRI	LLDFLFLDTE
90	100	110	120	130	140	150	160
CTYDYLFVYD	GDSPRGPLLA	SLSGSTRPPP	IEASSGKMLL	HLFSDANYNL	LGFNASFRFS	LCPGGCQSHG	QCQPPGVCAC
170	180	190	200	210	220	230	240
EPGWGGPDCG	LQECSAYCGS	HGTCASPLGP	CRCEPGFLGR	ACDLHLWENQ	GAGWWHNVSA	RDPAFSARIG	AAGAFLSPPG
250	260	270	280	290	300	310	320
LLAVFGGQDL	NNALGDLVLY	NFSANTWESW	DLSPAPAARH	SHVAVAWAGS	LVLMGGELAD	GSLTNDVWAF	SPLGRGHWEL
330	340	350	360	370	380	390	400
LAPPASSSSG	PPGLAGHAAA	LVDDVWLYVS	GGRTPHDLFS	SGLFRFRLDS	TSGGYWEQVI	PAGGRPPAAT	GHSMVFHAPS
410	420	430	440	450	460	470	480
RALLVHGGHR	PSTARFSVRV	NSTELFHVDR	HVWTTLKGRD	GLQGPRERAF	HTASVLGNYM	VVYGGNVHTH	YQEEKCYEDG
490	500	510	520	530	540	550	560
IFFYHLGCHQ	WVSGAELAPP	GTPEGRAAPP	SGRYSHVAAV	LGGSVLLVAG	GYSGRPRGDL	MAYKVPPFVF	QAPAPDYHLD
570	580	590	600	610	620	630	640
YCSMYTDHSV	CSRDPECSWC	QGACQAAPPP	GTPLGACPAA	SCLGLGRLLG	DCQACLAFSS	PTAPPRGPGT	LGWCVHNESC
650	660	670	680	690	700	710	720
LPRPEQARCR	GEQISGTVGW	WGPAPVFVTS	LEACVTQSFL	PGLHLLTFQQ	PPNTSQPDKE	EVGRWVAHQE	KETRRLQRPG
730	740	750	760	770	780	790	800
SARLFPLPGR	DHKYAVEIQG	QLNGSAGPGH	SELTLLWDRT	GVPGGSEISF	FFLEPYRSSS	CTSYSSCLGC	LADQGCGWCL
810	820	830	840	850	860	870	880
TSATCHLRQG	GAHCGDDGAG	GSLLVLVPTL	CPLCEEHRDC	HACTQDPFCE	WHQSTSRKGD	AACSRRGRGR	GALKSPEECP
890	900	910	920	930	940	950	960
PLCSQRLTCE	DCLANSSQCA	WCQSTHTCFL	FAAYLARYPH	GGCRGWDDSV	HSEPRCRSCD	GFLTCHECLQ	SHECGWCGNE
970	980	990	1000	1010	1020	1030	1040
DNPTLGRCLQ	GDFSGPLGGG	NCSLWVGEGL	GLPVALPARW	AYARCPDVDE	CRLGLARCHP	RATCLNTPLS	YECHCQRGYQ
1050	1060	1070	1080	1090	1100	1110	1120
GDGISHCNRT	CLEDCGHGVC	SGPPDFTCVC	DLGWTSDLPP	PTPAPGPPAP	RCSRDCGCSF	HSHCRKRGPG	FCDECQDWTW
1130	1140	1150	1160	1170	1180	1190	1200
GEHCERCRPG	SFGNATGSRG	CRPCQCNGHG	DPRRGHCDNL	SGLCFCQDHT	EGAHCQLCSP	GYYGDPRAGG	SCFRECGGRA
1210	1220	1230	1240	1250	1260	1270	1280
LLTNVSSVAL	GSRRVGGLLP	PGGGAARAGP	GLSYCVWVVS	ATEELQPCAP	GTLCPPLTLT	FSPDSSTPCT	LSYVLAFDGF
1290	1300	1310	1320	1330	1340	1350	1360
PRFLDTGVVQ	SDRSLIAAFC	GQRRDRPLTV	QALSGLLVLH	WEANGSSSWG	FNASVGSARC	GSGGPGSCPV	PQECVPQDGA
1370	1380	1390	1400	1410	1420	1430	1440
AGAGLCRCPQ	GWAGPHCRMA	LCPENCNAHT	GAGTCNQSLG	VCICAEGFGG	PDCATKLDGG	QLVWETLMDS	RLSADTASRF
1450	1460	1470	1480	1490	1500	1510	1520
LHRLGHTMVD	GPDATLWMFG	GLGLPQGLLG	NLYRYSVSER	RWTQMLAGAE	DGGPGPSPRS	FHAAAYVPAG	RGAMYLLGGL
1530	1540	1550	1560	1570	1580	1590	1600
TAGGVTRDFW	VLNLTTLQWR	QEKAPQTVEL	PAVAGHTLTA	RRGLSLLLVG	GYSPENGFNQ	QLLEYQLATG	TWVSGAQSGT
1610	1620	1630	1640	1650	1660	1670	1680
PPTGLYGHSA	VYHEATDSLY	VFGGFRFHVE	LAAPSPELYS	LHCPDRTWSL	LAPSQGAKRD	RMRNVRGSSR	GLGQVPGEQP
1690	1700	1710	1720	1730	1740	1750	1760
GSWGFREVRK	KMALWAALAG	TGGFLEEISP	HLKEPRPRLF	HASALLGDTM	VVLGGRSDPD	EFSSDVLLYQ	VNCNAWLLPD
1770	1780	1790	1800	1810	1820	1830	1840
LTRSASVGPP	MEESVAHAVA	AVGSRLYISG	GFGGVALGRL	LALTLPPDPC	RLLSSPEACN	QSGACTWCHG	ACLSGDQAHR
1850	1860	1870	1880	1890	1900	1910	1920
LGCGGSPCSP	MPRSPEECRR	LRTCSECLAR	HPRTLQPGDG	EASTPRCKWC	TNCPEGACIG	RNGSCTSEND	CRINQREVFW
1930	1940	1950	1960	1970	1980	1990	2000
AGNCSEAACG	AADCEQCTRE	GKCMWTRQFK	RTGETRRILS	VQPTYDWTCF	SHSLLNVSPM	PVESSPPLPC	PTPCHLLPNC
2010	2020	2030	2040	2050	2060	2070	2080
TSCLDSKGAD	GGWQHCVWSS	SLQQCLSPSY	LPLRCMAGGC	GRLLRGPESC	SLGCAQATQC	ALCLRRPHCG	WCAWGGQDGG
2090	2100	2110	2120	2130	2140	2150	2160
GRCMEGGLSG	PRDGLTCGRP	GASWAFLSCP	PEDECANGHH	DCNETQNCHD	QPHGYECSCK	TGYTMDNMTG	LCRPVCAQGC
2170	2180	2190	2200	2210	2220	2230	2240

Detailed Protein Report

Cmpd.	No. of Cmpds.	m/z meas.	Δ m/z [ppm]	z	Rt [min]	Score	P	Range	Sequence	Modification
2125	1	1023.8906	-13.46	3	57.0	12.4	2	1-31	-.MALGKVLAMALVLALAVLGSLSPGARAGDCK.G	Carbamidomethyl: 30; Oxidation: 1
1779	1	820.8211	-36.25	2	50.8	10.3	1	1000-1012	R.WAYARCPDVDECR.L	Carbamidomethyl: 12

Detailed Protein Report

Protein 483: **staphylococcal nuclease domain-containing protein 1 [Homo sapiens]**

**Accession:**	gi|77404397	**Score:**	22.7
**Database:**	refseq_human(refseq_human_20140103.fasta)	**MW [kDa]:**	101.9
**Database Date:**	2015-11-30	**pI:**	6.8
**Modification(s):**	Carbamidomethyl	**Sequence Coverage [%]:**	2.9
		**No. of unique Peptides:**	1

10	20	30	40	50	60	70	80
MASSAQSGGS	SGGPAVPTVQ	RGIIKMVLSG	CAIIVRGQPR	GGPPPERQIN	LSNIRAGNLA	RRAAATQPDA	KDTPDEPWAF
90	100	110	120	130	140	150	160
PAREFLRKKL	IGKEVCFTIE	NKTPQGREYG	MIYLGKDTNG	ENIAESLVAE	GLATRREGMR	ANNPEQNRLS	ECEEQAKAAK
170	180	190	200	210	220	230	240
KGMWSEGNGS	HTIRDLKYTI	ENPRHFVDSH	HQKPVNAIIE	HVRDGSVVRA	LLLPDYYLVT	VMLSGIKCPT	FRREADGSET
250	260	270	280	290	300	310	320
PEPFAAEAKF	FTESRLLQRD	VQIILESCHN	QNILGTILHP	NGNITELLLK	EGFARCVDWS	IAVYTRGAEK	LRAAERFAKE
330	340	350	360	370	380	390	400
RRLRIWRDYV	APTANLDQKD	KQFVAKVMQV	LNADAIVVKL	NSGDYKTIHL	SSIRPPRLEG	ENTQDKNKKL	RPLYDIPYMF
410	420	430	440	450	460	470	480
EAREFLRKKL	IGKKVNVTVD	YIRPASPATE	TVPAFSERTC	ATVTIGGINI	AEALVSKGLA	TVIRYRQDDD	QRSSHYDELL
490	500	510	520	530	540	550	560
AAEARAIKNG	KGLHSKKEVP	IHRVADISGD	TQKAKQFLPF	LQRAGRSEAV	VEYVFSGSRL	KLYLPKETCL	ITFLLAGIEC
570	580	590	600	610	620	630	640
PRGARNLPGL	VQEGEPFSEE	ATLFTKELVL	QREVEVEVES	MDKAGNFIGW	LHIDGANLSV	LLVEHALSKV	HFTAERSSYY
650	660	670	680	690	700	710	720
KSLLSAEEAA	KQKKEKVWAH	YEEQPVEEVM	PVLEEKERSA	SYKPVFVTEI	TDDLHFYVQD	VETGTQLEKL	MENMRNDIAS
730	740	750	760	770	780	790	800
HPPVEGSYAP	RRGEFCIAKF	VDGEWYRARV	EKVESPAKIH	VFYIDYGNRE	VLPSTRLGTL	SPAFSTRVLP	AQATEYAFAF
810	820	830	840	850	860	870	880
IQVPQDDDAR	TDAVDSVVRD	IQNTQCLLNV	EHLSAGCPHV	TLQFADSKGD	VGLGLVKEGL	VMVEVRKEKQ	FQKVITEYLN
890	900	910	920				
AQESAKSARL	NLWRYGDFRA	DDADEFGYSR					

Cmpd.	No. of Cmpds.	m/z meas.	Δ m/z [ppm]	z	Rt [min]	Score	P	Range	Sequence	Modification
349	1	626.3170	35.99	3	34.4	11.5	1	733-747	R.GEFCIAKFVDGEWYR.A	Carbamidomethyl: 4

Detailed Protein Report

Protein 484: azurocidin preproprotein [Homo sapiens]

Accession: gi|11342670
Database: refseq_human(refseq_human_20140103.fasta)
Database Date: 2015-11-30

Score: 22.6
MW [kDa]: 26.9
pI: 11.9
Sequence Coverage [%]: 5.2
No. of unique Peptides: 1

10	20	30	40	50	60	70	80
MTRLTVLALL	AGLLASSRAG	SSPLLDIVGG	RKARPRQFPF	LASIQNQGRH	FCGGALIHAR	FVMTAASCFQ	SQNPGVSTVV
**90**	**100**	**110**	**120**	**130**	**140**	**150**	**160**
LGAYDLRRRE	RQSRQTFSIS	SMSENGYDPQ	QNLNDLMLLQ	LDREANLTSS	VTILPLPLQN	ATVEAGTRCQ	VAGWGSQRSG
**170**	**180**	**190**	**200**	**210**	**220**	**230**	**240**
GRLSRFPRFV	NVTVTPEDQC	RPNNVCTGVL	TRRGGICNGD	GGTPLVCEGL	AHGVASFSLG	PCGRGPDFFT	RVALFRDWID
**250**	**260**						
GVLNNPGPGP	A						

Cmpd.	No. of Cmpds.	m/z meas.	Δ m/z [ppm]	z	Rt [min]	Score	P	Range	Sequence	Modification
2226	3	753.2849	-148.34	2	56.5	22.6	0	37-49	R.QFPFLASIQNQGR.H	

Detailed Protein Report

Protein 485: **PREDICTED: tyrosine-protein phosphatase non-receptor type 11 isoform X2 [Homo sapiens]**

Accession: gi|578824047
Database: refseq_human(refseq_human_20140103.fasta)
Database Date: 2015-11-30
Modification(s): Carbamidomethyl

Score: 22.6
MW [kDa]: 64.0
pI: 7.9
Sequence Coverage [%]: 3.4
No. of unique Peptides: 1

10	20	30	40	50	60	70	80
MTSRRWFHPN	ITGVEAENLL	LTRGVDGSFL	ARPSKSNPGD	FTLSVRRNGA	VTHIKIQNTG	DYYDLYGGEK	FATLAELVQY
90	100	110	120	130	140	150	160
YMEHHGQLKE	KNGDVIELKY	PLNCADPTSE	RWFHGHLSGK	EAEKLLTEKG	KHGSFLVRES	QSHPGDFVLS	VRTGDDKGES
170	180	190	200	210	220	230	240
NDGKSKVTHV	MIRCQELKYD	VGGGERFDSL	TDLVEHYKKN	PMVETLGTVL	QLKQTLQQQE	CKLLYSRKEG	QRQENKNKNR
250	260	270	280	290	300	310	320
YKNILPFDHT	RVVLHDGDPN	EPVSDYINAN	IIMPEFETKC	NNSKPKKSYI	ATQGCLQNTV	NDFWRMVFQE	NSRVIVMTTK
330	340	350	360	370	380	390	400
EVERGKSKCV	KYWPDEYALK	EYGVMRVRNV	KESAAHDYTL	RELKLSKVGQ	ALLQGNTERT	VWQYHFRTWP	DHGVPSDPGG
410	420	430	440	450	460	470	480
VLDFLEEVHH	KQESIMDAGP	VVVHCSAGIG	RTGTFIVIDI	LIDIIREKGV	DCDIDVPKTI	QMVRSQRSGM	VQTEAQYRFI
490	500	510	520	530	540	550	560
YMAVQHYIET	LQRRIEEEQK	SKRKGHEYTN	IKYSLADQTS	GDQSPLPPCT	PTPPCAEMRE	DSARVYENVG	LMQQQKSFR

Cmpd.	No. of Cmpds.	m/z meas.	Δ m/z [ppm]	z	Rt [min]	Score	P	Range	Sequence	Modification
360	1	559.1189	-258.27	2	33.2	10.9	0	449-458	K.GVDCDIDVPK.T	Carbamidomethyl: 4

BRUKER

Detailed Protein Report

Protein 486: zinc finger and SCAN domain-containing protein 29 [Homo sapiens]

**Accession:**	gi|109715825	**Score:**	22.6
**Database:**	refseq_human(refseq_human_20140103.fasta)	**MW [kDa]:**	96.7
**Database Date:**	2015-11-30	**pI:**	6.6
		**Sequence Coverage [%]:**	4.0
		**No. of unique Peptides:**	1

Quantitation

QD:QU **Median:** 0.45 **CV:** 0.00 % **No. of Peptides:** 1

Alias proteins:

Accession	Name	Description
gi|578826641	refseq_human (refseq_human_20140103.fasta)	PREDICTED: zinc finger and SCAN domain-containing protein 29 isoform X1 [Homo sapiens]

10	20	30	40	50	60	70	80
MMAKSALREN	GTNSETFRQR	FRRFHYQEVA	GPREAFSQLW	ELCCRWLRPE	VRTKEQIVEL	LVLEQFLTVL	PGEIQNWVQE
**90**	**100**	**110**	**120**	**130**	**140**	**150**	**160**
QCPENGEEAV	TLVEDLEREP	GRPRSSVTVS	VKGQEVRLEK	MTPPKSSQEL	LSVRQESVEP	QPRGVPKKER	ARSPDLGPQE
**170**	**180**	**190**	**200**	**210**	**220**	**230**	**240**
QMNPKEKLKP	FQRSGLPFPK	SGVVSRLEQG	EPWIPDLLGS	KEKELPSGSH	IGDRRVHADL	LPSKKDRRSW	VEQDHWSFED
**250**	**260**	**270**	**280**	**290**	**300**	**310**	**320**
EKVAGVHWGY	EETRTLLAIL	SQTEFYEALR	NCHRNSQVYG	AVAERLREYG	FLRTLEQCRT	KFKGLQKSYR	KVKSGHPPET
**330**	**340**	**350**	**360**	**370**	**380**	**390**	**400**
CPFFEEMEAL	MSAQVIALPS	NGLEAAASHS	GLVGSDAETE	EPGQRGWQHE	EGAEEAVAQE	SDSDDMDLEA	TPQDPNSAAP
**410**	**420**	**430**	**440**	**450**	**460**	**470**	**480**
VVFRSPGGVH	WGYEETKTYL	AILSETQFYE	ALRNCHRNSQ	LYGAVAERLW	EYGFLRTPEQ	CRTKFKSLQT	SYRKVKNGQA
**490**	**500**	**510**	**520**	**530**	**540**	**550**	**560**
PETCPFFEEM	DALVSVRVAA	PPNDGQEETA	SCPVQGTSEA	EAQKQAEEAD	EATEEDSDDD	EEDTEIPPGA	VITRAPVLFQ
**570**	**580**	**590**	**600**	**610**	**620**	**630**	**640**
SPRGFEAGFE	NEDNSKRDIS	EEVQLHRTLL	ARSERKIPRY	LHQGKGNESD	CRSGRQWAKT	SGEKRGKLTL	PEKSLSEVLS
**650**	**660**	**670**	**680**	**690**	**700**	**710**	**720**
QQRPCLGERP	YKYLKYSKSF	GPNSLLMHQV	SHQVENPYKC	ADCGKSFSRS	ARLIRHRRIH	TGEKPYKCLD	CGKSFRDSSN
**730**	**740**	**750**	**760**	**770**	**780**	**790**	**800**
FITHRRIHTG	EKPYQCGECG	KCFNQSSSLI	IHQRTHTGEK	PYQCEECGKS	FNNSSHFSAH	RRIHTGERPH	VCPDCGKSFS
**810**	**820**	**830**	**840**	**850**	**860**		
KSSDLRAHHR	THTGEKPYGC	HDCGKCFSKS	SALNKHGEIH	AREKLLTQSA	PK		

Cmpd.	No. of Cmpds.	m/z meas.	Δ m/z [ppm]	z	Rt [min]	Score	P	Range	Sequence	Modification	Ratios	
200	1	849.4012	-12.44	2	32.5	11.4	1	153-167	R.SPDLGPQEQMNPKEK.L		QD:QU	0.45

Detailed Protein Report

Protein 487: PREDICTED: short transient receptor potential channel 7 isoform X1 [Homo sapiens]

**Accession:**	gi|530380051	**Score:**	22.5
**Database:**	refseq_human(refseq_human_20140103.fasta)	**MW [kDa]:**	55.5
**Database Date:**	2015-11-30	**pI:**	9.0
		**Sequence Coverage [%]:**	8.8
		**No. of unique Peptides:**	1

10	20	30	40	50	60	70	80
MKFVAHAVSF	TIFLGLLVVN	ASDRFEGVKT	LPNETFTDYP	KQIFRVKTTQ	FSWTEMLIMK	WVLGMIWSEC	KEIWEEGPRE
**90**	**100**	**110**	**120**	**130**	**140**	**150**	**160**
YVLHLWNLLD	FGMLSIFVAS	FTARFMAFLK	ATEAQLYVDQ	HVQDDTLHNV	SLPPEVAYFT	YARDKWWPSD	PQIISEGLYA
**170**	**180**	**190**	**200**	**210**	**220**	**230**	**240**
IAVVLSFSRI	AYILPANESF	GPLQISLGRT	VKDIFKFMVI	FIMVFVAFMI	GMFNLYSYYR	GAKYNPAFTT	VEESFKTLFW
**250**	**260**	**270**	**280**	**290**	**300**	**310**	**320**
SIFGLSEVIS	VVLKYDHKFI	ENIGYVLYGV	YNVTMVVVLL	NMLIAMINNS	YQEIEEDADV	EWKFARAKLW	LSYFDEGRTL
**330**	**340**	**350**	**360**	**370**	**380**	**390**	**400**
PAPFNLVPSP	KSFYYLIMRI	KMCLIKLCKS	KAKSCENDLE	MGMLNSKFKK	TRYQAGMRNS	ENLTANNTLS	KPTRYQKIMK
**410**	**420**	**430**	**440**	**450**	**460**	**470**	**480**
RLIKRYVLKA	QVDRENDEVN	EGELKEIKQD	ISSLRYELLE	EKSQATGELA	DLIQQLSEKF	GKNLNKDHLR	VNKGKDI

Cmpd.	No. of Cmpds.	m/z meas.	Δ m/z [ppm]	z	Rt [min]	Score	P	Range	Sequence	Modification
398	1	879.0703	-93.51	3	35.0	10.2	1	1-24	-.MKFVAHAVSFTIFLGLLVVNASDR.F	

Detailed Protein Report

Protein 488: **PREDICTED: LIM and calponin homology domains-containing protein 1 isoform X14 [Homo sapiens]**

**Accession:**	gi|530376597	**Score:**	22.5
**Database:**	refseq_human(refseq_human_20140103.fasta)	**MW [kDa]:**	147.5
**Database Date:**	2015-11-30	**pI:**	6.3
**Modification(s):**	Oxidation	**Sequence Coverage [%]:**	3.1
		**No. of unique Peptides:**	1

10	20	30	40	50	60	70	80
MACPALGLEA	LQPLQPEPPP	EPAFSEAQKW	IEQVTGRSFG	DKDFRTGLEN	GILLCELLNA	IKPGLVKKIN	RLPTPIAGLD
90	100	110	120	130	140	150	160
NIILFLRGCK	ELGLKESQLF	DPSDLQDTSN	RVTVKSLDYS	RKLKNVLVTI	YWLGKAANSC	TSYSGTTLNL	KEFEGLLAQM
170	180	190	200	210	220	230	240
RKDTDDIESP	KRSIRDSGYI	DCWDSERSDS	LSPPRHGRDD	SFDSLDSFGS	RSRQTPSPDV	VLRGSSDGRG	SDSESDLPHR
250	260	270	280	290	300	310	320
KLPDVKKDDM	SARRTSHGEP	KSAVPFNQYL	PNKSNQTAYV	PAPLRKKKAE	REEYRKSWST	ATSPLGGERP	FSFPETIEEE
330	340	350	360	370	380	390	400
GSEVGSAGED	NPAGQMNPGW	KPSDGGCELP	DGSGKEHPSS	DGAVVAPAPK	SEEKDAAEIQ	KRKRLEQAGI	KVMPAAQRFA
410	420	430	440	450	460	470	480
SQKQLSEEKE	AIRDIVLRKE	NSFLTHQHGN	DSEAEGEVVC	RLPDLEKDDF	AARRARMNQT	KPMVPLNQLL	YGPYPKKGAE
490	500	510	520	530	540	550	560
KSDGSKQLSK	GISKKRSLEY	KRNQGHTEEV	KLIVTCNMRA	QESEPVEGGL	RKVPDLHKDD	LAQQRIQGSL	APHREPPSFI
570	580	590	600	610	620	630	640
TLSNITEADL	ETWERLKVSE	KARDGDVQHI	CASEPSPEIK	AETAIRDDFA	NRKARASKKA	SSPRQKFVHF	GPVTELDQQK
650	660	670	680	690	700	710	720
WKRLSIGKAG	PREDEEEVIC	HGSKIQMDSV	SPVSAATSSL	KGHQIFNRQN	DCRTMNCGRG	DYCRRASWLA	PVPESQEEWV
730	740	750	760	770	780	790	800
CSLGECPRGT	EEVTSKQLPQ	DGKEETESAP	RDSERLSKAE	RSEDSSQPLV	CPLASECEAS	GTEEKLEKMT	APAWSGSGLK
810	820	830	840	850	860	870	880
GQRKLDDSRK	DDMMARRTGM	SLRHTGSNPN	QFLPVPFAKQ	QDVEESSKGL	PMKDQRYGPR	TPVSDDAEST	SMFDMRCEEE
890	900	910	920	930	940	950	960
AAVQPHSRAR	QEQLQLINNQ	LREEDDKWQD	DLARWKSRRR	SVSQDLIKKE	EERKKMEKLL	AGEDGTSERR	KSIKTYREIV
970	980	990	1000	1010	1020	1030	1040
QEKERREREL	HEAYKNARSQ	EEAEGILQQY	IERFTISEAV	LERLEMPKIL	ERSHSTEPNL	SSFLNDPNPM	KYLRQQSLPP
1050	1060	1070	1080	1090	1100	1110	1120
PKFTATVETT	IARASVLDTS	MSAGSGSPSK	TVTPKAVPML	TPKPYSQPKN	SQDVLKTFKV	DGKVSVNGET	VHREEEKERE
1130	1140	1150	1160	1170	1180	1190	1200
CPTVAPAHSL	TKSQMFEGVA	RVHGSPLELK	QDNGSIEINI	KKPNSVPQEL	AATTEKTEPN	SQEDKNDGGK	SRKGNIELAS
1210	1220	1230	1240	1250	1260	1270	1280
SEPQHFTTTV	TRCSPTVAFV	EFPSSPQLKN	DVSEEKDQKK	PENEMSGKVE	LVLSQKVVKP	KSPEPEATLT	FPFLDKMPEA
1290	1300	1310	1320	1330			
NQLHLPNLNS	QESPGTASVP	LRVQNSWRRS	QFFSQSGKLH	TG			

Cmpd.	No. of Cmpds.	m/z meas.	Δ m/z [ppm]	z	Rt [min]	Score	P	Range	Sequence	Modification
1973	1	902.8715	-0.36	2	55.1	10.4	0	861-876	R.TPVSDDAESTSMFDMR.C	Oxidation: 12

Detailed Protein Report

Protein 489: dynein heavy chain 6, axonemal [Homo sapiens]

**Accession:**	gi|194353966	**Score:**	22.5
**Database:**	refseq_human(refseq_human_20140103.fasta)	**MW [kDa]:**	475.7
**Database Date:**	2015-11-30	**pI:**	5.6
		**Sequence Coverage [%]:**	0.6
		**No. of unique Peptides:**	1

Alias proteins:

Accession	Name	Description
gi|578802753	refseq_human (refseq_human_20140103.fasta)	PREDICTED: dynein heavy chain 6, axonemal isoform X1 [Homo sapiens]

Detailed Protein Report

10	20	30	40	50	60	70	80
MTFRATDSEF	DLTNIEEYAE	NSALSRLNNI	KAKQRVSYVT	STENESDTQI	LTFRHITKAQ	EKTRKRQQPI	KLEPLPVLKV
90	100	110	120	130	140	150	160
YQDHKQPEYI	HEQNRFQLMT	AGIIKRPVSI	AKKSFATSST	QFLEHQDAVK	KMQIHRPYVE	VFSPSPPKLP	HTGIGKRGLF
170	180	190	200	210	220	230	240
GTRSSAYPKY	TFHDREEVVK	ANIRDPLQII	KIIRENEHLG	FLYMIPAVPR	SSIEYDTYNL	KVVSYENINK	NDYYTISQRA
250	260	270	280	290	300	310	320
VTHIYNEDIE	FIEIDRWEQE	YLYHRELTKI	PIFSLFRKWK	AFSVWRKNVR	SKKITGCQKS	LQKNLFIVNP	HLRPALLKIN
330	340	350	360	370	380	390	400
ELCYHLSFMG	LCYIEKCHTY	TLQEFKAAQV	IRLAEVTERL	GEFRNEAKYV	VRRACRFALR	AAGFVPDDCA	FGPFEDYHKV
410	420	430	440	450	460	470	480
QSSGSFINTP	HELPTYGDSE	KMTYTEQASK	RHYCMRLTCF	IRLNDYLIEN	TMHILTVNAV	NSLLNHLTDK	LKRTPSADVI
490	500	510	520	530	540	550	560
QKWITEEKPE	VPDKKGTLMV	EKQEEDESLI	PMFLTELMLT	VQSLLFEPSL	EDFLDGILGA	VNHCQNTVLS	VPNLVPDSYF
570	580	590	600	610	620	630	640
DAFTSPYINN	KLEGKTCGTG	PSLAAVFEDD	KNFHTIISQI	KETIQAAFES	ARIYAATFEK	FQIFFKENES	LDLQALKLQE
650	660	670	680	690	700	710	720
PDINFFSEQL	EKYHKQHKDA	VALRPTRNVG	LLLIDTRLLR	EKLIPSPLRC	LEVLNFMLPR	QSKKKVDAII	FEAQDAEYKL
730	740	750	760	770	780	790	800
EFVPTTTTEY	VHSLLFLDEI	QERIESLEDE	GNIVTQMYKL	MEQYQVPTPP	EDFAVFATMK	PSIVAVRNAI	DKSVGDRESS
810	820	830	840	850	860	870	880
IKQFCVHLGS	DLEELNNEVN	EVKLQAQDPQ	ILDISADQDK	IRLILNNLQS	VLADLQKRAF	QYKSYQKNFK	VEVSKFEALE
890	900	910	920	930	940	950	960
EVSAELKLKQ	LLWDSFSEWD	KLQQEWLKSK	FDCLDPEVLN	GQVSKYAKFV	TQLEKGLPPN	SVVPQLKYKV	EKMKEKLPVI
970	980	990	1000	1010	1020	1030	1040
IDLRNPTLKA	RHWAAIEQTV	DATLVDAEIP	LTLERLSQLH	VFDFGQEIQD	ISGQASGEAA	LEAILKKVED	SWKTTEFVIL
1050	1060	1070	1080	1090	1100	1110	1120
PHRDSKDVFI	LGGTDDIQVL	LDDSTINVAT	LASSRYLGPL	KTRVDEWQKQ	LALFNQTLEE	WLTCQRNWLY	LESIFNAPDI
1130	1140	1150	1160	1170	1180	1190	1200
QRQLPAEAKM	FLQVDKSWKE	IMRKVNRLPN	ALRAATQPGL	LETFQNNNAL	LDQIQKCLEA	YLESKRVIFP	RFYFLSNDEL
1210	1220	1230	1240	1250	1260	1270	1280
LEILAQTRNP	QAVQPHLRKC	FDSISKLEFA	LMPPAEGKIP	GIDGEPEKVY	TNDILAMLSP	EGERVSLGKG	LKARGNVEEW
1290	1300	1310	1320	1330	1340	1350	1360
LGKVEEAMFT	SLRRLCKAAI	ADYQGKLRTD	WVVAGHPSQV	ILTVSQIMWC	RDLTECLETE	HSNHIQALKN	FEKVNFERLN
1370	1380	1390	1400	1410	1420	1430	1440
ALAAIVQGSL	PKLHRNILTA	LITIDVHARD	IVTELVQSKV	ETVESFDWQR	QLRYYWDIDL	DNCVARMALS	QYTYGYEYLG
1450	1460	1470	1480	1490	1500	1510	1520
ACPRLVITPL	TDRCYLCLMG	ALQLDLGGAP	AGPAGTGKTE	TTKDLAKALA	IQCVVFNCSD	GLDYKMMGRF	FSGLAQSGAW
1530	1540	1550	1560	1570	1580	1590	1600
CCFDEFNRID	IEVLSVIAQQ	LITIRNAKAA	KLSRFMFEGR	EIKLVMTCAA	FITMNPGYAG	RTELPDNLKA	LFRPFAMMVP
1610	1620	1630	1640	1650	1660	1670	1680
NYALIAEVIL	YSEGFESSKI	LARKMTQMYK	LCSEQLSQQD	HYDFGMRAVK	SVLVMAGSLK	RENPDLNEDV	VLIRALQDSN
1690	1700	1710	1720	1730	1740	1750	1760
LPKFLTDDAL	LFSGIISDLF	PGVQIPEHDY	GILQSTIVDV	MNRQNLQPEM	CMVRKVIQFY	ETMLVRHGVM	LVGPTGGGKT
1770	1780	1790	1800	1810	1820	1830	1840
TVYRILAETL	GNLQKLGIEN	SFYQAVKTYV	LNPKSITMGE	LYGEVNNLTL	EWKDGLMALS	VRAAVNDTSE	DHKWIISDGP
1850	1860	1870	1880	1890	1900	1910	1920
VDALWIENMN	TVLDDNKMLC	LANSERIKLT	PQIHMLFEVQ	DLRVASPATV	SRCGMVFVDP	EELKWMPYVK	TWMKGISKKL
1930	1940	1950	1960	1970	1980	1990	2000
TEETQEYILN	LFQRYVDEGL	HFINKKCSQA	IPQVDISKVT	TLCCLLESLI	LGKDGVNLAM	EQTKLNTILC	QTFVFCYLWS
2010	2020	2030	2040	2050	2060	2070	2080
LGGNLTENYY	DSFDTFIRTQ	FDDNPDARLP	NSGDLWSIHM	DFDTKRLDPW	ERIIPTFKYN	RDVPFFEMLV	PTTDTVRYGY
2090	2100	2110	2120	2130	2140	2150	2160
LMEKLLAVKH	SVLFTGITGV	GKSVIAKGLL	NKIQESAGYV	PVYLNFSAQT	SSARTQEIIE	SKLERKRKNI	LGAPGNKRIV
2170	2180	2190	2200	2210	2220	2230	2240

Detailed Protein Report

Cmpd.	No. of Cmpds.	m/z meas.	Δ m/z [ppm]	z	Rt [min]	Score	P	Range	Sequence	Modification
2662	1	698.0710	213.61	2	62.1	10.4	0	1359-1372	R.LNALAAIVQGSLPK.L	

Detailed Protein Report

Protein 490: zinc finger and SCAN domain-containing protein 16 [Homo sapiens]

Accession: gi|13376834
Database: refseq_human(refseq_human_20140103.fasta)
Database Date: 2015-11-30

Score: 22.5
MW [kDa]: 40.8
pI: 9.4
Sequence Coverage [%]: 4.6
No. of unique Peptides: 1

10	20	30	40	50	60	70	80
MTTALEPEDQ	KGLLIIKAED	HYWGQDSSSQ	KCSPHRRELY	RQHFRKLCYQ	DAPGPREALT	QLWELCRQWL	RPECHTKEQI
**90**	**100**	**110**	**120**	**130**	**140**	**150**	**160**
LDLLVLEQFL	SILPKDLQAW	VRAHHPETGE	EAVTVLEDLE	RELDEPGKQV	PGNSERRDIL	MDKLAPLGRP	YESLTVQLHP
**170**	**180**	**190**	**200**	**210**	**220**	**230**	**240**
KKTQLEQEAG	KPQRNGDKTR	TKNEELFQKE	DMPKDKEFLG	EINDRLNKDT	PQHPKSKDII	ENEGRSEWQQ	RERRRYKCDE
**250**	**260**	**270**	**280**	**290**	**300**	**310**	**320**
CGKSFSHSSD	LSKHRRTHTG	EKPYKCDECG	KAFIQRSHLI	GHHRVHTGVK	PYKCKECGKD	FSGRTGLIQH	QRIHTGEKPY
**330**	**340**	**350**					
ECDECGRPFR	VSSALIRHQR	IHTANKLY					

Cmpd.	No. of Cmpds.	m/z meas.	Δ m/z [ppm]	z	Rt [min]	Score	P	Range	Sequence	Modification
2161	2	1022.8836	-107.34	2	57.4	22.5	2	218-233	K.DIIENEGRSEWQQRER.R	

Detailed Protein Report

Protein 491: **PAS domain-containing protein 1 [Homo sapiens]**

**Accession:**	gi|157785548	**Score:**	22.5
**Database:**	refseq_human(refseq_human_20140103.fasta)	**MW [kDa]:**	87.4
**Database Date:**	2015-11-30	**pI:**	4.8
**Modification(s):**	Carbamidomethyl	**Sequence Coverage [%]:**	1.9
		**No. of unique Peptides:**	1

10	20	30	40	50	60	70	80
MKMRGEKRRD	KVNPKSSQRK	LNWIPSFPTY	DYFNQVTLQL	LDGFMITLST	DGVIICVAEN	ISSLLGHLPA	EIVGKKLLSL
**90**	**100**	**110**	**120**	**130**	**140**	**150**	**160**
LPDEEKDEVY	QKIILKFPLL	NSETHIEFCC	HLKRGNVEHG	DSSAYENVKF	IVNVRDICNE	FPVVFSGLFS	SHLCADFAAC
**170**	**180**	**190**	**200**	**210**	**220**	**230**	**240**
VPQEDRLYLV	GNVCILRTQL	LQQLYTSKAV	SDEAVLTQDS	DEEPFVGELS	SSQGQRGHTS	MKAVYVEPAA	AAAAAAISDD
**250**	**260**	**270**	**280**	**290**	**300**	**310**	**320**
QIDIAEVEQY	GPQENVHMFV	DSDSTYCSST	VFLDTMPESP	ALSLQDFRGE	PEVNPLYRAD	PVDLEFSVDQ	VDSVDQEGPM
**330**	**340**	**350**	**360**	**370**	**380**	**390**	**400**
DQQDPENPVA	PLDQAGLMDP	VDPEDSVDLG	AAGASAQPLQ	PSSPVAYDII	SQELELMKKL	KEQLEERTWL	LHDAIQNQQN
**410**	**420**	**430**	**440**	**450**	**460**	**470**	**480**
ALELMMDHLQ	KQPNTLRHVV	IPDLQSSEAV	PKKQQKQHAG	QVKRPLPHPK	DVKCFCGLSL	SNSLKNTGEL	QEPCVAFNQQ
**490**	**500**	**510**	**520**	**530**	**540**	**550**	**560**
QLVQQEQHLK	EQQRQLREQL	QQLREQRKVQ	KQKKMQEKKK	LQEQKMQEKK	KLQEQRRQKK	KKLQERKKWQ	GQMLQKEPEE
**570**	**580**	**590**	**600**	**610**	**620**	**630**	**640**
EQQKQQLQEQ	PLKHNVIVGN	ERVQICLQNP	RDVSVPLCNH	PVRFLQAQPI	VPVQRAAEQQ	PSGFYQDENC	GQQEDESQSF
**650**	**660**	**670**	**680**	**690**	**700**	**710**	**720**
YPEAYQGPPV	NQLPLIDTSN	SEAISSSSIP	QFPITSDSTI	STLETPQDYI	RLWQELSDSL	GPVVQVNTWS	CDEQGTLHGQ
**730**	**740**	**750**	**760**	**770**	**780**		
PTYHQVQVSE	VGVEGPPDPQ	AFQGPAAYQP	DQMRSAEQTR	LMPAEQRDSN	KPC		

Cmpd.	No. of Cmpds.	m/z meas.	Δ m/z [ppm]	z	Rt [min]	Score	P	Range	Sequence	Modification
2772	1	557.6353	40.20	3	65.8	22.5	1	451-465	K.DVKCFCGLSLSNSLK.N	Carbamidomethyl: 4

Detailed Protein Report

Protein 492: retinol dehydrogenase 14 [Homo sapiens]

Accession: gi|10190746
Database: refseq_human(refseq_human_20140103.fasta)
Database Date: 2015-11-30
Modification(s): Oxidation

Score: 22.4
MW [kDa]: 36.8
pI: 9.9
Sequence Coverage [%]: 10.4
No. of unique Peptides: 2

Quantitation

QD:QU **Median:** 0.73 **CV:** 0.00 % **No. of Peptides:** 1

10	20	30	40	50	60	70	80
MAVATAAAVL	AALGGALWLA	ARRFVGPRVQ	RLRRGGDPGL	MHGKTVLITG	ANSGLGRATA	AELLRLGARV	IMGCRDRARA
90	100	110	120	130	140	150	160
EEAAGQLRRE	LRQAAECGPE	PGVSGVGELI	VRELDLASLR	SVRAFCQEML	QEEPRLDVLI	NNAGIFQCPY	MKTEDGFEMQ
170	180	190	200	210	220	230	240
FGVNHLGHFL	LTNLLLGLLK	SSAPSRIVVV	SSKLYKYGDI	NFDDLNSEQS	YNKSFCYSRS	KLANILFTRE	LARRLEGTNV
250	260	270	280	290	300	310	320
TVNVLHPGIV	RTNLGRHIHI	PLLVKPLFNL	VSWAFFKTPV	EGAQTSIYLA	SSPEVEGVSG	RYFGDCKEEE	LLPKAMDESV
330	340						
ARKLWDISEV	MVGLLK						

Cmpd.	No. of Cmpds.	m/z meas.	Δ m/z [ppm]	z	Rt [min]	Score	P	Range	Sequence	Modification	Ratios
1653	1	698.3330	-119.22	3	50.9	11.2	1	2-23	M.AVATAAAVLAALGGALWLAARR.F		
865	1	705.2586	-165.25	2	40.9	11.2	2	32-44	R.LRRGGDPGLMHGK.T	Oxidation: 10	QD:QU 0.73

Detailed Protein Report

Protein 493: **E3 ubiquitin-protein ligase Topors isoform 2 [Homo sapiens]**

**Accession:**	gi|307344673	**Score:**	22.4
**Database:**	refseq_human(refseq_human_20140103.fasta)	**MW [kDa]:**	112.3
**Database Date:**	2015-11-30	**pI:**	10.1
		**Sequence Coverage [%]:**	1.9
		**No. of unique Peptides:**	1

10	20	30	40	50	60	70	80
MIMASAAKEF	KMDNFSPKAG	TSKLQQTVPA	DASPDSKCPI	CLDRFDNVSY	LDRCLHKFCF	RCVQEWSKNK	AECPLCKQPF
90	100	110	120	130	140	150	160
DSIFHSVRAE	DDFKEYVLRP	SYNGSFVTPD	RRFRYRTTLT	RERNASVYSP	SGPVNRRTTT	PPDSGVLFEG	LGISTRPRDV
170	180	190	200	210	220	230	240
EIPQFMRQIA	VRRPTTADER	SLRKIQEQDI	INFRRTLYRA	GARVRNIEDG	GRYRDISAEF	FRRNPACLHR	LVPWLKRELT
250	260	270	280	290	300	310	320
VLFGAHGSLV	NIVQHIIMSN	VTRYDLESQA	FVSDLRPFLL	NRTEHFIHEF	ISFARSPFNM	AAFDQHANYD	CPAPSYEEGS
330	340	350	360	370	380	390	400
HSDSSVITIS	PDEAETQELD	INVATVSQAP	WDDETPGPSY	SSSEQVHVTM	SSLLNTSDSS	DEELVTGGAT	SQIQGVQTND
410	420	430	440	450	460	470	480
DLNNDSDDSS	DNCVIVGFVK	PLAERTPELV	ELSSDSEDLG	SYEKMETVKT	QEQEQSYSSG	DSDVSRCSSP	HSVLGKDEQI
490	500	510	520	530	540	550	560
NKGHCDSSTR	IKSKKEEKRS	TSLSSPRNLN	SSVRGDRVYS	PYNHRHRKRG	RSRSSDSRSQ	SRSGHDQKNH	RKHHGKKRMK
570	580	590	600	610	620	630	640
SKRSRSRESS	RPRGRRDKKR	SRTRDSSWSR	RSQTLSLSSE	STSRSRSRSS	DHGKRRSRSR	NRDRYYLRNN	YGSRYKWEYT
650	660	670	680	690	700	710	720
YYSRNKDRDG	YESSYRRRTL	SRAHYSRQSS	SPEFRVQSFS	ERTNARKKNN	HSERKYYYYE	RHRSRSLSSN	RSRTASTGTD
730	740	750	760	770	780	790	800
RVRNEKPGGK	RKYKTRHLEG	TNEVAQPSRE	FASKAKDSHY	QKSSSKLDGN	YKNESDTFSD	SRSSDRETKH	KRRKRKTRSL
810	820	830	840	850	860	870	880
SVEIVYEGKA	TDTTKHHKKK	KKKHKKKHKK	HHGDNASRSP	VVITIDSDSD	KDSEVKEDTE	CDNSGPQDPL	QNEFLAPSLE
890	900	910	920	930	940	950	960
PFETKDVVTI	EAEFGVLDKE	CDIATLSNNL	NNANKTVDNI	PPLAASVEQT	LDVREESTFV	SDLENQPSNI	VSLQTEPSRQ
970	980	990					
LPSPRTSLMS	VCLGRDCDMS						

Cmpd.	No. of Cmpds.	m/z meas.	Δ m/z [ppm]	z	Rt [min]	Score	P	Range	Sequence	Modification
2563	1	625.3176	42.38	2	62.7	10.1	1	696-703	K.YYYYERHR.S	

Detailed Protein Report

Protein 494: **neuropathy target esterase isoform c [Homo sapiens]**

Accession: gi|260656039
Database: refseq_human(refseq_human_20140103.fasta)
Database Date: 2015-11-30

Score: 22.4
MW [kDa]: 143.3
pI: 8.8
Sequence Coverage [%]: 2.5
No. of unique Peptides: 1

10	20	30	40	50	60	70	80
MEAPLQTGMV	LGVMIGAGVA	VVVTAVLILL	VVRRLRVPKT	PAPDGPRYRF	RKRDKVLFYG	RKIMRKVSQS	TSSLVDTSVS
90	100	110	120	130	140	150	160
ATSRPRMRKK	LKMLNIAKKI	LRIQKETPTL	QRKEPPPAVL	EADLTEGDLA	NSHLPSEVLY	MLKNVRVLGH	FEKPLFLELC
170	180	190	200	210	220	230	240
RHMVFQRLGQ	GDYVFRPGQP	DASIYVVQDG	LLELCLPGPD	GKECVVKEVV	PGDSVNSLLS	ILDVITGHQH	PQRTVSARAA
250	260	270	280	290	300	310	320
RDSTVLRLPV	EAFSAVFTKY	PESLVRVVQI	IMVRLQRVTF	LALHNYLGLT	NELFSHEIQP	LRLFPSPGLP	TRTSPVRGSK
330	340	350	360	370	380	390	400
RMVSTSATDE	PRETPGRPPD	PTGAPLPGPT	GDPVKPTSLE	TPSAPLLSRC	VSMPGDISGL	QGGPRSDFDM	AYERGRISVS
410	420	430	440	450	460	470	480
LQEEASGGSL	AAPARTPTQE	PREQPAGACE	YSYCEDESAT	GGCPFGPYQG	RQTSSIFEAA	KQELAKLMRI	EDVSLHFVLW
490	500	510	520	530	540	550	560
GCLHVYQRMI	DKAEDVCLFV	AQPGELVGQL	AVLTGEPLIF	TLRAQRDCTF	LRISKSDFYE	IMRAQPSVVL	SAAHTVAARM
570	580	590	600	610	620	630	640
SPFVRQMDFA	IDWTAVEAGR	ALYRQGDRSD	CTYIVLNGRL	RSVIQRGSGK	KELVGEYGRG	DLIGVVEALT	RQPRATTVHA
650	660	670	680	690	700	710	720
VRDTELAKLP	EGTLGHIKRR	YPQVVTRLIH	LLSQKILGNL	QQLQGPFPGS	GLGVPPHSEL	TNPASNLATV	AILPVCAEVP
730	740	750	760	770	780	790	800
MVAFTLELQH	ALQAIGPTLL	LNSDIIRARL	GASALDSIQE	FRLSGWLAQQ	EDAHRIVLYQ	TDASLTPWTV	RCLRQADCIL
810	820	830	840	850	860	870	880
IVGLGDQEPT	LGQLEQMLEN	TAVRALKQLV	LLHREEGAGP	TRTVEWLNMR	SWCSGHLHLR	CPRRLFSRRS	PAKLHELYEK
890	900	910	920	930	940	950	960
VFSRRADRHS	DFSRLARVLT	GNTIALVLGG	GGARGCSHIG	VLKALEEAGV	PVDLVGGTSI	GSFIGALYAE	ERSASRTKQR
970	980	990	1000	1010	1020	1030	1040
AREWAKSMTS	VLEPVLDLTY	PVTSMFTGSA	FNRSIHRVFQ	DKQIEDLWLP	YFNVTTDITA	SAMRVHKDGS	LWRYVRASMT
1050	1060	1070	1080	1090	1100	1110	1120
LSGYLPPLCD	PKDGHLLMDG	GYINNLPADI	ARSMGAKTVI	AIDVGSQDET	DLSTYGDSLS	GWWLLWKRLN	PWADKVKVPD
1130	1140	1150	1160	1170	1180	1190	1200
MAEIQSRLAY	VSCVRQLEVV	KSSSYCEYLR	PPIDCFKTMD	FGKFDQIYDV	GYQYGKAVFG	GWSRGNVIEK	MLTDRRSTDL
1210	1220	1230	1240	1250	1260	1270	1280
NESRRADVLA	FPSSGFTDLA	EIVSRIEPPT	SYVSDGCADG	EESDCLTEYE	EDAGPDCSRD	EGGSPEGASP	STASEMEEEK
1290	1300	1310					
SILRQRRCLP	QEPPGSATDA						

Cmpd.	No. of Cmpds.	m/z meas.	Δ m/z [ppm]	z	Rt [min]	Score	P	Range	Sequence	Modification
55	1	699.0697	59.10	3	29.4	10.8	0	1206-1225	R.ADVLAFPSSGFTDLAEIVSR.I	

Detailed Protein Report

Protein 495: coiled-coil domain-containing protein 78 [Homo sapiens]

Accession: gi|199559954
Database: refseq_human(refseq_human_20140103.fasta)
Database Date: 2015-11-30
Modification(s): Oxidation

Score: 22.3
MW [kDa]: 48.5
pI: 9.3
Sequence Coverage [%]: 7.8
No. of unique Peptides: 1

10	20	30	40	50	60	70	80
MEHAATTGPR	PGPPSRRVEN	VVLRAKDWLP	GAPGGTAVWA	TSLEAEVPPD	LALNKEQQLQ	ISKELVDIQI	TTHHLHEQHE
**90**	**100**	**110**	**120**	**130**	**140**	**150**	**160**
AEIFQLKSEI	LRLESRVLEL	ELRGDGTSQG	CAVPVESDPR	HPRAAAQELR	HKAQVPGHSD	DHRFQVQPKN	TMNPENEQHR
**170**	**180**	**190**	**200**	**210**	**220**	**230**	**240**
LGSGLQGEVK	WALEHQEARQ	QALVTRVATL	GRQLQGAREE	ARAAGQRLAT	QAVVLCSCQG	QLRQAEAENA	RLQLQLKKLK
**250**	**260**	**270**	**280**	**290**	**300**	**310**	**320**
DEYVLRLQHC	AWQAVEHADG	AGQAPATTAL	RTFLEATLED	IRAAHRSREQ	QLARAARSYH	KRLVDLSRRH	EELLVAYRAP
**330**	**340**	**350**	**360**	**370**	**380**	**390**	**400**
GNPQAIFDIA	SLDLEPLPVP	LVTDFSHRED	QHGGPGALLS	SPKKRPGGAS	QGGTSEPQGL	DAASWAQIHQ	KLRDFSRSTQ
**410**	**420**	**430**	**440**				
SWNGSGHSCW	SGPRWLKSNF	LSYRSTWTST	WAGTSTKS				

Cmpd.	No. of Cmpds.	m/z meas.	Δ m/z [ppm]	z	Rt [min]	Score	P	Range	Sequence	Modification
1714	1	917.4108	-54.09	2	50.0	11.5	1	1-17	-.MEHAATTGPRPGPPSRR.V	Oxidation: 1

Detailed Protein Report

Protein 496: abnormal spindle-like microcephaly-associated protein isoform 2 [Homo sapiens]

**Accession:**	gi|332205965	**Score:**	22.2
**Database:**	refseq_human(refseq_human_20140103.fasta)	**MW [kDa]:**	217.7
**Database Date:**	2015-11-30	**pI:**	10.5
		**Sequence Coverage [%]:**	1.3
		**No. of unique Peptides:**	1

10	20	30	40	50	60	70	80
MANRRVGRGC	WEVSPTERRP	PAGLRGPAAE	EEASSPPVLS	LSHFCRSPFL	CFGDVLLGAS	RTLSLALDNP	NEEVAEVKIS
90	100	110	120	130	140	150	160
HFPAADLGFS	VSQRCFVLQP	KEKIVISVNW	TPLKEGRVRE	IMTFLVNDVL	KHQAILLGNA	EEQKKKKRSL	WDTIKKKKIS
170	180	190	200	210	220	230	240
ASTSHNRRVS	NIQNVNKTFS	VSQKVDRVRS	PLQACENLAM	NEGGPPTENN	SLILEENKIP	ISPISPAFNE	CHGATCLPLS
250	260	270	280	290	300	310	320
VRRSTTYSSL	HASENRELLN	VHSANVSKVS	FNEKAVTETS	FNSVNVNGQR	GENSKLSLTP	NCSSTLNITQ	SQIHFLSPDS
330	340	350	360	370	380	390	400
FVNNSHGANN	ELELVTCLSS	DMFMKDNSQP	VHLESTIAHE	IYQKILSPDS	FIKDNYGLNQ	DLESESVNPI	LSPNQFLKDN
410	420	430	440	450	460	470	480
MAYMCTSQQT	CKVPLSNENS	QVPQSPEDWR	KSEVSPRIPE	CQGSKSPKAI	FEELVEMKSN	YYSFIKQNNP	KFSAVQDISS
490	500	510	520	530	540	550	560
HSHNKQPKRR	PILSATVTKR	KATCTRENQT	EINKPKAKRC	LNSAVGEHEK	VINNQKEKED	FHSYLPIIDP	ILSKSKSYKN
570	580	590	600	610	620	630	640
EVTPSSTTAS	VARKRKSDGS	MEDANVRVAI	TEHTEVREIK	RIHFSPSEPK	TSAVKKTKNV	TTPISKRISN	REKLNLKKKT
650	660	670	680	690	700	710	720
DLSIFRTPIS	KTNKRTKPII	AVAQSSLTFI	KPLKTDIPRH	PMPFAAKNMF	YDERWKEKQE	QGFTWWLNFI	LTPDDFTVKT
730	740	750	760	770	780	790	800
NISEVNAATL	LLGIENQHKI	SVPRAPTKEE	MSLRAYTARC	RLNRLRRAAC	RLFTSEKMVK	AIKKLEIEIE	ARRLIVRKDR
810	820	830	840	850	860	870	880
HLWKDVGERQ	KVLNWLLSYN	PLWLRIGLET	TYGELISLED	NSDVTGLAMF	ILNRLLWNPD	IAAEYRHPTV	PHLYRDGHEE
890	900	910	920	930	940	950	960
ALSKFTLKKL	LLLVCFLDYA	KISRLIDHDP	CLFCKDAEFK	ASKEILLAFS	RDFLSGEGDL	SRHLGLLGLP	VNHVQTPFDE
970	980	990	1000	1010	1020	1030	1040
FDFAVTNLAV	DLQCGVRLVR	TMELLTQNWD	LSKKLRIPAI	SRLQKMHNVD	IVLQVLKSRG	IELSDEHGNT	ILSKDIVDRH
1050	1060	1070	1080	1090	1100	1110	1120
REKTLRLLWK	IAFAFQVDIS	LNLDQLKEEI	AFLKHTKSIK	KTISLLSCHS	DDLINKKKGK	RDSGSFEQYS	ENIKLLMDWV
1130	1140	1150	1160	1170	1180	1190	1200
NAVCAFYNKK	VENFTVSFSD	GRVLCYLIHH	YHPCYVPFDA	ICQRTTQTVE	CTQTGSVVLN	SSSESDDSSL	DMSLKAFDHE
1210	1220	1230	1240	1250	1260	1270	1280
NTSELYKELL	ENEKKNFHLV	RSAVRDLGGI	PAMINHSDMS	NTIPDEKVVI	TYLSFLCARL	LDLRKEIRAA	RLIQTTWRKY
1290	1300	1310	1320	1330	1340	1350	1360
KLKTDLKRHQ	EREKAARIIQ	LAVINFLAKQ	RLRKRVNAAL	VIQKYWRRVL	AQRKLLMLKK	EKLEKVQNKA	ASLIQAMWRR
1370	1380	1390	1400	1410	1420	1430	1440
YRAKKYLCKV	KAACKIQAWY	RCWRAHKEYL	AILKAVKIIQ	GCFYTKLERT	RFLNVRASAI	IIQRKWRAIL	PAKIAHEHFL
1450	1460	1470	1480	1490	1500	1510	1520
MIKRHRAACL	IQAHYRGYKG	RQVFLRQKSA	ALIIQKYIRA	REAGKHERIK	YIEFKKSTVI	LQALVRGWLV	RKRFLEQRAK
1530	1540	1550	1560	1570	1580	1590	1600
IRLLHFTAAA	YYHLNAVRIQ	RAYKLYLAVK	NANKQVNSVI	CIQRWFRARL	QEKRFIQKYH	SIKKIEHEGQ	ECLSQRNRAA
1610	1620	1630	1640	1650	1660	1670	1680
SVIQKAVRHF	LLRKKQEKFT	SGIIKIQALW	RGYSWRKKND	CTKIKAIRLS	LQVVNREIRE	ENKLYKRTAL	ALHYLLTYKH
1690	1700	1710	1720	1730	1740	1750	1760
LSAILEALKH	LEVVTRLSPL	CCENMAQSGA	ISKIFVLIRS	CNRSIPCMEV	IRYAVQVLLN	VSKYEKTTSA	VYDVENCIDI
1770	1780	1790	1800	1810	1820	1830	1840
LLELLQIYRE	KPGNKVADKG	GSIFTKTCCL	LAILLKTTNR	ASDVRSRSKV	VDRIYSLYKL	TAHKHKMNTE	RILYKQKKNS
1850	1860	1870	1880	1890	1900		
SISIPFIPET	PVRTRIVSRL	KPDWVLRRDN	MEEITNPLQA	IQMVMDTLGI	PY		

Detailed Protein Report

Cmpd.	No. of Cmpds.	m/z meas.	Δ m/z [ppm]	z	Rt [min]	Score	P	Range	Sequence	Modification
1490	1	719.8392	-53.72	2	48.8	11.2	1	1445-1456	R.HRAACLIQAHYR.G	

Detailed Protein Report

Protein 497: janus kinase and microtubule-interacting protein 3 [Homo sapiens]

**Accession:**	gi|157502225	**Score:**	22.2
**Database:**	refseq_human(refseq_human_20140103.fasta)	**MW [kDa]:**	98.5
**Database Date:**	2015-11-30	**pI:**	5.4
		**Sequence Coverage [%]:**	3.8
		**No. of unique Peptides:**	1

Alias proteins:

Accession	Name	Description
gi|578819295	refseq_human (refseq_human_20140103.fasta)	PREDICTED: janus kinase and microtubule-interacting protein 3 isoform X7 [Homo sapiens]

10	20	30	40	50	60	70	80
MSKRGMSSRA	KGDKAEALAA	LQAANEDLRA	KLTDIQIELQ	QEKSKVSKVE	REKNQELRQV	REHEQHKTAV	LLTELKTKLH
**90**	**100**	**110**	**120**	**130**	**140**	**150**	**160**
EEKMKELQAV	RETLLRQHEA	ELLRVIKIKD	NENQRLQALL	SALRDGGPEK	VKTVLLSEAK	EEAKKGFEVE	KVKMQQEISE
**170**	**180**	**190**	**200**	**210**	**220**	**230**	**240**
LKGAKRQVEE	ALTLVIQADK	IKAAEIRSVY	HLHQEEITRI	KKECEREIRR	LMEEIKFKDR	AVFVLERELG	VQAGHAQRLQ
**250**	**260**	**270**	**280**	**290**	**300**	**310**	**320**
LQKEALDEQL	SQVREADRHP	GSPRRELPHA	AGAGDASDHS	GSPEQQLDEK	DARRFQLKIA	ELSAIIRKLE	DRNALLSEER
**330**	**340**	**350**	**360**	**370**	**380**	**390**	**400**
NELLKRVREA	ESQYKPLLDK	NKRLSRKNED	LSHALRRMEN	KLKFVTQENI	EMRQRAGIIR	RPSSLNDLDQ	SQDEREVDFL
**410**	**420**	**430**	**440**	**450**	**460**	**470**	**480**
KLQIVEQQNL	IDELSKTLET	AGYVKSVLER	DKLLRFRKQR	KKMAKLPKPV	VVETFFGYDE	EASLESDGSS	VSYQTDRTDQ
**490**	**500**	**510**	**520**	**530**	**540**	**550**	**560**
TPCTPDDDLE	EGMAKEETEL	RFRQLTMEYQ	ALQRAYALLQ	EQVGGTLDAE	REVKTREQLQ	AEVQRAQARI	EDLEKALAEQ
**570**	**580**	**590**	**600**	**610**	**620**	**630**	**640**
GQDMKWIEEK	QALYRRNQEL	VEKIKQMETE	EARLRHEVQD	ARDQNELLEF	RILELEERER	KSPAISFHHT	PFVDGKSPLQ
**650**	**660**	**670**	**680**	**690**	**700**	**710**	**720**
VYCEAEGVTD	IVVAELMKKL	DILGDNANLT	NEEQVVVIQA	RTVLTLAEKW	LQQIEETEAA	LQRKMVDLES	EKELFSKQKG
**730**	**740**	**750**	**760**	**770**	**780**	**790**	**800**
YLDEELDYRK	QALDQANKHI	LELEAMLYDA	LQQEAGAKVA	ELLSEEEREK	LKVAVEQWKR	QVMSELRERD	AQILRERMEL
**810**	**820**	**830**	**840**	**850**			
LQLAQQRIKE	LEERIEAQKR	QIKELEEKFL	FLFLFFSLAF	ILWS			

Cmpd.	No. of Cmpds.	m/z meas.	Δ m/z [ppm]	z	Rt [min]	Score	P	Range	Sequence	Modification
2340	1	899.3808	-154.38	2	59.7	11.2	1	167-182	R.QVEEALTLVIQADKIK.A	

Detailed Protein Report

Protein 498: PREDICTED: sickle tail protein homolog isoform X17 [Homo sapiens]

**Accession:**	gi|530392291	**Score:**	22.1
**Database:**	refseq_human(refseq_human_20140103.fasta)	**MW [kDa]:**	162.3
**Database Date:**	2015-11-30	**pI:**	5.8
		**Sequence Coverage [%]:**	1.4
		**No. of unique Peptides:**	1

Quantitation

QD:QU **Median:** 6.19 **CV:** 0.00 % **No. of Peptides:** 1

10	20	30	40	50	60	70	80
MQRELVYARG	DGPGAPRPGS	TAHPPHAIPN	SPPSTPVPHS	MPPSPSRIPY	GGTRSMVVPG	NATIPRDRIS	SLPVSRPISP
**90**	**100**	**110**	**120**	**130**	**140**	**150**	**160**
SPSAILERRD	VKPDEDMSGK	NIAMYRNEGF	YADPYLYHEG	RMSIASSHGG	HPLDVPDHII	AYHRTAIRSA	SAYCNPSMQA
**170**	**180**	**190**	**200**	**210**	**220**	**230**	**240**
EMHMEQSLYR	QKSRKYPDSH	LPTLGSKTPP	ASPHRVSDLR	MIDMHAHYNA	HGPPHTMQPD	RASPSRQAFK	KEPGTLVYIE
**250**	**260**	**270**	**280**	**290**	**300**	**310**	**320**
KPRSAAGLSS	LVDLGPPLME	KQVFAYSTAT	IPKDRETSEK	MMKTTANRNH	TDSAGTPHVS	GGKMLSALES	TVPPSQPPPV
**330**	**340**	**350**	**360**	**370**	**380**	**390**	**400**
GTSAIHMSLL	EMRRSVAELR	LQLQQMRQLQ	LQNQELLRAM	MKKAELEISG	KVMETMKRLE	DPVQRQRVLV	EQERQKYLHE
**410**	**420**	**430**	**440**	**450**	**460**	**470**	**480**
EEKIVKKLCE	LEDFVEDLKK	DSTAASRLVT	LKDVEDGAFL	LRQVGEAVAT	LKGEFPTLQN	KMRAILRIEV	EAVRFLKEEP
**490**	**500**	**510**	**520**	**530**	**540**	**550**	**560**
HKLDSLLKRV	RSMTDVLTML	RRHVTDGLLK	GTDAAQAAQY	MAMEKATAAE	VLKSQEEAAH	TSGQPFHSTG	APGDAKSEVV
**570**	**580**	**590**	**600**	**610**	**620**	**630**	**640**
PLSGMMVRHA	QSSPVVIQPS	QHSVALLNPA	QNLPHVASSP	AVPQEATSTL	QMSQAPQSPQ	IPMNGSAMQS	LFIEEIHSVS
**650**	**660**	**670**	**680**	**690**	**700**	**710**	**720**
AKNRAVSIEK	AEKKWEEKRQ	NLDHYNGKEF	EKLLEEAQAN	IMKSIPNLEM	PPATGPLPRG	DAPVDKVELS	EDSPNSEQDL
**730**	**740**	**750**	**760**	**770**	**780**	**790**	**800**
EKLGGKSPPP	PPPPPRRSYL	PGSGLTTTRS	GDVVYTGRKE	NITAKASSED	AGPSPQTRAT	KYPAEEPASA	WTPSPPPVTT
**810**	**820**	**830**	**840**	**850**	**860**	**870**	**880**
SSSKDEEEEE	EEGDKIMAEL	QAFQKCSFMD	VNSNSHAEPS	RADSHVKDTR	SGATVPPKEK	KNLEFFHEDV	RKSDVEYENG
**890**	**900**	**910**	**920**	**930**	**940**	**950**	**960**
PQMEFQKVTT	GAVRPSDPPK	WERGMENSIS	DASRTSEYKT	EIIMKENSIS	NMSLLRDSRN	YSQETVPKAS	FGFSGISPLE
**970**	**980**	**990**	**1000**	**1010**	**1020**	**1030**	**1040**
DEINKGSKIS	GLQYSIPDTE	NQTLNYGKTK	EMEKQNTDKC	HVSSHTRLTE	SSVHDFKTED	QEVITTDFGQ	VVLRPKEARH
**1050**	**1060**	**1070**	**1080**	**1090**	**1100**	**1110**	**1120**
ANVNPNEDGE	SSSSSPTEEN	AATDNIAFMI	TETTVQVLSS	GEVHDIVSQK	GEDIQTVNID	ARKEMTPRQE	GTDNEDPVVC
**1130**	**1140**	**1150**	**1160**	**1170**	**1180**	**1190**	**1200**
LDKKPVIIIF	DEPMDIRSAY	KRLSTIFEEC	DEELERMMME	EKIEEEEEEE	NGDSVVQNNN	TSQMSHKKVA	PGNLRTGQQV
**1210**	**1220**	**1230**	**1240**	**1250**	**1260**	**1270**	**1280**
ETKSQPHSLA	TETRNPGGQE	MNRTELNKFS	HVDSPNSECK	GEDATDDQFE	SPKKKFKFKF	PKKQLAALTQ	AIRTGTKTGK
**1290**	**1300**	**1310**	**1320**	**1330**	**1340**	**1350**	**1360**
KTLQVVVYEE	EEEDGTLKQH	KEAKRFEIAR	SQPEDTPENT	VRRQEQPSIE	STSPISRTDE	IRKNTYRTLD	SLEQTIKQLE
**1370**	**1380**	**1390**	**1400**	**1410**	**1420**	**1430**	**1440**
NTISEMSPKA	LVDTSCSSNR	DSVASSSHIA	QEASPRPLLV	PDEGPTALEP	PTSIPSASRK	GSSGAPQTSR	MPVPMSAKNR
**1450**	**1460**	**1470**					
PGTLDKPGKQ	SKLQDPRQYR	QVVLP					

Cmpd.	No. of Cmpds.	m/z meas.	Δ m/z [ppm]	z	Rt [min]	Score	P	Range	Sequence	Modification	Ratios	
588	1	581.7946	6.95	2	35.9	10.0	0	1008-1017	R.LTESSVHDFK.T		QD:QU	6.19

Detailed Protein Report

Protein 499: PREDICTED: cyclin-dependent kinase 7 isoform X1 [Homo sapiens]

**Accession:**	gi|530379082	**Score:**	22.1
**Database:**	refseq_human(refseq_human_20140103.fasta)	**MW [kDa]:**	32.7
**Database Date:**	2015-11-30	**pI:**	9.8
		**Sequence Coverage [%]:**	8.7
		**No. of unique Peptides:**	1

Alias proteins:

Accession	Name	Description
gi|530430719	refseq_human (refseq_human_20140103.fasta)	PREDICTED: cyclin-dependent kinase 7 isoform X1 [Homo sapiens]

10	20	30	40	50	60	70	80
MRSWTSLGRD	SLPPFTRPEI	RTPTKLSPLR	KSNLDIDQKL	KMVIIKDNSL	VLTPSHIKAY	MLMTLQGLEY	LHQHWILHRD
90	100	110	120	130	140	150	160
LKPNNLLLDE	NGVLKLADFG	LAKSFGSPNR	AYTHQVVTRW	YRAPELLFGA	RMYGVGVDMW	AVGCILAELL	LRVPFLPGDS
170	180	190	200	210	220	230	240
DLDQLTRIFE	TLGTPTEEQW	PDMCSLPDYV	TFKSFPGIPL	HHIFSAAGDD	LLDLIQGLFL	FNPCARITAT	QALKMKYFSN
250	260	270	280	290			
RPGPTPGCQL	PRPNCPVETL	KEQSNPALAI	KRKRTEALEQ	GGLPKKLIF			

Cmpd.	No. of Cmpds.	m/z meas.	Δ m/z [ppm]	z	Rt [min]	Score	P	Range	Sequence	Modification
50	1	662.2443	-185.28	2	29.3	10.8	0	47-58	K.DNSLVLTPSHIK.A	

Detailed Protein Report

Protein 500: **PREDICTED: nascent polypeptide-associated complex subunit alpha isoform X6 [Homo sapiens]**

**Accession:**	gi|578823772	**Score:**	22.1
**Database:**	refseq_human(refseq_human_20140103.fasta)	**MW [kDa]:**	201.3
**Database Date:**	2015-11-30	**pI:**	10.2
		**Sequence Coverage [%]:**	1.1
		**No. of unique Peptides:**	1

Detailed Protein Report

10	20	30	40	50	60	70	80
MPGEATETVP	ATEQELPQPQ	AETAVLPMSS	ALSVTAALGQ	PGPTLPPPCS	PAPQQCPLSA	ANQASPFPSP	STIASTPLEV
90	100	110	120	130	140	150	160
PFPQSSSGTA	LPLGTAPEAP	TFLPNLIGPP	ISPAALALAS	PMIAPTLKGT	PSSSAPLALV	ALAPHSVQKS	SAFPPNLLTS
170	180	190	200	210	220	230	240
PPSVAVAESG	SVITLSAPIA	PSEPKTNLNK	VPSEVVPNPK	GTPSPPCIVS	TVPYHCVTPM	ASIQSGVASL	PQTTPTTTLA
250	260	270	280	290	300	310	320
IASPQVKDTT	ISSVLISPQN	PGSLSLKGPV	SPPAALSLST	QSLPVVTSSQ	KTAGPNTPPD	FPISLGSHLA	PLHQSSFGSV
330	340	350	360	370	380	390	400
QLLGQTGPSA	LSDPTVKTIS	VDHSSTGASY	PSQRSVIPPL	PSRNEVVPAT	VAAFPVVAPS	VDKGPSTISS	ITCSPSGSLN
410	420	430	440	450	460	470	480
VATSFSLSPT	TSLILKSSPN	ATYHYPLVAQ	MPVSSVGTTP	LVVTNPCTIA	AAPTTTFEVA	TCVSPPMSSG	PISNIEPTSP
490	500	510	520	530	540	550	560
AALVMAPVAP	KEPSTQVATT	LRIPVSPPLP	DPEDLKNLPS	SVLVKFPTQK	DLQTVPASLE	GAPFSPAQAG	LTTKKDPTVL
570	580	590	600	610	620	630	640
PLVQAAPKNS	PSFQSTSSSP	EIPLSPEATL	AKKSLGEPLP	IGKPASSMTS	PLGVNSSASV	IKTDSYAGPD	SAGPLLKSSL
650	660	670	680	690	700	710	720
ITPTVAAFPL	ESADPAGVAP	TTAKGTSTYT	TTASPFLEGT	VSLAPKNHPV	KEGTLTTLPL	VPTASENCPV	APSPQNTCAP
730	740	750	760	770	780	790	800
LATLVLAPEI	PKSVPSPSLP	PAGTPPGTKK	VDGISHTSAL	APVASSPKEC	PTEDSGASAT	ASSKGTLTYL	ADSPSPLGVS
810	820	830	840	850	860	870	880
VSPQTKRPPT	KKGSAGPDTP	IGNLSSPVSP	VEASFLPENS	LSFQGSKDSP	ATTHSPTPPS	PKGAPTPSAV	TPLSPKGVTL
890	900	910	920	930	940	950	960
PPKETPTPSV	VNLPFPKEGP	ATPAPKQAPA	LSMTSSSPKK	ARATPAPKGI	PASPSPKGAP	TPPAATPPSP	KGGPATPSPK
970	980	990	1000	1010	1020	1030	1040
WAPTPPAATP	PSPKGGPATP	SPKGAPTPPA	ATPPSPKGSP	AATPLPKGAP	TTPAATLPSP	KGGPATPSLK	GAPTPPAATP
1050	1060	1070	1080	1090	1100	1110	1120
PSPKGGPATP	SPKGAPMPPA	ATPPSPKGGL	ATPPHKGAPT	TPAATPPSPK	GGLATPPPKG	APTTPAATPP	SPKGGLATPP
1130	1140	1150	1160	1170	1180	1190	1200
PKGAPTTPAA	TPPSPKGGLA	TPSPKGAPTT	PAATPPSPKG	GLATPSPKGA	PTTPAATPPS	PKGGLATPSP	KGAPTTPAAT
1210	1220	1230	1240	1250	1260	1270	1280
PPSPKGGPAT	PPPKGAPTPP	AATPPSLKGG	LATPPHKGAP	NPAVVTPPSP	KGGPATSPPK	GAPTPPAATP	PSPKGSPGTP
1290	1300	1310	1320	1330	1340	1350	1360
PPKGAPTPPA	VTPPSPKGTP	TLPATTPSSK	GGPTTPSSKE	GPTPPAATPS	HKGGPAMTPP	SPKRGPAIPS	PKGDPTSPAV
1370	1380	1390	1400	1410	1420	1430	1440
IPLSPKKAPA	TPVTREGAAT	PSKGDLTPPA	VTPVSLKKAP	ATSAPKGGPA	TPSSKGDPTL	PAVTPPSPKE	PPAPKQVATS
1450	1460	1470	1480	1490	1500	1510	1520
SSPKKAPATP	APMGAPTLPA	VIPSSPKEVP	ATPSSRRDPI	APTATLLSKK	TPATLAPKEA	LIPPAMTVPS	PKKTPAIPTP
1530	1540	1550	1560	1570	1580	1590	1600
KEAPATPSSK	EASSPPAVTP	STYKGAPSPK	ELLIPPAVTS	PSPKEAPTPP	AVTPPSPEKG	PATPAPKGTP	TSPPVTPSSL
1610	1620	1630	1640	1650	1660	1670	1680
KDSPTSPASV	TCKMGATVPQ	ASKGLPAKKG	PTALKEVLVA	PAPESTPIIT	APTRKGPQTK	KSSATSPPIC	PDPSAKNGSK
1690	1700	1710	1720	1730	1740	1750	1760
GPLSTVAPAP	LLPVQKDSSK	TAKGKDASHS	PKGPLAPPES	KASTPLTAAA	FEKVLPKPES	ASVSAAPSPP	VSLPLAPSPV
1770	1780	1790	1800	1810	1820	1830	1840
PTLPPKQQFL	PSSPGLVLES	PSKPLAPADE	DELLPLIPPE	PISGGVPFQS	VLVNMPTPKS	AGIPVPTPSA	KQPVTKNNKG
1850	1860	1870	1880	1890	1900	1910	1920
SGTESDSDES	VPELEEQDST	QATTQQAQLA	AAAEIDEEPV	SKAKQSRSEK	KARKAMSKLG	LRQVTGVTRV	TIRKSKNILF
1930	1940	1950	1960	1970	1980	1990	2000
VITKPDVYKS	PASDTYIVFG	EAKIEDLSQQ	AQLAAAEKFK	VQGEAVSNIQ	ENTQTPTVQE	ESEEEEVDET	GVEVKDIELV
2010	2020	2030	2040				
MSQANVSRAK	AVRALKNNSN	DIVNAIMVSV	QAFVP				

Cmpd.	No. of Cmpds.	m/z meas.	Δ m/z [ppm]	z	Rt [min]	Score	P	Range	Sequence	Modification
52	1	717.7555	-144.48	2	30.5	11.1	0	1531-1544	K.EASSPPAVTPSTYK.G	

Detailed Protein Report

Protein 501: **PREDICTED: FERM domain-containing protein 4A isoform X7 [Homo sapiens]**

**Accession:**	gi|530392249	**Score:**	22.1
**Database:**	refseq_human(refseq_human_20140103.fasta)	**MW [kDa]:**	76.9
**Database Date:**	2015-11-30	**pI:**	9.6
**Modification(s):**	Carbamidomethyl	**Sequence Coverage [%]:**	4.1
		**No. of unique Peptides:**	1

```
        10         20         30         40         50         60         70         80
MFIASGSSLQ RFPNAPENDF LTWRSQESDS SQSAKKDMLA ALKSRQEALE ETLRQRLEEL KKLCLREAEL TGKLPVEYPL
        90        100        110        120        130        140        150        160
DPGEEPPIVR RRIGTAFKLD EQKILPKGEE AELERLEREF AIQSQITEAA RRLASDPNVS KKLKKQRKTS YLNALKKLQE
       170        180        190        200        210        220        230        240
IENAINENRI KSGKKPTQRA SLIIDDGNIA SEDSSLSDAL VLEDEDSQVT STISPLHSPH KGLPPRPPSH NRPPPPQSLE
       250        260        270        280        290        300        310        320
GLRQMHYHRN DYDKSPIKPK MWSESSLDEP YEKVKKRSSH SHSSSHKRFP STGSCAEAGG GSNSLQNSPI RGLPHWNSQS
       330        340        350        360        370        380        390        400
SMPSTPDLRV RSPHYVHSTR SVDISPTRLH SLALHFRHRS SSLESQGKLL GSENDTGSPD FYTPRTRSSN GSDPMDDCSS
       410        420        430        440        450        460        470        480
CTSHSSSEHY YPAQMNANYS TLAEDSPSKA RQRQRQRQRA AGALGSASSG SMPNLAARGG AGGAGGAGGG VYLHSQSQPS
       490        500        510        520        530        540        550        560
SQYRIKEYPL YIEGGATPVV VRSLESDQEG HYSVKAQFKT SNSYTAGGLF KESWRGGGGD EGDTGRLTPS RSQILRTPSL
       570        580        590        600        610        620        630        640
GREGAHDKGA GRAAVSDELR QWYQRSTASH KEHSRLSHTS STSSDSGSQY STSSQSTFVA HSRVTRMPQM CKATSAALPQ
       650        660        670        680        690        700        710
SQRSSTPSSE IGATPPSSPH HILTWQTGSY NDSCFLDSSL YPELADVQWY GQEKAKPGTL V
```

Cmpd.	No. of Cmpds.	m/z meas.	Δ m/z [ppm]	z	Rt [min]	Score	P	Range	Sequence	Modification
2251	1	952.9306	-31.49	2	56.8	10.2	1	627-643	R.MPQMCKATSAALPQSQR.S	Carbamidomethyl: 5

Detailed Protein Report

Protein 502: synaptonemal complex protein 2 [Homo sapiens]

**Accession:**	gi|38373673	**Score:**	22.0
**Database:**	refseq_human(refseq_human_20140103.fasta)	**MW [kDa]:**	175.5
**Database Date:**	2015-11-30	**pI:**	9.7
**Modification(s):**	Carbamidomethyl, Oxidation	**Sequence Coverage [%]:**	2.4
		**No. of unique Peptides:**	1

Alias proteins:

Accession	Name	Description
gi|530417767	refseq_human (refseq_human_20140103.fasta)	PREDICTED: synaptonemal complex protein 2 isoform X1 [Homo sapiens]

10	20	30	40	50	60	70	80
MPIRPDLQQL	EKCIDDALRK	NDFKPLKTLL	QIDICEDVKI	KCSKQFFHKV	DNLICRELNK	EDIHNVSAIL	VSVGRCGKNI
90	100	110	120	130	140	150	160
SVLGQAGLLT	MIKQGLIQKM	VAWFEKSKDI	IQSQGNSKDE	AVLNMIEDLV	DLLLVIHDVS	DEGKKQVVES	FVPRICSLVI
170	180	190	200	210	220	230	240
DSRVNICIQQ	EIIKKMNAML	DKMPQDARKI	LSNQEMLILM	SSMGERILDA	GDYDLQVGIV	EALCRMTTEK	QRQELAHQWF
250	260	270	280	290	300	310	320
SMDFIAKAFK	RIKDSEFETD	CRIFLNLVNG	MLGDKRRVFT	FPCLSAFLDK	YELQIPSDEK	LEEFWIDFNL	GSQTLSFYIA
330	340	350	360	370	380	390	400
GDNDDHQWEA	VTVPEEKVQI	YSIEVRESKK	LLTIILKNTV	KISKREGKEL	LLYFDASLEI	TNVTQKIFGA	TKHRESIRKQ
410	420	430	440	450	460	470	480
GISVAKTSLH	ILFDASGSQI	LVPESQISPV	GEELVSLKEK	SKSPKEFAKP	SKYIKNSDKG	NRNNSQLEKT	TPSKRKMSEA
490	500	510	520	530	540	550	560
SMIVSGADRY	TMRSPVLFSN	TSIPPRRRRI	KPPLQMTSSA	EKPSVSQTSE	NRVDNAASLK	SRSSEGRHRR	DNIDKHIKTA
570	580	590	600	610	620	630	640
KCVENTENKN	VEFPNQNFSE	LQDVIPDSQA	AEKRDHTILP	GVLDNICGNK	IHSKWACWTP	VTNIELCNNQ	RASTSSGDTL
650	660	670	680	690	700	710	720
NQDIVINKKL	TKQKSSSSIS	DHNSEGTGKV	KYKKEQTDHI	KIDKAEVEVC	KKHNQQQNHP	KYSGQKNTEN	AKQSDWPVES
730	740	750	760	770	780	790	800
ETTFKSVLLN	KTIEESLIYR	KKYILSKDVN	TATCDKNPSA	SKNVQSHRKA	EKELTSELNS	WDSKQKKMRE	KSKGKEFTNV
810	820	830	840	850	860	870	880
AESLISQINK	RYKTKDDIKS	TRKLKESLIN	SGFSNKPVVQ	LSKEKVQKKS	YRKLKTTFVN	VTSECPVNDV	YNFNLNGADD
890	900	910	920	930	940	950	960
PIIKLGIQEF	QATAKEACAD	RSIRLVGPRN	HDELKSSVKT	KDKKIITNHQ	KKNLFSDTET	EYRCDDSKTD	ISWLREPKSK
970	980	990	1000	1010	1020	1030	1040
PQLIDYSRNK	NVKNHKSGKS	RSSLEKGQPS	SKMTPSKNIT	KKMDKTIPEG	RIRLPRKATK	TKKNYKDLSN	SESECEQEFS
1050	1060	1070	1080	1090	1100	1110	1120
HSFKENIPVK	EENIHSRMKT	VKLPKKQQKV	FCAETEKELS	KQWKNSSLLK	DAIRDNCLDL	SPRSLSGSPS	SIEVTRCIEK
1130	1140	1150	1160	1170	1180	1190	1200
ITEKDFTQDY	DCITKSISPY	PKTSSLESLN	SNSGVGGTIK	SPKNNEKNFL	CASESCSPIP	RPLFLPRHTP	TKSNTIVNRK
1210	1220	1230	1240	1250	1260	1270	1280
KISSLVLTQE	TQNSNSYSDV	SSYSSEERFM	EIESPHINEN	YIQSKREESH	LASSLSKSSE	GREKTWFDMP	CDATHVSGPT
1290	1300	1310	1320	1330	1340	1350	1360
QHLSRKRIYI	EDNLSNSNEV	EMEEKGERRA	NLLPKKLCKI	EDADHHIHKM	SESVSSLSTN	DFSIPWETWQ	NEFAGIEMTY
1370	1380	1390	1400	1410	1420	1430	1440
ETYERLNSEF	KRRNNIRHKM	LSYFTTQSWK	TAQQHLRTMN	HQSQDSRIKK	LDKFQFIIIE	ELENFEKDSQ	SLKDLEKEFV
1450	1460	1470	1480	1490	1500	1510	1520
DFWEKIFQKF	SAYQKSEQQR	LHLLKTSLAK	SVFCNTDSEE	TVFTSEMCLM	KEDMKVLQDR	LLKDMLEEEL	LNVRRELMSV
1530	1540						
FMSHERNANV							

Cmpd.	No. of Cmpds.	m/z meas.	Δ m/z [ppm]	z	Rt [min]	Score	P	Range	Sequence	Modification
423	1	1294.0056	-68.64	2	35.3	11.4	1	1265-1286	K.TWFDMPCDATHVSGPTQHLSRK.R	Carbamidomethyl: 7; Oxidation: 5

Detailed Protein Report

Protein 503: PREDICTED: mucin-6 isoform X1 [Homo sapiens]

**Accession:**	gi|578840955	**Score:**	22.0
**Database:**	refseq_human(refseq_human_20140103.fasta)	**MW [kDa]:**	141.7
**Database Date:**	2015-11-30	**pI:**	5.7
**Modification(s):**	Carbamidomethyl	**Sequence Coverage [%]:**	2.1
		**No. of unique Peptides:**	1

```
        10         20         30         40         50         60         70         80
MVQRWLLLSC CGALLSAGLA NTSYTSPGLQ RLKDSPQTAP DKGQCSTWGA GHFSTFDHHV YDFSGTCNYI FAATCKDAFP
        90        100        110        120        130        140        150        160
TFSVQLRRGP DGSISRIIVE LGASVVTVSE AIISVKDIGV ISLPYTSNGL QITPFGQSVR LVAKQLELEL EVVWGPDSHL
       170        180        190        200        210        220        230        240
MVLVERKYMG QMCGLCGNFD GKVTNEFVSE EGKFLEPHKF AALQKLDDPG EICTFQDIPS THVRQAQHAR ICTQLLTLVA
       250        260        270        280        290        300        310        320
PECSVSKEPF VLSCQADVAA APQPGPQNSS CATLSEYSRQ CSMVGQPVRR WRSPGLCSVG QCPANQVYQE CGSACVKTCS
       330        340        350        360        370        380        390        400
NPQHSCSSSC TFGCFCPEGT VLNDLSNNHT CVPVTQCPCV LHGAMYAPGE VTIAACQTCR CTLGRWVCTE RPCPGHCSLE
       410        420        430        440        450        460        470        480
GGSFVTTFDA RPYRFHGTCT YILLQSPQLP EDGALMAVYD KSGVSHSETS LVAVVYLSRQ DKIVISQDEV VTNNGEAKWL
       490        500        510        520        530        540        550        560
PYKTRNITVF RQTSTHLQMA TSFGLELVVQ LRPIFQAYVT VGPQFRGQTR GLCGNFNGDT TDDFTTSMGI AEGTASLFVD
       570        580        590        600        610        620        630        640
SWRAGNCPAA LERETDPCSM SQLNKVCAET HCSMLLRTGT VFERCHATVN PAPFYKRCMY QACNYEETFP HICAALGDYV
       650        660        670        680        690        700        710        720
HACSLRGVLL WGWRSSVDNC TIPCTGNTTF SYNSQACERT CLSLSDRATE CHHSAVPVDG CNCPDGTYLN QKGECVRKAQ
       730        740        750        760        770        780        790        800
CPCILEGYKF ILAEQSTVIN GITCHCINGR LSCPQRPQMF LASCQAPKTF KSCSQSSENK FGAACAPTCQ MLATGVACVP
       810        820        830        840        850        860        870        880
TKCEPGCVCA EGLYENADGQ CVPPEECPCE FSGVSYPGGA ELHTDCRTCS CSRGRWACQQ GTHCPSTCTL YGEGHVITFD
       890        900        910        920        930        940        950        960
GQRFVFDGNC EYILATDVCG VNDSQPTFKI LTENVICGNS GVTCSRAIKI FLGGLSVVLA DRNYTVTGEE PHVQLGVTPG
       970        980        990       1000       1010       1020       1030       1040
ALSLVVDISI PGRYNLTLIW NRHMTILIRI ARASQDPLCG LCGNFNGNMK DDFETRSRYV ASSELELVNS WKESPLCGDV
      1050       1060       1070       1080       1090       1100       1110       1120
SFVTDPCSLN AFRRSWAERK CSVINSQTFA TCHSKVYHLP YYEACVRDAC GCDSGGDCEC LCDAVAAYAQ ACLDKGVCVD
      1130       1140       1150       1160       1170       1180       1190       1200
WRTPAFCPIY CGFYNTHTQD GHGEYQYTQE ANCTWHYQPC LCPSQPQSVP GSNIEGCYNC SQDEYFDHEE GVCVPCMPPT
      1210       1220       1230       1240       1250       1260       1270       1280
TPQPPTTPQL PTTGSRPTQV WPMTGTSTTI GLLSSTGPSP SSNHTPASPT QTPLLPATLT SSKPTASSGG KEPPAEPMER
      1290       1300       1310       1320
AAAGGPRYTS GVGLGMALLP GSHLPLSRFP A
```

Cmpd.	No. of Cmpds.	m/z meas.	Δ m/z [ppm]	z	Rt [min]	Score	P	Range	Sequence	Modification
2052	1	840.8203	-18.08	2	56.0	10.4	0	168-182	K.YMGQMCGLCGNFDGK.V	Carbamidomethyl: 6

Detailed Protein Report

Protein 504: metallothionein-4 [Homo sapiens]

Accession: gi|14269578
Database: refseq_human(refseq_human_20140103.fasta)
Database Date: 2015-11-30
Modification(s): Carbamidomethyl

Score: 22.0
MW [kDa]: 6.4
pI: 10.8
Sequence Coverage [%]: 25.8
No. of unique Peptides: 1

10	20	30	40	50	60	70
MDPRECVCMS	GGICMCGDNC	KCTTCNCKTC	RKSCCPCCPP	GCAKCARGCI	CKGGSDKCSC	CP

Cmpd.	No. of Cmpds.	m/z meas.	Δ m/z [ppm]	z	Rt [min]	Score	P	Range	Sequence	Modification
1709	1	892.3390	4.69	2	51.7	11.6	1	33-47	K.SCCPCCPPGCAKCAR.G	Carbamidomethyl: 2, 3, 6, 10, 13

Detailed Protein Report

Protein 505: **PREDICTED: splicing factor, suppressor of white-apricot homolog isoform X1 [Homo sapiens]**

**Accession:**	gi|578824119	**Score:**	22.0
**Database:**	refseq_human(refseq_human_20140103.fasta)	**MW [kDa]:**	96.4
**Database Date:**	2015-11-30	**pI:**	9.4
**Modification(s):**	Oxidation	**Sequence Coverage [%]:**	2.9
		**No. of unique Peptides:**	1

10	20	30	40	50	60	70	80
MYGASGGRAK	PERKSGAKEE	AGPGGAGGGG	SRVELLVFGY	ACKLFRDDER	ALAQEQGQHL	IPWMGDHKIL	IDRYDGRGHL
**90**	**100**	**110**	**120**	**130**	**140**	**150**	**160**
HDLSEYDAEY	STWNRDYQLS	EEEARIEALC	DEERYLALHT	DLLEEEARQE	EEYKRLSEAL	AEDGSYNAVG	FTYGSDYYDP
**170**	**180**	**190**	**200**	**210**	**220**	**230**	**240**
SEPTEEEEPS	KQREKNEAEN	LEENEEPFVA	PLGLSVPSDV	ELPPTAKMHA	IIERTASFVC	RQGAQFEIML	KAKQARNSQF
**250**	**260**	**270**	**280**	**290**	**300**	**310**	**320**
DFLRFDHYLN	PYYKFIQKAM	KEGRYTVLAE	NKSDEKKKSG	VSSDNEDDDD	EEDGNYLHPS	LFASKKCNRL	EELMKPLKVV
**330**	**340**	**350**	**360**	**370**	**380**	**390**	**400**
DPDHPLAALV	RKAQADSSTP	TPHNADGAPV	QPSQVEYTAD	STVAAMYYSY	YMLPDGTYCL	APPPPGIDVT	TYYSTLPAGV
**410**	**420**	**430**	**440**	**450**	**460**	**470**	**480**
TVSNSPGVTT	TAPPPPGTTP	LPPPTTAETS	SGATSTTTTT	SALAPVAAII	PPPPDVQPVI	DKLAEYVARN	GLKFETSVRA
**490**	**500**	**510**	**520**	**530**	**540**	**550**	**560**
KNDQRFEFLQ	PWHQYNAYYE	FKKQFFLQKE	GGDSMQAVSA	PEEAPTDSAP	EKPSDAGEDG	APEDAAEVGA	RAGSGGKKEA
**570**	**580**	**590**	**600**	**610**	**620**	**630**	**640**
SSSKTVPDGK	LVKAKQKLED	RLAAAAREKL	AQASKESKEK	QLQAERKRKA	ALFLQTLKNP	LPEAEAGKIE	ESPFSVEESS
**650**	**660**	**670**	**680**	**690**	**700**	**710**	**720**
TTPCPLLTGG	RPLPTLEVKP	PDRPSSKSKD	PPREEEKEKK	KKKHKKRSRT	RSRSPKYHSS	SKSRSRSHSK	AKHSLPSAYR
**730**	**740**	**750**	**760**	**770**	**780**	**790**	**800**
TVRRSRSRSR	SPRRRAHSPE	RRREERSVPT	AYRVSRSPGA	SRKRTRSRSP	HEKKKKRRSR	SRTKSKARSQ	SVSPSKQAAP
**810**	**820**	**830**	**840**	**850**	**860**	**870**	**880**
RPAAPAAHSA	HSASVSPVES	RGSSQERSRG	VSQEKEAQIS	SAIVSSVQSK	ITQDLMAKVR	AMLAASKNLQ	TSAS

Cmpd.	No. of Cmpds.	m/z meas.	Δ m/z [ppm]	z	Rt [min]	Score	P	Range	Sequence	Modification
2815	1	698.3601	23.69	2	64.3	10.1	1	1-13	-.MYGASGGRAKPER.K	Oxidation: 1

Detailed Protein Report

Protein 506: cullin-4A isoform 2 [Homo sapiens]

**Accession:**	gi|11140811	**Score:**	22.0
**Database:**	refseq_human(refseq_human_20140103.fasta)	**MW [kDa]:**	76.8
**Database Date:**	2015-11-30	**pI:**	6.8
**Modification(s):**	Oxidation	**Sequence Coverage [%]:**	3.3
		**No. of unique Peptides:**	2

Alias proteins:

Accession	Name	Description
gi|511772961	refseq_human (refseq_human_20140103.fasta)	cullin-4A isoform 2 [Homo sapiens]

10	20	30	40	50	60	70	80
MLYKQLRQAC	EDHVQAQILP	FREDSLDSVL	FLKKINTCWQ	DHCRQMIMIR	SIFLFLDRTY	VLQNSTLPSI	WDMGLELFRT
**90**	**100**	**110**	**120**	**130**	**140**	**150**	**160**
HIISDKMVQS	KTIDGILLLI	ERERSGEAVD	RSLLRSLLGM	LSDLQVYKDS	FELKFLEETN	CLYAAEGQRL	MQEREVPEYL
**170**	**180**	**190**	**200**	**210**	**220**	**230**	**240**
NHVSKRLEEE	GDRVITYLDH	STQKPLIACV	EKQLLGEHLT	AILQKGLDHL	LDENRVPDLA	QMYQLFSRVR	GGQQALLQHW
**250**	**260**	**270**	**280**	**290**	**300**	**310**	**320**
SEYIKTFGTA	IVINPEKDKD	MVQDLLDFKD	KVDHVIEVCF	QKNERFVNLM	KESFETFINK	RPNKPAELIA	KHVDSKLRAG
**330**	**340**	**350**	**360**	**370**	**380**	**390**	**400**
NKEATDEELE	RTLDKIMILF	RFIHGKDVFE	AFYKKDLAKR	LLVGKSASVD	AEKSMLSKLK	HECGAAFTSK	LEGMFKDMEL
**410**	**420**	**430**	**440**	**450**	**460**	**470**	**480**
SKDIMVHFKQ	HMQNQSDSGP	IDLTVNILTM	GYWPTYTPME	VHLTPEMIKL	QEVFKAFYLG	KHSGRKLQWQ	TTLGHAVLKA
**490**	**500**	**510**	**520**	**530**	**540**	**550**	**560**
EFKEGKKEFQ	VSLFQTLVLL	MFNEGDGFSF	EEIKMATGIE	DSELRRTLQS	LACGKARVLI	KSPKGKEVED	GDKFIFNGEF
**570**	**580**	**590**	**600**	**610**	**620**	**630**	**640**
KHKLFRIKIN	QIQMKETVEE	QVSTTERVFQ	DRQYQIDAAI	VRIMKMRKTL	GHNLLVSELY	NQLKFPVKPG	DLKKRIESLI
**650**	**660**						
DRDYMERDKD	NPNQYHYVA						

Cmpd.	No. of Cmpds.	m/z meas.	Δ m/z [ppm]	z	Rt [min]	Score	P	Range	Sequence	Modification
2598	1	625.3169	-85.65	2	63.1	10.6	1	332-341	R.TLDKIMILFR.F	
2723	1	774.4242	-43.85	2	63.0	11.3	2	564-575	K.LFRIKINQIQMK.E	Oxidation: 11

Detailed Protein Report

Protein 507: mothers against decapentaplegic homolog 3 isoform 1 [Homo sapiens]

**Accession:**	gi|5174513	**Score:**	21.9
**Database:**	refseq_human(refseq_human_20140103.fasta)	**MW [kDa]:**	48.0
**Database Date:**	2015-11-30	**pI:**	6.8
**Modification(s):**	Carbamidomethyl, Oxidation	**Sequence Coverage [%]:**	6.4
		**No. of unique Peptides:**	2

Quantitation

QD:QU **Median:** 1.36 **CV:** 0.00 % **No. of Peptides:** 1

```
        10         20         30         40         50         60         70         80
MSSILPFTPP IVKRLLGWKK GEQNGQEEKW CEKAVKSLVK KLKKTGQLDE LEKAITTQNV NTKCITIPRS LDGRLQVSHR
        90        100        110        120        130        140        150        160
KGLPHVIYCR LWRWPDLHSH HELRAMELCE FAFNMKKDEV CVNPYHYQRV ETPVLPPVLV PRHTEIPAEF PPLDDYSHSI
       170        180        190        200        210        220        230        240
PENTNFPAGI EPQSNIPETP PPGYLSEDGE TSDHQMNHSM DAGSPNLSPN PMSPAHNNLD LQPVTYCEPA FWCSISYYEL
       250        260        270        280        290        300        310        320
NQRVGETFHA SQPSMTVDGF TDPSNSERFC LGLLSNVNRN AAVELTRRHI GRGVRLYYIG GEVFAECLSD SAIFVQSPNC
       330        340        350        360        370        380        390        400
NQRYGWHPAT VCKIPPGCNL KIFNNQEFAA LLAQSVNQGF EAVYQLTRMC TIRMSFVKGW GAEYRRQTVT STPCWIELHL
       410        420        430
NGPLQWLDKV LTQMGSPSIR CSSVS
```

Cmpd.	No. of Cmpds.	m/z meas.	Δ m/z [ppm]	z	Rt [min]	Score	P	Range	Sequence	Modification	Ratios
1190	3	644.3159	-45.97	2	45.0	10.9	1	64-74	K.CITIPRSLDGR.L	Carbamidomethyl: 1	QD:QU 1.36
2171	1	862.9449	30.12	2	57.5	11.0	1	410-425	K.VLTQMGSPSIRCSSVS.-	Carbamidomethyl: 12; Oxidation: 5	

Detailed Protein Report

Protein 508: rho guanine nucleotide exchange factor 19 [Homo sapiens]

Accession: gi|40255149
Database: refseq_human(refseq_human_20140103.fasta)
Database Date: 2015-11-30

Score: 21.9
MW [kDa]: 89.1
pI: 8.0
Sequence Coverage [%]: 4.0
No. of unique Peptides: 1

10	20	30	40	50	60	70	80
MDCGPPATLQ	PHLTGPPGTA	HHPVAVCQQE	SLSFAELPAL	KPPSPVCLDL	FPVAPEELRA	PGSRWSLGTP	APLQGLLWPL
**90**	**100**	**110**	**120**	**130**	**140**	**150**	**160**
SPGGSDTEIT	SGGMRPSRAG	SWPHCPGAQP	PALEGPWSPR	HTQPQRRASH	GSEKKSAWRK	MRVYQREEVP	GCPEAHAVFL
**170**	**180**	**190**	**200**	**210**	**220**	**230**	**240**
EPGQVVQEQA	LSTEEPRVEL	SGSTRVSLEG	PERRRFSASE	LMTRLHSSLR	LGRNSAARAL	ISGSGTGAAR	EGKASGMEAR
**250**	**260**	**270**	**280**	**290**	**300**	**310**	**320**
SVEMSGDRVS	RPAPGDSREG	DWSEPRLDTQ	EEPPLGSRST	NERRQSRFLL	NSVLYQEYSD	VASARELRRQ	QREEEGPGDE
**330**	**340**	**350**	**360**	**370**	**380**	**390**	**400**
AEGAEEGPGP	PRANLSPSSS	FRAQRSARGS	TFSLWQDIPD	VRGSGVLATL	SLRDCKLQEA	KFELITSEAS	YIHSLSVAVG
**410**	**420**	**430**	**440**	**450**	**460**	**470**	**480**
HFLGSAELSE	CLGAQDKQWL	FSKLPEVKST	SERFLQDLEQ	RLEADVLRFS	VCDVVLDHCP	AFRRVYLPYV	TNQAYQERTY
**490**	**500**	**510**	**520**	**530**	**540**	**550**	**560**
QRLLLENPRF	PGILARLEES	PVCQRLPLTS	FLILPFQRIT	RLKMLVENIL	KRTAQGSEDE	DMATKAFNAL	KELVQECNAS
**570**	**580**	**590**	**600**	**610**	**620**	**630**	**640**
VQSMKRTEEL	IHLSKKIHFE	GKIFPLISQA	RWLVRHGELV	ELAPLPAAPP	AKLKLSSKAV	YLHLFNDCLL	LSRRKELGKF
**650**	**660**	**670**	**680**	**690**	**700**	**710**	**720**
AVFVHAKMAE	LQVRDLSLKL	QGIPGHVFLL	QLLHGQHMKH	QFLLRARTES	EKQRWISALC	PSSPQEDKEV	ISEGEDCPQV
**730**	**740**	**750**	**760**	**770**	**780**	**790**	**800**
QCVRTYKALH	PDELTLEKTD	ILSVRTWTSD	GWLEGVRLAD	GEKGWVPQAY	VEEISSLSAR	LRNLRENKRV	TSATSKLGEA
**810**							
PV							

Cmpd.	No. of Cmpds.	m/z meas.	Δ m/z [ppm]	z	Rt [min]	Score	P	Range	Sequence	Modification
2498	1	673.3379	-69.90	2	61.8	10.5	1	636-647	K.ELGKFAVFVHAK.M	

Detailed Protein Report

Protein 509: PREDICTED: zinc finger protein basonuclin-1 isoform X2 [Homo sapiens]

Accession: gi|578827289
Database: refseq_human(refseq_human_20140103.fasta)
Database Date: 2015-11-30
Modification(s): Carbamidomethyl

Score: 21.9
MW [kDa]: 110.6
pI: 6.5
Sequence Coverage [%]: 3.2
No. of unique Peptides: 1

10	20	30	40	50	60	70	80
MCYCIGPQSR	CSNNFFKNIF	FSSKRQDFGL	TAISCTLNCS	CQSFKPGKIN	HRQCDQCKHG	WVAHALSKLR	IPPMYPTSQV
90	100	110	120	130	140	150	160
EIVQSNVVFD	ISSLMLYGTQ	AIPVRLKILL	DRLFSVLKQD	EVLQILHALD	WTLQDYIRGY	VLQDASGKVL	DHWSIMTSEE
170	180	190	200	210	220	230	240
EVATLQQFLR	FGETKSIVEL	MAIQEKEEQS	IIIPPSTANV	DIRAFIESCS	HRSSSLPTPV	DKGNPSSIHP	FENLISNMTF
250	260	270	280	290	300	310	320
MLPFQFFNPL	PPALIGSLPE	QYMLEQGHDQ	SQDPKQEVHG	PFPDSSFLTS	SSTPFQVEKD	QCLNCPDAIT	KKEDSTHLSD
330	340	350	360	370	380	390	400
SSSYNIVTKF	ERTQLSPEAK	VKPERNSLGT	KKGRVFCTAC	EKTFYDKGTL	KIHYNAVHLK	IKHKCTIEGC	NMVFSSLRSR
410	420	430	440	450	460	470	480
NRHSANPNPR	LHMPMNRNNR	DKDLRNSLNL	ASSENYKCPG	FTVTSPDCRP	PPSYPGSGED	SKGQPAFPNI	GQNGVLFPNL
490	500	510	520	530	540	550	560
KTVQPVLPFY	RSPATPAEVA	NTPGILPSLP	LLSSSIPEQL	ISNEMPFDAL	PKKKSRKSSM	PIKIEKEAVE	IANEKRHNLS
570	580	590	600	610	620	630	640
SDEDMPLQVV	SEDEQEACSP	QSHRVSEEQH	VQSGGLGKPF	PEGERPCHRE	SVIESSGAIS	QTPEQATHNS	ERETEQTPAL
650	660	670	680	690	700	710	720
IMVPREVEDG	GHEHYFTPGM	EPQVPFSDYM	ELQQRLLAGG	LFSALSNRGM	AFPCLEDSKE	LEHVGQHALA	RQIEENRFQC
730	740	750	760	770	780	790	800
DICKKTFKNA	CSVKIHHKNM	HVKEMHTCTV	EGCNATFPSR	RSRDRHSSNL	NLHQKALSQE	ALESSEDHFR	AAYLLKDVAK
810	820	830	840	850	860	870	880
EAYQDVAFTQ	QASQTSVIFK	GTSRMGSLVY	PITQVHSASL	ESYNSGPLSE	GTILDLSTTS	SMKSESSSHS	SWDSDGVSEE
890	900	910	920	930	940	950	960
GTVLMEDSDG	NCEGSSLVPG	EDEYPICVLM	EKADQSLASL	PSGLPITCHL	CQKTYSNKGT	FRAHYKTVHL	RQLHKCKVPG
970	980	990	1000				
CNTMFSSVRS	RNRHSQNPNL	HKSLASSPSH	LQ				

Cmpd.	No. of Cmpds.	m/z meas.	Δ m/z [ppm]	z	Rt [min]	Score	P	Range	Sequence	Modification
1802	1	990.7899	-140.63	2	51.0	10.5	1	2-17	M.CYCIGPQSRCSNNFFK.N	Carbamidomethyl: 1, 10

Detailed Protein Report

Protein 510: rho-associated protein kinase 2 [Homo sapiens]

Accession: gi|41872583
Database: refseq_human(refseq_human_20140103.fasta)
Database Date: 2015-11-30
Modification(s): Oxidation

Score: 21.8
MW [kDa]: 160.8
pI: 5.7
Sequence Coverage [%]: 2.0
No. of unique Peptides: 2

10	20	30	40	50	60	70	80
MSRPPPTGKM	PGAPETAPGD	GAGASRQRKL	EALIRDPRSP	INVESLLDGL	NSLVLDLDFP	ALRKNKNIDN	FLNRYEKIVK
90	100	110	120	130	140	150	160
KIRGLQMKAE	DYDVVKVIGR	GAFGEVQLVR	HKASQKVYAM	KLLSKFEMIK	RSDSAFFWEE	RDIMAFANSP	WVVQLFYAFQ
170	180	190	200	210	220	230	240
DDRYLYMVME	YMPGGDLVNL	MSNYDVPEKW	AKFYTAEVVL	ALDAIHSMGL	IHRDVKPDNM	LLDKHGHLKL	ADFGTCMKMD
250	260	270	280	290	300	310	320
ETGMVHCDTA	VGTPDYISPE	VLKSQGGDGF	YGRECDWWSV	GVFLYEMLVG	DTPFYADSLV	GTYSKIMDHK	NSLCFPEDAE
330	340	350	360	370	380	390	400
ISKHAKNLIC	AFLTDREVRL	GRNGVEEIRQ	HPFFKNDQWH	WDNIRETAAP	VVPELSSDID	SSNFDDIEDD	KGDVETFPIP
410	420	430	440	450	460	470	480
KAFVGNQLPF	IGFTYYRENL	LLSDSPSCRE	TDSIQSRKNE	ESQEIQKKLY	TLEEHLSNEM	QAKEELEQKC	KSVNTRLEKT
490	500	510	520	530	540	550	560
AKELEEEITL	RKSVESALRQ	LEREKALLQH	KNAEYQRKAD	HEADKKRNLE	NDVNSLKDQL	EDLKKRNQNS	QISTEKVNQL
570	580	590	600	610	620	630	640
QRQLDETNAL	LRTESDTAAR	LRKTQAESSK	QIQQLESNNR	DLQDKNCLLE	TAKLKLEKEF	INLQSALESE	RRDRTHGSEI
650	660	670	680	690	700	710	720
INDLQGRICG	LEEDLKNGKI	LLAKVELEKR	QLQERFTDLE	KEKSNMEIDM	TYQLKVIQQS	LEQEEAEHKA	TKARLADKNK
730	740	750	760	770	780	790	800
IYESIEEAKS	EAMKEMEKKL	LEERTLKQKV	ENLLLEAEKR	CSLLDCDLKQ	SQQKINELLK	QKDVLNEDVR	NLTLKIEQET
810	820	830	840	850	860	870	880
QKRCLTQNDL	KMQTQQVNTL	KMSEKQLKQE	NNHLMEMKMN	LEKQNAELRK	ERQDADGQMK	ELQDQLEAEQ	YFSTLYKTQV
890	900	910	920	930	940	950	960
RELKEECEEK	TKLGKELQQK	KQELQDERDS	LAAQLEITLT	KADSEQLARS	IAEEQYSDLE	KEKIMKELEI	KEMMARHKQE
970	980	990	1000	1010	1020	1030	1040
LTEKDATIAS	LEETNRTLTS	DVANLANEKE	ELNNKLKDVQ	EQLSRLKDEE	ISAAAIKAQF	EKQLLTERTL	KTQAVNKLAE
1050	1060	1070	1080	1090	1100	1110	1120
IMNRKEPVKR	GNDTDVRRKE	KENRKLHMEL	KSEREKLTQQ	MIKYQKELNE	MQAQIAEESQ	IRIELQMTLD	SKDSDIEQLR
1130	1140	1150	1160	1170	1180	1190	1200
SQLQALHIGL	DSSSIGSGPG	DAEADDGFPE	SRLEGWLSLP	VRNNTKKFGW	VKKYVIVSSK	KILFYDSEQD	KEQSNPYMVL
1210	1220	1230	1240	1250	1260	1270	1280
DIDKLFHVRP	VTQTDVYRAD	AKEIPRIFQI	LYANEGESKK	EQEFPVEPVG	EKSNYICHKG	HEFIPTLYHF	PTNCEACMKP
1290	1300	1310	1320	1330	1340	1350	1360
LWHMFKPPPA	LECRRCHIKC	HKDHMDKKEE	IIAPCKVYYD	ISTAKNLLLL	ANSTEEQQKW	VSRLVKKIPK	KPPAPDPFAR
1370	1380	1390					
SSPRTSMKIQ	QNQSIRRPSR	QLAPNKPS					

Cmpd.	No. of Cmpds.	m/z meas.	Δ m/z [ppm]	z	Rt [min]	Score	P	Range	Sequence	Modification
2066	1	1044.9156	-114.45	2	56.2	11.5	2	583-600	R.KTQAESSKQIQQLESNNR.D	
302	1	409.4394	-221.40	3	32.5	10.3	2	730-739	K.SEAMKEMEKK.L	Oxidation: 7

Detailed Protein Report

Protein 511: **transcription factor SOX-6 isoform 3 [Homo sapiens]**

**Accession:**	gi|224967050	**Score:**	21.8
**Database:**	refseq_human(refseq_human_20140103.fasta)	**MW [kDa]:**	88.9
**Database Date:**	2015-11-30	**pI:**	6.9
		**Sequence Coverage [%]:**	3.5
		**No. of unique Peptides:**	2

10	20	30	40	50	60	70	80
MSSKQATSPF	ACAADGEDAM	TQDLTSREKE	EGSDQHVASH	LPLHPIMHNK	PHSEELPTLV	STIQQDADWD	SVLSSQQRME
**90**	**100**	**110**	**120**	**130**	**140**	**150**	**160**
SENNKLCSLY	SFRNTSTSPH	KPDEGSRDRE	IMTSVTFGTP	ERRKGSLADV	VDTLKQKKLE	EMTRTEQEDS	SCMEKLLSKD
**170**	**180**	**190**	**200**	**210**	**220**	**230**	**240**
WKEKMERLNT	SELLGEIKGT	PESLAEKERQ	LSTMITQLIS	LREQLLAAHD	EQKKLAASQI	EKQRQQMDLA	RQQQEQIARQ
**250**	**260**	**270**	**280**	**290**	**300**	**310**	**320**
QQQLLQQQHK	INLLQQQIQV	QGHMPPLMIP	IFPHDQRTLA	AAAAAQQGFL	FPPGITYKPG	DNYPVQFIPS	TMAAAAASGL
**330**	**340**	**350**	**360**	**370**	**380**	**390**	**400**
SPLQLQQLYA	AQLASMQVSP	GAKMPSTPQP	PNTAGTVSPT	GIKNEKRGTS	PVTQVKDEAA	AQPLNLSSRP	KTAEPVKSPT
**410**	**420**	**430**	**440**	**450**	**460**	**470**	**480**
SPTQNLFPAS	KTSPVNLPNK	SSIPSPIGGS	LGRGSSLGKW	KSQHQEETYE	LDILSSLNSP	ALFGDQDTVM	KAIQEARKMR
**490**	**500**	**510**	**520**	**530**	**540**	**550**	**560**
EQIQREQQQQ	QPHGVDGKLS	SINNMGLNSC	RNEKERTRFE	NLGPQLTGKS	NEDGKLGPGV	IDLTRPEDAE	GSKAMNGSAA
**570**	**580**	**590**	**600**	**610**	**620**	**630**	**640**
KLQQYYCWPT	GGATVAEARV	YRDARGRASS	EPHIKRPMNA	FMVWAKDERR	KILQAFPDMH	NSNISKILGS	RWKSMSNQEK
**650**	**660**	**670**	**680**	**690**	**700**	**710**	**720**
QPYYEEQARL	SKIHLEKYPN	YKYKPRPKRT	CIVDGKKLRI	GEYKQLMRSR	RQEMRQFFTV	GQQPQIPITT	GTGVVYPGAI
**730**	**740**	**750**	**760**	**770**	**780**	**790**	**800**
TMATTTPSPQ	MTSDCSSTSA	SPEPSLPVIQ	STYGMKTDGG	SLAGNEMING	EDEMEMYDDY	EDDPKSDYSS	ENEAPEAVSA
**810**							
N							

Cmpd.	No. of Cmpds.	m/z meas.	Δ m/z [ppm]	z	Rt [min]	Score	P	Range	Sequence	Modification
1137	4	500.6373	-249.35	2	42.7	11.7	0	232-239	R.QQQEQIAR.Q	
1327	1	660.9680	-58.76	3	46.7	10.1	0	344-363	K.MPSTPQPPNTAGTVSPTGIK.N	

Detailed Protein Report

Protein 512: nuclear mitotic apparatus protein 1 isoform 2 [Homo sapiens]

**Accession:**	gi|557440899	**Score:**	21.8
**Database:**	refseq_human(refseq_human_20140103.fasta)	**MW [kDa]:**	236.4
**Database Date:**	2015-11-30	**pI:**	5.5
**Modification(s):**	Oxidation	**Sequence Coverage [%]:**	1.6
		**No. of unique Peptides:**	2

Quantitation

QD:QU	**Median:** 0.82	**CV:** 0.00 %	**No. of Peptides:** 1

Alias proteins:

Accession	Name	Description
gi|578821456	refseq_human (refseq_human_20140103.fasta)	PREDICTED: nuclear mitotic apparatus protein 1 isoform X9 [Homo sapiens]

Detailed Protein Report

10	20	30	40	50	60	70	80
MTLHATRGAA	LLSWVNSLHV	ADPVEAVLQL	QDCSIFIKII	DRIHGTEEGQ	QILKQPVSER	LDFVCSFLQK	NRKHPSSPEC
90	100	110	120	130	140	150	160
LVSAQKVLEG	SELELAKMTM	LLLYHSTMSS	KSPRDWEQFE	YKIQAELAVI	LKFVLDHEDG	LNLNEDLENF	LQKAPVPSTC
170	180	190	200	210	220	230	240
SSTFPEELSP	PSHQAKREIR	FLELQKVASS	SSGNNFLSGS	PASPMGDILQ	TPQFQMRRLK	KQLADERSNR	DELELELAEN
250	260	270	280	290	300	310	320
RKLLTEKDAQ	IAMMQQRIDR	LALLNEKQAA	SPLEPKELEE	LRDKNESLTM	RLHETLKQCQ	DLKTEKSQMD	RKINQLSEEN
330	340	350	360	370	380	390	400
GDLSFKLREF	ASHLQQLQDA	LNELTEEHSK	ATQEWLEKQA	QLEKELSAAL	QDKKCLEEKN	EILQGKLSQL	EEHLSQLQDN
410	420	430	440	450	460	470	480
PPQEKGEVLG	DVLQLETLKQ	EAATLAANNT	QLQARVEMLE	TERGQQEAKL	LAERGHFEEE	KQQLSSLITD	LQSSISNLSQ
490	500	510	520	530	540	550	560
AKEELEQASQ	AHGARLTAQV	ASLTSELTTL	NATIQQQDQE	LAGLKQQAKE	KQAQLAQTLQ	QQEQASQGLR	HQVEQLSSSL
570	580	590	600	610	620	630	640
KQKEQQLKEV	AEKQEATRQD	HAQQLATAAE	EREASLRERD	AALKQLEALE	KEKAAKLEIL	QQQLQVANEA	RDSAQTSVTQ
650	660	670	680	690	700	710	720
AQREKAELSR	KVEELQACVE	TARQEQHEAQ	AQVAELELQL	RSEQQKATEK	ERVAQEKDQL	QEQLQALKES	LKVTKGSLEE
730	740	750	760	770	780	790	800
EKRRAADALE	EQQRCISELK	AETRSLVEQH	KRERKELEEE	RAGRKGLEAR	LQQLGEAHQA	ETEVLRRELA	EAMAAQHTAE
810	820	830	840	850	860	870	880
SECEQLVKEV	AAWRERYEDS	QQEEAQYGAM	FQEQLMTLKE	ECEKARQELQ	EAKEKVAGIE	SHSELQISRQ	QNELAELHAN
890	900	910	920	930	940	950	960
LARALQQVQE	KEVRAQKLAD	DLSTLQEKMA	ATSKEVARLE	TLVRKAGEQQ	ETASRELVKE	PARAGDRQPE	WLEEQQGRQF
970	980	990	1000	1010	1020	1030	1040
CSTQAALQAM	EREAEQMGNE	LERLRAALME	SQGQQQEERG	QQEREVARLT	QERGRAQADL	ALEKAARAEL	EMRLQNALNE
1050	1060	1070	1080	1090	1100	1110	1120
QRVEFATLQE	ALAHALTEKE	GKDQELAKLR	GLEAAQIKEL	EELRQTVKQL	KEQLAKKEKE	HASGSGAQSE	AAGRTEPTGP
1130	1140	1150	1160	1170	1180	1190	1200
KLEALRAEVS	KLEQQCQKQQ	EQADSLERSL	EAERASRAER	DSALETLQGQ	LEEKAQELGH	SQSALASAQR	ELAAFRTKVQ
1210	1220	1230	1240	1250	1260	1270	1280
DHSKAEDEWK	AQVARGRQEA	ERKNSLISSL	EEEVSILNRQ	VLEKEGESKE	LKRLVMAESE	KSQKLEERLR	LLQAETASNS
1290	1300	1310	1320	1330	1340	1350	1360
ARAAERSSAL	REEVQSLREE	AEKQRVASEN	LRQELTSQAE	RAEELGQELK	AWQEKFFQKE	QALSTLQLEH	TSTQALVSEL
1370	1380	1390	1400	1410	1420	1430	1440
LPAKHLCQQL	QAEQAAAEKR	HREELEQSKQ	AAGGLRAELL	RAQRELGELI	PLRQKVAEQE	RTAQQLRAEK	ASYAEQLSML
1450	1460	1470	1480	1490	1500	1510	1520
KKAHGLLAEE	NRGLGERANL	GRQFLEVELD	QAREKYVQEL	AAVRADAETR	LAEVQREAQS	TARELEVMTA	KYEGAKVKVL
1530	1540	1550	1560	1570	1580	1590	1600
EERQRFQEER	QKLTAQVEEL	SKKLADSDQA	SKVQQQKLKA	VQAQGGESQQ	EAQRLQAQLN	ELQAQLSQKE	QAAEHYKLQM
1610	1620	1630	1640	1650	1660	1670	1680
EKAKTHYDAK	KQQNQELQEQ	LRSLEQLQKE	NKELRAEAER	LGHELQQAGL	KTKEAEQTCR	HLTAQVRSLE	AQVAHADQQL
1690	1700	1710	1720	1730	1740	1750	1760
RDLGKFQVAT	DALKSREPQA	KPQLDLSIDS	LDLSCEEGTP	LSITSKLPRT	QPDGTSVPGE	PASPISQRLP	PKVESLESLY
1770	1780	1790	1800	1810	1820	1830	1840
FTPIPARSQA	PLESSLDSLG	DVFLDSGRKT	RSARRRTTQI	INITMTKKLD	VEEPDSANSS	FYSTRSAPAS	QASLRATSST
1850	1860	1870	1880	1890	1900	1910	1920
QSLARLGSPD	YGNSALLSLP	GYRPTTRSSA	RRSQAGVSSG	APPGRNSFYM	GTCQDEPEQL	DDWNRIAELQ	QRNRVCPPHL
1930	1940	1950	1960	1970	1980	1990	2000
KTCYPLESRP	SLSLGTITDE	EMKTGDPQET	LRRASMQPIQ	IAEGTGITTR	QQRKRVSLEP	HQGPGTPESK	KATSCFPRPM
2010	2020	2030	2040	2050	2060	2070	2080
TPRDRHEGRK	QSTTEAQKKA	APASTKQADR	RQSMAFSILN	TPKKLGNSLL	RRGASKKALS	KASPNTRSGT	RRSPRIATTT
2090	2100	2110					
ASAATAAAIG	ATPRAKGKAK	H					

Detailed Protein Report

Cmpd.	No. of Cmpds.	m/z meas.	Δ m/z [ppm]	z	Rt [min]	Score	P	Range	Sequence	Modification	Ratios	
500	1	630.6311	-18.35	3	36.2	11.0	1	984-999	R.LRAALMESQGQQQEER.G	Oxidation: 6	QD:QU	0.82
1859	1	682.2687	-75.42	3	53.6	10.7	1	1808-1825	K.KLDVEEPDSANSSFYSTR.S			

Detailed Protein Report

Protein 513: 2-oxoglutarate dehydrogenase-like, mitochondrial isoform c [Homo sapiens]

Accession: gi|221316669
Database: refseq_human(refseq_human_20140103.fasta)
Database Date: 2015-11-30

Score: 21.7
MW [kDa]: 91.2
pI: 6.4
Sequence Coverage [%]: 2.7
No. of unique Peptides: 1

Quantitation

QD:QU **Median:** 1.31 **CV:** 0.00 % **No. of Peptides:** 1

10	20	30	40	50	60	70	80
MFINDVEQCQ	WIRQKFETPG	VMQFSSEEKR	TLLARLVRSM	RFEDFLARKW	SSEKRFGLEG	CEVMIPALKT	IIDKSSEMGI
90	100	110	120	130	140	150	160
ENVILGMPHR	GRLNVLANVI	RKDLEQIFCQ	FDPKLEAADE	GSGDVKYHLG	MYHERINRVT	NRNITLSLVA	NPSHLEAVDP
170	180	190	200	210	220	230	240
VVQGKTKAEQ	FYRGDAQGKK	VMSILVHGDA	AFAGQGVVYE	TFHLSDLPSY	TTNGTVHVVV	NNQIGFTTDP	RMARSSPYPT
250	260	270	280	290	300	310	320
DVARVVNAPI	FHVNADDPEA	VIYVCSVAAE	WRNTFNKDVV	VDLVCYRRRG	HNEMDEPMFT	QPLMYKQIHR	QVPVLKKYAD
330	340	350	360	370	380	390	400
KLIAEGTVTL	QEFEEEIAKY	DRICEEAYGR	SKDKKILHIK	HWLDSPWPGF	FNVDGEPKSM	TCPATGIPED	MLTHIGSVAS
410	420	430	440	450	460	470	480
SVPLEDFKIH	TGLSRILRGR	ADMTKNRTVD	WALAEYMAFG	SLLKEGIHVR	LSGQDVERGT	FSHRHHVLHD	QEVDRRTCVP
490	500	510	520	530	540	550	560
MNHLWPDQAP	YTVCNSSLSE	YGVLGFELGY	AMASPNALVL	WEAQFGDFHN	TAQCIIDQFI	STGQAKWVRH	NGIVLLLPHG
570	580	590	600	610	620	630	640
MEGMGPEHSS	ARPERFLQMS	NDDSDAYPAF	TKDFEVSQLY	DCNWIVVNCS	TPANYFHVLR	RQILLPFRKP	LIIFTPKSLL
650	660	670	680	690	700	710	720
RHPEAKSSFD	QMVSGTSFQR	VIPEDGAAAR	APEQVQRLIF	CTGKVYYDLV	KERSSQDLEE	KVAITRLEQI	SPFPFDLIKQ
730	740	750	760	770	780	790	800
EAEKYPGAEL	AWCQEEHKNM	GYYDYISPRF	MTILRRARPI	WYVGRDPAAA	PATGNRNTHL	VSLKKFLDTA	FNLQAFEGKT
810							
F							

Cmpd.	No. of Cmpds.	m/z meas.	Δ m/z [ppm]	z	Rt [min]	Score	P	Range	Sequence	Modification	Ratios
865	1	676.8710	-83.95	2	39.3	10.8	2	91-102	R.GRLNVLANVIRK.D		QD:QU 1.31

Detailed Protein Report

Protein 514: protein MRVI1 isoform b [Homo sapiens]

**Accession:**	gi|154091332	**Score:**	21.6
**Database:**	refseq_human(refseq_human_20140103.fasta)	**MW [kDa]:**	89.7
**Database Date:**	2015-11-30	**pI:**	5.7
		**Sequence Coverage [%]:**	3.7
		**No. of unique Peptides:**	1

10	20	30	40	50	60	70	80
MTGDATSPEG	ETDKNLANRV	HSPHKRLSHR	HLKVSTASLT	SVDPAGHIID	LVNDQLPDIS	ISEEDKKKNL	ALLEEAKLVS
**90**	**100**	**110**	**120**	**130**	**140**	**150**	**160**
ERFLTRRGRK	SRSSPGDSPS	AVSPNLSPSA	SPTSSRSNSL	TVPTPPGLDV	CSGPPSPLPG	APPQKGDEAD	VSSPHPGEPN
**170**	**180**	**190**	**200**	**210**	**220**	**230**	**240**
VPKGLADRKQ	NDQRKVSQGR	LAPRPPPVEK	SKEIAIEQKE	NFDPLQYPET	TPKGLAPVTN	SSGKMALNSP	QPGPVESELG
**250**	**260**	**270**	**280**	**290**	**300**	**310**	**320**
KQLLKTGWEG	SPLPRSPTQD	AAGVGPPASQ	GRGPAGEPMG	PEAGSKAELP	PTVSRPPLLR	GLSWDSGPEE	PGPRLQKVLA
**330**	**340**	**350**	**360**	**370**	**380**	**390**	**400**
KLPLAEEEKR	FAGKAGGKLA	KAPGLKDFQI	QVQPVRMQKL	TKLREEHILM	RNQNLVGLKL	PDLSEAAEQE	KGLPSELSPA
**410**	**420**	**430**	**440**	**450**	**460**	**470**	**480**
IEEEESKSGL	DVMPNISDVL	LRKLRVHRSL	PGSAPPLTEK	EVENVFVQLS	LAFRNDSYTL	ESRINQAERE	RNLTEENTEK
**490**	**500**	**510**	**520**	**530**	**540**	**550**	**560**
ELENFKASIT	SSASLWHHCE	HRETYQKLLE	DIAVLHRLAA	RLSSRAEVVG	AVRQEKRMSK	ATEVMMQYVE	NLKRTYEKDH
**570**	**580**	**590**	**600**	**610**	**620**	**630**	**640**
AELMEFKKLA	NQNSSRSCGP	SEDGVPRTAR	SMSLTLGKNM	PRRRVSVAVV	PKFNALNLPG	QTPSSSSIPS	LPALSESPNG
**650**	**660**	**670**	**680**	**690**	**700**	**710**	**720**
KGSLPVTSAL	PALLENGKTN	GDPDCEASAP	ALTLSCLEEL	SQETKARMEE	EAYSKGFQEG	LKKTKELQDL	KEEEEEQKSE
**730**	**740**	**750**	**760**	**770**	**780**	**790**	**800**
SPEEPEEVEE	TEEEEKGPRS	SKLEELVHFL	QVMYPKLCQH	WQVIWMMAAV	MLVLTVVLGL	YNSYNSCAEQ	ADGPLGRSTC
**810**	**820**	**830**					
SAAQRDSWWS	SGLQHEQPTE	Q					

Cmpd.	No. of Cmpds.	m/z meas.	Δ m/z [ppm]	z	Rt [min]	Score	P	Range	Sequence	Modification
2109	1	1065.5116	-99.60	2	54.9	11.2	2	181-199	R.LAPRPPPVEKSKEIAIEQK.E	

Detailed Protein Report

Protein 515: nischarin isoform 1 [Homo sapiens]

**Accession:**	gi|66472382	**Score:**	21.6
**Database:**	refseq_human(refseq_human_20140103.fasta)	**MW [kDa]:**	166.5
**Database Date:**	2015-11-30	**pI:**	4.9
		**Sequence Coverage [%]:**	2.3
		**No. of unique Peptides:**	1

10	20	30	40	50	60	70	80
MATARTFGPE	REAEPAKEAR	VVGSELVDTY	TVYIIQVTDG	SHEWTVKHRY	SDFHDLHEKL	VAERKIDKNL	LPPKKIIGKN
90	100	110	120	130	140	150	160
SRSLVEKREK	DLEVYLQKLL	AAFPGVTPRV	LAHFLHFHFY	EINGITAALA	EELFEKGEQL	LGAGEVFAIG	PLQLYAVTEQ
170	180	190	200	210	220	230	240
LQQGKPTCAS	GDAKTDLGHI	LDFTCRLKYL	KVSGTEGPFG	TSNIQEQLLP	FDLSIFKSLH	QVEISHCDAK	HIRGLVASKP
250	260	270	280	290	300	310	320
TLATLSVRFS	ATSMKEVLVP	EASEFDEWEP	EGTTLEGPVT	AVIPTWQALT	TLDLSHNSIS	EIDESVKLIP	KIEFLDLSHN
330	340	350	360	370	380	390	400
GLLVVDNLQH	LYNLVHLDLS	YNKLSSLEGL	HTKLGNIKTL	NLAGNLLESL	SGLHKLYSLV	NLDLRDNRIE	QMEEVRSIGS
410	420	430	440	450	460	470	480
LPCLEHVSLL	NNPLSIIPDY	RTKVLAQFGE	RASEVCLDDT	VTTEKELDTV	EVLKAIQKAK	EVKSKLSNPE	KKGGEDSRLS
490	500	510	520	530	540	550	560
AAPCIRPSSS	PPTVAPASAS	LPQPILSNQG	IMFVQEEALA	SSLSSTDSLT	PEHQPIAQGC	SDSLESIPAG	QAASDDLRDV
570	580	590	600	610	620	630	640
PGAVGGASPE	HAEPEVQVVP	GSGQIIFLPF	TCIGYTATNQ	DFIQRLSTLI	RQAIERQLPA	WIEAANQREE	GQGEQGEEED
650	660	670	680	690	700	710	720
EEEEEEEDVA	ENRYFEMGPP	DVEEEEGGGQ	GEEEEEEEED	EEAEEERLAL	EWALGADEDF	LLEHIRILKV	LWCFLIHVQG
730	740	750	760	770	780	790	800
SIRQFAACLV	LTDFGIAVFE	IPHQESRGSS	QHILSSLRFV	FCFPHGDLTE	FGFLMPELCL	VLKVRHSENT	LFIISDAANL
810	820	830	840	850	860	870	880
HEFHADLRSC	FAPQHMAMLC	SPILYGSHTS	LQEFLRQLLT	FYKVAGGCQE	RSQGCFPVYL	VYSDKRMVQT	AAGDYSGNIE
890	900	910	920	930	940	950	960
WASCTLCSAV	RRSCCAPSEA	VKSAAIPYWL	LLTPQHLNVI	KADFNPMPNR	GTHNCRNRNS	FKLSRVPLST	VLLDPTRSCT
970	980	990	1000	1010	1020	1030	1040
QPRGAFADGH	VLELLVGYRF	VTAIFVLPHE	KFHFLRVYNQ	LRASLQDLKT	VVIAKTPGTG	GSPQGSFADG	QPAERRASND
1050	1060	1070	1080	1090	1100	1110	1120
QRPQEVPAEA	LAPAPAEVPA	PAPAAASASG	PAKTPAPAEA	STSALVPEET	PVEAPAPPPA	EAPAQYPSEH	LIQATSEENQ
1130	1140	1150	1160	1170	1180	1190	1200
IPSHLPACPS	LRHVASLRGS	AIIELFHSSI	AEVENEELRH	LMWSSVVFYQ	TPGLEVTACV	LLSTKAVYFV	LHDGLRRYFS
1210	1220	1230	1240	1250	1260	1270	1280
EPLQDFWHQK	NTDYNNSPFH	ISQCFVLKLS	DLQSVNVGLF	DQHFRLTGST	PMQVVTCLTR	DSYLTHCFLQ	HLMVVLSSLE
1290	1300	1310	1320	1330	1340	1350	1360
RTPSPEPVDK	DFYSEFGNKT	TGKMENYELI	HSSRVKFTYP	SEEEIGDLTF	TVAQKMAEPE	KAPALSILLY	VQAFQVGMPP
1370	1380	1390	1400	1410	1420	1430	1440
PGCCRGPLRP	KTLLLTSSEI	FLLDEDCVHY	PLPEFAKEPP	QRDRYRLDDG	RRVRDLDRVL	MGYQTYPQAL	TLVFDDVQGH
1450	1460	1470	1480	1490	1500	1510	
DLMGSVTLDH	FGEVPGGPAR	ASQGREVQWQ	VFVPSAESRE	KLISLLARQW	EALCGRELPV	ELTG	

Cmpd.	No. of Cmpds.	m/z meas.	Δ m/z [ppm]	z	Rt [min]	Score	P	Range	Sequence	Modification
2021	1	890.3901	-125.48	2	55.7	10.8	0	359-375	K.TLNLAGNLLESLSGLHK.L	

Detailed Protein Report

Protein 516: phosphatidylinositol 3,4,5-trisphosphate 5-phosphatase 1 isoform b [Homo sapiens]

Accession: gi|40254823
Database: refseq_human(refseq_human_20140103.fasta)
Database Date: 2015-11-30

Score: 21.6
MW [kDa]: 133.1
pI: 7.9
Sequence Coverage [%]: 0.8
No. of unique Peptides: 1

Quantitation

QD:QU **Median:** 0.90 **CV:** 0.00 % **No. of Peptides:** 1

10	20	30	40	50	60	70	80
MVPCWNHGNI	TRSKAEELLS	RTGKDGSFLV	RASESISRAY	ALCVLYRNCV	YTYRILPNED	DKFTVQASEG	VSMRFFTKLD
90	100	110	120	130	140	150	160
QLIEFYKKEN	MGLVTHLQYP	VPLEEEDTGD	DPEEDTESVV	SPPELPPRNI	PLTASSCEAK	EVPFSNENPR	ATETSRPSLS
170	180	190	200	210	220	230	240
ETLFQRLQSM	DTSGLPEEHL	KAIQDYLSTQ	LAQDSEFVKT	GSSSLPHLKK	LTTLLCKELY	GEVIRTLPSL	ESLQRLFDQQ
250	260	270	280	290	300	310	320
LSPGLRPRPQ	VPGEANPINM	VSKLSQLTSL	LSSIEDKVKA	LLHEGPESPH	RPSLIPPVTF	EVKAESLGIP	QKMQLKVDVE
330	340	350	360	370	380	390	400
SGKLIIKKSK	DGSEDKFYSH	KKILQLIKSQ	KFLNKLVILV	ETEKEKILRK	EYVFADSKKR	EGFCQLLQQM	KNKHSEQPEP
410	420	430	440	450	460	470	480
DMITIFIGTW	NMGNAPPPKK	ITSWFLSKGQ	GKTRDDSADY	IPHDIYVIGT	QEDPLSEKEW	LEILKHSLQE	ITSVTFKTVA
490	500	510	520	530	540	550	560
IHTLWNIRIV	VLAKPEHENR	ISHICTDNVK	TGIANTLGNK	GAVGVSFMFN	GTSLGFVNSH	LTSGSEKKLR	RNQNYMNILR
570	580	590	600	610	620	630	640
FLALGDKKLS	PFNITHRFTH	LFWFGDLNYR	VDLPTWEAET	IIQKIKQQQY	ADLLSHDQLL	TERREQKVFL	HFEEEEITFA
650	660	670	680	690	700	710	720
PTYRFERLTR	DKYAYTKQKA	TGMKYNLPSW	CDRVLWKSYP	LVHVVCQSYG	STSDIMTSDH	SPVFATFEAG	VTSQFVSKNG
730	740	750	760	770	780	790	800
PGTVDSQGQI	EFLRCYATLK	TKSQTKFYLE	FHSSCLESFV	KSQEGENEEG	SEGELVVKFG	ETLPKLKPII	SDPEYLLDQH
810	820	830	840	850	860	870	880
ILISIKSSDS	DESYGEGCIA	LRLEATETQL	PIYTPLTHHG	ELTGHFQGEI	KLQTSQGKTR	EKLYDFVKTE	RDESSGPKTL
890	900	910	920	930	940	950	960
KSLTSHDPMK	QWEVTSRAPP	CSGSSITEII	NPNYMGVGPF	GPPMPLHVKQ	TLSPDQQPTA	WSYDQPPKDS	PLGPCRGESP
970	980	990	1000	1010	1020	1030	1040
PTPPGQPPIS	PKKFLPSTAN	RGLPPRTQES	RPSDLGKNAG	DTLPQEDLPL	TKPEMFENPL	YGSLSSFPKP	APRKDQESPK
1050	1060	1070	1080	1090	1100	1110	1120
MPRKEPPPCP	EPGILSPSIV	LTKAQEADRG	EGPGKQVPAP	RLRSFTCSSS	AEGRAAGGDK	SQGKPKTPVS	SQAPVPAKRP
1130	1140	1150	1160	1170	1180	1190	
IKPSRSEINQ	QTPPTPTPRP	PLPVKSPAVL	HLQHSKGRDY	RDNTELPHHG	KHRPEEGPPG	PLGRTAMQ	

Cmpd.	No. of Cmpds.	m/z meas.	Δ m/z [ppm]	z	Rt [min]	Score	P	Range	Sequence	Modification	Ratios	
623	1	584.8181	3.43	2	36.4	21.6	0	79-87	K.LDQLIEFYK.K		QD:QU	0.90

Detailed Protein Report

Protein 517: spindle and kinetochore-associated protein 3 isoform 2 [Homo sapiens]

**Accession:**	gi|260763912	**Score:**	21.6
**Database:**	refseq_human(refseq_human_20140103.fasta)	**MW [kDa]:**	43.9
**Database Date:**	2015-11-30	**pI:**	4.7
		**Sequence Coverage [%]:**	7.7
		**No. of unique Peptides:**	1

10	20	30	40	50	60	70	80
MDPIRSFCGK	LRSLASTLDC	ETARLQRALD	GEESDFEDYP	MRILYDLHSE	VQTLKDDVNI	LLDKARLENQ	EGIDFIKATK
**90**	**100**	**110**	**120**	**130**	**140**	**150**	**160**
VLMEKNSMDI	MKIREYFQKY	GYSPRVKKNS	VHEQEAINSD	PELSNCENFQ	KTDVKDDLSD	PPVASSCISE	KSPRSPQLSD
**170**	**180**	**190**	**200**	**210**	**220**	**230**	**240**
FGLERYIVSQ	VLPNPPQAVN	NYKEEPVIVT	PPTKQSLVKV	LKTPKCALKM	DDFECVTPKL	EHFGISEYTM	CLNEDYTMGL
**250**	**260**	**270**	**280**	**290**	**300**	**310**	**320**
KNARNNKSEE	AIDTESRLND	NVFATPSPII	QQLEKSDAEY	TNSPLVPTFC	TPGLKIPSTK	NSIALVSTNY	PLSKTNSSSN
**330**	**340**	**350**	**360**	**370**	**380**	**390**	
DLEVEDRTSL	VLNSDTCFEN	LTDPSSPTIS	SYENLLRTPT	PPEVTKIPED	ILQKFQWIYP	TQKLNKMR	

Cmpd.	No. of Cmpds.	m/z meas.	Δ m/z [ppm]	z	Rt [min]	Score	P	Range	Sequence	Modification
1765	1	917.4139	-26.15	2	50.6	11.4	2	242-257	K.NARNNKSEEAIDTESR.L	

Detailed Protein Report

Protein 518: aprataxin and PNK-like factor [Homo sapiens]

**Accession:**	gi|27734905	**Score:**	21.6
**Database:**	refseq_human(refseq_human_20140103.fasta)	**MW [kDa]:**	56.9
**Database Date:**	2015-11-30	**pI:**	4.8
**Modification(s):**	Carbamidomethyl, Oxidation	**Sequence Coverage [%]:**	4.9
		**No. of unique Peptides:**	1

10	20	30	40	50	60	70	80
MSGGFELQPR	DGGPRVALAP	GETVIGRGPL	LGITDKRVSR	RHAILEVAGG	QLRIKPIHTN	PCFYQSSEKS	QLLPLKPNLW
**90**	**100**	**110**	**120**	**130**	**140**	**150**	**160**
CYLNPGDSFS	LLVDKYIFRI	LSIPSEVEMQ	CTLRNSQVLD	EDNILNETPK	SPVINLPHET	TGASQLEGST	EIAKTQMTPT
**170**	**180**	**190**	**200**	**210**	**220**	**230**	**240**
NSVSFLGENR	DCNKQQPILA	ERKRILPTWM	LAEHLSDQNL	SVPAISGGNV	IQGSGKEEIC	KDKSQLNTTQ	QGRRQLISSG
**250**	**260**	**270**	**280**	**290**	**300**	**310**	**320**
SSENTSAEQD	TGEECKNTDQ	EESTISSKEM	PQSFSAITLS	NTEMNNIKTN	AQRNKLPIEE	LGKVSKHKIA	TKRTPHKEDE
**330**	**340**	**350**	**360**	**370**	**380**	**390**	**400**
AMSCSENCSS	AQGDSLQDES	QGSHSESSSN	PSNPETLHAK	ATDSVLQGSE	GNKVKRTSCM	YGANCYRKNP	VHFQHFSHPG
**410**	**420**	**430**	**440**	**450**	**460**	**470**	**480**
DSDYGGVQIV	GQDETDDRPE	CPYGPSCYRK	NPQHKIEYRH	NTLPVRNVLD	EDNDNVGQPN	EYDLNDSFLD	DEEEDYEPTD
**490**	**500**	**510**	**520**				
EDSDWEPGKE	DEEKEDVEEL	LKEAKRFMKR	K				

Cmpd.	No. of Cmpds.	m/z meas.	Δ m/z [ppm]	z	Rt [min]	Score	P	Range	Sequence	Modification
1865	1	813.2646	-108.33	2	53.7	10.6	2	376-388	K.RTSCMYGANCYRK.N	Carbamidomethyl: 10; Oxidation: 5

Detailed Protein Report

Protein 519: **PREDICTED: cleavage and polyadenylation specificity factor subunit 7 isoform X5 [Homo sapiens]**

Accession: gi|530397403
Database: refseq_human(refseq_human_20140103.fasta)
Database Date: 2015-11-30

Score: 21.6
MW [kDa]: 41.9
pI: 10.1
Sequence Coverage [%]: 7.1
No. of unique Peptides: 2

Alias proteins:

Accession	Name	Description
gi|530397405	refseq_human (refseq_human_20140103.fasta)	PREDICTED: cleavage and polyadenylation specificity factor subunit 7 isoform X6 [Homo sapiens]

10	20	30	40	50	60	70	80
MWTTDQQLIQ	VIRSIGVYDV	VELKFAENRA	NGQSKGYAEV	VVASENSVHK	LLELLPGKVL	NGEKVDVRPA	TRQNLSQFEA
**90**	**100**	**110**	**120**	**130**	**140**	**150**	**160**
QARKRECVRV	PRGGIPPRAH	SRDSSDSADG	RATPSENLVP	SSARVDKPPS	VLPYFNRPPS	ALPLMGLPPP	PIPPPPPLSS
**170**	**180**	**190**	**200**	**210**	**220**	**230**	**240**
SFGVPPPPPG	IHYQHLMPPP	PRLPPHLAVP	PPGAIPPALH	LNPAFFPPPN	ATVGPPPDTY	MKASAPYNHH	GSRDSGPPPS
**250**	**260**	**270**	**280**	**290**	**300**	**310**	**320**
TVSEAEFEDI	MKRNRAISSS	AISKAVSGAS	AGDYSDAIET	LLTAIAVIKQ	SRVANDERCR	VLISSLKDCL	HGIEAKSYSV
**330**	**340**	**350**	**360**	**370**	**380**	**390**	
GASGSSSRKR	HRSRERSPSR	SRESSRRHRD	LLHNEDRHDD	YFQERNREHE	RHRDRERDRH	H	

Cmpd.	No. of Cmpds.	m/z meas.	Δ m/z [ppm]	z	Rt [min]	Score	P	Range	Sequence	Modification
1937	1	794.7610	-177.32	2	54.6	10.1	0	36-50	K.GYAEVVVASENSVHK.L	
2417	1	572.6438	-211.80	2	58.9	11.5	0	317-328	K.SYSVGASGSSSR.K	

Detailed Protein Report

Protein 520: protein FAM83F [Homo sapiens]

Accession: gi|156564372
Database: refseq_human(refseq_human_20140103.fasta)
Database Date: 2015-11-30

Score: 21.6
MW [kDa]: 55.5
pI: 9.2
Sequence Coverage [%]: 7.0
No. of unique Peptides: 1

10	20	30	40	50	60	70	80
MAESQLNCLD	EAHVNEKVTE	AQAAFYYCER	RRAALEALLG	GGEQAYRERL	KEEQLRDFLS	SPERQALRAA	WSPYEDAVPA
**90**	**100**	**110**	**120**	**130**	**140**	**150**	**160**
ANARGKSKAK	AKAPAPAPAE	SGESLAYWPD	RSDTEVPPLD	LGWTDTGFYR	GVSRVTLFTH	PPKDEKAPHL	KQVVRQMIQQ
**170**	**180**	**190**	**200**	**210**	**220**	**230**	**240**
AQKVIAVVMD	LFTDGDIFQD	IVDAACKRRV	PVYIILDEAG	VKYFLEMCQD	LQLTDFRIRN	IRVRSVTGVG	FYMPMGRIKG
**250**	**260**	**270**	**280**	**290**	**300**	**310**	**320**
TLSSRFLMVD	GDKVATGSYR	FTWSSSHVDR	NLLLLLTGQN	VEPFDTEFRE	LYAISEEVDL	YRQLSLAGRV	GLHYSSTVAR
**330**	**340**	**350**	**360**	**370**	**380**	**390**	**400**
KLINPKYALV	SGCRHPPGEM	MRWAARQQRE	AGGNPEGQEE	GASGGESAWR	LESFLKDLVT	VEQVLPPVEP	IPLGELSQKD
**410**	**420**	**430**	**440**	**450**	**460**	**470**	**480**
GRMVSHMHRD	LKPKSREAPS	RNGMGEAARG	EAAPARRFSS	RLFSRRAKRP	AAPNGMASSV	STETSEVEFL	TGKRPNENSS
**490**	**500**	**510**					
ADISGKTSPS	SAKPSNCVIS						

Cmpd.	No. of Cmpds.	m/z meas.	Δ m/z [ppm]	z	Rt [min]	Score	P	Range	Sequence	Modification
1194	1	628.4650	156.14	3	43.4	11.0	2	310-326	R.VGLHYSSTVARKLINPK.Y	

Detailed Protein Report

Protein 521: inhibitor of nuclear factor kappa-B kinase subunit alpha [Homo sapiens]

**Accession:**	gi|62241001	**Score:**	21.5
**Database:**	refseq_human(refseq_human_20140103.fasta)	**MW [kDa]:**	84.6
**Database Date:**	2015-11-30	**pI:**	6.3
**Modification(s):**	Carbamidomethyl	**Sequence Coverage [%]:**	1.6
		**No. of unique Peptides:**	1

10	20	30	40	50	60	70	80
MERPPGLRPG	AGGPWEMRER	LGTGGFGNVC	LYQHRELDLK	IAIKSCRLEL	STKNRERWCH	EIQIMKKLNH	ANVVKACDVP
90	100	110	120	130	140	150	160
EELNILIHDV	PLLAMEYCSG	GDLRKLLNKP	ENCCGLKESQ	ILSLLSDIGS	GIRYLHENKI	IHRDLKPENI	VLQDVGGKII
170	180	190	200	210	220	230	240
HKIIDLGYAK	DVDQGSLCTS	FVGTLQYLAP	ELFENKPYTA	TVDYWSFGTM	VFECIAGYRP	FLHHLQPFTW	HEKIKKKDPK
250	260	270	280	290	300	310	320
CIFACEEMSG	EVRFSSHLPQ	PNSLCSLVVE	PMENWLQLML	NWDPQQRGGP	VDLTLKQPRC	FVLMDHILNL	KIVHILNMTS
330	340	350	360	370	380	390	400
AKIISFLLPP	DESLHSLQSR	IERETGINTG	SQELLSETGI	SLDPRKPASQ	CVLDGVRGCD	SYMVYLFDKS	KTVYEGPFAS
410	420	430	440	450	460	470	480
RSLSDCVNYI	VQDSKIQLPI	IQLRKVWAEA	VHYVSGLKED	YSRLFQGQRA	AMLSLLRYNA	NLTKMKNTLI	SASQQLKAKL
490	500	510	520	530	540	550	560
EFFHKSIQLD	LERYSEQMTY	GISSEKMLKA	WKEMEEKAIH	YAEVGVIGYL	EDQIMSLHAE	IMELQKSPYG	RRQGDLMESL
570	580	590	600	610	620	630	640
EQRAIDLYKQ	LKHRPSDHSY	SDSTEMVKII	VHTVQSQDRV	LKELFGHLSK	LLGCKQKIID	LLPKVEVALS	NIKEADNTVM
650	660	670	680	690	700	710	720
FMQGKRQKEI	WHLLKIACTQ	SSARSLVGSS	LEGAVTPQTS	AWLPPTSAEH	DHSLSCVVTP	QDGETSAQMI	EENLNCLGHL
730	740	750					
STIIHEANEE	QGNSMMNLDW	SWLTE					

Cmpd.	No. of Cmpds.	m/z meas.	Δ m/z [ppm]	z	Rt [min]	Score	P	Range	Sequence	Modification
2406	1	665.3146	-54.38	2	60.5	21.5	0	366-377	R.KPASQCVLDGVR.G	Carbamidomethyl: 6

Detailed Protein Report

Protein 522: **transcription initiation factor TFIID subunit 4B [Homo sapiens]**

**Accession:**	gi|148792970	**Score:**	21.4
**Database:**	refseq_human(refseq_human_20140103.fasta)	**MW [kDa]:**	91.0
**Database Date:**	2015-11-30	**pI:**	10.4
**Modification(s):**	Carbamidomethyl	**Sequence Coverage [%]:**	1.6
		**No. of unique Peptides:**	1

10	20	30	40	50	60	70	80
MPAGLTEPAG	AAPPAAVSAS	GTVTMAPAGA	LPVRVESTPV	ALGAVTKAPV	SVCVEPTASQ	PLRSPVGTLV	TKVAPVSAPP
**90**	**100**	**110**	**120**	**130**	**140**	**150**	**160**
KVSSGPRLPA	PQIVAVKAPN	TTTIQFPANL	QLPPGTVLIK	SNSGPLMLVS	PQQTVTRAET	TSNITSRPAV	PANPQTVKIC
**170**	**180**	**190**	**200**	**210**	**220**	**230**	**240**
TVPNSSSQLI	KKVAVTPVKK	LAQIGTTVVT	TVPKPSSVQS	VAVPTSVVTV	TPGKPLNTVT	TLKPSSLGAS	STPSNEPNLK
**250**	**260**	**270**	**280**	**290**	**300**	**310**	**320**
AENSAAVQIN	LSPTMLENVK	KCKNFLAMLI	KLACSGSQSP	EMGQNVKKLV	EQLLDAKIEA	EEFTRKLYVE	LKSSPQPHLV
**330**	**340**	**350**	**360**	**370**	**380**	**390**	**400**
PFLKKSVVAL	RQLLPNSQSF	IQQCVQQTSS	DMVIATCTTT	VTTSPVVTTT	VSSSQSEKSI	IVSGATAPRT	VSVQTLNPLA
**410**	**420**	**430**	**440**	**450**	**460**	**470**	**480**
GPVGAKAGVV	TLHSVGPTAA	TGGTTAGTGL	LQTSKPLVTS	VANTVTTVSL	QPEKPVVSGT	AVTLSLPAVT	FGETSGAAIC
**490**	**500**	**510**	**520**	**530**	**540**	**550**	**560**
LPSVKPVVSS	AGTTSDKPVI	GTPVQIKLAQ	PGPVLSQPAG	IPQAVQVKQL	VVQQPSGGNE	KQVTTISHSS	TLTIQKCGQK
**570**	**580**	**590**	**600**	**610**	**620**	**630**	**640**
TMPVNTIIPT	SQFPPASILK	QITLPGNKIL	SLQASPTQKN	RIKENVTSCF	RDEDDINDVT	SMAGVNLNEE	NACILATNSE
**650**	**660**	**670**	**680**	**690**	**700**	**710**	**720**
LVGTLIQSCK	DEPFLFIGAL	QKRILDIGKK	HDITELNSDA	VNLISQATQE	RLRGLLEKLT	AIAQHRMTTY	KASENYILCS
**730**	**740**	**750**	**760**	**770**	**780**	**790**	**800**
DTRSQLKFLE	KLDQLEKQRK	DLEEREMLLK	AAKSRSNKED	PEQLRLKQKA	KELQQLELAQ	IQHRDANLTA	LAAIGPRKKR
**810**	**820**	**830**	**840**	**850**	**860**	**870**	
PLESGIEGLK	DNLLASGTSS	LTATKQLHRP	RITRICLRDL	IFCMEQEREM	KYSRALYLAL	LK	

Cmpd.	No. of Cmpds.	m/z meas.	Δ m/z [ppm]	z	Rt [min]	Score	P	Range	Sequence	Modification
2538	3	913.4572	17.18	2	62.4	21.4	1	835-848	R.ICLRDLIFCMEQER.E	Carbamidomethyl: 2

Detailed Protein Report

Protein 523: PREDICTED: uncharacterized protein LOC102725205, partial [Homo sapiens]

**Accession:**	gi|578845222	**Score:**	21.4
**Database:**	refseq_human(refseq_human_20140103.fasta)	**MW [kDa]:**	95.5
**Database Date:**	2015-11-30	**pI:**	6.0
		**Sequence Coverage [%]:**	3.8
		**No. of unique Peptides:**	2

10	20	30	40	50	60	70	80
TLSHTLHPPT	HTPNLTHSTH	THTTPPTHTP	HPTHHSTPLT	HSTPHTSHTH	STPLTHSTPH	SWAAKSLERL	LWVVASSPPR
**90**	**100**	**110**	**120**	**130**	**140**	**150**	**160**
RTAVVGRIPG	AGMPAGAGGP	ACRCPCLSRV	GWRDLDAEPS	SQEFRQLSLD	SHQVAAFQTE	MECWARGSGM	DATEPLCCDC
**170**	**180**	**190**	**200**	**210**	**220**	**230**	**240**
QTSDLVCWSQ	QGHQGPERGA	LGSLEEPGLL	SAREAWPVSC	SSIFLEVSGR	RLESRMPPGG	AAINISPGDK	RTNQSPAGYR
**250**	**260**	**270**	**280**	**290**	**300**	**310**	**320**
GEDKSGKRLM	SELWLQVAAA	RDGLQDFPGL	LVAGRGSSSG	DWSERAYGWE	LEELMEKRGR	VGWSSREALW	SGEGEEEPNK
**330**	**340**	**350**	**360**	**370**	**380**	**390**	**400**
EKQQSTWNIS	IPPSKAVLEE	QPQSPIVPKS	GKSVKTFIRN	RYEPAKRCEE	LICAELIRMN	KVTTDSAMGI	ADKDLSEELQ
**410**	**420**	**430**	**440**	**450**	**460**	**470**	**480**
HRLQHALASL	QVAQGEDLTA	DIMYSPSASE	PLPFMQCDGE	QETGTASPIW	LWNLWAGESH	PHGVHALATL	GWEGPMVNAG
**490**	**500**	**510**	**520**	**530**	**540**	**550**	**560**
EKVQEEEGAP	ATGPSCGQHR	EGCCGGEDVG	VEGCGGGHLL	ALGCPSKTLT	ARGILPVGGA	ASTTSLQEAM	FVFRTTLIRA
**570**	**580**	**590**	**600**	**610**	**620**	**630**	**640**
VGCKEQRPLK	GEGGAVKDVE	SRRCAGLWFR	EPCGRQVKME	LHKSAGETPG	KLKVEYADGG	MPEARLWEGT	HIFETKVKLS
**650**	**660**	**670**	**680**	**690**	**700**	**710**	**720**
TVIHEAAVQW	PSGDIEKTHL	SSGLFSELIR	GLCSFDMFLP	DTVVCWPLPW	SAFHPVLLQT	MSAPGLSPAL	DPLCPQLLAD
**730**	**740**	**750**	**760**	**770**	**780**	**790**	**800**
LGSEKQSCPS	REFGWGLAEV	PSVSNLCKGG	LALQAADGGS	GGGVRTPSYY	LNECMGITAA	LYEHLKTTLS	NPSFSKEKES
**810**	**820**	**830**	**840**	**850**	**860**	**870**	**880**
VNFPFSMHEV	TGVCHSGGPV	FKKNREETVR	LSAGGAARQK	ELMLYPVVSF	PKGLHQLPSF	LALIMTISVV	TSLLSKESIF
**890**							
SSCQE							

Cmpd.	No. of Cmpds.	m/z meas.	Δ m/z [ppm]	z	Rt [min]	Score	P	Range	Sequence	Modification
1138	1	983.4246	-85.91	2	44.4	11.1	1	82-103	R.TAVVGRIPGAGMPAGAGGPACR.C	
992	1	647.6724	-181.96	2	40.9	10.3	0	614-625	K.VEYADGGMPEAR.L	

Detailed Protein Report

Protein 524: **ubiquitin-associated and SH3 domain-containing protein A isoform 3 [Homo sapiens]**

**Accession:**	gi|343478263	**Score:**	21.3
**Database:**	refseq_human(refseq_human_20140103.fasta)	**MW [kDa]:**	59.1
**Database Date:**	2015-11-30	**pI:**	9.3
**Modification(s):**	Oxidation	**Sequence Coverage [%]:**	8.6
		**No. of unique Peptides:**	2

10	20	30	40	50	60	70	80
MAAGETQLYA	KVSNKLKSRS	SPSLLEPLLA	MGFPVHTALK	ALAATGRKTA	EEALAWLHDH	CNDPSLDDPI	PQEYALFLCP
**90**	**100**	**110**	**120**	**130**	**140**	**150**	**160**
TGPLLEKLQE	FWRESKRQCA	KNRAHEVFPH	VTLCDFFTCE	DQKVECLYEA	LKRAGDRLLG	SFPTAVPLAL	HSSISYLGFF
**170**	**180**	**190**	**200**	**210**	**220**	**230**	**240**
VSGSPADVIR	EFAMTFATEA	SLLADCSVKP	CTKQLHLTLA	HKFYPHHQRT	LEQLARAIPL	GHSCQWTAAL	YSRDMRFVHY
**250**	**260**	**270**	**280**	**290**	**300**	**310**	**320**
QTLRALFQYK	PQNVDELTLS	PGDYIFVDPT	QQDEASEGWV	IGISQRTGCR	GFLPENYTDR	ASESDTWVKH	RMYTFSLATD
**330**	**340**	**350**	**360**	**370**	**380**	**390**	**400**
LNSRKDGEAS	SRCSGEFLPQ	TARSLSSLQA	LQATVARKSV	LVVRHGERVD	QIFGKAWLQQ	CSTPDGKYYR	PDLNFPCSLP
**410**	**420**	**430**	**440**	**450**	**460**	**470**	**480**
RRSRGIKDFE	NDPPLSSCGI	FQSRIAGDAL	LDSGIRISSV	FASPALRCVQ	TAKLILEELK	LEKKIKIRVE	PGIFEWTKWE
**490**	**500**	**510**	**520**	**530**			
AGKTTPTLMS	LEELKEANFN	IDTDYRSLPW	ACASVKKIKR	KENGSW			

Cmpd.	No. of Cmpds.	m/z meas.	Δ m/z [ppm]	z	Rt [min]	Score	P	Range	Sequence	Modification
869	1	955.4602	-71.25	3	39.3	10.1	1	20-47	R.SSPSLLEPLLAMGFPVHTALKALAATGR.K	Oxidation: 12
2807	1	937.4543	-19.47	2	64.2	11.3	0	217-233	R.AIPLGHSCQWTAALYSR.D	

Detailed Protein Report

Protein 525: T-complex protein 10A homolog [Homo sapiens]

**Accession:**	gi|105553088	**Score:**	21.3
**Database:**	refseq_human(refseq_human_20140103.fasta)	**MW [kDa]:**	35.6
**Database Date:**	2015-11-30	**pI:**	10.1
**Modification(s):**	Oxidation	**Sequence Coverage [%]:**	4.9
		**No. of unique Peptides:**	1

Quantitation

QD:QU	**Median:** 0.82	**CV:** 0.00 %	**No. of Peptides:** 1

10	20	30	40	50	60	70	80
MEKTPAAAEV	PREDSNAGEM	PSLQQQITSL	HQELGRQQSL	WADIHRKLQS	HMDALRKQNR	ELREELRGLQ	RQQWEAGKKP
**90**	**100**	**110**	**120**	**130**	**140**	**150**	**160**
AASPHAGRES	HTLALEPAFG	KISHLSADED	TTPKYAGRKS	QSATLLGQRW	SSNHLAPPKP	MSLKTERINS	GKTPPQEDRE
**170**	**180**	**190**	**200**	**210**	**220**	**230**	**240**
KSPPGRRQDR	SPAPTGRPTP	GAERRGVSED	GKIMHPSSRS	PQNSGGRKSP	VQASQATTLQ	EQTAAARGAD	RSSSVLGSSE
**250**	**260**	**270**	**280**	**290**	**300**	**310**	**320**
GGFLSRVQAD	EFASSAPDSA	ERQNLPVNPP	SSLEIAQAMD	TKMKKEEVQE	EKRHPKGKAD	DCRRSGFPSE	FPGALHAAPS
**330**							
RQDMGP							

Cmpd.	No. of Cmpds.	m/z meas.	Δ m/z [ppm]	z	Rt [min]	Score	P	Range	Sequence	Modification	Ratios	
88	1	877.8653	-84.40	2	31.0	21.3	2	193-208	K.IMHPSSRSPQNSGGRK.S	Oxidation: 2	QD:QU	0.82

Detailed Protein Report

Protein 526: **PREDICTED: CAS1 domain-containing protein 1 isoform X2 [Homo sapiens]**

**Accession:**	gi|578814529	**Score:**	21.3
**Database:**	refseq_human(refseq_human_20140103.fasta)	**MW [kDa]:**	72.0
**Database Date:**	2015-11-30	**pI:**	9.6
**Modification(s):**	Carbamidomethyl	**Sequence Coverage [%]:**	2.2
		**No. of unique Peptides:**	1

Quantitation

QD:QU **Median:** 1.91 **CV:** 0.00 % **No. of Peptides:** 1

10	20	30	40	50	60	70	80
MGRCVLFSRN	CSSIAPTWAQ	VAASKLGDSP	KARVRARRVP	LVLSPHTPMA	LPQPPPPAGA	RDPVTPEHWA	SGPPSQAQPL
**90**	**100**	**110**	**120**	**130**	**140**	**150**	**160**
LRQEAKEEEE	GEETGVQGAW	GTGTAEQRRR	GWGEAAESAA	AEEGQAEVGG	AAAAGSGSPA	GGAGGGLGSW	RPLLAWLQRR
**170**	**180**	**190**	**200**	**210**	**220**	**230**	**240**
QPQCCPCAAP	LSRSAAHCCH	GGTKMAALAY	NLGKREINHY	FSVRSAKVLA	LVAVLLLAAC	HLASRRYRGN	DSCEYLLSSG
**250**	**260**	**270**	**280**	**290**	**300**	**310**	**320**
RFLGEKVWQP	HSCMMHKYKI	SEAKNCLVDK	HIAFIGDSRI	RQLFYSFVKI	INPQFKEEGN	KHENIPFEDK	TASVKVDFLW
**330**	**340**	**350**	**360**	**370**	**380**	**390**	**400**
HPEVNGSMKQ	CIKVWTEDSI	AKPHVIVAGA	ATWSIKIHNG	SSEALSQYKM	NITSIAPLLE	KLAKTSDVYW	VLQDPVYEDL
**410**	**420**	**430**	**440**	**450**	**460**	**470**	**480**
LSENRKMITN	EKIDAYNEAA	VSILNSSTRN	SKSNVKMFSV	SKLIAQETIM	ESLDGLHLPE	SSRETTAMIL	MNVYCNKILK
**490**	**500**	**510**	**520**	**530**	**540**	**550**	**560**
PVDGSCCQPR	PPVTLIQKLA	ACFFTLSIIG	YLIFYIIHRN	AHRKNKPCTD	LESGEEKKNI	INTPVSSLEI	LLQSFCKLGL
**570**	**580**	**590**	**600**	**610**	**620**	**630**	**640**
IMAYFYMCDR	ANLFMKENKF	YTHSSFFIPI	IYILVLGVFY	NENTKETKVL	NREQTDEWKG	WMQLVILIYH	ISGASTIVGA
**650**	**660**						
IGTQEGEMTF	I						

Cmpd.	No. of Cmpds.	m/z meas.	Δ m/z [ppm]	z	Rt [min]	Score	P	Range	Sequence	Modification	Ratios	
175	2	793.8315	-49.90	2	32.2	21.3	1	160-173	R.RQPQCCPCAAPLSR.S	Carbamidomethyl: 8	QD:QU	1.91

Detailed Protein Report

Protein 527: PREDICTED: C2 domain-containing protein 3 isoform X5 [Homo sapiens]

**Accession:**	gi|530396574	**Score:**	21.3
**Database:**	refseq_human(refseq_human_20140103.fasta)	**MW [kDa]:**	186.3
**Database Date:**	2015-11-30	**pI:**	9.5
**Modification(s):**	Oxidation	**Sequence Coverage [%]:**	2.1
		**No. of unique Peptides:**	1

10	20	30	40	50	60	70	80
MKQRKGQGSG	GSRGRKKRGL	SDISPSTSLP	PLVEGQLRCF	LKLTVNRVIW	KIAKPPTCVL	VRVRWWGETS	DGTLFCPRDA
**90**	**100**	**110**	**120**	**130**	**140**	**150**	**160**
LQTEPKAVRT	TTRYAIRCGP	KQFTSYLTDM	AVLVLEVITK	LDGLPIGRVQ	INGLAQLSPT	HQINGFFTIV	SSTSKKLGEL
**170**	**180**	**190**	**200**	**210**	**220**	**230**	**240**
QVSLALEPLS	ETYDSYHPLP	TTDMTENVLL	SKQGFRENTE	PSSTQFQVPS	RPRDIHTIKI	DGKELAANSS	RSTTPRGKDH
**250**	**260**	**270**	**280**	**290**	**300**	**310**	**320**
VCFAENPDTI	KDSSFGLQHS	LNSGQSLESV	TLKGRAPRKQ	MSLLNSSEFQ	PQIRTVAKSH	SDSCILSSNN	LPTKDLLSAL
**330**	**340**	**350**	**360**	**370**	**380**	**390**	**400**
LEQGNKLRNA	MVISAMKSSP	ETSMLLDQVH	PPINEDSLRA	STQIRAFSRN	RFKDHIEDHL	LPSTENTFWR	HDTKADTRAI
**410**	**420**	**430**	**440**	**450**	**460**	**470**	**480**
QLLLGSAELS	QGNFWDGLGS	PPDSPSPGSD	VYCISELNDP	QYDQSLLENL	FYTAPKSDTS	ISDFLSEEDD	IVPSKKISQS
**490**	**500**	**510**	**520**	**530**	**540**	**550**	**560**
TALARSSKVL	ESSDHKLKKR	SAGKRNRNLV	EQQMLSETPE	DAQTMTLSVD	RLALLGRTHS	VRIIIETMGV	PPDSPQMTPG
**570**	**580**	**590**	**600**	**610**	**620**	**630**	**640**
KKSYAGPPPK	VTTAKKRTFF	VEYHFPVGFS	ESGLGKTALI	TEVVRLASSK	ITDGKVKFQQ	RFVFPVQFGG	PMIEHWWNSN
**650**	**660**	**670**	**680**	**690**	**700**	**710**	**720**
LTFQIYVKKT	PQKKPEVIGS	VSLSLRAVIQ	SELLSFSDQL	PVQQENGQSP	FGPLKVTMEL	ITDNKDFTGI	NTKLSGNTHY
**730**	**740**	**750**	**760**	**770**	**780**	**790**	**800**
TPLCAPTSPN	KALPELNQDM	TCTKNPQNLN	QIHEETAKKA	QNLVLPNRKS	PSPVAPHPST	FVATPASHNL	VNQTNGTTKE
**810**	**820**	**830**	**840**	**850**	**860**	**870**	**880**
SALLLHVLLM	VPDGKDFISG	ESEKQSPCNV	YLNCKLFSTE	EVTRSVIAWG	TTQPVFNFSQ	VIPVSLSSKY	LERLKNNVMV
**890**	**900**	**910**	**920**	**930**	**940**	**950**	**960**
IETWNKVRSP	GQDKLLGLVK	LPLHQFYMSF	KDAKISRLLL	DAQYPVVAVD	SYMPVIDVFS	GHQNGSLRVF	LAMGSSNQIM
**970**	**980**	**990**	**1000**	**1010**	**1020**	**1030**	**1040**
ALQRLKNEEG	TLPPFSPRPA	HFLDQPTAAS	VAMAEDRGNG	LMEHCFEIHI	EMVKGLAPLQ	ATVWGEADCY	VQYYFPVQHS
**1050**	**1060**	**1070**	**1080**	**1090**	**1100**	**1110**	**1120**
QSSVLKGPEF	LENGITLKPF	RTATTLCVPD	PIFNSEHHHS	LLLPAEVPVQ	RLLLSAFSAQ	GLVPGGGVQF	EIWCRYYYPN
**1130**	**1140**	**1150**	**1160**	**1170**	**1180**	**1190**	**1200**
VRDQKVAKGT	LPLSRICAMV	TTQHREDVGI	QTFNLPLTPR	IENRKELRNQ	SSGLLDVGLR	YRRSPRTAEG	VLAARTVSIS
**1210**	**1220**	**1230**	**1240**	**1250**	**1260**	**1270**	**1280**
VQIIRACGLQ	AAAKALAERE	PALQFSATVG	VNASVTTHLS	FLPQGEQRRT	HPVACSFCPE	FSHHVEFTCN	LVTQHCSGEA
**1290**	**1300**	**1310**	**1320**	**1330**	**1340**	**1350**	**1360**
CFLAELLEFA	EVIFAVYHEN	TKSASDIISI	ESCKEYLLGV	VKVPTKELLI	KRSGITGWYP	IILPEDGGLP	HGLELMQKIV
**1370**	**1380**	**1390**	**1400**	**1410**	**1420**	**1430**	**1440**
GGLELSISFT	HRGDRERVLE	AAEHLGWSFE	NSLKDFVRMD	EGEPATVTIS	TPRLWLPIHC	VLLAGHNHIH	KNTYCYLRYK
**1450**	**1460**	**1470**	**1480**	**1490**	**1500**	**1510**	**1520**
FYDHEAFWTP	LKKPKESVNK	KQIMVTFKAS	KRAEVTRGPS	LLWYFREERL	EIQVWRAYGN	DSVERPHQTD	SWIGSAYVDL
**1530**	**1540**	**1550**	**1560**	**1570**	**1580**	**1590**	**1600**
ARLGERSART	LTVSGVYPLF	GRNASNLSGA	ALRVHVVLSS	LSSHLEPTHE	LDSMDCSSHS	ESEQLPRRND	EVQLSPPEVI
**1610**	**1620**	**1630**	**1640**	**1650**	**1660**	**1670**	**1680**
SCHQKSPAST	QVPCSSTTAE	VRLTQEGPAD	LDGTFAVSIL	VERAMHLSLK	GYQKSCFWTH	NKPWSSKFGI	KEMRRG

Cmpd.	No. of Cmpds.	m/z meas.	Δ m/z [ppm]	z	Rt [min]	Score	P	Range	Sequence	Modification
1725	1	917.8011	39.11	3	51.9	10.3	0	508-531	R.NLVEQQMLSETPEDAQTMTLSVDR.L	Oxidation: 7

Detailed Protein Report

Protein 528: PREDICTED: zinc finger protein 451 isoform X2 [Homo sapiens]

Accession: gi|530381758
Database: refseq_human(refseq_human_20140103.fasta)
Database Date: 2015-11-30
Modification(s): Carbamidomethyl

Score: 21.3
MW [kDa]: 111.8
pI: 6.8
Sequence Coverage [%]: 2.3
No. of unique Peptides: 1

Quantitation

QD:QU **Median:** 0.71 **CV:** 0.00 % **No. of Peptides:** 1

10	20	30	40	50	60	70	80
MGDPGSEIIE	SVPPAGPEAS	ESTTDENEDD	IQFVSEGPLR	PVLEYIDLVS	SDDEEPSTSY	TDENIKRKDH	IDYQKDKVAL
**90**	**100**	**110**	**120**	**130**	**140**	**150**	**160**
TLARLARHVE	VEKQQKEEKN	RAFREKIDFQ	HAHGLQELEF	IRGHSDTEAA	RLCVDQWLKM	PGLKTGTINC	GTKSSFRRGG
**170**	**180**	**190**	**200**	**210**	**220**	**230**	**240**
HTWVSGKPIL	CPIMHCNKEF	DNGHLLLGHL	KRFDHSPCDP	TITLHGPFFS	SFACVVCYKK	FVTQQQYRDH	LFDKEATDDG
**250**	**260**	**270**	**280**	**290**	**300**	**310**	**320**
HNNNLLPQII	QCFACPNCFL	LFSRKEECSK	HMSGKNHFHQ	SFKLGDNKGI	AHPISFPSFA	KKLLISLCKD	VPFQVKCVAC
**330**	**340**	**350**	**360**	**370**	**380**	**390**	**400**
HKTLRSHMEL	TAHFRVHCRN	AGPVAVAEKS	ITQVAEKFIL	RGYCPDCNQV	FVDETSTQNH	KQNSGHKVRV	INSVEESVLL
**410**	**420**	**430**	**440**	**450**	**460**	**470**	**480**
YCHSSEGNKD	PSSDLHLLLD	QSKFSSLKRT	MSIKESSSLE	CIAIPKKKMN	LKDKSHEGVA	CVQKEKSVVK	TWFCECNQRF
**490**	**500**	**510**	**520**	**530**	**540**	**550**	**560**
PSEDAVEKHV	FSANTMGYKC	VVCGKVCDDS	GVIRLHMSRI	HGGAHLNNFL	FWCRTCKKEL	TRKDTIMAHV	TEFHNGHRYF
**570**	**580**	**590**	**600**	**610**	**620**	**630**	**640**
YEMDEVEGET	LPSSSTTLDN	LTANKPSSAI	TVIDHSPANS	SPRGKWQCRI	CEDMFDSQEY	VKQHCMSLAS	HKFHRYSCAH
**650**	**660**	**670**	**680**	**690**	**700**	**710**	**720**
CRKPFHKIET	LYRHCQDEHD	NEIKIKYFCG	LCDLIFNVEE	AFLSHYEEHH	SIDYVFVSEK	TETSIKTEDD	FPVIETSNQL
**730**	**740**	**750**	**760**	**770**	**780**	**790**	**800**
TCGCRESYIC	KVNRKEDYSR	CLQIMLDKGK	LWFRCSLCSA	TAQNLTDMNT	HIHQVHKEKS	DEEEQQYVIK	CGTCTKAFHD
**810**	**820**	**830**	**840**	**850**	**860**	**870**	**880**
PESAQQHFHR	KHCFLQKPSV	AHFGSEKSNL	YKFTASASHT	ERKLKQAINY	SKSLDMEKGV	ENDLSYQNIE	EEIVELPDLD
**890**	**900**	**910**	**920**	**930**	**940**	**950**	**960**
YLRTMTHIVF	VDFDNWSNFF	GHLPGHLNQG	TFIWGFQGGN	TNWKPPLNCK	IYNYLNRIGC	FFLHPRCSKR	KDAADFAICM
**970**	**980**						
HNVTVMITWV	PKILQ						

Cmpd.	No. of Cmpds.	m/z meas.	Δ m/z [ppm]	z	Rt [min]	Score	P	Range	Sequence	Modification	Ratios
2396	1	832.2427	-131.97	2	58.6	10.3	0	610-622	R.ICEDMFDSQEYVK.Q	Carbamidomethyl: 2	QD:QU 0.71

BRUKER

Detailed Protein Report

Protein 529: PDZ domain-containing RING finger protein 4 isoform 2 [Homo sapiens]

**Accession:**	gi|142976783	**Score:**	21.3
**Database:**	refseq_human(refseq_human_20140103.fasta)	**MW [kDa]:**	89.0
**Database Date:**	2015-11-30	**pI:**	5.0
		**Sequence Coverage [%]:**	2.6
		**No. of unique Peptides:**	1

Quantitation

QD:QU **Median:** 0.46 **CV:** 0.00 % **No. of Peptides:** 1

10	20	30	40	50	60	70	80
MGCNLCTFQK	REEHYKLLYE	VSQVNGKDLS	KATHEEAVEA	FRNAKEPIVV	QVLRRTPLSR	PAYGMASEVQ	LMNASTQTDI
90	100	110	120	130	140	150	160
TFEHIMALAK	LRPPTPPVPD	ICPFLLSDSC	HSLHPMEHEF	YEDNEYISSL	PADADRTEDF	EYEEVELCRV	SSQEKLGLTV
170	180	190	200	210	220	230	240
CYRTDDEEDT	GIYVSEVDPN	SIAAKDGRIR	EGDRILQING	EDVQNREEAV	ALLSNDECKR	IVLLVARPEI	QLDEGWLEDE
250	260	270	280	290	300	310	320
RNEFLEELNL	EMLEEEHNEA	MQPTANEVEQ	PKKQEEEEGT	TDTATSSSNN	HEKDSGVGRT	DESLRNDESS	EQENAAEDPN
330	340	350	360	370	380	390	400
STSLKSKRDL	GQSQDTLGSV	ELQYNESLVS	GEYIDSDCIG	NPDEDCERFR	QLLELKCKIR	NHGEYDLYYS	SSTIECNQGE
410	420	430	440	450	460	470	480
QEGVEHELQL	LNEELRNIEL	ECQNIMQAHR	LQKVTDQYGD	IWTLHDGGFR	NYNTSIDMQR	GKLDDIMEHP	EKSDKDSSSA
490	500	510	520	530	540	550	560
YNTAESCRST	PLTVDRSPDS	SLPRVINLTN	KKNLRSTMAA	TQSSSGQSSK	ESTSTKAKTT	EQGCSAESKE	KVLEGSKLPD
570	580	590	600	610	620	630	640
QEKAVSEHIP	YLSPYHSSSY	RYANIPAHAR	HYQSYMQLIQ	QKSAVEYAQS	QLSLVSMCKE	SQKCSEPKME	WKVKIRSDGT
650	660	670	680	690	700	710	720
RYITKRPVRD	RILKERALKI	KEERSGMTTD	DDTMSEMKMG	RYWSKEERKQ	HLVRAKEQRR	RREFMMRSRL	ECLKESPQSG
730	740	750	760	770	780		
SEGKKEINII	ELSHKKMMKK	RNKKILDNWM	TIQELMTHGA	KSPDGTRVHN	AFLSVTTV		

Cmpd.	No. of Cmpds.	m/z meas.	Δ m/z [ppm]	z	Rt [min]	Score	P	Range	Sequence	Modification	Ratios	
1046	1	639.3055	-106.40	2	41.6	11.1	2	369-378	R.FRQLLELKCK.I		QD:QU	0.46

Detailed Protein Report

Protein 530: **PREDICTED: heterogeneous nuclear ribonucleoprotein M isoform X6 [Homo sapiens]**

**Accession:**	gi|530427511	**Score:**	21.3
**Database:**	refseq_human(refseq_human_20140103.fasta)	**MW [kDa]:**	60.5
**Database Date:**	2015-11-30	**pI:**	9.8
**Modification(s):**	Oxidation	**Sequence Coverage [%]:**	6.5
		**No. of unique Peptides:**	1

10	20	30	40	50	60	70	80
MEESMKKAAE	VLNKHSLSGR	PLKVKEDPDG	EHARRAMQKA	GRLGSTVFVA	NLDYKVGWKK	LKEVFSMAGV	VVRADILEDK
**90**	**100**	**110**	**120**	**130**	**140**	**150**	**160**
DGKSRGIGTV	TFEQSIEAVQ	AISMFNGQLL	FDRPMHVKMD	ERALPKGDFF	PPERPQQLPH	GLGGIGMGLG	PGGQPIDANH
**170**	**180**	**190**	**200**	**210**	**220**	**230**	**240**
LNKGIGMGNI	GPAGMGMEGI	GFGINKMGGM	EGPFGGGMEN	MGRFGSGMNM	GRINEILSNA	LKRGEIIAKQ	GGGGGGGSVP
**250**	**260**	**270**	**280**	**290**	**300**	**310**	**320**
GIERMGPGID	RLGGAGMERM	GAGLGHGMDR	VGSEIERMGL	VMDRMGSVER	MGSGIERMGP	LGLDHMASSI	ERMGQTMERI
**330**	**340**	**350**	**360**	**370**	**380**	**390**	**400**
GSGVERMGAG	MGFGLERMAA	PIDRVGQTIE	RMGSGVERMG	PAIERMGLSM	ERMVPAGMGA	GLERMGPVMD	RMATGLERMG
**410**	**420**	**430**	**440**	**450**	**460**	**470**	**480**
ANNLERMGLE	RMGANSLERM	GLERMGANSL	ERMGPAMGPA	LGAGIERMGL	AMGGGGGASF	DRAIEMERGN	FGGSFAGSFG
**490**	**500**	**510**	**520**	**530**	**540**	**550**	**560**
GAGGHAPGVA	RKACQIFVRN	LPFDFTWKML	KDKFNECGHV	LYADIKMENG	KSKGCGVVKF	ESPEVAERAC	RMMNGMKLSG
**570**	**580**						
REIDVRIDRN	A						

Cmpd.	No. of Cmpds.	m/z meas.	Δ m/z [ppm]	z	Rt [min]	Score	P	Range	Sequence	Modification
814	1	737.3793	37.51	3	40.2	10.9	0	164-186	K.GIGMGNIGPAGMGMEGIGFGINK.M	Oxidation: 4, 12

Detailed Protein Report

Protein 531: noelin isoform 2 precursor [Homo sapiens]

Accession: gi|5453547
Database: refseq_human(refseq_human_20140103.fasta)
Database Date: 2015-11-30
Modification(s): Carbamidomethyl, Oxidation

Score: 21.2
MW [kDa]: 15.9
pI: 10.2
Sequence Coverage [%]: 20.7
No. of unique Peptides: 2

10	20	30	40	50	60	70	80
MPGRWRWQRD	MHPARKLLSL	LFLILMGTEL	TQVLPTNPEE	SWQVYSSAQD	SEGRCICTVV	APQQTMCSRD	ARTKQLRQLL
**90**	**100**	**110**	**120**	**130**	**140**		
EKVQNMSQSI	EVLDRRTQRD	LQYVEKMENQ	MKGLESKFKQ	VEESHKQHLA	RQFKG		

Cmpd.	No. of Cmpds.	m/z meas.	Δ m/z [ppm]	z	Rt [min]	Score	P	Range	Sequence	Modification
2540	1	856.9279	52.38	2	62.4	10.2	0	55-69	R.CICTVVAPQQTMCSR.D	Carbamidomethyl: 1; Oxidation: 12
1127	1	759.8346	-63.26	2	44.2	11.0	0	83-95	K.VQNMSQSIEVLDR.R	

Detailed Protein Report

Protein 532: protein FAM78B [Homo sapiens]

Accession: gi|63003928
Database: refseq_human(refseq_human_20140103.fasta)
Database Date: 2015-11-30

Score: 21.2
MW [kDa]: 29.8
pI: 10.2
Sequence Coverage [%]: 11.1
No. of unique Peptides: 1

Quantitation

QD:QU **Median:** 0.87 **CV:** 0.00 % **No. of Peptides:** 1

10	20	30	40	50	60	70	80
MGCIQSITCK	ARIRRENIVV	YDVCATIDQC	PTRIEETSPI	VLRYKTPYFK	ASARVVMPPI	PRHETWVVGW	IQACNQMEFF
**90**	**100**	**110**	**120**	**130**	**140**	**150**	**160**
NTYSDLGMSS	WELPDLREGR	VKAISDSDGV	SYPWYGNTTE	TVTLVGPTNK	ISRFSVSMND	NFYPSVTWAV	PVSDSNVPLL
**170**	**180**	**190**	**200**	**210**	**220**	**230**	**240**
TRIKRDQSFT	TWLVAMNTTT	KEKIILQTIK	WRMRVDIEVD	PLQLLGQRAR	LVGRTQQEQP	RILSRMEPIP	PNALVKPNAN
**250**	**260**	**270**					
DAQVLMWRPK	RGPPLVVIPP	K					

Cmpd.	No. of Cmpds.	m/z meas.	Δ m/z [ppm]	z	Rt [min]	Score	P	Range	Sequence	Modification	Ratios
1179	1	941.4167	-103.81	2	44.9	10.9	1	193-208	R.MRVDIEVDPLQLLGQR.A		QD:QU 0.87

Detailed Protein Report

Protein 533: splicing factor 3B subunit 3 [Homo sapiens]

Accession: gi|54112121
Database: refseq_human(refseq_human_20140103.fasta)
Database Date: 2015-11-30
Modification(s): Oxidation

Score: 21.2
MW [kDa]: 135.5
pI: 5.0
Sequence Coverage [%]: 2.5
No. of unique Peptides: 2

10	20	30	40	50	60	70	80
MFLYNLTLQR	ATGISFAIHG	NFSGTKQQEI	VVSRGKILEL	LRPDPNTGKV	HTLLTVEVFG	VIRSLMAFRL	TGGTKDYIVV
**90**	**100**	**110**	**120**	**130**	**140**	**150**	**160**
GSDSGRIVIL	EYQPSKNMFE	KIHQETFGKS	GCRRIVPGQF	LAVDPKGRAV	MISAIEKQKL	VYILNRDAAA	RLTISSPLEA
**170**	**180**	**190**	**200**	**210**	**220**	**230**	**240**
HKANTLVYHV	VGVDVGFENP	MFACLEMDYE	EADNDPTGEA	AANTQQTLTF	YELDLGLNHV	VRKYSEPLEE	HGNFLITVPG
**250**	**260**	**270**	**280**	**290**	**300**	**310**	**320**
GSDGPSGVLI	CSENYITYKN	FGDQPDIRCP	IPRRRNDLDD	PERGMIFVCS	ATHKTKSMFF	FLAQTEQGDI	FKITLETDED
**330**	**340**	**350**	**360**	**370**	**380**	**390**	**400**
MVTEIRLKYF	DTVPVAAAMC	VLKTGFLFVA	SEFGNHYLYQ	IAHLGDDDEE	PEFSSAMPLE	EGDTFFFQPR	PLKNLVLVDE
**410**	**420**	**430**	**440**	**450**	**460**	**470**	**480**
LDSLSPILFC	QIADLANEDT	PQLYVACGRG	PRSSLRVLRH	GLEVSEMAVS	ELPGNPNAVW	TVRRHIEDEF	DAYIIVSFVN
**490**	**500**	**510**	**520**	**530**	**540**	**550**	**560**
ATLVLSIGET	VEEVTDSGFL	GTTPTLSCSL	LGDDALVQVY	PDGIRHIRAD	KRVNEWKTPG	KKTIVKCAVN	QRQVVIALTG
**570**	**580**	**590**	**600**	**610**	**620**	**630**	**640**
GELVYFEMDP	SGQLNEYTER	KEMSADVVCM	SLANVPPGEQ	RSRFLAVGLV	DNTVRIISLD	PSDCLQPLSM	QALPAQPESL
**650**	**660**	**670**	**680**	**690**	**700**	**710**	**720**
CIVEMGGTEK	QDELGERGSI	GFLYLNIGLQ	NGVLLRTVLD	PVTGDLSDTR	TRYLGSRPVK	LFRVRMQGQE	AVLAMSSRSW
**730**	**740**	**750**	**760**	**770**	**780**	**790**	**800**
LSYSYQSRFH	LTPLSYETLE	FASGFASEQC	PEGIVAISTN	TLRILALEKL	GAVFNQVAFP	LQYTPRKFVI	HPESNNLIII
**810**	**820**	**830**	**840**	**850**	**860**	**870**	**880**
ETDHNAYTEA	TKAQRKQQMA	EEMVEAAGED	ERELAAEMAA	AFLNENLPES	IFGAPKAGNG	QWASVIRVMN	PIQGNTLDLV
**890**	**900**	**910**	**920**	**930**	**940**	**950**	**960**
QLEQNEAAFS	VAVCRFSNTG	EDWYVLVGVA	KDLILNPRSV	AGGFVYTYKL	VNNGEKLEFL	HKTPVEEVPA	AIAPFQGRVL
**970**	**980**	**990**	**1000**	**1010**	**1020**	**1030**	**1040**
IGVGKLLRVY	DLGKKKLLRK	CENKHIANYI	SGIQTIGHRV	IVSDVQESFI	WVRYKRNENQ	LIIFADDTYP	RWVTTASLLD
**1050**	**1060**	**1070**	**1080**	**1090**	**1100**	**1110**	**1120**
YDTVAGADKF	GNICVVRLPP	NTNDEVDEDP	TGNKALWDRG	LLNGASQKAE	VIMNYHVGET	VLSLQKTTLI	PGGSESLVYT
**1130**	**1140**	**1150**	**1160**	**1170**	**1180**	**1190**	**1200**
TLSGGIGILV	PFTSHEDHDF	FQHVEMHLRS	EHPPLCGRDH	LSFRSYYFPV	KNVIDGDLCE	QFNSMEPNKQ	KNVSEELDRT
**1210**	**1220**						
PPEVSKKLED	IRTRYAF						

Cmpd.	No. of Cmpds.	m/z meas.	Δ m/z [ppm]	z	Rt [min]	Score	P	Range	Sequence	Modification
2564	1	822.4530	49.65	2	62.7	10.1	0	329-343	K.YFDTVPVAAAMCVLK.T	Oxidation: 11
2787	1	619.0056	136.85	3	66.0	11.0	0	817-832	K.QQMAEEMVEAAGEDER.E	Oxidation: 3, 7

Detailed Protein Report

Protein 534: X-linked retinitis pigmentosa GTPase regulator-interacting protein 1 [Homo sapiens]

**Accession:**	gi|112734867	**Score:**	21.1
**Database:**	refseq_human(refseq_human_20140103.fasta)	**MW [kDa]:**	146.6
**Database Date:**	2015-11-30	**pI:**	5.4
		**Sequence Coverage [%]:**	2.2
		**No. of unique Peptides:**	1

10	20	30	40	50	60	70	80
MSHLVDPTSG	DLPVRDIDAI	PLVLPASKGK	NMKTQPPLSR	MNREELEDSF	FRLREDHMLV	KELSWKQQDE	IKRLRTTLLR
90	100	110	120	130	140	150	160
LTAAGRDLRV	AEEAAPLSET	ARRGQKAGWR	QRLSMHQRPQ	MHRLQGHFHC	VGPASPRRAQ	PRVQVGHRQL	HTAGAPVPEK
170	180	190	200	210	220	230	240
PKRGPRDRLS	YTAPPSFKEH	ATNENRGEVA	SKPSELVSGS	NSIISFSSVI	SMAKPIGLCM	PNSAHIMASN	TMQVEEPPKS
250	260	270	280	290	300	310	320
PEKMWPKDEN	FEQRSSLECA	QKAAELRASI	KEKVELIRLK	KLLHERNASL	VMTKAQLTEV	QEAYETLLQK	NQGILSAAHE
330	340	350	360	370	380	390	400
ALLKQVNELR	AELKEESKKA	VSLKSQLEDV	SILQMTLKEF	QERVEDLEKE	RKLLNDNYDK	LLESMLDSSD	SSSQPHWSNE
410	420	430	440	450	460	470	480
LIAEQLQQQV	SQLQDQLDAE	LEDKRKVLLE	LSREKAQNED	LKLEVTNILQ	KHKQEVELLQ	NAATISQPPD	RQSEPATHPA
490	500	510	520	530	540	550	560
VLQENTQIEP	SEPKNQEEKK	LSQVLNELQV	SHAETTLELE	KTRDMLILQR	KINVCYQEEL	EAMMTKADND	NRDHKEKLER
570	580	590	600	610	620	630	640
LTRLLDLKNN	RIKQLEGILR	SHDLPTSEQL	KDVAYGTRPL	SLCLETLPAH	GDEDKVDISL	LHQGENLFEL	HIHQAFLTSA
650	660	670	680	690	700	710	720
ALAQAGDTQP	TTFCTYSFYD	FETHCTPLSV	GPQPLYDFTS	QYVMETDSLF	LHYLQEASAR	LDIHQAMASE	HSTLAAGWIC
730	740	750	760	770	780	790	800
FDRVLETVEK	VHGLATLIGA	GGEEFGVLEY	WMRLRFPIKP	SLQACNKRKK	AQVYLSTDVL	GGRKAQEEEF	RSESWEPQNE
810	820	830	840	850	860	870	880
LWIEITKCCG	LRSRWLGTQP	SPYAVYRFFT	FSDHDTAIIP	ASNNPYFRDQ	ARFPVLVTSD	LDHYLRREAL	SIHVFDDEDL
890	900	910	920	930	940	950	960
EPGSYLGRAR	VPLLPLAKNE	SIKGDFNLTD	PAEKPNGSIQ	VQLDWKFPYI	PPESFLKPEA	QTKGKDTKDS	SKISSEEEKA
970	980	990	1000	1010	1020	1030	1040
SFPSQDQMAS	PEVPIEAGQY	RSKRKPPHGG	ERKEKEHQVV	SYSRRKHGKR	IGVQGKNRME	YLSLNILNGN	TPEQVNYTEW
1050	1060	1070	1080	1090	1100	1110	1120
KFSETNSFIG	DGFKNQHEEE	EMTLSHSALK	QKEPLHPVND	KESSEQGSEV	SEAQTTDSDD	VIVPPMSQKY	PKADSEKMCI
1130	1140	1150	1160	1170	1180	1190	1200
EIVSLAFYPE	AEVMSDENIK	QVYVEYKFYD	LPLSETETPV	SLRKPRAGEE	IHFHFSKVID	LDPQEQQGRR	RFLFDMLNGQ
1210	1220	1230	1240	1250	1260	1270	1280
DPDQGHLKFT	VVSDPLDEEK	KECEEVGYAY	LQLWQILESG	RDILEQELDI	VSPEDLATPI	GRLKVSLQAA	AVLHAIYKEM
1290							
TEDLFS							

Cmpd.	No. of Cmpds.	m/z meas.	Δ m/z [ppm]	z	Rt [min]	Score	P	Range	Sequence	Modification
1534	1	676.3317	-93.91	2	49.4	10.1	0	16-28	R.DIDAIPLVLPASK.G	

Detailed Protein Report

Protein 535: PREDICTED: apolipophorins-like [Homo sapiens]

Accession: gi|578797156
Database: refseq_human(refseq_human_20140103.fasta)
Database Date: 2015-11-30
Modification(s): Carbamidomethyl

Score: 21.0
MW [kDa]: 74.4
pI: 6.6
Sequence Coverage [%]: 2.5
No. of unique Peptides: 1

Alias proteins:

Accession	Name	Description
gi|578828430	refseq_human (refseq_human_20140103.fasta)	PREDICTED: apolipophorins-like [Homo sapiens]

10	20	30	40	50	60	70	80
MDADASSRPL	DTDLSFSLRM	CAKKEIKQPL	LDALPSCGTE	HCVGLMKELI	ASAAVEADEV	EAWLSSLAFL	PQPTDAMVHM
**90**	**100**	**110**	**120**	**130**	**140**	**150**	**160**
LLPLLQTPRA	SPGAFLGISA	LVHNLCASLD	GPCGQLPGVG	SLVRILGDAL	GENCTIQEPS	DDDKVRTSSV	LSTGFSAPSG
**170**	**180**	**190**	**200**	**210**	**220**	**230**	**240**
AEANQLQLVL	KAVGNAGLAA	MALTPTLSAC	ASLRSSTPEI	RLGAIQAFRR	VPCSADRSVL	SRLYQSLEED	AEIRINAYLA
**250**	**260**	**270**	**280**	**290**	**300**	**310**	**320**
LMRCPSEEVF	AQVRRTQAGE	LSTQVGSFVW	SHILQLLETH	DPLKRALRDT	LPEDILSQEF	HPEMWKHSSY	SDVTFRSVSG
**330**	**340**	**350**	**360**	**370**	**380**	**390**	**400**
SLGANLEGTL	LFSPASFLPR	SATVNLTIHT	MGRAFNLLEL	GLRLENAEEI	AHRLFGRKSF	WGQEDGREPE	PEEPPGPEPG
**410**	**420**	**430**	**440**	**450**	**460**	**470**	**480**
PAPQPASPEC	PGDRDRRMRY	LQQKVTRRRG	ARQALRCELS	VKLLGQELSF	VNCGATGSHV	NHWPLNLAEL	AIKLMKGQEV
**490**	**500**	**510**	**520**	**530**	**540**	**550**	**560**
QMNRRLSLAA	QELVFPTVSG	LPARLTLNAS	AAISIRVRGT	TDFQQRSDFS	VNGYVKPRYL	APGSWRRLGG	GIPSLQRAAE
**570**	**580**	**590**	**600**	**610**	**620**	**630**	**640**
AWVTSVWGPR	DAAGTCLSLS	ALLQISAQMG	TAGILGQAGL	RWVTSVRSAA	SLDGGIQVQK	GRVLKVHLNT	PEEAVELLSF
**650**	**660**	**670**	**680**	**690**			
SSQLYLITRD	GVRSLRHVPG	PSEVQSCTGE	EGRQQGLHDI	VPRWASQG			

Cmpd.	No. of Cmpds.	m/z meas.	Δ m/z [ppm]	z	Rt [min]	Score	P	Range	Sequence	Modification
2604	1	913.4894	86.61	2	63.2	21.0	0	657-673	R.HVPGPSEVQSCTGEEGR.Q	Carbamidomethyl: 11

Detailed Protein Report

Protein 536: kinesin-like protein KIF20B isoform 2 [Homo sapiens]

**Accession:**	gi|46049114	**Score:**	21.0
**Database:**	refseq_human(refseq_human_20140103.fasta)	**MW [kDa]:**	206.0
**Database Date:**	2015-11-30	**pI:**	5.5
**Modification(s):**	Carbamidomethyl, Oxidation	**Sequence Coverage [%]:**	2.0
		**No. of unique Peptides:**	1

10	20	30	40	50	60	70	80
MESNFNQEGV	PRPSYVFSAD	PIARPSEINF	DGIKLDLSHE	FSLVAPNTEA	NSFESKDYLQ	VCLRIRPFTQ	SEKELESEGC
90	100	110	120	130	140	150	160
VHILDSQTVV	LKEPQCILGR	LSEKSSGQMA	QKFSFSKVFG	PATTQKEFFQ	GCIMQPVKDL	LKGQSRLIFT	YGLTNSGKTY
170	180	190	200	210	220	230	240
TFQGTEENIG	ILPRTLNVLF	DSLQERLYTK	MNLKPHRSRE	YLRLSSEQEK	EEIASKSALL	RQIKEVTVHN	DSDDTLYGSL
250	260	270	280	290	300	310	320
TNSLNISEFE	ESIKDYEQAN	LNMANSIKFS	VWVSFFEIYN	EYIYDLFVPV	SSKFQKRKML	RLSQDVKGYS	FIKDLQWIQV
330	340	350	360	370	380	390	400
SDSKEAYRLL	KLGIKHQSVA	FTKLNNASSR	SHSIFTVKIL	QIEDSEMSRV	IRVSELSLCD	LAGSERTMKT	QNEGERLRET
410	420	430	440	450	460	470	480
GNINTSLLTL	GKCINVLKNS	EKSKFQQHVP	FRESKLTHYF	QSFFNGKGKI	CMIVNISQCY	LAYDETLNVL	KFSAIAQKVC
490	500	510	520	530	540	550	560
VPDTLNSSQE	KLFGPVKSSQ	DVSLDSNSNS	KILNVKRATI	SWENSLEDLM	EDEDLVEELE	NAEETQNVET	KLLDEDLDKT
570	580	590	600	610	620	630	640
LEENKAFISH	EEKRKLLDLI	EDLKKKLINE	KKEKLTLEFK	IREEVTQEFT	QYWAQREADF	KETLLQEREI	LEENAERRLA
650	660	670	680	690	700	710	720
IFKDLVGKCD	TREEAAKDIC	ATKVETEEAT	ACLELKFNQI	KAELAKTKGE	LIKTKEELKK	RENESDSLIQ	ELETSNKKII
730	740	750	760	770	780	790	800
TQNQRIKELI	NIIDQKEDTI	NEFQNLKSHM	ENTFKCNDKA	DTSSLIINNK	LICNETVEVP	KDSKSKICSE	RKRVNENELQ
810	820	830	840	850	860	870	880
QDEPPAKKGS	IHVSSAITED	QKKSEEVRPN	IAEIEDIRVL	QENNEGLRAF	LLTIENELKN	EKEEKAELNK	QIVHFQQELS
890	900	910	920	930	940	950	960
LSEKKNLTLS	KEVQQIQSNY	DIAIAELHVQ	KSKNQEQEEK	IMKLSNEIET	ATRSITNNVS	QIKLMHTKID	ELRTLDSVSQ
970	980	990	1000	1010	1020	1030	1040
ISNIDLLNLR	DLSNGSEEDN	LPNTQLDLLG	NDYLVSKQVK	EYRIQEPNRE	NSFHSSIEAI	WEECKEIVKA	SSKKSHQIEE
1050	1060	1070	1080	1090	1100	1110	1120
LEQQIEKLQA	EVKGYKDENN	RLKEKEHKNQ	DDLLKEKETL	IQQLKEELQE	KNVTLDVQIQ	HVVEGKRALS	ELTQGVTCYK
1130	1140	1150	1160	1170	1180	1190	1200
AKIKELETIL	ETQKVECSHS	AKLEQDILEK	ESIILKLERN	LKEFQEHLQD	SVKNTKDLNV	KELKLKEEIT	QLTNNLQDMK
1210	1220	1230	1240	1250	1260	1270	1280
HLLQLKEEEE	ETNRQETEKL	KEELSASSAR	TQNLKADLQR	KEEDYADLKE	KLTDAKKQIK	QVQKEVSVMR	DEDKLLRIKI
1290	1300	1310	1320	1330	1340	1350	1360
NELEKKKNQC	SQELDMKQRT	IQQLKEQLNN	QKVEEAIQQY	ERACKDLNVK	EKIIEDMRMT	LEEQEQTQVE	QDQVLEAKLE
1370	1380	1390	1400	1410	1420	1430	1440
EVERLATELE	KWKEKCNDLE	TKNNQRSNKE	HENNTDVLGK	LTNLQDELQE	SEQKYNADRK	KWLEEKMMLI	TQAKEAENIR
1450	1460	1470	1480	1490	1500	1510	1520
NKEMKKYAED	RERFFKQQNE	MEILTAQLTE	KDSDLQKWRE	ERDQLVAALE	IQLKALISSN	VQKDNEIEQL	KRIISETSKI
1530	1540	1550	1560	1570	1580	1590	1600
ETQIMDIKPK	RISSADPDKL	QTEPLSTSFE	ISRNKIEDGS	VVLDSCEVST	ENDQSTRFPK	PELEIQFTPL	QPNKMAVKHP
1610	1620	1630	1640	1650	1660	1670	1680
GCTTPVTVKI	PKARKRKSNE	MEEDLVKCEN	KKNATPRTNL	KFPISDDRNS	SVKKEQKVAI	RPSSKKTYSL	RSQASIIGVN
1690	1700	1710	1720	1730	1740	1750	1760
LATKKKEGTL	QKFGDFLQHS	PSILQSKAKK	IIETMSSSKL	SNVEASKENV	SQPKRAKRKL	YTSEISSPID	ISGQVILMDQ
1770	1780	1790					
KMKESDHQII	KRRLRTKTAK						

Cmpd.	No. of	m/z meas.	Δ m/z	z	Rt	Score	P	Range	Sequence	Modification

Detailed Protein Report

	Cmpds.		[ppm]		[min]					
1683	1	954.5011	22.43	3	51.3	10.8	1	448-471	K.GKICMIVNISQCYLAYDETLNVLK.F	Carbamidomethyl: 4, 12; Oxidation: 5

Detailed Protein Report

Protein 537: 2'-5'-oligoadenylate synthase 2 isoform 1 [Homo sapiens]

**Accession:**	gi|74229019	**Score:**	21.0
**Database:**	refseq_human(refseq_human_20140103.fasta)	**MW [kDa]:**	82.4
**Database Date:**	2015-11-30	**pI:**	9.4
		**Sequence Coverage [%]:**	3.1
		**No. of unique Peptides:**	2

10	20	30	40	50	60	70	80
MGNGESQLSS	VPAQKLGWFI	QEYLKPYEEC	QTLIDEMVNT	ICDVLQEPEQ	FPLVQGVAIG	GSYGRKTVLR	GNSDGTLVLF
**90**	**100**	**110**	**120**	**130**	**140**	**150**	**160**
FSDLKQFQDQ	KRSQRDILDK	TGDKLKFCLF	TKWLKNNFEI	QKSLDGFTIQ	VFTKNQRISF	EVLAAFNALS	LNDNPSPWIY
**170**	**180**	**190**	**200**	**210**	**220**	**230**	**240**
RELKRSLDKT	NASPGEFAVC	FTELQQKFFD	NRPGKLKDLI	LLIKHWHQQC	QKKIKDLPSL	SPYALELLTV	YAWEQGCRKD
**250**	**260**	**270**	**280**	**290**	**300**	**310**	**320**
NFDIAEGVRT	VLELIKCQEK	LCIYWMVNYN	FEDETIRNIL	LHQLQSARPV	ILDPVDPTNN	VSGDKICWQW	LKKEAQTWLT
**330**	**340**	**350**	**360**	**370**	**380**	**390**	**400**
SPNLDNELPA	PSWNVLPAPL	FTTPGHLLDK	FIKEFLQPNK	CFLEQIDSAV	NIIRTFLKEN	CFRQSTAKIQ	IVRGGSTAKG
**410**	**420**	**430**	**440**	**450**	**460**	**470**	**480**
TALKTGSDAD	LVVFHNSLKS	YTSQKNERHK	IVKEIHEQLK	AFWREKEEEL	EVSFEPPKWK	APRVLSFSLK	SKVLNESVSF
**490**	**500**	**510**	**520**	**530**	**540**	**550**	**560**
DVLPAFNALG	QLSSGSTPSP	EVYAGLIDLY	KSSDLPGGEF	STCFTVLQRN	FIRSRPTKLK	DLIRLVKHWY	KECERKLKPK
**570**	**580**	**590**	**600**	**610**	**620**	**630**	**640**
GSLPPKYALE	LLTIYAWEQG	SGVPDFDTAE	GFRTVLELVT	QYQQLCIFWK	VNYNFEDETV	RKFLLSQLQK	TRPVILDPAE
**650**	**660**	**670**	**680**	**690**	**700**	**710**	**720**
PTGDVGGGDR	WCWHLLAKEA	KEWLSSPCFK	DGTGNPIPPW	KVPTMQTPGS	CGARIHPIVN	EMFSSRSHRI	LNNNSKRNF

Cmpd.	No. of Cmpds.	m/z meas.	Δ m/z [ppm]	z	Rt [min]	Score	P	Range	Sequence	Modification
2142	2	596.7577	-225.65	2	57.2	10.2	2	556-566	R.KLKPKGSLPPK.Y	
1541	1	669.8176	-79.84	2	49.5	10.7	2	707-717	R.SHRILNNNSKR.N	

Detailed Protein Report

Protein 538: paraneoplastic antigen Ma2 [Homo sapiens]

Accession: gi|11464969
Database: refseq_human(refseq_human_20140103.fasta)
Database Date: 2015-11-30
Modification(s): Oxidation

Score: 20.9
MW [kDa]: 41.5
pI: 4.7
Sequence Coverage [%]: 4.7
No. of unique Peptides: 1

10	20	30	40	50	60	70	80
MALALLEDWC	RIMSVDEQKS	LMVTGIPADF	EEAEIQEVLQ	ETLKSLGRYR	LLGKIFRKQE	NANAVLLELL	EDTDVSAIPS
**90**	**100**	**110**	**120**	**130**	**140**	**150**	**160**
EVQGKGGVWK	VIFKTPNQDT	EFLERLNLFL	EKEGQTVSGM	FRALGQEGVS	PATVPCISPE	LLAHLLGQAM	AHAPQPLLPM
**170**	**180**	**190**	**200**	**210**	**220**	**230**	**240**
RYRKLRVFSG	SAVPAPEEES	FEVWLEQATE	IVKEWPVTEA	EKKRWLAESL	RGPALDLMHI	VQADNPSISV	EECLEAFKQV
**250**	**260**	**270**	**280**	**290**	**300**	**310**	**320**
FGSLESRRTA	QVRYLKTYQE	EGEKVSAYVL	RLETLLRRAV	EKRAIPRRIA	DQVRLEQVMA	GATLNQMLWC	RLRELKDQGP
**330**	**340**	**350**	**360**	**370**			
PPSFLELMKV	IREEEEEEAS	FENESIEEPE	ERDGYGRWNH	EGDD			

Cmpd.	No. of Cmpds.	m/z meas.	Δ m/z [ppm]	z	Rt [min]	Score	P	Range	Sequence	Modification
1364	1	660.7454	137.08	3	47.2	20.9	0	295-311	R.LEQVMAGATLNQMLWCR.L	Oxidation: 13

Detailed Protein Report

Protein 539: **PREDICTED: membrane-associated guanylate kinase, WW and PDZ domain-containing protein 2 isoform X1 [Homo sapiens]**

**Accession:**	gi|530386693	**Score:**	20.9
**Database:**	refseq_human(refseq_human_20140103.fasta)	**MW [kDa]:**	157.1
**Database Date:**	2015-11-30	**pI:**	5.9
		**Sequence Coverage [%]:**	1.7
		**No. of unique Peptides:**	1

10	20	30	40	50	60	70	80
MSKSLKKKSH	WTSKVHESVI	GRNPEGQLGF	ELKGGAENGQ	FPYLGEVKPG	KVAYESGSKL	VSEELLLEVN	ETPVAGLTIR
90	100	110	120	130	140	150	160
DVLAVIKHCK	DPLRLKCVKQ	GGIVDKDLRH	YLNLRFQKGS	VDHELQQIIR	DNLYLRTVPC	TTRPHKEGEV	PGVDYIFITV
170	180	190	200	210	220	230	240
EDFMELEKSG	ALLESGTYED	NYYGTPKPPA	EPAPLLLNVT	DQILPGATPS	AEGKRKRNKS	VSNMEKASIE	PPEEEEEERP
250	260	270	280	290	300	310	320
VVNGNGVVVT	PESSEHEDKS	AGASGEMPSQ	PYPAPVYSQP	EELKEQMDDT	KPTKPEDNEE	PDPLPDNWEM	AYTEKGEVYF
330	340	350	360	370	380	390	400
IDHNTKTTSW	LDPRLAKKAK	PPEECKENEL	PYGWEKIDDP	IYGTYYVDHI	NRRTQFENPV	LEAKRKLQQH	NMPHTELGTK
410	420	430	440	450	460	470	480
PLQAPGFREK	PLFTRDASQL	KGTFLSTTLK	KSNMGFGFTI	IGGDEPDEFL	QVKSVIPDGP	AAQDGKMETG	DVIVYINEVC
490	500	510	520	530	540	550	560
VLGHTHADVV	KLFQSVPIGQ	SVNLVLCRGY	PLPFDPEDPA	NSMVPPLAIM	ERPPPVMVNG	RHNYETYLEY	ISRTSQSVPD
570	580	590	600	610	620	630	640
ITDRPPHSLH	SMPTDGQLDG	TYPPPVHDDN	VSMASSGATQ	AELMTLTIVK	GAQGFGFTIA	DSPTGQRVKQ	ILDIQGCPGL
650	660	670	680	690	700	710	720
CEGDLIVEIN	QQNVQNLSHT	EVVDILKDCP	IGSETSLIIH	RGGFFSPWKT	PKPIMDRWEN	QGSPQTSLSA	PAIPQNLPFP
730	740	750	760	770	780	790	800
PALHRSSFPD	STEAFDPRKP	DPYELYEKSR	AIYESRRPDY	KELDVHLRRM	ESGFGFRILG	GDEPGQPILI	GAVIAMGSAD
810	820	830	840	850	860	870	880
RDGRLHPGDE	LVYVDGIPVA	GKTHRYVIDL	MHHAARNGQV	NLTVRRKVLC	GGEPCPENGR	SPGSVSTHHS	SPRSDYATYT
890	900	910	920	930	940	950	960
NSNHAAPSSN	ASPPEGFASH	SLQTSDVVIH	RKENEGFGFV	IISSLNRPES	GSTITVPHKI	GRIIDGSPAD	RCAKLKVGDR
970	980	990	1000	1010	1020	1030	1040
ILAVNGQSII	NMPHADIVKL	IKDAGLSVTL	RIIPQEELNS	PTSAPSSEKQ	SPMAQQSPLA	QQSPLAQPSP	ATPNSPIAQP
1050	1060	1070	1080	1090	1100	1110	1120
APPQPLQLQG	HENSYRSEVK	ARQDVKPDIR	QPPFTDYRQP	PLDYRQPPGG	DYQQPPPLDY	RQPPLLDYRQ	HSPDTRQYPL
1130	1140	1150	1160	1170	1180	1190	1200
SDYRQPQDFD	YFTVDMEKGA	KGFGFSIRGG	REYKMDLYVL	RLAEDGPAIR	NGRMRVGDQI	IEINGESTRD	MTHARAIELI
1210	1220	1230	1240	1250	1260	1270	1280
KSGGRRVRLL	LKRGTGQVPE	YDEPAPWSSP	AAAAPGLPEV	GVSLDDGLAP	FSPSHPAPPS	DPSHQISPGP	TWDIKREHDV
1290	1300	1310	1320	1330	1340	1350	1360
RKPKELSACG	QKKQRLGEQR	ERSASPQRAA	RPRLEEAPGG	QGRPEAGRPA	SEARAPGLAA	ADAADAARAG	GKEAPRAAAG
1370	1380	1390	1400	1410	1420	1430	1440
SELCRREGPG	AAPAFAGPGG	GGSGALEAEG	RAGARAGPRP	GPRPPGGAPA	RKAAVAPGPW	KVPGSDKLPS	VLKPGASAAS
1450							
R							

Cmpd.	No. of Cmpds.	m/z meas.	Δ m/z [ppm]	z	Rt [min]	Score	P	Range	Sequence	Modification
301	1	638.3361	5.51	2	33.8	10.3	0	374-384	R.TQFENPVLEAK.R	

Detailed Protein Report

Protein 540: **PREDICTED: ataxin-7-like protein 1 isoform X1 [Homo sapiens]**

**Accession:**	gi|530385637	**Score:**	20.9
**Database:**	refseq_human(refseq_human_20140103.fasta)	**MW [kDa]:**	92.6
**Database Date:**	2015-11-30	**pI:**	10.5
**Modification(s):**	Carbamidomethyl	**Sequence Coverage [%]:**	3.8
		**No. of unique Peptides:**	1

10	20	30	40	50	60	70	80
MTSERSRIPC	LSAAAAEGTG	KKQQEGRAMA	TLDRKVPSPE	AFLGKPWSSW	IDAAKLHCSD	NVDLEEAGKE	GGKSREVMRL
90	100	110	120	130	140	150	160
NKEDMHLFGH	YPAHDDFYLV	VCSACNQVVK	PQVFQSHCER	RHGSMCRPSP	SPVSPASNPR	TSLVQVKTKA	CLSGHHSASS
170	180	190	200	210	220	230	240
TSKPFKTPKD	NLLTSSSKQH	TVFPAKGSRD	KPCVPVPVVS	LEKIPNLVKA	DGANVKMNST	TTTAVSASST	SSSAVSTPPL
250	260	270	280	290	300	310	320
IKPVLMSKSV	PPSPEKILNG	KGILPTTIDK	KHQNGTKNSN	KPYRRLSERE	FDPNKHCGVL	DPETKKPCTR	SLTCKTHSLS
330	340	350	360	370	380	390	400
HRRAVPGRKK	QFDLLLAEHK	AKSREKEVKD	KEHLLTSTRE	ILPSQSGPAQ	DSLLGSSGSS	GPEPKVASPA	KSRPPNSVLP
410	420	430	440	450	460	470	480
RPSSANSISS	STSSNHSGHT	PEPPLPPVGG	DLASRLSSDE	GEMDGADESE	KLDCQFSTHH	PRPLAFCSFG	SRLMGRGYYV
490	500	510	520	530	540	550	560
FDRRWDRFRF	ALNSMVEKHL	NSQMWKKIPP	AADSPLPSPA	AHITTPVPAS	VLQPFSNPSA	VYLPSAPISS	RLTSSYIMTS
570	580	590	600	610	620	630	640
AMLSNAAFVT	SPDPSALMSH	TTAFPHVAAT	LSIMDSTFKA	PSAVSPIPAV	IPSPSHKPSK	TKTSKSSKVK	DLSTRSDESP
650	660	670	680	690	700	710	720
SNKKRKPQSS	TSSSSSSSSS	SLQTSLSSPL	SGPHKKNCVL	NASSALNSYQ	AAPPYNSLSV	HNSNNGVSPL	SAKLEPSGRT
730	740	750	760	770	780	790	800
SLPGGPADIV	RQVGAVGGSS	DSCPLSVPSL	ALHAGDLSLA	SHNAVSSLPL	SFDKSEGKKR	KNSSSSSKAC	KITKMPGMNS
810	820	830	840	850	860	870	880
VHKKNPPSLL	APVPDPVNST	SSRQVRDLLA	PPSPALGGRG	SWAAPLLSRA	APTGNTSLIP	VLVFPEGSFH	PLL

Cmpd.	No. of Cmpds.	m/z meas.	Δ m/z [ppm]	z	Rt [min]	Score	P	Range	Sequence	Modification
92	1	726.3490	-5.83	3	31.1	10.2	1	121-140	R.RHGSMCRPSPSPVSPASNPR.T	Carbamidomethyl: 6

Detailed Protein Report

Protein 541: glycolipid transfer protein domain-containing protein 2 precursor [Homo sapiens]

Accession: gi|304571945
Database: refseq_human(refseq_human_20140103.fasta)
Database Date: 2015-11-30
Modification(s): Carbamidomethyl

Score: 20.9
MW [kDa]: 31.6
pI: 11.6
Sequence Coverage [%]: 10.7
No. of unique Peptides: 2

Quantitation

QD:QU **Median:** 1.44 **CV:** 0.00 % **No. of Peptides:** 1

10	20	30	40	50	60	70	80
MGVAARPPAL	RHWFSHSIPL	AIFALLLLYL	SVRSLGARSG	CGPRAQPCVP	GETAPFQVRQ	ESGTLEAPER	KQPPCLGPRG
**90**	**100**	**110**	**120**	**130**	**140**	**150**	**160**
MLGRMMRRFH	ASLKPEGDVG	LSPYLAGWRA	LVEFLTPLGS	VFAFATREAF	TKVTDLEARV	HGPDAEHYWS	LVAMAAWERR
**170**	**180**	**190**	**200**	**210**	**220**	**230**	**240**
AGLLEQPGAA	PRDPTRSSGS	RTLLLLHRAL	RWSQLCLHRV	ATGALGGPEA	GVQCSDAYRA	ALGPHHPWLV	RQTARLAFLA
**250**	**260**	**270**	**280**	**290**	**300**		
FPGRRRLLEL	ACPGATEAEA	RAALLRAAGT	LEDVYNRTQS	LLAERGLLQL	A		

Cmpd.	No. of Cmpds.	m/z meas.	Δ m/z [ppm]	z	Rt [min]	Score	P	Range	Sequence	Modification	Ratios
2172	1	900.3349	-132.49	2	55.8	10.4	2	72-87	K.QPPCLGPRGMLGRMMR.R		
366	1	800.8792	-30.48	2	34.5	10.5	0	247-261	R.LLELACPGATEAEAR.A	Carbamidomethyl: 6	QD:QU 1.44

Detailed Protein Report

Protein 542: **zinc finger protein Helios isoform 2 [Homo sapiens]**

Accession: gi|119220592
Database: refseq_human(refseq_human_20140103.fasta)
Database Date: 2015-11-30

Score: 20.8
MW [kDa]: 56.7
pI: 6.1
Sequence Coverage [%]: 5.6
No. of unique Peptides: 1

10	20	30	40	50	60	70	80
METEAIDGYI	TCDNELSPER	EHSNMAIDLT	SSTPNGQHAS	PSHMTSTNSV	KLEMQSDEEC	DRKPLSREDE	IRGHDEGSSL
90	100	110	120	130	140	150	160
EEPLIESSEV	ADNRKVQELQ	GEGGIRLPNG	ERPFHCNQCG	ASFTQKGNLL	RHIKLHSGEK	PFKCPFCSYA	CRRRDALTGH
170	180	190	200	210	220	230	240
LRTHSVGKPH	KCNYCGRSYK	QRSSLEEHKE	RCHNYLQNVS	MEAAGQVMSH	HVPPMEDCKE	QEPIMDNNIS	LVPFERPAVI
250	260	270	280	290	300	310	320
EKLTGNMGKR	KSSTPQKFVG	EKLMRFSYPD	IHFDMNLTYE	KEAELMQSHM	MDQAINNAIT	YLGAEALHPL	MQHPPSTIAE
330	340	350	360	370	380	390	400
VAPVISSAYS	QVYHPNRIER	PISRETADSH	ENNMDGPISL	IRPKSRPQER	EASPSNSCLD	STDSESSHDD	HQSYQGHPAL
410	420	430	440	450	460	470	480
NPKRKQSPAY	MKEDVKALDT	TKAPKGSLKD	IYKVFNGEGE	QIRAFKCEHC	RVLFLDHVMY	TIHMGCHGYR	DPLECNICGY
490	500	510					
RSQDRYEFSS	HIVRGEHTFH						

Cmpd.	No. of Cmpds.	m/z meas.	Δ m/z [ppm]	z	Rt [min]	Score	P	Range	Sequence	Modification
2120	1	1052.4607	1.63	3	56.9	20.8	0	192-219	R.CHNYLQNVSMEAAGQVMSHHVPPMEDCK.E	

Detailed Protein Report

Protein 543: PREDICTED: centrosomal protein of 290 kDa isoform X4 [Homo sapiens]

**Accession:**	gi|530400856	**Score:**	20.8
**Database:**	refseq_human(refseq_human_20140103.fasta)	**MW [kDa]:**	234.7
**Database Date:**	2015-11-30	**pI:**	6.1
		**Sequence Coverage [%]:**	0.7
		**No. of unique Peptides:**	1

Quantitation

QD:QU	**Median:** 0.77	**CV:** 0.00 %	**No. of Peptides:** 1

Detailed Protein Report

10	20	30	40	50	60	70	80
MQKLSLRQKS	AIFCQQIHEN	RADMDKSQVA	TLEEEQVHSQ	VKYADINLKE	DIIKSEVPLQ	TEILKNKLKV	NLPDPVSITA
90	100	110	120	130	140	150	160
QSKLSQINSL	ENLIEQLRRE	LVFLRSQNEI	IAQEFLIKEA	ECRNADIELE	HHRSQAEQNE	FLSRELIEKE	RDLERSRTVI
170	180	190	200	210	220	230	240
AKFQNKLKEL	VEENKQLEEG	MKEILQAIKE	MQKDPDVKGG	ETSLIIPSLE	RLVNAIESKN	AEGIFDASLH	LKAQVDQLTG
250	260	270	280	290	300	310	320
RNEELRQELR	ESRKEAINYS	QQLAKANLKI	DHLEKETSLL	RQSEGSNVVF	KGIDLPDGIA	PSSASIINSQ	NEYLIHLLQE
330	340	350	360	370	380	390	400
LENKEKKLKN	LEDSLEDYNR	KFAVIRHQQS	LLYKEYLSEK	ETWKTESKTI	KEEKRKLEDQ	VQQDAIKVKE	YNNLLNALQM
410	420	430	440	450	460	470	480
DSDEMKKILA	ENSRKITVLQ	VNEKSLIRQY	TTLVELERQL	RKENEKQKNE	LLSMEAEVCE	KIGCLQRFKE	MAIFKIAALQ
490	500	510	520	530	540	550	560
KVVDNSVSLS	ELELANKQYN	ELTAKYRDIL	QKDNMLVQRT	SNLEHLECEN	ISLKEQVESI	NKELEITKEK	LHTIEQAWEQ
570	580	590	600	610	620	630	640
ETKLGNESSM	DKAKKSITNS	DIVSISKKIT	MLEMKELNER	QRAEHCQKMY	EHLRTSLKQM	EERNFELETK	FAELTKINLD
650	660	670	680	690	700	710	720
AQKVEQMLRD	ELADSVSKAV	SDADRQRILE	LEKNEMELKV	EVSKLREISD	IARRQVEILN	AQQQSRDKEV	ESLRMQLLDY
730	740	750	760	770	780	790	800
QAQSDEKSLI	AKLHQHNVSL	QLSEATALGK	LESITSKLQK	MEAYNLRLEQ	KLDEKEQALY	YARLEGRNRA	KHLRQTIQSL
810	820	830	840	850	860	870	880
RRQFSGALPL	AQQEKFSKTM	IQLQNDKLKI	MQEMKNSQQE	HRNMENKTLE	MELKLKGLEE	LISTLKDTKG	AQKVINWHMK
890	900	910	920	930	940	950	960
IEELRLQELK	LNRELVKDKE	EIKYLNNIIS	EYERTISSLE	EEIVQQNKFH	EERQMAWDQR	EVDLERQLDI	FDRQQNEILN
970	980	990	1000	1010	1020	1030	1040
AAQKFEEATG	SIPDPSLPLP	NQLEIALRKI	KENIRIILET	RATCKSLEEK	LKEKESALRL	AEQNILSRDK	VINELRLRLP
1050	1060	1070	1080	1090	1100	1110	1120
ATAEREKLIA	ELGRKEMEPK	SHHTLKIAHQ	TIANMQARLN	QKEEVLKKYQ	RLLEKAREEQ	REIVKKHEED	LHILHHRLEL
1130	1140	1150	1160	1170	1180	1190	1200
QADSSLNKFK	QTAWDLMKQS	PTPVPTNKHF	IRLAEMEQTV	AEQDDSLSSL	LVKLKKVSQD	LERQREITEL	KVKEFENIKL
1210	1220	1230	1240	1250	1260	1270	1280
QLQENHEDEV	KKVKAEVEDL	KYLLDQSQKE	SQCLKSELQA	QKEANSRAPT	TTMRNLVERL	KSQLALKEKQ	QKALSRALLE
1290	1300	1310	1320	1330	1340	1350	1360
LRAEMTAAAE	ERIISATSQK	EAHLNVQQIV	DRHTRELKTQ	VEDLNENLLK	LKEALKTSKN	RENSLTDNLN	DLNNELQKKQ
1370	1380	1390	1400	1410	1420	1430	1440
KAYNKILREK	EEIDQENDEL	KRQIKRLTSG	LQGKPLTDNK	QSLIEELQRK	VKKLENQLEG	KVEEVDLKPM	KEKNAKEELI
1450	1460	1470	1480	1490	1500	1510	1520
RWEEGKKWQA	KIEGIRNKLK	EKEGEVFTLT	KQLNTLKDLF	AKADKEKLTL	QRKLKTTGMT	VDQVLGIRAL	ESEKELEELK
1530	1540	1550	1560	1570	1580	1590	1600
KRNLDLENDI	LYMRAHQALP	RDSVVEDLHL	QNRYLQEKLH	ALEKQFSKDT	YSKPSISGIE	SDDHCQREQE	LQKENLKLSS
1610	1620	1630	1640	1650	1660	1670	1680
ENIELKFQLE	QANKDLPRLK	NQVRDLKEMC	EFLKKEKAEV	QRKLGHVRGS	GRSGKTIPEL	EKTIGLMKKV	VEKVQRENEQ
1690	1700	1710	1720	1730	1740	1750	1760
LKKASGILTS	EKMANIEQEN	EKLKAELEKL	KAHLGHQLSM	HYESKTKGTE	KIIAENERLR	KELKKETDAA	EKLRIAKNNL
1770	1780	1790	1800	1810	1820	1830	1840
EILNEKMTVQ	LEETGKRLQF	AESRGPQLEG	ADSKSWKSIV	VTRMYETKLK	ELETDIAKKN	QSITDLKQLV	KEATEREQKV
1850	1860	1870	1880	1890	1900	1910	1920
NKYNEDLEQQ	IKILKHVPEG	AETEQGLKRE	LQVLRLANHQ	LDKEKAELIH	QIEANKDQSG	AESTIPDADQ	LKEKIKDLET
1930	1940	1950	1960	1970	1980	1990	2000
QLKMSDLEKQ	HLKEEIKKLK	KELENFDPSF	FEEIEDLKYN	YKEEVKKNIL	LEEKVKKLSE	QLGVELTSPV	AASEEFEDEE
2010							
ESPVNFPIY							

Cmpd.	No. of Cmpds.	m/z meas.	Δ m/z [ppm]	z	Rt [min]	Score	P	Range	Sequence	Modification	Ratios

Detailed Protein Report

Cmpd.	No. of Cmpds.	m/z meas.	Δ m/z [ppm]	z	Rt [min]	Score	P	Range	Sequence	Modification	Ratios
1299	1	500.8071	-19.25	2	46.4	10.4	2	2-9	M.QKLSLRQK.S		QD:QU 0.77

Detailed Protein Report

Protein 544: PREDICTED: ribosomal protein S6 kinase-like 1 isoform X5 [Homo sapiens]

Accession: gi|530404733
Database: refseq_human(refseq_human_20140103.fasta)
Database Date: 2015-11-30
Modification(s): Carbamidomethyl

Score: 20.8
MW [kDa]: 57.3
pI: 7.0
Sequence Coverage [%]: 6.8
No. of unique Peptides: 1

10	20	30	40	50	60	70	80
MSLVACECLP	SPGLEPEPCS	RARSQAHVYL	EQIRNRVALG	VPDMTKRDYL	VDAATQIRLA	LERDVSEDYE	AAFNHYQNGV
90	100	110	120	130	140	150	160
DVLLRGIHVD	PNKERREAVK	LKITKYLRRA	EEIFNCHLQR	PLSSGASPSA	GFSSLRLRPI	RTLSSAVEQL	RGCRVVGVIE
170	180	190	200	210	220	230	240
KVQLVQDPAT	GGTFVVKSLP	RCHMVSRERL	TIIPHGVPYM	TKLLRYFVSE	DSIFLHLEHV	QGGTLWSHLL	SQAHSRHSGL
250	260	270	280	290	300	310	320
SSGSTQERMK	AQLNPHLNLL	TPARLPSGHA	PGQDRIALEP	PRTSPNLLLA	GEAPSTRPQR	EAEGEPTART	STSGSSDLPK
330	340	350	360	370	380	390	400
APGGHLHLQA	RRAGQNSDAG	PPRGLTWVPE	GAGPVLGGCG	RGMDQSCLSA	DGAGRGCGRA	TWSVREEQVK	QWAAEMLVAL
410	420	430	440	450	460	470	480
EALHEQGVLC	RDLHPGNLLL	DQAGHIRLTY	FGQWSEVEPQ	CCGEAVDNLY	SAPEVGGISE	LTEACDWWSF	GSLLYELLTG
490	500	510	520	530			
MALSQSHPSG	IQAHTQLQLP	EWLSRPAASL	LTEVRCSPAR	GSSCLA			

Cmpd.	No. of Cmpds.	m/z meas.	Δ m/z [ppm]	z	Rt [min]	Score	P	Range	Sequence	Modification
2112	1	1072.4083	-80.50	2	56.8	10.0	0	2-21	M.SLVACECLPSPGLEPEPCSR.A	Carbamidomethyl: 18

Detailed Protein Report

Protein 545: NF-X1-type zinc finger protein NFXL1 [Homo sapiens]

Accession: gi|89363020
Database: refseq_human(refseq_human_20140103.fasta)
Database Date: 2015-11-30
Modification(s): Carbamidomethyl

Score: 20.7
MW [kDa]: 101.3
pI: 10.5
Sequence Coverage [%]: 3.3
No. of unique Peptides: 2

Alias proteins:

Accession	Name	Description
gi|520261838	refseq_human (refseq_human_20140103.fasta)	NF-X1-type zinc finger protein NFXL1 [Homo sapiens]
gi|520261712	refseq_human (refseq_human_20140103.fasta)	NF-X1-type zinc finger protein NFXL1 [Homo sapiens]

```
        10         20         30         40         50         60         70         80
MEASWRQVAG GRGRSRGRAT AAPSGNGVHL RGAGGGREKG SVGAVPSGTS PGGVATTAAA GSRHSPAGSQ ALQTTAASEL
        90        100        110        120        130        140        150        160
MSQKKFEEIK KANQAAARKL VEEQFSSSSE EGDEDFEGKQ GKILANTFIT YTTQTDGDTR ELERTKQYVN EAFQAGAMTC
       170        180        190        200        210        220        230        240
LICIASVKRN QAVWSCSGCF CIFHMPCIQK WAKDSQFLVS SVTDDDFGKK DCPWPCPKCR FEYKRSETPS RYYCYCGKVE
       250        260        270        280        290        300        310        320
DPPLDPWLVP HSCGQVCERE FKPPCGHKCL LLCHPGPCPP CPKMVTTTCY CKKAKPIPRR CSAKEWSCQL PCGQKLLCGQ
       330        340        350        360        370        380        390        400
HKCENPCHAG SCQPCPRVSR QKCVCGKKVA ERSCASPLWH CDQVCGKTLP CGNHTCEQVC HVGACGECPR SGKRFCPCQK
       410        420        430        440        450        460        470        480
SKFSLPCTED VPTCGDSCDK VLECGIHRCS QRCHRGPCET CRQEVEKHCR CGKHTKRMPC HKPYLCETKC VKMRDCQKHQ
       490        500        510        520        530        540        550        560
CRRKCCPGNC PPCDQNCGRT LGCRNHKCPS VCHRGSCYPC PETVDVKCNC GNTKVTVPCG RERTTRPPKC KEQCSRPPTC
       570        580        590        600        610        620        630        640
HHTSQEKHRC HFGSCPPCHQ PCQKVLEKCG HLCPAPCHDQ ALIKQTGRHQ PTGPWEQPSE PAFIQTALPC PPCQVPIPME
       650        660        670        680        690        700        710        720
CLGKHEVSPL PCHAVGPYSC KRVCGRILDC QNHTCMKECH KVTKTDGCTG KNKAGPECLH CEEGCSKSRP LGCLHPCILR
       730        740        750        760        770        780        790        800
CHPGECPPCV QMLRIKCHCK ITSLYVECRK ITTADVNEKN LLSCCKNQCP KELPCGHRCK EMCHPGECPF NCNQKVKLRC
       810        820        830        840        850        860        870        880
PCKRIKKELQ CNKVRENQVS IECDTTCKEM KRKASEIKEA EAKAALEEEK RRQQAELEAF ENRLKGRRKK NRKRDEVAVE
       890        900        910        920
LSLWQKHKYY LISVCGVVVV VFAWYITHDV N
```

Cmpd.	No. of Cmpds.	m/z meas.	Δ m/z [ppm]	z	Rt [min]	Score	P	Range	Sequence	Modification
2093	1	732.3366	-79.70	2	56.6	10.4	1	17-31	R.GRATAAPSGNGVHLR.G	
1824	1	916.3237	-87.07	2	53.1	10.4	2	433-447	R.CHRGPCETCRQEVEK.H	Carbamidomethyl: 6

Detailed Protein Report

Protein 546: cytoskeleton-associated protein 2 isoform 3 [Homo sapiens]

Accession: gi|557878731
Database: refseq_human(refseq_human_20140103.fasta)
Database Date: 2015-11-30

Score: 20.7
MW [kDa]: 71.2
pI: 10.0
Sequence Coverage [%]: 3.8
No. of unique Peptides: 1

10	20	30	40	50	60	70	80
MLSSRDQRVV	TSEDQVQEGT	KVLKLKTKMA	DKENMKRPAE	SKNNTVVGKH	CIPLKPSNEL	TNSTVVIDTH	KPKDSNQTPH
90	100	110	120	130	140	150	160
LLLTEDDPQS	QHMTLSQAFH	LKNNSKKKQM	TTEKQKQDAN	MPKKPVLGSY	RGQIVQSKIN	SFRKPLQVKD	ESSAATKKLS
170	180	190	200	210	220	230	240
ATIPKATKPQ	PVNTSSVTVK	SNRSSNMTAT	TKFVSTTSQN	TQLVRPPIRS	HHSNTRDTVK	QGISRTSANV	TIRKGPHEKE
250	260	270	280	290	300	310	320
LLQSKTALSS	VKTSSSQGII	RNKTLSRSIA	SEVIARPASL	SNDKLMEKSE	PVDQRRHTAG	KAIVDSRSAQ	PKETSEERKA
330	340	350	360	370	380	390	400
RLSEWKAGKG	RVLKRPPNSV	VTQHEPAGQN	EKPVGSFWTT	MAEEDEQRLF	TEKVNNTFSE	CLNLINEGCP	KEDILVTLND
410	420	430	440	450	460	470	480
LIKNIPDAKK	LVKYWICLAL	IEPITSPIEN	IIAIYEKAIL	AGAQPIEEMR	HTIVDILTMK	SQEKANLGEN	MEKSCASKEE
490	500	510	520	530	540	550	560
VKEVSIEDTG	VDVDPEKLEM	ESKLHRNLLF	QDCEKEQDNK	TKDPTHDVKT	PNTETRTSCL	IKYNVSTTPY	LQSVKKKVQF
570	580	590	600	610	620	630	640
DGTNSAFKEL	KFLTPVRRSR	RLQEKTSKLP	DMLKDHYPCV	SSLEQLTELG	RETDAFVCRP	NAALCRVYYE	ADTT

Cmpd.	No. of Cmpds.	m/z meas.	Δ m/z [ppm]	z	Rt [min]	Score	P	Range	Sequence	Modification
2617	1	732.5346	128.71	1	63.5	10.7	0	572-577	K.FLTPVR.R	

Detailed Protein Report

Protein 547: Fanconi anemia group J protein [Homo sapiens]

Accession: gi|301897118
Database: refseq_human(refseq_human_20140103.fasta)
Database Date: 2015-11-30
Modification(s): Carbamidomethyl

Score: 20.6
MW [kDa]: 140.8
pI: 6.5
Sequence Coverage [%]: 1.8
No. of unique Peptides: 1

10	20	30	40	50	60	70	80
MSSMWSEYTI	GGVKIYFPYK	AYPSQLAMMN	SILRGLNSKQ	HCLLESPTGS	GKSLALLCSA	LAWQQSLSGK	PADEGVSEKA
**90**	**100**	**110**	**120**	**130**	**140**	**150**	**160**
EVQLSCCCAC	HSKDFTNNDM	NQGTSRHFNY	PSTPPSERNG	TSSTCQDSPE	KTTLAAKLSA	KKQASIYRDE	NDDFQVEKKR
**170**	**180**	**190**	**200**	**210**	**220**	**230**	**240**
IRPLETTQQI	RKRHCFGTEV	HNLDAKVDSG	KTVKLNSPLE	KINSFSPQKP	PGHCSRCCCS	TKQGNSQESS	NTIKKDHTGK
**250**	**260**	**270**	**280**	**290**	**300**	**310**	**320**
SKIPKIYFGT	RTHKQIAQIT	RELRRTAYSG	VPMTILSSRD	HTCVHPEVVG	NFNRNEKCME	LLDGKNGKSC	YFYHGVHKIS
**330**	**340**	**350**	**360**	**370**	**380**	**390**	**400**
DQHTLQTFQG	MCKAWDIEEL	VSLGKKLKAC	PYYTARELIQ	DADIIFCPYN	YLLDAQIRES	MDLNLKEQVV	ILDEAHNIED
**410**	**420**	**430**	**440**	**450**	**460**	**470**	**480**
CARESASYSV	TEVQLRFARD	ELDSMVNNNI	RKKDHEPLRA	VCCSLINWLE	ANAEYLVERD	YESACKIWSG	NEMLLTLHKM
**490**	**500**	**510**	**520**	**530**	**540**	**550**	**560**
GITTATFPIL	QGHFSAVLQK	EEKISPIYGK	EEAREVPVIS	ASTQIMLKGL	FMVLDYLFRQ	NSRFADDYKI	AIQQTYSWTN
**570**	**580**	**590**	**600**	**610**	**620**	**630**	**640**
QIDISDKNGL	LVLPKNKKRS	RQKTAVHVLN	FWCLNPAVAF	SDINGKVQTI	VLTSGTLSPM	KSFSSELGVT	FTIQLEANHI
**650**	**660**	**670**	**680**	**690**	**700**	**710**	**720**
IKNSQVWVGT	IGSGPKGRNL	CATFQNTETF	EFQDEVGALL	LSVCQTVSQG	ILCFLPSYKL	LEKLKERWLS	TGLWHNLELV
**730**	**740**	**750**	**760**	**770**	**780**	**790**	**800**
KTVIVEPQGG	EKTNFDELLQ	VYYDAIKYKG	EKDGALLVAV	CRGKVSEGLD	FSDDNARAVI	TIGIPFPNVK	DLQVELKRQY
**810**	**820**	**830**	**840**	**850**	**860**	**870**	**880**
NDHHSKLRGL	LPGRQWYEIQ	AYRALNQALG	RCIRHRNDWG	ALILVDDRFR	NNPSRYISGL	SKWVRQQIQH	HSTFESALES
**890**	**900**	**910**	**920**	**930**	**940**	**950**	**960**
LAEFSKKHQK	VLNVSIKDRT	NIQDNESTLE	VTSLKYSTSP	YLLEAASHLS	PENFVEDEAK	ICVQELQCPK	IITKNSPLPS
**970**	**980**	**990**	**1000**	**1010**	**1020**	**1030**	**1040**
SIISRKEKND	PVFLEEAGKA	EKIVISRSTS	PTFNKQTKRV	SWSSFNSLGQ	YFTGKIPKAT	PELGSSENSA	SSPPRFKTEK
**1050**	**1060**	**1070**	**1080**	**1090**	**1100**	**1110**	**1120**
MESKTVLPFT	DKCESSNLTV	NTSFGSCPQS	ETIISSLKID	ATLTRKNHSE	HPLCSEEALD	PDIELSLVSE	EDKQSTSNRD
**1130**	**1140**	**1150**	**1160**	**1170**	**1180**	**1190**	**1200**
FETEAEDESI	YFTPELYDPE	DTDEEKNDLA	ETDRGNRLAN	NSDCILAKDL	FEIRTIKEVD	SAREVKAEDC	IDTKLNGILH
**1210**	**1220**	**1230**	**1240**	**1250**			
IEESKIDDID	GNVKTTWINE	LELGKTHEIE	IKNFKPSPSK	NKGMFPGFK			

Cmpd.	No. of Cmpds.	m/z meas.	Δ m/z [ppm]	z	Rt [min]	Score	P	Range	Sequence	Modification
2372	1	637.6653	-235.01	2	60.1	10.2	0	941-950	K.ICVQELQCPK.I	Carbamidomethyl: 2, 8

Detailed Protein Report

Protein 548: **PREDICTED: transmembrane and coiled-coil domains protein 2 isoform X7 [Homo sapiens]**

Accession: gi|530365798
Database: refseq_human(refseq_human_20140103.fasta)
Database Date: 2015-11-30

Score: 20.5
MW [kDa]: 51.6
pI: 5.5
Sequence Coverage [%]: 7.7
No. of unique Peptides: 1

10	20	30	40	50	60	70	80
MKSKEEETAV	DKGDLVALSL	PAGHGDTDGP	ISLDVPDGAP	DPQRTKAAID	HLHQKILKIT	EQIKIEQEAR	DDNVAEYLKL
**90**	**100**	**110**	**120**	**130**	**140**	**150**	**160**
ANNADKQQVS	RIKQVFEKKN	QKSAQTIAQL	HKKLEHYRRR	LKEIEQNGPS	RQPKDVLRDM	QQGLKDVGAN	VRAGISGFGG
**170**	**180**	**190**	**200**	**210**	**220**	**230**	**240**
GVVEGVKGSL	SGLSQATHTA	VVSKPREFAS	LIRNKFGSAD	NIAHLKDPLE	DGPPEEAARA	LSGSATLVSS	PKYGSDDECS
**250**	**260**	**270**	**280**	**290**	**300**	**310**	**320**
SASASSAGAG	SNSGAGPGGA	LGSPKSNALY	GAPGNLDALL	EELREIKEGQ	SHLEDSMEDL	KTQLQRDYTY	MTQCLQEERY
**330**	**340**	**350**	**360**	**370**	**380**	**390**	**400**
RYERLEEQLN	DLTELHQNEM	TNLKQELASM	EEKVAYQSYE	RARDIQEAVE	SCLTRVTKLE	LQQQQQVVQ	LEGVENANAR
**410**	**420**	**430**	**440**	**450**	**460**	**470**	
ALLGKFINVI	LALMAVLLVF	VSTIANFITP	LMKTRLRITS	TTLLVLVLFL	LWKHWDSLTY	LLEHVLLPS	

Cmpd.	No. of Cmpds.	m/z meas.	Δ m/z [ppm]	z	Rt [min]	Score	P	Range	Sequence	Modification
1834	1	682.2742	-131.55	3	53.3	10.2	1	146-167	K.DVGANVRAGISGFGGGVVEGVK.G	

Detailed Protein Report

Protein 549: PREDICTED: monocarboxylate transporter 8 isoform X1 [Homo sapiens]

Accession: gi|530421807
Database: refseq_human(refseq_human_20140103.fasta)
Database Date: 2015-11-30
Modification(s): Carbamidomethyl

Score: 20.5
MW [kDa]: 45.8
pI: 5.6
Sequence Coverage [%]: 4.1
No. of unique Peptides: 1

10	20	30	40	50	60	70	80
MALQSQASEE	AKGPWQEADQ	EQQEPVGSPE	PESEPEPEPE	PEPVPVPPPE	PQPEPQPLPD	PAPLPELEFE	SERVHEPEPT
**90**	**100**	**110**	**120**	**130**	**140**	**150**	**160**
PTVETRGTAR	GFQPPEGGFG	WVVVFAATWC	NGSIFGIHNS	VGILYSMLLE	EEKEKNRQVE	FQAAWVGALA	MGMIFFCSPI
**170**	**180**	**190**	**200**	**210**	**220**	**230**	**240**
VSIFTDRLGC	RITATAGAAV	AFIGLHTSSF	TSSLSLRYFT	YGILFGCGCS	FAFQPSLVIL	GHYFQRRLGL	ANGVVSAGSS
**250**	**260**	**270**	**280**	**290**	**300**	**310**	**320**
IFSMSFPFLI	RMLGDKIKLA	QTFQVLSTFM	FVLMLLSLTY	RPLLPSSQDT	PSKRGVRTLH	QRFLAQLRKY	FNMRVFRQRT
**330**	**340**	**350**	**360**	**370**	**380**	**390**	**400**
YRIWAFGIAA	AALGYFVPYV	HLMKYVEEEF	SEIKETWVLL	VCIGATSGLG	RLVSGHISDS	IPGLKKIYLQ	AYSATVLGTT
**410**	**420**						
MWPSTLPVCP	PSSGL						

Cmpd.	No. of Cmpds.	m/z meas.	Δ m/z [ppm]	z	Rt [min]	Score	P	Range	Sequence	Modification
2201	1	916.4855	-5.38	2	56.1	20.5	0	355-371	K.ETWVLLVCIGATSGLGR.L	Carbamidomethyl: 8

Detailed Protein Report

Protein 550: nuclear factor of activated T-cells, cytoplasmic 2 isoform D [Homo sapiens]

**Accession:**	gi|209862843	**Score:**	20.3
**Database:**	refseq_human(refseq_human_20140103.fasta)	**MW [kDa]:**	97.6
**Database Date:**	2015-11-30	**pI:**	9.1
**Modification(s):**	Oxidation	**Sequence Coverage [%]:**	5.0
		**No. of unique Peptides:**	2

10	20	30	40	50	60	70	80
MQREAAFRLG	HCHPLRIMGS	VDQEEPNAHK	VASPPSGPAY	PDDVLDYGLK	PYSPLASLSG	EPPGRFGEPD	RVGPQKFLSA
**90**	**100**	**110**	**120**	**130**	**140**	**150**	**160**
AKPAGASGLS	PRIEITPSHE	LIQAVGPLRM	RDAGLLVEQP	PLAGVAASPR	FTLPVPGFEG	YREPLCLSPA	SSGSSASFIS
**170**	**180**	**190**	**200**	**210**	**220**	**230**	**240**
DTFSPYTSPC	VSPNNGGPDD	LCPQFQNIPA	HYSPRTSPIM	SPRTSLAEDS	CLGRHSPVPR	PASRSSSPGA	KRRHSCAEAL
**250**	**260**	**270**	**280**	**290**	**300**	**310**	**320**
VALPPGASPQ	RSRSPSPQPS	SHVAPQDHGS	PAGYPPVAGS	AVIMDALNSL	ATDSPCGIPP	KMWKTSPDPS	PVSAAPSKAG
**330**	**340**	**350**	**360**	**370**	**380**	**390**	**400**
LPRHIYPAVE	FLGPCEQGER	RNSAPESILL	VPPTWPKPLV	PAIPICSIPV	TASLPPLEWP	LSSQSGSYEL	RIEVQPKPHH
**410**	**420**	**430**	**440**	**450**	**460**	**470**	**480**
RAHYETEGSR	GAVKAPTGGH	PVVQLHGYME	NKPLGLQIFI	GTADERILKP	HAFYQVHRIT	GKTVTTTSYE	KIVGNTKVLE
**490**	**500**	**510**	**520**	**530**	**540**	**550**	**560**
IPLEPKNNMR	ATIDCAGILK	LRNADIELRK	GETDIGRKNT	RVRLVFRVHI	PESSGRIVSL	QTASNPIECS	QRSAHELPMV
**570**	**580**	**590**	**600**	**610**	**620**	**630**	**640**
ERQDTDSCLV	YGGQQMILTG	QNFTSESKVV	FTEKTTDGQQ	IWEMEATVDK	DKSQPNMLFV	EIPEYRNKHI	RTPVKVNFYV
**650**	**660**	**670**	**680**	**690**	**700**	**710**	**720**
INGKRKRSQP	QHFTYHPVPA	IKTEPTDEYD	PTLICSPTHG	GLGSQPYYPQ	HPMVAESPSC	LVATMAPCQQ	FRTGLSSPDA
**730**	**740**	**750**	**760**	**770**	**780**	**790**	**800**
RYQQQNPAAV	LYQRSKSLSP	SLLGYQQPAL	MAAPLSLADA	HRSVLVHAGS	QGQSSALLHP	SPTNQQASPV	IHYSPTNQQL
**810**	**820**	**830**	**840**	**850**	**860**	**870**	**880**
RCGSHQEFQH	IMYCENFAPG	TTRPGPPPVS	QGQRLSPGSY	PTVIQQQNAT	SQRAAKNGPP	VSDQKEVLPA	GVTIKQEQNL
**890**	**900**	**910**					
DQTYLDDELI	DTHLSWIQNI	L					

Cmpd.	No. of Cmpds.	m/z meas.	Δ m/z [ppm]	z	Rt [min]	Score	P	Range	Sequence	Modification
114	1	1234.5408	-47.71	2	31.4	10.3	1	9-30	R.LGHCHPLRIMGSVDQEEPNAHK.V	
644	1	852.1620	38.48	3	38.0	10.0	2	478-500	K.VLEIPLEPKNNMRATIDCAGILK.L	Oxidation: 12

Detailed Protein Report

Protein 551: **PREDICTED: zinc finger protein 717 isoform X8 [Homo sapiens]**

**Accession:**	gi|578805503	**Score:**	20.3
**Database:**	refseq_human(refseq_human_20140103.fasta)	**MW [kDa]:**	73.9
**Database Date:**	2015-11-30	**pI:**	9.6
**Modification(s):**	Carbamidomethyl	**Sequence Coverage [%]:**	6.3
		**No. of unique Peptides:**	1

10	20	30	40	50	60	70	80
MFPVFSGCFQ	ELQEKNKSLE	LVSFEEVAVH	FTWEEWQDLD	DAQRTLYRDV	MLETYSSLVS	LGHYITKPEM	IFKLEQGAEP
90	100	110	120	130	140	150	160
WIVEETPNLR	LSAVQIIDDL	IERSHESHDR	FFWQIVITNS	NTSTQERVEL	GKTFNLNSNH	VLNLIINNGN	SSGMKPGQFN
170	180	190	200	210	220	230	240
DCQNMLFPIK	PGETQSGEKP	HVCDITRRSH	RHHEHLTQHH	KIQTLLQTFQ	CNEQGKTFNT	EAMFFIHKRV	HIVQTFGKYN
250	260	270	280	290	300	310	320
EYEKACNNSA	VIVQVITQVG	QPTCCRKSDF	TKHQQTHTGE	KPYECVECEK	PSISKSDLML	QCKMPTEEKP	YACNWCEKLF
330	340	350	360	370	380	390	400
SYKSSLIIHQ	RIHTGEKPYG	CNECGKTFRR	KSFLTLHERT	HTGDKPYKCI	ECGKTFHCKS	LLTLHHRTHS	GEKPYQCSEC
410	420	430	440	450	460	470	480
GKTFSQKSYL	TIHHRTHTGE	KPYACDHCEE	AFSHKSRLTV	HQRTHTGEKP	YECNECGKPF	INKSNLRLHQ	RTHTGEKPYE
490	500	510	520	530	540	550	560
CNECGKTFHR	KSFLTIHQWT	HTGEKPYECN	ECGKTFRCKS	FLTVHQRTHA	GEKPYACNEC	GKTYSHKSYL	TVHHRTHTGE
570	580	590	600	610	620	630	640
KPFRCNECRK	TFSQKSGLSI	HQRTHTGEKP	YECKECGKTF	CQKSHLSRHQ	QTHIGEKSDV	AEAGYVFPQN	HSFFP

Cmpd.	No. of Cmpds.	m/z meas.	Δ m/z [ppm]	z	Rt [min]	Score	P	Range	Sequence	Modification
2173	1	1273.4977	-108.18	2	55.8	10.1	1	245-267	K.ACNNSAVIVQVITQVGQPTCCRK.S	Carbamidomethyl: 2, 21

Detailed Protein Report

Protein 552: **PREDICTED: serine/threonine-protein phosphatase 2A 65 kDa regulatory subunit A beta isoform isoform X5 [Homo sapiens]**

**Accession:**	gi|578822285	**Score:**	20.3
**Database:**	refseq_human(refseq_human_20140103.fasta)	**MW [kDa]:**	70.9
**Database Date:**	2015-11-30	**pI:**	5.3
		**Sequence Coverage [%]:**	4.7
		**No. of unique Peptides:**	2

10	20	30	40	50	60	70	80
MNPCPCFFRL	GKGRRGSIRF	LSPPVFLSSD	LQLLQWGSEG	SLNHRDRNAA	PQLRLNQRGG	LRPYCLGAGR	GLGAGREAVT
90	100	110	120	130	140	150	160
SSRRRKNMAG	ASELGTGPGA	AGGDGDDSLY	PIAVLIDELR	NEDVQLRLNS	IKKLSTIALA	LGVERTRSEL	LPFLTDTIYD
170	180	190	200	210	220	230	240
EDEVLLALAE	QLGNFTGLVG	GPDFAHCLLP	PLENLATVEE	TVVRDKAVES	LRQISQEHTP	VALEAYFVPL	VKRLASGDWF
250	260	270	280	290	300	310	320
TSRTSACGLF	SVCYPRASNA	VKAEIRQQFR	SLCSDDTPMV	RRAAASKLGE	FAKVLELDSV	KSEIVPLFTS	LASDEQDSVR
330	340	350	360	370	380	390	400
LLAVEACVSI	AQLLSQDDLE	TLVMPTLRQA	AEDKSWRVRY	MVADRFSELQ	KAMGPKITLN	DLIPAFQNLL	KDCEAEVRAA
410	420	430	440	450	460	470	480
AAHKVKELGE	NLPIEDRETI	IMNQILPYIK	CPDVRLNIIS	NLDCVNEVIG	IRQLSQSLLP	AIVELAEDAK	WRVRLAIIEY
490	500	510	520	530	540	550	560
MPLLAGQLGV	EFFDEKLNSL	CMAWLVDHVY	AIREAATNNL	MKLVQKFGTE	WAQNTIVPKV	LVMANDPNYL	HRMTTLFCIN
570	580	590	600	610	620	630	640
ALSEACGQEI	TTKQMLPIVL	KMAGDQVANV	RFNVAKSLQK	IGPILDTNAL	QGEVKPVLQK	LGQDEDMDVK	YFAQEAISVL
650							
ALA							

Cmpd.	No. of Cmpds.	m/z meas.	Δ m/z [ppm]	z	Rt [min]	Score	P	Range	Sequence	Modification
49	1	776.2880	-178.77	2	30.5	10.1	2	46-58	R.DRNAAPQLRLNQR.G	
2800	1	942.9802	-36.08	2	64.1	10.2	0	436-452	R.LNIISNLDCVNEVIGIR.Q	

Detailed Protein Report

Protein 553: **PREDICTED: lipopolysaccharide-responsive and beige-like anchor protein isoform X6 [Homo sapiens]**

**Accession:**	gi|530378481	**Score:**	20.3
**Database:**	refseq_human(refseq_human_20140103.fasta)	**MW [kDa]:**	173.9
**Database Date:**	2015-11-30	**pI:**	5.5
		**Sequence Coverage [%]:**	1.5
		**No. of unique Peptides:**	2

10	20	30	40	50	60	70	80
MASEDNRVPS	PPPTGDDGGG	GGREETPTEG	GALSLKPGLP	IRGIRMKFAV	LTGLVEVGEV	SNRDIVETVF	NLLVGGQFDL
**90**	**100**	**110**	**120**	**130**	**140**	**150**	**160**
EMNFIIQEGE	SINCMVDLLE	KCDITCQAEV	WSMFTAILKK	SIRNLQVCTE	VGLVEKVLGK	IEKVDNMIAD	LLVDMLGVLA
**170**	**180**	**190**	**200**	**210**	**220**	**230**	**240**
SYNLTVRELK	LFFSKLQGDK	GRWPPHAGKL	LSVLKHMPQK	YGPDAFFNFP	GKSAAAIALP	PIAKWPYQNG	FTFHTWLRMD
**250**	**260**	**270**	**280**	**290**	**300**	**310**	**320**
PVNNINVDKD	KPYLYCFRTS	KGLGYSAHFV	GGCLIVTSIK	SKGKGFQHCV	KFDFKPQKWY	MVTIVHIYNR	WKNSELRCYV
**330**	**340**	**350**	**360**	**370**	**380**	**390**	**400**
NGELASYGEI	TWFVNTSDTF	DKCFLGSSET	ADANRVFCGQ	MTAVYLFSEA	LNAAQIFAIY	QLGLGYKGTF	KFKAESDLFL
**410**	**420**	**430**	**440**	**450**	**460**	**470**	**480**
AEHHKLLLYD	GKLSSAIAFT	YNPRATDAQL	CLESSPKDNP	SIFVHSPHAL	MLQDVKAVLT	HSIQSAMHSI	GGVQVLFPLF
**490**	**500**	**510**	**520**	**530**	**540**	**550**	**560**
AQLDYRQYLS	DEIDLTICST	LLAFIMELLK	NSIAMQEQML	ACKGFLVIGY	SLEKSSKSHV	SRAVLELCLA	FSKYLSNLQN
**570**	**580**	**590**	**600**	**610**	**620**	**630**	**640**
GMPLLKQLCD	HVLLNPAIWI	HTPAKVQLML	YTYLSTEFIG	TVNIYNTIRR	VGTVLLIMHT	LKYYYWAVNP	QDRSGITPKG
**650**	**660**	**670**	**680**	**690**	**700**	**710**	**720**
LDGPRPNQKE	MLSLRAFLLM	FIKQLVMKDS	GVKEDELQAI	LNYLLTMHED	DNLMDVLQLL	VALMSEHPNS	MIPAFDQRNG
**730**	**740**	**750**	**760**	**770**	**780**	**790**	**800**
LRVIYKLLAS	KSEGIRVQAL	KAMGYFLKHL	APKRKAEVML	GHGLFSLLAE	RLMLQTNLIT	MTTYNVLFEI	LIEQIGTQVI
**810**	**820**	**830**	**840**	**850**	**860**	**870**	**880**
HKQHPDPDSS	VKIQNPQILK	VIATLLRNSP	QCPESMEVRR	AFLSDMIKLF	NNSRENRRSL	LQCSVWQEWM	LSLCYFNPKN
**890**	**900**	**910**	**920**	**930**	**940**	**950**	**960**
SDEQKITEMV	YAIFRILLYH	AVKYEWGGWR	VWVDTLSITH	SKVTFEIHKE	NLANIFREQQ	GKVDEEIGLC	SSTSVQAASG
**970**	**980**	**990**	**1000**	**1010**	**1020**	**1030**	**1040**
IRRDINVSVG	SQQPDTKDSP	VCPHFTTNGN	ENSSIEKTSS	LESASNIELQ	TTNTSYEEMK	AEQENQELPD	EGTLEETLTN
**1050**	**1060**	**1070**	**1080**	**1090**	**1100**	**1110**	**1120**
ETRNADDLEV	SSDIIEAVAI	SSNSFITTGK	DSMTVSEVTA	SISSPSEEDA	SEMPEFLDKS	IVEEEEDDDY	VELKVEGSPT
**1130**	**1140**	**1150**	**1160**	**1170**	**1180**	**1190**	**1200**
EEANLPTELQ	DNSLSPAASE	AGEKLDMFGN	DDKLIFQEGK	PVTEKQTDTE	TQDSKDSGIQ	TMTASGSSAM	SPETTVSQIA
**1210**	**1220**	**1230**	**1240**	**1250**	**1260**	**1270**	**1280**
VESDLGQMLE	EGKKATNLTR	ETKLINDCHG	SVSEASSEQK	IAKLDVSNVA	TDTERLELKA	SPNVEAPQPH	RHVLEISRQH
**1290**	**1300**	**1310**	**1320**	**1330**	**1340**	**1350**	**1360**
EQPGQGIAPD	AVNGQRRDSR	STVFRIPEFN	WSQMHQRLLT	DLLFSIETDI	QMWRSHSTKT	VMDFVNSSDN	VIFVHNTIHL
**1370**	**1380**	**1390**	**1400**	**1410**	**1420**	**1430**	**1440**
ISQVMDNMVM	ACGGILPLLS	AATSATHELE	NIEPTQGLSI	EASVTFLQRL	ISLVDVLIFA	SSLGFTEIEA	EKSMSSGGIL
**1450**	**1460**	**1470**	**1480**	**1490**	**1500**	**1510**	**1520**
RQCLRLVCAV	AVRNCLECQQ	HSQLKTRGDK	ALKPMHSLIP	LGKSAAKSPV	DIVTGGISPV	RDLDRLLQDM	DINRLRAVVF
**1530**	**1540**	**1550**	**1560**				
RDITKKSLHM	NLQNIIDFVR	SRITNMSKNC	IKWICSN				

Cmpd.	No. of Cmpds.	m/z meas.	Δ m/z [ppm]	z	Rt [min]	Score	P	Range	Sequence	Modification
1142	1	555.2290	-64.32	2	44.4	10.2	0	803-812	K.QHPDPDSSVK.I	
1642	1	698.8555	-54.14	2	50.8	10.1	0	1488-1501	K.SPVDIVTGGISPVR.D	

Detailed Protein Report

Protein 554: Down syndrome cell adhesion molecule-like protein 1 [Homo sapiens]

**Accession:**	gi|21359935	**Score:**	20.2
**Database:**	refseq_human(refseq_human_20140103.fasta)	**MW [kDa]:**	230.5
**Database Date:**	2015-11-30	**pI:**	9.3
		**Sequence Coverage [%]:**	0.9
		**No. of unique Peptides:**	1

Detailed Protein Report

10	20	30	40	50	60	70	80
MTGAGEPRRE	EPPPQPSAGL	RSRESGAPSA	GGWERAERGR	GAAARPATGP	PPRRIGPLYG	MWLVTFLLLL	DSLHKARPED
90	100	110	120	130	140	150	160
VGTSLYFVND	SLQQVTFSSS	VGVVVPCPAA	GSPSAALRWY	LATGDDIYDV	PHIRHVHANG	TLQLYPFSPS	AFNSFIHDND
170	180	190	200	210	220	230	240
YFCTAENAAG	KIRSPNIRVK	AVFREPYTVR	VEDQRSMRGN	VAVFKCLIPS	SVQEYVSVVS	WEKDTVSIIP	EHRFFITYHG
250	260	270	280	290	300	310	320
GLYISDVQKE	DALSTYRCIT	KHKYSGETRQ	SNGARLSVTD	PAESIPTILD	GFHSQEVWAG	HTVELPCTAS	GYPIPAIRWL
330	340	350	360	370	380	390	400
KDGRPLPADS	RWTKRITGLT	ISDLRTEDSG	TYICEVTNTF	GSAEATGILM	VIDPLHVTLT	PKKLKTGIGS	TVILSCALTG
410	420	430	440	450	460	470	480
SPEFTIRWYR	NTELVLPDEA	ISIRGLSNET	LLITSAQKSH	SGAYQCFATR	KAQTAQDFAI	IALEDGTPRI	VSSFSEKVVN
490	500	510	520	530	540	550	560
PGEQFSLMCA	AKGAPPPTVT	WALDDEPIVR	DGSHRTNQYT	MSDGTTISHM	NVTGPQIRDG	GVYRCTARNL	VGSAEYQARI
570	580	590	600	610	620	630	640
NVRGPPSIRA	MRNITAVAGR	DTLINCRVIG	YPYYSIKWYK	DALLLPDNHR	QVVFENGTLK	LTDVQKGMDE	GEYLCSVLIQ
650	660	670	680	690	700	710	720
PQLSISQSVH	VAVKVPPLIQ	PFEFPPASIG	QLLYIPCVVS	SGDMPIRITW	RKDGQVIISG	SGVTIESKEF	MSSLQISSVS
730	740	750	760	770	780	790	800
LKHNGNYTCI	ASNAAATVSR	ERQLIVRVPP	RFVVQPNNQD	GIYGKAGVLN	CSVDGYPPPK	VMWKHAKGSG	NPQQYHPVPL
810	820	830	840	850	860	870	880
TGRIQILPNS	SLLIRHVLEE	DIGYYLCQAS	NGVGTDISKS	MFLTVKIPAM	ITSHPNTTIA	IKGHAKELNC	TARGERPIII
890	900	910	920	930	940	950	960
RWEKGDTVID	PDRVMRYAIA	TKDNGDEVVS	TLKLKPADRG	DSVFFSCHAI	NSYGEDRGLI	QLTVQEPPDP	PELEIREVKA
970	980	990	1000	1010	1020	1030	1040
RSMNLRWTQR	FDGNSIITGF	DIEYKNKSDS	WDFKQSTRNI	SPTINQANIV	DLHPASVYSI	RMYSFNKIGR	SEPSKELTIS
1050	1060	1070	1080	1090	1100	1110	1120
TEEAAPDGPP	MDVTLQPVTS	QSIQVTWKAP	KKELQNGVIR	GYQIGYRENS	PGSNGQYSIV	EMKATGDSEV	YTLDNLKKFA
1130	1140	1150	1160	1170	1180	1190	1200
QYGVVVQAFN	RAGTGPSSSE	INATTLEDVP	SQPPENVRAL	SITSDVAVIS	WSEPPRSTLN	GVLKGYRVIF	WSLYVDGEWG
1210	1220	1230	1240	1250	1260	1270	1280
EMQNITTTRE	RVELRGMEKF	TNYSVQVLAY	TQAGDGVRSS	VLYIQTKEDV	PGPPAGIKAV	PSSASSVVVS	WLPPTKPNGV
1290	1300	1310	1320	1330	1340	1350	1360
IRKYTIFCSS	PGSGQPAPSE	YETSPEQLFY	RIAHLNRGQQ	YLLWVAAVTS	AGRGNSSEKV	TIEPAGKAPA	KIISFGGTVT
1370	1380	1390	1400	1410	1420	1430	1440
TPWMKDVRLP	CNSVGDPAPA	VKWTKDSEDS	AIPVSMDGHR	LIHTNGTLLL	RAVKAEDSGY	YTCTATNTGG	FDTIIVNLLV
1450	1460	1470	1480	1490	1500	1510	1520
QVPPDQPRLT	VSKTSASSIT	LTWIPGDNGG	SSIRGFVLQY	SVDNSEEWKD	VFISSSERSF	KLDSLKCGTW	YKVKLAAKNS
1530	1540	1550	1560	1570	1580	1590	1600
VGSGRISEII	EAKTHGREPS	FSKDQHLFTH	INSTHARLNL	QGWNNGGCPI	TAIVLEYRPK	GTWAWQGLRA	NSSGEVFLTE
1610	1620	1630	1640	1650	1660	1670	1680
LREATWYELR	MRACNSAGCG	NETAQFATLD	YDGSTIPPIK	SAQGEGDDVK	KLFTIGCPVI	LATLGVALLF	IVRKKRKEKR
1690	1700	1710	1720	1730	1740	1750	1760
LKRLRDAKSL	AEMLISKNNR	SFDTPVKGPP	QGPRLHIDIP	RVQLLIEDKE	GIKQLGDDKA	TIPVTDAEFS	QAVNPQSFCT
1770	1780	1790	1800	1810	1820	1830	1840
GVSLHHPTLI	QSTGPLIDMS	DIRPGTNPVS	RKNVKSAHST	RNRYSSQWTL	TKCQASTPAR	TLTSDWRTVG	SQHGVTVTES
1850	1860	1870	1880	1890	1900	1910	1920
DSYSASLSQD	TDKGRNSMVS	TESASSTYEE	LARAYEHAKL	EEQLQHAKFE	ITECFISDSS	SDQMTTGTNE	NADSMTSMST
1930	1940	1950	1960	1970	1980	1990	2000
PSEPGICRFT	ASPPKPQDAD	RGKNVAVPIP	HRANKSDYCN	LPLYAKSEAF	FRKADGREPC	PVVPPREASI	RNLARTYHTQ
2010	2020	2030	2040	2050	2060	2070	2080
ARHLTLDPAS	KSLGLPHPGA	PAAASTATLP	QRTLAMPAPP	AGTAPPAPGP	TPAEPPTAPS	AAPPAPSTEP	PRAGGPHTKM
2090	2100	2110	2120				
GGSRDSLLEM	STSGVGRSQK	QGAGAYSKSY	TLV				

Detailed Protein Report

Cmpd.	No. of Cmpds.	m/z meas.	Δ m/z [ppm]	z	Rt [min]	Score	P	Range	Sequence	Modification
2598	1	728.7090	-26.65	3	61.1	20.2	2	867-884	K.ELNCTARGERPIIIRWEK.G	

Detailed Protein Report

Protein 555: PREDICTED: uncharacterized protein C4orf50 [Homo sapiens]

Accession: gi|530358255
Database: refseq_human(refseq_human_20140103.fasta)
Database Date: 2015-11-30

Score: 20.2
MW [kDa]: 97.6
pI: 5.4
Sequence Coverage [%]: 1.2
No. of unique Peptides: 1

10	20	30	40	50	60	70	80
MPLLQGEASV	SKEGPESLSR	RERVEGYVWG	LRGGLSSENE	EVAPPATFSR	AHETKEPWPT	DSQLLAGKVR	ATGRTARGKE
**90**	**100**	**110**	**120**	**130**	**140**	**150**	**160**
ENQVWLGNAL	LLQGGSLEDK	GLEEEDEMPH	QEASGLGCRG	APEEPDSQEH	ESKEMLFFAG	ETGLPLFPRF	ALSVEGAEPT
**170**	**180**	**190**	**200**	**210**	**220**	**230**	**240**
DHGHPQAVSK	GHDRCALTID	ELAQDVEACF	QQLSTLQPGS	RGWQCSASAC	RGENWSFAQK	WHSGWERAHS	QQVWGNWGIC
**250**	**260**	**270**	**280**	**290**	**300**	**310**	**320**
SNEEAKSKES	GEGDKPGKTT	ALGTSEVPGN	PGTLPHWDEA	SPNPPQGPAE	PWGALERVRS	RFHQLISGLK	KQRSQILHDN
**330**	**340**	**350**	**360**	**370**	**380**	**390**	**400**
TKLHGDQERF	HERVCALERE	REREVTKISR	LERDNHRLVG	DISQLKKELD	QYLQAISDLE	DCNGKSYCKI	LELEEENETL
**410**	**420**	**430**	**440**	**450**	**460**	**470**	**480**
KGNLGQLQKA	TSESVRKSKD	TMEQVTLENW	KLQTLISELG	VSYKELIKDI	VLGIEDMIRA	LSGENEHLLR	RVHVLEREVT
**490**	**500**	**510**	**520**	**530**	**540**	**550**	**560**
LQRSTDQGRL	VRGREHLQGK	AKMHALDKEV	QVTPLTGQLL	SRACGPPLEE	EMSLAAGQTG	PSTGTGNSRR	GADSPPPSLV
**570**	**580**	**590**	**600**	**610**	**620**	**630**	**640**
WRNTGVANAL	QGNVSGAEVK	EAHLEKEEKR	PRCSVAQGQA	LSSLSNGPML	RDSEAEVTEE	DPRLRAQQLH	HRVLTLQCQL
**650**	**660**	**670**	**680**	**690**	**700**	**710**	**720**
RDQGAAHQAS	LDEATRLQEE	LQAKLEELQK	KQHEAKLAVT	PLKAKIASLV	RKCRERNRLI	THLLQELHRH	GLGNLLLSEL
**730**	**740**	**750**	**760**	**770**	**780**	**790**	**800**
AQNMLNDVAL	AEYTATFLAP	GVPETSHHLD	VKSEMTAALR	AQTYLLNPEM	DSVLQSSLSS	ESWPVPEPEW	PAQTAQLDSL
**810**	**820**	**830**	**840**	**850**	**860**	**870**	**880**
KLPLSLVSTL	DPGTCLAAVT	VEPGLPAQRL	QEKGGMPCPA	LQVDNVPAPS	ELLSPARILA	FHQELRQSIC	SNSQVHKSPL
**890**							
ELEM							

Cmpd.	No. of Cmpds.	m/z meas.	Δ m/z [ppm]	z	Rt [min]	Score	P	Range	Sequence	Modification
1707	5	1273.8380	122.41	1	51.6	20.2	0	449-459	K.DIVLGIEDMIR.A	

Detailed Protein Report

Protein 556: **PREDICTED: Down syndrome cell adhesion molecule isoform X1 [Homo sapiens]**

**Accession:**	gi|578836469	**Score:**	20.1
**Database:**	refseq_human(refseq_human_20140103.fasta)	**MW [kDa]:**	210.7
**Database Date:**	2015-11-30	**pI:**	9.3
		**Sequence Coverage [%]:**	0.7
		**No. of unique Peptides:**	1

10	20	30	40	50	60	70	80
MIIYILPLCS	NLFYQPIKIE	QRKISLKVTF	SKGKKHFCPL	KLTLIFDLNV	GPFCSTEALP	VKIRGSRFLI	TSTGALYIKD
90	100	110	120	130	140	150	160
VQNEDGLYNY	RCITRHRYTG	ETRQSNSARL	FVSDPANSAP	SILDGFDHRK	AMAGQRVELP	CKALGHPEPD	YRWLKDNMPL
170	180	190	200	210	220	230	240
ELSGRFQKTV	TGLLIENIRP	SDSGSYVCEV	SNRYGTAKVI	GRLYVKQPLK	ATISPRKVKS	SVGSQVSLSC	SVTGTEDQEL
250	260	270	280	290	300	310	320
SWYRNGEILN	PGKNVRITGI	NHENLIMDHM	VKSDGGAYQC	FVRKDKLSAQ	DYVQVVLEDG	TPKIISAFSE	KVVSPAEPVS
330	340	350	360	370	380	390	400
LMCNVKGTPL	PTITWTLDDD	PILKGGSHRI	SQMITSEGNV	VSYLNISSSQ	VRDGGVYRCT	ANNSAGVVLY	QARINVRGPA
410	420	430	440	450	460	470	480
SIRPMKNITA	IAGRDTYIHC	RVIGYPYYSI	KWYKNSNLLP	FNHRQVAFEN	NGTLKLSDVQ	KEVDEGEYTC	NVLVQPQLST
490	500	510	520	530	540	550	560
SQSVHVTVKV	PPFIQPFEFP	RFSIGQRVFI	PCVVVSGDLP	ITITWQKDGR	PIPGSLGVTI	DNIDFTSSLR	ISNLSLMHNG
570	580	590	600	610	620	630	640
NYTCIARNEA	AAVEHQSQLI	VRVPPKFVVQ	PRDQDGIYGK	AVILNCSAEG	YPVPTIVWKF	SKGAGVPQFQ	PIALNGRIQV
650	660	670	680	690	700	710	720
LSNGSLLIKH	VVEEDSGYYL	CKVSNDVGAD	VSKSMYLTVK	IPAMITSYPN	TTLATQGQKK	EMSCTAHGEK	PIIVRWEKED
730	740	750	760	770	780	790	800
RIINPEMARY	LVSTKEVGEE	VISTLQILPT	VREDSGFFSC	HAINSYGEDR	GIIQLTVQEP	PDPPEIEIKD	VKARTITLRW
810	820	830	840	850	860	870	880
TMGFDGNSPI	TGYDIECKNK	SDSWDSAQRT	KDVSPQLNSA	TIIDIHPSST	YSIRMYAKNR	IGKSEPSNEL	TITADEAAPD
890	900	910	920	930	940	950	960
GPPQEVHLEP	ISSQSIRVTW	KAPKKHLQNG	IIRGYQIGYR	EYSTGGNFQF	NIISVDTSGD	SEVYTLDNLN	KFTQYGLVVQ
970	980	990	1000	1010	1020	1030	1040
ACNRAGTGPS	SQEIITTTLE	DVPSYPPENV	QAIATSPESI	SISWSTLSKE	ALNGILQGFR	VIYWANLMDG	ELGEIKNITT
1050	1060	1070	1080	1090	1100	1110	1120
TQPSLELDGL	EKYTNYSIQV	LAFTRAGDGV	RSEQIFTRTK	EDVPGPPAGV	KAAAASASMV	FVSWLPPLKL	NGIIRKYTVF
1130	1140	1150	1160	1170	1180	1190	1200
CSHPYPTVIS	EFEASPDSFS	YRIPNLSRNR	QYSVWVVAVT	SAGRGNSSEI	ITVEPLAKAP	ARILTFSGTV	TTPWMKDIVL
1210	1220	1230	1240	1250	1260	1270	1280
PCKAVGDPSP	AVKWMKDSNG	TPSLVTIDGR	RSIFSNGSFI	IRTVKAEDSG	YYSCIANNNW	GSDEIILNLQ	VQVPPDQPRL
1290	1300	1310	1320	1330	1340	1350	1360
TVSKTTSSSI	TLSWLPGDNG	GSSIRGYILQ	YSEDNSEQWG	SFPISPSERS	YRLENLKCGT	WYKFTLTAQN	GVGPGRISEI
1370	1380	1390	1400	1410	1420	1430	1440
IEAKTLGKEP	QFSKEQELFA	SINTTRVRLN	LIGWNDGGCP	ITSFTLEYRP	FGTTVWTTAQ	RTSLSKSYIL	YDLQEATWYE
1450	1460	1470	1480	1490	1500	1510	1520
LQMRVCNSAG	CAEKQANFAT	LNYDGSTIPP	LIKSVVQNEE	GLTTNEGLKM	LVTISCILVG	VLLLFVLLLV	VRRRRREQRL
1530	1540	1550	1560	1570	1580	1590	1600
KRLRDAKSLA	EMLMSKNTRT	SDTLSKQQQT	LRMHIDIPRA	QLLIEERDTM	ETIDDRSTVL	LTDADFGEAA	KQKSLTVTHT
1610	1620	1630	1640	1650	1660	1670	1680
VHYQSVSQAT	GPLVDVSDAR	PGTNPTTRRN	AKAGPTARNR	YASQWTLNRP	HPTISAHTLT	TDWRLPTPRA	AGSVDKESDS
1690	1700	1710	1720	1730	1740	1750	1760
YSVSPSQDTD	RARSSMVSTE	SASSTYEELA	RAYEHAKMEE	QLRHAKFTIT	ECFISDTSSE	QLTAGTNEYT	DSLTSSTPSE
1770	1780	1790	1800	1810	1820	1830	1840
SGICRFTASP	PKPQDGGRVM	NMAVPKAHRP	GDLIHLPPYL	RMDFLLNRGG	PGTSRDLSLG	QACLEPQKSR	TLKRPTVLEP
1850	1860	1870	1880	1890	1900	1910	
IPMEAASSAS	STREGQSWQP	GAVATLPQRE	GAELGQAAKM	SSSQESLLDS	RGHLKGNNPY	AKSYTLV	

Detailed Protein Report

Cmpd.	No. of Cmpds.	m/z meas.	Δ m/z [ppm]	z	Rt [min]	Score	P	Range	Sequence	Modification
432	1	719.8247	-131.20	2	35.4	20.1	1	394-406	R.INVRGPASIRPMK.N	

Detailed Protein Report

Protein 557: **uromodulin-like 1 isoform 4 [Homo sapiens]**

**Accession:**	gi|338753361	**Score:**	20.0
**Database:**	refseq_human(refseq_human_20140103.fasta)	**MW [kDa]:**	136.4
**Database Date:**	2015-11-30	**pI:**	5.4
**Modification(s):**	Carbamidomethyl, Oxidation	**Sequence Coverage [%]:**	1.6
		**No. of unique Peptides:**	1

10	20	30	40	50	60	70	80
MVYRTQYLVV	EVPESRNVTD	CCEGYEQLGL	YCVLPLNQSG	QFTSRPGACP	AEGPEPSTSP	CSLDIDCPGL	EKCCPWSGGR
90	100	110	120	130	140	150	160
YCMAPAPQAP	ERDPVGSWYN	VTILVKMDFK	ELQQVDPRLL	NHMRLLHSLV	TSALQPMAST	VHHLHSAPGN	ASTTVSRLLL
170	180	190	200	210	220	230	240
GLPRPLPVAD	VSTLLGDIAK	RVYEVISVQV	QDVNECFYEE	LNACSGRELC	ANLEGSYWCV	CHQEAPATSP	RKLNLEWEDC
250	260	270	280	290	300	310	320
PPVSDYVVLN	VTSDSFQVSW	RLNSTQNHTF	HVRVYRGMEL	LRSARTQSQA	LAVAGLEAGV	LYRVKTSYQG	CGADVSTTLT
330	340	350	360	370	380	390	400
IKTNAQVFEV	TIKIVNHNLT	EKLLNRSSVE	YQDFSRQLLH	EVESSFPPVV	SDLYRSGKLR	MQIVSLQAGS	VVVRLKLTVQ
410	420	430	440	450	460	470	480
DPGFPMGIST	LAPILQPLLA	STVFQIDRQG	TRVQDWDECV	DSAEHDCSPA	AWCINLEGSY	TCQCRTTRDA	TPSRAGRACE
490	500	510	520	530	540	550	560
GDLVSPTGGG	LSAATGVTVP	GLGTGTAALG	LENFTLSPSP	GYPQGTPAAG	QAWTPEPSPR	RGGSNVVGYD	RNNTGKGVEQ
570	580	590	600	610	620	630	640
ELQGNSIMEP	PSWPSPTEDP	TGHFLWHATR	STRETLLNPT	WLRNEDSGPS	GSVDLPLTST	LTALKTPACV	PVSIGRIMVS
650	660	670	680	690	700	710	720
NVTSTGFHLA	WEADLAMDST	FQLTLTSMWS	PAVVLETWNT	SVTLSGLEPG	VLHLVEIMAK	ACGKEGARAH	LKVRTAARKL
730	740	750	760	770	780	790	800
IGKVRIKNVR	YSESFRNASS	QEYRDFLELF	FRMVRGSLPA	TMCQHMDAGG	VRMEVVSVTN	GSIVVEFHLL	IIADVDVQEV
810	820	830	840	850	860	870	880
SAAFLTAFQT	VPLLEVIRGD	TFIQDYDECE	RKEDDCVPGT	SCRNTLGSFT	CSCEGGAPDF	PVEYSERPCE	GDSPGNETWA
890	900	910	920	930	940	950	960
TSPERPLTTA	GTKAAFVQGT	SPTPQGLPQR	LNLTGAVRVL	CEIEKVVVAI	QKRFLQQESI	PESSLYLSHP	SCNVSHSNGT
970	980	990	1000	1010	1020	1030	1040
HVLLEAGWSE	CGTLMQSNMT	NTVVRTTLRN	DLSQEGIIHH	LKILSPIYCA	FQNDLLTSSG	FTLEWGVYTI	IEDLHGAGNF
1050	1060	1070	1080	1090	1100	1110	1120
VTEMQLFIGD	SPIPQNYSVS	ASDDVRIEVG	LYRQKSNLKV	VLTECWATPS	SNARDPITFS	FINNSCPVPN	TYTNVIENGN
1130	1140	1150	1160	1170	1180	1190	1200
SNKAQFKLRI	FSFINDSIVY	LHCKLRVCME	SPGATCKINC	NNFRLLQNSE	TSATHQMSWG	PLIRSEGEPP	HAEAGLGAGY
1210	1220	1230	1240	1250			
VVLIVVAIFV	LVAGTATLLI	VRYQRMNGRY	NFKIQSNNFS	YQVFYE			

Cmpd.	No. of Cmpds.	m/z meas.	Δ m/z [ppm]	z	Rt [min]	Score	P	Range	Sequence	Modification
24	1	1103.5077	4.39	2	30.0	20.0	1	753-772	R.MVRGSLPATMCQHMDAGGVR.M	Carbamidomethyl: 11; Oxidation: 10, 14

Detailed Protein Report

Protein 558: replication factor C subunit 4 [Homo sapiens]

**Accession:**	gi|4506491	**Score:**	19.9
**Database:**	refseq_human(refseq_human_20140103.fasta)	**MW [kDa]:**	39.7
**Database Date:**	2015-11-30	**pI:**	9.3
		**Sequence Coverage [%]:**	3.3
		**No. of unique Peptides:**	1

Alias proteins:

Accession	Name	Description
gi|31881687	refseq_human (refseq_human_20140103.fasta)	replication factor C subunit 4 [Homo sapiens]

```
        10         20         30         40         50         60         70         80
MQAFLKGTSI STKPPLTKDR GVAASAGSSG ENKKAKPVPW VEKYRPKCVD EVAFQEEVVA VLKKSLEGAD LPNLLFYGPP
        90        100        110        120        130        140        150        160
GTGKTSTILA AARELFGPEL FRLRVLELNA SDERGIQVVR EKVKNFAQLT VSGSRSDGKP CPPFKIVILD EADSMTSAAQ
       170        180        190        200        210        220        230        240
AALRRTMEKE SKTTRFCLIC NYVSRIIEPL TSRCSKFRFK PLSDKIQQQR LLDIAKKENV KISDEGIAYL VKVSEGDLRK
       250        260        270        280        290        300        310        320
AITFLQSATR LTGGKEITEK VITDIAGVIP AEKIDGVFAA CQSGSFDKLE AVVKDLIDEG HAATQLVNQL HDVVVENNLS
       330        340        350        360        370
DKQKSIITEK LAEVDKCLAD GADEHLQLIS LCATVMQQLS QNC
```

Cmpd.	No. of Cmpds.	m/z meas.	Δ m/z [ppm]	z	Rt [min]	Score	P	Range	Sequence	Modification
1817	2	727.2971	-204.84	2	51.2	19.9	2	206-217	K.IQQQRLLDIAKK.E	

Detailed Protein Report

Protein 559: thymopoietin isoform alpha [Homo sapiens]

**Accession:**	gi|4507555	**Score:**	19.9
**Database:**	refseq_human(refseq_human_20140103.fasta)	**MW [kDa]:**	75.4
**Database Date:**	2015-11-30	**pI:**	8.5
		**Sequence Coverage [%]:**	1.3
		**No. of unique Peptides:**	1

10	20	30	40	50	60	70	80
MPEFLEDPSV	LTKDKLKSEL	VANNVTLPAG	EQRKDVYVQL	YLQHLTARNR	PPLPAGTNSK	GPPDFSSDEE	REPTPVLGSG
**90**	**100**	**110**	**120**	**130**	**140**	**150**	**160**
AAAAGRSRAA	VGRKATKKTD	KPRQEDKDDL	DVTELTNEDL	LDQLVKYGVN	PGPIVGTTRK	LYEKKLLKLR	EQGTESRSST
**170**	**180**	**190**	**200**	**210**	**220**	**230**	**240**
PLPTISSSAE	NTRQNGSNDS	DRYSDNEEGK	KKEHKKVKST	RDIVPFSELG	TTPSGGGFFQ	GISFPEISTR	PPLGSTELQA
**250**	**260**	**270**	**280**	**290**	**300**	**310**	**320**
AKKVHTSKGD	LPREPLVATN	LPGRGQLQKL	ASERNLFISC	KSSHDRCLEK	SSSSSSQPEH	SAMLVSTAAS	PSLIKETTTG
**330**	**340**	**350**	**360**	**370**	**380**	**390**	**400**
YYKDIVENIC	GREKSGIQPL	CPERSHISDQ	SPLSSKRKAL	EESESSQLIS	PPLAQAIRDY	VNSLLVQGGV	GSLPGTSNSM
**410**	**420**	**430**	**440**	**450**	**460**	**470**	**480**
PPLDVENIQK	RIDQSKFQET	EFLSPPRKVP	RLSEKSVEER	DSGSFVAFQN	IPGSELMSSF	AKTVVSHSLT	TLGLEVAKQS
**490**	**500**	**510**	**520**	**530**	**540**	**550**	**560**
QHDKIDASEL	SFPFHESILK	VIEEEWQQVD	RQLPSLACKY	PVSSREATQI	LSVPKVDDEI	LGFISEATPL	GGIQAASTES
**570**	**580**	**590**	**600**	**610**	**620**	**630**	**640**
CNQQLDLALC	RAYEAAASAL	QIATHTAFVA	KAMQADISQA	AQILSSDPSR	THQALGILSK	TYDAASYICE	AAFDEVKMAA
**650**	**660**	**670**	**680**	**690**	**700**		
HTMGNATVGR	RYLWLKDCKI	NLASKNKLAS	TPFKGGTLFG	GEVCKVIKKR	GNKH		

Cmpd.	No. of Cmpds.	m/z meas.	Δ m/z [ppm]	z	Rt [min]	Score	P	Range	Sequence	Modification
1398	2	991.6129	89.59	1	47.6	19.9	1	657-665	K.DCKINLASK.N	

Detailed Protein Report

Protein 560: fructose-bisphosphate aldolase A isoform 1 [Homo sapiens]

Accession: gi|4557305
Database: refseq_human(refseq_human_20140103.fasta)
Database Date: 2015-11-30

Score: 19.8
MW [kDa]: 39.4
pI: 9.2
Sequence Coverage [%]: 3.3
No. of unique Peptides: 1

Alias proteins:

Accession	Name	Description
gi|193794814	refseq_huma (refseq_human_20140103.fasta)	fructose-bisphosphate aldolase A isoform 1 [Homo sapiens]
gi|34577112	refseq_huma (refseq_human_20140103.fasta)	fructose-bisphosphate aldolase A isoform 1 [Homo sapiens]
gi|34577110	refseq_huma (refseq_human_20140103.fasta)	fructose-bisphosphate aldolase A isoform 1 [Homo sapiens]

```
        10         20         30         40         50         60         70         80
MPYQYPALTP EQKKELSDIA HRIVAPGKGI LAADESTGSI AKRLQSIGTE NTEENRRFYR QLLLTADDRV NPCIGGVILF
        90        100        110        120        130        140        150        160
HETLYQKADD GRPFPQVIKS KGGVVGIKVD KGVVPLAGTN GETTTQGLDG LSERCAQYKK DGADFAKWRC VLKIGEHTPS
       170        180        190        200        210        220        230        240
ALAIMENANV LARYASICQQ NGIVPIVEPE ILPDGDHDLK RCQYVTEKVL AAVYKALSDH HIYLEGTLLK PNMVTPGHAC
       250        260        270        280        290        300        310        320
TQKFSHEEIA MATVTALRRT VPPAVTGITF LSGGQSEEEA SINLNAINKC PLLKPWALTF SYGRALQASA LKAWGGKKEN
       330        340        350        360        370
LKAAQEEYVK RALANSLACQ GKYTPSGQAG AAASESLFVS NHAY
```

Cmpd.	No. of Cmpds.	m/z meas.	Δ m/z [ppm]	z	Rt [min]	Score	P	Range	Sequence	Modification
476	1	717.8845	24.70	2	35.9	19.8	0	2-13	M.PYQYPALTPEQK.K	

Detailed Protein Report

Protein 561: **PREDICTED: myotubularin-related protein 12 isoform X5 [Homo sapiens]**

**Accession:**	gi|530378841	**Score:**	19.7
**Database:**	refseq_human(refseq_human_20140103.fasta)	**MW [kDa]:**	42.8
**Database Date:**	2015-11-30	**pI:**	6.6
**Modification(s):**	Oxidation	**Sequence Coverage [%]:**	1.6
		**No. of unique Peptides:**	1

10	20	30	40	50	60	70	80
MLGKGVVGGG	GGTKAPKPSF	VSYVRPEEIH	TNEKEVTEKE	VTLHLLPGEQ	LLCEASTVLK	YVQEDSCQHG	VYGRLVCTDF
**90**	**100**	**110**	**120**	**130**	**140**	**150**	**160**
KIAFLGDDES	ALDNDETQFK	NKVIGENDIT	LHCVDQIYGV	FDEKKKTLFG	QLKKYPEKLI	IHCKDLRVFQ	FCLRYTKEEE
**170**	**180**	**190**	**200**	**210**	**220**	**230**	**240**
VKRIVSGIIH	HTQAPKLLKR	LFLFSYATAA	QNNTVTDPKN	HTVMFDTLKD	WCWELERTKG	NMKYKAVSVN	EGYKVCERLP
**250**	**260**	**270**	**280**	**290**	**300**	**310**	**320**
AYFVVPTPLP	EENVQRFQGH	GIPIWCWSCH	NGSALLKMSA	LPKEQDDGIL	QIQKSFLDGI	YKTIHRPPYE	IVKTEDLSSN
**330**	**340**	**350**	**360**	**370**	**380**		
FLSLQEIQTA	YSKFKQLFLI	GFPSPPPYCG	SDHTATLLFS	PSSAYHLPSG	EHFGFFL		

Cmpd.	No. of Cmpds.	m/z meas.	Δ m/z [ppm]	z	Rt [min]	Score	P	Range	Sequence	Modification
1197	1	662.2827	-107.94	1	45.1	19.7	0	278-283	K.MSALPK.E	Oxidation: 1

Detailed Protein Report

Protein 562: choline-phosphate cytidylyltransferase B isoform 2 [Homo sapiens]

**Accession:**	gi|253795514	**Score:**	19.7
**Database:**	refseq_human(refseq_human_20140103.fasta)	**MW [kDa]:**	40.2
**Database Date:**	2015-11-30	**pI:**	7.0
**Modification(s):**	Oxidation	**Sequence Coverage [%]:**	6.0
		**No. of unique Peptides:**	1

10	20	30	40	50	60	70	80
MVGHQECIME	EDNRAPQLWR	KTLTAPAPFA	DETNCQCQAP	HEKLTIAQAR	LGTPADRPVR	VYADGIFDLF	HSGHARALMQ
**90**	**100**	**110**	**120**	**130**	**140**	**150**	**160**
AKTLFPNSYL	LVGVCSDDLT	HKFKGFTVMN	EAERYEALRH	CRYVDEVIRD	APWTLTPEFL	EKHKIDFVAH	DDIPYSSAGS
**170**	**180**	**190**	**200**	**210**	**220**	**230**	**240**
DDVYKHIKEA	GMFVPTQRTE	GISTSDIITR	IVRDYDVYAR	RNLQRGYTAK	ELNVSFINEK	RYRFQNQVDK	MKEKVKNVEE
**250**	**260**	**270**	**280**	**290**	**300**	**310**	**320**
RSKEFVNRVE	EKSHDLIQKW	EEKSREFIGN	FLELFGPDGA	WKQMFQERSS	RMLQALSPKQ	SPVSSPTRSR	SPSRSPSPTF
**330**	**340**	**350**	**360**				
SWLPLKTSPP	SSPKAASASI	SSMSEGDEDE	K				

Cmpd.	No. of Cmpds.	m/z meas.	Δ m/z [ppm]	z	Rt [min]	Score	P	Range	Sequence	Modification
125	1	1301.5081	-71.85	2	31.5	19.7	2	1-21	-.MVGHQECIMEEDNRAPQLWRK.T	Oxidation: 1, 9

Detailed Protein Report

Protein 563: integrin beta-like protein 1 isoform 3 [Homo sapiens]

**Accession:**	gi|418203907	**Score:**	19.5
**Database:**	refseq_human(refseq_human_20140103.fasta)	**MW [kDa]:**	43.9
**Database Date:**	2015-11-30	**pI:**	5.0
		**Sequence Coverage [%]:**	3.0
		**No. of unique Peptides:**	1

Quantitation

QD:QU **Median:** 0.96 **CV:** 0.00 % **No. of Peptides:** 1

10	20	30	40	50	60	70	80
MNLVAVMGAF	NKGHGKCDCG	KCKCDQGWYG	DACQYPTNCD	LTKKKSNQMC	KNSQDIICSN	AGTCHCGRCK	CDNSDGSGLV
**90**	**100**	**110**	**120**	**130**	**140**	**150**	**160**
YGKFCECDDR	ECIDDETEEI	CGGHGKCYCG	NCYCKAGWHG	DKCEFQCDIT	PWESKRRCTS	PDGKICSNRG	TCVCGECTCH
**170**	**180**	**190**	**200**	**210**	**220**	**230**	**240**
DVDPTGDWGD	IHGDTCECDE	RDCRAVYDRY	SDDFCSGHGQ	CNCGRCDCKA	GWYGKKCEHP	QSCTLSAEES	IRKCQGSSDL
**250**	**260**	**270**	**280**	**290**	**300**	**310**	**320**
PCSGRGKCEC	GKCTCYPPGD	RRVYGKTCEC	DDRRCEDLDG	VVCGGHGTCS	CGRCVCERGW	FGKLCQHPRK	CNMTEEQSKN
**330**	**340**	**350**	**360**	**370**	**380**	**390**	**400**
LCESADGILC	SGKGSCHCGK	CICSAEEWYI	SGEFCDCDDR	DCDKHDGLIC	TGNGICSCGN	CECWDGWNGN	ACEIWLGSEY
**410**							
P							

Cmpd.	No. of Cmpds.	m/z meas.	Δ m/z [ppm]	z	Rt [min]	Score	P	Range	Sequence	Modification	Ratios
864	1	647.7154	-186.04	2	40.9	19.5	0	1-12	-.MNLVAVMGAFNK.G		QD:QU 0.96

Detailed Protein Report

Protein 564: PREDICTED: alpha-(1,3)-fucosyltransferase 11 isoform X4 [Homo sapiens]

**Accession:**	gi|578819000	**Score:**	19.5
**Database:**	refseq_human(refseq_human_20140103.fasta)	**MW [kDa]:**	51.4
**Database Date:**	2015-11-30	**pI:**	5.7
		**Sequence Coverage [%]:**	1.5
		**No. of unique Peptides:**	1

10	20	30	40	50	60	70	80
MAAGPIRVVL	VLLGVLSVCA	ASGHGSVAER	EAGGEAEWAE	PWDGAVFRPP	SALGAVGVTR	SSGTPRPGRE	EAGDLPVLLW
**90**	**100**	**110**	**120**	**130**	**140**	**150**	**160**
WSPGLFPHFP	GDSERIECAR	GACVASRNRR	ALRDSRTRAL	LFYGTDFRAS	AAPLPRLAHQ	SWALLHEESP	LNNFLLSHGP
**170**	**180**	**190**	**200**	**210**	**220**	**230**	**240**
GIRLFNLTST	FSRHSDYPLS	LQWLPGTAYL	RRPVPPPMER	AEWRRRGYAP	LLYLQSHCDV	PADRDRYVRE	LMRHIPVDSY
**250**	**260**	**270**	**280**	**290**	**300**	**310**	**320**
GKCLQNRELP	TARLQDTATA	TTEDPELLAF	LSRYKFHLAL	ENAICNDYMT	EKLWRPMHLG	AVPVYRGSPS	VRDWMPNNHS
**330**	**340**	**350**	**360**	**370**	**380**	**390**	**400**
VILIDDFESP	QKLAEFIDFL	DKNDEEYMKY	LAYKQPGGIT	NQFLLDSLKH	REWGVNDPLL	PNYLNGFECF	VCDYELARLD
**410**	**420**	**430**	**440**	**450**	**460**		
AEKAHAASPG	DSPVFEPHIA	QPSHMDCPVP	TPGFGNVEEI	PENDRYWMLL	HFDAPSF		

Cmpd.	No. of Cmpds.	m/z meas.	Δ m/z [ppm]	z	Rt [min]	Score	P	Range	Sequence	Modification
508	2	464.6701	-41.63	2	35.0	19.5	0	343-349	K.NDEEYMK.Y	

BRUKER

Detailed Protein Report

Protein 565: **calcium/calmodulin-dependent protein kinase type II subunit delta isoform 2 [Homo sapiens]**

**Accession:**	gi|26667189	**Score:**	19.4
**Database:**	refseq_human(refseq_human_20140103.fasta)	**MW [kDa]:**	54.1
**Database Date:**	2015-11-30	**pI:**	6.7
**Modification(s):**	Oxidation	**Sequence Coverage [%]:**	3.6
		**No. of unique Peptides:**	1

Quantitation

QD:QU **Median:** 0.40 **CV:** 0.00 % **No. of Peptides:** 1

10	20	30	40	50	60	70	80
MASTTTCTRF	TDEYQLFEEL	GKGAFSVVRR	CMKIPTGQEY	AAKIINTKKL	SARDHQKLER	EARICRLLKH	PNIVRLHDSI
90	100	110	120	130	140	150	160
SEEGFHYLVF	DLVTGGELFE	DIVAREYYSE	ADASHCIQQI	LESVNHCHLN	GIVHRDLKPE	NLLLASKSKG	AAVKLADFGL
170	180	190	200	210	220	230	240
AIEVQGDQQA	WFGFAGTPGY	LSPEVLRKDP	YGKPVDMWAC	GVILYILLVG	YPPFWDEDQH	RLYQQIKAGA	YDFPSPEWDT
250	260	270	280	290	300	310	320
VTPEAKDLIN	KMLTINPAKR	ITASEALKHP	WICQRSTVAS	MMHRQETVDC	LKKFNARRKL	KGAILTTMLA	TRNFSAAKSL
330	340	350	360	370	380	390	400
LKKPDGVKES	TESSNTTIED	EDVKARKQEI	IKVTEQLIEA	INNGDFEAYT	KICDPGLTAF	EPEALGNLVE	GMDFHRFYFE
410	420	430	440	450	460	470	480
NALSKSNKPI	HTIILNPHVH	LVGDDAACIA	YIRLTQYMDG	SGMPKTMQSE	ETRVWHRRDG	KWQNVHFHRS	GSPTVPIN

Cmpd.	No. of Cmpds.	m/z meas.	Δ m/z [ppm]	z	Rt [min]	Score	P	Range	Sequence	Modification	Ratios	
681	1	625.0022	-34.99	3	38.5	19.4	2	252-268	K.MLTINPAKRITASEALK.H	Oxidation: 1	QD:QU	0.40

Detailed Protein Report

Protein 566: protein S100-A9 [Homo sapiens]

Accession: gi|4506773
Database: refseq_human(refseq_human_20140103.fasta)
Database Date: 2015-11-30

Score: 19.4
MW [kDa]: 13.2
pI: 5.7
Sequence Coverage [%]: 11.4
No. of unique Peptides: 1

Quantitation

QD:QU **Median:** 11.07 **CV:** 0.00 % **No. of Peptides:** 1

10	20	30	40	50	60	70	80
MTCKMSQLER	NIETIINTFH	QYSVKLGHPD	TLNQGEFKEL	VRKDLQNFLK	KENKNEKVIE	HIMEDLDTNA	DKQLSFEEFI
90	100	110	120				
MLMARLTWAS	HEKMHEGDEG	PGHHHKPGLG	EGTP				

Cmpd.	No. of Cmpds.	m/z meas.	Δ m/z [ppm]	z	Rt [min]	Score	P	Range	Sequence	Modification	Ratios	
217	2	728.3427	-30.59	2	32.7	19.4	0	26-38	K.LGHPDTLNQGEFK.E		QD:QU	11.07

Detailed Protein Report

Protein 567: PREDICTED: zinc finger transcription factor Trps1 isoform X4 [Homo sapiens]

Accession: gi|530389293
Database: refseq_human(refseq_human_20140103.fasta)
Database Date: 2015-11-30
Modification(s): Carbamidomethyl, Oxidation

Score: 19.3
MW [kDa]: 141.4
pI: 8.3
Sequence Coverage [%]: 1.9
No. of unique Peptides: 1

10	20	30	40	50	60	70	80
MVRKKNPPLR	NVASEGEGQI	LEPIGTESKV	SGKNKEFSAD	QMSENTDQSD	AAELNHKEEH	SLHVQDPSSS	SKKDLKSAVL
**90**	**100**	**110**	**120**	**130**	**140**	**150**	**160**
SEKAGFNYES	PSKGGNFPSF	PHDEVTDRNM	LAFSSPAAGG	VCEPLKSPQR	AEADDPQDMA	CTPSGDSLET	KEDQKMSPKA
**170**	**180**	**190**	**200**	**210**	**220**	**230**	**240**
TEETGQAQSG	QANCQGLSPV	SVASKNPQVP	SDGGVRLNKS	KTDLLVNDNP	DPAPLSPELQ	DFKCNICGYG	YYGNDPTDLI
**250**	**260**	**270**	**280**	**290**	**300**	**310**	**320**
KHFRKYHLGL	HNRTRQDAEL	DSKILALHNM	VQFSHSKDFQ	KVNRSVFSGV	LQDINSSRPV	LLNGTYDVQV	TSGGTFIGIG
**330**	**340**	**350**	**360**	**370**	**380**	**390**	**400**
RKTPDCQGNT	KYFRCKFCNF	TYMGNSSTEL	EQHFLQTHPN	KIKASLPSSE	VAKPSEKNSN	KSIPALQSSD	SGDLGKWQDK
**410**	**420**	**430**	**440**	**450**	**460**	**470**	**480**
ITVKAGDDTP	VGYSVPIKPL	DSSRQNGTEA	TSYYWCKFCS	FSCESSSSLK	LLEHYGKQHG	AVQSGGLNPE	LNDKLSRGSV
**490**	**500**	**510**	**520**	**530**	**540**	**550**	**560**
INQNDLAKSS	EGETMTKTDK	SSSGAKKKDF	SSKGAEDNMV	TSYNCQFCDF	RYSKSHGPDV	IVVGPLLRHY	QQLHNIHKCT
**570**	**580**	**590**	**600**	**610**	**620**	**630**	**640**
IKHCPFCPRG	LCSPEKHLGE	ITYPFACRKS	NCSHCALLLL	HLSPGAAGSS	RVKHQCHQCS	FTTPDVDVLL	FHYESVHESQ
**650**	**660**	**670**	**680**	**690**	**700**	**710**	**720**
ASDVKQEANH	LQGSDGQQSV	KESKEHSCTK	CDFITQVEEE	ISRHYRRAHS	CYKCRQCSFT	AADTQSLLEH	FNTVHCQEQD
**730**	**740**	**750**	**760**	**770**	**780**	**790**	**800**
ITTANGEEDG	HAISTIKEEP	KIDFRVYNLL	TPDSKMGEPV	SESVVKREKL	EEKDGLKEKV	WTESSSDDLR	NVTWRGADIL
**810**	**820**	**830**	**840**	**850**	**860**	**870**	**880**
RGSPSYTQAS	LGLLTPVSGT	QEQTKTLRDS	PNVEAAHLAR	PIYGLAVETK	GFLQGAPAGG	EKSGALPQQY	PASGENKSKD
**890**	**900**	**910**	**920**	**930**	**940**	**950**	**960**
ESQSLLRRRR	GSGVFCANCL	TTKTSLWRKN	ANGGYVCNAC	GLYQKLHSTP	RPLNIIKQNN	GEQIIRRRTR	KRLNPEALQA
**970**	**980**	**990**	**1000**	**1010**	**1020**	**1030**	**1040**
EQLNKQQRGS	NEEQVNGSPL	ERRSEDHLTE	SHQREIPLPS	LSKYEAQGSL	TKSHSAQQPV	LVSQTLDIHK	RMQPLHIQIK
**1050**	**1060**	**1070**	**1080**	**1090**	**1100**	**1110**	**1120**
SPQESTGDPG	NSSSVSEGKG	SSERGSPIEK	YMRPAKHPNY	SPPGSPIEKY	QYPLFGLPFV	HNDFQSEADW	LRFWSKYKLS
**1130**	**1140**	**1150**	**1160**	**1170**	**1180**	**1190**	**1200**
VPGNPHYLSH	VPGLPNPCQN	YVPYPTFNLP	PHFSAVGSDN	DIPLDLAIKH	SRPGPTANGA	SKEKTKAPPN	VKNEGPLNVV
**1210**	**1220**	**1230**	**1240**	**1250**	**1260**	**1270**	**1280**
KTEKVDRSTQ	DELSTKCVHC	GIVFLDEVMY	ALHMSCHGDS	GPFQCSICQH	LCTDKYDFTT	HIQRGLHRNN	AQVEKNGKPK
**1290**							
E							

Cmpd.	No. of Cmpds.	m/z meas.	Δ m/z [ppm]	z	Rt [min]	Score	P	Range	Sequence	Modification
1488	1	955.7431	8.20	3	47.1	19.3	2	508-531	K.KDFSSKGAEDNMVTSYNCQFCDFR.Y	Carbamidomethyl: 21; Oxidation: 12

Detailed Protein Report

Protein 568: zinc finger protein 19 [Homo sapiens]

Accession: gi|38045956
Database: refseq_human(refseq_human_20140103.fasta)
Database Date: 2015-11-30
Modification(s): Carbamidomethyl

Score: 19.2
MW [kDa]: 52.4
pI: 9.1
Sequence Coverage [%]: 3.3
No. of unique Peptides: 1

10	20	30	40	50	60	70	80
MAAMPLKAQY	QEMVTFEDVA	VHFTKTEWTG	LSPAQRALYR	SVMLENFGNL	TALGYPVPKP	ALISLLERGD	MAWGLEAQDD
**90**	**100**	**110**	**120**	**130**	**140**	**150**	**160**
PPAERTKNVC	KDVETNIDSE	STLIQGISEE	RDGMMSHGQL	KSVPQRTDFP	ETRNVEKHQD	IPTVKNIQGK	VPRIPCARKP
**170**	**180**	**190**	**200**	**210**	**220**	**230**	**240**
FICEECGKSF	SYFSYYARHQ	RIHTGEKPFE	CSECGKAFNG	NSSLIRHQRI	HTGERPYQCE	ECGRAFNDNA	NLIRHQRIHS
**250**	**260**	**270**	**280**	**290**	**300**	**310**	**320**
GDRPYYCTEC	GNSFTSSSEF	VIHQRIHTGE	KPYECNECGK	AFVGNSPLLR	HQKIHTGEKP	YECNECGKSF	GRTSHLSQHQ
**330**	**340**	**350**	**360**	**370**	**380**	**390**	**400**
RIHTGEKPYS	CKVCGQAFNF	HTKLTRHQRI	HSEEKPFDCV	DCGKAFSAQE	QLKRHLRIHT	QESSYVCDEC	GKALTSKRNL
**410**	**420**	**430**	**440**	**450**	**460**		
HQHQRIHTGE	KPYECSKYEK	AFGTSSQLGH	LEHVYSGEKP	VLDICRFGLP	EFFTPFYW		

Cmpd.	No. of Cmpds.	m/z meas.	Δ m/z [ppm]	z	Rt [min]	Score	P	Range	Sequence	Modification
2142	1	882.4021	13.71	2	55.4	19.2	0	350-364	R.IHSEEKPFDCVDCGK.A	Carbamidomethyl: 13

Detailed Protein Report

Protein 569: uncharacterized protein KIAA1107 [Homo sapiens]

**Accession:**	gi|197245440	**Score:**	19.1
**Database:**	refseq_human(refseq_human_20140103.fasta)	**MW [kDa]:**	149.4
**Database Date:**	2015-11-30	**pI:**	5.8
**Modification(s):**	Carbamidomethyl	**Sequence Coverage [%]:**	1.3
		**No. of unique Peptides:**	1

10	20	30	40	50	60	70	80
MSSTADLLDT	ILKAIEENIT	TENSCSLLMA	LDTLLNSDST	KEMGFTCKIQ	ALRDKLWIFL	VQSFYAVRHT	ESWKLMSTDD
90	100	110	120	130	140	150	160
QQKIQAAAFD	KGDDRRLGKK	PIFSSSQQRK	QVSDSGDIKI	KSWRGNNKKE	CWSYLSTNKK	MKSDGLGASG	HSSSTNRNSI
170	180	190	200	210	220	230	240
NKTLKQDDVK	EKDGTKIASK	ITKELKTGGK	NVSGKPKTVT	KSKTENGDKA	RLENMSPRQV	VERSATAAAA	ATGQKNLLNG
250	260	270	280	290	300	310	320
KGVRNQEGQI	SGARPKVLTG	NLNVQAKAKP	LKKATGKDSP	CLSIAGPSSR	STDSSMEFSI	STECLDEPKE	NGSTEEEKPS
330	340	350	360	370	380	390	400
GHKLSFCDSP	GQMMKNSVDS	VKNSTVAIKS	RPVSRVTNGT	SNKKSIHEQD	TNVNNSVLKK	VSGKGCSEPV	PQAILKKRGT
410	420	430	440	450	460	470	480
SNGCTAAQQR	TKSTPSNLTK	TQGSQGESPN	SVKSSVSSRQ	SDENVAKLDH	NTTTEKQAPK	RKMVKQVHTA	LPKVNAKIVA
490	500	510	520	530	540	550	560
MPKNLNQSKK	GETLNNKDSK	QKMPPGQVIS	KTQPSSQRPL	KHETSTVQKS	MFHDVRDNNN	KDSVSEQKPH	KPLINLASEI
570	580	590	600	610	620	630	640
SDAEALQSSC	RPDPQKPLND	QEKEKLALEC	QNISKLDKSL	KHELESKQIC	LDKSETKFPN	HKETDDCDAA	NICCHSVGSD
650	660	670	680	690	700	710	720
NVNSKFYSTT	ALKYMVSNPN	ENSLNSNPVC	DLDSTSAGQI	HLISDRENQV	GRKDTNKQSS	IKCVEDVSLC	NPERTNGTLN
730	740	750	760	770	780	790	800
SAQEDKKSKV	PVEGLTIPSK	LSDESAMDED	KHATADSDVS	SKCFSGQLSE	KNSPKNMETS	ESPESHETPE	TPFVGHWNLS
810	820	830	840	850	860	870	880
TGVLHQRESP	ESDTGSATTS	SDDIKPRSED	YDAGGSQDDD	GSNDRGISKC	GTMLCHDFLG	RSSSDTSTPE	ELKIYDSNLR
890	900	910	920	930	940	950	960
IEVKMKKQSN	NDLFQVNSTS	DDEIPRKRPE	IWSRSAIVHS	RERENIPRGS	VQFAQEIDQV	SSSADETEDE	RSEAENVAEN
970	980	990	1000	1010	1020	1030	1040
FSISNPAPQQ	FQGIINLAFE	DATENECREF	SATKKFKRSV	LLSVDECEEL	GSDEGEVHTP	FQASVDSFSP	SDVFDGISHE
1050	1060	1070	1080	1090	1100	1110	1120
HHGRTCYSRF	SRESEDNILE	CKQNKGNSVC	KNESTVLDLS	SIDSSRKNKQ	SVSATEKKNT	IDVLSSRSRQ	LLREDKKVNN
1130	1140	1150	1160	1170	1180	1190	1200
GSNVENDIQQ	RSKFLDSDVK	SQERPCHLDL	HQREPNSDIP	KNSSTKSLDS	FRSQVLPQEG	PVKESHSTTT	EKANIALSAG
1210	1220	1230	1240	1250	1260	1270	1280
DIDDCDTLAQ	TRMYDHRPSK	TLSPIYEMDV	IEAFEQKVES	ETHVTDMDFE	DDQHFAKQDW	TLLKQLLSEQ	DSNLDVTNSV
1290	1300	1310	1320	1330	1340	1350	1360
PEDLSLAQYL	INQTLLLARD	SSKPQGITHI	DTLNRWSELT	SPLDSSASIT	MASFSSEDCS	PQGEWTILEL	ETQH

Cmpd.	No. of Cmpds.	m/z meas.	Δ m/z [ppm]	z	Rt [min]	Score	P	Range	Sequence	Modification
2225	1	916.3247	-158.70	2	56.5	19.1	2	273-290	K.KATGKDSPCLSIAGPSSR.S	Carbamidomethyl: 9

Detailed Protein Report

Protein 570: **putative sodium-coupled neutral amino acid transporter 10 isoform a [Homo sapiens]**

**Accession:**	gi|83921602	**Score:**	19.1
**Database:**	refseq_human(refseq_human_20140103.fasta)	**MW [kDa]:**	119.7
**Database Date:**	2015-11-30	**pI:**	5.4
		**Sequence Coverage [%]:**	1.8
		**No. of unique Peptides:**	1

10	20	30	40	50	60	70	80
MTAAAASNWG	LITNIVNSIV	GVSVLTMPFC	FKQCGIVLGA	LLLVFCSWMT	HQSCMFLVKS	ASLSKRRTYA	GLAFHAYGKA
**90**	**100**	**110**	**120**	**130**	**140**	**150**	**160**
GKMLVETSMI	GLMLGTCIAF	YVVIGDLGSN	FFARLFGFQV	GGTFRMFLLF	AVSLCIVLPL	SLQRNMMASI	QSFSAMALLF
**170**	**180**	**190**	**200**	**210**	**220**	**230**	**240**
YTVFMFVIVL	SSLKHGLFSG	QWLRRVSYVR	WEGVFRCIPI	FGMSFACQSQ	VLPTYDSLDE	PSVKTMSSIF	ASSLNVVTTF
**250**	**260**	**270**	**280**	**290**	**300**	**310**	**320**
YVMVGFFGYV	SFTEATAGNV	LMHFPSNLVT	EMLRVGFMMS	VAVGFPMMIL	PCRQALSTLL	CEQQQKDGTF	AAGGYMPPLR
**330**	**340**	**350**	**360**	**370**	**380**	**390**	**400**
FKALTLSVVF	GTMVGGILIP	NVETILGLTG	ATMGSLICFI	CPALIYKKIH	KNALSSQVVL	WVGLGVLVVS	TVTTLSVSEE
**410**	**420**	**430**	**440**	**450**	**460**	**470**	**480**
VPEDLAEEAP	GGRLGEAEGL	MKVEAARLSA	QDPVVAVAED	GREKPKLPKE	REELEQAQIK	GPVDVPGRED	GKEAPEEAQL
**490**	**500**	**510**	**520**	**530**	**540**	**550**	**560**
DRPGQGIAVP	VGEAHRHEPP	VPHDKVVVDE	GQDREVPEEN	KPPSRHAGGK	APGVQGQMAP	PLPDSEREKQ	EPEQGEVGKR
**570**	**580**	**590**	**600**	**610**	**620**	**630**	**640**
PGQAQALEEA	GDLPEDPQKV	PEADGQPAVQ	PAKEDLGPGD	RGLHPRPQAV	LSEQQNGLAV	GGGEKAKGGP	PPGNAAGDTG
**650**	**660**	**670**	**680**	**690**	**700**	**710**	**720**
QPAEDSDHGG	KPPLPAEKPA	PGPGLPPEPR	EQRDVERAGG	NQAASQLEEA	GRAEMLDHAV	LLQVIKEQQV	QQKRLLDQQE
**730**	**740**	**750**	**760**	**770**	**780**	**790**	**800**
KLLAVIEEQH	KEIHQQRQED	EEDKPRQVEV	HQEPGAAVPR	GQEAPEGKAR	ETVENLPPLP	LDPVLRAPGG	RPAPSQDLNQ
**810**	**820**	**830**	**840**	**850**	**860**	**870**	**880**
RSLEHSEGPV	GRDPAGPPDG	GPDTEPRAAQ	AKLRDGQKDA	APRAAGTVKE	LPKGPEQVPV	PDPAREAGGP	EERLAEEFPG
**890**	**900**	**910**	**920**	**930**	**940**	**950**	**960**
QSQDVTGGSQ	DRKKPGKEVA	ATGTSILKEA	NWLVAGPGAE	TGDPRMKPKQ	VSRDLGLAAD	LPGGAEGAAA	QPQAVLRQPE
**970**	**980**	**990**	**1000**	**1010**	**1020**	**1030**	**1040**
LRVISDGEQG	GQQGHRLDHG	GHLEMRKARG	GDHVPVSHEQ	PRGGEDAAVQ	EPRQRPEPEL	GLKRAVPGGQ	RPDNAKPNRD
**1050**	**1060**	**1070**	**1080**	**1090**	**1100**	**1110**	**1120**
LKLQAGSDLR	RRRRDLGPHA	EGQLAPRDGV	IIGLNPLPDV	QVNDLRGALD	AQLRQAAGGA	LQVVHSRQLR	QAPGPPEES

Cmpd.	No. of Cmpds.	m/z meas.	Δ m/z [ppm]	z	Rt [min]	Score	P	Range	Sequence	Modification
203	1	1043.9225	-84.97	2	32.5	19.1	1	854-873	K.GPEQVPVPDPAREAGGPEER.L	

Detailed Protein Report

Protein 571: PREDICTED: nucleolar complex protein 3 homolog isoform X1 [Homo sapiens]

**Accession:**	gi|530394138	**Score:**	19.1
**Database:**	refseq_human(refseq_human_20140103.fasta)	**MW [kDa]:**	58.7
**Database Date:**	2015-11-30	**pI:**	10.1
		**Sequence Coverage [%]:**	4.2
		**No. of unique Peptides:**	1

10	20	30	40	50	60	70	80
MKARRNKKQI	PSFRKLIKTS	KVKLENKLKN	KQFKQQSTLK	KYRKEQRKLR	QAVKDAVSKK	PIPLENPKEK	RPGKRIEREE
**90**	**100**	**110**	**120**	**130**	**140**	**150**	**160**
EEEEEALPLD	MMDEDDLQLM	KDLGQRVSFL	TRDLSSSEPV	HAKKRKHERI	IDKYEKIPRT	LQTAPEKELI	HLLPIKDKSG
**170**	**180**	**190**	**200**	**210**	**220**	**230**	**240**
IIPQTREKPV	TDSNKDEEDQ	EEERELEEEI	IEDPIQELTI	EEHLIERKKK	LQEKKMHIAA	LASAILSDPE	NNIKKLKELR
**250**	**260**	**270**	**280**	**290**	**300**	**310**	**320**
SMLMEQDPDV	AVTVRKLVIV	SLMELFKDIT	PSYKIRPLTE	AEKSTKTRKE	TQKLREFEEG	LVSQYKFYLE	NLEQMVKDWK
**330**	**340**	**350**	**360**	**370**	**380**	**390**	**400**
QRKLKKSNVV	SLKAYKGLAE	VAVKSLCELL	VALPHFNFHN	NIIVLIVPLM	NDMSKLISEM	CCEAVKKLFK	QDKLGQASLG
**410**	**420**	**430**	**440**	**450**	**460**	**470**	**480**
VIKVISGFVK	GRNYEVRPEM	LKTFLCLRIK	EVEVKKDTED	INKPKKFMTF	KEKRKSLSRM	QRKWKKAEEK	LERELREAEA
**490**	**500**	**510**					
SESTEKKLKL	VCSPYKCGIF						

Cmpd.	No. of Cmpds.	m/z meas.	Δ m/z [ppm]	z	Rt [min]	Score	P	Range	Sequence	Modification
2876	1	1132.6192	-5.27	2	65.1	19.1	2	215-235	K.KMHIAALASAILSDPENNIKK.L	

Detailed Protein Report

Protein 572: zinc finger protein 2 isoform b [Homo sapiens]

Accession: gi|62865883
Database: refseq_human(refseq_human_20140103.fasta)
Database Date: 2015-11-30
Modification(s): Carbamidomethyl

Score: 18.6
MW [kDa]: 43.9
pI: 10.2
Sequence Coverage [%]: 5.2
No. of unique Peptides: 1

10	20	30	40	50	60	70	80
MLENYNSIVS	LGLPVPQPDV	IFQLKRGDKP	WMVDLHGSEE	REWPESVSLD	WETKPEIHDA	SDKKSEGSLR	ECLGRQSPLC
**90**	**100**	**110**	**120**	**130**	**140**	**150**	**160**
PKFEVHTPNG	RMGTEKQSPS	GETRKKSLSR	DKGLRRRSAL	SREILTKERH	QECSDCGKTF	FDHSSLTRHQ	RTHTGEKPYD
**170**	**180**	**190**	**200**	**210**	**220**	**230**	**240**
CRECGKAFSH	RSSLSRHLMS	HTGESPYECS	VCSKAFFDRS	SLTVHQRIHT	GEKPFQCNEC	GKAFFDRSSL	TRHQRIHTGE
**250**	**260**	**270**	**280**	**290**	**300**	**310**	**320**
SPYECHQCGK	AFSQKSILTR	HQLIHTGRKP	YECNECGKAF	YGVSSLNRHQ	KAHAGDPRYQ	CNECGKAFFD	RSSLTQHQKI
**330**	**340**	**350**	**360**	**370**	**380**	**390**	
HTGDKPYECS	ECGKAFSQRC	RLTRHQRVHT	GEKPFECTVC	GKVFSSKSSV	IQHQRRYAKQ	GID	

Cmpd.	No. of Cmpds.	m/z meas.	Δ m/z [ppm]	z	Rt [min]	Score	P	Range	Sequence	Modification
1080	1	795.3895	26.37	3	43.6	18.6	1	208-227	R.IHTGEKPFQCNECGKAFFDR.S	Carbamidomethyl: 10

Detailed Protein Report

Protein 573: glycogenin-1 isoform 3 [Homo sapiens]

**Accession:**	gi|296040507	**Score:**	18.6
**Database:**	refseq_human(refseq_human_20140103.fasta)	**MW [kDa]:**	31.4
**Database Date:**	2015-11-30	**pI:**	6.1
		**Sequence Coverage [%]:**	5.4
		**No. of unique Peptides:**	1

10	20	30	40	50	60	70	80
MTDQAFVTLT	TNDAYAKGAL	VLGSSLKQHR	TTRRLVVLAT	PQVSDSMRKV	LETVFDEVIM	VDVLDSGDSA	HLTLMKRPEL
**90**	**100**	**110**	**120**	**130**	**140**	**150**	**160**
GVTLTKLHCW	SLTQYSKCVF	MDADTLVLAN	IDDLFDREEL	SAAPDPGWPD	CFNSGVFVYQ	PSVETYNQLL	HLASEQGSFD
**170**	**180**	**190**	**200**	**210**	**220**	**230**	**240**
GGDQGILNTF	FSSWATTDIR	KHLPFIYNLS	SISIYSYLPA	FKVKMSQEPY	HICPLGRSQL	WHSRLYPRKN	GRNDGNRARL
**250**	**260**	**270**	**280**				
IIWEQIPLTT	SRGNLTLTSS	RNTAFFCEHI	HFTSLVSDT				

Cmpd.	No. of Cmpds.	m/z meas.	Δ m/z [ppm]	z	Rt [min]	Score	P	Range	Sequence	Modification
2695	1	822.4608	-0.36	2	64.8	18.6	1	35-49	R.LVVLATPQVSDSMRK.V	

Detailed Protein Report

Protein 574: tripartite motif-containing protein 6 isoform 3 [Homo sapiens]

**Accession:**	gi|310772221	**Score:**	18.4
**Database:**	refseq_human(refseq_human_20140103.fasta)	**MW [kDa]:**	36.4
**Database Date:**	2015-11-30	**pI:**	9.4
**Modification(s):**	Carbamidomethyl	**Sequence Coverage [%]:**	8.0
		**No. of unique Peptides:**	1

Alias proteins:

Accession	Name	Description
gi|310772223	r e f s e q _ h u m a n (refseq_human_20140103.fasta)	tripartite motif-containing protein 6 isoform 3 [Homo sapiens]

10	20	30	40	50	60	70	80
MEPERCRIQT	EFNQLRNILD	RVEQRELKKL	EQEEKKGLRI	IEEAENDLVH	QTQSLRELIS	DLERRCQGST	MELLQDVSDV
**90**	**100**	**110**	**120**	**130**	**140**	**150**	**160**
TERSEFWTLR	KPEALPTKLR	SMFRAPDLKR	MLRVCRELTD	VQSYWVDVTL	NPHTANLNLV	LAKNRRQVRF	VGAKVSGPSC
**170**	**180**	**190**	**200**	**210**	**220**	**230**	**240**
LEKHYDCSVL	GSQHFSSGKH	YWEVDVAKKT	AWILGVCSNS	LGPTFSFNHF	AQNHSAYSRY	QPQSGYWVIG	LQHNHEYRAY
**250**	**260**	**270**	**280**	**290**	**300**	**310**	**320**
EDSSPSLLLS	MTVPPRRVGV	FLDYEAGTVS	FYNVTNHGFP	IYTFSKYYFP	TTLCPYFNPC	NCVIPMTLRR	PSS

Cmpd.	No. of Cmpds.	m/z meas.	Δ m/z [ppm]	z	Rt [min]	Score	P	Range	Sequence	Modification
1236	1	903.7473	-4.54	3	45.6	18.4	1	155-179	K.VSGPSCLEKHYDCSVLGSQHFSSGK.H	Carbamidomethyl: 13

Detailed Protein Report

Protein 575: PREDICTED: orofacial cleft 1 candidate gene 1 protein [Homo sapiens]

**Accession:**	gi|530358739	**Score:**	18.3
**Database:**	refseq_human(refseq_human_20140103.fasta)	**MW [kDa]:**	48.0
**Database Date:**	2015-11-30	**pI:**	5.4
		**Sequence Coverage [%]:**	2.9
		**No. of unique Peptides:**	1

Alias proteins:

Accession	Name	Description
gi|530435542	refseq_human (refseq_human_20140103.fasta)	PREDICTED: orofacial cleft 1 candidate gene 1 protein [Homo sapiens]
gi|530382966	refseq_human (refseq_human_20140103.fasta)	PREDICTED: orofacial cleft 1 candidate gene 1 protein [Homo sapiens]

```
        10         20         30         40         50         60         70         80
MRETGLKSVH CQGQEAENME REKFQQKALK QTKQKKSKSA EFLMVKEDRE ATEGTGNPAF NMSSPDLSAC QTAEKKVIRH
        90        100        110        120        130        140        150        160
DMPDRTLAAH QQKFRLPASA EPKGNEYGRN YFDPLMDEEI NPRQCATEVS REGAPSKEKW SIPKKIGPCS MDYDPNLLEE
       170        180        190        200        210        220        230        240
DDELHSQGDS LTDHSVKGKS TVWRIGEAED YSQDISYLEE LEEHRFSVCC SSVADSRYGD FFKHLHFVLV SASSELQLSQ
       250        260        270        280        290        300        310        320
WQSQGFWYII LLMFFLWFLR LYLHYLGQWL FLQAISTPVT KFHFSLHIVE LCYPTSSLHI GEELPVVVMG PLMLNAILLL
       330        340        350        360        370        380        390        400
LVLIRWGCQL LFASCPDVLS KLIITMGLWT ILDPLAVFIL DTLLGRLTDN EETPVADAAK LYWMFVRTVQ PGILGVVITV
       410        420        430
LLYILLFVIS SLILYLYCLR
```

Cmpd.	No. of Cmpds.	m/z meas.	Δ m/z [ppm]	z	Rt [min]	Score	P	Range	Sequence	Modification
48	1	630.8632	146.24	2	29.3	18.3	0	206-217	R.FSVCCSSVADSR.Y	

Detailed Protein Report

Protein 576: PREDICTED: period circadian protein homolog 1 isoform X2 [Homo sapiens]

**Accession:**	gi|530410420	**Score:**	18.2
**Database:**	refseq_human(refseq_human_20140103.fasta)	**MW [kDa]:**	130.2
**Database Date:**	2015-11-30	**pI:**	5.7
**Modification(s):**	Oxidation	**Sequence Coverage [%]:**	1.4
		**No. of unique Peptides:**	1

10	20	30	40	50	60	70	80
MSGPLEGADG	GGDPRPGESF	CPGGVPSPGP	PQHRPCPGPS	LADDTDANSN	GSSGNESNGH	ESRGASQRSS	HSSSSGNGKD
90	100	110	120	130	140	150	160
SALLETTESS	KSTNSQSPSP	PSSSIAYSLL	SASSEQDNPS	TSGCSSEQSA	RARTQKELMT	ALRELKLRLP	PERRGKGRSG
170	180	190	200	210	220	230	240
TLATLQYALA	CVKQVQANQE	YYQQWSLEEG	EPCSMDMSTY	TLEELEHITS	EYTLQNQDTF	SVAVSFLTGR	IVYISEQAAV
250	260	270	280	290	300	310	320
LLRCKRDVFR	GTRFSELLAP	QDVGVFYGST	APSRLPTWGT	GASAGSGLRD	FTQEKSVFCR	IRGGPDRDPG	PRYQPFRLTP
330	340	350	360	370	380	390	400
YVTKIRVSDG	APAQPCCLLI	AERIHSGYEA	PRIPPDKRIF	TTRHTPSCLF	QDVDERAAPL	LGYLPQDLLG	APVLLFLHPE
410	420	430	440	450	460	470	480
DRPLMLAIHK	KILQLAGQPF	DHSPIRFCAR	NGEYVTMDTS	WAGFVHPWSR	KVAFVLGRHK	VRTAPLNEDV	FTPPAPSPAP
490	500	510	520	530	540	550	560
SLDTDIQELS	EQIHRLLLQP	VHSPSPTGLC	GVGAVTSPGP	LHSPGSSSDS	NGGDAEGPGP	PAPVTFQQIC	KDVHLVKHQG
570	580	590	600	610	620	630	640
QQLFIESRAR	PQSRPRLPAT	GTFKAKALPC	QSPDPELEAG	SAPVQAPLAL	VPEEAERKEA	SSCSYQQINC	LDSILRYLES
650	660	670	680	690	700	710	720
CNLPSTTKRK	CASSSSYTTS	SASDDDRQRT	GPVSVGTKKD	IIMMEDLPGL	APGPAPSPAP	SPTVAPDPAP	DAYRPVGLTK
730	740	750	760	770	780	790	800
AVLSLHTQKE	EQAFLSRFRD	LGRLRGLDSS	STAPSALGER	GCHHGPAPPS	RRHHCRSKAK	RSRHHQNPRA	EAPCYVSHPS
810	820	830	840	850	860	870	880
PVPPSTPWPT	PPATTPFPAV	VQPYPLPVFS	PRGGPQPLPP	APTSVPPAAF	PAPLVTPMVA	LVLPNYLFPT	PSSYPYGALQ
890	900	910	920	930	940	950	960
TPAEGPPTPA	SHSPSPSLPA	LAPSPPHRPD	SPLFNSRCSS	PLQLNLLQLE	ELPRAEGAAV	AGGPGSSAGP	PPPSAEAAEP
970	980	990	1000	1010	1020	1030	1040
EARLAEVTES	SNQDALSGSS	DLLELLLQED	SRSGTGSAAS	GSLGSGLGSG	SGSGSHEGGS	TSASITRSSQ	SSHTSKYFGS
1050	1060	1070	1080	1090	1100	1110	1120
IDSSEAEAGA	ARGGAEPGDQ	VIKYVLQDPI	WLLMANADQR	VMMTYQVPSR	DMTSVLKQDR	ERLRAMQKQQ	PRFSEDQRRE
1130	1140	1150	1160	1170	1180	1190	1200
LGAVHSWVRK	GQLPRALDVM	ACVDCGSSTQ	DPGHPDDPLF	SELDGLGLEP	MEEGGGEQGS	SGGGSGEGEG	CEEAQGGAKA
1210	1220	1230	1240				
SSSQDLAMEE	EEEGRSSSSP	ALPTAGNCTS					

Cmpd.	No. of Cmpds.	m/z meas.	Δ m/z [ppm]	z	Rt [min]	Score	P	Range	Sequence	Modification
2488	1	673.3201	-14.80	3	61.7	18.2	1	1081-1097	R.VMMTYQVPSRDMTSVLK.Q	Oxidation: 2, 12

Detailed Protein Report

Protein 577: macrophage mannose receptor 1 precursor [Homo sapiens]

**Accession:**	gi|4505245	**Score:**	18.2
**Database:**	refseq_human(refseq_human_20140103.fasta)	**MW [kDa]:**	165.9
**Database Date:**	2015-11-30	**pI:**	6.1
		**Sequence Coverage [%]:**	1.2
		**No. of unique Peptides:**	1

10	20	30	40	50	60	70	80
MRLPLLLVFA	SVIPGAVLLL	DTRQFLIYNE	DHKRCVDAVS	PSAVQTAACN	QDAESQKFRW	VSESQIMSVA	FKLCLGVPSK
**90**	**100**	**110**	**120**	**130**	**140**	**150**	**160**
TDWVAITLYA	CDSKSEFQKW	ECKNDTLLGI	KGEDLFFNYG	NRQEKNIMLY	KGSGLWSRWK	IYGTTDNLCS	RGYEAMYTLL
**170**	**180**	**190**	**200**	**210**	**220**	**230**	**240**
GNANGATCAF	PFKFENKWYA	DCTSAGRSDG	WLWCGTTTDY	DTDKLFGYCP	LKFEGSESLW	NKDPLTSVSY	QINSKSALTW
**250**	**260**	**270**	**280**	**290**	**300**	**310**	**320**
HQARKSCQQQ	NAELLSITEI	HEQTYLTGLT	SSLTSGLWIG	LNSLSFNSGW	QWSDRSPFRY	LNWLPGSPSA	EPGKSCVSLN
**330**	**340**	**350**	**360**	**370**	**380**	**390**	**400**
PGKNAKWENL	ECVQKLGYIC	KKGNTTLNSF	VIPSESDVPT	HCPSQWWPYA	GHCYKIHRDE	KKIQRDALTT	CRKEGGDLTS
**410**	**420**	**430**	**440**	**450**	**460**	**470**	**480**
IHTIEELDFI	ISQLGYEPND	ELWIGLNDIK	IQMYFEWSDG	TPVTFTKWLR	GEPSHENNRQ	EDCVVMKGKD	GYWADRGCEW
**490**	**500**	**510**	**520**	**530**	**540**	**550**	**560**
PLGYICKMKS	RSQGPEIVEV	EKGCRKGWKK	HHFYCYMIGH	TLSTFAEANQ	TCNNENAYLT	TIEDRYEQAF	LTSFVGLRPE
**570**	**580**	**590**	**600**	**610**	**620**	**630**	**640**
KYFWTGLSDI	QTKGTFQWTI	EEEVRFTHWN	SDMPGRKPGC	VAMRTGIAGG	LWDVLKCDEK	AKFVCKHWAE	GVTHPPKPTT
**650**	**660**	**670**	**680**	**690**	**700**	**710**	**720**
TPEPKCPEDW	GASSRTSLCF	KLYAKGKHEK	KTWFESRDFC	RALGGDLASI	NNKEEQQTIW	RLITASGSYH	KLFWLGLTYG
**730**	**740**	**750**	**760**	**770**	**780**	**790**	**800**
SPSEGFTWSD	GSPVSYENWA	YGEPNNYQNV	EYCGELKGDP	TMSWNDINCE	HLNNWICQIQ	KGQTPKPEPT	PAPQDNPPVT
**810**	**820**	**830**	**840**	**850**	**860**	**870**	**880**
EDGWVIYKDY	QYYFSKEKET	MDNARAFCKR	NFGDLVSIQS	ESEKKFLWKY	VNRNDAQSAY	FIGLLISLDK	KFAWMDGSKV
**890**	**900**	**910**	**920**	**930**	**940**	**950**	**960**
DYVSWATGEP	NFANEDENCV	TMYSNSGFWN	DINCGYPNAF	ICQRHNSSIN	ATTVMPTMPS	VPSGCKEGWN	FYSNKCFKIF
**970**	**980**	**990**	**1000**	**1010**	**1020**	**1030**	**1040**
GFMEEERKNW	QEARKACIGF	GGNLVSIQNE	KEQAFLTYHM	KDSTFSAWTG	LNDVNSEHTF	LWTDGRGVHY	TNWGKGYPGG
**1050**	**1060**	**1070**	**1080**	**1090**	**1100**	**1110**	**1120**
RRSSLSYEDA	DCVVIIGGAS	NEAGKWMDDT	CDSKRGYICQ	TRSDPSLTNP	PATIQTDGFV	KYGKSSYSLM	RQKFQWHEAE
**1130**	**1140**	**1150**	**1160**	**1170**	**1180**	**1190**	**1200**
TYCKLHNSLI	ASILDPYSNA	FAWLQMETSN	ERVWIALNSN	LTDNQYTWTD	KWRVRYTNWA	ADEPKLKSAC	VYLDLDGYWK
**1210**	**1220**	**1230**	**1240**	**1250**	**1260**	**1270**	**1280**
TAHCNESFYF	LCKRSDEIPA	TEPPQLPGRC	PESDHTAWIP	FHGHCYYIES	SYTRNWGQAS	LECLRMGSSL	VSIESAAESS
**1290**	**1300**	**1310**	**1320**	**1330**	**1340**	**1350**	**1360**
FLSYRVEPLK	SKTNFWIGLF	RNVEGTWLWI	NNSPVSFVNW	NTGDPSGERN	DCVALHASSG	FWSNIHCSSY	KGYICKRPKI
**1370**	**1380**	**1390**	**1400**	**1410**	**1420**	**1430**	**1440**
IDAKPTHELL	TTKADTRKMD	PSKPSSNVAG	VVIIVILLIL	TGAGLAAYFF	YKKRRVHLPQ	EGAFENTLYF	NSQSSPGTSD
**1450**	**1460**						
MKDLVGNIEQ	NEHSVI						

Cmpd.	No. of Cmpds.	m/z meas.	Δ m/z [ppm]	z	Rt [min]	Score	P	Range	Sequence	Modification
1638	1	1069.9824	-53.73	2	49.0	18.2	2	324-341	K.NAKWENLECVQKLGYICK.K	

Detailed Protein Report

Protein 578: protein phosphatase 1 regulatory subunit 26 [Homo sapiens]

**Accession:**	gi|45387958	**Score:**	18.1
**Database:**	refseq_human(refseq_human_20140103.fasta)	**MW [kDa]:**	127.3
**Database Date:**	2015-11-30	**pI:**	9.8
**Modification(s):**	Carbamidomethyl	**Sequence Coverage [%]:**	0.9
		**No. of unique Peptides:**	1

Alias proteins:

Accession	Name	Description
gi|578818032	refseq_human (refseq_human_20140103.fasta)	PREDICTED: protein phosphatase 1 regulatory subunit 26 isoform X5 [Homo sapiens]
gi|578818030	refseq_human (refseq_human_20140103.fasta)	PREDICTED: protein phosphatase 1 regulatory subunit 26 isoform X4 [Homo sapiens]
gi|578818028	refseq_human (refseq_human_20140103.fasta)	PREDICTED: protein phosphatase 1 regulatory subunit 26 isoform X3 [Homo sapiens]
gi|530426639	refseq_human (refseq_human_20140103.fasta)	PREDICTED: protein phosphatase 1 regulatory subunit 26 isoform X2 [Homo sapiens]
gi|530426637	refseq_human (refseq_human_20140103.fasta)	PREDICTED: protein phosphatase 1 regulatory subunit 26 isoform X1 [Homo sapiens]

10	20	30	40	50	60	70	80
MFLMNASPVV	ALQSKWEAFG	PPGSCRFPRC	FSEADEGVES	ASVSARVQML	ISTLQRDGAA	RGTSDERAAQ	RGHRAEGCHD
90	100	110	120	130	140	150	160
ARPAAKPTVH	KEPPALAVCG	LVADFDPMGE	EETTDFGPLV	LDSDSDDSVD	RDIEEAIQEY	LKAKSGAAQP	GAGGAQPGAA
170	180	190	200	210	220	230	240
QPSRAAGGGS	RCKPEPAHGS	APTALCPPKL	VPGSGGGPGS	QVGSSKDQGS	ASPVSVSSDD	SFEQSIRAEI	EQFLNEKRQH
250	260	270	280	290	300	310	320
ETQKCDGSVE	KKPDTNENSA	KSLLKSHQEP	PTKVVHRQGL	LGVQKEFAFR	KPPRLAKMNV	QPRSLRSKVT	TTQENEGSTK
330	340	350	360	370	380	390	400
PATPCRPSEA	AQNKGGIKRS	ASAARRGKRV	MSAAQASEAS	DSSSDDGIEE	AIQLYQLQKT	RKEADGDLPQ	RVQLREERAP
410	420	430	440	450	460	470	480
DPPAHSTSSA	TKSALPETHR	KTPSKKKLVA	TKTMDPGPGG	LDTDHAPKLL	KETKAPPPAS	PASRSEFVER	SSCRADTSAE
490	500	510	520	530	540	550	560
LMCAEAILDI	SKTILPAPVE	GSDGSLSASP	LFYSPNVPSR	SDGDSSSVDS	DDSIEQEIRT	FLALKAQSGS	LLARGESCPQ
570	580	590	600	610	620	630	640
AAQGPLLPPG	LNSQTGGHKT	PLSKTPDPLL	GCKRKRRGGG	HVRPSTPKKM	QEVVKDGSQD	ADHSQGRAEP	GHERRDLPIQ
650	660	670	680	690	700	710	720
GKASEALGGE	GTARGPGDTR	MSQGQGKTDE	ARRLDEKESS	EDKSSSLDSD	EDLDTAIKDL	LRSKRKLKKR	CREPRAACRK
730	740	750	760	770	780	790	800
KVRFSTAQTH	FLEQLGGLRR	DWKDRGPPVL	KSCLSKSKRD	SGEGPGKKPP	SVFGSTAERM	RQEGAASQDA	ALAFRVRRPA
810	820	830	840	850	860	870	880
SASASEGNPF	PRESQGPAPS	PGSLSDDSSS	VDSNDSIELE	IRKFLAEKAK	ESVSSSEVQA	EGPTALGTGG	PARPEVLCRK
890	900	910	920	930	940	950	960
EPAPPPGVCT	RSQRARGVPH	LAEGLRGTES	AGAQGTAGLF	SQGGKGLPAA	PARGDPVPPR	STSGGVSAKG	LSVSRRNVYV
970	980	990	1000	1010	1020	1030	1040
HKDQSPRGAE	PAAKSAFGQL	PSCATAGTEA	GGARGTFHMG	CGSPSFLTPS	PGAERDAGAQ	ADRTPPWSDF	AHQSRLPSPW
1050	1060	1070	1080	1090	1100	1110	1120
VLRSEGRDAV	WRGGVGSERD	KGSEGPARGL	PSLPLAGFSP	LLSTQLFHFG	KGVSWGGRQA	GLFSPHLGLP	LQGPSFSAFR
1130	1140	1150	1160	1170	1180	1190	1200
EAQAGPSPVF	GSPHLLAKKD	GGPWPTRKAQ	AGLSLHDRRS	SGSEESILDL	RYRRRVNRDD	QEQDALGSDA	SDFSDTSTED
1210							
SGGSSVVKV							

Cmpd.	No. of Cmpds.	m/z meas.	Δ m/z [ppm]	z	Rt [min]	Score	P	Range	Sequence	Modification
1803	1	590.7798	-21.71	2	52.9	18.1	0	881-891	K.EPAPPPGVCTR.S	Carbamidomethyl: 9

Detailed Protein Report

Protein 579: PREDICTED: transmembrane 7 superfamily member 3 isoform X1 [Homo sapiens]

Accession: gi|530399194
Database: refseq_human(refseq_human_20140103.fasta)
Database Date: 2015-11-30
Modification(s): Oxidation

Score: 18.1
MW [kDa]: 58.7
pI: 6.4
Sequence Coverage [%]: 3.3
No. of unique Peptides: 1

10	20	30	40	50	60	70	80
MGFLQLLVVA	VLASEHRVAG	AAEVFGNSSE	GLIEFSVGKF	RYFELNRPFP	EEAILHDISS	NVTFLIFQIH	SQYQNTTVSF
**90**	**100**	**110**	**120**	**130**	**140**	**150**	**160**
SPTLLSNSSE	TGTASGLVFI	LRPEQSTCTW	YLGTSGIQPV	QNMAILLSYS	ERDPVPGGCN	LEFDLDIDPN	IYLEYNFFET
**170**	**180**	**190**	**200**	**210**	**220**	**230**	**240**
TIKFAPANLG	YARGVDPPPC	DAGTDQDSRW	RLQYDVYQYF	LPENDLTEEM	LLKHLQRMVS	VPQVKASALK	VVTLTANDKT
**250**	**260**	**270**	**280**	**290**	**300**	**310**	**320**
SVSFSSLPGQ	GVIYNVIVWD	PFLNTSAAYI	PAHTYACSFE	AGEGSCASLG	RVSSKVFFTL	FALLGFFICF	FGHRFWKTEL
**330**	**340**	**350**	**360**	**370**	**380**	**390**	**400**
FFIGFIIMGF	FFYILITRLT	PIKYDGNLKI	FHDDGVFWVT	FSCIAILIPV	VFMGCLRILN	ILTCGVIGSY	SVVLAIDSYW
**410**	**420**	**430**	**440**	**450**	**460**	**470**	**480**
STSLSYITLN	VLKRALNKDF	HRAFTNVPFQ	TNDFIILAVW	GMLAVSGITL	QIRRERGRPF	FPPHPYKLWK	QERERRVTNI
**490**	**500**	**510**	**520**				
LDPSYHIPPL	RERLYGRLTQ	IKGLFQKEQP	AGERTPLLL				

Cmpd.	No. of Cmpds.	m/z meas.	Δ m/z [ppm]	z	Rt [min]	Score	P	Range	Sequence	Modification
2409	1	954.4178	-134.28	2	58.8	18.1	2	214-230	K.HLQRMVSVPQVKASALK.V	Oxidation: 5

Detailed Protein Report

Protein 580: PREDICTED: putative L-type amino acid transporter 1-like protein MLAS-like [Homo sapiens]

**Accession:**	gi|578846041	**Score:**	18.1
**Database:**	refseq_human(refseq_human_20140103.fasta)	**MW [kDa]:**	23.1
**Database Date:**	2015-11-30	**pI:**	8.9
		**Sequence Coverage [%]:**	2.7
		**No. of unique Peptides:**	1

```
        10         20         30         40         50         60         70         80
MAGAGPKRRA LAAPVAEEKE EAREKMLASK RADGAAPAGE GEGVTLQRNI TLLNGVAIIV GAIIGSGIFV TPTGVLKEAG
        90        100        110        120        130        140        150        160
SPGLALVMWA ACGVFSIVGA LCYAELGTTI SKSGGDYAYM LDVYGSLPAF LKLWIELLVI RPSSQYIVAL VFATYLLKPL
       170        180        190        200        210        220        230
FPSCPVPEEA AKLMACHCVR EYGARAGGWE VVVPGSLHPC ITTPLAPTSL HFCVPGPSRQ F
```

Cmpd.	No. of Cmpds.	m/z meas.	Δ m/z [ppm]	z	Rt [min]	Score	P	Range	Sequence	Modification
1084	1	705.1875	-311.96	1	42.0	18.1	1	26-31	K.MLASKR.A	

Detailed Protein Report

Protein 581: PREDICTED: protein DBF4 homolog B isoform X7 [Homo sapiens]

Accession: gi|578831452
Database: refseq_human(refseq_human_20140103.fasta)
Database Date: 2015-11-30
Modification(s): Carbamidomethyl

Score: 18.1
MW [kDa]: 48.4
pI: 10.4
Sequence Coverage [%]: 2.0
No. of unique Peptides: 1

10	20	30	40	50	60	70	80
MSEPGKGDDC	LELESSMAES	RLRAPDLGVS	RCLGKCQKNS	PGARKHPFSG	KSFYLDLPAG	KNLQFLTGAI	QQLGGVIEGF
90	100	110	120	130	140	150	160
LSKEVSYIVS	SRREVKAESS	GKSHRGCPSP	SPSEVRVETS	AMVDPKGSHP	RPSRKPVDSV	PLSRGKELLQ	KAIRNQGSIS
170	180	190	200	210	220	230	240
GGGSGGSSSL	LTNARSWGVR	ILHVDEMMMH	VQQLSLASLC	VKKQQPKKPE	GTCPAAESRT	RKVARLKAPF	LKIEDESRKF
250	260	270	280	290	300	310	320
RPFHHQFKSF	PEISFLGPKD	ASPFEAPTTL	GSMHHTRESK	DGEPSPRSAA	HTMPRRKKGY	CECCQEAFEE	LHVHLQSAQH
330	340	350	360	370	380	390	400
RSFALEAHLY	AEVDRIIAQL	SHSFADIPFQ	AGLPSDPRQG	CGPAAMDRIT	RWCDGTSCKS	HMCECHNPPA	GLAQGLQGAG
410	420	430	440	450			
LPLSLPSLLY	PVSSGHFLGS	AAWGVVACRG	HAPPSLPRKL	LCPGGHSC			

Cmpd.	No. of Cmpds.	m/z meas.	Δ m/z [ppm]	z	Rt [min]	Score	P	Range	Sequence	Modification
1527	2	472.2297	42.07	2	49.3	18.1	0	440-448	K.LLCPGGHSC.-	Carbamidomethyl: 9

Detailed Protein Report

Protein 582: RING finger and transmembrane domain-containing protein 1 [Homo sapiens]

**Accession:**	gi|109134327	**Score:**	18.0
**Database:**	refseq_human(refseq_human_20140103.fasta)	**MW [kDa]:**	49.7
**Database Date:**	2015-11-30	**pI:**	9.8
**Modification(s):**	Oxidation	**Sequence Coverage [%]:**	5.5
		**No. of unique Peptides:**	1

10	20	30	40	50	60	70	80
MPLFLLSLPT	PPSASGHERR	QRPEAKTSGS	EKKYLRAMQA	NRSQLHSPPG	TGSSEDASTP	QCVHTRLTGE	GSCPHSGDVH
**90**	**100**	**110**	**120**	**130**	**140**	**150**	**160**
IQINSIPKEC	AENASSRNIR	SGVHSCAHGC	VHSRLRGHSH	SEARLTDDTA	AESGDHGSSS	FSEFRYLFKW	LQKSLPYILI
**170**	**180**	**190**	**200**	**210**	**220**	**230**	**240**
LSVKLVMQHI	TGISLGIGLL	TTFMYANKSI	VNQVFLRERS	SKIQCAWLLV	FLAGSSVLLY	YTFHSQSLYY	SLIFLNPTLD
**250**	**260**	**270**	**280**	**290**	**300**	**310**	**320**
HLSFWEVFWI	VGITDFILKF	FFMGLKCLIL	LVPSFIMPFK	SKGYWYMLLE	ELCQYYRTFV	PIPVWFRYLI	SYGEFGNVTR
**330**	**340**	**350**	**360**	**370**	**380**	**390**	**400**
WSLGILLALL	YLILKLLEFF	GHLRTFRQVL	RIFFTQPSYG	VAASKRQCSD	VDDICSICQA	EFQKPILLIC	QHIFCEECMT
**410**	**420**	**430**	**440**				
LWFNREKTCP	LCRTVISDHI	NKWKDGATSS	HLQIY				

Cmpd.	No. of Cmpds.	m/z meas.	Δ m/z [ppm]	z	Rt [min]	Score	P	Range	Sequence	Modification
241	1	879.7331	-87.01	3	33.0	18.0	0	165-188	K.LVMQHITGISLGIGLLTTFMYANK.S	Oxidation: 20

Detailed Protein Report

Protein 583: dysferlin isoform 9 [Homo sapiens]

**Accession:**	gi|195976754	**Score:**	17.9
**Database:**	refseq_human(refseq_human_20140103.fasta)	**MW [kDa]:**	235.8
**Database Date:**	2015-11-30	**pI:**	5.4
**Modification(s):**	Carbamidomethyl	**Sequence Coverage [%]:**	0.8
		**No. of unique Peptides:**	1

Detailed Protein Report

10	20	30	40	50	60	70	80
MLRVFILYAE	NVHTPDTDIS	DAYCSAVFAG	VKKRTKVIKN	SVNPVWNEGF	EWDLKGIPLD	QGSELHVVVK	DHETMGRNRF
90	100	110	120	130	140	150	160
LGEAKVPLRE	VLATPSLSAS	FNAPLLDTKK	QPTGASLVLQ	VSYTPLPGAV	PLFPPPTPLE	PSPTLPDLDV	VADTGGEEDT
170	180	190	200	210	220	230	240
EDQGLTGDEA	EPFLDQSGGP	GAPTTPRKLP	SRPPPHYPGI	KRKRSAPTSR	KLLSDKPQDF	QIRVQVIEGR	QLPGVNIKPV
250	260	270	280	290	300	310	320
VKVTAAGQTK	RTRIHKGNSP	LFNETLFFNL	FDSPGELFDE	PIFITVVDSR	SLRTDALLGE	FRMDVGTIYR	EPRHAYLRKW
330	340	350	360	370	380	390	400
LLLSDPDDFS	AGARGYLKTS	LCVLGPGDEA	PLERKDPSED	KEDIESNLLR	PTGVALRGAH	FCLKVFRAED	LPQMDDAVMD
410	420	430	440	450	460	470	480
NVKQIFGFES	NKKNLVDPFV	EVSFAGKMLC	SKILEKTANP	QWNQNITLPA	MFPSMCEKMR	IRIIDWDRLT	HNDIVATTYL
490	500	510	520	530	540	550	560
SMSKISAPGG	EIEVDDYLGF	LPTFGPCYIN	LYGSPREFTG	FPDPYTELNT	GKGEGVAYRG	RLLLSLETKL	VEHSEQKVED
570	580	590	600	610	620	630	640
LPADDILRVE	KYLRRRKYSL	FAAFYSATML	QDVDDAIQFE	VSIGNYGNKF	DMTCLPLAST	TQYSRAVFDG	CHYYYLPWGN
650	660	670	680	690	700	710	720
VKPVVVLSSY	WEDISHRIET	QNQLLGIADR	LEAGLEQVHL	ALKAQCSTED	VDSLVAQLTD	ELIAGCSQPL	GDIHETPSAT
730	740	750	760	770	780	790	800
HLDQYLYQLR	THHLSQITEA	ALALKLGHSE	LPAALEQAED	WLLRLRALAE	EPQNSLPDIV	IWMLQGDKRV	AYQRVPAHQV
810	820	830	840	850	860	870	880
LFSRRGANYC	GKNCGKLQTI	FLKYPMEKVP	GARMPVQIRV	KLWFGLSVDE	KEFNQFAEGK	LSVFAETYEN	ETKLALVGNW
890	900	910	920	930	940	950	960
GTTGLTYPKF	SDVTGKIKLP	KDSFRPSAGW	TWAGDWFVCP	EKTLLHDMDA	GHLSFVEEVF	ENQTRLPGGQ	WIYMSDNYTD
970	980	990	1000	1010	1020	1030	1040
VNGEKVLPKD	DIECPLGWKW	EDEEWSTDLN	RAVDEQGWEY	SITIPPERKP	KHWVPAEKMY	YTHRRRRWVR	LRRRDLSQME
1050	1060	1070	1080	1090	1100	1110	1120
ALKRHRQAEA	EGEGWEYASL	FGWKFHLEYR	KTDAFRRRRW	RRRMEPLEKT	GPAAVFALEG	ALGGVMDDKS	EDSMSVSTLS
1130	1140	1150	1160	1170	1180	1190	1200
FGVNRPTISC	IFDYGNRYHL	RCYMYQARDL	AAMDKDSFSD	PYAIVSFLHQ	SQKTVVVKNT	LNPTWDQTLI	FYEIEIFGEP
1210	1220	1230	1240	1250	1260	1270	1280
ATVAEQPPSI	VVELYDHDTY	GADEFMGRCI	CQPSLERMPR	LAWFPLTRGS	QPSGELLASF	ELIQREKPAI	HHIPGFEVQE
1290	1300	1310	1320	1330	1340	1350	1360
TSRILDESED	TDLPYPPPQR	EANIYMVPQN	IKPALQRTAI	EILAWGLRNM	KSYQLANISS	PSLVVECGGQ	TVQSCVIRNL
1370	1380	1390	1400	1410	1420	1430	1440
RKNPNFDICT	LFMEVMLPRE	ELYCPPITVK	VIDNRQFGRR	PVVGQCTIRS	LESFLCDPYS	AESPSPQGGP	DDVSLLSPGE
1450	1460	1470	1480	1490	1500	1510	1520
DVLIDIDDKE	PLIPIQEEEF	IDWWSKFFAS	IGEREKCGSY	LEKDFDTLKV	YDTQLENVEA	FEGLSDFCNT	FKLYRGKTQE
1530	1540	1550	1560	1570	1580	1590	1600
ETEDPSVIGE	FKGLFKIYPL	PEDPAIPMPP	RQFHQLAAQG	PQECLVRIYI	VRAFGLQPKD	PNGKCDPYIK	ISIGKKSVSD
1610	1620	1630	1640	1650	1660	1670	1680
QDNYIPCTLE	PVFGKMFELT	CTLPLEKDLK	ITLYDYDLLS	KDEKIGETVV	DLENRLLSKF	GARCGLPQTY	CVSGPNQWRD
1690	1700	1710	1720	1730	1740	1750	1760
QLRPSQLLHL	FCQQHRVKAP	VYRTDRVMFQ	DKEYSIEEIE	AGRIPNPHLG	PVEERLALHV	LQQQGLVPEH	VESRPLYSPL
1770	1780	1790	1800	1810	1820	1830	1840
QPDIEQGKLQ	MWVDLFPKAL	GRPGPPFNIT	PRRARRFFLR	CIIWNTRDVI	LDDLSLTGEK	MSDIYVKGWM	IGFEEHKQKT
1850	1860	1870	1880	1890	1900	1910	1920
DVHYRSLGGE	GNFNWRFIFP	FDYLPAEQVC	TIAKKDAFWR	LDKTESKIPA	RVVFQIWDND	KFSFDDFLGS	LQLDLNRMPK
1930	1940	1950	1960	1970	1980	1990	2000
PAKTAKKCSL	DQLDDAFHPE	WFVSLFEQKT	VKGWWPCVAE	EGEKKILAGK	LEMTLEIVAE	SEHEERPAGQ	GRDEPNMNPK
2010	2020	2030	2040	2050	2060	2070	
LEDPRRPDTS	FLWFTSPYKT	MKFILWRRFR	WAIILFIILF	ILLLFLAIFI	YAFPNYAAMK	LVKPFS	

Cmpd.	No. of Cmpds.	m/z meas.	Δ m/z [ppm]	z	Rt [min]	Score	P	Range	Sequence	Modification

Detailed Protein Report

Cmpd.	No. of Cmpds.	m/z meas.	Δ m/z [ppm]	z	Rt [min]	Score	P	Range	Sequence	Modification
2537	1	941.5642	96.79	2	62.3	17.9	0	1552-1567	R.QFHQLAAQGPQECLVR.I	Carbamidomethyl: 13

Detailed Protein Report

Protein 584: **PREDICTED: SCAN domain-containing protein 3 isoform X1 [Homo sapiens]**

**Accession:**	gi|578840800	**Score:**	17.9
**Database:**	refseq_human(refseq_human_20140103.fasta)	**MW [kDa]:**	133.8
**Database Date:**	2015-11-30	**pI:**	7.5
**Modification(s):**	Oxidation	**Sequence Coverage [%]:**	1.4
		**No. of unique Peptides:**	1

10	20	30	40	50	60	70	80
MEEMIPLDSA	KESLGTQLQS	MEDRMECESP	EPHPLQDNGS	FLWFSMMSQS	MGGDNLSSLD	TNEAEIEPEN	MREKFFRSLA
90	100	110	120	130	140	150	160
RLLENKSNNT	KIFSKAKYCQ	LIKEVKEAKA	KAKKESVDYR	RLARFDVILV	QGNEKLIEAV	NGETDKIRYY	LHSEDLFDIL
170	180	190	200	210	220	230	240
HNTHLSIGHG	GRTRMEKELQ	AKYKNITKEV	IMLYLTLCKP	CQQKNSKLKK	VLTSKSIKEV	SSRCQVDLID	MQLNPDGEYR
250	260	270	280	290	300	310	320
FILHYQDLCT	KLTFLRSLKS	KRPTEVAHAL	LDIFTIIGAP	SVLQSDNGRE	FSSQVVSELS	NIWPELKIVH	GKSQTCQSQS
330	340	350	360	370	380	390	400
SAEQTEDIRK	RIFSWMQTNN	SSHWTEFLWF	IQMSQNQPYH	RSMQQTPCES	AFSSEAKLGL	SHSQLTEELV	ASLHTENELD
410	420	430	440	450	460	470	480
QADKELENTL	RAQYEENIET	GTDSSDIEEN	LSVTPKVAEK	SPPESRLRFL	SCVVCEKECT	GVNSCISCDG	NIHAICGVPS
490	500	510	520	530	540	550	560
QHGTEGCGRQ	ITCSLCYETS	TMKRKHDEIQ	RSLPVKPSKM	LKPSGTPFSP	DKVGDWMAKQ	ASLDFFVKKR	HAFSEHSSSN
570	580	590	600	610	620	630	640
KRNVNNRSYP	EEGKTKRVHA	SFTRKYDPSY	IEFGFVAVID	GEVLKPQCII	CGDVLANEAM	KPSKLKRHLY	SKHKEISSQP
650	660	670	680	690	700	710	720
KEFFERKSSE	LKSQPKQVFN	VSHINISALR	ASYKVALPVA	KSKTPYTIAE	TLVKDCIKEV	CLEMLGESAA	KKVAQVPLSN
730	740	750	760	770	780	790	800
DTIARRIQEL	ANDMEDQLIE	QIKLAKYFSL	QLDECRDIAN	MIILLVYVRF	EHDDDIKEEF	FFSASLPTNT	TSSELYEAVK
810	820	830	840	850	860	870	880
NYIVNKCGLE	FKFCVGVCSD	GAASMTGKHS	EVVTQIKELA	PECKTTHCFI	HRESLAMKKI	SAELNSVLND	IVKIVNYIKS
890	900	910	920	930	940	950	960
NSLNSRLFSL	LCDNMEADHK	QLLLHAEIRW	LSRGKVLSRM	FEIRNELLVF	LQGKKPMWSQ	LFKDVNWTAR	LAYLSDIFSI
970	980	990	1000	1010	1020	1030	1040
FNDLNASMQG	KNATYFSMAD	KVEGQKQKLE	AWKNRISTDC	YDMFHNLTTI	INEVGNDLDI	AHLRKVISEH	LTNLLECFEF
1050	1060	1070	1080	1090	1100	1110	1120
YFPSKEDPRI	GNLWIQNPFL	SSKDNLNLTV	TLQDKLLKLA	TDEGLKISFE	NTASLPSFWI	KAKNDYPELA	EIALKLLLLF
1130	1140	1150	1160	1170	1180		
PSTYLCETGF	STLSVIKTKH	RNSLNIHYPL	RVALSSIQPR	LDKLTSKKQA	HLSH		

Cmpd.	No. of Cmpds.	m/z meas.	Δ m/z [ppm]	z	Rt [min]	Score	P	Range	Sequence	Modification
1614	1	873.9372	77.88	2	48.7	17.9	0	362-377	R.SMQQTPCESAFSSEAK.L	Oxidation: 2

Detailed Protein Report

Protein 585: nicotinate phosphoribosyltransferase isoform 2 [Homo sapiens]

Accession: gi|558472750
Database: refseq_human(refseq_human_20140103.fasta)
Database Date: 2015-11-30

Score: 17.8
MW [kDa]: 56.1
pI: 5.4
Sequence Coverage [%]: 1.9
No. of unique Peptides: 1

10	20	30	40	50	60	70	80
MAAEQDPEAR	AAARPLLTDL	YQATMALGYW	RAGRARDAAE	FELFFRRCPF	GGAFALAAGL	RDCVRFLRAF	RLRDADVQFL
**90**	**100**	**110**	**120**	**130**	**140**	**150**	**160**
ASVLPPDTDP	AFFEHLRALD	CSEVTVRALP	EGSLAFPGVP	LLQVSGPLLV	VQLLETPLLC	LVSYASLVAT	NAARLRLIAG
**170**	**180**	**190**	**200**	**210**	**220**	**230**	**240**
PEKRLLEMGL	RRAQGPDGGL	TASTYSYLGG	FDSSSNVLAG	QLRGVPVAGT	LAHSFVTSFS	GSEVPPDPML	APAAGEGPGV
**250**	**260**	**270**	**280**	**290**	**300**	**310**	**320**
DLAAKAQVWL	EQVCAHLGLG	VQEPHPGERA	AFVAYALAFP	RAFQGLLDTY	SVWRSGLPNF	LAVALALGEL	GYRAVGVRLD
**330**	**340**	**350**	**360**	**370**	**380**	**390**	**400**
SGDLLQQAQE	IRKVFRAAAA	QFQVPWLESV	LIVVSNNIDE	EALARLAQEG	SEVNVIGIGT	SVVTCPQQPS	LGGVYKLVAV
**410**	**420**	**430**	**440**	**450**	**460**	**470**	**480**
GGQPRMKLTE	DPEKQTLPGS	KAAFRLLGSD	GSPLMDMLQL	AEEPVPQAGQ	ELRVWPPGAQ	EPCTVRPAQL	CEPLPSLAES
**490**	**500**	**510**	**520**	**530**			
RALAQLSLSR	LSPEHRRLRS	PAQYQVVLSE	RLQALVNSLC	AGQSP			

Cmpd.	No. of Cmpds.	m/z meas.	Δ m/z [ppm]	z	Rt [min]	Score	P	Range	Sequence	Modification
1733	1	576.7624	-176.53	2	52.0	17.8	2	155-164	R.LRLIAGPEKR.L	

Detailed Protein Report

Protein 586: mesencephalic astrocyte-derived neurotrophic factor precursor [Homo sapiens]

**Accession:**	gi|299523086	**Score:**	17.7
**Database:**	refseq_human(refseq_human_20140103.fasta)	**MW [kDa]:**	20.7
**Database Date:**	2015-11-30	**pI:**	9.8
		**Sequence Coverage [%]:**	10.4
		**No. of unique Peptides:**	1

```
        10         20         30         40         50         60         70         80
MRRMWATQGL AVALALSVLP GSRALRPGDC EVCISYLGRF YQDLKDRDVT FSPATIENEL IKFCREARGK ENRLCYYIGA
        90        100        110        120        130        140        150        160
TDDAATKIIN EVSKPLAHHI PVEKICEKLK KKDSQICELK YDKQIDLSTV DLKKLRVKEL KKILDDWGET CKGCAEKSDY
       170        180        190
IRKINELMPK YAPKAASART DL
```

Cmpd.	No. of Cmpds.	m/z meas.	Δ m/z [ppm]	z	Rt [min]	Score	P	Range	Sequence	Modification
2038	2	696.9685	-53.02	3	55.9	17.7	2	69-87	R.GKENRLCYYIGATDDAATK.I	

Detailed Protein Report

Protein 587: PREDICTED: uncharacterized protein LOC101930405 [Homo sapiens]

Accession: gi|530356052
Database: refseq_human(refseq_human_20140103.fasta)
Database Date: 2015-11-30
Modification(s): Oxidation

Score: 17.7
MW [kDa]: 34.1
pI: 10.4
Sequence Coverage [%]: 8.7
No. of unique Peptides: 1

10	20	30	40	50	60	70	80
MTHSRRQGRS	EPPWGTQDTR	GTQETRGTSG	PSGLQGALRT	LGNTQNHQEA	LRTTRGPSGH	QSVLRTSGGA	QDLQRVIRTP
90	100	110	120	130	140	150	160
EGAQNTRSCL	RNFWMKTSPP	LTLLLWERSP	SLGIPKCLHS	VIRTEHTGLT	REFVLSWVFL	VAILKCVQCE	DQLVESGGGL
170	180	190	200	210	220	230	240
VQPGGSLRPS	CAASGFAFSS	YVLHWVRRAP	GKGPEWVSAI	GTGGDTYYAD	SVMGRFTISR	DNAKKSLYLQ	MNSLIAEDMA
250	260	270	280	290	300	310	
VYYCARDTVR	GSQSPSSVLC	GDYLGTNVDN	RSEGDELAWT	KWDVDVKFIL	CCTNNHRFNF	LTSSIRKRV	

Cmpd.	No. of Cmpds.	m/z meas.	Δ m/z [ppm]	z	Rt [min]	Score	P	Range	Sequence	Modification
2764	1	920.1235	28.38	3	65.7	17.7	1	189-215	R.APGKGPEWVSAIGTGGDTYYADSVMGR.F	Oxidation: 25

Detailed Protein Report

Protein 588: PREDICTED: transcription regulator protein BACH1 isoform X4 [Homo sapiens]

**Accession:**	gi|578836626	**Score:**	17.7
**Database:**	refseq_human(refseq_human_20140103.fasta)	**MW [kDa]:**	69.8
**Database Date:**	2015-11-30	**pI:**	4.9
**Modification(s):**	Carbamidomethyl, Oxidation	**Sequence Coverage [%]:**	2.6
		**No. of unique Peptides:**	1

10	20	30	40	50	60	70	80
MSLSENSVFA	YESSVHSTNV	LLSLNDQRKK	DVLCDVTIFV	EGQRFRAHRS	VLAACSSYFH	SRIVGQADGE	LNITLPEEVT
**90**	**100**	**110**	**120**	**130**	**140**	**150**	**160**
VKGFEPLIQF	AYTAKLILSK	ENVDEVCKCV	EFLSVHNIEE	SCFQFLKFKF	LDSTADQQEC	PRKKCFSSHC	QKTDLKLSLL
**170**	**180**	**190**	**200**	**210**	**220**	**230**	**240**
DQRDLETDEV	EEFLENKNVQ	TPQCKLRRYQ	GNAKASPPLQ	DSASQTYESM	CLEKDAALAL	PSLCPKYRKF	QKAFGTDRVR
**250**	**260**	**270**	**280**	**290**	**300**	**310**	**320**
TGESSVKDIH	ASVQPNERSE	NECLGGVPEC	RDLQVMLKCD	ESKLAMEPEE	TKKDPASQCP	TEKSEVTPFP	HNSSIDPHGL
**330**	**340**	**350**	**360**	**370**	**380**	**390**	**400**
YSLSLLHTYD	QYGDLNFAGM	QNTTVLTEKP	LSGTDVQEKT	FGESQDLPLK	SDLGTREDSS	VASSDRSSVE	REVAEHLAKG
**410**	**420**	**430**	**440**	**450**	**460**	**470**	**480**
FWSDICSTDT	PCQMQLSPAV	AKDGSEQISQ	KRSECPWLGI	RISESPEPGQ	RTFTTLSSVN	CPFISTLSTE	GCSSNLEIGN
**490**	**500**	**510**	**520**	**530**	**540**	**550**	**560**
DDYVSEPQQE	PCPYACVISL	GDDSETDTEG	DSESCSAREQ	ECEVKLPFNA	QRIISLSRND	FQSLLKMHKL	TPEQLDCIHD
**570**	**580**	**590**	**600**	**610**	**620**	**630**	
IRRRSKNRIA	AQRCRKRKLD	CIQNLESEIE	KLGSVENLMC	QEVFHTILRT	LDTCSDSSVS	AKQ	

Cmpd.	No. of Cmpds.	m/z meas.	Δ m/z [ppm]	z	Rt [min]	Score	P	Range	Sequence	Modification
1691	1	1011.6416	141.51	2	51.4	17.7	1	547-562	K.MHKLTPEQLDCIHDIR.R	Carbamidomethyl: 11; Oxidation: 1

Detailed Protein Report

Protein 589: aldo-keto reductase family 1 member C2 isoform 2 [Homo sapiens]

Accession: gi|207028673
Database: refseq_human(refseq_human_20140103.fasta)
Database Date: 2015-11-30

Score: 17.7
MW [kDa]: 15.7
pI: 8.9
Sequence Coverage [%]: 17.3
No. of unique Peptides: 1

10	20	30	40	50	60	70	80
MDSKYQCVKL	NDGHFMPVLG	FGTYAPAEVP	KSKALEAVKL	AIEAGFHHID	SAHVYNNEEQ	VGLAIRSKIA	DGSVKREDIF
**90**	**100**	**110**	**120**	**130**	**140**		
YTSKLWSNSH	RPELVRPALE	RSLKNLQLDY	VDLYLIHFPV	SVKEDIGILT	WKKSPKHNS		

Cmpd.	No. of Cmpds.	m/z meas.	Δ m/z [ppm]	z	Rt [min]	Score	P	Range	Sequence	Modification
2935	1	859.1202	17.35	3	65.9	17.7	1	10-33	K.LNDGHFMPVLGFGTYAPAEVPKSK.A	

Detailed Protein Report

Protein 590: potassium channel subfamily K member 10 isoform 2 [Homo sapiens]

**Accession:**	gi|20143944	**Score:**	17.6
**Database:**	refseq_human(refseq_human_20140103.fasta)	**MW [kDa]:**	60.3
**Database Date:**	2015-11-30	**pI:**	9.8
		**Sequence Coverage [%]:**	1.5
		**No. of unique Peptides:**	1

10	20	30	40	50	60	70	80
MKFPIETPRK	QVNWDPKVAV	PAAAPVCQPK	SATNGQPPAP	APTPTPRLSI	SSRATVVARM	EGTSQGGLQT	VMKWKTVVAI
**90**	**100**	**110**	**120**	**130**	**140**	**150**	**160**
FVVVVVYLVT	GGLVFRALEQ	PFESSQKNTI	ALEKAEFLRD	HVCVSPQELE	TLIQHALDAD	NAGVSPIGNS	SNNSSHWDLG
**170**	**180**	**190**	**200**	**210**	**220**	**230**	**240**
SAFFFAGTVI	TTIGYGNIAP	STEGGKIFCI	LYAIFGIPLF	GFLLAGIGDQ	LGTIFGKSIA	RVEKVFRKKQ	VSQTKIRVIS
**250**	**260**	**270**	**280**	**290**	**300**	**310**	**320**
TILFILAGCI	VFVTIPAVIF	KYIEGWTALE	SIYFVVVTLT	TVGFGDFVAG	GNAGINYREW	YKPLVWFWIL	VGLAYFAAVL
**330**	**340**	**350**	**360**	**370**	**380**	**390**	**400**
SMIGDWLRVL	SKKTKEEVGE	IKAHAAEWKA	NVTAEFRETR	RRLSVEIHDK	LQRAATIRSM	ERRRLGLDQR	AHSLDMLSPE
**410**	**420**	**430**	**440**	**450**	**460**	**470**	**480**
KRSVFAALDT	GRFKASSQES	INNRPNNLRL	KGPEQLNKHG	QGASEDNIIN	KFGSTSRLTK	RKNKDLKKTL	PEDVQKIYKT
**490**	**500**	**510**	**520**	**530**	**540**	**550**	
FRNYSLDEEK	KEEETEKMCN	SDNSSTAMLT	DCIQQHAELE	NGMIPTDTKD	REPENNSLLE	DRN	

Cmpd.	No. of Cmpds.	m/z meas.	Δ m/z [ppm]	z	Rt [min]	Score	P	Range	Sequence	Modification
1557	2	494.2527	-64.89	2	49.7	17.6	1	3-10	K.FPIETPRK.Q	

Detailed Protein Report

Protein 591: **PREDICTED: suppression of tumorigenicity 18 protein isoform X4 [Homo sapiens]**

**Accession:**	gi|578815754	**Score:**	17.6
**Database:**	refseq_human(refseq_human_20140103.fasta)	**MW [kDa]:**	106.1
**Database Date:**	2015-11-30	**pI:**	5.8
**Modification(s):**	Oxidation	**Sequence Coverage [%]:**	1.0
		**No. of unique Peptides:**	1

10	20	30	40	50	60	70	80
MDAEAEDKTL	RTRSKGTEVP	MDSLIQELSV	AYDCSMAKKR	TAEDQALGVP	VNKRKSLLMK	PRHYSPKADC	QEDRSDRTED
**90**	**100**	**110**	**120**	**130**	**140**	**150**	**160**
DGPLETHGHS	TAEEIMIKPM	DESLLSTAQE	NSSRKEDRYS	CYQELMVKSL	MHLGKFEKNV	SVQTVSENLN	DSGIQSLKAE
**170**	**180**	**190**	**200**	**210**	**220**	**230**	**240**
SDEADECFLI	HSDDGRDKID	DSQPPFCSSD	DNESNSESAE	NGWDSGSNFS	EETKPPRVPK	YVLTDHKKDL	LEVPEIKTEG
**250**	**260**	**270**	**280**	**290**	**300**	**310**	**320**
DKFIPCENRC	DSETERKDPQ	NALAEPLDGN	AQPSFPDVEE	EDSESLAVMT	EEGSDLEKAK	GNLSLLEQAI	ALQAERGCVF
**330**	**340**	**350**	**360**	**370**	**380**	**390**	**400**
HNTYKELDRF	LLEHLAGERR	QTKVIDMGGR	QIFNNKHSPR	PEKRETKCPI	PGCDGTGHVT	GLYPHHRSLS	GCPHKVRVPL
**410**	**420**	**430**	**440**	**450**	**460**	**470**	**480**
EILAMHENVL	KCPTPGCTGR	GHVNSNRNTH	RSLSGCPIAA	AEKLAMSQDK	NQLDSPQTGQ	CPDQAHRTSL	VKQIEFNFPS
**490**	**500**	**510**	**520**	**530**	**540**	**550**	**560**
QAITSPRATV	SKEQEKFGKV	PFDYASFDAQ	VFGKRPLIQT	VQGRKTPPFP	ESKHFPNPVK	FPNRLPSAGA	HTQSPGRASS
**570**	**580**	**590**	**600**	**610**	**620**	**630**	**640**
YSYGQCSEDT	HIAAAAAILN	LSTRCREATD	ILSNKPQSLH	AKKDPVSSLE	NLEEKKFPGE	ASIPSPKPKL	HARDLKKELI
**650**	**660**	**670**	**680**	**690**	**700**	**710**	**720**
TCPTPGCDGS	GHVTGNYASH	RSVSGCPLAD	KTLKSLMAAN	SQELKCPTPG	CDGSGHVTGN	YASHRSLSGC	PRARKGGVKM
**730**	**740**	**750**	**760**	**770**	**780**	**790**	**800**
TPTKEEKEDP	ELKCPVIGCD	GQGHISGKYT	SHRTASGCPL	AAKRQKENPL	NGASLSWKLN	KQELPHCPLP	GCNGLGHVNN
**810**	**820**	**830**	**840**	**850**	**860**	**870**	**880**
VFVTHRSLSG	CPLNAQVIKK	GKVSEELMTI	KLKATGGIES	DEEIRHLDEE	IKELNESNLK	IEADMMKLQT	QITSMESNLK
**890**	**900**	**910**	**920**	**930**	**940**	**950**	**960**
TIEEENKLIE	QNNESLLKEL	AGLSQALISS	LADIQLPQMG	PISEQNFEAY	VNTLTDMYSN	LERDYSPECK	ALLESIKQAV
**970**							
KGIHV							

Cmpd.	No. of Cmpds.	m/z meas.	Δ m/z [ppm]	z	Rt [min]	Score	P	Range	Sequence	Modification
1743	1	560.7234	-123.45	2	52.1	17.6	1	341-350	R.QTKVIDMGGR.Q	Oxidation: 7

Detailed Protein Report

Protein 592: differential display clone 8 isoform 1 [Homo sapiens]

**Accession:**	gi|343780900	**Score:**	17.4
**Database:**	refseq_human(refseq_human_20140103.fasta)	**MW [kDa]:**	63.6
**Database Date:**	2015-11-30	**pI:**	10.5
		**Sequence Coverage [%]:**	2.5
		**No. of unique Peptides:**	1

Alias proteins:

Accession	Name	Description
gi|530411299	refseq_human (refseq_human_20140103.fasta)	PREDICTED: differential display clone 8 isoform X1 [Homo sapiens]

10	20	30	40	50	60	70	80
MDGAMWLSLC	PDNEDLLWRK	KHKLLQARGK	GDLALQRRAD	AKLWKNYQLQ	RLAEELRRGY	QEAQHLHVGG	LDRLQSARLL
**90**	**100**	**110**	**120**	**130**	**140**	**150**	**160**
GWGGGRAREN	EPDSQGPIQR	RSARPPRAKE	KHRAALSEER	SCREELGQQH	PRHSRPRKTA	ASPEKPQTTK	ATGRMNSHLA
**170**	**180**	**190**	**200**	**210**	**220**	**230**	**240**
PPEKRKGRPE	PSTKSGGGRC	AIHPRRSKGA	DLERSNPLVA	AVGEIGLVEE	KEKGTARAGR	RQLGKGAVCF	VPALTSRSQG
**250**	**260**	**270**	**280**	**290**	**300**	**310**	**320**
QSLEGKLRDL	GQLWPADSSC	RREAVSPASQ	CTLREKNKWQ	KELELAFEEL	FNINRKLKKH	LCLYLALKPR	MDQRPGEGHA
**330**	**340**	**350**	**360**	**370**	**380**	**390**	**400**
FSEMQECGAG	TPRGKKMADP	EMLPAGEPRS	PAEEEAQQAA	SKTDLKTFMG	KAQNQKYQGT	VKPTFRNGSQ	TLSPEAGIFI
**410**	**420**	**430**	**440**	**450**	**460**	**470**	**480**
NKEDSLLYST	ESGQETPKLG	TLAEGSLQLH	LQDQADRVGS	TASRQRQKAE	MEQRRQKQLE	SLEQMEHPDM	SLEIHYKAEL
**490**	**500**	**510**	**520**	**530**	**540**	**550**	**560**
EKERREQRRA	RLAHLKSSST	RAQERERGSE	LSTTSPSGTS	LADDDRHSQM	IRDQQQQILQ	QNRLHKQFLE	EARKCLREFQ
**570**							
NIC							

Cmpd.	No. of Cmpds.	m/z meas.	Δ m/z [ppm]	z	Rt [min]	Score	P	Range	Sequence	Modification
2733	1	848.4051	-142.97	2	65.3	17.4	2	297-310	K.LKKHLCLYLALKPR.M	

Detailed Protein Report

Protein 593: uncharacterized protein KIAA0930 isoform b [Homo sapiens]

**Accession:**	gi|57863295	**Score:**	17.2
**Database:**	refseq_human(refseq_human_20140103.fasta)	**MW [kDa]:**	45.8
**Database Date:**	2015-11-30	**pI:**	9.0
		**Sequence Coverage [%]:**	5.9
		**No. of unique Peptides:**	1

Quantitation

QD:QU **Median:** 1.83 **CV:** 0.00 % **No. of Peptides:** 1

10	20	30	40	50	60	70	80
MLRAIAEERG	RLSLRREVCG	LGCFKDDRIV	FWTWMFSTYF	MEKWAPRQDD	MLFYVRRKLA	YSGSESGADG	RKAAEPEVEV
**90**	**100**	**110**	**120**	**130**	**140**	**150**	**160**
EVYRRDSKKL	PGLGDPDIDW	EESVCLNLIL	QKLDYMVTCA	VCTRADGGDI	HIHKKKSQQV	FASPSKHPMD	SKGEESKISY
**170**	**180**	**190**	**200**	**210**	**220**	**230**	**240**
PNIFFMIDSF	EEVFSDMTVG	EGEMVCVELV	ASDKTNTFQG	VIFQGSIRYE	ALKKVYDNRV	SVAARMAQKM	SFGFYKYSNM
**250**	**260**	**270**	**280**	**290**	**300**	**310**	**320**
EFVRMKGPQG	KGHAEMAVSR	VSTGDTSPCG	TEEDSSPASP	MHERVTSFST	PPTPERNNRP	AFFSPSLKRK	VPRNRIAEMK
**330**	**340**	**350**	**360**	**370**	**380**	**390**	**400**
KSHSANDSEE	FFREDDGGAD	LHNATNLRSR	SLSGTGRSLV	GSWLKLNRAD	GNFLLYAHLT	YVTLPLHRIL	TDILEVRQKP
**410**							
ILMT							

Cmpd.	No. of Cmpds.	m/z meas.	Δ m/z [ppm]	z	Rt [min]	Score	P	Range	Sequence	Modification	Ratios
169	1	826.3632	22.55	3	30.9	17.2	0	261-284	R. VSTGDTSPCGTEEDSSPASPMHE V		QD:QU 1.83

Detailed Protein Report

Protein 594: histone demethylase UTY isoform 1 [Homo sapiens]

**Accession:**	gi|33188429	**Score:**	17.2
**Database:**	refseq_human(refseq_human_20140103.fasta)	**MW [kDa]:**	118.0
**Database Date:**	2015-11-30	**pI:**	9.1
**Modification(s):**	Carbamidomethyl, Oxidation	**Sequence Coverage [%]:**	1.9
		**No. of unique Peptides:**	1

10	20	30	40	50	60	70	80
MKSCAVSLTT	AAVAFGDEAK	KMAEGKASRE	SEEESVSLTV	EEREALGGMD	SRLFGFVRLH	EDGARTKTLL	GKAVRCYESL
**90**	**100**	**110**	**120**	**130**	**140**	**150**	**160**
ILKAEGKVES	DFFCQLGHFN	LLLEDYSKAL	SAYQRYYSLQ	ADYWKNAAFL	YGLGLVYFYY	NAFHWAIKAF	QDVLYVDPSF
**170**	**180**	**190**	**200**	**210**	**220**	**230**	**240**
CRAKEIHLRL	GLMFKVNTDY	KSSLKHFQLA	LIDCNPCTLS	NAEIQFHIAH	LYETQRKYHS	AKEAYEQLLQ	TENLPAQVKA
**250**	**260**	**270**	**280**	**290**	**300**	**310**	**320**
TVLQQLGWMH	HNMDLVGDKA	TKESYAIQYL	QKSLEADPNS	GQSWYFLGRC	YSSIGKVQDA	FISYRQSIDK	SEASADTWCS
**330**	**340**	**350**	**360**	**370**	**380**	**390**	**400**
IGVLYQQQNQ	PMDALQAYIC	AVQLDHGHAA	AWMDLGTLYE	SCNQPQDAIK	CYLNAARSKR	CSNTSTLAAR	IKFLQNGSDN
**410**	**420**	**430**	**440**	**450**	**460**	**470**	**480**
WNGGQSLSHH	PVQQVYSLCL	TPQKLQHLEQ	LRANRDNLNP	AQKHQLEQLE	SQFVLMQQMR	HKEVAQVRTT	GIHNGAITDS
**490**	**500**	**510**	**520**	**530**	**540**	**550**	**560**
SLPTNSVSNR	QPHGALTRVS	SVSQPGVRPA	CVEKLLSSGA	FSAGCIPCGT	SKILGSTDTI	LLGSNCIAGS	ESNGNVPYLQ
**570**	**580**	**590**	**600**	**610**	**620**	**630**	**640**
QNTHTLPHNH	TDLNSSTEEP	WRKQLSNSAQ	GLHKSQSSCL	SGPNEEQPLF	STGSAQYHQA	TSTGIKKANE	HLTLPSNSVP
**650**	**660**	**670**	**680**	**690**	**700**	**710**	**720**
QGDADSHLSC	HTATSGGQQG	IMFTKESKPS	KNRSLVPETS	RHTGDTSNGC	ADVKGLSNHV	HQLIADAVSS	PNHGDSPNLL
**730**	**740**	**750**	**760**	**770**	**780**	**790**	**800**
IADNPQLSAL	LIGKANGNVG	TGTCDKVNNI	HPAVHTKTDH	SVASSPSSAI	STATPSPKST	EQRSINSVTS	LNSPHSGLHT
**810**	**820**	**830**	**840**	**850**	**860**	**870**	**880**
VNGEGLGKSQ	SSTKVDLPLA	SHRSTSQILP	SMSVSICPSS	TEVLKACRNP	GKNGLSNSCI	LLDKCPPPRP	PTSPYPPLPK
**890**	**900**	**910**	**920**	**930**	**940**	**950**	**960**
DKLNPPTPSI	YLENKRDAFF	PPLHQFCTNP	KNPVTVIRGL	AGALKLDLGL	FSTKTLVEAN	NEHMVEVRTQ	LLQPADENWD
**970**	**980**	**990**	**1000**	**1010**	**1020**	**1030**	**1040**
PTGTKKIWRC	ESNRSHTTIA	KYAQYQASSF	QESLRAGMQW	CDLSSLQPPP	PGFKRFSHLS	LPNSWNYRHL	PSCPTNFCIF
**1050**	**1060**	**1070**	**1080**				
VETGFHHVGQ	ACLELLTSGG	LLASASQSAG	ITGVSHHAR				

Cmpd.	No. of Cmpds.	m/z meas.	Δ m/z [ppm]	z	Rt [min]	Score	P	Range	Sequence	Modification
1890	1	1036.8846	-114.40	2	54.0	17.2	1	1-20	-.MKSCAVSLTTAAVAFGDEAK.K	Carbamidomethyl: 4; Oxidation: 1

Detailed Protein Report

Protein 595: **PREDICTED: dephospho-CoA kinase domain-containing protein isoform X4 [Homo sapiens]**

Accession: gi|530412893
Database: refseq_human(refseq_human_20140103.fasta)
Database Date: 2015-11-30
Modification(s): Oxidation

Score: 17.2
MW [kDa]: 14.9
pI: 10.2
Sequence Coverage [%]: 9.1
No. of unique Peptides: 1

Quantitation

QD:QU **Median:** 0.43 **CV:** 0.00 % **No. of Peptides:** 1

Alias proteins:

Accession	Name	Description
gi|578831425	refseq_human (refseq_human_20140103.fasta)	PREDICTED: dephospho-CoA kinase domain-containing protein isoform X7 [Homo sapiens]
gi|578831423	refseq_human (refseq_human_20140103.fasta)	PREDICTED: dephospho-CoA kinase domain-containing protein isoform X6 [Homo sapiens]

10	20	30	40	50	60	70	80
MFLVGLTGGI	ASGKSSVIQV	FQQLGCAVID	VDVMARHVVQ	PGYPAHRRIV	EVFGTEVLLE	NGDINRKVLG	DLIFNQPDRR
**90**	**100**	**110**	**120**	**130**	**140**		
QLLNAITHPE	IRKEMMKETF	KYFLREPRTS	PRGKKHVPSA	LKEADSLMRR	DT		

Cmpd.	No. of Cmpds.	m/z meas.	Δ m/z [ppm]	z	Rt [min]	Score	P	Range	Sequence	Modification	Ratios
1	1	819.8504	-66.08	2	29.4	17.2	2	94-105	K.EMMKETFKYFLR.E	Oxidation: 2	QD:QU 0.43

Detailed Protein Report

Protein 596: probable RNA-binding protein 46 isoform 3 [Homo sapiens]

Accession: gi|461496493
Database: refseq_human(refseq_human_20140103.fasta)
Database Date: 2015-11-30
Modification(s): Carbamidomethyl

Score: 17.2
MW [kDa]: 53.2
pI: 6.9
Sequence Coverage [%]: 2.8
No. of unique Peptides: 1

Alias proteins:

Accession	Name	Description
gi|578808850	refseq_human (refseq_human_20140103.fasta)	PREDICTED: probable RNA-binding protein 46 isoform X2 [Homo sapiens]

```
        10         20         30         40         50         60         70         80
MNEENIDGTN GCSKVRTGIQ NEAALLALME KTGYNMVQEN GQRKFGGPPP GWEGPPPPRG CEVFVGKIPR DMYEDELVPV
        90        100        110        120        130        140        150        160
FERAGKIYEF RLMMEFSGEN RGYAFVMYTT KEEAQLAIRI LNNYEIRPGK FIGVCVSLDN CRLFIGAIPK EKKKEEILDE
       170        180        190        200        210        220        230        240
MKKVTEGVVD VIVYPSATDK TKNRGFAFVE YESHRAAAMA RRKLIPGTFQ LWGHTIQVDW ADPEKEVDEE TMQRVKVLYV
       250        260        270        280        290        300        310        320
RNLMISTTEE TIKAEFNKFK PGAVERVKKL RDYAFVHFFN REDAVAAMSV MNGKCIDGAS IEVTLAKPVN KENTWRQHLN
       330        340        350        360        370        380        390        400
GQISPNSENL IVFANKEESH PKTLGKLPTL PARLNGQHSP SPPEVERCTY PFYPGTKLTP ISMYSLKSNH FNSAVMHLDY
       410        420        430        440        450        460        470        480
YCNKNNWAPP EYYLYSTTSQ DGKVLLVYKI VIPAIANGSQ SYFMPDKLCT TLEDAKELAA QFTLLHLGPF
```

Cmpd.	No. of Cmpds.	m/z meas.	Δ m/z [ppm]	z	Rt [min]	Score	P	Range	Sequence	Modification
2007	2	719.2958	-4.73	2	55.5	17.2	0	2-14	M.NEENIDGTNGCSK.V	Carbamidomethyl: 11

Detailed Protein Report

Protein 597: probable G-protein coupled receptor 179 precursor [Homo sapiens]

**Accession:**	gi|93352554	**Score:**	17.2
**Database:**	refseq_human(refseq_human_20140103.fasta)	**MW [kDa]:**	257.2
**Database Date:**	2015-11-30	**pI:**	5.4
		**Sequence Coverage [%]:**	0.4
		**No. of unique Peptides:**	1

Quantitation

QD:QU	**Median:** 0.42	**CV:** 0.00 %	**No. of Peptides:** 1

Detailed Protein Report

10	20	30	40	50	60	70	80
MGTRGAVMPP	PMWGLLGCCF	VCAWALGGPR	PIRSLPPLSS	QVKPGSVPMQ	VPLEGAEAAL	AYLYSGDAQQ	LSQVNCSERY
90	100	110	120	130	140	150	160
EARGAGAMPG	LPPSLQGAAG	TLAQAANFLN	MLLQANDIRE	SSVEEDVEWY	QALVRSVAEG	DPRVYRALLT	FNPPPGASHL
170	180	190	200	210	220	230	240
QLALQATRTG	EETILQDLSG	NWVQEENPPG	DLDTPALKKR	VLTNDLGSLG	SPKWPQADGY	VGDTQQVRLS	PPFLECQEGR
250	260	270	280	290	300	310	320
LRPGWLITLS	ATFYGLKPDL	SPEVRGQVQM	DVDLQSVDIN	QCASGPGWYS	NTHLCDLNST	QCVPLESQGF	VLGRYLCRCR
330	340	350	360	370	380	390	400
PGFYGASPSG	GLEESDFQTT	GQFGFPEGRS	GRLLQCLPCP	EGCTSCMDAT	PCLVEEAAVL	RAAVLACQAC	CMLAIFLSML
410	420	430	440	450	460	470	480
VSYRCRRNKR	IWASGVVLLE	TVLFGFLLLY	FPVFILYFKP	SVFRCIALRW	VRLLGFAIVY	GTIILKLYRV	LQLFLSRTAQ
490	500	510	520	530	540	550	560
RSALLSSGRL	LRRLGLLLLP	VLGFLAVWTV	GALERGIQHA	PLVIRGHTPS	GRHFYLCHHD	RWDYIMVVAE	LLLLCWGSFL
570	580	590	600	610	620	630	640
CYATRAVLSA	FHEPRYMGIA	LHNELLLSAA	FHTARFVLVP	SLHPDWTLLL	FFFHTHSTVT	TTLALIFIPK	FWKLGAPPRE
650	660	670	680	690	700	710	720
EMVDEVCEDE	LDLQHSGSYL	GSSIASAWSE	HSLDPGDIRD	ELKKLYAQLE	VHKTKEMAAN	NPHLPKKRGS	SCQGLGRSFM
730	740	750	760	770	780	790	800
RYLAEFPEAL	ARQHSRDSGS	PGHGSLPGSS	RRRLLSSSLQ	EPEGTPALHK	SRSTYDQRRE	QDPPLLDSLL	RRKLAKKASR
810	820	830	840	850	860	870	880
TESRESVEGP	PALGFRSASA	HNLTVGERLP	RARPASLQKS	LSVASSREKA	LLMASQAYLE	ETYRQAKERE	ERKKAKAAMA
890	900	910	920	930	940	950	960
SLVRRPSARR	LERPRGAPLS	APPSPAKSSS	VDSSHTSGRL	HEEARRRLPH	PPIRHQVSTP	ILALSGGLGE	PRMLSPTSTL
970	980	990	1000	1010	1020	1030	1040
APALLPALAP	TPAPALAPVP	VSPQSPNLLT	YICPWENAEL	PAKQENVPQE	GPSGPERGHH	SPAPARARLW	RALSVAVEKS
1050	1060	1070	1080	1090	1100	1110	1120
RAGENEMDAE	DAHHQREAND	VDEDRPKIFP	KSHSLKAPVQ	QGSMRSLGLA	IKALTRSRST	YREKESVEES	PEGQNSGTAG
1130	1140	1150	1160	1170	1180	1190	1200
ESMGAPSRSP	RLGRPKAVSK	QAALIPSDDK	ESLQNQQNAH	TSRMLQVCQR	EGSREQEDRG	RRMTQGLGER	KAERAGKTGL
1210	1220	1230	1240	1250	1260	1270	1280
AMLRQVSRDK	NIKQSKETPV	GWQELPKAGL	QSLGSADHRV	AEVCPWEVTE	SETRQPDSGN	KAEICPWETS	EGAPESRALR
1290	1300	1310	1320	1330	1340	1350	1360
QDPGDSQKKR	GEARGKSEPI	DVVPMMRKKP	ERLVREQEAV	CPWESADRGG	LSPGSAPQDP	GRIRDKSEAG	DSVEARKVEK
1370	1380	1390	1400	1410	1420	1430	1440
PGWEAAGPEA	HTPDITKAEP	CPWEASEGGE	DGKPAQEAVK	DLPQEKQKTR	KATFWKEQKP	GGDLESLCPW	ESTDFRGPSA
1450	1460	1470	1480	1490	1500	1510	1520
VSIQAPGSSE	CSGSLGSGIA	EVCLWEAGDA	PAIQKAEICP	WELDDNVMGQ	EMLSLGTGRE	SLQEKEKASR	KGSFGEMGEQ
1530	1540	1550	1560	1570	1580	1590	1600
TVKAVQKLSQ	QQESVCPRES	TVPGHSSPCL	DNSSSKAGSQ	FLCNGGSRAT	QVCPQEDLRP	EAQEATPAKT	EICPWEVNER
1610	1620	1630	1640	1650	1660	1670	1680
TREEWTSAQV	PRGGESQKDK	EKMPGKSEIE	DVTAWEKPEG	QIQKQEAVGP	WESVDPGSFS	PQPRPQDTER	PQTLLQMSGS
1690	1700	1710	1720	1730	1740	1750	1760
VGSKAADICP	LDVEENLTAG	KAEICPWEVG	AGAGEERALG	AEAIRKSPND	TGKVSADLGP	RERAVTAPEK	PQKPTPEWEV
1770	1780	1790	1800	1810	1820	1830	1840
ACPWGSVGPG	ACSQHPGTLD	ADGPKAGFQE	LDHMGCRPGE	VCPWEAQEAA	TSEKAKICPW	EVSEGTTGKG	LDQKAGSESA
1850	1860	1870	1880	1890	1900	1910	1920
EQREKALEKG	RLTSLGEDVS	KGMAKLCQQQ	ETICIWENKD	LRESPAQAPK	ISDLPSSMSS	EVAEGHSLEA	TEKGDLRQDP
1930	1940	1950	1960	1970	1980	1990	2000
KTGSFPEHIT	QEKAPAADTE	EFTTEDGEKT	SHELQSVCPW	ETTAPADSVS	HLDRQRPDQP	KASSQRLVST	GGRAADVCPW
2010	2020	2030	2040	2050	2060	2070	2080
DVPDAGVYKS	DSSAKAETCP	WEVTERIPVK	GVSRQDGKGD	SQEEKGRAPE	KSEPKGVPVQ	KKPEMADFRQ	QEAVCPWESQ
2090	2100	2110	2120	2130	2140	2150	2160
DGKGLSPQPA	PDASDRSRGS	SEAAGSVETR	VAEVCLWEVV	EAPSAKKAEI	CPWEAGGGAA	EEGEQERESQ	GQGEMFLQKA
2170	2180	2190	2200	2210	2220	2230	2240

Detailed Protein Report

Cmpd.	No. of Cmpds.	m/z meas.	Δ m/z [ppm]	z	Rt [min]	Score	P	Range	Sequence	Modification	Ratios
881	4	509.2453	-102.22	2	41.1	17.2	1	1195-1204	R.AGKTGLAMLR.Q		QD:QU 0.42

Detailed Protein Report

Protein 598: ankyrin repeat domain-containing protein SOWAHB [Homo sapiens]

**Accession:**	gi|71274172	**Score:**	17.1
**Database:**	refseq_human(refseq_human_20140103.fasta)	**MW [kDa]:**	85.7
**Database Date:**	2015-11-30	**pI:**	10.4
		**Sequence Coverage [%]:**	2.6
		**No. of unique Peptides:**	1

10	20	30	40	50	60	70	80
MARELSQEAL	LDFLCQAGGR	VTNAALLSHF	KSFLRDPDAS	PSQHQHRREL	FKGFVNSVAA	VRQDPDGTKY	VVLKRRYRDL
**90**	**100**	**110**	**120**	**130**	**140**	**150**	**160**
LGEEGLQRPR	EPPAAAPSAG	GAAPCSPRGA	RRGEPPQQQP	RRRRREKEPE	EEPAGAAARA	ADAACNGLPG	SDSRRAPGKG
**170**	**180**	**190**	**200**	**210**	**220**	**230**	**240**
GGSKGSPGQR	PPVPAAAAAG	AQARASCAAA	KTQGRCCWEC	LQNNLAVLPG	ELGALPHSAT	AEEKPARALP	AQDDRGASRE
**250**	**260**	**270**	**280**	**290**	**300**	**310**	**320**
REEGALAEPA	PVPAVAHSPP	ATVEAATSRA	SPPALLPGPA	PRGDRPELLT	PSSLHYSTLQ	QQQQRTREWV	ARHPQVPEAR
**330**	**340**	**350**	**360**	**370**	**380**	**390**	**400**
DQGPIRAWSV	LPDNFLQLPL	EPGSTEPNSE	PPDPCLSSHS	LFPVVPDESW	ESWAGNPSLT	VFRSIRCQLS	LQDLDDFVDQ
**410**	**420**	**430**	**440**	**450**	**460**	**470**	**480**
ESDGSEESSS	GPKDSPGASE	EGLQVVLGTP	DRGKLRNPAG	GLSVSRKEGS	PSRSPQGLRN	RGDGHISQQV	PAGANGLAGH
**490**	**500**	**510**	**520**	**530**	**540**	**550**	**560**
PLKPLPWPVP	KLRRSLRRSS	LAGRAKLSSS	DEEYLDEGLL	KRSRRPPRSR	KPSKAGTAPS	PRVDAGLSLK	LAEVKAVVAE
**570**	**580**	**590**	**600**	**610**	**620**	**630**	**640**
RGWRHSLWVP	SGEGSAALAP	HRTSEHKSSL	VPLDAREHEW	IVKLASGSWI	QVWTLFWEDP	QLALHKDFLT	GYTALHWIAK
**650**	**660**	**670**	**680**	**690**	**700**	**710**	**720**
HGDLRALQDL	VSGAKKAGIV	LDVNVRSSCG	YTPLHLAAIH	GHQGVIKLLV	QRLASRVNVR	DSSGKKPWQY	LTSNTSGEIW
**730**	**740**	**750**	**760**	**770**	**780**	**790**	**800**
QLLGAPRGKP	IFPVYPLVGS	SSPTRKAKSK	EISRSVTRKT	SFAALLKSQH	NKWKLANQYE	KFHSPREREE	YSD

Cmpd.	No. of Cmpds.	m/z meas.	Δ m/z [ppm]	z	Rt [min]	Score	P	Range	Sequence	Modification
1748	1	945.9280	-39.73	2	50.4	17.1	1	91-111	R.EPPAAAPSAGGAAPCSPRGAR.R	

Detailed Protein Report

Protein 599: PREDICTED: SH3 domain-containing RING finger protein 3 isoform X2 [Homo sapiens]

**Accession:**	gi|578804249	**Score:**	17.1
**Database:**	refseq_human(refseq_human_20140103.fasta)	**MW [kDa]:**	63.9
**Database Date:**	2015-11-30	**pI:**	9.8
**Modification(s):**	Carbamidomethyl	**Sequence Coverage [%]:**	1.8
		**No. of unique Peptides:**	1

Quantitation

QD:QU **Median:** 0.35 **CV:** 0.00 % **No. of Peptides:** 1

10	20	30	40	50	60	70	80
MLLGASWLCA	SKAAAAAAQS	EGDEDRPGER	RRRRAAATAA	GAGEDMDESS	LLDLLECSVC	LERLDTTAKV	LPCQHTFCRR
90	100	110	120	130	140	150	160
CLESIVCSRH	ELRCPECRIL	VGCGVDELPA	NILLVRLLDG	IRQRPRAGTS	PGGSPPARPI	PGQSAAPTLA	GGGGGAAGST
170	180	190	200	210	220	230	240
PGSPVFLSAA	AGSTAGSLRE	LATSRTAPAA	KNPCLLPYGK	ALYSYEGKEP	GDLKFNKGDI	IVLRRKVDEQ	WYHGELHGTQ
250	260	270	280	290	300	310	320
GFLPASYIQC	IQPLPHAPPQ	GKALYDFEMK	DKDQDKDCLT	FTKDEILTVL	RRVDENWAEG	MLGDKIGIFP	LLYVELNDSA
330	340	350	360	370	380	390	400
KQLIEMDKPC	PAAASSCNAS	LPSDSGAVAS	VAPSPTLSSS	GAVSAFQRRV	DGKKNTKKRH	SFTALSVTHR	SSQAASHRHS
410	420	430	440	450	460	470	480
MEISAPVLIS	SSDPRAAARI	GDLAHLSCAA	PTQDVSSSAG	STPTAVPRAA	SVSGEQGTPP	KVQLPLNVYL	ALYAYKPQKS
490	500	510	520	530	540	550	560
DELELHKGEM	YRVLEKCQDG	WFKGASLRTG	VSGVFPGNYV	TPVSRVPAGG	AGPPRNNVVG	GSPLAKGITT	TMHPGSGSLS
570	580	590	600	610	620		
SLATATRPAL	PITTPQAHAQ	HPTASPPTGS	CLRHSAQPTA	SQARSTISTG			

Cmpd.	No. of Cmpds.	m/z meas.	Δ m/z [ppm]	z	Rt [min]	Score	P	Range	Sequence	Modification	Ratios	
738	1	705.1981	-212.15	2	37.8	17.1	1	493-503	R.VLEKCQDGWFK.G	Carbamidomethyl: 5	QD:QU	0.35

Detailed Protein Report

Protein 600: centromere protein X isoform 3 [Homo sapiens]

**Accession:**	gi|401871068	**Score:**	17.0
**Database:**	refseq_human(refseq_human_20140103.fasta)	**MW [kDa]:**	6.5
**Database Date:**	2015-11-30	**pI:**	6.1
		**Sequence Coverage [%]:**	17.2
		**No. of unique Peptides:**	1

Quantitation

QD:QU **Median:** 0.49 **CV:** 0.00 % **No. of Peptides:** 1

```
        10         20         30         40         50         60
MEGAGAGSGF RKELVSRLLH LHFKDDKTKE AAVRGVRQAQ AEDALRVDVD QLEKLLDF
```

Cmpd.	No. of Cmpds.	m/z meas.	Δ m/z [ppm]	z	Rt [min]	Score	P	Range	Sequence	Modification	Ratios	
685	1	566.7912	-18.08	2	37.1	17.0	2	25-34	K.DDKTKEAAVR.G		QD:QU	0.49

Detailed Protein Report

Protein 601: zinc finger protein 586 isoform b [Homo sapiens]

Accession: gi|116812602
Database: refseq_human(refseq_human_20140103.fasta)
Database Date: 2015-11-30
Modification(s): Oxidation

Score: 17.0
MW [kDa]: 22.8
pI: 10.5
Sequence Coverage [%]: 6.7
No. of unique Peptides: 1

10	20	30	40	50	60	70	80
MAAAAALRAP	AQVVGMEGKM	RQHLLSRARV	YIYTKTREVI	VEKGLMSVGN	IGNYLRTSPA	SLNPEEITNT	GMFALEKGLM
**90**	**100**	**110**	**120**	**130**	**140**	**150**	**160**
SAANMGNYFT	KSLHSIFMRD	FILGKRPMSA	VSVENHFTKA	LHSCSVRHFT	LEKGLMSVLN	VGKPLLKSPV	SLTTGKFTLE
**170**	**180**	**190**	**200**	**210**	**220**		
QSVMNAMNVG	SPLLIHLVSL	NTGGFTLERG	LMSAVNVGDP	LLKTPVLLNT			

Cmpd.	No. of Cmpds.	m/z meas.	Δ m/z [ppm]	z	Rt [min]	Score	P	Range	Sequence	Modification
2516	1	715.3738	-22.06	2	62.0	17.0	0	190-203	R.GLMSAVNVGDPLLK.T	Oxidation: 3

Detailed Protein Report

Protein 602: **conserved oligomeric Golgi complex subunit 1 [Homo sapiens]**

Accession: gi|21237783
Database: refseq_human(refseq_human_20140103.fasta)
Database Date: 2015-11-30

Score: 16.9
MW [kDa]: 108.9
pI: 7.1
Sequence Coverage [%]: 1.8
No. of unique Peptides: 1

10	20	30	40	50	60	70	80
MATAATSPAL	KRLDLRDPAA	LFETHGAEEI	RGLERQVRAE	IEHKKEELRQ	MVGERYRDLI	EAADTIGQMR	RCAVGLVDAV
**90**	**100**	**110**	**120**	**130**	**140**	**150**	**160**
KATDQYCARL	RQAGSAAPRP	PRAQQPQQPS	QEKFYSMAAQ	IKLLLEIPEK	IWSSMEASQC	LHATQLYLLC	CHLHSLLQLD
**170**	**180**	**190**	**200**	**210**	**220**	**230**	**240**
SSSSRYSPVL	SRFPILIRQV	AAASHFRSTI	LHESKMLLKC	QGVSDQAVAE	ALCSIMLLEE	SSPRQALTDF	LLARKATIQK
**250**	**260**	**270**	**280**	**290**	**300**	**310**	**320**
LLNQPHHGAG	IKAQICSLVE	LLATTLKQAH	ALFYTLPEGL	LPDPALPCGL	LFSTLETITG	QHPAGKGTGV	LQEEMKLCSW
**330**	**340**	**350**	**360**	**370**	**380**	**390**	**400**
FKHLPASIVE	FQPTLRTLAH	PISQEYLKDT	LQKWIHMCNE	DIKNGITNLL	MYVKSMKGLA	GIRDAMWELL	TNESTNHSWD
**410**	**420**	**430**	**440**	**450**	**460**	**470**	**480**
VLCRRLLEKP	LLFWEDMMQQ	LFLDRLQTLT	KEGFDSISSS	SKELLVSALQ	ELESSTSNSP	SNKHIHFEYN	MSLFLWSESP
**490**	**500**	**510**	**520**	**530**	**540**	**550**	**560**
NDLPSDAAWV	SVANRGQFAS	SGLSMKAQAI	SPCVQNFCSA	LDSKLKVKLD	DLLAYLPSDD	SSLPKDVSPT	QAKSSAFDRY
**570**	**580**	**590**	**600**	**610**	**620**	**630**	**640**
ADAGTVQEML	RTQSVACIKH	IVDCIRAELQ	SIEEGVQGQQ	DALNSAKLHS	VLFMARLCQS	LGELCPHLKQ	CILGKSESSE
**650**	**660**	**670**	**680**	**690**	**700**	**710**	**720**
KPAREFRALR	KQGKVKTQEI	IPTQAKWQEV	KEVLLQQSVM	GYQVWSSAVV	KVLIHGFTQS	LLLDDAGSVL	ATATSWDELE
**730**	**740**	**750**	**760**	**770**	**780**	**790**	**800**
IQEEAESGSS	VTSKIRLPAQ	PSWYVQSFLF	SLCQEINRVG	GHALPKVTLQ	EMLKSCMVQV	VAAYEKLSEE	KQIKKEGAFP
**810**	**820**	**830**	**840**	**850**	**860**	**870**	**880**
VTQNRALQLL	YDLRYLNIVL	TAKGDEVKSG	RSKPDSRIEK	VTDHLEALID	PFDLDVFTPH	LNSNLHRLVQ	RTSVLFGLVT
**890**	**900**	**910**	**920**	**930**	**940**	**950**	**960**
GTENQLAPRS	STFNSQEPHN	ILPLASSQIR	FGLLPLSMTS	TRKAKSTRNI	ETKAQVVPPA	RSTAGDPTVP	GSLFRQLVSE
**970**	**980**	**990**					
EDNTSAPSLF	KLGWLSSMTK						

Cmpd.	No. of Cmpds.	m/z meas.	Δ m/z [ppm]	z	Rt [min]	Score	P	Range	Sequence	Modification
2511	1	941.9690	8.39	2	62.0	16.9	1	72-89	R.CAVGLVDAVKATDQYCAR.L	

Detailed Protein Report

Protein 603: probable G-protein coupled receptor 116 precursor [Homo sapiens]

**Accession:**	gi|148719671	**Score:**	16.9
**Database:**	refseq_human(refseq_human_20140103.fasta)	**MW [kDa]:**	149.4
**Database Date:**	2015-11-30	**pI:**	6.2
**Modification(s):**	Carbamidomethyl	**Sequence Coverage [%]:**	0.7
		**No. of unique Peptides:**	1

Alias proteins:

Accession	Name	Description
gi|148719673	refseq_human (refseq_human_20140103.fasta)	probable G-protein coupled receptor 116 precursor [Homo sapiens]

10	20	30	40	50	60	70	80
MKSPRRTTLC	LMFIVIYSSK	AALNWNYEST	IHPLSLHEHE	PAGEEALRQK	RAVATKSPTA	EEYTVNIEIS	FENASFLDPI
90	100	110	120	130	140	150	160
KAYLNSLSFP	IHGNNTDQIT	DILSINVTTV	CRPAGNEIWC	SCETGYGWPR	ERCLHNLICQ	ERDVFLPGHH	CSCLKELPPN
170	180	190	200	210	220	230	240
GPFCLLQEDV	TLNMRVRLNV	GFQEDLMNTS	SALYRSYKTD	LETAFRKGYG	ILPGFKGVTV	TGFKSGSVVV	TYEVKTTPPS
250	260	270	280	290	300	310	320
LELIHKANEQ	VVQSLNQTYK	MDYNSFQAVT	INESNFFVTP	EIIFEGDTVS	LVCEKEVLSS	NVSWRYEEQQ	LEIQNSSRFS
330	340	350	360	370	380	390	400
IYTALFNNMT	SVSKLTIHNI	TPGDAGEYVC	KLILDIFEYE	CKKKIDVMPI	QILANEEMKV	MCDNNPVSLN	CCSQGNVNWS
410	420	430	440	450	460	470	480
KVEWKQEGKI	NIPGTPETDI	DSSCSRYTLK	ADGTQCPSGS	SGTTVIYTCE	FISAYGARGS	ANIKVTFISV	ANLTITPDPI
490	500	510	520	530	540	550	560
SVSEGQNFSI	KCISDVSNYD	EVYWNTSAGI	KIYQRFYTTR	RYLDGAESVL	TVKTSTREWN	GTYHCIFRYK	NSYSIATKDV
570	580	590	600	610	620	630	640
IVHPLPLKLN	IMVDPLEATV	SCSGSHHIKC	CIEEDGDYKV	TFHTGSSSLP	AAKEVNKKQV	CYKHNFNASS	VSWCSKTVDV
650	660	670	680	690	700	710	720
CCHFTNAANN	SVWSPSMKLN	LVPGENITCQ	DPVIGVGEPG	KVIQKLCRFS	NVPSSPESPI	GGTITYKCVG	SQWEEKRNDC
730	740	750	760	770	780	790	800
ISAPINSLLQ	MAKALIKSPS	QDEMLPTYLK	DLSISIDKAE	HEISSSPGSL	GAIINILDLL	STVPTQVNSE	MMTHVLSTVN
810	820	830	840	850	860	870	880
VILGKPVLNT	WKVLQQQWTN	QSSQLLHSVE	RFSQALQSGD	SPPLSFSQTN	VQMSSMVIKS	SHPETYQQRF	VFPYFDLWGN
890	900	910	920	930	940	950	960
VVIDKSYLEN	LQSDSSIVTM	AFPTLQAILA	QDIQENNFAE	SLVMTTTVSH	NTTMPFRISM	TFKNNSPSGG	ETKCVFWNFR
970	980	990	1000	1010	1020	1030	1040
LANNTGGWDS	SGCYVEEGDG	DNVTCICDHL	TSFSILMSPD	SPDPSSLLGI	LLDIISYVGV	GFSILSLAAC	LVVEAVVWKS
1050	1060	1070	1080	1090	1100	1110	1120
VTKNRTSYMR	HTCIVNIAAS	LLVANTWFIV	VAAIQDNRYI	LCKTACVAAT	FFIHFFYLSV	FFWMLTLGLM	LFYRLVFILH
1130	1140	1150	1160	1170	1180	1190	1200
ETSRSTQKAI	AFCLGYGCPL	AISVITLGAT	QPREVYTRKN	VCWLNWEDTK	ALLAFAIPAL	IIVVVNITIT	IVVITKILRP
1210	1220	1230	1240	1250	1260	1270	1280
SIGDKPCKQE	KSSLFQISKS	IGVLTPLLGL	TWGFGLTTVF	PGTNLVFHII	FAILNVFQGL	FILLFGCLWD	LKVQEALLNK
1290	1300	1310	1320	1330	1340	1350	
FSLSRWSSQH	SKSTSLGSST	PVFSMSSPIS	RRFNNLFGKT	GTYNVSTPEA	TSSSLENSSS	ASSLLN	

Cmpd.	No. of Cmpds.	m/z meas.	Δ m/z [ppm]	z	Rt [min]	Score	P	Range	Sequence	Modification
2911	1	671.8322	16.14	2	65.6	16.9	0	133-142	R.CLHNLICQER.D	Carbamidomethyl: 1, 7

Detailed Protein Report

Protein 604: leucine-rich repeat-containing protein 69 [Homo sapiens]

**Accession:**	gi|193788651	**Score:**	16.9
**Database:**	refseq_human(refseq_human_20140103.fasta)	**MW [kDa]:**	39.6
**Database Date:**	2015-11-30	**pI:**	9.9
**Modification(s):**	Carbamidomethyl	**Sequence Coverage [%]:**	4.9
		**No. of unique Peptides:**	1

10	20	30	40	50	60	70	80
MTERLLIKAL	SGGKNTKIIT	LNGKKMTKMP	SALGKLPGLK	TLVLQNNLIP	KVCPELCNLT	QLTTLNLGNN	LLEEVPEEMK
**90**	**100**	**110**	**120**	**130**	**140**	**150**	**160**
YLTSLKNLHL	SGNRICRFAP	GACDGLQNLI	LLNLNNNHLT	QLPQEVSRLK	SLTYMSINYN	QLASIPRELC	FLENLVELQL
**170**	**180**	**190**	**200**	**210**	**220**	**230**	**240**
NYNQLICIPE	EIKFLKKLQK	LLLARNNIGV	LPEELCDLKK	LRILDIAGNI	IQIFPSGFQD	LKLREFYCEG	NPLFLQQPVI
**250**	**260**	**270**	**280**	**290**	**300**	**310**	**320**
STQQENVWSL	QEITSRFVMN	QLAENNPFLM	DDIERYPQVR	SMISQGKTCA	ICGQYFITVW	LECVRFVPPP	KDWKISKNLK
**330**	**340**	**350**					
LVPLQVLICS	YKCFTQRDPN	LFGIAQV					

Cmpd.	No. of Cmpds.	m/z meas.	Δ m/z [ppm]	z	Rt [min]	Score	P	Range	Sequence	Modification
2170	1	682.3011	-106.73	3	57.5	16.9	2	81-97	K.YLTSLKNLHLSGNRICR.F	Carbamidomethyl: 16

Detailed Protein Report

Protein 605: **PREDICTED: iron-sulfur cluster assembly enzyme ISCU, mitochondrial isoform X1 [Homo sapiens]**

**Accession:**	gi|530400013	**Score:**	16.9
**Database:**	refseq_human(refseq_human_20140103.fasta)	**MW [kDa]:**	16.9
**Database Date:**	2015-11-30	**pI:**	10.5
**Modification(s):**	Oxidation	**Sequence Coverage [%]:**	10.3
		**No. of unique Peptides:**	1

10	20	30	40	50	60	70	80
MAAAGAFRLR	RAASALLLRS	PRLPARELSA	PARLYHKKVV	DHYENPRNVG	SLDKTSKNVG	TGLVGAPACG	DVMKLQIQVD
**90**	**100**	**110**	**120**	**130**	**140**	**150**	**160**
EKGKIVDARF	KTFGCGSAIA	SSSLATEWVK	GKTVEEALTI	KNTDIAKELC	LPPVKLHCSR	KEGMRNISLN	ASMEVY

Cmpd.	No. of Cmpds.	m/z meas.	Δ m/z [ppm]	z	Rt [min]	Score	P	Range	Sequence	Modification
2964	1	619.9693	5.46	3	66.2	16.9	2	141-156	R.KEGMRNISLNASMEVY.-	Oxidation: 4

Detailed Protein Report

Protein 606: dynein heavy chain 12, axonemal isoform 1 [Homo sapiens]

**Accession:**	gi|194440727	**Score:**	16.9
**Database:**	refseq_human(refseq_human_20140103.fasta)	**MW [kDa]:**	356.7
**Database Date:**	2015-11-30	**pI:**	5.8
		**Sequence Coverage [%]:**	0.4
		**No. of unique Peptides:**	1

Detailed Protein Report

10	20	30	40	50	60	70	80
MSDANKAAIA	AEKEALNLKL	PPIVHLPENI	GVDTPTQSKL	LKYRRSKEQQ	QKINQLVIDG	AKRNLDRTLG	KRTPLLPPPD
90	100	110	120	130	140	150	160
YPQTMTSEMK	KKGFNYIYMK	QCVESSPLVP	IQQEWLDHML	RLIPESLKEG	KEREELLESL	INEVSSDFEN	SMKRYLVQSV
170	180	190	200	210	220	230	240
LVKPPVKSLE	DEGGPLPESP	VGLDYSNPWH	SSYVQARNQI	FSNLHIIHPT	MKMLLDLGYT	TFADTVLLDF	TGIRAKGPID
250	260	270	280	290	300	310	320
CESLKTDLSI	QTRNAEEKIM	NTWYPKVINL	FTKKEALEGV	KPEKLDAFYS	CVSTLMSNQL	KDLLRRTVEG	FVKLFDPKDQ
330	340	350	360	370	380	390	400
QRLPIFKIEL	TFDDDKMEFY	PTFQDLEDNV	LSLVERIAEA	LQNVQTIPSW	LSGTSTPVNL	DTELPEHVLH	WAVDTLKAAV
410	420	430	440	450	460	470	480
HRNLEGARKH	YETYVEKYNW	LLDGTAVENI	ETFQTEDHTF	DEYTEFIEKF	LSLASEIMLL	PQWIHYTMVR	LDCEDLKTGL
490	500	510	520	530	540	550	560
TNKAKAFANI	LLNDIASKYR	KENECICSEF	EAIKEHALKV	PETTEEMMDL	ISYVEKARTV	GIEELILRIQ	ESKRQMSYFL
570	580	590	600	610	620	630	640
DVFLFPQEDL	ALNATVLMWP	RKINPIFDEN	DELIENAKHK	KENELMAKRE	KLILEIEKES	RRMEEFTEFA	ELERMQQYVT
650	660	670	680	690	700	710	720
DVRQLQKRIQ	ESEEAVQFIN	KEEELFKWEL	TKYPELDKLK	VNIEPYQKFF	NFVLKWQRSE	KRWMDGGFLD	LNGESMEADV
730	740	750	760	770	780	790	800
EEFSREIFKT	LKFFQTKLKK	ELQEKRKAAR	KRSLEEEKIE	EEPKDNATIT	MCRMRARHWK	QISEIVGYDL	TPDSGTTLRK
810	820	830	840	850	860	870	880
VLKLNLTPYL	EQFEVISAGA	SKEFSLEKAM	NTMIGTWEDI	AFHISLYRDT	GVCILSSVDE	IQAILDDQII	KTQTMRGSPF
890	900	910	920	930	940	950	960
IKPFEHEIKA	WEDRLIRIQE	TIDEWLKVQA	QWLYLEPIFC	SEDIMQQMPE	EGRQFQTVDR	HWRDIMKFCA	KDPKVLAATS
970	980	990	1000	1010	1020	1030	1040
LTGLLEKLQN	CNELLEKIMK	GLNAYLEKKR	LFFPRFFFLS	NDEMLEILSE	TKDPLRVQPH	LKKCFEGIAK	LEFLPNLDIK
1050	1060	1070	1080	1090	1100	1110	1120
AMYSSEGERV	ELIALISTSA	ARGAVEKWLI	QVEDLMLRSV	HDVIAAARLA	YPESARRDWV	REWPGQVVLC	ISQMFWTSET
1130	1140	1150	1160	1170	1180	1190	1200
QEVISGGTEG	LKKYYKELQN	QLNEIVELVR	GKLSKQTRTT	LGALVTIDVH	ARDVVMDMIK	MGVSHDTDFL	WLAQLRYYWE
1210	1220	1230	1240	1250	1260	1270	1280
NENARVRIIN	CNVKYAYEYL	GNSPRLVITP	LTDRCYRTLI	GAFYLNLGGA	PEGPAGTGKT	ETTKDLAKAL	AVQCVVFNCS
1290	1300	1310	1320	1330	1340	1350	1360
DGLDYLAMGK	FFKGLASSGA	WACFDEFNRI	ELEVLSVVAQ	QILCIQRAIQ	QKLVVFVFEG	TELKLNPNCF	VAITMNPGYA
1370	1380	1390	1400	1410	1420	1430	1440
GRSELPDNLK	VLFRTVAMMV	PNYALIAEIS	LYSYGFLNAR	PLSVKIVMTY	RLCSEQLSSQ	FHYDYGMRAV	KAVLVAAGNL
1450	1460	1470	1480	1490	1500	1510	1520
KLKYPNENED	ILLLRSIKDV	NEPKFLSHDI	PLFNGITSDL	FPGIKLPEAD	YHEFLECAHE	ACNVHNLQPV	KFFLEKIIQT
1530	1540	1550	1560	1570	1580	1590	1600
YEMMIVRHGF	MLVGEPFAAK	TKVLHVLADT	LTLMNEHGYG	EEEKVIYRTV	NPKSITMGQL	FGQFDPVSHE	WTDGIVANTF
1610	1620	1630	1640	1650	1660	1670	1680
REFALSETPD	RKWVVFDGPI	DTLWIESMNT	VLDDNKKLCL	MSGEIIQMSP	QMSLIFETMD	LSQASPATVS	RCGMIYLEPS
1690	1700	1710	1720	1730	1740	1750	1760
QLGWEPLVSS	WLNSLKGPLC	EPEYQALLRG	LFAWLIPPSL	NQRVELFQLN	YLYTTIVSKI	LKILITFRIS	NYFKYVPLKT
1770	1780	1790	1800	1810	1820	1830	1840
QCTFIKFFLH	QQACFIFSLI	WSIGGSCDTD	GRRVFDTFIR	LIILGKDDEN	PVPDSVGKWE	CPFDEKGLVY	DYMYELKNKG
1850	1860	1870	1880	1890	1900	1910	1920
RWVHWNELIK	NTNLGDKQIK	IQDIIVPTMD	TIRYTFLMDL	SITYAKPLLF	VGPTGTGKSV	YVKDKLMNHL	EKDQYFPFYI
1930	1940	1950	1960	1970	1980	1990	2000
NLSARTSANQ	VQNIIMARLD	KRRKGVFGPP	MGKKCIIFID	DMNMPALEKY	GAQPPIELLR	QFFDCGHWYD	LKDTSKITLV
2010	2020	2030	2040	2050	2060	2070	2080
DIELIAAMGP	PGGGRNPVTP	RCIRHFNICS	INSFSDETMV	RIFSSIVAFY	LRTHEFPPEY	FVIGNQIVNG	TMEIYKQSVE
2090	2100	2110	2120	2130	2140	2150	2160
NLLPTPTKSH	YTFNLRDFSR	VIRGCLLIER	DAVANKHTMI	RLFVHEVLRV	FYDRLINDDD	RRWLFQLTKT	VIKDHFKESF
2170	2180	2190	2200	2210	2220	2230	2240

Detailed Protein Report

Cmpd.	No. of Cmpds.	m/z meas.	Δ m/z [ppm]	z	Rt [min]	Score	P	Range	Sequence	Modification
1721	1	646.7566	-109.88	2	51.8	16.9	1	1602-1612	R.EFALSETPDRK.W	

Detailed Protein Report

Protein 607: protein GNAS isoform GNASS [Homo sapiens]

Accession: gi|18426900
Database: refseq_human(refseq_human_20140103.fasta)
Database Date: 2015-11-30
Modification(s): Oxidation

Score: 16.9
MW [kDa]: 44.2
pI: 5.9
Sequence Coverage [%]: 4.2
No. of unique Peptides: 1

10	20	30	40	50	60	70	80
MGCLGNSKTE	DQRNEEKAQR	EANKKIEKQL	QKDKQVYRAT	HRLLLLGAGE	SGKSTIVKQM	RILHVNGFNG	DSEKATKVQD
**90**	**100**	**110**	**120**	**130**	**140**	**150**	**160**
IKNNLKEAIE	TIVAAMSNLV	PPVELANPEN	QFRVDYILSV	MNVPDFDFPP	EFYEHAKALW	EDEGVRACYE	RSNEYQLIDC
**170**	**180**	**190**	**200**	**210**	**220**	**230**	**240**
AQYFLDKIDV	IKQADYVPSD	QDLLRCRVLT	SGIFETKFQV	DKVNFHMFDV	GGQRDERRKW	IQCFNDVTAI	IFVVASSSYN
**250**	**260**	**270**	**280**	**290**	**300**	**310**	**320**
MVIREDNQTN	RLQEALNLFK	SIWNNRWLRT	ISVILFLNKQ	DLLAEKVLAG	KSKIEDYFPE	FARYTTPEDA	TPEPGEDPRV
**330**	**340**	**350**	**360**	**370**	**380**	**390**	
TRAKYFIRDE	FLRISTASGD	GRHYCYPHFT	CAVDTENIRR	VFNDCRDIIQ	RMHLRQYELL		

Cmpd.	No. of Cmpds.	m/z meas.	Δ m/z [ppm]	z	Rt [min]	Score	P	Range	Sequence	Modification
50	1	930.9411	-14.59	2	30.5	16.9	1	59-74	K.QMRILHVNGFNGDSEK.A	Oxidation: 2

Detailed Protein Report

Protein 608: zinc finger protein 383 [Homo sapiens]

**Accession:**	gi|23097321	**Score:**	16.8
**Database:**	refseq_human(refseq_human_20140103.fasta)	**MW [kDa]:**	54.6
**Database Date:**	2015-11-30	**pI:**	9.3
		**Sequence Coverage [%]:**	5.1
		**No. of unique Peptides:**	1

Alias proteins:

Accession	Name	Description
gi|530415862	refseq_human (refseq_human_20140103.fasta)	PREDICTED: zinc finger protein 383 isoform X4 [Homo sapiens]
gi|530415860	refseq_human (refseq_human_20140103.fasta)	PREDICTED: zinc finger protein 383 isoform X3 [Homo sapiens]
gi|530415856	refseq_human (refseq_human_20140103.fasta)	PREDICTED: zinc finger protein 383 isoform X1 [Homo sapiens]

10	20	30	40	50	60	70	80
MAEGSVMFSD	VSIDFSQEEW	DCLDPVQRDL	YRDVMLENYG	NLVSMGLYTP	KPQVISLLEQ	GKEPWMVGRE	LTRGLCSDLE
**90**	**100**	**110**	**120**	**130**	**140**	**150**	**160**
SMCETKLLSL	KKEVYEIELC	QREIMGLTKH	GLEYSSFGDV	LEYRSHLAKQ	LGYPNGHFSQ	EIFTPEYMPT	FIQQTFLTLH
**170**	**180**	**190**	**200**	**210**	**220**	**230**	**240**
QIINNEDRPY	ECKKCGKAFS	QNSQFIQHQR	IHIGEKSYEC	KECGKFFSCG	SHVTRHLKIH	TGEKPFECKE	CGKAFSCSSY
**250**	**260**	**270**	**280**	**290**	**300**	**310**	**320**
LSQHQRIHTG	KKPYECKECG	KAFSYCSNLI	DHQRIHTGEK	PYECKVCGKA	FTKSSQLFQH	ARIHTGEKPY	ECKECGKAFT
**330**	**340**	**350**	**360**	**370**	**380**	**390**	**400**
QSSKLVQHQR	IHTGEKPYEC	KECGKAFSSG	SALTNHQRIH	TGEKPYDCKE	CGKAFTQSSQ	LRQHQRIHAG	EKPFECLECG
**410**	**420**	**430**	**440**	**450**	**460**	**470**	**480**
KAFTQNSQLF	QHQRIHTDEK	PYECNECGKA	FNKCSNLTRH	LRIHTGEKPY	NCKECGKAFS	SGSDLIRHQG	IHTNK

Cmpd.	No. of Cmpds.	m/z meas.	Δ m/z [ppm]	z	Rt [min]	Score	P	Range	Sequence	Modification
156	1	930.1966	69.57	3	31.9	16.8	2	206-229	K.FFSCGSHVTRHLKIHTGEKPFECK.E	

Detailed Protein Report

Protein 609: **PREDICTED: N-acetylgalactosamine-6-sulfatase isoform X2 [Homo sapiens]**

**Accession:**	gi|530424709	**Score:**	16.8
**Database:**	refseq_human(refseq_human_20140103.fasta)	**MW [kDa]:**	59.0
**Database Date:**	2015-11-30	**pI:**	6.6
		**Sequence Coverage [%]:**	3.4
		**No. of unique Peptides:**	1

10	20	30	40	50	60	70	80
MQMLVSGWEE	ADGHIPHLKT	KQKWRRKTAW	ADRGAAPSCT	HAGDAEMGWG	DLGVYGEPSR	ETPNLDRMAA	EGLLFPNFYS
**90**	**100**	**110**	**120**	**130**	**140**	**150**	**160**
ANPLCSPSRA	ALLTGRLPIR	NGFYTTNAHA	RNAYTPQEIV	GGIPDSEQLL	PELLKKAGYV	SKIVGKWHLG	HRPQFHPLKH
**170**	**180**	**190**	**200**	**210**	**220**	**230**	**240**
GFDEWFGSPN	CHFGPYDNKA	RPNIPVYRDW	EMVGRYYEEF	PINLKTGEAN	LTQIYLQEAL	DFIKRQARHH	PFFLYWAVDA
**250**	**260**	**270**	**280**	**290**	**300**	**310**	**320**
THAPVYASKP	FLGTSQRGRY	GDAVREIDDS	IGKILELLQD	LHVADNTFVF	FTSDNGAALI	SAPEQGGSNG	PFLCGKQTTF
**330**	**340**	**350**	**360**	**370**	**380**	**390**	**400**
EGGMREPALA	WWPGHVTAGQ	VSHQLGSIMD	LFTTSLALAG	LTPPSDRAID	GLNLLPTLLQ	GRLMDRPIFY	YRGDTLMAAT
**410**	**420**	**430**	**440**	**450**	**460**	**470**	**480**
LGQHKAHFWT	WTNSWENFRQ	GIDFCPGQNV	SGVTTHNLED	HTKLPLIFHL	GRDPGERFPL	SFASAEYQEA	LSRITSVVQQ
**490**	**500**	**510**	**520**	**530**			
HQEALVPAQP	QLNVCNWAVM	NWAPPGCEKL	GKCLTPPESI	PKKCLWSH			

Cmpd.	No. of Cmpds.	m/z meas.	Δ m/z [ppm]	z	Rt [min]	Score	P	Range	Sequence	Modification
2101	1	1023.8833	-120.72	2	56.7	16.8	0	2-19	M.QMLVSGWEEADGHIPHLK.T	

Detailed Protein Report

Protein 610: tubby-related protein 4 isoform 1 [Homo sapiens]

**Accession:**	gi|55953100	**Score:**	16.8
**Database:**	refseq_human(refseq_human_20140103.fasta)	**MW [kDa]:**	168.9
**Database Date:**	2015-11-30	**pI:**	8.3
		**Sequence Coverage [%]:**	1.2
		**No. of unique Peptides:**	1

Alias proteins:

Accession	Name	Description
gi|530383880	refseq_human(refseq_human_20140103.fasta)	PREDICTED: tubby-related protein 4 isoform X1 [Homo sapiens]

10	20	30	40	50	60	70	80
MYAAVEHGPV	LCSDSNILCL	SWKGRVPKSE	KEKPVCRRRY	YEEGWLATGN	GRGVVGVTFT	SSHCRRDRST	PQRINFNLRG
90	100	110	120	130	140	150	160
HNSEVVLVRW	NEPYQKLATC	DADGGIFVWI	QYEGRWSVEL	VNDRGAQVSD	FTWSHDGTQA	LISYRDGFVL	VGSVSGQRHW
170	180	190	200	210	220	230	240
SSEINLESQI	TCGIWTPDDQ	QVLFGTADGQ	VIVMDCHGRM	LAHVLLHESD	GVLGMSWNYP	IFLVEDSSES	DTDSDDYAPP
250	260	270	280	290	300	310	320
QDGPAAYPIP	VQNIKPLLTV	SFTSGDISLM	NNYDDLSPTV	IRSGLKEVVA	QWCTQGDLLA	VAGMERQTQL	GELPNGPLLK
330	340	350	360	370	380	390	400
SAMVKFYNVR	GEHIFTLDTL	VQRPIISICW	GHRDSRLLMA	SGPALYVVRV	EHRVSSLQLL	CQQAIASTLR	EDKDVSKLTL
410	420	430	440	450	460	470	480
PPRLCSYLST	AFIPTIKPPI	PDPNNMRDFV	SYPSAGNERL	HCTMKRTEDD	PEVGGPCYTL	YLEYLGGLVP	ILKGRRISKL
490	500	510	520	530	540	550	560
RPEFVIMDPR	TDSKPDEIYG	NSLISTVIDS	CNCSDSSDIE	LSDDWAAKKS	PKISRASKSP	KLPRISIEAR	KSPKLPRAAQ
570	580	590	600	610	620	630	640
ELSRSPRLPL	RKPSVGSPSL	TRREFPFEDI	TQHNYLAQVT	SNIWGTKFKI	VGLAAFLPTN	LGAVIYKTSL	LHLQPRQMTI
650	660	670	680	690	700	710	720
YLPEVRKISM	DYINLPVFNP	NVFSEDEDDL	PVTGASGVPE	NSPPCTVNIP	IAPIHSSAQA	MSPTQSIGLV	QSLLANQNVQ
730	740	750	760	770	780	790	800
LDVLTNQTTA	VGTAEHAGDS	ATQYPVSNRY	SNPGQVIFGS	VEMGRIIQNP	PPLSLPPPPQ	GPMQLSTVGH	GDRDHEHLQK
810	820	830	840	850	860	870	880
SAKALRPTPQ	LAAEGDAVVF	SAPQEVQVTK	INPPPPYPGT	IPAAPTTAAP	PPPLPPPQPP	VDVCLKKGDF	SLYPTSVHYQ
890	900	910	920	930	940	950	960
TPLGYERITT	FDSSGNVEEV	CRPRTRMLCS	QNTYTLPGPG	SSATLRLTAT	EKKVPQPCSS	ATLNRLTVPR	YSIPTGDPPP
970	980	990	1000	1010	1020	1030	1040
YPEIASQLAQ	GRGAAQRSDN	SLIHATLRRN	NREATLKMAQ	LADSPRAPLQ	PLAKSKGGPG	GVVTQLPARP	PPALYTCSQC
1050	1060	1070	1080	1090	1100	1110	1120
SGTGPSSQPG	ASLAHTASAS	PLASQSSYSL	LSPPDSARDR	TDYVNSAFTE	DEALSQHCQL	EKPLRHPPLP	EAAVTLKRPP
1130	1140	1150	1160	1170	1180	1190	1200
PYQWDPMLGE	DVWVPQERTA	QTSGPNPLKL	SSLMLSQGQH	LDVSRLPFIS	PKSPASPTAT	FQTGYGMGVP	YPGSYNNPPL
1210	1220	1230	1240	1250	1260	1270	1280
PGVQAPCSPK	DALSPTQFAQ	QEPAVVLQPL	YPPSLSYCTL	PPMYPGSSTC	SSLQLPPVAL	HPWSSYSACP	PMQNPQGTLP
1290	1300	1310	1320	1330	1340	1350	1360
PKPHLVVEKP	LVSPPPADLQ	SHLGTEVMVE	TADNFQEVLS	LTESPVPQRT	EKFGKKNRKR	LDSRAEEGSV	QAITEGKVKK
1370	1380	1390	1400	1410	1420	1430	1440
EARTLSDFNS	LISSPHLGRE	KKKVKSQKDQ	LKSKKLNKTN	EFQDSSESEP	ELFISGDELM	NQSQGSRKGW	KSKRSPRAAG
1450	1460	1470	1480	1490	1500	1510	1520
ELEEAKCRRA	SEKEDGRLGS	QGFVYVMANK	QPLWNEATQV	YQLDFGGRVT	QESAKNFQIE	LEGRQVMQFG	RIDGSAYILD
1530	1540	1550					
FQYPFSAVQA	FAVALANVTQ	RLK					

Cmpd.	No. of Cmpds.	m/z meas.	Δ m/z [ppm]	z	Rt [min]	Score	P	Range	Sequence	Modification
2366	1	649.2906	-70.12	3	60.0	16.8	2	1340-1357	K.RLDSRAEEGSVQAITEGK.V	

Detailed Protein Report

Protein 611: SH2 domain-containing protein 4A isoform b [Homo sapiens]

**Accession:**	gi|292658785	**Score:**	16.8
**Database:**	refseq_human(refseq_human_20140103.fasta)	**MW [kDa]:**	46.9
**Database Date:**	2015-11-30	**pI:**	7.8
**Modification(s):**	Oxidation	**Sequence Coverage [%]:**	5.1
		**No. of unique Peptides:**	1

10	20	30	40	50	60	70	80
MWRVIEPPCP	GAPSTENGKS	VHWKLGADKE	VWVWVMGEHH	LDKPYDVLCN	EIIAERARLK	AEQEAEEPRK	THSEEFTNSL
**90**	**100**	**110**	**120**	**130**	**140**	**150**	**160**
KTKSQYHDLQ	APDNQQTKDI	WKKVAEKEEL	EQGSRPAPTL	EEEKIRSLSS	SSRNIQQMLA	DSINRMKAYA	FHQKKESMKK
**170**	**180**	**190**	**200**	**210**	**220**	**230**	**240**
KQDEEINQIE	EERTKQICKS	WKEDSEWQAS	LRKSKAADEK	RRSLAKQARE	DYKRLSLGAQ	KGRGGERLQS	PLRVPQKPER
**250**	**260**	**270**	**280**	**290**	**300**	**310**	**320**
PPLPPKPQFL	NSGAYPQKPL	RNQGVVRTLS	SSAQEDIIRW	FKEEQLPLRA	GYQKTSDTIA	PWFHGILTLK	KANELLLSTG
**330**	**340**	**350**	**360**	**370**	**380**	**390**	**400**
MPGSFLIRVS	ERIKGYALSY	LSEDGCKHFL	IDASADAYSF	LGVDQLQHAT	LADLVEYHKE	EPITSLGKEL	LLYPCGQQDQ
**410**							
LPDYLELFE							

Cmpd.	No. of Cmpds.	m/z meas.	Δ m/z [ppm]	z	Rt [min]	Score	P	Range	Sequence	Modification
1206	1	798.1046	26.39	3	45.2	16.8	2	125-145	K.IRSLSSSSRNIQQMLADSINR.M	Oxidation: 14

Detailed Protein Report

Protein 612: E3 ubiquitin-protein ligase RNF128 isoform 2 precursor [Homo sapiens]

**Accession:**	gi|37588871	**Score:**	16.8
**Database:**	refseq_human(refseq_human_20140103.fasta)	**MW [kDa]:**	44.6
**Database Date:**	2015-11-30	**pI:**	5.7
**Modification(s):**	Oxidation	**Sequence Coverage [%]:**	5.5
		**No. of unique Peptides:**	1

10	20	30	40	50	60	70	80
MNQENRSSFF	WLLVIFTFLL	KITASFSMSA	YVTVTYYNET	SNYTAIETCE	CGVYGLASPV	ANAMGVVGIP	KNNNYQACDH
**90**	**100**	**110**	**120**	**130**	**140**	**150**	**160**
NTEFSNTKKP	WIALIERGNC	TFSEKIQTAG	RRNADAVVIY	NAPETGNQTI	QMANFGAVDI	VAIMIGNLKG	TKILQSIQRG
**170**	**180**	**190**	**200**	**210**	**220**	**230**	**240**
IQVTMVIEVG	KKHGPWVNHY	SIFFVSVSFF	IITAATVGYF	IFYSARRLRN	ARAQSRKQRQ	LKADAKKAIG	RLQLRTLKQG
**250**	**260**	**270**	**280**	**290**	**300**	**310**	**320**
DKEIGPDGDS	CAVCIELYKP	NDLVRILTCN	HIFHKTCVDP	WLLEHRTCPM	CKCDILKALG	IEVDVEDGSV	SLQVPVSNEI
**330**	**340**	**350**	**360**	**370**	**380**	**390**	**400**
SNSASSHEED	NRSETASSGY	ASVQGTDEPP	LEEHVQSTNE	SLQLVNHEAN	SVAVDVIPHV	DNPTFEEDET	PNQETAVREI
**410**							
KS							

Cmpd.	No. of Cmpds.	m/z meas.	Δ m/z [ppm]	z	Rt [min]	Score	P	Range	Sequence	Modification
1366	2	1207.8238	107.80	2	45.6	16.8	2	150-171	K.GTKILQSIQRGIQVTMVIEVGK.K	Oxidation: 16

Detailed Protein Report

Protein 613: **von Willebrand factor D and EGF domain-containing protein precursor [Homo sapiens]**

**Accession:**	gi|209571555	**Score:**	16.8
**Database:**	refseq_human(refseq_human_20140103.fasta)	**MW [kDa]:**	176.7
**Database Date:**	2015-11-30	**pI:**	5.3
**Modification(s):**	Carbamidomethyl	**Sequence Coverage [%]:**	1.1
		**No. of unique Peptides:**	1

10	20	30	40	50	60	70	80
MPGGACVLVI	ALMFLAWGEA	QECSPGGHQF	LRSPYRSVRF	DSWHLQQSAV	QDLICDHSLS	PGWYRFLILD	RPAEMPTKCV
**90**	**100**	**110**	**120**	**130**	**140**	**150**	**160**
EMNHCGTQAP	IWLSLRDSET	LPSPGEIKQL	TACATWQFLF	STTKDCCLFQ	IPVSVRNCGN	FSVYLLQPTQ	GCMGYCAEAI
**170**	**180**	**190**	**200**	**210**	**220**	**230**	**240**
SDARLHPCGS	DETETGGDCV	RQLAASLPPP	PAGRPEVLVE	LIESRLFCRC	SFDVPATKNS	VGFHIAWSRL	SSQEVKEELT
**250**	**260**	**270**	**280**	**290**	**300**	**310**	**320**
QETTVQAFSL	LELDGINLRL	GDRIFCSASV	FFLENPHVQS	VAIESQEFFA	GFKLQPELST	ISEDGKEYYL	RIESTVPIIC
**330**	**340**	**350**	**360**	**370**	**380**	**390**	**400**
SEFSELDQEC	KISLKLKTIG	QGREHLGLNL	ALSSCHVDLL	QTSSCANGTC	SHTFVYYTAV	TDFSRDGDRV	SNIVVQPIVN
**410**	**420**	**430**	**440**	**450**	**460**	**470**	**480**
EDFLWNNYIP	DSIQIKVKDV	PTAYCYTFTD	PHIITFDGRV	YDNFKTGTFV	LYKSMSRDFE	VHVRQWDCRS	LHYPVSCNCG
**490**	**500**	**510**	**520**	**530**	**540**	**550**	**560**
FVAQEGGDIV	TFDMCNGQLR	ESQPYLFIKS	QDVTRNIKIS	ESYLGRKVTI	WFSSGAFIRA	DLGEWGMSLT	IRAPSVDYRN
**570**	**580**	**590**	**600**	**610**	**620**	**630**	**640**
TLGLCGTFDE	NPENDFHDKN	GMQIDQNFNN	YVAFINEWRI	LPGKSMSDTL	PVSMTSPGKP	SYCSCSLDTA	AYPSSEDLDS
**650**	**660**	**670**	**680**	**690**	**700**	**710**	**720**
VSRSEIALGC	KDLNHVSLSS	LIPELDVTSE	YINSDTLVRE	INKHTSPEEY	NLNLFLQEKK	HINLTKLGLN	VQKHPGNEKE
**730**	**740**	**750**	**760**	**770**	**780**	**790**	**800**
DSLQYLANKK	YTQGRGSHSQ	EMRYNRQNRW	KRQNFHEFPP	LFAFPSLSQT	DLEELTYFFP	EDHAEDVQQE	FFPSWPTPSG
**810**	**820**	**830**	**840**	**850**	**860**	**870**	**880**
LTEYSTLTLC	QETLANSSIG	RLCLAFLGKR	LDSVIEMCVK	DVLLKDDLSW	AEAGVALLEN	ECEKRIVEEG	KYNTEEYGTS
**890**	**900**	**910**	**920**	**930**	**940**	**950**	**960**
IEDILSVLKC	PNLCSGNGQC	MEWGCACSPS	FSSYDCSDSY	DKAPEITELG	NAGFCDVQKY	NCMMVRVFGK	GFKELPSIKC
**970**	**980**	**990**	**1000**	**1010**	**1020**	**1030**	**1040**
EVTKLQYNSS	EWMPGEPIYT	QTVFHNSRAV	DCQLPTDVQQ	FDTMDLVGGK	PTGKWQLKVS	NDGYKFSNPK	ITVIYDGACQ
**1050**	**1060**	**1070**	**1080**	**1090**	**1100**	**1110**	**1120**
VCGLYKNDSC	TIKENVCIID	GLCYVEGDKN	PTSPCLICRP	KISRFTWSFL	ENNQPPVIQA	LQDKLQTFYG	ENFEYQFVAF
**1130**	**1140**	**1150**	**1160**	**1170**	**1180**	**1190**	**1200**
DPEGSDIHFT	LDSGPEGASV	SSAGLFMWKT	DLLTTQQITV	RLNDDCDAET	RVTIEVTVKS	CDCLNGGSCV	SDRNFSPGSG
**1210**	**1220**	**1230**	**1240**	**1250**	**1260**	**1270**	**1280**
VYLCVCLPGF	HGSLCEVDIS	GCQSNPCGLG	SYISGFHSYS	CDCPPELKVE	TQFVNQFTTQ	TVVLTRSDKS	VNKEEDDKNA
**1290**	**1300**	**1310**	**1320**	**1330**	**1340**	**1350**	**1360**
QGRKRHVKPT	SGNAFTICKY	PCGKSRECVA	PNICKCKPGY	IGSNCQTALC	DPDCKNHGKC	IKPNICQCLP	GHGGATCDEE
**1370**	**1380**	**1390**	**1400**	**1410**	**1420**	**1430**	**1440**
HCNPPCQHGG	TCLAGNLCTC	PYGFVGPRCE	TMVCNRHCEN	GGQCLTPDIC	QCKPGWYGPT	CSTALCDPVC	LNGGSCNKPN
**1450**	**1460**	**1470**	**1480**	**1490**	**1500**	**1510**	**1520**
TCLCPNGFFG	EHCQNAFCHP	PCKNGGHCMR	NNVCVCREGY	TGRRFQKSIC	DPTCMNGGKC	VGPSTCSCPS	GWSGKRCNTP
**1530**	**1540**	**1550**	**1560**	**1570**	**1580**	**1590**	**1600**
ICLQKCKNGG	ECIAPSICHC	PSSWEGVRCQ	IPICNPKCLY	GGRCIFPNVC	SCRTEYSGVK	CEKKIQIRRH	

Cmpd.	No. of Cmpds.	m/z meas.	Δ m/z [ppm]	z	Rt [min]	Score	P	Range	Sequence	Modification
2213	1	916.8887	8.94	2	56.3	16.8	0	165-181	R.LHPCGSDETETGGDCVR.Q	Carbamidomethyl: 15

Detailed Protein Report

Protein 614: PREDICTED: vesicle-associated membrane protein 1 isoform X4 [Homo sapiens]

**Accession:**	gi|578822686	**Score:**	16.7
**Database:**	refseq_human(refseq_human_20140103.fasta)	**MW [kDa]:**	12.6
**Database Date:**	2015-11-30	**pI:**	9.7
		**Sequence Coverage [%]:**	15.5
		**No. of unique Peptides:**	1

```
        10         20         30         40         50         60         70         80
MSAPAQPPAE GTEGTAPGGG PPGPPPNMTS NRRLQQTQAQ VEEVVDIIRV NVDKVLERDQ KLSELDDRAD ALQAGASQFE
        90        100        110        120
SSAAKLKRKY WWKNCKMMIM LGAICAIIVV VIVRRG
```

Cmpd.	No. of Cmpds.	m/z meas.	Δ m/z [ppm]	z	Rt [min]	Score	P	Range	Sequence	Modification
2520	1	973.1016	37.36	2	62.1	16.7	0	97-114	K.MMIMLGAICAIIVVVIVR.R	

Detailed Protein Report

Protein 615: **PREDICTED: LOW QUALITY PROTEIN: uncharacterized protein LOC100129528 [Homo sapiens]**

Accession: gi|530401977
Database: refseq_human(refseq_human_20140103.fasta)
Database Date: 2015-11-30

Score: 16.7
MW [kDa]: 37.6
pI: 12.2
Sequence Coverage [%]: 5.4
No. of unique Peptides: 1

Alias proteins:

Accession	Name	Description
gi|530437161	refseq_human (refseq_human_20140103.fasta)	PREDICTED: LOW QUALITY PROTEIN: uncharacterized protein LOC100129528 [Homo sapiens]

10	20	30	40	50	60	70	80
MPALTEPVHV	HHELPTSSPG	RDPGPRAAHV	LSRKGPGSTS	CPRPLQEGTP	GSRAAHALSR	RGHRVHELPT	SSPGGDTGFM
**90**	**100**	**110**	**120**	**130**	**140**	**150**	**160**
SCPRPFQEGT	PGSRAAHVLS	RRGHRGPRVH	ELPTSSPGRD	PGSTSCPRPL	QEGTRVTNCP	RPLQEGTPGS	RAAHVLSRRG
**170**	**180**	**190**	**200**	**210**	**220**	**230**	**240**
HRVHELPTPS	PGRDPGFMSC	PRPLQEGTRV	TNCPRPLQEG	TRVTSCPRRL	QEGTRVTSCP	RPLQEGTRVT	NCPRALQEGT
**250**	**260**	**270**	**280**	**290**	**300**	**310**	**320**
PGSRAAHALS	RKGPRVHELP	TSSPGGDTGF	TSCPRPLQEG	TPGSRAAHAL	SRRGHRVHEL	PTSSPGRDPG	HELPTSSPGG
**330**	**340**	**350**	**360**				
DTGFTSCPRT	FQEGTPGSGL	LPAHIVPLCK	SEER				

Cmpd.	No. of Cmpds.	m/z meas.	Δ m/z [ppm]	z	Rt [min]	Score	P	Range	Sequence	Modification
2039	1	942.3107	-153.18	2	54.0	16.7	0	35-53	K.GPGSTSCPRPLQEGTPGSR.A	

Detailed Protein Report

Protein 616: **PREDICTED: E3 ubiquitin-protein ligase Hakai isoform X1 [Homo sapiens]**

**Accession:**	gi|578814630	**Score:**	16.7
**Database:**	refseq_human(refseq_human_20140103.fasta)	**MW [kDa]:**	40.8
**Database Date:**	2015-11-30	**pI:**	9.6
		**Sequence Coverage [%]:**	5.4
		**No. of unique Peptides:**	1

10	20	30	40	50	60	70	80
MIPCKHVFCY	DCAILHEKKG	DKMCPGCSDP	VQRIEQCTRG	SLFMCSIVQG	CKRTYLSQRD	LQAHINHRHM	RAGKPVTRAS
**90**	**100**	**110**	**120**	**130**	**140**	**150**	**160**
LENVHPPIAP	PPTEIPERFI	MPPDKHHMSH	IPPKQHIMMP	PPPLQHVPHE	HYNQPHEDIR	APPAELSMAP	PPPRSVSQET
**170**	**180**	**190**	**200**	**210**	**220**	**230**	**240**
FRISTRKHSN	LITVPIQDDS	NSGAREPPPP	APAPAHHHPE	YQGQPVVSHP	HHIMPPQQHY	APPPPPPPPI	SHPMPHPPQA
**250**	**260**	**270**	**280**	**290**	**300**	**310**	**320**
AGTPHLVYSQ	APPPPMTSAP	PPITPPPGHI	IAQMPPYMNH	PPPGPPPPQH	GGPPVTAPPP	HHYNPNSLPQ	FTEDQGTLSP
**330**	**340**	**350**	**360**	**370**	**380**		
PFTQPGGMSP	GIWPAPRGPP	PPPRLQGPPS	QTPLPGPHHP	DQTRYRPYYQ			

Cmpd.	No. of Cmpds.	m/z meas.	Δ m/z [ppm]	z	Rt [min]	Score	P	Range	Sequence	Modification
2201	1	753.7596	78.65	3	57.9	16.7	2	34-53	R.IEQCTRGSLFMCSIVQGCKR.T	

Detailed Protein Report

Protein 617: **poly(A)-specific ribonuclease PARN-like domain-containing protein 1 isoform 2 precursor [Homo sapiens]**

Accession: gi|27735155
Database: refseq_human(refseq_human_20140103.fasta)
Database Date: 2015-11-30
Modification(s): Carbamidomethyl

Score: 16.6
MW [kDa]: 60.1
pI: 9.6
Sequence Coverage [%]: 1.2
No. of unique Peptides: 1

10	20	30	40	50	60	70	80
MFCTRGLLFF	AFLAGLDIEF	TGLRSNLSGP	QQISLFDLPS	EWYLKTRQSV	QQFTVCQIGL	SVFSAIEGEA	NKYIAHSCNF
90	100	110	120	130	140	150	160
YLFPTTFGIL	DSEFSFQASS	VQFLNQYGFN	YNKFLKNGIP	YMNEEQEKKI	RHDILTGNWR	VRSSPDKDQI	KVVIDEVTRW
170	180	190	200	210	220	230	240
LELAKEGDWM	TLPGITGFQA	FEVQLVLRQA	LPNIWTVLKD	EGVVVKKVSK	QHRWYLQNTS	CDRESCWKEN	ILLSARGFSV
250	260	270	280	290	300	310	320
FFQMLVKAQK	PLVGHNMMMD	LLHLHEKFFR	PLPESYDQFK	QNIHSLFPVL	IDTKSVTKDI	WKEMNFPRVS	NLSEVYEVLN
330	340	350	360	370	380	390	400
SDLNPTKNSG	PEIVHASRCE	KYVETKCPHE	AAYDAFLCGS	VLLKVAHLLL	QKIYHIDPVP	ESSFPQYLDV	LAPYVNQVNL
410	420	430	440	450	460	470	480
IRAGVPKINF	SGPDYPSIRP	PILILSVKRW	PGVSEQQVYH	KFQNLCKFDV	RRLTRSQFLL	LTNKFKDARN	ILKEYRDHPT
490	500	510	520	530			
LCISLYRYWR	HSPNVNCLLQ	VCGIVTAWAL	LAFILGRSGT				

Cmpd.	No. of Cmpds.	m/z meas.	Δ m/z [ppm]	z	Rt [min]	Score	P	Range	Sequence	Modification
1449	1	405.2085	15.16	2	48.3	16.6	0	442-447	K.FQNLCK.F	Carbamidomethyl: 5

Detailed Protein Report

Protein 618: prolargin precursor [Homo sapiens]

**Accession:**	gi|4506041	**Score:**	16.6
**Database:**	refseq_human(refseq_human_20140103.fasta)	**MW [kDa]:**	43.8
**Database Date:**	2015-11-30	**pI:**	10.2
**Modification(s):**	Carbamidomethyl	**Sequence Coverage [%]:**	8.6
		**No. of unique Peptides:**	1

Alias proteins:

Accession	Name	Description
gi|41349454	refseq_human (refseq_human_20140103.fasta)	prolargin precursor [Homo sapiens]

10	20	30	40	50	60	70	80
MRSPLCWLLP	LLILASVAQG	QPTRRPRPGT	GPGRRPRPRP	RPTPSFPQPD	EPAEPTDLPP	PLPPGPPSIF	PDCPRECYCP
**90**	**100**	**110**	**120**	**130**	**140**	**150**	**160**
PDFPSALYCD	SRNLRKVPVI	PPRIHYLYLQ	NNFITELPVE	SFQNATGLRW	INLDNNRIRK	IDQRVLEKLP	GLVFLYMEKN
**170**	**180**	**190**	**200**	**210**	**220**	**230**	**240**
QLEEVPSALP	RNLEQLRLSQ	NHISRIPPGV	FSKLENLLLL	DLQHNRLSDG	VFKPDTFHGL	KNLMQLNLAH	NILRKMPPRV
**250**	**260**	**270**	**280**	**290**	**300**	**310**	**320**
PTAIHQLYLD	SNKIETIPNG	YFKSFPNLAF	IRLNYNKLTD	RGLPKNSFNI	SNLLVLHLSH	NRISSVPAIN	NRLEHLYLNN
**330**	**340**	**350**	**360**	**370**	**380**	**390**	
NSIEKINGTQ	ICPNDLVAFH	DFSSDLENVP	HLRYLRLDGN	YLKPPIPLDL	MMCFRLLQSV	VI	

Cmpd.	No. of Cmpds.	m/z meas.	Δ m/z [ppm]	z	Rt [min]	Score	P	Range	Sequence	Modification
1136	1	1207.6290	-47.21	3	44.3	16.6	2	2-34	M.RSPLCWLLPLLILASVAQGQPTRRPRPGTGPGR.R	Carbamidomethyl: 5

Detailed Protein Report

Protein 619: olfactory receptor 51A2 [Homo sapiens]

**Accession:**	gi|52317146	**Score:**	16.5
**Database:**	refseq_human(refseq_human_20140103.fasta)	**MW [kDa]:**	35.1
**Database Date:**	2015-11-30	**pI:**	10.0
		**Sequence Coverage [%]:**	3.5
		**No. of unique Peptides:**	1

10	20	30	40	50	60	70	80
MSIINTSYVE	ITTFFLVGMP	GLEYAHIWIS	IPICSMYLIA	ILGNGTILFI	IKTEPSLHGP	MYYFLSMLAM	SDLGLSLSSL
**90**	**100**	**110**	**120**	**130**	**140**	**150**	**160**
PTVLSIFLFN	APETSSSACF	AQEFFIHGFS	VLESSVLLIM	SFDRFLAIHN	PLRYTSILTT	VRVAQIGIVF	SFKSMLLVLP
**170**	**180**	**190**	**200**	**210**	**220**	**230**	**240**
FPFTLRSLRY	CKKNQLSHSY	CLHQDVMKLA	CSDNRIDVIY	GFFGALCLMV	DFILIAVSYT	LILKTVPGIA	SKKEELKALN
**250**	**260**	**270**	**280**	**290**	**300**	**310**	**320**
TCVSHICAVI	IFYLPIINLA	VVHRFAGHVS	PLINVLMANV	LLLVPPLMKP	IVYCVKTKQI	RVRVVAKLCQ	WKI

Cmpd.	No. of Cmpds.	m/z meas.	Δ m/z [ppm]	z	Rt [min]	Score	P	Range	Sequence	Modification
2802	1	665.3571	-56.63	2	64.1	16.5	2	302-312	R.VRVVAKLCQWK.I	

Detailed Protein Report

Protein 620: SLAM family member 7 isoform g [Homo sapiens]

**Accession:**	gi|543583727	**Score:**	16.5
**Database:**	refseq_human(refseq_human_20140103.fasta)	**MW [kDa]:**	16.5
**Database Date:**	2015-11-30	**pI:**	9.7
**Modification(s):**	Carbamidomethyl	**Sequence Coverage [%]:**	12.1
		**No. of unique Peptides:**	1

```
        10         20         30         40         50         60         70         80
MAGSPTCLTL IYILWQLTEH LSKPKVTMGL QSNKNGTCVT NLTCCMEHGE EDVIYTWKAL GQAANESHNG SILPISWRWG
        90        100        110        120        130        140        150
ESDMTFICVA RNPVSRNFSS PILARKLCEE NNPKGRSSKY GLLHCGNTEK DGKSPLTAHD ARHTKAICL
```

Cmpd.	No. of Cmpds.	m/z meas.	Δ m/z [ppm]	z	Rt [min]	Score	P	Range	Sequence	Modification
1671	1	1058.9456	-92.93	2	51.2	16.5	2	97-114	R.NFSSPILARKLCEENNPK.G	Carbamidomethyl: 12

Detailed Protein Report

Protein 621: **interferon-related developmental regulator 2 [Homo sapiens]**

**Accession:**	gi|197333755	**Score:**	16.5
**Database:**	refseq_human(refseq_human_20140103.fasta)	**MW [kDa]:**	54.8
**Database Date:**	2015-11-30	**pI:**	9.3
		**Sequence Coverage [%]:**	5.1
		**No. of unique Peptides:**	1

10	20	30	40	50	60	70	80
MAWNSPSRRP	VWQGGAPRED	GGARGVWLPS	SGQVSAQRTG	RRLVGLEPTP	TGSLTPRPPR	PVPGMPRARK	GNTLRKGGQR
**90**	**100**	**110**	**120**	**130**	**140**	**150**	**160**
RGGGARSSAQ	ADSGSSDDEA	ASEARSTASE	CPSLLSTTAE	DSLGGDVVDE	QGQQEDLEEK	LKEYVDCLTD	KSAKTRQGAL
**170**	**180**	**190**	**200**	**210**	**220**	**230**	**240**
ESLRLALASR	LLPDFLLERR	LTLADALEKC	LKKGKGEEQA	LAAAVLGLLC	VQLGPGPKGE	ELFHSLQPLL	VSVLSDSTAS
**250**	**260**	**270**	**280**	**290**	**300**	**310**	**320**
PAARLHCASA	LGLGCYVAAA	DIQDLVSCLA	CLESVFSRFY	GLGGSSTSPV	VPASLHGLLS	AALQAWALLL	TICPSTQISH
**330**	**340**	**350**	**360**	**370**	**380**	**390**	**400**
ILDRQLPRLP	QLLSSESVNL	RIAAGETIAL	LFELARDLEE	EFVYEDMEAL	CSVLRTLATD	SNKYRAKADR	RRQRSTFRAV
**410**	**420**	**430**	**440**	**450**	**460**	**470**	**480**
LHSVEGGECE	EEIVRFGFEV	LYMDSWARHR	IYAAFKEVLG	SGMHHHLQNN	ELLRDIFGLG	PVLLLDATAL	KACKVPRFEK
**490**	**500**	**510**					
HLYNAAAFKA	RTKARSRVRD	KRADIL					

Cmpd.	No. of Cmpds.	m/z meas.	Δ m/z [ppm]	z	Rt [min]	Score	P	Range	Sequence	Modification
1054	1	849.7307	-100.08	3	41.7	16.5	2	193-218	K.KGKGEEQALAAAVLGLLCVQLGPGPK.G	

Detailed Protein Report

Protein 622: eukaryotic translation initiation factor 6 isoform c [Homo sapiens]

**Accession:**	gi|31563374	**Score:**	16.5
**Database:**	refseq_human(refseq_human_20140103.fasta)	**MW [kDa]:**	23.7
**Database Date:**	2015-11-30	**pI:**	4.9
		**Sequence Coverage [%]:**	10.6
		**No. of unique Peptides:**	1

10	20	30	40	50	60	70	80
MAVRASFENN	CEIGCFAKLT	NTYCLVAIGG	SENFYRCGGS	PGAYGGGEAC	AGVKSSGSGR	VPAPLPRHHR	VHVPTVCSRA
**90**	**100**	**110**	**120**	**130**	**140**	**150**	**160**
SSPIPSPWCT	RLSPAAASSG	ACVWETEEIL	ADVLKVEVFR	QTVADQVLVG	SYCVFSNQGG	LVHPKTSIED	QDELSSLLQV
**170**	**180**	**190**	**200**	**210**	**220**	**230**	
PLVAGTVNRG	SEVIAAGMVV	NDWCAFCGLD	TTSTELSVVE	SVFKLNEAQP	STIATSMRDS	LIDSLT	

Cmpd.	No. of Cmpds.	m/z meas.	Δ m/z [ppm]	z	Rt [min]	Score	P	Range	Sequence	Modification
2446	1	862.1749	63.34	3	61.1	16.5	0	146-169	K.TSIEDQDELSSLLQVPLVAGTVNR.G	

Detailed Protein Report

Protein 623: PREDICTED: zinc finger protein 646 isoform X3 [Homo sapiens]

**Accession:**	gi|530409439	**Score:**	16.5
**Database:**	refseq_human(refseq_human_20140103.fasta)	**MW [kDa]:**	198.5
**Database Date:**	2015-11-30	**pI:**	6.8
**Modification(s):**	Carbamidomethyl	**Sequence Coverage [%]:**	0.4
		**No. of unique Peptides:**	1

10	20	30	40	50	60	70	80
MEDTPPSLSC	SDCQRHFPSL	PELSRHRELL	HPSPNQDSEE	ADSIPRPYRC	QQCGRGYRHP	GSLVNHRRTH	ETGLFPCTTC
90	100	110	120	130	140	150	160
GKDFSNPMAL	KSHMRTHAPE	GRRRHRPPRP	KEATPHLQGE	TVSTDSWGQR	LGSSEGWENQ	TKHTEETPDC	ESVPDPRAAS
170	180	190	200	210	220	230	240
GTWEDLPTRQ	REGLASHPGP	EDGADGWGPS	TNSARAPPLP	IPASSLLSNL	EQYLAESVVN	FTGGQEPTQS	PPAEEERRYK
250	260	270	280	290	300	310	320
CSQCGKTYKH	AGSLTNHRQS	HTLGIYPCAI	CFKEFSNLMA	LKNHSRLHAQ	YRPYHCPHCP	RVFRLPRELL	EHQQSHEGER
330	340	350	360	370	380	390	400
QEPRWEEKGM	PTTNGHTDES	SQDQLPSAQM	LNGSAELSTS	GELEDSGLEE	YRPFRCGDCG	RTYRHAGSLI	NHRKSHQTGV
410	420	430	440	450	460	470	480
YPCSLCSKQL	FNAAALKNHV	RAHHRPRQGV	GENGQPSVPP	APLLLAETTH	KEEEDPTTTL	DHRPYKCSEC	GRAYRHRGSL
490	500	510	520	530	540	550	560
VNHRHSHRTG	EYQCSLCPRK	YPNLMALRNH	VRVHCKAARR	SADIGAEGAP	SHLKVELPPD	PVEAEAAPHT	DQDHVCKHEE
570	580	590	600	610	620	630	640
EATDITPAAD	KTAAHICSIC	GLLFEDAESL	ERHGLTHGAG	EKENSRTETT	MSPPRAFACR	DCGKSYRHSG	SLINHRQTHQ
650	660	670	680	690	700	710	720
TGDFSCGACA	KHFHTMAAMK	NHLRRHSRRR	SRRHRKRAGG	ASGGREAKLL	AAESWTRELE	DNEGLESPQD	PSGESPHGAE
730	740	750	760	770	780	790	800
GNLESDGDCL	QAESEGDKCG	LERDETHFQG	DKESGGTGEG	LERKDASLLD	NLDIPGEEGG	GTHFCDSLTG	VDEDQKPATG
810	820	830	840	850	860	870	880
QPNSSSHSAN	AVTGWQAGAA	HTCSDCGHSF	PHATGLLSHR	PCHPPGIYQC	SLCPKEFDSL	PALRSHFQNH	RPGEATSAQP
890	900	910	920	930	940	950	960
FLCCLCGMIF	PGRAGYRLHR	RQAHSSSGMT	EGSEEEGEEE	GVAEAAPARS	PPLQLSEAEL	LNQLQREVEA	LDSAGYGHIC
970	980	990	1000	1010	1020	1030	1040
GCCGQTYDDL	GSLERHHQSQ	SSGTTADKAP	SPLGVAGDAM	EMVVDSVLED	IVNSVSGEGG	DAKSQEGAGT	PLGDSLCIQG
1050	1060	1070	1080	1090	1100	1110	1120
GESLLEAQPR	PFRCNQCGKT	YRHGGSLVNH	RKIHQTGDFL	CPVCSRCYPN	LAAYRNHLRN	HPRCKGSEPQ	VGPIPEAAGS
1130	1140	1150	1160	1170	1180	1190	1200
SELQVGPIPE	GGSNKPQHMA	EEGPGQAEVE	KLQEELKVEP	LEEVARVKEE	VWEETTVKGE	EIEPRLETAE	KGCQTEASSE
1210	1220	1230	1240	1250	1260	1270	1280
RPFSCEVCGR	SYKHAGSLIN	HRQSHQTGHF	GCQACSKGFS	NLMSLKNHRR	IHADPRRFRC	SECGKAFRLR	KQLASHQRVH
1290	1300	1310	1320	1330	1340	1350	1360
MERRGGGGTR	KATREDRPFR	CGQCGRTYRH	AGSLLNHRRS	HETGQYSCPT	CPKTYSNRMA	LKDHQRLHSE	NRRRRAGRSR
1370	1380	1390	1400	1410	1420	1430	1440
RTAVRCALCG	RSFPGRGSLE	RHLREHEETE	REPANGQGGL	DGTAASEANL	TGSQGLETQL	GGAEPVPHLE	DGVPRPGERS
1450	1460	1470	1480	1490	1500	1510	1520
QSPIRAASSE	APEPLSWGAG	KAGGWPVGGG	LGNHSGGWVP	QFLTRSEEPE	DSVHRSPCHA	GDCQLNGPTL	SHMDSWDNRD
1530	1540	1550	1560	1570	1580	1590	1600
NSSQLQPGSH	SSCSQCGKTY	CQSGSLLNHN	TNKTDRHYCL	LCSKEFLNPV	ATKSHSHNHI	DAQTFACPDC	GKAFESHQEL
1610	1620	1630	1640	1650	1660	1670	1680
ASHLQAHARG	HSQVPAQMEE	ARDPKAGTGE	DQVVLPGQGK	AQEAPSETPR	GPGESVERAR	GGQAVTSMAA	EDKERPFRCT
1690	1700	1710	1720	1730	1740	1750	1760
QCGRSYRHAG	SLLNHQKAHT	TGLYPCSLCP	KLLPNLLSLK	NHSRTHTDPK	RHCCSICGKA	FRTAARLEGH	GRVHAPREGP
1770	1780	1790	1800	1810			
FTCPHCPRHF	RRRISFVQHQ	QQHQEEWTVA	GSGRGHEGSQ	EEEPQWHQ			

Cmpd.	No. of	m/z meas.	Δ m/z	z	Rt	Score	P	Range	Sequence	Modification

Detailed Protein Report

	Cmpds.		[ppm]		[min]					
1417	7	1023.6578	178.56	1	47.9	16.5	0	1557-1564	R.HYCLLCSK.E	Carbamidomethyl: 3

Detailed Protein Report

Protein 624: **PREDICTED: nuclear pore complex protein Nup214 isoform X4 [Homo sapiens]**

**Accession:**	gi|530427224	**Score:**	16.5
**Database:**	refseq_human(refseq_human_20140103.fasta)	**MW [kDa]:**	209.0
**Database Date:**	2015-11-30	**pI:**	9.0
		**Sequence Coverage [%]:**	0.9
		**No. of unique Peptides:**	1

Detailed Protein Report

10	20	30	40	50	60	70	80
MRGQLRAAGA	EGRKFAVERP	GFRGQGRGRQ	RWLLRHTEGG	AMGDEMDAMI	PEREMKDFQF	RALKKVRIFD	SPEELPKERS
90	100	110	120	130	140	150	160
SLLAVSNKYG	LVFAGGASGL	QIFPTKNLLI	QNKPGDDPNK	IVDKVQGLLV	PMKFPIHHLA	LSCDNLTLSA	CMMSSEYGSI
170	180	190	200	210	220	230	240
IAFFDVRTFS	NEAKQQKRPF	AYHKLLKDAG	GMVIDMKWNP	TVPSMVAVCL	ADGSIAVLQV	TETVKVCATL	PSTVAVTSVC
250	260	270	280	290	300	310	320
WSPKGKQLAV	GKQNGTVVQY	LPTLQEKKVI	PCPPFYESDH	PVRVLDVLWI	GTYVFAIVYA	AADGTLETSP	DVVMALLPKK
330	340	350	360	370	380	390	400
EEKHPEIFVN	FMEPCYGSCT	ERQHHYYLSY	IEEWDLVLAA	SAASTEVSIL	ARQSDQINWE	SWLLEDSSRA	ELPVTDKSDD
410	420	430	440	450	460	470	480
SLPMGVVVDY	TNQVEITISD	EKTLPPAPVL	MLLSTDGVLC	PFYMINQNPG	VKSLIKTPER	LSLEGERQPK	SPASLAPTPA
490	500	510	520	530	540	550	560
ASPVAPSAAS	FSFGSSGFKP	TLESTPVPSV	SAPNIAMKPS	FPPSTSAVKV	NLSEKFTAAA	TSTPVSSSQS	APPMSPFSSA
570	580	590	600	610	620	630	640
SKPAASGPLS	HPTPLSAPPS	SVPLKSSVLP	SPSAGRSAQG	SSSPVPSMVQ	KSPRITPPAA	KPGSPQAKSL	QPAVAEKQGH
650	660	670	680	690	700	710	720
QWKDSDPVMA	GIGEEIAHFQ	KELEELKART	SKACFQVGTS	EEMKMLRTES	DDLHTFLLEI	KETTESLHGD	ISSLKTTLLE
730	740	750	760	770	780	790	800
GFAGVEEARE	QNERNRDSGY	LHLLYKRPLD	PKSEAQLQEI	RRLHQYVKFA	VQDVNDVLDL	EWDQHLEQKK	KQRHLLVPER
810	820	830	840	850	860	870	880
ETLFNTLANN	REIINQQRKR	LNHLVDSLQQ	LRLYKQTSLW	SLSSAVPSQS	SIHSFDSDLE	SLCNALLKTT	IESHTKSLPK
890	900	910	920	930	940	950	960
VPAKLSPMKQ	AQLRNFLAKR	KTPPVRSTAP	ASLSRSAFLS	QRYYEDLDEV	SSTSSVSQSL	ESEDARTSCK	DDEAVVQAPR
970	980	990	1000	1010	1020	1030	1040
HAPVVRTPSI	QPSLLPHAAP	FAKSHLVHGS	SPGVMGTSVA	TSASKIIPQG	ADSTMLATKT	VKHGAPSPSH	PISAPQAAAA
1050	1060	1070	1080	1090	1100	1110	1120
AALRRQMASQ	APAVNTLTES	TLKNVPQVVN	VQELKNNPAT	PSTAMGSSVP	YSTAKTPHPV	LTPVAANQAK	QGSLINSLKP
1130	1140	1150	1160	1170	1180	1190	1200
SGPTPASGQL	SSGDKASGTA	KIETAVTSTP	SASGQFSKPF	SFSPSGTGFN	FGIITPTPSS	NFTAAQGATP	STKESSQPDA
1210	1220	1230	1240	1250	1260	1270	1280
FSSGGGSKPS	YEAIPESSPP	SGITSASNTT	PGEPAASSSR	PVAPSGTALS	TTSSKLETPP	SKLGELLFPS	SLAGETLGSF
1290	1300	1310	1320	1330	1340	1350	1360
SGLRVGQADD	STKPTNKASS	TSLTSTQPTK	TSGVPSGFNF	TAPPVLGKHT	EPPVTSSATT	TSVAPPAATS	TSSTAVFGSL
1370	1380	1390	1400	1410	1420	1430	1440
PVTSAGSSGV	ISFGGTSLSA	GKTSFSFGSQ	QTNSTVPPSA	PPPTTAATPL	PTSFPTLSFG	SLLSSATTPS	LPMSAGRSTE
1450	1460	1470	1480	1490	1500	1510	1520
EATSSALPEK	PGDSEVSASA	ASLLEEQQSA	QLPQAPPQTS	DSVKKEPVLA	QPAVSNSGTA	ASSTSLVALS	AEATPATTGV
1530	1540	1550	1560	1570	1580	1590	1600
PDARTEAVPP	ASSFSVPGQT	AVTAAAISSA	GPVAVETSST	PIASSTTSIV	APGPSAEAAA	FGTVTSGSSV	FAQPPAASSS
1610	1620	1630	1640	1650	1660	1670	1680
SAFNQLTNNT	ATAPSATPVF	GQVAASTAPS	LFGQQTGSTA	STAAATPQVS	SSGFSSPAFG	TTAPGVFGQT	TFGQASVFGQ
1690	1700	1710	1720	1730	1740	1750	1760
SASSAASVFS	FSQPGFSSVP	AFGQPASSTP	TSTSGSVFGA	ASSTSSSSSF	SFGQSSPNTG	GGLFGQSNAP	AFGQSPGFGQ
1770	1780	1790	1800	1810	1820	1830	1840
GGSVFGGTSA	ATTTAATSGF	SFCQASGFGS	SNTGSVFGQA	ASTGGIVFGQ	QSSSSSGSVF	GSGNTGRGGG	FFSGLGGKPS
1850	1860	1870	1880	1890	1900	1910	1920
QDAANKNPFS	SASGGFGSTA	TSNTSNLFGN	SGAKTFGGFA	SSSFGEQKPT	GTFSSGGGSV	ASQGFGFSSP	NKTGGFGAAP
1930	1940	1950	1960	1970	1980	1990	2000
VFGSPPTFGG	SPGFGGVPAF	GSAPAFTSPL	GSTGGKVFGE	GTAAASAGGF	GFGSSSNTTS	FGTLASQNAP	TFGSLSQQTS
2010	2020	2030	2040				
GFGTQSSGFS	GFGSGTGGFS	FGSNNSSVQG	FGGWRS				

Cmpd.	No. of Cmpds.	m/z meas.	Δ m/z [ppm]	z	Rt [min]	Score	P	Range	Sequence	Modification

Detailed Protein Report

Cmpd.	No. of Cmpds.	m/z meas.	Δ m/z [ppm]	z	Rt [min]	Score	P	Range	Sequence	Modification
1945	2	1023.3932	-98.93	2	54.7	16.5	2	670-687	R.TSKACFQVGTSEEMKMLR.T	

Detailed Protein Report

Protein 625: lipocalin-1 isoform 3 precursor [Homo sapiens]

Accession: gi|357933621
Database: refseq_human(refseq_human_20140103.fasta)
Database Date: 2015-11-30

Score: 16.5
MW [kDa]: 25.1
pI: 9.9
Sequence Coverage [%]: 5.7
No. of unique Peptides: 1

10	20	30	40	50	60	70	80
MKPLLLAVSL	GLIAALQAHH	LLASDEEIQD	VSGTWYLKAM	TVDREFPEMN	LESVTPMTLT	TLEGGNLEAK	VTMLISGRCQ
**90**	**100**	**110**	**120**	**130**	**140**	**150**	**160**
EVKAVLEKTD	EPGKYTADGG	KHVAYIIRSH	VKDHYIFYCE	GELHGKPVRG	AETPRTTWKP	WRTLRKPQEP	ADSARRASSS
**170**	**180**	**190**	**200**	**210**	**220**	**230**	**240**
PGRAKPALQG	AIRGRGHLGS	SAAQGRHHPA	PPSFTGTWKK	LPTPAERGWL	HPFLPPPAFP	LPCAPSPGSP	

Cmpd.	No. of Cmpds.	m/z meas.	Δ m/z [ppm]	z	Rt [min]	Score	P	Range	Sequence	Modification
362	1	529.2876	15.71	3	34.5	16.5	1	130-142	R.GAETPRTTWKPWR.T	

Detailed Protein Report

Protein 626: ribosomal RNA-processing protein 8 [Homo sapiens]

**Accession:**	gi|12758125	**Score:**	16.5
**Database:**	refseq_human(refseq_human_20140103.fasta)	**MW [kDa]:**	50.7
**Database Date:**	2015-11-30	**pI:**	10.4
		**Sequence Coverage [%]:**	2.6
		**No. of unique Peptides:**	1

10	20	30	40	50	60	70	80
MFEEPEWAEA	APVAAGLGPV	ISRPPPAASS	QNKGSKRRQL	LATLRALEAA	SLSQHPPSLC	ISDSEEEEEE	RKKKCPKKAS
90	100	110	120	130	140	150	160
FASASAEVGK	KGKKKCQKQG	PPCSDSEEEV	ERKKKCHKQA	LVGSDSAEDE	KRKRKCQKHA	PINSAQHLDN	VDQTGPKAWK
170	180	190	200	210	220	230	240
GSTTNDPPKQ	SPGSTSPKPP	HTLSRKQWRN	RQKNKRRCKN	KFQPPQVPDQ	APAEAPTEKT	EVSPVPRTDS	HEARAGALRA
250	260	270	280	290	300	310	320
RMAQRLDGAR	FRYLNEQLYS	GPSSAAQRLF	QEDPEAFLLY	HRGFQSQVKK	WPLQPVDRIA	RDLRQRPASL	VVADFGCGDC
330	340	350	360	370	380	390	400
RLASSIRNPV	HCFDLASLDP	RVTVCDMAQV	PLEDESVDVA	VFCLSLMGTN	IRDFLEEANR	VLKPGGLLKV	AEVSSRFEDV
410	420	430	440	450	460		
RTFLRAVTKL	GFKIVSKDLT	NSHFFLFDFQ	KTGPPLVGPK	AQLSGLQLQP	CLYKRR		

Cmpd.	No. of Cmpds.	m/z meas.	Δ m/z [ppm]	z	Rt [min]	Score	P	Range	Sequence	Modification
719	1	690.8999	-31.62	2	39.0	16.5	2	402-413	R.TFLRAVTKLGFK.I	

Detailed Protein Report

Protein 627: unconventional myosin-Ia [Homo sapiens]

**Accession:**	gi|4885503	**Score:**	16.4
**Database:**	refseq_human(refseq_human_20140103.fasta)	**MW [kDa]:**	118.3
**Database Date:**	2015-11-30	**pI:**	9.9
**Modification(s):**	Oxidation	**Sequence Coverage [%]:**	1.8
		**No. of unique Peptides:**	1

Alias proteins:

Accession	Name	Description
gi|365733629	refseq_human (refseq_human_20140103.fasta)	unconventional myosin-Ia [Homo sapiens]

```
        10         20         30         40         50         60         70         80
MPLLEGSVGV EDLVLLEPLV EESLLKNLQL RYENKEIYTY IGNVVISVNP YQQLPIYGPE FIAKYQDYTF YELKPHIYAL
        90        100        110        120        130        140        150        160
ANVAYQSLRD RDRDQCILIT GESGSGKTEA SKLVMSYVAA VCGKGEQVNS VKEQLLQSNP VLEAFGNAKT IRNNNSSRFG
       170        180        190        200        210        220        230        240
KYMDIEFDFK GSPLGGVITN YLLEKSRLVK QLKGERNFHI FYQLLAGADE QLLKALKLER DTTGYAYLNH EVSRVDGMDD
       250        260        270        280        290        300        310        320
ASSFRAVQSA MAVIGFSEEE IRQVLEVTSM VLKLGNVLVA DEFQASGIPA SGIRDGRGVR EIGEMVGLNS EEVERALCSR
       330        340        350        360        370        380        390        400
TMETAKEKVV TALNVMQAQY ARDALAKNIY SRLFDWIVNR INESIKVGIG EKKKVMGVLD IYGFEILEDN SFEQFVINYC
       410        420        430        440        450        460        470        480
NEKLQQVFIE MTLKEEQEEY KREGIPWTKV DYFDNGIICK LIEHNQRGIL AMLDEECLRP GVVSDSTFLA KLNQLFSKHG
       490        500        510        520        530        540        550        560
HYESKVTQNA QRQYDHTMGL SCFRICHYAG KVTYNVTSFI DKNNDLLFRD LLQAMWKAQH PLLRSLFPEG NPKQASLKRP
       570        580        590        600        610        620        630        640
PTAGAQFKSS VAILMKNLYS KSPNYIRCIK PNEHQQRGQF SSDLVATQAR YLGLLENVRV RRAGYAHRQG YGPFLERYRL
       650        660        670        680        690        700        710        720
LSRSTWPHWN GGDREGVEKV LGELSMSSGE LAFGKTKIFI RSPKTLFYLE EQRRLRLQQL ATLIQKIYRG WRCRTHYQLM
       730        740        750        760        770        780        790        800
RKSQILISSW FRGNMQKKCY GKIKASVLLI QAFVRGWKAR KNYRKYFRSE AALTLADFIY KSMVQKFLLG LKNNLPSTNV
       810        820        830        840        850        860        870        880
LDKTWPAAPY KCLSTANQEL QQLFYQWKCK RFRDQLSPKQ VEILREKLCA SELFKGKKAS YPQSVPIPFC GDYIGLQGNP
       890        900        910        920        930        940        950        960
KLQKLKGGEE GPVLMAEAVK KVNRGNGKTS SRILLLTKGH VILTDTKKSQ AKIVIGLDNV AGVSVTSLKD GLFSLHLSEM
       970        980        990       1000       1010       1020       1030       1040
SSVGSKGDFL LVSEHVIELL TKMYRAVLDA TQRQLTVTVT EKFSVRFKEN SVAVKVVQGP AGGDNSKLRY KKKGSHCLEV
      1050
TVQ
```

Cmpd.	No. of Cmpds.	m/z meas.	Δ m/z [ppm]	z	Rt [min]	Score	P	Range	Sequence	Modification
717	1	1100.6526	53.81	2	38.9	16.4	2	569-587	K.SSVAILMKNLYSKSPNYIR.C	Oxidation: 7

Detailed Protein Report

Protein 628: AN1-type zinc finger protein 5 [Homo sapiens]

**Accession:**	gi|5174755	**Score:**	16.4
**Database:**	refseq_human(refseq_human_20140103.fasta)	**MW [kDa]:**	23.1
**Database Date:**	2015-11-30	**pI:**	10.0
**Modification(s):**	Carbamidomethyl, Oxidation	**Sequence Coverage [%]:**	16.4
		**No. of unique Peptides:**	1

Alias proteins:

Accession	Name	Description
gi|503774511	refseq_human (refseq_human_20140103.fasta)	AN1-type zinc finger protein 5 [Homo sapiens]
gi|503774491	refseq_human (refseq_human_20140103.fasta)	AN1-type zinc finger protein 5 [Homo sapiens]
gi|503774489	refseq_human (refseq_human_20140103.fasta)	AN1-type zinc finger protein 5 [Homo sapiens]
gi|156231049	refseq_human (refseq_human_20140103.fasta)	AN1-type zinc finger protein 5 [Homo sapiens]
gi|156231047	refseq_human (refseq_human_20140103.fasta)	AN1-type zinc finger protein 5 [Homo sapiens]

10	20	30	40	50	60	70	80
MAQETNQTPG	PMLCSTGCGF	YGNPRTNGMC	SVCYKEHLQR	QQNSGRMSPM	GTASGSNSPT	SDSASVQRAD	TSLNNCEGAA
**90**	**100**	**110**	**120**	**130**	**140**	**150**	**160**
GSTSEKSRNV	PVAALPVTQQ	MTEMSISRED	KITTPKTEVS	EPVVTQPSPS	VSQPSTSQSE	EKAPELPKPK	KNRCFMCRKK
**170**	**180**	**190**	**200**	**210**	**220**		
VGLTGFDCRC	GNLFCGLHRY	SDKHNCPYDY	KAEAAAKIRK	ENPVVVAEKI	QRI		

Cmpd.	No. of Cmpds.	m/z meas.	Δ m/z [ppm]	z	Rt [min]	Score	P	Range	Sequence	Modification
1730	1	1273.8446	-16.45	3	51.9	16.4	1	1-35	-.MAQETNQTPGPMLCSTGCGFYGNPRTNGMCSVCYK.E	Carbamidomethyl: 30; Oxidation: 29

Detailed Protein Report

Protein 629: **round spermatid basic protein 1 [Homo sapiens]**

**Accession:**	gi|38683851	**Score:**	16.4
**Database:**	refseq_human(refseq_human_20140103.fasta)	**MW [kDa]:**	90.0
**Database Date:**	2015-11-30	**pI:**	9.7
		**Sequence Coverage [%]:**	1.9
		**No. of unique Peptides:**	1

10	20	30	40	50	60	70	80
MFISGRRTAD	KWRAEERLQC	PAGSARAALA	RCADGGAVGP	FKCVFVGEMA	AQVGAVRVVR	AVAAQEEPDK	EGKEKPHAGV
90	100	110	120	130	140	150	160
SPRGVKRQRR	SSSGGSQEKR	GRPSQEPPLA	PPHRRRRSRQ	HPGPLPPTNA	APTVPGPVEP	LLLPPPPPPS	LAPAGPAVAA
170	180	190	200	210	220	230	240
PLPAPSTSAL	FTFSPLTVSA	AGPKHKGHKE	RHKHHHHRGP	DGDPSSCGTD	LKHKDKQENG	ERTGGVPLIK	APKRETPDEN
250	260	270	280	290	300	310	320
GKTQRADDFV	LKKIKKKKKK	KHREDMRGRR	LKMYNKEVQT	VCAGLTRISK	EILTQGQINS	TSGLNKESFR	YLKDEQLCRL
330	340	350	360	370	380	390	400
NLGMQEYRVP	QGVQTPFMTH	QEHSIRRNFL	KTGTKFSNFI	HEEHQSNGGA	LVLHAYMDEL	SFLSPMEMER	FSEEFLALTF
410	420	430	440	450	460	470	480
SENEKNAAYY	ALAIVHGAAA	YLPDFLDYFA	FNFPNTPVKM	EILGKKDIET	TTISNFHTQV	NRTYCCGTYR	AGPMRQISLV
490	500	510	520	530	540	550	560
GAVDEEVGDY	FPEFLDMLEE	SPFLKMTLPW	GTLSSLRLQC	RSQSDDGPIM	WVRPGEQMIP	TADMPKSPFK	RRRSMNEIKN
570	580	590	600	610	620	630	640
LQYLPRTSEP	REVLFEDRTR	AHADHVGQGF	DWQSTAAVGV	LKAVQFGEWS	DQPRITKDVI	CFHAEDFTDV	VQRLQLDLHE
650	660	670	680	690	700	710	720
PPVSQCVQWV	DEAKLNQMRR	EGIRYARIQL	CDNDIYFIPR	NVIHQFKTVS	AVCSLAWHIR	LKQYHPVVEA	TQNTESNSNM
730	740	750	760	770	780	790	800
DCGLTGKREL	EVDSQCVRIK	TESEEACTEI	QLLTTASSSF	PPASELNLQQ	DQKTQPIPVL	KVESRLDSDQ	QHNLQEHSTT
810							
SV							

Cmpd.	No. of Cmpds.	m/z meas.	Δ m/z [ppm]	z	Rt [min]	Score	P	Range	Sequence	Modification
1924	1	768.8264	-78.40	2	54.4	16.4	0	43-57	K.CVFVGEMAAQVGAVR.V	

Detailed Protein Report

Protein 630: zinc finger and SCAN domain-containing protein 30 isoform 2 [Homo sapiens]

**Accession:**	gi|569026709	**Score:**	16.4
**Database:**	refseq_human(refseq_human_20140103.fasta)	**MW [kDa]:**	34.5
**Database Date:**	2015-11-30	**pI:**	10.1
		**Sequence Coverage [%]:**	5.9
		**No. of unique Peptides:**	1

10	20	30	40	50	60	70	80
MVAGKVLMAK	QEIVECVASA	AMISPGKLPG	ETHSQRIAEE	ALGGLDNSKK	QKGNAAGNKI	SQLPSQDRHF	SLATFNRRIP
**90**	**100**	**110**	**120**	**130**	**140**	**150**	**160**
TEHSVLESHE	SEGSFSMNSN	DITQQSVDTR	EKLYECFDCG	KAFCQSSKLI	RHQRIHTGER	PYACKECGKA	FSLSSDLVRH
**170**	**180**	**190**	**200**	**210**	**220**	**230**	**240**
QRIHSGEKPY	ECCECGKAFR	GSSELIRHRR	IHTGEKPYEC	GECGKAFSRS	SALIQHKKIH	TGDKSYECIA	CGKAFGRSSI
**250**	**260**	**270**	**280**	**290**	**300**	**310**	
LIEHQRIHTG	EKPYECNECG	KSFNQSSALT	QHQRIHTGEK	PYECSECRKT	FRHRSGLMQH	QRTHTRV	

Cmpd.	No. of Cmpds.	m/z meas.	Δ m/z [ppm]	z	Rt [min]	Score	P	Range	Sequence	Modification
1677	1	956.4573	-48.17	2	49.5	16.4	2	51-68	K.QKGNAAGNKISQLPSQDR.H	

Detailed Protein Report

Protein 631: bone morphogenetic protein 1 isoform 1 precursor [Homo sapiens]

**Accession:**	gi|4502421	**Score:**	16.4
**Database:**	refseq_human(refseq_human_20140103.fasta)	**MW [kDa]:**	82.8
**Database Date:**	2015-11-30	**pI:**	9.7
		**Sequence Coverage [%]:**	1.8
		**No. of unique Peptides:**	1

10	20	30	40	50	60	70	80
MPGVARLPLL	LGLLLLPRPG	RPLDLADYTY	DLAEEDDSEP	LNYKDPCKAA	AFLGDIALDE	EDLRAFQVQQ	AVDLRRHTAR
90	100	110	120	130	140	150	160
KSSIKAAVPG	NTSTPSCQST	NGQPQRGACG	RWRGRSRSRR	AATSRPERVW	PDGVIPFVIG	GNFTGSQRAV	FRQAMRHWEK
170	180	190	200	210	220	230	240
HTCVTFLERT	DEDSYIVFTY	RPCGCCSYVG	RRGGGPQAIS	IGKNCDKFGI	VVHELGHVVG	FWHEHTRPDR	DRHVSIVREN
250	260	270	280	290	300	310	320
IQPGQEYNFL	KMEPQEVESL	GETYDFDSIM	HYARNTFSRG	IFLDTIVPKY	EVNGVKPPIG	QRTRLSKGDI	AQARKLYKCP
330	340	350	360	370	380	390	400
ACGETLQDST	GNFSSPEYPN	GYSAHMHCVW	RISVTPGEKI	ILNFTSLDLY	RSRLCWYDYV	EVRDGFWRKA	PLRGRFCGSK
410	420	430	440	450	460	470	480
LPEPIVSTDS	RLWVEFRSSS	NWVGKGFFAV	YEAICGGDVK	KDYGHIQSPN	YPDDYRPSKV	CIWRIQVSEG	FHVGLTFQSF
490	500	510	520	530	540	550	560
EIERHDSCAY	DYLEVRDGHS	ESSTLIGRYC	GYEKPDDIKS	TSSRLWLKFV	SDGSINKAGF	AVNFFKEVDE	CSRPNRGGCE
570	580	590	600	610	620	630	640
QRCLNTLGSY	KCSCDPGYEL	APDKRRCEAA	CGGFLTKLNG	SITSPGWPKE	YPPNKNCIWQ	LVAPTQYRIS	LQFDFFETEG
650	660	670	680	690	700	710	720
NDVCKYDFVE	VRSGLTADSK	LHGKFCGSEK	PEVITSQYNN	MRVEFKSDNT	VSKKGFKAHF	FSEKRPALQP	PRGRPHQLKF
730	740						
RVQKRNRTPQ							

Cmpd.	No. of Cmpds.	m/z meas.	Δ m/z [ppm]	z	Rt [min]	Score	P	Range	Sequence	Modification
2634	3	753.8426	-101.90	2	61.7	16.4	1	525-537	R.LWLKFVSDGSINK.A	

Detailed Protein Report

Protein 632: **protein-glutamine gamma-glutamyltransferase 5 isoform 2 [Homo sapiens]**

**Accession:**	gi|4759230	**Score:**	16.4
**Database:**	refseq_human(refseq_human_20140103.fasta)	**MW [kDa]:**	71.9
**Database Date:**	2015-11-30	**pI:**	5.9
**Modification(s):**	Carbamidomethyl	**Sequence Coverage [%]:**	2.5
		**No. of unique Peptides:**	1

10	20	30	40	50	60	70	80
MAQGLEVALT	DLQSSRNNVR	HHTEEITVDH	LLVRRGQAFN	LTLYFRNRSF	QPGLDNIIFV	VETEDAVYLD	SEPQRQEYVM
**90**	**100**	**110**	**120**	**130**	**140**	**150**	**160**
NDYGFIYQGS	KNWIRPCPWN	YGQFEDKIID	ICLKLLDKSL	HFQTDPATDC	ALRGSPVYVS	RVVCAMINSN	DDNGVLNGNW
**170**	**180**	**190**	**200**	**210**	**220**	**230**	**240**
SENYTDGANP	AEWTGSVAIL	KQWNATGCQP	VRYGQCWVFA	AVMCTVMRCL	GIPTRVITNF	DSGHDTDGNL	IIDEYYDNTG
**250**	**260**	**270**	**280**	**290**	**300**	**310**	**320**
RILGNKKKDT	IWNFHVWNEC	WMARKDLPPA	YGGWQVLDAT	PQEMSNGVYC	CGPASVRAIK	EGEVDLNYDT	PFVFSMVNAD
**330**	**340**	**350**	**360**	**370**	**380**	**390**	**400**
CMSWLVQGGK	EQKLHQDTSS	VGNFISTKSI	QSDERDDITE	NYKYEEGSLQ	ERQVFLKALQ	KLKARSFHGS	QRGAELQPSR
**410**	**420**	**430**	**440**	**450**	**460**	**470**	**480**
PTSLSQDSPR	SLHTPSLRPS	DVVQVSLKFK	LLDPPNMGQD	ICFVLLALNM	SSQFKDLKVN	LSAQSLLHDG	SPLSPFWQDT
**490**	**500**	**510**	**520**	**530**	**540**	**550**	**560**
AFITLSPKEA	KTYPCKISYS	QYSQYLSTDK	LIRISALGEE	KSSPEKILVN	KIITLSYPSI	TINVLGAAVV	NQPLSIQVIF
**570**	**580**	**590**	**600**	**610**	**620**	**630**	**640**
SNPLSEQVED	CVLTVEGSGL	FKKQQKVFLG	VLKPQHQASI	ILETVPFKSG	QRQIQANMRS	NKFKDIKGYR	NVYVDFAL

Cmpd.	No. of Cmpds.	m/z meas.	Δ m/z [ppm]	z	Rt [min]	Score	P	Range	Sequence	Modification
2196	1	961.3531	-82.76	2	56.1	16.4	0	193-208	R.YGQCWVFAAVMCTVMR.C	Carbamidomethyl: 4

Detailed Protein Report

Protein 633: PREDICTED: 5-hydroxytryptamine receptor 2B isoform X1 [Homo sapiens]

Accession: gi|530370281
Database: refseq_human(refseq_human_20140103.fasta)
Database Date: 2015-11-30

Score: 16.4
MW [kDa]: 41.1
pI: 10.3
Sequence Coverage [%]: 5.0
No. of unique Peptides: 1

10	20	30	40	50	60	70	80
MWPLPLVLCP	AWLFLDVLFS	TASIMHLCAI	SVDRYIAIKK	PIQANQYNSR	ATAFIKITVV	WLISIGIAIP	VPIKGIETDV
**90**	**100**	**110**	**120**	**130**	**140**	**150**	**160**
DNPNNITCVL	TKERFGDFML	FGSLAAFFTP	LAIMIVTYFL	TIHALQKKAY	LVKNKPPQRL	TWLTVSTVFQ	RDETPCSSPE
**170**	**180**	**190**	**200**	**210**	**220**	**230**	**240**
KVAMLDGSRK	DKALPNSGDE	TLMRRTSTIG	KKSVQTISNE	QRASKVLGIV	FFLFLLMWCP	FFITNITLVL	CDSCNQTTLQ
**250**	**260**	**270**	**280**	**290**	**300**	**310**	**320**
MLLEIFVWIG	YVSSGVNPLV	YTLFNKTFRD	AFGRYITCNY	RATKSVKTLR	KRSSKIYFRN	PMAENSKFFK	KHGIRNGINP
**330**	**340**	**350**	**360**	**370**			
AMYQSPMRLR	SSTIQSSSII	LLDTLLLTEN	EGDKTEEQVS	YV			

Cmpd.	No. of Cmpds.	m/z meas.	Δ m/z [ppm]	z	Rt [min]	Score	P	Range	Sequence	Modification
2778	3	973.5049	25.18	2	65.9	16.4	0	75-92	K.GIETDVDNPNNITCVLTK.E	

Detailed Protein Report

Protein 634: nucleolar complex protein 2 homolog [Homo sapiens]

Accession: gi|157694511
Database: refseq_human(refseq_human_20140103.fasta)
Database Date: 2015-11-30
Modification(s): Carbamidomethyl

Score: 16.3
MW [kDa]: 84.9
pI: 5.3
Sequence Coverage [%]: 2.3
No. of unique Peptides: 1

10	20	30	40	50	60	70	80
MAAAGSRKRR	LAELTVDEFL	ASGFDSESES	ESENSPQAET	REAREAARSP	DKPGGSPSAS	RRKGRASEHK	DQLSRLKDRD
**90**	**100**	**110**	**120**	**130**	**140**	**150**	**160**
PEFYKFLQEN	DQSLLNFSDS	DSSEEEEGPF	HSLPDVLEEA	SEEEDGAEEG	EDGDRVPRGL	KGKKNSVPVT	VAMVERWKQA
**170**	**180**	**190**	**200**	**210**	**220**	**230**	**240**
AKQRLTPKLF	HEVVQAFRAA	VATTRGDQES	AEANKFQVTD	SAAFNALVTF	CIRDLIGCLQ	KLLFGKVAKD	SSRMLQPSSS
**250**	**260**	**270**	**280**	**290**	**300**	**310**	**320**
PLWGKLRVDI	KAYLGSAIQL	VSCLSETTVL	AAVLRHISVL	VPCFLTFPKQ	CRMLLKRMVV	VWSTGEESLR	VLAFLVLSRV
**330**	**340**	**350**	**360**	**370**	**380**	**390**	**400**
CRHKKDTFLG	PVLKQMYITY	VRNCKFTSPG	ALPFISFMQW	TLTELLALEP	GVAYQHAFLY	IRQLAIHLRN	AMTTRKKETY
**410**	**420**	**430**	**440**	**450**	**460**	**470**	**480**
QSVYNWQYVH	CLFLWCRVLS	TAGPSEALQP	LVYPLAQVII	GCIKLIPTAR	FYPLRMHCIR	ALTLLSGSSG	AFIPVLPFIL
**490**	**500**	**510**	**520**	**530**	**540**	**550**	**560**
EMFQQVDFNR	KPGRMSSKPI	NFSVILKLSN	VNLQEKAYRD	GLVEQLYDLT	LEYLHSQAHC	IGFPELVLPV	VLQLKSFLRE
**570**	**580**	**590**	**600**	**610**	**620**	**630**	**640**
CKVANYCRQV	QQLLGKVQEN	SAYICSRRQR	VSFGVSEQQA	VEAWEKLTRE	EGTPLTLYYS	HWRKLRDREI	QLEISGKERL
**650**	**660**	**670**	**680**	**690**	**700**	**710**	**720**
EDLNFPEIKR	RKMADRKDED	RKQFKDLFDL	NSSEEDDTEG	FSERGILRPL	STRHGVEDDE	EDEEEGEEDS	SNSEDGDPDA
**730**	**740**	**750**					
EAGLAPGELQ	QLAQGPEDEL	EDLQLSEDD					

Cmpd.	No. of Cmpds.	m/z meas.	Δ m/z [ppm]	z	Rt [min]	Score	P	Range	Sequence	Modification
1281	1	1023.0326	-11.94	2	46.1	16.3	1	276-292	R.HISVLVPCFLTFPKQCR.M	Carbamidomethyl: 16

Detailed Protein Report

Protein 635: PREDICTED: synaptojanin-2 isoform X3 [Homo sapiens]

**Accession:**	gi|530384175	**Score:**	16.3
**Database:**	refseq_human(refseq_human_20140103.fasta)	**MW [kDa]:**	122.5
**Database Date:**	2015-11-30	**pI:**	6.9
		**Sequence Coverage [%]:**	1.1
		**No. of unique Peptides:**	1

10	20	30	40	50	60	70	80
MNCLDCLDRT	NTVQSFIALE	VLHLQLKTLG	LSSKPIVDRF	VESFKAMWSL	NGHSLSKVFT	GSRALEGKAK	VGKLKDGARS
90	100	110	120	130	140	150	160
MSRTIQSNFF	DGVKQEAIKL	LLVGDVYGEE	VADKGGMLLD	STALLVTPRI	LKAMTERQSE	FTNFKRIRIA	MGTWNVNGGK
170	180	190	200	210	220	230	240
QFRSNVLRTA	ELTDWLLDSP	QLSGATDSQD	DSSPADIFAV	GFEEMVELSA	GNIVNASTTN	KKMWGEQLQK	AISRSHRYIL
250	260	270	280	290	300	310	320
LTSAQLVGVC	LYIFVRPYHV	PFIRDVAIDT	VKTGMGGKAG	NKGAVGIRFQ	FHSTSFCFIC	SHLTAGQSQV	KERNEDYKEI
330	340	350	360	370	380	390	400
TQKLCFPMGR	NVFSHDYVFW	CGDFNYRIDL	TYEEVFYFVK	RQDWKKLLEF	DQLQLQKSSG	KIFKDFHEGA	INFGPTYKYD
410	420	430	440	450	460	470	480
VGSAAYDTSD	KCRTPAWTDR	VLWWRKKHPF	DKTAGELNLL	DSDLDVDTKV	RHTWSPGALQ	YYGRAELQAS	DHRPVLAIVE
490	500	510	520	530	540	550	560
VEVQEVDVGA	RERVFQEVSS	FQGPLDATVV	VNLQSPTLEE	KNEFPEDLRT	ELMQTLGSYG	TIVLVRINQG	QMLVTFADSH
570	580	590	600	610	620	630	640
SALSVLDVDG	MKVKGRAVKI	RPKTKDWLKG	LREEIIRKRD	SMAPVSPTAN	SCLLEENFDF	TSLDYESEGD	ILEDDEDYLV
650	660	670	680	690	700	710	720
DEFNQPGVSD	SELGGDDLSD	VPGPTALAPP	SKSPALTKKK	QHPTYKDDAD	LVELKRELEA	VGEFRHRSPS	RSLSVPNRPR
730	740	750	760	770	780	790	800
PPQPPQRPPP	PTGLMVKKSA	SDASISSGTH	GQYSILQTAR	LLPGAPQQPP	KARTGISKPY	NVKQIKTTNA	QEAEAAIRCL
810	820	830	840	850	860	870	880
LEARGGASEE	ALSAVAPRDL	EASSEPEPTP	GAAKPETPQA	PPLLPRRPPP	RVPAIKKPTL	RRTGKPLSPE	EQFEQQTVHF
890	900	910	920	930	940	950	960
TIGPPETSVE	APPVVTAPRV	PPVPKPRTFQ	PGKAAERPSH	RKPASDEAPP	GAGASVPPPL	EAPPLVPKVP	PRRKKSAPAA
970	980	990	1000	1010	1020	1030	1040
FHLQVLQSNS	QLLQGLTYNS	SDSPSGHPPA	AGTVFPQGDF	LSTSSATSPD	SDGTKAMKPE	AAPLLGDYQD	PFWNLLHHPK
1050	1060	1070	1080	1090	1100	1110	1120
LLNNTWLSKS	SDPLDSGTRS	PKRDPIDPVS	AGASAAKAEL	PPDHEHKTLG	HWVTISDQEK	RTALQVFDPL	AKT

Cmpd.	No. of Cmpds.	m/z meas.	Δ m/z [ppm]	z	Rt [min]	Score	P	Range	Sequence	Modification
1895	1	643.2981	-123.24	2	54.1	16.3	0	28-39	K.TLGLSSKPIVDR.F	

Detailed Protein Report

Protein 636: zinc finger C3H1 domain-containing protein [Homo sapiens]

**Accession:**	gi|87116683	**Score:**	16.3
**Database:**	refseq_human(refseq_human_20140103.fasta)	**MW [kDa]:**	226.2
**Database Date:**	2015-11-30	**pI:**	9.1
**Modification(s):**	Oxidation	**Sequence Coverage [%]:**	1.2
		**No. of unique Peptides:**	1

Detailed Protein Report

10	20	30	40	50	60	70	80
MATADTPAPA	SSGLSPKEEG	ELEDGEISDD	DNNSQIRSRS	SSSSSGGGLL	PYPRRRPPHS	ARGGGSGGGG	GSSSSSSSSQ
90	100	110	120	130	140	150	160
QQLRNFSRSR	HASERGHLRG	PSSYRPKEPF	RSHPPSVRMP	SSSLSESSPR	PSFWERSHLA	LDRFRFRGRP	YRGGSRWSRG
170	180	190	200	210	220	230	240
RGVGERGGKP	GCRPPLGGGA	GSGFSSSQSW	REPSPPRKSS	KSFGRSPSRK	QNYSSKNENC	VEETFEDLLL	KYKQIQLELE
250	260	270	280	290	300	310	320
CINKDEKLAL	SSKEENVQED	PKTLNFEDQT	STDNVSITKD	SSKEVAPEEK	TQVKTFQAFE	LKPLRQKLTL	PGDKNRLKKV
330	340	350	360	370	380	390	400
KDGAKPLSLK	SDTTDSSQGL	QDKEQNLTRR	ISTSDILSEK	KLGEDEEELS	ELQLRLLALQ	SASKKWQQKE	QQVMKESKEK
410	420	430	440	450	460	470	480
LTKTKTVQQK	VKTSTKTHSA	KKVSTTAKQA	LRKQQTKAWK	KLQQQKEQER	QKEEDQRKQA	EEEERRKREE	EIRKIRDLSN
490	500	510	520	530	540	550	560
QEEQYNRFMK	LVGGKRRSRS	KSSDPDLRRS	LDKQPTDSGG	GIYQYDNYEE	VAMDTDSETS	SPAPSPVQPP	FFSECSLGYF
570	580	590	600	610	620	630	640
SPAPSLSLPP	PPQVSSLPPL	SQPYVEGLCV	SLEPLPPLPP	LPPLPPEDPE	QPPKPPFADE	EEEEEMLLRE	ELLKSLANKR
650	660	670	680	690	700	710	720
AFKPEETSSN	SDPPSPPVLN	NSHPVPRSNL	SIVSINTVSQ	PRIQNPKFHR	GPRLPRTVIS	LPKHKSVVVT	LNDSDDSESD
730	740	750	760	770	780	790	800
GEASKSTNSV	FGGLESMIKE	ARRTAEQASK	PKVPPKSEKE	NDPLRTPEAL	PEEKKIEYRL	LKEEIANREK	QRLIKSDQLK
810	820	830	840	850	860	870	880
TSSSSPANSD	VEIDGIGRIA	MVTKQVTDAE	SKLKKHRILL	MKDESVLKNL	VQQEAKKKES	VRNAEAKITK	LTEQLQATEK
890	900	910	920	930	940	950	960
ILNVNRMFLK	KLQEQIHRVQ	QRVTIKKALT	LKYGEELARA	KAVASKEIGK	RKLEQDRFGP	NKMMRLDSSP	VSSPRKHSAE
970	980	990	1000	1010	1020	1030	1040
LIAMEKRRLQ	KLEYEYALKI	QKLKEARALK	AKEQQNISPV	VEEEPEFSLP	QPSLHDLTQD	KLTLDTEEND	VDDEILSGSS
1050	1060	1070	1080	1090	1100	1110	1120
RERRRSFLES	NYFTKPNLKH	TDTANKECIN	KLNKNTVEKP	ELFLGLKIGE	LQKLYSKADS	LKQLILKTTT	GITEKVLHGQ
1130	1140	1150	1160	1170	1180	1190	1200
EISVDVDFVT	AQSKTMEVKP	CPFRPYHSPL	LVFKSYRFSP	YYRTKEKLPL	SSVSYSNMIE	PDQCFCRFDL	TGTCNDDDCQ
1210	1220	1230	1240	1250	1260	1270	1280
WQHIQDYTLS	RKQLFQDILS	YNLSLIGCAE	TSTNEEITAS	AEKYVEKLFG	VNKDRMSMDQ	MAVLLVSNIN	ESKGHTPPFT
1290	1300	1310	1320	1330	1340	1350	1360
TYKDKRKWKP	KFWRKPISDN	SFSSDEEQST	GPIKYAFQPE	NQINVPALDT	VVTPDDVRYF	TNETDDIANL	EASVLENPSH
1370	1380	1390	1400	1410	1420	1430	1440
VQLWLKLAYK	YLNQNEGECS	ESLDSALNVL	ARALENNKDN	PEIWCHYLRL	FSKRGTKDEV	QEMCETAVEY	APDYQSFWTF
1450	1460	1470	1480	1490	1500	1510	1520
LHLESTFEEK	DYVCERMLEF	LMGAAKQETS	NILSFQLLEA	LLFRVQLHIF	TGRCQSALAI	LQNALKSAND	GIVAEYLKTS
1530	1540	1550	1560	1570	1580	1590	1600
DRCLAWLAYI	HLIEFNILPS	KFYDPSNDNP	SRIVNTESFV	MPWQAVQDVK	TNPDMLLAVF	EDAVKACTDE	SLAVEERIEA
1610	1620	1630	1640	1650	1660	1670	1680
CLPLYTNMIA	LHQLLERYEA	AMELCKSLLE	SCPINCQLLE	ALVALYLQTN	QHDKARAVWL	TAFEKNPQNA	EVFYHMCKFF
1690	1700	1710	1720	1730	1740	1750	1760
ILQNRGDNLL	PFLRKFIASF	FKPGFEKYNN	LDLFRYLLNI	PGPIDIPSRL	CKGNFDDDMF	NHQVPYLWLI	YCLCHPLQSS
1770	1780	1790	1800	1810	1820	1830	1840
IKETVEAYEA	ALGVAMRCDI	VQKIWMDYLV	FANNRAAGSR	NKVQEFKFFT	DLVNRCLVTV	PARYPIPFSS	ADYWSNYEFH
1850	1860	1870	1880	1890	1900	1910	1920
NRVIFFYLSC	VPKTQHSKTL	ERFCSVMPAN	SGLALRLLQH	EWEESNVQIL	KLQAKMFTYN	IPTCLATWKI	AIAAEIVLKG
1930	1940	1950	1960	1970	1980	1990	
QREVHRLYQR	ALQKLPLCAS	LWKDQLLFEA	SEGGKTDNLR	KLVSKCQEIG	VSLNELLNLN	SNKTESKNH	

Cmpd.	No. of Cmpds.	m/z meas.	Δ m/z [ppm]	z	Rt [min]	Score	P	Range	Sequence	Modification
750	1	937.7415	-84.31	3	37.9	16.3	1	1135-1157	K.TMEVKPCPFRPYHSPLLVFKSYR.F	Oxidation: 2

Detailed Protein Report

Protein 637: soluble lamin-associated protein of 75 kDa [Homo sapiens]

**Accession:**	gi|153792633	**Score:**	16.3
**Database:**	refseq_human(refseq_human_20140103.fasta)	**MW [kDa]:**	74.9
**Database Date:**	2015-11-30	**pI:**	4.4
		**Sequence Coverage [%]:**	2.7
		**No. of unique Peptides:**	1

Alias proteins:

Accession	Name	Description
gi|530379249	refseq_human (refseq_human_20140103.fasta)	PREDICTED: soluble lamin-associated protein of 75 kDa isoform X2 [Homo sapiens]

10	20	30	40	50	60	70	80
MAFPVDMLEN	CSHEELENSA	EDYMSDLRCG	DPENPECFSL	LNITIPISLS	NVGFVPLYGG	DQTQKILALF	APEDSLTAVA
90	100	110	120	130	140	150	160
LYLADQWWAI	DDIVKTSVPS	REGLKQVSTL	GERVVLYVLN	RIIYRKQEME	RNEIPFLCHS	STDYAKILWK	KGEAIGFYSV
170	180	190	200	210	220	230	240
KPTGSICASF	LTQSYQLPVL	DTMFLRKKYR	GKDFGLHMLE	DFVDSFTEDA	LGLRYPLSSL	MYTACKQYFE	KYPGDHELLW
250	260	270	280	290	300	310	320
EVEGVGHWYQ	RIPVTRALQR	EALKILALSQ	NEPKRPMSGE	YGPASVPEYE	ARTEDNQSSE	MQLTIDSLKD	AFASTSEGHD
330	340	350	360	370	380	390	400
KTSVSTHTRS	GNLKRPKIGK	RFQDSEFSSS	QGEDEKTSQT	SLTASINKLE	STARPSESSE	EFLEEEPEQR	GIEFEDESSD
410	420	430	440	450	460	470	480
RDARPALETQ	PQQEKQDGEK	ESELEPMNGE	IMDDSLKTSL	ITEEEDSTSE	VLDEELKLQP	FNSSEDSTNL	VPLVVESSKP
490	500	510	520	530	540	550	560
PEVDAPDKTP	RIPDSEMLMD	EGTSDEKGHM	EEKLSLLPRK	KAHLGSSDNV	ATMSNEERSD	GGFPNSVIAE	FSEEPVSENL
570	580	590	600	610	620	630	640
SPNTTSSLED	QGEEGVSEPQ	ETSTALPQSS	LIEVELEDVP	FSQNAGQKNQ	SEEQSEASSE	QLDQFTQSAE	KAVDSSSEEI
650	660	670	680				
EVEVPVVDRR	NLRRKAKGHK	GPAKKKAKLT					

Cmpd.	No. of Cmpds.	m/z meas.	Δ m/z [ppm]	z	Rt [min]	Score	P	Range	Sequence	Modification
2565	1	973.4946	40.45	2	62.7	16.3	1	521-538	K.KAHLGSSDNVATMSNEER.S	

Detailed Protein Report

Protein 638: peptidyl-prolyl cis-trans isomerase D [Homo sapiens]

Accession: gi|4826932
Database: refseq_human(refseq_human_20140103.fasta)
Database Date: 2015-11-30

Score: 16.2
MW [kDa]: 40.7
pI: 6.9
Sequence Coverage [%]: 3.2
No. of unique Peptides: 1

10	20	30	40	50	60	70	80
MSHPSPQAKP	SNPSNPRVFF	DVDIGGERVG	RIVLELFADI	VPKTAENFRA	LCTGEKGIGH	TTGKPLHFKG	CPFHRIIKKF
**90**	**100**	**110**	**120**	**130**	**140**	**150**	**160**
MIQGGDFSNQ	NGTGGESIYG	EKFEDENFHY	KHDREGLLSM	ANAGRNTNGS	QFFITTVPTP	HLDGKHVVFG	QVIKGIGVAR
**170**	**180**	**190**	**200**	**210**	**220**	**230**	**240**
ILENVEVKGE	KPAKLCVIAE	CGELKEGDDG	GIFPKDGSGD	SHPDFPEDAD	IDLKDVDKIL	LITEDLKNIG	NTFFKSQNWE
**250**	**260**	**270**	**280**	**290**	**300**	**310**	**320**
MAIKKYAEVL	RYVDSSKAVI	ETADRAKLQP	IALSCVLNIG	ACKLKMSNWQ	GAIDSCLEAL	ELDPSNTKAL	YRRAQGWQGL
**330**	**340**	**350**	**360**	**370**	**380**		
KEYDQALADL	KKAQGIAPED	KAIQAELLKV	KQKIKAQKDK	EKAVYAKMFA			

Cmpd.	No. of Cmpds.	m/z meas.	Δ m/z [ppm]	z	Rt [min]	Score	P	Range	Sequence	Modification
920	1	678.9535	63.37	2	40.0	16.2	0	32-43	R.IVLELFADIVPK.T	

Detailed Protein Report

Protein 639: **nutritionally-regulated adipose and cardiac enriched protein homolog isoform b [Homo sapiens]**

**Accession:**	gi|56605684	**Score:**	16.2
**Database:**	refseq_human(refseq_human_20140103.fasta)	**MW [kDa]:**	18.0
**Database Date:**	2015-11-30	**pI:**	12.3
		**Sequence Coverage [%]:**	8.8
		**No. of unique Peptides:**	1

Alias proteins:

Accession	Name	Description
gi|556695443	refseq_huma (refseq_human_20140103.fasta)	nutritionally-regulated adipose and cardiac enriched protein homolog isoform b [Homo sapiens]

10	20	30	40	50	60	70	80
MRTAAGAVSP	DSRPETRRQT	RKNEEAAWGP	RVCRAEREDN	RKCPPSILKR	SRPEHHRPEA	KPQRTSRRVW	FREPPAVTVH
**90**	**100**	**110**	**120**	**130**	**140**	**150**	**160**
YIADKNATAT	VRVPGRPRPH	GGSLLLQLCV	CVLLVLALGL	YCGRAKPVAT	ALEDLRARLL	GLVLHLRHVA	LTCWRGLLRL
**170**							

Cmpd.	No. of Cmpds.	m/z meas.	Δ m/z [ppm]	z	Rt [min]	Score	P	Range	Sequence	Modification
2183	1	862.9063	-57.86	2	57.7	16.2	0	51-64	R.SRPEHHRPEAKPQR.T	

Detailed Protein Report

Protein 640: cyclin-dependent kinase 4 [Homo sapiens]

**Accession:**	gi|4502735	**Score:**	16.2
**Database:**	refseq_human(refseq_human_20140103.fasta)	**MW [kDa]:**	33.7
**Database Date:**	2015-11-30	**pI:**	6.6
		**Sequence Coverage [%]:**	4.3
		**No. of unique Peptides:**	1

Quantitation

QD:QU **Median:** 1.06 **CV:** 0.00 % **No. of Peptides:** 1

10	20	30	40	50	60	70	80
MATSRYEPVA	EIGVGAYGTV	YKARDPHSGH	FVALKSVRVP	NGGGGGGGLP	ISTVREVALL	RRLEAFEHPN	VVRLMDVCAT
90	100	110	120	130	140	150	160
SRTDREIKVT	LVFEHVDQDL	RTYLDKAPPP	GLPAETIKDL	MRQFLRGLDF	LHANCIVHRD	LKPENILVTS	GGTVKLADFG
170	180	190	200	210	220	230	240
LARIYSYQMA	LTPVVVTLWY	RAPEVLLQST	YATPVDMWSV	GCIFAEMFRR	KPLFCGNSEA	DQLGKIFDLI	GLPPEDDWPR
250	260	270	280	290	300	310	
DVSLPRGAFP	PRGPRPVQSV	VPEMEESGAQ	LLLEMLTFNP	HKRISAFRAL	QHSYLHKDEG	NPE	

Cmpd.	No. of Cmpds.	m/z meas.	Δ m/z [ppm]	z	Rt [min]	Score	P	Range	Sequence	Modification	Ratios
463	1	717.8711	-17.46	2	35.8	16.2	1	23-35	K.ARDPHSGHFVALK.S		QD:QU 1.06

Detailed Protein Report

Protein 641: **PREDICTED: spermidine/spermine N(1)-acetyltransferase-like protein 1 isoform X1 [Homo sapiens]**

**Accession:**	gi|578838360	**Score:**	16.2
**Database:**	refseq_human(refseq_human_20140103.fasta)	**MW [kDa]:**	75.8
**Database Date:**	2015-11-30	**pI:**	5.0
		**Sequence Coverage [%]:**	1.9
		**No. of unique Peptides:**	1

10	20	30	40	50	60	70	80
MNQSGTNQSS	LSDSNQAGIN	QPSTNSLGMN	QMDMNQGSAS	LYEMNQVDMK	QPSMSQAGMR	QSGTNLPDIN	QPDMKQPDTW
90	100	110	120	130	140	150	160
QLGRSQPGML	QQELSQLVLS	KAGISQPDPS	QPGPSQSGPS	QSRMRQIGTN	QSGMSQPVMQ	QLDSQSGGSQ	PSMRQVGTSQ
170	180	190	200	210	220	230	240
LGTSQIGMSQ	PGTWQTGLSQ	PVLRQPNMSP	PGMWQPGVQQ	PGISQQVPSH	PDMSQPGMSQ	QVPSQPGIRQ	PDTSQSCKNQ
250	260	270	280	290	300	310	320
TDMSQPDANQ	SSLSDSNQTG	IIQPSPSLLG	MNQMDMNQWS	ASLYEMNQVD	MKQPSMSQAG	MRQSGTNLPD	INQPGMKQPG
330	340	350	360	370	380	390	400
TWQLGRSQPG	MWPQSLSELV	LSEASISQPG	PPQRAPSQSG	PRQSSTSQAG	TNQSGISQPV	MWQLDMRQSG	GSQPSMRQVG
410	420	430	440	450	460	470	480
TSQSGTSQIG	MSQPGTWQTG	LSQPVPRQPN	KSPPGMWQRG	MWQPGMSQQV	PSQLGMRQPG	TSQSSKNQTG	MSHPGRGQPG
490	500	510	520	530	540	550	560
IWEPGPSQPG	LSQQDLNQLV	LSQPGLSQPG	RSQPSVSQMG	MRQTSMDYFQ	IRHAEAGDCP	EILRLIKELA	ACENMLDAME
570	580	590	600	610	620	630	640
LTAADLLRDG	FGDNPLFYCL	IAEVNDQQKP	SGKLTVGFAM	YYFTYDSWTG	KVLYLEDFYV	TQAYQGLGIG	AEMLKRLSQI
650	660	670	680	690	700		
AITTQCNCMH	FLVVIWNQAS	INYYTSRGAL	DLSSEEGWHL	FRFNREELLD	MAWEE		

Cmpd.	No. of Cmpds.	m/z meas.	Δ m/z [ppm]	z	Rt [min]	Score	P	Range	Sequence	Modification
2493	1	841.3809	1.11	2	61.7	16.2	1	683-695	R.FNREELLDMAWEE.-	

Detailed Protein Report

Protein 642: PREDICTED: zinc finger protein 831 isoform X4 [Homo sapiens]

**Accession:**	gi|530417824	**Score:**	16.2
**Database:**	refseq_human(refseq_human_20140103.fasta)	**MW [kDa]:**	132.1
**Database Date:**	2015-11-30	**pI:**	9.2
**Modification(s):**	Carbamidomethyl	**Sequence Coverage [%]:**	1.5
		**No. of unique Peptides:**	1

10	20	30	40	50	60	70	80
MEVPEPTCPA	PPARDQPAPT	PGPPGAPGGQ	ASPHLTLGPV	LLPPEQGLAP	PTVFLKALPI	PLYHTVPPGG	LQPRAPLVTG
90	100	110	120	130	140	150	160
SLDGGNVPFI	LSPVLQPEGP	GPTQVGKPAA	PTLTVNIVGT	LPVLSPGLGP	TLGSPGKVRN	AGKYLCPHCG	RDCLKPSVLE
170	180	190	200	210	220	230	240
KHIRSHTGER	PFPCATCGIA	FKTQSNLYKH	RRTQTHLNNS	RLSSESEGAG	GGLLEEGDKA	GEPPRPEGRG	ESRCQGMHEG
250	260	270	280	290	300	310	320
ASERPLSPGA	HVPLLAKNLD	VRTEAAPCPG	SAFADREAPW	DSAPMASPGL	PAASTQPWRK	LPEQKSPTAG	KPCALQRQQA
330	340	350	360	370	380	390	400
TAAEKPWDAK	APEGRLRKCE	STDSGYLSRS	DSAEQPHAPC	SPLHSLSEHS	AESEGEGGPG	PGPGVAGAEP	GAREAGLELE
410	420	430	440	450	460	470	480
KKRLEERIAQ	LISHNQAVVD	DAQLDNVRPR	KTGLSKQGSI	DLPTPYTYKD	SFHFDIRALE	PGRRRAPGPV	RSTWTPPDKS
490	500	510	520	530	540	550	560
RPLFFHSVPT	QLSTTVECVP	VTRSNSLPFV	EGSRTWLEPR	EPRDPWSRTQ	KPLSPRPGPA	RLGCRSGLSS	TDVPSGHPRA
570	580	590	600	610	620	630	640
LVRQAAVEDL	PGTPIGDALV	PAEDTDAKRT	AAREAMAGKG	RAGGRKCGQR	RLKMFSQEKW	QVYGDETFKR	IYQKMKASPH
650	660	670	680	690	700	710	720
GGKKAREVGM	GSGAELGFPL	QKEAAGSSGT	VPTQDRRTPV	HEDISAGATP	EPWGNPPALE	ASLVTEPTKH	GETVARRGDS
730	740	750	760	770	780	790	800
DRPRVEEAVS	SPALGGRDSP	CSGSRSPLVS	PNGRLELGWQ	MPPAPGPLKG	GDVEAPRPVW	PDPKLEGGAR	GVGDVQETCL
810	820	830	840	850	860	870	880
WAQTVLRWPS	RGSGEDKLPS	ERKKLKVEDL	HSWKQPEPVS	AETPGGPTQP	ASLSSQKQDA	DPGEVPGGSK	ESARQVGEPL
890	900	910	920	930	940	950	960
ESSGASLAAA	SVALKRVGPR	DKATPLHPAA	PAPAEHPSLA	TPPQAPRVLS	ALADNAFSPK	YLLRLPQAET	PLPLPIPWGP
970	980	990	1000	1010	1020	1030	1040
RHSQDSLCSS	GWPEERASFV	GSGLGTPLSP	SPASGPSPGE	ADSILEDPSC	SRPQDGRKGA	QLGGDKGDRM	ATSRPAAREL
1050	1060	1070	1080	1090	1100	1110	1120
PISAPGAPRE	ATSSPPTPTC	EAHLVQDMEG	DSHRIHRLCM	GSTLARARLS	GDVLNPWVPN	WELGEPPGNA	PEDPSSGPLV
1130	1140	1150	1160	1170	1180	1190	1200
GPDPCSPLQP	GSFLTALTRP	QGVPPGWPEL	ALSSHSGTSR	SHSTRSPHST	QNPFPSLKAE	PRLTWCCLSR	SVPLPAEQKA
1210	1220	1230	1240	1250	1260		
KAASVYLAVH	FPGSSLRDEG	PNGPPGSNGG	WTWTSPGEGG	PAQMSKSENP	LEFSSS		

Cmpd.	No. of Cmpds.	m/z meas.	Δ m/z [ppm]	z	Rt [min]	Score	P	Range	Sequence	Modification
1981	3	1023.8811	-103.16	2	55.2	16.2	1	258-276	K.NLDVRTEAAPCPGSAFADR.E	Carbamidomethyl: 11

Detailed Protein Report

Protein 643: neuroplastin isoform a precursor [Homo sapiens]

**Accession:**	gi|9257240	**Score:**	16.1
**Database:**	refseq_human(refseq_human_20140103.fasta)	**MW [kDa]:**	31.3
**Database Date:**	2015-11-30	**pI:**	6.5
**Modification(s):**	Oxidation	**Sequence Coverage [%]:**	5.7
		**No. of unique Peptides:**	1

10	20	30	40	50	60	70	80
MSGSSLPSAL	ALSLLLVSGS	LLPGPGAAQN	EPRIVTSEEV	IIRDSPVLPV	TLQCNLTSSS	HTLTYSYWTK	NGVELSATRK
**90**	**100**	**110**	**120**	**130**	**140**	**150**	**160**
NASNMEYRIN	KPRAEDSGEY	HCVYHFVSAP	KANATIEVKA	APDITGHKRS	ENKNEGQDAT	MYCKSVGYPH	PDWIWRKKEN
**170**	**180**	**190**	**200**	**210**	**220**	**230**	**240**
GMPMDIVNTS	GRFFIINKEN	YTELNIVNLQ	ITEDPGEYEC	NATNAIGSAS	VVTVLRVRSH	LAPLWPFLGI	LAEIIILVVI
**250**	**260**	**270**	**280**	**290**			
IVVYEKRKRP	DEVPDDDEPA	GPMKTNSTNN	HKDKNLRQRN	TN			

Cmpd.	No. of Cmpds.	m/z meas.	Δ m/z [ppm]	z	Rt [min]	Score	P	Range	Sequence	Modification
1720	1	892.3075	-96.70	2	51.8	16.1	0	249-264	K.RPDEVPDDDEPAGPMK.T	Oxidation: 15

Detailed Protein Report

Protein 644: cytoplasmic phosphatidylinositol transfer protein 1 isoform b [Homo sapiens]

Accession: gi|32307142
Database: refseq_human(refseq_human_20140103.fasta)
Database Date: 2015-11-30

Score: 16.1
MW [kDa]: 31.7
pI: 5.3
Sequence Coverage [%]: 5.6
No. of unique Peptides: 1

10	20	30	40	50	60	70	80
MLLKEYRICM	PLTVDEYKIG	QLYMISKHSH	EQSDRGEGVE	VVQNEPFEDP	HHGNGQFTEK	RVYLNSKLPS	WARAVVPKIF
**90**	**100**	**110**	**120**	**130**	**140**	**150**	**160**
YVTEKAWNYY	PYTITEYTCS	FLPKFSIHIE	TKYEDNKGSN	DTIFDNEAKD	VEREVCFIDI	ACDEIPERYY	KESEDPKHFK
**170**	**180**	**190**	**200**	**210**	**220**	**230**	**240**
SEKTGRGQLR	EGWRDSHQPI	MCSYKLVTVK	FEVWGLQTRV	EQFVHKVVRD	ILLIGHRQAF	AWVDEWYDMT	MDDVREYEKN
**250**	**260**	**270**					
MHEQTNIKVC	NQHSSPVDDI	ESHAQTST					

Cmpd.	No. of Cmpds.	m/z meas.	Δ m/z [ppm]	z	Rt [min]	Score	P	Range	Sequence	Modification
38	1	918.9134	2.10	2	29.2	16.1	1	171-185	R.EGWRDSHQPIMCSYK.L	

Detailed Protein Report

Protein 645: PREDICTED: UHRF1-binding protein 1-like isoform X5 [Homo sapiens]

**Accession:**	gi|578823458	**Score:**	16.1
**Database:**	refseq_human(refseq_human_20140103.fasta)	**MW [kDa]:**	113.2
**Database Date:**	2015-11-30	**pI:**	5.4
**Modification(s):**	Carbamidomethyl	**Sequence Coverage [%]:**	1.3
		**No. of unique Peptides:**	1

10	20	30	40	50	60	70	80
MSSSVVVRLA	DFNIYQVSTA	EQCRSSPKSM	ICCNKKSLYL	PQEMSAVYIE	FTEYYYPDGK	DFPIPSPNLY	SQLNALQFTV
90	100	110	120	130	140	150	160
DERSILWLNQ	FLLDLKQSLN	QFMAVYKLND	NSKSDEHVDV	RVDGLMLKFV	IPSEVKSECH	QDQPRAISIQ	SSEMIATNTR
170	180	190	200	210	220	230	240
HCPNCRHSDL	EALFQDFKDC	DFFSKTYTSF	PKSCDNFNLL	HPIFQRHAHE	QDTKMHEIYK	GNITPQLNKN	TLKTSAATDV
250	260	270	280	290	300	310	320
WAVYFSQFWI	DYEGMKSGKG	RPISFVDSFP	LSIWICQPTR	YAESQKEPQT	CNQVSLNTSQ	SESSDLAGRL	KRKKLLKEYY
330	340	350	360	370	380	390	400
STESEPLTNG	GQKPSSSDTF	FRFSPSSSEA	DIHLLVHVHK	HVSMQINHYQ	YLLLLFLHES	LILLSENLRK	DVEAVTGSPA
410	420	430	440	450	460	470	480
SQTSICIGIL	LRSAELALLL	HPVDQANTLK	SPVSESVSPV	VPDYLPTENG	DFLSSKRKQI	SRDINRIRSV	TVNHMSDNRS
490	500	510	520	530	540	550	560
MSVDLSHIPL	KDPLLFKSAS	DTNLQKGISF	MDYLSDKHLG	KISEDESSGL	VYKSGSGEIG	SETSDKKDSF	YTDSSSILNY
570	580	590	600	610	620	630	640
REDSNILSFD	SDGNQNILSS	TLTSKGNETI	ESIFKAEDLL	PEAASLSENL	DISKEETPPV	RTLKSQSSLS	GKPKERCPPN
650	660	670	680	690	700	710	720
LAPLCVSYKN	MKRSSSQMSL	DTISLDSMIL	EEQLLESDGS	DSHMFLEKGN	KKNSTTNYRG	TAESVNAGAN	LQNYGETSPD
730	740	750	760	770	780	790	800
AISTNSEGAQ	ENHDDLMSVV	VFKITGVNGE	IDIRGEDTEI	CLQVNQVTPD	QLGNISLRHY	LCNRPVGSDQ	KAVIHSKSSP
810	820	830	840	850	860	870	880
EISLRFESGP	GAVIHSLLAE	KNGFLQCHIE	NFSTEFLTSS	LMNIQHFLED	ETVATVMPMK	IQVSNTKINL	KDDSPRSSTV
890	900	910	920	930	940	950	960
SLEPAPVTVH	IDHLVVERSD	DGSFHIRDSH	MLNTGNDLKE	NVKSDSVLLT	SGKYDLKKQR	SVTQATQTSP	GVPWPSQSAN
970	980	990	1000	1010	1020		
FPEFSFDFTR	EQLMEENESL	KQELAKAKMA	LAEAHLEKDA	LLHHIKKMTV	E		

Cmpd.	No. of Cmpds.	m/z meas.	Δ m/z [ppm]	z	Rt [min]	Score	P	Range	Sequence	Modification
2057	1	731.3545	-14.44	2	56.1	16.1	0	637-649	R.CPPNLAPLCVSYK.N	Carbamidomethyl: 1

Detailed Protein Report

Protein 646: PREDICTED: guanine nucleotide exchange factor VAV3 isoform X4 [Homo sapiens]

**Accession:**	gi|530361574	**Score:**	16.1
**Database:**	refseq_human(refseq_human_20140103.fasta)	**MW [kDa]:**	72.1
**Database Date:**	2015-11-30	**pI:**	6.9
		**Sequence Coverage [%]:**	1.9
		**No. of unique Peptides:**	1

Quantitation

QD:QU **Median:** 1.40 **CV:** 0.00 % **No. of Peptides:** 1

10	20	30	40	50	60	70	80
MEPWKQCAQW	LIHCKVLPTN	HRVTWDSAQV	FDLAQTLRDG	VLLCQLLNNL	RAHSINLKEI	NLRPQMSQFL	CLKNIRTFLT
**90**	**100**	**110**	**120**	**130**	**140**	**150**	**160**
ACCETFGMRK	SELFEAFDLF	DVRDFGKVIE	TLSRLSRTPI	ALATGIRPFP	TEESINDEDI	YKGLPDLIDE	TLVEDEEDLY
**170**	**180**	**190**	**200**	**210**	**220**	**230**	**240**
DCVYGEDEGG	EVYEDLMKAE	EAHQPKCPEN	DIRSCCLAEI	KQTEEKYTET	LESIEKYFMA	PLKRFLTAAE	FDSVFINIPE
**250**	**260**	**270**	**280**	**290**	**300**	**310**	**320**
LVKLHRNLMQ	EIHDSIVNKN	DQNLYQVFIN	YKERLVIYGQ	YCSGVESAIS	SLDYISKTKE	DVKLKLEECS	KRANNGKFTL
**330**	**340**	**350**	**360**	**370**	**380**	**390**	**400**
RDLLVVPMQR	VLKYHLLLQE	LVKHTTDPTE	KANLKLALDA	MKDLAQYVNE	VKRDNETLRE	IKQFQLSIEN	LNQPVLLFGR
**410**	**420**	**430**	**440**	**450**	**460**	**470**	**480**
PQGDGEIRIT	TLDKHTKQER	HIFLFDLAVI	VCKRKGDNYE	MKEIIDLQQY	KIANNPTTDK	ENKKWSYGFY	LIHTQGQNGL
**490**	**500**	**510**	**520**	**530**	**540**	**550**	**560**
EFYCKTKDLK	KKWLEQFEMA	LSNIRPDYAD	SNFHDFKMHT	FTRVTSCKVC	QMLLRGTFYQ	GYLCFKCGAR	AHKECLGRVD
**570**	**580**	**590**	**600**	**610**	**620**	**630**	
NCGRVNSGEQ	GTLKLPEKRT	NGLRRTPKQV	DPDVPCLLHF	FISMAPATRS	IVKSQKKNKK	F	

Cmpd.	No. of Cmpds.	m/z meas.	Δ m/z [ppm]	z	Rt [min]	Score	P	Range	Sequence	Modification	Ratios
877	1	705.9834	77.48	2	41.0	16.1	1	322-333	R.DLLVVPMQRVLK.Y		QD:QU 1.40

Detailed Protein Report

Protein 647: ras and Rab interactor 2 isoform 2 [Homo sapiens]

**Accession:**	gi|18466802	**Score:**	16.1
**Database:**	refseq_human(refseq_human_20140103.fasta)	**MW [kDa]:**	100.1
**Database Date:**	2015-11-30	**pI:**	6.1
		**Sequence Coverage [%]:**	1.5
		**No. of unique Peptides:**	1

Alias proteins:

Accession	Name	Description
gi|578835435	refseq_human (refseq_human_20140103.fasta)	PREDICTED: ras and Rab interactor 2 isoform X10 [Homo sapiens]
gi|578835433	refseq_human (refseq_human_20140103.fasta)	PREDICTED: ras and Rab interactor 2 isoform X9 [Homo sapiens]
gi|578835431	refseq_human (refseq_human_20140103.fasta)	PREDICTED: ras and Rab interactor 2 isoform X8 [Homo sapiens]
gi|578835429	refseq_human (refseq_human_20140103.fasta)	PREDICTED: ras and Rab interactor 2 isoform X7 [Homo sapiens]
gi|530425753	refseq_human (refseq_human_20140103.fasta)	PREDICTED: ras and Rab interactor 2 isoform X3 [Homo sapiens]

10	20	30	40	50	60	70	80
MTAWTMGARG	LDKRGSFFKL	IDTIASEIGE	LKQEMVRTDV	NLENGLEPAE	THSMVRHKDG	GYSEEEDVKT	CARDSGYDSL
90	100	110	120	130	140	150	160
SNRLSILDRL	LHTHPIWLQL	SLSEEEAAEV	LQAQPPGIFL	VHKSTKMQKK	VLSLRLPCEF	GAPLKEFAIK	ESTYTFSLEG
170	180	190	200	210	220	230	240
SGISFADLFR	LIAFYCISRD	VLPFTLKLPY	AISTAKSEAQ	LEELAQMGLN	FWSSPADSKP	PNLPPPHRPL	SSDGVCPASL
250	260	270	280	290	300	310	320
RQLCLINGVH	SIKTRTPSEL	ECSQTNGALC	FINPLFLKVH	SQDLSGGLKR	PSTRTPNANG	TERTRSPPPR	PPPPAINSLH
330	340	350	360	370	380	390	400
TSPRLARTET	QTSMPETVNH	NKHGNVALPG	TKPTPIPPPR	LKKQASFLEA	EGGAKTLSGG	RPGAGPELEL	GTAGSPGGAP
410	420	430	440	450	460	470	480
PEAAPGDCTR	APPPSSESRP	PCHGGRQRLS	DMSISTSSSD	SLEFDRSMPL	FGYEADTNSS	LEDYEGESDQ	ETMAPPIKSK
490	500	510	520	530	540	550	560
KKRSSSFVLP	KLVKSQLQKV	SGVFSSFMTP	EKRMVRRIAE	LSRDKCTYFG	CLVQDYVSFL	QENKECHVSS	TDMLQTIRQF
570	580	590	600	610	620	630	640
MTQVKNYLSQ	SSELDPPIES	LIPEDQIDVV	LEKAMHKCIL	KPLKGHVEAM	LKDFHMADGS	WKQLKENLQL	VRQRNPQELG
650	660	670	680	690	700	710	720
VFAPTPDFVD	VEKIKVKFMT	MQKMYSPEKK	VMLLLRVCKL	IYTVMENNSG	RMYGADDFLP	VLTYVIAQCD	MLELDTEIEY
730	740	750	760	770	780	790	800
MMELLDPSLL	HGEGGYYLTS	AYGALSLIKN	FQEEQAARLL	SSETRDTLRQ	WHKRRTTNRT	IPSVDDFQNY	LRVAFQEVNS
810	820	830	840	850	860	870	880
GCTGKTLLVR	PYITTEDVCQ	ICAEKFKVGD	PEEYSLFLFV	DETWQQLAED	TYPQKIKAEL	HSRPQPHIFH	FVYKRIKNDP
890	900						
YGIIFQNGEE	DLTTS						

Cmpd.	No. of Cmpds.	m/z meas.	Δ m/z [ppm]	z	Rt [min]	Score	P	Range	Sequence	Modification
2319	1	719.3506	-2.64	2	57.7	16.1	1	1-13	-.MTAWTMGARGLDK.R	

Detailed Protein Report

Protein 648: phosphatidylethanolamine N-methyltransferase isoform 4 [Homo sapiens]

Accession: gi|388890235
Database: refseq_human(refseq_human_20140103.fasta)
Database Date: 2015-11-30
Modification(s): Oxidation

Score: 16.1
MW [kDa]: 23.7
pI: 9.4
Sequence Coverage [%]: 8.4
No. of unique Peptides: 1

Quantitation

QD:QU **Median:** 0.88 **CV:** 0.00 % **No. of Peptides:** 1

10	20	30	40	50	60	70	80
MKRSGNPGAE	ADFCVMTRLL	GYVDPLDPSF	VAAVITITFN	PLYWNVVARW	EHKTRKLSRA	FGSPYLACYS	LSVTILLLNF
**90**	**100**	**110**	**120**	**130**	**140**	**150**	**160**
LRSHCFTQAM	LSQPRMESLD	TPAAYSLGLA	LLGLGVVLVL	SSFFALGFAG	TFLGDYFGIL	KEARVTVFPF	NILDNPMYWG
**170**	**180**	**190**	**200**	**210**	**220**		
STANYLGWAI	MHASPTGLLL	TVLVALTYIV	ALLYEEPFTA	EIYRQKASGS	HKRS		

Cmpd.	No. of Cmpds.	m/z meas.	Δ m/z [ppm]	z	Rt [min]	Score	P	Range	Sequence	Modification	Ratios	
1867	3	993.3614	-92.34	2	51.9	16.1	2	1-18	-.MKRSGNPGAEADFCVMTR.L	Oxidation: 1	QD:QU	0.88

Detailed Protein Report

Protein 649: cation channel sperm-associated protein 2 isoform 2 [Homo sapiens]

**Accession:**	gi|26051223	**Score:**	16.0
**Database:**	refseq_human(refseq_human_20140103.fasta)	**MW [kDa]:**	62.0
**Database Date:**	2015-11-30	**pI:**	7.0
**Modification(s):**	Oxidation	**Sequence Coverage [%]:**	3.6
		**No. of unique Peptides:**	1

10	20	30	40	50	60	70	80
MAAYQQEEQM	QLPRADAIRS	RLIDTFSLIE	HLQGLSQAVP	RHTIRELLDP	SRQKKLVLGD	QHQLVRFSIK	PQRIEQISHA
90	100	110	120	130	140	150	160
QRLLSRLHVR	CSQRPPLSLW	AGWVLECPLF	KNFIIFLVFL	NTIILMVEIE	LLESTNTKLW	PLKLTLEVAA	WFILLIFILE
170	180	190	200	210	220	230	240
ILLKWLSNFS	VFWKSAWNVF	DFVVTMLSLL	PEVVVLVGVT	GQSVWLQLLR	ICRVLRSLKL	LAQFRQIQII	ILVLVRALKS
250	260	270	280	290	300	310	320
MTFLLMLLLI	FFYIFAVTGV	YVFSEYTRSP	RQDLEYHVFF	SDLPNSLVTV	FILFTLDHWY	ALLQDVWKVP	EVSRIFSSIY
330	340	350	360	370	380	390	400
FILWLLLGSI	IFRSIIVAMM	VTNFQNIRKE	LNEEMARREV	QLKADMFKRQ	IIQRRKNMSH	EALTSSHSKI	EDSSRGASQQ
410	420	430	440	450	460	470	480
RESLDLSEVS	EVESNYGATE	EDLITSASKT	EETLSKKREY	QSSSCVSSTS	SSYSSSSESR	FSESIGRLDW	ETLVHENLPG
490	500	510	520	530	540		
LMEMDQDDRV	WPRDSLFRYF	ELLEKLQYNL	EERKKLQEFA	VQALMNLEDK			

Cmpd.	No. of Cmpds.	m/z meas.	Δ m/z [ppm]	z	Rt [min]	Score	P	Range	Sequence	Modification
2773	1	711.3671	55.94	3	65.8	16.0	1	377-395	K.NMSHEALTSSHSKIEDSSR.G	Oxidation: 2

Detailed Protein Report

Protein 650: **PREDICTED: cGMP-inhibited 3',5'-cyclic phosphodiesterase B isoform X2 [Homo sapiens]**

**Accession:**	gi|530395463	**Score:**	16.0
**Database:**	refseq_human(refseq_human_20140103.fasta)	**MW [kDa]:**	115.5
**Database Date:**	2015-11-30	**pI:**	5.6
**Modification(s):**	Carbamidomethyl	**Sequence Coverage [%]:**	1.7
		**No. of unique Peptides:**	1

10	20	30	40	50	60	70	80
MRRDERDAKA	MRSLQPPDGA	GSPPESLRNG	YVKSCVSPLR	QDPPRGFFFH	LCRFCNVELR	PPPASPQQPR	RCSPFCRARL
**90**	**100**	**110**	**120**	**130**	**140**	**150**	**160**
SLGALAAFVL	ALLLGAEPES	WAAGAAWLRT	LLSVCSHSLS	PLFSIACAFF	FLTCFLTRTK	RGPGPGRSCG	SWWLLALPAC
**170**	**180**	**190**	**200**	**210**	**220**	**230**	**240**
CYLGDFLVWQ	WWSWPWGDGD	AGSAAPHTPP	EAAAGRLLLV	LSCVGLLLTL	AHPLRLRHCV	LVLLLASFVW	WVSFTSLGSL
**250**	**260**	**270**	**280**	**290**	**300**	**310**	**320**
PSALRPLLSG	LVGGAGCLLA	LGLDHFFQIR	EAPLHPRLSS	AAEEKVPVIR	PRRRSSCVSL	GETAASYYGS	CKIFRRPSLP
**330**	**340**	**350**	**360**	**370**	**380**	**390**	**400**
CISREQMILW	DWDLKQWYKP	HYQGLNRNSL	PTPQLRRSSG	TSGLLPVEQS	SRWDRNNGKR	PHQEFGISSQ	GCYLNGPFNS
**410**	**420**	**430**	**440**	**450**	**460**	**470**	**480**
NLLTIPKQRS	SSVSLTHHVG	LRRAGVLSSL	SPVNSSNHGP	VSTGSLTNRS	PIEFPDTADF	LNKPSVILQR	SLGNAPNTPD
**490**	**500**	**510**	**520**	**530**	**540**	**550**	**560**
FYQQLRNSDS	NLCNSCGHQM	LKYVSTSESD	GTDCCSGKSG	EEENIFSKES	FKLMETQQEE	ETEKKDSRKL	FQEGDKWLTE
**570**	**580**	**590**	**600**	**610**	**620**	**630**	**640**
EAQSEQQTNI	EQEVSLDLIL	VEEYDSLIEK	MSNWNFPIFE	LVEKMGEKSG	RILSQVMYTL	FQDTGLLEIF	KIPTQQFMNY
**650**	**660**	**670**	**680**	**690**	**700**	**710**	**720**
FRALENGYRD	IPYHNRIHAT	DVLHAVWYLT	TRPVPGLQQI	HNGCGTGNET	DSDGRINHGR	IAYISSKSCS	NPDESYGCLS
**730**	**740**	**750**	**760**	**770**	**780**	**790**	**800**
SNIPALELMA	LYVAAAMHDY	DHPGRTNAFL	VATNAPQAVL	YNDRSVLENH	HAASAWNLYL	SRPEYNFLLH	LDHVEFKRFR
**810**	**820**	**830**	**840**	**850**	**860**	**870**	**880**
FLVIEAILAT	DLKKHFDFLA	EFNAKANDVN	SNGIEWSNEN	DRLLVCQVCI	KLADINGPAK	VRDLHLKWTE	GIVNEFYEQG
**890**	**900**	**910**	**920**	**930**	**940**	**950**	**960**
DEEANLGLPI	SPFMDRSSPQ	LAKLQESFIT	HIVGPLCNSY	DAAGLLPGQW	LEAEEDNDTE	SGDDEDGEEL	DTEDEEMENN
**970**	**980**	**990**	**1000**	**1010**	**1020**	**1030**	
LNPKPPRRKS	RRRIFCQLMH	HLTENHKIWK	EIVEEEEKCK	ADGNKLQVEN	SSLPQADEIQ	VIEEADEEE	

Cmpd.	No. of Cmpds.	m/z meas.	Δ m/z [ppm]	z	Rt [min]	Score	P	Range	Sequence	Modification
1602	1	978.1827	141.31	2	50.3	16.0	1	843-860	R.LLVCQVCIKLADINGPAK.V	Carbamidomethyl: 4

Detailed Protein Report

Protein 651: mucin-12 precursor [Homo sapiens]

**Accession:**	gi|256600257	**Score:**	16.0
**Database:**	refseq_human(refseq_human_20140103.fasta)	**MW [kDa]:**	542.4
**Database Date:**	2015-11-30	**pI:**	5.2
**Modification(s):**	Carbamidomethyl	**Sequence Coverage [%]:**	0.2
		**No. of unique Peptides:**	1

10	20	30	40	50	60	70	80
MLVIWILTLA	LRLCASVTTV	TPGSTVNTSI	GGNTTSASTP	SSSDPFTTFS	DYGVSVTFIT	GSTATKHFLD	SSTNSGHSEE
90	100	110	120	130	140	150	160
STVSHSGPGA	TGTTLFPSHS	ATSVFVGEPK	TSPITSASME	TTALPGSTTT	AGLSEKSTTF	YSSPRSPDRT	LSPARTTSSG
170	180	190	200	210	220	230	240
VSEKSTTSHS	RPGPTHTIAF	PDSTTMPGVS	QESTASHSIP	GSTDTTLSPG	TTTPSSLGPE	STTFHSSPGY	TKTTRLPDNT
250	260	270	280	290	300	310	320
TTSGLLEAST	PVHSSTGSPH	TTLSPSSSTT	HEGEPTTFQS	WPSSKDTSPA	PSGTTSAFVK	LSTTYHSSPS	STPTTHFSAS
330	340	350	360	370	380	390	400
STTLGHSEES	TPVHSSPVAT	ATTPPPARSA	TSGHVEESTA	YHRSPGSTQT	MHFPESSTTS	GHSEESATFH	GSTTHTKSST
410	420	430	440	450	460	470	480
PSTTAALAHT	SYHSSLGSTE	TTHFRDSSTI	SGRSEESKAS	HSSPDAMATT	VLPAGSTPSV	LVGDSTPSPI	SSGSMETTAL
490	500	510	520	530	540	550	560
PGSTTKPGLS	EKSTTFYSSP	RSPDTTHLPA	SMTSSGVSEE	STTSHSRPGS	THTTAFPGST	TMPGLSQEST	ASHSSPGPTD
570	580	590	600	610	620	630	640
TTLSPGSTTA	SSLGPEYTTF	HSRPGSTETT	LLPDNTTASG	LLEASMPVHS	STRSPHTTLS	PAGSTTRQGE	STTFHSWPSS
650	660	670	680	690	700	710	720
KDTRPAPPTT	TSAFVEPSTT	SHGSPSSIPT	THISARSTTS	GLVEESTTYH	SSPGSTQTMH	FPESDTTSGR	GEESTTSHSS
730	740	750	760	770	780	790	800
TTHTISSAPS	TTSALVEEPT	SYHSSPGSTA	TTHFPDSSTT	SGRSEESTAS	HSSQDATGTI	VLPARSTTSV	LLGESTTSPI
810	820	830	840	850	860	870	880
SSGSMETTAL	PGSTTTPGLS	ERSTTFHSSP	RSPATTLSPA	STTSSGVSEE	STTSRSRPGS	THTTAFPDST	TTPGLSRHST
890	900	910	920	930	940	950	960
TSHSSPGSTD	TTLLPASTTT	SGPSQESTTS	HSSSGSTDTA	LSPGSTTALS	FGQESTTFHS	NPGSTHTTLF	PDSTTSSGIV
970	980	990	1000	1010	1020	1030	1040
EASTRVHSST	GSPRTTLSPA	SSTSPGLQGE	STAFQTHPAS	THTTPSPPST	ATAPVEESTT	YHRSPGSTPT	THFPASSTTS
1050	1060	1070	1080	1090	1100	1110	1120
GHSEKSTIFH	SSPDASGTTP	SSAHSTTSGR	GESTTSRISP	GSTEITTLPG	STTTPGLSEA	STTFYSSPRS	PTTTLSPASM
1130	1140	1150	1160	1170	1180	1190	1200
TSLGVGEEST	TSRSQPGSTH	STVSPASTTT	PGLSEESTTV	YSSSRGSTET	TVFPHSTTTS	VHGEEPTTFH	SRPASTHTTL
1210	1220	1230	1240	1250	1260	1270	1280
FTEDSTTSGL	TEESTAFPGS	PASTQTGLPA	TLTTADLGEE	STTFPSSSGS	TGTKLSPARS	TTSGLVGEST	PSRLSPSSTE
1290	1300	1310	1320	1330	1340	1350	1360
TTTLPGSPTT	PSLSEKSTTF	YTSPRSPDAT	LSPATTTSSG	VSEESSTSHS	QPGSTHTTAF	PDSTTTSDLS	QEPTTSHSSQ
1370	1380	1390	1400	1410	1420	1430	1440
GSTEATLSPG	STTASSLGQQ	STTFHSSPGD	TETTLLPDDT	ITSGLVEAST	PTHSSTGSLH	TTLTPASSTS	AGLQEESTTF
1450	1460	1470	1480	1490	1500	1510	1520
QSWPSSSDTT	PSPPGTTAAP	VEVSTTYHSR	PSSTPTTHFS	ASSTTLGRSE	ESTTVHSSPG	ATGTALFPTR	SATSVLVGEP
1530	1540	1550	1560	1570	1580	1590	1600
TTSPISSGST	ETTALPGSTT	TAGLSEKSTT	FYSSPRSPDT	TLSPASTTSS	GVSEESTTSH	SRPGSTHTTA	FPGSTTMPGV
1610	1620	1630	1640	1650	1660	1670	1680
SQESTASHSS	PGSTDTTLSP	GSTTASSLGP	ESTTFHSSPG	STETTLLPDN	TTASGLLEAS	TPVHSSTGSP	HTTLSPAGST
1690	1700	1710	1720	1730	1740	1750	1760
TRQGESTTFQ	SWPSSKDTMP	APPTTTSAFV	ELSTTSHGSP	SSTPTTHFSA	SSTTLGRSEE	STTVHSSPVA	TATTPSPARS
1770	1780	1790	1800	1810	1820	1830	1840
TTSGLVEEST	AYHSSPGSTQ	TMHFPESSTA	SGRSEESRTS	HSSTTHTISS	PPSTTSALVE	EPTSYHSSPG	STATTHFPDS
1850	1860	1870	1880	1890	1900	1910	1920
STTSGRSEES	TASHSSQDAT	GTIVLPARST	TSVLLGESTT	SPISSGSMET	TALPGSTTTP	GLSEKSTTFH	SSPRSPATTL
1930	1940	1950	1960	1970	1980	1990	2000
SPASTTSSGV	SEESTTSHSR	PGSTHTTAFP	DSTTTPGLSR	HSTTSHSSPG	STDTTLLPAS	TTTSGPSQES	TTSHSSPGST
2010	2020	2030	2040	2050	2060	2070	2080
DTALSPGSTT	ALSFGQESTT	FHSSPGSTHT	TLFPDSTTSS	GIVEASTRVH	SSTGSPRTTL	SPASSTSPGL	QGESTAFQTH
2090	2100	2110	2120	2130	2140	2150	2160
PASTHTTPSP	PSTATAPVEE	STTYHRSPGS	TPTTHFPASS	TTSGHSEKST	IFHSSPDASG	TTPSSAHSTT	SGRGESTTSR
2170	2180	2190	2200	2210	2220	2230	2240
ISPGSTEITT	LPGSTTTPGL	SEASTTFYSS	PRSPTTTLSP	ASMTSLGVGE	ESTTSRSQPG	STHSTVSPAS	TTTPGLSEES
2250	2260	2270	2280	2290	2300	2310	2320
TTVYSSSPGS	TETTVFPRTP	TTSVRGEEPT	TFHSRPASTH	TTLFTEDSTT	SGLTEESTAF	PGSPASTQTG	LPATLTTADL
2330	2340	2350	2360	2370	2380	2390	2400

Detailed Protein Report

Cmpd.	No. of Cmpds.	m/z meas.	Δ m/z [ppm]	z	Rt [min]	Score	P	Range	Sequence	Modification
710	1	661.8474	-3.38	2	38.9	16.0	2	5182-5193	K.LACVNKCTKGTK.S	Carbamidomethyl: 7

Detailed Protein Report

Protein 652: intraflagellar transport protein 80 homolog isoform a [Homo sapiens]

**Accession:**	gi|46409657	**Score:**	16.0
**Database:**	refseq_human(refseq_human_20140103.fasta)	**MW [kDa]:**	88.0
**Database Date:**	2015-11-30	**pI:**	8.4
**Modification(s):**	Carbamidomethyl	**Sequence Coverage [%]:**	1.7
		**No. of unique Peptides:**	1

Quantitation

QD:QU	**Median:** 1.47	**CV:** 0.00 %	**No. of Peptides:** 1

10	20	30	40	50	60	70	80
MRLKISLLKE	PKHQELVSCV	GWTTAEELYS	CSDDHQIVKW	NLLTSETTQI	VKLPDDIYPI	DFHWFPKSLG	VKKQTQAESF
**90**	**100**	**110**	**120**	**130**	**140**	**150**	**160**
VLTSSDGKFH	LISKLGRVEK	SVEAHCGAVL	AGRWNYEGTA	LVTVGEDGQI	KIWSKTGMLR	STLAQQGTPV	YSVAWGPDSE
**170**	**180**	**190**	**200**	**210**	**220**	**230**	**240**
KVLYTAGKQL	IIKPLQPNAK	VLQWKAHDGI	ILKVDWNSVN	DLILSAGEDC	KYKVWDSYGR	PLYNSQPHEH	PITSVAWAPD
**250**	**260**	**270**	**280**	**290**	**300**	**310**	**320**
GELFAVGSFH	TLRLCDKTGW	SYALEKPNTG	SIFNIAWSID	GTQIAGACGN	GHVVFAHVVE	QHWEWKNFQV	TLTKRRAMQV
**330**	**340**	**350**	**360**	**370**	**380**	**390**	**400**
RNVLNDAVDL	LEFRDRVIKA	SLNYAHLVVS	TSLQCYVFST	KNWNTPIIFD	LKEGTVSLIL	QAERHFLLVD	GSSIYLYSYE
**410**	**420**	**430**	**440**	**450**	**460**	**470**	**480**
GRFISSPKFP	GMRTDILNAQ	TVSLSNDTIA	IRDKADEKII	FLFEASTGKP	LGDGKFLSHK	NEILEIALDQ	KGLTNDRKIA
**490**	**500**	**510**	**520**	**530**	**540**	**550**	**560**
FIDKNRDLCI	TSVKRFGKEE	QIIKLGTMVH	TLAWNDTCNI	LCGLQDTRFI	VWYYPNTVYV	DRDILPKTLY	ERDASEFSKN
**570**	**580**	**590**	**600**	**610**	**620**	**630**	**640**
PHIVSFVGNQ	VTIRRADGSL	VHISITPYPA	ILHEYVSSSK	WEDAVRLCRF	VKEQTMWACL	AAMAVANRDM	TTAEIAYAAI
**650**	**660**	**670**	**680**	**690**	**700**	**710**	**720**
GEIDKVQYIN	SIKNLPSKES	KMAHILLFSG	NIQEAEIVLL	QAGLVYQAIQ	ININLYNWER	ALELAVKYKT	HVDTVLAYRQ
**730**	**740**	**750**	**760**	**770**	**780**		
KFLETFGKQE	TNKRYLHYAE	GLQIDWEKIK	AKIEMEITKE	REQSSSSQSS	KSIGLKP		

Cmpd.	No. of Cmpds.	m/z meas.	Δ m/z [ppm]	z	Rt [min]	Score	P	Range	Sequence	Modification	Ratios
870	1	663.6930	-210.56	2	40.9	16.0	0	101-113	K.SVEAHCGAVLAGR.W	Carbamidomethyl: 6	QD:QU 1.47

Detailed Protein Report

Protein 653: transcription intermediary factor 1-alpha isoform b [Homo sapiens]

Accession: gi|47419909
Database: refseq_human(refseq_human_20140103.fasta)
Database Date: 2015-11-30
Score: 16.0
MW [kDa]: 112.9
pI: 6.4
Sequence Coverage [%]: 1.1
No. of unique Peptides: 1

10	20	30	40	50	60	70	80
MEVAVEKAVA	AAAAASAAAS	GGPSAAPSGE	NEAESRQGPD	SERGGEAARL	NLLDTCAVCH	QNIQSRAPKL	LPCLHSFCQR
90	100	110	120	130	140	150	160
CLPAPQRYLM	LPAPMLGSAE	TPPPVPAPGS	PVSGSSPFAT	QVGVIRCPVC	SQECAERHII	DNFFVKDTTE	VPSSTVEKSN
170	180	190	200	210	220	230	240
QVCTSCEDNA	EANGFCVECV	EWLCKTCIRA	HQRVKFTKDH	TVRQKEEVSP	EAVGVTSQRP	VFCPFHKKEQ	LKLYCETCDK
250	260	270	280	290	300	310	320
LTCRDCQLLE	HKEHRYQFIE	EAFQNQKVII	DTLITKLMEK	TKYIKFTGNQ	IQNRIIEVNQ	NQKQVEQDIK	VAIFTLMVEI
330	340	350	360	370	380	390	400
NKKGKALLHQ	LESLAKDHRM	KLMQQQQEVA	GLSKQLEHVM	HFSKWAVSSG	SSTALLYSKR	LITYRLRHLL	RARCDASPVT
410	420	430	440	450	460	470	480
NNTIQFHCDP	SFWAQNIINL	GSLVIEDKES	QPQMPKQNPV	VEQNSQPPSG	LSSNQLSKFP	TQISLAQLRL	QHMQQQQPPP
490	500	510	520	530	540	550	560
RLINFQNHSP	KPNGPVLPPH	PQQLRYPPNQ	NIPRQAIKPN	PLQMAFLAQQ	AIKQWQISSG	QGTPSTTNST	SSTPSSPTIT
570	580	590	600	610	620	630	640
SAAGYDGKAF	GSPMIDLSSP	VGGSYNLPSL	PDIDCSSTIM	LDNIVRKDTN	IDHGQPRPPS	NRTVQSPNSS	VPSPGLAGPV
650	660	670	680	690	700	710	720
TMTSVHPPIR	SPSASSVGSR	GSSGSSSKPA	GADSTHKVPV	VMLEPIRIKQ	ENSGPPENYD	FPVVIVKQES	DEESRPQNAN
730	740	750	760	770	780	790	800
YPRSILTSLL	LNSSQSSTSE	ETVLRSDAPD	STGDQPGLHQ	DNSSNGKSEW	LDPSQKSPLH	VGETRKEDDP	NEDWCAVCQN
810	820	830	840	850	860	870	880
GGELLCCEKC	PKVFHLSCHV	PTLTNFPSGE	WICTFCRDLS	KPEVEYDCDA	PSHNSEKKKT	EGLVKLTPID	KRKCERLLLF
890	900	910	920	930	940	950	960
LYCHEMSLAF	QDPVPLTVPD	YYKIIKNPMD	LSTIKKRLQE	DYSMYSKPED	FVADFRLIFQ	NCAEFNEPDS	EVANAGIKLE
970	980	990	1000	1010	1020		
NYFEELLKNL	YPEKRFPKPE	FRNESEDNKF	SDDSDDDFVQ	PRKKRLKSIE	ERQLLK		

Cmpd.	No. of Cmpds.	m/z meas.	Δ m/z [ppm]	z	Rt [min]	Score	P	Range	Sequence	Modification
1407	1	637.4446	122.09	2	46.1	16.0	0	459-469	K.FPTQISLAQLR.L	

Detailed Protein Report

Protein 654: **PREDICTED: E3 ubiquitin-protein ligase RNF126 isoform X1 [Homo sapiens]**

Accession: gi|530425318
Database: refseq_human(refseq_human_20140103.fasta)
Database Date: 2015-11-30
Modification(s): Carbamidomethyl

Score: 15.9
MW [kDa]: 30.9
pI: 4.7
Sequence Coverage [%]: 7.4
No. of unique Peptides: 1

10	20	30	40	50	60	70	80
MAEASPHPGR	YFCHCCSVEI	VPRLPDYICP	RCESGFIEEL	PEETRSTENG	SAPSTAPTDQ	SRPPLEHVDQ	HLFTLPQGYG
**90**	**100**	**110**	**120**	**130**	**140**	**150**	**160**
QFAFGIFDDS	FEIPTFPPGA	QADDGRDPES	RRERDHPSRH	RIIQQLVNGI	ITPATIPSLG	PWGVLHSNPM	DYAWGANGLD
**170**	**180**	**190**	**200**	**210**	**220**	**230**	**240**
AIITQLLNQF	ENTGPPPADK	EKIQALPTVP	VTEEHVGSGL	ECPVCKDDYA	LGERVRQLPC	NHLFHDGCIV	PWLEQHDSCP
**250**	**260**	**270**	**280**	**290**			
VCRKSLTGQN	TATNPPGLTG	VSFSSSSSSS	SSSSPSNENA	TSNS			

Cmpd.	No. of Cmpds.	m/z meas.	Δ m/z [ppm]	z	Rt [min]	Score	P	Range	Sequence	Modification
2345	1	857.4373	45.18	3	59.8	15.9	1	11-31	R.YFCHCCSVEIVPRLPDYICPR.C	Carbamidomethyl: 6

Detailed Protein Report

Protein 655: AP-1 complex subunit mu-2 [Homo sapiens]

Accession: gi|9506797
Database: refseq_human(refseq_human_20140103.fasta)
Database Date: 2015-11-30
Modification(s): Carbamidomethyl

Score: 15.9
MW [kDa]: 48.1
pI: 8.9
Sequence Coverage [%]: 3.3
No. of unique Peptides: 1

10	20	30	40	50	60	70	80
MSASAVFILD	VKGKPLISRN	YKGDVAMSKI	EHFMPLLVQR	EEEGALAPLL	SHGQVHFLWI	KHSNLYLVAT	TSKNANASLV
**90**	**100**	**110**	**120**	**130**	**140**	**150**	**160**
YSFLYKTIEV	FCEYFKELEE	ESIRDNFVIV	YELLDELMDF	GFPQTTDSKI	LQEYITQQSN	KLETGKSRVP	PTVTNAVSWR
**170**	**180**	**190**	**200**	**210**	**220**	**230**	**240**
SEGIKYKKNE	VFIDVIESVN	LLVNANGSVL	LSEIVGTIKL	KVFLSGMPEL	RLGLNDRVLF	ELTGRSKNKS	VELEDVKFHQ
**250**	**260**	**270**	**280**	**290**	**300**	**310**	**320**
CVRLSRFDND	RTISFIPPDG	DFELMSYRLS	TQVKPLIWIE	SVIEKFSHSR	VEIMVKAKGQ	FKKQSVANGV	EISVPVPSDA
**330**	**340**	**350**	**360**	**370**	**380**	**390**	**400**
DSPRFKTSVG	SAKYVPERNV	VIWSIKSFPG	GKEYLMRAHF	GLPSVEKEEV	EGRPPIGVKF	EIPYFTVSGI	QVRYMKIIEK
**410**	**420**	**430**					
SGYQALPWVR	YITQSGDYQL	RTS					

Cmpd.	No. of Cmpds.	m/z meas.	Δ m/z [ppm]	z	Rt [min]	Score	P	Range	Sequence	Modification
1641	1	873.4079	-31.68	2	49.0	15.9	1	230-243	K.SVELEDVKFHQCVR.L	Carbamidomethyl: 12

Detailed Protein Report

Protein 656: cadherin-related family member 4 precursor [Homo sapiens]

**Accession:**	gi|203097724	**Score:**	15.9
**Database:**	refseq_human(refseq_human_20140103.fasta)	**MW [kDa]:**	85.8
**Database Date:**	2015-11-30	**pI:**	5.1
**Modification(s):**	Oxidation	**Sequence Coverage [%]:**	2.3
		**No. of unique Peptides:**	1

10	20	30	40	50	60	70	80
MVLLRLLVFL	FAPVVSDLCS	LPCFINVSES	QGPGTVLQFL	SFNCSSYTPT	PTLELLNVQP	PTTFFNPPSL	ARWQGTYVGK
**90**	**100**	**110**	**120**	**130**	**140**	**150**	**160**
LTLSSSAQLD	ALMVNHYKVQ	LKFTCGNHVM	EGSLSVDVQR	DLSHIQCAGQ	FASPAGEMIQ	VPETVTPGAR	LYTLLLPGLE
**170**	**180**	**190**	**200**	**210**	**220**	**230**	**240**
LHGAQMSIIS	AQDLPHFPGP	FSINEQGWLQ	APSQGLLGQA	QKVFQLQISV	SFGQRQSCQG	MVIVKVLPVP	SSQVSFLEQA
**250**	**260**	**270**	**280**	**290**	**300**	**310**	**320**
QNITIPENLA	PGSEVVQVQA	RGVDLRYEIL	SPVPSPLFSI	GRADGVVRTT	TPLELARTSG	TAVSRLQVKA	FEQGQLWASA
**330**	**340**	**350**	**360**	**370**	**380**	**390**	**400**
KLNLTMNVQL	VNLWPPRCLP	ALLVSQIPET	APVGTVLNTL	TCEDPDSVGA	TLDYKLWFRS	SSNPASLCLY	DRVLEVNATL
**410**	**420**	**430**	**440**	**450**	**460**	**470**	**480**
DCDTPGACFQ	HAASILVLDG	GQPQMTTEVP	VLVMVTPINE	FSPACAPRTF	RVQEDAAPHT	LLGSVVGTDM	DYPHDNIEYY
**490**	**500**	**510**	**520**	**530**	**540**	**550**	**560**
TSGGPTTFAV	DRLSGEVHLL	GPLDYEQQRL	YRLTVLVIDH	GQDQNPNHHL	SGSCTITIEV	EDVNDHAPEC	EPPFQELTIY
**570**	**580**	**590**	**600**	**610**	**620**	**630**	**640**
APLGRSVEVT	KMSCQIPQEP	QRLIYSYSIV	GGNSQNRFIL	QGAILVHSDL	VLGPFWPEQP	RTYELLICVA	DAGPSTPHLS
**650**	**660**	**670**	**680**	**690**	**700**	**710**	**720**
TTATIIVHLV	PRRASTVATS	THRTTVPSTM	TPMLVTDTEA	FWQPQPWFVV	VLTATGALLL	LALGWLLGRL	LQGLAQLLQA
**730**	**740**	**750**	**760**	**770**	**780**	**790**	
PSKPAQALLL	NSIQGTEGSI	EGFLEAPKME	MSQAPSSVMS	LHFDGRAQDS	RTGRDYLFNT	HTGARRWL	

Cmpd.	No. of Cmpds.	m/z meas.	Δ m/z [ppm]	z	Rt [min]	Score	P	Range	Sequence	Modification
1960	1	1029.3192	-117.25	2	53.0	15.9	0	749-766	K.MEMSQAPSSVMSLHFDGR.A	Oxidation: 1, 3, 11

Detailed Protein Report

Protein 657: **oxysterol-binding protein-related protein 10 isoform 2 [Homo sapiens]**

Accession: gi|291327480
Database: refseq_human(refseq_human_20140103.fasta)
Database Date: 2015-11-30

Score: 15.9
MW [kDa]: 77.2
pI: 8.5
Sequence Coverage [%]: 3.1
No. of unique Peptides: 1

10	20	30	40	50	60	70	80
MERAVQGTDG	GGGSNSSSRS	SSRATSAGSS	PSCSLAGRGV	SSRSAAAGLG	GGGSRSSPGS	VAASPSGGGG	RRREPALEGV
90	100	110	120	130	140	150	160
LSKYTNLLQG	WQNRYFVLDF	EAGILQYFVN	EQSKHQKPRG	VLSLSGAIVS	LSDEAPHMLV	VYSANGEMFK	LRAADAKEKQ
170	180	190	200	210	220	230	240
FWVTQLRACA	KYHMEMNSKM	MNQVEGQQKN	LVHAIESLPG	SGPLTALDQD	LLLLKATSAA	TLSCLGECLN	LLQQSVHQAG
250	260	270	280	290	300	310	320
QPSQKPGASE	NILGWHGSKS	HSTEQLKNGT	LGSLPSASAN	ITWAILPNSA	EDEQTSQPEP	EPNSGSELVL	SEDEKSDNED
330	340	350	360	370	380	390	400
KEETELGVME	DQRSIILHLI	SQLKLGMDLT	KVVLPTFILE	KRSLLEMYAD	FMAHPDLLLA	ITAGATPEER	VICFVEYYLT
410	420	430	440	450	460	470	480
AFHEGRKGAL	AKKPYNPIIG	ETFHCSWEVP	KDRVKPKRTA	SRSPASCHEH	PMADDPSKSY	KLRFVAEQVS	HHPPISCFYC
490	500	510	520	530	540	550	560
ECEEKRLCVN	THVWTKSKFM	GMSVGVSMIG	EGVLRLLEHG	EEYVFTLPSA	YARSILTIPW	VELGGKVSIN	CAKTGYSATV
570	580	590	600	610	620	630	640
IFHTKPFYGG	KVHRVTAEVK	HNPTNTIVCK	AHGEWNGTLE	FTYNNGETKV	IDTTTLPVYP	KKIRPLEKQG	PMESRNLWRE
650	660	670	680	690	700	710	
VTRYLRLGDI	DAATEQKRHL	EEKQRVEERK	RENLRTPWKP	KYFIQEGDGW	VYFNPLWKAH		

Cmpd.	No. of Cmpds.	m/z meas.	Δ m/z [ppm]	z	Rt [min]	Score	P	Range	Sequence	Modification
1650	1	1070.0254	31.14	2	49.1	15.9	2	2-23	M.ERAVQGTDGGGGSNSSSRSSSR.A	

Detailed Protein Report

Protein 658: beta-defensin 118 precursor [Homo sapiens]

Accession: gi|16905373
Database: refseq_human(refseq_human_20140103.fasta)
Database Date: 2015-11-30
Modification(s): Carbamidomethyl

Score: 15.9
MW [kDa]: 13.6
pI: 7.8
Sequence Coverage [%]: 10.6
No. of unique Peptides: 1

10	20	30	40	50	60	70	80
MKLLLLALPM	LVLLPQVIPA	YSGEKKCWNR	SGHCRKQCKD	GEAVKDTCKN	LRACCIPSNE	DHRRVPATSP	TPLSDSTPGI
90	100	110	120	130			
IDDILTVRFT	TDYFEVSSKK	DMVEESEAGR	GTETSLPNVH	HSS			

Cmpd.	No. of Cmpds.	m/z meas.	Δ m/z [ppm]	z	Rt [min]	Score	P	Range	Sequence	Modification
2473	1	753.2385	-177.83	2	59.6	15.9	2	40-52	K.DGEAVKDTCKNLR.A	Carbamidomethyl: 9

Detailed Protein Report

Protein 659: isoleucine--tRNA ligase, cytoplasmic [Homo sapiens]

**Accession:**	gi|94721239	**Score:**	15.8
**Database:**	refseq_human(refseq_human_20140103.fasta)	**MW [kDa]:**	144.4
**Database Date:**	2015-11-30	**pI:**	5.8
		**Sequence Coverage [%]:**	1.3
		**No. of unique Peptides:**	1

Alias proteins:

Accession	Name	Description
gi|94721241	refseq_huma (refseq_human_20140103.fasta)	isoleucine--tRNA ligase, cytoplasmic [Homo sapiens]

```
        10         20         30         40         50         60         70         80
MLQQVPENIN FPAEEEKILE FWTEFNCFQE CLKQSKHKPK FTFYDGPPFA TGLPHYGHIL AGTIKDIVTR YAHQSGFHVD
        90        100        110        120        130        140        150        160
RRFGWDCHGL PVEYEIDKTL GIRGPEDVAK MGITEYNNQC RAIVMRYSAE WKSTVSRLGR WIDFDNDYKT LYPQFMESVW
       170        180        190        200        210        220        230        240
WVFKQLYDKG LVYRGVKVMP FSTACNTPLS NFESHQNYKD VQDPSVFVTF PLEEDETVSL VAWTTTPWTL PSNLAVCVNP
       250        260        270        280        290        300        310        320
EMQYVKIKDV ARGRLLILME ARLSALYKLE SDYEILERFP GAYLKGKKYR PLFDYFLKCK ENGAFTVLVD NYVKEEEGTG
       330        340        350        360        370        380        390        400
VVHQAPYFGA EDYRVCMDFN IIRKDSLPVC PVDASGCFTT EVTDFAGQYV KDADKSIIRT LKEQGRLLVA TTFTHSYPFC
       410        420        430        440        450        460        470        480
WRSDTPLIYK AVPSWFVRVE NMVDQLLRNN DLCYWVPELV REKRFGNWLK DARDWTISRN RYWGTPIPLW VSDDFEEVVC
       490        500        510        520        530        540        550        560
IGSVAELEEL SGAKISDLHR ESVDHLTIPS RCGKGSLHRI SEVFDCWFES GSMPYAQVHY PFENKREFED AFPADFIAEG
       570        580        590        600        610        620        630        640
IDQTRGWFYT LLVLATALFG QPPFKNVIVN GLVLASDGQK MSKRKKNYPD PVSIIQKYGA DALRLYLINS PVVRAENLRF
       650        660        670        680        690        700        710        720
KEEGVRDVLK DVLLPWYNAY RFLIQNVLRL QKEEEIEFLY NENTVRESPN ITDRWILSFM QSLIGFFETE MAAYRLYTVV
       730        740        750        760        770        780        790        800
PRLVKFVDIL TNWYVRMNRR RLKGENGMED CVMALETLFS VLLSLCRLMA PYTPFLTELM YQNLKVLIDP VSVQDKDTLS
       810        820        830        840        850        860        870        880
IHYLMLPRVR EELIDKKTES AVSQMQSVIE LGRVIRDRKT IPIKYPLKEI VVIHQDPEAL KDIKSLEKYI IEELNVRKVT
       890        900        910        920        930        940        950        960
LSTDKNKYGI RLRAEPDHMV LGKRLKGAFK AVMTSIKQLS SEELEQFQKT GTIVVEGHEL HDEDIRLMYT FDQATGGTAQ
       970        980        990       1000       1010       1020       1030       1040
FEAHSDAQAL VLLDVTPDQS MVDEGMAREV INRIQKLRKK CNLVPTDEIT VYYKAKSEGT YLNSVIESHT EFIFTTIKAP
      1050       1060       1070       1080       1090       1100       1110       1120
LKPYPVSPSD KVLIQEKTQL KGSELEITLT RGSSLPGPAC AYVNLNICAN GSEQGGVLLL ENPKGDNRLD LLKLKSVVTS
      1130       1140       1150       1160       1170       1180       1190       1200
IFGVKNTELA VFHDETEIQN QTDLLSLSGK TLCVTAGSAP SLINSSSTLL CQYINLQLLN AKPQECLMGT VGTLLLENPL
      1210       1220       1230       1240       1250       1260       1270
GQNGLTHQGL LYEAAKVFGL RSRKLKLFLN ETQTQEITED IPVKTLNMKT VYVSVLPTTA DF
```

Cmpd.	No. of Cmpds.	m/z meas.	Δ m/z [ppm]	z	Rt [min]	Score	P	Range	Sequence	Modification
1672	1	942.9746	4.27	2	51.2	15.8	0	2-17	M.LQQVPENINFPAEEEK.I	

Detailed Protein Report

Protein 660: olfactory receptor 6Y1 [Homo sapiens]

Accession: gi|52353280
Database: refseq_human(refseq_human_20140103.fasta)
Database Date: 2015-11-30

Score: 15.8
MW [kDa]: 36.6
pI: 9.4
Sequence Coverage [%]: 4.6
No. of unique Peptides: 1

10	20	30	40	50	60	70	80
MTTIILEVDN	HTVTTRFILL	GFPTRPAFQL	LFFSIFLATY	LLTLLENLLI	ILAIHSDGQL	HKPMYFFLSH	LSFLEMWYVT
90	100	110	120	130	140	150	160
VISPKMLVDF	LSHDKSISFN	GCMTQLYFFV	TFVCTEYILL	AIMAFDRYVA	ICNPLRYPVI	MTNQLCGTLA	GGCWFCGLMT
170	180	190	200	210	220	230	240
AMIKMVFIAQ	LHYCGMPQIN	HYFCDISPLL	NVSCEDASQA	EMVDFFLALM	VIAIPLCVVV	ASYAAILATI	LRIPSAQGRQ
250	260	270	280	290	300	310	320
KAFSTCASHL	TVVILFYSMT	LFTYARPKLM	YAYNSNKVVS	VLYTVIVPLL	NPIIYCLRNH	EVKAALRKTI	HCRGSGPQGN
330							
GAFSS							

Cmpd.	No. of Cmpds.	m/z meas.	Δ m/z [ppm]	z	Rt [min]	Score	P	Range	Sequence	Modification
2724	1	856.9336	-33.77	2	65.2	15.8	0	2-16	M.TTIILEVDNHTVTTR.F	

Detailed Protein Report

Protein 661: IGF-like family receptor 1 precursor [Homo sapiens]

Accession: gi|13375913
Database: refseq_human(refseq_human_20140103.fasta)
Database Date: 2015-11-30

Score: 15.8
MW [kDa]: 37.9
pI: 6.9
Sequence Coverage [%]: 5.1
No. of unique Peptides: 1

10	20	30	40	50	60	70	80
MGPGRCLLTA	LLLLALAPPP	EASQYCGRLE	YWNPDNKCCS	SCLQRFGPPP	CPDYEFRENC	GLNDHGDFVT	PPFRKCSSGQ
**90**	**100**	**110**	**120**	**130**	**140**	**150**	**160**
CNPDGAELCS	PCGGGAVTPT	PAAGGGRTPW	RCRERPVPAK	GHCPLTPGNP	GAPSSQERSS	PASSIAWRTP	EPVPQQAWPN
**170**	**180**	**190**	**200**	**210**	**220**	**230**	**240**
FLPLVVLVLL	LTLAVIAILL	FILLWHLCWP	KEKADPYPYP	GLVCGVPNTH	TPSSSHLSSP	GALETGDTWK	EASLLPLLSR
**250**	**260**	**270**	**280**	**290**	**300**	**310**	**320**
ELSSLASQPL	SRLLDELEVL	EELIVLLDPE	PGPGGGMAHG	TTRHLAARYG	LPAAWSTFAY	SLRPSRSPLR	ALIEMVVARE
**330**	**340**	**350**	**360**				
PSASLGQLGT	HLAQLGRADA	LRVLSKLGSS	GVCWA				

Cmpd.	No. of Cmpds.	m/z meas.	Δ m/z [ppm]	z	Rt [min]	Score	P	Range	Sequence	Modification
1323	1	918.0778	93.29	2	46.7	15.8	0	320-337	R.EPSASLGQLGTHLAQLGR.A	

Detailed Protein Report

Protein 662: G-protein coupled receptor 55 [Homo sapiens]

**Accession:**	gi|33695107	**Score:**	15.8
**Database:**	refseq_human(refseq_human_20140103.fasta)	**MW [kDa]:**	36.6
**Database Date:**	2015-11-30	**pI:**	10.0
		**Sequence Coverage [%]:**	6.3
		**No. of unique Peptides:**	1

Alias proteins:

Accession	Name	Description
gi|530371161	refseq_human (refseq_human_20140103.fasta)	PREDICTED: G-protein coupled receptor 55 isoform X1 [Homo sapiens]

10	20	30	40	50	60	70	80
MSQQNTSGDC	LFDGVNELMK	TLQFAVHIPT	FVLGLLLNLL	AIHGFSTFLK	NRWPDYAATS	IYMINLAVFD	LLLVLSLPFK
**90**	**100**	**110**	**120**	**130**	**140**	**150**	**160**
MVLSQVQSPF	PSLCTLVECL	YFVSMYGSVF	TICFISMDRF	LAIRYPLLVS	HLRSPRKIFG	ICCTIWVLVW	TGSIPIYSFH
**170**	**180**	**190**	**200**	**210**	**220**	**230**	**240**
GKVEKYMCFH	NMSDDTWSAK	VFFPLEVFGF	LLPMGIMGFC	CSRSIHILLG	RRDHTQDWVQ	QKACIYSIAA	SLAVFVVSFL
**250**	**260**	**270**	**280**	**290**	**300**	**310**	**320**
PVHLGFFLQF	LVRNSFIVEC	RAKQSISFFL	QLSMCFSNVN	CCLDVFCYYF	VIKEFRMNIR	AHRPSRVQLV	LQDTTISRG

Cmpd.	No. of Cmpds.	m/z meas.	Δ m/z [ppm]	z	Rt [min]	Score	P	Range	Sequence	Modification
2405	1	739.6886	40.20	3	58.8	15.8	0	1-20	-.MSQQNTSGDCLFDGVNELMK.T	

Detailed Protein Report

Protein 663: **MAM domain-containing glycosylphosphatidylinositol anchor protein 2 isoform 2 [Homo sapiens]**

Accession: gi|38158013
Database: refseq_human(refseq_human_20140103.fasta)
Database Date: 2015-11-30

Score: 15.8
MW [kDa]: 81.3
pI: 6.6
Sequence Coverage [%]: 2.1
No. of unique Peptides: 1

10	20	30	40	50	60	70	80
MVSFRLSNKT	ASPSIKLLVD	DPIVVNPGEA	ITLVCVTTGG	EPAPSLTWVR	SFGTLPEKTV	LNGGTLTIPA	ITSDDAGTYS
**90**	**100**	**110**	**120**	**130**	**140**	**150**	**160**
CIANNNVGNP	AKKSTNIIVR	ALKKGRFWIT	PDPYHKDDNI	QIGREVKISC	QVEAVPSEEL	TFSWFKNGRP	LRSSERMVIT
**170**	**180**	**190**	**200**	**210**	**220**	**230**	**240**
QTDPDVSPGT	TNLDIIDLKF	TDFGTYTCVA	SLKGGGISDI	SIDVNISSST	VPPNLTVPQE	KSPLVTREGD	TIELQCQVTG
**250**	**260**	**270**	**280**	**290**	**300**	**310**	**320**
KPKPIILWSR	ADKEVAMPDG	SMQMESYDGT	LRIVNVSREM	SGMYRCQTSQ	YNGFNVKPRE	ALVQLIVQYP	PAVEPAFLEI
**330**	**340**	**350**	**360**	**370**	**380**	**390**	**400**
RQGQDRSVTM	SCRVLRAYPI	RVLTYEWRLG	NKLLRTGQFD	SQEYTEYAVK	SLSNENYGVY	NCSIINEAGA	GRCSFLVTGK
**410**	**420**	**430**	**440**	**450**	**460**	**470**	**480**
AYAPEFYYDT	YNPVWQNRHR	VYSYSLQWTQ	MNPDAVDRIV	AYRLGIRQAG	QQRWWEQEIK	INGNIQKGEL	ITYNLTELIK
**490**	**500**	**510**	**520**	**530**	**540**	**550**	**560**
PEAYEVRLTP	LTKFGEGDST	IRVIKYSAPV	NPHLREFHCG	FEDGNICLFT	QDDTDNFDWT	KQSTATRNTK	YTPNTGPNAD
**570**	**580**	**590**	**600**	**610**	**620**	**630**	**640**
RSGSKEGFYM	YIETSRPRLE	GEKARLLSPV	FSIAPKNPYG	PTNTAYCFSF	FYHMYGQHIG	VLNVYLRLKG	QTTIENPLWS
**650**	**660**	**670**	**680**	**690**	**700**	**710**	**720**
SSGNKGQRWN	EAHVNIYPIT	SFQLIFEGIR	GPGIEGDIAI	DDVSIAEGEC	AKQDLATKNS	VDGAVGILVH	IWLFPIIVLI
**730**							
SILSPRR							

Cmpd.	No. of Cmpds.	m/z meas.	Δ m/z [ppm]	z	Rt [min]	Score	P	Range	Sequence	Modification
134	1	876.6061	150.69	2	30.4	15.8	2	327-341	R.SVTMSCRVLRAYPIR.V	

Detailed Protein Report

Protein 664: **protein FAM154A isoform b [Homo sapiens]**

**Accession:**	gi|559098458	**Score:**	15.7
**Database:**	refseq_human(refseq_human_20140103.fasta)	**MW [kDa]:**	46.8
**Database Date:**	2015-11-30	**pI:**	9.4
**Modification(s):**	Carbamidomethyl	**Sequence Coverage [%]:**	4.6
		**No. of unique Peptides:**	1

10	20	30	40	50	60	70	80
MEGLTTSRRD	FGPHKVAPVK	VHQYDQFVPS	EENMDLLTTY	KKDYNPYPVC	RVDPIKPRDS	KYPCSDKMEC	LPTYKADYLP
**90**	**100**	**110**	**120**	**130**	**140**	**150**	**160**
WNQPRREPLR	LEHKYQPASV	RFDNRTTHQD	DYPIKGLVKT	ISCKPLAMPK	LCNIPLEDVT	NYKMSYVAHP	VEKRFVHEAE
**170**	**180**	**190**	**200**	**210**	**220**	**230**	**240**
KFRPCEIPFE	SLTTQKQSYR	GLMGEPAKSL	KPLARPPGLD	MPFCNTTEFR	DKYQAWPMPR	MFSKAPITYV	PPEDRMDLLT
**250**	**260**	**270**	**280**	**290**	**300**	**310**	**320**
TVQAHYTCPK	GAPAQSCRPA	LQIKKCGRFE	GSSTTKDDYK	QWSSMRTEPV	KPVPQLDLPT	EPLDCLTTTR	AHYVPHLPIN
**330**	**340**	**350**	**360**	**370**	**380**	**390**	**400**
TKSCKPHWSG	PRGNVPVESQ	TTYTISFTPK	EMGRCLASYP	EPPGYTFEEV	DALGHRIYKP	VSQAGSQQSS	HLSVDDSENP
**410**							
NQRELEVLA							

Cmpd.	No. of Cmpds.	m/z meas.	Δ m/z [ppm]	z	Rt [min]	Score	P	Range	Sequence	Modification
2413	1	1160.1933	96.65	2	58.9	15.7	2	43-61	K.DYNPYPVCRVDPIKPRDSK.Y	Carbamidomethyl: 8

Detailed Protein Report

Protein 665: PREDICTED: transcription factor HES-4 isoform X1 [Homo sapiens]

Accession: gi|530360364
Database: refseq_human(refseq_human_20140103.fasta)
Database Date: 2015-11-30

Score: 15.7
MW [kDa]: 19.8
pI: 11.9
Sequence Coverage [%]: 7.9
No. of unique Peptides: 1

10	20	30	40	50	60	70	80
MAADTPGKPS	ASPMAGAPAS	ASRTPDKPRS	AAEHRKSSRH	SKLEKADILE	MTVRHLRSLR	RVQVTAALSA	DPAVLGKYRA
90	100	110	120	130	140	150	160
GFHECLAEVN	RFLAGCEGVP	ADVRSRLLGH	LAACLRQLGP	SRRPASLSPA	APAEAPAPEV	YAGRPLLPSL	GGPFPLLAPP
170	180	190					
LLPGLTRALP	AAPRAGPQGP	GGPWRPWLR					

Cmpd.	No. of Cmpds.	m/z meas.	Δ m/z [ppm]	z	Rt [min]	Score	P	Range	Sequence	Modification
2181	1	885.4041	-88.63	2	57.7	15.7	2	40-54	R.HSKLEKADILEMTVR.H	

Detailed Protein Report

Protein 666: olfactory receptor 9G9 [Homo sapiens]

**Accession:**	gi|513788269	**Score:**	15.7
**Database:**	refseq_human(refseq_human_20140103.fasta)	**MW [kDa]:**	34.0
**Database Date:**	2015-11-30	**pI:**	9.2
		**Sequence Coverage [%]:**	3.0
		**No. of unique Peptides:**	1

10	20	30	40	50	60	70	80
MQRSNHTVTE	FILLGFTTDP	GMQLGLFVVF	LGVYSLTVVG	NSTLIVLICN	DSHLHTPMYF	VVGNLSFLDL	WYSSVYTPKI
**90**	**100**	**110**	**120**	**130**	**140**	**150**	**160**
LVICISEDKS	ISFAGCLCQF	FFSAGLAYSE	CCLLAAMAYD	RYVAISKPLL	YAQAMSIKLC	ALLVAVSYCG	GFINSSIITK
**170**	**180**	**190**	**200**	**210**	**220**	**230**	**240**
KTFSFNFCCE	NIIDDFFCDL	LPLVKLACGE	KGGYKFLMYF	LLASNVICPA	VLILASYLFI	ITSVLRISSS	QGRLKAFSTC
**250**	**260**	**270**	**280**	**290**	**300**	**310**	
SSHLTSVTLY	YGSILYIYAL	PRSSYSFDMD	KIVSTFYTEV	LPMLNPMIYS	LRNKDVKEAL	KKLLP	

Cmpd.	No. of Cmpds.	m/z meas.	Δ m/z [ppm]	z	Rt [min]	Score	P	Range	Sequence	Modification
2624	1	488.2210	-126.35	2	61.5	15.7	1	227-235	R.ISSSQGRLK.A	

Detailed Protein Report

Protein 667: **putative pre-mRNA-splicing factor ATP-dependent RNA helicase DHX15 [Homo sapiens]**

Accession: gi|68509926
Database: refseq_human(refseq_human_20140103.fasta)
Database Date: 2015-11-30
Modification(s): Carbamidomethyl

Score: 15.7
MW [kDa]: 90.9
pI: 7.4
Sequence Coverage [%]: 1.4
No. of unique Peptides: 1

Quantitation

QD:QU **Median:** 1.41 **CV:** 0.00 % **No. of Peptides:** 1

10	20	30	40	50	60	70	80
MSKRHRLDLG	EDYPSGKKRA	GTDGKDRDRD	RDREDRSKDR	DRERDRGDRE	REREKEKEKE	LRASTNAMLI	SAGLPPLKAS
90	100	110	120	130	140	150	160
HSAHSTHSAH	STHSTHSAHS	THAGHAGHTS	LPQCINPFTN	LPHTPRYYDI	LKKRLQLPVW	EYKDRFTDIL	VRHQSFVLVG
170	180	190	200	210	220	230	240
ETGSGKTTQI	PQWCVEYMRS	LPGPKRGVAC	TQPRRVAAMS	VAQRVADEMD	VMLGQEVGYS	IRFEDCSSAK	TILKYMTDGM
250	260	270	280	290	300	310	320
LLREAMNDPL	LERYGVIILD	EAHERTLATD	ILMGVLKEVV	RQRSDLKVIV	MSATLDAGKF	QIYFDNCPLL	TIPGRTHPVE
330	340	350	360	370	380	390	400
IFYTPEPERD	YLEAAIRTVI	QIHMCEEEEG	DLLLFLTGQE	EIDEACKRIK	REVDDLGPEV	GDIKIIPLYS	TLPPQQQQRI
410	420	430	440	450	460	470	480
FEPPPPKKQN	GAIGRKVVVS	TNIAETSLTI	DGVVFVIDPG	FAKQKVYNPR	IRVESLLVTA	ISKASAQQRA	GRAGRTRPGK
490	500	510	520	530	540	550	560
CFRLYTEKAY	KTEMQDNTYP	EILRSNLGSV	VLQLKKLGID	DLVHFDFMDP	PAPETLMRAL	ELLNYLAALN	DDGDLTELGS
570	580	590	600	610	620	630	640
MMAEFPLDPQ	LAKMVIASCD	YNCSNEVLSI	TAMLSVPQCF	VRPTEAKKAA	DEAKMRFAHI	DGDHLTLLNV	YHAFKQNHES
650	660	670	680	690	700	710	720
VQWCYDNFIN	YRSLMSADNV	RQQLSRIMDR	FNLPRRSTDF	TSRDYYINIR	KALVTGYFMQ	VAHLERTGHY	LTVKDNQVVQ
730	740	750	760	770	780	790	800
LHPSTVLDHK	PEWVLYNEFV	LTTKNYIRTC	TDIKPEWLVK	IAPQYYDMSN	FPQCEAKRQL	DRIIAKLQSK	EYSQY

Cmpd.	No. of Cmpds.	m/z meas.	Δ m/z [ppm]	z	Rt [min]	Score	P	Range	Sequence	Modification	Ratios	
226	1	739.8495	-36.72	2	32.8	15.7	2	481-491	K.CFRLYTEKAYK.T	Carbamidomethyl: 1	QD:QU	1.41

Detailed Protein Report

Protein 668: PREDICTED: coiled-coil domain-containing protein 158 isoform X8 [Homo sapiens]

**Accession:**	gi|578809093	**Score:**	15.7
**Database:**	refseq_human(refseq_human_20140103.fasta)	**MW [kDa]:**	122.8
**Database Date:**	2015-11-30	**pI:**	6.4
**Modification(s):**	Oxidation	**Sequence Coverage [%]:**	1.2
		**No. of unique Peptides:**	1

10	20	30	40	50	60	70	80
MESKAWESNN	EDLLSSSGVT	SNGGSSSSFF	VSSIRGTIIE	NTSSAGTLTQ	VPFFPKYEVE	LDSPRKIIPS	PGKEHFERVL
90	100	110	120	130	140	150	160
EEYSHQVKDL	QRRLNESNEL	HEKQKFYLRQ	SVIDLQTKLQ	EMQMERDAMA	DIRRRESQSQ	EDLRNQLQNT	VHELEAAKCL
170	180	190	200	210	220	230	240
KEDMLKDSNT	QIEQLRKMML	SHEGVLQEIR	SILVDFEEAS	GKKICEHDSM	STLHFRSLGS	AISKILRELD	TEISYLKGRI
250	260	270	280	290	300	310	320
FPVEDQLEAL	KSESQNKIEL	LLQQHQDRIE	QLISEHEVEI	TGLTEKASSA	RSQANSIQSQ	MEIIQEQARN	QNSMYMRQLS
330	340	350	360	370	380	390	400
DLESTVSQLR	SELREAKRMY	EDKTEELEKQ	LVLANSELTE	ARTERDQFSQ	ESGNLDDQLQ	KLLADLHKRE	KELSLEKEQN
410	420	430	440	450	460	470	480
KRLWDRDTGN	SITIDHLRRE	LDNRNMEVQR	LEALLKALKS	ECQGQMERQM	AAIQGKNESL	EKVSSLTAQL	ESTKEMLRKV
490	500	510	520	530	540	550	560
VEELTAKKMT	LESSERTISD	LTTSLQEKER	AIEATNAEIT	KLRSRVDLKL	QELQHLKNEG	DHLRNVQTEC	EALKLQMTEK
570	580	590	600	610	620	630	640
DKVIEILRQQ	IENMTQLVGQ	HGRTAGAMQV	EKAQLEKEIN	DRRMELKELK	ILKDKKDAKI	RELEARVSDL	ELEKVKLVNA
650	660	670	680	690	700	710	720
GSERLRAVKD	IKQERDQLLN	EVKTSRSELN	NLSAMKVAMG	MQKQITAKRG	QIDALQSKIQ	FLEEAMTNAN	KEKHFLKEEK
730	740	750	760	770	780	790	800
SKLSQELSTV	ATEKNKMAGE	LEVLRSQERR	LKEKVTNMEV	ALDKASLQFA	ECQDIIQRQE	QESVRLKLQH	TLDIKELQGP
810	820	830	840	850	860	870	880
GYTSNSSLKP	RLLQPASVTR	SHSNVPSSQS	TASFLSHHST	KANTLKEDPT	RDLKQLLQEL	RSVINEEPAV	SLSKTEEDGR
890	900	910	920	930	940	950	960
TSLGALYVAV	EDRVRDCITE	SSLRSDMCHR	SNNSLRDSTE	GSKSSETLSR	EPVTLHAGDR	EDPSGCFTFT	SAASPSVKNS
970	980	990	1000	1010	1020	1030	1040
ASRSFNSSPK	KSPVHSLLTS	SVEGSIGSTS	QYRSAKPIHS	SDSVKVSANR	NNRKNMQEAS	EQTRKLANSG	RRFTAEEPSN
1050	1060	1070	1080				
VFNDQKSRKE	DTESKRPGKN	VTKMTRCLPI	LYRGMRPN				

Cmpd.	No. of Cmpds.	m/z meas.	Δ m/z [ppm]	z	Rt [min]	Score	P	Range	Sequence	Modification
1840	1	781.3073	-162.78	2	53.4	15.7	2	475-487	K.EMLRKVVEELTAK.K	Oxidation: 2

Detailed Protein Report

Protein 669: vacuolar protein sorting-associated protein 33A [Homo sapiens]

**Accession:**	gi|18105056	**Score:**	15.7
**Database:**	refseq_human(refseq_human_20140103.fasta)	**MW [kDa]:**	67.6
**Database Date:**	2015-11-30	**pI:**	6.5
		**Sequence Coverage [%]:**	2.2
		**No. of unique Peptides:**	1

Quantitation

QD:QU **Median:** 7.65 **CV:** 0.00 % **No. of Peptides:** 1

10	20	30	40	50	60	70	80
MAAHLSYGRV	NLNVLREAVR	RELREFLDKC	AGSKAIVWDE	YLTGPFGLIA	QYSLLKEHEV	EKMFTLKGNR	LPAADVKNII
90	100	110	120	130	140	150	160
FFVRPRLELM	DIIAENVLSE	DRRGPTRDFH	ILFVPRRSLL	CEQRLKDLGV	LGSFIHREEY	SLDLIPFDGD	LLSMESEGAF
170	180	190	200	210	220	230	240
KECYLEGDQT	SLYHAAKGLM	TLQALYGTIP	QIFGKGECAR	QVANMMIRMK	REFTGSQNSI	FPVFDNLLLL	DRNVDLLTPL
250	260	270	280	290	300	310	320
ATQLTYEGLI	DEIYGIQNSY	VKLPPEKFAP	KKQGDGGKDL	PTEAKKLQLN	SAEELYAEIR	DKNFNAVGSV	LSKKAKIISA
330	340	350	360	370	380	390	400
AFEERHNAKT	VGEIKQFVSQ	LPHMQAARGS	LANHTSIAEL	IKDVTTSEDF	FDKLTVEQEF	MSGIDTDKVN	NYIEDCIAQK
410	420	430	440	450	460	470	480
HSLIKVLRLV	CLQSVCNSGL	KQKVLDYYKR	EILQTYGYEH	ILTLHNLEKA	GLLKPQTGGR	NNYPTIRKTL	RLWMDDVNEQ
490	500	510	520	530	540	550	560
NPTDISYVYS	GYAPLSVRLA	QLLSRPGWRS	IEEVLRILPG	PHFEERQPLP	TGLQKKRQPG	ENRVTLIFFL	GGVTFAEIAA
570	580	590	600				
LRFLSQLEDG	GTEYVIATTK	LMNGTSWIEA	LMEKPF				

Cmpd.	No. of Cmpds.	m/z meas.	Δ m/z [ppm]	z	Rt [min]	Score	P	Range	Sequence	Modification	Ratios	
770	1	689.8846	21.29	2	38.1	15.7	1	301-313	R.DKNFNAVGSVLSK.K		QD:QU	7.65

Detailed Protein Report

Protein 670: **pappalysin-2 isoform 2 precursor [Homo sapiens]**

**Accession:**	gi|116174734	**Score:**	15.6
**Database:**	refseq_human(refseq_human_20140103.fasta)	**MW [kDa]:**	92.1
**Database Date:**	2015-11-30	**pI:**	5.9
		**Sequence Coverage [%]:**	1.5
		**No. of unique Peptides:**	1

10	20	30	40	50	60	70	80
MMCLKILRIS	LAILAGWALC	SANSELGWTR	KKSLVEREHL	NQVLLEGERC	WLGAKVRRPR	ASPQHHLFGV	YPSRAGNYLR
**90**	**100**	**110**	**120**	**130**	**140**	**150**	**160**
PYPVGEQEIH	HTGRSKPDTE	GNAVSLVPPD	LTENPAGLRG	AVEEPAAPWV	GDSPIGQSEL	LGDDDAYLGN	QRSKESLGEA
**170**	**180**	**190**	**200**	**210**	**220**	**230**	**240**
GIQKGSAMAA	TTTTAIFTTL	NEPKPETQRR	GWAKSRQRRQ	VWKRRAEDGQ	GDSGISSHFQ	PWPKHSLKHR	VKKSPPEESN
**250**	**260**	**270**	**280**	**290**	**300**	**310**	**320**
QNGGEGSYRE	AETFNSQVGL	PILYFSGRRE	RLLLRPEVLA	EIPREAFTVE	AWVKPEGGQN	NPAIIAGVFD	NCSHTVSDKG
**330**	**340**	**350**	**360**	**370**	**380**	**390**	**400**
WALGIRSGKD	KGKRDARFFF	SLCTDRVKKA	TILISHSRYQ	PGTWTHVAAT	YDGRHMALYV	DGTQVASSLD	QSGPLNSPFM
**410**	**420**	**430**	**440**	**450**	**460**	**470**	**480**
ASCRSLLLGG	DSSEDGHYFR	GHLGTLVFWS	TALPQSHFQH	SSQHSSGEEE	ATDLVLTASF	EPVNTEWVPF	RDEKYPRLEV
**490**	**500**	**510**	**520**	**530**	**540**	**550**	**560**
LQGFEPEPEI	LSPLQPPLCG	QTVCDNVELI	SQYNGYWPLR	GEKVIRYQVV	NICDDEGLNP	IVSEEQIRLQ	HEALNEAFSR
**570**	**580**	**590**	**600**	**610**	**620**	**630**	**640**
YNISWQLSVH	QVHNSTLRHR	VVLVNCEPSK	IGNDHCDPEC	EHPLTGYDGG	DCRLQGRCYS	WNRRDGLCHV	ECNNMLNDFD
**650**	**660**	**670**	**680**	**690**	**700**	**710**	**720**
DGDCCDPQVA	DVRKTCFDPD	SPKRAYMSVK	ELKEALQLNS	THFLNIYFAS	SVREDLAGAA	TWPWDKDAVT	HLGGIVLSPA
**730**	**740**	**750**	**760**	**770**	**780**	**790**	**800**
YYGMPGHTDT	MIHEVGHVLG	LYHVFKGVSE	RESCNDPCKE	TVPSMETGDL	CADTAPTPKS	ELCREPEPTS	DTCGFTRFPG
**810**	**820**	**830**					
APFTNYMSYT	GITTVLFCFL	LRIHGGL					

Cmpd.	No. of Cmpds.	m/z meas.	Δ m/z [ppm]	z	Rt [min]	Score	P	Range	Sequence	Modification
694	6	676.8503	-106.32	2	38.7	15.6	2	347-358	R.VKKATILISHSR.Y	

Detailed Protein Report

Protein 671: pre-mRNA-processing factor 17 [Homo sapiens]

**Accession:**	gi|7706657	**Score:**	15.6
**Database:**	refseq_human(refseq_human_20140103.fasta)	**MW [kDa]:**	65.5
**Database Date:**	2015-11-30	**pI:**	6.7
		**Sequence Coverage [%]:**	2.1
		**No. of unique Peptides:**	1

10	20	30	40	50	60	70	80
MSAAIAALAA	SYGSGSGSES	DSDSESSRCP	LPAADSLMHL	TKSPSSKPSL	AVAVDSAPEV	AVKEDLETGV	HLDPAVKEVQ
**90**	**100**	**110**	**120**	**130**	**140**	**150**	**160**
YNPTYETMFA	PEFGPENPFR	TQQMAAPRNM	LSGYAEPAHI	NDFMFEQQRR	TFATYGYALD	PSLDNHQVSA	KYIGSVEEAE
**170**	**180**	**190**	**200**	**210**	**220**	**230**	**240**
KNQGLTVFET	GQKKTEKRKK	FKENDASNID	GFLGPWAKYV	DEKDVAKPSE	EEQKELDEIT	AKRQKKGKQE	EEKPGEEKTI
**250**	**260**	**270**	**280**	**290**	**300**	**310**	**320**
LHVKEMYDYQ	GRSYLHIPQD	VGVNLRSTMP	PEKCYLPKKQ	IHVWSGHTKG	VSAVRLFPLS	GHLLLSCSMD	CKIKLWEVYG
**330**	**340**	**350**	**360**	**370**	**380**	**390**	**400**
ERRCLRTFIG	HSKAVRDICF	NTAGTQFLSA	AYDRYLKLWD	TETGQCISRF	TNRKVPYCVK	FNPDEDKQNL	FVAGMSDKKI
**410**	**420**	**430**	**440**	**450**	**460**	**470**	**480**
VQWDIRSGEI	VQEYDRHLGA	VNTIVFVDEN	RRFVSTSDDK	SLRVWEWDIP	VDFKYIAEPS	MHSMPAVTLS	PNGKWLACQS
**490**	**500**	**510**	**520**	**530**	**540**	**550**	**560**
MDNQILIFGA	QNRFRLNKKK	IFKGHMVAGY	ACQVDFSPDM	SYVISGDGNG	KLNIWDWKTT	KLYSRFKAHD	KVCIGAVWHP
**570**	**580**						
HETSKVITCG	WDGLIKLWD						

Cmpd.	No. of Cmpds.	m/z meas.	Δ m/z [ppm]	z	Rt [min]	Score	P	Range	Sequence	Modification
1673	1	694.3984	86.12	2	51.2	15.6	1	227-238	K.GKQEEEKPGEEK.T	

Detailed Protein Report

Protein 672: PREDICTED: mono [ADP-ribose] polymerase PARP16 isoform X3 [Homo sapiens]

Accession: gi|530406133
Database: refseq_human(refseq_human_20140103.fasta)
Database Date: 2015-11-30

Score: 15.6
MW [kDa]: 28.5
pI: 10.2
Sequence Coverage [%]: 6.7
No. of unique Peptides: 1

10	20	30	40	50	60	70	80
MQPSGWAAAR	EAAGRDMLAA	DLRCSLFASA	LQSYKRDSVL	RPFPASYARG	DCKDFEALLA	DASKLPNLKE	LLQSSGDNHK
**90**	**100**	**110**	**120**	**130**	**140**	**150**	**160**
RAWDLVSWIL	SSKVLTIHSA	GKAETSLFGE	GTYLTSDLSL	ALIYSPHGHG	WQHSLLGPIL	SCVAVCEVID	HPDVKCQTKK
**170**	**180**	**190**	**200**	**210**	**220**	**230**	**240**
KDSKEIDRRR	ARIKHSEGGD	IPPKYFVVTN	NQLLRVKYLL	VYSQKPPKSR	ASSQLSWFSS	HWFTVMISLY	LLLLLIVSVI
**250**	**260**						
NSSAFQHFWN	RAKR						

Cmpd.	No. of Cmpds.	m/z meas.	Δ m/z [ppm]	z	Rt [min]	Score	P	Range	Sequence	Modification
2526	1	941.5692	108.04	2	62.2	15.6	1	37-53	R.DSVLRPFPASYARGDCK.D	

Detailed Protein Report

Protein 673: G protein-regulated inducer of neurite outgrowth 1 [Homo sapiens]

**Accession:**	gi|112821681	**Score:**	15.6
**Database:**	refseq_human(refseq_human_20140103.fasta)	**MW [kDa]:**	102.3
**Database Date:**	2015-11-30	**pI:**	9.3
		**Sequence Coverage [%]:**	1.5
		**No. of unique Peptides:**	1

Alias proteins:

Accession	Name	Description
gi|530380833	refseq_human (refseq_human_20140103.fasta)	PREDICTED: G protein-regulated inducer of neurite outgrowth 1 isoform X1 [Homo sapiens]

10	20	30	40	50	60	70	80
MDTAEDPAWL	QLLQKDSSPP	GPRPTAFFCP	QDGSLGAGSS	AMRDYCPSQQ	KASPAPPRHT	PDQSPGMESR	HRSPSGAGEG
**90**	**100**	**110**	**120**	**130**	**140**	**150**	**160**
ASCSDGPRGS	LACPSPTCFS	PQESPSKETL	EAHGASISGT	PEATTSGKPE	PVSSVKTEPK	SSDDRNPMFL	EKMDFKSSKQ
**170**	**180**	**190**	**200**	**210**	**220**	**230**	**240**
ADSTSIGKED	PGSSRKADPM	FTGKAEPEIL	GKGDPVAPGR	MDPMTVRKED	LGSLGKVDPL	CSSKTYTVSP	RKEDPGSLRK
**250**	**260**	**270**	**280**	**290**	**300**	**310**	**320**
VDPVSSDKVD	PVFPRKEEPR	YSGKEHPVSS	EKVAPTSAEK	VDLVLSGKRD	PGPSGKADPM	PLESMDSAST	GKTEPGLLGK
**330**	**340**	**350**	**360**	**370**	**380**	**390**	**400**
LIPGSSGKNG	PVSSGTGAPG	SLGRLDPTCL	GMADPASVGN	VETVPATKED	SRFLGKMDPA	SSGEGRPVSG	HTDTTASAKT
**410**	**420**	**430**	**440**	**450**	**460**	**470**	**480**
DLTSLKNVDP	MSSGKVDPVS	LGKMDPMCSG	KPELLSPGQA	ERVSVGKAGT	VSPGKEDPVS	SRREDPISAG	SRKTSSEKVN
**490**	**500**	**510**	**520**	**530**	**540**	**550**	**560**
PESSGKTNPV	SSGPGDPRSL	GTAGPPSAVK	AEPATGGKGD	PLSSEKAGLV	ASGKAAPTAS	GKAEPLAVGK	EDPVSKGKAD
**570**	**580**	**590**	**600**	**610**	**620**	**630**	**640**
AGPSGQGDSV	SIGKVVSTPG	KTVPVPSGKV	DPVSLGKAEA	IPEGKVGSLP	LEKGSPVTTT	KADPRASGKA	QPQSGGKAET
**650**	**660**	**670**	**680**	**690**	**700**	**710**	**720**
KLPGQEGAAA	PGEAGAVCLK	KETPQASEKV	DPGSCRKAEP	LASGKGEPVS	LGKADSAPSR	KTESPSLGKV	VPLSLEKTKP
**730**	**740**	**750**	**760**	**770**	**780**	**790**	**800**
SSSSRQLDRK	ALGSARSPEG	ARGSEGRVEP	KAEPVSSTEA	SSLGQKDLEA	AGAERSPCPE	AAAPPPGPRT	RDNFTKAPSW
**810**	**820**	**830**	**840**	**850**	**860**	**870**	**880**
EASAPPPPRE	DAGTQAGAQA	CVSVAVSPMS	PQDGAGGSAF	SFQAAPRAPS	PPSRRDAGLQ	VSLGAAETRS	VATGPMTPQA
**890**	**900**	**910**	**920**	**930**	**940**	**950**	**960**
AAPPAFPEVR	VRPGSALAAA	VAPPEPAEPV	RDVSWDEKGM	TWEVYGAAME	VEVLGMAIQK	HLERQIEEHG	RQGAPAPPPA
**970**	**980**	**990**	**1000**	**1010**			
ARAGPGRSGS	VRTAPPDGAA	KRPPGLFRAL	LQSVRRPRCC	SRAGPTAE			

Cmpd.	No. of Cmpds.	m/z meas.	Δ m/z [ppm]	z	Rt [min]	Score	P	Range	Sequence	Modification
2305	1	853.3930	-63.26	2	57.5	15.6	2	225-239	K.TYTVSPRKEDPGSLR.K	

Detailed Protein Report

Protein 674: inactive heparanase-2 isoform 3 [Homo sapiens]

**Accession:**	gi|261878505	**Score:**	15.6
**Database:**	refseq_human(refseq_human_20140103.fasta)	**MW [kDa]:**	53.9
**Database Date:**	2015-11-30	**pI:**	10.6
		**Sequence Coverage [%]:**	3.3
		**No. of unique Peptides:**	1

10	20	30	40	50	60	70	80
MRVLCAFPEA	MPSSNSRPPA	CLAPGALYLA	LLLHLSLSSQ	AGDRRPLPVD	RAAGLKEKTL	ILLDVSTKNP	VRTVNENFLS
**90**	**100**	**110**	**120**	**130**	**140**	**150**	**160**
LQLDPSIIHD	GWLDFLSSKR	LVTLARGLSP	AFLRFGGKRT	DFLQFQNLRN	PAKSRGGPGP	DYYLKNYEDE	PNNYRTMHGR
**170**	**180**	**190**	**200**	**210**	**220**	**230**	**240**
AVNGSQLGKD	YIQLKSLLQP	IRIYSRASLY	GPNIGRPRKN	VIALLDGFMK	VAGSTVDAVT	WQHCYIDGRV	VKVMDFLKTR
**250**	**260**	**270**	**280**	**290**	**300**	**310**	**320**
LLDTLSDQIR	KIQKVVNTYT	PGKKIWLEGV	VTTSAGGTNN	LSDSYAAGFL	WLNTLGMLAN	QGIDVVIRHS	FFDHGYNHLV
**330**	**340**	**350**	**360**	**370**	**380**	**390**	**400**
DQNFNPLPDY	WLSLLYKRLI	GPKVLAVHVA	GLQRKPRPGR	VIRDKLRIYA	HCTNHHNHNY	VRGSITLFII	NLHRSRKKIK
**410**	**420**	**430**	**440**	**450**	**460**	**470**	**480**
LAGTLRDKLV	HQYLLQPYGQ	EGLKSKSVQL	NGQPLVMVDD	GTLPELKPRP	LRAGRTLVIP	PVTMGFYVVK	NVNALACRYR
**490**							

Cmpd.	No. of Cmpds.	m/z meas.	Δ m/z [ppm]	z	Rt [min]	Score	P	Range	Sequence	Modification
2517	1	913.4734	-73.94	2	62.1	15.6	2	57-72	K.EKTLILLDVSTKNPVR.T	

Detailed Protein Report

Protein 675: PREDICTED: kelch-like protein 18 isoform X4 [Homo sapiens]

**Accession:**	gi|578805908	**Score:**	15.6
**Database:**	refseq_human(refseq_human_20140103.fasta)	**MW [kDa]:**	57.4
**Database Date:**	2015-11-30	**pI:**	5.3
		**Sequence Coverage [%]:**	2.3
		**No. of unique Peptides:**	1

10	20	30	40	50	60	70	80
MFTNDMMECK	QDEIVMQGMD	PSALEALINF	AYNGNLAIDQ	QNVQSLLMGA	SFLQLQSIKD	ACCTFLRERL	HPKNCLGVRQ
90	100	110	120	130	140	150	160
FAETMMCAVL	YDAANSFIHQ	HFVEVSMSEE	FLALPLEDVL	ELVSRDELNV	KSEEQVFEAA	LAWVRYDREQ	RGPYLPELLS
170	180	190	200	210	220	230	240
NIRLPLCRPQ	FLSDRVQQDD	LVRCCHKCRD	LVDEAKDYHL	MPERRPHLPA	FRTRPRCCTS	IAGLIYAVGG	LNSAANFYAG
250	260	270	280	290	300	310	320
DSLNVVEVFD	PIANCWERCR	PMTTARSRVG	VAVVNGLLYA	IGGYDGQLRL	STVEAYNPET	DTWTRVGSMN	SKRSSVCFSA
330	340	350	360	370	380	390	400
MGTVVLDGQI	YVCGGYDGNS	SLSSVETYSP	ETDKWTVVTS	MSSNRSAAGV	TVFEGRIYVS	GGHDGLQIFS	SVEHYNHHTA
410	420	430	440	450	460	470	480
TWHPAAGMLN	KRCRHGAASL	GSKMFVCGGY	DGSGFLSIAE	MYSSVADQWC	LIVPMHTRRS	RVSLVASCGR	LYAVGGYDGQ
490	500	510	520				
SNLSSVEMYD	PETDCWTFMA	PMACHEGGVG	VGCIPLLTI				

Cmpd.	No. of Cmpds.	m/z meas.	Δ m/z [ppm]	z	Rt [min]	Score	P	Range	Sequence	Modification
2688	1	645.8319	-38.25	2	64.7	15.6	2	459-470	R.RSRVSLVASCGR.L	

Detailed Protein Report

Protein 676: PREDICTED: NAD-dependent protein deacetylase sirtuin-7 isoform X3 [Homo sapiens]

**Accession:**	gi|578832025	**Score:**	15.6
**Database:**	refseq_human(refseq_human_20140103.fasta)	**MW [kDa]:**	35.7
**Database Date:**	2015-11-30	**pI:**	10.8
		**Sequence Coverage [%]:**	6.0
		**No. of unique Peptides:**	1

10	20	30	40	50	60	70	80
MSSSNREKLA	FKAALRFFLL	KKRNDQNLPK	AASIPDYRGP	NGVWTLLQKG	RSVSAADLSE	AEPTLTHMSI	TRLHEQKLVQ
**90**	**100**	**110**	**120**	**130**	**140**	**150**	**160**
HVVSQNCDGL	HLRSGLPRTA	ISELHGNMYI	EVCTSCVPNR	EYVRVFDVTE	RTALHRHQTG	RTCHKCGTQL	RDTIVHFGER
**170**	**180**	**190**	**200**	**210**	**220**	**230**	**240**
GTLGQPLNWE	AATEAASRAD	TILCLGSSLK	VLKKYPRLWC	MTKPPSRRPK	LYIVNLQWTP	KDDWAALKLH	GKCDDVMRLL
**250**	**260**	**270**	**280**	**290**	**300**	**310**	**320**
MAELGLEIPA	YSRWQDPIFS	LATPLRAGEE	GSHSRKSLCR	SREEAPPGDR	GAPLSSAPIL	GGWFGRGCTK	RTKRKKVT

Cmpd.	No. of Cmpds.	m/z meas.	Δ m/z [ppm]	z	Rt [min]	Score	P	Range	Sequence	Modification
192	1	1043.5843	25.50	2	32.4	15.6	1	31-49	K.AASIPDYRGPNGVWTLLQK.G	

Detailed Protein Report

Protein 677: PREDICTED: coiled-coil domain-containing protein 92-like [Homo sapiens]

Accession: gi|578830217
Database: refseq_human(refseq_human_20140103.fasta)
Database Date: 2015-11-30

Score: 15.6
MW [kDa]: 47.8
pI: 11.9
Sequence Coverage [%]: 2.5
No. of unique Peptides: 1

10	20	30	40	50	60	70	80
MVSTCSQRSP	QAKGPPLAEI	KGLFGWLDAV	AHARGAQAWV	AMDTVSLEHQ	IQSVQRHISF	LKKEQMALLR	DLHLEILRLQ
**90**	**100**	**110**	**120**	**130**	**140**	**150**	**160**
KRCSELTHDL	EMREAQSHQQ	EAASRELESK	CRALESQLEA	RAAANAELRR	EVAQREALVS	ALRCSLRTEE	RRFLEELRRR
**170**	**180**	**190**	**200**	**210**	**220**	**230**	**240**
SHRATVLGTE	LQKHTEAAAY	LSCQLHAARQ	RLQAPRPGPG	ATAEPRPRRR	ALRARRPPAA	HEAAAKGPGR	DWAAWDRGAG
**250**	**260**	**270**	**280**	**290**	**300**	**310**	**320**
ALDDADPMPD	PALFLYARRP	LRPSARSPRQ	PPPQEPPDRA	GPQPAPSQPS	APGDPEQVPG	RRAAAPPPGS	GARRPRLPPN
**330**	**340**	**350**	**360**	**370**	**380**	**390**	**400**
PRTASLLYFP	TFPKLRALSS	RESSSAGPSG	GTFSTERTRR	PDPGLVPPAC	ETGGAGATPF	PGKADNPWWG	GEGWARLRKG
**410**	**420**	**430**	**440**				
TQSGIGAPGP	LAPHPPPPPP	PRRRPSRQAF	FLPRRGPG				

Cmpd.	No. of Cmpds.	m/z meas.	Δ m/z [ppm]	z	Rt [min]	Score	P	Range	Sequence	Modification
454	1	646.8079	-121.57	2	35.7	15.6	1	259-269	R.RPLRPSARSPR.Q	

Detailed Protein Report

Protein 678: **PREDICTED: LOW QUALITY PROTEIN: ubiquitin-conjugating enzyme E2 variant 1-like [Homo sapiens]**

Accession: gi|578818188
Database: refseq_human(refseq_human_20140103.fasta)
Database Date: 2015-11-30
Modification(s): Carbamidomethyl

Score: 15.6
MW [kDa]: 15.9
pI: 10.2
Sequence Coverage [%]: 0.0
No. of unique Peptides: 1

Alias proteins:

Accession	Name	Description
gi|578844793	refseq_human (refseq_human_20140103.fasta)	PREDICTED: LOW QUALITY PROTEIN: ubiquitin-conjugating enzyme E2 variant 1-like [Homo sapiens]

10	20	30	40	50	60	70	80
MLGENQNPLN	VSLPFYIRNL	GLWTLLWAQT	TYENRTHSLK	IECRPKTPKN	TXLCKIVTKI	NTNGVDSSNV	VVDPKAILVL
90	100	110	120	130	140	150	
AKGQNSHSII	VVLQELWHLV	MSKADIKLPQ	PPEGQCCSNS	SKRKTTGPSF	LPFDLTGLHF	PQ	

Cmpd.	No. of Cmpds.	m/z meas.	Δ m/z [ppm]	z	Rt [min]	Score	P	Range	Sequence	Modification
1326	1	946.6005	108.67	2	46.7	15.6	2	0-0	.IECRPKTPKNTFLCK.	Carbamidomethyl: 3, 14

Detailed Protein Report

Protein 679: **PREDICTED: heterogeneous nuclear ribonucleoproteins A2/B1 isoform X5 [Homo sapiens]**

**Accession:**	gi|578813467	**Score:**	15.5
**Database:**	refseq_human(refseq_human_20140103.fasta)	**MW [kDa]:**	33.9
**Database Date:**	2015-11-30	**pI:**	9.4
		**Sequence Coverage [%]:**	5.4
		**No. of unique Peptides:**	1

10	20	30	40	50	60	70	80
MEKTLETVPL	ERKKREKEQF	RKLFIGGLSF	ETTEESLRNY	YEQWGKLTDC	VVMRDPASKR	SRGFGFVTFS	SMAEVDAAMA
**90**	**100**	**110**	**120**	**130**	**140**	**150**	**160**
ARPHSIDGRV	VEPKRAVARE	ESGKPGAHVT	VKKLFVGGIK	EDTEEHHLRD	YFEEYGKIDT	IEIITDRQSG	KKRGFGFVTF
**170**	**180**	**190**	**200**	**210**	**220**	**230**	**240**
DDHDPVDKIV	LQKYHTINGH	NAEVRKALSR	QEMQEVQSSR	SGRGGNFGFG	DSRGGGGNFG	PGPGSNFRGG	SDGYGSGRGF
**250**	**260**	**270**	**280**	**290**	**300**	**310**	**320**
GDGYNGYGGG	PGGNYGSGNY	NDFGNYNQQP	SNYGPMKSGN	FGGSRNMGGP	YGGGNYGPGG	SGGSGGYGGR	SRY

Cmpd.	No. of Cmpds.	m/z meas.	Δ m/z [ppm]	z	Rt [min]	Score	P	Range	Sequence	Modification
1128	1	983.3895	-134.59	2	44.2	15.5	2	174-190	K.YHTINGHNAEVRKALSR.Q	

Detailed Protein Report

Protein 680: ER lumen protein retaining receptor 1 [Homo sapiens]

**Accession:**	gi|5803048	**Score:**	15.5
**Database:**	refseq_human(refseq_human_20140103.fasta)	**MW [kDa]:**	24.5
**Database Date:**	2015-11-30	**pI:**	9.4
		**Sequence Coverage [%]:**	5.7
		**No. of unique Peptides:**	1

10	20	30	40	50	60	70	80
MNLFRFLGDL	SHLLAIILLL	LKIWKSRSCA	GISGKSQVLF	AVVFTARYLD	LFTNYISLYN	TCMKVVYIAC	SFTTVWLIYS
**90**	**100**	**110**	**120**	**130**	**140**	**150**	**160**
KFKATYDGNH	DTFRVEFLVV	PTAILAFLVN	HDFTPLEILW	TFSIYLESVA	ILPQLFMVSK	TGEAETITSH	YLFALGVYRT
**170**	**180**	**190**	**200**	**210**	**220**		
LYLFNWIWRY	HFEGFFDLIA	IVAGLVQTVL	YCDFFYLYIT	KVLKGKKLSL	PA		

Cmpd.	No. of Cmpds.	m/z meas.	Δ m/z [ppm]	z	Rt [min]	Score	P	Range	Sequence	Modification
453	1	669.3289	-79.98	2	35.6	15.5	0	36-47	K.SQVLFAVVFTAR.Y	

Detailed Protein Report

Protein 681: **DNA-(apurinic or apyrimidinic site) lyase 2 isoform 1 [Homo sapiens]**

**Accession:**	gi|18375507	**Score:**	15.5
**Database:**	refseq_human(refseq_human_20140103.fasta)	**MW [kDa]:**	57.4
**Database Date:**	2015-11-30	**pI:**	9.8
		**Sequence Coverage [%]:**	2.5
		**No. of unique Peptides:**	1

Quantitation

QD:QU **Median:** 0.83 **CV:** 0.00 % **No. of Peptides:** 1

10	20	30	40	50	60	70	80
MLRVVSWNIN	GIRRPLQGVA	NQEPSNCAAV	AVGRILDELD	ADIVCLQETK	VTRDALTEPL	AIVEGYNSYF	SFSRNRSGYS
**90**	**100**	**110**	**120**	**130**	**140**	**150**	**160**
GVATFCKDNA	TPVAAEEGLS	GLFATQNGDV	GCYGNMDEFT	QEELRALDSE	GRALLTQHKI	RTWEGKEKTL	TLINVYCPHA
**170**	**180**	**190**	**200**	**210**	**220**	**230**	**240**
DPGRPERLVF	KMRFYRLLQI	RAEALLAAGS	HVIILGDLNT	AHRPIDHWDA	VNLECFEEDP	GRKWMDSLLS	NLGCQSASHV
**250**	**260**	**270**	**280**	**290**	**300**	**310**	**320**
GPFIDSYRCF	QPKQEGAFTC	WSAVTGARHL	NYGSRLDYVL	GDRTLVIDTF	QASFLLPEVM	GSDHCPVGAV	LSVSSVPAKQ
**330**	**340**	**350**	**360**	**370**	**380**	**390**	**400**
CPPLCTRFLP	EFAGTQLKIL	RFLVPLEQSP	VLEQSTLQHN	NQTRVQTCQN	KAQVRSTRPQ	PSQVGSSRGQ	KNLKSYFQPS
**410**	**420**	**430**	**440**	**450**	**460**	**470**	**480**
PSCPQASPDI	ELPSLPLMSA	LMTPKTPEEK	AVAKVVKGQA	KTSEAKDEKE	LRTSFWKSVL	AGPLRTPLCG	GHREPCVMRT
**490**	**500**	**510**	**520**				
VKKPGPNLGR	RFYMCARPRG	PPTDPSSRCN	FFLWSRPS				

Cmpd.	No. of Cmpds.	m/z meas.	Δ m/z [ppm]	z	Rt [min]	Score	P	Range	Sequence	Modification	Ratios
1941	1	695.2967	-51.70	2	54.6	15.5	1	75-87	R.NRSGYSGVATFCK.D		QD:QU 0.83

Detailed Protein Report

Protein 682: endothelial cell-specific molecule 1 isoform b precursor [Homo sapiens]

Accession: gi|208022668
Database: refseq_human(refseq_human_20140103.fasta)
Database Date: 2015-11-30

Score: 15.5
MW [kDa]: 14.5
pI: 6.4
Sequence Coverage [%]: 9.0
No. of unique Peptides: 1

10	20	30	40	50	60	70	80
MKSVLLLTTL	LVPAHLVAAW	SNNYAVDCPQ	HCDSSECKSS	PRCKRTVLDD	CGCCRVCAAG	RGETCYRTVS	GMDGMKCGPG
**90**	**100**	**110**	**120**	**130**	**140**		
LRCQPSNGED	PFGEEFGICK	EHDMASGDGN	IVREEVVKEN	AAGSPVMRKW	LNPR		

Cmpd.	No. of Cmpds.	m/z meas.	Δ m/z [ppm]	z	Rt [min]	Score	P	Range	Sequence	Modification
1903	1	643.3133	32.48	2	54.2	15.5	1	56-67	R.VCAAGRGETCYR.T	

Detailed Protein Report

Protein 683: protein SOGA2 [Homo sapiens]

**Accession:**	gi|163644316	**Score:**	15.5
**Database:**	refseq_human(refseq_human_20140103.fasta)	**MW [kDa]:**	177.8
**Database Date:**	2015-11-30	**pI:**	5.6
		**Sequence Coverage [%]:**	1.1
		**No. of unique Peptides:**	1

10	20	30	40	50	60	70	80
MEEMRDSYLE	EDVYQLQELR	RELDRANKNC	RILQYRLRKA	EQKSLKVAET	GQVDGELIRS	LEQDLKVAKD	VSVRLHHELK
90	100	110	120	130	140	150	160
TVEEKRAKAE	DENETLRQQM	IEVEISKQAL	QNELERLKES	SLKRRSTREM	YKEKKTFNQD	DSADLRCQLQ	FAKEEAFLMR
170	180	190	200	210	220	230	240
KKMAKLGREK	DELEQELQKY	KSLYGDVDSP	LPTGEAGGPP	STREAELKLR	LKLVEEEANI	LGRKIVELEV	ENRGLKAEME
250	260	270	280	290	300	310	320
DMRGQQEREG	PGRDHAPSIP	TSPFGDSLES	STELRRHLQF	VEEEAELLRR	SISEIEDHNR	QLTHELSKFK	FEPPREPGWL
330	340	350	360	370	380	390	400
GEGASPGAGG	GAPLQEELKS	ARLQISELSG	KVLKLQHENH	ALLSNIQRCD	LAAHLGLRAP	SPRDSDAESD	AGKKESDGEE
410	420	430	440	450	460	470	480
SRLPQPKREG	PVGGESDSEE	MFEKTSGFGS	GKPSEASEPC	PTELLKARED	SEYLVTLKHE	AQRLERTVER	LITDTDSFLH
490	500	510	520	530	540	550	560
DAGLRGGAPL	PGPGLQGEEE	QGEGDQQEPQ	LLGTINAKMK	AFKKELQAFL	EQVNRIGDGL	SPLPHLTESS	SFLSTVTSVS
570	580	590	600	610	620	630	640
RDSPIGNLGK	ELGPDLQSRL	KEQLEWQLGP	ARGDERESLR	LRAARELHRR	ADGDTGSHGL	GGQTCFSLEL	RGPPVLPEQS
650	660	670	680	690	700	710	720
VSIEELQGQL	VQAARLHQEE	TETFTNKIHK	MEEEHLYALR	WKELEMHSLA	LQNTLHERTW	SDEKNLMQQE	LRSLKQNIFL
730	740	750	760	770	780	790	800
FYVKLRWLLK	HWRQGKQMEE	EGEEFTEGEH	PETLSRLGEL	GVQGGHQADG	PDHDSDRGCG	FPVGEHSPHS	RVQIGDHSLR
810	820	830	840	850	860	870	880
LQTADRGQPH	KQVVENQQLF	SAFKALLEDF	RAELREDERA	RLRLQQQYAS	DKAAWDVEWA	VLKCRLEQLE	EKTENKLGEL
890	900	910	920	930	940	950	960
GSSAESKGAL	KKEREVHQKL	LADSHSLVMD	LRWQIHHSEK	NWNREKVELL	DRLDRDRQEW	ERQKKEFLWR	IEQLQKENSP
970	980	990	1000	1010	1020	1030	1040
RRGGSFLCDQ	KDGNVRPFPH	QGSLRMPRPV	AMWPCADADS	IPFEDRPLSK	LKESDRCSAS	ENLYLDALSL	DDEPEEPPAH
1050	1060	1070	1080	1090	1100	1110	1120
RPEREFRNRL	PEEEENHKGN	LQRAVSVSSM	SEFQRLMDIS	PFLPEKGLPS	TSSKEDVTPP	LSPDDLKYIE	EFNKSWDYTP
1130	1140	1150	1160	1170	1180	1190	1200
NRGHNGGGPD	LWADRTEVGR	AGHEDSTEPF	PDSSWYLTTS	VTMTTDTMTS	PEHCQKQPLR	SHVLTEQSGL	RVLHSPPAVR
1210	1220	1230	1240	1250	1260	1270	1280
RVDSITAAGG	EGPFPTSRAR	GSPGDTKGGP	PEPMLSRWPC	TSPRHSRDYV	EGARRPLDSP	LCTSLGFASP	LHSLEMSKNL
1290	1300	1310	1320	1330	1340	1350	1360
SDDMKEVAFS	VRNAICSGPG	ELQVKDMACQ	TNGSRTMGTQ	TVQTISVGLQ	TEALRGSGVT	SSPHKCLTPK	AGGGATPVSS
1370	1380	1390	1400	1410	1420	1430	1440
PSRSLRSRQV	APAIEKVQAK	FERTCCSPKY	GSPKLQRKPL	PKADQPNNRT	SPGMAQKGYS	ESAWARSTTT	RESPVHTTIN
1450	1460	1470	1480	1490	1500	1510	1520
DGLSSLFNII	DHSPVVQDPF	QKGLRAGSRS	RSAEPRPELG	PGQETGTNSR	GRSPSPIGVG	SEMCREEGGE	GTPVKQDLSA
1530	1540	1550	1560	1570	1580	1590	
PPGYTLTENV	ARILNKKLLE	HALKEERRQA	AHGPPGLHSD	SHSLGDTAEP	GPMENQTVLL	TAPWGL	

Cmpd.	No. of Cmpds.	m/z meas.	Δ m/z [ppm]	z	Rt [min]	Score	P	Range	Sequence	Modification
1525	1	1022.9446	-77.80	2	49.3	15.5	2	447-463	K.AREDSEYLVTLKHEAQR.L	

Detailed Protein Report

Protein 684: PREDICTED: WD repeat-containing protein 19 isoform X3 [Homo sapiens]

**Accession:**	gi|578808579	**Score:**	15.5
**Database:**	refseq_human(refseq_human_20140103.fasta)	**MW [kDa]:**	144.7
**Database Date:**	2015-11-30	**pI:**	6.1
		**Sequence Coverage [%]:**	1.5
		**No. of unique Peptides:**	1

10	20	30	40	50	60	70	80
MKRIFSLLEK	TWLGAPIQFA	WQKTSGNYLA	VTGADYIVKI	FDRHGQKRSE	INLPGNCVAM	DWDKDGDVLA	VIAEKSSCIY
90	100	110	120	130	140	150	160
LWDANTNKTS	QLDNGMRDQM	SFLLWSKVGS	FLAVGTVKGN	LLIYNHQTSR	KIPVLGKHTK	RITCGCWNAE	NLLALGGEDK
170	180	190	200	210	220	230	240
MITVSNQEGD	TIRQTQVRSE	PSNMQFFLMK	MDDRTSAAES	MISVVLGKKT	LFFLNLNEPD	NPADLEFQQD	FGNIVCYNWY
250	260	270	280	290	300	310	320
GDGRIMIGFS	CGHFVVISTH	TGELGQEIFQ	ARNHKDNLTS	IAVSQTLNKV	ATCGDNCIKI	QDLVDLKDMY	VILNLDEENK
330	340	350	360	370	380	390	400
GLGTLSWTDD	GQLLALSTQR	GSLHVFLTKL	PILGDACSTR	IAYLTSLLEV	TVANPVEGEL	PITVSVDVEP	NFVAVGLYHL
410	420	430	440	450	460	470	480
AVGMNNRAWF	YVLGENAVKK	LKDMEYLGTV	ASICLHSDYA	AALFEGKVQL	HLIESEILDA	QEERETRLFP	AVDDKCRILC
490	500	510	520	530	540	550	560
HALTSDFLIY	GTDTGVVQYF	YIEDWQFVND	YRHPVSVKKI	FPDPNGTRLV	FIDEKSDGFV	YCPVNDATYE	IPDFSPTIKG
570	580	590	600	610	620	630	640
VLWENWPMDK	GVFIAYDDDK	VYTYVFHKDT	IQGAKVILAG	STKVPFAHKP	LLLYNGELTC	QTQSGKVNNI	YLSTHGFLSN
650	660	670	680	690	700	710	720
LKDTGPDELR	PMLAQNLMLK	RFSDAWEMCR	ILNDEAAWNE	LARACLHHME	VEFAIRVYRR	IGNVGIVMSL	EQIKGIEDYN
730	740	750	760	770	780	790	800
LLAGHLAMFT	NDYNLAQDLY	LASSCPIAAL	EMRRDLQHWD	SALQLAKHLA	PDQIPFISKE	YAIQLEFAGD	YVNALAHYEK
810	820	830	840	850	860	870	880
GITGDNKEHD	EACLAGVAQM	SIRMGDIRRG	VNQALKHPSR	VLKRDCGAIL	ENMKQFSEAA	QLYEKGLYYD	KAASVYIRSK
890	900	910	920	930	940	950	960
NWAKVGDLLP	HVSSPKIHLQ	YAKAKEADGR	YKEAVVAYEN	AKQWQSVIRI	YLDHLNNPEK	AVNIVRETQS	LDGAKMVARF
970	980	990	1000	1010	1020	1030	1040
FLQLGDYGSA	IQFLVMSKCN	NEAFTLAQQH	NKMEIYADII	GSEDTTNEDY	QSIALYFEGE	KRYLQAGKFF	LLCGQYSRAL
1050	1060	1070	1080	1090	1100	1110	1120
KHFLKCPSSE	DNVAIEMAIE	TVGQAKDELL	TNQLIDHLLG	ENDGMPKDAK	YLFRLYMALK	QYREAAQTAI	IIAREEQSAG
1130	1140	1150	1160	1170	1180	1190	1200
NYRNAHDVLF	SMYAELKSQK	IKIPSEMATN	LMILHSYILV	KIHVKNGDHM	KGARMLIRVA	NNISKFPSHI	VPILTSTVIE
1210	1220	1230	1240	1250	1260	1270	1280
CHRAGLKNSA	FSFAAMLMRP	EYRSKIDAKY	KKKIEGMVRE	RQRENTKLTT	PVAQEERRPQ	DLPMLSAVEC	AMNSSSQRWP
1290							
M							

Cmpd.	No. of Cmpds.	m/z meas.	Δ m/z [ppm]	z	Rt [min]	Score	P	Range	Sequence	Modification
690	1	733.4795	128.10	3	38.6	15.5	1	643-661	K.DTGPDELRPMLAQNLMLKR.F	

Detailed Protein Report

Protein 685: PREDICTED: zinc finger protein 280C isoform X1 [Homo sapiens]

Accession: gi|578838699
Database: refseq_human(refseq_human_20140103.fasta)
Database Date: 2015-11-30
Modification(s): Oxidation

Score: 15.4
MW [kDa]: 77.3
pI: 10.2
Sequence Coverage [%]: 2.5
No. of unique Peptides: 1

10	20	30	40	50	60	70	80
MDDDKPFQPK	NISKMAELFM	ECEEEELEPW	QKKVEETQDE	DDDELIFVGE	ISSSKPAISN	ILNRGHSSSS	SKGIKSEPHS
90	100	110	120	130	140	150	160
PGIPEIFRTA	SQRCRDPPSN	PVAASPRFHL	VSKSSQSSVT	VENASKPDFT	KNSQVGSDNS	SILLFDSTQE	SLPPSQDIPA
170	180	190	200	210	220	230	240
IFREGMKNTS	YVLKHPSTSK	VNSVTPKKPK	TSEDVPQINP	STSLPLIGSP	PVTSSQVMLS	KGTNTSSPYD	AGADYLRACP
250	260	270	280	290	300	310	320
[truncated: 450,759 more chars]
