# Supplementary material for: Exploration of key factors in Gingival Crevicular fluids from patients undergoing Periodontally Accelerated Osteogenic Orthodontics (PAOO) using proteome analysis
Source: BMC Oral Health. 2023 Nov 27;23:934. doi: 10.1186/s12903-023-03606-7 (PMC10683118; doi:10.1186/s12903-023-03606-7)
Supplement: Supplementary file 2 — Supplementary Material 2 [file 12903_2023_3606_MOESM2_ESM.pdf]

Detailed Protein Report

Project Info

Name: teeth-151208 **Date:** Dec 8, 2015
Note:

Sample Info & Protocols

Name: ID-W **Date:** Dec 8, 2015
Fraction:
Volume: **Concentration:**
Note:
Buffer: **Organism:**

Search Result Info

Search Result	Location	Search Engine	Database
ZT_human_PE_2015-12-08 22:06:17	/teeth-151208/ID-W/"Combined MS/MS spectra"	Mascot	refseq_human

Protein 1: serum albumin preproprotein [Homo sapiens]

Accession: gi|4502027
Database: refseq_human(refseq_human_20140103.fasta)
Database Date: 2015-11-30
Modification(s): Carbamidomethyl, Oxidation

Score: 1403.0
MW [kDa]: 69.3
pI: 5.9
Sequence Coverage [%]: 57.1
No. of unique Peptides: 30

Quantitation

WD:WU **Median:** 0.80 **CV:** 33.46 % **No. of Peptides:** 13

10	20	30	40	50	60	70	80
MKWVTFISLL	FLFSSAYSRG	VFRRDAHKSE	VAHRFKDLGE	ENFKALVLIA	FAQYLQQCPF	EDHVKLVNEV	TEFAKTCVAD
90	100	110	120	130	140	150	160
ESAENCDKSL	HTLFGDKLCT	VATLRETYGE	MADCCAKQEP	ERNECFLQHK	DDNPNLPRLV	RPEVDVMCTA	FHDNEETFLK
170	180	190	200	210	220	230	240
KYLYEIARRH	PYFYAPELLF	FAKRYKAAFT	ECCQAADKAA	CLLPKLDELR	DEGKASSAKQ	RLKCASLQKF	GERAFKAWAV
250	260	270	280	290	300	310	320
ARLSQRFPKA	EFAEVSKLVT	DLTKVHTECC	HGDLLECADD	RADLAKYICE	NQDSISSKLK	ECCEKPLLEK	SHCIAEVEND
330	340	350	360	370	380	390	400
EMPADLPSLA	ADFVESKDVC	KNYAEAKDVF	LGMFLYEYAR	RHPDYSVVLL	LRLAKTYETT	LEKCCAAADP	HECYAKVFDE
410	420	430	440	450	460	470	480
FKPLVEEPQN	LIKQNCELFE	QLGEYKFQNA	LLVRYTKKVP	QVSTPTLVEV	SRNLGKVGSK	CCKHPEAKRM	PCAEDYLSVV
490	500	510	520	530	540	550	560
LNQLCVLHEK	TPVSDRVTKC	CTESLVNRRP	CFSALEVDET	YVPKEFNAET	FTFHADICTL	SEKERQIKKQ	TALVELVKHK
570	580	590	600	610			
PKATKEQLKA	VMDDFAAFVE	KCCKADDKET	CFAEEGKKLV	AASQAALGL			

Cmpd.	No. of Cmpds.	m/z meas.	Δ m/z [ppm]	z	Rt [min]	Score	P	Range	Sequence	Modification	Ratios
151	7	613.6605	-237.38	2	30.8	67.2	1	35-44	R.FKDLGEENFK.A		
514	8	575.2390	-125.46	2	34.9	63.4	0	66-75	K.LVNEVTEFAK.T		WD:WU 0.89
345	3	509.1558	-227.88	2	33.4	45.7	0	89-97	K.SLHTLFGDK.L		WD:WU 0.61
171	1	659.8150	4.22	4	31.4	26.5	2	118-138	K.QEPERNECFLQHKDDNPNLPR.L	Carbamidomethyl: 8	
144	1	666.2970	-26.44	3	30.7	28.6	1	123-138	R.NECFLQHKDDNPNLPR.L	Carbamidomethyl: 3	WD:WU 0.65
1425	1	695.3249	-29.21	4	46.6	14.9	1	139-161	R.LVRPEVDVMCTAFHDNEETFLKK.Y	Carbamidomethyl: 10	
326	8	464.1239	-272.44	2	32.8	30.1	0	162-168	K.YLYEIAR.R		WD:WU 1.28

Detailed Protein Report

Cmpd.	No. of Cmpds.	m/z meas.	Δ m/z [ppm]	z	Rt [min]	Score	P	Range	Sequence	Modification	Ratios
33	3	686.2794	-11.06	2	29.6	70.0	0	187-198	K.AAFTECCQAADK.A	Carbamidomethyl: 6, 7	
20	4	537.7465	-52.90	2	29.5	44.4	1	206-214	K.LDELRDEGK.A		WD:WU 0.60
102	2	696.2880	5.70	3	30.5	50.5	0	265-281	K.VHTECCHGDLLECADDR.A	Carbamidomethyl: 5, 6, 13	WD:WU 0.85
15	6	722.3161	-11.90	2	29.1	70.8	0	287-298	K.YICENQDSISSK.L	Carbamidomethyl: 3	WD:WU 0.63
75	1	773.8989	-4.03	2	30.2	28.9	1	299-310	K.LKECCEKPLLEK.S	Carbamidomethyl: 4, 5	WD:WU 0.62
2459	1	997.4538	2.48	3	59.0	27.7	0	311-337	K. SHCIAEVENDEMPADLPSLAADFV D	Carbamidomethyl: 3; Oxidation: 12	
2988	1	992.1219	2.21	3	66.1	32.9	0	311-337	K. SHCIAEVENDEMPADLPSLAADFV D	Carbamidomethyl: 3	
2516	5	820.4094	17.73	2	59.7	54.5	0	348-360	K.DVFLGMFLYEYAR.R	Oxidation: 6	
42	1	492.7365	-23.11	2	29.7	28.1	0	376-383	K.TYETTLEK.C		WD:WU 0.72
8	3	776.7890	-17.37	2	29.1	42.3	0	384-396	K.CCAAADPHECYAK.V	Carbamidomethyl: 1, 2, 10	
2246	54	682.3456	-35.78	3	56.6	62.3	0	397-413	K.VFDEFKPLVEEPQNLIK.Q		
1301	9	829.3216	-70.31	2	45.0	69.3	0	414-426	K.QNCELFEQLGEYK.F	Carbamidomethyl: 3	WD:WU 0.81
565	19	480.6987	-179.31	2	35.9	50.6	0	427-434	K.FQNALLVR.Y		
783	8	820.4638	-10.68	2	38.6	67.6	1	438-452	K.KVPQVSTPTLVEVSR.N		
1019	3	756.4151	-13.09	2	41.5	37.1	0	439-452	K.VPQVSTPTLVEVSR.N		WD:WU 0.54
33	3	569.7395	-23.07	2	29.3	55.6	0	500-508	K.CCTESLVNR.R	Carbamidomethyl: 1, 2	WD:WU 1.44
1168	2	637.6273	-33.66	3	43.3	58.3	0	509-524	R.RPCFSALEVDETYVPK.E	Carbamidomethyl: 3	
2419	14	753.9221	-119.82	3	58.5	64.2	0	525-543	K.EFNAETFTFHADICTLSEK.E	Carbamidomethyl: 14	
2154	1	848.9827	-91.45	3	55.4	11.2	1	525-545	K.EFNAETFTFHADICTLSEKER. Q	Carbamidomethyl: 14	
860	13	500.8124	13.72	2	39.6	52.8	0	550-558	K.QTALVELVK.H		
2105	11	671.7288	-137.21	2	54.9	98.5	0	570-581	K.AVMDDFAAFVEK.C		
15	1	543.2576	19.22	3	29.4	27.1	2	585-598	K.ADDKETCFAEEGKK.L	Carbamidomethyl: 7	
895	1	571.3207	-52.32	2	39.6	22.1	1	598-609	K.KLVAASQAALGL.-		WD:WU 1.39

Detailed Protein Report

Protein 2: hemoglobin subunit beta [Homo sapiens]

**Accession:**	gi|4504349	**Score:**	728.2
**Database:**	refseq_human(refseq_human_20140103.fasta)	**MW [kDa]:**	16.0
**Database Date:**	2015-11-30	**pI:**	6.9
**Modification(s):**	Carbamidomethyl, Oxidation	**Sequence Coverage [%]:**	83.0
		**No. of unique Peptides:**	12

Quantitation

WD:WU **Median:** 0.67 **CV:** 60.02 % **No. of Peptides:** 8

```
        10         20         30         40         50         60         70         80
MVHLTPEEKS AVTALWGKVN VDEVGGEALG RLLVVYPWTQ RFFESFGDLS TPDAVMGNPK VKAHGKKVLG AFSDGLAHLD
        90        100        110        120        130        140        150
NLKGTFATLS ELHCDKLHVD PENFRLLGNV LVCVLAHHFG KEFTPPVQAA YQKVVAGVAN ALAHKYH
```

Cmpd.	No. of Cmpds.	m/z meas.	Δ m/z [ppm]	z	Rt [min]	Score	P	Range	Sequence	Modification	Ratios	
47	3	476.7443	-29.87	2	29.8	25.8	0	2-9	M.VHLTPEEK.S		WD:WU	0.85
629	11	466.7591	-9.74	2	36.3	58.8	0	10-18	K.SAVTALWGK.V		WD:WU	1.30
696	8	657.8312	-7.38	2	37.5	40.3	0	19-31	K.VNVDEVGGEALGR.L		WD:WU	0.90
2284	23	637.8143	-81.79	2	56.9	61.8	0	32-41	R.LLVVYPWTQR.F			
2307	8	1029.9789	1.35	2	57.1	52.0	0	42-60	R.FFESFGDLSTPDAVMGNPK.V			
1677	1	1037.9101	-62.48	2	49.6	60.1	0	42-60	R.FFESFGDLSTPDAVMGNPK.V	Oxidation: 15		
2072	35	599.9066	-155.81	3	54.5	102.5	1	67-83	K.KVLGAFSDGLAHLDNLK.G		WD:WU	0.18
2515	93	835.4601	13.21	2	59.7	95.3	0	68-83	K.VLGAFSDGLAHLDNLK.G			
595	4	739.7263	-168.37	2	36.3	53.2	0	84-96	K.GTFATLSELHCDK.L	Carbamidomethyl: 11	WD:WU	0.58
187	15	563.6579	-226.49	2	31.2	45.5	0	97-105	K.LHVDPENFR.L		WD:WU	0.82
563	2	689.7251	-186.44	2	35.9	45.0	0	122-133	K.EFTPPVQAAYQK.V		WD:WU	0.82
213	4	575.2065	-233.10	2	31.9	88.0	0	134-145	K.VVAGVANALAHK.Y		WD:WU	0.60

Detailed Protein Report

Protein 3: apolipoprotein A-I preproprotein [Homo sapiens]

**Accession:**	gi|4557321	**Score:**	515.4
**Database:**	refseq_human(refseq_human_20140103.fasta)	**MW [kDa]:**	30.8
**Database Date:**	2015-11-30	**pI:**	5.5
		**Sequence Coverage [%]:**	39.3
		**No. of unique Peptides:**	11

Quantitation

WD:WU	**Median:** 0.58	**CV:** 22.73 %	**No. of Peptides:** 4

Alias proteins:

Accession	Name	Description
gi|530398069	refseq_human (refseq_human_20140103.fasta)	PREDICTED: apolipoprotein A-I isoform X2 [Homo sapiens]
gi|530398067	refseq_human (refseq_human_20140103.fasta)	PREDICTED: apolipoprotein A-I isoform X1 [Homo sapiens]

```
        10         20         30         40         50         60         70         80
MKAAVLTLAV LFLTGSQARH FWQQDEPPQS PWDRVKDLAT VYVDVLKDSG RDYVSQFEGS ALGKQLNLKL LDNWDSVTST
        90        100        110        120        130        140        150        160
FSKLREQLGP VTQEFWDNLE KETEGLRQEM SKDLEEVKAK VQPYLDDFQK KWQEEMELYR QKVEPLRAEL QEGARQKLHE
       170        180        190        200        210        220        230        240
LQEKLSPLGE EMRDRARAHV DALRTHLAPY SDELRQRLAA RLEALKENGG ARLAEYHAKA TEHLSTLSEK AKPALEDLRQ
       250        260        270
GLLPVLESFK VSFLSALEEY TKKLNTQ
```

Cmpd.	No. of Cmpds.	m/z meas.	Δ m/z [ppm]	z	Rt [min]	Score	P	Range	Sequence	Modification	Ratios	
1873	8	731.8942	-48.16	2	52.1	53.6	1	35-47	R.VKDLATVYVDVLK.D			
2093	4	618.2734	-120.22	2	54.7	58.3	0	37-47	K.DLATVYVDVLK.D			
805	1	908.4284	-0.73	2	38.9	11.7	1	48-64	K.DSGRDYVSQFEGSALGK.Q			
893	3	700.8159	-31.87	2	39.6	45.2	0	52-64	R.DYVSQFEGSALGK.Q		WD:WU	0.67
1807	11	806.8737	-27.99	2	51.3	94.1	0	70-83	K.LLDNWDSVTSTFSK.L			
2426	1	966.9435	-27.92	2	58.6	21.1	0	86-101	R.EQLGPVTQEFWDNLEK.E			
497	2	626.6875	-201.93	2	34.7	29.3	0	121-130	K.VQPYLDDFQK.K		WD:WU	0.43
257	1	690.8471	-20.89	2	32.0	53.5	1	121-131	K.VQPYLDDFQKK.W		WD:WU	0.76
182	2	651.3208	-10.84	2	31.5	40.3	0	185-195	R.THLAPYSDELR.Q		WD:WU	0.50
2876	20	615.8555	-4.42	2	64.7	47.2	0	240-250	R.QGLLPVLESFK.V			
2300	9	693.8302	-44.61	2	57.2	61.0	0	251-262	K.VSFLSALEEYTK.K			

Detailed Protein Report

Protein 4: hemoglobin subunit delta [Homo sapiens]

Accession: gi|4504351
Database: refseq_human(refseq_human_20140103.fasta)
Database Date: 2015-11-30
Modification(s): Carbamidomethyl, Oxidation

Score: 483.6
MW [kDa]: 16.0
pI: 9.1
Sequence Coverage [%]: 59.9
No. of unique Peptides: 2

Quantitation

WD:WU **Median:** 3.02 **CV:** 0.00 % **No. of Peptides:** 1

10	20	30	40	50	60	70	80
MVHLTPEEKT	AVNALWGKVN	VDAVGGEALG	RLLVVYPWTQ	RFFESFGDLS	SPDAVMGNPK	VKAHGKKVLG	AFSDGLAHLD
**90**	**100**	**110**	**120**	**130**	**140**	**150**	
NLKGTFSQLS	ELHCDKLHVD	PENFRLLGNV	LVCVLARNFG	KEFTPQMQAA	YQKVVAGVAN	ALAHKYH	

Cmpd.	No. of Cmpds.	m/z meas.	Δ m/z [ppm]	z	Rt [min]	Score	P	Range	Sequence	Modification	Ratios
2232	1	1030.8450	-118.44	2	56.4	33.3	0	42-60	R.FFESFGDLSSPDAVMGNPK.V	Oxidation: 15	
1086	2	761.3593	7.32	2	42.3	31.4	0	84-96	K.GTFSQLSELHCDK.L	Carbamidomethyl: 11	WD:WU 3.02

Detailed Protein Report

Protein 5: hemoglobin subunit alpha [Homo sapiens]

**Accession:**	gi|4504345	**Score:**	453.5
**Database:**	refseq_human(refseq_human_20140103.fasta)	**MW [kDa]:**	15.2
**Database Date:**	2015-11-30	**pI:**	9.4
**Modification(s):**	Oxidation	**Sequence Coverage [%]:**	50.7
		**No. of unique Peptides:**	8

Quantitation

WD:WU	**Median:** 2.18	**CV:** 58.48 %	**No. of Peptides:** 4

Alias proteins:

Accession	Name	Description
gi|4504347	refseq_human (refseq_human_20140103.fasta)	hemoglobin subunit alpha [Homo sapiens]

```
         10         20         30         40         50         60         70         80
MVLSPADKTN VKAAWGKVGA HAGEYGAEAL ERMFLSFPTT KTYFPHFDLS HGSAQVKGHG KKVADALTNA VAHVDDMPNA
         90        100        110        120        130        140        150
LSALSDLHAH KLRVDPVNFK LLSHCLLVTL AAHLPAEFTP AVHASLDKFL ASVSTVLTSK YR
```

Cmpd.	No. of Cmpds.	m/z meas.	Δ m/z [ppm]	z	Rt [min]	Score	P	Range	Sequence	Modification	Ratios
4	1	729.4098	-5.99	1	29.0	28.6	0	2-8	M.VLSPADK.T		
39	2	586.3399	3.78	2	29.7	55.0	1	2-12	M.VLSPADKTNVK.A		WD:WU 2.39
111	10	765.3757	6.39	2	30.7	80.1	0	18-32	K.VGAHAGEYGAEALER.M		
1126	3	544.1344	-264.30	2	42.3	39.7	0	33-41	R.MFLSFPTTK.T	Oxidation: 1	WD:WU 1.65
1845	35	536.1590	-227.09	2	51.4	57.9	0	33-41	R.MFLSFPTTK.T		
1032	5	917.4300	-21.34	2	41.7	79.5	0	42-57	K.TYFPHFDLSHGSAQVK.G		WD:WU 1.16
446	2	544.2062	-202.71	2	34.1	32.4	1	92-100	K.LRVDPVNFK.L		WD:WU 5.00
1648	44	626.8403	-33.03	2	48.9	80.4	0	129-140	K.FLASVSTVLTSK.Y		

Detailed Protein Report

Protein 6: alpha-2-macroglobulin precursor [Homo sapiens]

**Accession:**	gi|66932947	**Score:**	380.7
**Database:**	refseq_human(refseq_human_20140103.fasta)	**MW [kDa]:**	163.2
**Database Date:**	2015-11-30	**pI:**	6.0
**Modification(s):**	Carbamidomethyl	**Sequence Coverage [%]:**	11.9
		**No. of unique Peptides:**	13

Quantitation

WD:WU	**Median:** 0.91	**CV:** 30.93 %	**No. of Peptides:** 9

10	20	30	40	50	60	70	80
MGKNKLLHPS	LVLLLLVLLP	TDASVSGKPQ	YMVLVPSLLH	TETTEKGCVL	LSYLNETVTV	SASLESVRGN	RSLFTDLEAE
90	100	110	120	130	140	150	160
NDVLHCVAFA	VPKSSSNEEV	MFLTVQVKGP	TQEFKKRTTV	MVKNEDSLVF	VQTDKSIYKP	GQTVKFRVVS	MDENFHPLNE
170	180	190	200	210	220	230	240
LIPLVYIQDP	KGNRIAQWQS	FQLEGGLKQF	SFPLSSEPFQ	GSYKVVVQKK	SGGRTEHPFT	VEEFVLPKFE	VQVTVPKIIT
250	260	270	280	290	300	310	320
ILEEEMNVSV	CGLYTYGKPV	PGHVTVSICR	KYSDASDCHG	EDSQAFCEKF	SGQLNSHGCF	YQQVKTKVFQ	LKRKEYEMKL
330	340	350	360	370	380	390	400
HTEAQIQEEG	TVVELTGRQS	SEITRTITKL	SFVKVDSHFR	QGIPFFGQVR	LVDGKGVPIP	NKVIFIRGNE	ANYYSNATTD
410	420	430	440	450	460	470	480
EHGLVQFSIN	TTNVMGTSLT	VRVNYKDRSP	CYGYQWVSEE	HEEAHHTAYL	VFSPSKSFVH	LEPMSHELPC	GHTQTVQAHY
490	500	510	520	530	540	550	560
ILNGGTLLGL	KKLSFYYLIM	AKGGIVRTGT	HGLLVKQEDM	KGHFSISIPV	KSDIAPVARL	LIYAVLPTGD	VIGDSAKYDV
570	580	590	600	610	620	630	640
ENCLANKVDL	SFSPSQSLPA	SHAHLRVTAA	PQSVCALRAV	DQSVLLMKPD	AELSASSVYN	LLPEKDLTGF	PGPLNDQDDE
650	660	670	680	690	700	710	720
DCINRHNVYI	NGITYTPVSS	TNEKDMYSFL	EDMGLKAFTN	SKIRKPKMCP	QLQQYEMHGP	EGLRVGFYES	DVMGRGHARL
730	740	750	760	770	780	790	800
VHVEEPHTET	VRKYFPETWI	WDLVVVNSAG	VAEVGVTVPD	TITEWKAGAF	CLSEDAGLGI	SSTASLRAFQ	PFFVELTMPY
810	820	830	840	850	860	870	880
SVIRGEAFTL	KATVLNYLPK	CIRVSVQLEA	SPAFLAVPVE	KEQAPHCICA	NGRQTVSWAV	TPKSLGNVNF	TVSAEALESQ
890	900	910	920	930	940	950	960
ELCGTEVPSV	PEHGRKDTVI	KPLLVEPEGL	EKETTFNSLL	CPSGGEVSEE	LSLKLPPNVV	EESARASVSV	LGDILGSAMQ
970	980	990	1000	1010	1020	1030	1040
NTQNLLQMPY	GCGEQNMVLF	APNIYVLDYL	NETQQLTPEI	KSKAIGYLNT	GYQRQLNYKH	YDGSYSTFGE	RYGRNQGNTW
1050	1060	1070	1080	1090	1100	1110	1120
LTAFVLKTFA	QARAYIFIDE	AHITQALIWL	SQRQKDNGCF	RSSGSLLNNA	IKGGVEDEVT	LSAYITIALL	EIPLTVTHPV
1130	1140	1150	1160	1170	1180	1190	1200
VRNALFCLES	AWKTAQEGDH	GSHVYTKALL	AYAFALAGNQ	DKRKEVLKSL	NEEAVKKDNS	VHWERPQKPK	APVGHFYEPQ
1210	1220	1230	1240	1250	1260	1270	1280
APSAEVEMTS	YVLLAYLTAQ	PAPTSEDLTS	ATNIVKWITK	QQNAQGGFSS	TQDTVVALHA	LSKYGAATFT	RTGKAAQVTI
1290	1300	1310	1320	1330	1340	1350	1360
QSSGTFSSKF	QVDNNNRLLL	QQVSLPELPG	EYSMKVTGEG	CVYLQTSLKY	NILPEKEEFP	FALGVQTLPQ	TCDEPKAHTS
1370	1380	1390	1400	1410	1420	1430	1440
FQISLSVSYT	GSRSASNMAI	VDVKMVSGFI	PLKPTVKMLE	RSNHVSRTEV	SSNHVLIYLD	KVSNQTLSLF	FTVLQDVPVR
1450	1460	1470	1480				
DLKPAIVKVY	DYYETDEFAI	AEYNAPCSKD	LGNA				

Cmpd.	No. of Cmpds.	m/z meas.	Δ m/z [ppm]	z	Rt [min]	Score	P	Range	Sequence	Modification	Ratios
1683	6	802.8311	-117.18	2	49.7	41.2	0	175-188	R.IAQWQSFQLEGGLK.Q		WD:WU 0.76
2299	2	924.9110	-35.46	2	57.0	20.1	0	189-204	K.QFSFPLSSEPFQGSYK.V		
2052	4	574.7391	-130.69	2	54.0	26.2	0	361-370	R.QGIPFFGQVR.L		
898	2	542.6752	-250.41	2	39.6	25.9	0	522-531	K.GHFSISIPVK.S		WD:WU 0.97
2454	1	922.9898	-34.93	2	59.0	33.4	0	540-557	R.LLIYAVLPTGDVIGDSAK.Y		
174	1	636.8164	-37.15	2	31.0	23.9	0	587-598	R.VTAAPQSVCALR.A	Carbamidomethyl: 9	WD:WU 0.62
											WD:WU 0.98

Detailed Protein Report

Cmpd.	No. of Cmpds.	m/z meas.	Δ m/z [ppm]	z	Rt [min]	Score	P	Range	Sequence	Modification	Ratios	
1177	1	509.7828	-34.15	2	43.4	37.2	0	812-820	K.ATVLNYLPK.C			
2461	2	942.5232	-4.52	2	59.1	21.9	0	824-841	R.VSVQLEASPAFLAVPVEK.E			
305	1	558.6600	-261.38	2	32.5	48.3	0	854-863	R.QTVSWAVTPK.S		WD:WU	1.34
189	1	605.6838	-232.96	2	31.2	17.9	0	935-945	K.LPPNVVEESAR.A		WD:WU	0.64
2462	1	669.8332	5.93	2	59.1	43.0	0	1123-1133	R.NALFCLESAWK.T	Carbamidomethyl: 5	WD:WU	1.62
1151	1	796.4102	8.78	3	43.1	23.4	0	1241-1263	K.QQNAQGGFSSTQDTVVALHALSKY		WD:WU	0.78
891	2	809.4242	-4.39	2	39.9	18.3	0	1408-1421	R.TEVSSNHVLIYLDK.V		WD:WU	0.90

Detailed Protein Report

Protein 7: keratin, type II cytoskeletal 6C [Homo sapiens]

Accession: gi|155969697
Database: refseq_human(refseq_human_20140103.fasta)
Database Date: 2015-11-30

Score: 324.2
MW [kDa]: 60.0
pI: 8.9
Sequence Coverage [%]: 15.4
No. of unique Peptides: 7

Quantitation

WD:WU **Median:** 0.90 **CV:** 54.88 % **No. of Peptides:** 4

10	20	30	40	50	60	70	80
MASTSTTIRS	HSSSRRGFSA	NSARLPGVSR	SGFSSISVSR	SRGSGGLGGA	CGGAGFGSRS	LYGLGGSKRI	SIGGGSCAIS
**90**	**100**	**110**	**120**	**130**	**140**	**150**	**160**
GGYGSRAGGS	YGFGGAGSGF	GFGGGAGIGF	GLGGGAGLAG	GFGGPGFPVC	PPGGIQEVTV	NQSLLTPLNL	QIDPAIQRVR
**170**	**180**	**190**	**200**	**210**	**220**	**230**	**240**
AEEREQIKTL	NNKFASFIDK	VRFLEQQNKV	LDTKWTLLQE	QGTKTVRQNL	EPLFEQYINN	LRRQLDSIVG	ERGRLDSELR
**250**	**260**	**270**	**280**	**290**	**300**	**310**	**320**
NMQDLVEDLK	NKYEDEINKR	TAAENEFVTL	KKDVDAAYMN	KVELQAKADT	LTDEINFLRA	LYDAELSQMQ	THISDTSVVL
**330**	**340**	**350**	**360**	**370**	**380**	**390**	**400**
SMDNNRNLDL	DSIIAEVKAQ	YEEIAQRSRA	EAESWYQTKY	EELQVTAGRH	GDDLRNTKQE	IAEINRMIQR	LRSEIDHVKK
**410**	**420**	**430**	**440**	**450**	**460**	**470**	**480**
QCASLQAAIA	DAEQRGEMAL	KDAKNKLEGL	EDALQKAKQD	LARLLKEYQE	LMNVKLALDV	EIATYRKLLE	GEECRLNGEG
**490**	**500**	**510**	**520**	**530**	**540**	**550**	**560**
VGQVNVSVVQ	STISSGYGGA	SGVGSGLGLG	GGSSYSYGSG	LGIGGGFSSS	SGRAIGGGLS	SVGGGSSTIK	YTTTSSSSRK
**570**							
SYKH							

Cmpd.	No. of Cmpds.	m/z meas.	Δ m/z [ppm]	z	Rt [min]	Score	P	Range	Sequence	Modification	Ratios
709	1	602.2745	-78.87	2	37.7	32.3	0	195-204	K.WTLLQEQGTK.T		
2908	5	945.9990	10.55	2	65.1	35.8	0	208-222	R.QNLEPLFEQYINNLR.R		
1994	4	704.2662	-132.28	2	53.6	55.7	0	288-299	K.ADTLTDEINFLR.A		WD:WU 0.91
2923	16	665.3592	-11.18	2	65.3	86.6	0	327-338	R.NLDLDSIIAEVK.A		
145	2	583.2816	-24.68	2	31.1	63.8	0	360-369	K.YEELQVTAGR.H		WD:WU 0.49
1680	1	632.3373	-21.31	2	49.3	33.5	0	456-466	K.LALDVEIATYR.K		WD:WU 0.74
359	1	724.3637	-37.90	2	33.6	16.6	0	534-550	R.AIGGGLSSVGGGSSTIK.Y		WD:WU 2.01

Detailed Protein Report

Protein 8: keratin, type I cytoskeletal 13 isoform a [Homo sapiens]

Accession: gi|131412225
Database: refseq_human(refseq_human_20140103.fasta)
Database Date: 2015-11-30
Modification(s): Carbamidomethyl

Score: 313.8
MW [kDa]: 49.5
pI: 4.8
Sequence Coverage [%]: 16.6
No. of unique Peptides: 6

Quantitation

WD:WU **Median:** 0.87 **CV:** 31.32 % **No. of Peptides:** 6

10	20	30	40	50	60	70	80
MSLRLQSSSA	SYGGGFGGGS	CQLGGGRGVS	TCSTRFVSGG	SAGGYGGGVS	CGFGGGAGSG	FGGGYGGGLG	GGYGGGLGGG
**90**	**100**	**110**	**120**	**130**	**140**	**150**	**160**
FGGGFAGGFV	DFGACDGGLL	TGNEKITMQN	LNDRLASYLE	KVRALEEANA	DLEVKIRDWH	LKQSPASPER	DYSPYYKTIE
**170**	**180**	**190**	**200**	**210**	**220**	**230**	**240**
ELRDKILTAT	IENNRVILEI	DNARLAADDF	RLKYENELAL	RQSVEADING	LRRVLDELTL	SKTDLEMQIE	SLNEELAYMK
**250**	**260**	**270**	**280**	**290**	**300**	**310**	**320**
KNHEEEMKEF	SNQVVGQVNV	EMDATPGIDL	TRVLAEMREQ	YEAMAERNRR	DAEEWFHAKS	AELNKEVSTN	TAMIQTSKTE
**330**	**340**	**350**	**360**	**370**	**380**	**390**	**400**
ITELRRTLQG	LEIELQSQLS	MKAGLENTVA	ETECRYALQL	QQIQGLISSI	EAQLSELRSE	MECQNQEYKM	LLDIKTRLEQ
**410**	**420**	**430**	**440**	**450**	**460**		
EIATYRSLLE	GQDAKMIGFP	SSAGSVSPRS	TSVTTTSSAS	VTTTSNASGR	RTSDVRRP		

Cmpd.	No. of Cmpds.	m/z meas.	Δ m/z [ppm]	z	Rt [min]	Score	P	Range	Sequence	Modification	Ratios	
260	1	651.3006	-49.51	2	32.4	59.5	0	124-135	R.ALEEANADLEVK.I		WD:WU	0.89
539	1	521.6610	-263.02	2	35.6	53.1	0	176-184	R.VILEIDNAR.L		WD:WU	0.48
342	2	624.7261	-199.84	2	33.4	46.8	1	192-201	R.LKYENELALR.Q		WD:WU	0.86
101	1	725.3298	-7.92	2	30.2	62.2	0	343-355	K.AGLENTVAETECR.Y	Carbamidomethyl: 12	WD:WU	0.92
258	1	690.3402	-39.52	2	32.4	52.8	1	396-406	K.TRLEQEIATYR.S		WD:WU	0.90
17	1	1001.9627	-17.06	2	29.4	39.4	0	430-450	R.STSVTTTSSASVTTTSNASGR.R		WD:WU	1.38

Detailed Protein Report

Protein 9: serotransferrin precursor [Homo sapiens]

Accession: gi|4557871
Database: refseq_human(refseq_human_20140103.fasta)
Database Date: 2015-11-30
Modification(s): Carbamidomethyl

Score: 308.3
MW [kDa]: 77.0
pI: 7.0
Sequence Coverage [%]: 13.9
No. of unique Peptides: 8

Quantitation

WD:WU **Median:** 0.65 **CV:** 59.99 % **No. of Peptides:** 6

10	20	30	40	50	60	70	80
MRLAVGALLV	CAVLGLCLAV	PDKTVRWCAV	SEHEATKCQS	FRDHMKSVIP	SDGPSVACVK	KASYLDCIRA	IAANEADAVT
**90**	**100**	**110**	**120**	**130**	**140**	**150**	**160**
LDAGLVYDAY	LAPNNLKPVV	AEFYGSKEDP	QTFYYAVAVV	KKDSGFQMNQ	LRGKKSCHTG	LGRSAGWNIP	IGLLYCDLPE
**170**	**180**	**190**	**200**	**210**	**220**	**230**	**240**
PRKPLEKAVA	NFFSGSCAPC	ADGTDFPQLC	QLCPGCGCST	LNQYFGYSGA	FKCLKDGAGD	VAFVKHSTIF	ENLANKADRD
**250**	**260**	**270**	**280**	**290**	**300**	**310**	**320**
QYELLCLDNT	RKPVDEYKDC	HLAQVPSHTV	VARSMGGKED	LIWELLNQAQ	EHFGKDKSKE	FQLFSSPHGK	DLLFKDSAHG
**330**	**340**	**350**	**360**	**370**	**380**	**390**	**400**
FLKVPPRMDA	KMYLGYEYVT	AIRNLREGTC	PEAPTDECKP	VKWCALSHHE	RLKCDEWSVN	SVGKIECVSA	ETTEDCIAKI
**410**	**420**	**430**	**440**	**450**	**460**	**470**	**480**
MNGEADAMSL	DGGFVYIAGK	CGLVPVLAEN	YNKSDNCEDT	PEAGYFAVAV	VKKSASDLTW	DNLKGKKSCH	TAVGRTAGWN
**490**	**500**	**510**	**520**	**530**	**540**	**550**	**560**
IPMGLLYNKI	NHCRFDEFFS	EGCAPGSKKD	SSLCKLCMGS	GLNLCEPNNK	EGYYGYTGAF	RCLVEKGDVA	FVKHQTVPQN
**570**	**580**	**590**	**600**	**610**	**620**	**630**	**640**
TGGKNPDPWA	KNLNEKDYEL	LCLDGTRKPV	EEYANCHLAR	APNHAVVTRK	DKEACVHKIL	RQQQHLFGSN	VTDCSGNFCL
**650**	**660**	**670**	**680**	**690**	**700**		
FRSETKDLLF	RDDTVCLAKL	HDRNTYEKYL	GEEYVKAVGN	LRKCSTSSLL	EACTFRRP		

Cmpd.	No. of Cmpds.	m/z meas.	Δ m/z [ppm]	z	Rt [min]	Score	P	Range	Sequence	Modification	Ratios	
1759	1	815.3781	-41.02	2	50.7	25.1	0	108-121	K.EDPQTFYYAVAVVK.K			
172	2	761.3492	-29.85	2	31.0	78.7	1	372-384	R.LKCDEWSVNSVGK.I	Carbamidomethyl: 3		
708	2	625.2611	-72.75	2	37.7	48.9	0	454-464	K.SASDLTWDNLK.G		WD:WU	0.24
817	1	789.3225	-12.58	2	39.0	49.5	0	495-508	R.FDEFFSEGCAPGSK.K	Carbamidomethyl: 9	WD:WU	0.71
448	1	642.1562	-205.50	2	34.1	34.3	0	531-541	K.EGYYGYTGAFR.C		WD:WU	1.50
1439	1	783.3662	-47.33	2	46.8	10.9	1	647-659	K.DLLFRDDTVCLAK.L	Carbamidomethyl: 10	WD:WU	0.58
232	2	500.6172	-271.08	2	32.1	36.9	0	669-676	K.YLGEEYVK.A		WD:WU	0.58
915	2	766.3408	-8.88	2	40.2	24.0	0	684-696	K.CSTSSLLEACTFR.R	Carbamidomethyl: 1, 10	WD:WU	0.91

Detailed Protein Report

Protein 10: alpha-1-antitrypsin precursor [Homo sapiens]

**Accession:**	gi|50363217	**Score:**	228.4
**Database:**	refseq_human(refseq_human_20140103.fasta)	**MW [kDa]:**	46.7
**Database Date:**	2015-11-30	**pI:**	5.3
		**Sequence Coverage [%]:**	23.0
		**No. of unique Peptides:**	5

Quantitation

WD:WU **Median:** 0.95 **CV:** 65.16 % **No. of Peptides:** 3

Alias proteins:

Accession	Name	Description
gi|189163542	r e f s e q _ h u m a (refseq_human_20140103.fasta)	alpha-1-antitrypsin precursor [Homo sapiens]
gi|189163540	r e f s e q _ h u m a (refseq_human_20140103.fasta)	alpha-1-antitrypsin precursor [Homo sapiens]
gi|189163538	r e f s e q _ h u m a (refseq_human_20140103.fasta)	alpha-1-antitrypsin precursor [Homo sapiens]
gi|189163536	r e f s e q _ h u m a (refseq_human_20140103.fasta)	alpha-1-antitrypsin precursor [Homo sapiens]
gi|189163534	r e f s e q _ h u m a (refseq_human_20140103.fasta)	alpha-1-antitrypsin precursor [Homo sapiens]
gi|189163532	r e f s e q _ h u m a (refseq_human_20140103.fasta)	alpha-1-antitrypsin precursor [Homo sapiens]
gi|189163530	r e f s e q _ h u m a (refseq_human_20140103.fasta)	alpha-1-antitrypsin precursor [Homo sapiens]
gi|189163528	r e f s e q _ h u m a (refseq_human_20140103.fasta)	alpha-1-antitrypsin precursor [Homo sapiens]
gi|50363221	r e f s e q _ h u m a (refseq_human_20140103.fasta)	alpha-1-antitrypsin precursor [Homo sapiens]
gi|50363219	r e f s e q _ h u m a (refseq_human_20140103.fasta)	alpha-1-antitrypsin precursor [Homo sapiens]

10	20	30	40	50	60	70	80
MPSSVSWGIL	LLAGLCCLVP	VSLAEDPQGD	AAQKTDTSHH	DQDHPTFNKI	TPNLAEFAFS	LYRQLAHQSN	STNIFFSPVS
**90**	**100**	**110**	**120**	**130**	**140**	**150**	**160**
IATAFAMLSL	GTKADTHDEI	LEGLNFNLTE	IPEAQIHEGF	QELLRTLNQP	DSQLQLTTGN	GLFLSEGLKL	VDKFLEDVKK
**170**	**180**	**190**	**200**	**210**	**220**	**230**	**240**
LYHSEAFTVN	FGDTEEAKKQ	INDYVEKGTQ	GKIVDLVKEL	DRDTVFALVN	YIFFKGKWER	PFEVKDTEEE	DFHVDQVTTV
**250**	**260**	**270**	**280**	**290**	**300**	**310**	**320**
KVPMMKRLGM	FNIQHCKKLS	SWVLLMKYLG	NATAIFFLPD	EGKLQHLENE	LTHDIITKFL	ENEDRRSASL	HLPKLSITGT
**330**	**340**	**350**	**360**	**370**	**380**	**390**	**400**
YDLKSVLGQL	GITKVFSNGA	DLSGVTEEAP	LKLSKAVHKA	VLTIDEKGTE	AAGAMFLEAI	PMSIPPEVKF	NKPFVFLMIE
**410**	**420**						
QNTKSPLFMG	KVVNPTQK						

Cmpd.	No. of Cmpds.	m/z meas.	Δ m/z [ppm]	z	Rt [min]	Score	P	Range	Sequence	Modification	Ratios
2391	1	1287.6505	-18.34	2	58.2	20.0	0	126-149	R. TLNQPDSQLQLTTGNGLFLSEGLK L		
722	1	729.3434	-11.09	3	37.9	16.3	1	161-179	K.LYHSEAFTVNFGDTEEAKK.Q		
762	1	601.9916	0.24	3	38.4	25.3	0	284-298	K.LQHLENELTHDIITK.F		WD:WU 0.63
725	2	555.8033	-4.33	2	37.9	69.3	0	315-324	K.LSITGTYDLK.S		WD:WU 0.61
1068	2	508.1796	-258.32	2	42.1	41.2	0	325-334	K.SVLGQLGITK.V		WD:WU 2.19

Detailed Protein Report

Protein 11: keratin, type I cytoskeletal 14 [Homo sapiens]

Accession: gi|15431310
Database: refseq_human(refseq_human_20140103.fasta)
Database Date: 2015-11-30
Modification(s): Oxidation

Score: 208.6
MW [kDa]: 51.6
pI: 4.9
Sequence Coverage [%]: 11.7
No. of unique Peptides: 3

Quantitation

WD:WU **Median:** 0.77 **CV:** 16.45 % **No. of Peptides:** 2

10	20	30	40	50	60	70	80
MTTCSRQFTS	SSSMKGSCGI	GGGIGGGSSR	ISSVLAGGSC	RAPSTYGGGL	SVSSSRFSSG	GAYGLGGGYG	GGFSSSSSSF
**90**	**100**	**110**	**120**	**130**	**140**	**150**	**160**
GSGFGGGYGG	GLGAGLGGGF	GGGFAGGDGL	LVGSEKVTMQ	NLNDRLASYL	DKVRALEEAN	ADLEVKIRDW	YQRQRPAEIK
**170**	**180**	**190**	**200**	**210**	**220**	**230**	**240**
DYSPYFKTIE	DLRNKILTAT	VDNANVLLQI	DNARLAADDF	RTKYETELNL	RMSVEADING	LRRVLDELTL	ARADLEMQIE
**250**	**260**	**270**	**280**	**290**	**300**	**310**	**320**
SLKEELAYLK	KNHEEEMNAL	RGQVGGDVNV	EMDAAPGVDL	SRILNEMRDQ	YEKMAEKNRK	DAEEWFFTKT	EELNREVATN
**330**	**340**	**350**	**360**	**370**	**380**	**390**	**400**
SELVQSGKSE	ISELRRTMQN	LEIELQSQLS	MKASLENSLE	ETKGRYCMQL	AQIQEMIGSV	EEQLAQLRCE	MEQQNQEYKI
**410**	**420**	**430**	**440**	**450**	**460**	**470**	**480**
LLDVKTRLEQ	EIATYRRLLE	GEDAHLSSSQ	FSSGSQSSRD	VTSSSRQIRT	KVMDVHDGKV	VSTHEQVLRT	KN

Cmpd.	No. of Cmpds.	m/z meas.	Δ m/z [ppm]	z	Rt [min]	Score	P	Range	Sequence	Modification	Ratios
121	2	713.3373	-20.71	2	30.8	66.0	0	42-56	R.APSTYGGGLSVSSSR.F		
1027	2	636.9803	-7.37	3	41.6	10.2	0	337-352	R.TMQNLEIELQSQLSMK.A	Oxidation: 15	WD:WU 0.65
134	1	512.5910	-45.89	3	30.6	20.2	2	406-417	K.TRLEQEIATYRR.L		WD:WU 0.90

Detailed Protein Report

Protein 12: keratin, type II cytoskeletal 75 [Homo sapiens]

**Accession:**	gi|153791158	**Score:**	195.3
**Database:**	refseq_human(refseq_human_20140103.fasta)	**MW [kDa]:**	59.5
**Database Date:**	2015-11-30	**pI:**	8.7
		**Sequence Coverage [%]:**	8.2
		**No. of unique Peptides:**	1

Quantitation

WD:WU **Median:** 0.76 **CV:** 0.00 % **No. of Peptides:** 1

10	20	30	40	50	60	70	80
MSRQSSITFQ	SGSRRGFSTT	SAITPAAGRS	RFSSVSVARS	AAGSGGLGRI	SSAGASFGSR	SLYNLGGAKR	VSINGCGSSC
**90**	**100**	**110**	**120**	**130**	**140**	**150**	**160**
RSGFGGRASN	RFGVNSGFGY	GGGVGGGFSG	PSFPVCPPGG	IQEVTVNQSL	LTPLHLQIDP	TIQRVRAEER	EQIKTLNNKF
**170**	**180**	**190**	**200**	**210**	**220**	**230**	**240**
ASFIDKVRFL	EQQNKVLETK	WALLQEQGSR	TVRQNLEPLF	DSYTSELRRQ	LESITTERGR	LEAELRNMQD	VVEDFKVRYE
**250**	**260**	**270**	**280**	**290**	**300**	**310**	**320**
DEINKRTAAE	NEFVALKKDV	DAAYMNKVEL	EAKVKSLPEE	INFIHSVFDA	ELSQLQTQVG	DTSVVLSMDN	NRNLDLDSII
**330**	**340**	**350**	**360**	**370**	**380**	**390**	**400**
AEVKAQYEDI	ANRSRAEAES	WYQTKYEELQ	VTAGRHGDDL	RNTKQEISEM	NRMIQRLRAE	IDSVKKQCSS	LQTAIADAEQ
**410**	**420**	**430**	**440**	**450**	**460**	**470**	**480**
RGELALKDAR	AKLVDLEEAL	QKAKQDMARL	LREYQELMNI	KLALDVEIAT	YRKLLEGEEC	RLSGEGVSPV	NISVVTSTLS
**490**	**500**	**510**	**520**	**530**	**540**	**550**	**560**
SGYGSGSSIG	GGNLGLGGGS	GYSFTTSGGH	SLGAGLGGSG	FSATSNRGLG	GSGSSVKFVS	TTSSSQKSYT	H

Cmpd.	No. of Cmpds.	m/z meas.	Δ m/z [ppm]	z	Rt [min]	Score	P	Range	Sequence	Modification	Ratios
300	1	678.9411	74.83	2	32.5	11.4	1	413-424	K.LVDLEEALQKAK.Q		WD:WU 0.76

Detailed Protein Report

Protein 13: haptoglobin isoform 2 preproprotein [Homo sapiens]

**Accession:**	gi|186910296	**Score:**	169.3
**Database:**	refseq_human(refseq_human_20140103.fasta)	**MW [kDa]:**	38.4
**Database Date:**	2015-11-30	**pI:**	6.1
**Modification(s):**	Carbamidomethyl	**Sequence Coverage [%]:**	12.7
		**No. of unique Peptides:**	3

Quantitation

WD:WU **Median:** 0.91 **CV:** 32.14 % **No. of Peptides:** 3

```
        10         20         30         40         50         60         70         80
MSALGAVIAL LLWGQLFAVD SGNDVTDIAD DGCPKPPEIA HGYVEHSVRY QCKNYYKLRT EGDGVYTLNN EKQWINKAVG
        90        100        110        120        130        140        150        160
DKLPECEAVC GKPKNPANPV QRILGGHLDA KGSFPWQAKM VSHHNLTTGA TLINEQWLLT TAKNLFLNHS ENATAKDIAP
       170        180        190        200        210        220        230        240
TLTLYVGKKQ LVEIEKVVLH PNYSQVDIGL IKLKQKVSVN ERVMPICLPS KDYAEVGRVG YVSGWGRNAN FKFTDHLKYV
       250        260        270        280        290        300        310        320
MLPVADQDQC IRHYEGSTVP EKKTPKSPVG VQPILNEHTF CAGMSKYQED TCYGDAGSAF AVHDLEEDTW YATGILSFDK
       330        340        350
SCAVAEYGVY VKVTSIQDWV QKTIAEN
```

Cmpd.	No. of Cmpds.	m/z meas.	Δ m/z [ppm]	z	Rt [min]	Score	P	Range	Sequence	Modification	Ratios	
136	2	854.8916	-43.33	2	31.0	42.7	1	58-72	K.LRTEGDGVYTLNNEK.Q		WD:WU	0.63
73	2	619.9745	-5.86	3	29.9	61.3	1	78-94	K.AVGDKLPECEAVCGKPK.N	Carbamidomethyl: 9, 13	WD:WU	1.35
1754	4	645.8334	-54.84	2	50.3	65.3	0	157-168	K.DIAPTLTLYVGK.K		WD:WU	0.87

Detailed Protein Report

Protein 14: annexin A1 [Homo sapiens]

**Accession:**	gi|4502101	**Score:**	145.1
**Database:**	refseq_human(refseq_human_20140103.fasta)	**MW [kDa]:**	38.7
**Database Date:**	2015-11-30	**pI:**	6.7
**Modification(s):**	Oxidation	**Sequence Coverage [%]:**	13.9
		**No. of unique Peptides:**	4

10	20	30	40	50	60	70	80
MAMVSEFLKQ	AWFIENEEQE	YVQTVKSSKG	GPGSAVSPYP	TFNPSSDVAA	LHKAIMVKGV	DEATIIDILT	KRNNAQRQQI
**90**	**100**	**110**	**120**	**130**	**140**	**150**	**160**
KAAYLQETGK	PLDETLKKAL	TGHLEEVVLA	LLKTPAQFDA	DELRAAMKGL	GTDEDTLIEI	LASRTNKEIR	DINRVYREEL
**170**	**180**	**190**	**200**	**210**	**220**	**230**	**240**
KRDLAKDITS	DTSGDFRNAL	LSLAKGDRSE	DFGVNEDLAD	SDARALYEAG	ERRKGTDVNV	FNTILTTRSY	PQLRRVFQKY
**250**	**260**	**270**	**280**	**290**	**300**	**310**	**320**
TKYSKHDMNK	VLDLELKGDI	EKCLTAIVKC	ATSKPAFFAE	KLHQAMKGVG	TRHKALIRIM	VSRSEIDMND	IKAFYQKMYG
**330**	**340**	**350**					
ISLCQAILDE	TKGDYEKILV	ALCGGN					

Cmpd.	No. of Cmpds.	m/z meas.	Δ m/z [ppm]	z	Rt [min]	Score	P	Range	Sequence	Modification
2994	1	973.5095	-39.14	2	66.2	10.2	1	54-71	K.AIMVKGVDEATIIDILTK.R	Oxidation: 3
2228	1	694.3075	-115.27	2	56.3	17.4	0	59-71	K.GVDEATIIDILTK.R	
2959	5	851.9284	-21.25	2	65.8	81.3	0	129-144	K.GLGTDEDTLIEILASR.T	
2388	2	775.8834	-37.22	2	58.2	36.2	0	215-228	K.GTDVNVFNTILTTR.S	

Detailed Protein Report

Protein 15: fibrinogen gamma chain isoform gamma-A precursor [Homo sapiens]

Accession: gi|70906437
Database: refseq_human(refseq_human_20140103.fasta)
Database Date: 2015-11-30

Score: 139.8
MW [kDa]: 49.5
pI: 5.7
Sequence Coverage [%]: 10.8
No. of unique Peptides: 4

Quantitation

WD:WU **Median:** 0.89 **CV:** 12.14 % **No. of Peptides:** 2

10	20	30	40	50	60	70	80
MSWSLHPRNL	ILYFYALLFL	SSTCVAYVAT	RDNCCILDER	FGSYCPTTCG	IADFLSTYQT	KVDKDLQSLE	DILHQVENKT
90	100	110	120	130	140	150	160
SEVKQLIKAI	QLTYNPDESS	KPNMIDAATL	KSRKMLEEIM	KYEASILTHD	SSIRYLQEIY	NSNNQKIVNL	KEKVAQLEAQ
170	180	190	200	210	220	230	240
CQEPCKDTVQ	IHDITGKDCQ	DIANKGAKQS	GLYFIKPLKA	NQQFLVYCEI	DGSGNGWTVF	QKRLDGSVDF	KKNWIQYKEG
250	260	270	280	290	300	310	320
FGHLSPTGTT	EFWLGNEKIH	LISTQSAIPY	ALRVELEDWN	GRTSTADYAM	FKVGPEADKY	RLTYAYFAGG	DAGDAFDGFD
330	340	350	360	370	380	390	400
FGDDPSDKFF	TSHNGMQFST	WDNDNDKFEG	NCAEQDGSGW	WMNKCHAGHL	NGVYYQGGTY	SKASTPNGYD	NGIIWATWKT
410	420	430	440				
RWYSMKKTTM	KIIPFNRLTI	GEGQQHHLGG	AKQAGDV				

Cmpd.	No. of Cmpds.	m/z meas.	Δ m/z [ppm]	z	Rt [min]	Score	P	Range	Sequence	Modification	Ratios
127	2	757.2233	-190.62	2	30.5	61.4	0	135-146	R.YLQEIYNSNNQK.I		WD:WU 1.00
1191	1	647.3732	-13.52	2	43.1	26.2	0	189-199	K.QSGLYFIKPLK.A		
2104	2	841.8525	-155.05	2	54.7	23.4	0	259-273	K.IHLISTQSAIPYALR.V		
329	1	559.1487	-212.08	2	33.2	28.9	0	274-282	R.VELEDWNGR.T		WD:WU 0.79

Detailed Protein Report

Protein 16: titin isoform N2-A [Homo sapiens]

**Accession:**	gi|291045225	**Score:**	136.7
**Database:**	refseq_human(refseq_human_20140103.fasta)	**MW [kDa]:**	3711.3
**Database Date:**	2015-11-30	**pI:**	6.1
**Modification(s):**	Carbamidomethyl, Oxidation	**Sequence Coverage [%]:**	0.4
		**No. of unique Peptides:**	10

Quantitation

WD:WU	**Median:** 0.95	**CV:** 23.37 %	**No. of Peptides:** 3

Detailed Protein Report

10	20	30	40	50	60	70	80
MTTQAPTFTQ	PLQSVVVLEG	STATFEAHIS	GFPVPEVSWF	RDGQVISTST	LPGVQISFSD	GRAKLTIPAV	TKANSGRYSL
90	100	110	120	130	140	150	160
KATNGSGQAT	STAELLVKAE	TAPPNFVQRL	QSMTVRQGSQ	VRLQVRVTGI	PTPVVKFYRD	GAEIQSSLDF	QISQEGDLYS
170	180	190	200	210	220	230	240
LLIAEAYPED	SGTYSVNATN	SVGRATSTAE	LLVQGEEEVP	AKKTKTIVST	AQISESRQTR	IEKKIEAHFD	ARSIATVEMV
250	260	270	280	290	300	310	320
IDGAAGQQLP	HKTPPRIPPK	PKSRSPTPPS	IAAKAQLARQ	QSPSPIRHSP	SPVRHVRAPT	PSPVRSVSPA	ARISTSPIRS
330	340	350	360	370	380	390	400
VRSPLLMRKT	QASTVATGPE	VPPPWKQEGY	VASSSEAEMR	ETTLTTSTQI	RTEERWEGRY	GVQEQVTISG	AAGAAASVSA
410	420	430	440	450	460	470	480
SASYAAEAVA	TGAKEVKQDA	DKSAAVATVV	AAVDMARVRE	PVISAVEQTA	QRTTTTAVHI	QPAQEQVRKE	AEKTAVTKVV
490	500	510	520	530	540	550	560
VAADKAKEQE	LKSRTKEVIT	TKQEQMHVTH	EQIRKETEKT	FVPKVVISAA	KAKEQETRIS	EEITKKQKQV	TQEAIRQETE
570	580	590	600	610	620	630	640
ITAASMVVVA	TAKSTKLETV	PGAQEETTTQ	QDQMHLSYEK	IMKETRKTVV	PKVIVATPKV	KEQDLVSRGR	EGITTKREQV
650	660	670	680	690	700	710	720
QITQEKMRKE	AEKTALSTIA	VATAKAKEQE	TILRTRETMA	TRQEQIQVTH	GKVDVGKKAE	AVATVVAAVD	QARVREPREP
730	740	750	760	770	780	790	800
GHLEESYAQQ	TTLEYGYKER	ISAAKVAEPP	QRPASEPHVV	PKAVKPRVIQ	APSETHIKTT	DQKGMHISSQ	IKKTTDLTTE
810	820	830	840	850	860	870	880
RLVHVDKRPR	TASPHFTVSK	ISVPKTEHGY	EASIAGSAIA	TLQKELSATS	SAQKITKSVK	APTVKPSETR	VRAEPTPLPQ
890	900	910	920	930	940	950	960
FPFADTPDTY	KSEAGVEVKK	EVGVSITGTT	VREERFEVLH	GREAKVTETA	RVPAPVEIPV	TPPTLVSGLK	NVTVIEGESV
970	980	990	1000	1010	1020	1030	1040
TLECHISGYP	SPTVTWYRED	YQIESSIDFQ	ITFQSGIARL	MIREAFAEDS	GRFTCSAVNE	AGTVSTSCYL	AVQVSEEFEK
1050	1060	1070	1080	1090	1100	1110	1120
ETTAVTEKFT	TEEKRFVESR	DVVMTDTSLT	EEQAGPGEPA	APYFITKPVV	QKLVEGGSVV	FGCQVGGNPK	PHVYWKKSGV
1130	1140	1150	1160	1170	1180	1190	1200
PLTTGYRYKV	SYNKQTGECK	LVISMTFADD	AGEYTIVVRN	KHGETSASAS	LLEEADYELL	MKSQQEMLYQ	TQVTAFVQEP
1210	1220	1230	1240	1250	1260	1270	1280
KVGETAPGFV	YSEYEKEYEK	EQALIRKKMA	KDTVVVRTYV	EDQEFHISSF	EERLIKEIEY	RIIKTTLEEL	LEEDGEEKMA
1290	1300	1310	1320	1330	1340	1350	1360
VDISESEAVE	SGFDSRIKNY	RILEGMGVTF	HCKMSGYPLP	KIAWYKDGKR	IKHGERYQMD	FLQDGRASLR	IPVVLPEDEG
1370	1380	1390	1400	1410	1420	1430	1440
IYTAFASNIK	GNAICSGKLY	VEPAAPLGAP	TYIPTLEPVS	RIRSLSPRSV	SRSPIRMSPA	RMSPARMSPA	RMSPARMSPG
1450	1460	1470	1480	1490	1500	1510	1520
RRLEETDESQ	LERLYKPVFV	LKPVSFKCLE	GQTARFDLKV	VGRPMPETFW	FHDGQQIVND	YTHKVVIKED	GTQSLIIVPA
1530	1540	1550	1560	1570	1580	1590	1600
TPSDSGEWTV	VAQNRAGRSS	ISVILTVEAV	EHQVKPMFVE	KLKNVNIKEG	SRLEMKVRAT	GNPNPDIVWL	KNSDIIVPHK
1610	1620	1630	1640	1650	1660	1670	1680
YPKIRIEGTK	GEAALKIDST	VSQDSAWYTA	TAINKAGRDT	TRCKVNVEVE	FAEPEPERKL	IIPRGTYRAK	EIAAPELEPL
1690	1700	1710	1720	1730	1740	1750	1760
HLRYGQEQWE	EGDLYDKEKQ	QKPFFKKKLT	SLRLKRFGPA	HFECRLTPIG	DPTMVVEWLH	DGKPLEAANR	LRMINEFGYC
1770	1780	1790	1800	1810	1820	1830	1840
SLDYGVAYSR	DSGIITCRAT	NKYGTDHTSA	TLIVKDEKSL	VEESQLPEGR	KGLQRIEELE	RMAHEGALTG	VTTDQKEKQK
1850	1860	1870	1880	1890	1900	1910	1920
PDIVLYPEPV	RVLEGETARF	RCRVTGYPQP	KVNWYLNGQL	IRKSKRFRVR	YDGIHYLDIV	DCKSYDTGEV	KVTAENPEGV
1930	1940	1950	1960	1970	1980	1990	2000
IEHKVKLEIQ	QREDFRSVLR	RAPEPRPEFH	VHEPGKLQFE	VQKVDRPVDT	TETKEVVKLK	RAERITHEKV	PEESEELRSK
2010	2020	2030	2040	2050	2060	2070	2080
FKRRTEEGYY	EAITAVELKS	RKKDESYEEL	LRKTKDELLH	WTKELTEEEK	KALAEEGKIT	IPTFKPDKIE	LSPSMEAPKI
2090	2100	2110	2120	2130	2140	2150	2160
FERIQSQTVG	QGSDAHFRVR	VVGKPDPECE	WYKNGVKIER	SDRIYWYWPE	DNVCELVIRD	VTAEDSASIM	VKAINIAGET
2170	2180	2190	2200	2210	2220	2230	2240

Detailed Protein Report

Cmpd.	No. of Cmpds.	m/z meas.	Δ m/z [ppm]	z	Rt [min]	Score	P	Range	Sequence	Modification	Ratios	
1331	1	640.2131	-156.95	2	45.0	10.2	1	9719-9728	K.EVTIMEEKER.A	Oxidation: 5	WD:WU	0.85
881	2	555.3353	87.65	2	39.8	14.2	0	11321-11331	K.GTAIFACDIAK.D		WD:WU	1.31
627	8	481.1600	-205.16	2	36.2	13.1	0	13135-13142	K.TWVLATDR.A			
1108	1	540.7821	22.96	2	42.5	15.6	1	15065-15073	K.EIREGADYK.L			
2888	1	900.4707	-22.65	2	64.8	13.7	0	15435-15450	K.NETVIEKPTDALQITK.E			
2673	3	813.4051	24.69	2	61.8	13.6	0	17073-17085	R.VPDLLEGCQYEFR.V	Carbamidomethyl: 8		
2745	1	656.8669	67.42	2	62.7	10.9	0	28426-28437	K.YTLTVENNSGSK.S			
98	1	529.8207	-183.86	3	30.5	11.1	1	30355-30368	K.DDGGSRVTGYYIER.K			
2593	1	616.1731	-223.16	2	60.7	11.0	1	31970-31979	R.MPYDVPEPRK.Y			
27	2	731.6483	-99.39	3	29.3	11.9	2	32802-32820	K.TNLQFMGQAFKSIHEKVSK.I		WD:WU	0.77

Detailed Protein Report

Protein 17: heat shock protein beta-1 [Homo sapiens]

Accession: gi|4504517
Database: refseq_human(refseq_human_20140103.fasta)
Database Date: 2015-11-30

Score: 113.8
MW [kDa]: 22.8
pI: 6.0
Sequence Coverage [%]: 21.5
No. of unique Peptides: 3

Quantitation

WD:WU **Median:** 0.49 **CV:** 0.00 % **No. of Peptides:** 1

10	20	30	40	50	60	70	80
MTERRVPFSL	LRGPSWDPFR	DWYPHSRLFD	QAFGLPRLPE	EWSQWLGGSS	WPGYVRPLPP	AAIESPAVAA	PAYSRALSRQ
**90**	**100**	**110**	**120**	**130**	**140**	**150**	**160**
LSSGVSEIRH	TADRWRVSLD	VNHFAPDELT	VKTKDGVVEI	TGKHEERQDE	HGYISRCFTR	KYTLPPGVDP	TQVSSSLSPE
**170**	**180**	**190**	**200**	**210**			
GTLTVEAPMP	KLATQSNEIT	IPVTFESRAQ	LGGPEAAKSD	ETAAK			

Cmpd.	No. of Cmpds.	m/z meas.	Δ m/z [ppm]	z	Rt [min]	Score	P	Range	Sequence	Modification	Ratios
1772	4	582.3088	-9.00	2	50.5	62.7	0	28-37	R.LFDQAFGLPR.L		
1666	1	953.4317	-71.06	2	49.5	35.6	0	172-188	K.LATQSNEITIPVTFESR.A		WD:WU 0.49
2886	2	822.4678	63.76	2	64.8	15.6	1	189-205	R.AQLGGPEAAKSDETAAK.-		

Detailed Protein Report

Protein 18: PREDICTED: POTE ankyrin domain family member E isoform X1 [Homo sapiens]

**Accession:**	gi|578804361	**Score:**	106.7
**Database:**	refseq_human(refseq_human_20140103.fasta)	**MW [kDa]:**	76.6
**Database Date:**	2015-11-30	**pI:**	4.9
		**Sequence Coverage [%]:**	7.0
		**No. of unique Peptides:**	3

Quantitation

WD:WU **Median:** 0.44 **CV:** 20.64 % **No. of Peptides:** 3

10	20	30	40	50	60	70	80
MSQELEINKD	GDREVEEEMK	KHESNNVGLL	ENLTNGVTAG	NGDNGLIPQR	KSRTPENQQF	PDNESEEYHR	ICELLSDYKE
**90**	**100**	**110**	**120**	**130**	**140**	**150**	**160**
KQMPKYSSEN	SNPEQDLKLT	SEEESQRLKG	SENGQPEKRS	QEPEINKDGD	RELENFMAIE	EMKKHGSTHV	GFPENLTNGA
**170**	**180**	**190**	**200**	**210**	**220**	**230**	**240**
TAGNGDDGLI	PPRKSRTPES	QQFPDTENEE	YHSDEQNDTQ	KQFCEEQNTG	ILHDEILIHE	EKQIEVVEKM	NSELSLSCKK
**250**	**260**	**270**	**280**	**290**	**300**	**310**	**320**
EKDVLHENST	LREEIAMLRL	ELDTMKHQSQ	LREKKYLEDI	ESVKKKNDNL	LKALQLNELT	MDDDTAVLVI	DNGSGMCKAG
**330**	**340**	**350**	**360**	**370**	**380**	**390**	**400**
FAGDDAPRAV	FPSIVGRPRQ	QGMMGGMHQK	ESYVGKEAQS	KRGILTLKYP	MEHGIITNWD	DMEKIWHHTF	YNELRVAPEE
**410**	**420**	**430**	**440**	**450**	**460**	**470**	**480**
HPILLTEAPL	NPKANREKMT	QIMFETFNTP	AMYVAIQAVP	SLYTSGRTTG	IVMDSGDGVT	HTVPIYEGNA	LPHATLRLDL
**490**	**500**	**510**	**520**	**530**	**540**	**550**	**560**
AGRELPDYLM	KILTERGYRF	TTMAEREIVR	DIKEKLCYVA	LDFEQEMATA	ASSSSLEKSY	ELPDGQVITI	GNERFRCPEA
**570**	**580**	**590**	**600**	**610**	**620**	**630**	**640**
LFQPCFLGME	SCGIHETTFN	SIMKSDVDIR	KDLYTNTVLS	GGTTMYPGMA	HRMQKEIAAL	APSMMKIRII	APPKRKYSVW
**650**	**660**	**670**	**680**				
VGGSILASLS	TFQQMWISKQ	EYDESGPSIV	HRKCF				

Cmpd.	No. of Cmpds.	m/z meas.	Δ m/z [ppm]	z	Rt [min]	Score	P	Range	Sequence	Modification	Ratios	
1146	1	984.4853	-59.25	2	43.0	11.3	0	396-413	R.VAPEEHPILLTEAPLNPK.A		WD:WU	0.47
1411	4	895.9110	-43.08	2	46.4	42.4	0	539-554	K.SYELPDGQVITIGNER.F		WD:WU	0.54
74	2	758.8545	-0.57	2	30.1	53.0	0	660-672	K.QEYDESGPSIVHR.K		WD:WU	0.33

Detailed Protein Report

Protein 19: complement C3 precursor [Homo sapiens]

Accession: gi|115298678
Database: refseq_human(refseq_human_20140103.fasta)
Database Date: 2015-11-30
Modification(s): Carbamidomethyl

Score: 79.4
MW [kDa]: 187.0
pI: 6.0
Sequence Coverage [%]: 3.5
No. of unique Peptides: 4

Quantitation

WD:WU **Median:** 4.52 **CV:** 0.00 % **No. of Peptides:** 1

10	20	30	40	50	60	70	80
MGPTSGPSLL	LLLLTHLPLA	LGSPMYSIIT	PNILRLESEE	TMVLEAHDAQ	GDVPVTVTVH	DFPGKKLVLS	SEKTVLTPAT
90	100	110	120	130	140	150	160
NHMGNVTFTI	PANREFKSEK	GRNKFVTVQA	TFGTQVVEKV	VLVSLQSGYL	FIQTDKTIYT	PGSTVLYRIF	TVNHKLLPVG
170	180	190	200	210	220	230	240
RTVMVNIENP	EGIPVKQDSL	SSQNQLGVLP	LSWDIPELVN	MGQWKIRAYY	ENSPQQVFST	EFEVKEYVLP	SFEVIVEPTE
250	260	270	280	290	300	310	320
KFYYIYNEKG	LEVTITARFL	YGKKVEGTAF	VIFGIQDGEQ	RISLPESLKR	IPIEDGSGEV	VLSRKVLLDG	VQNPRAEDLV
330	340	350	360	370	380	390	400
GKSLYVSATV	ILHSGSDMVQ	AERSGIPIVT	SPYQIHFTKT	PKYFKPGMPF	DLMVFVTNPD	GSPAYRVPVA	VQGEDTVQSL
410	420	430	440	450	460	470	480
TQGDGVAKLS	INTHPSQKPL	SITVRTKKQE	LSEAEQATRT	MQALPYSTVG	NSNNYLHLSV	LRTELRPGET	LNVNFLLRMD
490	500	510	520	530	540	550	560
RAHEAKIRYY	TYLIMNKGRL	LKAGRQVREP	GQDLVVLPLS	ITTDFIPSFR	LVAYYTLIGA	SGQREVVADS	VWVDVKDSCV
570	580	590	600	610	620	630	640
GSLVVKSGQS	EDRQPVPGQQ	MTLKIEGDHG	ARVVLVAVDK	GVFVLNKKNK	LTQSKIWDVV	EKADIGCTPG	SGKDYAGVFS
650	660	670	680	690	700	710	720
DAGLTFTSSS	GQQTAQRAEL	QCPQPAARRR	RSVQLTEKRM	DKVGKYPKEL	RKCCEDGMRE	NPMRFSCQRR	TRFISLGEAC
730	740	750	760	770	780	790	800
KKVFLDCCNY	ITELRRQHAR	ASHLGLARSN	LDEDIIAEEN	IVSRSEFPES	WLWNVEDLKE	PPKNGISTKL	MNIFLKDSIT
810	820	830	840	850	860	870	880
TWEILAVSMS	DKKGICVADP	FEVTVMQDFF	IDLRLPYSVV	RNEQVEIRAV	LYNYRQNQEL	KVRVELLHNP	AFCSLATTKR
890	900	910	920	930	940	950	960
RHQQTVTIPP	KSSLSVPYVI	VPLKTGLQEV	EVKAAVYHHF	ISDGVRKSLK	VVPEGIRMNK	TVAVRTLDPE	RLGREGVQKE
970	980	990	1000	1010	1020	1030	1040
DIPPADLSDQ	VPDTESETRI	LLQGTPVAQM	TEDAVDAERL	KHLIVTPSGC	GEQNMIGMTP	TVIAVHYLDE	TEQWEKFGLE
1050	1060	1070	1080	1090	1100	1110	1120
KRQGALELIK	KGYTQQLAFR	QPSSAFAAFV	KRAPSTWLTA	YVVKVFSLAV	NLIAIDSQVL	CGAVKWLILE	KQKPDGVFQE
1130	1140	1150	1160	1170	1180	1190	1200
DAPVIHQEMI	GGLRNNNEKD	MALTAFVLIS	LQEAKDICEE	QVNSLPGSIT	KAGDFLEANY	MNLQRSYTVA	IAGYALAQMG
1210	1220	1230	1240	1250	1260	1270	1280
RLKGPLLNKF	LTTAKDKNRW	EDPGKQLYNV	EATSYALLAL	LQLKDFDFVP	PVVRWLNEQR	YYGGGYGSTQ	ATFMVFQALA
1290	1300	1310	1320	1330	1340	1350	1360
QYQKDAPDHQ	ELNLDVSLQL	PSRSSKITHR	IHWESASLLR	SEETKENEGF	TVTAEGKGQG	TLSVVTMYHA	KAKDQLTCNK
1370	1380	1390	1400	1410	1420	1430	1440
FDLKVTIKPA	PETEKRPQDA	KNTMILEICT	RYRGDQDATM	SILDISMMTG	FAPDTDDLKQ	LANGVDRYIS	KYELDKAFSD
1450	1460	1470	1480	1490	1500	1510	1520
RNTLIIYLDK	VSHSEDDCLA	FKVHQYFNVE	LIQPGAVKVY	AYYNLEESCT	RFYHPEKEDG	KLNKLCRDEL	CRCAEENCFI
1530	1540	1550	1560	1570	1580	1590	1600
QKSDDKVTLE	ERLDKACEPG	VDYVYKTRLV	KVQLSNDFDE	YIMAIEQTIK	SGSDEVQVGQ	QRTFISPIKC	REALKLEEKK
1610	1620	1630	1640	1650	1660	1670	
HYLMWGLSSD	FWGEKPNLSY	IIGKDTWVEH	WPEEDECQDE	ENQKQCQDLG	AFTESMVVFG	CPN	

Cmpd.	No. of Cmpds.	m/z meas.	Δ m/z [ppm]	z	Rt [min]	Score	P	Range	Sequence	Modification	Ratios
2933	1	940.0026	13.02	2	65.4	22.8	0	226-241	K.EYVLPSFEVIVEPTEK.F		

Detailed Protein Report

Cmpd.	No. of Cmpds.	m/z meas.	Δ m/z [ppm]	z	Rt [min]	Score	P	Range	Sequence	Modification	Ratios
2727	4	701.3852	-51.38	2	62.5	16.6	0	892-904	K.SSLSVPYVIVPLK.T		
1782	5	1086.9427	-40.31	2	50.9	20.1	2	1505-1522	K.LCRDELCRCAEENCFIQK.S		WD:WU 4.52
192	1	650.8121	22.36	2	31.2	19.9	0	1536-1546	K.ACEPGVDYVYK.T	Carbamidomethyl: 2	

Detailed Protein Report

Protein 20: hemoglobin subunit epsilon [Homo sapiens]

**Accession:**	gi|4885393	**Score:**	77.9
**Database:**	refseq_human(refseq_human_20140103.fasta)	**MW [kDa]:**	16.2
**Database Date:**	2015-11-30	**pI:**	9.4
		**Sequence Coverage [%]:**	15.0
		**No. of unique Peptides:**	1

10	20	30	40	50	60	70	80
MVHFTAEEKA	AVTSLWSKMN	VEEAGGEALG	RLLVVYPWTQ	RFFDSFGNLS	SPSAILGNPK	VKAHGKKVLT	SFGDAIKNMD
**90**	**100**	**110**	**120**	**130**	**140**	**150**	
NLKPAFAKLS	ELHCDKLHVD	PENFKLLGNV	MVIILATHFG	KEFTPEVQAA	WQKLVSAVAI	ALAHKYH	

Cmpd.	No. of Cmpds.	m/z meas.	Δ m/z [ppm]	z	Rt [min]	Score	P	Range	Sequence	Modification
2297	8	596.8075	-111.81	2	57.0	16.1	0	134-145	K.LVSAVAIALAHK.Y	

Detailed Protein Report

Protein 21: glyceraldehyde-3-phosphate dehydrogenase isoform 2 [Homo sapiens]

**Accession:**	gi|378404908	**Score:**	77.6
**Database:**	refseq_human(refseq_human_20140103.fasta)	**MW [kDa]:**	31.5
**Database Date:**	2015-11-30	**pI:**	7.9
**Modification(s):**	Carbamidomethyl	**Sequence Coverage [%]:**	15.7
		**No. of unique Peptides:**	3

Quantitation

WD:WU **Median:** 1.30 **CV:** 5.57 % **No. of Peptides:** 2

10	20	30	40	50	60	70	80
MVYMFQYDST	HGKFHGTVKA	ENGKLVINGN	PITIFQERDP	SKIKWGDAGA	EYVVESTGVF	TTMEKAGAHL	QGGAKRVIIS
**90**	**100**	**110**	**120**	**130**	**140**	**150**	**160**
APSADAPMFV	MGVNHEKYDN	SLKIISNASC	TTNCLAPLAK	VIHDNFGIVE	GLMTTVHAIT	ATQKTVDGPS	GKLWRDGRGA
**170**	**180**	**190**	**200**	**210**	**220**	**230**	**240**
LQNIIPASTG	AAKAVGKVIP	ELNGKLTGMA	FRVPTANVSV	VDLTCRLEKP	AKYDDIKKVV	KQASEGPLKG	ILGYTEHQVV
**250**	**260**	**270**	**280**	**290**	**300**		
SSDFNSDTHS	STFDAGAGIA	LNDHFVKLIS	WYDNEFGYSN	RVVDLMAHMA	SKE		

Cmpd.	No. of Cmpds.	m/z meas.	Δ m/z [ppm]	z	Rt [min]	Score	P	Range	Sequence	Modification	Ratios
703	1	917.4101	-58.22	2	37.6	39.4	0	104-120	K.IISNASCTTNCLAPLAK.V	Carbamidomethyl: 7, 11	WD:WU 1.23
904	2	706.3818	-24.09	2	40.1	26.3	0	159-173	R.GALQNIIPASTGAAK.A		WD:WU 1.37
1642	1	882.2784	-143.30	2	48.8	12.0	0	268-281	K.LISWYDNEFGYSNR.V		

Detailed Protein Report

Protein 22: immunoglobulin lambda-like polypeptide 5 isoform 2 [Homo sapiens]

Accession: gi|372466586
Database: refseq_human(refseq_human_20140103.fasta)
Database Date: 2015-11-30

Score: 76.9
MW [kDa]: 14.7
pI: 7.7
Sequence Coverage [%]: 24.5
No. of unique Peptides: 2

Quantitation

WD:WU **Median:** 0.70 **CV:** 7.71 % **No. of Peptides:** 2

```
        10         20         30         40         50         60         70         80
MACCAQWLHR KAGTQTLEPQ LEAADPACGA CGAGQPKANP TVTLFPPSSE ELQANKATLV CLISDFYPGA VTVAWKADGS
        90        100        110        120        130        140
PVKAGVETTK PSKQSNNKYA ASSYLSLTPE QWKSHRSYSC QVTHEGSTVE KTVAPTECS
```

Cmpd.	No. of Cmpds.	m/z meas.	Δ m/z [ppm]	z	Rt [min]	Score	P	Range	Sequence	Modification	Ratios	
1500	1	1022.0151	-8.03	2	47.1	12.8	0	38-56	K.ANPTVTLFPPSSEELQANK.A		WD:WU	0.65
1789	11	872.4180	-17.25	2	50.7	64.0	0	99-113	K.YAASSYLSLTPEQWK.S		WD:WU	0.76

Detailed Protein Report

Protein 23: transthyretin precursor [Homo sapiens]

Accession: gi|4507725
Database: refseq_human(refseq_human_20140103.fasta)
Database Date: 2015-11-30

Score: 76.1
MW [kDa]: 15.9
pI: 5.4
Sequence Coverage [%]: 8.8
No. of unique Peptides: 1

Quantitation

WD:WU **Median:** 1.69 **CV:** 0.00 % **No. of Peptides:** 1

10	20	30	40	50	60	70	80
MASHRLLLLC	LAGLVFVSEA	GPTGTGESKC	PLMVKVLDAV	RGSPAINVAV	HVFRKAADDT	WEPFASGKTS	ESGELHGLTT
90	100	110	120	130	140	150	
EEEFVEGIYK	VEIDTKSYWK	ALGISPFHEH	AEVVFTANDS	GPRRYTIAAL	LSPYSYSTTA	VVTNPKE	

Cmpd.	No. of Cmpds.	m/z meas.	Δ m/z [ppm]	z	Rt [min]	Score	P	Range	Sequence	Modification	Ratios
1401	2	683.8722	-15.95	2	45.9	76.1	0	42-54	R.GSPAINVAVHVFR.K		WD:WU 1.69

Detailed Protein Report

Protein 24: c-myc promoter-binding protein-1 isoform MBP-1 [Homo sapiens]

**Accession:**	gi|319996655	**Score:**	71.8
**Database:**	refseq_human(refseq_human_20140103.fasta)	**MW [kDa]:**	36.9
**Database Date:**	2015-11-30	**pI:**	5.9
**Modification(s):**	Carbamidomethyl	**Sequence Coverage [%]:**	7.9
		**No. of unique Peptides:**	2

Quantitation

WD:WU **Median:** 0.81 **CV:** 20.36 % **No. of Peptides:** 2

```
        10         20         30         40         50         60         70         80
MIEMDGTENK SKFGANAILG VSLAVCKAGA VEKGVPLYRH IADLAGNSEV ILPVPAFNVI NGGSHAGNKL AMQEFMILPV
        90        100        110        120        130        140        150        160
GAANFREAMR IGAEVYHNLK NVIKEKYGKD ATNVGDEGGF APNILENKEG LELLKTAIGK AGYTDKVVIG MDVAASEFFR
       170        180        190        200        210        220        230        240
SGKYDLDFKS PDDPSRYISP DQLADLYKSF IKDYPVVSIE DPFDQDDWGA WQKFTASAGI QVVGDDLTVT NPKRIAKAVN
       250        260        270        280        290        300        310        320
EKSCNCLLLK VNQIGSVTES LQACKLAQAN GWGVMVSHRS GETEDTFIAD LVVGLCTGQI KTGAPCRSER LAKYNQLLRI
       330        340        350
EEELGSKAKF AGRNFRNPLA K
```

Cmpd.	No. of Cmpds.	m/z meas.	Δ m/z [ppm]	z	Rt [min]	Score	P	Range	Sequence	Modification	Ratios	
1599	2	713.3423	-34.09	2	48.3	38.9	0	177-188	R.YISPDQLADLYK.S		WD:WU	0.67
584	1	817.3947	-24.01	2	36.2	33.0	0	251-265	K.VNQIGSVTESLQACK.L	Carbamidomethyl: 14	WD:WU	1.00

Detailed Protein Report

Protein 25: stAR-related lipid transfer protein 9 [Homo sapiens]

**Accession:**	gi|270265793	**Score:**	69.8
**Database:**	refseq_human(refseq_human_20140103.fasta)	**MW [kDa]:**	516.0
**Database Date:**	2015-11-30	**pI:**	5.9
		**Sequence Coverage [%]:**	1.3
		**No. of unique Peptides:**	4

Quantitation

WD:WU	**Median:** 0.73	**CV:** 0.00 %	**No. of Peptides:** 1

Detailed Protein Report

10	20	30	40	50	60	70	80
MANVQVAVRV	RPLSKRETKE	GGRIIVEVDG	KVAKIRNLKV	DNRPDGFGDS	REKVMAFGFD	YCYWSVNPED	PQYASQDVVF
90	100	110	120	130	140	150	160
QDLGMEVLSG	VAKGYNICLF	AYGQTGSGKT	YTMLGTPASV	GLTPRICEGL	FVREKDCASL	PSSCRIKVSF	LEIYNERVRD
170	180	190	200	210	220	230	240
LLKQSGQKKS	YTLRVREHPE	MGPYVQGLSQ	HVVTNYKQVI	QLLEEGIANR	ITAATHVHEA	SSRSHAIFTI	HYTQAILENN
250	260	270	280	290	300	310	320
LPSEMASKIN	LVDLAGSERA	DPSYCKDRIA	EGANINKSLV	TLGIVISTLA	QNSQVFSSCQ	SLNSSVSNGG	DSGILSSPSG
330	340	350	360	370	380	390	400
TSSGGAPSRR	QSYIPYRDSV	LTWLLKDSLG	GNSKTIMVAT	VSPAHTSYSE	TMSTLRYASS	AKNIINKPRV	NEDANLKLIR
410	420	430	440	450	460	470	480
ELREEIERLK	ALLLSFELRN	FSSLSDENLK	ELVLQNELKI	DQLTKDWTQK	WNDWQALMEH	YSVDINRRRA	GVVIDSSLPH
490	500	510	520	530	540	550	560
LMALEDDVLS	TGVVLYHLKE	GTTKIGRIDS	DQEQDIVLQG	QWIERDHCTI	TSACGVVVLR	PARGARCTVN	GREVTASCRL
570	580	590	600	610	620	630	640
TQGAVITLGK	AQKFRFNHPA	EAAVLRQRRQ	VGEAAAGRGS	LEWLDLDGDL	AASRLGLSPL	LWKERRALEE	QCDEDHQTPR
650	660	670	680	690	700	710	720
DGETSHRAQI	QQQQSYVEDL	RHQILAEEIR	AAKELEFDQA	WISQQIKENQ	QCLLREETWL	ASLQQQQQED	QVAEKELEAS
730	740	750	760	770	780	790	800
VALDAWLQTD	PEIQPSPFVQ	SQKRVVHLQL	LRRHTLRAAE	RNVRRKKVSF	QLERIIKKQR	LLEAQKRLEK	LTTLCWLQDD
810	820	830	840	850	860	870	880
STQEPPYQVL	SPDATVPRPP	CRSKLTSCSS	LSPQRLCSKH	MPQLHSIFLS	WDPSTTLPPR	PDPTHQTSEK	TSSEEHLPQA
890	900	910	920	930	940	950	960
ASYPARTGCL	RKNGLHSSGH	GQPCTARAAL	ARKGASAPDA	CLTMSPNSVG	IQEMEMGVKQ	PHQMVSQGLA	SLRKSANKLK
970	980	990	1000	1010	1020	1030	1040
PRHEPKIFTS	TTQTRGAKGL	ADPSHTQAGW	RKEGNLGTHK	AAKGASCNSL	YPHGPRQTAG	HGKAVKTFWT	EYKPPSPSRA
1050	1060	1070	1080	1090	1100	1110	1120
SKRHQRVLAT	RVRNITKKSS	HLPLGSPLKR	QQNTRDPDTM	VPLTDFSPVM	DHSREKDNDL	SDTDSNYSLD	SLSCVYAKAL
1130	1140	1150	1160	1170	1180	1190	1200
IEPLKPEERK	WDFPEPENSE	SDDSQLSEDS	LAEKRYQSPK	NRLGGNRPTN	NRGQPRTRTR	ASVRGFTAAS	DSDLLAQTHR
1210	1220	1230	1240	1250	1260	1270	1280
SFSLDSLIDA	EEELGEDQQE	EPFPGSADEI	PTETFWHLED	SSLPVMDQEA	ICRLGPINYR	TAARLDAVLP	MSSSFYLDPQ
1290	1300	1310	1320	1330	1340	1350	1360
FQPHCELQPH	CELQPHCELQ	PHCEQAESQV	EPSYSEQADS	LQGMQLSRES	PLMSMDSWFS	CDSKINPSSP	PGIVGSLCPS
1370	1380	1390	1400	1410	1420	1430	1440
PDMQEFHSCK	GERPGYWPNT	EELKPSDAET	VLPYSSKLHQ	GSTELLCSAR	DEHTASAADT	SRLSLWGIQR	LIQPGADGTF
1450	1460	1470	1480	1490	1500	1510	1520
QGRCIPDMTQ	QGSSEASHNS	SVSNVLAASA	TTLTHVGSTH	ERDWSALQQK	YLLELSCPVL	EAIGAPKPAY	PYLEEDSGSL
1530	1540	1550	1560	1570	1580	1590	1600
AQASSKGGDT	LLPVGPRVSS	NLNLNNFPVH	LSRIRRLRAE	KEQDSLNAKL	EGVSDFFSTS	EKEASYDETY	SADLESLSAS
1610	1620	1630	1640	1650	1660	1670	1680
RSTNAQVFAT	ENAIPDSMTE	ACEVKQNNLE	ECLQSCRKPG	LMTSSDEDFF	QKNACHSNVT	TATKADHWSQ	GWAPLRKNSA
1690	1700	1710	1720	1730	1740	1750	1760
VQPGQLSPDS	HYPLEEEKTD	CQESSKEAVR	RHINVSFALP	SGPELYLHSA	PWNPLSSSLQ	PPLLETFYVT	KSRDALTETA
1770	1780	1790	1800	1810	1820	1830	1840
LEIPACREVR	VPSPPPREAW	GFGHNHQALQ	GAYLKNNLPV	LLQNQNSKIA	SSQQVTAEIP	VDLNTREVIR	ESGKCPGNIT
1850	1860	1870	1880	1890	1900	1910	1920
EESHDSVYSS	VTQNRHFLPS	TSTKVCEFEN	QVVILNKKHS	FPALEGGEVT	AQSCCGASSD	STESGKSLLF	RESEAREEEE
1930	1940	1950	1960	1970	1980	1990	2000
LDQNTVLRQT	INVSLEKDMP	GESAVSLKSR	SVDRRVSSPV	MVAQGGGPTP	KWEGKNETGL	LEKGLRPKDS	SEEFKLPGTK
2010	2020	2030	2040	2050	2060	2070	2080
PAYERFQLVA	CPQERNPSEC	KSQEMLNPNR	EPSGKKQNKR	VNNTDEMARL	IRSVMQLENG	ILEIESKQNK	QVHASHTPGT
2090	2100	2110	2120	2130	2140	2150	2160
DKELVFQDQK	EQEKTDHAFR	PDSSGNPLPS	KDQPSSPRQT	DDTVFRDSEA	GAMEVNSIGN	HPQVQKITPN	PFRSREGVRE
2170	2180	2190	2200	2210	2220	2230	2240

Detailed Protein Report

Cmpd.	No. of Cmpds.	m/z meas.	Δ m/z [ppm]	z	Rt [min]	Score	P	Range	Sequence	Modification	Ratios	
2859	3	648.3692	122.78	2	64.5	15.6	1	134-145	R.EKDCASLPSSCR.I			
2847	1	665.3691	57.41	2	64.1	17.6	0	2192-2202	R.EFTNTSLHPQR.M			
405	1	565.3234	16.33	2	34.1	11.7	0	2310-2319	R.QETVSPLLSR.T			
1886	1	863.9273	-37.56	2	52.2	12.3	1	2739-2754	K.KVVAALPSQAPYDDPR.V		WD:WU	0.73

Detailed Protein Report

Protein 26: keratin, type II cytoskeletal 1 [Homo sapiens]

Accession: gi|119395750
Database: refseq_human(refseq_human_20140103.fasta)
Database Date: 2015-11-30

Score: 69.5
MW [kDa]: 66.0
pI: 8.8
Sequence Coverage [%]: 3.7
No. of unique Peptides: 2

10	20	30	40	50	60	70	80
MSRQFSSRSG	YRSGGGFSSG	SAGIINYQRR	TTSSSTRRSG	GGGGRFSSCG	GGGGSFGAGG	GFGSRSLVNL	GGSKSISISV
**90**	**100**	**110**	**120**	**130**	**140**	**150**	**160**
ARGGGRGSGF	GGGYGGGGFG	GGGFGGGGFG	GGGIGGGGFG	GFGSGGGGFG	GGGFGGGGYG	GGYGPVCPPG	GIQEVTINQS
**170**	**180**	**190**	**200**	**210**	**220**	**230**	**240**
LLQPLNVEID	PEIQKVKSRE	REQIKSLNNQ	FASFIDKVRF	LEQQNQVLQT	KWELLQQVDT	STRTHNLEPY	FESFINNLRR
**250**	**260**	**270**	**280**	**290**	**300**	**310**	**320**
RVDQLKSDQS	RLDSELKNMQ	DMVEDYRNKY	EDEINKRTNA	ENEFVTIKKD	VDGAYMTKVD	LQAKLDNLQQ	EIDFLTALYQ
**330**	**340**	**350**	**360**	**370**	**380**	**390**	**400**
AELSQMQTQI	SETNVILSMD	NNRSLDLDSI	IAEVKAQYED	IAQKSKAEAE	SLYQSKYEEL	QITAGRHGDS	VRNSKIEISE
**410**	**420**	**430**	**440**	**450**	**460**	**470**	**480**
LNRVIQRLRS	EIDNVKKQIS	NLQQSISDAE	QRGENALKDA	KNKLNDLEDA	LQQAKEDLAR	LLRDYQELMN	TKLALDLEIA
**490**	**500**	**510**	**520**	**530**	**540**	**550**	**560**
TYRTLLEGEE	SRMSGECAPN	VSVSVSTSHT	TISGGGSRGG	GGGGYGSGGS	SYGSGGGSYG	SGGGGGGGRG	SYGSGGSSYG
**570**	**580**	**590**	**600**	**610**	**620**	**630**	**640**
SGGGSYGSGG	GGGGHGSYGS	GSSSGGYRGG	SGGGGGGSSG	GRGSGGGSSG	GSIGGRGSSS	GGVKSSGGSS	SVKFVSTTYS
**650**							
GVTR							

Cmpd.	No. of Cmpds.	m/z meas.	Δ m/z [ppm]	z	Rt [min]	Score	P	Range	Sequence	Modification
1307	1	738.3615	-22.47	2	45.1	53.5	0	212-223	K.WELLQQVDTSTR.T	
2843	2	651.8509	-15.86	2	64.1	16.0	0	344-355	R.SLDLDSIIAEVK.A	

Detailed Protein Report

Protein 27: endothelin-2 preproprotein [Homo sapiens]

**Accession:**	gi|4503463	**Score:**	57.5
**Database:**	refseq_human(refseq_human_20140103.fasta)	**MW [kDa]:**	19.9
**Database Date:**	2015-11-30	**pI:**	12.1
**Modification(s):**	Carbamidomethyl	**Sequence Coverage [%]:**	14.0
		**No. of unique Peptides:**	3

Quantitation

WD:WU **Median:** 0.64 **CV:** 0.00 % **No. of Peptides:** 1

```
        10         20         30         40         50         60         70         80
MVSVPTTWCS VALALLVALH EGKGQAAATL EQPASSSHAQ GTHLRLRRCS CSSWLDKECV YFCHLDIIWV NTPEQTAPYG
        90        100        110        120        130        140        150        160
LGNPPRRRRR SLPRRCQCSS ARDPACATFC LRRPWTEAGA VPSRKSPADV FQTGKTGATT GELLQRLRDI STVKSLFAKR
       170        180
QQEAMREPRS THSRWRKR
```

Cmpd.	No. of Cmpds.	m/z meas.	Δ m/z [ppm]	z	Rt [min]	Score	P	Range	Sequence	Modification	Ratios
2946	1	1051.5610	95.12	2	65.6	10.9	2	95-112	R.RCQCSSARDPACATFCLR.R	Carbamidomethyl: 4, 12	
2155	1	1051.4608	-0.17	2	55.5	16.2	2	95-112	R.RCQCSSARDPACATFCLR.R	Carbamidomethyl: 12, 16	
587	1	459.6056	-275.62	2	35.7	17.8	1	160-166	K.RQQEAMR.E		WD:WU 0.64

Detailed Protein Report

Protein 28: keratin, type II cytoskeletal 4 [Homo sapiens]

Accession: gi|331999954
Database: refseq_human(refseq_human_20140103.fasta)
Database Date: 2015-11-30

Score: 57.2
MW [kDa]: 56.1
pI: 6.2
Sequence Coverage [%]: 3.3
No. of unique Peptides: 2

Quantitation

WD:WU **Median:** 0.82 **CV:** 0.00 % **No. of Peptides:** 1

10	20	30	40	50	60	70	80
MIARQQCVRG	GPRGFSCGSA	IVGGGKRGAF	SSVSMSGGAG	RCSSGGFGSR	SLYNLRGNKS	ISMSVAGSRQ	GACFGGAGGF
**90**	**100**	**110**	**120**	**130**	**140**	**150**	**160**
GTGGFGGGFG	GSFSGKGGPG	FPVCPAGGIQ	EVTINQSLLT	PLHVEIDPEI	QKVRTEEREQ	IKLLNNKFAS	FIDKVQFLEQ
**170**	**180**	**190**	**200**	**210**	**220**	**230**	**240**
QNKVLETKWN	LLQQQTTTTS	SKNLEPLFET	YLSVLRKQLD	TLGNDKGRLQ	SELKTMQDSV	EDFKTKYEEE	INKRTAAEND
**250**	**260**	**270**	**280**	**290**	**300**	**310**	**320**
FVVLKKDVDA	AYLNKVELEA	KVDSLNDEIN	FLKVLYDAEL	SQMQTHVSDT	SVVLSMDNNR	NLDLDSIIAE	VRAQYEEIAQ
**330**	**340**	**350**	**360**	**370**	**380**	**390**	**400**
RSKAEAEALY	QTKVQQLQIS	VDQHGDNLKN	TKSEIAELNR	MIQRLRAEIE	NIKKQCQTLQ	VSVADAEQRG	ENALKDAHSK
**410**	**420**	**430**	**440**	**450**	**460**	**470**	**480**
RVELEAALQQ	AKEELARMLR	EYQELMSVKL	ALDIEIATYR	KLLEGEEYRM	SGECQSAVSI	SVVSGSTSTG	GISGGLGSGS
**490**	**500**	**510**	**520**	**530**			
GFGLSSGFGS	GSGSGFGFGG	SVSGSSSSKI	ISTTTLNKRR				

Cmpd.	No. of Cmpds.	m/z meas.	Δ m/z [ppm]	z	Rt [min]	Score	P	Range	Sequence	Modification	Ratios
365	1	678.3483	-55.17	2	33.6	39.9	1	401-412	K.RVELEAALQQAK.E		WD:WU 0.82
1796	1	599.9815	-22.51	3	51.1	17.3	1	402-417	R.VELEAALQQAKEELAR.M		

Detailed Protein Report

Protein 29: centromere protein F [Homo sapiens]

**Accession:**	gi|55770834	**Score:**	56.6
**Database:**	refseq_human(refseq_human_20140103.fasta)	**MW [kDa]:**	357.3
**Database Date:**	2015-11-30	**pI:**	4.9
		**Sequence Coverage [%]:**	1.4
		**No. of unique Peptides:**	2

Detailed Protein Report

10	20	30	40	50	60	70	80
MSWALEEWKE	GLPTRALQKI	QELEGQLDKL	KKEKQQRQFQ	LDSLEAALQK	QKQKVENEKT	EGTNLKRENQ	RLMEICESLE
90	100	110	120	130	140	150	160
KTKQKISHEL	QVKESQVNFQ	EGQLNSGKKQ	IEKLEQELKR	CKSELERSQQ	AAQSADVSLN	PCNTPQKIFT	TPLTPSQYYS
170	180	190	200	210	220	230	240
GSKYEDLKEK	YNKEVEERKR	LEAEVKALQA	KKASQTLPQA	TMNHRDIARH	QASSSVFSWQ	QEKTPSHLSS	NSQRTPIRRD
250	260	270	280	290	300	310	320
FSASYFSGEQ	EVTPSRSTLQ	IGKRDANSSF	FDNSSSPHLL	DQLKAQNQEL	RNKINELELR	LQGHEKEMKG	QVNKFQELQL
330	340	350	360	370	380	390	400
QLEKAKVELI	EKEKVLNKCR	DELVRTTAQY	DQASTKYTAL	EQKLKKLTED	LSCQRQNAES	ARCSLEQKIK	EKEKEFQEEL
410	420	430	440	450	460	470	480
SRQQRSFQTL	DQECIQMKAR	LTQELQQAKN	MHNVLQAELD	KLTSVKQQLE	NNLEEFKQKL	CRAEQAFQAS	QIKENELRRS
490	500	510	520	530	540	550	560
MEEMKKENNL	LKSHSEQKAR	EVCHLEAELK	NIKQCLNQSQ	NFAEEMKAKN	TSQETMLRDL	QEKINQQENS	LTLEKLKLAV
570	580	590	600	610	620	630	640
ADLEKQRDCS	QDLLKKREHH	IEQLNDKLSK	TEKESKALLS	ALELKKKEYE	ELKEEKTLFS	CWKSENEKLL	TQMESEKENL
650	660	670	680	690	700	710	720
QSKINHLETC	LKTQQIKSHE	YNERVRTLEM	DRENLSVEIR	NLHNVLDSKS	VEVETQKLAY	MELQQKAEFS	DQKHQKEIEN
730	740	750	760	770	780	790	800
MCLKTSQLTG	QVEDLEHKLQ	LLSNEIMDKD	RCYQDLHAEY	ESLRDLLKSK	DASLVTNEDH	QRSLLAFDQQ	PAMHHSFANI
810	820	830	840	850	860	870	880
IGEQGSMPSE	RSECRLEADQ	SPKNSAILQN	RVDSLEFSLE	SQKQMNSDLQ	KQCEELVQIK	GEIEENLMKA	EQMHQSFVAE
890	900	910	920	930	940	950	960
TSQRISKLQE	DTSAHQNVVA	ETLSALENKE	KELQLLNDKV	ETEQAEIQEL	KKSNHLLEDS	LKELQLLSET	LSLEKKEMSS
970	980	990	1000	1010	1020	1030	1040
IISLNKREIE	ELTQENGTLK	EINASLNQEK	MNLIQKSESF	ANYIDEREKS	ISELSDQYKQ	EKLILLQRCE	ETGNAYEDLS
1050	1060	1070	1080	1090	1100	1110	1120
QKYKAAQEKN	SKLECLLNEC	TSLCENRKNE	LEQLKEAFAK	EHQEFLTKLA	FAEERNQNLM	LELETVQQAL	RSEMTDNQNN
1130	1140	1150	1160	1170	1180	1190	1200
SKSEAGGLKQ	EIMTLKEEQN	KMQKEVNDLL	QENEQLMKVM	KTKHECQNLE	SEPIRNSVKE	RESERNQCNF	KPQMDLEVKE
1210	1220	1230	1240	1250	1260	1270	1280
ISLDSYNAQL	VQLEAMLRNK	ELKLQESEKE	KECLQHELQT	IRGDLETSNL	QDMQSQEISG	LKDCEIDAEE	KYISGPHELS
1290	1300	1310	1320	1330	1340	1350	1360
TSQNDNAHLQ	CSLQTTMNKL	NELEKICEIL	QAEKYELVTE	LNDSRSECIT	ATRKMAEEVG	KLLNEVKILN	DDSGLLHGEL
1370	1380	1390	1400	1410	1420	1430	1440
VEDIPGGEFG	EQPNEQHPVS	LAPLDESNSY	EHLTLSDKEV	QMHFAELQEK	FLSLQSEHKI	LHDQHCQMSS	KMSELQTYVD
1450	1460	1470	1480	1490	1500	1510	1520
SLKAENLVLS	TNLRNFQGDL	VKEMQLGLEE	GLVPSLSSSC	VPDSSSLSSL	GDSSFYRALL	EQTGDMSLLS	NLEGAVSANQ
1530	1540	1550	1560	1570	1580	1590	1600
CSVDEVFCSS	LQEENLTRKE	TPSAPAKGVE	ELESLCEVYR	QSLEKLEEKM	ESQGIMKNKE	IQELEQLLSS	ERQELDCLRK
1610	1620	1630	1640	1650	1660	1670	1680
QYLSENEQWQ	QKLTSVTLEM	ESKLAAEKKQ	TEQLSLELEV	ARLQLQGLDL	SSRSLLGIDT	EDAIQGRNES	CDISKEHTSE
1690	1700	1710	1720	1730	1740	1750	1760
TTERTPKHDV	HQICDKDAQQ	DLNLDIEKIT	ETGAVKPTGE	CSGEQSPDTN	YEPPGEDKTQ	GSSECISELS	FSGPNALVPM
1770	1780	1790	1800	1810	1820	1830	1840
DFLGNQEDIH	NLQLRVKETS	NENLRLLHVI	EDRDRKVESL	LNEMKELDSK	LHLQEVQLMT	KIEACIELEK	IVGELKKENS
1850	1860	1870	1880	1890	1900	1910	1920
DLSEKLEYFS	CDHQELLQRV	ETSEGLNSDL	EMHADKSSRE	DIGDNVAKVN	DSWKERFLDV	ENELSRIRSE	KASIEHEALY
1930	1940	1950	1960	1970	1980	1990	2000
LEADLEVVQT	EKLCLEKDNE	NKQKVIVCLE	EELSVVTSER	NQLRGELDTM	SKKTTALDQL	SEKMKEKTQE	LESHQSECLH
2010	2020	2030	2040	2050	2060	2070	2080
CIQVAEAEVK	EKTELLQTLS	SDVSELLKDK	THLQEKLQSL	EKDSQALSLT	KCELENQIAQ	LNKEKELLVK	ESESLQARLS
2090	2100	2110	2120	2130	2140	2150	2160
ESDYEKLNVS	KALEAALVEK	GEFALRLSST	QEEVHQLRRG	IEKLRVRIEA	DEKKQLHIAE	KLKERERERND	SLKDKVENLE
2170	2180	2190	2200	2210	2220	2230	2240

Detailed Protein Report

Cmpd.	No. of Cmpds.	m/z meas.	Δ m/z [ppm]	z	Rt [min]	Score	P	Range	Sequence	Modification
104	2	1043.8924	-98.05	2	30.5	15.3	0	128-147	R.SQQAAQSADVSLNPCNTPQK.I	
552	1	600.8199	-67.28	2	35.3	13.6	1	2316-2325	K.KQLCVLQQLK.E	

Detailed Protein Report

Protein 30: PREDICTED: nebulin isoform X22 [Homo sapiens]

**Accession:**	gi|530370465	**Score:**	56.4
**Database:**	refseq_human(refseq_human_20140103.fasta)	**MW [kDa]:**	961.5
**Database Date:**	2015-11-30	**pI:**	9.5
**Modification(s):**	Oxidation	**Sequence Coverage [%]:**	0.6
		**No. of unique Peptides:**	4

Quantitation

WD:WU	**Median:** 0.74	**CV:** 5.98 %	**No. of Peptides:** 2

Detailed Protein Report

10	20	30	40	50	60	70	80
MADDEDYEEV	VEYYTEEVVY	EEVPGETITK	IYETTTTRTS	DYEQSETSKP	ALAQPALAQP	ASAKPVERRK	VIRKKVDPSK
90	100	110	120	130	140	150	160
FMTPYIAHSQ	KMQDLFSPNK	YKEKFEKTKG	QPYASTTDTP	ELRRIKKVQD	QLSEVKYRMD	GDVAKTICHV	DEKAKDIEHA
170	180	190	200	210	220	230	240
KKVSQQVSKV	LYKQNWEDTK	DKYLLPPDAP	ELVQAVKNTA	MFSKKLYTED	WEADKSLFYP	YNDSPELRRV	AQAQKALSDV
250	260	270	280	290	300	310	320
AYKKGLAEQQ	AQFTPLADPP	DIEFAKKVTN	QVSKQKYKED	YENKIKGKWS	ETPCFEVANA	RMNADNISTR	KYQEDFENMK
330	340	350	360	370	380	390	400
DQIYFMQTET	PEYKMNKKAG	VAASKVKYKE	DYEKNKGKAD	YNVLPASENP	QLRQLKAAGD	ALSDKLYKEN	YEKTKAKSIN
410	420	430	440	450	460	470	480
YCETPKFKLD	TVLQNFSSDK	KYKDSYLKDI	LGHYVGSFED	PYHSHCMKVT	AQNSDKNYKA	EYEEDRGKGF	FPQTITQEYE
490	500	510	520	530	540	550	560
AIKKLDQCKD	HTYKVHPDKT	KFTQVTDSPV	LLQAQVNSKQ	LSDLNYKAKH	ESEKFKCHIP	PDTPAFIQHK	VNAYNLSDNL
570	580	590	600	610	620	630	640
YKQDWEKSKA	KKFDIKVDAI	PLLAAKANTK	NTSDVMYKKD	YEKNKGKMIG	VLSINDDPKM	LHSLKVAKNQ	SDRLYKENYE
650	660	670	680	690	700	710	720
KTKAKSMNYC	ETPKYQLDTQ	LKNFSEARYK	DLYVKDVLGH	YVGSMEDPYH	THCMKVAAQN	SDKSYKAEYE	EDKGKCYFPQ
730	740	750	760	770	780	790	800
TITQEYEAIK	KLDQCKDHTY	KVHPDKTKFT	AVTDSPVLLQ	AQLNTKQLSD	LNYKAKHEGE	KFKCHIPADA	PQFIQHRVNA
810	820	830	840	850	860	870	880
YNLSDNVYKQ	DWEKSKAKKF	DIKVDAIPLL	AAKANTKNTS	DVMYKKDYEK	SKGKMIGALS	INDDPKMLHS	LKTAKNQSDR
890	900	910	920	930	940	950	960
EYRKDYEKSK	TIYTAPLDML	QVTQAKKSQA	IASDVDYKHI	LHSYSYPPDS	INVDLAKKAY	ALQSDVEYKA	DYNSWMKGCG
970	980	990	1000	1010	1020	1030	1040
WVPFGSLEME	KAKRASDILN	EKKYRQHPDT	LKFTSIEDAP	ITVQSKINQA	QRSDIAYKAK	GEEIIHKYNL	PPDLPQFIQA
1050	1060	1070	1080	1090	1100	1110	1120
KVNAYNISEN	MYKADLKDLS	KKGYDLRTDA	IPIRAAKAAR	QAASDVQYKK	DYEKAKGKMV	GFQSLQDDPK	LVHYMNVAKI
1130	1140	1150	1160	1170	1180	1190	1200
QSDREYKKDY	EKTKSKYNTP	HDMFNVVAAK	KAQDVVSNVN	YKHSLHHYTY	LPDAMDLELS	KNMMQIQSDN	VYKEDYNNWM
1210	1220	1230	1240	1250	1260	1270	1280
KGIGWIPIGS	LDVEKVKKAG	DALNEKKYRQ	HPDTLKFTSI	VDSPVMVQAK	QNTKQVSDIL	YKAKGEDVKH	KYTMSPDLPQ
1290	1300	1310	1320	1330	1340	1350	1360
FLQAKCNAYN	ISDVCYKRDW	YDLIAKGNNV	LGDAIPITAA	KASRNIASDY	KYKEAYEKSK	GKHVGFRSLQ	DDPKLVHYMN
1370	1380	1390	1400	1410	1420	1430	1440
VAKLQSDREY	KKNYENTKTS	YHTPGDMVSI	TAAKMAQDVA	TNVNYKQPLH	HYTYLPDAMS	LEHTRNVNQI	QSDNVYKDEY
1450	1460	1470	1480	1490	1500	1510	1520
NSFLKGIGWI	PIGSLEVEKV	KKAGDALNER	KYRQHPDTVK	FTSVPDSMGM	VLAQHNTKQL	SDLNYKVEGE	KLKHKYTIDP
1530	1540	1550	1560	1570	1580	1590	1600
ELPQFIQAKV	NALNMSDAHY	KADWKKTIAK	GYDLRPDAIP	IVAAKSSRNI	ASDCKYKEAY	EKAKGKQVGF	LSLQDDPKLV
1610	1620	1630	1640	1650	1660	1670	1680
HYMNVAKIQS	DREYKKGYEA	SKTKYHTPLD	MVSVTAAKKS	QEVATNANYR	QSYHHYTLLP	DALNVEHSRN	AMQIQSDNLY
1690	1700	1710	1720	1730	1740	1750	1760
KSDFTNWMKG	IGWVPIESLE	VEKAKKAGEI	LSEKKYRQHP	EKLKFTYAMD	TMEQALNKSN	KLNMDKRLYT	EKWNKDKTTI
1770	1780	1790	1800	1810	1820	1830	1840
HVMPDTPDIL	LSRVNQITMS	DKLYKAGWEE	EKKKGYDLRP	DAIAIKAARA	SRDIASDYKY	KKAYEQAKGK	HIGFRSLEDD
1850	1860	1870	1880	1890	1900	1910	1920
PKLVHFMQVA	KMQSDREYKK	GYEKSKTSFH	TPVDMLSVVA	AKKSQEVATN	ANYRNVIHTY	NMLPDAMSFE	LAKNMMQIQS
1930	1940	1950	1960	1970	1980	1990	2000
DNQYKADYAD	FMKGIGWLPL	GSLEAEKNKK	AMEIISEKKY	RQHPDTLKYS	TLMDSMNMVL	AQNNAKIMNE	HLYKQAWEAD
2010	2020	2030	2040	2050	2060	2070	2080
KTKVHIMPDI	PQIILAKANA	INMSDKLYKL	SLEESKKKGY	DLRPDAIPIK	AAKASRDIAS	DYKYKYNYEK	GKGKMVGFRS
2090	2100	2110	2120	2130	2140	2150	2160
LEDDPKLVHS	MQVAKMQSDR	EYKKNYENTK	TSYHTPADML	SVTAAKDAQA	NITNTNYKHL	IHKYILLPDA	MNIELTRNMN
2170	2180	2190	2200	2210	2220	2230	2240

Detailed Protein Report

Cmpd.	No. of Cmpds.	m/z meas.	Δ m/z [ppm]	z	Rt [min]	Score	P	Range	Sequence	Modification	Ratios
415	4	544.7701	86.52	2	34.2	12.0	0	646-654	K.SMNYCETPK.Y	Oxidation: 2	
1176	2	729.3599	-9.93	2	43.4	18.3	1	3617-3628	R.KAYDLQSDNLYK.S		WD:WU 0.69
993	1	662.3199	-62.62	2	41.2	12.9	2	7204-7214	K.VKDEVSDLKYK.E		WD:WU 0.78
2801	1	994.5013	20.67	2	63.5	13.2	1	8000-8015	K.LNQENFSSVLYKENMR.K	Oxidation: 15	

Detailed Protein Report

Protein 31: PREDICTED: splicing factor, proline- and glutamine-rich isoform X5 [Homo sapiens]

Accession: gi|530363148
Database: refseq_human(refseq_human_20140103.fasta)
Database Date: 2015-11-30
Modification(s): Oxidation

Score: 54.9
MW [kDa]: 72.2
pI: 9.7
Sequence Coverage [%]: 5.1
No. of unique Peptides: 3

Quantitation

WD:WU **Median:** 1.47 **CV:** 0.00 % **No. of Peptides:** 1

Alias proteins:

Accession	Name	Description
gi|530363150	refseq_human (refseq_human_20140103.fasta)	PREDICTED: splicing factor, proline- and glutamine-rich isoform X6 [Homo sapiens]

10	20	30	40	50	60	70	80
MSRDRFRSRG	GGGGGFHRRG	GGGGRGGLHD	FRSPPPGMGL	NQNRGPMGPG	PGQSGPKPPI	PPPPPHQQQQ	QPPPQQPPPQ
**90**	**100**	**110**	**120**	**130**	**140**	**150**	**160**
QPPPHQPPPH	PQPHQQQQPP	PPPQDSSKPV	VAQGPGPAPG	VGSAPPASSS	APPATPPTSG	APPGSGPGPT	PTPPPAVTSA
**170**	**180**	**190**	**200**	**210**	**220**	**230**	**240**
PPGAPPPTPP	SSGVPTTPPQ	AGGPPPPPAA	VPGPGPGPKQ	GPGPGGPKGG	KMPGGPKPGG	GPGLSTPGGH	PKPPHRGGGE
**250**	**260**	**270**	**280**	**290**	**300**	**310**	**320**
PRGGRQHHPP	YHQQHHQGPP	PGGPGGRSEE	KISDSEGFKA	NLSLLRRPGE	KTYTQRCRLF	VGNLPADITE	DEFKRLFAKY
**330**	**340**	**350**	**360**	**370**	**380**	**390**	**400**
GEPGEVFINK	GKGFGFIKLE	SRALAEIAKA	ELDDTPMRGR	QLRVRFATHA	AALSVRNLSP	YVSNELLEEA	FSQFGPIERA
**410**	**420**	**430**	**440**	**450**	**460**	**470**	**480**
VVIVDDRGRS	TGKGIVEFAS	KPAARKAFER	CSEGVFLLTT	TPRPVIVEPL	EQLDDEDGLP	EKLAQKNPMY	QKERETPPRF
**490**	**500**	**510**	**520**	**530**	**540**	**550**	**560**
AQHGTFEYEY	SQRWKSLDEM	EKQQREQVEK	NMKDAKDKLE	SEMEDAYHEH	QANLLRQDLM	RRQEELRRME	ELHNQEMQKR
**570**	**580**	**590**	**600**	**610**	**620**	**630**	**640**
KEMQLRQEEE	RRRREEEMMI	RQREMEEQMR	RQREESYSRM	GYMDPRERDM	RMGGGGAMNM	GDPYGSGGQK	FPPLGGGGGI
**650**	**660**	**670**					
GYEANPGVPP	ATMSGSMMGS	DMVRMIDVG					

Cmpd.	No. of Cmpds.	m/z meas.	Δ m/z [ppm]	z	Rt [min]	Score	P	Range	Sequence	Modification	Ratios
378	2	678.1836	-200.95	2	33.8	28.3	1	268-279	R.SEEKISDSEGFK.A		
18	2	736.0042	54.34	3	29.4	11.7	1	609-630	R. DMRMGGGGAMNMGDPYGSGGQ F	Oxidation: 2, 10	
8	2	736.0117	64.51	3	29.3	14.9	1	609-630	R. DMRMGGGGAMNMGDPYGSGGQ F	Oxidation: 4, 12	WD:WU 1.47

Detailed Protein Report

Protein 32: CDK5 regulatory subunit-associated protein 2 isoform c [Homo sapiens]

Accession: gi|440309853
Database: refseq_human(refseq_human_20140103.fasta)
Database Date: 2015-11-30
Modification(s): Oxidation

Score: 54.8
MW [kDa]: 189.5
pI: 5.4
Sequence Coverage [%]: 2.8
No. of unique Peptides: 4

Quantitation

WD:WU **Median:** 0.89 **CV:** 5.29 % **No. of Peptides:** 2

10	20	30	40	50	60	70	80
MMDLVLEEDV	TVPGTLSGCS	GLVPSVPDDL	DGINPNAGLG	NGLLPNVSEE	TVSPTRARNM	KDFENQITEL	KKENFNLKLR
90	100	110	120	130	140	150	160
IYFLEERMQQ	EFHGPTEHIY	KTNIELKVEV	ESLKRELQER	EQLLIKASKA	VESLAEAGGS	EIQRVKEDAR	KKVQQVEDLL
170	180	190	200	210	220	230	240
TKRILLLEKD	VTAAQAELEK	AFAGTETEKA	LRLRLESKLS	EMKKMHEGDL	AMALVLDEKD	RLIEELKLSL	KSKEALIQCL
250	260	270	280	290	300	310	320
KEEKSQMACP	DENVSSGELR	GLCAAPREEK	ERETEAAQME	HQKERNSFEE	RIQALEEDLR	EKEREIATEK	KNSLKRDKAI
330	340	350	360	370	380	390	400
QGLTMALKSK	EKKVEELNSE	IEKLSAAFAK	AREALQKAQT	QEFQGSEDYE	TALSGKEALS	AALRSQNLTK	STENHRLRRS
410	420	430	440	450	460	470	480
IKKITQELSD	LQQERERLEK	DLEEAHREKS	KGDCTIRDLR	NEVEKLRNEV	NEREKAMENR	YKSLLSESNK	KLHNQEQVIK
490	500	510	520	530	540	550	560
HLTESTNQKD	VLLQKFNEKD	LEVIQQNCYL	MAAEDLELRS	EGLITEKCSS	QQPPGSKTIF	SKEKKQSSDY	EELIQVLKKE
570	580	590	600	610	620	630	640
QDIYTHLVKS	LQESDSINNL	QAELNKIFAL	RKQLEQDVLS	YQNLRKTLEE	QISEIRRREE	SFSLYSDQTS	YLSICLEENN
650	660	670	680	690	700	710	720
RFQVEHFSQE	ELKKKVSDLI	QLVKELYTDN	QHLKKTIFDL	SCMGFQGNGF	PDRLASTEQT	EEAKKSRLPI	LIKPSRSLGN
730	740	750	760	770	780	790	800
MYRLPATQEV	VTQLQSQILE	LQGELKEFKT	CNKQLHQKLI	LAEAVMEGRP	TPDKTLLNAQ	PPVGAAYQDS	PGEQKGIKTT
810	820	830	840	850	860	870	880
SSVWRDKEMD	SDQQRSYEID	SEICPPDDLA	SLPSCKENPE	DVLSPTSVAT	YLSSKSQPSA	KVSVMGTDQS	ESINTSNETE
890	900	910	920	930	940	950	960
YLKQKIHDLE	TELEGYQNFI	FQLQKHSQCS	EAIITVLCGT	EGAQDGLSKP	KNGSDGEEMT	FSSLHQVRYV	KHVKILGPLA
970	980	990	1000	1010	1020	1030	1040
PEMIDSRVLE	NLKQQLEEQE	YKLQKEQNLN	MQLFSEIHNL	QNKFRDLSPP	RYDSLVQSQA	RELSLQRQQI	KDGHGICVIS
1050	1060	1070	1080	1090	1100	1110	1120
RQHMNTMIKA	FEELLQASDV	DYCVAEGFQE	QLNQCAELLE	KLEKLFLNGK	SVGVEMNTQN	ELMERIEEDN	LTYQHLLPES
1130	1140	1150	1160	1170	1180	1190	1200
PEPSASHALS	DYETSEKSFF	SRDQKQDNET	EKTSVMVNSF	SQDLLMEHIQ	EIRTLRKRLE	ESIKTNEKLR	KQLERQGSEF
1210	1220	1230	1240	1250	1260	1270	1280
VQGSTSIFAS	GSELHSSLTS	EIHFLRKQNQ	ALNAMLIKGS	RDKQKENDKL	RESLSRKTVS	LEHLQREYAS	VKEENERLQK
1290	1300	1310	1320	1330	1340	1350	1360
EGSEKERHNQ	QLIQEVRCSG	QELSRVQEEV	KLRQQLLSQN	DKLLQSLRVE	LKAYEKLDEE	HRRLREASGE	GWKGQDPFRD
1370	1380	1390	1400	1410	1420	1430	1440
LHSLLMEIQA	LRLQLERSIE	TSSTLQSRLK	EQLARGAEKA	QEGALTLAVQ	AVSIPEVPLQ	PDKHDGDKYP	MESDNSFDLF
1450	1460	1470	1480	1490	1500	1510	1520
DSSQAVTPKS	VSETPPLSGN	DTDSLSCDSG	SSATSTPCVS	RLVTGHHLWA	SKNGRHVLGL	IEDYEALLKQ	ISQGQRLLAE
1530	1540	1550	1560	1570	1580	1590	1600
MDIQTQEAPS	STSQELGTKG	PHPAPLSKFV	SSVSTAKLTL	EEAYRRLKLL	WRVSLPEDGQ	CPLHCEQIGE	MKAEVTKLHK
1610	1620	1630	1640	1650	1660	1670	
KLFEQEKKLQ	NTMKLLQLSK	RQEKVIFDQL	VVTHKILRKA	RGNLELRPGG	AHPGTCSPSR	PGS	

Cmpd.	No. of Cmpds.	m/z meas.	Δ m/z [ppm]	z	Rt [min]	Score	P	Range	Sequence	Modification	Ratios
93	1	529.5810	3.65	3	30.1	13.6	1	271-283	K.ERETEAAQMEHQK.E		WD:WU 0.84

Detailed Protein Report

Cmpd.	No. of Cmpds.	m/z meas.	Δ m/z [ppm]	z	Rt [min]	Score	P	Range	Sequence	Modification	Ratios
2305	1	937.4652	-77.24	2	57.1	11.9	1	708-723	R.LPILIKPSRSLGNMYR.L	Oxidation: 14	
268	3	501.1673	-209.52	2	32.1	13.8	1	1391-1399	K.EQLARGAEK.A		WD:WU 0.94
578	1	575.8071	-12.69	2	36.1	15.6	1	1558-1566	K.LTLEEAYRR.L		

Detailed Protein Report

Protein 33: PREDICTED: ceruloplasmin isoform X4 [Homo sapiens]

**Accession:**	gi|578807068	**Score:**	54.0
**Database:**	refseq_human(refseq_human_20140103.fasta)	**MW [kDa]:**	103.1
**Database Date:**	2015-11-30	**pI:**	5.4
**Modification(s):**	Carbamidomethyl	**Sequence Coverage [%]:**	1.6
		**No. of unique Peptides:**	1

10	20	30	40	50	60	70	80
MKILILGIFL	FLCSTPAWAK	EKHYYIGIIE	TTWDYASDHG	EKKLISVDTE	HSNIYLQNGP	DRIGRLYKKA	LYLQYTDETF
**90**	**100**	**110**	**120**	**130**	**140**	**150**	**160**
RTTIEKPVWL	GFLGPIIKAE	TGDKVYVHLK	NLASRPYTFH	SHGITYYKEH	EGAIYPDNTT	DFQRADDKVY	PGEQYTYMLL
**170**	**180**	**190**	**200**	**210**	**220**	**230**	**240**
ATEEQSPGEG	DGNCVTRIYH	SHIDAPKDIA	SGLIGPLIIC	KKDSLDKEKE	KHIDREFVVM	FSVVDENFSW	YLEDNIKTYC
**250**	**260**	**270**	**280**	**290**	**300**	**310**	**320**
SEPEKVDKDN	EDFQESNRMY	SVNGYTFGSL	PGLSMCAEDR	VKWYLFGMGN	EVDVHAAFFH	GQALTNKNYR	IDTINLFPAT
**330**	**340**	**350**	**360**	**370**	**380**	**390**	**400**
LFDAYMVAQN	PGEWMLSCQN	LNHLKAGLQA	FFQVQECNKS	SSKDNIRGKH	VRHYYIAAEE	IIWNYAPSGI	DIFTKENLTA
**410**	**420**	**430**	**440**	**450**	**460**	**470**	**480**
PGSDSAVFFE	QGTTRIGGSY	KKLVYREYTD	ASFTNRKERG	PEEEHLGILG	PVIWAEVGDT	IRVTFHNKGA	YPLSIEPIGV
**490**	**500**	**510**	**520**	**530**	**540**	**550**	**560**
RFNKNNEGTY	YSPNYNPQSR	SVPPSASHVA	PTETFTYEWT	VPKEVGPTNA	DPVCLAKMYY	SAVEPTKDIF	TGLIGPMKIC
**570**	**580**	**590**	**600**	**610**	**620**	**630**	**640**
KKGSLHANGR	QKDVDKEFYL	FPTVFDENES	LLLEDNIRMF	TTAPDQVDKE	DEDFQESNKM	HSMNGFMYGN	QPGLTMCKGD
**650**	**660**	**670**	**680**	**690**	**700**	**710**	**720**
SVVWYLFSAG	NEADVHGIYF	SGNTYLWRGE	RRDTANLFPQ	TSLTLHMWPD	TEGTFNVECL	TTDHYTGGMK	QKYTVNQCRR
**730**	**740**	**750**	**760**	**770**	**780**	**790**	**800**
QSEDSTFYLG	ERTYYIAAVE	VEWDYSPQRE	WEKELHHLQE	QNVSNAFLDK	GEFYIGSKYK	KVVYRQYTDS	TFRVPVERKA
**810**	**820**	**830**	**840**	**850**	**860**	**870**	**880**
EEEHLGILGP	QLHADVGDKV	KIIFKNMATR	PYSIHAHGVQ	TESSTVTPTL	PGETLTYVWK	IPERSGAGTE	DSACIPWAYY
**890**	**900**	**910**					
STVDQVKDLY	CKEGEKKWSR	L					

Cmpd.	No. of Cmpds.	m/z meas.	Δ m/z [ppm]	z	Rt [min]	Score	P	Range	Sequence	Modification
2949	1	735.4039	-26.64	2	65.6	54.0	0	188-201	K.DIASGLIGPLIICK.K	Carbamidomethyl: 13

Detailed Protein Report

Protein 34: cadherin EGF LAG seven-pass G-type receptor 3 precursor [Homo sapiens]

**Accession:**	gi|145309304	**Score:**	53.0
**Database:**	refseq_human(refseq_human_20140103.fasta)	**MW [kDa]:**	358.0
**Database Date:**	2015-11-30	**pI:**	6.2
		**Sequence Coverage [%]:**	1.2
		**No. of unique Peptides:**	3

Detailed Protein Report

10	20	30	40	50	60	70	80
MMARRPPWRG	LGGRSTPILL	LLLLSLFPLS	QEELGGGGHQ	GWDPGLAATT	GPRAHIGGGA	LALCPESSGV	REDGGPGLGV
90	100	110	120	130	140	150	160
REPIFVGLRG	RRQSARNSRG	PPEQPNEELG	IEHGVQPLGS	RERETGQGPG	SVLYWRPEVS	SCGRTGPLQR	GSLSPGALSS
170	180	190	200	210	220	230	240
GVPGSGNSSP	LPSDFLIRHH	GPKPVSSQRN	AGTGSRKRVG	TARCCGELWA	TGSKGQGERA	TTSGAERTAP	RRNCLPGASG
250	260	270	280	290	300	310	320
SGPELDSAPR	TARTAPASGS	APRESRTAPE	PAPKRMRSRG	LFRCRFLPQR	PGPRPPGLPA	RPEARKVTSA	NRARFRRAAN
330	340	350	360	370	380	390	400
RHPQFPQYNY	QTLVPENEAA	GTAVLRVVAQ	DPDAGEAGRL	VYSLAALMNS	RSLELFSIDP	QSGLIRTAAA	LDRESMERHY
410	420	430	440	450	460	470	480
LRVTAQDHGS	PRLSATTMVA	VTVADRNDHS	PVFEQAQYRE	TLRENVEEGY	PILQLRATDG	DAPPNANLRY	RFVGPPAARA
490	500	510	520	530	540	550	560
AAAAAFEIDP	RSGLISTSGR	VDREHMESYE	LVVEASDQGQ	EPGPRSATVR	VHITVLDEND	NAPQFSEKRY	VAQVREDVRP
570	580	590	600	610	620	630	640
HTVVLRVTAT	DRDKDANGLV	HYNIISGNSR	GHFAIDSLTG	EIQVVAPLDF	EAEREYALRI	RAQDAGRPPL	SNNTGLASIQ
650	660	670	680	690	700	710	720
VVDINDHIPI	FVSTPFQVSV	LENAPLGHSV	IHIQAVDADH	GENARLEYSL	TGVAPDTPFV	INSATGWVSV	SGPLDRESVE
730	740	750	760	770	780	790	800
HYFFGVEARD	HGSPPLSASA	SVTVTVLDVN	DNRPEFTMKE	YHLRLNEDAA	VGTSVVSVTA	VDRDANSAIS	YQITGGNTRN
810	820	830	840	850	860	870	880
RFAISTQGGV	GLVTLALPLD	YKQERYFKLV	LTASDRALHD	HCYVHINITD	ANTHRPVFQS	AHYSVSVNED	RPMGSTIVVI
890	900	910	920	930	940	950	960
SASDDDVGEN	ARITYLLEDN	LPQFRIDADS	GAITLQAPLD	YEDQVTYTLA	ITARDNGIPQ	KADTTYVEVM	VNDVNDNAPQ
970	980	990	1000	1010	1020	1030	1040
FVASHYTGLV	SEDAPPFTSV	LQISATDRDA	HANGRVQYTF	QNGEDGDGDF	TIEPTSGIVR	TVRRLDREAV	SVYELTAYAV
1050	1060	1070	1080	1090	1100	1110	1120
DRGVPPLRTP	VSIQVMVQDV	NDNAPVFPAE	EFEVRVKENS	IVGSVVAQIT	AVDPDEGPNA	HIMYQIVEGN	IPELFQMDIF
1130	1140	1150	1160	1170	1180	1190	1200
SGELTALIDL	DYEARQEYVI	VVQATSAPLV	SRATVHVRLV	DQNDNSPVLN	NFQILFNNYV	SNRSDTFPSG	IIGRIPAYDP
1210	1220	1230	1240	1250	1260	1270	1280
DVSDHLFYSF	ERGNELQLLV	VNQTSGELRL	SRKLDNNRPL	VASMLVTVTD	GLHSVTAQCV	LRVVIITEEL	LANSLTVRLE
1290	1300	1310	1320	1330	1340	1350	1360
NMWQERFLSP	LLGRFLEGVA	AVLATPAEDV	FIFNIQNDTD	VGGTVLNVSF	SALAPRGAGA	GAAGPWFSSE	ELQEQLYVRR
1370	1380	1390	1400	1410	1420	1430	1440
AALAARSLLD	VLPFDDNVCL	REPCENYMKC	VSVLRFDSSA	PFLASASTLF	RPIQPIAGLR	CRCPPGFTGD	FCETELDLCY
1450	1460	1470	1480	1490	1500	1510	1520
SNPCRNGGAC	ARREGGYTCV	CRPRFTGEDC	ELDTEAGRCV	PGVCRNGGTC	TDAPNGGFRC	QCPAGGAFEG	PRCEVAARSF
1530	1540	1550	1560	1570	1580	1590	1600
PPSSFVMFRG	LRQRFHLTLS	LSFATVQQSG	LLFYNGRLNE	KHDFLALELV	AGQVRLTYST	GESNTVVSPT	VPGGLSDGQW
1610	1620	1630	1640	1650	1660	1670	1680
HTVHLRYYNK	PRTDALGGAQ	GPSKDKVAVL	SVDDCDVAVA	LQFGAEIGNY	SCAAAGVQTS	SKKSLDLTGP	LLLGGVPNLP
1690	1700	1710	1720	1730	1740	1750	1760
ENFPVSHKDF	IGCMRDLHID	GRRVDMAAFV	ANNGTMAGCQ	AKLHFCDSGP	CKNSGFCSER	WGSFSCDCPV	GFGGKDCQLT
1770	1780	1790	1800	1810	1820	1830	1840
MAHPHHFRGN	GTLSWNFGSD	MAVSVPWYLG	LAFRTRATQG	VLMQVQAGPH	STLLCQLDRG	LLSVTVTRGS	GRASHLLLDQ
1850	1860	1870	1880	1890	1900	1910	1920
VTVSDGRWHD	LRLELQEEPG	GRRGHHVLMV	SLDFSLFQDT	MAVGSELQGL	KVKQLHVGGL	PPGSAEEAPQ	GLVGCIQGVW
1930	1940	1950	1960	1970	1980	1990	2000
LGSTPSGSPA	LLPPSHRVNA	EPGCVVTNAC	ASGPCPPHAD	CRDLWQTFSC	TCQPGYYGPG	CVDACLLNPC	QNQGSCRHLP
2010	2020	2030	2040	2050	2060	2070	2080
GAPHGYTCDC	VGGYFGHHCE	HRMDQQCPRG	WWGSPTCGPC	NCDVHKGFDP	NCNKTNGQCH	CKEFHYRPRG	SDSCLPCDCY
2090	2100	2110	2120	2130	2140	2150	2160
PVGSTSRSCA	PHSGQCPCRP	GALGRQCNSC	DSPFAEVTAS	GCRVLYDACP	KSLRSGVWWP	QTKFGVLATV	PCPRGALGAA
2170	2180	2190	2200	2210	2220	2230	2240

Detailed Protein Report

Cmpd.	No. of Cmpds.	m/z meas.	Δ m/z [ppm]	z	Rt [min]	Score	P	Range	Sequence	Modification
1390	1	942.4814	27.90	2	46.2	11.4	1	232-250	R.RNCLPGASGSGPELDSAPR.T	
2841	1	648.3701	87.56	1	64.1	13.5	0	1513-1518	R.CEVAAR.S	
942	1	470.6329	-182.64	2	40.6	10.3	0	2331-2338	R.MEHPSSPR.G	

Detailed Protein Report

Protein 35: testis-specific serine/threonine-protein kinase 2 [Homo sapiens]

Accession: gi|194294513
Database: refseq_human(refseq_human_20140103.fasta)
Database Date: 2015-11-30
Modification(s): Carbamidomethyl

Score: 52.5
MW [kDa]: 40.9
pI: 9.8
Sequence Coverage [%]: 5.3
No. of unique Peptides: 2

Quantitation

WD:WU **Median:** 0.95 **CV:** 0.00 % **No. of Peptides:** 1

10	20	30	40	50	60	70	80
MDDATVLRKK	GYIVGINLGK	GSYAKVKSAY	SERLKFNVAV	KIIDRKKTPT	DFVERFLPRE	MDILATVNHG	SIIKTYEIFE
90	100	110	120	130	140	150	160
TSDGRIYIIM	ELGVQGDLLE	FIKCQGALHE	DVARKMFRQL	SSAVKYCHDL	DIVHRDLKCE	NLLLDKDFNI	KLSDFGFSKR
170	180	190	200	210	220	230	240
CLRDSNGRII	LSKTFCGSAA	YAAPEVLQSI	PYQPKVYDIW	SLGVILYIMV	CGSMPYDDSD	IRKMLRIQKE	HRVDFPRSKN
250	260	270	280	290	300	310	320
LTCECKDLIY	RMLQPDVSQR	LHIDEILSHS	WLQPPKPKAT	SSASFKREGE	GKYRAECKLD	TKTGLRPDHR	PDHKLGAKTQ
330	340	350	360				
HRLLVVPENE	NRMEDRLAET	SRAKDHHISG	AEVGKAST				

Cmpd.	No. of Cmpds.	m/z meas.	Δ m/z [ppm]	z	Rt [min]	Score	P	Range	Sequence	Modification	Ratios
1388	1	570.1598	-180.58	2	45.7	10.7	1	238-246	R.SKNLTCECK.D	Carbamidomethyl: 6, 8	
174	3	642.2924	-49.65	2	31.4	20.1	2	293-302	K.YRAECKLDTK.T	Carbamidomethyl: 5	WD:WU 0.95

Detailed Protein Report

Protein 36: glycerol-3-phosphate dehydrogenase, mitochondrial precursor [Homo sapiens]

Accession: gi|285002231
Database: refseq_human(refseq_human_20140103.fasta)
Database Date: 2015-11-30
Modification(s): Carbamidomethyl, Oxidation

Score: 51.7
MW [kDa]: 80.8
pI: 8.4
Sequence Coverage [%]: 5.8
No. of unique Peptides: 4

Quantitation

WD:WU **Median:** 0.94 **CV:** 0.00 % **No. of Peptides:** 1

Alias proteins:

Accession	Name	Description
gi|530370176	refseq_human (refseq_human_20140103.fasta)	PREDICTED: glycerol-3-phosphate dehydrogenase, mitochondrial isoform X1 [Homo sapiens]
gi|285002233	refseq_human (refseq_human_20140103.fasta)	glycerol-3-phosphate dehydrogenase, mitochondrial precursor [Homo sapiens]

```
        10         20         30         40         50         60         70         80
MAFQKAVKGT ILVGGGALAT VLGLSQFAHY RRKQMNLAYV KAADCISEPV NREPPSREAQ LLTLQNTSEF DILVIGGGAT
        90        100        110        120        130        140        150        160
GSGCALDAVT RGLKTALVER DDFSSGTSSR STKLIHGGVR YLQKAIMKLD IEQYRMVKEA LHERANLLEI APHLSAPLPI
       170        180        190        200        210        220        230        240
MLPVYKWWQL PYYWVGIKLY DLVAGSNCLK SSYVLSKSRA LEHFPMLQKD KLVGAIVYYD GQHNDARMNL AIALTAARYG
       250        260        270        280        290        300        310        320
AATANYMEVV SLLKKTDPQT GKVRVSGARC KDVLTGQEFD VRAKCVINAT GPFTDSVRKM DDKDAAAICQ PSAGVHIVMP
       330        340        350        360        370        380        390        400
GYYSPESMGL LDPATSDGRV IFFLPWQKMT IAGTTDTPTD VTHHPIPSEE DINFILNEVR NYLSCDVEVR RGDVLAAWSG
       410        420        430        440        450        460        470        480
IRPLVTDPKS ADTQSISRNH VVDISESGLI TIAGGKWTTY RSMAEDTINA AVKTHNLKAG PSRTVGLFLQ GGKDWSPTLY
       490        500        510        520        530        540        550        560
IRLVQDYGLE SEVAQHLAAT YGDKAFEVAK MASVTGKRWP IVGVRLVSEF PYIEAEVKYG IKEYACTAVD MISRRTRLAF
       570        580        590        600        610        620        630        640
LNVQAAEEAL PRIVELMGRE LNWDDYKKQE QLETARKFLY YEMGYKSRSE QLTDRSEISL LPSDIDRYKK RFHKFDADQK
       650        660        670        680        690        700        710        720
GFITIVDVQR VLESINVQMD ENTLHEILNE VDLNKNGQVE LNEFLQLMSA IQKGRVSGSR LAILMKTAEE NLDRRVPIPV
       730
DRSCGGL
```

Cmpd.	No. of Cmpds.	m/z meas.	Δ m/z [ppm]	z	Rt [min]	Score	P	Range	Sequence	Modification	Ratios
810	2	491.6440	-225.52	2	38.5	17.3	0	34-41	K.QMNLAYVK.A	Oxidation: 2	WD:WU 0.94
1028	1	863.9268	-97.49	2	41.6	10.0	2	114-128	K.LIHGGVRYLQKAIMK.L		
2820	1	676.8699	33.04	2	63.9	12.5	0	179-190	K.LYDLVAGSNCLK.S	Carbamidomethyl: 10	
29	1	706.3122	-81.53	3	29.6	11.9	1	179-197	K.LYDLVAGSNCLKSSYVLSK.S	Carbamidomethyl: 10	

Detailed Protein Report

Protein 37: Fanconi anemia group I protein isoform 1 [Homo sapiens]

**Accession:**	gi|164607124	**Score:**	51.0
**Database:**	refseq_human(refseq_human_20140103.fasta)	**MW [kDa]:**	149.2
**Database Date:**	2015-11-30	**pI:**	6.3
		**Sequence Coverage [%]:**	3.2
		**No. of unique Peptides:**	3

10	20	30	40	50	60	70	80
MDQKILSLAA	EKTADKLQEF	LQTLREGDLT	NLLQNQAVKG	KVAGALLRAI	FKGSPCSEEA	GTLRRRKIYT	CCIQLVESGD
90	100	110	120	130	140	150	160
LQKEIASEII	GLLMLEAHHF	PGPLLVELAN	EFISAVREGS	LVNGKSLELL	PIILTALATK	KENLAYGKGV	LSGEECKKQL
170	180	190	200	210	220	230	240
INTLCSGRWD	QQYVIQLTSM	FKDVPLTAEE	VEFVVEKALS	MFSKMNLQEI	PPLVYQLLVL	SSKGSRKSVL	EGIIAFFSAL
250	260	270	280	290	300	310	320
DKQHNEEQSG	DELLDVVTVP	SGELRHVEGT	IILHIVFAIK	LDYELGRELV	KHLKVGQQGD	SNNNLSPFSI	ALLLSVTRIQ
330	340	350	360	370	380	390	400
RFQDQVLDLL	KTSVVKSFKD	LQLLQGSKFL	QNLVPHRSYV	STMILEVVKN	SVHSWDHVTQ	GLVELGFILM	DSYGPKKVLD
410	420	430	440	450	460	470	480
GKTIETSPSL	SRMPNQHACK	LGANILLETF	KIHEMIRQEI	LEQVLNRVVT	RASSPISHFL	DLLSNIVMYA	PLVLQSCSSK
490	500	510	520	530	540	550	560
VTEAFDYLSF	LPLQTVQRLL	KAVQPLLKVS	MSMRDCLILV	LRKAMFANQL	DARKSAVAGF	LLLLKNFKVL	GSLSSSQCSQ
570	580	590	600	610	620	630	640
SLSVSQVHVD	VHSHYNSVAN	ETFCLEIMDS	LRRCLSQQAD	VRLMLYEGFY	DVLRRNSQLA	NSVMQTLLSQ	LKQFYEPKPD
650	660	670	680	690	700	710	720
LLPPLKLEAC	ILTQGDKISL	QEPLDYLLCC	IQHCLAWYKN	TVIPLQQGEE	EEEEEEAFYE	DLDDILESIT	NRMIKSELED
730	740	750	760	770	780	790	800
FELDKSADFS	QSTSIGIKNN	ICAFLVMGVC	EVLIEYNFSI	SSFSKNRFED	ILSLFMCYKK	LSDILNEKAG	KAKTKMANKT
810	820	830	840	850	860	870	880
SDSLLSMKFV	SSLLTALFRD	SIQSHQESLS	VLRSSNEFMR	YAVNVALQKV	QQLKETGHVS	GPDGQNPEKI	FQNLCDITRV
890	900	910	920	930	940	950	960
LLWRYTSIPT	SVEESGKKEK	GKSISLLCLE	GLQKIFSAVQ	QFYQPKIQQF	LRALDVTDKE	GEEREDADVS	VTQRTAFQIR
970	980	990	1000	1010	1020	1030	1040
QFQRSLLNLL	SSQEEDFNSK	EALLLVTVLT	SLSKLLEPSS	PQFVQMLSWT	SKICKENSRE	DALFCKSLMN	LLFSLHVSYK
1050	1060	1070	1080	1090	1100	1110	1120
SPVILLRDLS	QDIHGHLGDI	DQDVEVEKTN	HFAIVNLRTA	APTVCLLVLS	QAEKVLEEVD	WLITKLKGQV	SQETLSEEAS
1130	1140	1150	1160	1170	1180	1190	1200
SQATLPNQPV	EKAIIMQLGT	LLTFFHELVQ	TALPSGSCVD	TLLKDLCKMY	TTLTALVRYY	LQVCQSSGGI	PKNMEKLVKL
1210	1220	1230	1240	1250	1260	1270	1280
SGSHLTPLCY	SFISYVQNKS	KSLNYTGEKK	EKPAAVATAM	ARVLRETKPI	PNLIFAIEQY	EKFLIHLSKK	SKVNLMQHMK
1290	1300	1310	1320	1330			
LSTSRDFKIK	GNILDMVLRE	DGEDENEEGT	ASEHGGQNKE	PAKKKRKK			

Cmpd.	No. of Cmpds.	m/z meas.	Δ m/z [ppm]	z	Rt [min]	Score	P	Range	Sequence	Modification
426	4	658.3901	11.29	2	34.4	22.4	2	780-791	K.KLSDILNEKAGK.A	
1909	2	918.3239	-154.66	2	52.5	13.3	1	945-960	R.EDADVSVTQRTAFQIR.Q	
13	1	814.3623	-84.24	2	29.4	15.2	1	1165-1178	K.DLCKMYTTLTALVR.Y	

Detailed Protein Report

Protein 38: peroxiredoxin-2 [Homo sapiens]

Accession: gi|32189392
Database: refseq_human(refseq_human_20140103.fasta)
Database Date: 2015-11-30

Score: 50.1
MW [kDa]: 21.9
pI: 5.6
Sequence Coverage [%]: 5.6
No. of unique Peptides: 1

Quantitation

WD:WU **Median:** 3.50 **CV:** 0.00 % **No. of Peptides:** 1

```
        10         20         30         40         50         60         70         80
MASGNARIGK PAPDFKATAV VDGAFKEVKL SDYKGKYVVL FFYPLDFTFV CPTEIIAFSN RAEDFRKLGC EVLGVSVDSQ
        90        100        110        120        130        140        150        160
FTHLAWINTP RKEGGLGPLN IPLLADVTRR LSEDYGVLKT DEGIAYRGLF IIDGKGVLRQ ITVNDLPVGR SVDEALRLVQ
       170        180        190        200
AFQYTDEHGE VCPAGWKPGS DTIKPNVDDS KEYFSKHN
```

Cmpd.	No. of Cmpds.	m/z meas.	Δ m/z [ppm]	z	Rt [min]	Score	P	Range	Sequence	Modification	Ratios
766	1	606.3268	-23.04	2	38.4	50.1	0	140-150	R.QITVNDLPVGR.S		WD:WU 3.50

Detailed Protein Report

Protein 39: PREDICTED: serine/threonine-protein kinase 31 isoform X3 [Homo sapiens]

Accession: gi|578813577
Database: refseq_human(refseq_human_20140103.fasta)
Database Date: 2015-11-30
Modification(s): Oxidation

Score: 49.5
MW [kDa]: 95.7
pI: 4.8
Sequence Coverage [%]: 3.8
No. of unique Peptides: 3

Quantitation

WD:WU **Median:** 0.61 **CV:** 0.00 % **No. of Peptides:** 1

10	20	30	40	50	60	70	80
MWVQGHSSRA	SATESVSFSG	IVQMDEDTHY	DKVEDVVGSH	IEDAVTFWAQ	SINRNKDIMK	IGCSLSEVCP	QASSVLGNLD
90	100	110	120	130	140	150	160
PNKIYGGLFS	EDQCWYRCKV	LKIISVEKCL	VRYIDYGNTE	ILNRSDIVEI	PLELQFSSVA	KKYKLWGLHI	PSDQEVTQFD
170	180	190	200	210	220	230	240
QQEKAAAVDL	TNHLEYTLKT	YIDTRMKNLA	AKMEILKEMR	HVDISVRFGK	DLSDAIQVLD	EGCFTTPASL	NGLEIIWAEY
250	260	270	280	290	300	310	320
SLAQENIKTC	EYVSEGNILI	AQRNEMQQKL	YMSVEDFILE	VDESSLNKRL	KTLQDLSVSL	EAVYGQAKEG	ANSDEILKKF
330	340	350	360	370	380	390	400
YDWKCDKREE	FTSVRSETDA	SLHRLVAWFQ	RTLKVFDLSV	EGSLISEDAM	DNIDEILEKT	ESSVCKELEI	ALVDQGDADK
410	420	430	440	450	460	470	480
EIISNTYSQV	LQKIHSEERL	IATVQAKYKD	SIEFKKQLIE	YLNKSPSVDH	LLSIKKTLKS	LKALLRWKLV	EKSNLEESDD
490	500	510	520	530	540	550	560
PDGSQIEKIK	EEITQLRNNV	FQEIYHEREE	YEMLTSLAQK	WFPELPLLHP	EIGLLKYMNS	GGLLTMSLER	DLLDAEPMKE
570	580	590	600	610	620	630	640
LSSKRPLVRS	EVNGQIILLK	GYSVDVDTEA	KVIERAATYH	RAWREAEGDS	GLLPLIFLFL	CKSDPMAYLM	VPYYPRANLN
650	660	670	680	690	700	710	720
AVQANMPLNS	EETLKVMKGV	AQGLHTLHKA	DIIHGSLHQN	NVFALNREQG	IVGDFDFTKS	VSQRASVNMM	VGDLSLMSPE
730	740	750	760	770	780	790	800
LKMGKPASPG	SDLYAYGCLL	LWLSVQNQEF	EINKDGIPKV	DQFHLDDKVK	SLLCSLICYR	SSMTAEQVLN	AECFLMPKEQ
810	820	830	840	850			
SVPNPEKDTE	YTLYKKEEEI	KTENLDKCME	KTRNGEANFD	C			

Cmpd.	No. of Cmpds.	m/z meas.	Δ m/z [ppm]	z	Rt [min]	Score	P	Range	Sequence	Modification	Ratios
452	1	524.1225	-252.65	2	34.6	17.8	0	551-559	R.DLLDAEPMK.E	Oxidation: 8	
181	7	592.2959	31.07	2	31.1	21.2	0	581-591	K.GYSVDVDTEAK.V		WD:WU 0.61
1907	1	734.2871	-85.93	2	52.2	10.5	2	822-833	K.TENLDKCMEKTR.N		

Detailed Protein Report

Protein 40: fibrillin-1 precursor [Homo sapiens]

**Accession:**	gi|281485550	**Score:**	49.3
**Database:**	refseq_human(refseq_human_20140103.fasta)	**MW [kDa]:**	312.1
**Database Date:**	2015-11-30	**pI:**	4.7
**Modification(s):**	Carbamidomethyl	**Sequence Coverage [%]:**	1.4
		**No. of unique Peptides:**	3

Quantitation

WD:WU	**Median:** 0.75	**CV:** 0.00 %	**No. of Peptides:** 1

Detailed Protein Report

10	20	30	40	50	60	70	80
MRRGRLLEIA	LGFTVLLASY	TSHGADANLE	AGNVKETRAS	RAKRRGGGGH	DALKGPNVCG	SRYNAYCCPG	WKTLPGGNQC
90	100	110	120	130	140	150	160
IVPICRHSCG	DGFCSRPNMC	TCPSGQIAPS	CGSRSIQHCN	IRCMNGGSCS	DDHCLCQKGY	IGTHCGQPVC	ESGCLNGGRC
170	180	190	200	210	220	230	240
VAPNRCACTY	GFTGPQCERD	YRTGPCFTVI	SNQMCQGQLS	GIVCTKTLCC	ATVGRAWGHP	CEMCPAQPHP	CRRGFIPNIR
250	260	270	280	290	300	310	320
TGACQDVDEC	QAIPGLCQGG	NCINTVGSFE	CKCPAGHKLN	EVSQKCEDID	ECSTIPGICE	GGECTNTVSS	YFCKCPPGFY
330	340	350	360	370	380	390	400
TSPDGTRCID	VRPGYCYTAL	TNGRCSNQLP	QSITKMQCCC	DAGRCWSPGV	TVAPEMCPIR	ATEDFNKLCS	VPMVIPGRPE
410	420	430	440	450	460	470	480
YPPPPLGPIP	PVLPVPPGFP	PGPQIPVPRP	PVEYLYPSRE	PPRVLPVNVT	DYCQLVRYLC	QNGRCIPTPG	SYRCECNKGF
490	500	510	520	530	540	550	560
QLDLRGECID	VDECEKNPCA	GGECINNQGS	YTCQCRAGYQ	STLTRTECRD	IDECLQNGRI	CNNGRCINTD	GSFHCVCNAG
570	580	590	600	610	620	630	640
FHVTRDGKNC	EDMDECSIRN	MCLNGMCINE	DGSFKCICKP	GFQLASDGRY	CKDINECETP	GICMNGRCVN	TDGSYRCECF
650	660	670	680	690	700	710	720
PGLAVGLDGR	VCVDTHMRST	CYGGYKRGQC	IKPLFGAVTK	SECCCASTEY	AFGEPCQPCP	AQNSAEYQAL	CSSGPGMTSA
730	740	750	760	770	780	790	800
GSDINECALD	PDICPNGICE	NLRGTYKCIC	NSGYEVDSTG	KNCVDINECV	LNSLLCDNGQ	CRNTPGSFVC	TCPKGFIYKP
810	820	830	840	850	860	870	880
DLKTCEDIDE	CESSPCINGV	CKNSPGSFIC	ECSSESTLDP	TKTICIETIK	GTCWQTVIDG	RCEININGAT	LKSQCCSSLG
890	900	910	920	930	940	950	960
AAWGSPCTLC	QVDPICGKGY	SRIKGTQCED	IDECEVFPGV	CKNGLCVNTR	GSFKCQCPSG	MTLDATGRIC	LDIRLETCFL
970	980	990	1000	1010	1020	1030	1040
RYEDEECTLP	IAGRHRMDAC	CCSVGAAWGT	EECEECPMRN	TPEYEELCPR	GPGFATKEIT	NGKPFFKDIN	ECKMIPSLCT
1050	1060	1070	1080	1090	1100	1110	1120
HGKCRNTIGS	FKCRCDSGFA	LDSEERNCTD	IDECRISPDL	CGRGQCVNTP	GDFECKCDEG	YESGFMMMKN	CMDIDECQRD
1130	1140	1150	1160	1170	1180	1190	1200
PLLCRGGVCH	NTEGSYRCEC	PPGHQLSPNI	SACIDINECE	LSAHLCPNGR	CVNLIGKYQC	ACNPGYHSTP	DRLFCVDIDE
1210	1220	1230	1240	1250	1260	1270	1280
CSIMNGGCET	FCTNSEGSYE	CSCQPGFALM	PDQRSCTDID	ECEDNPNICD	GGQCTNIPGE	YRCLCYDGFM	ASEDMKTCVD
1290	1300	1310	1320	1330	1340	1350	1360
VNECDLNPNI	CLSGTCENTK	GSFICHCDMG	YSGKKGKTGC	TDINECEIGA	HNCGKHAVCT	NTAGSFKCSC	SPGWIGDGIK
1370	1380	1390	1400	1410	1420	1430	1440
CTDLDECSNG	THMCSQHADC	KNTMGSYRCL	CKEGYTGDGF	TCTDLDECSE	NLNLCGNGQC	LNAPGGYRCE	CDMGFVPSAD
1450	1460	1470	1480	1490	1500	1510	1520
GKACEDIDEC	SLPNICVFGT	CHNLPGLFRC	ECEIGYELDR	SGGNCTDVNE	CLDPTTCISG	NCVNTPGSYI	CDCPPDFELN
1530	1540	1550	1560	1570	1580	1590	1600
PTRVGCVDTR	SGNCYLDIRP	RGDNGDTACS	NEIGVGVSKA	SCCCSLGKAW	GTPCEMCPAV	NTSEYKILCP	GGEGFRPNPI
1610	1620	1630	1640	1650	1660	1670	1680
TVILEDIDEC	QELPGLCQGG	KCINTFGSFQ	CRCPTGYYLN	EDTRVCDDVN	ECETPGICGP	GTCYNTVGNY	TCICPPDYMQ
1690	1700	1710	1720	1730	1740	1750	1760
VNGGNNCMDM	RRSLCYRNYY	ADNQTCDGEL	LFNMTKKMCC	CSYNIGRAWN	KPCEQCPIPS	TDEFATLCGS	QRPGFVIDIY
1770	1780	1790	1800	1810	1820	1830	1840
TGLPVDIDEC	REIPGVCENG	VCINMVGSFR	CECPVGFFYN	DKLLVCEDID	ECQNGPVCQR	NAECINTAGS	YRCDCKPGYR
1850	1860	1870	1880	1890	1900	1910	1920
FTSTGQCNDR	NECQEIPNIC	SHGQCIDTVG	SFYCLCHTGF	KTNDDQTMCL	DINECERDAC	GNGTCRNTIG	SFNCRCNHGF
1930	1940	1950	1960	1970	1980	1990	2000
ILSHNNDCID	VDECASGNGN	LCRNGQCINT	VGSFQCQCNE	GYEVAPDGRT	CVDINECLLE	PRKCAPGTCQ	NLDGSYRCIC
2010	2020	2030	2040	2050	2060	2070	2080
PPGYSLQNEK	CEDIDECVEE	PEICALGTCS	NTEGSFKCLC	PEGFSLSSSG	RRCQDLRMSY	CYAKFEGGKC	SSPKSRNHSK
2090	2100	2110	2120	2130	2140	2150	2160
QECCCALKGE	GWGDPCELCP	TEPDEAFRQI	CPYGSGIIVG	PDDSAVDMDE	CKEPDVCKHG	QCINTDGSYR	CECPFGYILA
2170	2180	2190	2200	2210	2220	2230	2240

Detailed Protein Report

Cmpd.	No. of Cmpds.	m/z meas.	Δ m/z [ppm]	z	Rt [min]	Score	P	Range	Sequence	Modification	Ratios	
25	1	796.8073	-13.58	2	29.5	12.2	0	166-179	R.CACTYGFTGPQCER.D	Carbamidomethyl: 1	WD:WU	0.75
2800	1	913.4636	99.50	2	63.5	13.3	0	1178-1192	K.YQCACNPGYHSTPDR.L	Carbamidomethyl: 3, 5		
748	6	635.3242	75.75	2	37.8	23.9	1	2482-2492	R.SCKDLDECATK.Q	Carbamidomethyl: 8		

Detailed Protein Report

Protein 41:	PREDICTED: ankyrin repeat domain-containing protein 11 isoform X3 [Homo sapiens]		
**Accession:**	gi|578828957	**Score:**	48.9
**Database:**	refseq_human(refseq_human_20140103.fasta)	**MW [kDa]:**	293.2
**Database Date:**	2015-11-30	**pI:**	6.6
**Modification(s):**	Oxidation	**Sequence Coverage [%]:**	2.1
		**No. of unique Peptides:**	3

Detailed Protein Report

10	20	30	40	50	60	70	80
MPPAQSLLWC	CKAGTLLEAP	WHEMEVPRSK	KKEKQGPERK	RIKKEPVTRK	AGLLFGMGLS	GIRAGYPLSE	RQQVALLMQM
90	100	110	120	130	140	150	160
TAEESANSPV	DTTPKHPSQS	TVCQKGTPNS	ASKTKDKVNK	RNERGETRLH	RAAIRGDARR	IKELISEGAD	VNVKDFAGWT
170	180	190	200	210	220	230	240
ALHEACNRGY	YDVAKQLLAA	GAEVNTKGLD	DDTPLHDAAN	NGHYKVVKLL	LRYGGNPQQS	NRKGETPLKV	ANSPTMVNLL
250	260	270	280	290	300	310	320
LGKGTYTSSE	ESSTESSEEE	DAPSFAPSSS	VDGNNTDSEF	EKGLKHKAKN	PEPQKATAPV	KDEYEFDEDD	EQDRVPPVDD
330	340	350	360	370	380	390	400
KHLLKKDYRK	ETKSNSFISI	PKMEVKSYTK	NNTIAPKKAS	HRILSDTSDE	EDASVTVGTG	EKLRLSAHTI	LPGSKTREPS
410	420	430	440	450	460	470	480
NAKQQKEKNK	VKKKRKKETK	GREVRFGKRS	DKFCSSESES	ESSESGEDDR	DSLGSSGCLK	GSPLVLKDPS	LFSSLSASST
490	500	510	520	530	540	550	560
SSHGSSAAQK	QNPSHTDQHT	KHWRTDNWKT	ISSPAWSEVS	SLSDSTRTRL	TSESDYSSEG	SSVESLKPVR	KRQEHRKRAS
570	580	590	600	610	620	630	640
LSEKKSPFLS	SAEGAVPKLD	KEGKVVKKHK	TKHKHKNKEK	GQCSISQELK	LKSFTYEYED	SKQKSDKAIL	LENDLSTENK
650	660	670	680	690	700	710	720
LKVLKHDRDH	FKKEEKLSKM	KLEEKEWLFK	DEKSLKRIKD	TNKDISRSFR	EEKDRSNKAE	KERSLKEKSP	KEEKLRLYKE
730	740	750	760	770	780	790	800
ERKKKSKDRP	SKLEKKNDLK	EDKISKEKEK	IFKEDKEKLK	KEKVYREDSA	FDEYCNKNQF	LENEDTKFSL	SDDQRDRWFS
810	820	830	840	850	860	870	880
DLSDSSFDFK	GEDSWDSPVT	DYRDMKSDSV	AKLILETVKE	DSKERRRDSR	AREKRDYREP	FFRKKDRDYL	DKNSEKRKEQ
890	900	910	920	930	940	950	960
TEKHKSVPGY	LSEKDKKRRE	SAEAGRDRKD	ALESCKERRD	GRAKPEEAHR	EELKECGCES	GFKDKSDGDF	GKGLEPWERH
970	980	990	1000	1010	1020	1030	1040
HPAREKEKKD	GPDKERKEKT	KPERYKEKSS	DKDKSEKSIL	EKCQKDKEFD	KCFKEKKDTK	EKHKDTHGKD	KERKASLDQG
1050	1060	1070	1080	1090	1100	1110	1120
KEKKEKAFPG	IISEDFSEKK	DDKKGKEKSW	YIADIFTDES	EDDRDSCMGS	GFKMGEASDL	PRTDGLQEKE	EGREAYASDR
1130	1140	1150	1160	1170	1180	1190	1200
HRKSSDKQHP	ERQKDKEPRD	RRKDRGAADA	GRDKKEKVFE	KHKEKKDKES	TEKYKDRKDR	ASVDSTQDKK	NKQKLPEKAE
1210	1220	1230	1240	1250	1260	1270	1280
KKHAAEDKAK	SKHKEKSDKE	HSKERKSSRS	ADAEKSLLEK	LEEEALHEYR	EDSNDKISEV	SSDSFTDRGQ	EPGLTAFLEV
1290	1300	1310	1320	1330	1340	1350	1360
SFTEPPGDDK	PRESACLPEK	LKEKERHRHS	SSSSKKSHDR	ERAKKEKAEK	KEKGEDYKEG	GSRKDSGQYE	KDFLEADAYG
1370	1380	1390	1400	1410	1420	1430	1440
VSYNMKADIE	DELDKTIELF	STEKKDKNDS	EREPSKKIEK	ELKPYGSSAI	NILKEKKKRE	KHREKWRDEK	ERHRDRHADG
1450	1460	1470	1480	1490	1500	1510	1520
LLRHHRDELL	RHHRDEQKPA	TRDKDSPPRV	LKDKSRDEGP	RLGDAKLKEK	FKDGAEKEKG	DPVKMSNGND	KVAPSKDPGK
1530	1540	1550	1560	1570	1580	1590	1600
KDARPREKLL	GDGDLMMTSF	ERMLSQKDLE	IEERHKRHKE	RMKQMEKLRH	RSGDPKLKEK	AKPADDGRKK	GLDIPAKKPP
1610	1620	1630	1640	1650	1660	1670	1680
GLDPPFKDKK	LKESTPIPPA	AENKLHPASG	ADSKDWLAGP	HMKEVLPASP	RPDQSRPTGV	PTPTSVLSCP	SYEEVMHTPR
1690	1700	1710	1720	1730	1740	1750	1760
TPSCSADDYA	DLVFDCADSQ	HSTPVPTAPT	SACSPSFFDR	FSVASSGLSE	NASQAPARPL	STNLYRSVSV	DIRRTPEEEF
1770	1780	1790	1800	1810	1820	1830	1840
SVGDKLFRQQ	SVPAASSYDS	PMPPSMEDRA	PLPPVPAEKF	ACLSPGYYSP	DYGLPSPKVD	ALHCPPAAVV	TVTPSPEGVF
1850	1860	1870	1880	1890	1900	1910	1920
SSLQAKPSPS	PRAELLVPSL	EGALPPDLDT	SEDQQATAAI	IPPEPSYLEP	LDEGPFSAVI	TEEPVEWAHP	SEQALASSLI
1930	1940	1950	1960	1970	1980	1990	2000
GGTSENPVSW	PVGSDLLLKS	PQRFPESPKR	FCPADPLHSA	APGPFSASEA	PYPAPPASPA	PYALPVAEPG	LEDVKDGVDA
2010	2020	2030	2040	2050	2060	2070	2080
VPAAISTSEA	APYAPPSGLE	SFFSNCKSLP	EAPLDVAPEP	ACVAAVAQVE	ALGPLENSFL	DGSRGLSHLG	QVEPVPWADA
2090	2100	2110	2120	2130	2140	2150	2160
FAGPEDDLDL	GPFSLPELPL	QTKDAADGEA	EPVEESLAPP	EEMPPGAPGV	INGGDVSTVV	AEEPPALPPD	QASTRLPAEL
2170	2180	2190	2200	2210	2220	2230	2240

Detailed Protein Report

Cmpd.	No. of Cmpds.	m/z meas.	Δ m/z [ppm]	z	Rt [min]	Score	P	Range	Sequence	Modification
2918	1	1155.0468	-67.97	2	65.2	13.4	2	50-71	R.KAGLLFGMGLSGIRAGYPLSER.Q	Oxidation: 8
462	1	475.6124	-248.29	2	34.3	11.4	1	1227-1235	K.SSRSADAEK.S	
2267	1	937.3359	-108.02	2	56.7	10.6	1	1527-1542	R.EKLLGDGDLMMTSFER.M	Oxidation: 10, 11

Detailed Protein Report

Protein 42: neurogenic locus notch homolog protein 1 preproprotein [Homo sapiens]

**Accession:**	gi|148833508	**Score:**	48.8
**Database:**	refseq_human(refseq_human_20140103.fasta)	**MW [kDa]:**	272.3
**Database Date:**	2015-11-30	**pI:**	4.8
**Modification(s):**	Carbamidomethyl	**Sequence Coverage [%]:**	1.3
		**No. of unique Peptides:**	4

10	20	30	40	50	60	70	80
MPPLLAPLLC	LALLPALAAR	GPRCSQPGET	CLNGGKCEAA	NGTEACVCGG	AFVGPRCQDP	NPCLSTPCKN	AGTCHVVDRR
90	100	110	120	130	140	150	160
GVADYACSCA	LGFSGPLCLT	PLDNACLTNP	CRNGGTCDLL	TLTEYKCRCP	PGWSGKSCQQ	ADPCASNPCA	NGGQCLPFEA
170	180	190	200	210	220	230	240
SYICHCPPSF	HGPTCRQDVN	ECGQKPGLCR	HGGTCHNEVG	SYRCVCRATH	TGPNCERPYV	PCSPSPCQNG	GTCRPTGDVT
250	260	270	280	290	300	310	320
HECACLPGFT	GQNCEENIDD	CPGNNCKNGG	ACVDGVNTYN	CRCPPEWTGQ	YCTEDVDECQ	LMPNACQNGG	TCHNTHGGYN
330	340	350	360	370	380	390	400
CVCVNGWTGE	DCSENIDDCA	SAACFHGATC	HDRVASFYCE	CPHGRTGLLC	HLNDACISNP	CNEGSNCDTN	PVNGKAICTC
410	420	430	440	450	460	470	480
PSGYTGPACS	QDVDECSLGA	NPCEHAGKCI	NTLGSFECQC	LQGYTGPRCE	IDVNECVSNP	CQNDATCLDQ	IGEFQCICMP
490	500	510	520	530	540	550	560
GYEGVHCEVN	TDECASSPCL	HNGRCLDKIN	EFQCECPTGF	TGHLCQYDVD	ECASTPCKNG	AKCLDGPNTY	TCVCTEGYTG
570	580	590	600	610	620	630	640
THCEVDIDEC	DPDPCHYGSC	KDGVATFTCL	CRPGYTGHHC	ETNINECSSQ	PCRHGGTCQD	RDNAYLCFCL	KGTTGPNCEI
650	660	670	680	690	700	710	720
NLDDCASSPC	DSGTCLDKID	GYECACEPGY	TGSMCNINID	ECAGNPCHNG	GTCEDGINGF	TCRCPEGYHD	PTCLSEVNEC
730	740	750	760	770	780	790	800
NSNPCVHGAC	RDSLNGYKCD	CDPGWSGTNC	DINNNECESN	PCVNGGTCKD	MTSGYVCTCR	EGFSGPNCQT	NINECASNPC
810	820	830	840	850	860	870	880
LNQGTCIDDV	AGYKCNCLLP	YTGATCEVVL	APCAPSPCRN	GGECRQSEDY	ESFSCVCPTG	WQGQTCEVDI	NECVLSPCRH
890	900	910	920	930	940	950	960
GASCQNTHGG	YRCHCQAGYS	GRNCETDIDD	CRPNPCHNGG	SCTDGINTAF	CDCLPGFRGT	FCEEDINECA	SDPCRNGANC
970	980	990	1000	1010	1020	1030	1040
TDCVDSYTCT	CPAGFSGIHC	ENNTPDCTES	SCFNGGTCVD	GINSFTCLCP	PGFTGSYCQH	DVNECDSQPC	LHGGTCQDGC
1050	1060	1070	1080	1090	1100	1110	1120
GSYRCTCPQG	YTGPNCQNLV	HWCDSSPCKN	GGKCWQTHTQ	YRCECPSGWT	GLYCDVPSVS	CEVAAQRQGV	DVARLCQHGG
1130	1140	1150	1160	1170	1180	1190	1200
LCVDAGNTHH	CRCQAGYTGS	YCEDLVDECS	PSPCQNGATC	TDYLGGYSCK	CVAGYHGVNC	SEEIDECLSH	PCQNGGTCLD
1210	1220	1230	1240	1250	1260	1270	1280
LPNTYKCSCP	RGTQGVHCEI	NVDDCNPPVD	PVSRSPKCFN	NGTCVDQVGG	YSCTCPPGFV	GERCEGDVNE	CLSNPCDARG
1290	1300	1310	1320	1330	1340	1350	1360
TQNCVQRVND	FHCECRAGHT	GRRCESVING	CKGKPCKNGG	TCAVASNTAR	GFICKCPAGF	EGATCENDAR	TCGSLRCLNG
1370	1380	1390	1400	1410	1420	1430	1440
GTCISGPRSP	TCLCLGPFTG	PECQFPASSP	CLGGNPCYNQ	GTCEPTSESP	FYRCLCPAKF	NGLLCHILDY	SFGGGAGRDI
1450	1460	1470	1480	1490	1500	1510	1520
PPPLIEEACE	LPECQEDAGN	KVCSLQCNNH	ACGWDGGDCS	LNFNDPWKNC	TQSLQCWKYF	SDGHCDSQCN	SAGCLFDGFD
1530	1540	1550	1560	1570	1580	1590	1600
CQRAEGQCNP	LYDQYCKDHF	SDGHCDQGCN	SAECEWDGLD	CAEHVPERLA	AGTLVVVVLM	PPEQLRNSSF	HFLRELSRVL
1610	1620	1630	1640	1650	1660	1670	1680
HTNVVFKRDA	HGQQMIFPYY	GREEELRKHP	IKRAAEGWAA	PDALLGQVKA	SLLPGGSEGG	RRRRELDPMD	VRGSIVYLEI
1690	1700	1710	1720	1730	1740	1750	1760
DNRQCVQASS	QCFQSATDVA	AFLGALASLG	SLNIPYKIEA	VQSETVEPPP	PAQLHFMYVA	AAAFVLLFFV	GCGVLLSRKR
1770	1780	1790	1800	1810	1820	1830	1840
RRQHGQLWFP	EGFKVSEASK	KKRREPLGED	SVGLKPLKNA	SDGALMDDNQ	NEWGDEDLET	KKFRFEEPVV	LPDLDDQTDH
1850	1860	1870	1880	1890	1900	1910	1920
RQWTQQHLDA	ADLRMSAMAP	TPPQGEVDAD	CMDVNVRGPD	GFTPLMIASC	SGGGLETGNS	EEEEDAPAVI	SDFIYQGASL
1930	1940	1950	1960	1970	1980	1990	2000
HNQTDRTGET	ALHLAARYSR	SDAAKRLLEA	SADANIQDNM	GRTPLHAAVS	ADAQGVFQIL	IRNRATDLDA	RMHDGTTPLI
2010	2020	2030	2040	2050	2060	2070	2080
LAARLAVEGM	LEDLINSHAD	VNAVDDLGKS	ALHWAAAVNN	VDAAVVLLKN	GANKDMQNNR	EETPLFLAAR	EGSYETAKVL
2090	2100	2110	2120	2130	2140	2150	2160
LDHFANRDIT	DHMDRLPRDI	AQERMHHDIV	RLLDEYNLVR	SPQLHGAPLG	GTPTLSPPLC	SPNGYLGSLK	PGVQGKKVRK

Detailed Protein Report

Cmpd.	No. of Cmpds.	m/z meas.	Δ m/z [ppm]	z	Rt [min]	Score	P	Range	Sequence	Modification
1878	2	716.0566	111.85	3	52.1	10.3	1	1331-1350	R.GFICKCPAGFEGATCENDAR.T	Carbamidomethyl: 6
1825	2	715.9982	30.20	3	51.5	11.6	1	1331-1350	R.GFICKCPAGFEGATCENDAR.T	Carbamidomethyl: 4
1618	4	697.0380	98.52	3	48.9	15.1	1	1331-1350	R.GFICKCPAGFEGATCENDAR.T	
2039	1	698.2787	-136.84	2	54.1	11.8	0	1992-2004	R.MHDGTTPLILAAR.L	

Detailed Protein Report

Protein 43: PREDICTED: neurobeachin-like protein 1 isoform X8 [Homo sapiens]

**Accession:**	gi|578804777	**Score:**	48.3
**Database:**	refseq_human(refseq_human_20140103.fasta)	**MW [kDa]:**	305.4
**Database Date:**	2015-11-30	**pI:**	6.0
**Modification(s):**	Carbamidomethyl, Oxidation	**Sequence Coverage [%]:**	1.7
		**No. of unique Peptides:**	3

Quantitation

WD:WU	**Median:** 0.18	**CV:** 0.00 %	**No. of Peptides:** 1

Detailed Protein Report

10	20	30	40	50	60	70	80
MASRERLFEL	WMLYCTKKDP	DYLKLWLDTF	VSSYEQFLDV	DFEKLPTRVD	DMPPGISLLP	DNILQVLRIQ	LLQCVQKMAD
90	100	110	120	130	140	150	160
GLEEQQQALS	ILLVKFFIIL	CRNLSNVEEI	GTCSYINYVI	TMTTLYIQQL	KSKKKEKEMA	DQTCIEEFVI	HALAFCESLY
170	180	190	200	210	220	230	240
DPYRNWRHRI	SGRILSTVEK	SRQKYKPASL	TVEFVPFFYQ	CFQESEHLKE	SLKCCLLHLF	GAIVAGGQRN	ALQAISPATM
250	260	270	280	290	300	310	320
EVLMRVLADC	DSWEDGDPEE	VGRKAELTLK	CLTEVVHILL	SSNSDQRQVE	TSTILENYFK	LLNSDHSALP	NQRRSRQWEN
330	340	350	360	370	380	390	400
RFIALQIKML	NTITAMLDCT	DRPVLQAIFL	NSNCFEHLIR	LLQNCKEVFK	ERIGYTHMLE	VLKSLGQPPL	ELLKELMNMA
410	420	430	440	450	460	470	480
VEGDHTSVGI	LGISNVQPLL	LLIQWLPELQ	SHDLQIFISD	WLKRICCINR	QSRTTCVNAN	MGIRIIETLD	LHSSLHQTCA
490	500	510	520	530	540	550	560
ENLIAIHGSL	GSQSVSSEEI	RRLLRLLRVD	ESESVHPYVT	PVTRAILTMA	RKLSLESALQ	YFNLSHSMAG	ISVPPIQKWP
570	580	590	600	610	620	630	640
GSAFSFSAWF	CLDQDQLTLG	IANKGGKRKQ	LYSFFTGSGM	GFEAFITHSG	MLVVAVCTKR	EYATVMLPDH	SFCDSLWHNI
650	660	670	680	690	700	710	720
TVVHMPGKRP	FGQSFVYIYD	NGQQKVSAPL	RFPAMNEPFT	SCCIGSAGQR	TTTPPPSQIP	DPPFSSPITP	HRTSFGGILS
730	740	750	760	770	780	790	800
SASWGGTIEK	SKLITKLISA	GTQDSEWGCP	TSLEGQLGSV	IIFYEPLQPP	QVKALYLAGP	NCLSPWKCQE	SDMADLPGNI
810	820	830	840	850	860	870	880
LLYYTAKACK	NSICLDLSTN	CLHGRLTGNK	VVNWDIKDII	NCIGGLNVLF	PLLEQISHFS	EGQIPEEKNE	STVPESVTPV
890	900	910	920	930	940	950	960
EGDWLVWTST	KASESRLERN	LVATFILIVK	HFIQRHPINQ	GNLIHSHGVA	TLGALLQKVP	STLMDVNVLM	AVQLLIEQVS
970	980	990	1000	1010	1020	1030	1040
LEKNMQLLQQ	MYQYLLFDFR	IWNRGDFPFR	IGHIQYLSTI	IKDSRRVFRK	KYGVQFLLDT	LRIYYGNGCK	YNELSLDDIR
1050	1060	1070	1080	1090	1100	1110	1120
TIRTSLYGLI	KYFLCKGGSH	EEIQSIMGYI	AATNEEEQLF	GILDVLFSLL	RTSPTRGQLF	LLLFEPGNAD	ILYALLLNQK
1130	1140	1150	1160	1170	1180	1190	1200
YSDRLREIIF	KIMEQMLKCT	NVYERSKQHI	RLREVGYSGL	GLLLNEALVN	TSLIKNLTHQ	IINTDPVINF	KDLLSVVYIS
1210	1220	1230	1240	1250	1260	1270	1280
HRAHINVRVA	ICRKVLQILQ	FQPDAAHQIS	QQVGWQDTLV	RLFLKAKFEN	GNTLHKHSRA	VLMKDNDKNM	STEDTKKNSD
1290	1300	1310	1320	1330	1340	1350	1360
EKTDEEKITS	FASANVSSDQ	WSLEDRHSLD	SNTPLFPEDS	SVGELSFKSE	NQEEFWHSNP	SHLSLDLSGI	DSCEMSDSGS
1370	1380	1390	1400	1410	1420	1430	1440
QVPDSLPSTP	SPVESTKSFS	VHSDRESSIT	NDMGFSDDFS	LLESQERCEE	ELLQLLTHIL	NYVMCKGLEK	SDDDTWIERG
1450	1460	1470	1480	1490	1500	1510	1520
QVFSALSKPG	ISSELLRPSD	EIKLTLLQKM	LEWAISENRE	AKTNPVTAEN	AFRLVLIIQD	FLQSEGLVNS	NMWTEKLLED
1530	1540	1550	1560	1570	1580	1590	1600
MMLLFDCLSV	CYSESPVWVK	LSQIQIQLLL	GFIGRGNLQV	CAMASAKLNT	LLQTKVIENQ	DEACYILGKL	EHVLSQSIKE
1610	1620	1630	1640	1650	1660	1670	1680
QTEIYSFLIP	LVRTLVSKIY	ELLFMNLHLP	SLPFTNGSSS	FFEDFQEYCN	SNEWQVYIEK	YIVPYMKQYE	AHTFYDGHEN
1690	1700	1710	1720	1730	1740	1750	1760
MALYWKDCYE	ALMVNMHKRD	REGGESKLKF	QELFVEPFNR	KARQENLRYN	NMLKQLSSQQ	LATLRRWKAI	QLYLTCERGP
1770	1780	1790	1800	1810	1820	1830	1840
WAKRKQNPIH	WKLANVENYS	RMRLKLVPNY	NFKTHEEASA	LRDNLGIQHS	QPSSDTLLLE	VVKQVKVSDM	VEDKLDLPEE
1850	1860	1870	1880	1890	1900	1910	1920
DITARVNVDE	KEEQDQKEKL	VLMEDCELIT	IIDVIPGRLE	ITTQHIYFYD	GSIEKEDGVG	FDFKWPHSQI	REIHLRRYNL
1930	1940	1950	1960	1970	1980	1990	2000
RRSALEIFHV	DQSNYFLNFK	KEVRNKIYSR	LLSLHSPNSY	YGSRSPQELF	KASGLTQKWV	NREISNFDYL	IQINTMAGRT
2010	2020	2030	2040	2050	2060	2070	2080
YNDLAQYPVF	PWILQDYTSE	ELDLNNPAVF	RDLSKPIGVV	NEKNAKAMRE	KYENFEDPMG	TIDKFHYGTH	YSNSAGVMHY
2090	2100	2110	2120	2130	2140	2150	2160
LIRVEPFTTL	HIQLQSGRFD	CADRQFHSIP	ATWQALMDNP	YDVKELIPEF	FYFPEFLENQ	NQFNLGRLQI	SKELVNDVIL
2170	2180	2190	2200	2210	2220	2230	2240

Detailed Protein Report

Cmpd.	No. of Cmpds.	m/z meas.	Δ m/z [ppm]	z	Rt [min]	Score	P	Range	Sequence	Modification	Ratios
1799	1	1045.0496	94.89	2	50.8	15.5	0	672-690	R.FPAMNEPFTSCCIGSAGQR.T	Carbamidomethyl: 12; Oxidation: 4	
2060	1	899.3817	-81.00	2	54.1	19.6	0	713-730	R.TSFGGILSSASWGGTIEK.S		WD:WU 0.18
864	1	471.1407	-120.89	2	39.2	13.3	0	1269-1276	K.NMSTEDTK.K	Oxidation: 2	

Detailed Protein Report

Protein 44: PREDICTED: pleckstrin homology domain-containing family A member 4 isoform X3 [Homo sapiens]

**Accession:**	gi|530416942	**Score:**	47.8
**Database:**	refseq_human(refseq_human_20140103.fasta)	**MW [kDa]:**	65.5
**Database Date:**	2015-11-30	**pI:**	10.7
		**Sequence Coverage [%]:**	9.6
		**No. of unique Peptides:**	4

Quantitation

WD:WU **Median:** 0.98 **CV:** 0.00 % **No. of Peptides:** 1

```
        10         20         30         40         50         60         70         80
MEGSRPRSSL SLASSASTIS SLSSLSPKKP TRAVNKIHAF GKRGNALRRD PNLPVHIRGW LHKQDSSGLR LWKRRWFVLS
        90        100        110        120        130        140        150        160
GHCLFYYKDS REESVLGSVL LPSYNIRPDG PGAPRGRRFT FTAEHPGMRT YVLAADTLED LRGWLRALGR ASRAEGDDYG
       170        180        190        200        210        220        230        240
QPRSPARPQP GEGPGGPGGP PEVSRGEEGR ISESPEVTRL SRGRGRPRLL TPSPTTDLHS GLQMRRARSP DLFTPLSRPP
       250        260        270        280        290        300        310        320
SPLSLPRPRS APARRPPAPS GDTAPPARPH TPLSRIDVRP PLDWGPQRQT LSRPPTPRRG PPSEAGGGKP PRSPQHWSQE
       330        340        350        360        370        380        390        400
PRTQAHSGSP TYLQLPPRPP GTRASMVLLP GPPLESTFHQ SLETDTLLTK LCGQDRLLRR LQEEIDQKQE EKEQLEAALE
       410        420        430        440        450        460        470        480
LTRQQLGQAT REAGAPGRAW GRQRLLQDRL VSVRATLCHL TQERERVWDT YSGLEQELGT LRETLEYLLH LGSPQDRVSA
       490        500        510        520        530        540        550        560
QQQLWMVEDT LAGLGGPQKP PPHTEPDSPS PVLQGEESSE RESLPESLEL SSPRSPETDW GRPPGGDKDL ASPHLGLGSP
       570        580        590        600
RVSRASSPEG RHLPSPQLGT KKYGSITDWP ALRSM
```

Cmpd.	No. of Cmpds.	m/z meas.	Δ m/z [ppm]	z	Rt [min]	Score	P	Range	Sequence	Modification	Ratios
361	1	711.1755	-208.54	2	33.6	11.6	1	151-163	R.ASRAEGDDYGQPR.S		WD:WU 0.98
375	1	603.6767	-228.84	2	33.8	10.1	0	300-312	R.GPPSEAGGGKPPR.S		
20	1	706.3988	42.59	2	29.2	15.0	2	412-424	R.EAGAPGRAWGRQR.L		
1037	1	945.4829	-32.03	2	41.7	11.1	2	565-582	R.ASSPEGRHLPSPQLGTKK.Y		

Detailed Protein Report

Protein 45: protein FAM45A [Homo sapiens]

Accession: gi|46275835
Database: refseq_human(refseq_human_20140103.fasta)
Database Date: 2015-11-30
Modification(s): Carbamidomethyl

Score: 47.4
MW [kDa]: 40.5
pI: 6.2
Sequence Coverage [%]: 10.4
No. of unique Peptides: 4

10	20	30	40	50	60	70	80
MAAAEVADTQ	LMLGVGLIEK	DTNGEVLWVW	CYPSTTATLR	NLLLRKCCLT	DENKLLHPFV	FGQYRRTWFY	ITTIEVPDSS
**90**	**100**	**110**	**120**	**130**	**140**	**150**	**160**
ILKKVTHFSI	VLTAKDFNPE	KYAAFTRILC	RMYLKHGSPV	KMMESYIAVL	TKGICQSEEN	GSFLSKDFDA	RKAYLAGSIK
**170**	**180**	**190**	**200**	**210**	**220**	**230**	**240**
DIVSQFGMET	VILHTALMLK	KRIVVYHPKI	EAVQEFTRTL	PALVWHRQDW	TILHSYVHLN	ADELEALQMC	TGYVAGFVDL
**250**	**260**	**270**	**280**	**290**	**300**	**310**	**320**
EVSNRPDLYD	VFVNLAESEI	TIAPLAKEAM	AMGKLHKEMG	QLIVQSAEDP	EKSESHVIQD	IALKTREIFT	NLAPFSEVSA
**330**	**340**	**350**	**360**				
DGEKRVLNLE	ALKQKRFPPA	TENFLYHLAA	AEQMLKI				

Cmpd.	No. of Cmpds.	m/z meas.	Δ m/z [ppm]	z	Rt [min]	Score	P	Range	Sequence	Modification
2766	1	804.4142	11.70	3	63.0	10.7	2	46-65	R.KCCLTDENKLLHPFVFGQYR.R	
837	1	491.6643	-76.81	2	38.9	11.9	0	47-54	K.CCLTDENK.L	Carbamidomethyl: 1
917	2	491.6624	-80.76	2	40.3	12.6	0	47-54	K.CCLTDENK.L	Carbamidomethyl: 2
2881	2	973.5257	3.04	2	64.8	12.2	1	85-101	K.VTHFSIVLTAKDFNPEK.Y	

Detailed Protein Report

Protein 46: pyruvate kinase PKM isoform d [Homo sapiens]

Accession: gi|332164777
Database: refseq_human(refseq_human_20140103.fasta)
Database Date: 2015-11-30

Score: 46.7
MW [kDa]: 49.9
pI: 9.0
Sequence Coverage [%]: 5.3
No. of unique Peptides: 2

Quantitation

WD:WU **Median:** 0.70 **CV:** 0.00 % **No. of Peptides:** 1

10	20	30	40	50	60	70	80
MSKPHSEAGT	AFIQTQQLHA	AMADTFLEHM	CRLDIDSPPI	TARNTGIICT	IGPASRSVEL	KKGATLKITL	DNAYMEKCDE
90	100	110	120	130	140	150	160
NILWLDYKNI	CKVVEVGSKI	YVDDGLISLQ	VKQKGADFLV	TEVENGGSLG	SKKGVNLPGA	AVDLPAVSEK	DIQDLKFGVE
170	180	190	200	210	220	230	240
QDVDMVFASF	IRKASDVHEV	RKVLGEKGKN	IKIISKIENH	EGVRRFDEIL	EASDGIMVAR	GDLGIEIPAE	KVFLAQKMMI
250	260	270	280	290	300	310	320
GRCNRAGKPV	ICATQMLESM	IKKPRPTRAE	GSDVANAVLD	GADCIMLSGE	TAKGDYPLEA	VRMQHLIARE	AEAAIYHLQL
330	340	350	360	370	380	390	400
FEELRRLAPI	TSDPTEATAV	GAVEASFKCC	SGAIIVLTKS	GRSAHQVARY	RPRAPIIAVT	RNPQTARQAH	LYRGIFPVLC
410	420	430	440	450	460		
KDPVQEAWAE	DVDLRVNFAM	NVGKARGFFK	KGDVVIVLTG	WRPGSGFTNT	MRVVPVP		

Cmpd.	No. of Cmpds.	m/z meas.	Δ m/z [ppm]	z	Rt [min]	Score	P	Range	Sequence	Modification	Ratios
721	1	599.3228	-7.49	2	37.8	35.2	0	33-43	R.LDIDSPPITAR.N		WD:WU 0.70
2063	1	731.7933	-161.16	2	54.4	11.5	0	100-112	K.IYVDDGLISLQVK.Q		

Detailed Protein Report

Protein 47: alpha-1-acid glycoprotein 2 precursor [Homo sapiens]

**Accession:**	gi|4505529	**Score:**	46.2
**Database:**	refseq_human(refseq_human_20140103.fasta)	**MW [kDa]:**	23.6
**Database Date:**	2015-11-30	**pI:**	4.9
		**Sequence Coverage [%]:**	5.0
		**No. of unique Peptides:**	1

Quantitation

WD:WU **Median:** 1.59 **CV:** 0.00 % **No. of Peptides:** 1

10	20	30	40	50	60	70	80
MALSWVLTVL	SLLPLLEAQI	PLCANLVPVP	ITNATLDRIT	GKWFYIASAF	RNEEYNKSVQ	EIQATFFYFT	PNKTEDTIFL
**90**	**100**	**110**	**120**	**130**	**140**	**150**	**160**
REYQTRQNQC	FYNSSYLNVQ	RENGTVSRYE	GGREHVAHLL	FLRDTKTLMF	GSYLDDEKNW	GLSFYADKPE	TTKEQLGEFY
**170**	**180**	**190**	**200**	**210**			
EALDCLCIPR	SDVMYTDWKK	DKCEPLEKQH	EKERKQEEGE	S			

Cmpd.	No. of Cmpds.	m/z meas.	Δ m/z [ppm]	z	Rt [min]	Score	P	Range	Sequence	Modification	Ratios
1023	3	617.8379	-30.01	2	41.5	46.2	0	114-123	R.EHVAHLLFLR.D		WD:WU 1.59

Detailed Protein Report

Protein 48: tetratricopeptide repeat protein 37 [Homo sapiens]

Accession: gi|7662078
Database: refseq_human(refseq_human_20140103.fasta)
Database Date: 2015-11-30
Modification(s): Carbamidomethyl

Score: 45.9
MW [kDa]: 175.4
pI: 8.1
Sequence Coverage [%]: 2.4
No. of unique Peptides: 2

Quantitation

WD:WU **Median:** 0.52 **CV:** 0.00 % **No. of Peptides:** 1

10	20	30	40	50	60	70	80
MSSKEVKTAL	KSARDAIRNK	EYKEALKHCK	TVLKQEKNNY	NAWVFIGVAA	AELEQPDQAQ	SAYKKAAELE	PDQLLAWQGL
90	100	110	120	130	140	150	160
ANLYEKYNHI	NAKDDLPGVY	QKLLDLYESV	DKQKWCDVCK	KLVDLYYQEK	KHLEVARTWH	KLIKTRQEQG	AENEELHQLW
170	180	190	200	210	220	230	240
RKLTQFLAES	TEDQNNETQQ	LLFTAFENAL	GLSDKIPSED	HQVLYRHFIQ	SLSKFPHESA	RLKKACEGMI	NIYPTVQYPL
250	260	270	280	290	300	310	320
EVLCLHLIES	GNLTDEGQQY	CCRLVEMDSK	SGPGLIGLGI	KALQDKKYED	AVRNLTEGLK	ESPVCTSGWY	HLAEAQVKMH
330	340	350	360	370	380	390	400
RPKEAVLSCS	QALKIVDNLG	ASGNSLYQRN	LCLHLKAEAL	IKLSDYDSSE	EAIRTLDQIS	DADNIPGLLV	LKSLAYRNKG
410	420	430	440	450	460	470	480
SFDEAAKIME	DLLSSYPDLA	EVHALEALIH	FTKKDYLQAE	KCFQRALEKD	TEVAEYHYQL	GLTYWFMGEE	TRKDKTKALT
490	500	510	520	530	540	550	560
HFLKAARLDT	YMGKVFCYLG	HYYRDVVGDK	NRARGCYRKA	FELDDTDAES	GAAAVDLSVE	LEDMEMALAI	LTTVTQKASA
570	580	590	600	610	620	630	640
GTAKWAWLRR	GLYYLKAGQH	SQAVADLQAA	LRADPKDFNC	WESLGEAYLS	RGGYTTALKS	FTKASELNPE	SIYSVFKVAA
650	660	670	680	690	700	710	720
IQQILGKYKE	AVAQYQMIIK	KKEDYVPALK	GLGECHLMMA	KAALVDYLDG	KAVDYIEKAL	EYFTCALQHR	ADVSCLWKLA
730	740	750	760	770	780	790	800
GDACTCLYAV	APSKVNVHVL	GVLLGQKEGK	QVLKKNELLH	LGGRCYGRAL	KLMSTSNTWC	DLGINYYRQA	QHLAETGSNM
810	820	830	840	850	860	870	880
NDLKELLEKS	LHCLKKAVRL	DSNNHLYWNA	LGVVACYSGI	GNYALAQHCF	IKSIQSEQIN	AVAWTNLGVL	YLTNENIEQA
890	900	910	920	930	940	950	960
HEAFKMAQSL	DPSYLMCWIG	QALIAEAVGS	YDTMDLFRHT	TELNMHTEGA	LGYAYWVCTT	LQDKSNRETE	LYQYNILQMN
970	980	990	1000	1010	1020	1030	1040
AIPAAQVILN	KYVERIQNYA	PAFTMLGYLN	EHLQLKKEAA	NAYQRAILLL	QTAEDQDTYN	VAIRNYGRLL	CSTGEYDKAI
1050	1060	1070	1080	1090	1100	1110	1120
QAFKSTPLEV	LEDIIGFALA	LFMKGLYKES	SKAYERALSI	VESEQDKAHI	LTALAITEYK	QGKTDVAKTL	LFKCSILKEP
1130	1140	1150	1160	1170	1180	1190	1200
TTESLQALCA	LGLAMQDATL	SKAALNELLK	HIKHKDSNYQ	RCLLTSAIYA	LQGRSVAVQK	QISKAVHSNP	GDPALWSLLS
1210	1220	1230	1240	1250	1260	1270	1280
RVVAQYAQRN	AKGGVVAGNV	AHILDSNHGK	KALLYTAVNQ	LAMGSSSAED	EKNTALKTIQ	KAALLSPGDP	AIWAGLMAAC
1290	1300	1310	1320	1330	1340	1350	1360
HADDKLALVN	NTQPKRIDLY	LALLSAVSAS	IKDEKFFENY	NQSLEKWSLS	QAVTGLIDTG	RISEAETLCT	KNLKSNPDQP
1370	1380	1390	1400	1410	1420	1430	1440
AVILLLRQVQ	CKPLLESQKP	LPDAVLEELQ	KTVMSNSTSV	PAWQWLAHVY	QSQGMMRAAE	MCYRKSLQLA	SQRGSWSGKL
1450	1460	1470	1480	1490	1500	1510	1520
SSLLRLALLA	LKVCMANISN	DHWPSLVQEA	TTEALKLCFC	PLAVLLQALL	QFKRKMGARE	TRRLLERVVY	QPGYPKSIAS
1530	1540	1550	1560	1570			
TARWYLLRHL	YAKDDYELID	VLVNNAKTHG	DTRALELNQR	LSSQ			

Cmpd.	No. of Cmpds.	m/z meas.	Δ m/z [ppm]	z	Rt [min]	Score	P	Range	Sequence	Modification	Ratios
1224	1	533.6546	-167.61	2	44.0	14.5	1	113-120	K.QKWCDVCK.K	Carbamidomethyl: 7	WD:WU 0.52
728	1	705.9560	-25.50	2	37.6	14.3	1	1440-1452	K.LSSLLRLALLALK.V		

Detailed Protein Report

Protein 49: serine/threonine-protein kinase Nek1 isoform 5 [Homo sapiens]

**Accession:**	gi|313661434	**Score:**	45.8
**Database:**	refseq_human(refseq_human_20140103.fasta)	**MW [kDa]:**	138.0
**Database Date:**	2015-11-30	**pI:**	5.4
		**Sequence Coverage [%]:**	5.2
		**No. of unique Peptides:**	1

10	20	30	40	50	60	70	80
MEKYVRLQKI	GEGSFGKAIL	VKSTEDGRQY	VIKEINISRM	SSKEREESRR	EVAVLANMKH	PNIVQYRESF	EENGSLYIVM
90	100	110	120	130	140	150	160
DYCEGGDLFK	RINAQKGVLF	QEDQILDWFV	QICLALKHVH	DRKILHRDIK	SQNIFLTKDG	TVQLGDFGIA	RVLNSTVELA
170	180	190	200	210	220	230	240
RTCIGTPYYL	SPEICENKPY	NNKSDIWALG	CVLYELCTLK	HAFEAGSMKN	LVLKIISGSF	PPVSLHYSYD	LRSLVSQLFK
250	260	270	280	290	300	310	320
RNPRDRPSVN	SILEKGFIAK	RIEKFLSPQL	IAEEFCLKTF	SKFGSQPIPA	KRPASGQNSI	SVMPAQKITK	PAAKYGIPLA
330	340	350	360	370	380	390	400
YKKYGDKKLH	EKKPLQKHKQ	AHQTPEKRVN	TGEERRKISE	EAARKRRLEF	IEKEKKQKDQ	IISLMKAEQM	KRQEKERLER
410	420	430	440	450	460	470	480
INRAREQGWR	NVLSAGGSGE	VKAPFLGSGG	TIAPSSFSSR	GQYEHYHAIF	DQMQQQRAED	NEAKWKREIY	GRGLPERQKG
490	500	510	520	530	540	550	560
QLAVERAKQV	EEFLQRKREA	MQNKARAEGH	MVYLARLRQI	RLQNFNERQQ	IKAKLRGEKK	EANHSEGQEG	SEEADMRRKK
570	580	590	600	610	620	630	640
IESLKAHANA	RAAVLKEQLE	RKRKEAYERE	KKVWEEHLVA	KGVKSSDVSP	PLGQHETGGS	PSKQQMRSVI	SVTSALKEVG
650	660	670	680	690	700	710	720
VDSSLTDTRE	TSEEMQKTNN	AISSKREILR	RLNENLKAQE	DEKGKQNLSD	TFEINVHEDA	KEHEKEKSVS	SDRKKWEAGG
730	740	750	760	770	780	790	800
QLVIPLDELT	LDTSFSTTER	HTVGEVIKLG	PNGSPRRAWG	KSPTDSVLKI	LGEAELQLQT	ELLENTTIRS	EISPEGEKYK
810	820	830	840	850	860	870	880
PLITGEKKVQ	CISHEINPSA	IVDSPVETKS	PEFSEASPQM	SLKLEGNLEE	PDDLETEILQ	EPSGTNKDES	LPCTITDVWI
890	900	910	920	930	940	950	960
SEEKETKETQ	SADRITIQEN	EVSEDGVSST	VDQLSDIHIE	PGTNDSQHSK	CDVDKSVQPE	PFFHKVVHSE	HLNLVPQVQS
970	980	990	1000	1010	1020	1030	1040
VQCSPEESFA	FRSHSHLPPK	NKNKNSLLIG	LSTGLFDANN	PKMLRTCSLP	DLSKLFRTLM	DVPTVGDVRQ	DNLEIDEIED
1050	1060	1070	1080	1090	1100	1110	1120
ENIKEGPSDS	EDIVFEETDT	DLQELQASME	QLLREQPGEE	YSEEEESVLK	NSDVEPTANG	TDVADEDDNP	SSESALNEEW
1130	1140	1150	1160	1170	1180	1190	1200
HSDNSDGEIA	SECECDSVFN	HLEELRLHLE	QEMGFEKFFE	VYEKIKAIHE	DEDENIEICS	KIVQNILGNE	HQHLYAKILH
1210	1220						
LVMADGAYQE	DNDE						

Cmpd.	No. of Cmpds.	m/z meas.	Δ m/z [ppm]	z	Rt [min]	Score	P	Range	Sequence	Modification
894	1	946.4617	-25.43	3	40.0	16.1	1	411-440	R.NVLSAGGSGEVKAPFLGSGGTIAPSSFSSR.G	

Detailed Protein Report

Protein 50: **transitional endoplasmic reticulum ATPase [Homo sapiens]**

**Accession:**	gi|6005942	**Score:**	45.4
**Database:**	refseq_human(refseq_human_20140103.fasta)	**MW [kDa]:**	89.3
**Database Date:**	2015-11-30	**pI:**	5.0
**Modification(s):**	Carbamidomethyl	**Sequence Coverage [%]:**	3.8
		**No. of unique Peptides:**	2

```
        10         20         30         40         50         60         70         80
MASGADSKGD DLSTAILKQK NRPNRLIVDE AINEDNSVVS LSQPKMDELQ LFRGDTVLLK GKKRREAVCI VLSDDTCSDE
        90        100        110        120        130        140        150        160
KIRMNRVVRN NLRVRLGDVI SIQPCPDVKY GKRIHVLPID DTVEGITGNL FEVYLKPYFL EAYRPIRKGD IFLVRGGMRA
       170        180        190        200        210        220        230        240
VEFKVVETDP SPYCIVAPDT VIHCEGEPIK REDEEESLNE VGYDDIGGCR KQLAQIKEMV ELPLRHPALF KAIGVKPPRG
       250        260        270        280        290        300        310        320
ILLYGPPGTG KTLIARAVAN ETGAFFFLIN GPEIMSKLAG ESESNLRKAF EEAEKNAPAI IFIDELDAIA PKREKTHGEV
       330        340        350        360        370        380        390        400
ERRIVSQLLT LMDGLKQRAH VIVMAATNRP NSIDPALRRF GRFDREVDIG IPDATGRLEI LQIHTKNMKL ADDVDLEQVA
       410        420        430        440        450        460        470        480
NETHGHVGAD LAALCSEAAL QAIRKKMDLI DLEDETIDAE VMNSLAVTMD DFRWALSQSN PSALRETVVE VPQVTWEDIG
       490        500        510        520        530        540        550        560
GLEDVKRELQ ELVQYPVEHP DKFLKFGMTP SKGVLFYGPP GCGKTLLAKA IANECQANFI SIKGPELLTM WFGESEANVR
       570        580        590        600        610        620        630        640
EIFDKARQAA PCVLFFDELD SIAKARGGNI GDGGGAADRV INQILTEMDG MSTKKNVFII GATNRPDIID PAILRPGRLD
       650        660        670        680        690        700        710        720
QLIYIPLPDE KSRVAILKAN LRKSPVAKDV DLEFLAKMTN GFSGADLTEI CQRACKLAIR ESIESEIRRE RERQTNPSAM
       730        740        750        760        770        780        790        800
EVEEDDPVPE IRRDHFEEAM RFARRSVSDN DIRKYEMFAQ TLQQSRGFGS FRFPSGNQGG AGPSQGSGGG TGGSVYTEDN
       810
DDDLYG
```

Cmpd.	No. of Cmpds.	m/z meas.	Δ m/z [ppm]	z	Rt [min]	Score	P	Range	Sequence	Modification
2051	4	1051.4305	-44.06	2	54.3	14.5	1	678-696	K.MTNGFSGADLTEICQRACK.L	Carbamidomethyl: 18
1970	4	1051.4242	-50.05	2	53.3	20.7	1	678-696	K.MTNGFSGADLTEICQRACK.L	Carbamidomethyl: 14

Detailed Protein Report

Protein 51: PREDICTED: zinc finger protein 665 isoform X5 [Homo sapiens]

**Accession:**	gi|578834915	**Score:**	45.3
**Database:**	refseq_human(refseq_human_20140103.fasta)	**MW [kDa]:**	76.3
**Database Date:**	2015-11-30	**pI:**	10.3
**Modification(s):**	Carbamidomethyl, Oxidation	**Sequence Coverage [%]:**	6.9
		**No. of unique Peptides:**	3

10	20	30	40	50	60	70	80
MVCPCSGLLL	AVGGMPPRCQ	GRTLEEEEEE	GKGVRDGSSS	DISCKCVNTD	LPPKGKNNMG	EAFYTVKLER	LESCDTVGLS
90	100	110	120	130	140	150	160
FQEVQKNTYD	FECQWKDDEG	NYKTVLMLQK	ENLPGRRAQR	DRRAAGNRHI	ENQLGVSFQS	HLPELQQFQH	EGKIYEYNQV
170	180	190	200	210	220	230	240
EKSPNNRGKH	YKCDECGKVF	SQNSRLTSHK	RIHTGEKPYQ	CNKCGKAFTV	RSNLTIHQVI	HTGEKPYKCN	ECGKVFSQPS
250	260	270	280	290	300	310	320
NLAGHQRIHT	GEKPYKCNEC	GKAFRAHSKL	TTHQVIHTGE	KPYKCKECGK	CFTQNSHLAS	HRRIHTGEKP	YKCNECGKAF
330	340	350	360	370	380	390	400
SVRSSLTTHQ	TIHTGEKPYK	CNECGKVFRH	NSYLAKHRRI	HTGEKPYKCN	ECGKAFSMHS	NLTKHQIIHT	GEKPFKCNEC
410	420	430	440	450	460	470	480
VKVFTQYSHL	ANHRRIHTGE	KPYRCDECGK	AFSVRSSLTT	HQAIHTGEKP	YKCNDCGKVF	TQNSHLASHR	GIHSGEKPYK
490	500	510	520	530	540	550	560
CDECGKAFSQ	TSQLARHWRV	HTGEKPYKCN	ECGKAFSVHS	SLTIHQTIHT	GQKPYKCNDC	GKVFRHNSYL	AIHQRIHTGE
570	580	590	600	610	620	630	640
KPYKCNECGK	AFSVHSNLAT	HQVIHTGEKP	YKCNECGKVF	TQNSHLANHR	RIHTGEKPYR	CNECGKAFSV	RSTLTTHMAV
650	660	670	680				
HTGDKPYKCN	QCGKVFTQNS	NLAKHRRIHS	G				

Cmpd.	No. of Cmpds.	m/z meas.	Δ m/z [ppm]	z	Rt [min]	Score	P	Range	Sequence	Modification
2293	1	937.4457	-16.78	2	57.0	19.3	0	1-18	-.MVCPCSGLLLAVGGMPPR.C	Carbamidomethyl: 3; Oxidation: 15
1877	2	1057.5274	21.10	3	52.1	10.3	2	453-480	K.CNDCGKVFTQNSHLASHRGIHSGEKPYK.C	Carbamidomethyl: 4
1830	2	1057.5034	-1.56	3	51.5	15.7	2	453-480	K.CNDCGKVFTQNSHLASHRGIHSGEKPYK.C	Carbamidomethyl: 1

Detailed Protein Report

Protein 52: **PREDICTED: pyridine nucleotide-disulfide oxidoreductase domain-containing protein 2 isoform X1 [Homo sapiens]**

Accession: gi|578819959
Database: refseq_human(refseq_human_20140103.fasta)
Database Date: 2015-11-30
Modification(s): Oxidation

Score: 45.1
MW [kDa]: 61.0
pI: 6.3
Sequence Coverage [%]: 8.6
No. of unique Peptides: 3

10	20	30	40	50	60	70	80
MAASGRGLCK	AVAASPFPAW	RRDNTEARGG	LKPEYDAVVI	GAGHNGLVAA	AYLQRLGVNT	AVFERRHVIG	GAAVTEEIIP
**90**	**100**	**110**	**120**	**130**	**140**	**150**	**160**
GFKFSRASYL	LSLLRPQIYT	DLELKKHGLR	LHLRNPYSFT	PMLEEGAGSK	VPRCLLLGTD	MAENQKQIAQ	FSQKDAQVFP
**170**	**180**	**190**	**200**	**210**	**220**	**230**	**240**
KYEEFMHRLA	LAIDPLLDAA	PVDMAAFQHG	SLLQRMRSLS	TLKPLLKAGR	ILGAQLPRYY	EVLTAPITKV	LDQWFESEPL
**250**	**260**	**270**	**280**	**290**	**300**	**310**	**320**
KATLATDAVI	GAMTSPHTPG	SGYVLLHHVM	GGLEGMQGAW	GYVQGGMGAL	SDAIASSATT	HGASIFTEKT	VAKVQVNSEG
**330**	**340**	**350**	**360**	**370**	**380**	**390**	**400**
CVQGVVLEDG	TEVRSKMVLS	NTSPQITFLK	LTPQEWLPEE	FLERISQLDT	RSPVTKINVA	VDRLPSFLAA	PNAPRGQPLP
**410**	**420**	**430**	**440**	**450**	**460**	**470**	**480**
HHQCSIHLNC	EDTLLLHQAF	EDAMDGLPSH	RPVIELCIPS	SLDPTLAPPG	CHVVSLFTQY	MPYTLAGGKA	WDEQERDAYA
**490**	**500**	**510**	**520**	**530**	**540**	**550**	**560**
DRVFDCIEVY	APGFKDSVVG	RDILTPPDLE	RIFGLPGGNI	FHCAMSLDQL	YFARPVPLHS	GYRCPLQGLY	LCGSGAHPGF
**570**							

Cmpd.	No. of Cmpds.	m/z meas.	Δ m/z [ppm]	z	Rt [min]	Score	P	Range	Sequence	Modification
2627	1	869.6010	138.74	2	61.1	14.0	0	67-83	R.HVIGGAAVTEEIIPGFK.F	
2482	1	836.1552	175.38	2	59.3	13.8	2	196-210	R.MRSLSTLKPLLKAGR.I	
2198	1	905.3670	-138.71	2	56.0	17.3	1	335-350	R.SKMVLSNTSPQITFLK.L	Oxidation: 3

Detailed Protein Report

Protein 53: PREDICTED: rootletin-like [Homo sapiens]

**Accession:**	gi|578805488	**Score:**	44.8
**Database:**	refseq_human(refseq_human_20140103.fasta)	**MW [kDa]:**	147.5
**Database Date:**	2015-11-30	**pI:**	5.5
		**Sequence Coverage [%]:**	4.9
		**No. of unique Peptides:**	2

10	20	30	40	50	60	70	80
MRAAIERRWR	REQELCLQLK	SSQALVASLQ	EQLSESRREL	WAAQKLQQER	AREQAREREA	LRGQLEAQRL	EVQQCRASCK
90	100	110	120	130	140	150	160
LLGREKAALE	MVVEELKGKA	DAADAEKQGL	EAEAAELQRS	LLLQAERREE	LALRRERSCR	ALETSQGRLQ	QLEEKVSGLR
170	180	190	200	210	220	230	240
EELASVREAL	STAQLQRDVV	ESEREGLRSA	LARAECSNAD	LELLVRRLKS	EGVEQRDSLA	AMAALMEGLA	QDKSALNHLA
250	260	270	280	290	300	310	320
LQLEQERDQL	REQRKTLEQE	RARAGEQLAQ	AEQQLALERA	ERRGLQQACG	RLEQRQEQLE	GQAALLGREK	AQLQEQVGQV
330	340	350	360	370	380	390	400
TCQKQALEEQ	LAQSLQDQEA	QMGTLQQALQ	GKDALSEERA	QLLAKQEALE	RQGRLAAEEA	ADLRVERDSL	ESSLLEAQQL
410	420	430	440	450	460	470	480
ATKLQEQLEE	EARSAGLARQ	ALQVEMEQLQ	SDWEVQEMKL	RQDTVRLQRQ	VAQQEREAQR	ALESQALAHR	EALAQLQREK
490	500	510	520	530	540	550	560
ETLSLTLAEE	KEVARCQLEQ	EKELVTKSAA	EREALKGEIQ	SLKQERDESL	LQLEHKMQQA	LSLKETERSL	LSEELSRARR
570	580	590	600	610	620	630	640
TLERVQQEAQ	SQQEQAQATI	SATTEELKAL	QAQFEDAITA	HQRETTALRE	SLQDLAAERG	DVEREVRGRA	GGLLGMPEDR
650	660	670	680	690	700	710	720
RKQGRRKVSF	QQISKSGPVS	DHTGKQNEIA	KLGAAGGGGG	GGVERAERLR	AQLTVAQEGL	AALRQELQGV	EESREGLHRE
730	740	750	760	770	780	790	800
AQEARRALSD	EAREKDVLLL	FNSELRATIC	RAEQEKASFK	RSKEEKEQKL	LILEEAQAAL	QQEASALRAH	LWELEQAGGD
810	820	830	840	850	860	870	880
ARQELRELHR	QVLGLQRKLA	EVEAAGEAHG	QRLQEHLRES	RGAEQTLRAE	LHSVTRKLQE	ASGVADALQA	RLDQACHRIH
890	900	910	920	930	940	950	960
SLEQELAQAE	GARQDAEAQL	GRLCSTLRRG	LGLQRQSPWA	SPEQPGSPTK	GSDSSQALPG	QQGTSPPARP	HSPLRWPSPT
970	980	990	1000	1010	1020	1030	1040
PGGRSSELMD	VATVQDILRD	FVQKLREAQR	ERDDSRIQMA	TLSSRLSEAE	CRCARAQSRV	GQLQKALAEA	EEGQRRVEGA
1050	1060	1070	1080	1090	1100	1110	1120
LSSARAARAL	QKEALRRLEL	EHLASVRAAG	QEKRRLQEQL	ETLRQALEES	RRHSQGLAKQ	GKLLEEQLTN	LEHRCQKAEV
1130	1140	1150	1160	1170	1180	1190	1200
SLEPLRQVLC	RPQRLSGGQE	AAEAQAERRV	LQEQTAALRT	ERARLQGELA	ALRAQLAQME	QETLKREEDV	ARLGAEKEQL
1210	1220	1230	1240	1250	1260	1270	1280
DQSLNSLHQE	VDGALRQNQQ	LQAQMTEMEQ	AHTQRLQDLT	AQHQRDLATE	AERLHGARPQ	ATQALESQEW	THQQQVKVLE
1290	1300	1310	1320				
EQVASLKEQL	DQEVQWRQQA	HLGQAFQTGQ					

Cmpd.	No. of Cmpds.	m/z meas.	Δ m/z [ppm]	z	Rt [min]	Score	P	Range	Sequence	Modification
2904	4	973.5046	-21.62	2	65.1	13.6	2	479-495	R.EKETLSLTLAEEKEVAR.C	
1510	1	899.4718	8.92	2	47.2	10.7	1	666-685	K.QNEIAKLGAAGGGGGGGVER.A	

Detailed Protein Report

Protein 54: PREDICTED: nesprin-2 isoform X6 [Homo sapiens]

**Accession:**	gi|530403362	**Score:**	44.8
**Database:**	refseq_human(refseq_human_20140103.fasta)	**MW [kDa]:**	795.8
**Database Date:**	2015-11-30	**pI:**	5.1
		**Sequence Coverage [%]:**	0.5
		**No. of unique Peptides:**	2

10	20	30	40	50	60	70	80
MASSPELPTE	DEQGSWGIDD	LHISLQAEQE	DTQKKAFTCW	INSQLARHTS	PSVISDLFTD	IKKGHVLLDL	LEVLSGQQLP
90	100	110	120	130	140	150	160
RDKGSNTFQC	RINIEHALTF	LRNRSIKLIN	IHVTDIIDGN	PSIILGLIWT	IILHFHIEKL	AQTLSCNYNQ	PSLDDVSVVD
170	180	190	200	210	220	230	240
SSPASSPPAK	KCSKVQARWQ	MSARKALLLW	AQEQCATYES	VNVTDFKSSW	RNGMAFLAII	HALRPDLIDM	KSVKHRSNKD
250	260	270	280	290	300	310	320
NLREAFRIAE	QELKIPRLLE	PEDVDVVDPD	EKSIMTYVAQ	FLQYSKDAPG	TGEEAQGKVK	DAMGWLTLQK	EKLQKLLKDS
330	340	350	360	370	380	390	400
ENDTYFKKYN	SLLSFMESFN	EEKKSFLDVL	SIKRDLDELD	KDHLQLREAW	DGLDHQINAW	KIKLNYALPP	PLHQTEAWLQ
410	420	430	440	450	460	470	480
EVEELMDEDL	SASQDHSQAV	TLIQEKMTLF	KSLMDRFEHH	SNILLTFENK	DENHLPLVPP	NKLEEMKRRI	NNILEKKFIL
490	500	510	520	530	540	550	560
LLEFHYYKCL	VLGLVDEVKS	KLDIWNIKYG	SRESVELLLE	DWHKFIEEKE	FLARLDTSFQ	KCGEIYKNLA	GECQNINKQY
570	580	590	600	610	620	630	640
MMVKSDVCMY	RKNIYNVKST	LQKVLACWAT	YVENLRLLRA	CFEETKKEEI	KEVPFETLAQ	WNLEHATLNE	AGNFLVEVSN
650	660	670	680	690	700	710	720
DVVGSSISKE	LRRLNKRWRK	LVSKTQLEMN	LPLMIKKQDQ	PTFDNSGNIL	SKEEKATVEF	STDMSVELPE	NYNQNIKAGE
730	740	750	760	770	780	790	800
KHEKENEEFT	GQLKVAKDVE	KLIGQVEIWE	AEAKSVLDQD	DVDTSMEESL	KHLIAKGSMF	DELMARSEDM	LQMDIQNISS
810	820	830	840	850	860	870	880
QESFQHVLTT	GLQAKIQEAK	EKVQINVVKL	IAALKNLTDV	SPDLDIRLKM	EESQKELESY	MMRAQQLLGQ	RESPGELISK
890	900	910	920	930	940	950	960
HKEALIISNT	KSLAKYLKAV	EELKNNVTED	IKMSLEEKSR	DVCAKWESLH	HELSLYVQQL	KIDIEKGKLS	DNILKLEKQI
970	980	990	1000	1010	1020	1030	1040
NKEKKLIRRG	RTKGLIKEHE	ACFSEEGCLY	QLNHHMEVLR	ELCEELPSQK	SQQEVKRLLK	DYEQKIERLL	KCASEIHMTL
1050	1060	1070	1080	1090	1100	1110	1120
QPTAGGTSKN	EGTITTSENR	GGDPHSEAPF	AKSDNQPSTE	KAMEPTMKFS	LASVLRPLQE	ESIMEKDYSA	SINSLLERYD
1130	1140	1150	1160	1170	1180	1190	1200
TYRDILEHHL	QNNKFRITSD	FSSEEDRSSS	CLQAKLTDLQ	VIKNETDARW	KEFEIISLKL	ENHVNDIKKP	FVIKERDTLK
1210	1220	1230	1240	1250	1260	1270	1280
ERERELQMTL	NTRMESLETA	LRLVLPVEKA	SLLLCGSDLP	LHKMAIQGFH	LIDADRIYQH	LRNIQDSIAK	QIEICNRLEE
1290	1300	1310	1320	1330	1340	1350	1360
PGNFVLKELH	PFDLHAMQNI	ILKYKTQFEG	MNHRVQRSED	TLKALEDFLA	SLRTAKLSAE	PVTDLSASDT	QVAQENTLTV
1370	1380	1390	1400	1410	1420	1430	1440
KNKEGEIHLM	KDKAKHLDKC	LKMLDMSFKD	AERGDDTSCE	NLLDAFSIKL	SETHGYGVQE	EFTEENKLLE	ACIFKNNELL
1450	1460	1470	1480	1490	1500	1510	1520
KNIQDVQSQI	SKIGLKDPTV	PAVKHRKKSL	IRLDKVLDEY	EEEKRHLQEM	ANSLPHFKDG	REKTVNQQCQ	NTVVLWENTK
1530	1540	1550	1560	1570	1580	1590	1600
ALVTECLEQC	GRVLELLKQY	QNFKSILTTL	IQKEESVISL	QASYMGKENL	KKRIAEIEIV	KEEFNEHLEV	VDKINQVCKN
1610	1620	1630	1640	1650	1660	1670	1680
LQFYLNKMKT	FEEPPFEKEA	NIIVDRWLDI	NEKTEDYYEN	LGRALALWDK	LFNLKNVIDE	WTEKALQKME	LHQLTEEDRE
1690	1700	1710	1720	1730	1740	1750	1760
RLKEELQVHE	QKTSEFSRRV	AEIQFLLQSS	EIPLELQVME	SSILNKMEHV	QKCLTGESNC	HALSGSTAEL	REDLDQAKTQ
1770	1780	1790	1800	1810	1820	1830	1840
IGMTESLLKA	LSPSDSLEIF	TKLEEIQQQI	LQQKHSMILL	ENQIGCLTPE	LSELKKQYES	VSDLFNTKKS	VLQDHFSKLL
1850	1860	1870	1880	1890	1900	1910	1920
NDQCKNFNDW	FSNIKVNLKE	CFESSETKKS	VEQKLQKLSD	FLTLEGRNSK	IKQVDSVLKH	VKKHLPKAHV	KELISWLVGQ
1930	1940	1950	1960	1970	1980	1990	2000
EFELEKMESI	CQARAKELED	SLQQLLRLQD	DHRNLRKWLT	NQEEKWKGME	EPGEKTELFC	QALARKREQF	ESVAQLNNSL
2010	2020	2030	2040	2050	2060	2070	2080
KEYGFTEEEE	IIMEATCLMD	RYQTLLRQLS	EIEEEDKLLP	TEDQSFNDLA	HDVIHWIKEI	KESLMVLNSS	EGKMPLEERI
2090	2100	2110	2120	2130	2140	2150	2160
QKIKEIILLK	PEGDARIETI	MKQAESSEAP	LVQKTLTDIS	NQWDNTLHLA	STYLSHQEKL	LLEGEKYLQS	KEDLRLMLIE
2170	2180	2190	2200	2210	2220	2230	2240
LKKKQEAGFA	LQHGLQEKKA	QLKIYKKFLK	KAQDLTSLLK	ELKSQGNYLL	ECTKNPSFSE	EPWLEIKHLH	ESLLQQLQDS
2250	2260	2270	2280	2290	2300	2310	2320
VQNLDGHVRE	HDSYQVCVTD	LNTTLDNFSK	EFVSFSDKPV	DQIAVEEKLQ	KLQELENRLS	LQDGTLKKIL	ALAKSVKQNT
2330	2340	2350	2360	2370	2380	2390	2400

Detailed Protein Report

Cmpd.	No. of Cmpds.	m/z meas.	Δ m/z [ppm]	z	Rt [min]	Score	P	Range	Sequence	Modification
2961	1	927.4882	-20.92	2	65.8	15.8	1	864-880	R.AQQLLGQRESPGELISK.H	
1911	1	568.7711	-62.35	2	52.2	11.0	1	4627-4636	K.SLKAGLDYNR.S	

Detailed Protein Report

Protein 55: **PREDICTED: chromodomain-helicase-DNA-binding protein 4 isoform X6 [Homo sapiens]**

**Accession:**	gi|578822562	**Score:**	44.0
**Database:**	refseq_human(refseq_human_20140103.fasta)	**MW [kDa]:**	216.7
**Database Date:**	2015-11-30	**pI:**	5.7
**Modification(s):**	Carbamidomethyl	**Sequence Coverage [%]:**	1.5
		**No. of unique Peptides:**	2

Detailed Protein Report

10	20	30	40	50	60	70	80
MASGLGSPSP	CSAGSEEEDM	DALLNNSLPP	PHPENEEDPE	EDLSETETPK	LKKKKKPKKP	RDPKIPKSKR	QKKERMLLCR
90	100	110	120	130	140	150	160
QLGDSSGEGP	EFVEEEEEVA	LRSDSEGSDY	TPGKKKKKKL	GPKKEKKSKS	KRKEEEEEED	DDDDSKEPKS	SAQLLEDWGM
170	180	190	200	210	220	230	240
EDIDHVFSEE	DYRTLTNYKA	FSQFVRPLIA	AKNPKIAVSK	MMMVLGAKWR	EFSTNNPFKG	SSGASVAAAA	AAAVAVVESM
250	260	270	280	290	300	310	320
VTATEVAPPP	PPVEVPIRKA	KTKEGKGPNA	RRKPKGSPRV	PDAKKPKPKK	VAPLKIKLGG	FGSKRKRSSS	EDDDLDVESD
330	340	350	360	370	380	390	400
FDDASINSYS	VSDGSTSRSS	RSRKKLRTTK	KKKKDHQDYC	EVCQQGGEII	LCDTCPRAYH	MVCLDPDMEK	APEGKWSCPH
410	420	430	440	450	460	470	480
CEKEGIQWEA	KEDNSEGEEI	LEEVGGDLEE	EDDHHMEFCR	VCKDGGELLC	CDTCPSSYHI	HCLNPPLPEI	PNGEWLCPRC
490	500	510	520	530	540	550	560
TCPALKGKVQ	KILIWKWGQP	PSPTPVPRPP	DADPNTPSPK	PLEGRPERQF	FVKWQGMSYW	HCSWVSELQL	ELHCQVMFRN
570	580	590	600	610	620	630	640
YQRKNDMDEP	PSGDFGGDEE	KSRKRKNKDP	KFAEMEERFY	RYGIKPEWMM	IHRILNHSVD	KKGHVHYLIK	WRDLPYDQAS
650	660	670	680	690	700	710	720
WESEDVEIQD	YDLFKQSYWN	HRELMRGEEG	RPGKKLKKVK	LRKLERPPET	PTVDPTVKYE	RQPEYLDATG	GTLHPYQMEG
730	740	750	760	770	780	790	800
LNWLRFSWAQ	GTDTILADEM	GLGKTVQTAV	FLYSLYKEGH	SKGPFLVSAP	LSTIINWERE	FEMWAPDMYV	VTYVGDKDSR
810	820	830	840	850	860	870	880
AIIRENEFSF	EDNAIRGGKK	ASRMKKEASV	KFHVLLTSYE	LITIDMAILG	SIDWACLIVD	EAHRLKNNQS	KFFRVLNGYS
890	900	910	920	930	940	950	960
LQHKLLLTGT	PLQNNLEELF	HLLNFLTPER	FHNLEGFLEE	FADIAKEDQI	KKLHDMLGPH	MLRRLKADVF	KNMPSKTELI
970	980	990	1000	1010	1020	1030	1040
VRVELSPMQK	KYYKYILTRN	FEALNARGGG	NQVSLLNVVM	DLKKCCNHPY	LFPVAAMEAP	KMPNGMYDGS	ALIRASGKLL
1050	1060	1070	1080	1090	1100	1110	1120
LLQKMLKNLK	EGGHRVLIFS	QMTKMLDLLE	DFLEHEGYKY	ERIDGGITGN	MRQEAIDRFN	APGAQQFCFL	LSTRAGGLGI
1130	1140	1150	1160	1170	1180	1190	1200
NLATADTVII	YDSDWNPHND	IQAFSRAHRI	GQNKKVMIYR	FVTRASVEER	ITQVAKKKMM	LTHLVVRPGL	GSKTGSMSKQ
1210	1220	1230	1240	1250	1260	1270	1280
ELDDILKFGT	EELFKDEATD	GGGDNKEGED	SSVIHYDDKA	IERLLDRNQD	ETEDTELQGM	NEYLSSFKVA	QYVVREEEMG
1290	1300	1310	1320	1330	1340	1350	1360
EEEEVEREII	KQEESVDPDY	WEKLLRHHYE	QQQEDLARNL	GKGKRIRKQV	NYNDGSQEDR	DWQDDQSDNQ	SDYSVASEEG
1370	1380	1390	1400	1410	1420	1430	1440
DEDFDERSEA	PRRPSRKGLR	NDKDKPLPPL	LARVGGNIEV	LGFNARQRKA	FLNAIMRYGM	PPQDAFTTQW	LVRDLRGKSE
1450	1460	1470	1480	1490	1500	1510	1520
KEFKAYVSLF	MRHLCEPGAD	GAETFADGVP	REGLSRQHVL	TRIGVMSLIR	KKVQEFEHVN	GRWSMPELAE	VEENKKMSQP
1530	1540	1550	1560	1570	1580	1590	1600
GSPSPKTPTP	STPGDTQPNT	PAPVPPAEDG	IKIEENSLKE	EESIEGEKEV	KSTAPETAIE	CTQAPAPASE	DEKVVVEPPE
1610	1620	1630	1640	1650	1660	1670	1680
GEEKVEKAEV	KERTEEPMET	EPKGKGAADV	EKVEEKSAID	LTPIVVEDKE	EKKEEEEKKE	VMLQNGETPK	DLNDEKQKKN
1690	1700	1710	1720	1730	1740	1750	1760
IKQRFMFNIA	DGGFTELHSL	WQNEERAATV	TKKTYEIWHR	RHDYWLLAGI	INHGYARWQD	IQNDPRYAIL	NEPFKGEMNR
1770	1780	1790	1800	1810	1820	1830	1840
GNFLEIKNKF	LARRFKLLEQ	ALVIEEQLRR	AAYLNMSEDP	SHPSMALNTR	FAEVECLAES	HQHLSKESMA	GNKPANAVLH
1850	1860	1870	1880	1890	1900	1910	
KGILKQLEEL	LSDMKADVTR	LPATIARIPP	VAVRLQMSER	NILSRLANRA	PEPTPQQVAQ	QQ	

Cmpd.	No. of Cmpds.	m/z meas.	Δ m/z [ppm]	z	Rt [min]	Score	P	Range	Sequence	Modification
2537	2	649.8872	-125.27	3	60.0	13.8	0	1005-1021	K.CCNHPYLFPVAAMEAPK.M	Carbamidomethyl: 2
2522	2	649.8743	-145.17	3	59.8	13.9	0	1005-1021	K.CCNHPYLFPVAAMEAPK.M	Carbamidomethyl: 1

Detailed Protein Report

**Protein 56:**	**voltage-dependent P/Q-type calcium channel subunit alpha-1A isoform 3 [Homo sapiens]**		
**Accession:**	gi|187828880	**Score:**	43.7
**Database:**	refseq_human(refseq_human_20140103.fasta)	**MW [kDa]:**	256.6
**Database Date:**	2015-11-30	**pI:**	9.3
		**Sequence Coverage [%]:**	1.9
		**No. of unique Peptides:**	3

Detailed Protein Report

10	20	30	40	50	60	70	80
MARFGDEMPA	RYGGGGSGAA	AGVVVGSGGG	RGAGGSRQGG	QPGAQRMYKQ	SMAQRARTMA	LYNPIPVRQN	CLTVNRSLFL
90	100	110	120	130	140	150	160
FSEDNVVRKY	AKKITEWPPF	EYMILATIIA	NCIVLALEQH	LPDDDKTPMS	ERLDDTEPYF	IGIFCFEAGI	KIIALGFAFH
170	180	190	200	210	220	230	240
KGSYLRNGWN	VMDFVVVLTG	ILATVGTEFD	LRTLRAVRVL	RPLKLVSGIP	SLQVVLKSIM	KAMIPLLQIG	LLLFFAILIF
250	260	270	280	290	300	310	320
AIIGLEFYMG	KFHTTCFEEG	TDDIQGESPA	PCGTEEPART	CPNGTKCQPY	WEGPNNGITQ	FDNILFAVLT	VFQCITMEGW
330	340	350	360	370	380	390	400
TDLLYNSNDA	SGNTWNWLYF	IPLIIIGSFF	MLNLVLGVLS	GEFAKERERV	ENRRAFLKLR	RQQQIERELN	GYMEWISKAE
410	420	430	440	450	460	470	480
EVILAEDETD	GEQRHPFDGA	LRRTTIKKSK	TDLLNPEEAE	DQLADIASVG	SPFARASIKS	AKLENSTFFH	KKERRMRFYI
490	500	510	520	530	540	550	560
RRMVKTQAFY	WTVLSLVALN	TLCVAIVHYN	QPEWLSDFLY	YAEFIFLGLF	MSEMFIKMYG	LGTRPYFHSS	FNCFDCGVII
570	580	590	600	610	620	630	640
GSIFEVIWAV	IKPGTSFGIS	VLRALRLLRI	FKVTKYWASL	RNLVVSLLNS	MKSIISLLFL	LFLFIVVFAL	LGMQLFGGQF
650	660	670	680	690	700	710	720
NFDEGTPPTN	FDTFPAAIMT	VFQILTGEDW	NEVMYDGIKS	QGGVQGGMVF	SIYFIVLTLF	GNYTLLNVFL	AIAVDNLANA
730	740	750	760	770	780	790	800
QELTKDEQEE	EEAANQKLAL	QKAKEVAEVS	PLSAANMSIA	VKEQQKNQKP	AKSVWEQRTS	EMRKQNLLAS	REALYNEMDP
810	820	830	840	850	860	870	880
DERWKAAYTR	HLRPDMKTHL	DRPLVVDPQE	NRNNNTNKSR	AAEPTVDQRL	GQQRAEDFLR	KQARYHDRAR	DPSGSAGLDA
890	900	910	920	930	940	950	960
RRPWAGSQEA	ELSREGPYGR	ESDHHAREGS	LEQPGFWEGE	AERGKAGDPH	RRHVHRQGGS	RESRSGSPRT	GADGEHRRHR
970	980	990	1000	1010	1020	1030	1040
AHRRPGEEGP	EDKAERRARH	REGSRPARGG	EGEGEGPDGG	ERRRRHRHGA	PATYEGDARR	EDKERRHRRR	KENQGSGVPV
1050	1060	1070	1080	1090	1100	1110	1120
SGPNLSTTRP	IQQDLGRQDP	PLAEDIDNMK	NNKLATAESA	APHGSLGHAG	LPQSPAKMGN	STDPGPMLAI	PAMATNPQNA
1130	1140	1150	1160	1170	1180	1190	1200
ASRRTPNNPG	NPSNPGPPKT	PENSLIVTNP	SGTQTNSAKT	ARKPDHTTVD	IPPACPPPLN	HTVVQVNKNA	NPDPLPKKEE
1210	1220	1230	1240	1250	1260	1270	1280
EKKEEEEDDR	GEDGPKPMPP	YSSMFILSTT	NPLRRLCHYI	LNLRYFEMCI	LMVIAMSSIA	LAAEDPVQPN	APRNNVLRYF
1290	1300	1310	1320	1330	1340	1350	1360
DYVFTGVFTF	EMVIKMIDLG	LVLHQGAYFR	DLWNILDFIV	VSGALVAFAF	TGNSKGKDIN	TIKSLRVLRV	LRPLKTIKRL
1370	1380	1390	1400	1410	1420	1430	1440
PKLKAVFDCV	VNSLKNVFNI	LIVYMLFMFI	FAVVAVQLFK	GKFFHCTDES	KEFEKDCRGK	YLLYEKNEVK	ARDREWKKYE
1450	1460	1470	1480	1490	1500	1510	1520
FHYDNVLWAL	LTLFTVSTGE	GWPQVLKHSV	DATFENQGPS	PGYRMEMSIF	YVVYFVVFPF	FFVNIFVALI	IITFQEQGDK
1530	1540	1550	1560	1570	1580	1590	1600
MMEEYSLEKN	ERACIDFAIS	AKPLTRHMPQ	NKQSFQYRMW	QFVVSPPFEY	TIMAMIALNT	IVLMMKFYGA	SVAYENALRV
1610	1620	1630	1640	1650	1660	1670	1680
FNIVFTSLFS	LECVLKVMAF	GILNYFRDAW	NIFDFVTVLG	SITDILVTEF	GNNFINLSFL	RLFRAARLIK	LLRQGYTIRI
1690	1700	1710	1720	1730	1740	1750	1760
LLWTFVQSFK	ALPYVCLLIA	MLFFIYAIIG	MQVFGNIGID	VEDEDSDEDE	FQITEHNNFR	TFFQALMLLF	RSATGEAWHN
1770	1780	1790	1800	1810	1820	1830	1840
IMLSCLSGKP	CDKNSGILTR	ECGNEFAYFY	FVSFIFLCSF	LMLNLFVAVI	MDNFEYLTRD	SSILGPHHLD	EYVRVWAEYD
1850	1860	1870	1880	1890	1900	1910	1920
PAACGRIHYK	DMYSLLRVIS	PPLGLGKKCP	HRVACKRLLR	MDLPVADDNT	VHFNSTLMAL	IRTALDIKIA	KGGADKQQMD
1930	1940	1950	1960	1970	1980	1990	2000
AELRKEMMAI	WPNLSQKTLD	LLVTPHKSTD	LTVGKIYAAM	MIMEYYRQSK	AKKLQAMREE	QDRTPLMFQR	MEPPSPTQEG
2010	2020	2030	2040	2050	2060	2070	2080
GPGQNALPST	QLDPGGALMA	HESGLKESPS	WVTQRAQEMF	QKTGTWSPEQ	GPPTDMPNSQ	PNSQSVEMRE	MGRDGYSDSE
2090	2100	2110	2120	2130	2140	2150	2160
HYLPMEGQGR	AASMPRLPAE	NQRRRGRPRG	NNLSTISDTS	PMKRSASVLG	PKARRLDDYS	LERVPPEENQ	RHHQRRRDRS
2170	2180	2190	2200	2210	2220	2230	2240

Detailed Protein Report

Cmpd.	No. of Cmpds.	m/z meas.	Δ m/z [ppm]	z	Rt [min]	Score	P	Range	Sequence	Modification
35	1	796.7935	-124.10	2	29.4	12.9	0	12-31	R.YGGGGSGAAAGVVVGSGGGR.G	
2300	2	822.4329	20.49	2	57.1	15.3	1	1124-1139	R.RTPNNPGNPSNPGPPK.T	
2837	1	913.4693	21.67	1	64.1	15.5	1	1869-1876	K.CPHRVACK.R	

Detailed Protein Report

Protein 57: serine/arginine repetitive matrix protein 2 [Homo sapiens]

**Accession:**	gi|118572613	**Score:**	43.2
**Database:**	refseq_human(refseq_human_20140103.fasta)	**MW [kDa]:**	299.4
**Database Date:**	2015-11-30	**pI:**	12.6
		**Sequence Coverage [%]:**	1.4
		**No. of unique Peptides:**	3

Alias proteins:

Accession	Name	Description
gi|578828051	refseq_human (refseq_human_20140103.fasta)	PREDICTED: serine/arginine repetitive matrix protein 2 isoform X6 [Homo sapiens]
gi|530407855	refseq_human (refseq_human_20140103.fasta)	PREDICTED: serine/arginine repetitive matrix protein 2 isoform X1 [Homo sapiens]

Detailed Protein Report

10	20	30	40	50	60	70	80
MYNGIGLPTP	RGSGTNGYVQ	RNLSLVRGRR	GERPDYKGEE	ELRRLEAALV	KRPNPDILDH	ERKRRVELRC	LELEEMMEEQ
90	100	110	120	130	140	150	160
GYEEQQIQEK	VATFRLMLLE	KDVNPGGKEE	TPGQRPAVTE	THQLAELNEK	KNERLRAAFG	ISDSYVDGSS	FDPQRRAREA
170	180	190	200	210	220	230	240
KQPAPEPPKP	YSLVRESSSS	RSPTPKQKKK	KKKKDRGRRS	ESSSPRRERK	KSSKKKKHRS	ESESKKRKHR	SPTPKSKRKS
250	260	270	280	290	300	310	320
KDKKRKRSRS	TTPAPKSRRA	HRSTSADSAS	SSDTSRSRSR	SAAAKTHTTA	LAGRSPSPAS	GRRGEGDAPF	SEPGTTSTQR
330	340	350	360	370	380	390	400
PSSPETATKQ	PSSPYEDKDK	DKKEKSATRP	SPSPERSSTG	PEPPAPTPLL	AERHGGSPQP	LATTPLSQEP	VNPPSEASPT
410	420	430	440	450	460	470	480
RDRSPPKSPE	KLPQSSSSES	SPPSPQPTKV	SRHASSSPES	PKPAPAPGSH	REISSSPTSK	NRSHGRAKRD	KSHSHTPSRR
490	500	510	520	530	540	550	560
MGRSRSPATA	KRGRSRSRTP	TKRGHSRSRS	PQWRRSRSAQ	RWGRSRSPQR	RGRSRSPQRP	GWSRSRNTQR	RGRSRSARRG
570	580	590	600	610	620	630	640
RSHSRSPATR	GRSRSRTPAR	RGRSRSRTPA	RRRSRSRTPT	RRRSRSRTPA	RRGRSRSRTP	ARRRSRTRSP	VRRRSRSRSP
650	660	670	680	690	700	710	720
ARRSGRSRSR	TPARRGRSRS	RTPARRGRSR	SRTPARRSGR	SRSRTPARRG	RSRSRTPRRG	RSRSRSLVRR	GRSHSRTPQR
730	740	750	760	770	780	790	800
RGRSGSSSER	KNKSRTSQRR	SRSNSSPEMK	KSRISSRRSR	SLSSPRSKAK	SRLSLRRSLS	GSSPCPKQKS	QTPPRRSRSG
810	820	830	840	850	860	870	880
SSQPKAKSRT	PPRRSRSSSS	PPPKQKSKTP	SRQSHSSSSP	HPKVKSGTPP	RQGSITSPQA	NEQSVTPQRR	SCFESSPDPE
890	900	910	920	930	940	950	960
LKSRTPSRHS	CSGSSPPRVK	SSTPPRQSPS	RSSSPQPKVK	AIISPRQRSH	SGSSSPSPSR	VTSRTTPRRS	RSVSPCSNVE
970	980	990	1000	1010	1020	1030	1040
SRLLPRYSHS	GSSSPDTKVK	PETPPRQSHS	GSISPYPKVK	AQTPPGPSLS	GSKSPCPQEK	SKDSLVQSCP	GSLSLCAGVK
1050	1060	1070	1080	1090	1100	1110	1120
SSTPPGESYF	GVSSLQLKGQ	SQTSPDHRSD	TSSPEVRQSH	SESPSLQSKS	QTSPKGGRSR	SSSPVTELAS	RSPIRQDRGE
1130	1140	1150	1160	1170	1180	1190	1200
FSASPMLKSG	MSPEQSRFQS	DSSSYPTVDS	NSLLGQSRLE	TAESKEKMAL	PPQEDATASP	PRQKDKFSPF	PVQDRPESSL
1210	1220	1230	1240	1250	1260	1270	1280
VFKDTLRTPP	RERSGAGSSP	ETKEQNSALP	TSSQDEELME	VVEKSEEPAG	QILSHLSSEL	KEMSTSNFES	SPEVEERPAV
1290	1300	1310	1320	1330	1340	1350	1360
SLTLDQSQSQ	ASLEAVEVPS	MASSWGGPHF	SPEHKELSNS	PLRENSFGSP	LEFRNSGPLG	TEMNTGFSSE	VKEDLNGPFL
1370	1380	1390	1400	1410	1420	1430	1440
NQLETDPSLD	MKEQSTRSSG	HSSSELSPDA	VEKAGMSSNQ	SISSPVLDAV	PRTPSRERSS	SASSPEMKDG	LPRTPSRRSR
1450	1460	1470	1480	1490	1500	1510	1520
SGSSPGLRDG	SGTPSRHSLS	GSSPGMKDIP	RTPSRGRSEC	DSSPEPKALP	QTPRPRSRSP	SSPELNNKCL	TPQRERSGSE
1530	1540	1550	1560	1570	1580	1590	1600
SSVDQKTVAR	TPLGQRSRSG	SSQELDVKPS	ASPQERSESD	SSPDSKAKTR	TPLRQRSRSG	SSPEVDSKSR	LSPRRSRSGS
1610	1620	1630	1640	1650	1660	1670	1680
SPEVKDKPRA	APRAQSGSDS	SPEPKAPAPR	ALPRRSRSGS	SSKGRGPSPE	GSSSTESSPE	HPPKSRTARR	GSRSSPEPKT
1690	1700	1710	1720	1730	1740	1750	1760
KSRTPPRRRS	SRSSPELTRK	ARLSRRSRSA	SSSPETRSRT	PPRHRRSPSV	SSPEPAEKSR	SSRRRRSASS	PRTKTTSRRG
1770	1780	1790	1800	1810	1820	1830	1840
RSPSPKPRGL	QRSRSRSRRE	KTRTTRRRDR	SGSSQSTSRR	RQRSRSRSRV	TRRRRGGSGY	HSRSPARQES	SRTSSRRRRG
1850	1860	1870	1880	1890	1900	1910	1920
RSRTPPTSRK	RSRSRTSPAP	WKRSRSRASP	ATHRRSRSRT	PLISRRRSRS	RTSPVSRRRS	RSRTSVTRRR	SRSRASPVSR
1930	1940	1950	1960	1970	1980	1990	2000
RRSRSRTPPV	TRRRSRSRTP	TTRRRSRSRT	PPVTRRRSRS	RTPPVTRRRS	RSRTSPITRR	RSRSRTSPVT	RRRSRSRTSP
2010	2020	2030	2040	2050	2060	2070	2080
VTRRRSRSRT	SPVTRRRSRS	RTPPAIRRRS	RSRTPLLPRK	RSRSRSPLAI	RRRSRSRTPR	TARGKRSLTR	SPPAIRRRSA
2090	2100	2110	2120	2130	2140	2150	2160
SGSSSDRSRS	ATPPATRNHS	GSRTPPVALN	SSRMSCFSRP	SMSPTPLDRC	RSPGMLEPLG	SSRTPMSVLQ	QAGGSMMDGP
2170	2180	2190	2200	2210	2220	2230	2240

Detailed Protein Report

Cmpd.	No. of Cmpds.	m/z meas.	Δ m/z [ppm]	z	Rt [min]	Score	P	Range	Sequence	Modification
2951	7	703.3519	-15.42	2	65.6	16.8	1	833-845	R.QSHSSSSPHPKVK.S	
2324	1	841.5088	116.64	2	57.3	10.3	1	1614-1630	R.AQSGSDSSPEPKAPAPR.A	
873	1	491.7878	51.39	2	39.7	16.0	1	1866-1874	R.SRASPATHR.R	

Detailed Protein Report

Protein 58: fibrillin-2 precursor [Homo sapiens]

**Accession:**	gi|66346695	**Score:**	43.2
**Database:**	refseq_human(refseq_human_20140103.fasta)	**MW [kDa]:**	314.6
**Database Date:**	2015-11-30	**pI:**	4.6
**Modification(s):**	Carbamidomethyl	**Sequence Coverage [%]:**	1.5
		**No. of unique Peptides:**	3

Quantitation

WD:WU	**Median:** 1.22	**CV:** 20.64 %	**No. of Peptides:** 2

Detailed Protein Report

10	20	30	40	50	60	70	80
MGRRRRLCLQ	LYFLWLGCVV	LWAQGTAGQP	QPPPPKPPRP	QPPPPQQVRSA	TAGSEGGFLA	PEYREEGAAV	ASRVRRRGQQ
90	100	110	120	130	140	150	160
DVLRGPNVCG	SRFHSYCCPG	WKTLPGGNQC	IVPICRNSCG	DGFCSRPNMC	TCSSGQISST	CGSKSIQQCS	VRCMNGGTCA
170	180	190	200	210	220	230	240
DDHCQCQKGY	IGTYCGQPVC	ENGCQNGGRC	IGPNRCACVY	GFTGPQCERD	YRTGPCFTQV	NNQMCQGQLT	GIVCTKTLCC
250	260	270	280	290	300	310	320
ATIGRAWGHP	CEMCPAQPQP	CRRGFIPNIR	TGACQDVDEC	QAIPGICQGG	NCINTVGSFE	CRCPAGHKQS	ETTQKCEDID
330	340	350	360	370	380	390	400
ECSIIPGICE	TGECSNTVGS	YFCVCPRGYV	TSTDGSRCID	QRTGMCFSGL	VNGRCAQELP	GRMTKMQCCC	EPGRCWGIGT
410	420	430	440	450	460	470	480
IPEACPVRGS	EEYRRLCMDG	LPMGGIPGSA	GSRPGGTGGN	GFAPSGNGNG	YGPGGTGFIP	IPGGNGFSPG	VGGAGVGAGG
490	500	510	520	530	540	550	560
QGPIITGLTI	LNQTIDICKH	HANLCLNGRC	IPTVSSYRCE	CNMGYKQDAN	GDCIDVDECT	SNPCTNGDCV	NTPGSYYCKC
570	580	590	600	610	620	630	640
HAGFQRTPTK	QACIDIDECI	QNGVLCKNGR	CVNTDGSFQC	ICNAGFELTT	DGKNCVDHDE	CTTTNMCLNG	MCINEDGSFK
650	660	670	680	690	700	710	720
CICKPGFVLA	PNGRYCTDVD	ECQTPGICMN	GHCINSEGSF	RCDCPPGLAV	GMDGRVCVDT	HMRSTCYGGI	KKGVCVRPFP
730	740	750	760	770	780	790	800
GAVTKSECCC	ANPDYGFGEP	CQPCPAKNSA	EFHGLCSSGV	GITVDGRDIN	ECALDPDICA	NGICENLRGS	YRCNCNSGYE
810	820	830	840	850	860	870	880
PDASGRNCID	IDECLVNRLL	CDNGLCRNTP	GSYSCTCPPG	YVFRTETETC	EDINECESNP	CVNGACRNNL	GSFNCECSPG
890	900	910	920	930	940	950	960
SKLSSTGLIC	IDSLKGTCWL	NIQDSRCEVN	INGATLKSEC	CATLGAAWGS	PCERCELDTA	CPRGLARIKG	VTCEDVNECE
970	980	990	1000	1010	1020	1030	1040
VFPGVCPNGR	CVNSKGSFHC	ECPEGLTLDG	TGRVCLDIRM	EQCYLKWDED	ECIHPVPGKF	RMDACCCAVG	AAWGTECEEC
1050	1060	1070	1080	1090	1100	1110	1120
PKPGTKEYET	LCPRGAGFAN	RGDVLTGRPF	YKDINECKAF	PGMCTYGKCR	NTIGSFKCRC	NSGFALDMEE	RNCTDIDECR
1130	1140	1150	1160	1170	1180	1190	1200
ISPDLCGSGI	CVNTPGSFEC	ECFEGYESGF	MMMKNCMDID	ECERNPLLCR	GGTCVNTEGS	FQCDCPLGHE	LSPSREDCVD
1210	1220	1230	1240	1250	1260	1270	1280
INECSLSDNL	CRNGKCVNMI	GTYQCSCNPG	YQATPDRQGC	TDIDECMIMN	GGCDTQCTNS	EGSYECSCSE	GYALMPDGRS
1290	1300	1310	1320	1330	1340	1350	1360
CADIDECENN	PDICDGGQCT	NIPGEYRCLC	YDGFMASMDM	KTCIDVNECD	LNSNICMFGE	CENTKGSFIC	HCQLGYSVKK
1370	1380	1390	1400	1410	1420	1430	1440
GTTGCTDVDE	CEIGAHNCDM	HASCLNIPGS	FKCSCREGWI	GNGIKCIDLD	ECSNGTHQCS	INAQCVNTPG	SYRCACSEGF
1450	1460	1470	1480	1490	1500	1510	1520
TGDGFTCSDV	DECAENINLC	ENGQCLNVPG	AYRCECEMGF	TPASDSRSCQ	DIDECSFQNI	CVFGTCNNLP	GMFHCICDDG
1530	1540	1550	1560	1570	1580	1590	1600
YELDRTGGNC	TDIDECADPI	NCVNGLCVNT	PGRYECNCPP	DFQLNPTGVG	CVDNRVGNCY	LKFGPRGDGS	LSCNTEIGVG
1610	1620	1630	1640	1650	1660	1670	1680
VSRSSCCCSL	GKAWGNPCET	CPPVNSTEYY	TLCPGGEGFR	PNPITIILED	IDECQELPGL	CQGGNCINTF	GSFQCECPQG
1690	1700	1710	1720	1730	1740	1750	1760
YYLSEDTRIC	EDIDECFAHP	GVCGPGTCYN	TLGNYTCICP	PEYMQVNGGH	NCMDMRKSFC	YRSYNGTTCE	NELPFNVTKR
1770	1780	1790	1800	1810	1820	1830	1840
MCCCTYNVGK	AWNKPCEPCP	TPGTADFKTI	CGNIPGFTFD	IHTGKAVDID	ECKEIPGICA	NGVCINQIGS	FRCECPTGFS
1850	1860	1870	1880	1890	1900	1910	1920
YNDLLLVCED	IDECSNGDNL	CQRNADCINS	PGSYRCECAA	GFKLSPNGAC	VDRNECLEIP	NVCSHGLCVD	LQGSYQCICH
1930	1940	1950	1960	1970	1980	1990	2000
NGFKASQDQT	MCMDVDECER	HPCGNGTCKN	TVGSYNCLCY	PGFELTHNND	CLDIDECSSF	FGQVCRNGRC	FNEIGSFKCL
2010	2020	2030	2040	2050	2060	2070	2080
CNEGYELTPD	GKNCIDTNEC	VALPGSCSPG	TCQNLEGSFR	CICPPGYEVK	SENCIDINEC	DEDPNICLFG	SCTNTPGGFQ
2090	2100	2110	2120	2130	2140	2150	2160
CLCPPGFVLS	DNGRRCFDTR	QSFCFTNFEN	GKCSVPKAFN	TTKAKCCCSK	MPGEGWGDPC	ELCPKDDEVA	FQDLCPYGHG
2170	2180	2190	2200	2210	2220	2230	2240

Detailed Protein Report

Cmpd.	No. of Cmpds.	m/z meas.	Δ m/z [ppm]	z	Rt [min]	Score	P	Range	Sequence	Modification	Ratios	
186	1	806.9057	27.18	2	31.2	11.0	0	49-64	R.SATAGSEGGFLAPEYR.E		WD:WU	0.99
2112	1	600.2270	-134.67	3	54.8	15.6	2	77-92	R.RGQQDVLRGPNVCGSR.F	Carbamidomethyl: 13		
291	2	642.2098	-115.38	2	32.8	16.5	1	2529-2539	K.TCKDLDECQTK.Q		WD:WU	1.50

Detailed Protein Report

Protein 59: PREDICTED: AP-3 complex subunit beta-2 isoform X2 [Homo sapiens]

Accession: gi|578827454
Database: refseq_human(refseq_human_20140103.fasta)
Database Date: 2015-11-30
Modification(s): Oxidation

Score: 43.0
MW [kDa]: 114.4
pI: 5.4
Sequence Coverage [%]: 2.9
No. of unique Peptides: 3

Quantitation

WD:WU	**Median:** 1.35	**CV:** 0.00 %	**No. of Peptides:** 1

Alias proteins:

Accession	Name	Description
gi|578827456	refseq_human (refseq_human_20140103.fasta)	PREDICTED: AP-3 complex subunit beta-2 isoform X3 [Homo sapiens]

10	20	30	40	50	60	70	80
MLDTNKDSLK	LEAMKRIVAM	IARGKNASDL	FPAVVKNVAC	KNIEVKKLVY	VYLVRYAEEQ	QDLALLSIST	FQRGLKDPNQ
90	100	110	120	130	140	150	160
LIRASALRVL	SSIRVPIIVP	IMMLAIKEAA	SDMSPYVRKT	AAHAIPKLYS	LDSDQKDQLI	EVIEKLLADK	TTLVAGSVVM
170	180	190	200	210	220	230	240
AFEEVCPERI	DLIHKNYRKL	CNLLIDVEEW	GQVVIISMLT	RYARTQFLSP	TQNESLLEEN	AEKAFYGSEE	DEAKGAGSEE
250	260	270	280	290	300	310	320
TAAAAAPSRK	PYVMDPDHRL	LLRNTKPLLQ	SRSAAVVMAV	AQLYFHLAPK	AEVGVIAKAL	VRLLRSHSEV	QYVVLQNVAT
330	340	350	360	370	380	390	400
MSIKRRGMFE	PYLKSFYIRS	TDPTQIKILK	LEVLTNLANE	TNIPTVLREF	QTYIRSMDKD	FVAATIQAIG	RCATNIGRVR
410	420	430	440	450	460	470	480
DTCLNGLVQL	LSNRDELVVA	ESVVVIKKLL	QMQPAQHGEI	IKHLAKLTDN	IQVPMARASI	LWLIGEYCEH	VPRIAPDVLR
490	500	510	520	530	540	550	560
KMAKSFTAEE	DIVKLQVINL	AAKLYLTNSK	QTKLLTQYVL	SLAKYDQNYD	IRDRARFTRQ	LIVPSEQGGA	LSRHAKKLFL
570	580	590	600	610	620	630	640
APKPAPVLES	SFKDRDHFQL	GSLSHLLNAK	ATGYQELPDW	PEEAPDPSVR	NVEVPEWTKC	SNREKRKEKE	KPFYSDSEGE
650	660	670	680	690	700	710	720
SGPTESADSD	PESESESDSK	SSSESGSGES	SSESDNEDQD	EDEEKGRGSE	SEQSEEDGKR	KTKKKVPERK	GEASSSDEGS
730	740	750	760	770	780	790	800
DSSSSSSESE	MTSESEEEQL	EPASWSRKTP	PSSKSAPATK	EISLLDLEDF	TPPSVQPVSP	PAIVSTSLAA	DLEGLTLTDS
810	820	830	840	850	860	870	880
TLVPSLLSPV	SGVGRQELLH	RVAGEGLAVD	YTFSRQPFSG	DPHMVSVHIH	FSNSSDTPIK	GLHVGTPKLP	AGISIQEFPE
890	900	910	920	930	940	950	960
IESLAPGESA	TAVMGINFCD	STQAANFQLC	TQTRQFYVSI	QPPVGELMAP	VFMSENEFKK	EQGKLMGMNE	ITEKLMLPDT
970	980	990	1000	1010	1020	1030	1040
CRSDHIVVQK	VTATANLGRV	PCGTSDEYRF	AGRTLTGGSL	VLLTLDARPA	GAAQLTVNSE	KMVIGTMLVK	DVIQALTQ

Cmpd.	No. of Cmpds.	m/z meas.	Δ m/z [ppm]	z	Rt [min]	Score	P	Range	Sequence	Modification	Ratios
2503	1	974.2875	-301.77	1	59.6	11.1	1	17-25	R.IVAMIARGK.N	Oxidation: 4	
619	1	677.3962	18.75	2	36.6	11.7	1	77-88	K.DPNQLIRASALR.V		
768	2	495.2668	-4.57	2	38.4	20.2	1	392-400	R.CATNIGRVR.D		WD:WU 1.35

Detailed Protein Report

Protein 60: PREDICTED: methylcytosine dioxygenase TET3 isoform X1 [Homo sapiens]

**Accession:**	gi|530367326	**Score:**	43.0
**Database:**	refseq_human(refseq_human_20140103.fasta)	**MW [kDa]:**	193.6
**Database Date:**	2015-11-30	**pI:**	9.0
**Modification(s):**	Oxidation	**Sequence Coverage [%]:**	2.8
		**No. of unique Peptides:**	2

Alias proteins:

Accession	Name	Description
gi|566559863	refseq_human (refseq_human_20140103.fasta)	methylcytosine dioxygenase TET3 [Homo sapiens]

Detailed Protein Report

10	20	30	40	50	60	70	80
MSQFQVPLAV	QPDLPGLYDF	PQRQVMVGSF	PGSGLSMAGS	ESQLRGGGDG	RKKRKRCGTC	EPCRRLENCG	ACTSCTNRRT
90	100	110	120	130	140	150	160
HQICKLRKCE	VLKKKVGLLK	EVEIKAGEGA	GPWGQGAAVK	TGSELSPVDG	PVPGQMDSGP	VYHGDSRQLS	ASGVPVNGAR
170	180	190	200	210	220	230	240
EPAGPSLLGT	GGPWRVDQKP	DWEAAPGPAH	TARLEDAHDL	VAFSAVAEAV	SSYGALSTRL	YETFNREMSR	EAGNNSRGPR
250	260	270	280	290	300	310	320
PGPEGCSAGS	EDLDTLQTAL	ALARHGMKPP	NCNCDGPECP	DYLEWLEGKI	KSVVMEGGEE	RPRLPGPLPP	GEAGLPAPST
330	340	350	360	370	380	390	400
RPLLSSEVPQ	ISPQEGLPLS	QSALSIAKEK	NISLQTAIAI	EALTQLSSAL	PQPSHSTPQA	SCPLPEALSP	PAPFRSPQSY
410	420	430	440	450	460	470	480
LRAPSWPVVP	PEEHSSFAPD	SSAFPPATPR	TEFPEAWGTD	TPPATPRSSW	PMPRPSPDPM	AELEQLLGSA	SDYIQSVFKR
490	500	510	520	530	540	550	560
PEALPTKPKV	KVEAPSSSPA	PAPSPVLQRE	APTPSSEPDT	HQKAQTALQQ	HLHHKRSLFL	EQVHDTSFPA	PSEPSAPGWW
570	580	590	600	610	620	630	640
PPPSSPVPRL	PDRPPKEKKK	KLPTPAGGPV	GTEKAAPGIK	PSVRKPIQIK	KSRPREAQPL	FPPVRQIVLE	GLRSPASQEV
650	660	670	680	690	700	710	720
QAHPPAPLPA	SQGSAVPLPP	EPSLALFAPS	PSRDSLLPPT	QEMRSPSPMT	ALQPGSTGPL	PPADDKLEEL	IRQFEAEFGD
730	740	750	760	770	780	790	800
SFGLPGPPSV	PIQDPENQQT	CLPAPESPFA	TRSPKQIKIE	SSGAVTVLST	TCFHSEEGGQ	EATPTKAENP	LTPTLSGFLE
810	820	830	840	850	860	870	880
SPLKYLDTPT	KSLLDTPAKR	AQAEFPTCDC	VEQIVEKDEG	PYYTHLGSGP	TVASIRELME	ERYGEKGKAI	RIEKVIYTGK
890	900	910	920	930	940	950	960
EGKSSRGCPI	AKWVIRRHTL	EEKLLCLVRH	RAGHHCQNAV	IVILILAWEG	IPRSLGDTLY	QELTDTLRKY	GNPTSRRCGL
970	980	990	1000	1010	1020	1030	1040
NDDRTCACQG	KDPNTCGASF	SFGCSWSMYF	NGCKYARSKT	PRKFRLAGDN	PKEEEVLRKS	FQDLATEVAP	LYKRLAPQAY
1050	1060	1070	1080	1090	1100	1110	1120
QNQVTNEEIA	IDCRLGLKEG	RPFAGVTACM	DFCAHAHKDQ	HNLYNGCTVV	CTLTKEDNRC	VGKIPEDEQL	HVLPLYKMAN
1130	1140	1150	1160	1170	1180	1190	1200
TDEFGSEENQ	NAKVGSGAIQ	VLTAFPREVR	RLPEPAKSCR	QRQLEARKAA	AEKKKIQKEK	LSTPEKIKQE	ALELAGITSD
1210	1220	1230	1240	1250	1260	1270	1280
PGLSLKGGLS	QQGLKPSLKV	EPQNHFSSFK	YSGNAVVESY	SVLGNCRPSD	PYSMNSVYSY	HSYYAQPSLT	SVNGFHSKYA
1290	1300	1310	1320	1330	1340	1350	1360
LPSFSYYGFP	SSNPVFPSQF	LGPGAWGHSG	SSGSFEKKPD	LHALHNSLSP	AYGGAEFAEL	PSQAVPTDAH	HPTPHHQQPA
1370	1380	1390	1400	1410	1420	1430	1440
YPGPKEYLLP	KAPLLHSVSR	DPSPFAQSSN	CYNRSIKQEP	VDPLTQAEPV	PRDAGKMGKT	PLSEVSQNGG	PSHLWGQYSG
1450	1460	1470	1480	1490	1500	1510	1520
GPSMSPKRTN	GVGGSWGVFS	SGESPAIVPD	KLSSFGASCL	APSHFTDGQW	GLFPGEGQQA	ASHSGGRLRG	KPWSPCKFGN
1530	1540	1550	1560	1570	1580	1590	1600
STSALAGPSL	TEKPWALGAG	DFNSALKGSP	GFQDKLWNPM	KGEEGRIPAA	GASQLDRAWQ	SFGLPLGSSE	KLFGALKSEE
1610	1620	1630	1640	1650	1660	1670	1680
KLWDPFSLEE	GPAEEPPSKG	AVKEEKGGGG	AEEEEEELWS	DSEHNFLDEN	IGGVAVAPAH	GSILIECARR	ELHATTPLKK
1690	1700	1710	1720	1730	1740	1750	1760
PNRCHPTRIS	LVFYQHKNLN	QPNHGLALWE	AKMKQLAERA	RARQEEAARL	GLGQQEAKLY	GKKRKWGGTV	VAEPQQKEKK
1770	1780	1790	1800				
GVVPTRQALA	VPTDSAVTVS	SYAYTKVTGP	YSRWI				

Cmpd.	No. of Cmpds.	m/z meas.	Δ m/z [ppm]	z	Rt [min]	Score	P	Range	Sequence	Modification
2885	1	1129.0588	22.81	2	64.8	17.8	0	24-45	R.QVMVGSFPGSGLSMAGSESQLR.G	Oxidation: 3, 14
1833	2	697.0345	-7.09	3	51.3	12.9	2	1724-1742	R.QEEAARLGLGQQEAKLYGK.K	

BRUKER

Detailed Protein Report

Protein 61: ATP-binding cassette sub-family A member 3 [Homo sapiens]

**Accession:**	gi|116734710	**Score:**	42.8
**Database:**	refseq_human(refseq_human_20140103.fasta)	**MW [kDa]:**	191.2
**Database Date:**	2015-11-30	**pI:**	8.2
**Modification(s):**	Carbamidomethyl	**Sequence Coverage [%]:**	1.5
		**No. of unique Peptides:**	2

Quantitation

WD:WU **Median:** 1.60 **CV:** 0.00 % **No. of Peptides:** 1

10	20	30	40	50	60	70	80
MAVLRQLALL	LWKNYTLQKR	KVLVTVLELF	LPLLFSGILI	WLRLKIQSEN	VPNATIYPGQ	SIQELPLFFT	FPPPGDTWEL
90	100	110	120	130	140	150	160
AYIPSHSDAA	KTVTETVRRA	LVINMRVRGF	PSEKDFEDYI	RYDNCSSSVL	AAVVFEHPFN	HSKEPLPLAV	KYHLRFSYTR
170	180	190	200	210	220	230	240
RNYMWTQTGS	FFLKETEGWH	TTSLFPLFPN	PGPREPTSPD	GGEPGYIREG	FLAVQHAVDR	AIMEYHADAA	TRQLFQRLTV
250	260	270	280	290	300	310	320
TIKRFPYPPF	IADPFLVAIQ	YQLPLLLLLS	FTYTALTIAR	AVVQEKERRL	KEYMRMMGLS	SWLHWSAWFL	LFFLFLLIAA
330	340	350	360	370	380	390	400
SFMTLLFCVK	VKPNVAVLSR	SDPSLVLAFL	LCFAISTISF	SFMVSTFFSK	ANMAAAFGGF	LYFFTYIPYF	FVAPRYNWMT
410	420	430	440	450	460	470	480
LSQKLCSCLL	SNVAMAMGAQ	LIGKFEAKGM	GIQWRDLLSP	VNVDDDFCFG	QVLGMLLLDS	VLYGLVTWYM	EAVFPGQFGV
490	500	510	520	530	540	550	560
PQPWYFFIMP	SYWCGKPRAV	AGKEEEDSDP	EKALRNEYFE	AEPEDLVAGI	KIKHLSKVFR	VGNKDRAAVR	DLNLNLYEGQ
570	580	590	600	610	620	630	640
ITVLLGHNGA	GKTTTLSMLT	GLFPPTSGRA	YISGYEISQD	MVQIRKSLGL	CPQHDILFDN	LTVAEHLYFY	AQLKGLSRQK
650	660	670	680	690	700	710	720
CPEEVKQMLH	IIGLEDKWNS	RSRFLSGGMR	RKLSIGIALI	AGSKVLILDE	PTSGMDAISR	RAIWDLLQRQ	KSDRTIVLTT
730	740	750	760	770	780	790	800
HFMDEADLLG	DRIAIMAKGE	LQCCGSSLFL	KQKYGAGYHM	TLVKEPHCNP	EDISQLVHHH	VPNATLESSA	GAELSFILPR
810	820	830	840	850	860	870	880
ESTHRFEGLF	AKLEKKQKEL	GIASFGASIT	TMEEVFLRVG	KLVDSSMDIQ	AIQLPALQYQ	HERRASDWAV	DSNLCGAMDP
890	900	910	920	930	940	950	960
SDGIGALIEE	ERTAVKLNTG	LALHCQQFWA	MFLKKAAYSW	REWKMVAAQV	LVPLTCVTLA	LLAINYSSEL	FDDPMLRLTL
970	980	990	1000	1010	1020	1030	1040
GEYGRTVVPF	SVPGTSQLGQ	QLSEHLKDAL	QAEGQEPREV	LGDLEEFLIF	RASVEGGGFN	ERCLVAASFR	DVGERTVVNA
1050	1060	1070	1080	1090	1100	1110	1120
LFNNQAYHSP	ATALAVVDNL	LFKLLCGPHA	SIVVSNFPQP	RSALQAAKDQ	FNEGRKGFDI	ALNLLFAMAF	LASTFSILAV
1130	1140	1150	1160	1170	1180	1190	1200
SERAVQAKHV	QFVSGVHVAS	FWLSALLWDL	ISFLIPSLLL	LVVFKAFDVR	AFTRDGHMAD	TLLLLLLYGW	AIIPLMYLMN
1210	1220	1230	1240	1250	1260	1270	1280
FFFLGAATAY	TRLTIFNILS	GIATFLMVTI	MRIPAVKLEE	LSKTLDHVFL	VLPNHCLGMA	VSSFYENYET	RRYCTSSEVA
1290	1300	1310	1320	1330	1340	1350	1360
AHYCKKYNIQ	YQENFYAWSA	PGVGRFVASM	AASGCAYLIL	LFLIETNLLQ	RLRGILCALR	RRRTLTELYT	RMPVLPEDQD
1370	1380	1390	1400	1410	1420	1430	1440
VADERTRILA	PSPDSLLHTP	LIIKELSKVY	EQRVPLLAVD	RLSLAVQKGE	CFGLLGFNGA	GKTTTFKMLT	GEESLTSGDA
1450	1460	1470	1480	1490	1500	1510	1520
FVGGHRISSD	VGKVRQRIGY	CPQFDALLDH	MTGREMLVMY	ARLRGIPERH	IGACVENTLR	GLLLEPHANK	LVRTYSGGNK
1530	1540	1550	1560	1570	1580	1590	1600
RKLSTGIALI	GEPAVIFLDE	PSTGMDPVAR	RLLWDTVARA	RESGKAIIIT	SHSMEECEAL	CTRLAIMVQG	QFKCLGSPQH
1610	1620	1630	1640	1650	1660	1670	1680
LKSKFGSGYS	LRAKVQSEGQ	QEALEEFKAF	VDLTFPGSVL	EDEHQGMVHY	HLPGRDLSWA	KVFGILEKAK	EKYGVDDYSV
1690	1700	1710					
SQISLEQVFL	SFAHLQPPTA	EEGR					

Detailed Protein Report

Cmpd.	No. of Cmpds.	m/z meas.	Δ m/z [ppm]	z	Rt [min]	Score	P	Range	Sequence	Modification	Ratios
32	1	877.8644	-76.86	2	29.3	10.4	1	739-753	K.GELQCCGSSLFLKQK.Y	Carbamidomethyl: 5, 6	
448	1	607.1484	-239.81	2	34.6	32.5	0	988-998	K.DALQAEGQEPR.E		WD:WU 1.60

Detailed Protein Report

Protein 62: glutathione S-transferase P [Homo sapiens]

Accession: gi|4504183
Database: refseq_human(refseq_human_20140103.fasta)
Database Date: 2015-11-30

Score: 42.5
MW [kDa]: 23.3
pI: 5.3
Sequence Coverage [%]: 7.6
No. of unique Peptides: 1

Quantitation

WD:WU **Median:** 0.72 **CV:** 0.00 % **No. of Peptides:** 1

10	20	30	40	50	60	70	80
MPPYTVVYFP	VRGRCAALRM	LLADQGQSWK	EEVVTVETWQ	EGSLKASCLY	GQLPKFQDGD	LTLYQSNTIL	RHLGRTLGLY
**90**	**100**	**110**	**120**	**130**	**140**	**150**	**160**
GKDQQEAALV	DMVNDGVEDL	RCKYISLIYT	NYEAGKDDYV	KALPGQLKPF	ETLLSQNQGG	KTFIVGDQIS	FADYNLLDLL
**170**	**180**	**190**	**200**	**210**	**220**		
LIHEVLAPGC	LDAFPLLSAY	VGRLSARPKL	KAFLASPEYV	NLPINGNGKQ			

Cmpd.	No. of Cmpds.	m/z meas.	Δ m/z [ppm]	z	Rt [min]	Score	P	Range	Sequence	Modification	Ratios
1632	2	942.4899	12.06	2	49.1	42.5	0	56-71	K.FQDGDLTLYQSNTILR.H		WD:WU 0.72

Detailed Protein Report

Protein 63: serine/threonine-protein phosphatase 2A 55 kDa regulatory subunit B beta isoform isoform e [Homo sapiens]

Accession: gi|430727947
Database: refseq_human(refseq_human_20140103.fasta)
Database Date: 2015-11-30

Score: 42.5
MW [kDa]: 62.5
pI: 6.4
Sequence Coverage [%]: 6.6
No. of unique Peptides: 1

10	20	30	40	50	60	70	80
MHQPPPASCS	SSSSSSSSSC	ECARVGVRVS	ALAPAAAPCP	APRQLPYPRL	PEPPSRGTST	LIPARLGPWC	SPTGSPAPLS
90	100	110	120	130	140	150	160
CETGCGEGSW	ILVCRLLVPT	QVSLLSMEED	IDTRKINNSF	LRDHSYATEA	DIISTVEFNH	TGELLATGDK	GGRVVIFQRE
170	180	190	200	210	220	230	240
QESKNQVHRR	GEYNVYSTFQ	SHEPEFDYLK	SLEIEEKINK	IRWLPQQNAA	YFLLSTNDKT	VKLWKVSERD	KRPEGYNLKD
250	260	270	280	290	300	310	320
EEGRLRDPAT	ITTLRVPVLR	PMDLMVEATP	RRVFANAHTY	HINSISVNSD	YETYMSADDL	RINLWNFEIT	NQSFNIVDIK
330	340	350	360	370	380	390	400
PANMEELTEV	ITAAEFHPHH	CNTFVYSSSK	GTIRLCDMRA	SALCDRHTKF	FEEPEDPSNR	SFFSEIISSI	SDVKFSHSGR
410	420	430	440	450	460	470	480
YIMTRDYLTV	KVWDLNMENR	PIETYQVHDY	LRSKLCSLYE	NDCIFDKFEC	VWNGSDSVIM	TGSYNNFFRM	FDRNTKRDVT
490	500	510	520	530	540	550	
LEASRENSKP	RAILKPRKVC	VGGKRRKDEI	SVDSLDFSKK	ILHTAWHPSE	NIIAVAATNN	LYIFQDKVN	

Cmpd.	No. of Cmpds.	m/z meas.	Δ m/z [ppm]	z	Rt [min]	Score	P	Range	Sequence	Modification
1632	1	1073.5401	-40.07	2	48.7	20.8	1	29-49	R.VSALAPAAAPCPAPRQLPYPR.L	

Detailed Protein Report

Protein 64: E3 ubiquitin-protein ligase BRE1A [Homo sapiens]

**Accession:**	gi|34878777	**Score:**	42.3
**Database:**	refseq_human(refseq_human_20140103.fasta)	**MW [kDa]:**	113.6
**Database Date:**	2015-11-30	**pI:**	5.6
**Modification(s):**	Carbamidomethyl, Oxidation	**Sequence Coverage [%]:**	5.5
		**No. of unique Peptides:**	3

10	20	30	40	50	60	70	80
MSGIGNKRAA	GEPGTSMPPE	KKAAVEDSGT	TVETIKLGGV	SSTEELDIRT	LQTKNRKLAE	MLDQRQAIED	ELREHIEKLE
**90**	**100**	**110**	**120**	**130**	**140**	**150**	**160**
RRQATDDASL	LIVNRYWSQF	DENIRIILKR	YDLEQGLGDL	LTERKALVVP	EPEPDSDSNQ	ERKDDRERGE	GQEPAFSFLA
**170**	**180**	**190**	**200**	**210**	**220**	**230**	**240**
TLASSSSEEM	ESQLQERVES	SRRAVSQIVT	VYDKLQEKVE	LLSRKLNSGD	NLIVEEAVQE	LNSFLAQENM	RLQELTDLLQ
**250**	**260**	**270**	**280**	**290**	**300**	**310**	**320**
EKHRTMSQEF	SKLQSKVETA	ESRVSVLESM	IDDLQWDIDK	IRKREQRLNR	HLAEVLERVN	SKGYKVYGAG	SSLYGGTITI
**330**	**340**	**350**	**360**	**370**	**380**	**390**	**400**
NARKFEEMNA	ELEENKELAQ	NRLCELEKLR	QDFEEVTTQN	EKLKVELRSA	VEQVVKETPE	YRCMQSQFSV	LYNESLQLKA
**410**	**420**	**430**	**440**	**450**	**460**	**470**	**480**
HLDEARTLLH	GTRGTHQHQV	ELIERDEVSL	HKKLRTEVIQ	LEDTLAQVRK	EYEMLRIEFE	QTLAANEQAG	PINREMRHLI
**490**	**500**	**510**	**520**	**530**	**540**	**550**	**560**
SSLQNHNHQL	KGEVLRYKRK	LREAQSDLNK	TRLRSGSALL	QSQSSTEDPK	DEPAELKPDS	EDLSSQSSAS	KASQEDANEI
**570**	**580**	**590**	**600**	**610**	**620**	**630**	**640**
KSKRDEEERE	RERREKERER	EREREKEKER	EREKQKLKES	EKERDSAKDK	EKGKHDDGRK	KEAEIIKQLK	IELKKAQESQ
**650**	**660**	**670**	**680**	**690**	**700**	**710**	**720**
KEMKLLLDMY	RSAPKEQRDK	VQLMAAEKKS	KAELEDLRQR	LKDLEDKEKK	ENKKMADEDA	LRKIRAVEEQ	IEYLQKKLAM
**730**	**740**	**750**	**760**	**770**	**780**	**790**	**800**
AKQEEEALLS	EMDVTGQAFE	DMQEQNIRLM	QQLREKDDAN	FKLMSERIKS	NQIHKLLKEE	KEELADQVLT	LKTQVDAQLQ
**810**	**820**	**830**	**840**	**850**	**860**	**870**	**880**
VVRKLEEKEH	LLQSNIGTGE	KELGLRTQAL	EMNKRKAMEA	AQLADDLKAQ	LELAQKKLHD	FQDEIVENSV	TKEKDMFNFK
**890**	**900**	**910**	**920**	**930**	**940**	**950**	**960**
RAQEDISRLR	RKLETTKKPD	NVPKCDEILM	EEIKDYKARL	TCPCCNMRKK	DAVLTKCFHV	FCFECVKTRY	DTRQRKCPKC
**970**	**980**						
NAAFGANDFH	RIYIG						

Cmpd.	No. of Cmpds.	m/z meas.	Δ m/z [ppm]	z	Rt [min]	Score	P	Range	Sequence	Modification
1337	1	926.7938	6.01	3	45.5	12.7	1	257-280	K.VETAESRVSVLESMIDDLQWDIDK.I	
2961	1	788.3914	-25.63	2	65.8	15.7	2	835-848	K.RKAMEAAQLADDLK.A	Oxidation: 4
1957	2	918.3405	-92.02	2	52.8	13.9	2	956-971	R.KCPKCNAAFGANDFHR.I	Carbamidomethyl: 5

Detailed Protein Report

Protein 65: frizzled-10 precursor [Homo sapiens]

**Accession:**	gi|6005762	**Score:**	42.2
**Database:**	refseq_human(refseq_human_20140103.fasta)	**MW [kDa]:**	65.3
**Database Date:**	2015-11-30	**pI:**	9.9
**Modification(s):**	Carbamidomethyl, Oxidation	**Sequence Coverage [%]:**	6.4
		**No. of unique Peptides:**	2

10	20	30	40	50	60	70	80
MQRPGPRLWL	VLQVMGSCAA	ISSMDMERPG	DGKCQPIEIP	MCKDIGYNMT	RMPNLMGHEN	QREAAIQLHE	FAPLVEYGCH
**90**	**100**	**110**	**120**	**130**	**140**	**150**	**160**
GHLRFFLCSL	YAPMCTEQVS	TPIPACRVMC	EQARLKCSPI	MEQFNFKWPD	SLDCRKLPNK	NDPNYLCMEA	PNNGSDEPTR
**170**	**180**	**190**	**200**	**210**	**220**	**230**	**240**
GSGLFPPLFR	PQRPHSAQEH	PLKDGGPGRG	GCDNPGKFHH	VEKSASCAPL	CTPGVDVYWS	REDKRFAVVW	LAIWAVLCFF
**250**	**260**	**270**	**280**	**290**	**300**	**310**	**320**
SSAFTVLTFL	IDPARFRYPE	RPIIFLSMCY	CVYSVGYLIR	LFAGAESIAC	DRDSGQLYVI	QEGLESTGCT	LVFLVLYYFG
**330**	**340**	**350**	**360**	**370**	**380**	**390**	**400**
MASSLWWVVL	TLTWFLAAGK	KWGHEAIEAN	SSYFHLAAWA	IPAVKTILIL	VMRRVAGDEL	TGVCYVGSMD	VNALTGFVLI
**410**	**420**	**430**	**440**	**450**	**460**	**470**	**480**
PLACYLVIGT	SFILSGFVAL	FHIRRVMKTG	GENTDKLEKL	MVRIGLFSVL	YTVPATCVIA	CYFYERLNMD	YWKILAAQHK
**490**	**500**	**510**	**520**	**530**	**540**	**550**	**560**
CKMNNQTKTL	DCLMAASIPA	VEIFMVKIFM	LLVVGITSGM	WIWTSKTLQS	WQQVCSRRLK	KKSRRKPASV	ITSGGIYKKA
**570**	**580**	**590**					
QHPQKTHHGK	YEIPAQSPTC	V					

Cmpd.	No. of Cmpds.	m/z meas.	Δ m/z [ppm]	z	Rt [min]	Score	P	Range	Sequence	Modification
1958	2	655.2730	-62.48	2	52.8	10.4	0	281-292	R.LFAGAESIACDR.D	Carbamidomethyl: 10
2186	1	928.7350	-68.15	3	55.7	17.1	1	483-507	K.MNNQTKTLDCLMAASIPAVEIFMVK.I	Oxidation: 23

Detailed Protein Report

Protein 66: PREDICTED: dynein heavy chain 12, axonemal isoform X1 [Homo sapiens]

**Accession:**	gi|578805765	**Score:**	42.2
**Database:**	refseq_human(refseq_human_20140103.fasta)	**MW [kDa]:**	454.9
**Database Date:**	2015-11-30	**pI:**	5.5
		**Sequence Coverage [%]:**	1.1
		**No. of unique Peptides:**	2

Quantitation

WD:WU	**Median:** 0.56	**CV:** 15.68 %	**No. of Peptides:** 2

Detailed Protein Report

10	20	30	40	50	60	70	80
MSDANKAAIA	AEKEALNLKL	PPIVHLPENI	GVDTPTQSKL	LKYRRSKEQQ	QKINQLVIDG	AKRNLDRTLG	KRTPLLPPPD
90	100	110	120	130	140	150	160
YPQTMTSEMK	KKGFNYIYMK	QCVESSPLVP	IQQEWLDHML	RLIPESLKEG	KEREELLESL	INEVSSDFEN	SMKRYLVQSV
170	180	190	200	210	220	230	240
LVKPPVKSLE	DEGGPLPESP	VGLDYSNPWH	SSYVQARNQI	FSNLHIIHPT	MKMLLDLGYT	TFADTVLLDF	TGIRAKGPID
250	260	270	280	290	300	310	320
CESLKTDLSI	QTRNAEEKIM	NTWYPKVINL	FTKKEALEGV	KPEKLDAFYS	CVSTLMSNQL	KDLLRRTVEG	FVKLFDPKDQ
330	340	350	360	370	380	390	400
QRLPIFKIEL	TFDDDKMEFY	PTFQDLEDNV	LSLVERIAEA	LQNVQTIPSW	LSGTSTPVNL	DTELPEHVLH	WAVDTLKAAV
410	420	430	440	450	460	470	480
HRNLEGARKH	YETYVEKYNW	LLDGTAVENI	ETFQTEDHTF	DEYTEFIEKF	LSLASEIMLL	PQWIHYTMVR	LDCEDLKTGL
490	500	510	520	530	540	550	560
TNKAKAFANI	LLNDIASKYR	KENECICSEF	EAIKEHALKV	PETTEEMMDL	ISYVEKARTV	GIEELILRIQ	ESKRQMSYFL
570	580	590	600	610	620	630	640
DVFLFPQEDL	ALNATVLMWP	RKINPIFDEN	DELIENAKHK	KENELMAKRE	KLILEIEKES	RRMEEFTEFA	ELERMQQYVT
650	660	670	680	690	700	710	720
DVRQLQKRIQ	ESEEAVQFIN	KEEELFKWEL	TKYPELDKLK	VNIEPYQKFF	NFVLKWQRSE	KRWMDGGFLD	LNGESMEADV
730	740	750	760	770	780	790	800
EEFSREIFKT	LKFFQTKLKK	ELQEKRKAAR	KRSLEEEKIE	EEPKDNATIT	MCSTVMEQIK	AFKEYIPTVS	ILCNPGMRAR
810	820	830	840	850	860	870	880
HWKQISEIVG	YDLTPDSGTT	LRKVLKLNLT	PYLEQFEVIS	AGASKEFSLE	KAMNTMIGTW	EDIAFHISLY	RDTGVCILSS
890	900	910	920	930	940	950	960
VDEIQAILDD	QIIKTQTMRG	SPFIKPFEHE	IKAWEDRLIR	IQETIDEWLK	VQAQWLYLEP	IFCSEDIMQQ	MPEEGRQFQT
970	980	990	1000	1010	1020	1030	1040
VDRHWRDIMK	FCAKDPKVLA	ATSLTGLLEK	LQNCNELLEK	IMKGLNAYLE	KKRLFFPRFF	FLSNDEMLEI	LSETKDPLRV
1050	1060	1070	1080	1090	1100	1110	1120
QPHLKKCFEG	IAKLEFLPNL	DIKAMYSSEG	ERVELIALIS	TSAARGAVEK	WLIQVEDLML	RSVHDVIAAA	RLAYPESARR
1130	1140	1150	1160	1170	1180	1190	1200
DWVREWPGQV	VLCISQMFWT	SETQEVISGG	TEGLKKYYKE	LQNQLNEIVE	LVRGKLSKQT	RTTLGALVTI	DVHARDVVMD
1210	1220	1230	1240	1250	1260	1270	1280
MIKMGVSHDT	DFLWLAQLRY	YWENENARVR	IINCNVKYAY	EYLGNSPRLV	ITPLTDRCYR	TLIGAFYLNL	GGAPEGPAGT
1290	1300	1310	1320	1330	1340	1350	1360
GKTETTKDLA	KALAVQCVVF	NCSDGLDYLA	MGKFFKGLAS	SGAWACFDEF	NRIELEVLSV	VAQQILCIQR	AIQQKLVVFV
1370	1380	1390	1400	1410	1420	1430	1440
FEGTELKLNP	NCFVAITMNP	GYAGRSELPD	NLKVLFRTVA	MMVPNYALIA	EISLYSYGFL	NARPLSVKIV	MTYRLCSEQL
1450	1460	1470	1480	1490	1500	1510	1520
SSQFHYDYGM	RAVKAVLVAA	GNLKLKYPNE	NEDILLLRSI	KDVNEPKFLS	HDIPLFNGIT	SDLFPGIKLP	EADYHEFLEC
1530	1540	1550	1560	1570	1580	1590	1600
AHEACNVHNL	QPVKFFLEKI	IQTYEMMIVR	HGFMLVGEPF	AAKTKVLHVL	ADTLTLMNEH	GYGEEEKVIY	RTVNPKSITM
1610	1620	1630	1640	1650	1660	1670	1680
GQLFGQFDPV	SHEWTDGIVA	NTFREFALSE	TPDRKWVVFD	GPIDTLWIES	MNTVLDDNKK	LCLMSGEIIQ	MSPQMSLIFE
1690	1700	1710	1720	1730	1740	1750	1760
TMDLSQASPA	TVSRCGMIYL	EPSQLGWEPL	VSSWLNSLKG	PLCEPEYQAL	LRGLFAWLIP	PSLNQRVELF	QLNYLYTTIV
1770	1780	1790	1800	1810	1820	1830	1840
SKILKILITF	RISNYFKYVP	LKTQCTFIKF	FLHQQACFIF	SLIWSIGGSC	DTDGRRVFDT	FIRLIILGKD	DENPVPDSVG
1850	1860	1870	1880	1890	1900	1910	1920
KWECPFDEKG	LVYDYMYELK	NKGRWVHWNE	LIKNTNLGDK	QIKIQDIIVP	TMDTIRYTFL	MDLSITYAKP	LLFVGPTGTG
1930	1940	1950	1960	1970	1980	1990	2000
KSVYVKDKLM	NHLEKDQYFP	FYINLSARTS	ANQVQNIIMA	RLDKRRKGVF	GPPMGKKCII	FIDDMNMPAL	EKYGAQPPIE
2010	2020	2030	2040	2050	2060	2070	2080
LLRQFFDCGH	WYDLKDTSKI	TLVDIELIAA	MGPPGGGRNP	VTPRCIRHFN	ICSINSFSDE	TMVRIFSSIV	AFYLRTHEFP
2090	2100	2110	2120	2130	2140	2150	2160
PEYFVIGNQI	VNGTMEIYKQ	SVENLLPTPT	KSHYTFNLRD	FSRVIRGCLL	IERDAVANKH	TMIRLFVHEV	LRVFYDRLIN
2170	2180	2190	2200	2210	2220	2230	2240

Detailed Protein Report

Cmpd.	No. of Cmpds.	m/z meas.	Δ m/z [ppm]	z	Rt [min]	Score	P	Range	Sequence	Modification	Ratios	
1482	1	681.7771	64.45	3	47.3	18.6	2	267-284	K.VINLFTKKEALEGVKPEK.L		WD:WU	0.65
1201	1	1070.0173	-20.70	2	43.7	12.3	1	583-600	K.INPIFDENDELIENAKHK.K		WD:WU	0.48

Detailed Protein Report

Protein 67: PREDICTED: tetratricopeptide repeat protein 13 isoform X7 [Homo sapiens]

Accession: gi|578802328
Database: refseq_human(refseq_human_20140103.fasta)
Database Date: 2015-11-30

Score: 41.4
MW [kDa]: 57.4
pI: 9.3
Sequence Coverage [%]: 8.2
No. of unique Peptides: 1

10	20	30	40	50	60	70	80
MMLYHHGSLQ	EALKNFKRCL	QLEPYNEVCQ	YMKGLSHVAM	GQFYEGIKAQ	TKVMLNDPLP	GQKASPEYLK	VKYLREYSRY
90	100	110	120	130	140	150	160
LHAHLDTPLT	EYNIDVDLPG	SFKDHWAKNL	PFLIEDYEEQ	PGLQPHIKDV	LHQNFESYKP	EVQELICVAD	RLGSLMQYET
170	180	190	200	210	220	230	240
PGFLPNKRIH	RAMGLAALEV	MQAVQRTWTN	SKVRMNGKTR	LMQWRDMFDI	AVKWRRIADP	DQPVLWLDQM	PARSLSRGFN
250	260	270	280	290	300	310	320
NHINLIRGQV	INMRYLEYFE	KILHFIKDRI	LVYHGANNPK	GLLEVREALE	KVHKVEDLLP	IMKQFNTKTK	DGFTVNTKVP
330	340	350	360	370	380	390	400
SLKDQGKEYD	GFTITITGDK	VGNILFSVET	QTTEERTQLY	HAEIDALYKD	LTAKGKVLIL	SSEFGEADAV	CNLILSLVYY
410	420	430	440	450	460	470	480
FYNLMPLSRG	SSVIAYSVIV	GALMASGKEV	AGKIPKGKLV	DFEAMTAPGS	EAFSKVAKSW	MNLKSISPSY	KTLPSVSETF
490	500	510					
PTLRSMIEVL	NTDSSPRCLK	KL					

Cmpd.	No. of Cmpds.	m/z meas.	Δ m/z [ppm]	z	Rt [min]	Score	P	Range	Sequence	Modification
1682	2	820.8728	-97.18	2	49.4	15.9	1	234-247	R.SLSRGFNNHINLIR.G	

Detailed Protein Report

Protein 68: DNA-directed RNA polymerase, mitochondrial precursor [Homo sapiens]

Accession: gi|110618253
Database: refseq_human(refseq_human_20140103.fasta)
Database Date: 2015-11-30
Modification(s): Carbamidomethyl

Score: 41.3
MW [kDa]: 138.5
pI: 10.1
Sequence Coverage [%]: 1.2
No. of unique Peptides: 1

10	20	30	40	50	60	70	80
MSALCWGRGA	AGLKRALRPC	GRPGLPGKEG	TAGGVCGPRR	SSSASPQEQD	QDRRKDWGHV	ELLEVLQARV	RQLQAESVSE
**90**	**100**	**110**	**120**	**130**	**140**	**150**	**160**
VVVNRVDVAR	LPECGSGDGS	LQPPRKVQMG	AKDATPVPCG	RWAKILEKDK	RTQQMRMQRL	KAKLQMPFQS	GEFKALTRRL
**170**	**180**	**190**	**200**	**210**	**220**	**230**	**240**
QVEPRLLSKQ	MAGCLEDCTR	QAPESPWEEQ	LARLLQEAPG	KLSLDVEQAP	SGQHSQAQLS	GQQQRLLAFF	KCCLLTDQLP
**250**	**260**	**270**	**280**	**290**	**300**	**310**	**320**
LAHHLLVVHH	GQRQKRKLLT	LDMYNAVMLG	WARQGAFKEL	VYVLFMVKDA	GLTPDLLSYA	AALQCMGRQD	QDAGTIERCL
**330**	**340**	**350**	**360**	**370**	**380**	**390**	**400**
EQMSQEGLKL	QALFTAVLLS	EEDRATVLKA	VHKVKPTFSL	PPQLPPPVNT	SKLLRDVYAK	DGRVSYPKLH	LPLKTLQCLF
**410**	**420**	**430**	**440**	**450**	**460**	**470**	**480**
EKQLHMELAS	RVCVVSVEKP	TLPSKEVKHA	RKTLKTLRDQ	WEKALCRALR	ETKNRLEREV	YEGRFSLYPF	LCLLDEREVV
**490**	**500**	**510**	**520**	**530**	**540**	**550**	**560**
RMLLQVLQAL	PAQGESFTTL	ARELSARTFS	RHVVQRQRVS	GQVQALQNHY	RKYLCLLASD	AEVPEPCLPR	QYWEELGAPE
**570**	**580**	**590**	**600**	**610**	**620**	**630**	**640**
ALREQPWPLP	VQMELGKLLA	EMLVQATQMP	CSLDKPHRSS	RLVPVLYHVY	SFRNVQQIGI	LKPHPAYVQL	LEKAAEPTLT
**650**	**660**	**670**	**680**	**690**	**700**	**710**	**720**
FEAVDVPMLC	PPLPWTSPHS	GAFLLSPTKL	MRTVEGATQH	QELLETCPPT	ALHGALDALT	QLGNCAWRVN	GRVLDLVLQL
**730**	**740**	**750**	**760**	**770**	**780**	**790**	**800**
FQAKGCPQLG	VPAPPSEAPQ	PPEAHLPHSA	APARKAELRR	ELAHCQKVAR	EMHSLRAEAL	YRLSLAQHLR	DRVFWLPHNM
**810**	**820**	**830**	**840**	**850**	**860**	**870**	**880**
DFRGRTYPCP	PHFNHLGSDV	ARALLEFAQG	RPLGPHGLDW	LKIHLVNLTG	LKKREPLRKR	LAFAEEVMDD	ILDSADQPLT
**890**	**900**	**910**	**920**	**930**	**940**	**950**	**960**
GRKWWMGAEE	PWQTLACCME	VANAVRASDP	AAYVSHLPVH	QDGSCNGLQH	YAALGRDSVG	AASVNLEPSD	VPQDVYSGVA
**970**	**980**	**990**	**1000**	**1010**	**1020**	**1030**	**1040**
AQVEVFRRQD	AQRGMRVAQV	LEGFITRKVV	KQTVMTVVYG	VTRYGGRLQI	EKRLRELSDF	PQEFVWEASH	YLVRQVFKSL
**1050**	**1060**	**1070**	**1080**	**1090**	**1100**	**1110**	**1120**
QEMFSGTRAI	QHWLTESARL	ISHMGSVVEW	VTPLGVPVIQ	PYRLDSKVKQ	IGGGIQSITY	THNGDISRKP	NTRKQKNGFP
**1130**	**1140**	**1150**	**1160**	**1170**	**1180**	**1190**	**1200**
PNFIHSLDSS	HMMLTALHCY	RKGLTFVSVH	DCYWTHAADV	SVMNQVCREQ	FVRLHSEPIL	QDLSRFLVKR	FCSEPQKILE
**1210**	**1220**	**1230**	**1240**				
ASQLKETLQA	VPKPGAFDLE	QVKRSTYFFS					

Cmpd.	No. of Cmpds.	m/z meas.	Δ m/z [ppm]	z	Rt [min]	Score	P	Range	Sequence	Modification
125	2	793.8591	-42.14	2	30.8	18.8	1	107-121	K.VQMGAKDATPVPCGR.W	Carbamidomethyl: 13

Detailed Protein Report

Protein 69: sarcoplasmic/endoplasmic reticulum calcium ATPase 2 isoform a [Homo sapiens]

**Accession:**	gi|4502285	**Score:**	41.2
**Database:**	refseq_human(refseq_human_20140103.fasta)	**MW [kDa]:**	109.6
**Database Date:**	2015-11-30	**pI:**	5.1
**Modification(s):**	Oxidation	**Sequence Coverage [%]:**	3.4
		**No. of unique Peptides:**	2

10	20	30	40	50	60	70	80
MENAHTKTVE	EVLGHFGVNE	STGLSLEQVK	KLKERWGSNE	LPAEEGKTLL	ELVIEQFEDL	LVRILLLAAC	ISFVLAWFEE
90	100	110	120	130	140	150	160
GEETITAFVE	PFVILLILVA	NAIVGVWQER	NAENAIEALK	EYEPEMGKVY	RQDRKSVQRI	KAKDIVPGDI	VEIAVGDKVP
170	180	190	200	210	220	230	240
ADIRLTSIKS	TTLRVDQSIL	TGESVSVIKH	TDPVPDPRAV	NQDKKNMLFS	GTNIAAGKAM	GVVVATGVNT	EIGKIRDEMV
250	260	270	280	290	300	310	320
ATEQERTPLQ	QKLDEFGEQL	SKVISLICIA	VWIINIGHFN	DPVHGGSWIR	GAIYYFKIAV	ALAVAAIPEG	LPAVITTCLA
330	340	350	360	370	380	390	400
LGTRRMAKKN	AIVRSLPSVE	TLGCTSVICS	DKTGTLTTNQ	MSVCRMFILD	RVEGDTCSLN	EFTITGSTYA	PIGEVHKDDK
410	420	430	440	450	460	470	480
PVNCHQYDGL	VELATICALC	NDSALDYNEA	KGVYEKVGEA	TETALTCLVE	KMNVFDTELK	GLSKIERANA	CNSVIKQLMK
490	500	510	520	530	540	550	560
KEFTLEFSRD	RKSMSVYCTP	NKPSRTSMSK	MFVKGAPEGV	IDRCTHIRVG	STKVPMTSGV	KQKIMSVIRE	WGSGSDTLRC
570	580	590	600	610	620	630	640
LALATHDNPL	RREEMHLEDS	ANFIKYETNL	TFVGCVGMLD	PPRIEVASSV	KLCRQAGIRV	IMITGDNKGT	AVAICRRIGI
650	660	670	680	690	700	710	720
FGQDEDVTSK	AFTGREFDEL	NPSAQRDACL	NARCFARVEP	SHKSKIVEFL	QSFDEITAMT	GDGVNDAPAL	KKAEIGIAMG
730	740	750	760	770	780	790	800
SGTAVAKTAS	EMVLADDNFS	TIVAAVEEGR	AIYNNMKQFI	RYLISSNVGE	VVCIFLTAAL	GFPEALIPVQ	LLWVNLVTDG
810	820	830	840	850	860	870	880
LPATALGFNP	PDLDIMNKPP	RNPKEPLISG	WLFFRYLAIG	CYVGAATVGA	AAWWFIADGG	GPRVSFYQLS	HFLQCKEDNP
890	900	910	920	930	940	950	960
DFEGVDCAIF	ESPYPMTMAL	SVLVTIEMCN	ALNSLSENQS	LLRMPPWENI	WLVGSICLSM	SLHFLILYVE	PLPLIFQITP
970	980	990	1000				
LNVTQWLMVL	KISLPVILMD	ETLKFVARNY	LEPAILE				

Cmpd.	No. of Cmpds.	m/z meas.	Δ m/z [ppm]	z	Rt [min]	Score	P	Range	Sequence	Modification
1921	1	781.2702	-187.08	2	52.7	14.2	0	219-234	K.AMGVVVATGVNTEIGK.I	Oxidation: 2
1931	1	709.7683	-152.59	2	52.8	13.6	1	511-523	K.MFVKGAPEGVIDR.C	

Detailed Protein Report

Protein 70: dynein heavy chain 6, axonemal [Homo sapiens]

**Accession:**	gi|194353966	**Score:**	41.2
**Database:**	refseq_human(refseq_human_20140103.fasta)	**MW [kDa]:**	475.7
**Database Date:**	2015-11-30	**pI:**	5.6
**Modification(s):**	Oxidation	**Sequence Coverage [%]:**	1.1
		**No. of unique Peptides:**	3

Alias proteins:

Accession	Name	Description
gi|578802753	refseq_human (refseq_human_20140103.fasta)	PREDICTED: dynein heavy chain 6, axonemal isoform X1 [Homo sapiens]

Detailed Protein Report

10	20	30	40	50	60	70	80
MTFRATDSEF	DLTNIEEYAE	NSALSRLNNI	KAKQRVSYVT	STENESDTQI	LTFRHITKAQ	EKTRKRQQPI	KLEPLPVLKV
90	100	110	120	130	140	150	160
YQDHKQPEYI	HEQNRFQLMT	AGIIKRPVSI	AKKSFATSST	QFLEHQDAVK	KMQIHRPYVE	VFSPSPPKLP	HTGIGKRGLF
170	180	190	200	210	220	230	240
GTRSSAYPKY	TFHDREEVVK	ANIRDPLQII	KIIRENEHLG	FLYMIPAVPR	SSIEYDTYNL	KVVSYENINK	NDYYTISQRA
250	260	270	280	290	300	310	320
VTHIYNEDIE	FIEIDRWEQE	YLYHRELTKI	PIFSLFRKWK	AFSVWRKNVR	SKKITGCQKS	LQKNLFIVNP	HLRPALLKIN
330	340	350	360	370	380	390	400
ELCYHLSFMG	LCYIEKCHTY	TLQEFKAAQV	IRLAEVTERL	GEFRNEAKYV	VRRACRFALR	AAGFVPDDCA	FGPFEDYHKV
410	420	430	440	450	460	470	480
QSSGSFINTP	HELPTYGDSE	KMTYTEQASK	RHYCMRLTCF	IRLNDYLIEN	TMHILTVNAV	NSLLNHLTDK	LKRTPSADVI
490	500	510	520	530	540	550	560
QKWITEEKPE	VPDKKGTLMV	EKQEEDESLI	PMFLTELMLT	VQSLLFEPSL	EDFLDGILGA	VNHCQNTVLS	VPNLVPDSYF
570	580	590	600	610	620	630	640
DAFTSPYINN	KLEGKTCGTG	PSLAAVFEDD	KNFHTIISQI	KETIQAAFES	ARIYAATFEK	FQIFFKENES	LDLQALKLQE
650	660	670	680	690	700	710	720
PDINFFSEQL	EKYHKQHKDA	VALRPTRNVG	LLLIDTRLLR	EKLIPSPLRC	LEVLNFMLPR	QSKKKVDAII	FEAQDAEYKL
730	740	750	760	770	780	790	800
EFVPTTTTEY	VHSLLFLDEI	QERIESLEDE	GNIVTQMYKL	MEQYQVPTPP	EDFAVFATMK	PSIVAVRNAI	DKSVGDRESS
810	820	830	840	850	860	870	880
IKQFCVHLGS	DLEELNNEVN	EVKLQAQDPQ	ILDISADQDK	IRLILNNLQS	VLADLQKRAF	QYKSYQKNFK	VEVSKFEALE
890	900	910	920	930	940	950	960
EVSAELKLKQ	LLWDSFSEWD	KLQQEWLKSK	FDCLDPEVLN	GQVSKYAKFV	TQLEKGLPPN	SVVPQLKYKV	EKMKEKLPVI
970	980	990	1000	1010	1020	1030	1040
IDLRNPTLKA	RHWAAIEQTV	DATLVDAEIP	LTLERLSQLH	VFDFGQEIQD	ISGQASGEAA	LEAILKKVED	SWKTTEFVIL
1050	1060	1070	1080	1090	1100	1110	1120
PHRDSKDVFI	LGGTDDIQVL	LDDSTINVAT	LASSRYLGPL	KTRVDEWQKQ	LALFNQTLEE	WLTCQRNWLY	LESIFNAPDI
1130	1140	1150	1160	1170	1180	1190	1200
QRQLPAEAKM	FLQVDKSWKE	IMRKVNRLPN	ALRAATQPGL	LETFQNNNAL	LDQIQKCLEA	YLESKRVIFP	RFYFLSNDEL
1210	1220	1230	1240	1250	1260	1270	1280
LEILAQTRNP	QAVQPHLRKC	FDSISKLEFA	LMPPAEGKIP	GIDGEPEKVY	TNDILAMLSP	EGERVSLGKG	LKARGNVEEW
1290	1300	1310	1320	1330	1340	1350	1360
LGKVEEAMFT	SLRRLCKAAI	ADYQGKLRTD	WVVAGHPSQV	ILTVSQIMWC	RDLTECLETE	HSNHIQALKN	FEKVNFERLN
1370	1380	1390	1400	1410	1420	1430	1440
ALAAIVQGSL	PKLHRNILTA	LITIDVHARD	IVTELVQSKV	ETVESFDWQR	QLRYYWDIDL	DNCVARMALS	QYTYGYEYLG
1450	1460	1470	1480	1490	1500	1510	1520
ACPRLVITPL	TDRCYLCLMG	ALQLDLGGAP	AGPAGTGKTE	TTKDLAKALA	IQCVVFNCSD	GLDYKMMGRF	FSGLAQSGAW
1530	1540	1550	1560	1570	1580	1590	1600
CCFDEFNRID	IEVLSVIAQQ	LITIRNAKAA	KLSRFMFEGR	EIKLVMTCAA	FITMNPGYAG	RTELPDNLKA	LFRPFAMMVP
1610	1620	1630	1640	1650	1660	1670	1680
NYALIAEVIL	YSEGFESSKI	LARKMTQMYK	LCSEQLSQQD	HYDFGMRAVK	SVLVMAGSLK	RENPDLNEDV	VLIRALQDSN
1690	1700	1710	1720	1730	1740	1750	1760
LPKFLTDDAL	LFSGIISDLF	PGVQIPEHDY	GILQSTIVDV	MNRQNLQPEM	CMVRKVIQFY	ETMLVRHGVM	LVGPTGGGKT
1770	1780	1790	1800	1810	1820	1830	1840
TVYRILAETL	GNLQKLGIEN	SFYQAVKTYV	LNPKSITMGE	LYGEVNNLTL	EWKDGLMALS	VRAAVNDTSE	DHKWIISDGP
1850	1860	1870	1880	1890	1900	1910	1920
VDALWIENMN	TVLDDNKMLC	LANSERIKLT	PQIHMLFEVQ	DLRVASPATV	SRCGMVFVDP	EELKWMPYVK	TWMKGISKKL
1930	1940	1950	1960	1970	1980	1990	2000
TEETQEYILN	LFQRYVDEGL	HFINKKCSQA	IPQVDISKVT	TLCCLLESLI	LGKDGVNLAM	EQTKLNTILC	QTFVFCYLWS
2010	2020	2030	2040	2050	2060	2070	2080
LGGNLTENYY	DSFDTFIRTQ	FDDNPDARLP	NSGDLWSIHM	DFDTKRLDPW	ERIIPTFKYN	RDVPFFEMLV	PTTDTVRYGY
2090	2100	2110	2120	2130	2140	2150	2160
LMEKLLAVKH	SVLFTGITGV	GKSVIAKGLL	NKIQESAGYV	PVYLNFSAQT	SSARTQEIIE	SKLERKRKNI	LGAPGNKRIV
2170	2180	2190	2200	2210	2220	2230	2240

Detailed Protein Report

Cmpd.	No. of Cmpds.	m/z meas.	Δ m/z [ppm]	z	Rt [min]	Score	P	Range	Sequence	Modification
2264	1	1011.7667	170.21	1	56.8	10.2	1	2148-2157	R.KNILGAPGNK.R	
1872	1	973.5013	-53.30	2	51.7	13.0	2	2694-2710	K.NGLTKLLETNILVDKMK.L	Oxidation: 16
2924	1	1015.5128	-43.32	2	65.3	18.0	2	2905-2922	K.LRAAQAELDITMATLREK.Q	

Detailed Protein Report

Protein 71: **PREDICTED: LOW QUALITY PROTEIN: ubiquitin carboxyl-terminal hydrolase 17-like protein 20 [Homo sapiens]**

Accession: gi|578843530
Database: refseq_human(refseq_human_20140103.fasta)
Database Date: 2015-11-30

Score: 41.1
MW [kDa]: 38.1
pI: 9.8
Sequence Coverage [%]: 6.0
No. of unique Peptides: 2

10	20	30	40	50	60	70	80
MEDDSLYLGA	SAKVLILVLK	RFSDVTGNKI	AKNVQYPECL	DMQPYMSQPN	TGPLVYVLYA	VLVHAGWSCH	NGHYFSYVKA
**90**	**100**	**110**	**120**	**130**	**140**	**150**	**160**
QEGQWYKMDD	AEVTASSITS	VLSQQAYVLF	YIQKSEWERH	SESVSRGREP	RALGAEDTDR	RATQGELKRD	HPCLQAPELD
**170**	**180**	**190**	**200**	**210**	**220**	**230**	**240**
EHLVERATQE	STLDHWKFLQ	EQNKTKPEFN	VRKVEGTLPP	DVLVIHQSXY	KCGMKNHHPE	QQSSLLNLSS	TTPTHQESMN
**250**	**260**	**270**	**280**	**290**	**300**	**310**	**320**
TGTLASLRGR	ARRSKGKNKH	SKRALLVCQX	SQWKYRPTRT	VIKVDQPQRK	AAQGTTQGSV	EPQNLGRNPA	QAPKCAYEQG
**330**	**340**						
LRVTTLYIRH	RKPSDG						

Cmpd.	No. of Cmpds.	m/z meas.	Δ m/z [ppm]	z	Rt [min]	Score	P	Range	Sequence	Modification
1896	1	599.9585	-51.71	3	52.4	13.5	2	260-274	K.HSKRALLVCQNSQWK.Y	
1491	1	981.6005	60.92	2	47.4	15.8	1	264-279	R.ALLVCQISQWKYRPTR.T	

Detailed Protein Report

Protein 72: PREDICTED: WD repeat-containing protein 88 isoform X1 [Homo sapiens]

Accession: gi|578833944
Database: refseq_human(refseq_human_20140103.fasta)
Database Date: 2015-11-30
Modification(s): Carbamidomethyl

Score: 41.0
MW [kDa]: 31.0
pI: 6.8
Sequence Coverage [%]: 13.7
No. of unique Peptides: 3

Quantitation

WD:WU **Median:** 0.74 **CV:** 25.19 % **No. of Peptides:** 2

```
        10         20         30         40         50         60         70         80
MASPPRCSPT AHDRECKLPP PSAPASEYCP GKLSWGTMAR ALGRFKLSIP HTHLLATLDP LALDREPPPH LLPEKHQVPE
        90        100        110        120        130        140        150        160
KLIWGDQDPL SKIPFKILSG HEHAVSTCHF CVDDTKLLSG SYDCTVKLWD PVDGSVVRDF EHRPKAPVVE CSITGDSSRV
       170        180        190        200        210        220        230        240
IAASYDKTVR AWDLETGKLL WKVRYDTFIV SCKFSPDGKY VVSGFDVDHG ICIMDAENIT TVSVIKDHHT RSITSCCFDP
       250        260        270        280
DSQRVASVSL DRCIKIWDVT SQATLLTITK LFSHFWRV
```

Cmpd.	No. of Cmpds.	m/z meas.	Δ m/z [ppm]	z	Rt [min]	Score	P	Range	Sequence	Modification	Ratios
573	1	472.1766	-62.66	2	36.0	10.2	0	7-14	R.CSPTAHDR.E	Carbamidomethyl: 1	
2091	1	757.2566	-151.54	2	54.7	16.0	0	18-32	K.LPPPSAPASEYCPGK.L		WD:WU 0.95
1628	1	882.4156	-3.95	2	48.7	14.8	1	185-199	R.YDTFIVSCKFSPDGK.Y	Carbamidomethyl: 8	WD:WU 0.58

Detailed Protein Report

Protein 73: protein scribble homolog isoform b [Homo sapiens]

Accession: gi|355390315
Database: refseq_human(refseq_human_20140103.fasta)
Database Date: 2015-11-30
Modification(s): Carbamidomethyl

Score: 40.8
MW [kDa]: 174.8
pI: 4.9
Sequence Coverage [%]: 1.7
No. of unique Peptides: 2

10	20	30	40	50	60	70	80
MLKCIPLWRC	NRHVESVDKR	HCSLQAVPEE	IYRYSRSLEE	LLLDANQLRE	LPKPFFRLLN	LRKLGLSDNE	IQRLPPEVAN
90	100	110	120	130	140	150	160
FMQLVELDVS	RNDIPEIPES	IKFCKALEIA	DFSGNPLSRL	PDGFTQLRSL	AHLALNDVSL	QALPGDVGNL	ANLVTLELRE
170	180	190	200	210	220	230	240
NLLKSLPASL	SFLVKLEQLD	LGGNDLEVLP	DTLGALPNLR	ELWLDRNQLS	ALPPELGNLR	RLVCLDVSEN	RLEELPAELG
250	260	270	280	290	300	310	320
GLVLLTDLLL	SQNLLRRLPD	GIGQLKQLSI	LKVDQNRLCE	VTEAIGDCEN	LSELILTENL	LMALPRSLGK	LTKLTNLNVD
330	340	350	360	370	380	390	400
RNHLEALPPE	IGGCVALSVL	SLRDNRLAVL	PPELAHTTEL	HVLDVAGNRL	QSLPFALTHL	NLKALWLAEN	QAQPMLRFQT
410	420	430	440	450	460	470	480
EDDARTGEKV	LTCYLLPQQP	PPSLEDAGQQ	GSLSETWSDA	PPSRVSVIQF	LEAPIGDEDA	EEAAAEKRGL	QRRATPHPSE
490	500	510	520	530	540	550	560
LKVMKRSIEG	RRSEACPCQP	DSGSPLPAEE	EKRLSAESGL	SEDSRPSAST	VSEAEPEGPS	AEAQGGSQQE	ATTAGGEEDA
570	580	590	600	610	620	630	640
EEDYQEPTVH	FAEDALLPGD	DREIEEGQPE	APWTLPGGRQ	RLIRKDTPHY	KKHFKISKLP	QPEAVVALLQ	GMQPDGEGPV
650	660	670	680	690	700	710	720
APGGWHNGPH	APWAPRAQKE	EEEEEEGSPQ	EEEEEEEEEN	RAEEEEASTE	EEDKEGAVVS	APSVKGVSFD	QANNLLIEPA
730	740	750	760	770	780	790	800
RIEEEELTLT	ILRQTGGLGI	SIAGGKGSTP	YKGDDEGIFI	SRVSEEGPAA	RAGVRVGDKL	LEVNGVALQG	AEHHEAVEAL
810	820	830	840	850	860	870	880
RGAGTAVQMR	VWRERMVEPE	NAVTITPLRP	EDDYSPRERR	GGGLRLPLLP	PESPGPLRQR	HVACLARSER	GLGFSIAGGK
890	900	910	920	930	940	950	960
GSTPYRAGDA	GIFVSRIAEG	GAAHRAGTLQ	VGDRVLSING	VDVTEARHDH	AVSLLTAASP	TIALLLEREA	GGPLPPSPLP
970	980	990	1000	1010	1020	1030	1040
HSSPPTAAVA	TTSITTATPG	VPGLPSLAPS	LLAAALEGPY	PVEEIRLPRA	GGPLGLSIVG	GSDHSSHPFG	VQEPGVFISK
1050	1060	1070	1080	1090	1100	1110	1120
VLPRGLAARS	GLRVGDRILA	VNGQDVRDAT	HQEAVSALLR	PCLELSLLVR	RDPAPPGLRE	LCIQKAPGER	LGISIRGGAR
1130	1140	1150	1160	1170	1180	1190	1200
GHAGNPRDPT	DEGIFISKVS	PTGAAGRDGR	LRVGLRLLEV	NQQSLLGLTH	GEAVQLLRSV	GDTLTVLVCD	GFEASTDAAL
1210	1220	1230	1240	1250	1260	1270	1280
EVSPGVIANP	FAAGIGHRNS	LESISSIDRE	LSPEGPGKEK	ELPGQTLHWG	PEATEAAGRG	LQPLKLDYRA	LAAVPSAGSV
1290	1300	1310	1320	1330	1340	1350	1360
QRVPSGAAGG	KMAESPCSPS	GQQPPSPPSP	DELPANVKQA	YRAFAAVPTS	HPPEDAPAQP	PTPGPAASPE	QLSFRERQKY
1370	1380	1390	1400	1410	1420	1430	1440
FELEVRVPQA	EGPPKRVSLV	GADDLRKMQE	EEARKLQQKR	AQMLREAAEA	GAEARLALDG	ETLGEEEQED	EQPPWASPSP
1450	1460	1470	1480	1490	1500	1510	1520
TSRQSPASPP	PLGGGAPVRT	AKAERRHQER	LRVQSPEPPA	PERALSPAEL	RALEAEKRAL	WRAARMKSLE	QDALRAQMVL
1530	1540	1550	1560	1570	1580	1590	1600
SRSQEGRGTR	GPLERLAEAP	SPAPTPSPTP	VEDLGPQTST	SPGRLSPDFA	EELRSLEPSP	SPGPQEEDGE	VALVLLGRPS
1610	1620	1630	1640				
PGAVGPEDVA	LCSSRRPVRP	GRRGLGPVPS					

Cmpd.	No. of Cmpds.	m/z meas.	Δ m/z [ppm]	z	Rt [min]	Score	P	Range	Sequence	Modification
707	1	450.6514	-240.33	2	37.3	16.3	2	838-845	R.ERRGGGLR.L	
92	2	696.1357	141.31	3	30.4	24.5	2	861-880	R.HVACLARSERGLGFSIAGGK.G	Carbamidomethyl: 4

Detailed Protein Report

Protein 74: Alstrom syndrome protein 1 [Homo sapiens]

**Accession:**	gi|110349786	**Score:**	40.7
**Database:**	refseq_human(refseq_human_20140103.fasta)	**MW [kDa]:**	460.9
**Database Date:**	2015-11-30	**pI:**	5.8
**Modification(s):**	Carbamidomethyl	**Sequence Coverage [%]:**	1.1
		**No. of unique Peptides:**	2

Detailed Protein Report

10	20	30	40	50	60	70	80
MEPEDLPWPG	ELEEEEEEEE	EEEEEEEEEA	AAAAAAANVDD	VVVVEEVEEE	AGRELDSDSH	YGPQHLESID	DEEDEEAKAW
90	100	110	120	130	140	150	160
LQAHPGRILP	PLSPPQHRYS	EGERTSLEKI	VPLTCHVWQQ	IVYQGNSRTQ	ISDTNVVCLE	TTAQRGSGDD	QKTESWHCLP
170	180	190	200	210	220	230	240
QEMDSSQTLD	TSQTRFNVRT	EDTEVTDFPS	LEEGILTQSE	NQVKEPNRDL	FCSPLLVIQD	SFASPDLPLL	TCLTQDQEFA
250	260	270	280	290	300	310	320
PDSLFHQSEL	SFAPLRGIPD	KSEDTEWSSR	PSEVSEALFQ	ATAEVASDLA	SSRFSVSQHP	LIGSTAVGSQ	CPFLPSEQGN
330	340	350	360	370	380	390	400
NEETISSVDE	LKIPKDCDRY	DDLCSYMSWK	TRKDTQWPEN	NLADKDQVSV	ATSFDITDEN	IATKRSDHFD	AARSYGQYWT
410	420	430	440	450	460	470	480
QEDSSKQAET	YLTKGLQGKV	ESDVITLDGL	NENAVVCSER	VAELQRKPTR	ESEYHSSDLR	MLRMSPDTVP	KAPKHLKAGD
490	500	510	520	530	540	550	560
TSKGGIAKVT	QSNLKSGITT	TPVDSDIGSH	LSLSLEDLSQ	LAVSSPLETT	TGQHTDTLNQ	KTLADTHLTE	ETLKVTAIPE
570	580	590	600	610	620	630	640
PADQKTATPT	VLSSSHSHRG	KPSIFYQQGL	PDSHLTEEAL	KVSAAPGLAD	QTTGMSTLTS	TSYSHREKPG	TFYQQELPES
650	660	670	680	690	700	710	720
NLTEEPLEVS	AAPGPVEQKT	GIPTVSSTSH	SHVEDLLFFY	RQTLPDGHLT	DQALKVSAVS	GPADQKTGTA	TVLSTPHSHR
730	740	750	760	770	780	790	800
EKPGIFYQQE	FADSHQTEET	LTKVSATPGP	ADQKTEIPAV	QSSSYSQREK	PSILYPQDLA	DSHLPEEGLK	VSAVAGPADQ
810	820	830	840	850	860	870	880
KTGLPTVPSS	AYSHREKLLV	FYQQALLDSH	LPEEALKVSA	VSGPADGKTG	TPAVTSTSSA	SSSLGEKPSA	FYQQTLPNSH
890	900	910	920	930	940	950	960
LTEEALKVSI	VPGPGDQKTG	IPSAPSSFYS	HREKPIIFSQ	QTLPDFLFPE	EALKVSAVSV	LAAQKTGTPT	VSSNSHSHSE
970	980	990	1000	1010	1020	1030	1040
KSSVFYQQEL	PDSDLPRESL	KMSAIPGLTD	QKTVPTPTVP	SGSFSHREKP	SIFYQQEWPD	SYATEKALKV	STGPGPADQK
1050	1060	1070	1080	1090	1100	1110	1120
TEIPAVQSSS	YPQREKPSVL	YPQVLSDSHL	PEESLKVSAF	PGPADQMTDT	PAVPSTFYSQ	REKPGIFYQQ	TLPESHLPKE
1130	1140	1150	1160	1170	1180	1190	1200
ALKISVAPGL	ADQKTGTPTV	TSTSYSQHRE	KPSIFHQQAL	PGTHIPEEAQ	KVSAVTGPGN	QKTWIPRVLS	TFYSQREKPG
1210	1220	1230	1240	1250	1260	1270	1280
IFYQQTLPGS	HIPEEAQKVS	PVLGPADQKT	GTPTPTSASY	SHTEKPGIFY	QQVLPDNHPT	EEALKISVAS	EPVDQTTGTP
1290	1300	1310	1320	1330	1340	1350	1360
AVTSTSYSQY	REKPSIFYQQ	SLPSSHLTEE	AKNVSAVPGP	ADQKTVIPIL	PSTFYSHTEK	PGVFYQQVLP	HSHPTEEALK
1370	1380	1390	1400	1410	1420	1430	1440
ISVASEPVDQ	TTGTPTVTST	SYSQHTEKPS	IFYQQSLPGS	HLTEEAKNVS	AVPGPGDRKT	GIPTLPSTFY	SHTEKPGSFY
1450	1460	1470	1480	1490	1500	1510	1520
QQVLPHSHLP	EEALEVSVAP	GPVDQTIGTP	TVTSPSSSFG	EKPIVIYKQA	FPEGHLPEES	LKVSVAPGPV	GQTTGAPTIT
1530	1540	1550	1560	1570	1580	1590	1600
SPSYSQHRAK	SGSFYQLALL	GSQIPEEALR	VSSAPGPADQ	TTGIPTITST	SYSFGEKPIV	NYKQAFPDGH	LPEEALKVSI
1610	1620	1630	1640	1650	1660	1670	1680
VSGPTEKKTD	IPAGPLGSSA	LGEKPITFYR	QALLDSPLNK	EVVKVSAAPG	PADQKTETLP	VHSTSYSNRG	KPVIFYQQTL
1690	1700	1710	1720	1730	1740	1750	1760
SDSHLPEEAL	KVPPVPGPDA	QKTETPSVSS	SLYSYREKPI	VFYQQALPDS	ELTQEALKVS	AVPQPADQKT	GLSTVTSSFY
1770	1780	1790	1800	1810	1820	1830	1840
SHTEKPNISY	QQELPDSHLT	EEALKVSNVP	GPADQKTGVS	TVTSTSYSHR	EKPIVSYQRE	LPHFTEAGLK	ILRVPGPADQ
1850	1860	1870	1880	1890	1900	1910	1920
KTGINILPSN	SYPQREHSVI	SYEQELPDLT	EVTLKAIGVP	GPADQKTGIQ	IASSSSYSNR	EKASIFHQQE	LPDVTEEALN
1930	1940	1950	1960	1970	1980	1990	2000
VFVVPGQGDR	KTEIPTVPLS	YYSRREKPSV	ISQQELPDSH	LTEEALKVSP	VSIPAEQKTG	IPIGLSSSYS	HSHKEKLKIS
2010	2020	2030	2040	2050	2060	2070	2080
TVHIPDDQKT	EFPAATLSSY	SQIEKPKIST	VIGPNDQKTP	SQTAFHSSYS	QTVKPNILFQ	QQLPDRDQSK	GILKISAVPE
2090	2100	2110	2120	2130	2140	2150	2160
LTDVNTGKPV	SLSSSYFHRE	KSNIFSPQEL	PGSHVTEDVL	KVSTIPGPAG	QKTVLPTALP	SSFSHREKPD	IFYQKDLPDR
2170	2180	2190	2200	2210	2220	2230	2240

Detailed Protein Report

Cmpd.	No. of Cmpds.	m/z meas.	Δ m/z [ppm]	z	Rt [min]	Score	P	Range	Sequence	Modification
312	1	862.8548	-22.20	2	33.0	10.7	1	340-352	R.YDDLCSYMSWKTR.K	Carbamidomethyl: 5
1836	4	863.4125	-60.24	2	51.6	18.6	1	3791-3805	R.LIQAFGHERVCLSPR.R	

Detailed Protein Report

Protein 75: PREDICTED: ADAMTS-like protein 3 isoform X3 [Homo sapiens]

Accession: gi|530406914
Database: refseq_human(refseq_human_20140103.fasta)
Database Date: 2015-11-30
Modification(s): Carbamidomethyl

Score: 40.4
MW [kDa]: 186.1
pI: 9.6
Sequence Coverage [%]: 3.4
No. of unique Peptides: 1

10	20	30	40	50	60	70	80
MASWTSPWWV	LIGMVFMHSP	LPQTTAEKSP	GAYFLPEFAL	SPQGSFLEDT	TGEQFLTYRY	DDQTSRNTRS	DEDKDGNWDA
**90**	**100**	**110**	**120**	**130**	**140**	**150**	**160**
WGDWSDCSRT	CGGGASYSLR	RCLTGRNCEG	QNIRYKTCSN	HDCPPDAEDF	RAQQCSAYND	VQYQGHYYEW	LPRYNDPAAP
**170**	**180**	**190**	**200**	**210**	**220**	**230**	**240**
CALKCHAQGQ	NLVVELAPKV	LDGTRCNTDS	LDMCISGICQ	AVGCDRQLGS	NAKEDNCGVC	AGDGSTCRLV	RGQSKSHVSP
**250**	**260**	**270**	**280**	**290**	**300**	**310**	**320**
EKREENVIAV	PLGSRSVRIT	VKGPAHLFIE	SKTLQGSKGE	HSFNSPGVFL	VENTTVEFQR	GSERQTFKIP	GPLMADFIFK
**330**	**340**	**350**	**360**	**370**	**380**	**390**	**400**
TRYTAAKDSV	VQFFFYQPIS	HQWRQTDFFP	CTVTCGGGYQ	LNSAECVDIR	LKRVVPDHYC	HYYPENVKPK	PKLKECSMDP
**410**	**420**	**430**	**440**	**450**	**460**	**470**	**480**
CPSSDGFKEI	MPYDHFQPLP	RWEHNPWTAC	SVSCGGGIQR	RSFVCVEESM	HGEILQVEEW	KCMYAPKPKV	MQTCNLFDCP
**490**	**500**	**510**	**520**	**530**	**540**	**550**	**560**
KWIAMEWSQC	TVTCGRGLRY	RVVLCINHRG	EHVGGCNPQL	KLHIKEECVI	PIPCYKPKEK	SPVEAKLPWL	KQAQELEETR
**570**	**580**	**590**	**600**	**610**	**620**	**630**	**640**
IATEEPTFIP	EPWSACSTTC	GPGVQVREVK	CRVLLTFTQT	ETELPEEECE	GPKLPTERPC	LLEACDESPA	SRELDIPLPE
**650**	**660**	**670**	**680**	**690**	**700**	**710**	**720**
DSETTYDWEY	AGFTPCTATC	VGGHQEAIAV	CLHIQTQQTV	NDSLCDMVHR	PPAMSQACNT	EPCPPRWHVG	SWGPCSATCG
**730**	**740**	**750**	**760**	**770**	**780**	**790**	**800**
VGIQTRDVYC	LHPGETPAPP	EECRDEKPHA	LQACNQFDCP	PGWHIEEWQQ	CSRTCGGGTQ	NRRVTCRQLL	TDGSFLNLSD
**810**	**820**	**830**	**840**	**850**	**860**	**870**	**880**
ELCQGPKASS	HKSCARTDCP	PHLAVGDWSK	CSVSCGVGIQ	RRKQVCQRLA	AKGRRIPLSE	MMCRDLPGLP	LVRSCQMPEC
**890**	**900**	**910**	**920**	**930**	**940**	**950**	**960**
SKIKSEMKTK	LGEQGPQILS	VQRVYIQTRE	EKRINLTIGS	RAYLLPNTSV	IIKCPVRRFQ	KSLIQWEKDG	RCLQNSKRLG
**970**	**980**	**990**	**1000**	**1010**	**1020**	**1030**	**1040**
ITKSGSLKIH	GLAAPDIGVY	RCIAGSAQET	VVLKLIGTDN	RLIARPALRE	PMREYPGMDH	SEANSLGVTW	HKMRQMWNNK
**1050**	**1060**	**1070**	**1080**	**1090**	**1100**	**1110**	**1120**
NDLYLDDDHI	SNQPFLRALL	GHCSNSAGST	NSWELKNKQF	EAAVKQGAYS	MDTAQFDELI	RNMSQLMETG	EVSDDLASQL
**1130**	**1140**	**1150**	**1160**	**1170**	**1180**	**1190**	**1200**
IYQLVAELAK	AQPTHMQWRG	IQEETPPAAQ	LRGETGSVSQ	SSHAKNSGKL	TFKPKGPVLM	RQSQPPSISF	NKTINSRIGN
**1210**	**1220**	**1230**	**1240**	**1250**	**1260**	**1270**	**1280**
TVYITKRTEV	INILCDLITP	SEATYTWTKD	GTLLQPSVKI	ILDGTGKIQI	QNPTRKEQGI	YECSVANHLG	SDVESSSVLY
**1290**	**1300**	**1310**	**1320**	**1330**	**1340**	**1350**	**1360**
AEAPVILSVE	RNITKPEHNH	LSVVVGGIVE	AALGANVTIR	CPVKGVPQPN	ITWLKRGGSL	SGNVSLLFNG	SLLLQNVSLE
**1370**	**1380**	**1390**	**1400**	**1410**	**1420**	**1430**	**1440**
NEGTYVCIAT	NALGKAVATS	VLHLLERRWP	ESRIVFLQGH	KKYILQATNT	RTNSNDPTGE	PPPQEPFWEP	GNWSHCSATC
**1450**	**1460**	**1470**	**1480**	**1490**	**1500**	**1510**	**1520**
GHLGARIQRP	QCVMANGQEV	SEALCDHLQK	PLAGFEPCNI	RDCPARWFTS	VWSQCSVSCG	EGYHSRQVTC	KRTKANGTVQ
**1530**	**1540**	**1550**	**1560**	**1570**	**1580**	**1590**	**1600**
VVSPRACAPK	DRPLGRKPCF	GHPCVQWEPG	NRCPGRCMGR	AVRMQQRHTA	CQHNSSDSNC	DDRKRPTLRR	NCTSGACDVC
**1610**	**1620**	**1630**	**1640**	**1650**	**1660**	**1670**	**1680**
WHTGPWKPCT	AACGRGFQSR	KVDCIHTRSC	KPVAKRHCVQ	KKKPISWRHC	LGPSCDNRYK	QRCCQSCQEG	

Cmpd.	No. of Cmpds.	m/z meas.	Δ m/z [ppm]	z	Rt [min]	Score	P	Range	Sequence	Modification
445	1	475.6547	-114.30	2	34.1	10.8	0	774-782	R.TCGGGTQNR.R	Carbamidomethyl: 2

Detailed Protein Report

Protein 76: kinesin-like protein KIF3B [Homo sapiens]

Accession: gi|4758646
Database: refseq_human(refseq_human_20140103.fasta)
Database Date: 2015-11-30

Score: 40.2
MW [kDa]: 85.1
pI: 7.8
Sequence Coverage [%]: 5.9
No. of unique Peptides: 2

10	20	30	40	50	60	70	80
MSKLKSSESV	RVVVRCRPMN	GKEKAASYDK	VVDVDVKLGQ	VSVKNPKGTA	HEMPKTFTFD	AVYDWNAKQF	ELYDETFRPL
90	100	110	120	130	140	150	160
VDSVLQGFNG	TIFAYGQTGT	GKTYTMEGIR	GDPEKRGVIP	NSFDHIFTHI	SRSQNQQYLV	RASYLEIYQE	EIRDLLSKDQ
170	180	190	200	210	220	230	240
TKRLELKERP	DTGVYVKDLS	SFVTKSVKEI	EHVMNVGNQN	RSVGATNMNE	HSSRSHAIFV	ITIECSEVGL	DGENHIRVGK
250	260	270	280	290	300	310	320
LNLVDLAGSE	RQAKTGAQGE	RLKEATKINL	SLSALGNVIS	ALVDGKSTHI	PYRDSKLTRL	LQDSLGGNAK	TVMVANVGPA
330	340	350	360	370	380	390	400
SYNVEETLTT	LRYANRAKNI	KNKPRVNEDP	KDALLREFQE	EIARLKAQLE	KRSIGRRKRR	EKRREGGGSG	GGGEEEEEEG
410	420	430	440	450	460	470	480
EEGEEEGDDK	DDYWREQQEK	LEIEKRAIVE	DHSLVAEEKM	RLLKEKEKKM	EDLRREKDAA	EMLGAKIKAM	ESKLLVGGKN
490	500	510	520	530	540	550	560
IVDHTNEQQK	ILEQKRQEIA	EQKRREREIQ	QQMESRDEET	LELKETYSSL	QQEVDIKTKK	LKKLFSKLQA	VKAEIHDLQE
570	580	590	600	610	620	630	640
EHIKERQELE	QTQNELTREL	KLKHLIIENF	IPLEEKSKIM	NRAFFDEEED	HWKLHPITRL	ENQQMMKRPV	SAVGYKRPLS
650	660	670	680	690	700	710	720
QHARMSMMIR	PEARYRAENI	VLLELDMPSR	TTRDYEGPAI	APKVQAALDA	ALQDEDEIQV	DASSFESTAN	KKSKARPKSG
730	740	750					
RKSGSSSSSS	GTPASQLYPQ	SRGLVPK					

Cmpd.	No. of Cmpds.	m/z meas.	Δ m/z [ppm]	z	Rt [min]	Score	P	Range	Sequence	Modification
850	1	911.1296	-3.32	3	39.4	14.3	2	178-201	K.DLSSFVTKSVKEIEHVMNVGNQNR.S	
182	2	795.4018	9.53	2	31.1	14.3	2	505-516	R.REREIQQQMESR.D	

Detailed Protein Report

Protein 77: DNA polymerase zeta catalytic subunit isoform b [Homo sapiens]

**Accession:**	gi|556695361	**Score:**	40.1
**Database:**	refseq_human(refseq_human_20140103.fasta)	**MW [kDa]:**	343.9
**Database Date:**	2015-11-30	**pI:**	9.5
**Modification(s):**	Carbamidomethyl, Oxidation	**Sequence Coverage [%]:**	1.4
		**No. of unique Peptides:**	3

Alias proteins:

Accession	Name	Description
gi|578812913	refseq_human (refseq_human_20140103.fasta)	PREDICTED: DNA polymerase zeta catalytic subunit isoform X6 [Homo sapiens]
gi|556695404	refseq_human (refseq_human_20140103.fasta)	DNA polymerase zeta catalytic subunit isoform b [Homo sapiens]

Detailed Protein Report

10	20	30	40	50	60	70	80
MAFSIDRALN	VALGNPSSTA	QHVFKVSLVS	GMPFYGYHEK	ERHFMKIYLY	NPTMVKRICE	LLQSGAIMNK	FYQPHEAHIP
90	100	110	120	130	140	150	160
YLLQLFIDYN	LYGMNLINLA	AVKFRKARRK	SNTLHATGSC	KNHLSGNSLA	DTLFRWEQDE	IPSSLILEGV	EPQSTCELEV
170	180	190	200	210	220	230	240
DAVAADILNR	LDIEAQIGGN	PGLQAIWEDE	KQRRRNRNET	SQMSQPESQD	HRFVPATESE	KKFQKRLQEI	LKQNDFSVTL
250	260	270	280	290	300	310	320
SGSVDYSDGS	QEFSAELTLH	SEVLSPEMLQ	CTPANMVEVH	KDKESSKGHT	RHKVEEALIN	EEAILNLMEN	SQTFQPLTQR
330	340	350	360	370	380	390	400
LSESPVFMDS	SPDEALVHLL	AGLESDGYRG	ERNRMPSPCR	SFGNNKYPQN	SDDEENEPQI	EKEEMELSLV	MSQRWDSNIE
410	420	430	440	450	460	470	480
EHCAKKRSLC	RNTHRSSTED	DDSSSGEEME	WSDNSLLLAS	LSIPQLDGTA	DENSDNPLNN	ENSRTHSSVI	ATSKLSVKPS
490	500	510	520	530	540	550	560
IFHKDAATLE	PSSSAKITFQ	CKHTSALSSH	VLNKEDLIED	LSQTNKNTEK	GLDNSVTSFT	NESTYSMKYP	GSLSSTVHSE
570	580	590	600	610	620	630	640
NSHKENSKKE	ILPVSSCESS	IFDYEEDIPS	VTRQVPSRKY	TNIRKIEKDS	PFIHMHRHPN	ENTLGKNSFN	FSDLNHSKNK
650	660	670	680	690	700	710	720
VSSEGNEKGN	STALSSLFPS	SFTENCELLS	CSGENRTMVH	SLNSTADESG	LNKLKIRYEE	FQEHKTEKPS	LSQQAAHYMF
730	740	750	760	770	780	790	800
FPSVVLSNCL	TRPQKLSPVT	YKLQPGNKPS	RLKLNKRKLA	GHQETSTKSS	ETGSTKDNFI	QNNPCNSNPE	KDNALASDLT
810	820	830	840	850	860	870	880
KTTRGAFENK	TPTDGFIDCH	FGDGTLETEQ	SFGLYGNKYT	LRAKRKVNYE	TEDSESSFVT	HNSKISLPHP	MEIGESLDGT
890	900	910	920	930	940	950	960
LKSRKRRKMS	KKLPPVIIKY	IIINRFRGRK	NMLVKLGKID	SKEKQVILTE	EKMELYKKLA	PLKDFWPKVP	DSPATKYPIY
970	980	990	1000	1010	1020	1030	1040
PLTPKKSHRR	KSKHKSAKKK	TGKQQRTNNE	NIKRTLSFRK	KRSHAILSPP	SPSYNAETED	CDLNYSDVMS	KLGFLSERST
1050	1060	1070	1080	1090	1100	1110	1120
SPINSSPPRC	WSPTDPRAEE	IMAAAEKEAM	LFKGPNVYKK	TVNSRIGKTS	RARAQIKKSK	AKLANPSIVT	KKRNKRNQTN
1130	1140	1150	1160	1170	1180	1190	1200
KLVDDGKKKP	RAKQKTNEKG	TSRKHTTLKD	EKIKSQSGAE	VKFVLKHQNV	SEFASSSGGS	QLLFKQKDMP	LMGSAVDHPL
1210	1220	1230	1240	1250	1260	1270	1280
SASLPTGINA	QQKLSGCFSS	FLESKKSVDL	QTFPSSRDDL	HPSVVCNSIG	PGVSKINVQR	PHNQSAMFTL	KESTLIQKNI
1290	1300	1310	1320	1330	1340	1350	1360
FDLSNHLSQV	AQNTQISSGM	SSKIEDNANN	IQRNYLSSIG	KLSEYRNSLE	SKLDQAYTPN	FLHCKDSQQQ	IVCIAEQSKH
1370	1380	1390	1400	1410	1420	1430	1440
SETCSPGNTA	SEESQMPNNC	FVTSLRSPIK	QIAWEQKQRG	FILDMSNFKP	ERVKPRSLSE	AISQTKALSQ	CKNRNVSTPS
1450	1460	1470	1480	1490	1500	1510	1520
AFGEGQSGLA	VLKELLQKRQ	QKAQNANTTQ	DPLSNKHQPN	KNISGSLEHN	KANKRTRSVT	SPRKPRTPRS	TKQKEKIPKL
1530	1540	1550	1560	1570	1580	1590	1600
LKVDSLNLQN	SSQLDNSVSD	DSPIFFSDPG	FESCYSLEDS	LSPEHNYNFD	INTIGQTGFC	SFYSGSQFVP	ADQNLPQKFL
1610	1620	1630	1640	1650	1660	1670	1680
SDAVQDLFPG	QAIEKNEFLS	HDNQKCDEDK	HHTTDSASWI	RSGTLSPEIF	EKSTIDSNEN	RRHNQWKNSF	HPLTTRSNSI
1690	1700	1710	1720	1730	1740	1750	1760
MDSFCVQQAE	DCLSEKSRLN	RSSVSKEVFL	SLPQPNNSDW	IQGHTRKEMG	QSLDSANTSF	TAILSSPDGE	LVDVACEDLE
1770	1780	1790	1800	1810	1820	1830	1840
LYVSRNNDML	TPTPDSSPRS	TSSPSQSKNG	SFTPRTANIL	KPLMSPPSRE	EIMATLLDHD	LSETIYQEPF	CSNPSDVPEK
1850	1860	1870	1880	1890	1900	1910	1920
PREIGGRLLM	VETRLANDLA	EFEGDFSLEG	LRLWKTAFSA	MTQNPRPGSP	LRSGQGVVNK	GSSNSPKMVE	DKKIVIMPCK
1930	1940	1950	1960	1970	1980	1990	2000
CAPSRQLVQV	WLQAKEEYER	SKKLPKTKPT	GVVKSAENFS	SSVNPDDKPV	VPPKMDVSPC	ILPTTAHTKE	DVDNSQIALQ
2010	2020	2030	2040	2050	2060	2070	2080
APTTGCSQTA	SESQMLPPVA	SASDPEKDED	DDDNYYISYS	SPDSPVIPPW	QQPISPDSKA	LNGDDRPSSP	VEELPSLAFE
2090	2100	2110	2120	2130	2140	2150	2160
NFLKPIKDGI	QKSPCSEPQE	PLVISPINTR	ARTGKCESLC	FHSTPIIQRK	LLERLPEAPG	LSPLSTEPKT	QKLSNKKGSN
2170	2180	2190	2200	2210	2220	2230	2240

Detailed Protein Report

Cmpd.	No. of Cmpds.	m/z meas.	Δ m/z [ppm]	z	Rt [min]	Score	P	Range	Sequence	Modification
727	2	517.1500	-168.10	2	37.5	14.5	1	353-360	R.NRMPSPCR.S	Carbamidomethyl: 7; Oxidation: 3
671	1	648.3571	-103.32	2	36.8	12.0	2	1943-1954	K.KLPKTKPTGVVK.S	
1743	1	786.4023	-29.21	3	50.5	13.6	1	2643-2664	R.QLGLKLIANVTFGYTSANFSGR.M	

Detailed Protein Report

Protein 78: von Willebrand factor preproprotein [Homo sapiens]

**Accession:**	gi|89191868	**Score:**	40.0
**Database:**	refseq_human(refseq_human_20140103.fasta)	**MW [kDa]:**	309.1
**Database Date:**	2015-11-30	**pI:**	5.2
**Modification(s):**	Carbamidomethyl	**Sequence Coverage [%]:**	1.0
		**No. of unique Peptides:**	3

Quantitation

WD:WU	**Median:** 0.69	**CV:** 0.00 %	**No. of Peptides:** 1

Detailed Protein Report

10	20	30	40	50	60	70	80
MIPARFAGVL	LALALILPGT	LCAEGTRGRS	STARCSLFGS	DFVNTFDGSM	YSFAGYCSYL	LAGGCQKRSF	SIIGDFQNGK
90	100	110	120	130	140	150	160
RVSLSVYLGE	FFDIHLFVNG	TVTQGDQRVS	MPYASKGLYL	ETEAGYYKLS	GEAYGFVARI	DGSGNFQVLL	SDRYFNKTCG
170	180	190	200	210	220	230	240
LCGNFNIFAE	DDFMTQEGTL	TSDPYDFANS	WALSSGEQWC	ERASPPSSSC	NISSGEMQKG	LWEQCQLLKS	TSVFARCHPL
250	260	270	280	290	300	310	320
VDPEPFVALC	EKTLCECAGG	LECACPALLE	YARTCAQEGM	VLYGWTDHSA	CSPVCPAGME	YRQCVSPCAR	TCQSLHINEM
330	340	350	360	370	380	390	400
CQERCVDGCS	CPEGQLLDEG	LCVESTECPC	VHSGKRYPPG	TSLSRDCNTC	ICRNSQWICS	NEECPGECLV	TGQSHFKSFD
410	420	430	440	450	460	470	480
NRYFTFSGIC	QYLLARDCQD	HSFSIVIETV	QCADDRDAVC	TRSVTVRLPG	LHNSLVKLKH	GAGVAMDGQD	VQLPLLKGDL
490	500	510	520	530	540	550	560
RIQHTVTASV	RLSYGEDLQM	DWDGRGRLLV	KLSPVYAGKT	CGLCGNYNGN	QGDDFLTPSG	LAEPRVEDFG	NAWKLHGDCQ
570	580	590	600	610	620	630	640
DLQKQHSDPC	ALNPRMTRFS	EEACAVLTSP	TFEACHRAVS	PLPYLRNCRY	DVCSCSDGRE	CLCGALASYA	AACAGRGVRV
650	660	670	680	690	700	710	720
AWREPGRCEL	NCPKGQVYLQ	CGTPCNLTCR	SLSYPDEECN	EACLEGCFCP	PGLYMDERGD	CVPKAQCPCY	YDGEIFQPED
730	740	750	760	770	780	790	800
IFSDHHTMCY	CEDGFMHCTM	SGVPGSLLPD	AVLSSPLSHR	SKRSLSCRPP	MVKLVCPADN	LRAEGLECTK	TCQNYDLECM
810	820	830	840	850	860	870	880
SMGCVSGCLC	PPGMVRHENR	CVALERCPCF	HQGKEYAPGE	TVKIGCNTCV	CRDRKWNCTD	HVCDATCSTI	GMAHYLTFDG
890	900	910	920	930	940	950	960
LKYLFPGECQ	YVLVQDYCGS	NPGTFRILVG	NKGCSHPSVK	CKKRVTILVE	GGEIELFDGE	VNVKRPMKDE	THFEVVESGR
970	980	990	1000	1010	1020	1030	1040
YIILLLGKAL	SVVWDRHLSI	SVVLKQTYQE	KVCGLCGNFD	GIQNNDLTSS	NLQVEEDPVD	FGNSWKVSSQ	CADTRKVPLD
1050	1060	1070	1080	1090	1100	1110	1120
SSPATCHNNI	MKQTMVDSSC	RILTSDVFQD	CNKLVDPEPY	LDVCIYDTCS	CESIGDCACF	CDTIAAYAHV	CAQHGKVVTW
1130	1140	1150	1160	1170	1180	1190	1200
RTATLCPQSC	EERNLRENGY	ECEWRYNSCA	PACQVTCQHP	EPLACPVQCV	EGCHAHCPPG	KILDELLQTC	VDPEDCPVCE
1210	1220	1230	1240	1250	1260	1270	1280
VAGRRFASGK	KVTLNPSDPE	HCQICHCDVV	NLTCEACQEP	GGLVVPPTDA	PVSPTTLYVE	DISEPPLHDF	YCSRLLDLVF
1290	1300	1310	1320	1330	1340	1350	1360
LLDGSSRLSE	AEFEVLKAFV	VDMMERLRIS	QKWVRVAVVE	YHDGSHAYIG	LKDRKRPSEL	RRIASQVKYA	GSQVASTSEV
1370	1380	1390	1400	1410	1420	1430	1440
LKYTLFQIFS	KIDRPEASRI	TLLLMASQEP	QRMSRNFVRY	VQGLKKKKVI	VIPVGIGPHA	NLKQIRLIEK	QAPENKAFVL
1450	1460	1470	1480	1490	1500	1510	1520
SSVDELEQQR	DEIVSYLCDL	APEAPPPTLP	PDMAQVTVGP	GLLGVSTLGP	KRNSMVLDVA	FVLEGSDKIG	EADFNRSKEF
1530	1540	1550	1560	1570	1580	1590	1600
MEEVIQRMDV	GQDSIHVTVL	QYSYMVTVEY	PFSEAQSKGD	ILQRVREIRY	QGGNRTNTGL	ALRYLSDHSF	LVSQGDREQA
1610	1620	1630	1640	1650	1660	1670	1680
PNLVYMVTGN	PASDEIKRLP	GDIQVVPIGV	GPNANVQELE	RIGWPNAPIL	IQDFETLPRE	APDLVLQRCC	SGEGLQIPTL
1690	1700	1710	1720	1730	1740	1750	1760
SPAPDCSQPL	DVILLLDGSS	SFPASYFDEM	KSFAKAFISK	ANIGPRLTQV	SVLQYGSITT	IDVPWNVVPE	KAHLLSLVDV
1770	1780	1790	1800	1810	1820	1830	1840
MQREGGPSQI	GDALGFAVRY	LTSEMHGARP	GASKAVVILV	TDVSVDSVDA	AADAARSNRV	TVFPIGIGDR	YDAAQLRILA
1850	1860	1870	1880	1890	1900	1910	1920
GPAGDSNVVK	LQRIEDLPTM	VTLGNSFLHK	LCSGFVRICM	DEDGNEKRPG	DVWTLPDQCH	TVTCQPDGQT	LLKSHRVNCD
1930	1940	1950	1960	1970	1980	1990	2000
RGLRPSCPNS	QSPVKVEETC	GCRWTCPCVC	TGSSTRHIVT	FDGQNFKLTG	SCSYVLFQNK	EQDLEVILHN	GACSPGARQG
2010	2020	2030	2040	2050	2060	2070	2080
CMKSIEVKHS	ALSVELHSDM	EVTVNGRLVS	VPYVGGNMEV	NVYGAIMHEV	RFNHLGHIFT	FTPQNNEFQL	QLSPKTFASK
2090	2100	2110	2120	2130	2140	2150	2160
TYGLCGICDE	NGANDFMLRD	GTVTTDWKTL	VQEWTVQRPG	QTCQPILEEQ	CLVPDSSHCQ	VLLLPLFAEC	HKVLAPATFY
2170	2180	2190	2200	2210	2220	2230	2240

Detailed Protein Report

Cmpd.	No. of Cmpds.	m/z meas.	Δ m/z [ppm]	z	Rt [min]	Score	P	Range	Sequence	Modification	Ratios
1145	4	729.1568	-199.23	2	42.6	14.5	0	1944-1956	R.WTCPCVCTGSSTR.H	Carbamidomethyl: 5	WD:WU 0.69
1133	2	729.1918	-151.33	2	42.4	15.1	0	1944-1956	R.WTCPCVCTGSSTR.H	Carbamidomethyl: 3	
1951	1	918.3471	-96.34	2	53.1	10.5	2	2365-2379	K.EECKRVSPPSCPPHR.L	Carbamidomethyl: 3, 11	

Detailed Protein Report

Protein 79: DNA-dependent protein kinase catalytic subunit isoform 2 [Homo sapiens]

**Accession:**	gi|126032350	**Score:**	40.0
**Database:**	refseq_human(refseq_human_20140103.fasta)	**MW [kDa]:**	465.2
**Database Date:**	2015-11-30	**pI:**	6.9
**Modification(s):**	Carbamidomethyl	**Sequence Coverage [%]:**	1.0
		**No. of unique Peptides:**	3

Quantitation

WD:WU	**Median:** 1.65	**CV:** 0.00 %	**No. of Peptides:** 1

Detailed Protein Report

10	20	30	40	50	60	70	80
MAGSGAGVRC	SLLRLQETLS	AADRCGAALA	GHQLIRGLGQ	ECVLSSSPAV	LALQTSLVFS	RDFGLLVFVR	KSLNSIEFRE
90	100	110	120	130	140	150	160
CREEILKFLC	IFLEKMGQKI	APYSVEIKNT	CTSVYTKDRA	AKCKIPALDL	LIKLLQTFRS	SRLMDEFKIG	ELFSKFYGEL
170	180	190	200	210	220	230	240
ALKKKIPDTV	LEKVYELLGL	LGEVHPSEMI	NNAENLFRAF	LGELKTQMTS	AVREPKLPVL	AGCLKGLSSL	LCNFTKSMEE
250	260	270	280	290	300	310	320
DPQTSREIFN	FVLKAIRPQI	DLKRYAVPSA	GLRLFALHAS	QFSTCLLDNY	VSLFEVLLKW	CAHTNVELKK	AALSALESFL
330	340	350	360	370	380	390	400
KQVSNMVAKN	AEMHKNKLQY	FMEQFYGIIR	NVDSNNKELS	IAIRGYGLFA	GPCKVINAKD	VDFMYVELIQ	RCKQMFLTQT
410	420	430	440	450	460	470	480
DTGDDRVYQM	PSFLQSVASV	LLYLDTVPEV	YTPVLEHLVV	MQIDSFPQYS	PKMQLVCCRA	IVKVFLALAA	KGPVLRNCIS
490	500	510	520	530	540	550	560
TVVHQGLIRI	CSKPVVLPKG	PESESEDHRA	SGEVRTGKWK	VPTYKDYVDL	FRHLLSSDQM	MDSILADEAF	FSVNSSSESL
570	580	590	600	610	620	630	640
NHLLYDEFVK	SVLKIVEKLD	LTLEIQTVGE	QENGDEAPGV	WMIPTSDPAA	NLHPAKPKDF	SAFINLVEFC	REILPEKQAE
650	660	670	680	690	700	710	720
FFEPWVYSFS	YELILQSTRL	PLISGFYKLL	SITVRNAKKI	KYFEGVSPKS	LKHSPEDPEK	YSCFALFVKF	GKEVAVKMKQ
730	740	750	760	770	780	790	800
YKDELLASCL	TFLLSLPHNI	IELDVRAYVP	ALQMAFKLGL	SYTPLAEVGL	NALEEWSIYI	DRHVMQPYYK	DILPCLDGYL
810	820	830	840	850	860	870	880
KTSALSDETK	NNWEVSALSR	AAQKGFNKVV	LKHLKKTKNL	SSNEAISLEE	IRIRVVQMLG	SLGGQINKNL	LTVTSSDEMM
890	900	910	920	930	940	950	960
KSYVAWDREK	RLSFAVPFRE	MKPVIFLDVF	LPRVTELALT	ASDRQTKVAA	CELLHSMVMF	MLGKATQMPE	GGQGAPPMYQ
970	980	990	1000	1010	1020	1030	1040
LYKRTFPVLL	RLACDVDQVT	RQLYEPLVMQ	LIHWFTNNKK	FESQDTVALL	EAILDGIVDP	VDSTLRDFCG	RCIREFLKWS
1050	1060	1070	1080	1090	1100	1110	1120
IKQITPQQQE	KSPVNTKSLF	KRLYSLALHP	NAFKRLGASL	AFNNIYREFR	EEESLVEQFV	FEALVIYMES	LALAHADEKS
1130	1140	1150	1160	1170	1180	1190	1200
LGTIQQCCDA	IDHLCRIIEK	KHVSLNKAKK	RRLPRGFPPS	ASLCLLDLVK	WLLAHCGRPQ	TECRHKSIEL	FYKFVPLLPG
1210	1220	1230	1240	1250	1260	1270	1280
NRSPNLWLKD	VLKEEGVSFL	INTFEGGGCG	QPSGILAQPT	LLYLRGPFSL	QATLCWLDLL	LAALECYNTF	IGERTVGALQ
1290	1300	1310	1320	1330	1340	1350	1360
VLGTEAQSSL	LKAVAFFLES	IAMHDIIAAE	KCFGTGAAGN	RTSPQEGERY	NYSKCTVVVR	IMEFTTTLLN	TSPEGWKLLK
1370	1380	1390	1400	1410	1420	1430	1440
KDLCNTHLMR	VLVQTLCEPA	SIGFNIGDVQ	VMAHLPDVCV	NLMKALKMSP	YKDILETHLR	EKITAQSIEE	LCAVNLYGPD
1450	1460	1470	1480	1490	1500	1510	1520
AQVDRSRLAA	VVSACKQLHR	AGLLHNILPS	QSTDLHHSVG	TELLSLVYKG	IAPGDERQCL	PSLDLSCKQL	ASGLLELAFA
1530	1540	1550	1560	1570	1580	1590	1600
FGGLCERLVS	LLLNPAVLST	ASLGSSQGSV	IHFSHGEYFY	SLFSETINTE	LLKNLDLAVL	ELMQSSVDNT	KMVSAVLNGM
1610	1620	1630	1640	1650	1660	1670	1680
LDQSFRERAN	QKHQGLKLAT	TILQHWKKCD	SWWAKDSPLE	TKMAVLALLA	KILQIDSSVS	FNTSHGSFPE	VFTTYISLLA
1690	1700	1710	1720	1730	1740	1750	1760
DTKLDLHLKG	QAVTLLPFFT	SLTGGSLEEL	RRVLEQLIVA	HFPMQSREFP	PGTPRFNNYV	DCMKKFLDAL	ELSQSPMLLE
1770	1780	1790	1800	1810	1820	1830	1840
LMTEVLCREQ	QHVMEELFQS	SFRRIARRGS	CVTQVGLLES	VYEMFRKDDP	RLSFTRQSFV	DRSLLTLLWH	CSLDALREFF
1850	1860	1870	1880	1890	1900	1910	1920
STIVVDAIDV	LKSRFTKLNE	STFDTQITKK	MGYYKILDVM	YSRLPKDDVH	AKESKINQVF	HGSCITEGNE	LTKTLIKLCY
1930	1940	1950	1960	1970	1980	1990	2000
DAFTENMAGE	NQLLERRRLY	HCAAYNCAIS	VICCVFNELK	FYQGFLFSEK	PEKNLLIFEN	LIDLKRRYNF	PVEVEVPMER
2010	2020	2030	2040	2050	2060	2070	2080
KKKYIEIRKE	AREAANGDSD	GPSYMSSLSY	LADSTLSEEM	SQFDFSTGVQ	SYSYSSQDPR	PATGRFRRRE	QRDPTVHDDV
2090	2100	2110	2120	2130	2140	2150	2160
LELEMDELNR	HECMAPLTAL	VKHMHRSLGP	PQGEEDSVPR	DLPSWMKFLH	GKLGNPIVPL	NIRLFLAKLV	INTEEVFRPY
2170	2180	2190	2200	2210	2220	2230	2240

Detailed Protein Report

Cmpd.	No. of Cmpds.	m/z meas.	Δ m/z [ppm]	z	Rt [min]	Score	P	Range	Sequence	Modification	Ratios	
9	1	798.7834	5.94	3	29.1	17.8	2	2314-2334	K.EVYAAAAEVLGLILRYVMERK.N			
698	1	512.2692	-22.73	2	37.6	10.8	0	2434-2441	K.VCLDIIYK.M	Carbamidomethyl: 2	WD:WU	1.65
2214	1	722.7106	-205.39	2	56.0	11.5	2	3236-3247	K.FSMKMKMIDSAR.K			

Detailed Protein Report

Protein 80: PREDICTED: lethal(3)malignant brain tumor-like protein 4 isoform X7 [Homo sapiens]

Accession: gi|578832232
Database: refseq_human(refseq_human_20140103.fasta)
Database Date: 2015-11-30
Modification(s): Carbamidomethyl

Score: 40.0
MW [kDa]: 55.5
pI: 7.9
Sequence Coverage [%]: 4.1
No. of unique Peptides: 1

Quantitation

WD:WU **Median:** 0.55 **CV:** 0.00 % **No. of Peptides:** 1

10	20	30	40	50	60	70	80
MKQPNRKRKL	NMDSKERLDQ	DGRLEQAEEE	KKPKDSTTPL	SHVPSAAAQG	AWSWEWYLKE	QKAVAAPVEL	FSKDQSFPEH
**90**	**100**	**110**	**120**	**130**	**140**	**150**	**160**
ENGFQIGMRL	EGIDPRHPSV	FCVLSVAEVC	GYRLRLHFDG	YLSCYDFWTN	AGSPDIHPVG	WCEKTKHELH	IPKGYRKDKF
**170**	**180**	**190**	**200**	**210**	**220**	**230**	**240**
VWMDYLKACK	LQNAPKKLFR	NRSPNGPMSK	EFQVGMKLEA	VDRKNPSLVC	VATIADIVED	RLLVHFDNWD	DSYDYWCDVN
**250**	**260**	**270**	**280**	**290**	**300**	**310**	**320**
SPYVQPVGWC	QENGRTLIAP	QGYPNPENFS	WTEYLEATQT	NAVPAKVFKM	RLPHGFLPNM	KLEVVDKRNP	RLIRVATIVD
**330**	**340**	**350**	**360**	**370**	**380**	**390**	**400**
VDDQRVKVHF	DGWDHKYDYW	VEADSPDIHP	IGWCDVTGHP	LEVPQRTNDL	KILPGQAVCP	TPGCRGIGHI	RGPRYSGHHS
**410**	**420**	**430**	**440**	**450**	**460**	**470**	**480**
AFGCPYSDMN	LKKEATLHDR	LREQTQANLE	SDSSHSKSKS	LCSLNFNGKH	EKVNSQPRLV	QQAKCLKIKG	KEDIDLDNLF
**490**							
RDGTV							

Cmpd.	No. of Cmpds.	m/z meas.	Δ m/z [ppm]	z	Rt [min]	Score	P	Range	Sequence	Modification	Ratios
2406	3	701.3175	-82.26	3	58.4	16.5	1	372-391	K.ILPGQAVCPTPGCRGIGHIR.G	Carbamidomethyl: 13	WD:WU 0.55

Detailed Protein Report

Protein 81: zinc finger protein 700 isoform 1 [Homo sapiens]

**Accession:**	gi|55742736	**Score:**	40.0
**Database:**	refseq_human(refseq_human_20140103.fasta)	**MW [kDa]:**	86.2
**Database Date:**	2015-11-30	**pI:**	10.1
**Modification(s):**	Oxidation	**Sequence Coverage [%]:**	6.1
		**No. of unique Peptides:**	2

10	20	30	40	50	60	70	80
MPCCSHRSCR	EDPGTSESRE	MDPVAFEDVA	VNFTQEEWTL	LDISQKNLFR	EVMLETFRNL	TSIGKKWSDQ	NIEYEYQNPR
**90**	**100**	**110**	**120**	**130**	**140**	**150**	**160**
RSFRSLIEEK	VNEIKEDSHC	GETFTQVPDD	RLNFQEKKAS	PEVKSCDSFV	CAEVGIGNSS	FNMSIRGDTG	HKAYEYQEYG
**170**	**180**	**190**	**200**	**210**	**220**	**230**	**240**
PKPYKCQQPK	NKKAFRYRPS	IRTQERDHTG	EKPYACKVCG	KTFIFHSSIR	RHMVMHSGDG	TYKCKFCGKA	FHSFSLYLIH
**250**	**260**	**270**	**280**	**290**	**300**	**310**	**320**
ERTHTGEKPY	ECKQCGKSFT	YSATLQIHER	THTGEKPYEC	SKCDKAFHSS	SSYHRHERSH	MGEKPYQCKE	CGKAFAYTSS
**330**	**340**	**350**	**360**	**370**	**380**	**390**	**400**
LRRHERTHSG	KKPYECKQYG	EGLSYLISFQ	THIRMNSGER	PYKCKICGKG	FYSAKSFQTH	EKTHTGEKRY	KCKQCGKAFN
**410**	**420**	**430**	**440**	**450**	**460**	**470**	**480**
LSSSFRYHER	IHTGEKPYEC	KQCGKAFRSA	SQLRVHGGTH	TGEKPYECKE	CGKAFRSTSH	LRVHGRTHTG	EKPYECKECG
**490**	**500**	**510**	**520**	**530**	**540**	**550**	**560**
KAFRYVKHLQ	IHERTEKHIR	MPSGERPYKC	SICEKGFYSA	KSFQTHEKTH	TGEKPYECNQ	CGKAFRCCNS	LRYHERTHTG
**570**	**580**	**590**	**600**	**610**	**620**	**630**	**640**
EKPYECKQCG	KAFRSASHLR	MHERTHTGEK	PYECKQCGKA	FSCASNLRKH	GRTHTGEKPY	ECKQCGKAFR	SASNLQMHER
**650**	**660**	**670**	**680**	**690**	**700**	**710**	**720**
THTGEKPYEC	KECEKAFCKF	SSFQIHERKH	RGEKPYECKH	CGNGFTSAKI	LQIHARTHIG	EKHYECKECG	KAFNYFSSLH
**730**	**740**	**750**					
IHARTHMGEK	PYECKDCGKA	FS					

Cmpd.	No. of Cmpds.	m/z meas.	Δ m/z [ppm]	z	Rt [min]	Score	P	Range	Sequence	Modification
2885	1	1023.0269	78.47	2	64.8	13.1	2	212-229	R.HMVMHSGDGTYKCKFCGK.A	Oxidation: 2
2660	4	484.7692	71.54	2	61.6	15.8	0	600-608	K.AFSCASNLR.K	

Detailed Protein Report

Protein 82: **PREDICTED: sphingosine-1-phosphate phosphatase 1 isoform X1 [Homo sapiens]**

**Accession:**	gi|578826162	**Score:**	39.5
**Database:**	refseq_human(refseq_human_20140103.fasta)	**MW [kDa]:**	45.6
**Database Date:**	2015-11-30	**pI:**	9.9
		**Sequence Coverage [%]:**	7.5
		**No. of unique Peptides:**	2

10	20	30	40	50	60	70	80
MSLRQRLAQL	VGRLQDPQKV	ARFQRLCGVE	APPRRSADRR	EDEKAEAPLA	GDPRLRGRQP	GAPGGPQPPG	SDRNQCPAKP
**90**	**100**	**110**	**120**	**130**	**140**	**150**	**160**
DGGGAPNGVR	NGLAAELGPA	SPRRAGALRR	NSLTGEEGQL	ARVSNWPLYC	LFCFGTELGN	ELFYILFFPF	WIWNLDPLVG
**170**	**180**	**190**	**200**	**210**	**220**	**230**	**240**
RRLVVIWVLV	MYLGQCTKDI	IRWPRPASPP	VVKLEVFYNS	EYSMPSTHAM	SGTAIPISMV	LLTYGRWQDI	IAGFLYTILI
**250**	**260**	**270**	**280**	**290**	**300**	**310**	**320**
LAVFYPFVDL	IDNFNQTHKY	APFIIIGLHL	ALGIFSFTLD	TWSTSRGDTA	EILGSGAGIA	CGSHVTYNMG	LVLDPSLDTL
**330**	**340**	**350**	**360**	**370**	**380**	**390**	**400**
PLAGPPITVT	LFGKAILRIL	IGMVFVLIIR	DVMKKITIPL	ACKIFNIPCD	DIRKARQHME	VELPYRYITY	GMVGFSITFF
**410**	**420**						
VPYIFFFIGI	S						

Cmpd.	No. of Cmpds.	m/z meas.	Δ m/z [ppm]	z	Rt [min]	Score	P	Range	Sequence	Modification
2846	1	626.8511	24.33	2	64.3	11.3	0	91-103	R.NGLAAELGPASPR.R	
2736	1	1023.0146	-34.37	2	62.6	14.4	1	356-373	K.ITIPLACKIFNIPCDDIR.K	

Detailed Protein Report

Protein 83: RNA polymerase II transcription factor SIII subunit A2 [Homo sapiens]

**Accession:**	gi|45439357	**Score:**	39.4
**Database:**	refseq_human(refseq_human_20140103.fasta)	**MW [kDa]:**	83.9
**Database Date:**	2015-11-30	**pI:**	10.5
**Modification(s):**	Carbamidomethyl	**Sequence Coverage [%]:**	4.9
		**No. of unique Peptides:**	2

Quantitation

WD:WU **Median:** 1.94 **CV:** 0.00 % **No. of Peptides:** 1

10	20	30	40	50	60	70	80
MAAGSTTLHA	VEKLQVRLAT	KTEPKKLEKY	LQKLSALPMT	ADILAETGIR	KTVKRLRKHQ	HVGDFARDLA	ARWKKLVLVD
90	100	110	120	130	140	150	160
RNTRPGPQDP	EESASRQRFG	EALQDQEKAW	GFPENATAPR	SPSHSPEHRR	TARRTPPGQQ	RPHPRSHSRE	PRAERKCPRI
170	180	190	200	210	220	230	240
APADSGRYRA	SPTRTAPLRM	PEGPEPAAPG	KQPGRGHTHA	AQGGPLLCPG	CQGQPQGKAV	VSHSKGHKSS	RQEKRPLCAQ
250	260	270	280	290	300	310	320
GDWHSPTLIR	EKSCGACLRE	ETPRMPSWAS	ARDRQPSDFK	TDKEGGQAGS	GQRVPALEEA	PDSHQKRPQH	SHSNKKRPSL
330	340	350	360	370	380	390	400
DGRDPGNGTH	GLSPEEKEQL	SNDRETQEGK	PPTAHLDRTS	VSSLSEVEEV	DMAEEFEQPT	LSCEKYLTYD	QLRKQKKKTG
410	420	430	440	450	460	470	480
KSATTALGDK	QRKANESKGT	RESWDSAKKL	PPVQESQSER	LQAAGADSAG	PKTVPSHVFS	ELWDLSEAWM	QANYDPLSDS
490	500	510	520	530	540	550	560
DSMTSQAKPE	ALSSPKFREE	AAFPGRRVNA	KMPVYSGSRP	ACQLQVPTLR	QQCAQVLRNN	PDALSDVGEV	PYWVLEPVLE
570	580	590	600	610	620	630	640
GWRPDQLYRR	KKDNHALVRE	TDELRRNHCF	QDFKEEKPQE	NKTWREQYLR	LPDAPEQRLR	VMTTNIRSAR	GNNPNGREAK
650	660	670	680	690	700	710	720
MICFKSVAKT	PYDTSRRQEK	SAGDADPENG	EIKPASKPAG	SSHTPSSQSS	SGGGRDSSSS	ILRWLPEKRA	NPCLSSSNEH
730	740	750	760				
AAPAAKTRKQ	AAKKVAPLMA	KAIRDYKRRF	SRR				

Cmpd.	No. of Cmpds.	m/z meas.	Δ m/z [ppm]	z	Rt [min]	Score	P	Range	Sequence	Modification	Ratios
687	1	512.1344	-204.56	2	37.4	13.3	1	251-259	R.EKSCGACLR.E	Carbamidomethyl: 4	WD:WU 1.94
436	1	734.3141	-70.58	3	34.5	10.6	2	422-440	R.ESWDSAKKLPPVQESQSER.L		

Detailed Protein Report

Protein 84: peroxisome biogenesis factor 1 isoform 3 [Homo sapiens]

**Accession:**	gi|544186104	**Score:**	39.4
**Database:**	refseq_human(refseq_human_20140103.fasta)	**MW [kDa]:**	119.5
**Database Date:**	2015-11-30	**pI:**	5.7
		**Sequence Coverage [%]:**	1.9
		**No. of unique Peptides:**	1

10	20	30	40	50	60	70	80
MMKELQTKQL	QSNTVGITES	NENESEIPVD	SSSVASLWTM	IGSIFSFQSE	KKQETSWGLT	EINAFKNMQS	KVVPLDNIFR
**90**	**100**	**110**	**120**	**130**	**140**	**150**	**160**
VCKSQPPSIY	NASATSVFHK	HCAIHVFPWD	QEYFDVEPSF	TVTYGKLVKL	LSPKQQQSKT	KQNVLSPEKE	KQMSEPLDQK
**170**	**180**	**190**	**200**	**210**	**220**	**230**	**240**
KIRSDHNEED	EKACVLQVVW	NGLEELNNAI	KYTKNVEVLH	LGKVWIPDDL	RKRLNIEMHA	VVRITPVEVT	PKIPRSLKLQ
**250**	**260**	**270**	**280**	**290**	**300**	**310**	**320**
PRENLPKDIS	EEDIKTVFYS	WLQQSTTTML	PLVISEEEFI	KLETKDGLKE	FSLSIVHSWE	KEKDKNIFLL	SPNLLQKTTI
**330**	**340**	**350**	**360**	**370**	**380**	**390**	**400**
QVLLDPMVKE	ENSEEIDFIL	PFLKLSSLGG	VNSLGVSSLE	HITHSLLGRP	LSRQLMSLVA	GLRNGALLLT	GGKGSGKSTL
**410**	**420**	**430**	**440**	**450**	**460**	**470**	**480**
AKAICKEAFD	KLDAHVERVD	CKALRGKRLE	NIQKTLEVAF	SEAVWMQPSV	VLLDDLDLIA	GLPAVPEHEH	SPDAVQSQRL
**490**	**500**	**510**	**520**	**530**	**540**	**550**	**560**
AHALNDMIKE	FISMGSLVAL	IATSQSQQSL	HPLLVSAQGV	HIFQCVQHIQ	PPNQEQRCEI	LCNVIKNKLD	CDINKFTDLD
**570**	**580**	**590**	**600**	**610**	**620**	**630**	**640**
LQHVAKETGG	FVARDFTVLV	DRAIHSRLSR	QSISTREKLV	LTTLDFQKAL	RGFLPASLRS	VNLHKPRDLG	WDKIGGLHEV
**650**	**660**	**670**	**680**	**690**	**700**	**710**	**720**
RQILMDTIQL	PAKYPELFAN	LPIRQRTGIL	LYGPPGTGKT	LLAGVIARES	RMNFISVKGP	ELLSKYIGAS	EQAVRDIFIR
**730**	**740**	**750**	**760**	**770**	**780**	**790**	**800**
AQAAKPCILF	FDEFESIAPR	RGHDNTGVTD	RVVNQLLTQL	DGVEGLQGVY	VLAATSRPDL	IDPALLRPGR	LDKCVYCPPP
**810**	**820**	**830**	**840**	**850**	**860**	**870**	**880**
DQVSRLEILN	VLSDSLPLAD	DVDLQHVASV	TDSFTGADLK	ALLYNAQLEA	LHGMLLSSGL	QDGSSSSDSD	LSLSSMVFLN
**890**	**900**	**910**	**920**	**930**	**940**	**950**	**960**
HSSGSDDSAG	DGECGLDQSL	VSLEMSEILP	DESKFNMYRL	YFGSSYESEL	GNGTSSDLSS	QCLSAPSSMT	QDLPGVPGKD
**970**	**980**	**990**	**1000**	**1010**	**1020**	**1030**	**1040**
QLFSQPPVLR	TASQEGCQEL	TQEQRDQLRA	DISIIKGRYR	SQSGEDESMN	QPGPIKTRLA	ISQSHLMTAL	GHTRPSISED
**1050**	**1060**	**1070**	**1080**				
DWKNFAELYE	SFQNPKRRKN	QSGTMFRPGQ	KVTLA				

Cmpd.	No. of Cmpds.	m/z meas.	Δ m/z [ppm]	z	Rt [min]	Score	P	Range	Sequence	Modification
1127	4	1207.8838	99.74	1	42.8	28.3	2	233-242	K.IPRSLKLQPR.E	

Detailed Protein Report

Protein 85: E3 ubiquitin-protein ligase TRIP12 isoform c [Homo sapiens]

**Accession:**	gi|10863903	**Score:**	39.3
**Database:**	refseq_human(refseq_human_20140103.fasta)	**MW [kDa]:**	220.3
**Database Date:**	2015-11-30	**pI:**	9.6
		**Sequence Coverage [%]:**	2.5
		**No. of unique Peptides:**	2

Quantitation

WD:WU	**Median:** 0.74	**CV:** 0.00 %	**No. of Peptides:** 1

Detailed Protein Report

10	20	30	40	50	60	70	80
MSNRPNNNPG	GSLRRSQRNT	AGAQPQDDSI	GGRSCSSSSA	VIVPQPEDPD	RANTSERQKT	GQVPKKDNSR	GVKRSASPDY
90	100	110	120	130	140	150	160
NRTNSPSSAK	KPKALQHTES	PSETNKPHSK	SKKRHLDQEQ	QLKSAQSPST	SKAHTRKSGA	TGGSRSQKRK	RTESSCVKSG
170	180	190	200	210	220	230	240
SGSESTGAEE	RSAKPTKLAS	KSATSAKAGC	STITDSSSAA	STSSSSSAVA	SASSTVPPGA	RVKQGKDQNK	ARRSRSASSP
250	260	270	280	290	300	310	320
SPRRSSREKE	QSKTGGSSKF	DWAARFSPKV	SLPKTKLSLP	GSSKSETSKP	GPSGLQAKLA	SLRKSTKKRS	ESPPAELPSL
330	340	350	360	370	380	390	400
RRSTRQKTTG	SCASTSRRGS	GLGKRGAAEA	RRQEKMADPE	SNQEAVNSSA	ARTDEAPQGA	AGAVGMTTSG	ESESDDSEMG
410	420	430	440	450	460	470	480
RLQALLEARG	LPPHLFGPLG	PRMSQLFHRT	IGSGASSKAQ	QLLQGLQASD	ESQQLQAVIE	MCQLLVMGNE	ETLGGFPVKS
490	500	510	520	530	540	550	560
VVPALITLLQ	MEHNFDIMNH	ACRALTYMME	ALPRSSAVVV	DAIPVFLEKL	QVIQCIDVAE	QALTALEMLS	RRHSKAILQA
570	580	590	600	610	620	630	640
GGLADCLLYL	EFFSINAQRN	ALAIAANCCQ	SITPDEFHFV	ADSLPLLTQR	LTHQDKKSVE	STCLCFARLV	DNFQHEENLL
650	660	670	680	690	700	710	720
QQVASKDLLT	NVQQLLVVTP	PILSSGMFIM	VVRMFSLMCS	NCPTLAVQLM	KQNIAETLHF	LLCGASNGSC	QEQIDLVPRS
730	740	750	760	770	780	790	800
PQELYELTSL	ICELMPCLPK	EGIFAVDTML	KKGNAQNTDG	AIWQWRDDRG	LWHPYNRIDS	RIIEQINEDT	GTARAIQRKP
810	820	830	840	850	860	870	880
NPLANSNTSG	YSESKKDDAR	AQLMKEDPEL	AKSFIKTLFG	VLYEVYSSSA	GPAVRHKCLR	AILRIIYFAD	AELLKDVLKN
890	900	910	920	930	940	950	960
HAVSSHIASM	LSSQDLKIVV	GALQMAEILM	QKLPDIFSVY	FRREGVMHQV	KHLAESESLL	TSPPKACTNG	SGSMGSTTSV
970	980	990	1000	1010	1020	1030	1040
SSGTATAATH	AAADLGSPSL	QHSRDDSLDL	SPQGRLSDVL	KRKRLPKRGP	RRPKYSPPRD	DDKVDNQAKS	PTTTQSPKSS
1050	1060	1070	1080	1090	1100	1110	1120
FLASLNPKTW	GRLSTQSNSN	NIEPARTAGG	SGLARAASKD	TISNNREKIK	GWIKEQAHKF	VERYFSSENM	DGSNPALNVL
1130	1140	1150	1160	1170	1180	1190	1200
QRLCAATEQL	NLQVDGGAEC	LVEIRSIVSE	SDVSSFEIQH	SGFVKQLLLY	LTSKSEKDAV	SREIRLKRFL	HVFFSSPLPG
1210	1220	1230	1240	1250	1260	1270	1280
EEPIGRVEPV	GNAPLLALVH	KMNNCLSQME	QFPVKVHDFP	SGNGTGGSFS	LNRGSQALKF	FNTHQLKCQL	QRHPDCANVK
1290	1300	1310	1320	1330	1340	1350	1360
QWKGGPVKID	PLALVQAIER	YLVVRGYGRV	REDDEDSDDD	GSDEEIDESL	AAQFLNSGNV	RHRLQFYIGE	HLLPYNMTVY
1370	1380	1390	1400	1410	1420	1430	1440
QAVRQFSIQA	EDERESTDDE	SNPLGRAGIW	TKTHTIWYKP	VREDEESNKD	CVGGKRGRAQ	TAPTKTSPRN	AKKHDELWHD
1450	1460	1470	1480	1490	1500	1510	1520
GVCPSVSNPL	EVYLIPTPPE	NITFEDPSLD	VILLLRVLHA	ISRYWYYLYD	NAMCKEIIPT	SEFINSKLTA	KANRQLQDPL
1530	1540	1550	1560	1570	1580	1590	1600
VIMTGNIPTW	LTELGKTCPF	FFPFDTRQML	FYVTAFDRDR	AMQRLLDTNP	EINQSDSQDS	RVAPRLDRKK	RTVNREELLK
1610	1620	1630	1640	1650	1660	1670	1680
QAESVMQDLG	SSRAMLEIQY	ENEVGTGLGP	TLEFYALVSQ	ELQRADLGLW	RGEEVTLSNP	KGSQEGTKYI	QNLQGLFALP
1690	1700	1710	1720	1730	1740	1750	1760
FGRTAKPAHI	AKVKMKFRFL	GKLMAKAIMD	FRLVDLPLGL	PFYKWMLRQE	TSLTSHDLFD	IDPVVARSVY	HLEDIVRQKK
1770	1780	1790	1800	1810	1820	1830	1840
RLEQDKSQTK	ESLQYALETL	TMNGCSVEDL	GLDFTLPGFP	NIELKKGGKD	IPVTIHNLEE	YLRLVIFWAL	NEGVSRQFDS
1850	1860	1870	1880	1890	1900	1910	1920
FRDGFESVFP	LSHLQYFYPE	ELDQLLCGSK	ADTWDAKTLM	ECCRPDHGYT	HDSRAVKFLF	EILSSFDNEQ	QRLFLQFVTG
1930	1940	1950	1960	1970	1980	1990	2000
SPRLPVGGFR	SLNPPLTIVR	KTFESTENPD	DFLPSVMTCV	NYLKLPDYSS	IEIMREKLLI	AAREGQQSFH	LS

Cmpd.	No. of Cmpds.	m/z meas.	Δ m/z [ppm]	z	Rt [min]	Score	P	Range	Sequence	Modification	Ratios
1453	1	929.4464	3.04	2	46.5	10.5	1	16-33	R.SQRNTAGAQPQDDSIGGR.S		WD:WU 0.74
2056	1	973.8963	-110.34	2	54.1	16.5	2	1067-1086	R.TAGGSGLARAASKDTISNNR.E		

Detailed Protein Report

Protein 86: relaxin receptor 1 isoform 7 [Homo sapiens]

Accession: gi|359279881
Database: refseq_human(refseq_human_20140103.fasta)
Database Date: 2015-11-30
Modification(s): Carbamidomethyl, Oxidation

Score: 38.8
MW [kDa]: 69.5
pI: 9.9
Sequence Coverage [%]: 1.5
No. of unique Peptides: 2

Quantitation

WD:WU **Median:** 1.34 **CV:** 79.73 % **No. of Peptides:** 2

10	20	30	40	50	60	70	80
MVTCTQNMKA	ALKSLLYHSR	NLGYLQNNKI	TSISIYAFRG	LNSLTKLYLS	HNRITFLKPG	VFEDLHRLEW	LIIEDNHLSR
**90**	**100**	**110**	**120**	**130**	**140**	**150**	**160**
ISPPTFYGLN	SLILLVLMNN	VLTRLPDKPL	CQHMPRLHWL	VMRKNKINHL	NENTFAPLQK	LDELDLGSNK	IENLPPLIFK
**170**	**180**	**190**	**200**	**210**	**220**	**230**	**240**
DLKELSQLNL	SYNPIQKIQA	NQFDYLVKLK	SLSLEGIEIS	NIQQRMFRPL	MNLSHIYFKK	FQYCGYAPHV	RSCKPNTDGI
**250**	**260**	**270**	**280**	**290**	**300**	**310**	**320**
SSLENLLASI	IQRVFVWVVS	AVTCFGNIFV	ICMRPYIRSE	NKLYAMSIIS	LCCADCLMGI	YLFVIGGFDL	KFRGEYNKHA
**330**	**340**	**350**	**360**	**370**	**380**	**390**	**400**
QLWMESTHCQ	LVGSLAILST	EVSVLLLTFL	TLEKYICIVY	PFRCVRPGKC	RTITVLILIW	ITGFIVAFIP	LSNKEFFKNY
**410**	**420**	**430**	**440**	**450**	**460**	**470**	**480**
YGTNGVCFPL	HSEDTESIGA	QIYSVAIFLG	INLAAFIIIV	FSYGSMFYSV	HQSAITATEI	RNQVKKEMIL	AKRFFFIVFT
**490**	**500**	**510**	**520**	**530**	**540**	**550**	**560**
DALCWIPIFV	VKFLSLLQVE	IPGTITSWVV	IFILPINSAL	NPILYTLTTR	PFKEMIHRFW	YNYRQRKSMD	SKGQKTYAPS
**570**	**580**	**590**	**600**	**610**			
FIWVEMWPLQ	EMPPELMKPD	LFTYPCEMSL	ISQSTRLNSY	S			

Cmpd.	No. of Cmpds.	m/z meas.	Δ m/z [ppm]	z	Rt [min]	Score	P	Range	Sequence	Modification	Ratios	
386	6	544.1543	-144.04	2	33.4	14.3	0	1-9	-.MVTCTQNMK.A	Oxidation: 1, 8	WD:WU	2.71
468	2	572.6830	-105.55	2	34.3	24.6	0	1-9	-.MVTCTQNMK.A	Carbamidomethyl: 4; Oxidation: 1, 8	WD:WU	0.67

Detailed Protein Report

Protein 87: G2/mitotic-specific cyclin-B3 isoform 3 [Homo sapiens]

**Accession:**	gi|90669307	**Score:**	38.7
**Database:**	refseq_human(refseq_human_20140103.fasta)	**MW [kDa]:**	157.8
**Database Date:**	2015-11-30	**pI:**	6.3
		**Sequence Coverage [%]:**	3.4
		**No. of unique Peptides:**	3

Quantitation

WD:WU **Median:** 2.71 **CV:** 0.00 % **No. of Peptides:** 1

10	20	30	40	50	60	70	80
MLLPLPPQSS	KPVPKKSQSS	KIVPSHHDPS	EKTGENCQTK	ISPSSLQESP	SSLQGALKKR	SAFEDLTNAS	QCQPVQPKKE
90	100	110	120	130	140	150	160
ANKEFVKVVS	KKINRNTHAL	GLAKKNKRNL	KWHKLEVTPV	VASTTVVPNI	MEKPLILDIS	TTSKTPNTEE	ASLFRKPLVL
170	180	190	200	210	220	230	240
KEEPTIEDET	LINKSLSLKK	CSNHEEVSLL	EKLQPLQEES	DSDDAFVIEP	MTFKKTHKTE	EAAITKKTLS	LKKKMCASQR
250	260	270	280	290	300	310	320
KQSCQEESLA	VQDVNMEEDS	FFMESMSFKK	KPKTEESIPT	HKLSSLKKKC	TIYGKICHFR	KPPVLQTTIC	GAMSSIKKPT
330	340	350	360	370	380	390	400
TEKETLFQEL	SVLQEKHTTE	HEMSILKKSL	ALQKTNFKED	SLVKESLAFK	KKPSTEEAIM	MPVILKEQCM	TEGKRSRLKP
410	420	430	440	450	460	470	480
LVLQEITSGE	KSLIMKPLSI	KEKPSTEKES	FSQEPSALQK	KHTTQEEVSI	LKEPSSLLKS	PTEESPFDEA	LAFTKKCTIE
490	500	510	520	530	540	550	560
EAPPTKKPLI	LKRKHATQGT	MSHLKKPLIL	QTTSGEKSLI	KEPLPFKKEEK	VSLKKKCTTQ	EMMSICPELL	DFQDMIGEDK
570	580	590	600	610	620	630	640
NSFFMEPMSF	RKNPTTEETV	LTKTSLSLQE	KKITQGKMSH	LKKPLVLQKI	TSEEESFYKK	LLPFKMKSTT	EEKFLSQEPS
650	660	670	680	690	700	710	720
ALKEKHTTLQ	EVSLSKESLA	IQEKATTEEE	FSQELFSLHV	KHTNKSGSLF	QEALVLQEKT	DAEEDSLKNL	LALQEKSTME
730	740	750	760	770	780	790	800
EESLINKLLA	LKEELSAEAA	TNIQTQLSLK	KKSTSHGKVF	FLKKQLALNE	TINEEEFLNK	QPLALEGYPS	IAEGETLFKK
810	820	830	840	850	860	870	880
LLAMQEEPSI	EKEAVLKEPT	IDTEAHFKEP	LALQEEPSTE	KEAVLKEPSV	DTEAHFKETL	ALQEKPSIEQ	EALFKRHSAL
890	900	910	920	930	940	950	960
WEKPSTEKET	IFKESLDLQE	KPSIKKETLL	KKPLALKMST	INEAVLFEDM	IALNEKPTTG	KELSFKEPLA	LQESPTYKED
970	980	990	1000	1010	1020	1030	1040
TFLKTLLVPQ	VGTSPNVSST	APESITSKSS	IATMTSVGKS	GTINEAFLFE	DMITLNEKPT	TGKELSFKEP	LALQESPTCK
1050	1060	1070	1080	1090	1100	1110	1120
EDTFLETFLI	PQIGTSPYVF	STTPESITEK	SSIATMTSVG	KSRTTTESSA	CESASDKPVS	PQAKGTPKEI	TPREDIDEDS
1130	1140	1150	1160	1170	1180	1190	1200
SDPSFNPMYA	KEIFSYMKER	EEQFILTDYM	NRQIEITSDM	RAILVDWLVE	VQVSFEMTHE	TLYLAVKLVD	LYLMKAVCKK
1210	1220	1230	1240	1250	1260	1270	1280
DKLQLLGATA	FMIAAKFEEH	NSPRVDDFVY	ICDDNYQRSE	VLSMEININ	VLKCDINIPI	AYHFLRRYAR	CIHTNMKTLT
1290	1300	1310	1320	1330	1340	1350	1360
LSRYICEMTL	QEYHYVQEKA	SKLAAASLLL	ALYMKKLGYW	VPFLEHYSGY	SISELHPLVR	QLNKLLTFSS	YDSLKAVYYK
1370	1380	1390	1400				
YSHPVFFEVA	KIPALDMLKL	EEILNCDCEA	QGLVL				

Cmpd.	No. of Cmpds.	m/z meas.	Δ m/z [ppm]	z	Rt [min]	Score	P	Range	Sequence	Modification	Ratios
1030	1	670.7981	-53.16	2	41.6	10.6	1	290-300	K.CTIYGKICHFR.K		
2723	1	639.3585	-37.42	2	62.4	12.0	2	348-358	K.KSLALQKTNFK.E		WD:WU 2.71
1809	2	1011.7628	-33.19	3	51.3	16.1	2	535-560	K. KKCTTQEMMSICPELLDFQDMIGE N		

Detailed Protein Report

Protein 88: **PREDICTED: N-alpha-acetyltransferase 15, NatA auxiliary subunit isoform X1 [Homo sapiens]**

**Accession:**	gi|530378196	**Score:**	38.5
**Database:**	refseq_human(refseq_human_20140103.fasta)	**MW [kDa]:**	101.1
**Database Date:**	2015-11-30	**pI:**	7.8
**Modification(s):**	Oxidation	**Sequence Coverage [%]:**	3.6
		**No. of unique Peptides:**	1

10	20	30	40	50	60	70	80
MPAVSLPPKE	NALFKRILRC	YEHKQYRNGL	KFCKQILSNP	KFAEHGETLA	MKGLTLNCLG	KKEEAYELVR	RGLRNDLKSH
**90**	**100**	**110**	**120**	**130**	**140**	**150**	**160**
VCWHVYGLLQ	RSDKKYDEAI	KCYRNALKWD	KDNLQILRDL	SLLQIQMRDL	EGYRETRYQL	LQLRPAQRAS	WIGYAIAYHL
**170**	**180**	**190**	**200**	**210**	**220**	**230**	**240**
LEDYEMAAKI	LEEFRKTQQT	SPDKVDYEYS	ELLLYQNQVL	REAGLYREAL	EHLCTYEKQI	CDKLAVEETK	GELLLQLCRL
**250**	**260**	**270**	**280**	**290**	**300**	**310**	**320**
EDAADVYRGL	QERNPENWAY	YKGLEKALKP	ANMLERLKIY	EEAWTKYPRG	LVPRRLPLNF	LSGEKFKECL	DKFLRMNFSK
**330**	**340**	**350**	**360**	**370**	**380**	**390**	**400**
GCPPVFNTLR	SLYKDKEKVA	IIEELVVGYE	TSLKSCRLFN	PNDDGKEEPP	TTLLWVQYYL	AQHYDKIGQP	SIALEYINTA
**410**	**420**	**430**	**440**	**450**	**460**	**470**	**480**
IESTPTLIEL	FLVKAKIYKH	AGNIKEAARW	MDEAQALDTA	DRFINSKCAK	YMLKANLIKE	AEEMCSKFTR	EGTSAVENLN
**490**	**500**	**510**	**520**	**530**	**540**	**550**	**560**
EMQCMWFQTE	CAQAYKAMNK	FGEALKKCHE	IERHFIEITD	DQFDFHTYCM	RKITLRSYVD	LLKLEDVLRQ	HPFYFKAARI
**570**	**580**	**590**	**600**	**610**	**620**	**630**	**640**
AIEIYLKLHD	NPLTDENKEH	EADTANMSDK	ELKKLRNKQR	RAQKKAQIEE	EKKNAEKEKQ	QRNQKKKKDD	DDEEIGGPKE
**650**	**660**	**670**	**680**	**690**	**700**	**710**	**720**
ELIPEKLAKV	ETPLEEAIKF	LTPLKNLVKN	KIETHLFAFE	IYFRKEKFLL	MLQSVKRAFA	IDSSHPWLHE	CMIRLFNTVC
**730**	**740**	**750**	**760**	**770**	**780**	**790**	**800**
ESKDLSDTVR	TVLKQEMNRL	FGATNPKNFN	ETFLKRNSDS	LPHRLSAAKM	VYYLDPSSQK	RAIELATTLD	ESLTNRNLQT
**810**	**820**	**830**	**840**	**850**	**860**	**870**	
CMEVLEALYD	GSLGDCKEAA	EIYRANCHKL	FPYALAFMPP	GYEEDMKITV	NGDSSAEAEE	LANEI	

Cmpd.	No. of Cmpds.	m/z meas.	Δ m/z [ppm]	z	Rt [min]	Score	P	Range	Sequence	Modification
2297	1	863.8705	-61.74	2	57.2	20.5	1	316-330	R.MNFSKGCPPVFNTLR.S	Oxidation: 1

Detailed Protein Report

Protein 89: **probable ATP-dependent RNA helicase DDX20 [Homo sapiens]**

Accession: gi|256223453
Database: refseq_human(refseq_human_20140103.fasta)
Database Date: 2015-11-30

Score: 38.4
MW [kDa]: 92.2
pI: 6.5
Sequence Coverage [%]: 3.3
No. of unique Peptides: 2

10	20	30	40	50	60	70	80
MAAAFEASGA	LAAVATAMPA	EHVAVQVPAP	EPTPGPVRIL	RTAQDLSSPR	TRTGDVLLAE	PADFESLLLS	RPVLEGLRAA
**90**	**100**	**110**	**120**	**130**	**140**	**150**	**160**
GFERPSPVQL	KAIPLGRCGL	DLIVQAKSGT	GKTCVFSTIA	LDSLVLENLS	TQILILAPTR	EIAVQIHSVI	TAIGIKMEGL
**170**	**180**	**190**	**200**	**210**	**220**	**230**	**240**
ECHVFIGGTP	LSQDKTRLKK	CHIAVGSPGR	IKQLIELDYL	NPGSIRLFIL	DEADKLLEEG	SFQEQINWIY	SSLPASKQML
**250**	**260**	**270**	**280**	**290**	**300**	**310**	**320**
AVSATYPEFL	ANALTKYMRD	PTFVRLNSSD	PSLIGLKQYY	KVVNSYPLAH	KVFEEKTQHL	QELFSRIPFN	QALVFSNLHS
**330**	**340**	**350**	**360**	**370**	**380**	**390**	**400**
RAQHLADILS	SKGFPAECIS	GNMNQNQRLD	AMAKLKHFHC	RVLISTDLTS	RGIDAEKVNL	VVNLDVPLDW	ETYMHRIGRA
**410**	**420**	**430**	**440**	**450**	**460**	**470**	**480**
GRFGTLGLTV	TYCCRGEEEN	MMMRIAQKCN	INLLPLPDPI	PSGLMEECVD	WDVEVKAAVH	TYGIASVPNQ	PLKKQIQKIE
**490**	**500**	**510**	**520**	**530**	**540**	**550**	**560**
RTLQIQKAHG	DHMASSRNNS	VSGLSVKSKN	NTKQKLPVKS	HSECGIIEKA	TSPKELGCDR	QSEEQMKNSV	QTPVENSTNS
**570**	**580**	**590**	**600**	**610**	**620**	**630**	**640**
QHQVKEALPV	SLPQIPCLSS	FKIHQPYTLT	FAELVEDYEH	YIKEGLEKPV	EIIRHYTGPG	DQTVNPQNGF	VRNKVIEQRV
**650**	**660**	**670**	**680**	**690**	**700**	**710**	**720**
PVLASSSQSG	DSESDSDSYS	SRTSSQSKGN	KSYLEGSSDN	QLKDSESTPV	DDRISLEQPP	NGSDTPNPEK	YQESPGIQMK
**730**	**740**	**750**	**760**	**770**	**780**	**790**	**800**
TRLKEGASQR	AKQSRRNLPR	RSSFRLQTEA	QEDDWYDCHR	EIRLSFSDTY	QDYEEYWRAY	YRAWQEYYAA	ASHSYYWNAQ
**810**	**820**	**830**					
RHPSWMAAYH	MNTIYLQEMM	HSNQ					

Cmpd.	No. of Cmpds.	m/z meas.	Δ m/z [ppm]	z	Rt [min]	Score	P	Range	Sequence	Modification
1828	1	807.3949	-77.75	2	51.2	11.4	2	39-52	R.ILRTAQDLSSPRTR.T	
827	1	683.3392	-78.48	2	39.2	27.0	2	178-190	R.LKKCHIAVGSPGR.I	

Detailed Protein Report

Protein 90: protein FAM104A isoform 3 [Homo sapiens]

Accession: gi|574956994
Database: refseq_human(refseq_human_20140103.fasta)
Database Date: 2015-11-30

Score: 38.3
MW [kDa]: 9.8
pI: 12.7
Sequence Coverage [%]: 40.8
No. of unique Peptides: 1

```
        10         20         30         40         50         60         70         80
MGGRGADAGS SGGTGPTEGY SPPAASTRAA ARAKARGGGR GGRRNTTPSV PSLRGAAPRS FHPPAAMSER LRPSLQAVTV
        90        100        110
VGAAAAAAAA SIARTGPAGR KAA
```

Cmpd.	No. of Cmpds.	m/z meas.	Δ m/z [ppm]	z	Rt [min]	Score	P	Range	Sequence	Modification
2165	5	974.4048	-53.21	3	55.4	21.1	2	1-32	-.MGGRGADAGSSGGTGPTEGYSPPAASTRAAAR.A	

Detailed Protein Report

Protein 91: small proline-rich protein 2E [Homo sapiens]

Accession: gi|83582817
Database: refseq_human(refseq_human_20140103.fasta)
Database Date: 2015-11-30
Modification(s): Carbamidomethyl

Score: 38.2
MW [kDa]: 7.8
pI: 10.6
Sequence Coverage [%]: 18.1
No. of unique Peptides: 2

Quantitation

WD:WU **Median:** 1.04 **CV:** 0.00 % **No. of Peptides:** 1

```
         10         20         30         40         50         60         70         80
MSYQQQQCKQ PCQPPPVCPT PKCPEPCPPP KCPEPCPPPK CPQPCPPQQC QQKCPPVTPS PPCQPKCPPK SK
```

Cmpd.	No. of Cmpds.	m/z meas.	Δ m/z [ppm]	z	Rt [min]	Score	P	Range	Sequence	Modification	Ratios
2928	1	724.8814	36.56	2	65.4	11.5	0	10-22	K.QPCQPPPVCPTPK.C	Carbamidomethyl: 3	
68	2	753.3471	-24.59	2	30.1	26.7	0	10-22	K.QPCQPPPVCPTPK.C	Carbamidomethyl: 3, 9	WD:WU 1.04

Detailed Protein Report

Protein 92: DNA repair and recombination protein RAD54B isoform 1 [Homo sapiens]

Accession: gi|6912622
Database: refseq_human(refseq_human_20140103.fasta)
Database Date: 2015-11-30
Modification(s): Oxidation

Score: 38.0
MW [kDa]: 102.9
pI: 9.4
Sequence Coverage [%]: 4.6
No. of unique Peptides: 2

10	20	30	40	50	60	70	80
MRRSAAPSQL	QGNSFKKPKF	IPPGRSNPGL	NEEITKLNPD	IKLFEGVAIN	NTFLPSQNDL	RICSLNLPSE	ESTREINNRD
90	100	110	120	130	140	150	160
NCSGKYCFEA	PTLATLDPPH	TVHSAPKEVA	VSKEQEEKSD	SLVKYFSVVW	CKPSKKKHKK	WEGDAVLIVK	GKSFILKNLE
170	180	190	200	210	220	230	240
GKDIGRGIGY	KFKELEKIEE	GQTLMICGKE	IEVMGVISPD	DFSSGRCFQL	GGGSTAISHS	SQVARKCFSN	PFKSVCKPSS
250	260	270	280	290	300	310	320
KENRQNDFQN	CKPRHDPYTP	NSLVMPRPDK	NHQWVFNKNC	FPLVDVVIDP	YLVYHLRPHQ	KEGIIFLYEC	VMGMRMNGRC
330	340	350	360	370	380	390	400
GAILADEMGL	GKTLQCISLI	WTLQCQGPYG	GKPVIKKTLI	VTPGSLVNNW	KKEFQKWLGS	ERIKIFTVDQ	DHKVEEFIKS
410	420	430	440	450	460	470	480
IFYSVLIISY	EMLLRSLDQI	KNIKFDLLIC	DEGHRLKNSA	IKTTTALISL	SCEKRIILTG	TPIQNDLQEF	FALIDFVNPG
490	500	510	520	530	540	550	560
ILGSLSSYRK	IYEEPIILSR	EPSASEEEKE	LGERRAAELT	CLTGLFILRR	TQEIINKYLP	PKIENVVFCR	PGALQIELYR
570	580	590	600	610	620	630	640
KLLNSQVVRF	CLQGLLENSP	HLICIGALKK	LCNHPCLLFN	SIKEKECSST	CDKNEEKSLY	KGLLSVFPAD	YNPLLFTEKE
650	660	670	680	690	700	710	720
SGKLQVLSKL	LAVIHELRPT	EKVVLVSNYT	QTLNILQEVC	KRHGYAYTRL	DGQTPISQRQ	QIVDGFNSQH	SSFFIFLLSS
730	740	750	760	770	780	790	800
KAGGVGLNLI	GGSHLILYDI	DWNPATDIQA	MSRVWRDGQK	YPVHIYRLLT	TGTIEEKIYQ	RQISKQGLCG	AVVDLTKTSE
810	820	830	840	850	860	870	880
HIQFSVEELK	NLFTLHESSD	CVTHDLLDCE	CTGEEVHTGD	SLEKFIVSRD	CQLGPHHQKS	NSLKPLSMSQ	LKQWKHFSGD
890	900	910	920				
HLNLTDPFLE	RITENVSFIF	QNITTQATGT					

Cmpd.	No. of Cmpds.	m/z meas.	Δ m/z [ppm]	z	Rt [min]	Score	P	Range	Sequence	Modification
1860	2	599.9988	-42.55	3	51.9	12.1	2	357-372	K.KTLIVTPGSLVNNWKK.E	
920	1	945.9532	-58.91	2	39.9	10.3	1	860-875	K.SNSLKPLSMSQLKQWK.H	Oxidation: 9

Detailed Protein Report

Protein 93: histone H4 [Homo sapiens]

Accession: gi|4504301
Database: refseq_human(refseq_human_20140103.fasta)
Database Date: 2015-11-30

Score: 37.9
MW [kDa]: 11.4
pI: 11.8
Sequence Coverage [%]: 7.8
No. of unique Peptides: 1

Quantitation

WD:WU **Median:** 1.43 **CV:** 0.00 % **No. of Peptides:** 1

Alias proteins:

Accession	Name	Description
gi|77539758	refseq_human (refseq_human_20140103.fasta)	histone H4 [Homo sapiens]
gi|28173560	refseq_human (refseq_human_20140103.fasta)	histone H4 [Homo sapiens]
gi|11415030	refseq_human (refseq_human_20140103.fasta)	histone H4 [Homo sapiens]
gi|4504323	refseq_human (refseq_human_20140103.fasta)	histone H4 [Homo sapiens]
gi|4504321	refseq_human (refseq_human_20140103.fasta)	histone H4 [Homo sapiens]
gi|4504317	refseq_human (refseq_human_20140103.fasta)	histone H4 [Homo sapiens]
gi|4504315	refseq_human (refseq_human_20140103.fasta)	histone H4 [Homo sapiens]
gi|4504313	refseq_human (refseq_human_20140103.fasta)	histone H4 [Homo sapiens]
gi|4504311	refseq_human (refseq_human_20140103.fasta)	histone H4 [Homo sapiens]
gi|4504309	refseq_human (refseq_human_20140103.fasta)	histone H4 [Homo sapiens]
gi|4504307	refseq_human (refseq_human_20140103.fasta)	histone H4 [Homo sapiens]
gi|4504305	refseq_human (refseq_human_20140103.fasta)	histone H4 [Homo sapiens]
gi|4504303	refseq_human (refseq_human_20140103.fasta)	histone H4 [Homo sapiens]

```
        10         20         30         40         50         60         70         80
MSGRGKGGKG LGKGGAKRHR KVLRDNIQGI TKPAIRRLAR RGGVKRISGL IYEETRGVLK VFLENVIRDA VTYTEHAKRK
        90        100        110
TVTAMDVVYA LKRQGRTLYG FGG
```

Cmpd.	No. of Cmpds.	m/z meas.	Δ m/z [ppm]	z	Rt [min]	Score	P	Range	Sequence	Modification	Ratios
1347	4	495.2734	-38.66	2	45.6	37.9	0	61-68	K.VFLENVIR.D		WD:WU 1.43

Detailed Protein Report

Protein 94: protein FAM115C isoform B [Homo sapiens]

Accession: gi|293651621
Database: refseq_human(refseq_human_20140103.fasta)
Database Date: 2015-11-30
Modification(s): Oxidation

Score: 37.9
MW [kDa]: 90.6
pI: 8.9
Sequence Coverage [%]: 3.8
No. of unique Peptides: 3

10	20	30	40	50	60	70	80
MKIFQKEFKF	ILKKCTPIQK	YREENGNSSS	SPHHQLQSHA	SGPRYGEDVR	QDQQQLLEGI	SELDIRTGGV	PSQLLVHGAL
**90**	**100**	**110**	**120**	**130**	**140**	**150**	**160**
AFPLGLDASL	NCFLAAAHYG	RGRVVLAAHE	CLLCAPKMGP	FLLNAVRWLA	RGQTGKVGVN	TNLKDLCPLL	SEHGLQCSLE
**170**	**180**	**190**	**200**	**210**	**220**	**230**	**240**
PHLNSDLCVY	CCKAYSDKEA	KQLQEFVAEG	GGLLIGGQAW	WWASQNPGHC	PLAGFPGNII	LNCFGLSILP	QTLKAGCFPV
**250**	**260**	**270**	**280**	**290**	**300**	**310**	**320**
PTPEMRSYHF	RKALSQFQAI	LNHENGNLEK	SCLAKLRVDG	AAFLQIPAEG	VPAYISLHRL	LRKMLRGSGL	PAVSRENPVA
**330**	**340**	**350**	**360**	**370**	**380**	**390**	**400**
SDSYEAAVLS	LATGLAHSGT	DCSQLAQGLG	TWTCSSSLYP	SKHPITVEIN	GINPESHSVI	QVGMQWRDLS	SCNLHLLGLS
**410**	**420**	**430**	**440**	**450**	**460**	**470**	**480**
NSSLSASCVA	GTTGTRHHAW	LIFVFLVERE	FHRKGNNDCW	VSTGLYLLEG	QNAEVSLSEA	AASAGLRVQI	GCHTDDLTKA
**490**	**500**	**510**	**520**	**530**	**540**	**550**	**560**
RKLSRAPVVT	HQCWMDRTER	SVSCLWGGLL	YVIVPKGSQL	GPVPVTIRGA	VPAPYYKLGK	TSLEEWKRQM	QENLAPWGEL
**570**	**580**	**590**	**600**	**610**	**620**	**630**	**640**
ATDNIILTVP	TTNLQALKDP	EPVLRLWDEM	MQAVARLAAE	PFPFRRPERI	VADVQISAGW	MHSGYPIMCH	LESVKEIINE
**650**	**660**	**670**	**680**	**690**	**700**	**710**	**720**
MDMRSRGVWG	PIHELGHNQQ	RHGWEFPPHT	TEATCNLWSV	YVHETVLGIP	RAQAHEALSP	PERERRIKAH	LGKGAPLCDW
**730**	**740**	**750**	**760**	**770**	**780**	**790**	**800**
NVWTALETYL	QLQEAFGWEP	FTQLFAEYQT	LSHLPKDNTG	RMNLWVKKFS	EKVKKNLVPF	FEAWGWPIQK	EVADSLASLP
**810**	**820**						
EWQENPMQVY	LRARK						

Cmpd.	No. of Cmpds.	m/z meas.	Δ m/z [ppm]	z	Rt [min]	Score	P	Range	Sequence	Modification
7	1	689.3232	-60.71	1	29.3	10.0	0	15-20	K.CTPIQK.Y	
126	1	822.3680	-116.44	2	30.8	17.1	1	118-131	K.MGPFLLNAVRWLAR.G	
495	1	705.1921	-194.39	2	35.1	10.8	1	636-646	K.EIINEMDMRSR.G	Oxidation: 6

Detailed Protein Report

Protein 95: **PREDICTED: probable E3 ubiquitin-protein ligase MYCBP2 isoform X1 [Homo sapiens]**

**Accession:**	gi|530402182	**Score:**	37.7
**Database:**	refseq_human(refseq_human_20140103.fasta)	**MW [kDa]:**	521.8
**Database Date:**	2015-11-30	**pI:**	6.6
**Modification(s):**	Oxidation	**Sequence Coverage [%]:**	1.2
		**No. of unique Peptides:**	1

BRUKER

Detailed Protein Report

10	20	30	40	50	60	70	80
MMMCAATASP	AAASSGLGGD	GFYPAATFSS	SPAPGALFMP	VPDGSVAAAG	LGLGLPAADS	RGHYQLLLSG	RALADRYRRI
90	100	110	120	130	140	150	160
YTAALNDRDQ	GGGSAGHPAS	RNKKILNKKK	LKRKQKSKSK	VKTRSKSENL	ENTVIIPDIK	LHSNPSAFNI	YCNVRHCVLE
170	180	190	200	210	220	230	240
WQKKEISLAA	ASKNSVQSGE	SDSDEEEESK	EPPIKLPKII	EVGLCEVFEL	IKETRFSHPS	LCLRSLQALL	NVLQGQQPEG
250	260	270	280	290	300	310	320
LQSEPPEVLE	SLFQLLLEIT	VRSTGMNDST	GQSLTALSCA	CLFSLVASWG	ETGRTLQAIS	AILTNNGSHA	CQTIQVPTIL
330	340	350	360	370	380	390	400
NSLQRSVQAV	LVGKIQIQDW	FSNGIKKAAL	MHKWPLKEIS	VDEDDQCLLQ	NDGFFLYLLC	KDGLYKIGSG	YSGTVRGHIY
410	420	430	440	450	460	470	480
NSTSRIRNRK	EKKSWLGYAQ	GYLLYRDVNN	HSMTAIRISP	ETLEQDGTVM	LPDCHTEGQN	ILFTDGEYIN	QIAASRDDGF
490	500	510	520	530	540	550	560
VVRIFATSTE	PVLQQELQLK	LARKCLHACG	ISLFDLEKDL	HIISTGFDEE	SAILGAGREF	ALMKTANGKI	YYTGKYQSLG
570	580	590	600	610	620	630	640
IKQGGPSAGK	WVELPITKSP	KIVHFSVGHD	GSHALLVAED	GSIFFTGSAS	KGEDGESTKS	RRQSKPYKPK	KIIKMEGKIV
650	660	670	680	690	700	710	720
VYTACNNGSS	SVISKDGELY	MFGKDAIYSD	SSSLVTDLKG	HFVTQVAMGK	AHTCVLMKNG	EVWTFGVNNK	GQCGRDTGAM
730	740	750	760	770	780	790	800
NQGGKGFGVE	NMATAMDEDL	EEELDEKDEK	SMMCPPGMHK	WKLEQCMVCT	VCGDCTGYGA	SCVSSGRPDR	VPGGICGCGS
810	820	830	840	850	860	870	880
GESGCAVCGC	CKACARELDG	QEARQRGILD	AVKEMIPLDL	LLAVPVPGVN	IEEHLQLRQE	EKRQRVIRRH	RLEEGRGPLV
890	900	910	920	930	940	950	960
FAGPIFMNHR	EQALARLRSH	PAQLKHKRDK	HKDGSGERGE	KDASKITTYP	PGSVRFDCEL	RAVQVSCGFH	HSVVLMENGD
970	980	990	1000	1010	1020	1030	1040
VYTFGYGQHG	QLGHGDVNSR	GCPTLVQALP	GPSTQVTAGS	NHTAVLLMDG	QVFTFGSFSK	GQLGRPILDV	PYWNAKPAPM
1050	1060	1070	1080	1090	1100	1110	1120
PNIGSKYGRK	ATWIGASGDQ	TFLRIDEALI	NSHVLATSEI	FASKHIIGLV	PASISEPPPF	KCLLINKVDG	SCKTFNDSEQ
1130	1140	1150	1160	1170	1180	1190	1200
EDLQGFGVCL	DPVYDVIWRF	RPNTRELWCY	NAVVADARLP	SAADMQSRCS	ILSPELALPT	GSRALTTRSH	AALHILGCLD
1210	1220	1230	1240	1250	1260	1270	1280
TLAAMQDLKM	GVASTEEETQ	AVMKVYSKED	YSVVNRFESH	GGGWGYSAHS	VEAIRFSADT	DILLGGLGLF	GGRGEYTAKI
1290	1300	1310	1320	1330	1340	1350	1360
KLFELGPDGG	DHETDGDLLA	ETDVLAYDCA	AREKYAMMFD	EPVLLQAGWW	YVAWARVSGP	SSDCGSHGQA	SITTDDGVVF
1370	1380	1390	1400	1410	1420	1430	1440
QFKSSKKSNN	GTDVNAGQIP	QLLYRLPTSD	GSASKGKQQT	SEPVHILKRS	FARTVSVECF	ESLLSILHWS	WTTLVLGVEE
1450	1460	1470	1480	1490	1500	1510	1520
LRGLKGFQFT	ATLLDLERLR	FVGTCCLRLL	RVYTCEIYPV	SATGKAVVEE	TSKLAECIGK	TRTLLRKILS	EGVDHCMVKL
1530	1540	1550	1560	1570	1580	1590	1600
DNDPQGYLSQ	PLSLLEAVLQ	ECHNTFTACF	HSFYPTPALQ	WACLCDLLNC	LDQDIQEANF	KTSSSRLLAA	VMSALCHTSV
1610	1620	1630	1640	1650	1660	1670	1680
KLTSIFPIAY	DGEVLLRSIV	KQVSTENDST	LVHRFPLLVA	HMEKLSQSEE	NISGMTSFRE	VLEKMLVIVV	LPVRNSLRRE
1690	1700	1710	1720	1730	1740	1750	1760
NELFSSHLVS	NTCGLLASIV	SELTASALGS	EVDGLNSLHS	VKASANRFTK	TSQGRSWNTG	NGSPDAICFS	VDKPGIVVVG
1770	1780	1790	1800	1810	1820	1830	1840
FSVYGGGGIH	EYELEVLVDD	SEHAGDSTHS	HRWTSLELVK	GTYTTDDSPS	DIAEIRLDKV	VPLKENVKYA	VRLRNYGSRT
1850	1860	1870	1880	1890	1900	1910	1920
ANGDGGMTTV	QCPDGVTFTF	STCSLSSNGT	NQTRGQIPQI	LYYRSEFDGD	LQSQLLSKAN	EEDKNCSRAL	SVVSTVVRAS
1930	1940	1950	1960	1970	1980	1990	2000
KDLLHRALAV	DADDIPELLS	SSSLFSMLLP	LIIAYIGPVA	AAIPKVAVEV	FGLVQQLLPS	VAILNQKYAP	PAFNPNQSTD
2010	2020	2030	2040	2050	2060	2070	2080
STTGNQPEQG	LSACTTSSHY	AVIESEHPYK	PACVMHYKVT	FPECVRWMTI	EFDPQCGTAQ	SEDVLRLLIP	VRTVQNSGYG
2090	2100	2110	2120	2130	2140	2150	2160
PKLTSVHENL	NSWIELKKFS	GSSGWPTMVL	VLPGNEALFS	LETASDYVKD	DKASFYGFKC	FAIGYEFSPG	PDEGVIQLEK
2170	2180	2190	2200	2210	2220	2230	2240

Detailed Protein Report

Cmpd.	No. of Cmpds.	m/z meas.	Δ m/z [ppm]	z	Rt [min]	Score	P	Range	Sequence	Modification
1748	3	697.3510	1.93	3	50.2	15.7	1	680-698	K.GHFVTQVAMGKAHTCVLMK.N	Oxidation: 9, 18

Detailed Protein Report

Protein 96: unconventional myosin-IXb isoform 2 [Homo sapiens]

**Accession:**	gi|194272142	**Score:**	37.5
**Database:**	refseq_human(refseq_human_20140103.fasta)	**MW [kDa]:**	229.0
**Database Date:**	2015-11-30	**pI:**	9.4
		**Sequence Coverage [%]:**	1.8
		**No. of unique Peptides:**	1

Detailed Protein Report

10	20	30	40	50	60	70	80
MSVKEAGSSG	RREQAAYHLH	IYPQLSTTES	QASCRVTATK	DSTTSDVIKD	AIASLRLDGT	KCYVLVEVKE	SGGEEWVLDA
90	100	110	120	130	140	150	160
NDSPVHRVLL	WPRRAQDEHP	QEDGYYFLLQ	ERNADGTIKY	VHMQLVAQAT	ATRRLVERGL	LPRQQADFDD	LCNLPELTEG
170	180	190	200	210	220	230	240
NLLKNLKHRF	LQQKIYTYAG	SILVAINPFK	FLPIYNPKYV	KMYENQQLGK	LEPHVFALAD	VAYYTMLRKR	VNQCIVISGE
250	260	270	280	290	300	310	320
SGSGKTQSTN	FLIHCLTALS	QKGYASGVER	TILGAGPVLE	AFGNAKTAHN	NNSSRFGKFI	QVSYLESGIV	RGAVVEKYLL
330	340	350	360	370	380	390	400
EKSRLVSQEK	DERNYHVFYY	LLLGVSEEER	QEFQLKQPED	YFYLNQHNLK	IEDGEDLKHD	FERLKQAMEM	VGFLPATKKQ
410	420	430	440	450	460	470	480
IFAVLSAILY	LGNVTYKKRA	TGREEGLEVG	PPEVLDTLSQ	LLKVKREILV	EVLTKRKTVT	VNDKLILPYS	LSEAITARDS
490	500	510	520	530	540	550	560
MAKSLYSALF	DWIVLRINHA	LLNKKDVEEA	VSCLSIGVLD	IFGFEDFERN	SFEQFCINYA	NEQLQYYFNQ	HIFKLEQEEY
570	580	590	600	610	620	630	640
QGEGITWHNI	GYTDNVGCIH	LISKKPTGLF	YLLDEESNFP	HATSQTLLAK	FKQQHEDNKY	FLGTPVMEPA	FIIQHFAGKV
650	660	670	680	690	700	710	720
KYQIKDFREK	NMDYMRPDIV	ALLRGSDSSY	VRELIGMDPV	AVFRWAVLRA	AIRAMAVLRE	AGRLRAERAE	KAAGMSSPGA
730	740	750	760	770	780	790	800
QSHPEELPRG	ASTPSEKLYR	DLHNQMIKSI	KGLPWQGEDP	RSLLQSLSRL	QKPRAFILKS	KGIKQKQIIP	KNLLDSKSLK
810	820	830	840	850	860	870	880
LIISMTLHDR	TTKSLLHLHK	KKKPPSISAQ	FQTSLNKLLE	ALGKAEPFFI	RCIRSNAEKK	ELCFDDELVL	QQLRYTGMLE
890	900	910	920	930	940	950	960
TVRIRRSGYS	AKYTFQDFTE	QFQVLLPKDA	QPCREVISTL	LEKMKIDKRN	YQIGKTKVFL	KETERQALQE	TLHREVVRKI
970	980	990	1000	1010	1020	1030	1040
LLLQSWFRMV	LERRHFLQMK	RAAVTIQACW	RSYRVRRALE	RTQAAVYLQA	SWRGYWQRKL	YRHQKQSIIR	LQSLCRGHLQ
1050	1060	1070	1080	1090	1100	1110	1120
RKSFSQMISE	KQKAEEKERE	ALEAARAGAE	EGGQGQAAGG	QQVAEQGPEP	AEDGGHLASE	PEVQPSDRSP	LEHSSPEKEA
1130	1140	1150	1160	1170	1180	1190	1200
PSPEKTLPPQ	KTVAAESHEK	VPSSREKRES	RRQRGLEHVK	FQNKHIQSCK	EESALREPSR	RVTQEQGVSL	LEDKKESRED
1210	1220	1230	1240	1250	1260	1270	1280
ETLLVVETEA	ENTSQKQPTE	QPQAMAVGKV	SEETEKTLPS	GSPRPGQLER	PTSLALDSRV	SPPAPGSAPE	TPEDKSKPCG
1290	1300	1310	1320	1330	1340	1350	1360
SPRVQEKPDS	PGGSTQIQRY	LDAERLASAV	ELWRGKKLVA	AASPSAMLSQ	SLDLSDRHRA	TGAALTPTEE	RRTSFSTSDV
1370	1380	1390	1400	1410	1420	1430	1440
SKLLPSLAKA	QPAAETTDGE	RSAKKPAVQK	KKPGDASSLP	DAGLSPGSQV	DSKSTFKRLF	LHKTKDKKYS	LEGAEELENA
1450	1460	1470	1480	1490	1500	1510	1520
VSGHVVLEAT	TMKKGLEAPS	GQQHRHAAGE	KRTKEPGGKG	KKNRNVKIGK	ITVSEKWRES	VFRQITNANE	LKYLDEFLLN
1530	1540	1550	1560	1570	1580	1590	1600
KINDLRSQKT	PIESLFIEAT	EKFRSNIKTM	YSVPNGKIHV	GYKDLMENYQ	IVVSNLATER	GQKDTNLVLN	LFQSLLDEFT
1610	1620	1630	1640	1650	1660	1670	1680
RGYTKNDFEP	VKQSKAQKKK	RKQERAVQEH	NGHVFASYQV	SIPQSCEQCL	SYIWLMDKAL	LCSVCKMTCH	KKCVHKIQSH
1690	1700	1710	1720	1730	1740	1750	1760
CSYTYGRKGE	PGVEPGHFGV	CVDSLTSDKA	SVPIVLEKLL	EHVEMHGLYT	EGLYRKSGAA	NRTRELRQAL	QTDPAAVKLE
1770	1780	1790	1800	1810	1820	1830	1840
NFPIHAITGV	LKQWLRELPE	PLMTFAQYGD	FLRAVELPEK	QEQLAAIYAV	LEHLPEANHN	SLERLIFHLV	KVALLEDVNR
1850	1860	1870	1880	1890	1900	1910	1920
MSPGALAIIF	APCLLRCPDN	SDPLTSMKDV	LKITTCVEML	IKEQMRKYKV	KMEEISQLEA	AESIAFRRLS	LLRQNAPWPL
1930	1940	1950	1960	1970	1980	1990	2000
KLGFSSPYEG	VLNKSPKTRD	IQEEELEVLL	EEEAAGGDED	REKEILIERI	QSIKEEKEDI	TYRLPELDPR	GSDEENLDSE
2010	2020	2030					
TSASTESLLE	ERAGRGASEG	QY					

Cmpd.	No. of Cmpds.	m/z meas.	Δ m/z [ppm]	z	Rt [min]	Score	P	Range	Sequence	Modification
1465	1	671.4099	1.89	2	47.1	16.6	1	1964-1974	K.EILIERIQSIK.E	

Detailed Protein Report

Protein 97: PREDICTED: NEDD8 ultimate buster 1 isoform X3 [Homo sapiens]

**Accession:**	gi|530387034	**Score:**	37.1
**Database:**	refseq_human(refseq_human_20140103.fasta)	**MW [kDa]:**	67.7
**Database Date:**	2015-11-30	**pI:**	5.7
**Modification(s):**	Carbamidomethyl	**Sequence Coverage [%]:**	1.7
		**No. of unique Peptides:**	1

10	20	30	40	50	60	70	80
MKTNGGRCRI	RALCWSRREW	RGAGMAQKKY	LQAKLTQFLR	EDRIQLWKPP	YTDENKKVGL	ALKDLAKQYS	DRLECCENEV
**90**	**100**	**110**	**120**	**130**	**140**	**150**	**160**
EKVIEEIRCK	AIERGTGNDN	YRTTGIATIE	VFLPPRLKKD	RKNLLETRLH	ITGRELRSKI	AETFGLQENY	IKIVINKKQL
**170**	**180**	**190**	**200**	**210**	**220**	**230**	**240**
QLGKTLEEQG	VAHNVKAMVL	ELKQSEEDAR	KNFQLEEEEQ	NEAKLKEKQI	QRTKRGLEIL	AKRAAETVVD	PEMTPYLDIA
**250**	**260**	**270**	**280**	**290**	**300**	**310**	**320**
NQTGRSIRIP	PSERKALMLA	MGYHEKGRAF	LKRKEYGIAL	PCLLDADKYF	CECCRELLDT	VDNYAVLQLD	IVWCYFRLEQ
**330**	**340**	**350**	**360**	**370**	**380**	**390**	**400**
LECLDDAEKK	LNLAQKCFKN	CYGENHQRLV	HIKGNCGKEK	VLFLRLYLLQ	GIRNYHSGND	VEAYEYLNKA	RQLFKELYID
**410**	**420**	**430**	**440**	**450**	**460**	**470**	**480**
PSKVDNLLQL	GFTAQEARLG	LRACDGNVDH	AATHITNRRE	ILLSNPQMWW	LNDSNPETDN	RQESPSQENI	DRLVYMGFDA
**490**	**500**	**510**	**520**	**530**	**540**	**550**	**560**
LVAEAALRVF	RGNVQLAAQT	LAHNGGSLPP	ELPLSPEDSL	SPPATSPSDS	AGTSSASTDE	DMETEAVNEI	LEDIPEHEED
**570**	**580**	**590**	**600**				
YLDSTLEDEE	IIIAEYLSYV	ENRKSATKKN					

Cmpd.	No. of Cmpds.	m/z meas.	Δ m/z [ppm]	z	Rt [min]	Score	P	Range	Sequence	Modification
2062	2	625.2581	-159.60	2	54.4	16.1	2	330-339	K.KLNLAQKCFK.N	Carbamidomethyl: 8

Detailed Protein Report

Protein 98: ubiquitin carboxyl-terminal hydrolase 32 [Homo sapiens]

**Accession:**	gi|22550104	**Score:**	37.1
**Database:**	refseq_human(refseq_human_20140103.fasta)	**MW [kDa]:**	181.5
**Database Date:**	2015-11-30	**pI:**	6.0
		**Sequence Coverage [%]:**	2.4
		**No. of unique Peptides:**	3

10	20	30	40	50	60	70	80
MGAKESRIGF	LSYEEALRRV	TDVELKRLKD	AFKRTCGLSY	YMGQHCFIRE	VLGDGVPPKV	AEVIYCSFGG	TSKGLHFNNL
90	100	110	120	130	140	150	160
IVGLVLLTRG	KDEEKAKYIF	SLFSSESGNY	VIREEMERML	HVVDGKVPDT	LRKCFSEGEK	VNYEKFRNWL	FLNKDAFTFS
170	180	190	200	210	220	230	240
RWLLSGGVYV	TLTDDSDTPT	FYQTLAGVTH	LEESDIIDLE	KRYWLLKAQS	RTGRFDLETF	GPLVSPPIRP	SLSEGLFNAF
250	260	270	280	290	300	310	320
DENRDNHIDF	KEISCGLSAC	CRGPLAERQK	FCFKVFDVDR	DGVLSRVELR	DMVVALLEVW	KDNRTDDIPE	LHMDLSDIVE
330	340	350	360	370	380	390	400
GILNAHDTTK	MGHLTLEDYQ	IWSVKNVLAN	EFLNLLFQVC	HIVLGLRPAT	PEEEGQIIRG	WLERESRYGL	QAGHNWFIIS
410	420	430	440	450	460	470	480
MQWWQQWKEY	VKYDANPVVI	EPSSVLNGGK	YSFGTAAHPM	EQVEDRIGSS	LSYVNTTEEK	FSDNISTASE	ASETAGSGFL
490	500	510	520	530	540	550	560
YSATPGADVC	FARQHNTSDN	NNQCLLGANG	NILLHLNPQK	PGAIDNQPLV	TQEPVKATSL	TLEGGRLKRT	PQLIHGRDYE
570	580	590	600	610	620	630	640
MVPEPVWRAL	YHWYGANLAL	PRPVIKNSKT	DIPELELFPR	YLLFLRQQPA	TRTQQSNIWV	NMGNVPSPNA	PLKRVLAYTG
650	660	670	680	690	700	710	720
CFSRMQTIKE	IHEYLSQRLR	IKEEDMRLWL	YNSENYLTLL	DDEDHKLEYL	KIQDEQHLVI	EVRNKDMSWP	EEMSFIANSS
730	740	750	760	770	780	790	800
KIDRHKVPTE	KGATGLSNLG	NTCFMNSSIQ	CVSNTQPLTQ	YFISGRHLYE	LNRTNPIGMK	GHMAKCYGDL	VQELWSGTQK
810	820	830	840	850	860	870	880
NVAPLKLRWT	IAKYAPRFNG	FQQQDSQELL	AFLLDGLHED	LNRVHEKPYV	ELKDSDGRPD	WEVAAEAWDN	HLRRNRSIVV
890	900	910	920	930	940	950	960
DLFHGQLRSQ	VKCKTCGHIS	VRFDPFNFLS	LPLPMDSYMH	LEITVIKLDG	TTPVRYGLRL	NMDEKYTGLK	KQLSDLCGLN
970	980	990	1000	1010	1020	1030	1040
SEQILLAEVH	GSNIKNFPQD	NQKVRLSVSG	FLCAFEIPVP	VSPISASSPT	QTDFSSSPST	NEMFTLTTNG	DLPRPIFIPN
1050	1060	1070	1080	1090	1100	1110	1120
GMPNTVVPCG	TEKNFTNGMV	NGHMPSLPDS	PFTGYIIAVH	RKMMRTELYF	LSSQKNRPSL	FGMPLIVPCT	VHTRKKDLYD
1130	1140	1150	1160	1170	1180	1190	1200
AVWIQVSRLA	SPLPPQEASN	HAQDCDDSMG	YQYPFTLRVV	QKDGNSCAWC	PWYRFCRGCK	IDCGEDRAFI	GNAYIAVDWD
1210	1220	1230	1240	1250	1260	1270	1280
PTALHLRYQT	SQERVVDEHE	SVEQSRRAQA	EPINLDSCLR	AFTSEEELGE	NEMYYCSKCK	THCLATKKLD	LWRLPPILII
1290	1300	1310	1320	1330	1340	1350	1360
HLKRFQFVNG	RWIKSQKIVK	FPRESFDPSA	FLVPRDPALC	QHKPLTPQGD	ELSEPRILAR	EVKKVDAQSS	AGEEDVLLSK
1370	1380	1390	1400	1410	1420	1430	1440
SPSSLSANII	SSPKGSPSSS	RKSGTSCPSS	KNSSPNSSPR	TLGRSKGRLR	LPQIGSKNKL	SSSKENLDAS	KENGAGQICE
1450	1460	1470	1480	1490	1500	1510	1520
LADALSRGHV	LGGSQPELVT	PQDHEVALAN	GFLYEHEACG	NGYSNGQLGN	HSEEDSTDDQ	REDTRIKPIY	NLYAISCHSG
1530	1540	1550	1560	1570	1580	1590	1600
ILGGGHYVTY	AKNPNCKWYC	YNDSSCKELH	PDEIDTDSAY	ILFYEQQGID	YAQFLPKTDG	KKMADTSSMD	EDFESDYKKY
1610							
CVLQ							

Cmpd.	No. of Cmpds.	m/z meas.	Δ m/z [ppm]	z	Rt [min]	Score	P	Range	Sequence	Modification
362	1	539.2298	-162.41	2	33.2	10.4	1	549-557	K.RTPQLIHGR.D	
434	3	565.3154	-75.69	2	34.4	15.6	2	1292-1300	R.WIKSQKIVK.F	
2778	1	1023.5416	14.56	2	63.2	11.2	1	1361-1381	K.SPSSLSANIISSPKGSPSSSR.K	

Detailed Protein Report

Protein 99: probable ATP-dependent RNA helicase DDX10 [Homo sapiens]

Accession: gi|13514831
Database: refseq_human(refseq_human_20140103.fasta)
Database Date: 2015-11-30

Score: 37.1
MW [kDa]: 100.8
pI: 9.3
Sequence Coverage [%]: 4.6
No. of unique Peptides: 2

10	20	30	40	50	60	70	80
MGKTANSPGS	GARPDPVRSF	NRWKKKHSHR	QNKKKQLRKQ	LKKPEWQVER	ESISRLMQNY	EKINVNEITR	FSDFPLSKKT
**90**	**100**	**110**	**120**	**130**	**140**	**150**	**160**
LKGLQEAQYR	LVTEIQKQTI	GLALQGKDVL	GAAKTGSGKT	LAFLVPVLEA	LYRLQWTSTD	GLGVLIISPT	RELAYQTFEV
**170**	**180**	**190**	**200**	**210**	**220**	**230**	**240**
LRKVGKNHDF	SAGLIIGGKD	LKHEAERINN	INILVCTPGR	LLQHMDETVS	FHATDLQMLV	LDEADRILDM	GFADTMNAVI
**250**	**260**	**270**	**280**	**290**	**300**	**310**	**320**
ENLPKKRQTL	LFSATQTKSV	KDLARLSLKN	PEYVWVHEKA	KYSTPATLEQ	NYIVCELQQK	ISVLYSFLRS	HLKKKSIVFF
**330**	**340**	**350**	**360**	**370**	**380**	**390**	**400**
SSCKEVQYLY	RVFCRLRPGV	SILALHGRQQ	QMRRMEVYNE	FVRKRAAVLF	ATDIAARGLD	FPAVNWVLQF	DCPEDANTYI
**410**	**420**	**430**	**440**	**450**	**460**	**470**	**480**
HRAGRTARYK	EDGEALLILL	PSEKAMVQQL	LQKKVPVKEI	KINPEKLIDV	QKKLESILAQ	DQDLKERAQR	CFVSYVRSVY
**490**	**500**	**510**	**520**	**530**	**540**	**550**	**560**
LMKDKEVFDV	SKLPIPEYAL	SLGLAVAPRV	RFLQKMQKQP	TKELVRSQAD	KVIEPRAPSL	TNDEVEEFRA	YFNEKMSILQ
**570**	**580**	**590**	**600**	**610**	**620**	**630**	**640**
KGGKRLEGTE	HRQDNDTGNE	EQEEEEDDEE	EMEEKLAKAK	GSQAPSLPNT	SEAQKIKEVP	TQFLDRDEEE	EDADFLKVKR
**650**	**660**	**670**	**680**	**690**	**700**	**710**	**720**
HNVFGLDLKD	EKTLQKKEPS	KSSIKKKMTK	VAEAKKVMKR	NFKVNKKITF	TDEGELVQQW	PQMQKSAIKD	AEEDDDTGGI
**730**	**740**	**750**	**760**	**770**	**780**	**790**	**800**
NLHKAKERLQ	EEDKFDKEEY	RKKIKAKHRE	KRLKEREARR	EANKRQAKAK	DEEEAFLDWS	DDDDDDDDGF	DPSTLPDPDK
**810**	**820**	**830**	**840**	**850**	**860**	**870**	**880**
YRSSEDSDSE	DMENKISDTK	KKQGMKKRSN	SEVEDVGPTS	HNRKKARWDT	LEPLDTGLSL	AEDEELVLHL	LRSQS

Cmpd.	No. of Cmpds.	m/z meas.	Δ m/z [ppm]	z	Rt [min]	Score	P	Range	Sequence	Modification
1771	5	599.9726	0.40	3	50.8	16.0	1	1-18	-.MGKTANSPGSGARPDPVR.S	
2294	1	713.3369	-116.31	2	57.0	10.6	2	638-649	K.VKRHNVFGLDLK.D	

Detailed Protein Report

Protein 100: conserved oligomeric Golgi complex subunit 1 [Homo sapiens]

Accession: gi|21237783
Database: refseq_human(refseq_human_20140103.fasta)
Database Date: 2015-11-30
Modification(s): Oxidation

Score: 37.0
MW [kDa]: 108.9
pI: 7.1
Sequence Coverage [%]: 4.5
No. of unique Peptides: 2

Quantitation

WD:WU **Median:** 0.38 **CV:** 0.00 % **No. of Peptides:** 1

10	20	30	40	50	60	70	80
MATAATSPAL	KRLDLRDPAA	LFETHGAEEI	RGLERQVRAE	IEHKKEELRQ	MVGERYRDLI	EAADTIGQMR	RCAVGLVDAV
**90**	**100**	**110**	**120**	**130**	**140**	**150**	**160**
KATDQYCARL	RQAGSAAPRP	PRAQQPQQPS	QEKFYSMAAQ	IKLLLEIPEK	IWSSMEASQC	LHATQLYLLC	CHLHSLLQLD
**170**	**180**	**190**	**200**	**210**	**220**	**230**	**240**
SSSSRYSPVL	SRFPILIRQV	AAASHFRSTI	LHESKMLLKC	QGVSDQAVAE	ALCSIMLLEE	SSPRQALTDF	LLARKATIQK
**250**	**260**	**270**	**280**	**290**	**300**	**310**	**320**
LLNQPHHGAG	IKAQICSLVE	LLATTLKQAH	ALFYTLPEGL	LPDPALPCGL	LFSTLETITG	QHPAGKGTGV	LQEEMKLCSW
**330**	**340**	**350**	**360**	**370**	**380**	**390**	**400**
FKHLPASIVE	FQPTLRTLAH	PISQEYLKDT	LQKWIHMCNE	DIKNGITNLL	MYVKSMKGLA	GIRDAMWELL	TNESTNHSWD
**410**	**420**	**430**	**440**	**450**	**460**	**470**	**480**
VLCRRLLEKP	LLFWEDMMQQ	LFLDRLQTLT	KEGFDSISSS	SKELLVSALQ	ELESSTSNSP	SNKHIHFEYN	MSLFLWSESP
**490**	**500**	**510**	**520**	**530**	**540**	**550**	**560**
NDLPSDAAWV	SVANRGQFAS	SGLSMKAQAI	SPCVQNFCSA	LDSKLKVKLD	DLLAYLPSDD	SSLPKDVSPT	QAKSSAFDRY
**570**	**580**	**590**	**600**	**610**	**620**	**630**	**640**
ADAGTVQEML	RTQSVACIKH	IVDCIRAELQ	SIEEGVQGQQ	DALNSAKLHS	VLFMARLCQS	LGELCPHLKQ	CILGKSESSE
**650**	**660**	**670**	**680**	**690**	**700**	**710**	**720**
KPAREFRALR	KQGKVKTQEI	IPTQAKWQEV	KEVLLQQSVM	GYQVWSSAVV	KVLIHGFTQS	LLLDDAGSVL	ATATSWDELE
**730**	**740**	**750**	**760**	**770**	**780**	**790**	**800**
IQEEAESGSS	VTSKIRLPAQ	PSWYVQSFLF	SLCQEINRVG	GHALPKVTLQ	EMLKSCMVQV	VAAYEKLSEE	KQIKKEGAFP
**810**	**820**	**830**	**840**	**850**	**860**	**870**	**880**
VTQNRALQLL	YDLRYLNIVL	TAKGDEVKSG	RSKPDSRIEK	VTDHLEALID	PFDLDVFTPH	LNSNLHRLVQ	RTSVLFGLVT
**890**	**900**	**910**	**920**	**930**	**940**	**950**	**960**
GTENQLAPRS	STFNSQEPHN	ILPLASSQIR	FGLLPLSMTS	TRKAKSTRNI	ETKAQVVPPA	RSTAGDPTVP	GSLFRQLVSE
**970**	**980**	**990**					
EDNTSAPSLF	KLGWLSSMTK						

Cmpd.	No. of Cmpds.	m/z meas.	Δ m/z [ppm]	z	Rt [min]	Score	P	Range	Sequence	Modification	Ratios
2917	1	724.8590	4.01	2	65.2	12.8	0	58-70	R.DLIEAADTIGQMR.R	Oxidation: 12	
1712	1	1045.0032	-51.34	2	49.7	13.3	1	527-545	K.VKLDDLLAYLPSDDSSLPK.D		WD:WU 0.38

Detailed Protein Report

Protein 101: PREDICTED: F-box/WD repeat-containing protein 11 isoform X5 [Homo sapiens]

**Accession:**	gi|530380941	**Score:**	36.9
**Database:**	refseq_human(refseq_human_20140103.fasta)	**MW [kDa]:**	56.8
**Database Date:**	2015-11-30	**pI:**	8.6
**Modification(s):**	Carbamidomethyl	**Sequence Coverage [%]:**	9.7
		**No. of unique Peptides:**	2

Quantitation

WD:WU **Median:** 1.09 **CV:** 0.00 % **No. of Peptides:** 1

10	20	30	40	50	60	70	80
MISNGTSSVI	VSRKRPSEGN	YQKEKDLCIK	YFDQWSESDQ	VEFVEHLISR	MCHYQHGHIN	SYLKPMLQRD	FITALPEQGL
**90**	**100**	**110**	**120**	**130**	**140**	**150**	**160**
DHIAENILSY	LDARSLCAAE	LVCKEWQRVI	SEGMLWKKLI	ERMVRTDPLW	KGLSERRGWD	QYLFKNRPTD	GPPNSFYRSL
**170**	**180**	**190**	**200**	**210**	**220**	**230**	**240**
YPKIIQDIET	IESNWRCGRH	NLQRIQCRSE	NSKGVYCLQY	DDEKIISGLR	DNSIKIWDKT	SLECLKVLTG	HTGSVLCLQY
**250**	**260**	**270**	**280**	**290**	**300**	**310**	**320**
DERVIVTGSS	DSTVRVWDVN	TGEVLNTLIH	HNEAVLHLRF	SNGLMVTCSK	DRSIAVWDMA	SATDITLRRV	LVGHRAAVNV
**330**	**340**	**350**	**360**	**370**	**380**	**390**	**400**
VDFDDKYIVS	ASGDRTIKVW	STSTCEFVRT	LNGHKRGIAC	LQYRDRLVVS	GSSDNTIRLW	DIECGACLRV	LEGHEELVRC
**410**	**420**	**430**	**440**	**450**	**460**	**470**	**480**
IRFDNKRIVS	GAYDGKIKVW	DLQAALDPRA	PASTLCLRTL	VEHSGRVFRL	QFDEFQIISS	SHDDTILIWD	FLNVPPSAQN
**490**	**500**						
ETRSPSRTYT	YISR						

Cmpd.	No. of Cmpds.	m/z meas.	Δ m/z [ppm]	z	Rt [min]	Score	P	Range	Sequence	Modification	Ratios
891	1	630.9500	-54.02	3	39.5	12.1	0	227-243	K.VLTGHTGSVLCLQYDER.V		WD:WU 1.09
1138	1	751.8302	130.90	3	42.5	11.7	1	419-438	K.VWDLQAALDPRAPASTLCLR.T	Carbamidomethyl: 18	

Detailed Protein Report

Protein 102: uncharacterized protein C4orf21 [Homo sapiens]

**Accession:**	gi|254826809	**Score:**	36.7
**Database:**	refseq_human(refseq_human_20140103.fasta)	**MW [kDa]:**	236.5
**Database Date:**	2015-11-30	**pI:**	5.8
**Modification(s):**	Carbamidomethyl	**Sequence Coverage [%]:**	2.1
		**No. of unique Peptides:**	3

Alias proteins:

Accession	Name	Description
gi|530377937	refseq_human (refseq_human_20140103.fasta)	PREDICTED: uncharacterized protein C4orf21 isoform X1 [Homo sapiens]

Detailed Protein Report

10	20	30	40	50	60	70	80
MESQEFIVLY	THQKMKKSKV	WQDGILKITH	LGNKAILYDD	KGACLESLFL	KCLEVKPGDD	LESDRYLITV	EEVKVAGAIG
90	100	110	120	130	140	150	160
IVKQNVNKEA	PELNSRTFIS	SGRSLGCQPS	GLKRKFTGFQ	GPRQVPKKMV	IMESGESAAS	HEAKKTGPTI	FSPFCSMPPL
170	180	190	200	210	220	230	240
FPTVGKKDVN	NILADPENIV	TYKNRERNAM	DFSSVFSPSF	QINPEVLCEE	NYFCSPVNSG	NKLSDSLLTN	EPVKRDSLAS
250	260	270	280	290	300	310	320
HYSGVSQNIR	SKAQILALLK	SESSSSCEEL	NSEMTEHFPQ	KQPQGSLKIA	TKPKYLIQQE	ECAEMKSTEN	LYYQHQSENT
330	340	350	360	370	380	390	400
MRNKSRWAMY	LSSQSSPIHS	STVDGNDTER	KPKAQEDDVN	SNLKDLSLQK	IIQFVETYAE	ERKKYNVDQS	VGNNDPSWNQ
410	420	430	440	450	460	470	480
EVKLEIPSFN	ESSSLQVTCS	SAENDGILSE	SDIQEDNKIP	FNQNDKGCIK	GSVLIKENAQ	EVNTCGTLEK	EYEQSESSLP
490	500	510	520	530	540	550	560
ELKHLQIESS	NNSRISDDIT	DMISESKMDN	ESLNSIHESL	SNVTQPFLEV	TFNLNNFETS	DTEEESQESN	KISQDSESWV
570	580	590	600	610	620	630	640
KDILVNDGNS	CFQKRSENTN	CEEIEGEHLP	FLTSVSDKPT	VTFPVKETLP	SQFCDKTYVG	FDMGICKTEN	TGKEIEEYSD
650	660	670	680	690	700	710	720
TLSNFESFKW	TDAVYGDNKE	DANKPIQEVR	INYDFALPPN	KSKGINMNLH	IPHIQNQIAE	NSNLFSEDAQ	PQPFILGSDL
730	740	750	760	770	780	790	800
DKNDEHVLPS	TSSSDNSVQL	LNTNQNHYEC	IALDKSNTHI	SNSLFYPLGK	KHLISKDTEA	HISEPEDLGK	IRSPPPDHVE
810	820	830	840	850	860	870	880
VETAREGKQY	WNPRNSSELS	GLVNTISILK	SLCEHSTALD	SLEILKKKNT	VFQQGTQQTY	EPDSPPEVRK	PFITVVSPKS
890	900	910	920	930	940	950	960
PHLHKDSQQI	LKEDEVELSE	PLQSVQFSSS	GSKEETAFQA	VIPKQIERKT	CDPKPVEFQG	HQVKGSATSG	VMVRGHSSQL
970	980	990	1000	1010	1020	1030	1040
GCSQFPDSTE	YENFMTETPE	LPSTCMQIDF	LQVTSPEENI	STLSPVSTFS	LNSRDEDFMV	EFSETSLKAR	TLPDDLHFLN
1050	1060	1070	1080	1090	1100	1110	1120
LEGMKKSRSL	ENENLQRLSL	LSRTQVPLIT	LPRTDGPPDL	DSHSYMINSN	TYESSGSPML	NLCEKSAVLS	FSIEPEDQNE
1130	1140	1150	1160	1170	1180	1190	1200
TFFSEESREV	NPGDVSLNNI	STQSKWLKYQ	NTSQCNVATP	NRVDKRITDG	FFAEAVSGMH	FRDTSERQSD	AVNESSLDSV
1210	1220	1230	1240	1250	1260	1270	1280
HLQMIKGMLY	QQRQDFSSQD	SVSRKKVLSL	NLKQTSKTEE	IKNVLGGSTC	YNYSVKDLQE	ISGSELCFPS	GQKIKSAYLP
1290	1300	1310	1320	1330	1340	1350	1360
QRQIHIPAVF	QSPAHYKQTF	TSCLIEHLNI	LLFGLAQNLQ	KALSKVDISF	YTSLKGEKLK	NAENNVPSCH	HSQPAKLVMV
1370	1380	1390	1400	1410	1420	1430	1440
KKEGPNKGRL	FYTCDGPKAD	RCKFFKWLED	VTPGYSTQEG	ARPGMVLSDI	KSIGLYLRSQ	KIPLYEECQL	LVRKGFDFQR
1450	1460	1470	1480	1490	1500	1510	1520
KQYGKLKKFT	TVNPEFYNEP	KTKLYLKLSR	KERSSAYSKN	DLWVVSKTLD	FELDTFIACS	AFFGPSSINE	IEILPLKGYF
1530	1540	1550	1560	1570	1580	1590	1600
PSNWPTNMVV	HALLVCNAST	ELTTLKNIQD	YFNPATLPLT	QYLLTTSSPT	IVSNKRVSKR	KFIPPAFTNV	STKFELLSLG
1610	1620	1630	1640	1650	1660	1670	1680
ATLKLASELI	QVHKLNKDQA	TALIQIAQMM	ASHESIEEVK	ELQTHTFPIT	IIHGVFGAGK	SYLLAVVILF	FVQLFEKSEA
1690	1700	1710	1720	1730	1740	1750	1760
PTIGNARPWK	LLISSSTNVA	VDRVLLGLLS	LGFENFIRVG	SVRKIAKPIL	PYSLHAGSEN	ESEQLKELHA	LMKEDLTPTE
1770	1780	1790	1800	1810	1820	1830	1840
RVYVRKSIEQ	HKLGTNRTLL	KQVRVVGVTC	AACPFPCMND	LKFPVVVLDE	CSQITEPASL	LPIARFECEK	LILVGDPKQL
1850	1860	1870	1880	1890	1900	1910	1920
PPTIQGSDAA	HENGLEQTLF	DRLCLMGHKP	ILLRTQYRCH	PAISAIANDL	FYKGALMNGV	TEIERSPLLE	WLPTLCFYNV
1930	1940	1950	1960	1970	1980	1990	2000
KGLEQIERDN	SFHNVAEATF	TLKLIQSLIA	SGIAGSMIGV	ITLYKSQMYK	LCHLLSAVDF	HHPDIKTVQV	STVDAFQGAE
2010	2020	2030	2040	2050	2060	2070	2080
KEIIILSCVR	TRQVGFIDSE	KRMNVALTRG	KRHLLIVGNL	ACLRKNQLWG	RVIQHCEGRE	DGLQHANQYE	PQLNHLLKDY
2090	2100	2110					
FEKQVEEKQK	KKSEKEKSKD	KSHS					

Detailed Protein Report

Cmpd.	No. of Cmpds.	m/z meas.	Δ m/z [ppm]	z	Rt [min]	Score	P	Range	Sequence	Modification
2940	1	973.5071	-8.25	2	65.5	10.8	1	167-183	K.KDVNNILADPENIVTYK.N	
2069	1	698.2854	-192.09	2	54.5	11.7	1	282-294	K.QPQGSLKIATKPK.Y	
2882	1	938.5029	-0.62	2	64.8	14.3	1	1419-1433	R.SQKIPLYEECQLLVR.K	Carbamidomethyl: 10

Detailed Protein Report

Protein 103: apolipoprotein A-II preproprotein [Homo sapiens]

Accession: gi|4502149
Database: refseq_human(refseq_human_20140103.fasta)
Database Date: 2015-11-30

Score: 36.7
MW [kDa]: 11.2
pI: 7.1
Sequence Coverage [%]: 22.0
No. of unique Peptides: 1

10	20	30	40	50	60	70	80
MKLLAATVLL	LTICSLEGAL	VRRQAKEPCV	ESLVSQYFQT	VTDYGKDLME	KVKSPELQAE	AKSYFEKSKE	QLTPLIKKAG
90	100	110					
TELVNFLSYF	VELGTQPATQ						

Cmpd.	No. of Cmpds.	m/z meas.	Δ m/z [ppm]	z	Rt [min]	Score	P	Range	Sequence	Modification
2890	2	1193.0548	-39.81	2	64.9	36.7	0	79-100	K.AGTELVNFLSYFVELGTQPATQ.-	

Detailed Protein Report

Protein 104: regulator of G-protein signaling 9 isoform 3 [Homo sapiens]

**Accession:**	gi|260099635	**Score:**	36.7
**Database:**	refseq_human(refseq_human_20140103.fasta)	**MW [kDa]:**	56.7
**Database Date:**	2015-11-30	**pI:**	9.8
		**Sequence Coverage [%]:**	6.2
		**No. of unique Peptides:**	1

10	20	30	40	50	60	70	80
MTIRHQGQQY	RPRMAFLQKI	EALVKDMQNP	ETGVRMQNQR	VLVTSVPHAM	TGSDVLQWIV	QRLWISSLEA	QNLGNFIVRY
**90**	**100**	**110**	**120**	**130**	**140**	**150**	**160**
GYIYPLQDPK	NLILKPDGSL	YRFQTPYFWP	TQQWPAEDTD	YAIYLAKRNI	KKKGILEEYE	KENYNFLNQK	MNYKWDFVIM
**170**	**180**	**190**	**200**	**210**	**220**	**230**	**240**
QAKEQYRAGK	ERNKADRYAL	DCQEKAYWLV	HRCPPGMDNV	LDYGLDRVTN	PNEVKKQTVV	AVKKEIMYYQ	QALMRSTVKS
**250**	**260**	**270**	**280**	**290**	**300**	**310**	**320**
SVSLGGIVKY	SEQFSSNDAI	MSGCLPSNPW	ITDDTQFWDL	NAKLVEIPTK	MRVERWAFNF	SELIRDPKGR	QSFQYFLKKE
**330**	**340**	**350**	**360**	**370**	**380**	**390**	**400**
FSGENLGFWE	ACEDLKYGDQ	SKVKEKAEEI	YKLFLAPGAR	RWINIDGKTM	DITVKGLKHP	HRYVLDAAQT	HIYMLMKKDS
**410**	**420**	**430**	**440**	**450**	**460**	**470**	**480**
YARYLKSPIY	KDMLAKAIEP	QETTKKSSTL	PFMRRHLRSS	PSPVILRQLE	EEAKAREAAN	TVDITQVMSK	LDRRSQLQKE
**490**							
LPPK							

Cmpd.	No. of Cmpds.	m/z meas.	Δ m/z [ppm]	z	Rt [min]	Score	P	Range	Sequence	Modification
2782	1	681.4961	134.76	2	63.3	19.3	1	436-447	R.HLRSSPSPVILR.Q	

Detailed Protein Report

Protein 105: biorientation of chromosomes in cell division protein 1-like 1 [Homo sapiens]

**Accession:**	gi|87299628	**Score:**	36.6
**Database:**	refseq_human(refseq_human_20140103.fasta)	**MW [kDa]:**	330.3
**Database Date:**	2015-11-30	**pI:**	4.9
**Modification(s):**	Oxidation	**Sequence Coverage [%]:**	0.8
		**No. of unique Peptides:**	1

Detailed Protein Report

10	20	30	40	50	60	70	80
MATNPQPQPP	PPAPPPPPPQ	PQPQPPPPPP	GPGAGPGAGG	AGGAGAGAGD	PQLVAMIVNH	LKSQGLFDQF	RRDCLADVDT
90	100	110	120	130	140	150	160
KPAYQNLRQR	VDNFVANHLA	THTWSPHLNK	NQLRNNIRQQ	VLKSGMLESG	IDRIISQVVD	PKINHTFRPQ	VEKAVHEFLA
170	180	190	200	210	220	230	240
TLNHKEEGSG	NTAPDDEKPD	TSLITQGVPT	PGPSANVAND	AMSILETITS	LNQEASAARA	STETSNAKTS	ERASKKLPSQ
250	260	270	280	290	300	310	320
PTTDTSTDKE	RTSEDMADKE	KSTADSGGEG	LETAPKSEEF	SDLPCPVEEI	KNYTKEHNNL	ILLNKDVQQE	SSEQKNKSTD
330	340	350	360	370	380	390	400
KGEKKPDSNE	KGERKKEKKE	KTEKKFDHSK	KSEDTQKVKD	EKQAKEKEVE	SLKLPSEKNS	NKAKTVEGTK	EDFSLIDSDV
410	420	430	440	450	460	470	480
DGLTDITVSS	VHTSDLSSFE	EDTEEEVVTS	DSMEEGEITS	DDEEKNKQNK	TKTQTSDSSE	GKTKSVRHAY	VHKPYLYSKY
490	500	510	520	530	540	550	560
YSDSDDELTV	EQRRQSIAKE	KEERLLRRQI	NREKLEEKRK	QKAEKTKSSK	TKGQGRSSVD	LEESSTKSLE	PKAARIKEVL
570	580	590	600	610	620	630	640
KERKVLEKKV	ALSKKRKKDS	RNVEENSKKK	QQYEEDSKET	LKTSEHCEKE	KISSSKELKH	VHAKSEPSKP	ARRLSESLHV
650	660	670	680	690	700	710	720
VDENKNESKL	EREHKRRTST	PVIMEGVQEE	TDTRDVKRQV	ERSEICTEEP	QKQKSTLKNE	KHLKKDDSET	PHLKSLLKKE
730	740	750	760	770	780	790	800
VKSSKEKPER	EKTPSEDKLS	VKHKYKGDCM	HKTGDETELH	SSEKGLKVEE	NIQKQSQQTK	LSSDDKTERK	SKHRNERKLS
810	820	830	840	850	860	870	880
VLGKDGKPVS	EYIIKTDENV	RKENNKKERR	LSAEKTKAEH	KSRRSSDSKI	QKDSLGSKQH	GITLQRRSES	YSEDKCDMDS
890	900	910	920	930	940	950	960
TNMDSNLKPE	EVVHKEKRRT	KSLLEEKLVL	KSKSKTQGKQ	VKVVETELQE	GATKQATTPK	PDKEKNTEEN	DSEKQRKSKV
970	980	990	1000	1010	1020	1030	1040
EDKPFEETGV	EPVLETASSS	AHSTQKDSSH	RAKLPLAKEK	YKSDKDSTST	RLERKLSDGH	KSRSLKHSSK	DIKKKDENKS
1050	1060	1070	1080	1090	1100	1110	1120
DDKDGKEVDS	SHEKARGNSS	LMEKKLSRRL	CENRRGSLSQ	EMAKGEEKLA	ANTLSTPSGS	SLQRPKKSGD	MTLIPEQEPM
1130	1140	1150	1160	1170	1180	1190	1200
EIDSEPGVEN	VFEVSKTQDN	RNNNSQQDID	SENMKQKTSA	TVQKDELRTC	TADSKATAPA	YKPGRGTGVN	SNSEKHADHR
1210	1220	1230	1240	1250	1260	1270	1280
STLTKKMHIQ	SAVSKMNPGE	KEPIHRGTTE	VNIDSETVHR	MLLSAPSEND	RVQKNLKNTA	AEEHVAQGDA	TLEHSTNLDS
1290	1300	1310	1320	1330	1340	1350	1360
SPSLSSVTVV	PLRESYDPDV	IPLFDKRTVL	EGSTASTSPA	DHSALPNQSL	TVRESEVLKT	SDSKEGGEGF	TVDTPAKASI
1370	1380	1390	1400	1410	1420	1430	1440
TSKRHIPEAH	QATLLDGKQG	KVIMPLGSKL	TGVIVENENI	TKEGGLVDMA	KKENDLNAEP	NLKQTIKATV	ENGKKDGIAV
1450	1460	1470	1480	1490	1500	1510	1520
DHVVGLNTEK	YAETVKLKHK	RSPGKVKDIS	IDVERRNENS	EVDTSAGSGS	APSVLHQRNG	QTEDVATGPR	RAEKTSVATS
1530	1540	1550	1560	1570	1580	1590	1600
TEGKDKDVTL	SPVKAGPATT	TSSETRQSEV	ALPCTSIEAD	EGLIIGTHSR	NNPLHVGAEA	SECTVFAAAE	EGGAVVTEGF
1610	1620	1630	1640	1650	1660	1670	1680
AESETFLTST	KEGESGECAV	AESEDRAADL	LAVHAVKIEA	NVNSVVTEEK	DDAVTSAGSE	EKCDGSLSRD	SEIVEGTITF
1690	1700	1710	1720	1730	1740	1750	1760
ISEVESDGAV	TSAGTEIRAG	SISSEEVDGS	QGNMMRMGPK	KETEGTVTCT	GAEGRSDNFV	ICSVTGAGPR	EERMVTGAGV
1770	1780	1790	1800	1810	1820	1830	1840
VLGDNDAPPG	TSASQEGDGS	VNDGTEGESA	VTSTGITEDG	EGPASCTGSE	DSSEGFAISS	ESEENGESAM	DSTVAKEGTN
1850	1860	1870	1880	1890	1900	1910	1920
VPLVAAGPCD	DEGIVTSTGA	KEEDEEGEDV	VTSTGRGNEI	GHASTCTGLG	EESEGVLICE	SAEGDSQIGT	VVEHVEAEAG
1930	1940	1950	1960	1970	1980	1990	2000
AAIMNANENN	VDSMSGTEKG	SKDTDICSSA	KGIVESSVTS	AVSGKDEVTP	VPGGCEGPMT	SAASDQSDSQ	LEKVEDTTIS
2010	2020	2030	2040	2050	2060	2070	2080
TGLVGGSYDV	LVSGEVPECE	VAHTSPSEKE	DEDIITSVEN	EECDGLMATT	ASGDITNQNS	LAGGKNQGKV	LIISTSTTND
2090	2100	2110	2120	2130	2140	2150	2160
YTPQVSAITD	VEGGLSDALR	TEENMEGTRV	TTEEFEAPMP	SAVSGDDSQL	TASRSEEKDE	CAMISTSIGE	EFELPISSAT
2170	2180	2190	2200	2210	2220	2230	2240

Detailed Protein Report

Cmpd.	No. of Cmpds.	m/z meas.	Δ m/z [ppm]	z	Rt [min]	Score	P	Range	Sequence	Modification
2965	1	557.6376	40.39	3	65.8	10.0	2	2881-2894	K.YPVETTLKMKDDSK.T	Oxidation: 9

Detailed Protein Report

Protein 106: PREDICTED: myotubularin-related protein 5 isoform X5 [Homo sapiens]

**Accession:**	gi|530420851	**Score:**	36.3
**Database:**	refseq_human(refseq_human_20140103.fasta)	**MW [kDa]:**	208.2
**Database Date:**	2015-11-30	**pI:**	6.5
**Modification(s):**	Oxidation	**Sequence Coverage [%]:**	2.0
		**No. of unique Peptides:**	2

Quantitation

WD:WU	**Median:** 0.65	**CV:** 0.00 %	**No. of Peptides:** 1

Detailed Protein Report

10	20	30	40	50	60	70	80
MARLADYFVL	VAFGPHPRGS	GEGQGQILQR	FPEKDWEDNP	FPQGIELFCQ	PSGWQLCPER	NPPTFFVAVL	TDINSERHYC
90	100	110	120	130	140	150	160
ACLTFWEPAE	PSQETTRVED	ATEREEEGDE	GGQTHLSPTA	PAPSAQLFAP	KTLVLVSRLD	HTEVFRNSLG	LIYAIHVEGL
170	180	190	200	210	220	230	240
NVCLENVIGN	LLTCTVPLAG	GSQRTISLGA	GDRQVIQTPL	ADSLPVSRCS	VALLFRQLGI	TNVLSLFCAA	LTEHKVLFLS
250	260	270	280	290	300	310	320
RSYQRLADAC	RGLLALLFPL	RYSFTYVPIL	PAQLLEVLST	PTPFIIGVNA	AFQAETQELL	DVIVADLDGG	TVTIPECVHI
330	340	350	360	370	380	390	400
PPLPEPLQSQ	THSVLSMVLD	PELELADLAF	PPPTTSTSSL	KMQDKELRAV	FLRLFAQLLQ	GYRWCLHVVR	IHPEPVIRFH
410	420	430	440	450	460	470	480
KAAFLGQRGL	VEDDFLMKVL	EGMAFAGFVS	ERGVPYRPTD	LFDELVAHEV	ARMRADENHP	QRVLRHVQEL	AEQLYKNENP
490	500	510	520	530	540	550	560
YPAVAMHKVQ	RPGESSHLRR	VPRPFPRLDE	GTVQWIVDQA	AAKMQGAPPA	VKAERRTTVP	SGPPMTAILE	RCSGLHVNSA
570	580	590	600	610	620	630	640
RRLEVVRNCI	SYVFEGKMLE	AKKLLPAVLR	ALKGRAARRC	LAQELHLHVQ	QNRAVLDHQQ	FDFVVRMMNC	CLQDCTSLDE
650	660	670	680	690	700	710	720
HGIAAALLPL	VTAFCRKLSP	GVTQFAYSCV	QEHVVWSTPQ	FWEAMFYGDV	QTHIRALYLE	PTEDLAPAQE	VGEAPSQEDE
730	740	750	760	770	780	790	800
RSALDVASEQ	RRLWPTLSRE	KQQELVQKEE	STVFSQAIHY	ANRMSYLLLP	LDSSKSRLLR	ERAGLGDLES	ASNSLVTNSM
810	820	830	840	850	860	870	880
AGSVAESYDT	ESGFEDAETC	DVAGAVVRFI	NRFVDKVCTE	SGVTSDHLKG	LHVMVPDIVQ	MHIETLEAVQ	RESRRLPPIQ
890	900	910	920	930	940	950	960
KPKLLRPRLL	PGEECVLDGL	RVYLLPDGRE	EGAGGSAGGP	ALLPAEGAVF	LTTYRVIFTG	MPTDPLVGEQ	VVVRSFPVAA
970	980	990	1000	1010	1020	1030	1040
LTKEKRISVQ	TPVDQLLQDG	LQLRSCTFQL	LKMAFDEEVG	SDSAELFRKQ	LHKLRYPPDI	RATFAFTLGS	AHTPGRPPRV
1050	1060	1070	1080	1090	1100	1110	1120
TKDKGPSLRT	LSRNLVKNAK	KTIGRQHVTR	KKYNPPSWEH	RGQPPPEDQE	DEISVSEELE	PSTLTPSSAL	KPSDRMTMSS
1130	1140	1150	1160	1170	1180	1190	1200
LVERACCRDY	QRLGLGTLSS	SLSRAKSEPF	RISPVNRMYA	ICRSYPGLLI	VPQSVQDNAL	QRVSRCYRQN	RFPVVCWRSG
1210	1220	1230	1240	1250	1260	1270	1280
RSKAVLLRSG	GLHGKGVVGL	FKAQNAPSPG	QSQADSSSLE	QEKYLQAVVS	SMPRYADASG	RNTLSGFSSA	HMGSHGKWGS
1290	1300	1310	1320	1330	1340	1350	1360
VRTSGRSSGL	GTDVGSRLAG	RDALAPPQAN	GGPPDPGFLR	PQRAALYILG	DKAQLKGVRS	DPLQQWELVP	IEVFEARQVK
1370	1380	1390	1400	1410	1420	1430	1440
ASFKKLLKAC	VPGCPAAEPS	PASFLRSLED	SEWLIQIHKL	LQVSVLVVEL	LDSGSSVLVG	LEDGWDITTQ	VVSLVQLLSD
1450	1460	1470	1480	1490	1500	1510	1520
PFYRTLEGFR	LLVEKEWLSF	GHRFSHRGAH	TLAGQSSGFT	PVFLQFLDCV	HQVHLQFPME	FEFSQFYLKF	LGYHHVSRRF
1530	1540	1550	1560	1570	1580	1590	1600
RTFLLDSDYE	RIELGLLYEE	KGERRGQVPC	RSVWEYVDRL	SKRTPVFHNY	MYAPEDAEVL	RPYSNVSNLK	VWDFYTEETL
1610	1620	1630	1640	1650	1660	1670	1680
AEGPPYDWEL	AQGPPEPPEE	ERSDGGAPQS	RRRVVWPCYD	SCPRAQPDAI	SRLLEELQRL	ETELGQPAER	WKDTWDRVKA
1690	1700	1710	1720	1730	1740	1750	1760
AQRLEGRPDG	RGTPSSLLVS	TAPHHRRSLG	VYLQEGPVGS	TLSLSLDSDQ	SSGSTTSGSR	QAARRSTSTL	YSQFQTAESE
1770	1780	1790	1800	1810	1820	1830	1840
NRSYEGTLYK	KGAFMKPWKA	RWFVLDKTKH	QLRYYDHRVD	TECKGVIDLA	EVEAVAPGTP	TMGAPKTVDE	KAFFDVKTTR
1850	1860	1870					
RVYNFCAQDV	PSAQQWVDRI	QSCLSDA					

Cmpd.	No. of Cmpds.	m/z meas.	Δ m/z [ppm]	z	Rt [min]	Score	P	Range	Sequence	Modification	Ratios
280	1	591.8074	33.08	2	32.6	12.3	1	242-251	R.SYQRLADACR.G		WD:WU 0.65
1064	3	751.8788	61.27	2	42.0	11.0	1	1116-1128	R.MTMSSLVERACCR.D	Oxidation: 3	

Detailed Protein Report

Protein 107: TRIO and F-actin-binding protein isoform 1 [Homo sapiens]

**Accession:**	gi|88501740	**Score:**	36.2
**Database:**	refseq_human(refseq_human_20140103.fasta)	**MW [kDa]:**	74.0
**Database Date:**	2015-11-30	**pI:**	6.5
		**Sequence Coverage [%]:**	6.1
		**No. of unique Peptides:**	2

10	20	30	40	50	60	70	80
MGGWKGPGQR	RGKEGPEARR	RAAERGGGGG	GGGVPAPRSP	AREPRPRSCL	LLPPPWGAAM	TPDLLNFKKG	WMSILDEPGE
**90**	**100**	**110**	**120**	**130**	**140**	**150**	**160**
PPSPSLTTTS	TSQWKKHWFV	LTDSSLKYYR	DSTAEEADEL	DGEIDLRSCT	DVTEYAVQRN	YGFQIHTKDA	VYTLSAMTSG
**170**	**180**	**190**	**200**	**210**	**220**	**230**	**240**
IRRNWIEALR	KTVRPTSAPD	VTKLSDSNKE	NALHSYSTQK	GPLKAGEQRA	GSEVISRGGP	RKADGQRQAL	DYVELSPLTQ
**250**	**260**	**270**	**280**	**290**	**300**	**310**	**320**
ASPQRARTPA	RTPDRLAKQE	ELERDLAQRS	EERRKWFEAT	DSRTPEVPAG	EGPRRGLGAP	LTEDQQNRLS	EEIEKKWQEL
**330**	**340**	**350**	**360**	**370**	**380**	**390**	**400**
EKLPLRENKR	VPLTALLNQS	RGERRGPPSD	GHEALEKEVQ	ALRAQLEAWR	LQGEAPQSAL	RSQEDGHIPP	GYISQEACER
**410**	**420**	**430**	**440**	**450**	**460**	**470**	**480**
SLAEMESSHQ	QVMEELQRHH	ERELQRLQQE	KEWLLAEETA	ATASAIEAMK	KAYQEELSRE	LSKTRSLQQG	PDGLRKQHQS
**490**	**500**	**510**	**520**	**530**	**540**	**550**	**560**
DVEALKRELQ	VLSEQYSQKC	LEIGALMRQA	EEREHTLRRC	QQEGQELLRH	NQELHGRLSE	EIDQLRGFIA	SQGMGNGCGR
**570**	**580**	**590**	**600**	**610**	**620**	**630**	**640**
SNERSSCELE	VLLRVKENEL	QYLKKEVQCL	RDELQMMQKD	KRFTSGKYQD	VYVELSHIKT	RSEREIEQLK	EHLRLAMAAL
**650**	**660**						
QEKESMRNSL	AE						

Cmpd.	No. of Cmpds.	m/z meas.	Δ m/z [ppm]	z	Rt [min]	Score	P	Range	Sequence	Modification
1361	1	711.8629	-0.75	2	45.3	11.9	1	22-38	R.AAERGGGGGGGGVPAPR.S	
2143	1	1051.3799	-114.76	2	55.3	12.5	1	276-294	K.WFEATDSRTPEVPAGEGPR.R	

Detailed Protein Report

Protein 108: plectin isoform 1d [Homo sapiens]

**Accession:**	gi|41322910	**Score:**	36.2
**Database:**	refseq_human(refseq_human_20140103.fasta)	**MW [kDa]:**	512.3
**Database Date:**	2015-11-30	**pI:**	5.5
		**Sequence Coverage [%]:**	1.0
		**No. of unique Peptides:**	2

BRUKER

Detailed Protein Report

10	20	30	40	50	60	70	80
MKIVPDERDR	VQKKTFTKWV	NKHLIKAQRH	ISDLYEDLRD	GHNLISLLEV	LSGDSLPREK	GRMRFHKLQN	VQIALDYLRH
90	100	110	120	130	140	150	160
RQVKLVNIRN	DDIADGNPKL	TLGLIWTIIL	HFQISDIQVS	GQSEDMTAKE	KLLLWSQRMV	EGYQGLRCDN	FTSSWRDGRL
170	180	190	200	210	220	230	240
FNAIIHRHKP	LLIDMNKVYR	QTNLENLDQA	FSVAERDLGV	TRLLDPEDVD	VPQPDEKSII	TYVSSLYDAM	PRVPDVQDGV
250	260	270	280	290	300	310	320
RANELQLRWQ	EYRELVLLLL	QWMRHHTAAF	EERRFPSSFE	EIEILWSQFL	KFKEMELPAK	EADKNRSKGI	YQSLEGAVQA
330	340	350	360	370	380	390	400
GQLKVPPGYH	PLDVEKEWGK	LHVAILEREK	QLRSEFERLE	CLQRIVTKLQ	MEAGLCEEQL	NQADALLQSD	VRLLAAGKVP
410	420	430	440	450	460	470	480
QRAGEVERDL	DKADSMIRLL	FNDVQTLKDG	RHPQGEQMYR	RVYRLHERLV	AIRTEYNLRL	KAGVAAPATQ	VAQVTLQSVQ
490	500	510	520	530	540	550	560
RRPELEDSTL	RYLQDLLAWV	EENQHRVDGA	EWGVDLPSVE	AQLGSHRGLH	QSIEEFRAKI	ERARSDEGQL	SPATRGAYRD
570	580	590	600	610	620	630	640
CLGRLDLQYA	KLLNSSKARL	RSLESLHSFV	AAATKELMWL	NEKEEEEVGF	DWSDRNTNMT	AKKESYSALM	RELELKEKKI
650	660	670	680	690	700	710	720
KELQNAGDRL	LREDHPARPT	VESFQAALQT	QWSWMLQLCC	CIEAHLKENA	AYFQFFSDVR	EAEGQLQKLQ	EALRRKYSCD
730	740	750	760	770	780	790	800
RSATVTRLED	LLQDAQDEKE	QLNEYKGHLS	GLAKRAKAVV	QLKPRHPAHP	MRGRLPLLAV	CDYKQVEVTV	HKGDECQLVG
810	820	830	840	850	860	870	880
PAQPSHWKVL	SSSGSEAAVP	SVCFLVPPPN	QEAQEAVTRL	EAQHQALVTL	WHQLHVDMKS	LLAWQSLRRD	VQLIRSWSLA
890	900	910	920	930	940	950	960
TFRTLKPEEQ	RQALHSLELH	YQAFLRDSQD	AGGFGPEDRL	MAEREYGSCS	HHYQQLLQSL	EQGAQEESRC	QRCISELKDI
970	980	990	1000	1010	1020	1030	1040
RLQLEACETR	TVHRLRLPLD	KEPARECAQR	IAEQQKAQAE	VEGLGKGVAR	LSAEAEKVLA	LPEPSPAAPT	LRSELELTLG
1050	1060	1070	1080	1090	1100	1110	1120
KLEQVRSLSA	IYLEKLKTIS	LVIRGTQGAE	EVLRAHEEQL	KEAQAVPATL	PELEATKASL	KKLRAQAEAQ	QPTFDALRDE
1130	1140	1150	1160	1170	1180	1190	1200
LRGAQEVGER	LQQRHGERDV	EVERWRERVA	QLLERWQAVL	AQTDVRQREL	EQLGRQLRYY	RESADPLGAW	LQDARRRQEQ
1210	1220	1230	1240	1250	1260	1270	1280
IQAMPLADSQ	AVREQLRQEQ	ALLEEIERHG	EKVEECQRFA	KQYINAIKDY	ELQLVTYKAQ	LEPVASPAKK	PKVQSGSESV
1290	1300	1310	1320	1330	1340	1350	1360
IQEYVDLRTH	YSELTTLTSQ	YIKFISETLR	RMEEEERLAE	QQRAEEERERL	AEVEAALEKQ	RQLAEAHAQA	KAQAEREAKE
1370	1380	1390	1400	1410	1420	1430	1440
LQQRMQEEVV	RREEAAVDAQ	QQKRSIQEEL	QQLRQSSEAE	IQAKARQAEA	AERSRLRIEE	EIRVVRLQLE	ATERQRGGAE
1450	1460	1470	1480	1490	1500	1510	1520
GELQALRARA	EEAEAQKRQA	QEEAERLRRQ	VQDESQRKRQ	AEVELASRVK	AEAEAAREKQ	RALQALEELR	LQAEEAERRL
1530	1540	1550	1560	1570	1580	1590	1600
RQAEVERARQ	VQVALETAQR	SAEAELQSKR	ASFAEKTAQL	ERSLQEEHVA	VAQLREEAER	RAQQQAEAER	AREEAEREL E
1610	1620	1630	1640	1650	1660	1670	1680
RWQLKANEAL	RLRLQAEEVA	QQKSLAQAEA	EKQKEEAERE	ARRRGKAEEQ	AVRQRELAEQ	ELEKQRQLAE	GTAQQRLAAE
1690	1700	1710	1720	1730	1740	1750	1760
QELIRLRAET	EQGEQQRQLL	EEELARLQRE	AAAATQKRQE	LEAELAKVRA	EMEVLLASKA	RAEEESRSTS	EKSKQRLEAE
1770	1780	1790	1800	1810	1820	1830	1840
AGRFRELAEE	AARLRALAEE	AKRQRQLAEE	DAARQRAEAE	RVLAEKLAAI	GEATRLKTEA	EIALKEKEAE	NERLRRLAED
1850	1860	1870	1880	1890	1900	1910	1920
EAFQRRRLEE	QAAQHKADIE	ERLAQLRKAS	DSELERQKGL	VEDTLRQRRQ	VEEEILALKA	SFEKAAAGKA	ELELELGRIR
1930	1940	1950	1960	1970	1980	1990	2000
SNAEDTLRSK	EQAELEAARQ	RQLAAEEERR	RREAEERVQK	SLAAEEEAAR	QRKAALEEVE	RLKAKVEEAR	RLRERAEQES
2010	2020	2030	2040	2050	2060	2070	2080
ARQLQLAQEA	AQKRLQAEEK	AHAFAVQQKE	QELQQTLQQE	QSVLDQLRGE	AEAARRAAEE	AEEARVQAER	EAAQSRRQVE
2090	2100	2110	2120	2130	2140	2150	2160
EAERLKQSAE	EQAQARAQAQ	AAAEKLRKEA	EQEAARRAQA	EQAALRQKQA	ADAEMEKHKK	FAEQTLRQKA	QVEQELTTLR
2170	2180	2190	2200	2210	2220	2230	2240

Detailed Protein Report

Cmpd.	No. of Cmpds.	m/z meas.	Δ m/z [ppm]	z	Rt [min]	Score	P	Range	Sequence	Modification
1213	1	713.6809	-62.89	3	43.8	10.9	2	2243-2262	K.QVAEEAARLSVAAQEAARLR.Q	
1785	2	582.1343	-236.00	2	50.7	11.8	0	2343-2352	R.QLEMSAEAER.L	

Detailed Protein Report

Protein 109: **PDZ domain-containing RING finger protein 4 isoform 2 [Homo sapiens]**

**Accession:**	gi|142976783	**Score:**	36.1
**Database:**	refseq_human(refseq_human_20140103.fasta)	**MW [kDa]:**	89.0
**Database Date:**	2015-11-30	**pI:**	5.0
		**Sequence Coverage [%]:**	3.2
		**No. of unique Peptides:**	2

10	20	30	40	50	60	70	80
MGCNLCTFQK	REEHYKLLYE	VSQVNGKDLS	KATHEEAVEA	FRNAKEPIVV	QVLRRTPLSR	PAYGMASEVQ	LMNASTQTDI
**90**	**100**	**110**	**120**	**130**	**140**	**150**	**160**
TFEHIMALAK	LRPPTPPVPD	ICPFLLSDSC	HSLHPMEHEF	YEDNEYISSL	PADADRTEDF	EYEEVELCRV	SSQEKLGLTV
**170**	**180**	**190**	**200**	**210**	**220**	**230**	**240**
CYRTDDEEDT	GIYVSEVDPN	SIAAKDGRIR	EGDRILQING	EDVQNREEAV	ALLSNDECKR	IVLLVARPEI	QLDEGWLEDE
**250**	**260**	**270**	**280**	**290**	**300**	**310**	**320**
RNEFLEELNL	EMLEEEHNEA	MQPTANEVEQ	PKKQEEEEGT	TDTATSSSNN	HEKDSGVGRT	DESLRNDESS	EQENAAEDPN
**330**	**340**	**350**	**360**	**370**	**380**	**390**	**400**
STSLKSKRDL	GQSQDTLGSV	ELQYNESLVS	GEYIDSDCIG	NPDEDCERFR	QLLELKCKIR	NHGEYDLYYS	SSTIECNQGE
**410**	**420**	**430**	**440**	**450**	**460**	**470**	**480**
QEGVEHELQL	LNEELRNIEL	ECQNIMQAHR	LQKVTDQYGD	IWTLHDGGFR	NYNTSIDMQR	GKLDDIMEHP	EKSDKDSSSA
**490**	**500**	**510**	**520**	**530**	**540**	**550**	**560**
YNTAESCRST	PLTVDRSPDS	SLPRVINLTN	KKNLRSTMAA	TQSSSGQSSK	ESTSTKAKTT	EQGCSAESKE	KVLEGSKLPD
**570**	**580**	**590**	**600**	**610**	**620**	**630**	**640**
QEKAVSEHIP	YLSPYHSSSY	RYANIPAHAR	HYQSYMQLIQ	QKSAVEYAQS	QLSLVSMCKE	SQKCSEPKME	WKVKIRSDGT
**650**	**660**	**670**	**680**	**690**	**700**	**710**	**720**
RYITKRPVRD	RILKERALKI	KEERSGMTTD	DDTMSEMKMG	RYWSKEERKQ	HLVRAKEQRR	RREFMMRSRL	ECLKESPQSG
**730**	**740**	**750**	**760**	**770**	**780**		
SEGKKEINII	ELSHKKMMKK	RNKKILDNWM	TIQELMTHGA	KSPDGTRVHN	AFLSVTTV		

Cmpd.	No. of Cmpds.	m/z meas.	Δ m/z [ppm]	z	Rt [min]	Score	P	Range	Sequence	Modification
652	2	639.3334	-62.76	2	37.0	25.9	2	369-378	R.FRQLLELKCK.I	
2536	1	821.0789	154.65	2	60.0	10.2	1	497-511	R.SPDSSLPRVINLTNK.K	

Detailed Protein Report

Protein 110: dynein heavy chain 5, axonemal [Homo sapiens]

**Accession:**	gi|19115954	**Score:**	36.0
**Database:**	refseq_human(refseq_human_20140103.fasta)	**MW [kDa]:**	528.7
**Database Date:**	2015-11-30	**pI:**	5.7
**Modification(s):**	Oxidation	**Sequence Coverage [%]:**	0.8
		**No. of unique Peptides:**	2

Detailed Protein Report

10	20	30	40	50	60	70	80
MFRIGRRQLW	KHSVTRVLTQ	RLKGEKEAKR	ALLDARHNYL	FAIVASCLDL	NKTEVEDAIL	EGNQIERIDQ	LFAVGGLRHL
90	100	110	120	130	140	150	160
MFYYQDVEEA	ETGQLGSLGG	VNLVSGKIKK	PKVFVTEGND	VALTGVCVFF	IRTDPSKAIT	PDNIHQEVSF	NMLDAADGGL
170	180	190	200	210	220	230	240
LNSVRRLLSD	IFIPALRATS	HGWGELEGLQ	DAANIRQEFL	SSLEGFVNVL	SGAQESLKEK	VNLRKCDILE	LKTLKEPTDY
250	260	270	280	290	300	310	320
LTLANNPETL	GKIEDCMKVW	IKQTEQVLAE	NNQLLKEADD	VGPRAELEHW	KKRLSKFNYL	LEQLKSPDVK	AVLAVLAAAK
330	340	350	360	370	380	390	400
SKLLKTWREM	DIRITDATNE	AKDNVKYLYT	LEKCCDPLYS	SDPLSMMDAI	PTLINAIKMI	YSISHYYNTS	EKITSLFVKV
410	420	430	440	450	460	470	480
TNQIISACKA	YITNNGTASI	WNQPQDVVEE	KILSAIKLKQ	EYQLCFHKTK	QKLKQNPNAK	QFDFSEMYIF	GKFETFHRRL
490	500	510	520	530	540	550	560
AKIIDIFTTL	KTYSVLQDST	IEGLEDMATK	YQGIVATIKK	KEYNFLDQRK	MDFDQDYEEF	CKQTNDLHNE	LRKFMDVTFA
570	580	590	600	610	620	630	640
KIQNTNQALR	MLKKFERLNI	PNLGIDDKYQ	LILENYGADI	DMISKLYTKQ	KYDPPLARNQ	PPIAGKILWA	RQLFHRIQQP
650	660	670	680	690	700	710	720
MQLFQQHPAV	LSTAEAKPII	RSYNRMAKVL	LEFEVLFHRA	WLRQIEEIHV	GLEASLLVKA	PGTGELFVNF	DPQILILFRE
730	740	750	760	770	780	790	800
TECMAQMGLE	VSPLATSLFQ	KRDRYKRNFS	NMKMMLAEYQ	RVKSKIPAAI	EQLIVPHLAK	VDEALQPGLA	ALTWTSLNIE
810	820	830	840	850	860	870	880
AYLENTFAKI	KDLELLLDRV	NDLIEFRIDA	ILEEMSSTPL	CQLPQEEPLT	CEEFLQMTKD	LCVNGAQILH	FKSSLVEEAV
890	900	910	920	930	940	950	960
NELVNMLLDV	EVLSEEESEK	ISNENSVNYK	NESSAKREEG	NFDTLTSSIN	ARANALLLTT	VTRKKKETEM	LGEEARELLS
970	980	990	1000	1010	1020	1030	1040
HFNHQNMDAL	LKVTRNTLEA	IRKRIHSSHT	INFRDSNSAS	NMKQNSLPIF	RASVTLAIPN	IVMAPALEDV	QQTLNKAVEC
1050	1060	1070	1080	1090	1100	1110	1120
IISVPKGVRQ	WSSELLSKKK	IQERKMAALQ	SNEDSDSDVE	MGENELQDTL	EIASVNLPIP	VQTKNYYKNV	SENKEIVKLV
1130	1140	1150	1160	1170	1180	1190	1200
SVLSTIINST	KKEVITSMDC	FKRYNHIWQK	GKEEAIKTFI	TQSPLLSEFE	SQILYFQNLE	QEINAEPEYV	CVGSIALYTA
1210	1220	1230	1240	1250	1260	1270	1280
DLKFALTAET	KAWMVVIGRH	CNKKYRSEME	NIFMLIEEFN	KKLNRPIKDL	DDIRIAMAAL	KEIREEQISI	DFQVGPIEES
1290	1300	1310	1320	1330	1340	1350	1360
YALLNRYGLL	IAREEIDKVD	TLHYAWEKLL	ARAGEVQNKL	VSLQPSFKKE	LISAVEVFLQ	DCHQFYLDYD	LNGPMASGLK
1370	1380	1390	1400	1410	1420	1430	1440
PQEASDRLIM	FQNQFDNIYR	KYITYTGGEE	LFGLPATQYP	QLLEIKKQLN	LLQKIYTLYN	SVIETVNSYY	DILWSEVNIE
1450	1460	1470	1480	1490	1500	1510	1520
KINNELLEFQ	NRCRKLPRAL	KDWQAFLDLK	KIIDDFSECC	PLLEYMASKA	MMERHWERIT	TLTGHSLDVG	NESFKLRNIM
1530	1540	1550	1560	1570	1580	1590	1600
EAPLLKYKEE	IEDICISAVK	ERDIEQKLKQ	VINEWDNKTF	TFGSFKTRGE	LLLRGDSTSE	IIANMEDSLM	LLGSLLSNRY
1610	1620	1630	1640	1650	1660	1670	1680
NMPFKAQIQK	WVQYLSNSTD	IIESWMTVQN	LWIYLEAVFV	GGDIAKQLPK	EAKRFSNIDK	SWVKIMTRAH	EVPSVVQCCV
1690	1700	1710	1720	1730	1740	1750	1760
GDETLGQLLP	HLLDQLEICQ	KSLTGYLEKK	RLCFPRFFFV	SDPALLEILG	QASDSHTIQA	HLLNVFDNIK	SVKFHEKIYD
1770	1780	1790	1800	1810	1820	1830	1840
RILSISSQEG	ETIELDKPVM	AEGNVEVWLN	SLLEESQSSL	HLVIRQAAAN	IQETGFQLTE	FLSSFPAQVG	LLGIQMIWTR
1850	1860	1870	1880	1890	1900	1910	1920
DSEEALRNAK	FDKKIMQKTN	QAFLELLNTL	IDVTTRDLSS	TERVKYETLI	TIHVHQRDIF	DDLCHMHIKS	PMDFEWLKQC
1930	1940	1950	1960	1970	1980	1990	2000
RFYFNEDSDK	MMIHITDVAF	IYQNEFLGCT	DRLVITPLTD	RCYITLAQAL	GMSMGGAPAG	PAGTGKTETT	KDMGRCLGKY
2010	2020	2030	2040	2050	2060	2070	2080
VVVFNCSDQM	DFRGLGRIFK	GLAQSGSWGC	FDEFNRIDLP	VLSVAAQQIS	IILTCKKEHK	KSFIFTDGDN	VTMNPEFGLF
2090	2100	2110	2120	2130	2140	2150	2160
LTMNPGYAGR	QELPENLKIN	FRSVAMMVPD	RQIIIRVKLA	SCGFIDNVVL	ARKFFTLYKL	CEEQLSKQVH	YDFGLRNILS
2170	2180	2190	2200	2210	2220	2230	2240

Detailed Protein Report

Cmpd.	No. of Cmpds.	m/z meas.	Δ m/z [ppm]	z	Rt [min]	Score	P	Range	Sequence	Modification
78	1	529.5845	-42.73	3	29.9	11.4	2	3744-3756	R.THLMEDVTANKRR.M	Oxidation: 4
1942	2	709.7806	-178.71	2	52.9	13.6	0	4137-4148	K.QFPITLLQMSIK.F	

Detailed Protein Report

Protein 111: transcription initiation factor TFIID subunit 1-like [Homo sapiens]

**Accession:**	gi|24429572	**Score:**	35.7
**Database:**	refseq_human(refseq_human_20140103.fasta)	**MW [kDa]:**	207.2
**Database Date:**	2015-11-30	**pI:**	5.1
**Modification(s):**	Oxidation	**Sequence Coverage [%]:**	2.6
		**No. of unique Peptides:**	3

Quantitation

WD:WU	**Median:** 0.80	**CV:** 0.00 %	**No. of Peptides:** 1

Detailed Protein Report

10	20	30	40	50	60	70	80
MRPGCDLLLR	AAATVTAAIM	SDSDSEEDSS	GGGPFTLAGI	LFGNISGAGQ	LEGESVLDDE	CKKHLAGLGA	LGLGSLITEL
90	100	110	120	130	140	150	160
TANEELTGTG	GALVNDEGWI	RSTEDAVDYS	DINEVAEDES	QRHQQTMGSL	QPLYHSDYDE	DDYDADCEDI	DCKLMPPPPP
170	180	190	200	210	220	230	240
PPGPMKKDKD	QDAITCVSES	GEDIILPSII	APSFLASEKV	DFSSYSDSES	EMGPQEATQA	ESEDGKLTLP	LAGIMQHDAT
250	260	270	280	290	300	310	320
KLLPSVTELF	PEFRPGKVLR	FLHLFGPGKN	VPSVWRSARR	KRKKHRELIQ	EEQIQEVECS	VESEVSQKSL	WNYDYAPPPP
330	340	350	360	370	380	390	400
PEQCLADDEI	TMMVPVESKF	SQSTGDVDKV	TDTKPRVAEW	RYGPARLWYD	MLGVSEDGSG	FDYGFKLRKT	QHEPVIKSRM
410	420	430	440	450	460	470	480
MEEFRKLEES	NGTDLLADEN	FLMVTQLHWE	DSIIWDGEDI	KHKGTKPQGA	SLAGWLPSIK	TRNVMAYNVQ	QGFAPTLDDD
490	500	510	520	530	540	550	560
KPWYSIFPID	NEDLVYGRWE	DNIIWDAQAM	PRLLEPPVLA	LDPNDENLIL	EIPDEKEEAT	SNSPSKESKK	ESSLKKSRIL
570	580	590	600	610	620	630	640
LGKTGVIREE	PQQNMSQPEV	KDPWNLSNDE	YYFPKQQGLR	GTFGGNIIQH	SIPAMELWQP	FFPTHMGPIK	IRQFHRPPLK
650	660	670	680	690	700	710	720
KYSFGALSQP	GPHSVQPLLK	HIKKKAKMRE	QERQASGGGE	LFFMRTPQDL	TGKDGDLILA	EYSEENGPLM	MQVGMATKIK
730	740	750	760	770	780	790	800
NYYKRKPGKD	PGAPDCKYGE	TVYCHTSPFL	GSLHPGQLLQ	ALENNLFRAP	VYLHKMPETD	FLIIRTRQGY	YIRELVDIFV
810	820	830	840	850	860	870	880
VGQQCPLFEV	PGPNSRRANM	HIRDFLQVFI	YRLFWKSKDR	PRRIRMEDIK	KAFPSHSESS	IRKRLKLCAD	FKRTGMDSNW
890	900	910	920	930	940	950	960
WVLKSDFRLP	TEEEIRAKVS	PEQCCAYYSM	IAAKQRLKDA	GYGEKSFFAP	EEENEEDFQM	KIDDEVHAAP	WNTTRAFIAA
970	980	990	1000	1010	1020	1030	1040
MKGKCLLEVT	GVADPTGCGE	GFSYVKIPNK	PTQQKDDKEP	QAVKKTVTGT	DADLRRLSLK	NAKQLLRKFG	VPEEEIKKLS
1050	1060	1070	1080	1090	1100	1110	1120
RWEVIDVVRT	MSTEQAHSGE	GPMSKFARGS	RFSVAEHQER	YKEECQRIFD	LQNKVLSSTE	VLSTDTDSIS	AEDSDFEEMG
1130	1140	1150	1160	1170	1180	1190	1200
KNIENMLQNK	KTSSQLSREW	EEQERKELRR	MLLVAGSAAS	GNNHRDDVTA	SMTSLKSSAT	GHCLKIYRTF	RDEEGKEYVR
1210	1220	1230	1240	1250	1260	1270	1280
CETVRKPAVI	DAYVRIRTTK	DEKFIQKFAL	FDEKHREEMR	KERRRIQEQL	RRLKRNQEKE	KLKGPPEKKP	KKMKERPDLK
1290	1300	1310	1320	1330	1340	1350	1360
LKCGACGAIG	HMRTNKFCPL	YYQTNVPPSK	PVAMTEEQEE	ELEKTVIHND	NEELIKVEGT	KIVFGKQLIE	NVHEVRRKSL
1370	1380	1390	1400	1410	1420	1430	1440
VLKFPKQQLP	PKKKRRVGTT	VHCDYLNIPH	KSIHRRRTDP	MVTLSSILES	IINDMRDLPN	THPFHTPVNA	KVVKDYYKII
1450	1460	1470	1480	1490	1500	1510	1520
TRPMDLQTLR	ENVRKCLYPS	REEFREHLEL	IVKNSATYNG	PKHSLTQISQ	SMLDLCDEKL	KEKEDKLARL	EKAINPLLDD
1530	1540	1550	1560	1570	1580	1590	1600
DDQVAFSFIL	DNIVTQKMMA	VPDSWPFHHP	VNKKFVPDYY	KMIVNPVDLE	TIRKNISKHK	YQSRESFLDD	VNLILANSVK
1610	1620	1630	1640	1650	1660	1670	1680
YNGPESQYTK	TAQEIVNICY	QTITEYDEHL	TQLEKDICTA	KEAALEEAEL	ESLDPMTPGP	YTSQPPDMYD	TNTSLSTSRD
1690	1700	1710	1720	1730	1740	1750	1760
ASVFQDESNL	SVLDISTATP	EKQMCQGQGR	LGEEDSDVDV	EGYDDEEEDG	KPKPPAPEGG	DGDLADEEEG	TVQQPEASVL
1770	1780	1790	1800	1810	1820	1830	
YEDLLISEGE	DDEEDAGSDE	EGDNPFSAIQ	LSESGSDSDV	GYGGIRPKQP	FMLQHASGEH	KDGHGK	

Cmpd.	No. of Cmpds.	m/z meas.	Δ m/z [ppm]	z	Rt [min]	Score	P	Range	Sequence	Modification	Ratios
2709	1	813.0615	149.49	2	62.3	13.8	0	227-241	K.LTLPLAGIMQHDATK.L	Oxidation: 9	
1092	1	839.4008	43.81	2	42.4	10.2	0	1050-1065	R.TMSTEQAHSGEGPMSK.F		WD:WU 0.80
2649	1	835.4339	3.24	2	61.4	11.7	1	1150-1165	R.RMLLVAGSAASGNNHR.D	Oxidation: 2	

Detailed Protein Report

Protein 112: **PREDICTED: cylicin-1 isoform X1 [Homo sapiens]**

**Accession:**	gi|530421958	**Score:**	35.7
**Database:**	refseq_human(refseq_human_20140103.fasta)	**MW [kDa]:**	74.1
**Database Date:**	2015-11-30	**pI:**	10.2
		**Sequence Coverage [%]:**	4.2
		**No. of unique Peptides:**	1

10	20	30	40	50	60	70	80
MSLPRLKVNI	RTYDNSIPIS	ESSRKSWNQK	HFALTFPKPL	QRGTNDKSRP	LKSQITVTRH	DKRKLEEGQK	PAHKWIRHSF
**90**	**100**	**110**	**120**	**130**	**140**	**150**	**160**
RKILQWPPIY	TAAREQTPFR	HLYTSKTHLK	KAEYKKSKDE	KGGTPLKKDS	KKKGGSYATN	PESKQIVEEK	TKRQNEADKT
**170**	**180**	**190**	**200**	**210**	**220**	**230**	**240**
PLKSSHENEQ	SKKSKSSSET	NPESQNSKTV	SKNCSQKDKK	DSKNSKKTNT	EFLHTKNNPK	KDLKRSKTSN	DPISEICSEN
**250**	**260**	**270**	**280**	**290**	**300**	**310**	**320**
SLNVDFLMLV	GQSDDESINF	DAWLRNYSQN	NSKNYSLKYT	KYTKKDTKKN	AKKSSDAESE	DSKDAKKDSK	KVKKNVKKDD
**330**	**340**	**350**	**360**	**370**	**380**	**390**	**400**
KKKDVKKDTE	STDAESGDSK	DERKDTKKDK	KKLKKDDKKK	DTKKYPESTD	TESGDAKDAR	NDSRNLKKAS	KNDDKKKDAK
**410**	**420**	**430**	**440**	**450**	**460**	**470**	**480**
KITFSTDSES	ELESKESQKD	EKKDKKDSKT	DNKKSVKNDE	ESTDADSEPK	GDSKKGKKDE	KKGKKDSKKD	DKKKDAKKNA
**490**	**500**	**510**	**520**	**530**	**540**	**550**	**560**
ESTEMESDLE	LKKDKKHSKE	KKGSKKDIKK	DARKDTESTD	AEFDESSKTG	FKTSTKIKGS	DTESEESLYK	PGAKKKIDES
**570**	**580**	**590**	**600**	**610**	**620**	**630**	**640**
DGTSANSKME	GLESKRGFRM	SSKKTTFNEK	GEKASTGRVP	PSREKPPLPA	CEPSLPSPKV	RRLCWCKMPP	PPPKPRYAPL
**650**	**660**						
PEAPWIHKLL							

Cmpd.	No. of Cmpds.	m/z meas.	Δ m/z [ppm]	z	Rt [min]	Score	P	Range	Sequence	Modification
547	1	472.6490	-226.33	2	35.3	16.3	1	119-127	K.DEKGGTPLK.K	

Detailed Protein Report

Protein 113: SURP and G-patch domain-containing protein 2 [Homo sapiens]

**Accession:**	gi|224282117	**Score:**	35.6
**Database:**	refseq_human(refseq_human_20140103.fasta)	**MW [kDa]:**	120.1
**Database Date:**	2015-11-30	**pI:**	7.0
**Modification(s):**	Carbamidomethyl	**Sequence Coverage [%]:**	4.4
		**No. of unique Peptides:**	2

Quantitation

WD:WU	**Median:**	0.67	**CV:**	0.00 %	**No. of Peptides:**	1	

Alias proteins:

**Accession**	**Name**	**Description**
gi|224282119	refseq_human (refseq_human_20140103.fasta)	SURP and G-patch domain-containing protein 2 [Homo sapiens]

10	20	30	40	50	60	70	80
MAARRITQET	FDAVLQEKAK	RYHMDASGEA	VSETLQFKAQ	DLLRAVPRSR	AEMYDDVHSD	GRYSLSGSVA	HSRDAGREGL
**90**	**100**	**110**	**120**	**130**	**140**	**150**	**160**
RSDVFPGPSF	RSSNPSISDD	SYFRKECGRD	LEFSHSDSRD	QVIGHRKLGH	FRSQDWKFAL	RGSWEQDFGH	PVSQESSWSQ
**170**	**180**	**190**	**200**	**210**	**220**	**230**	**240**
EYSFGPSAVL	GDFGSSRLIE	KECLEKESRD	YDVDHPGEAD	SVLRGGSQVQ	ARGRALNIVD	QEGSLLGKGE	TQGLLTAKGG
**250**	**260**	**270**	**280**	**290**	**300**	**310**	**320**
VGKLVTLRNV	STKKIPTVNR	ITPKTQGTNQ	IQKNTPSPDV	TLGTNPGTED	IQFPIQKIPL	GLDLKNLRLP	RRKMSFDIID
**330**	**340**	**350**	**360**	**370**	**380**	**390**	**400**
KSDVFSRFGI	EIIKWAGFHT	IKDDIKFSQL	FQTLFELETE	TCAKMLASFK	CSLKPEHRDF	CFFTIKFLKH	SALKTPRVDN
**410**	**420**	**430**	**440**	**450**	**460**	**470**	**480**
EFLNMLLDKG	AVKTKNCFFE	IIKPFDKYIM	RLQDRLLKSV	TPLLMACNAY	ELSVKMKTLS	NPLDLALALE	TTNSLCRKSL
**490**	**500**	**510**	**520**	**530**	**540**	**550**	**560**
ALLGQTFSLA	SSFRQEKILE	AVGLQDIAPS	PAAFPNFEDS	TLFGREYIDH	LKAWLVSSGC	PLQVKKAEPE	PMREEEKMIP
**570**	**580**	**590**	**600**	**610**	**620**	**630**	**640**
PTKPEIQAKA	PSSLSDAVPQ	RADHRVVGTI	DQLVKRVIEG	SLSPKERTLL	KEDPAYWFLS	DENSLEYKYY	KLKLAEMQRM
**650**	**660**	**670**	**680**	**690**	**700**	**710**	**720**
SENLRGADQK	PTSADCAVRA	MLYSRAVRNL	KKKLLPWQRR	GLLRAQGLRG	WKARRATTGT	QTLLSSGTRL	KHHGRQAPGL
**730**	**740**	**750**	**760**	**770**	**780**	**790**	**800**
SQAKPSLPDR	NDAAKDCPPD	PVGPSPQDPS	LEASGPSPKP	AGVDISEAPQ	TSSPCPSADI	DMKTMETAEK	LARFVAQVGP
**810**	**820**	**830**	**840**	**850**	**860**	**870**	**880**
EIEQFSIENS	TDNPDLWFLH	DQNSSAFKFY	RKKVFELCPS	ICFTSSPHNL	HTGGGDTTGS	QESPVDLMEG	EAEFEDEPPP
**890**	**900**	**910**	**920**	**930**	**940**	**950**	**960**
REAELESPEV	MPEEEDEDDE	DGGEEAPAPG	GAGKSEGSTP	ADGLPGEAAE	DDLAGAPALS	QASSGTCFPR	KRISSKSLKV
**970**	**980**	**990**	**1000**	**1010**	**1020**	**1030**	**1040**
GMIPAPKRVC	LIQEPKVHEP	VRIAYDRPRG	RPMSKKKKPK	DLDFAQQKLT	DKNLGFQMLQ	KMGWKEGHGL	GSLGKGIREP
**1050**	**1060**	**1070**	**1080**	**1090**			
VSVGTPSEGE	GLGADGQEHK	EDTFDVFRQR	MMQMYRHKRA	NK			

Cmpd.	No. of Cmpds.	m/z meas.	Δ m/z [ppm]	z	Rt [min]	Score	P	Range	Sequence	Modification	Ratios
2696	1	835.4525	-15.26	2	62.1	11.4	1	213-228	R.GRALNIVDQEGSLLGK.G		
1571	1	948.4992	16.11	2	48.0	14.2	0	439-455	K.SVTPLLMACNAYELSVK.M	Carbamidomethyl: 9	WD:WU 0.67

Detailed Protein Report

Protein 114: zinc finger protein 540 isoform a [Homo sapiens]

**Accession:**	gi|22749241	**Score:**	35.6
**Database:**	refseq_human(refseq_human_20140103.fasta)	**MW [kDa]:**	77.0
**Database Date:**	2015-11-30	**pI:**	10.5
		**Sequence Coverage [%]:**	6.5
		**No. of unique Peptides:**	3

Alias proteins:

Accession	Name	Description
gi|288557351	refseq_human (refseq_human_20140103.fasta)	zinc finger protein 540 isoform a [Homo sapiens]

10	20	30	40	50	60	70	80
MAHALVTFRD	VAIDFSQKEW	ECLDTTQRKL	YRDVMLENYN	NLVSLGYSGS	KPDVITLLEQ	GKEPCVVARD	VTGRQCPGLL
**90**	**100**	**110**	**120**	**130**	**140**	**150**	**160**
SRHKTKKLSS	EKDIHEISLS	KESIIEKSKT	LRLKGSIFRN	EWQNKSEFEG	QQGLKERSIS	QKKIVSKKMS	TDRKRPSFTL
**170**	**180**	**190**	**200**	**210**	**220**	**230**	**240**
NQRIHNSEKS	CDSHLVQHGK	IDSDVKHDCK	ECGSTFNNVY	QLTLHQKIHT	GEKSCKCEKC	GKVFSHSYQL	TLHQRFHTGE
**250**	**260**	**270**	**280**	**290**	**300**	**310**	**320**
KPYECQECGK	TFTLYPQLNR	HQKIHTGKKP	YMCKKCDKGF	FSRLELTQHK	RIHTGKKSYE	CKECGKVFQL	IFYFKEHERI
**330**	**340**	**350**	**360**	**370**	**380**	**390**	**400**
HTGKKPYECK	ECGKAFSVCG	QLTRHQKIHT	GVKPYECKEC	GKTFRLSFYL	TEHRRTHAGK	KPYECKECGK	SFNVRGQLNR
**410**	**420**	**430**	**440**	**450**	**460**	**470**	**480**
HKTIHTGIKP	FACKVCEKAF	SYSGDLRVHS	RIHTGEKPYE	CKECGKAFML	RSVLTEHQRL	HTGVKPYECK	ECGKTFRVRS
**490**	**500**	**510**	**520**	**530**	**540**	**550**	**560**
QISLHKKIHT	DVKPYKCVRC	GKTFRFGFYL	TEHQRIHTGE	KPYKCKECGK	AFIRRGNLKE	HLKIHSGLKP	YDCKECGKSF
**570**	**580**	**590**	**600**	**610**	**620**	**630**	**640**
SRRGQFTEHQ	KIHTGVKPYK	CKECGKAFSR	SVDLRIHQRI	HTGEKPYECK	QCGKAFRLNS	HLTEHQRIHT	GEKPYECKVC
**650**	**660**	**670**					
RKAFRQYSHL	YQHQKTHNVI						

Cmpd.	No. of Cmpds.	m/z meas.	Δ m/z [ppm]	z	Rt [min]	Score	P	Range	Sequence	Modification
2641	1	701.3872	28.83	2	61.3	12.6	1	70-82	R.DVTGRQCPGLLSR.H	
765	1	541.3095	42.61	3	38.4	11.9	1	303-315	K.ECGKVFQLIFYFK.E	
848	2	946.4705	3.74	2	39.4	11.1	2	331-347	K.ECGKAFSVCGQLTRHQK.I	

Detailed Protein Report

Protein 115: laminin subunit alpha-2 isoform b precursor [Homo sapiens]

**Accession:**	gi|119466532	**Score:**	35.5
**Database:**	refseq_human(refseq_human_20140103.fasta)	**MW [kDa]:**	343.3
**Database Date:**	2015-11-30	**pI:**	6.0
**Modification(s):**	Carbamidomethyl	**Sequence Coverage [%]:**	1.2
		**No. of unique Peptides:**	1

Detailed Protein Report

10	20	30	40	50	60	70	80
MPGAAGVLLL	LLLSGGLGGV	QAQRPQQQRQ	SQAHQQRGLF	PAVLNLASNA	LITTNATCGE	KGPEMYCKLV	EHVPGQPVRN
90	100	110	120	130	140	150	160
PQCRICNQNS	SNPNQRHPIT	NAIDGKNTWW	QSPSIKNGIE	YHYVTITLDL	QQVFQIAYVI	VKAANSPRPG	NWILERSLDD
170	180	190	200	210	220	230	240
VEYKPWQYHA	VTDTECLTLY	NIYPRTGPPS	YAKDDEVICT	SFYSKIHPLE	NGEIHISLIN	GRPSADDPSP	ELLEFTSARY
250	260	270	280	290	300	310	320
IRLRFQRIRT	LNADLMMFAH	KDPREIDPIV	TRRYYYSVKD	ISVGGMCICY	GHARACPLDP	ATNKSRCECE	HNTCGDSCDQ
330	340	350	360	370	380	390	400
CCPGFHQKPW	RAGTFLTKTE	CEACNCHGKA	EECYYDENVA	RRNLSLNIRG	KYIGGGVCIN	CTQNTAGINC	ETCTDGFFRP
410	420	430	440	450	460	470	480
KGVSPNYPRP	CQPCHCDPIG	SLNEVCVKDE	KHARRGLAPG	SCHCKTGFGG	VSCDRCARGY	TGYPDCKACN	CSGLGSKNED
490	500	510	520	530	540	550	560
PCFGPCICKE	NVEGGDCSRC	KSGFFNLQED	NWKGCDECFC	SGVSNRCQSS	YWTYGKIQDM	SGWYLTDLPG	RIRVAPQQDD
570	580	590	600	610	620	630	640
LDSPQQISIS	NAEARQALPH	SYYWSAPAPY	LGNKLPAVGG	QLTFTISYDL	EEEEEDTERV	LQLMIILEGN	DLSISTAQDE
650	660	670	680	690	700	710	720
VYLHPSEEHT	NVLLLKEESF	TIHGTHFPVR	RKEFMTVLAN	LKRVLLQITY	SFGMDAIFRL	SSVNLESAVS	YPTDGSIAAA
730	740	750	760	770	780	790	800
VEVCQCPPGY	TGSSCESCWP	RHRRVNGTIF	GGICEPCQCF	GHAESCDDVT	GECLNCKDHT	GGPYCDKCLP	GFYGEPTKGT
810	820	830	840	850	860	870	880
SEDCQPCACP	LNIPSNNFSP	TCHLDRSLGL	ICDGCPVGYT	GPRCERCAEG	YFGQPSVPGG	SCQPCQCNDN	LDFSIPGSCD
890	900	910	920	930	940	950	960
SLSGSCLICK	PGTTGRYCEL	CADGYFGDAV	DAKNCQPCRC	NAGGSFSEVC	HSQTGQCECR	ANVQGQRCDK	CKAGTFGLQS
970	980	990	1000	1010	1020	1030	1040
ARGCVPCNCN	SFGSKSFDCE	ESGQCWCQPG	VTGKKCDRCA	HGYFNFQEGG	CTACECSHLG	NNCDPKTGRC	ICPPNTIGEK
1050	1060	1070	1080	1090	1100	1110	1120
CSKCAPNTWG	HSITTGCKAC	NCSTVGSLDF	QCNVNTGQCN	CHPKFSGAKC	TECSRGHWNY	PRCNLCDCFL	PGTDATTCDS
1130	1140	1150	1160	1170	1180	1190	1200
ETKKCSCSDQ	TGQCTCKVNV	EGIHCDRCRP	GKFGLDAKNP	LGCSSCYCFG	TTTQCSEAKG	LIRTWVTLKA	EQTILPLVDE
1210	1220	1230	1240	1250	1260	1270	1280
ALQHTTTKGI	VFQHPEIVAH	MDLMREDLHL	EPFYWKLPEQ	FEGKKLMAYG	GKLKYAIYFE	AREETGFSTY	NPQVIIRGGT
1290	1300	1310	1320	1330	1340	1350	1360
PTHARIIVRH	MAAPLIGQLT	RHEIEMTEKE	WKYYGDDPRV	HRTVTREDFL	DILYDIHYIL	IKATYGNFMR	QSRISEISME
1370	1380	1390	1400	1410	1420	1430	1440
VAEQGRGTTM	TPPADLIEKC	DCPLGYSGLS	CEACLPGFYR	LRSQPGGRTP	GPTLGTCVPC	QCNGHSSLCD	PETSICQNCQ
1450	1460	1470	1480	1490	1500	1510	1520
HHTAGDFCER	CALGYYGIVK	GLPNDCQQCA	CPLISSSNNF	SPSCVAEGLD	DYRCTACPRG	YEGQYCERCA	PGYTGSPGNP
1530	1540	1550	1560	1570	1580	1590	1600
GGSCQECECD	PYGSLPVPCD	PVTGFCTCRP	GATGRKCDGC	KHWHAREGWE	CVFCGDECTG	LLLGDLARLE	QMVMSINLTG
1610	1620	1630	1640	1650	1660	1670	1680
PLPAPYKMLY	GLENMTQELK	HLLSPQRAPE	RLIQLAEGNL	NTLVTEMNEL	LTRATKVTAD	GEQTGQDAER	TNTRAKSLGE
1690	1700	1710	1720	1730	1740	1750	1760
FIKELARDAE	AVNEKAIKLN	ETLGTRDEAF	ERNLEGLQKE	IDQMIKELRR	KNLETQKEIA	EDELVAAEAL	LKKVKKLFGE
1770	1780	1790	1800	1810	1820	1830	1840
SRGENEEMEK	DLREKLADYK	NKVDDAWDLL	REATDKIREA	NRLFAVNQKN	MTALEKKKEA	VESGKRQIEN	TLKEGNDILD
1850	1860	1870	1880	1890	1900	1910	1920
EANRLADEIN	SIIDYVEDIQ	TKLPPMSEEL	NDKIDDLSQE	IKDRKLAEKV	SQAESHAAQL	NDSSAVLDGI	LDEAKNISFN
1930	1940	1950	1960	1970	1980	1990	2000
ATAAFKAYSN	IKDYIDEAEK	VAKEAKDLAH	EATKLATGPR	GLLKEDAKGC	LQKSFRILNE	AKKLANDVKE	NEDHLNGLKT
2010	2020	2030	2040	2050	2060	2070	2080
RIENADARNG	DLLRTLNDTL	GKLSAIPNDT	AAKLQAVKDK	ARQANDTAKD	VLAQITELHQ	NLDGLKKNYN	KLADSVAKTN
2090	2100	2110	2120	2130	2140	2150	2160
AVVKDPSKNK	IIADADATVK	NLEQEADRLI	DKLKPIKELE	DNLKKNISEI	KELINQARKQ	ANSIKVSVSS	GGDCIRTYKP
2170	2180	2190	2200	2210	2220	2230	2240
EIKKGSYNNI	VVNVKTAVAD	NLLFYLGSAK	FIDFLAIEMR	KGKVSFLWDV	GSGVGRVEYP	DLTIDDSYWY	RIVASRTGRN
2250	2260	2270	2280	2290	2300	2310	2320
GTISVRALDG	PKASIVPSTH	HSTSPPGYTI	LDVDANAMLF	VGGLTGKLKK	ADAVRVITFT	GCMGETYFDN	KPIGLWNFRE
2330	2340	2350	2360	2370	2380	2390	2400

Detailed Protein Report

Cmpd.	No. of Cmpds.	m/z meas.	Δ m/z [ppm]	z	Rt [min]	Score	P	Range	Sequence	Modification
892	1	739.3524	121.81	2	39.5	10.1	0	1125-1137	K.CSCSDQTGQCTCK.V	Carbamidomethyl: 1, 10

Detailed Protein Report

Protein 116: transmembrane protein 169 [Homo sapiens]

**Accession:**	gi|19923913	**Score:**	35.4
**Database:**	refseq_human(refseq_human_20140103.fasta)	**MW [kDa]:**	33.6
**Database Date:**	2015-11-30	**pI:**	4.6
		**Sequence Coverage [%]:**	6.4
		**No. of unique Peptides:**	1

Alias proteins:

Accession	Name	Description
gi|578805184	refseq_human (refseq_human_20140103.fasta)	PREDICTED: transmembrane protein 169 isoform X1 [Homo sapiens]
gi|214831834	refseq_human (refseq_human_20140103.fasta)	transmembrane protein 169 [Homo sapiens]
gi|214831799	refseq_human (refseq_human_20140103.fasta)	transmembrane protein 169 [Homo sapiens]
gi|214831780	refseq_human (refseq_human_20140103.fasta)	transmembrane protein 169 [Homo sapiens]

10	20	30	40	50	60	70	80
MEEPTAVEGQ	VQLPSPHQGS	LRKAVAAALA	LDGESTMGHR	KKKRKESRPE	SIIIYRSDNE	KTDEEPGESE	GGDQPKEEEG
**90**	**100**	**110**	**120**	**130**	**140**	**150**	**160**
DDFLDYPVDD	DMWNLPLDSR	YVTLTGTITR	GKKKGQMVDI	HVTLTEKELQ	ELTKPKESSR	ETTPEGRMAC	QMGADRGPHV
**170**	**180**	**190**	**200**	**210**	**220**	**230**	**240**
VLWTLICLPV	VFILSFVVSF	YYGTITWYNI	FLVYNEERTF	WHKISYCPCL	VLFYPVLIMA	MASSLGLYAA	VVQLSWSWEA
**250**	**260**	**270**	**280**	**290**	**300**		
WWQAARDMEK	GFCGWLCSKL	GLEDCSPYSI	VELLESDNIS	STLSNKDPIQ	EVETSTV		

Cmpd.	No. of Cmpds.	m/z meas.	Δ m/z [ppm]	z	Rt [min]	Score	P	Range	Sequence	Modification
1888	1	899.4768	10.43	2	52.3	12.3	1	24-41	K.AVAAALALDGESTMGHRK.K	

Detailed Protein Report

Protein 117: keratin, type II cuticular Hb5 [Homo sapiens]

**Accession:**	gi|4504935	**Score:**	35.2
**Database:**	refseq_human(refseq_human_20140103.fasta)	**MW [kDa]:**	55.8
**Database Date:**	2015-11-30	**pI:**	6.3
**Modification(s):**	Carbamidomethyl	**Sequence Coverage [%]:**	6.5
		**No. of unique Peptides:**	1

10	20	30	40	50	60	70	80
MSCRSYRISS	GCGVTRNFSS	CSAVAPKTGN	RCCISAAPYR	GVSCYRGLTG	FGSRSLCNLG	SCGPRIAVGG	FRAGSCGRSF
**90**	**100**	**110**	**120**	**130**	**140**	**150**	**160**
GYRSGGVCGP	SPPCITTVSV	NESLLTPLNL	EIDPNAQCVK	QEEKEQIKSL	NSRFAAFIDK	VRFLEQQNKL	LETKWQFYQN
**170**	**180**	**190**	**200**	**210**	**220**	**230**	**240**
QRCCESNLEP	LFSGYIETLR	REAECVEADS	GRLASELNHV	QEVLEGYKKK	YEEEVALRAT	AENEFVVLKK	DVDCAYLRKS
**250**	**260**	**270**	**280**	**290**	**300**	**310**	**320**
DLEANVEALV	EESSFLRRLY	EEEIRVLQAH	ISDTSVIVKM	DNSRDLNMDC	IIAEIKAQYD	DVASRSRAEA	ESWYRSKCEE
**330**	**340**	**350**	**360**	**370**	**380**	**390**	**400**
MKATVIRHGE	TLRRTKEEIN	ELNRMIQRLT	AEIENAKCQR	AKLEAAVAEA	EQQGEAALSD	ARCKLAELEG	ALQKAKQDMA
**410**	**420**	**430**	**440**	**450**	**460**	**470**	**480**
CLLKEYQEVM	NSKLGLDIEI	ATYRRLLEGE	EHRLCEGVGS	VNVCVSSSRG	GVSCGGLSYS	TTPGRQITSG	PSAIGGSITV
**490**	**500**	**510**					
VAPDSCAPCQ	PRSSSFSCGS	SRSVRFA					

Cmpd.	No. of Cmpds.	m/z meas.	Δ m/z [ppm]	z	Rt [min]	Score	P	Range	Sequence	Modification
953	1	531.2210	2.88	2	40.3	11.1	0	493-502	R.SSSFSCGSSR.S	Carbamidomethyl: 6

Detailed Protein Report

Protein 118: PREDICTED: regulator of G-protein signaling 3 isoform X1 [Homo sapiens]

**Accession:**	gi|578817731	**Score:**	35.1
**Database:**	refseq_human(refseq_human_20140103.fasta)	**MW [kDa]:**	120.9
**Database Date:**	2015-11-30	**pI:**	5.9
**Modification(s):**	Carbamidomethyl	**Sequence Coverage [%]:**	4.1
		**No. of unique Peptides:**	3

10	20	30	40	50	60	70	80
MEWLSPDIAL	PRRDEWTQTS	PARKRITHAK	VQGAGQLRLS	IDAQDRVLLL	HIIEGKGLIS	KQPGTCDPYV	KISLIPEDSR
**90**	**100**	**110**	**120**	**130**	**140**	**150**	**160**
LRHQKTQTVP	DCRDPAFHEH	FFFPVQEEDD	QKRLLVTVWN	RASQSRQSGL	IGCMSFGVKS	LLTPDKEISG	WYYLLGEHLG
**170**	**180**	**190**	**200**	**210**	**220**	**230**	**240**
RTKHLKVARR	RLRPLRDPLL	RMPGGGDTEN	GKKLKITIPR	GKDGFGFTIC	CDSPVRVQAV	DSGGPAERAG	LQQLDTVLQL
**250**	**260**	**270**	**280**	**290**	**300**	**310**	**320**
NERPVEHWKC	VELAHEIRSC	PSEIILLVWR	MVPQVKPGPD	GGVLRRASCK	STHDLQSPPN	KREKNCTHGV	QARPEQRHSC
**330**	**340**	**350**	**360**	**370**	**380**	**390**	**400**
HLVCDSSDGL	LLGGWERYTE	VAKRGGQHTL	PALSRATAPT	DPNYIILAPL	NPGSQLLRPV	YQEDTIPEES	GSPSKGKSYT
**410**	**420**	**430**	**440**	**450**	**460**	**470**	**480**
GLGKKSRLMK	TVQTMKGHGN	YQNCPVVRPH	ATHSSYGTYV	TLAPKVLVFP	VFVQPLDLCN	PARTLLLSEE	LLLYEGRNKA
**490**	**500**	**510**	**520**	**530**	**540**	**550**	**560**
AEVTLFAYSD	LLLFTKEDEP	GRCDVLRNPL	YLQSVKLQEG	SSEDLKFCVL	YLAEKAECLF	TLEAHSQEQK	KRVCWCLSEN
**570**	**580**	**590**	**600**	**610**	**620**	**630**	**640**
IAKQQQLAAS	PPDSKMFETE	ADEKREMALE	EGKGPGAEDS	PPSKEPSPGQ	ELPPGQDLPP	NKDSPSGQEP	APSQEPLSSK
**650**	**660**	**670**	**680**	**690**	**700**	**710**	**720**
DSATSEGSPP	GPDAPPSKDV	PPCQEPPPAQ	DLSPCQDLPA	GQEPLPHQDP	LLTKDLPAIQ	ESPTRDLPPC	QDLPPSQVSL
**730**	**740**	**750**	**760**	**770**	**780**	**790**	**800**
PAKALTEDTM	SSGDLLAATG	DPPAAPRPAF	VIPEVRLDST	YSQKAGAEQG	CSGDEEDAEE	AEEVEEGEEG	EEDEDEDTSD
**810**	**820**	**830**	**840**	**850**	**860**	**870**	**880**
DNYGERSEAK	RSSMIETGQG	AEGGLSLRVQ	NSLRRRTHSE	GSLLQEPRGP	CFASDTTLHC	SDGEGAASTW	GMPSPSTLKK
**890**	**900**	**910**	**920**	**930**	**940**	**950**	**960**
ELGRNGGSMH	HLSLFFTGHR	KMSGADTVGD	DDEASRKRKS	KNLAKDMKNK	LGIFRRRNES	PGAPPAGKAD	KMMKSFKPTS
**970**	**980**	**990**	**1000**	**1010**	**1020**	**1030**	**1040**
EEALKWGESL	EKLLVHKYGL	AVFQAFLRTE	FSEENLEFWL	ACEDFKKVKS	QSKMASKAKK	IFAEYIAIQA	CKEVNLDSYT
**1050**	**1060**	**1070**	**1080**	**1090**	**1100**		
REHTKDNLQS	VTRGCFDLAQ	KRIFGLMEKD	SYPRFLRSDL	YLDLINQKKM	SPPL		

Cmpd.	No. of Cmpds.	m/z meas.	Δ m/z [ppm]	z	Rt [min]	Score	P	Range	Sequence	Modification
2171	1	576.2313	-138.85	3	55.7	10.6	1	31-46	K.VQGAGQLRLSIDAQDR.V	
1	3	819.8417	-105.55	2	28.8	11.8	2	81-93	R.LRHQKTQTVPDCR.D	Carbamidomethyl: 12
1949	1	599.9043	-89.98	3	52.7	12.8	0	885-900	R.NGGSMHHLSLFFTGHR.K	

Detailed Protein Report

Protein 119: PREDICTED: CAS1 domain-containing protein 1 isoform X2 [Homo sapiens]

Accession: gi|578814529
Database: refseq_human(refseq_human_20140103.fasta)
Database Date: 2015-11-30
Modification(s): Carbamidomethyl

Score: 35.1
MW [kDa]: 72.0
pI: 9.6
Sequence Coverage [%]: 2.2
No. of unique Peptides: 1

Quantitation

WD:WU **Median:** 0.75 **CV:** 0.00 % **No. of Peptides:** 1

10	20	30	40	50	60	70	80
MGRCVLFSRN	CSSIAPTWAQ	VAASKLGDSP	KARVRARRVP	LVLSPHTPMA	LPQPPPPAGA	RDPVTPEHWA	SGPPSQAQPL
90	100	110	120	130	140	150	160
LRQEAKEEEE	GEETGVQGAW	GTGTAEQRRR	GWGEAAESAA	AEEGQAEVGG	AAAAGSGSPA	GGAGGGLGSW	RPLLAWLQRR
170	180	190	200	210	220	230	240
QPQCCPCAAP	LSRSAAHCCH	GGTKMAALAY	NLGKREINHY	FSVRSAKVLA	LVAVLLLAAC	HLASRRYRGN	DSCEYLLSSG
250	260	270	280	290	300	310	320
RFLGEKVWQP	HSCMMHKYKI	SEAKNCLVDK	HIAFIGDSRI	RQLFYSFVKI	INPQFKEEGN	KHENIPFEDK	TASVKVDFLW
330	340	350	360	370	380	390	400
HPEVNGSMKQ	CIKVWTEDSI	AKPHVIVAGA	ATWSIKIHNG	SSEALSQYKM	NITSIAPLLE	KLAKTSDVYW	VLQDPVYEDL
410	420	430	440	450	460	470	480
LSENRKMITN	EKIDAYNEAA	VSILNSSTRN	SKSNVKMFSV	SKLIAQETIM	ESLDGLHLPE	SSRETTAMIL	MNVYCNKILK
490	500	510	520	530	540	550	560
PVDGSCCQPR	PPVTLIQKLA	ACFFTLSIIG	YLIFYIIHRN	AHRKNKPCTD	LESGEEKKNI	INTPVSSLEI	LLQSFCKLGL
570	580	590	600	610	620	630	640
IMAYFYMCDR	ANLFMKENKF	YTHSSFFIPI	IYILVLGVFY	NENTKETKVL	NREQTDEWKG	WMQLVILIYH	ISGASTIVGA
650	660						
IGTQEGEMTF	I						

Cmpd.	No. of Cmpds.	m/z meas.	Δ m/z [ppm]	z	Rt [min]	Score	P	Range	Sequence	Modification	Ratios
117	1	793.7232	-186.24	2	30.4	10.2	1	160-173	R.RQPQCCPCAAPLSR.S	Carbamidomethyl: 8	WD:WU 0.75

Detailed Protein Report

Protein 120: hephaestin-like protein 1 precursor [Homo sapiens]

Accession: gi|148886707
Database: refseq_human(refseq_human_20140103.fasta)
Database Date: 2015-11-30
Modification(s): Oxidation

Score: 35.1
MW [kDa]: 131.5
pI: 6.3
Sequence Coverage [%]: 1.9
No. of unique Peptides: 2

10	20	30	40	50	60	70	80
MPRKQPAGCI	FLLTFLGLSG	LVGTVTRTYY	IGIVEEYWNY	VPQGKNVITG	KSFTEDKLAT	LFLERGPNRI	GSIYKKAVYR
**90**	**100**	**110**	**120**	**130**	**140**	**150**	**160**
RFTDGTYSIE	IPKPPWLGFL	GPILRAEVGD	VIVIHLKNFA	SRPYSLHPHG	VFYNKDSEGA	LYPDGTSGRN	KNDDMVPPGK
**170**	**180**	**190**	**200**	**210**	**220**	**230**	**240**
NYTYVWPVRE	EYAPTPADAN	CLTWVYHSHI	DAPKDICSGL	IGPLLVCKEG	ILNRYSGTRN	DVDREFVIMF	TLVDENQSWY
**250**	**260**	**270**	**280**	**290**	**300**	**310**	**320**
LNENIKHFCT	NPDSVDKKDA	VFQRSNKMHA	LNGYLFGNFP	EPDMCVGESV	SWHLFGMGNE	IDIHSIYFYG	NTFISRGHRT
**330**	**340**	**350**	**360**	**370**	**380**	**390**	**400**
DVVNLFPATF	LTTEMIAENP	GKWMITCQVS	DHLQAGMLGQ	YNVDNCKSDI	FYPKMKGQQR	RYFIAAEKIL	WDYAPQGYNK
**410**	**420**	**430**	**440**	**450**	**460**	**470**	**480**
FSGLPLNASG	SDSDLYFTQG	DNRIGGKYWK	VRYTEFVDAT	FTKRKRLSAE	EAHLGILGPV	IKAEVGDTLL	VTFANKADKV
**490**	**500**	**510**	**520**	**530**	**540**	**550**	**560**
YSILPHGVIY	DKASDAAPNL	DGFVKPGAHV	KPGETFTYKW	TVPESVSPTA	GDPPCLTYLY	FSAVDPIKDT	SSGLVGPLLV
**570**	**580**	**590**	**600**	**610**	**620**	**630**	**640**
CKKGVLNADG	TQKGIDKEFY	LLFTVFDENL	SRYFDENIQK	FIWHPFSIDK	EDKEFVKSNR	MHAVNGYMYG	NQPGLNMCKR
**650**	**660**	**670**	**680**	**690**	**700**	**710**	**720**
DRVSWHLIGL	GTDTDMHGIV	FQGNTIHLRG	THRDSLALFP	HMATTAFMQP	DHAGIFRVFC	ATMPHLSRGM	GQIYEVSSCD
**730**	**740**	**750**	**760**	**770**	**780**	**790**	**800**
NRDPSEQRYG	MIRTFYIAAE	EVEWDYAPNK	NWEFEKQHVD	ARGERHGDIF	MNRTENWIGS	QYKKVVYREY	TDGEFVEIKA
**810**	**820**	**830**	**840**	**850**	**860**	**870**	**880**
RPPREEHLEL	LGPMIHAEVG	NTVLIIFKNK	ASRPYSISAQ	GVEEMDSGKQ	FQVPMTKPGE	VKTYRWNIPK	RSGPGPSDPN
**890**	**900**	**910**	**920**	**930**	**940**	**950**	**960**
CIPWVYYSTV	NFVKDTYSGL	MGPLITCRKG	VLNEKGRRSD	VDYEFALLFL	VFNENESWYL	DDNIKKYLNK	DPRDFKRTDD
**970**	**980**	**990**	**1000**	**1010**	**1020**	**1030**	**1040**
FEESNRMHAI	NGKIFGNLHG	LIMNEDTMTN	WYLLGIGSEV	DIHTIHYHAE	SFLFKIDKSY	REDVYDLFPG	TFQTIELFAD
**1050**	**1060**	**1070**	**1080**	**1090**	**1100**	**1110**	**1120**
HPGTWLLHCH	VSDHIHAGME	TTYTVLRNID	NRIPYSTTSP	GVASHPATVP	SNERPGKEQL	YFFGKNLGPT	GAKAALVILF
**1130**	**1140**	**1150**	**1160**				
IIGLLLLITT	VILSLRLCSA	MKQTDYQQVQ	SCALPTDAL				

Cmpd.	No. of Cmpds.	m/z meas.	Δ m/z [ppm]	z	Rt [min]	Score	P	Range	Sequence	Modification
2733	1	639.3478	58.98	2	62.6	13.4	0	698-708	R.VFCATMPHLSR.G	Oxidation: 6
2243	1	666.2803	-55.46	2	56.5	21.7	1	763-773	R.GERHGDIFMNR.T	

Detailed Protein Report

Protein 121: collagen alpha-2(XI) chain isoform 3 preproprotein [Homo sapiens]

**Accession:**	gi|111118968	**Score:**	35.0
**Database:**	refseq_human(refseq_human_20140103.fasta)	**MW [kDa]:**	159.8
**Database Date:**	2015-11-30	**pI:**	9.5
		**Sequence Coverage [%]:**	1.7
		**No. of unique Peptides:**	1

10	20	30	40	50	60	70	80
MERCSRCHRL	LLLLPLVLGL	SAAPGWAGAP	PVDVLRALRF	PSLPDGVRRA	KGICPADVAY	RVARPAQLSA	PTRQLFPGGF
90	100	110	120	130	140	150	160
PKDFSLLTVV	RTRPGLQAPL	LTLYSAQGVR	QLGLELGRPV	RFLYEDQTGR	PQPPSQPVFR	GLSLADGKWH	RVAVAVKGQS
170	180	190	200	210	220	230	240
VTLIVDCKKR	VTRPLPRSAR	PVLDTHGVII	FGARILDEEV	FEGDVQELAI	VPGVQAAYES	CEQKELECEG	GQRERPQNQQ
250	260	270	280	290	300	310	320
PHRAQRSPQQ	QPSRLHRPQN	QEPQSQAAHG	PRGLKGEKGE	PAVLEPGMLV	EGPPGPEGPA	GLIGPPGIQG	NPGPVGDPGE
330	340	350	360	370	380	390	400
RGPPGRAGLP	GSDGAPGPPG	TSLMLPFRFG	SGGGDKGPVV	AAQEAQAQAI	LQQARLALRG	PPGPMGYTGR	PGPLGQPGSP
410	420	430	440	450	460	470	480
GLKGESGDLG	PQGPRGPQGL	TGPPGKAGRR	GRAGADGARG	MPGDPGVKGD	RGFDGLPGLP	GEKGHRGDTG	AQGLPGPPGE
490	500	510	520	530	540	550	560
DGERGDDGEI	GPRGLPGESG	PRGLLGPKGP	PGIPGPPGVR	GMDGPQGPKG	SLGPQGEPGP	PGQQGTPGTQ	GLPGPQGAIG
570	580	590	600	610	620	630	640
PHGEKGPQGK	PGLPGMPGSD	GPPGHPGKEG	PPGTKGNQGP	SGPQGPLGYP	GPRGVKGVDG	IRGLKGHKGE	KGEDGFPGFK
650	660	670	680	690	700	710	720
GDIGVKGDRG	EVGVPGSRGE	DGPEGPKGRT	GPTGDPGPPG	LMGEKGKLGV	PGLPGYPGRQ	GPKGSLGFPG	FPGASGEKGA
730	740	750	760	770	780	790	800
RGLSGKSGPR	GERGPTGPRG	QRGPRGATGK	SGAKGTSGGD	GPHGPPGERG	LPGPQGPNGF	PGPKGPPGPP	GKDGLPGHPG
810	820	830	840	850	860	870	880
QRGEVGFQGK	TGPPGPPGVV	GPQGAAGETG	PMGERGHPGP	PGPPGEQGLP	GTAGKEGTKG	DPGPPGAPGK	DGPAGLRGFP
890	900	910	920	930	940	950	960
GERGLPGTAG	GPGLKGNEGP	SGPPGPAGSP	GERGAAGSGG	PIGPPGRPGP	QGPPGAAGEK	GVPGEKGPIG	PTGRDGVQGP
970	980	990	1000	1010	1020	1030	1040
VGLPGPAGPP	GVAGEDGDKG	EVGDPGQKGT	KGNKGEHGPP	GPPGPIGPVG	QPGAAGADGE	PGARGPQGHF	GAKGDEGTRG
1050	1060	1070	1080	1090	1100	1110	1120
FNGPPGPIGL	QGLPGPSGEK	GETGDVGPMG	PPGPPGPRGP	AGPNGADGPQ	GPPGGVGNLG	PPGEKGEPGE	SGSPGIQGEP
1130	1140	1150	1160	1170	1180	1190	1200
GVKGPRGERG	EKGESGQPGE	PGPPGPKGPT	GDDGPKGNPG	PVGFPGDPGP	PGEGGPRGQD	GAKGDRGEDG	EPGQPGSPGP
1210	1220	1230	1240	1250	1260	1270	1280
TGENGPPGPL	GKRGPAGSPG	SEGRQGGKGA	KGDPGAIGAP	GKTGPVGPAG	PAGKPGPDGL	RGLPGSVGQQ	GRPGATGQAG
1290	1300	1310	1320	1330	1340	1350	1360
PPGPVGPPGL	PGLRGDAGAK	GEKGHPGLIG	LIGPPGEQGE	KGDRGLPGPQ	GSPGQKGEMG	IPGASGPIGP	GGPPGLPGPA
1370	1380	1390	1400	1410	1420	1430	1440
GPKGAKGATG	PGGPKGEKGV	QGPPGHPGPP	GEVIQPLPIQ	MPKKTRRSVD	GSRLMQEDEA	IPTGGAPGSP	GGLEEIFGSL
1450	1460	1470	1480	1490	1500	1510	1520
DSLREEIEQM	RRPTGTQDSP	ARTCQDLKLC	HPELPDGEYW	VDPNQGCARD	AFRVFCNFTA	GGETCVTPRD	DVTQFSYVDS
1530	1540	1550	1560	1570	1580	1590	1600
EGSPVGVVQL	TFLRLLSVSA	HQDVSYPCSG	AARDGPLRLR	GANEDELSPE	TSPYVKEFRD	GCQTQQGRTV	LEVRTPVLEQ
1610	1620	1630					
LPVLDASFSD	LGAPPRRGGV	LLGPVCFMG					

Cmpd.	No. of Cmpds.	m/z meas.	Δ m/z [ppm]	z	Rt [min]	Score	P	Range	Sequence	Modification
2871	4	645.8309	-41.94	2	64.6	23.7	1	158-169	K.GQSVTLIVDCKK.R	

Detailed Protein Report

Protein 122: PREDICTED: sacsin isoform X1 [Homo sapiens]

**Accession:**	gi|530402261	**Score:**	34.9
**Database:**	refseq_human(refseq_human_20140103.fasta)	**MW [kDa]:**	521.9
**Database Date:**	2015-11-30	**pI:**	6.7
**Modification(s):**	Carbamidomethyl	**Sequence Coverage [%]:**	0.9
		**No. of unique Peptides:**	2

Detailed Protein Report

10	20	30	40	50	60	70	80
METKENRWVP	VTVLPGCVGC	RTVAALASWT	VRDVKERIFA	ETGFPVSEQR	LWRGGRELSD	WIKIGDLTSK	NCHLFVNLQS
90	100	110	120	130	140	150	160
KGLKGGGRFG	QTTPPLVDFL	KDILRRYPEG	GQILKELIQN	AEDAGATEVK	FLYDETQYGT	ETLWSKDMAP	YQGPALYVYN
170	180	190	200	210	220	230	240
NAVFTPEDWH	GIQEIARSRK	KDDPLKVGRF	GIGFNSVYHI	TDVPCIFSGD	QIGMLDPHQT	LFGPHESGQC	WNLKDDSKEI
250	260	270	280	290	300	310	320
SELSDQFAPF	VGIFGSTKET	FINGNFPGTF	FRFPLRLQPS	QLSSNLYNKQ	KVLELFESFR	ADADTVLLFL	KSVQDVSLYV
330	340	350	360	370	380	390	400
READGTEKLV	FRVTSSESKA	LKHERPNSIK	ILGTAISNYC	KKTPSNNITC	VTYHVNIVLE	EESTKDAQKT	SWLVCNSVGG
410	420	430	440	450	460	470	480
RGISSKLDSL	ADELKFVPII	GIAMPLSSRD	DEAKGATSDF	SGKAFCFLPL	PPGEESSTGL	PVHISGFFGL	TDNRRSIKWR
490	500	510	520	530	540	550	560
ELDQWRDPAA	LWNEFLVMNV	VPKAYATLIL	DSIKRLEMEK	SSDFPLSVDV	IYKLWPEASK	VKVHWQPVLE	PLFSELLQNA
570	580	590	600	610	620	630	640
VIYSISCDWV	RLEQVYFSEL	DENLEYTKTV	LNYLQSSGKQ	IAKVPGNVDA	AVQLTAASGT	TPVRKVTPAW	VRQVLRKCAH
650	660	670	680	690	700	710	720
LGCAEEKLHL	LEFVLSDQAY	SELLGLELLP	LQNGNFVPFS	SSVSDQDVIY	ITSAEYPRSL	FPSLEGRFIL	DNLKPHLVAA
730	740	750	760	770	780	790	800
LKEAAQTRAQ	QQLWGYSGRP	CTQLQLLNPE	RFARLIKEVM	NTFWPGRELI	VQWYPFDENR	NHPSVSWLKM	VWKNLYIHFS
810	820	830	840	850	860	870	880
EDLTLFDEMP	LIPRTILEEG	QTCVELIRLR	IPSLVILDDE	SEAQLPEFLA	DIVQKLGGFV	LKKLDASIQH	PLIKKYIHSP
890	900	910	920	930	940	950	960
LPSAVLQIME	KMPLQKLCNQ	ITSLLPTHKD	ALRKFLASLT	DSSEKEKRII	QELAIFKRIN	HSSDQGISSY	TKLKGCKVLH
970	980	990	1000	1010	1020	1030	1040
HTAKLPADLR	LSISVIDSSD	EATIRLANML	KIEQLKTTSC	LKLVLKDIEN	AFYSHEEVTQ	LMLWVLENLS	SLKNENPNVL
1050	1060	1070	1080	1090	1100	1110	1120
EWLTPLKFIQ	ISQEQMVSAG	ELFDPDIEVL	KDLFCNEEGT	YFPPSVFTSP	DILHSLRQIG	LKNEASLKEK	DVVQVAKKIE
1130	1140	1150	1160	1170	1180	1190	1200
ALQVGACPDQ	DVLLKKAKTL	LLVLNKNHTL	LQSSEGKMTL	KKIKWVPACK	ERPPNYPGSL	VWKGDLCNLC	APPDMCDVGH
1210	1220	1230	1240	1250	1260	1270	1280
AILIGSSLPL	VESIHVNLEK	ALGIFTKPSL	SAVLKHFKIV	VDWYSSKTFS	DEDYYQFQHI	LLEIYGFMHD	HLNEGKDSFR
1290	1300	1310	1320	1330	1340	1350	1360
ALKFPWVWTG	KKFCPLAQAV	IKPIHDLDLQ	PYLHNVPKTM	AKFHQLFKVC	GSIEELTSDH	ISMVIQKIYL	KSDQDLSEQE
1370	1380	1390	1400	1410	1420	1430	1440
SKQNLHLMLN	IIRWLYSNQI	PASPNTPVPI	HHSKNPSKLI	MKPIHECCYC	DIKVDDLNDL	LEDSVEPIIL	VHEDIPMKTA
1450	1460	1470	1480	1490	1500	1510	1520
EWLKVPCLST	RLINPENMGF	EQSGQREPLT	VRIKNILEEY	PSVSDIFKEL	LQNADDANAT	ECSFLIDMRR	NMDIRENLLD
1530	1540	1550	1560	1570	1580	1590	1600
PGMAACHGPA	LWSFNNSQFS	DSDFVNITRL	GESLKRGEVD	KVGKFGLGFN	SVYHITDIPI	IMSREFMIMF	DPNINHISKH
1610	1620	1630	1640	1650	1660	1670	1680
IKDKSNPGIK	INWSKQQKRL	RKFPNQFKPF	IDVFGCQLPL	TVEAPYSYNG	TLFRLSFRTQ	QEAKVSEVSS	TCYNTADIYS
1690	1700	1710	1720	1730	1740	1750	1760
LVDEFSLCGH	RLIIFTQSVK	SMYLKYLKIE	ETNPSLAQDT	VIIKKKSCSS	KALNTPVLSV	LKEAAKLMKT	CSSSNKKLPS
1770	1780	1790	1800	1810	1820	1830	1840
DEPKSSCILQ	ITVEEFHHVF	RRIADLQSPL	FRGPDDDPAA	LFEMAKSGQS	KKPSDELSQK	TVECTTWLLC	TCMDTGEALK
1850	1860	1870	1880	1890	1900	1910	1920
FSLSESGRRL	GLVPCGAVGV	QLSEIQDQKW	TVKPHIGEVF	CYLPLRIKTG	LPVHINGCFA	VTSNRKEIWK	TDTKGRWNTT
1930	1940	1950	1960	1970	1980	1990	2000
FMRHVIVKAY	LQVLSVLRDL	ATSGELMDYT	YYAVWPDPDL	VHDDFSVICQ	GFYEDIAHGK	GKELTKVFSD	GSTWVSMKNV
2010	2020	2030	2040	2050	2060	2070	2080
RFLDDSILKR	RDVGSAAFKI	FLKYLKKTGS	KNLCAVELPS	SVKLGFEEAG	CKQILLENTF	SEKQFFSEVF	FPNIQEIEAE
2090	2100	2110	2120	2130	2140	2150	2160
LRDPLMIFVL	NEKVDEFSGV	LRVTPCIPCS	LEGHPLVLPS	RLIHPEGRVA	KLFDIKDGRF	PYGSTQDYLN	PIILIKLVQL
2170	2180	2190	2200	2210	2220	2230	2240

Detailed Protein Report

Cmpd.	No. of Cmpds.	m/z meas.	Δ m/z [ppm]	z	Rt [min]	Score	P	Range	Sequence	Modification
1798	1	916.4469	-78.82	2	50.8	10.5	2	1726-1742	K.KSCSSKALNTPVLSVLK.E	Carbamidomethyl: 3
1779	1	516.2947	105.86	2	50.6	10.7	0	2492-2499	K.YCHADIPR.E	Carbamidomethyl: 2

Detailed Protein Report

Protein 123: niban-like protein 1 isoform 2 [Homo sapiens]

**Accession:**	gi|79750824	**Score:**	34.9
**Database:**	refseq_human(refseq_human_20140103.fasta)	**MW [kDa]:**	82.6
**Database Date:**	2015-11-30	**pI:**	5.8
		**Sequence Coverage [%]:**	2.5
		**No. of unique Peptides:**	1

Quantitation

WD:WU **Median:** 1.73 **CV:** 0.00 % **No. of Peptides:** 1

10	20	30	40	50	60	70	80
MGWMGEKTGK	ILTEFLQFYE	DQYGVALFNS	MRHEIEGTGL	PQAQLLWRKV	PLDERIVFSG	NLFQHQEDSK	KWRNRFSLVP
90	100	110	120	130	140	150	160
HNYGLVLYEN	KAAYERQVPP	RAVINSAGYK	ILTSVDQYLE	LIGNSLPGTT	AKSGSAPILK	CPTQFPLILW	HPYARHYYFC
170	180	190	200	210	220	230	240
MMTEAEQDKW	QAVLQDCIRH	CNNGIPEDSK	VEGPAFTDAI	RMYRQSKELY	GTWEMLCGNE	VQILSNLVME	ELGPELKAEL
250	260	270	280	290	300	310	320
GPRLKGKPQE	RQRQWIQISD	AVYHMVYEQA	KARFEEVLSK	VQQVQPAMQA	VIRTDMDQII	TSKEHLASKI	RAFILPKAEV
330	340	350	360	370	380	390	400
CVRNHVQPYI	PSILEALMVP	TSQGFTEVRD	VFFKEVTDMN	LNVINEGGID	KLGEYMEKLS	RLAYHPLKMQ	SCYEKMESLR
410	420	430	440	450	460	470	480
LDGLQQRFDV	SSTSVFKQRA	QIHMREQMDN	AVYTFETLLH	QELGKGPTKE	ELCKSIQRVL	ERVLKKYDYD	SSSVRKRFFR
490	500	510	520	530	540	550	560
EALLQISIPF	LLKKLAPTCK	SELPRFQELI	FEDFARFILV	ENTYEEVVLQ	TVMKDILQAV	KEAAVQRKHN	LYRDSMVMHN
570	580	590	600	610	620	630	640
SDPNLHLLAE	GAPIDWGEEY	SNSGGGGSPS	PSTPESATLS	EKRRRAKQVV	SVVQDEEVGL	PFEASPESPP	PASPDGVTEI
650	660	670	680	690	700	710	720
RGLLAQGLRP	ESPPPAGPLL	NGAPAGESPQ	PKAAPEASSP	PASPLQHLLP	GKAVDLGPPK	PSDQETGEQV	SSPSSHPALH
730	740						
TTTEDSAGVQ	TEF						

Cmpd.	No. of Cmpds.	m/z meas.	Δ m/z [ppm]	z	Rt [min]	Score	P	Range	Sequence	Modification	Ratios
367	3	607.8374	9.31	2	33.2	19.6	1	495-505	K.LAPTCKSELPR.F		WD:WU 1.73

Detailed Protein Report

Protein 124: PREDICTED: lysosomal-trafficking regulator isoform X4 [Homo sapiens]

**Accession:**	gi|530365992	**Score:**	34.8
**Database:**	refseq_human(refseq_human_20140103.fasta)	**MW [kDa]:**	313.5
**Database Date:**	2015-11-30	**pI:**	6.1
**Modification(s):**	Oxidation	**Sequence Coverage [%]:**	1.5
		**No. of unique Peptides:**	2

Detailed Protein Report

10	20	30	40	50	60	70	80
MSTDSNSLAR	EFLTDVNRLC	NAVVQRVEAR	EEEEEETHMA	TLGQYLVHGR	GFLLLTKLNS	IIDQALTCRE	ELLTLLLSLL
90	100	110	120	130	140	150	160
PLVWKIPVQE	EKATDFNLPL	SADIILTKEK	NSSSQRSTQE	KLHLEGSALS	SQVSAKVNVF	RKSRRQRKIT	HRYSVRDARK
170	180	190	200	210	220	230	240
TQLSTSDSEA	NSDEKGIAMN	KHRRPHLLHH	FLTSFPKQDH	PKAKLDRLAT	KEQTPPDAMA	LENSREIIPR	QGSNTDILSE
250	260	270	280	290	300	310	320
PAALSVISNM	NNSPFDLCHV	LLSLLEKVCK	FDVTLNHNSP	LAASVVPTLT	EFLAGFGDCC	SLSDNLESRV	VSAGWTEEPV
330	340	350	360	370	380	390	400
ALIQRMLFRT	VLHLLSVDVS	TAEMMPENLR	KNLTELLRAA	LKIRICLEKQ	PDPFAPRQKK	TLQEVQEDFV	FSKYRHRALL
410	420	430	440	450	460	470	480
LPELLEGVLQ	ILICCLQSAA	SNPFYFSQAM	DLVQEFIQHH	GFNLFETAVL	QMEWLVLRDG	VPPEASEHLK	ALINSVMKIM
490	500	510	520	530	540	550	560
STVKKVKSEQ	LHHSMCTRKR	HRRCEYSHFM	HHHRDLSGLL	VSAFKNQVSK	NPFEETADGD	VYYPERCCCI	AVCAHQCLRL
570	580	590	600	610	620	630	640
LQQASLSSTC	VQILSGVHNI	GICCCMDPKS	VIIPLLHAFK	LPALKNFQQH	ILNILNKLIL	DQLGGAEISP	KIKKAACNIC
650	660	670	680	690	700	710	720
TVDSDQLAQL	EETLQGNLCD	AELSSSLSSP	SYRFQGILPS	SGSEDLLWKW	DALKAYQNFV	FEEDRLHSIQ	IANHICNLIQ
730	740	750	760	770	780	790	800
KGNIVVQWKL	YNYIFNPVLQ	RGVELAHHCQ	HLSVTSAQSH	VCSHHNQCLP	QDVLQIYVKT	LPILLKSRVI	RDLFLSCNGV
810	820	830	840	850	860	870	880
SQIIELNCLN	GIRSHSLKAF	ETLIISLGEQ	QKDASVPDID	GIDIEQKELS	SVHVGTSFHH	QQAYSDSPQS	LSKFYAGLKE
890	900	910	920	930	940	950	960
AYPKRRKTVN	QDVHINTINL	FLCVAFLCVS	KEAESDRESA	NDSEDTSGYD	STASEPLSHM	LPCISLESLV	LPSPEHMHQA
970	980	990	1000	1010	1020	1030	1040
ADIWSMCRWI	YMLSSVFQKQ	FYRLGGFRVC	HKLIFMIIQK	LFRSHKEEQG	KKEGDTSVNE	NQDLNRISQP	KRTMKEDLLS
1050	1060	1070	1080	1090	1100	1110	1120
LAIKSDPIPS	ELGSLKKSAD	SLGKLELQHI	SSINVEEVSA	TEAAPEEAKL	FTSQESETSL	QSIRLLEALL	AICLHGARTS
1130	1140	1150	1160	1170	1180	1190	1200
QQKMELELPN	QNLSVESILF	EMRDHLSQSK	VIETQLAKPL	FDALLRVALG	NYSADFEHND	AMTEKSHQSA	EELSSQPGDF
1210	1220	1230	1240	1250	1260	1270	1280
SEEAEDSQCC	SFKLLVEEEG	YEADSESNPE	DGETQDDGVD	LKSETEGFSA	SSSPNDLLEN	LTQGEIIYPE	ICMLELNLLS
1290	1300	1310	1320	1330	1340	1350	1360
ASKAKLDVLA	HVFESFLKII	RQKEKNVFLL	MQQGTVKNLL	GGFLSILTQD	DSDFQACQRV	LVDLLVSLMS	SRTCSEELTL
1370	1380	1390	1400	1410	1420	1430	1440
LLRIFLEKSP	CTKILLLGIL	KIIESDTTMS	PSQYLTFPLL	HAPNLSNGVS	SQKYPGILNS	KAMGLLRRAR	VSRSKKEADR
1450	1460	1470	1480	1490	1500	1510	1520
ESFPHRLLSS	WHIAPVHLPL	LGQNCWPHLS	EGFSVSLWFN	VECIHEAEST	TEKGKKIKKR	NKSLILPDSS	FDGTESDRPE
1530	1540	1550	1560	1570	1580	1590	1600
GAEYINPGER	LIEEGCIHII	SLGSKALMIQ	VWADPHNATL	IFRVCMDSND	DMKAVLLAQV	ESQENIFLPS	KWQHLVLTYL
1610	1620	1630	1640	1650	1660	1670	1680
QQPQGKRRIH	GKISIWVSGQ	RKPDVTLDFM	LPRKTSLSSD	SNKTFCMIGH	CLSSQEEFLQ	LAGKWDLGNL	LLFNGAKVGS
1690	1700	1710	1720	1730	1740	1750	1760
QEAFYLYACG	PNHTSVMPCK	YGKPVNDYSK	YINKEILRCE	QIRELFMTKK	DVDIGLLIES	LSVVYTTYCP	AQYTIYEPVI
1770	1780	1790	1800	1810	1820	1830	1840
RLKGQMKTQL	SQRPFSSKEV	QSILLEPHHL	KNLQPTEYKT	IQGILHEIGG	TGIFVFLFAR	VVELSSCEET	QALALRVILS
1850	1860	1870	1880	1890	1900	1910	1920
LIKYNQQRVH	ELENCNGLSM	IHQVLIKQKC	IVGFYILKTL	LEGCCGEDII	YMNENGEFKL	DVDSNAIIQD	VKLLEELLLD
1930	1940	1950	1960	1970	1980	1990	2000
WKIWSKAEQG	VWETLLAALE	VLIRADHHQQ	MFNIKQLLKA	QVVHHFLLTC	QVLQEYKEGQ	LTPMPREVCR	SFVKIIAEVL
2010	2020	2030	2040	2050	2060	2070	2080
GSPPDLELLT	IIFNFLLAVH	PPTNTYVCHN	PTNFYFSLHI	DGKIFQEKVR	SIMYLRHSSS	GGRSLMSPGF	MVISPSGFTA
2090	2100	2110	2120	2130	2140	2150	2160
SPYEGENSSN	IIPQQMAAHM	LRSRSLPAFP	TSSLLTQSQK	LTGSLGCSID	RLQNIADTYV	ATQSKKQNSL	GSSDTLKKGK
2170	2180	2190	2200	2210	2220	2230	2240

Detailed Protein Report

Cmpd.	No. of Cmpds.	m/z meas.	Δ m/z [ppm]	z	Rt [min]	Score	P	Range	Sequence	Modification
1683	1	853.4016	-89.30	2	49.4	11.6	1	515-530	R.DLSGLLVSAFKNQVSK.N	
2340	1	953.1627	116.28	2	57.7	13.1	2	989-1003	R.VCHKLIFMIIQKLFR.S	Oxidation: 8

Detailed Protein Report

Protein 125: dystrophin Dp260-1 isoform [Homo sapiens]

**Accession:**	gi|150036268	**Score:**	34.7
**Database:**	refseq_human(refseq_human_20140103.fasta)	**MW [kDa]:**	271.2
**Database Date:**	2015-11-30	**pI:**	5.8
**Modification(s):**	Carbamidomethyl	**Sequence Coverage [%]:**	1.7
		**No. of unique Peptides:**	2

Detailed Protein Report

10	20	30	40	50	60	70	80
MTEMILLIFF	PAYFLNAVRR	QKLLEQSIQS	AQETEKSLHL	IQESLTFIDK	QLAAYIADKV	DAAQMPQEAQ	KIQSDLTSHE
90	100	110	120	130	140	150	160
ISLEEMKKHN	QGKEAAQRVL	SQIDVAQKKL	QDVSMKFRLF	QKPANFEQRL	QESKMILDEV	KMHLPALETK	SVEQEVVQSQ
170	180	190	200	210	220	230	240
LNHCVNLYKS	LSEVKSEVEM	VIKTGRQIVQ	KKQTENPKEL	DERVTALKLH	YNELGAKVTE	RKQQLEKCLK	LSRKMRKEMN
250	260	270	280	290	300	310	320
VLTEWLAATD	MELTKRSAVE	GMPSNLDSEV	AWGKATQKEI	EKQKVHLKSI	TEVGEALKTV	LGKKETLVED	KLSLLNSNWI
330	340	350	360	370	380	390	400
AVTSRAEEWL	NLLLEYQKHM	ETFDQNVDHI	TKWIIQADTL	LDESEKKKPQ	QKEDVLKRLK	AELNDIRPKV	DSTRDQAANL
410	420	430	440	450	460	470	480
MANRGDHCRK	LVEPQISELN	HRFAAISHRI	KTGKASIPLK	ELEQFNSDIQ	KLLEPLEAEI	QQGVNLKEED	FNKDMNEDNE
490	500	510	520	530	540	550	560
GTVKELLQRG	DNLQQRITDE	RKREEIKIKQ	QLLQTKHNAL	KDLRSQRRKK	ALEISHQWYQ	YKRQADDLLK	CLDDIEKKLA
570	580	590	600	610	620	630	640
SLPEPRDERK	IKEIDRELQK	KKEELNAVRR	QAEGLSEDGA	AMAVEPTQIQ	LSKRWREIES	KFAQFRRLNF	AQIHTVREET
650	660	670	680	690	700	710	720
MMVMTEDMPL	EISYVPSTYL	TEITHVSQAL	LEVEQLLNAP	DLCAKDFEDL	FKQEESLKNI	KDSLQQSSGR	IDIIHSKKTA
730	740	750	760	770	780	790	800
ALQSATPVER	VKLQEALSQL	DFQWEKVNKM	YKDRQGRFDR	SVEKWRRFHY	DIKIFNQWLT	EAEQFLRKTQ	IPENWEHAKY
810	820	830	840	850	860	870	880
KWYLKELQDG	IGQRQTVVRT	LNATGEEIIQ	QSSKTDASIL	QEKLGSLNLR	WQEVCKQLSD	RKKRLEEQKN	ILSEFQRDLN
890	900	910	920	930	940	950	960
EFVLWLEEAD	NIASIPLEPG	KEQQLKEKLE	QVKLLVEELP	LRQGILKQLN	ETGGPVLVSA	PISPEEQDKL	ENKLKQTNLQ
970	980	990	1000	1010	1020	1030	1040
WIKVSRALPE	KQGEIEAQIK	DLGQLEKKLE	DLEEQLNHLL	LWLSPIRNQL	EIYNQPNQEG	PFDVQETEIA	VQAKQPDVEE
1050	1060	1070	1080	1090	1100	1110	1120
ILSKGQHLYK	EKPATQPVKR	KLEDLSSEWK	AVNRLLQELR	AKQPDLAPGL	TTIGASPTQT	VTLVTQPVVT	KETAISKLEM
1130	1140	1150	1160	1170	1180	1190	1200
PSSLMLEVPA	LADFNRAWTE	LTDWLSLLDQ	VIKSQRVMVG	DLEDINEMII	KQKATMQDLE	QRRPQLEELI	TAAQNLKNKT
1210	1220	1230	1240	1250	1260	1270	1280
SNQEARTIIT	DRIERIQNQW	DEVQEHLQNR	RQQLNEMLKD	STQWLEAKEE	AEQVLGQARA	KLESWKEGPY	TVDAIQKKIT
1290	1300	1310	1320	1330	1340	1350	1360
ETKQLAKDLR	QWQTNVDVAN	DLALKLLRDY	SADDTRKVHM	ITENINASWR	SIHKRVSERE	AALEETHRLL	QQFPLDLEKF
1370	1380	1390	1400	1410	1420	1430	1440
LAWLTEAETT	ANVLQDATRK	ERLLEDSKGV	KELMKQWQDL	QGEIEAHTDV	YHNLDENSQK	ILRSLEGSDD	AVLLQRRLDN
1450	1460	1470	1480	1490	1500	1510	1520
MNFKWSELRK	KSLNIRSHLE	ASSDQWKRLH	LSLQELLVWL	QLKDDELSRQ	APIGGDFPAV	QKQNDVHRAF	KRELKTKEPV
1530	1540	1550	1560	1570	1580	1590	1600
IMSTLETVRI	FLTEQPLEGL	EKLYQEPREL	PPEERAQNVT	RLLRKQAEEV	NTEWEKLNLH	SADWQRKIDE	TLERLQELQE
1610	1620	1630	1640	1650	1660	1670	1680
ATDELDLKLR	QAEVIKGSWQ	PVGDLLIDSL	QDHLEKVKAL	RGEIAPLKEN	VSHVNDLARQ	LTTLGIQLSP	YNLSTLEDLN
1690	1700	1710	1720	1730	1740	1750	1760
TRWKLLQVAV	EDRVRQLHEA	HRDFGPASQH	FLSTSVQGPW	ERAISPNKVP	YYINHETQTT	CWDHPKMTEL	YQSLADLNNV
1770	1780	1790	1800	1810	1820	1830	1840
RFSAYRTAMK	LRRLQKALCL	DLLSLSAACD	ALDQHNLKQN	DQPMDILQII	NCLTTIYDRL	EQEHNNLVNV	PLCVDMCLNW
1850	1860	1870	1880	1890	1900	1910	1920
LLNVYDTGRT	GRIRVLSFKT	GIISLCKAHL	EDKYRYLFKQ	VASSTGFCDQ	RRLGLLLHDS	IQIPRQLGEV	ASFGGSNIEP
1930	1940	1950	1960	1970	1980	1990	2000
SVRSCFQFAN	NKPEIEAALF	LDWMRLEPQS	MVWLPVLHRV	AAAETAKHQA	KCNICKECPI	IGFRYRSLKH	FNYDICQSCF
2010	2020	2030	2040	2050	2060	2070	2080
FSGRVAKGHK	MHYPMVEYCT	PTTSGEDVRD	FAKVLKNKFR	TKRYFAKHPR	MGYLPVQTVL	EGDNMETPVT	LINFWPVDSA
2090	2100	2110	2120	2130	2140	2150	2160
PASSPQLSHD	DTHSRIEHYA	SRLAEMENSN	GSYLNDSISP	NESIDDEHLL	IQHYCQSLNQ	DSPLSQPRSP	AQILISLESE
2170	2180	2190	2200	2210	2220	2230	2240

Detailed Protein Report

Cmpd.	No. of Cmpds.	m/z meas.	Δ m/z [ppm]	z	Rt [min]	Score	P	Range	Sequence	Modification
129	1	619.6434	29.86	3	30.9	11.2	2	395-410	R.DQAANLMANRGDHCRK.L	Carbamidomethyl: 14
1813	1	785.8684	-31.80	2	51.3	11.2	0	1318-1330	K.VHMITENINASWR.S	

Detailed Protein Report

Protein 126: tumor protein D54 isoform g [Homo sapiens]

**Accession:**	gi|345197262	**Score:**	34.7
**Database:**	refseq_human(refseq_human_20140103.fasta)	**MW [kDa]:**	19.5
**Database Date:**	2015-11-30	**pI:**	6.2
		**Sequence Coverage [%]:**	9.9
		**No. of unique Peptides:**	1

10	20	30	40	50	60	70	80
MDSAGQDINL	NSPNKGLLSD	SMTDVPVDTG	VEEEIVTLRQ	VLAAKERHCG	ELKRRLGLST	LGELKQNLSR	SWHDVQVSSA
**90**	**100**	**110**	**120**	**130**	**140**	**150**	**160**
YVKTSEKLGE	WNEKVTQSDL	YKKTQETLSQ	AGQKTSAALS	TVGSAISRKL	GDMRNSATFK	SFEDRVGTIK	SKVVGDRENG
**170**	**180**	**190**					
SDNLPSSAGS	GDKPLSDPAP	F					

Cmpd.	No. of Cmpds.	m/z meas.	Δ m/z [ppm]	z	Rt [min]	Score	P	Range	Sequence	Modification
2523	1	1052.4103	-115.77	2	59.8	34.7	1	66-83	K.QNLSRSWHDVQVSSAYVK.T	

Detailed Protein Report

Protein 127: PREDICTED: rho guanine nucleotide exchange factor 5-like, partial [Homo sapiens]

**Accession:**	gi|578844357	**Score:**	34.6
**Database:**	refseq_human(refseq_human_20140103.fasta)	**MW [kDa]:**	165.0
**Database Date:**	2015-11-30	**pI:**	5.4
		**Sequence Coverage [%]:**	2.3
		**No. of unique Peptides:**	1

10	20	30	40	50	60	70	80
FSIIPEAPMR	SSQVSALGLE	AQEDEDPSYK	WREEHRLSAT	QQSELRDVCD	YAIETMPSFP	KEGSADVEPN	QESLVAEACD
90	100	110	120	130	140	150	160
TPEHWEAVPQ	SLAGRQARTL	APPELWACPI	QSEHLDMAPF	SSDLGSEEEE	VEFWPGLTSL	TLGSGQAEEE	EETSSDNSGQ
170	180	190	200	210	220	230	240
TRYYSPCEEH	PAETNQNEGS	ESGTIRQGEE	LPPEELQESQ	GLLHPQEVQV	LEEQGQQEAG	FRGEGTLRED	VCADGLLGEE
250	260	270	280	290	300	310	320
QMIEQVNDEK	GEQKQKQEQV	QDVMLGRQGE	RMGLTGEPEG	LNDGEWEQED	MERKAQGQGG	PEQGEERKRE	LQVPEENRAD
330	340	350	360	370	380	390	400
SQDEKSQIFL	GKSEEVTGKQ	EDHGIKEKGV	PVSGQEAKEP	ESWDGGRLGA	VGRARSREEE	NEHHGPSMPA	LIAPEDSPHC
410	420	430	440	450	460	470	480
DLFPGASYLM	TQIPGTQTES	RAEELSPAAL	SPSLEPIRCS	HQPISLLGSF	LTEESPDKEI	DQNSQQEGSR	LRKGTVSSQG
490	500	510	520	530	540	550	560
TEVVFASASV	TPPRTPDSAP	PSPAEAYPIT	PASVSARPPV	AFPRRETSCA	ARAPETASAP	LSMDDPSPCG	TSEMCPAALY
570	580	590	600	610	620	630	640
GFPSTGTSPP	RPPANSTGTV	QHLRSDSFPG	SHRTEQTPDL	VGMLLSYSHS	ELPQRPPKPA	IYSSVTPRRD	RRSGRDYSTV
650	660	670	680	690	700	710	720
SASPTALSTL	KQDSQESISN	LERPSSPPSI	QPWVSPHNPA	FATESPAYGS	SPSFVSMEDV	RIHEPLPPPP	PQRRDTHPSV
730	740	750	760	770	780	790	800
VETDGHARVV	VPTLKQHSHP	PPLALGSGLH	APHKGPLPQA	SDPAVARQHR	PLPSTPDSSH	HAQATPRWRY	NKPLPPTPDL
810	820	830	840	850	860	870	880
PQPHLPPISA	PGSSRIYRPL	PPLPIIDPPT	EPPPLPPKSR	GRSRSTRGGH	MNSGGHAKTR	PACQDWTVPL	PASAGRTSWP
890	900	910	920	930	940	950	960
PATARSTESF	TSTSRSKSEV	SPGMAFSNMT	NFLCPSSPTT	PWTPELQGPT	SKDEAGVSEH	PEAPAREPLR	RTTPQQGASG
970	980	990	1000	1010	1020	1030	1040
PGRSPVGQAR	QPEKPSHLHL	EKASSWPHRR	DSGRPPGDSS	GQAVAPSEGA	NKHKGWSRQG	LRRPSILPEG	SSDSRGPAVE
1050	1060	1070	1080	1090	1100	1110	1120
KHPGPSDTVV	FREKKPKEVM	GGFSRRCSKL	INSSQLLYQE	YSDVVLNKEI	QSQQRLESLS	ETPGPSSPRQ	PRKALVSSES
1130	1140	1150	1160	1170	1180	1190	1200
YLQRLSMASS	GSLWQEIPVV	RNSTVLLSMT	HEDQKLQEVK	FELIVSEASY	LRSLNIAVDH	FQLSTSLRAT	LSNQEHQWLF
1210	1220	1230	1240	1250	1260	1270	1280
SRLQDVRDVS	ATFLSDLEEN	FENNIFSFQV	CDVVLNHAPD	FRRVYLPYVT	NQTYQERTFQ	SLMNSNSNFR	EVLEKLESDP
1290	1300	1310	1320	1330	1340	1350	1360
VCQRLSLKSF	LILPFQRITR	LKLLLQNILK	RTQPGSSEEA	EATKAHHALE	QLIRDCNNNV	QSMRRTEELI	YLSQKIEFEC
1370	1380	1390	1400	1410	1420	1430	1440
KIFPLISQSR	WLVKSGELTA	LEFSASPGLR	RKLNTRPVHL	HLFNDCLLLS	RPREGSRFLV	FDHAPFSSIR	GEKCEMKLHG
1450	1460	1470	1480	1490	1500		
PHKNLFRLFL	RQNTQGAQAE	FLFRTETQSE	KLRWISALAM	PREELDLLEC	YTM		

Cmpd.	No. of Cmpds.	m/z meas.	Δ m/z [ppm]	z	Rt [min]	Score	P	Range	Sequence	Modification
1626	1	1058.4994	-29.00	2	49.0	12.0	2	933-951	K.DEAGVSEHPEAPAREPLRR.T	

Detailed Protein Report

Protein 128: retinoic acid-induced protein 1 [Homo sapiens]

**Accession:**	gi|40807477	**Score:**	34.5
**Database:**	refseq_human(refseq_human_20140103.fasta)	**MW [kDa]:**	203.2
**Database Date:**	2015-11-30	**pI:**	9.9
		**Sequence Coverage [%]:**	0.8
		**No. of unique Peptides:**	1

Quantitation

WD:WU	**Median:** 0.84	**CV:** 0.00 %	**No. of Peptides:** 1

Detailed Protein Report

10	20	30	40	50	60	70	80
MQSFRERCGF	HGKQQNYQQT	SQETSRLENY	RQPSQAGLSC	DRQRLLAKDY	YNPQPYPSYE	GGAGTPSGTA	AAVAADKYHR
90	100	110	120	130	140	150	160
GSKALPTQQG	LQGRPAFPGY	GVQDSSPYPG	RYAGEESLQA	WGAPQPPPPQ	PQPLPAGVAK	YDENLMKKTA	VPPSRQYAEQ
170	180	190	200	210	220	230	240
GAQVPFRTHS	LHVQQPPPPQ	QPLAYPKLQR	QKLQNDIASP	LPFPQGTHFP	QHSQSFPTSS	TYSSSVQGGG	QGAHSYKSCT
250	260	270	280	290	300	310	320
APTAQPHDRP	LTASSSLAPG	QRVQNLHAYQ	SGRLSYDQQQ	QQQQQQQQQQ	QALQSRHHAQ	ETLHYQNLAK	YQHYGQQGQG
330	340	350	360	370	380	390	400
YCQPDAAVRT	PEQYYQTFSP	SSSHSPARSV	GRSPSYSSTP	SPLMPNLENF	PYSQQPLSTG	AFPAGITDHS	HFMPLLNPSP
410	420	430	440	450	460	470	480
TDATSSVDTQ	AGNCKPLQKD	KLPENLLSDL	SLQSLTALTS	QVENISNTVQ	QLLLSKAAVP	QKKGVKNLVS	RTPEQHKSQH
490	500	510	520	530	540	550	560
CSPEGSGYSA	EPAGTPLSEP	PSSTPQSTHA	EPQEADYLSG	SEDPLERSFL	YCNQARGSPA	RVNSNSKAKP	ESVSTCSVTS
570	580	590	600	610	620	630	640
PDDMSTKSDD	SFQSLHGSLP	LDSFSKFVAG	ERDCPRLLLS	ALAQEDLASE	ILGLQEAIGE	KADKAWAEAP	SLVKDSSKPP
650	660	670	680	690	700	710	720
FSLENHSACL	DSVAKSAWPR	PGEPEALPDS	LQLDKGGNAK	DFSPGLFEDP	SVAFATPDPK	KTTGPLSFGT	KPTLGVPAPD
730	740	750	760	770	780	790	800
PTTAAFDCFP	DTTAASSADS	ANPFAWPEEN	LGDACPRWGL	HPGELTKGLE	QGGKASDGIS	KGDTHEASAC	LGFQEEDPPG
810	820	830	840	850	860	870	880
EKVASLPGDF	KQEEVGGVKE	EAGGLLQCPE	VAKADRWLED	SRHCCSTADF	GDLPLLPPTS	RKEDLEAEEE	YSSLCELLGS
890	900	910	920	930	940	950	960
PEQRPGMQDP	LSPKAPLICT	KEEVEEVLDS	KAGWGSPCHL	SGESVILLGP	TVGTESKVQS	WFESSLSHMK	PGEEGPDGER
970	980	990	1000	1010	1020	1030	1040
APGDSTTSDA	SLAQKPNKPA	VPEAPIAKKE	PVPRGKSLRS	RRVHRGLPEA	EDSPCRAPVL	PKDLLLPESC	TGPPQGQMEG
1050	1060	1070	1080	1090	1100	1110	1120
AGAPGRGASE	GLPRMCTRSL	TALSEPRTPG	PPGLTTTPAP	PDKLGGKQRA	AFKSGKRVGK	PSPKAASSPS	NPAALPVASD
1130	1140	1150	1160	1170	1180	1190	1200
SSPMGSKTKE	TDSPSTPGKD	QRSMILRSRT	KTQEIFHSKR	RRPSEGRLPN	CRATKKLLDN	SHLPATFKVS	SSPQKEGRVS
1210	1220	1230	1240	1250	1260	1270	1280
QRARVPKPGA	GSKLSDRPLH	ALKRKSAFMA	PVPTKKRNLV	LRSRSSSSSN	ASGNGGDGKE	ERPEGSPTLF	KRMSSPKKAK
1290	1300	1310	1320	1330	1340	1350	1360
PTKGNGEPAT	KLPPPETPDA	CLKLASRAAF	QGAMKTKVLP	PRKGRGLKLE	AIVQKITSPS	LKKFACKAPG	ASPGNPLSPS
1370	1380	1390	1400	1410	1420	1430	1440
LSDKDRGLKG	AGGSPVGVEE	GLVNVGTGQK	LPTSGADPLC	RNPTNRSLKG	KLMNSKKLSS	TDCFKTEAFT	SPEALQPGGT
1450	1460	1470	1480	1490	1500	1510	1520
ALAPKKRSRK	GRAGAHGLSK	GPLEKRPYLG	PALLLTPRDR	ASGTQGASED	NSGGGGKKPK	MEELGLASQP	PEGRPCQPQT
1530	1540	1550	1560	1570	1580	1590	1600
RAQKQPGHTN	YSSYSKRKRL	TRGRAKNTTS	SPCKGRAKRR	RQQQVLPLDP	AEPEIRLKYI	SSCKRLRSDS	RTPAFSPFVR
1610	1620	1630	1640	1650	1660	1670	1680
VEKRDAFTTI	CTVVNSPGDA	PKPHRKPSSS	ASSSSSSSSF	SLDAAGASLA	TLPGGSILQP	RPSLPLSSTM	HLGPVVSKAL
1690	1700	1710	1720	1730	1740	1750	1760
STSCLVCCLC	QNPANFKDLG	DLCGPYYPEH	CLPKKKPKLK	EKVRPEGTCE	EASLPLERTL	KGPECAAAAT	AGKPPRPDGP
1770	1780	1790	1800	1810	1820	1830	1840
ADPAKQGPLR	TSARGLSRRL	QSCYCCDGRE	DGGEEAAPAD	KGRKHECSKE	APAEPGGEAQ	EHWVHEACAV	WTGGVYLVAG
1850	1860	1870	1880	1890	1900	1910	
KLFGLQEAMK	VAVDMMCSSC	QEAGATIGCC	HKGCLHTYHY	PCASDAGCIF	IEENFSLKCP	KHKRLP	

Cmpd.	No. of Cmpds.	m/z meas.	Δ m/z [ppm]	z	Rt [min]	Score	P	Range	Sequence	Modification	Ratios
95	1	510.4790	-193.63	3	30.4	18.7	2	1790-1804	R.EDGGEEAAPADKGRK.H		WD:WU 0.84

Detailed Protein Report

Protein 129: tyrosine-protein kinase Fgr [Homo sapiens]

**Accession:**	gi|4885235	**Score:**	34.2
**Database:**	refseq_human(refseq_human_20140103.fasta)	**MW [kDa]:**	59.4
**Database Date:**	2015-11-30	**pI:**	5.3
**Modification(s):**	Carbamidomethyl, Oxidation	**Sequence Coverage [%]:**	6.6
		**No. of unique Peptides:**	3

Alias proteins:

Accession	Name	Description
gi|578798632	refseq_human (refseq_human_20140103.fasta)	PREDICTED: tyrosine-protein kinase Fgr isoform X1 [Homo sapiens]
gi|112382244	refseq_human (refseq_human_20140103.fasta)	tyrosine-protein kinase Fgr [Homo sapiens]
gi|112382241	refseq_human (refseq_human_20140103.fasta)	tyrosine-protein kinase Fgr [Homo sapiens]

```
        10         20         30         40         50         60         70         80
MGCVFCKKLE PVATAKEDAG LEGDFRSYGA ADHYGPDPTK ARPASSFAHI PNYSNFSSQA INPGFLDSGT IRGVSGIGVT
        90        100        110        120        130        140        150        160
LFIALYDYEA RTEDDLTFTK GEKFHILNNT EGDWWEARSL SSGKTGCIPS NYVAPVDSIQ AEEWYFGKIG RKDAERQLLS
       170        180        190        200        210        220        230        240
PGNPQGAFLI RESETTKGAY SLSIRDWDQT RGDHVKHYKI RKLDMGGYYI TTRVQFNSVQ ELVQHYMEVN DGLCNLLIAP
       250        260        270        280        290        300        310        320
CTIMKPQTLG LAKDAWEISR SSITLERRLG TGCFGDVWLG TWNGSTKVAV KTLKPGTMSP KAFLEEAQVM KLLRHDKLVQ
       330        340        350        360        370        380        390        400
LYAVVSEEPI YIVTEFMCHG SLLDFLKNPE GQDLRLPQLV DMAAQVAEGM AYMERMNYIH RDLRAANILV GERLACKIAD
       410        420        430        440        450        460        470        480
FGLARLIKDD EYNPCQGSKF PIKWTAPEAA LFGRFTIKSD VWSFGILLTE LITKGRIPYP GMNKREVLEQ VEQGYHMPCP
       490        500        510        520        530
PGCPASLYEA MEQTWRLDPE ERPTFEYLQS FLEDYFTSAE PQYQPGDQT
```

Cmpd.	No. of Cmpds.	m/z meas.	Δ m/z [ppm]	z	Rt [min]	Score	P	Range	Sequence	Modification
1840	1	899.4842	30.62	2	51.7	11.7	2	1-16	-.MGCVFCKKLEPVATAK.E	Carbamidomethyl: 3; Oxidation: 1
2000	2	899.3791	-86.20	2	53.4	11.8	2	1-16	-.MGCVFCKKLEPVATAK.E	Carbamidomethyl: 6; Oxidation: 1
1458	1	1022.6515	107.92	2	46.6	10.7	0	73-91	R.GVSGIGVTLFIALYDYEAR.T	

Detailed Protein Report

Protein 130: PREDICTED: exostosin-like 1 isoform X3 [Homo sapiens]

**Accession:**	gi|578798624	**Score:**	34.1
**Database:**	refseq_human(refseq_human_20140103.fasta)	**MW [kDa]:**	53.0
**Database Date:**	2015-11-30	**pI:**	9.6
		**Sequence Coverage [%]:**	6.0
		**No. of unique Peptides:**	2

Quantitation

WD:WU **Median:** 0.62 **CV:** 0.00 % **No. of Peptides:** 1

10	20	30	40	50	60	70	80
MQSWRRRKSL	WLALSASWLL	LVLLGGFSLL	RLALPPRPRP	GASQGWPRWL	DAELLQSFSQ	PGELPEDAVS	PPQAPHGGSC
**90**	**100**	**110**	**120**	**130**	**140**	**150**	**160**
NWESCFDTSK	CRGDGLKVFV	YPAVGTISET	HRRILASIEG	SRFYTFSPAG	ACLLLLLSLD	AQTGECSSMP	LQWNRGRNHL
**170**	**180**	**190**	**200**	**210**	**220**	**230**	**240**
VLRLHPAPCP	RTFQLGQAMV	AEASPTVDSF	RPGFDVALPF	LPEAHPLRGG	APGQLRQHSP	QPGVALLALE	EERGGWRTAD
**250**	**260**	**270**	**280**	**290**	**300**	**310**	**320**
TGSSACPWDG	RCEQDPGPGQ	TQRQETLPNA	TFCLISGHRP	EAASRFLQAL	QAGCIPVLLS	PRWELPFSEV	IDWTKAAIVA
**330**	**340**	**350**	**360**	**370**	**380**	**390**	**400**
DERLPLQVLA	ALQEMSPARV	LALRQQTQFL	WDAYFSSVEK	VIHTTLEVIQ	DRIFGTSAHP	SLLWNSPPGA	LLALSTFSTS
**410**	**420**	**430**	**440**	**450**	**460**	**470**	**480**
PQDFPFYYLQ	QGSRPEGRFS	ALIWVGPPGQ	PPLKLIQAVA	GSQHCAQFTK	LTSQTSSTLR	KITPAPRNPP	SAPDPRLQEQ
**490**							
PPIL							

Cmpd.	No. of Cmpds.	m/z meas.	Δ m/z [ppm]	z	Rt [min]	Score	P	Range	Sequence	Modification	Ratios
2276	1	937.4372	-58.45	2	56.9	14.5	0	217-233	R.QHSPQPGVALLALEEER.G		
933	1	712.4616	88.10	2	40.0	19.7	0	361-372	K.VIHTTLEVIQDR.I		WD:WU 0.62

Detailed Protein Report

Protein 131: kinesin light chain 4 isoform d [Homo sapiens]

**Accession:**	gi|573459761	**Score:**	34.1
**Database:**	refseq_human(refseq_human_20140103.fasta)	**MW [kDa]:**	59.6
**Database Date:**	2015-11-30	**pI:**	5.8
		**Sequence Coverage [%]:**	8.5
		**No. of unique Peptides:**	2

10	20	30	40	50	60	70	80
MSGLVLGQRD	EPAGHRLSQE	EILGSTRLVS	QGLEALRSEH	QAVLQSLSQT	IECLQQGGHE	EGLVHEKARQ	LRRSMENIEL
**90**	**100**	**110**	**120**	**130**	**140**	**150**	**160**
GLSEAQEEKE	GDATKDSLDD	LFPNEEEEDP	SNGLSRGQGA	TAAQQGGYEI	PARLRTLHNL	VIQYAAQGRY	EVAVPLCKQA
**170**	**180**	**190**	**200**	**210**	**220**	**230**	**240**
LEDLERTSGR	GHPDVATMLN	ILALVYRDQN	KYKEAAHLLN	DALSIRESTL	GPDHPAVAAT	LNNLAVLYGK	RGKYKEAEPL
**250**	**260**	**270**	**280**	**290**	**300**	**310**	**320**
CQRALEIREK	VLGTNHPDVA	KQLNNLALLC	QNQGKYEAVE	RYYQRALAIY	EGQLGPDNPN	VARTKNNLAS	CYLKQGKYAE
**330**	**340**	**350**	**360**	**370**	**380**	**390**	**400**
AETLYKEILT	RAHVQEFGSV	DDDHKPIWMH	AEEREEMSKS	RHHEGGTPYA	EYGGWYKACK	VSSPTVNTTL	RNLGALYRRQ
**410**	**420**	**430**	**440**	**450**	**460**	**470**	**480**
GKLEAAETLE	ECALRSRRQG	TDPISQTKVA	ELLGESDGRR	TSQEGPGDSV	KFEGGEDASV	AVEWSGDGSG	TLQRSGSLGK
**490**	**500**	**510**	**520**	**530**	**540**	**550**	
IRDVLRRSSE	LLVRKLQGTE	PRPSSSNMKR	AASLNYLNQP	SAAPLQVSRG	LSASTMDLSS	SS	

Cmpd.	No. of Cmpds.	m/z meas.	Δ m/z [ppm]	z	Rt [min]	Score	P	Range	Sequence	Modification
1691	1	1293.5862	-85.42	2	49.8	17.1	1	136-158	R.TLHNLVIQYAAQGRYEVAVPLCK.Q	
2084	1	900.3300	-81.09	3	54.4	16.9	2	355-377	R.EEMSKSRHHEGGTPYAEYGGWYK.A	

Detailed Protein Report

Protein 132: **PREDICTED: TFIIH basal transcription factor complex helicase XPB subunit isoform X1 [Homo sapiens]**

**Accession:**	gi|530369093	**Score:**	34.1
**Database:**	refseq_human(refseq_human_20140103.fasta)	**MW [kDa]:**	81.9
**Database Date:**	2015-11-30	**pI:**	8.9
		**Sequence Coverage [%]:**	4.7
		**No. of unique Peptides:**	1

10	20	30	40	50	60	70	80
MPLKDDHTSR	PLWVAPDGHI	FLEAFSPVYK	YAQDFLVAIA	EPVCRPTHVH	EYKLTAYSLY	AAVSVGLQTS	DITEYLRKLS
**90**	**100**	**110**	**120**	**130**	**140**	**150**	**160**
KTGVPDGIMQ	FIKLCTVSYG	KVKLVLKHNR	YFVESCHPDV	IQHLLQDPVI	RECRLRNSEG	EATELITETF	TSKSAISKTA
**170**	**180**	**190**	**200**	**210**	**220**	**230**	**240**
ESSGGPSTSR	VTDPQGKSDI	PMDLFDFYEQ	MDKDEEEEEE	TQTVSFEVKQ	EMIEELQKRC	IHLEYPLLAE	YDFRNDSVNP
**250**	**260**	**270**	**280**	**290**	**300**	**310**	**320**
DINIDLKPTA	VLRPYQEKSL	RKMFGNGRAR	SGVIVLPCGA	GKSLVGVTAA	CTVRKRCLVL	GNSAVSVEQW	KAQFKMWSTI
**330**	**340**	**350**	**360**	**370**	**380**	**390**	**400**
DDSQICRFTS	DAKDKPIGCS	VAISTYSMLG	HTTKRSWEAE	RVMEWLKTQE	WGLMILDEVH	TIPAKMFRRV	LTIVQAHCKL
**410**	**420**	**430**	**440**	**450**	**460**	**470**	**480**
GLTATLVRED	DKIVDLNFLI	GPKLYEANWM	ELQNNGYIAK	VQCAEVWCPM	SPEFYREYVA	IKTKKRILLY	TMNPNKFRAC
**490**	**500**	**510**	**520**	**530**	**540**	**550**	**560**
QFLIKFHERR	NDKIIVFADN	VFALKEYAIR	LNKPYIYGPT	SQGERMQILQ	NFKHNPKINT	IFISKVGDTS	FDLPEANVLI
**570**	**580**	**590**	**600**	**610**	**620**	**630**	**640**
QISSHGGSRR	QEAQRLGRVL	RAKKGMVAEE	YNAFFYSLVS	QDTQEMAYST	KRQRFLVDQG	YSFKVITKLA	GMEEEDLAFS
**650**	**660**	**670**	**680**	**690**	**700**	**710**	**720**
TKEEQQQLLQ	KVLAATDLDA	EEEVVAGEFG	SRSSQASRRF	GTMSSMSGAD	DTVYMEYHSS	RSKAPSKHVH	PLFKRFRK

Cmpd.	No. of Cmpds.	m/z meas.	Δ m/z [ppm]	z	Rt [min]	Score	P	Range	Sequence	Modification
141	2	822.3919	-53.97	2	31.0	11.9	2	612-624	K.RQRFLVDQGYSFK.V	

Detailed Protein Report

Protein 133: PREDICTED: complement receptor type 1 isoform X4 [Homo sapiens]

**Accession:**	gi|578800553	**Score:**	34.0
**Database:**	refseq_human(refseq_human_20140103.fasta)	**MW [kDa]:**	224.1
**Database Date:**	2015-11-30	**pI:**	6.7
**Modification(s):**	Carbamidomethyl	**Sequence Coverage [%]:**	1.7
		**No. of unique Peptides:**	1

Quantitation

WD:WU	**Median:** 0.66	**CV:** 0.00 %	**No. of Peptides:** 1

10	20	30	40	50	60	70	80
MCLGRMGASS	PRSPEPVGPP	APGLPFCCGG	SLLAVVVLLA	LPVAWGQCNA	PEWLPFARPT	NLTDEFEFPI	GTYLNYECRP
90	100	110	120	130	140	150	160
GYSGRPFSII	CLKNSVWTGA	KDRCRRKSCR	NPPDPVNGMV	HVIKGIQFGS	QIKYSCTKGY	RLIGSSSATC	IISGDTVIWD
170	180	190	200	210	220	230	240
NETPICDRIP	CGLPPTITNG	DFISTNRENF	HYGSVVTYRC	NPGSGGRKVF	ELVGEPSIYC	TSNDDQVGIW	SGPAPQCIIP
250	260	270	280	290	300	310	320
NKCTPPNVEN	GILVSDNRSL	FSLNEVVEFR	CQPGFVMKGP	RRVKCQALNK	WEPELPSCSR	VCQPPPDVLH	AERTQRDKDN
330	340	350	360	370	380	390	400
FSPGQEVFYS	CEPGYDLRGA	ASMRCTPQGD	WSPAAPTCEV	KSCDDFMGQL	LNGRVLFPVN	LQLGAKVDFV	CDEGFQLKGS
410	420	430	440	450	460	470	480
SASYCVLAGM	ESLWNSSVPV	CEQIFCPSPP	VIPNGRHTGK	PLEVFPFGKT	VNYTCDPHPD	RGTSFDLIGE	STIRCTSDPQ
490	500	510	520	530	540	550	560
GNGVWSSPAP	RCGILGHCQA	PDHFLFAKLK	TQTNASDFPI	GTSLKYECRP	EYYGRPFSIT	CLDNLVWSSP	KDVCKRKSCK
570	580	590	600	610	620	630	640
TPPDPVNGMV	HVITDIQVGS	RINYSCTTGH	RLIGHSSAEC	ILSGNAAHWS	TKPPICQRIP	CGLPPTIANG	DFISTNRENF
650	660	670	680	690	700	710	720
HYGSVVTYRC	NPGSGGRKVF	ELVGEPSIYC	TSNDDQVGIW	SGPAPQCIIP	NKCTPPNVEN	GILVSDNRSL	FSLNEVVEFR
730	740	750	760	770	780	790	800
CQPGFVMKGP	RRVKCQALNK	WEPELPSCSR	VCQPPPDVLH	AERTQRDKDN	FSPGQEVFYS	CEPGYDLRGA	ASMRCTPQGD
810	820	830	840	850	860	870	880
WSPAAPTCEV	KSCDDFMGQL	LNGRVLFPVN	LQLGAKVDFV	CDEGFQLKGS	SASYCVLAGM	ESLWNSSVPV	CEQIFCPSPP
890	900	910	920	930	940	950	960
VIPNGRHTGK	PLEVFPFGKT	VNYTCDPHPD	RGTSFDLIGE	STIRCTSDPQ	GNGVWSSPAP	RCGILGHCQA	PDHFLFAKLK
970	980	990	1000	1010	1020	1030	1040
TQTNASDFPI	GTSLKYECRP	EYYGRPFSIT	CLDNLVWSSP	KDVCKRKSCK	TPPDPVNGMV	HVITDIQVGS	RINYSCTTGH
1050	1060	1070	1080	1090	1100	1110	1120
RLIGHSSAEC	ILSGNAAHWS	TKPPICQRIP	CGLPPTIANG	DFISTNRENF	HYGSVVTYRC	NPGSGGRKVF	ELVGEPSIYC
1130	1140	1150	1160	1170	1180	1190	1200
TSNDDQVGIW	SGPAPQCIIP	NKCTPPNVEN	GILVSDNRSL	FSLNEVVEFR	CQPGFVMKGP	RRVKCQALNK	WEPELPSCSR
1210	1220	1230	1240	1250	1260	1270	1280
VCQPPPDVLH	AERTQRDKDN	FSPGQEVFYS	CEPGYDLRGA	ASLHCTPQGD	WSPEAPRCAV	KSCDDFLGQL	PHGRVLFPLN
1290	1300	1310	1320	1330	1340	1350	1360
LQLGAKVSFV	CDEGFRLKGS	SVSHCVLVGM	RSLWNNSVPV	CEHIFCPNPP	AILNGRHTGT	PSGDIPYGKE	ISYTCDPHPD
1370	1380	1390	1400	1410	1420	1430	1440
RGMTFNLIGE	STIRCTSDPH	GNGVWSSPAP	RCELSVRAGH	CKTPEQFPFA	SPTIPINDFE	FPVGTSLNYE	CRPGYFGKMF
1450	1460	1470	1480	1490	1500	1510	1520
SISCLENLVW	SSVEDNCRRK	SCGPPPEPFN	GMVHINTDTQ	FGSTVNYSCN	EGFRLIGSPS	TTCLVSGNNV	TWDKKAPICE
1530	1540	1550	1560	1570	1580	1590	1600
IISCEPPPTI	SNGDFYSNNR	TSFHNGTVVT	YQCHTGPDGE	QLFELVGERS	IYCTSKDDQV	GVWSSPPPRC	ISTNKCTAPE
1610	1620	1630	1640	1650	1660	1670	1680
VENAIRVPGN	RSFFTLTEII	RFRCQPGFVM	VGSHTVQCQT	NGRWGPKLPH	CSRVCQPPPE	ILHGEHTLSH	QDNFSPGQEV
1690	1700	1710	1720	1730	1740	1750	1760
FYSCEPSYDL	RGAASLHCTP	QGDWSPEAPR	CTVKSCDDFL	GQLPHGRVLL	PLNLQLGAKV	SFVCDEGFRL	KGRSASHCVL
1770	1780	1790	1800	1810	1820	1830	1840
AGMKALWNSS	VPVCEQIFCP	NPPAILNGRH	TGTPFGDIPY	GKEISYACDT	HPDRGMTFNL	IGESSIRCTS	DPQGNGVWSS
1850	1860	1870	1880	1890	1900	1910	1920
PAPRCELSVP	AACPHPPKIQ	NGHYIGGHVS	LYLPGMTISY	ICDPGYLLVG	KGFIFCTDQG	IWSQLDHYCK	EVNCSFPLFM
1930	1940	1950	1960	1970	1980	1990	2000
NGISKELEMK	KVYHYGDYVT	LKCEDGYTLE	GSPWSQCQAD	DRWDPPLAKC	TSRTHDALIV	GTLSGTIFFI	LLIIFLSWII
2010	2020	2030	2040	2050			
LKHRKGNNAH	ENPKEVAIHL	HSQGGSSVHP	RTLQTNEENS	RVLP			

Cmpd.	No. of Cmpds.	m/z meas.	Δ m/z [ppm]	z	Rt [min]	Score	P	Range	Sequence	Modification	Ratios	
39	2	826.3468	-30.22	3	29.4	21.7	1	339-361	R.GAASMRCTPQGDWSPAAPTCEV S	Carbamidomethyl: 7, 20	WD:WU	0.66

Detailed Protein Report

Protein 134: PREDICTED: histone-lysine N-methyltransferase ASH1L isoform X3 [Homo sapiens]

Accession: gi|530365072
Database: refseq_human(refseq_human_20140103.fasta)
Database Date: 2015-11-30
Modification(s): Carbamidomethyl

Score: 33.7
MW [kDa]: 191.3
pI: 10.4
Sequence Coverage [%]: 2.9
No. of unique Peptides: 2

10	20	30	40	50	60	70	80
MDPRNTAMLG	LGSDSEGFSR	KSPSAISTGT	LVSKREVELE	KNTKEEEDLR	KRNRERNIEA	GKDDGLTDAQ	QQFSVKETNF
90	100	110	120	130	140	150	160
SEGNLKLKIG	LQAKRTKKPP	KNLENYVCRP	AIKTTIKHPR	KALKSGKMTD	EKNEHCPSKR	DPSKLYKKAD	DVAAIECQSE
170	180	190	200	210	220	230	240
EVIRLHSQGE	NNPLSKKLSP	VHSEMADYIN	ATPSTLLGSR	DPDLKDRALL	NGGTSVTEKL	AQLIATCPPS	KSSKTKPKKL
250	260	270	280	290	300	310	320
GTGTTAGLVS	KDLIRKAGVG	SVAGIIHKDL	IKKPTISTAV	GLVTKDPGKK	PVFNAAVGLV	NKDSVKKLGT	GTTAVFINKN
330	340	350	360	370	380	390	400
LGKKPGTITT	VGLLSKDSGK	KLGIGIVPGL	VHKESGKKLG	LGTVVGLVNK	DLGKKLGSTV	GLVAKDCAKK	IVASSAMGLV
410	420	430	440	450	460	470	480
NKDIGKKLMS	CPLAGLISKD	AINLKAEALL	PTQEPLKASC	STNINNQESQ	ELSESLKDSA	TSKTFEKNVV	RQNKESILEK
490	500	510	520	530	540	550	560
FSVRKEIINL	EKEMFNEGTC	IQQDSFSSSE	KGSYETSKHE	KQPPVYCTSP	DFKMGGASDV	STAKSPFSAV	GESNLPSPSP
570	580	590	600	610	620	630	640
TVSVNPLTRS	PPETSSQLAP	NPLLLSSTTE	LIEEISESVG	KNQFTSESTH	LNVGHRSVGH	SISIECKGID	KEVNDSKTTH
650	660	670	680	690	700	710	720
IDIPRISSSL	GKKPSLTSES	SIHTITPSVV	NFTSLFSNKP	FLKLGAVSAS	DKHCQVAESL	STSLQSKPLK	KRKGRKPRWT
730	740	750	760	770	780	790	800
KVVARSTCRS	PKGLELERSE	LFKNVSCSSL	SNSNSEPAKF	MKNIGPPSFV	DHDFLKRRLP	KLSKSTAPSL	ALLADSEKPS
810	820	830	840	850	860	870	880
HKSFATHKLS	SSMCVSSDLL	SDIYKPKRGR	PKSKEMPQLE	GPPKRTLKIP	ASKVFSLQSK	EEQEPPILQP	EIEIPSFKQG
890	900	910	920	930	940	950	960
LSVSPFPKKR	GRPKRQMRSP	VKMKPPVLSV	APFVATESPS	KLESESDNHR	SSSDFFESED	QLQDPDDLDD	SHRPSVCSMS
970	980	990	1000	1010	1020	1030	1040
DLEMEPDKKI	TKRNNGQLMK	TIIRKINKMK	TLKRKKLLNQ	ILSSSVESSN	KGKVQSKLHN	TVSSLAATFG	SKLGQQINVS
1050	1060	1070	1080	1090	1100	1110	1120
KKGTIYIGKR	RGRKPKTVLN	GILSGSPTSL	AVLEQTAQQA	AGSALGQILP	PLLPSSASSS	EILPSPICSQ	SSGTSGGQSP
1130	1140	1150	1160	1170	1180	1190	1200
VSSDAGFVEP	SSVPYLHLHS	RQGSMIQTLA	MKKASKGRRR	LSPPTLLPNS	PSHLSELTSL	KEATPSPISE	SHSDETIPSD
1210	1220	1230	1240	1250	1260	1270	1280
SGIGTDNNST	SDRAEKFCGQ	KKRRHSFEHV	SLIPPETSTV	LSSLKEKHKH	KCKRRNHDYL	SYDKMKRQKR	KRKKKYPQLR
1290	1300	1310	1320	1330	1340	1350	1360
NRQDPDFIAE	LEELISRLSE	IRITHRSHHF	IPRDLLPTIF	RINFNSFYTH	PSFPLDPLHY	IRKPDLKKKR	GRPPKMREAM
1370	1380	1390	1400	1410	1420	1430	1440
AEMPFMHSLS	FPLSSTGFYP	SYGMPYSPSP	LTAAPIGLGY	YGRYPPTLYP	PPPSPSFTTP	LPPPSYMHAG	HLLLNPAKYH
1450	1460	1470	1480	1490	1500	1510	1520
KKKHKLLRQE	AFLTTSRTPL	LSMSTYPSVP	PEMAYGWMVE	HKHRHRHKHR	EHRSSEQPQV	SMDTGSSRSV	LESLKRYRFG
1530	1540	1550	1560	1570	1580	1590	1600
KDAVGERYKH	KEKHRCHMSC	PHLSPSKSLI	NREEQWVHRE	PSESSPLALG	LQTPLQIDCS	ESSPSLSLGG	FTPNSEPASS
1610	1620	1630	1640	1650	1660	1670	1680
DEHTNLFTSA	IGSCRVSNPN	SSGRKKLTDS	PGLFSAQDTS	LNRLHRKESL	PSNERAVQTL	AGSQPTSDKP	SQRPSESTNC
1690	1700	1710	1720	1730	1740	1750	
SPTRKRSSSE	STSSTDKTED	WYEYLEVTYI	LMIIETMRIN	MFPVGNKRSS	LSKSKISCFW	G	

Cmpd.	No. of Cmpds.	m/z meas.	Δ m/z [ppm]	z	Rt [min]	Score	P	Range	Sequence	Modification
1736	1	853.3885	-153.79	2	50.1	10.2	2	256-272	R.KAGVGSVAGIIHKDLIK.K	
1687	1	696.2420	-115.78	3	49.4	11.7	1	744-762	K.NVSCSSLSNSNSEPAKFMK.N	Carbamidomethyl: 4

Detailed Protein Report

Protein 135: stromal interaction molecule 2 isoform 2 precursor [Homo sapiens]

**Accession:**	gi|281182822	**Score:**	33.7
**Database:**	refseq_human(refseq_human_20140103.fasta)	**MW [kDa]:**	83.9
**Database Date:**	2015-11-30	**pI:**	6.3
**Modification(s):**	Carbamidomethyl, Oxidation	**Sequence Coverage [%]:**	3.8
		**No. of unique Peptides:**	2

10	20	30	40	50	60	70	80
MLVLGLLVAG	AADGCELVPR	HLRGRRATGS	AATAASSPAA	AAGDSPALMT	DPCMSLSPPC	FTEEDRFSLE	ALQTIHKQMD
**90**	**100**	**110**	**120**	**130**	**140**	**150**	**160**
DDKDGGIEVE	ESDEFIREDM	KYKDATNKHS	HLHREDKHIT	IEDLWKRWKT	SEVHNWTLED	TLQWLIEFVE	LPQYEKNFRD
**170**	**180**	**190**	**200**	**210**	**220**	**230**	**240**
NNVKGTTLPR	IAVHEPSFMI	SQLKISDRSH	RQKLQLKALD	VVLFGPLTRP	PHNWMKDFIL	TVSIVIGVGG	CWFAYTQNKT
**250**	**260**	**270**	**280**	**290**	**300**	**310**	**320**
SKEHVAKMMK	DLESLQTAEQ	SLMDLQERLE	KAQEENRNVA	VEKQNLERKM	MDEINYAKEE	ACRLRELREG	AECELSRRQY
**330**	**340**	**350**	**360**	**370**	**380**	**390**	**400**
AEQELEQVRM	ALKKAEKEFE	LRSSWSVPDA	LQKWLQLTHE	VEVQYYNIKR	QNAEMQLAIA	KDEAEKIKKK	RSTVFGTLHV
**410**	**420**	**430**	**440**	**450**	**460**	**470**	**480**
AHSSSLDEVD	HKILEAKKAL	SELTTCLRER	LFRWQQIEKI	CGFQIAHNSG	LPSLTSSLYS	DHSWVVMPRV	SIPPYPIAGG
**490**	**500**	**510**	**520**	**530**	**540**	**550**	**560**
VDDLDEDTPP	IVSQFPGTMA	KPPGSLARSS	SLCRSRRSIV	PSSPQPQRAQ	LAPHAPHPSH	PRHPHHPQHT	PHSLPSPDPD
**570**	**580**	**590**	**600**	**610**	**620**	**630**	**640**
ILSVSSCPAL	YRNEEEEEAI	YFSAEKQWEV	PDTASECDSL	NSSIGRKQSP	PLSLEIYQTL	SPRKISRDEV	SLEDSSRGDS
**650**	**660**	**670**	**680**	**690**	**700**	**710**	**720**
PVTVDVSWGS	PDCVGLTETK	SMIFSPASKV	YNGILEKSCS	MNQLSSGIPV	PKPRHTSCSS	AGNDSKPVQE	APSVARISSI
**730**	**740**	**750**					
PHDLCHNGEK	SKKPSKIKSL	FKKKSK					

Cmpd.	No. of Cmpds.	m/z meas.	Δ m/z [ppm]	z	Rt [min]	Score	P	Range	Sequence	Modification
2262	1	937.3682	-104.97	2	56.7	12.8	0	678-694	K.SCSMNQLSSGIPVPKPR.H	Carbamidomethyl: 2; Oxidation: 4
1467	1	619.9415	-65.11	3	46.7	10.7	0	678-694	K.SCSMNQLSSGIPVPKPR.H	Carbamidomethyl: 2

Detailed Protein Report

Protein 136: PREDICTED: ubiquitin thioesterase ZRANB1 isoform X3 [Homo sapiens]

**Accession:**	gi|530393888	**Score:**	33.7
**Database:**	refseq_human(refseq_human_20140103.fasta)	**MW [kDa]:**	44.2
**Database Date:**	2015-11-30	**pI:**	4.7
		**Sequence Coverage [%]:**	5.5
		**No. of unique Peptides:**	2

Quantitation

WD:WU **Median:** 1.22 **CV:** 41.38 % **No. of Peptides:** 2

10	20	30	40	50	60	70	80
MLAILLTEVS	QQAAKCIPAM	VCPELTEQIR	REIAASLHQR	KGDFACYFLT	DLVTFTLPAD	IEDLPPTVQE	KLFDEVLDRD
**90**	**100**	**110**	**120**	**130**	**140**	**150**	**160**
VQKELEEESP	IINWSLELAT	RLDSRLYALW	NRTAGDCLLD	SVLQATWGIY	DKDSVLRKAL	HDSLHDCSHW	FYTRWKDWES
**170**	**180**	**190**	**200**	**210**	**220**	**230**	**240**
WYSQSFGLHF	SLREEQWQED	WAFILSLASQ	PGASLEQTHI	FVLAHILRRP	IIVYGVKYYK	SFRGETLGYT	RFQGVYLPLL
**250**	**260**	**270**	**280**	**290**	**300**	**310**	**320**
WEQSFCWKSP	IALGYTRGHF	SALVAMENDG	YGNRGAGANL	NTDDDVTITF	LPLVDSERKL	LHVHFLSAQE	LGNEEQQEKL
**330**	**340**	**350**	**360**	**370**	**380**	**390**	
LREWLDCCVT	EGGVLVAMQK	SSRRRNHPLV	TQMVEKWLDR	YRQIRPCTSL	SDGEEDEDDE	DE	

Cmpd.	No. of Cmpds.	m/z meas.	Δ m/z [ppm]	z	Rt [min]	Score	P	Range	Sequence	Modification	Ratios	
1033	2	590.8060	-42.13	2	41.7	16.3	1	31-40	R.REIAASLHQR.K		WD:WU	0.82
2848	4	648.3529	16.16	2	64.3	17.3	0	346-356	R.NHPLVTQMVEK.W		WD:WU	1.81

Detailed Protein Report

Protein 137: replication factor C subunit 1 isoform 1 [Homo sapiens]

**Accession:**	gi|32528306	**Score:**	33.6
**Database:**	refseq_human(refseq_human_20140103.fasta)	**MW [kDa]:**	128.1
**Database Date:**	2015-11-30	**pI:**	9.9
**Modification(s):**	Carbamidomethyl	**Sequence Coverage [%]:**	4.6
		**No. of unique Peptides:**	3

Quantitation

WD:WU **Median:** 1.66 **CV:** 0.00 % **No. of Peptides:** 1

10	20	30	40	50	60	70	80
MDIRKFFGVI	PSGKKLVSET	VKKNEKTKSD	EETLKAKKGI	KEIKVNSSRK	EDDFKQKQPS	KKKRIIYDSD	SESEETLQVK
90	100	110	120	130	140	150	160
NAKKPPEKLP	VSSKPGKISR	QDPVTYISET	DEEDDFMCKK	AASKSKENGR	STNSHLGTSN	MKKNEENTKT	KNKPLSPIKL
170	180	190	200	210	220	230	240
TPTSVLDYFG	TGSVQRSNKK	MVASKRKELS	QNTDESGLND	EAIAKQLQLD	EDAELERQLH	EDEEFARTLA	MLDEEPKTKK
250	260	270	280	290	300	310	320
ARKDTEAGET	FSSVQANLSK	AEKHKYPHKV	KTAQVSDERK	SYSPRKQSKY	ESSKESQQHS	KSSADKIGEV	SSPKASSKLA
330	340	350	360	370	380	390	400
IMKRKEESSY	KEIEPVASKR	KENAIKLKGE	TKTPKKTKSS	PAKKESVSPE	DSEKKRTNYQ	AYRSYLNREG	PKALGSKEIP
410	420	430	440	450	460	470	480
KGAENCLEGL	IFVITGVLES	IERDEAKSLI	ERYGGKVTGN	VSKKTNYLVM	GRDSGQSKSD	KAAALGTKII	DEDGLLNLIR
490	500	510	520	530	540	550	560
TMPGKKSKYE	IAVETEMKKE	SKLERTPQKN	VQGKRKISPS	KKESESKKSR	PTSKRDSLAK	TIKKETDVFW	KSLDFKEQVA
570	580	590	600	610	620	630	640
EETSGDSKAR	NLADDSSENK	VENLLWVDKY	KPTSLKTIIG	QQGDQSCANK	LLRWLRNWQK	SSSEDKKHAK	FGKFSGKDDG
650	660	670	680	690	700	710	720
SSFKAALLSG	PPGVGKTTTA	SLVCQELGYS	YVELNASDTR	SKSSLKAIVA	ESLNNTSIKG	FYSNGAASSV	STKHALIMDE
730	740	750	760	770	780	790	800
VDGMAGNEDR	GGIQELIGLI	KHTKIPIICM	CNDRNHPKIR	SLVHYCFDLR	FQRPRVEQIK	GAMMSIAFKE	GLKIPPPAMN
810	820	830	840	850	860	870	880
EIILGANQDI	RQVLHNLSMW	CARSKALTYD	QAKADSHRAK	KDIKMGPFDV	ARKVFAAGEE	TAHMSLVDKS	DLFFHDYSIA
890	900	910	920	930	940	950	960
PLFVQENYIH	VKPVAAGGDM	KKHLMLLSRA	ADSICDGDLV	DSQIRSKQNW	SLLPAQAIYA	SVLPGELMRG	YMTQFPTFPS
970	980	990	1000	1010	1020	1030	1040
WLGKHSSTGK	HDRIVQDLAL	HMSLRTYSSK	RTVNMDYLSL	LRDALVQPLT	SQGVDGVQDV	VALMDTYYLM	KEDFENIMEI
1050	1060	1070	1080	1090	1100	1110	1120
SSWGGKPSPF	SKLDPKVKAA	FTRAYNKEAH	LTPYSLQAIK	ASRHSTSPSL	DSEYNEELNE	DDSQSDEKDQ	DAIETDAMIK
1130	1140	1150					
KKTKSSKPSK	PEKDKEPRKG	KGKSSKK					

Cmpd.	No. of Cmpds.	m/z meas.	Δ m/z [ppm]	z	Rt [min]	Score	P	Range	Sequence	Modification	Ratios
967	2	530.7788	-57.17	2	40.4	11.1	2	42-50	K.EIKVNSSRK.E		WD:WU 1.66
1884	1	974.4729	4.34	2	51.9	11.6	2	125-142	K.SKENGRSTNSHLGTSNMK.K		
2929	1	965.1417	7.65	3	65.4	10.9	1	657-682	K. TTTASLVCQELGYSYVELNASDTR S	Carbamidomethyl: 8	

Detailed Protein Report

Protein 138: PREDICTED: extracellular matrix protein FRAS1 isoform X2 [Homo sapiens]

**Accession:**	gi|578809381	**Score:**	33.5
**Database:**	refseq_human(refseq_human_20140103.fasta)	**MW [kDa]:**	438.5
**Database Date:**	2015-11-30	**pI:**	5.2
**Modification(s):**	Carbamidomethyl	**Sequence Coverage [%]:**	1.1
		**No. of unique Peptides:**	2

Detailed Protein Report

10	20	30	40	50	60	70	80
MGVLKVWLGL	ALALAEFAVL	PHHSEGACVY	QDSLLADATI	WKPDSCQSCR	CHGDIVICKP	AVCRNPQCAF	EKGEVLQIAA
90	100	110	120	130	140	150	160
NQCCPECVLR	TPGSCHHEKK	IHEHGTEWAS	SPCSVCSCNH	GEVRCTPQPC	PPLSCGHQEL	AFIPEGSCCP	VCVGLGKPCS
170	180	190	200	210	220	230	240
YEGHVFQDGE	DWRLSRCAKC	LCRNGVAQCF	TAQCQPLFCN	QDETVVRVPG	KCCPQCSARS	CSAAGQVYEH	GEQWSENACT
250	260	270	280	290	300	310	320
TCICDRGEVR	CHKQACLPLR	CGKGQSRARR	HGQCCEECVS	PAGSCSYDGV	VRYQDEMWKG	SACEFCMCDH	GQVTCQTGEC
330	340	350	360	370	380	390	400
AKVECARDEE	LIHLDGKCCP	ECISRNGYCV	YEETGEFMSS	NASEVKRIPE	GEKWEDGPCK	VCECRGAQVT	CYEPSCPPCP
410	420	430	440	450	460	470	480
VGTLALEVKG	QCCPDCTSVH	CHPDCLTCSQ	SPDHCDLCQD	PTKLLQNGWC	VHSCGLGFYQ	AGSLCLACQP	QCSTCTSGLE
490	500	510	520	530	540	550	560
CSSCQPPLLM	RHGQCVPTCG	DGFYQDRHSC	AVCHESCAGC	WGPTEKHCLA	CRDPLHVLRD	GGCESSCGKG	FYNRQGTCSA
570	580	590	600	610	620	630	640
CDQSCDSCGP	SSPRCLTCTE	KTVLHDGKCM	SECPGGYYAD	ATGRCKACHS	SCLACMGPAP	SHCTGCKKPE	EGLQVEQLSD
650	660	670	680	690	700	710	720
VGIPSGECLA	QCRAHFYLES	TGICEACHQS	CFRCAGKSPH	NCTDCGPSHV	LLDGQCLSQC	PDGYFHQEGS	CTECHPTCRQ
730	740	750	760	770	780	790	800
CHGPLESDCI	SCYPHISLTN	GNCRTSCREE	QFLNLVGYCA	DCHHLCQHCA	ADLHNTGSIC	LRCQNAHYLL	LGDHCVPDCP
810	820	830	840	850	860	870	880
SGYYAERGAC	KKCHSSCRTC	QGRGPFSCSS	CDTNLVLSHT	GTCSTTCFPG	HYLDDNHVCQ	PCNTHCGSCD	SQASCTSCRD
890	900	910	920	930	940	950	960
PNKVLLFGEC	QYESCAPQYY	LDFSTNTCKE	CDWSCSACSG	PLKTDCLQCM	DGYVLQDGAC	VEQCLSSFYQ	DSGLCKNCDS
970	980	990	1000	1010	1020	1030	1040
YCLQCQGPHE	CTRCKGPFLL	LEAQCVQECG	KGYFADHAKH	KCTACPQGCL	QCSHRDRCHL	CDHGFFLKSG	LCVYNCVPGF
1050	1060	1070	1080	1090	1100	1110	1120
SVHTSNETCS	GKIHTPSLHV	NGSLILPIGS	IKPLDFSLLN	VQDQEGRVED	LLFHVVSTPT	NGQLVLSRNG	KEVQLDKAGR
1130	1140	1150	1160	1170	1180	1190	1200
FSWKDVNEKK	VRFVHSKEKL	RKGYLFLKIS	DQQFFSEPQL	INIQAFSTQA	PYVLRNEVLH	ISRGERATIT	TQMLDIRDDD
1210	1220	1230	1240	1250	1260	1270	1280
NPQDVVIEII	DPPLHGQLLQ	TLQSPATPIY	QFQLDELSRG	LLHYAHDGSD	STSDVAVLQA	NDGHSFHNIL	FQVKTVPQND
1290	1300	1310	1320	1330	1340	1350	1360
RGLQLVANSM	VWVPEGGMLQ	ITNRILQAEA	PGASAEEIIY	KITQDYPQFG	EVVLLVNMPA	DSPADEGQHL	PDGRTATPTS
1370	1380	1390	1400	1410	1420	1430	1440
TFTQQDINEG	IVWYRHSGAP	AQSDSFRFEV	SSASNAQTRL	ESHMFNIAIL	PQTPEAPKVS	LEASLHMTAR	EDGLTVIQPH
1450	1460	1470	1480	1490	1500	1510	1520
SLSFINSEKP	SGKIVYNITL	PLHPNQGIIE	HRDHPHSPIR	YFTQEDINQG	KVMYRPPPAA	PHLQELMAFS	FAGLPESVKF
1530	1540	1550	1560	1570	1580	1590	1600
HFTVSDGEHT	SPEMVLTIHL	LPSDQQLPVF	QVTAPRLAVS	PGGSTSVGLQ	VVVRDAETAP	KELFFELRRP	PQHGVLLKHT
1610	1620	1630	1640	1650	1660	1670	1680
AEFRRPMATG	DTFTYEDVEK	NALQYIHDGS	STREDSMEIS	VTDGLTVTML	EVRVEVSLSE	DRGPRLAAGS	SLSITVASKS
1690	1700	1710	1720	1730	1740	1750	1760
TAIITRSHLA	YVDDSSPDPE	IWIQLNYLPS	YGTLLRISGS	EVEELSEVSN	FTMEDINNKK	IRYSAVFETD	GHLVTDSFYF
1770	1780	1790	1800	1810	1820	1830	1840
SVSDMDHNHL	DNQIFTIMIT	PAENPPPVIA	FADLITVDEG	GRAPLSFHHF	FATDDDDNLQ	RDAIIKLSAL	PKYGCIENTG
1850	1860	1870	1880	1890	1900	1910	1920
TGDRFGPETA	SDLEASFPIQ	DVLENYIYYF	QSVHESIEPT	HDIFSFYVSD	GTSRSEIHSI	NITIERKNDE	PPRMTLQPLR
1930	1940	1950	1960	1970	1980	1990	2000
VQLSSGVVIS	NSSLSLQDLD	TPDNELIFVL	TKKPDHGHVL	WRQTASEPLE	NGRVLVQGST	FTYQDILAGL	VGYVPSVPGM
2010	2020	2030	2040	2050	2060	2070	2080
VVDEFQFSLT	DGLHVDTGRM	KIYTELPASD	TPHLAINQGL	QLSAGSVARI	TEQHLKVTDI	DSDDHQVMYI	MKEDPGAGRL
2090	2100	2110	2120	2130	2140	2150	2160
QMMKHGNLEQ	ISIKGPIRSF	TQADISQGHV	EYSHGTGEPG	GSFAFKFDVV	DGEGNRLIDK	SFSISILEDK	SPPVITTNKG
2170	2180	2190	2200	2210	2220	2230	2240

Detailed Protein Report

Cmpd.	No. of Cmpds.	m/z meas.	Δ m/z [ppm]	z	Rt [min]	Score	P	Range	Sequence	Modification
2609	1	1176.3241	-122.97	2	60.9	16.9	1	605-627	R.CKACHSSCLACMGPAPSHCTGCK.K	Carbamidomethyl: 1
30	1	1096.9812	-111.34	2	29.6	16.6	2	2080-2098	R.LQMMKHGNLEQISIKGPIR.S	

Detailed Protein Report

Protein 139: PREDICTED: putative tripartite motif-containing protein 61 isoform X1 [Homo sapiens]

**Accession:**	gi|578809113	**Score:**	33.5
**Database:**	refseq_human(refseq_human_20140103.fasta)	**MW [kDa]:**	55.4
**Database Date:**	2015-11-30	**pI:**	9.5
		**Sequence Coverage [%]:**	6.4
		**No. of unique Peptides:**	1

Alias proteins:

Accession	Name	Description
gi|578809117	refseq_human (refseq_human_20140103.fasta)	PREDICTED: putative tripartite motif-containing protein 61 isoform X3 [Homo sapiens]
gi|578809115	refseq_human (refseq_human_20140103.fasta)	PREDICTED: putative tripartite motif-containing protein 61 isoform X2 [Homo sapiens]

10	20	30	40	50	60	70	80
MEFVTALADL	RAEASCPICL	DYLKDPVTIS	CGHNFCLSCI	IMSWKDLHDS	FPCPFCHFCC	PERKFISNPQ	LGSLTEIAKQ
**90**	**100**	**110**	**120**	**130**	**140**	**150**	**160**
LQIRSKKRKR	QEEKHVCKKH	NQVLTFFCQK	DLELLCPRCS	LSTDHQHHCV	WPIKKAASYH	RKKLEEYNAP	WKERVELIEK
**170**	**180**	**190**	**200**	**210**	**220**	**230**	**240**
VITMQTRKSL	ELKKKVKHRA	EEVKSEFEQL	RLFLQNEQET	VLRQLQDEEM	DILAQLNESL	TKFSDYTSSL	KYLLKEIESI
**250**	**260**	**270**	**280**	**290**	**300**	**310**	**320**
YVKSELELLA	NVKDIYHRYE	NLKFPELFLF	KLKKYGYHLP	PQYSGLDKII	KRFQVDVILD	PETAHRKLIV	SEDRKTVRYG
**330**	**340**	**350**	**360**	**370**	**380**	**390**	**400**
NTTQNVPHNP	RRFYLLPAVL	GSKGYSCGRQ	YWEVEVKDKP	EWILGVCNDC	LPRRRKSQPI	LVQDGLWGIW	RSSQNNYIVL
**410**	**420**	**430**	**440**	**450**	**460**	**470**	**480**
GHREIILLPQ	VIPSKIGIFL	DYEMNEVSFY	NLNDRSLLYT	FNDNFTGALW	PYFYTGTDSK	PLKISTVTDS	E

Cmpd.	No. of Cmpds.	m/z meas.	Δ m/z [ppm]	z	Rt [min]	Score	P	Range	Sequence	Modification
1483	1	620.0670	154.80	3	47.3	17.2	0	358-373	K.DKPEWILGVCNDCLPR.R	

Detailed Protein Report

Protein 140: wee1-like protein kinase 2 [Homo sapiens]

Accession: gi|157738687
Database: refseq_human(refseq_human_20140103.fasta)
Database Date: 2015-11-30
Modification(s): Carbamidomethyl

Score: 32.9
MW [kDa]: 62.9
pI: 6.1
Sequence Coverage [%]: 4.9
No. of unique Peptides: 2

10	20	30	40	50	60	70	80
MDDKDIDKEL	RQKLNFSYCE	ETEIEGQKKV	EESREASSQT	PEKGEVQDSE	AKGTPPWTPL	SNVHELDTSS	EKDKESPDQI
**90**	**100**	**110**	**120**	**130**	**140**	**150**	**160**
LRTPVSHPLK	CPETPAQPDS	RSKLLPSDSP	STPKTMLSRL	VISPTGKLPS	RGPKHLKLTP	APLKDEMTSL	ALVNINPFTP
**170**	**180**	**190**	**200**	**210**	**220**	**230**	**240**
ESYKKLFLQS	GGKRKIRGDL	EEAGPEEGKG	GLPAKRCVLR	ETNMASRYEK	EFLEVEKIGV	GEFGTVYKCI	KRLDGCVYAI
**250**	**260**	**270**	**280**	**290**	**300**	**310**	**320**
KRSMKTFTEL	SNENSALHEV	YAHAVLGHHP	HVVRYYSSWA	EDDHMIIQNE	YCNGGSLQAA	ISENTKSGNH	FEEPKLKDIL
**330**	**340**	**350**	**360**	**370**	**380**	**390**	**400**
LQISLGLNYI	HNSSMVHLDI	KPSNIFICHK	MQSESSGVIE	EVENEADWFL	SANVMYKIGD	LGHATSINKP	KVEEGDSRFL
**410**	**420**	**430**	**440**	**450**	**460**	**470**	**480**
ANEILQEDYR	HLPKADIFAL	GLTIAVAAGA	ESLPTNGAAW	HHIRKGNFPD	VPQELSESFS	SLLKNMIQPD	AEQRPSAAAL
**490**	**500**	**510**	**520**	**530**	**540**	**550**	**560**
ARNTVLRPSL	GKTEELQQQL	NLEKFKTATL	ERELREAQQA	QSPQGYTHHG	DTGVSGTHTG	SRSTKRLVGG	KSARSSSFTS
**570**							
GEREPLH							

Cmpd.	No. of Cmpds.	m/z meas.	Δ m/z [ppm]	z	Rt [min]	Score	P	Range	Sequence	Modification
1865	1	785.9040	-18.42	2	51.7	15.7	1	218-231	K.IGVGEFGTVYKCIK.R	Carbamidomethyl: 12
135	1	725.9700	90.61	2	30.9	17.2	0	378-391	K.IGDLGHATSINKPK.V	

Detailed Protein Report

Protein 141: H/ACA ribonucleoprotein complex subunit 4 isoform 3 [Homo sapiens]

Accession: gi|570700851
Database: refseq_human(refseq_human_20140103.fasta)
Database Date: 2015-11-30
Modification(s): Oxidation

Score: 32.8
MW [kDa]: 47.6
pI: 10.0
Sequence Coverage [%]: 6.7
No. of unique Peptides: 2

10	20	30	40	50	60	70	80
MADAEVIILP	KKHKKKKERK	SLPEEDVAEI	QHAEEFLIKP	ESKVAKLDTS	QWPLLLKNFD	KLNVRTTHYT	PLACGSNPLK
**90**	**100**	**110**	**120**	**130**	**140**	**150**	**160**
REIGDYIRTG	FINLDKPSNP	SSHEVVAWIR	RILRVEKTGH	SGTLDPKVTG	CLIVCIERAT	RLVKSQQSAG	KEYVGIVRLH
**170**	**180**	**190**	**200**	**210**	**220**	**230**	**240**
NAIEGGTQLS	RALETLTGAL	FQRPPLIAAV	KRQLRVRTIY	ESKMIEYDPE	RRLGIFWVSC	EAGTYIRTLC	VHLGLLLGVG
**250**	**260**	**270**	**280**	**290**	**300**	**310**	**320**
GQMQELRRVR	SGVMSEKDHM	VTMHDVLDAQ	WLYDNHKDES	YLRRVVYPLE	KLLTSHKRLV	MKDSAVNAIC	YGAKIMLPGV
**330**	**340**	**350**	**360**	**370**	**380**	**390**	**400**
LRYEDGIEVN	QEIVVITTKG	EAICMAIALM	TTAVISTCDH	GIVAKIKRVI	MERDTYPRKW	GLGPKASQKK	LMIKQGLLDK
**410**	**420**	**430**					
HGKPTDSTPA	TWKQEYVDYR						

Cmpd.	No. of Cmpds.	m/z meas.	Δ m/z [ppm]	z	Rt [min]	Score	P	Range	Sequence	Modification
1944	1	618.9252	-105.31	3	53.0	18.6	2	298-314	K.RLVMKDSAVNAICYGAK.I	Oxidation: 4
2864	1	651.8581	-55.52	2	64.5	14.1	2	390-400	K.KLMIKQGLLDK.H	Oxidation: 3

Detailed Protein Report

Protein 142: protein unc-13 homolog C [Homo sapiens]

**Accession:**	gi|122937514	**Score:**	32.8
**Database:**	refseq_human(refseq_human_20140103.fasta)	**MW [kDa]:**	250.8
**Database Date:**	2015-11-30	**pI:**	5.6
**Modification(s):**	Carbamidomethyl, Oxidation	**Sequence Coverage [%]:**	1.5
		**No. of unique Peptides:**	2

Quantitation

WD:WU	**Median:** 0.62	**CV:** 0.00 %	**No. of Peptides:** 1

Alias proteins:

Accession	Name	Description
gi|530405876	refseq_human (refseq_human_20140103.fasta)	PREDICTED: protein unc-13 homolog C isoform X1 [Homo sapiens]

Detailed Protein Report

10	20	30	40	50	60	70	80
MVANFFKSLI	LPYIHKLCKG	MFTKKLGNTN	KNKEYRQQKK	DQDFPTAGQT	KSPKFSYTFK	STVKKIAKCS	STHNLSTEED
90	100	110	120	130	140	150	160
EASKEFSLSP	TFSYRVAIAN	GLQKNAKVTN	SDNEDLLQEL	SSIESSYSES	LNELRSSTEN	QAQSTHTMPV	RRNRKSSSSL
170	180	190	200	210	220	230	240
APSEGSSDGE	RTLHGLKLGA	LRKLRKWKKS	QECVSSDSEL	STMKKSWGIR	SKSLDRTVRN	PKTNALEPGF	SSSGCISQTH
250	260	270	280	290	300	310	320
DVMEMIFKEL	QGISQIETEL	SELRGHVNAL	KHSIDEISSS	VEVVQSEIEQ	LRTGFVQSRR	ETRDIHDYIK	HLGHMGSKAS
330	340	350	360	370	380	390	400
LRFLNVTEER	FEYVESVVYQ	ILIDKMGFSD	APNAIKIEFA	QRIGHQRDCP	NAKPRPILVY	FETPQQRDSV	LKKSYKLKGT
410	420	430	440	450	460	470	480
GIGISTDILT	HDIRERKEKG	IPSSQTYESM	AIKLSTPEPK	IKKNNWQSPD	DSDEDLESDL	NRNSYAVLSK	SELLTKGSTS
490	500	510	520	530	540	550	560
KPSSKSHSAR	SKNKTANSSR	ISNKSDYDKI	SSQLPESDIL	EKQTTTHYAD	ATPLWHSQSD	FFTAKLSRSE	SDFSKLCQSY
570	580	590	600	610	620	630	640
SEDFSENQFF	TRTNGSSLLS	SSDRELWQRK	QEGTATLYDS	PKDQHLNGGV	QGIQGQTETE	NTETVDSGMS	NGMVCASGDR
650	660	670	680	690	700	710	720
SHYSDSQLSL	HEDLSPWKEW	NQGADLGLDS	STQEGFDYET	NSLFDQQLDV	YNKDLEYLGK	CHSDLQDDSE	SYDLTQDDNS
730	740	750	760	770	780	790	800
SPCPGLDNEP	QGQWVGQYDS	YQGANSNELY	QNQNQLSMMY	RSQSELQSDD	SEDAPPKSWH	SRLSIDLSDK	TFSFPKFGST
810	820	830	840	850	860	870	880
LQRAKSALEV	VWNKSTQSLS	GYEDSGSSLM	GRFRTLSQST	ANESSTTLDS	DVYTEPYYYK	AEDEEDYTEP	VADNETDYVE
890	900	910	920	930	940	950	960
VMEQVLAKLE	NRTSITETDE	QMQAYDHLSY	ETPYETPQDE	GYDGPADDMV	SEEGLEPLNE	TSAEMEIRED	ENQNIPEQPV
970	980	990	1000	1010	1020	1030	1040
EITKPKRIRP	SFKEAALRAY	KKQMAELEEK	ILAGDSSSVD	EKARIVSGND	LDASKFSALQ	VCGGAGGGLY	GIDSMPDLRR
1050	1060	1070	1080	1090	1100	1110	1120
KKTLPIVRDV	AMTLAARKSG	LSLAMVIRTS	LNNEELKMHV	FKKTLQALIY	PMSSTIPHNF	EVWTATTPTY	CYECEGLLWG
1130	1140	1150	1160	1170	1180	1190	1200
IARQGMKCLE	CGVKCHEKCQ	DLLNADCLQR	AAEKSSKHGA	EDKTQTIITA	MKERMKIREK	NRPEVFEVIQ	EMFQISKEDF
1210	1220	1230	1240	1250	1260	1270	1280
VQFTKAAKQS	VLDGTSKWSA	KITITVVSAQ	GLQAKDKTGS	SDPYVTVQVG	KNKRRTKTIF	GNLNPVWDEK	FYFECHNSTD
1290	1300	1310	1320	1330	1340	1350	1360
RIKVRVWDED	DDIKSRVKQH	FKKESDDFLG	QTIVEVRTLS	GEMDVWYNLE	KRTDKSAVSG	AIRLKINVEI	KGEEKVAPYH
1370	1380	1390	1400	1410	1420	1430	1440
IQYTCLHENL	FHYLTEVKSN	GGVKIPEVKG	DEAWKVFFDD	ASQEIVDEFA	MRYGIESIYQ	AMTHFSCLSS	KYMCPGVPAV
1450	1460	1470	1480	1490	1500	1510	1520
MSTLLANINA	FYAHTTVSTN	IQVSASDRFA	ATNFGREKFI	KLLDQLHNSL	RIDLSKYREN	FPASNTERLQ	DLKSTVDLLT
1530	1540	1550	1560	1570	1580	1590	1600
SITFFRMKVL	ELQSPPKASM	VVKDCVRACL	DSTYKYIFDN	CHELYSQLTD	PSKKQDIPRE	DQGPTTKNLD	FWPQLITLMV
1610	1620	1630	1640	1650	1660	1670	1680
TIIDEDKTAY	TPVLNQFPQE	LNMGKISAEI	MWTLFALDMK	YALEEHENQR	LCKSTDYMNL	HFKVKWFYNE	YVRELPAFKD
1690	1700	1710	1720	1730	1740	1750	1760
AVPEYSLWFE	PFVMQWLDEN	EDVSMEFLHG	ALGRDKKDGF	QQTSEHALFS	CSVVDVFAQL	NQSFEIIKKL	ECPNPEALSH
1770	1780	1790	1800	1810	1820	1830	1840
LMRRFAKTIN	KVLLQYAAIV	SSDFSSHCDK	ENVPCILMNN	IQQLRVQLEK	MFESMGGKEL	DSEASTILKE	LQVKLSGVLD
1850	1860	1870	1880	1890	1900	1910	1920
ELSVTYGESF	QVIIEECIKQ	MSFELNQMRA	NGNTTSNKNS	AAMDAEIVLR	SLMDFLDKTL	SLSAKICEKT	VLKRVLKELW
1930	1940	1950	1960	1970	1980	1990	2000
KLVLNKIEKQ	IVLPPLTDQT	GPQMIFIAAK	DLGQLSKLKE	HMIREDARGL	TPRQCAIMEV	VLATIKQYFH	AGGNGLKKNF
2010	2020	2030	2040	2050	2060	2070	2080
LEKSPDLQSL	RYALSLYTQT	TDALIKKFID	TQTSQSRSSK	DAVGQISVHV	DITATPGTGD	HKVTVKVIAI	NDLNWQTTAM
2090	2100	2110	2120	2130	2140	2150	2160
FRPFVEVCIL	GPNLGDKKRK	QGTKTKSNTW	SPKYNETFQF	ILGKENRPGA	YELHLSVKDY	CFAREDRIIG	MTVIQLQNIA
2170	2180	2190	2200	2210	2220		

Detailed Protein Report

Cmpd.	No. of Cmpds.	m/z meas.	Δ m/z [ppm]	z	Rt [min]	Score	P	Range	Sequence	Modification	Ratios
1046	1	742.4006	68.40	2	41.8	12.0	1	1497-1508	K.YRENFPASNTER.L		WD:WU 0.62
1482	1	619.9673	-23.43	3	46.9	10.7	0	1791-1805	K.ENVPCILMNNIQQLR.V	Carbamidomethyl: 5; Oxidation: 8	

Detailed Protein Report

Protein 143: **PREDICTED: MAGUK p55 subfamily member 6 isoform X2 [Homo sapiens]**

**Accession:**	gi|530384825	**Score:**	32.6
**Database:**	refseq_human(refseq_human_20140103.fasta)	**MW [kDa]:**	48.7
**Database Date:**	2015-11-30	**pI:**	9.5
**Modification(s):**	Oxidation	**Sequence Coverage [%]:**	10.0
		**No. of unique Peptides:**	2

10	20	30	40	50	60	70	80
MNNSSINNQL	LPVDAIRILG	IHKRAGEPLG	VTFRVENNDL	VIARILHGGM	IDRQGLLHVG	DIIKEVNGHE	VGNNPKELQE
**90**	**100**	**110**	**120**	**130**	**140**	**150**	**160**
LLKNISGSVT	LKILPSYRDT	ITPQQVFVKC	HFDYNPYNDN	LIPCKEAGLK	FSKGEILQIV	NREDPNWWQA	SHVKEGGSAG
**170**	**180**	**190**	**200**	**210**	**220**	**230**	**240**
LIPSQFLEEK	RKAFVRRDWD	NSGPFCGTIS	SKKKKKMMYL	TTRNAEFDRH	EIQIYEEVAK	MPPFQRKTLV	LIGAQGVGRR
**250**	**260**	**270**	**280**	**290**	**300**	**310**	**320**
SLKNRFIVLN	PTRFGTTVPF	TSRKPREDEK	DGQAYKFVSR	SEMEADIKAG	KYLEHGEYEG	NLYGTKIDSI	LEVVQTGRTC
**330**	**340**	**350**	**360**	**370**	**380**	**390**	**400**
ILDVNPQALK	VLRTSEFMPY	VVFIAAPELE	TLRAMHKAVV	DAGITTKLLT	DSDLKKTVDE	SARIQRAYNH	YFDLIIINDN
**410**	**420**	**430**					
LDKAFEKLQT	AIEKLRMEPQ	WVPISWVY					

Cmpd.	No. of Cmpds.	m/z meas.	Δ m/z [ppm]	z	Rt [min]	Score	P	Range	Sequence	Modification
2335	1	938.2188	84.20	3	57.5	10.4	1	334-357	R.TSEFMPYVVFIAAPELETLRAMHK.A	Oxidation: 5, 22
2850	1	994.4934	-74.35	2	64.3	22.2	2	358-376	K.AVVDAGITTKLLTDSDLKK.T	

Detailed Protein Report

Protein 144: usherin isoform B [Homo sapiens]

**Accession:**	gi|219842266	**Score:**	32.4
**Database:**	refseq_human(refseq_human_20140103.fasta)	**MW [kDa]:**	575.2
**Database Date:**	2015-11-30	**pI:**	6.4
		**Sequence Coverage [%]:**	0.5
		**No. of unique Peptides:**	1

Detailed Protein Report

10	20	30	40	50	60	70	80
MNCPVLSLGS	GFLFQVIEML	IFAYFASISL	TESRGLFPRL	ENVGAFKKVS	IVPTQAVCGL	PDRSTFCHSS	AAAESIQFCT
90	100	110	120	130	140	150	160
QRFCIQDCPY	RSSHPTYTAL	FSAGLSSCIT	PDKNDLHPNA	HSNSASFIFG	NHKSCFSSPP	SPKLMASFTL	AVWLKPEQQG
170	180	190	200	210	220	230	240
VMCVIEKTVD	GQIVFKLTIS	EKETMFYYRT	VNGLQPPIKV	MTLGRILVKK	WIHLSVQVHQ	TKISFFINGV	EKDHTPFNAR
250	260	270	280	290	300	310	320
TLSGSITDFA	SGTVQIGQSL	NGLEQFVGRM	QDFRLYQVAL	TNREILEVFS	GDLLRLHAQS	HCRCPGSHPR	VHPLAQRYCI
330	340	350	360	370	380	390	400
PNDAGDTADN	RVSRLNPEAH	PLSFVNDNDV	GTSWVSNVFT	NITQLNQGVT	ISVDLENGQY	QVFYIIIQFF	SPQPTEIRIQ
410	420	430	440	450	460	470	480
RKKENSLDWE	DWQYFARNCG	AFGMKNNGDL	EKPDSVNCLQ	LSNFTPYSRG	NVTFSILTPG	PNYRPGYNNF	YNTPSLQEFV
490	500	510	520	530	540	550	560
KATQIRFHFH	GQYYTTETAV	NLRHRYYAVD	EITISGRCQC	HGHADNCDTT	SQPYRCLCSQ	ESFTEGLHCD	RCLPLYNDKP
570	580	590	600	610	620	630	640
FRQGDQVYAF	NCKPCQCNSH	SKSCHYNISV	DPFPFEHFRG	GGGVCDDCEH	NTTGRNCELC	KDYFFRQVGA	DPSAIDVCKP
650	660	670	680	690	700	710	720
CDCDTVGTRN	GSILCDQIGG	QCNCKRHVSG	RQCNQCQNGF	YNLQELDPDG	CSPCNCNTSG	TVDGDITCHQ	NSGQCKCKAN
730	740	750	760	770	780	790	800
VIGLRCDHCN	FGFKFLRSFN	DVGCEPCQCN	LHGSVNKFCN	PHSGQCECKK	EAKGLQCDTC	RENFYGLDVT	NCKACDCDTA
810	820	830	840	850	860	870	880
GSLPGTVCNA	KTGQCICKPN	VEGRQCNKCL	EGNFYLRQNN	SFLCLPCNCD	KTGTINGSLL	CNKSTGQCPC	KLGVTGLRCN
890	900	910	920	930	940	950	960
QCEPHRYNLT	IDNFQHCQMC	ECDSLGTLPG	TICDPISGQC	LCVPNRQGRR	CNQCQPGFYI	SPGNATGCLP	CSCHTTGAVN
970	980	990	1000	1010	1020	1030	1040
HICNSLTGQC	VCQDASIAGQ	RCDQCKDHYF	GFDPQTGRCQ	PCNCHLSGAL	NETCHLVTGQ	CFCKQFVTGS	KCDACVPSAS
1050	1060	1070	1080	1090	1100	1110	1120
HLDVNNLLGC	SKTPFQQPPP	RGQVQSSSAI	NLSWSPPDSP	NAHWLTYSLL	RDGFEIYTTE	DQYPYSIQYF	LDTDLLPYTK
1130	1140	1150	1160	1170	1180	1190	1200
YSYYIETTNV	HGSTRSVAVT	YKTKPGVPEG	NLTLSYIIPI	GSDSVTLTWT	TLSNQSGPIE	KYILSCAPLA	GGQPCVSYEG
1210	1220	1230	1240	1250	1260	1270	1280
HETSATIWNL	VPFAKYDFSV	QACTSGGCLH	SLPITVTTAQ	APPQRLSPPK	MQKISSTELH	VEWSPPAELN	GIIIRYELYM
1290	1300	1310	1320	1330	1340	1350	1360
RRLRSTKETT	SEESRVFQSS	GWLSPHSFVE	SANENALKPP	QTMTTITGLE	PYTKYEFRVL	AVNMAGSVSS	AWVSERTGES
1370	1380	1390	1400	1410	1420	1430	1440
APVFMIPPSV	FPLSSYSLNI	SWEKPADNVT	RGKVVGYDIN	MLSEQSPQQS	IPMAFSQLLH	TAKSQELSYT	VEGLKPYRIY
1450	1460	1470	1480	1490	1500	1510	1520
EFTITLCNSV	GCVTSASGAG	QTLAAAPAQL	RPPLVKGINS	TTIHLKWFPP	EELNGPSPIY	QLERRESSLP	ALMTTMMKGI
1530	1540	1550	1560	1570	1580	1590	1600
RFIGNGYCKF	PSSTHPVNTD	FTGIKASFRT	KVPEGLIVFA	ASPGNQEEYF	ALQLKKGRLY	FLFDPQGSPV	EVTTTNDHGK
1610	1620	1630	1640	1650	1660	1670	1680
QYSDGKWHEI	IAIRHQAFGQ	ITLDGIYTGS	SAILNGSTVI	GDNTGVFLGG	LPRSYTILRK	DPEIIQKGFV	GCLKDVHFMK
1690	1700	1710	1720	1730	1740	1750	1760
NYNPSAIWEP	LDWQSSEEQI	NVYNSWEGCP	ASLNEGAQFL	GAGFLELHPY	MFHGGMNFEI	SFKFRTDQLN	GLLLFVYNKD
1770	1780	1790	1800	1810	1820	1830	1840
GPDFLAMELK	SGILTFRLNT	SLAFTQVDLL	LGLSYCNGKW	NKVIIKKEGS	FISASVNGLM	KHASESGDQP	LVVNSPVYVG
1850	1860	1870	1880	1890	1900	1910	1920
GIPQELLNSY	QHLCLEQGFG	GCMKDVKFTR	GAVVNLASVS	SGAVRVNLDG	CLSTDSAVNC	RGNDSILVYQ	GKEQSVYEGG
1930	1940	1950	1960	1970	1980	1990	2000
LQPFTEYLYR	VIASHEGGSV	YSDWSRGRTT	GAAPQSVPTP	SRVRSLNGYS	IEVTWDEPVV	RGVIEKYILK	AYSEDSTRPP
2010	2020	2030	2040	2050	2060	2070	2080
RMPSASAEFV	NTSNLTGILT	GLLPFKNYAV	TLTACTLAGC	TESSHALNIS	TPQEAPQEVQ	PPVAKSLPSS	LLLSWNPPKK
2090	2100	2110	2120	2130	2140	2150	2160
ANGIITQYCL	YMDGRLIYSG	SEENYTVTDL	AVFTPHQFLL	SACTHVGCTN	SSWVLLYTAQ	LPPEHVDSPV	LTVLDSRTIH
2170	2180	2190	2200	2210	2220	2230	2240

Detailed Protein Report

Cmpd.	No. of Cmpds.	m/z meas.	Δ m/z [ppm]	z	Rt [min]	Score	P	Range	Sequence	Modification
2191	1	863.3484	-20.01	2	55.8	14.2	0	794-811	K.ACDCDTAGSLPGTVCNAK.T	

Detailed Protein Report

Protein 145: macrophage mannose receptor 1 precursor [Homo sapiens]

Accession: gi|4505245
Database: refseq_human(refseq_human_20140103.fasta)
Database Date: 2015-11-30
Modification(s): Oxidation

Score: 32.1
MW [kDa]: 165.9
pI: 6.1
Sequence Coverage [%]: 1.6
No. of unique Peptides: 1

10	20	30	40	50	60	70	80
MRLPLLLVFA	SVIPGAVLLL	DTRQFLIYNE	DHKRCVDAVS	PSAVQTAACN	QDAESQKFRW	VSESQIMSVA	FKLCLGVPSK
**90**	**100**	**110**	**120**	**130**	**140**	**150**	**160**
TDWVAITLYA	CDSKSEFQKW	ECKNDTLLGI	KGEDLFFNYG	NRQEKNIMLY	KGSGLWSRWK	IYGTTDNLCS	RGYEAMYTLL
**170**	**180**	**190**	**200**	**210**	**220**	**230**	**240**
GNANGATCAF	PFKFENKWYA	DCTSAGRSDG	WLWCGTTTDY	DTDKLFGYCP	LKFEGSESLW	NKDPLTSVSY	QINSKSALTW
**250**	**260**	**270**	**280**	**290**	**300**	**310**	**320**
HQARKSCQQQ	NAELLSITEI	HEQTYLTGLT	SSLTSGLWIG	LNSLSFNSGW	QWSDRSPFRY	LNWLPGSPSA	EPGKSCVSLN
**330**	**340**	**350**	**360**	**370**	**380**	**390**	**400**
PGKNAKWENL	ECVQKLGYIC	KKGNTTLNSF	VIPSESDVPT	HCPSQWWPYA	GHCYKIHRDE	KKIQRDALTT	CRKEGGDLTS
**410**	**420**	**430**	**440**	**450**	**460**	**470**	**480**
IHTIEELDFI	ISQLGYEPND	ELWIGLNDIK	IQMYFEWSDG	TPVTFTKWLR	GEPSHENNRQ	EDCVVMKGKD	GYWADRGCEW
**490**	**500**	**510**	**520**	**530**	**540**	**550**	**560**
PLGYICKMKS	RSQGPEIVEV	EKGCRKGWKK	HHFYCYMIGH	TLSTFAEANQ	TCNNENAYLT	TIEDRYEQAF	LTSFVGLRPE
**570**	**580**	**590**	**600**	**610**	**620**	**630**	**640**
KYFWTGLSDI	QTKGTFQWTI	EEEVRFTHWN	SDMPGRKPGC	VAMRTGIAGG	LWDVLKCDEK	AKFVCKHWAE	GVTHPPKPTT
**650**	**660**	**670**	**680**	**690**	**700**	**710**	**720**
TPEPKCPEDW	GASSRTSLCF	KLYAKGKHEK	KTWFESRDFC	RALGGDLASI	NNKEEQQTIW	RLITASGSYH	KLFWLGLTYG
**730**	**740**	**750**	**760**	**770**	**780**	**790**	**800**
SPSEGFTWSD	GSPVSYENWA	YGEPNNYQNV	EYCGELKGDP	TMSWNDINCE	HLNNWICQIQ	KGQTPKPEPT	PAPQDNPPVT
**810**	**820**	**830**	**840**	**850**	**860**	**870**	**880**
EDGWVIYKDY	QYYFSKEKET	MDNARAFCKR	NFGDLVSIQS	ESEKKFLWKY	VNRNDAQSAY	FIGLLISLDK	KFAWMDGSKV
**890**	**900**	**910**	**920**	**930**	**940**	**950**	**960**
DYVSWATGEP	NFANEDENCV	TMYSNSGFWN	DINCGYPNAF	ICQRHNSSIN	ATTVMPTMPS	VPSGCKEGWN	FYSNKCFKIF
**970**	**980**	**990**	**1000**	**1010**	**1020**	**1030**	**1040**
GFMEEERKNW	QEARKACIGF	GGNLVSIQNE	KEQAFLTYHM	KDSTFSAWTG	LNDVNSEHTF	LWTDGRGVHY	TNWGKGYPGG
**1050**	**1060**	**1070**	**1080**	**1090**	**1100**	**1110**	**1120**
RRSSLSYEDA	DCVVIIGGAS	NEAGKWMDDT	CDSKRGYICQ	TRSDPSLTNP	PATIQTDGFV	KYGKSSYSLM	RQKFQWHEAE
**1130**	**1140**	**1150**	**1160**	**1170**	**1180**	**1190**	**1200**
TYCKLHNSLI	ASILDPYSNA	FAWLQMETSN	ERVWIALNSN	LTDNQYTWTD	KWRVRYTNWA	ADEPKLKSAC	VYLDLDGYWK
**1210**	**1220**	**1230**	**1240**	**1250**	**1260**	**1270**	**1280**
TAHCNESFYF	LCKRSDEIPA	TEPPQLPGRC	PESDHTAWIP	FHGHCYYIES	SYTRNWGQAS	LECLRMGSSL	VSIESAAESS
**1290**	**1300**	**1310**	**1320**	**1330**	**1340**	**1350**	**1360**
FLSYRVEPLK	SKTNFWIGLF	RNVEGTWLWI	NNSPVSFVNW	NTGDPSGERN	DCVALHASSG	FWSNIHCSSY	KGYICKRPKI
**1370**	**1380**	**1390**	**1400**	**1410**	**1420**	**1430**	**1440**
IDAKPTHELL	TTKADTRKMD	PSKPSSNVAG	VVIIVILLIL	TGAGLAAYFF	YKKRRVHLPQ	EGAFENTLYF	NSQSSPGTSD
**1450**	**1460**						
MKDLVGNIEQ	NEHSVI						

Cmpd.	No. of Cmpds.	m/z meas.	Δ m/z [ppm]	z	Rt [min]	Score	P	Range	Sequence	Modification
2976	1	928.0036	17.04	2	66.0	14.3	2	126-140	K.NIMLYKGSGLWSRWK.I	Oxidation: 3

Detailed Protein Report

Protein 146: unconventional myosin-Ie [Homo sapiens]

**Accession:**	gi|55956916	**Score:**	32.1
**Database:**	refseq_human(refseq_human_20140103.fasta)	**MW [kDa]:**	127.0
**Database Date:**	2015-11-30	**pI:**	9.5
		**Sequence Coverage [%]:**	1.6
		**No. of unique Peptides:**	1

10	20	30	40	50	60	70	80
MGSKGVYQYH	WQSHNVKHSG	VDDMVLLSKI	TENSIVENLK	KRYMDDYIFT	YIGSVLISVN	PFKQMPYFGE	KEIEMYQGAA
**90**	**100**	**110**	**120**	**130**	**140**	**150**	**160**
QYENPPHIYA	LADNMYRNMI	IDRENQCVII	SGESGAGKTV	AAKYIMSYIS	RVSGGGTKVQ	HVKDIILQSN	PLLEAFGNAK
**170**	**180**	**190**	**200**	**210**	**220**	**230**	**240**
TVRNNNSSRF	GKYFEIQFSP	GGEPDGGKIS	NFLLEKSRVV	MRNPGERSFH	IFYQLIEGAS	AEQKHSLGIT	SMDYYYYLSL
**250**	**260**	**270**	**280**	**290**	**300**	**310**	**320**
SGSYKVDDID	DRREFQETLH	AMNVIGIFAE	EQTLVLQIVA	GILHLGNISF	KEVGNYAAVE	SEEFLAFPAY	LLGINQDRLK
**330**	**340**	**350**	**360**	**370**	**380**	**390**	**400**
EKLTSRQMDS	KWGGKSESIH	VTLNVEQACY	TRDALAKALH	ARVFDFLVDS	INKAMEKDHE	EYNIGVLDIY	GFEIFQKNGF
**410**	**420**	**430**	**440**	**450**	**460**	**470**	**480**
EQFCINFVNE	KLQQIFIELT	LKAEQEEYVQ	EGIRWTPIEY	FNNKIVCDLI	ENKVNPPGIM	SILDDVCATM	HAVGEGADQT
**490**	**500**	**510**	**520**	**530**	**540**	**550**	**560**
LLQKLQMQIG	SHEHFNSWNQ	GFIIHHYAGK	VSYDMDGFCE	RNRDVLFMDL	IELMQSSELP	FIKSLFPENL	QADKKGRPTT
**570**	**580**	**590**	**600**	**610**	**620**	**630**	**640**
AGSKIKKQAN	DLVSTLMKCT	PHYIRCIKPN	ETKKPRDWEE	SRVKHQVEYL	GLKENIRVRR	AGYAYRRIFQ	KFLQRYAILT
**650**	**660**	**670**	**680**	**690**	**700**	**710**	**720**
KATWPSWQGE	EKQGVLHLLQ	SVNMDSDQFQ	LGRSKVFIKA	PESLFLLEEM	RERKYDGYAR	VIQKSWRKFV	ARKKYVQMRE
**730**	**740**	**750**	**760**	**770**	**780**	**790**	**800**
EASDLLLNKK	ERRRNSINRN	FIGDYIGMEE	HPELQQFVGK	REKIDFADTV	TKYDRRFKGV	KRDLLLTPKC	LYLIGREKVK
**810**	**820**	**830**	**840**	**850**	**860**	**870**	**880**
QGPDKGLVKE	VLKRKIEIER	ILSVSLSTMQ	DDIFILHEQE	YDSLLESVFK	TEFLSLLAKR	YEEKTQKQLP	LKFSNTLELK
**890**	**900**	**910**	**920**	**930**	**940**	**950**	**960**
LKKENWGPWS	AGGSRQVQFH	QGFGDLAVLK	PSNKVLQVSI	GPGLPKNSRP	TRRNTTQNTG	YSSGTQNANY	PVRAAPPPPG
**970**	**980**	**990**	**1000**	**1010**	**1020**	**1030**	**1040**
YHQNGVIRNQ	YVPYPHAPGS	QRSNQKSLYT	SMARPPLPRQ	QSTSSDRVSQ	TPESLDFLKV	PDQGAAGVRR	QTTSRPPPAG
**1050**	**1060**	**1070**	**1080**	**1090**	**1100**	**1110**	
GRPKPQPKPK	PQVPQCKALY	AYDAQDTDEL	SFNANDIIDI	IKEDPSGWWT	GRLRGKQGLF	PNNYVTKI	

Cmpd.	No. of Cmpds.	m/z meas.	Δ m/z [ppm]	z	Rt [min]	Score	P	Range	Sequence	Modification
1182	1	478.1928	-225.01	2	43.0	13.4	1	782-789	K.RDLLLTPK.C	

Detailed Protein Report

Protein 147: cystatin-B [Homo sapiens]

**Accession:**	gi|4503117	**Score:**	31.9
**Database:**	refseq_human(refseq_human_20140103.fasta)	**MW [kDa]:**	11.1
**Database Date:**	2015-11-30	**pI:**	7.9
		**Sequence Coverage [%]:**	12.2
		**No. of unique Peptides:**	1

Quantitation

WD:WU **Median:** 0.65 **CV:** 0.00 % **No. of Peptides:** 1

```
         10         20         30         40         50         60         70         80
MMCGAPSATQ PATAETQHIA DQVRSQLEEK ENKKFPVFKA VSFKSQVVAG TNYFIKVHVG DEDFVHLRVF QSLPHENKPL
         90        100
TLSNYQTNKA KHDELTYF
```

Cmpd.	No. of Cmpds.	m/z meas.	Δ m/z [ppm]	z	Rt [min]	Score	P	Range	Sequence	Modification	Ratios
695	1	663.8458	-15.71	2	37.1	31.9	0	45-56	K.SQVVAGTNYFIK.V		WD:WU 0.65

Detailed Protein Report

Protein 148: PREDICTED: uncharacterized protein C7orf63 isoform X1 [Homo sapiens]

**Accession:**	gi|530386429	**Score:**	31.7
**Database:**	refseq_human(refseq_human_20140103.fasta)	**MW [kDa]:**	101.8
**Database Date:**	2015-11-30	**pI:**	6.2
		**Sequence Coverage [%]:**	3.1
		**No. of unique Peptides:**	1

Quantitation

WD:WU **Median:** 2.21 **CV:** 0.00 % **No. of Peptides:** 1

10	20	30	40	50	60	70	80
MWTEEAGATA	EAQESGIRNK	SSSSSQIPVV	GVVTEDDEAQ	DVFKPMDLNR	VIKLLEETDK	DGLEEKQLKF	VKKLVQCYQN
**90**	**100**	**110**	**120**	**130**	**140**	**150**	**160**
GLPLRDLAQI	FKILNLCSGK	IKNQPRFIES	AYDIIKLCGL	PFLKKKVSDE	ITYAEDTANS	IALLGDLMKI	PSSELRIQIC
**170**	**180**	**190**	**200**	**210**	**220**	**230**	**240**
KCIVDFYHAE	PPKKHIPGYQ	QASSSYKIQM	AEVGGLAKTM	VQSMTLLENQ	LVEKLWVLKV	LQHLSTSEVN	CTIMMKAQAA
**250**	**260**	**270**	**280**	**290**	**300**	**310**	**320**
SGICTHLNDP	DPSGQLLFRS	SEILWNLLEK	SSKEEVIQQL	SNLECLLALK	EVFKNLFMRG	FSHYDRQLRN	DILVITTIIA
**330**	**340**	**350**	**360**	**370**	**380**	**390**	**400**
QNPEAPMIEC	GFTKDLILFA	TFNEVKSQNL	LVKGLKLSNS	YEDFELKKLL	FNVIVILCKD	LPTVQLLIDG	KVILALFTYV
**410**	**420**	**430**	**440**	**450**	**460**	**470**	**480**
KKPEKQKIID	WSAAQHEELQ	LHAIATLSSV	APLLIEEYMS	CQGNARVLAF	LEWCESEDPF	FSHGNSFHGT	GGRGNKFAQM
**490**	**500**	**510**	**520**	**530**	**540**	**550**	**560**
RYSLRLLRAV	VYLEDETVNK	DLCEKGTIQQ	MIGIFKNIIS	KPNEKEEAIV	LEIQSDILLI	LSGLCENHIQ	RKEIFGTEGV
**570**	**580**	**590**	**600**	**610**	**620**	**630**	**640**
DIVLHVMKTD	PRKLQSGLGY	NVLLFSTLDS	IWCCILGCYP	SEDYFLEKEG	IFLLLDLLAL	NQKKFCNLIL	GIMVEFCDNP
**650**	**660**	**670**	**680**	**690**	**700**	**710**	**720**
KTAAHVNAWQ	GKKDQTAASL	LIKLWRKEEK	ELGVKRDKNG	KIIDTKKPLF	TSFQEEQKII	PLPANCPSIA	VMDVSENIRA
**730**	**740**	**750**	**760**	**770**	**780**	**790**	**800**
KIYAILGKLD	FENLPGLSAE	DFVTLCIIHR	YLDFKIGEIW	NEIYEEIKLE	KLRPVTTDKK	ALEAITTASE	NIGKMVASLQ
**810**	**820**	**830**	**840**	**850**	**860**	**870**	**880**
SDIIESQACQ	DMQNEQKVYA	KIQATHKQRE	LANKSWEDFL	ARTSNAKTLK	VPSGGVVTVE	STPARLVGGP	LVDTDIALKK
**890**	**900**	**910**					
LPIRGGALQR	VKAVKIVDAP	KKSIPT					

Cmpd.	No. of Cmpds.	m/z meas.	Δ m/z [ppm]	z	Rt [min]	Score	P	Range	Sequence	Modification	Ratios
1060	1	540.7960	-72.96	2	41.5	14.8	1	881-890	K.LPIRGGALQR.V		WD:WU 2.21

Detailed Protein Report

Protein 149: estrogen sulfotransferase [Homo sapiens]

Accession: gi|4885617
Database: refseq_human(refseq_human_20140103.fasta)
Database Date: 2015-11-30
Modification(s): Oxidation

Score: 31.5
MW [kDa]: 35.1
pI: 6.2
Sequence Coverage [%]: 7.8
No. of unique Peptides: 1

10	20	30	40	50	60	70	80
MNSELDYYEK	FEEVHGILMY	KDFVKYWDNV	EAFQARPDDL	VIATYPKSGT	TWVSEIVYMI	YKEGDVEKCK	EDVIFNRIPF
**90**	**100**	**110**	**120**	**130**	**140**	**150**	**160**
LECRKENLMN	GVKQLDEMNS	PRIVKTHLPP	ELLPASFWEK	DCKIIYLCRN	AKDVAVSFYY	FFLMVAGHPN	PGSFPEFVEK
**170**	**180**	**190**	**200**	**210**	**220**	**230**	**240**
FMQGQVPYGS	WYKHVKSWWE	KGKSPRVLFL	FYEDLKEDIR	KEVIKLIHFL	ERKPSEELVD	RIIHHTSFQE	MKNNPSTNYT
**250**	**260**	**270**	**280**	**290**	**300**		
TLPDEIMNQK	LSPFMRKGIT	GDWKNHFTVA	LNEKFDKHYE	QQMKESTLKF	RTEI		

Cmpd.	No. of Cmpds.	m/z meas.	Δ m/z [ppm]	z	Rt [min]	Score	P	Range	Sequence	Modification
2974	1	826.4375	3.18	2	65.9	13.7	2	251-264	K.LSPFMRKGITGDWK.N	Oxidation: 5

Detailed Protein Report

Protein 150: **PREDICTED: sodium bicarbonate transporter-like protein 11 isoform X2 [Homo sapiens]**

**Accession:**	gi|530426013	**Score:**	31.4
**Database:**	refseq_human(refseq_human_20140103.fasta)	**MW [kDa]:**	96.0
**Database Date:**	2015-11-30	**pI:**	8.9
**Modification(s):**	Oxidation	**Sequence Coverage [%]:**	2.8
		**No. of unique Peptides:**	1

10	20	30	40	50	60	70	80
MSQNGYFEDS	SYYKCDTDDT	FEAREEILGD	EAFDTANSSI	VSGESIRFFV	NVNLEMQATN	TENEATSGGC	VLLHTSRKYL
90	100	110	120	130	140	150	160
KLKNFKEEIR	AHRDLDGFLA	QASIVLNETA	TSLDNVLRTM	LRRFARDPDN	NEPNCNLDLL	MAMLFTDAGA	PMRGKVHLLS
170	180	190	200	210	220	230	240
DTIQGVTATV	TGVRYQQSWL	CIICTMKALQ	KRHVCISRLV	RPQNWGENSC	EVRFVILVLA	PPKMKSTKTA	MEVARTFATM
250	260	270	280	290	300	310	320
FSDIAFRQKL	LETRTEEEFK	EALVHQRQLL	TMVSHGPVAP	RTKERSTVSL	PAHRHPEPPK	CKDFVPFGKG	IREDIARRFP
330	340	350	360	370	380	390	400
LYPLDFTDGI	IGKNKAVGKY	ITTTLFLYFA	CLLPTIAFGS	LNDENTDGAI	DVQKTIAGQS	IGGLLYALFS	GQPLVILLTT
410	420	430	440	450	460	470	480
APLALYIQVI	RVICDDYDLD	FNSFYAWTGL	WNSFFLALYA	FFNLSLVMSL	FKRSTEEIIA	LFISITFVLD	AVKGTVKIFW
490	500	510	520	530	540	550	560
KYYYGHYLDD	YHTKRTSSLV	SLSGLGASLN	ASLHTALNAS	FLASPTELPS	ATHSGQATAV	LSLLIMLGTL	WLGYTLYQFK
570	580	590	600	610	620	630	640
KSPYLHPCVR	EILSDCALPI	AVLAFSLISS	HGFREIEMSK	FRYNPSESPF	AMAQIQSLSL	RAVSGAMGLG	FLLSMLFFIE
650	660	670	680	690	700	710	720
QNLVAALVNA	PENRLVKGTA	YHWDLLLLAI	INTGLSLFGL	PWIHAAYPHS	PLHVRALALV	EERVENGHIY	DTIVNVKETR
730	740	750	760	770	780	790	800
LTSLGASVLV	GLSLLLLPVP	LQWIPKPVLY	GLFLYIALTS	LDGNQLVQRV	ALLLKEQTAY	PPTHYIRRVP	QRKIHYFTGL
810	820	830	840	850	860		
QVLQLLLLCA	FGMSSLPYMK	MIFPLIMIAM	IPIRYILLPR	IIEAKYLDVM	DAEHRP		

Cmpd.	No. of Cmpds.	m/z meas.	Δ m/z [ppm]	z	Rt [min]	Score	P	Range	Sequence	Modification
823	1	711.9737	188.22	2	38.7	20.7	0	236-247	R.TFATMFSDIAFR.Q	Oxidation: 5

Detailed Protein Report

Protein 151: archaemetzincin-1 isoform 1 [Homo sapiens]

**Accession:**	gi|55741645	**Score:**	31.4
**Database:**	refseq_human(refseq_human_20140103.fasta)	**MW [kDa]:**	54.9
**Database Date:**	2015-11-30	**pI:**	5.6
		**Sequence Coverage [%]:**	4.4
		**No. of unique Peptides:**	1

Quantitation

WD:WU **Median:** 0.54 **CV:** 0.00 % **No. of Peptides:** 1

10	20	30	40	50	60	70	80
MLQCRPAQEF	SFGPRALKDA	LVSTDAALQQ	LYVSAFSPAE	RLFLAEAYNP	QRTLFCTLLI	RTGFDWLLSR	PEAPEDFQTF
**90**	**100**	**110**	**120**	**130**	**140**	**150**	**160**
HASLQHRKPR	LARKHIYLQP	IDLSEEPVGS	SLLHQLCSCT	EAFFLGLRVK	CLPSVAAASI	RCSSRPSRDS	DRLQLHTDGI
**170**	**180**	**190**	**200**	**210**	**220**	**230**	**240**
LSFLKNNKPG	DALCVLGLTL	SDLYPHEAWS	FTFSKFLPGH	EVGVCSFARF	SGEFPKSGPS	APDLALVEAA	ADGPEAPLQD
**250**	**260**	**270**	**280**	**290**	**300**	**310**	**320**
RGWALCFSAL	GMVQCCKVTC	HELCHLLGLG	NCRWLRCLMQ	GALSLDEALR	RPLDLCPICL	RKLQHVLGFR	LIERYQRLYT
**330**	**340**	**350**	**360**	**370**	**380**	**390**	**400**
WTQAVVGTWP	SQEAGEPSVW	EDTPPASADS	GMCCESDSEP	GTSVSEPLTP	DAGSHTFASG	PEEGLSYLAA	SEAPLPPGGP
**410**	**420**	**430**	**440**	**450**	**460**	**470**	**480**
AEAIKEHERW	LAMCIQALQR	EVAEEDLVQV	DRAVDALDRW	EMFTGQLPAT	RQDPPSSRDS	VGLRKVLGDK	FSSLRRKLSA
**490**	**500**						
RKLARAESAP	RPWDGEES						

Cmpd.	No. of Cmpds.	m/z meas.	Δ m/z [ppm]	z	Rt [min]	Score	P	Range	Sequence	Modification	Ratios
704	1	457.7465	-113.16	2	37.6	18.2	2	478-485	K.LSARKLAR.A		WD:WU 0.54

Detailed Protein Report

Protein 152: PREDICTED: kelch-like protein 17 isoform X1 [Homo sapiens]

**Accession:**	gi|530360301	**Score:**	31.4
**Database:**	refseq_human(refseq_human_20140103.fasta)	**MW [kDa]:**	66.6
**Database Date:**	2015-11-30	**pI:**	6.9
**Modification(s):**	Carbamidomethyl	**Sequence Coverage [%]:**	4.6
		**No. of unique Peptides:**	1

10	20	30	40	50	60	70	80
MEGAVQLLSR	EGHSVAHNSK	RHYHDAFVAM	SRMRQRGLLC	DIVLHVAAKE	IRAHKVVLAS	CSPYFHAMFT	NEMSESRQTH
**90**	**100**	**110**	**120**	**130**	**140**	**150**	**160**
VTLHDIDPQA	LDQLVQFAYT	AEIVVGEGNV	QTLLPAASLL	QLNGVRDACC	KFLLSQLDPS	NCLGIRGFAD	AHSCSDLLKA
**170**	**180**	**190**	**200**	**210**	**220**	**230**	**240**
AHRYVLQHFV	DVAKTEEFML	LPLKQASSGL	PGSTGPSPDL	RSEDPHSQVL	ELVSSDSLNV	PSEEEVYRAV	LSWVKHDVDA
**250**	**260**	**270**	**280**	**290**	**300**	**310**	**320**
RRQHVPRLMK	CVRLPLLSRD	FLLGHVDAES	LVRHHPDCKD	LLIEALKFHL	LPEQRGVLGT	SRTRPRRCEG	AGPVLFAVGG
**330**	**340**	**350**	**360**	**370**	**380**	**390**	**400**
GSLFAIHGDC	EAYDTRTDRW	HVVASMSTRR	ARVGVAAVGN	RLYAVGGYDG	TSDLATVESY	DPVTNTWQPE	VSMGTRRSCL
**410**	**420**	**430**	**440**	**450**	**460**	**470**	**480**
GVAALHGLLY	SAGGYDGASC	LNSAERYDPL	TGTWTSVAAM	STRRRYVRVA	TLDGNLYAVG	GYDSSSHLAT	VEKYEPQVNV
**490**	**500**	**510**	**520**	**530**	**540**	**550**	**560**
WSPVASMLSR	RSSAGVAVLE	GALYVAGGND	GTSCLNSVER	YSPKAGAWES	VAPMNIRRST	HDLVAMDGWL	YAVGGNDGSS
**570**	**580**	**590**	**600**	**610**	**620**		
SLNSIEKYNP	RTNKWVAASC	MFTRRSSVGV	AVLELLNFPP	PSSPTLSVSS	TSL		

Cmpd.	No. of Cmpds.	m/z meas.	Δ m/z [ppm]	z	Rt [min]	Score	P	Range	Sequence	Modification
2931	1	1051.5566	47.56	2	65.4	17.0	2	568-584	K.YNPRTNKWVAASCMFTR.R	Carbamidomethyl: 13

Detailed Protein Report

Protein 153: beta-crystallin B1 [Homo sapiens]

Accession: gi|4503061
Database: refseq_human(refseq_human_20140103.fasta)
Database Date: 2015-11-30

Score: 31.3
MW [kDa]: 28.0
pI: 9.2
Sequence Coverage [%]: 13.5
No. of unique Peptides: 2

10	20	30	40	50	60	70	80
MSQAAKASAS	ATVAVNPGPD	TKGKGAPPAG	TSPSPGTTLA	PTTVPITSAK	AAELPPGNYR	LVVFELENFQ	GRRAEFSGEC
**90**	**100**	**110**	**120**	**130**	**140**	**150**	**160**
SNLADRGFDR	VRSIIVSAGP	WVAFEQSNFR	GEMFILEKGE	YPRWNTWSSS	YRSDRLMSFR	PIKMDAQEHK	ISLFEGANFK
**170**	**180**	**190**	**200**	**210**	**220**	**230**	**240**
GNTIEIQGDD	APSLWVYGFS	DRVGSVKVSS	GTWVGYQYPG	YRGYQYLLEP	GDFRHWNEWG	AFQPQMQSLR	RLRDKQWHLE
**250**	**260**						
GSFPVLATEP	PK						

Cmpd.	No. of Cmpds.	m/z meas.	Δ m/z [ppm]	z	Rt [min]	Score	P	Range	Sequence	Modification
2177	1	1051.4295	-96.54	2	55.7	20.8	1	1-22	-.MSQAAKASASATVAVNPGPDTK.G	
2974	1	729.3714	20.28	2	65.9	10.5	0	203-214	R.GYQYLLEPGDFR.H	

Detailed Protein Report

Protein 154: PREDICTED: pulmonary surfactant-associated protein C isoform X1 [Homo sapiens]

Accession: gi|530387966
Database: refseq_human(refseq_human_20140103.fasta)
Database Date: 2015-11-30

Score: 31.2
MW [kDa]: 28.6
pI: 10.8
Sequence Coverage [%]: 14.4
No. of unique Peptides: 1

10	20	30	40	50	60	70	80
MAPRDAHRDP	EMPAVLGSPA	GQLPTWLRPS	THPGHTWERR	RGEHSTCSKM	DVGSKEVLME	SPPDYSAAPR	GRFGIPCCPV
**90**	**100**	**110**	**120**	**130**	**140**	**150**	**160**
HLKRLLIVVV	VVVLIVVVIV	GALLMGLHMS	QKHTEMVLEM	SIGAPEAQQR	LALSEHLVTT	ATFSIGSTGL	VVYDYQQLLI
**170**	**180**	**190**	**200**	**210**	**220**	**230**	**240**
AYKPAPGTCC	YIMKIAPESI	PSLEALTRKV	HNFQGQWKPQ	RERKRPGQRV	FCSFCRRARS	CFCPHRRDKP	WRNGSLGRGW
**250**	**260**						
EWAEVAPRGP	GTPATTE						

Cmpd.	No. of Cmpds.	m/z meas.	Δ m/z [ppm]	z	Rt [min]	Score	P	Range	Sequence	Modification
1479	1	1006.6165	136.56	2	46.9	11.4	1	239-257	R.GWEWAEVAPRGPGTPATTE.-	

Detailed Protein Report

Protein 155: **RNA polymerase II subunit A C-terminal domain phosphatase isoform 3 [Homo sapiens]**

**Accession:**	gi|321267522	**Score:**	31.2
**Database:**	refseq_human(refseq_human_20140103.fasta)	**MW [kDa]:**	92.3
**Database Date:**	2015-11-30	**pI:**	4.9
		**Sequence Coverage [%]:**	4.9
		**No. of unique Peptides:**	1

Quantitation

WD:WU **Median:** 0.82 **CV:** 0.00 % **No. of Peptides:** 1

10	20	30	40	50	60	70	80
MKGLCAECGQ	DLTQLQSKNG	KQQVPLSTAT	VSMVHSVPEL	MVSSEQAEQL	GREDQQRLHR	NRKLVLMVDL	DQTLIHTTEQ
90	100	110	120	130	140	150	160
HCQQMSNKGI	FHFQLGRGEP	MLHTRLRPHC	KDFLEKIAKL	YELHVFTFGS	RLYAHTIAGF	LDPEKKLFSH	RILSRDECID
170	180	190	200	210	220	230	240
PFSKTGNLRN	LFPCGDSMVC	IIDDREDVWK	FAPNLITVKK	YVYFQGTGDM	NAPPGSRESQ	TRKKVNHSRG	TEVSEPSPPV
250	260	270	280	290	300	310	320
RDPEGVTQAP	GVEPSNGLEK	PARELNGSEA	ATPRDSPRPG	KPDERDIWPP	AQAPTSSQEL	AGAPEPQGSC	AQGGRVAPGQ
330	340	350	360	370	380	390	400
RPAQGATGTD	LDFDLSSDSE	SSSESEGTKS	SSSASDGESE	GKRGRQKPKA	APEGAGALAQ	GSSLEPGRPA	APSLPGEAEP
410	420	430	440	450	460	470	480
GAHAPDKEPE	LGGQEEGERD	GLCGLGNGCA	DRKEAETESQ	NSELSGVTAG	ESLDQSMEEE	EEEDTDEDDH	LIYLEEILVR
490	500	510	520	530	540	550	560
VHTDYYAKYD	RYLNKEIEEA	PDIRKIVPEL	KSKVLADVAI	IFSGLHPTNF	PIEKTREHYH	ATALGAKILT	RLVLSPDAPD
570	580	590	600	610	620	630	640
RATHLIAARA	GTEKVLQAQE	CGHLHVVNPD	WLWSCLERWD	KVEEQLFPLR	DDHTKAQREN	SPAAFPDREG	VPPTALFHPM
650	660	670	680	690	700	710	720
PVLPKAQPGP	EVRIYDSNTG	KLIRTGARGP	PAPSSSLPIR	QEPSSFRAVP	PPQPQMFGEE	LPDAQDGEQP	GPSRRKRQPS
730	740	750	760	770	780	790	800
MSETMPLYTL	CKEDLESMDK	EVDDILGEGS	DDSDSEKRRP	EEQEEEPQPR	KPGTRRERTL	GAPASSERSA	AGGRGPRGHK
810	820	830	840	850			
RKLNEEDAAS	ESSRESSNED	EGSSSEADEM	AKALEAELND	LM			

Cmpd.	No. of Cmpds.	m/z meas.	Δ m/z [ppm]	z	Rt [min]	Score	P	Range	Sequence	Modification	Ratios
1965	2	899.3643	-116.81	2	52.9	16.3	2	779-797	R.TLGAPASSERSAAGGRGPR.G		WD:WU 0.82

Detailed Protein Report

Protein 156: PREDICTED: autoimmune regulator isoform X1 [Homo sapiens]

**Accession:**	gi|530419216	**Score:**	31.1
**Database:**	refseq_human(refseq_human_20140103.fasta)	**MW [kDa]:**	36.5
**Database Date:**	2015-11-30	**pI:**	6.4
		**Sequence Coverage [%]:**	6.9
		**No. of unique Peptides:**	1

10	20	30	40	50	60	70	80
MWLVYSSGAP	GTQQPARNRV	FFPIGMAPGG	VCWRPDGWGT	GGQGRISGPG	SMGAGQRLGS	SGTQRCCWGS	CFGKEVALRR
**90**	**100**	**110**	**120**	**130**	**140**	**150**	**160**
VLHPSPVCMG	VSCLCQKNED	ECAVCRDGGE	LICCDGCPRA	FHLACLSPPL	REIPSGTWRC	SSCLQATVQE	VQPRAEEPRP
**170**	**180**	**190**	**200**	**210**	**220**	**230**	**240**
QEPPVETPLP	PGLRSAGEEV	RGPPGEPLAG	MDTTLVYKHL	PAPPSAAPLP	GLDSSALHPL	LCVGPEGQQN	LAPGARCGVC
**250**	**260**	**270**	**280**	**290**	**300**	**310**	**320**
GDGTDVLRCT	HCAAAFHWRC	HFPAGTSRPG	TGLRCRSCSG	DVTPAPVEGV	LAPSPARLAP	GPAKDDTASH	EPALHRDDLE
**330**	**340**	**350**					
SLLSEHTFDG	ILQWAIQSMA	RPAAPFPS					

Cmpd.	No. of Cmpds.	m/z meas.	Δ m/z [ppm]	z	Rt [min]	Score	P	Range	Sequence	Modification
1351	1	669.2815	20.48	2	45.7	14.6	0	107-119	R.DGGELICCDGCPR.A	

Detailed Protein Report

Protein 157: PREDICTED: Golgin subfamily A member 8A isoform X1 [Homo sapiens]

**Accession:**	gi|578826741	**Score:**	31.1
**Database:**	refseq_human(refseq_human_20140103.fasta)	**MW [kDa]:**	67.1
**Database Date:**	2015-11-30	**pI:**	6.4
**Modification(s):**	Oxidation	**Sequence Coverage [%]:**	3.2
		**No. of unique Peptides:**	1

Quantitation

WD:WU **Median:** 0.66 **CV:** 0.00 % **No. of Peptides:** 1

10	20	30	40	50	60	70	80
MAEETGQSKL	AAAKKKFKEY	WQRNRPGVPA	AAKRNTKANG	SSPETAASGG	CHSSEASSSA	SSSLHARQSP	CQEQAAVLNS
**90**	**100**	**110**	**120**	**130**	**140**	**150**	**160**
RSIKISRLND	TIKSLKQQKK	QVEHQLEEEK	KANNEKQKAE	RELEGQIQRL	NTEKKKLNTD	LYHMKHSLRY	FEEESKDLAG
**170**	**180**	**190**	**200**	**210**	**220**	**230**	**240**
RLQRSSQRIG	ELEWSLCAVA	ATQKKKPDGF	SSRSKALLKR	QLEQSIREQI	LLKGHVTQLK	ESLKEVQLER	DQYAEQIKGE
**250**	**260**	**270**	**280**	**290**	**300**	**310**	**320**
RAQWQQRMRK	MSQEVCTLKE	EKKHDTHRVE	ELERSLSRLK	NQMAEPLPPD	APAVSSEVEL	QDLRKELERV	AGELQAQVEN
**330**	**340**	**350**	**360**	**370**	**380**	**390**	**400**
NQCISLLNRG	QKERLREQEE	RLQEQQERLR	EREKRLQQLA	EPQSDLEELH	ENKSALQLEQ	QVKELQEKLG	QVMETLTSAE
**410**	**420**	**430**	**440**	**450**	**460**	**470**	**480**
KEPEAAVPAS	GTGGESSGLM	DLLEEKADLR	EHVEKLELGF	IQYRRERCHQ	KVHRLLTEPG	DSAKDASPGG	GHHQAGPGQG
**490**	**500**	**510**	**520**	**530**	**540**	**550**	**560**
GEEGEAAGAA	GDGVAACGSY	SEGHGKFLAA	ARNPAAEPSP	GAPAPQELGA	ADKHGDLCEA	SLTNSVEPAQ	GEAREGSSQD
**570**	**580**	**590**	**600**	**610**			
NPTAQPVVQL	LGEMQDHQEH	PGLGSNCCVP	CFCWAWLPRR	RR			

Cmpd.	No. of Cmpds.	m/z meas.	Δ m/z [ppm]	z	Rt [min]	Score	P	Range	Sequence	Modification	Ratios
103	1	822.3979	-26.54	2	30.5	11.3	1	137-149	K.LNTDLYHMKHSLR.Y	Oxidation: 8	WD:WU 0.66

Detailed Protein Report

Protein 158: keratin, type II cytoskeletal 73 [Homo sapiens]

**Accession:**	gi|28173564	**Score:**	30.7
**Database:**	refseq_human(refseq_human_20140103.fasta)	**MW [kDa]:**	58.9
**Database Date:**	2015-11-30	**pI:**	7.6
		**Sequence Coverage [%]:**	4.6
		**No. of unique Peptides:**	1

10	20	30	40	50	60	70	80
MSRQFTYKSG	AAAKGGFSGC	SAVLSGGSSS	SYRAGGKGLS	GGFSSRSLYS	LGGARSISFN	VASGSGWAGG	YGFGRGRASG
90	100	110	120	130	140	150	160
FAGSMFGSVA	LGSVCPSLCP	PGGIHQVTIN	KSLLAPLNVE	LDPEIQKVRA	QEREQIKVLN	NKFASFIDKV	RFLEQQNQVL
170	180	190	200	210	220	230	240
ETKWELLQQL	DLNNCKNNLE	PILEGYISNL	RKQLETLSGD	RVRLDSELRS	VREVVEDYKK	RYEEEINKRT	TAENEFVVLK
250	260	270	280	290	300	310	320
KDVDAAYTSK	VELQAKVDAL	DGEIKFFKCL	YEGETAQIQS	HISDTSIILS	MDNNRNLDLD	SIIAEVRAQY	EEIARKSKAE
330	340	350	360	370	380	390	400
AEALYQTKFQ	ELQLAAGRHG	DDLKHTKNEI	SELTRLIQRL	RSEIESVKKQ	CANLETAIAD	AEQRGDCALK	DARAKLDELE
410	420	430	440	450	460	470	480
GALQQAKEEL	ARMLREYQEL	LSVKLSLDIE	IATYRKLLEG	EECRMSGEYT	NSVSISVINS	SMAGMAGTGA	GFGFSNAGTY
490	500	510	520	530	540	550	
GYWPSSVSGG	YSMLPGGCVT	GSGNCSPRGE	ARTRLGSASE	FRDSQGKTLA	LSSPTKKTMR		

Cmpd.	No. of Cmpds.	m/z meas.	Δ m/z [ppm]	z	Rt [min]	Score	P	Range	Sequence	Modification
2930	1	899.4860	22.51	2	65.4	12.0	1	329-344	K.FQELQLAAGRHGDDLK.H	

Detailed Protein Report

Protein 159: PREDICTED: kinesin-like protein KIF13B isoform X2 [Homo sapiens]

**Accession:**	gi|530387685	**Score:**	30.4
**Database:**	refseq_human(refseq_human_20140103.fasta)	**MW [kDa]:**	195.4
**Database Date:**	2015-11-30	**pI:**	5.4
**Modification(s):**	Oxidation	**Sequence Coverage [%]:**	0.9
		**No. of unique Peptides:**	1

Alias proteins:

Accession	Name	Description
gi|578815259	refseq_human (refseq_human_20140103.fasta)	PREDICTED: kinesin-like protein KIF13B isoform X4 [Homo sapiens]
gi|578815257	refseq_human (refseq_human_20140103.fasta)	PREDICTED: kinesin-like protein KIF13B isoform X3 [Homo sapiens]

Detailed Protein Report

10	20	30	40	50	60	70	80
MDESVKEKYA	GQDIVFKCLG	ENILQNAFDG	YNACIFAYGQ	TGSGKSYTMM	GTADQPGLIP	RLCSGLFERT	QKEENEEQSF
90	100	110	120	130	140	150	160
KVEVSYMEIY	NEKVRDLLDP	KGSRQTLKVR	EHSVLGPYVD	GLSKLAVTSY	KDIESLMSEG	NKSRTVAATN	MNEESSRSHA
170	180	190	200	210	220	230	240
VFKITLTHTL	YDVKSGTSGE	KVGKLSLVDL	AGSERATKTG	AAGDRLKEGS	NINKSLTTLG	LVISALADQS	AGKNKNKFVP
250	260	270	280	290	300	310	320
YRDSVLTWLL	KDSLGGNSKT	AMVATVSPAA	DNYDETLSTL	RYADRAKHIV	NHAVVNEDPN	ARIIRDLREE	VEKLREQLTK
330	340	350	360	370	380	390	400
AEAMKSPELK	DRLEESEKLI	QEMTVTWEEK	LRKTEEIAQE	RQKQLESLGI	SLQSSGIKVG	DDKCFLVNLN	ADPALNELLV
410	420	430	440	450	460	470	480
YYLKEHTLIG	SANSQDIQLC	GMGILPEHCI	IDITSEGQVM	LTPQKNTRTF	VNGSSVSSPI	QLHHGDRILW	GNNHFFRLNL
490	500	510	520	530	540	550	560
PKKKKKAERE	DEDQDPSMKN	ENSSEQLDVD	GDSSSEVSSE	VNFNYEYAQM	EVTMKALGSN	DPMQSILNSL	EQQHEEEKRS
570	580	590	600	610	620	630	640
ALERQRLMYE	HELEQLRRRL	SPEKQNCRSM	DRFSFHSPSA	QQRLRQWAEE	REATLNNSLM	RLREQIVKAN	LLVREANYIA
650	660	670	680	690	700	710	720
EELDKRTEYK	VTLQIPASSL	DANRKRGSLL	SEPAIQVRRK	GKGKQIWSLE	KLDNRLLDMR	DLYQEWKECE	EDNPVIRSYF
730	740	750	760	770	780	790	800
KRADPFYDEQ	ENHSLIGVAN	VFLESLFYDV	KLQYAVPIIN	QKGEVAGRLH	VEVMRLSGDV	GERIAGGDEV	AEVSFEKETQ
810	820	830	840	850	860	870	880
ENKLVCMVKI	LQATGLPQHL	SHFVFCKYSF	WDQQEPVIVA	PEVDTSSSSV	SKEPHCMVVF	DHCNEFSVNI	TEDFIEHLSE
890	900	910	920	930	940	950	960
GALAIEVYGH	KINDPRKNPA	LWDLGIIQAK	TRSLRDRWSE	VTRKLEFWVQ	ILEQNENGEY	CPVEVISAKD	VPTGGIFQLR
970	980	990	1000	1010	1020	1030	1040
QGQSRRVQVE	VKSVQESGTL	PLMEECILSV	GIGCVKVRPL	RAPRTHETFH	EEEEDMDSYQ	DRDLERLRRK	WLNALTKRQE
1050	1060	1070	1080	1090	1100	1110	1120
YLDQQLQKLV	SKRDKTEDDA	DREAQLLEMR	LTLTEERNAV	MVPSAGSGIP	GAPAEWTPVP	GMETHIPVIF	LDLNADDFSS
1130	1140	1150	1160	1170	1180	1190	1200
QDNLDDPEAG	GWDATLTGEE	EEEFFELQIV	KQHDGEVKAE	ASWDSAVHGC	PQLSRGTPVD	ERLFLIVRVT	VQLSHPADMQ
1210	1220	1230	1240	1250	1260	1270	1280
LVLRKRICVN	VHGRQGFAQS	LLKKMSHRSS	IPGCGVTFEI	VSNIPEDAQG	VEEREALARM	AANVENPASA	DSEAYIEKYL
1290	1300	1310	1320	1330	1340	1350	1360
RSVLAVENLL	TLDRLRQEVA	VKEQLTGKGK	LSRRSISSPN	VNRLSGSRQD	LIPSYSLGSN	KGRWESQQDV	SQTTVSRGIA
1370	1380	1390	1400	1410	1420	1430	1440
PAPALSVSPQ	NNHSPDPGLS	NLAASYLNPV	KSFVPQMPKL	LKSLFPVRDE	KRGKRPSPLA	HQPVPRIMVQ	SASPDIRVTR
1450	1460	1470	1480	1490	1500	1510	1520
MEEAQPEMGP	DVLVQTMGAP	ALKICDKPAK	VPSPPPVIAV	TAVTPAPEAQ	DGPPSPLSEA	SSGYFSHSVS	TATLSDALGP
1530	1540	1550	1560	1570	1580	1590	1600
GLDAAAPPGS	MPTAPEAEPE	APISHPPPPT	AVPAEEPPGP	QQLVSPGRER	PDLEAPAPGS	PFRVRRVRAS	ELRSFSRMLA
1610	1620	1630	1640	1650	1660	1670	1680
GDPGCSPGAE	GNAPAPGAGG	QALASDSEEA	DEVPEWLREG	EFVTVGAHKT	GVVRYVGPAD	FQEGTWVGVE	LDLPSGKNDG
1690	1700	1710	1720	1730	1740	1750	1760
SIGGKQYFRC	NPGYGLLVRP	SRVRRATGPV	RRRSTGLRLG	APEARRSATL	SGSATNLASL	TAALAKADRS	HKNPENRKSW
1770							
AS							

Cmpd.	No. of Cmpds.	m/z meas.	Δ m/z [ppm]	z	Rt [min]	Score	P	Range	Sequence	Modification
1541	1	513.1056	-215.54	2	48.0	12.4	1	585-592	K.QNCRSMDR.F	Oxidation: 6

Detailed Protein Report

Protein 160:	PREDICTED: beta/gamma crystallin domain-containing protein 3 isoform X1 [Homo sapiens]		
**Accession:**	gi|530373918	**Score:**	30.4
**Database:**	refseq_human(refseq_human_20140103.fasta)	**MW [kDa]:**	298.2
**Database Date:**	2015-11-30	**pI:**	5.0
**Modification(s):**	Oxidation	**Sequence Coverage [%]:**	1.3
		**No. of unique Peptides:**	1

Detailed Protein Report

10	20	30	40	50	60	70	80
MKGNLLEGPL	EDSDCSKTSF	NKENSLTNNP	ELQNIASSNN	LLNKNAWGSI	ERNRSSPSSV	TNSSYDGESD	SQHHLSCEPV
90	100	110	120	130	140	150	160
SQTNRNLVCS	ALLTGSNHRK	VPCSPDFQRV	TTTENTIKEN	STVMSNRTLV	QREELVEPQG	PAISDFSCSK	SDGSDTTEQE
170	180	190	200	210	220	230	240
STNLPSPNKS	IRHEHLQLPE	SECSDKQTID	SSSKQAATHT	NIIALQRHAV	TDTEFVNEGK	RLSAQDSQKN	VAVREIRRET
250	260	270	280	290	300	310	320
ESASAGESIA	SSHVKAPEDK	IESLPKDTDQ	YFETKAKKLD	FRSHDKIPHI	RMNKKDLASL	NYISESAVVA	SLGNENAPEL
330	340	350	360	370	380	390	400
KFELNRSHIS	ETPLDSESPQ	QAEVSPDAKT	SLSLDCKKLN	FSISPPTFVS	GVGMLSKLDI	PDLMNEGSPV	PIETGNVNIV
410	420	430	440	450	460	470	480
GISYQPRKCK	EENVKNHVEA	AGRKSPPPSF	CLEYTSAIFE	FKEVLSNSEK	CQVLPGSEAS	GPHLTGLELL	SFDSGNLSKD
490	500	510	520	530	540	550	560
CSSILSQDPN	RVELVSSNTK	ANMSIIEKSD	SLSLEAKTAN	IVSKAEIDGQ	NNVLVESHSG	RGKTISLSKV	SLSKVEPRNI
570	580	590	600	610	620	630	640
SQDKMSSFPL	KITHVPEKPI	LSELTFLEVE	QGKRFQSINH	NEIGEKCSDA	GLKENCQAEL	SPAASKYEDK	PEPEVDALGS
650	660	670	680	690	700	710	720
PPALLKSNIS	WILPPIHDEK	ISRQMAQNCE	AHTCVFHQSL	DICGTKKISG	HSEMAELSLT	NISPKFQETG	SMKVNSPFLD
730	740	750	760	770	780	790	800
SDSSLEKNSS	ASEDSSFLKV	PSVLKLEKKS	SSYRKKENIH	FLNGGIDSVS	SSSSYPEEVS	MIVNSHKPQN	NLDSIQVTKD
810	820	830	840	850	860	870	880
LTHEGTSVTN	LLYPTTSYLE	FETSVSIGTE	VTPFQEHFGI	YTGKISIDFP	TAAQFDNLVE	AETGAVAGPA	ASVNSSGQQC
890	900	910	920	930	940	950	960
SEASAEHIEA	RRRAHDQLLD	LKSSLLKKAD	TLIGEIFNSV	REELKFKHTV	STCQEHIAIE	GIMNLGTLKE	DISEKNPSEV
970	980	990	1000	1010	1020	1030	1040
TLTEIQQTEG	LEEQGMENMS	EVKEKPCVSP	TVGEKNLLVD	PNSMNVSCLL	EDKARELVNE	IIYVAQEKLR	NDTFEDTEDT
1050	1060	1070	1080	1090	1100	1110	1120
WDSELQANTS	KILNSDSVKP	HDVVREFLVS	EQPVNQSTQI	SENKVLNEFF	SLSNLASGTE	SIKGGEIVLY	QKSLFSGNGS
1130	1140	1150	1160	1170	1180	1190	1200
GLSDSINLQE	SDTVLLAEDM	SHKRLDDRVK	THLFRSEDCN	ETMEIENVDN	NKTETEDRRT	LVLNFKWPPL	VNDDIHAPGT
1210	1220	1230	1240	1250	1260	1270	1280
SKSSLSDSLV	CISEKNLPGH	SKNTPLAMSD	VGKVHKKDNE	INIGKIELIP	SMLETGKTNK	KDAELNILKY	EAVPPMIEMG
1290	1300	1310	1320	1330	1340	1350	1360
RIHKMDAELN	VTKTEPKANV	FKMGEVYQMD	AESCIEKTEG	SAVILGMEKA	YKMKDTEGDI	GKIEVIPMMP	EVKNIHQKDA
1370	1380	1390	1400	1410	1420	1430	1440
EGDIVKTEMT	PVTVDMENIY	QTHAEGDIGK	TGTIALSEVE	NIHQKGGEGI	SEKAEVIPVT	LAMENTYQKD	AEGDIGKAEV
1450	1460	1470	1480	1490	1500	1510	1520
MPVRLEMENT	YPKDTERDGG	KTEVMPLALE	VVNTYQKNAK	GFTGNTEGSV	LKMEATYRKT	AEEVIKNTEI	VPCVLKVKEA
1530	1540	1550	1560	1570	1580	1590	1600
HETAPAPLEM	EKACKRDVKE	TIGATVSTPS	VIEMEKISPE	DRGENIGKHK	VLPAVVDIEK	IHGTGLELTT	KQGEAMLPAF
1610	1620	1630	1640	1650	1660	1670	1680
ESKTPQEYAE	GSVEETKEEP	TEIKEGLIAH	ENRLPTYFRG	YESPTLSKDY	EGYPAPAMPD	FQPGDTTVRL	DKRMSLTAIY
1690	1700	1710	1720	1730	1740	1750	1760
DKRRETDYSD	KGYNLAFVSQ	DEQENSSFTI	LYEEPLQEED	KYASAEARQT	QSVLFHDTSA	DSMPVLACER	SESRTDLVHH
1770	1780	1790	1800	1810	1820	1830	1840
FEKGTKLGET	FDSDSSEMFL	SVEAKRYKIY	PLALSPIYED	DSSQEDILSS	EVSPGHHGPR	KSRDSENQSS	SVLSLLQSVS
1850	1860	1870	1880	1890	1900	1910	1920
ERLKMNFDED	DREAADEEEE	EEEAAVLHKG	DLRAGSGERV	TFQLPDPSIT	FYPDDQESVG	ISKNSYVMPN	EPTTSNLQVG
1930	1940	1950	1960	1970	1980	1990	2000
LWPEKTSFLQ	KSDLTSKLHS	SLKSAYHQYL	QTSQSHSSEK	GARFGGIFQE	PVSKYFRVQD	SPGRLSPFIE	NVDKQTLRCN
2010	2020	2030	2040	2050	2060	2070	2080
PRPGKMVIYD	LHESTYKQEV	YCNIPDATSW	SFPNGVLIKV	VRGCWILYEK	PHFRGQKCVL	EEGEKVLNRD	WILQNRRHPQ
2090	2100	2110	2120	2130	2140	2150	2160
RNFILGSLKR	VLKDCSIPEI	ELFPQSDPAC	CPVYIQRAVP	NLEELNISKS	VSFTVKSGVW	LAYPDINFKG	QATVLEEDHG
2170	2180	2190	2200	2210	2220	2230	2240

Detailed Protein Report

Cmpd.	No. of Cmpds.	m/z meas.	Δ m/z [ppm]	z	Rt [min]	Score	P	Range	Sequence	Modification
1726	1	900.4572	47.30	3	49.9	14.8	1	1649-1672	K.DYEGYPAPAMPDFQPGDTTVRLDK.R	Oxidation: 10

Detailed Protein Report

Protein 161: PREDICTED: OTU domain-containing protein 7B isoform X2 [Homo sapiens]

**Accession:**	gi|530432124	**Score:**	30.4
**Database:**	refseq_human(refseq_human_20140103.fasta)	**MW [kDa]:**	84.0
**Database Date:**	2015-11-30	**pI:**	7.0
**Modification(s):**	Oxidation	**Sequence Coverage [%]:**	3.8
		**No. of unique Peptides:**	1

10	20	30	40	50	60	70	80
MPPSVILNSY	VKSMLETYPH	PLVRGVVAPG	PLKKGFLTES	LLALPDPSSS	GRMTSFKLPD	LTVYNEDFRS	FIERDLIEQS
**90**	**100**	**110**	**120**	**130**	**140**	**150**	**160**
MLVALEQAGR	LNWWVSVDPT	SQRLLPLATT	GDGNCLLHAA	SLGMWGFHDR	DLMLRKALYA	LMEKGVEKEA	LKRRWRWQQT
**170**	**180**	**190**	**200**	**210**	**220**	**230**	**240**
QQNKESGLVY	TEDEWQKEWN	ELIKLASSEP	RMHLGTNGAN	CGGVESSEEP	VYESLEEFHV	FVLAHVLRRP	IVVVADTMLR
**250**	**260**	**270**	**280**	**290**	**300**	**310**	**320**
DSGGEAFAPI	PFGGIYLPLE	VPASQCHRSP	LVLAYDQAHF	SALVSMEQKE	NTKEQAVIPL	TDSEYKLLPL	HFAVDPGKGW
**330**	**340**	**350**	**360**	**370**	**380**	**390**	**400**
EWGKDDSDNV	RLASVILSLE	VKLHLLHSYM	NVKWIPLSSD	AQAPLAQPES	PTASAGDEPR	STPESGDSDK	ESVGSSSTSN
**410**	**420**	**430**	**440**	**450**	**460**	**470**	**480**
EGGRRKEKSK	RDREKDKKRA	DSVANKLGSF	GKTLGSKLKK	NMGGLMHSKG	SKPGGVGTGL	GGSSGTETLE	KKKKNSLKSW
**490**	**500**	**510**	**520**	**530**	**540**	**550**	**560**
KGGKEEAAGD	GPVSEKPPAE	SVGNGGSKYS	QEVMQSLSIL	RTAMQGEGKF	IFVGTLKMGH	RHQYQEEMIQ	RYLSDAEERF
**570**	**580**	**590**	**600**	**610**	**620**	**630**	**640**
LAEQKQKEAE	RKIMNGGIGG	GPPPAKKPEP	DAREEQPTGP	PAESRAMAFS	TGYPGDFTIP	RPSGGGVHCQ	EPRRQLAGGP
**650**	**660**	**670**	**680**	**690**	**700**	**710**	**720**
CVGGLPPYAT	FPRQCPPGRP	YPHQDSIPSL	EPGSHSKDGL	HRGALLPPPY	RVADSYSNGY	REPPEPDGWA	GGLRGLPPTQ
**730**	**740**	**750**	**760**	**770**			
TKCKQPNCSF	YGHPETNNFC	SCCYREELRR	REREPDGELL	VHRF			

Cmpd.	No. of Cmpds.	m/z meas.	Δ m/z [ppm]	z	Rt [min]	Score	P	Range	Sequence	Modification
2718	1	1175.5515	-33.60	2	62.4	11.6	0	269-289	R.SPLVLAYDQAHFSALVSMEQK.E	Oxidation: 18

Detailed Protein Report

Protein 162: **PREDICTED: calcium-binding mitochondrial carrier protein SCaMC-3 isoform X1 [Homo sapiens]**

**Accession:**	gi|578833593	**Score:**	30.4
**Database:**	refseq_human(refseq_human_20140103.fasta)	**MW [kDa]:**	45.9
**Database Date:**	2015-11-30	**pI:**	6.0
		**Sequence Coverage [%]:**	6.8
		**No. of unique Peptides:**	2

Quantitation

WD:WU **Median:** 0.81 **CV:** 0.00 % **No. of Peptides:** 1

10	20	30	40	50	60	70	80
MRGSPGDAER	RQRWGRLFEE	LDSNKDGRVD	VHELRQGLAR	LGGGNPDPGA	QQGISSEGDA	DPDGGLDLEE	FSRYLQEREQ
**90**	**100**	**110**	**120**	**130**	**140**	**150**	**160**
RLLLMFHSLD	RNQDGHIDVS	EIQQSFRALG	ISISLEQAEK	ILHSMDRDGT	MTIDWQEWRD	HFLLHSLENV	EDVLYFWKHS
**170**	**180**	**190**	**200**	**210**	**220**	**230**	**240**
TVLDIGECLT	VPDEFSKQEK	LTGMWWKQLV	AGAVAGAVSR	TGTAPLDRLK	VFMQVHASKT	NRLNILGGLR	SMVLEGGIRS
**250**	**260**	**270**	**280**	**290**	**300**	**310**	**320**
LWRGNGINVL	KIAPESAIKF	MAYEQIKRAI	LGQQETLHVQ	ERFVAGSLAG	ATAQTIIYPM	ETLKNWWLQQ	YSHDSADPGI
**330**	**340**	**350**	**360**	**370**	**380**	**390**	**400**
LVLLACGTIS	STCGQIASYP	LALVRTRMQA	QASIEGGPQL	SMLGLLRHIL	SQEGMRGLYR	GIAPNFMKVI	PAVSISYVVY
**410**	**420**						
ENMKQALGVT	SR						

Cmpd.	No. of Cmpds.	m/z meas.	Δ m/z [ppm]	z	Rt [min]	Score	P	Range	Sequence	Modification	Ratios
1152	1	644.3310	-53.87	2	43.1	13.1	1	209-219	R.LKVFMQVHASK.T		WD:WU 0.81
1852	4	899.4901	-35.49	2	51.8	17.3	1	223-239	R.LNILGGLRSMVLEGGIR.S		

Detailed Protein Report

Protein 163: PREDICTED: nuclear pore complex protein Nup214 isoform X4 [Homo sapiens]

**Accession:**	gi|530427224	**Score:**	30.4
**Database:**	refseq_human(refseq_human_20140103.fasta)	**MW [kDa]:**	209.0
**Database Date:**	2015-11-30	**pI:**	9.0
		**Sequence Coverage [%]:**	1.5
		**No. of unique Peptides:**	2

Quantitation

WD:WU	**Median:** 1.48	**CV:** 0.00 %	**No. of Peptides:** 1

Detailed Protein Report

10	20	30	40	50	60	70	80
MRGQLRAAGA	EGRKFAVERP	GFRGQGRGRQ	RWLLRHTEGG	AMGDEMDAMI	PEREMKDFQF	RALKKVRIFD	SPEELPKERS
90	100	110	120	130	140	150	160
SLLAVSNKYG	LVFAGGASGL	QIFPTKNLLI	QNKPGDDPNK	IVDKVQGLLV	PMKFPIHHLA	LSCDNLTLSA	CMMSSEYGSI
170	180	190	200	210	220	230	240
IAFFDVRTFS	NEAKQQKRPF	AYHKLLKDAG	GMVIDMKWNP	TVPSMVAVCL	ADGSIAVLQV	TETVKVCATL	PSTVAVTSVC
250	260	270	280	290	300	310	320
WSPKGKQLAV	GKQNGTVVQY	LPTLQEKKVI	PCPPFYESDH	PVRVLDVLWI	GTYVFAIVYA	AADGTLETSP	DVVMALLPKK
330	340	350	360	370	380	390	400
EEKHPEIFVN	FMEPCYGSCT	ERQHHYYLSY	IEEWDLVLAA	SAASTEVSIL	ARQSDQINWE	SWLLEDSSRA	ELPVTDKSDD
410	420	430	440	450	460	470	480
SLPMGVVVDY	TNQVEITISD	EKTLPPAPVL	MLLSTDGVLC	PFYMINQNPG	VKSLIKTPER	LSLEGERQPK	SPASLAPTPA
490	500	510	520	530	540	550	560
ASPVAPSAAS	FSFGSSGFKP	TLESTPVPSV	SAPNIAMKPS	FPPSTSAVKV	NLSEKFTAAA	TSTPVSSSQS	APPMSPFSSA
570	580	590	600	610	620	630	640
SKPAASGPLS	HPTPLSAPPS	SVPLKSSVLP	SPSAGRSAQG	SSSPVPSMVQ	KSPRITPPAA	KPGSPQAKSL	QPAVAEKQGH
650	660	670	680	690	700	710	720
QWKDSDPVMA	GIGEEIAHFQ	KELEELKART	SKACFQVGTS	EEMKMLRTES	DDLHTFLLEI	KETTESLHGD	ISSLKTTLLE
730	740	750	760	770	780	790	800
GFAGVEEARE	QNERNRDSGY	LHLLYKRPLD	PKSEAQLQEI	RRLHQYVKFA	VQDVNDVLDL	EWDQHLEQKK	KQRHLLVPER
810	820	830	840	850	860	870	880
ETLFNTLANN	REIINQQRKR	LNHLVDSLQQ	LRLYKQTSLW	SLSSAVPSQS	SIHSFDSDLE	SLCNALLKTT	IESHTKSLPK
890	900	910	920	930	940	950	960
VPAKLSPMKQ	AQLRNFLAKR	KTPPVRSTAP	ASLSRSAFLS	QRYYEDLDEV	SSTSSVSQSL	ESEDARTSCK	DDEAVVQAPR
970	980	990	1000	1010	1020	1030	1040
HAPVVRTPSI	QPSLLPHAAP	FAKSHLVHGS	SPGVMGTSVA	TSASKIIPQG	ADSTMLATKT	VKHGAPSPSH	PISAPQAAAA
1050	1060	1070	1080	1090	1100	1110	1120
AALRRQMASQ	APAVNTLTES	TLKNVPQVVN	VQELKNNPAT	PSTAMGSSVP	YSTAKTPHPV	LTPVAANQAK	QGSLINSLKP
1130	1140	1150	1160	1170	1180	1190	1200
SGPTPASGQL	SSGDKASGTA	KIETAVTSTP	SASGQFSKPF	SFSPSGTGFN	FGIITPTPSS	NFTAAQGATP	STKESSQPDA
1210	1220	1230	1240	1250	1260	1270	1280
FSSGGGSKPS	YEAIPESSPP	SGITSASNTT	PGEPAASSSR	PVAPSGTALS	TTSSKLETPP	SKLGELLFPS	SLAGETLGSF
1290	1300	1310	1320	1330	1340	1350	1360
SGLRVGQADD	STKPTNKASS	TSLTSTQPTK	TSGVPSGFNF	TAPPVLGKHT	EPPVTSSATT	TSVAPPAATS	TSSTAVFGSL
1370	1380	1390	1400	1410	1420	1430	1440
PVTSAGSSGV	ISFGGTSLSA	GKTSFSFGSQ	QTNSTVPPSA	PPPTTAATPL	PTSFPTLSFG	SLLSSATTPS	LPMSAGRSTE
1450	1460	1470	1480	1490	1500	1510	1520
EATSSALPEK	PGDSEVSASA	ASLLEEQQSA	QLPQAPPQTS	DSVKKEPVLA	QPAVSNSGTA	ASSTSLVALS	AEATPATTGV
1530	1540	1550	1560	1570	1580	1590	1600
PDARTEAVPP	ASSFSVPGQT	AVTAAAISSA	GPVAVETSST	PIASSTTSIV	APGPSAEAAA	FGTVTSGSSV	FAQPPAASSS
1610	1620	1630	1640	1650	1660	1670	1680
SAFNQLTNNT	ATAPSATPVF	GQVAASTAPS	LFGQQTGSTA	STAAATPQVS	SSGFSSPAFG	TTAPGVFGQT	TFGQASVFGQ
1690	1700	1710	1720	1730	1740	1750	1760
SASSAASVFS	FSQPGFSSVP	AFGQPASSTP	TSTSGSVFGA	ASSTSSSSSF	SFGQSSPNTG	GGLFGQSNAP	AFGQSPGFGQ
1770	1780	1790	1800	1810	1820	1830	1840
GGSVFGGTSA	ATTTAATSGF	SFCQASGFGS	SNTGSVFGQA	ASTGGIVFGQ	QSSSSSGSVF	GSGNTGRGGG	FFSGLGGKPS
1850	1860	1870	1880	1890	1900	1910	1920
QDAANKNPFS	SASGGFGSTA	TSNTSNLFGN	SGAKTFGGFA	SSSFGEQKPT	GTFSSGGGSV	ASQGFGFSSP	NKTGGFGAAP
1930	1940	1950	1960	1970	1980	1990	2000
VFGSPPTFGG	SPGFGGVPAF	GSAPAFTSPL	GSTGGKVFGE	GTAAASAGGF	GFGSSSNTTS	FGTLASQNAP	TFGSLSQQTS
2010	2020	2030	2040				
GFGTQSSGFS	GFGSGTGGFS	FGSNNSSVQG	FGGWRS				

Cmpd.	No. of Cmpds.	m/z meas.	Δ m/z [ppm]	z	Rt [min]	Score	P	Range	Sequence	Modification	Ratios

Detailed Protein Report

Cmpd.	No. of Cmpds.	m/z meas.	Δ m/z [ppm]	z	Rt [min]	Score	P	Range	Sequence	Modification	Ratios
2741	1	715.3112	-107.27	2	62.7	17.4	1	66-77	K.VRIFDSPEELPK.E		WD:WU 1.48
2086	1	1023.4014	-90.92	2	54.7	13.0	2	670-687	R.TSKACFQVGTSEEMKMLR.T		

Detailed Protein Report

Protein 164: sortilin-related receptor preproprotein [Homo sapiens]

**Accession:**	gi|4507157	**Score:**	30.4
**Database:**	refseq_human(refseq_human_20140103.fasta)	**MW [kDa]:**	248.3
**Database Date:**	2015-11-30	**pI:**	5.2
**Modification(s):**	Carbamidomethyl	**Sequence Coverage [%]:**	0.7
		**No. of unique Peptides:**	1

Quantitation

WD:WU	**Median:**	1.02	**CV:**	0.00 %	**No. of Peptides:**	1

Detailed Protein Report

10	20	30	40	50	60	70	80
MATRSSRRES	RLPFLFTLVA	LLPPGALCEV	WTQRLHGGSA	PLPQDRGFLV	VQGDPRELRL	WARGDARGAS	RADEKPLRRK
90	100	110	120	130	140	150	160
RSAALQPEPI	KVYGQVSLND	SHNQMVVHWA	GEKSNVIVAL	ARDSLALARP	KSSDVYVSYD	YGKSFKKISD	KLNFGLGNRS
170	180	190	200	210	220	230	240
EAVIAQFYHS	PADNKRYIFA	DAYAQYLWIT	FDFCNTLQGF	SIPFRAADLL	LHSKASNLLL	GFDRSHPNKQ	LWKSDDFGQT
250	260	270	280	290	300	310	320
WIMIQEHVKS	FSWGIDPYDK	PNTIYIERHE	PSGYSTVFRS	TDFFQSRENQ	EVILEEVRDF	QLRDKYMFAT	KVVHLLGSEQ
330	340	350	360	370	380	390	400
QSSVQLWVSF	GRKPMRAAQF	VTRHPINEYY	IADASEDQVF	VCVSHSNNRT	NLYISEAEGL	KFSLSLENVL	YYSPGGAGSD
410	420	430	440	450	460	470	480
TLVRYFANEP	FADFHRVEGL	QGVYIATLIN	GSMNEENMRS	VITFDKGGTW	EFLQAPAFTG	YGEKINCELS	QGCSLHLAQR
490	500	510	520	530	540	550	560
LSQLLNLQLR	RMPILSKESA	PGLIIATGSV	GKNLASKTNV	YISSSAGARW	REALPGPHYY	TWGDHGGIIT	AIAQGMETNE
570	580	590	600	610	620	630	640
LKYSTNEGET	WKTFIFSEKP	VFVYGLLTEP	GEKSTVFTIF	GSNKENVHSW	LILQVNATDA	LGVPCTENDY	KLWSPSDERG
650	660	670	680	690	700	710	720
NECLLGHKTV	FKRRTPHATC	FNGEDFDRPV	VVSNCSCTRE	DYECDFGFKM	SEDLSLEVCV	PDPEFSGKSY	SPPVPCPVGS
730	740	750	760	770	780	790	800
TYRRTRGYRK	ISGDTCSGGD	VEARLEGELV	PCPLAEENEF	ILYAVRKSIY	RYDLASGATE	QLPLTGLRAA	VALDFDYEHN
810	820	830	840	850	860	870	880
CLYWSDLALD	VIQRLCLNGS	TGQEVIINSG	LETVEALAFE	PLSQLLYWVD	AGFKKIEVAN	PDGDFRLTIV	NSSVLDRPRA
890	900	910	920	930	940	950	960
LVLVPQEGVM	FWTDWGDLKP	GIYRSNMDGS	AAYHLVSEDV	KWPNGISVDD	QWIYWTDAYL	ECIERITFSG	QQRSVILDNL
970	980	990	1000	1010	1020	1030	1040
PHPYAIAVFK	NEIYWDDWSQ	LSIFRASKYS	GSQMEILANQ	LTGLMDMKIF	YKGKNTGSNA	CVPRPCSLLC	LPKANNSRSC
1050	1060	1070	1080	1090	1100	1110	1120
RCPEDVSSSV	LPSGDLMCDC	PQGYQLKNNT	CVKEENTCLR	NQYRCSNGNC	INSIWWCDFD	NDCGDMSDER	NCPTTICDLD
1130	1140	1150	1160	1170	1180	1190	1200
TQFRCQESGT	CIPLSYKCDL	EDDCGDNSDE	SHCEMHQCRS	DEYNCSSGMC	IRSSWVCDGD	NDCRDWSDEA	NCTAIYHTCE
1210	1220	1230	1240	1250	1260	1270	1280
ASNFQCRNGH	CIPQRWACDG	DTDCQDGSDE	DPVNCEKKCN	GFRCPNGTCI	PSSKHCDGLR	DCSDGSDEQH	CEPLCTHFMD
1290	1300	1310	1320	1330	1340	1350	1360
FVCKNRQQCL	FHSMVCDGII	QCRDGSDEDA	AFAGCSQDPE	FHKVCDEFGF	QCQNGVCISL	IWKCDGMDDC	GDYSDEANCE
1370	1380	1390	1400	1410	1420	1430	1440
NPTEAPNCSR	YFQFRCENGH	CIPNRWKCDR	ENDCGDWSDE	KDCGDSHILP	FSTPGPSTCL	PNYYRCSSGT	CVMDTWVCDG
1450	1460	1470	1480	1490	1500	1510	1520
YRDCADGSDE	EACPLLANVT	AASTPTQLGR	CDRFEFECHQ	PKTCIPNWKR	CDGHQDCQDG	RDEANCPTHS	TLTCMSREFQ
1530	1540	1550	1560	1570	1580	1590	1600
CEDGEACIVL	SERCDGFLDC	SDESDEKACS	DELTVYKVQN	LQWTADFSGD	VTLTWMRPKK	MPSASCVYNV	YYRVVGESIW
1610	1620	1630	1640	1650	1660	1670	1680
KTLETHSNKT	NTVLKVLKPD	TTYQVKVQVQ	CLSKAHNTND	FVTLRTPEGL	PDAPRNLQLS	LPREAEGVIV	GHWAPPIHTH
1690	1700	1710	1720	1730	1740	1750	1760
GLIREYIVEY	SRSGSKMWAS	QRAASNFTEI	KNLLVNTLYT	VRVAAVTSRG	IGNWSDSKSI	TTIKGKVIPP	PDIHIDSYGE
1770	1780	1790	1800	1810	1820	1830	1840
NYLSFTLTME	SDIKVNGYVV	NLFWAFDTHK	QERRTLNFRG	SILSHKVGNL	TAHTSYEISA	WAKTDLGDSP	LAFEHVMTRG
1850	1860	1870	1880	1890	1900	1910	1920
VRPPAPSLKA	KAINQTAVEC	TWTGPRNVVY	GIFYATSFLD	LYRNPKSLTT	SLHNKTVIVS	KDEQYLFLVR	VVVPYQGPSS
1930	1940	1950	1960	1970	1980	1990	2000
DYVVVKMIPD	SRLPPRHLHV	VHTGKTSVVI	KWESPYDSPD	QDLLYAIAVK	DLIRKTDRSY	KVKSRNSTVE	YTLNKLEPGG
2010	2020	2030	2040	2050	2060	2070	2080
KYHIIVQLGN	MSKDSSIKIT	TVSLSAPDAL	KIITENDHVL	LFWKSLALKE	KHFNESRGYE	IHMFDSAMNI	TAYLGNTTDN
2090	2100	2110	2120	2130	2140	2150	2160
FFKISNLKMG	HNYTFTVQAR	CLFGNQICGE	PAILLYDELG	SGADASATQA	ARSTDVAAVV	VPILFLILLS	LGVGFAILYT
2170	2180	2190	2200	2210	2220		

Detailed Protein Report

Cmpd.	No. of Cmpds.	m/z meas.	Δ m/z [ppm]	z	Rt [min]	Score	P	Range	Sequence	Modification	Ratios	
1196	1	610.6448	-210.75	2	43.2	10.3	0	1244-1254	R.CPNGTCIPSSK.H	Carbamidomethyl: 1, 6	WD:WU	1.02

Detailed Protein Report

Protein 165: **CMP-N-acetylneuraminate-beta-1,4-galactoside alpha-2,3-sialyltransferase isoform n [Homo sapiens]**

**Accession:**	gi|394953753	**Score:**	30.3
**Database:**	refseq_human(refseq_human_20140103.fasta)	**MW [kDa]:**	26.7
**Database Date:**	2015-11-30	**pI:**	10.2
**Modification(s):**	Carbamidomethyl	**Sequence Coverage [%]:**	10.4
		**No. of unique Peptides:**	2

Quantitation

WD:WU **Median:** 0.77 **CV:** 0.00 % **No. of Peptides:** 1

10	20	30	40	50	60	70	80
MGLLVFVRNL	LLALCLFLVL	GFLYYSAWKL	HLLQWEEDSK	YDRLGFLLNL	DSKLFSKPAP	MFLDDSFRKW	ARIREFVPPF
**90**	**100**	**110**	**120**	**130**	**140**	**150**	**160**
GIKGQDNLIK	AILSVTKEYR	LTPALDSLRC	RRCIIVGNGG	VLANKSLGSR	IDDYDIVVRL	NSAPVKGFEK	DVGSKTTLRI
**170**	**180**	**190**	**200**	**210**	**220**	**230**	**240**
TYPEGAMQRP	EQYERDSLFV	LAGFKWQDFK	WLKYIVYKER	VSWTHNIQRE	KEFLRKLVKA	RVITDLSSGI	

Cmpd.	No. of Cmpds.	m/z meas.	Δ m/z [ppm]	z	Rt [min]	Score	P	Range	Sequence	Modification	Ratios	
146	1	657.8165	-71.56	2	31.1	10.8	0	113-125	R.CIIVGNGGVLANK.S	Carbamidomethyl: 1	WD:WU	0.77
2295	3	1131.4541	-161.50	1	57.1	19.5	1	220-230	K.ARVITDLSSGI.-			

Detailed Protein Report

Protein 166: protein FAM83G [Homo sapiens]

Accession: gi|115392150
Database: refseq_human(refseq_human_20140103.fasta)
Database Date: 2015-11-30

Score: 30.2
MW [kDa]: 90.8
pI: 5.9
Sequence Coverage [%]: 2.3
No. of unique Peptides: 2

10	20	30	40	50	60	70	80
MAFSQVQCLD	DNHVNWRSSE	SKPEFFYSEE	QRLALEALVA	RGRDAFYEVL	KRENIRDFLS	ELELKRILET	IEVYDPGSED
90	100	110	120	130	140	150	160
PRGTGPSQGP	EDNGVGDGEE	ASGADGVPIE	AEPLPSLEYW	PQKSDRSIPQ	LDLGWPDTIA	YRGVTRASVY	MQPPIDGQAH
170	180	190	200	210	220	230	240
IKEVVRKMIS	QAQKVIAVVM	DMFTDVDIFK	DLLDAGFKRK	VAVYIIVDES	NVKYFLHMCE	RACMHLGHLK	NLRVRSSGGT
250	260	270	280	290	300	310	320
EFFTRSATKF	KGALAQKFMF	VDGDRAVCGS	YSFTWSAART	DRNVISVLSG	QVVEMFDRQF	QELYLMSHSV	SLKGIPMEKE
330	340	350	360	370	380	390	400
PEPEPIVLPS	VVPLVPAGTV	AKKLVNPKYA	LVKAKSVDEI	AKISSEKQEA	KKPLGLKGPA	LAEHPGELPE	LLPPIHPGLL
410	420	430	440	450	460	470	480
HLERANMFEY	LPTWVEPDPE	PGSDILGYIN	IIDPNIWNPQ	PSQMNRIKIR	DTSQASAQHQ	LWKQSQDSRP	RPEPCPPPEP
490	500	510	520	530	540	550	560
SAPQDGVPAE	NGLPQGDPEP	LPPVPKPRTV	PVADVLARDS	SDIGWVLELP	KEEAPQNGTD	HRLPRMAGPG	HAPLQRQLSV
570	580	590	600	610	620	630	640
TQDDPESLGV	GLPNGLDGVE	EEDDDDYVTL	SDQDSHSGSS	GRGPGPRRPS	VASSVSEEYF	EVREHSVPLR	RRHSEQVANG
650	660	670	680	690	700	710	720
PTPPPRRQLS	APHITRGTFV	GPQGGSPWAQ	SRGREEADAL	KRMQAQRSTD	KEAQGQQFHH	HRVPASGTRD	KDGFPGPPRY
730	740	750	760	770	780	790	800
RSAADSVQSS	TRNAGPAMAG	PHHWQAKGGQ	VPRLLPDPGS	PRLAQNARPM	TDGRATEEHP	SPFGIPYSKL	SQSKHLKART
810	820	830					
GGSQWASSDS	KRRAQAPRDR	KDP					

Cmpd.	No. of Cmpds.	m/z meas.	Δ m/z [ppm]	z	Rt [min]	Score	P	Range	Sequence	Modification
1617	1	712.0414	-43.20	3	48.9	13.8	2	33-51	R.LALEALVARGRDAFYEVLK.R	
509	4	492.6651	-183.49	2	34.8	16.4	0	44-51	R.DAFYEVLK.R	

Detailed Protein Report

Protein 167: cGMP-specific 3',5'-cyclic phosphodiesterase isoform 2 [Homo sapiens]

**Accession:**	gi|61744430	**Score:**	30.1
**Database:**	refseq_human(refseq_human_20140103.fasta)	**MW [kDa]:**	94.7
**Database Date:**	2015-11-30	**pI:**	5.7
**Modification(s):**	Oxidation	**Sequence Coverage [%]:**	3.0
		**No. of unique Peptides:**	1

10	20	30	40	50	60	70	80
MLPFGDKTRE	MVNAWFAERV	HTIPVCKEGI	RGHTESCSCP	LQQSPRADNS	APGTPTRKIS	ASEFDRPLRP	IVVKDSEGTV
90	100	110	120	130	140	150	160
SFLSDSEKKE	QMPLTPPRFD	HDEGDQCSRL	LELVKDISSH	LDVTALCHKI	FLHIHGLISA	DRYSLFLVCE	DSSNDKFLIS
170	180	190	200	210	220	230	240
RLFDVAEGST	LEEVSNNCIR	LEWNKGIVGH	VAALGEPLNI	KDAYEDPRFN	AEVDQITGYK	TQSILCMPIK	NHREEVVGVA
250	260	270	280	290	300	310	320
QAINKKSGNG	GTFTEKDEKD	FAAYLAFCGI	VLHNAQLYET	SLLENKRNQV	LLDLASLIFE	EQQSLEVILK	KIAATIISFM
330	340	350	360	370	380	390	400
QVQKCTIFIV	DEDCSDSFSS	VFHMECEELE	KSSDTLTREH	DANKINYMYA	QYVKNTMEPL	NIPDVSKDKR	FPWTTENTGN
410	420	430	440	450	460	470	480
VNQQCIRSLL	CTPIKNGKKN	KVIGVCQLVN	KMEENTGKVK	PFNRNDEQFL	EAFVIFCGLG	IQNTQMYEAV	ERAMAKQMVT
490	500	510	520	530	540	550	560
LEVLSYHASA	AEEETRELQS	LAAAVVPSAQ	TLKITDFSFS	DFELSDLETA	LCTIRMFTDL	NLVQNFQMKH	EVLCRWILSV
570	580	590	600	610	620	630	640
KKNYRKNVAY	HNWRHAFNTA	QCMFAALKAG	KIQNKLTDLE	ILALLIAALS	HDLDHRGVNN	SYIQRSEHPL	AQLYCHSIME
650	660	670	680	690	700	710	720
HHHFDQCLMI	LNSPGNQILS	GLSIEEYKTT	LKIIKQAILA	TDLALYIKRR	GEFFELIRKN	QFNLEDPHQK	ELFLAMLMTA
730	740	750	760	770	780	790	800
CDLSAITKPW	PIQQRIAELV	ATEFFDQGDR	ERKELNIEPT	DLMNREKKNK	IPSMQVGFID	AICLQLYEAL	THVSEDCFPL
810	820	830	840				
LDGCRKNRQK	WQALAEQQEK	MLINGESGQA	KRN				

Cmpd.	No. of Cmpds.	m/z meas.	Δ m/z [ppm]	z	Rt [min]	Score	P	Range	Sequence	Modification
2288	1	937.3710	-111.47	2	57.1	12.3	2	751-765	R.ERKELNIEPTDLMNR.E	Oxidation: 13

Detailed Protein Report

Protein 168: PREDICTED: dedicator of cytokinesis protein 7 isoform X2 [Homo sapiens]

**Accession:**	gi|530363514	**Score:**	30.1
**Database:**	refseq_human(refseq_human_20140103.fasta)	**MW [kDa]:**	209.0
**Database Date:**	2015-11-30	**pI:**	6.1
**Modification(s):**	Oxidation	**Sequence Coverage [%]:**	2.0
		**No. of unique Peptides:**	1

10	20	30	40	50	60	70	80
MAERRAFAQK	ISRTVAAEVR	KQISGQYSGS	PQLLKNLNIV	GNISHHTTVP	LTEAVDPVDL	EDYLITHPLA	VDSGPLRDLI
90	100	110	120	130	140	150	160
EFPPDDIEVV	YSPRDCRTLV	SAVPEESEMD	PHVRDCIRSY	TEDWAIVIRK	YHKLGTGFNP	NTLDKQKERQ	KGLPKQVFES
170	180	190	200	210	220	230	240
DEAPDGNSYQ	DDQDDLKRRS	MSIDDTPRGS	WACSIFDLKN	SLPDALLPNL	LDRTPNEEID	RQNDDQRKSN	RHKELFALHP
250	260	270	280	290	300	310	320
SPDEEEPIER	LSVPDIPKEH	FGQRLLVKCL	SLKFEIEIEP	IFASLALYDV	KEKKKISENF	YFDLNSEQMK	GLLRPHVPPA
330	340	350	360	370	380	390	400
AITTLARSAI	FSITYPSQDV	FLVIKLEKVL	QQGDIGECAE	PYMIFKEADA	TKNKEKLEKL	KSQADQFCQR	LGKYRMPFAW
410	420	430	440	450	460	470	480
TAIHLMNIVS	SAGSLERDST	EVEISTGERK	GSWSERRNSS	IVGRRSLERT	TSGDDACNLT	SFRPATLTVT	NFFKQEGDRL
490	500	510	520	530	540	550	560
SDEDLYKFLA	DMRRPSSVLR	RLRPITAQLK	IDISPAPENP	HYCLTPELLQ	VKLYPDSRVR	PTREILEFPA	RDVYVPNTTY
570	580	590	600	610	620	630	640
RNLLYIYPQS	LNFANRQGSA	RNITVKVQFM	YGEDPSNAMP	VIFGKSSCSE	FSKEAYTAVV	YHNRSPDFHE	EIKVKLPATL
650	660	670	680	690	700	710	720
TDHHHLLFTF	YHVSCQQKQN	TPLETPVGYT	WIPMLQNGRL	KTGQFCLPVS	LEKPPQAYSV	LSPEVPLPGM	KWVDNHKGVF
730	740	750	760	770	780	790	800
NVEVVAVSSI	HTQDPYLDKF	FALVNALDEH	LFPVRIGDMR	IMENNLENEL	KSSISALNSS	QLEPVVRFLH	LLLDKLILLV
810	820	830	840	850	860	870	880
IRPPVIAGQI	VNLGQASFEA	MASIINRLHK	NLEGNHDQHG	RNSLLASYIH	YVFRLPNTYP	NSSSPGPGGL	GGSVHYATMA
890	900	910	920	930	940	950	960
RSAVRPASLN	LNRSRSLSNS	NPDISGTPTS	PDDEVRSIIG	SKGLDRSNSW	VNTGGPKAAP	WGSNPSPSAE	STQAMDRSCN
970	980	990	1000	1010	1020	1030	1040
RMSSHTETSS	FLQTLTGRLP	TKKLFHEELA	LQWVVCSGSV	RESALQQAWF	FFELMVKSMV	HHLYFNDKLE	APRKSRFPER
1050	1060	1070	1080	1090	1100	1110	1120
FMDDIAALVS	TIASDIVSRF	QKDTEMVERL	NTSLAFFLND	LLSVMDRGFV	FSLIKSCYKQ	VSSKLYSLPN	PSVLVSLRLD
1130	1140	1150	1160	1170	1180	1190	1200
FLRIICSHEH	YVTLNLPCSL	LTPPASPSPS	VSSATSQSSG	FSTNVQDQKI	ANMFELSVPF	RQQHYLAGLV	LTELAVILDP
1210	1220	1230	1240	1250	1260	1270	1280
DAEGLFGLHK	KVINMVHNLL	SSHDSDPRYS	DPQIKARVAM	LYLPLIGIIM	ETVPQLYDFT	ETHNQRGRPI	CIATDDYESE
1290	1300	1310	1320	1330	1340	1350	1360
SGSMISQTVA	MAIAGTSVPQ	LTRPGSFLLT	STSGRQHTTF	SAESSRSLLI	CLLWVLKNAD	ETVLQKWFTD	LSVLQLNRLL
1370	1380	1390	1400	1410	1420	1430	1440
DLLYLCVSCF	EYKGKKVFER	MNSLTFKKSK	DMRAKLEEAI	LGSIGARQEM	VRRSRGQLER	SPSGSAFGSQ	ENLRWRKDMT
1450	1460	1470	1480	1490	1500	1510	1520
HWRQNTEKLD	KSRAEIEHEA	LIDGNLATEA	NLIILDTLEI	VVQTVSVTES	KESILGGVLK	VLLHSMACNQ	SAVYLQHCFA
1530	1540	1550	1560	1570	1580	1590	1600
TQRALVSKFP	ELLFEEETEQ	CADLCLRLLR	HCSSSIGTIR	SHASASLYLL	MRQNFEIGNN	FARVKMQVTM	SLSSLVGTSQ
1610	1620	1630	1640	1650	1660	1670	1680
NFNEEFLRRS	LKTILTYAEE	DLELRETTFP	DQVQDLVFNL	HMILSDTVKM	KEHQEDPEML	IDLMYRIAKG	YQTSPDLRLT
1690	1700	1710	1720	1730	1740	1750	1760
WLQNMAGKHS	ERSNHAEAAQ	CLVHSAALVA	EYLSMLEDRK	YLPVGCVTFQ	NISSNVLEES	AVSDDVVSPD	EEGICSGKYF
1770	1780	1790	1800	1810	1820	1830	1840
TESGLVGLLE	QAAASFSMAG	MYEAVNEVYK	VLIPIHEANR	DAKKLSTIHG	KLQEAFSKIV	HQSTGWEFYL	VYIFLFDSMK
1850	1860						
TSVILFFLKA	MFPS						

Cmpd.	No. of Cmpds.	m/z meas.	Δ m/z [ppm]	z	Rt [min]	Score	P	Range	Sequence	Modification

Detailed Protein Report

Cmpd.	No. of Cmpds.	m/z meas.	Δ m/z [ppm]	z	Rt [min]	Score	P	Range	Sequence	Modification
122	1	879.4101	-14.16	3	30.5	16.1	0	1501-1523	K.VLLHSMACNQSAVYLQHCFATQR.A	Oxidation: 6

Detailed Protein Report

Protein 169: kinesin-like protein KIF1C [Homo sapiens]

**Accession:**	gi|40254834	**Score:**	29.7
**Database:**	refseq_human(refseq_human_20140103.fasta)	**MW [kDa]:**	122.9
**Database Date:**	2015-11-30	**pI:**	6.5
**Modification(s):**	Oxidation	**Sequence Coverage [%]:**	2.7
		**No. of unique Peptides:**	1

Alias proteins:

Accession	Name	Description
gi|530409731	refseq_human (refseq_human_20140103.fasta)	PREDICTED: kinesin-like protein KIF1C isoform X1 [Homo sapiens]

10	20	30	40	50	60	70	80
MAGASVKVAV	RVRPFNARET	SQDAKCVVSM	QGNTTSIINP	KQSKDAPKSF	TFDYSYWSHT	STEDPQFASQ	QQVYRDIGEE
**90**	**100**	**110**	**120**	**130**	**140**	**150**	**160**
MLLHAFEGYN	VCIFAYGQTG	AGKSYTMMGR	QEPGQQGIVP	QLCEDLFSRV	SENQSAQLSY	SVEVSYMEIY	CERVRDLLNP
**170**	**180**	**190**	**200**	**210**	**220**	**230**	**240**
KSRGSLRVRE	HPILGPYVQD	LSKLAVTSYA	DIADLMDCGN	KARTVAATNM	NETSSRSHAV	FTIVFTQRCH	DQLTGLDSEK
**250**	**260**	**270**	**280**	**290**	**300**	**310**	**320**
VSKISLVDLA	GSERADSSGA	RGMRLKEGAN	INKSLTTLGK	VISALADMQS	KKRKSDFIPY	RDSVLTWLLK	ENLGGNSRTA
**330**	**340**	**350**	**360**	**370**	**380**	**390**	**400**
MIAALSPADI	NYEETLSTLR	YADRTKQIRC	NAIINEDPNA	RLIRELQEEV	ARLRELLMAQ	GLSASALEGL	KTEEGSVRGA
**410**	**420**	**430**	**440**	**450**	**460**	**470**	**480**
LPAVSSPPAP	VSPSSPTTHN	GELEPSFSPN	TESQIGPEEA	MERLQETEKI	IAELNETWEE	KLRKTEALRM	EREALLAEMG
**490**	**500**	**510**	**520**	**530**	**540**	**550**	**560**
VAVREDGGTV	GVFSPKKTPH	LVNLNEDPLM	SECLLYHIKD	GVTRVGQVDM	DIKLTGQFIR	EQHCLFRSIP	QPDGEVVVTL
**570**	**580**	**590**	**600**	**610**	**620**	**630**	**640**
EPCEGAETYV	NGKLVTEPLV	LKSGNRIVMG	KNHVFRFNHP	EQARLERERG	VPPPPGPPSE	PVDWNFAQKE	LLEQQGIDIK
**650**	**660**	**670**	**680**	**690**	**700**	**710**	**720**
LEMEKRLQDL	ENQYRKEKEE	ADLLLEQQRL	YADSDSGDDS	DKRSCEESWR	LISSLREQLP	PTTVQTIVKR	CGLPSSGKRR
**730**	**740**	**750**	**760**	**770**	**780**	**790**	**800**
APRRVYQIPQ	RRRLQGKDPR	WATMADLKMQ	AVKEICYEVA	LADFRHGRAE	IEALAALKMR	ELCRTYGKPD	GPGDAWRAVA
**810**	**820**	**830**	**840**	**850**	**860**	**870**	**880**
RDVWDTVGEE	EGGGAGSGGG	SEEGARGAEV	EDLRAHIDKL	TGILQEVKLQ	NSSKDRELQA	LRDRMLRMER	VIPLAQDHED
**890**	**900**	**910**	**920**	**930**	**940**	**950**	**960**
ENEEGGEVPW	APPEGSEAAE	EAAPSDRMPS	ARPPSPPLSS	WERVSRLMEE	DPAFRRGRLR	WLKQEQLRLQ	GLQGSGGRGG
**970**	**980**	**990**	**1000**	**1010**	**1020**	**1030**	**1040**
GLRRPPARFV	PPHDCKLRFP	FKSNPQHRES	WPGMGSGEAP	TPLQPPEEVT	PHPATPARRP	PSPRRSHHPR	RNSLDGGGRS
**1050**	**1060**	**1070**	**1080**	**1090**	**1100**	**1110**	
RGAGSAQPEP	QHFQPKKHNS	YPQPPQPYPA	QRPPGPRYPP	YTTPPRMRRQ	RSAPDLKESG	AAV	

Cmpd.	No. of Cmpds.	m/z meas.	Δ m/z [ppm]	z	Rt [min]	Score	P	Range	Sequence	Modification
1261	1	1044.6916	131.82	2	44.1	13.7	2	630-646	K.ELLEQQGIDIKLEMEKR.L	Oxidation: 14

Detailed Protein Report

Protein 170: **PREDICTED: putative uncharacterized protein LOC388900 isoform X1 [Homo sapiens]**

**Accession:**	gi|530420293	**Score:**	29.7
**Database:**	refseq_human(refseq_human_20140103.fasta)	**MW [kDa]:**	55.6
**Database Date:**	2015-11-30	**pI:**	10.1
**Modification(s):**	Oxidation	**Sequence Coverage [%]:**	4.8
		**No. of unique Peptides:**	2

Quantitation

WD:WU **Median:** 0.04 **CV:** 0.00 % **No. of Peptides:** 1

```
        10         20         30         40         50         60         70         80
MLFSWVKRQR KSQYSCKGSE LRHARSSVIK RKTADKNLLA ELYQYSNFNS SKPNKLPNGV DFCDMVGNVV RAERDCLSGK
        90        100        110        120        130        140        150        160
HFCSGRELEK FLSSSSPRAI WLDSFWWIFH ERYQPNKELQ NNLFDRIAQH YALLLFRVPK SHSEEALLKR LPSLLSKAVY
       170        180        190        200        210        220        230        240
TSFCCCFPQS WFDTHEFKSD ICNTMSLWIS GTYPSPQSYD SWDYSELDPE RFRREELMLY RRRLTKGREF SLFAGKRAFS
       250        260        270        280        290        300        310        320
QKPAQSRKFY HPQSSSANSP SEKTSSAKQN SEKSLRMQNT AKEHHCQTLV LKKPTQEVKR ISEARECENM FPKKSCAACK
       330        340        350        360        370        380        390        400
SPELTSNLFN IYGKSPLIVY FLQNYASLQQ HGKNVLIVRR EKTTSTPDCT PTYTDVISET LCSMKKRKDN LNQLYQHHWT
       410        420        430        440        450        460        470        480
EWNYFDKHLK ELQDNFSREM KNIDPKAADT KKANHMFIPP SAVNEESPDK KTKEGKGGEG KRRETEVEHF FPLTSKP
```

Cmpd.	No. of Cmpds.	m/z meas.	Δ m/z [ppm]	z	Rt [min]	Score	P	Range	Sequence	Modification	Ratios
1201	2	507.1525	-225.20	2	43.3	10.5	0	141-149	K.SHSEEALLK.R		WD:WU 0.04
1811	4	863.4321	30.21	2	51.3	19.2	2	300-313	K.RISEARECENMFPK.K	Oxidation: 11	

Detailed Protein Report

Protein 171: **PREDICTED: sushi, nidogen and EGF-like domain-containing protein 1 isoform X4 [Homo sapiens]**

**Accession:**	gi|578804038	**Score:**	29.7
**Database:**	refseq_human(refseq_human_20140103.fasta)	**MW [kDa]:**	142.7
**Database Date:**	2015-11-30	**pI:**	6.4
**Modification(s):**	Carbamidomethyl	**Sequence Coverage [%]:**	1.4
		**No. of unique Peptides:**	2

10	20	30	40	50	60	70	80
MRHGVAWALL	VAAALGLGAR	GVRGAVALAD	FYPFGAERGD	AVTPKQDDGG	SGLRPLSVPF	PFFGAEHSGL	YVNNNGIISF
90	100	110	120	130	140	150	160
LKEVSQFTPV	AFPIAKDRCV	VAAFWADVDN	RRAGDVYYRE	ATDPAMLRRA	TEDVRHYFPE	LLDFNATWVF	VATWYRVTFF
170	180	190	200	210	220	230	240
GGSSSSPVNT	FQTVLITDGK	LSFTIFNYES	IVWTTGTHAS	SGGNATGLGG	IAAQAGFNAG	DGQRYFSIPG	SRTADMAEVE
250	260	270	280	290	300	310	320
TTTNVGVPGR	WAFRIDDAQV	RVGGCGHTTS	VCLALRPCLN	GGKCIDDCVT	GNPSYTCSCL	SGFTGRRCHL	DVNECASQPC
330	340	350	360	370	380	390	400
QNGGTCTHGI	NSFRCQCPAG	FGGPTCETAQ	SPCDTKECQH	GGQCQVENGS	AVCVCQAGYT	GAACEMDVDD	CSPDPCLNGG
410	420	430	440	450	460	470	480
SCVDLVGNYT	CLCAEPFKGL	RCETGDHPVP	DACLSAPCHN	GGTCVDADQG	YVCECPEGFM	GLDCRERVPD	DCECRNGGRC
490	500	510	520	530	540	550	560
LGANTTLCQC	PLGFFGLLCE	FEITAMPCNM	NTQCPDGGYC	MEHGGSYLCV	CHTDHNASHS	LPSPCDSDPC	FNGGSCDAHD
570	580	590	600	610	620	630	640
DSYTCECPRG	FHGKHCEKAR	PHLCSSGPCR	NGGTCKEAGG	EYHCSCPYRF	TGRHCEIGKP	DSCASGPCHN	GGTCFHYIGK
650	660	670	680	690	700	710	720
YKCDCPPGFS	GRHCEIAPSP	CFRSPCVNGG	TCEDRDTDFF	CHCQAGYMGR	RCQAEVDCGP	PEEVKHATLR	FNGTRLGAVA
730	740	750	760	770	780	790	800
LYACDRGYSL	SAPSRIRVCQ	PHGVWSEPPQ	CLEIDECRSQ	PCLHGGSCQD	RVAGYLCLCS	TGYEGAHCEL	ERDECRAHPC
810	820	830	840	850	860	870	880
RNGGSCRNLP	GAYVCRCPAG	FVGVHCETEV	DACDSSPCQH	GGRCESGGGA	YLCVCPESFF	GYHCETVSDP	CFSSPCGGRG
890	900	910	920	930	940	950	960
YCLASNGSHS	CTCKVGYTGE	DCAKELFPPT	ALKMERVEES	GVSISWNPPN	GPAARQMLDG	YAVTYVSSDG	SYRRTDFVDR
970	980	990	1000	1010	1020	1030	1040
TRSSHQLQAL	AAGRAYNISV	FSVKRNSNNK	NDISRPAVLL	ARTRPRPVEG	FEVTNVTAST	ISVQWALHRI	RHATVSGVRV
1050	1060	1070	1080	1090	1100	1110	1120
SIRHPEALRD	QATDVDRSVD	RFTFRALLPG	KRYTIQLTTL	SGLRGEEHPT	ESLATAPTHV	WTRPLPPANL	TAARVTATSA
1130	1140	1150	1160	1170	1180	1190	1200
HVVWDAPTPG	SLLEAYVINV	TTSQSTKSRY	VPNGKLASYT	VRDLLPGRRY	QLSVIAVQST	ELGPQHSEPA	HLYIITSPRD
1210	1220	1230	1240	1250	1260	1270	1280
GADRRWHQGG	HHPRVLKNRP	PPARLPELRL	LNDHSAPETP	TQPPRFSELV	DGRGRVSARF	GGSPSKAATV	RSQPTASAQL
1290	1300	1310	1320	1330			
ENMEEAPKRV	SLALQLPEHG	SKDIGSLDLL	LAPVSTRRTG	LCIRRE			

Cmpd.	No. of Cmpds.	m/z meas.	Δ m/z [ppm]	z	Rt [min]	Score	P	Range	Sequence	Modification
1667	1	1073.2934	-127.64	2	49.2	19.6	1	591-609	R.NGGTCKEAGGEYHCSCPYR.F	Carbamidomethyl: 5, 16
2019	1	715.9639	11.03	3	53.6	10.1	1	591-609	R.NGGTCKEAGGEYHCSCPYR.F	Carbamidomethyl: 14, 16

Detailed Protein Report

Protein 172: structural maintenance of chromosomes protein 4 isoform 2 [Homo sapiens]

**Accession:**	gi|570700827	**Score:**	29.6
**Database:**	refseq_human(refseq_human_20140103.fasta)	**MW [kDa]:**	144.4
**Database Date:**	2015-11-30	**pI:**	6.7
		**Sequence Coverage [%]:**	1.2
		**No. of unique Peptides:**	1

10	20	30	40	50	60	70	80
MPRKGTQPST	ARRREEGPPP	PSPDGASSDA	EPEPPSGRTE	SPATAAAMTN	EAGAPRLMIT	HIVNQNFKSY	AGEKILGPFH
90	100	110	120	130	140	150	160
KRFSCIIGPN	GSGKSNVIDS	MLFVFGYRAQ	KIRSKKLSVL	IHNSDEHKDI	QSCTVEVHFQ	KIIDKEGDDY	EVIPNSNFYV
170	180	190	200	210	220	230	240
SRTACRDNTS	VYHISGKKKT	FKDVGNLLRS	HGIDLDHNRF	LILQGEVEQI	AMMKPKGQTE	HDEGMLEYLE	DIIGCGRLNE
250	260	270	280	290	300	310	320
PIKVLCRRVE	ILNEHRGEKL	NRVKMVEKEK	DALEGEKNIA	IEFLTLENEI	FRKKNHVCQY	YIYELQKRIA	EMETQKEKIH
330	340	350	360	370	380	390	400
EDTKEINEKS	NILSNEMKAK	NKDVKDTEKK	LNKITKFIEE	NKEKFTQLDL	EDVQVREKLK	HATSKAKKLE	KQLQKDKEKV
410	420	430	440	450	460	470	480
EEFKSIPAKS	NNIINETTTR	NNALEKEKEK	EEKKLKEVMD	SLKQETQGLQ	KEKESREKEL	MGFSKSVNEA	RSKMDVAQSE
490	500	510	520	530	540	550	560
LDIYLSRHNT	AVSQLTKAKE	ALIAASETLK	ERKAAIRDIE	GKLPQTEQEL	KEKEKELQKL	TQEETNFKSL	VHDLFQKVEE
570	580	590	600	610	620	630	640
AKSSLAMNRS	RGKVLDAIIQ	EKKSGRIPGI	YGRLGDLGAI	DEKYDVAISS	CCHALDYIVV	DSIDIAQECV	NFLKRQNIGV
650	660	670	680	690	700	710	720
ATFIGLDKMA	VWAKKMTEIQ	TPENTPRLFD	LVKVKDEKIR	QAFYFALRDT	LVADNLDQAT	RVAYQKDRRW	RVVTLQGQII
730	740	750	760	770	780	790	800
EQSGTMTGGG	SKVMKGRMGS	SLVIEISEEE	VNKMESQLQN	DSKKAMQIQE	QKVQLEERVV	KLRHSEREMR	NTLEKFTASI
810	820	830	840	850	860	870	880
QRLIEQEEYL	NVQVKELEAN	VLATAPDKKK	QKLLEENVSA	FKTEYDAVAE	KAGKVEAEVK	RLHNTIVEIN	NHKLKAQQDK
890	900	910	920	930	940	950	960
LDKINKQLDE	CASAITKAQV	AIKTADRNLQ	KAQDSVLRTE	KEIKDTEKEV	DDLTAELKSL	EDKAAEVVKN	TNAAEESLPE
970	980	990	1000	1010	1020	1030	1040
IQKEHRNLLQ	ELKVIQENEH	ALQKDALSIK	LKLEQIDGHI	AEHNSKIKYW	HKEISKISLH	PIEDNPIEEI	SVLSPEDLEA
1050	1060	1070	1080	1090	1100	1110	1120
IKNPDSITNQ	IALLEARCHE	MKPNLGAIAE	YKKKEELYLQ	RVAELDKITY	ERDSFRQAYE	DLRKQRLNEF	MAGFYIITNK
1130	1140	1150	1160	1170	1180	1190	1200
LKENYQMLTL	GGDAELELVD	SLDPFSEGIM	FSVRPPKKSW	KKIFNLSGGE	KTLSSLALVF	ALHHYKPTPL	YFMDEIDAAL
1210	1220	1230	1240	1250	1260	1270	
DFKNVSIVAF	YIYEQTKNAQ	FIIISLRNNM	FEISDRLIGI	YKTYNITKSV	AVNPKEIASK	GLC	

Cmpd.	No. of Cmpds.	m/z meas.	Δ m/z [ppm]	z	Rt [min]	Score	P	Range	Sequence	Modification
282	2	862.8833	-60.41	2	32.7	29.6	2	457-471	R.EKELMGFSKSVNEAR.S	

Detailed Protein Report

Protein 173: zinc finger protein 622 [Homo sapiens]

Accession: gi|15529978
Database: refseq_human(refseq_human_20140103.fasta)
Database Date: 2015-11-30
Modification(s): Carbamidomethyl

Score: 29.6
MW [kDa]: 54.2
pI: 5.8
Sequence Coverage [%]: 6.3
No. of unique Peptides: 2

10	20	30	40	50	60	70	80
MATYTCITCR	VAFRDADMQR	AHYKTDWHRY	NLRRKVASMA	PVTAEGFQER	VRAQRAVAEE	ESKGSATYCT	VCSKKFASFN
90	100	110	120	130	140	150	160
AYENHLKSRR	HVELEKKAVQ	AVNRKVEMMN	EKNLEKGLGV	DSVDKDAMNA	AIQQAIKAQP	SMSPKKAPPA	PAKEARNVVA
170	180	190	200	210	220	230	240
VGTGGRGTHD	RDPSEKPPRL	QWFEQQAKKL	AKQQEEDSEE	EEEDLDGDDW	EDIDSDEELE	CEDTEAMDDV	VEQDAEEEEA
250	260	270	280	290	300	310	320
EEGPPLGAIP	ITDCLFCSHH	SSSLMKNVAH	MTKDHSFFIP	DIEYLSDIKG	LIKYLGEKVG	VGKICLWCNE	KGKSFYSTEA
330	340	350	360	370	380	390	400
VQAHMNDKSH	CKLFTDGDAA	LEFADFYDFR	SSYPDHKEGE	DPNKAEELPS	EKNLEYDDET	MELILPSGAR	VGHRSLMRYY
410	420	430	440	450	460	470	480
KQRFGLSRAV	AVAKNRKAVG	RVLQQYRALG	WTGSTGAALM	RERDMQYVQR	MKSKWMLKTG	MKNNATKQMH	FRVQVRF

Cmpd.	No. of Cmpds.	m/z meas.	Δ m/z [ppm]	z	Rt [min]	Score	P	Range	Sequence	Modification
1830	3	938.9541	-36.71	2	51.2	19.3	2	34-50	R.RKVASMAPVTAEGFQER.V	
2237	1	753.3462	-49.90	2	56.3	10.4	1	299-311	K.VGVGKICLWCNEK.G	Carbamidomethyl: 7

Detailed Protein Report

Protein 174: polycystin-2 [Homo sapiens]

**Accession:**	gi|4505835	**Score:**	29.5
**Database:**	refseq_human(refseq_human_20140103.fasta)	**MW [kDa]:**	109.6
**Database Date:**	2015-11-30	**pI:**	5.4
**Modification(s):**	Carbamidomethyl, Oxidation	**Sequence Coverage [%]:**	3.9
		**No. of unique Peptides:**	2

10	20	30	40	50	60	70	80
MVNSSRVQPQ	QPGDAKRPPA	PRAPDPGRLM	AGCAAVGASL	AAPGGLCEQR	GLEIEMQRIR	QAAARDPPAG	AAASPSPPLS
**90**	**100**	**110**	**120**	**130**	**140**	**150**	**160**
SCSRQAWSRD	NPGFEAEEEE	EEVEGEEGGM	VVEMDVEWRP	GSRRSAASSA	VSSVGARSRG	LGGYHGAGHP	SGRRRRREDQ
**170**	**180**	**190**	**200**	**210**	**220**	**230**	**240**
GPPCPSPVGG	GDPLHRHLPL	EGQPPRVAWA	ERLVRGLRGL	WGTRLMEESS	TNREKYLKSV	LRELVTYLLF	LIVLCILTYG
**250**	**260**	**270**	**280**	**290**	**300**	**310**	**320**
MMSSNVYYYT	RMMSQLFLDT	PVSKTEKTNF	KTLSSMEDFW	KFTEGSLLDG	LYWKMQPSNQ	TEADNRSFIF	YENLLLGVPR
**330**	**340**	**350**	**360**	**370**	**380**	**390**	**400**
IRQLRVRNGS	CSIPQDLRDE	IKECYDVYSV	SSEDRAPFGP	RNGTAWIYTS	EKDLNGSSHW	GIIATYSGAG	YYLDLSRTRE
**410**	**420**	**430**	**440**	**450**	**460**	**470**	**480**
ETAAQVASLK	KNVWLDRGTR	ATFIDFSVYN	ANINLFCVVR	LLVEFPATGG	VIPSWQFQPL	KLIRYVTTFD	FFLAACEIIF
**490**	**500**	**510**	**520**	**530**	**540**	**550**	**560**
CFFIFYYVVE	EILEIRIHKL	HYFRSFWNCL	DVVIVVLSVV	AIGINIYRTS	NVEVLLQFLE	DQNTFPNFEH	LAYWQIQFNN
**570**	**580**	**590**	**600**	**610**	**620**	**630**	**640**
IAAVTVFFVW	IKLFKFINFN	RTMSQLSTTM	SRCAKDLFGF	AIMFFIIFLA	YAQLAYLVFG	TQVDDFSTFQ	ECIFTQFRII
**650**	**660**	**670**	**680**	**690**	**700**	**710**	**720**
LGDINFAEIE	EANRVLGPIY	FTTFVFFMFF	ILLNMFLAII	NDTYSEVKSD	LAQQKAEMEL	SDLIRKGYHK	ALVKLKLKKN
**730**	**740**	**750**	**760**	**770**	**780**	**790**	**800**
TVDDISESLR	QGGGKLNFDE	LRQDLKGKGH	TDAEIEAIFT	KYDQDGDQEL	TEHEHQQMRD	DLEKEREDLD	LDHSSLPRPM
**810**	**820**	**830**	**840**	**850**	**860**	**870**	**880**
SSRSFPRSLD	DSEEDDDEDS	GHSSRRRGSI	SSGVSYEEFQ	VLVRRVDRME	HSIGSIVSKI	DAVIVKLEIM	ERAKLKRREV
**890**	**900**	**910**	**920**	**930**	**940**	**950**	**960**
LGRLLDGVAE	DERLGRDSEI	HREQMERLVR	EELERWESDD	AASQISHGLG	TPVGLNGQPR	PRSSRPSSSQ	STEGMEGAGG
**970**							
NGSSNVHV							

Cmpd.	No. of Cmpds.	m/z meas.	Δ m/z [ppm]	z	Rt [min]	Score	P	Range	Sequence	Modification
418	1	686.6239	-116.77	3	33.8	12.4	2	400-417	R.EETAAQVASLKKNVWLDR.G	
5	1	1205.0356	-34.52	2	29.0	17.2	2	576-595	K.FINFNRTMSQLSTTMSRCAK.D	Carbamidomethyl: 18; Oxidation: 8

Detailed Protein Report

Protein 175: **PREDICTED: protein FAM214A isoform X6 [Homo sapiens]**

**Accession:**	gi|578827201	**Score:**	29.5
**Database:**	refseq_human(refseq_human_20140103.fasta)	**MW [kDa]:**	111.5
**Database Date:**	2015-11-30	**pI:**	9.2
		**Sequence Coverage [%]:**	2.3
		**No. of unique Peptides:**	2

Alias proteins:

Accession	Name	Description
gi|578827203	refseq_human (refseq_human_20140103.fasta)	PREDICTED: protein FAM214A isoform X7 [Homo sapiens]

10	20	30	40	50	60	70	80
MVEMMLLPDC	CYSDDGPTTE	GIDLNDPAIK	QDALLLERWI	LEPVPRQNGD	RFIEEKTLLL	AVRSFVFFSQ	LSAWLSVSHG
**90**	**100**	**110**	**120**	**130**	**140**	**150**	**160**
AIPRNILYRI	SAADVDLQWN	FSQTPIEHVF	PVPNVSHNVA	LKVSVQSLPR	QSNYPVLTCS	IHTNIGLYEK	RIQQHKLKTH
**170**	**180**	**190**	**200**	**210**	**220**	**230**	**240**
QHHNPNEAEQ	CGTNSSQRLC	SKQTWTMAPE	SVLHAKSGPS	PEYTAAVKNI	KLYPGTGSKS	DHGTSQANIL	GFSGIGDIKS
**250**	**260**	**270**	**280**	**290**	**300**	**310**	**320**
QETSVRTLKS	FSMVDSSISN	RQSFWQSAGE	TNPLIGSLIQ	ERQEIIARIA	QHLIHCDPST	SHVSGRPFNT	QESSSLHSKL
**330**	**340**	**350**	**360**	**370**	**380**	**390**	**400**
FRVSQENENV	GKGKEAFSMT	FGSPEFSSPE	DTNEGKIRLK	PETPRSETCI	SNDFYSHMPV	GETNPLIGSL	LQERQDVIAR
**410**	**420**	**430**	**440**	**450**	**460**	**470**	**480**
IAQHLEHIDP	TASHIPRQSF	NMHDSSSVAS	KVFRSSYEDK	NLLKKNKDES	SVSISHTKCS	LLGDISDGKN	LVPNKCFTSF
**490**	**500**	**510**	**520**	**530**	**540**	**550**	**560**
KNNSKEKCSL	KHQTRNQCQN	NPSEIIQSTY	QETQNKSSSL	STSSILSQHK	ENNLDLTSRF	KEQEMSNGID	KQYSNCTTID
**570**	**580**	**590**	**600**	**610**	**620**	**630**	**640**
KQICTNKYKE	KIINENYNPK	FFGNLQSDDS	KKNDSKIKVT	VLEMSEYLNK	YESMSSNKDS	KRPKTCEQNT	QLNSIENYLN
**650**	**660**	**670**	**680**	**690**	**700**	**710**	**720**
KDNEGFKCKK	SDQLKNEQDK	QEDPTNEKSQ	NYSQRRSIKD	CLSTCEQPKN	TEVLRTTLKH	SNVWRKHNFH	SLDGTSTRAF
**730**	**740**	**750**	**760**	**770**	**780**	**790**	**800**
HPQTGLPLLS	SPVPQRKTQS	GCFDLDSSLL	HLKSFSSRSP	RPCLNIEDDP	DIHEKPFLSS	SAPPITSLSL	LGNFEESVLN
**810**	**820**	**830**	**840**	**850**	**860**	**870**	**880**
YRFDPLGIVD	GFTAEVGASG	AFCPTHLTLP	VEVSFYSVSD	DNAPSPYMGV	ITLESLGKRG	YRVPPSGTIQ	VTLFNPNKTV
**890**	**900**	**910**	**920**	**930**	**940**	**950**	**960**
VKMFVVIYDL	RDMPANHQTF	LRQRTFSVPV	KQEVKRSVNK	ENIRHTEERL	LRYLIHLRFQ	SSKSGKIYLH	RDVRLLFSRK
**970**	**980**	**990**					
SMEVDSGAAY	ELKSYTESPT	NPQFSPRC					

Cmpd.	No. of Cmpds.	m/z meas.	Δ m/z [ppm]	z	Rt [min]	Score	P	Range	Sequence	Modification
2952	1	557.9715	37.67	3	65.7	10.1	1	247-261	R.TLKSFSMVDSSISNR.Q	
1649	3	480.7758	122.24	2	48.9	19.4	0	661-668	K.QEDPTNEK.S	

Detailed Protein Report

Protein 176: squalene monooxygenase [Homo sapiens]

**Accession:**	gi|62865635	**Score:**	29.4
**Database:**	refseq_human(refseq_human_20140103.fasta)	**MW [kDa]:**	63.9
**Database Date:**	2015-11-30	**pI:**	9.4
**Modification(s):**	Carbamidomethyl	**Sequence Coverage [%]:**	1.9
		**No. of unique Peptides:**	1

Quantitation

WD:WU **Median:** 2.62 **CV:** 0.00 % **No. of Peptides:** 1

10	20	30	40	50	60	70	80
MWTFLGIATF	TYFYKKFGDF	ITLANREVLL	CVLVFLSLGL	VLSYRCRHRN	GGLLGRQQSG	SQFALFSDIL	SGLPFIGFFW
**90**	**100**	**110**	**120**	**130**	**140**	**150**	**160**
AKSPPESENK	EQLEARRRRK	GTNISETSLI	GTAACTSTSS	QNDPEVIIVG	AGVLGSALAA	VLSRDGRKVT	VIERDLKEPD
**170**	**180**	**190**	**200**	**210**	**220**	**230**	**240**
RIVGEFLQPG	GYHVLKDLGL	GDTVEGLDAQ	VVNGYMIHDQ	ESKSEVQIPY	PLSENNQVQS	GRAFHHGRFI	MSLRKAAMAE
**250**	**260**	**270**	**280**	**290**	**300**	**310**	**320**
PNAKFIEGVV	LQLLEEDDVV	MGVQYKDKET	GDIKELHAPL	TVVADGLFSK	FRKSLVSNKV	SVSSHFVGFL	MKNAPQFKAN
**330**	**340**	**350**	**360**	**370**	**380**	**390**	**400**
HAELILANPS	PVLIYQISSS	ETRVLVDIRG	EMPRNLREYM	VEKIYPQIPD	HLKEPFLEAT	DNSHLRSMPA	SFLPPSSVKK
**410**	**420**	**430**	**440**	**450**	**460**	**470**	**480**
RGVLLLGDAY	NMRHPLTGGG	MTVAFKDIKL	WRKLLKGIPD	LYDDAAIFEA	KKSFYWARKT	SHSFVVNILA	QALYELFSAT
**490**	**500**	**510**	**520**	**530**	**540**	**550**	**560**
DDSLHQLRKA	CFLYFKLGGE	CVAGPVGLLS	VLSPNPLVLI	GHFFAVAIYA	VYFCFKSEPW	ITKPRALLSS	GAVLYKACSV
**570**	**580**						
IFPLIYSEMK	YMVH						

Cmpd.	No. of Cmpds.	m/z meas.	Δ m/z [ppm]	z	Rt [min]	Score	P	Range	Sequence	Modification	Ratios
1572	3	648.3277	-29.30	2	48.0	29.4	2	46-56	R.CRHRNGGLLGR.Q	Carbamidomethyl: 1	WD:WU 2.62

Detailed Protein Report

Protein 177: tenascin-X isoform 1 precursor [Homo sapiens]

**Accession:**	gi|188528648	**Score:**	29.4
**Database:**	refseq_human(refseq_human_20140103.fasta)	**MW [kDa]:**	457.9
**Database Date:**	2015-11-30	**pI:**	4.9
		**Sequence Coverage [%]:**	0.8
		**No. of unique Peptides:**	2

Detailed Protein Report

10	20	30	40	50	60	70	80
MMPAQYALTS	SLVLLVLLST	ARAGPFSSRS	NVTLPAPRPP	PQPGGHTVGA	GVGSPSSQLY	EHTVEGGEKQ	VVFTHRINLP
90	100	110	120	130	140	150	160
PSTGCGCPPG	TEPPVLASEV	QALRVRLEIL	EELVKGLKEQ	CTGGCCPASA	QAGTGQTDVR	TLCSLHGVFD	LSRCTCSCEP
170	180	190	200	210	220	230	240
GWGGPTCSDP	TDAEIPPSSP	PSASGSCPDD	CNDQGRCVRG	RCVCFPGYTG	PSCGWPSCPG	DCQGRGRCVQ	GVCVCRAGFS
250	260	270	280	290	300	310	320
GPDCSQRSCP	RGCSQRGRCE	GGRCVCDPGY	TGDDCGMRSC	PRGCSQRGRC	ENGRCVCNPG	YTGEDCGVRS	CPRGCSQRGR
330	340	350	360	370	380	390	400
CKDGRCVCDP	GYTGEDCGTR	SCPWDCGEGG	RCVDGRCVCW	PGYTGEDCST	RTCPRDCRGR	GRCEDGECIC	DTGYSGDDCG
410	420	430	440	450	460	470	480
VRSCPGDCNQ	RGRCEDGRCV	CWPGYTGTDC	GSRACPRDCR	GRGRCENGVC	VCNAGYSGED	CGVRSCPGDC	RGRGRCESGR
490	500	510	520	530	540	550	560
CMCWPGYTGR	DCGTRACPGD	CRGRGRCVDG	RCVCNPGFTG	EDCGSRRCPG	DCRGHGLCED	GVCVCDAGYS	GEDCSTRSCP
570	580	590	600	610	620	630	640
GGCRGRGQCL	DGRCVCEDGY	SGEDCGVRQC	PNDCSQHGVC	QDGVCICWEG	YVSEDCSIRT	CPSNCHGRGR	CEEGRCLCDP
650	660	670	680	690	700	710	720
GYTGPTCATR	MCPADCRGRG	RCVQGVCLCH	VGYGGEDCGQ	EEPPASACPG	GCGPRELCRA	GQCVCVEGFR	GPDCAIQTCP
730	740	750	760	770	780	790	800
GDCRGRGECH	DGSCVCKDGY	AGEDCGEEVP	TIEGMRMHLL	EETTVRTEWT	PAPGPVDAYE	IQFIPTTEGA	SPPFTARVPS
810	820	830	840	850	860	870	880
SASAYDQRGL	APGQEYQVTV	RALRGTSWGL	PASKTITTMI	DGPQDLRVVA	VTPTTLELGW	LRPQAEVDRF	VVSYVSAGNQ
890	900	910	920	930	940	950	960
RVRLEVPPEA	DGTLLTDLMP	GVEYVVTVTA	ERGRAVSYPA	SVRANTGSSP	LGLLGTTDEP	PPSGPSTTQG	AQAPLLQQRP
970	980	990	1000	1010	1020	1030	1040
QELGELRVLG	RDETGRLRVV	WTAQPDTFAY	FQLRMRVPEG	PGAHEEVLPG	DVRQALVPPP	PPGTPYELSL	HGVPPGGKPS
1050	1060	1070	1080	1090	1100	1110	1120
DPIIYQGIMD	KDEEKPGKSS	GPPRLGELTV	TDRTSDSLLL	RWTVPEGEFD	SFVIQYKDRD	GQPQVVPVEG	PQRSAVITSL
1130	1140	1150	1160	1170	1180	1190	1200
DPGRKYKFVL	YGFVGKKRHG	PLVAEAKILP	QSDPSPGTPP	HLGNLWVTDP	TPDSLHLSWT	VPEGQFDTFM	VQYRDRDGRP
1210	1220	1230	1240	1250	1260	1270	1280
QVVPVEGPER	SFVVSSLDPD	HKYRFTLFGI	ANKKRYGPLT	ADGTTAPERK	EEPPRPEFLE	QPLLGELTVT	GVTPDSLRLS
1290	1300	1310	1320	1330	1340	1350	1360
WTVAQGPFDS	FMVQYKDAQG	QPQAVPVAGD	ENEVTVPGLD	PDRKYKMNLY	GLRGRQRVGP	ESVVAKTAPQ	EDVDETPSPT
1370	1380	1390	1400	1410	1420	1430	1440
ELGTEAPESP	EEPLLGELTV	TGSSPDSLSL	FWTVPQGSFD	SFTVQYKDRD	GRPRAVRVGG	KESEVTVGGL	EPGHKYKMHL
1450	1460	1470	1480	1490	1500	1510	1520
YGLHEGQRVG	PVSAVGVTAP	QQEETPPATE	SPLEPRLGEL	TVTDVTPNSV	GLSWTVPEGQ	FDSFIVQYKD	KDGQPQVVPV
1530	1540	1550	1560	1570	1580	1590	1600
AADQREVTVY	NLEPERKYKM	NMYGLHDGQR	MGPLSVVIVT	APLPPAPATE	ASKPPLEPRL	GELTVTDITP	DSVGLSWTVP
1610	1620	1630	1640	1650	1660	1670	1680
EGEFDSFVVQ	YKDRDGQPQV	VPVAADQREV	TIPDLEPSRK	YKFLLFGIQD	GKRRSPVSVE	AKTVARGDAS	PGAPPRLGEL
1690	1700	1710	1720	1730	1740	1750	1760
WVTDPTPDSL	RLSWTVPEGQ	FDSFVVQFKD	KDGPQVVPVE	GHERSVTVTP	LDAGRKYRFL	LYGLLGKKRH	GPLTADGTTE
1770	1780	1790	1800	1810	1820	1830	1840
ARSAMDDTGT	KRPPKPRLGE	ELQVTTVTQN	SVGLSWTVPE	GQFDSFVVQY	KDRDGQPQVV	PVEGSLREVS	VPGLDPAHRY
1850	1860	1870	1880	1890	1900	1910	1920
KLLLYGLHHG	KRVGPISAVA	ITAGREETET	ETTAPTPPAP	EPHLGELTVE	EATSHTLHLS	WMVTEGEFDS	FEIQYTDRDG
1930	1940	1950	1960	1970	1980	1990	2000
QLQMVRIGGD	RNDITLSGLE	SDHRYLVTLY	GFSDGKHVGP	VHVEALTVPE	EEKPSEPPTA	TPEPPIKPRL	GELTVTDATP
2010	2020	2030	2040	2050	2060	2070	2080
DSLSLSWTVP	EGQFDHFLVQ	YRNGDGQPKA	VRVPGHEEGV	TISGLEPDHK	YKMNLYGFHG	GQRMGPVSVV	GVTAAEEETP
2090	2100	2110	2120	2130	2140	2150	2160
SPTEPSMEAP	EPAEEPLLGE	LTVTGSSPDS	LSLSWTVPQG	RFDSFTVQYK	DRDGRPQVVR	VGGEESEVTV	GGLEPGRKYK
2170	2180	2190	2200	2210	2220	2230	2240

Detailed Protein Report

Cmpd.	No. of Cmpds.	m/z meas.	Δ m/z [ppm]	z	Rt [min]	Score	P	Range	Sequence	Modification
1929	1	890.3591	-119.35	2	52.8	13.5	1	1418-1435	R.VGGKESEVTVGGLEPGHK.Y	
2906	2	557.9675	41.54	3	65.1	15.9	0	2141-2157	R.VGGEESEVTVGGLEPGR.K	

Detailed Protein Report

Protein 178: probable G-protein coupled receptor 179 precursor [Homo sapiens]

**Accession:**	gi|93352554	**Score:**	29.4
**Database:**	refseq_human(refseq_human_20140103.fasta)	**MW [kDa]:**	257.2
**Database Date:**	2015-11-30	**pI:**	5.4
		**Sequence Coverage [%]:**	0.9
		**No. of unique Peptides:**	1

Quantitation

WD:WU	**Median:** 1.45	**CV:** 0.00 %	**No. of Peptides:** 1

Detailed Protein Report

10	20	30	40	50	60	70	80
MGTRGAVMPP	PMWGLLGCCF	VCAWALGGPR	PIRSLPPLSS	QVKPGSVPMQ	VPLEGAEAAL	AYLYSGDAQQ	LSQVNCSERY
90	100	110	120	130	140	150	160
EARGAGAMPG	LPPSLQGAAG	TLAQAANFLN	MLLQANDIRE	SSVEEDVEWY	QALVRSVAEG	DPRVYRALLT	FNPPPGASHL
170	180	190	200	210	220	230	240
QLALQATRTG	EETILQDLSG	NWVQEENPPG	DLDTPALKKR	VLTNDLGSLG	SPKWPQADGY	VGDTQQVRLS	PPFLECQEGR
250	260	270	280	290	300	310	320
LRPGWLITLS	ATFYGLKPDL	SPEVRGQVQM	DVDLQSVDIN	QCASGPGWYS	NTHLCDLNST	QCVPLESQGF	VLGRYLCRCR
330	340	350	360	370	380	390	400
PGFYGASPSG	GLEESDFQTT	GQFGFPEGRS	GRLLQCLPCP	EGCTSCMDAT	PCLVEEAAVL	RAAVLACQAC	CMLAIFLSML
410	420	430	440	450	460	470	480
VSYRCRRNKR	IWASGVVLLE	TVLFGFLLLY	FPVFILYFKP	SVFRCIALRW	VRLLGFAIVY	GTIILKLYRV	LQLFLSRTAQ
490	500	510	520	530	540	550	560
RSALLSSGRL	LRRLGLLLLP	VLGFLAVWTV	GALERGIQHA	PLVIRGHTPS	GRHFYLCHHD	RWDYIMVVAE	LLLLCWGSFL
570	580	590	600	610	620	630	640
CYATRAVLSA	FHEPRYMGIA	LHNELLLSAA	FHTARFVLVP	SLHPDWTLLL	FFFHTHSTVT	TTLALIFIPK	FWKLGAPPRE
650	660	670	680	690	700	710	720
EMVDEVCEDE	LDLQHSGSYL	GSSIASAWSE	HSLDPGDIRD	ELKKLYAQLE	VHKTKEMAAN	NPHLPKKRGS	SCQGLGRSFM
730	740	750	760	770	780	790	800
RYLAEFPEAL	ARQHSRDSGS	PGHGSLPGSS	RRRLLSSSLQ	EPEGTPALHK	SRSTYDQRRE	QDPPLLDSLL	RRKLAKKASR
810	820	830	840	850	860	870	880
TESRESVEGP	PALGFRSASA	HNLTVGERLP	RARPASLQKS	LSVASSREKA	LLMASQAYLE	ETYRQAKERE	ERKKAKAAMA
890	900	910	920	930	940	950	960
SLVRRPSARR	LERPRGAPLS	APPSPAKSSS	VDSSHTSGRL	HEEARRRLPH	PPIRHQVSTP	ILALSGGLGE	PRMLSPTSTL
970	980	990	1000	1010	1020	1030	1040
APALLPALAP	TPAPALAPVP	VSPQSPNLLT	YICPWENAEL	PAKQENVPQE	GPSGPERGHH	SPAPARARLW	RALSVAVEKS
1050	1060	1070	1080	1090	1100	1110	1120
RAGENEMDAE	DAHHQREAND	VDEDRPKIFP	KSHSLKAPVQ	QGSMRSLGLA	IKALTRSRST	YREKESVEES	PEGQNSGTAG
1130	1140	1150	1160	1170	1180	1190	1200
ESMGAPSRSP	RLGRPKAVSK	QAALIPSDDK	ESLQNQQNAH	TSRMLQVCQR	EGSREQEDRG	RRMTQGLGER	KAERAGKTGL
1210	1220	1230	1240	1250	1260	1270	1280
AMLRQVSRDK	NIKQSKETPV	GWQELPKAGL	QSLGSADHRV	AEVCPWEVTE	SETRQPDSGN	KAEICPWETS	EGAPESRALR
1290	1300	1310	1320	1330	1340	1350	1360
QDPGDSQKKR	GEARGKSEPI	DVVPMMRKKP	ERLVREQEAV	CPWESADRGG	LSPGSAPQDP	GRIRDKSEAG	DSVEARKVEK
1370	1380	1390	1400	1410	1420	1430	1440
PGWEAAGPEA	HTPDITKAEP	CPWEASEGGE	DGKPAQEAVK	DLPQEKQKTR	KATFWKEQKP	GGDLESLCPW	ESTDFRGPSA
1450	1460	1470	1480	1490	1500	1510	1520
VSIQAPGSSE	CSGSLGSGIA	EVCLWEAGDA	PAIQKAEICP	WELDDNVMGQ	EMLSLGTGRE	SLQEKEKASR	KGSFGEMGEQ
1530	1540	1550	1560	1570	1580	1590	1600
TVKAVQKLSQ	QQESVCPRES	TVPGHSSPCL	DNSSSKAGSQ	FLCNGGSRAT	QVCPQEDLRP	EAQEATPAKT	EICPWEVNER
1610	1620	1630	1640	1650	1660	1670	1680
TREEWTSAQV	PRGGESQKDK	EKMPGKSEIE	DVTAWEKPEG	QIQKQEAVGP	WESVDPGSFS	PQPRPQDTER	PQTLLQMSGS
1690	1700	1710	1720	1730	1740	1750	1760
VGSKAADICP	LDVEENLTAG	KAEICPWEVG	AGAGEERALG	AEAIRKSPND	TGKVSADLGP	RERAVTAPEK	PQKPTPEWEV
1770	1780	1790	1800	1810	1820	1830	1840
ACPWGSVGPG	ACSQHPGTLD	ADGPKAGFQE	LDHMGCRPGE	VCPWEAQEAA	TSEKAKICPW	EVSEGTTGKG	LDQKAGSESA
1850	1860	1870	1880	1890	1900	1910	1920
EQREKALEKG	RLTSLGEDVS	KGMAKLCQQQ	ETICIWENKD	LRESPAQAPK	ISDLPSSMSS	EVAEGHSLEA	TEKGDLRQDP
1930	1940	1950	1960	1970	1980	1990	2000
KTGSFPEHIT	QEKAPAADTE	EFTTEDGEKT	SHELQSVCPW	ETTAPADSVS	HLDRQRPDQP	KASSQRLVST	GGRAADVCPW
2010	2020	2030	2040	2050	2060	2070	2080
DVPDAGVYKS	DSSAKAETCP	WEVTERIPVK	GVSRQDGKGD	SQEEKGRAPE	KSEPKGVPVQ	KKPEMADFRQ	QEAVCPWESQ
2090	2100	2110	2120	2130	2140	2150	2160
DGKGLSPQPA	PDASDRSRGS	SEAAGSVETR	VAEVCLWEVV	EAPSAKKAEI	CPWEAGGGAA	EEGEQERESQ	GQGEMFLQKA
2170	2180	2190	2200	2210	2220	2230	2240

Detailed Protein Report

Cmpd.	No. of Cmpds.	m/z meas.	Δ m/z [ppm]	z	Rt [min]	Score	P	Range	Sequence	Modification	Ratios
813	6	509.1525	-284.31	2	38.6	17.2	1	1195-1204	R.AGKTGLAMLR.Q		WD:WU 1.45

Detailed Protein Report

Protein 179: cyclic AMP-dependent transcription factor ATF-5 [Homo sapiens]

**Accession:**	gi|12597625	**Score:**	29.3
**Database:**	refseq_human(refseq_human_20140103.fasta)	**MW [kDa]:**	30.7
**Database Date:**	2015-11-30	**pI:**	4.7
**Modification(s):**	Carbamidomethyl	**Sequence Coverage [%]:**	6.4
		**No. of unique Peptides:**	2

Quantitation

WD:WU **Median:** 1.40 **CV:** 0.00 % **No. of Peptides:** 1

Alias proteins:

Accession	Name	Description
gi|302370956	refseq_human (refseq_human_20140103.fasta)	cyclic AMP-dependent transcription factor ATF-5 [Homo sapiens]

```
        10         20         30         40         50         60         70         80
MSLLATLGLE LDRALLPASG LGWLVDYGKL PPAPAPLAPY EVLGGALEGG LPVGGEPLAG DGFSDWMTER VDFTALLPLE
        90        100        110        120        130        140        150        160
PPLPPGTLPQ PSPTPPDLEA MASLLKKELE QMEDFFLDAP PLPPPSPPPL PPPPLPPAPS LPLSLPSFDL PQPPVLDTLD
       170        180        190        200        210        220        230        240
LLAIYCRNEA GQEEVGMPPL PPPQQPPPPS PPQPSRLAPY PHPATTRGDR KQKKRDQNKS AALRYRQRKR AEGEALEGEC
       250        260        270        280        290
QGLEARNREL KERAESVERE IQYVKDLLIE VYKARSQRTR SC
```

Cmpd.	No. of Cmpds.	m/z meas.	Δ m/z [ppm]	z	Rt [min]	Score	P	Range	Sequence	Modification	Ratios
2748	1	1002.0026	18.34	2	62.8	15.8	2	229-246	R.KRAEGEALEGECQGLEAR.N	Carbamidomethyl: 12	
533	1	625.6275	0.96	3	35.1	13.5	1	230-246	K.RAEGEALEGECQGLEAR.N	Carbamidomethyl: 11	WD:WU 1.40

Detailed Protein Report

Protein 180: multicilin [Homo sapiens]

**Accession:**	gi|299782571	**Score:**	29.2
**Database:**	refseq_human(refseq_human_20140103.fasta)	**MW [kDa]:**	41.7
**Database Date:**	2015-11-30	**pI:**	5.5
**Modification(s):**	Carbamidomethyl, Oxidation	**Sequence Coverage [%]:**	2.9
		**No. of unique Peptides:**	1

10	20	30	40	50	60	70	80
MQACGGGAAG	RRAFDSICPN	RMLALPGRAL	LCKPGKPERK	FAPPRKFFPG	CTGGSPVSVY	EDPPDAEPTA	LPALTTIDLQ
**90**	**100**	**110**	**120**	**130**	**140**	**150**	**160**
DLADCSSLLG	SDAPPGGDLA	ASQNHSHQTE	ADFNLQDFRD	TVDDLISDSS	SMMSPTLASG	DFPFSPCDIS	PFGPCLSPPL
**170**	**180**	**190**	**200**	**210**	**220**	**230**	**240**
DPRALQSPPL	RPPDVPPPEQ	YWKEVADQNQ	RALGDALVEN	NQLHVTLTQK	QEEIASLKER	NVQLKELASR	TRHLASVLDK
**250**	**260**	**270**	**280**	**290**	**300**	**310**	**320**
LMITQSRDCG	AAAEPFLLKA	KAKRSLEELV	SAAGQDCAEV	DAILREISER	CDEALQSRDP	KRPRLLPEPA	NTDTRPGNLH
**330**	**340**	**350**	**360**	**370**	**380**	**390**	
GAFRGLRTDC	SRSALNLSHS	ELEEGGSFST	RIRSHSTIRT	LAFPQGNAFT	IRTANGGYKF	RWVPS	

Cmpd.	No. of Cmpds.	m/z meas.	Δ m/z [ppm]	z	Rt [min]	Score	P	Range	Sequence	Modification
809	1	526.2922	129.61	2	38.5	29.2	0	1-11	-.MQACGGGAAGR.R	Carbamidomethyl: 4; Oxidation: 1

Detailed Protein Report

Protein 181: **PREDICTED: phosphatidylinositol-glycan-specific phospholipase D isoform X3 [Homo sapiens]**

**Accession:**	gi|530381810	**Score:**	29.2
**Database:**	refseq_human(refseq_human_20140103.fasta)	**MW [kDa]:**	73.7
**Database Date:**	2015-11-30	**pI:**	5.7
		**Sequence Coverage [%]:**	2.5
		**No. of unique Peptides:**	1

Quantitation

WD:WU **Median:** 0.78 **CV:** 0.00 % **No. of Peptides:** 1

10	20	30	40	50	60	70	80
MSAGIVWALN	KDSLGPWELY	VPVKDLLGIY	EKLYGRKVIT	ENVIVDCSHI	QFLEMYGEML	AVSKLYPTYS	TKSPFLVEQF
90	100	110	120	130	140	150	160
QEYFLGGLDD	MAFWSTNIYH	LTSFMLENGT	SDCNLPENPL	FIACGGQQNH	TQGSKMQKND	FHRNLTTSLT	ESVDRNINYT
170	180	190	200	210	220	230	240
ERGVFFSVNS	WTPDSMSFIY	KALERNIRTM	FIGGSQLSQK	HVSSPLASYF	LSFPYARLGW	AMTSADLNQD	GHGDLVVGAP
250	260	270	280	290	300	310	320
GYSRPGHIHI	GRVYLIYGND	LGLPPVDLDL	DKEAHRILEG	FQPSGRFGSA	LAVLDFNVDG	VPDLAVGAPS	VGSEQLTYKG
330	340	350	360	370	380	390	400
AVYVYFGSKQ	GGMSSSPNIT	ISCQDIYCNL	GWTLLAADVN	GDSEPDLVIG	SPFAPGGGKQ	KGIVAAFYSG	PSLSDKEKLN
410	420	430	440	450	460	470	480
VEAANWTVRG	EEDFSWFGYS	LHGVTVDNRT	LLLVGSPTWK	NASRLGHLLH	IRDEKKSLGR	VYGYFPPNGQ	SWFTISGDKA
490	500	510	520	530	540	550	560
MGKLGTSLSS	GHVLMNGTLK	QVLLVGAPTY	DDVSKVAFLT	VTLHQGGATR	MYALTSDAQP	LLLSTFSGDR	RFSRFGGVLH
570	580	590	600	610	620	630	640
LSDLDDDGLD	EIIMAAPLRI	ADVTSGLIGG	EDGRVYVYNG	KETTLGDMTG	KCKSWITPCP	EEKAQYVLIS	PEASSRFGSS
650	660	670	680				
LITVRSKAKN	QVVIAAGRSS	LGARLSGALH	VYSLGSD				

Cmpd.	No. of Cmpds.	m/z meas.	Δ m/z [ppm]	z	Rt [min]	Score	P	Range	Sequence	Modification	Ratios
257	1	625.9672	-15.19	3	32.4	29.2	1	595-611	R.VYVYNGKETTLGDMTGK.C		WD:WU 0.78

Detailed Protein Report

Protein 182: PREDICTED: condensin-2 complex subunit H2 isoform X1 [Homo sapiens]

**Accession:**	gi|530420802	**Score:**	29.2
**Database:**	refseq_human(refseq_human_20140103.fasta)	**MW [kDa]:**	65.2
**Database Date:**	2015-11-30	**pI:**	4.6
		**Sequence Coverage [%]:**	4.1
		**No. of unique Peptides:**	1

10	20	30	40	50	60	70	80
MSQRWGRCWL	DQICISFDEG	KTTMNFIEAA	LLIQGSACVY	SKKVEYLYSL	VYQALDFISG	KRRAKQLSSV	QEDRANGVAS
**90**	**100**	**110**	**120**	**130**	**140**	**150**	**160**
SGVPQEAENE	FLSLDDFPDS	RTNVDLKNDQ	TPSEVLIIPL	LPMALVAPDE	MEKNNNPLYS	RQGEVLASRK	DFRMNTCVPH
**170**	**180**	**190**	**200**	**210**	**220**	**230**	**240**
PRGAFMLEPE	GMSPMEPAGV	SPMPGTQKDT	GRTEEQPMEV	SVCRSPVPAL	GFSQEPGPSP	EGPMPLGGGE	DEDAEEAVEL
**250**	**260**	**270**	**280**	**290**	**300**	**310**	**320**
PEASAPKAAL	EPKESRSPQQ	SAALPRRYML	REREGAPEPA	SCVKETPDPW	QSLDPFDSLE	SKPFKKGRPY	SVPPCVEEAL
**330**	**340**	**350**	**360**	**370**	**380**	**390**	**400**
GQKRKRKGAA	KLQDFHQWYL	AAYADHADSR	RLRRKGPSFA	DMEVLYWTHV	KEQLETLRKL	QRREVAEQWL	RPAEEDHLED
**410**	**420**	**430**	**440**	**450**	**460**	**470**	**480**
SLEDLGAAAD	DFLEPEEYME	PEGADPREAA	DLDAVPMSLS	YEELVRRNVE	LFIATSQKFV	QETELSQRIR	DWEDTVQPLL
**490**	**500**	**510**	**520**	**530**	**540**	**550**	**560**
QEQEQHVPFD	IHTYGDQLVS	RFPQLNEWCP	FAELVAGQPA	FEVCRSMLAS	LQLANDYTVE	ITQQPGLEMA	VDTMSLRLLT
**570**	**580**						
HQRAHKRFQT	YAAPSMAQP						

Cmpd.	No. of Cmpds.	m/z meas.	Δ m/z [ppm]	z	Rt [min]	Score	P	Range	Sequence	Modification
1785	1	734.4108	74.56	2	51.0	11.4	1	567-579	K.RFQTYAAPSMAQP.-	

Detailed Protein Report

Protein 183: unconventional myosin-Id [Homo sapiens]

**Accession:**	gi|51100974	**Score:**	29.2
**Database:**	refseq_human(refseq_human_20140103.fasta)	**MW [kDa]:**	116.1
**Database Date:**	2015-11-30	**pI:**	10.0
		**Sequence Coverage [%]:**	1.8
		**No. of unique Peptides:**	1

10	20	30	40	50	60	70	80
MAEQESLEFG	KADFVLMDTV	SMPEFMANLR	LRFEKGRIYT	FIGEVVVSVN	PYKLLNIYGR	DTIEQYKGRE	LYERPPHLFA
90	100	110	120	130	140	150	160
IADAAYKAMK	RRSKDTCIVI	SGESGAGKTE	ASKYIMQYIA	AITNPSQRAE	VERVKNMLLK	SNCVLEAFGN	AKTNRNDNSS
170	180	190	200	210	220	230	240
RFGKYMDINF	DFKGDPIGGH	INNYLLEKSR	VIVQQPGERS	FHSFYQLLQG	GSEQMLRSLH	LQKSLSSYNY	IHVGAQLKSS
250	260	270	280	290	300	310	320
INDAAEFRVV	ADAMKVIGFK	PEEIQTVYKI	LAAILHLGNL	KFVVDGDTPL	IENGKVVSII	AELLSTKTDM	VEKALLYRTV
330	340	350	360	370	380	390	400
ATGRDIIDKQ	HTEQEASYGR	DAFAKAIYER	LFCWIVTRIN	DIIEVKNYDT	TIHGKNTVIG	VLDIYGFEIF	DNNSFEQFCI
410	420	430	440	450	460	470	480
NYCNEKLQQL	FIQLVLKQEQ	EEYQREGIPW	KHIDYFNNQI	IVDLVEQQHK	GIIAILDDAC	MNVGKVTDEM	FLEALNSKLG
490	500	510	520	530	540	550	560
KHAHFSSRKL	CASDKILEFD	RDFRIRHYAG	DVVYSVIGFI	DKNKDTLFQD	FKRLMYNSSN	PVLKNMWPEG	KLSITEVTKR
570	580	590	600	610	620	630	640
PLTAATLFKN	SMIALVDNLA	SKEPYYVRCI	KPNDKKSPQI	FDDERCRHQV	EYLGLLENVR	VRRAGFAFRQ	TYEKFLHRYK
650	660	670	680	690	700	710	720
MISEFTWPNH	DLPSDKEAVK	KLIERCGFQD	DVAYGKTKIF	IRTPRTLFTL	EELRAQMLIR	IVLFLQKVWR	GTLARMRYKR
730	740	750	760	770	780	790	800
TKAALTIIRY	YRRYKVKSYI	HEVARRFHGV	KTMRDYGKHV	KWPSPPKVLR	RFEEALQTIF	NRWRASQLIK	SIPASDLPQV
810	820	830	840	850	860	870	880
RAKVAAVEML	KGQRADLGLQ	RAWEGNYLAS	KPDTPQTSGT	FVPVANELKR	KDKYMNVLFS	CHVRKVNRFS	KVEDRAIFVT
890	900	910	920	930	940	950	960
DRHLYKMDPT	KQYKVMKTIP	LYNLTGLSVS	NGKDQLVVFH	TKDNKDLIVC	LFSKQPTHES	RIGELVGVLV	NHFKSEKRHL
970	980	990	1000	1010			
QVNVTNPVQC	SLHGKKCTVS	VETRLNQPQP	DFTKNRSGFI	LSVPGN			

Cmpd.	No. of Cmpds.	m/z meas.	Δ m/z [ppm]	z	Rt [min]	Score	P	Range	Sequence	Modification
2982	3	729.3741	-5.85	2	66.0	18.2	1	985-996	R.LNQPQPDFTKNR.S	

Detailed Protein Report

Protein 184: **PREDICTED: forkhead box protein P3 isoform X2 [Homo sapiens]**

**Accession:**	gi|578838031	**Score:**	29.1
**Database:**	refseq_human(refseq_human_20140103.fasta)	**MW [kDa]:**	56.2
**Database Date:**	2015-11-30	**pI:**	10.7
		**Sequence Coverage [%]:**	7.1
		**No. of unique Peptides:**	1

10	20	30	40	50	60	70	80
MTRKGQRKKP	KISKFPFKSH	NQEKEKHRER	EKKNYENPSP	PRDYQRTHSS	KKIWIIRRER	SAASTPYSVV	FLLGIKAKLF
**90**	**100**	**110**	**120**	**130**	**140**	**150**	**160**
LIRDSFPQAR	LILFCQSTSP	SLPLDKDPMP	NPRPGKPSAP	SLALGPSPGA	SPSWRAAPKA	SDLLGARGPG	GTFQGRDLRG
**170**	**180**	**190**	**200**	**210**	**220**	**230**	**240**
GAHASSSSLN	PMPPSQLQLS	TVDAHARTPV	LQVHPLESPA	MISLTPPTTA	TGVFSLKARP	GLPPGINVAS	LEWVSREPAL
**250**	**260**	**270**	**280**	**290**	**300**	**310**	**320**
LCTFPNPSAP	RKDSTLSAVP	QSSYPLLANG	VCKWPGCEKV	FEEPEDFLKH	CQADHLLDEK	GRAQCLLQRE	MVQSLEQQLV
**330**	**340**	**350**	**360**	**370**	**380**	**390**	**400**
LEKEKLSAMQ	AHLAGKMALT	KASSVASSDK	GSCCIVAAGS	QGPVVPAWSG	PREAPDSLFA	VRRHLWGSHG	NSTFPEFLHN
**410**	**420**	**430**	**440**	**450**	**460**	**470**	**480**
MDYFKFHNMR	PPFTYATLIR	WAILEAPEKQ	RTLNEIYHWF	TRMFAFFRNH	PATWKNAIRH	NLSLHKCFVR	VESEKGAVWT
**490**	**500**	**510**					
VDELEFRKKR	SQRPSRCSNP	TPGP					

Cmpd.	No. of Cmpds.	m/z meas.	Δ m/z [ppm]	z	Rt [min]	Score	P	Range	Sequence	Modification
14	1	712.9876	-118.49	3	29.1	16.9	2	59-78	R.ERSAASTPYSVVFLLGIKAK.L	

Detailed Protein Report

Protein 185: PREDICTED: rap guanine nucleotide exchange factor 3 isoform X2 [Homo sapiens]

**Accession:**	gi|530399600	**Score:**	29.1
**Database:**	refseq_human(refseq_human_20140103.fasta)	**MW [kDa]:**	87.5
**Database Date:**	2015-11-30	**pI:**	7.2
**Modification(s):**	Carbamidomethyl, Oxidation	**Sequence Coverage [%]:**	3.3
		**No. of unique Peptides:**	2

Quantitation

WD:WU **Median:** 2.90 **CV:** 0.00 % **No. of Peptides:** 1

Alias proteins:

Accession	Name	Description
gi|530399602	refseq_human (refseq_human_20140103.fasta)	PREDICTED: rap guanine nucleotide exchange factor 3 isoform X3 [Homo sapiens]

10	20	30	40	50	60	70	80
MGSWPWDLGS	IPGAKLWESA	RCCWMKVPSA	MDRDAQFYRF	PGPEPEPVRT	HEMEEELAEA	VALLSQRGPD	ALLTVALRKP
**90**	**100**	**110**	**120**	**130**	**140**	**150**	**160**
PGQRTDEELD	LIFEELLHIK	AVAHLSNSVK	RELAAVLLFE	PHSKAGTVLF	SQGDKGTSWY	IIWKGSVNVV	THGKGLVTTL
**170**	**180**	**190**	**200**	**210**	**220**	**230**	**240**
HEGDDFGQLA	LVNDAPRAAT	IILREDNCHF	LRVDKQDFNR	IIKDVEAKTM	RLEEHGKVVL	VLERASQGAG	PSRPPTPGRN
**250**	**260**	**270**	**280**	**290**	**300**	**310**	**320**
RYTVMSGTPE	KILELLLEAM	GPDSSAHDPT	ETFLSDFLLT	HRVFMPSAQL	CAALLHHFHV	EPAGGSEQER	STYVCNKRQQ
**330**	**340**	**350**	**360**	**370**	**380**	**390**	**400**
ILRLVSQWVA	LYGSMLHTDP	VATSFLQKLS	DLVGRDTRLS	NLLREQWPER	RRCHRLENGC	GNASPQMKAR	NLPVWLPNQD
**410**	**420**	**430**	**440**	**450**	**460**	**470**	**480**
EPLPGSSCAI	QVGDKVPYDI	CRPDHSVLTL	QLPVTASVRE	VMAALAQEDG	WTKGQVLVKV	NSAGDAIGLQ	PDARGVATSL
**490**	**500**	**510**	**520**	**530**	**540**	**550**	**560**
GLNERLFVVN	PQEVHELIPH	PDQLGPTVGS	AEGLDLVSAK	DLAGQLTDHD	WSLFNSIHQV	ELIHYVLGPQ	HLRDVTTANL
**570**	**580**	**590**	**600**	**610**	**620**	**630**	**640**
ERFMRRFNEL	QYWVATELCL	CPVPGPRAQL	LRKFIKLAAH	LKEQKNLNSF	FAVMFGLSNS	AISRLAHTWE	RLPHKVRKLY
**650**	**660**	**670**	**680**	**690**	**700**	**710**	**720**
SALERLLDPS	WNHRVYRLAL	AKLSPPVIPF	MPLLLKDMTF	IHEGNHTLVE	NLINFEKMRM	MARAARMLHH	CRSHNPVPLS
**730**	**740**	**750**	**760**	**770**	**780**		
PLRSRVSHLH	EDSQVARIST	CSEQSLSTRS	PASTWAYVQQ	LKVIDNQREL	SRLSRELEP		

Cmpd.	No. of Cmpds.	m/z meas.	Δ m/z [ppm]	z	Rt [min]	Score	P	Range	Sequence	Modification	Ratios
1402	1	727.3976	-11.71	2	45.9	15.2	0	112-124	R.ELAAVLLFEPHSK.A		
870	1	711.3337	31.93	2	39.7	13.9	0	376-388	R.LENGCGNASPQMK.A	Carbamidomethyl: 5; Oxidation: 12	WD:WU 2.90

Detailed Protein Report

Protein 186: deoxyribonuclease gamma isoform 2 precursor [Homo sapiens]

Accession: gi|375151549
Database: refseq_human(refseq_human_20140103.fasta)
Database Date: 2015-11-30
Modification(s): Carbamidomethyl, Oxidation

Score: 29.0
MW [kDa]: 31.8
pI: 9.7
Sequence Coverage [%]: 9.1
No. of unique Peptides: 2

10	20	30	40	50	60	70	80
MSRELAPLLL	LLLSIHSALA	MRICSFNVRS	FGESKQEDKN	AMDVIVKVIK	RCDIILVMEI	KDSNNRICPI	LMEKLNREKL
**90**	**100**	**110**	**120**	**130**	**140**	**150**	**160**
VSVKRSYHYH	DYQDGDADVF	SREPFVVWFQ	SPHTAVKDFV	IIPLHTTPET	SVKEIDELVE	VYTDVKHRWK	AENFIFMGDF
**170**	**180**	**190**	**200**	**210**	**220**	**230**	**240**
NAGCSYVPKK	AWKNIRLRTD	PRFVWLIGDQ	EDTTVKKSTN	CAYDRIVLRG	QEIVSSVVPK	SNSVFDFQKA	YKLTEEEALD
**250**	**260**	**270**	**280**				
VSDHFPVEFK	LQSSRAFTNS	KKSVTLRKKT	KSKRS				

Cmpd.	No. of Cmpds.	m/z meas.	Δ m/z [ppm]	z	Rt [min]	Score	P	Range	Sequence	Modification
2460	2	873.4565	-23.34	2	59.0	11.5	2	36-50	K.QEDKNAMDVIVKVIK.R	Oxidation: 7
2658	1	625.2870	-69.08	2	61.5	17.6	0	52-61	R.CDIILVMEIK.D	Carbamidomethyl: 1; Oxidation: 7

Detailed Protein Report

Protein 187: poly [ADP-ribose] polymerase 4 [Homo sapiens]

**Accession:**	gi|112789550	**Score:**	29.0
**Database:**	refseq_human(refseq_human_20140103.fasta)	**MW [kDa]:**	192.5
**Database Date:**	2015-11-30	**pI:**	5.3
		**Sequence Coverage [%]:**	2.1
		**No. of unique Peptides:**	1

10	20	30	40	50	60	70	80
MVMGIFANCI	FCLKVKYLPQ	QQKKKLQTDI	KENGGKFSFS	LNPQCTHIIL	DNADVLSQYQ	LNSIQKNHVH	IANPDFIWKS
90	100	110	120	130	140	150	160
IREKRLLDVK	NYDPYKPLDI	TPPPDQKASS	SEVKTEGLCP	DSATEEEDTV	ELTEFGMQNV	EIPHLPQDFE	VAKYNTLEKV
170	180	190	200	210	220	230	240
GMEGGQEAVV	VELQCSRDSR	DCPFLISSHF	LLDDGMETRR	QFAIKKTSED	ASEYFENYIE	ELKKQGFLLR	EHFTPEATQL
250	260	270	280	290	300	310	320
ASEQLQALLL	EEVMNSSTLS	QEVSDLVEMI	WAEALGHLEH	MLLKPVNRIS	LNDVSKAEGI	LLLVKAALKN	GETAEQLQKM
330	340	350	360	370	380	390	400
MTEFYRLIPH	KGTMPKEVNL	GLLAKKADLC	QLIRDMVNVC	ETNLSKPNPP	SLAKYRALRC	KIEHVEQNTE	EFLRVRKEVL
410	420	430	440	450	460	470	480
QNHHSKSPVD	VLQIFRVGRV	NETTEFLSKL	GNVRPLLHGS	PVQNIVGILC	RGLLLPKVVE	DRGVQRTDVG	NLGSGIYFSD
490	500	510	520	530	540	550	560
SLSTSIKYSH	PGETDGTRLL	LICDVALGKC	MDLHEKDFSL	TEAPPGYDSV	HGVSQTASVT	TDFEDDEFVV	YKTNQVKMKY
570	580	590	600	610	620	630	640
IIKFSMPGDQ	IKDFHPSDHT	ELEEYRPEFS	NFSKVEDYQL	PDAKTSSSTK	AGLQDASGNL	VPLEDVHIKG	RIIDTVAQVI
650	660	670	680	690	700	710	720
VFQTYTNKSH	VPIEAKYIFP	LDDKAAVCGF	EAFINGKHIV	GEIKEKEEAQ	QEYLEAVTQG	HGAYLMSQDA	PDVFTVSVGN
730	740	750	760	770	780	790	800
LPPKAKVLIK	ITYITELSIL	GTVGVFFMPA	TVAPWQQDKA	LNENLQDTVE	KICIKEIGTK	QSFSLTMSIE	MPYVIEFIFS
810	820	830	840	850	860	870	880
DTHELKQKRT	DCKAVISTME	GSSLDSSGFS	LHIGLSAAYL	PRMWVEKHPE	KESEACMLVF	QPDLDVDLPD	LASESEVIIC
890	900	910	920	930	940	950	960
LDCSSSMEGV	TFLQAKQIAL	HALSLVGEKQ	KVNIIQFGTG	YKELFSYPKH	ITSNTMAAEF	IMSATPTMGN	TDFWKTLRYL
970	980	990	1000	1010	1020	1030	1040
SLLYPARGSR	NILLVSDGHL	QDESLTLQLV	KRSRPHTRLF	ACGIGSTANR	HVLRILSQCG	AGVFEYFNAK	SKHSWRKQIE
1050	1060	1070	1080	1090	1100	1110	1120
DQMTRLCSPS	CHSVSVKWQQ	LNPDVPEALQ	APAQVPSLFL	NDRLLVYGFI	PHCTQATLCA	LIQEKEFRTM	VSTTELQKTT
1130	1140	1150	1160	1170	1180	1190	1200
GTMIHKLAAR	ALIRDYEDGI	LHENETSHEM	KKQTLKSLII	KLSKENSLIT	QFTSFVAVEK	RDENESPFPD	IPKVSELIAK
1210	1220	1230	1240	1250	1260	1270	1280
EDVDFLPYMS	WQGEPQEAVR	NQSLLASSEW	PELRLSKRKH	RKIPFSKRKM	ELSQPEVSED	FEEDGLGVLP	AFTSNLERGG
1290	1300	1310	1320	1330	1340	1350	1360
VEKLLDLSWT	ESCKPTATEP	LFKKVSPWET	STSSFFPILA	PAVGSYLPPT	ARAHSPASLS	FASYRQVASF	GSAAPPRQFD
1370	1380	1390	1400	1410	1420	1430	1440
ASQFSQGPVP	GTCADWIPQS	ASCPTGPPQN	PPSSPYCGIV	FSGSSLSSAQ	SAPLQHPGGF	TTRPSAGTFP	ELDSPQLHFS
1450	1460	1470	1480	1490	1500	1510	1520
LPTDPDPIRG	FGSYHPSASS	PFHFQPSAAS	LTANLRLPMA	SALPEALCSQ	SRTTPVDLCL	LEESVGSLEG	SRCPVFAFQS
1530	1540	1550	1560	1570	1580	1590	1600
SDTESDELSE	VLQDSCFLQI	KCDTKDDSIL	CFLEVKEEDE	IVCIQHWQDA	VPWTELLSLQ	TEDGFWKLTP	ELGLILNLNT
1610	1620	1630	1640	1650	1660	1670	1680
NGLHSFLKQK	GIQSLGVKGR	ECLLDLIATM	LVLQFIRTRL	EKEGIVFKSL	MKMDDASISR	NIPWAFEAIK	QASEWVRRTE
1690	1700	1710	1720	1730			
GQYPSICPRL	ELGNDWDSAT	KQLLGLQPIS	TVSPLHRVLH	YSQG			

Cmpd.	No. of Cmpds.	m/z meas.	Δ m/z [ppm]	z	Rt [min]	Score	P	Range	Sequence	Modification
122	1	981.4639	-29.75	2	30.8	17.4	1	1015-1032	R.ILSQCGAGVFEYFNAKSK.H	

Detailed Protein Report

Protein 188: nucleolin [Homo sapiens]

Accession: gi|55956788
Database: refseq_human(refseq_human_20140103.fasta)
Database Date: 2015-11-30

Score: 28.9
MW [kDa]: 76.6
pI: 4.4
Sequence Coverage [%]: 3.9
No. of unique Peptides: 1

Quantitation

WD:WU **Median:** 1.03 **CV:** 0.00 % **No. of Peptides:** 1

10	20	30	40	50	60	70	80
MVKLAKAGKN	QGDPKKMAPP	PKEVEEDSED	EEMSEDEEDD	SSGEEVVIPQ	KKGKKAAATS	AKKVVVSPTK	KVAVATPAKK
**90**	**100**	**110**	**120**	**130**	**140**	**150**	**160**
AAVTPGKKAA	ATPAKKTVTP	AKAVTTPGKK	GATPGKALVA	TPGKKGAAIP	AKGAKNGKNA	KKEDSDEEED	DDSEEDEEDD
**170**	**180**	**190**	**200**	**210**	**220**	**230**	**240**
EDEDEDEDEI	EPAAMKAAAA	APASEDEDDE	DDEDDEDDDD	DEEDDSEEEA	METTPAKGKK	AAKVVPVKAK	NVAEDEDEEE
**250**	**260**	**270**	**280**	**290**	**300**	**310**	**320**
DDEDEDDDDD	EDDEDDDDED	DEEEEEEEEE	EPVKEAPGKR	KKEMAKQKAA	PEAKKQKVEG	TEPTTAFNLF	VGNLNFNKSA
**330**	**340**	**350**	**360**	**370**	**380**	**390**	**400**
PELKTGISDV	FAKNDLAVVD	VRIGMTRKFG	YVDFESAEDL	EKALELTGLK	VFGNEIKLEK	PKGKDSKKER	DARTLLAKNL
**410**	**420**	**430**	**440**	**450**	**460**	**470**	**480**
PYKVTQDELK	EVFEDAAEIR	LVSKDGKSKG	IAYIEFKTEA	DAEKTFEEKQ	GTEIDGRSIS	LYYTGEKGQN	QDYRGGKNST
**490**	**500**	**510**	**520**	**530**	**540**	**550**	**560**
WSGESKTLVL	SNLSYSATEE	TLQEVFEKAT	FIKVPQNQNG	KSKGYAFIEF	ASFEDAKEAL	NSCNKREIEG	RAIRLELQGP
**570**	**580**	**590**	**600**	**610**	**620**	**630**	**640**
RGSPNARSQP	SKTLFVKGLS	EDTTEETLKE	SFDGSVRARI	VTDRETGSSK	GFGFVDFNSE	EDAKAAKEAM	EDGEIDGNKV
**650**	**660**	**670**	**680**	**690**	**700**	**710**	**720**
TLDWAKPKGE	GGFGGRGGGR	GGFGGRGGGR	GGRGGFGGRG	RGGFGGRGGF	RGGRGGGGDH	KPQGKKTKFE	

Cmpd.	No. of Cmpds.	m/z meas.	Δ m/z [ppm]	z	Rt [min]	Score	P	Range	Sequence	Modification	Ratios
165	1	635.8027	-108.85	2	31.3	12.6	1	97-109	K.TVTPAKAVTTPGK.K		WD:WU 1.03

Detailed Protein Report

Protein 189: PREDICTED: protein TBATA isoform X13 [Homo sapiens]

**Accession:**	gi|578819092	**Score:**	28.9
**Database:**	refseq_human(refseq_human_20140103.fasta)	**MW [kDa]:**	35.1
**Database Date:**	2015-11-30	**pI:**	6.4
		**Sequence Coverage [%]:**	9.2
		**No. of unique Peptides:**	1

10	20	30	40	50	60	70	80
MGIPTISVPI	GDPQSNRNPQ	LSSAWKKELK	ELASRVAFLT	KEDELKKKED	TTSLNWNCKS	RRPQAPGLSG	TDLGAAELGI
**90**	**100**	**110**	**120**	**130**	**140**	**150**	**160**
KKEQKEEPLR	EQGAKYSAET	GRLIPASTRA	VGRRRSHQGQ	QSQSSSRHEG	VQAFLLQDQE	LLVLELLCRI	LETDLLSAIQ
**170**	**180**	**190**	**200**	**210**	**220**	**230**	**240**
FWLLYAPPKE	KDLALGLLQT	AVAQLLPQPL	VSIPTEKLLS	QLPEVHEPPQ	EKQEPPCRWA	PHPALPFCSL	LSSQSIPEEN
**250**	**260**	**270**	**280**	**290**	**300**	**310**	**320**
EDITFYKKRK	TRVHWRSSSP	PDAFKPEHRE	EDIEAEGREL	RGPNTCTCLA	QEQPQGFRGV	SVSTTFTAVP	DSLP

Cmpd.	No. of Cmpds.	m/z meas.	Δ m/z [ppm]	z	Rt [min]	Score	P	Range	Sequence	Modification
2897	1	655.6487	-212.02	2	65.0	11.2	0	49-59	K.EDTTSLNWNCK.S	

Detailed Protein Report

Protein 190: zinc finger protein 581 [Homo sapiens]

Accession: gi|7705495
Database: refseq_human(refseq_human_20140103.fasta)
Database Date: 2015-11-30
Modification(s): Carbamidomethyl

Score: 28.8
MW [kDa]: 22.0
pI: 10.6
Sequence Coverage [%]: 13.7
No. of unique Peptides: 2

Alias proteins:

Accession	Name	Description
gi|578834534	refseq_human (refseq_human_20140103.fasta)	PREDICTED: zinc finger protein 581 isoform X1 [Homo sapiens]

10	20	30	40	50	60	70	80
MLVLPSPCPQ	PLAFSSVETM	EGPPRRTCRS	PEPGPSSSIG	SPQASSPPRP	NHYLLIDTQG	VPYTVLVDEE	SQREPGASGA
**90**	**100**	**110**	**120**	**130**	**140**	**150**	**160**
PGQKKCYSCP	VCSRVFEYMS	YLQRHSITHS	EVKPFECDIC	GKAFKRASHL	ARHHSIHLAG	GGRPHGCPLC	PRRFRDAGEL
**170**	**180**	**190**	**200**				
AQHSRVHSGE	RPFQCPHCPR	RFMEQNTLQK	HTRWKHP				

Cmpd.	No. of Cmpds.	m/z meas.	Δ m/z [ppm]	z	Rt [min]	Score	P	Range	Sequence	Modification
1072	2	984.1823	6.75	3	42.1	11.2	2	127-153	R.ASHLARHHSIHLAGGGRPHGCPLCPRR.F	Carbamidomethyl: 24
1056	2	984.2136	38.53	3	41.9	17.6	2	127-153	R.ASHLARHHSIHLAGGGRPHGCPLCPRR.F	Carbamidomethyl: 21

Detailed Protein Report

Protein 191: **PREDICTED: focal adhesion kinase 1 isoform X12 [Homo sapiens]**

**Accession:**	gi|530389222	**Score:**	28.6
**Database:**	refseq_human(refseq_human_20140103.fasta)	**MW [kDa]:**	108.1
**Database Date:**	2015-11-30	**pI:**	5.7
**Modification(s):**	Carbamidomethyl, Oxidation	**Sequence Coverage [%]:**	2.7
		**No. of unique Peptides:**	2

10	20	30	40	50	60	70	80
MEMLLMSGYE	LRIRYLPKGF	LNQFTEDKPT	LNFFYQQVKS	DYMLEIADQV	DQEIALKLGC	LEIRRSYWEM	RGNALEKKSN
**90**	**100**	**110**	**120**	**130**	**140**	**150**	**160**
YEVLEKDVGL	KRFFPKSLLD	SVKAKTLRKL	IQQTFRQFAN	LNREESILKF	FEILSPVYRF	DKECFKCALG	SSWIISVELA
**170**	**180**	**190**	**200**	**210**	**220**	**230**	**240**
IGPEEGISYL	TDKGCNPTHL	ADFTQVQTIQ	YSNSEDKDRK	GMLQLKIAGA	PEPLTVTAPS	LTIAENMADL	IDGYCRLVNG
**250**	**260**	**270**	**280**	**290**	**300**	**310**	**320**
TSQSFIIRPQ	KEGERALPSI	PKLANSEKQG	MRTHAVSVSD	EISGDETDDY	AEIIDEEDTY	TMPSKSYGID	EARDYEIQRE
**330**	**340**	**350**	**360**	**370**	**380**	**390**	**400**
RIELGRCIGE	GQFGDVHQGI	YMSPENPALA	VAIKTCKNCT	SDSVREKFLQ	EALTMRQFDH	PHIVKLIGVI	TENPVWIIME
**410**	**420**	**430**	**440**	**450**	**460**	**470**	**480**
LCTLGELRSF	LQVRKYSLDL	ASLILYAYQL	STALAYLESK	RFVHRDIAAR	NVLVSSNDCV	KLGDFGLSRY	MEDSTYYKAS
**490**	**500**	**510**	**520**	**530**	**540**	**550**	**560**
KGKLPIKWMA	PESINFRRFT	SASDVWMFGV	CMWEILMHGV	KPFQGVKNND	VIGRIENGER	LPMPPNCPPT	LYSLMTKCWA
**570**	**580**	**590**	**600**	**610**	**620**	**630**	**640**
YDPSRRPRFT	ELKAQLSTIL	EEEKAQQEER	MRMESRRQAT	VSWDSGGSDE	APPKPSRPGY	PSPRSSEGFY	PSPQHMVQTN
**650**	**660**	**670**	**680**	**690**	**700**	**710**	**720**
HYQVSGYPGS	HGITAMAGSI	YPGQASLLDQ	TDSWNHRPQE	IAMWQPNVED	STVLDLRGIG	QVLPTHLMEE	RLIRQQQEME
**730**	**740**	**750**	**760**	**770**	**780**	**790**	**800**
EDQRWLEKEE	RFLKPDVRLS	RGSIDREDGS	LQGPIGNQHI	YQPVGKPDPA	APPKKPPRPG	APGHLGSLAS	LSSPADSYNE
**810**	**820**	**830**	**840**	**850**	**860**	**870**	**880**
GVKPWRLQPQ	EISPPPTANL	DRSNDKVYEN	VTGLVKAVIE	MSSKIQPAPP	EEYVPMVKEV	GLALRTLLAT	VDETIPLLPA
**890**	**900**	**910**	**920**	**930**	**940**	**950**	**960**
STHREIEMAQ	KLLNSDLGEL	INKMKLAQQY	VMTSLQQEYK	KQMLTAAHAL	AVDAKNLLDV	IDQARLKMLG	QTRPH

Cmpd.	No. of Cmpds.	m/z meas.	Δ m/z [ppm]	z	Rt [min]	Score	P	Range	Sequence	Modification
153	2	635.8187	61.87	2	31.1	14.9	1	355-365	K.TCKNCTSDSVR.E	Carbamidomethyl: 2
2249	1	937.4207	-94.13	2	56.6	13.7	2	484-498	K.LPIKWMAPESINFRR.F	Oxidation: 6

Detailed Protein Report

Protein 192: ankyrin repeat and zinc finger domain-containing protein 1 isoform 1 [Homo sapiens]

**Accession:**	gi|109150425	**Score:**	28.6
**Database:**	refseq_human(refseq_human_20140103.fasta)	**MW [kDa]:**	80.9
**Database Date:**	2015-11-30	**pI:**	9.6
		**Sequence Coverage [%]:**	2.9
		**No. of unique Peptides:**	1

Alias proteins:

Accession	Name	Description
gi|109150435	refseq_human (refseq_human_20140103.fasta)	ankyrin repeat and zinc finger domain-containing protein 1 isoform 1 [Homo sapiens]

10	20	30	40	50	60	70	80
MSPAPDAAPA	PASISLFDLS	ADAPVFQGLS	LVSHAPGEAL	ARAPRTSCSG	SGERESPERK	LLQGPMDISE	KLFCSTCDQT
90	100	110	120	130	140	150	160
FQNHQEQREH	YKLDWHRFNL	KQRLKDKPLL	SALDFEKQSS	TGDLSSISGS	EDSDSASEED	LQTLDRERAT	FEKLSRPPGF
170	180	190	200	210	220	230	240
YPHRVLFQNA	QGQFLYAYRC	VLGPHQDPPE	EAELLLQNLQ	SRGPRDCVVL	MAAAGHFAGA	IFQGREVVTH	KTFHRYTVRA
250	260	270	280	290	300	310	320
KRGTAQGLRD	ARGGPSHSAG	ANLRRYNEAT	LYKDVRDLLA	GPSWAKALEE	AGTILLRAPR	SGRSLFFGGK	GAPLQRGDPR
330	340	350	360	370	380	390	400
LWDIPLATRR	PTFQELQRVL	HKLTTLHVYE	EDPREAVRLH	SPQTHWKTVR	EERKKPTEEE	IRKICRDEKE	ALGQNEESPK
410	420	430	440	450	460	470	480
QGSGSEGEDG	FQVELELVEL	TVGTLDLCES	EVLPKRRRRK	RNKKEKSRDQ	EAGAHRTLLQ	QTQEEEPSTQ	SSQAVAAPLG
490	500	510	520	530	540	550	560
PLLDEAKAPG	QPELWNALLA	ACRAGDVGVL	KLQLAPSPAD	PRVLSLLSAP	LGSGGFTLLH	AAAAAGRGSV	VRLLLEAGAD
570	580	590	600	610	620	630	640
PTVQDSRARP	PYTVAADKST	RNEFRRFMEK	NPDAYDYNKA	QVPGPLTPEM	EARQATRKRE	QKAARRQEE	QQQRQQEQEE
650	660	670	680	690	700	710	720
REREEQRRFA	ALSDREKRAL	AAERRLAAQL	GAPTSPIPDS	AIVNTRRCWS	CGASLQGLTP	FHYLDFSFCS	TRCLQDHRRQ
730							
AGRPSS							

Cmpd.	No. of Cmpds.	m/z meas.	Δ m/z [ppm]	z	Rt [min]	Score	P	Range	Sequence	Modification
883	4	529.1891	-185.79	2	39.4	16.5	0	277-286	R.DLLAGPSWAK.A	

Detailed Protein Report

Protein 193: **activating signal cointegrator 1 complex subunit 3 isoform c [Homo sapiens]**

**Accession:**	gi|546232111	**Score:**	28.6
**Database:**	refseq_human(refseq_human_20140103.fasta)	**MW [kDa]:**	83.6
**Database Date:**	2015-11-30	**pI:**	6.1
**Modification(s):**	Carbamidomethyl, Oxidation	**Sequence Coverage [%]:**	3.6
		**No. of unique Peptides:**	2

10	20	30	40	50	60	70	80
MALPRLTGAL	RSFSNVTKQD	NYNEEVADLK	IKRSKLHEQV	LDLGLTWKKI	IKFLNEKLEK	SKMQSINEDL	KDILHAAKQI
**90**	**100**	**110**	**120**	**130**	**140**	**150**	**160**
VGTDNGREAI	ESGAAFLFMT	FHLKDSVGHK	ETKAIKQMFG	PFPSSSATAA	CNATNRIISH	FSQDDLTALV	QMTEKEHGDR
**170**	**180**	**190**	**200**	**210**	**220**	**230**	**240**
VFFGKNLAFS	FDMHDLDHFD	ELPINGETQK	TISLDYKKFL	NEHLQEACTP	ELKPVEKTNG	SFLWCEVEKY	LNSTLKEMTE
**250**	**260**	**270**	**280**	**290**	**300**	**310**	**320**
VPRVEDLCCT	LYDMLASIKS	GDELQDELFE	LLGPEGLELI	EKLLQNRITI	VDRFLNSSND	HRFQALQDNC	KKILGENAKP
**330**	**340**	**350**	**360**	**370**	**380**	**390**	**400**
NYGCQVTIQS	EQEKQLMKQY	RREEKRIARR	EKKAGEDLEV	SEGLMCFDPK	ELRIQREQAL	LNARSVPILS	RQRDADVEKI
**410**	**420**	**430**	**440**	**450**	**460**	**470**	**480**
HYPHVYDSQA	EAMKTSAFIA	GAKMILPEGI	QRENNKLYEE	VRIPYSEPMP	LSFEEKPVYI	QDLDEIGQLA	FKGMKRLNRI
**490**	**500**	**510**	**520**	**530**	**540**	**550**	**560**
QSIVFETAYN	TNENMLICAP	TGAGKTNIAM	LTVLHEIRQH	FQQGVIKKNE	FKIVYVAPMK	ALAAEMTDYF	SRRLEPLGII
**570**	**580**	**590**	**600**	**610**	**620**	**630**	**640**
VKELTGDMQL	SKSEILRTQM	LVTTPEKWDV	VTRKSVGDVA	LSQIVRLLIL	DEVHLLHEDR	GPVLESIVAR	TLRQVESTQS
**650**	**660**	**670**	**680**	**690**	**700**	**710**	**720**
MIRILGLSAT	LPNYLDVATF	LHVNPYIGLF	FFDGRFRPVP	LGQTFLGIKC	ANKMQQLNNM	DEVCYENVLK	QVKAGHQVHL
**730**	**740**						
FYLLLHLFIC	F						

Cmpd.	No. of Cmpds.	m/z meas.	Δ m/z [ppm]	z	Rt [min]	Score	P	Range	Sequence	Modification
330	1	539.2345	-58.27	2	32.8	12.1	0	63-71	K.MQSINEDLK.D	
106	1	715.2726	-66.45	3	30.6	16.5	0	694-710	K.MQQLNNMDEVCYENVLK.Q	Carbamidomethyl: 11; Oxidation: 7

Detailed Protein Report

Protein 194: PREDICTED: ankyrin repeat domain-containing protein 24 isoform X6 [Homo sapiens]

Accession: gi|578833058
Database: refseq_human(refseq_human_20140103.fasta)
Database Date: 2015-11-30

Score: 28.4
MW [kDa]: 121.1
pI: 4.8
Sequence Coverage [%]: 2.6
No. of unique Peptides: 1

10	20	30	40	50	60	70	80
MKTLRARFKK	TESQDWGKSD	ERLLQAVENN	DAPRVAALIA	RKGLVPTKLD	PEGKSAFHLA	AMRGAASCLE	VMIAHGSNVM
90	100	110	120	130	140	150	160
SADGAGYNAL	HLAAKYGHPQ	CLKQLLQASC	VVDVVDSSGW	TALHHAAAGG	CLSCSEVLCS	FKAHLNPQDR	SGATPLIIAA
170	180	190	200	210	220	230	240
QMCHTDLCRL	LLQQGAAAND	QDLQGRTALM	LACEGASPET	VEVLLQGGAQ	PGITDALGQD	AAHYGALAGD	KLILHLLQEA
250	260	270	280	290	300	310	320
AQRPSPPSAL	TEDDSGEASS	QNSMSSHGKQ	GAPKKRKAPP	PPASIPMPDD	RDAYEEIVRL	RQERGRLLQK	IRGLEQHKER
330	340	350	360	370	380	390	400
RQQESPEASS	LHILERQVQE	LQQLLVERQE	EKESLGREVE	SLQSRLSLLE	NERENTSYDV	TTLQDEEGEL	PDLPGAEVLL
410	420	430	440	450	460	470	480
SRQLSPSAQE	HLASLQEQVA	VLTRQNQELM	EKVQILENFE	KDETQMEVEA	LAEVIPLALY	DSLRAEFDQL	RRQHAEALQA
490	500	510	520	530	540	550	560
LRQQETREVP	REEGAACGES	EVAGATATKN	GPTHMELNGS	VAPETKVNGA	ETIDEEAAGD	ETMEARTMEA	EATGAEATGA
570	580	590	600	610	620	630	640
EATGAKVTET	KPTGAEVREM	ETTEEEANME	TKPTGAQATD	TETTGVEAMG	VEATKTKAEE	AEMQAYGVGA	GQAEPPVTGT
650	660	670	680	690	700	710	720
TNMEATGSRA	TGMESTGVSA	TGVENPGVEA	TVPGISAGPI	LHPGAAEASE	KLQVELETRI	RGLEEALRQR	EREAAAELEA
730	740	750	760	770	780	790	800
ALGKCEAAEA	EAGRLRERVR	EAEGSGASGG	GGGDTTQLRA	ALEQAREDLR	DRDSRLRELE	AASACLDEAR	ASRLLAEEEA
810	820	830	840	850	860	870	880
RGLRAELAQR	EEARLEQSRE	LEVLREQLAT	ARATGEQQRT	AAAELGRARD	AAEARVAELP	AACEEARQGL	AELREASEAL
890	900	910	920	930	940	950	960
RQSVVPASEH	RRLQEEALEL	RGRAASLEQE	VVATGKEAAR	LRAELERERV	CSVALSEHER	IVGTLQANVA	QLEGQLEELG
970	980	990	1000	1010	1020	1030	1040
RRHEKTSAEV	FQVQREALFM	KSERHAAEAQ	LATAEQQLRG	LRTEAERARQ	AQSRAQEALD	KAKEKDKKIT	ELSKEVFNLK
1050	1060	1070	1080	1090	1100	1110	1120
EALKEQPAAL	ATPEVEALRD	QVKDLQQQLQ	EAARDHSSVV	ALYRSHLLYA	IQGQMDEDVQ	RILSQILQMQ	RLQAQGR

Cmpd.	No. of Cmpds.	m/z meas.	Δ m/z [ppm]	z	Rt [min]	Score	P	Range	Sequence	Modification
1938	1	780.7925	-113.31	2	52.9	10.6	2	725-738	K.CEAAEAEAGRLRER.V	

Detailed Protein Report

Protein 195: **PREDICTED: SNW domain-containing protein 1 isoform X2 [Homo sapiens]**

**Accession:**	gi|530403266	**Score:**	28.3
**Database:**	refseq_human(refseq_human_20140103.fasta)	**MW [kDa]:**	65.4
**Database Date:**	2015-11-30	**pI:**	10.2
		**Sequence Coverage [%]:**	6.0
		**No. of unique Peptides:**	1

10	20	30	40	50	60	70	80
MALTSFLPAP	TQLSQDQLEA	EEKARSQRSR	QTSLVSSRRE	PPPYGYRKGW	IPRLLEDFGD	GGAFPEIHVA	QYPLDMGRKK
90	100	110	120	130	140	150	160
KMSNALAIQV	DSEGKIKYDA	IARQGQSKDK	VIYSKYTDLV	PKEVMNADDP	DLQRPDEEAI	KEITEKTRVA	LEKSVSQKVA
170	180	190	200	210	220	230	240
AAMPVRAADK	LAPAQYIRYT	PSQQGVAFNS	GAKQRVIRMV	EMQKDPMEPP	RFKINKKIPR	GPPSPPAPVM	HSPSRKMTVK
250	260	270	280	290	300	310	320
EQQEWKIPPC	ISNWKNAKGY	TIPLDKRLAA	DGRGLQTVHI	NENFAKLAEA	LYIADRKARE	AVEMRAQVER	KMAQKEKEKH
330	340	350	360	370	380	390	400
EEKLREMAQK	ARERRAGIKT	HVEKEDGEAR	ERDEIRHDRR	KERQHDRNLS	RAAPDKRSKL	QRNENRDISE	VIALGVPNPR
410	420	430	440	450	460	470	480
TSNEVQYDQR	LFNQSKGMDS	GFAGGEDEIY	NVYDQAWRGG	KDMAQSIYRP	SKNLDKDMYG	DDLEARIKTN	RCQAIQLNFS
490	500	510	520	530	540	550	560
VYTGESKVVH	SFFSFSLDLF	PTRSFLVQTV	DREAEKDQCS	LRKILLVWTS	FWKKPNSMVA	LKDPQIAAAP	RNTSMKARRG
570	580						
GRNRHRSLQS	E						

Cmpd.	No. of Cmpds.	m/z meas.	Δ m/z [ppm]	z	Rt [min]	Score	P	Range	Sequence	Modification
2279	3	863.8727	-94.70	2	57.0	13.5	2	543-558	K.DPQIAAAPRNTSMKAR.R	

Detailed Protein Report

Protein 196: **glycogenin-1 isoform 3 [Homo sapiens]**

**Accession:**	gi|296040507	**Score:**	28.2
**Database:**	refseq_human(refseq_human_20140103.fasta)	**MW [kDa]:**	31.4
**Database Date:**	2015-11-30	**pI:**	6.1
		**Sequence Coverage [%]:**	5.4
		**No. of unique Peptides:**	1

10	20	30	40	50	60	70	80
MTDQAFVTLT	TNDAYAKGAL	VLGSSLKQHR	TTRRLVVLAT	PQVSDSMRKV	LETVFDEVIM	VDVLDSGDSA	HLTLMKRPEL
**90**	**100**	**110**	**120**	**130**	**140**	**150**	**160**
GVTLTKLHCW	SLTQYSKCVF	MDADTLVLAN	IDDLFDREEL	SAAPDPGWPD	CFNSGVFVYQ	PSVETYNQLL	HLASEQGSFD
**170**	**180**	**190**	**200**	**210**	**220**	**230**	**240**
GGDQGILNTF	FSSWATTDIR	KHLPFIYNLS	SISIYSYLPA	FKVKMSQEPY	HICPLGRSQL	WHSRLYPRKN	GRNDGNRARL
**250**	**260**	**270**	**280**				
IIWEQIPLTT	SRGNLTLTSS	RNTAFFCEHI	HFTSLVSDT				

Cmpd.	No. of Cmpds.	m/z meas.	Δ m/z [ppm]	z	Rt [min]	Score	P	Range	Sequence	Modification
2959	2	822.4635	2.95	2	65.8	28.2	1	35-49	R.LVVLATPQVSDSMRK.V	

Detailed Protein Report

Protein 197: keratin-like protein KRT222 [Homo sapiens]

**Accession:**	gi|22748757	**Score:**	28.2
**Database:**	refseq_human(refseq_human_20140103.fasta)	**MW [kDa]:**	34.1
**Database Date:**	2015-11-30	**pI:**	5.6
		**Sequence Coverage [%]:**	7.8
		**No. of unique Peptides:**	2

10	20	30	40	50	60	70	80
MELSQLLNEI	RANYEKILTR	NQIETVLSTR	IQLEEDISKK	MDKDEEALKA	AQAELKEARR	QWHHLQVEIE	SLHAVERGLE
90	100	110	120	130	140	150	160
NSLHASEQHY	QMQLQDLETV	IEGLEKELQE	VRRGIEKQLQ	EHEMLLNTKM	RLEQEIATYR	HLLEKEEIRY	YGCIQGGKKD
170	180	190	200	210	220	230	240
KKPTTSRVGF	VLPSAIINEI	SFTTKVPQKY	ENENVETVTK	QAILNGSIVK	ESTEAHGTIQ	TEKVDEVIKE	WEGSFFKDNP
250	260	270	280	290	300		
RLRKKSVSLR	FDLHLAATDE	GCLETKQDNL	PDIEVRLIMR	RSCSIPSIKP	PSTAN		

Cmpd.	No. of Cmpds.	m/z meas.	Δ m/z [ppm]	z	Rt [min]	Score	P	Range	Sequence	Modification
2910	1	822.4663	-11.24	2	65.1	15.1	1	17-30	K.ILTRNQIETVLSTR.I	
1053	2	530.7772	-60.09	2	41.4	13.2	2	159-167	K.KDKKPTTSR.V	

Detailed Protein Report

Protein 198: **PREDICTED: collagen alpha-2(I) chain-like [Homo sapiens]**

**Accession:**	gi|530375734	**Score:**	28.2
**Database:**	refseq_human(refseq_human_20140103.fasta)	**MW [kDa]:**	28.8
**Database Date:**	2015-11-30	**pI:**	12.9
		**Sequence Coverage [%]:**	12.6
		**No. of unique Peptides:**	1

10	20	30	40	50	60	70	80
MAPFHLSHAP	AGARKVKLRH	PRKWSPSGSP	QPPAQLQPLE	APRLPGEVRT	SGRLATWFCR	LLARRSAAQR	KSKSRKDLNR
**90**	**100**	**110**	**120**	**130**	**140**	**150**	**160**
RVQSPLTPHS	FHYFSSQGIG	GGGGGRDKFA	GRLAWSNPRG	WDRPRGSGGA	LPRPEGDCGG	ARPPAGGAGR	WASPGAAGRW
**170**	**180**	**190**	**200**	**210**	**220**	**230**	**240**
SLPGRVCGAF	AQPAPCPHLT	SSPPRPLGGP	LRCAAQARSV	AARAVRPRSL	GQGPGAQGVG	RAGRERGRPS	TASGSARAGA
**250**	**260**	**270**	**280**				
ALPAAVRPRE	GPRGPSACAL	GRRRLCVGRP	GGPRGTD				

Cmpd.	No. of Cmpds.	m/z meas.	Δ m/z [ppm]	z	Rt [min]	Score	P	Range	Sequence	Modification
1759	1	881.8978	-107.69	2	50.4	14.1	1	204-221	R.AVRPRSLGQGPGAQGVGR.A	

Detailed Protein Report

Protein 199: protein fem-1 homolog C [Homo sapiens]

Accession: gi|17864094
Database: refseq_human(refseq_human_20140103.fasta)
Database Date: 2015-11-30
Modification(s): Carbamidomethyl, Oxidation

Score: 28.1
MW [kDa]: 68.6
pI: 8.5
Sequence Coverage [%]: 4.5
No. of unique Peptides: 2

Alias proteins:

Accession	Name	Description
gi|530380049	refseq_human (refseq_human_20140103.fasta)	PREDICTED: protein fem-1 homolog C isoform X1 [Homo sapiens]

10	20	30	40	50	60	70	80
MDLKTAVFNA	ARDGKLRLLT	KLLASKSKEE	VSSLISEKTN	GATPLLMAAR	YGHLDMVEFL	LEQCSASIEV	GGSVNFDGET
**90**	**100**	**110**	**120**	**130**	**140**	**150**	**160**
IEGAPPLWAA	SAAGHLKVVQ	SLLNHGASVN	NTTLTNSTPL	RAACFDGHLE	IVKYLVEHKA	DLEVSNRHGH	TCLMISCYKG
**170**	**180**	**190**	**200**	**210**	**220**	**230**	**240**
HKEIAQYLLE	KGADVNRKSV	KGNTALHDCA	ESGSLDIMKM	LLMYCAKMEK	DGYGMTPLLS	ASVTGHTNIV	DFLTHHAQTS
**250**	**260**	**270**	**280**	**290**	**300**	**310**	**320**
KTERINALEL	LGATFVDKKR	DLLGALKYWK	KAMNMRYSDR	TNIISKPVPQ	TLIMAYDYAK	EVNSAEELEG	LIADPDEMRM
**330**	**340**	**350**	**360**	**370**	**380**	**390**	**400**
QALLIRERIL	GPSHPDTSYY	IRYRGAVYAD	SGNFKRCINL	WKYALDMQQS	NLDPLSPMTA	SSLLSFAELF	SFMLQDRAKG
**410**	**420**	**430**	**440**	**450**	**460**	**470**	**480**
LLGTTVTFDD	LMGILCKSVL	EIERAIKQTQ	CPADPLQLNK	ALSIILHLIC	LLEKVPCTLE	QDHFKKQTIY	RFLKLHPRGK
**490**	**500**	**510**	**520**	**530**	**540**	**550**	**560**
NNFSPLHLAV	DKNTTCVGRY	PVCKFPSLQV	TAILIECGAD	VNVRDSDDNS	PLHIAALNNH	PDIMNLLIKS	GAHFDATNLH
**570**	**580**	**590**	**600**	**610**	**620**		
KQTASDLLDE	KEIAKNLIQP	INHTTLQCLA	ARVIVNHRIY	YKGHIPEKLE	TFVSLHR		

Cmpd.	No. of Cmpds.	m/z meas.	Δ m/z [ppm]	z	Rt [min]	Score	P	Range	Sequence	Modification
2809	1	676.8768	34.84	2	63.7	12.6	1	1-12	-.MDLKTAVFNAAR.D	Oxidation: 1
2824	1	912.9573	-30.84	2	63.8	15.5	1	425-440	R.AIKQTQCPADPLQLNK.A	Carbamidomethyl: 7

Detailed Protein Report

Protein 200: PREDICTED: general transcription factor 3C polypeptide 1 isoform X1 [Homo sapiens]

**Accession:**	gi|578828542	**Score:**	27.9
**Database:**	refseq_human(refseq_human_20140103.fasta)	**MW [kDa]:**	231.4
**Database Date:**	2015-11-30	**pI:**	6.3
**Modification(s):**	Carbamidomethyl, Oxidation	**Sequence Coverage [%]:**	1.6
		**No. of unique Peptides:**	2

Detailed Protein Report

10	20	30	40	50	60	70	80
MDALESLLDE	VALEGLDGLC	LPALWSRLET	RVPPFPLPLE	PCTQEFLWRA	LATHPGISFY	EEPRERPDLQ	LQDRYEEIDL
90	100	110	120	130	140	150	160
ETGILESRRD	PVALEDVYPI	HMILENKDGI	QGSCRYFKER	KNITNDIRTK	SLQPRCTMVE	AFDRWGKKLI	IVASQAMRYR
170	180	190	200	210	220	230	240
ALIGQEGDPD	LKLPDFSYCI	LERLGRSRWQ	GELQRDLHTT	AFKVDAGKLH	YHRKILNKNG	LITMQSHVIR	LPTGAQQHSI
250	260	270	280	290	300	310	320
LLLLNRFHVD	RRSKYDILME	KLSVMLSTRT	NHIETLGKLR	EELGLCERTF	KRLYQYMLNA	GLAKVVSLRL	QEIHPECGPC
330	340	350	360	370	380	390	400
KTKKGTDVMV	RCLKLLKEFK	RNDHDDDEDE	EVISKTVPPV	DIVFERDMLT	QTYDLIERRG	TKGISQAEIR	VAMNVGKLEA
410	420	430	440	450	460	470	480
RMLCRLLQRF	KVVKGFMEDE	GRQRTTKYIS	CVFAEESDLS	RQYQREKARS	ELLTTVSLAS	MQEESLLPEG	EDTFLSESDS
490	500	510	520	530	540	550	560
EEERSSSKRR	GRGSQKDTRA	SANLRPKTQP	HHSTPTKGGW	KVVNLHPLKK	QPPSFPGAAE	ERACQSLASR	DSLLDTSSVS
570	580	590	600	610	620	630	640
EPNVSFVSHC	ADSNSGDIAV	IEEVRMENPK	ESSSSLKTGR	HSSGQDKPHE	TYRLLKRRNL	IIEAVTNLRL	IESLFTIQKM
650	660	670	680	690	700	710	720
IMDQEKQEGV	STKCCKKSIV	RLVRNLSEEG	LLRLYRTTVI	QDGIKKKVDL	VVHPSMDQND	PLVRSAIEQV	RFRISNSSTA
730	740	750	760	770	780	790	800
NRVKTSQPPV	PQGEAEEDSQ	GKEGPSGSGD	SQLSASSRSE	SGRMKKSDNK	MGITPLRNYH	PIVVPGLGRS	LGFLPKMPRL
810	820	830	840	850	860	870	880
RVVHMFLWYL	IYGHPASNTV	EKPSFISERR	TIKQESGRAG	VRPSSSGSAW	EACSEAPSKG	SQDGVTWEAE	VELATETVYV
890	900	910	920	930	940	950	960
DDASWMRYIP	PIPVHRDFGF	GWALVSDILL	CLPLSIFIQI	VQVSYKVDNL	EEFLNDPLKK	HTLIRFLPRP	IRQQLLYKRR
970	980	990	1000	1010	1020	1030	1040
YIFSVVENLQ	RLCYMGLLQF	GPTEKFQDKD	QVFIFLKKNA	VIVDTTICDP	HYNLARSSRP	FERRLYVLNS	MQDVENYWFD
1050	1060	1070	1080	1090	1100	1110	1120
LQCVCLNTPL	GVVRCPRVRK	NSSTDQGSDE	EGSLQKEQES	AMDKHNLERK	CAMLEYTTGS	REVVDEGLIP	GDGLGAAGLD
1130	1140	1150	1160	1170	1180	1190	1200
SSFYGHLKRN	WIWTSYIINQ	AKKENTAAEN	GLTVRLQTFL	SKRPMPLSAR	GEFPGEKSKR	LRYHDEADQS	ALQRMTRLRV
1210	1220	1230	1240	1250	1260	1270	1280
TWSMQEDGLL	VLCRIASNVL	NTKVKGPFVT	WQVVRDILHA	TFEESLDKTS	HSVGRRARYI	VKNPQAYLNY	KVCLAEVYQD
1290	1300	1310	1320	1330	1340	1350	1360
KALVGDFMNR	RGDYDDPKVC	ANEFKEFVEK	LKEKFSSALR	NSNLEIPDTL	QELFARYRVL	AIGDEKDQTR	KEDELNSVDD
1370	1380	1390	1400	1410	1420	1430	1440
IHFLVLQNLI	QSTLALSDSQ	MKSYQSFQTF	RLYREYKDHV	LVKAFMECQK	RSLVNRRRVN	HTLGPKKNRA	LPFVPMSYQL
1450	1460	1470	1480	1490	1500	1510	1520
SQTYYRIFTW	RFPSTICTES	FQFLDRMRAA	GKLDQPDRFS	FKDQDNNEPT	NDMVAFSLDG	PGGNCVAVLT	LFSLGLISVD
1530	1540	1550	1560	1570	1580	1590	1600
VRIPEQIIVV	DSSMVENEVI	KSLGKDGSLE	DDEDEEDDLD	EGVGGKRRSM	EVKPAQASHT	NYLLMRGYYS	PGIVSTRNLN
1610	1620	1630	1640	1650	1660	1670	1680
PNDSIVVNSC	QMKFQLRCTP	VPARLRPAAA	PLEELTMGTS	CLPDTFTKLI	NPQENTCSLE	EFVLQLELSG	YSPEDLTAAL
1690	1700	1710	1720	1730	1740	1750	1760
EILEAIIATG	CFGIDKEELR	RRFSALEKAG	GGRTRTFADC	IQALLEQHQV	LEVGGNTARL	VAMGSAWPWL	LHSVRLKDRE
1770	1780	1790	1800	1810	1820	1830	1840
DADIQREDPQ	ARPLEGSSSE	DSPPEGQAPP	SHSPRGTKRR	ASWASENGET	DAEGTQMTPA	KRPALQDSNL	APSLGPGAED
1850	1860	1870	1880	1890	1900	1910	1920
GAEAQAPSPP	PALEDTAAAG	AAQEDQEGVG	EFSSPGQEQL	SGQAQPPEGS	EDPRGFTESF	GAANISQAAR	ERDCESVCFI
1930	1940	1950	1960	1970	1980	1990	2000
GRPWRVVDGH	LNLPVCKGMM	EAMLYHIMTR	PGIPESSLLR	HYQGVLQPVA	VLELLQGLES	LGCIRKRWLR	KPRPVSLFST
2010	2020	2030	2040	2050			
PVVEEVEVPS	SLDESPMAFY	EPTLDCTLRL	GRVFPHEVNW	NKWIHL			

Cmpd.	No. of Cmpds.	m/z meas.	Δ m/z [ppm]	z	Rt [min]	Score	P	Range	Sequence	Modification
2710	1	941.5667	103.70	2	62.3	14.6	2	129-144	R.TKSLQPRCTMVEAFDR.W	
2192	1	1052.3938	-65.32	2	55.9	13.3	2	640-656	K.MIMDQEKQEGVSTKCCK.K	Carbamidomethyl: 15, 16; Oxidation: 1, 3

Detailed Protein Report

Protein 201: Na(+)/H(+) exchange regulatory cofactor NHE-RF3 isoform 2 [Homo sapiens]

Accession: gi|318037207
Database: refseq_human(refseq_human_20140103.fasta)
Database Date: 2015-11-30

Score: 27.9
MW [kDa]: 44.6
pI: 4.9
Sequence Coverage [%]: 7.4
No. of unique Peptides: 1

10	20	30	40	50	60	70	80
MTSTFNPREC	KLSKQEGQNY	GFFLRIEKDT	EGHLVRVVEK	CSPAEKAGLQ	DGDRVLRING	VFVDKEEHMQ	VVDLVRKSGN
90	100	110	120	130	140	150	160
SVTLLVLDGD	SYEKAVKTRV	DLKELGQSQK	EQGLSDNILS	PVMNGGVQTW	TQPRLCYLVK	EGGSYGFSLK	TVQGQIIKDI
170	180	190	200	210	220	230	240
DSGSPAEEAG	LKNNDLVVAV	NGESVETLDH	DSVVEMIRKG	GDQTSLLVVD	KETDNMYRLA	HFSPFLYYQS	QELPNGSVKE
250	260	270	280	290	300	310	320
APAPTPTSLE	VSSPPDTTEE	VDHKPKLCRL	AKGENGYGFH	LNAIRGLPGS	FIKEVQKGGP	ADLAGLEDED	VIIEVNGVNV
330	340	350	360	370	380	390	400
LDEPYEKVVD	RIQSSGKNVT	LLVCGKKAYD	YFQAKKIPIV	SSLADPLDTP	PDSKEGIVVE	SNHDSHMAKE	RAHSTASHSS
410							
SNSEDTEM							

Cmpd.	No. of Cmpds.	m/z meas.	Δ m/z [ppm]	z	Rt [min]	Score	P	Range	Sequence	Modification
2317	1	951.3323	-147.80	2	57.4	13.6	2	37-54	R.VVEKCSPAEKAGLQDGDR.V	

Detailed Protein Report

Protein 202: trafficking protein particle complex subunit 9 isoform a [Homo sapiens]

**Accession:**	gi|238624122	**Score:**	27.9
**Database:**	refseq_human(refseq_human_20140103.fasta)	**MW [kDa]:**	139.3
**Database Date:**	2015-11-30	**pI:**	6.9
		**Sequence Coverage [%]:**	2.7
		**No. of unique Peptides:**	2

10	20	30	40	50	60	70	80
MVPAGDQDRA	PHRGKPAQAG	ARTSRASRAL	RSWRRSQAAR	ATVTHPRGGH	DRGSHGGYRE	GHRGCRRDPQ	WASAGPPPLS
90	100	110	120	130	140	150	160
FTEEVKFELR	ALKDWDFKMS	VPDYMQCAED	HQTLLVVVQP	VGIVSEENFF	RIYKRICSVS	QISVRDSQRV	LYIRYRHHYP
170	180	190	200	210	220	230	240
PENNEWGDFQ	THRKVVGLIT	ITDCFSAKDW	PQTFEKFHVQ	KEIYGSTLYD	SRLFVFGLQG	EIVEQPRTDV	AFYPNYEDCQ
250	260	270	280	290	300	310	320
TVEKRIEDFI	ESLFIVLESK	RLDRATDKSG	DKIPLLCVPF	EKKDFVGLDT	DSRHYKKRCQ	GRMRKHVGDL	CLQAGMLQDS
330	340	350	360	370	380	390	400
LVHYHMSVEL	LRSVNDFLWL	GAALEGLCSA	SVIYHYPGGT	GGKSGARRFQ	GSTLPAEAAN	RHRPGAQEVL	IDPGALTTNG
410	420	430	440	450	460	470	480
INPDTSTEIG	RAKNCLSPED	IIDKYKEAIS	YYSKYKNAGV	IELEACIKAV	RVLAIQKRSM	EASEFLQNAV	YINLRQLSEE
490	500	510	520	530	540	550	560
EKIQRYSILS	ELYELIGFHR	KSAFFKRVAA	MQCVAPSIAE	PGWRACYKLL	LETLPGYSLS	LDPKDFSRGT	HRGWAAVQMR
570	580	590	600	610	620	630	640
LLHELVYASR	RMGNPALSVR	HLSFLLQTML	DFLSDQEKKD	VAQSLENYTS	KCPGTMEPIA	LPGGLTLPPV	PFTKLPIVRH
650	660	670	680	690	700	710	720
VKLLNLPASL	RPHKMKSLLG	QNVSTKSPFI	YSPIIAHNRG	EERNKKIDFQ	WVQGDVCEVQ	LMVYNPMPFE	LRVENMGLLT
730	740	750	760	770	780	790	800
SGVEFESLPA	ALSLPAESGL	YPVTLVGVPQ	TTGTITVNGY	HTTVFGVFSD	CLLDNLPGIK	TSGSTVEVIP	ALPRLQISTS
810	820	830	840	850	860	870	880
LPRSAHSLQP	SSGDEISTNV	SVQLYNGESQ	QLIIKLENIG	MEPLEKLEVT	SKVLTTKEKL	YGDFLSWKLE	ETLAQFPLQP
890	900	910	920	930	940	950	960
GKVATFTINI	KVKLDFSCQE	NLLQDLSDDG	ISVSGFPLSS	PFRQVVRPRV	EGKPVNPPES	NKAGDYSHVK	TLEAVLNFKY
970	980	990	1000	1010	1020	1030	1040
SGGPGHTEGY	YRNLSLGLHV	EVEPSVFFTR	VSTLPATSTR	QCHLLLDVFN	STEHELTVST	RSSEALILHA	GECQRMAIQV
1050	1060	1070	1080	1090	1100	1110	1120
DKFNFESFPE	SPGEKGQFAN	PKQLEEERRE	ARGLEIHSKL	GICWRIPSLK	RSGEASVEGL	LNQLVLEHLQ	LAPLQWDVLV
1130	1140	1150	1160	1170	1180	1190	1200
DGQPCDREAV	AACQVGDPVR	LEVRLTNRSP	RSVGPFALTV	VPFQDHQNGV	HNYDLHDTVS	FVGSSTFYLD	AVQPSGQSAC
1210	1220	1230	1240	1250			
LGALLFLYTG	DFFLHIRFHE	DSTSKELPPS	WFCLPSVHVC	ALEAQA			

Cmpd.	No. of Cmpds.	m/z meas.	Δ m/z [ppm]	z	Rt [min]	Score	P	Range	Sequence	Modification
1485	1	600.4356	169.47	2	47.3	15.9	1	14-25	R.GKPAQAGARTSR.A	
69	1	820.3460	-71.39	3	30.1	12.0	1	951-972	K.TLEAVLNFKYSGGPGHTEGYYR.N	

Detailed Protein Report

Protein 203: **PREDICTED: zinc finger protein 778 isoform X4 [Homo sapiens]**

**Accession:**	gi|578828903	**Score:**	27.8
**Database:**	refseq_human(refseq_human_20140103.fasta)	**MW [kDa]:**	66.5
**Database Date:**	2015-11-30	**pI:**	9.6
		**Sequence Coverage [%]:**	4.1
		**No. of unique Peptides:**	2

10	20	30	40	50	60	70	80
MAAPDLAHGG	HVSRDSVCLH	EEQTQAAGMV	AGWLINCYQD	AVTFDDVAVD	FTQEEWTLLD	PSQRDLYRDV	MLENYENLAS
**90**	**100**	**110**	**120**	**130**	**140**	**150**	**160**
VGHHLFQPSV	IYWLEQEEEL	RAGRRAVLQE	WRLKTKGPAL	RQDRSWFRAS	NETQTARSHN	GGQLCDRTQC	GEAFSEHSGL
**170**	**180**	**190**	**200**	**210**	**220**	**230**	**240**
STHVRTQNTG	DSCVSNHYER	DFFIPCQKTL	FKIGEQFSVL	GQCGKAFSST	PNVVSQQACT	RDRSLDYSSC	GEVFLNQSYL
**250**	**260**	**270**	**280**	**290**	**300**	**310**	**320**
QARAGSHNGE	ETWKWKPCGK	ALTHSMGCAT	PVEMHAVRNP	HVCRECGKAF	RYTAYLTGRV	QVHPGEKPCE	LEECGKASPV
**330**	**340**	**350**	**360**	**370**	**380**	**390**	**400**
SSSLTQHVRI	HAAEKPCECK	ECGKAFTGLS	GLSKHVQTDP	GQKPYECKDC	GKAYNRVYLL	NEHVKTHTEE	KPFICTVCRK
**410**	**420**	**430**	**440**	**450**	**460**	**470**	**480**
SFRNSSCLNK	HIQIHTGIKP	YECKDCGKTF	TVSSSLTEHI	RTHTGEKPYE	CKVCGKAFTT	SSHLIVHIRT	HTGEKPYICK
**490**	**500**	**510**	**520**	**530**	**540**	**550**	**560**
ECGKAFASSS	HLIEHRRTHT	GEKPYICNEC	GKAFRASSHL	HKHGRIHTGQ	KPYKCKECGK	AYNRFYLLKE	HLKTYTEEQV
**570**	**580**	**590**					
FVCKDCGKSF	KNSSCLNHHT	QIHTDEKPF					

Cmpd.	No. of Cmpds.	m/z meas.	Δ m/z [ppm]	z	Rt [min]	Score	P	Range	Sequence	Modification
1559	1	977.1043	137.72	2	48.2	11.4	2	122-137	R.QDRSWFRASNETQTAR.S	
559	1	472.6946	-107.46	2	35.8	16.4	0	292-299	R.YTAYLTGR.V	

Detailed Protein Report

Protein 204: probable guanine nucleotide exchange factor MCF2L2 [Homo sapiens]

Accession: gi|540344580
Database: refseq_human(refseq_human_20140103.fasta)
Database Date: 2015-11-30

Score: 27.8
MW [kDa]: 126.9
pI: 6.0
Sequence Coverage [%]: 3.1
No. of unique Peptides: 2

10	20	30	40	50	60	70	80
MLSCLKEEMP	PQELTRRLAT	VITHVDEIMQ	QEVRPLMAVE	IIEQLHRQFA	ILSGGRGEDG	APIITFPEFS	GFKHIPDEDF
90	100	110	120	130	140	150	160
LNVMTYLTSI	PSVEAASIGF	IVVIDRRRDK	WSSVKASLTR	IAVAFPGNLQ	LIFILRPSRF	IQRTFTDIGI	KYYRNEFKTK
170	180	190	200	210	220	230	240
VPIIMVNSVS	DLHGYIDKSQ	LTRELGGTLE	YRHGQWVNHR	TAIENFALTL	KTTAQMLQTF	GSCLATAELP	RSMLSTEDLL
250	260	270	280	290	300	310	320
MSHTRQRDKL	QDELKLLGKQ	GTTLLSCIQE	PATKCPNSKL	NLNQLENVTT	MERLLVQLDE	TEKAFSHFWS	EHHLKLNQCL
330	340	350	360	370	380	390	400
QLQHFEHDFC	KAKLALDNLL	EEQAEFTGIG	DSVMHVEQIL	KEHKKLEEKS	QEPLEKAQLL	ALVGDQLIQS	HHYAADAIRP
410	420	430	440	450	460	470	480
RCVELRHLCD	DFINGNKKKW	DILGKSLEFH	RQLDKVSQWC	EAGIYLLASQ	AVDKCQSREG	VDIALNDIAT	FLGTVKEYPL
490	500	510	520	530	540	550	560
LSPKEFYNEF	ELLLTLDAKA	KAQKVLQRLD	DVQEIFHKRQ	VSLMKLAAKQ	TRPVQPVAPH	PESSPKWVSS	KTSQPSTSVP
570	580	590	600	610	620	630	640
LARPLRTSEE	PYTETELNSR	GKEDDETKFE	VKSEEIFESH	HERGNPELEQ	QARLGDLSPR	RRIIRDLLET	EEIYIKEIKS
650	660	670	680	690	700	710	720
IIDGYITPMD	FIWLKHLIPD	VLQNNKDFLF	GNIRELYEFH	NRTFLKELEK	CAENPELLAH	CFLKRKEDLQ	IYFKYHKNLP
730	740	750	760	770	780	790	800
RARAIWQECQ	DCAYFGVCQR	QLDHNLPLFK	YLKGPSQRLI	KYQMLLKGLL	DFESPEDMEI	DPGELGGSAK	DGPKRTKDSA
810	820	830	840	850	860	870	880
FSTELQQALA	VIEDLIKSCE	LAVDLAAVTE	CPDDIGKLGK	LLLHGPFSVW	TIHKDRYKMK	DLIRFKPSQR	QIYLFERGIV
890	900	910	920	930	940	950	960
FCKIRMEPGD	QGLSPHYSFK	KAMKLMTLSI	RQLGRGSHRK	FEIASRNGLE	KYILQAASKE	IRDCWFSEIS	KLLMEQQNNI
970	980	990	1000	1010	1020	1030	1040
KDQGNPQFEM	STSKGSGAGS	GPWIKNMERA	TTSKEDPASS	TGGIKGCSSR	EFSSMDTFED	CEGAEDMEKE	SSALSLAGLF
1050	1060	1070	1080	1090	1100	1110	1120
QSDDSHETCS	SKSAFLERGE	SSQGEKEERD	EEETATRSTE	EERAGASTGR	LAPAGATAGF	QARALRPRTS	AQES

Cmpd.	No. of Cmpds.	m/z meas.	Δ m/z [ppm]	z	Rt [min]	Score	P	Range	Sequence	Modification
2332	1	952.9342	-37.03	2	57.6	11.4	1	232-247	R.SMLSTEDLLMSHTRQR.D	
2845	1	938.5013	-5.79	2	64.1	16.3	0	459-476	R.EGVDIALNDIATFLGTVK.E	

Detailed Protein Report

Protein 205: chromosome-associated kinesin KIF4A [Homo sapiens]

**Accession:**	gi|116686122	**Score:**	27.7
**Database:**	refseq_human(refseq_human_20140103.fasta)	**MW [kDa]:**	139.8
**Database Date:**	2015-11-30	**pI:**	5.9
**Modification(s):**	Carbamidomethyl	**Sequence Coverage [%]:**	1.5
		**No. of unique Peptides:**	1

10	20	30	40	50	60	70	80
MKEEVKGIPV	RVALRCRPLV	PKEISEGCQM	CLSFVPGEPQ	VVVGTDKSFT	YDFVFDPSTE	QEEVFNTAVA	PLIKGVFKGY
90	100	110	120	130	140	150	160
NATVLAYGQT	GSGKTYSMGG	AYTAEQENEP	TVGVIPRVIQ	LLFKEIDKKS	DFEFTLKVSY	LEIYNEEILD	LLCPSREKAQ
170	180	190	200	210	220	230	240
INIREDPKEG	IKIVGLTEKT	VLVALDTVSC	LEQGNNSRTV	ASTAMNSQSS	RSHAIFTISL	EQRKKSDKNS	SFRSKLHLVD
250	260	270	280	290	300	310	320
LAGSERQKKT	KAEGDRLKEG	ININRGLLCL	GNVISALGDD	KKGGFVPYRD	SKLTRLLQDS	LGGNSHTLMI	ACVSPADSNL
330	340	350	360	370	380	390	400
EETLNTLRYA	DRARKIKNKP	IVNIDPQTAE	LNHLKQQVQQ	LQVLLLQAHG	GTLPGSITVE	PSENLQSLME	KNQSLVEENE
410	420	430	440	450	460	470	480
KLSRGLSEAA	GQTAQMLERI	ILTEQANEKM	NAKLEELRQH	AACKLDLQKL	VETLEDQELK	ENVEIICNLQ	QLITQLSDET
490	500	510	520	530	540	550	560
VACMAAAIDT	AVEQEAQVET	SPETSRSSDA	FTTQHALRQA	QMSKELVELN	KALALKEALA	RKMTQNDSQL	QPIQYQYQDN
570	580	590	600	610	620	630	640
IKELELEVIN	LQKEKEELVL	ELQTAKKDAN	QAKLSERRRK	RLQELEGQIA	DLKKKLNEQS	KLLKLKESTE	RTVSKLNQEI
650	660	670	680	690	700	710	720
RMMKNQRVQL	MRQMKEDAEK	FRQWKQKKDK	EVIQLKERDR	KRQYELLKLE	RNFQKQSNVL	RRKTEEAAAA	NKRLKDALQK
730	740	750	760	770	780	790	800
QREVADKRKE	TQSRGMEGTA	ARVKNWLGNE	IEVMVSTEEA	KRHLNDLLED	RKILAQDVAQ	LKEKKESGEN	PPPKLRRRTF
810	820	830	840	850	860	870	880
SLTEVRGQVS	ESEDSITKQI	ESLETEMEFR	SAQIADLQQK	LLDAESEDRP	KQRWENIATI	LEAKCALKYL	IGELVSSKIQ
890	900	910	920	930	940	950	960
VSKLESSLKQ	SKTSCADMQK	MLFEERNHFA	EIETELQAEL	VRMEQQHQEK	VLYLLSQLQQ	SQMAEKQLEE	SVSEKEQQLL
970	980	990	1000	1010	1020	1030	1040
STLKCQDEEL	EKMREVCEQN	QQLLRENEII	KQKLTLLQVA	SRQKHLPKDT	LLSPDSSFEY	VPPKPKPSRV	KEKFLEQSMD
1050	1060	1070	1080	1090	1100	1110	1120
IEDLKYCSEH	SVNEHEDGDG	DDDEGDDEEW	KPTKLVKVSR	KNIQGCSCKG	WCGNKQCGCR	KQKSDCGVDC	CCDPTKCRNR
1130	1140	1150	1160	1170	1180	1190	1200
QQGKDSLGTV	ERTQDSEGSF	KLEDPTEVTP	GLSFFNPVCA	TPNSKILKEM	CDVEQVLSKK	TPPAPSPFDL	PELKHVATEY
1210	1220	1230	1240				
QENKAPGKKK	KRALASNTSF	FSGCSPIEEE	AH				

Cmpd.	No. of Cmpds.	m/z meas.	Δ m/z [ppm]	z	Rt [min]	Score	P	Range	Sequence	Modification
2387	1	1051.5184	80.34	2	58.2	16.1	2	1082-1100	K.NIQGCSCKGWCGNKQCGCR.K	Carbamidomethyl: 11

Detailed Protein Report

Protein 206: **PREDICTED: leucine-rich repeat-containing protein 16C isoform X1 [Homo sapiens]**

**Accession:**	gi|530423607	**Score:**	27.7
**Database:**	refseq_human(refseq_human_20140103.fasta)	**MW [kDa]:**	148.1
**Database Date:**	2015-11-30	**pI:**	6.1
**Modification(s):**	Oxidation	**Sequence Coverage [%]:**	1.9
		**No. of unique Peptides:**	1

```
        10         20         30         40         50         60         70         80
MAQTPDGISC ELRGEITRFL WPKEVELLLK TWLPGEGAVQ NHVLALLRWR AYLLHTTCLP LRVDCTFSYL EVQAMALQET
        90        100        110        120        130        140        150        160
PPQVTFELES LRELVLEFPG VAALEQLAQH VAAAIKKVFP RSTLGKLFRR PTPASMLARL ERSSPSESTD PCSPCGGFLE
       170        180        190        200        210        220        230        240
TYEALCDYNG FPFREEIQWD VDTIYHRQGC RHFSLGDFSH LGSRDLALSV AALSYNLWFR CLSCVDMKLS LEVSEQILHM
       250        260        270        280        290        300        310        320
MSQSSHLEEL VLETCSLRGD FVRRLAQALA GHSSSGLREL SLAGNLLDDR GMTALSRHLE RCPGALRRLS LAQTGLTPRG
       330        340        350        360        370        380        390        400
MRALGRALAT NAAFDSTLTH LDLSGNPGAL GASEDSGGLY SFLSRPNVLS FLNLAGTDTA LDTLFAAVSR GCCTSLTHLD
       410        420        430        440        450        460        470        480
ASRNVFSRTK SRAAPAALQL FLSRARTLRH LGLAGCKLPP DALRALLDGL ALNTHLRDLH LDLSACELRS AGAQVIQDLV
       490        500        510        520        530        540        550        560
CDAGAVSSLD LADNGFGSDM VTLVLAIGRS RSLRHVALGR NFNVRCKETL DDVLHRIVQL MQDDDCPLQS LSVAESRLKL
       570        580        590        600        610        620        630        640
GASVLLRALA TNPNLTALDI SGNAMGDAGA KLLAKALRVN SRLRSVVWDR NHTSALGLLD VAQALEQNHS LKAMPLPLND
       650        660        670        680        690        700        710        720
VAQAQRSRPE LTARAVHQIQ ACLLRNNRAD PASSDHTTRL QPLGLVSDPS EQEVNELCQS VQEHVELLGC GAGPQGEAAV
       730        740        750        760        770        780        790        800
RQAEDAIQNA NFSLSILPIL YEAGSSPSHH WQLGQKLEGL LRQVGEVCRQ DIQDFTQATL DTARSLCPQM LQGSSWREQL
       810        820        830        840        850        860        870        880
EGVLAGSRGL PELLPEQLLQ DAFTRLRDMR LSITGTLAES IVAQALAGLS AARDQLVESL AQQATVTMPP ALPAPDGGEP
       890        900        910        920        930        940        950        960
SLLEPGELEG LFFPEEKEEE KEKDDSPPQK WPELSHGLHL VPFIHSAAEE AEPEPELAAP GEDAEPQAGP SARGSPSPAA
       970        980        990       1000       1010       1020       1030       1040
PGPPAGPLPR MDLPLAGQPL RHPTRARPRP RRQHHHRPPP GGPQVPPALP QEGNGLSARV DEGVEEFFSK RLIQQDRLWA
      1050       1060       1070       1080       1090       1100       1110       1120
PEEDPATEGG ATPVPRTLRK KLGTLFAFKK PRSTRGPRTD LETSPGAAPR TRKTTFGDLL RPPTRPSRGE ELGGAEGDTS
      1130       1140       1150       1160       1170       1180       1190       1200
SPDPAGRSRP RYTRDSKAYS MILLPAEEEA TLGARPDKRR PLERGETELA PSFEQRVQVM LQRIGVSRGS GGAEGKRKQS
      1210       1220       1230       1240       1250       1260       1270       1280
KDGEIKKAGS DGDIMDSSTE APPISIKSRT HSVSADPSCR PGPGSQGPES ATWKTLGQQL NAELRSRGWG QQDGPGPPSP
      1290       1300       1310       1320       1330       1340       1350       1360
GQSPSPCRTS PSPDSLGLPE DPCLGPRNEE RPLRLQRSPV LKRRPKLEAP PSPSLGSGLG TEPLPPQPTE PSSPERSPPS
      1370       1380
PATDQRGGGP NP
```

Cmpd.	No. of Cmpds.	m/z meas.	Δ m/z [ppm]	z	Rt [min]	Score	P	Range	Sequence	Modification
2909	1	682.3620	-10.00	3	65.1	17.7	2	1177-1196	R.VQVMLQRIGVSRGSGGAEGK.R	Oxidation: 4

Detailed Protein Report

Protein 207: PREDICTED: midasin isoform X3 [Homo sapiens]

**Accession:**	gi|578812559	**Score:**	27.7
**Database:**	refseq_human(refseq_human_20140103.fasta)	**MW [kDa]:**	627.0
**Database Date:**	2015-11-30	**pI:**	5.4
		**Sequence Coverage [%]:**	0.3
		**No. of unique Peptides:**	1

Detailed Protein Report

10	20	30	40	50	60	70	80
MEHFLLEVAA	APLRLIAAKN	EKSRSELGRF	LAKQVWTPQD	RQCVLSTLAQ	LLLDKDCTVL	VGRQLRPLLL	DLLERNAEAI
90	100	110	120	130	140	150	160
KAGGQINHDL	HERLCVSMSK	LIGNHPDVLP	FALRYFKDTS	PVFQRLFLES	SDANPVRYGR	RRMKLRDLME	AAFKFLQQEQ
170	180	190	200	210	220	230	240
SVFRELWDWS	VCVPLLRSHD	TLVRWYTANC	LALVTCMNEE	HKLSFLKKIF	NSDELIHFRL	RLLEEAQLQD	LEKALVLANP
250	260	270	280	290	300	310	320
EVSLWRKQKE	LQYLQGHLVS	SDLSPRVTAV	CGVVLPGQLP	APGELGGNRS	SSREQELALR	SYVLVESVCK	SLQTLAMAVA
330	340	350	360	370	380	390	400
SQNAVLLEGP	IGCGKTSLVE	YLAAVTGRTK	PPQLLKVQLG	DQTDSKMLLG	MYRCTDVPGE	FVWQPGTLTQ	AATMGHWILL
410	420	430	440	450	460	470	480
EDIDYAPLDV	VSVLIPLLEN	GELLIPGRGD	CLKVAPGFQF	FATRRLLSCG	GNWYRPLNSH	ATLLDKYWTK	IHLDNLDKRE
490	500	510	520	530	540	550	560
LNEVLQSRYP	SLLAVVDHLL	DIYIQLTGEK	HHSWSDSSVG	CEQAPEEVSE	ARRENKRPTL	EGRELSLRDL	LNWCNRIAHS
570	580	590	600	610	620	630	640
FDSSSLSASL	NIFQEALDCF	TAMLSEHTSK	LKMAEVIGSK	LNISRKKAEF	FCQLYKPEIV	INELDLQVGR	VRLLRKQSEA
650	660	670	680	690	700	710	720
VHLQREKFTF	AATRPSSVLI	EQLAVCVSKG	EPVLLVGETG	TGKTSTIQYL	AHITGHRLRV	VNMNQQSDTA	DLLGGYKPVD
730	740	750	760	770	780	790	800
HKLIWLPLRE	AFEELFAQTF	SKKQNFTFLG	HIQTCYRQKR	WHDLLRLMQH	VHKSAVNKDG	KDSETGLLIK	EKWEAFGLRL
810	820	830	840	850	860	870	880
NHAQQQMKMT	ENTLLFAFVE	GTLAQAVKKG	EWILLDEINL	AAPEILECLS	GLLEGSSGSL	VLLDRGDTEP	LVRHPDFRLF
890	900	910	920	930	940	950	960
ACMNPATDVG	KRNLPPGIRN	RFTELYVEEL	ESKEDLQVLI	VDYLKGLSVN	KNTVQGIINF	YTALRKESGT	KLVDGTGHRP
970	980	990	1000	1010	1020	1030	1040
HYSLRTLCRA	LRFAASNPCG	NIQRSLYEGF	CLGFLTQLDR	ASHPIVQKLI	CQHIVPGNVK	SLLKQPIPEP	KGGRLIQVEG
1050	1060	1070	1080	1090	1100	1110	1120
YWIAVGDKEP	TIDETYILTS	SVKLNLRDIV	RVVSAGTYPV	LIQGETSVGK	TSLIQWLAAA	TGNHCVRINN	HEHTDIQEYI
1130	1140	1150	1160	1170	1180	1190	1200
GCYTSDSSGK	LVFKEGVLID	AMRKGYWIIL	DELNLAPTDV	LEALNRLLDD	NRELLVTETQ	EVVKAHPRFM	LFATQNPPGL
1210	1220	1230	1240	1250	1260	1270	1280
YGGRKVLSRA	FRNRFVELHF	DELPSSELET	ILHKRCSLPP	SYCSKLVKVM	LDLQSYRRSS	SVFAGKQGFI	TLRDLFRWAE
1290	1300	1310	1320	1330	1340	1350	1360
RYRLAEPTEK	EYDWLQHLAN	DGYMLLAGRV	RKQEEIDVIQ	EVLEKHFKKK	LCPQSLFSKE	NVLKLLGKLS	TQISTLECNF
1370	1380	1390	1400	1410	1420	1430	1440
GHIVWTEGMR	RLAMLVGRAL	EFGEPVLLVG	DTGCGKTTIC	QVFAALANQK	LYSVSCHLHM	ETSDFLGGLR	PVRQKPNDKE
1450	1460	1470	1480	1490	1500	1510	1520
EIDTSRLFEW	HDGPLVQAMK	EDGFFLLDEI	SLADDSVLER	LNSVLEVEKS	LVLAEKGSPE	DKDSEIELLT	AGKKFRILAT
1530	1540	1550	1560	1570	1580	1590	1600
MNPGGDFGKK	ELSPALRNRF	TEIWCPQSTS	REDLIQIISH	NLRPGLCLGR	IDPKGSDIPE	VMLDFIDWLT	HQEFGRKCVV
1610	1620	1630	1640	1650	1660	1670	1680
SIRDILSWVN	FMNKMGEEAA	LKRPEIISTV	TSFVHAACLV	YIDGIGSGVT	SSGFGTALLA	RKECLKFLIK	RLAKIVRLTE
1690	1700	1710	1720	1730	1740	1750	1760
YQKNELKIYD	RMKAKEFTGI	DNLWGIHPFF	IPRGPVLHRN	NIADYALSAG	TTAMNAQRLL	RATKLKKPIL	LEGSPGVGKT
1770	1780	1790	1800	1810	1820	1830	1840
SLVGALAKAS	GNTLVRINLS	EQTDITDLFG	ADLPVEGGKG	GEFAWRDGPL	LAALKAGHWV	VLDELNLASQ	SVLEGLNACF
1850	1860	1870	1880	1890	1900	1910	1920
DHRGEIYVPE	LGMSFQVQHE	KTKIFGCQNP	FRQGGGRKGL	PRSFLNRFTQ	VFVDPLTVID	MEFIASTLFP	AIEKNIVKKM
1930	1940	1950	1960	1970	1980	1990	2000
VAFNNQIDHE	VTVEKKWGQK	GGPWEFNLRD	LFRWCQLMLV	DQSPGCYDPG	QHVFLVYGER	MRTEEDKKKV	IAVFKDVFGS
2010	2020	2030	2040	2050	2060	2070	2080
NSNPYMGTRL	FRITPYDVQL	GYSVLSRGSC	VPHPSRHPLL	LLHQSFQPLE	SIMKCVQMSW	MVILVGPASV	GKTSLVQLLA
2090	2100	2110	2120	2130	2140	2150	2160
HLTGHTLKIM	AMNSAMDTTE	LLGGFEQVDL	IRPWRRLLEK	VEGTVRALLR	DSLLISADDA	EVVLRAWSHF	LLTYKPKCLG
2170	2180	2190	2200	2210	2220	2230	2240

Detailed Protein Report

Cmpd.	No. of Cmpds.	m/z meas.	Δ m/z [ppm]	z	Rt [min]	Score	P	Range	Sequence	Modification
558	1	512.6910	-181.41	2	35.4	13.3	2	1688-1695	K.IYDRMKAK.E	

Detailed Protein Report

Protein 208: protein FAM65B isoform 2 [Homo sapiens]

Accession: gi|14277690
Database: refseq_human(refseq_human_20140103.fasta)
Database Date: 2015-11-30
Modification(s): Carbamidomethyl, Oxidation

Score: 27.7
MW [kDa]: 65.7
pI: 5.1
Sequence Coverage [%]: 4.7
No. of unique Peptides: 2

10	20	30	40	50	60	70	80
MLVGSQSFSP	GGPNGIIRSQ	SFAGFSGLQE	RRSRCNSFIE	NSSALKKPQA	KLKKMHNLGH	KNNNPPKEPQ	PKRVEEVYRA
**90**	**100**	**110**	**120**	**130**	**140**	**150**	**160**
LKNGLDEYLE	VHQTELDKLT	AQLKDMKRNS	RLGVLYDLDK	QIKTIERYMR	RLEFHISKVD	ELYEAYCIQR	RLQDGASKMK
**170**	**180**	**190**	**200**	**210**	**220**	**230**	**240**
QAFATSPASK	AARESLTEIN	RSFKEYTENM	CTIEVELENL	LGEFSIKMKG	LAGFARLCPG	DQYEIFMKYG	RQRWKLKGKI
**250**	**260**	**270**	**280**	**290**	**300**	**310**	**320**
EVNGKQSWDG	EETVFLPLIV	GFISIKVTEL	KGLATHILVG	SVTCETKELF	AARPQVVAVD	INDLGTIKLN	LEITWYPFDV
**330**	**340**	**350**	**360**	**370**	**380**	**390**	**400**
EDMTASSGAG	NKAAALQRRM	SMYSQGTPET	PTFKDHSFFS	NLPDDIFENG	KAAEEKMPLS	LSFSDLPNGD	CALTSHSTGS
**410**	**420**	**430**	**440**	**450**	**460**	**470**	**480**
PSNSTNPEIT	ITPAEFNLSS	LASQNEGMDD	TSSASSRNSL	GEGQEPKSHL	KEEDPEEPRK	PASAPSEACR	RQSSGAGAEH
**490**	**500**	**510**	**520**	**530**	**540**	**550**	**560**
LFLENDVAEA	LLQESEEASE	LKPVELDTSE	GNITKQLVKR	LTSAEVPMAT	DRLLSEGSVG	GESEGCRSFL	DGSLEDAFNG
**570**	**580**	**590**	**600**				
LLLALEPHKE	QYKEFQDLNQ	EVMNLDDILK	K				

Cmpd.	No. of Cmpds.	m/z meas.	Δ m/z [ppm]	z	Rt [min]	Score	P	Range	Sequence	Modification
1567	1	994.1375	-4.03	3	47.9	10.8	2	520-547	K.RLTSAEVPMATDRLLSEGSVGGESEGCR.S	Carbamidomethyl: 27; Oxidation: 9
625	1	740.4433	138.33	2	36.7	16.9	0	533-547	R.LLSEGSVGGESEGCR.S	

Detailed Protein Report

Protein 209: queuine tRNA-ribosyltransferase [Homo sapiens]

**Accession:**	gi|13654276	**Score:**	27.7
**Database:**	refseq_human(refseq_human_20140103.fasta)	**MW [kDa]:**	44.0
**Database Date:**	2015-11-30	**pI:**	7.0
**Modification(s):**	Oxidation	**Sequence Coverage [%]:**	6.5
		**No. of unique Peptides:**	1

Quantitation

WD:WU **Median:** 0.94 **CV:** 0.00 % **No. of Peptides:** 1

10	20	30	40	50	60	70	80
MAGAATQASL	ESAPRIMRLV	AECSRSRARA	GELWLPHGTV	ATPVFMPVGT	QATMKGITTE	QLDALGCRIC	LGNTYHLGLR
**90**	**100**	**110**	**120**	**130**	**140**	**150**	**160**
PGPELIQKAN	GLHGFMNWPH	NLLTDSGGFQ	MVSLVSLSEV	TEEGVRFRSP	YDGNETLLSP	EKSVQIQNAL	GSDIIMQLDD
**170**	**180**	**190**	**200**	**210**	**220**	**230**	**240**
VVSSTVTGPR	VEEAMYRSIR	WLDRCIAAHQ	RPDKQNLFAI	IQGGLDADLR	ATCLEEMTKR	DVPGFAIGGL	SGGESKSQFW
**250**	**260**	**270**	**280**	**290**	**300**	**310**	**320**
RMVALSTSRL	PKDKPRYLMG	VGYATDLVVC	VALGCDMFDC	VFPTRTARFG	SALVPTGNLQ	LRKKVFEKDF	GPIDPECTCP
**330**	**340**	**350**	**360**	**370**	**380**	**390**	**400**
TCQKHSRAFL	HALLHSDNTA	ALHHLTVHNI	AYQLQLMSAV	RTSIVEKRFP	DFVRDFMGAM	YGDPTLCPTW	ATDALASVGI
**410**							
TLG							

Cmpd.	No. of Cmpds.	m/z meas.	Δ m/z [ppm]	z	Rt [min]	Score	P	Range	Sequence	Modification	Ratios
512	1	719.0204	199.94	2	35.3	10.4	2	16-27	R.IMRLVAECSRSR.A	Oxidation: 2	WD:WU 0.94

Detailed Protein Report

Protein 210: glyoxalase domain-containing protein 4 [Homo sapiens]

Accession: gi|217330598
Database: refseq_human(refseq_human_20140103.fasta)
Database Date: 2015-11-30

Score: 27.6
MW [kDa]: 33.2
pI: 5.3
Sequence Coverage [%]: 8.4
No. of unique Peptides: 1

Quantitation

WD:WU **Median:** 0.86 **CV:** 0.00 % **No. of Peptides:** 1

10	20	30	40	50	60	70	80
MAARRALHFV	FKVGNRFQTA	RFYRDVLGMK	VLRHEEFEEG	CKAACNGPYD	GKWSKTMVGF	GPEDDHFVAE	LTYNYGVGDY
**90**	**100**	**110**	**120**	**130**	**140**	**150**	**160**
KLGNDFMGIT	LASSQAVSNA	RKLEWPLTEV	AEGVFETEAP	GGYKFYLQNR	SLPQSDPVLK	VTLAVSDLQK	SLNYWCNLLG
**170**	**180**	**190**	**200**	**210**	**220**	**230**	**240**
MKIYEKDEEK	QRALLGYADN	QCKLELQGVK	GGVDHAAAFG	RIAFSCPQKE	LPDLEDLMKR	ENQKILTPLV	SLDTPGKATV
**250**	**260**	**270**	**280**	**290**	**300**		
QVVILADPDG	HEICFVGDEA	FRELSKMDPE	GSKLLDDAMA	ADKSDEWFAK	HNKPKASG		

Cmpd.	No. of Cmpds.	m/z meas.	Δ m/z [ppm]	z	Rt [min]	Score	P	Range	Sequence	Modification	Ratios
1097	3	631.1089	141.12	3	42.0	17.0	2	6-21	R.ALHFVFKVGNRFQTAR.F		WD:WU 0.86

Detailed Protein Report

Protein 211: dnaJ homolog subfamily A member 3, mitochondrial isoform 3 [Homo sapiens]

**Accession:**	gi|557357763	**Score:**	27.6
**Database:**	refseq_human(refseq_human_20140103.fasta)	**MW [kDa]:**	33.1
**Database Date:**	2015-11-30	**pI:**	10.1
**Modification(s):**	Oxidation	**Sequence Coverage [%]:**	9.3
		**No. of unique Peptides:**	1

10	20	30	40	50	60	70	80
MAEPQAERPR	LCVFPDLLRP	PSAADIETWC	QPYRKIFGEF	SSSSFGDFQT	VFDQPQEYFM	ELTFNQAAKG	VNKEFTVNIM
**90**	**100**	**110**	**120**	**130**	**140**	**150**	**160**
DTCERCNGKG	NEPGTKVQHC	HYCGGSGMET	INTGPFVMRS	TCRRCGGRGS	IIISPCVVCR	GAGQAKQKKR	VMIPVPAGVE
**170**	**180**	**190**	**200**	**210**	**220**	**230**	**240**
DGQTVRMPVG	KREIFITFRV	QKSPVFRRDG	ADIHSDLFIS	IAQALLGGTA	RAQGLYETIN	VTIPPGTQTD	QKIRMGGKGI
**250**	**260**	**270**	**280**	**290**	**300**	**310**	
PRINSYGYGD	HYIHIKIRVP	KRLTSRQQSL	ILSYAEDETD	VEGTVNGVTL	TSSGKRSTGN		

Cmpd.	No. of Cmpds.	m/z meas.	Δ m/z [ppm]	z	Rt [min]	Score	P	Range	Sequence	Modification
2705	1	842.5419	120.53	2	62.2	10.1	0	151-166	R.VMIPVPAGVEDGQTVR.M	Oxidation: 2

Detailed Protein Report

Protein 212: **PREDICTED: leucine-rich PPR motif-containing protein, mitochondrial isoform X2 [Homo sapiens]**

**Accession:**	gi|578802653	**Score:**	27.6
**Database:**	refseq_human(refseq_human_20140103.fasta)	**MW [kDa]:**	119.1
**Database Date:**	2015-11-30	**pI:**	5.9
		**Sequence Coverage [%]:**	2.9
		**No. of unique Peptides:**	1

10	20	30	40	50	60	70	80
MAALLRSARW	LLRAGAAPRL	PLSLRLLPGG	PGRLHAASYL	PAARAGPVAG	GLLSPARLYA	IAAKEKDIQE	ESTFSSRKIS
90	100	110	120	130	140	150	160
NQFDWALMRL	DLSVRRTGRI	PKKLLQKVFN	DTCRSGGLGG	SHALLLLRSC	GSLLPELKLE	ERTEFAHRIW	DTLQKLGAVY
170	180	190	200	210	220	230	240
DVSHYNALLK	VYLQNEYKFS	PTDFLAKMEE	ANIQPNRVTY	QRLIASYCNV	GDIEGASKIL	GFMKTKDLPV	TEAVFSALVT
250	260	270	280	290	300	310	320
GHARAGDMEN	AENILTVMRD	AGIEPGPDTY	LALLNAYAEK	GDIDHVKQTL	EKVEKSELHL	MDRDLLQIIF	SFSKAGYPQY
330	340	350	360	370	380	390	400
VSEILEKVTC	ERRYIPDAMN	LILLLVTEKL	EDVALQILLA	CPVSKEDGPS	VFGSFFLQHC	VTMNTPVEKL	TDYCKKLKEV
410	420	430	440	450	460	470	480
QMHSFPLQFT	LHCALLANKT	DLAKALMKAV	KEEGFPIRPH	YFWPLLVGRR	KEKNVQGIIE	ILKGMQELGV	HPDQETYTDY
490	500	510	520	530	540	550	560
VIPCFDSVNS	ARAILQENGC	LSDSDMFSQA	GLRSEAANGN	LDFVLSFLKS	NTLPISLQSI	RSSLLLGFRR	SMNINLWSEI
570	580	590	600	610	620	630	640
TELLYKDGRY	CQEPRGPTEA	VGYFLYNLID	SMSDSEVQAK	EEHLRQYFHQ	LEKMNVKIPE	NIYRGIRNLL	ESYHVPELIK
650	660	670	680	690	700	710	720
DAHLLVESKN	LDFQKTVQLT	SSELESTLET	LKAENQPIRD	VLKQLILVLC	SEENMQKALE	LKAKYESDMV	TGGYAALINL
730	740	750	760	770	780	790	800
CCRHDKVEDA	LNLKEEFDRL	DSSAVLDTGK	YVGLVRVLAK	HGKLQDAINI	LKEMKEKDVL	IKDTTALSFF	HMLNGAALRG
810	820	830	840	850	860	870	880
EIETVKQLHE	AIVTLGLAEP	STNISFPLVT	VHLEKGDLST	ALEVAIDCYE	KYKVLPRIHD	VLCKLVEKGE	TDLIQKAMDF
890	900	910	920	930	940	950	960
VSQEQGEMVM	LYDLFFAFLQ	TGNYKEAKKI	IETPGIRARS	ARLQWFCDRC	VANNQVETLE	KLVELTQKLF	ECDRDQMYYN
970	980	990	1000	1010	1020	1030	1040
LLKLYKINGD	WQRADAVWNK	IQEENVIPRE	KTLRLLAEIL	REGNQEVPFD	VPELWYEDEK	HSLNSSSAST	TEPDFQKDIL
1050	1060						
IACRLNQKKR	GI						

Cmpd.	No. of Cmpds.	m/z meas.	Δ m/z [ppm]	z	Rt [min]	Score	P	Range	Sequence	Modification
235	2	627.3257	-99.42	2	32.1	15.2	2	909-919	K.KIIETPGIRAR.S	

Detailed Protein Report

Protein 213: **PREDICTED: calpastatin isoform X10 [Homo sapiens]**

**Accession:**	gi|578810591	**Score:**	27.6
**Database:**	refseq_human(refseq_human_20140103.fasta)	**MW [kDa]:**	80.1
**Database Date:**	2015-11-30	**pI:**	4.9
		**Sequence Coverage [%]:**	4.3
		**No. of unique Peptides:**	1

10	20	30	40	50	60	70	80
MSQPGQKPAA	SPRPRRAAAA	RRTHEHVSEK	TSESPSKPGE	KKGSDEKKAA	SLGSSQSSRT	YAGGTASATK	VSASSGATSK
**90**	**100**	**110**	**120**	**130**	**140**	**150**	**160**
SSSMNPTETK	AVKTEPEKKS	QSTKPKSLPK	QASDTGSNDA	HNKKAVSRSA	EQQPSEKSTE	PKTKPQDMIS	AGGESVAGIT
**170**	**180**	**190**	**200**	**210**	**220**	**230**	**240**
AISGKPGDKK	KEKKSLTPAV	PVESKPDKPS	GKSGMDAALD	DLIDTLGGPE	ETEEENTTYT	GPEVSDPMSS	TYIEELGKRE
**250**	**260**	**270**	**280**	**290**	**300**	**310**	**320**
VTIPPKYREL	LAKKEGITGP	PADSSKPIGP	DDAIDALSSD	FTCGSPTAAG	KKTEKEESTE	VLKAQSAGTV	RSAAPPQEKK
**330**	**340**	**350**	**360**	**370**	**380**	**390**	**400**
RKVEKDTMSD	QALEALSASL	GTRQAEPELD	LRSIKEVDEA	KAKEEKLEKC	GEDDETIPSE	YRLKPATDKD	GKPLLPEPEE
**410**	**420**	**430**	**440**	**450**	**460**	**470**	**480**
KPKPRSESEL	IDELSEDFDR	SECKEKPSKP	TEKTEESKAA	APAPVSEAVC	RTSMCSIQSA	PPEPATLKGT	VPDDAVEALA
**490**	**500**	**510**	**520**	**530**	**540**	**550**	**560**
DSLGKKEADP	EDGKPVMDKV	KEKAKEEDRE	KLGEKEETIP	PDYRLEEVKD	KDGKPLLPKE	SKEQLPPMSE	DFLLDALSED
**570**	**580**	**590**	**600**	**610**	**620**	**630**	**640**
FSGPQNASSL	KFEDAKLAAA	ISEVVSQTPA	STTQAGAPPR	DTSSDKDLDD	ALDKLSDSLG	QRQPDPDENK	PMEDKVKEKA
**650**	**660**	**670**	**680**	**690**	**700**	**710**	**720**
KAEHRDKLGE	RDDTIPPEYR	HLLDDNGQDK	PVKPPTKKSE	DSKKPADDQD	PIDALSGDLD	SCPSTTETSQ	NTAKDKCKKA
**730**	**740**	**750**					
ASSSKAPKNG	GKAKDSAKTT	EETSKPKDD					

Cmpd.	No. of Cmpds.	m/z meas.	Δ m/z [ppm]	z	Rt [min]	Score	P	Range	Sequence	Modification
2971	1	816.9085	-57.77	2	65.9	16.1	1	2-16	M.SQPGQKPAASPRPRR.A	

Detailed Protein Report

Protein 214: **dehydrogenase/reductase SDR family member on chromosome X precursor [Homo sapiens]**

Accession: gi|193804850
Database: refseq_human(refseq_human_20140103.fasta)
Database Date: 2015-11-30

Score: 27.5
MW [kDa]: 36.4
pI: 9.7
Sequence Coverage [%]: 11.2
No. of unique Peptides: 1

10	20	30	40	50	60	70	80
MSPLSAARAA	LRVYAVGAAV	ILAQLLRRCR	GGFLEPVFPP	RPDRVAIVTG	GTDGIGYSTA	KHLARLGMHV	IIAGNNDSKA
**90**	**100**	**110**	**120**	**130**	**140**	**150**	**160**
KQVVSKIKEE	TLNDKVEFLY	CDLASMTSIR	QFVQKFKMKK	IPLHVLINNA	GVMMVPQRKT	RDGFEEHFGL	NYLGHFLLTN
**170**	**180**	**190**	**200**	**210**	**220**	**230**	**240**
LLLDTLKESG	SPGHSARVVT	VSSATHYVAE	LNMDDLQSSA	CYSPHAAYAQ	SKLALVLFTY	HLQRLLAAEG	SHVTANVVDP
**250**	**260**	**270**	**280**	**290**	**300**	**310**	**320**
GVVNTDVYKH	VFWATRLAKK	LLGWLLFKTP	DEGAWTSIYA	AVTPELEGVG	GHYLYNEKET	KSLHVTYNQK	LQQQLWSKSC
**330**	**340**						
EMTGVLDVTL							

Cmpd.	No. of Cmpds.	m/z meas.	Δ m/z [ppm]	z	Rt [min]	Score	P	Range	Sequence	Modification
1586	1	1073.1340	41.86	2	48.5	14.7	2	62-81	K.HLARLGMHVIIAGNNDSKAK.Q	

Detailed Protein Report

Protein 215: ephrin type-A receptor 3 isoform b precursor [Homo sapiens]

**Accession:**	gi|32967314	**Score:**	27.5
**Database:**	refseq_human(refseq_human_20140103.fasta)	**MW [kDa]:**	60.9
**Database Date:**	2015-11-30	**pI:**	5.5
**Modification(s):**	Carbamidomethyl	**Sequence Coverage [%]:**	3.0
		**No. of unique Peptides:**	1

10	20	30	40	50	60	70	80
MDCQLSILLL	LSCSVLDSFG	ELIPQPSNEV	NLLDSKTIQG	ELGWISYPSH	GWEEISGVDE	HYTPIRTYQV	CNVMDHSQNN
**90**	**100**	**110**	**120**	**130**	**140**	**150**	**160**
WLRTNWVPRN	SAQKIYVELK	FTLRDCNSIP	LVLGTCKETF	NLYYMESDDD	HGVKFREHQF	TKIDTIAADE	SFTQMDLGDR
**170**	**180**	**190**	**200**	**210**	**220**	**230**	**240**
ILKLNTEIRE	VGPVNKKGFY	LAFQDVGACV	ALVSVRVYFK	KCPFTVKNLA	MFPDTVPMDS	QSLVEVRGSC	VNNSKEEDPP
**250**	**260**	**270**	**280**	**290**	**300**	**310**	**320**
RMYCSTEGEW	LVPIGKCSCN	AGYEERGFMC	QACRPGFYKA	LDGNMKCAKC	PPHSSTQEDG	SMNCRCENNY	FRADKDPPSM
**330**	**340**	**350**	**360**	**370**	**380**	**390**	**400**
ACTRPPSSPR	NVISNINETS	VILDWSWPLD	TGGRKDVTFN	IICKKCGWNI	KQCEPCSPNV	RFLPRQFGLT	NTTVTVTDLL
**410**	**420**	**430**	**440**	**450**	**460**	**470**	**480**
AHTNYTFEID	AVNGVSELSS	PPRQFAAVSI	TTNQAAPSPV	LTIKKDRTSR	NSISLSWQEP	EHPNGIILDY	EVKYYEKQEQ
**490**	**500**	**510**	**520**	**530**	**540**		
ETSYTILRAR	GTNVTISSLK	PDTIYVFQIR	ARTAAGYGTN	SRKFEFETSP	DCMYYFNAV		

Cmpd.	No. of Cmpds.	m/z meas.	Δ m/z [ppm]	z	Rt [min]	Score	P	Range	Sequence	Modification
2832	2	945.9883	67.92	2	64.0	16.1	1	366-381	K.CGWNIKQCEPCSPNVR.F	Carbamidomethyl: 8

Detailed Protein Report

Protein 216: **PREDICTED: uncharacterized protein LOC101927628 [Homo sapiens]**

**Accession:**	gi|578827891	**Score:**	27.5
**Database:**	refseq_human(refseq_human_20140103.fasta)	**MW [kDa]:**	109.0
**Database Date:**	2015-11-30	**pI:**	12.3
**Modification(s):**	Carbamidomethyl	**Sequence Coverage [%]:**	1.8
		**No. of unique Peptides:**	1

10	20	30	40	50	60	70	80
MEESAEQAQR	RDETRVGRRR	HSWRSRQSRQ	TKEFIFSELL	SNLYSRRNLQ	TLVEESAEQA	QRCDEMLLLR	AAVQPALAWE
**90**	**100**	**110**	**120**	**130**	**140**	**150**	**160**
REDTRGGVGR	AGRPRSSSSQ	SCCPTCTHVG	TSRHSWRRRQ	SRQTKEFIFS	ELLSNLHSRG	NEKTLVKASA	EKADQGVHLL
**170**	**180**	**190**	**200**	**210**	**220**	**230**	**240**
RAAVQPALAW	EREDTRGGVG	RAGRPRSSSS	QSCCPNCTRV	GTRRHSWRSR	QSRQTKEFIF	SELLSNLYSR	RNLQTLVEES
**250**	**260**	**270**	**280**	**290**	**300**	**310**	**320**
AEQAQRCDEM	LLLRAAVQPA	LAWEREDTRG	GVGRAGRPRS	SSSQSCCPTC	TRVGTRRHSW	RRRQSRQTKE	FIFSELLSNL
**330**	**340**	**350**	**360**	**370**	**380**	**390**	**400**
HSRGNEKTLM	EESAEQAQRR	DETRVGRRRH	SWRSRQSRQT	KEFIFSELLS	NLYSHRNLQT	LVEESAEQAQ	RCDEMLLLRA
**410**	**420**	**430**	**440**	**450**	**460**	**470**	**480**
AVQPALAWER	EDTRGGVGRA	GRPRSSSSQS	CCPTCTHVGT	SRHSWRRRQS	RQTKEFIFSE	LLSNLHSRGN	EKTLVKASAE
**490**	**500**	**510**	**520**	**530**	**540**	**550**	**560**
KADQGVHLLR	AAVQPALAWE	REDTRGGVGR	AGRPRSSSSQ	SCCPNCTRVG	TRRHSWRSRQ	SRHRGVTRLA	WEEEDTPGGV
**570**	**580**	**590**	**600**	**610**	**620**	**630**	**640**
GRAGRPRSSS	SRSCCPTCTR	VGTCRHLWRS	RQSRHSGATR	CCFSELLSNL	HSRGNKKTLV	EALAEQADQG	VHLLRAAVQP
**650**	**660**	**670**	**680**	**690**	**700**	**710**	**720**
VLAWEREDTR	GGVGRAGRPR	SSSSQSCCPT	CTRVGTRRHS	CRSRQSRHSG	VTRLAWEEED	IPGGVGRAGR	ARSSSSRSCC
**730**	**740**	**750**	**760**	**770**	**780**	**790**	**800**
PTCTRVGTCR	HLWRSRQSRH	SGATRCCFSE	LLSNLHSRGN	QKTLVEASAE	QADQGVHLLR	AAVQPVLAWE	REDTRGGVGR
**810**	**820**	**830**	**840**	**850**	**860**	**870**	**880**
AGRPRSSSSQ	SCCPTCTRVG	TRRHSWRSRP	SRHSGVTRLA	WEEEDTPGGV	GRAGRPRSSS	SRSCCPTCTR	VGTCRHLWRS
**890**	**900**	**910**	**920**	**930**	**940**	**950**	**960**
RQSRHSGATR	CCFSELLSNL	HWRGNEKTLV	EASAEQADQG	VHLLRAAVQP	VLAWERADTR	GGVGRAGRPR	NSSSQSCCPT
**970**	**980**						
CTRVGMRRHS							

Cmpd.	No. of Cmpds.	m/z meas.	Δ m/z [ppm]	z	Rt [min]	Score	P	Range	Sequence	Modification
2316	1	937.2798	-119.93	2	57.4	14.7	1	951-967	R.NSSSQSCCPTCTRVGMR.R	Carbamidomethyl: 7

Detailed Protein Report

Protein 217: PREDICTED: insulin-like growth factor 1 receptor isoform X2 [Homo sapiens]

**Accession:**	gi|530407102	**Score:**	27.4
**Database:**	refseq_human(refseq_human_20140103.fasta)	**MW [kDa]:**	141.1
**Database Date:**	2015-11-30	**pI:**	5.4
**Modification(s):**	Carbamidomethyl, Oxidation	**Sequence Coverage [%]:**	2.4
		**No. of unique Peptides:**	2

Quantitation

WD:WU **Median:** 0.98 **CV:** 0.00 % **No. of Peptides:** 1

10	20	30	40	50	60	70	80
MTNLKDIGLY	NLRNITRGAI	RIEKNADLCY	LSTVDWSLIL	DAVSNNYIVG	NKPPKECGDL	CPGTMEEKPM	CEKTTINNEY
90	100	110	120	130	140	150	160
NYRCWTTNRC	QKMCPSTCGK	RACTENNECC	HPECLGSCSA	PDNDTACVAC	RHYYYAGVCV	PACPPNTYRF	EGWRCVDRDF
170	180	190	200	210	220	230	240
CANILSAESS	DSEGFVIHDG	ECMQECPSGF	IRNGSQSMYC	IPCEGPCPKV	CEEEKKTKTI	DSVTSAQMLQ	GCTIFKGNLL
250	260	270	280	290	300	310	320
INIRRGNNIA	SELENFMGLI	EVVTGYVKIR	HSHALVSLSF	LKNLRLILGE	EQLEGNYSFY	VLDNQNLQQL	WDWDHRNLTI
330	340	350	360	370	380	390	400
KAGKMYFAFN	PKLCVSEIYR	MEEVTGTKGR	QSKGDINTRN	NGERASCESD	VLHFTSTTTS	KNRIIITWHR	YRPPDYRDLI
410	420	430	440	450	460	470	480
SFTVYYKEAP	FKNVTEYDGQ	DACGSNSWNM	VDVDLPPNKD	VEPGILLHGL	KPWTQYAVYV	KAVTLTMVEN	DHIRGAKSEI
490	500	510	520	530	540	550	560
LYIRTNASVP	SIPLDVLSAS	NSSSQLIVKW	NPPSLPNGNL	SYYIVRWQRQ	PQDGYLYRHN	YCSKDKIPIR	KYADGTIDIE
570	580	590	600	610	620	630	640
EVTENPKTEV	CGGEKGPCCA	CPKTEAEKQA	EKEEAEYRKV	FENFLHNSIF	VPRPERKRRD	VMQVANTTMS	SRSRNTTAAD
650	660	670	680	690	700	710	720
TYNITDPEEL	ETEYPFFESR	VDNKERTVIS	NLRPFTLYRI	DIHSCNHEAE	KLGCSASNFV	FARTMPAEGA	DDIPGPVTWE
730	740	750	760	770	780	790	800
PRPENSIFLK	WPEPENPNGL	ILMYEIKYGS	QVEDQRECVS	RQEYRKYGGA	KLNRLNPGNY	TARIQATSLS	GNGSWTDPVF
810	820	830	840	850	860	870	880
FYVQAKTGYE	NFIHLIIALP	VAVLLIVGGL	VIMLYVFHRK	RNNSRLGNGV	LYASVNPEYF	SAADVYVPDE	WEVAREKITM
890	900	910	920	930	940	950	960
SRELGQGSFG	MVYEGVAKGV	VKDEPETRVA	IKTVNEAASM	RERIEFLNEA	SVMKEFNCHH	VVRLLGVVSQ	GQPTLVIMEL
970	980	990	1000	1010	1020	1030	1040
MTRGDLKSYL	RSLRPEMENN	PVLAPPSLSK	MIQMAGEIAD	GMAYLNANKF	VHRDLAARNC	MVAEDFTVKI	GDFGMTRDIY
1050	1060	1070	1080	1090	1100	1110	1120
ETDYYRKGGK	GLLPVRWMSP	ESLKDGVFTT	YSDVWSFGVV	LWEIATLAEQ	PYQGLSNEQV	LRFVMEGGLL	DKPDNCPDML
1130	1140	1150	1160	1170	1180	1190	1200
FELMRMCWQY	NPKMRPSFLE	IISSIKEEME	PGFREVSFYY	SEENKLPEPE	ELDLEPENME	SVPLDPSASS	SSLPLPDRHS
1210	1220	1230	1240	1250			
GHKAENGPGP	GVLVLRASFD	ERQPYAHMNG	GRKNERALPL	PQSSTC			

Cmpd.	No. of Cmpds.	m/z meas.	Δ m/z [ppm]	z	Rt [min]	Score	P	Range	Sequence	Modification	Ratios
1849	2	1045.0301	118.60	2	51.5	16.2	0	56-73	K.ECGDLCPGTMEEKPMCEK.T	Carbamidomethyl: 16; Oxidation: 10, 15	
1420	1	669.8545	-38.10	2	46.5	11.2	0	271-282	R.HSHALVSLSFLK.N		WD:WU 0.98

Detailed Protein Report

Protein 218: **PREDICTED: suppressor of tumorigenicity 7 protein-like isoform X10 [Homo sapiens]**

Accession: gi|530362849
Database: refseq_human(refseq_human_20140103.fasta)
Database Date: 2015-11-30
Modification(s): Oxidation

Score: 27.4
MW [kDa]: 35.9
pI: 9.9
Sequence Coverage [%]: 5.6
No. of unique Peptides: 2

Quantitation

WD:WU **Median:** 3.90 **CV:** 0.00 % **No. of Peptides:** 1

10	20	30	40	50	60	70	80
MADRGGVGEA	AAVGASPASV	PGLNPTLGWR	ERLRAGLAGT	GASLWFVAGL	GLLYALRIPL	RLCENLAAVT	VFLNSLTPKF
**90**	**100**	**110**	**120**	**130**	**140**	**150**	**160**
YVALTGTSSL	ISGLIFIFEW	WYFHKHGTSF	IEQVSVSHLQ	PLMGGTESSI	SEPGSPSRNR	ENETSRQNLS	ECKVWRNPLN
**170**	**180**	**190**	**200**	**210**	**220**	**230**	**240**
LFRGAEYRRY	TWVTGKEPLT	YYDMNLSAQD	HQTFFTCDTD	FLRPSDTVMQ	KAWRERNPPA	RIKAAYQALE	LNNDCATAYV
**250**	**260**	**270**	**280**	**290**	**300**	**310**	**320**
LLAEEEATTI	VDAERLFKQA	LKAGETIYRQ	SQQCQHQSPQ	HEAQLRRDTN	VLVYIKRRLA	MCARKLGRIR	EAVKIMRDI

Cmpd.	No. of Cmpds.	m/z meas.	Δ m/z [ppm]	z	Rt [min]	Score	P	Range	Sequence	Modification	Ratios
2047	4	625.2681	-128.78	2	54.2	13.8	1	259-269	K.QALKAGETIYR.Q		WD:WU 3.90
2581	2	836.1729	-298.97	1	60.5	13.6	1	298-304	R.RLAMCAR.K	Oxidation: 4	

Detailed Protein Report

Protein 219: PREDICTED: zinc finger protein 462 isoform X10 [Homo sapiens]

**Accession:**	gi|578817729	**Score:**	27.4
**Database:**	refseq_human(refseq_human_20140103.fasta)	**MW [kDa]:**	192.3
**Database Date:**	2015-11-30	**pI:**	6.4
**Modification(s):**	Oxidation	**Sequence Coverage [%]:**	1.9
		**No. of unique Peptides:**	1

10	20	30	40	50	60	70	80
MEVLQCDGCD	FRAPSYEDLK	AHIQDVHTAF	LQPTDVAEDN	VNELRCGSVN	ASNQTEVEFS	SIKDEFAIAE	DLSGQNATSL
**90**	**100**	**110**	**120**	**130**	**140**	**150**	**160**
GTGGYYGHSP	GYYGQHIAAN	PKPTNKFFQC	KFCVRYFRSK	NLLIEHTRKV	HGAQAEGSSS	GPPVPGSLNY	NIMMHEGFGK
**170**	**180**	**190**	**200**	**210**	**220**	**230**	**240**
VFSCQFCTYK	SPRRARIIKH	QKMYHKNNLK	ETTAPPPAPA	PMPDPVVPPV	SLQDPCKELP	AEVVERSILE	SMVKPLTKSR
**250**	**260**	**270**	**280**	**290**	**300**	**310**	**320**
GNFCCEWCSY	QTPRRERWCD	HMMKKHRSMV	KILSSLRQQQ	EGTNLPDVPN	KSAPSPTSNS	TYLTMNAASR	EIPNTTVSNF
**330**	**340**	**350**	**360**	**370**	**380**	**390**	**400**
RGSMGNSIMR	PNSSASKFSP	MSYPQMKPKS	PHNSGLVNLT	ERSRYGMTDM	TNSSADLETN	SMLNDSSSDE	ELNEIDSENG
**410**	**420**	**430**	**440**	**450**	**460**	**470**	**480**
LSAMDHQTSG	LSAEQLMGSD	GNKLLETKGI	PFRRFMNRFQ	CPFCPFLTMH	RRSISRHIEN	IHLSGKTAVY	KCDECPFTCK
**490**	**500**	**510**	**520**	**530**	**540**	**550**	**560**
SSLKLGAHKQ	CHTGTTSDWD	AVNSQSESIS	SSLNEGVVSY	ESSSINGRKS	GVMLDPLQQQ	QPPQPPPPPP	PPPPSQPQPL
**570**	**580**	**590**	**600**	**610**	**620**	**630**	**640**
QQPQPPQLQP	PHQVPPQPQT	QPPPTQQPQP	PTQAAPLHPY	KCTMCNYSTT	TLKGLRVHQQ	HKHSFCDNLP	KFEGQPSSLP
**650**	**660**	**670**	**680**	**690**	**700**	**710**	**720**
LENETDSHPS	SSNTVKKSQT	SILGLSSKNN	FVAKASRKLA	NDFPLDLSPV	KKRTRIDEIA	SNLQSKINQT	KQQEDAVINV
**730**	**740**	**750**	**760**	**770**	**780**	**790**	**800**
EDDEEEEEDN	EVEIEVELDR	EEEPTEPIIE	VPTSFSAQQI	WVRDTSEPQK	EPNFRNITHD	YNATNGAEIE	LTLSEDEEDY
**810**	**820**	**830**	**840**	**850**	**860**	**870**	**880**
YGSSTNLKDH	QVSNTALLNT	QTPIYGTEHN	SENTDFGDSG	RLYYCKHCDF	NNKSARSVST	HYQRMHPYIK	FSFRYILDPN
**890**	**900**	**910**	**920**	**930**	**940**	**950**	**960**
DHSAVYRCLE	CYIDYTNFED	LQQHYGEHHP	EAMNVLNFDH	SDLIYRCRFC	SYTSPNVRSL	MPHYQRMHPT	VKINNAMIFS
**970**	**980**	**990**	**1000**	**1010**	**1020**	**1030**	**1040**
SYVVEQQEGL	NTESQTLREI	LNSAPKNMAT	STPVARGGGL	PATFNKNTPK	TFTPECENQK	DPLVNTVVVY	DCDVCSFASP
**1050**	**1060**	**1070**	**1080**	**1090**	**1100**	**1110**	**1120**
NMHSVLVHYQ	KKHPEEKASY	FRIQKTMRMV	SVDRGSALSQ	LSFEPFGHLE	EVPKIKERKV	VGYKCKFCVE	VHPTLRAICN
**1130**	**1140**	**1150**	**1160**	**1170**	**1180**	**1190**	**1200**
HLRKHVQYGN	VPAVSAAVKQ	EADDPAHLFL	DGLEAAKDAS	GALVGRVDGE	HCLLDGMLED	ETRPGGYHCS	QCDRVLMSMQ
**1210**	**1220**	**1230**	**1240**	**1250**	**1260**	**1270**	**1280**
GLRSHERSHL	ALAMFTREDK	YSCQYCSFVS	AFRHNLDRHM	QTHHGHHKPF	RCKLCSFKSS	YNSRLKTHIL	KAHAGEHAYK
**1290**	**1300**	**1310**	**1320**	**1330**	**1340**	**1350**	**1360**
CSWCSFSTMT	ISQLKEHSLK	VHGKALTLPR	PRIVSLLSSH	SHHSSQKATP	AEEVEDSNDS	SYSEPPDVQQ	QLNHYQSAAL
**1370**	**1380**	**1390**	**1400**	**1410**	**1420**	**1430**	**1440**
ARNNSRVSPV	PLSGAAAGTE	QKTEAVLHCE	FCEFSSGYIQ	SIRRHYRDKH	GGKKLFKCKD	CSFYTGFKSA	FTMHVEAGHS
**1450**	**1460**	**1470**	**1480**	**1490**	**1500**	**1510**	**1520**
AVPEEGPKDL	RCPLCLYHTK	YKRNMIDHIV	LHREERVVPI	EVCRSKLSKY	LQGVVFRCDK	CTFTCSSDES	LQQHIEKHNE
**1530**	**1540**	**1550**	**1560**	**1570**	**1580**	**1590**	**1600**
LKPYKCQLCY	YETKHTEELD	SHLRDEHKVS	RNFELVGRVN	LDQLEQMKEK	MESSSSDDED	KEEEMNSKAE	DRELMRFSDH
**1610**	**1620**	**1630**	**1640**	**1650**	**1660**	**1670**	**1680**
GAALNTEKRF	PCEFCGRAFS	QGSEWERHVL	RHGMALNDTK	QVSREEIHPK	EIMENSVKMP	SIEEKEDDEA	IGIDFSLKNE
**1690**	**1700**	**1710**					
TVAICVVTAD	KSLLENAEAK	KE					

Cmpd.	No. of Cmpds.	m/z meas.	Δ m/z [ppm]	z	Rt [min]	Score	P	Range	Sequence	Modification
2878	1	869.4050	22.02	2	64.7	13.3	0	1281-1295	K.CSWCSFSTMTISQLK.E	Oxidation: 9

Detailed Protein Report

Protein 220: ankyrin repeat and fibronectin type-III domain-containing protein 1 [Homo sapiens]

**Accession:**	gi|114842396	**Score:**	27.3
**Database:**	refseq_human(refseq_human_20140103.fasta)	**MW [kDa]:**	87.5
**Database Date:**	2015-11-30	**pI:**	9.5
		**Sequence Coverage [%]:**	4.7
		**No. of unique Peptides:**	1

10	20	30	40	50	60	70	80
MEASLTRRLL	FKDRHFTCSK	IIGRRFACFA	QRLSHRRKQS	QCDLLNESTG	QLPTTCSSAA	SNSINWNCRV	KMTQQMQNLH
90	100	110	120	130	140	150	160
LCQSKKHSAP	SSPNAAKRLY	RNLSEKLKGS	HSSFDEAYFR	TRTDRLSLRK	TSVNFQGNEA	MFEAVEQQDM	DAVQILLYQY
170	180	190	200	210	220	230	240
TPEELDLNTP	NSEGLTPLDI	AIMTNNVPIA	RILLRTGARE	SPHFVSLESR	AMHLNTLVQE	AQERVSELSA	QVENEGFTLD
250	260	270	280	290	300	310	320
NTEKEKQLKA	WEWRYRLYRR	MKTGFEHARA	PEMPTNVCLM	VTSSTSLTVS	FQEPLSVNAA	VVTRYKVEWS	MSEDFSPLAG
330	340	350	360	370	380	390	400
EIIMDNLQTL	RCTITGLTMG	QQYFVQVSAY	NMKGWGPAQT	TTPACASPSN	WKDYDDREPR	HKGQSEVLEG	LLQQVRALHQ
410	420	430	440	450	460	470	480
HYSCRESTKL	QTTGRKQSVS	RSLKHLFHSS	NKFVKTLKRG	LYIAVIFYYK	DNILVTNEDQ	VPIVEIDDSH	TSSITQDFLW
490	500	510	520	530	540	550	560
FTKLSCMWED	IRWLRQSIPI	SSSSSTVLQT	RQKMLAATAQ	LQNLLGTHNL	GRVYYEPIKD	RHGNILIVTI	REVEMLYSFF
570	580	590	600	610	620	630	640
NGKWMQISKL	QSQRKSLSTP	EEPTALDILL	ITIQDILSYH	KRSHQRLFPG	LYLGYLKLCS	SVDQIKVLVT	QKLPNILCHV
650	660	670	680	690	700	710	720
KIRENNNISR	EEWEWIQKLS	GSESMESVDH	TSDCPMQLFF	YELQMAVKAL	LQQINIPLHQ	ARNFRLYTQE	VLEMGHNVSF
730	740	750	760	770			
LLLLPASDDV	CTAPGQNNPY	TPHSGFLNLP	LQMFELGIVA	CFT			

Cmpd.	No. of Cmpds.	m/z meas.	Δ m/z [ppm]	z	Rt [min]	Score	P	Range	Sequence	Modification
2705	1	716.0445	-19.86	3	62.3	12.3	1	493-511	R.WLRQSIPISSSSSTVLQTR.Q	

Detailed Protein Report

Protein 221: **PREDICTED: rho guanine nucleotide exchange factor 10-like protein isoform X17 [Homo sapiens]**

**Accession:**	gi|578799376	**Score:**	27.2
**Database:**	refseq_human(refseq_human_20140103.fasta)	**MW [kDa]:**	114.3
**Database Date:**	2015-11-30	**pI:**	6.5
		**Sequence Coverage [%]:**	1.5
		**No. of unique Peptides:**	2

10	20	30	40	50	60	70	80
MSLSTFAQMT	QLMKAAKSGT	KDGLEKTRMA	VMRKVSFLHR	KDVLGDSEEE	DMGLLEVSVS	DIKPPAPELG	PMPEGLSPQQ
**90**	**100**	**110**	**120**	**130**	**140**	**150**	**160**
VVRRHILGSI	VQSEGSYVES	LKRILQDYRN	PLMEMEPKAL	SARKCQVVFF	RVKEILHCHS	MFQIALSSRV	AEWDSTEKIG
**170**	**180**	**190**	**200**	**210**	**220**	**230**	**240**
DLFVASFSKS	MVLDVYSDYV	NNFTSAMSII	KKACLTKPAF	LEFLKRRQVC	SPDRVTLYGL	MVKPIQRFPQ	FILLLQDMLK
**250**	**260**	**270**	**280**	**290**	**300**	**310**	**320**
NTPRGHPDRL	SLQLALTELE	TLAEKLNEQK	RLADQVAEIQ	QLTKSVSDRS	SLNKLLTSGQ	RQLLLCETLT	ETVYGDRGQL
**330**	**340**	**350**	**360**	**370**	**380**	**390**	**400**
IKSKERRVFL	LNDMLVCANI	NFKGQLEISS	LVPLGPKYVV	KWNTALPQVQ	VVEVGQDGGT	YDKDNVLIQH	SGAKKASASG
**410**	**420**	**430**	**440**	**450**	**460**	**470**	**480**
QAQNKVYLGP	PRLFQELQDL	QKDLAVVEQI	TLLISTLHGT	YQNLNMTVAQ	DWCLALQRLM	RVKEEEIHSA	NKCRLRLLLP
**490**	**500**	**510**	**520**	**530**	**540**	**550**	**560**
GKPDKSGRPI	SFMVVFITPN	PLSKISWVNR	LHLAKIGLRE	ENQPGWLCPD	EDKKSKAPFW	CPILACCIPA	FSSRALSLQL
**570**	**580**	**590**	**600**	**610**	**620**	**630**	**640**
GALVHSPVNC	PLLGFSAVST	SLPQGYLWVG	GGQEGAGGQV	EIFSLNRPSP	RTVKSFPLAA	PVLCMEYIPE	LEEEAESRDE
**650**	**660**	**670**	**680**	**690**	**700**	**710**	**720**
SPTVADPSAT	VHPTICLGLQ	DGSILLYSSV	DTGTQCLVSC	RSPGLQPVLC	LRHSPFHLLA	GLQDGTLAAY	PRTSGGVLWD
**730**	**740**	**750**	**760**	**770**	**780**	**790**	**800**
LESPPVCLTV	GPGPVRTLLS	LEDAVWASCG	PRVTVLEATT	LQPQQSFEAH	QDEAVSVTHM	VKAGSGVWMA	FSSGTSIRLF
**810**	**820**	**830**	**840**	**850**	**860**	**870**	**880**
HTETLEHLQE	INIATRTTFL	LPGQKHLCVT	SLLICQGLLW	VGTDQGVIVL	LPVPRLEGIP	KITGKGMVSL	NGHCGPVAFL
**890**	**900**	**910**	**920**	**930**	**940**	**950**	**960**
AVATSILAPD	ILRSDQEEAE	GPRAEEDKPD	GQAHEPMPDS	HVGRELTRKK	GILLQYRLRS	TAHLPGPLLS	MREPAPADGA
**970**	**980**	**990**	**1000**	**1010**	**1020**	**1030**	**1040**
ALEHSEEDGS	IYEMADDPDI	WVRSRPCARD	AHRKEICSVA	IISGGQGYRN	FGSALGSSGR	QAPCGETDST	LLIWQVPLML
**1050**							

Cmpd.	No. of Cmpds.	m/z meas.	Δ m/z [ppm]	z	Rt [min]	Score	P	Range	Sequence	Modification
2999	4	840.9055	-41.65	2	66.4	15.4	1	994-1009	R.KEICSVAIISGGQGYR.N	
63	1	518.2825	34.79	3	30.0	11.8	0	995-1009	K.EICSVAIISGGQGYR.N	

Detailed Protein Report

Protein 222: PREDICTED: putative GTP-binding protein 6 isoform X1 [Homo sapiens]

**Accession:**	gi|578837777	**Score:**	27.2
**Database:**	refseq_human(refseq_human_20140103.fasta)	**MW [kDa]:**	42.1
**Database Date:**	2015-11-30	**pI:**	10.2
**Modification(s):**	Carbamidomethyl	**Sequence Coverage [%]:**	7.2
		**No. of unique Peptides:**	1

Quantitation

WD:WU	**Median:** 0.84	**CV:** 0.00 %	**No. of Peptides:** 1

Alias proteins:

Accession	Name	Description
gi|578838996	refseq_human (refseq_human_20140103.fasta)	PREDICTED: putative GTP-binding protein 6 isoform X2 [Homo sapiens]

10	20	30	40	50	60	70	80
MVVSTKTPDR	KLIFGKGNFE	HLTEKIRGSP	DITCVFLNVE	RMAAPTKKEL	EAAWGVEVFD	RFTVVLHIFR	CNARTKEARL
**90**	**100**	**110**	**120**	**130**	**140**	**150**	**160**
QVALAEMPLH	RSNLKRDVAH	LYRGVGSRYI	MGSGESFMQL	QQRLLREKEA	KIRKALDRLR	KKRHLLRRQR	TRREFPVISV
**170**	**180**	**190**	**200**	**210**	**220**	**230**	**240**
VGYTNCGKTT	LIKALTGDAA	IQPRDQLFAT	LDVTAHAGTL	PSRMTVLYVD	TIGFLSQLPH	GLIESFSATL	EDVAHSDLIL
**250**	**260**	**270**	**280**	**290**	**300**	**310**	**320**
HVRDVSHPEA	ELQKCSVLST	LRGLQLPAPL	LDSMVEVHNK	VDLVPGYSPT	EPNVVPVSAL	RGHGLQELKA	ELDAAVLKAT
**330**	**340**	**350**	**360**	**370**	**380**		
GRQILTLRVR	LAGAQLSWLY	KEATVQEVDV	IPEDGAADVR	VIISNSAYGK	FRKLFPG		

Cmpd.	No. of Cmpds.	m/z meas.	Δ m/z [ppm]	z	Rt [min]	Score	P	Range	Sequence	Modification	Ratios
2802	1	913.4638	-3.45	2	63.6	10.3	1	153-168	R.REFPVISVVGYTNCGK.T	Carbamidomethyl: 14	WD:WU 0.84

Detailed Protein Report

Protein 223: poly [ADP-ribose] polymerase 8 isoform 2 [Homo sapiens]

**Accession:**	gi|295844832	**Score:**	27.2
**Database:**	refseq_human(refseq_human_20140103.fasta)	**MW [kDa]:**	91.1
**Database Date:**	2015-11-30	**pI:**	9.5
		**Sequence Coverage [%]:**	3.1
		**No. of unique Peptides:**	1

Quantitation

WD:WU **Median:** 1.25 **CV:** 0.00 % **No. of Peptides:** 1

10	20	30	40	50	60	70	80
MGMCSRQERI	QKDIDVVIQK	SRAEKDCLFA	DFRYSDSTFT	FTYVGGPRSV	SYSVHVSEDY	PDNTYVSSSE	NDEDVLVTTE
90	100	110	120	130	140	150	160
PIPVIFHRIA	TELRKTNDIN	CCLSIKSKLQ	KENGEESRQN	STVEEDSEGD	NDSEEFYYGG	QVNYDGELHK	HPQLEADLSA
170	180	190	200	210	220	230	240
VREIYGPHAV	SLREYGAIDD	VDIDLHIDVS	FLDEEIAVAW	EVIRTEPIIV	RLHCSLTQYL	NGPVPTVDVF	QISTKERFGL
250	260	270	280	290	300	310	320
GHQLKKIMQT	FVTQQWKQSK	EKSNCLHNKK	LSEKKVKSPL	HLFSTLRRSP	SYPPPGCGKS	KSKLKSEQDG	ISKTHKLLRR
330	340	350	360	370	380	390	400
TCSSTVKTDD	VCVTKSHRTF	GRSLSSDPRA	EQAMTAIKSH	KLLNRPCPAA	VKSEECLTLK	SHRLLTRSCS	GDPRCEHNTN
410	420	430	440	450	460	470	480
LKPHKLLSRS	YSSNLRMEEL	YGLKNHKLLS	KSYSSAPKSS	KTELFKEPNA	EGRRLSLTSG	LIGILTPSSS	SSSQLAPNGA
490	500	510	520	530	540	550	560
KCIPVRDRGF	LVQTIEFAEQ	RIPVLNEYCV	VCDEPHVFQN	GPMLRPTVCE	RELCVFAFQT	LGVMNEAADE	IATGAQKKNY
570	580	590	600	610	620	630	640
DRVMKALDSI	TSIREMTQAP	YLEIKKQMDK	QDPLAHPLLQ	WVISSNRSHI	VKLPVNRQLK	FMHTPHQFLL	LSSPPAKESN
650	660	670	680	690	700	710	720
FRAAKKLFGS	TFAFHGSHIE	NWHSILRNGL	VVASNTRLQL	HGAMYGSGIY	LSPMSSISFG	YSGMNKKQKV	SAKDEPASSS
730	740	750	760	770	780	790	800
KSSNTSQSQK	KGQQSQFLQS	RNLKCIALCE	VITSSDLHKH	GEIWVVPNTD	HVCTRFFFVY	EDGQVGDANI	NTQEGGIHKE
810	820						
ILRVIGNQTA	TG						

Cmpd.	No. of Cmpds.	m/z meas.	Δ m/z [ppm]	z	Rt [min]	Score	P	Range	Sequence	Modification	Ratios
1355	1	719.8571	2.88	2	45.3	12.6	1	96-108	K.TNDINCCLSIKSK.L		WD:WU 1.25

Detailed Protein Report

Protein 224: matrix metalloproteinase-27 precursor [Homo sapiens]

Accession: gi|73808268
Database: refseq_human(refseq_human_20140103.fasta)
Database Date: 2015-11-30
Modification(s): Carbamidomethyl

Score: 27.2
MW [kDa]: 59.0
pI: 9.4
Sequence Coverage [%]: 4.3
No. of unique Peptides: 2

10	20	30	40	50	60	70	80
MKRLLLLFLF	FITFSSAFPL	VRMTENEENM	QLAQAYLNQF	YSLEIEGNHL	VQSKNRSLID	DKIREMQAFF	GLTVTGKLDS
**90**	**100**	**110**	**120**	**130**	**140**	**150**	**160**
NTLEIMKTPR	CGVPDVGQYG	YTLPGWRKYN	LTYRIINYTP	DMARAAVDEA	IQEGLEVWSK	VTPLKFTKIS	KGIADIMIAF
**170**	**180**	**190**	**200**	**210**	**220**	**230**	**240**
RTRVHGRCPR	YFDGPLGVLG	HAFPPGPGLG	GDTHFDEDEN	WTKDGAGFNL	FLVAAHEFGH	ALGLSHSNDQ	TALMFPNYVS
**250**	**260**	**270**	**280**	**290**	**300**	**310**	**320**
LDPRKYPLSQ	DDINGIQSIY	GGLPKEPAKP	KEPTIPHACD	PDLTFDAITT	FRREVMFFKG	RHLWRIYYDI	TDVEFELIAS
**330**	**340**	**350**	**360**	**370**	**380**	**390**	**400**
FWPSLPADLQ	AAYENPRDKI	LVFKDENFWM	IRGYAVLPDY	PKSIHTLGFP	GRVKKIDAAV	CDKTTRKTYF	FVGIWCWRFD
**410**	**420**	**430**	**440**	**450**	**460**	**470**	**480**
EMTQTMDKGF	PQRVVKHFPG	ISIRVDAAFQ	YKGFFFFSRG	SKQFEYDIKT	KNITRIMRTN	TWFQCKEPKN	SSFGFDINKE
**490**	**500**	**510**	**520**				
KAHSGGIKIL	YHKSLSLFIF	GIVHLLKNTS	IYQ				

Cmpd.	No. of Cmpds.	m/z meas.	Δ m/z [ppm]	z	Rt [min]	Score	P	Range	Sequence	Modification
2670	1	625.3045	-14.98	2	61.7	12.8	1	376-386	K.IDAAVCDKTTR.K	Carbamidomethyl: 6
2792	4	626.8670	-20.54	2	63.5	14.4	1	414-424	R.VVKHFPGISIR.V	

Detailed Protein Report

Protein 225: **PREDICTED: U2 snRNP-associated SURP motif-containing protein isoform X3 [Homo sapiens]**

**Accession:**	gi|530374190	**Score:**	27.1
**Database:**	refseq_human(refseq_human_20140103.fasta)	**MW [kDa]:**	111.5
**Database Date:**	2015-11-30	**pI:**	6.5
**Modification(s):**	Oxidation	**Sequence Coverage [%]:**	2.1
		**No. of unique Peptides:**	2

10	20	30	40	50	60	70	80
MADKTPGGSQ	KASSKTRSSD	VHSSGSSDAH	MDASGPSDSD	MPSRTRPKSP	RKHNYRNESA	RESLCDSPHQ	NLSRPLLENK
**90**	**100**	**110**	**120**	**130**	**140**	**150**	**160**
LKAFSIGKMS	TAKRTLSKKE	QEELKKKEDE	KAAAEIYEEF	LAAFEGSDGN	KVKTFVRGGV	VNAAKEEHET	DEKRGKIYKP
**170**	**180**	**190**	**200**	**210**	**220**	**230**	**240**
SSRFADQKNP	PNQSSNERPP	SLLVIETKKP	PLKKGEKEKK	KSNLELFKEE	LKQIQEERDE	RHKTKGRLSR	FEPPQSDSDG
**250**	**260**	**270**	**280**	**290**	**300**	**310**	**320**
QRRSMDAPSR	RNRSSGVLDD	YAPGSHDVGD	PSTTNLYLGN	INPQMNEEML	CQEFGRFGPL	ASVKIMWPRT	DEERARERNC
**330**	**340**	**350**	**360**	**370**	**380**	**390**	**400**
GFVAFMNRRD	AERALKNLNG	KMIMSFEMKL	GWGKAVPIPP	HPIYIPPSMM	EHTLPPPPSG	LPFNAQPRER	LKNPNAPMLP
**410**	**420**	**430**	**440**	**450**	**460**	**470**	**480**
PPKNKEDFEK	TLSQAIVKVV	IPTERNLLAL	IHRMIEFVVR	EGPMFEAMIM	NREINNPMFR	FLFENQTPAH	VYYRWKLYSI
**490**	**500**	**510**	**520**	**530**	**540**	**550**	**560**
LQGDSPTKWR	TEDFRMFKNG	SFWRPPPLNP	YLHGMSEEQE	TEAFVEEPSK	KGALKEEQRD	KLEEILRGLT	PRKNDIGDAM
**570**	**580**	**590**	**600**	**610**	**620**	**630**	**640**
VFCLNNAEAA	EEIVDCITES	LSILKTPLPK	KIARLYLVSD	VLYNSSAKVA	NASYYRKFFE	TKLCQIFSDL	NATYRTIQGH
**650**	**660**	**670**	**680**	**690**	**700**	**710**	**720**
LQSENFKQRV	MTCFRAWEDW	AIYPEPFLIK	LQNIFLGLVN	IIEEKETEDV	PDDLDGAPIE	EELDGAPLED	VDGIPIDATP
**730**	**740**	**750**	**760**	**770**	**780**	**790**	**800**
IDDLDGVPIK	SLDDDLDGVP	LDATEDSKKN	EPIFKVAPSK	WEAVDESELE	AQAVTTSKWE	LFDQHEESEE	EENQNQEEES
**810**	**820**	**830**	**840**	**850**	**860**	**870**	**880**
EDEEDTQSSK	SEEHHLYSNP	IKEEMTESKF	SKYSEMSEEK	RAKLREIELK	VMKFQDELES	GKRPKKPGQS	FQEQVEHYRD
**890**	**900**	**910**	**920**	**930**	**940**	**950**	**960**
KLLQREKEKE	LERERERDKK	DKEKLESRSK	DKKEKDECTP	TRKERSPSGS	RTPKRSRRSR	SRSPKKSGKK	SRSQSRSPHR
**970**	**980**						
SHKKSKKNKH							

Cmpd.	No. of Cmpds.	m/z meas.	Δ m/z [ppm]	z	Rt [min]	Score	P	Range	Sequence	Modification
662	1	460.1225	-221.33	2	37.1	12.9	1	244-251	R.SMDAPSRR.N	
2921	1	729.3717	78.12	2	65.3	14.3	0	441-452	R.EGPMFEAMIMNR.E	Oxidation: 4, 10

Detailed Protein Report

Protein 226: nuclear receptor corepressor 1 isoform 1 [Homo sapiens]

**Accession:**	gi|22538461	**Score:**	27.1
**Database:**	refseq_human(refseq_human_20140103.fasta)	**MW [kDa]:**	270.0
**Database Date:**	2015-11-30	**pI:**	6.7
**Modification(s):**	Oxidation	**Sequence Coverage [%]:**	1.5
		**No. of unique Peptides:**	2

Quantitation

WD:WU	**Median:** 0.92	**CV:** 0.00 %	**No. of Peptides:** 1

Detailed Protein Report

10	20	30	40	50	60	70	80
MSSSGYPPNQ	GAFSTEQSRY	PPHSVQYTFP	NTRHQQEFAV	PDYRSSHLEV	SQASQLLQQQ	QQQQLRRRPS	LLSEFHPGSD
90	100	110	120	130	140	150	160
RPQERRTSYE	PFHPGPSPVD	HDSLESKRPR	LEQVSDSHFQ	RVSAAVLPLV	HPLPEGLRAS	ADAKKDPAFG	GKHEAPSSPI
170	180	190	200	210	220	230	240
SGQPCGDDQN	ASPSKLSKEE	LIQSMDRVDR	EIAKVEQQIL	KLKKKQQQLE	EEAAKPPEPE	KPVSPPPVEQ	KHRSIVQIIY
250	260	270	280	290	300	310	320
DENRKKAEEA	HKIFEGLGPK	VELPLYNQPS	DTKVYHENIK	TNQVMRKKLI	LFFKRRNHAR	KQREQKICQR	YDQLMEAWEK
330	340	350	360	370	380	390	400
KVDRIENNPR	RKAKESKTRE	YYEKQFPEIR	KQREQQERFQ	RVGQRGAGLS	ATIARSEHEI	SEIIDGLSEQ	ENNEKQMRQL
410	420	430	440	450	460	470	480
SVIPPMMFDA	EQRRVKFINM	NGLMEDPMKV	YKDRQFMNVW	TDHEKEIFKD	KFIQHPKNFG	LIASYLERKS	VPDCVLYYYL
490	500	510	520	530	540	550	560
TKKNENYKAL	VRRNYGKRRG	RNQQIARPSQ	EEKVEEKEED	KAEKTEKKEE	EKKDEEEKDE	KEDSKENTKE	KDKIDGTAEE
570	580	590	600	610	620	630	640
TEEREQATPR	GRKTANSQGR	RKGRITRSMT	NEAAAASAAA	AAATEEPPPP	LPPPPEPIST	EPVETSRWTE	EEMEVAKKGL
650	660	670	680	690	700	710	720
VEHGRNWAAI	AKMVGTKSEA	QCKNFYFNYK	RRHNLDNLLQ	QHKQKTSRKP	REERDVSQCE	SVASTVSAQE	DEDIEASNEE
730	740	750	760	770	780	790	800
ENPEDSEVEA	VKPSEDSPEN	ATSRGNTEPA	VELEPTTETA	PSTSPSLAVP	STKPAEDESV	ETQVNDSISA	ETAEQMDVDQ
810	820	830	840	850	860	870	880
QEHSAEEGSV	CDPPPATKAD	SVDVEVRVPE	NHASKVEGDN	TKERDLDRAS	EKVEPRDEDL	VVAQQINAQR	PEPQSDNDSS
890	900	910	920	930	940	950	960
ATCSADEDVD	GEPERQRMFP	MDSKPSLLNP	TGSILVSSPL	KPNPLDLPQL	QHRAAVIPPM	VSCTPCNIPI	GTPVSGYALY
970	980	990	1000	1010	1020	1030	1040
QRHIKAMHES	ALLEEQRQRQ	EQIDLECRSS	TSPCGTSKSP	NREWEVLQPA	PHQVITNLPE	GVRLPTTRPT	RPPPPLIPSS
1050	1060	1070	1080	1090	1100	1110	1120
KTTVASEKPS	FIMGGSISQG	TPGTYLTSHN	QASYTQETPK	PSVGSISLGL	PRQQESAKSA	TLPYIKQEEF	SPRSQNSQPE
1130	1140	1150	1160	1170	1180	1190	1200
GLLVRAQHEG	VVRGTAGAIQ	EGSITRGTPT	SKISVESIPS	LRGSITQGTP	ALPQTGIPTE	ALVKGSISRM	PIEDSSPEKG
1210	1220	1230	1240	1250	1260	1270	1280
REEAASKGHV	IYEGKSGHIL	SYDNIKNARE	GTRSPRTAHE	ISLKRSYESV	EGNIKQGMSM	RESPVSAPLE	GLICRALPRG
1290	1300	1310	1320	1330	1340	1350	1360
SPHSDLKERT	VLSGSIMQGT	PRATTESFED	GLKYPKQIKR	ESPPIRAFEG	AITKGKPYDG	ITTIKEMGRS	IHEIPRQDIL
1370	1380	1390	1400	1410	1420	1430	1440
TQESRKTPEV	VQSTRPIIEG	SISQGTPIKF	DNNSGQSAIK	HNVKSLITGP	SKLSRGMPPL	EIVPENIKVV	ERGKYEDVKA
1450	1460	1470	1480	1490	1500	1510	1520
GETVRSRHTS	VVSSGPSVLR	STLHEAPKAQ	LSPGIYDDTS	ARRTPVSYQN	TMSRGSPMMN	RTSDVTISSN	KSTNHERKST
1530	1540	1550	1560	1570	1580	1590	1600
LTPTQRESIP	AKSPVPGVDP	VVSHSPFDPH	HRGSTAGEVY	RSHLPTHLDP	AMPFHRALDP	AAAAYLFQRQ	LSPTPGYPSQ
1610	1620	1630	1640	1650	1660	1670	1680
YQLYAMENTR	QTILNDYITS	QQMQVNLRPD	VARGLSPREQ	PLGLPYPATR	GIIDLTNMPP	TILVPHPGGT	STPPMDRITY
1690	1700	1710	1720	1730	1740	1750	1760
IPGTQITFPP	RPYNSASMSP	GHPTHLAAAA	SAEREREREr	EKERERERIA	AASSDLYLRP	GSEQPGRPGS	HGYVRSPSPS
1770	1780	1790	1800	1810	1820	1830	1840
VRTQETMLQQ	RPSVFQGTNG	TSVITPLDPT	AQLRIMPLPA	GGPSISQGLP	ASRYNTAADA	LAALVDAAAS	APQMDVSKTK
1850	1860	1870	1880	1890	1900	1910	1920
ESKHEAARLE	ENLRSRSAAV	SEQQQLEQKT	LEVEKRSVQC	LYTSSAFPSG	KPQPHSSVVY	SEAGKDKGPP	PKSRYEEELR
1930	1940	1950	1960	1970	1980	1990	2000
TRGKTTITAA	NFIDVIITRQ	IASDKDARER	GSQSSDSSSS	LSSHRYETPS	DAIEVISPAS	SPAPPQEKLQ	TYQPEVVKAN
2010	2020	2030	2040	2050	2060	2070	2080
QAENDPTRQY	EGPLHHYRPQ	QESPSPQQQL	PPSSQAEGMG	QVPRTHRLIT	LADHICQIIT	QDFARNQVSS	QTPQQPPTST
2090	2100	2110	2120	2130	2140	2150	2160
FQNSPSALVS	TPVRTKTSNR	YSPESQAQSV	HHQRPGSRVS	PENLVDKSRG	SRPGKSPERS	HVSSEPYEPI	SPPQVPVVHE
2170	2180	2190	2200	2210	2220	2230	2240

Detailed Protein Report

Cmpd.	No. of Cmpds.	m/z meas.	Δ m/z [ppm]	z	Rt [min]	Score	P	Range	Sequence	Modification	Ratios
2870	1	973.5098	46.41	2	64.6	11.5	1	417-432	K.FINMNGLMEDPMKVYK.D	Oxidation: 8	
41	3	1068.4648	-20.43	2	29.7	15.6	2	1946-1965	K.DARERGSQSSDSSSSLSSHR.Y		WD:WU 0.92

Detailed Protein Report

Protein 227: PREDICTED: thrombopoietin isoform X2 [Homo sapiens]

**Accession:**	gi|578807693	**Score:**	27.0
**Database:**	refseq_human(refseq_human_20140103.fasta)	**MW [kDa]:**	56.2
**Database Date:**	2015-11-30	**pI:**	10.3
		**Sequence Coverage [%]:**	1.5
		**No. of unique Peptides:**	1

Quantitation

WD:WU	**Median:** 1.07	**CV:** 0.00 %	**No. of Peptides:** 1

Alias proteins:

Accession	Name	Description
gi|578807695	refseq_human (refseq_human_20140103.fasta)	PREDICTED: thrombopoietin isoform X3 [Homo sapiens]

10	20	30	40	50	60	70	80
MSCQIPPGKK	LLLLSPSRSQ	VAHVQEKMDP	PIQIFSVVCV	GGGVDPGPGR	GSREEKASLP	GAFTSVWWLP	SLIGQKWPRQ
**90**	**100**	**110**	**120**	**130**	**140**	**150**	**160**
AYDLLLWRGC	APPPHVFLPI	CSPEGCLLCT	WVLEPFSTRI	DSSPLARLCP	TLLCPEVQEP	KPPPWPQEGF	RGEAPNREPR
**170**	**180**	**190**	**200**	**210**	**220**	**230**	**240**
QPDTPARMEL	TELLLVVMLL	LTARLTLSSP	APPACDLRVL	SKLLRDSHVL	HSRLSQCPEV	HPLPTPVLLP	AVDFSLGEWK
**250**	**260**	**270**	**280**	**290**	**300**	**310**	**320**
TQMEETKAQD	ILGAVTLLLE	GVMAARGQLG	PTCLSSLLGQ	LSGQVRLLLG	ALQSLLGTQL	PPQGRTTAHK	DPNAIFLSFQ
**330**	**340**	**350**	**360**	**370**	**380**	**390**	**400**
HLLRGKVRFL	MLVGGSTLCV	RRAPPTTAVP	SRTSLVLTLN	ELPNRTSGLL	ETNFTASART	TGSGLLKWQQ	GFRAKIPGLL
**410**	**420**	**430**	**440**	**450**	**460**	**470**	**480**
NQTSRSLDQI	PGYLNRIHEL	LNGTRGLFPG	PSRRTLGAPD	ISSGTSDTGS	LPPNLQPGYS	PSPTHPPTGQ	YTLFPLPPTL
**490**	**500**	**510**	**520**	**530**			
PTPVVQLHPL	LPDPSAPTPT	PTSPLLNTSY	THSQNLSQEG				

Cmpd.	No. of Cmpds.	m/z meas.	Δ m/z [ppm]	z	Rt [min]	Score	P	Range	Sequence	Modification	Ratios
2610	1	449.6838	-235.36	2	60.9	27.0	0	11-18	K.LLLLSPSR.S		WD:WU 1.07

Detailed Protein Report

Protein 228: hemicentin-1 precursor [Homo sapiens]

**Accession:**	gi|118572606	**Score:**	27.0
**Database:**	refseq_human(refseq_human_20140103.fasta)	**MW [kDa]:**	613.0
**Database Date:**	2015-11-30	**pI:**	6.1
**Modification(s):**	Carbamidomethyl	**Sequence Coverage [%]:**	0.6
		**No. of unique Peptides:**	1

Detailed Protein Report

10	20	30	40	50	60	70	80
MISWEVVHTV	FLFALLYSSL	AQDASPQSEI	RAEEIPEGAS	TLAFVFDVTG	SMYDDLVQVI	EGASKILETS	LKRPKRPLFN
90	100	110	120	130	140	150	160
FALVPFHDPE	IGPVTITTDP	KKFQYELREL	YVQGGGDCPE	MSIGAIKIAL	EISLPGSFIY	VFTDARSKDY	RLTHEVLQLI
170	180	190	200	210	220	230	240
QQKQSQVVFV	LTGDCDDRTH	IGYKVYEEIA	STSSGQVFHL	DKKQVNEVLK	WVEEAVQASK	VHLLSTDHLE	QAVNTWRIPF
250	260	270	280	290	300	310	320
DPSLKEVTVS	LSGPSPMIEI	RNPLGKLIKK	GFGLHELLNI	HNSAKVVNVK	EPEAGMWTVK	TSSSGRHSVR	ITGLSTIDFR
330	340	350	360	370	380	390	400
AGFSRKPTLD	FKKTVSRPVQ	GIPTYVLLNT	SGISTPARID	LLELLSISGS	SLKTIPVKYY	PHRKPYGIWN	ISDFVPPNEA
410	420	430	440	450	460	470	480
FFLKVTGYDK	DDYLFQRVSS	VSFSSIVPDA	PKVTMPEKTP	GYYLQPGQIP	CSVDSLLPFT	LSFVRNGVTL	GVDQYLKESA
490	500	510	520	530	540	550	560
SVNLDIAKVT	LSDEGFYECI	AVSSAGTGRA	QTFFDVSEPP	PVIQVPNNVT	VTPGERAVLT	CLIISAVDYN	LTWQRNDRDV
570	580	590	600	610	620	630	640
RLAEPARIRT	LANLSLELKS	VKFNDAGEYH	CMVSSEGGSS	AASVFLTVQE	PPKVTVMPKN	QSFTGGSEVS	IMCSATGYPK
650	660	670	680	690	700	710	720
PKIAWTVNDM	FIVGSHRYRM	TSDGTLFIKN	AAPKDAGIYG	CLASNSAGTD	KQNSTLRYIE	APKLMVVQSE	LLVALGDITV
730	740	750	760	770	780	790	800
MECKTSGIPP	PQVKWFKGDL	ELRPSTFLII	DPLLGLLKIQ	ETQDLDAGDY	TCVAINEAGR	ATGKITLDVG	SPPVFIQEPA
810	820	830	840	850	860	870	880
DVSMEIGSNV	TLPCYVQGYP	EPTIKWRRLD	NMPIFSRPFS	VSSISQLRTG	ALFILNLWAS	DKGTYICEAE	NQFGKIQSET
890	900	910	920	930	940	950	960
TVTVTGLVAP	LIGISPSVAN	VIEGQQLTLP	CTLLAGNPIP	ERRWIKNSAM	LLQNPYITVR	SDGSLHIERV	QLQDGGEYTC
970	980	990	1000	1010	1020	1030	1040
VASNVAGTNN	KTTSVVVHVL	PTIQHGQQIL	STIEGIPVTL	PCKASGNPKP	SVIWSKKGEL	ISTSSAKFSA	GADGSLYVVS
1050	1060	1070	1080	1090	1100	1110	1120
PGGEESGEYV	CTATNTAGYA	KRKVQLTVYV	RPRVFGDQRG	LSQDKPVEIS	VLAGEEVTLP	CEVKSLPPPI	ITWAKETQLI
1130	1140	1150	1160	1170	1180	1190	1200
SPFSPRHTFL	PSGSMKITET	RTSDSGMYLC	VATNIAGNVT	QAVKLNVHVP	PKIQRGPKHL	KVQVGQRVDI	PCNAQGTPLP
1210	1220	1230	1240	1250	1260	1270	1280
VITWSKGGST	MLVDGEHHVS	NPDGTLSIDQ	ATPSDAGIYT	CVATNIAGTD	ETEITLHVQE	PPTVEDLEPP	YNTTFQERVA
1290	1300	1310	1320	1330	1340	1350	1360
NQRIEFPCPA	KGTPKPTIKW	LHNGRELTGR	EPGISILEDG	TLLVIASVTP	YDNGEYICVA	VNEAGTTERK	YNLKVHVPPV
1370	1380	1390	1400	1410	1420	1430	1440
IKDKEQVTNV	SVLLNQLTNL	FCEVEGTPSP	IIMWYKDNVQ	VTESSTIQTV	NNGKILKLFR	ATPEDAGRYS	CKAINIAGTS
1450	1460	1470	1480	1490	1500	1510	1520
QKYFNIDVLV	PPTIIGTNFP	NEVSVVLNRD	VALECQVKGT	PFPDIHWFKD	GKPLFLGDPN	VELLDRGQVL	HLKNARRNDK
1530	1540	1550	1560	1570	1580	1590	1600
GRYQCTVSNA	AGKQAKDIKL	TIYIPPSIKG	GNVTTDISVL	INSLIKLECE	TRGLPMPAIT	WYKDGQPIMS	SSQALYIDKG
1610	1620	1630	1640	1650	1660	1670	1680
QYLHIPRAQV	SDSATYTCHV	ANVAGTAEKS	FHVDVYVPPM	IEGNLATPLN	KQVVIAHSLT	LECKAAGNPS	PILTWLKDGV
1690	1700	1710	1720	1730	1740	1750	1760
PVKANDNIRI	EAGGKKLEIM	SAQEIDRGQY	ICVATSVAGE	KEIKYEVDVL	VPPAIEGGDE	TSYFIVMVNN	LLELDCHVTG
1770	1780	1790	1800	1810	1820	1830	1840
SPPPTIMWLK	DGQLIDERDG	FKILLNGRKL	VIAQAQVSNT	GLYRCMAANT	AGDHKKEFEV	TVHVPPTIKS	SGLSERVVVK
1850	1860	1870	1880	1890	1900	1910	1920
YKPVALQCIA	NGIPNPSITW	LKDDQPVNTA	QGNLKIQSSG	RVLQIAKTLL	EDAGRYTCVA	TNAAGETQQH	IQLHVHEPPS
1930	1940	1950	1960	1970	1980	1990	2000
LEDAGKMLNE	TVLVSNPVQL	ECKAAGNPVP	VITWYKDNRL	LSGSTSMTFL	NRGQIIDIES	AQISDAGIYK	CVAINSAGAT
2010	2020	2030	2040	2050	2060	2070	2080
ELFYSLQVHV	APSISGSNNM	VAVVVNNPVR	LECEARGIPA	PSLTWLKDGS	PVSSFSNGLQ	VLSGGRILAL	TSAQISDTGR
2090	2100	2110	2120	2130	2140	2150	2160
YTCVAVNAAG	EKQRDIDLRV	YVPPNIMGEE	QNVSVLISQA	VELLCQSDAI	PPPTLTWLKD	GHPLLKKPGL	SISENRSVLK
2170	2180	2190	2200	2210	2220	2230	2240

Detailed Protein Report

Cmpd.	No. of Cmpds.	m/z meas.	Δ m/z [ppm]	z	Rt [min]	Score	P	Range	Sequence	Modification
1984	1	883.9757	-72.13	3	53.5	15.0	1	5183-5205	R.RTSDGLSCQDINECQESSPCHQR.C	Carbamidomethyl: 8

Detailed Protein Report

Protein 229: N-acetyl-D-glucosamine kinase [Homo sapiens]

**Accession:**	gi|295844834	**Score:**	27.0
**Database:**	refseq_human(refseq_human_20140103.fasta)	**MW [kDa]:**	42.0
**Database Date:**	2015-11-30	**pI:**	6.2
		**Sequence Coverage [%]:**	6.4
		**No. of unique Peptides:**	1

10	20	30	40	50	60	70	80
MRTRTGSQLA	AREVTGSGAV	PRQLEGRRCQ	AGRDANGGTS	SDGSSSMAAI	YGGVEGGGTR	SEVLLVSEDG	KILAEADGLS
**90**	**100**	**110**	**120**	**130**	**140**	**150**	**160**
TNHWLIGTDK	CVERINEMVN	RAKRKAGVDP	LVPLRSLGLS	LSGGDQEDAG	RILIEELRDR	FPYLSESYLI	TTDAAGSIAT
**170**	**180**	**190**	**200**	**210**	**220**	**230**	**240**
ATPDGGVVLI	SGTGSNCRLI	NPDGSESGCG	GWGHMMGDEG	SAYWIAHQAV	KIVFDSIDNL	EAAPHDIGYV	KQAMFHYFQV
**250**	**260**	**270**	**280**	**290**	**300**	**310**	**320**
PDRLGILTHL	YRDFDKCRFA	GFCRKIAEGA	QQGDPLSRYI	FRKAGEMLGR	HIVAVLPEID	PVLFQGKIGL	PILCVGSVWK
**330**	**340**	**350**	**360**	**370**	**380**	**390**	**400**
SWELLKEGFL	LALTQGREIQ	AQNFFSSFTL	MKLRHSSALG	GASLGARHIG	HLLPMDYSAN	AIAFYSYTFS	

Cmpd.	No. of Cmpds.	m/z meas.	Δ m/z [ppm]	z	Rt [min]	Score	P	Range	Sequence	Modification
1617	1	781.3954	24.46	2	48.5	14.0	0	116-131	R.SLGLSLSGGDQEDAGR.I	

Detailed Protein Report

Protein 230: PREDICTED: UPF0501 protein KIAA1430 isoform X2 [Homo sapiens]

**Accession:**	gi|530378018	**Score:**	26.9
**Database:**	refseq_human(refseq_human_20140103.fasta)	**MW [kDa]:**	50.5
**Database Date:**	2015-11-30	**pI:**	7.9
		**Sequence Coverage [%]:**	4.9
		**No. of unique Peptides:**	1

10	20	30	40	50	60	70	80
MDQFGDILEG	EVDHSFFDSD	FEEGKKCETN	SVFDKQNDDP	KERIDKDTKN	VNSNTGMQTT	ENYLTEKGNE	RNVKFPPEHP
**90**	**100**	**110**	**120**	**130**	**140**	**150**	**160**
VENDVTQTVS	SFSLPASSRS	KKLCDVTTGL	KIHVSIPNRI	PKIVKEGEDD	YYTDGEESSD	DGKKYHVKSK	SAKPSTNVKK
**170**	**180**	**190**	**200**	**210**	**220**	**230**	**240**
SIRKKYCKVS	SSSSSSLSSS	SSGSGTDCLD	AGSDSHLSDS	SPSSKSSKKH	VSGITLLSPK	HKYKSGIKST	ETQPSSTTPK
**250**	**260**	**270**	**280**	**290**	**300**	**310**	**320**
CGHYPEESED	TVTDVSPLST	PDISPLQSFE	LGIANDQKVK	IKKQENVSQE	IYEDVEDLKN	NSKYLKAAKK	GKEKHEPDVS
**330**	**340**	**350**	**360**	**370**	**380**	**390**	**400**
SKSSSVLDSS	LDHRHKQKVL	HDTMDLNHLL	KAFLQLDKKG	PQKHHFDQPS	VAPGKNYSFT	REEVRQIDRE	NQRLLKELSR
**410**	**420**	**430**	**440**	**450**	**460**		
QAEKPGSKST	IPRSADHPPK	LYHSALNRQK	EQQRIERENL	TISLCHPGWS	AVT		

Cmpd.	No. of Cmpds.	m/z meas.	Δ m/z [ppm]	z	Rt [min]	Score	P	Range	Sequence	Modification
2833	1	651.8526	53.29	2	63.9	14.8	0	323-334	K.SSSVLDSSLDHR.H	

Detailed Protein Report

Protein 231: catenin alpha-2 isoform 2 [Homo sapiens]

**Accession:**	gi|259013216	**Score:**	26.9
**Database:**	refseq_human(refseq_human_20140103.fasta)	**MW [kDa]:**	95.5
**Database Date:**	2015-11-30	**pI:**	5.6
		**Sequence Coverage [%]:**	2.4
		**No. of unique Peptides:**	1

Quantitation

WD:WU **Median:** 7.25 **CV:** 0.00 % **No. of Peptides:** 1

```
        10         20         30         40         50         60         70         80
MTSATSPIIL KWDPKSLEIR TLTVERLLEP LVTQVTTLVN TSNKGPSGKK KGRSKKAHVL AASVEQATQN FLEKGEQIAK
        90        100        110        120        130        140        150        160
ESQDLKEELV AAVEDVRKQG ETMRIASSEF ADDPCSSVKR GTMVRAARAL LSAVTRLLIL ADMADVMRLL SHLKIVEEAL
       170        180        190        200        210        220        230        240
EAVKNATNEQ DLANRFKEFG KEMVKLNYVA ARRQQELKDP HCRDEMAAAR GALKKNATML YTASQAFLRH PDVAATRANR
       250        260        270        280        290        300        310        320
DYVFKQVQEA IAGISNAAQA TSPTDEAKGH TGIGELAAAL NEFDNKIILD PMTFSEARFR PSLEERLESI ISGAALMADS
       330        340        350        360        370        380        390        400
SCTRDDRRER IVAECNAVRQ ALQDLLSEYM NNTGRKEKGD PLNIAIDKMT KKTRDLRRQL RKAVMDHISD SFLETNVPLL
       410        420        430        440        450        460        470        480
VLIEAAKSGN EKEVKEYAQV FREHANKLVE VANLACSISN NEEGVKLVRM AATQIDSLCP QVINAALTLA ARPQSKVAQD
       490        500        510        520        530        540        550        560
NMDVFKDQWE KQVRVLTEAV DDITSVDDFL SVSENHILED VNKCVIALQE GDVDTLDRTA GAIRGRAARV IHIINAEMEN
       570        580        590        600        610        620        630        640
YEAGVYTEKV LEATKLLSET VMPRFAEQVE VAIEALSANV PQPFEENEFI DASRLVYDGV RDIRKAVLMI RTPEELEDDS
       650        660        670        680        690        700        710        720
DFEQEDYDVR SRTSVQTEDD QLIAGQSARA IMAQLPQEEK AKIAEQVEIF HQEKSKLDAE VAKWDDSGND IIVLAKQMCM
       730        740        750        760        770        780        790        800
IMMEMTDFTR GKGPLKNTSD VINAAKKIAE AGSRMDKLAR AVADQLDSAT SLIQAAKNLM NAVVLTVKAS YVASTKYQKV
       810        820        830        840        850        860        870
YGTAAVNSPV VSWKMKAPEK KPLVKREKPE EFQTRVRRGS QKKHISPVQA LSEFKAMDSF
```

Cmpd.	No. of Cmpds.	m/z meas.	Δ m/z [ppm]	z	Rt [min]	Score	P	Range	Sequence	Modification	Ratios
747	1	516.6531	-225.60	2	37.8	14.1	0	737-746	K.NTSDVINAAK.K		WD:WU 7.25

Detailed Protein Report

Protein 232: protein diaphanous homolog 2 isoform 12C [Homo sapiens]

**Accession:**	gi|6382071	**Score:**	26.9
**Database:**	refseq_human(refseq_human_20140103.fasta)	**MW [kDa]:**	124.7
**Database Date:**	2015-11-30	**pI:**	6.0
		**Sequence Coverage [%]:**	3.2
		**No. of unique Peptides:**	1

10	20	30	40	50	60	70	80
MEQPGAAASG	AGGGSEEPGG	GRSNKRSAGN	RAANEEETKN	KPKLNIQIKT	LADDVRDRIT	SFRKSTVKKE	KPLIQHPIDS
**90**	**100**	**110**	**120**	**130**	**140**	**150**	**160**
QVAMSEFPAA	QPLYDERSLN	LSEKEVLDLF	EKMMEDMNLN	EEKKAPLRNK	DFTTKREMVV	QYISATAKSG	GLKNSKHECT
**170**	**180**	**190**	**200**	**210**	**220**	**230**	**240**
LSSQEYVHEL	RSGISDEKLL	NCLESLRVSL	TSNPVSWVNN	FGHEGLGLLL	DELEKLLDKK	QQENIDKKNQ	YKLIQCLKAF
**250**	**260**	**270**	**280**	**290**	**300**	**310**	**320**
MNNKFGLQRI	LGDERSLLLL	ARAIDPKQPN	MMTEIVKILS	AICIVGEENI	LDKLLGAITT	AAERNNRERF	SPIVEGLENQ
**330**	**340**	**350**	**360**	**370**	**380**	**390**	**400**
EALQLQVACM	QFINALVTSP	YELDFRIHLR	NEFLRSGLKT	MLPDLKEKEN	DELDIQLKVF	DENKEDDLTE	LSHRLNDIRA
**410**	**420**	**430**	**440**	**450**	**460**	**470**	**480**
EMDDMNEVYH	LLYNMLKDTA	AENYFLSILQ	HFLLIRNDYY	IRPQYYKIIE	ECVSQIVLHC	SGMDPDFKYR	QRLDIDLTHL
**490**	**500**	**510**	**520**	**530**	**540**	**550**	**560**
IDSCVNKAKV	EESEQKAAEF	SKKFDEEFTA	RQEAQAELQK	RDEKIKELEA	EIQQLRTQAQ	VLSSSSGIPG	PPAAPPLPGV
**570**	**580**	**590**	**600**	**610**	**620**	**630**	**640**
GPPPPPPAPP	LPGGAPLPPP	PPPLPGMMGI	PPPPPPPLLF	GGPPPPPPLG	GVPPPPGISL	NLPYGMKQKK	MYKPEVSMKR
**650**	**660**	**670**	**680**	**690**	**700**	**710**	**720**
INWSKIEPTE	LSENCFWLRV	KEDKFENPDL	FAKLALNFAT	QIKVQKNAEA	LEEKKTGPTK	KKVKELRILD	PKTAQNLSIF
**730**	**740**	**750**	**760**	**770**	**780**	**790**	**800**
LGSYRMPYED	IRNVILEVNE	DMLSEALIQN	LVKHLPEQKI	LNELAELKNE	YDDLCEPEQF	GVVMSSVKML	QPRLSSILFK
**810**	**820**	**830**	**840**	**850**	**860**	**870**	**880**
LTFEEHINNI	KPSIIAVTLA	CEELKKSESF	NRLLELVLLV	GNYMNSGSRN	AQSLGFKINF	LCKIRDTKSA	DQKTTLLHFI
**890**	**900**	**910**	**920**	**930**	**940**	**950**	**960**
ADICEEKYRD	ILKFPEELEH	VESASKVSAQ	ILKSNLASME	QQIVHLERDI	KKFPQAENQH	DKFVEKMTSF	TKTAREQYEK
**970**	**980**	**990**	**1000**	**1010**	**1020**	**1030**	**1040**
LSTMHNNMMK	LYENLGEYFI	FDSKTVSIEE	FFGDLNNFRT	LFLEAVRENN	KRREMEEKTR	RAKLAKEKAE	QEKLERQKKK
**1050**	**1060**	**1070**	**1080**	**1090**	**1100**		
KQLIDINKEG	DETGVMDNLL	EALQSGAAFR	DRRKRIPRNP	VVNHPCATRA	NPRSAT		

Cmpd.	No. of Cmpds.	m/z meas.	Δ m/z [ppm]	z	Rt [min]	Score	P	Range	Sequence	Modification
2360	1	1151.7502	128.51	2	57.8	15.9	2	469-487	K.YRQRLDIDLTHLIDSCVNK.A	

Detailed Protein Report

Protein 233: proteoglycan 4 isoform D precursor [Homo sapiens]

Accession: gi|189181724
Database: refseq_human(refseq_human_20140103.fasta)
Database Date: 2015-11-30

Score: 26.8
MW [kDa]: 136.5
pI: 10.1
Sequence Coverage [%]: 1.4
No. of unique Peptides: 2

Quantitation

WD:WU **Median:** 2.06 **CV:** 9.19 % **No. of Peptides:** 2

10	20	30	40	50	60	70	80
MAWKTLPIYL	LLLLSVFVIQ	QVSSQELSCK	GRCFESFERG	RECDCDAQCK	KYDKCCPDYE	SFCAEVKDNK	KNRTKKKPTP
**90**	**100**	**110**	**120**	**130**	**140**	**150**	**160**
KPPVVDEAGS	GLDNGDFKVT	TPDTSTTQHN	KVSTSPKITT	AKPINPRPSL	PPNSDTSKET	SLTVNKETTV	ETKETTTTNK
**170**	**180**	**190**	**200**	**210**	**220**	**230**	**240**
QTSTDGKEKT	TSAKETQSIE	KTSAKDLAPT	SKVLAKPTPK	AETTTKGPAL	TTPKEPTPTT	PKEPASTTPK	EPTPTTIKSA
**250**	**260**	**270**	**280**	**290**	**300**	**310**	**320**
PTTPKEPAPT	TTKSAPTTPK	EPAPTTTKEP	APTTPKEPAP	TTTKEPAPTT	TKSAPTTPKE	PAPTTPKKPA	PTTPKEPAPT
**330**	**340**	**350**	**360**	**370**	**380**	**390**	**400**
TPKEPTPTTP	KEPAPTTKEP	APTTPKEPAP	TAPKKPAPTT	PKEPAPTTPK	EPAPTTTKEP	SPTTPKEPAP	TTTKSAPTTT
**410**	**420**	**430**	**440**	**450**	**460**	**470**	**480**
KEPAPTTTKS	APTTPKEPSP	TTTKEPAPTT	PKEPAPTTPK	KPAPTTPKEP	APTTPKEPAP	TTTKKPAPTT	PKEPAPTTPK
**490**	**500**	**510**	**520**	**530**	**540**	**550**	**560**
ETAPTTPKKL	TPTTPEKLAP	TTPEKPAPTT	PEELAPTTPE	EPTPTTPEEP	APTTPKAAAP	NTPKEPAPTT	PKEPAPTTPK
**570**	**580**	**590**	**600**	**610**	**620**	**630**	**640**
EPAPTTPKET	APTTPKGTAP	TTLKEPAPTT	PKKPAPKELA	PTTTKEPTST	TCDKPAPTTP	KGTAPTTPKE	PAPTTPKEPA
**650**	**660**	**670**	**680**	**690**	**700**	**710**	**720**
PTTPKGTAPT	TLKEPAPTTP	KKPAPKELAP	TTTKGPTSTT	SDKPAPTTPK	ETAPTTPKEP	APTTPKKPAP	TTPETPPPTT
**730**	**740**	**750**	**760**	**770**	**780**	**790**	**800**
SEVSTPTTTK	EPTTIHKSPD	ESTPELSAEP	TPKALENSPK	EPGVPTTKTP	AATKPEMTTT	AKDKTTERDL	RTTPETTTAA
**810**	**820**	**830**	**840**	**850**	**860**	**870**	**880**
PKMTKETATT	TEKTTESKIT	ATTTQVTSTT	TQDTTPFKIT	TLKTTTLAPK	VTTTKKTITT	TEIMNKPEET	AKPKDRATNS
**890**	**900**	**910**	**920**	**930**	**940**	**950**	**960**
KATTPKPQKP	TKAPKKPTST	KKPKTMPRVR	KPKTTPTPRK	MTSTMPELNP	TSRIAEAMLQ	TTTRPNQTPN	SKLVEVNPKS
**970**	**980**	**990**	**1000**	**1010**	**1020**	**1030**	**1040**
EDAGGAEGET	PHMLLRPHVF	MPEVTPDMDY	LPRVPNQGII	INPMLSDETN	ICNGKPVDGL	TTLRNGTLVA	FRGHYFWMLS
**1050**	**1060**	**1070**	**1080**	**1090**	**1100**	**1110**	**1120**
PFSPPSPARR	ITEVWGIPSP	IDTVFTRCNC	EGKTFFFKDS	QYWRFTNDIK	DAGYPKPIFK	GFGGLTGQIV	AALSTAKYKN
**1130**	**1140**	**1150**	**1160**	**1170**	**1180**	**1190**	**1200**
WPESVYFFKR	GGSIQQYIYK	QEPVQKCPGR	RPALNYPVYG	ETTQVRRRRF	ERAIGPSQTH	TIRIQYSPAR	LAYQDKGVLH
**1210**	**1220**	**1230**	**1240**	**1250**	**1260**	**1270**	**1280**
NEVKVSILWR	GLPNVVTSAI	SLPNIRKPDG	YDYYAFSKDQ	YYNIDVPSRT	ARAITTRSGQ	TLSKVWYNCP	

Cmpd.	No. of Cmpds.	m/z meas.	Δ m/z [ppm]	z	Rt [min]	Score	P	Range	Sequence	Modification	Ratios
2386	1	994.9816	-34.55	2	58.2	14.8	2	785-802	K.TTERDLRTTPETTTAAPK.M		WD:WU 1.88
285	2	559.1440	-261.71	2	32.7	12.0	0	792-802	R.TTPETTTAAPK.M		WD:WU 2.25

Detailed Protein Report

Protein 234: long-chain fatty acid transport protein 6 [Homo sapiens]

**Accession:**	gi|13325055	**Score:**	26.8
**Database:**	refseq_human(refseq_human_20140103.fasta)	**MW [kDa]:**	70.1
**Database Date:**	2015-11-30	**pI:**	9.5
		**Sequence Coverage [%]:**	3.6
		**No. of unique Peptides:**	1

Alias proteins:

Accession	Name	Description
gi|530379865	refseq_human (refseq_human_20140103.fasta)	PREDICTED: long-chain fatty acid transport protein 6 isoform X5 [Homo sapiens]
gi|530379863	refseq_human (refseq_human_20140103.fasta)	PREDICTED: long-chain fatty acid transport protein 6 isoform X4 [Homo sapiens]
gi|530379861	refseq_human (refseq_human_20140103.fasta)	PREDICTED: long-chain fatty acid transport protein 6 isoform X3 [Homo sapiens]
gi|530379859	refseq_human (refseq_human_20140103.fasta)	PREDICTED: long-chain fatty acid transport protein 6 isoform X2 [Homo sapiens]
gi|530379857	refseq_human (refseq_human_20140103.fasta)	PREDICTED: long-chain fatty acid transport protein 6 isoform X1 [Homo sapiens]
gi|62865631	refseq_human (refseq_human_20140103.fasta)	long-chain fatty acid transport protein 6 [Homo sapiens]

10	20	30	40	50	60	70	80
MLLSWLTVLG	AGMVVLHFLQ	KLLFPYFWDD	FWFVLKVVLI	IIRLKKYEKR	GELVTVLDKF	LSHAKRQPRK	PFIIYEGDIY
90	100	110	120	130	140	150	160
TYQDVDKRSS	RVAHVFLNHS	SLKKGDTVAL	LMSNEPDFVH	VWFGLAKLGC	VVAFLNTNIR	SNSLLNCIRA	CGPRALVVGA
170	180	190	200	210	220	230	240
DLLGTVEEIL	PSLSENISVW	GMKDSVPQGV	ISLKEKLSTS	PDEPVPRSHH	VVSLLKSTCL	YIFTSGTTGL	PKAAVISQLQ
250	260	270	280	290	300	310	320
VLRGSAVLWA	FGCTAHDIVY	ITLPLYHSSA	AILGISGCVE	LGATCVLKKK	FSASQFWSDC	KKYDVTVFQY	IGELCRYLCK
330	340	350	360	370	380	390	400
QSKREGEKDH	KVRLAIGNGI	RSDVWREFLD	RFGNIKVCEL	YAATESSISF	MNYTGRIGAI	GRTNLFYKLL	STFDLIKYDF
410	420	430	440	450	460	470	480
QKDEPMRNEQ	GWCIHVKKGE	PGLLISRVNA	KNPFFGYAGP	YKHTKDKLLC	DVFKKGDVYL	NTGDLIVQDQ	DNFLYFWDRT
490	500	510	520	530	540	550	560
GDTFRWKGEN	VATTEVADVI	GMLDFIQEAN	VYGVAISGYE	GRAGMASIIL	KPNTSLDLEK	VYEQVVTFLP	AYACPRFLRI
570	580	590	600	610	620		
QEKMEATGTF	KLLKHQLVED	GFNPLKISEP	LYFMDNLKKS	YVLLTRELYD	QIMLGEIKL		

Cmpd.	No. of Cmpds.	m/z meas.	Δ m/z [ppm]	z	Rt [min]	Score	P	Range	Sequence	Modification
2869	1	724.8749	-70.28	2	64.6	15.4	2	329-341	K.DHKVRLAIGNGIR.S	

Detailed Protein Report

Protein 235: PREDICTED: zinc finger protein 124 isoform X3 [Homo sapiens]

Accession: gi|578802322
Database: refseq_human(refseq_human_20140103.fasta)
Database Date: 2015-11-30

Score: 26.7
MW [kDa]: 35.5
pI: 10.4
Sequence Coverage [%]: 10.0
No. of unique Peptides: 1

10	20	30	40	50	60	70	80
MQETFRNLAS	IGNKGEDQSI	EDQYKNSSRN	LRHIISHSGN	NPYGCEECGK	KPCTCKQCQK	TSLSVTRVHR	DTVMHTGNGH
**90**	**100**	**110**	**120**	**130**	**140**	**150**	**160**
YGCTICEKVF	NIPSSFQIHQ	RNHTGEKPYE	CMECGKALGF	SRSLNRHKRI	HTGEKRYECK	QCGKAFSRSS	HLRDHERTHT
**170**	**180**	**190**	**200**	**210**	**220**	**230**	**240**
GEKPYECKHC	GKAFRYSNCL	HYHERTHTGE	KPYVCMECGK	AFSCLSSLQG	HIKAHAGEEP	YPCKQCGKAF	RYASSLQKHE
**250**	**260**	**270**	**280**	**290**	**300**	**310**	**320**
KTHIAQKPYV	CNNCGKGFRC	SSSLRDHERT	HTGEKPYECQ	KCGKAFSRAS	TLWKHKKTHT	GEKPYKCKKM	

Cmpd.	No. of Cmpds.	m/z meas.	Δ m/z [ppm]	z	Rt [min]	Score	P	Range	Sequence	Modification
2539	1	787.0551	174.57	2	60.0	14.7	0	89-101	K.VFNIPSSFQIHQR.N	

Detailed Protein Report

Protein 236: IgGFc-binding protein precursor [Homo sapiens]

**Accession:**	gi|154146262	**Score:**	26.7
**Database:**	refseq_human(refseq_human_20140103.fasta)	**MW [kDa]:**	571.6
**Database Date:**	2015-11-30	**pI:**	5.0
		**Sequence Coverage [%]:**	0.5
		**No. of unique Peptides:**	1

Detailed Protein Report

10	20	30	40	50	60	70	80
MGALWSWWIL	WAGATLLWGL	TQEASVDLKN	TGREEFLTAF	LQNYQLAYSK	AYPRLLISSL	SESPASVSIL	SQADNTSKKV
90	100	110	120	130	140	150	160
TVRPGESVMV	NISAKAEMIG	SKIFQHAVVI	HSDYAISVQA	LNAKPDTAEL	TLLRPIQALG	TEYFVLTPPG	TSARNVKEFA
170	180	190	200	210	220	230	240
VVAGAAGASV	SVTLKGSVTF	NGKFYPAGDV	LRVTLQPYNV	AQLQSSVDLS	GSKVTASSPV	AVLSGHSCAQ	KHTTCNHVVE
250	260	270	280	290	300	310	320
QLLPTSAWGT	HYVVPTLASQ	SRYDLAFVVA	SQATKLTYNH	GGITGSRGLQ	AGDVVEFEVR	PSWPLYLSAN	VGIQVLLFGT
330	340	350	360	370	380	390	400
GAIRNEVTYD	PYLVLIPDVA	AYCPAYVVKS	VPGCEGVALV	VAQTKAISGL	TIDGHAVGAK	LTWEAVPGSE	FSYAEVELGT
410	420	430	440	450	460	470	480
ADMIHTAEAT	TNLGLLTFGL	AKAIGYATAA	DCGRTVLSPV	EPSCEGMQCA	AGQRCQVVGG	KAGCVAESTA	VCRAQGDPHY
490	500	510	520	530	540	550	560
TTFDGRRYDM	MGTCSYTMVE	LCSEDDTLPA	FSVEAKNEHR	GSRRVSYVGL	VTVRAYSHSV	SLTRGEVGFV	LVDNQRSRLP
570	580	590	600	610	620	630	640
VSLSEGRLRV	YQSGPRAVVE	LVFGLVVTYD	WDCQLALSLP	ARFQDQVCGL	CGNYNGDPAD	DFLTPDGALA	PDAVEFASSW
650	660	670	680	690	700	710	720
KLDDGDYLCE	DGCQNNCPAC	TPGQAQHYEG	DRLCGMLTKL	DGPFAVCHDT	LDPRPFLEQC	VYDLCVVGGE	RLSLCRGLSA
730	740	750	760	770	780	790	800
YAQACLELGI	SVGDWRSPAN	CPLSCPANSR	YELCGPACPT	SCNGAAAPSN	CSGRPCVEGC	VCLPGFVASG	GACVPASSCG
810	820	830	840	850	860	870	880
CTFQGLQLAP	GQEVWADELC	QRRCTCNGAT	HQVTCRDKQS	CPAGERCSVQ	NGLLGCYPDR	FGTCQGSGDP	HYVSFDGRRF
890	900	910	920	930	940	950	960
DFMGTCTYLL	VGSCGQNAAL	PAFRVLVENE	HRGSQTVSYT	RAVRVEARGV	KVAVRREYPG	QVLVDDVLQY	LPFQAADGQV
970	980	990	1000	1010	1020	1030	1040
QVFRQGRDAV	VRTDFGLTVT	YDWNARVTAK	VPSSYAEALC	GLCGNFNGDP	ADDLALRGGG	QAANALAFGN	SWQEETRPGC
1050	1060	1070	1080	1090	1100	1110	1120
GATEPGDCPK	LDSLVAQQLQ	SKNECGILAD	PKGPFRECHS	KLDPQGAVRD	CVYDRCLLPG	QSGPLCDALA	TYAAACQAAG
1130	1140	1150	1160	1170	1180	1190	1200
ATVHPWRSEE	LCPLSCPPHS	HYEACSYGCP	LSCGDLPVPG	GCGSECHEGC	VCDEGFALSG	ESCLPLASCG	CVHQGTYHPP
1210	1220	1230	1240	1250	1260	1270	1280
GQTFYPGPGC	DSLCHCQEGG	LVSCESSSCG	PHEACQPSGG	SLGCVAVGSS	TCQASGDPHY	TTFDGRRFDF	MGTCVYVLAQ
1290	1300	1310	1320	1330	1340	1350	1360
TCGTRPGLHR	FAVLQENVAW	GNGRVSVTRV	ITVQVANFTL	RLEQRQWKVT	VNGVDMKLPV	VLANGQIRAS	QHGSDVVIET
1370	1380	1390	1400	1410	1420	1430	1440
DFGLRVAYDL	VYYVRVTVPG	NYYQQMCGLC	GNYNGDPKDD	FQKPNGSQAG	NANEFGNSWE	EVVPDSPCLP	PTPCPPGSED
1450	1460	1470	1480	1490	1500	1510	1520
CIPSHKCPPE	LEKKYQKEEF	CGLLSSPTGP	LSSCHKLVDP	QGPLKDCIFD	LCLGGGNLSI	LCSNIHAYVS	ACQAAGGHVE
1530	1540	1550	1560	1570	1580	1590	1600
PWRTETFCPM	ECPPNSHYEL	CADTCSLGCS	ALSAPPQCQD	GCAEGCQCDS	GFLYNGQACV	PIQQCGCYHN	GVYYEPEQTV
1610	1620	1630	1640	1650	1660	1670	1680
LIDNCRQQCT	CHAGKGMVCQ	EHSCKPGQVC	QPSGGILSCV	TKDPCHGVTC	RPQETCKEQG	GQGVCLPNYE	ATCWLWGDPH
1690	1700	1710	1720	1730	1740	1750	1760
YHSFDGRKFD	FQGTCNYVLA	TTGCPGVSTQ	GLTPFTVTTK	NQNRGNPAVS	YVRVVTVAAL	GTNISIHKDE	IGKVRVNGVL
1770	1780	1790	1800	1810	1820	1830	1840
TALPVSVADG	RISVTQGASK	ALLVADFGLQ	VSYDWNWRVD	VTLPSSYHGA	VCGLCGNMDR	NPNNDQVFPN	GTLAPSIPIW
1850	1860	1870	1880	1890	1900	1910	1920
GGSWRAPGWD	PLCWDECRGS	CPTCPEDRLE	QYEGPGFCGP	LAPGTGGPFT	TCHAHVPPES	FFKGCVLDVC	MGGGDRDILC
1930	1940	1950	1960	1970	1980	1990	2000
KALASYVAAC	QAAGVVIEDW	RAQVGCEITC	PENSHYEVCG	SPCPASCPSP	APLTTPAVCE	GPCVEGCQCD	AGFVLSADRC
2010	2020	2030	2040	2050	2060	2070	2080
VPLNNGCGCW	ANGTYHEAGS	EFWADGTCSQ	WCRCGPGGGS	LVCTPASCGL	GEVCGLLPSG	QHGCQPVSTA	ECQAWGDPHY
2090	2100	2110	2120	2130	2140	2150	2160
VTLDGHRFNF	QGTCEYLLSA	PCHGPPLGAE	NFTVTVANEH	RGSQAVSYTR	SVTLQIYNHS	LTLSARWPRK	LQVDGVFVTL
2170	2180	2190	2200	2210	2220	2230	2240

Detailed Protein Report

Cmpd.	No. of Cmpds.	m/z meas.	Δ m/z [ppm]	z	Rt [min]	Score	P	Range	Sequence	Modification
2667	1	665.3404	79.29	2	61.7	11.0	1	5395-5405	K.WRAQDFSPCYG.-	

Detailed Protein Report

Protein 237: ectodysplasin-A isoform 2 [Homo sapiens]

**Accession:**	gi|54112101	**Score:**	26.7
**Database:**	refseq_human(refseq_human_20140103.fasta)	**MW [kDa]:**	41.0
**Database Date:**	2015-11-30	**pI:**	9.6
		**Sequence Coverage [%]:**	8.7
		**No. of unique Peptides:**	2

10	20	30	40	50	60	70	80
MGYPEVERRE	LLPAAAPRER	GSQGCGCGGA	PARAGEGNSC	LLFLGFFGLS	LALHLLTLCC	YLELRSELRR	ERGAESRLGG
**90**	**100**	**110**	**120**	**130**	**140**	**150**	**160**
SGTPGTSGTL	SSLGGLDPDS	PITSHLGQPS	PKQQPLEPGE	AALHSDSQDG	HQMALLNFFF	PDEKPYSEEE	SRRVRRNKRS
**170**	**180**	**190**	**200**	**210**	**220**	**230**	**240**
KSNEGADGPV	KNKKKGKKAG	PPGPNGPPGP	PGPPGPQGPP	GIPGIPGIPG	TTVMGPPGPP	GPPGPQGPPG	LQGPSGAADK
**250**	**260**	**270**	**280**	**290**	**300**	**310**	**320**
AGTRENQPAV	VHLQGQGSAI	QVKNDLSGGV	LNDWSRITMN	PKVFKLHPRS	GELEVLVDGT	YFIYSQVYYI	NFTDFASYEV
**330**	**340**	**350**	**360**	**370**	**380**	**390**	
VVDEKPFLQC	TRSIETGKTN	YNTCYTAGVC	LLKARQKIAV	KMVHADISIN	MSKHTTFFGA	IRLGEAPAS	

Cmpd.	No. of Cmpds.	m/z meas.	Δ m/z [ppm]	z	Rt [min]	Score	P	Range	Sequence	Modification
1964	4	703.2668	-56.59	2	52.9	13.6	1	19-33	R.ERGSQGCGCGGAPAR.A	
1562	2	1059.0865	57.30	2	48.2	13.1	1	264-282	K.NDLSGGVLNDWSRITMNPK.V	

Detailed Protein Report

Protein 238: **copper-transporting ATPase 1 isoform 2 [Homo sapiens]**

**Accession:**	gi|532691752	**Score:**	26.7
**Database:**	refseq_human(refseq_human_20140103.fasta)	**MW [kDa]:**	154.3
**Database Date:**	2015-11-30	**pI:**	5.7
		**Sequence Coverage [%]:**	2.3
		**No. of unique Peptides:**	1

10	20	30	40	50	60	70	80
MDPSMGVNSV	TISVEGMTCN	SCVWTIEQQI	GKVNGVHHIK	VSLEEKNATI	IYDPKLQTPK	TLQEAIDDMG	FDAVIHNPDP
90	100	110	120	130	140	150	160
LPVLTDTLFL	TVTASLTLPW	DHIQSTLLKT	KGVTDIKIYP	QKRTVAVTII	PSIVNANQIK	ELVPELSLDT	GTLEKKSGAC
170	180	190	200	210	220	230	240
EDHSMAQAGE	VVLKMKVEGM	TCHSCTSTIE	GKIGKLQGVQ	RIKVSLDNQE	ATIVYQPHLI	SVEEMKKQIE	AMGFPAFVKK
250	260	270	280	290	300	310	320
QPKYLKLGAI	DVERLKNTPV	KSSEGSQQRS	PSYTNDSTAT	FIIDGMHCKS	CVSNIESTLS	ALQYVSSIVV	SLENRSAIVK
330	340	350	360	370	380	390	400
YNASSVTPES	LRKAIEAVSP	GLYRVSITSE	VESTSNSPSS	SSLQKIPLNV	VSQPLTQETV	INIDGMTCNS	CVQSIEGVIS
410	420	430	440	450	460	470	480
KKPGVKSIRV	SLANSNGTVE	YDPLLTSPET	LRGAIEDMGF	DATLSDTNEP	LVVIAQPSSE	MPLLTSTNEF	YTKGMTPVQD
490	500	510	520	530	540	550	560
KEEGKNSSKC	YIQVTGMTCA	SCVANIERNL	RREEGIYSIL	VALMAGKAEV	RYNPAVIQPP	MIAEFIRELG	FGATVIENAD
570	580	590	600	610	620	630	640
EGDGVLELVV	RGMTCASCVH	KIESSLTKHR	GILYCSVALA	TNKAHIKYDP	EIIGPRDIIH	TIESLGFEAS	LVKKDRSASH
650	660	670	680	690	700	710	720
LDHKREIRQW	RRSFLVSLFF	CIPVMGLMIY	MMVMDHHFAT	LHHNQNMSKE	EMINLHSSMF	LERQILPGLS	VMNLLSFLLC
730	740	750	760	770	780	790	800
VPVQGKTSEA	LAKLISLQAT	EATIVTLDSD	NILLSEEQVD	VELVQRGDII	KVVPGGKFPV	DGRVIEGHSM	VDESLITGEA
810	820	830	840	850	860	870	880
MPVAKKPGST	VIAGSINQNG	SLLICATHVG	ADTTLSQIVK	LVEEAQTSKA	PIQQFADKLS	GYFVPFIVFV	SIATLLVWIV
890	900	910	920	930	940	950	960
IGFLNFEIVE	TYFPGYNRSI	SRTETIIRFA	FQASITVLCI	ACPCSLGLAT	PTAVMVGTGV	GAQNGILIKG	GEPLEMAHKV
970	980	990	1000	1010	1020	1030	1040
KVVVFDKTGT	ITHGTPVVNQ	VKVLTESNRI	SHHKILAIVG	TAESNSEHPL	GTAITKYCKQ	ELDTETLGTC	IDFQVVPGCG
1050	1060	1070	1080	1090	1100	1110	1120
ISCKVTNIEG	LLHKNNWNIE	DNNIKNASLV	QIDASNEQSS	TSSSMIIDAQ	ISNALNAQQY	KVLIGNREWM	IRNGLVINND
1130	1140	1150	1160	1170	1180	1190	1200
VNDFMTEHER	KGRTAVLVAV	DDELCGLIAI	ADTVKPEAEL	AIHILKSMGL	EVVLMTGDNS	KTARSIASQV	GITKVFAEVL
1210	1220	1230	1240	1250	1260	1270	1280
PSHKVAKVKQ	LQEEGKRVAM	VGDGINDSPA	LAMANVGIAI	GTGTDVAIEA	ADVVLIRNDL	LDVVASIDLS	RKTVKRIRIN
1290	1300	1310	1320	1330	1340	1350	1360
FVFALIYNLV	GIPIAAGVFM	PIGLVLQPWM	GSAAMAASSV	SVVLSSLFLK	LYRKPTYESY	ELPARSQIGQ	KSPSEISVHV
1370	1380	1390	1400	1410	1420	1430	
GIDDTSRNSP	KLGLLDRIVN	YSRASINSLL	SDKRSLNSVV	TSEPDKHSLL	VGDFREDDDT	AL	

Cmpd.	No. of Cmpds.	m/z meas.	Δ m/z [ppm]	z	Rt [min]	Score	P	Range	Sequence	Modification
2946	1	822.4713	37.34	2	65.6	13.4	0	141-155	K.ELVPELSLDTGTLEK.K	

Detailed Protein Report

Protein 239: uncharacterized protein KIAA1751 [Homo sapiens]

Accession: gi|122937416
Database: refseq_human(refseq_human_20140103.fasta)
Database Date: 2015-11-30

Score: 26.6
MW [kDa]: 86.9
pI: 5.3
Sequence Coverage [%]: 2.5
No. of unique Peptides: 1

10	20	30	40	50	60	70	80
MEDDGSLLPE	DELLADALLL	EDERDELEDP	EFDIKCLLQE	AEDDVDPGHS	SSVKELDTDA	DKLKKKTAED	RTQAFHLRQN
**90**	**100**	**110**	**120**	**130**	**140**	**150**	**160**
LSALDKMHEE	QELFTEKMRG	ELRACRQRRD	LIDKQQEAVA	AEIATEEEAG	NMAAVGRLQA	VSRRLFAELE	NERDLQSRTE
**170**	**180**	**190**	**200**	**210**	**220**	**230**	**240**
AVLKESENTM	WHIEIQEGRL	EAFRTADREE	VEATGRRLQV	RAAEQLCREQ	EALGKVERNR	LLRIRKSLNT	QKELGLRHQK
**250**	**260**	**270**	**280**	**290**	**300**	**310**	**320**
LLEDARKNHK	VAVRFLKASL	GRIREQEKKE	EMECHEYMRR	RMDAVVALKG	SISANRDTLR	KFQAWDRAKA	ELAEQRVQAE
**330**	**340**	**350**	**360**	**370**	**380**	**390**	**400**
KKAILAQGRD	AFRHLVHQRR	RQELEAQKRA	FEEEQKLRKQ	EIISRILKEE	AEEEKRKKQH	PPTSARHRLT	LRDKTWNYIS
**410**	**420**	**430**	**440**	**450**	**460**	**470**	**480**
DFCKKTTVPT	NTYTLDYEAA	AGPGPSRLLE	VVSSELIQGD	PGASSEEETL	AEPEISGLWN	EDYKPYQVPK	EDVDRKPVGG
**490**	**500**	**510**	**520**	**530**	**540**	**550**	**560**
TKMDKDILER	TVERLRSRVV	HKQVVWGREF	QGRPFNSKPE	LLHFQDFDIG	KVYKKKITLV	NTTYTINYCK	LVGVEEHLRD
**570**	**580**	**590**	**600**	**610**	**620**	**630**	**640**
FIHVDFDPPG	PLSAGMSCEV	LVTFKPMINK	DLEGNISFLA	QTGEFSVPLK	CSTKKCSLSL	DKELIDFGSY	VVGETTSRTI
**650**	**660**	**670**	**680**	**690**	**700**	**710**	**720**
TLTNVGGLGT	TFKFLPASEP	CEMDDSQSAL	KLSSLLTYED	KSLYDKAATS	FSEQQLEGTE	SSQADMQSRK	ELEKLDKEQE
**730**	**740**	**750**	**760**	**770**			
EEQPAGEPGC	QAKPGWAGGE	GLRAAFQGAE	GPRGGLESRE	FS			

Cmpd.	No. of Cmpds.	m/z meas.	Δ m/z [ppm]	z	Rt [min]	Score	P	Range	Sequence	Modification
2144	1	687.4001	47.83	2	55.2	15.0	2	366-376	R.ILKEEAEEEKR.K	

Detailed Protein Report

Protein 240: protein canopy homolog 4 precursor [Homo sapiens]

**Accession:**	gi|22749479	**Score:**	26.6
**Database:**	refseq_human(refseq_human_20140103.fasta)	**MW [kDa]:**	28.3
**Database Date:**	2015-11-30	**pI:**	4.4
**Modification(s):**	Oxidation	**Sequence Coverage [%]:**	13.7
		**No. of unique Peptides:**	1

10	20	30	40	50	60	70	80
MGPVRLGILL	FLFLAVHEAW	AGMLKEEDDD	TERLPSKCEV	CKLLSTELQA	ELSRTGRSRE	VLELGQVLDT	GKRKRHVPYS
**90**	**100**	**110**	**120**	**130**	**140**	**150**	**160**
VSETRLEEAL	ENLCERILDY	SVHAERKGSL	RYAKGQSQTM	ATLKGLVQKG	VKVDLGIPLE	LWDEPSVEVT	YLKKQCETML
**170**	**180**	**190**	**200**	**210**	**220**	**230**	**240**
EEFEDIVGDW	YFHHQEQPLQ	NFLCEGHVLP	AAETACLQET	WTGKEITDGE	EKTEGEEEQE	EEEEEEEEEG	GDKMTKTGSH
**250**							
PKLDREDL							

Cmpd.	No. of Cmpds.	m/z meas.	Δ m/z [ppm]	z	Rt [min]	Score	P	Range	Sequence	Modification
2841	1	945.5312	3.89	2	64.2	12.2	2	115-132	K.GQSQTMATLKGLVQKGVK.V	Oxidation: 6

Detailed Protein Report

Protein 241: nuclear receptor coactivator 4 isoform 3 [Homo sapiens]

**Accession:**	gi|14149617	**Score:**	26.6
**Database:**	refseq_human(refseq_human_20140103.fasta)	**MW [kDa]:**	69.7
**Database Date:**	2015-11-30	**pI:**	5.7
**Modification(s):**	Carbamidomethyl	**Sequence Coverage [%]:**	3.1
		**No. of unique Peptides:**	1

Alias proteins:

Accession	Name	Description
gi|223890287	refseq_human (refseq_human_20140103.fasta)	nuclear receptor coactivator 4 isoform 3 [Homo sapiens]
gi|223890284	refseq_human (refseq_human_20140103.fasta)	nuclear receptor coactivator 4 isoform 3 [Homo sapiens]

10	20	30	40	50	60	70	80
MNTFQDQSGS	SSNREPLLRC	SDARRDLELA	IGGVLRAEQQ	IKDNLREVKA	QIHSCISRHL	ECLRSREVWL	YEQVDLIYQL
**90**	**100**	**110**	**120**	**130**	**140**	**150**	**160**
KEETLQQQAQ	QLYSLLGQFN	CLTHQLECTQ	NKDLANQVSV	CLERLGSLTL	KPEDSTVLLF	EADTITLRQT	ITTFGSLKTI
**170**	**180**	**190**	**200**	**210**	**220**	**230**	**240**
QIPEHLMAHA	SSANIGPFLE	KRGCISMPEQ	KSASGIVAVP	FSEWLLGSKP	ASGYQAPYIP	STDPQDWLTQ	KQTLENSQTS
**250**	**260**	**270**	**280**	**290**	**300**	**310**	**320**
SRACNFFNNV	GGNLKGLENW	LLKSEKSSYQ	KCNSHSTTSS	FSIEMEKVGD	QELPDQDEMD	LSDWLVTPQE	SHKLRKPENG
**330**	**340**	**350**	**360**	**370**	**380**	**390**	**400**
SRETSEKFKL	LFQSYNVNDW	LVKTDSCTNC	QGNQPKGVEI	ENLGNLKCLN	DHLEAKKPLS	TPSMVTEDWL	VQNHQDPCKV
**410**	**420**	**430**	**440**	**450**	**460**	**470**	**480**
EEVCRANEPC	TSFAECVCDE	NCEKEALYKW	LLKKEGKDKN	GMPVEPKPEP	EKHKDSLNMW	LCPRKEVIEQ	TKAPKAMTPS
**490**	**500**	**510**	**520**	**530**	**540**	**550**	**560**
RIADSFQVIK	NSPLSEWLIR	PPYKEGSPKE	VPGTEDRAGK	QKFKSPMNTS	WCSFNTADWV	LPGKKMGNLS	QLSSGEDKWL
**570**	**580**	**590**	**600**	**610**	**620**		
LRKKAQEVLL	NSPLQEEHNF	PPDHYGLPAV	CDLFACMQLK	VDKEKWLYRT	PLQM		

Cmpd.	No. of Cmpds.	m/z meas.	Δ m/z [ppm]	z	Rt [min]	Score	P	Range	Sequence	Modification
24	1	1103.4962	75.19	2	29.2	26.6	0	406-424	R.ANEPCTSFAECVCDENCEK.E	Carbamidomethyl: 5, 17

Detailed Protein Report

Protein 242: ubiquitin carboxyl-terminal hydrolase 12 [Homo sapiens]

Accession: gi|301500675
Database: refseq_human(refseq_human_20140103.fasta)
Database Date: 2015-11-30
Modification(s): Carbamidomethyl

Score: 26.6
MW [kDa]: 42.8
pI: 6.2
Sequence Coverage [%]: 7.0
No. of unique Peptides: 1

Quantitation

WD:WU **Median:** 0.64 **CV:** 0.00 % **No. of Peptides:** 1

10	20	30	40	50	60	70	80
MEILMTVSKF	ASICTMGANA	SALEKEIGPE	QFPVNEHYFG	LVNFGNTCYC	NSVLQALYFC	RPFREKVLAY	KSQPRKKESL
**90**	**100**	**110**	**120**	**130**	**140**	**150**	**160**
LTCLADLFHS	IATQKKKVGV	IPPKKFITRL	RKENELFDNY	MQQDAHEFLN	YLLNTIADIL	QEERKQEKQN	GRLPNGNIDN
**170**	**180**	**190**	**200**	**210**	**220**	**230**	**240**
ENNNSTPDPT	WVHEIFQGTL	TNETRCLTCE	TISSKDEDFL	DLSVDVEQNT	SITHCLRGFS	NTETLCSEYK	YYCEECRSKQ
**250**	**260**	**270**	**280**	**290**	**300**	**310**	**320**
EAHKRMKVKK	LPMILALHLK	RFKYMDQLHR	YTKLSYRVVF	PLELRLFNTS	GDATNPDRMY	DLVAVVVHCG	SGPNRGHYIA
**330**	**340**	**350**	**360**	**370**	**380**		
IVKSHDFWLL	FDDDIVEKID	AQAIEEFYGL	TSDISKNSES	GYILFYQSRD			

Cmpd.	No. of Cmpds.	m/z meas.	Δ m/z [ppm]	z	Rt [min]	Score	P	Range	Sequence	Modification	Ratios
620	1	647.6390	-191.68	2	36.2	14.2	1	231-239	K.YYCEECRSK.Q	Carbamidomethyl: 3, 6	WD:WU 0.64

Detailed Protein Report

Protein 243: PREDICTED: zinc finger protein 737 isoform X4 [Homo sapiens]

Accession: gi|530414419
Database: refseq_human(refseq_human_20140103.fasta)
Database Date: 2015-11-30
Modification(s): Carbamidomethyl

Score: 26.5
MW [kDa]: 60.3
pI: 10.4
Sequence Coverage [%]: 3.4
No. of unique Peptides: 1

10	20	30	40	50	60	70	80
MGPLQFRDVA	IEFSLEEWHC	LDTAQRNLYR	NVMLENYRNL	VFLGIVVSKP	DLITCLEQGK	KPLTMKKHEM	VANPSVTCSH
**90**	**100**	**110**	**120**	**130**	**140**	**150**	**160**
FARDLWPEQS	IKDSFQKVTL	RRYENYGHDN	LQFKKGCESV	DECKVHKRGY	NGLNQYLTTT	QSKIFQCDKY	VKVIHKFSNS
**170**	**180**	**190**	**200**	**210**	**220**	**230**	**240**
NRHKIRHTGK	KPFKCIECGK	AFNQSSTLTT	HKKIHTGEKP	FKCEECGKAF	NWSSHLTTHK	RIHTGEKRYK	CEDCGKAFSR
**250**	**260**	**270**	**280**	**290**	**300**	**310**	**320**
FSYLTAHKII	HSGEKPYKCE	ECGKAFKRSS	NLTTHKIIHT	GEKPYKCEEC	GKAFKRSSIL	TAHKIIHSGE	KPYKCEECGK
**330**	**340**	**350**	**360**	**370**	**380**	**390**	**400**
AFKHPSVLTT	HKRIHTGEKP	YKCEECGKAF	NWSSHLTTHK	RIHTGEKPYK	CEECGEAFKY	SSSLTTHKII	HTGQQPFKCE
**410**	**420**	**430**	**440**	**450**	**460**	**470**	**480**
ECGKAFKCFS	ILTTHKRIHT	GEKPYKCEEC	GKAFNSSSHL	TAHKRIHTGE	KPYKCERCGK	AFKRSFILTR	HKRIHTGEKP
**490**	**500**	**510**	**520**	**530**			
YKCEECGKGF	KCPSTLTTHK	RSGCPTRKLL	LSSVSTLNNS	EWK			

Cmpd.	No. of Cmpds.	m/z meas.	Δ m/z [ppm]	z	Rt [min]	Score	P	Range	Sequence	Modification
2022	1	1051.4074	-85.21	2	53.9	10.6	2	399-416	K.CEECGKAFKCFSILTTHK.R	Carbamidomethyl: 10

Detailed Protein Report

Protein 244: PREDICTED: hedgehog-interacting protein isoform X1 [Homo sapiens]

**Accession:**	gi|530378067	**Score:**	26.5
**Database:**	refseq_human(refseq_human_20140103.fasta)	**MW [kDa]:**	76.8
**Database Date:**	2015-11-30	**pI:**	9.5
**Modification(s):**	Carbamidomethyl	**Sequence Coverage [%]:**	5.9
		**No. of unique Peptides:**	1

10	20	30	40	50	60	70	80
MLKMLSFKLL	LLAVALGFFE	GDAKFGERNE	GSGARRRRCL	NGNPPKRLKR	RDRRMMSQLE	LLSGGEMLCG	GFYPRLSCCL
90	100	110	120	130	140	150	160
RSDSPGLGRL	ENKIFSVTNN	TECGKLLEEI	KCALCSPHSQ	SLFHSPEREV	LERDLVLPLL	CKDYCKEFFY	TCRGHIPGFL
170	180	190	200	210	220	230	240
QTTADEFCFY	YARKDGGLCF	PDFPRKQVRG	PASNYLDQME	EYDKVEEISR	KHKHNCFCIQ	EVVSGLRQPV	GALHSGDGSQ
250	260	270	280	290	300	310	320
RLFILEKEGY	VKILTPEGEI	FKEPYLDIHK	LVQSGIKGGD	ERGLLSLAFH	PNYKKNGKLY	VSYTTNQERW	AIGPHDHILR
330	340	350	360	370	380	390	400
VVEYTVSRKN	PHQVDLRTAR	VFLEVAELHR	KHLGGQLLFG	PDGFLYIILG	DGMITLDDME	EMDGLSDFTG	SVLRLDVDTD
410	420	430	440	450	460	470	480
MCNVPYSIPR	SNPHFNSTNQ	PPEVFAHGLH	DPGRCAVDRH	PTDININLTI	LCSDSNGKNR	SSARILQIIK	GKDYESEPSL
490	500	510	520	530	540	550	560
LEFKPFSNGP	LVGGFVYRGC	QSERLYGSYV	FGDRNGNFLT	LQQSPVTKQW	QEKPLCLGTS	GSCRGYFSGH	ILGFGEDELG
570	580	590	600	610	620	630	640
EVYILSSSKS	MTQTHNGKLY	KIVDPKRPLM	PEECRATVQP	AQTLTSECSR	LCRNGYCTPT	GKCCCSPGWE	GDFCRTAKCE
650	660	670	680	690			
PACRHGGVCV	RPNKCLCKKG	YLGPQCEQVD	RNIRRVTRAD	IT			

Cmpd.	No. of Cmpds.	m/z meas.	Δ m/z [ppm]	z	Rt [min]	Score	P	Range	Sequence	Modification
1709	1	938.7505	-122.25	2	49.7	10.2	1	623-638	K.CCCSPGWEGDFCRTAK.C	Carbamidomethyl: 3, 12

Detailed Protein Report

Protein 245: **tumor necrosis factor receptor superfamily member 10B isoform 2 precursor [Homo sapiens]**

**Accession:**	gi|224494026	**Score:**	26.5
**Database:**	refseq_human(refseq_human_20140103.fasta)	**MW [kDa]:**	45.1
**Database Date:**	2015-11-30	**pI:**	5.4
**Modification(s):**	Carbamidomethyl	**Sequence Coverage [%]:**	4.6
		**No. of unique Peptides:**	2

Quantitation

WD:WU **Median:** 0.58 **CV:** 0.00 % **No. of Peptides:** 1

```
        10         20         30         40         50         60         70         80
MEQRGQNAPA ASGARKRHGP GPREARGARP GPRVPKTLVL VVAAVLLLVS AESALITQQD LAPQQRAAPQ QKRSSPSEGL
        90        100        110        120        130        140        150        160
CPPGHHISED GRDCISCKYG QDYSTHWNDL LFCLRCTRCD SGEVELSPCT TTRNTVCQCE EGTFREEDSP EMCRKCRTGC
       170        180        190        200        210        220        230        240
PRGMVKVGDC TPWSDIECVH KESGIIIGVT VAAVVLIVAV FVCKSLLWKK VLPYLKGICS GGGGDPERVD RSSQRPGAED
       250        260        270        280        290        300        310        320
NVLNEIVSIL QPTQVPEQEM EVQEPAEPTG VNMLSPGESE HLLEPAEAER SQRRRLLVPA NEGDPTETLR QCFDDFADLV
       330        340        350        360        370        380        390        400
PFDSWEPLMR KLGLMDNEIK VAKAEAAGHR DTLYTMLIKW VNKTGRDASV HTLLDALETL GERLAKQKIE DHLLSSGKFM
       410        420
YLEGNADSAM S
```

Cmpd.	No. of Cmpds.	m/z meas.	Δ m/z [ppm]	z	Rt [min]	Score	P	Range	Sequence	Modification	Ratios
2927	3	973.5285	17.13	2	65.4	12.3	2	210-228	K.KVLPYLKGICSGGGGDPER.V		WD:WU 0.58
2235	1	937.9180	-60.88	2	56.4	14.1	1	211-228	K.VLPYLKGICSGGGGDPER.V	Carbamidomethyl: 9	

Detailed Protein Report

Protein 246: cytoplasmic phosphatidylinositol transfer protein 1 isoform a [Homo sapiens]

Accession: gi|32307140
Database: refseq_human(refseq_human_20140103.fasta)
Database Date: 2015-11-30
Modification(s): Carbamidomethyl

Score: 26.4
MW [kDa]: 38.4
pI: 6.0
Sequence Coverage [%]: 10.8
No. of unique Peptides: 1

10	20	30	40	50	60	70	80
MLLKEYRICM	PLTVDEYKIG	QLYMISKHSH	EQSDRGEGVE	VVQNEPFEDP	HHGNGQFTEK	RVYLNSKLPS	WARAVVPKIF
90	100	110	120	130	140	150	160
YVTEKAWNYY	PYTITEYTCS	FLPKFSIHIE	TKYEDNKGSN	DTIFDNEAKD	VEREVCFIDI	ACDEIPERYY	KESEDPKHFK
170	180	190	200	210	220	230	240
SEKTGRGQLR	EGWRDSHQPI	MCSYKLVTVK	FEVWGLQTRV	EQFVHKVVRD	ILLIGHRQAF	AWVDEWYDMT	MDEVREFERA
250	260	270	280	290	300	310	320
TQEATNKKIG	IFPPAISISS	IPLLPSSVRS	APSSAPSTPL	STDAPEFLSV	PKDRPRKKSA	PETLTLPDPE	KKATLNLPGM
330	340						
HSSDKPCRPK	SE						

Cmpd.	No. of Cmpds.	m/z meas.	Δ m/z [ppm]	z	Rt [min]	Score	P	Range	Sequence	Modification
26	1	712.9803	-81.39	3	29.3	11.2	1	312-330	K.KATLNLPGMHSSDKPCRPK.S	Carbamidomethyl: 16

Detailed Protein Report

Protein 247: PREDICTED: oleosin-B4-like [Homo sapiens]

Accession: gi|410170263
Database: refseq_human(refseq_human_20140103.fasta)
Database Date: 2015-11-30
Modification(s): Carbamidomethyl

Score: 26.4
MW [kDa]: 49.8
pI: 12.2
Sequence Coverage [%]: 7.5
No. of unique Peptides: 1

10	20	30	40	50	60	70	80
MSSPVLEPSP	GPRSPYLHGP	YLLLGEVLGH	SALGAQHAQP	ANGDADELLE	LPALLQHPTG	CDPCASLRDS	RVTPATALLL
**90**	**100**	**110**	**120**	**130**	**140**	**150**	**160**
LSHRGRAQRR	SIQAAAAQEQ	STGRGCLGKE	PPSQESWTRS	DPRRCLSQDA	VRSGSRVCRS	RPQTRGGGGK	QPRRQKAAVA
**170**	**180**	**190**	**200**	**210**	**220**	**230**	**240**
GAKSRGGEGK	KPQRRGQKAA	TAGAKSRGGG	VRSLGGKKMR	RGQKGAAGAK	RCGGGKQPRR	RGQKASKSGG	VRCQTPQKAA
**250**	**260**	**270**	**280**	**290**	**300**	**310**	**320**
AQGVKSRKKL	RQRGQKAAAA	GQEAAAGKTC	GGGGEKHRGG	GDKKPLRKNS	RWRGQKAAAA	GGKKPQKAAA	AGGERRKKLR
**330**	**340**	**350**	**360**	**370**	**380**	**390**	**400**
RQKAAVARAK	SPKKPRWRGQ	NSGNGVEGQR	SLAFLEFDVE	GKVQRKTKIP	GGVVKQFVPA	DQGPLWQKAA	NLHKMSLPHQ
**410**	**420**	**430**	**440**	**450**	**460**	**470**	
LALKMEWNGI	IIEWNRMELS	SNGINRDHHR	MEPNGVIIEW	NRKASSCNGI	EWHHQRMDSK	GIIIE	

Cmpd.	No. of Cmpds.	m/z meas.	Δ m/z [ppm]	z	Rt [min]	Score	P	Range	Sequence	Modification
1321	1	1045.0547	21.73	2	44.8	13.5	2	90-109	R.RSIQAAAAQEQSTGRGCLGK.E	Carbamidomethyl: 17

Detailed Protein Report

Protein 248: **PREDICTED: ninein isoform X5 [Homo sapiens]**

**Accession:**	gi|578825856	**Score:**	26.4
**Database:**	refseq_human(refseq_human_20140103.fasta)	**MW [kDa]:**	231.1
**Database Date:**	2015-11-30	**pI:**	4.8
**Modification(s):**	Carbamidomethyl, Oxidation	**Sequence Coverage [%]:**	1.4
		**No. of unique Peptides:**	2

Detailed Protein Report

10	20	30	40	50	60	70	80
MDEVEQDQHE	ARLKELFDSF	DTTGTGSLGQ	EELTDLCHML	SLEEVAPVLQ	QTLLQDNLLG	RVHFDQFKEA	LILILSRTLS
90	100	110	120	130	140	150	160
NEEHFQEPDC	SLEAQPKYVR	GGKRYGRRSL	PEFQESVEEF	PEVTVIEPLD	EEARPSHIPA	GDCSEHWKTQ	RSEEYEAEGQ
170	180	190	200	210	220	230	240
LRFWNPDDLN	ASQSGSSPPQ	DWIEEKLQEV	CEDLGITRDG	HLNRKKLVSI	CEQYGLQNVD	GEMLEEVFHN	LDPDGTMSVE
250	260	270	280	290	300	310	320
DFFYGLFKNG	KSLTPSASTP	YRQLKRHLSM	QSFDESGRRT	TTSSAMTSTI	GFRVFSCLDD	GMGHASVERI	LDTWQEEGIE
330	340	350	360	370	380	390	400
NSQEILKALD	FSLDGNINLT	ELTLALENEL	LVTKNSIHQA	ALASFKAEIR	HLLERVDQVV	REKEKLRSDL	DKAEKLKSLM
410	420	430	440	450	460	470	480
ASEVDDHHAA	IERRNEYNLR	KLDEEYKERI	AALKNELRKE	REQILQQAGK	QRLELEQEIE	KAKTEENYIR	DRLALSLKEN
490	500	510	520	530	540	550	560
SRLENELLEN	AEKLAEYENL	TNKLQRNLEN	VLAEKFGDLD	PSSAEFFLQE	ERLTQMRNEY	ERQCRVLQDQ	VDELQSELEE
570	580	590	600	610	620	630	640
YRAQGRVLRL	PLKNSPSEEV	EANSGGIEPE	HGLGSEECNP	LNMSIEAELV	IEQMKEQHHR	DICCLRLELE	DKVRHYEKQL
650	660	670	680	690	700	710	720
DETVVSCKKA	QENMKQRHEN	ETHTLEKQIS	DLKNEIAELQ	GQAAVLKEAH	HEATCRHEEE	KKQLQVKLEE	EKTHLQEKLR
730	740	750	760	770	780	790	800
LQHEMELKAR	LTQAQASFER	EREGLQSSAW	TEEKVRGLTQ	ELEQFHQEQL	TSLVEKHTLE	KEELRKELLE	KHQRELQEGR
810	820	830	840	850	860	870	880
EKMETECNRR	TSQIEAQFQS	DCQKVTERCE	SALQSLEGRY	RQELKDLQEQ	QREEKSQWEF	EKDELTQECA	EAQELLKETL
890	900	910	920	930	940	950	960
KREKTTSLVL	TQEREMLEKT	YKEHLNSMVV	ERQQLLQDLE	DLRNVSETQQ	SLLSDQILEL	KSSHKRELRE	REEVLCQAGA
970	980	990	1000	1010	1020	1030	1040
SEQLASQRLE	RLEMEHDQER	QEMMSKLLAM	ENIHKATCET	ADRERAEMST	EISRLQSKIK	EMQQATSPLS	MLQSGCQVIG
1050	1060	1070	1080	1090	1100	1110	1120
EEEVEGDGAL	SLLQQGEQLL	EENGDVLLSL	QRAHEQAVKE	NVKMATEISR	LQQRLQKLEP	GLVMSSCLDE	PATEFFGNTA
1130	1140	1150	1160	1170	1180	1190	1200
EQTEQFLQQN	RTKQVEGVTR	RHVLSDLEDD	EVRDLGSTGT	SSVQRQEVKI	EESEASVEGF	SELENSEETR	TESWELKNQI
1210	1220	1230	1240	1250	1260	1270	1280
SQLQEQLMML	CADCDRASEK	KQDLLFDVSV	LKKKLKMLER	IPEASPKYKL	LYEDVSREND	CLQEELRMME	TRYDEALENN
1290	1300	1310	1320	1330	1340	1350	1360
KELTAEVFRL	QDELKKMEEV	TETFLSLEKS	YDEVKIENEG	LNVLVLRLQG	KIEKLQESVV	QRCDCCLWEA	SLENLEIEPD
1370	1380	1390	1400	1410	1420	1430	1440
GNILQLNQTL	EECVPRVRSV	HHVIEECKQE	NQYLEGNTQL	LEKVKAHEIA	WLHGTIQTHQ	ERPRVQNQVI	LEENTTLLGF
1450	1460	1470	1480	1490	1500	1510	1520
QDKHFQHQAT	IAELELEKTK	LQELTRKLKE	RVTILVKQKD	VLSHGEKEEE	LKAMMHDLQI	TCSEMQQKVE	LLRYESEKLQ
1530	1540	1550	1560	1570	1580	1590	1600
QENSILRNEI	TTLNEEDSIS	NLKLGTLNGS	QEEMWQKTET	VKQENAAVQK	MVENLKKQIS	ELKIKNQQLD	LENTELSQKN
1610	1620	1630	1640	1650	1660	1670	1680
SQNQEKLQEL	NQRLTEMLCQ	KEKEPGNSAL	EEREQEKFNL	KEELERCKVQ	SSTLVSSLEA	ELSEVKIQTH	IVQQENHLLK
1690	1700	1710	1720	1730	1740	1750	1760
DELEKMKQLH	RCPDLSDFQQ	KISSVLSYNE	KLLKEKEALS	EELNSCVDKL	AKSSLLEHRI	ATMKQEQKSW	EHQSASLKSQ
1770	1780	1790	1800	1810	1820	1830	1840
LVASQEKVQN	LEDTVQNVNL	QMSRMKSDLR	VTQQEKEALK	QEVMSLHKQL	QNAGGKSWAP	EIATHPSGLH	NQQKRLSWDK
1850	1860	1870	1880	1890	1900	1910	1920
LDHLMNEEQQ	LLWQENERLQ	TMVQNTKAEL	THSREKVRQL	ESNLLPKHQK	HLNPSGTMNP	TEQEKLSLKR	ECDQFQKEQS
1930	1940	1950	1960	1970	1980	1990	
PANRKVSQMN	SLEQELETIH	LENEGLKKKQ	VKLDEQLMEN	SVVGSSREGC	SSLPEIVCEG	RTFSFNELS	

Cmpd.	No. of Cmpds.	m/z meas.	Δ m/z [ppm]	z	Rt [min]	Score	P	Range	Sequence	Modification
1578	1	685.8860	27.18	2	48.0	10.3	0	913-923	R.QQLLQDLEDLR.N	
2071	5	1023.8928	-100.81	2	54.5	16.0	2	1686-1701	K.MKQLHRCPDLSDFQQK.I	Carbamidomethyl: 7; Oxidation: 1

Detailed Protein Report

Protein 249: huntingtin [Homo sapiens]

**Accession:**	gi|90903231	**Score:**	26.3
**Database:**	refseq_human(refseq_human_20140103.fasta)	**MW [kDa]:**	347.6
**Database Date:**	2015-11-30	**pI:**	5.8
		**Sequence Coverage [%]:**	0.9
		**No. of unique Peptides:**	1

Detailed Protein Report

10	20	30	40	50	60	70	80
MATLEKLMKA	FESLKSFQQQ	QQQQQQQQQQ	QQQQQQQQQQ	PPPPPPPPPP	PQLPQPPPQA	QPLLPQPQPP	PPPPPPPPGP
90	100	110	120	130	140	150	160
AVAEEPLHRP	KKELSATKKD	RVNHCLTICE	NIVAQSVRNS	PEFQKLLGIA	MELFLLCSDD	AESDVRMVAD	ECLNKVIKAL
170	180	190	200	210	220	230	240
MDSNLPRLQL	ELYKEIKKNG	APRSLRAALW	RFAELAHLVR	PQKCRPYLVN	LLPCLTRTSK	RPEESVQETL	AAAVPKIMAS
250	260	270	280	290	300	310	320
FGNFANDNEI	KVLLKAFIAN	LKSSSPTIRR	TAAGSAVSIC	QHSRRTQYFY	SWLLNVLLGL	LVPVEDEHST	LLILGVLLTL
330	340	350	360	370	380	390	400
RYLVPLLQQQ	VKDTSLKGSF	GVTRKEMEVS	PSAEQLVQVY	ELTLHHTQHQ	DHNVVTGALE	LLQQLFRTPP	PELLQTLTAV
410	420	430	440	450	460	470	480
GGIGQLTAAK	EESGGRSRSG	SIVELIAGGG	SSCSPVLSRK	QKGKVLLGEE	EALEDDSESR	SDVSSSALTA	SVKDEISGEL
490	500	510	520	530	540	550	560
AASSGVSTPG	SAGHDIITEQ	PRSQHTLQAD	SVDLASCDLT	SSATDGDEED	ILSHSSSQVS	AVPSDPAMDL	NDGTQASSPI
570	580	590	600	610	620	630	640
SDSSQTTTEG	PDSAVTPSDS	SEIVLDGTDN	QYLGLQIGQP	QDEDEEATGI	LPDEASEAFR	NSSMALQQAH	LLKNMSHCRQ
650	660	670	680	690	700	710	720
PSDSSVDKFV	LRDEATEPGD	QENKPCRIKG	DIGQSTDDDS	APLVHCVRLL	SASFLLTGGK	NVLVPDRDVR	VSVKALALSC
730	740	750	760	770	780	790	800
VGAAVALHPE	SFFSKLYKVP	LDTTEYPEEQ	YVSDILNYID	HGDPQVRGAT	AILCGTLICS	ILSRSRFHVG	DWMGTIRTLT
810	820	830	840	850	860	870	880
GNTFSLADCI	PLLRKTLKDE	SSVTCKLACT	AVRNCVMSLC	SSSYSELGLQ	LIIDVLTLRN	SSYWLVRTEL	LETLAEIDFR
890	900	910	920	930	940	950	960
LVSFLEAKAE	NLHRGAHHYT	GLLKLQERVL	NNVVIHLLGD	EDPRVRHVAA	ASLIRLVPKL	FYKCDQGQAD	PVVAVARDQS
970	980	990	1000	1010	1020	1030	1040
SVYLKLLMHE	TQPPSHFSVS	TITRIYRGYN	LLPSITDVTM	ENNLSRVIAA	VSHELITSTT	RALTFGCCEA	LCLLSTAFPV
1050	1060	1070	1080	1090	1100	1110	1120
CIWSLGWHCG	VPPLSASDES	RKSCTVGMAT	MILTLLSSAW	FPLDLSAHQD	ALILAGNLLA	ASAPKSLRSS	WASEEEANPA
1130	1140	1150	1160	1170	1180	1190	1200
ATKQEEVWPA	LGDRALVPMV	EQLFSHLLKV	INICAHVLDD	VAPGPAIKAA	LPSLTNPPSL	SPIRRKGKEK	EPGEQASVPL
1210	1220	1230	1240	1250	1260	1270	1280
SPKKGSEASA	ASRQSDTSGP	VTTSKSSSLG	SFYHLPSYLK	LHDVLKATHA	NYKVTLDLQN	STEKFGGFLR	SALDVLSQIL
1290	1300	1310	1320	1330	1340	1350	1360
ELATLQDIGK	CVEEILGYLK	SCFSREPMMA	TVCVQQLLKT	LFGTNLASQF	DGLSSNPSKS	QGRAQRLGSS	SVRPGLYHYC
1370	1380	1390	1400	1410	1420	1430	1440
FMAPYTHFTQ	ALADASLRNM	VQAEQENDTS	GWFDVLQKVS	TQLKTNLTSV	TKNRADKNAI	HNHIRLFEPL	VIKALKQYTT
1450	1460	1470	1480	1490	1500	1510	1520
TTCVQLQKQV	LDLLAQLVQL	RVNYCLLDSD	QVFIGFVLKQ	FEYIEVGQFR	ESEAIIPNIF	FFLVLLSYER	YHSKQIIGIP
1530	1540	1550	1560	1570	1580	1590	1600
KIIQLCDGIM	ASGRKAVTHA	IPALQPIVHD	LFVLRGTNKA	DAGKELETQK	EVVVSMLLRL	IQYHQVLEMF	ILVLQQCHKE
1610	1620	1630	1640	1650	1660	1670	1680
NEDKWKRLSR	QIADIILPML	AKQQMHIDSH	EALGVLNTLF	EILAPSSLRP	VDMLLRSMFV	TPNTMASVST	VQLWISGILA
1690	1700	1710	1720	1730	1740	1750	1760
ILRVLISQST	EDIVLSRIQE	LSFSPYLISC	TVINRLRDGD	STSTLEEHSE	GKQIKNLPEE	TFSRFLLQLV	GILLEDIVTK
1770	1780	1790	1800	1810	1820	1830	1840
QLKVEMSEQQ	HTFYCQELGT	LLMCLIHIFK	SGMFRRITAA	ATRLFRSDGC	GGSFYTLDSL	NLRARSMITT	HPALVLLWCQ
1850	1860	1870	1880	1890	1900	1910	1920
ILLLVNHTDY	RWWAEVQQTP	KRHSLSSTKL	LSPQMSGEEE	DSDLAAKLGM	CNREIVRRGA	LILFCDYVCQ	NLHDSEHLTW
1930	1940	1950	1960	1970	1980	1990	2000
LIVNHIQDLI	SLSHEPPVQD	FISAVHRNSA	ASGLFIQAIQ	SRCENLSTPT	MLKKTLQCLE	GIHLSQSGAV	LTLYVDRLLC
2010	2020	2030	2040	2050	2060	2070	2080
TPFRVLARMV	DILACRRVEM	LLAANLQSSM	AQLPMEELNR	IQEYLQSSGL	AQRHQRLYSL	LDRFRLSTMQ	DSLSPSPPVS
2090	2100	2110	2120	2130	2140	2150	2160
SHPLDGDGHV	SLETVSPDKD	WYVHLVKSQC	WTRSDSALLE	GAELVNRIPA	EDMNAFMMNS	EFNLSLLAPC	LSLGMSEISG
2170	2180	2190	2200	2210	2220	2230	2240

Detailed Protein Report

Cmpd.	No. of Cmpds.	m/z meas.	Δ m/z [ppm]	z	Rt [min]	Score	P	Range	Sequence	Modification
347	1	524.3773	160.20	2	33.0	15.3	2	93-101	K.ELSATKKDR.V	

Detailed Protein Report

Protein 250: sodium-driven chloride bicarbonate exchanger isoform 2 [Homo sapiens]

Accession: gi|155722998
Database: refseq_human(refseq_human_20140103.fasta)
Database Date: 2015-11-30

Score: 26.3
MW [kDa]: 122.6
pI: 5.9
Sequence Coverage [%]: 1.8
No. of unique Peptides: 1

10	20	30	40	50	60	70	80
MEIKDQGAQM	EPLLPTRNDE	EAVVDRGGTR	SILKTHFEKE	DLEGHRTLFI	GVHVPLGGRK	SHRRHRHRGH	KHRKRDRERD
90	100	110	120	130	140	150	160
SGLEDGRESP	SFDTPSQRVQ	FILGTEDDDE	EHIPHDLFTE	LDEICWREGE	DAEWRETARW	LKFEEDVEDG	GERWSKPYVA
170	180	190	200	210	220	230	240
TLSLHSLFEL	RSCILNGTVL	LDMHANTLEE	IADMVLDQQV	SSGQLNEDVR	HRVHEALMKQ	HHHQNQKKLT	NRIPIVRSFA
250	260	270	280	290	300	310	320
DIGKKQSEPN	SMDKNAGQVV	SPQSAPACVE	NKNDVSRENS	TVDFSKVDLH	FMKKIPPGAE	ASNILVGELE	FLDRTVVAFV
330	340	350	360	370	380	390	400
RLSPAVLLQG	LAEVPIPTRF	LFILLGPLGK	GQQYHEIGRS	IATLMTDEVF	HDVAYKAKDR	NDLVSGIDEF	LDQVTVLPPG
410	420	430	440	450	460	470	480
EWDPSIRIEP	PKNVPSQEKR	KIPAVPNGTA	AHGEAEPHGG	HSGPELQRTG	RIFGGLILDI	KRKAPYFWSD	FRDAFSLQCL
490	500	510	520	530	540	550	560
ASFLFLYCAC	MSPVITFGGL	LGEATEGRIS	AIESLFGASM	TGIAYSLFGG	QPLTILGSTG	PVLVFEKILF	KFCKEYGLSY
570	580	590	600	610	620	630	640
LSLRASIGLW	TATLCIILVA	TDASSLVCYI	TRFTEEAFAS	LICIIFIYEA	LEKLFELSEA	YPINMHNDLE	LLTQYSCNCV
650	660	670	680	690	700	710	720
EPHNPSNGTL	KEWRESNISA	SDIIWENLTV	SECKSLHGEY	VGRACGHDHP	YVPDVLFWSV	ILFFSTVTLS	ATLKQFKTSR
730	740	750	760	770	780	790	800
YFPTKVRSIV	SDFAVFLTIL	CMVLIDYAIG	IPSPKLQVPS	VFKPTRDDRG	WFVTPLGPNP	WWTVIAAIIP	ALLCTILIFM
810	820	830	840	850	860	870	880
DQQITAVIIN	RKEHKLKKGC	GYHLDLLMVA	VMLGVCSIMG	LPWFVAATVL	SITHVNSLKL	ESECSAPGEQ	PKFLGIREQR
890	900	910	920	930	940	950	960
VTGLMIFILM	GSSVFMTSIL	KFIPMPVLYG	VFLYMGASSL	KGIQFFDRIK	LFWMPAKHQP	DFIYLRHVPL	RKVHLFTIIQ
970	980	990	1000	1010	1020	1030	1040
MSCLGLLWII	KVSRAAIVFP	MMVLALVFVR	KLMDLLFTKR	ELSWLDDLMP	ESKKKKLEDA	EKEEEQSMLA	MEDEGTVQLP
1050	1060	1070	1080	1090			
LEGHYRDDPS	VINISDEMSK	TALWRNLLIT	ADNSKDKESS	FPSKSSPS			

Cmpd.	No. of Cmpds.	m/z meas.	Δ m/z [ppm]	z	Rt [min]	Score	P	Range	Sequence	Modification
63	1	724.6832	-193.81	2	29.7	11.3	1	128-139	R.EGEDAEWRETAR.W	

Detailed Protein Report

Protein 251: potassium voltage-gated channel subfamily KQT member 5 isoform 5 [Homo sapiens]

**Accession:**	gi|236462242	**Score:**	26.2
**Database:**	refseq_human(refseq_human_20140103.fasta)	**MW [kDa]:**	89.9
**Database Date:**	2015-11-30	**pI:**	10.2
		**Sequence Coverage [%]:**	3.8
		**No. of unique Peptides:**	2

```
        10         20         30         40         50         60         70         80
MPRHHAGGEE GGAAGLWVKS GAAAAAAGGG RLGSGMKDVE SGRGRVLLNS AAARGDGLLL LGTRAATLGG GGGGLRESRR
        90        100        110        120        130        140        150        160
GKQGARMSLL GKPLSYTSSQ SCRRNVKYRR VQNYLYNVLE RPRGWAFIYH AFVFLLVFGC LILSVFSTIP EHTKLASSCL
       170        180        190        200        210        220        230        240
LILEFVMIVV FGLEFIIRIW SAGCCCRYRG WQGRLRFARK PFCVIDTIVL IASIAVVSAK TQGNIFATSA LRSLRFLQIL
       250        260        270        280        290        300        310        320
RMVRMDRRGG TWKLLGSVVY AHSKELITAW YIGFLVLIFS SFLVYLVEKD ANKEFSTYAD ALWWGTITLT TIGYGDKTPL
       330        340        350        360        370        380        390        400
TWLGRLLSAG FALLGISFFA LPAGILGSGF ALKVQEQHRQ KHFEKRRNPA ANLIQCVWRS YAADEKSVSI ATWKPHLKAL
       410        420        430        440        450        460        470        480
HTCSPTKKEQ GEASSRIMKF HVAKRKFKET LRPYDVKDVI EQYSAGHLDM LCRIKSLQTR VDQILGKGQI TSDKKSREKI
       490        500        510        520        530        540        550        560
TAEHETTDDL SMLGRVVKVE KQVQSIESKL DCLLDIYQQV LRKGSASALA LASFQIPPFE CEQTSDYQSP VDSKDLSGSA
       570        580        590        600        610        620        630        640
QNSGCLSRST SANISRGLQF ILTPNEFSAQ TFYALSPTMH SQATQVPISQ SDGSAVAATN TIANQINTAP KPAAPTTLQI
       650        660        670        680        690        700        710        720
PPPLPAIKHL PRPETLHPNP AGLQESISDV TTCLVASKEN VQVAQSNLTK DRSMRKSFDM GGETLLSVCP MVPKDLGKSL
       730        740        750        760        770        780        790        800
SVQNLIRSTE ELNIQLSGSE SSGSRGSQDF YPKWRESKLF ITDEEVGPEE TETDTFDAAP QPAREAAFAS DSLRTGRSRS
       810        820        830
SQSICKAGES TDALSLPHVK LK
```

Cmpd.	No. of Cmpds.	m/z meas.	Δ m/z [ppm]	z	Rt [min]	Score	P	Range	Sequence	Modification
2983	1	965.4581	-18.53	2	66.1	10.6	2	399-416	K.ALHTCSPTKKEQGEASSR.I	
316	1	690.7007	-211.65	2	33.0	15.6	1	785-797	R.EAAFASDSLRTGR.S	

Detailed Protein Report

Protein 252: PREDICTED: spidroin-1-like [Homo sapiens]

**Accession:**	gi|530356740	**Score:**	26.2
**Database:**	refseq_human(refseq_human_20140103.fasta)	**MW [kDa]:**	27.8
**Database Date:**	2015-11-30	**pI:**	12.6
		**Sequence Coverage [%]:**	17.2
		**No. of unique Peptides:**	2

10	20	30	40	50	60	70	80
MLRPRARADC	GVRGRGALGG	VPGRAASRGA	GSRGGRGPGE	GGVPGGRGGA	WGALSGGRVS	RGEGLRLLGA	TGGGCGAWGR
**90**	**100**	**110**	**120**	**130**	**140**	**150**	**160**
PGLPAAAPKG	LSGGSGGRGS	SQGLSYGRTR	GEAAKSRRAW	TGPRADRTVL	GELRGAAGDR	GVRLPRGDAQ	RPGQNLDGAE
**170**	**180**	**190**	**200**	**210**	**220**	**230**	**240**
SPARAPAAST	WEDTQGPGAR	SGRRAASAVS	TDGQKPQRVP	SCSRRGSITI	GPGLPGGASE	GSMLRQELGT	AGDEMRLFGQ
**250**	**260**	**270**	**280**				
GVCFRAMRQP	GSWATGGRPG	VRALQPVPAP	AFHGRRVLP				

Cmpd.	No. of Cmpds.	m/z meas.	Δ m/z [ppm]	z	Rt [min]	Score	P	Range	Sequence	Modification
1604	2	978.1044	-91.99	3	48.4	15.7	2	59-89	R.VSRGEGLRLLGATGGGCGAWGRPGLPAAAPK.G	
2694	1	913.4475	-91.49	2	62.1	10.6	2	263-279	R.ALQPVPAPAFHGRRVLP.-	

Detailed Protein Report

Protein 253: interleukin-33 isoform 2 [Homo sapiens]

Accession: gi|313851030
Database: refseq_human(refseq_human_20140103.fasta)
Database Date: 2015-11-30
Modification(s): Oxidation

Score: 26.2
MW [kDa]: 25.9
pI: 10.2
Sequence Coverage [%]: 11.0
No. of unique Peptides: 1

10	20	30	40	50	60	70	80
MKPKMKYSTN	KISTAKWKNT	ASKALCFKLG	KSQQKAKEVC	PMYFMKLRSG	LMIKKEACYF	RRETTKRPSL	KTGRKHKRHL
**90**	**100**	**110**	**120**	**130**	**140**	**150**	**160**
VLAACQQQST	VECFAFGISG	VQKYTRALHD	SSITDKVLLS	YYESQHPSNE	SGDGVDGKML	MVTLSPTKDF	WLHANNKEHS
**170**	**180**	**190**	**200**	**210**	**220**	**230**	
VELHKCEKPL	PDQAFFVLHN	MHSNCVSFEC	KTDPGVFIGV	KDNHLALIKV	DSSENLCTEN	ILFKLSET	

Cmpd.	No. of Cmpds.	m/z meas.	Δ m/z [ppm]	z	Rt [min]	Score	P	Range	Sequence	Modification
1897	1	568.7721	-60.19	2	52.1	15.8	0	139-148	K.MLMVTLSPTK.D	Oxidation: 1

Detailed Protein Report

Protein 254: PREDICTED: sarcolemmal membrane-associated protein isoform X16 [Homo sapiens]

**Accession:**	gi|530373158	**Score:**	26.2
**Database:**	refseq_human(refseq_human_20140103.fasta)	**MW [kDa]:**	90.8
**Database Date:**	2015-11-30	**pI:**	5.1
		**Sequence Coverage [%]:**	3.3
		**No. of unique Peptides:**	1

10	20	30	40	50	60	70	80
MPSALAIFTC	RPNSHPFQER	HVYLDEPIKI	GRSVARCRPA	QNNATFDCKV	LSRNHALVWF	DHKTGKFYLQ	DTKSSNGTFI
**90**	**100**	**110**	**120**	**130**	**140**	**150**	**160**
NSQRLSRGSE	ESPPCEILSG	DIIQFGVDVT	ENTRKVTHGC	IVSTIKLFLP	DGMEARLRSD	VIHAPLPSPV	DKVAANTPSM
**170**	**180**	**190**	**200**	**210**	**220**	**230**	**240**
YSQELFQLSQ	YLQEALHREQ	MLEQKLATLQ	RLLAITQEAS	DTSWQALIDE	DRLLSRLEVM	GNQLQACSKN	QTEDSLRKEL
**250**	**260**	**270**	**280**	**290**	**300**	**310**	**320**
IALQEDKHNY	ETTAKESLRR	VLQEKIEVVR	KLSEVERSLS	NTEDECTHLK	EMNERTQEEL	RELANKYNGA	VNEIKDLSDK
**330**	**340**	**350**	**360**	**370**	**380**	**390**	**400**
LKVAEGKQEE	IQQKGQAEKK	ELQHKIDEME	EKEQELQAKI	EALQADNDFT	NERLTALQEK	LIVEGHLTKA	VEETKLSKEN
**410**	**420**	**430**	**440**	**450**	**460**	**470**	**480**
QTRAKESDFS	DTLSPSKEKS	SDDTTDAQMD	EQDLNEPLAK	VSLLKDDLQG	AQSEIEAKQE	IQHLRKELIE	AQELARTSKQ
**490**	**500**	**510**	**520**	**530**	**540**	**550**	**560**
KCFELQALLE	EERKAYRNQV	EESTKQIQVL	QAQLQRLHID	TENLREEKDS	EITSTRDELL	SARDEILLLH	QAAAKVASER
**570**	**580**	**590**	**600**	**610**	**620**	**630**	**640**
DTDIASLQEE	LKKVRAELER	WRKAASEYEK	EITSLQNSFQ	LRCQQCEDQQ	REEATRLQGE	LEKLRKEWNA	LETECHSLKR
**650**	**660**	**670**	**680**	**690**	**700**	**710**	**720**
ENVLLSSELQ	RQEKELHNSQ	KQSLELTSDL	SILQMSRKEL	ENQVGSLKEQ	HLRDSADLKT	LLSKAENQAK	DVQKEYEKTQ
**730**	**740**	**750**	**760**	**770**	**780**	**790**	**800**
TVLSELKLKF	EMTEQEKQSI	TDELKQCKNN	LKLLREKGNN	KPWPWMPMLA	ALVAVTAIVL	YVPGLARASP	

Cmpd.	No. of Cmpds.	m/z meas.	Δ m/z [ppm]	z	Rt [min]	Score	P	Range	Sequence	Modification
1042	1	586.1730	-249.89	2	41.3	10.5	0	467-476	K.ELIEAQELAR.T	

Detailed Protein Report

Protein 255: nuclear receptor subfamily 1 group D member 1 [Homo sapiens]

Accession: gi|13430848
Database: refseq_human(refseq_human_20140103.fasta)
Database Date: 2015-11-30
Modification(s): Carbamidomethyl, Oxidation

Score: 26.1
MW [kDa]: 66.8
pI: 9.9
Sequence Coverage [%]: 2.9
No. of unique Peptides: 1

10	20	30	40	50	60	70	80
MTTLDSNNNT	GGVITYIGSS	GSSPSRTSPE	SLYSDNSNGS	FQSLTQGCPT	YFPPSPTGSL	TQDPARSFGS	IPPSLSDDGS
**90**	**100**	**110**	**120**	**130**	**140**	**150**	**160**
PSSSSSSSSS	SSSFYNGSPP	GSLQVAMEDS	SRVSPSKSTS	NITKLNGMVL	LCKVCGDVAS	GFHYGVHACE	GCKGFFRRSI
**170**	**180**	**190**	**200**	**210**	**220**	**230**	**240**
QQNIQYKRCL	KNENCSIVRI	NRNRCQQCRF	KKCLSVGMSR	DAVRFGRIPK	REKQRMLAEM	QSAMNLANNQ	LSSQCPLETS
**250**	**260**	**270**	**280**	**290**	**300**	**310**	**320**
PTQHPTPGPM	GPSPPPAPVP	SPLVGFSQFP	QQLTPPRSPS	PEPTVEDVIS	QVARAHREIF	TYAHDKLGSS	PGNFNANHAS
**330**	**340**	**350**	**360**	**370**	**380**	**390**	**400**
GSPPATTPHR	WENQGCPPAP	NDNNTLAAQR	HNEALNGLRQ	APSSYPPTWP	PGPAHHSCHQ	SNSNGHRLCP	THVYAAPEGK
**410**	**420**	**430**	**440**	**450**	**460**	**470**	**480**
APANSPRQGN	SKNVLLACPM	NMYPHGRSGR	TVQEIWEDFS	MSFTPAVREV	VEFAKHIPGF	RDLSQHDQVT	LLKAGTFEVL
**490**	**500**	**510**	**520**	**530**	**540**	**550**	**560**
MVRFASLFNV	KDQTVMFLSR	TTYSLQELGA	MGMGDLLSAM	FDFSEKLNSL	ALTEEELGLF	TAVVLVSADR	SGMENSASVE
**570**	**580**	**590**	**600**	**610**	**620**		
QLQETLLRAL	RALVLKNRPL	ETSRFTKLLL	KLPDLRTLNN	MHSEKLLSFR	VDAQ		

Cmpd.	No. of Cmpds.	m/z meas.	Δ m/z [ppm]	z	Rt [min]	Score	P	Range	Sequence	Modification
116	1	1044.8604	-131.76	2	30.7	26.1	1	413-430	K.NVLLACPMNMYPHGRSGR.T	Carbamidomethyl: 6; Oxidation: 10

Detailed Protein Report

Protein 256: PREDICTED: bromodomain-containing protein 1 isoform X12 [Homo sapiens]

Accession: gi|578837129
Database: refseq_human(refseq_human_20140103.fasta)
Database Date: 2015-11-30
Modification(s): Carbamidomethyl

Score: 26.1
MW [kDa]: 110.4
pI: 9.9
Sequence Coverage [%]: 3.9
No. of unique Peptides: 1

Quantitation

WD:WU **Median:** 0.87 **CV:** 0.00 % **No. of Peptides:** 1

10	20	30	40	50	60	70	80
MKSFTAPWER	VGFQGLLVVR	ATQDFAVEGK	MTGNHYQMRR	KGRCHRGSAA	RHPSSPCSVK	HSPTRETLTY	AQAQRMVEIE
**90**	**100**	**110**	**120**	**130**	**140**	**150**	**160**
IEGRLHRISI	FDPLEIILED	DLTAQEMSEC	NSNKENSERP	PVCLRTKRHK	NNRVKKKNEA	LPSAHGTPAS	ASALPEPKVR
**170**	**180**	**190**	**200**	**210**	**220**	**230**	**240**
IVEYSPPSAP	RRPPVYYKFI	EKSAEELDNE	VEYDMDEEDY	AWLEIVNEKR	KGDCVPAVSQ	SMFEFLMDRF	EKESHCENQK
**250**	**260**	**270**	**280**	**290**	**300**	**310**	**320**
QGEQQSLIDE	DAVCCICMDG	ECQNSNVILF	CDMCNLAVHQ	ECYGVPYIPE	GQWLCRHCLQ	SRARPADCVL	CPNKGGAFKK
**330**	**340**	**350**	**360**	**370**	**380**	**390**	**400**
TDDDRWGHVV	CALWIPEVGF	ANTVFIEPID	GVRNIPPARW	KLTCYLCKQK	GVGACIQCHK	ANCYTAFHVT	CAQKAGLYMK
**410**	**420**	**430**	**440**	**450**	**460**	**470**	**480**
MEPVKELTGG	GTTFSVRKTA	YCDVHTPPGC	TRRPLNIYGD	VEMKNGVCRK	ESSVKTVRST	SKVRKKAKKA	KKALAEPCAV
**490**	**500**	**510**	**520**	**530**	**540**	**550**	**560**
LPTVCAPYIP	PQRLNRIANQ	VAIQRKKQFV	ERAHSYWLLK	RLSRNGAPLL	RRLQSSLQSQ	RSSQQRENDE	EMKAAKEKLK
**570**	**580**	**590**	**600**	**610**	**620**	**630**	**640**
YWQRLRHDLE	RARLLIELLR	KREKLKREQV	KVEQVAMELR	LTPLTVLLRS	VLDQLQDKDP	ARIFAQPVSL	KEVPDYLDHI
**650**	**660**	**670**	**680**	**690**	**700**	**710**	**720**
KHPMDFATMR	KRLEAQGYKN	LHEFEEDFDL	IIDNCMKYNA	RDTVFYRAAV	RLRDQGGVVL	RQARREVDSI	GLEEASGMHL
**730**	**740**	**750**	**760**	**770**	**780**	**790**	**800**
PERPAAAPRR	PFSWEDVDRL	LDPANRAHLG	LEEQLRELLD	MLDLTCAMKS	SGSRSKRAKL	LKKEIALLRN	KLSQQHSQPL
**810**	**820**	**830**	**840**	**850**	**860**	**870**	**880**
PTGPGLEGFE	EDGAALGPEA	GEEGDKSPPK	LEPSDALPLP	SNSETNSEPP	TLKPVELNPE	QSKLFKRVTF	DNESHSACTQ
**890**	**900**	**910**	**920**	**930**	**940**	**950**	**960**
SALVSGRPPE	PTRASSGDVP	AAAASAVAEP	ASDVNRRTSV	LFCKSKSSFR	GWRLFCSQGK	GRGAHAETPR	WRRSPQESAW
**970**	**980**						
TQVLTLTTNV	LGSSLFGP						

Cmpd.	No. of Cmpds.	m/z meas.	Δ m/z [ppm]	z	Rt [min]	Score	P	Range	Sequence	Modification	Ratios
832	1	902.3468	-117.98	2	39.2	13.7	1	303-319	R.ARPADCVLCPNKGGAFK.K	Carbamidomethyl: 6	WD:WU 0.87

Detailed Protein Report

Protein 257: long-chain-fatty-acid--CoA ligase 3 [Homo sapiens]

**Accession:**	gi|42794752	**Score:**	26.1
**Database:**	refseq_human(refseq_human_20140103.fasta)	**MW [kDa]:**	80.4
**Database Date:**	2015-11-30	**pI:**	9.4
		**Sequence Coverage [%]:**	4.9
		**No. of unique Peptides:**	1

Alias proteins:

Accession	Name	Description
gi|42794754	refseq_human (refseq_human_20140103.fasta)	long-chain-fatty-acid--CoA ligase 3 [Homo sapiens]

10	20	30	40	50	60	70	80
MNNHVSSKPS	TMKLKHTINP	ILLYFIHFLI	SLYTILTYIP	FYFFSESRQE	KSNRIKAKPV	NSKPDSAYRS	VNSLDGLASV
**90**	**100**	**110**	**120**	**130**	**140**	**150**	**160**
LYPGCDTLDK	VFTYAKNKFK	NKRLLGTREV	LNEEDEVQPN	GKIFKKVILG	QYNWLSYEDV	FVRAFNFGNG	LQMLGQKPKT
**170**	**180**	**190**	**200**	**210**	**220**	**230**	**240**
NIAIFCETRA	EWMIAAQACF	MYNFQLVTLY	ATLGGPAIVH	ALNETEVTNI	ITSKELLQTK	LKDIVSLVPR	LRHIITVDGK
**250**	**260**	**270**	**280**	**290**	**300**	**310**	**320**
PPTWSEFPKG	IIVHTMAAVE	ALGAKASMEN	QPHSKPLPSD	IAVIMYTSGS	TGLPKGVMIS	HSNIIAGITG	MAERIPELGE
**330**	**340**	**350**	**360**	**370**	**380**	**390**	**400**
EDVYIGYLPL	AHVLELSAEL	VCLSHGCRIG	YSSPQTLADQ	SSKIKKGSKG	DTSMLKPTLM	AAVPEIMDRI	YKNVMNKVSE
**410**	**420**	**430**	**440**	**450**	**460**	**470**	**480**
MSSFQRNLFI	LAYNYKMEQI	SKGRNTPLCD	SFVFRKVRSL	LGGNIRLLLC	GGAPLSATTQ	RFMNICFCCP	VGQGYGLTES
**490**	**500**	**510**	**520**	**530**	**540**	**550**	**560**
AGAGTISEVW	DYNTGRVGAP	LVCCEIKLKN	WEEGGYFNTD	KPHPRGEILI	GGQSVTMGYY	KNEAKTKADF	FEDENGQRWL
**570**	**580**	**590**	**600**	**610**	**620**	**630**	**640**
CTGDIGEFEP	DGCLKIIDRK	KDLVKLQAGE	YVSLGKVEAA	LKNLPLVDNI	CAYANSYHSY	VIGFVVPNQK	ELTELARKKG
**650**	**660**	**670**	**680**	**690**	**700**	**710**	**720**
LKGTWEELCN	SCEMENEVLK	VLSEAAISAS	LEKFEIPVKI	RLSPEPWTPE	TGLVTDAFKL	KRKELKTHYQ	ADIERMYGRK
**730**							

Cmpd.	No. of Cmpds.	m/z meas.	Δ m/z [ppm]	z	Rt [min]	Score	P	Range	Sequence	Modification
1801	1	785.8979	-30.88	2	51.2	15.7	1	2-15	M.NNHVSSKPSTMKLK.H	

Detailed Protein Report

Protein 258: PREDICTED: zinc finger protein 449 isoform X1 [Homo sapiens]

Accession: gi|530422388
Database: refseq_human(refseq_human_20140103.fasta)
Database Date: 2015-11-30
Modification(s): Carbamidomethyl

Score: 26.1
MW [kDa]: 32.2
pI: 10.5
Sequence Coverage [%]: 11.6
No. of unique Peptides: 1

10	20	30	40	50	60	70	80
METMYPFIVT	LEGNALQGPI	LQKDYVQLEN	QWETPPEDLQ	TDLAKLVDQQ	NPTLGETPEN	SNLEEPLNPK	PHKKKSPGEK
**90**	**100**	**110**	**120**	**130**	**140**	**150**	**160**
PHRCPQCGKC	FARKSQLTGH	QRIHSGEEPH	KCPECGKRFL	RSSDLYRHQR	LHTGERPYEC	TVCKKRFTRR	SHLIGHQRTH
**170**	**180**	**190**	**200**	**210**	**220**	**230**	**240**
SEEETYKCLE	CGKSFCHGSS	LKRHLKTHTG	EKPHRCHNCG	KSFSRLTALT	LHQRTHTEER	PFKCNYCGKS	FRQRPSLVIH
**250**	**260**	**270**	**280**				
LRIHTGEKPY	KCTHCSKSFR	QRAGLIMHQV	THFRGLI				

Cmpd.	No. of Cmpds.	m/z meas.	Δ m/z [ppm]	z	Rt [min]	Score	P	Range	Sequence	Modification
2874	1	964.7996	7.38	3	64.7	11.1	2	122-144	R.SSDLYRHQRLHTGERPYECTVCK.K	Carbamidomethyl: 19, 22

Detailed Protein Report

Protein 259: treacle protein isoform b [Homo sapiens]

**Accession:**	gi|57164975	**Score:**	26.0
**Database:**	refseq_human(refseq_human_20140103.fasta)	**MW [kDa]:**	144.2
**Database Date:**	2015-11-30	**pI:**	9.6
		**Sequence Coverage [%]:**	1.9
		**No. of unique Peptides:**	1

10	20	30	40	50	60	70	80
MAEARKRREL	LPLIYHHLLR	AGYVRAAREV	KEQSGQKCFL	AQPVTLLDIY	THWQQTSELG	RKRKAEEDAA	LQAKKTRVSD
90	100	110	120	130	140	150	160
PISTSESSEE	EEEAEAETAK	ATPRLASTNS	SVLGADLPSS	MKEKAKAETE	KAGKTGNSMP	HPATGKTVAN	LLSGKSPRKS
170	180	190	200	210	220	230	240
AEPSANTTLV	SETEEEGSVP	AFGAAAKPGM	VSAGQADSSS	EDTSSSSDET	DVEVKASEKI	LQVRAASAPA	KGTPGKGATP
250	260	270	280	290	300	310	320
APPGKAGAVA	SQTKAGKPEE	DSESSSEESS	DSEEETPAAK	ALLQAKASGK	TSQVGAASAP	AKESPRKGAA	PAPPGKTGPA
330	340	350	360	370	380	390	400
VAKAQAGKRE	EDSQSSSEES	DSEEEAPAQA	KPSGKAPQVR	AASAPAKESP	RKGAAPAPPR	KTGPAAAQVQ	VGKQEEDSRS
410	420	430	440	450	460	470	480
SSEESDSDRE	ALAAMNAAQV	KPLGKSPQVK	PASTMGMGPL	GKGAGPVPPG	KVGPATPSAQ	VGKWEEDSES	SSEESSDSSD
490	500	510	520	530	540	550	560
GEVPTAVAPA	QEKSLGNILQ	AKPTSSPAKG	PPQKAGPVAV	QVKAEKPMDN	SESSEESSDS	ADSEEAPAAM	TAAQAKPALK
570	580	590	600	610	620	630	640
IPQTKACPKK	TNTTASAKVA	PVRVGTQAPR	KAGTATSPAG	SSPAVAGGTQ	RPAEDSSSSE	ESDSEEEKTG	LAVTVGQAKS
650	660	670	680	690	700	710	720
VGKGLQVKAA	SVPVKGSLGQ	GTAPVLPGKT	GPTVTQVKAE	KQEDSESSEE	ESDSEEAAAS	PAQVKTSVKK	TQAKANPAAA
730	740	750	760	770	780	790	800
RAPSAKGTIS	APGKVVTAAA	QAKQRSPSKV	KPPVRNPQNS	TVLARGPASV	PSVGKAVATA	AQAQTGPEED	SGSSEEESDS
810	820	830	840	850	860	870	880
EEEAETLAQV	KPSGKTHQIR	AALAPAKESP	RKGAAPTPPG	KTGPSAAQAG	KQDDSGSSSE	ESDSDGEAPA	AVTSAQVIKP
890	900	910	920	930	940	950	960
PLIFVDPNRS	PAGPAATPAQ	AQAASTPRKA	RASESTARSS	SSESEDEDVI	PATQCLTPGI	RTNVVTMPTA	HPRIAPKASM
970	980	990	1000	1010	1020	1030	1040
AGASSSKESS	RISDGKKQEG	PATQVSKKNP	ASLPLTQAAL	KVLAQKASEA	QPPVARTQPS	SGVDSAVGTL	PATSPQSTSV
1050	1060	1070	1080	1090	1100	1110	1120
QAKGTNKLRK	PKLPEVQQAT	KAPESSDDSE	DSSDSSSGSE	EDGEGPQGAK	SAHTLGPTPS	RTETLVEETA	AESSEDDVVA
1130	1140	1150	1160	1170	1180	1190	1200
PSQSLLSGYM	TPGLTPANSQ	ASKATPKLDS	SPSVSSTLAA	KDDPDGKQEA	KPQQAAGMLS	PKTGGKEAAS	GTTPQKSRKP
1210	1220	1230	1240	1250	1260	1270	1280
KKGAGNPQAS	TLALQSNITQ	CLLGQPWPLN	EAQVQASVVK	VLTELLEQER	KKVVDTTKES	SRKGWESRKR	KLSGDQPAAR
1290	1300	1310	1320	1330	1340	1350	1360
TPRSKKKKKL	GAGEGGEASV	SPEKTSTTSK	GKAKRDKASG	DVKEKKGKGS	LGSQGAKDEP	EEELQKGMGT	VEGGDQSNPK
1370	1380	1390	1400	1410	1420		
SKKEKKKSDK	RKKDKEKKEK	KKKAKKASTK	DSESPSQKKK	KKKKKTAEQT	V		

Cmpd.	No. of Cmpds.	m/z meas.	Δ m/z [ppm]	z	Rt [min]	Score	P	Range	Sequence	Modification
2600	1	625.2017	-222.77	2	60.8	10.7	2	1252-1262	K.KVVDTTKESSR.K	

Detailed Protein Report

Protein 260: **polyadenylate-binding protein 4-like [Homo sapiens]**

Accession: gi|291084655
Database: refseq_human(refseq_human_20140103.fasta)
Database Date: 2015-11-30

Score: 26.0
MW [kDa]: 48.1
pI: 9.9
Sequence Coverage [%]: 6.3
No. of unique Peptides: 1

10	20	30	40	50	60	70	80
MRVLPELGES	CLNSIAWPLC	GDPEASTLEP	GQSCDLVSPH	RDCSKNSRGQ	THSGKDKEMN	VAAKYRMASL	YVGDLHADVT
**90**	**100**	**110**	**120**	**130**	**140**	**150**	**160**
EDLLFRKFST	VGPVLSIRIC	RDQVTRRSLG	YAYVNFLQLA	DAQKALDTMN	FDIIKGKSIR	LMWSQRDAYL	RRSGIGNVFI
**170**	**180**	**190**	**200**	**210**	**220**	**230**	**240**
KNLDKSIDNK	TLYEHFSAFG	KILSSKVMSD	DQGSKGYAFV	HFQNQSAADR	AIEEMNGKLL	KGCKVFVGRF	KNRKDREAEL
**250**	**260**	**270**	**280**	**290**	**300**	**310**	**320**
RSKASEFTNV	YIKNFGGDMD	DERLKDVFSK	YGKTLSVKVM	TDSSGKSKGF	GFVSFDSHEA	AKKAVEEMNG	RDINGQLIFV
**330**	**340**	**350**	**360**	**370**	**380**	**390**	**400**
GRAQKKVERQ	AELKQMFEQL	KRERIRGCQG	VKLYIKNLDD	TIDDEKLRNE	FSSFGSISRV	KVMQEEGQSK	GFGLICFSSP
**410**	**420**	**430**					
EDATKAMTEM	NGRILGSKPL	SIALAQRH					

Cmpd.	No. of Cmpds.	m/z meas.	Δ m/z [ppm]	z	Rt [min]	Score	P	Range	Sequence	Modification
1937	1	847.8487	-93.34	2	52.6	15.3	1	353-366	K.LYIKNLDDTIDDEK.L	

Detailed Protein Report

Protein 261: **PREDICTED: mediator of RNA polymerase II transcription subunit 1 isoform X1 [Homo sapiens]**

**Accession:**	gi|530412429	**Score:**	26.0
**Database:**	refseq_human(refseq_human_20140103.fasta)	**MW [kDa]:**	149.1
**Database Date:**	2015-11-30	**pI:**	9.6
**Modification(s):**	Carbamidomethyl	**Sequence Coverage [%]:**	2.8
		**No. of unique Peptides:**	2

10	20	30	40	50	60	70	80
MYLALQSLEQ	DLSKMAIMYW	KATNAGPLDK	ILHGSVGYLT	PRSGGHLMNL	KYYVSPSDLL	DDKTASPIIL	HENNVSRSLG
90	100	110	120	130	140	150	160
MNASVTIEGT	SAVYKLPIAP	LIMGSHPVDN	KWTPSFSSIT	SANSVDLPAC	FFLKFPQPIP	VSRAFVQKLQ	NCTGIPLFET
170	180	190	200	210	220	230	240
QPTYAPLYEL	ITQFELSKDP	DPIPLNHNMR	FYAALPGQQH	CYFLNKDAPL	PDGRSLQGTL	VSKITFQHPG	RVPLILNLIR
250	260	270	280	290	300	310	320
HQVAYNTLIG	SCVKRTILKE	DSPGLLQFEV	CPLSESRFSV	SFQHPVNDSL	VCVVMDVQDS	THVSCKLYKG	LSDALICTDD
330	340	350	360	370	380	390	400
FIAKVVQRCM	SIPVTMRAIR	RKAETIQADT	PALSLIAETV	EDMVKKNLPP	ASSPGYGMTT	GNNPMSGTTT	PTNTFPGGPI
410	420	430	440	450	460	470	480
TTLFNMSMSI	KDRHESVGHG	EDFSKVSQNP	ILTSLLQITG	NGGSTIGSSP	TPPHHTPPPV	SSMAGNTKNH	PMLMNLLKDN
490	500	510	520	530	540	550	560
PAQDFSTLYG	SSPLERQNSS	SGSPRMEICS	GSNKTKKKKS	SRLPPEKPKH	QTEDDFQREL	FSMDVDSQNP	IFDVNMTADT
570	580	590	600	610	620	630	640
LDTPHITPAP	SQCSTPPTTY	PQPVPHPQPS	IQRMVRLSSS	DSIGPDVTDI	LSDIAEEASK	LPSTSDDCPA	IGTPLRDSSS
650	660	670	680	690	700	710	720
SGHSQSTLFD	SDVFQTNNNE	NPYTDPADLI	ADAAGSPSSD	SPTNHFFHDG	VDFNPDLLNS	QSQSGFGEEY	FDESSQSGDN
730	740	750	760	770	780	790	800
DDFKGFASQA	LNTLGVPMLG	GDNGETKFKG	NNQADTVDFS	IISVAGKALA	PADLMEHHSG	SQGPLLTTGD	LGKEKTQKRV
810	820	830	840	850	860	870	880
KEGNGTSNST	LSGPGLDSKP	GKRSRTPSND	GKSKDKPPKR	KKADTEGKSP	SHSSSNRPFT	PPTSTGGSKS	PGSAGRSQTP
890	900	910	920	930	940	950	960
PGVATPPIPK	ITIQIPKGTV	MVGKPSSHSQ	YTSSGSVSSS	GSKSHHSHSS	SSSSSASTSG	KMKSSKSEGS	SSSKLSSSMY
970	980	990	1000	1010	1020	1030	1040
SSQGSSGSSQ	SKNSSQSGGK	PGSSPITKHG	LSSGSSSTKM	KPQGKPSSLM	NPSLSKPNIS	PSHSRPPGGS	DKLASPMKPV
1050	1060	1070	1080	1090	1100	1110	1120
PGTPPSSKAK	SPISSGSGGS	HMSGTSSSSG	MKSSSGLGSS	GSLSQKTPPS	SNSCTASSSS	FSSSGSSMSS	SQNQHGSSKG
1130	1140	1150	1160	1170	1180	1190	1200
KSPSRNKKPS	LTAVIDKLKH	GVVTSGPGGE	DPLDGQMGVS	TNSSSHPMSS	KHNMSGGEFQ	GKREKSDKDK	SKVSTSGSSV
1210	1220	1230	1240	1250	1260	1270	1280
DSSKKTSESK	NVGSTGVAKI	IISKHDGGSP	SIKAKVTLQK	PGESSGEGLR	PQMASSKNYG	SPLISGSTPK	HERGSPSHSK
1290	1300	1310	1320	1330	1340	1350	1360
SPAYTPQNLD	SESESGSSIA	EKSYQNSPSS	DDGIRPLPEY	STEKHKKHKK	EKKKVKDKDR	DRDRDKDRDK	KKSHSIKPES
1370	1380	1390	1400	1410			
WSKSPISSDQ	SLSMTSNTIL	SADRPSRLSP	DFMIGEEDDD	LMDVALIGN			

Cmpd.	No. of Cmpds.	m/z meas.	Δ m/z [ppm]	z	Rt [min]	Score	P	Range	Sequence	Modification
2702	1	842.5321	94.33	3	62.2	11.9	2	307-328	K.LYKGLSDALICTDDFIAKVVQR.C	Carbamidomethyl: 11
1993	1	918.3565	-113.08	2	53.3	14.1	0	750-767	K.GNNQADTVDFSIISVAGK.A	

Detailed Protein Report

Protein 262: putative hexokinase HKDC1 [Homo sapiens]

Accession: gi|156151420
Database: refseq_human(refseq_human_20140103.fasta)
Database Date: 2015-11-30
Modification(s): Oxidation

Score: 26.0
MW [kDa]: 102.4
pI: 7.2
Sequence Coverage [%]: 3.3
No. of unique Peptides: 2

10	20	30	40	50	60	70	80
MFAVHLMAFY	FSKLKEDQIK	KVDRFLYHMR	LSDDTLLDIM	RRFRAEMEKG	LAKDTNPTAA	VKMLPTFVRA	IPDGSENGEF
90	100	110	120	130	140	150	160
LSLDLGGSKF	RVLKVQVAEE	GKRHVQMESQ	FYPTPNEIIR	GNGTELFEYV	ADCLADFMKT	KDLKHKKLPL	GLTFSFPCRQ
170	180	190	200	210	220	230	240
TKLEEGVLLS	WTKKFKARGV	QDTDVVSRLT	KAMRRHKDMD	VDILALVNDT	VGTMMTCAYD	DPYCEVGVII	GTGTNACYME
250	260	270	280	290	300	310	320
DMSNIDLVEG	DEGRMCINTE	WGAFGDDGAL	EDIRTEFDRE	LDLGSLNPGK	QLFEKMISGL	YLGELVRLIL	LKMAKAGLLF
330	340	350	360	370	380	390	400
GGEKSSALHT	KGKIETRHVA	AMEKYKEGLA	NTREILVDLG	LEPSEADCIA	VQHVCTIVSF	RSANLCAAAL	AAILTRLREN
410	420	430	440	450	460	470	480
KKVERLRTTV	GMDGTLYKIH	PQYPKRLHKV	VRKLVPSCDV	RFLLSESGST	KGAAMVTAVA	SRVQAQRKQI	DRVLALFQLT
490	500	510	520	530	540	550	560
REQLVDVQAK	MRAELEYGLK	KKSHGLATVR	MLPTYVCGLP	DGTEKGKFLA	LDLGGTNFRV	LLVKIRSGRR	SVRMYNKIFA
570	580	590	600	610	620	630	640
IPLEIMQGTG	EELFDHIVQC	IADFLDYMGL	KGASLPLGFT	FSFPCRQMSI	DKGTLIGWTK	GFKATDCEGE	DVVDMLREAI
650	660	670	680	690	700	710	720
KRRNEFDLDI	VAVVNDTVGT	MMTCGYEDPN	CEIGLIAGTG	SNMCYMEDMR	NIEMVEGGEG	KMCINTEWGG	FGDNGCIDDI
730	740	750	760	770	780	790	800
RTRYDTEVDE	GSLNPGKQRY	EKMTSGMYLG	EIVRQILIDL	TKQGLLFRGQ	ISERLRTRGI	FETKFLSQIE	SDRLALLQVR
810	820	830	840	850	860	870	880
RILQQLGLDS	TCEDSIVVKE	VCGAVSRRAA	QLCGAGLAAI	VEKRREDQGL	EHLRITVGVD	GTLYKLHPHF	SRILQETVKE
890	900	910	920				
LAPRCDVTFM	LSEDGSGKGA	ALITAVAKRL	QQAQKEN				

Cmpd.	No. of Cmpds.	m/z meas.	Δ m/z [ppm]	z	Rt [min]	Score	P	Range	Sequence	Modification
2718	1	648.3573	22.96	2	62.4	12.9	2	43-53	R.FRAEMEKGLAK.D	Oxidation: 5
2984	5	973.5068	-23.83	2	66.1	13.0	2	313-331	K.MAKAGLLFGGEKSSALHTK.G	

Detailed Protein Report

Protein 263: **PREDICTED: zinc finger protein with KRAB and SCAN domains 4 isoform X3 [Homo sapiens]**

**Accession:**	gi|530381975	**Score:**	25.9
**Database:**	refseq_human(refseq_human_20140103.fasta)	**MW [kDa]:**	49.6
**Database Date:**	2015-11-30	**pI:**	9.3
**Modification(s):**	Carbamidomethyl	**Sequence Coverage [%]:**	7.5
		**No. of unique Peptides:**	2

Alias proteins:

Accession	Name	Description
gi|530381977	refseq_human (refseq_human_20140103.fasta)	PREDICTED: zinc finger protein with KRAB and SCAN domains 4 isoform X4 [Homo sapiens]

10	20	30	40	50	60	70	80
MAREPRKNAA	LDAQSAEDQT	GLLTVKVEKE	EASALTAEVP	VGDQGQELLC	CKMALLTQTQ	GSQSSQCQPM	KALFKHESLG
**90**	**100**	**110**	**120**	**130**	**140**	**150**	**160**
SQPLHDRVLQ	VPGLAQGGCC	REDAMVASRL	TPGSQGLLKM	EDVALTLTPG	WTQLDSSQVN	LYRDEKQENH	SSLVSLGGEI
**170**	**180**	**190**	**200**	**210**	**220**	**230**	**240**
QTKSRDLPPV	KKLPEKEHGK	ICHLREDIAQ	IPTHAEAGEQ	EGRLQRKQKN	AIGSRRHYCH	ECGKSFAQSS	GLTKHRRIHT
**250**	**260**	**270**	**280**	**290**	**300**	**310**	**320**
GEKPYECEDC	GKTFIGSSAL	VIHQRVHTGE	KPYECEECGK	VFSHSSNLIK	HQRTHTGEKP	YECDDCGKTF	SQSCSLLEHH
**330**	**340**	**350**	**360**	**370**	**380**	**390**	**400**
KIHTGEKPYQ	CNMCGKAFRR	NSHLLRHQRI	HGDKNVQNPE	HGESWESQGR	TESQWENTEA	PVSYKCNECE	RSFTRNRSLI
**410**	**420**	**430**	**440**	**450**			
EHQKIHTGEK	PYQCDTCGKG	FTRTSYLVQH	QRSHVGKKTL	SQ			

Cmpd.	No. of Cmpds.	m/z meas.	Δ m/z [ppm]	z	Rt [min]	Score	P	Range	Sequence	Modification
2714	2	973.1242	130.42	2	62.3	15.6	0	8-26	K.NAALDAQSAEDQTGLLTVK.V	
2444	1	729.3737	3.44	2	58.9	10.3	0	88-101	R.VLQVPGLAQGGCCR.E	Carbamidomethyl: 12

Detailed Protein Report

Protein 264: PREDICTED: sp110 nuclear body protein isoform X3 [Homo sapiens]

**Accession:**	gi|530370295	**Score:**	25.9
**Database:**	refseq_human(refseq_human_20140103.fasta)	**MW [kDa]:**	58.5
**Database Date:**	2015-11-30	**pI:**	10.2
		**Sequence Coverage [%]:**	6.3
		**No. of unique Peptides:**	1

10	20	30	40	50	60	70	80
MNAEEDSEEM	PSLLTSTVQV	ASDNLIPQIR	DKEDPQEMPH	SPLGSMPEIR	DNSPEPNDPE	EPQEVSSTPS	DKKGKKRKRC
**90**	**100**	**110**	**120**	**130**	**140**	**150**	**160**
IWSTPKRRHK	KKSLPGGTAS	SRHGIQKKLK	RVDQVPQKKD	DSTCNSTVET	RAQKARTECA	RKSRSEEIID	GTSEMNEGKR
**170**	**180**	**190**	**200**	**210**	**220**	**230**	**240**
SQKTPSTPRR	VTQGAASPGH	GIQEKLQVVD	KVTQRKDDST	WNSEVMMRVQ	KARTKCARKS	RLKEKKKEKD	ICSSSKRRFQ
**250**	**260**	**270**	**280**	**290**	**300**	**310**	**320**
KNIHRRGKPK	SDTVDFHCSK	LPVTCGEAKG	ILYKKKMKHG	SSVKCIRNED	GTWLTPNEFE	VEGKGRNAKN	WKRNIRCEGM
**330**	**340**	**350**	**360**	**370**	**380**	**390**	**400**
TLGELLKRKN	SDECEVCCQG	GQLLCCGTCP	RVFHEDCHIP	PVEAKRMLWS	CTFCRMKRSS	GSQQCHHVSK	TLERQMQPQD
**410**	**420**	**430**	**440**	**450**	**460**	**470**	**480**
QLKCEFLLLK	AYCHPQSSFF	TGIPFNIRDY	GEPFQEAMWL	DLVKERLITE	MYTVAWFVRD	MRLMFRNHKT	FYKASDFGQV
**490**	**500**	**510**	**520**				
GLDLEAEFEK	DLKDVLGFHE	ANDGGFWTLP					

Cmpd.	No. of Cmpds.	m/z meas.	Δ m/z [ppm]	z	Rt [min]	Score	P	Range	Sequence	Modification
1618	1	882.8999	95.52	3	48.5	11.3	2	171-195	R.VTQGAASPGHGIQEKLQVVDKVTQR.K	

Detailed Protein Report

Protein 265: phosphatidylinositol phosphatase PTPRQ precursor [Homo sapiens]

**Accession:**	gi|222537743	**Score:**	25.8
**Database:**	refseq_human(refseq_human_20140103.fasta)	**MW [kDa]:**	257.1
**Database Date:**	2015-11-30	**pI:**	5.4
**Modification(s):**	Oxidation	**Sequence Coverage [%]:**	1.2
		**No. of unique Peptides:**	2

Detailed Protein Report

10	20	30	40	50	60	70	80
MDFLIIFLLL	FIGTSETQVD	VSNVVPGTRY	DITISSISTT	YTSPVTRIVT	TNVTKPGPPV	FLAGERVGSA	GILLSWNTPP
90	100	110	120	130	140	150	160
NPNGRIISYI	VKYKEVCPWM	QTVYTQVRSK	PDSLEVLLTN	LNPGTTYEIK	VAAENSAGIG	VFSDPFLFQT	AESAPGKVVN
170	180	190	200	210	220	230	240
LTVEAYNASA	VKLIWYLPRQ	PNGKITSFKI	SVKHARSGIV	VKDVSIRVED	ILTGKLPECN	ENSESFLWST	ASPSPTLGRV
250	260	270	280	290	300	310	320
TPPSRTTHSS	STLTQNEISS	VWKEPISFVV	THLRPYTTYL	FEVSAATTEA	GYIDSTIVRT	PESVPEGPPQ	NCVTGNITGK
330	340	350	360	370	380	390	400
SFSILWDPPT	IVTGKFSYRV	ELYGPSGRIL	DNSTKDLKFA	FTNLTPFTMY	DVYIAAETSA	GTGPKSNISV	FTPPDVPGAV
410	420	430	440	450	460	470	480
FDLQLAEVES	TQVRITWKKP	RQPNGIINQY	RVKVLVPETG	IILENTLLTG	NNEYINDPMA	PEIVNIVEPM	VGLYEGSAEM
490	500	510	520	530	540	550	560
SSDLHSLATF	IYNSHPDKNF	PARNRAEDQT	SPVVTTRNQY	ITDIAAEQLS	YVIRRLVPFT	EHMISVSAFT	IMGEGPPTVL
570	580	590	600	610	620	630	640
SVRTRQQVPS	SIKIINYKNI	SSSSILLYWD	PPEYPNGKIT	HYTIYAMELD	TNRAFQITTI	DNSFLITGLK	KYTKYKMRVA
650	660	670	680	690	700	710	720
ASTHVGESSL	SEENDIFVRT	SEDEPESSPQ	DVEVIDVTAD	EIRLKWSPPE	KPNGIIIAYE	VLYKNIDTLY	MKNTSTTDII
730	740	750	760	770	780	790	800
LRNLRPHTLY	NISVRSYTRF	GHGNQVSSLL	SVRTSETVPD	SAPENITYKN	ISSGEIELSF	LPPSSPNGII	QKYTIYLKRS
810	820	830	840	850	860	870	880
NGNEERTINT	TSLTQNIKVL	KKYTQYIIEV	SASTLKGEGV	RSAPISILTE	EDAPDSPPQD	FSVKQLSGVT	VKLSWQPPLE
890	900	910	920	930	940	950	960
PNGIILYYTV	YVWNRSSLKT	INVTETSLEL	SDLDYNVEYS	AYVTASTRFG	DGKTRSNIIS	FQTPEGAPSD	PPKDVYYANL
970	980	990	1000	1010	1020	1030	1040
SSSSIILFWT	PPSKPNGIIQ	YYSVYYRNTS	GTFMQNFTLH	EVTNDFDNMT	VSTIIDKLTI	FSYYTFWLTA	STSVGNGNKS
1050	1060	1070	1080	1090	1100	1110	1120
SDIIEVYTDQ	DIPEGFVGNL	TYESISSTAI	NVSWVPPAQP	NGLVFYYVSL	ILQQTPRHVR	PPLVTYERSI	YFDNLEKYTD
1130	1140	1150	1160	1170	1180	1190	1200
YILKITPSTE	KGFSDTYTAQ	LYIKTEEDVP	ETSPIINTFK	NLSSTSVLLS	WDPPVKPNGA	IISYDLTLQG	PNENYSFITS
1210	1220	1230	1240	1250	1260	1270	1280
DNYIILEELS	PFTLYSFFAA	ARTRKGLGPS	SILFFYTDES	VPLAPPQNLT	LINCTSDFVW	LKWSPSPLPG	GIVKVYSFKI
1290	1300	1310	1320	1330	1340	1350	1360
HEHETDTIYY	KNISGFKTEA	KLVGLEPVST	YSIRVSAFTK	VGNGNQFSNV	VKFTTQESVP	DVVQNMQCMA	TSWQSVLVKW
1370	1380	1390	1400	1410	1420	1430	1440
DPPKKANGII	TQYMVTVERN	STKVSPQDHM	YTFIKLLANT	SYVFKVRAST	SAGEGDESTC	HVSTLPETVP	SVPTNIAFSD
1450	1460	1470	1480	1490	1500	1510	1520
VQSTSATLTW	IRPDTILGYF	QNYKITTQLR	AQKCKEWESE	ECVEYQKIQY	LYEAHLTEET	VYGLKKFRWY	RFQVAASTNA
1530	1540	1550	1560	1570	1580	1590	1600
GYGNASNWIS	TKTLPGPPDG	PPENVHVVAT	SPFSISISWS	EPAVITGPTC	YLIDVKSVDN	DEFNISFIKS	NEENKTIEIK
1610	1620	1630	1640	1650	1660	1670	1680
DLEIFTRYSV	VITAFTGNIS	AAYVEGKSSA	EMIVTTLESA	PKDPPNNMTF	QKIPDEVTKF	QLTFLPPSQP	NGNIQVYQAL
1690	1700	1710	1720	1730	1740	1750	1760
VYREDDPTAV	QIHNLSIIQK	TNTFVIAMLE	GLKGGHTYNI	SVYAVNSAGA	GPKVPMRITM	DIKAPARPKT	KPTPIYDATG
1770	1780	1790	1800	1810	1820	1830	1840
KLLVTSTTIT	IRMPICYYSD	DHGPIKNVQV	LVTETGAQHD	GNVTKWYDAY	FNKARPYFTN	EGFPNPPCTE	GKTKFSGNEE
1850	1860	1870	1880	1890	1900	1910	1920
IYIIGADNAC	MIPGNEDKIC	NGPLKPKKQY	LFKFRATNIM	GQFTDSDYSD	PVKTLGEGLS	ERTVEIILSV	TLCILSIILL
1930	1940	1950	1960	1970	1980	1990	2000
GTAIFAFARI	RQKQKEGGTY	SPQDAEIIDT	KLKLDQLITV	ADLELKDERL	TRLLSYRKSI	KPISKKSFLQ	HVEELCTNNN
2010	2020	2030	2040	2050	2060	2070	2080
LKFQEEFSEL	PKFLQDLSST	DADLPWNRAK	NRFPNIKPYN	NNRVKLIADA	SVPGSDYINA	SYISGYLCPN	EFIATQGPLP
2090	2100	2110	2120	2130	2140	2150	2160
GTVGDFWRMV	WETRAKTLVM	LTQCFEKGRI	RCHQYWPEDN	KPVTVFGDIV	ITKLMEDVQI	DWTIRDLKIE	RHGDCMTVRQ
2170	2180	2190	2200	2210	2220	2230	2240

Detailed Protein Report

Cmpd.	No. of Cmpds.	m/z meas.	Δ m/z [ppm]	z	Rt [min]	Score	P	Range	Sequence	Modification
13	1	1002.9641	-45.61	2	29.1	11.9	0	30-47	R.YDITISSISTTYTSPVTR.I	
627	1	604.2948	34.08	2	36.7	13.9	0	1643-1652	K.DPPNNMTFQK.I	Oxidation: 6

Detailed Protein Report

Protein 266: sushi domain-containing protein 1 isoform 3 precursor [Homo sapiens]

**Accession:**	gi|544063419	**Score:**	25.8
**Database:**	refseq_human(refseq_human_20140103.fasta)	**MW [kDa]:**	80.4
**Database Date:**	2015-11-30	**pI:**	6.0
**Modification(s):**	Carbamidomethyl	**Sequence Coverage [%]:**	4.0
		**No. of unique Peptides:**	1

Quantitation

WD:WU **Median:** 1.11 **CV:** 0.00 % **No. of Peptides:** 1

10	20	30	40	50	60	70	80
MGRGPWDAGP	SRRLLPLLLL	LGLARGAAGA	PGPDGLDVCA	TCHEHATCQQ	REGKKICICN	YGFVGNGRTQ	CVDKNECQFG
90	100	110	120	130	140	150	160
ATLVCGNHTS	CHNTPGGFYC	ICLEGYRATN	NNKTFIPNDG	TFCTDIDECE	VSGLCRHGGR	CVNTHGSFEC	YCMDGYLPRN
170	180	190	200	210	220	230	240
GPEPFHPTTD	ATSCTEIDCG	TPPEVPDGYI	IGNYTSSLGS	QVRYACREGF	FSVPEDTVSS	CTGLGTWESP	KLHCQEINCG
250	260	270	280	290	300	310	320
NPPEMRHAIL	VGNHSSRLGG	VARYVCQEGF	ESPGGKITSV	CTEKGTWRES	TLTCTEILTK	INDVSLFNDT	CVRWQINSRR
330	340	350	360	370	380	390	400
INPKISYVIS	IKGQRLDPME	SVREETVNLT	TDSRTPEVCL	ALYPGTNYTV	NISTAPPRRS	MPAVIGFQTA	EVDLLEDDGS
410	420	430	440	450	460	470	480
FNISIFNETC	LKLNRRSRKV	GSEHMYQFTV	LGQRWYLANF	SHATSFNFTT	REQVPVVCLD	LYPTTDYTVN	VTLLRSPKRH
490	500	510	520	530	540	550	560
SVQITIATPP	AVKQTISNIS	GFNETCLRWR	SIKTADMEEM	YLFHIWGQRW	YQKEFAQEMT	FNISSSSRDP	EVCLDLRPGT
570	580	590	600	610	620	630	640
NYNVSLRALS	SELPVVISLT	TQITEPPLPE	VEFFTVHRGP	LPRLRLRKAK	EKNGPISSYQ	VLVLPLALQS	TFSCDSEGAS
650	660	670	680	690	700	710	720
SFFSNASDAD	GYVAAELLAK	DVPDDAMEIP	IGDRLYYGEY	YNAPLKRGSD	YCIILRITSE	WNKIRHSCCC	RWRVLDWVPW
730							
LL							

Cmpd.	No. of Cmpds.	m/z meas.	Δ m/z [ppm]	z	Rt [min]	Score	P	Range	Sequence	Modification	Ratios
844	1	776.9364	79.23	2	39.3	10.6	0	301-313	K.INDVSLFNDTCVR.W	Carbamidomethyl: 11	WD:WU 1.11

Detailed Protein Report

Protein 267: nucleus accumbens-associated protein 1 [Homo sapiens]

**Accession:**	gi|16418383	**Score:**	25.8
**Database:**	refseq_human(refseq_human_20140103.fasta)	**MW [kDa]:**	57.2
**Database Date:**	2015-11-30	**pI:**	5.4
		**Sequence Coverage [%]:**	4.9
		**No. of unique Peptides:**	1

Quantitation

WD:WU **Median:** 1.11 **CV:** 0.00 % **No. of Peptides:** 1

Alias proteins:

Accession	Name	Description
gi|578832952	refseq_human (refseq_human_20140103.fasta)	PREDICTED: nucleus accumbens-associated protein 1 isoform X2 [Homo sapiens]
gi|530414464	refseq_human (refseq_human_20140103.fasta)	PREDICTED: nucleus accumbens-associated protein 1 isoform X1 [Homo sapiens]

10	20	30	40	50	60	70	80
MAQTLQMEIP	NFGNSILECL	NEQRLQGLYC	DVSVVVKGHA	FKAHRAVLAA	SSSYFRDLFN	NSRSAVVELP	AAVQPQSFQQ
**90**	**100**	**110**	**120**	**130**	**140**	**150**	**160**
ILSFCYTGRL	SMNVGDQFLL	MYTAGFLQIQ	EIMEKGTEFF	LKVSSPSCDS	QGLHAEEAPS	SEPQSPVAQT	SGWPACSTPL
**170**	**180**	**190**	**200**	**210**	**220**	**230**	**240**
PLVSRVKTEQ	QESDSVQCMP	VAKRLWDSGQ	KEAGGGGNGS	RKMAKFSTPD	LAANRPHQPP	PPQQAPVVAA	AQPAVAAGAG
**250**	**260**	**270**	**280**	**290**	**300**	**310**	**320**
QPAGGVAAAG	GVVSGPSTSE	RTSPGTSSAY	TSDSPGSYHN	EEDEEEDGGE	EGMDEQYRQI	CNMYTMYSMM	NVGQTAEKVE
**330**	**340**	**350**	**360**	**370**	**380**	**390**	**400**
ALPEQVAPES	RNRIRVRQDL	ASLPAELINQ	IGNRCHPKLY	DEGDPSEKLE	LVTGTNVYIT	RAQLMNCHVS	AGTRHKVLLR
**410**	**420**	**430**	**440**	**450**	**460**	**470**	**480**
RLLASFFDRN	TLANSCGTGI	RSSTNDPRRK	PLDSRVLHAV	KYYCQNFAPN	FKESEMNAIA	ADMCTNARRV	VRKSWMPKVK
**490**	**500**	**510**	**520**	**530**			
VLKAEDDAYT	TFISETGKIE	PDMMGVEHGF	ETASHEGEAG	PSAEALQ			

Cmpd.	No. of Cmpds.	m/z meas.	Δ m/z [ppm]	z	Rt [min]	Score	P	Range	Sequence	Modification	Ratios
1836	4	916.4485	5.61	2	51.3	14.2	2	184-201	K.RLWDSGQKEAGGGGNGSR.K		WD:WU 1.11

Detailed Protein Report

Protein 268: lysine-specific demethylase 4C isoform 3 [Homo sapiens]

Accession: gi|226442893
Database: refseq_human(refseq_human_20140103.fasta)
Database Date: 2015-11-30
Modification(s): Carbamidomethyl

Score: 25.7
MW [kDa]: 91.8
pI: 5.3
Sequence Coverage [%]: 3.2
No. of unique Peptides: 2

10	20	30	40	50	60	70	80
MEVAEVESPL	NPSCKIMTFR	PSMEEFREFN	KYLAYMESKG	AHRAGLAKVI	PPKEWKPRQC	YDDIDNLLIP	APIQQMVTGQ
90	100	110	120	130	140	150	160
SGLFTQYNIQ	KKAMTVKEFR	QLANSGKYCT	PRYLDYEDLE	RKYWKNLTFV	APIYGADING	SIYDEGVDEW	NIARLNTVLD
170	180	190	200	210	220	230	240
VVEEECGISI	EGVNTPYLYF	GMWKTTFAWH	TEDMDLYSIN	YLHFGEPKSW	YAIPPEHGKR	LERLAQGFFP	SSSQGCDAFL
250	260	270	280	290	300	310	320
RHKMTLISPS	VLKKYGIPFD	KITQEAGEFM	ITFPYGYHAG	FNHGFNCAES	TNFATVRWID	YGKVAKLCTC	RKDMVKISMD
330	340	350	360	370	380	390	400
IFVRKFQPDR	YQLWKQGKDI	YTIDHTKPTP	ASTPEVKAWL	QRRRKVRKAS	RSFQCARSTS	KRPKADEEEE	VSDEVDGAEV
410	420	430	440	450	460	470	480
PNPDSVTDDL	KVSEKSEAAV	KLRNTEASSE	EESSASRMQV	EQNLSDHIKL	SGNSCLSTSV	TEDIKTEDDK	AYAYRSVPSI
490	500	510	520	530	540	550	560
SSEADDSIPL	SSGYEKPEKS	DPSELSWPKS	PESCSSVAES	NGVLTEGEES	DVESHGNGLE	PGEIPAVPSG	ERNSFKVPSI
570	580	590	600	610	620	630	640
AEGENKTSKS	WRHPLSRPPA	RSPMTLVKQQ	APSDEELPEV	LSIEEEVEET	ESWAKPLIHL	WQTKSPNFAA	EQEYNATVAR
650	660	670	680	690	700	710	720
MKPHCAICTL	LMPYHKPDSS	NEENDARWET	KLDEVVTSEG	KTKPLIPEMC	FIYSEENIEY	SPPNAFLEED	GTSLLISCAK
730	740	750	760	770	780	790	800
CCVRVHASCY	GIPSHEICDG	WLCARCKRNA	WTAECCLCNL	RGGALKQTKN	NKWAHVMCAV	AVPEVRFTNV	PERTQIDVGR
810	820						
IPLQRLKLGR	LGI						

Cmpd.	No. of Cmpds.	m/z meas.	Δ m/z [ppm]	z	Rt [min]	Score	P	Range	Sequence	Modification
1358	1	727.3750	-7.81	2	45.7	15.7	1	313-324	K.DMVKISMDIFVR.K	
149	1	855.2460	-164.97	2	31.1	10.1	1	748-761	K.RNAWTAECCLCNLR.G	Carbamidomethyl: 8

Detailed Protein Report

Protein 269: PREDICTED: ribonuclease 3 isoform X4 [Homo sapiens]

Accession: gi|530378791
Database: refseq_human(refseq_human_20140103.fasta)
Database Date: 2015-11-30
Modification(s): Carbamidomethyl

Score: 25.6
MW [kDa]: 151.2
pI: 8.0
Sequence Coverage [%]: 2.2
No. of unique Peptides: 1

Quantitation

WD:WU **Median:** 0.82 **CV:** 0.00 % **No. of Peptides:** 1

10	20	30	40	50	60	70	80
MMQGNTCHRM	SFHPGRGCPR	GRGGHGARPS	APSFRPQNLR	LLHPQQPPVQ	YQYEPPSAPS	TTFSNSPAPN	FLPPRPDFVP
90	100	110	120	130	140	150	160
FPPPMPPSAQ	GPLPPCPIRP	PFPNHQMRHP	FPVPPCFPPM	PPPMPCPNNP	PVPGAPPGQG	TFPFMMPPPS	MPHPPPPPVM
170	180	190	200	210	220	230	240
PQQVNYQYPP	GYSHHNFPPP	SFNSFQNNPS	SFLPSANNSS	SPHFRHLPPY	PLPKAPSERR	SPERLKHYDD	HRHRDHSHGR
250	260	270	280	290	300	310	320
GERHRSLDRR	ERGRSPDRRR	QDSRYRSDYD	RGRTPSRHRS	YERSSRSPSR	EKKRARWEEE	KDRWSDNQSS	GKDKNYTSIK
330	340	350	360	370	380	390	400
EKEPEETMPD	KNEEEEEELL	KPVWIRCTHS	ENYYSSDPMD	QVGDSTVVGT	SRLRDLYDKF	EEELGSRQEK	AKAARPPWEP
410	420	430	440	450	460	470	480
PKTKLDEDLE	SSSESECESD	EDSTCSSSSD	SEVFDVIAEI	KRKKAHPDRL	HDELWYNDPG	QMNDGPLCKC	SAKARRTGIR
490	500	510	520	530	540	550	560
HSIYPGEEAI	KPCRPMTNNA	GRLFHYRITV	SPPTNFLTDR	PTVIEYDDHE	YIFEGFSMFA	HAPLTNIPLC	KVIRFNIDYT
570	580	590	600	610	620	630	640
IHFIEEMMPE	NFCVKGLELF	SLFLFRDILE	LYDWNLKGPL	FEDSPPCCPR	FHFMPRFVRF	LPDGGKEVLS	MHQILLYLLR
650	660	670	680	690	700	710	720
CSKALVPEEE	IANMLQWEEL	EWQKYAEECK	GMIVTNPGTK	PSSVRIDQLD	REQFNPDVIT	FPIIVHFGIR	PAQLSYAGDP
730	740	750	760	770	780	790	800
QYQKLWKSYV	KLRHLLANSP	KVKQTDKQKL	AQREEALQKI	RQKNTMRREV	TVELSSQGFW	KTGIRSDVCQ	HAMMLPVLTH
810	820	830	840	850	860	870	880
HIRYHQCLMH	LDKLIGYTFQ	DRCLLQLAMT	HPSHHLNFGM	NPDHARNSLS	NCGIRQPKYG	DRKVHHMHMR	KKGINTLINI
890	900	910	920	930	940	950	960
MSRLGQDDPT	PSRINHNERL	EFLGDAVVEF	LTSVHLYYLF	PSLEEGGLAT	YRTAIVQNQH	LAMLAKKLEL	DRFMLYAHGP
970	980	990	1000	1010	1020	1030	1040
DLCRESDLRH	AMANCFEALI	GAVYLEGSLE	EAKQLFGRLL	FNDPDLREVW	LNYPLHPLQL	QEPNTDRQLI	ETSPVLQKLT
1050	1060	1070	1080	1090	1100	1110	1120
EFEEAIGVIF	THVRLLARAF	TLRTVGFNHL	TLGHNQRMEF	LGDSIMQLVA	TEYLFIHFPD	HHEGHLTLLR	SSLVNNRTQA
1130	1140	1150	1160	1170	1180	1190	1200
KVAEELGMQE	YAITNDKTKR	PVALRTKTLA	DLLESFIAAL	YIDKDLEYVH	TFMNVCFFPR	LKEFILNQDW	NDPKSQLQQC
1210	1220	1230	1240	1250	1260	1270	1280
CLTLRTEGKE	PDIPLYKTLQ	TVGPSHARTY	TVAVYFKGER	IGCGKGPSIQ	QAEMGAAMDA	LEKYNFPQMA	HQKRFIERKY
1290	1300	1310					
RQELKEMRWE	REHQEREPDE	TEDIKK					

Cmpd.	No. of Cmpds.	m/z meas.	Δ m/z [ppm]	z	Rt [min]	Score	P	Range	Sequence	Modification	Ratios
787	1	1115.0348	37.23	2	38.7	10.4	2	2-20	M.MQGNTCHRMSFHPGRGCPR.G	Carbamidomethyl: 6	WD:WU 0.82

Detailed Protein Report

Protein 270: protein strawberry notch homolog 2 isoform 2 [Homo sapiens]

**Accession:**	gi|154355004	**Score:**	25.6
**Database:**	refseq_human(refseq_human_20140103.fasta)	**MW [kDa]:**	144.1
**Database Date:**	2015-11-30	**pI:**	6.2
**Modification(s):**	Oxidation	**Sequence Coverage [%]:**	3.0
		**No. of unique Peptides:**	1

10	20	30	40	50	60	70	80
MREPLPGSAS	WGTPGPPSAG	TMSQLQLWLQ	FEALNKDSSY	FEDFSNISIF	SSSVDSLSDI	VDTPDFLPAD	SLNQVSTIWD
**90**	**100**	**110**	**120**	**130**	**140**	**150**	**160**
DNPAPSTHDK	LFQLSRPFAG	FEDFLPSHST	PLLVSYQEQS	VQSQPEEEDE	AEEEEAEELG	HTETYADYVP	SKSKIGKQHP
**170**	**180**	**190**	**200**	**210**	**220**	**230**	**240**
DRVVETSTLS	SVPPPDITYT	LALPSDSGAL	SALQLEAITY	ACQQHEVLLP	SGQRAGFLIG	DGAGVGKGRT	VAGVILENHL
**250**	**260**	**270**	**280**	**290**	**300**	**310**	**320**
RGRKKALWFS	VSNDLKYDAE	RDLRDIEATG	IAVHALSKIK	YGDTTTSEGV	LFATYSALIG	ESQAGGQHRT	RLRQILDWCG
**330**	**340**	**350**	**360**	**370**	**380**	**390**	**400**
EAFEGVIVFD	ECHKAKNAGS	TKMGKAVLDL	QNKLPLARVV	YASATGASEP	RNMIYMSRLG	IWGEGTPFRN	FEEFLHAIEK
**410**	**420**	**430**	**440**	**450**	**460**	**470**	**480**
RGVGAMEIVA	MDMKVSGMYI	ARQLSFSGVT	FRIEEIPLAP	AFECVYNRAA	LLWAEALNVF	QQAADWIGLE	SRKSLWGQFW
**490**	**500**	**510**	**520**	**530**	**540**	**550**	**560**
SAHQRFFKYL	CIAAKVRRLV	ELAREELARD	KCVVIGLQST	GEARTREVLG	ENDGHLNCFV	SAAEGVFLSL	IQKHFPSTKR
**570**	**580**	**590**	**600**	**610**	**620**	**630**	**640**
KRDRGAGSKR	KRRPRGRGAK	APRLACETAG	VIRISDDSST	ESDPGLDSDF	NSSPESLVDD	DVVIVDAVGL	PSDDRGPLCL
**650**	**660**	**670**	**680**	**690**	**700**	**710**	**720**
LQRDPHGPGV	LERVERLKQD	LLDKVRRLGR	ELPVNTLDEL	IDQLGGPQRV	AEMTGRKGRV	VSRPDGTVAF	ESRAEQGLSI
**730**	**740**	**750**	**760**	**770**	**780**	**790**	**800**
DHVNLREKQR	FMSGEKLVAI	ISEASSSGVS	LQADRRVQNQ	RRRVHMTLEL	PWSADRAIQQ	FGRTHRSNQV	SAPEYVFLIS
**810**	**820**	**830**	**840**	**850**	**860**	**870**	**880**
ELAGERRFAS	IVAKRLESLG	ALTHGDRRAT	ESRDLSKYNF	ENKYGTRALH	CVLTTILSQT	ENKVPVPQGY	PGGVPTFFRD
**890**	**900**	**910**	**920**	**930**	**940**	**950**	**960**
MKQGLLSVGI	GGRESRNGCL	DVEKDCSITK	FLNRILGLEV	HKQNALFQYF	SDTFDHLIEM	DKREGKYDMG	ILDLAPGIEE
**970**	**980**	**990**	**1000**	**1010**	**1020**	**1030**	**1040**
IYEESQQVFL	APGHPQDGQV	VFYKISVDRG	LKWEDAFAKS	LALTGPYDGF	YLSYKVRGNK	PSCLLAEQNR	GQFFTVYKPN
**1050**	**1060**	**1070**	**1080**	**1090**	**1100**	**1110**	**1120**
IGRQSQLEAL	DSLRRKFHRV	TAEEAKEPWE	SGYALSLTHC	SHSAWNRHCR	LAQEGKDCLQ	GLRLRHHYML	CGALLRVWGR
**1130**	**1140**	**1150**	**1160**	**1170**	**1180**	**1190**	**1200**
IAAVMADVSS	SSYLQIVRLK	TKDRKKQVGI	KIPEGCVRRV	LQELRLMDAD	VKRRQAPALG	CPAPPAPRPL	ALPCGPGEVL
**1210**	**1220**	**1230**	**1240**	**1250**	**1260**	**1270**	**1280**
DLTYSPPAEA	FPPPPHFSFP	APLSLDAGPG	VVPLGTPDAQ	ADPAALAHQG	CDINFKEVLE	DMLRSLHAGP	PSEGALGEGA
**1290**	**1300**	**1310**					
GAGGAAGGGP	ERQSVIQFSP	PFPGAQAPL					

Cmpd.	No. of Cmpds.	m/z meas.	Δ m/z [ppm]	z	Rt [min]	Score	P	Range	Sequence	Modification
2599	1	665.2392	-138.50	2	60.7	12.3	0	1106-1116	R.HHYMLCGALLR.V	Oxidation: 4

Detailed Protein Report

Protein 271: prolow-density lipoprotein receptor-related protein 1 precursor [Homo sapiens]

**Accession:**	gi|126012562	**Score:**	25.6
**Database:**	refseq_human(refseq_human_20140103.fasta)	**MW [kDa]:**	504.3
**Database Date:**	2015-11-30	**pI:**	5.0
**Modification(s):**	Carbamidomethyl, Oxidation	**Sequence Coverage [%]:**	0.7
		**No. of unique Peptides:**	1

Detailed Protein Report

10	20	30	40	50	60	70	80
MLTPPLLLLL	PLLSALVAAA	IDAPKTCSPK	QFACRDQITC	ISKGWRCDGE	RDCPDGSDEA	PEICPQSKAQ	RCQPNEHNCL
90	100	110	120	130	140	150	160
GTELCVPMSR	LCNGVQDCMD	GSDEGPHCRE	LQGNCSRLGC	QHHCVPTLDG	PTCYCNSSFQ	LQADGKTCKD	FDECSVYGTC
170	180	190	200	210	220	230	240
SQLCTNTDGS	FICGCVEGYL	LQPDNRSCKA	KNEPVDRPPV	LLIANSQNIL	ATYLSGAQVS	TITPTSTRQT	TAMDFSYANE
250	260	270	280	290	300	310	320
TVCWVHVGDS	AAQTQLKCAR	MPGLKGFVDE	HTINISLSLH	HVEQMAIDWL	TGNFYFVDDI	DDRIFVCNRN	GDTCVTLLDL
330	340	350	360	370	380	390	400
ELYNPKGIAL	DPAMGKVFFT	DYGQIPKVER	CDMDGQNRTK	LVDSKIVFPH	GITLDLVSRL	VYWADAYLDY	IEVVDYEGKG
410	420	430	440	450	460	470	480
RQTIIQGILI	EHLYGLTVFE	NYLYATNSDN	ANAQQKTSVI	RVNRFNSTEY	QVVTRVDKGG	ALHIYHQRRQ	PRVRSHACEN
490	500	510	520	530	540	550	560
DQYGKPGGCS	DICLLANSHK	ARTCRCRSGF	SLGSDGKSCK	KPEHELFLVY	GKGRPGIIRG	MDMGAKVPDE	HMIPIENLMN
570	580	590	600	610	620	630	640
PRALDFHAET	GFIYFADTTS	YLIGRQKIDG	TERETILKDG	IHNVEGVAVD	WMGDNLYWTD	DGPKKTISVA	RLEKAAQTRK
650	660	670	680	690	700	710	720
TLIEGKMTHP	RAIVVDPLNG	WMYWTDWEED	PKDSRRGRLE	RAWMDGSHRD	IFVTSKTVLW	PNGLSLDIPA	GRLYWVDAFY
730	740	750	760	770	780	790	800
DRIETILLNG	TDRKIVYEGP	ELNHAFGLCH	HGNYLFWTEY	RSGSVYRLER	GVGGAPPTVT	LLRSERPPIF	EIRMYDAQQQ
810	820	830	840	850	860	870	880
QVGTNKCRVN	NGGCSSLCLA	TPGSRQCACA	EDQVLDADGV	TCLANPSYVP	PPQCQPGEFA	CANSRCIQER	WKCDGDNDCL
890	900	910	920	930	940	950	960
DNSDEAPALC	HQHTCPSDRF	KCENNRCIPN	RWLCDGDNDC	GNSEDESNAT	CSARTCPPNQ	FSCASGRCIP	ISWTCDLDDD
970	980	990	1000	1010	1020	1030	1040
CGDRSDESAS	CAYPTCFPLT	QFTCNNGRCI	NINWRCDNDN	DCGDNSDEAG	CSHSCSSTQF	KCNSGRCIPE	HWTCDGDNDC
1050	1060	1070	1080	1090	1100	1110	1120
GDYSDETHAN	CTNQATRPPG	GCHTDEFQCR	LDGLCIPLRW	RCDGDTDCMD	SSDEKSCEGV	THVCDPSVKF	GCKDSARCIS
1130	1140	1150	1160	1170	1180	1190	1200
KAWVCDGDND	CEDNSDEENC	ESLACRPPSH	PCANNTSVCL	PPDKLCDGND	DCGDGSDEGE	LCDQCSLNNG	GCSHNCSVAP
1210	1220	1230	1240	1250	1260	1270	1280
GEGIVCSCPL	GMELGPDNHT	CQIQSYCAKH	LKCSQKCDQN	KFSVKCSCYE	GWVLEPDGES	CRSLDPFKPF	IIFSNRHEIR
1290	1300	1310	1320	1330	1340	1350	1360
RIDLHKGDYS	VLVPGLRNTI	ALDFHLSQSA	LYWTDVVEDK	IYRGKLLDNG	ALTSFEVVIQ	YGLATPEGLA	VDWIAGNIYW
1370	1380	1390	1400	1410	1420	1430	1440
VESNLDQIEV	AKLDGTLRTT	LLAGDIEHPR	AIALDPRDGI	LFWTDWDASL	PRIEAASMSG	AGRRTVHRET	GSGGWPNGLT
1450	1460	1470	1480	1490	1500	1510	1520
VDYLEKRILW	IDARSDAIYS	ARYDGSGHME	VLRGHEFLSH	PFAVTLYGGE	VYWTDWRTNT	LAKANKWTGH	NVTVVQRTNT
1530	1540	1550	1560	1570	1580	1590	1600
QPFDLQVYHP	SRQPMAPNPC	EANGGQGPCS	HLCLINYNRT	VSCACPHLMK	LHKDNTTCYE	FKKFLLYARQ	MEIRGVDLDA
1610	1620	1630	1640	1650	1660	1670	1680
PYYNYIISFT	VPDIDNVTVL	DYDAREQRVY	WSDVRTQAIK	RAFINGTGVE	TVVSADLPNA	HGLAVDWVSR	NLFWTSYDTN
1690	1700	1710	1720	1730	1740	1750	1760
KKQINVARLD	GSFKNAVVQG	LEQPHGLVVH	PLRGKLYWTD	GDNISMANMD	GSNRTLLFSG	QKGPVGLAID	FPESKLYWIS
1770	1780	1790	1800	1810	1820	1830	1840
SGNHTINRCN	LDGSGLEVID	AMRSQLGKAT	ALAIMGDKLW	WADQVSEKMG	TCSKADGSGS	VVLRNSTTLV	MHMKVYDESI
1850	1860	1870	1880	1890	1900	1910	1920
QLDHKGTNPC	SVNNGDCSQL	CLPTSETTRS	CMCTAGYSLR	SGQQACEGVG	SFLLYSVHEG	IRGIPLDPND	KSDALVPVSG
1930	1940	1950	1960	1970	1980	1990	2000
TSLAVGIDFH	AENDTIYWVD	MGLSTISRAK	RDQTWREDVV	TNGIGRVEGI	AVDWIAGNIY	WTDQGFDVIE	VARLNGSFRY
2010	2020	2030	2040	2050	2060	2070	2080
VVISQGLDKP	RAITVHPEKG	YLFWTEWGQY	PRIERSRLDG	TERVVLVNVS	ISWPNGISVD	YQDGKLYWCD	ARTDKIERID
2090	2100	2110	2120	2130	2140	2150	2160
LETGENREVV	LSSNNMDMFS	VSVFEDFIYW	SDRTHANGSI	KRGSKDNATD	SVPLRTGIGV	QLKDIKVFNR	DRQKGTNVCA
2170	2180	2190	2200	2210	2220	2230	2240

Detailed Protein Report

Cmpd.	No. of Cmpds.	m/z meas.	Δ m/z [ppm]	z	Rt [min]	Score	P	Range	Sequence	Modification
127	2	849.3809	-43.54	2	30.9	15.1	1	1560-1573	R.TVSCACPHLMKLHK.D	Carbamidomethyl: 4, 6; Oxidation: 10

Detailed Protein Report

Protein 272: PREDICTED: tolloid-like protein 1 isoform X2 [Homo sapiens]

**Accession:**	gi|530378097	**Score:**	25.6
**Database:**	refseq_human(refseq_human_20140103.fasta)	**MW [kDa]:**	82.0
**Database Date:**	2015-11-30	**pI:**	5.5
		**Sequence Coverage [%]:**	3.9
		**No. of unique Peptides:**	1

10	20	30	40	50	60	70	80
MISTVSCTMP	GTPSQGWSLR	GMFLDTILPS	RDDNGIRPAI	GQRTRLSKGD	IAQARKLYRC	PACGETLQES	NGNLSSPGFP
**90**	**100**	**110**	**120**	**130**	**140**	**150**	**160**
NGYPSYTHCI	WRVSVTPGEK	IVLNFTTMDL	YKSSLCWYDY	IEVRDGYWRK	SPLLGRFCGD	KLPEVLTSTD	SRMWIEFRSS
**170**	**180**	**190**	**200**	**210**	**220**	**230**	**240**
SNWVGKGFAA	VYEAICGGEI	RKNEGQIQSP	NYPDDYRPMK	ECVWKITVSE	SYHVGLTFQS	FEIERHDNCA	YDYLEVRDGT
**250**	**260**	**270**	**280**	**290**	**300**	**310**	**320**
SENSPLIGRF	CGYDKPEDIR	STSNTLWMKF	VSDGTVNKAG	FAANFFKEED	ECAKPDRGGC	EQRCLNTLGS	YQCACEPGYE
**330**	**340**	**350**	**360**	**370**	**380**	**390**	**400**
LGPDRRSCEA	ACGGLLTKLN	GTITTPGWPK	EYPPNKNCVW	QVVAPTQYRI	SVKFEFFELE	GNEVCKYDYV	EIWSGLSSES
**410**	**420**	**430**	**440**	**450**	**460**	**470**	**480**
KLHGKFCGAE	VPEVITSQFN	NMRIEFKSDN	TVSKKGFKAH	FFSDKDECSK	DNGGCQHECV	NTMGSYMCQC	RNGFVLHDNK
**490**	**500**	**510**	**520**	**530**	**540**	**550**	**560**
HDCKEAECEQ	KIHSPSGLIT	SPNWPDKYPS	RKECTWEISA	TPGHRIKLAF	SEFEIEQHQE	CAYDHLEVFD	GETEKSPILG
**570**	**580**	**590**	**600**	**610**	**620**	**630**	**640**
RLCGNKIPDP	LVATGNKMFV	RFVSDASVQR	KGFQATHSTE	CGGRLKAESK	PRDLYSHAQF	GDNNYPGQVD	CEWLLVSERG
**650**	**660**	**670**	**680**	**690**	**700**	**710**	**720**
SRLELSFQTF	EVEEEADCGY	DYVELFDGLD	STAVGLGRFC	GSGPPEEIYS	IGDSVLIHFH	TDDTINKKGF	HIRYKSIRYP
**730**							
DTTHTKK							

Cmpd.	No. of Cmpds.	m/z meas.	Δ m/z [ppm]	z	Rt [min]	Score	P	Range	Sequence	Modification
556	1	658.3907	22.79	2	35.8	11.5	2	44-55	R.TRLSKGDIAQAR.K	

Detailed Protein Report

Protein 273: PREDICTED: glucose-6-phosphate 1-dehydrogenase isoform X2 [Homo sapiens]

**Accession:**	gi|530422749	**Score:**	25.5
**Database:**	refseq_human(refseq_human_20140103.fasta)	**MW [kDa]:**	59.4
**Database Date:**	2015-11-30	**pI:**	6.6
		**Sequence Coverage [%]:**	4.8
		**No. of unique Peptides:**	1

10	20	30	40	50	60	70	80
MAEQVALSRT	QVCGILREEL	FQGDAFHQSD	THIFIIMGAS	GDLAKKKIYP	TIWWLFRDGL	LPENTFIVGY	ARSRLTVADI
**90**	**100**	**110**	**120**	**130**	**140**	**150**	**160**
RKQSEPFFKA	TPEEKLKLED	FFARNSYVAG	QYDDAASYQR	LNSHMNALHL	GSQANRLFYL	ALPPTVYEAV	TKNIHESCMS
**170**	**180**	**190**	**200**	**210**	**220**	**230**	**240**
QIRGWNRIIV	EKPFGRDLQS	SDRLSNHISS	LFREDQIYRI	DHYLGKEMVQ	NLMVLRFANR	IFGPIWNRDN	IACVILTFKE
**250**	**260**	**270**	**280**	**290**	**300**	**310**	**320**
PFGTEGRGGY	FDEFGIIRDV	MQNHLLQMLC	LVAMEKPAST	NSDDVRDEKV	KVLKCISEVQ	ANNVVLGQYV	GNPDGEGEAT
**330**	**340**	**350**	**360**	**370**	**380**	**390**	**400**
KGYLDDPTVP	RGSTTATFAA	VVLYVENERW	DGVPFILRCG	KALNERKAEV	RLQFHDVAGD	IFHQQCKRNE	LVIRVQPNEA
**410**	**420**	**430**	**440**	**450**	**460**	**470**	**480**
VYTKMMTKKP	GMFFNPEESE	LDLTYGNRYK	NVKLPDAYER	LILDVFCGSQ	MHFVRSDELR	EAWRIFTPLL	HQIELEKPKP
**490**	**500**	**510**	**520**				
IPYIYGSRGP	TEADELMKRV	GFQYEGTYKW	VNPHKL				

Cmpd.	No. of Cmpds.	m/z meas.	Δ m/z [ppm]	z	Rt [min]	Score	P	Range	Sequence	Modification
2614	1	659.1695	-206.35	2	61.0	15.2	0	153-163	K.NIHESCMSQIR.G	

Detailed Protein Report

Protein 274: scaffold attachment factor B1 isoform 4 [Homo sapiens]

Accession: gi|321267473
Database: refseq_human(refseq_human_20140103.fasta)
Database Date: 2015-11-30

Score: 25.5
MW [kDa]: 95.1
pI: 6.0
Sequence Coverage [%]: 2.6
No. of unique Peptides: 1

Quantitation

WD:WU **Median:** 1.08 **CV:** 0.00 % **No. of Peptides:** 1

10	20	30	40	50	60	70	80
MAETLSGLGD	SGAAGAAALS	SASSETGTRR	LSDLRVIDLR	AELRKRNVDS	SGNKSVLMER	LKKAIEDEGG	NPDEIEITSE
90	100	110	120	130	140	150	160
GNKKTSKRSS	KGRKPEEEGV	EDNGLEENSG	DGQIEDKETI	NNLDTSSSDF	TILQEIEEPS	LEPENEKILD	ILGETCKSEP
170	180	190	200	210	220	230	240
VKEESSELEQ	PFAQDTSSVG	PDRKLAEEED	LFDSAHPEEG	DLDLASESTA	HAQSSKADSL	LAVVKREPAE	QPGDGERTDC
250	260	270	280	290	300	310	320
EPVGLEPAVE	QSSAASELAE	ASSEELAEAP	TEAPSPEARD	SKEDGRKFDF	DACNEVPPAP	KESSTSEGAD	QKMSSPEDDS
330	340	350	360	370	380	390	400
DTKRLSKEEK	GRSSCGRNFW	VSGLSSTTRA	TDLKNLFSKY	GKVVGAKVVT	NARSPGARCY	GFVTMSTAEE	ATKCINHLHK
410	420	430	440	450	460	470	480
TELHGKMISV	EKAKNEPVGK	KTSDKRDSDG	KKEKSSNSDR	STNLKRDDKC	DRKDDAKKGD	DGSGEKSKDQ	DDQKPGPSER
490	500	510	520	530	540	550	560
SRATKSGSRG	TERTVVMDKS	KGVPVISVKT	SGSKERASKS	QDRKSASREK	RSVVSFDKVK	EPRKSRDSES	HSRVRERSER
570	580	590	600	610	620	630	640
EQRMQAQWER	EERERLEIAR	ERLAFQRQRL	ERERMERERL	ERERMHVEHE	RRREQERIHR	EREELRRQQE	LRYEQERRPA
650	660	670	680	690	700	710	720
VRRPYDLDRR	DDAYWPEAKR	AALDERYHSD	FNRQDRFHDF	DHRDRGRYPD	HSVDRREGSR	SMMGEREGQH	YPERHGGPER
730	740	750	760	770	780	790	800
HGRDSRDGWG	GYGSDKRMSE	GRGLPPPPRG	RRDWGDHGRR	EDDRSWQGTA	DGGMMDRDHK	RWQGGERSMS	GHSGPGHMMN
810	820	830	840	850			
RGGMSGRGSF	APGGASRGHP	IPHGGMQGGF	GGQSRGSRPS	DARFTRRY			

Cmpd.	No. of Cmpds.	m/z meas.	Δ m/z [ppm]	z	Rt [min]	Score	P	Range	Sequence	Modification	Ratios
980	2	530.6065	-255.21	2	40.6	14.1	1	545-553	K.SRDSESHSR.V		WD:WU 1.08

Detailed Protein Report

Protein 275: catenin delta-2 isoform 4 [Homo sapiens]

**Accession:**	gi|570359551	**Score:**	25.5
**Database:**	refseq_human(refseq_human_20140103.fasta)	**MW [kDa]:**	87.2
**Database Date:**	2015-11-30	**pI:**	9.4
**Modification(s):**	Carbamidomethyl	**Sequence Coverage [%]:**	5.8
		**No. of unique Peptides:**	1

10	20	30	40	50	60	70	80
MRSLSQSQGD	PLPPAHTGTY	RTSTAPSSPG	VDSVPLQRTG	SQHGPQNAAA	ATFQRASYAA	GPASNYADPY	RQLQYCPSVE
90	100	110	120	130	140	150	160
SPYSKSGPAL	PPEGTLARSP	SIDSIQKDPR	EFGWRDPELP	EVIQMLQHQF	PSVQSNAAAY	LQHLCFGDNK	IKAEIRRQGG
170	180	190	200	210	220	230	240
IQLLVDLLDH	RMTEVHRSAC	GALRNLVYGK	ANDDNKIALK	NCGGIPALVR	LLRKTTDLEI	RELVTGVLWN	LSSCDALKMP
250	260	270	280	290	300	310	320
IIQDALAVLT	NAVIIPHSGW	ENSPLQDDRK	IQLHSSQVLR	NATGCLRNVS	SAGEEARRRM	RECDGLTDAL	LYVIQSALGS
330	340	350	360	370	380	390	400
SEIDSKTVEN	CVCILRNLSY	RLAAETSQGQ	HMGTDELDGL	LCGEANGKDA	ESSGCWGKKK	KKKKSQDQWD	GVGPLPDCAE
410	420	430	440	450	460	470	480
PPKGIQMLWH	PSIVKPYLTL	LSECSNPDTL	EGAAGALQNL	AAGSWKWSVY	IRAAVRKEKG	LPILVELLRI	DNDRVVCAVA
490	500	510	520	530	540	550	560
TALRNMALDV	RNKELIGKYA	MRDLVHRLPG	GNNSNNTASK	AMSDDTVTAV	CCTLHEVITK	NMENAKALRD	AGGIEKLVGI
570	580	590	600	610	620	630	640
SKSKGDKHSP	KVVKAASQVL	NSMWQYRDLR	SLYKKDGWSQ	YHFVASSSTI	ERDRQRPYSS	SRTPSISPVR	VSPNNRSASA
650	660	670	680	690	700	710	720
PASPREMISL	KERKTDYECT	GSNATYHGAK	GEHTSRKDAM	TAQNTGISTL	YRNSYGAPAE	DIKHNQVSAQ	PVPQEPSRKD
730	740	750	760	770	780	790	800
YETYQPFQNS	TRNYDESFFE	DQVHHRPPAS	EYTMHLGLKS	TGNYVDFYSA	ARPYSELNYE	TSHYPASPDS	WV

Cmpd.	No. of Cmpds.	m/z meas.	Δ m/z [ppm]	z	Rt [min]	Score	P	Range	Sequence	Modification
292	1	1293.8398	-46.50	3	32.8	12.9	1	342-378	R.LAAETSQGQHMGTDELDGLLCGEANGKDAESSGCWGK.K	Carbamidomethyl: 21, 34

Detailed Protein Report

Protein 276: vacuole membrane protein 1 [Homo sapiens]

Accession: gi|20070349
Database: refseq_human(refseq_human_20140103.fasta)
Database Date: 2015-11-30
Modification(s): Carbamidomethyl, Oxidation

Score: 25.4
MW [kDa]: 46.2
pI: 6.5
Sequence Coverage [%]: 7.1
No. of unique Peptides: 2

Quantitation

WD:WU **Median:** 1.62 **CV:** 0.00 % **No. of Peptides:** 1

10	20	30	40	50	60	70	80
MAENGKNCDQ	RRVAMNKEHH	NGNFTDPSSV	NEKKRREREE	RQNIVLWRQP	LITLQYFSLE	ILVILKEWTS	KLWHRQSIVV
**90**	**100**	**110**	**120**	**130**	**140**	**150**	**160**
SFLLLLAVLI	ATYYVEGVHQ	QYVQRIEKQF	LLYAYWIGLG	ILSSVGLGTG	LHTFLLYLGP	HIASVTLAAY	ECNSVNFPEP
**170**	**180**	**190**	**200**	**210**	**220**	**230**	**240**
PYPDQIICPD	EEGTEGTISL	WSIISKVRIE	ACMWGIGTAI	GELPPYFMAR	AARLSGAEPD	DEEYQEFEEM	LEHAESAQDF
**250**	**260**	**270**	**280**	**290**	**300**	**310**	**320**
ASRAKLAVQK	LVQKVGFFGI	LACASIPNPL	FDLAGITCGH	FLVPFWTFFG	ATLIGKAIIK	MHIQKIFVII	TFSKHIVEQM
**330**	**340**	**350**	**360**	**370**	**380**	**390**	**400**
VAFIGAVPGI	GPSLQKPFQE	YLEAQRQKLH	HKSEMGTPQG	ENWLSWMFEK	LVVVMVCYFI	LSIINSMAQS	YAKRIQQRLN
**410**							
SEEKTK							

Cmpd.	No. of Cmpds.	m/z meas.	Δ m/z [ppm]	z	Rt [min]	Score	P	Range	Sequence	Modification	Ratios
2411	1	669.6353	-215.87	2	58.5	12.4	1	1-11	-.MAENGKNCDQR.R	Carbamidomethyl: 8; Oxidation: 1	WD:WU 1.62
443	2	699.2695	-93.64	3	34.5	13.0	2	18-35	K.EHHNGNFTDPSSVNEKKR.R		

Detailed Protein Report

Protein 277: PREDICTED: peroxisomal acyl-coenzyme A oxidase 2 isoform X3 [Homo sapiens]

Accession: gi|578806607
Database: refseq_human(refseq_human_20140103.fasta)
Database Date: 2015-11-30
Modification(s): Oxidation

Score: 25.4
MW [kDa]: 65.2
pI: 7.3
Sequence Coverage [%]: 5.5
No. of unique Peptides: 2

10	20	30	40	50	60	70	80
MKQVGEKISR	ALSGDVALNI	HRVFVRALRS	LGSEEQIAKW	DPLCKNIQII	ATYAQTELGH	GTYLQGLETE	ATYDAATQEF
**90**	**100**	**110**	**120**	**130**	**140**	**150**	**160**
VIHSPTLTAT	KWWPGDLGRS	ATHALVQAQL	ICSGARRGMH	AFIVPIRSLQ	DHTPLPGIII	GDIGPKMDFD	QTDNGFLQLN
**170**	**180**	**190**	**200**	**210**	**220**	**230**	**240**
HVRVPRENML	SRFAQVLPDG	TYVKLGTAQS	NYLPMVVVRV	ELLSGEILPI	LQKACVIAMR	YSVIRRQSRL	RPSDPEAKVL
**250**	**260**	**270**	**280**	**290**	**300**	**310**	**320**
DYQTQQQKLF	PQLAISYAFH	FLAVSLLEFF	QHSYTAILNQ	DFSFLPELHA	LSTGMKAMMS	EFCTQGAEMC	RRACGGHGYS
**330**	**340**	**350**	**360**	**370**	**380**	**390**	**400**
KLSGLPSLVT	KLSASCTYEG	ENTVLYLQVA	RFLVKSYLQT	QMSPGSTPQR	SLSPSVAYLT	APDLARCPAQ	RAADFLCPEL
**410**	**420**	**430**	**440**	**450**	**460**	**470**	**480**
YTTAWAHVAV	RLIKDSVQHL	QTLTQSGADQ	HEAWNQTTVI	HLQAAKVHCY	YVTVKGFTEA	LEKLENEPAI	QQVLKRLCDL
**490**	**500**	**510**	**520**	**530**	**540**	**550**	**560**
HAIHGILTNS	GDFLHDAFLS	GAQVDMARTA	YLDLLRLIRK	DAILLTDAFD	FTDQCLNSAL	GCYDGNVYER	LFQWAQKSPT
**570**	**580**	**590**					
NTQENPAYEE	YIRPLLQSWR	SKL					

Cmpd.	No. of Cmpds.	m/z meas.	Δ m/z [ppm]	z	Rt [min]	Score	P	Range	Sequence	Modification
2421	1	863.4315	-29.41	2	58.6	10.1	0	100-116	R.SATHALVQAQLICSGAR.R	
2448	1	863.7544	-77.93	2	58.9	15.3	0	297-311	K.AMMSEFCTQGAEMCR.R	Oxidation: 2, 13

Detailed Protein Report

Protein 278: **PREDICTED: uncharacterized protein C6orf201 isoform X6 [Homo sapiens]**

**Accession:**	gi|578811686	**Score:**	25.4
**Database:**	refseq_human(refseq_human_20140103.fasta)	**MW [kDa]:**	15.9
**Database Date:**	2015-11-30	**pI:**	12.3
		**Sequence Coverage [%]:**	10.5
		**No. of unique Peptides:**	1

```
        10         20         30         40         50         60         70         80
MASGPLGPGA RPTRLHPPFP PPAHIKPGAP PGENPELSGL ERILARHQLP KEINLTPKPN RMPPWKRKII NNVTDGWKKC
        90        100        110        120        130        140        150
HLLKRNTKEP PMSTIVVRKL IQKNVPRRHS LRNTSRKLRN LPTTAKGTQT GKSQCLLGIS EPT
```

Cmpd.	No. of Cmpds.	m/z meas.	Δ m/z [ppm]	z	Rt [min]	Score	P	Range	Sequence	Modification
1772	1	793.3983	-70.98	2	50.8	25.4	2	118-132	K.LRNLPTTAKGTQTGK.S	

Detailed Protein Report

Protein 279: brain-specific angiogenesis inhibitor 1 precursor [Homo sapiens]

**Accession:**	gi|111118994	**Score:**	25.3
**Database:**	refseq_human(refseq_human_20140103.fasta)	**MW [kDa]:**	173.4
**Database Date:**	2015-11-30	**pI:**	9.5
**Modification(s):**	Carbamidomethyl	**Sequence Coverage [%]:**	1.8
		**No. of unique Peptides:**	2

Quantitation

WD:WU	**Median:** 0.46	**CV:** 0.00 %	**No. of Peptides:** 1

10	20	30	40	50	60	70	80
MRGQAAAPGP	VWILAPLLLL	LLLLGRRARA	AAGADAGPGP	EPCATLVQGK	FFGYFSAAAV	FPANASRCSW	TLRNPDPRRY
90	100	110	120	130	140	150	160
TLYMKVAKAP	VPCSGPGRVR	TYQFDSFLES	TRTYLGVESF	DEVLRLCDPS	APLAFLQASK	QFLQMRRQQP	PQHDGLRPRA
170	180	190	200	210	220	230	240
GPPGPTDDFS	VEYLVVGNRN	PSRAACQMLC	RWLDACLAGS	RSSHPCGIMQ	TPCACLGGEA	GGPAAGPLAP	RGDVCLRDAV
250	260	270	280	290	300	310	320
AGGPENCLTS	LTQDRGGHGA	TGGWKLWSLW	GECTRDCGGG	LQTRTRTCLP	APGVEGGGCE	GVLEEGRQCN	REACGPAGRT
330	340	350	360	370	380	390	400
SSRSQSLRST	DARRREELGD	ELQQFGFPAP	QTGDPAAEEW	SPWSVCSSTC	GEGWQTRTRF	CVSSSYSTQC	SGPLREQRLC
410	420	430	440	450	460	470	480
NNSAVCPVHG	AWDEWSPWSL	CSSTCGRGFR	DRTRTCRPPQ	FGGNPCEGPE	KQTKFCNIAL	CPGRAVDGNW	NEWSSWSACS
490	500	510	520	530	540	550	560
ASCSQGRQQR	TRECNGPSYG	GAECQGHWVE	TRDCFLQQCP	VDGKWQAWAS	WGSCSVTCGA	GSQRRERVCS	GPFFGGAACQ
570	580	590	600	610	620	630	640
GPQDEYRQCG	TQRCPEPHEI	CDEDNFGAVI	WKETPAGEVA	AVRCPRNATG	LILRRCELDE	EGIAYWEPPT	YIRCVSIDYR
650	660	670	680	690	700	710	720
NIQMMTREHL	AKAQRGLPGE	GVSEVIQTLV	EISQDGTSYS	GDLLSTIDVL	RNMTEIFRRA	YYSPTPGDVQ	NFVQILSNLL
730	740	750	760	770	780	790	800
AEENRDKWEE	AQLAGPNAKE	LFRLVEDFVD	VIGFRMKDLR	DAYQVTDNLV	LSIHKLPASG	ATDISFPMKG	WRATGDWAKV
810	820	830	840	850	860	870	880
PEDRVTVSKS	VFSTGLTEAD	EASVFVVGTV	LYRNLGSFLA	LQRNTTVLNS	KVISVTVKPP	PRSLRTPLEI	EFAHMYNGTT
890	900	910	920	930	940	950	960
NQTCILWDET	DVPSSSAPPQ	LGPWSWRGCR	TVPLDALRTR	CLCDRLSTFA	ILAQLSADAN	MEKATLPSVT	LIVGCGVSSL
970	980	990	1000	1010	1020	1030	1040
TLLMLVIIYV	SVWRYIRSER	SVILINFCLS	IISSNALILI	GQTQTRNKVV	CTLVAAFLHF	FFLSSFCWVL	TEAWQSYMAV
1050	1060	1070	1080	1090	1100	1110	1120
TGHLRNRLIR	KRFLCLGWGL	PALVVAISVG	FTKAKGYSTM	NYCWLSLEGG	LLYAFVGPAA	AVVLVNMVIG	ILVFNKLVSK
1130	1140	1150	1160	1170	1180	1190	1200
DGITDKKLKE	RAGASLWSSC	VVLPLLALTW	MSAVLAVTDR	RSALFQILFA	VFDSLEGFVI	VMVHCILRRE	VQDAVKCRVV
1210	1220	1230	1240	1250	1260	1270	1280
DRQEEGNGDS	GGSFQNGHAQ	LMTDFEKDVD	LACRSVLNKD	IAACRTATIT	GTLKRPSLPE	EEKLKLAHAK	GPPTNFNSLP
1290	1300	1310	1320	1330	1340	1350	1360
ANVSKLHLHG	SPRYPGGPLP	DFPNHSLTLK	RDKAPKSSFV	GDGDIFKKLD	SELSRAQEKA	LDTSYVILPT	ATATLRPKPK
1370	1380	1390	1400	1410	1420	1430	1440
EEPKYSIHID	QMPQTRLIHL	STAPEASLPA	RSPPSRQPPS	GGPPEAPPAQ	PPPPPPPPPP	PPQQPLPPPP	NLEPAPPSLG
1450	1460	1470	1480	1490	1500	1510	1520
DPGEPAAHPG	PSTGPSTKNE	NVATLSVSSL	ERRKSRYAEL	DFEKIMHTRK	RHQDMFQDLN	RKLQHAAEKD	KEVLGPDSKP
1530	1540	1550	1560	1570	1580	1590	
EKQQTPNKRP	WESLRKAHGT	PTWVKKELEP	LQPSPLELRS	VEWERSGATI	PLVGQDIIDL	QTEV	

Cmpd.	No. of Cmpds.	m/z meas.	Δ m/z [ppm]	z	Rt [min]	Score	P	Range	Sequence	Modification	Ratios
273	1	442.6382	-283.34	2	32.1	14.2	0	911-918	R.TVPLDALR.T		WD:WU 0.46
1629	1	711.6687	-83.42	3	49.1	11.1	2	1235-1254	R.SVLNKDIAACRTATITGTLK.R	Carbamidomethyl: 10	

Detailed Protein Report

Protein 280: **E3 ubiquitin-protein ligase parkin isoform 2 [Homo sapiens]**

Accession: gi|169790971
Database: refseq_human(refseq_human_20140103.fasta)
Database Date: 2015-11-30

Score: 25.3
MW [kDa]: 48.7
pI: 7.9
Sequence Coverage [%]: 9.4
No. of unique Peptides: 1

10	20	30	40	50	60	70	80
MIVFVRFNSS	HGFPVEVDSD	TSIFQLKEVV	AKRQGVPADQ	LRVIFAGKEL	RNDWTVQNCD	LDQQSIVHIV	QRPWRKGQEM
90	100	110	120	130	140	150	160
NATGGDDPRN	AAGGCEREPQ	SLTRVDLSSS	VLPGDSVGLA	VILHTDSRKD	SPPAGSPAGR	SIYNSFYVYC	KGPCQRVQPG
170	180	190	200	210	220	230	240
KLRVQCSTCR	QATLTLTQEF	FFKCGAHPTS	DKETSVALHL	IATNSRNITC	ITCTDVRSPV	LVFQCNSRHV	ICLDCFHLYC
250	260	270	280	290	300	310	320
VTRLNDRQFV	HDPQLGYSLP	CVAGCPNSLI	KELHHFRILG	EEQYNRYQQY	GAEECVLQMG	GVLCPRPGCG	AGLLPEPDQR
330	340	350	360	370	380	390	400
KVTCEGGNGL	GCGFAFCREC	KEAYHEGECS	AVFEASGTTT	QAYRVDERAA	EQARWEAASK	ETIKKTTKPC	PRCHVPVEKN
410	420	430	440				
GGCMHMKCPQ	PQCRLEWCWN	CGCEWNRVCM	GDHWFDV				

Cmpd.	No. of Cmpds.	m/z meas.	Δ m/z [ppm]	z	Rt [min]	Score	P	Range	Sequence	Modification
2156	1	836.3125	-111.36	3	55.3	11.7	2	129-151	R.KDSPPAGSPAGRSIYNSFYVYCK.G	

Detailed Protein Report

Protein 281: protein AKNAD1 [Homo sapiens]

**Accession:**	gi|91754185	**Score:**	25.3
**Database:**	refseq_human(refseq_human_20140103.fasta)	**MW [kDa]:**	92.8
**Database Date:**	2015-11-30	**pI:**	6.4
**Modification(s):**	Carbamidomethyl, Oxidation	**Sequence Coverage [%]:**	3.7
		**No. of unique Peptides:**	1

10	20	30	40	50	60	70	80
MDEADFSEHT	TYKQEDLPYD	GDLSQIKIGN	DYSFTSKKDG	LEVLNQIIFI	ADDPQEKAMH	SETCGNTAVT	IPLGKITENA
90	100	110	120	130	140	150	160
ANKKDEKEKQ	CTAALHIPAN	EGDASKSSIS	DILLHHLSKE	PFLRGQGIDC	ETLPEISNAD	SFEEEAIIKS	IISCYNKNSW
170	180	190	200	210	220	230	240
PKEQTPELTD	QLNPKRDGEN	SNKPGSATTT	EENTSDLEGP	VAAGDSSHQE	NVNVLTKTKG	PGDKQKSYQG	QSPQKQQTEK
250	260	270	280	290	300	310	320
ANSGNTFKYG	QGQVHYQLPD	FSKIAPKVKI	PKNKIINKPL	AIAKQASFSS	KSRDKPTLVQ	DSLETTPESN	CVEKQHQEQK
330	340	350	360	370	380	390	400
GKITEPSQQI	QMEPIVHIHQ	ELLTGIESEA	SLSKLSPTSQ	KGTSSSSSYI	FQKISQGKQM	CQKLKEQTDQ	LKTKVQEFSK
410	420	430	440	450	460	470	480
RIKQDSPYHL	QDKKLVLEKL	QGHLELLEQN	FLATKDKHLT	LQQQVHKHES	TIVGDFDPER	KVEGEIFKLE	MLLEDVKEKM
490	500	510	520	530	540	550	560
DESKYTSAPS	LPVSSPVTLD	DLASTFSSLS	NEIPKEHPGH	PSGPRGSGGS	EVTGTPQGGP	QEAPNEELCE	LAPQTYLNGH
570	580	590	600	610	620	630	640
YGDAAAQNKP	DQVAMRLSSN	SGEDPNGTPR	RQDCAEMTAP	SPSCAFCRRL	LEWKQNVEKK	GHGRINCGRF	SIVLHEKAPH
650	660	670	680	690	700	710	720
SDSTPNSDTG	HSFCSDSGTE	MQSNKCQDCG	TKIPTSRRAC	RKEPTKEFHY	RYNTPGQNYS	NHSKRGAFVQ	PHSLDESKNS
730	740	750	760	770	780	790	800
SPSFLKPKRI	CSQRVNSKSF	KGEHEPTPGK	KKLQAFMTYS	SDPATPSPHF	YSCRISGSKS	LCDFDSTEEI	KSEILNSALD
810	820	830	840				
HALRTATILK	ETTDQMIKTI	AEDLAKAQRW	RNRLKY				

Cmpd.	No. of Cmpds.	m/z meas.	Δ m/z [ppm]	z	Rt [min]	Score	P	Range	Sequence	Modification
2161	1	1023.4064	-11.81	2	55.6	13.9	1	592-609	R.QDCAEMTAPSPSCAFCRR.L	Carbamidomethyl: 16; Oxidation: 6

Detailed Protein Report

Protein 282: **PREDICTED: uncharacterized aarF domain-containing protein kinase 1 isoform X2 [Homo sapiens]**

**Accession:**	gi|530404248	**Score:**	25.2
**Database:**	refseq_human(refseq_human_20140103.fasta)	**MW [kDa]:**	52.0
**Database Date:**	2015-11-30	**pI:**	9.7
		**Sequence Coverage [%]:**	7.9
		**No. of unique Peptides:**	1

10	20	30	40	50	60	70	80
MARKALKLAS	WTSMALAASG	IYFYSNKYLD	PNDFGAVRVG	RAVATTAVIS	YDYLTSLKSV	PYGSEEYLQL	RSKVHLRSAR
**90**	**100**	**110**	**120**	**130**	**140**	**150**	**160**
RLCELCCANR	GTFIKVGQHL	GALDYLLPEE	YTSTLKVLHS	QAPQSSMQEI	RQVIREDLGK	EIHDLFQSFD	DTPLGTASLA
**170**	**180**	**190**	**200**	**210**	**220**	**230**	**240**
QVHKAVLHDG	RTVAVKVQHP	KVRAQSSKDI	LLMEVLVLAV	KQLFPEFEFM	WLVDEAKKNL	PLELDFLNEG	RNAEKVSQML
**250**	**260**	**270**	**280**	**290**	**300**	**310**	**320**
RHFDFLKVPR	IHWDLSTERV	LLMEFVDGGQ	VNDRDYMERN	KIDVNEISRH	LGKMYSEMIF	VNGFVHCDPH	PGNVLVRKHP
**330**	**340**	**350**	**360**	**370**	**380**	**390**	**400**
GTGKAEIVLL	DHGLYQDLEI	RNNAANYLPQ	ISHLLNHVPR	QMLLILKTND	LLRGIEAALG	TRASASSFLN	MSRCCIRALA
**410**	**420**	**430**	**440**	**450**	**460**		
EHKKKNTCSF	FRRTQISFSE	AFNLWQINLH	ELILRVKGLK	LADRVLALIC	WLFPAPL		

Cmpd.	No. of Cmpds.	m/z meas.	Δ m/z [ppm]	z	Rt [min]	Score	P	Range	Sequence	Modification
2197	1	863.8529	-90.52	2	56.0	13.6	2	81-95	R.RLCELCCANRGTFIK.V	

Detailed Protein Report

Protein 283: zinc finger protein 358 [Homo sapiens]

**Accession:**	gi|133922582	**Score:**	25.2
**Database:**	refseq_human(refseq_human_20140103.fasta)	**MW [kDa]:**	59.2
**Database Date:**	2015-11-30	**pI:**	6.1
		**Sequence Coverage [%]:**	2.6
		**No. of unique Peptides:**	1

Alias proteins:

Accession	Name	Description
gi|530427462	refseq_human (refseq_human_20140103.fasta)	PREDICTED: zinc finger protein 358 isoform X1 [Homo sapiens]

10	20	30	40	50	60	70	80
MRRSVLVRNP	GHKGLRPVYE	ELDSDSEDLD	PNPEDLDPVS	EDPEPDPEDL	NTVPEDVDPS	YEDLEPVSED	LDPDAEAPGS
**90**	**100**	**110**	**120**	**130**	**140**	**150**	**160**
EPQDPDPMSS	SFDLDPDVIG	PVPLILDPNS	DTLSPGDPKV	DPISSGLTAT	PQVLATSPAV	LPAPASPPRP	FSCPDCGRAF
**170**	**180**	**190**	**200**	**210**	**220**	**230**	**240**
RRSSGLSQHR	RTHSGEKPYR	CPDCGKSFSH	GATLAQHRGI	HTGARPYQCA	ACGKAFGWRS	TLLKHRSSHS	GEKPHHCPVC
**250**	**260**	**270**	**280**	**290**	**300**	**310**	**320**
GKAFGHGSLL	AQHLRTHGGP	RPHKCPVCAK	GFGQGSALLK	HLRTHTGERP	YPCPQCGKAF	GQSSALLQHQ	RTHTAERPYR
**330**	**340**	**350**	**360**	**370**	**380**	**390**	**400**
CPHCGKAFGQ	SSNLQHHLRI	HTGERPYACP	HCSKAFGQSS	ALLQHLHVHS	GERPYRCQLC	GKAFGQASSL	TKHKRVHEGA
**410**	**420**	**430**	**440**	**450**	**460**	**470**	**480**
AAAAAAAAAA	AAAAAAGLGL	GPGLSPASMM	RPGQVSLLGP	DAVSVLGSGL	GLSPGTSSGR	NPDPGSGPGT	LPDPSSKPLP
**490**	**500**	**510**	**520**	**530**	**540**	**550**	**560**
GSRSTPSPTP	VESSDPKAGH	DAGPDLVPSP	DLDPVPSPDP	DPVPSPDPNP	VSCPDPCSPT	RGTVSPALPT	GESPEWVQEQ
**570**							
GALLGPDG							

Cmpd.	No. of Cmpds.	m/z meas.	Δ m/z [ppm]	z	Rt [min]	Score	P	Range	Sequence	Modification
770	1	839.4295	62.27	2	38.4	25.2	1	172-186	R.THSGEKPYRCPDCGK.S	

Detailed Protein Report

Protein 284: PREDICTED: fibrillin-3 isoform X1 [Homo sapiens]

**Accession:**	gi|578833689	**Score:**	25.2
**Database:**	refseq_human(refseq_human_20140103.fasta)	**MW [kDa]:**	301.1
**Database Date:**	2015-11-30	**pI:**	4.8
**Modification(s):**	Carbamidomethyl	**Sequence Coverage [%]:**	1.1
		**No. of unique Peptides:**	2

Quantitation

WD:WU	**Median:** 0.85	**CV:** 0.00 %	**No. of Peptides:** 1

Detailed Protein Report

10	20	30	40	50	60	70	80
MTLEGLYLAR	GPLARLLLAW	SALLCMAGGQ	GRWDGALEAA	GPGRVRRRGS	PGILQGPNVC	GSRFHAYCCP	GWRTFPGRSQ
90	100	110	120	130	140	150	160
CVVRSGCSVS	CMNGGTCRGA	SCLCQKGYTG	TVCGQPICDR	GCHNGGRCIG	PNRCACVYGF	MGPQCERDYR	TGPCFGQVGP
170	180	190	200	210	220	230	240
EGCQHQLTGL	VCTKALCCAT	VGRAWGLPCE	LCPAQPHPCR	RGFIPNIHTG	ACQDVDECQA	VPGLCQGGSC	VNMVGSFHCR
250	260	270	280	290	300	310	320
CPVGHRLSDS	SAACEDVNEC	LSLSGLCSGG	DCTNTVGSYV	CTCSQGFASS	LDGTHCLNYR	AGACFSVLFG	GRCAGDLAGH
330	340	350	360	370	380	390	400
YTRRQCCCDR	GRCWAAGPVP	ELCPPRGSNE	FQQLCAQRLP	LLPGHPGLFP	GLLGFGSNGM	GPPLGPARLN	PHGSDARGIP
410	420	430	440	450	460	470	480
SLGPGNSNIG	TATLNQTIDI	CRHFTNLCLN	GRCLPTPSSY	RCECNVGYTQ	DVRGECIDVD	ECTSSPCHHG	DCVNIPGTYH
490	500	510	520	530	540	550	560
CRCYPGFQAT	PTRQACVDVD	ECIVSGGLCH	LGRCVNTEGS	FQCVCNAGFE	LSPDGKNCVD	HNECATSTMC	VNGVCLNEDG
570	580	590	600	610	620	630	640
SFSCLCKPGF	LLAPGGHYCM	DIDECQTPGI	CVNGHCTNTE	GSFRCQCLGG	LAVGTDGRVC	VDTHVRSTCY	GAIEKGSCAR
650	660	670	680	690	700	710	720
PFPGTVTKSE	CCCANPDHGF	GEPCQLCPAK	DSAEFQALCS	SGLGITTDGR	DINECALDPE	VCANGVCENL	RGSYRCVCNL
730	740	750	760	770	780	790	800
GYEAGASGKD	CTDVDECALN	SLLCDNGWCQ	NSPGSYSCSC	PPGFHFWQDT	EICKDVDECL	SSPCVSGVCR	NLAGSYTCKC
810	820	830	840	850	860	870	880
GPGSRLDPSG	TFCLDSTKGT	CWLKIQESRC	EVNLQGASLR	SECCATLGAA	WGSPCERCEI	DPACARGFAR	MTGVTCDDVN
890	900	910	920	930	940	950	960
ECESFPGVCP	NGRCVNTAGS	FRCECPEGLM	LDASGRLCVD	VRLEPCFLRW	DEDECGVTLP	GKYRMDVCCC	SIGAVWGVEC
970	980	990	1000	1010	1020	1030	1040
EACPDPESLE	FASLCPRGLG	FASRDFLSGR	PFYKDVNECK	VFPGLCTHGT	CRNTVGSFHC	ACAGGFALDA	QERNCTDIDE
1050	1060	1070	1080	1090	1100	1110	1120
CRISPDLCGQ	GTCVNTPGSF	ECECFPGYES	GFMLMKNCMD	VDECARDPLL	CRGGTCTNTD	GSYKCQCPPG	HELTAKGTAC
1130	1140	1150	1160	1170	1180	1190	1200
EDIDECSLSD	GLCPHGQCVN	VIGAFQCSCH	AGFQSTPDRQ	GCVDINECRV	QNGGCDVHCI	NTEGSYRCSC	GQGYSLMPDG
1210	1220	1230	1240	1250	1260	1270	1280
RACADVDECE	ENPRVCDQGH	CTNMPGGHRC	LCYDGFMATP	DMRTCVDVDE	CDLNPHICLH	GDCENTKGSF	VCHCQLGYMV
1290	1300	1310	1320	1330	1340	1350	1360
RKGATGCSDV	DECEVGGHNC	DSHASCLNIP	GSFSCRCLPG	WVGDGFECHD	LDECVSQEHR	CSPRGDCLNV	PGSYRCTCRQ
1370	1380	1390	1400	1410	1420	1430	1440
GFAGDGFFCE	DRDECAENVD	LCDNGQCLNA	PGGYRCECEM	GFDPTEDHRA	CQDVDECAQG	NLCAFGSCEN	LPGMFRCICN
1450	1460	1470	1480	1490	1500	1510	1520
GGYELDRGGG	NCTDINECAD	PVNCINGVCI	NTPGSYLCSC	PQDFELNPSG	VGCVDTRAGN	CFLETHDRGD	SGISCSAEIG
1530	1540	1550	1560	1570	1580	1590	1600
VGVTRASCCC	SLGRAWGNPC	ELCPMANTTE	YRTLCPGGEG	FQPNRITVIL	EDIDECQELP	GLCQGGDCVN	TFGSFQCECP
1610	1620	1630	1640	1650	1660	1670	1680
PGYHLSEHTR	ICEDIDECST	HSGICGPGTC	YNTLGNYTCV	CPAEYLQVNG	GNNCMDMRKS	VCFRHYNGTC	QNELAFNVTR
1690	1700	1710	1720	1730	1740	1750	1760
KMCCCSYNIG	QAWNRPCEAC	PTPISPDYQI	LCGNQAPGFL	TDIHTGKPLD	IDECGEIPAI	CANGICINQI	GSFRCECPAG
1770	1780	1790	1800	1810	1820	1830	1840
FNYNSILLAC	EDVDECGSRE	SPCQQNADCI	NIPGSYRCKC	TRGYKLSPGG	ACVGRNECRE	IPNVCSHGDC	MDTEGSYMCL
1850	1860	1870	1880	1890	1900	1910	1920
CHRGFQASAD	QTLCMDIDEC	DRQPCGNGTC	KNIIGSYNCL	CFPGFVVTHN	GDCVDFDECT	TLVGQVCRFG	HCLNTAGSFH
1930	1940	1950	1960	1970	1980	1990	2000
CLCQDGFELT	ADGKNCVDTN	ECLSLAGTCL	PGTCQNLEGS	FRCICPPGFQ	VQSDHCIDID	ECSEEPNLCL	FGTCTNSPGS
2010	2020	2030	2040	2050	2060	2070	2080
FQCLCPPGFV	LSDNGHRCFD	TRQSFCFTRF	EAGKCSVPKA	FNTTKTRCCC	SKRPGEGWGD	PCELCPQEGS	AAFQELCPFG
2090	2100	2110	2120	2130	2140	2150	2160
HGAVPGPDDS	REDVNECAEN	PGVCTNGVCV	NTDGSFRCEC	PFGYSLDFTG	INCVDTDECS	VGHPCGQGTC	TNVIGGFECA
2170	2180	2190	2200	2210	2220	2230	2240

Detailed Protein Report

Cmpd.	No. of Cmpds.	m/z meas.	Δ m/z [ppm]	z	Rt [min]	Score	P	Range	Sequence	Modification	Ratios
147	1	492.5609	80.32	3	31.1	13.5	0	85-98	R.SGCSVSCMNGGTCR.G	Carbamidomethyl: 3, 7	WD:WU 0.85
2042	1	733.5869	-72.63	3	53.9	11.7	2	313-330	R.CAGDLAGHYTRRQCCCDR.G	Carbamidomethyl: 14, 15, 16	

Detailed Protein Report

Protein 285: **epidermal growth factor receptor kinase substrate 8-like protein 1 isoform b [Homo sapiens]**

**Accession:**	gi|148539855	**Score:**	25.1
**Database:**	refseq_human(refseq_human_20140103.fasta)	**MW [kDa]:**	66.4
**Database Date:**	2015-11-30	**pI:**	5.9
		**Sequence Coverage [%]:**	4.0
		**No. of unique Peptides:**	1

10	20	30	40	50	60	70	80
MNRTWPRRIW	GSSQDEAELI	REDIQGALHN	YRSGRGERRA	AALRATQEEL	QRDRSPAAET	PPLQRRPSVR	AVISTVERGA
**90**	**100**	**110**	**120**	**130**	**140**	**150**	**160**
GRGRPQAKPI	PEAEEAQRPE	PVGTSSNADS	ASPDLGPRGP	DLAVLQAERE	VDILNHVFDD	VESFVSRLQK	SAEAARVLEH
**170**	**180**	**190**	**200**	**210**	**220**	**230**	**240**
RERGRRSRRR	AAGEGLLTLR	AKPPSEAEYT	DVLQKIKYAF	SLLARLRGNI	ADPSSPELLH	FLFGPLQMIV	NTSGGPEFAS
**250**	**260**	**270**	**280**	**290**	**300**	**310**	**320**
SVRRPHLTSD	AVALLRDNVT	PRENELWTSL	GDSWTRPGLE	LSPEEGPPYR	PEFFSGWEPP	VTDPQSRAWE	DPVEKQLQHE
**330**	**340**	**350**	**360**	**370**	**380**	**390**	**400**
RRRRQQSAPQ	VAVNGHRDLE	PESEPQLESE	TAGKWVLCNY	DFQARNSSEL	SVKQRDVLEV	LDDSRKWWKV	RDPAGQEGYV
**410**	**420**	**430**	**440**	**450**	**460**	**470**	**480**
PYNILTPYPG	PRLHHSQSPA	RSLNSTPPPP	PAPAPAPPPA	LARPRWDRPR	WDSCDSLNGL	DPSEKEKFSQ	MLIVNEELQA
**490**	**500**	**510**	**520**	**530**	**540**	**550**	**560**
RLAQGRSGPS	RAVPGPRAPE	PQLSPGSDAS	EVRAWLQAKG	FSSGTVDALG	VLTGAQLFSL	QKEELRAVSP	EEGARVYSQV
**570**	**580**	**590**	**600**				
TVQRSLLEDK	EKVSELEAVM	EKQKKKVEGE	VEMEVI				

Cmpd.	No. of Cmpds.	m/z meas.	Δ m/z [ppm]	z	Rt [min]	Score	P	Range	Sequence	Modification
2109	1	696.2869	-105.74	2	54.9	11.9	0	325-337	R.QQSAPQVAVNGHR.D	

Detailed Protein Report

Protein 286: **zinc finger protein 92 isoform 4 [Homo sapiens]**

**Accession:**	gi|566559828	**Score:**	25.1
**Database:**	refseq_human(refseq_human_20140103.fasta)	**MW [kDa]:**	59.4
**Database Date:**	2015-11-30	**pI:**	10.3
		**Sequence Coverage [%]:**	1.8
		**No. of unique Peptides:**	1

Quantitation

WD:WU **Median:** 0.46 **CV:** 0.00 % **No. of Peptides:** 1

Alias proteins:

Accession	Name	Description
gi|566559924	refseq_human (refseq_human_20140103.fasta)	zinc finger protein 92 isoform 4 [Homo sapiens]

10	20	30	40	50	60	70	80
MCSHFAQDVW	PEHSIKDSFQ	KVILRTYGKY	GHENLQLRKD	HKSVDACKVY	KGGYNGLNQC	LTTTDSKIFQ	CDKYVKVFHK
**90**	**100**	**110**	**120**	**130**	**140**	**150**	**160**
FPNVNRNKIR	HTGKKPFKCK	NRGKSFCMLS	QLTQHKKIHT	REYSYKCEEC	GKAFNWSSTL	TKHKIIHTGE	KPYKCEECGK
**170**	**180**	**190**	**200**	**210**	**220**	**230**	**240**
AFNRSSNLTK	HKIIHTGEKP	YKCEECGKAF	NRSSTLTKHK	RIHTEEKPYK	CEECGKAFNQ	FSILNKHKRI	HMEDKPYKCE
**250**	**260**	**270**	**280**	**290**	**300**	**310**	**320**
ECGKAFRVFS	ILKKHKIIHT	GEKPYKCEEC	GKAFNQFSNL	TKHKIIHTGE	KPYKCDECGK	AFNQSSTLTK	HKRIHTGEKP
**330**	**340**	**350**	**360**	**370**	**380**	**390**	**400**
YKCEECGKAF	KQSSTLTEHK	IIHTGEKPYK	CEKCGKAFSW	SSAFTKHKRN	HMEDKPYKCE	ECGKAFSVFS	TLTKHKIIHT
**410**	**420**	**430**	**440**	**450**	**460**	**470**	**480**
REKPYKCEEC	GKAFNQSSIF	TKHKIIHTEG	KSYKCEKCGN	AFNQSSNLTA	RKIIYTGEKP	YKYEECDKAF	NKFSTLITHQ
**490**	**500**	**510**	**520**				
IIYTGEKPCK	HECGRAFNKS	SNYTKEKLQT					

Cmpd.	No. of Cmpds.	m/z meas.	Δ m/z [ppm]	z	Rt [min]	Score	P	Range	Sequence	Modification	Ratios
1095	1	540.7909	-75.68	2	41.9	25.1	1	245-253	K.AFRVFSILK.K		WD:WU 0.46

Detailed Protein Report

Protein 287: GH3 domain-containing protein isoform 2 precursor [Homo sapiens]

**Accession:**	gi|217330592	**Score:**	25.1
**Database:**	refseq_human(refseq_human_20140103.fasta)	**MW [kDa]:**	53.5
**Database Date:**	2015-11-30	**pI:**	10.0
		**Sequence Coverage [%]:**	3.9
		**No. of unique Peptides:**	1

10	20	30	40	50	60	70	80
MLLWPLLLLL	LLLPTLALLR	QQRSQDARLS	WLAGLQHRVA	WGALVWAATW	QRRRLEQSTL	HVHQSQQQAL	RWCLQGAQRP
90	100	110	120	130	140	150	160
HCSLRRSTDI	STFRNHLPLT	KAYPEVLAQG	RTARVTLTSP	WPRPLPWPGN	TLGQVGTPGT	KDPRALLLDA	LRSPGLRALE
170	180	190	200	210	220	230	240
AGTAVELLDV	FLGLETDGEE	LAGAIAAGNP	GAPLRERAAE	LREALEQGPR	GLALRLWPKL	QVVVTLDAGG	QAEAVAALGA
250	260	270	280	290	300	310	320
LWCQGLAFFS	PAYAASGGVL	GLNLQPEQPH	GLYLLPPGAP	FIELLPVKEG	TQEEAASTLL	LAEAQQGKEY	ELVLTDRASL
330	340	350	360	370	380	390	400
TRCRLGDVVR	VVGAYNQCPV	VRFICRLDQT	LSVRGEDIGE	DLFSEALGRA	VGQWAGAKLL	DHGCVESSIL	DSSAGSAPHY
410	420	430	440	450	460	470	480
EVFVALRGLR	NLSEENRDKL	DHCLQEASPR	YKSLRFWGSV	GPARVHLVGQ	GAFRALRAAL	AACPSSPFPP	AMPRVLRHRH
490	500						
LAQCLQERVV	S						

Cmpd.	No. of Cmpds.	m/z meas.	Δ m/z [ppm]	z	Rt [min]	Score	P	Range	Sequence	Modification
2063	1	634.7944	-18.66	2	54.2	10.9	0	420-430	K.LDHCLQEASPR.Y	

Detailed Protein Report

Protein 288: PREDICTED: glutamine-rich protein 2 isoform X2 [Homo sapiens]

**Accession:**	gi|578831523	**Score:**	25.1
**Database:**	refseq_human(refseq_human_20140103.fasta)	**MW [kDa]:**	72.2
**Database Date:**	2015-11-30	**pI:**	9.9
**Modification(s):**	Oxidation	**Sequence Coverage [%]:**	5.5
		**No. of unique Peptides:**	1

10	20	30	40	50	60	70	80
MRSRPAKPTW	RRSSSCWHRW	VERLQRILEG	EGNQEAGKEL	KAGELRLQLG	VLRVTVADIE	KELAELRESQ	DRGKAAMENS
**90**	**100**	**110**	**120**	**130**	**140**	**150**	**160**
VSEASLYLQD	QLDKLRMIIE	SMLTSSSTLL	SMSMAPHKAH	TLAPGQIDPE	ATCPACSLDV	SHQVSTLVRR	YEQLQDMVNS
**170**	**180**	**190**	**200**	**210**	**220**	**230**	**240**
LAVSRPSKKA	KLQRQDEELL	GRVQSAILQV	QGDCEKLNIT	TSNLIEDHRQ	KQKDIAMLYQ	GLEKLEKEKA	NREHLEMEID
**250**	**260**	**270**	**280**	**290**	**300**	**310**	**320**
VKADKSALAT	KVSRVQFDAT	TEQLNHMMQE	LVAKMSGQEQ	DWQKMLDRLL	TEMDNKLDRL	ELDPVKQLLE	DRWKSLRQQL
**330**	**340**	**350**	**360**	**370**	**380**	**390**	**400**
RERPPLYQAD	EAAAMRRQLL	AHFHCLSCDR	PLETPVTGHA	IPVTPAGPGL	PGHHSIRPYT	VFELEQVRQH	SRNLKLGSAF
**410**	**420**	**430**	**440**	**450**	**460**	**470**	**480**
PRGDLAQMEQ	SVGRLRSMHS	KMLMNIEKVQ	IHFGGSTKAS	SQIIRELLHA	QCLGSPCYKR	VTDMADYTYS	TVPRRCGGSH
**490**	**500**	**510**	**520**	**530**	**540**	**550**	**560**
TLTYPYHRSR	PQHLPRGLYP	TEEIQIAMKH	DEVDILGLDG	HIYKGRMDTR	LPGILRKDSS	GTSKRKSQQP	RPHVHRPPSL
**570**	**580**	**590**	**600**	**610**	**620**	**630**	**640**
SSNGQLPSRP	QSAQISAGNT	SVSSRQQKDR	PSSEGRLSQP	NTAHPPSSAA	VANRGLERHV	DMPPGEGLEE	PTRGPRSSTA
**650**							
Q							

Cmpd.	No. of Cmpds.	m/z meas.	Δ m/z [ppm]	z	Rt [min]	Score	P	Range	Sequence	Modification
592	1	1105.2058	124.92	2	35.8	10.7	1	151-169	R.YEQLQDMVNSLAVSRPSKK.A	Oxidation: 7

Detailed Protein Report

Protein 289: retinoblastoma-associated protein [Homo sapiens]

**Accession:**	gi|108773787	**Score:**	25.1
**Database:**	refseq_human(refseq_human_20140103.fasta)	**MW [kDa]:**	106.1
**Database Date:**	2015-11-30	**pI:**	8.9
**Modification(s):**	Carbamidomethyl, Oxidation	**Sequence Coverage [%]:**	1.8
		**No. of unique Peptides:**	2

Quantitation

WD:WU **Median:** 5.87 **CV:** 0.00 % **No. of Peptides:** 1

10	20	30	40	50	60	70	80
MPPKTPRKTA	ATAAAAAAEP	PAPPPPPPPE	EDPEQDSGPE	DLPLVRLEFE	ETEEPDFTAL	CQKLKIPDHV	RERAWLTWEK
90	100	110	120	130	140	150	160
VSSVDGVLGG	YIQKKKELWG	ICIFIAAVDL	DEMSFTFTEL	QKNIEISVHK	FFNLLKEIDT	STKVDNAMSR	LLKKYDVLFA
170	180	190	200	210	220	230	240
LFSKLERTCE	LIYLTQPSSS	ISTEINSALV	LKVSWITFLL	AKGEVLQMED	DLVISFQLML	CVLDYFIKLS	PPMLLKEPYK
250	260	270	280	290	300	310	320
TAVIPINGSP	RTPRRGQNRS	ARIAKQLEND	TRIIEVLCKE	HECNIDEVKN	VYFKNFIPFM	NSLGLVTSNG	LPEVENLSKR
330	340	350	360	370	380	390	400
YEEIYLKNKD	LDARLFLDHD	KTLQTDSIDS	FETQRTPRKS	NLDEEVNVIP	PHTPVRTVMN	TIQQLMMILN	SASDQPSENL
410	420	430	440	450	460	470	480
ISYFNNCTVN	PKESILKRVK	DIGYIFKEKF	AKAVGQGCVE	IGSQRYKLGV	RLYYRVMESM	LKSEEERLSI	QNFSKLLNDN
490	500	510	520	530	540	550	560
IFHMSLLACA	LEVVMATYSR	STSQNLDSGT	DLSFPWILNV	LNLKAFDFYK	VIESFIKAEG	NLTREMIKHL	ERCEHRIMES
570	580	590	600	610	620	630	640
LAWLSDSPLF	DLIKQSKDRE	GPTDHLESAC	PLNLPLQNNH	TAADMYLSPV	RSPKKKGSTT	RVNSTANAET	QATSAFQTQK
650	660	670	680	690	700	710	720
PLKSTSLSLF	YKKVYRLAYL	RLNTLCERLL	SEHPELEHII	WTLFQHTLQN	EYELMRDRHL	DQIMMCSMYG	ICKVKNIDLK
730	740	750	760	770	780	790	800
FKIIVTAYKD	LPHAVQETFK	RVLIKEEEYD	SIIVFYNSVF	MQRLKTNILQ	YASTRPPTLS	PIPHIPRSPY	KFPSSPLRIP
810	820	830	840	850	860	870	880
GGNIYISPLK	SPYKISEGLP	TPTKMTPRSR	ILVSIGESFG	TSEKFQKINQ	MVCNSDRVLK	RSAEGSNPPK	PLKKLRFDIE
890	900	910	920	930			
GSDEADGSKH	LPGESKFQQK	LAEMTSTRTR	MQKQKMNDSM	DTSNKEEK			

Cmpd.	No. of Cmpds.	m/z meas.	Δ m/z [ppm]	z	Rt [min]	Score	P	Range	Sequence	Modification	Ratios
1645	2	1045.0357	59.07	2	49.3	10.1	1	699-715	R.HLDQIMMCSMYGICKVK.N	Carbamidomethyl: 14; Oxidation: 6, 7	
1658	2	1045.0497	72.41	2	49.4	15.0	1	699-715	R.HLDQIMMCSMYGICKVK.N	Carbamidomethyl: 14; Oxidation: 6, 10	WD:WU 5.87

Detailed Protein Report

Protein 290: PREDICTED: vinexin isoform X5 [Homo sapiens]

**Accession:**	gi|578815141	**Score:**	25.0
**Database:**	refseq_human(refseq_human_20140103.fasta)	**MW [kDa]:**	73.7
**Database Date:**	2015-11-30	**pI:**	9.9
		**Sequence Coverage [%]:**	4.7
		**No. of unique Peptides:**	2

10	20	30	40	50	60	70	80
MQGPPRSLRA	GLSLDDFIPG	HLQSHIGSSS	RGTRFHDPAP	RTVCNGGYTP	RRDASQHPDP	AWYQTWPGPG	SKPSASTKIP
**90**	**100**	**110**	**120**	**130**	**140**	**150**	**160**
ASQHTQNWSA	TWTKDSKRRD	KRWVKYEGIG	PVDESGMPIA	PRSSVDRPRD	WYRRMFQQIH	RKMPDLQLDW	TFEEPPRDPR
**170**	**180**	**190**	**200**	**210**	**220**	**230**	**240**
HLGAQQRPAH	RPGPATSSSG	RSWDHSEELP	RSTFNYRPGA	FSTVLQPSNQ	VLRRREKVDN	VWTEESWNQF	LQELETGQRP
**250**	**260**	**270**	**280**	**290**	**300**	**310**	**320**
KKPLVDDPGE	KPSQPIEVLL	ERELAELSAE	LDKDLRAIET	RLPSPKSSPA	PRRAPEQRPP	AGPASAWSSS	YPHAPYLGSA
**330**	**340**	**350**	**360**	**370**	**380**	**390**	**400**
RSLSPHKMAD	GGSPFLGRRD	FVYPSSTRDP	SASNGGGSPA	RREEKKRKAA	RLKFDFQAQS	PKELTLQKGD	IVYIHKEVDK
**410**	**420**	**430**	**440**	**450**	**460**	**470**	**480**
NWLEGEHHGR	LGIFPANYVE	VLPADEIPKP	IKPPTYQVLE	YGEAVAQYTF	KGDLEVELSF	RKGEHICLIR	KVNENWYEGR
**490**	**500**	**510**	**520**	**530**	**540**	**550**	**560**
ITGTGRQGIF	PASYVQVSRE	PRLRLCDDGP	QLPTSPRLTA	AARSARHPSS	PSALRSPADP	IDLGGQTSPR	RTGFSFPTQE
**570**	**580**	**590**	**600**	**610**	**620**	**630**	**640**
PRPQTQNLGT	PGPALSHSRG	PSHPLDLGTS	SPNTSQIHWT	PYRAMYQYRP	QNEDELELRE	GDRVDVMQQC	DDGWFVGVSR
**650**	**660**						
RTQKFGTFPG	NYVAPV						

Cmpd.	No. of Cmpds.	m/z meas.	Δ m/z [ppm]	z	Rt [min]	Score	P	Range	Sequence	Modification
2813	1	944.5069	60.70	2	63.6	12.9	1	35-51	R.FHDPAPRTVCNGGYTPR.R	
1731	1	803.8844	-67.43	2	50.0	12.1	2	369-382	K.AARLKFDFQAQSPK.E	

Detailed Protein Report

Protein 291: PREDICTED: E3 ubiquitin-protein ligase RNF220 isoform X7 [Homo sapiens]

**Accession:**	gi|578799381	**Score:**	25.0
**Database:**	refseq_human(refseq_human_20140103.fasta)	**MW [kDa]:**	35.4
**Database Date:**	2015-11-30	**pI:**	6.8
		**Sequence Coverage [%]:**	6.8
		**No. of unique Peptides:**	1

10	20	30	40	50	60	70	80
MDLHRAAFKM	ENSSYLPNPL	ASPALMVLAS	TAEASRDASI	PCQQPRPFGV	PVSVDKDVHI	PFTNGSYTFA	SMYHRQGGVP
**90**	**100**	**110**	**120**	**130**	**140**	**150**	**160**
GTFANRDFPP	SLLHLHPQFA	PPNLDCTPIS	MLNHSGVGAF	RPFASTEDRE	SYQSAFTPAK	RLKNCHDTES	PHLRFSDADG
**170**	**180**	**190**	**200**	**210**	**220**	**230**	**240**
KEYDFGTQLP	SSSPGSLKVD	DTGKKIFAVS	GLISDREASS	SPEDRNDRCK	KKAAALFDSQ	APICPICQVL	LRPSELQEHM
**250**	**260**	**270**	**280**	**290**	**300**	**310**	**320**
EQELEQLAQL	PSSKNSLLKD	AMAPGTPKSL	LLSASIKREG	ESPTASPHSS	ATDDLHHSDR	YQTFLRVRAN	RQTRLNEGRL
**330**							
LHG							

Cmpd.	No. of Cmpds.	m/z meas.	Δ m/z [ppm]	z	Rt [min]	Score	P	Range	Sequence	Modification
2277	1	775.3245	-72.44	2	56.8	11.4	1	142-154	R.LKNCHDTESPHLR.F	

Detailed Protein Report

Protein 292: beta-defensin 119 isoform a precursor [Homo sapiens]

**Accession:**	gi|37704382	**Score:**	24.9
**Database:**	refseq_human(refseq_human_20140103.fasta)	**MW [kDa]:**	9.8
**Database Date:**	2015-11-30	**pI:**	10.1
**Modification(s):**	Carbamidomethyl	**Sequence Coverage [%]:**	20.2
		**No. of unique Peptides:**	1

Quantitation

WD:WU **Median:** 0.88 **CV:** 0.00 % **No. of Peptides:** 1

10	20	30	40	50	60	70	80
MKLLYLFLAI	LLAIEEPVIS	GKRHILRCMG	NSGICRASCK	KNEQPYLYCR	NCQSCCLQSY	MRISISGKEE	NTDWSYEKQW
90							
PRLP							

Cmpd.	No. of Cmpds.	m/z meas.	Δ m/z [ppm]	z	Rt [min]	Score	P	Range	Sequence	Modification	Ratios	
1509	1	981.9713	5.72	2	47.6	13.7	2	24-40	R.HILRCMGNSGICRASCK.K	Carbamidomethyl: 12, 16	WD:WU	0.88

Detailed Protein Report

Protein 293: zinc finger protein with KRAB and SCAN domains 1 isoform c [Homo sapiens]

**Accession:**	gi|559098399	**Score:**	24.9
**Database:**	refseq_human(refseq_human_20140103.fasta)	**MW [kDa]:**	39.3
**Database Date:**	2015-11-30	**pI:**	10.0
**Modification(s):**	Carbamidomethyl	**Sequence Coverage [%]:**	8.3
		**No. of unique Peptides:**	1

10	20	30	40	50	60	70	80
MASALFTADS	QAMVKIEDMA	VSLILEEWGC	QNLARRNLSR	DNRQENYGSA	FPQGGENRNE	NEESTSKAET	SEDSASRGET
**90**	**100**	**110**	**120**	**130**	**140**	**150**	**160**
TGRSQKEFGE	KRDQEGKTGE	RQQKNPEEKT	RKEKRDSGPA	IGKDKKTITG	ERGPREKGKG	LGRSFSLSSN	FTTPEEVPTG
**170**	**180**	**190**	**200**	**210**	**220**	**230**	**240**
TKSHRCDECG	KCFTRSSSLI	RHKIIHTGEK	PYECSECGKA	FSLNSNLVLH	QRIHTGEKPH	ECNECGKAFS	HSSNLILHQR
**250**	**260**	**270**	**280**	**290**	**300**	**310**	**320**
IHSGEKPYEC	NECGKAFSQS	SDLTKHQRIH	TGEKPYECSE	CGKAFNRNSY	LILHRRIHTR	EKPYKCTKCG	KAFTRSSTLT
**330**	**340**	**350**	**360**				
LHHRIHARER	ASEYSPASLD	AFGAFLKSCV					

Cmpd.	No. of Cmpds.	m/z meas.	Δ m/z [ppm]	z	Rt [min]	Score	P	Range	Sequence	Modification
1971	1	1079.8531	-128.17	2	53.3	10.5	1	266-283	K.HQRIHTGEKPYECSECGK.A	Carbamidomethyl: 16

Detailed Protein Report

Protein 294: **protein lin-7 homolog C [Homo sapiens]**

Accession: gi|8922944
Database: refseq_human(refseq_human_20140103.fasta)
Database Date: 2015-11-30
Modification(s): Carbamidomethyl

Score: 24.9
MW [kDa]: 21.8
pI: 9.3
Sequence Coverage [%]: 11.2
No. of unique Peptides: 1

10	20	30	40	50	60	70	80
MAALGEPVRL	ERDICRAIEL	LEKLQRSGEV	PPQKLQALQR	VLQSEFCNAV	REVYEHVYET	VDISSSPEVR	ANATAKATVA
**90**	**100**	**110**	**120**	**130**	**140**	**150**	**160**
AFAASEGHSH	PRVVELPKTE	EGLGFNIMGG	KEQNSPIYIS	RIIPGGIADR	HGGLKRGDQL	LSVNGVSVEG	EHHEKAVELL
**170**	**180**	**190**	**200**				
KAAQGKVKLV	VRYTPKVLEE	MESRFEKMRS	AKRRQQT				

Cmpd.	No. of Cmpds.	m/z meas.	Δ m/z [ppm]	z	Rt [min]	Score	P	Range	Sequence	Modification
2712	1	878.0523	93.56	2	62.4	10.8	2	2-16	M.AALGEPVRLERDICR.A	Carbamidomethyl: 14

Detailed Protein Report

Protein 295: myosin-13 [Homo sapiens]

**Accession:**	gi|110624781	**Score:**	24.9
**Database:**	refseq_human(refseq_human_20140103.fasta)	**MW [kDa]:**	223.5
**Database Date:**	2015-11-30	**pI:**	5.4
		**Sequence Coverage [%]:**	1.4
		**No. of unique Peptides:**	1

Quantitation

WD:WU	**Median:** 0.84	**CV:** 0.00 %	**No. of Peptides:** 1

Detailed Protein Report

10	20	30	40	50	60	70	80
MSSDAEMAIF	GEAAPYLRKP	EKERIEAQNR	PFDSKKACFV	ADNKEMYVKG	MIQTRENDKV	IVKTLDDRML	TLNNDQVFPM
90	100	110	120	130	140	150	160
NPPKFDKIED	MAMMTHLHEP	AVLYNLKERY	AAWMIYTYSG	LFCVTVNPYK	WLPVYKPEVV	AAYRGKKRQE	APPHIFSISD
170	180	190	200	210	220	230	240
NAYQFMLTDR	DNQSILITGE	SGAGKTVNTK	RVIQYFATIA	VTGDKKKETQ	PGKMQGTLED	QIIQANPLLE	AFGNAKTVRN
250	260	270	280	290	300	310	320
DNSSRFGKFI	RIHFGATGKL	ASADIETYLL	EKSRVTFQLS	SERSYHIFYQ	IMSNKKPELI	DLLLISTNPF	DFPFVSQGEV
330	340	350	360	370	380	390	400
TVASIDDSEE	LLATDNAIDI	LGFSSEEKVG	IYKLTGAVMH	YGNMKFKQKQ	REEQAEPDGT	EVADKAGYLM	GLNSAEMLKG
410	420	430	440	450	460	470	480
LCCPRVKVGN	EYVTKGQNVQ	QVTNSVGALA	KAVYEKMFLW	MVTRINQQLD	TKQPRQYFIG	VLDIAGFEIF	DFNSLEQLCI
490	500	510	520	530	540	550	560
NFTNEKLQQF	FNHHMFVLEQ	EEYKKEGIEW	EFIDFGMDLA	ACIELIEKPM	GIFSILEEEC	MFPKATDTSF	KNKLYDQHLG
570	580	590	600	610	620	630	640
KSNNFQKPKP	AKGKAEAHFS	LVHYAGTVDY	NIAGWLDKNK	DPLNETVVGL	YQKSSLKLLS	FLFSNYAGAE	TGDSGGSKKG
650	660	670	680	690	700	710	720
GKKKGSSFQT	VSAVFRENLN	KLMTNLRSTH	PHFVRCLIPN	ETKTPGVMDH	YLVMHQLRCN	GVLEGIRICR	KGFPSRILYA
730	740	750	760	770	780	790	800
DFKQRYRILN	ASAIPEGQFI	DSKNASEKLL	NSIDVDREQF	RFGNTKVFFK	AGLLGLLEEM	RDEKLVTLMT	STQAVCRGYL
810	820	830	840	850	860	870	880
MRVEFKKMME	RRDSIFCIQY	NIRSFMNVKH	WPWMNLFFKI	KPLLKSAEAE	KEMATMKEDF	ERTKEELARS	EARRKELEEK
890	900	910	920	930	940	950	960
MVSLLQEKND	LQLQVQSETE	NLMDAEERCE	GLIKSKILLE	AKVKELTERL	EEEEEMNSEL	VAKKRNLEDK	CSSLKRDIDD
970	980	990	1000	1010	1020	1030	1040
LELTLTKVEK	EKHATENKVK	NLSEEMTALE	ENISKLTKEK	KSLQEAHQQT	LDDLQVEEDK	VNGLIKINAK	LEQQTDDLEG
1050	1060	1070	1080	1090	1100	1110	1120
SLEQEKKLRA	DLERAKRKLE	GDLKMSQESI	MDLENDKQQI	EEKLKKKEFE	LSQLQAKIDD	EQVHSLQFQK	KIKELQARIE
1130	1140	1150	1160	1170	1180	1190	1200
ELEEEIEAEH	TLRAKIEKQR	SDLARELEEI	SERLEEASGA	TSAQIEMNKK	REAEFQKMRR	DLEEATLQHE	ATAATLRKKQ
1210	1220	1230	1240	1250	1260	1270	1280
ADSVAELGEQ	IDNLQRVKQK	LEKEKSELKM	EIDDMASNIE	ALSKSKSNIE	RTCRTVEDQF	SEIKAKDEQQ	TQLIHDLNMQ
1290	1300	1310	1320	1330	1340	1350	1360
KARLQTQNGE	LSHRVEEKES	LISQLTKSKQ	ALTQQLEELK	RQMEEETKAK	NAMAHALQSS	RHDCDLLREQ	YEEEQEAKAE
1370	1380	1390	1400	1410	1420	1430	1440
LQRALSKANS	EVAQWRTKYE	TDAIQRTEEL	EEAKKKLAQR	LQEAEENTET	ANSKCASLEK	TKQRLQGEVE	DLMRDLERSH
1450	1460	1470	1480	1490	1500	1510	1520
TACATLDKKQ	RNFDKVLAEW	KQKLDESQAE	LEAAQKESRS	LSTELFKMRN	AYEEVVDQLE	TLRRENKNLQ	EEISDLTEQI
1530	1540	1550	1560	1570	1580	1590	1600
AETGKNLQEA	EKTKKLVEQE	KSDLQVALEE	VEGSLEHEES	KILRVQLELS	QVKSELDRKV	IEKDEEIEQL	KRNSQRAAEA
1610	1620	1630	1640	1650	1660	1670	1680
LQSVLDAEIR	SRNDALRLKK	KMEGDLNEME	IQLGHSNRQM	AETQKHLRTV	QGQLKDSQLH	LDDALRSNED	LKEQLAIVER
1690	1700	1710	1720	1730	1740	1750	1760
RNGLLLEELE	EMKVALEQTE	RTRRLSEQEL	LDASDRVQLL	HSQNTSLINT	KKKLEADIAQ	CQAEVENSIQ	ESRNAEEKAK
1770	1780	1790	1800	1810	1820	1830	1840
KAITDAAMMA	EELKKEQDTS	AHLERMKKNL	EQTVKDLQHR	LDEAEQLALK	GGKKQIQKLE	NRVRELENEL	DVEQKRGAEA
1850	1860	1870	1880	1890	1900	1910	1920
LKGAHKYERK	VKEMTYQAEE	DHKNILRLQD	LVDKLQAKVK	SYKRQAEEAE	EQANTQLSRC	RRVQHELEEA	AERADIAESQ
1930	1940						
VNKLRAKSRD	VGSQKMEE						

Cmpd.	No. of Cmpds.	m/z meas.	Δ m/z [ppm]	z	Rt [min]	Score	P	Range	Sequence	Modification	Ratios
112	1	793.8850	-11.43	2	30.7	10.0	0	1098-1110	K.IDDEQVHSLQFQK.K		WD:WU 0.84

Detailed Protein Report

Protein 296: PREDICTED: autophagy-related protein 2 homolog B isoform X1 [Homo sapiens]

**Accession:**	gi|578825925	**Score:**	24.8
**Database:**	refseq_human(refseq_human_20140103.fasta)	**MW [kDa]:**	232.5
**Database Date:**	2015-11-30	**pI:**	5.4
		**Sequence Coverage [%]:**	1.0
		**No. of unique Peptides:**	1

Detailed Protein Report

10	20	30	40	50	60	70	80
MPWPFSESIK	KRACRYLLQR	YLGHFLQEKL	SLEQLSLDLY	QGTGSLAQVP	LDKWCLNEIL	ESADAPLEVT	EGFIQSISLS
90	100	110	120	130	140	150	160
VPWGSLLQDN	CALEVRGLEM	VFRPRPRPAT	GSEPMYWSSF	MTSSMQLAKE	CLSQKLTDEQ	GEGSQPFEGL	EKFAETIETV
170	180	190	200	210	220	230	240
LRRVKVTFID	TVLRIEHVPE	NSKTGTALEI	RIERTVYCDE	TADESSGINV	HQPTAFAHKL	LQLSGVSLFW	DEFSASAKSS
250	260	270	280	290	300	310	320
PVCSTAPVET	EPKLSPSWNP	KIIYEPHPQL	TRNLPEIAPS	DPVQIGRLIG	RLELSLTLKQ	NEVLPGAKLD	VDGQIDSIHL
330	340	350	360	370	380	390	400
LLSPRQVHLL	LDMLAAIAGP	ENSSKIGLAN	KDRKNRPMQQ	EDEYRIQMEL	NRYYLRKDSL	SVGVSSEQSF	YETETARTPS
410	420	430	440	450	460	470	480
SREEVFFSMA	DMDMSHSLSS	LPPLGDPPNM	DLELSLTSTY	TNTPAGSPLS	ATVLQPTWGE	FLDHHKEQPV	RGSTFPSNLV
490	500	510	520	530	540	550	560
HPTPLQKTSL	PSRSVSVDES	RPELIFRLAV	GTFSISVLHI	DPLSPPETSQ	NLNPLTPMAV	AFFTCIEKID	PARFSTEDFK
570	580	590	600	610	620	630	640
SFRAVFAEAC	SHDHLRFIGT	GIKVSYEQRQ	RSASRYFSTD	MSIGQMEFLE	CLFPTDFHSV	PPHYTELLTF	HSKEETGSHS
650	660	670	680	690	700	710	720
PVCLQLHYKH	SENRGPQGNQ	ARLSSVPHKA	ELQIKLNPVC	CELDISIVDR	LNSLLQPQKL	ATVEMMASHM	YTSYNKHISL
730	740	750	760	770	780	790	800
HKAFTEVFLD	DSHSPANCRI	SVQVATPALN	LSVRFPIPDL	RSDQERGPWF	KKSLQKEILY	LAFTDLEFKT	EFIGGSTPEQ
810	820	830	840	850	860	870	880
IKLELTFREL	IGSFQEEKGD	PSIKFFHVSS	GVDGDTTSSD	DFDWPRIVLK	INPPAMHSIL	ERIAAEEEEE	NDGHYQEEEE
890	900	910	920	930	940	950	960
GGAHSLKDVC	DLRRPAPSPF	SSRRVMFENE	QMVMPGDPVE	MTEFQDKAIS	NSHYVLELTL	PNIYVTLPNK	SFYEKLYNRI
970	980	990	1000	1010	1020	1030	1040
FNDLLLWEPT	APSPVETFEN	ISYGIGLSVA	SQLINTFNKD	SFSAFKSAVH	YDEESGSEEE	TLQYFSTVDP	NYRSRRKKKL
1050	1060	1070	1080	1090	1100	1110	1120
DSQNKNSQSF	LSVLLNINHG	LIAVFTDVKQ	DNGDLLENKH	GEFWLEFNSG	SLFCVTKYEG	FDDKHYICLH	SSSFSLYHKG
1130	1140	1150	1160	1170	1180	1190	1200
IVNGVILPTE	TRLPSSTRPH	WLEPTIYSSE	EDGLSKTSSD	GVGGDSLNML	SVAVKILSDK	SESNTKEFLI	AVGLKGATLQ
1210	1220	1230	1240	1250	1260	1270	1280
HRMLPSGLSW	HEQILYFLNI	ADEPVLGYNP	PTSFTTFHVH	LWSCALDYRP	LYLPIRSLLT	VETFSVSSSV	ALDKSSSTLR
1290	1300	1310	1320	1330	1340	1350	1360
IILDEAALHL	SDKCNTVTIN	LSRDYVRVMD	MGLLELTITA	VKSDSDGEQT	EPRFELHCSS	DVVHIRTCSD	SCAALMNLIQ
1370	1380	1390	1400	1410	1420	1430	1440
YIASYGDLQT	PNKADMKPGA	FQRRSKVDSS	GRSSSRGPVL	PEADQQMLRD	LMSDAMEEID	MQQGTSSVKP	QANGVLDEKS
1450	1460	1470	1480	1490	1500	1510	1520
QIQEPCCSDL	FLFPDESGNV	SQESGPTYAS	FSHHFISDAM	TGVPTENDDF	CILFAPKAAM	QEKEEEPVIK	IMVDDAIVIR
1530	1540	1550	1560	1570	1580	1590	1600
DNYFSLPVNK	TDTSKAPLHF	PIPVIRYVVK	EVSLVWHLYG	GKDFGIVPPT	SPAKSYISPH	SSPSHTPTRH	GRNTVCGGKG
1610	1620	1630	1640	1650	1660	1670	1680
RNHDFLMEIQ	LSKVKFQHEV	YPPCKPDCDS	SLSEHPVSRQ	VFIVQDLEIR	DRLATSQMNK	FLYLYCSKEM	PRKAHSNMLT
1690	1700	1710	1720	1730	1740	1750	1760
VKALHVCPES	GRSPQECCLR	VSLMPLRLNI	DQDALFFLKD	FFTSLSAEVE	LQMTPDPEVK	KSPGADVTCS	LPRHLSTSKE
1770	1780	1790	1800	1810	1820	1830	1840
PNLVISFSGP	KQPSQNDSAN	SVEVVNGMEE	KNFSAEEASF	RDQPVFFREF	RFTSEVPIRL	DYHGKHVSMD	QGTLAGILIG
1850	1860	1870	1880	1890	1900	1910	1920
LAQLNCSELK	LKRLSYRHGL	LGVDKLFSYA	ITEWLNDIKK	NQLPGILGGV	GPMHSLVQLV	QGLKDLVWLP	IEQYRKDGRI
1930	1940	1950	1960	1970	1980	1990	2000
VRGFQRGAAS	FGTSTAMAAL	ELTNRMVQTI	QAAAETAYDM	VSPGTLSIEP	KKTKRFPHHR	LAHQPVDLRE	GVAKAYSVVK
2010	2020	2030	2040	2050	2060	2070	2080
EGITDTAQTI	YETAAREHES	RGVTGAVGEV	LRQIPPAVVK	PLIVATEATS	NVLGGMRNQI	RPDVRQDESQ	KWRHGDD

Cmpd.	No. of Cmpds.	m/z meas.	Δ m/z [ppm]	z	Rt [min]	Score	P	Range	Sequence	Modification

Detailed Protein Report

Cmpd.	No. of Cmpds.	m/z meas.	Δ m/z [ppm]	z	Rt [min]	Score	P	Range	Sequence	Modification
2278	1	713.2212	-186.29	2	56.8	10.0	1	549-560	K.IDPARFSTEDFK.S	

Detailed Protein Report

Protein 297: leucine-rich repeat neuronal protein 2 precursor [Homo sapiens]

**Accession:**	gi|42544231	**Score:**	24.8
**Database:**	refseq_human(refseq_human_20140103.fasta)	**MW [kDa]:**	78.8
**Database Date:**	2015-11-30	**pI:**	7.3
		**Sequence Coverage [%]:**	4.6
		**No. of unique Peptides:**	1

Quantitation

WD:WU **Median:** 1.32 **CV:** 0.00 % **No. of Peptides:** 1

Alias proteins:

Accession	Name	Description
gi|578800423	refseq_human (refseq_human_20140103.fasta)	PREDICTED: leucine-rich repeat neuronal protein 2 isoform X3 [Homo sapiens]
gi|578800421	refseq_human (refseq_human_20140103.fasta)	PREDICTED: leucine-rich repeat neuronal protein 2 isoform X2 [Homo sapiens]
gi|530364022	refseq_human (refseq_human_20140103.fasta)	PREDICTED: leucine-rich repeat neuronal protein 2 isoform X1 [Homo sapiens]
gi|42544233	refseq_human (refseq_human_20140103.fasta)	leucine-rich repeat neuronal protein 2 precursor [Homo sapiens]

10	20	30	40	50	60	70	80
MRLLVAPLLL	AWVAGATAAV	PVVPWHVPCP	PQCACQIRPW	YTPRSSYREA	TTVDCNDLFL	TAVPPALPAG	TQTLLLQSNS
**90**	**100**	**110**	**120**	**130**	**140**	**150**	**160**
IVRVDQSELG	YLANLTELDL	SQNSFSDARD	CDFHALPQLL	SLHLEENQLT	RLEDHSFAGL	ASLQELYLNH	NQLYRIAPRA
**170**	**180**	**190**	**200**	**210**	**220**	**230**	**240**
FSGLSNLLRL	HLNSNLLRAI	DSRWFEMLPN	LEILMIGGNK	VDAILDMNFR	PLANLRSLVL	AGMNLREISD	YALEGLQSLE
**250**	**260**	**270**	**280**	**290**	**300**	**310**	**320**
SLSFYDNQLA	RVPRRALEQV	PGLKFLDLNK	NPLQRVGPGD	FANMLHLKEL	GLNNMEELVS	IDKFALVNLP	ELTKLDITNN
**330**	**340**	**350**	**360**	**370**	**380**	**390**	**400**
PRLSFIHPRA	FHHLPQMETL	MLNNNALSAL	HQQTVESLPN	LQEVGLHGNP	IRCDCVIRWA	NATGTRVRFI	EPQSTLCAEP
**410**	**420**	**430**	**440**	**450**	**460**	**470**	**480**
PDLQRLPVRE	VPFREMTDHC	LPLISPRSFP	PSLQVASGES	MVLHCRALAE	PEPEIYWVTP	AGLRLTPAHA	GRRYRVYPEG
**490**	**500**	**510**	**520**	**530**	**540**	**550**	**560**
TLELRRVTAE	EAGLYTCVAQ	NLVGADTKTV	SVVVGRALLQ	PGRDEGQGLE	LRVQETHPYH	ILLSWVTPPN	TVSTNLTWSS
**570**	**580**	**590**	**600**	**610**	**620**	**630**	**640**
ASSLRGQGAT	ALARLPRGTH	SYNITRLLQA	TEYWACLQVA	FADAHTQLAC	VWARTKEATS	CHRALGDRPG	LIAILALAVL
**650**	**660**	**670**	**680**	**690**	**700**	**710**	**720**
LLAAGLAAHL	GTGQPRKGVG	GRRPLPPAWA	FWGWSAPSVR	VVSAPLVLPW	NPGRKLPRSS	EGETLLPPLS	QNS

Cmpd.	No. of Cmpds.	m/z meas.	Δ m/z [ppm]	z	Rt [min]	Score	P	Range	Sequence	Modification	Ratios
753	1	541.2892	-37.06	3	38.3	12.1	1	170-183	R.LHLNSNLLRAIDSR.W		WD:WU 1.32

Detailed Protein Report

Protein 298: PREDICTED: LOW QUALITY PROTEIN: spidroin-1-like [Homo sapiens]

Accession: gi|578798093
Database: refseq_human(refseq_human_20140103.fasta)
Database Date: 2015-11-30

Score: 24.8
MW [kDa]: 23.4
pI: 12.7
Sequence Coverage [%]: 4.4
No. of unique Peptides: 2

10	20	30	40	50	60	70	80
MRGLDQMEIR	GGPYLLLGEV	VGHSALGAQP	AQAADEVLEL	PALLQRPAGR	RPCASHRGRR	ITPATALLLL	SHRGRAEXAA
**90**	**100**	**110**	**120**	**130**	**140**	**150**	**160**
AAKAGSPGRR	RVGKGPPIRE	SWTRSAPQRC	PSQDAGRAGS	RVCRSRPQSR	GGQKAAASGA	KRRGVGGKEL	WRRGQRAATA
**170**	**180**	**190**	**200**	**210**	**220**	**230**	
KTRGGGGKKP	RGQKPQKATA	AGAKSRGGGG	KKPRRQKTAA	AGAKSRGGGG	NKPQKAAAAG	ICCYQR	

Cmpd.	No. of Cmpds.	m/z meas.	Δ m/z [ppm]	z	Rt [min]	Score	P	Range	Sequence	Modification
2067	1	643.2889	-61.34	2	54.2	12.7	1	0-0	.AEEAAAAKAGSPGR.	
1162	2	500.6748	-221.98	2	43.2	12.1	2	74-83	R.GRAERAAAAK.A	

Detailed Protein Report

Protein 299: **PREDICTED: lipopolysaccharide-responsive and beige-like anchor protein isoform X5 [Homo sapiens]**

**Accession:**	gi|530378479	**Score:**	24.8
**Database:**	refseq_human(refseq_human_20140103.fasta)	**MW [kDa]:**	315.5
**Database Date:**	2015-11-30	**pI:**	5.3
		**Sequence Coverage [%]:**	1.1
		**No. of unique Peptides:**	2

Detailed Protein Report

10	20	30	40	50	60	70	80
MASEDNRVPS	PPPTGDDGGG	GGREETPTEG	GALSLKPGLP	IRGIRMKFAV	LTGLVEVGEV	SNRDIVETVF	NLLVGGQFDL
90	100	110	120	130	140	150	160
EMNFIIQEGE	SINCMVDLLE	KCDITCQAEV	WSMFTAILKK	SIRNLQVCTE	VGLVEKVLGK	IEKVDNMIAD	LLVDMLGVLA
170	180	190	200	210	220	230	240
SYNLTVRELK	LFFSKLQGDK	GRWPPHAGKL	LSVLKHMPQK	YGPDAFFNFP	GKSAAAIALP	PIAKWPYQNG	FTFHTWLRMD
250	260	270	280	290	300	310	320
PVNNINVDKD	KPYLYCFRTS	KGLGYSAHFV	GGCLIVTSIK	SKGKGFQHCV	KFDFKPQKWY	MVTIVHIYNR	WKNSELRCYV
330	340	350	360	370	380	390	400
NGELASYGEI	TWFVNTSDTF	DKCFLGSSET	ADANRVFCGQ	MTAVYLFSEA	LNAAQIFAIY	QLGLGYKGTF	KFKAESDLFL
410	420	430	440	450	460	470	480
AEHHKLLLYD	GKLSSAIAFT	YNPRATDAQL	CLESSPKDNP	SIFVHSPHAL	MLQDVKAVLT	HSIQSAMHSI	GGVQVLFPLF
490	500	510	520	530	540	550	560
AQLDYRQYLS	DEIDLTICST	LLAFIMELLK	NSIAMQEQML	ACKGFLVIGY	SLEKSSKSHV	SRAVLELCLA	FSKYLSNLQN
570	580	590	600	610	620	630	640
GMPLLKQLCD	HVLLNPAIWI	HTPAKVQLML	YTYLSTEFIG	TVNIYNTIRR	VGTVLLIMHT	LKYYYWAVNP	QDRSGITPKG
650	660	670	680	690	700	710	720
LDGPRPNQKE	MLSLRAFLLM	FIKQLVMKDS	GVKEDELQAI	LNYLLTMHED	DNLMDVLQLL	VALMSEHPNS	MIPAFDQRNG
730	740	750	760	770	780	790	800
LRVIYKLLAS	KSEGIRVQAL	KAMGYFLKHL	APKRKAEVML	GHGLFSLLAE	RLMLQTNLIT	MTTYNVLFEI	LIEQIGTQVI
810	820	830	840	850	860	870	880
HKQHPDPDSS	VKIQNPQILK	VIATLLRNSP	QCPESMEVRR	AFLSDMIKLF	NNSRENRRSL	LQCSVWQEWM	LSLCYFNPKN
890	900	910	920	930	940	950	960
SDEQKITEMV	YAIFRILLYH	AVKYEWGGWR	VWVDTLSITH	SKVTFEIHKE	NLANIFREQQ	GKVDEEIGLC	SSTSVQAASG
970	980	990	1000	1010	1020	1030	1040
IRRDINVSVG	SQQPDTKDSP	VCPHFTTNGN	ENSSIEKTSS	LESASNIELQ	TTNTSYEEMK	AEQENQELPD	EGTLEETLTN
1050	1060	1070	1080	1090	1100	1110	1120
ETRNADDLEV	SSDIIEAVAI	SSNSFITTGK	DSMTVSEVTA	SISSPSEEDA	SEMPEFLDKS	IVEEEEDDDY	VELKVEGSPT
1130	1140	1150	1160	1170	1180	1190	1200
EEANLPTELQ	DNSLSPAASE	AGEKLDMFGN	DDKLIFQEGK	PVTEKQTDTE	TQDSKDSGIQ	TMTASGSSAM	SPETTVSQIA
1210	1220	1230	1240	1250	1260	1270	1280
VESDLGQMLE	EGKKATNLTR	ETKLINDCHG	SVSEASSEQK	IAKLDVSNVA	TDTERLELKA	SPNVEAPQPH	RHVLEISRQH
1290	1300	1310	1320	1330	1340	1350	1360
EQPGQGIAPD	AVNGQRRDSR	STVFRIPEFN	WSQMHQRLLT	DLLFSIETDI	QMWRSHSTKT	VMDFVNSSDN	VIFVHNTIHL
1370	1380	1390	1400	1410	1420	1430	1440
ISQVMDNMVM	ACGGILPLLS	AATSATHELE	NIEPTQGLSI	EASVTFLQRL	ISLVDVLIFA	SSLGFTEIEA	EKSMSSGGIL
1450	1460	1470	1480	1490	1500	1510	1520
RQCLRLVCAV	AVRNCLECQQ	HSQLKTRGDK	ALKPMHSLIP	LGKSAAKEDS	KQAQFLALAV	VYFISVLMVS	KYRDILEPQN
1530	1540	1550	1560	1570	1580	1590	1600
ERHSQSCTET	GSENENVSLS	EITPAAFSTL	TTASVEESES	TSSARRRDSG	IGEETATGLG	SHVEVTPHTA	PPGVSAGPDA
1610	1620	1630	1640	1650	1660	1670	1680
ISEVLSTLSL	EVNKSPETKN	DRGNDLDTKA	TPSVSVSKNV	NVKDILRSLV	NIPADGVTVD	PALLPPACLG	ALGDLSVEQP
1690	1700	1710	1720	1730	1740	1750	1760
VQFRSFDRSV	IVAAKKSAVS	PSTFNTSIPT	NAVSVVSSVD	SAQASDMGGE	SPGSRSSNAK	LPSVPTVDSV	SQDPVSNMSI
1770	1780	1790	1800	1810	1820	1830	1840
TERLEHALEK	AAPLLREIFV	DFAPFLSRTL	LGSHGQELLI	EGTSLVCMKS	SSSVVELVML	LCSQEWQNSI	QKNAGLAFIE
1850	1860	1870	1880	1890	1900	1910	1920
LVNEGRLLSQ	TMKDHLVRVA	NEAEFILSRQ	RAEDIHRHAE	FESLCAQYSA	DKREDEKMCD	HLIRAAKYRD	HVTATQLIQK
1930	1940	1950	1960	1970	1980	1990	2000
IINILTDKHG	AWGNSAVSRP	LEFWRLDYWE	DDLRRRRRFV	RNPLGSTHPE	ATLKTAVEHV	CIFKLRENSK	ATDEDILAKG
2010	2020	2030	2040	2050	2060	2070	2080
KQSIRSQALG	NQNSENEILL	EGDDDTLSSV	DEKDLENLAG	PVSLSTPAQL	VAPSVVVKGT	LSVTSSELYF	EVDEEDPNFK
2090	2100	2110	2120	2130	2140	2150	2160
KIDPKILAYT	EGLHGKWLFT	EIRSIFSRRY	LLQNTALEIF	MANRVAVMFN	FPDPATVKKV	VNYLPRVGVG	TSFGLPQTRR
2170	2180	2190	2200	2210	2220	2230	2240

Detailed Protein Report

Cmpd.	No. of Cmpds.	m/z meas.	Δ m/z [ppm]	z	Rt [min]	Score	P	Range	Sequence	Modification
1230	1	715.1053	93.99	3	43.7	12.4	1	821-839	K.VIATLLRNSPQCPESMEVR.R	
2402	1	682.7983	-93.44	2	58.4	12.3	0	1962-1974	R.NPLGSTHPEATLK.T	

Detailed Protein Report

Protein 300: transcription factor HES-4 isoform 1 [Homo sapiens]

**Accession:**	gi|216548627	**Score:**	24.7
**Database:**	refseq_human(refseq_human_20140103.fasta)	**MW [kDa]:**	26.1
**Database Date:**	2015-11-30	**pI:**	12.0
		**Sequence Coverage [%]:**	14.2
		**No. of unique Peptides:**	2

```
        10         20         30         40         50         60         70         80
MAADTPGKPS ASPMAGAPAS ASRTPDKPRS AAEHRKVGSR PGVRGATGGR EGRGTQPVPD PQSSKPVMEK RRRARINESL
        90        100        110        120        130        140        150        160
AQLKTLILDA LRKESSRHSK LEKADILEMT VRHLRSLRRV QVTAALSADP AVLGKYRAGF HECLAEVNRF LAGCEGVPAD
       170        180        190        200        210        220        230        240
VRSRLLGHLA ACLRQLGPSR RPASLSPAAP AEAPAPEVYA GRPLLPSLGG PFPLLAPPLL PGLTRALPAA PRAGPQGPGG
       250
PWRPWLR
```

Cmpd.	No. of Cmpds.	m/z meas.	Δ m/z [ppm]	z	Rt [min]	Score	P	Range	Sequence	Modification
2708	1	835.0993	174.40	2	62.3	10.6	2	37-53	K.VGSRPGVRGATGGREGR.G	
1118	1	974.4533	-104.11	2	42.7	14.1	2	163-180	R.SRLLGHLAACLRQLGPSR.R	

Detailed Protein Report

Protein 301: rho GTPase-activating protein 11A isoform 3 [Homo sapiens]

**Accession:**	gi|557128992	**Score:**	24.7
**Database:**	refseq_human(refseq_human_20140103.fasta)	**MW [kDa]:**	92.6
**Database Date:**	2015-11-30	**pI:**	9.9
		**Sequence Coverage [%]:**	3.7
		**No. of unique Peptides:**	2

Alias proteins:

Accession	Name	Description
gi|578827655	refseq_human (refseq_human_20140103.fasta)	PREDICTED: rho GTPase-activating protein 11A isoform X3 [Homo sapiens]
gi|557129007	refseq_human (refseq_human_20140103.fasta)	rho GTPase-activating protein 11A isoform 3 [Homo sapiens]

```
        10         20         30         40         50         60         70         80
MDSSNLAVIF APNLLQTSEG HEKMSSNTEK KLRLQAAVVQ TLIDYASDIG RVPDFILEKI PAMLGIDGLC ATPSLEGFEE
        90        100        110        120        130        140        150        160
GEYETPGEYK RKRRQSVGDF VSGALNKFKP NRTPSITPQE ERIAQLSESP VILTPNAKRT LPVDSSHGFS SKKRKSIKHN
       170        180        190        200        210        220        230        240
FNFELLPSNL FNSSSTPVSV HIDTSSEGSS QSSLSPVLIG GNHLITAGVP RRSKRIAGKK VCRVESGKAG CFSPKISHKE
       250        260        270        280        290        300        310        320
KVRRSLRLKF NLGKNGREVN GCSGVNRYES VGWRLANQQS LKNRIESVKT GLLFSPDVDE KLPKKGSEKI SKSEETLLTP
       330        340        350        360        370        380        390        400
ERLVGTNYRM SWTGPNNSSF QEVDANEASS MVENLEVENS LEPDIMVEKS PATSCELTPS NLNNKHNSNI TSSPLSGDEN
       410        420        430        440        450        460        470        480
NMTKETLVKV QKAFSESGSN LHALMNQRQS SVTNVGKVKL TEPSYLEDSP EENLFETNDL TIVESKEKYE HHTGKGEKCF
       490        500        510        520        530        540        550        560
SERDFSPLQT QTFNRETTIK CYSTQMKMEH EKDIHSNMPK DYLSKQEFSS DEEIKKQQSP KDKLNNKLKE NENMMEGNLP
       570        580        590        600        610        620        630        640
KCAAHSKDEA RSSFSQQSTC VVTNLSKPRP MRIAKQQSLE TCEKTVSESS QMTEHRKVSD HIQWFNKLSL NEPNRIKVKS
       650        660        670        680        690        700        710        720
PLKFQRTPVR QSVRRINSLL EYSRQPTGHK LASLGDTASP LVKSVSCDGA LSSCIESASK DSSVSCIKSG PKEQKSMSCE
       730        740        750        760        770        780        790        800
ESNIGAISKS SMELPSKSFL KMRKHPDSVN ASLRSTTVYK QKILSDGQVK VPLDDLTNHD IVKPVVNNNM GISSGINNRV
       810        820        830        840
LRRPSERGRA WYKGSPKHPI GKTQLLPTSK PVDL
```

Cmpd.	No. of Cmpds.	m/z meas.	Δ m/z [ppm]	z	Rt [min]	Score	P	Range	Sequence	Modification
2089	2	682.6083	-53.17	3	54.7	11.6	0	386-404	K.HNSNITSSPLSGDENNMTK.E	
2120	2	703.2439	-91.13	2	54.9	13.2	0	550-561	K.ENENMMEGNLPK.C	

Detailed Protein Report

Protein 302: PREDICTED: basic proline-rich protein-like [Homo sapiens]

Accession: gi|578837749
Database: refseq_human(refseq_human_20140103.fasta)
Database Date: 2015-11-30
Modification(s): Carbamidomethyl

Score: 24.7
MW [kDa]: 40.4
pI: 13.1
Sequence Coverage [%]: 8.7
No. of unique Peptides: 2

Alias proteins:

Accession	Name	Description
gi|578847054	refseq_human (refseq_human_20140103.fasta)	PREDICTED: basic proline-rich protein-like [Homo sapiens]

10	20	30	40	50	60	70	80
MPGPCPASAP	PALLQVHLQG	KCPAPAQHPS	PGPPPGSPPE	QMPGPCPASA	PPALLQVHLQ	GKCPAPAQHP	CPRPSSRFTS
**90**	**100**	**110**	**120**	**130**	**140**	**150**	**160**
RANARPLPSI	HAPGPPPGSP	PGQVPGPCPA	SMPPALLQVH	LQSKCPAPAQ	HPCPRPSSRF	TSRANARPLP	SVRAPGPPPG
**170**	**180**	**190**	**200**	**210**	**220**	**230**	**240**
SPPEQMPGPC	PASMPPALLQ	VHLQSKCPAP	AQRLRPRPSS	RFTSRANARP	LPSIHAPGPP	PGSPPGQMPG	PCPASMPPAL
**250**	**260**	**270**	**280**	**290**	**300**	**310**	**320**
LQVHLQGKCL	APAQRPRPRP	SSRFTSRANA	RPLPSIHAPG	PPPGSPPEQM	PGPCPASMPP	ALLQVHLQSK	CPAPAQRPRP
**330**	**340**	**350**	**360**	**370**	**380**	**390**	**400**
QPSSRFTSRA	NARPLPSIHA	PGPPPGSPPE	QMPGPCPASA	PPALLQVHLQ	SKCPAPAQCP	RPLPSSRTLL	FE

Cmpd.	No. of Cmpds.	m/z meas.	Δ m/z [ppm]	z	Rt [min]	Score	P	Range	Sequence	Modification
407	1	717.9592	-76.97	3	34.1	11.6	1	63-81	K.CPAPAQHPCPRPSSRFTSR.A	Carbamidomethyl: 1
2994	1	846.4470	-21.67	2	66.2	13.1	1	187-201	K.CPAPAQRLRPRPSSR.F	

Detailed Protein Report

Protein 303: PREDICTED: keratin, type II cuticular Hb3 isoform X1 [Homo sapiens]

Accession: gi|530400232
Database: refseq_human(refseq_human_20140103.fasta)
Database Date: 2015-11-30
Modification(s): Carbamidomethyl

Score: 24.7
MW [kDa]: 49.6
pI: 5.3
Sequence Coverage [%]: 7.4
No. of unique Peptides: 1

10	20	30	40	50	60	70	80
MTCGFNSIGC	GFRPGNFSCV	SACGPRPSRC	CITAAPYRGI	SCYRGLTGGF	GSHSVCGGFR	AGSCGRSFGY	RSGGVCGPSP
**90**	**100**	**110**	**120**	**130**	**140**	**150**	**160**
PCITTVSVNE	SLLTPLNLEI	DPNAQCVKQE	EKEQIKSLNS	RFAAFIDKVR	FLEQQNKLLE	TKLQFYQNRE	CCQSNLEPLF
**170**	**180**	**190**	**200**	**210**	**220**	**230**	**240**
AGYIETLRRE	AECVEADSGR	LASELNHVQE	VLEGYKKKYE	EEVALRATAE	NEFVALKKDV	DCAYLRKSDL	EANVEALIQE
**250**	**260**	**270**	**280**	**290**	**300**	**310**	**320**
IDFLRRLYEE	EIRILQSHIS	DTSVVVKLDN	SRDLNMDCIV	AEIKAQYDDI	ATRSRAEAES	WYRSKCEEMK	ATVIRHGETL
**330**	**340**	**350**	**360**	**370**	**380**	**390**	**400**
RRTKEEINEL	NRMIQRLTAE	VENAKCQNSK	LEAAVAQSEQ	QGEAALSDAR	CKLAELEGAL	QKAKQDMACL	IREYQEVMNS
**410**	**420**	**430**	**440**	**450**			
KLGLDIEIAT	YRRLLEGEEQ	RLCEGVEAAA	PGVGLCAGIS	ACRAPGR			

Cmpd.	No. of Cmpds.	m/z meas.	Δ m/z [ppm]	z	Rt [min]	Score	P	Range	Sequence	Modification
2096	1	1052.4199	-78.76	2	54.8	12.5	0	422-443	R.LCEGVEAAAPGVGLCAGISACR.A	Carbamidomethyl: 2

Detailed Protein Report

Protein 304: TPR and ankyrin repeat-containing protein 1 [Homo sapiens]

**Accession:**	gi|257467636	**Score:**	24.7
**Database:**	refseq_human(refseq_human_20140103.fasta)	**MW [kDa]:**	336.0
**Database Date:**	2015-11-30	**pI:**	6.3
**Modification(s):**	Carbamidomethyl	**Sequence Coverage [%]:**	1.1
		**No. of unique Peptides:**	1

Detailed Protein Report

10	20	30	40	50	60	70	80
MWDPRAARVP	PRDLAVLLCN	KSNAFFSLGK	WNEAFVAAKE	CLQWDPTYVK	GYYRAGYSLL	RLHQPYEAAR	MFFEGLRLVQ
90	100	110	120	130	140	150	160
RSQDQAPVAD	FLVGVFTTMS	SDSIVLQSFL	PCFDHIFTTG	FPTEVWQSVI	EKLAKKGLWH	SFLLLSAKKD	RLPRNIHVPE
170	180	190	200	210	220	230	240
LSLKSLFEKY	VFIGLYEKME	QVPKLVQWLI	SIGASVETIG	PYPLHALMRL	CIQARENHLF	RWLMDHKPEW	KGRINQKDGD
250	260	270	280	290	300	310	320
GCTVLHVVAA	HSPGYLVKRQ	TEDVQMLLRF	GADPTLLDRQ	SRSVVDVLKR	NKNFKAIEKI	NSHLEKLATC	SKDLSGFSNG
330	340	350	360	370	380	390	400
DGPTSENDIF	RKVLEQLVKY	MNSGNRLLHK	NFLKQEVVQR	FLRLLSTLQE	IPPDLVCDIN	QDCATTVFKF	LLEKQRWPEV
410	420	430	440	450	460	470	480
LLLLTRKVSG	EPPLGDCLIK	DCNFSDLDIC	TIIPHLSTWD	QRKKQLLGCL	IDSGALPDGL	QESQERPVVT	CLKHEDFELA
490	500	510	520	530	540	550	560
FLLLTKGADP	RAISLTEGDT	PLHAALHIFL	EIKADIGFSF	LSHLLDLFWS	NPTEFDYLNP	NVQDSNGNTL	MHILFQKGML
570	580	590	600	610	620	630	640
KRVKKLLDLL	VKFDINFNLK	NKEGKDARHR	IKKNDSLLLA	WNKALMENRR	RSRQDSAAHL	GKLSKSTAPG	HTSQLKSQGS
650	660	670	680	690	700	710	720
FKSVPCGATA	RTLPEGSAVP	DSWETLPGTQ	VTRKEPGALR	PCSLRDCLMQ	DITVLIQQVE	VDPSFPEDCL	QSSEPLEAGA
730	740	750	760	770	780	790	800
GKEGKKDDKP	TLGAGAPDCS	EVGEGHAQVG	LGALQLVPDD	NRGKEGNDDQ	DDWSTQEIEA	CLQDFDNMTW	EIECTSEMLK
810	820	830	840	850	860	870	880
KLSSKVMTKV	IKKKIILAIQ	QLGNGEWTQG	LQKRLKHLKG	SIQLFEAKLD	KGARMLWELA	IDFSPRCSEN	PEKIIATEQN
890	900	910	920	930	940	950	960
TCAMEKSGRI	YTEIIRIWDI	VLDHCKLADS	IKAICNAYNR	GLSCVLRKKL	KGINKGQVSA	NMKIQKRIPR	CYVEDTEAEK
970	980	990	1000	1010	1020	1030	1040
GREHVNPEYF	PPASAVETEY	NIMKFHSFST	NMAFNILNDT	TATVEYPFRV	GELEYAVIDL	NPRPLEPIIL	IGRSGTGKTT
1050	1060	1070	1080	1090	1100	1110	1120
CCLYRLWKKF	HVYWEKAEQA	GSPLLAKQVW	LKRRLEVEPG	KESPGGEEEE	EEEDEEEEDS	IEVETVESID	EQEYEACAGG
1130	1140	1150	1160	1170	1180	1190	1200
AGVEPAGDGQ	AAEVCAPEHP	HQLEHLHQIF	VTKNHVLCQE	VQRNFIELSK	STKATSHYKP	LDPNIHKLQD	LRDENFPLFV
1210	1220	1230	1240	1250	1260	1270	1280
TSKQLLLLLD	ASLPKPFFLR	NEDGSLKRTI	IGWSAQEEST	IPSWQEDEEE	AEVDGDYSEE	DKAVEMRTGD	SDPRVYVTFE
1290	1300	1310	1320	1330	1340	1350	1360
VFKNEIWPKM	TKGRTAYNPA	LIWKEIKSFL	KGSFEALSCP	HGRLTEEVYK	KLGRKRCPNF	KEDRSEIYSL	FSLYQQIRSQ
1370	1380	1390	1400	1410	1420	1430	1440
KGYFDEEDVL	YNISRRLSKL	RVLPWSIHEL	YGDEIQDFTQ	AELALLMKCI	NDPNSMFLTG	DTAQSIMKGV	AFRFSDLRSL
1450	1460	1470	1480	1490	1500	1510	1520
FHYASRNTID	KQCAVRKPKK	IHQLYQNYRS	HSGILNLASG	VVDLLQFYFP	ESFDRLPRDS	GLFDGPKPTV	LESCSVSDLA
1530	1540	1550	1560	1570	1580	1590	1600
ILLRGNKRKT	QPIEFGAHQV	ILVANETAKE	KIPEELGLAL	VLTIYEAKGL	EFDDVLLYNF	FTDSEAYKEW	KIISSFTPTS
1610	1620	1630	1640	1650	1660	1670	1680
TDSREENRPL	VEVPLDKPGS	SQGRSLMVNP	EMYKLLNGEL	KQLYTAITRA	RVNLWIFDEN	REKRAPAFKY	FIRRDFVQVV
1690	1700	1710	1720	1730	1740	1750	1760
KTDENKDFDD	SMFVKTSTPA	EWIAQGDYYA	KHQCWKVAAK	CYQKGGAFEK	EKLALAHDTA	LSMKSKKVSP	KEKQLEYLEL
1770	1780	1790	1800	1810	1820	1830	1840
AKTYLECKEP	TLSLKCLSYA	KEFQLSAQLC	ERLGKIRDAA	YFYKRSQCYK	DAFRCFEQIQ	EFDLALKMYC	QEELFEEAAI
1850	1860	1870	1880	1890	1900	1910	1920
AVEKYEEMLK	TKTLPISKLS	YSASQFYLEA	AAKYLSANKM	KEMMAVLSKL	DIEDQLVFLK	SRKRLAEAAD	LLNREGRREE
1930	1940	1950	1960	1970	1980	1990	2000
AALLMKQHGC	LLEAARLTAD	KDFQASCLLG	AARLNVARDS	DIEHTKDILR	EALDICYQTG	QLSGIAEAHF	LQGVILRDFQ
2010	2020	2030	2040	2050	2060	2070	2080
KLRDAFFKFD	TLNHSAGVVE	ALYEAASQCE	AEPEKILGLA	PGGLEILLSL	VRALKRVTNN	AEKEMVKSCF	EFFGISQVDA
2090	2100	2110	2120	2130	2140	2150	2160
KYCQIAQNDP	GPILRIIFDL	DLNLREKKTK	DHFLIMTDQV	KLALNKHLLG	RLCQITRSLL	GKTYRGVCMR	FIVGLKCEDE
2170	2180	2190	2200	2210	2220	2230	2240

Detailed Protein Report

Cmpd.	No. of Cmpds.	m/z meas.	Δ m/z [ppm]	z	Rt [min]	Score	P	Range	Sequence	Modification
1592	1	919.4423	-40.82	2	48.6	13.0	2	913-928	K.AICNAYNRGLSCVLRK.K	Carbamidomethyl: 3

Detailed Protein Report

Protein 305: PREDICTED: protein FAM186A isoform X1 [Homo sapiens]

**Accession:**	gi|578823289	**Score:**	24.6
**Database:**	refseq_human(refseq_human_20140103.fasta)	**MW [kDa]:**	262.1
**Database Date:**	2015-11-30	**pI:**	9.1
		**Sequence Coverage [%]:**	0.9
		**No. of unique Peptides:**	1

Detailed Protein Report

10	20	30	40	50	60	70	80
MFFKMKNEID	NDPESEKCIK	DSTIMRREPQ	NILSPLMLPN	LEIPFSVKDI	ISRIERAQLH	RAREDIDMQL	SEIMNNVHRI
90	100	110	120	130	140	150	160
MTRYTLVFNS	SSERNVSLTE	HKKKQRTNFL	EKMATYAKTI	EIREKTLANI	LAWLEEWNDV	LSEMTLMDVD	EHHHWIAQME
170	180	190	200	210	220	230	240
LLPDTLKAIE	NNVKILSRFS	TSFLDEKKKQ	KKKILSRGTL	WKSWKERVIK	RPSTARALRP	DQMISDQLAT	NTKVSEIQGM
250	260	270	280	290	300	310	320
LQELIGTTMF	STLENNAIKY	ISSTIVNLST	ALSMLNDELK	CVNFQSSTVY	AHETSEAEKE	LSLKIIRDLS	NENEMLQQKL
330	340	350	360	370	380	390	400
QDAEEKCEQL	IRSKIVIEQL	YAKLSTSSTL	KVLPGPSPQS	SRAIIKVGDT	EDNMDNILDK	ELENIVDEVQ	RKETKDSGIK
410	420	430	440	450	460	470	480
WDSTISYTAQ	AERTPDLTEL	RQQPVASEDI	SEDSTKDNVS	LKKGDFYQED	ETDEYQSWKR	SHKKATYVYE	TSGPNLSDNK
490	500	510	520	530	540	550	560
SGQKVSEAKP	SQYYELQVLK	KKRKEMKSFS	EDKSKSPTEA	KRKHLSLTET	KSQGGKSGTS	MMMLEQFRKV	KRESPFDKRP
570	580	590	600	610	620	630	640
TAAEIKVEPT	TESLDKEGKG	EIRSLVEPLS	MIQFDDTAEP	QKGKIKGKKH	HISSGTITSK	EEKTEEKEEL	TKQVKSHQLV
650	660	670	680	690	700	710	720
KSLSRVAKET	SESTRVLESP	DGKSEQSNLE	EFQEAIMAFL	KQKIDNIGKA	FDKKTVPKEE	ELLKRAEAEK	LGIIKAKMEE
730	740	750	760	770	780	790	800
YFQKVAETVT	KILRKYKDTK	KEEQVGEKPI	KQKKVVSFMP	GLHFQKSPIS	AKSESSTLLS	YESTDPVINN	LIQMILAEIE
810	820	830	840	850	860	870	880
SERDIPTVST	VQKDHKEKEK	QRQEQYLQEG	QEQMSGMSLK	QQLLGERNLL	KEHYEKISEN	WEEKKAWLQM	KEGKQEQQSQ
890	900	910	920	930	940	950	960
KQWQEEEMWK	EEQKQATPKQ	AEQEEKQKQR	GQEEEELPKS	SLQRLEEGTQ	KMKTQGLLLE	KENGQMRQIQ	KEAKHLGPHR
970	980	990	1000	1010	1020	1030	1040
RREKGKEKQK	PERGLEDLER	QIKTKDQMQM	KETQPKELEK	MVIQTPMTLS	PRWKSVLKDV	QRSYEGKEFQ	RNLKTLENLP
1050	1060	1070	1080	1090	1100	1110	1120
DEKEPISITP	PPSLQYSLPG	ALPISGQPLT	KCIHLTPQQA	QEVGITLTPQ	QAQAQGITLT	LQQAQELGIP	LTPQQAQALE
1130	1140	1150	1160	1170	1180	1190	1200
ILFTPQQAQA	LGIPLTPQQT	QVQGITLTPQ	QDQAPGISLT	TQQAQKLGIP	LTPQQAQALG	IPLTPQQAQE	LGIPLTPQQA
1210	1220	1230	1240	1250	1260	1270	1280
QALRVSLTPQ	QAQELGIPLT	PQQAQALGIT	LTLQQAQQLG	IPLTPQQAQA	LGITLTPKQV	QELGIPLTPQ	QAQALGITLT
1290	1300	1310	1320	1330	1340	1350	1360
PKQAQELGIP	LNPQQAQTLG	IPLTPKQAQA	LGIPFTPQQA	QALGIPLTPQ	QAQTQEITLT	PQQAQALGMP	LTTQQAQELG
1370	1380	1390	1400	1410	1420	1430	1440
IPLTPQHAQA	LGMPLTTQQA	QELGIPLTPQ	QAQALGMPLT	TQQAQELGIP	LTPQQAQELG	IPFTPQQAQA	QEITLTPQQA
1450	1460	1470	1480	1490	1500	1510	1520
QALGMPLTAQ	QAQELGITLT	PQQAQELGIP	LTPQQAQALG	IPLIPPQAQE	LGIPLTPQQA	QALGILLIPP	QAQELGIPLT
1530	1540	1550	1560	1570	1580	1590	1600
PQQAQALGIP	LIPPQAQELG	IPLTPQQVQA	LGIPLIPPQA	QELEIPLTPQ	QAQALGIPLT	PQQAQELGIP	LTPQQAQELG
1610	1620	1630	1640	1650	1660	1670	1680
IPLTPQQAQA	QGIPLTPQQA	QALGISLTPQ	QAQAQGITLT	PQQAQALGVP	ITPVNAWVSA	VTLTSEQTHA	LESPMNLEQA
1690	1700	1710	1720	1730	1740	1750	1760
QEQLLKLGVP	LTLDKAHTLG	SPLTLKQVQW	SHRPFQKSKA	SLPTGQSIIS	RLSPSLRLSL	ASSAPTAEKS	SIFGVSSTPL
1770	1780	1790	1800	1810	1820	1830	1840
QISRVPLNQG	PFAPGKPLEM	GILSEPGKLG	APQTLRSSGQ	TLVYGGQSTS	AQFPAPQAPP	SPGQLPISRA	PPTPGQPFIA
1850	1860	1870	1880	1890	1900	1910	1920
GVPPTSGQIP	SLWAPLSPGQ	PLVPEASSIP	GDLLESGPLT	FSEQLQEFQP	PATAEQSPYL	QAPSTPGQHL	ATWTLPGRAS
1930	1940	1950	1960	1970	1980	1990	2000
SLWIPPTSRH	PPTLWPSPAP	GKPQKSWSPS	VAKKRLAIIS	SLKSKSVLIH	PSAPDFKVAQ	VPFTTKKFQM	SEVSDTSEET
2010	2020	2030	2040	2050	2060	2070	2080
QILRDTFAIE	SFRTFQSHFT	KYRTPVYQTP	YTDERALLTL	MKPTTSPSSL	TTLLRTSQIS	PLEWYQKSRF	PPIDKPWILS
2090	2100	2110	2120	2130	2140	2150	2160
SVSDTKKPKV	MVPPSSPQEL	EEKRYFVDVE	AQKKNLILLN	QAIKTCGLPS	QLHTMARTLI	IEILHMDTVQ	LGYLFRKYIA
2170	2180	2190	2200	2210	2220	2230	2240

Detailed Protein Report

Cmpd.	No. of Cmpds.	m/z meas.	Δ m/z [ppm]	z	Rt [min]	Score	P	Range	Sequence	Modification
82	1	724.8160	-30.23	2	30.3	10.0	0	308-319	R.DLSNENEMLQQK.L	

Detailed Protein Report

Protein 306: 60S ribosomal protein L9 [Homo sapiens]

**Accession:**	gi|15431303	**Score:**	24.6
**Database:**	refseq_human(refseq_human_20140103.fasta)	**MW [kDa]:**	21.8
**Database Date:**	2015-11-30	**pI:**	10.5
		**Sequence Coverage [%]:**	13.5
		**No. of unique Peptides:**	1

Alias proteins:

Accession	Name	Description
gi|530376503	refseq_human (refseq_human_20140103.fasta)	PREDICTED: 60S ribosomal protein L9 isoform X1 [Homo sapiens]
gi|67944630	refseq_human (refseq_human_20140103.fasta)	60S ribosomal protein L9 [Homo sapiens]

```
        10         20         30         40         50         60         70         80
MKTILSNQTV DIPENVDITL KGRTVIVKGP RGTLRRDFNH INVELSLLGK KKKRLRVDKW WGNRKELATV RTICSHVQNM
        90        100        110        120        130        140        150        160
IKGVTLGFRY KMRSVYAHFP INVVIQENGS LVEIRNFLGE KYIRRVRMRP GVACSVSQAQ KDELILEGND IELVSNSAAL
       170        180        190        200
IQQATTVKNK DIRKFLDGIY VSEKGTVQQA DE
```

Cmpd.	No. of Cmpds.	m/z meas.	Δ m/z [ppm]	z	Rt [min]	Score	P	Range	Sequence	Modification
2187	1	618.9601	-95.70	3	55.8	10.7	2	37-52	R.DFNHINVELSLLGKKK.K	

Detailed Protein Report

Protein 307: laminin subunit gamma-1 precursor [Homo sapiens]

**Accession:**	gi|145309326	**Score:**	24.6
**Database:**	refseq_human(refseq_human_20140103.fasta)	**MW [kDa]:**	177.5
**Database Date:**	2015-11-30	**pI:**	4.9
**Modification(s):**	Carbamidomethyl	**Sequence Coverage [%]:**	1.9
		**No. of unique Peptides:**	1

10	20	30	40	50	60	70	80
MRGSHRAAPA	LRPRGRLWPV	LAVLAAAAAA	GCAQAAMDEC	TDEGGRPQRC	MPEFVNAAFN	VTVVATNTCG	TPPEEYCVQT
90	100	110	120	130	140	150	160
GVTGVTKSCH	LCDAGQPHLQ	HGAAFLTDYN	NQADTTWWQS	QTMLAGVQYP	SSINLTLHLG	KAFDITYVRL	KFHTSRPESF
170	180	190	200	210	220	230	240
AIYKRTREDG	PWIPYQYYSG	SCENTYSKAN	RGFIRTGGDE	QQALCTDEFS	DISPLTGGNV	AFSTLEGRPS	AYNFDNSPVL
250	260	270	280	290	300	310	320
QEWVTATDIR	VTLNRLNTFG	DEVFNDPKVL	KSYYYAISDF	AVGGRCKCNG	HASECMKNEF	DKLVCNCKHN	TYGVDCEKCL
330	340	350	360	370	380	390	400
PFFNDRPWRR	ATAESASECL	PCDCNGRSQE	CYFDPELYRS	TGHGGHCTNC	QDNTDGAHCE	RCRENFFRLG	NNEACSSCHC
410	420	430	440	450	460	470	480
SPVGSLSTQC	DSYGRCSCKP	GVMGDKCDRC	QPGFHSLTEA	GCRPCSCDPS	GSIDECNIET	GRCVCKDNVE	GFNCERCKPG
490	500	510	520	530	540	550	560
FFNLESSNPR	GCTPCFCFGH	SSVCTNAVGY	SVYSISSTFQ	IDEDGWRAEQ	RDGSEASLEW	SSERQDIAVI	SDSYFPRYFI
570	580	590	600	610	620	630	640
APAKFLGKQV	LSYGQNLSFS	FRVDRRDTRL	SAEDLVLEGA	GLRVSVPLIA	QGNSYPSETT	VKYVFRLHEA	TDYPWRPALT
650	660	670	680	690	700	710	720
PFEFQKLLNN	LTSIKIRGTY	SERSAGYLDD	VTLASARPGP	GVPATWVESC	TCPVGYGGQF	CEMCLSGYRR	ETPNLGPYSP
730	740	750	760	770	780	790	800
CVLCACNGHS	ETCDPETGVC	NCRDNTAGPH	CEKCSDGYYG	DSTAGTSSDC	QPCPCPGGSS	CAVVPKTKEV	VCTNCPTGTT
810	820	830	840	850	860	870	880
GKRCELCDDG	YFGDPLGRNG	PVRLCRLCQC	SDNIDPNAVG	NCNRLTGECL	KCIYNTAGFY	CDRCKDGFFG	NPLAPNPADK
890	900	910	920	930	940	950	960
CKACNCNLYG	TMKQQSSCNP	VTGQCECLPH	VTGQDCGACD	PGFYNLQSGQ	GCERCDCHAL	GSTNGQCDIR	TGQCECQPGI
970	980	990	1000	1010	1020	1030	1040
TGQHCERCEV	NHFGFGPEGC	KPCDCHPEGS	LSLQCKDDGR	CECREGFVGN	RCDQCEENYF	YNRSWPGCQE	CPACYRLVKD
1050	1060	1070	1080	1090	1100	1110	1120
KVADHRVKLQ	ELESLIANLG	TGDEMVTDQA	FEDRLKEAER	EVMDLLREAQ	DVKDVDQNLM	DRLQRVNNTL	SSQISRLQNI
1130	1140	1150	1160	1170	1180	1190	1200
RNTIEETGNL	AEQARAHVEN	TERLIEIASR	ELEKAKVAAA	NVSVTQPEST	GDPNNMTLLA	EEARKLAERH	KQEADDIVRV
1210	1220	1230	1240	1250	1260	1270	1280
AKTANDTSTE	AYNLLLRTLA	GENQTAFEIE	ELNRKYEQAK	NISQDLEKQA	ARVHEEAKRA	GDKAVEIYAS	VAQLSPLDSE
1290	1300	1310	1320	1330	1340	1350	1360
TLENEANNIK	MEAENLEQLI	DQKLKDYEDL	REDMRGKELE	VKNLLEKGKT	EQQTADQLLA	RADAAKALAE	EAAKKGRDTL
1370	1380	1390	1400	1410	1420	1430	1440
QEANDILNNL	KDFDRRVNDN	KTAAEEALRK	IPAINQTITE	ANEKTREAQQ	ALGSAAADAT	EAKNKAHEAE	RIASAVQKNA
1450	1460	1470	1480	1490	1500	1510	1520
TSTKAEAERT	FAEVTDLDNE	VNNMLKQLQE	AEKELKRKQD	DADQDMMMAG	MASQAAQEAE	INARKAKNSV	TSLLSIINDL
1530	1540	1550	1560	1570	1580	1590	1600
LEQLGQLDTV	DLNKLNEIEG	TLNKAKDEMK	VSDLDRKVSD	LENEAKKQEA	AIMDYNRDIE	EIMKDIRNLE	DIRKTLPSGC
1610							
FNTPSIEKP							

Cmpd.	No. of Cmpds.	m/z meas.	Δ m/z [ppm]	z	Rt [min]	Score	P	Range	Sequence	Modification
2687	1	941.9781	66.10	2	62.0	14.3	1	303-318	K.LVCNCKHNTYGVDCEK.C	Carbamidomethyl: 14

Detailed Protein Report

Protein 308: **GDP-Man:Man(3)GlcNAc(2)-PP-Dol alpha-1,2-mannosyltransferase [Homo sapiens]**

**Accession:**	gi|304434672	**Score:**	24.6
**Database:**	refseq_human(refseq_human_20140103.fasta)	**MW [kDa]:**	55.6
**Database Date:**	2015-11-30	**pI:**	9.6
**Modification(s):**	Oxidation	**Sequence Coverage [%]:**	4.5
		**No. of unique Peptides:**	1

10	20	30	40	50	60	70	80
MAAGERSWCL	CKLLRFFYSL	FFPGLIVCGT	LCVCLVIVLW	GIRLLLQRKK	KLVSTSKNGK	NQMVIAFFHP	YCNAGGGGER
**90**	**100**	**110**	**120**	**130**	**140**	**150**	**160**
VLWCALRALQ	KKYPEAVYVV	YTGDVNVNGQ	QILEGAFRRF	NIRLIHPVQF	VFLRKRYLVE	DSLYPHFTLL	GQSLGSIFLG
**170**	**180**	**190**	**200**	**210**	**220**	**230**	**240**
WEALMQCVPD	VYIDSMGYAF	TLPLFKYIGG	CQVGSYVHYP	TISTDMLSVV	KNQNIGFNNA	AFITRNPFLS	KVKLIYYYLF
**250**	**260**	**270**	**280**	**290**	**300**	**310**	**320**
AFIYGLVGSC	SDVVMVNSSW	TLNHILSLWK	VGNCTNIVYP	PCDVQTFLDI	PLHEKKMTPG	HLLVSVGQFR	PEKNHPLQIR
**330**	**340**	**350**	**360**	**370**	**380**	**390**	**400**
AFAKLLNKKM	VESPPSLKLV	LIGGCRNKDD	ELRVNQLRRL	SEDLGVQEYV	EFKINIPFDE	LKNYLSEATI	GLHTMWNEHF
**410**	**420**	**430**	**440**	**450**	**460**	**470**	**480**
GIGVVECMAA	GTIILAHNSG	GPKLDIVVPH	EGDITGFLAE	SEEDYAETIA	HILSMSAEKR	LQIRKSARAS	VSRFSDQEFE
**490**	**500**						
VTFLSSVEKL	FK						

Cmpd.	No. of Cmpds.	m/z meas.	Δ m/z [ppm]	z	Rt [min]	Score	P	Range	Sequence	Modification
2001	1	876.8626	-87.10	2	53.7	10.1	2	1-15	-.MAAGERSWCLCKLLR.F	Oxidation: 1

Detailed Protein Report

Protein 309: spectrin alpha chain, non-erythrocytic 1 isoform 3 [Homo sapiens]

**Accession:**	gi|306966132	**Score:**	24.6
**Database:**	refseq_human(refseq_human_20140103.fasta)	**MW [kDa]:**	282.1
**Database Date:**	2015-11-30	**pI:**	5.1
		**Sequence Coverage [%]:**	1.0
		**No. of unique Peptides:**	1

Detailed Protein Report

10	20	30	40	50	60	70	80
MDPSGVKVLE	TAEDIQERRQ	QVLDRYHRFK	ELSTLRRQKL	EDSYRFQFFQ	RDAEELEKWI	QEKLQIASDE	NYKDPTNLQG
90	100	110	120	130	140	150	160
KLQKHQAFEA	EVQANSGAIV	KLDETGNLMI	SEGHFASETI	RTRLMELHRQ	WELLLEKMRE	KGIKLLQAQK	LVQYLRECED
170	180	190	200	210	220	230	240
VMDWINDKEA	IVTSEELGQD	LEHVEVLQKK	FEEFQTDMAA	HEERVNEVNQ	FAAKLIQEQH	PEEELIKTKQ	DEVNAAWQRL
250	260	270	280	290	300	310	320
KGLALQRQGK	LFGAAEVQRF	NRDVDETISW	IKEKEQLMAS	DDFGRDLASV	QALLRKHEGL	ERDLAALEDK	VKALCAEADR
330	340	350	360	370	380	390	400
LQQSHPLSAT	QIQVKREELI	TNWEQIRTLA	AERHARLNDS	YRLQRFLADF	RDLTSWVTEM	KALINADELA	SDVAGAEALL
410	420	430	440	450	460	470	480
DRHQEHKGEI	DAHEDSFKSA	DESGQALLAA	GHYASDEVRE	KLTVLSEERA	ALLELWELRR	QQYEQCMDLQ	LFYRDTEQVD
490	500	510	520	530	540	550	560
NWMSKQEAFL	LNEDLGDSLD	SVEALLKKHE	DFEKSLSAQE	EKITALDEFA	TKLIQNNHYA	MEDVATRRDA	LLSRRNALHE
570	580	590	600	610	620	630	640
RAMRRRAQLA	DSFHLQQFFR	DSDELKSWVN	EKMKTATDEA	YKDPSNLQGK	VQKHQAFEAE	LSANQSRIDA	LEKAGQKLID
650	660	670	680	690	700	710	720
VNHYAKDEVA	ARMNEVISLW	KKLLEATELK	GIKLREANQQ	QQFNRNVEDI	ELWLYEVEGH	LASDDYGKDL	TNVQNLQKKH
730	740	750	760	770	780	790	800
ALLEADVAAH	QDRIDGITIQ	ARQFQDAGHF	DAENIKKKQE	ALVARYEALK	EPMVARKQKL	ADSLRLQQLF	RDVEDEETWI
810	820	830	840	850	860	870	880
REKEPIAAST	NRGKDLIGVQ	NLLKKHQALQ	AEIAGHEPRI	KAVTQKGNAM	VEEGHFAAED	VKAKLHELNQ	KWEALKAKAS
890	900	910	920	930	940	950	960
QRRQDLEDSL	QAQQYFADAN	EAESWMREKE	PIVGSTDYGK	DEDSAEALLK	KHEALMSDLS	AYGSSIQALR	EQAQSCRQQV
970	980	990	1000	1010	1020	1030	1040
APTDDETGKE	LVLALYDYQE	KSPREVTMKK	GDILTLLNST	NKDWWKVEVN	DRQGFVPAAY	VKKLDPAQSA	SRENLLEEQG
1050	1060	1070	1080	1090	1100	1110	1120
SIALRQEQID	NQYHSLLELG	EKRKGMLEKS	CKKFMLFREA	NELQQWINEK	EAALTSEEVG	ADLEQVEVLQ	KKFDDFQKDL
1130	1140	1150	1160	1170	1180	1190	1200
KANESRLKDI	NKVAEDLESE	GLMAEEVQAV	QQQEVYGMMP	RDETDSKTAS	PWKSARLMVH	TVATFNSIKE	LNERWRSLQQ
1210	1220	1230	1240	1250	1260	1270	1280
LAEERSQLLG	SAHEVQRFHR	DADETKEWIE	EKNQALNTDN	YGHDLASVQA	LQRKHEGFER	DLAALGDKVN	SLGETAERLI
1290	1300	1310	1320	1330	1340	1350	1360
QSHPESAEDL	QEKCTELNQA	WSSLGKRADQ	RKAKLGDSHD	LQRFLSDFRD	LMSWINGIRG	LVSSDELAKD	VTGAEALLER
1370	1380	1390	1400	1410	1420	1430	1440
HQEHRTEIDA	RAGTFQAFEQ	FGQQLLAHGH	YASPEIKQKL	DILDQERADL	EKAWVQRRMM	LDQCLELQLF	HRDCEQAENW
1450	1460	1470	1480	1490	1500	1510	1520
MAAREAFLNT	EDKGDSLDSV	EALIKKHEDF	DKAINVQEEK	IAALQAFADQ	LIAAGHYAKG	DISSRRNEVL	DRWRRLKAQM
1530	1540	1550	1560	1570	1580	1590	1600
IEKRSKLGES	QTLQQFSRDV	DEIEAWISEK	LQTASDESYK	DPTNIQSKHQ	KHQAFEAELH	ANADRIRGVI	DMGNSLIERG
1610	1620	1630	1640	1650	1660	1670	1680
ACAGSEDAVK	ARLAALADQW	QFLVQKSAEK	SQKLKEANKQ	QNFNTGIKDF	DFWLSEVEAL	LASEDYGKDL	ASVNNLLKKH
1690	1700	1710	1720	1730	1740	1750	1760
QLLEADISAH	EDRLKDLNSQ	ADSLMTSSAF	DTSQVKDKRD	TINGRFQKIK	SMAASRRAKL	NESHRLHQFF	RDMDDEESWI
1770	1780	1790	1800	1810	1820	1830	1840
KEKKLLVGSE	DYGRDLTGVQ	NLRKKHKRLE	AELAAHEPAI	QGVLDTGKKL	SDDNTIGKEE	IQQRLAQFVE	HWKELKQLAA
1850	1860	1870	1880	1890	1900	1910	1920
ARGQRLEESL	EYQQFVANVE	EEEAWINEKM	TLVASEDYGD	TLAAIQGLLK	KHEAFETDFT	VHKDRVNDVC	TNGQDLIKKN
1930	1940	1950	1960	1970	1980	1990	2000
NHHEENISSK	MKGLNGKVSD	LEKAAAQRKA	KLDENSAFLQ	FNWKADVVES	WIGEKENSLK	TDDYGRDLSS	VQTLLTKQET
2010	2020	2030	2040	2050	2060	2070	2080
FDAGLQAFQQ	EGIANITALK	DQLLAAKHVQ	SKAIEARHAS	LMKRWSQLLA	NSAARKKKLL	EAQSHFRKVE	DLFLTFAKKA
2090	2100	2110	2120	2130	2140	2150	2160
SAFNSWFENA	EEDLTDPVRC	NSLEEIKALR	EAHDAFRSSL	SSAQADFNQL	AELDRQIKSF	RVASNPYTWF	TMEALEETWR
2170	2180	2190	2200	2210	2220	2230	2240

Detailed Protein Report

Cmpd.	No. of Cmpds.	m/z meas.	Δ m/z [ppm]	z	Rt [min]	Score	P	Range	Sequence	Modification
2410	1	873.4266	-11.74	2	58.4	10.4	1	1810-1824	K.LSDDNTIGKEEIQQR.L	

Detailed Protein Report

Protein 310: PREDICTED: uncharacterized protein KIAA1109 isoform X12 [Homo sapiens]

**Accession:**	gi|578809467	**Score:**	24.5
**Database:**	refseq_human(refseq_human_20140103.fasta)	**MW [kDa]:**	451.7
**Database Date:**	2015-11-30	**pI:**	6.4
		**Sequence Coverage [%]:**	0.8
		**No. of unique Peptides:**	1

Detailed Protein Report

10	20	30	40	50	60	70	80
MFINCLYKLA	CLLEWGQTFV	FHVVCREYEL	ERPKSVIICQ	HGIDRRFCES	KLSCIPGPCP	TSDDLKYTMI	RLAVDGADIY
90	100	110	120	130	140	150	160
IVEHGCATNI	KMGAIRVANC	NLHNQSVGEG	ISAAIQDFQV	RQYIEQLNNC	RIGLQPAVLR	RAYWLEAGSA	NLGLITVDIA
170	180	190	200	210	220	230	240
LAADHHSKHE	AQRHFLETHD	ARTKRLWFLW	PDDILKNKRC	RNKCGCLGGC	RFFGGTVTGL	DFFKLEELTP	SSSSAFSSTS
250	260	270	280	290	300	310	320
AESDMYYGQS	LLQPGEWIIT	KEIPKIIDGN	VNGMKRKEWE	NKSVGIEVER	KTQHLSLQVP	LRSHSSSSSS	EENSSSSAAQ
330	340	350	360	370	380	390	400
PLLAGEKESP	SSVADDHLVQ	KEFLHGTKRD	DGQASIPTEI	SGNSPVSPNT	QDKSVGQSPL	RSPLKRQASV	CSTRLGSTKS
410	420	430	440	450	460	470	480
LTAAFYGDKQ	PVTVGVQFSS	DVSRSDENVL	DSPKQRRSFG	SFPYTPSADS	NSFHQYRSMD	SSMSMADSEA	YFSAAEEFEP
490	500	510	520	530	540	550	560
ISSDEGPGTY	PGRKKKKKQT	QQIDYSRGSI	YHSVEGPLTG	HGESIQDSRT	LPFKTHPSQA	SFVSALGGED	DVIEHLYIVE
570	580	590	600	610	620	630	640
GEKTVESEQI	TPQQPVMNCY	QTYLTQFQVI	NWSVKHPTNK	RTSKSSLHRP	LDLDTPTSEE	SSSSFEQLSV	PTFKVIKQGL
650	660	670	680	690	700	710	720
TANSLLDRGM	QLSGSTSNTP	YTPLEKKLAD	NTDDETLTEE	WTLDQPVSQT	RTTAIVEVKG	TVDIVLTPLV	AEALDRYIEA
730	740	750	760	770	780	790	800
MVHCASTRHP	AAIVDDLHAK	VLREAVQNSK	TTFSENLSSK	QDIRGTKTEQ	STIGTTNQGQ	AQTNLTMKQD	NVTIKGLQTN
810	820	830	840	850	860	870	880
VSIPKVNLCL	LQASVEESPT	TAPSRSVTHV	SLVALCFDRI	ATQVRMNRGV	VEETSNNAEP	GRTSNFDRYV	HATKMQPQSS
890	900	910	920	930	940	950	960
GSLRSNAGAE	KGKEIAAKLN	IHRVHGQLRG	LDTTDIGTCA	ITAIPFEKSK	VLFTLEELDE	FTFVDETDQQ	AVPDVTRIGP
970	980	990	1000	1010	1020	1030	1040
SQEKWGWIMF	ECGLENLTIK	GGRQSGAVLY	NSFGIMGKAS	DTERGGVLTS	NNSSDSPTGS	GYNTDVSDDN	LPCDRTSPSS
1050	1060	1070	1080	1090	1100	1110	1120
DLNGNSVSDE	QDEGVESDDL	KKDLPLMPPP	PDSCSMKLTI	KEIWFSFAAP	TNVRSHTHAF	SRQLNLLSTA	TPAVGAWLVP
1130	1140	1150	1160	1170	1180	1190	1200
IDQLKSSLNK	LETEGTLRIC	AVMGCIMTEA	LENKSVHFPL	RSKYNRLTKV	ARFLQENPSC	LLCNILHHYL	HQANYSIIDD
1210	1220	1230	1240	1250	1260	1270	1280
ATMSDGLPAL	VTLKKGLVAL	ARQWMKFIVV	TPAFKGVSLH	RPAQPLKPQI	AMDHEHEDGL	GLDNGGGLQS	DTSADGAEFE
1290	1300	1310	1320	1330	1340	1350	1360
FDAATVSEHT	MLLEGTANRP	PPGSSGPVTG	AEIMRKLSKT	HTHSDSALKI	KGIHPYHSLS	YTSGDTATDS	PVHVGRAGMP
1370	1380	1390	1400	1410	1420	1430	1440
VKDSPRKESL	LSYLTGSFPS	LHNLLEGTPQ	RSSAAVKSSS	LTRTGNTVAT	DMLSEHPLLS	EPSSVSFYNW	MSNAVGNRGS
1450	1460	1470	1480	1490	1500	1510	1520
VLQESPVTKS	GHNSLPTGVA	PNLPTIPSAS	DFNTVLSSDQ	NTLDGTHSQH	STSQDDVAGV	EEANQGFPAV	QLADAQVVFK
1530	1540	1550	1560	1570	1580	1590	1600
PLLSHTGIQS	QDTMPFCYRM	YFGEHLSFSG	TLDCLRADIV	DSDTAKERKG	KRARRQGHVN	LPPLEFKPAL	MLGTFSISAV
1610	1620	1630	1640	1650	1660	1670	1680
VMEKSVCTPQ	NSTSALSFHD	LSKRYYNTFH	CNFTISCQSI	SQHVDMALVR	LIHQFSTMID	DIKATQTDIK	LSRYTAGSAS
1690	1700	1710	1720	1730	1740	1750	1760
PTPTFKTRKH	RDFRSSDFSR	SSRGSLNGGN	RVNNAKNKRT	NNENNKKESR	NKNSLGRSER	RTSKVSRKGS	KDVVDHMTIH
1770	1780	1790	1800	1810	1820	1830	1840
MDDSDSITVS	EQSEPSAECW	QNMYKLLNFY	SLISDPTGIL	EKSSETFGPA	GVRSPTEPTC	KVVFENEQDN	SSLTKTQRKR
1850	1860	1870	1880	1890	1900	1910	1920
SLVTSEPQHV	TLIVFGIGMV	NRTHLEADIG	GLTMESELKR	IHGSFTLKEK	MKDVLHQKMT	ETCATAHIGG	VNIVLLEGIT
1930	1940	1950	1960	1970	1980	1990	2000
PNIQLEDFPT	SPTSTAKQEF	LTVVKCSIAK	SQALYSAQRG	LKTNNAAVFK	VGAISINIPQ	HPATLHSMMV	RSSHQLSKQI
2010	2020	2030	2040	2050	2060	2070	2080
SDLIRQPSTA	PQPVKEDIAT	PLPSEKTPTS	VNQTPVETNE	FPQLPEGLEK	KPIVLKFSAM	LDGIAIGAAL	LPSLKAEYKM
2090	2100	2110	2120	2130	2140	2150	2160
GRMRSHGMTG	AQTRFTFELP	NHRLRFTSKV	SATDMSTIPP	SASLNLPPVT	MSGKYIMEEH	DSYSDQVWSI	DELPSKQGYY
2170	2180	2190	2200	2210	2220	2230	2240

Detailed Protein Report

Cmpd.	No. of Cmpds.	m/z meas.	Δ m/z [ppm]	z	Rt [min]	Score	P	Range	Sequence	Modification
2265	1	618.9864	-97.88	3	56.8	10.2	2	4077-4093	K.VLSVLIKKLGTALQDEK.E	

Detailed Protein Report

Protein 311: protein CASP isoform a [Homo sapiens]

**Accession:**	gi|148277064	**Score:**	24.5
**Database:**	refseq_human(refseq_human_20140103.fasta)	**MW [kDa]:**	164.1
**Database Date:**	2015-11-30	**pI:**	5.6
**Modification(s):**	Carbamidomethyl	**Sequence Coverage [%]:**	1.4
		**No. of unique Peptides:**	1

Quantitation

WD:WU **Median:** 1.11 **CV:** 0.00 % **No. of Peptides:** 1

10	20	30	40	50	60	70	80
MLCVAGARLK	RELDATATVL	ANRQDESEQS	RKRLIEQSRE	FKKNTPEDLR	KQVAPLLKSF	QGEIDALSKR	SKEAEAAFLN
90	100	110	120	130	140	150	160
VYKRLIDVPD	PVPALDLGQQ	LQLKVQRLHD	IETENQKLRE	TLEEYNKEFA	EVKNQEVTIK	ALKEKIREYE	QTLKNQAETI
170	180	190	200	210	220	230	240
ALEKEQKLQN	DFAEKERKLQ	ETQMSTTSKL	EEAEHKVQSL	QTALEKTRTE	LFDLKTKYDE	ETTAKADEIE	MIMTDLERAN
250	260	270	280	290	300	310	320
QRAEVAQREA	ETLREQLSSA	NHSLQLASQI	QKAPDVEQAI	EVLTRSSLEV	ELAAKEREIA	QLVEDVQRLQ	ASLTKLRENS
330	340	350	360	370	380	390	400
ASQISQLEQQ	LSAKNSTLKQ	LEEKLKGQAD	YEEVKKELNI	LKSMEFAPSE	GAGTQDAAKP	LEVLLLEKNR	SLQSENAALR
410	420	430	440	450	460	470	480
ISNSDLSGSA	RRKGKDQPES	RRPGSLPAPP	PSQLPRNPGE	QASNTNGTHQ	FSPAGLSQDF	FSSSLASPSL	PLASTGKFAL
490	500	510	520	530	540	550	560
NSLLQRQLMQ	SFYSKAMQEA	GSTSMIFSTG	PYSTNSISSQ	SPLQQSPDVN	GMAPSPSQSE	SAGSVSEGEE	MDTAEIARQV
570	580	590	600	610	620	630	640
KEQLIKHNIG	QRIFGHYVLG	LSQGSVSEIL	ARPKPWNKLT	VRGKEPFHKM	KQFLSDEQNI	LALRSIQGRQ	RENPGQSLNR
650	660	670	680	690	700	710	720
LFQEVPKRRN	GSEGNITTRI	RASETGSDEA	IKSILEQAKR	ELQVQKTAEP	AQPSSASGSG	NSDDAIRSIL	QQARREMEAQ
730	740	750	760	770	780	790	800
QAALDPALKQ	APLSQSDITI	LTPKLLSTSP	MPTVSSYPPL	AISLKKPSAA	PEAGASALPN	PPALKKEAQD	APGLDPQGAA
810	820	830	840	850	860	870	880
DCAQGVLRQV	KNEVGRSGAW	KDHWWSAVQP	ERRNAASSEE	AKAEETGGGK	EKGSGGSGGG	SQPRAERSQL	QGPSSSEYWK
890	900	910	920	930	940	950	960
EWPSAESPYS	QSSELSLTGA	SRSETPQNSP	LPSSPIVPMS	KPTKPSVPPL	TPEQYEVYMY	QEVDTIELTR	QVKEKLAKNG
970	980	990	1000	1010	1020	1030	1040
ICQRIFGEKV	LGLSQGSVSD	MLSRPKPWSK	LTQKGREPFI	RMQLWLNGEL	GQGVLPVQGQ	QQGPVLHSVT	SLQDPLQQGC
1050	1060	1070	1080	1090	1100	1110	1120
VSSESTPKTS	ASCSPAPESP	MSSSESVKSL	TELVQQPCPP	IEASKDSKPP	EPSDPPASDS	QPTTPLPLSG	HSALSIQELV
1130	1140	1150	1160	1170	1180	1190	1200
AMSPELDTYG	ITKRVKEVLT	DNNLGQRLFG	ETILGLTQGS	VSDLLARPKP	WHKLSLKGRE	PFVRMQLWLN	DPNNVEKLMD
1210	1220	1230	1240	1250	1260	1270	1280
MKRMEKKAYM	KRRHSSVSDS	QPCEPPSVGT	EYSQGASPQP	QHQLKKPRVV	LAPEEKEALK	RAYQQKPYPS	PKTIEDLATQ
1290	1300	1310	1320	1330	1340	1350	1360
LNLKTSTVIN	WFHNYRSRIR	RELFIEEIQA	GSQGQAGASD	SPSARSGRAA	PSSEGDSCDG	VEATEGPGSA	DTEEPKSQGE
1370	1380	1390	1400	1410	1420	1430	1440
AEREEVPRPA	EQTEPPPSGT	PGPDDARDDD	HEGGPVEGPG	PLPSPASATA	TAAPAAPEDA	ATSAAAAPGE	GPAAPSSAPP
1450	1460	1470	1480	1490	1500	1510	
PSNSSSSSAP	RRPSSLQSLF	GLPEAAGARD	SRDNPLRKKK	AANLNSIIHR	LEKAASREEP	IEWEF	

Cmpd.	No. of Cmpds.	m/z meas.	Δ m/z [ppm]	z	Rt [min]	Score	P	Range	Sequence	Modification	Ratios
561	2	661.2196	-178.61	2	35.9	14.0	1	959-969	K.NGICQRIFGEK.V	Carbamidomethyl: 4	WD:WU 1.11

Detailed Protein Report

Protein 312: **PREDICTED: UDP-glucose:glycoprotein glucosyltransferase 1 isoform X3 [Homo sapiens]**

**Accession:**	gi|578804605	**Score:**	24.5
**Database:**	refseq_human(refseq_human_20140103.fasta)	**MW [kDa]:**	176.2
**Database Date:**	2015-11-30	**pI:**	5.3
**Modification(s):**	Carbamidomethyl	**Sequence Coverage [%]:**	2.1
		**No. of unique Peptides:**	1

10	20	30	40	50	60	70	80
MGCKGDASGV	CYKMGVLVVL	TVLWLFSSVK	ADSKAITTSL	TTKWFSTPLL	LEASEFLAED	SQEKFWNFVE	ASQNIGSSDH
**90**	**100**	**110**	**120**	**130**	**140**	**150**	**160**
DGTDYSYYHA	ILEAAFQFLS	PLQQNLFKFC	LSLRSYSATI	QAFQQIAADE	PPPEGCNSFF	SVHGKKTCES	DTLEALLLTA
**170**	**180**	**190**	**200**	**210**	**220**	**230**	**240**
SERPKPLLFK	GDHRYPSSNP	ESPVVIFYSE	IGSEEFSNFH	RQLISKSNAG	KINYVFRHYI	FNPRKEPVYL	SGYGVELAIK
**250**	**260**	**270**	**280**	**290**	**300**	**310**	**320**
STEYKAKDDT	QVKGTEVNTT	VIGENDPIDE	VQGFLFGKLR	DLHPDLEGQL	KELRKHLVES	TNEMAPLKVW	QLQDLSFQTA
**330**	**340**	**350**	**360**	**370**	**380**	**390**	**400**
ARILASPVEL	ALVVMKDLSQ	NFPTKARAIT	KTAVSSELRT	EVEENQKYFK	GTLGLQPGDS	ALFINGLHMD	LDTQDIFSLF
**410**	**420**	**430**	**440**	**450**	**460**	**470**	**480**
DVLRNEARVM	EGLHRLGIEG	LSLHNVLKLN	IQPSEADYAV	DIRSPAISWV	NNLEVDSRYN	SWPSSLQELL	RPTFPGVIRQ
**490**	**500**	**510**	**520**	**530**	**540**	**550**	**560**
IRKNLHNMVF	IVDPAHETTA	ELMNTAEMFL	SNHIPLRIGF	IFVVNDSEDV	DGMQDAGVAV	LRAYNYVAQE	VDDYHAFQTL
**570**	**580**	**590**	**600**	**610**	**620**	**630**	**640**
THIYNKVRTG	EKVKVEHVVS	VLEKKYPYVE	VNSILGIDSA	YDRNRKEARG	YYEQTGVGPL	PVVLFNGMPF	EREQLDPDEL
**650**	**660**	**670**	**680**	**690**	**700**	**710**	**720**
ETITMHKILE	TTTFFQRAVY	LGELPHDQDV	VEYIMNQPNV	VPRINSRILT	AERDYLDLTA	SNNFFVDDYA	RFTILDSQGK
**730**	**740**	**750**	**760**	**770**	**780**	**790**	**800**
TAAVANSMNY	LTKKGMSSKE	IYDDSFIRPV	TFWIVGDFDS	PSGRQLLYDA	IKHQKSSNNV	RISMINNPAK	EISYENTQIS
**810**	**820**	**830**	**840**	**850**	**860**	**870**	**880**
RAIWAALQTQ	TSNAAKNFIT	KMAKEGAAEA	LAAGADIAEF	SVGGMDFSLF	KEVFESSKMD	FILSHAVYCR	DVLKLKKGQR
**890**	**900**	**910**	**920**	**930**	**940**	**950**	**960**
AVISNGRIIG	PLEDSELFNQ	DDFHLLENII	LKTSGQKIKS	HIQQLRVEED	VASDLVMKVD	ALLSAQPKGD	PRIEYQFFED
**970**	**980**	**990**	**1000**	**1010**	**1020**	**1030**	**1040**
RHSAIKLRPK	EGETYFDVVA	VVDPVTREAQ	RLAPLLLVLA	QLINMNLRVF	MNCQSKLSDM	PLKSFYRYVL	EPEISFTSDN
**1050**	**1060**	**1070**	**1080**	**1090**	**1100**	**1110**	**1120**
SFAKGPIAKF	LDMPQSPLFT	LNLNTPESWM	VESVRTPYDL	DNIYLEEVDS	VVAAEYELEY	LLLEGHCYDI	TTGQPPRGLQ
**1130**	**1140**	**1150**	**1160**	**1170**	**1180**	**1190**	**1200**
FTLGTSANPV	IVDTIVMANL	GYFQLKANPG	AWILRLRKGR	SEDIYRIYSH	DGTDSPPDAD	EVVIVLNNFK	SKIIKVKVQK
**1210**	**1220**	**1230**	**1240**	**1250**	**1260**	**1270**	**1280**
KADMVNEDLL	SDGTSENESG	FWDSFKWGFT	GQKTEEVKQD	KDDIINIFSV	ASGHLYERFL	RIMMLSVLKN	TKTPVKFWFL
**1290**	**1300**	**1310**	**1320**	**1330**	**1340**	**1350**	**1360**
KNYLSPTFKE	FIPYMANEYN	FQYELVQYKW	PRWLHQQTEK	QRIIWGYKIL	FLDVLFPLVV	DKFLFVDADQ	IVRTDLKELR
**1370**	**1380**	**1390**	**1400**	**1410**	**1420**	**1430**	**1440**
DFNLDGAPYG	YTPFCDSRRE	MDGYRFWKSG	YWASHLAGRK	YHISALYVVD	LKKFRKIAAG	DRLRGQYQGL	SQDPNSLSNL
**1450**	**1460**	**1470**	**1480**	**1490**	**1500**	**1510**	**1520**
DQDLPNNMIH	QVPIKSLPQE	WLWCETWCDD	ASKKRAKTID	LCNNPMTKEP	KLEAAVRIVP	EWQDYDQEIK	QLQIRFQKEK
**1530**	**1540**	**1550**					
ETGALYKEKT	KEPSREGPQK	REEL					

Cmpd.	No. of Cmpds.	m/z meas.	Δ m/z [ppm]	z	Rt [min]	Score	P	Range	Sequence	Modification
2009	1	599.9210	-67.30	3	53.5	11.7	1	1009-1023	R.VFMNCQSKLSDMPLK.S	Carbamidomethyl: 5

Detailed Protein Report

Protein 313: PREDICTED: disks large homolog 2 isoform X10 [Homo sapiens]

**Accession:**	gi|578821149	**Score:**	24.5
**Database:**	refseq_human(refseq_human_20140103.fasta)	**MW [kDa]:**	73.1
**Database Date:**	2015-11-30	**pI:**	6.7
**Modification(s):**	Oxidation	**Sequence Coverage [%]:**	5.1
		**No. of unique Peptides:**	1

Quantitation

WD:WU **Median:** 2.30 **CV:** 0.00 % **No. of Peptides:** 1

10	20	30	40	50	60	70	80
MNAYLTKQHS	CSRGSDGMDA	VRSAPTLIRD	AHCACGWQRN	CQGLGYSSQT	MPSSGPGGPA	SNRTGGSSFN	RTLWDSVRKS
**90**	**100**	**110**	**120**	**130**	**140**	**150**	**160**
PHKTSTKGKG	TCGEHCTCPH	GWFSPAQASP	APIIVNTDTL	DTIPYVNGTE	IEYEFEEITL	ERGNSGLGFS	IAGGTDNPHI
**170**	**180**	**190**	**200**	**210**	**220**	**230**	**240**
GDDPGIFITK	IIPGGAAAED	GRLRVNDCIL	RVNEVDVSEV	SHSKAVEALK	EAGSIVRLYV	RRRRPILETV	VEIKLFKGPK
**250**	**260**	**270**	**280**	**290**	**300**	**310**	**320**
GLGFSIAGGV	GNQHIPGDNS	IYVTKIIDGG	AAQKDGRLQV	GDRLLMVNNY	SLEEVTHEEA	VAILKNTSEV	VYLKVGKPTT
**330**	**340**	**350**	**360**	**370**	**380**	**390**	**400**
IYMTDPYGPP	DITHSYSPPM	ENHLLSGNNG	TLEYKTSLPP	ISPGRYSPIP	KHMLVDDDYT	RPPEPVYSTV	NKLCDKPASP
**410**	**420**	**430**	**440**	**450**	**460**	**470**	**480**
RHYSPVECDK	SFLLSAPYSH	YHLGLLPDSE	MTSHSQHSTA	TRQPSMTLQR	AVSLEGEPRK	VVLHKGSTGL	GFNIVGGEDG
**490**	**500**	**510**	**520**	**530**	**540**	**550**	**560**
EGIFVSFILA	GGPADLSGEL	QRGDQILSVN	GIDLRGASHE	QAAAALKGAG	QTVTIIAQYQ	PEDYARFEAK	IHDLREQMMN
**570**	**580**	**590**	**600**	**610**	**620**	**630**	**640**
HSMSSGSGSL	RTNQKRSLYV	RAMFDYDKSK	DSGLPSQGLS	FKYGDILHVI	NASDDEWWQA	RRVMLEGDSE	EMGVIPSKRR
**650**	**660**	**670**	**680**				
VERKERARLK	TVKFNAKPGV	IDSKGLLEFK	YA				

Cmpd.	No. of Cmpds.	m/z meas.	Δ m/z [ppm]	z	Rt [min]	Score	P	Range	Sequence	Modification	Ratios
56	1	877.9030	41.74	2	29.9	12.3	0	556-571	R.EQMMNHSMSSGSGSLR.T	Oxidation: 8	WD:WU 2.30

Detailed Protein Report

Protein 314: inner centromere protein isoform 2 [Homo sapiens]

**Accession:**	gi|102467242	**Score:**	24.5
**Database:**	refseq_human(refseq_human_20140103.fasta)	**MW [kDa]:**	104.9
**Database Date:**	2015-11-30	**pI:**	10.0
		**Sequence Coverage [%]:**	2.2
		**No. of unique Peptides:**	1

10	20	30	40	50	60	70	80
MGTTAPGPIH	LLELCDQKLM	EFLCNMDNKD	LVWLEEIQEE	AERMFTREFS	KEPELMPKTP	SQKNRRKKRR	ISYVQDENRD
**90**	**100**	**110**	**120**	**130**	**140**	**150**	**160**
PIRRRLSRRK	SRSSQLSSRR	LRSKDSVEKL	ATVVGENGSV	LRRVTRAAAA	AAAATMALAA	PSSPTPESPT	MLTKKPEDNH
**170**	**180**	**190**	**200**	**210**	**220**	**230**	**240**
TQCQLVPVVE	IGISERQNAE	QHVTQLMSTE	PLPRTLSPTP	ASATAPTSQG	IPTSDEESTP	KKSKARILES	ITVSSLMATP
**250**	**260**	**270**	**280**	**290**	**300**	**310**	**320**
QDPKGQGVGT	GRSASKLRIA	QVSPGPRDSP	AFPDSPWRER	VLAPILPDNF	STPTGSRTDS	QSVRHSPIAP	SSPSPQVLAQ
**330**	**340**	**350**	**360**	**370**	**380**	**390**	**400**
KYSLVAKQES	VVRRASRRLA	KKTAEEPAAS	GRIICHSYLE	RLLNVEVPQK	VGSEQKEPPE	EAEPVAAAEP	EVPENNGNNS
**410**	**420**	**430**	**440**	**450**	**460**	**470**	**480**
WPHNDTEIAN	STPNPKPAAS	SPETPSAGQQ	EAKTDQADGP	REPPQSARRK	RSYKQAVSEL	DEEQHLEDEE	LQPPRSKTPS
**490**	**500**	**510**	**520**	**530**	**540**	**550**	**560**
SPCPASKVVR	PLRTFLHTVQ	RNQMLMTPTS	APRSVMKSFI	KRNTPLRMDP	KEKERQRLEN	LRRKEEAEQL	RRQKVEEDKR
**570**	**580**	**590**	**600**	**610**	**620**	**630**	**640**
RRLEEVKLKR	EERLRKVLQA	RERVEQMKEE	KKKQIEQKFA	QIDEKTEKAK	EERLAEEKAK	KKAAAKKMEE	VEARRKQEEE
**650**	**660**	**670**	**680**	**690**	**700**	**710**	**720**
ARRLRWLQQE	EEERRHQELL	QKKKEEEQER	LRKAAEAKRL	AEQREQERRE	QERREQERRE	QERREQERRE	QERQLAEQER
**730**	**740**	**750**	**760**	**770**	**780**	**790**	**800**
RREQERLQAE	RELQEREKAL	RLQKEQLQRE	LEEKKKKEEQ	QRLAERQLQE	EQEKKAKEAA	GASKALNVTV	DVQSPACTSY
**810**	**820**	**830**	**840**	**850**	**860**	**870**	**880**
QMTPQGHRAP	PKINPDNYGM	DLNSDDSTDD	EAHPRKPIPT	WARGTPLSQA	IIHQYYHPPN	LLELFGTILP	LDLEDIFKKS
**890**	**900**	**910**	**920**				
KPRYHKRTSS	AVWNSPPLQG	ARVPSSLAYS	LKKH				

Cmpd.	No. of Cmpds.	m/z meas.	Δ m/z [ppm]	z	Rt [min]	Score	P	Range	Sequence	Modification
1737	3	502.2720	31.12	2	50.1	13.7	1	611-618	K.EERLAEEK.A	

Detailed Protein Report

Protein 315: histone deacetylase complex subunit SAP18 [Homo sapiens]

Accession: gi|215490089
Database: refseq_human(refseq_human_20140103.fasta)
Database Date: 2015-11-30
Modification(s): Oxidation

Score: 24.4
MW [kDa]: 19.5
pI: 10.3
Sequence Coverage [%]: 7.0
No. of unique Peptides: 1

Quantitation

WD:WU **Median:** 1.13 **CV:** 0.00 % **No. of Peptides:** 1

10	20	30	40	50	60	70	80
MLAAGVGGQG	ERLAGRRRKM	AVESRVTQEE	IKKEPEKPID	REKTCPLLLR	VFTTNNGRHH	RMDEFSRGNV	PSSELQIYTW
**90**	**100**	**110**	**120**	**130**	**140**	**150**	**160**
MDATLKELTS	LVKEVYPEAR	KKGTHFNFAI	VFTDVKRPGY	RVKEIGSTMS	GRKGTDDSMT	LQSQKFQIGD	YLDIAITPPN
**170**	**180**						
RAPPPSGRMR	PY						

Cmpd.	No. of Cmpds.	m/z meas.	Δ m/z [ppm]	z	Rt [min]	Score	P	Range	Sequence	Modification	Ratios
2664	1	541.3186	91.64	2	61.6	14.2	1	124-133	K.EIGSTMSGRK.G	Oxidation: 6	WD:WU 1.13

Detailed Protein Report

Protein 316: PREDICTED: neuroblast differentiation-associated protein AHNAK isoform X10 [Homo sapiens]

**Accession:**	gi|578821732	**Score:**	24.4
**Database:**	refseq_human(refseq_human_20140103.fasta)	**MW [kDa]:**	368.2
**Database Date:**	2015-11-30	**pI:**	6.1
		**Sequence Coverage [%]:**	0.7
		**No. of unique Peptides:**	1

Quantitation

WD:WU	**Median:** 0.64	**CV:** 0.00 %	**No. of Peptides:** 1

Detailed Protein Report

10	20	30	40	50	60	70	80
MEKEETTREL	LLPNWQGSGS	HGLTIAQRDD	GVFVQEVTQN	SPAARTGVVK	EGDQIVGATI	YFDNLQSGEV	TQLLNTMGHH
90	100	110	120	130	140	150	160
TVGLKLHRKG	DRSPEPGQTW	TREVFSSCSS	EVVLSGDDEE	YQRIYTTKIK	PRLKSEDGVE	GDLGETQSRT	ITVTRRVTAY
170	180	190	200	210	220	230	240
TVDVTGREGA	KDIDISSPEF	KIKIPRHELT	EISNVDVETQ	SGKTVIRLPS	GSGAASPTGS	AVDIRAGAIS	ASGPELQGAG
250	260	270	280	290	300	310	320
HSKLQVTMPG	IKVGGSGVNV	NAKGLDLGGR	GGVQVPAVDI	SSSLGGRAVE	VQGPSLESGD	HGKIKFPTMK	VPKFGVSTGR
330	340	350	360	370	380	390	400
EGQTPKAGLR	VSAPEVSVGH	KGGKPGLTIQ	APQLEVSVPS	ANIEGLEGKL	KGPQITGPSL	EGDLGLKGAK	PQGHIGVDAS
410	420	430	440	450	460	470	480
APQIGGSITG	PSVEVQAPDI	DVQGPGSKLN	VPKMKVPKFS	VSGAKGEETG	IDVTLPTGEV	TVPGVSGDVS	LPEIATGGLE
490	500	510	520	530	540	550	560
GKMKGTKVKT	PEMIIQKPKI	SMQDVDLSLG	SPKLKGDIKV	SAPGVQGDVK	GPQVALKGSR	VDIETPNLEG	TLTGPRLGSP
570	580	590	600	610	620	630	640
SGKTGTCRIS	MSEVDLNVAA	PKVKGGVDVT	LPRVEGKVKV	PEVDVRGPKV	DVSAPDVEAH	GPEWNLKMPK	MKMPTFSTPG
650	660	670	680	690	700	710	720
AKGEGPDVHM	TLPKGDISIS	GPKVNVEAPD	VNLEGLGGKL	KGPDVKLPDM	SVKTPKISMP	DVDLHVKGTK	VKGEYDVTVP
730	740	750	760	770	780	790	800
KLEGELKGPK	VDIDAPDVDV	HGPDWHLKMP	KMKMPKFSVP	GFKAEGPEVD	VNLPKADLDV	SGPKVDVDVP	DVNIEGPDAK
810	820	830	840	850	860	870	880
LKGPKFKMPE	MNIKAPKISM	PDLDLNLKGP	KMKGEVDVSL	ANVEGDLKGP	ALDIKGPKID	VDAPDIDIHG	PDAKLKGPKL
890	900	910	920	930	940	950	960
KMPDMHVNMP	KISMPEIDLN	LKGSKLKGDV	DVSGPKLEGD	IKAPSLDIKG	PEVDVSGPKL	NIEGKSKKSR	FKLPKFNFSG
970	980	990	1000	1010	1020	1030	1040
SKVQTPEVDV	KGKKPDIDIT	GPKVDINAPD	VEVQGKVKGS	KFKMPFLSIS	SPKVSMPDVE	LNLKSPKVKG	DLDIAGPNLE
1050	1060	1070	1080	1090	1100	1110	1120
GDFKGPKVDI	KAPEVNLNAP	DVDVHGPDWN	LKMPKMKMPK	FSVSGLKAEG	PDVAVDLPKG	DINIEGPSMN	IEGPDLNVEG
1130	1140	1150	1160	1170	1180	1190	1200
PEGGLKGPKF	KMPDMNIKAP	KISMPDIDLN	LKGPKVKGDV	DISLPKLEGD	LKGPEVDIKG	PKVDINAPDV	DVHGPDWHLK
1210	1220	1230	1240	1250	1260	1270	1280
MPKVKMPKFS	MPGFKGEGPE	VDVTLPKADI	DISGPNVDVD	VPDVNIEGPD	AKLKGPKFKM	PEMNIKAPKI	SMPDFDLNLK
1290	1300	1310	1320	1330	1340	1350	1360
GPKMKGDVVV	SLPKVEGDLK	GPEVDIKGPK	VDIDTPDINI	EGSEGKFKGP	KFKIPEMHLK	APKISMPDID	LNLKGPKVKG
1370	1380	1390	1400	1410	1420	1430	1440
DVDVSLPKME	GDLKGPEVDI	KGPKVDINAP	DVDVQGPDWH	LKMPKVKMPK	FSMPGFKGEG	PDVDVNLPKA	DLDVSGPKVD
1450	1460	1470	1480	1490	1500	1510	1520
IDVPDVNIEG	PEGKLKGPKF	KMPEMNIKAP	KISMPDIDLN	LKGPKVKGDM	DVSLPKVEGD	MQVPDLDIKG	PKVDINAPDV
1530	1540	1550	1560	1570	1580	1590	1600
DVRGPDWHLK	MPKIKMPKIS	MPGFKGEGPE	VDVNLPKADL	DVSGPKVDVD	VPDVNIEGPD	AKLKGPKFKM	PEMNIKAPKI
1610	1620	1630	1640	1650	1660	1670	1680
SMPDFDLHLK	GPKVKGDVDV	SLPKMEGDLK	APEVDIKGPK	VDIDAPDVDV	HGPDWHLKMP	KVKMPKFSMP	GFKGEGPEVD
1690	1700	1710	1720	1730	1740	1750	1760
VNLPKADIDV	SGPKVDIDTP	DIDIHGPEGK	LKGPKFKMPD	LHLKAPKISM	PEVDLNLKGP	KMKGDVDVSL	PKVEGDLKGP
1770	1780	1790	1800	1810	1820	1830	1840
EVDIKGPKVD	IDVPDVDVQG	PDWHLKMPKV	KMPKFSMPGF	KGEGPDVDVN	LPKADLDVSG	PKVDIDVPDV	NIEGPDAKLK
1850	1860	1870	1880	1890	1900	1910	1920
GPKFKMPEMN	IKAPKISMPD	FDLHLKGPKV	KGDVDVSLPK	VEGDLKGPEV	DIKGPKVDID	APDVDVHGPD	WHLKMPKVKM
1930	1940	1950	1960	1970	1980	1990	2000
PKFSMPGFKG	EGPDVDVTLP	KADIEISGPK	VDIDAPDVSI	EGPDAKLKGP	KFKMPEMNIK	APKISMPDID	FNLKGPKVKG
2010	2020	2030	2040	2050	2060	2070	2080
DVDVSLPKVE	GDLKGPEIDI	KGPSLDIDTP	DVNIEGPEGK	LKGPKFKMPE	MNIKAPKISM	PDFDLHLKGP	KVKGDVDVSL
2090	2100	2110	2120	2130	2140	2150	2160
PKVESDLKGP	EVDIEGPEGK	LKGPKFKMPD	VHFKSPQISM	SDIDLNLKGP	KIKGDMDISV	PKLEGDLKGP	KVDVKGPKVG
2170	2180	2190	2200	2210	2220	2230	2240

Detailed Protein Report

Cmpd.	No. of Cmpds.	m/z meas.	Δ m/z [ppm]	z	Rt [min]	Score	P	Range	Sequence	Modification	Ratios	
1546	1	506.2878	21.02	2	47.6	11.8	1	2351-2360	K.GPEADIKGPK.V		WD:WU	0.64

Detailed Protein Report

Protein 317: noggin precursor [Homo sapiens]

**Accession:**	gi|4885523	**Score:**	24.4
**Database:**	refseq_human(refseq_human_20140103.fasta)	**MW [kDa]:**	25.8
**Database Date:**	2015-11-30	**pI:**	10.2
**Modification(s):**	Carbamidomethyl, Oxidation	**Sequence Coverage [%]:**	9.9
		**No. of unique Peptides:**	2

```
        10         20         30         40         50         60         70         80
MERCPSLGVT LYALVVVLGL RATPAGGQHY LHIRPAPSDN LPLVDLIEHP DPIFDPKEKD LNETLLRSLL GGHYDPGFMA
        90        100        110        120        130        140        150        160
TSPPEDRPGG GGGAAGGAED LAELDQLLRQ RPSGAMPSEI KGLEFSEGLA QGKKQRLSKK LRRKLQMWLW SQTFCPVLYA
       170        180        190        200        210        220        230        240
WNDLGSRFWP RYVKVGSCFS KRSCSVPEGM VCKPSKSVHL TVLRWRCQRR GGQRCGWIPI QYPIISECKC SC
```

Cmpd.	No. of Cmpds.	m/z meas.	Δ m/z [ppm]	z	Rt [min]	Score	P	Range	Sequence	Modification
2199	1	869.3168	-104.52	2	56.0	10.2	1	182-196	K.RSCSVPEGMVCKPSK.S	Carbamidomethyl: 3, 11; Oxidation: 9
1566	1	862.7593	-26.29	3	47.9	14.3	2	182-204	K.RSCSVPEGMVCKPSKSVHLTVLR.W	Carbamidomethyl: 11; Oxidation: 9

Detailed Protein Report

Protein 318: catenin alpha-2 isoform 5 [Homo sapiens]

Accession: gi|543583701
Database: refseq_human(refseq_human_20140103.fasta)
Database Date: 2015-11-30
Modification(s): Oxidation

Score: 24.4
MW [kDa]: 65.1
pI: 5.4
Sequence Coverage [%]: 4.8
No. of unique Peptides: 1

10	20	30	40	50	60	70	80
MDGWRRPLQS	VGKVCEKNMK	TSEMHTMSGA	QTGRKEKGDP	LNIAIDKMTK	KTRDLRRQLR	KAVMDHISDS	FLETNVPLLV
90	100	110	120	130	140	150	160
LIEAAKSGNE	KEVKEYAQVF	REHANKLVEV	ANLACSISNN	EEGVKLVRMA	ATQIDSLCPQ	VINAALTLAA	RPQSKVAQDN
170	180	190	200	210	220	230	240
MDVFKDQWEK	QVRVLTEAVD	DITSVDDFLS	VSENHILEDV	NKCVIALQEG	DVDTLDRTAG	AIRGRAARVI	HIINAEMENY
250	260	270	280	290	300	310	320
EAGVYTEKVL	EATKLLSETV	MPRFAEQVEV	AIEALSANVP	QPFEENEFID	ASRLVYDGVR	DIRKAVLMIR	TPEELEDDSD
330	340	350	360	370	380	390	400
FEQEDYDVRS	RTSVQTEDDQ	LIAGQSARAI	MAQLPQEEKA	KIAEQVEIFH	QEKSKLDAEV	AKWDDSGNDI	IVLAKQMCMI
410	420	430	440	450	460	470	480
MMEMTDFTRG	KGPLKNTSDV	INAAKKIAEA	GSRMDKLARA	VADQCPDSAC	KQDLLAYLQR	IALYCHQLNI	CSKVKAEVQN
490	500	510	520	530	540	550	560
LGGELIVSGL	DSATSLIQAA	KNLMNAVVLT	VKASYVASTK	YQKVYGTAAV	NSPVVSWKMK	APEKKPLVKR	EKPEEFQTRV
570	580	590					
RRGSQKKHIS	PVQALSEFKA	MDSF					

Cmpd.	No. of Cmpds.	m/z meas.	Δ m/z [ppm]	z	Rt [min]	Score	P	Range	Sequence	Modification
1343	1	1021.9669	10.42	2	45.6	10.3	2	18-35	K.NMKTSEMHTMSGAQTGRK.E	Oxidation: 2, 7, 10

Detailed Protein Report

Protein 319: PREDICTED: centrosome-associated protein CEP250 isoform X4 [Homo sapiens]

Accession: gi|530417804
Database: refseq_human(refseq_human_20140103.fasta)
Database Date: 2015-11-30
Modification(s): Oxidation

Score: 24.4
MW [kDa]: 208.3
pI: 4.9
Sequence Coverage [%]: 2.2
No. of unique Peptides: 2

10	20	30	40	50	60	70	80
MEAAEQARNA	LQVDLAEAEK	RREALWEKNT	HLEAQLQKAE	EAGAELQADL	RDIQEEKEEI	QKKLSESRHQ	QEAATTQLEQ
90	100	110	120	130	140	150	160
LHQEAKRQEE	VLARAVQEKE	ALVREKAALE	VRLQAVERDR	QDLAEQLQGL	SSAKELLESS	LFEAQQQNSV	IEVTKGQLEV
170	180	190	200	210	220	230	240
QIQTVTQAKE	VIQGEVRCLK	LELDTERSQA	EQERDAAARQ	LAQAEQEGKT	ALEQQKAAHE	KEVNQLREKW	EKERSWHQQE
250	260	270	280	290	300	310	320
LAKALESLER	EKMELEMRLK	EQQTEMEAIQ	AQREEERTQA	ESALCQMQLE	TEKERVSLLE	TLLQTQKELA	DASQQLERLR
330	340	350	360	370	380	390	400
QDMKVQKLKE	QETTGILQTQ	LQEAQRELKE	AARQHRDDLA	ALQEESSSLL	QDKMDLQKQV	EDLKSQLVAQ	DDSQRLVEQE
410	420	430	440	450	460	470	480
VQEKLRETQE	YNRIQKELER	EKASLTLSLM	EKEQRLLVLQ	EADSIRQQEL	SALRQDMQEA	QGEQKELSAQ	MELLRQEVKE
490	500	510	520	530	540	550	560
KEADFLAQEA	QLLEELEASH	ITEQQLRASL	WAQEAKAAQL	QLRLRSTESQ	LEALAAEQQP	GNQAQAQAQL	ASLYSALQQA
570	580	590	600	610	620	630	640
LGSVCESRPE	LSGGGDSAPS	VWGLEPDQNG	ARSLFKRGPL	LTALSAEAVA	SALHKLHQDL	WKTQQTRDVL	RDQVQKLEER
650	660	670	680	690	700	710	720
LTDTEAEKSQ	VHTELQDLQR	QLSQNQEEKS	KWEGKQNSLE	SELMELHETM	ASLQSRLRRA	ELQRMEAQGE	RELLQAAKEN
730	740	750	760	770	780	790	800
LTAQVEHLQA	AVVEARAQAS	AAGILEEDLR	TARSALKLKN	EEVESERERA	QALQEQGELK	VAQGKALQEN	LALLTQTLAE
810	820	830	840	850	860	870	880
REEEVETLRG	QIQELEKQRE	MQKAALELLS	LDLKKRNQEV	DLQQEQIQEL	EKCRSVLEHL	PMAVQEREQK	LTVQREQIRE
890	900	910	920	930	940	950	960
LEKDRETQRN	VLEHQLLELE	KKDQMIESQR	GQVQDLKKQL	VTLECLALEL	EENHHKMECQ	QKLIKELEGQ	RETQRVALTH
970	980	990	1000	1010	1020	1030	1040
LTLDLEERSQ	ELQAQSSQIH	DLESHSTVLA	RELQERDQEV	KSQREQIEEL	QRQKEHLTQD	LERRDQELML	QKERIQVLED
1050	1060	1070	1080	1090	1100	1110	1120
QRTRQTKILE	EDLEQIKLSL	RERGRELTTQ	RQLMQERAEE	GKGPSKAQRG	SLEHMKLILR	DKEKEVECQQ	EHIHELQELK
1130	1140	1150	1160	1170	1180	1190	1200
DQLEQQLQGL	HRKVGETSLL	LSQREQEIVV	LQQQLQEARE	QGELKEQSLQ	SQLDEAQRAL	AQRDQELEAL	QQEQQQAQGQ
1210	1220	1230	1240	1250	1260	1270	1280
EERVKEKADA	LQGALEQAHM	TLKERHGELQ	DHKEQARRLE	EELAVEGRRV	QALEEVLGDL	RAESREQEKA	LLALQQQCAE
1290	1300	1310	1320	1330	1340	1350	1360
QAQEHEVETR	ALQDSWLQAQ	AVLKERDQEL	EALRAESQSS	RHQEEAARAR	AEALQEALGK	AHAALQGKEQ	HLLEQAELSR
1370	1380	1390	1400	1410	1420	1430	1440
SLEASTATLQ	ASLDACQAHS	RQLEEALRIQ	EGEIQDQDLR	YQEDVQQLQQ	ALAQRDEELR	HQQEREQLLE	KSLAQRVQEN
1450	1460	1470	1480	1490	1500	1510	1520
MIQEKQNLGQ	EREEEEIRGL	HQSVRELQLT	LAQKEQEILE	LRETQQRNNL	EALPHSHKTS	PMEEQSLKLD	SLEPRLQREL
1530	1540	1550	1560	1570	1580	1590	1600
ERLQAALRQT	EAREIEWREK	AQDLALSLAQ	TKASVSSLQE	VAMFLQASVL	ERDSEQQRLQ	DELELTRRAL	EKERLHSPGA
1610	1620	1630	1640	1650	1660	1670	1680
TSTAELGSRG	EQGVQLGEVS	GVEAEPSPDG	MEKQSWRQRL	EHLQQAVARL	EIDRSRLQRH	NVQLRSTLEQ	VERERRKLKR
1690	1700	1710	1720	1730	1740	1750	1760
EAMRAAQAGS	LEISKATASS	PTQQDGRGQK	NSDAKCVAEL	QKEVVLLQAQ	LTLERKQKQD	YITRSAQTSR	ELAGLHHSLS
1770	1780	1790	1800	1810	1820		
HSLLAVAQAP	EATVLEAETR	RLDESLTQSL	TSPGPVLLHP	SPSTTQAASR			

Cmpd.	No. of	m/z meas.	Δ m/z	z	Rt	Score	P	Range	Sequence	Modification

Detailed Protein Report

	Cmpds.		[ppm]		[min]					
813	1	683.3344	-49.61	2	39.0	12.1	1	421-432	R.EKASLTLSLMEK.E	Oxidation: 10
1397	1	926.5056	55.39	3	46.2	12.2	2	1681-1707	R.EAMRAAQAGSLEISKATASSPTQQDGR.G	Oxidation: 3

Detailed Protein Report

Protein 320: **PREDICTED: p21-activated protein kinase-interacting protein 1 isoform X2 [Homo sapiens]**

**Accession:**	gi|578811766	**Score:**	24.4
**Database:**	refseq_human(refseq_human_20140103.fasta)	**MW [kDa]:**	41.4
**Database Date:**	2015-11-30	**pI:**	10.1
**Modification(s):**	Carbamidomethyl	**Sequence Coverage [%]:**	7.3
		**No. of unique Peptides:**	1

10	20	30	40	50	60	70	80
MLEQQWTLVA	DFTHHAHTAS	LSAVAVNSRF	VVTGSKDETI	HIYDMKKKIE	HGALVHHSGT	ITCLKFYGNR	HLISGAEDGL
**90**	**100**	**110**	**120**	**130**	**140**	**150**	**160**
ICIWDAKKWE	CLKSIKAHKG	QVTFLSIHPS	GKLALSVGTD	KTLRTWNLVE	GRSAFIKNIK	QNAHIVEWSP	RGEQYVVIIQ
**170**	**180**	**190**	**200**	**210**	**220**	**230**	**240**
NKIDIYQLDT	ASISGTITNE	KRISSVKFLS	ESVLAVAGDE	EVIRFFDCDS	LVCLCEFKAH	ENRVKDMFSF	EIPEHHVIVS
**250**	**260**	**270**	**280**	**290**	**300**	**310**	**320**
ASSDGFIKMW	KLKQDKKVPP	SLLCEINTNA	RLTCLGVWLD	KVADMKESLP	PAAEPSPVSK	EQSKIGKKEP	GDTVHKEEKR
**330**	**340**	**350**	**360**	**370**			
SKPNTKKRGL	TGDSKKATKE	SGLISTKKRK	MVEMLEKKRK	KKKIKTMQ			

Cmpd.	No. of Cmpds.	m/z meas.	Δ m/z [ppm]	z	Rt [min]	Score	P	Range	Sequence	Modification
2184	1	778.2730	-90.09	3	55.8	13.6	1	205-223	R.FFDCDSLVCLCEFKAHENR.V	Carbamidomethyl: 11

Detailed Protein Report

Protein 321: uncharacterized aarF domain-containing protein kinase 2 [Homo sapiens]

Accession: gi|32261307
Database: refseq_human(refseq_human_20140103.fasta)
Database Date: 2015-11-30
Modification(s): Oxidation

Score: 24.3
MW [kDa]: 68.9
pI: 10.0
Sequence Coverage [%]: 4.8
No. of unique Peptides: 2

10	20	30	40	50	60	70	80
MVAPWRVSVR	VCLSHLRCFE	LRQGLSLLRP	SECPRDARLC	WLLLGTLPKV	VSLCGDVGEG	APDVLSRRRV	RCSGAAGAGP
**90**	**100**	**110**	**120**	**130**	**140**	**150**	**160**
AESLPRAGPL	GGVFLHLRLW	LRAGALLVKF	FPLLLLYPLT	YLAPSVSTLW	LHLLLKATET	SGPTYIKLGQ	WASTRRDLFS
**170**	**180**	**190**	**200**	**210**	**220**	**230**	**240**
EAFCAQFSKL	HVRVTPHPWT	HTERFLRQAF	GDDWGSILSF	ENREPVGSGC	VAQVYKAYAN	TAFLETDSVQ	RLGRASCLPP
**250**	**260**	**270**	**280**	**290**	**300**	**310**	**320**
FSHTGAVGGL	RELFGYLGNG	RKPPENLADQ	SFLERLLLPK	ADLVGSNAGV	SRAQVPGHQP	EATNLISVAV	KVLHPGLLAQ
**330**	**340**	**350**	**360**	**370**	**380**	**390**	**400**
VHMDLLLMKI	GSRVLGVLPG	IKWLSLPEIV	EEFEKLMVQQ	IDLRYEAQNL	EHFQVNFRNV	KAVKFPTPLR	PFVTREVLVE
**410**	**420**	**430**	**440**	**450**	**460**	**470**	**480**
TYEESVPVSS	YQQAGIPVDL	KRKIARLGIN	MLLKMIFVDN	FVHADLHPGN	ILVQGANGLS	SSQEAQLQQA	DICDTLVVAV
**490**	**500**	**510**	**520**	**530**	**540**	**550**	**560**
PSSLCPLRLV	LLDAGIVAEL	QAPDLRNFRA	VFMAVVMGQG	QRVAELILHH	ARASECRDVE	GFKTEMAMLV	TQARKNTITL
**570**	**580**	**590**	**600**	**610**	**620**	**630**	
EKLHVSSLLS	SVFKLLMTHK	VKLESNFASI	VFAIMVLEGL	GRSLDPKLDI	LEAARPFLLT	GPVCPP	

Cmpd.	No. of Cmpds.	m/z meas.	Δ m/z [ppm]	z	Rt [min]	Score	P	Range	Sequence	Modification
2782	2	835.4333	6.80	2	63.2	13.6	0	235-251	R.ASCLPPFSHTGAVGGLR.E	
2880	1	713.3447	-10.93	2	64.7	10.8	0	510-522	R.AVFMAVVMGQGQR.V	Oxidation: 4, 8

Detailed Protein Report

Protein 322: **PREDICTED: exosome complex exonuclease RRP44 isoform X1 [Homo sapiens]**

Accession: gi|530402172
Database: refseq_human(refseq_human_20140103.fasta)
Database Date: 2015-11-30

Score: 24.3
MW [kDa]: 90.5
pI: 6.5
Sequence Coverage [%]: 3.4
No. of unique Peptides: 1

10	20	30	40	50	60	70	80
MSADNQLQVI	FITNDRRNKE	KAIEEGIPAF	TCEEYVKSLT	ANPELIDRLA	CLSEEGNEIE	SGKIIFSEHL	PLSKLQQGIK
**90**	**100**	**110**	**120**	**130**	**140**	**150**	**160**
SGTYLQGTFR	ASRENYLEAT	VWIHGDNEEN	KEIILQGLKH	LNRAVHEDIV	AVELLPKSQW	VAPSSVVLHD	EGQNEEDVEK
**170**	**180**	**190**	**200**	**210**	**220**	**230**	**240**
EEETERMLKT	AVSEKMLKPT	GRVVGIIKRN	WRPYCGMLSK	SDIKESRRHL	FTPADKRIPR	IRIETRQAST	LEGRRIIVAI
**250**	**260**	**270**	**280**	**290**	**300**	**310**	**320**
DGWPRNSRYP	NGHFVRNLGD	VGEKETETEV	LLLEHDVPHQ	PFSQAVLSFL	PKMPWSITEK	DMKNREDLRH	LCICSVDPPG
**330**	**340**	**350**	**360**	**370**	**380**	**390**	**400**
CTDIDDALHC	RELENGNLEV	GVHIADVSHF	IRPGNALDQE	SARRGTTVYL	CEKRIDMVPE	LLSSNLCSLK	CDVDRLAFSC
**410**	**420**	**430**	**440**	**450**	**460**	**470**	**480**
IWEMNHNAEI	LKTKFTKSVI	NSKASLTYAE	AQLRIDSANM	NDDITTSLRG	LNKLAKILKK	RRIEKGALTL	SSPEVRFHMD
**490**	**500**	**510**	**520**	**530**	**540**	**550**	**560**
SETHDPIDLQ	TKELRETNSM	VEEFMLLANI	SVAKKIHEEF	SEHALLRKHP	APPPSNYEIL	VKAARSRNLE	IKTDTAKSLA
**570**	**580**	**590**	**600**	**610**	**620**	**630**	**640**
ESLDQAESPT	FPYLNTLLRI	LATRCMMQAV	YFCSGMDNDF	HHYGLASPIY	THFTSPIRRY	ADVIVHRLLA	VAIGADCTYP
**650**	**660**	**670**	**680**	**690**	**700**	**710**	**720**
ELTDKHKLAD	ICKNLNFRHK	MAQYAQRASV	AFHTQLFFKS	KGIVSEEAYI	LFVRKNAIVV	LIPKYGLEGT	VFFEEKDKPN
**730**	**740**	**750**	**760**	**770**	**780**	**790**	**800**
PQLIYDDEIP	SLKIEDTVFH	VFDKVKVKIM	LDSSNLQHQK	IRMSLVEPQI	PGISIPTDTS	NMDLNGPKKK	KMKLGK

Cmpd.	No. of Cmpds.	m/z meas.	Δ m/z [ppm]	z	Rt [min]	Score	P	Range	Sequence	Modification
2612	1	588.6601	-226.00	2	60.9	13.0	2	301-309	K.DMKNREDLR.H	

Detailed Protein Report

Protein 323: fibrocystin isoform 2 precursor [Homo sapiens]

**Accession:**	gi|126131104	**Score:**	24.3
**Database:**	refseq_human(refseq_human_20140103.fasta)	**MW [kDa]:**	371.4
**Database Date:**	2015-11-30	**pI:**	5.9
**Modification(s):**	Carbamidomethyl, Oxidation	**Sequence Coverage [%]:**	0.9
		**No. of unique Peptides:**	2

Detailed Protein Report

10	20	30	40	50	60	70	80
MTAWLISLMS	IEVLLLAVRH	LSLHIEPEEG	SLAGGTWITV	IFDGLELGVL	YPNNGSQLEI	HLVNVNMVVP	ALRSVPCDVF
90	100	110	120	130	140	150	160
PVFLDLPVVT	CRTRSVLSEA	HEGLYFLEAY	FGGQLVSSPN	PGPRDSCTFK	FSKAQTPIVH	QVYPPSGVPG	KLIHVYGWII
170	180	190	200	210	220	230	240
TGRLETFDFD	AEYIDSPVIL	EAQGDKWVTP	CSLINRQMGS	CYPIQEDHGL	GTLQCHVEGD	YIGSQNVSFS	VFNKGKSMVH
250	260	270	280	290	300	310	320
KKAWLISAKQ	DLFLYQTHSE	ILSVFPETGS	LGGRTNITIT	GDFFDNSAQV	TIAGIPCDIR	HVSPRKIECT	TRAPGKDVRL
330	340	350	360	370	380	390	400
TTPQPGNRGL	LFEVGDAVEG	LELTEATPGY	RWQIVPNASS	PFGFWSQEGQ	PFRARLSGFF	VAPETNNYTF	WIQADSQASL
410	420	430	440	450	460	470	480
HFSWSEEPRT	KVKVASISVG	TADWFDSWEQ	NRDEGTWQQK	TPKLELLGGA	MYYLEAEHHG	IAPSRGMRIG	VQIHNTWLNP
490	500	510	520	530	540	550	560
DVVTTYLREK	HQIRVRAQRL	PEVQVLNVSG	RGNFFLTWDN	VSSQPIPANA	TAHLIQTTIE	ELLAVKCKLE	PLWSNILLRL
570	580	590	600	610	620	630	640
GFERGPEVSN	SDGDLTSGTE	PFCGRFSLRQ	PRHLVLTPPA	AQKGYRLDQY	THLCLAYKGH	MNKILKMIVS	FTIGFQNMVK
650	660	670	680	690	700	710	720
NTTCDWSLTR	TSPESWQFDC	TDLWETCVRC	FGDLQPPPAN	SPVLVHQINL	LPLAQETGLF	YVDEIIIADT	NVTVSQADSG
730	740	750	760	770	780	790	800
TARPGGNLVE	SVSVVGSPPV	YSVTSWLAGC	GTELPLITAR	SVPTEGTEEG	SGLVLVTTQR	RQRTSPPLGG	HFRIQLPNTV
810	820	830	840	850	860	870	880
ISDVPVQISA	HHLHQLLQNN	ADDFTSRYLN	ASDFTVKEDL	YTCYEHVWTL	SWSTQIGDLP	NFIRVSDENL	TGVNPAAATR
890	900	910	920	930	940	950	960
VVYDGGVFLG	PIFGDMLATA	NQHTQVVVRV	NDVPAHCPGS	CSFQYLQGST	PCVHSVWYSI	DGDINLMIYI	TGTGFSGDSQ
970	980	990	1000	1010	1020	1030	1040
FLQVTVNKTS	CKVIFSNQTN	VVCQTDLLPV	GMHRILMLVR	PSGLAISATG	EDLFLNVKPR	LDMVEPSRAA	DIGGLWATIR
1050	1060	1070	1080	1090	1100	1110	1120
GSSLEGVSLI	LFGSYSCAIN	VATSNSSRIQ	CKVPPRGKDG	RIVNVTVIRG	DYSAVLPRAF	TYVSSLNPVI	VTLSRNISNI
1130	1140	1150	1160	1170	1180	1190	1200
AGGETLVIGV	ARLMNYTDLD	VEVHVQDALA	PVHTQSAWGL	EVALPPLPAG	LHRISVSING	VSIHSQGVDL	HIQYLTEVFS
1210	1220	1230	1240	1250	1260	1270	1280
IEPCCGSLLG	GTILSISGIG	FSRDPALVWV	LVGNRSCDIV	NLTEASIWCE	TLPAPQIPDA	GAPTVPAAVE	VWAGNRFFAR
1290	1300	1310	1320	1330	1340	1350	1360
GPSPSLVGKG	FTFMYEAAAT	PVVTAMQGEI	TNSSLSLHVG	GSNLSNSVIL	LGNLNCDVET	QSFQGNVSLS	GCSIPLHSLE
1370	1380	1390	1400	1410	1420	1430	1440
AGIYPLQVRQ	KQMGFANMSV	VLQQFAVMPR	IMAIFPSQGS	ACGGTILTVR	GLLLNSRRRS	VRVDLSGPFT	CVILSLGDHT
1450	1460	1470	1480	1490	1500	1510	1520
ILCQVSLEGD	PLPGASFSLN	VTVLVNGLTS	ECQGNCTLFI	REEASPVMDA	LSTNTSGSLT	TVLIRGQRLA	TTADEPMVFV
1530	1540	1550	1560	1570	1580	1590	1600
DDQLPCNVTF	FNASHVVCQT	RDLAPGPHYL	SVFYTRNGYA	CSGNVSRHFY	IMPQVFHYFP	KNFSLHGGSL	LTIEGTGLRG
1610	1620	1630	1640	1650	1660	1670	1680
QNTTSVYIDQ	QTCLTVNIGA	ELIRCIVPTG	NGSVALEIEV	DGLWYHIGVI	GYNKAFTPEL	ISISQSDDIL	TFAVAQISGA
1690	1700	1710	1720	1730	1740	1750	1760
ANIDIFIGMS	PCVGVSGNHT	VLQCVVPSLP	AGEYHVRGYD	CIRGWASSAL	VFTSRVIITA	VTENFGCLGG	RLVHVFGAGF
1770	1780	1790	1800	1810	1820	1830	1840
SPGNVSAAVC	GAPCRVLANA	TVSAFSCLVL	PLDVSLAFLC	GLKREEDSCE	AARHTYVQCD	LTVAMATEQL	LESWPYLYIC
1850	1860	1870	1880	1890	1900	1910	1920
EESSQCLFVP	DHWAESMFPS	FSGLFISPKL	ERDEVLIYNS	SCNITMETEA	EMECETPNQP	ITVKITEIRK	RWGQNTQGNF
1930	1940	1950	1960	1970	1980	1990	2000
SLQFCRRWSR	THSWFPERLP	QDGDNVTVEN	GQLLLLDTNT	SILNLLHIKG	GKLIFMAPGP	IELRAHAILV	SDGGELRIGS
2010	2020	2030	2040	2050	2060	2070	2080
EDKPFQGRAQ	ITLYGSSYST	PFFPYGVKFL	AVRNGTLSLH	GSLPEVIVTC	LRATAHALDT	VLALEDAVDW	NPGDEVVIIS
2090	2100	2110	2120	2130	2140	2150	2160
GTGVKGAKPM	EEIVTVETVQ	DTDLYLKSPL	RYSHNFTENW	VAGEHHILKA	TVALLSRSIT	IQGNLTNERE	KLLVSCQEAN
2170	2180	2190	2200	2210	2220	2230	2240

Detailed Protein Report

Cmpd.	No. of Cmpds.	m/z meas.	Δ m/z [ppm]	z	Rt [min]	Score	P	Range	Sequence	Modification
2756	1	1023.5380	40.37	2	63.0	11.3	1	1718-1735	R.GYDCIRGWASSALVFTSR.V	Carbamidomethyl: 4
2847	1	686.8886	50.79	2	64.3	13.0	1	3263-3274	K.VRNDHSISGIMK.L	Oxidation: 11

Detailed Protein Report

Protein 324: hematopoietic lineage cell-specific protein [Homo sapiens]

Accession: gi|167234422
Database: refseq_human(refseq_human_20140103.fasta)
Database Date: 2015-11-30

Score: 24.3
MW [kDa]: 54.0
pI: 4.6
Sequence Coverage [%]: 8.0
No. of unique Peptides: 2

10	20	30	40	50	60	70	80
MWKSVVGHDV	SVSVETQGDD	WDTDPDFVND	ISEKEQRWGA	KTIEGSGRTE	HINIHQLRNK	VSEEHDVLRK	KEMESGPKAS
**90**	**100**	**110**	**120**	**130**	**140**	**150**	**160**
HGYGGRFGVE	RDRMDKSAVG	HEYVAEVEKH	SSQTDAAKGF	GGKYGVERDR	ADKSAVGFDY	KGEVEKHTSQ	KDYSRGFGGR
**170**	**180**	**190**	**200**	**210**	**220**	**230**	**240**
YGVEKDKWDK	AALGYDYKGE	TEKHESQRDY	AKGFGGQYGI	QKDRVDKSAV	GFNEMEAPTT	AYKKTTPIEA	ASSGTRGLKA
**250**	**260**	**270**	**280**	**290**	**300**	**310**	**320**
KFESMAEEKR	KREEEEKAQQ	VARRQQERKA	VTKRSPEAPQ	PVIAMEEPAV	PAPLPKKISS	EAWPPVGTPP	SSESEPVRTS
**330**	**340**	**350**	**360**	**370**	**380**	**390**	**400**
REHPVPLLPI	RQTLPEDNEE	PPALPPRTLE	GLQVEEEPVY	EAEPEPEPEP	EPEPENDYED	VEEMDRHEQE	DEPEGDYEEV
**410**	**420**	**430**	**440**	**450**	**460**	**470**	**480**
LEPEDSSFSS	ALAGSSGCPA	GAGAGAVALG	ISAVALYDYQ	GEGSDELSFD	PDDVITDIEM	VDEGWWRGRC	HGHFGLFPAN
**490**							
YVKLLE							

Cmpd.	No. of Cmpds.	m/z meas.	Δ m/z [ppm]	z	Rt [min]	Score	P	Range	Sequence	Modification
2470	1	1131.1745	0.11	3	59.2	13.4	0	4-34	K.SVVGHDVSVSVETQGDDWDTDPDFVNDISEK.E	
2838	1	485.7928	164.40	2	64.0	10.8	0	242-249	K.FESMAEEK.R	

Detailed Protein Report

Protein 325: **PREDICTED: latent-transforming growth factor beta-binding protein 1 isoform X5 [Homo sapiens]**

**Accession:**	gi|530367595	**Score:**	24.3
**Database:**	refseq_human(refseq_human_20140103.fasta)	**MW [kDa]:**	134.0
**Database Date:**	2015-11-30	**pI:**	9.1
**Modification(s):**	Carbamidomethyl	**Sequence Coverage [%]:**	2.9
		**No. of unique Peptides:**	1

10	20	30	40	50	60	70	80
MAGAWLRWGL	LLWAGLLASS	AHGRLRRITY	VVHPGPGLAA	GALPLSGPPR	SRTFNVALNA	RYSRSSAAAG	APSRASPGVP
90	100	110	120	130	140	150	160
SERTRRTSKP	GGAALQGLRP	PPPPPPEPAR	PAVPGGQLHP	NPGGHPAAAP	FTKQGRQVVR	SKVPQETQSG	GGSRLQVHQK
170	180	190	200	210	220	230	240
QQLQGVNVCG	GRCCHGWSKA	PGSQRCTKPS	CVPPCQNGGM	CLRPQLCVCK	PGTKGKACET	IAAQDTSSPV	FGGQSPGAAS
250	260	270	280	290	300	310	320
SWGPPEQAAK	HTSSKKADTL	PRVSPVAQMT	LTLKPKPSVG	LPQQIHSQVT	PLSSQSVVIH	HGQTQEYVLK	PKYFPAQKGI
330	340	350	360	370	380	390	400
SGEQSTEGSF	PLRYVQDQVA	APFQLSNHTG	RIKVVFTPSI	CKVTCTKGSC	QNSCEKGNTT	TLISENGHAA	DTLTATNFRV
410	420	430	440	450	460	470	480
VICHLPCMNG	GQCSSRDKCQ	CPPNFTGKLC	QIPVHGASVP	KLYQHSQQPG	KALGTHVIHS	THTLPLTVTS	QQGVKVKFPP
490	500	510	520	530	540	550	560
NIVNIHVKHP	PEASVQIHQV	SRIDGPTGQK	TKEAQPGQSQ	VSYQGLPVQK	TQTIHSTYSH	QQVIPHVYPV	AAKTQLGRCF
570	580	590	600	610	620	630	640
QETIGSQCGK	ALPGLSKQED	CCGTVGTSWG	FNKCQKCPKK	PSYHGYNQMM	ECLPGYKRVN	NTFCQDINEC	QLQGVCPNGE
650	660	670	680	690	700	710	720
CLNTMGSYRC	TCKIGFGPDP	TFSSCVPDPP	VISEEKGPCY	RLVSSGRQCM	HPLSVHLTKQ	LCCCSVGKAW	GPHCEKCPLP
730	740	750	760	770	780	790	800
GTAAFKEICP	GGMGYTVSGV	HRRRPIHHHV	GKGPVFVKPK	NTQPVAKSTH	PPPLPAKEEP	VEALTFSREH	GPGVAEPEVA
810	820	830	840	850	860	870	880
TAPPEKEIPS	LDQEKTKLEP	GQPQLSPGIS	TIHLHPQFPV	VIEKTSPPVP	VEVAPEASTS	SASQVIAPTQ	VTEINECTVN
890	900	910	920	930	940	950	960
PDICGAGHCI	NLPVRYTCIC	YEGYRFSEQQ	RKCVDIDECT	QVQHLCSQGR	CENTEGSFLC	ICPAGFMASE	EGTNCIDVDE
970	980	990	1000	1010	1020	1030	1040
CLRPDVCGEG	HCVNTVGAFR	CEYCDSGYRM	TQRGRCEDID	ECLNPSTCPD	EQCVNSPGSY	QCVPCTEGFR	GWNGQCLDVD
1050	1060	1070	1080	1090	1100	1110	1120
ECLEPNVCAN	GDCSNLEGSY	MCSCHKGYTR	TPDHKHCRDI	DECQQGNLCV	NGQCKNTEGS	FRCTCGQGYQ	LSAAKDQCED
1130	1140	1150	1160	1170	1180	1190	1200
IDECQHRHLC	AHGQCRNTEG	SFQCVCDQGY	RASGLGDHCE	DINECLEDKS	VCQRGDCINT	AGSYDCTCPD	GFQLDDNKTC
1210	1220	1230	1240	1250			
QDINECEHPG	LCGPQGECLN	TEGSFHCVCQ	QGFSISADGR	TCEDVNE			

Cmpd.	No. of Cmpds.	m/z meas.	Δ m/z [ppm]	z	Rt [min]	Score	P	Range	Sequence	Modification
1842	1	1074.5505	92.79	2	51.7	12.2	1	578-596	K.QEDCCGTVGTSWGFNKCQK.C	Carbamidomethyl: 4

Detailed Protein Report

Protein 326: **PREDICTED: pleckstrin homology domain-containing family B member 2 isoform X1 [Homo sapiens]**

**Accession:**	gi|578804522	**Score:**	24.2
**Database:**	refseq_human(refseq_human_20140103.fasta)	**MW [kDa]:**	16.1
**Database Date:**	2015-11-30	**pI:**	10.3
**Modification(s):**	Carbamidomethyl, Oxidation	**Sequence Coverage [%]:**	23.9
		**No. of unique Peptides:**	1

10	20	30	40	50	60	70	80
MAFVKSGWLL	RQSTILKRWK	KNWFDLWSDG	HLIYYDDQTR	QNIEDKVHMP	MDCINIRTGQ	ECRDTQPPDG	KSKDCMLQIV
**90**	**100**	**110**	**120**	**130**	**140**		
CRDGKTISLC	AESTDDCFRL	MAMGHTVVRT	RQELKLSTLR	MGRRMPCPTS	THMQRPSS		

Cmpd.	No. of Cmpds.	m/z meas.	Δ m/z [ppm]	z	Rt [min]	Score	P	Range	Sequence	Modification
2213	1	1103.9435	-65.96	2	56.2	14.1	2	64-82	R.DTQPPDGKSKDCMLQIVCR.D	Carbamidomethyl: 12; Oxidation: 13

Detailed Protein Report

Protein 327: **spermine synthase isoform 2 [Homo sapiens]**

**Accession:**	gi|386643030	**Score:**	24.2
**Database:**	refseq_human(refseq_human_20140103.fasta)	**MW [kDa]:**	35.3
**Database Date:**	2015-11-30	**pI:**	4.7
**Modification(s):**	Oxidation	**Sequence Coverage [%]:**	9.3
		**No. of unique Peptides:**	2

10	20	30	40	50	60	70	80
MAAARHSTLD	FMLGAKADGE	TILKGLQSIF	QEQGMAESVH	TWQDHGYLAT	YTNKNGRLPP	IVRGGAIDRY	WPTADGRLVE
**90**	**100**	**110**	**120**	**130**	**140**	**150**	**160**
YDIDEVVYDE	DSPYQNIKIL	HSKQFGNILI	LSGDVNLAES	DLAYTRAIMG	SGKEDYTGKD	VLILGGGDGG	ILCEIVKLKP
**170**	**180**	**190**	**200**	**210**	**220**	**230**	**240**
KMVTMVEIDQ	MVIDGCKKYM	RKTCGDVLDN	LKGDCYQVLI	EDCIPVLKRY	AKEGREFDYV	INDLTAVPIS	TSPEEDSTWE
**250**	**260**	**270**	**280**	**290**	**300**	**310**	**320**
FLRLILDLSM	KVLKQDGKYF	TQGNCVNLTE	ALSLYEEQLG	RLYCPVEFSK	EIVCVPSYLE	LWVFYTVWKK	AKP

Cmpd.	No. of Cmpds.	m/z meas.	Δ m/z [ppm]	z	Rt [min]	Score	P	Range	Sequence	Modification
143	1	796.0835	151.41	2	30.7	10.9	2	55-69	K.NGRLPPIVRGGAIDR.Y	
2926	5	557.9598	27.25	3	65.3	13.3	2	179-192	K.YMRKTCGDVLDNLK.G	Oxidation: 2

Detailed Protein Report

Protein 328: mis18-binding protein 1 [Homo sapiens]

**Accession:**	gi|42415492	**Score:**	24.2
**Database:**	refseq_human(refseq_human_20140103.fasta)	**MW [kDa]:**	129.0
**Database Date:**	2015-11-30	**pI:**	9.9
**Modification(s):**	Oxidation	**Sequence Coverage [%]:**	3.3
		**No. of unique Peptides:**	2

Alias proteins:

Accession	Name	Description
gi|530404134	refseq_human (refseq_human_20140103.fasta)	PREDICTED: mis18-binding protein 1 isoform X1 [Homo sapiens]

10	20	30	40	50	60	70	80
MIATPLKHSR	IYLPPEASSQ	RRNLPMDAIF	FDSIPSGTLT	PVKDLVKYQN	SSLKLNDHKK	NQFLKMTTFN	NKNIFQSTML
**90**	**100**	**110**	**120**	**130**	**140**	**150**	**160**
TEATTSNSSL	DISAIKPNKD	GLKNKANYES	PGKIFLRMKE	KVLRDKQEQP	SRNSSLLEPQ	KSGNNETFTP	NRVEKKKLQH
**170**	**180**	**190**	**200**	**210**	**220**	**230**	**240**
TYLCEEKENN	KSFQSDDSSL	RASVQGVPLE	SSNNDIFLPV	KQKIQCQQEK	KAPLHNLTYE	LPTLNQEQEN	FLAVEARNKT
**250**	**260**	**270**	**280**	**290**	**300**	**310**	**320**
LTRAQLAKQI	FHSKESIVAT	TKSKKDTFVL	ESVDSADEQF	QNTNAETLST	NCIPIKNGSL	LMVSDSERTT	EGTSQQKVKE
**330**	**340**	**350**	**360**	**370**	**380**	**390**	**400**
GNGKTVPGET	GLPGSMKDTC	KIVLATPRLH	ITIPRRSKRN	ISKLSPPRIF	QTVTNGLKKN	QVVQLQEWMI	KSINNNTAIC
**410**	**420**	**430**	**440**	**450**	**460**	**470**	**480**
VEGKLIDVTN	IYWHSNVIIE	RIEHNKLRTI	SGNVYILKGM	IDQISMKEAG	YPNYLIRKFM	FGFPENWKEH	IDNFLEQLRA
**490**	**500**	**510**	**520**	**530**	**540**	**550**	**560**
GEKNREKTKQ	KQKTGRSVRD	IRKSMKNDAR	ENQTDTAQRA	TTTYDFDCDN	LELKSNKHSE	SPGATELNMC	HSNCQNKPTL
**570**	**580**	**590**	**600**	**610**	**620**	**630**	**640**
RFPDDQVNNT	IQNGGGDDLS	NQELIGKKEY	KMSSKKLKIG	ERTNERIIKS	QKQETTEELD	VSIDILTSRE	QFFSDEERKY
**650**	**660**	**670**	**680**	**690**	**700**	**710**	**720**
MAINQKKAYI	LVTPLKSRKV	IEQRCMRYNL	SAGTIKAVTD	FVIPECQKKS	PISKSMGTLE	NTFEGHKSKN	KEDCDERDLL
**730**	**740**	**750**	**760**	**770**	**780**	**790**	**800**
TVNRKIKISN	LEKEQMLTSD	FKKNTRLLPK	LKKIENQVAM	SFYKHQSSPD	LSSEESETEK	EIKRKAEVKK	TKAGNTKEAV
**810**	**820**	**830**	**840**	**850**	**860**	**870**	**880**
VHLRKSTRNT	SNIPVILEPE	TEESENEFYI	KQKKARPSVK	ETLQKSGVRK	EFPITEAVGS	DKTNRHPLEC	LPGLIQDKEW
**890**	**900**	**910**	**920**	**930**	**940**	**950**	**960**
NEKELQKLHC	AFASLPKHKP	GFWSEVAAAV	GSRSPEECQR	KYMENPRGKG	SQKHVTKKKP	ANSKGQNGKR	GDADQKQTIK
**970**	**980**	**990**	**1000**	**1010**	**1020**	**1030**	**1040**
ITAKVGTLKR	KQQMREFLEQ	LPKDDHDDFF	STTPLQHQRI	LLPSFQDSED	DDDILPNMDK	NPTTPSSVIF	PLVKTPQCQH
**1050**	**1060**	**1070**	**1080**	**1090**	**1100**	**1110**	**1120**
VSPGMLGSIN	RNDCDKYVFR	MQKYHKSNGG	IVWGNIKKKL	VETDFSTPTP	RRKTPFNTDL	GENSGIGKLF	TNAVESLDEE
**1130**	**1140**						
EKDYYFSNSD	SA						

Cmpd.	No. of Cmpds.	m/z meas.	Δ m/z [ppm]	z	Rt [min]	Score	P	Range	Sequence	Modification
1865	1	1057.5367	-22.09	2	52.0	12.7	0	182-201	R.ASVQGVPLESSNNDIFLPVK.Q	
2837	1	868.9237	11.97	2	64.0	11.5	1	325-341	K.TVPGETGLPGSMKDTCK.I	Oxidation: 12

Detailed Protein Report

Protein 329: AT-hook DNA-binding motif-containing protein 1 [Homo sapiens]

**Accession:**	gi|71274144	**Score:**	24.2
**Database:**	refseq_human(refseq_human_20140103.fasta)	**MW [kDa]:**	168.2
**Database Date:**	2015-11-30	**pI:**	10.0
**Modification(s):**	Oxidation	**Sequence Coverage [%]:**	2.2
		**No. of unique Peptides:**	1

Alias proteins:

Accession	Name	Description
gi|530361060	refseq_human (refseq_human_20140103.fasta)	PREDICTED: AT-hook DNA-binding motif-containing protein 1 isoform X6 [Homo sapiens]
gi|530361058	refseq_human (refseq_human_20140103.fasta)	PREDICTED: AT-hook DNA-binding motif-containing protein 1 isoform X5 [Homo sapiens]
gi|530361056	refseq_human (refseq_human_20140103.fasta)	PREDICTED: AT-hook DNA-binding motif-containing protein 1 isoform X4 [Homo sapiens]
gi|530361054	refseq_human (refseq_human_20140103.fasta)	PREDICTED: AT-hook DNA-binding motif-containing protein 1 isoform X3 [Homo sapiens]
gi|530361052	refseq_human (refseq_human_20140103.fasta)	PREDICTED: AT-hook DNA-binding motif-containing protein 1 isoform X2 [Homo sapiens]
gi|530361050	refseq_human (refseq_human_20140103.fasta)	PREDICTED: AT-hook DNA-binding motif-containing protein 1 isoform X1 [Homo sapiens]

Detailed Protein Report

10	20	30	40	50	60	70	80
MRVKPQGLVV	TSSAVCSSPD	YLREPKYYPG	GPPTPRPLLP	TRPPASPPDK	AFSTHAFSEN	PRPPPRRDPS	TRRPPVLAKG
90	100	110	120	130	140	150	160
DDPLPPRAAR	PVSQARCPTP	VGDGSSSRRC	WDNGRVNLRP	VVQLIDIMKD	LTRLSQDLQH	SGVHLDCGGL	RLSRPPAPPP
170	180	190	200	210	220	230	240
GDLQYSFFSS	PSLANSIRSP	EERATPHAKS	ERPSHPLYEP	EPEPRDSPQP	GQGHSPGATA	AATGLPPEPE	PDSTDYSELA
250	260	270	280	290	300	310	320
DADILSELAS	LTCPEAQLLE	AQALEPPSPE	PEPQLLDPQP	RFLDPQALEP	LGEALELPPL	QPLADPLGLP	GLALQALDTL
330	340	350	360	370	380	390	400
PDSLESQLLD	PQALDPLPKL	LDVPGRRLEP	QQPLGHCPLA	EPLRLDLCSP	HGPPGPEGHP	KYALRRTDRP	KILCRRRKAG
410	420	430	440	450	460	470	480
RGRKADAGPE	GRLLPLPMPT	GLVAALAEPP	PPPPPPPPAL	PGPGPVSVPE	LKPESSQTPV	VSTRKGKCRG	VRRMVVKMAK
490	500	510	520	530	540	550	560
IPVSLGRRNK	TTYKVSSLSS	SLSVEGKELG	LRVSAEPTPL	LKMKNNGRNV	VVVFPPGEMP	IILKRKRGRP	PKNLLLGPGK
570	580	590	600	610	620	630	640
PKEPAVVAAE	AATVAAATMA	MPEVKKRRRR	KQKLASPQPS	YAADANDSKA	EYSDVLAKLA	FLNRQSQCAG	RCSPPRCWTP
650	660	670	680	690	700	710	720
SEPESVHQAP	DTQSISHFLH	RVQGFRRRGG	KAGGFGGRGG	GHAAKSARCS	FSDFFEGIGK	KKKVVAVAAA	GVGGPGLTEL
730	740	750	760	770	780	790	800
GHPRKRGRGE	VDAVTGKPKR	KRRSRKNGTL	FPEQVPSGPG	FGEAGAEWAG	DKGGGWAPHH	GHPGGQAGRN	CGFQGTEARA
810	820	830	840	850	860	870	880
FASTGLESGA	SGRGSYYSTG	APSGQTELSQ	ERQNLFTGYF	RSLLDSDDSS	DLLDFALSAS	RPESRKASGT	YAGPPTSALP
890	900	910	920	930	940	950	960
AQRGLATFPS	RGAKASPVAV	GSSGAGADPS	FQPVLSARQT	FPPGRAASYG	LTPAASDCRA	AETFPKLVPP	PSAMARSPTT
970	980	990	1000	1010	1020	1030	1040
HPPANTYLPQ	YGGYGAGQSV	FAPTKPFTGQ	DCANSKDCSF	AYGSGNSLPA	SPSSAHSAGY	APPPTGGPCL	PPSKASFFSS
1050	1060	1070	1080	1090	1100	1110	1120
SEGAPFSGSA	PTPLRCDSRA	STVSPGGYMV	PKGTTASATS	AASAASSSSS	SFQPSPENCR	QFAGASQWPF	RQGYGGLDWA
1130	1140	1150	1160	1170	1180	1190	1200
SEAFSQLYNP	SFDCHVSEPN	VILDISNYTP	QKVKQQTAVS	ETFSESSSDS	TQFNQPVGGG	GFRRANSEAS	SSEGQSSLSS
1210	1220	1230	1240	1250	1260	1270	1280
LEKLMMDWNE	ASSAPGYNWN	QSVLFQSSSK	PGRGRRKKVD	LFEASHLGFP	TSASAAASGY	PSKRSTGPRQ	PRGGRGGGAC
1290	1300	1310	1320	1330	1340	1350	1360
SAKKERGGAA	AKAKFIPKPQ	PVNPLFQDSP	DLGLDYYSGD	SSMSPLPSQS	RAFGVGERDP	CDFIGPYSMN	PSTPSDGTFG
1370	1380	1390	1400	1410	1420	1430	1440
QGFHCDSPSL	GAPELDGKHF	PPLAHPPTVF	DAGLQKAYSP	TCSPTLGFKE	ELRPPPTKLA	ACEPLKHGLQ	GASLGHAAAA
1450	1460	1470	1480	1490	1500	1510	1520
QAHLSCRDLP	LGQPHYDSPS	CKGTAYWYPP	GSAARSPPYE	GKVGTGLLAD	FLGRTEAACL	SAPHLASPPA	TPKADKEPLE
1530	1540	1550	1560	1570	1580	1590	1600
MARPPGPPRG	PAAAAAGYGC	PLLSDLTLSP	VPRDSLLPLQ	DTAYRYPGFM	PQAHPGLGGG	PKSGFLGPMA	EPHPEDTFTV
1610							
TSL							

Cmpd.	No. of Cmpds.	m/z meas.	Δ m/z [ppm]	z	Rt [min]	Score	P	Range	Sequence	Modification
2595	1	665.2784	-146.69	2	60.7	10.1	1	513-524	R.VSAEPTPLLKMK.N	Oxidation: 11

Detailed Protein Report

Protein 330: zinc finger protein 568 isoform 3 [Homo sapiens]

Accession: gi|325651958
Database: refseq_human(refseq_human_20140103.fasta)
Database Date: 2015-11-30

Score: 24.1
MW [kDa]: 67.0
pI: 10.0
Sequence Coverage [%]: 1.6
No. of unique Peptides: 1

Alias proteins:

Accession	Name	Description
gi|325651960	refseq_human (refseq_human_20140103.fasta)	zinc finger protein 568 isoform 3 [Homo sapiens]

10	20	30	40	50	60	70	80
MKPAQRNLYR	DVMLENYSNL	VTVGCQVTKP	DVIFKLEQEE	EPWVMEEEMF	GRHCPEVWEV	DEQIKKQQET	LVRKVTSISK
**90**	**100**	**110**	**120**	**130**	**140**	**150**	**160**
KILIKEKVIE	CKKVAKIFPL	SSDIVTSRQS	FYDCDSLDKG	LEHNLDLLRY	EKGCVREKQS	NEFGKPFYHC	ASYVVTPFKC
**170**	**180**	**190**	**200**	**210**	**220**	**230**	**240**
NQCGQDFSHK	FDLIRHERIH	AGEKPYECKE	CGKAFSRKEN	LITHQKIHTG	EKPYKCNECG	KAFIQMSNLI	RHHRIHTGEK
**250**	**260**	**270**	**280**	**290**	**300**	**310**	**320**
PYACKDCWKA	FSQKSNLIEH	ERIHTGEKPY	ECKECGKSFS	QKQNLIEHEK	IHTGEKPYAC	NECGRAFSRM	SSVTLHMRSH
**330**	**340**	**350**	**360**	**370**	**380**	**390**	**400**
TGEKPYKCNK	CGKAFSQCSV	FIIHMRSHTG	EKPYVCSECG	KAFSQSSSLT	VHMRNHTAEK	PYECKECGKA	FSRKENLITH
**410**	**420**	**430**	**440**	**450**	**460**	**470**	**480**
QKIHTGEKPY	ECSECGKAFI	QMSNLIRHQR	IHTGEKPYAC	TVCGKAFSQK	SNLTEHEKIH	TGEKPYHCNQ	CGKAFSQRQN
**490**	**500**	**510**	**520**	**530**	**540**	**550**	**560**
LLEHEKIHTG	EKPFKCNECG	KAFSRISSLT	LHVRSHTGEK	PYECNKCGKA	FSQCSLLIIH	MRSHTGEKPF	ECNECGKAFS
**570**	**580**	**590**					
QRASLSIHKR	GHTGERHQVY						

Cmpd.	No. of Cmpds.	m/z meas.	Δ m/z [ppm]	z	Rt [min]	Score	P	Range	Sequence	Modification
858	1	501.2115	-157.63	2	39.1	12.8	1	66-73	K.KQQETLVR.K	

Detailed Protein Report

Protein 331: ribonucleoside-diphosphate reductase subunit M2 isoform 1 [Homo sapiens]

**Accession:**	gi|260064013	**Score:**	24.0
**Database:**	refseq_human(refseq_human_20140103.fasta)	**MW [kDa]:**	51.1
**Database Date:**	2015-11-30	**pI:**	6.1
**Modification(s):**	Oxidation	**Sequence Coverage [%]:**	2.9
		**No. of unique Peptides:**	2

10	20	30	40	50	60	70	80
MGRVGGMAQP	MGRAGAPKPM	GRAGSARRGR	FKGCWSEGSP	VHPVPAVLSW	LLALLRCAST	MLSLRVPLAP	ITDPQQLQLS
**90**	**100**	**110**	**120**	**130**	**140**	**150**	**160**
PLKGLSLVDK	ENTPPALSGT	RVLASKTARR	IFQEPTEPKT	KAAAPGVEDE	PLLRENPRRF	VIFPIEYHDI	WQMYKKAEAS
**170**	**180**	**190**	**200**	**210**	**220**	**230**	**240**
FWTAEEVDLS	KDIQHWESLK	PEERYFISHV	LAFFAASDGI	VNENLVERFS	QEVQITEARC	FYGFQIAMEN	IHSEMYSLLI
**250**	**260**	**270**	**280**	**290**	**300**	**310**	**320**
DTYIKDPKER	EFLFNAIETM	PCVKKKADWA	LRWIGDKEAT	YGERVVAFAA	VEGIFFSGSF	ASIFWLKKRG	LMPGLTFSNE
**330**	**340**	**350**	**360**	**370**	**380**	**390**	**400**
LISRDEGLHC	DFACLMFKHL	VHKPSEERVR	EIIINAVRIE	QEFLTEALPV	KLIGMNCTLM	KQYIEFVADR	LMLELGFSKV
**410**	**420**	**430**	**440**	**450**			
FRVENPFDFM	ENISLEGKTN	FFEKRVGEYQ	RMGVMSSPTE	NSFTLDADF			

Cmpd.	No. of Cmpds.	m/z meas.	Δ m/z [ppm]	z	Rt [min]	Score	P	Range	Sequence	Modification
517	8	690.2024	-171.27	2	34.9	11.7	1	1-13	-.MGRVGGMAQPMGR.A	Oxidation: 7, 11
653	5	690.4099	129.21	2	37.0	12.3	1	1-13	-.MGRVGGMAQPMGR.A	Oxidation: 1, 11

Detailed Protein Report

Protein 332: protein FAM216B [Homo sapiens]

**Accession:**	gi|32698855	**Score:**	24.0
**Database:**	refseq_human(refseq_human_20140103.fasta)	**MW [kDa]:**	16.4
**Database Date:**	2015-11-30	**pI:**	12.1
		**Sequence Coverage [%]:**	14.4
		**No. of unique Peptides:**	1

Alias proteins:

Accession	Name	Description
gi|530402103	refseq_human (refseq_human_20140103.fasta)	PREDICTED: protein FAM216B isoform X1 [Homo sapiens]

```
        10         20         30         40         50         60         70         80
MGQNWKRQQK LWNVPQLPFI RVPPSIYDTS LLKALNQGQQ RYFYSIMRIY NSRPQWEALQ TRYIHSLQHQ QLLGYITQRE
        90        100        110        120        130        140
ALSYALVLRD STKRASAKVA PQRTIPRKTS AMTRRCPSVL PVSVVLPRAQ SKRRQVLRN
```

Cmpd.	No. of Cmpds.	m/z meas.	Δ m/z [ppm]	z	Rt [min]	Score	P	Range	Sequence	Modification
421	1	666.9204	69.47	2	34.3	10.7	0	22-33	R.VPPSIYDTSLLK.A	

Detailed Protein Report

Protein 333: PREDICTED: serine/threonine-protein kinase MRCK alpha isoform X7 [Homo sapiens]

**Accession:**	gi|530366603	**Score:**	24.0
**Database:**	refseq_human(refseq_human_20140103.fasta)	**MW [kDa]:**	198.5
**Database Date:**	2015-11-30	**pI:**	5.8
		**Sequence Coverage [%]:**	2.1
		**No. of unique Peptides:**	1

Quantitation

WD:WU	**Median:** 0.47	**CV:** 0.00 %	**No. of Peptides:** 1

10	20	30	40	50	60	70	80
MSGEVRLRQL	EQFILDGPAQ	TNGQCFSVET	LLDILICLYD	ECNNSPLRRE	KNILEYLEWA	KPFTSKVKQM	RLHREDFEIL
90	100	110	120	130	140	150	160
KVIGRGAFGE	VAVVKLKNAD	KVFAMKILNK	WEMLKRAETA	CFREERDVLV	NGDNKWITTL	HYAFQDDNNL	YLVMDYYVGG
170	180	190	200	210	220	230	240
DLLTLLSKFE	DRLPEDMARF	YLAEMVIAID	SVHQLHYVHR	DIKPDNILMD	MNGHIRLADF	GSCLKLMEDG	TVQSSVAVGT
250	260	270	280	290	300	310	320
PDYISPEILQ	AMEDGKGRYG	PECDWWSLGV	CMYEMLYGET	PFYAESLVET	YGKIMNHKER	FQFPAQVTDV	SENAKDLIRR
330	340	350	360	370	380	390	400
LICSREHRLG	QNGIEDFKKH	PFFSGIDWDN	IRNCEAPYIP	EVSSPTDTSN	FDVDDDCLKN	SETMPPPTHT	AFSGHHLPFV
410	420	430	440	450	460	470	480
GFTYTSSCVL	SDRSCLRVTA	GPTSLDLDVN	VQRTLDNNLA	TEAYERRIKR	LEQEKLELSR	KLQESTQTVQ	ALQYSTVDGP
490	500	510	520	530	540	550	560
LTASKDLEIK	NLKEEIEKLR	KQVTESSHLE	QQLEEANAVR	QELDDAFRQI	KAYEKQIKTL	QQEREDLNKL	EVHTEALAAE
570	580	590	600	610	620	630	640
ASKDRKLREQ	SEHYSKQLEN	ELEGLKQKQI	SYSPGVCSIE	HQQEITKLKT	DLEKKSIFYE	EELSKREGIH	ANEIKNLKKE
650	660	670	680	690	700	710	720
LHDSEGQQLA	LNKEIMILKD	KLEKTRRESQ	SEREEFESEF	KQQYEREKVL	LTEENKKLTS	ELDKLTTLYE	NLSIHNQQLE
730	740	750	760	770	780	790	800
EEVKDLADKK	ESVAHWEAQI	TEIIQWVSDE	KDARGYLQAL	ASKMTEELEA	LRNSSLGTRA	TDMPWKMRRF	AKLDMSARLE
810	820	830	840	850	860	870	880
LQSALDAEIR	AKQAIQEELN	KVKASNIITE	CKLKDSEKKN	LELLSEIEQL	IKDTEELRSE	KGIEHQDSQH	SFLAFLNTPT
890	900	910	920	930	940	950	960
DALDQFEDSF	SSSSSSLIDF	LDDTDPVENT	YYWNPSVKFH	IQSRSTSPST	SSEAEPVKTV	DSTPLSVHTP	TLRKKGCPGS
970	980	990	1000	1010	1020	1030	1040
TGFPPKRKTH	QFFVKSFTTP	TKCHQCTSLM	VGLIRQGCSC	EVCGFSCHIT	CVNKAPTTCP	VPPEQTKGPL	GIDPQKGIGT
1050	1060	1070	1080	1090	1100	1110	1120
AYEGHVRIPK	PAGVKKGWQR	ALAIVCDFKL	FLYDIAEGKA	SQPSVVISQV	IDMRDEEFSV	SSVLASDVIH	ASRKDIPCIF
1130	1140	1150	1160	1170	1180	1190	1200
RVTASQLSAS	NNKCSILMLA	DTENEKNKWV	GVLSELHKIL	KKNKFRDRSV	YVPKEAYDST	LPLIKTTQAA	AIIDHERIAL
1210	1220	1230	1240	1250	1260	1270	1280
GNEEGLFVVH	VTKDEIIRVG	DNKKIHQIEL	IPNDQLVAVI	SGRNRHVRLF	PMSALDGRET	DFYKLSETKG	CQTVTSGKVR
1290	1300	1310	1320	1330	1340	1350	1360
HGALTCLCVA	MKRQVLCYEL	FQSKTRHRKF	KEIQVPYNVQ	WMAIFSEQLC	VGFQSGFLRY	PLNGEGNPYS	MLHSNDHTLS
1370	1380	1390	1400	1410	1420	1430	1440
FIAHQPMDAI	CAVEISSKEY	LLCFNSIGIY	TDCQGRRSRQ	QELMWPANPS	SCCYNAPYLS	VYSENAVDIF	DVNSMEWIQT
1450	1460	1470	1480	1490	1500	1510	1520
LPLKKVRPLN	NEGSLNLLGL	ETIRLIYFKN	KMAEGDELVV	PETSDNSRKQ	MVRNINNKRR	YSFRVPEEER	MQQRREMLRD
1530	1540	1550	1560	1570	1580	1590	1600
PEMRNKLISN	PTNFNHIAHM	GPGDGIQILK	DLPMPGFPYP	SPHHHSGLIS	SPINFEHIYH	MTVNSAEKFL	SPDSINPEYS
1610	1620	1630	1640	1650	1660	1670	1680
PSLRSVPGTP	SFMTLRNPRP	QESRTVFSGS	VSIPSITKSR	PEPGRSMSAS	SGLSARSSAQ	NGSALKREFS	GGSYSAKRQP
1690	1700	1710	1720	1730	1740	1750	1760
MPSPSEGSLS	SGGMDQGSDA	PARDFDGEDS	DSPRHSTASN	SSNLSSPPSP	ASPRKTKSLS	LESTDRGSWD	P

Detailed Protein Report

Cmpd.	No. of Cmpds.	m/z meas.	Δ m/z [ppm]	z	Rt [min]	Score	P	Range	Sequence	Modification	Ratios	
2023	3	703.7681	-96.27	2	53.7	13.5	0	925-938	R.STSPSTSSEAEPVK.T		WD:WU	0.47

Detailed Protein Report

Protein 334: ubiquitin carboxyl-terminal hydrolase 34 [Homo sapiens]

**Accession:**	gi|110347427	**Score:**	24.0
**Database:**	refseq_human(refseq_human_20140103.fasta)	**MW [kDa]:**	404.0
**Database Date:**	2015-11-30	**pI:**	5.4
**Modification(s):**	Oxidation	**Sequence Coverage [%]:**	1.0
		**No. of unique Peptides:**	1

Detailed Protein Report

10	20	30	40	50	60	70	80
MCENCADLVE	VLNEISDVEG	GDGLQLRKEH	TLKIFTYINS	WTQRQCLCCF	KEYKHLEIFN	QVVCALINLV	IAQVQVLRDQ
90	100	110	120	130	140	150	160
LCKHCTTINI	DSTWQDESNQ	AEEPLNIDRE	CNEGSTERQK	SIEKKSNSTR	ICNLTEEESS	KSSDPFSLWS	TDEKEKLLLC
170	180	190	200	210	220	230	240
VAKIFQIQFP	LYTAYKHNTH	PTIEDISTQE	SNILGAFCDM	NDVEVPLHLL	RYVCLFCGKN	GLSLMKDCFE	YGTPETLPFL
250	260	270	280	290	300	310	320
IAHAFITVVS	NIRIWLHIPA	VMQHIIPFRT	YVIRYLCKLS	DQELRQSAAR	NMADLMWSTV	KEPLDTTLCF	DKESLDLAFK
330	340	350	360	370	380	390	400
YFMSPTLTMR	LAGLSQITNQ	LHTFNDVCNN	ESLVSDTETS	IAKELADWLI	SNNVVEHIFG	PNLHIEIIKQ	CQVILNFLAA
410	420	430	440	450	460	470	480
EGRLSTQHID	CIWAAAQLKH	CSRYIHDLFP	SLIKNLDPVP	LRHLLNLVSA	LEPSVHTEQT	LYLASMLIKA	LWNNALAAKA
490	500	510	520	530	540	550	560
QLSKQSSFAS	LLNTNIPIGN	KKEEEELRRT	APSPWSPAAS	PQSSDNSDTH	QSGGSDIEMD	EQLINRTKHV	QQRLSDTEES
570	580	590	600	610	620	630	640
MQGSSDETAN	SGEDGSSGPG	SSSGHSDGSS	NEVNSSHASQ	SAGSPGSEVQ	SEDIADIEAL	KEEDEDDDHG	HNPPKSSCGT
650	660	670	680	690	700	710	720
DLRNRKLESQ	AGICLGDSQG	MSERNGTSSG	TGKDLVFNTE	SLPSVDNRMR	MLDACSHSED	PEHDISGEMN	ATHIAQGSQE
730	740	750	760	770	780	790	800
SCITRTGDFL	GETIGNELFN	CRQFIGPQHH	HHHHHHHHHH	DGHMVDDMLS	ADDVSCSSSQ	VSAKSEKNMA	DFDGEESGCE
810	820	830	840	850	860	870	880
EELVQINSHA	ELTSHLQQHL	PNLASIYHEH	LSQGPVVHKH	QFNSNAVTDI	NLDNVCKKGN	TLLWDIVQDE	DAVNLSEGLI
890	900	910	920	930	940	950	960
NEAEKLLCSL	VCWFTDRQIR	MRFIEGCLEN	LGNNRSVVIS	LRLLPKLFGT	FQQFGSSYDT	HWITMWAEKE	LNMMKLFFDN
970	980	990	1000	1010	1020	1030	1040
LVYYIQTVRE	GRQKHALYSH	SAEVQVRLQF	LTCVFSTLGS	PDHFRLSLEQ	VDILWHCLVE	DSECYDDALH	WFLNQVRSKD
1050	1060	1070	1080	1090	1100	1110	1120
QHAMGMETYK	HLFLEKMPQL	KPETISMTGL	NLFQHLCNLA	RLATSAYDGC	SNSELCGMDQ	FWGIALRAQS	GDVSRAAIQY
1130	1140	1150	1160	1170	1180	1190	1200
INSYYINGKT	GLEKEQEFIS	KCMESLMIAS	SSLEQESHSS	LMVIERGLLM	LKTHLEAFRR	RFAYHLRQWQ	IEGTGISSHL
1210	1220	1230	1240	1250	1260	1270	1280
KALSDKQSLP	LRVVCQPAGL	PDKMTIEMYP	SDQVADLRAE	VTHWYENLQK	EQINQQAQLQ	EFGQSNRKGE	FPGGLMGPVR
1290	1300	1310	1320	1330	1340	1350	1360
MISSGHELTT	DYDEKALHEL	GFKDMQMVFV	SLGAPRRERK	GEGVQLPASC	LPPPQKDNIP	MLLLLQEPHL	TTLFDLLEML
1370	1380	1390	1400	1410	1420	1430	1440
ASFKPPSGKV	AVDDSESLRC	EELHLHAENL	SRRVWELLML	LPTCPNMLMA	FQNISDEQSN	DGFNWKELLK	IKSAHKLLYA
1450	1460	1470	1480	1490	1500	1510	1520
LEIIEALGKP	NRRIRRESTG	SYSDLYPDSD	DSSEDQVENS	KNSWSCKFVA	AGGLQQLLEI	FNSGILEPKE	QESWTVWQLD
1530	1540	1550	1560	1570	1580	1590	1600
CLACLLKLIC	QFAVDPSDLD	LAYHDVFAWS	GIAESHRKRT	WPGKSRKAAG	DHAKGLHIPR	LTEVFLVLVQ	GTSLIQRLMS
1610	1620	1630	1640	1650	1660	1670	1680
VAYTYDNLAP	RVLKAQSDHR	SRHEVSHYSM	WLLVSWAHCC	SLVKSSLADS	DHLQDWLKKL	TLLIPETAVR	HESCSGLYKL
1690	1700	1710	1720	1730	1740	1750	1760
SLSGLDGGDS	INRSFLLLAA	STLLKFLPDA	QALKPIRIDD	YEEEPILKPG	CKEYFWLLCK	LVDNIHIKDA	SQTTLLDLDA
1770	1780	1790	1800	1810	1820	1830	1840
LARHLADCIR	SREILDHQDG	NVEDDGLTGL	LRLATSVVKH	KPPFKFSREG	QEFLRDIFNL	LFLLPSLKDR	QQPKCKSHSS
1850	1860	1870	1880	1890	1900	1910	1920
RAAAYDLLVE	MVKGSVENYR	LIHNWVMAQH	MQSHAPYKWD	YWPHEDVRAE	CRFVGLTNLG	ATCYLASTIQ	QLYMIPEARQ
1930	1940	1950	1960	1970	1980	1990	2000
AVFTAKYSED	MKHKTTLLEL	QKMFTYLMES	ECKAYNPRPF	CKTYTMDKQP	LNTGEQKDMT	EFFTDLITKI	EEMSPELKNT
2010	2020	2030	2040	2050	2060	2070	2080
VKSLFGGVIT	NNVVSLDCEH	VSQTAEEFYT	VRCQVADMKN	IYESLDEVTI	KDTLEGDNMY	TCSHCGKKVR	AEKRACFKKL
2090	2100	2110	2120	2130	2140	2150	2160
PRILSFNTMR	YTFNMVTMMK	EKVNTHFSFP	LRLDMTPYTE	DFLMGKSERK	EGFKEVSDHS	KDSESYEYDL	IGVTVHTGTA
2170	2180	2190	2200	2210	2220	2230	2240

Detailed Protein Report

Cmpd.	No. of Cmpds.	m/z meas.	Δ m/z [ppm]	z	Rt [min]	Score	P	Range	Sequence	Modification
1545	1	948.9999	79.00	2	47.6	10.2	0	647-664	K.LESQAGICLGDSQGMSER.N	Oxidation: 15

Detailed Protein Report

Protein 335: regulator of G-protein signaling 22 isoform 2 [Homo sapiens]

Accession: gi|557878614
Database: refseq_human(refseq_human_20140103.fasta)
Database Date: 2015-11-30
Modification(s): Carbamidomethyl

Score: 23.9
MW [kDa]: 145.7
pI: 8.7
Sequence Coverage [%]: 2.6
No. of unique Peptides: 2

10	20	30	40	50	60	70	80
MPEKRLTAEP	PTITEEEFED	SLATDDFLVD	YFNEFLSLPT	FSEAIRFNAD	YGVFEVANDA	PQFLEKQLKK	ILQNQQPRNP
**90**	**100**	**110**	**120**	**130**	**140**	**150**	**160**
IYDVVRKGKN	EVKPVQMNAP	DEDETINVNY	NIMCLSREEG	IKWIKKERLP	AFLESDCYFE	YRLAKLVSQV	RWSKSGMNFT
**170**	**180**	**190**	**200**	**210**	**220**	**230**	**240**
VGSNFSPWIV	KKPPSLPPPA	TEEDNLASYT	QTKDWFALAK	QSQQTVSTFS	LPCCVPYNKL	KSPAISSVSE	NFIFDDGVHP
**250**	**260**	**270**	**280**	**290**	**300**	**310**	**320**
RTKKDPSKTN	KLISEFEEEE	GEEEEVSVSL	QDTPSQALLR	VYLEKKQDVD	ESLTMHFSTC	EEFLSSYIYF	ILRGAIQQIV
**330**	**340**	**350**	**360**	**370**	**380**	**390**	**400**
GKPVGETPDY	INFNNITKVS	FDDCFESIHG	KNFLSELVQT	TKERSEEIEQ	TSLSSKNESA	GPESRADWCI	SHRTYDIGNR
**410**	**420**	**430**	**440**	**450**	**460**	**470**	**480**
KEFERFKKFI	KGTLGERYWW	LWMDIERLKV	LKDPGRHQRH	LEKMKKCYLV	SNGDYYLSAE	ILSKFKLLDG	SQWNEEHLRN
**490**	**500**	**510**	**520**	**530**	**540**	**550**	**560**
IQSEVLKPLL	LYWAPRFCVT	HSASTKYASA	ELKFWHLRQA	KPRKDIDPFP	QMATLLPLRP	KSCIPQIPEI	QKEEFSLSQP
**570**	**580**	**590**	**600**	**610**	**620**	**630**	**640**
PKSPNKSPEV	KTATQKPWKR	ELLYPGSSKD	DVIEKGSKYM	SESSKVIHLT	SFTDISECLK	PQLDRRYAYT	EEPRVKTVSD
**650**	**660**	**670**	**680**	**690**	**700**	**710**	**720**
VGALGGSDME	NLLQSLYVEN	RAGFFFTKFC	EHSGNKLWKN	SVYFWFDLQA	YHQLFYQETL	QPFKVCKQAQ	YLFATYVAPS
**730**	**740**	**750**	**760**	**770**	**780**	**790**	**800**
ATLDIGLQQE	KKKEIYMKIQ	PPFEDLFDTA	EEYILLLLLE	PWTKMVKSDQ	IAYKKVELVE	ETRQLDSTYF	RKLQALHKET
**810**	**820**	**830**	**840**	**850**	**860**	**870**	**880**
FSKKAEDTTC	EIGTGILSLS	NVSKRTEYWD	NVPAEYKHFK	FSDLLNNKLE	FEHFRQFLET	HSSSMDLMCW	TDIEQFRRIT
**890**	**900**	**910**	**920**	**930**	**940**	**950**	**960**
YRDRNQRKAK	SIYIKNKYLN	KKYFFGPNSP	ASLYQQNQVM	HLSGGWGKIL	HEQLDAPVLV	EIQKHVQNRL	ENVWLPLFLA
**970**	**980**	**990**	**1000**	**1010**	**1020**	**1030**	**1040**
SEQFAARQKI	KVQMKDIAEE	LLLQKAEKKI	GVWKPVESKW	ISSSCKIIAF	RKALLNPVTS	RQFQRFVALK	GDLLENGLLF
**1050**	**1060**	**1070**	**1080**	**1090**	**1100**	**1110**	**1120**
WQEVQKYKDL	CHSHCDESVI	QKKITTIINC	FINSSIPPAL	QIDIPVEQAQ	KIIEHRKELG	PYVFREAQMT	IFGVLFKFWP
**1130**	**1140**	**1150**	**1160**	**1170**	**1180**	**1190**	**1200**
QFCEFRKNLT	DENIMSVLER	RQEYNKQKKK	LAVLEDEKSG	KDGIKQYANT	SVPAIKTALL	SDSFLGLQPY	GRQPTWCYSK
**1210**	**1220**	**1230**	**1240**	**1250**	**1260**		
YIEALEQERI	LLKIQEELEK	KLFAGLQPLT	NFKASSSTMS	LKKNMSAHSS	QK		

Cmpd.	No. of Cmpds.	m/z meas.	Δ m/z [ppm]	z	Rt [min]	Score	P	Range	Sequence	Modification
2902	1	1192.7378	113.29	2	65.0	10.5	0	172-193	K.KPPSLPPPATEEDNLASYTQTK.D	
2013	1	703.2744	-90.02	2	53.5	13.4	1	669-679	K.FCEHSGNKLWK.N	Carbamidomethyl: 2

Detailed Protein Report

Protein 336: zinc finger and BTB domain-containing protein 21 isoform L [Homo sapiens]

**Accession:**	gi|50345873	**Score:**	23.9
**Database:**	refseq_human(refseq_human_20140103.fasta)	**MW [kDa]:**	118.8
**Database Date:**	2015-11-30	**pI:**	9.6
		**Sequence Coverage [%]:**	1.9
		**No. of unique Peptides:**	2

Quantitation

WD:WU **Median:** 0.61 **CV:** 0.00 % **No. of Peptides:** 1

Alias proteins:

Accession	Name	Description
gi|530419238	refseq_human (refseq_human_20140103.fasta)	PREDICTED: zinc finger and BTB domain-containing protein 21 isoform X1 [Homo sapiens]
gi|148491088	refseq_human (refseq_human_20140103.fasta)	zinc finger and BTB domain-containing protein 21 isoform L [Homo sapiens]

```
        10         20         30         40         50         60         70         80
MEGLLHYINP AHAISLLSAL NEERLKGQLC DVLLIVGDQK FRAHKNVLAA SSEYFQSLFT NKENESQTVF QLDFCEPDAF
        90        100        110        120        130        140        150        160
DNVLNYIYSS SLFVEKSSLA AVQELGYSLG ISFLTNIVSK TPQAPFPTCP NRKKVFVEDD ENSSQKRSVI VCQSRNEAQG
       170        180        190        200        210        220        230        240
KTVSQNQPDV SHTSRPSPSI AVKANTNKPH VPKPIEPLHN LSLTEKSWPK DSSVVYAKSL EHSGSLDDPN RISLVKRNAV
       250        260        270        280        290        300        310        320
LPSKPLQDRE AMDDKPGVSG QLPKGKALEL ALKRPRPPVL SVCSSSETPY LLKETNKGNG QGEDRNLLYY SKLGLVIPSS
       330        340        350        360        370        380        390        400
GSGSGNQSID RSGPLVKSLL RRSLSMDSQV PVYSPSIDLK SSQGSSSVSS DAPGNVLCAL SQKSSLKDCS EKTALDDRPQ
       410        420        430        440        450        460        470        480
VLQPHRLRSF SASQSTDREG ASPVTEVRIK TEPSSPLSDP SDIIRVTVGD AATTAAASSS SVTRDLSLKT EDDQKDMSRL
       490        500        510        520        530        540        550        560
PAKRRFQADR RLPFKKLKVN EHGSPVSEDN FEEGSSPTLL DADFPDSDLN KDEFGELEGT RPNKKFKCKH CLKIFRSTAG
       570        580        590        600        610        620        630        640
LHRHVNMYHN PEKPYACDIC HKRFHTNFKV WTHCQTQHGI VKNPSPASSS HAVLDEKFQR KLIDIVRERE IKKALIIKLR
       650        660        670        680        690        700        710        720
RGKPGFQGQS SSQAQQVIKR NLRSRAKGAY ICTYCGKAYR FLSQFKQHIK MHPGEKPLGV NKVAKPKEHA PLASPVENKE
       730        740        750        760        770        780        790        800
VYQCRLCNAK LSSLLEQGSH ERLCRNAAVC PYCSLRFFSP ELKQEHESKC EYKKLTCLEC MRTFKSSFSI WRHQVEVHNQ
       810        820        830        840        850        860        870        880
NNMAPTENFS LPVLDHNGDV TGSSRPQSQP EPNKVNHIVT TKDDNVFSDS SEQVNFDSED SSCLPEDLSL SKQLKIQVKE
       890        900        910        920        930        940        950        960
EPVEEAEEEA PEASTAPKEA GPSKEASLWP CEKCGKMFTV HKQLERHQEL LCSVKPFICH VCNKAFRTNF RLWSHFQSHM
       970        980        990       1000       1010       1020       1030       1040
SQASEESAHK ESEVCPVPTN SPSPPPLPPP PPLPKIQPLE PDSPTGLSEN PTPATEKLFV PQESDTLFYH APPLSAITFK
      1050       1060       1070
RQFMCKLCHR TFKTAFSLWS HEQTHN
```

Cmpd.	No. of Cmpds.	m/z meas.	Δ m/z [ppm]	z	Rt [min]	Score	P	Range	Sequence	Modification	Ratios
1308	3	698.8804	93.56	2	44.7	13.3	0	135-146	K.VFVEDDENSSQK.R		
861	1	491.8136	-69.69	2	39.6	10.6	2	634-641	K.ALIIKLRR.G		WD:WU 0.61

Detailed Protein Report

Protein 337: A disintegrin and metalloproteinase with thrombospondin motifs 6 preproprotein [Homo sapiens]

Accession: gi|64276808
Database: refseq_human(refseq_human_20140103.fasta)
Database Date: 2015-11-30
Modification(s): Carbamidomethyl, Oxidation

Score: 23.9
MW [kDa]: 125.2
pI: 6.3
Sequence Coverage [%]: 3.8
No. of unique Peptides: 2

Quantitation

WD:WU **Median:** 1.11 **CV:** 0.00 % **No. of Peptides:** 1

10	20	30	40	50	60	70	80
MEILWKTLTW	ILSLIMASSE	FHSDHRLSYS	SQEEFLTYLE	HYQLTIPIRV	DQNGAFLSFT	VKNDKHSRRR	RSMDPIDPQQ
90	100	110	120	130	140	150	160
AVSKLFFKLS	AYGKHFHLNL	TLNTDFVSKH	FTVEYWGKDG	PQWKHDFLDN	CHYTGYLQDQ	RSTTKVALSN	CVGLHGVIAT
170	180	190	200	210	220	230	240
EDEEYFIEPL	KNTTEDSKHF	SYENGHPHVI	YKKSALQQRH	LYDHSHCGVS	DFTRSGKPWW	LNDTSTVSYS	LPINNTHIHH
250	260	270	280	290	300	310	320
RQKRSVSIER	FVETLVVADK	MMVGYHGRKD	IEHYILSVMN	IVAKLYRDSS	LGNVVNIIVA	RLIVLTEDQP	NLEINHHADK
330	340	350	360	370	380	390	400
SLDSFCKWQK	SILSHQSDGN	TIPENGIAHH	DNAVLITRYD	ICTYKNKPCG	TLGLASVAGM	CEPERSCSIN	EDIGLGSAFT
410	420	430	440	450	460	470	480
IAHEIGHNFG	MNHDGIGNSC	GTKGHEAAKL	MAAHITANTN	PFSWSACSRD	YITSFLDSGR	GTCLDNEPPK	RDFLYPAVAP
490	500	510	520	530	540	550	560
GQVYDADEQC	RFQYGATSRQ	CKYGEVCREL	WCLSKSNRCV	TNSIPAAEGT	LCQTGNIEKG	WCYQGDCVPF	GTWPQSIDGG
570	580	590	600	610	620	630	640
WGPWSLWGEC	SRTCGGGVSS	SLRHCDSPAP	SGGGKYCLGE	RKRYRSCNTD	PCPLGSRDFR	EKQCADFDNM	PFRGKYYNWK
650	660	670	680	690	700	710	720
PYTGGGVKPC	ALNCLAEGYN	FYTERAPAVI	DGTQCNADSL	DICINGECKH	VGCDNILGSD	AREDRCRVCG	GDGSTCDAIE
730	740	750	760	770	780	790	800
GFFNDSLPRG	GYMEVVQIPR	GSVHIEVREV	AMSKNYIALK	SEGDDYYING	AWTIDWPRKF	DVAGTAFHYK	RPTDEPESLE
810	820	830	840	850	860	870	880
ALGPTSENLI	VMVLLQEQNL	GIRYKFNVPI	TRTGSGDNEV	GFTWNHQPWS	ECSATCAGGV	QRQEVVCKRL	DDNSIVQNNY
890	900	910	920	930	940	950	960
CDPDSKPPEN	QRACNTEPCP	PEWFIGDWLE	CSKTCDGGMR	TRAVLCIRKI	GPSEEETLDY	SGCLTHRPVE	KEPCNNQSCP
970	980	990	1000	1010	1020	1030	1040
PQWVALDWSE	CTPKCGPGFK	HRIVLCKSSD	LSKTFPAAQC	PEESKPPVRI	RCSLGRCPPP	RWVTGDWGQC	SAQCGLGQQM
1050	1060	1070	1080	1090	1100	1110	1120
RTVQCLSYTG	QASSDCLETV	RPPSMQQCES	KCDSTPISNT	EECKDVNKVA	YCPLVLKFKF	CSRAYFRQMC	CKTCQGH

Cmpd.	No. of Cmpds.	m/z meas.	Δ m/z [ppm]	z	Rt [min]	Score	P	Range	Sequence	Modification	Ratios
885	1	649.9950	65.11	3	39.5	10.1	1	584-601	R.HCDSPAPSGGGKYCLGER.K	Carbamidomethyl: 2, 14	WD:WU 1.11
2995	1	964.4577	34.33	3	66.2	13.8	1	1017-1041	R. CPPPRWVTGDWGQCSAQCGLG[illegible] T	Carbamidomethyl: 14, 18; Oxidation: 24	

Detailed Protein Report

Protein 338: prosaposin receptor GPR37 precursor [Homo sapiens]

**Accession:**	gi|4885323	**Score:**	23.8
**Database:**	refseq_human(refseq_human_20140103.fasta)	**MW [kDa]:**	67.1
**Database Date:**	2015-11-30	**pI:**	9.9
		**Sequence Coverage [%]:**	2.1
		**No. of unique Peptides:**	1

10	20	30	40	50	60	70	80
MRAPGALLAR	MSRLLLLLLL	KVSASSALGV	APASRNETCL	GESCAPTVIQ	RRGRDAWGPG	NSARDVLRAR	APREEQGAAF
**90**	**100**	**110**	**120**	**130**	**140**	**150**	**160**
LAGPSWDLPA	APGRDPAAGR	GAEASAAGPP	GPPTRPPGPW	RWKGARGQEP	SETLGRGNPT	ALQLFLQISE	EEEKGPRGAG
**170**	**180**	**190**	**200**	**210**	**220**	**230**	**240**
ISGRSQEQSV	KTVPGASDLF	YWPRRAGKLQ	GSHHKPLSKT	ANGLAGHEGW	TIALPGRALA	QNGSLGEGIH	EPGGPRRGNS
**250**	**260**	**270**	**280**	**290**	**300**	**310**	**320**
TNRRVRLKNP	FYPLTQESYG	AYAVMCLSVV	IFGTGIIGNL	AVMCIVCHNY	YMRSISNSLL	ANLAFWDFLI	IFFCLPLVIF
**330**	**340**	**350**	**360**	**370**	**380**	**390**	**400**
HELTKKWLLE	DFSCKIVPYI	EVASLGVTTF	TLCALCIDRF	RAATNVQMYY	EMIENCSSTT	AKLAVIWVGA	LLLALPEVVL
**410**	**420**	**430**	**440**	**450**	**460**	**470**	**480**
RQLSKEDLGF	SGRAPAERCI	IKISPDLPDT	IYVLALTYDS	ARLWWYFGCY	FCLPTLFTIT	CSLVTARKIR	KAEKACTRGN
**490**	**500**	**510**	**520**	**530**	**540**	**550**	**560**
KRQIQLESQM	NCTVVALTIL	YGFCIIPENI	CNIVTAYMAT	GVSQQTMDLL	NIISQFLLFF	KSCVTPVLLF	CLCKPFSRAF
**570**	**580**	**590**	**600**	**610**	**620**		
MECCCCCCEE	CIQKSSTVTS	DDNDNEYTTE	LELSPFSTIR	REMSTFASVG	THC		

Cmpd.	No. of Cmpds.	m/z meas.	Δ m/z [ppm]	z	Rt [min]	Score	P	Range	Sequence	Modification
2716	2	715.3661	-44.05	2	62.4	11.7	2	1-13	-.MRAPGALLARMSR.L	

Detailed Protein Report

Protein 339: telomerase reverse transcriptase isoform 2 [Homo sapiens]

**Accession:**	gi|301129200	**Score:**	23.8
**Database:**	refseq_human(refseq_human_20140103.fasta)	**MW [kDa]:**	120.0
**Database Date:**	2015-11-30	**pI:**	11.9
**Modification(s):**	Oxidation	**Sequence Coverage [%]:**	2.8
		**No. of unique Peptides:**	1

10	20	30	40	50	60	70	80
MPRAPRCRAV	RSLLRSHYRE	VLPLATFVRR	LGPQGWRLVQ	RGDPAAFRAL	VAQCLVCVPW	DARPPPAAPS	FRQVSCLKEL
90	100	110	120	130	140	150	160
VARVLQRLCE	RGAKNVLAFG	FALLDGARGG	PPEAFTTSVR	SYLPNTVTDA	LRGSGAWGLL	LRRVGDDVLV	HLLARCALFV
170	180	190	200	210	220	230	240
LVAPSCAYQV	CGPPLYQLGA	ATQARPPPHA	SGPRRRLGCE	RAWNHSVREA	GVPLGLPAPG	ARRRGGSASR	SLPLPKRPRR
250	260	270	280	290	300	310	320
GAAPEPERTP	VGQGSWAHPG	RTRGPSDRGF	CVVSPARPAE	EATSLEGALS	GTRHSHPSVG	RQHHAGPPST	SRPPRPWDTP
330	340	350	360	370	380	390	400
CPPVYAETKH	FLYSSGDKEQ	LRPSFLLSSL	RPSLTGARRL	VETIFLGSRP	WMPGTPRRLP	RLPQRYWQMR	PLFLELLGNH
410	420	430	440	450	460	470	480
AQCPYGVLLK	THCPLRAAVT	PAAGVCAREK	PQGSVAAPEE	EDTDPRRLVQ	LLRQHSSPWQ	VYGFVRACLR	RLVPPGLWGS
490	500	510	520	530	540	550	560
RHNERRFLRN	TKKFISLGKH	AKLSLQELTW	KMSVRDCAWL	RRSPGVGCVP	AAEHRLREEI	LAKFLHWLMS	VYVVELLRSF
570	580	590	600	610	620	630	640
FYVTETTFQK	NRLFFYRKSV	WSKLQSIGIR	QHLKRVQLRE	LSEAEVRQHR	EARPALLTSR	LRFIPKPDGL	RPIVNMDYVV
650	660	670	680	690	700	710	720
GARTFRREKR	AERLTSRVKA	LFSVLNYERA	RRPGLLGASV	LGLDDIHRAW	RTFVLRVRAQ	DPPPELYFVK	VDVTGAYDTI
730	740	750	760	770	780	790	800
PQDRLTEVIA	SIIKPQNTYC	VRRYAVVQKA	AHGHVRKAFK	SHVSTLTDLQ	PYMRQFVAHL	QETSPLRDAV	VIEQSSSLNE
810	820	830	840	850	860	870	880
ASSGLFDVFL	RFMCHHAVRI	RGKSYVQCQG	IPQGSILSTL	LCSLCYGDME	NKLFAGIRRD	GLLLRLVDDF	LLVTPHLTHA
890	900	910	920	930	940	950	960
KTFLSYARTS	IRASLTFNRG	FKAGRNMRRK	LFGVLRLKCH	SLFLDLQVNS	LQTVCTNIYK	ILLLQAYRFH	ACVLQLPFHQ
970	980	990	1000	1010	1020	1030	1040
QVWKNPTFFL	RVISDTASLC	YSILKAKNAG	MSLGAKGAAG	PLPSEAVQWL	CHQAFLLKLT	RHRVTYVPLL	GSLRTAQTQL
1050	1060	1070					
SRKLPGTTLT	ALEAAANPAL	PSDFKTILD					

Cmpd.	No. of Cmpds.	m/z meas.	Δ m/z [ppm]	z	Rt [min]	Score	P	Range	Sequence	Modification
2746	1	626.8425	68.04	2	62.7	12.4	1	512-521	K.MSVRDCAWLR.R	Oxidation: 1

Detailed Protein Report

Protein 340: GTPase HRas isoform 2 [Homo sapiens]

**Accession:**	gi|34222246	**Score:**	23.8
**Database:**	refseq_human(refseq_human_20140103.fasta)	**MW [kDa]:**	18.9
**Database Date:**	2015-11-30	**pI:**	4.7
		**Sequence Coverage [%]:**	10.6
		**No. of unique Peptides:**	1

Alias proteins:

Accession	Name	Description
gi|578839846	refseq_human (refseq_human_20140103.fasta)	PREDICTED: GTPase HRas isoform X1 [Homo sapiens]
gi|578820498	refseq_human (refseq_human_20140103.fasta)	PREDICTED: GTPase HRas isoform X3 [Homo sapiens]

```
        10         20         30         40         50         60         70         80
MTEYKLVVVG AGGVGKSALT IQLIQNHFVD EYDPTIEDSY RKQVVIDGET CLLDILDTAG QEEYSAMRDQ YMRTGEGFLC
        90        100        110        120        130        140        150        160
VFAINNTKSF EDIHQYREQI KRVKDSDDVP MVLVGNKCDL AARTVESRQA QDLARSYGIP YIETSAKTRQ GSRSGSSSSS
       170        180
GTLWDPPGPM
```

Cmpd.	No. of Cmpds.	m/z meas.	Δ m/z [ppm]	z	Rt [min]	Score	P	Range	Sequence	Modification
2770	1	1002.0175	9.51	2	63.1	10.3	2	118-135	K.CDLAARTVESRQAQDLAR.S	

Detailed Protein Report

Protein 341: **PREDICTED: nascent polypeptide-associated complex subunit alpha isoform X6 [Homo sapiens]**

**Accession:**	gi|578823772	**Score:**	23.8
**Database:**	refseq_human(refseq_human_20140103.fasta)	**MW [kDa]:**	201.3
**Database Date:**	2015-11-30	**pI:**	10.2
		**Sequence Coverage [%]:**	1.1
		**No. of unique Peptides:**	1

Detailed Protein Report

10	20	30	40	50	60	70	80
MPGEATETVP	ATEQELPQPQ	AETAVLPMSS	ALSVTAALGQ	PGPTLPPPCS	PAPQQCPLSA	ANQASPFPSP	STIASTPLEV
90	100	110	120	130	140	150	160
PFPQSSSGTA	LPLGTAPEAP	TFLPNLIGPP	ISPAALALAS	PMIAPTLKGT	PSSSAPLALV	ALAPHSVQKS	SAFPPNLLTS
170	180	190	200	210	220	230	240
PPSVAVAESG	SVITLSAPIA	PSEPKTNLNK	VPSEVVPNPK	GTPSPPCIVS	TVPYHCVTPM	ASIQSGVASL	PQTTPTTTLA
250	260	270	280	290	300	310	320
IASPQVKDTT	ISSVLISPQN	PGSLSLKGPV	SPPAALSLST	QSLPVVTSSQ	KTAGPNTPPD	FPISLGSHLA	PLHQSSFGSV
330	340	350	360	370	380	390	400
QLLGQTGPSA	LSDPTVKTIS	VDHSSTGASY	PSQRSVIPPL	PSRNEVVPAT	VAAFPVVAPS	VDKGPSTISS	ITCSPSGSLN
410	420	430	440	450	460	470	480
VATSFSLSPT	TSLILKSSPN	ATYHYPLVAQ	MPVSSVGTTP	LVVTNPCTIA	AAPTTTFEVA	TCVSPPMSSG	PISNIEPTSP
490	500	510	520	530	540	550	560
AALVMAPVAP	KEPSTQVATT	LRIPVSPPLP	DPEDLKNLPS	SVLVKFPTQK	DLQTVPASLE	GAPFSPAQAG	LTTKKDPTVL
570	580	590	600	610	620	630	640
PLVQAAPKNS	PSFQSTSSSP	EIPLSPEATL	AKKSLGEPLP	IGKPASSMTS	PLGVNSSASV	IKTDSYAGPD	SAGPLLKSSL
650	660	670	680	690	700	710	720
ITPTVAAFPL	ESADPAGVAP	TTAKGTSTYT	TTASPFLEGT	VSLAPKNHPV	KEGTLTTLPL	VPTASENCPV	APSPQNTCAP
730	740	750	760	770	780	790	800
LATLVLAPEI	PKSVPSPSLP	PAGTPPGTKK	VDGISHTSAL	APVASSPKEC	PTEDSGASAT	ASSKGTLTYL	ADSPSPLGVS
810	820	830	840	850	860	870	880
VSPQTKRPPT	KKGSAGPDTP	IGNLSSPVSP	VEASFLPENS	LSFQGSKDSP	ATTHSPTPPS	PKGAPTPSAV	TPLSPKGVTL
890	900	910	920	930	940	950	960
PPKETPTPSV	VNLPFPKEGP	ATPAPKQAPA	LSMTSSSPKK	ARATPAPKGI	PASPSPKGAP	TPPAATPPSP	KGGPATPSPK
970	980	990	1000	1010	1020	1030	1040
WAPTPPAATP	PSPKGGPATP	SPKGAPTPPA	ATPPSPKGSP	AATPLPKGAP	TTPAATLPSP	KGGPATPSLK	GAPTPPAATP
1050	1060	1070	1080	1090	1100	1110	1120
PSPKGGPATP	SPKGAPMPPA	ATPPSPKGGL	ATPPHKGAPT	TPAATPPSPK	GGLATPPPKG	APTTPAATPP	SPKGGLATPP
1130	1140	1150	1160	1170	1180	1190	1200
PKGAPTTPAA	TPPSPKGGLA	TPSPKGAPTT	PAATPPSPKG	GLATPSPKGA	PTTPAATPPS	PKGGLATPSP	KGAPTTPAAT
1210	1220	1230	1240	1250	1260	1270	1280
PPSPKGGPAT	PPPKGAPTPP	AATPPSLKGG	LATPPHKGAP	NPAVVTPPSP	KGGPATSPPK	GAPTPPAATP	PSPKGSPGTP
1290	1300	1310	1320	1330	1340	1350	1360
PPKGAPTPPA	VTPPSPKGTP	TLPATTPSSK	GGPTTPSSKE	GPTPPAATPS	HKGGPAMTPP	SPKRGPAIPS	PKGDPTSPAV
1370	1380	1390	1400	1410	1420	1430	1440
IPLSPKKAPA	TPVTREGAAT	PSKGDLTPPA	VTPVSLKKAP	ATSAPKGGPA	TPSSKGDPTL	PAVTPPSPKE	PPAPKQVATS
1450	1460	1470	1480	1490	1500	1510	1520
SSPKKAPATP	APMGAPTLPA	VIPSSPKEVP	ATPSSRRDPI	APTATLLSKK	TPATLAPKEA	LIPPAMTVPS	PKKTPAIPTP
1530	1540	1550	1560	1570	1580	1590	1600
KEAPATPSSK	EASSPPAVTP	STYKGAPSPK	ELLIPPAVTS	PSPKEAPTPP	AVTPPSPEKG	PATPAPKGTP	TSPPVTPSSL
1610	1620	1630	1640	1650	1660	1670	1680
KDSPTSPASV	TCKMGATVPQ	ASKGLPAKKG	PTALKEVLVA	PAPESTPIIT	APTRKGPQTK	KSSATSPPIC	PDPSAKNGSK
1690	1700	1710	1720	1730	1740	1750	1760
GPLSTVAPAP	LLPVQKDSSK	TAKGKDASHS	PKGPLAPPES	KASTPLTAAA	FEKVLPKPES	ASVSAAPSPP	VSLPLAPSPV
1770	1780	1790	1800	1810	1820	1830	1840
PTLPPKQQFL	PSSPGLVLES	PSKPLAPADE	DELLPLIPPE	PISGGVPFQS	VLVNMPTPKS	AGIPVPTPSA	KQPVTKNNKG
1850	1860	1870	1880	1890	1900	1910	1920
SGTESDSDES	VPELEEQDST	QATTQQAQLA	AAAEIDEEPV	SKAKQSRSEK	KARKAMSKLG	LRQVTGVTRV	TIRKSKNILF
1930	1940	1950	1960	1970	1980	1990	2000
VITKPDVYKS	PASDTYIVFG	EAKIEDLSQQ	AQLAAAEKFK	VQGEAVSNIQ	ENTQTPTVQE	ESEEEEVDET	GVEVKDIELV
2010	2020	2030	2040				
MSQANVSRAK	AVRALKNNSN	DIVNAIMVSV	QAFVP				

Cmpd.	No. of Cmpds.	m/z meas.	Δ m/z [ppm]	z	Rt [min]	Score	P	Range	Sequence	Modification

Detailed Protein Report

Cmpd.	No. of Cmpds.	m/z meas.	Δ m/z [ppm]	z	Rt [min]	Score	P	Range	Sequence	Modification
2894	2	724.8707	-70.24	2	64.9	11.4	0	1551-1564	K.ELLIPPAVTSPSPK.E	

Detailed Protein Report

Protein 342: PREDICTED: multimerin-1 isoform X1 [Homo sapiens]

**Accession:**	gi|530377406	**Score:**	23.7
**Database:**	refseq_human(refseq_human_20140103.fasta)	**MW [kDa]:**	134.3
**Database Date:**	2015-11-30	**pI:**	9.2
**Modification(s):**	Carbamidomethyl	**Sequence Coverage [%]:**	2.7
		**No. of unique Peptides:**	1

10	20	30	40	50	60	70	80
MKGARLFVLL	SSLWSGGIGL	NNSKHSWTIP	EDGNSQKTMP	SASVPPNKIQ	SLQILPTTRV	MSAEIATTPE	ARTSEDSLLK
90	100	110	120	130	140	150	160
STLPPSETSA	PAEGVRNQTL	TSTEKAEGVV	KLQNLTLPTN	ASIKFNPGAE	SVVLSNSTLK	FLQSFARKSN	EQATSLNTVG
170	180	190	200	210	220	230	240
GTGGIGGVGG	TGGVGNRAPR	ETYLSRGDSS	SSQRTDYQKS	NFETTRGKNW	CAYVHTRLSP	TVILDNQVTY	VPGGKGPCGW
250	260	270	280	290	300	310	320
TGGSCPQRSQ	KISNPVYRMQ	HKIVTSLDWR	CCPGYSGPKC	QLREVMQKMT	DQVNYQAMKL	TLLQKKIDNI	SLTVNDVRNT
330	340	350	360	370	380	390	400
YSSLEGKVSE	DKSREFQSLL	KGLKSKSINV	LIRDIVREQF	KIFQNDMQET	VAQLFKTVSS	LSEDLESTRQ	IIQKVNESVV
410	420	430	440	450	460	470	480
SIAAQQKFVL	VQENRPTLTD	IVELRNHIVN	VRQEMTLTCE	KPIKELEVKQ	THLEGALEQE	HSRSILYYES	LNKTLSKLKE
490	500	510	520	530	540	550	560
VHEQLLSTEQ	VSDQKNAPAA	ESVSNNVTEY	MSTLHENIKK	QSLMMLQMFE	DLHIQESKIN	NLTVSLEMEK	ESLRGECEDM
570	580	590	600	610	620	630	640
LSKCRNDFKF	QLKDTEENLH	VLNQTLAEVL	FPMDNKMDKM	SEQLNDLTYD	MEILQPLLEQ	GASLRQTMTY	EQPKEAIVIR
650	660	670	680	690	700	710	720
KKIENLTSAV	NSLNFIIKEL	TKRHNLLRNE	VQGRDDALER	RINEYALEME	DGLNKTMTII	NNAIDFIQDN	YALKETLSTI
730	740	750	760	770	780	790	800
KDNSEIHHKC	TSDMETILTF	IPQFHRLNDS	IQTLVNDNQR	YNFVLQVAKT	LAGIPRDEKL	NQSNFQKMYQ	MFNETTSQVR
810	820	830	840	850	860	870	880
KYQQNMSHLE	EKLLLTTKIS	KNFETRLQDI	ESKVTQTLIP	YYISVKKGSV	VTNERDQALQ	LQVLNSRFKA	LEAKSIHLSI
890	900	910	920	930	940	950	960
NFFSLNKTLH	EVLTMCHNAS	TSVSELNATI	PKWIKHSLPD	IQLLQKGLTE	FVEPIIQIKT	QAALSNLTCC	IDRSLPGSLA
970	980	990	1000	1010	1020	1030	1040
NVVKSQKQVK	SLPKKINALK	KPTVNLTTVL	IGRTQRNTDN	IIYPEEYSSC	SRHPCQNGGT	CINGRTSFTC	ACRHPFTGDN
1050	1060	1070	1080	1090	1100	1110	1120
CTIKLVEENA	LAPDFSKGSY	RYAPMVAFFA	SHTYGMTIPG	PILFNNLDVN	YGASYTPRTG	KFRIPYLGVY	VFKYTIESFS
1130	1140	1150	1160	1170	1180	1190	1200
AHISGFLVVD	GIDKLAFESE	NINSEIHCDR	VLTGDALLEL	NYGQEVWLRL	AKGTIPAKFP	PVTTFSGYLL	YRT

Cmpd.	No. of Cmpds.	m/z meas.	Δ m/z [ppm]	z	Rt [min]	Score	P	Range	Sequence	Modification
1058	1	974.4611	39.05	2	41.5	12.5	0	997-1012	R.NTDNIIYPEEYSSCSR.H	Carbamidomethyl: 14

Detailed Protein Report

Protein 343: protein unc-45 homolog A isoform 3 [Homo sapiens]

**Accession:**	gi|89179321	**Score:**	23.7
**Database:**	refseq_human(refseq_human_20140103.fasta)	**MW [kDa]:**	101.6
**Database Date:**	2015-11-30	**pI:**	5.7
		**Sequence Coverage [%]:**	2.6
		**No. of unique Peptides:**	1

10	20	30	40	50	60	70	80
MTASSVEQLR	KEGNELFKCG	DYGGALAAYT	QALGLDATPQ	DQAVLHRNRA	ACHLKLEDYD	KAETEASKAI	EKDGGDVKAL
90	100	110	120	130	140	150	160
YRRSQALEKL	GRLDQAVLDL	QRCVSLEPKN	KVFQEALRNI	GGQIQEKVRY	MSSTDAKVEQ	MFQILLDPEE	KGTEKKQKAS
170	180	190	200	210	220	230	240
QNLVVLARED	AGAEKIFRSN	GVQLLQRLLD	MGETDLMLAA	LRTLVGICSE	HQSRTVATLS	ILGTRRVVSI	LGVESQAVSL
250	260	270	280	290	300	310	320
AACHLLQVMF	DALKEGVKKG	FRGKEGAIIV	DPARELKVLI	SNLLDLLTEV	GVSGQGRDNA	LTLLIKAVPR	KSLKDPNNSL
330	340	350	360	370	380	390	400
TLWVIDQGLK	KILEVGGSLQ	DPPGELAVTA	NSRMSASILL	SKLFDDLKCD	AERENFHRLC	ENYIKSWFEG	QGLAGKLRAI
410	420	430	440	450	460	470	480
QTVSCLLQGP	CDAGNRALEL	SGVMESVIAL	CASEQEEEQL	VAVEALIHAA	GKAKRASFIT	ANGVSLLKDL	YKCSEKDSIR
490	500	510	520	530	540	550	560
IRALVGLCKL	GSAGGTDFSM	KQFAEGSTLK	LAKQCRKWLC	NDQIDAGTRR	WAVEGLAYLT	FDADVKEEFV	EDAAALKALF
570	580	590	600	610	620	630	640
QLSRLEERSV	LFAVASALVN	CTNSYDYEEP	DPKMVELAKY	AKQHVPEQHP	KDKPSFVRAR	VKKLLAAGVV	SAMVCMVKTE
650	660	670	680	690	700	710	720
SPVLTSSCRE	LLSRVFLALV	EEVEDRGTVV	AQGGGRALIP	LALEGTDVGQ	TKAAQALAKL	TITSNPEMTF	PGERIYEVVR
730	740	750	760	770	780	790	800
PLVSLLHLNC	SGLQNFEALM	ALTNLAGISE	RLRQKILKEK	AVPMIEGYMF	EEHEMIRRAA	TECMCNLAMS	KEVQDLFEAQ
810	820	830	840	850	860	870	880
GNDRLKLLVL	YSGEDDELLQ	RAAAGGLAML	TSMRPTLCSR	IPQVTTHWLE	ILQALLLSSN	QELQHRGAVV	VLNMVEASRE
890	900	910	920	930			
IASTLMESEM	MEILSVLAKG	DHSPVTRAAA	ACLDKAVEYG	LIQPNQDGE			

Cmpd.	No. of Cmpds.	m/z meas.	Δ m/z [ppm]	z	Rt [min]	Score	P	Range	Sequence	Modification
2777	1	703.4893	208.47	2	63.3	10.5	1	50-61	R.AACHLKLEDYDK.A	

Detailed Protein Report

Protein 344: PREDICTED: ubiquitin carboxyl-terminal hydrolase 24 isoform X1 [Homo sapiens]

**Accession:**	gi|530362258	**Score:**	23.7
**Database:**	refseq_human(refseq_human_20140103.fasta)	**MW [kDa]:**	275.5
**Database Date:**	2015-11-30	**pI:**	6.1
**Modification(s):**	Carbamidomethyl, Oxidation	**Sequence Coverage [%]:**	1.1
		**No. of unique Peptides:**	2

Quantitation

WD:WU	**Median:** 0.78	**CV:** 0.00 %	**No. of Peptides:** 1

Detailed Protein Report

10	20	30	40	50	60	70	80
MDRCMPEAFK	KLLTSSAVHK	WGTEIHEGIY	NMLMLLIELV	AERIKQDPIP	TGLLGVLTMA	FNPDNEYHFK	NRMKVSQRNW
90	100	110	120	130	140	150	160
AEVFGEGNMF	AVSPVSTFQK	EPHGWVVDLV	NKFGELGGFA	AIQAKLHSED	IELGAVSALI	QPLGVCAEYL	NSSVVQPMLD
170	180	190	200	210	220	230	240
PVILTTIQDV	RSVEEKDLKD	KRLVSIPELL	SAVKLLCMRF	QPDLVTIVDD	LRLDILLRML	KSPHFSAKMN	SLKEVTKLIE
250	260	270	280	290	300	310	320
DSTLSKSVKN	AIDTDRLLDW	LVENSVLSIA	LEGNIDQAQY	CDRIKGIIEL	LGSKLSLDEL	TKIWKIQSGQ	SSTVIENIHT
330	340	350	360	370	380	390	400
IIAAAAVKFN	SDQLNHLFVL	IQKSWETESD	RVRQKLLSLI	GRIGREARFE	TTSGKVLDVL	WELAHLPTLP	SSLIQQALEE
410	420	430	440	450	460	470	480
HLTILSDAYA	VKEAIKRSYI	IKCIEDIKRP	GEWSGLEKNK	KDGFKSSQLN	NPQFVWVVPA	LRQLHEITRS	FIKQTYQKQD
490	500	510	520	530	540	550	560
KSIIQDLKKN	FEIVKLVTGS	LIACHRLAAA	VAGPGGLSGS	TLVDGRYTYR	EYLEAHLKFL	AFFLQEATLY	LGWNRAKEIW
570	580	590	600	610	620	630	640
ECLVTGQDVC	ELDREMCFEW	FTKGQHDLES	DVQQQLFKEK	ILKLESYEIT	MNGFNLFKTF	FENVNLCDHR	LKRQGAQLYV
650	660	670	680	690	700	710	720
EKLELIGMDF	IWKIAMESPD	EEIANEAIQL	IINYSYINLN	PRLKKDSVSL	HKKFIADCYT	RLEAASSALG	GPTLTHAVTR
730	740	750	760	770	780	790	800
ATKMLTATAM	PTVATSVQSP	YRSTKLVIIE	RLLLLAERYV	ITIEDFYSVP	RTILPHGASF	HGHLLTLNVT	YESTKDTFTV
810	820	830	840	850	860	870	880
EAHSNETIGS	VRWKIAKQLC	SPVDNIQIFT	NDSLLTVNKD	QKLLHQLGFS	DEQILTVKTS	GSGTPSGSSA	DSSTSSSSSS
890	900	910	920	930	940	950	960
SGVFSSSYAM	EQEKSLPGVV	MALVCNVFDM	LYQLANLEEP	RITLRVRKLL	LLIPTDPAIQ	EALDQLDSLG	RKKTLLSESS
970	980	990	1000	1010	1020	1030	1040
SQSSKSPSLS	SKQQHQPSAS	SILESLFRSF	APGMSTFRVL	YNLEVLSSKL	MPTADDDMAR	SCAKSFCENF	LKAGGLSLVV
1050	1060	1070	1080	1090	1100	1110	1120
NVMQRDSIPS	EVDYETRQGV	YSICLQLARF	LLVGQTMPTL	LDEDLTKDGI	EALSSRPFRN	VSRQTSRQMS	LCGTPEKSSY
1130	1140	1150	1160	1170	1180	1190	1200
RQLSVSDRSS	IRVEEIIPAA	RVAIQTMEVS	DFTSTVACFM	RLSWAAAAGR	LDLVGSSQPI	KESNSLCPAG	IRNRLSSSGS
1210	1220	1230	1240	1250	1260	1270	1280
NCSSGSEGEP	VALHAGICVR	QQSVSTKDSL	IAGEALSLLV	TCLQLRSQQL	ASFYNLPCVA	DFIIDILLGS	PSAEIRRVAC
1290	1300	1310	1320	1330	1340	1350	1360
DQLYTLSQTD	TSAHPDVQKP	NQFLLGVILT	AQLPLWSPTS	IMRGVNQRLL	SQCMEYFDLR	CQLLDDLTTS	EMEQLRISPA
1370	1380	1390	1400	1410	1420	1430	1440
TMLEDEITWL	DNFEPNRTAE	CETSEADNIL	LAGHLRLIKT	LLSLCGAEKE	MLGSSLIKPL	LDDFLFRASR	IILNSHSPAG
1450	1460	1470	1480	1490	1500	1510	1520
SAAISQQDFH	PKCSTANSRL	AAYEVLVMLA	DSSPSNLQII	IKELLSMHHQ	PDPALTKEFD	YLPPVDSRSS	SGFVGLRNGG
1530	1540	1550	1560	1570	1580	1590	1600
ATCYMNAVFQ	QLYMQPGLPE	SLLSVDDDTD	NPDDSVFYQV	QSLFGHLMES	KLQYYVPENF	WKIFKMWNKE	LYVREQQDAY
1610	1620	1630	1640	1650	1660	1670	1680
EFFTSLIDQM	DEYLKKMGRD	QIFKNTFQGI	YSDQKICKDC	PHRYEREEAF	MALNLGVTSC	QSLEISLDQF	VRGEVLEGSN
1690	1700	1710	1720	1730	1740	1750	1760
AYYCEKCKEK	RITVKRTCIK	SLPSVLVIHL	MRFGFDWESG	RSIKYDEQIR	FPWMLNMEPY	TVSGMARQDS	SSEVGENGRS
1770	1780	1790	1800	1810	1820	1830	1840
VDQGGGGSPR	KKVALTENYE	LVGVIVHSGQ	AHAGHYYSFI	KDRRGCGKGK	WYKFNDTVIE	EFDLNDETLE	YECFGGEYRP
1850	1860	1870	1880	1890	1900	1910	1920
KVYDQTNPYT	DVRRRYWNAY	MLFYQRVSDQ	NSPVLPKKSR	VSVVRQEAED	LSLSAPSSPE	ISPQSSPRPH	RPNNDRLSIL
1930	1940	1950	1960	1970	1980	1990	2000
TKLVKKGEKK	GLFVEKMPAR	IYQMVRDENL	KFMKNRDVYS	SDYFSFVLSL	ASLNATKLKH	PYYPCMAKVS	LQLAIQFLFQ
2010	2020	2030	2040	2050	2060	2070	2080
TYLRTKKKLR	VDTEEWIATI	EALLSKSFDA	CQWLVEYFIS	SEGRELIKIF	LLECNVREVR	VAVATILEKT	LDSALFYQDK
2090	2100	2110	2120	2130	2140	2150	2160
LKSLHQLLEV	LLALLDKDVP	ENCKNCAQYF	FLFNTFVQKQ	GIRAGDLLLR	HSALRHMISF	LLGASRQNNQ	IRRWSSAQAR
2170	2180	2190	2200	2210	2220	2230	2240

Detailed Protein Report

Cmpd.	No. of Cmpds.	m/z meas.	Δ m/z [ppm]	z	Rt [min]	Score	P	Range	Sequence	Modification	Ratios
2676	1	585.8808	7.47	2	61.8	11.9	1	284-294	R.IKGIIELLGSK.L		
143	1	849.3662	-2.35	2	31.0	11.7	1	1010-1024	K.LMPTADDDMARSCAK.S	Carbamidomethyl: 13; Oxidation: 2	WD:WU 0.78

Detailed Protein Report

Protein 345: PREDICTED: CAD protein isoform X4 [Homo sapiens]

**Accession:**	gi|530368098	**Score:**	23.6
**Database:**	refseq_human(refseq_human_20140103.fasta)	**MW [kDa]:**	235.9
**Database Date:**	2015-11-30	**pI:**	6.1
		**Sequence Coverage [%]:**	1.4
		**No. of unique Peptides:**	1

Detailed Protein Report

10	20	30	40	50	60	70	80
MAALVLEDGS	VLRGQPFGAA	VSTAGEVVFQ	TGMVGYPEAL	TDPSYKAQIL	VLTYPLIGNY	GIPPDEMDEF	GLCKWFESSG
90	100	110	120	130	140	150	160
IHVAALVVGE	CCPTPSHWSA	TRTLHEWLQQ	HGIPGLQGVD	TRELTKKLRE	QGSLLGKLVQ	NGTEPSSLPF	LDPNARPLVP
170	180	190	200	210	220	230	240
EVSIKTPRVF	NTGGAPRILA	LDCGLKYNQI	RCLCQRGAEV	TVVPWDHALD	SQEYEGLFLS	NGPGDPASYP	SVVSTLSRVL
250	260	270	280	290	300	310	320
SEPNPRPVFG	ICLGHQLLAL	AIGAKTYKMR	YGNRGHNQPC	LLVGSGRCFL	TSQNHGFAVE	TDSLPADWAP	LFTNANDGSN
330	340	350	360	370	380	390	400
EGIVHNSLPF	FSVQFHPEHQ	AGPSDMELLF	DIFLETVKEA	TAGNPGGQTV	RERLTERLCP	PGIPTPGSGL	PPPRKVLILG
410	420	430	440	450	460	470	480
SGGLSIGQAG	EFDYSGSQAI	KALKEENIQT	LLINPNIATV	QTSQGLADKV	YFLPITPHYV	TQVIRNERPD	GVLLTFGGQT
490	500	510	520	530	540	550	560
ALNCGVELTK	AGVLARYGVR	VLGTPVETIE	LTEDRRAFAA	RMAEIGEHVA	PSEAANSLEQ	AQAAAERLGY	PVLVRAAFAL
570	580	590	600	610	620	630	640
GGLGSGFASN	REELSALVAP	AFAHTSQVLV	DKSLKGWKEI	EYEVVRDAYG	NCVTYYIIEV	NARLSRSSAL	ASKATGYPLA
650	660	670	680	690	700	710	720
YVAAKLALGI	PLPELRNSVT	GGTAAFEPSV	DYCVVKIPRW	DLSKFLRVST	KIGSCMKSVG	EVMGIGRSFE	EAFQKALRMV
730	740	750	760	770	780	790	800
DENCVGFDHT	VKPVSDMELE	TPTDKRIFVV	AAALWAGYSV	DRLYELTRID	RWFLHRMKRI	IAHAQLLEQH	RGQPLPPDLL
810	820	830	840	850	860	870	880
QQAKCLGFSD	KQIALAVLST	ELAVRKLRQE	LGICPAVKQI	DTVAAEWPAQ	TNYLYLTYWG	TTHDLTFRTP	HVLVLGSGVY
890	900	910	920	930	940	950	960
RIGSSVEFDW	CAVGCIQQLR	KMGYKTIMVN	YNPETVSTDY	DMCDRLYFDE	ISFEVVMDIY	ELENPEGVIL	SMGGQLPNNM
970	980	990	1000	1010	1020	1030	1040
AMALHRQQCR	VLGTSPEAID	SAENRFKFSR	LLDTIGISQP	QWRELSDLES	ARQFCQTVGY	PCVVRPSYVL	SGAAMNVAYT
1050	1060	1070	1080	1090	1100	1110	1120
DGDLERFLSS	AAAVSKEHPV	VISKFIQEAK	EIDVDAVASD	GVVAAIAISE	HVENAGVHSG	DATLVTPPQD	ITAKTLERIK
1130	1140	1150	1160	1170	1180	1190	1200
AIVHAVGQEL	QVTGPFNLQL	IAKDDQLKVI	ECNVRVSRSF	PFVSKTLGVD	LVALATRVIM	GEEVEPVGLM	TGSGVVGVKV
1210	1220	1230	1240	1250	1260	1270	1280
PQFSFSRLAG	ADVVLGVEMT	STGEVAGFGE	SRCEAYLKAM	LSTGFKIPKK	NILLTIGSYK	NKSELLPTVR	LLESLGYSLY
1290	1300	1310	1320	1330	1340	1350	1360
ASLGTADFYT	EHGVKVTAVD	WHFEEAVDGE	CPPQRSILEQ	LAEKNFELVI	NLSMRGAGGR	RLSSFVTKGY	RTRRLAADFS
1370	1380	1390	1400	1410	1420	1430	1440
VPLIIDIKCT	KLFVEALGQI	GPAPPLKVHV	DCMTSQKLVR	LPGLIDVHVH	LREPGGTHKE	DFASGTAAAL	AGGITMVCAM
1450	1460	1470	1480	1490	1500	1510	1520
PNTRPPIIDA	PALALAQKLA	EAGARCDFAL	FLGASSENAG	TLGTVAGSAA	GLKLYLNETF	SELRLDSVVQ	WMEHFETWPS
1530	1540	1550	1560	1570	1580	1590	1600
HLPIVAHAEQ	QTVAAVLMVA	QLTQRSVHIC	HVARKEEILL	IKAAKARGLP	VTCEVAPHHL	FLSHDDLERL	GPGKGEVRPE
1610	1620	1630	1640	1650	1660	1670	1680
LGSRQDVEAL	WENMAVIDCF	ASDHAPHTLE	EKCGSRPPPG	FPGLETMLPL	LLTAVSEGRL	SLDDLLQRLH	HNPRRIFHLP
1690	1700	1710	1720	1730	1740	1750	1760
PQEDTYVEVD	LEHEWTIPSH	MPFSKAHWTP	FEGQKVKGTV	RRVVLRGEVA	YIDGQVLVPP	GYGQDVRKWP	QGAVPQLPPS
1770	1780	1790	1800	1810	1820	1830	1840
APATSEMTTT	PERPRRGIPG	LPDGRFHLPP	RIHRASDPGL	PAEEPKEKSS	RKVAEPELMG	TPDGTCYPPP	PVPRQASPQN
1850	1860	1870	1880	1890	1900	1910	1920
LGTPGLLHPQ	TSPLLHSLVG	QHILSVQQFT	KDQMSHLFNV	AHTLRMMVQK	ERSLDILKGK	VMASMFYEVS	TRTSSSFAAA
1930	1940	1950	1960	1970	1980	1990	2000
MARLGGAVLS	FSEATSSVQK	GESLADSVQT	MSCYADVVVL	RHPQPGAVEL	AAKHCRRPVI	NAGDGVGEHP	TQALLDIFTI
2010	2020	2030	2040	2050	2060	2070	2080
REELGTVNGM	TITMVGDLKH	GRTVHSLACL	LTQYRVSLRY	VAPPSLRMPP	TVRAFVASRG	TKQEEFESIE	EALPDTDVLY
2090	2100	2110	2120	2130	2140	2150	2160
MTRIQKERFG	STQEYEACFG	QFILTPHIMT	RAKKKMVVMH	PMPRVNEISV	EVDSDPRAAY	FRQAENGMYI	RMALLATVLG
2170							

Detailed Protein Report

Cmpd.	No. of Cmpds.	m/z meas.	Δ m/z [ppm]	z	Rt [min]	Score	P	Range	Sequence	Modification
2542	1	836.1207	149.60	2	60.1	10.3	1	1354-1368	R.RLAADFSVPLIIDIK.C	

Detailed Protein Report

Protein 346: **melanotransferrin isoform 1 precursor [Homo sapiens]**

**Accession:**	gi|134244281	**Score:**	23.6
**Database:**	refseq_human(refseq_human_20140103.fasta)	**MW [kDa]:**	80.2
**Database Date:**	2015-11-30	**pI:**	5.5
**Modification(s):**	Oxidation	**Sequence Coverage [%]:**	3.8
		**No. of unique Peptides:**	1

10	20	30	40	50	60	70	80
MRGPSGALWL	LLALRTVLGG	MEVRWCATSD	PEQHKCGNMS	EAFREAGIQP	SLLCVRGTSA	DHCVQLIAAQ	EADAITLDGG
**90**	**100**	**110**	**120**	**130**	**140**	**150**	**160**
AIYEAGKEHG	LKPVVGEVYD	QEVGTSYYAV	AVVRRSSHVT	IDTLKGVKSC	HTGINRTVGW	NVPVGYLVES	GRLSVMGCDV
**170**	**180**	**190**	**200**	**210**	**220**	**230**	**240**
LKAVSDYFGG	SCVPGAGETS	YSESLCRLCR	GDSSGEGVCD	KSPLERYYDY	SGAFRCLAEG	AGDVAFVKHS	TVLENTDGKT
**250**	**260**	**270**	**280**	**290**	**300**	**310**	**320**
LPSWGQALLS	QDFELLCRDG	SRADVTEWRQ	CHLARVPAHA	VVVRADTDGG	LIFRLLNEGQ	RLFSHEGSSF	QMFSSEAYGQ
**330**	**340**	**350**	**360**	**370**	**380**	**390**	**400**
KDLLFKDSTS	ELVPIATQTY	EAWLGHEYLH	AMKGLLCDPN	RLPPYLRWCV	LSTPEIQKCG	DMAVAFRRQR	LKPEIQCVSA
**410**	**420**	**430**	**440**	**450**	**460**	**470**	**480**
KSPQHCMERI	QAEQVDAVTL	SGEDIYTAGK	TYGLVPAAGE	HYAPEDSSNS	YYVVAVVRRD	SSHAFTLDEL	RGKRSCHAGF
**490**	**500**	**510**	**520**	**530**	**540**	**550**	**560**
GSPAGWDVPV	GALIQRGFIR	PKDCDVLTAV	SEFFNASCVP	VNNPKNYPSS	LCALCVGDEQ	GRNKCVGNSQ	ERYYGYRGAF
**570**	**580**	**590**	**600**	**610**	**620**	**630**	**640**
RCLVENAGDV	AFVRHTTVFD	NTNGHNSEPW	AAELRSEDYE	LLCPNGARAE	VSQFAACNLA	QIPPHAVMVR	PDTNIFTVYG
**650**	**660**	**670**	**680**	**690**	**700**	**710**	**720**
LLDKAQDLFG	DDHNKNGFKM	FDSSNYHGQD	LLFKDATVRA	VPVGEKTTYR	GWLGLDYVAA	LEGMSSQQCS	GAAAPAPGAP
**730**	**740**						
LLPLLLPALA	ARLLPPAL						

Cmpd.	No. of Cmpds.	m/z meas.	Δ m/z [ppm]	z	Rt [min]	Score	P	Range	Sequence	Modification
2932	1	713.3656	28.08	2	65.4	11.9	2	379-390	K.CGDMAVAFRRQR.L	Oxidation: 4

Detailed Protein Report

Protein 347: vacuolar protein sorting-associated protein 13B isoform 1 [Homo sapiens]

**Accession:**	gi|35493701	**Score:**	23.5
**Database:**	refseq_human(refseq_human_20140103.fasta)	**MW [kDa]:**	445.7
**Database Date:**	2015-11-30	**pI:**	6.0
**Modification(s):**	Oxidation	**Sequence Coverage [%]:**	1.0
		**No. of unique Peptides:**	1

10	20	30	40	50	60	70	80
MLESYVTPIL	MSYVNRYIKN	LKPSDLQLSL	WGGDVVLSKL	ELKLDVLEQE	LKLPFTFLSG	HIHELRIHVP	WTKLGSEPVV
90	100	110	120	130	140	150	160
ITINTMECIL	KLKDGIQDDH	ESCGSNSTNR	STAESTKSSI	KPRRMQQAAP	TDPDLPPGYV	QSLIRRVVNN	VNIVINNLIL
170	180	190	200	210	220	230	240
KYVEDDIVLS	VNITSAECYT	VGELWDRAFM	DISATDLVLR	KVINFSDCTV	CLDKRNASGK	IEFYQDPLLY	KCSFRTRLHF
250	260	270	280	290	300	310	320
TYENLNSKMP	SVIKIHTLVE	SLKLSITDQQ	LPMFIRIMQL	GIALYYGEIG	NFKEGEIEDL	TCHNKDMLGN	ITGSEDETRI
330	340	350	360	370	380	390	400
DMQYPAQHKG	QELYSQQDEE	QPQGWVSWAW	SFVPAIVSYD	DGEEDFVGND	PASTMHQQKA	QTLKDPIVSI	GFYCTKATVT
410	420	430	440	450	460	470	480
FKLTEMQVES	SYYSPQKVKS	KEVLCWEQEG	TTVEALMMGE	PFFDCQIGFV	GCRAMCLKGI	MGVKDFEENM	NRSETEACFF
490	500	510	520	530	540	550	560
ICGDNLSTKG	FTYLTNSLFD	YRSPENNGTR	AEFILDSTHH	KETYTEIAGM	QRFGAFYMDY	LYTMENTSGK	GSTNQQDFSS
570	580	590	600	610	620	630	640
GKSEDLGTVQ	EKSTKSLVIG	PLDFRLDSSA	VHRILKMIVC	ALEHEYEPYS	RLKSDIKDEN	ETILNPEEVA	LLEEYIPTRH
650	660	670	680	690	700	710	720
TSVTLLKCTC	TISMAEFNLL	DHLLPVIMGE	KNSSNFMNTT	NFQSLRPLPS	IRILVDKINL	EHSVPMYAEQ	LVHVVSSLTQ
730	740	750	760	770	780	790	800
PSDNLLHYCY	VHCYLKIFGF	QAGLTSLDCS	GSYCLPVPVI	PSFSTALYGK	LLKLPTCWTK	RSQIAITEGI	FELPNLTIQA
810	820	830	840	850	860	870	880
TRAQTLLLQA	IYQSWSHLGN	VSSSAVIEAL	INEIFLSIGV	KSKNPLPTLE	GSIQNVELKY	CSTSLVKCAS	GTMGSIKICA
890	900	910	920	930	940	950	960
KAPVDSGKEK	LIPLLQGPSD	TKDLHSTKWL	NESRKPESLL	APDLMAFTIQ	VPQYIDYCHN	SGAVLLCSIQ	GLAVNIDPIL
970	980	990	1000	1010	1020	1030	1040
YTWLIYQPQK	RTSRHMQQQP	VVAVPLVMPV	CRRKEDEVSI	GSAPLAKQQS	YQASEYASSP	VKTKTVTESR	PLSVPVKAML
1050	1060	1070	1080	1090	1100	1110	1120
NISESCRSPE	ERMKEFIGIV	WNAVKHLTLQ	LEVQSCCVFI	PNDSLPSPST	IVSGDIPGTV	RSWYHGQTSM	PGTLVLCLPQ
1130	1140	1150	1160	1170	1180	1190	1200
IKIISAGHKY	MEPLQEIPFV	IPRPILEEGD	AFPWTISLHN	FSIYTLLGKQ	VTLCLVEPMG	CTSTLAVTSQ	KLLATGPDTR
1210	1220	1230	1240	1250	1260	1270	1280
HSFVVCLHVD	LESLEIKCSN	PQVQLFYELT	DIMNKVWNKI	QKRGNLNLSP	TSPETMAGPV	PTSPVRSSIG	TAPPDTSTCS
1290	1300	1310	1320	1330	1340	1350	1360
PSADIGTTTE	GDSIQAGEES	PFSDSVTLEQ	TTSNIGGTSG	RVSLWMQWVL	PKITIKLFAP	DPENKGTEVC	MVSELEDLSA
1370	1380	1390	1400	1410	1420	1430	1440
SIDVQDVYTK	VKCKIESFNI	DHYRSRPGEG	WQSGHFEGVF	LQCKEKSVTT	TKLLDGTHQQ	HGFLSLTYTK	AVTKNVRHKL
1450	1460	1470	1480	1490	1500	1510	1520
TSRNERRSFH	KLSEGLMDGS	PHFLHEILLS	AQAFDIVLYF	PLLNAIASIF	QAKLPKTQKE	KRKSPGQPMR	THTLTSRNLP
1530	1540	1550	1560	1570	1580	1590	1600
LIYVNTSVIR	IFIPKTEEMQ	PTVEANQAAK	EDTVVLKIGS	VAMAPQADNP	LGRSVLRKDI	YQRALNLGIL	RDPGSEIEDR
1610	1620	1630	1640	1650	1660	1670	1680
QYQIDLQSIN	IGTAQWHQLK	PEKESVSGGV	VTETERNSQN	PALEWNMASS	IRRHQERRAI	LTPVLTDFSV	RITGAPAVIF
1690	1700	1710	1720	1730	1740	1750	1760
TKVVSPENLH	TEEILVCGHS	LEVNITTNLD	FFLSVAQVQL	LHQLIVANMT	GLEPSNKAAE	ISKQEQKKVD	IFDGGMAETS
1770	1780	1790	1800	1810	1820	1830	1840
SRYSGAQDSG	IGSDSVKIRI	VQIEQHSGAS	QHRIARPSRQ	SSIVKNLNFI	PFDIFITASR	ISLMTYSCMA	LSKSKSQEQK
1850	1860	1870	1880	1890	1900	1910	1920
NNEKTDKSSL	NLPEVDSDVA	KPNQACISTV	TAEDLLRSSI	SFPSGKKIGV	LSLESLHAST	RSSARQALGI	TIVRQPGRRG
1930	1940	1950	1960	1970	1980	1990	2000
TGDLQLEPFL	YFIVSQPSLL	LSCHHRKQRV	EVSIFDAVLK	GVASDYKCID	PGKTLPEALD	YCTVWLQTVP	GEIDSKSGIP
2010	2020	2030	2040	2050	2060	2070	2080
PSFITLQIKD	FLNGPADVNL	DISKPLKANL	SFTKLDQINL	FLKKIKNAHS	LAHSEETSAM	SNTMVNKDDL	PVSKYYRGKL
2090	2100	2110	2120	2130	2140	2150	2160
SKPKIHGDGV	QKISAQENMW	RAVSCFQKIS	VQTTQIVISM	ETVPHTSKPC	LLASLSNLNG	SLSVKATQKV	PGIILGSSFL
2170	2180	2190	2200	2210	2220	2230	2240
LSINDFLLKT	SLKERSRILI	GPCCATANLE	AKWCKHSGNP	GPEQSIPKIS	IDLRGGLLQV	FWGQEHLNCL	VLLHELLNGY
2250	2260	2270	2280	2290	2300	2310	2320
LNEEGNFEVQ	VSEPVPQMSS	PVEKNQTFKS	EQSSDDLRTG	LFQYVQDAES	LKLPGVYEVL	FYNETEDCPG	MMLWRYPEPR
2330	2340	2350	2360	2370	2380	2390	2400

Detailed Protein Report

Cmpd.	No. of Cmpds.	m/z meas.	Δ m/z [ppm]	z	Rt [min]	Score	P	Range	Sequence	Modification
1842	1	1273.5438	-34.83	2	51.4	10.7	1	3110-3132	K.EYFRVPDSATFSICPGGEQPAMK.S	Oxidation: 22

Detailed Protein Report

Protein 348: obscurin isoform a [Homo sapiens]

**Accession:**	gi|403501448	**Score:**	23.5
**Database:**	refseq_human(refseq_human_20140103.fasta)	**MW [kDa]:**	721.1
**Database Date:**	2015-11-30	**pI:**	5.4
**Modification(s):**	Oxidation	**Sequence Coverage [%]:**	0.6
		**No. of unique Peptides:**	2

Detailed Protein Report

10	20	30	40	50	60	70	80
MDQPQFSGAP	RFLTRPKAFV	VSVGKDATLS	CQIVGNPTPQ	VSWEKDQQPV	AAGARFRLAQ	DGDLYRLTIL	DLALGDSGQY
90	100	110	120	130	140	150	160
VCRARNAIGE	AFAAVGLQVD	AEAACAEQAP	HFLLRPTSIR	VREGSEATFR	CRVGGSPRPA	VSWSKDGRRL	GEPDGPRVRV
170	180	190	200	210	220	230	240
EELGEASALR	IRAARPRDGG	TYEVRAENPL	GAASAAAALV	VDSDAADTAS	RPGTSTAALL	AHLQRRREAM	RAEGAPASPP
250	260	270	280	290	300	310	320
STGTRTCTVT	EGKHARLSCY	VTGEPKPETV	WKKDGQLVTE	GRRHVVYEDA	QENFVLKILF	CKQSDRGLYT	CTASNLVGQT
330	340	350	360	370	380	390	400
YSSVLVVVRE	PAVPFKKRLQ	DLEVREKESA	TFLCEVPQPS	TEAAWFKEET	RLWASAKYGI	EEEGTERRLT	VRNVSADDDA
410	420	430	440	450	460	470	480
VYICETPEGS	RTVAELAVQG	NLLRKLPRKT	AVRVGDTAMF	CVELAVPVGP	VHWLRNQEEV	VAGGRVAISA	EGTRHTLTIS
490	500	510	520	530	540	550	560
QCCLEDVGQV	AFMAGDCQTS	TQFCVSAPRK	PPLQPPVDPV	VKARMESSVI	LSWSPPPHGE	RPVTIDGYLV	EKKKLGTYTW
570	580	590	600	610	620	630	640
IRCHEAEWVA	TPELTVADVA	EEGNFQFRVS	ALNSFGQSPY	LEFPGTVHLA	PKLAVRTPLK	AVQAVEGGEV	TFSVDLTVAS
650	660	670	680	690	700	710	720
AGEWFLDGQA	LKASSVYEIH	CDRTRHTLTI	REVPASLHGA	QLKFVANGIE	SSIRMEVRAA	PGLTANKPPA	AAAREVLARL
730	740	750	760	770	780	790	800
HEEAQLLAEL	SDQAAAVTWL	KDGRTLSPGP	KYEVQASAGR	RVLLVRDVAR	DDAGLYECVS	RGGRIAYQLS	VQGLARFLHK
810	820	830	840	850	860	870	880
DMAGSCVDAV	AGGPAQFECE	TSEAHVHVHW	YKDGMELGHS	GERFLQEDVG	TRHRLVAATV	TRQDEGTYSC	RVGEDSVDFR
890	900	910	920	930	940	950	960
LRVSEPKVVF	AKEQLARRKL	QAEAGASATL	SCEVAQAQTE	VTWYKDGKKL	SSSSKVCMEA	TGCTRRLVVQ	QAGQADAGEY
970	980	990	1000	1010	1020	1030	1040
SCEAGGQRLS	FHLDVKEPKV	VFAKDQVAHS	EVQAEAGASA	TLSCEVAQAQ	TEVMWYKDGK	KLSSSLKVHV	EAKGCRRRLV
1050	1060	1070	1080	1090	1100	1110	1120
VQQAGKTDAG	DYSCEARGQR	VSFRLHITEP	KMMFAKEQSV	HNEVQAEAGA	SAMLSCEVAQ	AQTEVTWYKD	GKKLSSSSKV
1130	1140	1150	1160	1170	1180	1190	1200
GMEVKGCTRR	LVLPQAGKAD	AGEYSCEAGG	QRVSFHLHIT	EPKGVFAKEQ	SVHNEVQAEA	GTTAMLSCEV	AQPQTEVTWY
1210	1220	1230	1240	1250	1260	1270	1280
KDGKKLSSSS	KVRMEVKGCT	RRLVVQQVGK	ADAGEYSCEA	GGQRVSFQLH	ITEPKAVFAK	EQLVHNEVRT	EAGASATLSC
1290	1300	1310	1320	1330	1340	1350	1360
EVAQAQTEVT	WYKDGKKLSS	SSKVRIEAAG	CMRQLVVQQA	GQADAGEYTC	EAGGQRLSFH	LDVSEPKAVF	AKEQLAHRKV
1370	1380	1390	1400	1410	1420	1430	1440
QAEAGAIATL	SCEVAQAQTE	VTWYKDGKKL	SSSSKVRMEA	VGCTRRLVVQ	QACQADTGEY	SCEAGGQRLS	FSLDVAEPKV
1450	1460	1470	1480	1490	1500	1510	1520
VFAKEQPVHR	EVQAQAGAST	TLSCEVAQAQ	TEVMWYKDGK	KLSFSSKVRM	EAVGCTRRLV	VQQAGQAVAG	EYSCEAGSQR
1530	1540	1550	1560	1570	1580	1590	1600
LSFHLHVAEP	KAVFAKEQPA	SREVQAEAGT	SATLSCEVAQ	AQTEVTWYKD	GKKLSSSSKV	RMEAVGCTRR	LVVQEAGQAD
1610	1620	1630	1640	1650	1660	1670	1680
AGEYSCKAGD	QRLSFHLHVA	EPKVVFAKEQ	PAHREVQAEA	GASATLSCEV	AQAQTEVTWY	KDGKKLSSSS	KVRVEAVGCT
1690	1700	1710	1720	1730	1740	1750	1760
RRLVVQQAGQ	AEAGEYSCEA	GGQQLSFRLQ	VAELEPQISE	RPCRREPLVV	KEHEDIILTA	TLATPSAATV	TWLKDGVEIR
1770	1780	1790	1800	1810	1820	1830	1840
RSKRHETASQ	GDTHTLTVHG	AQVLDSAIYS	CRVGAEGQDF	PVQVEEVAAK	FCRLLEPVCG	ELGGTVTLAC	ELSPACAEVV
1850	1860	1870	1880	1890	1900	1910	1920
WRCGNTQLRV	GKRFQMVAEG	PVRSLTVLGL	RAEDAGEYVC	ESRDDHTSAQ	LTVSVPRVVK	FMSGLSTVVA	EEGGEATFQC
1930	1940	1950	1960	1970	1980	1990	2000
VVSPSDVAVV	WFRDGALLQP	SEKFAISQSG	ASHSLTISDL	VLEDAGQITV	EAEGASSSAA	LRVREAPVLF	KKKLEPQTVE
2010	2020	2030	2040	2050	2060	2070	2080
ERSSVTLEVE	LTRPWPELRW	TRNATALAPG	KNVEIHAEGA	RHRLVLHNVG	FADRGFFGCE	TPDDKTQAKL	TVEMRQVRLV
2090	2100	2110	2120	2130	2140	2150	2160
RGLQAVEARE	QGTATMEVQL	SHADVDGSWT	RDGLRFQQGP	TCHLAVRGPM	HTLTLSGLRP	EDSGLMVFKA	EGVHTSARLV
2170	2180	2190	2200	2210	2220	2230	2240
VTELPVSFSR	PLQDVVTTEK	EKVTLECELS	RPNVDVRWLK	DGVELRAGKT	MAIAAQGACR	SLTIYRCEFA	DQGVYVCDAH
2250	2260	2270	2280	2290	2300	2310	2320
DAQSSASVKV	QGRTYTLIYR	RVLAEDAGEI	QFVAENAESR	AQLRVKELPV	TLVRPLRDKI	AMEKHRGVLE	CQVSRASAQV
2330	2340	2350	2360	2370	2380	2390	2400

Detailed Protein Report

Cmpd.	No. of Cmpds.	m/z meas.	Δ m/z [ppm]	z	Rt [min]	Score	P	Range	Sequence	Modification
2872	1	686.8855	-49.05	2	64.6	10.5	2	881-892	R.LRVSEPKVVFAK.E	
1563	1	927.4401	-8.97	3	48.2	13.0	1	5326-5351	R.EDSGQYAAYISNAMGAAYSSARLLVR.G	Oxidation: 14

Detailed Protein Report

Protein 349: neuropeptides B/W receptor type 1 [Homo sapiens]

**Accession:**	gi|53828924	**Score:**	23.5
**Database:**	refseq_human(refseq_human_20140103.fasta)	**MW [kDa]:**	36.1
**Database Date:**	2015-11-30	**pI:**	10.1
		**Sequence Coverage [%]:**	3.4
		**No. of unique Peptides:**	1

10	20	30	40	50	60	70	80
MDNASFSEPW	PANASGPDPA	LSCSNASTLA	PLPAPLAVAV	PVVYAVICAV	GLAGNSAVLY	VLLRAPRMKT	VTNLFILNLA
**90**	**100**	**110**	**120**	**130**	**140**	**150**	**160**
IADELFTLVL	PINIADFLLR	QWPFGELMCK	LIVAIDQYNT	FSSLYFLTVM	SADRYLVVLA	TAESRRVAGR	TYSAARAVSL
**170**	**180**	**190**	**200**	**210**	**220**	**230**	**240**
AVWGIVTLVV	LPFAVFARLD	DEQGRRQCVL	VFPQPEAFWW	RASRLYTLVL	GFAIPVSTIC	VLYTTLLCRL	HAMRLDSHAK
**250**	**260**	**270**	**280**	**290**	**300**	**310**	**320**
ALERAKKRVT	FLVVAILAVC	LLCWTPYHLS	TVVALTTDLP	QTPLVIAISY	FITSLSYANS	CLNPFLYAFL	DASFRRNLRQ
**330**							
LITCRAAA							

Cmpd.	No. of Cmpds.	m/z meas.	Δ m/z [ppm]	z	Rt [min]	Score	P	Range	Sequence	Modification
408	2	604.3472	17.95	2	34.1	23.5	2	146-156	R.RVAGRTYSAAR.A	

Detailed Protein Report

Protein 350: plasminogen isoform 1 precursor [Homo sapiens]

Accession: gi|4505881
Database: refseq_human(refseq_human_20140103.fasta)
Database Date: 2015-11-30
Modification(s): Carbamidomethyl

Score: 23.5
MW [kDa]: 90.5
pI: 7.3
Sequence Coverage [%]: 3.1
No. of unique Peptides: 1

Quantitation

WD:WU **Median:** 1.93 **CV:** 0.00 % **No. of Peptides:** 1

10	20	30	40	50	60	70	80
MEHKEVVLLL	LLFLKSGQGE	PLDDYVNTQG	ASLFSVTKKQ	LGAGSIEECA	AKCEEDEEFT	CRAFQYHSKE	QQCVIMAENR
90	100	110	120	130	140	150	160
KSSIIIRMRD	VVLFEKKVYL	SECKTGNGKN	YRGTMSKTKN	GITCQKWSST	SPHRPRFSPA	THPSEGLEEN	YCRNPDNDPQ
170	180	190	200	210	220	230	240
GPWCYTTDPE	KRYDYCDILE	CEEECMHCSG	ENYDGKISKT	MSGLECQAWD	SQSPHAHGYI	PSKFPNKNLK	KNYCRNPDRE
250	260	270	280	290	300	310	320
LRPWCFTTDP	NKRWELCDIP	RCTTPPPSSG	PTYQCLKGTG	ENYRGNVAVT	VSGHTCQHWS	AQTPHTHNRT	PENFPCKNLD
330	340	350	360	370	380	390	400
ENYCRNPDGK	RAPWCHTTNS	QVRWEYCKIP	SCDSSPVSTE	QLAPTAPPEL	TPVVQDCYHG	DGQSYRGTSS	TTTTGKKCQS
410	420	430	440	450	460	470	480
WSSMTPHRHQ	KTPENYPNAG	LTMNYCRNPD	ADKGPWCFTT	DPSVRWEYCN	LKKCSGTEAS	VVAPPPVVLL	PDVETPSEED
490	500	510	520	530	540	550	560
CMFGNGKGYR	GKRATTVTGT	PCQDWAAQEP	HRHSIFTPET	NPRAGLEKNY	CRNPDGDVGG	PWCYTTNPRK	LYDYCDVPQC
570	580	590	600	610	620	630	640
AAPSFDCGKP	QVEPKKCPGR	VVGGCVAHPH	SWPWQVSLRT	RFGMHFCGGT	LISPEWVLTA	AHCLEKSPRP	SSYKVILGAH
650	660	670	680	690	700	710	720
QEVNLEPHVQ	EIEVSRLFLE	PTRKDIALLK	LSSPAVITDK	VIPACLPSPN	YVVADRTECF	ITGWGETQGT	FGAGLLKEAQ
730	740	750	760	770	780	790	800
LPVIENKVCN	RYEFLNGRVQ	STELCAGHLA	GGTDSCQGDS	GGPLVCFEKD	KYILQGVTSW	GLGCARPNKP	GVYVRVSRFV
810	820						
TWIEGVMRNN							

Cmpd.	No. of Cmpds.	m/z meas.	Δ m/z [ppm]	z	Rt [min]	Score	P	Range	Sequence	Modification	Ratios	
1599	1	1073.5631	49.12	2	48.7	12.7	1	236-252	R.NPDRELRPWCFTTDPNK.R	Carbamidomethyl: 10	WD:WU	1.93

Detailed Protein Report

Protein 351: vacuolar protein sorting-associated protein 13D isoform 1 [Homo sapiens]

**Accession:**	gi|54607139	**Score:**	23.4
**Database:**	refseq_human(refseq_human_20140103.fasta)	**MW [kDa]:**	491.6
**Database Date:**	2015-11-30	**pI:**	6.1
**Modification(s):**	Oxidation	**Sequence Coverage [%]:**	0.8
		**No. of unique Peptides:**	1

Detailed Protein Report

10	20	30	40	50	60	70	80
MLEGLVAWVL	NTYLGKYVNN	LNTDQLSVAL	LKGAVELENL	PLKKDALKEL	ELPFEVKAGF	IGKVTLQIPF	YRPHVDPWVI
90	100	110	120	130	140	150	160
SISSLHLIGA	PEKIQDFNDE	KEKLLERERK	KALLQALEEK	WKNDRQQKGE	SYWYSVTASV	VTRIVENIEL	KIQDVHLRFE
170	180	190	200	210	220	230	240
DGVTNPSHPF	AFGICIKNVS	MQNAVNEPVQ	KLMRKKQLDV	AEFSIYWDVD	CTLLGDLPQM	ELQEAMARSM	ESRSHHYVLE
250	260	270	280	290	300	310	320
PVFASALLKR	NCSKKPLRSR	HSPRIDCDIQ	LETIPLKLSQ	LQYRQIMEFL	KELERKERQV	KFRRWKPKVA	ISKNCREWWY
330	340	350	360	370	380	390	400
FALNANLYEI	REQRKRCTWD	FMLHRARDAV	SYTDKYFNKL	KGGLLSTDDK	EEMCRIEEEQ	SFEELKILRE	LVHDRFHKQE
410	420	430	440	450	460	470	480
ELAESLREPQ	FDSPGACPGA	PEPGGGSGML	QYLQSWFPGW	GGWYGQQTPE	GNVVEGLSAE	QQEQWIPEEI	LGTEEFFDPT
490	500	510	520	530	540	550	560
ADASCMNTYT	KRDHVFAKLN	LQLQRGTVTL	LHKEQGTPQM	NESAFMQLEF	SDVKLLAESL	PRRNSSLLSV	RLGGLFLRDL
570	580	590	600	610	620	630	640
ATEGTMFPLL	VFPNPQKEVG	RVSQSFGLQT	TSADRSDHYP	AADPDGPVFE	MLYERNPAHS	HFERRLNVST	RPLNIIYNPQ
650	660	670	680	690	700	710	720
AIKKVADFFY	KGKVHTSGFG	YQSELELRVA	EAARRQYNKL	KMQTKAEIRQ	TLDRLLVGDF	IEESKRWTVR	LDISAPQVIF
730	740	750	760	770	780	790	800
PDDFKFKNPV	LVVVDLGRML	LTNTQDNSRR	KSRDGSASEE	TQFSDDEYKT	PLATPPNTPP	PESSSSNGEK	TPPFSGVEFS
810	820	830	840	850	860	870	880
EEQLQAHLMS	TKMYERYSLS	FMDLQIMVGR	VKDNWKHVQD	IDVGPTHVVE	KFNVHLQLER	RLIYTSDPKY	PGAVLSGNLP
890	900	910	920	930	940	950	960
DLKIHINEDK	ISALKNCFAL	LTTPEMKTSD	TQIKEKIFPQ	EEQRGSLQDS	VMNLTQSIVL	LEQHTREVLV	ESQLLLAEFK
970	980	990	1000	1010	1020	1030	1040
VNCMQLGVES	NGRYISVLKV	FGTNAHFVKR	PYDAEVSLTV	HGLLLVDTMQ	TYGADFDLLM	ASHKNLSFDI	PTGSLRDSRA
1050	1060	1070	1080	1090	1100	1110	1120
QSPVSGPNVA	HLTDGATLND	RSATSVSLDK	ILTKEQESLI	KLEYQFVSSE	CPSMNLDSTL	QVISLQVNNL	DIILNPETIV
1130	1140	1150	1160	1170	1180	1190	1200
ELIGFLQKSF	PKEKDDLSPQ	PLMTDFERSF	REQGTYQSTY	EQNTEVAVEI	HRLNLLLLRT	VGMANREKYG	RKIATASIGG
1210	1220	1230	1240	1250	1260	1270	1280
TKVNVSMGST	FDMNGSLGCL	QLMDLTQDNV	KNQYVVSIGN	SVGYENIISD	IGYFESVFVR	MEDAALTEAL	SFTFVERSKQ
1290	1300	1310	1320	1330	1340	1350	1360
ECFLNLKMAS	LHYNHSAKFL	KELTLSMDEL	EENFRGMLKS	AATKVTTVLA	TKTAEYSEMV	SLFETPRKTR	EPFILEENEI
1370	1380	1390	1400	1410	1420	1430	1440
YGFDLASSHL	DTVKLILNIN	IESPVVSIPR	KPGSPELLVG	HLGQIFIQNF	VAGDDESRSD	RLQVEIKDIK	LYSLNCTQLA
1450	1460	1470	1480	1490	1500	1510	1520
GREAVGSEGS	RMFCPPSGSG	SANSQEEAHF	TRHDFFESLH	RGQAFHILNN	TTIQFKLEKI	PIERESELTF	SLSPDDLGTS
1530	1540	1550	1560	1570	1580	1590	1600
SIMKIEGKFV	NPVQVVLAKH	VYEQVLQTLD	NLVYSEDLNK	YPASATSSPC	PDSPLPPLST	CGESSVERKE	NGLFSHSSLS
1610	1620	1630	1640	1650	1660	1670	1680
NTSQKSLSVK	EVKSFTQIQA	TFCISELQVQ	LSGDLTLGAQ	GLVSLKFQDF	EVEFSKDHPQ	TLSIQIALHS	LLMEDLLEKN
1690	1700	1710	1720	1730	1740	1750	1760
PDSKYKNLMV	SRGAPKPSSL	AQKEYLSQSC	PSVSNVEYPD	MPRSLPSHME	EAPNVFQLYQ	RPTSASRKKQ	KEVQDKDYPL
1770	1780	1790	1800	1810	1820	1830	1840
TPPPSPTVDE	PKILVGKSKF	DDSLVHINIF	LVDKKHPEFS	SSYNRVNRSI	DVDFNCLDVL	ITLQTWVVIL	DFFGIGSTAD
1850	1860	1870	1880	1890	1900	1910	1920
NHAMRLPPEG	ILHNVKLEPH	ASMESGLQDP	VNTKLDLKVH	SLSLVLNKTT	SELAKANVSK	LVAHLEMIEG	DLALQGSIGS
1930	1940	1950	1960	1970	1980	1990	2000
LSLSDLTCHG	EFYRERFTTS	GEEALIFQTF	KYGRPDPLLR	REHDIRVSLR	MASVQYVHTQ	RFQAEVVAFI	QHFTQLQDVL
2010	2020	2030	2040	2050	2060	2070	2080
GRQRAAIEGQ	TVRDQAQRCS	RVLLDIEAGA	PVLLIPESSR	SNNLIVANLG	KLKVKNKFLF	AGFPGTFSLQ	DKESVPSASP
2090	2100	2110	2120	2130	2140	2150	2160
TGIPKHSLRK	TTSTEEPRGT	HSQGQFTMPL	AGMSLGSLKS	EFVPSTSTKQ	QGPQPTLSVG	QESSSPEDHV	CLLDCVVVDL
2170	2180	2190	2200	2210	2220	2230	2240
QDMDIFAAER	HPREYSKAPE	DSSGDLIFPS	YFVRQTGGSL	LTEPCRLKLQ	VERNLDKEIS	HTVPDISIHG	NLSSVHCSLD
2250	2260	2270	2280	2290	2300	2310	2320
LYKYKLIRGL	LENNLGEPIE	EFMRPYDLQD	PRIHTVLSGE	VYTCMCFLID	MVNVSLELKD	PKRKEGAGSL	ARFDFKKCKL
2330	2340	2350	2360	2370	2380	2390	2400

Detailed Protein Report

Cmpd.	No. of Cmpds.	m/z meas.	Δ m/z [ppm]	z	Rt [min]	Score	P	Range	Sequence	Modification
2118	1	900.3217	-110.13	3	54.8	10.2	0	2835-2858	K.SEDWMGSSVDPPCFGQSLPLVYLR.T	Oxidation: 5

Detailed Protein Report

Protein 352: regulator of G-protein signaling 11 isoform 3 [Homo sapiens]

**Accession:**	gi|557129067	**Score:**	23.4
**Database:**	refseq_human(refseq_human_20140103.fasta)	**MW [kDa]:**	51.5
**Database Date:**	2015-11-30	**pI:**	9.9
**Modification(s):**	Oxidation	**Sequence Coverage [%]:**	7.7
		**No. of unique Peptides:**	2

10	20	30	40	50	60	70	80
MAAGPAPPPG	RPRAQMPHLR	KMERVVVSMQ	DPDQGVKMRS	QRLLVTVIPH	AVTGSDVVQW	LAQKFCVSEE	EALHLGAVLV
**90**	**100**	**110**	**120**	**130**	**140**	**150**	**160**
QHGYIYPLRD	PRSLMLRPDE	TPYRFQVRLG	GAAIYLAKKN	IRKRGTLVDY	EKDCYDRLHK	KINHAWDLVL	MQAREQLRAA
**170**	**180**	**190**	**200**	**210**	**220**	**230**	**240**
KQRSKGDRLV	IACQEQTYWL	VNRPPPGAPD	VLEQGPGRGS	CAASRVLMTK	SADFHKREIE	YFRKALGRTR	VKSSVCLEAY
**250**	**260**	**270**	**280**	**290**	**300**	**310**	**320**
LSFCGQRGPH	DPLVSGCLPS	NPWISDNDAY	WVMNAPTVAA	PTKLRVERWG	FSFRELLEDP	VGRAHFMDFL	GKEFSGENLS
**330**	**340**	**350**	**360**	**370**	**380**	**390**	**400**
FWEACEELRY	GAQAQVPTLV	DAVYEQFLAP	GAAHWVNIDS	RTMEQTLEGL	RQPHRYVLDD	AQLHIYMLMK	KDSYPRFLKS
**410**	**420**	**430**	**440**	**450**	**460**		
DMYKALLAEA	GIPLEMKRRV	FPFTWRPRHS	SPSPALLPTP	VEPTAACGPG	GGDGVA		

Cmpd.	No. of Cmpds.	m/z meas.	Δ m/z [ppm]	z	Rt [min]	Score	P	Range	Sequence	Modification
2916	1	973.5104	15.53	2	65.2	12.7	2	21-37	R.KMERVVVSMQDPDQGVK.M	
1806	1	697.3618	15.85	3	50.9	10.7	1	295-312	R.ELLEDPVGRAHFMDFLGK.E	Oxidation: 13

Detailed Protein Report

Protein 353: PREDICTED: E3 ubiquitin-protein ligase LRSAM1 isoform X2 [Homo sapiens]

**Accession:**	gi|578817973	**Score:**	23.4
**Database:**	refseq_human(refseq_human_20140103.fasta)	**MW [kDa]:**	79.5
**Database Date:**	2015-11-30	**pI:**	5.5
**Modification(s):**	Carbamidomethyl, Oxidation	**Sequence Coverage [%]:**	2.6
		**No. of unique Peptides:**	2

10	20	30	40	50	60	70	80
MPLFFRKRKP	SEEARKRLEY	QMCLAKEAGA	DDILDISKCE	LSEIPFGAFA	TCKVLQKKVL	IVHTNHLTSL	LPKSCSLLSL
**90**	**100**	**110**	**120**	**130**	**140**	**150**	**160**
ATIKVLDLHD	NQLTALPDDL	GQLTALQVLN	VERNQLMQLP	RSIGNLTQLQ	TLNVKDNKLK	ELPDTVGELR	SLRTLNISGN
**170**	**180**	**190**	**200**	**210**	**220**	**230**	**240**
EIQRLPQMLA	HVRTLEMLSL	DASAMVYPPR	EVCGAGTAAI	LQFLCKESGL	EYYPPSQYLL	PILEQDGIEN	SRDSPDGPTD
**250**	**260**	**270**	**280**	**290**	**300**	**310**	**320**
RFSREELEWQ	NRFSDYEKRK	EQKMLEKLEF	ERRLELGQRE	HTQLLQQSSS	QKDEILQTVK	EEQSRLEQGL	SEHQRHLNAE
**330**	**340**	**350**	**360**	**370**	**380**	**390**	**400**
RQRLQEQLKQ	TEQNISSRIQ	KLLQDNQRQK	KSSEILKSLE	NERIRMEQLM	SITQEETESL	RRDVASAMQ	QMLTESCKNR
**410**	**420**	**430**	**440**	**450**	**460**	**470**	**480**
LIQMAYESQR	QNLVQQACSS	MAEMDERFQQ	ILSWQQMDQN	KAISQILQES	AMQKAAFEAL	QVKKDLMHRQ	IRSQIKLIET
**490**	**500**	**510**	**520**	**530**	**540**	**550**	**560**
ELLQLTQLEL	KRKSLDTESL	QEMISEQRWA	LSSLLQQLLK	EKQQREEELR	EILEEGMERQ	LVALLEELSA	EHYLPIFAHH
**570**	**580**	**590**	**600**	**610**	**620**	**630**	**640**
RLSLDLLSQM	SPGDLAKVGV	SEAGLQHEIL	RRVQELLDAA	RIQPELKPPM	GEVVTPTAPQ	EPPESVRPSA	PPAELEVQAS
**650**	**660**	**670**	**680**	**690**	**700**		
ECVVCLEREA	QMIFLNCGHV	CCCQQCCQPL	RTCPLCRQDI	AQRLRIYHSS			

Cmpd.	No. of Cmpds.	m/z meas.	Δ m/z [ppm]	z	Rt [min]	Score	P	Range	Sequence	Modification
1755	1	689.9840	-17.28	3	50.6	11.1	2	383-400	R.RDVASAMQQMLTESCKNR.L	
2902	1	992.7980	-150.93	2	65.0	12.3	1	384-400	R.DVASAMQQMLTESCKNR.L	Carbamidomethyl: 14; Oxidation: 6

Detailed Protein Report

Protein 354: acetylcholine receptor subunit delta isoform 2 precursor [Homo sapiens]

Accession: gi|375331911
Database: refseq_human(refseq_human_20140103.fasta)
Database Date: 2015-11-30

Score: 23.4
MW [kDa]: 57.0
pI: 6.6
Sequence Coverage [%]: 5.4
No. of unique Peptides: 2

Quantitation

WD:WU **Median:** 1.45 **CV:** 0.00 % **No. of Peptides:** 1

10	20	30	40	50	60	70	80
MEGPVLTLGL	LAALAVCGSW	GLNEEERLIR	HLFQEKGYNK	ELRPVAHKEE	SVDVALALTL	SNLISLGWTD	NRLKWNAEEF
**90**	**100**	**110**	**120**	**130**	**140**	**150**	**160**
GNISVLRLPP	DMVWLPEIVL	ENNNDGSFQI	SYSCNVLVYH	YGFVYWLPPA	IFRSSCPISV	TYFPFDWQNC	SLKFSSLKYT
**170**	**180**	**190**	**200**	**210**	**220**	**230**	**240**
AKEITLSLKQ	DAKENRTYPV	EWIIIDPEGF	TENGEWEIVH	RPARVNVDPR	APLDSPSRQD	ITFYLIIRRK	PLFYIINILV
**250**	**260**	**270**	**280**	**290**	**300**	**310**	**320**
PCVLISFMVN	LVFYLPADSG	EKTSVAISVL	LAQSVFLLLI	SKRLPATSMA	IPLIGKFLLF	GMVLVTMVVV	ICVIVLNIHF
**330**	**340**	**350**	**360**	**370**	**380**	**390**	**400**
RTPSTHVLSE	GVKKLFLETL	PELLHMSRPA	EDGPSPGALV	RRSSSLGYIS	KAEEYFLLKS	RSDLMFEKQS	ERHGLARRLT
**410**	**420**	**430**	**440**	**450**	**460**	**470**	**480**
TARRPPASSE	QAQQELFNEL	KPAVDGANFI	VNHMRDQNNY	NEEKDSWNRV	ARTVDRLCLF	VVTPVMVVGT	AWIFLQGVYN
**490**	**500**	**510**					
QPPPQPFPGD	PYSYNVQDKR	FI					

Cmpd.	No. of Cmpds.	m/z meas.	Δ m/z [ppm]	z	Rt [min]	Score	P	Range	Sequence	Modification	Ratios
1638	2	823.4155	-56.77	2	48.8	12.6	2	28-40	R.LIRHLFQEKGYNK.E		
1931	1	734.3090	-177.58	2	52.5	10.8	1	283-296	K.RLPATSMAIPLIGK.F		WD:WU 1.45

Detailed Protein Report

Protein 355: PREDICTED: polycystic kidney disease protein 1-like 1 isoform X1 [Homo sapiens]

**Accession:**	gi|578813318	**Score:**	23.3
**Database:**	refseq_human(refseq_human_20140103.fasta)	**MW [kDa]:**	223.0
**Database Date:**	2015-11-30	**pI:**	6.1
		**Sequence Coverage [%]:**	1.3
		**No. of unique Peptides:**	2

Quantitation

WD:WU	**Median:** 0.71	**CV:** 0.00 %	**No. of Peptides:** 1

Detailed Protein Report

10	20	30	40	50	60	70	80
MAEEAAQNIS	DDQERCLQAA	CCLSFGGELS	VSTDKSWGLH	LCSCSPPGGG	LWVEVYANHV	LLMSDGKCGC	PWCALNGKAE
90	100	110	120	130	140	150	160
DRESQSPSSS	ASRQKNIWKT	TSEAALSVVN	EKTQAVVNEK	TQAPLDCDNS	ADRIPHKPFI	IIARAWSSGG	PRFHHRRLCA
170	180	190	200	210	220	230	240
TGTADSTFSA	LLQLQGTTSA	AAPCSLKMEA	SCCVLRLLCC	AEDVATGLLP	GTVTMETPTK	VARPTQTSSQ	RVPLWPISHF
250	260	270	280	290	300	310	320
PTSPRSSHGL	PPGIPRTPSF	TASQSGSEIL	YPPTQHPPVA	ILARNSDNFM	NPVLNCSLEV	EARAPPNLGF	RVHMASGEAL
330	340	350	360	370	380	390	400
CLMMDFGDSS	GVEMRLHNMS	EAMAVTAYHQ	YSKEDWQRNS	QEGVYMLKAV	IYNEFHGTEV	ELGPYYVEIG	HEAVSAFMNS
410	420	430	440	450	460	470	480
SSVHEDEVLV	FADSQVNQKS	TVVIHHFPSI	PSYNVSFISQ	TQVGDSQAWH	SMTVWYKMQS	VSVYTNGTVF	ATDTDITFTA
490	500	510	520	530	540	550	560
VTKETIPLEF	EWYFGEDPPV	RTTSRSIKKR	LSIPQWYRVM	VKASNRMSSV	VSEPHVIRVQ	KKIVANRLTS	PSSALVNASV
570	580	590	600	610	620	630	640
AFECWINFGT	DVAYLWDFGD	GTVSLGSSSS	SHVYSREGEF	TVEVLAFNNV	SASTLRQQLF	IVCEPCQPPL	VKNMGPGKVQ
650	660	670	680	690	700	710	720
IWRSQPVRLG	VTFEAAVFCD	ISQGLSYTWN	LMDSEGLPVS	LPAAVDTHRQ	TLILPSHTLE	YGNYTALAKV	QIEGSVVYSN
730	740	750	760	770	780	790	800
YCVGLEVRAQ	APVSVISEGT	HLFFSRTTSS	PIVLRGTQSF	DPDDPGATLR	YHWECATAGS	PAHPCFDSST	AHQLDAAAPT
810	820	830	840	850	860	870	880
VSFEAQWLSD	SYDQFLVMLR	VSSGGRNSSE	TRVFLSPYPD	SAFRFVHISW	VSFKDTFVNW	NDELSLQAMC	EDCSEIPNLS
890	900	910	920	930	940	950	960
YSWDLFLVNA	TEKNRIEVPF	CRVVGLLGSL	GLGAISESSQ	LNLLPTEPGT	ADPDATTTPF	SREPSPVTLG	QPATSAPRGT
970	980	990	1000	1010	1020	1030	1040
PTEPMTGVYW	IPPAGDSAVL	GEAPEEGSLD	LEPGPQSKGS	LMTGRSERSQ	PTHSPDPHLS	DFEAYYSDIQ	EAIPSGGRQP
1050	1060	1070	1080	1090	1100	1110	1120
AKDTSFPGSG	PSLSAEESPG	DGDNLVDPSL	SAGRAEPVLM	IDWPKALLGR	AVFQGYSSSG	ITEQTVTIKP	YSLSSGETYV
1130	1140	1150	1160	1170	1180	1190	1200
LQVSVASKHG	LLGKAQLYLT	VNPAPRDMAC	QVQPHHGLEA	HTVFSVFCMS	GKPDFHYEFS	YQIGNTSKHT	LYHGRDTQYY
1210	1220	1230	1240	1250	1260	1270	1280
FVLPAGEHLD	NYKVMVSTEI	TDGKGSKVQP	CTVVVTVLPR	YHGNDCLGED	LYNSSLKNLS	TLQLMGSYTE	IRNYITVITR
1290	1300	1310	1320	1330	1340	1350	1360
ILSRLSKEDK	TASCNQWSRI	QDALISSVCR	LAFVDQEEMI	GSVLMLRDLV	SFSNKLGFMS	AVLILKYTRA	LLAQGQFSGP
1370	1380	1390	1400	1410	1420	1430	1440
FVIDKGVRLE	LIGLISRVWE	VSEQENSKEE	VYRHEEGITV	ISDLLLGCLS	LNHVSTGQME	FRTLLHYNLQ	SSVQSLGSVQ
1450	1460	1470	1480	1490	1500	1510	1520
VHLPGDLAGH	SPAGAETQSP	CYISQLILFK	KNPYPGSQAP	GQIGGVVGLN	LYTCSSRRPI	NRQWLRKPVM	VEFGEEDGLD
1530	1540	1550	1560	1570	1580	1590	1600
NRRNKTTFVL	LRDKVNLHQF	TELSENPQES	LQIEIEFSKP	VTRAFPVMLL	VRFSEKPTPS	DFLVKQIYFW	DESIVQIYIP
1610	1620	1630	1640	1650	1660	1670	1680
AASQKDASVG	YLSLLDADYD	RKPPNRYLAK	AVNYTVHFQW	IRCLFWDKRE	WKSERFSPQP	GTSPEKVNCS	YHRLAAFALL
1690	1700	1710	1720	1730	1740	1750	1760
RRKLKASFEV	SDISKLQSHP	ENLLPSIFIM	GSVILYGFLV	AKSRQVDHHE	KKKAGYIFLQ	EASLPGHQLY	AVVIDTGFRA
1770	1780	1790	1800	1810	1820	1830	1840
PARLTSKVYI	VLCGDNGLSE	TKELSCPEKP	LFERNSRHTF	ILSAPAQLGL	LRKIRLWHDS	RGPSPGWFIS	HVMVKELHTG
1850	1860	1870	1880	1890	1900	1910	1920
QGWFFPAQCW	LSAGRHDGRV	ERELTCLQGG	LGFRKLFYCK	FTEYLEDFHV	WLSVYSRPSS	SRYLHTPRLT	VSFSLLCVYA
1930	1940	1950	1960	1970	1980	1990	2000
CLTALVAAGG	QEQPHLDVSP	TLGSFRVGLL	CTLLASPGAQ	LLSLLFRLSK	EAPGSARVEP	HSPLRGGAQT	EAPHGPNSWG
2010	2020	2030					
RIPDAQEPRK	VNQSLCAAAN	LLA					

Cmpd.	No. of Cmpds.	m/z meas.	Δ m/z [ppm]	z	Rt [min]	Score	P	Range	Sequence	Modification	Ratios

Detailed Protein Report

Cmpd.	No. of Cmpds.	m/z meas.	Δ m/z [ppm]	z	Rt [min]	Score	P	Range	Sequence	Modification	Ratios
465	1	667.6915	-184.62	2	34.3	10.3	0	1378-1388	R.VWEVSEQENSK.E		WD:WU 0.71
714	1	541.1775	-140.74	3	37.4	13.0	0	1986-2001	R.GGAQTEAPHGPNSWGR.I		

Detailed Protein Report

Protein 356: coiled-coil domain-containing protein 168 [Homo sapiens]

**Accession:**	gi|226246554	**Score:**	23.3
**Database:**	refseq_human(refseq_human_20140103.fasta)	**MW [kDa]:**	801.4
**Database Date:**	2015-11-30	**pI:**	9.6
		**Sequence Coverage [%]:**	0.5
		**No. of unique Peptides:**	2

Detailed Protein Report

10	20	30	40	50	60	70	80
MSKQYYSFKK	GVGSGLEDNT	FMTLWDFLES	WIIQNDWVAI	FFIILLGIIF	EIILMKACAS	FWKKPTLPEK	GSSDVQETED
90	100	110	120	130	140	150	160
SCPKSRKLAP	ENWSVINSSS	GERVGTFLEK	RITSSLTSEE	KECNFEDRIL	FSREILWSGT	SESEDQVSPS	SESHVPSSNG
170	180	190	200	210	220	230	240
ISSSLPLFYS	EVEETCLSHT	EHPDREYETI	QFSSKKLFSM	MKTNKNKNSG	FSSDLSFSAS	RFTVENEDLD	VAPCPLAHLF
250	260	270	280	290	300	310	320
LSRDQVRLLE	ENVRNQIPSK	PKTKLGSRTT	YQCSRSQESL	NQNQPSVGMV	ISVQAQDSFP	GQNAFQNQGL	YEVQFTSQAQ
330	340	350	360	370	380	390	400
YINHNQESIK	SQPESKASNF	AQPEDVMKKP	FSSSTQDSFQ	SQDLDRNQHF	VEVPSIVEAK	YSVKGLESDE	HLGEDQHCVW
410	420	430	440	450	460	470	480
FIDSNKVKYS	IKGQDTIFKN	AEFLVLTLNP	NLVTEDMPQL	RSVKAQGQQQ	IVSSELNQDS	VYSSVPLLST	IKGQKNRRKT
490	500	510	520	530	540	550	560
PDSKSKLSLN	VPSLKAKKTP	TSQVFQITVC	HTLKNRNELG	CKNNTEKKEL	HERKDISDIA	LHLISVSKLI	LPYVKNYSRK
570	580	590	600	610	620	630	640
QLVKVMPGLI	KCGHFLQKQN	KSPDTEKINY	AGPLEETGIS	DITKKEKEYD	KENKRLKNIS	PKMLPQLEQS	FMVNTVQLKA
650	660	670	680	690	700	710	720
PCLLVETNGK	SKESLKDSIT	QAKGIGITEF	HVLNSKKPFD	LHIPKHKTSL	EEAISKPMQK	LVSSPEMESN	NRMKIQEDLQ
730	740	750	760	770	780	790	800
SSENSHLQLS	NGEELPTSTP	KTQRCFPREN	TQKQKDFLEL	VLELSNVGLL	ISPGSKMHKS	SEELEAIKIQ	VNTESVNLKE
810	820	830	840	850	860	870	880
SKPLILNVTE	DSDLRESEEL	ECNTGSNITN	MHQDKETSDA	FHSATYTTIS	QLPDTETHSI	SKAKADTLRI	IRLSHSASKQ
890	900	910	920	930	940	950	960
EKLPDEKETQ	NAEYIDKSCT	FKKPQQCDRK	EQEKEANSEL	TQGFRFSIHL	KQKPKYVKFQ	MEQISSGSSK	APNKEQEVQP
970	980	990	1000	1010	1020	1030	1040
QTLSTQTILE	NSPCPMMDPF	QVEKVKQSTD	RPTDRESAGD	PKNPLTMPEN	LPVGELLIET	TEYSVPFGGN	LQKTTDSHIA
1050	1060	1070	1080	1090	1100	1110	1120
EEKEDVKRYL	PAVALGSFNN	HLLTLPYFKR	QEIKKKLSET	KSVLSVKYVI	MKVKKPAISL	MPYINICGTS	NHRKKMGGNF
1130	1140	1150	1160	1170	1180	1190	1200
EIIIKQILQD	KIAAGMLLNV	IYPPMSILPN	TRMYSRLNAE	NHSHIKLVQE	ESQIEREEKY	PYFINEGNES	QNTLDAKLQD
1210	1220	1230	1240	1250	1260	1270	1280
EVKGVKETLP	KAVLHDSCNL	GLDAHLEKEI	KTEKEMHQPI	PFTETIIESV	VSPIMELSHA	ENVKSTQKTQ	TDCKCTADSE
1290	1300	1310	1320	1330	1340	1350	1360
TPSPISGKSL	IGDPLNQTRE	SYIPSNGSDT	REMGYCFAEE	KTEIPKDLPA	TSPETFNYCT	PVLSCSKVMK	KRVTFALTTS
1370	1380	1390	1400	1410	1420	1430	1440
TAKPKCVNTK	AVKPSISETV	SVTSHRKKSE	LDFKTKFKKI	NQTKGLVPEC	LNTLCSPMHS	RLQREFCLPA	SQLKQGETAD
1450	1460	1470	1480	1490	1500	1510	1520
KTYTDVFAKN	SISHDREEKL	QDGKEEEHKV	LLEAAPQLSQ	HLGSEAGQMK	EIHLESDPVL	NCLTLELHIN	GQRLQHQTGF
1530	1540	1550	1560	1570	1580	1590	1600
EQTTLETSLQ	MGPLEAEELQ	KANETENDIK	VLGGPKIPPP	KALQALENSD	GLILNAYQKD	NELVKSDEEL	NQPGSTNIQV
1610	1620	1630	1640	1650	1660	1670	1680
QPQTHFTQTI	LKSTSCPTLD	QFPFEKVESH	VRFSPLKSGE	AKVDEIIFYA	REGGISSDSS	HQKEQAGGTE	KKETAIFGSC
1690	1700	1710	1720	1730	1740	1750	1760
MPALSTPKTT	RNLKQFSDMK	TLVNPKCGII	KAKKPSISYM	LNIRAGAGPK	RRKELSCNLT	TKMKELHQGK	KGVDETYAFL
1770	1780	1790	1800	1810	1820	1830	1840
TMTPDINKYS	KVETEKDTLR	EKRLSSTQVK	QDTSPHEDSI	TSRDIKETLL	QDEEQEERKQ	EALLKVIPQH	LQHFMFRSGQ
1850	1860	1870	1880	1890	1900	1910	1920
GKDLDFHKLE	NQGSRKILFV	TKQDVPQQLQ	PAEPIQREET	KKCLQTQNGT	ICTVNSKLLP	LKSEDSVNGE	VLTGAIKRGV
1930	1940	1950	1960	1970	1980	1990	2000
PTDRKCMGEQ	HNSGKGEKAE	FNKDLQATVL	ELQKSPHGGE	AQKANLTDME	SGSSNAMNMN	VQHEREDKNI	QKMLTESVPC
2010	2020	2030	2040	2050	2060	2070	2080
YSQHLRFSTH	QMKDPDPCKS	GSEPKSPEGR	SWNLSHIVQK	TKQETHFRET	VLEPISGYMM	KQSPHMQEGI	KCMEGLKTSF
2090	2100	2110	2120	2130	2140	2150	2160
PKTGKSKIGS	IPRDTPWDEN	PRRKWDSSIS	EKTAWNQKNL	QTVLKPLDFS	SLMSSEYESR	SYTLEFIGKK	SMSPKCVTLK
2170	2180	2190	2200	2210	2220	2230	2240

Detailed Protein Report

Cmpd.	No. of Cmpds.	m/z meas.	Δ m/z [ppm]	z	Rt [min]	Score	P	Range	Sequence	Modification
2780	1	701.4146	-7.24	2	63.2	11.0	2	2385-2396	R.KIILNPKCLTMK.E	
2180	4	974.2038	45.87	3	55.6	12.3	2	5658-5682	R.DEDIYFTGFGTIRSGKRPEWLFTGK.K	

Detailed Protein Report

Protein 357: G protein-regulated inducer of neurite outgrowth 1 [Homo sapiens]

**Accession:**	gi|112821681	**Score:**	23.3
**Database:**	refseq_human(refseq_human_20140103.fasta)	**MW [kDa]:**	102.3
**Database Date:**	2015-11-30	**pI:**	9.3
		**Sequence Coverage [%]:**	3.5
		**No. of unique Peptides:**	1

Alias proteins:

Accession	Name	Description
gi|530380833	refseq_human (refseq_human_20140103.fasta)	PREDICTED: G protein-regulated inducer of neurite outgrowth 1 isoform X1 [Homo sapiens]

10	20	30	40	50	60	70	80
MDTAEDPAWL	QLLQKDSSPP	GPRPTAFFCP	QDGSLGAGSS	AMRDYCPSQQ	KASPAPPRHT	PDQSPGMESR	HRSPSGAGEG
90	100	110	120	130	140	150	160
ASCSDGPRGS	LACPSPTCFS	PQESPSKETL	EAHGASISGT	PEATTSGKPE	PVSSVKTEPK	SSDDRNPMFL	EKMDFKSSKQ
170	180	190	200	210	220	230	240
ADSTSIGKED	PGSSRKADPM	FTGKAEPEIL	GKGDPVAPGR	MDPMTVRKED	LGSLGKVDPL	CSSKTYTVSP	RKEDPGSLRK
250	260	270	280	290	300	310	320
VDPVSSDKVD	PVFPRKEEPR	YSGKEHPVSS	EKVAPTSAEK	VDLVLSGKRD	PGPSGKADPM	PLESMDSAST	GKTEPGLLGK
330	340	350	360	370	380	390	400
LIPGSSGKNG	PVSSGTGAPG	SLGRLDPTCL	GMADPASVGN	VETVPATKED	SRFLGKMDPA	SSGEGRPVSG	HTDTTASAKT
410	420	430	440	450	460	470	480
DLTSLKNVDP	MSSGKVDPVS	LGKMDPMCSG	KPELLSPGQA	ERVSVGKAGT	VSPGKEDPVS	SRREDPISAG	SRKTSSEKVN
490	500	510	520	530	540	550	560
PESSGKTNPV	SSGPGDPRSL	GTAGPPSAVK	AEPATGGKGD	PLSSEKAGLV	ASGKAAPTAS	GKAEPLAVGK	EDPVSKGKAD
570	580	590	600	610	620	630	640
AGPSGQGDSV	SIGKVVSTPG	KTVPVPSGKV	DPVSLGKAEA	IPEGKVGSLP	LEKGSPVTTT	KADPRASGKA	QPQSGGKAET
650	660	670	680	690	700	710	720
KLPGQEGAAA	PGEAGAVCLK	KETPQASEKV	DPGSCRKAEP	LASGKGEPVS	LGKADSAPSR	KTESPSLGKV	VPLSLEKTKP
730	740	750	760	770	780	790	800
SSSSRQLDRK	ALGSARSPEG	ARGSEGRVEP	KAEPVSSTEA	SSLGQKDLEA	AGAERSPCPE	AAAPPPGPRT	RDNFTKAPSW
810	820	830	840	850	860	870	880
EASAPPPPRE	DAGTQAGAQA	CVSVAVSPMS	PQDGAGGSAF	SFQAAPRAPS	PPSRRDAGLQ	VSLGAAETRS	VATGPMTPQA
890	900	910	920	930	940	950	960
AAPPAFPEVR	VRPGSALAAA	VAPPEPAEPV	RDVSWDEKGM	TWEVYGAAME	VEVLGMAIQK	HLERQIEEHG	RQGAPAPPPA
970	980	990	1000	1010			
ARAGPGRSGS	VRTAPPDGAA	KRPPGLFRAL	LQSVRRPRCC	SRAGPTAE			

Cmpd.	No. of Cmpds.	m/z meas.	Δ m/z [ppm]	z	Rt [min]	Score	P	Range	Sequence	Modification
2330	1	682.5941	-86.77	3	57.6	11.6	0	424-442	K.MDPMCSGKPELLSPGQAER.V	

Detailed Protein Report

Protein 358: PREDICTED: sperm flagellar protein 2 isoform X4 [Homo sapiens]

**Accession:**	gi|578809999	**Score:**	23.3
**Database:**	refseq_human(refseq_human_20140103.fasta)	**MW [kDa]:**	202.4
**Database Date:**	2015-11-30	**pI:**	5.2
**Modification(s):**	Oxidation	**Sequence Coverage [%]:**	2.0
		**No. of unique Peptides:**	2

10	20	30	40	50	60	70	80
MSEILCQWLN	KELKVSRTVS	PKSFAKAFSS	GYLLGEVLHK	FELQDDFSEF	LDSRVSSAKL	NNFSRLEPTL	NLLGVQFDQN
90	100	110	120	130	140	150	160
VAHGIITEKP	GVATKLLYQL	YIALQKKKKS	GLTGVEMQTM	QRLTNLRLQN	MKSDTFQEQY	LNRRRQNEIM	AKIQAAIIQI
170	180	190	200	210	220	230	240
PKPASNRTLK	ALEAQKMMKK	KKEAEDVADE	IKKFEALIKK	DLQAKESASK	TSLDTAGQTT	TDLLNTYSDD	EYIKKIQKRL
250	260	270	280	290	300	310	320
EEDAFAREQR	EKRRRKLLMD	QLIAHEAQEE	AYREEQLINR	LMRQSQQERR	IAVQLMHVRH	EKEVLWQNRI	FREKQHEERR
330	340	350	360	370	380	390	400
LKDFQDALDR	EAALAKQAKI	DFEEQFLKEK	RFHDQIAVER	AQARYEKHYS	VCAEILDQIV	DLSTKVADYR	MLTNNLIPYK
410	420	430	440	450	460	470	480
LMHDWKELFF	NAKPIYEQAS	VKTLPANPSR	EQLTELEKRD	LLDTNDYEEY	KNMVGEWALP	EEMVDNLPPS	NNCILGHILH
490	500	510	520	530	540	550	560
RLAEKSLPPR	AESTTPELPS	FAVKGCLLGK	TLSGKTTILR	SLQKDFPIQI	LSIDTLVQEA	IQAFHDNEKV	SEVLPIQKND
570	580	590	600	610	620	630	640
EEDALPVLQE	EIKESQDPQH	VFSAGPVSDE	VLPETEGETM	LSANADKTPK	AEEVKSSDSF	LKLTTRAQLG	AKSEQLLKKG
650	660	670	680	690	700	710	720
KSIPDVLLVD	IIVNAINEIP	VNQDCILDGF	PMTLNQAQLL	EEALTGCNRN	LTEVERKKAQ	KSTLAIDPAT	SKEIPLPSPA
730	740	750	760	770	780	790	800
FDFVILLDVS	DTSSMSRMND	IIAEELSYKT	AHEDISQRVA	AENQDKDGDQ	NLRDQIQHRI	IGFLDNWPLL	EQWFSEPENI
810	820	830	840	850	860	870	880
LIKINAEIDK	ESLCEKVKEI	LTTEIAKKKN	KVEKKLEEKE	AEKKAAASLA	ELPLPTPPPA	PPPEPEKEKE	IHQSHVASKT
890	900	910	920	930	940	950	960
PTAKGKPQSE	APHGKQESLQ	EGKGKKGETA	LKRKGSPKGK	SSGGKVPVKK	SPADSTDTSP	VAIVPQPPKP	GSEEWVYVNE
970	980	990	1000	1010	1020	1030	1040
PVPEEMPLFL	VPYWELIENS	YINTIKTVLR	HLREDQHTVL	AYLYEIRTSF	QEFLKRPDHK	QDFVAQWQAD	FNSLPDDLWD
1050	1060	1070	1080	1090	1100	1110	1120
DEETKAELHQ	RVNDLRDRLW	DICDARKEEA	EQERLDIINE	SWLQDTLGMT	MNHFFSLMQA	ELNRFQDTKR	LLQDYYWGME
1130	1140	1150	1160	1170	1180	1190	1200
SKIPVEDNKR	FTRIPLVQLD	SKDNSESQLR	IPLVPRISIS	LETVTPKPKT	KSVLKGKMDN	SLENVESNFE	ADEKLVMDTW
1210	1220	1230	1240	1250	1260	1270	1280
QQASLAVSHM	VAAEIHQRLM	EEEKENQPAD	PKEKSPQMGA	NKKVKKEPPK	KKQEDKKPKG	KSPPMAEATP	VIVTTEEIAE
1290	1300	1310	1320	1330	1340	1350	1360
IKRKNELRVK	IKEEHLAALQ	FEEIATQFRL	ELIKTKALAL	LEDLVTKVVD	VYKLMEKWLG	ERYLNEMAST	EKLTDVARYH
1370	1380	1390	1400	1410	1420	1430	1440
IETSTKIQNE	LYLSQEDFFI	NGNIKVFPDP	PPSIRPPPVE	KEEDGTLTIE	QLDSLRDQFL	DMAPKGIIGN	KAFTDILIDL
1450	1460	1470	1480	1490	1500	1510	1520
VTLNLGTNNF	PSNWMHLTQP	ELQELTSLLT	VNSEFVDWRK	FLLVTSMPWP	IPLEEELLET	LQKFKAVDKE	QLGTITFEQY
1530	1540	1550	1560	1570	1580	1590	1600
MQAGLWFTGD	EDIKIPENPL	EPLPFNRQEH	LIEFFFRLFA	DYEKDPPQLD	YTQMLLYFAC	HPDTVEGVYR	ALSVAVGTHV
1610	1620	1630	1640	1650	1660	1670	1680
FQQVKASIPS	AEKTSSTDAG	PAEEFPEPEE	NAAREERKLK	DDTEKREQKD	EEIPENANNE	KMSMETLLKV	FKGGSEAQDS
1690	1700	1710	1720	1730	1740	1750	1760
NRFASHLKIE	NIYAEGFIKT	FQDLGAKNLE	PIEVAVLLKH	PFIQDLISNY	SDYKFPVHII	YVVEMIVCAK	GWAPLPSGKQ
1770							
CARWRN							

Cmpd.	No. of	m/z meas.	Δ m/z	z	Rt	Score	P	Range	Sequence	Modification

Detailed Protein Report

	Cmpds.		[ppm]		[min]					
1496	1	1006.5889	17.49	3	47.4	12.9	2	1143-1169	K.DNSESQLRIPLVPRISISLETVTPKPK.T	
2688	1	484.7720	59.46	2	62.0	10.3	0	1662-1669	K.MSMETLLK.V	Oxidation: 1

Detailed Protein Report

Protein 359: **TBC1 domain family member 8B isoform b [Homo sapiens]**

Accession: gi|38570103
Database: refseq_human(refseq_human_20140103.fasta)
Database Date: 2015-11-30

Score: 23.3
MW [kDa]: 72.6
pI: 5.9
Sequence Coverage [%]: 4.0
No. of unique Peptides: 1

10	20	30	40	50	60	70	80
MWLKPEEVLL	KNALKLWLME	RSNDYFVLQR	RRGYGEEGGG	GLTGLLVGTL	DSVLDSTAKV	APFRILHQTP	DSQVYLSIAC
**90**	**100**	**110**	**120**	**130**	**140**	**150**	**160**
GANREEITKH	WDWLEQNIMK	TLSVFDSNED	ITNFVQGKIR	GLIAEEGKHC	FAKEDDPEKF	REALLKFEKC	FGLPEKEKLV
**170**	**180**	**190**	**200**	**210**	**220**	**230**	**240**
TYYSCSYWKG	RVPCQGWLYL	STNFLSFYSF	LLGSEIKLII	SWDEVSKLEK	TSNVILTESI	HVCSQGENHY	FSMFLHINQT
**250**	**260**	**270**	**280**	**290**	**300**	**310**	**320**
YLLMEQLANY	AIRRLFDKET	FDNDPVLYNP	LQITKRGLEN	RAHSEQFNAF	FRLPKGESLK	EVHECFLWVP	FSHFNTHGKM
**330**	**340**	**350**	**360**	**370**	**380**	**390**	**400**
CISENYICFA	SQDGNQCSVI	IPLREVLAID	KTNDSSKSVI	ISIKGKTAFR	FHEVKDFEQL	VAKLRLRCGA	ASTQYHDIST
**410**	**420**	**430**	**440**	**450**	**460**	**470**	**480**
ELAISSESTE	PSDNFEVQSL	TSQRECSKTV	NTEALMTVFH	PQNLETLNSK	MLKEKMKEQS	WKILFAECGR	GVSMFRTKKT
**490**	**500**	**510**	**520**	**530**	**540**	**550**	**560**
RDLVVRGIPE	TLRGELWMLF	SGAVNDMATN	PDYYTEVVEQ	SLGTCNLATE	EIERDLRRSL	PEHPAFQSDT	GISALRRVLT
**570**	**580**	**590**	**600**	**610**	**620**	**630**	**640**
AYAYRNPKIG	YCQAMNILTS	VLLLYAKEEE	AFWLLVAVCE	RMLPDYFNRR	IIGSDDFMPL	VRIQGQCVIG	EK

Cmpd.	No. of Cmpds.	m/z meas.	Δ m/z [ppm]	z	Rt [min]	Score	P	Range	Sequence	Modification
2720	1	941.9748	14.19	2	62.5	10.1	2	157-171	K.EKLVTYYSCSYWKGR.V	

Detailed Protein Report

Protein 360: fibronectin type-III domain-containing protein 3A isoform 2 [Homo sapiens]

**Accession:**	gi|118918397	**Score:**	23.2
**Database:**	refseq_human(refseq_human_20140103.fasta)	**MW [kDa]:**	125.7
**Database Date:**	2015-11-30	**pI:**	6.8
**Modification(s):**	Carbamidomethyl, Oxidation	**Sequence Coverage [%]:**	2.9
		**No. of unique Peptides:**	2

10	20	30	40	50	60	70	80
MSGPAQVPMM	SPNGSVPPIY	VPPGYAPQVI	EDNGVRRVVV	VPQAPEFHPG	SHTVLHRSPH	PPLPGFIPVP	TMMPPPPRHM
**90**	**100**	**110**	**120**	**130**	**140**	**150**	**160**
YSPVTGAGDM	TTQYMPQYQS	SQVYGDVDAH	STHGRSNFRD	ERSSKTYERL	QKKLKDRQGT	QKDKMSSPPS	SPQKCPSPIN
**170**	**180**	**190**	**200**	**210**	**220**	**230**	**240**
EHNGLIKGQI	AGGINTGSAK	IKSGKGKGGT	QVDTEIEEKD	EETKAFEALL	SNIVKPVASD	IQARTVVLTW	SPPSSLINGE
**250**	**260**	**270**	**280**	**290**	**300**	**310**	**320**
TDESSVPELY	GYEVLISSTG	KDGKYKSVYV	GEETNITLND	LKPAMDYHAK	VQAEYNSIKG	TPSEAEIFTT	LSCEPDIPNP
**330**	**340**	**350**	**360**	**370**	**380**	**390**	**400**
PRIANRTKNS	LTLQWKAPSD	NGSKIQNFVL	EWDEGKGNGE	FCQCYMGSQK	QFKITKLSPA	MGCKFRLSAR	NDYGTSGFSE
**410**	**420**	**430**	**440**	**450**	**460**	**470**	**480**
EVLYYTSGCA	PSMPASPVLT	KAGITWLSLQ	WSKPSGTPSD	EGISYILEME	EETSGYGFKP	KYDGEDLAYT	VKNLRRSTKY
**490**	**500**	**510**	**520**	**530**	**540**	**550**	**560**
KFKVIAYNSE	GKSNPSEVVE	FTTCPDKPGI	PVKPSVKGKI	HSHSFKITWD	PPKDNGGATI	NKYVVEMAEG	SNGNKWEMIY
**570**	**580**	**590**	**600**	**610**	**620**	**630**	**640**
SGATREHLCD	RLNPGCFYRL	RVYCISDGGQ	SAVSESLLVQ	TPAVPPGPCL	PPRLQGRPKA	KEIQLRWGPP	LVDGGSPISC
**650**	**660**	**670**	**680**	**690**	**700**	**710**	**720**
YSVEMSPIEK	DEPREVYQGS	EVECTVSSLL	PGKTYSFRLR	AANKMGFGPF	SEKCDITTAP	GPPDQCKPPQ	VTCRSATCAQ
**730**	**740**	**750**	**760**	**770**	**780**	**790**	**800**
VNWEVPLSNG	TDVTEYRLEW	GGVEGSMQIC	YCGPGLSYEI	KGLSPATTYY	CRVQALSVVG	AGPFSEVVAC	VTPPSVPGIV
**810**	**820**	**830**	**840**	**850**	**860**	**870**	**880**
TCLQEISDDE	IENPHYSPST	CLAISWEKPC	DHGSEILAYS	IDFGDKQSLT	VGKVTSYIIN	NLQPDTTYRI	RIQALNSLGA
**890**	**900**	**910**	**920**	**930**	**940**	**950**	**960**
GPFSHMIKLK	TKPLPPDPPR	LECVAFSHQN	LKLKWGEGTP	KTLSTDSIQY	HLQMEDKNGR	FVSLYRGPCH	TYKVQRLNES
**970**	**980**	**990**	**1000**	**1010**	**1020**	**1030**	**1040**
TSYKFCIQAC	NEAGEGPLSQ	EYIFTTPKSV	PAALKAPKIE	KVNDHICEIT	WECLQPMKGD	PVIYSLQVML	GKDSEFKQIY
**1050**	**1060**	**1070**	**1080**	**1090**	**1100**	**1110**	**1120**
KGPDSSFRYS	SLQLNCEYRF	RVCAIRQCQD	SLGHQDLVGP	YSTTVLFISQ	RTEPPASTNR	DTVESTRTRR	ALSDEQCAAV
**1130**	**1140**	**1150**					
ILVLFAFFSI	LIAFIIQYFV	IK					

Cmpd.	No. of Cmpds.	m/z meas.	Δ m/z [ppm]	z	Rt [min]	Score	P	Range	Sequence	Modification
2160	1	734.3496	-60.68	2	55.4	13.1	2	374-386	K.ITKLSPAMGCKFR.L	Oxidation: 8
1739	1	626.2821	-30.30	4	50.1	10.2	1	999-1018	K.IEKVNDHICEITWECLQPMK.G	Carbamidomethyl: 15; Oxidation: 19

Detailed Protein Report

Protein 361: PREDICTED: focadhesin isoform X1 [Homo sapiens]

Accession: gi|578816557
Database: refseq_human(refseq_human_20140103.fasta)
Database Date: 2015-11-30
Modification(s): Carbamidomethyl

Score: 23.2
MW [kDa]: 199.9
pI: 6.2
Sequence Coverage [%]: 1.4
No. of unique Peptides: 2

10	20	30	40	50	60	70	80
MSDDIRKRFE	FPNSLIQSQA	VGHLIAAVLK	ENGFSEKIHQ	STNQTPALNL	LWEKCCSDNV	VVRTACCEGL	VALVAQDHAE
90	100	110	120	130	140	150	160
FSYVLNGILN	LIPSTRNTHG	LIKAIMHLLQ	MQALKEGQGG	EKNIQSIYTI	RNHPHPLITV	LEHRPDCWPV	FLQQLTAFFQ
170	180	190	200	210	220	230	240
QCPERLEVSC	IQIMAPFLWY	LYCEPSQLQE	YAKLRLALLK	VLLQPQVLCD	KDQPSILEQQ	ILQLCCDIVP	CLQVKDLIQT
250	260	270	280	290	300	310	320
TEAMMFIEEV	CLSLLRHPVF	WKIQLTQMSL	QLLCVSEVSL	KITGECSSSI	HLLEHSVELL	KEDFPVELVI	IGIALLLLQT
330	340	350	360	370	380	390	400
PASQQKPILN	LALKLLSVTE	DQKIPKSSLL	LVMPILQILS	STALEDCISV	DEEGPSRQQL	ALNLLEMIQQ	ECYRDDHQKL
410	420	430	440	450	460	470	480
SYKLVCPVTS	MYGTIFTAWR	ILEVMTDSSA	ASDWLASVES	LLPITAVIPA	PAFLLLAHLL	VEDKGQNLHQ	ILKVTTELAQ
490	500	510	520	530	540	550	560
ADSSQVPNLI	PVLMFKLGRP	LEPILYNDIL	YTLPKLGVHK	VCIGQILRII	QLLGTTPRLR	AVTLRLLTSL	WEKQDRVYPE
570	580	590	600	610	620	630	640
LQRFMAVSDV	PSLSVGKEVQ	WEKLIAKAAS	IRDICKQRPY	QHGADMLAAI	SQVLNECTKP	DQATPAALVL	QGLHALCQAE
650	660	670	680	690	700	710	720
VVCIRSTWNA	LSPKLSCDTR	PLILKTLSEL	FSLVPSLTVN	TTEYENFKVQ	VLSFLWTHTQ	NKDPIVANAA	YRSLANFSAG
730	740	750	760	770	780	790	800
EHTILHLPEK	IRPEIPIPEE	LDDDEDVEDV	DLSVPGSCYL	KLLSLTPPLV	LPALEEFFTS	LVKQEMVNMP	RGIYHSALKG
810	820	830	840	850	860	870	880
GARSDQGKTV	AGIPNFILKM	YETNKQPGLK	PGLAGGMLFC	YDVSMYQSKD	GKPLNRLMAS	RGRSFKQTSL	ALVHEVHIQL
890	900	910	920	930	940	950	960
SEWHRAIFLP	QAWLAYMNRA	YHAILQGRLG	ELELQLKHGK	EEPEEVQYKK	STAWLWVRDM	LTDEITKAAA	KESPVVKGNA
970	980	990	1000	1010	1020	1030	1040
LLALSSLAVV	VSRHEASLSS	DSDGLLEVQP	NFLSMKEWVS	MVLDTLLVIV	DSHYQPRGQL	LSWFYYKSYS	GENTASAIAR
1050	1060	1070	1080	1090	1100	1110	1120
SAAATALSLL	VPVFIISCKE	KVEEILNMLT	ARLPGKPSAD	ESQAVQIHMG	LALGMFLSRL	CEEKLSDISG	QEMNLLLMKS
1130	1140	1150	1160	1170	1180	1190	1200
LDALENCCFD	TSLEYNTGCI	LGVGLVLSLM	SHSSQMQSRV	HVAALLRKLS	AHVDDSGSQS	RTFQEVLAYT	LSCVCTSAFS
1210	1220	1230	1240	1250	1260	1270	1280
AGIIEATEAE	DVMNKLRLLV	ENSQQTSGFA	LALGNIVHGL	SVCGHGKAED	LGSKLLPAWI	RIVLTEGTPT	MLCLAALHGM
1290	1300	1310	1320	1330	1340	1350	1360
VALVGSEGDV	MQLKSEAIQT	SHFQGRLNEV	IRTLTQVISV	SGVIGLQSNA	VWLLGHLHLS	TLSSSQSRAS	VPTDYSYLPE
1370	1380	1390	1400	1410	1420	1430	1440
SSFIGAAIGF	FITGGKKGPE	SVPPSLLKVV	MKPIATVGES	YQYPPVNWAA	LLSPLMRLNF	GEEIQQLCLE	IMVTQAQSSQ
1450	1460	1470	1480	1490	1500	1510	1520
NAAALLGLWV	TPPLIHSLSL	NTKRYLLISA	PLWIKHISDE	QILGFVENLM	VAVFKAASPL	GSPELCPSAL	HGLSQAMKLP
1530	1540	1550	1560	1570	1580	1590	1600
SPAHHLWSLL	SEATGKIFDL	LPNKIRRKDL	ELYISIAKCL	LEMTDDDANR	IAQVTKSNIE	KAAFVKLYLV	SQGRFPLVNL
1610	1620	1630	1640	1650	1660	1670	1680
TDMLSVAVQH	REKEVLAWMI	LHSLYQARIV	SHANTGVLKR	MEWLLELMGY	IRNVAYQSTS	FHNTALDKAL	DFFLLIFATA
1690	1700	1710	1720	1730	1740	1750	1760
VVAWADHTAP	LLLGLSASWL	PWHQENGPAG	PVPSFLGRSP	MHRVTLQEVL	TLLPNSMALL	LQKEPWKEQT	QKFIDWLFSI
1770	1780	1790	1800	1810			
MESPKEALSA	QSRDLLKATL	LSLRVLPEFK	KKAVWTRAYG	W			

Cmpd.	No. of	m/z meas.	Δ m/z	z	Rt	Score	P	Range	Sequence	Modification

Detailed Protein Report

	Cmpds.		[ppm]		[min]					
2532	1	729.9581	43.06	2	59.9	12.6	2	584-596	K.LIAKAASIRDICK.Q	Carbamidomethyl: 12
882	1	647.8007	-127.22	2	39.8	10.6	1	1629-1640	R.IVSHANTGVLKR.M	

Detailed Protein Report

Protein 362: PREDICTED: inositol 1,4,5-trisphosphate receptor type 2 isoform X1 [Homo sapiens]

**Accession:**	gi|578822836	**Score:**	23.1
**Database:**	refseq_human(refseq_human_20140103.fasta)	**MW [kDa]:**	212.9
**Database Date:**	2015-11-30	**pI:**	6.3
**Modification(s):**	Oxidation	**Sequence Coverage [%]:**	1.3
		**No. of unique Peptides:**	1

Quantitation

WD:WU	**Median:** 0.82	**CV:** 0.00 %	**No. of Peptides:** 1

Detailed Protein Report

10	20	30	40	50	60	70	80
MTEKMSSFLY	IGDIVSLYAE	GSVNGFISTL	GLVDDRCVVH	PEAGDLANPP	KKFRDCLFKV	CPMNRYSAQK	QYWKAKQAKQ
90	100	110	120	130	140	150	160
GNHTEAALLK	KLQHAAELEQ	KQNESENKKL	LGEIVKYSNV	IQLLHIKSNK	YLTVNKRLPA	LLEKNAMRVS	LDAAGNEGSW
170	180	190	200	210	220	230	240
FYIHPFWKLR	SEGDNIVVGD	KVVLMPVNAG	QPLHASNIEL	LDNPGCKEVN	AVNCNTSWKI	TLFMKYSSYR	EDVLKGGDVV
250	260	270	280	290	300	310	320
RLFHAEQEKF	LTCDEYEKKQ	HIFLRTTLRQ	SATSATSSKA	LWEIEVVHHD	PCRGGAGQWN	SLFRFKHLAT	GNYLAAELNP
330	340	350	360	370	380	390	400
DYRDAQNEGK	NVRDGVPPTS	KKKRQAGEKI	MYTLVSVPHG	NDIASLFELD	ATTLQRADCL	VPRNSYVRLR	HLCTNTWVTS
410	420	430	440	450	460	470	480
TSIPIDTDEE	RPVMLKIGTC	QTKEDKEAFA	IVSVPLSEVR	DLDFANDANK	VLATTVKKLE	NGTITQNERR	FVTKLLEDLI
490	500	510	520	530	540	550	560
FFVADVPNNG	QEVLDVVITK	PNRERQKLMR	EQNILAQVFG	ILKAPFKEKA	GEGSMLRLED	LGDQRYAPYK	YMLRLCYRVL
570	580	590	600	610	620	630	640
RHSQQDYRKN	QEYIAKNFCV	MQSQIGYDIL	AEDTITALLH	NNRKLLEKHI	TAKEIETFVS	LLRRNREPRF	LDYLSDLCVS
650	660	670	680	690	700	710	720
NTTAIPVTQE	LICKFMLSPG	NADILIQTKV	VSMQADNPME	SSILSDDIDD	EEVWLYWIDS	NKEPHGKAIR	HLAQEAKEGT
730	740	750	760	770	780	790	800
KADLEVLTYY	RYQLNLFARM	CLDRQYLAIN	QISTQLSVDL	ILRCVSDESL	PFDLRASFCR	LMLHMHVDRD	PQESVVPVRY
810	820	830	840	850	860	870	880
ARLWTEIPTK	ITIHEYDSIT	DSSRNDMKRK	FALTMEFVEE	YLKEVVNQPF	PFGDKEKNKL	TFEVVHLARN	LIYFGFYSFS
890	900	910	920	930	940	950	960
ELLRLTRTLL	AILDIVQAPM	SSYFERLSKF	QDGGNNVMRT	IHGVGEMMTQ	MVLSRGSIFP	MSVPDVPPSI	HPSKQGSPTE
970	980	990	1000	1010	1020	1030	1040
HEDVTVMDTK	LKIIEILQFI	LSVRLDYRIS	YMLSIYKKEF	GEDNDNAETS	ASGSPDTLLP	SAIVPDIDEI	AAQAETMFAG
1050	1060	1070	1080	1090	1100	1110	1120
RKEKNPVQLD	DEGGRTFLRV	LIHLIMHDYP	PLLSGALQLL	FKHFSQRAEV	LQAFKQVQLL	VSNQDVDNYK	QIKADLDQLR
1130	1140	1150	1160	1170	1180	1190	1200
LTVEKSELWV	EKSSNYENGE	IGESQVKGGE	EPIEESNILS	PVQDGTKKPQ	IDSNKSNNYR	IVKEILIRLS	KLCVQNKKCR
1210	1220	1230	1240	1250	1260	1270	1280
NQHQRLLKNM	GAHSVVLDLL	QIPYEKNDEK	MNEVMNLAHT	FLQNFCRGNP	QNQVLLHKHL	NLFLTPGLLE	AETMRHIFMN
1290	1300	1310	1320	1330	1340	1350	1360
NYHLCNEISE	RVVQHFVHCI	ETHGRHVEYL	RFLQTIVKAD	GKYVKKCQDM	VMTELINGGE	DVLIFYNDRA	SFPILLHMMC
1370	1380	1390	1400	1410	1420	1430	1440
SERDRGDESG	PLAYHITLVE	LLAACTEGKN	VYTEIKCNSL	LPLDDIVRVV	THDDCIPEVK	IAYVNFVNHC	YVDTEVEMKE
1450	1460	1470	1480	1490	1500	1510	1520
IYTSNHIWKL	FENFLVDMAR	VCNTTTDRKH	ADIFLEKCVT	ESIMNIVSGF	FNSPFSDNST	SLQTHQPVFI	QLLQSAFRIY
1530	1540	1550	1560	1570	1580	1590	1600
NCTWPNPAQK	ASVESCIRTL	AEVAKNRGIA	IPVDLDSQVN	TLFMKSHSNM	VQRAAMGWRL	SARSGPRFKE	ALGGPAWDYR
1610	1620	1630	1640	1650	1660	1670	1680
NIIEKLQDVV	ASLEHQFSPM	MQAEFSVLVD	VLYSPELLFP	EGSDARIRCG	AFMSKLINHT	KKLMEKEEKL	CIKILQTLRE
1690	1700	1710	1720	1730	1740	1750	1760
MLEKKDSFVE	EGNTLRKILL	NRYFKGDYSI	GVNGHLSGAY	SKTAQVGGSF	SGQDSDKMGI	SMSDIQCLLD	KEGASELVID
1770	1780	1790	1800	1810	1820	1830	1840
VIVNTKNDRI	FSEGIFLGIA	LLEGGNTQTQ	YSFYQQLHEQ	KKSEKFFKVL	YDRMKAAQKE	IRSTVTVNTI	DLGNKKRDDD
1850	1860	1870					
NELMTSGPRM	REFWSWGDPK	TALQD					

Cmpd.	No. of Cmpds.	m/z meas.	Δ m/z [ppm]	z	Rt [min]	Score	P	Range	Sequence	Modification	Ratios
1156	1	867.9132	-54.78	2	43.1	10.9	2	1647-1661	R.IRCGAFMSKLINHTK.K	Oxidation: 7	WD:WU 0.82

Detailed Protein Report

Protein 363: metallothionein-1A [Homo sapiens]

**Accession:**	gi|71274113	**Score:**	23.0
**Database:**	refseq_human(refseq_human_20140103.fasta)	**MW [kDa]:**	6.1
**Database Date:**	2015-11-30	**pI:**	10.5
**Modification(s):**	Carbamidomethyl, Oxidation	**Sequence Coverage [%]:**	32.8
		**No. of unique Peptides:**	2

```
         10         20         30         40         50         60         70
MDPNCSCATG GSCTCTGSCK CKECKCTSCK KSCCSCCPMS CAKCAQGCIC KGASEKCSCC A
```

Cmpd.	No. of Cmpds.	m/z meas.	Δ m/z [ppm]	z	Rt [min]	Score	P	Range	Sequence	Modification
2067	2	701.2708	34.48	3	54.4	11.5	1	32-51	K.SCCSCCPMSCAKCAQGCICK.G	Carbamidomethyl: 6; Oxidation: 8
2158	1	701.2771	43.49	3	55.5	11.5	1	32-51	K.SCCSCCPMSCAKCAQGCICK.G	Carbamidomethyl: 19; Oxidation: 8

Detailed Protein Report

Protein 364: glycogen phosphorylase, liver form isoform 2 [Homo sapiens]

Accession: gi|255653002
Database: refseq_human(refseq_human_20140103.fasta)
Database Date: 2015-11-30
Modification(s): Oxidation

Score: 23.0
MW [kDa]: 93.1
pI: 7.0
Sequence Coverage [%]: 2.8
No. of unique Peptides: 1

10	20	30	40	50	60	70	80
MAKPLTDQEK	RRQISIRGIV	GVENVAELKK	SFNRHLHFTL	VKDRNVATTR	DYYFALAHTV	RDHLVGRWIR	TQQHYYDKCP
90	100	110	120	130	140	150	160
KLGLDIEELE	EIEEDAGLGN	GGLGRLAACF	LDSMATLGLA	AYGYGIRYEY	GIFNQKIRDG	WQVEEADDWL	RYGNPWEKSR
170	180	190	200	210	220	230	240
PEFMLPVHFY	GKVEHTNTGT	KWIDTQVVLA	LPYDTPVPGY	MNNTVNTMRL	WSARAPNDFN	LRDFNVGDYI	QAVLDRNLAE
250	260	270	280	290	300	310	320
NISRVLYPND	NFFEGKELRL	KQEYFVVAAT	LQDIIRRFKA	SKFGSTRGAG	TVFDAFPDQV	AIQLNDTHPA	LAIPELMRIF
330	340	350	360	370	380	390	400
VDIEKLPWSK	AWELTQKTFA	YTNHTVLPEA	LERWPVDLVE	KLLPRHLEII	YEINQKHLDR	IVALFPKDVD	RLRRMSLIEE
410	420	430	440	450	460	470	480
EGSKRINMAH	LCIVGSHAVN	GVAKIHSDIV	KTKVFKDFSE	LEPDKFQNKT	NGITPRRWLL	LCNPGLAELI	AEKIGEDYVK
490	500	510	520	530	540	550	560
DLSQLTKLHS	FLGDDVFLRE	LAKVKQENKL	KFSQFLETEY	KVKINPSSMF	DVQVKRIHEY	KRQLLNCLHV	ITMYNRIKKD
570	580	590	600	610	620	630	640
PKKLFVPRTV	IIGGKAAPGY	HMAKMIIKLI	TSVADVVNND	PMVGSKLKVI	FLENYRVSLA	EKVIPATDLS	EQISTAGTEA
650	660	670	680	690	700	710	720
SGTGNMKFML	NGALTIGTMD	GANVEMAEEA	GEENLFIFGM	RIDDVAALDK	KGYEAKEYYE	ALPELKLVID	QIDNGFFSPK
730	740	750	760	770	780	790	800
QPDLFKDIIN	MLFYHDRFKV	FADYEAYVKC	QDKVSQLYMN	PKAWNTMVLK	NIAASGKFSS	DRTIKEYAQN	IWNVEPSDLK
810	820						
ISLSNESNKV	NGN						

Cmpd.	No. of Cmpds.	m/z meas.	Δ m/z [ppm]	z	Rt [min]	Score	P	Range	Sequence	Modification
772	1	647.8341	23.34	2	38.5	10.2	1	394-404	R.RMSLIEEEGSK.R	Oxidation: 2

Detailed Protein Report

**Protein 365:**	**1-phosphatidylinositol 4,5-bisphosphate phosphodiesterase eta-1 isoform c [Homo sapiens]**

**Accession:**	gi|195972873	**Score:**	23.0
**Database:**	refseq_human(refseq_human_20140103.fasta)	**MW [kDa]:**	114.4
**Database Date:**	2015-11-30	**pI:**	8.3
**Modification(s):**	Carbamidomethyl, Oxidation	**Sequence Coverage [%]:**	4.0
		**No. of unique Peptides:**	2

10	20	30	40	50	60	70	80
MADLEVYKNL	SPEKVERCMS	VMQSGTQMIK	LKRGTKGLVR	LFYLDEHRTR	LRWRPSRKSE	KAKILIDSIY	KVTEGRQSEI
**90**	**100**	**110**	**120**	**130**	**140**	**150**	**160**
FHRQAEGNFD	PSCCFTIYHG	NHMESLDLIT	SNPEEARTWI	TGLKYLMAGI	SDEDSLAKRQ	RTHDQWVKQT	FEEADKNGDG
**170**	**180**	**190**	**200**	**210**	**220**	**230**	**240**
LLNIEEIHQL	MHKLNVNLPR	RKVRQMFQEA	DTDENQGTLT	FEEFCVFYKM	MSLRRDLYLL	LLSYSDKKDH	LTVEELAQFL
**250**	**260**	**270**	**280**	**290**	**300**	**310**	**320**
KVEQKMNNVT	TDYCLDIIKK	FEVSEENKVK	NVLGIEGFTN	FMRSPACDIF	NPLHHEVYQD	MDQPLCNYYI	ASSHNTYLTG
**330**	**340**	**350**	**360**	**370**	**380**	**390**	**400**
DQLLSQSKVD	MYARVLQEGC	RCVEVDCWDG	PDGEPVVHHG	YTLTSKILFR	DVVETINKHA	FVKNEFPVIL	SIENHCSIQQ
**410**	**420**	**430**	**440**	**450**	**460**	**470**	**480**
QRKIAQYLKG	IFGDKLDLSS	VDTGECKQLP	SPQSLKGKIL	VKGKKLPYHL	GDDAEEGEVS	DEDSADEIED	ECKFKLHYSN
**490**	**500**	**510**	**520**	**530**	**540**	**550**	**560**
GTTEHQVESF	IRKKLESLLK	ESQIRDKEDP	DSFTVRALLK	ATHEGLNAHL	KQSPDVKESG	KKSHGRSLMT	NFGKHKKTTK
**570**	**580**	**590**	**600**	**610**	**620**	**630**	**640**
SRSKSYSTDD	EEDTQQSTGK	EGGQLYRLGR	RRKTMKLCRE	LSDLVVYTNS	VAAQDIVDDG	TTGNVLSFSE	TRAHQVVQQK
**650**	**660**	**670**	**680**	**690**	**700**	**710**	**720**
SEQFMIYNQK	QLTRIYPSAY	RIDSSNFNPL	PYWNAGCQLV	ALNYQSEGRM	MQLNRAKFKA	NGNCGYVLKP	QQMCKGTFNP
**730**	**740**	**750**	**760**	**770**	**780**	**790**	**800**
FSGDPLPANP	KKQLILKVIS	GQQLPKPPDS	MFGDRGEIID	PFVEVEIIGL	PVDCCKDQTR	VVDDNGFNPV	WEETLTFTVH
**810**	**820**	**830**	**840**	**850**	**860**	**870**	**880**
MPEIALVRFL	VWDHDPIGRD	FVGQRTVTFS	SLVPGYRHVY	LEGLTEASIF	VHITINEIYG	KWSPLILNPS	YTILHFLGAT
**890**	**900**	**910**	**920**	**930**	**940**	**950**	**960**
KNRQLQGLKG	LFNKNPRHSS	SENNSHYVRK	RSIGDRILRR	TASAPAKGRK	KSKMGFQEMV	EIKDSVSEAT	RDQDGVLRRT
**970**	**980**	**990**	**1000**	**1010**			
TRSLQARPVS	MPVDRNLLGA	LSLPVSETAK	DIEGKENSLV	QI			

Cmpd.	No. of Cmpds.	m/z meas.	Δ m/z [ppm]	z	Rt [min]	Score	P	Range	Sequence	Modification
2076	1	649.2852	-47.07	3	54.3	10.8	2	18-33	R.CMSVMQSGTQMIKLKR.G	Carbamidomethyl: 1; Oxidation: 2, 5, 11
845	1	1419.3283	69.70	2	39.4	12.2	1	379-402	K.HAFVKNEFPVILSIENHCSIQQQR.K	

Detailed Protein Report

Protein 366: dynein heavy chain 11, axonemal [Homo sapiens]

**Accession:**	gi|459642365	**Score:**	23.0
**Database:**	refseq_human(refseq_human_20140103.fasta)	**MW [kDa]:**	520.0
**Database Date:**	2015-11-30	**pI:**	6.0
		**Sequence Coverage [%]:**	0.5
		**No. of unique Peptides:**	1

Quantitation

WD:WU	**Median:** 0.42	**CV:** 0.00 %	**No. of Peptides:** 1

BRUKER

Detailed Protein Report

10	20	30	40	50	60	70	80
MAAQVAAREA	RDFREAPTLR	LTSGAGLEAV	GAVELEEEEE	NEEEAAARRA	RSFAQDARVR	FLGGRLAMML	GFTEEKWSQY
90	100	110	120	130	140	150	160
LESEDNRQVL	GEFLESTSPA	CLVFSFAASG	RLAASQEIPR	DANHKLVFIS	KKITESIGVN	DFSQVVLFGE	LPALSLGHVS
170	180	190	200	210	220	230	240
AFLDEILVPV	LSNKNNHKSW	SCFTSQDMEY	HIEVMKKKMY	IFRGKMSRRT	LLPIPTVAGK	MDLDQNCSEN	KPPSNERIIL
250	260	270	280	290	300	310	320
HAIESVVIEW	SHQIQEIIER	DSVQRLLNGL	HLSPQAELDF	WMMRRENLSC	IYDQLQAPVV	LKMVKILTTK	QSSYFPTLKD
330	340	350	360	370	380	390	400
IFLAVENALL	EAQDVELYLR	PLRRHIQCLQ	ETEFPQTRIL	IAPLFHTICL	IWSHSKFYNT	PARVIVLLQE	FCNLFINQAT
410	420	430	440	450	460	470	480
AYLSPEDLLR	GEIEESLEKV	QVAVNILKTF	KNSFFNYRKK	LASYFMGRKL	RPWDFQSHLV	FCRFDKFLDR	LIKIEDIFAT
490	500	510	520	530	540	550	560
TLEFEKLERL	EFGGTKGAIL	NGQVHEMSEE	LMELCKLFKQ	STYDPSDCTN	MEFESDYVAF	KSKTLEFDRR	LGTIICEAFF
570	580	590	600	610	620	630	640
NCNGLEAAFK	LLTIFGNFLE	KPVVMEIFSL	HYSTLVHMFN	TELDVCKQLY	NEHMKQIECG	HVVLNKNMPF	TSGNMKWAQQ
650	660	670	680	690	700	710	720
VLQRLQMFWS	NFASLRYLFL	GNPDHALVYQ	KYVEMTTLLD	QFESRIYNEW	KSNVDEICEF	NLNQPLVKFS	AINGLLCVNF
730	740	750	760	770	780	790	800
DPKLVAVLRE	VKYLLMLKKQ	DIPDSALAIF	KKRNTILKYI	GNLDLLVQGY	NKLKQTLLEV	EYPLIEDELR	AIDEQLTAAT
810	820	830	840	850	860	870	880
TWLTWQDDCW	GYIERVRAAT	SELEHRVERT	QKNVKVIQQT	MRGWARCVLP	PRREHRREAA	FTLEDKGDLF	TKKYKLIQGD
890	900	910	920	930	940	950	960
GCKIHNLVEE	NRKLFKANPS	LDTWKIYVEF	IDDIVVEGFF	QAIMHDLDFF	LKNTEKQLKP	APFFQAQMIL	LPPEIVFKPS
970	980	990	1000	1010	1020	1030	1040
LDREAGDGFY	DLVEEMLCNS	FRMSAQMNRI	ATHLEIKNYQ	NDMDNMLGLA	EVRQEIMNRV	VNVINKVLDF	RNTLETHTYL
1050	1060	1070	1080	1090	1100	1110	1120
WVDDRAEFMK	HFLLYGHAVS	SDEMDAHANE	EIPEQPPTLE	QFKEQIDIYE	ALYVQMSKFE	DFRVFDSWFK	VDMKPFKVSL
1130	1140	1150	1160	1170	1180	1190	1200
LTIIKKWSWM	FQEHLLRFVI	DSLNELQEFI	KETDSGLQRE	LNEGDHDGLV	DIMVHLLAVR	SRQRATDELF	EPLKETITLL
1210	1220	1230	1240	1250	1260	1270	1280
ESYGQKMPEQ	VYIQLEELPE	RWETTKKIAA	TVRHEVSPLH	NAEVTLIRKK	CILFDAKQAE	FRERFRHYAP	LGFNAENPYT
1290	1300	1310	1320	1330	1340	1350	1360
ALDKANEELE	ALEEEMLQMQ	ESTRLFEVAL	PEYKQMKQCR	KEIKLLKGLW	DVIIYVRRSI	DNWTKTQWRQ	IHVEQMDVEL
1370	1380	1390	1400	1410	1420	1430	1440
RRFAKEIWSL	NKEVRVWDAY	TGLEGTVKDM	TASLRAITEL	QSPALRDRHW	HQLMKAIGVK	FLINEATTLA	DLLALRLHRV
1450	1460	1470	1480	1490	1500	1510	1520
EDDVRRIVDK	AVKELGTEKV	ITEISQTWAT	MKFSYEVHYR	TGIPLLKSDE	QLFETLEHNQ	VQLQTLLQSK	YVEYFIEQVL
1530	1540	1550	1560	1570	1580	1590	1600
SWQNKLNIAD	LVIFTWMEVQ	RTWSHLESIF	VCSEDIRIQL	VKDARRFDGV	DAEFKELMFK	TAKVENVLEA	TCRPNLYEKL
1610	1620	1630	1640	1650	1660	1670	1680
KDLQSRLSLC	EKALAEYLET	KRIAFPRFYF	VSSADLLDIL	SKGAQPKQVT	CHLAKLFDSI	ADLQFEDNQD	VSAHRAVGMY
1690	1700	1710	1720	1730	1740	1750	1760
SKEKEYVPFQ	AECECVGHVE	TWLLQLEQTM	QETVRHSITE	AIVAYEEKPR	ELWIFDFPAQ	VALTSSQIWW	TTDVGIAFSR
1770	1780	1790	1800	1810	1820	1830	1840
LEEGYETALK	DFHKKQISQL	NTLITLLLGE	LPPGDRQKIM	TICTIDVHAR	DVVAKLISQK	VVSPQAFTWL	SQLRHRWEDT
1850	1860	1870	1880	1890	1900	1910	1920
QKHCFVNICD	AQFQYFYEYL	GNSPRLVITP	LTDRCYITLT	QSLHLTMSGA	PAGPAGTGKT	ETTKDLGRAL	GMMVYVFNCS
1930	1940	1950	1960	1970	1980	1990	2000
EQMDYKSIGN	IYKGLVQTGA	WGCFDEFNRI	SVEVLSVVAV	QVKMIHDAIR	NRKKRFVFLG	EAITLKPSVG	IFITMNPGYA
2010	2020	2030	2040	2050	2060	2070	2080
GRTELPENLK	ALFRPCAMVA	PDIELICEIL	LVAEGFVDAR	ALARKFITLY	TLCKELLSKQ	DHYDWGLRAI	KSVLVVAGSL
2090	2100	2110	2120	2130	2140	2150	2160
KRGDKNRPED	QVLMRALRDF	NMPKIVTDDI	PVFLGLVGDL	FPALDVPRRR	KLHFEQMVRQ	STLELRLQPE	ESFILKVVQL
2170	2180	2190	2200	2210	2220	2230	2240

Detailed Protein Report

Cmpd.	No. of Cmpds.	m/z meas.	Δ m/z [ppm]	z	Rt [min]	Score	P	Range	Sequence	Modification	Ratios
36	1	779.3158	-100.01	2	29.7	10.1	1	2083-2095	R.GDKNRPEDQVLMR.A		WD:WU 0.42

Detailed Protein Report

Protein 367: condensin complex subunit 1 [Homo sapiens]

**Accession:**	gi|178056552	**Score:**	22.9
**Database:**	refseq_human(refseq_human_20140103.fasta)	**MW [kDa]:**	157.1
**Database Date:**	2015-11-30	**pI:**	6.2
**Modification(s):**	Carbamidomethyl	**Sequence Coverage [%]:**	2.9
		**No. of unique Peptides:**	2

10	20	30	40	50	60	70	80
MAPQMYEFHL	PLSPEELLKS	GGVNQYVVQE	VLSIKHLPPQ	LRAFQAAFRA	QGPLAMLQHF	DTIYSILHHF	RSIDPGLKED
**90**	**100**	**110**	**120**	**130**	**140**	**150**	**160**
TLQFLIKVVS	RHSQELPAIL	DDTTLSGSDR	NAHLNALKMN	CYALIRLLES	FETMASQTNL	VDLDLGGKGK	KARTKAAHGF
**170**	**180**	**190**	**200**	**210**	**220**	**230**	**240**
DWEEERQPIL	QLLTQLLQLD	IRHLWNHSII	EEEFVSLVTG	CCYRLLENPT	INHQKNRPTR	EAITHLLGVA	LTRYNHMLSA
**250**	**260**	**270**	**280**	**290**	**300**	**310**	**320**
TVKIIQMLQH	FEHLAPVLVA	AVSLWATDYG	MKSIVGEIVR	EIGQKCPQEL	SRDPSGTKGF	AAFLTELAER	VPAILMSSMC
**330**	**340**	**350**	**360**	**370**	**380**	**390**	**400**
ILLDHLDGEN	YMMRNAVLAA	MAEMVLQVLS	GDQLEAAARD	TRDQFLDTLQ	AHGHDVNSFV	RSRVLQLFTR	IVQQKALPLT
**410**	**420**	**430**	**440**	**450**	**460**	**470**	**480**
RFQAVVALAV	GRLADKSVLV	CKNAIQLLAS	FLANNPFSCK	LSDADLAGPL	QKETQKLQEM	RAQRRTAAAS	AVLDPEEEWE
**490**	**500**	**510**	**520**	**530**	**540**	**550**	**560**
AMLPELKSTL	QQLLQLPQGE	EEIPEQIANT	ETTEDVKGRI	YQLLAKASYK	KAIILTREAT	GHFQESEPFS	HIDPEESEET
**570**	**580**	**590**	**600**	**610**	**620**	**630**	**640**
RLLNILGLIF	KGPAASTQEK	NPRESTGNMV	TGQTVCKNKP	NMSDPEESRG	NDELVKQEML	VQYLQDAYSF	SRKITEAIGI
**650**	**660**	**670**	**680**	**690**	**700**	**710**	**720**
ISKMMYENTT	TVVQEVIEFF	VMVFQFGVPQ	ALFGVRRMLP	LIWSKEPGVR	EAVLNAYRQL	YLNPKGDSAR	AKAQALIQNL
**730**	**740**	**750**	**760**	**770**	**780**	**790**	**800**
SLLLVDASVG	TIQCLEEILC	EFVQKDELKP	AVTQLLWERA	TEKVACCPLE	RCSSVMLLGM	MARGKPEIVG	SNLDTLVSIG
**810**	**820**	**830**	**840**	**850**	**860**	**870**	**880**
LDEKFPQDYR	LAQQVCHAIA	NISDRRKPSL	GKRHPPFRLP	QEHRLFERLR	ETVTKGFVHP	DPLWIPFKEV	AVTLIYQLAE
**890**	**900**	**910**	**920**	**930**	**940**	**950**	**960**
GPEVICAQIL	QGCAKQALEK	LEEKRTSQED	PKESPAMLPT	FLLMNLLSLA	GDVALQQLVH	LEQAVSGELC	RRRVLREEQE
**970**	**980**	**990**	**1000**	**1010**	**1020**	**1030**	**1040**
HKTKDPKEKN	TSSETTMEEE	LGLVGATADD	TEAELIRGIC	EMELLDGKQT	LAAFVPLLLK	VCNNPGLYSN	PDLSAAASLA
**1050**	**1060**	**1070**	**1080**	**1090**	**1100**	**1110**	**1120**
LGKFCMISAT	FCDSQLRLLF	TMLEKSPLPI	VRSNLMVATG	DLAIRFPNLV	DPWTPHLYAR	LRDPAQQVRK	TAGLVMTHLI
**1130**	**1140**	**1150**	**1160**	**1170**	**1180**	**1190**	**1200**
LKDMVKVKGQ	VSEMAVLLID	PEPQIAALAK	NFFNELSHKG	NAIYNLLPDI	ISRLSDPELG	VEEEPFHTIM	KQLLSYITKD
**1210**	**1220**	**1230**	**1240**	**1250**	**1260**	**1270**	**1280**
KQTESLVEKL	CQRFRTSRTE	RQQRDLAYCV	SQLPLTERGL	RKMLDNFDCF	GDKLSDESIF	SAFLSVVGKL	RRGAKPEGKA
**1290**	**1300**	**1310**	**1320**	**1330**	**1340**	**1350**	**1360**
IIDEFEQKLR	ACHTRGLDGI	KELEIGQAGS	QRAPSAKKPS	TGSRYQPLAS	TASDNDFVTP	EPRRTTRRHP	NTQQRASKKK
**1370**	**1380**	**1390**	**1400**	**1410**			
PKVVFSSDES	SEEDLSAEMT	EDETPKKTTP	ILRASARRHR	S			

Cmpd.	No. of Cmpds.	m/z meas.	Δ m/z [ppm]	z	Rt [min]	Score	P	Range	Sequence	Modification
1660	1	927.4336	-29.14	3	49.5	11.3	2	286-310	K.CPQELSRDPSGTKGFAAFLTELAER.V	Carbamidomethyl: 1
2825	1	913.4475	-27.71	2	63.9	11.6	0	1086-1100	R.FPNLVDPWTPHLYAR.L	

Detailed Protein Report

Protein 368: thymidine phosphorylase isoform 1 proprotein [Homo sapiens]

**Accession:**	gi|4503445	**Score:**	22.9
**Database:**	refseq_human(refseq_human_20140103.fasta)	**MW [kDa]:**	49.9
**Database Date:**	2015-11-30	**pI:**	5.2
**Modification(s):**	Oxidation	**Sequence Coverage [%]:**	6.4
		**No. of unique Peptides:**	2

Quantitation

WD:WU **Median:** 1.63 **CV:** 0.00 % **No. of Peptides:** 1

Alias proteins:

Accession	Name	Description
gi|384229049	refseq_huma (refseq_human_20140103.fasta)	thymidine phosphorylase isoform 1 proprotein [Homo sapiens]
gi|166158925	refseq_huma (refseq_human_20140103.fasta)	thymidine phosphorylase isoform 1 proprotein [Homo sapiens]
gi|166158922	refseq_huma (refseq_human_20140103.fasta)	thymidine phosphorylase isoform 1 proprotein [Homo sapiens]

```
        10         20         30         40         50         60         70         80
MAALMTPGTG APPAPGDFSG EGSQGLPDPS PEPKQLPELI RMKRDGGRLS EADIRGFVAA VVNGSAQGAQ IGAMLMAIRL
        90        100        110        120        130        140        150        160
RGMDLEETSV LTQALAQSGQ QLEWPEAWRQ QLVDKHSTGG VGDKVSLVLA PALAACGCKV PMISGRGLGH TGGTLDKLES
       170        180        190        200        210        220        230        240
IPGFNVIQSP EQMQVLLDQA GCCIVGQSEQ LVPADGILYA ARDVTATVDS LPLITASILS KKLVEGLSAL VVDVKFGGAA
       250        260        270        280        290        300        310        320
VFPNQEQARE LAKTLVGVGA SLGLRVAAAL TAMDKPLGRC VGHALEVEEA LLCMDGAGPP DLRDLVTTLG GALLWLSGHA
       330        340        350        360        370        380        390        400
GTQAQGAARV AAALDDGSAL GRFERMLAAQ GVDPGLARAL CSGSPAERRQ LLPRAREQEE LLAPADGTVE LVRALPLALV
       410        420        430        440        450        460        470        480
LHELGAGRSR AGEPLRLGVG AELLVDVGQR LRRGTPWLRV HRDGPALSGP QSRALQEALV LSDRAPFAAP SPFAELVLPP
       490
QQ
```

Cmpd.	No. of Cmpds.	m/z meas.	Δ m/z [ppm]	z	Rt [min]	Score	P	Range	Sequence	Modification	Ratios	
41	1	906.3675	-118.89	2	29.5	10.9	1	140-157	K.VPMISGRGLGHTGGTLDK.L	Oxidation: 3	WD:WU	1.63
1488	1	671.3911	-17.76	2	47.0	12.0	0	223-235	K.LVEGLSALVVDVK.F			

Detailed Protein Report

Protein 369: PREDICTED: stabilin-1 isoform X3 [Homo sapiens]

**Accession:**	gi|530372115	**Score:**	22.9
**Database:**	refseq_human(refseq_human_20140103.fasta)	**MW [kDa]:**	273.1
**Database Date:**	2015-11-30	**pI:**	6.1
**Modification(s):**	Carbamidomethyl	**Sequence Coverage [%]:**	0.9
		**No. of unique Peptides:**	1

Detailed Protein Report

10	20	30	40	50	60	70	80
MAGPRGLLPL	CLLAFCLAGF	SFVRGQVLFK	GCDVKTTFVT	HVPCTSCAAI	KKQTCPSGWL	RELPDQITQD	CRYEVQLGGS
90	100	110	120	130	140	150	160
MVSMSGCRRK	CRKQVVQKAC	CPGYWGSRCH	ECPGGAETPC	NGHGTCLDGM	DRNGTCVCQE	NFRGSACQEC	QDPNRFGPDC
170	180	190	200	210	220	230	240
QSVCSCVHGV	CNHGPRGDGS	CLCFAGYTGP	HCDQELPVCQ	ELRCPQNTQC	SAEAPSCRCL	PGYTQQGSEC	RAPNPCWPSP
250	260	270	280	290	300	310	320
CSLLAQCSVS	PKGQAQCHCP	ENYHGDGMVC	LPKDPCTDNL	GGCPSNSTLC	VYQKPGQAFC	TCRPGLVSIN	SNASAGCFAF
330	340	350	360	370	380	390	400
CSPFSCDRSA	TCQVTADGKT	SCVCRESEVG	DGRACYGHLL	HEVQKATQTG	RVFLQLRVAV	AMMDQGCREI	LTTAGPFTVL
410	420	430	440	450	460	470	480
VPSVSSFSSR	TMNASLAQQL	CRQHIIAGQH	ILEDTRTQQT	RRWWTLAGQE	ITVTFNQFTK	YSYKYKDQPQ	QTFNIYKANN
490	500	510	520	530	540	550	560
IAANGVFHVV	TGLRWQAPSG	TPGDPKRTIG	QILASTEAFS	RFETILENCG	LPSILDGPGP	FTVFAPSNEA	VDSLRDGRLI
570	580	590	600	610	620	630	640
YLFTAGLSKL	QELVRYHIYN	HGQLTVEKLI	SKGRILTMAN	QVLAVNISEE	GRILLGPEGV	PLQRVDVMAA	NGVIHMLDGI
650	660	670	680	690	700	710	720
LLPPTILPIL	PKHCSEEQHK	IVAGSCVDCQ	ALNTSTCPPN	SVKLDIFPKE	CVYIHDPTGL	NVLKKGCASY	CNQTIMEQGC
730	740	750	760	770	780	790	800
CKGFFGPDCT	QCPGGFSNPC	YGKGNCSDGI	QGNGACLCFP	DYKGIACHIC	SNPNKHGEQC	QEDCGCVHGL	CDNRPGSGGV
810	820	830	840	850	860	870	880
CQQGTCAPGF	SGRFCNESMG	DCGPTGLAQH	CHLHARCVSQ	EGVARCRCLD	GFEGDGFSCT	PSNPCSHPDR	GGCSENAECV
890	900	910	920	930	940	950	960
PGSLGTHHCT	CHKGWSGDGR	VCVAIDECEL	DMRGGCHTDA	LCSYVGPGQS	RCTCKLGFAG	DGYQCSPIDP	CRAGNGGCHG
970	980	990	1000	1010	1020	1030	1040
LATCRAVGGG	QRVCTCPPGF	GGDGFSCYGD	IFRELEANAH	FSIFYQWLKS	AGITLPADRR	VTALVPSEAA	VRQLSPEDRA
1050	1060	1070	1080	1090	1100	1110	1120
FWLQPRTLPN	LVRAHFLQGA	LFEEELARLG	GQEVATLNPT	TRWEIRNISG	RVWVQNASVD	VADLLATNGV	LHILSQVLLP
1130	1140	1150	1160	1170	1180	1190	1200
PRGDVPGGQG	LLQQLDLVPA	FSLFRELLQH	HGLVPQIEAA	TAYTIFVPTN	RSLEAQGNSS	HLDADTVRHH	VVLGEALSME
1210	1220	1230	1240	1250	1260	1270	1280
TLRKGGHRNS	LLGPAHWIVF	YNHSGQPEVN	HVPLEGPMLE	APGRSLIGLS	GVLTVGSSRC	LHSHAEALRE	KCVNCTRRFR
1290	1300	1310	1320	1330	1340	1350	1360
CTQGFQLQDT	PRKSCVYRSG	FSFSRGCSYT	CAKKIQVPDC	CPGFFGTLCE	PCPGGLGGVC	SGHGQCQDRF	LGSGECHCHE
1370	1380	1390	1400	1410	1420	1430	1440
GFHGTACEVC	ELGRYGPNCT	GVCDCAHGLC	QEGLQGDGSC	VCNVGWQGLR	CDQKITSPQC	PRKCDPNANC	VQDSAGASTC
1450	1460	1470	1480	1490	1500	1510	1520
ACAAGYSGNG	IFCSEVDPCA	HGHGGCSPHA	NCTKVAPGQR	TCTCQDGYMG	DGELCQEINS	CLIHHGGCHI	HAECIPTGPQ
1530	1540	1550	1560	1570	1580	1590	1600
QVSCSCREGY	SGDGIRTCEL	LDPCSKNNGG	CSPYATCKST	GDGQRTCTCD	TAHTVGDGLT	CRARVGLELL	RDKHASFFSL
1610	1620	1630	1640	1650	1660	1670	1680
RLLEYKELKG	DGPFTIFVPH	ADLMSNLSQD	ELARIRAHRQ	LVFRYHVVGC	RRLRSEDLLE	QGYATALSGH	PLRFSEREGS
1690	1700	1710	1720	1730	1740	1750	1760
IYLNDFARVV	SSDHEAVNGI	LHFIDRVLLP	PEALHWEPDD	APIPRRNVTA	AAQGFGYKIF	SGLLKVAGLL	PLLREASHRP
1770	1780	1790	1800	1810	1820	1830	1840
FTMLWPTDAA	FRALPPDRQA	WLYHEDHRDK	LAAILRGHMI	RNVEALASDL	PNLGPLRTMH	GTPISFSCSR	TRAGELMVGE
1850	1860	1870	1880	1890	1900	1910	1920
DDARIVQRHL	PFEGGLAYGI	DQLLEPPGLG	ARCDHFETRP	LRLNTCSICG	LEPPCPEGSQ	EQGSPEACWR	FYPKFWTSPP
1930	1940	1950	1960	1970	1980	1990	2000
LHSLGLRSVW	VHPSLWGRPQ	GLGRGCHRNC	VTTTWKPSCC	PGHYGSECQA	CRCTVHGRCD	EGLGGSGSCF	CDEGWTGPRC
2010	2020	2030	2040	2050	2060	2070	2080
EVQLELQPVC	TPPCAPEAVC	RAGNSCECSL	GYEGDGRVCT	VADLCQDGHG	GCSEHANCSQ	VGTMVTCTCL	PDYEGDGWSC
2090	2100	2110	2120	2130	2140	2150	2160
RARNPCTDGH	RGGCSEHANC	LSTGLNTRRC	ECHAGYVGDG	LQCLEESEPP	VDRCLGQPPP	CHSDAMCTDL	HFQEKRAGVF
2170	2180	2190	2200	2210	2220	2230	2240

Detailed Protein Report

Cmpd.	No. of Cmpds.	m/z meas.	Δ m/z [ppm]	z	Rt [min]	Score	P	Range	Sequence	Modification
2603	1	637.1527	-199.59	2	60.8	12.7	0	953-965	R.AGNGGCHGLATCR.A	Carbamidomethyl: 12

Detailed Protein Report

Protein 370: DNA repair protein XRCC1 [Homo sapiens]

Accession: gi|190684675
Database: refseq_human(refseq_human_20140103.fasta)
Database Date: 2015-11-30

Score: 22.9
MW [kDa]: 69.5
pI: 5.9
Sequence Coverage [%]: 5.5
No. of unique Peptides: 1

10	20	30	40	50	60	70	80
MPEIRLRHVV	SCSSQDSTHC	AENLLKADTY	RKWRAAKAGE	KTISVVLQLE	KEEQIHSVDI	GNDGSAFVEV	LVGSSAGGAG
**90**	**100**	**110**	**120**	**130**	**140**	**150**	**160**
EQDYEVLLVT	SSFMSPSESR	SGSNPNRVRM	FGPDKLVRAA	AEKRWDRVKI	VCSQPYSKDS	PFGLSFVRFH	SPPDKDEAEA
**170**	**180**	**190**	**200**	**210**	**220**	**230**	**240**
PSQKVTVTKL	GQFRVKEEDE	SANSLRPGAL	FFSRINKTSP	VTASDPAGPS	YAAATLQASS	AASSASPVSR	AIGSTSKPQE
**250**	**260**	**270**	**280**	**290**	**300**	**310**	**320**
SPKGKRKLDL	NQEEKKTPSK	PPAQLSPSVP	KRPKLPAPTR	TPATAPVPAR	AQGAVTGKPR	GEGTEPRRPR	AGPEELGKIL
**330**	**340**	**350**	**360**	**370**	**380**	**390**	**400**
QGVVVVLSGF	QNPFRSELRD	KALELGAKYR	PDWTRDSTHL	ICAFANTPKY	SQVLGLGGRI	VRKEWVLDCH	RMRRRLPSQR
**410**	**420**	**430**	**440**	**450**	**460**	**470**	**480**
YLMAGPGSSS	EEDEASHSGG	SGDEAPKLPQ	KQPQTKTKPT	QAAGPSSPQK	PPTPEETKAA	SPVLQEDIDI	EGVQSEGQDN
**490**	**500**	**510**	**520**	**530**	**540**	**550**	**560**
GAEDSGDTED	ELRRVAEQKE	HRLPPGQEEN	GEDPYAGSTD	ENTDSEEHQE	PPDLPVPELP	DFFQGKHFFL	YGEFPGDERR
**570**	**580**	**590**	**600**	**610**	**620**	**630**	**640**
KLIRYVTAFN	GELEDYMSDR	VQFVITAQEW	DPSFEEALMD	NPSLAFVRPR	WIYSCNEKQK	LLPHQLYGVV	PQA

Cmpd.	No. of Cmpds.	m/z meas.	Δ m/z [ppm]	z	Rt [min]	Score	P	Range	Sequence	Modification
2390	1	767.3810	-70.91	2	58.1	11.1	0	257-271	K.TPSKPPAQLSPSVPK.R	

Detailed Protein Report

Protein 371: testis- and ovary-specific PAZ domain-containing protein 1 [Homo sapiens]

**Accession:**	gi|222537754	**Score:**	22.9
**Database:**	refseq_human(refseq_human_20140103.fasta)	**MW [kDa]:**	190.8
**Database Date:**	2015-11-30	**pI:**	9.1
**Modification(s):**	Carbamidomethyl	**Sequence Coverage [%]:**	2.1
		**No. of unique Peptides:**	2

Quantitation

WD:WU **Median:** 0.65 **CV:** 0.00 % **No. of Peptides:** 1

10	20	30	40	50	60	70	80
MRRPPPLGPT	TASGPEGNVR	NLQKRQAPGP	GAAGGCGPEA	GGCRENKQKR	RMVARATPGR	GEVESDKSVA	ASGAGKAARR
90	100	110	120	130	140	150	160
QVEGRRGPVS	PSDSSDPRGL	EAAKEAELPL	QTERHTKEKR	KVTEASSDDP	QPGLDLVRKE	SLTSSESFQT	VECLQSLGKE
170	180	190	200	210	220	230	240
SIIEGIKRRI	RNKKLKSLEN	PPLKITENEA	TQNIKVEFQD	ELYKNTPKYS	CNILSPEVEN	NSVLKLRDCN	CFPHSKGCND
250	260	270	280	290	300	310	320
ENNLPYKPDG	GCMHVAENFS	KKENLRSLAE	KSDTNSIPQL	LQTEENVMGV	NKLLPEESDL	YQSKTNGLLS	CLQHEKNKYS
330	340	350	360	370	380	390	400
IEESSVGRKP	RKRMKLSEKA	DETVTEMNFS	NEYNKSELML	QENQMIADGK	EAETKSPLNV	LRKVSHNTVS	LMDHLLSVPE
410	420	430	440	450	460	470	480
TVEKETSSEH	HVNAVFQKTI	EPLLKEETEN	ASEPLGYESM	ASKEDFKSMK	SFIGKSPNEY	HIERRSSRED	LRSASEELKL
490	500	510	520	530	540	550	560
SCQRTIPMTG	KRTWPYYSCA	RISAWCWKKA	SLPESSYFLR	GSQESCRQVD	VPKHQTNQTH	LTDSKLLLQS	SLTETNTESS
570	580	590	600	610	620	630	640
SKEKLDSNSN	CLSSVSAVEP	TLMVIKEPII	KDDKKIKSEE	LSRRGSEVIS	NTTEDTQLTS	ETQSLTGNKK	KARGNLTKLN
650	660	670	680	690	700	710	720
LTATSKDGQE	ANNSAGKTIH	RKACIAQQTF	IVPDLVKILN	TGRLTNFKIP	LLKNKSEKRK	EVNAKSSERE	AYSPLELLDN
730	740	750	760	770	780	790	800
LSGADVRQNR	SKENVSMMML	GPQTLSIRNS	VTPVQASSDS	FYNKKSYSIS	PSFTKQGNNS	KPSNHVSEPG	NIVSNKEVAS
810	820	830	840	850	860	870	880
LTVENNAFSC	DPGYVEKSPS	FCCNEQETFR	PVSSEVRGRK	ITKNFSEVGF	PDILKAYEDD	VLLIDVIQDD	PDLFGVSNEG
890	900	910	920	930	940	950	960
ELSFTSEVPK	ISQEPNVAGE	HQSTDSKYME	TPVKKEPSDD	LRELPVLDCG	WIKPDICASN	SAESEIKRDP	KDVNTSLGEV
970	980	990	1000	1010	1020	1030	1040
ANETSENETL	GDFSEQIKGS	DLDEKHRFTD	KVITKEEKEN	IYEVCKSKDS	RNADFMVGEC	QFAVPVPKPL	CLLVPPLNLS
1050	1060	1070	1080	1090	1100	1110	1120
GRQEDTILNT	WMNDFRFLGK	HSVLKLQNPE	TCEIFKREKN	VGVFQKSLGL	MIPYKYCKFH	FNTLRGCERP	LCKFAHVPEQ
1130	1140	1150	1160	1170	1180	1190	1200
GDEKVCMDVF	KKYININELC	LLQRAVNIFM	EYYRKFPPGV	YFDLQVLNDL	LNSLLKHCLL	KEVFQIVNLS	IMVKMLPSLK
1210	1220	1230	1240	1250	1260	1270	1280
ILLNIFEYVA	TMKLRNAVPA	LIDIFCKLVE	AGMVLDPEHF	NYIVKLLYQV	QASKQEITAV	LEMKSRLQMR	RFKKNWKCDL
1290	1300	1310	1320	1330	1340	1350	1360
DSALNKLEHC	KEKGDWTKLG	KLYINVKMGC	EKFADFQTFC	ACIAETLTKN	YEDERPDIPF	CEFAETVSKD	PQNSKVDKGV
1370	1380	1390	1400	1410	1420	1430	1440
LGRIGISAMY	FYHKLLQWSK	GRKVLEKLYE	LKIHFTSLKG	LIGPEKLASR	CQIVNVAAEI	FLKSGSLDGA	IWVMRESEWI
1450	1460	1470	1480	1490	1500	1510	1520
INTPLWPCDR	LDVLNRHNLL	CTIAHEILAK	SLYRQTFEVL	QNLPGFQNSQ	ETVEVSQYSL	LFNKLLGSCI	ESSSLGMSSS
1530	1540	1550	1560	1570	1580	1590	1600
VAEFMISKSI	PIDFSFLRRL	ITSLGRSRLW	LKARAHYKSA	LSLGCYPPLE	GNLYRKLLLI	PSYLSEIEML	LAIEIFMVSN
1610	1620	1630	1640	1650	1660	1670	1680
ASSIQSPGTS	TQILQIVLKR	CEDNQSRSND	DYQAAVERLI	MAARISDPKL	FVKHMTVNVN	KEQVYSLEHC	SALKWLKENM
1690	1700						
KWAGKVWLFS	NH						

Detailed Protein Report

Cmpd.	No. of Cmpds.	m/z meas.	Δ m/z [ppm]	z	Rt [min]	Score	P	Range	Sequence	Modification	Ratios
2747	5	913.4548	46.11	2	62.8	12.3	1	25-44	K. RQAPGPGAAGGCGPEAGGCR. E	Carbamidomethyl: 19	
934	1	872.4494	41.89	2	40.5	10.6	0	749-764	R.NSVTPVQASSDSFYNK.K		WD:WU 0.65

Detailed Protein Report

Protein 372: TBC1 domain family member 2A isoform 2 [Homo sapiens]

Accession: gi|222352143
Database: refseq_human(refseq_human_20140103.fasta)
Database Date: 2015-11-30

Score: 22.9
MW [kDa]: 104.0
pI: 6.0
Sequence Coverage [%]: 2.9
No. of unique Peptides: 2

10	20	30	40	50	60	70	80
MEGAGENAPE	SSSSAPGSEE	SARDPQVPPP	EEESGDCARS	LEAVPKKLCG	YLSKFGGKGP	IRGWKSRWFF	YDERKCQLYY
90	100	110	120	130	140	150	160
SRTAQDANPL	DSIDLSSAVF	DCKADAEEGI	FEIKTPSRVI	TLKAATKQAM	LYWLQQLQMK	RWEFHNSPPA	PPATPDAALA
170	180	190	200	210	220	230	240
GNGPVLHLEL	GQEEAELEEF	LCPVKTPPGL	VGVAAALQPF	PALQNISLKH	LGTEIQNTMH	NIRGNKQAQG	TGHEPPGEDS
250	260	270	280	290	300	310	320
PQSGEPQREE	QPLASDASTP	GREPEDSPKP	APKPSLTISF	AQKAKRQNNT	FPFFSEGITR	NRTAQEKVAA	LEQQVLMLTK
330	340	350	360	370	380	390	400
ELKSQKELVK	ILHKALEAAQ	QEKRASSAYL	AAAEDKDRLE	LVRHKVRQIA	ELGRRVEALE	QERESLAHTA	SLREQQVQEL
410	420	430	440	450	460	470	480
QQHVQLLMDK	NHAKQQVICK	LSEKVTQDFT	HPPDQSPLRP	DAANRDFLSQ	QGKIEHLKDD	MEAYRTQNCF	LNSEIHQVTK
490	500	510	520	530	540	550	560
IWRKVAEKEK	ALLTKCAYLQ	ARNCQVESKY	LAGLRRLQEA	LGDEASECSE	LLRQLVQEAL	QWEAGEASSD	SIELSPISKY
570	580	590	600	610	620	630	640
DEYGFLTVPD	YEVEDLKLLA	KIQALESRSH	HLLGLEAVDR	PLRERWAALG	DLVPSAELKQ	LLRAGVPREH	RPRVWRWLVH
650	660	670	680	690	700	710	720
LRVQHLHTPG	CYQELLSRGQ	AREHPAARQI	ELDLNRTFPN	NKHFTCPTSS	FPDKLRRVLL	AFSWQNPTIG	YCQGLNRLAA
730	740	750	760	770	780	790	800
IALLVLEEEE	SAFWCLVAIV	ETIMPADYYC	NTLTASQVDQ	RVLQDLLSEK	LPRLMAHLGQ	HHVDLSLVTF	NWFLVVFADS
810	820	830	840	850	860	870	880
LISNILLRVW	DAFLYEGTKY	NEKEILRLQN	GLEIYQYLRF	FTKTISNSRK	LMNIAFNDMN	PFRMKQLRQL	RMVHRERLEA
890	900	910	920				
ELRELEQLKA	EYLERRASRR	RAVSEGCASE	DEVEGEA				

Cmpd.	No. of Cmpds.	m/z meas.	Δ m/z [ppm]	z	Rt [min]	Score	P	Range	Sequence	Modification
2337	1	729.3160	-42.50	2	57.6	12.5	0	249-262	R.EEQPLASDASTPGR.E	
2673	1	676.8390	-7.24	2	61.8	10.3	1	344-356	K.RASSAYLAAAEDK.D	

Detailed Protein Report

Protein 373: PREDICTED: zinc finger protein 155 isoform X1 [Homo sapiens]

Accession: gi|530417166
Database: refseq_human(refseq_human_20140103.fasta)
Database Date: 2015-11-30
Modification(s): Carbamidomethyl, Oxidation

Score: 22.8
MW [kDa]: 62.0
pI: 9.9
Sequence Coverage [%]: 5.8
No. of unique Peptides: 2

10	20	30	40	50	60	70	80
MTTFKEAVTF	KDVAVVFTEE	ELGLLDPAQR	KLYRDVMLEN	FRNLLSVGHQ	PFHQDTCHFL	REEKFWMMGT	ATQREGNSGG
**90**	**100**	**110**	**120**	**130**	**140**	**150**	**160**
KIQTELESVP	EAGAHEEWSC	QQIWEQIAKD	LTRSQDSIIN	NSQFFENGDV	PSQVEAGLPT	IHTGQKPSQG	GKCKQSISDV
**170**	**180**	**190**	**200**	**210**	**220**	**230**	**240**
PIFDLPQQLY	SEEKSYTCDE	CGKSICYISA	LHVHQRVHVG	EKLFMCDVCG	KEFSQSSHLQ	THQRVHTGEK	PFKCEQCGKG
**250**	**260**	**270**	**280**	**290**	**300**	**310**	**320**
FSRRSALNVH	RKLHTGEKPY	ICEACGKAFI	HDSQLKEHKR	IHTGEKPFKC	DICGKTFYFR	SRLKSHSMVH	TGEKPFRCDT
**330**	**340**	**350**	**360**	**370**	**380**	**390**	**400**
CDKSFHQRSA	LNRHCMVHTG	EKPYRCEQCG	KGFIGRLDFY	KHQVVHTGEK	PYNCKECGKS	FRWSSCLLNH	QRVHSGEKSF
**410**	**420**	**430**	**440**	**450**	**460**	**470**	**480**
KCEECGKGFY	TNSQLSSHQR	SHSGEKPYKC	EECGKGYVTK	FNLDLHQRVH	TGERPYNCKE	CGKNFSRASS	ILNHKRLHCQ
**490**	**500**	**510**	**520**	**530**	**540**		
KKPFKCEDCG	KRLVHRTYRK	DQPRDYSGEN	PSKCEDCGRR	YKRRLNLDIL	LSLFLNDT		

Cmpd.	No. of Cmpds.	m/z meas.	Δ m/z [ppm]	z	Rt [min]	Score	P	Range	Sequence	Modification
977	1	512.1767	-143.10	2	41.0	10.1	0	35-42	R.DVMLENFR.N	
1111	1	903.7510	4.42	3	42.6	12.7	2	334-356	R.HCMVHTGEKPYRCEQCGKGFIGR.L	Carbamidomethyl: 16; Oxidation: 3

BRUKER

Detailed Protein Report

Protein 374: ephrin type-A receptor 5 isoform e [Homo sapiens]

Accession: gi|528881091
Database: refseq_human(refseq_human_20140103.fasta)
Database Date: 2015-11-30
Modification(s): Carbamidomethyl

Score: 22.8
MW [kDa]: 110.9
pI: 6.3
Sequence Coverage [%]: 2.9
No. of unique Peptides: 2

Quantitation

WD:WU **Median:** 1.09 **CV:** 33.58 % **No. of Peptides:** 2

10	20	30	40	50	60	70	80
MRGSGPRGAG	RRRPPSGGGD	TPITPASLAG	CYSAPRRAPL	WTCLLLCAAL	RTLLASPSNE	VNLLDSRTVM	GDLGWIAFPK
90	100	110	120	130	140	150	160
NGWEEIGEVD	ENYAPIHTYQ	VCKVMEQNQN	NWLLTSWISN	EGASRIFIEL	KFTLRDCNSL	PGGLGTCKET	FNMYYFESDD
170	180	190	200	210	220	230	240
QNGRNIKENQ	YIKIDTIAAD	ESFTELDLGD	RVMKLNTEVR	DVGPLSKKGF	YLAFQDVGAC	IALVSVRVYY	KKCPSVVRHL
250	260	270	280	290	300	310	320
AVFPDTITGA	DSSQLLEVSG	SCVNHSVTDE	PPKMHCSAEG	EWLVPIGKCM	CKAGYEEKNG	TCQVCRPGFF	KASPHIQSCG
330	340	350	360	370	380	390	400
KCPPHSYTHE	EASTSCVCEK	DYFRRESDPP	TMACTRPPSA	PRNAISNVNE	TSVFLEWIPP	ADTGGRKDVS	YYIACKKCNS
410	420	430	440	450	460	470	480
HAGVCEECGG	HVRYLPRQSG	LKNTSVMMVD	LLAHTNYTFE	IEAVNGVSDL	SPGARQYVSV	NVTTNQAAPS	PVTNVKKGKI
490	500	510	520	530	540	550	560
AKNSISLSWQ	EPDRPNGIIL	EYEIKYFEKD	QETSYTIIKS	KETTITAEGL	KPASVYVFQI	RARTAAGYGV	FSRRFEFETT
570	580	590	600	610	620	630	640
PVSVAASSDQ	SQIPVIAVSV	TVGVILLAVV	IGVLLSGSCC	ECGCGRASSL	CAVAHPSLIW	RCGYSKAKQD	PEEEKMHFHN
650	660	670	680	690	700	710	720
GHIKLPGVRT	YIDPHTYEDP	NQAVHEFAKE	IEASCITIER	VIGAGEFGEV	CSGRLKLPGK	RELPVAIKTL	KVGYTEKQRR
730	740	750	760	770	780	790	800
DFLGEASIMG	QFDHPNIIHL	EGVVTKSKPV	MIVTEYMENG	SLDTFLKKND	GQFTVIQLVG	MLRGISAGMK	YLSDMGYVHR
810	820	830	840	850	860	870	880
DLAARNILIN	SNLVCKVSDF	GLSRVLEDDP	EAAYTTRGGK	IPIRWTAPEA	IAFRKFTSAS	DVWSYGIVMW	EVVSYGERPY
890	900	910	920	930	940	950	960
WEMTNQDVIK	AVEEGYRLPS	PMDCPAALYQ	LMLDCWQKER	NSRPKFDEIV	NMLDKLIRNP	SSLKTLVNAS	CRVSNLLAEH
970	980	990	1000	1010			
SPLGSGAYRS	VGEWLEAIKM	GRYTEIFMEN	GYSSMDAVAQ	VTLE			

Cmpd.	No. of Cmpds.	m/z meas.	Δ m/z [ppm]	z	Rt [min]	Score	P	Range	Sequence	Modification	Ratios	
2043	1	661.2613	-54.57	2	53.9	10.7	0	136-148	R.DCNSLPGGLGTCK.E	Carbamidomethyl: 2	WD:WU	1.51
51	1	857.9018	65.31	2	29.6	12.1	0	398-413	K.CNSHAGVCEECGGHVR.Y	Carbamidomethyl: 11	WD:WU	0.79

Detailed Protein Report

Protein 375: PREDICTED: rho GTPase-activating protein SYDE2 isoform X5 [Homo sapiens]

**Accession:**	gi|530363440	**Score:**	22.8
**Database:**	refseq_human(refseq_human_20140103.fasta)	**MW [kDa]:**	105.4
**Database Date:**	2015-11-30	**pI:**	6.6
**Modification(s):**	Carbamidomethyl	**Sequence Coverage [%]:**	4.6
		**No. of unique Peptides:**	1

10	20	30	40	50	60	70	80
MKGRELEELK	DNIEFRGHKP	LNSITVSKKR	NWLYQSTLRP	LNLEEENKKC	QDRSHLSISP	VSLPKHQLSQ	SFLKSSKEYC
**90**	**100**	**110**	**120**	**130**	**140**	**150**	**160**
TYVVCNATNS	SLSKNCALDF	NEENDADDEG	EIWYNPIPED	DDLGISSALS	FGEADSAVLK	LPAVNLSMLS	GSDLMKAERH
**170**	**180**	**190**	**200**	**210**	**220**	**230**	**240**
TEDSLCSSEH	AGDIQTTRSN	GMNPIHPAHS	TEFVQQYKQK	LGHKTQEGIM	VEDSPMLKSP	FAGSGILAAT	NSTELGIMEP
**250**	**260**	**270**	**280**	**290**	**300**	**310**	**320**
SSPNPSPVKK	GSSINWSLPD	KIKSPRTVRK	LSMKMKKLPE	FSRKLSVKGT	LNYINSPDNT	PSLSKYNCRE	VHHTDILPSG
**330**	**340**	**350**	**360**	**370**	**380**	**390**	**400**
NTTTAAKRNV	ISRYHLDTSV	SSQQSYQKKN	SMSSKYSCKG	GYLSDGDSPE	LTTKASKHGS	ENKFGKGKEI	ISNSCSKNEI
**410**	**420**	**430**	**440**	**450**	**460**	**470**	**480**
DIDAFRHYSF	SDQPKCSQYI	SGLMSVHFYG	AEDLKPPRID	SKDVFCAIQV	DSVNKARTAL	LTCRTTFLDM	DHTFNIEIEN
**490**	**500**	**510**	**520**	**530**	**540**	**550**	**560**
AQHLKLVVFS	WEPTPRKNRV	CCHGTVVLPT	LFRVTKTHQL	AVKLEPRGLI	YVKVTLMEQW	ENSLHGLDIN	QEPIIFGVDI
**570**	**580**	**590**	**600**	**610**	**620**	**630**	**640**
QKVVEKENIG	LMVPLLIQKC	IMEIEKRGCQ	VVGLYRLCGS	AAVKKELREA	FERDSKAVGL	CENQYPDINV	ITGVLKDYLR
**650**	**660**	**670**	**680**	**690**	**700**	**710**	**720**
ELPSPLITKQ	LYEAVLDAMA	KSPLKMSSNG	CENDPGDSKY	TVDLLDCLPE	IEKATLKMLL	DHLKLVASYH	EVNKMTCQNL
**730**	**740**	**750**	**760**	**770**	**780**	**790**	**800**
AVCFGPVLLS	QRQEPSTHNN	RVFTDSEELA	SALDFKKHIE	VLHYLLQLWP	VQRLTVKKST	DNLFPEQKSS	LNYLRQKKER
**810**	**820**	**830**	**840**	**850**	**860**	**870**	**880**
PHMLNLSGTD	SSGVLRPRQN	RLDSPLSNRY	AGDWSSCGEN	YFLNTKENLN	DVDYDDVPSE	DRKIGENYSK	MDGPEVMIEQ
**890**	**900**	**910**	**920**	**930**	**940**		
PIPMSKECTF	QTYLTMQTVE	STVDRKNNLK	DLQESIDTLI	GNLERELNKN	KLNMSF		

Cmpd.	No. of Cmpds.	m/z meas.	Δ m/z [ppm]	z	Rt [min]	Score	P	Range	Sequence	Modification
2968	1	964.7824	-62.98	3	65.9	11.8	2	50-74	K.CQDRSHLSISPVSLPKHQLSQSFLK.S	Carbamidomethyl: 1

Detailed Protein Report

Protein 376: keratin, type II cuticular Hb2 [Homo sapiens]

**Accession:**	gi|27477127	**Score:**	22.7
**Database:**	refseq_human(refseq_human_20140103.fasta)	**MW [kDa]:**	56.6
**Database Date:**	2015-11-30	**pI:**	6.4
**Modification(s):**	Carbamidomethyl	**Sequence Coverage [%]:**	5.7
		**No. of unique Peptides:**	1

10	20	30	40	50	60	70	80
MSYHSFQPGS	RCGSQSFSSY	SAVMPRMVTH	YAVSKGPCRP	GGGRGLRALG	CLGSRSLCNV	GFGRPRVASR	CGGTLPGFGY
**90**	**100**	**110**	**120**	**130**	**140**	**150**	**160**
RLGATCGPSA	CITPVTINES	LLVPLALEID	PTVQRVKRDE	KEQIKCLNNR	FASFINKVRF	LEQKNKLLET	KWNFMQQQRC
**170**	**180**	**190**	**200**	**210**	**220**	**230**	**240**
CQTNIEPIFE	GYISALRRQL	DCVSGDRVRL	ESELCSLQAA	LEGYKKKYEE	ELSLRPCVEN	EFVALKKDVD	TAFLMKADLE
**250**	**260**	**270**	**280**	**290**	**300**	**310**	**320**
TNAEALVQEI	DFLKSLYEEE	ICLLQSQISE	TSVIVKMDNS	RELDVDGIIA	EIKAQYDDIA	SRSKAEAEAW	YQCRYEELRV
**330**	**340**	**350**	**360**	**370**	**380**	**390**	**400**
TAGNHCDNLR	NRKNEILEMN	KLIQRLQQET	ENVKAQRCKL	EGAIAEAEQQ	GEAALNDAKC	KLAGLEEALQ	KAKQDMACLL
**410**	**420**	**430**	**440**	**450**	**460**	**470**	**480**
KEYQEVMNSK	LGLDIEIATY	RRLLEGEEHR	LCEGIGPVNI	SVSSSKGAFL	YEPCGVSTPV	LSTGVLRSNG	GCSIVGTGEL
**490**	**500**	**510**	**520**				
YVPCEPQGLL	SCGSGRKSSM	TLGAGGSSPS	HKH				

Cmpd.	No. of Cmpds.	m/z meas.	Δ m/z [ppm]	z	Rt [min]	Score	P	Range	Sequence	Modification
1841	1	1057.4972	-8.19	2	51.7	10.5	0	160-177	R.CCQTNIEPIFEGYISALR.R	Carbamidomethyl: 2

Detailed Protein Report

Protein 377: PREDICTED: rho GTPase-activating protein 32 isoform X2 [Homo sapiens]

**Accession:**	gi|578822484	**Score:**	22.6
**Database:**	refseq_human(refseq_human_20140103.fasta)	**MW [kDa]:**	156.7
**Database Date:**	2015-11-30	**pI:**	6.2
		**Sequence Coverage [%]:**	2.0
		**No. of unique Peptides:**	1

10	20	30	40	50	60	70	80
MKKSPVGSWR	SFFNLGKSSS	VSKRKLQRNE	SEPSEMKAMA	LKGGRAEGTL	RSAKSEESLT	SLHAVDGDSK	LFRPRRPRSS
90	100	110	120	130	140	150	160
SDALSASFNG	EMLGNRCNSY	DNLPHDNESE	EEGGLLHIPA	LMSPHSAEDV	DLSPPDIGVA	SLDFDPMSFQ	CSPPKAESEC
170	180	190	200	210	220	230	240
LESGASFLDS	PGYSKDKPSA	NKKDAETGSS	QCQTPGSTAS	SEPVSPLQEK	LSPFFTLDLS	PTEDKSSKPS	SFTEKVVYAF
250	260	270	280	290	300	310	320
SPKIGRKLSK	SPSMSISEPI	SVTLPPRVSE	VIGTVSNTTA	QNASSSTWDK	CVEERDATNR	SPTQIVKMKT	NETVAQEAYE
330	340	350	360	370	380	390	400
SEVQPLDQVA	AEEVELPGKE	DQSVSSSQSK	AVASGQTQTG	AVTHDPPQDS	VPVSSVSLIP	PPPPPKNVAR	MLALALAESA
410	420	430	440	450	460	470	480
QQASTQSLKR	PGTSQAGYTN	YGDIAVATTE	DNLSSSYSAV	ALDKAYFQTD	RPAEQFHLQN	NAPGNCDHPL	PETTATGDPT
490	500	510	520	530	540	550	560
HSNTTESGEQ	HHQVDLTGNQ	PHQAYLSGDP	EKARITSVPL	DSEKSDDHVS	FPEDQSGKNS	MPTVSFLDQD	QSPPRFYSGD
570	580	590	600	610	620	630	640
QPPSYLGASV	DKLHHPLEFA	DKSPTPPNLP	SDKIYPPSGS	PEENTSTATM	TYMTTTPATA	QMSTKEASWD	VAEQPTTADF
650	660	670	680	690	700	710	720
AAATLQRTHR	TNRPLPPPPS	QRSAEQPPVV	GQVQAATNIG	LNNSHKVQGV	VPVPERPPEP	RAMDDPASAF	ISDSGAAAAQ
730	740	750	760	770	780	790	800
CPMATAVQPG	LPEKVRDGAR	VPLLHLRAES	VPAHPCGFPA	PLPPTRMMES	KMIAAIHSSS	ADATSSSNYH	SFVTASSTSV
810	820	830	840	850	860	870	880
DDALPLPLPV	PQPKHASQKT	VYSSFARPDV	TTEPFGPDNC	LHFNMTPNCQ	YRPQSVPPHH	NKLEQHQVYG	ARSEPPASMG
890	900	910	920	930	940	950	960
LRYNTYVAPG	RNASGHHSKP	CSRVEYVSSL	SSSVRNTCYP	EDIPPYPTIR	RVQSLHAPPS	SMIRSVPISR	TEVPPDDEPA
970	980	990	1000	1010	1020	1030	1040
YCPRPLYQYK	PYQSSQARSD	YHVTQLQPYF	ENGRVHYRYS	PYSSSSSSYY	SPDGALCDVD	AYGTVQLRPL	HRLPNRDFAF
1050	1060	1070	1080	1090	1100	1110	1120
YNPRLQGKSL	YSYAGLAPRP	RANVTGYFSP	NDHNVVSMPP	AADVKHTYTS	WDLEDMEKYR	MQSIRRESRA	RQKVKGPVMS
1130	1140	1150	1160	1170	1180	1190	1200
QYDNMTPAVQ	DDLGGIYVIH	LRSKSDPGKT	GLLSVAEGKE	SRHAAKAISP	EGEDRFYRRH	PEAEMDRAHH	HGGHGSTQPE
1210	1220	1230	1240	1250	1260	1270	1280
KPSLPQKQSS	LRSRKLPDMG	CSLPEHRAHQ	EASHRQFCES	KNGPPYPQGA	GQLDYGSKGI	PDTSEPVSYH	NSGVKYAASG
1290	1300	1310	1320	1330	1340	1350	1360
QESLRLNHKE	VRLSKEMERP	WVRQPSAPEK	HSRDCYKEEE	HLTQSIVPPP	KPERSHSLKL	HHTQNVERDP	SVLYQYQPHG
1370	1380	1390	1400	1410	1420	1430	1440
KRQSSVTVVS	QYDNLEDYHS	LPQHQRGVFG	GGGMGTYVPP	GFPHPQSRTY	ATALGQGAFL	PAELSLQHPE	TQIHAE

Cmpd.	No. of Cmpds.	m/z meas.	Δ m/z [ppm]	z	Rt [min]	Score	P	Range	Sequence	Modification
2733	1	656.8584	26.75	2	62.6	11.3	0	904-915	R.VEYVSSLSSSVR.N	

Detailed Protein Report

Protein 378: PREDICTED: coiled-coil domain-containing protein 74B isoform X9 [Homo sapiens]

**Accession:**	gi|578805131	**Score:**	22.6
**Database:**	refseq_human(refseq_human_20140103.fasta)	**MW [kDa]:**	36.8
**Database Date:**	2015-11-30	**pI:**	12.0
**Modification(s):**	Carbamidomethyl	**Sequence Coverage [%]:**	10.2
		**No. of unique Peptides:**	2

10	20	30	40	50	60	70	80
MSGAGVAAGT	RPPSSPTPGS	RRRRQRPSVG	VQSLRPQSPQ	LRQSDPQKRN	LDLEKSLQFL	QQQHSEMLAK	LHEEIEHLKR
**90**	**100**	**110**	**120**	**130**	**140**	**150**	**160**
ENKGEPARGP	RPALPPQAHS	TLPLPQHRNT	AINSSTRLGS	GGTQDDLRYK	LIMNQTSQKK	DGPSGNHLSR	ASAPLGARWV
**170**	**180**	**190**	**200**	**210**	**220**	**230**	**240**
CINGVWVEPG	GPSPARLKEG	SSRTHRPGGK	HGRLAGGSAD	TVRSPADSLS	TSSFQSVKSI	SNSANSQGKA	RPQPGSFNKQ
**250**	**260**	**270**	**280**	**290**	**300**	**310**	**320**
DSKADVPQKA	DLEEEPLLHN	SKLDKVPGVQ	GQASPPHLGS	AKCSSASCGI	PTSCRPKSCS	TSSPSWKGAR	GPRQSRRKLA
**330**	**340**	**350**					
FPGTKKPRIS	PRSPPRASPR	NACF					

Cmpd.	No. of Cmpds.	m/z meas.	Δ m/z [ppm]	z	Rt [min]	Score	P	Range	Sequence	Modification
2681	1	945.9624	-37.93	2	61.9	11.2	2	140-158	K.KDGPSGNHLSRASAPLGAR.W	
1874	1	860.9002	-14.63	2	51.8	11.4	2	298-313	K.SCSTSSPSWKGARGPR.Q	Carbamidomethyl: 2

Detailed Protein Report

Protein 379: sphingosine 1-phosphate receptor 4 precursor [Homo sapiens]

Accession: gi|4503459
Database: refseq_human(refseq_human_20140103.fasta)
Database Date: 2015-11-30

Score: 22.5
MW [kDa]: 41.6
pI: 11.8
Sequence Coverage [%]: 7.3
No. of unique Peptides: 2

10	20	30	40	50	60	70	80
MNATGTPVAP	ESCQQLAAGG	HSRLIVLHYN	HSGRLAGRGG	PEDGGLGALR	GLSVAASCLV	VLENLLVLAA	ITSHMRSRRW
**90**	**100**	**110**	**120**	**130**	**140**	**150**	**160**
VYYCLVNITL	SDLLTGAAYL	ANVLLSGART	FRLAPAQWFL	REGLLFTALA	ASTFSLLFTA	GERFATMVRP	VAESGATKTS
**170**	**180**	**190**	**200**	**210**	**220**	**230**	**240**
RVYGFIGLCW	LLAALLGMLP	LLGWNCLCAF	DRCSSLLPLY	SKRYILFCLV	IFAGVLATIM	GLYGAIFRLV	QASGQKAPRP
**250**	**260**	**270**	**280**	**290**	**300**	**310**	**320**
AARRKARRLL	KTVLMILLAF	LVCWGPLFGL	LLADVFGSNL	WAQEYLRGMD	WILALAVLNS	AVNPIIYSFR	SREVCRAVLS
**330**	**340**	**350**	**360**	**370**	**380**	**390**	
FLCCGCLRLG	MRGPGDCLAR	AVEAHSGAST	TDSSLRPRDS	FRGSRSLSFR	MREPLSSISS	VRSI	

Cmpd.	No. of Cmpds.	m/z meas.	Δ m/z [ppm]	z	Rt [min]	Score	P	Range	Sequence	Modification
1600	1	853.3743	-147.58	2	48.3	12.4	2	229-244	R.LVQASGQKAPRPAARR.K	
94	1	707.7845	-110.73	2	30.1	10.1	2	359-370	R.DSFRGSRSLSFR.M	

Detailed Protein Report

Protein 380: transmembrane protein 194A isoform a [Homo sapiens]

**Accession:**	gi|195972881	**Score:**	22.5
**Database:**	refseq_human(refseq_human_20140103.fasta)	**MW [kDa]:**	50.6
**Database Date:**	2015-11-30	**pI:**	6.7
**Modification(s):**	Carbamidomethyl	**Sequence Coverage [%]:**	6.8
		**No. of unique Peptides:**	1

10	20	30	40	50	60	70	80
MAGGMKVAVS	PAVGPGPWGS	GVGGGGTVRL	LLILSGCLVY	GTAETDVNVV	MLQESQVCEK	RASQQFCYTN	VLIPKWHDIW
**90**	**100**	**110**	**120**	**130**	**140**	**150**	**160**
TRIQIRVNSS	RLVRVTQVEN	EEKLKELEQF	SIWNFFSSFL	KEKLNDTYVN	VGLYSTKTCL	KVEIIEKDTK	YSVIVIRRFD
**170**	**180**	**190**	**200**	**210**	**220**	**230**	**240**
PKLFLVFLLG	LMLFFCGDLL	SRSQIFYYST	GMTVGIVASL	LIIIFILSKF	MPKKSPIYVI	LVGGWSFSLY	LIQLVFKNLQ
**250**	**260**	**270**	**280**	**290**	**300**	**310**	**320**
EIWRCYWQYL	LSYVLTVGFM	SFAVCYKYGP	LENERSINLL	TWTLQLMGLC	FMYSGIQIPH	IALAIIIIAL	CTKNLEHPIQ
**330**	**340**	**350**	**360**	**370**	**380**	**390**	**400**
WLYITCRKVC	KGAEKPVPPR	LLTEEEYRIQ	GEVETRKALE	ELREFCNSPD	CSAWKTVSRI	QSPKRFADFV	EGSSHLTPNE
**410**	**420**	**430**	**440**	**450**			
VSVHEQEYGL	GSIIAQDEIY	EEASSEEEDS	YSRCPAITQN	NFLT			

Cmpd.	No. of Cmpds.	m/z meas.	Δ m/z [ppm]	z	Rt [min]	Score	P	Range	Sequence	Modification
1826	1	1045.0325	-2.71	2	51.2	10.5	1	124-141	K.LNDTYVNVGLYSTKTCLK.V	Carbamidomethyl: 16

Detailed Protein Report

Protein 381: PREDICTED: WD repeat-containing protein 11 isoform X1 [Homo sapiens]

**Accession:**	gi|530393962	**Score:**	22.5
**Database:**	refseq_human(refseq_human_20140103.fasta)	**MW [kDa]:**	108.0
**Database Date:**	2015-11-30	**pI:**	5.9
		**Sequence Coverage [%]:**	3.4
		**No. of unique Peptides:**	1

Alias proteins:

Accession	Name	Description
gi|530393964	refseq_human (refseq_human_20140103.fasta)	PREDICTED: WD repeat-containing protein 11 isoform X2 [Homo sapiens]

10	20	30	40	50	60	70	80
MLLLYPREIL	ILDLEVNQTV	GVIAIERTGV	PFLQVIPCFQ	RDGLFCLHEN	GCITLRVRRS	YNNIFTTSNE	EPDPDPVQEL
**90**	**100**	**110**	**120**	**130**	**140**	**150**	**160**
TYDLRSQCDA	IRVTKTVRPF	SMVCCPVNEN	AAALVVSDGR	VMIWELKSAV	CNRNSRNSSS	GVSPLYSPVS	FCGIPVGVLQ
**170**	**180**	**190**	**200**	**210**	**220**	**230**	**240**
NKLPDLSLDN	MIGQSAIAGE	EHPRGSILRE	VHLKFLLTGL	LSGLPAPQFA	IRMCPPLTTK	NIKMYQPLLA	VGTSNGSVLV
**250**	**260**	**270**	**280**	**290**	**300**	**310**	**320**
YHLTSGLLHK	ELSIHSCEVK	GIEWTSLTSF	LSFATSTPNN	MGLVRNELQL	VDLPTGRSIA	FRGERGNDES	AIEMIKVSHL
**330**	**340**	**350**	**360**	**370**	**380**	**390**	**400**
KQYLAVVFRD	KPLELWDVRT	CTLLREMSKN	FPTITALEWS	PSHNLKSLRK	KQLATREAMA	RQTVVSDTEL	SIVESSVISL
**410**	**420**	**430**	**440**	**450**	**460**	**470**	**480**
LQEAESKSEL	SQNISAREHF	VFTDIDGQVY	HLTVEGNSVK	DSARIPPDGS	MGSITCIAWK	GDTLVLGDMD	GNLNFWDLKG
**490**	**500**	**510**	**520**	**530**	**540**	**550**	**560**
RVSRGIPTHR	SWVRKIRFAP	GKGNQKLIAM	YNDGAEVWDT	KEVQMVSSLR	SGRNVTFRIL	DVDWCTSDKV	ILASDDGCIR
**570**	**580**	**590**	**600**	**610**	**620**	**630**	**640**
VLEMSMKSAC	FRMDEQELTE	PVWCPYLLVP	RASLALKAFL	LHQPWNGQYS	LDISHVDYPE	NEEIKNLLQE	QLNSLSNDIK
**650**	**660**	**670**	**680**	**690**	**700**	**710**	**720**
KLLLDPEFTL	LQRCLLVSRL	YGDESELHFW	TVAAHYLHSL	SQEKSASTTA	PKEAAPRDKL	SNPLDICYDV	LCENAYFQKF
**730**	**740**	**750**	**760**	**770**	**780**	**790**	**800**
QLERVNLQEV	KRSTYDHTRK	CTDQLLLLGQ	TDRAVQLLLE	TSADNQHYYC	DSLKACLVTT	VTSSGPSQST	IKLVATNMIA
**810**	**820**	**830**	**840**	**850**	**860**	**870**	**880**
NGKLAEGVQL	LCLIDKAADA	CRYLQTYGEW	NRAAWLAKVR	LNPEECADVL	RRWVDHLCSP	QVNQKSKALL	VLLSLGCFFS
**890**	**900**	**910**	**920**	**930**	**940**	**950**	**960**
VAETLHSMRY	FDRAALFVEA	CLKYGAFEVT	EDTEKLITAI	YADYARSLKN	LGFKQGAVLF	ASKAGAAGKD	LLNELESPKE
**970**							
EPIEE							

Cmpd.	No. of Cmpds.	m/z meas.	Δ m/z [ppm]	z	Rt [min]	Score	P	Range	Sequence	Modification
2753	1	973.5005	10.37	2	62.9	11.5	1	42-58	R.DGLFCLHENGCITLRVR.R	

Detailed Protein Report

Protein 382: protein Hook homolog 1 [Homo sapiens]

**Accession:**	gi|7705415	**Score:**	22.5
**Database:**	refseq_human(refseq_human_20140103.fasta)	**MW [kDa]:**	84.6
**Database Date:**	2015-11-30	**pI:**	5.0
**Modification(s):**	Oxidation	**Sequence Coverage [%]:**	3.7
		**No. of unique Peptides:**	1

Alias proteins:

Accession	Name	Description
gi|530362743	refseq_human (refseq_human_20140103.fasta)	PREDICTED: protein Hook homolog 1 isoform X1 [Homo sapiens]

10	20	30	40	50	60	70	80
MEETQPPPQP	KLPLCDSLMI	WLQTFNTASP	CQDVKQLTSG	VAMAQVLHQI	DAAWFNESWL	SRIKEDVGDN	WRIKASNVKK
**90**	**100**	**110**	**120**	**130**	**140**	**150**	**160**
VLQGIMSYYH	EFLGQQISEA	LIPDLNQITE	CSDPVELGRL	LQLILGCAIN	CEKKQEHIQN	IMTLEESVQH	VVMTAIQELM
**170**	**180**	**190**	**200**	**210**	**220**	**230**	**240**
SKEILSSPPN	DAVGELEQQL	KRALEELQEA	LAEKEELRQR	CEELDMQVTT	LQDEKNSLVS	ENEMMNEKLD	QLDGSFDDPN
**250**	**260**	**270**	**280**	**290**	**300**	**310**	**320**
TVVAKKYFHA	QLQLEQLQEE	NFRLEAAKDD	YRVHCEELEK	QLIEFQHRND	ELTSLAEETR	ALKDEIDVLR	ATSDKANKLE
**330**	**340**	**350**	**360**	**370**	**380**	**390**	**400**
STVEIYRQKL	QDLNDLRKQV	KTLQETNMMY	MHNTVSLEEE	LKKANAARTQ	LETYKRQVQD	LHVKLSSESK	RADTLAFEMK
**410**	**420**	**430**	**440**	**450**	**460**	**470**	**480**
RLEEKHEALL	KEKERLIEQR	DTLKETNEEL	RCSQVQQDHL	NQTDASATKS	YENLAAEIMP	VEYREVFIRL	QHENKMLRLQ
**490**	**500**	**510**	**520**	**530**	**540**	**550**	**560**
QEGSENERIE	ELQEQLEQKH	RKMNELETEQ	RLSKERIREL	QQQIEDLQKS	LQEQGSKSEG	ESSSKLKQKL	EAHMEKLTEV
**570**	**580**	**590**	**600**	**610**	**620**	**630**	**640**
HEELQKKQEL	IEDLQPDINQ	NVQKINELEA	ALQKKDEDMK	AMEERYKMYL	EKARNVIKTL	DPKLNPASAE	IMLLRKQLAE
**650**	**660**	**670**	**680**	**690**	**700**	**710**	**720**
KERRIEILES	ECKVAKFRDY	EEKLIVSAWY	NKSLAFQKLG	MESRLVSGGG	ACSDTGACTP	ARSFLAQQRH	ITNTRRNLSV
**730**							
KVPATTSD							

Cmpd.	No. of Cmpds.	m/z meas.	Δ m/z [ppm]	z	Rt [min]	Score	P	Range	Sequence	Modification
1597	1	685.8641	-18.30	2	48.3	10.6	2	546-556	K.LKQKLEAHMEK.L	Oxidation: 9

Detailed Protein Report

Protein 383: V-type proton ATPase subunit E 2 [Homo sapiens]

**Accession:**	gi|18087815	**Score:**	22.5
**Database:**	refseq_human(refseq_human_20140103.fasta)	**MW [kDa]:**	26.1
**Database Date:**	2015-11-30	**pI:**	9.4
**Modification(s):**	Oxidation	**Sequence Coverage [%]:**	10.6
		**No. of unique Peptides:**	1

Quantitation

WD:WU	**Median:** 0.61	**CV:** 0.00 %	**No. of Peptides:** 1

Alias proteins:

Accession	Name	Description
gi|530368262	refseq_human (refseq_human_20140103.fasta)	PREDICTED: V-type proton ATPase subunit E 2 isoform X2 [Homo sapiens]
gi|530368260	refseq_human (refseq_human_20140103.fasta)	PREDICTED: V-type proton ATPase subunit E 2 isoform X1 [Homo sapiens]

```
        10         20         30         40         50         60         70         80
MALSDVDVKK QIKHMMAFIE QEANEKAEEI DAKAEEEFNI EKGRLVQTQR LKIMEYYEKK EKQIEQQKKI LMSTMRNQAR
        90        100        110        120        130        140        150        160
LKVLRARNDL ISDLLSEAKL RLSRIVEDPE VYQGLLDKLV LQGLLRLLEP VMIVRCRPQD LLLVEAAVQK AIPEYMTISQ
       170        180        190        200        210        220        230
KHVEVQIDKE AYLAVNAAGG VEVYSGNQRI KVSNTLESRL DLSAKQKMPE IRMALFGANT NRKFFI
```

Cmpd.	No. of Cmpds.	m/z meas.	Δ m/z [ppm]	z	Rt [min]	Score	P	Range	Sequence	Modification	Ratios	
792	1	555.7879	29.14	2	38.3	11.6	0	213-222	R.MALFGANTNR.K	Oxidation: 1	WD:WU	0.61

Detailed Protein Report

Protein 384: **PREDICTED: replication factor C subunit 2 isoform X1 [Homo sapiens]**

**Accession:**	gi|578814487	**Score:**	22.4
**Database:**	refseq_human(refseq_human_20140103.fasta)	**MW [kDa]:**	24.5
**Database Date:**	2015-11-30	**pI:**	7.8
		**Sequence Coverage [%]:**	16.1
		**No. of unique Peptides:**	1

Quantitation

WD:WU **Median:** 0.62 **CV:** 0.00 % **No. of Peptides:** 1

10	20	30	40	50	60	70	80
MCPTSSLRMT	DGAQQALRRT	MEIYSKTTRF	ALACNASDKI	IEPIQSRCAV	LRYTKLTDAQ	ILTRLMNVIE	KERVPYTDDG
**90**	**100**	**110**	**120**	**130**	**140**	**150**	**160**
LEAIIFTAQG	DMRQALNNLQ	STFSGFGFIN	SENVFKVCDE	PHPLLVKEMI	QHCVNANIDE	AYKILAHLWH	LGYSPEDIIG
**170**	**180**	**190**	**200**	**210**	**220**		
NIFRVCKTFQ	MAEYLKLEFI	KEIGYTHMKI	AEGVNSLLQM	AGLLARLCQK	TMAPVAS		

Cmpd.	No. of Cmpds.	m/z meas.	Δ m/z [ppm]	z	Rt [min]	Score	P	Range	Sequence	Modification	Ratios
1044	1	983.4577	-11.26	2	41.8	10.1	1	1-18	-.MCPTSSLRMTDGAQQALR.R		WD:WU 0.62

Detailed Protein Report

Protein 385: PREDICTED: EH domain-binding protein 1 isoform X4 [Homo sapiens]

Accession: gi|530367409
Database: refseq_human(refseq_human_20140103.fasta)
Database Date: 2015-11-30

Score: 22.4
MW [kDa]: 108.6
pI: 5.2
Sequence Coverage [%]: 2.5
No. of unique Peptides: 2

Quantitation

WD:WU **Median:** 0.94 **CV:** 77.37 % **No. of Peptides:** 2

10	20	30	40	50	60	70	80
MASVWKRLQR	VGKHASKFQF	VASYQELMVE	CTKKWQPDKL	VVVWTRRSRR	KSSKAHSWQP	GIKNPYRGVV	VWPVPENIEI
90	100	110	120	130	140	150	160
TVTLFKDPHA	EEFEDKEWTF	VIENESPSGR	RKALATSSIN	MKQYASPMPT	QTDVKLKFKP	LSKKVVSAAL	QFSLSCIFLR
170	180	190	200	210	220	230	240
EGKATDEDMQ	SLASLMSMKQ	ADIGNLDDFE	EDNEDDDENR	VNQEEKAAKI	TEIVNQLNAL	SSLDEDQDDC	IKQANMRSAK
250	260	270	280	290	300	310	320
SASSSEELIN	KLNFLDEAEK	DLATVNSNPF	DDPDAAELNP	FGDPDSEEPI	TETASPRKTE	DSFYNNSYNP	FKEVQTPQYL
330	340	350	360	370	380	390	400
NPFDEPEAFV	TIKDSPPQST	KRKNIRPVDM	SKYLYADSSK	TEEEELDESN	PFYEPKSTPP	PNNLVNPVQE	LETERRVKRK
410	420	430	440	450	460	470	480
APAPPVLSPK	TGVLNENTVS	AGKDLSTSPK	PSPIPSPVLG	RKPNASQSLL	VWCKEVTKNY	RGVKITNFTT	SWRNGLSFCA
490	500	510	520	530	540	550	560
ILHHFRPDLI	DYKSLNPQDI	KENNKKAYDG	FASIGISRLL	EPSDMHRLLS	RQEELKERAR	VLLEQARRDA	ALKAGNKHNT
570	580	590	600	610	620	630	640
NTATPFCNRQ	LSDQQDEERR	RQLRERARQL	IAEARSGVKM	SELPSYGEMA	AEKLKERSKA	SGDENDNIEI	DTNEEIPEGF
650	660	670	680	690	700	710	720
VVGGGDELTN	LENDLDTPEQ	NSKLVDLKLK	KLLEVQPQVA	NSPSSAAQKA	VTESSEQDMK	SGTEDLRTER	LQKTTERFRN
730	740	750	760	770	780	790	800
PVVFSKDSTV	RKTQLQSFSQ	YIENRPEMKR	QRSIQEDTKK	GNEEKAAITE	TQRKPSEDEV	LNKGFKDTSQ	YVVGELAALE
810	820	830	840	850	860	870	880
NEQKQIDTRA	ALVEKRLRYL	MDTGRNTEEE	EAMMQEWFML	VNKKNALIRR	MNQLSLLEKE	HDLERRYELL	NRELRAMLAI
890	900	910	920	930	940	950	
EDWQKTEAQK	RREQLLLDEL	VALVNKRDAL	VRDLDAQEKQ	AEEEDEHLER	TLEQNKGKMA	KKEEKCVLQ	

Cmpd.	No. of Cmpds.	m/z meas.	Δ m/z [ppm]	z	Rt [min]	Score	P	Range	Sequence	Modification	Ratios	
804	1	660.3468	-45.46	2	38.5	12.3	2	111-122	R.RKALATSSINMK.Q		WD:WU	1.86
157	2	688.1811	-190.45	2	31.2	10.1	0	558-569	K.HNTNTATPFCNR.Q		WD:WU	0.47

Detailed Protein Report

Protein 386: PHD and RING finger domain-containing protein 1 isoform 4 [Homo sapiens]

Accession: gi|557440810
Database: refseq_human(refseq_human_20140103.fasta)
Database Date: 2015-11-30

Score: 22.4
MW [kDa]: 178.1
pI: 10.1
Sequence Coverage [%]: 1.8
No. of unique Peptides: 1

Quantitation

WD:WU **Median:** 1.82 **CV:** 0.00 % **No. of Peptides:** 1

10	20	30	40	50	60	70	80
MDDDSLDELV	ARSPGPDGHP	QVGPADPAES	SVGSSGDSGD	DSDSEHGDGT	DGEDEGASEE	EDLEDRSGSE	DSEDDGETLL
90	100	110	120	130	140	150	160
EVAGTQGKLE	AAGSFNSDDD	AESCPICLNA	FRDQAVGTPE	NCAHYFCLDC	IVEWSKNANS	CPVDRTLFKC	ICIRAQFGGK
170	180	190	200	210	220	230	240
ILRKIPVENT	KASEEEEDPT	FCEVCGRSDR	EDRLLLCDGC	DAGYHMECLD	PPLQEVPVDE	WFCPECAAPG	VVLAADAGPV
250	260	270	280	290	300	310	320
SEEEVSLLLA	DVVPTTSRLR	PRAGRTRAIA	RTRQSERVRA	TVNRNRISTA	RRVQHTPGRL	GSSLLDEAIE	AVATGLSTAV
330	340	350	360	370	380	390	400
YQRPLTPRTP	ARRKRKTRRR	KKVPGRKKTP	SGPSAKSKSS	ATRSKKRQHR	VKKRRGKKVK	SEATTRSRIA	RTLGLRRPVH
410	420	430	440	450	460	470	480
SSCIPSVLKP	VEPSLGLLRA	DIGAASLSLF	GDPYELDPFD	SSEELSANPL	SPLSAKRRAL	SRSALQSHQP	VARPVSVGLS
490	500	510	520	530	540	550	560
RRRLPAAVPE	PDLEEEPVPD	LLGSILSGQS	LLMLGSSDVI	IHRDGSLSAK	RAAPVSFQRN	SGSLSRGEEG	FKGCLQPRAL
570	580	590	600	610	620	630	640
PSGSPAQGPS	GNRPQSTGLS	CQGRSRTPAR	TAGAPVRLDL	PAAPGAVQAR	NLSNGSVPGF	RQSHSPWFNG	TNKHTLPLAS
650	660	670	680	690	700	710	720
AASKISSRDS	KPPCRSVVPG	PPLKPAPRRT	DISELPRIPK	IRRDDGGGRR	DAAPAHGQSI	EIPSACISRL	TGREGTGQPG
730	740	750	760	770	780	790	800
RGTRAESEAS	SRVPREPGVH	TGSSRPPAPS	SHGSLAPLGP	SRGKGVGSTF	ESFRINIPGN	MAHSSQLSSP	GFCNTFRPVD
810	820	830	840	850	860	870	880
DKEQRKENPS	PLFSIKKTKQ	LRSEVYDPSD	PTGSDSSAPG	SSPERSGPGL	LPSEITRTIS	INSPKAQTVQ	AVRCVTSYTV
890	900	910	920	930	940	950	960
ESIFGTEPEP	PLGPSSAMSK	LRGAVAAEGA	SDTEREEPTE	SQGLAARLRR	PSPPEPWDEE	DGASCSTFFG	SEERTVTCVT
970	980	990	1000	1010	1020	1030	1040
VVEPEAPPSP	DVLQAATHRV	VELRPPSRSR	STSSSRSRKK	AKRKRVSREH	GRTRSGTRSE	SRDRSSRSAS	PSVGEERPRR
1050	1060	1070	1080	1090	1100	1110	1120
QRSKAKSRRS	SSDRSSSRER	AKRKKAKDKS	REHRRGPWGH	SRRTSRSRSG	SPGSSSYEHY	ESRKKKKRRS	ASRPRGRECS
1130	1140	1150	1160	1170	1180	1190	1200
PTSSLERLCR	HKHQRERSHE	RPDRKESVAW	PRDRRKRRSR	SPSSEHRARE	HRRPRSREKW	PQTRSHSPER	KGAVREASPA
1210	1220	1230	1240	1250	1260	1270	1280
PLAQGEPGRE	DLPTRLPALG	EAHVSPEVAT	ADKAPLQAPP	VLEVAAECEP	DDLDLDYGDS	VEAGHVFDDF	SSDAVFIQLD
1290	1300	1310	1320	1330	1340	1350	1360
DMSSPPSPES	TDSSPERDFP	LKPALPPASL	AVAAIQREVS	LMHDEDPSQP	PPLPEGTQEP	HLLRPDAAEK	AEAPSSPDVA
1370	1380	1390	1400	1410	1420	1430	1440
PAGKEDSPSA	SGRVQEAARP	EEVVSQTPLL	RSRALVKRVT	WNLQESESSA	PAEDRAPRAP	LHRPQKPREG	AWDMEDVAPT
1450	1460	1470	1480	1490	1500	1510	1520
GVRQVFSELP	FPSHVLPEPG	FPDTDPSQVY	SPGLPPAPAQ	PSSIPPCALV	SQPTVQFILQ	GSLPLVGCGA	AQTLAPVPAA
1530	1540	1550	1560	1570	1580	1590	1600
LTPASEPASQ	ATAASNSEEK	TPAPRLAAEK	TKKEEYMKKL	HMQERAVEEV	KLAIKPFYQK	REVTKEEYKD	ILRKAVQKIC
1610	1620	1630	1640	1650			
HSKSGEINPV	KVANLVKAYV	DKYRHMRRHK	KPEAGEEPPT	QGAEG			

Cmpd.	No. of Cmpds.	m/z meas.	Δ m/z [ppm]	z	Rt [min]	Score	P	Range	Sequence	Modification	Ratios	
1190	1	500.7991	12.23	2	43.5	10.8	2	347-356	R.KKTPSGPSAK.S		WD:WU	1.82

Detailed Protein Report

Protein 387: PREDICTED: protein transport protein Sec16A isoform X5 [Homo sapiens]

**Accession:**	gi|530426857	**Score:**	22.3
**Database:**	refseq_human(refseq_human_20140103.fasta)	**MW [kDa]:**	240.9
**Database Date:**	2015-11-30	**pI:**	5.7
		**Sequence Coverage [%]:**	1.0
		**No. of unique Peptides:**	1

Quantitation

WD:WU	**Median:** 0.81	**CV:** 0.00 %	**No. of Peptides:** 1

10	20	30	40	50	60	70	80
MGAAGLGLGP	TTCSLTLFKQ	VRNLTPERQM	LAEQIGPGAT	SAGLKLSLHR	LRPCWRGTDI	TPSLTSRRPS	WGQGWLPPRE
90	100	110	120	130	140	150	160
DLSRCWELSL	PRCHRAGTPT	TAPALWKEQQ	RKAAALPCPC	QAGVQLQLRN	SYILLPSVSS	IDLCKGSVMQ	PPPQTVPSGM
170	180	190	200	210	220	230	240
AGPPPAGNPR	SVFWASSPYR	RRANNNAAVA	PTTCPLQPVT	DPFAFSRQAL	QSTPLGSSSK	SSPPVLQGPA	PAGFSQHPGL
250	260	270	280	290	300	310	320
LVPHTHARDS	SQGPCEPLPG	PLTQPRAHAS	PFSGALTPSA	PPGPEMNRSA	EVGPSSEPEV	QTLPYLPHYI	PGVDPETSHG
330	340	350	360	370	380	390	400
GHPHGNMPGL	DRPLSRQNPH	DGVVTPAASP	SLPQPGLQMP	GQWGPVQGGP	QPSGQHRSPC	PEGPVPSGVP	CATSVPHFPT
410	420	430	440	450	460	470	480
PSILHQGPGH	EQHSPLVAPP	AALPSDGRDE	VSHLQSGSHL	ANNSDPESTF	RQNPRIVNHW	ASPELRQNPG	VKNEHRPASA
490	500	510	520	530	540	550	560
LVNPLARGDS	PENRTHHPLG	AGAGSGCAPL	EADSGASGAL	AMFFQGGETE	NEENLSSEKA	GLSGQADFDD	FCSSPGLGRP
570	580	590	600	610	620	630	640
PAPTHVGAGS	LCQALLPGPS	NEAAGDVWGD	TASTGVPDAS	GSQYENVENL	EFVQNQEVLP	SEPLNLDPSS	PSDQFRYGPL
650	660	670	680	690	700	710	720
PGPAVPRHGA	VCHTGAPDAT	LHTVHPDSVS	SSYSSRSHGR	LSGSARPQEL	VGTFIQQEVG	KPEDEASGSF	FKQIDSSPVG
730	740	750	760	770	780	790	800
GETDETTVSQ	NYRGSVSQPS	TPSPPKPTGI	FQTSANSSFE	PVKSHLVGVK	PFEADRANVV	GEVRETCVRQ	KQCRPAAALP
810	820	830	840	850	860	870	880
DASPGNLEQP	PDNMETLCAP	QVCPLPLNST	TEAVHMLPHA	GAPPLDTVYP	APEKRPSART	QGPVKCESPA	TTLWAQSELP
890	900	910	920	930	940	950	960
DFGGNVLLAP	AAPALYVCAK	PQPPVVQPPE	EAMSGQQSRN	PSSAAPVQSR	GGIGASENLE	NPPKMGEEEA	LQSQVTKDAQ
970	980	990	1000	1010	1020	1030	1040
GQPGLERAQQ	ELVPPQQQAS	PPQLPKAMFS	ELSNPESLPA	QGQAQNSAQS	PASLVLVDAG	QQLPPRPPQS	SSVSLVSSGS
1050	1060	1070	1080	1090	1100	1110	1120
GQAAVPSEQP	WPQPVPALAP	GPPPQDLAAY	YYYRPLYDAY	QPQYSLPYPP	EPGAASLYYQ	DVYSLYEPRY	RPYDGAASAY
1130	1140	1150	1160	1170	1180	1190	1200
AQNYRYPEPE	RPSSRASHSS	ERPPPRQGYP	EGYYSSKSGW	SSQSDYYASY	YSSQYDYGDP	GHWDRYHYSA	RVRDPRTYDR
1210	1220	1230	1240	1250	1260	1270	1280
RYWCDAEYDA	YRREHSAFGD	RPEKRDNNWR	YDPRFTGSFD	DDPDPHRDPY	GEEVDRRSVH	SEHSARSLHS	AHSLASRRSS
1290	1300	1310	1320	1330	1340	1350	1360
LSSHSHQSQI	YRSHNVAAGS	YEAPLPPGSF	HGDFAYGTYR	SNFSSGPGFP	EYGYPADTVW	PAMEQVSSRP	TSPEKFSVPH
1370	1380	1390	1400	1410	1420	1430	1440
VCARFGPGGQ	LIKVIPNLPS	EGQPALVEVH	SMEALLQHTS	EQEEMRAFPG	PLAKDDTHKV	DVINFAQNKA	MKCLQNENLI
1450	1460	1470	1480	1490	1500	1510	1520
DKESASLLWN	FIVLLCRQNG	TVVGTDIAEL	LLRDHRTVWL	PGKSPNEANL	IDFTNEAVEQ	VEEEESGEAQ	LSFLTGGPAA
1530	1540	1550	1560	1570	1580	1590	1600
AASSLERETE	RFRELLLYGR	KKDALESAMK	NGLWGHALLL	ASKMDSRTHA	RVMTRFANSL	PINDPLQTVY	QLMSGRMPAA
1610	1620	1630	1640	1650	1660	1670	1680
STCCGDEKWG	DWRPHLAMVL	SNLNNNMDVE	SRTMATMGDT	LASRGLLDAA	HFCYLMAQAG	FGVYTKKTTK	LVLIGSNHSL
1690	1700	1710	1720	1730	1740	1750	1760
PFLKFATNEA	IQRTEAYEYA	QSLGAETCPL	PSFQVFKFIY	SCRLAEMGLA	TQAFHYCEAI	AKSILTQPHL	YSPVLISQLV
1770	1780	1790	1800	1810	1820	1830	1840
QMASQLRLFD	PQLKEKPEEE	SLAAPTWLVH	LQQVERQIKE	GAGVWHQDGA	LPQQCPGTPS	SEMEQLDRPG	LSQPGALGIA
1850	1860	1870	1880	1890	1900	1910	1920
NPLLAVPAPS	PEHSSPSVRL	LPSAPQTLPD	GPLASPARVP	MFPVPLPPGP	LEPGPGCVTP	GPALGFLEPS	GPGLPPGVPP
1930	1940	1950	1960	1970	1980	1990	2000
LQERRHLLQE	ARSPDPGIVP	QEAPVGNSLS	ELSEENFDGK	FANLTPSRTV	PDSEAPPGWD	RADSGPTQPP	LSLSPAPETK
2010	2020	2030	2040	2050	2060	2070	2080
RPGQAAKKET	KEPKKGESWF	FRWLPGKKKT	EAYLPDDKNK	SIVWDEKKNQ	WVNLNEPEEE	KKAPPPPPTS	MPKTVQAAPP
2090	2100	2110	2120	2130	2140	2150	2160
ALPGPPGAPV	NMYSRRAAGT	RARYVDVLNP	SGTQRSEPAL	APADFVAPLA	PLPIPSNLFV	PTPDAEEPQL	PDGTGREGPA
2170	2180	2190	2200	2210	2220	2230	2240

Detailed Protein Report

Cmpd.	No. of Cmpds.	m/z meas.	Δ m/z [ppm]	z	Rt [min]	Score	P	Range	Sequence	Modification	Ratios	
2722	2	607.3395	-24.29	2	62.5	12.3	2	2001-2011	K.RPGQAAKKETK.E		WD:WU	0.81

Detailed Protein Report

Protein 388: PREDICTED: microspherule protein 1 isoform X2 [Homo sapiens]

Accession: gi|530399608
Database: refseq_human(refseq_human_20140103.fasta)
Database Date: 2015-11-30

Score: 22.3
MW [kDa]: 42.5
pI: 9.7
Sequence Coverage [%]: 9.1
No. of unique Peptides: 1

10	20	30	40	50	60	70	80
MVKDYIEVSK	APSTPVPPSP	APAPGLTKRV	KKSKQPLQVT	KDLGRWKPAD	DLLLINAVLQ	TNDLTSVHLG	VKFSCRFTLR
90	100	110	120	130	140	150	160
EVQERWYALL	YDPVISKLAC	QAMRQLHPEA	IAAIQSKALF	SKAEEQLLSK	VGSTSQPTLE	TFQDLLHRHP	DAFYLARTAK
170	180	190	200	210	220	230	240
ALQAHWQLMK	QYYLLEDQTV	QPLPKGDQVL	NFSDAEDLID	DSKLKDMRDE	VLEHELMVAD	RRQKREIRQL	EQELHKWQVL
250	260	270	280	290	300	310	320
VDSITGMSSP	DFDNQTLAVL	RGRMVRYLMR	SREITLGRAT	KDNQIDVDLS	LEGPAWKISR	KQGVIKLKNN	GDFFIANEGR
330	340	350	360	370	380		
RPIYIDGRPV	LCGSKWRLSN	NSVVEIASLR	FVFLINQDLI	ALIRAEAAKI	TPQ		

Cmpd.	No. of Cmpds.	m/z meas.	Δ m/z [ppm]	z	Rt [min]	Score	P	Range	Sequence	Modification
1814	1	689.9700	-142.87	3	51.0	11.1	2	11-31	K.APSTPVPPSPAPAPGLTKRVK.K	

Detailed Protein Report

Protein 389: PREDICTED: olfactomedin-like protein 2A isoform X2 [Homo sapiens]

**Accession:**	gi|530390640	**Score:**	22.3
**Database:**	refseq_human(refseq_human_20140103.fasta)	**MW [kDa]:**	64.4
**Database Date:**	2015-11-30	**pI:**	9.5
**Modification(s):**	Carbamidomethyl	**Sequence Coverage [%]:**	6.0
		**No. of unique Peptides:**	1

10	20	30	40	50	60	70	80
MAAAALPPRP	LLLLPLVLLL	SGRPTRADSK	VFGDLDQVRM	TSEGSDCRCK	CIMRPLSKDA	CSRVRSGRAR	VEDFYTVETV
**90**	**100**	**110**	**120**	**130**	**140**	**150**	**160**
SSGTDCRCSC	TAPPSSLNPC	ENEWKMEKLK	KQAPELLKLQ	SMVDLLEGTL	YSMDLMKVHA	YVHKVASQMN	TLEESIKANL
**170**	**180**	**190**	**200**	**210**	**220**	**230**	**240**
SRENEVVKDS	VRHLSEQLRH	YENHSAIMLG	IKKELSRLGL	QLLQKDAAAA	PATPATGTGS	KAQDTARGKG	KDISKYGSVQ
**250**	**260**	**270**	**280**	**290**	**300**	**310**	**320**
KSFADRGLPK	PPKEKLLQVE	KLRKESGKGS	FLQPTAKPRA	LAQQQAVIRG	FTYYKAGKQE	VTEAVAGREA	SCEGTLRAVD
**330**	**340**	**350**	**360**	**370**	**380**	**390**	**400**
PPVRHHSYGR	HEGAWMKDPA	ARDDRIYVTN	YYYGNSLVEF	RNLENFKQGR	WSNMYKLPYN	WIGTGHVVYQ	GAFYYNRAFT
**410**	**420**	**430**	**440**	**450**	**460**	**470**	**480**
KNIIKYDLRQ	RFVASWALLP	DVVYEDTTPW	KWRGHSDIDF	AVDESGLWVI	YPAVDDRDEA	QPEVIVLSRL	DPGDLSVHRE
**490**	**500**	**510**	**520**	**530**	**540**	**550**	**560**
TTWKTRLRRN	SYGNCFLVCG	ILYAVDTYNQ	QEGQVAYAFD	THTGTDARPQ	LPFLNEHAYT	TQIDYNPKER	VLYAWDNGHQ
**570**							
LTYTLHFVV							

Cmpd.	No. of Cmpds.	m/z meas.	Δ m/z [ppm]	z	Rt [min]	Score	P	Range	Sequence	Modification
2186	1	1159.9541	-101.22	2	55.8	11.4	2	296-317	K.AGKQEVTEAVAGREASCEGTLR.A	Carbamidomethyl: 17

Detailed Protein Report

Protein 390: puratrophin-1 isoform 2 [Homo sapiens]

Accession: gi|193211603
Database: refseq_human(refseq_human_20140103.fasta)
Database Date: 2015-11-30
Modification(s): Oxidation

Score: 22.3
MW [kDa]: 122.8
pI: 5.6
Sequence Coverage [%]: 2.4
No. of unique Peptides: 2

10	20	30	40	50	60	70	80
MERPLENGDE	SPDSQGHATD	WRFAVCSFRD	AWEEEEPASQ	MHVKDPGPPR	PPAGATQDEE	LQGSPLSRKF	QLPPAADESG
90	100	110	120	130	140	150	160
DAQRGTVESS	SVLSEGPGPS	GVESLLCPMS	SHLSLAQGTR	DVQGRAVLLL	CAHSPAWLQS	ECSSQELIRL	LLYLRSIPRP
170	180	190	200	210	220	230	240
EVQALGLTVL	VDARICAPSS	SLFSGLSQLQ	EAAPGAVYQV	LLVGSTLLKE	VPSGLQLEQL	PSQSLLTHIP	TAGLPTSLGG
250	260	270	280	290	300	310	320
GLPYCHQAWL	DFRRRLEALL	QNCQAACALL	QGAIESVKAV	PQPMEPGEVG	QLLQQTEVLM	QQVLDSPWLA	WLQCQGGREL
330	340	350	360	370	380	390	400
TWLKQEVPEV	TLSPDYRTAM	DKADELYDRV	DGLLHQLTLQ	SNQRIQALEL	VQTLEARESG	LHQIEVWLQQ	VGWPALEEAG
410	420	430	440	450	460	470	480
EPSLDMLLQA	QGSFQELYQV	AQEQVRQGEK	FLQPLTGWEA	AELDPPGARF	LALRAQLTEF	SRALAQRCQR	LADAERLFQL
490	500	510	520	530	540	550	560
FREALTWAEE	GQRVLAELEQ	ERPGVVLQQL	QLHWTRHPDL	PPAHFRKMWA	LATGLGSEAI	RQECRWAWAR	CQDTWLALDQ
570	580	590	600	610	620	630	640
KLEASLKLPP	VGSTASLCVS	QVPAAPAHPP	LRKAYSFDRN	LGQSLSEPAC	HCHHAATIAA	CRRPEAGGGA	LPQASPTVPP
650	660	670	680	690	700	710	720
PGSSDPRSLN	RLQLVLAEMV	ATEREYVRAL	EYTMENYFPE	LDRPDVPQGL	RGQRAHLFGN	LEKLRDFHCH	FFLRELEACT
730	740	750	760	770	780	790	800
RHPPRVAYAF	LRHRVQFGMY	ALYSKNKPRS	DALMSSYGHT	FFKDKQQALG	DHLDLASYLL	KPIQRMGKYA	LLLQELARAC
810	820	830	840	850	860	870	880
GGPTQELSAL	REAQSLVHFQ	LRHGNDLLAM	DAIQGCDVNL	KEQGQLVRQD	EFVVRTGRHK	SVRRIFLFEE	LLLFSKPRHG
890	900	910	920	930	940	950	960
PTGVDTFAYK	RSFKMADLGL	TECCGNSNLR	FEIWFRRRKA	RDTFVLQASS	LAIKQAWTAD	ISHLLWRQAV	HNKEVRMAEM
970	980	990	1000	1010	1020	1030	1040
VSMGVGNKAF	RDIAPSEEAI	NDRTVNYVLK	CREVRSRASI	AVAPFDHDSL	YLGASNSLPG	DPASCSVLGS	LNLHLYRDPA
1050	1060	1070	1080	1090	1100	1110	1120
LLGLRCPLYP	SFPEEAALEA	EAELGGQPSL	TAEDSEISSQ	CPSASGSSGS	DSSCVSGQAL	GRGLEDLPCV	

Cmpd.	No. of Cmpds.	m/z meas.	Δ m/z [ppm]	z	Rt [min]	Score	P	Range	Sequence	Modification
1533	1	835.4469	69.38	2	47.9	10.1	1	954-968	K.EVRMAEMVSMGVGNK.A	Oxidation: 4, 7
2818	1	665.3626	67.46	2	63.7	12.3	0	972-983	R.DIAPSEEAINDR.T	

Detailed Protein Report

Protein 391: protocadherin beta-15 precursor [Homo sapiens]

Accession: gi|9256610
Database: refseq_human(refseq_human_20140103.fasta)
Database Date: 2015-11-30
Modification(s): Oxidation

Score: 22.3
MW [kDa]: 86.3
pI: 4.6
Sequence Coverage [%]: 2.8
No. of unique Peptides: 1

10	20	30	40	50	60	70	80
MEPAGERFPE	QRQVLILLLL	LEVTLAGWEP	RRYSVMEETE	RGSFVANLAN	DLGLGVGELA	ERGARVVSED	NEQGLQLDLQ
**90**	**100**	**110**	**120**	**130**	**140**	**150**	**160**
TGQLILNEKL	DREKLCGPTE	PCIMHFQVLL	KKPLEVFRAE	LLVTDINDHS	PEFPEREMTL	KIPETSSLGT	VFPLKKARDL
**170**	**180**	**190**	**200**	**210**	**220**	**230**	**240**
DVGSNNVQNY	NISPNSHFHV	STRTRGDGRK	YPELVLDTEL	DREEQAELRL	TLTAVDGGSP	PRSGTVQILI	LVLDANDNAP
**250**	**260**	**270**	**280**	**290**	**300**	**310**	**320**
EFVQALYEVQ	VPENSPVGSL	VVKVSARDLD	TGTNGEISYS	LYYSSQEIDK	PFELSSLSGE	IRLIKKLDFE	TMSSYDLDIE
**330**	**340**	**350**	**360**	**370**	**380**	**390**	**400**
ASDGGGLSGK	CSVSVKVLDV	NDNFPELSIS	SLTSPIPENS	PETEVALFRI	RDRDSGENGK	MICSIQDDVP	FKLKPSVENF
**410**	**420**	**430**	**440**	**450**	**460**	**470**	**480**
YRLVTEGALD	RETRAEYNIT	ITITDLGTPR	LKTEQSITVL	VSDVNDNAPA	FTQTSYTLFV	RENNSPALHI	GSVSATDRDS
**490**	**500**	**510**	**520**	**530**	**540**	**550**	**560**
GTNAQVTYSL	LPPRDPHLPL	TSLVSINTDN	GHLFALQSLD	YEALQAFEFR	VGATDRGFPA	LSSEALVRVL	VLDANDNSPF
**570**	**580**	**590**	**600**	**610**	**620**	**630**	**640**
VLYPLQNGSA	PCTELVPRAA	EPGYLVTKVV	AVDGDSGQNA	WLSYQLLKAT	EPGLFGVWAH	NGEVRTARLL	SERDVAKHRL
**650**	**660**	**670**	**680**	**690**	**700**	**710**	**720**
VVLVKDNGEP	PRSATATLQV	LLVDGFSQPY	LPLPEAAPAQ	AQADSLTVYL	VVALASVSSL	FLFSVFLFVA	VRLCRRSRAA
**730**	**740**	**750**	**760**	**770**	**780**	**790**	
SVGRCSVPEG	PFPGHLVDVS	GTGTLSQSYQ	YEVCLTGGSE	SNDFKFLKPI	FPNIVSQDSR	RKSEFLE	

Cmpd.	No. of Cmpds.	m/z meas.	Δ m/z [ppm]	z	Rt [min]	Score	P	Range	Sequence	Modification
270	1	862.8505	82.24	3	32.5	22.3	2	90-111	K.LDREKLCGPTEPCIMHFQVLLK.K	Oxidation: 15

Detailed Protein Report

Protein 392: **testis-specific protein 10-interacting protein [Homo sapiens]**

**Accession:**	gi|154759273	**Score:**	22.3
**Database:**	refseq_human(refseq_human_20140103.fasta)	**MW [kDa]:**	62.3
**Database Date:**	2015-11-30	**pI:**	10.9
**Modification(s):**	Carbamidomethyl	**Sequence Coverage [%]:**	4.3
		**No. of unique Peptides:**	2

10	20	30	40	50	60	70	80
MGQDTDMLNT	YQQLVRTPSV	RPGQDVRLQA	PGTRTGLLKL	LSTVSQDKQG	CLGSGDGVPN	QDLQQRSQSS	RQTAKKDRKP
**90**	**100**	**110**	**120**	**130**	**140**	**150**	**160**
RGQSKKGQGS	EESEDHFPLL	PRKPSFPFQW	AWESIATDVR	AVLQPSSPTP	GHQALPMPSS	FSQRQSRRKS	TANLPEAHGC
**170**	**180**	**190**	**200**	**210**	**220**	**230**	**240**
CWKTEAQNLK	ARQQLGAWGG	VSIPTGKGEL	GSEPPSGLQL	PGRRPGSGSA	SDKQVQLQSL	GAEEAERGLS	SGVLPQRPRR
**250**	**260**	**270**	**280**	**290**	**300**	**310**	**320**
GSISEEEQFS	EATEEAEEGE	HRTPCRRRAG	CQRKGQISGE	EASDEGEVQG	QSQGSSPSFN	NLRRRQWRKT	RAKELQGPWD
**330**	**340**	**350**	**360**	**370**	**380**	**390**	**400**
LEKLHRQLQR	DLDCGPQKLP	WKTLRAAFQA	SKRNGKAYAS	GYDETFVSAN	LPNRTFHKRQ	EATRSLLQAW	ERQRQEERQQ
**410**	**420**	**430**	**440**	**450**	**460**	**470**	**480**
AELRRARTQH	VQRQVAHCLA	AYAPRGSRGP	GAAQRKLEEL	RRQERQRFAE	YQAELQGIQH	RVQARPFLFQ	QAMQANARLT
**490**	**500**	**510**	**520**	**530**	**540**	**550**	**560**
VTRRFSQVLS	ALGLDEEQLL	SEAGKVDREG	TPRKPRSHRS	MGVRMEHSPQ	RPPRTEPTGS	QPDRHYNPSL	DPECSP

Cmpd.	No. of Cmpds.	m/z meas.	Δ m/z [ppm]	z	Rt [min]	Score	P	Range	Sequence	Modification
2105	1	707.2250	-208.16	2	54.7	11.8	1	312-323	R.AKELQGPWDLEK.L	
467	1	708.1877	-156.38	2	34.3	10.4	0	545-556	R.HYNPSLDPECSP.-	Carbamidomethyl: 10

Detailed Protein Report

Protein 393: **transient receptor potential cation channel subfamily M member 3 isoform d [Homo sapiens]**

**Accession:**	gi|154091318	**Score:**	22.3
**Database:**	refseq_human(refseq_human_20140103.fasta)	**MW [kDa]:**	176.1
**Database Date:**	2015-11-30	**pI:**	6.4
		**Sequence Coverage [%]:**	1.4
		**No. of unique Peptides:**	1

Quantitation

WD:WU **Median:** 0.46 **CV:** 0.00 % **No. of Peptides:** 1

10	20	30	40	50	60	70	80
MYVRVSFDTK	PDLLLHLMTK	EWQLELPKLL	ISVHGGLQNF	ELQPKLKQVF	GKGLIKAAMT	TGAWIFTGGV	NTGVIRHVGD
90	100	110	120	130	140	150	160
ALKDHASKSR	GKICTIGIAP	WGIVENQEDL	IGRDVVRPYQ	TMSNPMSKLT	VLNSMHSHFI	LADNGTTGKY	GAEVKLRRQL
170	180	190	200	210	220	230	240
EKHISLQKIN	TRIGQGVPVV	ALIVEGGPNV	ISIVLEYLRD	TPPVPVVVCD	GSGRASDILA	FGHKYSEEGG	LINESLRDQL
250	260	270	280	290	300	310	320
LVTIQKTFTY	TRTQAQHLFI	ILMECMKKKE	LITVFRMGSE	GHQDIDLAIL	TALLKGANAS	APDQLSLALA	WNRVDIARSQ
330	340	350	360	370	380	390	400
IFIYGQQWPV	GSLEQAMLDA	LVLDRVDFVK	LLIENGVSMH	RFLTISRLEE	LYNTRHGPSN	TLYHLVRDVK	KGNLPPDYRI
410	420	430	440	450	460	470	480
SLIDIGLVIE	YLMGGAYRCN	YTRKRFRTLY	HNLFGPKRDD	IPLRRGRKTT	KKREEEVDID	LDDPEINHFP	FPFHELMVWA
490	500	510	520	530	540	550	560
VLMKRQKMAL	FFWQHGEEAM	AKALVACKLC	KAMAHEASEN	DMVDDISQEL	NHNSRDFGQL	AVELLDQSYK	QDEQLAMKLL
570	580	590	600	610	620	630	640
TYELKNWSNA	TCLQLAVAAK	HRDFIAHTCS	QMLLTDMWMG	RLRMRKNSGL	KVILGILLPP	SILSLEFKNK	DDMPYMSQAQ
650	660	670	680	690	700	710	720
EIHLQEKEAE	EPEKPTKEKE	EEDMELTAML	GRNNGESSRK	KDEEEVQSKH	RLIPLGRKIY	EFYNAPIVKF	WFYTLAYIGY
730	740	750	760	770	780	790	800
LMLFNYIVLV	KMERWPSTQE	WIVISYIFTL	GIEKMREILM	SEPGKLLQKV	KVWLQEYWNV	TDLIAILLFS	VGMILRLQDQ
810	820	830	840	850	860	870	880
PFRSDGRVIY	CVNIIYWYIR	LLDIFGVNKY	LGPYVMMIGK	MMIDMMYFVI	IMLVVLMSFG	VARQAILFPN	EEPSWKLAKN
890	900	910	920	930	940	950	960
IFYMPYWMIY	GEVFADQIDP	PCGQNETRED	GKIIQLPPCK	TGAWIVPAIM	ACYLLVANIL	LVNLLIAVFN	NTFFEVKSIS
970	980	990	1000	1010	1020	1030	1040
NQVWKFQRYQ	LIMTFHERPV	LPPPLIIFSH	MTMIFQHLCC	RWRKHESDPD	ERDYGLKLFI	TDDELKKVHD	FEEQCIEEYF
1050	1060	1070	1080	1090	1100	1110	1120
REKDDRFNSS	NDERIRVTSE	RVENMSMRLE	EVNEREHSMK	ASLQTVDIRL	AQLEDLIGRM	ATALERLTGL	ERAESNKIRS
1130	1140	1150	1160	1170	1180	1190	1200
RTSSDCTDAA	YIVRQSSFNS	QEGNTFKLQE	SIDPAGEETM	SPTSPTLMPR	MRSHSFYSVN	MKDKGGIEKL	ESIFKERSLS
1210	1220	1230	1240	1250	1260	1270	1280
LHRATSSHSV	AKEPKAPAAP	ANTLAIVPDS	RRPSSCIDIY	VSAMDELHCD	IDPLDNSVNI	LGLGEPSFST	PVPSTAPSSS
1290	1300	1310	1320	1330	1340	1350	1360
AYATLAPTDR	PPSRSIDFED	ITSMDTRSFS	SDYTHLPECQ	NPWDSEPPMY	HTIERSKSSR	YLATTPFLLE	EAPIVKSHSF
1370	1380	1390	1400	1410	1420	1430	1440
MFSPSRSYYA	NFGVPVKTAE	YTSITDCIDT	RCVNAPQAIA	DRAAFPGGLG	DKVEDLTCCH	PEREAELSHP	SSDSEENEAK
1450	1460	1470	1480	1490	1500	1510	1520
GRRATIAISS	QEGDNSERTL	SNNITVPKIE	RANSYSAEEP	SAPYAHTRKS	FSISDKLDRQ	RNTASLRNPF	QRSKSSKPEG
1530	1540	1550					
RGDSLSMRRL	SRTSAFQSFE	SKHN					

Cmpd.	No. of Cmpds.	m/z meas.	Δ m/z [ppm]	z	Rt [min]	Score	P	Range	Sequence	Modification	Ratios	
317	1	442.6411	-248.40	2	33.0	10.9	1	438-444	K.RDDIPLR.R		WD:WU	0.46

Detailed Protein Report

Protein 394: dynein heavy chain 10, axonemal [Homo sapiens]

**Accession:**	gi|198442844	**Score:**	22.2
**Database:**	refseq_human(refseq_human_20140103.fasta)	**MW [kDa]:**	514.5
**Database Date:**	2015-11-30	**pI:**	5.6
**Modification(s):**	Oxidation	**Sequence Coverage [%]:**	0.7
		**No. of unique Peptides:**	1

Detailed Protein Report

10	20	30	40	50	60	70	80
MVPEEVEVEI	DEIPVLSEEG	EEEEETYSQK	VESVDKVRAK	RVSLRTESLG	QPLNREDEEM	DKEISEKLPS	KRTAKHIMEK
90	100	110	120	130	140	150	160
MHLHMLCTPL	PEEFLDQNVV	FFLRNTKEAI	SEATDMKEAM	EIMPETLEYG	IINANVLHFL	KNIICQVFLP	ALSFNQHRTS
170	180	190	200	210	220	230	240
TTVGVTSGEV	SNSSEHESDL	PPMPGEAVEY	HSIQLIRDEF	LMNVQKFASN	IQRTMQQLEG	EIKLEMPIIS	VEGEVSDLAA
250	260	270	280	290	300	310	320
DPETVDILEQ	CVINWLNQIS	TAVEAQLKKT	PQGKGPLAEI	EFWRERNATL	SALHEQTKLP	IVRKVLDVIK	ESDSMLVANL
330	340	350	360	370	380	390	400
QPVFTELFKF	HTEASDNVRF	LSTVERYFKN	ITHGSGFHVV	LDTIPAMMSA	LRMVWIISRH	YNKDERMIPL	MERIAWEIAE
410	420	430	440	450	460	470	480
RVCRVVNLRT	LFKENRASAQ	SKTLEARNTL	RLWKKAYFDT	RAKIEASGRE	DRWEFDRKRL	FERTDYMATI	CQDLSDVLQI
490	500	510	520	530	540	550	560
LEEFYNIFGP	ELKAVTGDPK	RIDDVLCRVD	GLVTPMENLT	FDPFSIKSSQ	FWKYVMDEFK	IEVLIDIINK	IFVQNLENPP
570	580	590	600	610	620	630	640
LYKNHPPVAG	AIYWERSLFF	RIKHTILRFQ	EVQEILDSDR	GQEVKQKYLE	VGRTMKEYED	RKYEQWMEVT	EQVLPALMKK
650	660	670	680	690	700	710	720
SLLTKSSIAT	EEPSTLERGA	VFAINFSPAL	REIINETKYL	EQLGFTVPEL	ARNVALQEDK	FLRYTAGIQR	MLDHYHMLIG
730	740	750	760	770	780	790	800
TLNDAESVLL	KDHSQELLRV	FRSGYKRLNW	NSLGIGDYIT	GCKQAIGKFE	SLVHQIHKNA	DDISSRLTLI	EAINLFKYPA
810	820	830	840	850	860	870	880
AKSEEELPGV	KEFFEHIERE	RASDVDHMVR	WYLAIGPLLT	KVEGLVVHTN	TGKAPKLASY	YKYWEKKIYE	VLTKLILKNL
890	900	910	920	930	940	950	960
QSFNSLILGN	VPLFHTETIL	TAPEIILHPN	TNEIDKMCFH	CVRNCVEITK	HFVRWMNGSC	IECPPQKGEE	EEVVIINFYN
970	980	990	1000	1010	1020	1030	1040
DISLNPQIIE	QAVMIPQNVH	RILINLMKYL	QKWKRYRPLW	KLDKAIVMEK	FAAKKPPCVA	YDEKLQFYSK	IAYEVMRHPL
1050	1060	1070	1080	1090	1100	1110	1120
IKDEHCIRLQ	LRHLANTVQE	NAKSWVISLG	KLLNESAKEE	LYNLHEEMEH	LAKNLRKIPN	TLEDLKFVLA	TIAEIRSKSL
1130	1140	1150	1160	1170	1180	1190	1200
VMELRYRDVQ	ERYRTMAMYN	LFPPDAEKEL	VDKIESIWSN	LFNDSVNVEH	ALGDIKRTFT	ELTRGEIMNY	RVQIEEFAKR
1210	1220	1230	1240	1250	1260	1270	1280
FYSEGPGSVG	DDLDKGVELL	GVYERELARH	EKSRQELANA	EKLFDLPITM	YPELLKVQKE	MSGLRMIYEL	YEGLKVAKEE
1290	1300	1310	1320	1330	1340	1350	1360
WSQTLWINLN	VQILQEGIEG	FLRALRKLPR	PVRGLSVTYY	LEAKMKAFKD	SIPLLLDLKN	EALRDRHWKE	LMEKTSVFFE
1370	1380	1390	1400	1410	1420	1430	1440
MTETFTLENM	FAMELHKHTD	VLNEIVTAAI	KEVAIEKAVK	EILDTWENMK	FTVVKYCKGT	QERGYILGSV	DEIIQSLDDN
1450	1460	1470	1480	1490	1500	1510	1520
TFNLQSISGS	RFVGPFLQTV	HKWEKTLSLI	GEVIEIWMLV	QRKWMYLESI	FIGGDIRSQL	PEEAKKFDNI	DKVFKRIMGE
1530	1540	1550	1560	1570	1580	1590	1600
TLKDPVIKRC	CEAPNRLSDL	QNVSEGLEKC	QKSLNDYLDS	KRNAFPRFFF	ISDDELLSIL	GSSDPLCVQE	HMIKMYDNIA
1610	1620	1630	1640	1650	1660	1670	1680
SLRFNDGDSG	EKLVSAMISA	EGEVMEFRKI	LRAEGRVEDW	MTAVLNEMRR	TNRLITKEAI	FRYCEDRSRV	DWMLLYQGMV
1690	1700	1710	1720	1730	1740	1750	1760
VLAASQVWWT	WEVEDVFHKA	QKGEKQAMKN	YGRKMHRQID	ELVTRITMPL	SKNDRKKYNT	VLIIDVHARD	IVDSFIRGSI
1770	1780	1790	1800	1810	1820	1830	1840
LEAREFDWES	QLRFYWDREP	DELNIRQCTG	TFGYGYEYMG	LNGRLVITPL	TDRIYLTLTQ	ALSMYLGGAP	AGPAGTGKTE
1850	1860	1870	1880	1890	1900	1910	1920
TTKDLAKALG	LLCVVTNCGE	GMDYRAVGKI	FSGLAQCGAW	GCFDEFNRID	ASVLSVISSQ	IQTIRNALIH	QLTTFQFEGQ
1930	1940	1950	1960	1970	1980	1990	2000
EISLDSRMGI	FITMNPGYAG	RTELPESVKA	LFRPVVVIVP	DLQQICEIML	FSEGFLEAKT	LAKKMTVLYK	LAREQLSKQY
2010	2020	2030	2040	2050	2060	2070	2080
HYDFGLRALK	SVLVMAGELK	RGSSDLREDV	VLMRALRDMN	LPKFVFEDVP	LFLGLISDLF	PGLDCPRVRY	PDFNDAVEQV
2090	2100	2110	2120	2130	2140	2150	2160
LEENGYAVLP	IQVDKVVQMF	ETMLTRHTTM	VVGPTRGGKS	VVINTLCQAQ	TKLGLTTKLY	ILNPKAVSVI	ELYGILDPTT
2170	2180	2190	2200	2210	2220	2230	2240

Detailed Protein Report

Cmpd.	No. of Cmpds.	m/z meas.	Δ m/z [ppm]	z	Rt [min]	Score	P	Range	Sequence	Modification
2662	1	835.4217	30.04	2	61.6	11.7	1	2516-2528	K.RLLVFMDDMNMPR.V	Oxidation: 6, 9

Detailed Protein Report

Protein 395: PREDICTED: protein LAP2 isoform X4 [Homo sapiens]

**Accession:**	gi|578810464	**Score:**	22.2
**Database:**	refseq_human(refseq_human_20140103.fasta)	**MW [kDa]:**	139.9
**Database Date:**	2015-11-30	**pI:**	5.1
**Modification(s):**	Carbamidomethyl, Oxidation	**Sequence Coverage [%]:**	2.5
		**No. of unique Peptides:**	2

Quantitation

WD:WU **Median:** 0.92 **CV:** 0.00 % **No. of Peptides:** 1

10	20	30	40	50	60	70	80
MTTKRSLFVR	LVPCRCLRGE	EETVTTLDYS	HCSLEQVPKE	IFTFEKTLEE	LYLDANQIEE	LPKQLFNCQS	LHKLSLPDND
90	100	110	120	130	140	150	160
LTTLPASIAN	LINLRELDVS	KNGIQEFPEN	IKNCKVLTIV	EASVNPISKL	PDGFSQLLNL	TQLYLNDAFL	EFLPANFGRL
170	180	190	200	210	220	230	240
TKLQILELRE	NQLKMLPKTM	NRLTQLERLD	LGSNEFTEVP	EVLEQLSGLK	EFWMDANRLT	FIPGFIGSLK	QLTYLDVSKN
250	260	270	280	290	300	310	320
NIEMVEEGIS	TCENLQDLLL	SSNSLQQLPE	TIGSLKNITT	LKIDENQLMY	LPDSIGGLIS	VEELDCSFNE	VEALPSSIGQ
330	340	350	360	370	380	390	400
LTNLRTFAAD	HNYLQQLPPE	IGSWKNITVL	FLHSNKLETL	PEEMGDMQKL	KVINLSDNRL	KNLPFSFTKL	QQLTAMWLSD
410	420	430	440	450	460	470	480
NQSKPLIPLQ	KETDSETQKM	VLTNYMFPQQ	PRTEDVMFIS	DNESFNPSLW	EEQRKQRAQV	AFECDEDKDE	REAPPREGNL
490	500	510	520	530	540	550	560
KRYPTPYPDE	LKNMVKTVQT	IVHRLKDEET	NEDSGRDLKP	HEDQQDINKD	VGVKTSESTT	TVKSKVDERE	KYMIGNSVQK
570	580	590	600	610	620	630	640
ISEPEAEISP	GSLPVTANMK	ASENLKHIVN	HDDVFEESEE	LSSDEEMKMA	EMRPPLIETS	INQPKVVALS	NNKKDDTKET
650	660	670	680	690	700	710	720
DSLSDEVTHN	SNQNNSNCSS	PSRMSDSVSL	NTDSSQDTSL	CSPVKQTHID	INSKIRQEDE	NFNSLLQNGD	ILNSSTEEKF
730	740	750	760	770	780	790	800
KAHDKKDFNL	PEYDLNVEER	LVLIEKSVDS	TATADDTHKL	DHINMNLNKL	ITNDTFQPEI	MERSKTQDIV	LGTSFLSINS
810	820	830	840	850	860	870	880
KEETEHLENG	NKYPNLESVN	KVNGHSEETS	QSPNRTEPHD	SDCSVDLGIS	KSTEDLSPQK	SGPVGSVVKS	HSITNMEIGG
890	900	910	920	930	940	950	960
LKIYDILSDN	GPQQPSTTVK	ITSAVDGKNI	VRSKSATLLY	DQPLQVFTGS	SSSSDLISGT	KAIFKFDSNH	NPEEPNIIRG
970	980	990	1000	1010	1020	1030	1040
PTSGPQSAPQ	IYGPPQYNIQ	YSSSAAVKDT	LWHSKQNPQI	DHASFPPQLL	PRSESTENQS	YAKHSANMNF	SNHNNVRANT
1050	1060	1070	1080	1090	1100	1110	1120
AYHLHQRLGP	ARHGEMWAIS	PNDRLIPAVT	RSTIQRQSSV	SSTASVNLGD	PGSTRRAQIP	EGDYLSYREF	HSAGRTPPMM
1130	1140	1150	1160	1170	1180	1190	1200
PGSQRPLSAR	TYSIDGPNAS	RPQSARPSIN	EIPERTMSVS	DFNYSRTSPS	KRPNARVGSE	HSLLDPPGKS	KVPRDWREQV
1210	1220	1230	1240	1250			
LRHIEAKKLE	KVFFSGTGNR	VPNLNSARTI	GMIMCVSILS	FTPHCF			

Cmpd.	No. of Cmpds.	m/z meas.	Δ m/z [ppm]	z	Rt [min]	Score	P	Range	Sequence	Modification	Ratios
486	1	668.2076	-211.28	2	34.5	10.8	0	1177-1189	R.VGSEHSLLDPPGK.S		WD:WU 0.92
1657	1	697.0355	61.97	3	49.4	11.4	0	1229-1246	R.TIGMIMCVSILSFTPHCF.-	Carbamidomethyl: 7; Oxidation: 4, 6	

Detailed Protein Report

Protein 396: **PREDICTED: ATP-binding cassette sub-family A member 1 isoform X4 [Homo sapiens]**

**Accession:**	gi|530390674	**Score:**	22.2
**Database:**	refseq_human(refseq_human_20140103.fasta)	**MW [kDa]:**	247.0
**Database Date:**	2015-11-30	**pI:**	6.5
**Modification(s):**	Oxidation	**Sequence Coverage [%]:**	2.0
		**No. of unique Peptides:**	2

Detailed Protein Report

10	20	30	40	50	60	70	80
MPSAGTLPWV	QGIICNANNP	CFRYPTPGEA	PGVVGNFNKS	IVARLFSDAR	RLLLYSQKDT	SMKDMRKVLR	TLQQIKKSSS
90	100	110	120	130	140	150	160
NLKLQDFLVD	NETFSGFLYH	NLSLPKSTVD	KMLRADVILH	KVFLQGYQLH	LTSLCNGSKS	EEMIQLGDQE	VSELCGLPRE
170	180	190	200	210	220	230	240
KLAAAERVLR	SNMDILKPIL	RTLNSTSPFP	SKELAEATKT	LLHSLGTLAQ	ELFSMRSWSD	MRQEVMFLTN	VNSSSSSTQI
250	260	270	280	290	300	310	320
YQAVSRIVCG	HPEGGGLKIK	SLNWYEDNNY	KALFGGNGTE	EDAETFYDNS	TTPYCNDLMK	NLESSPLSRI	IWKALKPLLV
330	340	350	360	370	380	390	400
GKILYTPDTP	ATRQVMAEVN	KTFQELAVFH	DLEGMWEELS	PKIWTFMENS	QEMDLVRMLL	DSRDNDHFWE	QQLDGLDWTA
410	420	430	440	450	460	470	480
QDIVAFLAKH	PEDVQSSNGS	VYTWREAFNE	TNQAIRTISR	FMECVNLNKL	EPIATEVWLI	NKSMELLDER	KFWAGIVFTG
490	500	510	520	530	540	550	560
ITPGSIELPH	HVKYKIRMDI	DNVERTNKIK	DGYWDPGPRA	DPFEDMRYVW	GGFAYLQDVV	EQAIIRVLTG	TEKKTGVYMQ
570	580	590	600	610	620	630	640
QMPYPCYVDD	IFLRVMSRSM	PLFMTLAWIY	SVAVIIKGIV	YEKEARLKET	MRIMGLDNSI	LWFSWFISSL	IPLLVSAGLL
650	660	670	680	690	700	710	720
VVILKLGNLL	PYSDPSVVFV	FLSVFAVVTI	LQCFLISTLF	SRANLAAACG	GIIYFTLYLP	YVLCVAWQDY	VGFTLKIFAS
730	740	750	760	770	780	790	800
LLSPVAFGFG	CEYFALFEEQ	GIGVQWDNLF	ESPVEEDGFN	LTTSVSMMLF	DTFLYGVMTW	YIEAVFPGQY	GIPRPWYFPC
810	820	830	840	850	860	870	880
TKSYWFGEES	DEKSHPGSNQ	KRISEICMEE	EPTHLKLGVS	IQNLVKVYRD	GMKVAVDGLA	LNFYEGQITS	FLGHNGAGKT
890	900	910	920	930	940	950	960
TTMSILTGLF	PPTSGTAYIL	GKDIRSEMST	IRQNLGVCPQ	HNVLFDMLTV	EEHIWFYARL	KGLSEKHVKA	EMEQMALDVG
970	980	990	1000	1010	1020	1030	1040
LPSSKLKSKT	SQLSGGMQRK	LSVALAFVGG	SKVVILDEPT	AGVDPYSRRG	IWELLLKYRQ	GRTIILSTHH	MDEADVLGDR
1050	1060	1070	1080	1090	1100	1110	1120
IAIISHGKLC	CVGSSLFLKN	QLGTGYYLTL	VKKDVESSLS	SCRNSSSTVS	YLKKVVPELP	PAGQSGGRGG	EVQRLVALTQ
1130	1140	1150	1160	1170	1180	1190	1200
DVSAISNLIR	KHVSEARLVE	DIGHELTYVL	PYEAAKEGAF	VELFHEIDDR	LSDLGISSYG	ISETTLEEIF	LKVAEESGVD
1210	1220	1230	1240	1250	1260	1270	1280
AETSDGTLPA	RRNRRAFGDK	QSCLRPFTED	DAADPNDSDI	DPESRETDLL	SGMDGKGSYQ	VKGWKLTQQQ	FVALLWKRLL
1290	1300	1310	1320	1330	1340	1350	1360
IARRSRKGFF	AQIVLPAVFV	CIALVFSLIV	PPFGKYPSLE	LQPWMYNEQY	TFVSNDAPED	TGTLELLNAL	TKDPGFGTRC
1370	1380	1390	1400	1410	1420	1430	1440
MEGNPIPDTP	CQAGEEEWTT	APVPQTIMDL	FQNGNWTMQN	PSPACQCSSD	KIKKMLPVCP	PGAGGLPPPQ	RKQNTADILQ
1450	1460	1470	1480	1490	1500	1510	1520
DLTGRNISDY	LVKTYVQIIA	KSLKNKIWVN	EFRYGGFSLG	VSNTQALPPS	QEVNDAIKQM	KKHLKLAKDS	SADRFLNSLG
1530	1540	1550	1560	1570	1580	1590	1600
RFMTGLDTKN	NVKVWFNNKG	WHAISSFLNV	INNAILRANL	QKGENPSHYG	ITAFNHPLNL	TKQQLSEVAL	MTTSVDVLVS
1610	1620	1630	1640	1650	1660	1670	1680
ICVIFAMSFV	PASFVVFLIQ	ERVSKAKHLQ	FISGVKPVIY	WLSNFVWDMC	NYVVPATLVI	IIFICFQQKS	YVSSTNLPVL
1690	1700	1710	1720	1730	1740	1750	1760
ALLLLLYGWS	ITPLMYPASF	VFKIPSTAYV	VLTSVNLFIG	INGSVATFVL	ELFTDNKLNN	INDILKSVFL	IFPHFCLGRG
1770	1780	1790	1800	1810	1820	1830	1840
LIDMVKNQAM	ADALERFGEN	RFVSPLSWDL	VGRNLFAMAV	EGVVFFLITV	LIQYRFFIRP	RPVNAKLSPL	NDEDEDVRRE
1850	1860	1870	1880	1890	1900	1910	1920
RQRILDGGGQ	NDILEIKELT	KIYRRKRKPA	VDRICVGIPP	GECFGLLGVN	GAGKSSTFKM	LTGDTTVTRG	DAFLNKNSIL
1930	1940	1950	1960	1970	1980	1990	2000
SNIHEVHQNM	GYCPQFDAIT	ELLTGREHVE	FFALLRGVPE	KEVGKVGEWA	IRKLGLVKYG	EKYAGNYSGG	NKRKLSTAMA
2010	2020	2030	2040	2050	2060	2070	2080
LIGGPPVVFL	DEPTTGMDPK	ARRFLWNCAL	SVVKEGRSVV	LTSHSMEECE	ALCTRMAIMV	NGRFRCLGSV	QHLKNRFGDG
2090	2100	2110	2120	2130	2140	2150	2160
YTIVVRIAGS	NPDLKPVQDF	FGLAFPGSVL	KEKHRNMLQY	QLPSSLSSLA	RIFSILSQSK	KRLHIEDYSV	SQTTLDQVFV
2170	2180	2190	2200	2210			

Detailed Protein Report

Cmpd.	No. of Cmpds.	m/z meas.	Δ m/z [ppm]	z	Rt [min]	Score	P	Range	Sequence	Modification
1993	1	1051.4220	-89.87	2	53.6	11.9	1	947-965	K.HVKAEMEQMALDVGLPSSK.L	Oxidation: 6, 9
2097	1	900.3873	-102.80	3	54.6	10.3	1	2174-2197	K.DLSLHKNQTVVDVAVLTSFLQDEK.V	

Detailed Protein Report

Protein 397: germ cell-less protein-like 1 [Homo sapiens]

**Accession:**	gi|30410765	**Score:**	22.2
**Database:**	refseq_human(refseq_human_20140103.fasta)	**MW [kDa]:**	58.6
**Database Date:**	2015-11-30	**pI:**	8.5
		**Sequence Coverage [%]:**	3.1
		**No. of unique Peptides:**	1

10	20	30	40	50	60	70	80
MGSLSSRVLR	QPRPALAQQA	QGARAGGSAR	RPDTGDDAAG	HGFCYCAGSH	KRKRSSGSFC	YCHPDSETDE	DEEEGDEQQR
**90**	**100**	**110**	**120**	**130**	**140**	**150**	**160**
LLNTPRRKKL	KSTSKYIYQT	LFLNGENSDI	KICALGEEWS	LHKIYLCQSG	YFSSMFSGSW	KESSMNIIEL	EIPDQNIDVE
**170**	**180**	**190**	**200**	**210**	**220**	**230**	**240**
ALQVAFGSLY	RDDVLIKPSR	VVAILAAACL	LQLDGLIQQC	GETMKETVNV	KTVCGYYTSA	GTYGLDSVKK	KCLEWLLNNL
**250**	**260**	**270**	**280**	**290**	**300**	**310**	**320**
MTHQNVELFK	ELSINVMKQL	IGSSNLFVMQ	VEMDIYTALK	KWMFLQLVPS	WNGSLKQLLT	ETDVWFSKQR	KDFEGMAFLE
**330**	**340**	**350**	**360**	**370**	**380**	**390**	**400**
TEQGKPFVSV	FRHLRLQYII	SDLASARIIE	QDAVVPSEWL	SSVYKQQWFA	MLRAEQDSEV	GPQEINKEEL	EGNSMRCGRK
**410**	**420**	**430**	**440**	**450**	**460**	**470**	**480**
LAKDGEYCWR	WTGFNFGFDL	LVTYTNRYII	FKRNTLNQPC	SGSVSLQPRR	SIAFRLRLAS	FDSSGKLICS	RTTGYQILTL
**490**	**500**	**510**	**520**				
EKDQEQVVMN	LDSRLLIFPL	YICCNFLYIS	PEKKN				

Cmpd.	No. of Cmpds.	m/z meas.	Δ m/z [ppm]	z	Rt [min]	Score	P	Range	Sequence	Modification
1992	1	584.9082	-132.40	3	53.5	10.2	2	456-471	R.LRLASFDSSGKLICSR.T	

Detailed Protein Report

Protein 398: PREDICTED: collagen alpha-1(XIV) chain isoform X1 [Homo sapiens]

**Accession:**	gi|530389313	**Score:**	22.2
**Database:**	refseq_human(refseq_human_20140103.fasta)	**MW [kDa]:**	191.8
**Database Date:**	2015-11-30	**pI:**	5.1
		**Sequence Coverage [%]:**	1.5
		**No. of unique Peptides:**	1

10	20	30	40	50	60	70	80
MKIFQRKMRY	WLLPPFLAIV	YFCTIVQGQV	APPTRLRYNV	ISHDSIQISW	KAPRGKFGGY	KLLVTPTSGG	KTNQLNLQNT
90	100	110	120	130	140	150	160
ATKAIIQGLM	PDQNYTVQII	AYNKDKESKP	AQGQFRIKDL	EKRKDPKPRV	KVVDRGNGSR	PSSPEEVKFV	CQTPAIADIV
170	180	190	200	210	220	230	240
ILVDGSWSIG	RFNFRLVRHF	LENLVTAFDV	GSEKTRIGLA	QYSGDPRIEW	HLNAFSTKDE	VIEAVRNLPY	KGGNTLTGLA
250	260	270	280	290	300	310	320
LNYIFENSFK	PEAGSRTGVS	KIGILITDGK	SQDDIIPPSR	NLRESGVELF	AIGVKNADVN	ELQEIASEPD	STHVYNVAEF
330	340	350	360	370	380	390	400
DLMHTVVESL	TRTLCSRVEE	QDREIKASAH	AITGPPTELI	TSEVTARSFM	VNWTHAPGNV	EKYRVVYYPT	RGGKPDEVVV
410	420	430	440	450	460	470	480
DGTVSSTVLK	NLMSLTEYQI	AVFAIYAHTA	SEGLRGTETT	LALPMASDLL	LYDVTENSMR	VKWDAVPGAS	GYLILYAPLT
490	500	510	520	530	540	550	560
EGLAGDEKEM	KIGETHTDIE	LSGLLPNTEY	TVTVYAMFGE	EASDPVTGQE	TTLALSPPRN	LRISNVGSNS	ARLTWDPTSR
570	580	590	600	610	620	630	640
QINGYRIVYN	NADGTEINEV	EVDPITTFPL	KGLTPLTEYT	IAIFSIYDEG	QSEPLTGVFT	TEEVPAQQYL	EIDEVTTDSF
650	660	670	680	690	700	710	720
RVTWHPLSAD	EGLHKLMWIP	VYGGKTEEVV	LKEEQDSHVI	EGLEPGTEYE	VSLLAVLDDG	SESEVVTAVG	TTLDSFWTEP
730	740	750	760	770	780	790	800
ATTIVPTTSV	TSVFQTGIRN	LVVGDETTSS	LRVKWDISDS	DVQQFRVTYM	TAQGDPEEEV	IGTVMVPGSQ	NNLLLKPLLP
810	820	830	840	850	860	870	880
DTEYKVTVTP	IYTDGEGVSV	SAPGKTLPSS	GPQNLRVSEE	WYNRLRITWD	PPSSPVKGYR	IVYKPVSVPG	PTLETFVGAD
890	900	910	920	930	940	950	960
INTILITNLL	SGMDYNVKIF	ASQASGFSDA	LTGMVKTLFL	GVTNLQAKHV	EMTSLCAHWQ	VHRHATAYRV	VIESLQDRQK
970	980	990	1000	1010	1020	1030	1040
QESTVGGGTT	RHCFYGLQPD	SEYKISVYTK	LQEIEGPSVS	IMEKTQSLPT	RPPTFPPTIP	PAKEVCKAAK	ADLVFMVDGS
1050	1060	1070	1080	1090	1100	1110	1120
WSIGDENFNK	IISFLYSTVG	ALNKIGTDGT	QVAMVQFTDD	PRTEFKLNAY	KTKETLLDAI	KHISYKGGNT	KTGKAIKYVR
1130	1140	1150	1160	1170	1180	1190	1200
DTLFTAESGT	RRGIPKVIVV	ITDGRSQDDV	NKISREMQLD	GYSIFAIGVA	DADYSELVSI	GSKPSARHVF	FVDDFDAFKK
1210	1220	1230	1240	1250	1260	1270	1280
IEDELITFVC	ETASATCPVV	HKDGIDLAGF	KMMEMFGLVE	KDFSSVEGVS	MEPGTFNVFP	CYQLHKDALV	SQPTRYLHPE
1290	1300	1310	1320	1330	1340	1350	1360
GLPSDYTISF	LFRILPDTPQ	EPFALWEILN	KNSDPLVGVI	LDNGGKTLTY	FNYDQSGDFQ	TVTFEGPEIR	KIFYGSFHKL
1370	1380	1390	1400	1410	1420	1430	1440
HIVVSETLVK	VVIDCKQVGE	KAMNASANIT	SDGVEVLGKM	VRSRGPGGNS	APFQLQMFDI	VCSTSWANTD	KCCELPGLRD
1450	1460	1470	1480	1490	1500	1510	1520
DESCPDLPHS	CSCSETNEVA	LGPAGPPGGP	GLRGPKGQQG	EPGPKGPDGP	RGEIGLPGPQ	GPPGPQGPSG	LSIQGMPGMP
1530	1540	1550	1560	1570	1580	1590	1600
GEKGEKGDTG	LPGPQGIPGG	VGSPGRDGSP	GQRGLPGKDG	SSGPPGPPGP	IGIPGTPGVP	GITGSMGPQG	ALGPPGVPGA
1610	1620	1630	1640	1650	1660	1670	1680
KGERGERGDL	QSQAMVRSVA	RQVCEQLIQS	HMARYTAILN	QIPSHSSSIR	TVQGPPGEPG	RPGSPGAPGE	QGPPGTPGFP
1690	1700	1710	1720	1730	1740	1750	1760
GNAGVPGTPG	ERGLTGIKGE	KGNPGVGTQG	PRGPPGPAGP	SGESRPGSPG	PPGSPGPRGP	PGHLGVPGPQ	GPSGQPGYCD
1770	1780	1790					
PSSCSAYGVR	DLIPYNDYQH						

Cmpd.	No. of	m/z meas.	Δ m/z	z	Rt	Score	P	Range	Sequence	Modification

Detailed Protein Report

	Cmpds.		[ppm]		[min]					
64	1	809.3817	-76.89	2	30.0	10.1	1	740-754	R.NLVVGDETTSSLRVK.W	

Detailed Protein Report

Protein 399: **methylthioribose-1-phosphate isomerase isoform 2 [Homo sapiens]**

**Accession:**	gi|23943880	**Score:**	22.2
**Database:**	refseq_human(refseq_human_20140103.fasta)	**MW [kDa]:**	34.5
**Database Date:**	2015-11-30	**pI:**	5.4
		**Sequence Coverage [%]:**	1.9
		**No. of unique Peptides:**	1

10	20	30	40	50	60	70	80
MTLEAIRYSR	GSLQILDQLL	LPKQSRYEAV	GSVHQAWEAI	RAMKVRGAPA	IALVGCLSLA	VELQAGAGGP	GLAALVAFVR
**90**	**100**	**110**	**120**	**130**	**140**	**150**	**160**
DKLSFLVTAR	PTAVNMARAA	RDLADVAARE	AEREGATEEA	VRERRETELC	EHWEEHTRQR	ELPLRGPLGG	TVLETRPYNQ
**170**	**180**	**190**	**200**	**210**	**220**	**230**	**240**
GARLTAFELV	YEQIPATLIT	DSMVAAAMAH	RGVSAVVVGA	DRVVANGDTA	NKVGTYQLAI	VAKHHGIPFY	VAAPSSSCDL
**250**	**260**	**270**	**280**	**290**	**300**	**310**	**320**
RLETGKEIII	EERPGQELTD	VNGVRIAAPG	IGVWNPAFDV	TPHDLITGGI	ITELGVFAPE	ELRTALTTTI	SSRDGTLDGP
**330**							
QM							

Cmpd.	No. of Cmpds.	m/z meas.	Δ m/z [ppm]	z	Rt [min]	Score	P	Range	Sequence	Modification
2639	1	702.1743	-341.86	1	61.3	22.2	0	2-7	M.TLEAIR.Y	

Detailed Protein Report

Protein 400: **PREDICTED: maestro heat-like repeat-containing protein family member 2A isoform X1 [Homo sapiens]**

Accession: gi|578804228
Database: refseq_human(refseq_human_20140103.fasta)
Database Date: 2015-11-30
Modification(s): Oxidation

Score: 22.1
MW [kDa]: 189.4
pI: 6.2
Sequence Coverage [%]: 1.7
No. of unique Peptides: 2

10	20	30	40	50	60	70	80
MTEAITEAAV	ASSEEVSEER	DDLGPLELHD	SGTFQQVVNL	LDIIDSESAK	TDTTGAGLDM	RKTLASVIIM	EKATTEPSVV
**90**	**100**	**110**	**120**	**130**	**140**	**150**	**160**
INTLIRCLQV	PEISTQRKVN	IYNILQDIIQ	QEGELEEQCV	QRLVAIASKE	MREIPEMEGY	MKAEVASDTL	VALSRNHFSL
**170**	**180**	**190**	**200**	**210**	**220**	**230**	**240**
VMYELQHHLK	PLNLTDEFVI	ITLAKLANGN	VFEFMPYMGI	TLATIFTMLR	LANEAKIRQA	ICSAMETFCE	TVQFYLKHLE
**250**	**260**	**270**	**280**	**290**	**300**	**310**	**320**
ESVYPVMTEE	EFALKVFPMY	RYFVTVWLRH	YNPEVKLGVI	KSLKPMLGLL	LPNDDLREQV	YDYIPLLLAE	YQGSLEVLFV
**330**	**340**	**350**	**360**	**370**	**380**	**390**	**400**
TQVLRQILEL	SVTTNTPVPQ	MQLHTIFTEL	HVQVCNKAPA	QHQYSSQNLM	EMVHCFVALA	RSYPKELMKF	FFSQMETNKE
**410**	**420**	**430**	**440**	**450**	**460**	**470**	**480**
AVRVGTLNLI	RAIVSADEPR	MSIRAIYLAI	RVVKNTISDT	RSKVRMAILH	IIGQLALCGY	QERIKGWGLK	YLSVQLTLST
**490**	**500**	**510**	**520**	**530**	**540**	**550**	**560**
YKLTNRREKF	YQRDLEERMV	HKVTMDTVKI	ITSSVSGMTT	EFWVRLLCYI	METDYVEALT	PICISLTNLA	EHQLHGQDVD
**570**	**580**	**590**	**600**	**610**	**620**	**630**	**640**
VSVAGKSRQV	DLPAPQKLLA	RLLVLMSSPY	KGEGRGIAML	NLLRTLSQSI	APSMADMWEL	EIALLVRYLE	EHTEFTWDQK
**650**	**660**	**670**	**680**	**690**	**700**	**710**	**720**
AWEDKLIQFL	RNSLKKTRGS	SWSLRLSKEL	NNQIASFDSP	SLEKGFLYRA	LGFTLATGLE	ASKVEVLLLE	LLYKTDYSND
**730**	**740**	**750**	**760**	**770**	**780**	**790**	**800**
FDSEGVIMCF	GLCARGQVKT	VLNVLHDFEE	RIQESEQSWQ	ISAWRKDHPW	RRETVKSALM	VMYSCVASYC	HPQLLLNLVD
**810**	**820**	**830**	**840**	**850**	**860**	**870**	**880**
SPITAKIIHH	YVSSCQDICL	KMAFMKSVVQ	VTKAINNIKD	LEDFHFAQKT	TLTSIIVAVI	KAEPTDNLVS	PVRALAMEAL
**890**	**900**	**910**	**920**	**930**	**940**	**950**	**960**
SHLSKLKPFY	STEENSELMD	ISIHSVISLQ	LPGEDNESIK	TLYANALSSL	EQLMESLLQR	QLDPKGLQEM	VQLLEKWILS
**970**	**980**	**990**	**1000**	**1010**	**1020**	**1030**	**1040**
EKEWEREKAV	SLHLYLMWIY	VHSTAVCIHL	KLGQFGTMVG	LIAPCTCDAH	QRTRMASMNV	LSSLLDLHAS	QTCSLWGPSK
**1050**	**1060**	**1070**	**1080**	**1090**	**1100**	**1110**	**1120**
QKELEKCKGD	LQSTDVEKIF	CASSRIAKVV	CMEFSCDEVV	SLIQKLCENT	GAMNLQHDKA	SVTWIAFFLQ	MRAKELEDKV
**1130**	**1140**	**1150**	**1160**	**1170**	**1180**	**1190**	**1200**
AEILSAILVH	LPVVDHPEVR	RLLIDGILLL	AHHHQETILT	SLLRQPLPME	SHLAEVWLAV	SENVPFARTM	LHSLMGRLQS
**1210**	**1220**	**1230**	**1240**	**1250**	**1260**	**1270**	**1280**
RLSPRISATS	KADIWRLAAV	DPLMTLCTIH	LLIQKLDEND	KLPDFLPDLI	YTLLLQLGSS	HRPEAAPPVL	KMWKLVHTTP
**1290**	**1300**	**1310**	**1320**	**1330**	**1340**	**1350**	**1360**
LPEEMNLQRV	TIKSMQLLFK	RVKSQHLAHT	LDEQAVWDLL	QDGGTFLEGV	SLLARLCMQH	VEGHRQRLAE	LVLRGMDSEV
**1370**	**1380**	**1390**	**1400**	**1410**	**1420**	**1430**	**1440**
LSCRISSTAV	CFMSGPVLYQ	EKLLKPAALL	LEKGADQEED	EALRVLSLRA	LGNMALGAPK	KVKQYRKVLL	EKCLGPLREP
**1450**	**1460**	**1470**	**1480**	**1490**	**1500**	**1510**	**1520**
VSNSVTAEGM	EALTKILAEL	REGDVGSSFD	AMSEQCRIFF	DNESELLRLK	AFILFGKLAR	VVGMSKKHFF	KGEVKKAWIP
**1530**	**1540**	**1550**	**1560**	**1570**	**1580**	**1590**	**1600**
LMLHSQDPCS	NAAQACMATM	FQCVHFWGWK	SLEHPSGPSD	TATDDKMTVF	QTTMCSILTR	KKPAVLYRFL	LETMAYVKNN
**1610**	**1620**	**1630**	**1640**	**1650**	**1660**	**1670**	**1680**
LSRIRIAACN	LAGIIMKQMS	THYLKKLDFP	ALRNSLQELQ	LDPDPGVRRA	ALETLTVLDS	CSQHGFLASP	QGMS

Cmpd.	No. of Cmpds.	m/z meas.	Δ m/z [ppm]	z	Rt [min]	Score	P	Range	Sequence	Modification
2646	1	616.8461	-35.09	2	61.4	11.5	1	62-72	R.KTLASVIIMEK.A	
288	1	636.2301	-155.37	3	32.3	10.6	1	1348-1364	R.LAELVLRGMDSEVLSCR.I	Oxidation: 9

Detailed Protein Report

Protein 401: activator of apoptosis harakiri [Homo sapiens]

Accession: gi|4504493
Database: refseq_human(refseq_human_20140103.fasta)
Database Date: 2015-11-30
Modification(s): Carbamidomethyl

Score: 22.1
MW [kDa]: 9.9
pI: 12.9
Sequence Coverage [%]: 34.1
No. of unique Peptides: 1

```
        10         20         30         40         50         60         70         80
MCPCPLHRGR GPPAVCACSA GRLGLRSSAA QLTAARLKAL GDELHQRTMW RRRARSRRAP APGALPTYWP WLCAAAQVAA
        90        100
LAAWLLGRRN L
```

Cmpd.	No. of Cmpds.	m/z meas.	Δ m/z [ppm]	z	Rt [min]	Score	P	Range	Sequence	Modification
1505	1	599.9721	-16.93	3	47.2	10.9	2	9-26	R.GRGPPAVCACSAGRLGLR.S	Carbamidomethyl: 8

Detailed Protein Report

Protein 402: olfactory receptor 2T27 [Homo sapiens]

Accession: gi|49227791
Database: refseq_human(refseq_human_20140103.fasta)
Database Date: 2015-11-30
Modification(s): Carbamidomethyl, Oxidation

Score: 22.1
MW [kDa]: 35.5
pI: 9.4
Sequence Coverage [%]: 10.7
No. of unique Peptides: 1

10	20	30	40	50	60	70	80
MEQSNYSVYA	DFILLGLFSN	ARFPWLLFAL	ILLVFLTSIA	SNVVKIILIH	IDSRLHTPMY	FLLSQLSLRD	ILYISTIVPK
**90**	**100**	**110**	**120**	**130**	**140**	**150**	**160**
MLVDQVMSQR	AISFAGCTAQ	HFLYLTLAGA	EFFLLGLMSY	DRYVAICNPL	HYPVLMSRKI	CWLIVAAAWL	GGSIDGFLLT
**170**	**180**	**190**	**200**	**210**	**220**	**230**	**240**
PVTMQFPFCA	SREINHFFCE	VPALLKLSCT	DTSAYETAMY	VCCIMMLLIP	FSVISGSYTR	ILITVYRMSE	AEGRGKAVAT
**250**	**260**	**270**	**280**	**290**	**300**	**310**	**320**
CSSHMVVVSL	FYGAAMYTYV	LPHSYHTPEQ	DKAVSAFYTI	LTPMLNPLIY	SLRNKDVTGA	LQKVVGRCVS	SGKVTTF

Cmpd.	No. of Cmpds.	m/z meas.	Δ m/z [ppm]	z	Rt [min]	Score	P	Range	Sequence	Modification
128	1	1319.6098	4.84	3	30.5	22.1	0	187-220	K.LSCTDTSAYETAMYVCCIMMLLIPFSVISGSYTR.I	Carbamidomethyl: 3, 16, 17; Oxidation: 13

Detailed Protein Report

Protein 403: desmin [Homo sapiens]

Accession: gi|55749932
Database: refseq_human(refseq_human_20140103.fasta)
Database Date: 2015-11-30

Score: 22.1
MW [kDa]: 53.5
pI: 5.1
Sequence Coverage [%]: 6.0
No. of unique Peptides: 1

10	20	30	40	50	60	70	80
MSQAYSSSQR	VSSYRRTFGG	APGFPLGSPL	SSPVFPRAGF	GSKGSSSSVT	SRVYQVSRTS	GGAGGLGSLR	ASRLGTTRTP
**90**	**100**	**110**	**120**	**130**	**140**	**150**	**160**
SSYGAGELLD	FSLADAVNQE	FLTTRTNEKV	ELQELNDRFA	NYIEKVRFLE	QQNAALAAEV	NRLKGREPTR	VAELYEEELR
**170**	**180**	**190**	**200**	**210**	**220**	**230**	**240**
ELRRQVEVLT	NQRARVDVER	DNLLDDLQRL	KAKLQEEIQL	KEEAENNLAA	FRADVDAATL	ARIDLERRIE	SLNEEIAFLK
**250**	**260**	**270**	**280**	**290**	**300**	**310**	**320**
KVHEEEIREL	QAQLQEQQVQ	VEMDMSKPDL	TAALRDIRAQ	YETIAAKNIS	EAEEWYKSKV	SDLTQAANKN	NDALRQAKQE
**330**	**340**	**350**	**360**	**370**	**380**	**390**	**400**
MMEYRHQIQS	YTCEIDALKG	TNDSLMRQMR	ELEDRFASEA	SGYQDNIARL	EEEIRHLKDE	MARHLREYQD	LLNVKMALDV
**410**	**420**	**430**	**440**	**450**	**460**	**470**	**480**
EIATYRKLLE	GEESRINLPI	QTYSALNFRE	TSPEQRGSEV	HTKKTVMIKT	IETRDGEVVS	EATQQQHEVL	

Cmpd.	No. of Cmpds.	m/z meas.	Δ m/z [ppm]	z	Rt [min]	Score	P	Range	Sequence	Modification
80	1	707.7702	-112.32	2	29.9	10.9	1	38-52	R.AGFGSKGSSSSVTSR.V	

Detailed Protein Report

Protein 404: protocadherin-20 precursor [Homo sapiens]

**Accession:**	gi|190194360	**Score:**	22.1
**Database:**	refseq_human(refseq_human_20140103.fasta)	**MW [kDa]:**	104.9
**Database Date:**	2015-11-30	**pI:**	4.8
		**Sequence Coverage [%]:**	2.4
		**No. of unique Peptides:**	1

10	20	30	40	50	60	70	80
MRGRGNARSS	QALGVSWCPA	TWHPRLDMGR	LHRPRSSTSY	RNLPHLFLFF	LFVGPFSCLG	SYSRATELLY	SLNEGLPAGV
**90**	**100**	**110**	**120**	**130**	**140**	**150**	**160**
LIGSLAEDLR	LLPRSAGRPD	PQSQLPERTG	AEWNPPLSFS	LASRGLSGQY	VTLDNRSGEL	HTSAQEIDRE	ALCVEGGGGT
**170**	**180**	**190**	**200**	**210**	**220**	**230**	**240**
AWSGSVSISS	SPSDSCLLLL	DVLVLPQEYF	RFVKVKIAIR	DINDNAPQFP	VSQISVWVPE	NAPVNTRLAI	EHPAVDPDVG
**250**	**260**	**270**	**280**	**290**	**300**	**310**	**320**
INGVQTYRLL	DYHGMFTLDV	EENENGERTP	YLIVMGALDR	ETQDQYVSII	IAEDGGSPPL	LGSATLTIGI	SDINDNCPLF
**330**	**340**	**350**	**360**	**370**	**380**	**390**	**400**
TDSQINVTVY	GNATVGTPIA	AVQAVDKDLG	TNAQITYSYS	QKVPQASKDL	FHLDENTGVI	KLFSKIGGSV	LESHKLTILA
**410**	**420**	**430**	**440**	**450**	**460**	**470**	**480**
NGPGCIPAVI	TALVSIIKVI	FRPPEIVPRY	IANEIDGVVY	LKELEPVNTP	IAFFTIRDPE	GKYKVNCYLD	GEGPFRLSPY
**490**	**500**	**510**	**520**	**530**	**540**	**550**	**560**
KPYNNEYLLE	TTKPMDYELQ	QFYEVAVVAW	NSEGFHVKRV	IKVQLLDDND	NAPIFLQPLI	ELTIEENNSP	NAFLTKLYAT
**570**	**580**	**590**	**600**	**610**	**620**	**630**	**640**
DADSEERGQV	SYFLGPDAPS	YFSLDSVTGI	LTVSTQLDRE	EKEKYRYTVR	AVDCGKPPRE	SVATVALTVL	DKNDNSPRFI
**650**	**660**	**670**	**680**	**690**	**700**	**710**	**720**
NKDFSFFVPE	NFPGYGEIGV	ISVTDADAGR	NGWVALSVVN	QSDIFVIDTG	KGMLRAKVSL	DREQQSSYTL	WVEAVDGGEP
**730**	**740**	**750**	**760**	**770**	**780**	**790**	**800**
ALSSTAKITI	LLLDINDNPP	LVLFPQSNMS	YLLVLPSTLP	GSPVTEVYAV	DKDTGMNAVI	AYSIIGRRGP	RPESFRIDPK
**810**	**820**	**830**	**840**	**850**	**860**	**870**	**880**
TGNITLEEAL	LQTDYGLHRL	LVKVSDHGYP	EPLHSTVMVN	LFVNDTVSNE	SYIESLLRKE	PEINIEEKEP	QISIEPTHRK
**890**	**900**	**910**	**920**	**930**	**940**	**950**	**960**
VESVSCMPTL	VALSVISLGS	ITLVTGMGIY	ICLRKGEKHP	REDENLEVQI	PLKGKIDLHM	RERKPMDISN	I

Cmpd.	No. of Cmpds.	m/z meas.	Δ m/z [ppm]	z	Rt [min]	Score	P	Range	Sequence	Modification
1727	1	1293.5590	-108.94	2	50.3	22.1	2	363-385	K.VPQASKDLFHLDENTGVIKLFSK.I	

Detailed Protein Report

Protein 405: thrombospondin type-1 domain-containing protein 7B [Homo sapiens]

**Accession:**	gi|122937257	**Score:**	22.0
**Database:**	refseq_human(refseq_human_20140103.fasta)	**MW [kDa]:**	175.5
**Database Date:**	2015-11-30	**pI:**	9.1
**Modification(s):**	Carbamidomethyl	**Sequence Coverage [%]:**	1.6
		**No. of unique Peptides:**	1

Quantitation

WD:WU **Median:** 1.31 **CV:** 0.00 % **No. of Peptides:** 1

10	20	30	40	50	60	70	80
MHNTADEVVL	YHKLAGPWGR	CTGDCGPGGV	QSRAVWCFHV	DGWTSHLSNC	GESNRPPKER	SCFRVCDWHS	DLFQWEVSDW
90	100	110	120	130	140	150	160
HHCVLVPYAR	GEVKPRTAEC	VTAQHGLQHR	MVRCIQKLNR	TVVANEICEH	FALQPPTEQA	CLIPCPRDCV	VSEFLPWSNC
170	180	190	200	210	220	230	240
SKGCGKKLQH	RTRAVIAPPL	FGGLQCPNLT	ESRACDAPIS	CPLGEEEYTF	SLKVGPWSKC	RLPHLKEINP	SGRTVLDFNS
250	260	270	280	290	300	310	320
DSNERVTFKH	QSYKAHHHSK	SWAIEIGYQT	RQVSCTRSDG	QNAMLSLCLQ	DSFPLTVQSC	IMPKDCETSQ	WSSWSPCSKT
330	340	350	360	370	380	390	400
CRSGSLLPGF	RSRSRNVKHM	AIGGGKECPE	LLEKEACIVE	GELLQQCPRY	SWRTSEWKEC	QVSLLLEQQD	PHWHVTGPVC
410	420	430	440	450	460	470	480
GGGIQTREVY	CAQSVPAAAA	LRAKEVSRPV	EKALCVGPAP	LPSQLCNIPC	STDCIVSSWS	AWGLCIHENC	HDPQGKKGFR
490	500	510	520	530	540	550	560
TRQRHVLMES	TGPAGHCPHL	VESVPCEDPM	CYRWLASEGI	CFPDHGKCGL	GHRILKAVCQ	NDRGEDVSGS	LCPVPPPPER
570	580	590	600	610	620	630	640
KSCEIPCRMD	CVLSEWTEWS	SCSQSCSNKN	SDGKQTRSRT	ILALAGEGGK	PCPPSQALQE	HRLCNDHSCM	QLHWETSPWG
650	660	670	680	690	700	710	720
PCSEDTLVTA	LNATIGWNGE	ATCGVGIQTR	RVFCVKSHVG	QVMTKRCPDS	TRPETVRPCF	LPCKKDCIVT	AFSEWTPCPR
730	740	750	760	770	780	790	800
MCQAGNATVK	QSRYRIIIQE	AANGGQECPD	TLYEERECED	VSLCPVYRWK	PQKWSPCILV	PESVWQGITG	SSEACGKGLQ
810	820	830	840	850	860	870	880
TRAVSCISDD	NRSAEMMECL	KQTNGMPLLV	QECTVPCRED	CTFTAWSKFT	PCSTNCEATK	SRRRQLTGKS	RKKEKCQDSD
890	900	910	920	930	940	950	960
LYPLVETELC	PCDEFISQPY	GNWSDCILPE	GRREPHRGLR	VQADSKECGE	GLRFRAVACS	DKNGRPVDPS	FCSSSGYIQE
970	980	990	1000	1010	1020	1030	1040
KCVIPCPFDC	KLSDWSSWGS	CSSSCGIGVR	IRSKWLKEKP	YNGGRPCPKL	DLKNQAQVHE	AVPCYSECNQ	YSWVVEHWSS
1050	1060	1070	1080	1090	1100	1110	1120
CKINNELRSL	RCGGGTQSRK	IRCVNTADGE	GGAVDSNLCN	QDEIPPETQS	CSLMCPNECV	MSEWGLWSKC	PQSCDPHTMQ
1130	1140	1150	1160	1170	1180	1190	1200
RRTRHLLRPS	LNSRTCAEDS	QVQPCLLNEN	CFQFQYNLTE	WSTCQLSENA	PCGQGVRTRL	LSCVCSDGKP	VSMDQCEQHN
1210	1220	1230	1240	1250	1260	1270	1280
LEKPQRMSIP	CLVECVVNCQ	LSGWTAWTEC	SQTCGHGGRM	SRTRFIIMPT	QGEGRPCPTE	LTQEKTCPVT	PCYSWVLGNW
1290	1300	1310	1320	1330	1340	1350	1360
SACKLEGGDC	GEGVQIRSLS	CMVHSGSISH	AAGRVEDALC	GEMPFQDSIL	KQLCSVPCPG	DCHLTEWSEW	STCELTCIDG
1370	1380	1390	1400	1410	1420	1430	1440
RSFETVGRQS	RSRTFIIQSF	ENQDSCPQQV	LETRPCTGGK	CYHYTWKASL	WNNNERTVWC	QRSDGVNVTG	GCSPQARPAA
1450	1460	1470	1480	1490	1500	1510	1520
IRQCIPACRK	PFSYCTQGGV	CGCEKGYTEI	MKSNGFLDYC	MKVPGSEDKK	ADVKNLSGKN	RPVNSKIHDI	FKGWSLQPLD
1530	1540	1550	1560	1570	1580		
PDGRVKIWVY	GVSGGAFLIM	IFLIFTSYLV	CKKPKPHQST	PPQQKPLTLA	YDGDLDM		

Cmpd.	No. of Cmpds.	m/z meas.	Δ m/z [ppm]	z	Rt [min]	Score	P	Range	Sequence	Modification	Ratios
417	1	475.6019	-263.42	2	33.8	10.3	1	1052-1060	R.CGGGTQSRK.I	Carbamidomethyl: 1	WD:WU 1.31

Detailed Protein Report

Protein 406: PREDICTED: dystonin isoform X15 [Homo sapiens]

**Accession:**	gi|530382446	**Score:**	22.0
**Database:**	refseq_human(refseq_human_20140103.fasta)	**MW [kDa]:**	825.5
**Database Date:**	2015-11-30	**pI:**	5.0
**Modification(s):**	Carbamidomethyl, Oxidation	**Sequence Coverage [%]:**	0.4
		**No. of unique Peptides:**	1

Detailed Protein Report

10	20	30	40	50	60	70	80
MHSSSYSYRS	SDSVFSNTTS	TRTSLDSNEN	LLLVHCGPTL	INSCISFGSE	SFDGHRLEML	QQIANRVQRD	SVICEDKLIL
90	100	110	120	130	140	150	160
AGNALQSDSK	RLESGVQFQN	EAEIAGYILE	CENLLRQHVI	DVQILIDGKY	YQADQLVQRV	AKLRDEIMAL	RNECSSVYSK
170	180	190	200	210	220	230	240
GRILTTEQTK	LMISGITQSL	NSGFAQTLHP	SLTSGLTQSL	TPSLTSSSMT	SGLSSGMTSR	LTPSVTPAYT	PGFPSGLVPN
250	260	270	280	290	300	310	320
FSSGVEPNSL	QTLKLMQIRK	PLLKSSLLDQ	NLTEEEINMK	FVQDLLNWVD	EMQVQLDRTE	WGSDLPSVES	HLENHKNVHR
330	340	350	360	370	380	390	400
AIEEFESSLK	EAKISEIQMT	APLKLTYAEK	LHRLESQYAK	LLNTSRNQER	HLDTLHNFVS	RATNELIWLN	EKEEEEVAYD
410	420	430	440	450	460	470	480
WSERNTNIAR	KKDYHAELMR	ELDQKEENIK	SVQEIAEQLL	LENHPARLTI	EAYRAAMQTQ	WSWILQLCQC	VEQHIKENTA
490	500	510	520	530	540	550	560
YFEFFNDAKE	ATDYLRNLKD	AIQRKYSCDR	SSSIHKLEDL	VQESMEEKEE	LLQYKSTIAN	LMGKAKTIIQ	LKPRNSDCPL
570	580	590	600	610	620	630	640
KTSIPIKAIC	DYRQIEITIY	KDDECVLANN	SHRAKWKVIS	PTGNEAMVPS	VCFTVPPPNK	EAVDLANRIE	QQYQNVLTLW
650	660	670	680	690	700	710	720
HESHINMKSV	VSWHYLINEI	DRIRASNVAS	IKTMLPGEHQ	QVLSNLQSRF	EDFLEDSQES	QVFSGSDITQ	LEKEVNVCKQ
730	740	750	760	770	780	790	800
YYQELLKSAE	REEQEESVYN	LYISEVRNIR	LRLENCEDRL	IRQIRTPLER	DDLHESVFRI	TEQEKLKKEL	ERLKDDLGTI
810	820	830	840	850	860	870	880
TNKCEEFFSQ	AAASSSVPTL	RSELNVVLQN	MNQVYSMSST	YIDKLKTVNL	VLKNTQAAEA	LVKLYETKLC	EEEAVIADKN
890	900	910	920	930	940	950	960
NIENLISTLK	QWRSEVDEKR	QVFHALEDEL	QKAKAISDEM	FKTYKERDLD	FDWHKEKADQ	LVERWQNVHV	QIDNRLRDLE
970	980	990	1000	1010	1020	1030	1040
GIGKSLKYYR	DTYHPLDDWI	QQVETTQRKI	QENQPENSKT	LATQLNQQKM	LVSEIEMKQS	KMDECQKYAE	QYSATVKDYE
1050	1060	1070	1080	1090	1100	1110	1120
LQTMTYRAMV	DSQQKSPVKR	RRMQSSADLI	IQEFMDLRTR	YTALVTLMTQ	YIKFAGDSLK	RLEEEEKSLE	EEKKEHVEKA
1130	1140	1150	1160	1170	1180	1190	1200
KELQKWVSNI	SKTLKDAEKA	GKPPFSKQKI	SSEEISTKKE	QLSEALQTIQ	LFLAKHGDKM	TDEERNELEK	QVKTLQESYN
1210	1220	1230	1240	1250	1260	1270	1280
LLFSESLKQL	QESQTSGDVK	VEEKLDKVIA	GTIDQTTGEV	LSVFQAVLRG	LIDYDTGIRL	LETQLMISGL	ISPELRKCFD
1290	1300	1310	1320	1330	1340	1350	1360
LKDAKSHGLI	DEQILCQLKE	LSKAKEIISA	ASPTTIPVLD	ALAQSMITES	MAIKVLEILL	STGSLVIPAT	GEQLTLQKAF
1370	1380	1390	1400	1410	1420	1430	1440
QQNLVSSALF	SKVLERQNMC	KDLIDPCTSE	KVSLIDMVQR	STLQENTGMW	LLPVRPQEGG	RITLKCGRNI	SILRAAHEGL
1450	1460	1470	1480	1490	1500	1510	1520
IDRETMFRLL	SAQLLSGGLI	NSNSGQRMTV	EEAVREGVID	RDTASSILTY	QVQTGGIIQS	NPAKRLTVDE	AVQCDLITSS
1530	1540	1550	1560	1570	1580	1590	1600
SALLVLEAQR	GYVGLIWPHS	GEIFPTSSSL	QQELITNELA	YKILNGRQKI	AALYIPESSQ	VIGLDAAKQL	GIIDNNTASI
1610	1620	1630	1640	1650	1660	1670	1680
LKNITLPDKM	PDLGDLEACK	NARRWLSFCK	FQPSTVHDYR	QEEDVFDGEE	PVTTQTSEET	KKLFLSYLMI	NSYMDANTGQ
1690	1700	1710	1720	1730	1740	1750	1760
RLLLYDGDLD	EAVGMLLEGC	HAEFDGNTAI	KECLDVLSSS	GVFLNNASGR	EKDECTATPS	SFNKCHCGEP	EHEETPENRK
1770	1780	1790	1800	1810	1820	1830	1840
CAIDEEFNEM	RNTVINSEFS	QSGKLASTIS	IDPKVNSSPS	VCVPSLISYL	TQTELADISM	LRSDSENILT	NYENQSRVET
1850	1860	1870	1880	1890	1900	1910	1920
NERANECSHS	KNIQNFPSDL	IENPIMKSKM	SKFCGVNETE	NEDNTNRDSP	IFDYSPRLSA	LLSHDKLMHS	QGSFNDTHTP
1930	1940	1950	1960	1970	1980	1990	2000
ESNGNKCEAP	ALSFSDKTML	SGQRIGEKFQ	DQFLGIAAIN	ISLPGEQYGQ	KSLNMISSNP	QVQYHNDKYI	SNTSGEDEKT
2010	2020	2030	2040	2050	2060	2070	2080
HPGFQQMPED	KEDESEIEEY	SCAVTPGGDT	DNAIVSLTCA	TPLLDETISA	SDYETSLLND	QQNNTGTDTD	SDDDFYDTPL
2090	2100	2110	2120	2130	2140	2150	2160
FEDDDHDSLL	LDGDDRDCLH	PEDYDTLQEE	NDETASPADV	FYDVSKENEN	SMVPQGAPVG	SLSVKNKAHC	LQDFLMDVEK
2170	2180	2190	2200	2210	2220	2230	2240
DELDSGEKIH	LNPVGSDKVN	GQSLETGSER	ECTNILEGDE	SDSLTDYDIV	GGKESFTASL	KFDDSGSWRG	RKEEYVTGQE
2250	2260	2270	2280	2290	2300	2310	2320
FHSDTDHLDS	MQSEESYGDY	IYDSNDQDDD	DDDGIDEEGG	GIRDENGKPR	CQNVAEDMDI	QLCASILNEN	SDENENINTM
2330	2340	2350	2360	2370	2380	2390	2400

Detailed Protein Report

Cmpd.	No. of Cmpds.	m/z meas.	Δ m/z [ppm]	z	Rt [min]	Score	P	Range	Sequence	Modification
1982	1	927.8655	-42.70	2	53.4	10.5	1	1377-1391	R.QNMCKDLIDPCTSEK.V	Carbamidomethyl: 4, 11; Oxidation: 3

Detailed Protein Report

Protein 407: **sedoheptulokinase [Homo sapiens]**

**Accession:**	gi|74315356	**Score:**	22.0
**Database:**	refseq_human(refseq_human_20140103.fasta)	**MW [kDa]:**	51.5
**Database Date:**	2015-11-30	**pI:**	6.4
		**Sequence Coverage [%]:**	7.3
		**No. of unique Peptides:**	1

10	20	30	40	50	60	70	80
MAARPITLGI	DLGTTSVKAA	LLRAAPDDPS	GFAVLASCAR	AARAEAAVES	AVAGPQGREQ	DVSRILQALH	ECLAALPRPQ
**90**	**100**	**110**	**120**	**130**	**140**	**150**	**160**
LRSVVGIGVS	GQMHGVVFWK	TGQGCEWTEG	GITPVFEPRA	VSHLVTWQDG	RCSSEFLASL	PQPKSHLSVA	TGFGCATIFW
**170**	**180**	**190**	**200**	**210**	**220**	**230**	**240**
LLKYRPEFLK	SYDAAGTIHD	YVVAMLCGLP	RPLMSDQNAA	SWGYFNTQSQ	SWNVETLRSS	GFPVHLLPDI	AEPGSVAGRT
**250**	**260**	**270**	**280**	**290**	**300**	**310**	**320**
SHMWFEIPKG	TQVGVALGDL	QASVYSCMAQ	RTDAVLNIST	SVQLAASMPS	GFQPAQTPDP	TAPVAYFPYF	NRTYLGVAAS
**330**	**340**	**350**	**360**	**370**	**380**	**390**	**400**
LNGGNVLATF	VHMLVQWMAD	LGLEVEESTV	YSRMIQAAVQ	QRDTHLTITP	TVLGERHLPD	QLASVTRISS	SDLSLGHVTR
**410**	**420**	**430**	**440**	**450**	**460**	**470**	**480**
ALCRGIVQNL	HSMLPIQQLQ	EWGVERVMGS	GSALSRNDVL	KQEVQRAFPL	PMSFGQDVDA	AVGAALVMLR	RHLNQKES

Cmpd.	No. of Cmpds.	m/z meas.	Δ m/z [ppm]	z	Rt [min]	Score	P	Range	Sequence	Modification
425	1	767.5397	173.85	2	33.9	10.6	1	427-441	R.VMGSGSALSRNDVLK.Q	

Detailed Protein Report

Protein 408: PREDICTED: stabilin-2 isoform X1 [Homo sapiens]

**Accession:**	gi|578823928	**Score:**	22.0
**Database:**	refseq_human(refseq_human_20140103.fasta)	**MW [kDa]:**	243.6
**Database Date:**	2015-11-30	**pI:**	6.1
**Modification(s):**	Carbamidomethyl	**Sequence Coverage [%]:**	1.1
		**No. of unique Peptides:**	2

Detailed Protein Report

10	20	30	40	50	60	70	80
MMLQHLVIFC	LGLVVQNFCS	PAETTGQARR	CDRKSLLTIR	TECRSCALNL	GVKCPDGYTM	ITSGSVGVRD	CRYTFEVRTY
90	100	110	120	130	140	150	160
SLSLPGCRHI	CRKDYLQPRC	CPGRWGPDCI	ECPGGAGSPC	NGRGSCAEGM	EGNGTCSCQE	GFGGTACETC	ADDNLFGPSC
170	180	190	200	210	220	230	240
SSVCNCVHGV	CNSGLDGDGT	CECYSAYTGP	KCDKPIPECA	ALLCPENSRC	SPSTEDENKL	ECKCLPNYRG	DGKYCDPINP
250	260	270	280	290	300	310	320
CLRKICHPHA	HCTYLGPNRH	SCTCQEGYRG	DGQVCLPVDP	CQINFGNCPT	KSTVCKYDGP	GQSHCECKEH	YQNFVPGVGC
330	340	350	360	370	380	390	400
SMTDICKSDN	PCHRNANCTT	VAPGRTECIC	QKGYVGDGLT	CYGNIMERLR	ELNTEPRGKW	QGRLTSFISL	LDKAYAWPLS
410	420	430	440	450	460	470	480
KLGPFTVLLP	TDKGLKGFNV	NELLVDNKAA	QYFVKLHIIA	GQMNIEYMNN	TDMFYTLTGK	SGEIFNSDKD	NQIKLKLHGG
490	500	510	520	530	540	550	560
KKKVKIIQGD	IIASNGLLHI	LDRAMDKLEP	TFESNNEQTI	MTMLQPRYSK	FRSLLEETNL	GHALDEDGVG	GPYTIFVPNN
570	580	590	600	610	620	630	640
EALNNMKDGT	LDYLLSPEGS	RKLLELVRYH	IVPFTQLEVA	TLISTPHIRS	MANQLIQFNT	TDNGQILAND	VAMEEIEITA
650	660	670	680	690	700	710	720
KNGRIYTLTG	VLIPPSIVPI	LPHRCDETKR	EMKLGTCVSC	SLVYWSRCPA	NSEPTALFTH	RCVYSGRFGS	LKSGCARYCN
730	740	750	760	770	780	790	800
ATVKIPKCCK	GFYGPDCNQC	PGGFSNPCSG	NGQCADSLGG	NGTCICEEGF	QGSQCQFCSD	PNKYGPRCNK	KCLCVHGTCN
810	820	830	840	850	860	870	880
NRIDSDGACL	TGTCRDGSAG	RLCDKQTSAC	GPYVQFCHIH	ATCEYSNGTA	SCICKAGYEG	DGTLCSEMDP	CTGLTPGGCS
890	900	910	920	930	940	950	960
RNAECIKTGT	GTHTCVCQQG	WTGNGRDCSE	INNCLLPSAG	GCHDNASCLY	VGPGQNECEC	KKGFRGNGID	CEPITSCLEQ
970	980	990	1000	1010	1020	1030	1040
TGKCHPLASC	QSTSSGVWSC	VCQEGYEGDG	FLCYGNAAVE	LSFLSEAAIF	NRWINNASLQ	PTLSATSNLT	VLVPSQQATE
1050	1060	1070	1080	1090	1100	1110	1120
DMDQDEKSFW	LSQSNIPALI	KYHMLLGTYR	VADLQTLSSS	DMLATSLQGN	FLHLAKVDGN	ITIEGASIVD	GDNAATNGVI
1130	1140	1150	1160	1170	1180	1190	1200
HIINKVLVPQ	RRLTGSLPNL	LMRLEQMPDY	SIFRGYIIQY	NLANAIEAAD	AYTVFAPNNN	AIENYIREKK	VLSLEEDVLR
1210	1220	1230	1240	1250	1260	1270	1280
YHVVLEEKLL	KNDLHNGMHR	ETMLGFSYFL	SFFLHNDQLY	VNEAPINYTN	VATDKGVIHG	LGKVLEIQKN	RCDNNDTTII
1290	1300	1310	1320	1330	1340	1350	1360
RGRCRTCSSE	LTCPFGTKSL	GNEKRRCIYT	SYFMGRRTLF	IGCQPKCVRT	VITRECCAGF	FGPQCQPCPG	NAQNVCFGNG
1370	1380	1390	1400	1410	1420	1430	1440
ICLDGVNGTG	VCECGEGFSG	TACETCTEGK	YGIHCDQACS	CVHGRCNQGP	LGDGSCDCDV	GWRGVHCDNA	TTEDNCNGTC
1450	1460	1470	1480	1490	1500	1510	1520
HTSANCLTNS	DGTASCKCAA	GFQGNGTICT	AINACEISNG	GCSAKADCKR	TTPGRRVCTC	KAGYTGDGIV	CLEINPCLEN
1530	1540	1550	1560	1570	1580	1590	1600
HGGCDKNAEC	TQTGPNQAAC	NCLPAYTGDG	KVCTLINVCL	TKNGGCSEFA	ICNHTGQVER	TCTCKPNYIG	DGFTCRGSIY
1610	1620	1630	1640	1650	1660	1670	1680
QELPKNPKTS	QYFFQLQEHF	VKDLVGPGPF	TVFAPLSAAF	DEEARVKDWD	KYGLMPQVLR	YHVVACHQLL	LENLKLISNA
1690	1700	1710	1720	1730	1740	1750	1760
TSLQGEPIVI	SVSQSTVYIN	NKAKIISSDI	ISTNGIVHII	DKLLSPKNLL	ITPKDNSGRI	LQNLTTLATN	NGYIKFSNLI
1770	1780	1790	1800	1810	1820	1830	1840
QDSGLLSVIT	DPIHTPVTLF	WPTDQALHAL	PAEQQDFLFN	QDNKDKLKEY	LKFHVIRDAK	VLAVDLPTST	AWKTLQGSEL
1850	1860	1870	1880	1890	1900	1910	1920
SVKCGAGRDI	GDLFLNGQTC	RIVQRELLFD	LGVAYGIDCL	LIDPTLGGRC	DTFTTFDASG	ECGSCVNTPS	CPRWSKPKGV
1930	1940	1950	1960	1970	1980	1990	2000
KQKCLYNLPF	KRNLEGCRER	CSLVIQIPRC	CKGYFGRDCQ	ACPGGPDAPC	NNRGVCLDQY	SATGECKCNT	GFNGTACEMC
2010	2020	2030	2040	2050	2060	2070	2080
WPGRFGPDCL	PCGCSDHGQC	DDGITGSGQC	LCETGWTGPS	CDTQAVLPAV	CTPPCSAHAT	CKENNTCECN	LDYEGDGITC
2090	2100	2110	2120	2130	2140	2150	2160
TVVDFCKQDN	GGCAKVARCS	QKGTKVSCSC	QKGYKGDGHS	CTEIDPCADG	LNGGCHEHAT	CKMTGPGKHK	CECKSHYVGD
2170	2180	2190	2200	2210	2220	2230	2240

Detailed Protein Report

Cmpd.	No. of Cmpds.	m/z meas.	Δ m/z [ppm]	z	Rt [min]	Score	P	Range	Sequence	Modification
2882	2	648.3630	73.51	2	64.8	11.7	1	220-229	K.LECKCLPNYR.G	Carbamidomethyl: 3
424	1	761.9985	164.95	2	34.3	10.3	0	568-581	K.DGTLDYLLSPEGSR.K	

Detailed Protein Report

Protein 409: CCR4-NOT transcription complex subunit 1 isoform c [Homo sapiens]

**Accession:**	gi|388454220	**Score:**	22.0
**Database:**	refseq_human(refseq_human_20140103.fasta)	**MW [kDa]:**	266.2
**Database Date:**	2015-11-30	**pI:**	6.6
		**Sequence Coverage [%]:**	0.9
		**No. of unique Peptides:**	1

Quantitation

WD:WU	**Median:** 0.63	**CV:** 0.00 %	**No. of Peptides:** 1

Detailed Protein Report

10	20	30	40	50	60	70	80
MNLDSLSLAL	SQISYLVDNL	TKKNYRASQQ	EIQHIVNRHG	PEADRHLLRC	LFSHVDFSGD	GKSSGKDFHQ	TQFLIQECAL
90	100	110	120	130	140	150	160
LITKPNFIST	LSYAIDNPLH	YQKSLKPAPH	LFAQLSKVLK	LSKVQEVIFG	LALLNSSSSD	LRGFAAQFIK	QKLPDLLRSY
170	180	190	200	210	220	230	240
IDADVSGNQE	GGFQDIAIEV	LHLLLSHLLF	GQKGAFGVGQ	EQIDAFLKTL	RRDFPQERCP	VVLAPLLYPE	KRDILMDRIL
250	260	270	280	290	300	310	320
PDSGGVAKTM	MESSLADFMQ	EVGYGFCASI	EECRNIIVQF	GVREVTAAQV	ARVLGMMART	HSGLTDGIPL	QSISAPGSGI
330	340	350	360	370	380	390	400
WSDGKDKSDG	AQAHTWNVEV	LIDVLKELNP	SLNFKEVTYE	LDHPGFQIRD	SKGLHNVVYG	IQRGLGMEVF	PVDLIYRPWK
410	420	430	440	450	460	470	480
HAEGQLSFIQ	HSLINPEIFC	FADYPCHTVA	TDILKAPPED	DNREIATWKS	LDLIESLLRL	AEVGQYEQVK	QLFSFPIKHC
490	500	510	520	530	540	550	560
PDMLVLALLQ	INTSWHTLRH	ELISTLMPIF	LGNHPNSAII	LHYAWHGQGQ	SPSIRQLIMH	AMAEWYMRGE	QYDQAKLSRI
570	580	590	600	610	620	630	640
LDVAQDLKAL	SMLLNGTPFA	FVIDLAALAS	RREYLKLDKW	LTDKIREHGE	PFIQACMTFL	KRRCPSILGG	LAPEKDQPKS
650	660	670	680	690	700	710	720
AQLPPETLAT	MLACLQACAG	SVSQELSETI	LTMVANCSNV	MNKARQPPPG	VMPKGRPPSA	SSLDAISPVQ	IDPLAGMTSL
730	740	750	760	770	780	790	800
SIGGSAAPHT	QSMQGFPPNL	GSAFSTPQSP	AKAFPPLSTP	NQTTAFSGIG	GLSSQLPVGG	LGTGSLTGIG	TGALGLPAVN
810	820	830	840	850	860	870	880
NDPFVQRKLG	TSGLNQPTFQ	QTDLSQVWPE	ANQHFSKEID	DEANSYFQRI	YNHPPHPTMS	VDEVLEMLQR	FKDSTIKRER
890	900	910	920	930	940	950	960
EVFNCMLRNL	FEEYRFFPQY	PDKELHITAC	LFGGIIEKGL	VTYMALGLAL	RYVLEALRKP	FGSKMYYFGI	AALDRFKNRL
970	980	990	1000	1010	1020	1030	1040
KDYPQYCQHL	ASISHFMQFP	HHLQEYIEYG	QQSRDPPVKM	QGSITTPGSI	ALAQAQAQAQ	VPAKAPLAGQ	VSTMVTTSTT
1050	1060	1070	1080	1090	1100	1110	1120
TTVAKTVTVT	RPTGVSFKKD	VPPSINTTNI	DTLLVATDQT	ERIVEPPENI	QEKIAFIFNN	LSQSNMTQKV	EELKETVKEE
1130	1140	1150	1160	1170	1180	1190	1200
FMPWVSQYLV	MKRVSIEPNF	HSLYSNFLDT	LKNPEFNKMV	LNETYRNIKV	LLTSDKAAAN	FSDRSLLKNL	GHWLGMITLA
1210	1220	1230	1240	1250	1260	1270	1280
KNKPILHTDL	DVKSLLLEAY	VKGQQELLYV	VPFVAKVLES	SIRSVVFRPP	NPWTMAIMNV	LAELHQEHDL	KLNLKFEIEV
1290	1300	1310	1320	1330	1340	1350	1360
LCKNLALDIN	ELKPGNLLKD	KDRLKNLDEQ	LSAPKKDVKQ	PEELPPITTT	TTSTTPATNT	TCTATVPPQP	QYSYHDINVY
1370	1380	1390	1400	1410	1420	1430	1440
SLAGLAPHIT	LNPTIPLFQA	HPQLKQCVRQ	AIERAVQELV	HPVVDRSIKI	AMTTCEQIVR	KDFALDSEES	RMRIAAHHMM
1450	1460	1470	1480	1490	1500	1510	1520
RNLTAGMAMI	TCREPLLMSI	STNLKNSFAS	ALRTASPQQR	EMMDQAAAQL	AQDNCELACC	FIQKTAVEKA	GPEMDKRLAT
1530	1540	1550	1560	1570	1580	1590	1600
EFELRKHARQ	EGRRYCDPVV	LTYQAERMPE	QIRLKVGGVD	PKQLAVYEEF	ARNVPGFLPT	NDLSQPTGFL	AQPMKQAWAT
1610	1620	1630	1640	1650	1660	1670	1680
DDVAQIYDKC	ITELEQHLHA	IPPTLAMNPQ	AQALRSLLEV	VVLSRNSRDA	IAALGLLQKA	VEGLLDATSG	ADADLLLRYR
1690	1700	1710	1720	1730	1740	1750	1760
ECHLLVLKAL	QDGRAYGSPW	CNKQITRCLI	ECRDEYKYNV	EAVELLIRNH	LVNMQQYDLH	LAQSMENGLN	YMAVAFAMQL
1770	1780	1790	1800	1810	1820	1830	1840
VKILLVDERS	VAHVTEADLF	HTIETLMRIN	AHSRGNAPEG	LPQLMEVVRS	NYEAMIDRAH	GGPNFMMHSG	ISQASEYDDP
1850	1860	1870	1880	1890	1900	1910	1920
PGLREKAEYL	LREWVNLYHS	AAAGRDSTKA	FSAFVGQMHQ	QGILKTDDLI	TRFFRLCTEM	CVEISYRAQA	EQQHNPAANP
1930	1940	1950	1960	1970	1980	1990	2000
TMIRAKCYHN	LDAFVRLIAL	LVKHSGEATN	TVTKINLLNK	VLGIVVGVLL	QDHDVRQSEF	QQLPYHRIFI	MLLLELNAPE
2010	2020	2030	2040	2050	2060	2070	2080
HVLETINFQT	LTAFCNTFHI	LRPTKAPGFV	YAWLELISHR	IFIARMLAHT	PQQKGWPMYA	QLLIDLFKYL	APFLRNVELT
2090	2100	2110	2120	2130	2140	2150	2160
KPMQILYKGT	LRVLLVLLHD	FPEFLCDYHY	GFCDVIPPNC	IQLRNLILSA	FPRNMRLPDP	FTPNLKVDML	SEINIAPRIL
2170	2180	2190	2200	2210	2220	2230	2240

Detailed Protein Report

Cmpd.	No. of Cmpds.	m/z meas.	Δ m/z [ppm]	z	Rt [min]	Score	P	Range	Sequence	Modification	Ratios	
585	1	603.8101	58.84	2	36.2	10.4	0	2360-2371	K.QAQQVMEGTGAS.-		WD:WU	0.63

Detailed Protein Report

Protein 410: PREDICTED: multiple PDZ domain protein isoform X3 [Homo sapiens]

**Accession:**	gi|530390235	**Score:**	21.9
**Database:**	refseq_human(refseq_human_20140103.fasta)	**MW [kDa]:**	213.7
**Database Date:**	2015-11-30	**pI:**	4.8
**Modification(s):**	Carbamidomethyl	**Sequence Coverage [%]:**	0.8
		**No. of unique Peptides:**	1

Detailed Protein Report

10	20	30	40	50	60	70	80
MLEAIDKNRA	LHAAERLQTK	LRERGDVANE	DKLSLLKSVL	QSPLFSQILS	LQTSVQQLKD	QVNIATSATS	NIEYAHVPHL
90	100	110	120	130	140	150	160
SPAVIPTLQN	ESFLLSPNNG	NLEALTGPGI	PHINGKPACD	EFDQLIKNMA	QGRHVEVFEL	LKPPSGGLGF	SVVGLRSENR
170	180	190	200	210	220	230	240
GELGIFVQEI	QEGSVAHRDG	RLKETDQILA	INGQALDQTI	THQQAISILQ	KAKDTVQLVI	ARGSLPQLVS	PIVSRSPSAA
250	260	270	280	290	300	310	320
STISAHSNPV	HWQHMETIEL	VNDGSGLGFG	IIGGKATGVI	VKTILPGGVA	DQHGRLCSGD	HILKIGDTDL	AGMSSEQVAQ
330	340	350	360	370	380	390	400
VLRQCGNRVK	LMIARGAIEE	RTAPTALGIT	LSSSPTSTPE	LRVDASTQKG	EESETFDVEL	TKNVQGLGIT	IAGYIGDKKL
410	420	430	440	450	460	470	480
EPSGIFVKSI	TKSSAVEHDG	RIQIGDQIIA	VDGTNLQGFT	NQQAVEVLRH	TGQTVLLTLM	RRGMKQEAEL	MSREDVTKDA
490	500	510	520	530	540	550	560
DLSPVNASII	KENYEKDEDF	LSSTRNTNIL	PTEEEGYPLL	SAEIEEIEDA	QKQEAALLTK	WQRIMGINYE	IVVAHVSKFS
570	580	590	600	610	620	630	640
ENSGLGISLE	ATVGHHFIRS	VLPEGPVGHS	GKLFSGDELL	EVNGITLLGE	NHQDVVNILK	ELPIEVTMVC	CRRTVPPTTQ
650	660	670	680	690	700	710	720
SELDSLDLCD	IELTEKPHVD	LGEFIGSSET	EDPVLAMTDA	GQSTEEVQAP	LAMWEAGIQH	IELEKGSKGL	GFSILDYQDP
730	740	750	760	770	780	790	800
IDPASTVIII	RSLVPGGIAE	KDGRLLPGDR	LMFVNDVNLE	NSSLEEAVEA	LKGAPSGTVR	IGVAKPLPLS	PEEGYVSAKE
810	820	830	840	850	860	870	880
DSFLYPPHSC	EEAGLADKPL	FRADLALVGT	NDADLVDEST	FESPYSPEND	SIYSTQASIL	SLHGSSCGDG	LNYGSSLPSS
890	900	910	920	930	940	950	960
PPKDVIENSC	DPVLDLHMSL	EELYTQNLLQ	RQDENTPSVD	ISMGPASGFT	INDYTPANAI	EQQYECENTI	VWTESHLPSE
970	980	990	1000	1010	1020	1030	1040
VISSAELPSV	LPDSAGKGSE	YLLEQSSLAC	NAECVMLQNV	SKESFERTIN	IAKGNSSLGM	TVSANKDGLG	MIVRSIIHGG
1050	1060	1070	1080	1090	1100	1110	1120
AISRDGRIAI	GDCILSINEE	STISVTNAQA	RAMLRRHSLI	GPDIKITYVP	AEHLEEFKIS	LGQQSGRVMA	LDIFSSYTGR
1130	1140	1150	1160	1170	1180	1190	1200
DIPELPEREE	GEGEESELQN	TAYSNWNQPR	RVELWREPSK	SLGISIVGGR	GMGSRLSNGE	VMRGIFIKHV	LEDSPAGKNG
1210	1220	1230	1240	1250	1260	1270	1280
TLKPGDRIVE	APSQSESEPE	KAPLCSVPPP	PPSAFAEMGS	DHTQSSASKI	SQDVDKEDEF	GYSWKNIRER	YGTLTGELHM
1290	1300	1310	1320	1330	1340	1350	1360
IELEKGHSGL	GLSLAGNKDR	SRMSVFIVGI	DPNGAAGKDG	RLQIADELLE	INGQILYGRS	HQNASSIIKC	APSKVKIIFI
1370	1380	1390	1400	1410	1420	1430	1440
RNKDAVNQMA	VCPGNAVEPL	PSNSENLQNK	ETEPTVTTSD	AAVDLSSFKN	VQHLELPKDQ	GGLGIAISEE	DTLSGVIIKS
1450	1460	1470	1480	1490	1500	1510	1520
LTEHGVAATD	GRLKVGDQIL	AVDDEIVVGY	PIEKFISLLK	TAKMTVKLTI	HAENPDSQAV	PSAAGAASGE	KKNSSQSLMV
1530	1540	1550	1560	1570	1580	1590	1600
PQSGSPEPES	IRNTSRSSTP	AIFASDPATC	PIIPGCETTI	EISKGRTGLG	LSIVGGSDTL	LGAIIIHEVY	EEGAACKDGR
1610	1620	1630	1640	1650	1660	1670	1680
LWAGDQILEV	NGIDLRKATH	DEAINVLRQT	PQRVRLTLYR	DEAPYKEEEV	CDTLTIELQK	KPGKGLGLSI	VGKRNDTGVF
1690	1700	1710	1720	1730	1740	1750	1760
VSDIVKGGIA	DADGRLMQGD	QILMVNGEDV	RNATQEAVAA	LLKCSLGTVT	LEVGRIKAGP	FHSERRPSQS	SQVSEGSLSS
1770	1780	1790	1800	1810	1820	1830	1840
FTFPLSGSST	SESLESSSKK	NALASEIQGL	RTVEMKKGPT	DSLGISIAGG	VGSPLGDVPI	FIAMMHPTGV	AAQTQKLRVG
1850	1860	1870	1880	1890	1900	1910	1920
DRIVTICGTS	TEGMTHTQAV	NLLKNASGSI	EMQVVAGGDV	SVVTGHQQEP	ASSSLSFTGL	TSSSIFQDDL	GPPQCKSITL
1930	1940	1950	1960	1970	1980	1990	2000
ERGPDGLGFS	IVGGYGSPHG	DLPIYVKTVF	AKGAASEDGR	LKRGDQIIAV	NGQSLEGVTH	EEAVAILKRT	KGTVTLMVLS
2010							

Cmpd.	No. of Cmpds.	m/z meas.	Δ m/z [ppm]	z	Rt [min]	Score	P	Range	Sequence	Modification

Detailed Protein Report

Cmpd.	No. of Cmpds.	m/z meas.	Δ m/z [ppm]	z	Rt [min]	Score	P	Range	Sequence	Modification
1954	1	618.9203	-128.74	3	53.1	10.4	2	1340-1356	R.SHQNASSIIKCAPSKVK.I	Carbamidomethyl: 11

Detailed Protein Report

Protein 411: **PREDICTED: transcriptional regulator ATRX isoform X8 [Homo sapiens]**

**Accession:**	gi|578838421	**Score:**	21.9
**Database:**	refseq_human(refseq_human_20140103.fasta)	**MW [kDa]:**	211.9
**Database Date:**	2015-11-30	**pI:**	6.7
		**Sequence Coverage [%]:**	0.8
		**No. of unique Peptides:**	1

10	20	30	40	50	60	70	80
MTAEPMSESK	LNTLVQKLHD	FLAHSSEESE	ETSSPPRLAM	NQNTDKISGS	GSNSDMMENS	KEEGTSSSEK	SKSSGSSRSK
90	100	110	120	130	140	150	160
RKPSIVTKYV	ESDDEKPLDD	ETVNEDASNE	NSENDITMQS	LPKGTVIVQP	EPVLNEDKDD	FKGPEFRSRS	KMKTENLKKR
170	180	190	200	210	220	230	240
GEDGLHGIVS	CTACGQQVNH	FQKDSIYRHP	SLQVLICKNC	FKYYMSDDIS	RDSDGMDEQC	RWCAEGGNLI	CCDFCHNAFC
250	260	270	280	290	300	310	320
KKCILRNLGR	KELSTIMDEN	NQWYCYICHP	EPLLDLVTAC	NSVFENLEQL	LQQNKKKIKV	DSEKSNKVYE	HTSRFSPKKT
330	340	350	360	370	380	390	400
SSNCNGEEKK	LDDSCSGSVT	YSYSALIVPK	EMIKKAKKLI	ETTANMNSSY	VKFLKQATDN	SEISSATKLR	QLKAFKSVLA
410	420	430	440	450	460	470	480
DIKKAHLALE	EDLNSEFRAM	DAVNKEKNTK	EHKVIDAKFE	TKARKGEKPC	ALEKKDISKS	EAKLSRKQVD	SEHMHQNVPT
490	500	510	520	530	540	550	560
EEQRTNKSTG	GEHKKSDRKE	EPQYEPANTS	EDLDMDIVSV	PSSVPEDIFE	NLETAMEVQS	SVDHQGDGSS	GTEQEVESSS
570	580	590	600	610	620	630	640
VKLNISSKDN	RGGIKSKTTA	KVTKELYVKL	TPVSLSNSPI	KGADCQEVPQ	DKDGYKSCGL	NPKLEKCGLG	QENSDNEHLV
650	660	670	680	690	700	710	720
ENEVSLLLEE	SDLRRSPRVK	TTPLRRPTET	NPVTSNSDEE	CNETVKEKQK	LSVPVRKKDK	RNSSDSAIDN	PKPNKLPKSK
730	740	750	760	770	780	790	800
QSETVDQNSD	SDEMLAILKE	VSRMSHSSSS	DTDINEIHTN	HKTLYDLKTQ	AGKDDKGKRK	RKSSTSGSDF	DTKKGKSAKS
810	820	830	840	850	860	870	880
SIISKKKRQT	QSESSNYDSE	LEKEIKSMSK	IGAARTTKKR	IPNTKDFDSS	EDEKHSKKGM	DNQGHKNLKT	SQEGSSDDAE
890	900	910	920	930	940	950	960
RKQERETFSS	AEGTVDKDTT	IMELRDRLPK	KQQASASTDG	VDKLSGKEES	FTSLEVRKVA	ETKEKSKHLK	TKTCKKVQDG
970	980	990	1000	1010	1020	1030	1040
LSDIAEKFLK	KDQSDETSED	DKKQSKKGTE	EKKKPSDFKK	KVIKMEQQYE	SSSDGTEKLP	EREEICHFPK	GIKQIKNGTT
1050	1060	1070	1080	1090	1100	1110	1120
DGEKKSKKIR	DKTSKKKDEL	SDYAEKSTGK	GDSCDSSEDK	KSKNGAYGRE	KKRCKLLGKS	SRKRQDCSSS	DTEKYSMKED
1130	1140	1150	1160	1170	1180	1190	1200
GCNSSDKRLK	RIELRERRNL	SSKRNTKEIQ	SGSSSSDAEE	SSEDNKKKKQ	RTSSKKKAVI	VKEKKRNSLR	TSTKRKQADI
1210	1220	1230	1240	1250	1260	1270	1280
TSSSSSDIED	DDQNSIGEGS	SDEQKIKPVT	ENLVLSSHTG	FCQSSGDEAL	SKSVPVTVDD	DDDDNDPENR	IAKKMLLEEI
1290	1300	1310	1320	1330	1340	1350	1360
KANLSSDEDG	SSDDEPEEGK	KRTGKQNEEN	PGDEEAKNQV	NSESDSDSEE	SKKPRYRHRL	LRHKLTVSDG	ESGEEKKTKP
1370	1380	1390	1400	1410	1420	1430	1440
KEHKEVKGRN	RRKVSSEDSE	DSDFQESGVS	EEVSESEDEQ	RPRTRSAKKA	ELEENQRSYK	QKKKRRRIKV	QEDSSSENKS
1450	1460	1470	1480	1490	1500	1510	1520
NSEEEEEEKE	EEEEEEEEEE	EEEEDENDDS	KSPGKGRKKI	RKILKDDKLR	TETQNALKEE	EERRKRIAER	EREREKLREV
1530	1540	1550	1560	1570	1580	1590	1600
IEIEDASPTK	CPITTKLVLD	EDEETKEPLV	QVHRNMVIKL	KPHQVDGVQF	MWDCCCESVK	KTKKSPGSGC	ILAHCMGLGK
1610	1620	1630	1640	1650	1660	1670	1680
TLQVVSFLHT	VLLCDKLDFS	TALVVCPLNT	ALNWMNEFEK	WQEGLKDDEK	LEVSELATVK	RPQERSYMLQ	RWQEDGGVMI
1690	1700	1710	1720	1730	1740	1750	1760
IGYEMYRNLA	QGRNVKSRKL	KEIFNKALVD	PGPDFVVCDE	GHILKNEASA	VSKAMNSIRS	RRRIILTGTP	LQNNLIEYHC
1770	1780	1790	1800	1810	1820	1830	1840
MVNFIKENLL	GSIKEFRNRF	INPIQNGQCA	DSTMVDVRVM	KKRAHILYEM	LAGCVQRKDY	TALTKFLPPK	HEYVLAVRMT
1850	1860	1870	1880				
SIQCKLYQYY	LDHLTVLHLN	KMGMLKSIKY	F				

Detailed Protein Report

Cmpd.	No. of Cmpds.	m/z meas.	Δ m/z [ppm]	z	Rt [min]	Score	P	Range	Sequence	Modification
2633	1	589.1691	-335.46	1	61.2	10.3	1	572-577	R.GGIKSK.T	

Detailed Protein Report

Protein 412: PREDICTED: brain-specific angiogenesis inhibitor 3 isoform X3 [Homo sapiens]

**Accession:**	gi|578812890	**Score:**	21.9
**Database:**	refseq_human(refseq_human_20140103.fasta)	**MW [kDa]:**	168.1
**Database Date:**	2015-11-30	**pI:**	6.7
**Modification(s):**	Carbamidomethyl	**Sequence Coverage [%]:**	2.1
		**No. of unique Peptides:**	2

10	20	30	40	50	60	70	80
MKAVRNLLIY	IFSTYLLVMF	GFNAAQDFWC	STLVKGVIYG	SYSVSEMFPK	NFTNCTWTLE	NPDPTKYSIY	LKFSKKDLSC
90	100	110	120	130	140	150	160
SNFSLLAYQF	DHFSHEKIKD	LLRKNHSIMQ	LCNSKNAFVF	LQYDKNFIQI	RRVFPTNFPG	LQKKGEEDQK	SFFEFLVLNK
170	180	190	200	210	220	230	240
VSPSQFGCHV	LCTWLESCLK	SENGRTESCG	IMYTKCTCPQ	HLGEWGIDDQ	SLILLNNVVL	PLNEQTEGCL	TQELQTTQVC
250	260	270	280	290	300	310	320
NLTREAKRPP	KEEFGMMGDH	TIKSQRPRSV	HEKRVPQEQA	DAAKFMAQTG	ESGVEEWSQW	STCSVTCGQG	SQVRTRTCVS
330	340	350	360	370	380	390	400
PYGTHCSGPL	RESRVCNNTA	LCPVHGVWEE	WSPWSLCSFT	CGRGQRTRTR	SCTPPQYGGR	PCEGPETHHK	PCNIALCPVD
410	420	430	440	450	460	470	480
GQWQEWSSWS	QCSVTCSNGT	QQRSRQCTAA	AHGGSECRGP	WAESRECYNP	ECTANGQWNQ	WGHWSGCSKS	CDGGWERRIR
490	500	510	520	530	540	550	560
TCQGAVITGQ	QCEGTGEEVR	RCNEQRCPAP	YEICPEDYLM	SMVWKRTPAG	DLAFNQCPLN	ATGTTSRRCS	LSLHGVAFWE
570	580	590	600	610	620	630	640
QPSFARCISN	EYRHLQHSIK	EHLAKGQRML	AGDGMSQVTK	TLLDLTQRKN	FYAGDLLMSV	EILRNVTDTF	KRASYIPASD
650	660	670	680	690	700	710	720
GVQNFFQIVS	NLLDEENKEK	WEDAQQIYPG	SIELMQVIED	FIHIVGMGMM	DFQNSYLMTG	NVVASIQKLP	AASVLTDINF
730	740	750	760	770	780	790	800
PMKGRKGMVD	WARNSEDRVV	IPKSIFTPVS	SKELDESSVF	VLGAVLYKNL	DLILPTLRNY	TVINSKIIVV	TIRPEPKTTD
810	820	830	840	850	860	870	880
SFLEIELAHL	ANGTLNPYCV	LWDDSKTNES	LGTWSTQGCK	TVLTDASHTK	CLCDRLSTFA	ILAQQPREII	MESSGTPSVT
890	900	910	920	930	940	950	960
LIVGSGLSCL	ALITLAVVYA	ALWRYIRSER	SIILINFCLS	IISSNILILV	GQTQTHNKSI	CTTTTAFLHF	FFLASFCWVL
970	980	990	1000	1010	1020	1030	1040
TEAWQSYMAV	TGKIRTRLIR	KRFLCLGWGL	PALVVATSVG	FTRTKGYGTD	HYCWLSLEGG	LLYAFVGPAA	AVVLVNMVIG
1050	1060	1070	1080	1090	1100	1110	1120
ILVFNKLVSR	DGILDKKLKH	RAGASLWSSC	VVLPLLALTW	MSAVLAMTDK	RSILFQILFA	VFDSLQGFVI	VMVHCILRRE
1130	1140	1150	1160	1170	1180	1190	1200
VQDAFRCRLR	NCQDPINADS	SSSFPNGHAQ	IMTDFEKDVD	IACRSVLHKD	IGPCRAATIT	GTLSRISLND	DEEEKGTNPE
1210	1220	1230	1240	1250	1260	1270	1280
GLSYSTLPGN	VISKVIIQQP	TGLHMPMSMN	ELSNPCLKKE	NSELRRTVYL	CTDDNLRGAD	MDIVHPQERM	MESDYIVMPR
1290	1300	1310	1320	1330	1340	1350	1360
SSVNNQPSMK	EESKMNIGME	TLPHERLLHY	KVNPEFNMNP	PVMDQFNMNL	EQHLAPQEHM	QNLPFEPRTA	VKNFMASELD
1370	1380	1390	1400	1410	1420	1430	1440
DNAGLSRSET	GSTISMSSLE	RRKSRYSDLD	FEKVMHTRKR	HMELFQELNQ	KFQTLDRFRD	IPNTSSMENP	APNKNPWDTF
1450	1460	1470	1480	1490			
KNPSEYPHYT	TINVLDTEAK	DALELRPAEW	EKCLNLPLDV	QEGDFQTEV			

Cmpd.	No. of Cmpds.	m/z meas.	Δ m/z [ppm]	z	Rt [min]	Score	P	Range	Sequence	Modification
205	1	850.2148	81.42	3	31.8	11.2	2	981-1003	R.KRFLCLGWGLPALVVATSVGFTR.T	Carbamidomethyl: 5
1966	1	590.7814	2.32	2	53.0	10.8	1	1120-1128	R.EVQDAFRCR.L	Carbamidomethyl: 8

Detailed Protein Report

Protein 413: nuclear receptor coactivator 3 isoform d [Homo sapiens]

Accession: gi|291490685
Database: refseq_human(refseq_human_20140103.fasta)
Database Date: 2015-11-30
Modification(s): Oxidation

Score: 21.9
MW [kDa]: 154.3
pI: 7.0
Sequence Coverage [%]: 2.9
No. of unique Peptides: 1

10	20	30	40	50	60	70	80
MSGLGENLDP	LASDSRKRKL	PCDTPGQGLT	CSGEKRREQ	ESKYIEELAE	LISANLSDID	NFNVKPDKCA	ILKETVRQIR
90	100	110	120	130	140	150	160
QIKEQGKTIS	NDDDVQKADV	SSTGQGVIDK	DSLGPLLLQA	LDGFLFVVNR	DGNIVFVSEN	VTQYLQYKQE	DLVNTSVYNI
170	180	190	200	210	220	230	240
LHEEDRKDFL	KNLPKSTVNG	VSWTNETQRQ	KSHTFNCRML	MKTPHDILED	INASPEMRQR	YETMQCFALS	QPRAMMEEGE
250	260	270	280	290	300	310	320
DLQSCMICVA	RRITTGERTF	PSNPESFITR	HDLSGKVVNI	DTNSLRSSMR	PGFEDIIRRC	IQRFFSLNDG	QSWSQKRHYQ
330	340	350	360	370	380	390	400
EVTSDGIFSP	TAYLNGHAET	PVYRFSLADG	TIVTAQTKSK	LFRNPVTNDR	HGFVSTHFLQ	REQNGYRPNP	NPVGQGIRPP
410	420	430	440	450	460	470	480
MAGCNSSVGG	MSMSPNQGLQ	MPSSRAYGLA	DPSTTGQMSG	ARYGGSSNIA	SLTPGPGMQS	PSSYQNNNYG	LNMSSPPHGS
490	500	510	520	530	540	550	560
PGLAPNQQNI	MISPRNRGSP	KIASHQFSPV	AGVHSPMASS	GNTGNHSFSS	SSLSALQAIS	EGVGTSLLST	LSSPGPKLDN
570	580	590	600	610	620	630	640
SPNMNITQPS	KVSNQDSKSP	LGFYCDQNPV	ESSMCQSNSR	DHLSDKESKE	SSVEGAENQR	GPLESKGHKK	LLQLLTCSSD
650	660	670	680	690	700	710	720
DRGHSSLTNS	PLDSSCKESS	VSVTSPSGVS	SSTSGGVSST	SNMHGSLLQE	KHRILHKLLQ	NGNSPAEVAK	ITAEATGKDT
730	740	750	760	770	780	790	800
SSITSCGDGN	VVKQEQLSPK	KKENNALLRY	LLDRDDPSDA	LSKELQPQVE	GVDNKMSQCT	SSTIPSSSQE	KDPKIKTETS
810	820	830	840	850	860	870	880
EEGSGDLDNL	DAILGDLTSS	DFYNNSISSN	GSHLGTKQQV	FQGTNSLGLK	SSQSVQSIRP	PYNRAVSLDS	PVSVGSSPPV
890	900	910	920	930	940	950	960
KNISAFPMLP	KQPMLGGNPR	MMDSQENYGS	SMGDWGLPNS	KAGRMEPMNS	NSMGRPGGDY	NTSLPRPALG	GSIPTLPLRS
970	980	990	1000	1010	1020	1030	1040
NSIPGARPVL	QQQQQMLQMR	PGEIPMGMGA	NPYGQAAASN	QLGSWPDGML	SMEQVSHGTQ	NRPLLRNSLD	DLVGPPSNLE
1050	1060	1070	1080	1090	1100	1110	1120
GQSDERALLD	QLHTLLSNTD	ATGLEEIDRA	LGIPELVNQG	QALEPKQDAF	QGQEAAVMMD	QKAGLYGQTY	PAQGPPMQGG
1130	1140	1150	1160	1170	1180	1190	1200
FHLQGQSPSF	NSMMNQMNQQ	GNFPLQGMHP	RANIMRPRTN	TPKQLRMQLQ	QRLQGQQFLN	QSRQALELKM	ENPTAGGAAV
1210	1220	1230	1240	1250	1260	1270	1280
MRPMMQPQQG	FLNAQMVAQR	SRELLSHHFR	QQRVAMMMQQ	QQQQQQQQQQ	QQQQQQQQQQ	QQQQQQQTQA	FSPPPNVTAS
1290	1300	1310	1320	1330	1340	1350	1360
PSMDGLLAGP	TMPQAPPQQF	PYQPNYGMGQ	QPDPAFGRVS	SPPNAMMSSR	MGPSQNPMMQ	HPQAASIYQS	SEMKGWPSGN
1370	1380	1390	1400	1410	1420		
LARNSSFSQQ	QFAHQGNPAV	YSMVHMNGSS	GHMGQMNMNP	MPMSGMPMGP	DQKYC		

Cmpd.	No. of Cmpds.	m/z meas.	Δ m/z [ppm]	z	Rt [min]	Score	P	Range	Sequence	Modification
286	1	881.1736	82.04	3	32.7	11.0	2	199-220	R.MLMKTPHDILEDINASPEMRQR.Y	Oxidation: 19

Detailed Protein Report

Protein 414: vesicular integral-membrane protein VIP36 precursor [Homo sapiens]

Accession: gi|5803023
Database: refseq_human(refseq_human_20140103.fasta)
Database Date: 2015-11-30
Modification(s): Carbamidomethyl

Score: 21.9
MW [kDa]: 40.2
pI: 6.5
Sequence Coverage [%]: 6.5
No. of unique Peptides: 1

10	20	30	40	50	60	70	80
MAAEGWIWRW	GWGRRCLGRP	GLLGPGPGPT	TPLFLLLLLG	SVTADITDGN	SEHLKREHSL	IKPYQGVGSS	SMPLWDFQGS
**90**	**100**	**110**	**120**	**130**	**140**	**150**	**160**
TMLTSQYVRL	TPDERSKEGS	IWNHQPCFLK	DWEMHVHFKV	HGTGKKNLHG	DGIALWYTRD	RLVPGPVFGS	KDNFHGLAIF
**170**	**180**	**190**	**200**	**210**	**220**	**230**	**240**
LDTYPNDETT	ERVFPYISVM	VNNGSLSYDH	SKDGRWTELA	GCTADFRNRD	HDTFLAVRYS	RGRLTVMTDL	EDKNEWKNCI
**250**	**260**	**270**	**280**	**290**	**300**	**310**	**320**
DITGVRLPTG	YYFGASAGTG	DLSDNHDIIS	MKLFQLMVEH	TPDEESIDWT	KIEPSVNFLK	SPKDNVDDPT	GNFRSGPLTG
**330**	**340**	**350**	**360**				
WRVFLLLLCA	LLGIVVCAVV	GAVVFQKRQE	RNKRFY				

Cmpd.	No. of Cmpds.	m/z meas.	Δ m/z [ppm]	z	Rt [min]	Score	P	Range	Sequence	Modification
470	1	524.1609	-200.81	2	34.4	11.0	0	238-246	K.NCIDITGVR.L	Carbamidomethyl: 2

Detailed Protein Report

Protein 415: protocadherin Fat 4 precursor [Homo sapiens]

**Accession:**	gi|165932370	**Score:**	21.9
**Database:**	refseq_human(refseq_human_20140103.fasta)	**MW [kDa]:**	542.4
**Database Date:**	2015-11-30	**pI:**	4.6
		**Sequence Coverage [%]:**	0.6
		**No. of unique Peptides:**	1

Detailed Protein Report

10	20	30	40	50	60	70	80
MDLAPDRATG	RPWLPLHTLS	VSQLLRVFWL	LSLLPGQAWV	HGAEPRQVFQ	VLEEQPPGTL	VGTIQTRPGF	TYRLSESHAL
90	100	110	120	130	140	150	160
FAINSSTGAL	YTTSTIDRES	LPSDVINLVV	LSSAPTYPTE	VRVLVRDLND	NAPVFPDPSI	VVTFKEDSSS	GRQVILDTAT
170	180	190	200	210	220	230	240
DSDIGSNGVD	HRSYRIIRGN	EAGRFRLDIT	LNPSGEGAFL	HLVSKGGLDR	EVTPQYQLLV	EVEDKGEPKR	RGYLQVNVTV
250	260	270	280	290	300	310	320
QDINDNPPVF	GSSHYQAGVP	EDAVVGSSVL	QVAAADADEG	TNADIRYRLQ	DEGTPFQMDP	ETGLITVREP	LDFEARRQYS
330	340	350	360	370	380	390	400
LTVQAMDRGV	PSLTGRAEAL	IQLLDVNDND	PVVKFRYFPA	TSRYASVDEN	AQVGTVVALL	TVTDADSPAA	NGNISVQILG
410	420	430	440	450	460	470	480
GNEQRHFEVQ	SSKVPNLSLI	KVASALDRER	IPSYNLTVSV	SDNYGAPPGA	AVQARSSVAS	LVIFVNDIND	HPPVFSQQVY
490	500	510	520	530	540	550	560
RVNLSEEAPP	GSYVSGISAT	DGDSGLNANL	RYSIVSGNGL	GWFHISEHSG	LVTTGSSGGL	DRELASQIVL	NISARDQGVH
570	580	590	600	610	620	630	640
PKVSYAQLVV	TLLDVNDEKP	VFSQPEGYDV	SVVENAPTGT	ELLMLRATDG	DLGDNGTVRF	SLQEAETDRR	SFRLDPVSGR
650	660	670	680	690	700	710	720
LSTISSLDRE	EQAFYSLLVL	ATDLGSPPQS	SMARINVSLL	DINDNSPVFY	PVQYFAHIKE	NEPGGSYITT	VSATDPDLGT
730	740	750	760	770	780	790	800
NGTVKYSISA	GDRSRFQVNA	QSGVISTRMA	LDREEKTAYQ	LQIVATDGGN	LQSPNQAIVT	ITVLDTQDNP	PVFSQVAYSF
810	820	830	840	850	860	870	880
VVFENVALGY	HVGSVSASTM	DLNSNISYLI	TTGDQKGMFA	INQVTGQLTT	ANVIDREEQS	FYQLKVVASG	GTVTGDTMVN
890	900	910	920	930	940	950	960
ITVKDLNDNS	PHFLQAIESV	NVVENWQAGH	SIFQAKAVDP	DEGVNGMVLY	SLKQNPKNLF	AINEKNGTIS	LLGPLDVHAG
970	980	990	1000	1010	1020	1030	1040
SYQIEILASD	MGVPQLSSSV	ILTVYVHDVN	DNSPVFDQLS	YEVTLSESEP	VNSRFFKVQA	SDKDSGANGE	IAYTIAEGNT
1050	1060	1070	1080	1090	1100	1110	1120
GDAFGIFPDG	QLYIKSELDR	ELQDRYVLMV	VASDRAVEPL	SATVNVTVIL	EDVNDNRPLF	NSTNYTFYFE	EEQRAGSFVG
1130	1140	1150	1160	1170	1180	1190	1200
KVSAVDKDFG	PNGEVRYSFE	MVQPDFELHA	ISGEITNTHQ	FDRESLMRRR	GTAVFSFTVI	ATDQGIPQPL	KDQATVHVYM
1210	1220	1230	1240	1250	1260	1270	1280
KDINDNAPKF	LKDFYQATIS	ESAANLTQVL	RVSASDVDEG	NNGLIHYSII	KGNEERQFAI	DSTSGQVTLI	GKLDYEATPA
1290	1300	1310	1320	1330	1340	1350	1360
YSLVIQAVDS	GTIPLNSTCT	LNIDILDEND	NTPSFPKSTL	FVDVLENMRI	GELVSSVTAT	DSDSGDNADL	YYSITGTNNH
1370	1380	1390	1400	1410	1420	1430	1440
GTFSISPNTG	SIFLAKKLDF	ETQSLYKLNI	TAKDQGRPPR	SSTMSVVIHV	RDFNDNPPSF	PPGDIFKSIV	ENIPIGTSVI
1450	1460	1470	1480	1490	1500	1510	1520
SVTAHDPDAD	INGQLSYTII	QQMPRGNHFT	IDEVKGTIYT	NAEIDREFAN	LFELTVKAND	QAVPIETRRY	ALKNVTILVT
1530	1540	1550	1560	1570	1580	1590	1600
DLNDNVPMFI	SQNALAADPS	AVIGSVLTTI	MAADPDEGAN	GEIEYEIING	DTDTFIVDRY	SGDLRVASAL	VPSQLIYNLI
1610	1620	1630	1640	1650	1660	1670	1680
VSATDLGPER	RKSTTELTII	LQGLDGPVFT	QPKYITILKE	GEPIGTNVIS	IEAASPRGSE	APVEYYIVSV	RCEEKTVGRL
1690	1700	1710	1720	1730	1740	1750	1760
FTIGRHTGII	QTAAILDREQ	GACLYLVDVY	AIEKSTAFPR	TQRAEVEITL	QDINDNPPVF	PTDMLDLTVE	ENIGDGSKIM
1770	1780	1790	1800	1810	1820	1830	1840
QLTAMDADEG	ANALVTYTII	SGADDSFRID	PESGDLIATR	RLDRERRSKY	SLLVRADDGL	QSSDMRINIT	VSDVNDHTPK
1850	1860	1870	1880	1890	1900	1910	1920
FSRPVYSFDI	PEDTIPGSLV	AAILATDDDS	GVNGEITYIV	NEDDEDGIFF	LNPITGVFNL	TRLLDYEVQQ	YYILTVRAED
1930	1940	1950	1960	1970	1980	1990	2000
GGGQFTTIRV	YFNILDVNDN	PPIFSLNSYS	TSLMENLPVG	STVLVFNVTD	ADDGINSQLT	YSIASGDSLG	QFTVDKNGVL
2010	2020	2030	2040	2050	2060	2070	2080
KVLKALDRES	QSFYNLVVQV	HDLPQIPASR	FTSTAQVSII	LLDVNDNPPT	FLSPKLTYIP	ENTPIDTVVF	KAQATDPDSG
2090	2100	2110	2120	2130	2140	2150	2160
PNSYIEYTLL	NPLGNKFSIG	TIDGEVRLTG	ELDREEVSNY	TLTVVATDKG	QPSLSSSTEV	VVMVLDINDN	NPIFAQALYK
2170	2180	2190	2200	2210	2220	2230	2240

Detailed Protein Report

Cmpd.	No. of Cmpds.	m/z meas.	Δ m/z [ppm]	z	Rt [min]	Score	P	Range	Sequence	Modification
654	1	602.1692	-235.73	2	37.0	11.0	1	3115-3125	R.DRDAAMNGLIK.Y	

Detailed Protein Report

Protein 416: PREDICTED: tetratricopeptide repeat protein 18 isoform X8 [Homo sapiens]

**Accession:**	gi|530392981	**Score:**	21.9
**Database:**	refseq_human(refseq_human_20140103.fasta)	**MW [kDa]:**	105.0
**Database Date:**	2015-11-30	**pI:**	5.6
		**Sequence Coverage [%]:**	2.3
		**No. of unique Peptides:**	1

Alias proteins:

Accession	Name	Description
gi|578818878	refseq_huma (refseq_human_20140103.fasta)	PREDICTED: tetratricopeptide repeat protein 18 isoform X16 [Homo sapiens]
gi|578818876	refseq_huma (refseq_human_20140103.fasta)	PREDICTED: tetratricopeptide repeat protein 18 isoform X15 [Homo sapiens]
gi|578818874	refseq_huma (refseq_human_20140103.fasta)	PREDICTED: tetratricopeptide repeat protein 18 isoform X14 [Homo sapiens]

```
        10         20         30         40         50         60         70         80
MVGLQVPSLG EKDYPILFKN GTLKLGGERE PVPRPKKWPI ANILAPGANN IPDAFIVGGP YEEEEGELNH PEDSEFRNQA
        90        100        110        120        130        140        150        160
ECIKKRIIWD LESRCYLDPS AVVSFQKRIA DCRLWPVEIT RVPLVTIPKG KAGKTEKTDE EAQLSFHGVA YVNMVPLLYP
       170        180        190        200        210        220        230        240
GVKRIRGAFH VYPYLDSVVH EKTKCLLSLF RDIGHHLIHN NKIGGINSLL SKQAVSKNLK EDKPVKEKDI DGRPRPGDVQ
       250        260        270        280        290        300        310        320
APSIKSQSSD TPLEGEPPLS HNPEGQQYVE AGTYIVLEIQ LDKALVPKRM PEELARRVKE MIPPRPPLTR RTGGAQKAVS
       330        340        350        360        370        380        390        400
DYHIQIKNIS RAILDEYYRM FGKQVAKLES DMDSETLEEQ KCQLSYELNC SGKYFAFKEQ LKHAVVKIVR DKYLKTTSFE
       410        420        430        440        450        460        470        480
SQEELQTFIS ELYVFLVDQM HVALNQTMPD DVQGTVATIY TSSEQLQLFA FEAEVNENFE MAAAYYKERL VREPQNLDHW
       490        500        510        520        530        540        550        560
LDYGAFCLLT EDNIKAQECF QKALSLNQSH IHSLLLCGVL AVLLENYEQA EIFFEDATCL EPTNVVAWTL LGLYYEIQNN
       570        580        590        600        610        620        630        640
DIRMEMAFHE ASKQLQARML QAQVTKQKST GVEDTEERGK RESSLGPWGI TNGSATAIKV EAPAGPGAAL SILDKFLEES
       650        660        670        680        690        700        710        720
SKLQSDSQEP ILTTQTWDPS ISQKPSNTFI KEIPTKKEAS KCQDSSALLH PGLHYGVSQT TTIFMETIHF LMKVKAVQYV
       730        740        750        760        770        780        790        800
HRVLAHELLC PQGGPSCEYY LVLAQTHILK KNFAKAEEYL QQAAQMDYLN PNVWGLKGHL YFLSGNHSEA KACYERTISF
       810        820        830        840        850        860        870        880
VVDASEMHFI FLRLGLIYLE EKEYEKAKKT YMQACKRSPS CLTWLGLGIA CYRLEELTEA EDALSEANAL NNYNAEVWAY
       890        900        910        920        930
LALVCLKVGR QLEAEQAYKY MIKLKLKDEA LLAEIHTLQE TVGFGNPSF
```

Cmpd.	No. of Cmpds.	m/z meas.	Δ m/z [ppm]	z	Rt [min]	Score	P	Range	Sequence	Modification
2801	1	813.9256	-11.47	2	63.6	11.1	1	814-826	R.LGLIYLEEKEYEK.A	

Detailed Protein Report

Protein 417: PREDICTED: protein eva-1 homolog C isoform X3 [Homo sapiens]

Accession: gi|530419006
Database: refseq_human(refseq_human_20140103.fasta)
Database Date: 2015-11-30

Score: 21.8
MW [kDa]: 37.5
pI: 5.9
Sequence Coverage [%]: 5.1
No. of unique Peptides: 1

10	20	30	40	50	60	70	80
MLLPGRARQP	PTPQPVQHPG	LRRQVEPPGQ	LLRLFYCTVL	VCSKEISALT	DFSDELKNKT	VCEDQELKLH	CHESKFLNIY
90	100	110	120	130	140	150	160
SATYGRRTQE	RDICSSKAER	LPPFDCLSYS	ALQVLSRRCY	GKQRCKIIVN	NHHFGSPCLP	GVKKYLTVTY	ACVPKNILTA
170	180	190	200	210	220	230	240
IDPAIANLKP	SLKQKDGEYG	INFDPSGSKV	LRKDGILVSN	SLAAFAYIRA	HPERAALLFV	SSVCIGLALT	LCALVIRESC
250	260	270	280	290	300	310	320
AKDFRDLQLG	REQLVPGSDK	VEEDSEDEEE	EEDPSESDFP	GELSGFCRTS	YPIYSSIEAA	ELAERIERRE	QIIQEIWMNS
330	340						
GLDTSLPRNM	GQFY						

Cmpd.	No. of Cmpds.	m/z meas.	Δ m/z [ppm]	z	Rt [min]	Score	P	Range	Sequence	Modification
1516	1	927.4902	37.95	2	47.7	21.8	1	176-192	K.DGEYGINFDPSGSKVLR.K	

Detailed Protein Report

Protein 418: PREDICTED: E3 ubiquitin-protein ligase UBR5 isoform X7 [Homo sapiens]

**Accession:**	gi|530389115	**Score:**	21.6
**Database:**	refseq_human(refseq_human_20140103.fasta)	**MW [kDa]:**	200.9
**Database Date:**	2015-11-30	**pI:**	5.2
		**Sequence Coverage [%]:**	1.6
		**No. of unique Peptides:**	1

10	20	30	40	50	60	70	80
MMRRSLRAAG	LGRHEAGASS	SDHQDPVSPP	IAPPSWVPDP	PAMDPDGDID	FILAPAVGSL	TTAATGTGQG	PSTSTIPGPS
90	100	110	120	130	140	150	160
TEPSVVESKD	RKANAHFILK	LLCDSVVLQP	YLRELLSAKD	ARGMTPFMSA	VSGRAYPAAI	TILETAQKIA	KAEISSSEKE
170	180	190	200	210	220	230	240
EDVFMGMVCP	SGTNPDDSPL	YVLCCNDTCS	FTWTGAEHIN	QDIFECRTCG	LLESLCCCTE	CARVCHKGHD	CKLKRTSPTA
250	260	270	280	290	300	310	320
YCDCWEKCKC	KTLIAGQKSA	RLDLLYRLLT	ATNLVTLPNS	RGEHLLLFLV	QTVARQTVEH	CQYRPPRIRE	DRNRKTASPE
330	340	350	360	370	380	390	400
DSDMPDHDLE	PPRFAQLALE	RVLQDWNALK	SMIMFGSQEN	KDPLSASSRI	GHLLPEEQVY	LNQQSGTIRL	DCFTHCLIVK
410	420	430	440	450	460	470	480
CTADILLLDT	LLGTLVKELQ	NKYTPGRREE	AIAVTMRFLR	SVARVFVILS	VEMASSKKKN	NFIPQPIGKC	KRVFQALLPY
490	500	510	520	530	540	550	560
AVEELCNVAE	SLIVPVRMGI	ARPTAPFTLA	STSIDAMQGS	EELFSVEPLP	PRPSSDQSSS	SSQSQSSYII	RNPQQRRISQ
570	580	590	600	610	620	630	640
SQPVRGRDEE	QDDIVSADVE	EVEVVEGVAG	EEDHHDEQEE	HGEENAEAEG	QHDEHDEDGS	DMELDLLAAA	ETESDSESNH
650	660	670	680	690	700	710	720
SNQDNASGRR	SVVTAATAGS	EAGASSVPAF	FSEDDSQSND	SSDSDSSSSQ	SDDIEQETFM	LDEPLERTTN	SSHANGAAQA
730	740	750	760	770	780	790	800
PRSMQWAVRN	TQHQRAASTA	PSSTSTPAAS	SAGLIYIDPS	NLRRSGTIST	SAAAAAAALE	ASNASSYLTS	ASSLARAYSI
810	820	830	840	850	860	870	880
VIRQISDLMG	LIPKYNHLVY	SQIPAAVKLT	YQDAVNLQNY	VEEKLIPTWN	WMVSIMDSTE	AQLRYGSALA	SAGDPGHPNH
890	900	910	920	930	940	950	960
PLHASQNSAR	RERMTAREEA	SLRTLEGRRR	ATLLSARQGM	MSARGDFLNY	ALSLMRSHND	EHSDVLPVLD	VCSLKHVAYV
970	980	990	1000	1010	1020	1030	1040
FQALIYWIKA	MNQQTTLDTP	QLERKRTREL	LELGIDNEDS	EHENDDDTNQ	SATLNDKDDD	SLPAETGQNH	PFFRRSDSMT
1050	1060	1070	1080	1090	1100	1110	1120
FLGCIPPNPF	EVPLAEAIPL	ADQPHLLQPN	ARKEDLFGRP	SQGLYSSSAS	SGKCLMEVTV	DRNCLEVLPT	KMSYAANLKN
1130	1140	1150	1160	1170	1180	1190	1200
VMNMQNRQKK	EGEEQPVLPE	ETESSKPGPS	AHDLAAQLKS	SLLAEIGLTE	SEGPPLTSFR	PQCSFMGMVI	SHDMLLGRWR
1210	1220	1230	1240	1250	1260	1270	1280
LSLELFGRVF	MEDVGAEPGS	ILTELGGFEV	KESKFRREME	KLRNQQSRDL	SLEVKVDRDR	DLLIQQTMRQ	LNNHFGRRCA
1290	1300	1310	1320	1330	1340	1350	1360
TTPMAVHRVK	VTFKDEPGEG	SGVARSFYTA	IAQAFLSNEK	LPNLECIQNA	NKGTHTSLMQ	RLRNRGERDR	ERERERERMRR
1370	1380	1390	1400	1410	1420	1430	1440
SSGLRAGSRR	DRDRDFRRQL	SIDTRPFRPA	SEGNPSDDPE	PLPAHRQALG	ERLYPRVQAM	QPAFASKITG	MLLELSPAQL
1450	1460	1470	1480	1490	1500	1510	1520
LLLLASEDSL	RARVDEAMEL	IIAHGRENGA	DSILDLGLVD	SSEKVQQENR	KRHGSSRSVV	DMDLDDTDDG	DDNAPLFYQP
1530	1540	1550	1560	1570	1580	1590	1600
GKRGFYTPRP	GKNTEARLNC	FRNIGRILGL	CLLQNELCPI	TLNRHVIKVL	LGRKVNWHDF	AFFDPVMYES	LRQLILASQS
1610	1620	1630	1640	1650	1660	1670	1680
SDADAVFSAM	DLAFAIDLCK	EEGGGQVELI	PNGVNIPVTP	QNVYEYVRKY	AEHRMLVVAE	QPLHAMRKGL	LDVLPKNSLE
1690	1700	1710	1720	1730	1740	1750	1760
DLTAEDFRLL	VNGCGEVNVQ	MLISFTSFND	ESGENAEKLL	QFKRWFWSIV	EKMSMTERQD	LVYFWTSSPS	LPASEEGFQP
1770	1780	1790	1800	1810	1820		
MPSITIRPPD	DQHLPTANTC	ISRLYVPLYS	SKQILKQKLL	LAIKTKNFGF	V		

Cmpd.	No. of	m/z meas.	Δ m/z	z	Rt	Score	P	Range	Sequence	Modification

Detailed Protein Report

	Cmpds.		[ppm]		[min]					
31	1	712.9884	0.22	3	29.6	11.4	1	315-333	R.KTASPEDSDMPDHDLEPPR.F	

Detailed Protein Report

Protein 419: PREDICTED: coiled-coil domain-containing protein 13 isoform X2 [Homo sapiens]

**Accession:**	gi|530371957	**Score:**	21.6
**Database:**	refseq_human(refseq_human_20140103.fasta)	**MW [kDa]:**	52.8
**Database Date:**	2015-11-30	**pI:**	9.0
		**Sequence Coverage [%]:**	7.1
		**No. of unique Peptides:**	1

Quantitation

WD:WU **Median:** 0.44 **CV:** 0.00 % **No. of Peptides:** 1

10	20	30	40	50	60	70	80
MAADESSQNT	LRLQFKAMQE	MQHKRLQKQM	EKKREKELSL	KSRADDQEEP	LEVSDGLSLL	HAGEPNSKNS	FEKRVLEDEI
90	100	110	120	130	140	150	160
EHLRNELRET	VDENGRLYKL	LKERDFEIKH	LKKKIEEDRF	AFTGTAGVAG	DVVATKIVEL	SKKNRLLMAE	SEGAKTRVKQ
170	180	190	200	210	220	230	240
LTNRIQELER	ELQTALTRLS	AKGATDAGAK	PPRAQMGDRA	LLETPEVKAL	QDRLVATNLK	MSDLRNQIQS	VKQELRMAQK
250	260	270	280	290	300	310	320
VLAREVGEDI	NVQQLLSSPG	TWRGRAQQIL	VLQSKVQELE	KQLGQARSQS	AGTASDELSV	YPDPRKLSAQ	EKNLLRIRSL
330	340	350	360	370	380	390	400
EREKQEGLEK	LASERDVLQR	ELEELKKKFE	GMRSRNKLLS	SEMKTLKSQM	GTLVEKGRHD	DELIDALMDQ	LKQLQEILGS
410	420	430	440	450	460	470	
LSLQEEKTRV	SQHHLDQQLN	SEAQRSNSLV	AQLQAMVAER	EAKVRQLEME	IGQLNVHASE	PKP	

Cmpd.	No. of Cmpds.	m/z meas.	Δ m/z [ppm]	z	Rt [min]	Score	P	Range	Sequence	Modification	Ratios
1302	1	857.8300	-157.01	2	45.0	11.4	0	393-407	K.QLQEILGSLSLQEEK.T		WD:WU 0.44

Detailed Protein Report

Protein 420: PREDICTED: PR domain zinc finger protein 16 isoform X3 [Homo sapiens]

**Accession:**	gi|530360370	**Score:**	21.5
**Database:**	refseq_human(refseq_human_20140103.fasta)	**MW [kDa]:**	129.7
**Database Date:**	2015-11-30	**pI:**	5.9
**Modification(s):**	Carbamidomethyl	**Sequence Coverage [%]:**	1.6
		**No. of unique Peptides:**	1

Quantitation

WD:WU **Median:** 1.19 **CV:** 0.00 % **No. of Peptides:** 1

10	20	30	40	50	60	70	80
MRSKARARKL	AKSDGDVVNN	MYEPNRDLLA	SHSAEDEAED	SAMSPIPVGP	PSPFPTSEDF	TPKEGSPYEA	PVYIPEDIPI
**90**	**100**	**110**	**120**	**130**	**140**	**150**	**160**
PADFELRESS	IPGAGLGVWA	KRKMEAGERL	GPCVVVPRAA	AKETDFGWEQ	ILTDVEVSPQ	EGCITKQISE	DLGSEKFCVD
**170**	**180**	**190**	**200**	**210**	**220**	**230**	**240**
ANQAGAGSWL	KYIRVACSCD	DQNLTMCQIS	EQIYYKVIKD	IEPGEELLVH	VKEGVYPLGT	VPPGLDEEPT	FRCDECDELF
**250**	**260**	**270**	**280**	**290**	**300**	**310**	**320**
QSKLDLRRHK	KYTCGSVGAA	LYEGLAEELK	PEGLGGGSGQ	AHECKDCERM	FPNKYSLEQH	MVIHTEEREY	KCDQCPKAFN
**330**	**340**	**350**	**360**	**370**	**380**	**390**	**400**
WKSNLIRHQM	SHDSGKRFEC	ENCVKVFTDP	SNLQRHIRSQ	HVGARAHACP	DCGKTFATSS	GLKQHKHIHS	TVKPFICEVC
**410**	**420**	**430**	**440**	**450**	**460**	**470**	**480**
HKSYTQFSNL	CRHKRMHADC	RTQIKCKDCG	QMFSTTSSLN	KHRRFCEGKN	HYTPGGIFAP	GLPLTPSPMM	DKAKPSPSLN
**490**	**500**	**510**	**520**	**530**	**540**	**550**	**560**
HASLGFNEYF	PSRPHPGSLP	FSTAPPTFPA	LTPGFPGIFP	PSLYPRPPLL	PPTSLLKSPL	NHTQDAKLPS	PLGNPALPLV
**570**	**580**	**590**	**600**	**610**	**620**	**630**	**640**
SAVSNSSQGT	TAAAGPEEKF	ESRLEDSCVE	KLKTRSSDMS	DGSDFEDVNT	TTGTDLDTTT	GTGSDLDSDV	DSDPDKDKGK
**650**	**660**	**670**	**680**	**690**	**700**	**710**	**720**
GKSAEGQPKF	GGGLAPPGAP	NSVAEVPVFY	SQHSFFPPPD	EQLLTATGAA	GDSIKAIASI	AEKYFGPGFM	GMQEKKLGSL
**730**	**740**	**750**	**760**	**770**	**780**	**790**	**800**
PYHSAFPFQF	LPNFPHSLYP	FTDRALAHNL	LVKAEPKSPR	DALKVGGPSA	ECPFDLTTKP	KDVKPILPMP	KGPSAPASGE
**810**	**820**	**830**	**840**	**850**	**860**	**870**	**880**
EQPLDLSIGS	RARASQNGGG	REPRKNHVYG	ERKLGAGEGL	PQVCPARMPQ	QPPLHYAKPS	PFFMDPIYSR	VEKRKVTDPV
**890**	**900**	**910**	**920**	**930**	**940**	**950**	**960**
GALKEKYLRP	SPLLFHPQMS	AIETMTEKLE	SFAAMKADSG	SSLQPLPHHP	FNFRSPPPTL	SDPILRKGKE	RYTCRYCGKI
**970**	**980**	**990**	**1000**	**1010**	**1020**	**1030**	**1040**
FPRSANLTRH	LRTHTGEQPY	RCKYCDRSFS	ISSNLQRHVR	NIHNKEKPFK	CHLCNRCFGQ	QTNLDRHLKK	HEHENAPVSQ
**1050**	**1060**	**1070**	**1080**	**1090**	**1100**	**1110**	**1120**
HPGVLTNHLG	TSASSPTSES	DNHALLDEKE	DSYFSEIRNF	IANSEMNQAS	TRTEKRADMQ	IVDGSAQCPG	LASEKQEDVE
**1130**	**1140**	**1150**	**1160**	**1170**	**1180**		
EEDDDDLEED	DEDSLAGKSQ	DDTVSPAPEP	QAAYEDEEDE	EPAASLAVGF	DHTRRHMQ		

Cmpd.	No. of Cmpds.	m/z meas.	Δ m/z [ppm]	z	Rt [min]	Score	P	Range	Sequence	Modification	Ratios
1511	1	1005.9710	-61.06	2	47.6	21.5	2	104-122	K.MEAGERLGPCVVVPRAAAK.E	Carbamidomethyl: 10	WD:WU 1.19

Detailed Protein Report

Protein 421: NACHT, LRR and PYD domains-containing protein 12 isoform 2 [Homo sapiens]

**Accession:**	gi|21955154	**Score:**	21.5
**Database:**	refseq_human(refseq_human_20140103.fasta)	**MW [kDa]:**	120.1
**Database Date:**	2015-11-30	**pI:**	6.6
**Modification(s):**	Carbamidomethyl	**Sequence Coverage [%]:**	2.5
		**No. of unique Peptides:**	1

10	20	30	40	50	60	70	80
MLRTAGRDGL	CRLSTYLEEL	EAVELKKFKL	YLGTATELGE	GKIPWGSMEK	AGPLEMAQLL	ITHFGPEEAW	RLALSTFERI
**90**	**100**	**110**	**120**	**130**	**140**	**150**	**160**
NRKDLWERGQ	REDLVRDTPP	GGPSSLGNQS	TCLLEVSLVT	PRKDPQETYR	DYVRRKFRLM	EDRNARLGEC	VNLSHRYTRL
**170**	**180**	**190**	**200**	**210**	**220**	**230**	**240**
LLVKEHSNPM	QVQQQLLDTG	RGHARTVGHQ	ASPIKIETLF	EPDEERPEPP	RTVVMQGAAG	IGKSMLAHKV	MLDWADGKLF
**250**	**260**	**270**	**280**	**290**	**300**	**310**	**320**
QGRFDYLFYI	NCREMNQSAT	ECSMQDLIFS	CWPEPSAPLQ	ELIRVPERLL	FIIDGFDELK	PSFHDPQGPW	CLCWEEKRPT
**330**	**340**	**350**	**360**	**370**	**380**	**390**	**400**
ELLLNSLIRK	KLLPELSLLI	TTRPTALEKL	HRLLEHPRHV	EILGFSEAER	KEYFYKYFHN	AEQAGQVFNY	VRDNEPLFTM
**410**	**420**	**430**	**440**	**450**	**460**	**470**	**480**
CFVPLVCWVV	CTCLQQQLEG	GGLLRQTSRT	TTAVYMLYLL	SLMQPKPGAP	RLQPPPNQRG	LCSLAADGLW	NQKILFEEQD
**490**	**500**	**510**	**520**	**530**	**540**	**550**	**560**
LRKHGLDGED	VSAFLNMNIF	QKDINCERYY	SFIHLSFQEF	FAAMYYILDE	GEGGAGPDQD	VTRLLTEYAF	SERSFLALTS
**570**	**580**	**590**	**600**	**610**	**620**	**630**	**640**
RFLFGLLNEE	TRSHLEKSLC	WKVSPHIKMD	LLQWIQSKAQ	SDGSTLQQGS	LEFFSCLYEI	QEEEFIQQAL	SHFQVIVVSN
**650**	**660**	**670**	**680**	**690**	**700**	**710**	**720**
IASKMEHMVS	SFCLKRCRSA	QVLHLYGATY	SADGEDRARC	SAGAHTLLVQ	LPERTVLLDA	YSEHLAAALC	TNPNLIELSL
**730**	**740**	**750**	**760**	**770**	**780**	**790**	**800**
YRNALGSRGV	KLLCQGLRHP	NCKLQNLRLK	RCRISSSACE	DLSAALIANK	NLTRMDLSGN	GVGFPGMMLL	CEGLRHPQCR
**810**	**820**	**830**	**840**	**850**	**860**	**870**	**880**
LQMIQLRKCQ	LESGACQEMA	SVLGTNPHLV	ELDLTGNALE	DLGLRLLCQG	LRHPVCRLRT	LWLKICRLTA	AACDELASTL
**890**	**900**	**910**	**920**	**930**	**940**	**950**	**960**
SVNQSLRELD	LSLNELGDLG	VLLLCEGLRH	PTCKLQTLRL	GICRLGSAAC	EGLSVVLQAN	HNLRELDLSF	NDLGDWGLWL
**970**	**980**	**990**	**1000**	**1010**	**1020**	**1030**	**1040**
LAEGLQHPAC	RLQKLWLDSC	GLTAKACENL	YFTLGINQTL	TDLYLTNNAL	GDTGVRLLCK	RLSHPGCKLR	VLWLFGMDLN
**1050**	**1060**	**1070**					
KMTHSRLAAL	RVTKPYLDIG	C					

Cmpd.	No. of Cmpds.	m/z meas.	Δ m/z [ppm]	z	Rt [min]	Score	P	Range	Sequence	Modification
2687	1	973.4884	-3.97	2	62.0	11.3	2	144-159	R.NARLGECVNLSHRYTR.L	Carbamidomethyl: 7

Detailed Protein Report

Protein 422: **PREDICTED: mediator of RNA polymerase II transcription subunit 19 isoform X1 [Homo sapiens]**

Accession: gi|530396419
Database: refseq_human(refseq_human_20140103.fasta)
Database Date: 2015-11-30

Score: 21.4
MW [kDa]: 28.0
pI: 10.4
Sequence Coverage [%]: 7.7
No. of unique Peptides: 1

10	20	30	40	50	60	70	80
MKITNGRHGD	SAGAEGTMEN	FTALFGAQAD	PPPPPTALGF	GPGKPPPPPP	PPAGGGPGTA	PPPTAATAPP	GADKSGAGCG
**90**	**100**	**110**	**120**	**130**	**140**	**150**	**160**
PFYLMRELPG	STELTGSTNL	ITHYNLEQAY	NKFCGKKVKE	KLSNFLPDLP	GMIDLPGSHD	NSSLRSLIEK	PPILSSSFNP
**170**	**180**	**190**	**200**	**210**	**220**	**230**	**240**
ITGTMLAGFR	LHTGPLPEQC	RLMHIQPPKK	KNKHKHKQSR	TQDPVPPETP	SDSDHKKKKK	KKEEDPERKR	KKKEKKKKKN
**250**	**260**	**270**					
RHSPDHPGMG	SSQASSSSSL	R					

Cmpd.	No. of Cmpds.	m/z meas.	Δ m/z [ppm]	z	Rt [min]	Score	P	Range	Sequence	Modification
1493	1	671.3930	137.00	3	47.4	21.4	0	242-261	R.HSPDHPGMGSSQASSSSSLR.-	

Detailed Protein Report

Protein 423: regulator of G-protein signaling 9-binding protein [Homo sapiens]

Accession: gi|115496700
Database: refseq_human(refseq_human_20140103.fasta)
Database Date: 2015-11-30

Score: 21.4
MW [kDa]: 25.1
pI: 7.8
Sequence Coverage [%]: 15.3
No. of unique Peptides: 1

10	20	30	40	50	60	70	80
MAREECKALL	DGLNKTTACY	HHLVLTVGGS	ADSQNLRQEL	QKTRQKAQEL	AVSTCARLTA	VLRDRGLAAD	ERAEFERLWV
**90**	**100**	**110**	**120**	**130**	**140**	**150**	**160**
AFSGCLDLLE	ADMRRALELG	AAFPLHAPRR	PLVRTGVAGA	SSGVAARALS	TRSLRLEAEG	DFDVADLREL	EREVLQVGEM
**170**	**180**	**190**	**200**	**210**	**220**	**230**	**240**
IDNMEMKVNV	PRWTVQARQA	AGAELLSTVS	AGPSSVVSLQ	ERGGGCDPRK	ALAAILFGAV	LLAAVALAVC	VAKLS

Cmpd.	No. of Cmpds.	m/z meas.	Δ m/z [ppm]	z	Rt [min]	Score	P	Range	Sequence	Modification
2371	1	994.7007	148.11	2	58.0	11.3	2	115-135	R.TGVAGASSGVAARALSTRSLR.L	

Detailed Protein Report

Protein 424: heat shock protein 75 kDa, mitochondrial isoform 2 [Homo sapiens]

**Accession:**	gi|440309857	**Score:**	21.4
**Database:**	refseq_human(refseq_human_20140103.fasta)	**MW [kDa]:**	74.2
**Database Date:**	2015-11-30	**pI:**	8.5
**Modification(s):**	Oxidation	**Sequence Coverage [%]:**	4.9
		**No. of unique Peptides:**	2

10	20	30	40	50	60	70	80
MARELRALLL	WGRRLRPLLR	APALAAVPGG	STSKHEFQAE	TKKLLDIVAR	SLYSEKEVFI	RELISNASDA	LEKLRHKLVS
90	100	110	120	130	140	150	160
DGQALPEMEI	HLQTNAEKGT	ITIQDTGIGM	TQEELVSNLG	TIARSGSKAF	LDALQNQAEA	SSKIIGQFGV	GFYSAFMVAD
170	180	190	200	210	220	230	240
RVEVYSRSAA	PGSLGYQWLS	DGSGVFEIAE	ASGVRTGTKI	IIHLKSDCKE	FSSEARVRDV	VTKYSNFVSF	PLYLNGRRMN
250	260	270	280	290	300	310	320
TLQAIWMMDP	KDVREWQHEE	FYRYVAQAHD	KPRYTLHYKT	DAPLNIRSIF	YVPDMKPSMF	DVSRELGSSV	ALYSRKVLIQ
330	340	350	360	370	380	390	400
TKATDILPKW	LRFIRGVVDS	EDIPLNLSRE	LLQESALIRK	LRDVLQQRLI	KFFIDQSKKD	AEKYAKFFED	YGLFMREGIV
410	420	430	440	450	460	470	480
TATEQEVKED	IAKLLRYESS	ALPSGQLTSL	SEYASRMRAG	TRNIYYLCAP	NRHLAEHSPY	YEAMKKKDTE	VLFCFEQFDE
490	500	510	520	530	540	550	560
LTLLHLREFD	KKKLISVETD	IVVDHYKEEK	FEDRSPAAEC	LSEKETEELM	AWMRNVLGSR	VTNVKVTLRL	DTHPAMVTVL
570	580	590	600	610	620	630	640
EMGAARHFLR	MQQLAKTQEE	RAQLLQPTLE	INPRHALIKK	LNQLRASEPG	LAQLLVDQIY	ENAMIAAGLV	DDPRAMVGRL
650	660						
NELLVKALER	H						

Cmpd.	No. of Cmpds.	m/z meas.	Δ m/z [ppm]	z	Rt [min]	Score	P	Range	Sequence	Modification
35	1	796.7750	-156.03	2	29.7	10.7	0	129-143	K.AFLDALQNQAEASSK.I	
2046	1	1073.4473	-50.14	2	53.9	10.7	2	380-396	K.DAEKYAKFFEDYGLFMR.E	Oxidation: 16

Detailed Protein Report

Protein 425: myotubularin-related protein 8 [Homo sapiens]

**Accession:**	gi|21361759	**Score:**	21.4
**Database:**	refseq_human(refseq_human_20140103.fasta)	**MW [kDa]:**	78.9
**Database Date:**	2015-11-30	**pI:**	6.2
**Modification(s):**	Oxidation	**Sequence Coverage [%]:**	2.8
		**No. of unique Peptides:**	1

10	20	30	40	50	60	70	80
MDHITVPKVE	NVKLVDRYVS	KKPANGILYL	TATHLIYVEA	SGAARKETWI	ALHHIATVEK	LPITSLGCPL	TLRCKNFRVA
**90**	**100**	**110**	**120**	**130**	**140**	**150**	**160**
HFVLDSDLVC	HEVYISLLKL	SQPALPEDLY	AFSYNPKSSK	EMRESGWKLI	DPISDFGRMG	IPNRNWTITD	ANRNYEICST
**170**	**180**	**190**	**200**	**210**	**220**	**230**	**240**
YPPEIVVPKS	VTLGTVVGSS	KFRSKERVPV	LSYLYKENNA	AICRCSQPLS	GFYTRCVDDE	LLLEAISQTN	PGSQFMYVVD
**250**	**260**	**270**	**280**	**290**	**300**	**310**	**320**
TRPKLNAMAN	RAAGKGYENE	DNYANIRFRF	MGIENIHVMR	SSLQKLLEVC	ELKTPTMSEF	LSGLESSGWL	RHIKAIMDAG
**330**	**340**	**350**	**360**	**370**	**380**	**390**	**400**
IFITKAVKVE	KASVLVHCSD	GWDRTAQVCS	VASILLDPFY	RTFKGLMILI	EKEWISMGHK	FSQRCGHLDG	DSKEVSPIFT
**410**	**420**	**430**	**440**	**450**	**460**	**470**	**480**
QFLDCIWQLM	EQFPCAFEFN	ENFLLEIHDH	VFSCQFGNFL	GNCQKDREDL	RVYEKTHSVW	PFLVQRKPDF	RNPLYKGFTM
**490**	**500**	**510**	**520**	**530**	**540**	**550**	**560**
YGVLNPSTVP	YNIQFWCGMY	NRFDKGLQPK	QSMLESLLEI	KKQRAMLETD	VHELEKKLKV	RDEPPEEICT	CSQLGNILSQ
**570**	**580**	**590**	**600**	**610**	**620**	**630**	**640**
HLGSPLTNPL	GFMGINGDLN	TLMENGTLSR	EGGLRAQMDQ	VKSQGADLHH	NCCEIVGSLR	AINISGDVGI	SEAMGISGDM
**650**	**660**	**670**	**680**	**690**	**700**	**710**	
CTFEATGFSK	DLGICGAMDI	SEATGISGNL	GISEARGFSG	DMGILGDTGI	SKASTKEADY	SKHQ	

Cmpd.	No. of Cmpds.	m/z meas.	Δ m/z [ppm]	z	Rt [min]	Score	P	Range	Sequence	Modification
1132	1	956.7332	257.45	1	42.4	10.9	0	1-8	-.MDHITVPK.V	Oxidation: 1

Detailed Protein Report

Protein 426: A disintegrin and metalloproteinase with thrombospondin motifs 2 isoform 1 preproprotein [Homo sapiens]

**Accession:**	gi|110825974	**Score:**	21.4
**Database:**	refseq_human(refseq_human_20140103.fasta)	**MW [kDa]:**	134.7
**Database Date:**	2015-11-30	**pI:**	6.8
**Modification(s):**	Carbamidomethyl	**Sequence Coverage [%]:**	2.3
		**No. of unique Peptides:**	2

Quantitation

WD:WU **Median:** 0.53 **CV:** 4.81 % **No. of Peptides:** 2

10	20	30	40	50	60	70	80
MDPPAGAARR	LLCPALLLLL	LLLPPPLLPP	PPPPANARLA	AAADPPGGPL	GHGAERILAV	PVRTDAQGRL	VSHVVSAATS
**90**	**100**	**110**	**120**	**130**	**140**	**150**	**160**
RAGVRARRAA	PVRTPSFPGG	NEEEPGSHLF	YNVTVFGRDL	HLRLRPNARL	VAPGATMEWQ	GEKGTTRVEP	LLGSCLYVGD
**170**	**180**	**190**	**200**	**210**	**220**	**230**	**240**
VAGLAEASSV	ALSNCDGLAG	LIRMEEEEFF	IEPLEKGLAA	QEAEQGRVHV	VYRRPPTSPP	LGGPQALDTG	ASLDSLDSLS
**250**	**260**	**270**	**280**	**290**	**300**	**310**	**320**
RALGVLEEHA	NSSRRRARRH	AADDDYNIEV	LLGVDDSVVQ	FHGKEHVQKY	LLTLMNIVNE	IYHDESLGAH	INVVLVRIIL
**330**	**340**	**350**	**360**	**370**	**380**	**390**	**400**
LSYGKSMSLI	EIGNPSQSLE	NVCRWAYLQQ	KPDTGHDEYH	DHAIFLTRQD	FGPSGMQGYA	PVTGMCHPVR	SCTLNHEDGF
**410**	**420**	**430**	**440**	**450**	**460**	**470**	**480**
SSAFVVAHET	GHVLGMEHDG	QGNRCGDEVR	LGSIMAPLVQ	AAFHRFHWSR	CSQQELSRYL	HSYDCLLDDP	FAHDWPALPQ
**490**	**500**	**510**	**520**	**530**	**540**	**550**	**560**
LPGLHYSMNE	QCRFDFGLGY	MMCTAFRTFD	PCKQLWCSHP	DNPYFCKTKK	GPPLDGTMCA	PGKHCFKGHC	IWLTPDILKR
**570**	**580**	**590**	**600**	**610**	**620**	**630**	**640**
DGSWGAWSPF	GSCSRTCGTG	VKFRTRQCDN	PHPANGGRTC	SGLAYDFQLC	SRQDCPDSLA	DFREEQCRQW	DLYFEHGDAQ
**650**	**660**	**670**	**680**	**690**	**700**	**710**	**720**
HHWLPHEHRD	AKERCHLYCE	SRETGEVVSM	KRMVHDGTRC	SYKDAFSLCV	RGDCRKVGCD	GVIGSSKQED	KCGVCGGDNS
**730**	**740**	**750**	**760**	**770**	**780**	**790**	**800**
HCKVVKGTFT	RSPKKHGYIK	MFEIPAGARH	LLIQEVDATS	HHLAVKNLET	GKFILNEEND	VDASSKTFIA	MGVEWEYRDE
**810**	**820**	**830**	**840**	**850**	**860**	**870**	**880**
DGRETLQTMG	PLHGTITVLV	IPVGDTRVSL	TYKYMIHEDS	LNVDDNNVLE	EDSVVYEWAL	KKWSPCSKPC	GGGSQFTKYG
**890**	**900**	**910**	**920**	**930**	**940**	**950**	**960**
CRRRLDHKMV	HRGFCAALSK	PKAIRRACNP	QECSQPVWVT	GEWEPCSQTC	GRTGMQVRSV	RCIQPLHDNT	TRSVHAKHCN
**970**	**980**	**990**	**1000**	**1010**	**1020**	**1030**	**1040**
DARPESRRAC	SRELCPGRWR	AGPWSQCSVT	CGNGTQERPV	LCRTADDSFG	ICQEERPETA	RTCRLGPCPR	NISDPSKKSY
**1050**	**1060**	**1070**	**1080**	**1090**	**1100**	**1110**	**1120**
VVQWLSRPDP	DSPIRKISSK	GHCQGDKSIF	CRMEVLSRYC	SIPGYNKLCC	KSCNLYNNLT	NVEGRIEPPP	GKHNDIDVFM
**1130**	**1140**	**1150**	**1160**	**1170**	**1180**	**1190**	**1200**
PTLPVPTVAM	EVRPSPSTPL	EVPLNASSTN	ATEDHPETNA	VDEPYKIHGL	EDEVQPPNLI	PRRPSPYEKT	RNQRIQELID
**1210**	**1220**						
EMRKKEMLGK	F						

Cmpd.	No. of Cmpds.	m/z meas.	Δ m/z [ppm]	z	Rt [min]	Score	P	Range	Sequence	Modification	Ratios	
1134	1	984.4501	-53.47	2	42.9	11.3	1	544-559	K.HCFKGHCIWLTPDILK.R	Carbamidomethyl: 2	WD:WU	0.56
2148	1	766.2823	-164.03	2	55.4	10.0	2	1183-1194	R.RPSPYEKTRNQR.I		WD:WU	0.51

Detailed Protein Report

Protein 427: integrin beta-1-binding protein 2 [Homo sapiens]

Accession: gi|6912438
Database: refseq_human(refseq_human_20140103.fasta)
Database Date: 2015-11-30

Score: 21.3
MW [kDa]: 38.4
pI: 4.9
Sequence Coverage [%]: 6.6
No. of unique Peptides: 1

10	20	30	40	50	60	70	80
MSLLCRNKGC	GQHFDPNTNL	PDSCCHHPGV	PIFHDALKGW	SCCRKRTVDF	SEFLNIKGCT	MGPHCAEKLP	EAPQPEGPAT
**90**	**100**	**110**	**120**	**130**	**140**	**150**	**160**
SSSLQEQKPL	NVIPKSAETL	RRERPKSELP	LKLLPLNISQ	ALEMALEQKE	LDQEPGAGLD	SLIRTGSSCQ	NPGCDAVYQG
**170**	**180**	**190**	**200**	**210**	**220**	**230**	**240**
PESDATPCTY	HPGAPRFHEG	MKSWSCCGIQ	TLDFGAFLAQ	PGCRVGRHDW	GKQLPASCRH	DWHQTDSLVV	VTVYGQIPLP
**250**	**260**	**270**	**280**	**290**	**300**	**310**	**320**
AFNWVKASQT	ELHVHIVFDG	NRVFQAQMKL	WGVINVEQSS	VFLMPSRVEI	SLVKADPGSW	AQLEHPDALA	KKARAGVVLE
**330**	**340**	**350**					
MDEEESDDSD	DDLSWTEEEE	EEEAMGE					

Cmpd.	No. of Cmpds.	m/z meas.	Δ m/z [ppm]	z	Rt [min]	Score	P	Range	Sequence	Modification
297	1	861.8427	115.33	3	32.8	21.3	2	46-68	K.RTVDFSEFLNIKGCTMGPHCAEK.L	

Detailed Protein Report

Protein 428: **protein-arginine deiminase type-2 [Homo sapiens]**

**Accession:**	gi|122939159	**Score:**	21.3
**Database:**	refseq_human(refseq_human_20140103.fasta)	**MW [kDa]:**	75.5
**Database Date:**	2015-11-30	**pI:**	5.3
		**Sequence Coverage [%]:**	5.6
		**No. of unique Peptides:**	1

10	20	30	40	50	60	70	80
MLRERTVRLQ	YGSRVEAVYV	LGTYLWTDVY	SAAPAGAQTF	SLKHSEHVWV	EVVRDGEAEE	VATNGKQRWL	LSPSTTLRVT
90	100	110	120	130	140	150	160
MSQASTEASS	DKVTVNYYDE	EGSIPIDQAG	LFLTAIEISL	DVDADRDGVV	EKNNPKKASW	TWGPEGQGAI	LLVNCDRETP
170	180	190	200	210	220	230	240
WLPKEDCRDE	KVYSKEDLKD	MSQMILRTKG	PDRLPAGYEI	VLYISMSDSD	KVGVFYVENP	FFGQRYIHIL	GRRKLYHVVK
250	260	270	280	290	300	310	320
YTGGSAELLF	FVEGLCFPDE	GFSGLVSIHV	SLLEYMAQDI	PLTPIFTDTV	IFRIAPWIMT	PNILPPVSVF	VCCMKDNYLF
330	340	350	360	370	380	390	400
LKEVKNLVEK	TNCELKVCFQ	YLNRGDRWIQ	DEIEFGYIEA	PHKGFPVVLD	SPRDGNLKDF	PVKELLGPDF	GYVTREPLFE
410	420	430	440	450	460	470	480
SVTSLDSFGN	LEVSPPVTVN	GKTYPLGRIL	IGSSFPLSGG	RRMTKVVRDF	LKAQQVQAPV	ELYSDWLTVG	HVDEFMSFVP
490	500	510	520	530	540	550	560
IPGTKKFLLL	MASTSACYKL	FREKQKDGHG	EAIMFKGLGG	MSSKRITINK	ILSNESLVQE	NLYFQRCLDW	NRDILKKELG
570	580	590	600	610	620	630	640
LTEQDIIDLP	ALFKMDEDHR	ARAFFPNMVN	MIVLDKDLGI	PKPFGPQVEE	ECCLEMHVRG	LLEPLGLECT	FIDDISAYHK
650	660	670					
FLGEVHCGTN	VRRKPFTFKW	WHMVP					

Cmpd.	No. of Cmpds.	m/z meas.	Δ m/z [ppm]	z	Rt [min]	Score	P	Range	Sequence	Modification
183	1	823.9125	-36.73	2	31.1	10.2	2	323-336	K.EVKNLVEKTNCELK.V	

Detailed Protein Report

Protein 429: NACHT, LRR and PYD domains-containing protein 2 isoform 3 [Homo sapiens]

Accession: gi|291463280
Database: refseq_human(refseq_human_20140103.fasta)
Database Date: 2015-11-30

Score: 21.3
MW [kDa]: 117.8
pI: 5.8
Sequence Coverage [%]: 1.9
No. of unique Peptides: 1

10	20	30	40	50	60	70	80
MVSSAQMGFN	LQALLEQLSQ	DELSKFKYLI	TTFSLAHELQ	KIPHKEMASL	QVFEKMHRMD	LSERAKDEVR	EAALKSFNKR
90	100	110	120	130	140	150	160
KPLSLGITRK	ERPPLDVDEM	LERFKTEAQA	FTETKGNVIC	LGKEVFKGKK	PDKDNRCRYI	LKTKFREMWK	SWPGDSKEVQ
170	180	190	200	210	220	230	240
VMAERYKMLI	PFSNPRVLPG	PFSYTVVLYG	PAGLGKTTLA	QKLMLDWAED	NLIHKFKYAF	YLSCRELSRL	GPCSFAELVF
250	260	270	280	290	300	310	320
RDWPELQDDI	PHILAQARKI	LFVIDGFDEL	GAAPGALIED	ICGDWEKKKP	VPVLLGSLLN	RVMLPKAALL	VTTRPRALRD
330	340	350	360	370	380	390	400
LRILAEEPIY	IRVEGFLEED	RRAYFLRHFG	DEDQAMRAFE	LMRSNAALFQ	LGSAPAVCWI	VCTTLKLQME	KGEDPVPTCL
410	420	430	440	450	460	470	480
TRTGLFLRFL	CSRFPQGAQL	RGALRTLSLL	AAQGLWAQTS	VLHREDLERL	GVQESDLRLF	LDGDILRQDR	VSKGCYSFIH
490	500	510	520	530	540	550	560
LSFQQFLTAL	FYTLEKEEEE	DRDGHTWDIG	DVQKLLSGVE	RLRNPDLIQA	GYYSFGLANE	KRAKELEATF	GCRMSPDIKQ
570	580	590	600	610	620	630	640
ELLRCDISCK	GGHSTVTDLQ	ELLGCLYESQ	EEELVKEVMA	QFKEISLHLN	AVDVVPSSFC	VKHCRNLQKM	SLQVIKENLP
650	660	670	680	690	700	710	720
ENVTASESDA	EVERSQDDQH	MLPFWTDLCS	IFGSNKDLMG	LAINDSFLSA	SLVRILCEQI	ASDTCHLQRV	VFKNISPADA
730	740	750	760	770	780	790	800
HRNLCLALRG	HKTVTYLTLQ	GNDQDDMFPA	LCEVLRHPEC	NLRYLGLVSC	SATTQQWADL	SLALEVNQSL	TCVNLSDNEL
810	820	830	840	850	860	870	880
LDEGAKLLYT	TLRHPKCFLQ	RLSLENCHLT	EANCKDLAAV	LVVSRELTHL	CLAKNPIGNT	GVKFLCEGLR	YPECKLQTLV
890	900	910	920	930	940	950	960
LWNCDITSDG	CCDLTKLLQE	KSSLLCLDLG	LNHIGVKGMK	FLCEALRKPL	CNLRCLWLWG	CSIPPFSCED	LCSALSCNQS
970	980	990	1000	1010	1020	1030	1040
LVTLDLGQNP	LGSSGVKMLF	ETLTCSSGTL	RTLRLKIDDF	NDELNKLLEE	IEEKNPQLII	DTEKHHPWAE	RPSSHDFMI

Cmpd.	No. of Cmpds.	m/z meas.	Δ m/z [ppm]	z	Rt [min]	Score	P	Range	Sequence	Modification
1270	1	419.0769	-336.69	2	44.6	11.0	0	864-870	K.FLCEGLR.Y	

Detailed Protein Report

Protein 430: sulfiredoxin-1 [Homo sapiens]

Accession: gi|22129778
Database: refseq_human(refseq_human_20140103.fasta)
Database Date: 2015-11-30

Score: 21.3
MW [kDa]: 14.3
pI: 9.1
Sequence Coverage [%]: 12.4
No. of unique Peptides: 1

10	20	30	40	50	60	70	80
MGLRAGGTLG	RAGAGRGAPE	GPGPSGGAQG	GSIHSGRIAA	VHNVPLSVLI	RPLPSVLDPA	KVQSLVDTIR	EDPDSVPPID
**90**	**100**	**110**	**120**	**130**	**140**		
VLWIKGAQGG	DYFYSFGGCH	RYAAYQQLQR	ETIPAKLVQS	TLSDLRVYLG	ASTPDLQ		

Cmpd.	No. of Cmpds.	m/z meas.	Δ m/z [ppm]	z	Rt [min]	Score	P	Range	Sequence	Modification
486	1	658.1368	-364.79	1	35.0	10.4	0	111-116	R.ETIPAK.L	

Detailed Protein Report

Protein 431: PREDICTED: zinc finger protein 556 isoform X1 [Homo sapiens]

Accession: gi|530425435
Database: refseq_human(refseq_human_20140103.fasta)
Database Date: 2015-11-30

Score: 21.3
MW [kDa]: 51.5
pI: 11.1
Sequence Coverage [%]: 7.5
No. of unique Peptides: 1

10	20	30	40	50	60	70	80
MDTVVFEDVV	VDFTLEEWAL	LNPAQRKLYR	DVMLETFKHL	ASVDNEAQLK	ASGSISQQDT	SGEKLSLKQK	IEKFTRKNIW
**90**	**100**	**110**	**120**	**130**	**140**	**150**	**160**
ASLLGKNWEE	HSVKDKHNTK	ERHLRNPRVE	RPCKSSKGNK	RGRTFRKTRN	CNRHLRKNCC	TSVRRYECSQ	CGKLFTHSSS
**170**	**180**	**190**	**200**	**210**	**220**	**230**	**240**
LIRHKRAHSG	QKLYKCKECG	KAFSRPSYLQ	THEKTHSGEK	PYACQSCGKT	FLRSHSLTEH	VRTHTGEKPY	ECGQCGKGFS
**250**	**260**	**270**	**280**	**290**	**300**	**310**	**320**
CPKSFRAHVM	MHAGGRPYEC	KHCGKAFRCQ	KSFRVHMIMH	AGGRPYECKQ	CGKAYCWATS	FQRHVRIHNG	EKPYKCGKCG
**330**	**340**	**350**	**360**	**370**	**380**	**390**	**400**
KAFGWPSSLH	KHARTHAKKK	PVSGGSVGKS	SARPRPSTDV	KSQTREKVYK	CETCGKTYGW	SSSLHKHERK	HTGEKPVNAA
**410**	**420**	**430**	**440**	**450**	**460**		
SVGKPSGGLC	SSKNVRTQIG	QKPSKCEKCG	KAFSCPKAFQ	GHVRSHTGKK	SCTSK		

Cmpd.	No. of Cmpds.	m/z meas.	Δ m/z [ppm]	z	Rt [min]	Score	P	Range	Sequence	Modification
1615	1	853.4020	-19.62	2	48.9	10.3	2	316-331	K.CGKCGKAFGWPSSLHK.H	

Detailed Protein Report

Protein 432: OTU domain-containing protein 7A [Homo sapiens]

**Accession:**	gi|18702331	**Score:**	21.3
**Database:**	refseq_human(refseq_human_20140103.fasta)	**MW [kDa]:**	100.6
**Database Date:**	2015-11-30	**pI:**	9.5
		**Sequence Coverage [%]:**	4.6
		**No. of unique Peptides:**	1

10	20	30	40	50	60	70	80
MVSSVLPNPT	SAECWAALLH	DPMTLDMDAV	LSDFVRSTGA	EPGLARDLLE	GKNWDLTAAL	SDYEQLRQVH	TANLPHVFNE
**90**	**100**	**110**	**120**	**130**	**140**	**150**	**160**
GRGPKQPERE	PQPGHKVERP	CLQRQDDIAQ	EKRLSRGISH	ASSAIVSLAR	SHVASECNNE	QFPLEMPIYT	FQLPDLSVYS
**170**	**180**	**190**	**200**	**210**	**220**	**230**	**240**
EDFRSFIERD	LIEQATMVAL	EQAGRLNWWS	TVCTSCKRLL	PLATTGDGNC	LLHAASLGMW	GFHDRDLVLR	KALYTMMRTG
**250**	**260**	**270**	**280**	**290**	**300**	**310**	**320**
AEREALKRRW	RWQQTQQNKE	EEWEREWTEL	LKLASSEPRT	HFSKNGGTGG	GVDNSEDPVY	ESLEEFHVFV	LAHILRRPIV
**330**	**340**	**350**	**360**	**370**	**380**	**390**	**400**
VVADTMLRDS	GGEAFAPIPF	GGIYLPLEVP	PNRCHCSPLV	LAYDQAHFSA	LVSMEQRDQQ	REQAVIPLTD	SEHKLLPLHF
**410**	**420**	**430**	**440**	**450**	**460**	**470**	**480**
AVDPGKDWEW	GKDDNDNARL	AHLILSLEAK	LNLLHSYMNV	TWIRIPSETR	APLAQPESPT	ASAGEDVQSL	ADSLDSDRDS
**490**	**500**	**510**	**520**	**530**	**540**	**550**	**560**
VCSNSNSNNG	KNGKDKEKEK	QRKEKDKTRA	DSVANKLGSF	SKTLGIKLKK	NMGGLGGLVH	GKMGRANSAN	GKNGDSAERG
**570**	**580**	**590**	**600**	**610**	**620**	**630**	**640**
KEKKAKSRKG	SKEESGASAS	TSPSEKTTPS	PTDKAAGASP	AEKGGGPRGD	AWKYSTDVKL	SLNILRAAMQ	GERKFIFAGL
**650**	**660**	**670**	**680**	**690**	**700**	**710**	**720**
LLTSHRHQFH	EEMIGYYLTS	AQERFSAEQE	QRRRDAATAA	AAAAAAAAAT	AKRPPRRPET	EGVPVPERAS	PGPPTQLVLK
**730**	**740**	**750**	**760**	**770**	**780**	**790**	**800**
LKERPSPGPA	AGRAARAAAG	GTASPGGGAR	RASASGPVPG	RSPPAPARQS	VIHVQASGAR	DEACAPAVGA	LRPCATYPQQ
**810**	**820**	**830**	**840**	**850**	**860**	**870**	**880**
NRSLSSQSYS	PARAAALRTV	NTVESLARAV	PGALPGAAGT	AGAAEHKSQT	YTNGFGALRD	GLEFADADAP	TARSNGECGR
**890**	**900**	**910**	**920**	**930**			
GGPGPVQRRC	QRENCAFYGR	AETEHYCSYC	YREELRRRRE	ARGARP			

Cmpd.	No. of Cmpds.	m/z meas.	Δ m/z [ppm]	z	Rt [min]	Score	P	Range	Sequence	Modification
2849	1	1001.0230	-1.34	2	64.2	10.3	1	68-85	R.QVHTANLPHVFNEGRGPK.Q	

Detailed Protein Report

Protein 433: **PREDICTED: probable global transcription activator SNF2L1 isoform X5 [Homo sapiens]**

**Accession:**	gi|578838748	**Score:**	21.3
**Database:**	refseq_human(refseq_human_20140103.fasta)	**MW [kDa]:**	121.1
**Database Date:**	2015-11-30	**pI:**	9.0
		**Sequence Coverage [%]:**	1.2
		**No. of unique Peptides:**	1

Quantitation

WD:WU **Median:** 2.90 **CV:** 0.00 % **No. of Peptides:** 1

10	20	30	40	50	60	70	80
MEQDTAAVAA	TVAAADATAT	IVVIEDEQPG	PSTSQEEGAA	AAATEATAAT	EKGEKKKEKN	VSSFQLKLAA	KAPKSEKEMD
90	100	110	120	130	140	150	160
PEYEEKMKAD	RAKRFEFLLK	QTELFAHFIQ	PSAQKSPTSP	LNMKLGRPRI	KKDEKQSLIS	AGDYRHRRTE	QEEDEELLSE
170	180	190	200	210	220	230	240
SRKTSNVCIR	FEVSPSYVKG	GPLRDYQIRG	LNWLISLYEN	GVNGILADEM	GLGKTLQTIA	LLGYLKHYRN	IPGPHMVLVP
250	260	270	280	290	300	310	320
KSTLHNWMNE	FKRWVPSLRV	ICFVGDKDAR	AAFIRDEMMP	GEWDVCVTSY	EMVIKEKSVF	KKFHWRYLVI	DEAHRIKNEK
330	340	350	360	370	380	390	400
SKLSEIVREF	KSTNRLLLTG	TPLQNNLHEL	WALLNFLLPD	VFNSADDFDS	WFDTKNCLGD	QKLVERLHAV	LKPFLLRRIK
410	420	430	440	450	460	470	480
TDVEKSLPPK	KEIKIYLGLS	KMQREWYTKI	LMKDIDVLNS	SGKMDKMRLL	NILMQLRKCC	NHPYLFDGAE	PGPPYTTDEH
490	500	510	520	530	540	550	560
IVSNSGKMVV	LDKLLAKLKE	QGSRVLIFSQ	MTRLLDILED	YCMWRGYEYC	RLDGQTPHEE	REEAIEAFNA	PNSSKFIFML
570	580	590	600	610	620	630	640
STRAGGLGIN	LASADVVILY	DSDWNPQVDL	QAMDRAHRIG	QKKPVRVFRL	ITDNTVEERI	VERAEIKLRL	DSIVIQQGRL
650	660	670	680	690	700	710	720
IDQQSNKLAK	EEMLQMIRHG	ATHVFASKES	ELTDEDITTI	LERGEKKTAE	MNERLQKMGE	SSLRNFRMDI	EQSLYKFEGE
730	740	750	760	770	780	790	800
DYREKQKLGM	VEWIEPPKRE	RKANYAVDAY	FREALRVSEP	KIPKAPRPPK	QPNVQDFQFF	PPRLFELLEK	EILYYRKTIG
810	820	830	840	850	860	870	880
YKVPRNPDIP	NPALAQREEQ	KKIDGAEPLT	PEETEEKEKL	LTQGFTNWTK	RDFNQFIKAN	EKYGRDDIDN	IAREVEGKSP
890	900	910	920	930	940	950	960
EEVMEYSAVF	WERCNELQDI	EKIMAQIERG	EARIQRRISI	KKALDAKIAR	YKAPFHQLRI	QYGTSKGKNY	TEEEDRFLIC
970	980	990	1000	1010	1020	1030	1040
MLHKMGFDRE	NVYEELRQCV	RNAPQFRFDW	FIKSRTAMEF	QRRCNTLISL	IEKENMEIEE	RERAEKKKRA	TKTPMVKFSA
1050							
FS							

Cmpd.	No. of Cmpds.	m/z meas.	Δ m/z [ppm]	z	Rt [min]	Score	P	Range	Sequence	Modification	Ratios
966	1	711.3525	2.57	2	40.4	21.3	1	434-446	K.DIDVLNSSGKMDK.M		WD:WU 2.90

Detailed Protein Report

Protein 434: deoxyuridine 5'-triphosphate nucleotidohydrolase, mitochondrial isoform 2 [Homo sapiens]

**Accession:**	gi|4503423	**Score:**	21.3
**Database:**	refseq_human(refseq_human_20140103.fasta)	**MW [kDa]:**	17.7
**Database Date:**	2015-11-30	**pI:**	6.2
		**Sequence Coverage [%]:**	20.1
		**No. of unique Peptides:**	2

Quantitation

WD:WU **Median:** 0.88 **CV:** 31.40 % **No. of Peptides:** 2

```
        10         20         30         40         50         60         70         80
MPCSEETPAI SPSKRARPAE VGGMQLRFAR LSEHATAPTR GSARAAGYDL YSAYDYTIPP MEKAVVKTDI QIALPSGCYG
        90        100        110        120        130        140        150        160
RVAPRSGLAA KHFIDVGAGV IDEDYRGNVG VVLFNFGKEK FEVKKGDRIA QLICERIFYP EIEEVQALDD TERGSGGFGS
       170
TGKN
```

Cmpd.	No. of Cmpds.	m/z meas.	Δ m/z [ppm]	z	Rt [min]	Score	P	Range	Sequence	Modification	Ratios	
1060	1	816.8541	-43.54	2	42.0	10.9	1	1-15	-.MPCSEETPAISPSKR.A		WD:WU	0.65
1576	1	671.0938	82.25	3	48.0	10.4	2	107-124	R.GNVGVVLFNFGKEKFEVK.K		WD:WU	1.19

Detailed Protein Report

Protein 435: DIS3-like exonuclease 1 isoform 2 [Homo sapiens]

**Accession:**	gi|19115966	**Score:**	21.2
**Database:**	refseq_human(refseq_human_20140103.fasta)	**MW [kDa]:**	111.0
**Database Date:**	2015-11-30	**pI:**	5.9
		**Sequence Coverage [%]:**	2.3
		**No. of unique Peptides:**	2

Quantitation

WD:WU **Median:** 2.22 **CV:** 0.00 % **No. of Peptides:** 1

Alias proteins:

Accession	Name	Description
gi|530405359	refseq_human (refseq_human_20140103.fasta)	PREDICTED: DIS3-like exonuclease 1 isoform X4 [Homo sapiens]
gi|530405355	refseq_human (refseq_human_20140103.fasta)	PREDICTED: DIS3-like exonuclease 1 isoform X2 [Homo sapiens]

10	20	30	40	50	60	70	80
MQTACQAVQH	QRGRRQYNKL	RNLLKDARHD	CILFANEFQQ	CCYLPRERGE	SMEKWQTRSI	YNAAVWYYHH	CQDRMPIVMV
**90**	**100**	**110**	**120**	**130**	**140**	**150**	**160**
TEDEEAIQQY	GSETEGVFVI	TFKNYLDNFW	PDLKAAHELC	DSILQSRRER	ENESQESHGK	EYPEHLPLEV	LEAGIKSGRY
**170**	**180**	**190**	**200**	**210**	**220**	**230**	**240**
IQGILNVNKH	RAQIEAFVRL	QGASSKDSDL	VSDILIHGMK	ARNRSIHGDV	VVVELLPKNE	WKGRTVALCE	NDCDDKASGE
**250**	**260**	**270**	**280**	**290**	**300**	**310**	**320**
SPSEPMPTGR	VVGILQKNWR	DYVVTFPSKE	EVQSQGKNAQ	KILVTPWDYR	IPKIRISTQQ	AETLQDFRVV	VRIDSWESTS
**330**	**340**	**350**	**360**	**370**	**380**	**390**	**400**
VYPNGHFVRV	LGRIGDLEGE	IATILVENSI	SVIPFSEAQM	CEMPVNTPES	PWKVSPEEEQ	KRKDLRKSHL	VFSIDPKGCE
**410**	**420**	**430**	**440**	**450**	**460**	**470**	**480**
DVDDTLSVRT	LNNGNLELGV	HIADVTHFVA	PNSYIDIEAR	TRATTYYLAD	RRYDMLPSVL	SADLCSLLGG	VDRYAVSIMW
**490**	**500**	**510**	**520**	**530**	**540**	**550**	**560**
ELDKASYEIK	KVWYGRTIIR	SAYKLFYEAA	QELLDGNLSV	VDDIPEFKDL	DEKSRQAKLE	ELVWAIGKLT	DIARHVRAKR
**570**	**580**	**590**	**600**	**610**	**620**	**630**	**640**
DGCGALELEG	VEVCVQLDDK	KNIHDLIPKQ	PLEVHETVAE	CMILANHWVA	KKIWESFPHQ	ALLRQHPPPH	QEFFSELREC
**650**	**660**	**670**	**680**	**690**	**700**	**710**	**720**
AKAKGFFIDT	RSNKTLADSL	DNANDPHDPI	VNRLLRSMAT	QAMSNALYFS	TGSCAEEEFH	HYGLALDKYT	HFTSPIRRYS
**730**	**740**	**750**	**760**	**770**	**780**	**790**	**800**
DIVVHRLLMA	AISKDKKMEI	KGNLFSNKDL	EELCRHINNR	NQAAQHSQKQ	STELFQCMYF	KDKDPATEER	CISDGVIYSI
**810**	**820**	**830**	**840**	**850**	**860**	**870**	**880**
RTNGVLLFIP	RFGIKGAAYL	KNKDGLVISC	GPDSCSEWKP	GSLQRFQNKI	TSTTTDGESV	TFHLFDHVTV	RISIQASRCH
**890**	**900**	**910**	**920**	**930**	**940**	**950**	**960**
SDTIRLEIIS	NKPYKIPNTE	LIHQSSPLLK	SELVKEVTKS	VEEAQLAQEV	KVNIIQEEYQ	EYRQTKGRSL	YTLLEEIRDL
**970**	**980**						
ALLDVSNNYG	I						

Cmpd.	No. of Cmpds.	m/z meas.	Δ m/z [ppm]	z	Rt [min]	Score	P	Range	Sequence	Modification	Ratios
2734	1	639.3382	-4.54	2	62.6	10.8	1	709-718	K.YTHFTSPIRR.Y		
1921	1	655.2832	-155.36	2	52.4	10.4	2	812-823	R.FGIKGAAYLKNK.D		WD:WU 2.22

Detailed Protein Report

Protein 436: PREDICTED: protein Shroom2 isoform X1 [Homo sapiens]

**Accession:**	gi|578837883	**Score:**	21.2
**Database:**	refseq_human(refseq_human_20140103.fasta)	**MW [kDa]:**	176.2
**Database Date:**	2015-11-30	**pI:**	6.7
		**Sequence Coverage [%]:**	2.5
		**No. of unique Peptides:**	1

10	20	30	40	50	60	70	80
MEGAEPRARP	ERLAEAETRA	ADGGRLVEVQ	LSGGAPWGFT	LKGGREHGEP	LVITKIEEGS	KAAAVDKLLA	GDEIVGINDI
90	100	110	120	130	140	150	160
GLSGFRQEAI	CLVKGSHKTL	KLVVKRRSEL	GWRPHSWHAT	KFSDSHPELA	ASPFTSTSGC	PSWSGRHHAS	SSSHDLSSSW
170	180	190	200	210	220	230	240
EQTNLQRTLD	HFSSLGSVDS	LDHPSSRLSV	AKSNSSIDHL	GSHSKRDSAY	GSFSTSSSTP	DHTLSKADTS	SAENILYTVG
250	260	270	280	290	300	310	320
LWEAPRQGGR	QAQAAGDPQG	SEEKLSCFPP	RVPGDSGKGP	RPEYNAEPKL	AAPGRSNFGP	VWYVPDKKKA	PSSPPPPPPP
330	340	350	360	370	380	390	400
LRSDSFAATK	SHEKAQGPVF	SEAAAAQHFT	ALAQAQPRGD	RRPELTDRPW	RSAHPGSLGK	GSGGPGCPQE	AHADGSWPPS
410	420	430	440	450	460	470	480
KDGASSRLQA	SLSSSDVRFP	QSPHSGRHPP	LYSDHSPLCA	DSLGQEPGAA	SFQNDSPPQV	RGLSSCDQKL	GSGWQGPRPC
490	500	510	520	530	540	550	560
VQGDLQAAQL	WAGCWPSDTA	LGALESLPPP	TVGQSPRHHL	PQPEGPPDAR	ETGRCYPLDK	GAEGCSAGAQ	EPPRASRAEK
570	580	590	600	610	620	630	640
ASQRLAASIT	WADGESSRIC	PQETPLLHSL	TQEGKRRPES	SPEDSATRPP	PFDAHVGKPT	RRSDRFATTL	RNEIQMHRAK
650	660	670	680	690	700	710	720
LQKSRSTVAL	TAAGEAEDGT	GRWRAGLGGG	TQEGPLAGTY	KDHLKEAQAR	VLRATSFKRR	DLDPNPGDLY	PESLEHRMGD
730	740	750	760	770	780	790	800
PDTVPHFWEA	GLAQPPSSTS	GGPHPPRIGG	RRRFTAEQKL	KSYSEPEKMN	EVGLTRGYSP	HQHPRTSEDT	VGTFADRWKF
810	820	830	840	850	860	870	880
FEETSKPVPQ	RPAQKQALHG	IPRDKPERPR	TAGRTCEGTE	PWSRTTSLGD	SLNAHSAAEK	AGTSDLPRRL	GTFAEYQASW
890	900	910	920	930	940	950	960
KEQRKPLEAR	SSGRCHSADD	ILDVSLDPQE	RPQHVHGRSR	SSPSTDHYKQ	EASVELRRQA	GDPGEPREEL	PSAVRAEEGQ
970	980	990	1000	1010	1020	1030	1040
STPRQADAQC	REGSPGSQQH	PPSQKAPNPP	TFSELSHCRG	APELPREGRG	RAGTLPRDYR	YSEESTPADL	GPRAQSPGSP
1050	1060	1070	1080	1090	1100	1110	1120
LHARGQDSWP	VSSALLSKRP	APQRPPPPKR	EPRRYRATDG	APADAPVGVL	GRPFPTPSPA	SLDVYVARLS	LSHSPSVFSS
1130	1140	1150	1160	1170	1180	1190	1200
AQPQDTPKAT	VCERGSQHVS	GDASRPLPEA	LLPPKQQHLR	LQTATMETSR	SPSPQFAPQK	LTDKPPLLIQ	DEDSTRIERV
1210	1220	1230	1240	1250	1260	1270	1280
MDNNTTVKMV	PIKIVHSESQ	PEKESRQSLA	CPAEPPALPH	GLEKDQIKTL	STSEQFYSRF	CLYTRQGAEP	EAPHRAQPAE
1290	1300	1310	1320	1330	1340	1350	1360
PQPLGTQVPP	EKDRCTSPPG	LSYMKAKEKT	VEDLKSEELA	REIVGKDKSL	ADILDPSVKI	KTTMDLMEGI	FPKDEHLLEE
1370	1380	1390	1400	1410	1420	1430	1440
AQQRRKLLPK	IPSPRSTEER	KEEPSVPAAV	SLATNSTYYS	TSAPKAELLI	KMKDLQEQQE	HEEDSGSDLD	HDLSVKKELI
1450	1460	1470	1480	1490	1500	1510	1520
ESISRKLQVL	REARESLLED	VQANTVLGAE	VEAIVKGVCK	PSEFDKFRMF	IGDLDKVVNL	LLSLSGRLAR	VENALNNLDD
1530	1540	1550	1560	1570	1580	1590	1600
GASPGDRQSL	LEKQRVLIQQ	HEDAKELKEN	LDRRERIVFD	ILANYLSEES	LADYEHFVKM	KSALIIEQRE	LEDKIHLGEE
1610	1620						
QLKCLLDSLQ	PERGK						

Cmpd.	No. of Cmpds.	m/z meas.	Δ m/z [ppm]	z	Rt [min]	Score	P	Range	Sequence	Modification
2357	1	994.6851	148.86	2	57.7	11.2	2	1197-1213	R.IERVMDNNTTVKMVPIK.I	

Detailed Protein Report

Protein 437: **supervillin isoform 1 [Homo sapiens]**

**Accession:**	gi|150417971	**Score:**	21.2
**Database:**	refseq_human(refseq_human_20140103.fasta)	**MW [kDa]:**	200.7
**Database Date:**	2015-11-30	**pI:**	6.5
**Modification(s):**	Oxidation	**Sequence Coverage [%]:**	1.8
		**No. of unique Peptides:**	1

10	20	30	40	50	60	70	80
MKRKERIARR	LEGIENDTQP	ILLQSCTGLV	THRLLEEDTP	RYMRASDPAS	PHIGRSNEEE	ETSDSSLEKQ	TRSKYCTETS
**90**	**100**	**110**	**120**	**130**	**140**	**150**	**160**
GVHGDSPYGS	GTMDTHSLES	KAERIARYKA	ERRRQLAEKY	GLTLDPEADS	EYLSRYTKSR	KEPDAVEKRG	GKSDKQEESS
**170**	**180**	**190**	**200**	**210**	**220**	**230**	**240**
RDASSLYPGT	ETMGLRTCAG	ESKDYALHVG	DGSSDPEVLL	NIENQRRGQE	LSATRQAHDL	SPAAESSSTF	SFSGRDSSFT
**250**	**260**	**270**	**280**	**290**	**300**	**310**	**320**
EVPRSPKHAH	SSSLQQAASR	SPSFGDPQLS	PEARPRCTSH	SETPTVDDEE	KVDERAKLSV	AAKRLLFREM	EKSFDEQNVP
**330**	**340**	**350**	**360**	**370**	**380**	**390**	**400**
KRRSRNTAVE	QRLRRLQDRS	LTQPITTEEV	VIAATLQASA	HQKALAKDQT	NEGKELAEQG	EPDSSTLSLA	EKLALFNKLS
**410**	**420**	**430**	**440**	**450**	**460**	**470**	**480**
QPVSKAISTR	NRIDTRQRRM	NARYQTQPVT	LGEVEQVQSG	KLIPFSPAVN	TSVSTVASTV	APMYAGDLRT	KPPLDHNASA
**490**	**500**	**510**	**520**	**530**	**540**	**550**	**560**
TDYKFSSSIE	NSDSPVRSIL	KSQAWQPLVE	GSENKGMLRE	YGETESKRAL	TGRDSGMEKY	GSFEEAEASY	PILNRAREGD
**570**	**580**	**590**	**600**	**610**	**620**	**630**	**640**
SHKESKYAVP	RRGSLERANP	PITHLGDEPK	EFSMAKMNAQ	GNLDLRDRLP	FEEKVEVENV	MKRKFSLRAA	EFGEPTSEQT
**650**	**660**	**670**	**680**	**690**	**700**	**710**	**720**
GTAAGKTIAQ	TTAPVSWKPQ	DSSEQPQEKL	CKNPCAMFAA	GEIKTPTGEG	LLDSPSKTMS	IKERLALLKK	SGEEDWRNRL
**730**	**740**	**750**	**760**	**770**	**780**	**790**	**800**
SRRQEGGKAP	ASSLHTQEAG	RSLIKKRVTE	SRESQMTIEE	RKQLITVREE	AWKTRGRGAA	NDSTQFTVAG	RMVKKGLASP
**810**	**820**	**830**	**840**	**850**	**860**	**870**	**880**
TAITPVASPI	CGKTRGTTPV	SKPLEDIEAR	PDMQLESDLK	LDRLETFLRR	LNNKVGGMHE	TVLTVTGKSV	KEVMKPDDDE
**890**	**900**	**910**	**920**	**930**	**940**	**950**	**960**
TFAKFYRSVD	YNMPRSPVEM	DEDFDVIFDP	YAPKLTSSVA	EHKRAVRPKR	RVQASKNPLK	MLAAREDLLQ	EYTEQRLNVA
**970**	**980**	**990**	**1000**	**1010**	**1020**	**1030**	**1040**
FMESKRMKVE	KMSSNSNFSE	VTLAGLASKE	NFSNVSLRSV	NLTEQNSNNS	AVPYKRLMLL	QIKGRRHVQT	RLVEPRASAL
**1050**	**1060**	**1070**	**1080**	**1090**	**1100**	**1110**	**1120**
NSGDCFLLLS	PHCCFLWVGE	FANVIEKAKA	SELATLIQTK	RELGCRATYI	QTIEEGINTH	THAAKDFWKL	LGGQTSYQSA
**1130**	**1140**	**1150**	**1160**	**1170**	**1180**	**1190**	**1200**
GDPKEDELYE	AAIIETNCIY	RLMDDKLVPD	DDYWGKIPKC	SLLQPKEVLV	FDFGSEVYVW	HGKEVTLAQR	KIAFQLAKHL
**1210**	**1220**	**1230**	**1240**	**1250**	**1260**	**1270**	**1280**
WNGTFDYENC	DINPLDPGEC	NPLIPRKGQG	RPDWAIFGRL	TEHNETILFK	EKFLDWTELK	RSNEKNPGEL	AQHKEDPRTD
**1290**	**1300**	**1310**	**1320**	**1330**	**1340**	**1350**	**1360**
VKAYDVTRMV	SMPQTTAGTI	LDGVNVGRGY	GLVEGHDRRQ	FEITSVSVDV	WHILEFDYSR	LPKQSIGQFH	EGDAYVVKWK
**1370**	**1380**	**1390**	**1400**	**1410**	**1420**	**1430**	**1440**
FMVSTAVGSR	QKGEHSVRAA	GKEKCVYFFW	QGRHSTVSEK	GTSALMTVEL	DEERGAQVQV	LQGKEPPCFL	QCFQGGMVVH
**1450**	**1460**	**1470**	**1480**	**1490**	**1500**	**1510**	**1520**
SGRREEEEEN	VQSEWRLYCV	RGEVPVEGNL	LEVACHCSSL	RSRTSMVVLN	VNKALIYLWH	GCKAQAHTKE	VGRTAANKIK
**1530**	**1540**	**1550**	**1560**	**1570**	**1580**	**1590**	**1600**
EQCPLEAGLH	SSSKVTIHEC	DEGSEPLGFW	DALGRRDRKA	YDCMLQDPGS	FNFAPRLFIL	SSSSGDFAAT	EFVYPARAPS
**1610**	**1620**	**1630**	**1640**	**1650**	**1660**	**1670**	**1680**
VVSSMPFLQE	DLYSAPQPAL	FLVDNHHEVY	LWQGWWPIEN	KITGSARIRW	ASDRKSAMET	VLQYCKGKNL	KKPAPKSYLI
**1690**	**1700**	**1710**	**1720**	**1730**	**1740**	**1750**	**1760**
HAGLEPLTFT	NMFPSWEHRE	DIAEITEMDT	EVSNQITLVE	DVLAKLCKTI	YPLADLLARP	LPEGVDPLKL	EIYLTDEDFE
**1770**	**1780**	**1790**					
FALDMTRDEY	NALPAWKQVN	LKKAKGLF					

Cmpd.	No. of	m/z meas.	Δ m/z	z	Rt	Score	P	Range	Sequence	Modification

Detailed Protein Report

	Cmpds.		[ppm]		[min]					
2904	1	974.3155	-109.14	2	65.1	10.9	0	1560-1576	K.AYDCMLQDPGSFNFAPR.L	Oxidation: 5

Detailed Protein Report

Protein 438: zinc finger protein 425 [Homo sapiens]

**Accession:**	gi|48717241	**Score:**	21.2
**Database:**	refseq_human(refseq_human_20140103.fasta)	**MW [kDa]:**	87.7
**Database Date:**	2015-11-30	**pI:**	10.6
		**Sequence Coverage [%]:**	3.7
		**No. of unique Peptides:**	1

10	20	30	40	50	60	70	80
MAEPASVTVT	FDDVALYFSE	QEWEILEKWQ	KQMYKQEMKT	NYETLDSLGY	AFSKPDLITW	MEQGRMLLIS	EQGCLDKTRR
**90**	**100**	**110**	**120**	**130**	**140**	**150**	**160**
TTSPPTDEQL	NMKNTGKLLC	FDDEGTPRTK	EEDCRLNGPQ	KQDLCAALRG	KERKILLAQT	ATFQSPSLRE	TEILNKKVSI
**170**	**180**	**190**	**200**	**210**	**220**	**230**	**240**
TAYDPDKKDL	RHKPRETPGR	LEIPTGPRCY	SCYVCRKVFQ	VRRDLLKHKR	SHSKSQLCRY	PKYKNSSRGK	SELRRTQRLL
**250**	**260**	**270**	**280**	**290**	**300**	**310**	**320**
CQKKRFQCSE	CEKSYFLKGS	LVTHQVVHTG	QRPYPCPECD	KTFRYRANLK	KHLCLHRGER	PFCCGECGRA	FVQQCELTEH
**330**	**340**	**350**	**360**	**370**	**380**	**390**	**400**
LRLHSGEKPF	QCPQCDRCFR	LKRGMKVHLT	QHSGKRPFHC	PECGRSFSRK	AALKTHQRTH	SEEKPFSCGE	CGRKFIYKIK
**410**	**420**	**430**	**440**	**450**	**460**	**470**	**480**
LDEHIRVHTG	EKPFSCPECN	KSFRLKRSLK	AHGLQHIGKR	PFQCPECSRG	FFWRNAMRAH	QRLHSEQKPF	PCAECGKRFT
**490**	**500**	**510**	**520**	**530**	**540**	**550**	**560**
RPSKLACHTR	VHDRQKEFPC	GECKKTFSQQ	SRLTQHLKVH	TTEKPFSCAE	CGRSFRRRAH	LTEHTRLHSG	EEPFQCPECD
**570**	**580**	**590**	**600**	**610**	**620**	**630**	**640**
KSFSWKASMK	FHQRMHRDEK	PFACGECDKT	YTHQSQLTEH	LRLHSGEKPY	QCPECEKTFR	LKGNLKSHLL	QHSGQKPFSC
**650**	**660**	**670**	**680**	**690**	**700**	**710**	**720**
VMCGKSFTQQ	YRLTEHIRVH	SGEKPFQCPE	CDKSYCIRGS	LKVHLYKHSG	ERPFQCPECG	KGFLQKRSLK	AHLCLHSGER
**730**	**740**	**750**	**760**				
PFSCDECGRS	FTYVGALKTH	IAVHAKEKPS	SL				

Cmpd.	No. of Cmpds.	m/z meas.	Δ m/z [ppm]	z	Rt [min]	Score	P	Range	Sequence	Modification
1689	1	1043.8593	-137.44	2	49.4	10.7	0	627-645	K.SHLLQHSGQKPFSCVMCGK.S	

Detailed Protein Report

Protein 439: **von Willebrand factor A domain-containing protein 8 isoform a precursor [Homo sapiens]**

**Accession:**	gi|57863271	**Score:**	21.1
**Database:**	refseq_human(refseq_human_20140103.fasta)	**MW [kDa]:**	214.7
**Database Date:**	2015-11-30	**pI:**	7.1
		**Sequence Coverage [%]:**	1.9
		**No. of unique Peptides:**	1

Detailed Protein Report

10	20	30	40	50	60	70	80
MQSRLLLLGA	PGGHGGPASR	RMRLLLRQVV	QRRPGGDRQR	PEVRLLHAGS	GADTGDTVNI	GDVSYKLKIP	KNPELVPQNY
90	100	110	120	130	140	150	160
ISDSLAQSVV	QHLRWIMQKD	LLGQDVFLIG	PPGPLRRSIA	MQYLELTKRE	VEYIALSRDT	TETDLKQRRE	IRAGTAFYID
170	180	190	200	210	220	230	240
QCAVRAATEG	RTLILEGLEK	AERNVLPVLN	NLLENREMQL	EDGRFLMSAE	RYDKLLRDHT	KKELDSWKIV	RVSENFRVIA
250	260	270	280	290	300	310	320
LGLPVPRYSG	NPLDPPLRSR	FQARDIYYLP	FKDQLKLLYS	IGANVSAEKV	SQLLSFATTL	CSQESSTLGL	PDFPLDSLAA
330	340	350	360	370	380	390	400
AVQILDSFPM	MPIKHAIQWL	YPYSILLGHE	GKMAVEGVLK	RFELQDSGSS	LLPKEIVKVE	KMMENHVSQA	SVTIRIADKE
410	420	430	440	450	460	470	480
VTIKVPAGTR	LLSQPCASDR	FIQTLSHKQL	QAEMMQSHMV	KDICLIGGKG	CGKTVIAKNF	ADTLGYNIEP	IMLYQDMTAR
490	500	510	520	530	540	550	560
DLLQQRYTLP	NGDTAWRSSP	LVNAALEGKL	VLLDGIHRVN	AGTLAVLQRL	IHDRELSLYD	GSRLLREDRY	MRLKEELQLS
570	580	590	600	610	620	630	640
DEQLQKRSIF	PIHPSFRIIA	LAEPPVIGST	AHQWLGPEFL	TMFFFHYMKP	LVKSEEIQVI	KEKVPNVPQE	ALDKLLSFTH
650	660	670	680	690	700	710	720
KLRETQDPTA	QSLAASLSTR	QLLRISRRLS	QYPNENLHSA	VTKACLSRFL	PSLARSALEK	NLADATIEIN	TDDNLEPELK
730	740	750	760	770	780	790	800
DYKCEVTSGT	LRIGAVSAPI	YNAHEKMKVP	DVLFYDNIQH	VIVMEDMLKD	FLLGEHLLLV	GNQGVGKNKI	VDRFLHLLNR
810	820	830	840	850	860	870	880
PREYIQLHRD	TTVQTLTLQP	SVKDGLIVYE	DSPLVKAVKL	GHILVVDEAD	KAPTNVTCIL	KTLVENGEMI	LADGRRIVAN
890	900	910	920	930	940	950	960
SANVNGRENV	VVIHPDFRMI	VLANRPGFPF	LGNDFFGTLG	DIFSCHAVDN	PKPHSELEML	RQYGPNVPEP	ILQKLVAAFG
970	980	990	1000	1010	1020	1030	1040
ELRSLADQGI	INYPYSTREV	VNIVKHLQKF	PTEGLSSVVR	NVFDFDSYNN	DMREILINTL	HKYGIPIGAK	PTSVQLAKEL
1050	1060	1070	1080	1090	1100	1110	1120
TLPEQTFMGY	WTIGQARSGM	QKLLCPVETH	HIDIKGPALI	NIQEYPIERH	EERSLNFTEE	CASWRIPLDE	INIICDIATS
1130	1140	1150	1160	1170	1180	1190	1200
HENEQNTLYV	VTCNPASLYF	MNMTGKSGFF	VDFFDIFPRT	ANGVWHPFVT	VAPLGSPLKG	QVVLHEQQSN	VILLLDTTGR
1210	1220	1230	1240	1250	1260	1270	1280
ALHRLILPSE	KFTSKKPFWW	NKEEAETYKM	CKEFSHKNWL	VFYKEKGNSL	TVLDVLEGRT	HTISLPINLK	TVFLVAEDKW
1290	1300	1310	1320	1330	1340	1350	1360
LLVESKTNQK	YLLTKPAHIE	SEGSGVCQLY	VLKEEPPSTG	FGVTQETEFS	IPHKISSDQL	SSEHLSSAVE	QKIASPNRIL
1370	1380	1390	1400	1410	1420	1430	1440
SDEKNYATIV	VGFPDLMSPS	EVYSWKRPSS	LHKRSGTDTS	FYRGKKKRGT	PKQSNCVTLL	DTNQVVRILP	PGEVPLKDIY
1450	1460	1470	1480	1490	1500	1510	1520
PKDVTPPQTS	GYIEVTDLQS	KKLRYIPIPR	SESLSPYTTW	LSTISDTDAL	LAEWDKSGVV	TVDMGGHIRL	WETGLERLQR
1530	1540	1550	1560	1570	1580	1590	1600
SLMEWRNMIG	QDDRNMQITI	NRDSGEDVSS	PKHGKEDPDN	MPHVGGNTWA	GGTGGRDTAG	LGGKGGPYRL	DAGHTVYQVS
1610	1620	1630	1640	1650	1660	1670	1680
QAEKDAVPEE	VKRAAREMGQ	RAFQQRLKEI	QMSEYDAATY	ERFSGAVRRQ	VHSLRIILDN	LQAKGKERQW	LRHQATGELD
1690	1700	1710	1720	1730	1740	1750	1760
DAKIIDGLTG	EKAIYKRRGE	LEPQLGSPQQ	KPKRLRLVVD	VSGSMYRFNR	MDGRLERTME	AVCMVMEAFE	NYEEKFQYDI
1770	1780	1790	1800	1810	1820	1830	1840
VGHSGDGYNI	GLVPMNKIPK	DNKQRLEILK	TMHAHSQFCM	SGDHTLEGTE	HAIKEIVKEE	ADEYFVIVLS	DANLSRYGIH
1850	1860	1870	1880	1890	1900	1910	
PAKFAQILTR	DPQVNAFAIF	IGSLGDQATR	LQRTLPAGRS	FVAMDTKDIP	QILQQIFTST	MLSSV	

Cmpd.	No. of Cmpds.	m/z meas.	Δ m/z [ppm]	z	Rt [min]	Score	P	Range	Sequence	Modification
2188	1	958.6176	79.28	2	55.7	10.0	2	5-23	R.LLLLGAPGGHGGPASRRMR.L	

Detailed Protein Report

Protein 440: PREDICTED: multiple epidermal growth factor-like domains protein 6 isoform X4 [Homo sapiens]

**Accession:**	gi|578798522	**Score:**	21.0
**Database:**	refseq_human(refseq_human_20140103.fasta)	**MW [kDa]:**	135.9
**Database Date:**	2015-11-30	**pI:**	5.9
**Modification(s):**	Carbamidomethyl	**Sequence Coverage [%]:**	3.0
		**No. of unique Peptides:**	1

10	20	30	40	50	60	70	80
MGASRDRGLA	ALWCLGLLGG	LARVAGTHYR	YLWRGCYPCH	LGQAGYPVSA	GDQRPDVDEC	RTHNGGCQHR	CVNTPGSYLC
90	100	110	120	130	140	150	160
ECKPGFRLHT	DSRTCLAINS	CALGNGGCQH	HCVQLTITRH	RCQCRPGFQL	QEDGRHCVRR	SPCANRNGSC	MHRCQVVRGL
170	180	190	200	210	220	230	240
ARCECHVGYQ	LAADGKACED	VDECAAGLAQ	CAHGCLNTQG	SFKCVCHAGY	ELGADGRQCY	RIEMEIVNSC	EANNGGCSHG
250	260	270	280	290	300	310	320
CSHTSAGPLC	TCPRGYELDT	DQRTCIDVDD	CADSPCCQQV	CTNNPGGYEC	GCYAGYRLSA	DGCGCEDVDE	CASSRGGCEH
330	340	350	360	370	380	390	400
HCTNLAGSFQ	CSCEAGYRLH	EDRRGCSPLE	EPMVDLDGEL	PFVRPLPHIA	VLQDELPQLF	QDDDVGADEE	EAELRGEHTL
410	420	430	440	450	460	470	480
TEKFVCLDDS	FGHDCSLTCD	DCRNGGTCLL	GLDGCDCPEG	WTGLICNETC	PPDTFGKNCS	FSCSCQNGGT	CDSVTGACRC
490	500	510	520	530	540	550	560
PPGVSGTNCE	DGCPKGYYGK	HCRKKCNCAN	RGRCHRLYGA	CLCDPGLYGR	FCHLTCPPWA	FGPGCSEECQ	CVQPHTQSCD
570	580	590	600	610	620	630	640
KRDGSCSCKA	GFRGERCQAE	CELGYFGPGC	WQACTCPVGV	ACDSVSGECG	KRCPAGFQGE	DCGQECPVGT	FGVNCSSSCS
650	660	670	680	690	700	710	720
CGGAPCHGVT	GQCRCPPGRT	GEDCEAECPQ	GHFGPGCEQR	CQCQHGAACD	HVSGACTCPA	GWRGTFCEHA	CPAGFFGLDC
730	740	750	760	770	780	790	800
RSACNCTAGA	ACDAVNGSCL	CPAGRRGPRC	AETCPAHTYG	HNCSQACACF	NGASCDPVHG	QCHCAPGWMG	PSCLQACPAG
810	820	830	840	850	860	870	880
LYGDNCRHSC	LCQNGGTCDP	VSGHCACPEG	WAGLACEKEC	LPRDVRAGCR	HSGGCLNGGL	CDPHTGRCLC	PAGWTGDKCQ
890	900	910	920	930	940	950	960
SPCLRGWFGE	ACAQRCSCPP	GAACHHVTGA	CRCPPGFTGS	GCEQACPPGS	FGEDCAQMCQ	CPGENPACHP	ATGTCSCAAG
970	980	990	1000	1010	1020	1030	1040
YHGPSCQQRC	PPGRYGPGCE	QLCGCLNGGS	CDAATGACRC	PTGFLGTDCN	LTCPQGRFGP	NCTHVCGCGQ	GAACDPVTGT
1050	1060	1070	1080	1090	1100	1110	1120
CLCPPGRAGV	RCERGCPQNR	FGVGCEHTCS	CRNGGLCHAS	NGSCSCGLGW	TGRHCELACP	PGRYGAACHL	ECSCHNNSTC
1130	1140	1150	1160	1170	1180	1190	1200
EPATGTCRCG	PGFYGQACEH	PCPPGFHGAG	CQGLCWCQHG	APCDPISGRC	LCPAGFHGHF	CERGCEPGSF	GEGCHQRCDC
1210	1220	1230	1240	1250	1260	1270	1280
DGGAPCDPVT	GLCLCPPGRS	GATCNLDCRR	GQFGPSCTLH	CDCGGGADCD	PVSGQCHCVD	GYMGPTCREG	GPLRLPENPS
1290	1300	1310					
LAQGSAGTLP	ASSRPTSRSG	GPARH					

Cmpd.	No. of Cmpds.	m/z meas.	Δ m/z [ppm]	z	Rt [min]	Score	P	Range	Sequence	Modification
12	1	1103.5102	57.86	2	29.4	10.6	0	1198-1219	R.CDCDGGAPCDPVTGLCLCPPGR.S	Carbamidomethyl: 16

Detailed Protein Report

Protein 441: voltage-dependent R-type calcium channel subunit alpha-1E isoform 2 [Homo sapiens]

**Accession:**	gi|329663531	**Score:**	21.0
**Database:**	refseq_human(refseq_human_20140103.fasta)	**MW [kDa]:**	254.4
**Database Date:**	2015-11-30	**pI:**	9.3
**Modification(s):**	Oxidation	**Sequence Coverage [%]:**	1.4
		**No. of unique Peptides:**	1

Detailed Protein Report

10	20	30	40	50	60	70	80
MARFGEAVVA	RPGSGDGDSD	QSRNRQGTPV	PASGQAAAYK	QTKAQRARTM	ALYNPIPVRQ	NCFTVNRSLF	IFGEDNIVRK
90	100	110	120	130	140	150	160
YAKKLIDWPP	FEYMILATII	ANCIVLALEQ	HLPEDDKTPM	SRRLEKTEPY	FIGIFCFEAG	IKIVALGFIF	HKGSYLRNGW
170	180	190	200	210	220	230	240
NVMDFIVVLS	GILATAGTHF	NTHVDLRTLR	AVRVLRPLKL	VSGIPSLQIV	LKSIMKAMVP	LLQIGLLLFF	AILMFAIIGL
250	260	270	280	290	300	310	320
EFYSGKLHRA	CFMNNSGILE	GFDPPHPCGV	QGCPAGYECK	DWIGPNDGIT	QFDNILFAVL	TVFQCITMEG	WTTVLYNTND
330	340	350	360	370	380	390	400
ALGATWNWLY	FIPLIIIGSF	FVLNLVLGVL	SGEFAKERER	VENRRAFMKL	RRQQQIEREL	NGYRAWIDKA	EEVMLAEENK
410	420	430	440	450	460	470	480
NAGTSALEVL	RRATIKRSRT	EAMTRDSSDE	HCVDISSVGT	PLARASIKSA	KVDGVSYFRH	KERLLRISIR	HMVKSQVFYW
490	500	510	520	530	540	550	560
IVLSLVALNT	ACVAIVHHNQ	PQWLTHLLYY	AEFLFLGLFL	LEMSLKMYGM	GPRLYFHSSF	NCFDFGVTVG	SIFEVVWAIF
570	580	590	600	610	620	630	640
RPGTSFGISV	LRALRLLRIF	KITKYWASLR	NLVVSLMSSM	KSIISLLFLL	FLFIVVFALL	GMQLFGGRFN	FNDGTPSANF
650	660	670	680	690	700	710	720
DTFPAAIMTV	FQILTGEDWN	EVMYNGIRSQ	GGVSSGMWSA	IYFIVLTLFG	NYTLLNVFLA	IAVDNLANAQ	ELTKDEQEEE
730	740	750	760	770	780	790	800
EAFNQKHALQ	KAKEVSPMSA	PNMPSIERER	RRRHHMSVWE	QRTSQLRKHM	QMSSQEALNR	EEAPTMNPLN	PLNPLSSLNP
810	820	830	840	850	860	870	880
LNAHPSLYRR	PRAIEGLALG	LALEKFEEER	ISRGGSLKGD	GGDRSSALDN	QRTPLSLGQR	EPPWLARPCH	GNCDPTQQEA
890	900	910	920	930	940	950	960
GGGEAVVTFE	DRARHRQSQR	RSRHRRVRTE	GKESSSASRS	RSASQERSLD	EAMPTEGEKD	HELRGNHGAK	EPTIQEERAQ
970	980	990	1000	1010	1020	1030	1040
DLRRTNSLMV	SRGSGLAGGL	DEADTPLVLP	HPELEVGKHV	VLTEQEPEGS	SEQALLGNVQ	LDMGRVISQS	EPDLSCITAN
1050	1060	1070	1080	1090	1100	1110	1120
TDKATTESTS	VTVAIPDVDP	LVDSTVVHIS	NKTDGEASPL	KEAEIREDEE	EVEKKKQKKE	KRETGKAMVP	HSSMFIFSTT
1130	1140	1150	1160	1170	1180	1190	1200
NPIRRACHYI	VNLRYFEMCI	LLVIAASSIA	LAAEDPVLTN	SERNKVLRYF	DYVFTGVFTF	EMVIKMIDQG	LILQDGSYFR
1210	1220	1230	1240	1250	1260	1270	1280
DLWNILDFVV	VVGALVAFAL	ANALGTNKGR	DIKTIKSLRV	LRVLRPLKTI	KRLPKLKAVF	DCVVTSLKNV	FNILIVYKLF
1290	1300	1310	1320	1330	1340	1350	1360
MFIFAVIAVQ	LFKGKFFYCT	DSSKDTEKEC	IGNYVDHEKN	KMEVKGREWK	RHEFHYDNII	WALLTLFTVS	TGEGWPQVLQ
1370	1380	1390	1400	1410	1420	1430	1440
HSVDVTEEDR	GPSRSNRMEM	SIFYVVYFVV	FPFFFVNIFV	ALIIITFQEQ	GDKMMEECSL	EKNERACIDF	AISAKPLTRY
1450	1460	1470	1480	1490	1500	1510	1520
MPQNRHTFQY	RVWHFVVSPS	FEYTIMAMIA	LNTVVLMMKY	YSAPCTYELA	LKYLNIAFTM	VFSLECVLKV	IAFGFLNYFR
1530	1540	1550	1560	1570	1580	1590	1600
DTWNIFDFIT	VIGSITEIIL	TDSKLVNTSG	FNMSFLKLFR	AARLIKLLRQ	GYTIRILLWT	FVQSFKALPY	VCLLIAMLFF
1610	1620	1630	1640	1650	1660	1670	1680
IYAIIGMQVF	GNIKLDEESH	INRHNNFRSF	FGSLMLLFRS	ATGEAWQEIM	LSCLGEKGCE	PDTTAPSGQN	ENERCGTDLA
1690	1700	1710	1720	1730	1740	1750	1760
YVYFVSFIFF	CSFLMLNLFV	AVIMDNFEYL	TRDSSILGPH	HLDEFVRVWA	EYDRAACGRI	HYTEMYEMLT	LMSPPLGLGK
1770	1780	1790	1800	1810	1820	1830	1840
RCPSKVAYKR	LVLMNMPVAE	DMTVHFTSTL	MALIRTALDI	KIAKGGADRQ	QLDSELQKET	LAIWPHLSQK	MLDLLVPMPK
1850	1860	1870	1880	1890	1900	1910	1920
ASDLTVGKIY	AAMMIMDYYK	QSKVKKQRQQ	LEEQKNAPMF	QRMEPSSLPQ	EIIANAKALP	YLQQDPVSGL	SGRSGYPSMS
1930	1940	1950	1960	1970	1980	1990	2000
PLSPQDIFQL	ACMDPADDGQ	FQERQSLVVT	DPSSMRRSFS	TIRDKRSNSS	WLEEFSMERS	SENTYKSRRR	SYHSSLRLSA
2010	2020	2030	2040	2050	2060	2070	2080
HRLNSDSGHK	SDTHRSGGRE	RGRSKERKHL	LSPDVSRCNS	EERGTQADWE	SPERRQSRSP	SEGRSQTPNR	QGTGSLSESS
2090	2100	2110	2120	2130	2140	2150	2160
IPSVSDTSTP	RRSRRQLPPV	PPKPRPLLSY	SSLIRHAGSI	SPPADGSEEG	SPLTSQALES	NNACLTESSN	SPHPQQSQHA
2170	2180	2190	2200	2210	2220	2230	2240

Detailed Protein Report

Cmpd.	No. of Cmpds.	m/z meas.	Δ m/z [ppm]	z	Rt [min]	Score	P	Range	Sequence	Modification
2785	1	973.5222	64.95	2	63.4	10.8	1	734-750	K.EVSPMSAPNMPSIERER.R	Oxidation: 10

Detailed Protein Report

Protein 442: glycosyltransferase-like domain-containing protein 1 isoform d [Homo sapiens]

**Accession:**	gi|545746399	**Score:**	21.0
**Database:**	refseq_human(refseq_human_20140103.fasta)	**MW [kDa]:**	37.7
**Database Date:**	2015-11-30	**pI:**	6.6
**Modification(s):**	Carbamidomethyl, Oxidation	**Sequence Coverage [%]:**	10.6
		**No. of unique Peptides:**	2

Quantitation

WD:WU **Median:** 0.72 **CV:** 0.00 % **No. of Peptides:** 1

10	20	30	40	50	60	70	80
MESFLTSMGK	FMKLIPDHRP	KDLESIIRPK	CQVIYFPIRF	PDVSRFMPKH	KTTHLKKMLG	LKGNGGAVLS	MALPFQPEQR
90	100	110	120	130	140	150	160
DSEDLLKNFN	SECDTHCGLD	TARQEYLGNS	LRQESDLKKS	TSSDNSSSHH	GENKQNLTVD	PCDILGGVDN	QQRLLHIVWP
170	180	190	200	210	220	230	240
HRWEHDKDPE	SFFKVLMHLK	DLGLNFHVSV	LGETFTDVPD	IFSEAKKALG	SSVLHWGYLP	SKDDYFQVLC	MADVVISTAK
250	260	270	280	290	300	310	320
HEFFGVAMLE	AVYCGCYPLC	PKDLVYPEIF	PAEYLYSTPE	QLSKRLQNFC	KRPDIIRKHL	YKGEIAPFSW	AALHGKFRSL
330							
LTTEPREDL							

Cmpd.	No. of Cmpds.	m/z meas.	Δ m/z [ppm]	z	Rt [min]	Score	P	Range	Sequence	Modification	Ratios
48	1	518.2542	17.55	3	29.5	10.4	1	1-13	-.MESFLTSMGKFMK.L	Oxidation: 1	WD:WU 0.72
2608	1	869.7489	29.96	3	60.9	10.6	0	241-262	K. HEFFGVAMLEAVYCGCYPLCPK. D	Carbamidomethyl: 14, 20; Oxidation: 8	

Detailed Protein Report

Protein 443: rho guanine nucleotide exchange factor 33 [Homo sapiens]

Accession: gi|290463441
Database: refseq_human(refseq_human_20140103.fasta)
Database Date: 2015-11-30

Score: 21.0
MW [kDa]: 97.5
pI: 6.4
Sequence Coverage [%]: 3.7
No. of unique Peptides: 2

Quantitation

WD:WU **Median:** 0.64 **CV:** 0.00 % **No. of Peptides:** 1

10	20	30	40	50	60	70	80
MEKTKTKQGE	NEHMPVNNPS	TQIYQLQALA	SELKTGFTEA	MQELSRIQHG	EYALEEKVKS	CRCSMEEKVT	EMKNSLNYFK
90	100	110	120	130	140	150	160
EELSNAMSMI	QAITSKQEEM	QQKIEQLQQE	KRRESRKVKA	KKTQKEEHSS	QAGPAQAQGS	PFRSINIPEP	VLPSEDFTNL
170	180	190	200	210	220	230	240
LPSQAYEKAQ	ESRSVHVGDS	NVKGMMGPGV	NPTTPEAEEN	LKSCLSADIQ	SKGHLPSGMW	RQPKDGKEWG	EEYVTKDHPD
250	260	270	280	290	300	310	320
KLKEAGQGRH	SSLENVLCET	SLAAKRQTVA	LELLESERKY	VINISLILKI	KATFQGSDGK	RNSKERSLFP	GSLRYLVQQH
330	340	350	360	370	380	390	400
LDLLHALQER	VLKWPRQGVL	GDLFLKLTND	ENNFLDYYVA	YLRDLPECIS	LVHVVVLKEG	DEEIKSDIYT	LFFHIVQRIP
410	420	430	440	450	460	470	480
EYLIHLQNVL	KFTEQEHPDY	YLLLVCVQRL	RVFISHYTLL	FQCNEDLLIQ	KRKKLKKSSM	AKLYKGLASQ	CANAGQDASP
490	500	510	520	530	540	550	560
TAGPEAVRDT	GIHSEELLQP	YPSAPSSGPA	ITHLMPPVKK	SQQQQSLMES	MQPGKPSDWE	LEGRKHERPE	SLLAPTQFCA
570	580	590	600	610	620	630	640
AEQDVKALAG	PLQAIPEMDF	ESSPAEPLGN	VERSLRAPAE	LLPDARGFVP	AAYEEFEYGG	EIFALPAPYD	EEPFQAPALF
650	660	670	680	690	700	710	720
ENCSPASSES	SLDICFLRPV	SFAMEAERPE	HPLQPLPKSA	TSPAGSSSAY	KLEAAAQAHG	KAKPLSRSLK	EFPRAPPADG
730	740	750	760	770	780	790	800
VAPRLYSTRS	SSGGRAPIKA	ERAAQAHGPA	AAAVAARGAS	RTFFPQQRSQ	SEKQTYLEVR	REMHLEDTTR	FCPKEERESE
810	820	830	840	850	860	870	880
QTSFSDQNPR	QDQKGGFRSS	FRKLFKKKSS	GSEYREKTNE	NPSMDPSPTK	QDFFRNRLAL	ANDLDQGTAV	

Cmpd.	No. of Cmpds.	m/z meas.	Δ m/z [ppm]	z	Rt [min]	Score	P	Range	Sequence	Modification	Ratios
2130	1	701.3007	-88.45	3	55.2	10.3	2	715-735	R.APPADGVAPRLYSTRSSSGGR.A		
856	1	647.8324	-5.87	2	39.5	10.7	1	758-768	R.GASRTFFPQQR.S		WD:WU 0.64

Detailed Protein Report

Protein 444: spermatogenesis-associated protein 31E1 [Homo sapiens]

Accession: gi|155029550
Database: refseq_human(refseq_human_20140103.fasta)
Database Date: 2015-11-30

Score: 20.9
MW [kDa]: 157.0
pI: 10.3
Sequence Coverage [%]: 1.7
No. of unique Peptides: 1

10	20	30	40	50	60	70	80
MGNLVIPLGK	GRAGRVESGQ	RIPPPAPRPS	VECTGDDIAL	QMEKMLFPLK	SPSATWLSPS	STPWMMDFIL	TSVCGLVLLF
90	100	110	120	130	140	150	160
LLLLYVHSDP	PSPPPGRKRS	SREPQRERSG	RSRSRKISAL	KACRILLREL	EETRDLNYLL	ESHLRKLAGE	GSSHLPLGGD
170	180	190	200	210	220	230	240
PLGDVCKPVP	AKAHQPHGKC	MQDPSPASLS	PPAPPAPLAS	TLSPGPMTFS	EPFGPHSTLS	ASGPPEPLLP	LKCPATQPHV
250	260	270	280	290	300	310	320
VFPPSPQPHG	PLASSPPPPD	SSLAGLQCGS	TTCPVPQSSP	LHNQVLPPPT	RVISGLGCSS	DPIWDLYCWR	EAATTWGLST
330	340	350	360	370	380	390	400
YSHGKSQPRH	LPDHTSEASF	WGDPTPKHME	VGGCTFIHPD	VQKLLETLIA	KRALMKMWQE	KERKRADHPH	MTSLGKEWDI
410	420	430	440	450	460	470	480
TTLNPFWNVS	TQPQQLPRPQ	QVSDATTVGN	HLQQKRSQLF	WDLPSLNSES	LATTVWVSRN	PSSQNAHSVP	LDKASTSLPG
490	500	510	520	530	540	550	560
EPEVEASSQL	SQAPPQPHHM	AQPQHFTPAW	PQSQPPPLAE	IQTQAHLSPP	VPSLGCSSPP	QIRGCGASYP	TSQERTQSVI
570	580	590	600	610	620	630	640
PTGKEYLEWP	LKKRPKWKRV	LPSLLKKSQA	VLSQPTAHLP	QERPASWSPK	SAPILPGVVT	SPELPEHWWQ	GRNAIHQEQS
650	660	670	680	690	700	710	720
CGPPSRLQAS	GDLLQPDGEF	PGRPQSQAED	TQQALLPSQP	SDFAGKGRKD	VQKTGFRSSG	RFSDKGCLGS	KLGPDPSRDQ
730	740	750	760	770	780	790	800
GSGRTSVKAL	DEDKEAEGDL	RRSWKYQSVS	STPRDPDKEH	LENKLQIHLA	RKVGEIKEGW	IPMPVRRSWL	MAKCAVPKSD
810	820	830	840	850	860	870	880
THRKPGKLAS	WRGGKAHVNT	SQELSFLHPC	TQQILEVHLV	RFCVRHSWGT	DLQSLEPINV	WSGEAQAPPF	PQSTFTPWAS
890	900	910	920	930	940	950	960
WVSRVESVPK	VPIFLGKRPQ	NGPGDNRTTS	KSVPTVSGPL	AAPPPEQEGV	QRPPRGSQSA	DTHGRSEAFP	TGHKGRGCSQ
970	980	990	1000	1010	1020	1030	1040
PPTCSLVGRT	WQSRTVLESG	KPKPRLEGSM	GSEMAGNEAW	LESESMSPGD	PCSSRALQVL	SIGSQWARAE	DALQALKVGE
1050	1060	1070	1080	1090	1100	1110	1120
KPPTWEVTLG	ASVRASSGSV	QEDLRSTGAL	GTTGNPSASS	VCVAQDPEQL	HLKAQVVSEI	ALIVQVDSEE	QLPGRAPGIL
1130	1140	1150	1160	1170	1180	1190	1200
LQDGATGLCL	PGRHMDMLTA	ADRLPTQAPL	STSQSVSGKN	MTASQGPCAL	LWKGGDSPGQ	QEPGSPKAKA	PQKSQKTLGC
1210	1220	1230	1240	1250	1260	1270	1280
ADKGEAHRRP	RTGEQGHRSK	GPRTSEASGR	SHPAQAREIG	DKQERKYNQL	QLEKGQTPPE	SHFQRKISHH	PQGLHPRKGG
1290	1300	1310	1320	1330	1340	1350	1360
TRWEDVLQKG	KPGADAFQSW	GSGPPRQFMD	CMADKAWTIS	RVVGQILVDK	LGLQWGRGPS	EVNRHKGDFR	AQENVPSCCH
1370	1380	1390	1400	1410	1420	1430	1440
RGHCHQERSR	EMRALACSPK	ATPKGHHCPV	KNRGIRDRDS	SWAPPPREPV	SPAGPHHHRP	RMASTSGGPH	PQLQELMSAQ
1450							
RCLAS							

Cmpd.	No. of Cmpds.	m/z meas.	Δ m/z [ppm]	z	Rt [min]	Score	P	Range	Sequence	Modification
2055	1	643.7814	-75.01	2	54.1	10.5	1	706-718	K.GCLGSKLGPDPSR.D	

Detailed Protein Report

Protein 445: PREDICTED: A-kinase anchor protein 12 isoform X3 [Homo sapiens]

**Accession:**	gi|530384249	**Score:**	20.9
**Database:**	refseq_human(refseq_human_20140103.fasta)	**MW [kDa]:**	180.9
**Database Date:**	2015-11-30	**pI:**	4.3
		**Sequence Coverage [%]:**	0.7
		**No. of unique Peptides:**	1

10	20	30	40	50	60	70	80
MVGQRDSEDV	SKRDSDKEMA	TKSAVVHDIT	DDGQEETPEI	IEQIPSSESN	LEELTQPTES	QANDIGFKKV	FKFVGFKFTV
90	100	110	120	130	140	150	160
KKDKTEKPDT	VQLLTVKKDE	GEGAAGAGDH	KDPSLGAGEA	ASKESEPKQS	TEKPEETLKR	EQSHAEISPP	AESGQAVEEC
170	180	190	200	210	220	230	240
KEEGEEKQEK	EPSKSAESPT	SPVTSETGST	FKKFFTQGWA	GWRKKTSFRK	PKEDEVEASE	KKKEQEPEKV	DTEEDGKAEV
250	260	270	280	290	300	310	320
ASEKLTASEQ	AHPQEPAESA	HEPRLSAEYE	KVELPSEEQV	SGSQGPSEEK	PAPLATEVFD	EKIEVHQEEV	VAEVHVSTVE
330	340	350	360	370	380	390	400
ERTEEQKTEV	EETAGSVPAE	ELVEMDAEPQ	EAEPAKELVK	LKETCVSGED	PTQGADLSPD	EKVLSKPPEG	VVSEVEMLSS
410	420	430	440	450	460	470	480
QERMKVQGSP	LKKLFTSTGL	KKLSGKKQKG	KRGGGDEESG	EHTQVPADSP	DSQEEQKGES	SASSPEEPEE	ITCLEKGLAE
490	500	510	520	530	540	550	560
VQQDGEAEEG	ATSDGEKKRE	GVTPWASFKK	MVTPKKRVRR	PSESDKEDEL	DKVKSATLSS	TESTASEMQE	EMKGSVEEPK
570	580	590	600	610	620	630	640
PEEPKRKVDT	SVSWEALICV	GSSKKRARRG	SSSDEEGGPK	AMGGDHQKAD	EAGKDKETGT	DGILAGSQEH	DPGQGSSSPE
650	660	670	680	690	700	710	720
QAGSPTEGEG	VSTWESFKRL	VTPRKKSKSK	LEEKSEDSIA	GSGVEHSTPD	TEPGKEESWV	SIKKFIPGRR	KKRPDGKQEQ
730	740	750	760	770	780	790	800
APVEDAGPTG	ANEDDSDVPA	VVPLSEYDAV	EREKMEAQQA	QKSAEQPEQK	AATEVSKELS	ESQVHMMAAA	VADGTRAATI
810	820	830	840	850	860	870	880
IEERSPSWIS	ASVTEPLEQV	EAEAALLTEE	VLEREVIAEE	EPPTVTEPLP	ENREARGDTV	VSEAELTPEA	VTAAETAGPL
890	900	910	920	930	940	950	960
GAEEGTEASA	AEETTEMVSA	VSQLTDSPDT	TEEATPVQEV	EGGVPDIEEQ	ERRTQEVLQA	VAEKVKEESQ	LPGTGGPEDV
970	980	990	1000	1010	1020	1030	1040
LQPVQRAEAE	RPEEQAEASG	LKKETDVVLK	VDAQEAKTEP	FTQGKVVGQT	TPESFEKAPQ	VTESIESSEL	VTTCQAETLA
1050	1060	1070	1080	1090	1100	1110	1120
GVKSQEMVME	QAIPPDSVET	PTDSETDGST	PVADFDAPGT	TQKDEIVEIH	EENEVASGTQ	SGGTEAEAVP	AQKERPPAPS
1130	1140	1150	1160	1170	1180	1190	1200
SFVFQEETKE	QSKMEDTLEH	TDKEVSVETV	SILSKTEGTQ	EADQYADEKT	KDVPFFEGLE	GSIDTGITVS	REKVTEVALK
1210	1220	1230	1240	1250	1260	1270	1280
GEGTEEAECK	KDDALELQSH	AKSPPSPVER	EMVVQVEREK	TEAEPTHVNE	EKLEHETAVT	VSEEVSKQLL	QTVNVPIIDG
1290	1300	1310	1320	1330	1340	1350	1360
AKEVSSLEGS	PPPCLGQEEA	VCTKIQVQSS	EASFTLTAAA	EEEKVLGETA	NILETGETLE	PAGAHLVLEE	KSSEKNEDFA
1370	1380	1390	1400	1410	1420	1430	1440
AHPGEDAVPT	GPDCQAKSTP	VIVSATTKKG	LSSDLEGEKT	TSLKWKSDEV	DEQVACQEVK	VSVAIEDLEP	ENGILELETK
1450	1460	1470	1480	1490	1500	1510	1520
SSKLVQNIIQ	TAVDQFVRTE	ETATEMLTSE	LQTQAHVIKA	DSQDAGQETE	KEGEEPQASA	QDETPITSAK	EESESTAVGQ
1530	1540	1550	1560	1570	1580	1590	1600
AHSDISKDMS	EASEKTMTVE	VEGSTVNDQQ	LEEVVLPSEE	EGGGAGTKSV	PEDDGHALLA	ERIEKSLVEP	KEDEKGDDVD
1610	1620	1630	1640	1650	1660	1670	1680
DPENQNSALA	DTDASGGLTK	ESPDTNGPKQ	KEKEDAQEVE	LQEGKVHSES	DKAITPQAQE	ELQKQERESA	KSELTES

Cmpd.	No. of Cmpds.	m/z meas.	Δ m/z [ppm]	z	Rt [min]	Score	P	Range	Sequence	Modification
2985	2	703.3668	-23.06	2	66.1	20.9	2	498-509	K.KREGVTPWASFK.K	

Detailed Protein Report

Protein 446: polycystic kidney disease protein 1-like 2 isoform d [Homo sapiens]

Accession: gi|509155833
Database: refseq_human(refseq_human_20140103.fasta)
Database Date: 2015-11-30

Score: 20.9
MW [kDa]: 197.3
pI: 6.0
Sequence Coverage [%]: 1.7
No. of unique Peptides: 1

10	20	30	40	50	60	70	80
MGEDSPVAMF	SWYLDNTPTE	QAEPLLDACR	LRGFWPRSLT	LLQSNTSTLL	LNSSFLQSRG	EVIRIRATAL	TRHAYGEDTY
90	100	110	120	130	140	150	160
VISTVPPREV	PACTIAPEEG	TVLTSFAIFC	NASTALGPLE	FCFCLESGSC	LHCGPEPALP	SVYLPLGEEN	NDFVLTVVIS
170	180	190	200	210	220	230	240
ATNRAGDTQQ	TQAMAKVALG	DTCVEDVAFQ	AAVSEKIPTA	LQGEGGPEQL	LQLAKAVSSM	LNQEHESQGS	GQSLSIDVRQ
250	260	270	280	290	300	310	320
KVREHVLGSL	SAVTTGLEDV	QRVQELAEVL	REVTCRSKEL	TPSAQWEASL	ALQHASEALL	TVSAKARPED	QRRQAATRDL
330	340	350	360	370	380	390	400
FQAVGSVLEA	SLSNRPEEPA	EASSSQIATV	LRLLRVMEHV	QTTLLLGKLP	GGLPAMLATP	SISVYTNRIQ	PWSWQGSSLR
410	420	430	440	450	460	470	480
PDAADSATFM	LPAASSLSSL	EGGQEPVDIK	IMSFPKSPFP	ARSHFDVSGT	VGGLRVTSPS	GQLIPVKNLS	ENIEILLPRH
490	500	510	520	530	540	550	560
SQRHSQPTVL	NLTSPEALWV	NVTSGEATLG	IQLHWRPDIA	LTLSLGYGYH	PNKSSYDAQT	HLVPMVAPDE	LPTWILSPQD
570	580	590	600	610	620	630	640
LRFGEGVYYL	TVVPESDLEP	APGRDLTVGI	TTFLSHCVFW	DEVQETWDDS	GCQVGPRTSP	YQTHCLCNHL	TFFGSTFLVM
650	660	670	680	690	700	710	720
SNAINIHQTA	ELFATFEDNP	VVVTTVGCLC	VVYVLVVIWA	RRKDAQDQAK	VKVTVLEDND	PFAQYHYLVT	VYTGHRRGAA
730	740	750	760	770	780	790	800
TSSKVTVTLY	GLDGEREPHH	LADPDTPVFE	RGAVDAFLLS	TLFPLGELRS	LRLWHDNSGD	RPSWYVSRVL	VYDLVMDRKW
810	820	830	840	850	860	870	880
YFLCNSWLSI	NVGDCVLDKV	FPVATEQDRK	QFSHLFFMKT	SAGFQDGHIW	YSIFSRCARS	SFTRVQRVSC	CFSLLLCTML
890	900	910	920	930	940	950	960
TSIMFWGVPK	DPAEQKMDLG	KIEFTWQEVM	IGLESSILMF	PINLLIVQIF	QNTRPRVAKE	QNTGKWDRGS	PNLTPSPQPM
970	980	990	1000	1010	1020	1030	1040
EDGLLTPEAV	TKDVSRIVSS	LFKALKVPSP	ALGWDSVNLM	DINSLLALVE	DVIYPQNTSG	QVFWEEAKKR	EDPVTLTLGS
1050	1060	1070	1080	1090	1100	1110	1120
SEMKEKSQCP	KPKAARSGPW	KDSAYRQCLY	LQLEHVEQEL	RLVGPRGFSQ	PHSHAQALRQ	LQTLKGGLGV	QPGTWAPAHA
1130	1140	1150	1160	1170	1180	1190	1200
SALQVSKPPQ	GLPWWCILVG	WLLVAATSGV	AAFFTMLYGL	HYGRASSLRW	LISMAVSFVE	SMFVTQPLKV	LGFAAFFALV
1210	1220	1230	1240	1250	1260	1270	1280
LKRVDDEEDT	VAPLPGHLLG	PDPYALFRAR	RNSSRDVYQP	PLTAAIEKMK	TTHLKEQKAF	ALIREILAYL	GFLWMLLLVA
1290	1300	1310	1320	1330	1340	1350	1360
YGQRDPSAYH	LNRHLQHSFT	RGFSGVLGFR	EFFKWANTTL	VSNLYGHPPG	FITDGNSKLV	GSAQIRQVRV	QESSCPLAQQ
1370	1380	1390	1400	1410	1420	1430	1440
PQAYLNGCRA	PYSLDAEDMA	DYGEGWNATT	LSEWQYQSQD	QRQGYPIWGK	LTVYRGGGYV	VPLGTDRQST	SRILRYLFDN
1450	1460	1470	1480	1490	1500	1510	1520
TWLDALTRAV	FVESTVYNAN	VNLFCIVTLT	LETSALGTFF	THAALQSLRL	YPFTDGWHPF	VVAAELIYFL	FLLYYMVVQG
1530	1540	1550	1560	1570	1580	1590	1600
KRMSKETWGY	FCSKWNLLEL	AIILASWSAL	AVFVKRAVLA	ERDLQRCRNH	REEGISFSET	AAADAALGYI	IAFLVLLSTV
1610	1620	1630	1640	1650	1660	1670	1680
KLWHLLRLNP	KMNMITAALR	RAWGDISGFM	IVILTMLLAY	SIASNLIFGW	KLRSYKTLFD	AAETMVSLQL	GIFNYEEVLD
1690	1700	1710	1720	1730	1740	1750	1760
YSPVLGSFLI	GSCIVFMTFV	VLNLFISVIL	VAFSEEQKYY	QLSEEGEIVD	LLLMKILSFL	GIKSKREEPG	SSREQPGSLS
1770	1780						
QTRHSRPAQA	LPKD						

Cmpd.	No. of	m/z meas.	Δ m/z	z	Rt	Score	P	Range	Sequence	Modification

Detailed Protein Report

	Cmpds.		[ppm]		[min]					
2783	1	616.3560	86.02	2	63.3	10.8	1	891-901	K.DPAEQKMDLGK.I	

Detailed Protein Report

Protein 447: PREDICTED: zinc finger homeobox protein 3 isoform X1 [Homo sapiens]

**Accession:**	gi|530423920	**Score:**	20.8
**Database:**	refseq_human(refseq_human_20140103.fasta)	**MW [kDa]:**	404.0
**Database Date:**	2015-11-30	**pI:**	5.8
		**Sequence Coverage [%]:**	0.8
		**No. of unique Peptides:**	1

Detailed Protein Report

10	20	30	40	50	60	70	80
MEGCDSPVVS	GKDNGCGIPQ	HQQWTELNST	HLPDKPSSME	QSTGESHGPL	DSLRAPFNER	LAESTASAGP	PSEPASKEVT
90	100	110	120	130	140	150	160
CNECSASFAS	LQTYMEHHCP	SARPPPPLRE	ESASDTGEEG	DEESDVENLA	GEIVYQPDGS	AYIVESLSQL	TQGGGACGSG
170	180	190	200	210	220	230	240
SGSGPLPSLF	LNSLPGAGGK	QGDPSCAAPV	YPQIINTFHI	ASSFGKWFEG	PDQAFPNTSA	LAGLSPVLHS	FRVFDVRHKS
250	260	270	280	290	300	310	320
NKDYLNSDGS	AKSSCVSKDV	PNNVDLSKFD	GFVLYGKRKP	ILMCFLCKLS	FGYVRSFVTH	AVHDHRMTLS	EDERKILSNK
330	340	350	360	370	380	390	400
NISAIIQGIG	KDKEPLVSFL	EPKNKNFQHP	LVSTANLIGP	GHSFYGKFSG	IRMEGEEALP	AGSAAGPEQP	QAGLLTPSTL
410	420	430	440	450	460	470	480
LNLGGLTSSV	LKTPITSVPL	GPLASSPTKS	SEGKDSGAAE	GEKQEVGDGD	CFSEKVEPAE	EEAEEEEEEE	EAEEEEEEEE
490	500	510	520	530	540	550	560
EEEEEEEDEG	CKGLFPSELD	EELEDRPHEE	PGAAAGSSSK	KDLALSNQSI	SNSPLMPNVL	QTLSRGTAST	SSNSASSFVV
570	580	590	600	610	620	630	640
FDGANRRNRL	SFNSEGVRAN	VAEGGRRLDF	ADESANKDNA	TAPEPNESTE	GDDGGFVPHH	QHAGSLCELG	VGECPSGSGV
650	660	670	680	690	700	710	720
ECPKCDTVLG	SSRSLGGHMT	MMHSRNSCKT	LKCPKCNWHY	KYQQTLEAHM	KEKHPEPGGS	CVYCKSGQPH	PRLARGESYT
730	740	750	760	770	780	790	800
CGYKPFRCEV	CNYSTTTKGN	LSIHMQSDKH	LNNMQNLQNG	GGEQVFSHTA	GAAAAAVAAA	AAAANISSSC	GAPSPTKPKT
810	820	830	840	850	860	870	880
KPTWRCEVCD	YETNVARNLR	IHMTSEKHMH	NMMLLQQNMT	QIQHNRHLGL	GSLPSPAEAE	LYQYYLAQNM	NLPNLKMDSA
890	900	910	920	930	940	950	960
ASDAQFMMSG	FQLDPAGPMA	AMTPALVGGE	IPLDMRLGGG	QLVSEELMNL	GESFIQTNDP	SLKLFQCAVC	NKFTTDNLDM
970	980	990	1000	1010	1020	1030	1040
LGLHMNVERS	LSEDEWKAVM	GDSYQCKLCR	YNTQLKANFQ	LHCKTDKHVQ	KYQLVAHIKE	GGKANEWRLK	CVAIGNPVHL
1050	1060	1070	1080	1090	1100	1110	1120
KCNACDYYTN	SLEKLRLHTV	NSRHEASLKL	YKHLQQHESG	VEGESCYYHC	VLCNYSTKAK	LNLIQHVRSM	KHQRSESLRK
1130	1140	1150	1160	1170	1180	1190	1200
LQRLQKGLPE	EDEDLGQIFT	IRRCPSTDPE	AIEDVEGPSE	TAADPEELAK	DQEGGASSSQ	AEKELTDSPA	TSKRISFPGS
1210	1220	1230	1240	1250	1260	1270	1280
SESPLSSKRP	KTAEEIKPEQ	MYQCPYCKYS	NADVNRLRVH	AMTQHSVQPM	LRCPLCQDML	NNKIHLQLHL	THLHSVAPDC
1290	1300	1310	1320	1330	1340	1350	1360
VEKLIMTVTT	PEMVMPSSMF	LPAAVPDRDG	NSNLEEAGKQ	PETSEDLGKN	ILPSASTEQS	GDLKPSPADP	GSVREDSGFI
1370	1380	1390	1400	1410	1420	1430	1440
CWKKGCNQVF	KTSAALQTHF	NEVHAKRPQL	PVSDRHVYKY	RCNQCSLAFK	TIEKLQLHSQ	YHVIRAATMC	CLCQRSFRTF
1450	1460	1470	1480	1490	1500	1510	1520
QALKKHLETS	HLELSEADIQ	QLYGGLLANG	DLLAMGDPTL	AEDHTIIVEE	DKEEESDLED	KQSPTGSDSG	SVQEDSGSEP
1530	1540	1550	1560	1570	1580	1590	1600
KRALPFRKGP	NFTMEKFLDP	SRPYKCTVCK	ESFTQKNILL	VHYNSVSHLH	KLKRALQESA	TGQPEPTSSP	DNKPFKCNTC
1610	1620	1630	1640	1650	1660	1670	1680
NVAYSQSSTL	EIHMRSVLHQ	TKARAAKLEA	ASGSSNGTGN	SSSISLSSST	PSPVSTSGSN	TFTTSNPSSA	GIAPSSNLLS
1690	1700	1710	1720	1730	1740	1750	1760
QVPTESVGMP	PLGNPIGANI	ASPSEPKEAN	RKKLADMIAS	RQQQQQQQQQ	QQQQQQQQQQ	AQTLAQAQAQ	VQAHLQQELQ
1770	1780	1790	1800	1810	1820	1830	1840
QQAALIQSQL	FNPTLLPHFP	MTTETLLQLQ	QQQHLLFPFY	IPSAEFQLNP	EVSLPVTSGA	LTLTGTGPGL	LEDLKAQVQV
1850	1860	1870	1880	1890	1900	1910	1920
PQQSHQQILP	QQQQNQLSIA	QSHSALLQPS	QHPEKKNKLV	IKEKEKESQR	ERDSAEGGEG	NTGPKETLPD	ALKAKEKKEL
1930	1940	1950	1960	1970	1980	1990	2000
APGGGSEPSM	LPPRIASDAR	GNATKALLEN	FGFELVIQYN	ENKQKVQKKN	GKTDQGENLE	KLECDSCGKL	FSNILILKSH
2010	2020	2030	2040	2050	2060	2070	2080
QEHVHQNYFP	FKQLERFAKQ	YRDHYDKLYP	LRPQTPEPPP	PPPPPPPPPL	PAAPPQPAST	PAIPASAPPI	TSPTIAPAQP
2090	2100	2110	2120	2130	2140	2150	2160
SVPLTQLSMP	MELPIFSPLM	MQTMPLQTLP	AQLPPQLGPV	EPLPADLAQL	YQHQLNPTLL	QQQNKRPRTR	ITDDQLRVLR
2170	2180	2190	2200	2210	2220	2230	2240
QYFDINNSPS	EEQIKEMADK	SGLPQKVIKH	WFRNTLFKER	QRNKDSPYNF	SNPPITSLEE	LKIDSRPPSP	EPPKQEYWGS
2250	2260	2270	2280	2290	2300	2310	2320
KRSSRTRFTD	YQLRVLQDFF	DANAYPKDDE	FEQLSNLLNL	PTRVIVVWFQ	NARQKARKNY	ENQGEGKDGE	RRELTNDRYI
2330	2340	2350	2360	2370	2380	2390	2400

Detailed Protein Report

Cmpd.	No. of Cmpds.	m/z meas.	Δ m/z [ppm]	z	Rt [min]	Score	P	Range	Sequence	Modification
2701	1	973.4995	-53.38	2	62.2	10.8	1	2643-2658	R.LRTTITPEQLEILYQK.Y	

Detailed Protein Report

Protein 448: transmembrane protein 70, mitochondrial isoform a [Homo sapiens]

**Accession:**	gi|34147498	**Score:**	20.8
**Database:**	refseq_human(refseq_human_20140103.fasta)	**MW [kDa]:**	29.0
**Database Date:**	2015-11-30	**pI:**	9.6
		**Sequence Coverage [%]:**	10.8
		**No. of unique Peptides:**	1

10	20	30	40	50	60	70	80
MLFLALGSPW	AVELPLCGRR	TALCAAAALR	GPRASVSRAS	SSSGPSGPVA	GWSTGPSGAA	RLLRRPGRAQ	IPVYWEGYVR
**90**	**100**	**110**	**120**	**130**	**140**	**150**	**160**
FLNTPSDKSE	DGRLIYTGNM	ARAVFGVKCF	SYSTSLIGLT	FLPYIFTQNN	AISESVPLPI	QIIFYGIMGS	FTVITPVLLH
**170**	**180**	**190**	**200**	**210**	**220**	**230**	**240**
FITKGYVIRL	YHEATTDTYK	AITYNAMLAE	TSTVFHQNDV	KIPDAKHVFT	TFYAKTKSLL	VNPVLFPNRE	DYIHLMGYDK
**250**	**260**	**270**					
EEFILYMEET	SEEKRHKDDK						

Cmpd.	No. of Cmpds.	m/z meas.	Δ m/z [ppm]	z	Rt [min]	Score	P	Range	Sequence	Modification
1713	1	820.3878	-79.60	2	50.1	10.2	1	94-108	R.LIYTGNMARAVFGVK.C	

Detailed Protein Report

Protein 449: serine/threonine-protein kinase WNK3 isoform 2 [Homo sapiens]

**Accession:**	gi|50845416	**Score:**	20.8
**Database:**	refseq_human(refseq_human_20140103.fasta)	**MW [kDa]:**	191.7
**Database Date:**	2015-11-30	**pI:**	5.4
		**Sequence Coverage [%]:**	1.2
		**No. of unique Peptides:**	1

Alias proteins:

Accession	Name	Description
gi|578838231	refseq_human (refseq_human_20140103.fasta)	PREDICTED: serine/threonine-protein kinase WNK3 isoform X2 [Homo sapiens]
gi|578838229	refseq_human (refseq_human_20140103.fasta)	PREDICTED: serine/threonine-protein kinase WNK3 isoform X1 [Homo sapiens]

BRUKER

Detailed Protein Report

10	20	30	40	50	60	70	80
MATDSGDPAS	TEDSEKPDGI	SFENRVPQVA	ATLTVEARLK	EKNSTFSASG	ETVERKRFFR	KSVEMTEDDK	VAESSPKDER
90	100	110	120	130	140	150	160
IKAAMNIPRV	DKLPSNVLRG	GQEVKYEQCS	KSTSEISKDC	FKEKNEKEME	EEAEMKAVAT	SPSGRFLKFD	IELGRGAFKT
170	180	190	200	210	220	230	240
VYKGLDTETW	VEVAWCELQD	RKLTKAEQQR	FKEEAEMLKG	LQHPNIVRFY	DSWESILKGK	KCIVLVTELM	TSGTLKTYLK
250	260	270	280	290	300	310	320
RFKVMKPKVL	RSWCRQILKG	LQFLHTRTPP	IIHRDLKCDN	IFITGPTGSV	KIGDLGLATL	MRTSFAKSVI	GTPEFMAPEM
330	340	350	360	370	380	390	400
YEEHYDESVD	VYAFGMCMLE	MATSEYPYSE	CQNAAQIYRK	VTSGIKPASF	NKVTDPEVKE	IIEGCIRQNK	SERLSIRDLL
410	420	430	440	450	460	470	480
NHAFFAEDTG	LRVELAEEDD	CSNSSLALRL	WVEDPKKLKG	KHKDNEAIEF	SFNLETDTPE	EVAYEMVKSG	FFHESDSKAV
490	500	510	520	530	540	550	560
AKSIRDRVTP	IKKTREKKPA	GCLEERRDSQ	CKSMGNVFPQ	PQNTTLPLAP	AQQTGAECEE	TEVDQHVRQQ	LLQRKPQQHC
570	580	590	600	610	620	630	640
SSVTGDNLSE	AGAASVIHSD	TSSQPSVAYS	SNQTMGSQMV	SNIPQAEVNV	PGQIYSSQQL	VGHYQQVSGL	QKHSKLTQPQ
650	660	670	680	690	700	710	720
ILPLVQGQST	VLPVHVLGPT	VVSQPQVSPL	TVQKVPQIKP	VSQPVGAEQQ	AALLKPDLVR	SLNQDVATTK	ENVSSPDNPS
730	740	750	760	770	780	790	800
GNGKQDRIKQ	RRASCPRPEK	GTKFQLTVLQ	VSTSGDNMVE	CQLETHNNKM	VTFKFDVDGD	APEDIADYMV	EDNFVLESEK
810	820	830	840	850	860	870	880
EKFVEELRAI	VGQAQEILHV	HFATERATGV	DSITVDSNSS	QTGSSEQVQI	NSTSTQTSNE	SAPQSSPVGR	WRFCINQTIR
890	900	910	920	930	940	950	960
NRETQSPPSL	QHSMSAVPGR	HPLPSPKNTS	NKEISRDTLL	TIENNPCHRA	LFTSKSEHKD	VVDGKISECA	SVETKQPAIL
970	980	990	1000	1010	1020	1030	1040
YQVEDNRQIM	APVTNSSSYS	TTSVRAVPAE	CEGLTKQASI	FIPVYPCHQT	ASQADALMSH	PGESTQTSGN	SLTTLAFDQK
1050	1060	1070	1080	1090	1100	1110	1120
PQTLSVQQPA	MDAEFISQEG	ETTVNTEASS	PKTVIPTQTP	GLEPTTLQPT	TVLESDGERP	PKLEFADNRI	KTLDEKLRNL
1130	1140	1150	1160	1170	1180	1190	1200
LYQEHSISSI	YPESQKDTQS	IDSPFSSSAE	DTLSCPVTEV	IAISHCGIKD	SPVQSPNFQQ	TGSKLLSNVA	ASQPANISVF
1210	1220	1230	1240	1250	1260	1270	1280
KRDLNVITSV	PSELCLHEMS	SDASLPGDPE	AYPAAVSSGG	AIHLQTGVET	EEMRSAIAPD	PIPLTRESTA	DTRALNRCKA
1290	1300	1310	1320	1330	1340	1350	1360
MSGSFQRGRF	QVITIPQQQS	AKMTSFGIEH	ISVFSETNHS	SEEAFIKTAK	SQLVEIEPAT	QNPKTSFSYE	KLQALQETCK
1370	1380	1390	1400	1410	1420	1430	1440
ENKGVPKQGD	NFLSFSAACE	TDVSSVTPEK	EFEETSATGS	SMQSGSELLL	KEREILTAGK	QPSSDSEFSA	SLAGSGKSVA
1450	1460	1470	1480	1490	1500	1510	1520
KTGPESNQCL	PHHEEQAYAQ	TQSSLFYSPS	SPMSSDDESE	IEDEDLKVEL	QRLREKHIQE	VVNLQTQQNK	ELQELYERLR
1530	1540	1550	1560	1570	1580	1590	1600
SIKDSKTQST	EIPLPPASPR	RPRSFKSKLR	SRPQSLTHVD	NGIVATDPLC	VESNAASCQQ	SPASKKGMFT	DDLHKLVDDW
1610	1620	1630	1640	1650	1660	1670	1680
TKEAVGNSLI	KPSLNQLKQS	QHKLETENWN	KVSENTPSTM	GYTSTWISSL	SQIRGAVPTS	LPQGLSLPSF	PGPLSSYGMP
1690	1700	1710	1720	1730	1740	1750	
HVCQYNAVAG	AGYPVQWVGI	SGTTQQSVVI	PAQSGGPFQP	GMNMQAFPTS	SVQNPATIPP	GPK	

Cmpd.	No. of Cmpds.	m/z meas.	Δ m/z [ppm]	z	Rt [min]	Score	P	Range	Sequence	Modification
1147	1	956.4746	-119.29	1	43.0	10.6	2	486-493	R.DRVTPIKK.T	

Detailed Protein Report

Protein 450: PREDICTED: uncharacterized protein LOC101928193 [Homo sapiens]

**Accession:**	gi|530427280	**Score:**	20.8
**Database:**	refseq_human(refseq_human_20140103.fasta)	**MW [kDa]:**	65.8
**Database Date:**	2015-11-30	**pI:**	10.1
**Modification(s):**	Oxidation	**Sequence Coverage [%]:**	3.8
		**No. of unique Peptides:**	2

10	20	30	40	50	60	70	80
MKVLVLGLAL	AVSWELGLEL	VPELILAVVL	GQRLALPLEF	VLELDLPPLM	VLELFLELLV	ALKLVLGPSV	ALQLVRELLL
**90**	**100**	**110**	**120**	**130**	**140**	**150**	**160**
ARALPLELMR	EPVLPLALPL	NLALGLAPCL	VVEQREELWL	QSPETENLKG	LPLYSRFTGF	TVIFQGPGVH	HHIPGVWGSL
**170**	**180**	**190**	**200**	**210**	**220**	**230**	**240**
SYSRCLGFIV	VFQVSGVHHC	IPGVWGSLSY	SRCLGFTVIF	QGPGVHHHIP	EVWSSPSYSR	CLAFTVVFQV	SGVHHCIPGV
**250**	**260**	**270**	**280**	**290**	**300**	**310**	**320**
WGSLSYSRCL	GFTVVFQVSG	VHCHIPGAWG	SPSYSRCLGL	TVVFQVSGVH	CHILGAWGSP	PYSRCLGFTV	VFQVSGVHRH
**330**	**340**	**350**	**360**	**370**	**380**	**390**	**400**
IPGAWVHCYI	PGVWDSLSYS	RCLGFTVIFQ	GPGVHHHIPG	VWGSLLYSRC	LGFTVIFQGP	GVHHHIPGVW	RSLSYSRCLG
**410**	**420**	**430**	**440**	**450**	**460**	**470**	**480**
FTIVFQVSGA	HCCIPGVWGS	LLYSRCLGFT	VIFQGPGVHH	HIPGVWRSLS	YSRCRVHRCI	PGVWGSLSYS	RCLGFTVIFQ
**490**	**500**	**510**	**520**	**530**	**540**	**550**	**560**
VPGFTVIFQV	SGIHCHIPGV	WGSLLYSRCL	GFTLIFQVSG	VHCCIPGVWA	HHRIPGAWGS	PSYSRGLGFI	IVFQGSGIHH
**570**	**580**	**590**	**600**				
HIPGAWGSLL	YSRCLRLTVI	FQVSAYNREQ	HTGAECMND				

Cmpd.	No. of Cmpds.	m/z meas.	Δ m/z [ppm]	z	Rt [min]	Score	P	Range	Sequence	Modification
2754	1	639.3520	54.35	2	62.8	10.3	0	265-276	H.IPGAWGSPSYSR.C	
2636	1	625.6343	-143.74	2	61.2	10.5	0	589-599	R.EQHTGAECMND.-	Oxidation: 9

Detailed Protein Report

Protein 451: latent-transforming growth factor beta-binding protein 4 isoform b [Homo sapiens]

**Accession:**	gi|110347412	**Score:**	20.8
**Database:**	refseq_human(refseq_human_20140103.fasta)	**MW [kDa]:**	169.3
**Database Date:**	2015-11-30	**pI:**	5.0
		**Sequence Coverage [%]:**	1.7
		**No. of unique Peptides:**	2

Quantitation

WD:WU **Median:** 0.86 **CV:** 0.00 % **No. of Peptides:** 1

10	20	30	40	50	60	70	80
MGDVKALLFV	AAARARRLGG	AAASESLAVS	EAFCRVRSCQ	PKKCAGPQRC	LNPVPAVPSP	SPSVRKRQVS	LNWQPLTLQE
90	100	110	120	130	140	150	160
ARALLKRRRP	RGPGGRGLLR	RRPPQRAPAG	KAPVLCPLIC	HNGGVCVKPD	RCLCPPDFAG	KFCQLHSSGA	RPPAPAVPGL
170	180	190	200	210	220	230	240
TRSVYTMPLA	NHRDDEHGVA	SMVSVHVEHP	QEASVVVHQV	ERVSGPWEEA	DAEAVARAEA	AARAEAAAPY	TVLAQSAPRE
250	260	270	280	290	300	310	320
DGYSDASGFG	YCFRELRGGE	CASPLPGLRT	QEVCCRGAGL	AWGVHDCQLC	SERLGNSERV	SAPDGPCPTG	FERVNGSCED
330	340	350	360	370	380	390	400
VDECATGGRC	QHGECANTRG	GYTCVCPDGF	LLDSSRSSCI	SQHVISEAKG	PCFRVLRDGG	CSLPILRNIT	KQICCCSRVG
410	420	430	440	450	460	470	480
KAWGRGCQLC	PPFGSEGFRE	ICPAGPGYHY	SASDLRYNTR	PLGQEPPRVS	LSQPRTLPAT	SRPSAGFLPT	HRLEPRPEPR
490	500	510	520	530	540	550	560
PDPRPGPELP	LPSIPAWTGP	EIPESGPSSG	MCQRNPQVCG	PGRCISRPSG	YTCACDSGFR	LSPQGTRCID	VDECRRVPPP
570	580	590	600	610	620	630	640
CAPGRCENSP	GSFRCVCGPG	FRAGPRAAEC	LDVDECHRVP	PPCDLGRCEN	TPGSFLCVCP	AGYQAAPHGA	SCQDVDECTQ
650	660	670	680	690	700	710	720
SPGLCGRGAC	KNLPGSFRCV	CPAGFRGSAC	EEDVDECAQE	PPPCGPGRCD	NTAGSFHCAC	PAGFRSRGPG	APCQDVDECA
730	740	750	760	770	780	790	800
RSPPPCTYGR	CENTEGSFQC	VCPMGFQPNT	AGSECEDVDE	CENHLACPGQ	ECVNSPGSFQ	CRTCPSGHHL	HRGRCTDVDE
810	820	830	840	850	860	870	880
CSSGAPPCGP	HGHCTNTEGS	FRCSCAPGYR	APSGRPGPCA	DVNECLEGDF	CFPHGECLNT	DGSFACTCAP	GYRPGPRGAS
890	900	910	920	930	940	950	960
CLDVDECSEE	DLCQSGICTN	TDGSFECICP	PGHRAGPDLA	SCLDVDECRE	RGPALCGSQR	CENSPGSYRC	VRDCDPGYHA
970	980	990	1000	1010	1020	1030	1040
GPEGTCDDVD	ECQEYGPEIC	GAQRCENTPG	SYRCTPACDP	GYQPTPGGGC	QDVDECRNRS	FCGAHAVCQN	LPGSFQCLCD
1050	1060	1070	1080	1090	1100	1110	1120
QGYEGARDGR	HCVDVNECET	LQGVCGAALC	ENVEGSFLCV	CPNSPEEFDP	MTGRCVPPRT	SAGTFPGSQP	QAPASPVLPA
1130	1140	1150	1160	1170	1180	1190	1200
RPPPPPLPRR	PSTPRQGPVG	SGRRECYFDT	AAPDACDNIL	ARNVTWQECC	CTVGEGWGSG	CRIQQCPGTE	TAEYQSLCPH
1210	1220	1230	1240	1250	1260	1270	1280
GRGYLAPSGD	LSLRRDVDEC	QLFRDQVCKS	GVCVNTAPGY	SCYCSNGYYY	HTQRLECIDN	DECADEEPAC	EGGRCVNTVG
1290	1300	1310	1320	1330	1340	1350	1360
SYHCTCEPPL	VLDGSQRRCV	SNESQSLDDN	LGVCWQEVGA	DLVCSHPRLD	RQATYTECCC	LYGEAWGMDC	ALCPAQDSDD
1370	1380	1390	1400	1410	1420	1430	1440
FEALCNVLRP	PAYSPPRPGG	FGLPYEYGPD	LGPPYQGLPY	GPELYPPPAL	PYDPYPPPPG	PFARREAPYG	APRFDMPDFE
1450	1460	1470	1480	1490	1500	1510	1520
DDGGPYGESE	APAPPGPGTR	WPYRSRDTRR	SFPEPEEPPE	GGSYAGSLAE	PYEELEAEEC	GILDGCTNGR	CVRVPEGFTC
1530	1540	1550	1560	1570	1580	1590	
RCFDGYRLDM	TRMACVDINE	CDEAEAASPL	CVNARCLNTD	GSFRCICRPG	FAPTHQPHHC	APARPRA	

Cmpd.	No. of Cmpds.	m/z meas.	Δ m/z [ppm]	z	Rt [min]	Score	P	Range	Sequence	Modification	Ratios
398	1	646.4124	27.70	2	34.0	10.5	2	89-100	R.RPRGPGGRGLLR.R		
407	1	846.5265	154.58	2	33.7	10.2	1	541-555	R.LSPQGTRCIDVDECR.R		WD:WU 0.86

Detailed Protein Report

Protein 452: zinc finger protein 467 [Homo sapiens]

Accession: gi|46409310
Database: refseq_human(refseq_human_20140103.fasta)
Database Date: 2015-11-30
Modification(s): Carbamidomethyl

Score: 20.7
MW [kDa]: 65.1
pI: 11.0
Sequence Coverage [%]: 4.0
No. of unique Peptides: 2

Alias proteins:

Accession	Name	Description
gi|578813959	refseq_human (refseq_human_20140103.fasta)	PREDICTED: zinc finger protein 467 isoform X6 [Homo sapiens]
gi|530386928	refseq_human (refseq_human_20140103.fasta)	PREDICTED: zinc finger protein 467 isoform X3 [Homo sapiens]
gi|530386926	refseq_human (refseq_human_20140103.fasta)	PREDICTED: zinc finger protein 467 isoform X2 [Homo sapiens]
gi|530386924	refseq_human (refseq_human_20140103.fasta)	PREDICTED: zinc finger protein 467 isoform X1 [Homo sapiens]

```
        10         20         30         40         50         60         70         80
MRETLEALSS LGFSVGQPEM APQSEPREGS HNAQEQMSSS REERALGVCS GHEAPTPEEG AHTEQAEAPC RGQACSAQKA
        90        100        110        120        130        140        150        160
QPVGTCPGEE WMIRKVKVED EDQEAEEEVE WPQHLSLLPS PFPAPDLGHL AAAYKLEPGA PGALSGLALS GWGPMPEKPY
       170        180        190        200        210        220        230        240
GCGECERRFR DQLTLRLHQR LHRGEGPCAC PDCGRSFTQR AHMLLHQRSH RGERPFPCSE CDKRFSKKAH LTRHLRTHTG
       250        260        270        280        290        300        310        320
ERPYPCAECG KRFSQKIHLG SHQKTHTGER PFPCTECEKR FRKKTHLIRH QRIHTGERPY QCAQCARSFT HKQHLVRHQR
       330        340        350        360        370        380        390        400
VHQTAGPARP SPDSSASPHS TAPSPTPSFP GPKPFACSDC GLSFGWKKNL ATHQCLHRSE GRPFGCDECA LGATVDAPAA
       410        420        430        440        450        460        470        480
KPLASAPGGP GCGPGSDPVV PQRAPSGERS FFCPDCGRGF SHGQHLARHP RVHTGERPFA CTQCDRRFGS RPNLVAHSRA
       490        500        510        520        530        540        550        560
HSGARPFACA QCGRRFSRKS HLGRHQAVHT GSRPHACAVC ARSFSSKTNL VRHQAIHTGS RPFSCPQCGK SFSRKTHLVR
       570        580        590        600
HQLIHGEAAH AAPDAALAAP AWSAPPEVAP PPLFF
```

Cmpd.	No. of Cmpds.	m/z meas.	Δ m/z [ppm]	z	Rt [min]	Score	P	Range	Sequence	Modification
1902	1	785.7933	-55.89	2	52.1	10.0	1	181-195	R.LHRGEGPCACPDCGR.S	
340	3	544.7855	123.91	2	32.9	10.7	0	430-438	R.SFFCPDCGR.G	Carbamidomethyl: 7

Detailed Protein Report

Protein 453: **trinucleotide repeat-containing gene 6B protein isoform 3 [Homo sapiens]**

**Accession:**	gi|67782330	**Score:**	20.7
**Database:**	refseq_human(refseq_human_20140103.fasta)	**MW [kDa]:**	109.3
**Database Date:**	2015-11-30	**pI:**	7.0
		**Sequence Coverage [%]:**	2.3
		**No. of unique Peptides:**	1

Quantitation

WD:WU **Median:** 1.32 **CV:** 0.00 % **No. of Peptides:** 1

10	20	30	40	50	60	70	80
MQTNEGEVSE	ESSSKVEQED	FVMEGHGKTP	PPGEESKQEK	EQEREEQLME	DKKRKKEDKK	KKEATQKVTE	QKTKVPEVTK
90	100	110	120	130	140	150	160
PSLSQPTAAS	PIGSSPSPPV	NGGNNAKRVA	VPNGQPPSAA	RYMPREVPPR	FRCQQDHKVL	LKRGQPPPPS	CMLLGGGAGP
170	180	190	200	210	220	230	240
PPCTAPGANP	NNAQVTGALL	QSESGTAPVW	SKSTPPAPDN	GTSAWGEPNE	SSPGWGEMDD	TGASTTGWGN	TPANAPNAMK
250	260	270	280	290	300	310	320
PNSKSMQDGW	GESDGPVTGA	RHPSWEEEED	GGVWNTTGSQ	GSASSHNSAS	WGQGGKKQMK	CSLKGGNNDS	WMNPLAKQFS
330	340	350	360	370	380	390	400
NMGLLSQTED	NPSSKMDLSV	GSLSDKKFDV	DKRAMNLGDF	NDIMRKDRSG	FRPPNSKDMG	TTDSGPYFEK	GGSHGLFGNS
410	420	430	440	450	460	470	480
TAQSRGLHTP	VQPLNSSPSL	RAQVPPQFIS	PQVSASMLKQ	FPNSGLSPGL	FNVGPQLSPQ	QIAMLSQLPQ	IPQFQLACQL
490	500	510	520	530	540	550	560
LLQQQQQQQL	LQNQRKISQA	VRQQQEQQLA	RMVSALQQQQ	QQQQRQPGMK	HSPSHPVGPK	PHLDNMVPNA	LNVGLPDLQT
570	580	590	600	610	620	630	640
KGPIPGYGSG	FSSGGMDYGM	VGGKEAGTES	RFKQWTSMME	GLPSVATQEA	NMHKNGAIVA	PGKTRGGSPY	NQFDIIPGDT
650	660	670	680	690	700	710	720
LGGHTGPAGD	SWLPAKSPPT	NKIGSKSSNA	SWPPEFQPGV	PWKGIQNIDP	ESDPYVTPGS	VLGGTATSPI	VDTDHQLLRD
730	740	750	760	770	780	790	800
NTTGSNSSLN	TSLPSPGAWP	YSASDNSFTN	VHSTSAKFPD	YKSTWSPDPI	GHNPTHLSNK	MWKNHISSRN	TTPLPRPPPG
810	820	830	840	850	860	870	880
LTNPKPSSPW	SSTAPRSVRG	WGTQDSRLAS	ASTWSDGGSV	RPSYWLVLHN	LTPQIDGSTL	RTICMQHGPL	LTFHLNLTQG
890	900	910	920	930	940	950	960
TALIRYSTKQ	EAAKAQTALH	MCVLGNTTIL	AEFATDDEVS	RFLAQAQPPT	PAATPSAPAA	GWQSLETGQN	QSDPVGPALN
970	980	990	1000	1010	1020	1030	
LFGGSTGLGQ	WSSSAGGSSG	ADLAGASLWG	PPNYSSSLWG	VPTVEDPHRM	GSPAPLLPGD	LLGGGSDSI	

Cmpd.	No. of Cmpds.	m/z meas.	Δ m/z [ppm]	z	Rt [min]	Score	P	Range	Sequence	Modification	Ratios
325	1	564.6741	-207.76	2	32.8	10.1	0	503-511	R.QQQEQQLAR.M		WD:WU 1.32

Detailed Protein Report

Protein 454: PREDICTED: diphthine--ammonia ligase isoform X1 [Homo sapiens]

**Accession:**	gi|530406619	**Score:**	20.6
**Database:**	refseq_human(refseq_human_20140103.fasta)	**MW [kDa]:**	40.1
**Database Date:**	2015-11-30	**pI:**	7.8
		**Sequence Coverage [%]:**	4.4
		**No. of unique Peptides:**	1

Quantitation

WD:WU **Median:** 2.21 **CV:** 0.00 % **No. of Peptides:** 1

10	20	30	40	50	60	70	80
MSLPQGQGAK	VPHPPRSGVP	QAAAGLAAPI	RGQAAERTPG	RGPAVRHASC	GATSWLPPAL	AARTRTAPSM	RVAALISGGK
90	100	110	120	130	140	150	160
DSCYNMMQCI	AAGHQIVALA	NLRPAENQVG	SDELDSYMYQ	TVGHHAIDLY	AEAMALPLYR	RTIRGRSLDT	RQVYTKCEGD
170	180	190	200	210	220	230	240
EVEDLYELLK	LVKEKEEVEG	ISVGAILSDY	QRIRVENVCK	RLNLQPLAYL	WQRNQEDLLR	EMISSNIQAM	IIKVAALGLD
250	260	270	280	290	300	310	320
PDKHLGKTLD	QMEPYLIELS	KKYGVHVCGE	GGEYETFTLD	CPLFKKKIIV	DSSEVVIHSA	DAFAPVAYLR	FLELHLEDKA
330	340	350	360	370			
KEQKSDLSRW	SKVFTSLKPN	AVKLNETTWG	LNGCWAVGTA	GSLLL			

Cmpd.	No. of Cmpds.	m/z meas.	Δ m/z [ppm]	z	Rt [min]	Score	P	Range	Sequence	Modification	Ratios
1742	1	480.7260	-88.12	2	50.1	10.3	1	193-200	R.IRVENVCK.R		WD:WU 2.21

Detailed Protein Report

Protein 455: RNA-binding protein 12B [Homo sapiens]

**Accession:**	gi|118722349	**Score:**	20.6
**Database:**	refseq_human(refseq_human_20140103.fasta)	**MW [kDa]:**	118.0
**Database Date:**	2015-11-30	**pI:**	6.3
		**Sequence Coverage [%]:**	0.7
		**No. of unique Peptides:**	1

Alias proteins:

Accession	Name	Description
gi|578815970	refseq_human (refseq_human_20140103.fasta)	PREDICTED: RNA-binding protein 12B isoform X5 [Homo sapiens]
gi|578815966	refseq_human (refseq_human_20140103.fasta)	PREDICTED: RNA-binding protein 12B isoform X4 [Homo sapiens]
gi|578815964	refseq_human (refseq_human_20140103.fasta)	PREDICTED: RNA-binding protein 12B isoform X3 [Homo sapiens]
gi|530389016	refseq_human (refseq_human_20140103.fasta)	PREDICTED: RNA-binding protein 12B isoform X2 [Homo sapiens]
gi|530389014	refseq_human (refseq_human_20140103.fasta)	PREDICTED: RNA-binding protein 12B isoform X1 [Homo sapiens]

10	20	30	40	50	60	70	80
MAVVIRLLGL	PFIAGPVDIR	HFFTGLTIPD	GGVHIIGGEI	GEAFIIFATD	EDARRAISRS	GGFIKDSSVE	LFLSSKAEMQ
90	100	110	120	130	140	150	160
KTIEMKRTDR	VGRGRPGSGT	SGVDSLSNFI	ESVKEEASNS	GYGSSINQDA	GFHTNGTGHG	NLRPRKTRPL	KAENPYLFLR
170	180	190	200	210	220	230	240
GLPYLVNEDD	VRVFFSGLCV	DGVIFLKHHD	GRNNGDAIVK	FASCVDASGG	LKCHRSFMGS	RFIEVMQGSE	QQWIEFGGNA
250	260	270	280	290	300	310	320
VKEGDVLRRS	EEHSPPRGIN	DRHFRKRSHS	KSPRRTRSRS	PLGFYVHLKN	LSLSIDERDL	RNFFRGTDLT	DEQIRFLYKD
330	340	350	360	370	380	390	400
ENRTRYAFVM	FKTLKDYNTA	LSLHKTVLQY	RPVHIDPISR	KQMLKFIARY	EKKRSGSLER	DRPGHVSQKY	SQEGNSGQKL
410	420	430	440	450	460	470	480
CIYIRNFPFD	VTKVEVQKFF	ADFLLAEDDI	YLLYDDKGVG	LGEALVKFKS	EEQAMKAERL	NRRRFLGTEV	LLRLISEAQI
490	500	510	520	530	540	550	560
QEFGVNFSVM	SSEKMQARSQ	SRERGDHSHL	FDSKDPPIYS	VGAFENFRHQ	LEDLRQLDNF	KHPQRDFRQP	DRHPPEDFRH
570	580	590	600	610	620	630	640
SSEDFRFPPE	DFRHSPEDFR	RPREEDFRRP	SEEDFRRPWE	EDFRRPPEDD	FRHPREEDWR	RPLEEDWRRP	LEEDFRRSPT
650	660	670	680	690	700	710	720
EDFRQLPEED	FRQPPEEDLR	WLPEEDFRRP	PEEDWRRPPE	EDFRRPLQGE	WRRPPEDDFR	RPPEEDFRHS	PEEDFRQSPQ
730	740	750	760	770	780	790	800
EHFRRPPQEH	FRRPPPEHFR	RPPPEHFRRP	PPEHFRRPPP	EHFRRPPPEH	FRRPPPEHFR	RPPQEHFRRP	PQEHFRRSRE
810	820	830	840	850	860	870	880
EDFRHPPDED	FRGPPDEDFR	HPPDEDFRSP	QEEDFRCPSD	EDFRQLPEED	LREAPEEDPR	LPDNFRPPGE	DFRSPPDDFR
890	900	910	920	930	940	950	960
SHRPFVNFGR	PEGGKFDFGK	HNMGSFPEGR	FMPDPKINCG	SGRVTPIKIM	NLPFKANVNE	ILDFFHGYRI	IPDSVSIQYN
970	980	990	1000	1010			
EQGLPTGEAI	VAMINYNEAM	AAIKDLNDRP	VGPRKVKLTL	L			

Cmpd.	No. of Cmpds.	m/z meas.	Δ m/z [ppm]	z	Rt [min]	Score	P	Range	Sequence	Modification
2646	1	439.1044	-203.15	2	61.4	20.6	0	560-566	R.HSSEDFR.F	

Detailed Protein Report

Protein 456: **latent-transforming growth factor beta-binding protein 4 isoform c precursor [Homo sapiens]**

**Accession:**	gi|110347437	**Score:**	20.6
**Database:**	refseq_human(refseq_human_20140103.fasta)	**MW [kDa]:**	165.6
**Database Date:**	2015-11-30	**pI:**	4.8
**Modification(s):**	Carbamidomethyl	**Sequence Coverage [%]:**	2.1
		**No. of unique Peptides:**	1

10	20	30	40	50	60	70	80
MAGGVRLLWV	SLLVLLAQLG	PQPGLGRLGE	RLRVRFTPVV	CGLRCVHGPT	GSRCTPTCAP	RNATSVDSGA	PGGAAPGGPG
90	100	110	120	130	140	150	160
FRAFLCPLIC	HNGGVCVKPD	RCLCPPDFAG	KFCQLHSSGA	RPPAPAVPGL	TRSVYTMPLA	NHRDDEHGVA	SMVSVHVEHP
170	180	190	200	210	220	230	240
QEASVVVHQV	ERVSGPWEEA	DAEAVARAEA	AARAEAAAPY	TVLAQSAPRE	DGYSDASGFG	YCFRELRGGE	CASPLPGLRT
250	260	270	280	290	300	310	320
QEVCCRGAGL	AWGVHDCQLC	SERLGNSERV	SAPDGPCPTG	FERVNGSCED	VDECATGGRC	QHGECANTRG	GYTCVCPDGF
330	340	350	360	370	380	390	400
LLDSSRSSCI	SQHVISEAKG	PCFRVLRDGG	CSLPILRNIT	KQICCCSRVG	KAWGRGCQLC	PPFGSEGFRE	ICPAGPGYHY
410	420	430	440	450	460	470	480
SASDLRYNTR	PLGQEPPRVS	LSQPRTLPAT	SRPSAGFLPT	HRLEPRPEPR	PDPRPGPELP	LPSIPAWTGP	EIPESGPSSG
490	500	510	520	530	540	550	560
MCQRNPQVCG	PGRCISRPSG	YTCACDSGFR	LSPQGTRCID	VDECRRVPPP	CAPGRCENSP	GSFRCVCGPG	FRAGPRAAEC
570	580	590	600	610	620	630	640
LDVDECHRVP	PPCDLGRCEN	TPGSFLCVCP	AGYQAAPHGA	SCQDVDECTQ	SPGLCGRGAC	KNLPGSFRCV	CPAGFRGSAC
650	660	670	680	690	700	710	720
EEDVDECAQE	PPPCGPGRCD	NTAGSFHCAC	PAGFRSRGPG	APCQDVDECA	RSPPPCTYGR	CENTEGSFQC	VCPMGFQPNT
730	740	750	760	770	780	790	800
AGSECEDVDE	CENHLACPGQ	ECVNSPGSFQ	CRTCPSGHHL	HRGRCTDVDE	CSSGAPPCGP	HGHCTNTEGS	FRCSCAPGYR
810	820	830	840	850	860	870	880
APSGRPGPCA	DVNECLEGDF	CFPHGECLNT	DGSFACTCAP	GYRPGPRGAS	CLDVDECSEE	DLCQSGICTN	TDGSFECICP
890	900	910	920	930	940	950	960
PGHRAGPDLA	SCLDVDECRE	RGPALCGSQR	CENSPGSYRC	VRDCDPGYHA	GPEGTCDDVD	ECQEYGPEIC	GAQRCENTPG
970	980	990	1000	1010	1020	1030	1040
SYRCTPACDP	GYQPTPGGGC	QDVDECRNRS	FCGAHAVCQN	LPGSFQCLCD	QGYEGARDGR	HCVDVNECET	LQGVCGAALC
1050	1060	1070	1080	1090	1100	1110	1120
ENVEGSFLCV	CPNSPEEFDP	MTGRCVPPRT	SAGTFPGSQP	QAPASPVLPA	RPPPPPLPRR	PSTPRQGPVG	SGRRECYFDT
1130	1140	1150	1160	1170	1180	1190	1200
AAPDACDNIL	ARNVTWQECC	CTVGEGWGSG	CRIQQCPGTE	TAEYQSLCPH	GRGYLAPSGD	LSLRRDVDEC	QLFRDQVCKS
1210	1220	1230	1240	1250	1260	1270	1280
GVCVNTAPGY	SCYCSNGYYY	HTQRLECIDN	DECADEEPAC	EGGRCVNTVG	SYHCTCEPPL	VLDGSQRRCV	SNESQSLDDN
1290	1300	1310	1320	1330	1340	1350	1360
LGVCWQEVGA	DLVCSHPRLD	RQATYTECCC	LYGEAWGMDC	ALCPAQDSDD	FEALCNVLRP	PAYSPPRPGG	FGLPYEYGPD
1370	1380	1390	1400	1410	1420	1430	1440
LGPPYQGLPY	GPELYPPPAL	PYDPYPPPPG	PFARREAPYG	APRFDMPDFE	DDGGPYGESE	APAPPGPGTR	WPYRSRDTRR
1450	1460	1470	1480	1490	1500	1510	1520
SFPEPEEPPE	GGSYAGSLAE	PYEELEAEEC	GILDGCTNGR	CVRVPEGFTC	RCFDGYRLDM	TRMACVDINE	CDEAEAASPL
1530	1540	1550	1560				
CVNARCLNTD	GSFRCICRPG	FAPTHQPHHC	APARPRA				

Cmpd.	No. of Cmpds.	m/z meas.	Δ m/z [ppm]	z	Rt [min]	Score	P	Range	Sequence	Modification
1901	1	900.3202	-98.12	2	52.4	10.3	1	45-61	R.CVHGPTGSRCTPTCAPR.N	Carbamidomethyl: 10

Detailed Protein Report

Protein 457: **receptor-type tyrosine-protein phosphatase-like N isoform 3 [Homo sapiens]**

**Accession:**	gi|315113881	**Score:**	20.5
**Database:**	refseq_human(refseq_human_20140103.fasta)	**MW [kDa]:**	96.2
**Database Date:**	2015-11-30	**pI:**	6.1
		**Sequence Coverage [%]:**	3.3
		**No. of unique Peptides:**	2

10	20	30	40	50	60	70	80
MSQGLSWHDD	LTQYVISQEM	ERIPRLRPPE	PRPRDRSGLA	PKRPGPAGEL	LLQDIPTGSA	PAAQHRLPQP	PVGKGGAGAS
**90**	**100**	**110**	**120**	**130**	**140**	**150**	**160**
SSLSPLQAEL	LPPLLEHLLL	PPQPPHPSLS	YEPALLQPYL	FHQFGSRDGS	RVSEGSPGMV	SVGPLPKAEA	PALFSRTASK
**170**	**180**	**190**	**200**	**210**	**220**	**230**	**240**
GIFGDHPGHS	YGDLPGPSPA	QLFQDSGLLY	LAQELPAPSR	ARVPRLPEQG	SSSRAEDSPE	GYEKEGLGDR	GEKPASPAVQ
**250**	**260**	**270**	**280**	**290**	**300**	**310**	**320**
PDAALQRLAA	VLAGYGVELR	QLTPEQLSTL	LTLLQLLPKG	AGRNPGGVVN	VGADIKKTME	GPVEGRDTAE	LPARTSPMPG
**330**	**340**	**350**	**360**	**370**	**380**	**390**	**400**
HPTASPTSSE	VQQVPSPVSS	EPPKAARPPV	TPVLLEKKSP	LGQSQPTVAG	QPSARPAAEE	YGYIVTDQKP	LSLAAGVKLL
**410**	**420**	**430**	**440**	**450**	**460**	**470**	**480**
EILAEHVHMS	SGSFINISVV	GPALTFRIRH	NEQNLSLADV	TQQAGLVKSE	LEAQTGLQIL	QTGVGQREEA	AAVLPQTAHS
**490**	**500**	**510**	**520**	**530**	**540**	**550**	**560**
TSPMRSVLLT	LVALAGVAGL	LVALAVALCV	RQHARQQDKE	RLAALGPEGA	HGDTTFEYQD	LCRQHMATKS	LFNRAEGPPE
**570**	**580**	**590**	**600**	**610**	**620**	**630**	**640**
PSRVSSVSSQ	FSDAAQASPS	SHSSTPSWCE	EPAQANMDIS	TGHMILAYME	DHLRNRDRLA	KEWQALCAYQ	AEPNTCATAQ
**650**	**660**	**670**	**680**	**690**	**700**	**710**	**720**
GEGNIKKNRH	PDFLPYDHAR	IKLKVESSPS	RSDYINASPI	IEHDPRMPAY	IATQGPLSHT	IADFWQMVWE	SGCTVIVMLT
**730**	**740**	**750**	**760**	**770**	**780**	**790**	**800**
PLVEDGVKQC	DRYWPDEGAS	LYHVYEVNLV	SEHIWCEDFL	VRSFYLKNVQ	TQETRTLTQF	HFLSWPAEGT	PASTRPLLDF
**810**	**820**	**830**	**840**	**850**	**860**	**870**	**880**
RRKVNKCYRG	RSCPIIVHCS	DGAGRTGTYI	LIDMVLNRMA	KGVKEIDIAA	TLEHVRDQRP	GLVRSKDQFE	FALTAVAEEV
**890**							
NAILKALPQ							

Cmpd.	No. of Cmpds.	m/z meas.	Δ m/z [ppm]	z	Rt [min]	Score	P	Range	Sequence	Modification
2983	1	863.9596	41.86	2	66.1	10.0	0	672-686	R.SDYINASPIIEHDPR.M	
531	1	472.2515	58.93	3	35.1	10.5	0	812-825	R.SCPIIVHCSDGAGR.T	

Detailed Protein Report

Protein 458: **PREDICTED: mitogen-activated protein kinase kinase kinase kinase 2 isoform X4 [Homo sapiens]**

**Accession:**	gi|578821648	**Score:**	20.5
**Database:**	refseq_human(refseq_human_20140103.fasta)	**MW [kDa]:**	71.1
**Database Date:**	2015-11-30	**pI:**	6.0
		**Sequence Coverage [%]:**	5.0
		**No. of unique Peptides:**	1

10	20	30	40	50	60	70	80
MLMSKSSFQP	PKLRDKTRWT	QNFHHFLKLA	LTKNPKKRPT	AEKLLQHPFT	TQQLPRALLT	QLLDKASDPH	LGTPSPEDCE
**90**	**100**	**110**	**120**	**130**	**140**	**150**	**160**
LETYDMFPDT	IHSRGQHGPA	ERTPSEIQFH	QVKFGAPRRK	ETDPLNEPWE	EEWTLLGKEE	LSGSLLQSVQ	EALEERSLTI
**170**	**180**	**190**	**200**	**210**	**220**	**230**	**240**
RSASEFQVPH	RGRLELGSRP	LQRVRLEWDV	VTGQPGNGRA	GPCPSYQELD	SPDDTMGTIK	RAPFLGPLPT	DPPAEEPLSS
**250**	**260**	**270**	**280**	**290**	**300**	**310**	**320**
PPGTLPPPPS	GPNSSPLLPT	AWATMKQRED	PERSSCHGLP	PTPKVHMGAC	FSKVFNGCPL	RIHAAVTWIH	PVTRDQFLVV
**330**	**340**	**350**	**360**	**370**	**380**	**390**	**400**
GAEEGIYTLN	LHELHEDTLE	KLISHRCSWL	YCVNNVLLSL	SGKSTHIWAH	DLPGLFEQRR	LQQQVPLSIP	TNRLTQRIIP
**410**	**420**	**430**	**440**	**450**	**460**	**470**	**480**
RRFALSTKIP	DTKGCLQCRV	VRNPYTGATF	LLAALPTSLL	LLQWYEPLQK	FLLLKNFSSP	LPSPAGMLEP	LVLDGKELPQ
**490**	**500**	**510**	**520**	**530**	**540**	**550**	**560**
VCVGAEGPEG	PGCRVLFHVL	PLEAGLTPDI	LIPPEGIPGS	AQQVIQVDRD	TILVSFERCV	RIVNMQGEPT	ATLAPELTFD
**570**	**580**	**590**	**600**	**610**	**620**	**630**	**640**
FPIETVVCLQ	DSVLAFWSHG	MQGRSLDTNE	VTQEITDETR	IFRVLGAHRD	IILESIPTDN	PEAHSNLYIL	TGHQSTY

Cmpd.	No. of Cmpds.	m/z meas.	Δ m/z [ppm]	z	Rt [min]	Score	P	Range	Sequence	Modification
1876	1	899.5084	102.10	2	52.1	10.3	0	477-494	K.ELPQVCVGAEGPEGPGCR.V	

Detailed Protein Report

Protein 459: succinate dehydrogenase assembly factor 1, mitochondrial [Homo sapiens]

Accession: gi|111038124
Database: refseq_human(refseq_human_20140103.fasta)
Database Date: 2015-11-30

Score: 20.4
MW [kDa]: 12.8
pI: 12.0
Sequence Coverage [%]: 29.6
No. of unique Peptides: 1

10	20	30	40	50	60	70	80
MSRHSRLQRQ	VLSLYRDLLR	AGRGKPGAEA	RVRAEFRQHA	GLPRSDVLRI	EYLYRRGRRQ	LQLLRSGHAT	AMGAFVRPRA
90	100	110	120				
PTGEPGGVGS	QPDDGDSPRN	PHDSTGAPET	RPDGR				

Cmpd.	No. of Cmpds.	m/z meas.	Δ m/z [ppm]	z	Rt [min]	Score	P	Range	Sequence	Modification
2984	1	729.3744	-1.00	2	66.1	10.3	0	66-79	R.SGHATAMGAFVRPR.A	

Detailed Protein Report

Protein 460: PREDICTED: kelch-like protein 8 isoform X3 [Homo sapiens]

**Accession:**	gi|530378014	**Score:**	20.2
**Database:**	refseq_human(refseq_human_20140103.fasta)	**MW [kDa]:**	47.9
**Database Date:**	2015-11-30	**pI:**	7.2
		**Sequence Coverage [%]:**	2.1
		**No. of unique Peptides:**	1

10	20	30	40	50	60	70	80
MASDSMSSKQ	ARNHITKGKR	QQQHQQIKNR	SSISDGDGED	SFIFEANEAW	KDFHGSLLRF	YENGELCDVT	LKVRLPLLPV
**90**	**100**	**110**	**120**	**130**	**140**	**150**	**160**
DFLMGVVAKE	QIVKQNLKCR	DLLDEARNYH	LHLSSRAVPD	FEYSIRTTPR	KHTAGVLFCV	GGRGGSGDPF	RSIECYSINK
**170**	**180**	**190**	**200**	**210**	**220**	**230**	**240**
NSWFFGPEMN	SRRRHVGVIS	VEGKVYAVGG	HDGNEHLGSM	EMFDPLTNKW	MMKASMNTKR	RGIALASLGG	PIYAIGGLDD
**250**	**260**	**270**	**280**	**290**	**300**	**310**	**320**
NTCFNDVERY	DIESDQWSTV	APMNTPRGGV	GSVALVNHVY	AVGGNDGMAS	LSSVERYDPH	LDKWIEVKEM	GQRRAGNGVS
**330**	**340**	**350**	**360**	**370**	**380**	**390**	**400**
KLHGCLYVVG	GFDDNSPLSS	VERYDPRSNK	WDYVAALTTP	RGGVGIATVM	GKIFAVGGHN	GNAYLNTVEA	FDPVLNRWEL
**410**	**420**	**430**	**440**				
VGSVSHCRAG	AGVAVCSCLT	SQIRDVGHGS	NNVVDCM				

Cmpd.	No. of Cmpds.	m/z meas.	Δ m/z [ppm]	z	Rt [min]	Score	P	Range	Sequence	Modification
490	1	472.1760	-43.61	2	34.6	20.2	0	1-9	-.MASDSMSSK.Q	

Detailed Protein Report

Protein 461: **PREDICTED: ubiquinone biosynthesis protein COQ7 homolog isoform X1 [Homo sapiens]**

**Accession:**	gi|530407440	**Score:**	20.1
**Database:**	refseq_human(refseq_human_20140103.fasta)	**MW [kDa]:**	22.8
**Database Date:**	2015-11-30	**pI:**	6.5
**Modification(s):**	Oxidation	**Sequence Coverage [%]:**	9.4
		**No. of unique Peptides:**	1

10	20	30	40	50	60	70	80
MGSQCHLPQM	AYGRRTSVRF	RSSGMTLDNI	SRAAVDRIIR	VDHAGEYGAN	RIYAGQMAVL	GRTSVGPVIQ	KMWDQEKDHL
**90**	**100**	**110**	**120**	**130**	**140**	**150**	**160**
KKFNELMVTF	RVRPTVLMPL	WNVLGFALGA	GTALLGKEGA	MACTVAVEES	IAHHYNNQIR	TLMEEDPEKY	EELLQLIKKF
**170**	**180**	**190**	**200**	**210**			
RDEELEHHDI	GLDHDAELAP	AYAVLKSIIQ	AGCRVAIYLS	ERL			

Cmpd.	No. of Cmpds.	m/z meas.	Δ m/z [ppm]	z	Rt [min]	Score	P	Range	Sequence	Modification
1373	1	1045.9338	-113.36	2	45.5	20.1	2	22-40	R.SSGMTLDNISRAAVDRIIR.V	Oxidation: 4

Detailed Protein Report

Protein 462: PREDICTED: ankyrin-3 isoform X15 [Homo sapiens]

**Accession:**	gi|578819314	**Score:**	20.1
**Database:**	refseq_human(refseq_human_20140103.fasta)	**MW [kDa]:**	386.8
**Database Date:**	2015-11-30	**pI:**	5.6
		**Sequence Coverage [%]:**	0.4
		**No. of unique Peptides:**	1

Quantitation

WD:WU	**Median:** 0.79	**CV:** 0.00 %	**No. of Peptides:** 1

Detailed Protein Report

10	20	30	40	50	60	70	80
MALPQSEDAM	TGDTDKYLGP	QDLKELGDDS	LPAEGYMGFS	LGARSASSDR	SYTLNRSSYA	RDSMMIEELL	VPSKEQHLTF
90	100	110	120	130	140	150	160
TREFDSDSLR	HYSWAADTLD	NVNLVSSPIH	SGFLVSFMVD	ARGGSMRGSR	HHGMRIIIPP	RKCTAPTRIT	CRLVKRHKLA
170	180	190	200	210	220	230	240
NPPPMVEGEG	LASRLVEMGP	AGAQFLGPVI	VEIPHFGSMR	GKERELIVLR	SENGETWKEH	QFDSKNEDLT	ELLNGMDEEL
250	260	270	280	290	300	310	320
DSPEELGKKR	ICRIITKDFP	QYFAVVSRIK	QESNQIGPEG	GILSSTTVPL	VQASFPEGAL	TKRIRVGLQA	QPVPDEIVKK
330	340	350	360	370	380	390	400
ILGNKATFSP	IVTVEPRRRK	FHKPITMTIP	VPPPSGEGVS	NGYKGDTTPN	LRLLCSITGG	TSPAQWEDIT	GTTPLTFIKD
410	420	430	440	450	460	470	480
CVSFTTNVSA	RFWLADCHQV	LETVGLATQL	YRELICVPYM	AKFVVFAKMN	DPVESSLRCF	CMTDDKVDKT	LEQQENFEEV
490	500	510	520	530	540	550	560
ARSKDIEVLE	GKPIYVDCYG	NLAPLTKGGQ	QLVFNFYSFK	ENRLPFSIKI	RDTSQEPCGR	LSFLKEPKTT	KGLPQTAVCN
570	580	590	600	610	620	630	640
LNITLPAHKK	ETESDQDDEI	EKTDRRQSFA	SLALRKRYSY	LTEPGMKTVE	RSTGATRSLP	TTYSYKPFFS	TRPYQSWTTA
650	660	670	680	690	700	710	720
PITVPGPAKS	GFTSLSSSSS	NTPSASPLKS	IWSVSTPSPI	KSTLGASTTS	SVKSISDVAS	PIRSFRTMSS	PIKTVVSQSP
730	740	750	760	770	780	790	800
YNIQVSSGTL	ARAPAVTEAT	PLKGLASNST	FSSRTSPVTT	AGSLLERSSI	TMTPPASPKS	NINMYSSSLP	FKSIITSAAP
810	820	830	840	850	860	870	880
LISSPLKSVV	SPVKSAVDVI	SSAKITMASS	LSSPVKQMPG	HAEVALVNGS	ISPLKYPSSS	TLINGCKATA	TLQEKISSAT
890	900	910	920	930	940	950	960
NSVSSVVSAA	TDTVEKVFST	TTAMPFSPLR	SYVSAAPSAF	QSLRTPSASA	LYTSLGSSIS	ATTSSVTSSI	ITVPVYSVVN
970	980	990	1000	1010	1020	1030	1040
VLPEPALKKL	PDSNSFTKSA	AALLSPIKTL	TTETHPQPHF	SRTSSPVKSS	LFLAPSALKL	STPSSLSSSQ	EILKDVAEMK
1050	1060	1070	1080	1090	1100	1110	1120
EDLMRMTAIL	QTDVPEEKPF	QPELPKEGRI	DDEEPFKIVE	KVKEDLVKVS	EILKKDVCVD	NKGSPKSPKS	DKGHSPEDDW
1130	1140	1150	1160	1170	1180	1190	1200
IEFSSEEIRE	ARQQAAASQS	PSLPERVQVK	AKAASEKDYN	LTKVIDYLTN	DIGSSSLTNL	KYKFEDAKKD	GEERQKRVLK
1210	1220	1230	1240	1250	1260	1270	1280
PAIALQEHKL	KMPPASMRTS	TSEKELCKMA	DSFFGTDTIL	ESPDDFSQHD	QDKSPLSDSG	FETRSEKTPS	APQSAESTGP
1290	1300	1310	1320	1330	1340	1350	1360
KPLFHEVPIP	PVITETRTEV	VHVIRSYDPS	AGDVPQTQPE	EPVSPKPSPT	FMELEPKPTT	SSIKEKVKAF	QMKASSEEDD
1370	1380	1390	1400	1410	1420	1430	1440
HNRVLSKGMR	VKEETHITTT	TRMVYHSPPG	GEGASERIEE	TMSVHDIMKA	FQSGRDPSKE	LAGLFEHKSA	VSPDVHKSAA
1450	1460	1470	1480	1490	1500	1510	1520
ETSAQHAEKD	NQMKPKLERI	IEVHIEKGNQ	AEPTEVIIRE	TKKHPEKEMY	VYQKDLSRGD	INLKDFLPEK	HDAFPCSEEQ
1530	1540	1550	1560	1570	1580	1590	1600
GQQEEEELTA	EESLPSYLES	SRVNTPVSQE	EDSRPSSAQL	ISDDSYKTLK	LLSQHSIEYH	DDELSELRGE	SYRFAEKMLL
1610	1620	1630	1640	1650	1660	1670	1680
SEKLDVSHSD	TEESVTDHAG	PPSSELQGSD	KRSREKIATA	PKKEILSKIY	KDVSENGVGK	VSKDEHFDKV	TVLHYSGNVS
1690	1700	1710	1720	1730	1740	1750	1760
SPKHAMWMRF	TEDRLDRGRE	KLIYEDRVDR	TVKEAEEKLT	EVSQFFRDKT	EKLNDELQSP	EKKARPKNGK	EYSSQSPTSS
1770	1780	1790	1800	1810	1820	1830	1840
SPEKVLLTEL	LASNDEWVKA	RQHGPDGQGF	PKAEEKAPSL	PSSPEKMVLS	QQTEDSKSTV	EAKGSISQSK	APDGPQSGFQ
1850	1860	1870	1880	1890	1900	1910	1920
LKQSKLSSIR	LKFEQGTHAK	SKDMSQEDRK	SDGQSRIPVK	KIQESKLPVY	QVFAREKQQK	AIDLPDESVS	VQKDFMVLKT
1930	1940	1950	1960	1970	1980	1990	2000
KDEHAQSNEI	VVNDSGSDNV	KKQRTEMSSK	AMPDSFSEQQ	AKDLACHITS	DLATRGPWDK	KVFRTWESSG	ATNNKSQKEK
2010	2020	2030	2040	2050	2060	2070	2080
LSHVLVHDVR	ENHIGHPESK	SVDQKNEFMS	VTERERKLLT	NGSLSEIKEM	TVKSPSKKVL	YREYVVKEGD	HPGGLLDQPS
2090	2100	2110	2120	2130	2140	2150	2160
RRSESSAVSH	IPVRVADERR	MLSSNIPDGF	CEQSAFPKHE	LSQKLSQSSM	SKETVETQHF	NSIEDEKVTY	SEISKVSKHQ
2170	2180	2190	2200	2210	2220	2230	2240

Detailed Protein Report

Cmpd.	No. of Cmpds.	m/z meas.	Δ m/z [ppm]	z	Rt [min]	Score	P	Range	Sequence	Modification	Ratios	
534	1	676.8481	-14.81	2	35.1	20.1	1	3029-3041	K.TKALTTSSCVDVK.S		WD:WU	0.79

Detailed Protein Report

Protein 463: uncharacterized protein KIAA0226-like isoform f [Homo sapiens]

**Accession:**	gi|557947998	**Score:**	20.0
**Database:**	refseq_human(refseq_human_20140103.fasta)	**MW [kDa]:**	50.5
**Database Date:**	2015-11-30	**pI:**	8.6
		**Sequence Coverage [%]:**	2.5
		**No. of unique Peptides:**	1

10	20	30	40	50	60	70	80
MIISAMEKMK	CNILSQQQTE	SWSKEVSGLL	GSDQPDSEMT	FDTNIKQESG	SSTSSYSGYE	GCAVLQVSPV	TETRTYHDVK
**90**	**100**	**110**	**120**	**130**	**140**	**150**	**160**
EICKCDVDEF	VILELGDFND	ITETCSCSCS	SSKSVTYEPD	FNSAELLAKE	LYRVFQKCWI	LSVVNSQLAG	SLSAAGSIVV
**170**	**180**	**190**	**200**	**210**	**220**	**230**	**240**
NEECVRKDFE	SSMNVVQEIK	FKSRIRGTED	WAPPRFQIIF	NIHPPLKRDL	VVAAQNFFCA	GCGTPVEPKF	VKRLRYCEYL
**250**	**260**	**270**	**280**	**290**	**300**	**310**	**320**
GKYFCDCCHS	YAESCIPARI	LMMWDFKKYY	VSNFSKQLLD	SIWHQPIFNL	LSIGQSLYAK	AKELDRVKEI	QEQLFHIKKL
**330**	**340**	**350**	**360**	**370**	**380**	**390**	**400**
LKTCRFANSA	LKEFEQVPGH	LTDELHLFSL	EDLVRIKKGL	LAPLLKDILK	ASLAHVAGCE	LCQGKGFICE	FCQNTTVIFP
**410**	**420**	**430**	**440**	**450**			
FQTATCRRCS	ACRACFHKQC	FQSSECPRCA	RITARRKLLE	SVASAAT			

Cmpd.	No. of Cmpds.	m/z meas.	Δ m/z [ppm]	z	Rt [min]	Score	P	Range	Sequence	Modification
2445	1	597.3363	-141.46	2	58.8	20.0	2	356-366	R.IKKGLLAPLLK.D	

Detailed Protein Report

Protein 464: ovochymase-2 precursor [Homo sapiens]

**Accession:**	gi|373838920	**Score:**	19.9
**Database:**	refseq_human(refseq_human_20140103.fasta)	**MW [kDa]:**	62.6
**Database Date:**	2015-11-30	**pI:**	6.2
		**Sequence Coverage [%]:**	2.5
		**No. of unique Peptides:**	1

10	20	30	40	50	60	70	80
MLISRNKLIL	LLGIVFFERG	KSATLSLPKA	PSCGQSLVKV	QPWNYFNIFS	RILGGSQVEK	GSYPWQVSLK	QRQKHICGGS
90	100	110	120	130	140	150	160
IVSPQWVITA	AHCIANRNIV	STLNVTAGEY	DLSQTDPGEQ	TLTIETVIIH	PHFSTKKPMD	YDIALLKMAG	AFQFGHFVGP
170	180	190	200	210	220	230	240
ICLPELREQF	EAGFICTTAG	WGRLTEGGVL	SQVLQEVNLP	ILTWEECVAA	LLTLKRPISG	KTFLCTGFPD	GGRDACQGDS
250	260	270	280	290	300	310	320
GGSLMCRNKK	GAWTLAGVTS	WGLGCGRGWR	NNVRKSDQGS	PGIFTDISKV	LPWIHEHIQT	GNRRKSSRAW	CSEQDVIVSG
330	340	350	360	370	380	390	400
AEGKLHFPES	LHLYYESKQR	CVWTLLVPEE	MHVLLSFSHL	DVESCHHSYL	SMYSLEDRPI	GKFCGESLPS	SILIGSNSLR
410	420	430	440	450	460	470	480
LKFVSDATDN	AAGFNLTYKA	LKPNYIPDSG	CSYLTVLFEE	GLIQSLNYPE	NYSDKANCDW	IFQASKHHLI	KLSFQSLEIE
490	500	510	520	530	540	550	560
ESGDCTSDYV	TVHSDVERKK	EIARLCGYDV	PTPVLSPSSI	MLISFQSDEN	GTCRGFQATV	SFIPKAVYPD	LNISISEDES
570							
MFLET							

Cmpd.	No. of Cmpds.	m/z meas.	Δ m/z [ppm]	z	Rt [min]	Score	P	Range	Sequence	Modification
1535	1	850.5690	134.04	2	47.5	19.9	0	290-303	K.VLPWIHEHIQTGNR.R	

Detailed Protein Report

Protein 465: inorganic pyrophosphatase [Homo sapiens]

**Accession:**	gi|11056044	**Score:**	19.8
**Database:**	refseq_human(refseq_human_20140103.fasta)	**MW [kDa]:**	32.6
**Database Date:**	2015-11-30	**pI:**	5.5
**Modification(s):**	Carbamidomethyl, Oxidation	**Sequence Coverage [%]:**	6.6
		**No. of unique Peptides:**	1

10	20	30	40	50	60	70	80
MSGFSTEERA	APFSLEYRVF	LKNEKGQYIS	PFHDIPIYAD	KDVFHMVVEV	PRWSNAKMEI	ATKDPLNPIK	QDVKKGKLRY
**90**	**100**	**110**	**120**	**130**	**140**	**150**	**160**
VANLFPYKGY	IWNYGAIPQT	WEDPGHNDKH	TGCCGDNDPI	DVCEIGSKVC	ARGEIIGVKV	LGILAMIDEG	ETDWKVIAIN
**170**	**180**	**190**	**200**	**210**	**220**	**230**	**240**
VDDPDAANYN	DINDVKRLKP	GYLEATVDWF	RRYKVPDGKP	ENEFAFNAEF	KDKDFAIDII	KSTHDHWKAL	VTKKTNGKGI
**250**	**260**	**270**	**280**	**290**			
SCMNTTLSES	PFKCDPDAAR	AIVDALPPPC	ESACTVPTDV	DKWFHHQKN			

Cmpd.	No. of Cmpds.	m/z meas.	Δ m/z [ppm]	z	Rt [min]	Score	P	Range	Sequence	Modification
1596	1	696.7599	139.50	3	48.7	19.8	1	235-253	K.TNGKGISCMNTTLSESPFK.C	Carbamidomethyl: 8; Oxidation: 9

Detailed Protein Report

Protein 466: retinol dehydrogenase 10 [Homo sapiens]

**Accession:**	gi|25282469	**Score:**	19.8
**Database:**	refseq_human(refseq_human_20140103.fasta)	**MW [kDa]:**	38.1
**Database Date:**	2015-11-30	**pI:**	7.9
		**Sequence Coverage [%]:**	2.9
		**No. of unique Peptides:**	1

10	20	30	40	50	60	70	80
MNIVVEFFVV	TFKVLWAFVL	AAARWLVRPK	EKSVAGQVCL	ITGAGSGLGR	LFALEFARRR	ALLVLWDINT	QSNEETAGMV
**90**	**100**	**110**	**120**	**130**	**140**	**150**	**160**
RHIYRDLEAA	DAAALQAGNG	EEEILPHCNL	QVFTYTCDVG	KRENVYLTAE	RVRKEVGEVS	VLVNNAGVVS	GHHLLECPDE
**170**	**180**	**190**	**200**	**210**	**220**	**230**	**240**
LIERTMMVNC	HAHFWTTKAF	LPTMLEINHG	HIVTVASSLG	LFSTAGVEDY	CASKFGVVGF	HESLSHELKA	AEKDGIKTTL
**250**	**260**	**270**	**280**	**290**	**300**	**310**	**320**
VCPYLVDTGM	FRGCRIRKEI	EPFLPPLKPD	YCVKQAMKAI	LTDQPMICTP	RLMYIVTFMK	SILPFEAVVC	MYRFLGADKC
**330**	**340**	**350**					
MYPFIAQRKQ	ATNNNEAKNG	I					

Cmpd.	No. of Cmpds.	m/z meas.	Δ m/z [ppm]	z	Rt [min]	Score	P	Range	Sequence	Modification
365	1	639.8907	24.31	2	33.2	19.8	2	51-60	R.LFALEFARRR.A	

Detailed Protein Report

Protein 467: tuberin isoform 4 [Homo sapiens]

**Accession:**	gi|116256350	**Score:**	19.8
**Database:**	refseq_human(refseq_human_20140103.fasta)	**MW [kDa]:**	193.1
**Database Date:**	2015-11-30	**pI:**	6.7
**Modification(s):**	Oxidation	**Sequence Coverage [%]:**	0.8
		**No. of unique Peptides:**	1

Quantitation

WD:WU **Median:** 0.90 **CV:** 0.00 % **No. of Peptides:** 1

10	20	30	40	50	60	70	80
MAKPTSKDSG	LKEKFKILLG	LGTPRPNPRS	AEGKQTEFII	TAEILRELSM	ECGLNNRIRM	IGQICEVAKT	KKFEEHAVEA
90	100	110	120	130	140	150	160
LWKAVADLLQ	PERPLEARHA	VLALLKAIVQ	GQGERLGVLR	ALFFKVIKDY	PSNEDLHERL	EVFKALTDNG	RHITYLEEEL
170	180	190	200	210	220	230	240
ADFVLQWMDV	GLSSEFLLVL	VNLVKFNSCY	LDEYIARMVQ	MICLLCVRTA	SSVDIEVSLQ	VLDAVVCYNC	LPAESLPLFI
250	260	270	280	290	300	310	320
VTLCRTINVK	ELCEPCWKLM	RNLLGTHLGH	SAIYNMCHLM	EDRAYMEDAP	LLRGAVFFVG	MALWGAHRLY	SLRNSPTSVL
330	340	350	360	370	380	390	400
PSFYQAMACP	NEVVSYEIVL	SITRLIKKYR	KELQVVAWDI	LLNIIERLLQ	QLQTLDSPEL	RTIVHDLLTT	VEELCDQNEF
410	420	430	440	450	460	470	480
HGSQERYFEL	VERCADQRPE	SSLLNLISYR	AQSIHPAKDG	WIQNLQALME	RFFRSESRGA	VRIKVLDVLS	FVLLINRQFY
490	500	510	520	530	540	550	560
EEELINSVVI	SQLSHIPEDK	DHQVRKLATQ	LLVDLAEGCH	THHFNSLLDI	IEKVMARSLS	PPPELEERDV	AAYSASLEDV
570	580	590	600	610	620	630	640
KTAVLGLLVI	LQTKLYTLPA	SHATRVYEML	VSHIQLHYKH	SYTLPIASSI	RLQAFDFLLL	LRADSLHRLG	LPNKDGVVRF
650	660	670	680	690	700	710	720
SPYCVCDYME	PERGSEKKTS	GPLSPPTGPP	GPAPAGPAVR	LGSVPYSLLF	RVLLQCLKQE	SDWKVLKLVL	GRLPESLRYK
730	740	750	760	770	780	790	800
VLIFTSPCSV	DQLCSALCSM	LSGPKTLERL	RGAPEGFSRT	DLHLAVVPVL	TALISYHNYL	DKTKQREMVY	CLEQGLIHRC
810	820	830	840	850	860	870	880
ASQCVVALSI	CSVEMPDIII	KALPVLVVKL	THISATASMA	VPLLEFLSTL	ARLPHLYRNF	AAEQYASVFA	ISLPYTNPSK
890	900	910	920	930	940	950	960
FNQYIVCLAH	HVIAMWFIRC	RLPFRKDFVP	FITKGLRSNV	LLSFDDTPEK	DSFRARSTSL	NERPKRIQTS	LTSASLGSAD
970	980	990	1000	1010	1020	1030	1040
ENSVAQADDS	LKNLHLELTE	TCLDMMARYV	FSNFTAVPKR	SPVGEFLLAG	GRTKTWLVGN	KLVTVTTSVG	TGTRSLLGLD
1050	1060	1070	1080	1090	1100	1110	1120
SGELQSGPES	SSSPGVHVRQ	TKEAPAKLES	QAGQQVSRGA	RDRVRSMSGG	HGLRVGALDV	PASQFLGSAT	SPGPRTAPAA
1130	1140	1150	1160	1170	1180	1190	1200
KPEKASAGTR	VPVQEKTNLA	AYVPLLTQGW	AEILVRRPTG	NTSWLMSLEN	PLSPFSSDIN	NMPLQELSNA	LMAAERFKEH
1210	1220	1230	1240	1250	1260	1270	1280
RDTALYKSLS	VPAASTAKPP	PLPRSNTDSA	VVMEEGSPGE	VPVLVEPPGL	EDVEAALGMD	RRTDAYSRSS	SVSSQEEKSL
1290	1300	1310	1320	1330	1340	1350	1360
HAEELVGRGI	PIERVVSSEG	GRPSVDLSFQ	PSQPLSKSSS	SPELQTLQDI	LGDPGDKADV	GRLSPEVKAR	SQSGTLDGES
1370	1380	1390	1400	1410	1420	1430	1440
AAWSASGEDS	RGQPEGPLPS	SSPRSPSGLR	PRGYTISDSA	PSRRGKRVER	DALKSRATAS	NAEKVPGINP	SFVFLQLYHS
1450	1460	1470	1480	1490	1500	1510	1520
PFFGDESNKP	ILLPNESQSF	ERSVQLLDQI	PSYDTHKIAV	LYVGEGQSNS	ELAILSNEHG	SYRYTEFLTG	LGRLIELKDC
1530	1540	1550	1560	1570	1580	1590	1600
QPDKVYLGGL	DVCGEDGQFT	YCWHDDIMQA	VFHIATLMPT	KDVDKHRCDK	KRHLGNDFVS	IVYNDSGEDF	KLGTIKGQFN
1610	1620	1630	1640	1650	1660	1670	1680
FVHVIVTPLD	YECNLVSLQC	RKDMEGLVDT	SVAKIVSDRN	LPFVARQMAL	HANMASQVHH	SRSNPTDIYP	SKWIARLRHI
1690	1700	1710	1720	1730	1740	1750	
KRLRQRICEE	AAYSNPSLPL	VHPPSHSKAP	AQTPAEPTPG	YEVGQRKRLI	SSVEDFTEFV		

Detailed Protein Report

Cmpd.	No. of Cmpds.	m/z meas.	Δ m/z [ppm]	z	Rt [min]	Score	P	Range	Sequence	Modification	Ratios	
52	4	768.2749	-173.91	2	29.9	19.8	2	1-14	-.MAKPTSKDSGLKEK.F	Oxidation: 1	WD:WU	0.90

Detailed Protein Report

Protein 468: **PREDICTED: DNA topoisomerase 2-binding protein 1 isoform X1 [Homo sapiens]**

**Accession:**	gi|530373836	**Score:**	19.8
**Database:**	refseq_human(refseq_human_20140103.fasta)	**MW [kDa]:**	170.0
**Database Date:**	2015-11-30	**pI:**	6.5
		**Sequence Coverage [%]:**	0.9
		**No. of unique Peptides:**	1

10	20	30	40	50	60	70	80
MSRNDKEPFF	VKFLKSSDNS	KCFFKALESI	KEFQSEEYLQ	IITEEEALKI	KENDRSLYIC	DPFSGVVFDH	LKKLGCRIVG
**90**	**100**	**110**	**120**	**130**	**140**	**150**	**160**
PQVVIFCMHH	QRCVPRAEHP	VYNMVMSDVT	ISCTSLEKEK	REEVHKYVQM	MGGRVYRDLN	VSVTHLIAGE	VGSKKYLVAA
**170**	**180**	**190**	**200**	**210**	**220**	**230**	**240**
NLKKPILLPS	WIKTLWEKSQ	EKKITRYTDI	NMEDFKCPIF	LGCIICVTGL	CGLDRKEVQQ	LTVKHGGQYM	GQLKMNECTH
**250**	**260**	**270**	**280**	**290**	**300**	**310**	**320**
LIVQEPKGQK	YECAKRWNVH	CVTTQWFFDS	IEKGFCQDES	IYKTEPRPEA	KTMPNSSTPT	SQINTIDNVS	NISNINASCV
**330**	**340**	**350**	**360**	**370**	**380**	**390**	**400**
SESICNSLNS	KLEPTLENLE	NLDVSAFQAP	EDLLDGCRIY	LCGFSGRKLD	KLRRLINSGG	GVRFNQLNED	VTHVIVGDYD
**410**	**420**	**430**	**440**	**450**	**460**	**470**	**480**
DELKQFWNKS	AHRPHVVGAK	WLLECFSKGY	MLSEEPYIHA	NYQPVEIPVS	HKPESKAALL	KKKNSSFSKK	DFAPSEKHEQ
**490**	**500**	**510**	**520**	**530**	**540**	**550**	**560**
ADEDLLSQYE	NGSSTVVEAK	TSEARPFNDS	THAEPLNDST	HISLQEENQS	SVSHCVPDVS	TITEEGLFSQ	KSFLVLGFSN
**570**	**580**	**590**	**600**	**610**	**620**	**630**	**640**
ENESNIANII	KENAGKIMSL	LSRTVADYAV	VPLLGCEVEA	TVGEVVTNTW	LVTCIDYQTL	FDPKSNPLFT	PVPVMTGMTP
**650**	**660**	**670**	**680**	**690**	**700**	**710**	**720**
LEDCVISFSQ	CAGAEKESLT	FLANLLGASV	QEYFVRKSNA	KKGMFASTHL	ILKERGGSKY	EAAKKWNLPA	VTIAWLLETA
**730**	**740**	**750**	**760**	**770**	**780**	**790**	**800**
RTGKRADESH	FLIENSTKEE	RSLETEITNG	INLNSDTAEH	PGTRLQTHRK	TVVTPLDMNR	FQSKAFRAVV	SQHARQVAAS
**810**	**820**	**830**	**840**	**850**	**860**	**870**	**880**
PAVGQPLQKE	PSLHLDTPSK	FLSKDKLFKP	SFDVKDALAA	LETPGRPSQQ	KRKPSTPLSE	VIVKNLQLAL	ANSSRNAVAL
**890**	**900**	**910**	**920**	**930**	**940**	**950**	**960**
SASPQLKEAQ	SEKEEAPKPL	HKVVVCVSKK	LSKKQSELNG	IAASLGADYR	WSFDETVTHF	IYQGRPNDTN	REYKSVKERG
**970**	**980**	**990**	**1000**	**1010**	**1020**	**1030**	**1040**
VHIVSEHWLL	DCAQECKHLP	ESLYPHTYNP	KMSLDISAVQ	DGRLCNSRLL	SAVSSTKDDE	PDPLILEEND	VDNMATNNKE
**1050**	**1060**	**1070**	**1080**	**1090**	**1100**	**1110**	**1120**
SAPSNGSGKN	DSKGVLTQTL	EMRENFQKQL	QEIMSATSIV	KPQGQRTSLS	RSGCNSASST	PDSTRSARSG	RSRVLEALRQ
**1130**	**1140**	**1150**	**1160**	**1170**	**1180**	**1190**	**1200**
SRQTVPDVNT	EPSQNEQIIW	DDPTAREERA	RLASNLQWPS	CPTQYSELQV	DIQNLEDSPF	QKPLHDSEIA	KQAVCDPGNI
**1210**	**1220**	**1230**	**1240**	**1250**	**1260**	**1270**	**1280**
RVTEAPKHPI	SEELETPIKD	SHLIPTPQAP	SIAFPLANPP	VAPHPREKII	TIEETHEELK	KQYIFQLSSL	NPQERIDYCH
**1290**	**1300**	**1310**	**1320**	**1330**	**1340**	**1350**	**1360**
LIEKLGGLVI	EKQCFDPTCT	HIVVGHPLRN	EKYLASVAAG	KWVLHRSYLE	ACRTAGHFVQ	EEDYEWGSSS	ILDVLTGINV
**1370**	**1380**	**1390**	**1400**	**1410**	**1420**	**1430**	**1440**
QQRRLALAAM	RWRKKIQQRQ	ESGIVEGAFS	GWKVILHVDQ	SREAGFKRLL	QSGGAKVLPG	HSVPLFKEAT	HLFSDLNKLK
**1450**	**1460**	**1470**	**1480**	**1490**	**1500**	**1510**	**1520**
PDDSGVNIAE	AAAQNVYCLR	TEYIADYLMQ	ESPPHVENYC	LPEAISFIQN	NKELGTGLSQ	KRKAPTEKNK	IKRPRVH

Cmpd.	No. of Cmpds.	m/z meas.	Δ m/z [ppm]	z	Rt [min]	Score	P	Range	Sequence	Modification
1880	1	697.5427	213.85	2	51.8	19.8	0	796-809	R.QVAASPAVGQPLQK.E	

Detailed Protein Report

Protein 469: alpha-2-HS-glycoprotein preproprotein [Homo sapiens]

Accession: gi|156523970
Database: refseq_human(refseq_human_20140103.fasta)
Database Date: 2015-11-30
Modification(s): Carbamidomethyl

Score: 19.8
MW [kDa]: 39.3
pI: 5.4
Sequence Coverage [%]: 6.5
No. of unique Peptides: 1

10	20	30	40	50	60	70	80
MKSLVLLLCL	AQLWGCHSAP	HGPGLIYRQP	NCDDPETEEA	ALVAIDYINQ	NLPWGYKHTL	NQIDEVKVWP	QQPSGELFEI
**90**	**100**	**110**	**120**	**130**	**140**	**150**	**160**
EIDTLETTCH	VLDPTPVARC	SVRQLKEHAV	EGDCDFQLLK	LDGKFSVVYA	KCDSSPDSAE	DVRKVCQDCP	LLAPLNDTRV
**170**	**180**	**190**	**200**	**210**	**220**	**230**	**240**
VHAAKAALAA	FNAQNNGSNF	QLEEISRAQL	VPLPPSTYVE	FTVSGTDCVA	KEATEAAKCN	LLAEKQYGFC	KATLSEKLGG
**250**	**260**	**270**	**280**	**290**	**300**	**310**	**320**
AEVAVTCMVF	QTQPVSSQPQ	PEGANEAVPT	PVVDPDAPPS	PPLGAPGLPP	AGSPPDSHVL	LAAPPGHQLH	RAHYDLRHTF
**330**	**340**	**350**	**360**	**370**			
MGVVSLGSPS	GEVSHPRKTR	TVVQPSVGAA	AGPVVPPCPG	RIRHFKV			

Cmpd.	No. of Cmpds.	m/z meas.	Δ m/z [ppm]	z	Rt [min]	Score	P	Range	Sequence	Modification
2906	1	860.4973	66.36	3	65.1	19.8	0	188-211	R.AQLVPLPPSTYVEFTVSGTDCVAK.E	Carbamidomethyl: 21

Detailed Protein Report

Protein 470: **PREDICTED: zinc finger CCCH domain-containing protein 7A isoform X4 [Homo sapiens]**

**Accession:**	gi|530407964	**Score:**	19.7
**Database:**	refseq_human(refseq_human_20140103.fasta)	**MW [kDa]:**	67.6
**Database Date:**	2015-11-30	**pI:**	9.6
		**Sequence Coverage [%]:**	1.6
		**No. of unique Peptides:**	1

10	20	30	40	50	60	70	80
MNGPGSLFAS	ENFLGISSQP	RNDFGNFFGS	AVTKPSSSVT	PRHPLEGTHE	LRQACQICFV	KSGPKLMDFT	YHANIDHKCK
90	100	110	120	130	140	150	160
KDILIGRIKN	VEDKSWKKIR	PRPTKTNYEG	PYYICKDVAA	EEECRYSGHC	TFAYCQEEID	VWTLERKGAF	SREAFFGGNG
170	180	190	200	210	220	230	240
KINLTVFKLL	QEHLGEFIFL	CEKCFDHKPR	MISKRNKDNS	TACSHPVTKH	EFEDNKCLVH	ILRETTVKYS	KIRSFHGQCQ
250	260	270	280	290	300	310	320
LDLCRHEVRY	GCLREDECFY	AHSLVELKVW	IMQNETGISH	DAIAQESKRY	WQNLEANVPG	AQVLGNQIMP	GFLNMKIKFV
330	340	350	360	370	380	390	400
CAQCLRNGQV	IEPDKNRKYC	SAKARHSWTK	DRRAMRVMSI	ERKKWMNIRP	LPTKKQMPLQ	FDLCNHIASG	KKCQYVGNCS
410	420	430	440	450	460	470	480
FAHSPEEREV	WTYMKENGIQ	DMEQFYELWL	KSQKNEKSED	IASQSNKENG	KQIHMPTDYA	EVTVDFHCWM	CGKNCNSEKQ
490	500	510	520	530	540	550	560
WQGHISSEKH	KEKVFHTEDD	QYCWQHRFPT	GYFSICDRYM	NGTCPEGNSC	KFAHGNAELH	EWEERRDALK	MKLNKARKDH
570	580	590					
LIGPNDNDFG	KYSFLFKDLN						

Cmpd.	No. of Cmpds.	m/z meas.	Δ m/z [ppm]	z	Rt [min]	Score	P	Range	Sequence	Modification
202	1	999.6888	178.38	1	31.7	19.7	0	327-335	R.NGQVIEPDK.N	

Detailed Protein Report

Protein 471: interferon regulatory factor 2-binding protein-like [Homo sapiens]

**Accession:**	gi|33457336	**Score:**	19.6
**Database:**	refseq_human(refseq_human_20140103.fasta)	**MW [kDa]:**	82.6
**Database Date:**	2015-11-30	**pI:**	9.6
		**Sequence Coverage [%]:**	1.5
		**No. of unique Peptides:**	1

10	20	30	40	50	60	70	80
MSAAQVSSSR	RQSCYLCDLP	RMPWAMIWDF	SEPVCRGCVN	YEGADRIEFV	IETARQLKRA	HGCFQDGRSP	GPPPPVGVKT
**90**	**100**	**110**	**120**	**130**	**140**	**150**	**160**
VALSAKEAAA	AAAAAAAAAA	AAQQQQQQQQ	QQQQQQQQQQ	QQQQQQQLNH	VDGSSKPAVL	AAPSGLERYG	LSAAAAAAAA
**170**	**180**	**190**	**200**	**210**	**220**	**230**	**240**
AAAAVEQRSR	FEYPPPPVSL	GSSSHTARLP	NGLGGPNGFP	KPTPEEGPPE	LNRQSPNSSS	AAASVASRRG	THGGLVTGLP
**250**	**260**	**270**	**280**	**290**	**300**	**310**	**320**
NPGGGGGPQL	TVPPNLLPQT	LLNGPASAAV	LPPPPPHALG	SRGPPTPAPP	GAPGGPACLG	GTPGVSATSS	SASSSTSSSV
**330**	**340**	**350**	**360**	**370**	**380**	**390**	**400**
AEVGVGAGGK	RPGSVSSTDQ	ERELKEKQRN	AEALAELSES	LRNRAEEWAS	KPKMVRDTLL	TLAGCTPYEV	RFKKDHSLLG
**410**	**420**	**430**	**440**	**450**	**460**	**470**	**480**
RVFAFDAVSK	PGMDYELKLF	IEYPTGSGNV	YSSASGVAKQ	MYQDCMKDFG	RGLSSGFKYL	EYEKKHGSGD	WRLLGDLLPE
**490**	**500**	**510**	**520**	**530**	**540**	**550**	**560**
AVRFFKEGVP	GADMLPQPYL	DASCPMLPTA	LVSLSRAPSA	PPGTGALPPA	APSGRGAAAS	LRKRKASPEP	PDSAEGALKL
**570**	**580**	**590**	**600**	**610**	**620**	**630**	**640**
GEEQQRQQWM	ANQSEALKLT	MSAGGFAAPG	HAAGGPPPPP	PPLGPHSNRT	TPPESAPQNG	PSPMAALMSV	ADTLGTAHSP
**650**	**660**	**670**	**680**	**690**	**700**	**710**	**720**
KDGSSVHSTT	ASARRNSSSP	VSPASVPGQR	RLASRNGDLN	LQVAPPPPSA	HPGMDQVHPQ	NIPDSPMANS	GPLCCTICHE
**730**	**740**	**750**	**760**	**770**	**780**	**790**	**800**
RLEDTHFVQC	PSVPSHKFCF	PCSRESIKAQ	GATGEVYCPS	GEKCPLVGSN	VPWAFMQGEI	ATILAGDVKV	KKERDP

Cmpd.	No. of Cmpds.	m/z meas.	Δ m/z [ppm]	z	Rt [min]	Score	P	Range	Sequence	Modification
2766	1	659.8617	61.81	2	63.1	19.6	0	331-342	K.RPGSVSSTDQER.E	

Detailed Protein Report

Protein 472: serine protease 58 precursor [Homo sapiens]

**Accession:**	gi|48255915	**Score:**	19.6
**Database:**	refseq_human(refseq_human_20140103.fasta)	**MW [kDa]:**	27.1
**Database Date:**	2015-11-30	**pI:**	6.4
**Modification(s):**	Carbamidomethyl	**Sequence Coverage [%]:**	11.6
		**No. of unique Peptides:**	1

10	20	30	40	50	60	70	80
MKFILLWALL	NLTVALAFNP	DYTVSSTPPY	LVYLKSDYLP	CAGVLIHPLW	VITAAHCNLP	KLRVILGVTI	PADSNEKHLQ
**90**	**100**	**110**	**120**	**130**	**140**	**150**	**160**
VIGYEKMIHH	PHFSVTSIDH	DIMLIKLKTE	AELNDYVKLA	NLPYQTISEN	TMCSVSTWSY	NVCDIYKEPD	SLQTVNISVI
**170**	**180**	**190**	**200**	**210**	**220**	**230**	**240**
SKPQCRDAYK	TYNITENMLC	VGIVPGRRQP	CKEVSAAPAI	CNGMLQGILS	FADGCVLRAD	VGIYAKIFYY	IPWIENVIQN
**250**							
N							

Cmpd.	No. of Cmpds.	m/z meas.	Δ m/z [ppm]	z	Rt [min]	Score	P	Range	Sequence	Modification
1245	1	1072.1826	-52.32	3	43.9	19.6	1	36-63	K.SDYLPCAGVLIHPLWVITAAHCNLPKLR.V	Carbamidomethyl: 6, 22

Detailed Protein Report

Protein 473: PREDICTED: 39S ribosomal protein L28, mitochondrial isoform X1 [Homo sapiens]

Accession: gi|530407456
Database: refseq_human(refseq_human_20140103.fasta)
Database Date: 2015-11-30
Modification(s): Oxidation

Score: 19.6
MW [kDa]: 33.8
pI: 9.4
Sequence Coverage [%]: 4.9
No. of unique Peptides: 1

10	20	30	40	50	60	70	80
MAFTASQRWE	EGQARQIGFR	FRWALNPERL	AMPLHKYPVW	LWKRLQLREG	ICSRLPGHYL	RSLEEERTPT	PVHYRPHGAK
**90**	**100**	**110**	**120**	**130**	**140**	**150**	**160**
FKINPKNGQR	ERVEDVPIPI	YFPPESQRGL	WGGEGWILGQ	IYANNDKLSK	RLKKVWKPQL	FEREFYSEIL	DKKFTVTVTM
**170**	**180**	**190**	**200**	**210**	**220**	**230**	**240**
RTLDLIDEAY	GLDFYILKTP	KEDLCSKFGM	DLKRGMLLRL	ARQDPQLHPE	DPERRAAIYD	KYKEFAIPEE	EAEWVGLTLE
**250**	**260**	**270**	**280**	**290**			
EAIEKQRLLE	EKDPVPLFKI	YVAELIQQLQ	QQALSEPAVV	QKRASGQ			

Cmpd.	No. of Cmpds.	m/z meas.	Δ m/z [ppm]	z	Rt [min]	Score	P	Range	Sequence	Modification
2394	1	899.3523	-161.20	2	58.3	19.6	1	30-43	R.LAMPLHKYPVWLWK.R	Oxidation: 3

Detailed Protein Report

Protein 474: coilin [Homo sapiens]

**Accession:**	gi|4758024	**Score:**	19.5
**Database:**	refseq_human(refseq_human_20140103.fasta)	**MW [kDa]:**	62.6
**Database Date:**	2015-11-30	**pI:**	10.0
		**Sequence Coverage [%]:**	2.3
		**No. of unique Peptides:**	1

10	20	30	40	50	60	70	80
MAASETVRLR	LQFDYPPPAT	PHCTAFWLLV	DLNRCRVVTD	LISLIRQRFG	FSSGAFLGLY	LEGGLLPPAE	SARLVRDNDC
**90**	**100**	**110**	**120**	**130**	**140**	**150**	**160**
LRVKLEERGV	AENSVVISNG	DINLSLRKAK	KRAFQLEEGE	ETEPDCKYSK	KHWKSRENNN	NNEKVLDLEP	KAVTDQTVSK
**170**	**180**	**190**	**200**	**210**	**220**	**230**	**240**
KNKRKNKATC	GTVGDDNEEA	KRKSPKKKEK	CEYKKKAKNP	KSPKVQAVKD	WANQRCSSPK	GSARNSLVKA	KRKGSVSVCS
**250**	**260**	**270**	**280**	**290**	**300**	**310**	**320**
KESPSSSSES	ESCDESISDG	PSKVTLEARN	SSEKLPTELS	KEEPSTKNTT	ADKLAIKLGF	SLTPSKGKTS	GTTSSSSDSS
**330**	**340**	**350**	**360**	**370**	**380**	**390**	**400**
AESDDQCLMS	SSTPECAAGF	LKTVGLFAGR	GRPGPGLSSQ	TAGAAGWRRS	GSNGGGQAPG	ASPSVSLPAS	LGRGWGREEN
**410**	**420**	**430**	**440**	**450**	**460**	**470**	**480**
LFSWKGAKGR	GMRGRGRGRG	HPVSCVVNRS	TDNQRQQQLN	DVVKNSSTII	QNPVETPKKD	YSLLPLLAAA	PQVGEKIAFK
**490**	**500**	**510**	**520**	**530**	**540**	**550**	**560**
LLELTSSYSP	DVSDYKEGRI	LSHNPETQQV	DIEILSSLPA	LREPGKFDLV	YHNENGAEVV	EYAVTQESKI	TVFWKELIDP
**570**	**580**						
RLIIESPSNT	SSTEPA						

Cmpd.	No. of Cmpds.	m/z meas.	Δ m/z [ppm]	z	Rt [min]	Score	P	Range	Sequence	Modification
1827	1	835.3577	-42.45	2	51.5	19.5	2	132-144	K.HWKSRENNNNNEK.V	

Detailed Protein Report

Protein 475: protein maestro isoform b [Homo sapiens]

**Accession:**	gi|187761377	**Score:**	19.5
**Database:**	refseq_human(refseq_human_20140103.fasta)	**MW [kDa]:**	23.0
**Database Date:**	2015-11-30	**pI:**	10.3
		**Sequence Coverage [%]:**	8.7
		**No. of unique Peptides:**	1

Quantitation

WD:WU **Median:** 1.09 **CV:** 0.00 % **No. of Peptides:** 1

Alias proteins:

Accession	Name	Description
gi|530413995	refseq_human (refseq_human_20140103.fasta)	PREDICTED: protein maestro isoform X4 [Homo sapiens]

```
        10         20         30         40         50         60         70         80
MDQRQRRILG QPLSIPTSQP KQKRTSMISF FSKVSWKLRF QKREPLKNVF FILAERARDP SAKKRHMAMR NLGTMAYEAP
        90        100        110        120        130        140        150        160
DKVRKYKKIV LDLLVYGLYD PVNLEVIHES MKTLTVVLGK IQGKGLGSFF IDITLQTRTL LDDACKTTFQ ACSPYLKLKE
       170        180        190        200
EYSFQSEEDQ RNTKLYQQLS HYHPEILQFF YANKIL
```

Cmpd.	No. of Cmpds.	m/z meas.	Δ m/z [ppm]	z	Rt [min]	Score	P	Range	Sequence	Modification	Ratios
1043	1	630.9810	-97.07	3	41.8	19.5	2	8-24	R.ILGQPLSIPTSQPKQKR.T		WD:WU 1.09

Detailed Protein Report

Protein 476: rho GTPase-activating protein 19 isoform 3 [Homo sapiens]

**Accession:**	gi|374093192	**Score:**	19.5
**Database:**	refseq_human(refseq_human_20140103.fasta)	**MW [kDa]:**	54.9
**Database Date:**	2015-11-30	**pI:**	10.1
		**Sequence Coverage [%]:**	2.1
		**No. of unique Peptides:**	1

10	20	30	40	50	60	70	80
MPHQKLSALI	DAICSFVICN	DSSLRGQPII	FNPDFFVEKL	RHEKPEIFTE	LVVSNITRLI	DLPGTELAQL	MGEVDLKLPG
**90**	**100**	**110**	**120**	**130**	**140**	**150**	**160**
GAGPASGFFR	SLMSLKRKEK	GVIFGSPLTE	EGIAQIYQLI	EYLHKNLRVE	GLFRVPGNSV	RQQILRDALN	NGTDIDLESG
**170**	**180**	**190**	**200**	**210**	**220**	**230**	**240**
EFHSNDVATL	LKMFLGELPE	PLLTHKHFNA	HLKIADLMQF	DDKGNKTNIP	DKDRQIEALQ	LLFLILPPPN	RNLLKLLLDL
**250**	**260**	**270**	**280**	**290**	**300**	**310**	**320**
LYQTAKKQDK	NKMSAYNLAL	MFAPHVLWPK	NVTANDLQEN	ITKLNSGMAF	MIKHSQKLFK	APAYIRECAR	LHYLGSRTQA
**330**	**340**	**350**	**360**	**370**	**380**	**390**	**400**
SKDDLDLIAS	CHTKSFQLAK	SQKRNRVDSC	PHQEETQHHT	EEALRELFQH	VHDMPESAKK	KQLIRQFNKQ	SLTQTPGREP
**410**	**420**	**430**	**440**	**450**	**460**	**470**	**480**
STSQVQKRAR	SRSFSGLIKR	KVLGNQMMSE	KKKKNPTPES	VAIGELKGTS	KENRNLLFSG	SPAVTMTPTR	LKWSEGKKEG
**490**							
KKGFL							

Cmpd.	No. of Cmpds.	m/z meas.	Δ m/z [ppm]	z	Rt [min]	Score	P	Range	Sequence	Modification
693	1	556.2788	-12.08	2	37.1	19.5	0	284-293	K.LNSGMAFMIK.H	

Detailed Protein Report

Protein 477: neuroendocrine convertase 1 isoform 3 [Homo sapiens]

Accession: gi|295424145
Database: refseq_human(refseq_human_20140103.fasta)
Database Date: 2015-11-30

Score: 19.5
MW [kDa]: 49.3
pI: 5.3
Sequence Coverage [%]: 5.2
No. of unique Peptides: 1

10	20	30	40	50	60	70	80
MLDGIVTDAI	EASSIGFNPG	HVDIYSASWG	PNDDGKTVEG	PGRLAQKAFE	YGVKQTSADL	HNDCTETHTG	TSASAPLAAG
**90**	**100**	**110**	**120**	**130**	**140**	**150**	**160**
IFALALEANP	NLTWRDMQHL	VVWTSEYDPL	ANNPGWKKNG	AGLMVNSRFG	FGLLNAKALV	DLADPRTWRS	VPEKKECVVK
**170**	**180**	**190**	**200**	**210**	**220**	**230**	**240**
DNDFEPRALK	ANGEVIIEIP	TRACEGQENA	IKSLEHVQFE	ATIEYSRRGD	LHVTLTSAAG	TSTVLLAERE	RDTSPNGFKN
**250**	**260**	**270**	**280**	**290**	**300**	**310**	**320**
WDFMSVHTWG	ENPIGTWTLR	ITDMSGRIQN	EGRIVNWKLI	LHGTSSQPEH	MKQPRVYTSY	NTVQNDRRGV	EKMVDPGEEQ
**330**	**340**	**350**	**360**	**370**	**380**	**390**	**400**
PTQENPKENT	LVSKSPSSSS	VGGRRDELEE	GAPSQAMLRL	LQSAFSKNSP	PKQSPKKSPS	AKLNIPYENF	YEALEKLNKP
**410**	**420**	**430**	**440**	**450**			
SQLKDSEDSL	YNDYVDVFYN	TKPYKHRDDR	LLQALVDILN	EEN			

Cmpd.	No. of Cmpds.	m/z meas.	Δ m/z [ppm]	z	Rt [min]	Score	P	Range	Sequence	Modification
1185	1	956.7150	-62.95	3	43.5	19.5	1	405-427	K.DSEDSLYNDYVDVFYNTKPYKHR.D	

Detailed Protein Report

Protein 478: mucin-17 precursor [Homo sapiens]

**Accession:**	gi|91982772	**Score:**	19.4
**Database:**	refseq_human(refseq_human_20140103.fasta)	**MW [kDa]:**	451.5
**Database Date:**	2015-11-30	**pI:**	3.9
		**Sequence Coverage [%]:**	0.2
		**No. of unique Peptides:**	1

Detailed Protein Report

10	20	30	40	50	60	70	80
MPRPGTMALC	LLTLVLSLLP	PQAAAEQDLS	VNRAVWDGGG	CISQGDVLNR	QCQQLSQHVR	TGSAANTATG	TTSTNVVEPR
90	100	110	120	130	140	150	160
MYLSCSTNPE	MTSIESSVTS	DTPGVSSTRM	TPTESRTTSE	STSDSTTLFP	SSTEDTSSPT	TPEGTDVPMS	TPSEESISST
170	180	190	200	210	220	230	240
MAFVSTAPLP	SFEAYTSLTY	KVDMSTPLTT	STQASSSPTT	PESTTIPKST	NSEGSTPLTS	MPASTMKVAS	SEAITLLTTP
250	260	270	280	290	300	310	320
VEISTPVTIS	AQASSSPTTA	EGPSLSNSAP	SGGSTPLTRM	PLSVMLVVSS	EASTLSTTPA	ATNIPVITST	EASSSPTTAE
330	340	350	360	370	380	390	400
GTSIPTSTYT	EGSTPLTSTP	ASTMPVATSE	MSTLSITPVD	TSTLVTTSTE	PSSLPTTAEA	TSMLTSTLSE	GSTPLTNMPV
410	420	430	440	450	460	470	480
STILVASSEA	STTSTIPVDS	KTFVTTASEA	SSSPTTAEDT	SIATSTPSEG	STPLTSMPVS	TTPVASSEAS	NLSTTPVDSK
490	500	510	520	530	540	550	560
TQVTTSTEAS	SSPPTAEVNS	MPTSTPSEGS	TPLTSMSVST	MPVASSEAST	LSTTPVDTST	PVTTSSEASS	SSTTPEGTSI
570	580	590	600	610	620	630	640
PTSTPSEGST	PLTNMPVSTR	LVVSSEASTT	STTPADSNTF	VTTSSEASSS	STTAEGTSMP	TSTYSERGTT	ITSMSVSTTL
650	660	670	680	690	700	710	720
VASSEASTLS	TTPVDSNTPV	TTSTEATSSS	TTAEGTSMPT	STYTEGSTPL	TSMPVNTTLV	ASSEASTLST	TPVDTSTPVT
730	740	750	760	770	780	790	800
TSTEASSSPT	TADGASMPTS	TPSEGSTPLT	SMPVSKTLLT	SSEASTLSTT	PLDTSTHITT	STEASCSPTT	TEGTSMPIST
810	820	830	840	850	860	870	880
PSEGSPLLTS	IPVSITPVTS	PEASTLSTTP	VDSNSPVTTS	TEVSSSPTPA	EGTSMPTSTY	SEGRTPLTSM	PVSTTLVATS
890	900	910	920	930	940	950	960
AISTLSTTPV	DTSTPVTNST	EARSSPTTSE	GTSMPTSTPG	EGSTPLTSMP	DSTTPVVSSE	ARTLSATPVD	TSTPVTTSTE
970	980	990	1000	1010	1020	1030	1040
ATSSPTTAEG	TSIPTSTPSE	GTTPLTSTPV	SHTLVANSEA	STLSTTPVDS	NTPLTTSTEA	SSPPPTAEGT	SMPTSTPSEG
1050	1060	1070	1080	1090	1100	1110	1120
STPLTRMPVS	TTMVASSETS	TLSTTPADTS	TPVTTYSQAS	SSSTTADGTS	MPTSTYSEGS	TPLTSVPVST	RLVVSSEAST
1130	1140	1150	1160	1170	1180	1190	1200
LSTTPVDTSI	PVTTSTEASS	SPTTAEGTSI	PTSPPSEGTT	PLASMPVSTT	LVVSSEANTL	STTPVDSKTQ	VATSTEASSP
1210	1220	1230	1240	1250	1260	1270	1280
PPTAEVTSMP	TSTPGERSTP	LTSMPVRHTP	VASSEASTLS	TSPVDTSTPV	TTSAETSSSP	TTAEGTSLPT	STTSEGSTLL
1290	1300	1310	1320	1330	1340	1350	1360
TSIPVSTTLV	TSPEASTLLT	TPVDTKGPVV	TSNEVSSSPT	PAEGTSMPTS	TYSEGRTPLT	SIPVNTTLVA	SSAISILSTT
1370	1380	1390	1400	1410	1420	1430	1440
PVDNSTPVTT	STEACSSPTT	SEGTSMPNSN	PSEGTTPLTS	IPVSTTPVVS	SEASTLSATP	VDTSTPGTTS	AEATSSPTTA
1450	1460	1470	1480	1490	1500	1510	1520
EGISIPTSTP	SEGKTPLKSI	PVSNTPVANS	EASTLSTTPV	DSNSPVVTST	AVSSSPTPAE	GTSIAISTPS	EGSTALTSIP
1530	1540	1550	1560	1570	1580	1590	1600
VSTTTVASSE	INSLSTTPAV	TSTPVTTYSQ	ASSSPTTADG	TSMQTSTYSE	GSTPLTSLPV	STMLVVSSEA	NTLSTTPIDS
1610	1620	1630	1640	1650	1660	1670	1680
KTQVTASTEA	SSSTTAEGSS	MTISTPSEGS	PLLTSIPVST	TPVASPEAST	LSTTPVDSNS	PVITSTEVSS	SPTPAEGTSM
1690	1700	1710	1720	1730	1740	1750	1760
PTSTYTEGRT	PLTSITVRTT	PVASSAISTL	STTPVDNSTP	VTTSTEARSS	PTTSEGTSMP	NSTPSEGTTP	LTSIPVSTTP
1770	1780	1790	1800	1810	1820	1830	1840
VLSSEASTLS	ATPIDTSTPV	TTSTEATSSP	TTAEGTSIPT	STLSEGMTPL	TSTPVSHTLV	ANSEASTLST	TPVDSNSPVV
1850	1860	1870	1880	1890	1900	1910	1920
TSTAVSSSPT	PAEGTSIATS	TPSEGSTALT	SIPVSTTTVA	SSETNTLSTT	PAVTSTPVTT	YAQVSSSPTT	ADGSSMPTST
1930	1940	1950	1960	1970	1980	1990	2000
PREGRPPLTS	IPVSTTTVAS	SEINTLSTTL	ADTRTPVTTY	SQASSSPTTA	DGTSMPTPAY	SEGSTPLTSM	PLSTTLVVSS
2010	2020	2030	2040	2050	2060	2070	2080
EASTLSTTPV	DTSTPATTST	EGSSSPTTAG	GTSIQTSTPS	ERTTPLAGMP	VSTTLVVSSE	GNTLSTTPVD	SKTQVTNSTE
2090	2100	2110	2120	2130	2140	2150	2160
ASSSATAEGS	SMTISAPSEG	SPLLTSIPLS	TTPVASPEAS	TLSTTPVDSN	SPVITSTEVS	SSPIPTEGTS	MQTSTYSDRR
2170	2180	2190	2200	2210	2220	2230	2240

Detailed Protein Report

Cmpd.	No. of Cmpds.	m/z meas.	Δ m/z [ppm]	z	Rt [min]	Score	P	Range	Sequence	Modification
138	2	613.7562	-82.11	2	31.0	19.4	0	51-60	R.QCQQLSQHVR.T	

Detailed Protein Report

Protein 479: PREDICTED: alpha-L-iduronidase isoform X1 [Homo sapiens]

**Accession:**	gi|578808149	**Score:**	19.4
**Database:**	refseq_human(refseq_human_20140103.fasta)	**MW [kDa]:**	78.7
**Database Date:**	2015-11-30	**pI:**	10.4
		**Sequence Coverage [%]:**	3.7
		**No. of unique Peptides:**	1

10	20	30	40	50	60	70	80
MRPLRPRAAL	LALLASLLAA	PPVAPAEAPH	LVHVDAARAL	WPLRRFWRST	GFCPPLPHSQ	ADQYVLSWDQ	QLNLAYVGAV
**90**	**100**	**110**	**120**	**130**	**140**	**150**	**160**
PHRGIKQVRT	HWLLELVTTR	GSTGRGLSYN	FTHLDGYLDL	LRENQLLPGF	ELMGSASGHF	TDFEDKQQVF	EWKDLVSSLA
**170**	**180**	**190**	**200**	**210**	**220**	**230**	**240**
RRYIGRYGLA	HVSKWNFETW	NEPDHHDFDN	VSMTMQGFLN	YYDACSEGLR	AASPALRLGG	PGDSFHTPPR	SPLSWGLLRH
**250**	**260**	**270**	**280**	**290**	**300**	**310**	**320**
CHDGTNFFTG	EAGVRLDYIS	LHRKVRPAPP	SAPVFCALSR	CAPGRADPGG	AEAAPPAGCA	QLHLHPGAGE	GRRAADPAAL
**330**	**340**	**350**	**360**	**370**	**380**	**390**	**400**
PQVRGHPHLQ	RRGGPAGGLV	PATAVEGGRD	LRGHGGEGGP	AQRPARPPAT	FLPRRDRRAV	AAPPGPSCPG	HPQVIAQHQN
**410**	**420**	**430**	**440**	**450**	**460**	**470**	**480**
LLLANTTSAF	PYALLSNDNA	FLSYHPHPFA	QRTLTARFQV	NNTRPPHVQL	LRKPVLTAMG	LLALLDEEQL	WAEVSQAGTV
**490**	**500**	**510**	**520**	**530**	**540**	**550**	**560**
LDSNHTVGVL	ASAHRPQGPA	DAWRAAVLIY	ASDDTRAHPN	RSVAVTLRLR	GVPPGPGLVY	VTRYLDNGLC	SPDGEWRRLG
**570**	**580**	**590**	**600**	**610**	**620**	**630**	**640**
RPVFPTAEQF	RRMRAAEDPV	AAAPRPLPAG	GRLTLRPALR	LPSLLLVHVC	ARPEKPPGQV	TRLRALPLTQ	GQLVLVWSDE
**650**	**660**	**670**	**680**	**690**	**700**	**710**	**720**
HVGSKCLWTY	EIQFSQDGKA	YTPVSRKPST	FNLFVFSPDT	GAVSGSYRVR	ALDYWARPGP	FSDPVPYLEV	PVPRGPPSPG
**730**							
NP							

Cmpd.	No. of Cmpds.	m/z meas.	Δ m/z [ppm]	z	Rt [min]	Score	P	Range	Sequence	Modification
1856	1	836.3130	-103.64	3	51.9	19.4	0	286-312	R.ADPGGAEAAPPAGCAQLHLHPGAGEGR.R	

Detailed Protein Report

Protein 480: PREDICTED: NT-3 growth factor receptor isoform X9 [Homo sapiens]

Accession: gi|578827025
Database: refseq_human(refseq_human_20140103.fasta)
Database Date: 2015-11-30
Modification(s): Oxidation

Score: 19.3
MW [kDa]: 55.2
pI: 6.6
Sequence Coverage [%]: 3.8
No. of unique Peptides: 1

10	20	30	40	50	60	70	80
MDVSLCPAKC	SFWRIFLLGS	VWLDYVGSVL	ACPANCVCSK	TEINCRRPDD	GNLFPLLEGQ	DSGNSNGNAS	INITDISRNI
**90**	**100**	**110**	**120**	**130**	**140**	**150**	**160**
TSIHIENWRS	LHTLNAVDME	LYTGLQKLTI	KNSGLRSIQP	RAFAKNPHLR	YINLSSNRLT	TLSWQLFQTL	SLRELQLEQN
**170**	**180**	**190**	**200**	**210**	**220**	**230**	**240**
FFNCSCDIRW	MQLWQEQGEA	KLNSQNLYCI	NADGSQLPLF	RMNISQCDLP	EISVSHVNLT	VREGDNAVIT	CNGSGSPLPD
**250**	**260**	**270**	**280**	**290**	**300**	**310**	**320**
VDWIVTGLQS	INTHQTNLNW	TNVHAINLTL	VNVTSEDNGF	TLTCIAENVV	GMSNASVALT	VYYPPRVVSL	EEPELRLEHC
**330**	**340**	**350**	**360**	**370**	**380**	**390**	**400**
IEFVVRGNPP	PTLHWLHNGQ	PLRESKIIHV	EYYQEGEISE	GCLLFNKPTH	YNNGNYTLIA	KNPLGTANQT	INGHFLKEPF
**410**	**420**	**430**	**440**	**450**	**460**	**470**	**480**
PVDEVSPTPP	ITVTHKPEED	TFGVSIAVGL	AAFACVLLVV	LFVMINKYGR	RSKFGMKDGL	ELLAVLLKCD	SKQLKNKEGN
**490**	**500**						
KMKNNNGKKK	RATSA						

Cmpd.	No. of Cmpds.	m/z meas.	Δ m/z [ppm]	z	Rt [min]	Score	P	Range	Sequence	Modification
342	2	699.2835	-131.98	3	32.9	19.3	2	454-472	K.FGMKDGLELLAVLLKCDSK.Q	Oxidation: 3

Detailed Protein Report

Protein 481: PREDICTED: protein SSX3 isoform X3 [Homo sapiens]

**Accession:**	gi|530421398	**Score:**	19.3
**Database:**	refseq_human(refseq_human_20140103.fasta)	**MW [kDa]:**	19.4
**Database Date:**	2015-11-30	**pI:**	10.2
**Modification(s):**	Carbamidomethyl	**Sequence Coverage [%]:**	15.3
		**No. of unique Peptides:**	1

10	20	30	40	50	60	70	80
MNGDDTFARR	PTVGAQIPEK	IQKAFDDIAK	YFSKEEWEKM	KVSEKIVYVY	MKRKYEAMTK	LGFKAILPSF	MRNKRVTDFQ
**90**	**100**	**110**	**120**	**130**	**140**	**150**	**160**
GNDFDNDPNR	GNQVQRPQMT	FGRLQGIFPK	IMPKKPAEEG	NVSKEVPEAS	GPQNDGKQLC	PPGKPTTSEK	INMISGVLQR
**170**	**180**						
YCRFGSRPLQ							

Cmpd.	No. of Cmpds.	m/z meas.	Δ m/z [ppm]	z	Rt [min]	Score	P	Range	Sequence	Modification
2926	4	992.7825	-63.37	3	65.3	19.3	2	138-163	K.QLCPPGKPTTSEKINMISGVLQRYCR.F	Carbamidomethyl: 25

Detailed Protein Report

Protein 482: PREDICTED: CD300c molecule-like isoform X1 [Homo sapiens]

**Accession:**	gi|530356604	**Score:**	19.2
**Database:**	refseq_human(refseq_human_20140103.fasta)	**MW [kDa]:**	18.8
**Database Date:**	2015-11-30	**pI:**	5.0
**Modification(s):**	Oxidation	**Sequence Coverage [%]:**	7.6
		**No. of unique Peptides:**	1

Alias proteins:

Accession	Name	Description
gi|578846497	refseq_human (refseq_human_20140103.fasta)	PREDICTED: CD300c molecule-like isoform X4 [Homo sapiens]
gi|578831945	refseq_human (refseq_human_20140103.fasta)	PREDICTED: CD300c molecule-like isoform X3 [Homo sapiens]

```
        10         20         30         40         50         60         70         80
MGAVGESLSV QCRYEEKYKT FNKYWCRQPC LPIWHEMVET GGSEGVVRSD QVIITDHPGD LTFTVTLENL TADDAGKYRC
        90        100        110        120        130        140        150        160
GIATILQEDG LSGFLPDPFF QVQVLVSSAS STENSVKTPA SPTRPSQCQG SLPSSTCFLL LPLLKVPLLL SILGAILWVN
       170        180
RPWRTPWTES
```

Cmpd.	No. of Cmpds.	m/z meas.	Δ m/z [ppm]	z	Rt [min]	Score	P	Range	Sequence	Modification
2788	2	676.8636	66.68	2	63.4	19.2	0	1-13	-.MGAVGESLSVQCR.Y	Oxidation: 1

Detailed Protein Report

Protein 483: thyroid peroxidase isoform e precursor [Homo sapiens]

**Accession:**	gi|28558990	**Score:**	18.9
**Database:**	refseq_human(refseq_human_20140103.fasta)	**MW [kDa]:**	84.5
**Database Date:**	2015-11-30	**pI:**	5.9
**Modification(s):**	Oxidation	**Sequence Coverage [%]:**	2.1
		**No. of unique Peptides:**	1

10	20	30	40	50	60	70	80
MRALAVLSVT	LVMACTEAFF	PFISRGKELL	WGKPEESRVS	SVLEESKRLV	DTAMYATMQR	NLKKRGILSP	AQLLSFSKLP
**90**	**100**	**110**	**120**	**130**	**140**	**150**	**160**
EPTSGVIARA	AEIMETSIQA	MKRKVNLKTQ	QSQHPTDALS	EDLLSIIANM	SGCLPYMLPP	KCPNTCLANK	YRPITGACNN
**170**	**180**	**190**	**200**	**210**	**220**	**230**	**240**
RDHPRWGASN	TALARWLPPV	YEDGFSQPRG	WNPGFLYNGF	PLPPVREVTR	HVIQVSNEVV	TDDDRYSDLL	MAWGQYIDHD
**250**	**260**	**270**	**280**	**290**	**300**	**310**	**320**
IAFTPQSTSK	AAFGGGADCQ	MTCENQNPCF	PIQIITLRDY	IPRILGPEAF	QQYVGPYEGY	DSTANPTVSN	VFSTAAFRFG
**330**	**340**	**350**	**360**	**370**	**380**	**390**	**400**
HATIHPLVRR	LDASFQEHPD	LPGLWLHQAF	FSPWTLLRGG	GLDPLIRGLL	ARPAKLQVQD	QLMNEELTER	LFVLSNSSTL
**410**	**420**	**430**	**440**	**450**	**460**	**470**	**480**
DLASINLQRG	RDHGLPGYNE	WREFCGLPRL	ETPADLSTAI	ASRSVADKIL	DLYKHPDNID	VWLGGLAENF	LPRARTGPLF
**490**	**500**	**510**	**520**	**530**	**540**	**550**	**560**
ACLIGKQMKA	LRDGDWFWWE	NSHVFTDAQR	RELEKHSLSR	VICDNTGLTR	VPMDAFQVGK	FPEDFESCDS	ITGMNLEAWR
**570**	**580**	**590**	**600**	**610**	**620**	**630**	**640**
ETFPQDDKCG	FPESVENGDF	VHCEESGRRV	LVYSCRHGYE	LQGREQLTCT	QEGWDFQPPL	CKDVNECADG	AHPPCHASAR
**650**	**660**	**670**	**680**	**690**	**700**	**710**	**720**
CRNTKGGFQC	LCADPYELGD	DGRTCVDSGR	LPRVTWISMS	LAALLIGGFA	GLTSTVICRW	TRTGTKSTLP	ISETGGGTPE
**730**	**740**	**750**	**760**	**770**			
LRCGKHQAVG	TSPQRAAAQD	SEQESAGMEG	RDTHRLPRAL				

Cmpd.	No. of Cmpds.	m/z meas.	Δ m/z [ppm]	z	Rt [min]	Score	P	Range	Sequence	Modification
2490	1	826.6956	-180.11	2	59.4	18.9	0	736-751	R.AAAQDSEQESAGMEGR.D	Oxidation: 13

BRUKER

Detailed Protein Report

Protein 484: PREDICTED: rho guanine nucleotide exchange factor 15 isoform X3 [Homo sapiens]

Accession: gi|578829770
Database: refseq_human(refseq_human_20140103.fasta)
Database Date: 2015-11-30

Score: 18.8
MW [kDa]: 85.0
pI: 10.3
Sequence Coverage [%]: 2.3
No. of unique Peptides: 1

10	20	30	40	50	60	70	80
MSAQSLPAAT	PPTQKPPRII	RPRPPSRSRA	AQSPGPPHNG	SSPQELPRNS	NDAPTPMCTP	IFWEPPAASL	KPPALLPPSA
90	100	110	120	130	140	150	160
SRASLDSQTS	PDSPSSTPTP	SPVSRRSASP	EPAPRSPVPP	PKPSGSPCTP	LLPMAGVLAQ	NGSASAPGTV	RRLAGRFEGG
170	180	190	200	210	220	230	240
AEGRAQDADA	PEPGLQARAD	VNGEREAPLT	GSGSQENGAP	DAGLACPPCC	PCVCHTTRPG	LELRWVPVGG	YEEVPRVPRR
250	260	270	280	290	300	310	320
ASPLRTSRSR	PHPPSIGHPA	VVLTSYRSTA	ERKLLPLLKP	PKPTRVRQDA	TIFGDPPQPD	LDLLSEDGIQ	TGDSPDEAPQ
330	340	350	360	370	380	390	400
NTPPATVEGR	EEEGLEVLKE	QNWELPLQDE	PLYQTYRAAV	LSEELWGVGE	DGSPSPANAG	DAPTFPRPPG	PRNTLWQELP
410	420	430	440	450	460	470	480
AVQASGLLDT	LSPQERRMQE	SLFEVVTSEA	SYLRSLRLLT	DTFVLSQALR	DTLTPRDHHT	LFSNVQRVQG	VSERFLATLL
490	500	510	520	530	540	550	560
SRVRSSPHIS	DLCDVVHAHA	VGPFSVYVDY	VRNQQYQEET	YSRLMDTNVR	FSAELRRLQS	LPKCERLPLP	SFLLLPFQRI
570	580	590	600	610	620	630	640
TRLRMLLQNI	LRQTEEGSSR	QENAQKALGA	VSKIIERCSA	EVGRMKQTEE	LIRLTQRLRF	HKVKALPLVS	WSRRLEFQGE
650	660	670	680	690	700	710	720
LTELGCRRGG	VLFASRPRFT	PLCLLLFSDL	LLITQPKRPS	RFRIHLDPLP	SASPFSATTR	AAPPTDYSKL	LPYQTCSAGW
730	740	750	760	770	780		
EPSQPQAPFP	APQTPSMRTV	TVPRNCVQSR	LHLPRLKDGV	WSPGLPPNTC	TRPLKVG		

Cmpd.	No. of Cmpds.	m/z meas.	Δ m/z [ppm]	z	Rt [min]	Score	P	Range	Sequence	Modification
1331	1	1023.0328	-98.25	2	45.4	18.8	2	268-285	R.STAERKLLPLLKPPKPTR.V	

Detailed Protein Report

Protein 485: T-complex protein 1 subunit gamma isoform c [Homo sapiens]

Accession: gi|58761484
Database: refseq_human(refseq_human_20140103.fasta)
Database Date: 2015-11-30

Score: 18.8
MW [kDa]: 56.4
pI: 6.1
Sequence Coverage [%]: 3.7
No. of unique Peptides: 1

10	20	30	40	50	60	70	80
MMGHRPVLVL	SQNTKRESGR	KVQSGNINAA	KIQVQHPAAK	SMIEISRTQD	EEVGDGTTSV	IILAGEMLSV	AEHFLEQQMH
**90**	**100**	**110**	**120**	**130**	**140**	**150**	**160**
PTVVISAYRK	ALDDMISTLK	KISIPVDISD	SDMMLNIINS	SITTKAISRW	SSLACNIALD	AVKMVQFEEN	GRKEIDIKKY
**170**	**180**	**190**	**200**	**210**	**220**	**230**	**240**
ARVEKIPGGI	IEDSCVLRGV	MINKDVTHPR	MRRYIKNPRI	VLLDSSLEYK	KGESQTDIEI	TREEDFTRIL	QMEEEYIQQL
**250**	**260**	**270**	**280**	**290**	**300**	**310**	**320**
CEDIIQLKPD	VVITEKGISD	LAQHYLMRAN	ITAIRRVRKT	DNNRIARACG	ARIVSRPEEL	REDDVGTGAG	LLEIKKIGDE
**330**	**340**	**350**	**360**	**370**	**380**	**390**	**400**
YFTFITDCKD	PKACTILLRG	ASKEILSEVE	RNLQDAMQVC	RNVLLDPQLV	PGGGASEMAV	AHALTEKSKA	MTGVEQWPYR
**410**	**420**	**430**	**440**	**450**	**460**	**470**	**480**
AVAQALEVIP	RTLIQNCGAS	TIRLLTSLRA	KHTQENCETW	GVNGETGTLV	DMKELGIWEP	LAVKLQTYKT	AVETAVLLLR
**490**	**500**	**510**					
IDDIVSGHKK	KGDDQSRQGG	APDAGQE					

Cmpd.	No. of Cmpds.	m/z meas.	Δ m/z [ppm]	z	Rt [min]	Score	P	Range	Sequence	Modification
93	1	1044.7083	124.99	2	30.4	18.8	2	333-351	K.ACTILLRGASKEILSEVER.N	

Detailed Protein Report

Protein 486: PREDICTED: protein Shroom3 isoform X5 [Homo sapiens]

**Accession:**	gi|578809297	**Score:**	18.7
**Database:**	refseq_human(refseq_human_20140103.fasta)	**MW [kDa]:**	212.8
**Database Date:**	2015-11-30	**pI:**	8.5
		**Sequence Coverage [%]:**	1.6
		**No. of unique Peptides:**	1

Detailed Protein Report

10	20	30	40	50	60	70	80
MLSHEARGLL	SLSNGGDLSQ	MVEEGGKADT	LSSKLQAGDE	VVHINEVTLS	SSRKEAVSLV	KGSYKTLRLV	VRRDVCTDPG
90	100	110	120	130	140	150	160
HADTGASNFV	SPEHLTSGPQ	HRKAAWSGGV	KLRLKHRRSE	PAGRPHSWHT	TKSGEKQPDA	SMMQISQGMI	GPPWHQSYHS
170	180	190	200	210	220	230	240
SSSTSDLSNY	DHAYLRRSPD	QCSSQGSMES	LEPSGAYPPC	HLSPAKSTGS	IDQLSHFHNK	RDSAYSSFST	SSSILEYPHP
250	260	270	280	290	300	310	320
GISGRERSGS	MDNTSARGGL	LEGMRQADIR	YVKTVYDTRR	GVSAEYEVNS	SALLLQGREA	RASANGQGYD	KWSNIPRGKG
330	340	350	360	370	380	390	400
VPPPSWSQQC	PSSLETATDN	LPPKVGAPLP	PARSDSYAAF	RHRERPSSWS	SLDQKRLCRP	QANSLGSLKS	PFIEEQLHTV
410	420	430	440	450	460	470	480
LEKSPENSPP	VKPKHNYTQK	AQPGQPLLPT	SIYPVPSLEP	HFAQVPQPSV	SSNGMLYPAL	AKESGYIAPQ	GACNKMATID
490	500	510	520	530	540	550	560
ENGNQNGSGR	PGFAFCQPLE	HDLLSPVEKK	PEATAKYVPS	KVHFCSVPEN	EEDASLKRHL	TPPQGNSPHS	NERKSTHSNK
570	580	590	600	610	620	630	640
PSSHPHSLKC	PQAQAWQAGE	DKRSSRLSEP	WEGDFQEDHN	ANLWRRLERE	GLGQSLSGNF	GKTKSAFSSL	QNIPESLRRH
650	660	670	680	690	700	710	720
SSLELGRGTQ	EGYPGGRPTC	AVNTKAEDPG	RKAAPDLGSH	LDRQVSYPRP	EGRTGASASF	NSTDPSPEEP	PAPSHPHTSS
730	740	750	760	770	780	790	800
LGRRGPGPGS	ASALQGFQYG	KPHCSVLEKV	SKFEQREQGS	QRPSVGGSGF	GHNYRPHRTV	STSSTSGNDF	EETKAHIRFS
810	820	830	840	850	860	870	880
ESAEPLGNGE	QHFKNGELKL	EEASRQPCGQ	QLSGGASDSG	RGPQRPDARL	LRSQSTFQLS	SEPEREPEWR	DRPGSPESPL
890	900	910	920	930	940	950	960
LDAPFSRAYR	NSIKDAQSRV	LGATSFRRRD	LELGAPVASR	SWRPRPSSAH	VGLRSPEASA	SASPHTPRER	HSVTPAEGDL
970	980	990	1000	1010	1020	1030	1040
ARPVPPAARR	GARRRLTPEQ	KKRSYSEPEK	MNEVGIVEEA	EPAPLGPQRN	GMRFPESSVA	DRRRLFERDG	KACSTLSLSG
1050	1060	1070	1080	1090	1100	1110	1120
PELKQFQQSA	LADYIQRKTG	KRPTSAAGCS	LQEPGPLRER	AQSAYLQPGP	AALEGSGLAS	ASSLSSLREP	SLQPRREATL
1130	1140	1150	1160	1170	1180	1190	1200
LPATVAETQQ	APRDRSSSFA	GGRRLGERRR	GDLLSGANGG	TRGTQRGDET	PREPSSWGAR	AGKSMSAEDL	LERSDVLAGP
1210	1220	1230	1240	1250	1260	1270	1280
VHVRSRSSPA	TADKRQDVLL	GQDSGFGLVK	DPCYLAGPGS	RSLSCSERGQ	EEMLPLFHHL	TPRWGGSGCK	AIGDSSVPSE
1290	1300	1310	1320	1330	1340	1350	1360
CPGTLDHQRQ	ASRTPCPRPP	LAGTQGLVTD	TRAAPLTPIG	TPLPSAIPSG	YCSQDGQTGR	QPLPPYTPAM	MHRSNGHTLT
1370	1380	1390	1400	1410	1420	1430	1440
QPPGPRGCEG	DGPEHGVEEG	TRKRVSLPQW	PPPSRAKWAH	AAREDSLPEE	SSAPDFANLK	HYQKQQSLPS	LCSTSDPDTP
1450	1460	1470	1480	1490	1500	1510	1520
LGAPSTPGRI	SLRISESVLR	DSPPPHEDYE	DEVFVRDPHP	KATSSPTFEP	LPPPPPPPPS	QETPVYSMDD	FPPPPPHTVC
1530	1540	1550	1560	1570	1580	1590	1600
EAQLDSEDPE	GPRPSFNKLS	KVTIARERHM	PGAAHVVGSQ	TLASRLQTSI	KGSEAESTPP	SFMSVHAQLA	GSLGGQPAPI
1610	1620	1630	1640	1650	1660	1670	1680
QTQSLSHDPV	SGTQGLEKKV	SPDPQKSSED	IRTEALAKEI	VHQDKSLADI	LDPDSRLKTT	MDLMEGLFPR	DVNLLKENSV
1690	1700	1710	1720	1730	1740	1750	1760
KRKAIQRTVS	SSGCEGKRNE	DKEAVSMLVN	CPAYYSVSAP	KAELLNKIKE	MPAEVNEEEE	QADVNEKKAE	LIGSLTHKLE
1770	1780	1790	1800	1810	1820	1830	1840
TLQEAKGSLL	TDIKLNNALG	EEVEALISEL	CKPNEFDKYR	MFIGDLDKVV	NLLLSLSGRL	ARVENVLSGL	GEDASNEERS
1850	1860	1870	1880	1890	1900	1910	1920
SLYEKRKILA	GQHEDARELK	ENLDRRERVV	LGILANYLSE	EQLQDYQHFV	KMKSTLLIEQ	RKLDDKIKLG	QEQVKCLLES
1930	1940	1950	1960	1970			
LPSDFIPKAG	ALALPPNLTS	EPIPAGGCTF	SGIFPTLTSP	L			

Cmpd.	No. of Cmpds.	m/z meas.	Δ m/z [ppm]	z	Rt [min]	Score	P	Range	Sequence	Modification
676	1	1106.2349	41.88	3	37.3	18.7	2	73-103	R.RDVCTDPGHADTGASNFVSPEHLTSGPQHRK.A	

Detailed Protein Report

Protein 487: IQ domain-containing protein H isoform 4 [Homo sapiens]

**Accession:**	gi|547235270	**Score:**	18.6
**Database:**	refseq_human(refseq_human_20140103.fasta)	**MW [kDa]:**	71.2
**Database Date:**	2015-11-30	**pI:**	9.8
		**Sequence Coverage [%]:**	3.7
		**No. of unique Peptides:**	1

10	20	30	40	50	60	70	80
MTKAMKVKTP	LRALKSLWDY	DFLIYDGVID	NTAPDFLAFK	EHFSLAWGGI	FSLLEHVEKF	LRNYAIPEVK	IKGNNLVALL
**90**	**100**	**110**	**120**	**130**	**140**	**150**	**160**
PEFELTNKLT	RYDLLSVLED	PAHVQMLINL	PGQRYKGQDG	NSEAAMKIQA	TWKCYKARKF	FLFYRQQKWA	SGVIAIAWLL
**170**	**180**	**190**	**200**	**210**	**220**	**230**	**240**
YCHKTRLKKI	LKESRQRHLE	NFRIRAKHLA	ANWNRIRTSR	RTIIHIPSLG	YSQPVREHIA	DFNTQQNMQL	GRLCDILDAN
**250**	**260**	**270**	**280**	**290**	**300**	**310**	**320**
VNVIYICSHH	MNDELVLYYK	KILSLHAAVK	SGNLEDRSDL	QDRFKIITPE	AVNIFPMIEQ	LSQLITDHLQ	IQRWLFKMDS
**330**	**340**	**350**	**360**	**370**	**380**	**390**	**400**
EFRGNGTAFC	DIPSYLKCYK	WVLKESSRYG	LEDWRKKWAQ	EPALVKISEE	LAGILAQHAQ	PVNEKRFPTW	RKFLQTFLSQ
**410**	**420**	**430**	**440**	**450**	**460**	**470**	**480**
GGVIEAFPPA	DNVTNLTVDM	LIEPNGKISV	LSTGDQLHAE	SPFISSGTTV	PQTSVDPQVL	TYLCLQIGKA	CRMRDVVGYF
**490**	**500**	**510**	**520**	**530**	**540**	**550**	**560**
SIDLVTFIDP	STLEQQVWAT	GLNLAYSDQL	ALTQLTLYLT	NGHLDCSLST	LEVPRFVPKE	RKKTKCMSAL	SMPMLATSRY
**570**	**580**	**590**	**600**	**610**	**620**	**630**	
AVMTTQLRHS	NLSLVFHYVF	LQICRAHGIG	YDLENNRRGS	PGGPHDLCSP	SLHHPSRNIS	T	

Cmpd.	No. of Cmpds.	m/z meas.	Δ m/z [ppm]	z	Rt [min]	Score	P	Range	Sequence	Modification
1719	1	882.4159	18.82	3	50.2	18.6	2	318-340	K.MDSEFRGNGTAFCDIPSYLKCYK.W	

Detailed Protein Report

Protein 488: **PREDICTED: SH3-containing GRB2-like protein 3-interacting protein 1 isoform X9 [Homo sapiens]**

**Accession:**	gi|530363468	**Score:**	18.5
**Database:**	refseq_human(refseq_human_20140103.fasta)	**MW [kDa]:**	68.2
**Database Date:**	2015-11-30	**pI:**	9.4
		**Sequence Coverage [%]:**	1.7
		**No. of unique Peptides:**	1

10	20	30	40	50	60	70	80
MMEGLKKRTR	KAFGIRKKEK	DTDSTGSPDR	DGIKKSNGAP	NGFYAEIDWE	RYNSPELDEE	GYSIRPEEPG	STKGKHFYSS
**90**	**100**	**110**	**120**	**130**	**140**	**150**	**160**
SESEEEEESH	KKFNIKIKPL	QSKDILKNAA	TVDELKASIG	NIALSPSPVG	AIKRNLSSEE	VARPRRSTPT	PELISKKPPD
**170**	**180**	**190**	**200**	**210**	**220**	**230**	**240**
DTTALAPLFG	PPLESAFDEQ	KTEVLLDQPE	IWGSGQPINP	SMESPKLTRP	FPTGTPPPLP	PKNVPATPPR	TGSPLTIGPG
**250**	**260**	**270**	**280**	**290**	**300**	**310**	**320**
ASSPARPATP	LVPCRSTTPP	PPPPRPPSRP	KLPPGKPGVG	DVSRPFSPPI	HSSSPPPIAP	LARAESTSSI	SSTNSLSAAT
**330**	**340**	**350**	**360**	**370**	**380**	**390**	**400**
TPTVENEQPS	LVWFDRGKFY	LTFEGSSRGP	SPLTMGAQDT	LPVAAAFTET	VNAYFKGADP	SKCIVKITGE	MVLSFPAGIT
**410**	**420**	**430**	**440**	**450**	**460**	**470**	**480**
RHFANNPSPA	ALTFRVINFS	RLEHVLPNPQ	LLCCDNTQND	ANTKEFWVNM	PNLMTHLKKV	SEQKPQATYY	NVDMLKYQVS
**490**	**500**	**510**	**520**	**530**	**540**	**550**	**560**
AQGIQSTPLN	LAVNWRCEPS	STDLRIDYKY	NTDAMTTAVA	LNNVQFLVPI	DGGVTKLQAV	LPPAVWNAEQ	QRILWKIPDI
**570**	**580**	**590**	**600**	**610**	**620**	**630**	
SQKSENGGVG	SLLARFQLSE	GPSKPSPLVV	QFTSEGSTLS	GCDIELVGAG	YRFSLIKKRF	AAGKYLADN	

Cmpd.	No. of Cmpds.	m/z meas.	Δ m/z [ppm]	z	Rt [min]	Score	P	Range	Sequence	Modification
363	3	629.3611	47.90	2	33.2	18.5	0	135-145	R.NLSSEEVARPR.R	

Detailed Protein Report

Protein 489: PREDICTED: probable G-protein coupled receptor 142 isoform X1 [Homo sapiens]

**Accession:**	gi|530412100	**Score:**	18.4
**Database:**	refseq_human(refseq_human_20140103.fasta)	**MW [kDa]:**	41.1
**Database Date:**	2015-11-30	**pI:**	10.3
**Modification(s):**	Oxidation	**Sequence Coverage [%]:**	4.8
		**No. of unique Peptides:**	1

10	20	30	40	50	60	70	80
MLTGSCGDPQ	KKPQVTQDSG	PQSMGLEGRE	TAGQPRVTLL	PTPHVSGLSQ	EFESHWPEIA	ERSPCVAGVI	PVIYYSVLLG
90	100	110	120	130	140	150	160
LGLPVSLLTA	VALARLATRT	RRPSYYYLLA	LTASDIIIQV	VIVFAGFLLQ	GAVLARQVPQ	AVVRTANILE	FAANHASVWI
170	180	190	200	210	220	230	240
AILLTVDRYT	ALCHPLHHRA	ASSPGRTRRA	IAAVLSAALL	TGIPFYWWLD	MWRDTDSPRT	LDEVLKWAHC	LTVYFIPCGV
250	260	270	280	290	300	310	320
FLVTNSAIIH	RLRRRGRSGL	QPRVGKSTAI	LLGITTLFTL	LWAPRVFVML	YHMYVAPVHR	DWRVHLALDV	ANMVAMLHTA
330	340	350	360	370	380		
ANFGLYCFVS	KTFRATVRQV	IHDAYLPCTL	ASQPEGMAAK	PVMEPPGLPT	GAEV		

Cmpd.	No. of Cmpds.	m/z meas.	Δ m/z [ppm]	z	Rt [min]	Score	P	Range	Sequence	Modification
2323	1	965.9652	-2.87	2	57.3	18.4	0	12-29	K.KPQVTQDSGPQSMGLEGR.E	Oxidation: 13

Detailed Protein Report

Protein 490: PREDICTED: anamorsin isoform X3 [Homo sapiens]

**Accession:**	gi|530424142	**Score:**	18.3
**Database:**	refseq_human(refseq_human_20140103.fasta)	**MW [kDa]:**	27.5
**Database Date:**	2015-11-30	**pI:**	7.8
		**Sequence Coverage [%]:**	5.9
		**No. of unique Peptides:**	1

10	20	30	40	50	60	70	80
MADFGISAGQ	FVAVVWDKSS	PVEALKGLVD	KLQALTGNEG	RVSVENIKQL	LQCLVPGSTT	LHSAEILAEI	ARILRPGGCL
**90**	**100**	**110**	**120**	**130**	**140**	**150**	**160**
FLKEPVETAV	DNNSKVKTAS	KLCSALTLSG	LVEVKELQRE	PLTPEEVQSV	REHLGHESDN	LLFVQITGKK	PNFEVGSSRQ
**170**	**180**	**190**	**200**	**210**	**220**	**230**	**240**
LKLSITKKSS	PSVKPAVDPA	AAKLWTLSAN	DMEDDSMHLW	PCRRTGKREV	KGTDELPTQV	SLWKLLPGRC	LPLCQLPLPW
**250**	**260**						
DASLQTWGKG	ASE						

Cmpd.	No. of Cmpds.	m/z meas.	Δ m/z [ppm]	z	Rt [min]	Score	P	Range	Sequence	Modification
1841	2	785.8992	-40.49	2	51.4	18.3	1	27-41	K.GLVDKLQALTGNEGR.V	

Detailed Protein Report

Protein 491: histone H1.2 [Homo sapiens]

**Accession:**	gi|4885375	**Score:**	18.3
**Database:**	refseq_human(refseq_human_20140103.fasta)	**MW [kDa]:**	21.4
**Database Date:**	2015-11-30	**pI:**	11.4
		**Sequence Coverage [%]:**	8.0
		**No. of unique Peptides:**	1

10	20	30	40	50	60	70	80
MSETAPAAPA	AAPPAEKAPV	KKKAAKKAGG	TPRKASGPPV	SELITKAVAA	SKERSGVSLA	ALKKALAAAG	YDVEKNNSRI
**90**	**100**	**110**	**120**	**130**	**140**	**150**	**160**
KLGLKSLVSK	GTLVQTKGTG	ASGSFKLNKK	AASGEAKPKV	KKAGGTKPKK	PVGAAKKPKK	AAGGATPKKS	AKKTPKKAKK
**170**	**180**	**190**	**200**	**210**	**220**		
PAAATVTKKV	AKSPKKAKVA	KPKKAAKSAA	KAVKPKAAKP	KVVKPKKAAP	KKK		

Cmpd.	No. of Cmpds.	m/z meas.	Δ m/z [ppm]	z	Rt [min]	Score	P	Range	Sequence	Modification
1937	1	805.3055	-115.03	2	52.9	18.3	0	1-17	-.MSETAPAAPAAAPPAEK.A	

Detailed Protein Report

Protein 492: protein FAM107A isoform a [Homo sapiens]

**Accession:**	gi|6005924	**Score:**	18.1
**Database:**	refseq_human(refseq_human_20140103.fasta)	**MW [kDa]:**	17.4
**Database Date:**	2015-11-30	**pI:**	10.3
		**Sequence Coverage [%]:**	12.5
		**No. of unique Peptides:**	1

Quantitation

WD:WU **Median:** 5.87 **CV:** 0.00 % **No. of Peptides:** 1

Alias proteins:

Accession	Name	Description
gi|530371819	refseq_human (refseq_human_20140103.fasta)	PREDICTED: protein FAM107A isoform X3 [Homo sapiens]
gi|115583665	refseq_human (refseq_human_20140103.fasta)	protein FAM107A isoform a [Homo sapiens]

```
        10         20         30         40         50         60         70         80
MYSEIQRERA DIGGLMARPE YREWNPELIK PKKLLNPVKA SRSHQELHRE LLMNHRRGLG VDSKPELQRV LEHRRRNQLI
        90        100        110        120        130        140        150
KKKKEELEAK RLQCPFEQEL LRRQQRLNQL EKPPEKEEDH APEFIKVREN LRRIATLTSE EREL
```

Cmpd.	No. of Cmpds.	m/z meas.	Δ m/z [ppm]	z	Rt [min]	Score	P	Range	Sequence	Modification	Ratios
2090	1	696.9638	-136.58	3	54.5	18.1	2	57-74	R.RGLGVDSKPELQRVLEHR.R		WD:WU 5.87

Detailed Protein Report

Protein 493: eosinophil peroxidase preproprotein [Homo sapiens]

Accession: gi|4503595
Database: refseq_human(refseq_human_20140103.fasta)
Database Date: 2015-11-30

Score: 18.0
MW [kDa]: 81.0
pI: 11.5
Sequence Coverage [%]: 2.2
No. of unique Peptides: 1

Alias proteins:

Accession	Name	Description
gi|578831504	refseq_human (refseq_human_20140103.fasta)	PREDICTED: eosinophil peroxidase isoform X1 [Homo sapiens]

10	20	30	40	50	60	70	80
MHLLPALAGV	LATLVLAQPC	EGTDPASPGA	VETSVLRDCI	AEAKLLVDAA	YNWTQKSIKQ	RLRSGSASPM	DLLSYFKQPV
**90**	**100**	**110**	**120**	**130**	**140**	**150**	**160**
AATRTVVRAA	DYMHVALGLL	EEKLQPQRSG	PFNVTDVLTE	PQLRLLSQAS	GCALRDQAER	CSDKYRTITG	RCNNKRRPLL
**170**	**180**	**190**	**200**	**210**	**220**	**230**	**240**
GASNQALARW	LPAEYEDGLS	LPFGWTPSRR	RNGFLLPLVR	AVSNQIVRFP	NERLTSDRGR	ALMFMQWGQF	IDHDLDFSPE
**250**	**260**	**270**	**280**	**290**	**300**	**310**	**320**
SPARVAFTAG	VDCERTCAQL	PPCFPIKIPP	NDPRIKNQRD	CIPFFRSAPS	CPQNKNRVRN	QINALTSFVD	ASMVYGSEVS
**330**	**340**	**350**	**360**	**370**	**380**	**390**	**400**
LSLRLRNRTN	YLGLLAINQR	FQDNGRALLP	FDNLHDDPCL	LTNRSARIPC	FLAGDTRSTE	TPKLAAMHTL	FMREHNRLAT
**410**	**420**	**430**	**440**	**450**	**460**	**470**	**480**
ELRRLNPRWN	GDKLYNEARK	IMGAMVQIIT	YRDFLPLVLG	KARARRTLGH	YRGYCSNVDP	RVANVFTLAF	RFGHTMLQPF
**490**	**500**	**510**	**520**	**530**	**540**	**550**	**560**
MFRLDSQYRA	SAPNSHVPLS	SAFFASWRIV	YEGGIDPILR	GLMATPAKLN	RQDAMLVDEL	RDRLFRQVRR	IGLDLAALNM
**570**	**580**	**590**	**600**	**610**	**620**	**630**	**640**
QRSRDHGLPG	YNAWRRFCGL	SQPRNLAQLS	RVLKNQDLAR	KFLNLYGTPD	NIDIWIGAIA	EPLLPGARVG	PLLACLFENQ
**650**	**660**	**670**	**680**	**690**	**700**	**710**	**720**
FRRARDGDRF	WWQKRGVFTK	RQRKALSRIS	LSRIICDNTG	ITTVSRDIFR	ANIYPRGFVN	CSRIPRLNLS	AWRGT

Cmpd.	No. of Cmpds.	m/z meas.	Δ m/z [ppm]	z	Rt [min]	Score	P	Range	Sequence	Modification
2217	1	927.3936	-145.10	2	56.1	18.0	2	547-562	R.QVRRIGLDLAALNMQR.S	

Detailed Protein Report

Protein 494: PREDICTED: epidermal growth factor-like protein 7 isoform X3 [Homo sapiens]

**Accession:**	gi|578817547	**Score:**	17.8
**Database:**	refseq_human(refseq_human_20140103.fasta)	**MW [kDa]:**	23.9
**Database Date:**	2015-11-30	**pI:**	10.4
		**Sequence Coverage [%]:**	8.2
		**No. of unique Peptides:**	1

Alias proteins:

Accession	Name	Description
gi|578817549	refseq_human (refseq_human_20140103.fasta)	PREDICTED: epidermal growth factor-like protein 7 isoform X4 [Homo sapiens]

10	20	30	40	50	60	70	80
MRGSQEVLLM	WLLVLAVGGT	EHAYRPGRRV	CAVRAHGDPV	SESFVQRVYQ	PFLTTCDGHR	ACSTYRTIYR	TAYRRSPGLA
**90**	**100**	**110**	**120**	**130**	**140**	**150**	**160**
PARPRYACCP	GWKRTSGLPG	ACGAAICQPP	CRNGGSCVQP	GRCRCPAGWR	GDTCQSGVDS	AMKEEVQRLQ	SRVDLLEEKL
**170**	**180**	**190**	**200**	**210**	**220**		
QLVLAPLHSL	ASQALEHGLP	DPGSLLVHSF	QQLGRIDSLS	EQISFLEEQL	GSCSCKKDS		

Cmpd.	No. of Cmpds.	m/z meas.	Δ m/z [ppm]	z	Rt [min]	Score	P	Range	Sequence	Modification
1851	2	851.2954	-119.49	2	51.8	17.8	0	95-112	R.TSGLPGACGAAICQPPCR.N	

Detailed Protein Report

Protein 495: transmembrane protein 151A [Homo sapiens]

**Accession:**	gi|23397566	**Score:**	17.8
**Database:**	refseq_human(refseq_human_20140103.fasta)	**MW [kDa]:**	51.2
**Database Date:**	2015-11-30	**pI:**	9.3
		**Sequence Coverage [%]:**	3.6
		**No. of unique Peptides:**	1

10	20	30	40	50	60	70	80
MPEDGAGDGG	EVPALIPDGE	PLREEQRPLK	QSLGSSLCRE	SHWKCLLLTL	LIHACGAVVA	WCRLATVPRL	VLGPEAALAR
90	100	110	120	130	140	150	160
GAGGPPPTYP	ASPCSDGYLY	IPLAFVSLLY	LLYLAECWHC	HVRSCQAPRT	DAHTVLALIR	RLQQAPPCVW	WKATSYHYVR
170	180	190	200	210	220	230	240
RTRQITRYRN	GDAYTTTQVY	HERADSRTAR	GEFDYSAHGV	RDVSKELVGL	AEHAATRLRF	TKCFSFGSAE	AEASYLTQRA
250	260	270	280	290	300	310	320
RFFSANEGLD	DYLEAREGMH	LKDVDFRESL	MVFADPRSPP	WYARAWVFWL	VSAATLSWPL	RVVAAYGTAH	VHYQVEKLFG
330	340	350	360	370	380	390	400
ASSPPPGAVP	SGPPLSRVAT	VDFTELEWHI	CSNRQLVPSY	SEAVVMGAGS	GAYLRGCQRC	RRSVSSNSLP	PARPSGPRLP
410	420	430	440	450	460	470	
FSRSRLSLGA	GGRATPGVFR	SLSGGPLGRR	GEDTEPLESP	PCYEDALYFP	VLIVHGDSGC	QGDGQGAL	

Cmpd.	No. of Cmpds.	m/z meas.	Δ m/z [ppm]	z	Rt [min]	Score	P	Range	Sequence	Modification
1863	1	864.4736	-15.26	2	52.0	17.8	2	414-430	R.ATPGVFRSLSGGPLGRR.G	

Detailed Protein Report

Protein 496: NUT family member 1 isoform 3 [Homo sapiens]

**Accession:**	gi|50233787	**Score:**	17.8
**Database:**	refseq_human(refseq_human_20140103.fasta)	**MW [kDa]:**	120.3
**Database Date:**	2015-11-30	**pI:**	5.2
		**Sequence Coverage [%]:**	1.6
		**No. of unique Peptides:**	1

10	20	30	40	50	60	70	80
MASDGASALP	GPDMSMKPSA	ALSPSPALPF	LPPTSDPPDH	PPREPPPQPI	MPSVFSPDNP	LMLSAFPSSL	LVTGDGGPCL
**90**	**100**	**110**	**120**	**130**	**140**	**150**	**160**
SGAGAGKVIV	KVKTEGGSAE	PSQTQNFILT	QTALNSTAPG	TPCGGLEGPA	PPFVTASNVK	TILPSKAVGV	SQEGPPGLPP
**170**	**180**	**190**	**200**	**210**	**220**	**230**	**240**
QPPPPVAQLV	PIVPLEKAWP	GPHGTTGEGG	PVATLSKPSL	GDRSKISKDV	YENFRQWQRY	KALARRHLSQ	SPDTEALSCF
**250**	**260**	**270**	**280**	**290**	**300**	**310**	**320**
LIPVLRSLAR	LKPTMTLEEG	LPLAVQEWEH	TSNFDRMIFY	EMAERFMEFE	AEEMQIQNTQ	LMNGSQGLSP	ATPLKLDPLG
**330**	**340**	**350**	**360**	**370**	**380**	**390**	**400**
PLASEVCQQP	VYIPKKAASK	TRAPRRRQRK	AQRPPAPEAP	KEIPPEAVKE	YVDIMEWLVG	THLATGESDG	KQEEEGQQQE
**410**	**420**	**430**	**440**	**450**	**460**	**470**	**480**
EEGMYPDPGL	LSYINELCSQ	KVFVSKVEAV	IHPQFLADLL	SPEKQRDPLA	LIEELEQEEG	LTLAQLVQKR	LMALEEEEDA
**490**	**500**	**510**	**520**	**530**	**540**	**550**	**560**
EAPPSFSGAQ	LDSSPSGSVE	DEDGDGRLRP	SPGLQGAGGA	ACLGKVSSSG	KRAREVHGGQ	EQALDSPRGM	HRDGNTLPSP
**570**	**580**	**590**	**600**	**610**	**620**	**630**	**640**
SSWDLQPELA	APQGTPGPLG	VERRGSGKVI	NQVSLHQDGH	LGGAGPPGHC	LVADRTSEAL	PLCWQGGFQP	ESTPSLDAGL
**650**	**660**	**670**	**680**	**690**	**700**	**710**	**720**
AELAPLQGQG	LEKQVLGLQK	GQQTGGRGVL	PQGKEPLAVP	WEGSSGAMWG	DDRGTPMAQS	YDQNPSPRAA	GERDDVCLSP
**730**	**740**	**750**	**760**	**770**	**780**	**790**	**800**
GVWLSSEMDA	VGLELPVQIE	EVIESFQVEK	CVTEYQEGCQ	GLGSRGNISL	GPGETLVPGD	TESSVIPCGG	TVAAAALEKR
**810**	**820**	**830**	**840**	**850**	**860**	**870**	**880**
NYCSLPGPLR	ANSPPLRSKE	NQEQSCETVG	HPSDLWAEGC	FPLLESGDST	LGSSKETLPP	TCQGNLLIMG	TEDASSLPEA
**890**	**900**	**910**	**920**	**930**	**940**	**950**	**960**
SQEAGSRGNS	FSPLLETIEP	VNILDVKDDC	GLQLRVSEDT	CPLNVHSYDP	QGEGRVDPDL	SKPKNLAPLQ	ESQESYTTGT
**970**	**980**	**990**	**1000**	**1010**	**1020**	**1030**	**1040**
PKATSSHQGL	GSTLPRRGTR	NAIVPRETSV	SKTHRSADRA	KGKEKKKKEA	EEEDEELSNF	AYLLASKLSL	SPREHPLSPH
**1050**	**1060**	**1070**	**1080**	**1090**	**1100**	**1110**	**1120**
HASGGQGSQR	ASHLLPAGAK	GPSKLPYPVA	KSGKRALAGG	PAPTEKTPHS	GAQLGVPREK	PLALGVVRPS	QPRKRRCDSF
**1130**	**1140**						
VTGRRKKRRR	SQ						

Cmpd.	No. of Cmpds.	m/z meas.	Δ m/z [ppm]	z	Rt [min]	Score	P	Range	Sequence	Modification
1503	1	671.1069	119.58	3	47.1	17.8	2	800-817	K.RNYCSLPGPLRANSPPLR.S	

Detailed Protein Report

Protein 497: ankyrin repeat domain-containing protein 40 [Homo sapiens]

**Accession:**	gi|16418357	**Score:**	17.8
**Database:**	refseq_human(refseq_human_20140103.fasta)	**MW [kDa]:**	41.1
**Database Date:**	2015-11-30	**pI:**	4.7
**Modification(s):**	Oxidation	**Sequence Coverage [%]:**	2.7
		**No. of unique Peptides:**	1

10	20	30	40	50	60	70	80
MNALLEQKEQ	QERLREAAAL	GDIREVQKLV	ESGVDVNSQN	EVNGWTCLHW	ACKRNHGQVV	SYLLKSGADK	EILTTKGEMP
**90**	**100**	**110**	**120**	**130**	**140**	**150**	**160**
VQLTSRREIR	KIMGVEEEDD	DDDDDDNLPQ	LKKESELPFV	PNYLANPAFP	FIYTPTAEDS	AQMQNGGPST	PPASPPADGS
**170**	**180**	**190**	**200**	**210**	**220**	**230**	**240**
PPLLPPGEPP	LLGTFPRDHT	SLALVQNGDV	SAPSAILRTP	ESTKPGPVCQ	PPVSQSRSLF	SSVPSKPPMS	LEPQNGTYAG
**250**	**260**	**270**	**280**	**290**	**300**	**310**	**320**
PAPAFQPFFF	TGAFPFNMQE	LVLKVRIQNP	SLRENDFIEI	ELDRQELTYQ	ELLRVCCCEL	GVNPDQVEKI	RKLPNTLLRK
**330**	**340**	**350**	**360**	**370**			
DKDVARLQDF	QELELVLMIS	ENNFLFRNAA	STLTERPCYN	RRASKLTY			

Cmpd.	No. of Cmpds.	m/z meas.	Δ m/z [ppm]	z	Rt [min]	Score	P	Range	Sequence	Modification
940	2	567.1435	-248.79	2	40.6	17.8	0	77-86	K.GEMPVQLTSR.R	Oxidation: 3

Detailed Protein Report

Protein 498: PREDICTED: 5-hydroxytryptamine receptor 2B isoform X1 [Homo sapiens]

**Accession:**	gi|530370281	**Score:**	17.7
**Database:**	refseq_human(refseq_human_20140103.fasta)	**MW [kDa]:**	41.1
**Database Date:**	2015-11-30	**pI:**	10.3
		**Sequence Coverage [%]:**	5.0
		**No. of unique Peptides:**	1

10	20	30	40	50	60	70	80
MWPLPLVLCP	AWLFLDVLFS	TASIMHLCAI	SVDRYIAIKK	PIQANQYNSR	ATAFIKITVV	WLISIGIAIP	VPIKGIETDV
**90**	**100**	**110**	**120**	**130**	**140**	**150**	**160**
DNPNNITCVL	TKERFGDFML	FGSLAAFFTP	LAIMIVTYFL	TIHALQKKAY	LVKNKPPQRL	TWLTVSTVFQ	RDETPCSSPE
**170**	**180**	**190**	**200**	**210**	**220**	**230**	**240**
KVAMLDGSRK	DKALPNSGDE	TLMRRTSTIG	KKSVQTISNE	QRASKVLGIV	FFLFLLMWCP	FFITNITLVL	CDSCNQTTLQ
**250**	**260**	**270**	**280**	**290**	**300**	**310**	**320**
MLLEIFVWIG	YVSSGVNPLV	YTLFNKTFRD	AFGRYITCNY	RATKSVKTLR	KRSSKIYFRN	PMAENSKFFK	KHGIRNGINP
**330**	**340**	**350**	**360**	**370**			
AMYQSPMRLR	SSTIQSSSII	LLDTLLLTEN	EGDKTEEQVS	YV			

Cmpd.	No. of Cmpds.	m/z meas.	Δ m/z [ppm]	z	Rt [min]	Score	P	Range	Sequence	Modification
2657	1	973.4534	-27.78	2	61.5	17.7	0	75-92	K.GIETDVDNPNNITCVLTK.E	

Detailed Protein Report

Protein 499: PREDICTED: Golgin-45 isoform X1 [Homo sapiens]

Accession: gi|530365531
Database: refseq_human(refseq_human_20140103.fasta)
Database Date: 2015-11-30

Score: 17.7
MW [kDa]: 30.1
pI: 9.8
Sequence Coverage [%]: 3.7
No. of unique Peptides: 1

10	20	30	40	50	60	70	80
MTTKNLETKV	TVTSSPIRGA	GDGMETEEPP	KSVEVTSGVQ	SRKHHSLQSP	WKKAVPSESP	GVLQLGKMLT	EKAMEVKAVR
90	100	110	120	130	140	150	160
ILVPKAAITH	DIPNKNTKVK	SLGHHKGEFL	GQSEGVIEPN	KELSEVKNVL	EKLKNSERRL	LQDKEGLSNQ	LRVQTEVNRE
170	180	190	200	210	220	230	240
LKKLLVASVG	DDLQYHFERL	AREKNQLILE	NEALGRNTAQ	LSEQLERMSI	QCDVWRSKFL	ASRVMADELT	NSRAALQRQN
250	260	270					
RDAHGAIQDL	LSEREQFRQE	MIATQKF					

Cmpd.	No. of Cmpds.	m/z meas.	Δ m/z [ppm]	z	Rt [min]	Score	P	Range	Sequence	Modification
131	2	608.2888	-81.31	2	30.9	17.7	1	153-162	R.VQTEVNRELK.K	

Detailed Protein Report

Protein 500: **PREDICTED: complement factor H-related protein 2 isoform X1 [Homo sapiens]**

**Accession:**	gi|530364612	**Score:**	17.7
**Database:**	refseq_human(refseq_human_20140103.fasta)	**MW [kDa]:**	22.9
**Database Date:**	2015-11-30	**pI:**	6.1
**Modification(s):**	Carbamidomethyl	**Sequence Coverage [%]:**	13.2
		**No. of unique Peptides:**	1

10	20	30	40	50	60	70	80
MWLLVSVILI	SRISSVGGEG	LCFFPFVENG	HSESSGQTHL	EGDTVQIICN	TGYRLQNNEN	NISCVERGWS	TPPKCRSTIS
**90**	**100**	**110**	**120**	**130**	**140**	**150**	**160**
AEKCGPPPPI	DNGDITSFLL	SVYAPGSSVE	YQCQNLYQLE	GNNQITCRNG	QWSEPPKCLD	PCVISQEIME	KYNIKLKWTN
**170**	**180**	**190**	**200**	**210**			
QQKLYSRTGD	IVEFVCKSGY	HPTKSHSFRA	MCQNGKLVYP	SCEEK			

Cmpd.	No. of Cmpds.	m/z meas.	Δ m/z [ppm]	z	Rt [min]	Score	P	Range	Sequence	Modification
11	1	1069.4808	-32.42	3	29.1	17.7	2	129-155	R.NGQWSEPPKCLDPCVISQEIMEKYNIK.L	Carbamidomethyl: 10

Detailed Protein Report

Protein 501: **PREDICTED: sprouty-related, EVH1 domain-containing protein 2 isoform X2 [Homo sapiens]**

**Accession:**	gi|530367354	**Score:**	17.6
**Database:**	refseq_human(refseq_human_20140103.fasta)	**MW [kDa]:**	25.4
**Database Date:**	2015-11-30	**pI:**	7.8
**Modification(s):**	Carbamidomethyl, Oxidation	**Sequence Coverage [%]:**	7.7
		**No. of unique Peptides:**	1

10	20	30	40	50	60	70	80
MPRPYRQVSF	PDDDEEIVRI	NPREKIWMTG	YEDYRHAPVR	GKYPDPSEDA	DSSYVRFAKG	EVPKHDYNYP	YVDSSDFGLG
**90**	**100**	**110**	**120**	**130**	**140**	**150**	**160**
EDPKGRGGSV	IKTQPSRGKS	RRRKEDGERS	RCVYCRDMFN	HEENRRGHCQ	DAPDSVRTCI	RRVSCMWCAD	SMLYHCMSDP
**170**	**180**	**190**	**200**	**210**	**220**	**230**	
EGDYTDPCSC	DTSDEKFCLR	WMALIALSFL	APCMCCYLPL	RACYHCGVMC	RCCGGKHKAA	A	

Cmpd.	No. of Cmpds.	m/z meas.	Δ m/z [ppm]	z	Rt [min]	Score	P	Range	Sequence	Modification
1522	2	993.5046	109.60	2	47.7	17.6	2	202-218	R.ACYHCGVMCRCCGGKHK.A	Carbamidomethyl: 2, 5; Oxidation: 8

Detailed Protein Report

Protein 502: E-selectin precursor [Homo sapiens]

**Accession:**	gi|187960042	**Score:**	17.6
**Database:**	refseq_human(refseq_human_20140103.fasta)	**MW [kDa]:**	66.6
**Database Date:**	2015-11-30	**pI:**	5.1
**Modification(s):**	Carbamidomethyl, Oxidation	**Sequence Coverage [%]:**	2.6
		**No. of unique Peptides:**	1

10	20	30	40	50	60	70	80
MIASQFLSAL	TLVLLIKESG	AWSYNTSTEA	MTYDEASAYC	QQRYTHLVAI	QNKEEIEYLN	SILSYSPSYY	WIGIRKVNNV
90	100	110	120	130	140	150	160
WVWVGTQKPL	TEEAKNWAPG	EPNNRQKDED	CVEIYIKREK	DVGMWNDERC	SKKKLALCYT	AACTNTSCSG	HGECVETINN
170	180	190	200	210	220	230	240
YTCKCDPGFS	GLKCEQIVNC	TALESPEHGS	LVCSHPLGNF	SYNSSCSISC	DRGYLPSSME	TMQCMSSGEW	SAPIPACNVV
250	260	270	280	290	300	310	320
ECDAVTNPAN	GFVECFQNPG	SFPWNTTCTF	DCEEGFELMG	AQSLQCTSSG	NWDNEKPTCK	AVTCRAVRQP	QNGSVRCSHS
330	340	350	360	370	380	390	400
PAGEFTFKSS	CNFTCEEGFM	LQGPAQVECT	TQGQWTQQIP	VCEAFQCTAL	SNPERGYMNC	LPSASGSFRY	GSSCEFSCEQ
410	420	430	440	450	460	470	480
GFVLKGSKRL	QCGPTGEWDN	EKPTCEAVRC	DAVHQPPKGL	VRCAHSPIGE	FTYKSSCAFS	CEEGFELHGS	TQLECTSQGQ
490	500	510	520	530	540	550	560
WTEEVPSCQV	VKCSSLAVPG	KINMSCSGEP	VFGTVCKFAC	PEGWTLNGSA	ARTCGATGHW	SGLLPTCEAP	TESNIPLVAG
570	580	590	600	610	620		
LSAAGLSLLT	LAPFLLWLRK	CLRKAKKFVP	ASSCQSLESD	GSYQKPSYIL			

Cmpd.	No. of Cmpds.	m/z meas.	Δ m/z [ppm]	z	Rt [min]	Score	P	Range	Sequence	Modification
2878	1	872.9387	57.19	2	64.7	17.6	0	502-517	K.INMSCSGEPVFGTVCK.F	Carbamidomethyl: 15; Oxidation: 3

Detailed Protein Report

Protein 503: amiloride-sensitive sodium channel subunit alpha isoform 1 [Homo sapiens]

Accession: gi|4506815
Database: refseq_human(refseq_human_20140103.fasta)
Database Date: 2015-11-30

Score: 17.6
MW [kDa]: 75.7
pI: 8.5
Sequence Coverage [%]: 2.4
No. of unique Peptides: 1

10	20	30	40	50	60	70	80
MEGNKLEEQD	SSPPQSTPGL	MKGNKREEQG	LGPEPAAPQQ	PTAEEEALIE	FHRSYRELFE	FFCNNTTIHG	AIRLVCSQHN
90	100	110	120	130	140	150	160
RMKTAFWAVL	WLCTFGMMYW	QFGLLFGEYF	SYPVSLNINL	NSDKLVFPAV	TICTLNPYRY	PEIKEELEEL	DRITEQTLFD
170	180	190	200	210	220	230	240
LYKYSSFTTL	VAGSRSRRDL	RGTLPHPLQR	LRVPPPPHGA	RRARSVASSL	RDNNPQVDWK	DWKIGFQLCN	QNKSDCFYQT
250	260	270	280	290	300	310	320
YSSGVDAVRE	WYRFHYINIL	SRLPETLPSL	EEDTLGNFIF	ACRFNQVSCN	QANYSHFHHP	MYGNCYTFND	KNNSNLWMSS
330	340	350	360	370	380	390	400
MPGINNGLSL	MLRAEQNDFI	PLLSTVTGAR	VMVHGQDEPA	FMDDGGFNLR	PGVETSISMR	KETLDRLGGD	YGDCTKNGSD
410	420	430	440	450	460	470	480
VPVENLYPSK	YTQQVCIHSC	FQESMIKECG	CAYIFYPRPQ	NVEYCDYRKH	SSWGYCYYKL	QVDFSSDHLG	CFTKCRKPCS
490	500	510	520	530	540	550	560
VTSYQLSAGY	SRWPSVTSQE	WVFQMLSRQN	NYTVNNKRNG	VAKVNIFFKE	LNYKTNSESP	SVTMVTLLSN	LGSQWSLWFG
570	580	590	600	610	620	630	640
SSVLSVVEMA	ELVFDLLVIM	FLMLLRRFRS	RYWSPGRGGR	GAQEVASTLA	SSPPSHFCPH	PMSLSLSQPG	PAPSPALTAP
650	660	670					
PPAYATLGPR	PSPGGSAGAS	SSTCPLGGP					

Cmpd.	No. of Cmpds.	m/z meas.	Δ m/z [ppm]	z	Rt [min]	Score	P	Range	Sequence	Modification
2868	1	873.7778	-171.39	2	64.6	17.6	0	477-492	R.KPCSVTSYQLSAGYSR.W	

Detailed Protein Report

Protein 504: PREDICTED: leucine-rich repeat and IQ domain-containing protein 1 isoform X6 [Homo sapiens]

**Accession:**	gi|578824314	**Score:**	17.6
**Database:**	refseq_human(refseq_human_20140103.fasta)	**MW [kDa]:**	191.8
**Database Date:**	2015-11-30	**pI:**	5.8
		**Sequence Coverage [%]:**	1.3
		**No. of unique Peptides:**	1

10	20	30	40	50	60	70	80
MDDDDAKLKA	EIEAELDKLS	ISSLEKEDIE	SDAKSETQSD	DSDTDSVELP	ESVLHCINII	KNRSKAVEEL	ILQDLEDTDI
90	100	110	120	130	140	150	160
LSCSYGAVSN	NHMHLRTGLS	TEYEESSEQL	IKILSEIEKE	EFMRSKTDCA	TPDFVPEPSP	HDLPMDEHVL	PDDADINFGY
170	180	190	200	210	220	230	240
CEVEEKCRQS	FEAWQEKQKE	LEDKEKQTLK	AQRDREEKQF	QEEEEKRHCW	MKQFKVEKKK	LENIQKQEQD	KMNDELYKEE
250	260	270	280	290	300	310	320
KIWKEKFKQH	EEYIRNLHLQ	MEEERTRFKD	QQEKEKNSLL	KQQNNAAVKI	QAKYKAFVAY	QKYGPIIKEQ	IESKKRKAQE
330	340	350	360	370	380	390	400
WKEKEAKIRQ	KEEENRKRLE	EEQRIKEERK	KQKEEERKRR	EKEYEEKKNI	VKQEREQLIS	KEKIILREDA	SQQLIISSAL
410	420	430	440	450	460	470	480
KKSGYNNKHL	SLEDISNDKG	DIAKNLVDEN	SKKQEDVLLW	LVEESNMKEN	VDRQTILKES	IQVKLKESIS	SQTILADFKM
490	500	510	520	530	540	550	560
EEKNENLAKK	RCSEELVKQE	RKYENTDNKT	ELGNSDLKGN	LKEQFPLQEL	KSDAQKEEKI	MKHVINENTG	QKTQIILGHN
570	580	590	600	610	620	630	640
QEISEVKTNE	EQKIIKDNQQ	KKIQKVEKEE	IQEQNGLLYK	DKDTLVISVK	QRSLSLTSEN	SKDVRENVIL	QEKEIYSKSK
650	660	670	680	690	700	710	720
EIEENPKDNA	WNSGIVIFNT	TDTMINIEGK	RNDQDYVLGR	HAPCEGLSNY	NAESSMVSKE	VNSLKSEIRN	ISEKCHENAP
730	740	750	760	770	780	790	800
EPDSMTCCVS	ESTLLYSIEE	RRLAWIKSFK	PWLEIFKQNQ	QKKIVRRKRP	VKCPANMTPA	LDKLEILRCG	PWDTLQQVTT
810	820	830	840	850	860	870	880
VTFQDLPGCV	LSTLAECTNL	QFLSLRRCGL	TSLHSLSNCK	KLKYIDAQEN	HIEAIECENL	ENLCVVLLNK	NQLTSLHGLD
890	900	910	920	930	940	950	960
GCTNIQCLEL	SYNKITRIGG	LESLKNLQQL	ILDHNQLINT	KGLCDTPTIV	YLDCSHNHLT	DVEGVENCGL	LQILKLQGNY
970	980	990	1000	1010	1020	1030	1040
LSELPSLENL	VLLRELHLDD	NSISTVEAFS	SYWLPLLQNI	TISQNSLTKI	VPLFHFVSLE	KLDVSHNCLS	DLKSAIKWFD
1050	1060	1070	1080	1090	1100	1110	1120
ACYSLHELSL	TGNPLLQETN	WRDSLLKVLP	ALRILNGNIL	NSNSESRTEE	HNQLGSAGFL	ALCQSQIREF	NLLIENYITG
1130	1140	1150	1160	1170	1180	1190	1200
KGDVFTLDTA	ENLCHYFKKL	MILSTEYRHA	HERGDVTITK	KDESEAQKNH	LAPTNSDSTL	QNGVFYSCAR	EGEPDSPDIP
1210	1220	1230	1240	1250	1260	1270	1280
EKWMDSVSSH	SPLSKSATCE	NMEGRHQEIL	VCQKREDSKA	SSIPTIRIPF	KEVVMTNSLL	RNHQNIEPSE	KIMAAVVIQS
1290	1300	1310	1320	1330	1340	1350	1360
YWRGYLMRRQ	THFSTRLHTA	ATEGLPNSSI	KNQTILKKGK	RENIVNIRKQ	REKAAILIQA	VWKGFILRKK	LTTALEAIKN
1370	1380	1390	1400	1410	1420	1430	1440
EESDEEYREI	DLEDFIFDEA	ALEEEWLALD	STRFPSQTLL	LSNQLHWPKI	PGNLKWDDTS	FNLPSNPAQA	WLCNDKENLS
1450	1460	1470	1480	1490	1500	1510	1520
SSEHTQFNSR	SENKTSSWTP	ESKTSRKSLL	KSEKEKKISE	EWGFKDISTA	QQMLKRAQKM	KSKKLKKKID	STVRLALFKN
1530	1540	1550	1560	1570	1580	1590	1600
NENKVSLPKS	PKMVQPRRDG	YFEGIEEDPI	HKDTTANEKL	ERNREYTYQW	LHTQVGVHET	TSSRNMKCNH	FLPELDPDVL
1610	1620	1630	1640	1650	1660		
NGGRVQLVAR	LVSREDTDLD	LFSMTNGSAL	SVNREKKNQA	HRHSAGSSSK	LWFPSKLI		

Cmpd.	No. of Cmpds.	m/z meas.	Δ m/z [ppm]	z	Rt [min]	Score	P	Range	Sequence	Modification
862	1	1198.5391	-67.11	2	39.2	17.6	1	1290-1311	R.QTHFSTRLHTAATEGLPNSSIK.N	

Detailed Protein Report

Protein 505: **PREDICTED: exosome complex component RRP4 isoform X6 [Homo sapiens]**

**Accession:**	gi|578817232	**Score:**	17.5
**Database:**	refseq_human(refseq_human_20140103.fasta)	**MW [kDa]:**	21.9
**Database Date:**	2015-11-30	**pI:**	10.0
		**Sequence Coverage [%]:**	11.1
		**No. of unique Peptides:**	1

10	20	30	40	50	60	70	80
MAMEMRLPVA	RKPLSERLGR	DTKKHLVVPG	DTITTDTGFM	RGHGTYMGEE	KLIASVAGSV	ERVNKLICVK	ALKTRYIGEV
**90**	**100**	**110**	**120**	**130**	**140**	**150**	**160**
GDIVVGRITE	VQQKRWKVET	NSRLDSVLLL	SSMNLPGGEL	RRRSAEDELA	MRGFLQEGDL	ISAEVQAVFS	DGAVSLHTRS
**170**	**180**	**190**	**200**				
LKYGKDVDLS	CPARTRHGFA	PSSTHLLTYP	EAHISQAR				

Cmpd.	No. of Cmpds.	m/z meas.	Δ m/z [ppm]	z	Rt [min]	Score	P	Range	Sequence	Modification
1784	1	807.3967	-14.46	3	51.0	17.5	0	177-198	R.HGFAPSSTHLLTYPEAHISQAR.-	

Detailed Protein Report

Protein 506: PREDICTED: ras association domain-containing protein 3 isoform X1 [Homo sapiens]

**Accession:**	gi|578823589	**Score:**	17.5
**Database:**	refseq_human(refseq_human_20140103.fasta)	**MW [kDa]:**	26.3
**Database Date:**	2015-11-30	**pI:**	8.8
		**Sequence Coverage [%]:**	4.8
		**No. of unique Peptides:**	1

10	20	30	40	50	60	70	80
MSHCAWPAIW	IFKSTLASSA	YSLPTLQDVE	KEKETHSYLS	KEEIKEKVHK	YNLAVTDKLK	MTLNSNGIYT	GFIKVQMELC
**90**	**100**	**110**	**120**	**130**	**140**	**150**	**160**
KPPQTSPNSG	KLSPSSNGCM	NTLHISSTNT	VGEVIEALLK	KFLVTESPAK	FALYKRCHRE	DQVYACKLSD	REHPLYLRLV
**170**	**180**	**190**	**200**	**210**	**220**	**230**	
AGPRTDTLSF	VLREHEIGEW	EAFSLPELQN	FLRILDKEED	EQLQNLKRRY	TAYRQKLEEA	LREVWKPD	

Cmpd.	No. of Cmpds.	m/z meas.	Δ m/z [ppm]	z	Rt [min]	Score	P	Range	Sequence	Modification
675	1	676.1711	-187.18	2	37.3	17.5	1	137-147	R.CHREDQVYACK.L	

Detailed Protein Report

Protein 507: uncharacterized protein KIAA0947 [Homo sapiens]

**Accession:**	gi|149363685	**Score:**	17.5
**Database:**	refseq_human(refseq_human_20140103.fasta)	**MW [kDa]:**	247.7
**Database Date:**	2015-11-30	**pI:**	5.2
**Modification(s):**	Oxidation	**Sequence Coverage [%]:**	1.1
		**No. of unique Peptides:**	1

Detailed Protein Report

10	20	30	40	50	60	70	80
MMPGETHSAA	PGTAADLSRC	QGCASLQQNL	NEYVEALITL	KQKIINTDNL	LTEYQKKCDE	LQFARRENSN	LHHQVEEMLQ
90	100	110	120	130	140	150	160
KISPLQKCQE	ELGSLKAELE	EKKSSLKLYQ	DTHQEYARVK	EECLKSDAQK	KKLEAKVKKL	QEAAVKQTQD	FKQLRNEKKI
170	180	190	200	210	220	230	240
LEKEFKKTQE	RLDEFSKQKN	EKELRHIGTQ	ISSDSYGSID	KRKVKLLLKE	LWLCVNTTHR	LPGEGSRCVP	EKPAKAITSS
250	260	270	280	290	300	310	320
RVPGEDGTLP	PTQGSPLRTS	NVQTCLTKLS	MEIKEDFLCQ	NVEKQSSSGT	NCSSDHVFNE	NGNLEVLVQS	HRDGGSTEFV
330	340	350	360	370	380	390	400
DHDHFFDEDL	QAAIDFFKLP	PPLLSPVPSP	PPMSSPHPGS	LPSSFAPETY	FGEYTDSSDN	DSVQLRNSAE	CVSEDDTTES
410	420	430	440	450	460	470	480
QNYFGSLRKN	KGSGTWEEKP	KSHEAIQALN	TWEVNKVTTS	GLETFTATLR	ESSATHSLVG	EKHWTTASRS	MSDRKRDILH
490	500	510	520	530	540	550	560
ETKTQMEVRE	MDKSVQTEKT	IHKLTRGLCI	ERLSASPAQE	KEAAPGKSEL	CSSPLGKRPL	NELMESEGKT	VLSKMMGSPK
570	580	590	600	610	620	630	640
SEFTKWTRIN	EITSEPDRIT	VSGHFHRLSR	ELEKEKEDTQ	GFTLGESPES	EDDDSGDGMD	VAGLDIETSF	SSSSTLVALS
650	660	670	680	690	700	710	720
VGSNPQSSSG	LDCGNDTDIT	TKVFSTEPHH	SEHKLQTKTL	NTLHLQSEPP	ECSIGGNNLE	NSLCALSPEL	GASNFNDQKS
730	740	750	760	770	780	790	800
SGIEYTKVVK	GLTKIHSLPR	SVFMKATKDG	QCESQDPRIE	LTLNKPDFTS	LIGSQAALIK	SGLGFVKSTS	WHHSDLLRKG
810	820	830	840	850	860	870	880
GEESLRAKSE	HEQKTSHQLQ	KAMPFLQNRG	PTPKPDLLRE	NNNPVEFKTT	ASVLPNQVSV	ITKQTRPEKV	QSAKLEHLRP
890	900	910	920	930	940	950	960
HRVEPTLVTE	NSGNKTGMST	VAKCDGERDD	TTQNITEVAA	VKSISPEVSA	SRRKLDFNSP	GGSSPVENSD	CSTNSRLSFS
970	980	990	1000	1010	1020	1030	1040
PENILIQNQD	IVREAAVQGD	GQKQRQPQAT	DLDSSGTHGS	EMLPATEVTV	SGGFSVEETS	CGDTGRSGGE	ALAVANDSTS
1050	1060	1070	1080	1090	1100	1110	1120
TPQNANGLWK	LKSTTPGGAL	PECFGTTDTT	FSSAFCRKHG	ETQDTSQSSL	PGTLHCYTGI	REGGDDTEVE	SEAFSCSEGS
1130	1140	1150	1160	1170	1180	1190	1200
EQQDAPDDSQ	KNLGDTDAAV	AEVRPSLEVG	YLTSALQDFN	ISTFSELDRL	STSEVVMFLE	SCQLGDYSSG	DSVSECSSKG
1210	1220	1230	1240	1250	1260	1270	1280
TLSKEMNKEL	KASEIGEKYR	KQPCEEETLG	TCEEWIESEE	DDYSLKNTSQ	LTQCSLETLS	EVLTKIRQEL	QTNSEDCNGK
1290	1300	1310	1320	1330	1340	1350	1360
DTGSLLLLNV	NNNMTTENLK	EKSPFRETTG	SSSHASEPTP	QAAALDTEGS	SPISGMPQNE	NPQSRPEARS	DAGRQTDGGE
1370	1380	1390	1400	1410	1420	1430	1440
EDLPEPVEPS	ALCSDSVMEP	SIEQSSNCEA	ETTFQCQIAT	VTSEVINVLI	NKDQNLVIEK	GDNWTIISGV	AVLPHVDQVT
1450	1460	1470	1480	1490	1500	1510	1520
LCDIPGDIPI	SQDQGELEAG	CIPVTSAEKS	PEASHTGPAF	QEAPCGNNLS	CPQEDVSSSG	QSTNFDKSRL	RNRPVKPSIW
1530	1540	1550	1560	1570	1580	1590	1600
ISSQIYDQNF	ETQIVASDHT	YYNSKLEPSG	KNKNRSKISN	KDQSNKPVKT	SASSRVETHQ	SEVAQSFSGE	KANTKTQRSQ
1610	1620	1630	1640	1650	1660	1670	1680
TQTILANADT	STPTDCSPDT	LSKIRQEVGP	PLPPLLAPLI	ATPPRTSQPL	SPLISSSSPS	SPASPVGQVS	PFRETPVPPA
1690	1700	1710	1720	1730	1740	1750	1760
MSPWPEDPRR	ASPPDPSPSP	SAASASERVV	PSPLQFCAAT	PKHALPVPGR	LPPCASGHAA	VGGPQENSVK	ILDTMYPELS
1770	1780	1790	1800	1810	1820	1830	1840
ARARTLNILK	GNIQLTRGPP	ADCKNLPGPA	SAMIGFKTIT	SAATAFVKTG	SSSGGDCNQD	KSRDLGTQQD	SSGKRTLSTS
1850	1860	1870	1880	1890	1900	1910	1920
TLRSAKRLRL	DTGSPEPETR	GVTAEGIHKN	LPGNLPPAEV	ATTNEERSCS	SPAVSAVSQL	PLSPKETVES	HDKAIANALK
1930	1940	1950	1960	1970	1980	1990	2000
KIAEFSFDLL	PVIRSHVYVG	NISKKPVMRD	QEKEVVYEFS	TTKKHLAECL	LHSILSELKI	QKISMDHNYI	HALCRVYVGI
2010	2020	2030	2040	2050	2060	2070	2080
CRQLGDLERA	RLFCYSLLKE	DFPESEKLTL	FIANMWHDIF	LSQSVINKAM	QLVARQRAKG	EVLNCLRAFL	NWEKNAPVDV
2090	2100	2110	2120	2130	2140	2150	2160
GFMVSKLLLT	IQLCPKTEFQ	PSEKFGEDLS	DNTWEYIFAI	DLLCCHQKWI	WTHDNIISKE	LWPVMDKWIK	YRKGHANIAY
2170	2180	2190	2200	2210	2220	2230	2240
TPDIIIASIL	RLIGRLGQLG	LKEGFPSAVK	NISSVIGMFI	QHAHDEDIPW	GIQLAAVYAL	CDLSPSNPAE	ISKILEAWRR
2250	2260	2270					
EASKSVPSAI	VSCLEEVSAL	STEELG					

Detailed Protein Report

Cmpd.	No. of Cmpds.	m/z meas.	Δ m/z [ppm]	z	Rt [min]	Score	P	Range	Sequence	Modification
9	1	803.7023	-79.81	3	29.3	17.5	1	1785-1808	K.NLPGPASAMIGFKTITSAATAFVK.T	Oxidation: 9

Detailed Protein Report

Protein 508: **histone deacetylase complex subunit SAP25 [Homo sapiens]**

Accession: gi|284004891
Database: refseq_human(refseq_human_20140103.fasta)
Database Date: 2015-11-30

Score: 17.5
MW [kDa]: 20.9
pI: 9.2
Sequence Coverage [%]: 8.5
No. of unique Peptides: 1

10	20	30	40	50	60	70	80
MTPLAPWDPK	YEAKAGPRPV	WGANCSSGAS	FSGRTLCHPS	FWPLYEAASG	RGLRPVAPAT	GHWNGQQAPP	DAGFPVVCCE
**90**	**100**	**110**	**120**	**130**	**140**	**150**	**160**
DVFLSDPLLP	RGQRVPLYLS	KAPQQMMGSL	KLLPPPPIMS	ARVLPRPSPS	RGPSTAWLSG	PELIALTGLL	QMSQGEPRPS
**170**	**180**	**190**	**200**				
SSAVGPPDHT	SDPPSPCGSP	SSSQGADLSL	PQTPDTHCP				

Cmpd.	No. of Cmpds.	m/z meas.	Δ m/z [ppm]	z	Rt [min]	Score	P	Range	Sequence	Modification
2810	2	945.9828	-31.58	2	63.7	17.5	1	95-111	R.VPLYLSKAPQQMMGSLK.L	

Detailed Protein Report

Protein 509: **sushi, von Willebrand factor type A, EGF and pentraxin domain-containing protein 1 precursor [Homo sapiens]**

**Accession:**	gi|148886654	**Score:**	17.4
**Database:**	refseq_human(refseq_human_20140103.fasta)	**MW [kDa]:**	389.9
**Database Date:**	2015-11-30	**pI:**	5.2
		**Sequence Coverage [%]:**	0.3
		**No. of unique Peptides:**	1

Quantitation

WD:WU	**Median:** 2.71	**CV:** 0.00 %	**No. of Peptides:** 1

Detailed Protein Report

10	20	30	40	50	60	70	80
MWPRLAFCCW	GLALVSGWAT	FQQMSPSRNF	SFRLFPETAP	GAPGSIPAPP	APGDEAAGSR	VERLGQAFRR	RVRLLRELSE
90	100	110	120	130	140	150	160
RLELVFLVDD	SSSVGEVNFR	SELMFVRKLL	SDFPVVPTAT	RVAIVTFSSK	NYVVPRVDYI	STRRARQHKC	ALLLQEIPAI
170	180	190	200	210	220	230	240
SYRGGGTYTK	GAFQQAAQIL	LHARENSTKV	VFLITDGYSN	GGDPRPIAAS	LRDSGVEIFT	FGIWQGNIRE	LNDMASTPKE
250	260	270	280	290	300	310	320
EHCYLLHSFE	EFEALARRAL	HEDLPSGSFI	QDDMVHCSYL	CDEGKDCCDR	MGSCKCGTHT	GHFECICEKG	YYGKGLQYEC
330	340	350	360	370	380	390	400
TACPSGTYKP	EGSPGGISSC	IPCPDENHTS	PPGSTSPEDC	VCREGYRASG	QTCELVHCPA	LKPPENGYFI	QNTCNNHFNA
410	420	430	440	450	460	470	480
ACGVRCHPGF	DLVGSSIILC	LPNGLWSGSE	SYCRVRTCPH	LRQPKHGHIS	CSTREMLYKT	TCLVACDEGY	RLEGSDKLTC
490	500	510	520	530	540	550	560
QGNSQWDGPE	PRCVERHCST	FQMPKDVIIS	PHNCGKQPAK	FGTICYVSCR	QGFILSGVKE	MLRCTTSGKW	NVGVQAAVCK
570	580	590	600	610	620	630	640
DVEAPQINCP	KDIEAKTLEQ	QDSANVTWQI	PTAKDNSGEK	VSVHVHPAFT	PPYLFPIGDV	AIVYTATDLS	GNQASCIFHI
650	660	670	680	690	700	710	720
KVIDAEPPVI	DWCRSPPPVQ	VSEKVHAASW	DEPQFSDNSG	AELVITRSHT	QGDLFPQGET	IVQYTATDPS	GNNRTCDIHI
730	740	750	760	770	780	790	800
VIKGSPCEIP	FTPVNGDFIC	TPDNTGVNCT	LTCLEGYDFT	EGSTDKYYCA	YEDGVWKPTY	TTEWPDCAKK	RFANHGFKSF
810	820	830	840	850	860	870	880
EMFYKAARCD	DTDLMKKFSE	AFETTLGKMV	PSFCSDAEDI	DCRLEENLTK	KYCLEYNYDY	ENGFAIGPGG	WGAANRLDYS
890	900	910	920	930	940	950	960
YDDFLDTVQE	TATSIGNAKS	SRIKRSAPLS	DYKIKLIFNI	TASVPLPDER	NDTLEWENQQ	RLLQTLETIT	NKLKRTLNKD
970	980	990	1000	1010	1020	1030	1040
PMYSFQLASE	ILIADSNSLE	TKKASPFCRP	GSVLRGRMCV	NCPLGTYYNL	EHFTCESCRI	GSYQDEEGQL	ECKLCPSGMY
1050	1060	1070	1080	1090	1100	1110	1120
TEYIHSRNIS	DCKAQCKQGT	YSYSGLETCE	SCPLGTYQPK	FGSRSCLSCP	ENTSTVKRGA	VNISACGVPC	PEGKFSRSGL
1130	1140	1150	1160	1170	1180	1190	1200
MPCHPCPRDY	YQPNAGKAFC	LACPFYGTTP	FAGSRSITEC	SSFSSTFSAA	EESVVPPASL	GHIKKRHEIS	SQVFHECFFN
1210	1220	1230	1240	1250	1260	1270	1280
PCHNSGTCQQ	LGRGYVCLCP	LGYTGLKCET	DIDECSPLPC	LNNGVCKDLV	GEFICECPSG	YTGQRCEENI	NECSSSPCLN
1290	1300	1310	1320	1330	1340	1350	1360
KGICVDGVAG	YRCTCVKGFV	GLHCETEVNE	CQSNPCLNNA	VCEDQVGGFL	CKCPPGFLGT	RCGKNVDECL	SQPCKNGATC
1370	1380	1390	1400	1410	1420	1430	1440
KDGANSFRCL	CAAGFTGSHC	ELNINECQSN	PCRNQATCVD	ELNSYSCKCQ	PGFSGKRCET	EQSTGFNLDF	EVSGIYGYVM
1450	1460	1470	1480	1490	1500	1510	1520
LDGMLPSLHA	LTCTFWMKSS	DDMNYGTPIS	YAVDNGSDNT	LLLTDYNGWV	LYVNGREKIT	NCPSVNDGRW	HHIAITWTSA
1530	1540	1550	1560	1570	1580	1590	1600
NGIWKVYIDG	KLSDGGAGLS	VGLPIPGGGA	LVLGQEQDKK	GEGFSPAESF	VGSISQLNLW	DYVLSPQQVK	SLATSCPEEL
1610	1620	1630	1640	1650	1660	1670	1680
SKGNVLAWPD	FLSGIVGKVK	IDSKSIFCSD	CPRLGGSVPH	LRTASEDLKP	GSKVNLFCDP	GFQLVGNPVQ	YCLNQGQWTQ
1690	1700	1710	1720	1730	1740	1750	1760
PLPHCERISC	GVPPPLENGF	HSADDFYAGS	TVTYQCNNGY	YLLGDSRMFC	TDNGSWNGVS	PSCLDVDECA	VGSDCSEHAS
1770	1780	1790	1800	1810	1820	1830	1840
CLNVDGSYIC	SCVPPYTGDG	KNCAEPIKCK	APGNPENGHS	SGEIYTVGAE	VTFSCQEGYQ	LMGVTKITCL	ESGEWNHLIP
1850	1860	1870	1880	1890	1900	1910	1920
YCKAVSCGKP	AIPENGCIEE	LAFTFGSKVT	YRCNKGYTLA	GDKESSCLAN	SSWSHSPPVC	EPVKCSSPEN	INNGKYILSG
1930	1940	1950	1960	1970	1980	1990	2000
LTYLSTASYS	CDTGYSLQGP	SIIECTASGI	WDRAPPACHL	VFCGEPPAIK	DAVITGNNFT	FRNTVTYTCK	EGYTLAGLDT
2010	2020	2030	2040	2050	2060	2070	2080
IECLADGKWS	RSDQQCLAVS	CDEPPIVDHA	SPETAHRLFG	DIAFYYCSDG	YSLADNSQLL	CNAQGKWVPP	EGQDMPRCIA
2090	2100	2110	2120	2130	2140	2150	2160
HFCEKPPSVS	YSILESVSKA	KFAAGSVVSF	KCMEGFVLNT	SAKIECMRGG	QWNPSPMSIQ	CIPVRCGEPP	SIMNGYASGS
2170	2180	2190	2200	2210	2220	2230	2240
NYSFGAMVAY	SCNKGFYIKG	EKKSTCEATG	QWSSPIPTCH	PVSCGEPPKV	ENGFLEHTTG	RIFESEVRYQ	CNPGYKSVGS
2250	2260	2270	2280	2290	2300	2310	2320
PVFVCQANRH	WHSESPLMCV	PLDCGKPPPI	QNGFMKGENF	EVGSKVQFFC	NEGYELVGDS	SWTCQKSGKW	NKKSNPKCMP
2330	2340	2350	2360	2370	2380	2390	2400

Detailed Protein Report

Cmpd.	No. of Cmpds.	m/z meas.	Δ m/z [ppm]	z	Rt [min]	Score	P	Range	Sequence	Modification	Ratios	
2764	2	639.3642	-0.12	2	63.1	17.4	2	903-913	R.IKRSAPLSDYK.I		WD:WU	2.71

Detailed Protein Report

Protein 510: **PREDICTED: chromodomain-helicase-DNA-binding protein 7 isoform X4 [Homo sapiens]**

**Accession:**	gi|578815680	**Score:**	17.4
**Database:**	refseq_human(refseq_human_20140103.fasta)	**MW [kDa]:**	214.1
**Database Date:**	2015-11-30	**pI:**	5.2
**Modification(s):**	Carbamidomethyl	**Sequence Coverage [%]:**	0.8
		**No. of unique Peptides:**	1

Detailed Protein Report

10	20	30	40	50	60	70	80
MILTDCPELR	NIPWRCVVID	EAHRLKNRNC	KLLEGLKMMD	LEHKVLLTGT	PLQNTVEELF	SLLHFLEPSR	FPSETTFMQE
90	100	110	120	130	140	150	160
FGDLKTEEQV	QKLQAILKPM	MLRRLKEDVE	KNLAPKEETI	IEVELTNIQK	KYYRAILEKN	FTFLSKGGGQ	ANVPNLLNTM
170	180	190	200	210	220	230	240
MELRKCCNHP	YLINGAEEKI	LEEFKETHNA	ESPDFQLQAM	IQAAGKLVLI	DKLLPKLKAG	GHRVLIFSQM	VRCLDILEDY
250	260	270	280	290	300	310	320
LIQRRYPYER	IDGRVRGNLR	QAAIDRFSKP	DSDRFVFLLC	TRAGGLGINL	TAADTCIIFD	SDWNPQNDLQ	AQARCHRIGQ
330	340	350	360	370	380	390	400
SKSVKIYRLI	TRNSYEREMF	DKASLKLGLD	KAVLQSMSGR	ENATNGVQQL	SKKEIEDLLR	KGAYGALMDE	EDEGSKFCEE
410	420	430	440	450	460	470	480
DIDQILLRRT	HTITIESEGK	GSTFAKASFV	ASGNRTDISL	DDPNFWQKWA	KKAELDIDAL	NGRNNLVIDT	PRVRKQTRLY
490	500	510	520	530	540	550	560
SAVKEDELME	FSDLESDSEE	KPCAKPRRPQ	DKSQGYARSE	CFRVEKNLLV	YGWGRWTDIL	SHGRYKRQLT	EQDVETICRT
570	580	590	600	610	620	630	640
ILVYCLNHYK	GDENIKSFIW	DLITPTADGQ	TRALVNHSGL	SAPVPRGRKG	KKVKAQSTQP	VVQDADWLAS	CNPDALFQED
650	660	670	680	690	700	710	720
SYKKHLKHHC	NKVLLRVRML	YYLRQEVIGD	QADKILEGAD	SSEADVWIPE	PFHAEVPADW	WDKEADKSLL	IGVFKHGYEK
730	740	750	760	770	780	790	800
YNSMRADPAL	CFLERVGMPD	AKAIAAEQRG	TDMLADGGDG	GEFDREDEDP	EYKPTRTPFK	DEIDEFANSP	SEDKEESMEI
810	820	830	840	850	860	870	880
HATGKHSESN	AELGQLYWPN	TSTLTTRLRR	LITAYQRSYK	RQQMRQEALM	KTDRRRRRPR	EEVRALEAER	EAIISEKRQK
890	900	910	920	930	940	950	960
WTRREEADFY	RVVSTFGVIF	DPVKQQFDWN	QFRAFARLDK	KSDESLEKYF	SCFVAMCRRV	CRMPVKPDDE	PPDLSSIIEP
970	980	990	1000	1010	1020	1030	1040
ITEERASRTL	YRIELLRKIR	EQVLHHPQLG	ERLKLCQPSL	DLPEWWECGR	HDRDLLVGAA	KHGVSRTDYH	ILNDPELSFL
1050	1060	1070	1080	1090	1100	1110	1120
DAHKNFAQNR	GAGNTSSLNP	LAVGFVQTPP	VISSAHIQDE	RVLEQAEGKV	EEPENPAAKE	KCEGKEEEEE	TDGSGKESKQ
1130	1140	1150	1160	1170	1180	1190	1200
ECEAEASSVK	NELKGVEVGA	DTGSKSISEK	GSEEDEEEKL	EDDDKSEESS	QPEAGAVSRG	KNFDEESNAS	MSTARDETRD
1210	1220	1230	1240	1250	1260	1270	1280
GFYMEDGDPS	VAQLLHERTF	AFSFWPKDRV	MINRLDNICE	AVLKGKWPVN	RRQMFDFQGL	IPGYTPTTVD	SPLQKRSFAE
1290	1300	1310	1320	1330	1340	1350	1360
LSMVGQASIS	GSEDITTSPQ	LSKEDALNLS	VPRQRRRRRR	KIEIEAERAA	KRRNLMEMVA	QLRESQVVSE	NGQEKVVDLS
1370	1380	1390	1400	1410	1420	1430	1440
KASREATSST	SNFSSLSSKF	ILPNVSTPVS	DAFKTQMELL	QAGLSRTPTR	HLLNGSLVDG	EPPMKRRRGR	RKNVEGLDLL
1450	1460	1470	1480	1490	1500	1510	1520
FMSHKRTSLS	AEDAEVTKAF	EEDIETPPTR	NIPSPGQLDP	DTRIPVINLE	DGTRLVGEDA	PKNKDLVEWL	KLHPTYTVDM
1530	1540	1550	1560	1570	1580	1590	1600
PSYVPKNADV	LFSSFQKPKQ	KRHRCRNPNK	LDINTLTGEE	RVPVVNKRNG	KKMGGAMAPP	MKDLPRWLEE	NPEFAVAPDW
1610	1620	1630	1640	1650	1660	1670	1680
TDIVKQSGFV	PESMFDRLLT	GPVVRGEGAS	RRGRRPKSEI	ARAAAAAAAV	ASTSGINPLL	VNSLFAGMDL	TSLQNLQNLQ
1690	1700	1710	1720	1730	1740	1750	1760
SLQLAGLMGF	PPGLATAATA	GGDAKNPAAV	LPLMLPGMAG	LPNVFGLGGL	LNNPLSAATG	NTTTASSQGE	PEDSTSKGEE
1770	1780	1790	1800	1810	1820	1830	1840
KGNENEDENK	DSEKSTDAVS	AADSANGSVG	AATAPAGLPS	NPLAFNPFLL	STMAPGLFYP	SMFLPPGLGG	LTLPGFPALA
1850	1860	1870	1880	1890	1900	1910	1920
GLQNAVGSSE	EKAADKAEGG	PFKDGETLEG	SDAEESLDKT	AESSLLEDEI	AQGEELDSLD	GGDEIENNEN	DE

Cmpd.	No. of Cmpds.	m/z meas.	Δ m/z [ppm]	z	Rt [min]	Score	P	Range	Sequence	Modification
2811	1	995.0043	51.30	2	63.6	17.4	0	995-1010	K.LCQPSLDLPEWWECGR.H	Carbamidomethyl: 14

Detailed Protein Report

Protein 511: t-SNARE domain-containing protein 1 [Homo sapiens]

**Accession:**	gi|223972648	**Score:**	17.4
**Database:**	refseq_human(refseq_human_20140103.fasta)	**MW [kDa]:**	55.9
**Database Date:**	2015-11-30	**pI:**	10.2
		**Sequence Coverage [%]:**	1.6
		**No. of unique Peptides:**	1

Quantitation

WD:WU **Median:** 1.10 **CV:** 0.00 % **No. of Peptides:** 1

10	20	30	40	50	60	70	80
MSYGSIARGG	GLGSRGPFGG	PSRQGCQPLE	CARCWTEYGI	RHFPCPSPES	KLQNRCVGKD	GEGDLGPAGT	PIVPRARKRG
90	100	110	120	130	140	150	160
PGVAPEGSRM	PEPTSSPTIG	PRKDSAAGPH	GRMAGPSTTR	AKKRKPNFCP	QETEVLVSKV	SKHHQLLFGT	GLLKAEPTRR
170	180	190	200	210	220	230	240
YRVWSRILQA	VNALGYCRRD	VVDLKHKWRD	LRAVVRRKLG	DLRKAAHGPS	PGSGKPQALA	LTPVEQVVAK	TFSCQALPSE
250	260	270	280	290	300	310	320
GFSLEPPRAT	QVDPCNLQEL	FQEMSANVFR	INSSVTSLER	SLQSLGTPSD	TQELRDSLHT	AQQETNKTIA	ASASSVKQMA
330	340	350	360	370	380	390	400
ELLRSSCPQE	RLQQERPQLD	RLKTQLSDAI	QCYGVVQKKI	AEKSRALLPM	AQRGSKQSPQ	APFAELADDE	KVFNGSDNMW
410	420	430	440	450	460	470	480
QGQEQALLPD	ITEEDLEAIR	LREEAILQME	SNLLDVNQII	KDLASMVSEQ	GEAVDSIEAS	LEAASSHAEA	ARQLLAGASR
490	500	510	520				
HQLQRHKIKC	CFLSAGVTAL	LVIIIIIATS	VRK				

Cmpd.	No. of Cmpds.	m/z meas.	Δ m/z [ppm]	z	Rt [min]	Score	P	Range	Sequence	Modification	Ratios
258	1	442.7344	36.28	2	32.0	17.4	0	1-8	-.MSYGSIAR.G		WD:WU 1.10

Detailed Protein Report

Protein 512: peptidyl-prolyl cis-trans isomerase A-like 4G [Homo sapiens]

**Accession:**	gi|178057341	**Score:**	17.3
**Database:**	refseq_human(refseq_human_20140103.fasta)	**MW [kDa]:**	18.2
**Database Date:**	2015-11-30	**pI:**	10.0
**Modification(s):**	Oxidation	**Sequence Coverage [%]:**	12.8
		**No. of unique Peptides:**	1

```
        10         20         30         40         50         60         70         80
MVNSVIFFDI TVDGKPLGRI SIKQFADKIP KTAENFRALS TGEKGFRYKG SCFHRIIPGF MCQGGDFTHP NGTGDKSIYG
        90        100        110        120        130        140        150        160
EKFDDENLIR KHTGSGILSM ANAGPNTNGS QFFICTAKTE WLDGKHVAFG KVKERVNIVE AMEHFGYRNS KTSKKITIAD
       170
CGQF
```

Cmpd.	No. of Cmpds.	m/z meas.	Δ m/z [ppm]	z	Rt [min]	Score	P	Range	Sequence	Modification
23	1	1104.5165	18.54	2	29.5	17.3	0	56-76	R.IIPGFMCQGGDFTHPNGTGDK.S	Oxidation: 6

Detailed Protein Report

Protein 513: PREDICTED: YLP motif-containing protein 1 isoform X1 [Homo sapiens]

**Accession:**	gi|530404193	**Score:**	17.2
**Database:**	refseq_human(refseq_human_20140103.fasta)	**MW [kDa]:**	231.6
**Database Date:**	2015-11-30	**pI:**	6.0
**Modification(s):**	Oxidation	**Sequence Coverage [%]:**	0.3
		**No. of unique Peptides:**	1

Detailed Protein Report

10	20	30	40	50	60	70	80
MYPNWGRYGG	SSHYPPPPVP	PPPPVALPEA	SPGPGYSSST	TPAAPSSSGF	MSFREQHLAQ	LQQLQQMHQK	QMQCVLQPHH
90	100	110	120	130	140	150	160
LPPPPLPPPP	VMPGGGYGDW	QPPPPPMPPP	PGPALSYQKQ	QQYKHQMLHH	QRDGPPGLVP	MELESPPESP	PVPPGSYMPP
170	180	190	200	210	220	230	240
SQSYMPPPQP	PPSYYPPTSS	QPYLPPAQPS	PSQSPPSQSY	LAPTPSYSSS	SSSSQSYLSH	SQSYLPSSQA	SPSRPSQGHS
250	260	270	280	290	300	310	320
KSQLLAPPPP	SAPPGNKTTV	QQEPLESGAK	NKSTEQQQAA	PEPDPSTMTP	QEQQQYWYRQ	HLLSLQQRTK	VHLPGHKKGP
330	340	350	360	370	380	390	400
VVAKDTPEPV	KEEVTVPATS	QVPESPSSEE	PPLPPPNEEV	PPPLPPEEPQ	SEDPEEDARL	KQLQAAAAHW	QQHQQHRVGF
410	420	430	440	450	460	470	480
QYQGIMQKHT	QLQQILQQYQ	QIIQPPPHIQ	TMSVDMQLRH	YEMQQQQFQH	LYQEWEREFQ	LWEEQLHSYP	HKDQLQEYEK
490	500	510	520	530	540	550	560
QWKTWQGHMK	ATQSYLQEKV	NSFQNMKNQY	MGNMSMPPPF	VPYSQMPPPL	PTMPPPVLPP	SLPPPVMPPA	LPATVPPPGM
570	580	590	600	610	620	630	640
PPPVMPPSLP	TSVPPPGMPP	SLSSAGPPPV	LPPPSLSSAG	PPPVLPPPSL	SSTAPPPVMP	LPPLSSATPP	PGIPPPGVPQ
650	660	670	680	690	700	710	720
GIPPQLTAAP	VPPASSSQSS	QVPEKPRPAL	LPTPVSFGSA	PPTTYHPPLQ	SAGPSEQVNS	KAPLSKSALP	YSSFSSDQGL
730	740	750	760	770	780	790	800
GESSAAPSQP	ITAVKDMPVR	SGGLLPDPPR	SSYLESPRGP	RFDGPRRFED	LGSRCEGPRP	KGPRFEGNRP	DGPRPRYEGH
810	820	830	840	850	860	870	880
PAEGTKSKWG	MIPRGPASQF	YITPSTSLSP	RQSGPQWKGP	KPAFGQQHQQ	QPKSQAEPLS	GNKEPLADTS	SNQQKNFKMQ
890	900	910	920	930	940	950	960
SAAFSIAADV	KDVKAAQSNE	NLSDSQQEPP	KSEVSEGPVE	PSNWDQNVQS	METQIDKAQA	VTQPVPLANK	PVPAQSTFPS
970	980	990	1000	1010	1020	1030	1040
KTGGMEGGTA	VATSSLTADN	DFKPVGIGLP	HSENNQDKGL	PRPDNRDNRL	EGNRGNSSSY	RGPGQSRMED	TRDKGLVNRG
1050	1060	1070	1080	1090	1100	1110	1120
RGQAISRGPG	LVKQEDFRDK	MMGRREDSRE	KMNRGEGSRD	RGLVRPGSSR	EKVPGGLQGS	QDRGAAGSRE	RGPPRRAGSQ
1130	1140	1150	1160	1170	1180	1190	1200
ERGPLRRAGS	RERIPPRRAG	SRERGPPRGP	GSRERGLGRS	DFGRDRGPFR	PEPGDGGEKM	YPYHRDEPPR	APWNHGEERG
1210	1220	1230	1240	1250	1260	1270	1280
HEEFPLDGRN	APMERERLDD	WDRERYWREC	ERDYQDDTLE	LYNREDRFSA	PPSRSHDGDR	RGPWWDDWER	DQDMDEDYNR
1290	1300	1310	1320	1330	1340	1350	1360
EMERDMDRDV	DRISRPMDMY	DRSLDNEWDR	DYGRPLDEQE	SQFRERDIPS	LPPLPPLPPL	PPLDRYRDDR	WREERNREHG
1370	1380	1390	1400	1410	1420	1430	1440
YDRDFRDRGE	LRIREYPERG	DTWREKRDYV	PDRMDWERER	LSDRWYPSDV	DRHSPMAEHM	PSSHHSSEMM	GSDASLDSDQ
1450	1460	1470	1480	1490	1500	1510	1520
GLGGVMVLSQ	RQHEIILKAA	QELKMLREQK	EQLQKMKDFG	SEPQMADHLP	PQESRLQNTS	SRPGMYPPPG	SYRPPPPMGK
1530	1540	1550	1560	1570	1580	1590	1600
PPGSIVRPSA	PPARSSVPVT	RPPVPIPPPP	PPPPLPPPPP	VIKPQTSAVE	QERWDEDSFY	GLWDTNDEQG	LNSEFKSETA
1610	1620	1630	1640	1650	1660	1670	1680
AIPSAPVLPP	PPVHSSIPPP	GPVPMGMPPM	SKPPPVQQTV	DYGHGRDIST	NKVEQIPYGE	RITLRPDPLP	ERSTFETEHA
1690	1700	1710	1720	1730	1740	1750	1760
GQRDRYDRER	DREPYFDRQS	NVIADHRDFK	RDRETHRDRD	RDRGVIDYDR	DRFDRERRPR	DDRAQSYRDK	KDHSSSRRGG
1770	1780	1790	1800	1810	1820	1830	1840
FDRPSYDRKS	DRPVYEGPSM	FGGERRTYPE	ERMPLPAPSL	SHQPPPAPRV	EKKPESKNVD	DILKPPGRES	RPERIVVIMR
1850	1860	1870	1880	1890	1900	1910	1920
GLPGSGKTHV	AKLIRDKEVE	FGGPAPRVLS	LDDYFITEVE	KEEKDPDSGK	KVKKKVMEYE	YEAEMEETYR	TSMFKTFKKT
1930	1940	1950	1960	1970	1980	1990	2000
LDDGFFPFII	LDAINDRVRH	FDQFWSAAKT	KGFEVYLAEM	SADNQTCGKR	NIHGRKLKEI	NKMADHWETA	PRHMMRLDIR
2010	2020	2030	2040	2050	2060	2070	
SLLQDAAIEE	VEMEDFDANI	EEQKEEKKDA	EEEESELGYI	PKSKWEMDTS	EAKLVCHVEE	V	

Cmpd.	No. of Cmpds.	m/z meas.	Δ m/z [ppm]	z	Rt [min]	Score	P	Range	Sequence	Modification
2835	3	746.4789	26.21	1	64.1	17.2	0	1835-1840	R.IVVIMR.G	Oxidation: 5

Detailed Protein Report

Protein 514: trichohyalin [Homo sapiens]

**Accession:**	gi|148746195	**Score:**	17.2
**Database:**	refseq_human(refseq_human_20140103.fasta)	**MW [kDa]:**	253.8
**Database Date:**	2015-11-30	**pI:**	5.6
		**Sequence Coverage [%]:**	0.8
		**No. of unique Peptides:**	1

Detailed Protein Report

10	20	30	40	50	60	70	80
MSPLLRSICD	ITEIFNQYVS	HDCDGAALTK	KDLKNLLERE	FGAVLRRPHD	PKTVDLILEL	LDLDSNGRVD	FNEFLLFIFK
90	100	110	120	130	140	150	160
VAQACYYALG	QATGLDEEKR	ARCDGKESLL	QDRRQEEDQR	RFEPRDRQLE	EEPGQRRRQK	RQEQERELAE	GEEQSEKQER
170	180	190	200	210	220	230	240
LEQRDRQRRD	EELWRQRQEW	QEREERRAEE	EQLQSCKGHE	TEEFPDEEQL	RRRELLELRR	KGREEKQQQR	RERQDRVFQE
250	260	270	280	290	300	310	320
EEEKEWRKRE	TVLRKEEEKL	QEEEPQRQRE	LQEEEEQLRK	LERQELRRER	QEEEQQQQRL	RREQQLRRKQ	EEERREQQEE
330	340	350	360	370	380	390	400
RREQQERREQ	QEERREQQLR	REQEERREQQ	LRREQEEERR	EQQLRREQEE	ERREQQLRRE	QQLRREQQLR	REQQLRREQQ
410	420	430	440	450	460	470	480
LRREQQLRRE	QQLRREQQLR	REQQLRREQE	EERHEQKHEQ	ERREQRLKRE	QEERRDWLKR	EEETERHEQE	RRKQQLKRDQ
490	500	510	520	530	540	550	560
EEERRERWLK	LEEEERREQQ	ERREQQLRRE	QEERREQRLK	RQEEEERLQQ	RLRSEQQLRR	EQEERREQLL	KREEEKRLEQ
570	580	590	600	610	620	630	640
ERREQRLKRE	QEERRDQLLK	REEERRQQRL	KREQEERLEQ	RLKREEVERL	EQEERREQRL	KREEPEEERR	QQLLKSEEQE
650	660	670	680	690	700	710	720
ERRQQQLRRE	QQERREQRLK	REEEEERLEQ	RLKREHEEER	REQELAEEEQ	EQARERIKSR	IPKWQWQLES	EADARQSKVY
730	740	750	760	770	780	790	800
SRPRKQEGQR	RRQEQEEKRR	RRESELQWQE	EERAHRQQQE	EEQRRDFTWQ	WQAEEKSERG	RQRLSARPPL	REQRERQLRA
810	820	830	840	850	860	870	880
EERQQREQRF	LPEEEEKEQR	RRQRREREKE	LQFLEEEEQL	QRERAQQLQ	EEEDGLQEDQ	ERRRSQEQRR	DQKWRWQLEE
890	900	910	920	930	940	950	960
ERKRRRHTLY	AKPALQEQLR	KEQQLLQEEE	EELQREEREK	RRRQEQERQY	REEEQLQQEE	EQLLREEREK	RRRQERERQY
970	980	990	1000	1010	1020	1030	1040
RKDKKLQQKE	EQLLGEEPEK	RRRQEREKKY	REEEELQQEE	EQLLREEREK	RRRQEWERQY	RKKDELQQEE	EQLLREEREK
1050	1060	1070	1080	1090	1100	1110	1120
RRLQERERQY	REEEELQQEE	EQLLGEERET	RRRQELERQY	RKEEELQQEE	EQLLREEPEK	RRRQERERQC	REEEELQQEE
1130	1140	1150	1160	1170	1180	1190	1200
EQLLREEREK	RRRQELERQY	REEEEVQQEE	EQLLREEPEK	RRRQELERQY	REEEELQQEE	EQLLREEQEK	RRQERERQYR
1210	1220	1230	1240	1250	1260	1270	1280
EEEELQRQKR	KQRYRDEDQR	SDLKWQWEPE	KENAVRDNKV	YCKGRENEQF	RQLEDSQLRD	RQSQQDLQHL	LGEQQERDRE
1290	1300	1310	1320	1330	1340	1350	1360
QERRRWQQRD	RHFPEEEQLE	REEQKEAKRR	DRKSQEEKQL	LREEREEKRR	RQETDRKFRE	EEQLLQEREE	QPLRRQERDR
1370	1380	1390	1400	1410	1420	1430	1440
KFREEELRHQ	EQGRKFLEEE	QRLRRQERER	KFLKEEQQLR	CQEREQQLRQ	DRDRKFREEE	QQLSRQERDR	KFREEEQQVR
1450	1460	1470	1480	1490	1500	1510	1520
RQERERKFLE	EEQQLRQERH	RKFREEEQLL	QEREEQQLHR	QERDRKFLEE	EQQLRRQERD	RKFREQELRS	QEPERKFLEE
1530	1540	1550	1560	1570	1580	1590	1600
EQQLHRQQRQ	RKFLQEEQQL	RRQERGQQRR	QDRDRKFREE	EQLRQEREEQ	QLSRQERDRK	FRLEEQKVRR	QEQERKFMED
1610	1620	1630	1640	1650	1660	1670	1680
EQQLRRQEGQ	QQLRQERDRK	FREDEQLLQE	REEQQLHRQE	RDRKFLEEEP	QLRRQEREQQ	LRHDRDRKFR	EEEQLLQEGE
1690	1700	1710	1720	1730	1740	1750	1760
EQQLRRQERD	RKFREEEQQL	RRQERERKFL	QEEQQLRRQE	LERKFREEEQ	LRQETEQEQL	RRQERYRKIL	EEEQLRPERE
1770	1780	1790	1800	1810	1820	1830	1840
EQQLRRQERD	RKFREEEQLR	QEREEQQLRS	QESDRKFREE	EQLRQEREEQ	QLRPQQRDGK	YRWEEEQLQL	EEQEQRLRQE
1850	1860	1870	1880	1890	1900	1910	1920
RDRQYRAEEQ	FATQEKSRRE	EQELWQEEEQ	KRRQERERKL	REEHIRRQQK	EEQRHRQVGE	IKSQEGKGHG	RLLEPGTHQF
1930	1940	1950					
ASVPVRSSPL	YEYIQEQRSQ	YRP					

Cmpd.	No. of Cmpds.	m/z meas.	Δ m/z [ppm]	z	Rt [min]	Score	P	Range	Sequence	Modification
2093	2	974.3559	-134.06	2	54.5	17.2	1	828-842	R.EKELQFLEEEEQLQR.R	

Detailed Protein Report

Protein 515: PREDICTED: uncharacterized protein KIAA1211 isoform X7 [Homo sapiens]

**Accession:**	gi|578808650	**Score:**	17.1
**Database:**	refseq_human(refseq_human_20140103.fasta)	**MW [kDa]:**	124.6
**Database Date:**	2015-11-30	**pI:**	5.5
		**Sequence Coverage [%]:**	1.3
		**No. of unique Peptides:**	1

10	20	30	40	50	60	70	80
MEEKVAPVKP	SRPKRHFSSA	GTIESVNLDA	IPLAIARLDN	SAAKHKLAVK	PKKQRVSKKH	RRLAQDPQHE	QGGLESRPCL
**90**	**100**	**110**	**120**	**130**	**140**	**150**	**160**
DQNGHPGEDK	PTWHEEEPNP	LDSEEERRRQ	EDYWRELEAK	CKRQKAEAAE	KRRLEEQRLQ	ALERRLWEEN	RRQELLEEEG
**170**	**180**	**190**	**200**	**210**	**220**	**230**	**240**
EGQEPPLEAE	RAPREEQQRS	LEAPGWEDAE	RREREERERL	EAEEERRRLQ	AQAQAEERRR	LEEDARLEER	RRQEEEEGRC
**250**	**260**	**270**	**280**	**290**	**300**	**310**	**320**
AEELKRQEEE	EAEGWEELEQ	QEAEVQGPPE	ALEETGEGRR	GAEEEDLGEE	EEEGQAHLED	WRGQLSELLN	DFEERLEDQE
**330**	**340**	**350**	**360**	**370**	**380**	**390**	**400**
RLKPEGQREH	SEEPGICEEQ	NPEAERRREQ	QGRSGDFQGA	DRPGPEEKRE	EGDTEPLLKQ	EGPVEAAQPP	VERKEAAALE
**410**	**420**	**430**	**440**	**450**	**460**	**470**	**480**
QGRKVEELRW	QEVDERQTMP	RPYTFQVSSG	GKQILFPKVN	LSPVTPAKDT	GLTAAPQEPK	APKASPVQHA	LPSSLSVPHT
**490**	**500**	**510**	**520**	**530**	**540**	**550**	**560**
AILVTGAQLC	GPAVNLSQIK	DTACKSLLGL	EEKKHAEAPA	GENPPRGPGD	ARAGSGKAKP	RQESPSSASA	LAEWASIRSR
**570**	**580**	**590**	**600**	**610**	**620**	**630**	**640**
ILKNAESDPR	SSERDQLRPG	DESTPRGRCD	SRGNQRKTPP	VNAKFSIMPA	WQKFSDGGTE	TSKQSTEAES	IRKRPMLGPS
**650**	**660**	**670**	**680**	**690**	**700**	**710**	**720**
EETAPQPPPA	GVRELGKGPE	KSEMHREPAD	TTEGCKFAKD	LPSFLVPSLP	YPPQKVVAHT	EFTTSSDSET	ANGIAKPDPV
**730**	**740**	**750**	**760**	**770**	**780**	**790**	**800**
MPGGEEKASP	FGIKLRRTNY	SLRFNCDQQA	EQKKKKRHSS	TGDSADAGPP	AAGSARGEKE	MEGVALKHGP	SLPQERKQAP
**810**	**820**	**830**	**840**	**850**	**860**	**870**	**880**
STRRDSAEPS	SSRSVPVAHP	GPPPASSQTP	APEHDKAANK	MPLAQKPALA	PKPTSQTPPA	SPLSKLSRPY	LVELLSRRAG
**890**	**900**	**910**	**920**	**930**	**940**	**950**	**960**
RPDPEPSEPS	KEDQESSDRR	PPSPPGPEER	KGQKRDEEEE	ATERKPASPP	LPATQQEKPS	QTPEAGRKEK	PMLQSRHSLD
**970**	**980**	**990**	**1000**	**1010**	**1020**	**1030**	**1040**
GSKLTEKVET	AQPLWITLAL	QKQKGFREQQ	ATREERKQAR	EAKQAEKLSK	ENVSVSVQPG	SSSVSRAGSL	HKSTALPEEK
**1050**	**1060**	**1070**	**1080**	**1090**	**1100**	**1110**	**1120**
RPETAVSRLE	RREQLKKANT	LPTSVTVEIS	DSAPPAPLVK	EVTKRFSTPD	AAPVSTEPAW	LALAKRKAKA	WSDCPQIIK

Cmpd.	No. of Cmpds.	m/z meas.	Δ m/z [ppm]	z	Rt [min]	Score	P	Range	Sequence	Modification
1846	3	734.2644	-176.08	2	51.4	17.1	1	1027-1040	R.AGSLHKSTALPEEK.R	

Detailed Protein Report

Protein 516: protein cramped-like [Homo sapiens]

**Accession:**	gi|223972612	**Score:**	17.1
**Database:**	refseq_human(refseq_human_20140103.fasta)	**MW [kDa]:**	134.6
**Database Date:**	2015-11-30	**pI:**	9.1
		**Sequence Coverage [%]:**	1.3
		**No. of unique Peptides:**	1

10	20	30	40	50	60	70	80
MTVKLGDGGS	GEDGLKKLGK	RAADEESLEG	EGAGGADAAE	ESSGTKRDEK	TPRAGADGPP	APPGAPQAPS	PPQGSPQDQH
**90**	**100**	**110**	**120**	**130**	**140**	**150**	**160**
HFLRSSVRPQ	SKRPRKDPPS	AVGSGNAGGS	GPRGKGAEGG	GSSSGNVSGV	APAAPAGGSR	SSSRNLGSSG	GEKEEGKKVR
**170**	**180**	**190**	**200**	**210**	**220**	**230**	**240**
RQWESWSTED	KNTFFEGLYE	HGKDFEAIQN	NIALKYKKKG	KPASMVKNKE	QVRHFYYRTW	HKITKYIDFD	HVFSRGLKKS
**250**	**260**	**270**	**280**	**290**	**300**	**310**	**320**
SQELYGLICY	GELRKKIGGC	MDDKNATKLN	ELIQVGATTV	RYKGRNLRIK	APMCRALKKL	CDPDGLSDEE	DQKPVRLPLK
**330**	**340**	**350**	**360**	**370**	**380**	**390**	**400**
VPIELQPRNN	HAWARVQSLA	QNPRLRMIVE	LHRKVSSLIE	FLKQKWALHE	VRVRKTLEER	QLQDSCSAPM	QEKVTLHLFP
**410**	**420**	**430**	**440**	**450**	**460**	**470**	**480**
GENCTLTPLP	GVARVVHSKA	FCTVHWQEGG	RCKQSAKDAH	VLPPAQILGI	QSGQGTARGQ	VKCPRSGAEG	KGVGRPPPAA
**490**	**500**	**510**	**520**	**530**	**540**	**550**	**560**
DALQSSGESS	PESAPGEGAA	LSLSSPDAPD	RPPPRHQDTG	PCLEKTPAEG	RDSPTREPGA	LPCACGQLPD	LEDELSLLDP
**570**	**580**	**590**	**600**	**610**	**620**	**630**	**640**
LPRYLKSCQD	LIVPEQCRCA	DTRPGSEQPP	LGGAASPEVL	APVSKEAADL	APTGPSPRPG	PGLLLDVCTK	DLADAPAEEL
**650**	**660**	**670**	**680**	**690**	**700**	**710**	**720**
QEKGSPAGPP	PSQGQPAARP	PKEVPASRLA	QQLREEGWNL	QTSESLTLAE	VYLMMGKPSK	LQLEYDWLGP	GRQDPRPGSL
**730**	**740**	**750**	**760**	**770**	**780**	**790**	**800**
PTALHKQRLL	SCLLKLISTE	VNPKLALEAN	TISTASVRPA	QEEQSMTPPG	KVVTVSSRSP	RCPRNQASLR	SSKTFPPSSA
**810**	**820**	**830**	**840**	**850**	**860**	**870**	**880**
PCSSGLRNPP	RPLLVPGPSS	TGSNDSDGGL	FAVPTTLPPN	SRHGKLFSPS	KEAELTFRQH	LNSISMQSDF	FLPKPRKLRN
**890**	**900**	**910**	**920**	**930**	**940**	**950**	**960**
RHLRKPLVVQ	RTLLPRPSEN	QSHNVCSFSI	LSNSSVTGRG	SFRPIQSSLT	KAALSRPIVP	KVLPPQATSH	LASAIDLAAT
**970**	**980**	**990**	**1000**	**1010**	**1020**	**1030**	**1040**
SAGILSGNPL	PALDTEGLSG	ISPLSSDEVT	GAISGQDSTG	THQDGDTLPT	VGGSDPFVSI	PSRPEQEPVA	DSFQGSSVLS
**1050**	**1060**	**1070**	**1080**	**1090**	**1100**	**1110**	**1120**
LSELPKAPLQ	NGLSIPLSSS	ESSSTRLSPP	DVSALLDISL	PGPPEDALSQ	GEPATHISDS	IIEIAISSGQ	YGEGVPLSPA
**1130**	**1140**	**1150**	**1160**	**1170**	**1180**	**1190**	**1200**
KLNGSDSSKS	LPSPSSSPQP	HWIASPTHDP	QWYPSDSTDS	SLSSLFASFI	SPEKSRKMLP	TPIGTNSGTS	LLGPSLLDGN
**1210**	**1220**	**1230**	**1240**	**1250**	**1260**	**1270**	
SRDSFVSRSL	ADVAEVVDSQ	LVCMMNENSI	DYISRFNDLA	QELSIAEPGR	REALFDGGGG	GPAVSDLSQ	

Cmpd.	No. of Cmpds.	m/z meas.	Δ m/z [ppm]	z	Rt [min]	Score	P	Range	Sequence	Modification
2985	1	846.4337	-1.84	2	66.1	17.1	2	1-17	-.MTVKLGDGGSGEDGLKK.L	

Detailed Protein Report

Protein 517: PREDICTED: mucin-3A [Homo sapiens]

**Accession:**	gi|578844260	**Score:**	17.1
**Database:**	refseq_human(refseq_human_20140103.fasta)	**MW [kDa]:**	21.4
**Database Date:**	2015-11-30	**pI:**	4.4
		**Sequence Coverage [%]:**	9.9
		**No. of unique Peptides:**	1

10	20	30	40	50	60	70	80
MLCADVVETE	VGMEVSVDQQ	FSPDLNDNTS	QAYRDFNKTF	WNQMQKIFAD	MQGFTFKGVE	ILSLRNGSIV	VDYLVLLEMP
**90**	**100**	**110**	**120**	**130**	**140**	**150**	**160**
FSPQLESEYE	QVKTTLKEGL	QNASQDANSC	QDSQTLCFKP	DSIKVNNNSK	TELTPEAICR	RAAPTGYEEF	YFPLVEATRL
**170**	**180**	**190**	**200**				
RCVTKCTSGV	DNAIDCHQGQ	CVLETSGPAC	R				

Cmpd.	No. of Cmpds.	m/z meas.	Δ m/z [ppm]	z	Rt [min]	Score	P	Range	Sequence	Modification
1444	1	1086.6084	26.40	2	46.4	17.1	1	47-65	K.IFADMQGFTFKGVEILSLR.N	

Detailed Protein Report

Protein 518: **alpha-1,3-mannosyl-glycoprotein 2-beta-N-acetylglucosaminyltransferase [Homo sapiens]**

**Accession:**	gi|167857778	**Score:**	17.1
**Database:**	refseq_human(refseq_human_20140103.fasta)	**MW [kDa]:**	50.8
**Database Date:**	2015-11-30	**pI:**	9.8
		**Sequence Coverage [%]:**	3.6
		**No. of unique Peptides:**	1

Alias proteins:

Accession	Name	Description
gi|578811120	refseq_human (refseq_human_20140103.fasta)	PREDICTED: alpha-1,3-mannosyl-glycoprotein 2-beta-N-acetylglucosaminyltransferase isoform X4 [Homo sapiens]
gi|578811118	refseq_human (refseq_human_20140103.fasta)	PREDICTED: alpha-1,3-mannosyl-glycoprotein 2-beta-N-acetylglucosaminyltransferase isoform X3 [Homo sapiens]
gi|530381058	refseq_human (refseq_human_20140103.fasta)	PREDICTED: alpha-1,3-mannosyl-glycoprotein 2-beta-N-acetylglucosaminyltransferase isoform X2 [Homo sapiens]
gi|530381056	refseq_human (refseq_human_20140103.fasta)	PREDICTED: alpha-1,3-mannosyl-glycoprotein 2-beta-N-acetylglucosaminyltransferase isoform X1 [Homo sapiens]
gi|167857786	refseq_human (refseq_human_20140103.fasta)	alpha-1,3-mannosyl-glycoprotein 2-beta-N-acetylglucosaminyltransferase [Homo sapiens]
gi|167857784	refseq_human (refseq_human_20140103.fasta)	alpha-1,3-mannosyl-glycoprotein 2-beta-N-acetylglucosaminyltransferase [Homo sapiens]
gi|167857782	refseq_human (refseq_human_20140103.fasta)	alpha-1,3-mannosyl-glycoprotein 2-beta-N-acetylglucosaminyltransferase [Homo sapiens]
gi|167857780	refseq_human (refseq_human_20140103.fasta)	alpha-1,3-mannosyl-glycoprotein 2-beta-N-acetylglucosaminyltransferase [Homo sapiens]

10	20	30	40	50	60	70	80
MLKKQSAGLV	LWGAILFVAW	NALLLLFFWT	RPAPGRPPSV	SALDGDPASL	TREVIRLAQD	AEVELERQRG	LLQQIGDALS
90	100	110	120	130	140	150	160
SQRGRVPTAA	PPAQPRVPVT	PAPAVIPILV	IACDRSTVRR	CLDKLLHYRP	SAELFPIIVS	QDCGHEETAQ	AIASYGSAVT
170	180	190	200	210	220	230	240
HIRQPDLSSI	AVPPDHRKFQ	GYYKIARHYR	WALGQVFRQF	RFPAAVVVED	DLEVAPDFFE	YFRATYPLLK	ADPSLWCVSA
250	260	270	280	290	300	310	320
WNDNGKEQMV	DASRPELLYR	TDFFPGLGWL	LLAELWAELE	PKWPKAFWDD	WMRRPEQRQG	RACIRPEISR	TMTFGRKGVS
330	340	350	360	370	380	390	400
HGQFFDQHLK	FIKLNQQFVH	FTQLDLSYLQ	REAYDRDFLA	RVYGAPQLQV	EKVRTNDRKE	LGEVRVQYTG	RDSFKAFAKA
410	420	430	440	450			
LGVMDDLKSG	VPRAGYRGIV	TFQFRGRRVH	LAPPLTWEGY	DPSWN			

Cmpd.	No. of Cmpds.	m/z meas.	Δ m/z [ppm]	z	Rt [min]	Score	P	Range	Sequence	Modification
1777	1	881.9118	17.60	2	50.6	17.1	0	231-246	K.ADPSLWCVSAWNDNGK.E	

Detailed Protein Report

Protein 519: deoxyribonuclease-1-like 2 precursor [Homo sapiens]

**Accession:**	gi|4503347	**Score:**	17.0
**Database:**	refseq_human(refseq_human_20140103.fasta)	**MW [kDa]:**	32.8
**Database Date:**	2015-11-30	**pI:**	4.6
		**Sequence Coverage [%]:**	5.4
		**No. of unique Peptides:**	1

Alias proteins:

Accession	Name	Description
gi|578828006	refseq_human (refseq_human_20140103.fasta)	PREDICTED: deoxyribonuclease-1-like 2 isoform X3 [Homo sapiens]
gi|530407699	refseq_human (refseq_human_20140103.fasta)	PREDICTED: deoxyribonuclease-1-like 2 isoform X2 [Homo sapiens]

```
        10         20         30         40         50         60         70         80
MGGPRALLAA LWALEAAGTA ALRIGAFNIQ SFGDSKVSDP ACGSIIAKIL AGYDLALVQE VRDPDLSAVS ALMEQINSVS
        90        100        110        120        130        140        150        160
EHEYSFVSSQ PLGRDQYKEM YLFVYRKDAV SVVDTYLYPD PEDVFSREPF VVKFSAPGTG ERAPPLPSRR ALTPPPLPAA
       170        180        190        200        210        220        230        240
AQNLVLIPLH AAPHQAVAEI DALYDVYLDV IDKWGTDDML FLGDFNADCS YVRAQDWAAI RLRSSEVFKW LIPDSADTTV
       250        260        270        280        290        300
GNSDCAYDRI VACGARLRRS LKPQSATVHD FQEEFGLDQT QALAISDHFP VEVTLKFHR
```

Cmpd.	No. of Cmpds.	m/z meas.	Δ m/z [ppm]	z	Rt [min]	Score	P	Range	Sequence	Modification
1326	1	939.1023	103.59	2	45.3	17.0	2	214-229	R.AQDWAAIRLRSSEVFK.W	

Detailed Protein Report

Protein 520: protocadherin alpha-1 isoform 2 precursor [Homo sapiens]

**Accession:**	gi|14165400	**Score:**	17.0
**Database:**	refseq_human(refseq_human_20140103.fasta)	**MW [kDa]:**	87.6
**Database Date:**	2015-11-30	**pI:**	4.6
		**Sequence Coverage [%]:**	1.1
		**No. of unique Peptides:**	1

Quantitation

WD:WU **Median:** 0.39 **CV:** 0.00 % **No. of Peptides:** 1

10	20	30	40	50	60	70	80
MVFSRRGGLG	ARDLLLWLLL	LAAWEVGSGQ	LHYSIPEEAK	HGTFVGRVAQ	DLGLELAELV	PRLFRVASKT	HRDLLEVNLQ
90	100	110	120	130	140	150	160
NGILFVNSRI	DREELCQWSA	ECSIHLELIA	DRPLQVFHVE	VKVKDINDNP	PVFRGREQII	FIPESRLLNS	RFPIEGAADA
170	180	190	200	210	220	230	240
DIGANALLTY	TLSPSDYFSL	DVEASDELSK	SLWLELRKYL	DREETPELHL	LLTATDGGKP	ELQGTVELLI	TVLDVNDNAP
250	260	270	280	290	300	310	320
LFDQAVYRVH	LLETTANGTL	VTTLNASDAD	EGVNGEVVFS	FDSGISRDIQ	EKFKVDSSSG	EIRLIDKLDY	EETKSYEIQV
330	340	350	360	370	380	390	400
KAVDKGSPPM	SNHCKVLVKV	LDVNDNAPEL	AVTSLYLPIR	EDAPLSTVIA	LITVSDRDSG	ANGQVTCSLM	PHVPFKLVST
410	420	430	440	450	460	470	480
FKNYYSLVLD	SALDRESLSV	YELVVTARDG	GSPSLWATAR	VSVEVADVND	NAPAFAQPEY	TVFVKENNPP	GCHIFTVSAR
490	500	510	520	530	540	550	560
DADAQENALV	SYSLVERRVG	ERALSNYVSV	HAESGKVYAL	QPLDHEELEL	LQFQVSARDA	GVPPLGSNVT	LQVFVLDEND
570	580	590	600	610	620	630	640
NAPALLAPRV	GGTIGAVSEL	VPRLVGAGHV	VAKVRAVDAD	SGYNAWLSYE	LQPAAGGARI	PFRVGLYTGE	ISTTRVLDEA
650	660	670	680	690	700	710	720
DLSRYRLLVL	VKDHGEPALT	ATATVLVSLV	ESGQAPKASS	RASVGVAGPE	AALVDVNVYL	IIAICAVSSL	LVLTLLLYTA
730	740	750	760	770	780	790	800
LRCSVPPTEG	AYVPGKPTLV	CSSALGSWSN	SQQRRQRVCS	SEGPPKTDLM	AFSPGLSPSL	NTSERNEQPE	ANLDLSGNVS
810							
PTFEFWL							

Cmpd.	No. of Cmpds.	m/z meas.	Δ m/z [ppm]	z	Rt [min]	Score	P	Range	Sequence	Modification	Ratios	
642	1	509.1279	-267.69	2	36.9	17.0	0	636-644	R.VLDEADLSR.Y		WD:WU	0.39

Detailed Protein Report

Protein 521: fibrinogen-like protein 1 precursor [Homo sapiens]

**Accession:**	gi|42544189	**Score:**	16.9
**Database:**	refseq_human(refseq_human_20140103.fasta)	**MW [kDa]:**	36.4
**Database Date:**	2015-11-30	**pI:**	5.5
		**Sequence Coverage [%]:**	3.5
		**No. of unique Peptides:**	1

Alias proteins:

Accession	Name	Description
gi|42544202	refseq_huma (refseq_human_20140103.fasta)	fibrinogen-like protein 1 precursor [Homo sapiens]
gi|42544200	refseq_huma (refseq_human_20140103.fasta)	fibrinogen-like protein 1 precursor [Homo sapiens]
gi|42544198	refseq_huma (refseq_human_20140103.fasta)	fibrinogen-like protein 1 precursor [Homo sapiens]

10	20	30	40	50	60	70	80
MAKVFSFILV	TTALTMGREI	SALEDCAQEQ	MRLRAQVRLL	ETRVKQQQVK	IKQLLQENEV	QFLDKGDENT	VIDLGSKRQY
**90**	**100**	**110**	**120**	**130**	**140**	**150**	**160**
ADCSEIFNDG	YKLSGFYKIK	PLQSPAEFSV	YCDMSDGGGW	TVIQRRSDGS	ENFNRGWKDY	ENGFGNFVQK	HGEYWLGNKN
**170**	**180**	**190**	**200**	**210**	**220**	**230**	**240**
LHFLTTQEDY	TLKIDLADFE	KNSRYAQYKN	FKVGDEKNFY	ELNIGEYSGT	AGDSLAGNFH	PEVQWWASHQ	RMKFSTWDRD
**250**	**260**	**270**	**280**	**290**	**300**	**310**	**320**
HDNYEGNCAE	EDQSGWWFNR	CHSANLNGVY	YSGPYTAKTD	NGIVWYTWHG	WWYSLKSVVM	KIRPNDFIPN	VI

Cmpd.	No. of Cmpds.	m/z meas.	Δ m/z [ppm]	z	Rt [min]	Score	P	Range	Sequence	Modification
1103	1	1355.1276	221.32	1	42.5	16.9	2	33-43	R.LRAQVRLLETR.V	

Detailed Protein Report

Protein 522: formin-1 isoform c [Homo sapiens]

**Accession:**	gi|472235321	**Score:**	16.9
**Database:**	refseq_human(refseq_human_20140103.fasta)	**MW [kDa]:**	71.8
**Database Date:**	2015-11-30	**pI:**	10.1
		**Sequence Coverage [%]:**	1.4
		**No. of unique Peptides:**	1

Quantitation

WD:WU **Median:** 0.98 **CV:** 0.00 % **No. of Peptides:** 1

10	20	30	40	50	60	70	80
MEGTHCTLQL	HKPITELCYI	SFCLPKGEVR	GFSYKGTVTL	DRSNKGFHNC	YQVREESDII	SLSQEPDEHP	GDIFFKQTPT
**90**	**100**	**110**	**120**	**130**	**140**	**150**	**160**
KDILTELYKL	TTERERLLTN	LLSSDHILGI	TMGNQEGKLQ	ELSVSLAPED	DCFQSAGDWQ	GELPVGPLNK	RSTHGNKKPR
**170**	**180**	**190**	**200**	**210**	**220**	**230**	**240**
RSSGRRESFG	ALPQKRTKRK	GRGGRESAPL	MGKDKICSSH	SLPLSRTRPN	LWVLEEKGNL	LPNGALACSL	QRRESCPPDI
**250**	**260**	**270**	**280**	**290**	**300**	**310**	**320**
PKTPDTDLGF	GSFETAFKDT	GLGREVLPPD	CSSTEAGGDG	IRRPPSGLEH	QQTGLSESHQ	DPEKHPEAEK	DEMEKPAKRT
**330**	**340**	**350**	**360**	**370**	**380**	**390**	**400**
CKQKPVSKVV	AKVQDLSSQV	QRVVKTHSKG	KETIAIRPAA	HAEFVPKADL	LTLPGAEAGA	HGSRRQGKER	QGDRSSQSPA
**410**	**420**	**430**	**440**	**450**	**460**	**470**	**480**
GETASISSVS	ASAEGAVNKV	PLKVIESEKL	DEAPEGKRLG	FPVHTSVPHT	RPETRNKRRA	GLPLGGHKSL	FLDLPHKVGP
**490**	**500**	**510**	**520**	**530**	**540**	**550**	**560**
DSSQPRGDKK	KPSPPAPAAL	GKVFNNSASQ	SSTHKQTSPV	PSPLSPRLPS	PQQHHRILRL	PALPGEREAA	LNDSPCRKSR
**570**	**580**	**590**	**600**	**610**	**620**	**630**	**640**
VFSGCVSADT	LEPPSSAKVT	ETKGASPAFL	RAGQPRLVPG	ETLEKSLGPG	KTTAEPQHQS	PPAFHWDLQQ	HFQEPVIRTV
**650**	**660**	**670**					
SISCASNLIK	EEAGKGKESR	SG					

Cmpd.	No. of Cmpds.	m/z meas.	Δ m/z [ppm]	z	Rt [min]	Score	P	Range	Sequence	Modification	Ratios
787	1	509.7081	-159.31	2	38.3	16.9	1	320-328	R.TCKQKPVSK.V		WD:WU 0.98

Detailed Protein Report

Protein 523: E3 ubiquitin-protein ligase ZNF645 [Homo sapiens]

**Accession:**	gi|22749189	**Score:**	16.9
**Database:**	refseq_human(refseq_human_20140103.fasta)	**MW [kDa]:**	48.8
**Database Date:**	2015-11-30	**pI:**	10.3
**Modification(s):**	Oxidation	**Sequence Coverage [%]:**	2.8
		**No. of unique Peptides:**	1

10	20	30	40	50	60	70	80
MNKMPAGEQE	CEYNKEGKYY	SKGVKLVRKK	KKIPGYRWGD	IKINIIGEKD	DLPIHFCDKC	DLPIKIYGRI	IPCKHAFCYH
**90**	**100**	**110**	**120**	**130**	**140**	**150**	**160**
CANLYDKVGY	KVCPRCRYPV	LRIEAHKRGS	VFMCSIVQQC	KRTYLSQKSL	QAHIKRRHKR	ARKQVTSASL	EKVRPHIAPP
**170**	**180**	**190**	**200**	**210**	**220**	**230**	**240**
QTEISDIPKR	LQDRDHLSYI	PPEQHTMVSL	PSVQHMLQEQ	HNQPHKDIQA	PPPELSLSLP	FPIQWETVSI	FTRKHGNLTV
**250**	**260**	**270**	**280**	**290**	**300**	**310**	**320**
DHIQNNSDSG	AKKPTPPDYY	PECQSQPAVS	SPHHIIPQKQ	HYAPPPSPSS	PVNHQMPYPP	QDVVTPNSVR	SQVPALTTTY
**330**	**340**	**350**	**360**	**370**	**380**	**390**	**400**
DPSSGYIIVK	VPPDMNSPPL	RAPQSQNGNP	SASEFASHHY	NLNILPQFTE	NQETLSPQFT	QTDAMDHRRW	PAWKRLSPCP
**410**	**420**	**430**					
PTRSPPPSTL	HGRSHHSHQR	RHRRY					

Cmpd.	No. of Cmpds.	m/z meas.	Δ m/z [ppm]	z	Rt [min]	Score	P	Range	Sequence	Modification
2100	1	707.7227	-87.06	2	54.8	16.9	0	4-15	K.MPAGEQECEYNK.E	Oxidation: 1

Detailed Protein Report

Protein 524: **PREDICTED: thrombospondin type-1 domain-containing protein 7A isoform X4 [Homo sapiens]**

**Accession:**	gi|578813343	**Score:**	16.9
**Database:**	refseq_human(refseq_human_20140103.fasta)	**MW [kDa]:**	169.7
**Database Date:**	2015-11-30	**pI:**	9.0
**Modification(s):**	Carbamidomethyl	**Sequence Coverage [%]:**	1.3
		**No. of unique Peptides:**	1

10	20	30	40	50	60	70	80
MGLQARRWAS	GSRGAAGPRR	GVLQLLPLPL	PLPLLLLLLL	RPGAGRAAAQ	GEAEAPTLYL	WKTGPWGRCM	GDECGPGGIQ
90	100	110	120	130	140	150	160
TRAVWCAHVE	GWTTLHTNCK	QAERPNNQQN	CFKVCDWHKE	LYDWRLGPWN	QCQPVISKSL	EKPLECIKGE	EGIQVREIAC
170	180	190	200	210	220	230	240
IQKDKDIPAE	DIICEYFEPK	PLLEQACLIP	CQQDCIVSEF	SAWSECSKTC	GSGLQHRTRH	VVAPPQFGGS	GCPNLTEFQV
250	260	270	280	290	300	310	320
CQSSPCEAEE	LRYSLHVGPW	STCSMPHSRQ	VRQARRRGKN	KEREKDRSKG	VKDPEARELI	KKKRNRNRQN	RQENKYWDIQ
330	340	350	360	370	380	390	400
IGYQTREVMC	INKTGKAADL	SFCQQEKLPM	TFQSCVITKE	CQVSEWSEWS	PCSKTCHDMV	SPAGTRVRTR	TIRQFPIGSE
410	420	430	440	450	460	470	480
KECPEFEEKE	PCLSQGDGVV	PCATYGWRTT	EWTECRVDPL	LSQQDKRRGN	QTALCGGGIQ	TREVYCVQAN	ENLLSQLSTH
490	500	510	520	530	540	550	560
KNKEASKPMD	LKLCTGPIPN	TTQLCHIPCP	TECEVSPWSA	WGPCTYENCN	DQQGKKGFKL	RKRRITNEPT	GGSGVTGNCP
570	580	590	600	610	620	630	640
HLLEAIPCEE	PACYDWKAVR	LGNCEPDNGK	ECGPGTQVQE	VVCINSDGEE	VDRQLCRDAI	FPIPVACDAP	CPKDCVLSTW
650	660	670	680	690	700	710	720
STWSSCSHTC	SGKTTEGKQI	RARSILAYAG	EEGGIRCPNS	SALQEVRSCN	EHPCTVYHWQ	TGPWGQCIED	TSVSSFNTTT
730	740	750	760	770	780	790	800
TWNGEASCSV	GMQTRKVICV	RVNVGQVGPK	KCPESLRPET	VRPCLLPCKK	DCIVTPYSDW	TSCPSSCKEG	DSSIRKQSRH
810	820	830	840	850	860	870	880
RVIIQLPANG	GRDCTDPLYE	EKACEAPQAC	QSYRWKTHKW	RRCQLVPWSV	QQDSPGAQEG	CGPGRQARAI	TCRKQDGGQA
890	900	910	920	930	940	950	960
GIHECLQYAG	PVPALTQACQ	IPCQDDCQLT	SWSKFSSCNG	DCGAVRTRKR	TLVGKSKKKE	KCKNSHLYPL	IETQYCPCDK
970	980	990	1000	1010	1020	1030	1040
YNAQPVGNWS	DCILPEGKVE	VLLGMKVQGD	IKECGQGYRY	QAMACYDQNG	RLVETSRCNS	HGYIEEACII	PCPSDCKLSE
1050	1060	1070	1080	1090	1100	1110	1120
WSNWSRCSKS	CGSGVKVRSK	WLREKPYNGG	RPCPKLDHVN	QAQVYEVVPC	HSDCNQYLWV	TEPWSICKVT	FVNMRENCGE
1130	1140	1150	1160	1170	1180	1190	1200
GVQTRKVRCM	QNTADGPSEH	VEDYLCDPEE	MPLGSRVCKL	PCPEDCVISE	WGPWTQCVLP	CNQSSFRQRS	ADPIRQPADE
1210	1220	1230	1240	1250	1260	1270	1280
GRSCPNAVEK	EPCNLNKNCY	HYDYNVTDWS	TCQLSEKAVC	GNGIKTRMLD	CVRSDGKSVD	LKYCEALGLE	KNWQMNTSCM
1290	1300	1310	1320	1330	1340	1350	1360
VECPVNCQLS	DWSPWSECSQ	TCGLTGKMIR	RRTVTQPFQG	DGRPCPSLMD	QSKPCPVKPC	YRWQYGQWSP	CQVQEAQCGE
1370	1380	1390	1400	1410	1420	1430	1440
GTRTRNISCV	VSDGSADDFS	KVVDEEFCAD	IELIIDGNKN	MVLEESCSQP	CPGDCYLKDW	SSWSLCQLTC	VNGEDLGFGG
1450	1460	1470	1480	1490	1500	1510	1520
IQVRSRPVII	QELENQHLCP	EQMLETKSCY	DGQCYEYKWM	ASAWKGSSRT	VWCQRSDGIN	VTAKSQRNPK	EGKTTD

Cmpd.	No. of Cmpds.	m/z meas.	Δ m/z [ppm]	z	Rt [min]	Score	P	Range	Sequence	Modification
2861	1	795.7251	11.18	3	64.4	16.9	1	253-272	R.YSLHVGPWSTCSMPHSRQVR.Q	Carbamidomethyl: 11

Detailed Protein Report

Protein 525: PREDICTED: uncharacterized protein KIAA1551 isoform X1 [Homo sapiens]

**Accession:**	gi|530399223	**Score:**	16.8
**Database:**	refseq_human(refseq_human_20140103.fasta)	**MW [kDa]:**	194.7
**Database Date:**	2015-11-30	**pI:**	9.7
		**Sequence Coverage [%]:**	0.6
		**No. of unique Peptides:**	1

10	20	30	40	50	60	70	80
MNWNEKPKSA	TLPPLYPKSQ	PPFLHQSLIN	QITTTSQSSF	SYPGSNQEAC	MYPGNSNPIS	QPLLNIQNYP	QQISVSDMHN
**90**	**100**	**110**	**120**	**130**	**140**	**150**	**160**
GTVVASHTSV	ERITYANVNG	PKQLTHNLQM	SSGVTQNVWL	NSPMRNPVHS	HIGATVSHQT	DFGANVPNMP	ALQSQLITSD
**170**	**180**	**190**	**200**	**210**	**220**	**230**	**240**
TYSMQMQMIP	SNSTRLPVAY	QGNQGLNQSF	SEQQVDWTQQ	CISKGLTYPD	YRPPPKLYRY	SPQSFLPDST	IQKQNFIPHT
**250**	**260**	**270**	**280**	**290**	**300**	**310**	**320**
SLQVKNSQLL	NSVLTLPSRQ	TSAVPSQQYA	TQTDKRPPPP	PYNCRYGSQP	LQSTQHITKH	LSMEVPQSRE	MLSSEIRTSF
**330**	**340**	**350**	**360**	**370**	**380**	**390**	**400**
QQQWQNPNEN	VSTIGNFTNL	KVNTNSKQPF	NSPIRSSVDG	VQTLAQTNEE	KIMDSCNPTS	NQVLDTSVAK	EKLVRDIKTL
**410**	**420**	**430**	**440**	**450**	**460**	**470**	**480**
VEIKQKFSEL	ARKIKINKDL	LMAAGCIKMT	NTSYSEPAQN	SKLSLKQTAK	IQSGPQITPV	MPENAERQTP	TVVESAETNK
**490**	**500**	**510**	**520**	**530**	**540**	**550**	**560**
TQCMLNSDIQ	EVNCRRFNQV	DSVLPNPVYS	EKRPMPDSSH	DVKVLTSKTS	AVEMTQAVLN	TQLSSENVTK	VEQNSPAVCE
**570**	**580**	**590**	**600**	**610**	**620**	**630**	**640**
TISVPKSMST	EEYKSKIQNE	NMLLLALLSQ	ARKTQKTVLK	DANQTIQDSK	PDSCEMNPNT	QMTGNQLNLK	NMETPSTSNV
**650**	**660**	**670**	**680**	**690**	**700**	**710**	**720**
SGRVLDNSFC	SGQESSTKGM	PAKSDSSCSM	EVLATCLSLW	KKQPSDTAKE	KECDKLRTNT	TAVGISKPAN	IHVKSPCSVV
**730**	**740**	**750**	**760**	**770**	**780**	**790**	**800**
GNSNSQNKIS	NPSQQTALSM	VMHNYESSGI	NITKGTELQI	AVVSPLVLSE	VKTLSVKGIT	PAVLPETVYP	VIKEGSVCSL
**810**	**820**	**830**	**840**	**850**	**860**	**870**	**880**
QNQLAENAKA	TAALKVDVSG	PVASTATSTK	IFPLTQKEKQ	NESTNGNSEV	TPNVNQGKHN	KLESAIHSPM	NDQQISQESR
**890**	**900**	**910**	**920**	**930**	**940**	**950**	**960**
NSTVVSSDTL	QIDNICSLVE	GDTSYNSQIA	KIFSSLPLKM	VEPQKPSLPN	QQGIGSREPE	KQLDNTTENK	DFGFQKDKPV
**970**	**980**	**990**	**1000**	**1010**	**1020**	**1030**	**1040**
QCTDVSHKIC	DQSKSEPPLE	SSFNNLETNR	VILEKSSLEH	ATEKSTANDT	CSSAAIQEDI	YPQEIDASSN	YTPQDPARNE
**1050**	**1060**	**1070**	**1080**	**1090**	**1100**	**1110**	**1120**
IHSDKAPVLY	LHDQLSELLK	EFPYGIEAVN	TREGSVGQQT	TYQTSEDQTA	DKTSSDSKDP	ADQIQITILS	SEQMKEIFPE
**1130**	**1140**	**1150**	**1160**	**1170**	**1180**	**1190**	**1200**
QDDQPYVVDK	LAEPQKEEPI	TEVVSQCDLQ	APAAGQSRDS	VILDSEKDDI	HCCALGWLSM	VYEGVPQCQC	NSIKNSSSEE
**1210**	**1220**	**1230**	**1240**	**1250**	**1260**	**1270**	**1280**
EKQKEQCSPL	DTNSCKQGER	TSDRDVTVVQ	FKSLVNNPKT	PPDGKSHFPE	LQDDSRKDTP	KTKHKSLPRT	EQELVAGQFS
**1290**	**1300**	**1310**	**1320**	**1330**	**1340**	**1350**	**1360**
SKCDKLNPLQ	NHKRKKLRFH	EVTFHSSNKM	TASYEQASQE	TRQKKHVTQN	SRPLKTKTAF	LPNKDVYKKH	SSLGQSLSPE
**1370**	**1380**	**1390**	**1400**	**1410**	**1420**	**1430**	**1440**
KIKLKLKSVS	FKQKRKLDQG	NVLDMEVKKK	KHDKQEQKGS	VGATFKLGDS	LSNPNERAIV	KEKMVSNTKS	VDTKASSSKF
**1450**	**1460**	**1470**	**1480**	**1490**	**1500**	**1510**	**1520**
SRILTPKEYL	QRQKHKEALS	NKASKKICVK	NVPCDSEHMR	PSKLAVQVES	CGKSNEKHSS	GVQTSKESLN	GLTSHGKNLK
**1530**	**1540**	**1550**	**1560**	**1570**	**1580**	**1590**	**1600**
IHHSQESKTY	NILRNVKEKV	GGKQPDKIWI	DKTKLDKLTN	ISNEAQFSQM	PPQVKDQKKL	YLNRVGFKCT	ERESISLTKL
**1610**	**1620**	**1630**	**1640**	**1650**	**1660**	**1670**	**1680**
ESSPRKLHKD	KRQENKHKTF	LPVKGNTEKS	NMLEFKLCPD	ILLKNTNSVE	ERKDVKPHPR	KEQAPLQVSG	IKSTKEDWLK
**1690**	**1700**	**1710**	**1720**	**1730**	**1740**	**1750**	
FVATKKRTQK	DSQERDNVNS	RLSKRSFSAD	GFEMLQNPVK	DSKEMFQTYK	QMYLEKRSRS	LGSSPVK	

Cmpd.	No. of Cmpds.	m/z meas.	Δ m/z [ppm]	z	Rt [min]	Score	P	Range	Sequence	Modification
120	1	585.2911	-66.24	2	30.4	16.8	0	1662-1672	K.EQAPLQVSGIK.S	

Detailed Protein Report

Protein 526: PREDICTED: M-phase phosphoprotein 8 isoform X2 [Homo sapiens]

**Accession:**	gi|530402464	**Score:**	16.8
**Database:**	refseq_human(refseq_human_20140103.fasta)	**MW [kDa]:**	68.4
**Database Date:**	2015-11-30	**pI:**	5.3
		**Sequence Coverage [%]:**	1.5
		**No. of unique Peptides:**	1

Quantitation

WD:WU **Median:** 0.56 **CV:** 0.00 % **No. of Peptides:** 1

10	20	30	40	50	60	70	80
MEQVAEGARV	TAVPVSAADS	TEELAEVEEG	VGVVGEDNDA	AARGAEAFGD	SEEDGEDVFE	VEKILDMKTE	GGKVLYKVRW
**90**	**100**	**110**	**120**	**130**	**140**	**150**	**160**
KGYTSDDDTW	EPEIHLEDCK	EVLLEFRKKI	AENKAKAVRK	DIQRLSLNND	IFEANSDSDQ	QSETKEDTSP	KKKKKKLRQR
**170**	**180**	**190**	**200**	**210**	**220**	**230**	**240**
EEKSPDDLKK	KKAKAGKLKD	KSKPDLESSL	ESLVFDLRTK	KRISEAKEEL	KESKKPKKDE	VKETKELKKV	KKGEIRDLKT
**250**	**260**	**270**	**280**	**290**	**300**	**310**	**320**
KTREDPKENR	KTKKEKFVES	QVESESSVLN	DSPFPEDDSE	GLHSDSREEK	QNTKSARERA	GQDMGLEHGF	EKPLDSAMSA
**330**	**340**	**350**	**360**	**370**	**380**	**390**	**400**
EEDTDVRGRR	KKKTPRKAED	TRENRKLENK	NAFLEKKTVP	KKQRNQDRSK	SAAELEKLMP	VSAQTPKGRR	LSGEERGLWS
**410**	**420**	**430**	**440**	**450**	**460**	**470**	**480**
TDSAEEDKET	KRNESKEKYQ	KRHDSDKEEK	GRKEPKGLKT	LKEIRNAFDL	FKLTPEEKND	VSENNRKREE	IPLDFKTIDD
**490**	**500**	**510**	**520**	**530**	**540**	**550**	**560**
HKTKENKQSL	KERRNTRDET	DTWAYIAAEG	DQEVLDSVCQ	ADENSDGRQQ	ILSLGMDLQL	EWMKLEDFQK	HLDGKDENFA
**570**	**580**	**590**	**600**				
ATDAIPSNVL	RDAVKNGDYI	TVKVALNSNE	EYNLDQE				

Cmpd.	No. of Cmpds.	m/z meas.	Δ m/z [ppm]	z	Rt [min]	Score	P	Range	Sequence	Modification	Ratios
236	1	495.7647	55.25	2	32.1	16.8	0	1-9	-.MEQVAEGAR.V		WD:WU 0.56

Detailed Protein Report

Protein 527: PREDICTED: HMG box transcription factor BBX isoform X10 [Homo sapiens]

**Accession:**	gi|578807613	**Score:**	16.7
**Database:**	refseq_human(refseq_human_20140103.fasta)	**MW [kDa]:**	98.5
**Database Date:**	2015-11-30	**pI:**	9.6
**Modification(s):**	Oxidation	**Sequence Coverage [%]:**	1.9
		**No. of unique Peptides:**	1

10	20	30	40	50	60	70	80
MKGSNRNKDH	SAEGEGVGKR	PKRKCLQWHP	LLAKKLLDFS	EEEEEEDEEE	DIDKVQLLGA	DGLEQDVGET	EDDESPEQRA
**90**	**100**	**110**	**120**	**130**	**140**	**150**	**160**
RRPMNAFLLF	CKRHRSLVRQ	EHPRLDNRGA	TKILADWWAV	LDPKEKQKYT	DMAKEYKDAF	MKANPGYKWC	PTTNKPVKSP
**170**	**180**	**190**	**200**	**210**	**220**	**230**	**240**
TPTVNPRKKL	WAFPSDSSRD	LPSPKKAKTE	EMPQLNFGMA	DPTQMGGLSM	LLLAGEHALG	TPEVSSGTCR	PDVSESPELR
**250**	**260**	**270**	**280**	**290**	**300**	**310**	**320**
QKSPLFQFAE	ISSSTSHSDA	STKQCQTSAL	FQFAEISSNT	SQLGGAEPVK	RCGKSALFQL	AEMCLASEGM	KMEESKLIKA
**330**	**340**	**350**	**360**	**370**	**380**	**390**	**400**
KESDGGRIKE	LEKGKEEKEI	KMEKTDETRL	QKEAEFEKSA	KENLRDSKEL	RNFEALQIDD	IMAIKMEDPK	EIRKEELEED
**410**	**420**	**430**	**440**	**450**	**460**	**470**	**480**
HKCSHFPDFS	YSASSKIIIS	DVPSRKDHMC	HPHGIMIIED	PAALNKPEKL	KKKKKKSKMD	RHGNDKSTPK	KTCKKRQSSE
**490**	**500**	**510**	**520**	**530**	**540**	**550**	**560**
SDIESVIYTI	EAVAKGDWGI	EKLGDTPRKK	VRTSSSGKGS	ILDAKPPKKK	VKSREKKMSK	EKSSDTTKES	RPPDFISISA
**570**	**580**	**590**	**600**	**610**	**620**	**630**	**640**
SKNISGETPE	GIKAEPLTPM	EDALPPSLSG	QAKPEDSDCH	RKIETCGSRK	SERSCKGALY	KTLVSEGMLT	SLRANVDRGK
**650**	**660**	**670**	**680**	**690**	**700**	**710**	**720**
RSSGKGNSSD	HEGCWNEESW	TFSQSGTSGS	KKFKKTKPKE	DCLLGSAKLD	EEFEKKFNSL	PQYSPVTFDR	KCVPVPRKKK
**730**	**740**	**750**	**760**	**770**	**780**	**790**	**800**
KTGNVSSEPT	KTSKGSGDKW	SNKQLFLDAI	HPTEEPTTTQ	EPLVGSQKRK	ARKTKITHLV	RTADGRVSPA	GGTLDDKPKE
**810**	**820**	**830**	**840**	**850**	**860**	**870**	**880**
QLQRSLPKAT	ETDCNDKCSH	NTEVGETRSS	TPEMPAVSAF	FSLAALAEVA	AMENVHRGQR	STPLTHDGQP	KEMPQAPVLI
**890**							
SCADQ							

Cmpd.	No. of Cmpds.	m/z meas.	Δ m/z [ppm]	z	Rt [min]	Score	P	Range	Sequence	Modification
2908	1	939.4942	3.35	2	65.1	16.7	1	622-638	K.TLVSEGMLTSLRANVDR.G	Oxidation: 7

Detailed Protein Report

Protein 528: HEAT repeat-containing protein 4 [Homo sapiens]

**Accession:**	gi|333805657	**Score:**	16.7
**Database:**	refseq_human(refseq_human_20140103.fasta)	**MW [kDa]:**	117.1
**Database Date:**	2015-11-30	**pI:**	9.9
		**Sequence Coverage [%]:**	1.4
		**No. of unique Peptides:**	1

Alias proteins:

Accession	Name	Description
gi|578825794	refseq_human (refseq_human_20140103.fasta)	PREDICTED: HEAT repeat-containing protein 4 isoform X1 [Homo sapiens]
gi|333805659	refseq_human (refseq_human_20140103.fasta)	HEAT repeat-containing protein 4 [Homo sapiens]

10	20	30	40	50	60	70	80
MTRTQKGKTF	LPHCFYQSLP	PRLGWGMILN	YSKLKGKEEC	ASVSSVPMVF	FSSQYRLHRK	SQYLKMAAAN	LTFSQEVVWQ
90	100	110	120	130	140	150	160
RGLPSIPYSQ	YSFDHLYNTN	DIIHTPQIRK	ARPQKPVSFK	FLGSSSPLTG	DTSLAVKTES	SANPEKKLKK	SKPASTVREA
170	180	190	200	210	220	230	240
PRPLIHHPCM	HPDMLGRPPS	LDVNLEEREA	WLLPPEKEAR	AWEATVLEKL	NERTARWIQS	KRPRRPGASP	NKWQSFLRQQ
250	260	270	280	290	300	310	320
YDWSHIRDEL	TSASDLELLK	QLEAEETAEF	EDQSVILPPQ	EKKKPELLLP	VYYRLPSYFQ	QAETVEIMPG	NKSTEDIHEK
330	340	350	360	370	380	390	400
TSLSQPQTQS	YFRQVTPRAG	KFAYSTDNTF	EQEIYFDEVQ	IIHQIGAKRD	QIVLENLNRY	NKQLSKVFPE	TPEKWSAQAI
410	420	430	440	450	460	470	480
PEASYRPVQG	ALRWTALPTP	AKDMLLQVGE	KDVPIKTRRL	KKQAKSLQED	VTWELVVLRR	MLKEWKTAWA	LIIEWHHETV
490	500	510	520	530	540	550	560
ENLLQSLGDL	HDDVRIKAIT	TCATAALERP	RIATSQRDSD	KTIQDLPEVL	LPALEAALCD	KNAHVRMAAA	ICQYAIQSHN
570	580	590	600	610	620	630	640
PLARNIMQTA	LLKGNSVDSW	AAAQCLALEG	TATYPVIKRI	LHQLFTKKNE	DTEEQSYILL	SYLSEKTTLI	HTMLAVELNS
650	660	670	680	690	700	710	720
CQWKNRIVAC	QAFSRISGNV	CLDMKHKLIQ	LMWNDWNKEV	RRAAAQALGQ	MSLGKEVHDI	IRVKLGQGNS	QERVEALYLI
730	740	750	760	770	780	790	800
GELKLMTAKL	LPSFLHCFSD	DFTAVRRAAC	LAAGALQIRD	KMVLECLLNL	MQRDPYWKIK	AFAIRALGQI	GQVSPELTDL
810	820	830	840	850	860	870	880
LLWAIHYEES	PGVRLEACRS	ILALKLQGDR	VRDTFLDVLL	LENHDAVLKE	MYQTMKILNL	GNEGNQEMLQ	EIKNRIKTLS
890	900	910	920	930	940	950	960
QKDLLTHKIL	KLEMVMGKVR	EEAKRVYLKP	KGEQGPLTLQ	TLLQETFQDE	MVLPRRPSEV	CDTEAVIKPV	KPRAPNPWLQ
970	980	990	1000	1010	1020	1030	
SSVPGLTTRS	KVRSSLVKDL	RTSPEKRIAV	GPFRSDYPAL	YLGKFSERTF	FSPIMSSPSG	KKGAHL	

Cmpd.	No. of Cmpds.	m/z meas.	Δ m/z [ppm]	z	Rt [min]	Score	P	Range	Sequence	Modification
2100	1	757.3120	-102.39	2	54.6	16.7	1	1009-1022	R.TFFSPIMSSPSGKK.G	

Detailed Protein Report

Protein 529: protein FAM122A [Homo sapiens]

Accession: gi|49472830
Database: refseq_human(refseq_human_20140103.fasta)
Database Date: 2015-11-30

Score: 16.6
MW [kDa]: 30.5
pI: 6.4
Sequence Coverage [%]: 3.5
No. of unique Peptides: 1

10	20	30	40	50	60	70	80
MAQEKMELDL	ELPPGTGGSP	AEGGGSGGGG	GLRRSNSAPL	IHGLSDTSPV	FQAEAPSARR	NSTTFPSRHG	LLLPASPVRM
90	100	110	120	130	140	150	160
HSSRLHQIKQ	EEGMDLINRE	TVHEREVQTA	MQISHSWEES	FSLSDNDVEK	SASPKRIDFI	PVSPAPSPTR	GIGKQCFSPS
170	180	190	200	210	220	230	240
LQSFVSSNGL	PPSPIPSPTT	RFTTRRSQSP	INCIRPSVLG	PLKRKCEMET	EYQPKRFFQG	ITNMLSSDVA	QLSDPGVCVS
250	260	270	280	290			
SDTLDGNSSS	AGSSCNSPAK	VSTTTDSPVS	PAQAASPFIP	LDELSSK			

Cmpd.	No. of Cmpds.	m/z meas.	Δ m/z [ppm]	z	Rt [min]	Score	P	Range	Sequence	Modification
588	1	602.8102	41.84	2	35.8	16.6	0	90-99	K.QEEGMDLINR.E	

Detailed Protein Report

Protein 530: PREDICTED: zinc finger protein 646 isoform X3 [Homo sapiens]

**Accession:**	gi|530409439	**Score:**	16.6
**Database:**	refseq_human(refseq_human_20140103.fasta)	**MW [kDa]:**	198.5
**Database Date:**	2015-11-30	**pI:**	6.8
**Modification(s):**	Carbamidomethyl, Oxidation	**Sequence Coverage [%]:**	0.8
		**No. of unique Peptides:**	1

10	20	30	40	50	60	70	80
MEDTPPSLSC	SDCQRHFPSL	PELSRHRELL	HPSPNQDSEE	ADSIPRPYRC	QQCGRGYRHP	GSLVNHRRTH	ETGLFPCTTC
**90**	**100**	**110**	**120**	**130**	**140**	**150**	**160**
GKDFSNPMAL	KSHMRTHAPE	GRRRHRPPRP	KEATPHLQGE	TVSTDSWGQR	LGSSEGWENQ	TKHTEETPDC	ESVPDPRAAS
**170**	**180**	**190**	**200**	**210**	**220**	**230**	**240**
GTWEDLPTRQ	REGLASHPGP	EDGADGWGPS	TNSARAPPLP	IPASSLLSNL	EQYLAESVVN	FTGGQEPTQS	PPAEEERRYK
**250**	**260**	**270**	**280**	**290**	**300**	**310**	**320**
CSQCGKTYKH	AGSLTNHRQS	HTLGIYPCAI	CFKEFSNLMA	LKNHSRLHAQ	YRPYHCPHCP	RVFRLPRELL	EHQQSHEGER
**330**	**340**	**350**	**360**	**370**	**380**	**390**	**400**
QEPRWEEKGM	PTTNGHTDES	SQDQLPSAQM	LNGSAELSTS	GELEDSGLEE	YRPFRCGDCG	RTYRHAGSLI	NHRKSHQTGV
**410**	**420**	**430**	**440**	**450**	**460**	**470**	**480**
YPCSLCSKQL	FNAAALKNHV	RAHHRPRQGV	GENGQPSVPP	APLLLAETTH	KEEEDPTTTL	DHRPYKCSEC	GRAYRHRGSL
**490**	**500**	**510**	**520**	**530**	**540**	**550**	**560**
VNHRHSHRTG	EYQCSLCPRK	YPNLMALRNH	VRVHCKAARR	SADIGAEGAP	SHLKVELPPD	PVEAEAAPHT	DQDHVCKHEE
**570**	**580**	**590**	**600**	**610**	**620**	**630**	**640**
EATDITPAAD	KTAAHICSIC	GLLFEDAESL	ERHGLTHGAG	EKENSRTETT	MSPPRAFACR	DCGKSYRHSG	SLINHRQTHQ
**650**	**660**	**670**	**680**	**690**	**700**	**710**	**720**
TGDFSCGACA	KHFHTMAAMK	NHLRRHSRRR	SRRHRKRAGG	ASGGREAKLL	AAESWTRELE	DNEGLESPQD	PSGESPHGAE
**730**	**740**	**750**	**760**	**770**	**780**	**790**	**800**
GNLESDGDCL	QAESEGDKCG	LERDETHFQG	DKESGGTGEG	LERKDASLLD	NLDIPGEEGG	GTHFCDSLTG	VDEDQKPATG
**810**	**820**	**830**	**840**	**850**	**860**	**870**	**880**
QPNSSSHSAN	AVTGWQAGAA	HTCSDCGHSF	PHATGLLSHR	PCHPPGIYQC	SLCPKEFDSL	PALRSHFQNH	RPGEATSAQP
**890**	**900**	**910**	**920**	**930**	**940**	**950**	**960**
FLCCLCGMIF	PGRAGYRLHR	RQAHSSSGMT	EGSEEEGEEE	GVAEAAPARS	PPLQLSEAEL	LNQLQREVEA	LDSAGYGHIC
**970**	**980**	**990**	**1000**	**1010**	**1020**	**1030**	**1040**
GCCGQTYDDL	GSLERHHQSQ	SSGTTADKAP	SPLGVAGDAM	EMVVDSVLED	IVNSVSGEGG	DAKSQEGAGT	PLGDSLCIQG
**1050**	**1060**	**1070**	**1080**	**1090**	**1100**	**1110**	**1120**
GESLLEAQPR	PFRCNQCGKT	YRHGGSLVNH	RKIHQTGDFL	CPVCSRCYPN	LAAYRNHLRN	HPRCKGSEPQ	VGPIPEAAGS
**1130**	**1140**	**1150**	**1160**	**1170**	**1180**	**1190**	**1200**
SELQVGPIPE	GGSNKPQHMA	EEGPGQAEVE	KLQEELKVEP	LEEVARVKEE	VWEETTVKGE	EIEPRLETAE	KGCQTEASSE
**1210**	**1220**	**1230**	**1240**	**1250**	**1260**	**1270**	**1280**
RPFSCEVCGR	SYKHAGSLIN	HRQSHQTGHF	GCQACSKGFS	NLMSLKNHRR	IHADPRRFRC	SECGKAFRLR	KQLASHQRVH
**1290**	**1300**	**1310**	**1320**	**1330**	**1340**	**1350**	**1360**
MERRGGGGTR	KATREDRPFR	CGQCGRTYRH	AGSLLNHRRS	HETGQYSCPT	CPKTYSNRMA	LKDHQRLHSE	NRRRRAGRSR
**1370**	**1380**	**1390**	**1400**	**1410**	**1420**	**1430**	**1440**
RTAVRCALCG	RSFPGRGSLE	RHLREHEETE	REPANGQGGL	DGTAASEANL	TGSQGLETQL	GGAEPVPHLE	DGVPRPGERS
**1450**	**1460**	**1470**	**1480**	**1490**	**1500**	**1510**	**1520**
QSPIRAASSE	APEPLSWGAG	KAGGWPVGGG	LGNHSGGWVP	QFLTRSEEPE	DSVHRSPCHA	GDCQLNGPTL	SHMDSWDNRD
**1530**	**1540**	**1550**	**1560**	**1570**	**1580**	**1590**	**1600**
NSSQLQPGSH	SSCSQCGKTY	CQSGSLLNHN	TNKTDRHYCL	LCSKEFLNPV	ATKSHSHNHI	DAQTFACPDC	GKAFESHQEL
**1610**	**1620**	**1630**	**1640**	**1650**	**1660**	**1670**	**1680**
ASHLQAHARG	HSQVPAQMEE	ARDPKAGTGE	DQVVLPGQGK	AQEAPSETPR	GPGESVERAR	GGQAVTSMAA	EDKERPFRCT
**1690**	**1700**	**1710**	**1720**	**1730**	**1740**	**1750**	**1760**
QCGRSYRHAG	SLLNHQKAHT	TGLYPCSLCP	KLLPNLLSLK	NHSRTHTDPK	RHCCSICGKA	FRTAARLEGH	GRVHAPREGP
**1770**	**1780**	**1790**	**1800**	**1810**			
FTCPHCPRHF	RRRISFVQHQ	QQHQEEWTVA	GSGRGHEGSQ	EEEPQWHQ			

Cmpd.	No. of Cmpds.	m/z meas.	Δ m/z [ppm]	z	Rt [min]	Score	P	Range	Sequence	Modification
2876	1	899.9560	111.44	2	64.7	16.6	0	1-15	-.MEDTPPSLSCSDCQR.H	Carbamidomethyl: 10, 13; Oxidation: 1

Detailed Protein Report

Protein 531: SKI/DACH domain-containing protein 1 [Homo sapiens]

Accession: gi|148762982
Database: refseq_human(refseq_human_20140103.fasta)
Database Date: 2015-11-30
Modification(s): Carbamidomethyl

Score: 16.6
MW [kDa]: 98.1
pI: 9.6
Sequence Coverage [%]: 1.9
No. of unique Peptides: 1

Alias proteins:

Accession	Name	Description
gi|578818410	refseq_human (refseq_human_20140103.fasta)	PREDICTED: SKI/DACH domain-containing protein 1 isoform X3 [Homo sapiens]
gi|530392158	refseq_human (refseq_human_20140103.fasta)	PREDICTED: SKI/DACH domain-containing protein 1 isoform X2 [Homo sapiens]
gi|530392156	refseq_human (refseq_human_20140103.fasta)	PREDICTED: SKI/DACH domain-containing protein 1 isoform X1 [Homo sapiens]

10	20	30	40	50	60	70	80
MGDLKSGFEE	VDGVRLGYLI	IKGKQMFALS	QVFTDLLKNI	PRTTVHKRMD	HLKVKKHHCD	LEELRKLKAI	NSIAFHAAKC
**90**	**100**	**110**	**120**	**130**	**140**	**150**	**160**
TLISREDVEA	LYTSCKTERV	LKTKRRRVGR	ALATKAPPPE	RAAAASPRPG	FWKDKHQLWR	GLSGAARPLP	ISAQSQRPGA
**170**	**180**	**190**	**200**	**210**	**220**	**230**	**240**
AAARPAAHLP	QIFSKYPGSH	YPEIVRSPCK	PPLNYETAPL	QGNYVAFPSD	PAYFRSLLCS	KHPAAAAAAA	AAAAAAAAAA
**250**	**260**	**270**	**280**	**290**	**300**	**310**	**320**
AAAAYYQVSA	AGPQPKAAAG	AGGPGSLSYR	CKRKRGGAKD	CLLAPHAGAR	RLLLLPRSYK	AKAAAAAAAA	AAAAAAAAGA
**330**	**340**	**350**	**360**	**370**	**380**	**390**	**400**
TCLERFHLVN	GFCPPPHHHH	HHHHHHHHHH	HRAQPPQQSH	HPPHHHRPQP	HLGSFPESCS	SDSESSSYSD	HAANDSDFGS
**410**	**420**	**430**	**440**	**450**	**460**	**470**	**480**
SLSSSSNSVS	SEEEEEEGEE	EEEEEEEEGG	SGASDSSEVS	SEEEDSSTES	DSSSGSSQVS	VQSIRFRRTS	FCKPPSVQAQ
**490**	**500**	**510**	**520**	**530**	**540**	**550**	**560**
ANFLYHLASA	AAATKPAAFE	DAGRLPDLKS	SVKAESPAEW	NLQSWAPKAS	PVYCPASLGS	CFAEIRNDRV	SEITFPHSEI
**570**	**580**	**590**	**600**	**610**	**620**	**630**	**640**
SNAVKRTDLT	INCLAEGASS	PSPKTNNAFP	QQRILREARK	CLQTTPTTHC	ADNNTIAARF	LNNDSSGAEA	NSEKYSKILH
**650**	**660**	**670**	**680**	**690**	**700**	**710**	**720**
CPEFATDLPS	SQTDPEVNAA	GAAATKAENP	CTDTGDKTLP	FLHNIKIKVE	DSSANEEYEP	HLFTNKLKCE	CNDTKGEFYS
**730**	**740**	**750**	**760**	**770**	**780**	**790**	**800**
VTESKEEDAL	LTTAKEGFAC	PEKETPSLNP	LAQSQGLSCT	LGSPKPEDGE	YKFGARVRKN	YRTLVLGKRP	VLQTPPVKPN
**810**	**820**	**830**	**840**	**850**	**860**	**870**	**880**
LKSARSPRPT	GKTETNEGTL	DDFTVINRRK	KVASNVASAV	KRPFHFMANF	PCPPSLIIGR	DGDLWPAYSL	NTTKDSQTPH
**890**	**900**	**910**					
KAHPIWKWQL	GGSAIPLPPS	HKFRKFNS					

Cmpd.	No. of Cmpds.	m/z meas.	Δ m/z [ppm]	z	Rt [min]	Score	P	Range	Sequence	Modification
2905	1	1023.0342	45.64	2	65.1	16.6	1	80-96	K.CTLISREDVEALYTSCK.T	Carbamidomethyl: 1, 16

Detailed Protein Report

Protein 532: **calcium/calmodulin-dependent protein kinase kinase 2 isoform 6 [Homo sapiens]**

**Accession:**	gi|27437023	**Score:**	16.6
**Database:**	refseq_human(refseq_human_20140103.fasta)	**MW [kDa]:**	54.1
**Database Date:**	2015-11-30	**pI:**	7.0
		**Sequence Coverage [%]:**	3.3
		**No. of unique Peptides:**	1

10	20	30	40	50	60	70	80
MSSCVSSQPS	SNRAAPQDEL	GGRGSSSSES	QKPCEALRGL	SSLSIHLGME	SFIVVTECEP	GCAVDLGLAR	DRPLEADGQE
90	100	110	120	130	140	150	160
VPLDTSGSQA	RPHLSGRKLS	LQERSQGGLA	AGGSLDMNGR	CICPSLPYSP	VSSPQSSPRL	PRRPTVESHH	VSITGMQDCV
170	180	190	200	210	220	230	240
QLNQYTLKDE	IGKGSYGVVK	LAYNENDNTY	YAMKVLSKKK	LIRQAGFPRR	PPPRGTRPAP	GGCIQPRGPI	EQVYQEIAIL
250	260	270	280	290	300	310	320
KKLDHPNVVK	LVEVLDDPNE	DHLYMVFELV	NQGPVMEVPT	LKPLSEDQAR	FYFQDLIKGI	EYLHYQKIIH	RDIKPSNLLV
330	340	350	360	370	380	390	400
GEDGHIKIAD	FGVSNEFKGS	DALLSNTVGT	PAFMAPESLS	ETRKIFSGKA	LDVWAMGVTL	YCFVFGQCPF	MDERIMCLHS
410	420	430	440	450	460	470	480
KIKSQALEFP	DQPDIAEDLK	DLITRMLDKN	PESRIVVPEI	KILVKTMIRK	RSFGNPFEGS	RREERSLSAP	GNLLTKKPTR
490	500						
ECESLSELKT							

Cmpd.	No. of Cmpds.	m/z meas.	Δ m/z [ppm]	z	Rt [min]	Score	P	Range	Sequence	Modification
1288	1	917.4243	-149.73	2	44.9	16.6	2	430-445	K.NPESRIVVPEIKILVK.T	

Detailed Protein Report

Protein 533: POC1 centriolar protein homolog A isoform 2 [Homo sapiens]

**Accession:**	gi|239787760	**Score:**	16.6
**Database:**	refseq_human(refseq_human_20140103.fasta)	**MW [kDa]:**	39.8
**Database Date:**	2015-11-30	**pI:**	9.5
		**Sequence Coverage [%]:**	3.3
		**No. of unique Peptides:**	1

10	20	30	40	50	60	70	80
MAAPCAEDPS	LERHFKGHRD	AVTCVDFSIN	TKQLASGSMD	SCLMVWHMKP	QSRAYRFTGH	KDAVTCVNFS	PSGHLLASGS
**90**	**100**	**110**	**120**	**130**	**140**	**150**	**160**
RDKTVRIWVP	NVKGESTVFR	AHTATVRSVH	FCSDGQSFVT	ASDDKTVKVW	ATHRQKFLFS	LSQHINWVRC	AKFSPDGRLI
**170**	**180**	**190**	**200**	**210**	**220**	**230**	**240**
VSASDDKTVK	LWDKSSRECV	HSYCEHGGFV	TYVDFHPSGT	CIAAAGMDNT	VKVWDVRTHR	LLQHYQLHSA	AVNGLSFHPS
**250**	**260**	**270**	**280**	**290**	**300**	**310**	**320**
GNYLITASSD	STLKILDLME	GRLLYTLHGH	QGPATTVAFS	RTGEYFASGG	SDEQVMVWKS	NFDIVDHGEV	TKVPRPPATL
**330**	**340**	**350**	**360**				
ASSMGNLTVS	ILEQRLTLTE	DKLKQCLENQ	QLIMQRATP				

Cmpd.	No. of Cmpds.	m/z meas.	Δ m/z [ppm]	z	Rt [min]	Score	P	Range	Sequence	Modification
2441	1	638.3753	21.87	2	58.8	16.6	1	159-170	R.LIVSASDDKTVK.L	

Detailed Protein Report

Protein 534: PREDICTED: HAUS augmin-like complex subunit 5 isoform X2 [Homo sapiens]

**Accession:**	gi|578834213	**Score:**	16.5
**Database:**	refseq_human(refseq_human_20140103.fasta)	**MW [kDa]:**	62.7
**Database Date:**	2015-11-30	**pI:**	10.1
		**Sequence Coverage [%]:**	4.5
		**No. of unique Peptides:**	1

10	20	30	40	50	60	70	80
MELAQEAREL	GCWAVEEMGV	PVAARAPEST	LRRLCLGQGA	DIWAYILQHV	HSQRTVKKIR	GNLLWYGHQD	SPQVRRKLEL
**90**	**100**	**110**	**120**	**130**	**140**	**150**	**160**
EAAVTRLRAE	IQELDQSLEL	MERDTEAQDT	AMEQARQHTQ	DTQRRALLLR	AQAGAMRRQQ	HTLRDPMQRL	QNQLRRLQDM
**170**	**180**	**190**	**200**	**210**	**220**	**230**	**240**
ERKAKVDVTF	GSLTSAALGL	EPVVLRDVRT	ACTLRAQFLQ	NLLLPQAKRG	SLPTPHDDHF	GTSYQQWLSS	VETLLTNHPP
**250**	**260**	**270**	**280**	**290**	**300**	**310**	**320**
GHVLAALEHL	AAEREAEIRS	LCSGDGLGDT	EISRPQAPDQ	SDSSQTLPSM	VHLIQEGWRT	VGVLVSQRST	LLKERQVLTQ
**330**	**340**	**350**	**360**	**370**	**380**	**390**	**400**
RLQGLVEEVE	RRVLGSSERQ	VLILGLRRCC	LWTELKALHD	QSQELQDAAG	HRQLLLRELQ	AKQQRILHWR	QLVEETQEQV
**410**	**420**	**430**	**440**	**450**	**460**	**470**	**480**
RLLIKGNSAS	KTRLCRSPGE	VLALVQRKVV	PTFEAVAPQS	RELLRCLEEE	VRHLPHILLG	TLLRHRPGEL	KPLPTVLPSI
**490**	**500**	**510**	**520**	**530**	**540**	**550**	**560**
HQLHPASPRG	SSFIALSHKL	GLPPGKASEL	LLPAAASLRQ	DLLLLQDQRS	LWCWDLLHMK	TSLPPGLPTQ	VPIVC

Cmpd.	No. of Cmpds.	m/z meas.	Δ m/z [ppm]	z	Rt [min]	Score	P	Range	Sequence	Modification
2816	1	964.8042	-49.50	3	63.7	16.5	2	33-57	R.RLCLGQGADIWAYILQHVHSQRTVK.K	

Detailed Protein Report

Protein 535: forkhead box protein J3 isoform 2 [Homo sapiens]

Accession: gi|311893307
Database: refseq_human(refseq_human_20140103.fasta)
Database Date: 2015-11-30

Score: 16.4
MW [kDa]: 65.5
pI: 6.8
Sequence Coverage [%]: 3.2
No. of unique Peptides: 1

Quantitation

WD:WU **Median:** 0.96 **CV:** 0.00 % **No. of Peptides:** 1

10	20	30	40	50	60	70	80
MGLYGQACPS	VTSLRMTSEL	ESSLTSMDWL	PQLTMRAAIQ	KSDATQNAHG	TGISKKNALL	DPNTTLDQEE	VQQHKDGKPP
**90**	**100**	**110**	**120**	**130**	**140**	**150**	**160**
YSYASLITFA	INSSPKKKMT	LSEIYQWICD	NFPYYREAGS	GWKNSIRHNL	SLNKCFLKVP	RSKDDPGKGS	YWAIDTNPKE
**170**	**180**	**190**	**200**	**210**	**220**	**230**	**240**
DVLPTRPKKR	ARSVERVTLY	NTDQDGSDSP	RSSLNNSLSD	QSLASVNLNS	VGSVHSYTPV	TSHPESVSQS	LTPQQQPQYN
**250**	**260**	**270**	**280**	**290**	**300**	**310**	**320**
LPERDKQLLF	SEYNFEDLSA	SFRSLYKSVF	EQSLSQQGLM	NIPSESSQQS	HTSCTYQHSP	SSTVSTHPHS	NQSSLSNSHG
**330**	**340**	**350**	**360**	**370**	**380**	**390**	**400**
SGLNTTGSNS	VAQVSLSHPQ	MHTQPSPHPP	HRPHGLPQHP	QRSPHPAPHP	QQHSQLQSPH	PQHPSPHQHI	QHHPNHQHQT
**410**	**420**	**430**	**440**	**450**	**460**	**470**	**480**
LTHQAPPPPQ	QVSCNSGVSN	DWYATLDMLK	ESCRIASSVN	WSDVDLSQFQ	GLMESMRQAD	LKNWSLDQVQ	FADLCSSLNQ
**490**	**500**	**510**	**520**	**530**	**540**	**550**	**560**
FFTQTGLIHS	QSNVQQNVCH	GAMHPTKPSQ	HIGTGNLYID	SRQNLPPSVM	PPPGYPHIPQ	ALSTPGTTMA	GHHRAMNQQH
**570**	**580**	**590**					
MMPSQAFQMR	RSLPPDDIQD	DFDWDSIV					

Cmpd.	No. of Cmpds.	m/z meas.	Δ m/z [ppm]	z	Rt [min]	Score	P	Range	Sequence	Modification	Ratios
36	1	1096.9864	-51.33	2	29.4	16.4	0	57-75	K.NALLDPNTTLDQEEVQQHK.D		WD:WU 0.96

Detailed Protein Report

Protein 536: **PREDICTED: TBC domain-containing protein kinase-like protein isoform X1 [Homo sapiens]**

**Accession:**	gi|578809649	**Score:**	16.4
**Database:**	refseq_human(refseq_human_20140103.fasta)	**MW [kDa]:**	84.9
**Database Date:**	2015-11-30	**pI:**	5.8
**Modification(s):**	Carbamidomethyl	**Sequence Coverage [%]:**	1.9
		**No. of unique Peptides:**	1

10	20	30	40	50	60	70	80
MFPLKDAEMG	AFTFFASALP	HDVCGSNGLP	LTPNSIKILG	RFQILKTITH	PRLCQYVDIS	RGKHERLVVV	AEHCERSLED
**90**	**100**	**110**	**120**	**130**	**140**	**150**	**160**
LLRERKPVSC	STVLCIAFEV	LQGLQYMNKH	GIVHRALSPH	NILLDRKGHI	KLAKFGLYHM	TAHGDDVDFP	IGYPSYLAPE
**170**	**180**	**190**	**200**	**210**	**220**	**230**	**240**
VIAQGIFKTT	DHMPSKKPLP	SGPKSDVWSL	GIILFELCVG	RKLFQSLDIS	ERLKFLLTLD	CVDDTLIVLA	EEHGCLDIIK
**250**	**260**	**270**	**280**	**290**	**300**	**310**	**320**
ELPETVIDLL	NKCLTFHPSK	RPTPDQLMKD	KVFSEVSPLY	TPFTKPASLF	SSSLRCADLT	LPEDISQLCK	DINNDYLAER
**330**	**340**	**350**	**360**	**370**	**380**	**390**	**400**
SIEEVYYLWC	LAGGDLEKEL	VNKEIIRSKP	PICTLPNFLF	EDGESFGQGR	DRSSLLDDTT	VTLSLCQLRN	RLKDVGGEAF
**410**	**420**	**430**	**440**	**450**	**460**	**470**	**480**
YPLLEDDQSN	LPHSNSNNEL	SAAATLPLII	REKDTEYQLN	RIILFDRLLK	AYPYKKNQIW	KEARVDIPPL	MRGLTWAALL
**490**	**500**	**510**	**520**	**530**	**540**	**550**	**560**
GVEGAIHAKY	DAIDKDTPIP	TDRQIEVDIP	RCHQYDELLS	SPEGHAKFRR	VLKAWVVSHP	DLVYWQGLDS	LCAPFLYLNF
**570**	**580**	**590**	**600**	**610**	**620**	**630**	**640**
NNEALAYACM	SAFIPKYLYN	FFLKDNSHVI	QEYLTVFSQM	IAFHDPELSN	HLNEIGFIPD	LYAIPWFLTM	FTHVFPLHKI
**650**	**660**	**670**	**680**	**690**	**700**	**710**	**720**
FHLWDTLLLG	NSSFPFCIGV	AILQQLRDRL	LANGFNECIL	LFSDLPEIDI	ERCVRESINL	FCWTPKSATY	RQHAQPPKPS
**730**	**740**	**750**					
SDSSGGRSSA	PYFSAECPDP	PKTDLKIH					

Cmpd.	No. of Cmpds.	m/z meas.	Δ m/z [ppm]	z	Rt [min]	Score	P	Range	Sequence	Modification
1977	3	876.8617	-79.82	2	53.4	16.4	1	693-706	R.CVRESINLFCWTPK.S	Carbamidomethyl: 10

Detailed Protein Report

Protein 537: PREDICTED: transmembrane protein 2 isoform X2 [Homo sapiens]

Accession: gi|578817261
Database: refseq_human(refseq_human_20140103.fasta)
Database Date: 2015-11-30
Modification(s): Carbamidomethyl

Score: 16.4
MW [kDa]: 136.2
pI: 9.0
Sequence Coverage [%]: 0.5
No. of unique Peptides: 1

10	20	30	40	50	60	70	80
MYATDSRGHS	PAFLQPQNGN	SRHPSGYVPG	KVVPLRPPPP	PKSQASAKFT	SIRREDRATF	AFSPEEQQAQ	RESQKQKRHK
**90**	**100**	**110**	**120**	**130**	**140**	**150**	**160**
NTFICFAITS	FSFFIALAII	LGISSKYAPD	ENCPDQNPRL	RNWDPGQDSA	KQVVIKEGDM	LRLTSDATVH	SIVIQDGGLL
**170**	**180**	**190**	**200**	**210**	**220**	**230**	**240**
VFGDNKDGSR	NITLRTHYIL	IQDGGALHIG	AEKCRYKSKA	TITLYGKSDE	GESMPTFGKK	FIGVEAGGTL	ELHGARKASW
**250**	**260**	**270**	**280**	**290**	**300**	**310**	**320**
TLLARTLNSS	GLPFGSYTFE	KDFSRGLNVR	VIDQDTAKIL	ESERFDTHEY	RNESRRLQEF	LRFQDPGRIV	AIAVGDSAAK
**330**	**340**	**350**	**360**	**370**	**380**	**390**	**400**
SLLQGTIQMI	QERLGSELIQ	GLGYRQAWAL	VGVIDGGSTS	CNESVRNYEN	HSSGGKALAQ	REFYTVDGQK	FSVTAYSEWI
**410**	**420**	**430**	**440**	**450**	**460**	**470**	**480**
EGVSLSGFRV	EVVDGVKLNL	LDDVSSWKPG	DQIVVASTDY	SMYQAEEFTL	LPCSECSHFQ	VKVKETPQFL	HMGEIIDGVD
**490**	**500**	**510**	**520**	**530**	**540**	**550**	**560**
MRAEVGILTR	NIVIQGEVED	SCYAENQCQF	FDYDTFGGHI	MIMKNFTSVH	LSYVELKHMG	QQQMGRYPVH	FHLCGDVDYK
**570**	**580**	**590**	**600**	**610**	**620**	**630**	**640**
GGYRHATFVD	GLSIHHSFSR	CITVHGTNGL	LIKDTIGFDT	LGHCFFLEDG	IEQRNTLFHN	LGLLTKPGTL	LPTDRNNSMC
**650**	**660**	**670**	**680**	**690**	**700**	**710**	**720**
TTMRDKVFGN	YIPVPATDCM	AVSTFWIAHP	NNNLINNAAA	GSQDAGIWYL	FHKEPTGESS	GLQLLAKPEL	TPLGIFYNNR
**730**	**740**	**750**	**760**	**770**	**780**	**790**	**800**
VHSNFKAGLF	IDKGVKTTNS	SAADPREYLC	LDNSARFRPH	QDANPEKPRV	AALIDRLIAF	KNNDNGAWVR	GGDIIVQNSA
**810**	**820**	**830**	**840**	**850**	**860**	**870**	**880**
FADNGIGLTF	ASDGSFPSDE	GSSQEVSESL	FVGESRNYGF	QGGQNKYVGT	GGIDQKPRTL	PRNRTFPIRG	FQIYDGPIHL
**890**	**900**	**910**	**920**	**930**	**940**	**950**	**960**
TRSTFKKYVP	TPDRYSSAIG	FLMKNSWQIT	PRNNISLVKF	GPHVSLNVFF	GKPGPWFEDC	EMDGDKNSIF	HDIDGSVTGY
**970**	**980**	**990**	**1000**	**1010**	**1020**	**1030**	**1040**
KDAYVGRMDN	YLIRHPSCVN	VSKWNAVICS	GTYAQVYVQT	WSTQNLSMTI	TRDEYPSNPM	VLRGINQKAA	FPQYQPVVML
**1050**	**1060**	**1070**	**1080**	**1090**	**1100**	**1110**	**1120**
EKGYTIHWNG	PAPRTTFLYL	VNFNKNDWIR	VGLCYPSNTS	FQVTFGYLQR	QNGSLSKIEE	YEPVHSLEEL	QRKQSERKFY
**1130**	**1140**	**1150**	**1160**	**1170**	**1180**	**1190**	**1200**
FDSSTGLLFL	YLKAKSHRHG	HSYCSSQGCE	RVKIQAATDS	KDISNCMAKA	YPQYYRKPSV	VKRMPAMLTG	LCQGCGTRQM
**1210**	**1220**						
AAYWISRKHC	NESEPKI						

Cmpd.	No. of Cmpds.	m/z meas.	Δ m/z [ppm]	z	Rt [min]	Score	P	Range	Sequence	Modification
2384	1	841.6330	235.44	1	58.2	16.4	2	194-199	K.CRYKSK.A	Carbamidomethyl: 1

Detailed Protein Report

Protein 538: PREDICTED: centrosomal protein CEP57L1 isoform X5 [Homo sapiens]

**Accession:**	gi|578812699	**Score:**	16.4
**Database:**	refseq_human(refseq_human_20140103.fasta)	**MW [kDa]:**	55.5
**Database Date:**	2015-11-30	**pI:**	9.8
**Modification(s):**	Oxidation	**Sequence Coverage [%]:**	5.2
		**No. of unique Peptides:**	1

Alias proteins:

Accession	Name	Description
gi|578812701	refseq_human (refseq_human_20140103.fasta)	PREDICTED: centrosomal protein CEP57L1 isoform X6 [Homo sapiens]

10	20	30	40	50	60	70	80
MDSELMHSIV	GSYHKPPERV	FVPSFTQNEP	SQNCHPANLE	VTSPKILHSP	NSQALILALK	TLQEKIHRLE	LERTQAEDNL
**90**	**100**	**110**	**120**	**130**	**140**	**150**	**160**
NILSREAAQY	KKALENETNE	RNLAHQELIK	QKKDISIQLS	SAQSRCTLLE	KQLEYTKRMV	LNVEREKNMI	LEQQAQLQRE
**170**	**180**	**190**	**200**	**210**	**220**	**230**	**240**
KEQDQMKLYA	KLEKLDVLEK	ECFRLTTTQK	TAEDKIKHLE	EKLKEEEHQR	KLFQDKASEL	QTGLEISKII	MSSVSNLKHS
**250**	**260**	**270**	**280**	**290**	**300**	**310**	**320**
KEKKKSSKKT	KCIKRRPPWQ	ICSKFGALPF	VAEKVRGDKM	KIVVQKNSCF	VMRQHRDPHI	LQKPFNVTET	RCLPKPSRTT
**330**	**340**	**350**	**360**	**370**	**380**	**390**	**400**
SWCKAIPPDS	EKSISICDNL	SELLMAMQDE	LDQMSMEHQE	LLKQMKETES	HSVCDDIECE	LECLLKKMEI	KGEQISKLKK
**410**	**420**	**430**	**440**	**450**	**460**	**470**	**480**
HQDSVCKLQQ	KVQNSKMSEA	SGIQQEDSYP	KGSKNIKNSP	RKCLTDTNLF	QKNSSFHPIR	VHNLQMKLRR	DDIMWEQ

Cmpd.	No. of Cmpds.	m/z meas.	Δ m/z [ppm]	z	Rt [min]	Score	P	Range	Sequence	Modification
1822	1	1023.8932	45.34	3	51.5	16.4	2	287-311	K.NSCFVMRQHRDPHILQKPFNVTETR.C	Oxidation: 6

Detailed Protein Report

Protein 539: PREDICTED: NACHT, LRR and PYD domains-containing protein 8 isoform X1 [Homo sapiens]

**Accession:**	gi|530415693	**Score:**	16.4
**Database:**	refseq_human(refseq_human_20140103.fasta)	**MW [kDa]:**	117.3
**Database Date:**	2015-11-30	**pI:**	9.5
**Modification(s):**	Carbamidomethyl, Oxidation	**Sequence Coverage [%]:**	1.7
		**No. of unique Peptides:**	1

Quantitation

WD:WU **Median:** 0.63 **CV:** 0.00 % **No. of Peptides:** 1

10	20	30	40	50	60	70	80
MSDVNPPSDT	PIPFSSSSTH	SSHIPPWTFS	CYPGSPCENG	VMLYMRNVSH	EELQRFKQLL	LTELSTGTMP	ITWDQVETAS
**90**	**100**	**110**	**120**	**130**	**140**	**150**	**160**
WAEVVHLLIE	RFPGRRAWDV	TSNIFAIMNC	DKMCVVVRRE	INAILPTLEP	EDLNVGETQV	NLEEGESGKI	RRYKSNVMEK
**170**	**180**	**190**	**200**	**210**	**220**	**230**	**240**
FFPIWDITTW	PGNQRDFFYQ	GVHRHEEYLP	CLLLPKRPQG	RQPKTVAIQG	APGIGKTILA	KKVMFEWARN	KFYAHKRWCA
**250**	**260**	**270**	**280**	**290**	**300**	**310**	**320**
FYFHCQEVNQ	TTDQSFSELI	EQKWPGSQDL	VSKIMSKPDQ	LLLLLDGFEE	LTSTLIDRLE	DLSEDWRQKL	PGSVLLSSLL
**330**	**340**	**350**	**360**	**370**	**380**	**390**	**400**
SKTMLPEATL	LIMIRFTSWQ	TCKPLLKCPS	LVTLPGFNTM	EKIKYFQMYF	GHTEEGDQVL	SFAMENTILF	SMCRVPVVCW
**410**	**420**	**430**	**440**	**450**	**460**	**470**	**480**
MVCSGLKQQM	ERGNNLTQSC	PNATSVFVRY	ISSLFPTRAE	NFSRKIHQAQ	LEGLCHLAAD	SMWHRKWVLG	KEDLEEAKLD
**490**	**500**	**510**	**520**	**530**	**540**	**550**	**560**
QTGVTAFLGM	SILRRIAGEE	DHYVFTLVTF	QEFFAALFYV	LCFPQRLKNF	HVLSHVNIQR	LIASPRGSKS	YLSHMGLFLF
**570**	**580**	**590**	**600**	**610**	**620**	**630**	**640**
GFLNEACASA	VEQSFQCKVS	FGNKRKLLKV	IPLLHKCDPP	SPGSGVPQLF	YCLHEIREEA	FVSQALNDYH	KVVLRIGNNK
**650**	**660**	**670**	**680**	**690**	**700**	**710**	**720**
EVQVSAFCLK	RCQYLHEVEL	TVTLNFMNVW	KLSSSSHPGS	EAPESNGLHR	WWQDLCSVFA	TNDKLEVLTM	TNSVLGPPFL
**730**	**740**	**750**	**760**	**770**	**780**	**790**	**800**
KALAAALRHP	QCKLQKLLLR	RVNSTMLNQD	LIGVLTGNQH	LRYLEIQHVE	VESKAVKLLC	RVLRSPRCRL	QCLRLEDCLA
**810**	**820**	**830**	**840**	**850**	**860**	**870**	**880**
TPRIWTDLGN	NLQGNGHLKT	LILRKNSLEN	CGAYYLSVAQ	LERLSQSKML	THLSLAENAL	KDEGAKHIWN	ALPHLRCPLQ
**890**	**900**	**910**	**920**	**930**	**940**	**950**	**960**
RLVLRKCDLT	FNCCQDMISA	LCKNKTLKSL	DLSFNSLKDD	GVILLCEALK	NPDCTLQILE	LENCLFTSIC	CQAMASMLRK
**970**	**980**	**990**	**1000**	**1010**	**1020**	**1030**	
NQHLRHLDLS	KNAIGVYGIL	TLCEAFSSQK	KREEVIFCIP	AWTRITSFSP	TPHPPDFTGK	SDCLSQINP	

Cmpd.	No. of Cmpds.	m/z meas.	Δ m/z [ppm]	z	Rt [min]	Score	P	Range	Sequence	Modification	Ratios
1190	1	660.9872	74.48	3	43.1	16.4	0	887-903	K.CDLTFNCCQDMISALCK.N	Carbamidomethyl: 16; Oxidation: 11	WD:WU 0.63

Detailed Protein Report

Protein 540: protein-methionine sulfoxide oxidase MICAL2 isoform e [Homo sapiens]

**Accession:**	gi|544063447	**Score:**	16.4
**Database:**	refseq_human(refseq_human_20140103.fasta)	**MW [kDa]:**	109.1
**Database Date:**	2015-11-30	**pI:**	9.2
**Modification(s):**	Oxidation	**Sequence Coverage [%]:**	1.8
		**No. of unique Peptides:**	1

10	20	30	40	50	60	70	80
MGENEDEKQA	QAGQVFENFV	QASTCKGTLQ	AFNILTRHLD	LDPLDHRNFY	SKLKSKVTTW	KAKALWYKLD	KRGSHKEYKR
**90**	**100**	**110**	**120**	**130**	**140**	**150**	**160**
GKSCTNTKCL	IVGGGPCGLR	TAIELAYLGA	KVVVVEKRDS	FSRNNVLHLW	PFTIHDLRGL	GAKKFYGKFC	AGSIDHISIR
**170**	**180**	**190**	**200**	**210**	**220**	**230**	**240**
QLQLILFKVA	LMLGVEIHVN	VEFVKVLEPP	EDQENQKIGW	RAEFLPTDHS	LSEFEFDVII	GADGRRNTLE	GFRRKEFRGK
**250**	**260**	**270**	**280**	**290**	**300**	**310**	**320**
LAIAITANFI	NRNSTAEAKV	EEISGVAFIF	NQKFFQDLKE	ETGIDLENIV	YYKDCTHYFV	MTAKKQSLLD	KGVIINDYID
**330**	**340**	**350**	**360**	**370**	**380**	**390**	**400**
TEMLLCAENV	NQDNLLSYAR	EAADFATNYQ	LPSLDFAMNH	YGQPDVAMFD	FTCMYASENA	ALVRERQAHQ	LLVALVGDSL
**410**	**420**	**430**	**440**	**450**	**460**	**470**	**480**
LEPFWPMGTG	CARGFLAAFD	TAWMVKSWNQ	GTPPLELLAE	RESLYRLLPQ	TTPENINKNF	EQYTLDPGTR	YPNLNSHCVR
**490**	**500**	**510**	**520**	**530**	**540**	**550**	**560**
PHQVKHLYIT	KELEHYPLER	LGSVRRSVNL	SRKESDIRPS	KLLTWCQQQT	EGYQHVNVTD	LTTSWRSGLA	LCAIIHRFRP
**570**	**580**	**590**	**600**	**610**	**620**	**630**	**640**
ELINFDSLNE	DDAVENNQLA	FDVAEREFGI	PPVTTGKEMA	SAQEPDKLSM	VMYLSKFYEL	FRGTPLRPVD	SWRKNYGENA
**650**	**660**	**670**	**680**	**690**	**700**	**710**	**720**
DLSLAKSSIS	NNYLNLTFPR	KRTPRVDGQT	GENDMNKRRR	KGFTNLDEPS	NFSSRSLGSN	QECGSSKEGG	NQNKVKSMAN
**730**	**740**	**750**	**760**	**770**	**780**	**790**	**800**
QLLAKFEEST	RNPSLMKQES	MRKSFPLNLG	GSDTCYFCKK	RVYVMERLSA	EGHFFHRECF	RCSICATTLR	LAAYTFDCDE
**810**	**820**	**830**	**840**	**850**	**860**	**870**	**880**
GKFYCKPHFI	HCKTNSKQRK	RRAELKQQRE	EEATWQEQEA	PRRDTPTESS	CAVAAIGTLE	GSPPGISTSF	FRKVLGWPLR
**890**	**900**	**910**	**920**	**930**	**940**	**950**	**960**
LPRDLCNWMQ	GLLQAAGLHI	RDNAYNYCYM	YELLSLGLPL	LWAFSEVLAA	MYRESEGSLE	SICNWVLRCF	PVKLR

Cmpd.	No. of Cmpds.	m/z meas.	Δ m/z [ppm]	z	Rt [min]	Score	P	Range	Sequence	Modification
2008	2	1051.4154	-66.88	2	53.8	16.4	2	726-742	K.FEESTRNPSLMKQESMR.K	Oxidation: 11, 16

Detailed Protein Report

Protein 541: PREDICTED: tyrosine-protein kinase BAZ1B isoform X1 [Homo sapiens]

**Accession:**	gi|530386606	**Score:**	16.2
**Database:**	refseq_human(refseq_human_20140103.fasta)	**MW [kDa]:**	121.5
**Database Date:**	2015-11-30	**pI:**	9.7
**Modification(s):**	Carbamidomethyl	**Sequence Coverage [%]:**	1.7
		**No. of unique Peptides:**	1

10	20	30	40	50	60	70	80
MAPLLGRKPF	PLVKPLPGEE	PLFTIPHTQE	AFRTREEYEA	RLERYSERIW	TCKSTGSSQL	THKEAWEEEQ	EVAELLKEEF
**90**	**100**	**110**	**120**	**130**	**140**	**150**	**160**
PAWYEKLVLE	MVHHNTASLE	KLVDTAWLEI	MTKYAVGEEC	DFEVGKEKML	KVKIVKIHPL	EKVDEEATEK	KSDGACDSPS
**170**	**180**	**190**	**200**	**210**	**220**	**230**	**240**
SDKENSSQIA	QDHQKKETVV	KEDEGRRESI	NDRARRSPRK	LPTSLKKGER	KWAPPKFLPH	KYDVKLQNED	KIISNVPADS
**250**	**260**	**270**	**280**	**290**	**300**	**310**	**320**
LIRTERPPNK	EIVRYFIRHN	ALRAGTGENA	PWVVEDELVK	KYSLPSKFSD	FLLDPYKYMT	LNPSTKRKNT	GSPDRKPSKK
**330**	**340**	**350**	**360**	**370**	**380**	**390**	**400**
SKTDNSSLSS	PLNPKLWCHV	HLKKSLSGSP	LKVKNSKNSK	SPEEHLEEMM	KMMSPNKLHT	NFHIPKKGPP	AKKPGKHSDK
**410**	**420**	**430**	**440**	**450**	**460**	**470**	**480**
PLKAKGRSKG	ILNGQKSTGN	SKSPKKGLKT	PKTKMKQMTL	LDMAKGTQKM	TRAPRNSGGT	PRTSSKPHKH	LPPAALHLIA
**490**	**500**	**510**	**520**	**530**	**540**	**550**	**560**
YYKENKDRED	KRSALSCVIS	KTARLLSSED	RARLPEELRS	LVQKRYELLE	HKKRWASMSE	EQRKEYLKKK	REELKKKLKE
**570**	**580**	**590**	**600**	**610**	**620**	**630**	**640**
KAKERREKEM	LERLEKQKRY	EDQELTGKNL	PAFRLVDTPE	GLPNTLFGDV	AMVVEFLSCY	SGLLLPDAQY	PITAVSLMEA
**650**	**660**	**670**	**680**	**690**	**700**	**710**	**720**
LSADKGGFLY	LNRVLVILLQ	TLLQDEIAED	YGELGMKLSE	IPLTLHSVSE	LVRLCLRRSD	VQEESEGSDT	DDNKDSAAFE
**730**	**740**	**750**	**760**	**770**	**780**	**790**	**800**
DNEVQDEFLE	KLETSEFFEL	TSEEKLQILT	ALCHRILMTY	SVQDHMETRQ	QMSAELWKER	LAVLKEENDK	KRAEKQKRKE
**810**	**820**	**830**	**840**	**850**	**860**	**870**	**880**
MEAKNKENGK	VENGLGKTDR	KKEIVKFEPQ	VDTEAEDMIS	AVKSRRLLAI	QAKKEREIQE	REMKVKLERQ	AEEERIRKHK
**890**	**900**	**910**	**920**	**930**	**940**	**950**	**960**
AAAEKAFQEG	IAKAKLVMRR	TPIGTDRNHN	RYWLFSDEVP	GLFIEKGWVH	DSIDYRFNHH	CKDHTVSGDE	DYCPRSKKAN
**970**	**980**	**990**	**1000**	**1010**	**1020**	**1030**	**1040**
LGKNASMNTQ	HGTATEVAVE	TTTPKQGQNL	WFLCDSQKEL	DELLNCLHPQ	GIRESQLKER	LEKRFDGREK	ENMIQYILYT
**1050**	**1060**						
IARLLRRTIG	TLY						

Cmpd.	No. of Cmpds.	m/z meas.	Δ m/z [ppm]	z	Rt [min]	Score	P	Range	Sequence	Modification
1692	1	711.3527	18.60	3	49.8	16.2	2	114-131	K.YAVGEECDFEVGKEKMLK.V	Carbamidomethyl: 7

Detailed Protein Report

Protein 542: cathepsin Z preproprotein [Homo sapiens]

**Accession:**	gi|22538442	**Score:**	16.2
**Database:**	refseq_human(refseq_human_20140103.fasta)	**MW [kDa]:**	33.8
**Database Date:**	2015-11-30	**pI:**	6.9
**Modification(s):**	Carbamidomethyl, Oxidation	**Sequence Coverage [%]:**	7.3
		**No. of unique Peptides:**	1

10	20	30	40	50	60	70	80
MARRGPGWRP	LLLLVLLAGA	AQGGLYFRRG	QTCYRPLRGD	GLAPLGRSTY	PRPHEYLSPA	DLPKSWDWRN	VDGVNYASIT
**90**	**100**	**110**	**120**	**130**	**140**	**150**	**160**
RNQHIPQYCG	SCWAHASTSA	MADRINIKRK	GAWPSTLLSV	QNVIDCGNAG	SCEGGNDLSV	WDYAHQHGIP	DETCNNYQAK
**170**	**180**	**190**	**200**	**210**	**220**	**230**	**240**
DQECDKFNQC	GTCNEFKECH	AIRNYTLWRV	GDYGSLSGRE	KMMAEIYANG	PISCGIMATE	RLANYTGGIY	AEYQDTTYIN
**250**	**260**	**270**	**280**	**290**	**300**	**310**	
HVVSVAGWGI	SDGTEYWIVR	NSWGEPWGER	GWLRIVTSTY	KDGKGARYNL	AIEEHCTFGD	PIV	

Cmpd.	No. of Cmpds.	m/z meas.	Δ m/z [ppm]	z	Rt [min]	Score	P	Range	Sequence	Modification
2	1	1244.4788	-73.78	2	28.9	16.2	1	200-221	R.EKMMAEIYANGPISCGIMATER.L	Carbamidomethyl: 15; Oxidation: 18

Detailed Protein Report

Protein 543: WAP four-disulfide core domain protein 3 precursor [Homo sapiens]

Accession: gi|32307109
Database: refseq_human(refseq_human_20140103.fasta)
Database Date: 2015-11-30
Modification(s): Carbamidomethyl, Oxidation

Score: 16.2
MW [kDa]: 24.7
pI: 9.4
Sequence Coverage [%]: 10.4
No. of unique Peptides: 1

10	20	30	40	50	60	70	80
MMLSCLFLLK	ALLALGSLES	WITAGEHAKE	GECPPHKNPC	KELCQGDELC	PAEQKCCTTG	CGRICRDIPK	GRKRDCPRVI
**90**	**100**	**110**	**120**	**130**	**140**	**150**	**160**
RKQSCLKRCI	TDETCPGVKK	CCTLGCNKSC	VVPISKQKLA	EFGGECPADP	LPCEELCDGD	ASCPQGHKCC	STGCGRTCLG
**170**	**180**	**190**	**200**	**210**	**220**	**230**	**240**
DIEGGRGGDC	PKVLVGLCIV	GCVMDENCQA	GEKCCKSGCG	RFCVPPVLPP	KLTMNPNWTV	RSDSELEIPV	P

Cmpd.	No. of Cmpds.	m/z meas.	Δ m/z [ppm]	z	Rt [min]	Score	P	Range	Sequence	Modification
1720	1	863.0927	43.31	3	50.2	16.2	1	173-196	K.VLVGLCIVGCVMDENCQAGEKCCK.S	Carbamidomethyl: 10; Oxidation: 12

Detailed Protein Report

Protein 544: otogelin-like protein precursor [Homo sapiens]

**Accession:**	gi|294774585	**Score:**	16.2
**Database:**	refseq_human(refseq_human_20140103.fasta)	**MW [kDa]:**	263.4
**Database Date:**	2015-11-30	**pI:**	4.9
**Modification(s):**	Carbamidomethyl	**Sequence Coverage [%]:**	0.8
		**No. of unique Peptides:**	1

Detailed Protein Report

10	20	30	40	50	60	70	80
MIPWSIFLLH	VLLFSLQEYI	CASSILMGTS	KNGFNENRQK	RALLAAQFEA	TSPRYFFHDA	INWGESKIKG	SCPYECLNGA
90	100	110	120	130	140	150	160
FCSKTGTCDC	QIFQALGTRC	QIIPNMGNGR	DGICKTWGQY	HFETFDGIYY	YFPGNCSYIF	AKDCGDLEPR	YTVWVHNSPK
170	180	190	200	210	220	230	240
CLGSVYSCYR	SISLFFSNQE	EIRIYGHEIK	KNGISLTLPQ	TIGQIFIEKL	ADYILVKTTF	GFSLAWDGIS	GIYLKLSEDH
250	260	270	280	290	300	310	320
KGKSCGLCGN	YNDIQSDDFI	ILQEDYTEDI	AMFANSWSVQ	TPDDTKCVLT	PSDFPNPCSS	GMPAFEAIFF	KCQILLQFPF
330	340	350	360	370	380	390	400
LSCHEYIDPY	LYIASCVNDL	CKTDDDETYC	RAATEYARAC	SHAGYPIQDW	RDDFPACTDK	CDDSFVHRDC	ISCCPPTCTF
410	420	430	440	450	460	470	480
EKQCLGSNLH	CLDGCYCPDG	LVMDNGTCIS	LENCPCGFHG	LAYSVGSKIE	QECTECVCVG	GVWNCTEQDC	PVQCSVVGDS
490	500	510	520	530	540	550	560
HFTTFDGRHY	SFIGMCQYIL	VKGTGKDKFT	ITLQKAPCEQ	NLGLVCLQSI	TLILEDDFNK	QVTLGRGGQI	LTSPNQGFNL
570	580	590	600	610	620	630	640
NGIVEIQTLS	SLFILLKTTF	GLKILFAIDG	ERIYIQLTSA	WKRRTLGLCG	TFNGNIRDDF	LSPSGMIEGT	PQLHANAWRV
650	660	670	680	690	700	710	720
SSTCFAPVHV	PVVDPCNINQ	QNIGYAAHCD	VIHQELFAPC	HIYISPGLYY	QLCRHDACKC	GSSCLCNALA	HYAYLCGQHG
730	740	750	760	770	780	790	800
VPIDFRTQIS	FCAVVCQKGM	LYHHCSSFCL	HSCISLSSPE	QCSDDCAEGC	NCPEGKFYED	TLNFCVPIFH	CRCHYRGSVY
810	820	830	840	850	860	870	880
QPGELIPTPS	GLCQCSNGTV	KCDELATPSA	VHICPEGKEY	FDCRFPDPEL	PAGGVNCETT	CANLAMNFTC	TPSSPCISGC
890	900	910	920	930	940	950	960
VCAPGMAEHR	GKCYVPESCP	CIWKDWEYLS	GEVIATPCYT	CVCRRGMFNC	TYYPCPAVCT	IYGDRHYYSF	DGLEYDYISD
970	980	990	1000	1010	1020	1030	1040
CQVFLIKSAD	DSDISVIAQN	KKCFDNDIVC	SKSVLISVGD	TEIYLNDTPY	KQKQSGFFLE	NKSTYQLWKA	GYYIVVYFPE
1050	1060	1070	1080	1090	1100	1110	1120
KDITILWDRK	TTIHIKVGPQ	WKNKLSGLCG	NFDKCTSNDM	TTSNNLEVRN	ARVFGDSWAL	GQCESPDETI	KPCEAHQNKF
1130	1140	1150	1160	1170	1180	1190	1200
PYAKKECSIL	YSDIFASCRN	VIDVTSFAKN	CHEDTCNCNL	GGDCECLCTS	IAAYAYKCCQ	EGISIHWRSS	TVCSLDCEYY
1210	1220	1230	1240	1250	1260	1270	1280
NEGLGEGPYM	LASYGQSGLV	LGANMTSRSV	FCLPRSSVHT	SLFFYFMITP	GLFKEKVSSL	ALVSLESAER	PNYFLYVHDN
1290	1300	1310	1320	1330	1340	1350	1360
DTLSLELWEA	NSAFHRRATF	FHHQGLWIPG	YSAFELYSKK	GFFIIFTDSS	VKASKYDDSE	EFKHSSSFSI	EEIQAAVPYR
1370	1380	1390	1400	1410	1420	1430	1440
KMCEWRYEPC	ATPCFKTCSD	PEALACKFLP	PVEGCLPYCP	KNMILDEVTL	KCVYPRDCIP	VIPTEPTLMP	PAKPTVPMFT
1450	1460	1470	1480	1490	1500	1510	1520
VWEMITPSDI	TVFDMLTPTT	GLECEPQKFD	PVYDCSQYIC	LNMEWQLYNW	SLNCPKDVEM	PDCGFRGRPV	QVNSDICCPE
1530	1540	1550	1560	1570	1580	1590	1600
WECPCRCSML	SELSIITFDG	NNAALYSMAS	YILVRIPGEI	IVAHIEKCSM	NQNGNSLKKL	APSGRISGLC	FKKLNVTTPI
1610	1620	1630	1640	1650	1660	1670	1680
HKIIVNRLAR	KVEVDSIVVP	LPFSSQELSI	EDSGSMYVIT	TPAGLIIKWS	HLTGIIDIHF	GFRFNLSSYT	EGLCGICNED
1690	1700	1710	1720	1730	1740	1750	1760
PDDDLRMQNG	TIITNMEDIG	LFIESWEIEK	SFEVTMRRPV	RNCTEHDCSQ	CIDLLNRRIF	IPCHDKVSPE	DFCEKMWINY
1770	1780	1790	1800	1810	1820	1830	1840
TYFWNYECDA	LSAYVALCNK	FDICIQWRTP	DYCSLSCPEG	KEYQPCVRPC	EARTCLNQWF	YGHTSCLNLR	EDCVCKVGTI
1850	1860	1870	1880	1890	1900	1910	1920
LHRPHSAQCI	PEKECACTDS	EDQPRTAGEI	WNGGIDECTL	YKCLENGSII	PIEPDCDEEP	TPVCEREAEV	VMGIIDKWTC
1930	1940	1950	1960	1970	1980	1990	2000
CSKEVCGCDT	TLCETSIPTC	TNSQKLIVGH	SPLSCCPQYK	CECDPLKCPS	ISTPECREDQ	FMIQVRQEEP	CCFSPFCVCE
2010	2020	2030	2040	2050	2060	2070	2080
SCTKPVPLCH	DGEFLTVDLN	STHFCCPQYY	CVCEPNLCPM	PLLNCAEDMN	LVKENVSGQC	CPTWHCECNC	ENLIMPTCEV
2090	2100	2110	2120	2130	2140	2150	2160
GEFTAIDHNF	QSDCGCIQYL	CEKDDVCVFQ	EVSVLNPGQS	MIKYLEEDFC	YAIECLEEKD	NHTGFHTLNF	TLVNCSKKCD
2170	2180	2190	2200	2210	2220	2230	2240
VHQVYTPSPS	DYGCCGTCKN	VSCKFHMENG	TSVVYAVGST	WHYNCTTYEC	VKTDEGAIIL	NYTMVCPPFN	ETECKMNEGI
2250	2260	2270	2280	2290	2300	2310	2320
VKLYNEGCCK	ICKREERICQ	KVIIKSVIRK	QDCMSQSPIN	VASCDGKCPS	ATIYNINIES	HLRFCKCCRE	NGVRNLSVPL
2330	2340	2350					

Detailed Protein Report

Cmpd.	No. of Cmpds.	m/z meas.	Δ m/z [ppm]	z	Rt [min]	Score	P	Range	Sequence	Modification
2111	1	1051.4432	-84.15	2	55.0	16.2	1	489-506	R.HYSFIGMCQYILVKGTGK.D	Carbamidomethyl: 8

Detailed Protein Report

Protein 545: interferon-inducible GTPase 5 [Homo sapiens]

**Accession:**	gi|10257429	**Score:**	16.1
**Database:**	refseq_human(refseq_human_20140103.fasta)	**MW [kDa]:**	50.3
**Database Date:**	2015-11-30	**pI:**	5.1
		**Sequence Coverage [%]:**	3.0
		**No. of unique Peptides:**	1

Alias proteins:

Accession	Name	Description
gi|578834645	refseq_human (refseq_human_20140103.fasta)	PREDICTED: interferon-inducible GTPase 5 isoform X2 [Homo sapiens]

10	20	30	40	50	60	70	80
MATSKLPVVP	GEEENTILMA	KERLEALRTA	FESGDLPQAA	SHLQELLAST	ESIRLEVGVT	GESGAGKSSL	INALRGLEAE
**90**	**100**	**110**	**120**	**130**	**140**	**150**	**160**
DPGAALTGVM	ETTMQPSPYP	HPQFPDVTLW	DLPGAGSPGC	PADKYLKQVD	FSRYDFFLLV	SPRRCGAVET	RLAAEILCQG
**170**	**180**	**190**	**200**	**210**	**220**	**230**	**240**
KKFYFVRTKV	DEDLAATRTQ	RPSGFREAAV	LQEIRDHCAE	RLREAGVADP	RIFLVSNLSP	ARYDFPTLVS	TWEHDLPSHR
**250**	**260**	**270**	**280**	**290**	**300**	**310**	**320**
RHAGLLSLPD	ISLEALQKKK	AMLQEQVLKT	ALVLGVIQAL	PVPGLAAAYD	DALLIHSLRG	YHRSFGLDDD	SLAKLAEQVG
**330**	**340**	**350**	**360**	**370**	**380**	**390**	**400**
KQAGDLRSVI	RSPLANEVSP	ETVLRLYSQS	SDGAMRVARA	FERGIPVFGT	LVAGGISFGA	VYTMLQGCLN	EMAEDAQRVR
**410**	**420**	**430**	**440**	**450**	**460**	**470**	
IKALEDDEPQ	PEVSLEVASD	NGVEKGGSGE	GGGEEAPLST	CRKLGLLLKY	ILDSWKKHDS	EEK	

Cmpd.	No. of Cmpds.	m/z meas.	Δ m/z [ppm]	z	Rt [min]	Score	P	Range	Sequence	Modification
123	1	504.5745	-64.47	3	30.8	16.1	0	332-345	R.SPLANEVSPETVLR.L	

Detailed Protein Report

Protein 546: protein phosphatase 1D [Homo sapiens]

Accession: gi|4505997
Database: refseq_human(refseq_human_20140103.fasta)
Database Date: 2015-11-30
Modification(s): Oxidation

Score: 16.1
MW [kDa]: 66.6
pI: 10.0
Sequence Coverage [%]: 2.1
No. of unique Peptides: 1

10	20	30	40	50	60	70	80
MAGLYSLGVS	VFSDQGGRKY	MEDVTQIVVE	PEPTAEEKPS	PRRSLSQPLP	PRPSPAALPG	GEVSGKGPAV	AAREARDPLP
**90**	**100**	**110**	**120**	**130**	**140**	**150**	**160**
DAGASPAPSR	CCRRRSSVAF	FAVCDGHGGR	EAAQFAREHL	WGFIKKQKGF	TSSEPAKVCA	AIRKGFLACH	LAMWKKLAEW
**170**	**180**	**190**	**200**	**210**	**220**	**230**	**240**
PKTMTGLPST	SGTTASVVII	RGMKMYVAHV	GDSGVVLGIQ	DDPKDDFVRA	VEVTQDHKPE	LPKERERIEG	LGGSVMNKSG
**250**	**260**	**270**	**280**	**290**	**300**	**310**	**320**
VNRVVWKRPR	LTHNGPVRRS	TVIDQIPFLA	VARALGDLWS	YDFFSGEFVV	SPEPDTSVHT	LDPQKHKYII	LGSDGLWNMI
**330**	**340**	**350**	**360**	**370**	**380**	**390**	**400**
PPQDAISMCQ	DQEEKKYLMG	EHGQSCAKML	VNRALGRWRQ	RMLRADNTSA	IVICISPEVD	NQGNFTNEDE	LYLNLTDSPS
**410**	**420**	**430**	**440**	**450**	**460**	**470**	**480**
YNSQETCVMT	PSPCSTPPVK	SLEEDPWPRV	NSKDHIPALV	RSNAFSENFL	EVSAEIAREN	VQGVVIPSKD	PEPLEENCAK
**490**	**500**	**510**	**520**	**530**	**540**	**550**	**560**
ALTLRIHDSL	NNSLPIGLVP	TNSTNTVMDQ	KNLKMSTPGQ	MKAQEIERTP	PTNFKRTLEE	SNSGPLMKKH	RRNGLSRSSG
**570**	**580**	**590**	**600**	**610**			
AQPASLPTTS	QRKNSVKLTM	RRRLRGQKKI	GNPLLHQHRK	TVCVC			

Cmpd.	No. of Cmpds.	m/z meas.	Δ m/z [ppm]	z	Rt [min]	Score	P	Range	Sequence	Modification
2103	1	734.3073	-43.91	2	54.7	16.1	1	336-348	K.KYLMGEHGQSCAK.M	Oxidation: 4

Detailed Protein Report

Protein 547: **PREDICTED: putative E3 ubiquitin-protein ligase SH3RF2 isoform X3 [Homo sapiens]**

**Accession:**	gi|578810803	**Score:**	16.1
**Database:**	refseq_human(refseq_human_20140103.fasta)	**MW [kDa]:**	64.0
**Database Date:**	2015-11-30	**pI:**	10.9
**Modification(s):**	Carbamidomethyl	**Sequence Coverage [%]:**	2.8
		**No. of unique Peptides:**	1

10	20	30	40	50	60	70	80
MDDLTLLDLL	ECPVCFEKLD	VTAKVLPCQH	TFCKPCLQRV	FKAHKELRCP	ECRTPVFSNI	EALPANLLLV	RLLDGVRSGQ
**90**	**100**	**110**	**120**	**130**	**140**	**150**	**160**
SSGRGGSFRR	PGTMTLQDGR	KSRTNPRRLQ	ASPFRLVPNV	RIHMDGVPRA	KALCNYRGQN	PGDLRFNKGD	IILLRRQLDE
**170**	**180**	**190**	**200**	**210**	**220**	**230**	**240**
NWYQGEINGI	SGNFPASSVE	VIKQLPQPPP	LCRALYNFDL	RGKDKSENQD	CLTFLKDDII	TVISRVDENW	AEGKLGDKVG
**250**	**260**	**270**	**280**	**290**	**300**	**310**	**320**
IFPILFVEPN	LTARHLLEKN	KGRQSSRTKN	LSLVSSSSRG	NTSTLRRGPG	SRRKVPGQFS	ITTALNTLNR	MVHSPSGRHM
**330**	**340**	**350**	**360**	**370**	**380**	**390**	**400**
VEISTPVLIS	SSNPSVITQP	MEKADVPSSC	VGQVSTYHPA	PVSPGHSTAV	VSLPGSQQHL	SANMFVALHS	YSAHGPDELD
**410**	**420**	**430**	**440**	**450**	**460**	**470**	**480**
LQKGEGVRVL	GKCQDGWLRG	VSLVTGRVGI	FPNNYVIPIF	RKTSSFPDSR	SPGLYTTWTL	STSSVSSQGS	ISEGDPRQSR
**490**	**500**	**510**	**520**	**530**	**540**	**550**	**560**
PFKSVFVPTA	IVNPVRSTAG	PGTLGQGSLR	KGRSSMRKSK	WWQRECNELL	IPQVTYRIYV	QWMGISCLPE	NLRGRNDLFL
**570**	**580**						
RQDHLNLKKK	KKGKEKQ						

Cmpd.	No. of Cmpds.	m/z meas.	Δ m/z [ppm]	z	Rt [min]	Score	P	Range	Sequence	Modification
1746	1	916.9097	-57.96	2	50.5	16.1	2	130-145	R.AKALCNYRGQNPGDLR.F	Carbamidomethyl: 5

Detailed Protein Report

Protein 548: PREDICTED: alpha-(1,3)-fucosyltransferase 11 isoform X4 [Homo sapiens]

**Accession:**	gi|578819000	**Score:**	16.1
**Database:**	refseq_human(refseq_human_20140103.fasta)	**MW [kDa]:**	51.4
**Database Date:**	2015-11-30	**pI:**	5.7
		**Sequence Coverage [%]:**	1.5
		**No. of unique Peptides:**	1

10	20	30	40	50	60	70	80
MAAGPIRVVL	VLLGVLSVCA	ASGHGSVAER	EAGGEAEWAE	PWDGAVFRPP	SALGAVGVTR	SSGTPRPGRE	EAGDLPVLLW
90	100	110	120	130	140	150	160
WSPGLFPHFP	GDSERIECAR	GACVASRNRR	ALRDSRTRAL	LFYGTDFRAS	AAPLPRLAHQ	SWALLHEESP	LNNFLLSHGP
170	180	190	200	210	220	230	240
GIRLFNLTST	FSRHSDYPLS	LQWLPGTAYL	RRPVPPPMER	AEWRRRGYAP	LLYLQSHCDV	PADRDRYVRE	LMRHIPVDSY
250	260	270	280	290	300	310	320
GKCLQNRELP	TARLQDTATA	TTEDPELLAF	LSRYKFHLAL	ENAICNDYMT	EKLWRPMHLG	AVPVYRGSPS	VRDWMPNNHS
330	340	350	360	370	380	390	400
VILIDDFESP	QKLAEFIDFL	DKNDEEYMKY	LAYKQPGGIT	NQFLLDSLKH	REWGVNDPLL	PNYLNGFECF	VCDYELARLD
410	420	430	440	450	460		
AEKAHAASPG	DSPVFEPHIA	QPSHMDCPVP	TPGFGNVEEI	PENDRYWMLL	HFDAPSF		

Cmpd.	No. of Cmpds.	m/z meas.	Δ m/z [ppm]	z	Rt [min]	Score	P	Range	Sequence	Modification
310	1	464.6377	-111.51	2	32.6	16.1	0	343-349	K.NDEEYMK.Y	

Detailed Protein Report

Protein 549: PREDICTED: terminal uridylyltransferase 4 isoform X7 [Homo sapiens]

**Accession:**	gi|530362244	**Score:**	16.0
**Database:**	refseq_human(refseq_human_20140103.fasta)	**MW [kDa]:**	173.2
**Database Date:**	2015-11-30	**pI:**	9.3
		**Sequence Coverage [%]:**	1.0
		**No. of unique Peptides:**	1

10	20	30	40	50	60	70	80
MEESKTLKSE	NHEPKKNVIC	EESKAVQVIG	NQTLKARNDK	SVKEIENSSP	NRNSSKKNKQ	NDICIEKTEV	KSCKVNAANL
90	100	110	120	130	140	150	160
PGPKDLGLVL	RDQSHCKAKK	FPNSPVKAEK	ATISQAKSEK	ATSLQAKAEK	SPKSPNSVKA	EKASSYQMKS	EKVPSSPAEA
170	180	190	200	210	220	230	240
EKGPSLLLKD	MRQKTELQQI	GKKIPSSFTS	VDKVNIEAVG	GEKCALQNSP	RSQKQQTCTD	NTGDSDDSAS	GIEDVSDDLS
250	260	270	280	290	300	310	320
KMKNDESNKE	NSSEMDYLEN	ATVIDESALT	PEQRLGLKQA	EERLERDHIF	RLEKRSPEYT	NCRYLCKLCL	IHIENIQGAH
330	340	350	360	370	380	390	400
KHIKEKRHKK	NILEKQEESE	LRSLPPPSPA	HLAALSVAVI	ELAKEHGITD	DDLRVRQEIV	EEMSKVITTF	LPECSLRLYG
410	420	430	440	450	460	470	480
SSLTRFALKS	SDVNIDIKFP	PKMNHPDLLI	KVLGILKKNV	LYVDVESDFH	AKVPVVVCRD	RKSGLLCRVS	AGNDMACLTT
490	500	510	520	530	540	550	560
DLLTALGKIE	PVFIPLVLAF	RYWAKSPLAL	ETPNRVSLGQ	LWLELLKFYT	LDFALEEYVI	CVRIQDILTR	ENKNWPKRRI
570	580	590	600	610	620	630	640
AIEDPFSVKR	NVARSLNSQL	VYEYVVERFR	AAYRYFACPQ	TKGGNKSTVD	FKKREKGKIS	NKKPVKSNNM	ATNGCILLGE
650	660	670	680	690	700	710	720
TTEKINAERE	QPVQCDEMDC	TSQRCIIDNN	NLLVNELDFA	DHGQDSSSLS	TSKSSEIEPK	LDKKQDDLAP	SETCLKKELS
730	740	750	760	770	780	790	800
QCNCIDLSKS	PDPDKSTGTD	CRSNLETESS	HQSVCTDTSA	TSCNCKATED	ASDLNDDDNL	PTQELYYVFD	KFILTSGKPP
810	820	830	840	850	860	870	880
TIVCSICKKD	GHSKNDCPED	FRKIDLKPLP	PMTNRFREIL	DLVCKRCFDE	LSPPCSEQHN	REQILIGLEK	FIQKEYDEKA
890	900	910	920	930	940	950	960
RLCLFGSSKN	GFGFRDSDLD	ICMTLEGHEN	AEKLNCKEII	ENLAKILKRH	PGLRNILPIT	TAKVPIVKFE	HRRSGLEGDI
970	980	990	1000	1010	1020	1030	1040
SLYNTLAQHN	TRMLATYAAI	DPRVQYLGYT	MKVFAKRCDI	GDASRGSLSS	YAYILMVLYF	LQQRKPPVIP	VLQEIFDGKQ
1050	1060	1070	1080	1090	1100	1110	1120
IPQRMVDGWN	AFFFDKTEEL	KKRLPSLGKN	TESLGELWLG	LLRFYTEEFD	FKEYVISIRQ	KKLLTTFEKQ	WTSKCIAIED
1130	1140	1150	1160	1170	1180	1190	1200
PFDLNHNLGA	GVSRKMTNFI	MKAFINGRKL	FGTPFYPLIG	REAEYFFDSR	VLTDGELAPN	DRCCRVCGKI	GHYMKDCPKR
1210	1220	1230	1240	1250	1260	1270	1280
KSSLLFRLKK	KDSEEEKEGN	EEEKDSRDVL	DPRDLHDTRD	FRDPRDLRCF	ICGDAGHVRR	ECPEVKLARQ	RNSSVAAAQL
1290	1300	1310	1320	1330	1340	1350	1360
VRNLVNAQQV	AGSAQQQGDQ	SIRTRQSSEC	SESPSYSPQP	QPFPQNSSQS	AAITQPSSQP	GSQPKLGPPQ	QGAQPPHQVQ
1370	1380	1390	1400	1410	1420	1430	1440
MPLYNFPQSP	PAQYSPMHNM	GLLPMHPLQI	PAPSWPIHGP	VIHSAPGSAP	SNIGLNDPSI	IFAQPAARPV	AIPNTSHDGH
1450	1460	1470	1480	1490	1500	1510	1520
WPRTVAPNSL	VNSGAVGNSE	PGFRGLTPPI	PWEHAPRPHF	PLVPASWPYG	LHQNFMHQGN	ARFQPNKPFY	TQAGLPMHSN
1530	1540	1550					
QPILLSQGYP	YLNVSYIQQK	K					

Cmpd.	No. of Cmpds.	m/z meas.	Δ m/z [ppm]	z	Rt [min]	Score	P	Range	Sequence	Modification
2583	1	840.6326	169.50	2	60.5	16.0	0	1025-1039	R.KPPVIPVLQEIFDGK.Q	

Detailed Protein Report

Protein 550: olfactory receptor 2AK2 [Homo sapiens]

Accession: gi|52317190
Database: refseq_human(refseq_human_20140103.fasta)
Database Date: 2015-11-30
Modification(s): Oxidation

Score: 15.9
MW [kDa]: 37.7
pI: 10.0
Sequence Coverage [%]: 3.0
No. of unique Peptides: 1

Quantitation

WD:WU **Median:** 0.57 **CV:** 0.00 % **No. of Peptides:** 1

10	20	30	40	50	60	70	80
MNISDVISFD	ILVSAMKTGN	QSFGTDFLLV	GLFQYGWINS	LLFVVIATLF	TVALTGNIML	IHLIRLNTRL	HTPMYFLLSQ
**90**	**100**	**110**	**120**	**130**	**140**	**150**	**160**
LSIVDLMYIS	TTVPKMAVSF	LSQSKTIRFL	GCEIQTYVFL	ALGGTEALLL	GFMSYDRYVA	ICHPLHYPML	MSKKICCLMV
**170**	**180**	**190**	**200**	**210**	**220**	**230**	**240**
ACAWASGSIN	AFIHTLYVFQ	LPFCRSRLIN	HFFCEVPALL	SLVCQDTSQY	EYTVLLSGLI	ILLLPFLAIL	ASYARVLIVV
**250**	**260**	**270**	**280**	**290**	**300**	**310**	**320**
FQMSSGKGQA	KAVSTCSSHL	IVASLFYATT	LFTYTRPHSL	RSPSRDKAVA	VFYTIVTPLL	NPFIYSLRNK	EVTGAVRRLL
**330**	**340**						
GYWICCRKYD	FRSLY						

Cmpd.	No. of Cmpds.	m/z meas.	Δ m/z [ppm]	z	Rt [min]	Score	P	Range	Sequence	Modification	Ratios
108	1	557.2530	-55.78	2	30.6	15.9	0	96-105	K.MAVSFLSQSK.T	Oxidation: 1	WD:WU 0.57

Detailed Protein Report

Protein 551: PREDICTED: ras-related GTP-binding protein D isoform X1 [Homo sapiens]

Accession: gi|530383216
Database: refseq_human(refseq_human_20140103.fasta)
Database Date: 2015-11-30

Score: 15.9
MW [kDa]: 34.2
pI: 4.1
Sequence Coverage [%]: 3.3
No. of unique Peptides: 1

Quantitation

WD:WU **Median:** 1.81 **CV:** 0.00 % **No. of Peptides:** 1

10	20	30	40	50	60	70	80
MSQVLGKPQP	QDEDDAEEEE	EEDELVGLAD	YGDGPDSSDA	DPDSGTEEGV	LDFSDPFSTE	VKPRILLMGL	RRSGKSSIQK
**90**	**100**	**110**	**120**	**130**	**140**	**150**	**160**
VVFHKMSPNE	TLFLESTNKI	CREDVSNSSF	VNFQIWDFPG	QIDFFDPTFD	YEMIFRGTGA	LIFVIDSQDD	YMEALARLHL
**170**	**180**	**190**	**200**	**210**	**220**	**230**	**240**
TVTRAYKVNT	DINFEVFIHK	VDGLSDDHKI	ETQRDIHQRA	NDDLADAGLE	KIHLSFYLTS	IYDHSIFEAF	SKVVQKLIPQ
**250**	**260**	**270**	**280**	**290**	**300**	**310**	
LPTLENLLNI	FISNSGIEKA	FLFDVVSKIY	IATDSTPVDM	QTYELCCDMI	DVVIDISCIY	GAN	

Cmpd.	No. of Cmpds.	m/z meas.	Δ m/z [ppm]	z	Rt [min]	Score	P	Range	Sequence	Modification	Ratios
2830	1	648.3562	18.22	2	63.9	15.9	1	190-199	K.IETQRDIHQR.A		WD:WU 1.81

Detailed Protein Report

Protein 552: uncharacterized protein KIAA0355 [Homo sapiens]

**Accession:**	gi|46276868	**Score:**	15.8
**Database:**	refseq_human(refseq_human_20140103.fasta)	**MW [kDa]:**	115.9
**Database Date:**	2015-11-30	**pI:**	6.8
**Modification(s):**	Carbamidomethyl, Oxidation	**Sequence Coverage [%]:**	1.3
		**No. of unique Peptides:**	1

Alias proteins:

Accession	Name	Description
gi|530417641	refseq_human (refseq_human_20140103.fasta)	PREDICTED: uncharacterized protein KIAA0355 isoform X1 [Homo sapiens]

10	20	30	40	50	60	70	80
MYCCSAQDSK	MDYKRRFLLG	GSKQKVQQHQ	QYPMPELGRA	LSAPLASTAT	TAPLGSLTAA	GSCHHAMPHT	TPIADIQQGI
**90**	**100**	**110**	**120**	**130**	**140**	**150**	**160**
SKYLDALNVF	CRASTFLTDL	FSTVFRNSHY	SKAATQLKDV	QEHVMEAASR	LTSAIKPEIA	KMLMELSAGA	ANFTDQKEFS
**170**	**180**	**190**	**200**	**210**	**220**	**230**	**240**
LQDIEVLGRC	FLTVVQVHFQ	FLTHALQKVQ	PVAHSCFAEV	IVPEKKNSGS	GGGLSGMGHT	PEVEEAVRSW	RGAAEATSRL
**250**	**260**	**270**	**280**	**290**	**300**	**310**	**320**
RERGCDGCLA	GIEVQQLFCS	QSAAIPEHQL	KELNIKIDSA	LQAYKIALES	LGHCEYAMKA	GFHLNPKAIE	ASLQGCCSEA
**330**	**340**	**350**	**360**	**370**	**380**	**390**	**400**
EAQQTGRRQT	PPQPMQCELP	TVPVQIGSHF	LKGVSFNESA	ADNLKLKTHT	MLQLMKEAGC	YNGITSRDDF	PVTEVLNQVC
**410**	**420**	**430**	**440**	**450**	**460**	**470**	**480**
PSTWRGACKT	AVQLLFGQAG	LVVVDTAQIE	NKEAYAPQIS	LEGSRIVVQV	PSTWCLKEDP	ATMSLLQRSL	DPEKTLGLVD
**490**	**500**	**510**	**520**	**530**	**540**	**550**	**560**
VLYTAVLDLN	RWRAGREQAL	PCIQIQLQRE	ICDFGNQADL	PSGNGNKSSG	GLQKTFSKLT	SRFTKKASCT	SSSSSTNYSI
**570**	**580**	**590**	**600**	**610**	**620**	**630**	**640**
QNTPSKNIFI	AGCSEEKAKM	PGNIDTRLQS	ILNIGNFPRT	TDPSQSAQNS	SNTVANGFLM	ERRENFLHGD	DGKDEKGMNL
**650**	**660**	**670**	**680**	**690**	**700**	**710**	**720**
PTDQEMQEVI	DFLSGFNMGQ	SHQGSPLVTR	HNSAATAMVT	EQKAGAMQPQ	QPSLPVPPPP	RAPQAGAHTP	LTPQPGLAPQ
**730**	**740**	**750**	**760**	**770**	**780**	**790**	**800**
QQSPKQQQPQ	VQYYQHLLQP	IGPQQPPPQP	RAPGKWVHGS	SQQPAQAVGA	GLSPLGQWPG	ISDLSSDLYS	LGLVSSYMDN
**810**	**820**	**830**	**840**	**850**	**860**	**870**	**880**
VMSEVLGQKP	QGPRNNTWPN	RDQSDGVFGM	LGEILPFDPA	VGSDPEFARY	VAGVSQAMQQ	KRQAQHGRRP	GNPRGNWPPM
**890**	**900**	**910**	**920**	**930**	**940**	**950**	**960**
DDAHRTWPFP	EFFTEGDGLH	GGWSGAQGDS	ASSSDETSSA	NGDSLFSMFS	GPDLVAAVKQ	RRKHSSGEQD	TSTLPSPPLL
**970**	**980**	**990**	**1000**	**1010**	**1020**	**1030**	**1040**
TTVEDVNQDN	KTKTWPPKAP	WQHPSPLPST	LPSPSAPLYA	VTSPGSQWND	TMQMLQSPVW	AATNDCSAAA	FSYVQTPPQP
**1050**	**1060**	**1070**	**1080**				
PPPPAHKAAP	KGFKAFPGKG	ERRPAYLPQY					

Cmpd.	No. of Cmpds.	m/z meas.	Δ m/z [ppm]	z	Rt [min]	Score	P	Range	Sequence	Modification
1382	1	873.4411	120.85	2	45.6	15.8	1	1-14	-.MYCCSAQDSKMDYK.R	Carbamidomethyl: 3; Oxidation: 11

Detailed Protein Report

Protein 553: **PREDICTED: disintegrin and metalloproteinase domain-containing protein 10 isoform X1 [Homo sapiens]**

**Accession:**	gi|530405298	**Score:**	15.8
**Database:**	refseq_human(refseq_human_20140103.fasta)	**MW [kDa]:**	80.5
**Database Date:**	2015-11-30	**pI:**	8.7
**Modification(s):**	Carbamidomethyl, Oxidation	**Sequence Coverage [%]:**	3.6
		**No. of unique Peptides:**	1

```
        10         20         30         40         50         60         70         80
MVLLRVLILL LSWAAGMGGQ YGNPLNKYIR HYEGLSYNVD SLHQKHQRAK RAVSHEDQFL RLDFHAHGRH FNLRMKRDTS
        90        100        110        120        130        140        150        160
LFSDEFKVET SNKVLDYDTS HIYTGHIYGE EGSFSHGSVI DGRFEGFIQT RGGTFYVEPA ERYIKDRTLP FHSVIYHEDD
       170        180        190        200        210        220        230        240
INYPHKYGPQ GGCADHSVFE RMRKYQMTGV EEVTQIPQEE HAANGPELLR KKRTTSAEKN TCQLYIQTDH LFFKYYGTRE
       250        260        270        280        290        300        310        320
AVIAQINTTA DEKDPTNPFR FPNIGVEKFL ELNSEQNHDD YCLAYVFTDR DFDDGVLGLA WVGAPSGSSG GICEKSKLYS
       330        340        350        360        370        380        390        400
DGKKKSLNTG IITVQNYGSH VPPKVSHITF AHEVGHNFGS PHDSGTECTP GESKNLGQKE NGNYIMYARA TSGDKLNNNK
       410        420        430        440        450        460        470        480
FSLCSIRNIS QVLEKKRNNC FVESGQPICG NGMVEQGEEC DCGYSDQCKD ECCFDANQPE GRKCKLKPGK QCSPSQGPCC
       490        500        510        520        530        540        550        560
TAQCAFKSKS EKCRDDSDCA REGICNGFTA LCPASDPKPN FTDCNRHTQV CINGQCAGSI CEKYGLEECT CASSDGKDDK
       570        580        590        600        610        620        630        640
ELCHVCCMKK MDPSTCASTG SVQWSRHFSG RTITLQPGSP CNDFRGYCDV FMRCRLVDAD GPLARLKKAI FSPELYENIA
       650        660        670        680        690        700        710        720
EWIVAHWWAV LLMGIALIML MAGFIKICSV HTPSSNPKLP PPKPLPGTLK RRRPPQPIQQ PQRQRPRESY QMGHMRR
```

Cmpd.	No. of Cmpds.	m/z meas.	Δ m/z [ppm]	z	Rt [min]	Score	P	Range	Sequence	Modification
1896	1	1056.6778	-73.07	3	52.0	15.8	2	544-569	K.YGLEECTCASSDGKDDKELCHVCCMK.K	Carbamidomethyl: 6, 8, 20, 23, 24; Oxidation: 25

Detailed Protein Report

Protein 554: PREDICTED: pre-B-cell leukemia transcription factor 3 isoform X3 [Homo sapiens]

**Accession:**	gi|578817531	**Score:**	15.7
**Database:**	refseq_human(refseq_human_20140103.fasta)	**MW [kDa]:**	38.7
**Database Date:**	2015-11-30	**pI:**	9.3
**Modification(s):**	Carbamidomethyl	**Sequence Coverage [%]:**	4.6
		**No. of unique Peptides:**	1

10	20	30	40	50	60	70	80
MDDQSRMLQT	LAGVNLAGHS	VQGGMALPPP	PHGHEGADGD	GRKQDIGDIL	HQIMTITDQS	LDEAQAKKHA	LNCHRMKPAL
**90**	**100**	**110**	**120**	**130**	**140**	**150**	**160**
FSVLCEIKEK	TGLSIRGAQE	EDPPDPQLMR	LDNMLLAEGV	SGPEKGGGSA	AAAAAAAASG	GSSDNSIEHS	DYRAKLTQIR
**170**	**180**	**190**	**200**	**210**	**220**	**230**	**240**
QIYHTELEKY	EQACNEFTTH	VMNLLREQSR	TRPISPKEIE	RMVGIIHRKF	SSIQMQLKQS	TCEAVMILRS	RFLDARRKRR
**250**	**260**	**270**	**280**	**290**	**300**	**310**	**320**
NFSKQATEIL	NEYFYSHLSN	PYPSEEAKEE	LAKKCSITVS	QVSNWFGNKR	IRYKKNIGKF	QEEANLYAAK	TAVTAAHAVA
**330**	**340**	**350**	**360**				
AAVQNNQTNS	PTTPNSGGYP	PSCYQSDGRL	Q				

Cmpd.	No. of Cmpds.	m/z meas.	Δ m/z [ppm]	z	Rt [min]	Score	P	Range	Sequence	Modification
1938	1	618.9208	-90.90	3	52.6	15.7	1	274-289	K.KCSITVSQVSNWFGNK.R	Carbamidomethyl: 2

Detailed Protein Report

Protein 555: **PREDICTED: high affinity cGMP-specific 3',5'-cyclic phosphodiesterase 9A isoform X2 [Homo sapiens]**

**Accession:**	gi|530419261	**Score:**	15.7
**Database:**	refseq_human(refseq_human_20140103.fasta)	**MW [kDa]:**	61.1
**Database Date:**	2015-11-30	**pI:**	6.1
		**Sequence Coverage [%]:**	2.1
		**No. of unique Peptides:**	1

10	20	30	40	50	60	70	80
MGSGSSSYRP	KAIYLDIDGR	IQKVIFSKYC	NSSDIMDLFC	IATGLPRNTT	ISLLTTDDAM	VSIDPTMPAN	SERTPYKVRP
**90**	**100**	**110**	**120**	**130**	**140**	**150**	**160**
VAIKQLSAGV	EDKRTTSRGQ	SAERPLRDRR	VVGLEQPRRE	GAFESGQVEP	RPREPQGCYQ	EGQRIPPERE	ELIQSVLAQV
**170**	**180**	**190**	**200**	**210**	**220**	**230**	**240**
AEQFSRAFKI	NELKAEVANH	LAVLEKRVEL	EGLKVVEIEK	CKSDIKKMRE	ELAARSSRTN	CPCKYSFLDN	HKKLTPRRDV
**250**	**260**	**270**	**280**	**290**	**300**	**310**	**320**
PTYPKYLLSP	ETIEALRKPT	FDVWLWEPNE	MLSCLEHMYH	DLGLVRDFSI	NPVTLRRWLF	CVHDNYRNNP	FHNFRHCFCV
**330**	**340**	**350**	**360**	**370**	**380**	**390**	**400**
AQMMYSMVWL	CSLQEKFSQT	DILILMTAAI	CHDLDHPGYN	NTYQINARTE	LAVRYNDISP	LENHHCAVAF	QILAEPECNI
**410**	**420**	**430**	**440**	**450**	**460**	**470**	**480**
FSNIPPDGFK	QIRQGMITLI	LATDMARHAE	IMDSFKEKME	NFDYSNEEHM	TLLKMILIKC	CDISNEVRPM	EVAEPWVDCL
**490**	**500**	**510**	**520**	**530**	**540**		
LEEYFMQPCQ	TNCLKVKKQG	LQKQEGAQEG	RDPAGQLTQM	ATGKVGDIEN	QAS		

Cmpd.	No. of Cmpds.	m/z meas.	Δ m/z [ppm]	z	Rt [min]	Score	P	Range	Sequence	Modification
756	1	647.6373	-216.64	2	38.3	15.7	0	134-144	R.EPQGCYQEGQR.I	

Detailed Protein Report

Protein 556: cytochrome P450 26C1 [Homo sapiens]

**Accession:**	gi|62952502	**Score:**	15.7
**Database:**	refseq_human(refseq_human_20140103.fasta)	**MW [kDa]:**	57.1
**Database Date:**	2015-11-30	**pI:**	10.3
		**Sequence Coverage [%]:**	3.3
		**No. of unique Peptides:**	1

10	20	30	40	50	60	70	80
MFPWGLSCLS	VLGAAGTALL	CAGLLLSLAQ	HLWTLRWMLS	RDRASTLPLP	KGSMGWPFFG	ETLHWLVQGS	RFHSSRRERY
**90**	**100**	**110**	**120**	**130**	**140**	**150**	**160**
GTVFKTHLLG	RPVIRVSGAE	NVRTILLGEH	RLVRSQWPQS	AHILLGSHTL	LGAVGEPHRR	RRKVLARVFS	RAALERYVPR
**170**	**180**	**190**	**200**	**210**	**220**	**230**	**240**
LQGALRHEVR	SWCAAGGPVS	VYDASKALTF	RMAARILLGL	RLDEAQCATL	ARTFEQLVEN	LFSLPLDVPF	SGLRKGIRAR
**250**	**260**	**270**	**280**	**290**	**300**	**310**	**320**
DQLHRHLEGA	ISEKLHEDKA	AEPGDALDLI	IHSARELGHE	PSMQELKESA	VELLFAAFFT	TASASTSLVL	LLLQHPAAIA
**330**	**340**	**350**	**360**	**370**	**380**	**390**	**400**
KIREELVAQG	LGRACGCAPG	AAGGSEGPPP	DCGCEPDLSL	AALGRLRYVD	CVVKEVLRLL	PPVSGGYRTA	LRTFELDGYQ
**410**	**420**	**430**	**440**	**450**	**460**	**470**	**480**
IPKGWSVMYS	IRDTHETAAV	YRSPPEGFDP	ERFGAAREDS	RGASSRFHYI	PFGGGARSCL	GQELAQAVLQ	LLAVELVRTA
**490**	**500**	**510**	**520**	**530**			
RWELATPAFP	AMQTVPIVHP	VDGLRLFFHP	LTPSVAGNGL	CL			

Cmpd.	No. of Cmpds.	m/z meas.	Δ m/z [ppm]	z	Rt [min]	Score	P	Range	Sequence	Modification
2715	1	928.5944	75.04	2	62.3	15.7	1	196-212	R.ILLGLRLDEAQCATLAR.T	

Detailed Protein Report

Protein 557: PREDICTED: cytoplasmic tyrosine-protein kinase BMX isoform X1 [Homo sapiens]

**Accession:**	gi|578837945	**Score:**	15.7
**Database:**	refseq_human(refseq_human_20140103.fasta)	**MW [kDa]:**	77.9
**Database Date:**	2015-11-30	**pI:**	9.3
**Modification(s):**	Oxidation	**Sequence Coverage [%]:**	2.7
		**No. of unique Peptides:**	1

10	20	30	40	50	60	70	80
MDTKSILEEL	LLKRSQQKKK	MSPNNYKERL	FVLTKTNLSY	YEYDKMKRGS	RKGSIEIKKI	RCVEKVNLEE	QTPVERQYPF
**90**	**100**	**110**	**120**	**130**	**140**	**150**	**160**
QIVYKDGLLY	VYASNEESRS	QWLKALQKEI	RGNPHLLVKY	HSGFFVDGKF	LCCQQSCKAA	PGCTLWEAYA	NLHTAVNEEK
**170**	**180**	**190**	**200**	**210**	**220**	**230**	**240**
HRVPTFPDRV	LKIPRAVPVL	KMDAPSSSTT	LAQYDNESKK	NYGSQPPSSS	TSLAQYDSNS	KKIYGSQPNF	NMQYIPREDF
**250**	**260**	**270**	**280**	**290**	**300**	**310**	**320**
PDWWQVRKLK	SSSSEDVASS	NQKERNVNHT	TSKISWEFPE	SSSSEEEENL	DDYDWFAGNI	SRSQSEQLLR	QKGKEGAFMV
**330**	**340**	**350**	**360**	**370**	**380**	**390**	**400**
RNSSQVGMYT	VSLFSKAVND	KKGTVKHYHV	HTNAENKLYL	AENYCFDSIP	KLIHYHQHNS	AGMITRLRHP	VSTKANKVPD
**410**	**420**	**430**	**440**	**450**	**460**	**470**	**480**
SVSLGNGIWE	LKREEITLLK	ELGSGQFGVV	QLGKWKGQYD	VAVKMIKEGS	MSEDEFFQEA	QTMMKLSHPK	LVKFYGVCSK
**490**	**500**	**510**	**520**	**530**	**540**	**550**	**560**
EYPIYIVTEY	ISNGCLLNYL	RSHGKGLEPS	QLLEMCYDVC	EGMAFLESHQ	FIHRDLAARN	CLVDRDLCVK	VSDFGMTRYV
**570**	**580**	**590**	**600**	**610**	**620**	**630**	**640**
LDDQYVSSVG	TKFPVKWSAP	EVFHYFKYSS	KSDVWAFGIL	MWEVFSLGKQ	PYDLYDNSQV	VLKVSQGHRL	YRPHLASDTI
**650**	**660**	**670**	**680**				
YQIMYSCWHE	LPEKRPTFQQ	LLSSIEPLRE	KDKH				

Cmpd.	No. of Cmpds.	m/z meas.	Δ m/z [ppm]	z	Rt [min]	Score	P	Range	Sequence	Modification
2432	1	701.1001	113.81	3	58.7	15.7	0	592-609	K.SDVWAFGILMWEVFSLGK.Q	Oxidation: 10

Detailed Protein Report

Protein 558: BTB/POZ domain-containing protein KCTD18 [Homo sapiens]

**Accession:**	gi|45387953	**Score:**	15.6
**Database:**	refseq_human(refseq_human_20140103.fasta)	**MW [kDa]:**	46.7
**Database Date:**	2015-11-30	**pI:**	9.9
		**Sequence Coverage [%]:**	2.1
		**No. of unique Peptides:**	1

Quantitation

WD:WU **Median:** 1.58 **CV:** 0.00 % **No. of Peptides:** 1

Alias proteins:

Accession	Name	Description
gi|530369815	refseq_human (refseq_human_20140103.fasta)	PREDICTED: BTB/POZ domain-containing protein KCTD18 isoform X1 [Homo sapiens]

10	20	30	40	50	60	70	80
MEGHKAEEEV	LDVLRLNVGG	CIYTARRESL	CRFKDSMLAS	MFSGRFPLKT	DESGACVIDR	DGRLFKYLLD	YLHGEVQIPT
**90**	**100**	**110**	**120**	**130**	**140**	**150**	**160**
DEQTRIALQE	EADYFGIPYP	YSLSDHLANE	METYSLRSNI	ELKKALTDFC	DSYGLVCNKP	TVWVLHYLNT	SGASCESRII
**170**	**180**	**190**	**200**	**210**	**220**	**230**	**240**
GVYATKTDGT	DAIEKQLGGR	IHSKGIFKRE	AGNNVQYIWS	YYSVAELKKM	MDAFDAWEGK	GVSYWRVPHE	LIECWTLEER
**250**	**260**	**270**	**280**	**290**	**300**	**310**	**320**
PLLGSLRHMA	PIRKRRLITF	NEADESVNYK	TGPKPVRFLG	PSTSTQIKVK	NSASVTVSPA	SAIQTSAGAT	ANRFQSGSRR
**330**	**340**	**350**	**360**	**370**	**380**	**390**	**400**
KAAQRSAPSR	ATALVGTGAP	GHPQASPGAA	SAENGGTHLP	PAKVLLSDKK	PTPQRVIKLK	RTPLCATAPC	LPSPTATRQA
**410**	**420**	**430**					
NSLKPLPGEA	ARALGVRTEN	GKNKGN					

Cmpd.	No. of Cmpds.	m/z meas.	Δ m/z [ppm]	z	Rt [min]	Score	P	Range	Sequence	Modification	Ratios
587	1	472.2712	30.05	2	36.2	15.6	1	322-330	K.AAQRSAPSR.A		WD:WU 1.58

Detailed Protein Report

Protein 559: extended synaptotagmin-1 isoform 2 [Homo sapiens]

Accession: gi|14149680
Database: refseq_human(refseq_human_20140103.fasta)
Database Date: 2015-11-30

Score: 15.5
MW [kDa]: 122.8
pI: 5.5
Sequence Coverage [%]: 1.1
No. of unique Peptides: 1

Quantitation

WD:WU **Median:** 5.86 **CV:** 0.00 % **No. of Peptides:** 1

10	20	30	40	50	60	70	80
MERSPGEGPS	PSPMDQPSAP	SDPTDQPPAA	HAKPDPGSGG	QPAGPGAAGE	ALAVLTSFGR	RLLVLIPVYL	AGAVGLSVGF
**90**	**100**	**110**	**120**	**130**	**140**	**150**	**160**
VLFGLALYLG	WRRVRDEKER	SLRAARQLLD	DEEQLTAKTL	YMSHRELPAW	VSFPDVEKAE	WLNKIVAQVW	PFLGQYMEKL
**170**	**180**	**190**	**200**	**210**	**220**	**230**	**240**
LAETVAPAVR	GSNPHLQTFT	FTRVELGEKP	LRIIGVKVHP	GQRKEQILLD	LNISYVGDVQ	IDVEVKKYFC	KAGVKGMQLH
**250**	**260**	**270**	**280**	**290**	**300**	**310**	**320**
GVLRVILEPL	IGDLPFVGAV	SMFFIRRPTL	DINWTGMTNL	LDIPGLSSLS	DTMIMDSIAA	FLVLPNRLLV	PLVPDLQDVA
**330**	**340**	**350**	**360**	**370**	**380**	**390**	**400**
QLRSPLPRGI	IRIHLLAARG	LSSKDKYVKG	LIEGKSDPYA	LVRLGTQTFC	SRVIDEELNP	QWGETYEVMV	HEVPGQEIEV
**410**	**420**	**430**	**440**	**450**	**460**	**470**	**480**
EVFDKDPDKD	DFLGRMKLDV	GKVLQASVLD	DWFPLQGGQG	QVHLRLEWLS	LLSDAEKLEQ	VLQWNWGVSS	RPDPPSAAIL
**490**	**500**	**510**	**520**	**530**	**540**	**550**	**560**
VVYLDRAQDL	PLKKGNKEPN	PMVQLSIQDV	TQESKAVYST	NCPVWEEAFR	FFLQDPQSQE	LDVQVKDDSR	ALTLGALTLP
**570**	**580**	**590**	**600**	**610**	**620**	**630**	**640**
LARLLTAPEL	ILDQWFQLSS	SGPNSRLYMK	LVMRILYLDS	SEICFPTVPG	CPGAWDVDSE	NPQRGSSVDA	PPRPCHTTPD
**650**	**660**	**670**	**680**	**690**	**700**	**710**	**720**
SQFGTEHVLR	IHVLEAQDLI	AKDRFLGGLV	KGKSDPYVKL	KLAGRSFRSH	VVREDLNPRW	NEVFEVIVTS	VPGQELEVEV
**730**	**740**	**750**	**760**	**770**	**780**	**790**	**800**
FDKDLDKDDF	LGRCKVRLTT	VLNSGFLDEW	LTLEDVPSGR	LHLRLERLTP	RPTAAELEEV	LQVNSLIQTQ	KSAELAAALL
**810**	**820**	**830**	**840**	**850**	**860**	**870**	**880**
SIYMERAEDL	PLRKGTKHLS	PYATLTVGDS	SHKTKTISQT	SAPVWDESAS	FLIRKPHTES	LELQVRGEGT	GVLGSLSLPL
**890**	**900**	**910**	**920**	**930**	**940**	**950**	**960**
SELLVADQLC	LDRWFTLSSG	QGQVLLRAQL	GILVSQHSGV	EAHSHSYSHS	SSSLSEEPEL	SGGPPHITSS	APELRQRLTH
**970**	**980**	**990**	**1000**	**1010**	**1020**	**1030**	**1040**
VDSPLEAPAG	PLGQVKLTLW	YYSEERKLVS	IVHGCRSLRQ	NGRDPPDPYV	SLLLLPDKNR	GTKRRTSQKK	RTLSPEFNER
**1050**	**1060**	**1070**	**1080**	**1090**	**1100**	**1110**	
FEWELPLDEA	QRRKLDVSVK	SNSSFMSRER	ELLGKVQLDL	AETDLSQGVA	RWYDLMDNKD	KGSS	

Cmpd.	No. of Cmpds.	m/z meas.	Δ m/z [ppm]	z	Rt [min]	Score	P	Range	Sequence	Modification	Ratios
2291	2	666.2683	-223.38	2	57.1	15.5	2	193-204	R.IIGVKVHPGQRK.E		WD:WU 5.86

Detailed Protein Report

Protein 560: gastric inhibitory polypeptide receptor precursor [Homo sapiens]

**Accession:**	gi|4503999	**Score:**	15.5
**Database:**	refseq_human(refseq_human_20140103.fasta)	**MW [kDa]:**	53.1
**Database Date:**	2015-11-30	**pI:**	10.1
**Modification(s):**	Carbamidomethyl, Oxidation	**Sequence Coverage [%]:**	1.5
		**No. of unique Peptides:**	1

```
        10         20         30         40         50         60         70         80
MTTSPILQLL LRLSLCGLLL QRAETGSKGQ TAGELYQRWE RYRRECQETL AAAEPPSGLA CNGSFDMYVC WDYAAPNATA
        90        100        110        120        130        140        150        160
RASCPWYLPW HHHVAAGFVL RQCGSDGQWG LWRDHTQCEN PEKNEAFLDQ RLILERLQVM YTVGYSLSLA TLLLALLILS
       170        180        190        200        210        220        230        240
LFRRLHCTRN YIHINLFTSF MLRAAAILSR DRLLPRPGPY LGDQALALWN QALAACRTAQ IVTQYCVGAN YTWLLVEGVY
       250        260        270        280        290        300        310        320
LHSLLVLVGG SEEGHFRYYL LLGWGAPALF VIPWVIVRYL YENTQCWERN EVKAIWWIIR TPILMTILIN FLIFIRILGI
       330        340        350        360        370        380        390        400
LLSKLRTRQM RCRDYRLRLA RSTLTLVPLL GVHEVVFAPV TEEQARGALR FAKLGFEIFL SSFQGFLVSV LYCFINKEVQ
       410        420        430        440        450        460        470
SEIRRGWHHC RLRRSLGEEQ RQLPERAFRA LPSGSGPGEV PTSRGLSSGT LPGPGNEASR ELESYC
```

Cmpd.	No. of Cmpds.	m/z meas.	Δ m/z [ppm]	z	Rt [min]	Score	P	Range	Sequence	Modification
1605	3	1023.6518	154.68	1	48.8	15.5	2	327-333	R.TRQMRCR.D	Carbamidomethyl: 6; Oxidation: 4

Detailed Protein Report

Protein 561: uncharacterized protein KIAA0408 [Homo sapiens]

**Accession:**	gi|59806363	**Score:**	15.5
**Database:**	refseq_human(refseq_human_20140103.fasta)	**MW [kDa]:**	79.1
**Database Date:**	2015-11-30	**pI:**	9.6
**Modification(s):**	Oxidation	**Sequence Coverage [%]:**	1.9
		**No. of unique Peptides:**	1

Quantitation

WD:WU **Median:** 2.23 **CV:** 0.00 % **No. of Peptides:** 1

10	20	30	40	50	60	70	80
MDLHKQWENT	ETNWHKEKME	LLDQFDNERK	EWESQWKIMQ	KKIEELCREV	KLWRKININE	SAKIIDLYHE	KTIPEKVIES
90	100	110	120	130	140	150	160
SPNYPDLGQS	EFIRTNHKDG	LRKENKREQS	LVSGGNQMCK	EQKATKKSKV	GFLDPLATDN	QKECEAWPDL	RTSEEDSKSC
170	180	190	200	210	220	230	240
SGALSTALEE	LAKVSEELCS	FQEEIRKRSN	HRRMKSDSFL	QEMPNVTNIP	HGDPMINNDQ	CILPISLEKE	KQKNRKNLSC
250	260	270	280	290	300	310	320
TNVLQSNSTK	KCGIDTIDLK	RNETPPVPPP	RSTSRNFPSS	DSEQAYERWK	ERLDHNSWVP	HEGRSKRNYN	PHFPLRQQEM
330	340	350	360	370	380	390	400
SMLYPNEGKT	SKDGIIFSSL	VPEVKIDSKP	PSNEDVGLSM	WSCDIGIGAK	RSPSTSWFQK	TCSTPSNPKY	EMVIPDHPAK
410	420	430	440	450	460	470	480
SHPDLHVSND	CSSSVAESSS	PLRNFSCGFE	RTTRNEKLAA	KTDEFNRTVF	RTDRNCQAIQ	QNHSCSKSSE	DLKPCDTSST
490	500	510	520	530	540	550	560
HTGSISQSND	VSGIWKTNAH	MPVPMENVPD	NPTKKSTTGL	VRQMQGHLSP	RSYRNMLHEH	DWRPSNLSGR	PRSADPRSNY
570	580	590	600	610	620	630	640
GVVEKLLKTY	ETATESALQN	SKCFQDNWTK	CNSDVSGGAT	LSQHLEMLQM	EQQFQQKTAV	WGGQEVKQGI	DPKKITEESM
650	660	670	680	690	700		
SVNASHGKGF	SRPARPANRR	LPSRWASRSP	SAPPALRRTT	HNYTISLRSE	ALMV		

Cmpd.	No. of Cmpds.	m/z meas.	Δ m/z [ppm]	z	Rt [min]	Score	P	Range	Sequence	Modification	Ratios
2682	5	841.8664	-25.81	2	61.9	15.5	1	17-29	K.EKMELLDQFDNER.K	Oxidation: 3	WD:WU 2.23

Detailed Protein Report

Protein 562: twist-related protein 1 [Homo sapiens]

**Accession:**	gi|4507741	**Score:**	15.5
**Database:**	refseq_human(refseq_human_20140103.fasta)	**MW [kDa]:**	20.9
**Database Date:**	2015-11-30	**pI:**	10.1
		**Sequence Coverage [%]:**	9.9
		**No. of unique Peptides:**	1

Quantitation

WD:WU	**Median:** 0.75	**CV:** 0.00 %	**No. of Peptides:** 1

Alias proteins:

Accession	Name	Description
gi|578813613	refseq_human (refseq_human_20140103.fasta)	PREDICTED: twist-related protein 1 isoform X1 [Homo sapiens]

10	20	30	40	50	60	70	80
MMQDVSSSPV	SPADDSLSNS	EEEPDRQQPP	SGKRGGRKRR	SSRRSAGGGA	GPGGAAGGGV	GGGDEPGSPA	QGKRGKKSAG
**90**	**100**	**110**	**120**	**130**	**140**	**150**	**160**
CGGGGGAGGG	GGSSSGGGSP	QSYEELQTQR	VMANVRERQR	TQSLNEAFAA	LRKIIPTLPS	DKLSKIQTLK	LAARYIDFLY
**170**	**180**	**190**	**200**	**210**			
QVLQSDELDS	KMASCSYVAH	ERLSYAFSVW	RMEGAWSMSA	SH			

Cmpd.	No. of Cmpds.	m/z meas.	Δ m/z [ppm]	z	Rt [min]	Score	P	Range	Sequence	Modification	Ratios
14	1	788.3704	-3.92	3	29.4	15.5	1	172-191	K.MASCSYVAHERLSYAFSVWR.M		WD:WU 0.75

Detailed Protein Report

Protein 563: mitochondrial tRNA-specific 2-thiouridylase 1 isoform f [Homo sapiens]

Accession: gi|544346335
Database: refseq_human(refseq_human_20140103.fasta)
Database Date: 2015-11-30
Modification(s): Carbamidomethyl, Oxidation

Score: 15.4
MW [kDa]: 26.9
pI: 10.3
Sequence Coverage [%]: 4.7
No. of unique Peptides: 1

Quantitation

WD:WU **Median:** 1.22 **CV:** 0.00 % **No. of Peptides:** 1

10	20	30	40	50	60	70	80
MPLPQVTMQE	LPWKMKKSLS	RSTLRSPKGF	SEIGLKLEMV	SQDALRRTIF	PLGGLTKEFV	KKIAAENRLH	HVLQKKESMG
90	100	110	120	130	140	150	160
MCFIGKRNFE	HFLLQYLQPR	PGHFISIEDN	KVLGTHKGWF	LYTLGQRANI	GGLREPWYVV	EKDSVKGDVF	VAPRTDHPAL
170	180	190	200	210	220	230	240
YRDLLRTSRV	HWIAEEPPAA	LVRDKMMECH	FRFRHQMALV	CCVLQGGRVP	GQREDPAAGA	VCLHAPEGPA	QSWDGH

Cmpd.	No. of Cmpds.	m/z meas.	Δ m/z [ppm]	z	Rt [min]	Score	P	Range	Sequence	Modification	Ratios
2031	1	652.2575	-58.70	2	54.0	15.4	1	76-86	K.KESMGMCFIGK.R	Carbamidomethyl: 7; Oxidation: 6	WD:WU 1.22

Detailed Protein Report

Protein 564: histone deacetylase 1 [Homo sapiens]

Accession: gi|13128860
Database: refseq_human(refseq_human_20140103.fasta)
Database Date: 2015-11-30

Score: 15.4
MW [kDa]: 55.1
pI: 5.2
Sequence Coverage [%]: 2.7
No. of unique Peptides: 1

Quantitation

WD:WU **Median:** 1.72 **CV:** 0.00 % **No. of Peptides:** 1

10	20	30	40	50	60	70	80
MAQTQGTRRK	VCYYYDGDVG	NYYYGQGHPM	KPHRIRMTHN	LLLNYGLYRK	MEIYRPHKAN	AEEMTKYHSD	DYIKFLRSIR
90	100	110	120	130	140	150	160
PDNMSEYSKQ	MQRFNVGEDC	PVFDGLFEFC	QLSTGGSVAS	AVKLNKQQTD	IAVNWAGGLH	HAKKSEASGF	CYVNDIVLAI
170	180	190	200	210	220	230	240
LELLKYHQRV	LYIDIDIHHG	DGVEEAFYTT	DRVMTVSFHK	YGEYFPGTGD	LRDIGAGKGK	YYAVNYPLRD	GIDDESYEAI
250	260	270	280	290	300	310	320
FKPVMSKVME	MFQPSAVVLQ	CGSDSLSGDR	LGCFNLTIKG	HAKCVEFVKS	FNLPMLMLGG	GGYTIRNVAR	CWTYETAVAL
330	340	350	360	370	380	390	400
DTEIPNELPY	NDYFEYFGPD	FKLHISPSNM	TNQNTNEYLE	KIKQRLFENL	RMLPHAPGVQ	MQAIPEDAIP	EESGDEDEDD
410	420	430	440	450	460	470	480
PDKRISICSS	DKRIACEEEF	SDSEEEGEGG	RKNSSNFKKA	KRVKTEDEKE	KDPEEKKEVT	EEEKTKEEKP	EAKGVKEEVK
490							
LA							

Cmpd.	No. of Cmpds.	m/z meas.	Δ m/z [ppm]	z	Rt [min]	Score	P	Range	Sequence	Modification	Ratios
184	1	530.6117	41.73	3	31.2	15.4	2	452-464	K.DPEEKKEVTEEEK.T		WD:WU 1.72

Detailed Protein Report

Protein 565: **PREDICTED: proline-rich protein PRCC isoform X2 [Homo sapiens]**

Accession: gi|530365026
Database: refseq_human(refseq_human_20140103.fasta)
Database Date: 2015-11-30

Score: 15.4
MW [kDa]: 48.9
pI: 5.3
Sequence Coverage [%]: 2.6
No. of unique Peptides: 1

10	20	30	40	50	60	70	80
MSLVAYASSD	ESEPDEAEPE	PEEEEAVAPT	SGPALGGLFA	SLPAPKGPAL	LPPPPQMLAP	AFPPPLLLPP	PTGDPRLQPP
**90**	**100**	**110**	**120**	**130**	**140**	**150**	**160**
PPLPFGLGGF	PPPPGVSPAE	AAGVGEGLGL	GLPSPRGPGL	NLPPPIGGAG	PPLGLPKPKK	RKEPVKIAAP	ELHKGDSDSE
**170**	**180**	**190**	**200**	**210**	**220**	**230**	**240**
EDEPTKKKTI	LQGSSEGTGL	SALLPQPKNL	TVKETNRLLL	PHAFSRKPSD	GSPDTKPSRL	ASKTKTSSLA	PVVGTTTTTP
**250**	**260**	**270**	**280**	**290**	**300**	**310**	**320**
SPSAIKAAAK	SAALQVTKQI	TQEEDDSDEE	VAPENFFSLP	EKAEPPGVEP	YPYPIPTVPE	ELPPGTEPEP	AFQDDAANAP
**330**	**340**	**350**	**360**	**370**	**380**	**390**	**400**
LEFKMAAGSS	GAPWMPKPGD	DYSYNQFSTY	GDANAAGAYY	QFKRLQGKRN	RGREEINFVE	IKGDDQLSGA	QQWMTKSLTE
**410**	**420**	**430**	**440**	**450**	**460**		
EKTMKSFSKK	KGEQPTGQQR	RKHQITYLIH	QAKERELELK	NTWSENKLSR	RQTQAKYGF		

Cmpd.	No. of Cmpds.	m/z meas.	Δ m/z [ppm]	z	Rt [min]	Score	P	Range	Sequence	Modification
2175	2	666.2454	-213.60	2	55.7	15.4	1	143-154	K.EPVKIAAPELHK.G	

Detailed Protein Report

Protein 566: WD repeat-containing protein 74 [Homo sapiens]

**Accession:**	gi|221219004	**Score:**	15.4
**Database:**	refseq_human(refseq_human_20140103.fasta)	**MW [kDa]:**	42.4
**Database Date:**	2015-11-30	**pI:**	9.7
**Modification(s):**	Carbamidomethyl	**Sequence Coverage [%]:**	6.5
		**No. of unique Peptides:**	1

Alias proteins:

Accession	Name	Description
gi|530396902	refseq_human (refseq_human_20140103.fasta)	PREDICTED: WD repeat-containing protein 74 isoform X1 [Homo sapiens]

10	20	30	40	50	60	70	80
MAAAAARWNH	VWVGTETGIL	KGVNLQRKQA	ANFTAGGQPR	REEAVSALCW	GTGGETQMLV	GCADRTVKHF	STEDGIFQGQ
**90**	**100**	**110**	**120**	**130**	**140**	**150**	**160**
RHCPGGEGMF	RGLAQADGTL	ITCVDSGILR	VWHDKDKDTS	SDPLLELRVG	PGVCRMRQDP	AHPHVVATGG	KENALKIWDL
**170**	**180**	**190**	**200**	**210**	**220**	**230**	**240**
QGSEEPVFRA	KNVRNDWLDL	RVPIWDQDIQ	FLPGSQKLVT	CTGYHQVRVY	DPASPQRRPV	LETTYGEYPL	TAMTLTPGGN
**250**	**260**	**270**	**280**	**290**	**300**	**310**	**320**
SVIVGNTHGQ	LAEIDLRQGR	LLGCLKGLAG	SVRGLQCHPS	KPLLASCGLD	RVLRIHRIQN	PRGLEHKVYL	KSQLNCLLLS
**330**	**340**	**350**	**360**	**370**	**380**	**390**	
GRDNWEDEPQ	EPQEPNKVPL	EDTETDELWA	SLEAAAKRKL	SGLEQPQGAL	QTRRRKKKRP	GSTSP	

Cmpd.	No. of Cmpds.	m/z meas.	Δ m/z [ppm]	z	Rt [min]	Score	P	Range	Sequence	Modification
1187	1	918.4360	17.92	3	43.1	15.4	1	41-65	R.REEAVSALCWGTGGETQMLVGCADR.T	Carbamidomethyl: 9, 22

Detailed Protein Report

Protein 567: PREDICTED: roundabout homolog 2 isoform X3 [Homo sapiens]

**Accession:**	gi|578806427	**Score:**	15.4
**Database:**	refseq_human(refseq_human_20140103.fasta)	**MW [kDa]:**	132.9
**Database Date:**	2015-11-30	**pI:**	6.9
		**Sequence Coverage [%]:**	0.7
		**No. of unique Peptides:**	1

Quantitation

WD:WU **Median:** 1.11 **CV:** 0.00 % **No. of Peptides:** 1

10	20	30	40	50	60	70	80
MARRHERVTR	RMWTWAPGLL	MMTVVFWGHQ	GNGQGQGSRL	RQEDFPPRIV	EHPSDVIVSK	GEPTTLNCKA	EGRPTPTIEW
90	100	110	120	130	140	150	160
YKDGERVETD	KDDPRSHRML	LPSGSLFFLR	IVHGRRSKPD	EGSYVCVARN	YLGEAVSRNA	SLEVALLRDD	FRQNPTDVVV
170	180	190	200	210	220	230	240
AAGEPAILEC	QPPRGHPEPT	IYWKKDKVRI	DDKEERISIR	GGKLMISNTR	KSDAGMYTCV	GTNMVGERDS	DPAELTVFER
250	260	270	280	290	300	310	320
PTFLRRPINQ	VVLEEEAVEF	RCQVQGDPQP	TVRWKKDDAD	LPRGRYDIKD	DYTLRIKKTM	STDEGTYMCI	AENRVGKMEA
330	340	350	360	370	380	390	400
SATLTVRARP	VAPPQFVVRP	RDQIVAQGRT	VTFPCETKGN	PQPAVFWQKE	GSQNLLFPNQ	PQQPNSRCSV	SPTGDLTITN
410	420	430	440	450	460	470	480
IQRSDAGYYI	CQALTVAGSI	LAKAQLEVTD	VLTDRPPPII	LQGPANQTLA	VDGTALLKCK	ATGDPLPVIS	WLKEGFTFPG
490	500	510	520	530	540	550	560
RDPRATIQEQ	GTLQIKNLRI	SDTGTYTCVA	TSSSGETSWS	AVLDVTESGA	TISKNYDLSD	LPGPPSKPQV	TDVTKNSVTL
570	580	590	600	610	620	630	640
SWQPGTPGTL	PASAYIIEAF	SQSVSNSWQT	VANHVKTTLY	TVRGLRPNTI	YLFMVRAINP	QGLSDPSPMS	DPVRTQDISP
650	660	670	680	690	700	710	720
PAQGVDHRQV	QKELGDVLVR	LHNPVVLTPT	TVQVTWTVDR	QPQFIQGYRV	MYRQTSGLQA	TSSWQNLDAK	VPTERSAVLV
730	740	750	760	770	780	790	800
NLKKGVTYEI	KVRPYFNEFQ	GMDSESKTVR	TTEEAPSAPP	QSVTVLTVGS	YNSTSISVSW	DPPPPDHQNG	IIQEYKIWCL
810	820	830	840	850	860	870	880
GNETRFHINK	TVDAAIRSVI	IGGLFPGIQY	RVEVAASTSA	GVGVKSEPQP	IIIGRRNEVV	ITENNNSITE	QITDVVKQPA
890	900	910	920	930	940	950	960
FIAGIGGACW	VILMGFSIWL	YWRRKKRKGL	SNYAVTFQRG	DGGLMSNGSR	PGLLNAGDPS	YPWLADSWPA	TSLPVNNSNS
970	980	990	1000	1010	1020	1030	1040
GPNEIGNFGR	GDVLPPVPGQ	GDKTATMLSD	GAIYSSIDFT	TKTSYNSSSQ	ITQATPYATT	QILHSNSIHE	LAVDLPDPQW
1050	1060	1070	1080	1090	1100	1110	1120
KSSIQQKTDL	MGFGYSLPDQ	NKGNNGGKGG	KKKKNKNSSK	PQKNNGSTWA	NVPLPPPPVQ	PLPGTELEHY	AVEQQENGYD
1130	1140	1150	1160	1170	1180	1190	1200
SDSWCPPLPV	QTYLHQGLED	ELEEDDDRVP	TPPVRGVASS	PAISFGQQST	ATLTPSPREE	MQPMLQAHLD	ELTRAYQFDI
1210							
AKQTW							

Cmpd.	No. of Cmpds.	m/z meas.	Δ m/z [ppm]	z	Rt [min]	Score	P	Range	Sequence	Modification	Ratios
588	1	469.2361	-75.04	2	36.2	15.4	1	724-731	K.KGVTYEIK.V		WD:WU 1.11

Detailed Protein Report

Protein 568: insulin receptor-related protein precursor [Homo sapiens]

**Accession:**	gi|31657140	**Score:**	15.3
**Database:**	refseq_human(refseq_human_20140103.fasta)	**MW [kDa]:**	143.6
**Database Date:**	2015-11-30	**pI:**	6.0
**Modification(s):**	Carbamidomethyl	**Sequence Coverage [%]:**	1.0
		**No. of unique Peptides:**	1

10	20	30	40	50	60	70	80
MAVPSLWPWG	ACLPVIFLSL	GFGLDTVEVC	PSLDIRSEVA	ELRQLENCSV	VEGHLQILLM	FTATGEDFRG	LSFPRLTQVT
90	100	110	120	130	140	150	160
DYLLLFRVYG	LESLRDLFPN	LAVIRGTRLF	LGYALVIFEM	PHLRDVALPA	LGAVLRGAVR	VEKNQELCHL	STIDWGLLQP
170	180	190	200	210	220	230	240
APGANHIVGN	KLGEECADVC	PGVLGAAGEP	CAKTTFSGHT	DYRCWTSSHC	QRVCPCPHGM	ACTARGECCH	TECLGGCSQP
250	260	270	280	290	300	310	320
EDPRACVACR	HLYFQGACLW	ACPPGTYQYE	SWRCVTAERC	ASLHSVPGRA	STFGIHQGSC	LAQCPSGFTR	NSSSIFCHKC
330	340	350	360	370	380	390	400
EGLCPKECKV	GTKTIDSIQA	AQDLVGCTHV	EGSLILNLRQ	GYNLEPQLQH	SLGLVETITG	FLKIKHSFAL	VSLGFFKNLK
410	420	430	440	450	460	470	480
LIRGDAMVDG	NYTLYVLDNQ	NLQQLGSWVA	AGLTIPVGKI	YFAFNPRLCL	EHIYRLEEVT	GTRGRQNKAE	INPRTNGDRA
490	500	510	520	530	540	550	560
ACQTRTLRFV	SNVTEADRIL	LRWERYEPLE	ARDLLSFIVY	YKESPFQNAT	EHVGPDACGT	QSWNLLDVEL	PLSRTQEPGV
570	580	590	600	610	620	630	640
TLASLKPWTQ	YAVFVRAITL	TTEEDSPHQG	AQSPIVYLRT	LPAAPTVPQD	VISTSNSSSH	LLVRWKPPTQ	RNGNLTYYLV
650	660	670	680	690	700	710	720
LWQRLAEDGD	LYLNDYCHRG	LRLPTSNNDP	RFDGEDGDPE	AEMESDCCPC	QHPPPGQVLP	PLEAQEASFQ	KKFENFLHNA
730	740	750	760	770	780	790	800
ITIPISPWKV	TSINKSPQRD	SGRHRRAAGP	LRLGGNSSDF	EIQEDKVPRE	RAVLSGLRHF	TEYRIDIHAC	NHAAHTVGCS
810	820	830	840	850	860	870	880
AATFVFARTM	PHREADGIPG	KVAWEASSKN	SVLLRWLEPP	DPNGLILKYE	IKYRRLGEEA	TVLCVSRLRY	AKFGGVHLAL
890	900	910	920	930	940	950	960
LPPGNYSARV	RATSLAGNGS	WTDSVAFYIL	GPEEEDAGGL	HVLLTATPVG	LTLLIVLAAL	GFFYGKKRNR	TLYASVNPEY
970	980	990	1000	1010	1020	1030	1040
FSASDMYVPD	EWEVPREQIS	IIRELGQGSF	GMVYEGLARG	LEAGEESTPV	ALKTVNELAS	PRECIEFLKE	ASVMKAFKCH
1050	1060	1070	1080	1090	1100	1110	1120
HVVRLLGVVS	QGQPTLVIME	LMTRGDLKSH	LRSLRPEAEN	NPGLPQPALG	EMIQMAGEIA	DGMAYLAANK	FVHRDLAARN
1130	1140	1150	1160	1170	1180	1190	1200
CMVSQDFTVK	IGDFGMTRDV	YETDYYRKGG	KGLLPVRWMA	PESLKDGIFT	THSDVWSFGV	VLWEIVTLAE	QPYQGLSNEQ
1210	1220	1230	1240	1250	1260	1270	1280
VLKFVMDGGV	LEELEGCPLQ	LQELMSRCWQ	PNPRLRPSFT	HILDSIQEEL	RPSFRLLSFY	YSPECRGARG	SLPTTDAEPD
1290	1300						
SSPTPRDCSP	QNGGPGH						

Cmpd.	No. of Cmpds.	m/z meas.	Δ m/z [ppm]	z	Rt [min]	Score	P	Range	Sequence	Modification
571	1	730.2246	-112.42	2	36.0	15.3	0	213-225	R.VCPCPHGMACTAR.G	Carbamidomethyl: 2, 4

Detailed Protein Report

Protein 569: PREDICTED: kazrin isoform X2 [Homo sapiens]

Accession: gi|530360946
Database: refseq_human(refseq_human_20140103.fasta)
Database Date: 2015-11-30

Score: 15.3
MW [kDa]: 51.7
pI: 6.6
Sequence Coverage [%]: 1.9
No. of unique Peptides: 1

Quantitation

WD:WU **Median:** 1.67 **CV:** 0.00 % **No. of Peptides:** 1

10	20	30	40	50	60	70	80
MTNTKDPKRA	MESTLATSNS	ATGPVTFSHV	FGQQCQLMQA	AVQSLHTLND	QISHFIVTKS	KALEEDKDPF	LPTEKETLKS
90	100	110	120	130	140	150	160
SMILMRHLLM	DAQAKILSMM	EDNKQLALRI	DGAVQSASQE	VTNLRAELTA	TNRRLAELSG	GGGPGPGPGA	AASASAAGDS
170	180	190	200	210	220	230	240
AATNMENPQL	GAQVLLREEV	SRLQEEVHLL	RQMKEMLAKD	LEESQGGKSS	EVLSATELRV	QLAQKEQELA	RAKEALQAMK
250	260	270	280	290	300	310	320
ADRKRLKGEK	TDLVSQMQQL	YATLESREEQ	LRDFIRNYEQ	HRKESEDAVK	ALAKEKDLLE	REKWELRRQA	KEATDHATAL
330	340	350	360	370	380	390	400
RSQLDLKDNR	MKELEAELAM	AKQSLATLTK	DVPKRHSLAM	PGETVLNGNQ	EWVVQADLPL	TAAIRQSQQT	LYHSHPPHPA
410	420	430	440	450	460	470	
DRQAVRVSPC	HSRQPSVISD	ASAAEGDRSS	TPSDINSPRH	RTHSLWRQSR	PSSEEPAQPY	CTVTRGS	

Cmpd.	No. of Cmpds.	m/z meas.	Δ m/z [ppm]	z	Rt [min]	Score	P	Range	Sequence	Modification	Ratios
989	2	540.6455	-205.92	2	40.7	15.3	0	96-104	K.ILSMMEDNK.Q		WD:WU 1.67

Detailed Protein Report

Protein 570: **PREDICTED: nischarin isoform X2 [Homo sapiens]**

**Accession:**	gi|578805595	**Score:**	15.3
**Database:**	refseq_human(refseq_human_20140103.fasta)	**MW [kDa]:**	110.7
**Database Date:**	2015-11-30	**pI:**	4.7
		**Sequence Coverage [%]:**	1.9
		**No. of unique Peptides:**	1

10	20	30	40	50	60	70	80
MKCGIMFVQE	EALASSLSST	DSLTPEHQPI	AQGCSDSLES	IPAGQAASDD	LRDVPGAVGG	ASPEHAEPEV	QVVPGSGQII
90	100	110	120	130	140	150	160
FLPFTCIGYT	ATNQDFIQRL	STLIRQAIER	QLPAWIEAAN	QREEGQGEQG	EEEDEEEEEE	EDVAENRYFE	MGPPDVEEEE
170	180	190	200	210	220	230	240
GGGQGEEEEE	EEEDEEAEEE	RLALEWALGA	DEDFLLEHIR	ILKVLWCFLI	HVQGSIRQFA	ACLVLTDFGI	AVFEIPHQES
250	260	270	280	290	300	310	320
RGSSQHILSS	LRFVFCFPHG	DLTEFGFLMP	ELCLVLKVRH	SENTLFIISD	AANLHEFHAD	LRSCFAPQHM	AMLCSPILYG
330	340	350	360	370	380	390	400
SHTSLQEFLR	QLLTFYKVAG	GCQERSQGCF	PVYLVYSDKR	MVQTAAGDYS	GNIEWASCTL	CSAVRRSCCA	PSEAVKSAAI
410	420	430	440	450	460	470	480
PYWLLLTPQH	LNVIKADFNP	MPNRGTHNCR	NRNSFKLSRV	PLSTVLLDPT	RSCTQPRGAF	ADGHVLELLV	GYRFVTAIFV
490	500	510	520	530	540	550	560
LPHEKFHFLR	VYNQLRASLQ	DLKTVVIAKT	PGTGGSPQGS	FADGQPAERR	ASNDQRPQEV	PAEALAPAPA	EVPAPAPAAA
570	580	590	600	610	620	630	640
SASGPAKTPA	PAEASTSALV	PEETPVEAPA	PPPAEAPAQY	PSEHLIQATS	EENQIPSHLP	ACPSLRHVAS	LRGSAIIELF
650	660	670	680	690	700	710	720
HSSIAEVENE	ELRHLMWSSV	VFYQTPGLEV	TACVLLSTKA	VYFVLHDGLR	RYFSEPLQDF	WHQKNTDYNN	SPFHISQCFV
730	740	750	760	770	780	790	800
LKLSDLQSVN	VGLFDQHFRL	TGSTPMQVVT	CLTRDSYLTH	CFLQHLMVVL	SSLERTPSPE	PVDKDFYSEF	GNKTTGKMEN
810	820	830	840	850	860	870	880
YELIHSSRVK	FTYPSEEEIG	DLTFTVAQKM	AEPEKAPALS	ILLYVQAFQV	GMPPPGCCRG	PLRPKTLLLT	SSEIFLLDED
890	900	910	920	930	940	950	960
CVHYPLPEFA	KEPPQRDRYR	LDDGRRVRDL	DRVLMGYQTY	PQALTLVFDD	VQGHDLMGSV	TLDHFGEVPG	GPARASQGRE
970	980	990	1000				
VQWQVFVPSA	ESREKLISLL	ARQWEALCGR	ELPVELTG				

Cmpd.	No. of Cmpds.	m/z meas.	Δ m/z [ppm]	z	Rt [min]	Score	P	Range	Sequence	Modification
1738	1	1087.9436	-77.77	2	50.4	15.3	0	811-829	K.FTYPSEEEIGDLTFTVAQK.M	

Detailed Protein Report

Protein 571: CUB and sushi domain-containing protein 1 precursor [Homo sapiens]

**Accession:**	gi|259013213	**Score:**	15.3
**Database:**	refseq_human(refseq_human_20140103.fasta)	**MW [kDa]:**	388.5
**Database Date:**	2015-11-30	**pI:**	5.6
**Modification(s):**	Carbamidomethyl	**Sequence Coverage [%]:**	0.3
		**No. of unique Peptides:**	1

Quantitation

WD:WU	**Median:** 0.80	**CV:** 0.00 %	**No. of Peptides:** 1

Detailed Protein Report

10	20	30	40	50	60	70	80
MTAWRRFQSL	LLLLGLLVLC	ARLLTAAKGQ	NCGGLVQGPN	GTIESPGFPH	GYPNYANCTW	IIITGERNRI	QLSFHTFALE
90	100	110	120	130	140	150	160
EDFDILSVYD	GQPQQGNLKV	RLSGFQLPSS	IVSTGSILTL	WFTTDFAVSA	QGFKALYEVL	PSHTCGNPGE	ILKGVLHGTR
170	180	190	200	210	220	230	240
FNIGDKIRYS	CLPGYILEGH	AILTCIVSPG	NGASWDFPAP	FCRAEGACGG	TLRGTSSSIS	SPHFPSEYEN	NADCTWTILA
250	260	270	280	290	300	310	320
EPGDTIALVF	TDFQLEEGYD	FLEISGTEAP	SIWLTGMNLP	SPVISSKNWL	RLHFTSDSNH	RRKGFNAQFQ	VKKAIELKSR
330	340	350	360	370	380	390	400
GVKMLPSKDG	SHKNSVLSQG	GVALVSDMCP	DPGIPENGRR	AGSDFRVGAN	VQFSCEDNYV	LQGSKSITCQ	RVTETLAAWS
410	420	430	440	450	460	470	480
DHRPICRART	CGSNLRGPSG	VITSPNYPVQ	YEDNAHCVWV	ITTTDPDKVI	KLAFEEFELE	RGYDTLTVGD	AGKVGDTRSV
490	500	510	520	530	540	550	560
LYVLTGSSVP	DLIVSMSNQM	WLHLQSDDSI	GSPGFKAVYQ	EIEKGGCGDP	GIPAYGKRTG	SSFLHGDTLT	FECPAAFELV
570	580	590	600	610	620	630	640
GERVITCQQN	NQWSGNKPSC	VFSCFFNFTA	SSGIILSPNY	PEEYGNNMNC	VWLIISEPGS	RIHLIFNDFD	VEPQFDFLAV
650	660	670	680	690	700	710	720
KDDGISDITV	LGTFSGNEVP	SQLASSGHIV	RLEFQSDHST	TGRGFNITYT	TFGQNECHDP	GIPINGRRFG	DRFLLGSSVS
730	740	750	760	770	780	790	800
FHCDDGFVKT	QGSESITCIL	QDGNVVWSST	VPRCEAPCGG	HLTASSGVIL	PPGWPGYYKD	SLHCEWIIEA	KPGHSIKITF
810	820	830	840	850	860	870	880
DRFQTEVNYD	TLEVRDGPAS	SSPLIGEYHG	TQAPQFLIST	GNFMYLLFTT	DNSRSSIGFL	IHYESVTLES	DSCLDPGIPV
890	900	910	920	930	940	950	960
NGHRHGGDFG	IRSTVTFSCD	PGYTLSDDEP	LVCERNHQWN	HALPSCDALC	GGYIQGKSGT	VLSPGFPDFY	PNSLNCTWTI
970	980	990	1000	1010	1020	1030	1040
EVSHGKGVQM	IFHTFHLESS	HDYLLITEDG	SFSEPVARLT	GSVLPHTIKA	GLFGNFTAQL	RFISDFSISY	EGFNITFSEY
1050	1060	1070	1080	1090	1100	1110	1120
DLEPCDDPGV	PAFSRRIGFH	FGVGDSLTFS	CFLGYRLEGA	TKLTCLGGGR	RVWSAPLPRC	VAECGASVKG	NEGTLLSPNF
1130	1140	1150	1160	1170	1180	1190	1200
PSNYDNNHEC	IYKIETEAGK	GIHLRTRSFQ	LFEGDTLKVY	DGKDSSSRPL	GTFTKNELLG	LILNSTSNHL	WLEFNTNGSD
1210	1220	1230	1240	1250	1260	1270	1280
TDQGFQLTYT	SFDLVKCEDP	GIPNYGYRIR	DEGHFTDTVV	LYSCNPGYAM	HGSNTLTCLS	GDRRVWDKPL	PSCIAECGGQ
1290	1300	1310	1320	1330	1340	1350	1360
IHAATSGRIL	SPGYPAPYDN	NLHCTWIIEA	DPGKTISLHF	IVFDTEMAHD	ILKVWDGPVD	SDILLKEWSG	SALPEDIHST
1370	1380	1390	1400	1410	1420	1430	1440
FNSLTLQFDS	DFFISKSGFS	IQFSTSIAAT	CNDPGMPQNG	TRYGDSREAG	DTVTFQCDPG	YQLQGQAKIT	CVQLNNRFFW
1450	1460	1470	1480	1490	1500	1510	1520
QPDPPTCIAA	CGGNLTGPAG	VILSPNYPQP	YPPGKECDWR	VKVNPDFVIA	LIFKSFNMEP	SYDFLHIYEG	EDSNSPLIGS
1530	1540	1550	1560	1570	1580	1590	1600
YQGSQAPERI	ESSGNSLFLA	FRSDASVGLS	GFAIEFKEKP	REACFDPGNI	MNGTRVGTDF	KLGSTITYQC	DSGYKILDPS
1610	1620	1630	1640	1650	1660	1670	1680
SITCVIGADG	KPSWDQVLPS	CNAPCGGQYT	GSEGVVLSPN	YPHNYTAGQI	CLYSITVPKE	FVVFGQFAYF	QTALNDLAEL
1690	1700	1710	1720	1730	1740	1750	1760
FDGTHAQARL	LSSLSGSHSG	ETLPLATSNQ	ILLRFSAKSG	ASARGFHFVY	QAVPRTSDTQ	CSSVPEPRYG	RRIGSEFSAG
1770	1780	1790	1800	1810	1820	1830	1840
SIVRFECNPG	YLLQGSTALH	CQSVPNALAQ	WNDTIPSCVV	PCSGNFTQRR	GTILSPGYPE	PYGNNLNCIW	KIIVTEGSGI
1850	1860	1870	1880	1890	1900	1910	1920
QIQVISFATE	QNWDSLEIHD	GGDVTAPRLG	SFSGTTVPAL	LNSTSNQLYL	HFQSDISVAA	AGFHLEYKTV	GLAACQEPAL
1930	1940	1950	1960	1970	1980	1990	2000
PSNSIKIGDR	YMVNDVLSFQ	CEPGYTLQGR	SHISCMPGTV	RRWNYPSPLC	IATCGGTLST	LGGVILSPGF	PGSYPNNLDC
2010	2020	2030	2040	2050	2060	2070	2080
TWRISLPIGY	GAHIQFLNFS	TEANHDFLEI	QNGPYHTSPM	IGQFSGTDLP	AALLSTTHET	LIHFYSDHSQ	NRQGFKLAYQ
2090	2100	2110	2120	2130	2140	2150	2160
AYELQNCPDP	PPFQNGYMIN	SDYSVGQSVS	FECYPGYILI	GHPVLTCQHG	INRNWNYPFP	RCDAPCGYNV	TSQNGTIYSP
2170	2180	2190	2200	2210	2220	2230	2240

Detailed Protein Report

Cmpd.	No. of Cmpds.	m/z meas.	Δ m/z [ppm]	z	Rt [min]	Score	P	Range	Sequence	Modification	Ratios	
714	1	512.2777	86.29	2	37.7	15.3	0	1100-1109	R.CVAECGASVK.G	Carbamidomethyl: 1	WD:WU	0.80

Detailed Protein Report

Protein 572: tumor necrosis factor alpha-induced protein 8-like protein 1 [Homo sapiens]

**Accession:**	gi|269846912	**Score:**	15.3
**Database:**	refseq_human(refseq_human_20140103.fasta)	**MW [kDa]:**	20.8
**Database Date:**	2015-11-30	**pI:**	10.4
		**Sequence Coverage [%]:**	5.4
		**No. of unique Peptides:**	1

Alias proteins:

Accession	Name	Description
gi|530425099	refseq_human (refseq_human_20140103.fasta)	PREDICTED: tumor necrosis factor alpha-induced protein 8-like protein 1 isoform X1 [Homo sapiens]
gi|269846918	refseq_human (refseq_human_20140103.fasta)	tumor necrosis factor alpha-induced protein 8-like protein 1 [Homo sapiens]

10	20	30	40	50	60	70	80
MDTFSTKSLA	LQAQKKLLSK	MASKAVVAVL	VDDTSSEVLD	ELYRATREFT	RSRKEAQKML	KNLVKVALKL	GLLLRGDQLG
**90**	**100**	**110**	**120**	**130**	**140**	**150**	**160**
GEELALLRRF	RHRARCLAMT	AVSFHQVDFT	FDRRVLAAGL	LECRDLLHQA	VGPHLTAKSH	GRINHVFGHL	ADCDFLAALY
**170**	**180**	**190**					
GPAEPYRSHL	RRICEGLGRM	LDEGSL					

Cmpd.	No. of Cmpds.	m/z meas.	Δ m/z [ppm]	z	Rt [min]	Score	P	Range	Sequence	Modification
1438	1	1095.6551	-96.87	1	46.4	15.3	1	66-75	K.VALKLGLLLR.G	

Detailed Protein Report

Protein 573: patatin-like phospholipase domain-containing protein 5 isoform 1 [Homo sapiens]

**Accession:**	gi|20304127	**Score:**	15.3
**Database:**	refseq_human(refseq_human_20140103.fasta)	**MW [kDa]:**	47.9
**Database Date:**	2015-11-30	**pI:**	6.3
**Modification(s):**	Carbamidomethyl	**Sequence Coverage [%]:**	4.2
		**No. of unique Peptides:**	1

10	20	30	40	50	60	70	80
MGFLEEEGRW	NLSFSGAGYL	GAHHVGATEC	LRQRAPRLLQ	GARRIYGSSS	GALNAVSIVC	GKSVDFCCSH	LLGMVGQLER
**90**	**100**	**110**	**120**	**130**	**140**	**150**	**160**
LSLSILHPAY	APIEHVKQQL	QDALPPDAHV	LASQRLGISL	TRWPDGRNFL	VTDFATCDEL	IQALVCTLYF	PFYCGLIPPE
**170**	**180**	**190**	**200**	**210**	**220**	**230**	**240**
FRGERYIDGA	LSNNLPFADC	PSTITVSPFH	GTVDICPQST	SPNLHELNVF	NFSFQISTEN	FFLGLICLIP	PSLEVVADNC
**250**	**260**	**270**	**280**	**290**	**300**	**310**	**320**
RQGYLDALRF	LERRGLTKEP	VLWTLVSKEP	PAPADGNWDA	GCDQRWKGGL	SLNWKVPHVQ	VKDVPNFEQL	SPELEAALKK
**330**	**340**	**350**	**360**	**370**	**380**	**390**	**400**
ACTRDPSRWA	RFWHSGPGQV	LTYLLLPCTL	PFEYIYFRSR	RLVVWLPDVP	ADLWWMQGLL	RNMALEVFSR	TKAQLLGPIS
**410**	**420**	**430**					
PPATRVLETS	PLQPQIAPHR	EELGPTHQA					

Cmpd.	No. of Cmpds.	m/z meas.	Δ m/z [ppm]	z	Rt [min]	Score	P	Range	Sequence	Modification
958	1	684.2638	-84.66	3	40.3	15.3	0	63-80	K.SVDFCCSHLLGMVGQLER.L	Carbamidomethyl: 5

Detailed Protein Report

Protein 574: mannan-binding lectin serine protease 2 isoform 1 preproprotein [Homo sapiens]

**Accession:**	gi|21264363	**Score:**	15.2
**Database:**	refseq_human(refseq_human_20140103.fasta)	**MW [kDa]:**	75.7
**Database Date:**	2015-11-30	**pI:**	5.3
		**Sequence Coverage [%]:**	2.8
		**No. of unique Peptides:**	1

10	20	30	40	50	60	70	80
MRLLTLLGLL	CGSVATPLGP	KWPEPVFGRL	ASPGFPGEYA	NDQERRWTLT	APPGYRLRLY	FTHFDLELSH	LCEYDFVKLS
90	100	110	120	130	140	150	160
SGAKVLATLC	GQESTDTERA	PGKDTFYSLG	SSLDITFRSD	YSNEKPFTGF	EAFYAAEDID	ECQVAPGEAP	TCDHHCHNHL
170	180	190	200	210	220	230	240
GGFYCSCRAG	YVLHRNKRTC	SALCSGQVFT	QRSGELSSPE	YPRPYPKLSS	CTYSISLEEG	FSVILDFVES	FDVETHPETL
250	260	270	280	290	300	310	320
CPYDFLKIQT	DREEHGPFCG	KTLPHRIETK	SNTVTITFVT	DESGDHTGWK	IHYTSTAQPC	PYPMAPPNGH	VSPVQAKYIL
330	340	350	360	370	380	390	400
KDSFSIFCET	GYELLQGHLP	LKSFTAVCQK	DGSWDRPMPA	CSIVDCGPPD	DLPSGRVEYI	TGPGVTTYKA	VIQYSCEETF
410	420	430	440	450	460	470	480
YTMKVNDGKY	VCEADGFWTS	SKGEKSLPVC	EPVCGLSART	TGGRIYGGQK	AKPGDFPWQV	LILGGTTAAG	ALLYDNWVLT
490	500	510	520	530	540	550	560
AAHAVYEQKH	DASALDIRMG	TLKRLSPHYT	QAWSEAVFIH	EGYTHDAGFD	NDIALIKLNN	KVVINSNITP	ICLPRKEAES
570	580	590	600	610	620	630	640
FMRTDDIGTA	SGWGLTQRGF	LARNLMYVDI	PIVDHQKCTA	AYEKPPYPRG	SVTANMLCAG	LESGGKDSCR	GDSGGALVFL
650	660	670	680	690			
DSETERWFVG	GIVSWGSMNC	GEAGQYGVYT	KVINYIPWIE	NIISDF			

Cmpd.	No. of Cmpds.	m/z meas.	Δ m/z [ppm]	z	Rt [min]	Score	P	Range	Sequence	Modification
16	1	1068.4952	-116.47	2	29.4	15.2	2	538-556	K.LNNKVVINSNITPICLPRK.E	

Detailed Protein Report

Protein 575: serine/threonine-protein kinase MRCK gamma [Homo sapiens]

Accession: gi|156766068
Database: refseq_human(refseq_human_20140103.fasta)
Database Date: 2015-11-30
Modification(s): Oxidation

Score: 15.2
MW [kDa]: 172.4
pI: 5.9
Sequence Coverage [%]: 1.2
No. of unique Peptides: 1

10	20	30	40	50	60	70	80
MERRLRALEQ	LARGEAGGCP	GLDGLLDLLL	ALHHELSSGP	LRRERSVAQF	LSWASPFVSK	VKELRLQRDD	FEILKVIGRG
90	100	110	120	130	140	150	160
AFGEVTVVRQ	RDTGQIFAMK	MLHKWEMLKR	AETACFREER	DVLVKGDSRW	VTTLHYAFQD	EEYLYLVMDY	YAGGDLLTLL
170	180	190	200	210	220	230	240
SRFEDRLPPE	LAQFYLAEMV	LAIHSLHQLG	YVHRDVKPDN	VLLDVNGHIR	LADFGSCLRL	NTNGMVDSSV	AVGTPDYISP
250	260	270	280	290	300	310	320
EILQAMEEGK	GHYGPQCDWW	SLGVCAYELL	FGETPFYAES	LVETYGKIMN	HEDHLQFPPD	VPDVPASAQD	LIRQLLCRQE
330	340	350	360	370	380	390	400
ERLGRGGLDD	FRNHPFFEGV	DWERLASSTA	PYIPELRGPM	DTSNFDVDDD	TLNHPGTLPP	PSHGAFSGHH	LPFVGFTYTS
410	420	430	440	450	460	470	480
GSHSPESSSE	AWAALERKLQ	CLEQEKVELS	RKHQEALHAP	TDHRELEQLR	KEVQTLRDRL	PEMLRDKASL	SQTDGPPAGS
490	500	510	520	530	540	550	560
PGQDSDLRQE	LDRLHRELAE	GRAGLQAQEQ	ELCRAQGQQE	ELLQRLQEAQ	EREAATASQT	RALSSQLEEA	RAAQRELEAQ
570	580	590	600	610	620	630	640
VSSLSRQVTQ	LQGQWEQRLE	ESSQAKTIHT	ASETNGMGPP	EGGPQEAQLR	KEVAALREQL	EQAHSHRPSG	KEEALCQLQE
650	660	670	680	690	700	710	720
ENRRLSREQE	RLEAELAQEQ	ESKQRLEGER	RETESNWEAQ	LADILSWVND	EKVSRGYLQA	LATKMAEELE	SLRNVGTQTL
730	740	750	760	770	780	790	800
PARPLDHQWK	ARRLQKMEAS	ARLELQSALE	AEIRAKQGLQ	ERLTQVQEAQ	LQAERRLQEA	EKQSQALQQE	LAMLREELRA
810	820	830	840	850	860	870	880
RGPVDTKPSN	SLIPFLSFRS	SEKDSAKDPG	ISGEATRHGG	EPDLRPEGRR	SLRMGAVFPR	APTANTASTE	GLPAKPGSHT
890	900	910	920	930	940	950	960
LRPRSFPSPT	KCLRCTSLML	GLGRQGLGCD	ACGYFCHTTC	APQAPPCPVP	PDLLRTALGV	HPETGTGTAY	EGFLSVPRPS
970	980	990	1000	1010	1020	1030	1040
GVRRGWQRVF	AALSDSRLLL	FDAPDLRLSP	PSGALLQVLD	LRDPQFSATP	VLASDVIHAQ	SRDLPRIFRV	TTSQLAVPPT
1050	1060	1070	1080	1090	1100	1110	1120
TCTVLLLAES	EGERERWLQV	LGELQRLLLD	ARPRPRPVYT	LKEAYDNGLP	LLPHTLCAAI	LDQDRLALGT	EEGLFVIHLR
1130	1140	1150	1160	1170	1180	1190	1200
SNDIFQVGEC	RRVQQLTLSP	SAGLLVVLCG	RGPSVRLFAL	AELENIEVAG	AKIPESRGCQ	VLAAGSILQA	RTPVLCVAVK
1210	1220	1230	1240	1250	1260	1270	1280
RQVLCYQLGP	GPGPWQRRIR	ELQAPATVQS	LGLLGDRLCV	GAAGGFALYP	LLNEAAPLAL	GAGLVPEELP	PSRGGLGEAL
1290	1300	1310	1320	1330	1340	1350	1360
GAVELSLSEF	LLLFTTAGIY	VDGAGRKSRG	HELLWPAAPM	GWGYAAPYLT	VFSENSIDVF	DVRRAEWVQT	VPLKKVRPLN
1370	1380	1390	1400	1410	1420	1430	1440
PEGSLFLYGT	EKVRLTYLRN	QLAEKDEFDI	PDLTDNSRRQ	LFRTKSKRRF	FFRVSEEQQK	QQRREMLKDP	FVRSKLISPP
1450	1460	1470	1480	1490	1500	1510	1520
TNFNHLVHVG	PANGRPGARD	KSPAPEEKGR	VARGSGPQRP	HSFSEALRRP	ASMGSEGLGG	DADPMKRKPW	TSLSSESVSC
1530	1540	1550	1560				
PQGSLSPATS	LMQVSERPRS	LPLSPELESS	P				

Cmpd.	No. of Cmpds.	m/z meas.	Δ m/z [ppm]	z	Rt [min]	Score	P	Range	Sequence	Modification
2153	1	974.3330	-124.42	2	55.3	15.2	1	1489-1507	R.RPASMGSEGLGGDADPMKR.K	Oxidation: 5

Detailed Protein Report

Protein 576: **PREDICTED: ATP-dependent RNA helicase DDX19B isoform X1 [Homo sapiens]**

**Accession:**	gi|578828798	**Score:**	15.2
**Database:**	refseq_human(refseq_human_20140103.fasta)	**MW [kDa]:**	52.0
**Database Date:**	2015-11-30	**pI:**	7.7
		**Sequence Coverage [%]:**	4.1
		**No. of unique Peptides:**	1

10	20	30	40	50	60	70	80
MLSNLHLKEE	KIKPDTNGAV	VKTNANAEKT	DEEEKEDRAA	QSLLNKLIRS	NLVDNTNQVE	VLQRDPNSPL	YSVKSFEELR
**90**	**100**	**110**	**120**	**130**	**140**	**150**	**160**
LKPQLLQGVY	AMGFNRPSKI	QENALPLMLA	EPPQNLIAQS	QSGTGKTAAF	VLAMLSQVEP	ANKYPQCLCL	SPTYELALQT
**170**	**180**	**190**	**200**	**210**	**220**	**230**	**240**
GKVIEQMGKF	YPELKLAYAV	RGNKLERGQK	ISEQIVIGTP	GTVLDWCSKL	KFIDPKKIKV	FVLDEADVMI	ATQGHQDQSI
**250**	**260**	**270**	**280**	**290**	**300**	**310**	**320**
RIQRMLPRNC	QMLLFSATFE	DSVWKFAQKV	VPDPNVIKLK	REEETLDTIK	QYYVLCSSRD	EKFQALCNLY	GAITIAQAMI
**330**	**340**	**350**	**360**	**370**	**380**	**390**	**400**
FCHTRKTASW	LAAELSKEGH	QVALLSGEMM	VEQRAAVIER	FREGKEKVLV	TTNVCARGID	VEQVSVVINF	DLPVDKDGNP
**410**	**420**	**430**	**440**	**450**	**460**	**470**	
DNETYLHRIG	RTGRFGKRGL	AVNMVDSKHS	MNILNRIQEH	FNKKIERLDT	DDLDEIEKIA	N	

Cmpd.	No. of Cmpds.	m/z meas.	Δ m/z [ppm]	z	Rt [min]	Score	P	Range	Sequence	Modification
1837	2	716.3485	-69.03	3	51.6	15.2	0	81-99	R.LKPQLLQGVYAMGFNRPSK.I	

Detailed Protein Report

Protein 577: polyadenylate-binding protein 4 isoform 3 [Homo sapiens]

**Accession:**	gi|208431836	**Score:**	15.2
**Database:**	refseq_human(refseq_human_20140103.fasta)	**MW [kDa]:**	69.5
**Database Date:**	2015-11-30	**pI:**	10.0
**Modification(s):**	Oxidation	**Sequence Coverage [%]:**	3.0
		**No. of unique Peptides:**	1

10	20	30	40	50	60	70	80
MNAAASSYPM	ASLYVGDLHS	DVTEAMLYEK	FSPAGPVLSI	RVCRDMITRR	SLGYAYVNFQ	QPADAERALD	TMNFDVIKGK
**90**	**100**	**110**	**120**	**130**	**140**	**150**	**160**
PIRIMWSQRD	PSLRKSGVGN	VFIKNLDKSI	DNKALYDTFS	AFGNILSCKV	VCDENGSKGY	AFVHFETQEA	ADKAIEKMNG
**170**	**180**	**190**	**200**	**210**	**220**	**230**	**240**
MLLNDRKVFV	GRFKSRKERE	AELGAKAKEF	TNVYIKNFGE	EVDDESLKEL	FSQFGKTLSV	KVMRDPNGKS	KGFGFVSYEK
**250**	**260**	**270**	**280**	**290**	**300**	**310**	**320**
HEDANKAVEE	MNGKEISGKI	IFVGRAQKKV	ERQAELKRKF	EQLKQERISR	YQGVNLYIKN	LDDTIDDEKL	RKEFSPFGSI
**330**	**340**	**350**	**360**	**370**	**380**	**390**	**400**
TSAKVMLEDG	RSKGFGFVCF	SSPEEATKAV	TEMNGRIVGS	KPLYVALAQR	KEERKAHLTN	QYMQRVAGMR	ALPANAILNQ
**410**	**420**	**430**	**440**	**450**	**460**	**470**	**480**
FQPAAGGYFV	PAVPQAQGRP	PYYTPNQLAQ	MRPNPRWQQG	GRPQGFQGMP	SAIRQSGPRP	TLRHLAPTGN	APASRGLPTT
**490**	**500**	**510**	**520**	**530**	**540**	**550**	**560**
TQRVGVPTAV	QNLAPRAAVA	AAAPRAVAPY	KYASSVRSPH	PAIQPLQAPQ	PAVHVQGQEP	LTASMLAAAP	PQEQKQMLGE
**570**	**580**	**590**	**600**	**610**	**620**	**630**	**640**
RLFPLIQTMH	SNLAGKITGM	LLEIDNSELL	HMLESPESLR	SKVDEAVAVL	QAHHAKKEAA	QKVGAVAAAT	S

Cmpd.	No. of Cmpds.	m/z meas.	Δ m/z [ppm]	z	Rt [min]	Score	P	Range	Sequence	Modification
2973	1	1051.5430	46.04	2	65.9	15.2	2	241-259	K.HEDANKAVEEMNGKEISGK.I	Oxidation: 11

Detailed Protein Report

Protein 578: GLIPR1-like protein 2 isoform 1 [Homo sapiens]

**Accession:**	gi|394025727	**Score:**	15.1
**Database:**	refseq_human(refseq_human_20140103.fasta)	**MW [kDa]:**	40.2
**Database Date:**	2015-11-30	**pI:**	4.7
**Modification(s):**	Oxidation	**Sequence Coverage [%]:**	4.7
		**No. of unique Peptides:**	1

10	20	30	40	50	60	70	80
MEAARPFARE	WRAQSLPLAV	GGVLKLRLCE	LWLLLLGSSL	NARFLPDEED	VDFINEYVNL	HNELRGDVIP	RGSNLRFMTW
**90**	**100**	**110**	**120**	**130**	**140**	**150**	**160**
DVALSRTARA	WGKKCLFTHN	IYLQDVQMVH	PKFYGIGENM	WVGPENEFTA	SIAIRSWHAE	KKMYNFENGS	CSGDCSNYIQ
**170**	**180**	**190**	**200**	**210**	**220**	**230**	**240**
LVWDHSYKVG	CAVTPCSKIG	HIIHAAIFIC	NYAPGGTLTR	RPYEPGIFCT	RCGRRDKCTD	FLCSNADRDQ	ATYYRFWYPK
**250**	**260**	**270**	**280**	**290**	**300**	**310**	**320**
WEMPRPVVCD	PLCTFILLLR	ILCFILCVIT	VLIVQSQFPN	ILLEQQMIFT	PEESEAGNEE	EEKEEEKKEK	EEMEMEIMEM
**330**	**340**	**350**					
EEEKEEREEE	EEETQKEKME	EEEK					

Cmpd.	No. of Cmpds.	m/z meas.	Δ m/z [ppm]	z	Rt [min]	Score	P	Range	Sequence	Modification
2538	1	1045.4188	-0.85	2	60.0	15.1	1	309-324	K.EKEEMEMEIMEMEEEK.E	Oxidation: 12

Detailed Protein Report

Protein 579: **PREDICTED: mitogen-activated protein kinase-binding protein 1 isoform X2 [Homo sapiens]**

**Accession:**	gi|578826739	**Score:**	15.1
**Database:**	refseq_human(refseq_human_20140103.fasta)	**MW [kDa]:**	99.8
**Database Date:**	2015-11-30	**pI:**	5.5
		**Sequence Coverage [%]:**	1.4
		**No. of unique Peptides:**	1

10	20	30	40	50	60	70	80
MISCGADKSI	YFRTAQKSGD	GVQFTRTHHV	VRKTTLYDMD	VEPSWKYTAI	GCQDRNIRIF	NISSGKQKKL	FKGSQGEDGT
**90**	**100**	**110**	**120**	**130**	**140**	**150**	**160**
LIKVQTDPSG	IYIATSCSDK	NLSIFDFSSG	ECVATMFGHS	EIVTGMKFSN	DCKHLISVSG	DSCIFVWRLS	SEMTISMRQR
**170**	**180**	**190**	**200**	**210**	**220**	**230**	**240**
LAELRQRQRG	GKQQGPSSPQ	RASGPNRHQA	PSMLSPGPAL	SSDSDKEGED	EGTEEELPAL	PVLAKSTKKA	LASVPSPALP
**250**	**260**	**270**	**280**	**290**	**300**	**310**	**320**
RSLSHWEMSR	AQESVGFLDP	APAANPGPRR	RGRWVQPGVE	LSVRSMLDLR	QLETLAPSLQ	DPSQDSLAII	PSGPRKHGQE
**330**	**340**	**350**	**360**	**370**	**380**	**390**	**400**
ALETSLTSQN	EKPPRPQASQ	PCSYPHIIRL	LSQEEGVFAQ	DLEPAPIEDG	IVYPEPSDNP	TMDTSEFQVQ	APARGTLGRV
**410**	**420**	**430**	**440**	**450**	**460**	**470**	**480**
YPGSRSSEKH	SPDSACSVDY	SSSCLSSPEH	PTEDSESTEP	LSVDGISSDL	EEPAEGDEEE	EEEEGGMGPY	GLQEGSPQTP
**490**	**500**	**510**	**520**	**530**	**540**	**550**	**560**
DQEQFLKQHF	ETLASGAAPG	APVQVPERSE	SRSISSRFLL	QVQTRPLREP	SPSSSSLALM	SRPAQVPQAS	GEQPRGNGAN
**570**	**580**	**590**	**600**	**610**	**620**	**630**	**640**
PPGAPPEVEP	SSGNPSPQQA	ASVLLPRCRL	NPDSSWAPKR	VATASPFSGL	QKAQSVHSLV	PQERHEASLQ	APSPGALLSR
**650**	**660**	**670**	**680**	**690**	**700**	**710**	**720**
EIEAQDGLGS	LPPADGRPSR	PHSYQNPTTS	SMAKISRSIS	VGENLGLVAE	PQAHAPIRVS	PLSKLALPSR	AHLVLDIPKP
**730**	**740**	**750**	**760**	**770**	**780**	**790**	**800**
LPDRPTLAAF	SPVTKGRAPG	EAEKPGFPVG	LGKAHSTTER	WACLGEGTTP	KPRTECQAHP	GPSSPCAQQL	PVSSLFQGPE
**810**	**820**	**830**	**840**	**850**	**860**	**870**	**880**
NLQPPPPEKT	PNPMECTKPG	AALSQDSEPA	VSLEQCEQLV	AELRGSVRQA	VRLYHSVAGC	KMPSAEQSRI	AQLLRDTFSS
**890**	**900**	**910**	**920**	**930**	**940**		
VRQELEAVAG	AVLSSPGSSP	GAVGAEQTQA	LLEQYSELLL	RAVERRMERK	L		

Cmpd.	No. of Cmpds.	m/z meas.	Δ m/z [ppm]	z	Rt [min]	Score	P	Range	Sequence	Modification
809	2	697.8381	-23.89	2	38.9	15.1	1	14-26	R.TAQKSGDGVQFTR.T	

Detailed Protein Report

Protein 580: **PREDICTED: ubiquitin-associated and SH3 domain-containing protein B isoform X1 [Homo sapiens]**

**Accession:**	gi|530398460	**Score:**	15.1
**Database:**	refseq_human(refseq_human_20140103.fasta)	**MW [kDa]:**	75.5
**Database Date:**	2015-11-30	**pI:**	6.1
**Modification(s):**	Carbamidomethyl, Oxidation	**Sequence Coverage [%]:**	2.2
		**No. of unique Peptides:**	1

Quantitation

WD:WU **Median:** 0.84 **CV:** 0.00 % **No. of Peptides:** 1

10	20	30	40	50	60	70	80
MICVVLCIHG	FCTEKKKARC	VASDCLVSRE	ETDKAATQQQ	LSTLSKCGNE	RVWVSVLPAF	SAPAVPSGAE	GSVLLLRNFI
**90**	**100**	**110**	**120**	**130**	**140**	**150**	**160**
NLQKALASTG	GRSVQAACDW	LFSHVGDPFL	DDPLPREYVL	YLRPTGPLAQ	KLSDFWQQSK	QICGKNKAHN	IFPHITLCQF
**170**	**180**	**190**	**200**	**210**	**220**	**230**	**240**
FMCEDSKVDA	LGEALQTTVS	RWKCKFSAPL	PLELYTSSNF	IGLFVKEDSA	EVLKKFAADF	AAEAASKTEV	HVEPHKKQLH
**250**	**260**	**270**	**280**	**290**	**300**	**310**	**320**
VTLAYHFQAS	HLPTLEKLAQ	NIDVKLGCDW	VATIFSRDIR	FANHETLQVI	YPYTPQNDDE	LELVPGDFIF	MSPMEQTSTS
**330**	**340**	**350**	**360**	**370**	**380**	**390**	**400**
EGWIYGTSLT	TGCSGLLPEN	YITKADECST	WIFHGSYSIL	NTSSSNSLTF	GDGVLERRPY	EDQGLGETTP	LTIICQPMQP
**410**	**420**	**430**	**440**	**450**	**460**	**470**	**480**
LRVNSQPGPQ	KRCLFVCRHG	ERMDVVFGKY	WLSQCFDAKG	RYIRTNLNMP	HSLPQRSGGF	RDYEKDAPIT	VFGCMQARLV
**490**	**500**	**510**	**520**	**530**	**540**	**550**	**560**
GEALLESNTI	IDHVYCSPSL	RCVQTAHNIL	KGLQQENHLK	IRVEPGLFEW	TKWVAGSTLP	AWIPPSELAA	ANLSVDTTYR
**570**	**580**	**590**	**600**	**610**	**620**	**630**	**640**
PHIPISKLVV	SESYDTYISR	SFQVTKEIIS	ECKSKGNNIL	IVAHASSLEA	CTCQLQGLSP	QNSKDFVQMV	RKIPYLGFCS
**650**	**660**	**670**	**680**				
CEELGETGIW	QLTDPPILPL	THGPTGGFNW	RETLLQE				

Cmpd.	No. of Cmpds.	m/z meas.	Δ m/z [ppm]	z	Rt [min]	Score	P	Range	Sequence	Modification	Ratios
2812	1	913.4675	44.29	2	63.6	15.1	0	1-15	-.MICVVLCIHGFCTEK.K	Carbamidomethyl: 7, 12; Oxidation: 1	WD:WU 0.84

Detailed Protein Report

Protein 581: armadillo repeat-containing X-linked protein 2 [Homo sapiens]

**Accession:**	gi|7662162	**Score:**	15.1
**Database:**	refseq_human(refseq_human_20140103.fasta)	**MW [kDa]:**	65.6
**Database Date:**	2015-11-30	**pI:**	9.3
		**Sequence Coverage [%]:**	2.5
		**No. of unique Peptides:**	1

Alias proteins:

Accession	Name	Description
gi|532691767	refseq_human (refseq_human_20140103.fasta)	armadillo repeat-containing X-linked protein 2 [Homo sapiens]
gi|530433311	refseq_human (refseq_human_20140103.fasta)	PREDICTED: armadillo repeat-containing X-linked protein 2 isoform X9 [Homo sapiens]
gi|530433309	refseq_human (refseq_human_20140103.fasta)	PREDICTED: armadillo repeat-containing X-linked protein 2 isoform X8 [Homo sapiens]
gi|530433307	refseq_human (refseq_human_20140103.fasta)	PREDICTED: armadillo repeat-containing X-linked protein 2 isoform X7 [Homo sapiens]
gi|530433305	refseq_human (refseq_human_20140103.fasta)	PREDICTED: armadillo repeat-containing X-linked protein 2 isoform X6 [Homo sapiens]
gi|530433303	refseq_human (refseq_human_20140103.fasta)	PREDICTED: armadillo repeat-containing X-linked protein 2 isoform X5 [Homo sapiens]
gi|530433299	refseq_human (refseq_human_20140103.fasta)	PREDICTED: armadillo repeat-containing X-linked protein 2 isoform X3 [Homo sapiens]
gi|530433297	refseq_human (refseq_human_20140103.fasta)	PREDICTED: armadillo repeat-containing X-linked protein 2 isoform X2 [Homo sapiens]
gi|530433295	refseq_human (refseq_human_20140103.fasta)	PREDICTED: armadillo repeat-containing X-linked protein 2 isoform X1 [Homo sapiens]
gi|29540562	refseq_human (refseq_human_20140103.fasta)	armadillo repeat-containing X-linked protein 2 [Homo sapiens]

10	20	30	40	50	60	70	80
MSRVRDAGCV	AAGIVIGAGA	WYCVYKYTRG	RDQTKKRMAK	PKNRAVAGTG	ARARAGLRAG	FTIDLGSGFS	PPTPVRAEAE
90	100	110	120	130	140	150	160
DRAQDEASAL	DTVGAEAVAP	AASSAEAQSG	AGSQAQEADG	AGVGPKAESV	VGAAMASAIA	PPPGVTEALG	AAEAPAMAGA
170	180	190	200	210	220	230	240
PKVAEAPREA	ETSRAAVPPG	TVVPTEAAAP	TEVTEGPGVA	APTKVAEAPG	VASPTEAAEA	PVPATPTGAA	APTGAAESPG
250	260	270	280	290	300	310	320
TSGSPRTAVV	PGTSAAKKAT	PGAHTGAIPK	ATSATGAVPK	GGGKGVTRSR	NGGKGKGKKS	KVEVDELGMG	FRPGDGAAAA
330	340	350	360	370	380	390	400
AAASANGGQA	FLAEVPDSEE	GESGWTDTES	DSDSEPETQR	RGRGRRPVAM	QKRPFPYEID	EILGVRDLRK	VLALLQKSDD
410	420	430	440	450	460	470	480
PFIQQVALLT	LSNNANYSCN	QETIRKLGGL	PIIANMINKT	DPHIKEKALM	AMNNLSENYE	NQGRLQVYMN	KVMDDIMASN
490	500	510	520	530	540	550	560
LNSAVQVVGL	KFLTNMTITN	DYQHLLVNSI	ANFFRLLSQG	GGKIKVEILK	ILSNFAENPD	MLKKLLSTQV	PASFSSLYNS
570	580	590	600	610	620	630	640
YVESEILINA	LTLFEIIYDN	LRAEVFNYRE	FNKGSLFYLC	TTSGVCVKKI	RALANHHDLL	VKVKVIKLVN	KF

Cmpd.	No. of Cmpds.	m/z meas.	Δ m/z [ppm]	z	Rt [min]	Score	P	Range	Sequence	Modification
2790	1	853.4116	-28.91	2	63.3	15.1	1	594-609	K.GSLFYLCTTSGVCVKK.I	

Detailed Protein Report

Protein 582: breast cancer anti-estrogen resistance protein 3 isoform 2 [Homo sapiens]

**Accession:**	gi|387157911	**Score:**	15.0
**Database:**	refseq_human(refseq_human_20140103.fasta)	**MW [kDa]:**	82.3
**Database Date:**	2015-11-30	**pI:**	9.4
**Modification(s):**	Carbamidomethyl, Oxidation	**Sequence Coverage [%]:**	1.9
		**No. of unique Peptides:**	1

10	20	30	40	50	60	70	80
MPKECSAFHA	LSAALCCFYH	RKSFIGVKFS	KERHIMDRTP	EKLKKELEEE	LLLSSEDLRS	HAWYHGRIPR	QVSENLVQRD
**90**	**100**	**110**	**120**	**130**	**140**	**150**	**160**
GDFLVRDSLS	SPGNFVLTCQ	WKNLAQHFKI	NRTVLRLSEA	YSRVQYQFEM	ESFDSIPGLV	RCYVGNRRPI	SQQSGAIIFQ
**170**	**180**	**190**	**200**	**210**	**220**	**230**	**240**
PINRTVPLRC	LEEHYGTSPG	QAREGSLTKG	RPDVAKRLSL	TMGGVQAREQ	NLPRGNLLRN	KEKSGSQPAC	LDHMQDRRAL
**250**	**260**	**270**	**280**	**290**	**300**	**310**	**320**
SLKAHQSESY	LPIGCKLPPQ	SSGVDTSPCP	NSPVFRTGSE	PALSPAVVRR	VSSDARAGEA	LRGSDSQLCP	KPPPKPCKVP
**330**	**340**	**350**	**360**	**370**	**380**	**390**	**400**
FLKVPSSPSA	WLNSEANYCE	LNPAFATGCG	RGAKLPSCAQ	GSHTELLTAK	QNEAPGPRNS	GVNYLILDDD	DRERPWEPAA
**410**	**420**	**430**	**440**	**450**	**460**	**470**	**480**
AQMEKGQWDK	GEFVTPLLET	VSSFRPNEFE	SKFLPPENKP	LETAMLKRAK	ELFTNNDPKV	IAQHVLSMDC	RVARILGVSE
**490**	**500**	**510**	**520**	**530**	**540**	**550**	**560**
EMRRNMGVSS	GLELITLPHG	HQLRLDIIER	HNTMAIGIAV	DILGCTGTLE	DRAATLSKII	QVAVELKDSM	GDLYSFSALM
**570**	**580**	**590**	**600**	**610**	**620**	**630**	**640**
KALEMPQITR	LEKTWTALRH	QYTQTAILYE	KQLKPFSKLL	HEGRESTCVP	PNNVSVPLLM	PLVTLMERQA	VTFEGTDMWE
**650**	**660**	**670**	**680**	**690**	**700**	**710**	**720**
KNDQSCEIML	NHLATARFMA	EAADSYRMNA	ERILAGFQPD	EEMNEICKTE	FQMRLLWGSK	GAQVNQTERY	EKFNQILTAL
**730**	**740**						
SRKLEPPPVK	QAEL						

Cmpd.	No. of Cmpds.	m/z meas.	Δ m/z [ppm]	z	Rt [min]	Score	P	Range	Sequence	Modification
46	1	809.3894	59.94	2	29.5	15.0	0	224-237	K.SGSQPACLDHMQDR.R	Carbamidomethyl: 7; Oxidation: 11

Detailed Protein Report

Protein 583: PREDICTED: microtubule-associated tumor suppressor 1 isoform X7 [Homo sapiens]

**Accession:**	gi|530387935	**Score:**	15.0
**Database:**	refseq_human(refseq_human_20140103.fasta)	**MW [kDa]:**	106.1
**Database Date:**	2015-11-30	**pI:**	9.1
**Modification(s):**	Oxidation	**Sequence Coverage [%]:**	1.5
		**No. of unique Peptides:**	1

10	20	30	40	50	60	70	80
MTDDNSDDKI	EDELQTFFTS	DKDGNTHAYN	PKSPPTQNSS	ASSVNWNSAN	PDDMVVDYET	DPAVVTGENI	SLSLQGVEVF
**90**	**100**	**110**	**120**	**130**	**140**	**150**	**160**
GHEKSSSDFI	SKQVLDMHKD	SICQCPALVG	TEKPKYLQHS	CHSLEAVEGQ	SVEPSLPFVW	KPNDNLNCAG	YCDALELNQT
**170**	**180**	**190**	**200**	**210**	**220**	**230**	**240**
FDMTVDKVNC	TFISHHAIGK	SQSFHTAGSL	PPTGRRSGST	SSLSYSTWTS	SHSDKTHARE	TTYDRESFEN	PQVTPSEAQD
**250**	**260**	**270**	**280**	**290**	**300**	**310**	**320**
MTYTAFSDVV	MQSEVFVSDI	GNQCACSSGK	VTSEYTDGSQ	QRLVGEKETQ	ALTPVSDGME	VPNDSALQEF	FCLSHDESNS
**330**	**340**	**350**	**360**	**370**	**380**	**390**	**400**
EPHSQSSYRH	KEMGQNLRET	VSYCLIDDEC	PLMVPAFDKS	EAQVLNPEHK	VTETEDTQMV	SKGKDLGTQN	HTSELILSSP
**410**	**420**	**430**	**440**	**450**	**460**	**470**	**480**
PGQKVGSSFG	LTWDANDMVI	STDKTMCMST	PVLEPTKVTF	SVSPIEATEK	CKKVEKGNRG	LKNIPDSKEA	PVNLCKPSLG
**490**	**500**	**510**	**520**	**530**	**540**	**550**	**560**
KSTIKTNTPI	GCKVRKTEII	SYPRPNFKNV	KAKVMSRAVL	QPKDAALSKV	TPRPQQTSAS	SPSSVNSRQQ	TVLSRTPRSD
**570**	**580**	**590**	**600**	**610**	**620**	**630**	**640**
LNADKKAEIL	INKTHKQQFN	KLITSQAVHV	TTHSKNASHR	VPRTTSAVKS	NQEDVDKASS	SNSACETGSV	SALFQKIKGI
**650**	**660**	**670**	**680**	**690**	**700**	**710**	**720**
LPVKMESAEC	LEMTYVPNID	RISPEKKGEK	ENGTSMEKQE	LKQEIMNETF	EYGSLFLGSA	SKTTTTSGRN	ISKPDSCGLR
**730**	**740**	**750**	**760**	**770**	**780**	**790**	**800**
QIAAPKAKVG	PPVSCLRRNS	DNRNPSADRA	VSPQRIRRVS	SSGKPTSLKT	AQSSWVNLPR	PLPKSKASLK	SPALRRTGST
**810**	**820**	**830**	**840**	**850**	**860**	**870**	**880**
PSIASTHSEL	STYSNNSGNA	AVIKYEEKPP	KPAFQNGSSG	SFYLKPLVSR	AHVHLMKTPP	KGPSRKNLFT	ALNAVEKSRQ
**890**	**900**	**910**	**920**	**930**	**940**	**950**	**960**
KNPRSLCIQP	QTAPDALPPE	KTLELTQYKT	KCENQSGFIL	QLKQLLACGN	TKFEALTVVI	QHLLSEREEA	LKQHKTLSQE
**970**							
LVNLRGELA							

Cmpd.	No. of Cmpds.	m/z meas.	Δ m/z [ppm]	z	Rt [min]	Score	P	Range	Sequence	Modification
2237	1	869.3795	-55.11	2	56.4	15.0	1	85-99	K.SSSDFISKQVLDMHK.D	Oxidation: 13

Detailed Protein Report

Protein 584: cyclin-dependent kinase 5 activator 1 [Homo sapiens]

**Accession:**	gi|4502737	**Score:**	15.0
**Database:**	refseq_human(refseq_human_20140103.fasta)	**MW [kDa]:**	34.0
**Database Date:**	2015-11-30	**pI:**	10.2
		**Sequence Coverage [%]:**	5.2
		**No. of unique Peptides:**	1

Quantitation

WD:WU **Median:** 0.88 **CV:** 0.00 % **No. of Peptides:** 1

10	20	30	40	50	60	70	80
MGTVLSLSPS	YRKATLFEDG	AATVGHYTAV	QNSKNAKDKN	LKRHSIISVL	PWKRIVAVSA	KKKNSKKVQP	NSSYQNNITH
90	100	110	120	130	140	150	160
LNNENLKKSL	SCANLSTFAQ	PPPAQPPAPP	ASQLSGSQTG	GSSSVKKAPH	PAVTSAGTPK	RVIVQASTSE	LLRCLGEFLC
170	180	190	200	210	220	230	240
RRCYRLKHLS	PTDPVLWLRS	VDRSLLLQGW	QDQGFITPAN	VVFLYMLCRD	VISSEVGSDH	ELQAVLLTCL	YLSYSYMGNE
250	260	270	280	290	300	310	
ISYPLKPFLV	ESCKEAFWDR	CLSVINLMSS	KMLQINADPH	YFTQVFSDLK	NESGQEDKKR	LLLGLDR	

Cmpd.	No. of Cmpds.	m/z meas.	Δ m/z [ppm]	z	Rt [min]	Score	P	Range	Sequence	Modification	Ratios
2666	1	945.9810	-33.71	2	61.6	15.0	1	168-183	K.HLSPTDPVLWLRSVDR.S		WD:WU 0.88

Detailed Protein Report

Protein 585: PDZ domain-containing protein MAGIX isoform d [Homo sapiens]

Accession: gi|532164745
Database: refseq_human(refseq_human_20140103.fasta)
Database Date: 2015-11-30

Score: 15.0
MW [kDa]: 26.7
pI: 11.6
Sequence Coverage [%]: 5.1
No. of unique Peptides: 1

10	20	30	40	50	60	70	80
MEPRTGDAAD	PRGSRGGRGP	SPLAGPSARQ	LLARLDARPL	AARAAVDVAA	LVRRAGATLR	LRRKEAVSVL	DSADIEVTDS
**90**	**100**	**110**	**120**	**130**	**140**	**150**	**160**
RLPHATIVDH	RPQVGDLVLH	INGESTQGLT	HAQAVERIRA	GGPQLHLVIR	RPLETHPGKP	RGVGEPRKGV	DRSPDPGGPE
**170**	**180**	**190**	**200**	**210**	**220**	**230**	**240**
VTGSRSSSTS	LVQHPPSRTT	LKKTRGSPEP	SPEAAADGPT	VSPPERRAED	PNDQIPGSPG	PWLVPSEERL	SRALGVRGAA
**250**	**260**						
QLAQEMAAGR	RRH						

Cmpd.	No. of Cmpds.	m/z meas.	Δ m/z [ppm]	z	Rt [min]	Score	P	Range	Sequence	Modification
1817	2	1273.5520	-62.61	1	51.4	15.0	0	238-250	R.GAAQLAQEMAAGR.R	

Detailed Protein Report

Protein 586: **PREDICTED: sterol regulatory element-binding protein cleavage-activating protein isoform X2 [Homo sapiens]**

Accession: gi|530372098
Database: refseq_human(refseq_human_20140103.fasta)
Database Date: 2015-11-30

Score: 14.9
MW [kDa]: 139.5
pI: 6.4
Sequence Coverage [%]: 0.7
No. of unique Peptides: 1

10	20	30	40	50	60	70	80
MTLTERLREK	ISRAFYNHGL	LCASYPIPII	LFTGFCILAC	CYPLLKLPLP	GTGPVEFTTP	VKDYSPPPVD	SDRKQGEPTE
90	100	110	120	130	140	150	160
QPEWYVGAPV	AYVQQIFVKS	SVFPWHKNLL	AVDVFRSPLS	RAFQLVEEIR	NHVLRDSSGI	RSLEELCLQV	TDLLPGLRKL
170	180	190	200	210	220	230	240
RNLLPEHGCL	LLSPGNFWQN	DWERFHADPD	IIGTIHQHEP	KTLQTSATLK	DLLFGVPGKY	SGVSLYTRKR	MVSYTITLVF
250	260	270	280	290	300	310	320
QHYHAKFLGS	LRARLMLLHP	SPNCSLRAES	LVHVHFKEEI	GVAELIPLVT	TYIILFAYIY	FSTRKIDMVK	SKWGLALAAV
330	340	350	360	370	380	390	400
VTVLSSLLMS	VGLCTLFGLT	PTLNGGEIFP	YLVVVIGLEN	VLVLTKSVVS	TPVDLEVKLR	IAQGLSSESW	SIMKNMATEL
410	420	430	440	450	460	470	480
GIILIGYFTL	VPAIQEFCLF	AVVGLVSDFF	LQMLFFTTVL	SIDIRRMELA	DLNKRLPPEA	CLPSAKPVGQ	PTRYERQLAV
490	500	510	520	530	540	550	560
RPSTPHTITL	QPSSFRNLRL	PKRLRVVYFL	ARTRLAQRLI	MAGTVVWIGI	LVYTDPAGLR	NYLAAQVTEQ	SPLGEGALAP
570	580	590	600	610	620	630	640
MPVPSGMLPP	SHPDPAFSIF	PPDAPKLPEN	QTSPGESPER	GGPAEVVHDS	PVPEVTWGPE	DEELWRKLSF	RHWPTLFSYY
650	660	670	680	690	700	710	720
NITLAKRYIS	LLPVIPVTLR	LNPREALEGR	HPQDGRSAWP	PPGPIPAGHW	EAGPKGPGGV	QAHGDVTLYK	VAALGLATGI
730	740	750	760	770	780	790	800
VLVLLLLCLY	RVLCPRNYGQ	LGGGPGRRRR	GELPCDDYGY	APPETEIVPL	VLRGHLMDIE	CLASDGMLLV	SCCLAGHVCV
810	820	830	840	850	860	870	880
WDAQTGDCLT	RIPRPGQRRD	SGVGSGLEAQ	ESWERLSDGG	KAGPEEPGDS	PPLRHRPRGP	PPPSLFGDQP	DLTCLIDTNF
890	900	910	920	930	940	950	960
SAQPRSSQPT	QPEPRHRAVC	GRSRDSPGYD	FSCLVQRVYQ	EEGLAAVCTP	ALRPPSPGPV	LSQAPEDEGG	SPEKGSPSLA
970	980	990	1000	1010	1020	1030	1040
WAPSAEGSIW	SLELQGNLIV	VGRSSGRLEV	WDAIEGVLCC	SSEEVSSGIT	ALVFLDKRIV	AARLNGSLDF	FSLETHTALS
1050	1060	1070	1080	1090	1100	1110	1120
PLQFRGTPGR	GSSPASPVYS	SSDTVACHLT	HTVPCAHQKP	ITALKAAAGR	LVTGSQDHTL	RVFRLEDSCC	LFTLQGHSGA
1130	1140	1150	1160	1170	1180	1190	1200
ITTVYIDQTM	VLASGGQDGA	ICLWDVLTGS	RVSHVFAHRG	DVTSLTCTTS	CVISSGLDDL	ISIWDRSTGI	KFYSIQQDLG
1210	1220	1230	1240	1250	1260	1270	1280
CGASLGVISD	NLLVTGGQGC	VSFWDLNYGD	LLQTVYLGKN	SEAQPARQIL	VLDNAAIVCN	FGSELSLVYV	PSVLEKLD

Cmpd.	No. of Cmpds.	m/z meas.	Δ m/z [ppm]	z	Rt [min]	Score	P	Range	Sequence	Modification
1688	1	1045.6717	134.35	1	49.8	14.9	0	220-228	K.YSGVSLYTR.K	

Detailed Protein Report

Protein 587: toll-like receptor 9 precursor [Homo sapiens]

**Accession:**	gi|8394456	**Score:**	14.9
**Database:**	refseq_human(refseq_human_20140103.fasta)	**MW [kDa]:**	115.8
**Database Date:**	2015-11-30	**pI:**	9.7
		**Sequence Coverage [%]:**	1.3
		**No. of unique Peptides:**	1

Quantitation

WD:WU **Median:** 0.91 **CV:** 0.00 % **No. of Peptides:** 1

10	20	30	40	50	60	70	80
MGFCRSALHP	LSLLVQAIML	AMTLALGTLP	AFLPCELQPH	GLVNCNWLFL	KSVPHFSMAA	PRGNVTSLSL	SSNRIHHLHD
**90**	**100**	**110**	**120**	**130**	**140**	**150**	**160**
SDFAHLPSLR	HLNLKWNCPP	VGLSPMHFPC	HMTIEPSTFL	AVPTLEELNL	SYNNIMTVPA	LPKSLISLSL	SHTNILMLDS
**170**	**180**	**190**	**200**	**210**	**220**	**230**	**240**
ASLAGLHALR	FLFMDGNCYY	KNPCRQALEV	APGALLGLGN	LTHLSLKYNN	LTVVPRNLPS	SLEYLLLSYN	RIVKLAPEDL
**250**	**260**	**270**	**280**	**290**	**300**	**310**	**320**
ANLTALRVLD	VGGNCRRCDH	APNPCMECPR	HFPQLHPDTF	SHLSRLEGLV	LKDSSLSWLN	ASWFRGLGNL	RVLDLSENFL
**330**	**340**	**350**	**360**	**370**	**380**	**390**	**400**
YKCITKTKAF	QGLTQLRKLN	LSFNYQKRVS	FAHLSLAPSF	GSLVALKELD	MHGIFFRSLD	ETTLRPLARL	PMLQTLRLQM
**410**	**420**	**430**	**440**	**450**	**460**	**470**	**480**
NFINQAQLGI	FRAFPGLRYV	DLSDNRISGA	SELTATMGEA	DGGEKVWLQP	GDLAPAPVDT	PSSEDFRPNC	STLNFTLDLS
**490**	**500**	**510**	**520**	**530**	**540**	**550**	**560**
RNNLVTVQPE	MFAQLSHLQC	LRLSHNCISQ	AVNGSQFLPL	TGLQVLDLSH	NKLDLYHEHS	FTELPRLEAL	DLSYNSQPFG
**570**	**580**	**590**	**600**	**610**	**620**	**630**	**640**
MQGVGHNFSF	VAHLRTLRHL	SLAHNNIHSQ	VSQQLCSTSL	RALDFSGNAL	GHMWAEGDLY	LHFFQGLSGL	IWLDLSQNRL
**650**	**660**	**670**	**680**	**690**	**700**	**710**	**720**
HTLLPQTLRN	LPKSLQVLRL	RDNYLAFFKW	WSLHFLPKLE	VLDLAGNQLK	ALTNGSLPAG	TRLRRLDVSC	NSISFVAPGF
**730**	**740**	**750**	**760**	**770**	**780**	**790**	**800**
FSKAKELREL	NLSANALKTV	DHSWFGPLAS	ALQILDVSAN	PLHCACGAAF	MDFLLEVQAA	VPGLPSRVKC	GSPGQLQGLS
**810**	**820**	**830**	**840**	**850**	**860**	**870**	**880**
IFAQDLRLCL	DEALSWDCFA	LSLLAVALGL	GVPMLHHLCG	WDLWYCFHLC	LAWLPWRGRQ	SGRDEDALPY	DAFVVFDKTQ
**890**	**900**	**910**	**920**	**930**	**940**	**950**	**960**
SAVADWVYNE	LRGQLEECRG	RWALRLCLEE	RDWLPGKTLF	ENLWASVYGS	RKTLFVLAHT	DRVSGLLRAS	FLLAQQRLLE
**970**	**980**	**990**	**1000**	**1010**	**1020**	**1030**	**1040**
DRKDVVVLVI	LSPDGRRSRY	VRLRQRLCRQ	SVLLWPHQPS	GQRSFWAQLG	MALTRDNHHF	YNRNFCQGPT	AE

Cmpd.	No. of Cmpds.	m/z meas.	Δ m/z [ppm]	z	Rt [min]	Score	P	Range	Sequence	Modification	Ratios
1392	1	698.8594	-48.59	2	45.8	14.9	0	235-247	K.LAPEDLANLTALR.V		WD:WU 0.91

Detailed Protein Report

Protein 588: PREDICTED: metal transporter CNNM4 isoform X2 [Homo sapiens]

Accession: gi|530368586
Database: refseq_human(refseq_human_20140103.fasta)
Database Date: 2015-11-30

Score: 14.9
MW [kDa]: 86.4
pI: 6.2
Sequence Coverage [%]: 0.9
No. of unique Peptides: 1

10	20	30	40	50	60	70	80
MAPVGGGGRP	VGGPARGRLL	LAAPVLLVLL	WALGARGQGS	PQQGTIVGMR	LASCNKSCGT	NPDGIIFVSE	GSTVNLRLYG
90	100	110	120	130	140	150	160
YSLGNISSNL	ISFTEVDDAE	TLHKSTSCLE	LTKDLVVQQL	VNVSRGNTSG	VLVVLTKFLR	RSESMKLYAL	CTRAQPDGPW
170	180	190	200	210	220	230	240
LKWTDKDSLL	FMVEEPGRFL	PLWLHILLIT	VLLVLSGIFS	GLNLGLMALD	PMELRIVQNC	GTEKERRYAR	KIEPIRRKGN
250	260	270	280	290	300	310	320
YLLCSLLLGN	VLVNTSLTIL	LDNLIGSGLM	AVASSTIGIV	IFGEILPQAL	CSRHGLAVGA	NTILLTKFFM	LLTFPLSFPI
330	340	350	360	370	380	390	400
SKLLDFFLGQ	EIRTVYNREK	LMEMLKVTEP	YNDLVKEELN	MIQGALELRT	KTVEDIMTQL	QDCFMIRSDA	ILDFNTMSEI
410	420	430	440	450	460	470	480
MESGYTRIPV	FEDEQSNIVD	ILYVKDLAFV	DPDDCTPLKT	ITRFYNHPVH	FVFHDTKLDA	MLEEFKKGKS	HLAIVQKVNN
490	500	510	520	530	540	550	560
EGEGDPFYEV	LGLVTLEDVI	EEIIKSEILD	ESDMYTDNRS	RKRVSEKNKR	DFSAFKDADN	ELKVKISPQL	LLAAHRFLAT
570	580	590	600	610	620	630	640
EVSQFSPSLI	SEKILLRLLK	YPDVIQELKF	DEHNKYYARH	YLYTRNKPAD	YFILILQGKV	EVEAGKENMK	FETGAFSYYG
650	660	670	680	690	700	710	720
TMALTSVPSD	RSPAHPTPLS	RSASLSYPDR	TDVSTAATLA	GSSNQFGSSV	LGQYISDFSV	RALVDLQYIK	PQSWPCFSRP
730	740	750	760	770	780		
GDRVCLPAPP	SMNSSLPHAD	HSAAVPERAA	GFSHGEQPSV	SHRRVHHPHG	ELGREV		

Cmpd.	No. of Cmpds.	m/z meas.	Δ m/z [ppm]	z	Rt [min]	Score	P	Range	Sequence	Modification
2779	1	870.4221	-28.45	1	63.2	14.9	1	530-536	K.RDFSAFK.D	

Detailed Protein Report

Protein 589: PREDICTED: pyruvate carboxylase, mitochondrial isoform X6 [Homo sapiens]

**Accession:**	gi|578821481	**Score:**	14.9
**Database:**	refseq_human(refseq_human_20140103.fasta)	**MW [kDa]:**	73.3
**Database Date:**	2015-11-30	**pI:**	5.6
		**Sequence Coverage [%]:**	2.1
		**No. of unique Peptides:**	1

10	20	30	40	50	60	70	80
MVNGPTTPIP	VKASPSPTDP	VVPAVPIGPP	PAGFRDILLR	EGPEGFARAV	RNHPGLLLMD	TTFRDAHQSL	LATRVRTHDL
**90**	**100**	**110**	**120**	**130**	**140**	**150**	**160**
KKIAPYVAHN	FSKLFSMENW	GGATFDVAMR	FLYECPWRRL	QELRELIPNI	PFQMLLRGAN	AVGYTNYPDN	VVFKFCEVAK
**170**	**180**	**190**	**200**	**210**	**220**	**230**	**240**
ENGMDVFRVF	DSLNYLPNML	LGMEAAGSAG	GVVEAAISYT	GDVADPSRTK	YSLQYYMGLA	EELVRAGTHI	LCIKDMAGLL
**250**	**260**	**270**	**280**	**290**	**300**	**310**	**320**
KPTACTMLVS	SLRDRFPDLP	LHIHTHDTSG	AGVAAMLACA	QAGADVVDVA	ADSMSGMTSQ	PSMGALVACT	RGTPLDTEVP
**330**	**340**	**350**	**360**	**370**	**380**	**390**	**400**
MERVFDYSEY	WEGARGLYAA	FDCTATMKSG	NSDVYENEIP	GGQYTNLHFQ	AHSMGLGSKF	KEVKKAYVEA	NQMLGDLIKV
**410**	**420**	**430**	**440**	**450**	**460**	**470**	**480**
TPSSKIVGDL	AQFMVQNGLS	RAEAEAQAEE	LSFPRSVVEF	LQGYIGVPHG	GFPEPFRSKV	LKDLPRVEGR	PGASLPPLDL
**490**	**500**	**510**	**520**	**530**	**540**	**550**	**560**
QALEKELVDR	HGEEVTPEDV	LSAAMYPDVF	AHFKDFTATF	GPLDSLNTRL	FLQGPKIAEE	FEVELERGKT	LHIKALAVSD
**570**	**580**	**590**	**600**	**610**	**620**	**630**	**640**
LNRAGQRQVF	FELNGQLRSI	LVKDTQAMKE	MHFHPKALKD	VKGQIGAPMP	GKVIDIKVVA	GAKVAKGQPL	CVLSAMKMET
**650**	**660**	**670**	**680**				
VVTSPMEGTV	RKVHVTKDMT	LEGDDLILEI	E				

Cmpd.	No. of Cmpds.	m/z meas.	Δ m/z [ppm]	z	Rt [min]	Score	P	Range	Sequence	Modification
511	1	706.0223	94.27	2	35.3	14.9	2	613-626	K.VIDIKVVAGAKVAK.G	

Detailed Protein Report

Protein 590: bisphosphoglycerate mutase [Homo sapiens]

**Accession:**	gi|4502445	**Score:**	14.9
**Database:**	refseq_human(refseq_human_20140103.fasta)	**MW [kDa]:**	30.0
**Database Date:**	2015-11-30	**pI:**	6.1
		**Sequence Coverage [%]:**	5.0
		**No. of unique Peptides:**	1

Quantitation

WD:WU **Median:** 0.93 **CV:** 0.00 % **No. of Peptides:** 1

Alias proteins:

Accession	Name	Description
gi|578814542	refseq_human (refseq_human_20140103.fasta)	PREDICTED: bisphosphoglycerate mutase isoform X1 [Homo sapiens]
gi|40353764	refseq_human (refseq_human_20140103.fasta)	bisphosphoglycerate mutase [Homo sapiens]

```
        10         20         30         40         50         60         70         80
MSKYKLIMLR HGEGAWNKEN RFCSWVDQKL NSEGMEEARN CGKQLKALNF EFDLVFTSVL NRSIHTAWLI LEELGQEWVP
        90        100        110        120        130        140        150        160
VESSWRLNER HYGALIGLNR EQMALNHGEE QVRLWRRSYN VTPPPIEESH PYYQEIYNDR RYKVCDVPLD QLPRSESLKD
       170        180        190        200        210        220        230        240
VLERLLPYWN ERIAPEVLRG KTILISAHGN SSRALLKHLE GISDEDIINI TLPTGVPILL ELDENLRAVG PHQFLGDQEA
       250        260
IQAAIKKVED QGKVKQAKK
```

Cmpd.	No. of Cmpds.	m/z meas.	Δ m/z [ppm]	z	Rt [min]	Score	P	Range	Sequence	Modification	Ratios
937	2	770.8595	-3.57	2	40.5	14.9	0	101-113	R.EQMALNHGEEQVR.L		WD:WU 0.93

Detailed Protein Report

Protein 591: **myosin-IIIb isoform 1 [Homo sapiens]**

**Accession:**	gi|284172510	**Score:**	14.9
**Database:**	refseq_human(refseq_human_20140103.fasta)	**MW [kDa]:**	148.5
**Database Date:**	2015-11-30	**pI:**	8.7
		**Sequence Coverage [%]:**	1.8
		**No. of unique Peptides:**	1

10	20	30	40	50	60	70	80
MKHLYGLFHY	NPMMLGLESL	PDPTDTWEII	ETIGKGTYGK	VYKVTNKRDG	SLAAVKILDP	VSDMDEEIEA	EYNILQFLPN
**90**	**100**	**110**	**120**	**130**	**140**	**150**	**160**
HPNVVKFYGM	FYKADHCVGG	QLWLVLELCN	GGSVTELVKG	LLRCGQRLDE	AMISYILYGA	LLGLQHLHNN	RIIHRDVKGN
**170**	**180**	**190**	**200**	**210**	**220**	**230**	**240**
NILLTTEGGV	KLVDFGVSAQ	LTSTRLRRNT	SVGTPFWMAP	EVIACEQQYD	SSYDARCDVW	SLGITAIELG	DGDPPLFDMH
**250**	**260**	**270**	**280**	**290**	**300**	**310**	**320**
PVKTLFKIPR	NPPPTLLHPE	KWCEEFNHFI	SQCLIKDFER	RPSVTHLLDH	PFIKGVHGKV	LFLQKQLAKV	LQDQKHQNPV
**330**	**340**	**350**	**360**	**370**	**380**	**390**	**400**
AKTRHERMHT	RRPYHVEDAE	KYCLEDDLVN	LEVLDEDTII	HQLQKRYADL	LIYTYVGDIL	IALNPFQNLS	IYSPQFSRLY
**410**	**420**	**430**	**440**	**450**	**460**	**470**	**480**
HGVKRASNPP	HIFASADAAY	QCMVTLSKDQ	CIVISGESGS	GKTESAHLIV	QHLTFLGKAN	NQTLREKILQ	VNSLVEAFGN
**490**	**500**	**510**	**520**	**530**	**540**	**550**	**560**
SCTAINDNSS	RFGKYLEMMF	TPTGVVMGAR	ISEYLLEKSR	VIKQAAREKN	FHIFYYIYAG	LHHQKKLSDF	RLPEEKPPRY
**570**	**580**	**590**	**600**	**610**	**620**	**630**	**640**
IADETGRVMH	DITSKESYRR	QFEAIQHCFR	IIGFTDKEVH	SVYRILAGIL	NIGNIEFAAI	SSQHQTDKSE	VPNAEALQNA
**650**	**660**	**670**	**680**	**690**	**700**	**710**	**720**
ASVLCISPEE	LQEALTSHCV	VTRGETIIRA	NTVDRAADVR	DAMSKALYGR	LFSWIVNRIN	TLLQPDENIC	SAGGGMNVGI
**730**	**740**	**750**	**760**	**770**	**780**	**790**	**800**
LDIFGFENFQ	RNSFEQLCIN	IANEQIQYYF	NQHVFALEQM	EYQNEGIDAV	PVEYEDNRPL	LDMFLQKPLG	LLALLDEESR
**810**	**820**	**830**	**840**	**850**	**860**	**870**	**880**
FPQATDQTLV	DKFEDNLRCK	YFWRPKGVEL	CFGIQHYAGK	VLYDASGVLE	KNRDTLPADV	VVVLRTSENK	LLQQLFSIPL
**890**	**900**	**910**	**920**	**930**	**940**	**950**	**960**
TKTGNLAQTR	ARITVASSSL	PPHFSAGKAK	VDTLEVIRHP	EETTNMKRQT	VASYFRYSLM	DLLSKMVVGQ	PHFVRCIKPN
**970**	**980**	**990**	**1000**	**1010**	**1020**	**1030**	**1040**
DDREALQFSR	ERVLAQLRST	GILETVSIRR	QGYSHRILFE	EFVKRYYYLA	FTAHQTPLAS	KESCVAILEK	SRLDHWVLGK
**1050**	**1060**	**1070**	**1080**	**1090**	**1100**	**1110**	**1120**
TKVFLKYYHV	EQLNLLLREV	IGRVVVLQAY	TKGWLGARRY	KRVREKREKG	AIAIQSGDTS	NQSSGPHSPV	AAGTRGSAEV
**1130**	**1140**	**1150**	**1160**	**1170**	**1180**	**1190**	**1200**
QDCSEPGDHK	VLRGSVHRRS	HSQAESNNGR	TQTSSNSPAV	TEKNGHSQAQ	SSPKGCDIFA	GHANKHSVSG	TDLLSSRICH
**1210**	**1220**	**1230**	**1240**	**1250**	**1260**	**1270**	**1280**
PAPDQQGLSL	WGAPQKPGSE	NGLAQKHRTP	RRRCQQPKML	SSPEDTMYYN	QLNGTLEYQG	SKRKPRKLGQ	IKVLDGEDEY
**1290**	**1300**	**1310**	**1320**				
YKSLSPVDCI	PEENNSAHPS	FFSSSSKGDS	FAQH				

Cmpd.	No. of Cmpds.	m/z meas.	Δ m/z [ppm]	z	Rt [min]	Score	P	Range	Sequence	Modification
1349	2	926.7733	-30.95	3	45.6	14.9	1	495-518	K.YLEMMFTPTGVVMGARISEYLLEK.S	

Detailed Protein Report

Protein 592: PREDICTED: RANBP2-like and GRIP domain-containing protein 5/6 isoform X5 [Homo sapiens]

**Accession:**	gi|530369376	**Score:**	14.9
**Database:**	refseq_human(refseq_human_20140103.fasta)	**MW [kDa]:**	165.9
**Database Date:**	2015-11-30	**pI:**	6.1
		**Sequence Coverage [%]:**	1.1
		**No. of unique Peptides:**	1

Alias proteins:

Accession	Name	Description
gi|530369509	refseq_human (refseq_human_20140103.fasta)	PREDICTED: RANBP2-like and GRIP domain-containing protein 5/6 isoform X5 [Homo sapiens]

10	20	30	40	50	60	70	80
MKGHFYMYAG	SLLLKMGQHG	NNVQWRALSE	LAALCYLIAF	QVPRPKIKLR	EGKAGQNLLE	MMACDRLSQS	GHMLLSLSRG
**90**	**100**	**110**	**120**	**130**	**140**	**150**	**160**
KQDFLKEVVE	TFANKIGQSA	LYDALFSSQS	PKDTSFLGSD	DIGKIDVQEP	ELEDLARYDV	GAIRAHNGSL	QHLTWLGLQW
**170**	**180**	**190**	**200**	**210**	**220**	**230**	**240**
NSLPALPGIR	KWLKQLFHRL	PHETSRLETN	APESICILDL	EVFLLGVVYT	SHLQLKEKCN	SHHSSYQPLC	LPFPVCKQLC
**250**	**260**	**270**	**280**	**290**	**300**	**310**	**320**
TERQKSWWDA	VCTLIHRKAV	PGNLAKLRLL	VQHEINTLRA	QEKHGLQPAL	LVHWAKYLQK	TGSGLNSFYG	QLEYIGRSVH
**330**	**340**	**350**	**360**	**370**	**380**	**390**	**400**
YWKKVLPLLK	IIKKNSIPEP	IDPLFKHFHS	VDIQASEIVE	YEEDAHITFA	MLDAVNGNIE	DAVTAFESIK	SVVSYWNLAL
**410**	**420**	**430**	**440**	**450**	**460**	**470**	**480**
IFHRKAEDIE	NDALSPEEQE	ECRNYLTKTR	DYLIKIIDDG	DSNLSVVKKL	PVPLESVKQM	LNSVMQELED	YSEGGPLYKN
**490**	**500**	**510**	**520**	**530**	**540**	**550**	**560**
GSLRNADSEI	KHSTPSPTKY	SLSPSKSYKY	SPETPPRWTE	DRNSLLNMIC	QQVEAIKKEM	QELKLNSSKS	ASRHRWPTEN
**570**	**580**	**590**	**600**	**610**	**620**	**630**	**640**
YGPDSVPDGY	QGSQTFHGAP	LTVATTGPSV	YYSQSPAYNS	QYLLRPAANV	TPTKGSSNTE	FKSTKEGFSI	PVSADGFKFG
**650**	**660**	**670**	**680**	**690**	**700**	**710**	**720**
ISEPGNQEKK	REKPLENDTG	FQAQDISGRK	KGRGVIFGQT	SSTFTFADVA	KSTSGEGFQF	GKKDLNFKGF	SGAGEKLFSS
**730**	**740**	**750**	**760**	**770**	**780**	**790**	**800**
RYGKMANKAN	TSGDFEKDDD	AYKTEDSDDI	HFEPVVQMPE	KVELVTGEEG	EKVLYSQGVK	LFRFDAEVRQ	WKERGLGNLK
**810**	**820**	**830**	**840**	**850**	**860**	**870**	**880**
ILKNEVNGKL	RMLMRREQVL	KVCANHWITT	TMNLKPLSGS	DRAWMWSASD	FSDGDAKLER	LAAKFKTPEL	AEEFKQKFEE
**890**	**900**	**910**	**920**	**930**	**940**	**950**	**960**
CQRLLLDIPL	QTPHKLVDTG	RAAKLIQRAE	EMKSGLKDFK	TFLTNDQTKV	TEEENKGSGT	GAAGASDTTI	KPNAENTGPT
**970**	**980**	**990**	**1000**	**1010**	**1020**	**1030**	**1040**
LEWDNYDLRE	DALDDSVSSS	SVHASPLASS	PVRKNLFRFD	ESTTGSNFSF	KSALSLSKSP	AKLNQSGTSV	GTDEESVVTQ
**1050**	**1060**	**1070**	**1080**	**1090**	**1100**	**1110**	**1120**
EEERDGQYFE	PVVPLPDLVE	VSSGEENEQV	VFSHRAEIYR	YDKDVGQWKE	RGIGDIKILQ	NYDNKQVRIV	MRRDQVLKLC
**1130**	**1140**	**1150**	**1160**	**1170**	**1180**	**1190**	**1200**
ANHRITPDMS	LQNMKGTERV	WVWTACDFAD	GERKVEHLAV	RFKLQDVADS	FKKIFDEAKT	AQEKDSLITP	HVSRSSTPRE
**1210**	**1220**	**1230**	**1240**	**1250**	**1260**	**1270**	**1280**
SPCGKIAVAI	LEETTRERTD	VIQGDDVADA	ASEVEVSSTS	ETTTKAVVSP	PKFVFVSESV	KRIFSSEKSK	PFVFGNSSAT
**1290**	**1300**	**1310**	**1320**	**1330**	**1340**	**1350**	**1360**
GSLFGFSFNA	PLKSNNSETS	SVAQSGSESK	VEPKKCELSK	NSDIEQSSDS	KVKNLSASFP	TEESSINYTF	KTPEKEPPLW
**1370**	**1380**	**1390**	**1400**	**1410**	**1420**	**1430**	**1440**
HAEFTKEELV	QKLRSTTKSA	DHLNGLLREI	EATNAVLMEQ	IKLLKSEIRR	LERNQEREKS	AANLEYLKNV	LLQFIFLKPG
**1450**	**1460**	**1470**	**1480**	**1490**			
SERERLLPVI	NTMLQLSPEE	KGKLAAVAQD	EEENASRSSG				

Cmpd.	No. of Cmpds.	m/z meas.	Δ m/z [ppm]	z	Rt [min]	Score	P	Range	Sequence	Modification
2775	1	557.2986	22.82	3	63.1	14.9	1	623-638	K.STKEGFSIPVSADGFK.F	

Detailed Protein Report

Protein 593: **ATP-dependent Clp protease ATP-binding subunit clpX-like, mitochondrial precursor [Homo sapiens]**

**Accession:**	gi|7242140	**Score:**	14.8
**Database:**	refseq_human(refseq_human_20140103.fasta)	**MW [kDa]:**	69.2
**Database Date:**	2015-11-30	**pI:**	8.5
**Modification(s):**	Carbamidomethyl	**Sequence Coverage [%]:**	3.9
		**No. of unique Peptides:**	1

Quantitation

WD:WU **Median:** 1.13 **CV:** 0.00 % **No. of Peptides:** 1

10	20	30	40	50	60	70	80
MPSCGACTCG	AAAVRLITSS	LASAQRGISG	GRIHMSVLGR	LGTFETQILQ	RAPLRSFTET	PAYFASKDGI	SKDGSGDGNK
**90**	**100**	**110**	**120**	**130**	**140**	**150**	**160**
KSASEGSSKK	SGSGNSGKGG	NQLRCPKCGD	LCTHVETFVS	STRFVKCEKC	HHFFVVLSEA	DSKKSIIKEP	ESAAEAVKLA
**170**	**180**	**190**	**200**	**210**	**220**	**230**	**240**
FQQKPPPPPK	KIYNYLDKYV	VGQSFAKKVL	SVAVYNHYKR	IYNNIPANLR	QQAEVEKQTS	LTPRELEIRR	REDEYRFTKL
**250**	**260**	**270**	**280**	**290**	**300**	**310**	**320**
LQIAGISPHG	NALGASMQQQ	VNQQIPQEKR	GGEVLDSSHD	DIKLEKSNIL	LLGPTGSGKT	LLAQTLAKCL	DVPFAICDCT
**330**	**340**	**350**	**360**	**370**	**380**	**390**	**400**
TLTQAGYVGE	DIESVIAKLL	QDANYNVEKA	QQGIVFLDEV	DKIGSVPGIH	QLRDVGGEGV	QQGLLKLLEG	TIVNVPEKNS
**410**	**420**	**430**	**440**	**450**	**460**	**470**	**480**
RKLRGETVQV	DTTNILFVAS	GAFNGLDRII	SRRKNEKYLG	FGTPSNLGKG	RRAAAAADLA	NRSGESNTHQ	DIEEKDRLLR
**490**	**500**	**510**	**520**	**530**	**540**	**550**	**560**
HVEARDLIEF	GMIPEFVGRL	PVVVPLHSLD	EKTLVQILTE	PRNAVIPQYQ	ALFSMDKCEL	NVTEDALKAI	ARLALERKTG
**570**	**580**	**590**	**600**	**610**	**620**	**630**	**640**
ARGLRSIMEK	LLLEPMFEVP	NSDIVCVEVD	KEVVEGKKEP	GYIRAPTKES	SEEEYDSGVE	EEGWPRQADA	ANS

Cmpd.	No. of Cmpds.	m/z meas.	Δ m/z [ppm]	z	Rt [min]	Score	P	Range	Sequence	Modification	Ratios	
524	1	817.7754	49.53	3	35.0	14.8	1	2-26	M. PSCGACTCGAAAVRLITSSLASAQ G	Carbamidomethyl: 3	WD:WU	1.13

Detailed Protein Report

Protein 594: MOSC domain-containing protein 1, mitochondrial precursor [Homo sapiens]

**Accession:**	gi|224600454	**Score:**	14.8
**Database:**	refseq_human(refseq_human_20140103.fasta)	**MW [kDa]:**	37.5
**Database Date:**	2015-11-30	**pI:**	10.0
		**Sequence Coverage [%]:**	2.7
		**No. of unique Peptides:**	1

10	20	30	40	50	60	70	80
MGAAGSSALA	RFVLLAQSRP	GWLGVAALGL	TAVALGAVAW	RRAWPTRRRR	LLQQVGTVAQ	LWIYPVKSCK	GVPVSEAECT
**90**	**100**	**110**	**120**	**130**	**140**	**150**	**160**
AMGLRSGNLR	DRFWLVINQE	GNMVTARQEP	RLVLISLTCD	GDTLTLSAAY	TKDLLLPIKT	PTTNAVHKCR	VHGLEIEGRD
**170**	**180**	**190**	**200**	**210**	**220**	**230**	**240**
CGEATAQWIT	SFLKSQPYRL	VHFEPHMRPR	RPHQIADLFR	PKDQIAYSDT	SPFLILSEAS	LADLNSRLEK	KVKATNFRPN
**250**	**260**	**270**	**280**	**290**	**300**	**310**	**320**
IVISGCDVYA	EDSWDELLIG	DVELKRVMAC	SRCILTTVDP	DTGVMSRKEP	LETLKSYRQC	DPSERKLYGK	SPLFGQYFVL
**330**	**340**						
ENPGTIKVGD	PVYLLGQ						

Cmpd.	No. of Cmpds.	m/z meas.	Δ m/z [ppm]	z	Rt [min]	Score	P	Range	Sequence	Modification
48	1	505.2491	-51.14	2	29.8	14.8	0	151-159	R.VHGLEIEGR.D	

Detailed Protein Report

Protein 595: PREDICTED: keratin, type I cuticular Ha1 isoform X1 [Homo sapiens]

**Accession:**	gi|530412192	**Score:**	14.7
**Database:**	refseq_human(refseq_human_20140103.fasta)	**MW [kDa]:**	42.2
**Database Date:**	2015-11-30	**pI:**	4.6
		**Sequence Coverage [%]:**	2.5
		**No. of unique Peptides:**	1

Quantitation

WD:WU **Median:** 1.94 **CV:** 0.00 % **No. of Peptides:** 1

Alias proteins:

Accession	Name	Description
gi|530431086	refseq_human (refseq_human_20140103.fasta)	PREDICTED: keratin, type I cuticular Ha1 isoform X1 [Homo sapiens]

10	20	30	40	50	60	70	80
MPYNFCLPSL	SCRTSCSSRP	CVPPSCHSCT	LPGACNIPAN	VSNCNWFCEG	SFNGSEKETM	QFLNDRLASY	LEKVRQLERD
90	100	110	120	130	140	150	160
NAELENLIRE	RSQQQEPLLC	PSYQSYFKTI	EELQQKILCT	KSENARLVVQ	IDNAKLAADD	FRTKYQTELS	LRQLVESDIN
170	180	190	200	210	220	230	240
GLRRILDELT	LCKSDLEAQV	ESLKEELLCL	KSNHEQEVNT	LRCQLGDRLN	VEVDAAPTVD	LNRVLNETRS	QYEALVETNR
250	260	270	280	290	300	310	320
REVEQWFTTQ	TEELNKQVVS	SSEQLQSYQA	EIIELRRTVN	ALEIELQAQH	NLRDSLENTL	TESEARYSSQ	LSQVQSLITN
330	340	350	360	370			
VESQLAEIRS	DLERQNQEYQ	VLLDVRARLE	CEINTYRSLL	ESEDCK			

Cmpd.	No. of Cmpds.	m/z meas.	Δ m/z [ppm]	z	Rt [min]	Score	P	Range	Sequence	Modification	Ratios
881	1	512.1602	-149.56	2	39.4	14.7	0	358-366	R.SLLESEDCK.-		WD:WU 1.94

Detailed Protein Report

Protein 596: transcription factor ETV7 isoform 8 [Homo sapiens]

Accession: gi|333470747
Database: refseq_human(refseq_human_20140103.fasta)
Database Date: 2015-11-30
Modification(s): Carbamidomethyl, Oxidation

Score: 14.7
MW [kDa]: 22.2
pI: 9.6
Sequence Coverage [%]: 9.5
No. of unique Peptides: 1

Quantitation

WD:WU **Median:** 0.90 **CV:** 0.00 % **No. of Peptides:** 1

10	20	30	40	50	60	70	80
MDTRRGHLLQ	PPDPGLTSNF	GHLDDPGLAR	WTPGKEESLN	LCHCAELGCR	TQGVCSFPAM	PQAPIDGRIA	DCRLLWDYVY
**90**	**100**	**110**	**120**	**130**	**140**	**150**	**160**
QLLLDTRYEP	YIKWEDKDAK	IFRVVDPNGL	ARLWGNHKNR	VNMTYEKMSR	ALRHYYKLNI	IKKEPGQKLL	FRFLKTPGKM
**170**	**180**	**190**	**200**				
VQDKHSHLEP	LESQEQDRIE	FKDKRPEISP					

Cmpd.	No. of Cmpds.	m/z meas.	Δ m/z [ppm]	z	Rt [min]	Score	P	Range	Sequence	Modification	Ratios
930	1	974.4715	15.67	2	40.4	14.7	0	51-68	R.TQGVCSFPAMPQAPIDGR.I	Carbamidomethyl: 5; Oxidation: 10	WD:WU 0.90

Detailed Protein Report

Protein 597: **DEP domain-containing protein 1B isoform 2 [Homo sapiens]**

**Accession:**	gi|223633997	**Score:**	14.7
**Database:**	refseq_human(refseq_human_20140103.fasta)	**MW [kDa]:**	54.7
**Database Date:**	2015-11-30	**pI:**	9.9
		**Sequence Coverage [%]:**	2.6
		**No. of unique Peptides:**	1

10	20	30	40	50	60	70	80
MEHRIVGPGP	YRATRLWNET	VELFRAKMPL	RKHRCRFKSY	EHCFTAAEAV	DWLHELLRCS	QNFGPEVTRK	QTVQLLKKFL
**90**	**100**	**110**	**120**	**130**	**140**	**150**	**160**
KNHVIEDIKG	KWGEEDFEDN	RHLYRFPPSS	PLKPYPKKPP	NQKDVIKFPE	WNDLPPGTSQ	ENIPVRPVVM	NSEMWYKRHS
**170**	**180**	**190**	**200**	**210**	**220**	**230**	**240**
IAIGEVPACR	LVHRRQLTEA	NVEEIWKSMT	LSYLQKILGL	DSLEEVLDVK	LVNSKFIIHN	VYSVSKQGVV	ILDDKSKELP
**250**	**260**	**270**	**280**	**290**	**300**	**310**	**320**
HWVLSAMKCL	ANWPNCSDLK	QPMYLGFEKD	VFKTIADYYG	HLKEPLLTFH	LFDAFVSVLG	LLQKEKVAVE	AFQICCLLLP
**330**	**340**	**350**	**360**	**370**	**380**	**390**	**400**
PENRRKLQLL	MRMMARICLN	KEMPPLCDGF	GTRTLMVQTF	SRCILCSKDE	VDLDELLAAR	LVTFLMDNYQ	EILKVPLALQ
**410**	**420**	**430**	**440**	**450**	**460**	**470**	
TSIEERVAHL	RRVQFQKSYP	EVYQERFPTP	ESAALLFPEK	PKPKPQLLMW	ALKKPFQPFQ	RTRSFRM	

Cmpd.	No. of Cmpds.	m/z meas.	Δ m/z [ppm]	z	Rt [min]	Score	P	Range	Sequence	Modification
2797	2	626.8488	34.86	2	63.4	14.7	0	159-170	R.HSIAIGEVPACR.L	

Detailed Protein Report

Protein 598: secretogranin-3 isoform 2 [Homo sapiens]

Accession: gi|259089433
Database: refseq_human(refseq_human_20140103.fasta)
Database Date: 2015-11-30

Score: 14.7
MW [kDa]: 26.9
pI: 4.8
Sequence Coverage [%]: 8.1
No. of unique Peptides: 1

10	20	30	40	50	60	70	80
MAAIQDGLAK	GENDETVSNT	LTLTNGLERR	TKTYSEDNFE	ELQYFPNFYA	LLKSIDSEKE	AKEKETLITI	MKTLIDFVKM
**90**	**100**	**110**	**120**	**130**	**140**	**150**	**160**
MVKYGTISPE	EGVSYLENLD	EMIALQTKNK	LEKNATDNIS	KLFPAPSEKS	HEETDSTKEE	AAKMEKEYGS	LKDSTKDDNS
**170**	**180**	**190**	**200**	**210**	**220**	**230**	**240**
NPGGKTDEPK	GKTEAYLEAI	RKNIEWLKKH	DKKGNKEDYD	LSKMRDFINK	QADAYVEKGI	LDKEEAEAIK	RIYSSL

Cmpd.	No. of Cmpds.	m/z meas.	Δ m/z [ppm]	z	Rt [min]	Score	P	Range	Sequence	Modification
1548	1	671.4028	135.82	3	48.0	14.7	2	147-165	K.EYGSLKDSTKDDNSNPGGK.T	

Detailed Protein Report

Protein 599: PREDICTED: uncharacterized protein CXorf65 isoform X1 [Homo sapiens]

Accession: gi|530421697
Database: refseq_human(refseq_human_20140103.fasta)
Database Date: 2015-11-30

Score: 14.7
MW [kDa]: 18.6
pI: 11.1
Sequence Coverage [%]: 8.8
No. of unique Peptides: 1

10	20	30	40	50	60	70	80
MFIFIKHGDN	QQFLVNTNCA	VVVLLYYIRS	KVKLPKTNTI	DLCEQTGKMK	MLFLMKPNHA	EYASKYLTAR	STYYVCKVER
**90**	**100**	**110**	**120**	**130**	**140**	**150**	**160**
GPPVALRIQC	DALERRRIQM	LKMKEAKKVV	IIEPPASVPS	KQSGRSDKKK	STRKSPTFRN	RPDFRKNKGR	QLNKTTKQKK
**170**							

Cmpd.	No. of Cmpds.	m/z meas.	Δ m/z [ppm]	z	Rt [min]	Score	P	Range	Sequence	Modification
2029	1	732.3134	-180.02	2	53.8	14.7	1	108-121	K.KVVIIEPPASVPSK.Q	

Detailed Protein Report

Protein 600: PDZ domain-containing protein 4 [Homo sapiens]

**Accession:**	gi|46559761	**Score:**	14.6
**Database:**	refseq_human(refseq_human_20140103.fasta)	**MW [kDa]:**	86.1
**Database Date:**	2015-11-30	**pI:**	5.7
**Modification(s):**	Oxidation	**Sequence Coverage [%]:**	4.0
		**No. of unique Peptides:**	1

10	20	30	40	50	60	70	80
MGCNMCVVQK	PEEQYKVMLQ	VNGKELSKLS	QEQTLQALRS	SKEPLVIQVL	RRSPRLRGDS	SCHDLQLVDS	GTQTDITFEH
**90**	**100**	**110**	**120**	**130**	**140**	**150**	**160**
IMALGKLRPP	TPPMVILEPP	PISHEYYDPA	EFMEGGPQEA	DRLDELEYEE	VELYKSSHRD	KLGLMVCYRT	DDEEDLGIYV
**170**	**180**	**190**	**200**	**210**	**220**	**230**	**240**
GEVNPNSIAA	KDGRIREGDR	IIQINGVDVQ	NREEAVAILS	QEENTNISLL	VARPESQLAK	RWKDSDRDDF	LDDFGSENEG
**250**	**260**	**270**	**280**	**290**	**300**	**310**	**320**
ELRARKLKSP	PAQQPGNEEE	KGAPDAGPGL	SNSQELDSGV	GRTDESTRNE	ESSEHDLLGD	EPPSSTNTPG	SLRKFGLQGD
**330**	**340**	**350**	**360**	**370**	**380**	**390**	**400**
ALQSRDFHFS	MDSLLAEGAG	LGGGDVPGLT	DEEYERYREL	LEIKCHLENG	NQLGLLFPRA	SGGNSALDVN	RNESLGHEMA
**410**	**420**	**430**	**440**	**450**	**460**	**470**	**480**
MLEEELRHLE	FKCRNILRAQ	KMQQLRERCM	KAWLLEEESL	YDLAASEPKK	HELSDISELP	EKSDKDSTSA	YNTGESCRST
**490**	**500**	**510**	**520**	**530**	**540**	**550**	**560**
PLLVEPLPES	PLRRAMAGNS	NLNRTPPGPA	VATPAKAAPP	PGSPAKFRSL	SRDPEAGRRQ	HAEERGRRNP	KTGLTLERVG
**570**	**580**	**590**	**600**	**610**	**620**	**630**	**640**
PESSPYLSRR	HRGQGQEGEH	YHSCVQLAPT	RGLEELGHGP	LSLAGGPRVG	GVAAAATEAP	RMEWKVKVRS	DGTRYVAKRP
**650**	**660**	**670**	**680**	**690**	**700**	**710**	**720**
VRDRLLKARA	LKIREERSGM	TTDDDAVSEM	KMGRYWSKEE	RKQHLIRARE	QRKRREFMMQ	SRLECLREQQ	NGDSKPELNI
**730**	**740**	**750**	**760**	**770**			
IALSHRKTMK	KRNKKILDNW	ITIQEMLAHG	ARSADGKRVY	NPLLSVTTV			

Cmpd.	No. of Cmpds.	m/z meas.	Δ m/z [ppm]	z	Rt [min]	Score	P	Range	Sequence	Modification
450	1	1100.7316	-85.85	3	34.6	14.6	0	326-356	R.DFHFSMDSLLAEGAGLGGGDVPGLTDEEYER.Y	Oxidation: 6

Detailed Protein Report

Protein 601: ATP-binding cassette sub-family A member 5 [Homo sapiens]

**Accession:**	gi|27262624	**Score:**	14.6
**Database:**	refseq_human(refseq_human_20140103.fasta)	**MW [kDa]:**	186.4
**Database Date:**	2015-11-30	**pI:**	6.5
		**Sequence Coverage [%]:**	0.9
		**No. of unique Peptides:**	1

Alias proteins:

Accession	Name	Description
gi|27262626	refseq_human (refseq_human_20140103.fasta)	ATP-binding cassette sub-family A member 5 [Homo sapiens]

10	20	30	40	50	60	70	80
MSTAIREVGV	WRQTRTLLLK	NYLIKCRTKK	SSVQEILFPL	FFLFWLILIS	MMHPNKKYEE	VPNIELNPMD	KFTLSNLILG
90	100	110	120	130	140	150	160
YTPVTNITSS	IMQKVSTDHL	PDVIITEEYT	NEKEMLTSSL	SKPSNFVGVV	FKDSMSYELR	FFPDMIPVSS	IYMDSRAGCS
170	180	190	200	210	220	230	240
KSCEAAQYWS	SGFTVLQASI	DAAIIQLKTN	VSLWKELEST	KAVIMGETAV	VEIDTFPRGV	ILIYLVIAFS	PFGYFLAIHI
250	260	270	280	290	300	310	320
VAEKEKKIKE	FLKIMGLHDT	AFWLSWVLLY	TSLIFLMSLL	MAVIATASLL	FPQSSSIVIF	LLFFLYGLSS	VFFALMLTPL
330	340	350	360	370	380	390	400
FKKSKHVGIV	EFFVTVAFGF	IGLMIILIES	FPKSLVWLFS	PFCHCTFVIG	IAQVMHLEDF	NEGASFSNLT	AGPYPLIITI
410	420	430	440	450	460	470	480
IMLTLNSIFY	VLLAVYLDQV	IPGEFGLRRS	SLYFLKPSYW	SKSKRNYEEL	SEGNVNGNIS	FSEIIEPVSS	EFVGKEAIRI
490	500	510	520	530	540	550	560
SGIQKTYRKK	GENVEALRNL	SFDIYEGQIT	ALLGHSGTGK	STLMNILCGL	CPPSDGFASI	YGHRVSEIDE	MFEARKMIGI
570	580	590	600	610	620	630	640
CPQLDIHFDV	LTVEENLSIL	ASIKGIPANN	IIQEVQKVLL	DLDMQTIKDN	QAKKLSGGQK	RKLSLGIAVL	GNPKILLLDE
650	660	670	680	690	700	710	720
PTAGMDPCSR	HIVWNLLKYR	KANRVTVFST	HFMDEADILA	DRKAVISQGM	LKCVGSSMFL	KSKWGIGYRL	SMYIDKYCAT
730	740	750	760	770	780	790	800
ESLSSLVKQH	IPGATLLQQN	DQQLVYSLPF	KDMDKFSGLF	SALDSHSNLG	VISYGVSMTT	LEDVFLKLEV	EAEIDQADYS
810	820	830	840	850	860	870	880
VFTQQPLEEE	MDSKSFDEME	QSLLILSETK	AALVSTMSLW	KQQMYTIAKF	HFFTLKRESK	SVRSVLLLLL	IFFTVQIFMF
890	900	910	920	930	940	950	960
LVHHSFKNAV	VPIKLVPDLY	FLKPGDKPHK	YKTSLLLQNS	ADSDISDLIS	FFTSQNIMVT	MINDSDYVSV	APHSAALNVM
970	980	990	1000	1010	1020	1030	1040
HSEKDYVFAA	VFNSTMVYSL	PILVNIISNY	YLYHLNVTET	IQIWSTPFFQ	EITDIVFKIE	LYFQAALLGI	IVTAMPPYFA
1050	1060	1070	1080	1090	1100	1110	1120
MENAENHKIK	AYTQLKLSGL	LPSAYWIGQA	VVDIPLFFII	LILMLGSLLA	FHYGLYFYTV	KFLAVVFCLI	GYVPSVILFT
1130	1140	1150	1160	1170	1180	1190	1200
YIASFTFKKI	LNTKEFWSFI	YSVAALACIA	ITEITFFMGY	TIATILHYAF	CIIIPIYPLL	GCLISFIKIS	WKNVRKNVDT
1210	1220	1230	1240	1250	1260	1270	1280
YNPWDRLSVA	VISPYLQCVL	WIFLLQYYEK	KYGGRSIRKD	PFFRNLSTKS	KNRKLPEPPD	NEDEDEDVKA	ERLKVKELMG
1290	1300	1310	1320	1330	1340	1350	1360
CQCCEEKPSI	MVSNLHKEYD	DKKDFLLSRK	VKKVATKYIS	FCVKKGEILG	LLGPNGAGKS	TIINILVGDI	EPTSGQVFLG
1370	1380	1390	1400	1410	1420	1430	1440
DYSSETSEDD	DSLKCMGYCP	QINPLWPDTT	LQEHFEIYGA	VKGMSASDMK	EVISRITHAL	DLKEHLQKTV	KKLPAGIKRK
1450	1460	1470	1480	1490	1500	1510	1520
LCFALSMLGN	PQITLLDEPS	TGMDPKAKQH	MWRAIRTAFK	NRKRAAILTT	HYMEEAEAVC	DRVAIMVSGQ	LRCIGTVQHL
1530	1540	1550	1560	1570	1580	1590	1600
KSKFGKGYFL	EIKLKDWIEN	LEVDRLQREI	QYIFPNASRQ	ESFSSILAYK	IPKEDVQSLS	QSFFKLEEAK	HAFAIEEYSF
1610	1620	1630	1640	1650			
SQATLEQVFV	ELTKEQEEED	NSCGTLNSTL	WWERTQEDRV	VF			

Cmpd.	No. of Cmpds.	m/z meas.	Δ m/z [ppm]	z	Rt [min]	Score	P	Range	Sequence	Modification
2136	1	805.8396	-128.42	2	55.1	14.6	1	1560-1573	R.QESFSSILAYKIPK.E	

Detailed Protein Report

Protein 602: **PREDICTED: conserved oligomeric Golgi complex subunit 3 isoform X9 [Homo sapiens]**

**Accession:**	gi|578825042	**Score:**	14.6
**Database:**	refseq_human(refseq_human_20140103.fasta)	**MW [kDa]:**	79.6
**Database Date:**	2015-11-30	**pI:**	6.0
		**Sequence Coverage [%]:**	2.4
		**No. of unique Peptides:**	1

10	20	30	40	50	60	70	80
MRDYLSGFQE	QCDAILNDVN	SALQHLESLQ	KQYLFVSNKT	GTLHEACEQL	LKEQSELVDL	AENIQQKLSY	FNELETINTK
**90**	**100**	**110**	**120**	**130**	**140**	**150**	**160**
LNSPTLSVNS	DGFIPMLAKL	DDCITYISSH	PNFKDYPIYL	LKFKQCLSKA	LHLMKTYTVN	TLQTLTSQLL	KRDPSSVPNA
**170**	**180**	**190**	**200**	**210**	**220**	**230**	**240**
DNAFTLFYVK	FRAAAPKVRT	LIEQIELRSE	KIPEYQQLLN	DIHQCYLDQR	ELLLGPSIAC	TVAELTSQNN	RDHCALVRSG
**250**	**260**	**270**	**280**	**290**	**300**	**310**	**320**
CAFMVHVCQD	EHQLYNEFFT	KPTSKLDELL	EKLCVSLYDV	FRPLIIHVIH	LETLSELCGI	LKNEVLEDHV	QNNAEQLGAF
**330**	**340**	**350**	**360**	**370**	**380**	**390**	**400**
AAGVKQMLED	VQERLVYRTH	IYIQTDITGY	KPAPGDLAYP	DKLVMMEQIA	QSLKDEQKKV	PSEASFSDVH	LEEGESNSLT
**410**	**420**	**430**	**440**	**450**	**460**	**470**	**480**
KSGSTESLNP	RPQTTISPAD	LHGMWYPTVR	RTLVCLSKLY	RCIDRAVFQG	LSQEALSACI	QSLLGASESI	SKNKTQIDGQ
**490**	**500**	**510**	**520**	**530**	**540**	**550**	**560**
LFLIKHLLIL	REQIAPFHTE	FTIKEISLDL	KKTRDAAFKI	LNPMTVPRFF	RLNSNNALIE	FLLEGTPEIR	EHYLDSKKDV
**570**	**580**	**590**	**600**	**610**	**620**	**630**	**640**
DRHLKSACEQ	FIQQQTKLFV	EQLEEFMTKV	SALKTMASQG	GPKYTLSQQP	WAQPAKVNDL	AATAYKTIKT	KLPVTLRSMS
**650**	**660**	**670**	**680**	**690**	**700**		
LYLSNKDTEF	ILFKPVRNNI	QQVFQKFHAL	LKEEFSPEDI	QIIACPSMEQ	LSLLLVSK		

Cmpd.	No. of Cmpds.	m/z meas.	Δ m/z [ppm]	z	Rt [min]	Score	P	Range	Sequence	Modification
1254	1	990.6132	53.89	2	44.3	14.6	1	136-152	K.TYTVNTLQTLTSQLLKR.D	

Detailed Protein Report

Protein 603: **PREDICTED: cGMP-inhibited 3',5'-cyclic phosphodiesterase A isoform X1 [Homo sapiens]**

**Accession:**	gi|578822894	**Score:**	14.5
**Database:**	refseq_human(refseq_human_20140103.fasta)	**MW [kDa]:**	118.5
**Database Date:**	2015-11-30	**pI:**	5.5
**Modification(s):**	Carbamidomethyl	**Sequence Coverage [%]:**	1.0
		**No. of unique Peptides:**	1

Quantitation

WD:WU **Median:** 0.68 **CV:** 0.00 % **No. of Peptides:** 1

10	20	30	40	50	60	70	80
MAVPGDAARV	RDKPVHSGVS	QAPTAGRDCH	HRADPASPRD	SGCRGCWGDL	VLQPLRSSRK	LSSALCAGSL	SFLLALLVRL
90	100	110	120	130	140	150	160
VRGEVGCDLE	QCKEAAAAEE	EEAAPGAEGG	VFPGPRGGAP	GGGARLSPWL	QPSALLFSLL	CAFFWMGLYL	LRAGVRLPLA
170	180	190	200	210	220	230	240
VALLAACCGG	EALVQIGLGV	GEDHLLSLPA	AGVVLSCLAA	ATWLVLRLRL	GVLMIALTSA	VRTVSLISLE	RFKVAWRPYL
250	260	270	280	290	300	310	320
AYLAGVLGIL	LARYVEQILP	QSAEAAPREH	LGSQLIAGTK	EDIPVFKRRR	RSSSVVSAEM	SGCSSKSHRR	TSLPCIPREQ
330	340	350	360	370	380	390	400
LMGHSEWDHK	RGPRGSQSSG	TSITVDIAVM	GEAHGLITDL	LADPSLPPNV	CTSLRAVSNL	LSTQLTFQAI	HKPRVNPVTS
410	420	430	440	450	460	470	480
LSENYTCSDS	EESSEKDKLA	IPKRLRRSLP	PGLLRRVSST	WTTTTSATGL	PTLEPAPVRR	DRSTSIKLQE	APSSSPDSWN
490	500	510	520	530	540	550	560
NPVMMTLTKS	RSFTSSYAIS	AANHVKAKKQ	SRPGALAKIS	PLSSPCSSPL	QGTPASSLVS	KISAVQFPES	ADTTAKQSLG
570	580	590	600	610	620	630	640
SHRALTYTQS	APDLSPQILT	PPVICSSCGR	PYSQGNPADE	PLERSGVATR	TPSRTDDTAQ	VTSDYETNNN	SDSSDIVQNE
650	660	670	680	690	700	710	720
DETECLREPL	RKASACSTYA	PETMMFLDKP	ILAPEPLVMD	NLDSIMEQLN	TWNFPIFDLV	ENIGRKCGRI	LSQVSYRLFE
730	740	750	760	770	780	790	800
DMGLFEAFKI	PIREFMNYFH	ALEIGYRDIP	YHNRIHATDV	LHAVWYLTTQ	PIPGLSTVIN	DHGSTSDSDS	DSGFTHGHMG
810	820	830	840	850	860	870	880
YVFSKTYNVT	DDKYGCLSGN	IPALELMALY	VAAAMHDYDH	PGRTNAFLVA	TSAPQAVLYN	DRSVLENHHA	AAAWNLFMSR
890	900	910	920	930	940	950	960
PEYNFLINLD	HVEFKHFRFL	VIEAILATDL	KKHFDFVAKF	NGKVNDDVGI	DWTNENDRLL	VCQMCIKLAD	INGPAKCKEL
970	980	990	1000	1010	1020	1030	1040
HLQWTDGIVN	EFYEQGDEEA	SLGLPISPFM	DRSAPQLANL	QESFISHIVG	PLCNSYDSAG	LMPGKWVEDS	DESGDTDDPE
1050	1060	1070	1080	1090			
EEEEEAPAPN	EEETCENNES	PSKYQMPLAY	RLEDTLQDKF	LKGGMLR			

Cmpd.	No. of Cmpds.	m/z meas.	Δ m/z [ppm]	z	Rt [min]	Score	P	Range	Sequence	Modification	Ratios	
645	1	647.6267	-227.08	2	36.5	14.5	0	83-93	R.GEVGCDLEQCK.E	Carbamidomethyl: 5, 10	WD:WU	0.68

Detailed Protein Report

Protein 604: inhibitor of nuclear factor kappa-B kinase subunit alpha [Homo sapiens]

Accession: gi|62241001
Database: refseq_human(refseq_human_20140103.fasta)
Database Date: 2015-11-30
Modification(s): Oxidation

Score: 14.5
MW [kDa]: 84.6
pI: 6.3
Sequence Coverage [%]: 1.1
No. of unique Peptides: 1

Quantitation

WD:WU **Median:** 0.52 **CV:** 0.00 % **No. of Peptides:** 1

10	20	30	40	50	60	70	80
MERPPGLRPG	AGGPWEMRER	LGTGGFGNVC	LYQHRELDLK	IAIKSCRLEL	STKNRERWCH	EIQIMKKLNH	ANVVKACDVP
**90**	**100**	**110**	**120**	**130**	**140**	**150**	**160**
EELNILIHDV	PLLAMEYCSG	GDLRKLLNKP	ENCCGLKESQ	ILSLLSDIGS	GIRYLHENKI	IHRDLKPENI	VLQDVGGKII
**170**	**180**	**190**	**200**	**210**	**220**	**230**	**240**
HKIIDLGYAK	DVDQGSLCTS	FVGTLQYLAP	ELFENKPYTA	TVDYWSFGTM	VFECIAGYRP	FLHHLQPFTW	HEKIKKKDPK
**250**	**260**	**270**	**280**	**290**	**300**	**310**	**320**
CIFACEEMSG	EVRFSSHLPQ	PNSLCSLVVE	PMENWLQLML	NWDPQQRGGP	VDLTLKQPRC	FVLMDHILNL	KIVHILNMTS
**330**	**340**	**350**	**360**	**370**	**380**	**390**	**400**
AKIISFLLPP	DESLHSLQSR	IERETGINTG	SQELLSETGI	SLDPRKPASQ	CVLDGVRGCD	SYMVYLFDKS	KTVYEGPFAS
**410**	**420**	**430**	**440**	**450**	**460**	**470**	**480**
RSLSDCVNYI	VQDSKIQLPI	IQLRKVWAEA	VHYVSGLKED	YSRLFQGQRA	AMLSLLRYNA	NLTKMKNTLI	SASQQLKAKL
**490**	**500**	**510**	**520**	**530**	**540**	**550**	**560**
EFFHKSIQLD	LERYSEQMTY	GISSEKMLKA	WKEMEEKAIH	YAEVGVIGYL	EDQIMSLHAE	IMELQKSPYG	RRQGDLMESL
**570**	**580**	**590**	**600**	**610**	**620**	**630**	**640**
EQRAIDLYKQ	LKHRPSDHSY	SDSTEMVKII	VHTVQSQDRV	LKELFGHLSK	LLGCKQKIID	LLPKVEVALS	NIKEADNTVM
**650**	**660**	**670**	**680**	**690**	**700**	**710**	**720**
FMQGKRQKEI	WHLLKIACTQ	SSARSLVGSS	LEGAVTPQTS	AWLPPTSAEH	DHSLSCVVTP	QDGETSAQMI	EENLNCLGHL
**730**	**740**	**750**					
STIIHEANEE	QGNSMMNLDW	SWLTE					

Cmpd.	No. of Cmpds.	m/z meas.	Δ m/z [ppm]	z	Rt [min]	Score	P	Range	Sequence	Modification	Ratios
1405	1	533.6158	-246.47	2	45.9	14.5	1	510-517	K.AWKEMEEK.A	Oxidation: 5	WD:WU 0.52

Detailed Protein Report

Protein 605: zinc finger and BTB domain-containing protein 5 [Homo sapiens]

**Accession:**	gi|7662074	**Score:**	14.5
**Database:**	refseq_human(refseq_human_20140103.fasta)	**MW [kDa]:**	74.2
**Database Date:**	2015-11-30	**pI:**	5.8
**Modification(s):**	Carbamidomethyl	**Sequence Coverage [%]:**	2.5
		**No. of unique Peptides:**	1

Alias proteins:

Accession	Name	Description
gi|530390257	refseq_human (refseq_human_20140103.fasta)	PREDICTED: zinc finger and BTB domain-containing protein 5 isoform X1 [Homo sapiens]

10	20	30	40	50	60	70	80
MDFPGHFEQI	FQQLNYQRLH	GQLCDCVIVV	GNRHFKAHRS	VLAACSTHFR	ALFSVAEGDQ	TMNMIQLDSE	VVTAEAFAAL
90	100	110	120	130	140	150	160
IDMMYTSTLM	LGESNVMDVL	LAASHLHLNS	VVKACKHYLT	TRTLPMSPPS	ERVQEQSARM	QRSFMLQQLG	LSIVSSALNS
170	180	190	200	210	220	230	240
SQNGEEQPAP	MSSSMRSNLD	QRTPFPMRRL	HKRKQSAEER	ARQRLRPSID	ESAISDVTPE	NGPSGVHSRE	EFFSPDSLKI
250	260	270	280	290	300	310	320
VDNPKADGMT	DNQEDSAIMF	DQSFGTQEDA	QVPSQSDNSA	GNMAQLSMAS	RATQVETSFD	QEAAPEKSSF	QCENPEVGLG
330	340	350	360	370	380	390	400
EKEHMRVVVK	SEPLSSPEPQ	DEVSDVTSQA	EGSESVEVEG	VVVSAEKIDL	SPESSDRSFS	DPQSSTDRVG	DIHILEVTNN
410	420	430	440	450	460	470	480
LEHKSTFSIS	NFLNKSRGNN	FTANQNNDDN	IPNTTSDCRL	ESEAPYLLSP	EAGPAGGPSS	APGSHVENPF	SEPADSHFVR
490	500	510	520	530	540	550	560
PMQEVMGLPC	VQTSGYQGGE	QFGMDFSRSG	LGLHSSFSRV	MIGSPRGGAS	NFPYYRRIAP	KMPVVTSVRS	SQIPENSTSS
570	580	590	600	610	620	630	640
QLMMNGATSS	FENGHPSQPG	PPQLTRASAD	VLSKCKKALS	EHNVLVVEGA	RKYACKICCK	TFLTLTDCKK	HIRVHTGEKP
650	660	670	680				
YACLKCGKRF	SQSSHLYKHS	KTTCLRWQSS	NLPSTLL				

Cmpd.	No. of Cmpds.	m/z meas.	Δ m/z [ppm]	z	Rt [min]	Score	P	Range	Sequence	Modification
1742	1	689.9841	-17.31	3	50.5	14.5	2	613-629	K.YACKICCKTFLTLTDCK.K	Carbamidomethyl: 6, 7

Detailed Protein Report

Protein 606: beta-1-syntrophin [Homo sapiens]

**Accession:**	gi|11321640	**Score:**	14.4
**Database:**	refseq_human(refseq_human_20140103.fasta)	**MW [kDa]:**	58.0
**Database Date:**	2015-11-30	**pI:**	9.5
		**Sequence Coverage [%]:**	2.6
		**No. of unique Peptides:**	1

10	20	30	40	50	60	70	80
MAVAAAAAAA	GPAGAGGGRA	QRSGLLEVLV	RDRWHKVLVN	LSEDALVLSS	EEGAAAYNGI	GTATNGSFCR	GAGAGHPGAG
**90**	**100**	**110**	**120**	**130**	**140**	**150**	**160**
GAQPPDSPAG	VRTAFTDLPE	QVPESISNQK	RGVKVLKQEL	GGLGISIKGG	KENKMPILIS	KIFKGLAADQ	TQALYVGDAI
**170**	**180**	**190**	**200**	**210**	**220**	**230**	**240**
LSVNGADLRD	ATHDEAVQAL	KRAGKEVLLE	VKYMREATPY	VKKGSPVSEI	GWETPPPESP	RLGGSTSDPP	SSQSFSFHRD
**250**	**260**	**270**	**280**	**290**	**300**	**310**	**320**
RKSIPLKMCY	VTRSMALADP	ENRQLEIHSP	DAKHTVILRS	KDSATAQAWF	SAIHSNVNDL	LTRVIAEVRE	QLGKTGIAGS
**330**	**340**	**350**	**360**	**370**	**380**	**390**	**400**
REIRHLGWLA	EKVPGESKKQ	WKPALVVLTE	KDLLIYDSMP	RRKEAWFSPV	HTYPLLATRL	VHSGPGKGSP	QAGVDLSFAT
**410**	**420**	**430**	**440**	**450**	**460**	**470**	**480**
RTGTRQGIET	HLFRAETSRD	LSHWTRSIVQ	GCHNSAELIA	EISTACTYKN	QECRLTIHYE	NGFSITTEPQ	EGAFPKTIIQ
**490**	**500**	**510**	**520**	**530**	**540**		
SPYEKLKMSS	DDGIRMLYLD	FGGKDGEIQL	DLHSCPKPIV	FIIHSFLSAK	ITRLGLVA		

Cmpd.	No. of Cmpds.	m/z meas.	Δ m/z [ppm]	z	Rt [min]	Score	P	Range	Sequence	Modification
2302	1	703.3364	-29.47	2	57.1	14.4	0	388-401	K.GSPQAGVDLSFATR.T	

Detailed Protein Report

Protein 607: granzyme A precursor [Homo sapiens]

**Accession:**	gi|5453676	**Score:**	14.4
**Database:**	refseq_human(refseq_human_20140103.fasta)	**MW [kDa]:**	28.9
**Database Date:**	2015-11-30	**pI:**	10.1
**Modification(s):**	Carbamidomethyl	**Sequence Coverage [%]:**	5.3
		**No. of unique Peptides:**	1

Quantitation

WD:WU **Median:** 0.78 **CV:** 0.00 % **No. of Peptides:** 1

10	20	30	40	50	60	70	80
MRNSYRFLAS	SLSVVVSLLL	IPEDVCEKII	GGNEVTPHSR	PYMVLLSLDR	KTICAGALIA	KDWVLTAAHC	NLNKRSQVIL
**90**	**100**	**110**	**120**	**130**	**140**	**150**	**160**
GAHSITREEP	TKQIMLVKKE	FPYPCYDPAT	REGDLKLLQL	TEKAKINKYV	TILHLPKKGD	DVKPGTMCQV	AGWGRTHNSA
**170**	**180**	**190**	**200**	**210**	**220**	**230**	**240**
SWSDTLREVN	ITIIDRKVCN	DRNHYNFNPV	IGMNMVCAGS	LRGGRDSCNG	DSGSPLLCEG	VFRGVTSFGL	ENKCGDPRGP
**250**	**260**	**270**					
GVYILLSKKH	LNWIIMTIKG	AV					

Cmpd.	No. of Cmpds.	m/z meas.	Δ m/z [ppm]	z	Rt [min]	Score	P	Range	Sequence	Modification	Ratios	
92	1	566.5853	-66.20	3	30.1	14.4	1	62-75	K.DWVLTAAHCNLNKR.S	Carbamidomethyl: 9	WD:WU	0.78

Detailed Protein Report

Protein 608: putative dehydrogenase/reductase SDR family member 4-like 2 [Homo sapiens]

Accession: gi|482628371
Database: refseq_human(refseq_human_20140103.fasta)
Database Date: 2015-11-30
Modification(s): Oxidation

Score: 14.4
MW [kDa]: 16.3
pI: 9.1
Sequence Coverage [%]: 8.7
No. of unique Peptides: 1

Quantitation

WD:WU **Median:** 1.24 **CV:** 0.00 % **No. of Peptides:** 1

10	20	30	40	50	60	70	80
MTGTVCHVGK	MKDWERLVAT	VSCREMGTEP	GGGKGSQPEP	PSLLSWTALG	FSPYNVSKTA	LLGLNKTLAI	ELAPRNIRVN
**90**	**100**	**110**	**120**	**130**	**140**	**150**	**160**
CLAPGLIKTS	FSRMLWMDKE	KEESMKETLR	IRRLGEPEDS	LGIVSFLCSE	DASYLTGETV	MVGGGTPSRL	

Cmpd.	No. of Cmpds.	m/z meas.	Δ m/z [ppm]	z	Rt [min]	Score	P	Range	Sequence	Modification	Ratios
717	1	866.9167	38.74	2	37.8	14.4	2	94-106	R.MLWMDKEKEESMK.E	Oxidation: 1, 4, 12	WD:WU 1.24

Detailed Protein Report

Protein 609: PREDICTED: catenin alpha-1 isoform X2 [Homo sapiens]

**Accession:**	gi|530379718	**Score:**	14.3
**Database:**	refseq_human(refseq_human_20140103.fasta)	**MW [kDa]:**	59.5
**Database Date:**	2015-11-30	**pI:**	5.3
**Modification(s):**	Carbamidomethyl	**Sequence Coverage [%]:**	2.2
		**No. of unique Peptides:**	1

Alias proteins:

Accession	Name	Description
gi|530379720	refseq_human (refseq_human_20140103.fasta)	PREDICTED: catenin alpha-1 isoform X3 [Homo sapiens]

10	20	30	40	50	60	70	80
MTKKTRDLRR	QLRKAVMDHV	SDSFLETNVP	LLVLIEAAKN	GNEKEVKEYA	QVFREHANKL	IEVANLACSI	SNNEEGVKLV
**90**	**100**	**110**	**120**	**130**	**140**	**150**	**160**
RMSASQLEAL	CPQVINAALA	LAAKPQSKLA	QENMDLFKEQ	WEKQVRVLTD	AVDDITSIDD	FLAVSENHIL	EDVNKCVIAL
**170**	**180**	**190**	**200**	**210**	**220**	**230**	**240**
QEKDVDGLDR	TAGAIRGRAA	RVIHVVTSEM	DNYEPGVYTE	KVLEATKLLS	NTVMPRFTEQ	VEAAVEALSS	DPAQPMDENE
**250**	**260**	**270**	**280**	**290**	**300**	**310**	**320**
FIDASRLVYD	GIRDIRKAVL	MIRTPEELDD	SDFETEDFDV	RSRTSVQTED	DQLIAGQSAR	AIMAQLPQEQ	KAKIAEQVAS
**330**	**340**	**350**	**360**	**370**	**380**	**390**	**400**
FQEEKSKLDA	EVSKWDDSGN	DIIVLAKQMC	MIMMEMTDFT	RGKGPLKNTS	DVISAAKKIA	EAGSRMDKLG	RTIADHCPDS
**410**	**420**	**430**	**440**	**450**	**460**	**470**	**480**
ACKQDLLAYL	QRIALYCHQL	NICSKVKAEV	QNLGGELVVS	GVDSAMSLIQ	AAKNLMNAVV	QTVKASYVAS	TKYQKSQGMA
**490**	**500**	**510**	**520**	**530**	**540**		
SLNLPAVSWK	MKAPEKKPLV	KREKQDETQT	KIKRASQKKH	VNPVQALSEF	KAMDSI		

Cmpd.	No. of Cmpds.	m/z meas.	Δ m/z [ppm]	z	Rt [min]	Score	P	Range	Sequence	Modification
2652	3	659.3500	103.51	2	61.5	14.3	0	392-403	R.TIADHCPDSACK.Q	Carbamidomethyl: 11

Detailed Protein Report

Protein 610: probable ATP-dependent RNA helicase DHX58 [Homo sapiens]

**Accession:**	gi|149408122	**Score:**	14.3
**Database:**	refseq_human(refseq_human_20140103.fasta)	**MW [kDa]:**	76.6
**Database Date:**	2015-11-30	**pI:**	7.1
		**Sequence Coverage [%]:**	2.4
		**No. of unique Peptides:**	1

10	20	30	40	50	60	70	80
MELRSYQWEV	IMPALEGKNI	IIWLPTGAGK	TRAAAYVAKR	HLETVDGAKV	VVLVNRVHLV	TQHGEEFRRM	LDGRWTVTTL
90	100	110	120	130	140	150	160
SGDMGPRAGF	GHLARCHDLL	ICTAELLQMA	LTSPEEEEHV	ELTVFSLIVV	DECHHTHKDT	VYNVIMSQYL	ELKLQRAQPL
170	180	190	200	210	220	230	240
PQVLGLTASP	GTGGASKLDG	AINHVLQLCA	NLDTWCIMSP	QNCCPQLQEH	SQQPCKQYNL	CHRRSQDPFG	DLLKKLMDQI
250	260	270	280	290	300	310	320
HDHLEMPELS	RKFGTQMYEQ	QVVKLSEAAA	LAGLQEQRVY	ALHLRRYNDA	LLIHDTVRAV	DALAALQDFY	HREHVTKTQI
330	340	350	360	370	380	390	400
LCAERRLLAL	FDDRKNELAH	LATHGPENPK	LEMLEKILQR	QFSSSNSPRG	IIFTRTRQSA	HSLLLWLQQQ	QGLQTVDIRA
410	420	430	440	450	460	470	480
QLLIGAGNSS	QSTHMTQRDQ	QEVIQKFQDG	TLNLLVATSV	AEEGLDIPHC	NVVVRYGLLT	NEISMVQARG	RARADQSVYA
490	500	510	520	530	540	550	560
FVATEGSREL	KRELINEALE	TLMEQAVAAV	QKMDQAEYQA	KIRDLQQAAL	TKRAAQAAQR	ENQRQQFPVE	HVQLLCINCM
570	580	590	600	610	620	630	640
VAVGHGSDLR	KVEGTHHVNV	NPNFSNYYNV	SRDPVVINKV	FKDWKPGGVI	SCRNCGEVWG	LQMIYKSVKL	PVLKVRSMLL
650	660	670	680				
ETPQGRIQAK	KWSRVPFSVP	DFDFLQHCAE	NLSDLSLD				

Cmpd.	No. of Cmpds.	m/z meas.	Δ m/z [ppm]	z	Rt [min]	Score	P	Range	Sequence	Modification
1926	1	618.9091	-113.75	3	52.4	14.3	1	614-629	R.NCGEVWGLQMIYKSVK.L	

Detailed Protein Report

Protein 611: probable ATP-dependent RNA helicase DHX36 isoform 2 [Homo sapiens]

**Accession:**	gi|167830436	**Score:**	14.3
**Database:**	refseq_human(refseq_human_20140103.fasta)	**MW [kDa]:**	113.1
**Database Date:**	2015-11-30	**pI:**	8.3
		**Sequence Coverage [%]:**	1.8
		**No. of unique Peptides:**	1

10	20	30	40	50	60	70	80
MSYDYHQNWG	RDGGPRSSGG	GYGGGPAGGH	GGNRGSGGGG	GGGGGGRGGR	GRHPGHLKGR	EIGMWYAKKQ	GQKNKEAERQ
90	100	110	120	130	140	150	160
ERAVVHMDER	REEQIVQLLN	SVQAKNDKES	EAQISWFAPE	DHGYGTEVST	KNTPCSENKL	DIQEKKLINQ	EKKMFRIRNR
170	180	190	200	210	220	230	240
SYIDRDSEYL	LQENEPDGTL	DQKLLEDLQK	KKNDLRYIEM	QHFREKLPSY	GMQKELVNLI	DNHQVTVISG	ETGCGKTTQV
250	260	270	280	290	300	310	320
TQFILDNYIE	RGKGSACRIV	CTQPRRISAI	SVAERVAAER	AESCGSGNST	GYQIRLQSRL	PRKQGSILYC	TTGIILQWLQ
330	340	350	360	370	380	390	400
SDPYLSSVSH	IVLDEIHERN	LQSDVLMTVV	KDLLNFRSDL	KVILMSATLN	AEKFSEYFGN	CPMIHIPGFT	FPVVEYLLED
410	420	430	440	450	460	470	480
VIEKIRYVPE	QKEHRSQFKR	GFMQGHVNRQ	EKEEKEAIYK	ERWPDYVREL	RRRYSASTVD	VIEMMEDDKV	DLNLIVALIR
490	500	510	520	530	540	550	560
YIVLEEEDGA	ILVFLPGWDN	ISTLHDLLMS	QVMFKSVNQT	QVFKRTPPGV	RKIVIATNIA	ETSITIDDVV	YVIDGGKIKE
570	580	590	600	610	620	630	640
THFDTQNNIS	TMSAEWVSKA	NAKQRKGRAG	RVQPGHCYHL	YNGLRASLLD	DYQLPEILRT	PLEELCLQIK	ILRLGGIAYF
650	660	670	680	690	700	710	720
LSRLMDPPSN	EAVLLSIRHL	MELNALDKQE	ELTPLGVHLA	RLPVEPHIGK	MILFGALFCC	LDPVLTIAAS	LSFKDPFVIP
730	740	750	760	770	780	790	800
LGKEKIADAR	RKELAKDTRS	DHLTVVNAFE	GWEEARRRGF	RYEKDYCWEY	FLSSNTLQML	HNMKGQFAEH	LLGAGFVSSR
810	820	830	840	850	860	870	880
NPKDPESNIN	SDNEKIIKAV	ICAGLYPKVA	KIRLNLGKKR	KMVKVYTKTD	GLVAVHPKSV	NVEQTDFHYN	WLIYHLKMRT
890	900	910	920	930	940	950	960
SSIYLYDCTE	VSPYCLLFFG	GDISIQKDND	QETIAVDEWI	VFQSPARIAH	LVKELRKELD	ILLQEKIESP	HPVDWNDTKS
970	980	990	1000				
RDCAVLSAII	DLIKTQEKAT	PRNFPPRFQD	GYYS				

Cmpd.	No. of Cmpds.	m/z meas.	Δ m/z [ppm]	z	Rt [min]	Score	P	Range	Sequence	Modification
338	2	686.9336	150.15	2	32.9	14.3	2	35-52	R.GSGGGGGGGGGGRGGRGR.H	

Detailed Protein Report

Protein 612: glypican-5 precursor [Homo sapiens]

Accession: gi|4758464
Database: refseq_human(refseq_human_20140103.fasta)
Database Date: 2015-11-30

Score: 14.3
MW [kDa]: 63.7
pI: 6.4
Sequence Coverage [%]: 3.0
No. of unique Peptides: 1

10	20	30	40	50	60	70	80
MDAQTWPVGF	RCLLLLALVG	SARSEGVQTC	EEVRKLFQWR	LLGAVRGLPD	SPRAGPDLQV	CISKKPTCCT	RKMEERYQIA
**90**	**100**	**110**	**120**	**130**	**140**	**150**	**160**
ARQDMQQFLQ	TSSSTLKFLI	SRNAAAFQET	LETLIKQAEN	YTSILFCSTY	RNMALEAAAS	VQEFFTDVGL	YLFGADVNPE
**170**	**180**	**190**	**200**	**210**	**220**	**230**	**240**
EFVNRFFDSL	FPLVYNHLIN	PGVTDSSLEY	SECIRMARRD	VSPFGNIPQR	VMGQMGRSLL	PSRTFLQALN	LGIEVINTTD
**250**	**260**	**270**	**280**	**290**	**300**	**310**	**320**
YLHFSKECSR	ALLKMQYCPH	CQGLALTKPC	MGYCLNVMRG	CLAHMAELNP	HWHAYIRSLE	ELSDAMHGTY	DIGHVLLNFH
**330**	**340**	**350**	**360**	**370**	**380**	**390**	**400**
LLVNDAVLQA	HLNGQKLLEQ	VNRICGRPVR	TPTQSPRCSF	DQSKEKHGMK	TTTRNSEETL	ANRRKEFINS	LRLYRSFYGG
**410**	**420**	**430**	**440**	**450**	**460**	**470**	**480**
LADQLCANEL	AAADGLPCWN	GEDIVKSYTQ	RVVGNGIKAQ	SGNPEVKVKG	IDPVINQIID	KLKHVVQLLQ	GRSPKPDKWE
**490**	**500**	**510**	**520**	**530**	**540**	**550**	**560**
LLQLGSGGGM	VEQVSGDCDD	EDGCGGSGSG	EVKRTLKITD	WMPDDMNFSD	VKQIHQTDTG	STLDTTGAGC	AVATESMTFT
**570**	**580**						
LISVVMLLPG	IW						

Cmpd.	No. of Cmpds.	m/z meas.	Δ m/z [ppm]	z	Rt [min]	Score	P	Range	Sequence	Modification
1972	1	973.3912	-90.24	2	53.0	14.3	2	367-383	K.HGMKTTTRNSEETLANR.R	

Detailed Protein Report

Protein 613: long-chain-fatty-acid--CoA ligase 1 isoform d [Homo sapiens]

Accession: gi|557878742
Database: refseq_human(refseq_human_20140103.fasta)
Database Date: 2015-11-30

Score: 14.3
MW [kDa]: 58.5
pI: 8.6
Sequence Coverage [%]: 2.3
No. of unique Peptides: 1

10	20	30	40	50	60	70	80
MVIVPLYDTL	GNEAITYIVN	KAELSLVFVD	KPEKAKLLLE	GVENKLIPGL	KIIVVMDAYG	SELVERGQRC	GVEVTSMKAM
90	100	110	120	130	140	150	160
EDLGRANRRK	PKPPAPEDLA	VICFTSGTTG	NPKGAMVTHR	NIVSDCSAFV	KATENTVNPC	PDDTLISFLP	LAHMFERVVE
170	180	190	200	210	220	230	240
CVMLCHGAKI	GFFQGDIRLL	MDDLKVLQPT	VFPVVPRLLN	RMFDRIFGQA	NTTLKRWLLD	FASKRKEAEL	RSGIIRNNSL
250	260	270	280	290	300	310	320
WDRLIFHKVQ	SSLGGRVRLM	VTGAAPVSAT	VLTFLRAALG	CQFYEGYGQT	ECTAGCCLTM	PGDWTAGHVG	APMPCNLIKL
330	340	350	360	370	380	390	400
VDVEEMNYMA	AEGEGEVCVK	GPNVFQGYLK	DPAKTAEALD	KDGWLHTGDI	GKWLPNGTLK	IIDRKKHIFK	LAQGEYIAPE
410	420	430	440	450	460	470	480
KIENIYMRSE	PVAQVFVHGE	SLQAFLIAIV	VPDVETLCSW	AQKRGFEGSF	EELCRNKDVK	KAILEDMVRL	GKDSGLKPFE
490	500	510	520	530			
QVKGITLHPE	LFSIDNGLLT	PTMKAKRPEL	RNYFRSQIDD	LYSTIKV			

Cmpd.	No. of Cmpds.	m/z meas.	Δ m/z [ppm]	z	Rt [min]	Score	P	Range	Sequence	Modification
707	1	647.7728	-66.33	2	37.7	14.3	1	67-78	R.GQRCGVEVTSMK.A	

Detailed Protein Report

Protein 614: SH3 domain-binding glutamic acid-rich-like protein [Homo sapiens]

**Accession:**	gi|4506925	**Score:**	14.3
**Database:**	refseq_human(refseq_human_20140103.fasta)	**MW [kDa]:**	12.8
**Database Date:**	2015-11-30	**pI:**	5.1
		**Sequence Coverage [%]:**	14.0
		**No. of unique Peptides:**	1

```
         10         20         30         40         50         60         70         80
MVIRVYIASS SGSTAIKKKQ QDVLGFLEAN KIGFEEKDIA ANEENRKWMR ENVPENSRPA TGYPLPPQIF NESQYRGDYD
         90        100        110        120
AFFEARENNA VYAFLGLTAP PGSKEAEVQA KQQA
```

Cmpd.	No. of Cmpds.	m/z meas.	Δ m/z [ppm]	z	Rt [min]	Score	P	Range	Sequence	Modification
2960	1	826.4281	-53.76	2	65.8	14.3	1	2-17	M.VIRVYIASSSGSTAIK.K	

Detailed Protein Report

Protein 615: **selenium-binding protein 1 isoform 2 [Homo sapiens]**

**Accession:**	gi|385137128	**Score:**	14.3
**Database:**	refseq_human(refseq_human_20140103.fasta)	**MW [kDa]:**	45.4
**Database Date:**	2015-11-30	**pI:**	5.2
**Modification(s):**	Oxidation	**Sequence Coverage [%]:**	4.9
		**No. of unique Peptides:**	1

10	20	30	40	50	60	70	80
MATKCGNCGP	GYSTPLEAMK	GPREEIVYLP	CIYRNTGTEA	PDYLATVDVD	PKSPQYCQVI	EPKDIHAKCE	LAFLHTSHCL
**90**	**100**	**110**	**120**	**130**	**140**	**150**	**160**
ASGEVMISSL	GDVKGNGKGG	FVLLDGETFE	VKGTWERPGG	AAPLGYDFWY	QPRHNVMIST	EWAAPNVLRD	GFNPADVEAG
**170**	**180**	**190**	**200**	**210**	**220**	**230**	**240**
LYGSHLYVWD	WQRHEIVQTL	SLKDGLIPLE	IRFLHNPDAA	QGFVGCALSS	TIQRFYKNEG	GTWSVEKVIQ	VPPKKVKGWL
**250**	**260**	**270**	**280**	**290**	**300**	**310**	**320**
LPEMPGLITD	ILLSLDDRFL	YFSNWLHGDL	RQYDISDPQR	PRLTGQLFLG	GSIVKGGPVQ	VLEDEELKSQ	PEPLVVKGKR
**330**	**340**	**350**	**360**	**370**	**380**	**390**	**400**
VAGGPQMIQL	SLDGKRLYIT	TSLYSAWDKQ	FYPDLIREGS	VMLQVDVDTV	KGGLKLNPNF	LVDFGKEPLG	PALAHELRYP
**410**	**420**						
GGDCSSDIWI							

Cmpd.	No. of Cmpds.	m/z meas.	Δ m/z [ppm]	z	Rt [min]	Score	P	Range	Sequence	Modification
1766	1	1045.8477	-101.94	2	50.4	14.3	1	1-20	-.MATKCGNCGPGYSTPLEAMK.G	Oxidation: 1, 19

Detailed Protein Report

Protein 616: **chymotrypsinogen B precursor [Homo sapiens]**

Accession: gi|118498341
Database: refseq_human(refseq_human_20140103.fasta)
Database Date: 2015-11-30
Modification(s): Carbamidomethyl, Oxidation

Score: 14.2
MW [kDa]: 27.7
pI: 7.7
Sequence Coverage [%]: 10.6
No. of unique Peptides: 1

Quantitation

WD:WU **Median:** 1.11 **CV:** 0.00 % **No. of Peptides:** 1

10	20	30	40	50	60	70	80
MASLWLLSCF	SLVGAAFGCG	VPAIHPVLSG	LSRIVNGEDA	VPGSWPWQVS	LQDKTGFHFC	GGSLISEDWV	VTAAHCGVRT
**90**	**100**	**110**	**120**	**130**	**140**	**150**	**160**
SDVVVAGEFD	QGSDEENIQV	LKIAKVFKNP	KFSILTVNND	ITLLKLATPA	RFSQTVSAVC	LPSADDDFPA	GTLCATTGWG
**170**	**180**	**190**	**200**	**210**	**220**	**230**	**240**
KTKYNANKTP	DKLQQAALPL	LSNAECKKSW	GRRITDVMIC	AGASGVSSCM	GDSGGPLVCQ	KDGAWTLVGI	VSWGSDTCST
**250**	**260**	**270**					
SSPGVYARVT	KLIPWVQKIL	AAN					

Cmpd.	No. of Cmpds.	m/z meas.	Δ m/z [ppm]	z	Rt [min]	Score	P	Range	Sequence	Modification	Ratios
323	1	939.4727	58.29	3	32.7	14.2	0	194-221	R.ITDVMICAGASGVSSCMGDSGGPD	Carbamidomethyl: 16, 26; Oxidation: 17	WD:WU 1.11

Detailed Protein Report

Protein 617: PREDICTED: putative uncharacterized protein FLJ46235-like [Homo sapiens]

**Accession:**	gi|530359082	**Score:**	14.2
**Database:**	refseq_human(refseq_human_20140103.fasta)	**MW [kDa]:**	24.4
**Database Date:**	2015-11-30	**pI:**	13.0
		**Sequence Coverage [%]:**	6.0
		**No. of unique Peptides:**	1

Alias proteins:

Accession	Name	Description
gi|530435845	refseq_human (refseq_human_20140103.fasta)	PREDICTED: putative uncharacterized protein FLJ46235-like [Homo sapiens]
gi|530386771	refseq_human (refseq_human_20140103.fasta)	PREDICTED: putative uncharacterized protein FLJ46235-like [Homo sapiens]

```
        10         20         30         40         50         60         70         80
MPSPSLQTAA SDSARAQIPA AFVRPKPPPA QLSRPTCGLA VASPGPDLAS RWPSPAVFLP ASQRPRQAHR MPLTGLWRSS
        90        100        110        120        130        140        150        160
SSLTVASPGP APARPDGVSR PRVSQVGPSR AQLLPPGRWP VRAQSVLKSA SPGPAPACRR PLDLRHGLCL LTVDPHQAQL
       170        180        190        200        210        220        230        240
LPQHGRLRPG SCSACGQPPR ARLSPLAILS RPRSVSSQPL QAQLSLPAAP AGPAPASQQP RSAQLLPSSW QPL
```

Cmpd.	No. of Cmpds.	m/z meas.	Δ m/z [ppm]	z	Rt [min]	Score	P	Range	Sequence	Modification
2544	1	694.4688	179.47	2	60.1	14.2	0	2-15	M.PSPSLQTAASDSAR.A	

Detailed Protein Report

Protein 618: **PREDICTED: probable E3 ubiquitin-protein ligase HECTD2 isoform X2 [Homo sapiens]**

**Accession:**	gi|530393122	**Score:**	14.2
**Database:**	refseq_human(refseq_human_20140103.fasta)	**MW [kDa]:**	76.8
**Database Date:**	2015-11-30	**pI:**	8.4
		**Sequence Coverage [%]:**	2.7
		**No. of unique Peptides:**	1

Quantitation

WD:WU **Median:** 0.88 **CV:** 0.00 % **No. of Peptides:** 1

10	20	30	40	50	60	70	80
MDASSSEMKA	PVLPEPILPI	QPKTVKDFQE	DVEKVKSSGD	WKAVHDFYLT	TFDSFPELNA	AFKKDATASF	NTIEDSGINA
**90**	**100**	**110**	**120**	**130**	**140**	**150**	**160**
KFVNAVYDTL	LNTPQDVQKT	VLKGIINSLL	REWKGPRTKD	DLRAYFILLQ	NPQFNNTSTY	VIYAHLLRQI	ATLVEADHHF
**170**	**180**	**190**	**200**	**210**	**220**	**230**	**240**
LVHWFKKLSQ	KRFKQLVERL	LQFISLRLFP	AKPEEFPPIT	KCSWWIPSAA	KVLALLNTAN	NLVHPPLIPY	TDFYNSTLDH
**250**	**260**	**270**	**280**	**290**	**300**	**310**	**320**
IDLMEEYHTW	QNFGNSHRFS	FCQYPFVISV	AAKKIIIQRD	SEQQMINIAR	QSLVDKVSRR	QRPDMNILFL	NMKVRRTHLV
**330**	**340**	**350**	**360**	**370**	**380**	**390**	**400**
SDSLDELTRK	RADLKKKLKV	TFVGEAGLDM	GGLTKEWFLL	LIRQIFHPDY	GMFTYHKDSH	CHWFSSFKCD	NYSEFRLVGI
**410**	**420**	**430**	**440**	**450**	**460**	**470**	**480**
LMGLAVYNSI	TLDIRFPPCC	YKKLLSPPII	PSDQNIPVGI	CNVTVDDLCQ	IMPELAHGLS	ELLSHEGNVE	EDFYSTFQVF
**490**	**500**	**510**	**520**	**530**	**540**	**550**	**560**
QEEFGIIKSY	NLKPGGDKIS	VTNQNRKEYV	QLYTDFLLNK	SIYKQFAAFY	YGFHSVCASN	ALMLLRPEEV	EILVCGSPDL
**570**	**580**	**590**	**600**	**610**	**620**	**630**	**640**
DMHALQRSTQ	YDGYAKTDLT	IKYFWDVVLG	FPLDLQKKLL	HFTTGSDRVP	VGGMADLNFK	ISKNETSTNC	LPVAHTCFNQ
**650**	**660**	**670**					
LCLPPYKSKK	DLKQKLIIGI	SNSEGFGLE					

Cmpd.	No. of Cmpds.	m/z meas.	Δ m/z [ppm]	z	Rt [min]	Score	P	Range	Sequence	Modification	Ratios
1946	1	918.3545	-158.85	2	52.7	14.2	1	338-355	K.LKVTFVGEAGLDMGGLTK.E		WD:WU 0.88

Detailed Protein Report

Protein 619: cardiomyopathy-associated protein 5 [Homo sapiens]

**Accession:**	gi|62241003	**Score:**	14.2
**Database:**	refseq_human(refseq_human_20140103.fasta)	**MW [kDa]:**	448.9
**Database Date:**	2015-11-30	**pI:**	4.6
		**Sequence Coverage [%]:**	0.3
		**No. of unique Peptides:**	1

Detailed Protein Report

10	20	30	40	50	60	70	80
MASRDSNHAG	ESFLGSDGDE	EATRELETEE	ESEGEEDETA	AESEEEPDSR	LSDQDEEGKI	KQEYIISDPS	FSMVTVQRED
90	100	110	120	130	140	150	160
SGITWETNSS	RSSTPWASEE	SQTSGVCSRE	GSTVNSPPGN	VSFIVDEVKK	VRKRTHKSKH	GSPSLRRKGN	RKRNSFESQD
170	180	190	200	210	220	230	240
VPTNKKGSPL	TSASQVLTTE	KEKSYTGIYD	KARKKKTTSN	TPPITGAIYK	EHKPLVLRPV	YIGTVQYKIK	MFNSVKEELI
250	260	270	280	290	300	310	320
PLQFYGTLPK	GYVIKEIHYR	KGKDASISLE	PDLDNSGSNT	VSKTRKLVAQ	SIEDKVKEVF	PPWRGALSKG	SESLTLMFSH
330	340	350	360	370	380	390	400
EDQKKIYADS	PLNATSALEH	TVPSYSSSGR	AEQGIQLRHS	QSVPQQPEDE	AKPHEVEPPS	VTPDTPATMF	LRTTKEECEL
410	420	430	440	450	460	470	480
ASPGTAASEN	DSSVSPSFAN	EVKKEDVYSA	HHSISLEAAS	PGLAASTQDG	LDPDQEQPDL	TSIERAEPVS	AKLTPTHPSV
490	500	510	520	530	540	550	560
KGEKEENMLE	PSISLSEPLM	LEEPEKEEIE	TSLPIAITPE	PEDSNLVEEE	IVELDYPESP	LVSEKPFPPH	MSPEVEHKEE
570	580	590	600	610	620	630	640
ELILPLLAAS	SPEHVALSEE	EREEIASVST	GSAFVSEYSV	PQDLNHELQE	QEGEPVPPSN	VEAIAEHAVL	SEEENEEFEA
650	660	670	680	690	700	710	720
YSPAAAPTSE	SSLSPSTTEK	TSENQSPLFS	TVTPEYMVLS	GDEASESGCY	TPDSTSASEY	SVPSLATKES	LKKTIDRKSP
730	740	750	760	770	780	790	800
LILKGVSEYM	IPSEEKEDTG	SFTPAVAPAS	EPSLSPSTTE	KTSECQSPLP	STATSEHVVP	SEGEDLGSER	FTPDSKLISK
810	820	830	840	850	860	870	880
YAAPLNATQE	SQKKIINEAS	QFKPKGISEH	TVLSVDGKEV	IGPSSPDLVV	ASEHSFPPHT	TEMTSECQAP	PLSATPSEYV
890	900	910	920	930	940	950	960
VLSDEEAVEL	ERYTPSSTSA	SEFSVPPYAT	PEAQEEEIVH	RSLNLKGASS	PMNLSEEDQE	DIGPFSPDSA	FVSEFSFPPY
970	980	990	1000	1010	1020	1030	1040
ATQEAEKREF	ECDSPICLTS	PSEHTILSDE	DTEEAELFSP	DSASQVSIPP	FRISETEKNE	LEPDSLLTAV	SASGYSCFSE
1050	1060	1070	1080	1090	1100	1110	1120
ADEEDIGSTA	ATPVSEQFSS	SQKQKAETFP	LMSPLEDLSL	PPSTDKSEKA	EIKPEIPTTS	TSVSEYLILA	QKQKTQAYLE
1130	1140	1150	1160	1170	1180	1190	1200
PESEDLIPSH	LTSEVEKGER	EASSSVAAIP	AALPAQSSIV	KEETKPASPH	SVLPDSVPAI	KKEQEPTAAL	TLKAADEQMA
1210	1220	1230	1240	1250	1260	1270	1280
LSKVRKEEIV	PDSQEATAHV	SQDQKMEPQP	PNVPESEMKY	SVLPDMVDEP	KKGVKPKLVL	NVTSELEQRK	LSKNEPEVIK
1290	1300	1310	1320	1330	1340	1350	1360
PYSPLKETSL	SGPEALSAVK	MEMKHDSKIT	TTPIVLHSAS	SGVEKQVEHG	PPALAFSALS	EEIKKEIEPS	SSTTTASVTK
1370	1380	1390	1400	1410	1420	1430	1440
LDSNLTRAVK	EEIPTDSSLI	TPVDRPVLTK	VGKGELGSGL	PPLVTSADEH	SVLAEEDKVA	IKGASPIETS	SKHLAWSEAE
1450	1460	1470	1480	1490	1500	1510	1520
KEIKFDSLPS	VSSIAEHSVL	SEVEAKEVKA	GLPVIKTSSS	QHSDKSEEAR	VEDKQDLLFS	TVCDSERLVS	SQKKSLMSTS
1530	1540	1550	1560	1570	1580	1590	1600
EVLEPEHELP	LSLWGEIKKK	ETELPSSQNV	SPASKHIIPK	GKDEETASSS	PELENLASGL	APTLLLLSDD	KNKPAVEVSS
1610	1620	1630	1640	1650	1660	1670	1680
TAQGDFPSEK	QDVALAELSL	EPEKKDKPHQ	PLELPNAGSE	FSSDLGRQSG	SIGTKQAKSP	ITETEDSVLE	KGPAELRSRE
1690	1700	1710	1720	1730	1740	1750	1760
GKEENRELCA	SSTMPAISEL	SSLLREESQN	EEIKPFSPKI	ISLESKEPPA	SVAEGGNPEE	FQPFTFSLKG	LSEEVSHPAD
1770	1780	1790	1800	1810	1820	1830	1840
FKKGGNQEIG	PLPPTGNLKA	QVMGDILDKL	SEETGHPNSS	QVLQSITEPS	KIAPSDLLVE	QKKTEKALHS	DQTVKLPDVS
1850	1860	1870	1880	1890	1900	1910	1920
TSSEDKQDLG	IKQFSLMREN	LPLEQSKSFM	TTKPADVKET	KMEEFFISPK	DENWMLGKPE	NVASQHEQRI	AGSVQLDSSS
1930	1940	1950	1960	1970	1980	1990	2000
SNELRPGQLK	AAVSSKDHTC	EVRKQVLPHS	AEESHLSSQE	AVSALDTSSG	NTETLSSKSY	SSEEVKLAEE	PKSLVLAGNV
2010	2020	2030	2040	2050	2060	2070	2080
ERNIAEGKEI	HSLMESESLL	LEKANTELSW	PSKEDSQEKI	KLPPERFFQK	PVSGLSVEQV	KSETISSSVK	TAHFPAEGVE
2090	2100	2110	2120	2130	2140	2150	2160
PALGNEKEAH	RSTPPFPEEK	PLEESKMVQS	KVIDDADEGK	KPSPEVKIPT	QRKPISSIHA	REPQSPESPE	VTQNPPTQPK
2170	2180	2190	2200	2210	2220	2230	2240

Detailed Protein Report

Cmpd.	No. of Cmpds.	m/z meas.	Δ m/z [ppm]	z	Rt [min]	Score	P	Range	Sequence	Modification
1989	3	703.2718	-183.94	2	53.2	14.2	2	251-261	K.GYVIKEIHYRK.G	

Detailed Protein Report

Protein 620: PREDICTED: uncharacterized protein C12orf42 isoform X5 [Homo sapiens]

**Accession:**	gi|578823684	**Score:**	14.2
**Database:**	refseq_human(refseq_human_20140103.fasta)	**MW [kDa]:**	25.8
**Database Date:**	2015-11-30	**pI:**	11.7
		**Sequence Coverage [%]:**	6.4
		**No. of unique Peptides:**	1

```
        10         20         30         40         50         60         70         80
MSTVICMKQR EEEFLLTIRP FANRMQKSPC YIPIVSSATL WDRSTPSAKH IPCYERTSVP CSRFINHMKN FSESPKFRSL
        90        100        110        120        130        140        150        160
HFLNFPGSAA RPSTAIGLCR RSQTPGALQS TGPSNTELEP EERMAVPAGA QAHPDDIQSR LLGASGNPVG KGAVAMAPEM
       170        180        190        200        210        220        230        240
LPKHPHTPRD RRPQADTSLH GNLAGAPLPL LAGASTHFPS KRLIKVCSSA PPRPTRRFHT VCSQALSRPV VNAHLH
```

Cmpd.	No. of Cmpds.	m/z meas.	Δ m/z [ppm]	z	Rt [min]	Score	P	Range	Sequence	Modification
2634	1	841.1201	156.92	2	61.2	14.2	2	202-216	K.RLIKVCSSAPPRPTR.R	

Detailed Protein Report

Protein 621: heat shock 70 kDa protein 13 precursor [Homo sapiens]

**Accession:**	gi|48928056	**Score:**	14.2
**Database:**	refseq_human(refseq_human_20140103.fasta)	**MW [kDa]:**	51.9
**Database Date:**	2015-11-30	**pI:**	5.4
		**Sequence Coverage [%]:**	3.6
		**No. of unique Peptides:**	1

10	20	30	40	50	60	70	80
MAREMTILGS	AVLTLLLAGY	LAQQYLPLPT	PKVIGIDLGT	TYCSVGVFFP	GTGKVKVIPD	ENGHISIPSM	VSFTDNDVYV
**90**	**100**	**110**	**120**	**130**	**140**	**150**	**160**
GYESVELADS	NPQNTIYDAK	RFIGKIFTAE	ELEAEIGRYP	FKVLNKNGMV	EFSVTSNETI	TVSPEYVGSR	LLLKLKEMAE
**170**	**180**	**190**	**200**	**210**	**220**	**230**	**240**
AYLGMPVANA	VISVPAEFDL	KQRNSTIEAA	NLAGLKILRV	INEPTAAAMA	YGLHKADVFH	VLVIDLGGGT	LDVSLLNKQG
**250**	**260**	**270**	**280**	**290**	**300**	**310**	**320**
GMFLTRAMSG	NNKLGGQDFN	QRLLQYLYKQ	IYQTYGFVPS	RKEEIHRLRQ	AVEMVKLNLT	LHQSAQLSVL	LTVEEQDRKE
**330**	**340**	**350**	**360**	**370**	**380**	**390**	**400**
PHSSDTELPK	DKLSSADDHR	VNSGFGRGLS	DKKSGESQVL	FETEISRKLF	DTLNEDLFQK	ILVPIQQVLK	EGHLEKTEID
**410**	**420**	**430**	**440**	**450**	**460**	**470**	**480**
EVVLVGGSTR	IPRIRQVIQE	FFGKDPNTSV	DPDLAVVTGV	AIQAGIDGGS	WPLQVSALEI	PNKHLQKTNF	N

Cmpd.	No. of Cmpds.	m/z meas.	Δ m/z [ppm]	z	Rt [min]	Score	P	Range	Sequence	Modification
1869	1	920.9474	-68.00	2	52.0	14.2	1	397-413	K.TEIDEVVLVGGSTRIPR.I	

BRUKER

Detailed Protein Report

Protein 622: mitogen-activated protein kinase kinase kinase 11 [Homo sapiens]

**Accession:**	gi|4505195	**Score:**	14.1
**Database:**	refseq_human(refseq_human_20140103.fasta)	**MW [kDa]:**	92.6
**Database Date:**	2015-11-30	**pI:**	9.4
		**Sequence Coverage [%]:**	2.6
		**No. of unique Peptides:**	1

10	20	30	40	50	60	70	80
MEPLKSLFLK	SPLGSWNGSG	SGGGGGGGGG	RPEGSPKAAG	YANPVWTALF	DYEPSGQDEL	ALRKGDRVEV	LSRDAAISGD
90	100	110	120	130	140	150	160
EGWWAGQVGG	QVGIFPSNYV	SRGGGPPPCE	VASFQELRLE	EVIGIGGFGK	VYRGSWRGEL	VAVKAARQDP	DEDISVTAES
170	180	190	200	210	220	230	240
VRQEARLFAM	LAHPNIIALK	AVCLEEPNLC	LVMEYAAGGP	LSRALAGRRV	PPHVLVNWAV	QIARGMHYLH	CEALVPVIHR
250	260	270	280	290	300	310	320
DLKSNNILLL	QPIESDDMEH	KTLKITDFGL	AREWHKTTQM	SAAGTYAWMA	PEVIKASTFS	KGSDVWSFGV	LLWELLTGEV
330	340	350	360	370	380	390	400
PYRGIDCLAV	AYGVAVNKLT	LPIPSTCPEP	FAQLMADCWA	QDPHRRPDFA	SILQQLEALE	AQVLREMPRD	SFHSMQEGWK
410	420	430	440	450	460	470	480
REIQGLFDEL	RAKEKELLSR	EEELTRAARE	QRSQAEQLRR	REHLLAQWEL	EVFERELTLL	LQQVDRERPH	VRRRRGTFKR
490	500	510	520	530	540	550	560
SKLRARDGGE	RISMPLDFKH	RITVQASPGL	DRRRNVFEVG	PGDSPTFPRF	RAIQLEPAEP	GQAWGRQSPR	RLEDSSNGER
570	580	590	600	610	620	630	640
RACWAWGPSS	PKPGEAQNGR	RRSRMDEATW	YLDSDDSSPL	GSPSTPPALN	GNPPRPSLEP	EEPKRPVPAE	RGSSSGTPKL
650	660	670	680	690	700	710	720
IQRALLRGTA	LLASLGLGRD	LQPPGGPGRE	RGESPTTPPT	PTPAPCPTEP	PPSPLICFSL	KTPDSPPTPA	PLLLDLGIPV
730	740	750	760	770	780	790	800
GQRSAKSPRR	EEEPRGGTVS	PPPGTSRSAP	GTPGTPRSPP	LGLISRPRPS	PLRSRIDPWS	FVSAGPRPSP	LPSPQPAPRR
810	820	830	840	850			
APWTLFPDSD	PFWDSPPANP	FQGGPQDCRA	QTKDMGAQAP	WVPEAGP			

Cmpd.	No. of Cmpds.	m/z meas.	Δ m/z [ppm]	z	Rt [min]	Score	P	Range	Sequence	Modification
1232	1	752.1726	114.82	3	44.0	14.1	0	702-723	K.TPDSPPTPAPLLLDLGIPVGQR.S	

Detailed Protein Report

Protein 623: dynein heavy chain 17, axonemal [Homo sapiens]

**Accession:**	gi|256542310	**Score:**	14.1
**Database:**	refseq_human(refseq_human_20140103.fasta)	**MW [kDa]:**	509.0
**Database Date:**	2015-11-30	**pI:**	5.4
		**Sequence Coverage [%]:**	0.3
		**No. of unique Peptides:**	1

Detailed Protein Report

10	20	30	40	50	60	70	80
MTMAPDVRLE	YLEEVASIVL	KFKPDKWSKL	IGAEENVALF	TEFFEKPDVQ	VLVLTLNAAG	MIIPCLGFPQ	SLKSKGVYFI
90	100	110	120	130	140	150	160
KTKSENINKD	NYRARLLYGD	ISPTPVDQLI	AVVEEVLSSL	LNQSENMAGW	PQVVSEDIVK	QVHRLKNEMF	VMSGKIKGKT
170	180	190	200	210	220	230	240
LLPIPEHLGS	LDGTLESMER	IPSSLDNLLL	HAIETTIIDW	SHQIRDVLSK	DSAQALLDGL	HPLPQVEFEF	WDTRLLNLKC
250	260	270	280	290	300	310	320
IHEQLNRPKV	NKIVEILEKA	KSCYWPALQN	VYTNVTEGLK	EANDIVLYLK	PLRILLEEME	QADFTMLPTF	IAKVLDTICF
330	340	350	360	370	380	390	400
IWATSEYYNT	PARIIVILQE	FCNQIIEMTR	TFLSPEEVLK	GLQGEIEEVL	SGISLAVNVL	KELYQTYDFC	CVNMKLFFKD
410	420	430	440	450	460	470	480
KEPVPWEFPS	SLAFSRINSF	FQRIQTIEEL	YKTAIEFLKL	EKIELGGVRG	NLLGSLVTRI	YDEVFELVKV	FADCKYDPLD
490	500	510	520	530	540	550	560
PGDSNFDRDY	ADFEIKIQDL	DRRLATIFCQ	GFDDCSCIKS	SAKLLYMCGG	LMERPLILAE	VAPRYSVMLE	LFDAELDNAK
570	580	590	600	610	620	630	640
ILYDAQMAAS	EEGNIPLIHK	NMPPVAGQLK	WSLELQERLE	VSMKHLKHVE	HPVMSGAEAK	LTYQKYDEMM	ELLRCHREKI
650	660	670	680	690	700	710	720
YQQWVAGVDQ	DCHFNLGQPL	ILRDAASNLI	HVNFSKALVA	VLREVKYLNF	QQQKEIPDSA	ESLFSENETF	RKFVGNLELI
730	740	750	760	770	780	790	800
VGWYNEIKTI	VKAVEFLLIK	SELEAIDVKL	LSAETTLFWN	GEGVFQYIQE	VREILHNLQN	RMQKAKQNIE	GISQAMKDWS
810	820	830	840	850	860	870	880
ANPLFERKDN	KKEALLDLDG	RIANLNKRYA	AVRDAGVKIQ	AMVAENAELF	RADTLSLPWK	DYVIYIDDMV	LDEFDQFIRK
890	900	910	920	930	940	950	960
SLSFLMDNMV	IDESIAPLFE	IRMELDEDGL	TFNPTLEVGS	DRGFLALIEG	LVNDIYNVAR	LIPRLAKDRM	NYKMDLEDNT
970	980	990	1000	1010	1020	1030	1040
DLIEMREEVS	SLVINAMKEA	EEYQDSFERY	SYLWTDNLQE	FMKNFLIYGC	AVTAEDLDTW	TDDTIPKTPP	TLAQFQEQID
1050	1060	1070	1080	1090	1100	1110	1120
SYEKLYEEVS	KCENTKVFHG	WLQCDCRPFK	QALLSTIRRW	GFMFKRHLSN	HVTNSLADLE	AFMKVARMGL	TKPLKEGDYD
1130	1140	1150	1160	1170	1180	1190	1200
GLVEVMGHLM	KVKERQAATD	NMFEPLKQTI	ELLKTYGEEM	PEEIHLKLQE	LPEHWANTKK	LAIQVKLTVA	PLQANEVSIL
1210	1220	1230	1240	1250	1260	1270	1280
RRKCQQFELK	QHEFRERFRR	EAPFSFSDPN	PYKSLNKQQK	SISAMEGIME	ALSKSGGLFE	VPVPDYKQLK	ACHREVRLLK
1290	1300	1310	1320	1330	1340	1350	1360
ELWDMVVVVN	TSIEDWKTTK	WKDINVEQMD	IDCKKFAKDM	RSLDKEMKTW	DAFVGLDNTV	KNVITSLRAV	SELQNPAIRE
1370	1380	1390	1400	1410	1420	1430	1440
RHWQQLMQAT	QVKFKMSEET	TLADLLQLNL	HSYEDEVRNI	VDKAVKESGM	EKVLKALDST	WSMMEFQHEP	HPRTGTMMLK
1450	1460	1470	1480	1490	1500	1510	1520
SSEVLVETLE	DNQVQLQNLM	MSKYLAHFLK	EVTSWQQKLS	TADSVISIWF	EVQRTWSHLE	SIFIGSEDIR	TQLPGDSQRF
1530	1540	1550	1560	1570	1580	1590	1600
DDINQEFKAL	MEDAVKTPNV	VEATSKPGLY	NKLEALKKSL	AICEKALAEY	LETKRLAFPR	FYFVSSADLL	DILSNGNDPV
1610	1620	1630	1640	1650	1660	1670	1680
EVSRHLSKLF	DSLCKLKFRL	DASDKPLKVG	LGMYSKEDEY	MVFDQECDLS	GQVEVWLNRV	LDRMCSTLRH	EIPEAVVTYE
1690	1700	1710	1720	1730	1740	1750	1760
EKPREQWILD	YPAQVALTCT	QIWWTTEVGL	AFARLEEGYE	NAIRDYNKKQ	ISQLNVLITL	LMGNLNAGDR	MKIMTICTID
1770	1780	1790	1800	1810	1820	1830	1840
VHARDVVAKM	IVAKVESSQA	FTWQAQLRHR	WDEEKRHCFA	NICDAQIQYS	YEYLGNTPRL	VITPLTDRCY	ITLTQSLHLI
1850	1860	1870	1880	1890	1900	1910	1920
MGGAPAGPAG	TGKTETTKDL	GRALGTMVYV	FNCSEQMDYK	SCGNIYKGLA	QTGAWGCFDE	FNRISVEVLS	VIAVQVKCVQ
1930	1940	1950	1960	1970	1980	1990	2000
DAIRAKKKAF	NFLGEIIGLI	PTVGIFITMN	PGYAGRAELP	ENLKALFRPC	AMVVPDFELI	CEIMLMAEGF	LEARLLARKF
2010	2020	2030	2040	2050	2060	2070	2080
ITLYTLCKEL	LSKQDHYDWG	LRAIKSVLVV	AGSLKRGDPS	RAEDQVLMRA	LRDFNIPKIV	TDDLPVFMGL	IGDLFPALDV
2090	2100	2110	2120	2130	2140	2150	2160
PRKRDLNFEK	IIKQSIVELK	LQAEDSFVLK	VVQLEELLQV	RHSVFIVGNA	GSGKSQVLKS	LNKTYQNLKR	KPVAVDLDPK
2170	2180	2190	2200	2210	2220	2230	2240

Detailed Protein Report

Cmpd.	No. of Cmpds.	m/z meas.	Δ m/z [ppm]	z	Rt [min]	Score	P	Range	Sequence	Modification
2850	1	869.4224	-91.80	2	64.2	14.1	0	3728-3742	K.LIFLAQVTFQVLSMK.K	

Detailed Protein Report

Protein 624: zinc finger imprinted 2 [Homo sapiens]

**Accession:**	gi|33354273	**Score:**	14.1
**Database:**	refseq_human(refseq_human_20140103.fasta)	**MW [kDa]:**	61.1
**Database Date:**	2015-11-30	**pI:**	6.3
**Modification(s):**	Carbamidomethyl	**Sequence Coverage [%]:**	2.8
		**No. of unique Peptides:**	1

Alias proteins:

Accession	Name	Description
gi|226423929	refseq_human (refseq_human_20140103.fasta)	zinc finger imprinted 2 [Homo sapiens]
gi|226423927	refseq_human (refseq_human_20140103.fasta)	zinc finger imprinted 2 [Homo sapiens]

10	20	30	40	50	60	70	80
MYQPEDDNNS	DVTSDDDMTR	NRRESSPPHS	VHSFSGDRDW	DRRGRSRDME	PRDRWSHTRN	PRSRMPPRDL	SLPVVAKTSF
90	100	110	120	130	140	150	160
EMDREDDRDS	RAYESRSQDA	ESYQNVVDLA	EDRKPHNTIQ	DNMENYRKLL	SLGFLAQDSV	PAEKRNTEML	DNLPSAGSQF
170	180	190	200	210	220	230	240
PDFKHLGTFL	VFEELVTFED	VLVDFSPEEL	SSLSAAQRNL	YREVMLENYR	NLVSLGHQFS	KPDIISRLEE	EESYAMETDS
250	260	270	280	290	300	310	320
RHTVICQGES	HDDPLEPHQG	NQEKLLTPIT	MNDPKTLTPE	RSYGSDEFER	SSNLSKQSKD	PLGKDPQEGT	APGICTSPQS
330	340	350	360	370	380	390	400
ASQENKHNRC	EFCKRTFSTQ	VALRRHERIH	TGKKPYECKQ	CAEAFYLMPH	LNRHQKTHSG	RKTSGCNEGR	KPSVQCANLC
410	420	430	440	450	460	470	480
ERVRIHSQED	YFECFQCGKA	FLQNVHLLQH	LKAHEAARVL	PPGLSHSKTY	LIRYQRKHDY	VGERACQCCD	CGRVFSRNSY
490	500	510	520	530			
LIQHYRTHTQ	ERPYQCQLCG	KCFGRPSYLT	QHYQLHSQEK	TVECDHC			

Cmpd.	No. of Cmpds.	m/z meas.	Δ m/z [ppm]	z	Rt [min]	Score	P	Range	Sequence	Modification
2843	1	616.8649	-144.67	3	64.2	14.1	0	487-501	R.THTQERPYQCQLCGK.C	Carbamidomethyl: 13

Detailed Protein Report

Protein 625: zinc finger protein 688 isoform b [Homo sapiens]

Accession: gi|67010013
Database: refseq_human(refseq_human_20140103.fasta)
Database Date: 2015-11-30

Score: 14.1
MW [kDa]: 28.9
pI: 11.6
Sequence Coverage [%]: 6.5
No. of unique Peptides: 1

10	20	30	40	50	60	70	80
MPRSPLRSSL	STEASGPRGP	FKCPASPRPI	TAQLLPGSSP	IIGRCVLRVQ	RGFPGPKPAL	ISWMEQESEA	WSPAAQDPEK
**90**	**100**	**110**	**120**	**130**	**140**	**150**	**160**
GERLGGARRG	DVPNRKEEEP	EEVPRAKGPR	KAPVKESPEV	LVERNPDPAI	SVAPARAQPP	KNAAWDPTTG	AQPPAPIPSM
**170**	**180**	**190**	**200**	**210**	**220**	**230**	**240**
DAQAGQRRHV	CTDCGRRFTY	PSLLVSHRRM	HSGERPFPCP	ECGMRFKRKF	AVEAHQWIHR	SCSGGRRGRR	PGIRAVPRAP
**250**	**260**	**270**					
VRGDRDPPVL	FRHYPDIFEE	CG					

Cmpd.	No. of Cmpds.	m/z meas.	Δ m/z [ppm]	z	Rt [min]	Score	P	Range	Sequence	Modification
1884	1	600.0016	18.70	3	52.2	14.1	2	2-18	M.PRSPLRSSLSTEASGPR.G	

Detailed Protein Report

Protein 626: PREDICTED: zinc finger protein 197 isoform X2 [Homo sapiens]

Accession: gi|578805510
Database: refseq_human(refseq_human_20140103.fasta)
Database Date: 2015-11-30
Modification(s): Carbamidomethyl, Oxidation

Score: 14.0
MW [kDa]: 96.1
pI: 10.1
Sequence Coverage [%]: 1.2
No. of unique Peptides: 1

10	20	30	40	50	60	70	80
MALMLLTAQP	QELVMFEEVS	VCFTSEEWAC	LGPIQRALYW	DVMLENYGNV	TSLEWETMTE	NEEVTSKPSS	SQRADSHKGT
**90**	**100**	**110**	**120**	**130**	**140**	**150**	**160**
SKRLQGSVPQ	VLDFEEECEW	QVLASQWGNE	TDERADTVKK	VSLCERDKKK	RTPPEKQGQK	WKELGDSLTF	GSAISESLIG
**170**	**180**	**190**	**200**	**210**	**220**	**230**	**240**
TEGKKFYKCD	MCCKHFNKIS	HLINHRRIHT	GEKPHKCKEC	GKGFIQRSSL	LMHLRNHSGE	KPYKCNECGK	AFSQSAYLLN
**250**	**260**	**270**	**280**	**290**	**300**	**310**	**320**
HQRIHTGEKP	YKCKECGKGF	YRHSGLIIHL	RRHSGERPYK	CNECGKVFSQ	NAYLIDHQRL	HKGEEPYKCN	KCQKAFILKK
**330**	**340**	**350**	**360**	**370**	**380**	**390**	**400**
SLILHQRIHS	GEKPYKCDEC	GKTFAQTTYL	IDHQRLHSAE	NPYKCKECGK	VFIRSKSLLL	HQRVHTEKKT	FGCKKCGKIF
**410**	**420**	**430**	**440**	**450**	**460**	**470**	**480**
SSKSNFIDHK	RMHSREKPYK	CTECGKAFTQ	SAYLFDHQRL	HNGEKPYECN	ECGKVFILKK	SLILHQRFHT	GENLYECKDC
**490**	**500**	**510**	**520**	**530**	**540**	**550**	**560**
GKVFGSNRNL	IDHERLHNGE	KPYECRECGK	TFIMSKSFMV	HQKLHTQEKA	YKCEDCGKAF	SYNSSLLVHR	RIHTGEKPFE
**570**	**580**	**590**	**600**	**610**	**620**	**630**	**640**
CSECGRAFSS	NRNLIEHKRI	HSGEKPYECD	ECGKCFILKK	SLIGHQRIHT	REKSYKCNDC	GKVFSYRSNL	IAHQRIHTGE
**650**	**660**	**670**	**680**	**690**	**700**	**710**	**720**
KPYACSECGK	GFTYNRNLIE	HQRIHSGEKT	YECHVCRKVL	TSSRNLMVHQ	RIHTGEKPYK	CNECGKDFSQ	NKNLVVHQRM
**730**	**740**	**750**	**760**	**770**	**780**	**790**	**800**
HTGEKPYECD	KCRKSFTSKR	NLVGHQRIHT	GEKPYGCNDC	SKVFRQRKNL	TVHQKIHTDE	KPCECDVSEK	EFSQTSNLHL
**810**	**820**	**830**					
QQKIHTIEEF	SWLQNTNESK	IEIQKI					

Cmpd.	No. of Cmpds.	m/z meas.	Δ m/z [ppm]	z	Rt [min]	Score	P	Range	Sequence	Modification
1461	1	671.4129	203.04	2	46.6	14.0	2	165-174	K.KFYKCDMCCK.H	Carbamidomethyl: 8; Oxidation: 7

Detailed Protein Report

Protein 627: ribosomal protein S6 kinase alpha-1 isoform b [Homo sapiens]

Accession: gi|55743134
Database: refseq_human(refseq_human_20140103.fasta)
Database Date: 2015-11-30

Score: 14.0
MW [kDa]: 83.9
pI: 9.5
Sequence Coverage [%]: 2.3
No. of unique Peptides: 1

10	20	30	40	50	60	70	80
MEQDPKPPRL	RLWALIPWLP	RKQRPRISQT	SLPVPGPGSG	PQRDSDEGVL	KEISITHHVK	AGSEKADPSH	FELLKVLGQG
**90**	**100**	**110**	**120**	**130**	**140**	**150**	**160**
SFGKVFLVRK	VTRPDSGHLY	AMKVLKKATL	KVRDRVRTKM	ERDILADVNH	PFVVKLHYAF	QTEGKLYLIL	DFLRGGDLFT
**170**	**180**	**190**	**200**	**210**	**220**	**230**	**240**
RLSKEVMFTE	EDVKFYLAEL	ALGLDHLHSL	GIIYRDLKPE	NILLDEEGHI	KLTDFGLSKE	AIDHEKKAYS	FCGTVEYMAP
**250**	**260**	**270**	**280**	**290**	**300**	**310**	**320**
EVVNRQGHSH	SADWWSYGVL	MFEMLTGSLP	FQGKDRKETM	TLILKAKLGM	PQFLSTEAQS	LLRALFKRNP	ANRLGSGPDG
**330**	**340**	**350**	**360**	**370**	**380**	**390**	**400**
AEEIKRHVFY	STIDWNKLYR	REIKPPFKPA	VAQPDDTFYF	DTEFTSRTPK	DSPGIPPSAG	AHQLFRGFSF	VATGLMEDDG
**410**	**420**	**430**	**440**	**450**	**460**	**470**	**480**
KPRAPQAPLH	SVVQQLHGKN	LVFSDGYVVK	ETIGVGSYSE	CKRCVHKATN	MEYAVKVIDK	SKRDPSEEIE	ILLRYGQHPN
**490**	**500**	**510**	**520**	**530**	**540**	**550**	**560**
IITLKDVYDD	GKHVYLVTEL	MRGGELLDKI	LRQKFFSERE	ASFVLHTIGK	TVEYLHSQGV	VHRDLKPSNI	LYVDESGNPE
**570**	**580**	**590**	**600**	**610**	**620**	**630**	**640**
CLRICDFGFA	KQLRAENGLL	MTPCYTANFV	APEVLKRQGY	DEGCDIWSLG	ILLYTMLAGY	TPFANGPSDT	PEEILTRIGS
**650**	**660**	**670**	**680**	**690**	**700**	**710**	**720**
GKFTLSGGNW	NTVSETAKDL	VSKMLHVDPH	QRLTAKQVLQ	HPWVTQKDKL	PQSQLSHQDL	QLVKGAMAAT	YSALNSSKPT
**730**	**740**	**750**					
PQLKPIESSI	LAQRRVRKLP	STTL					

Cmpd.	No. of Cmpds.	m/z meas.	Δ m/z [ppm]	z	Rt [min]	Score	P	Range	Sequence	Modification
1051	1	839.4103	-46.72	2	41.9	14.0	0	27-43	R.ISQTSLPVPGPGSGPQR.D	

Detailed Protein Report

Protein 628: cytokine receptor common subunit beta precursor [Homo sapiens]

**Accession:**	gi|4559408	**Score:**	14.0
**Database:**	refseq_human(refseq_human_20140103.fasta)	**MW [kDa]:**	97.3
**Database Date:**	2015-11-30	**pI:**	5.2
		**Sequence Coverage [%]:**	2.1
		**No. of unique Peptides:**	1

10	20	30	40	50	60	70	80
MVLAQGLLSM	ALLALCWERS	LAGAEETIPL	QTLRCYNDYT	SHITCRWADT	QDAQRLVNVT	LIRRVNEDLL	EPVSCDLSDD
90	100	110	120	130	140	150	160
MPWSACPHPR	CVPRRCVIPC	QSFVVTDVDY	FSFQPDRPLG	TRLTVTLTQH	VQPPEPRDLQ	ISTDQDHFLL	TWSVALGSPQ
170	180	190	200	210	220	230	240
SHWLSPGDLE	FEVVYKRLQD	SWEDAAILLS	NTSQATLGPE	HLMPSSTYVA	RVRTRLAPGS	RLSGRPSKWS	PEVCWDSQPG
250	260	270	280	290	300	310	320
DEAQPQNLEC	FFDGAAVLSC	SWEVRKEVAS	SVSFGLFYKP	SPDAGEEECS	PVLREGLGSL	HTRHHCQIPV	PDPATHGQYI
330	340	350	360	370	380	390	400
VSVQPRRAEK	HIKSSVNIQM	APPSLNVTKD	GDSYSLRWET	MKMRYEHIDH	TFEIQYRKDT	ATWKDSKTET	LQNAHSMALP
410	420	430	440	450	460	470	480
ALEPSTRYWA	RVRVRTSRTG	YNGIWSEWSE	ARSWDTESVL	PMWVLALIVI	FLTIAVLLAL	RFCGIYGYRL	RRKWEEKIPN
490	500	510	520	530	540	550	560
PSKSHLFQNG	SAELWPPGSM	SAFTSGSPPH	QGPWGSRFPE	LEGVFPVGFG	DSEVSPLTIE	DPKHVCDPPS	GPDTTPAASD
570	580	590	600	610	620	630	640
LPTEQPPSPQ	PGPPAASHTP	EKQASSFDFN	GPYLGPPHSR	SLPDILGQPE	PPQEGGSQKS	PPPGSLEYLC	LPAGGQVQLV
650	660	670	680	690	700	710	720
PLAQAMGPGQ	AVEVERRPSQ	GAAGSPSLES	GGGPAPPALG	PRVGGQDQKD	SPVAIPMSSG	DTEDPGVASG	YVSSADLVFT
730	740	750	760	770	780	790	800
PNSGASSVSL	VPSLGLPSDQ	TPSLCPGLAS	GPPGAPGPVK	SGFEGYVELP	PIEGRSPRSP	RNNPVPPEAK	SPVLNPGERP
810	820	830	840	850	860	870	880
ADVSPTSPQP	EGLLVLQQVG	DYCFLPGLGP	GPLSLRSKPS	SPGPGPEIKN	LDQAFQVKKP	PGQAVPQVPV	IQLFKALKQQ
890	900						
DYLSLPPWEV	NKPGEVC						

Cmpd.	No. of Cmpds.	m/z meas.	Δ m/z [ppm]	z	Rt [min]	Score	P	Range	Sequence	Modification
2606	1	495.0590	111.94	4	60.9	14.0	0	601-619	R.SLPDILGQPEPPQEGGSQK.S	

Detailed Protein Report

Protein 629: **PREDICTED: loss of heterozygosity 12 chromosomal region 1 protein isoform X1 [Homo sapiens]**

Accession: gi|530398989
Database: refseq_human(refseq_human_20140103.fasta)
Database Date: 2015-11-30

Score: 14.0
MW [kDa]: 20.2
pI: 9.0
Sequence Coverage [%]: 10.7
No. of unique Peptides: 1

10	20	30	40	50	60	70	80
MTPSPAKHRA	KMDDIVVVAQ	GSQASRNVSN	DPDVIKLQEI	PTFQPLLKGL	LSGQTSPTNA	KLEKLDSQQV	LQLCLRYQDH
**90**	**100**	**110**	**120**	**130**	**140**	**150**	**160**
LHQCAEAVAF	DQNALVKRIK	EMDLSVETLF	SFMQERQKRY	AKYAEQIQKV	NEMSAILRRI	QMGIDQTVPL	LDRLNSMLPE
**170**	**180**						
GERLEPFSMK	PDRELRL						

Cmpd.	No. of Cmpds.	m/z meas.	Δ m/z [ppm]	z	Rt [min]	Score	P	Range	Sequence	Modification
1730	1	690.1247	139.16	3	50.3	14.0	2	8-26	K.HRAKMDDIVVVAQGSQASR.N	

Detailed Protein Report

Protein 630: acyl-coenzyme A thioesterase 13 isoform 2 [Homo sapiens]

Accession: gi|231567183
Database: refseq_human(refseq_human_20140103.fasta)
Database Date: 2015-11-30
Modification(s): Oxidation

Score: 14.0
MW [kDa]: 12.4
pI: 9.6
Sequence Coverage [%]: 15.4
No. of unique Peptides: 1

10	20	30	40	50	60	70	80
MVRKITLVSA	APGKVICEMK	VEEEHTNAIG	TLHGGLTATL	VDNISTMALL	CTERGAPGVS	VDMNITYMSP	AKLGEDIVIT
**90**	**100**	**110**	**120**				
AHVLKQGKTL	AFTSVDLTNK	ATGKLIAQGR	HTKHLGN				

Cmpd.	No. of Cmpds.	m/z meas.	Δ m/z [ppm]	z	Rt [min]	Score	P	Range	Sequence	Modification
1761	1	927.4490	7.27	2	50.7	14.0	0	55-72	R.GAPGVSVDMNITYMSPAK.L	Oxidation: 9

Detailed Protein Report

Protein 631: PREDICTED: golgin subfamily A member 6-like protein 2-like [Homo sapiens]

**Accession:**	gi|578845630	**Score:**	14.0
**Database:**	refseq_human(refseq_human_20140103.fasta)	**MW [kDa]:**	23.1
**Database Date:**	2015-11-30	**pI:**	5.0
**Modification(s):**	Oxidation	**Sequence Coverage [%]:**	4.6
		**No. of unique Peptides:**	1

```
        10         20         30         40         50         60         70         80
MRGAGGEDAG DRRRRCGGQE EKMREQEEKM REQEEKMQGQ EEKMREQEEK MRGQEEKMRE QEEKMRGQEE KMWGQEEKMW
        90        100        110        120        130        140        150        160
GQEEKMWGQE EMMREKEERI RDQKEKMQER LPEHEERCSE PCLPPSKVLC NMSHTGSVEP AGGEAGEGSP QDNPTAQEIM
       170        180        190        200
QLFCGMKNAQ QCPGLGSTSC IPFFYRGDKR KMKIINI
```

Cmpd.	No. of Cmpds.	m/z meas.	Δ m/z [ppm]	z	Rt [min]	Score	P	Range	Sequence	Modification
1953	1	590.7822	0.87	2	52.8	14.0	2	51-59	K.MRGQEEKMR.E	Oxidation: 8

Detailed Protein Report

Protein 632: uncharacterized protein CXorf22 [Homo sapiens]

**Accession:**	gi|90963001	**Score:**	14.0
**Database:**	refseq_human(refseq_human_20140103.fasta)	**MW [kDa]:**	110.3
**Database Date:**	2015-11-30	**pI:**	9.3
**Modification(s):**	Oxidation	**Sequence Coverage [%]:**	2.3
		**No. of unique Peptides:**	1

10	20	30	40	50	60	70	80
MNTQKGSLTI	NVHRGSLAMS	IQRGSLVPRD	MDSSGRDMQL	RVIPAEVKFL	DTMAGRVYRL	PITVHNICRW	NQKIRFKEPV
**90**	**100**	**110**	**120**	**130**	**140**	**150**	**160**
KPQFKLMLTS	LDKELASGLQ	MTAMVEYHPD	KDEDTFDRLL	ISIENKTTEI	PLIGLIPSCQ	LEIESVVNFG	TLVANSKVYS
**170**	**180**	**190**	**200**	**210**	**220**	**230**	**240**
KEITITNHGK	APGIFKAEYH	GQLPILIFPT	SGIVDAKSSM	VIKVDFCADQ	PRIVDEEAIV	ILQGQPEMLL	SIKAHVVEQI
**250**	**260**	**270**	**280**	**290**	**300**	**310**	**320**
IELLSMSSDR	RLECIHFGPV	FFGSSKIKHA	RVYNNSPEPI	NWVAIIQDDA	VGEELGTDIQ	QRTDIALNNL	TYIRKIKNID
**330**	**340**	**350**	**360**	**370**	**380**	**390**	**400**
TTIIISCLPN	EGTLQPYQKT	VITFCFTPKL	MAVGKKDIGP	SYRQDYALFL	RFESVGSKDG	FLRDDDYKTI	KSERFQKVEL
**410**	**420**	**430**	**440**	**450**	**460**	**470**	**480**
ALTGTGLPVL	LQFDPGPVLN	FKPCFMGERS	EIQCIIKNQC	ELLPVTYHFK	KTANFEIDPE	KGKITGGGMV	DVMCSFVPHQ
**490**	**500**	**510**	**520**	**530**	**540**	**550**	**560**
LGVFKVKQMI	EIIGLVAEED	LQSLSVKSFH	HVYLAFNSIC	KASTKKVVMK	FDPGILPSIR	NPTGKFVVKD	LAKRKNYAPV
**570**	**580**	**590**	**600**	**610**	**620**	**630**	**640**
AMLQSAMTRT	HNHRSCEEPV	KDMLLAFPND	RAATIRSKDH	HKHFRPIFTK	VPRFNYVNHD	FAYTTFEKQQ	KKLHENYYAM
**650**	**660**	**670**	**680**	**690**	**700**	**710**	**720**
YLKYLRSVRL	QKKQAERERM	YSYDDTDIGL	EPGSGLKSPS	LSEAEIEEEL	SSAANSIRAN	RLLTTRGIAS	QEEESVRRKV
**730**	**740**	**750**	**760**	**770**	**780**	**790**	**800**
LKGLKSEPST	PQEKHDCSLM	LTPKQIHQVI	VGPSVLNFGN	ICVNSPNTHL	LHVINMLPMH	VLLQLDTDLE	ELQKTNQFSY
**810**	**820**	**830**	**840**	**850**	**860**	**870**	**880**
VILPTSSTYI	SMVFDSPTIG	KFWKSFTFTV	NNVPSGHILV	VAVVQPVTLE	LSSNELVLRP	RGFFMKTCFR	GTVRLYNRQN
**890**	**900**	**910**	**920**	**930**	**940**	**950**	**960**
CCAQFQWQPV	NTGRGIAFSI	CPAKGTVEAY	SSLECEVTWQ	QGFSSPEEGE	FILHVFQGNA	LKLKCVAHVI	IFLEHGFCFE
**970**	**980**						
GYEFVGYTLV	YIVTYI						

Cmpd.	No. of Cmpds.	m/z meas.	Δ m/z [ppm]	z	Rt [min]	Score	P	Range	Sequence	Modification
2562	1	1221.7289	102.41	2	60.3	14.0	2	723-744	K.GLKSEPSTPQEKHDCSLMLTPK.Q	Oxidation: 18

Detailed Protein Report

Protein 633: TGF-beta receptor type-1 isoform 1 precursor [Homo sapiens]

**Accession:**	gi|4759226	**Score:**	14.0
**Database:**	refseq_human(refseq_human_20140103.fasta)	**MW [kDa]:**	55.9
**Database Date:**	2015-11-30	**pI:**	8.7
		**Sequence Coverage [%]:**	1.6
		**No. of unique Peptides:**	1

Quantitation

WD:WU **Median:** 0.70 **CV:** 0.00 % **No. of Peptides:** 1

10	20	30	40	50	60	70	80
MEAAVAAPRP	RLLLLVLAAA	AAAAAALLPG	ATALQCFCHL	CTKDNFTCVT	DGLCFVSVTE	TTDKVIHNSM	CIAEIDLIPR
**90**	**100**	**110**	**120**	**130**	**140**	**150**	**160**
DRPFVCAPSS	KTGSVTTTYC	CNQDHCNKIE	LPTTVKSSPG	LGPVELAAVI	AGPVCFVCIS	LMLMVYICHN	RTVIHHRVPN
**170**	**180**	**190**	**200**	**210**	**220**	**230**	**240**
EEDPSLDRPF	ISEGTTLKDL	IYDMTTSGSG	SGLPLLVQRT	IARTIVLQES	IGKGRFGEVW	RGKWRGEEVA	VKIFSSREER
**250**	**260**	**270**	**280**	**290**	**300**	**310**	**320**
SWFREAEIYQ	TVMLRHENIL	GFIAADNKDN	GTWTQLWLVS	DYHEHGSLFD	YLNRYTVTVE	GMIKLALSTA	SGLAHLHMEI
**330**	**340**	**350**	**360**	**370**	**380**	**390**	**400**
VGTQGKPAIA	HRDLKSKNIL	VKKNGTCCIA	DLGLAVRHDS	ATDTIDIAPN	HRVGTKRYMA	PEVLDDSINM	KHFESFKRAD
**410**	**420**	**430**	**440**	**450**	**460**	**470**	**480**
IYAMGLVFWE	IARRCSIGGI	HEDYQLPYYD	LVPSDPSVEE	MRKVVCEQKL	RPNIPNRWQS	CEALRVMAKI	MRECWYANGA
**490**	**500**	**510**					
ARLTALRIKK	TLSQLSQQEG	IKM					

Cmpd.	No. of Cmpds.	m/z meas.	Δ m/z [ppm]	z	Rt [min]	Score	P	Range	Sequence	Modification	Ratios
684	1	450.6291	-320.69	2	37.0	14.0	0	109-116	K.IELPTTVK.S		WD:WU 0.70

Detailed Protein Report

Protein 634: PREDICTED: zinc finger protein 91 isoform X4 [Homo sapiens]

Accession: gi|578833582
Database: refseq_human(refseq_human_20140103.fasta)
Database Date: 2015-11-30

Score: 13.9
MW [kDa]: 101.7
pI: 10.3
Sequence Coverage [%]: 1.2
No. of unique Peptides: 1

10	20	30	40	50	60	70	80
MPGTPGSLEM	GLLTFRDVAI	EFSPEEWQCL	DTAQQNLYRN	VMLENYRNLA	FLGIALSKPD	LITYLEQGKE	PWNMKQHEMV
90	100	110	120	130	140	150	160
DEPTGICPHF	PQDFWPEQSM	EDSFQKVLLR	KYEKCGHENL	QLRKGCKSVD	ECKVHKEGYN	KLNQCLTTAQ	SKVFQCGKYL
170	180	190	200	210	220	230	240
KVFYKFLNSN	RHTIRHTGKK	CFKCKKCVKS	FCIRLHKTQH	KCVYITEKSC	KCKECEKTFH	WSSTLTNHKE	IHTEDKPYKC
250	260	270	280	290	300	310	320
EECGKAFKQL	STLTTHKIIC	AKEKIYKCEE	CGKAFLWSST	LTRHKRIHTG	EKPYKCEECG	KAFSHSSTLA	KHKRIHTGEK
330	340	350	360	370	380	390	400
PYKCEECGKA	FSRSSTLAKH	KRIHTGEKPY	KCKECGKAFS	NSSTLANHKI	THTEEKPYKC	KECDKAFKRL	STLTKHKIIH
410	420	430	440	450	460	470	480
AGEKLYKCEE	CGKAFNRSSN	LTIHKFIHTG	EKPYKCEECG	KAFNWSSSLT	KHKRFHTREK	PFKCKECGKA	FIWSSTLTRH
490	500	510	520	530	540	550	560
KRIHTGEKPY	KCEECGKAFR	QSSTLTKHKI	IHTGEKPYKF	EECGKAFRQS	LTLNKHKIIH	SREKPYKCKE	CGKAFKQFST
570	580	590	600	610	620	630	640
LTTHKIIHAG	KKLYKCEECG	KAFNHSSSLS	THKIIHTGEK	SYKCEECGKA	FLWSSTLRRH	KRIHTGEKPY	KCEECGKAFS
650	660	670	680	690	700	710	720
QSSTLTTHKI	IHTGEKPYKC	EECGKAFRKS	STLTEHKIIH	TGEKPYKCEE	CGKAFSQSST	LTRHTRMHTG	EKPYKCEECG
730	740	750	760	770	780	790	800
KAFNRSSKLT	THKIIHTGEK	PYKCEECGKA	FISSSTLNGH	KRIHTREKPY	KCEECGKAFS	QSSTLRHKR	LHTGEKPYKC
810	820	830	840	850	860	870	880
GECGKAFKES	SALTKHKIIH	TGEKPYKCEK	CGKAFNQSSI	LTNHKKIHTI	TPVIPLLWEA	EAGGSRGQEM	ETILANTVKP
890							
LLY							

Cmpd.	No. of Cmpds.	m/z meas.	Δ m/z [ppm]	z	Rt [min]	Score	P	Range	Sequence	Modification
2677	1	665.3406	42.21	2	61.8	13.9	2	263-273	K.EKIYKCEECGK.A	

Detailed Protein Report

Protein 635: testis anion transporter 1 isoform b [Homo sapiens]

Accession: gi|301601602
Database: refseq_human(refseq_human_20140103.fasta)
Database Date: 2015-11-30

Score: 13.9
MW [kDa]: 97.3
pI: 5.7
Sequence Coverage [%]: 1.8
No. of unique Peptides: 1

10	20	30	40	50	60	70	80
MAQLERSAIS	GFSSKSRRNS	FAYDVKREVY	NEETFQQEHK	RKASSSGNMN	INITTFRHHV	QCRCSWHRFL	RCVLTIFPFL
90	100	110	120	130	140	150	160
EWMCMYRLKD	WLLGDLLAGI	SVGLVQVPQG	LTLSLLARQL	IPPLNIAYAA	FCSSVIYVIF	GSCHQMSIGS	FFLVSALLIN
170	180	190	200	210	220	230	240
VLKVSPFNNG	QLVMGSFVKN	EFSAPSYLMG	YNKSLSVVAT	TTFLTGIIQI	IGFTVIANKI	SMATETSQTL	IDMIPYSFLL
250	260	270	280	290	300	310	320
PVTPDFSLLP	KIILQAFSLS	LVSSFLLIFL	GKKIASLHNY	SVNSNQDLIA	IGLCNVVSSF	FRSCVFTGAI	ARTIIQDKSG
330	340	350	360	370	380	390	400
GRQQFASLVG	AGVMLLLMVK	MGHFFYTLPN	AVLAGIILSN	VIPYLETISN	LPSLWRQDQY	DCALWMMTFS	SSIFLGLDIG
410	420	430	440	450	460	470	480
LIISVVSAFF	ITTVRSHRAK	ILLLGQIPNT	NIYRSINDYR	EIITIPGVKI	FQCCSSITFV	NVYYLKHKLL	KEVDMVKVPL
490	500	510	520	530	540	550	560
KEEEIFSLFN	SSDTNLQGGK	ICRCFCNCDD	LEPLPRILYT	ERFENKLDPE	ASSINLIHCS	HFESMNTSQT	ASEDQVPYTV
570	580	590	600	610	620	630	640
SSVSQKNQGQ	QYEEVEEVWL	PNNSSRNSSP	GLPDVAESQG	RRSLIPYSDA	SLLPSVHTII	LDFSMVHYVD	SRGLVVLRQI
650	660	670	680	690	700	710	720
CNAFQNANIL	ILIAGCHSSI	VRAFERNDFF	DAGITKTQLF	LSVHDAVLFA	LSRKVIGSSE	LSIDESETVI	RETYSETDKN
730	740	750	760	770	780	790	800
DNSRYKMSSS	FLGSQKNVSP	GFIKIQQPVE	EESELDLELE	SEQEAGLGLD	LDLDRELEPE	MEPKAETETK	TQTEMEPQPE
810	820	830	840	850	860	870	
TEPEMEPNPK	SRPRAHTFPQ	QRYWPMYHPS	MASTQSQTQT	RTWSVERRRH	PMDSYSPEGN	SNEDV	

Cmpd.	No. of Cmpds.	m/z meas.	Δ m/z [ppm]	z	Rt [min]	Score	P	Range	Sequence	Modification
2795	1	835.4427	31.63	2	63.4	13.9	1	587-602	R.NSSPGLPDVAESQGRR.S	

Detailed Protein Report

Protein 636: **OTU domain-containing protein 4 isoform 3 [Homo sapiens]**

**Accession:**	gi|156630992	**Score:**	13.9
**Database:**	refseq_human(refseq_human_20140103.fasta)	**MW [kDa]:**	117.0
**Database Date:**	2015-11-30	**pI:**	6.2
		**Sequence Coverage [%]:**	2.0
		**No. of unique Peptides:**	1

10	20	30	40	50	60	70	80
MACIHYLREN	REKFEAFIEG	SFEEYLKRLE	NPQEWVGQVE	ISALSLMYRK	DFIIYREPNV	SPSQVTENNF	PEKVLLCFSN
**90**	**100**	**110**	**120**	**130**	**140**	**150**	**160**
GNHYDIVYPI	KYKESSAMCQ	SLLYELLYEK	VFKTDVSKIV	MELDTLEVAD	EDNSEISDSE	DDSCKSKTAA	AAADVNGFKP
**170**	**180**	**190**	**200**	**210**	**220**	**230**	**240**
LSGNEQLKNN	GNSTSLPLSR	KVLKSLNPAV	YRNVEYEIWL	KSKQAQQKRD	YSIAAGLQYE	VGDKCQVRLD	HNGKFLNADV
**250**	**260**	**270**	**280**	**290**	**300**	**310**	**320**
QGIHSENGPV	LVEELGKKHT	SKNLKAPPPE	SWNTVSGKKM	KKPSTSGQNF	HSDVDYRGPK	NPSKPIKAPS	ALPPRLQHPS
**330**	**340**	**350**	**360**	**370**	**380**	**390**	**400**
GVRQHAFSSH	SSGSQSQKFS	SEHKNLSRTP	SQIIRKPDRE	RVEDFDHTSR	ESNYFGLSPE	ERREKQAIEE	SRLLYEIQNR
**410**	**420**	**430**	**440**	**450**	**460**	**470**	**480**
DEQAFPALSS	SSVNQSASQS	SNPCVQRKSS	HVGDRKGSRR	RMDTEERKDK	DSIHGHSQLD	KRPEPSTLEN	ITDDKYATVS
**490**	**500**	**510**	**520**	**530**	**540**	**550**	**560**
SPSKSKKLEC	PSPAEQKPAE	HVSLSNPAPL	LVSPEVHLTP	AVPSLPATVP	AWPSEPTTFG	PTGVPAPIPV	LSVTQTLTTG
**570**	**580**	**590**	**600**	**610**	**620**	**630**	**640**
PDSAVSQAHL	TPSPVPVSIQ	AVNQPLMPLP	QTLSLYQDPL	YPGFPCNEKG	DRAIVPPYSL	CQTGEDLPKD	KNILRFFFNL
**650**	**660**	**670**	**680**	**690**	**700**	**710**	**720**
GVKAYSCPMW	APHSYLYPLH	QAYLAACRMY	PKVPVPVYPH	NPWFQEAPAA	QNESDCTCTD	AHFPMQTEAS	VNGQMPQPEI
**730**	**740**	**750**	**760**	**770**	**780**	**790**	**800**
GPPTFSSPLV	IPPSQVSESH	GQLSYQADLE	SETPGQLLHA	DYEESLSGKN	MFPQPSFGPN	PFLGPVPIAP	PFFPHVWYGY
**810**	**820**	**830**	**840**	**850**	**860**	**870**	**880**
PFQGFIENPV	MRQNIVLPSD	EKGELDLSLE	NLDLSKDCGS	VSTVDEFPEA	RGEHVHSLPE	ASVSSKPDEG	RTEQSSQTRK
**890**	**900**	**910**	**920**	**930**	**940**	**950**	**960**
ADTALASIPP	VAEGKAHPPT	QILNRERETV	PVELEPKRTI	QSLKEKTEKV	KDPKTAADVV	SPGANSVDSR	VQRPKEESSE
**970**	**980**	**990**	**1000**	**1010**	**1020**	**1030**	**1040**
DENEVSNILR	SGRSKQFYNQ	TYGSRKYKSD	WGYSGRGGYQ	HVRSEESWKG	QPSRSRDEGY	QYHRNVRGRP	FRGDRRRSGM
**1050**							
GDGHRGQHT							

Cmpd.	No. of Cmpds.	m/z meas.	Δ m/z [ppm]	z	Rt [min]	Score	P	Range	Sequence	Modification
2038	1	701.2778	-127.81	3	54.1	13.9	0	148-168	K.TAAAAADVNGFKPLSGNEQLK.N	

Detailed Protein Report

Protein 637: **PREDICTED: zinc finger CCHC domain-containing protein 10 isoform X4 [Homo sapiens]**

Accession: gi|530380033
Database: refseq_human(refseq_human_20140103.fasta)
Database Date: 2015-11-30

Score: 13.9
MW [kDa]: 13.8
pI: 11.3
Sequence Coverage [%]: 6.1
No. of unique Peptides: 1

10	20	30	40	50	60	70	80
MATPMHRLIA	RRQAFDTELQ	PVKTFWILIQ	PSIVISEANK	QHVRCQKCLE	FGHWTYECTG	KRKYLHRPSR	TAELKKALKE
90	100	110	120				
KENRLLLQQR	SFFPPRVYQH	WRNQCRKKGQ	EKKV				

Cmpd.	No. of Cmpds.	m/z meas.	Δ m/z [ppm]	z	Rt [min]	Score	P	Range	Sequence	Modification
422	1	464.6450	-245.69	2	34.3	13.9	0	64-70	K.YLHRPSR.T	

Detailed Protein Report

Protein 638: protein FAM222B isoform 1 [Homo sapiens]

**Accession:**	gi|568786296	**Score:**	13.9
**Database:**	refseq_human(refseq_human_20140103.fasta)	**MW [kDa]:**	59.8
**Database Date:**	2015-11-30	**pI:**	10.3
**Modification(s):**	Oxidation	**Sequence Coverage [%]:**	2.5
		**No. of unique Peptides:**	1

Alias proteins:

Accession	Name	Description
gi|578831131	refseq_human (refseq_human_20140103.fasta)	PREDICTED: protein FAM222B isoform X1 [Homo sapiens]

10	20	30	40	50	60	70	80
MRSSFPLASA	KKNLRVGTNL	EQRDSSTHQG	DTTQKMRTAH	YPTPAELDAY	AKKVANNPLT	IKIFPNSVKV	PQRKHVRRTV
**90**	**100**	**110**	**120**	**130**	**140**	**150**	**160**
NGLDTSAQRY	SPYPTQAATK	AGLLAIVKVP	AKSILKDFDG	TRARLLPEAI	MNPPVAPYAT	VAPSTLAHPQ	AQALARQQAL
**170**	**180**	**190**	**200**	**210**	**220**	**230**	**240**
QHAQTLAHAP	PQTLQHPQGI	PPPQALSHPQ	SLQQPQGLGH	PQPMAQTQGL	VHPQALAHQG	LQHPHNPLLH	GGRKMPDSDA
**250**	**260**	**270**	**280**	**290**	**300**	**310**	**320**
PPNVTVSTST	IPLSMAATLQ	HSQPPDLSSI	VHQINQFCQT	RAGISTTSVC	EGQIANPSPI	SRSLLINAST	RVSTHSVPTP
**330**	**340**	**350**	**360**	**370**	**380**	**390**	**400**
MPSCVVNPME	HTHAATAALP	AAGPVNLPTG	ISRVPTGYPS	DLKPVTWNQH	QLAHLQQMCS	EASGTPAPGL	TGKHAAGREL
**410**	**420**	**430**	**440**	**450**	**460**	**470**	**480**
AGPGFVGKAP	AYPQELCLAQ	SFHLKPPLEK	PTPSPPVNGM	AAPLAYPNGH	YFQPLWNNIL	PTPNSDSSGS	QDLAMPFHGG
**490**	**500**	**510**	**520**	**530**	**540**	**550**	**560**
QPTGAPLDCA	AAPGAHYRAG	TGGGPVASQN	SLMQTVDYLS	GDFQQACFRE	QSLAMLSKAH	RAPGNRAPDP	TESRSLHIQH
**570**							
PGYR							

Cmpd.	No. of Cmpds.	m/z meas.	Δ m/z [ppm]	z	Rt [min]	Score	P	Range	Sequence	Modification
7	1	804.3526	-6.18	2	29.0	13.9	1	24-37	R.DSSTHQGDTTQKMR.T	Oxidation: 13

Detailed Protein Report

Protein 639: **PREDICTED: zinc finger and BTB domain-containing protein 2 isoform X3 [Homo sapiens]**

**Accession:**	gi|530383922	**Score:**	13.9
**Database:**	refseq_human(refseq_human_20140103.fasta)	**MW [kDa]:**	48.3
**Database Date:**	2015-11-30	**pI:**	5.5
		**Sequence Coverage [%]:**	3.7
		**No. of unique Peptides:**	1

10	20	30	40	50	60	70	80
MYTGKMAPQL	IDPVRLEQGI	KFLHAYPLIQ	EASLASQGAF	SHPDQVFPLA	SSLYGIQIAD	HQLRQATKIA	SAPEKLGRDP
**90**	**100**	**110**	**120**	**130**	**140**	**150**	**160**
RPQTSRISQE	QVPEASQLSQ	LTSNLAQVNR	TNMTPSDPLQ	TSLSPELVST	PVPPPPPGEE	TNLEASSSDE	QPASLTIAHV
**170**	**180**	**190**	**200**	**210**	**220**	**230**	**240**
KPSIMKRNGS	FPKYYACHLC	GRRFTLRSSL	REHLQIHTGV	PFTSSQQGES	RVPLTLCSNA	ADLGKDAMEV	PEAGMISDSE
**250**	**260**	**270**	**280**	**290**	**300**	**310**	**320**
LQHISDSPII	DGQQQSETPP	PSDIADIDNL	EQADQEREVK	RRKYECTICG	RKFIQKSHWR	EHMYIHTGKP	FKCSTCDKSF
**330**	**340**	**350**	**360**	**370**	**380**	**390**	**400**
CRANQAARHV	CLNQSIDTYT	MVDKQTLELC	TFEEGSQMDN	MLVQTNKPYK	CNLCDKTFST	PNEVVKHSCQ	NQNSDVFALD
**410**	**420**	**430**	**440**				
EGRSILLGSG	DSEVTEPDHP	VLASIKKEQE	TVLLD				

Cmpd.	No. of Cmpds.	m/z meas.	Δ m/z [ppm]	z	Rt [min]	Score	P	Range	Sequence	Modification
1963	1	899.3605	-76.28	2	53.2	13.9	1	371-386	K.CNLCDKTFSTPNEVVK.H	

Detailed Protein Report

Protein 640: protein phosphatase 1 regulatory subunit 26 [Homo sapiens]

**Accession:**	gi|45387958	**Score:**	13.9
**Database:**	refseq_human(refseq_human_20140103.fasta)	**MW [kDa]:**	127.3
**Database Date:**	2015-11-30	**pI:**	9.8
		**Sequence Coverage [%]:**	1.5
		**No. of unique Peptides:**	1

Alias proteins:

Accession	Name	Description
gi|578818032	refseq_human (refseq_human_20140103.fasta)	PREDICTED: protein phosphatase 1 regulatory subunit 26 isoform X5 [Homo sapiens]
gi|578818030	refseq_human (refseq_human_20140103.fasta)	PREDICTED: protein phosphatase 1 regulatory subunit 26 isoform X4 [Homo sapiens]
gi|578818028	refseq_human (refseq_human_20140103.fasta)	PREDICTED: protein phosphatase 1 regulatory subunit 26 isoform X3 [Homo sapiens]
gi|530426639	refseq_human (refseq_human_20140103.fasta)	PREDICTED: protein phosphatase 1 regulatory subunit 26 isoform X2 [Homo sapiens]
gi|530426637	refseq_human (refseq_human_20140103.fasta)	PREDICTED: protein phosphatase 1 regulatory subunit 26 isoform X1 [Homo sapiens]

10	20	30	40	50	60	70	80
MFLMNASPVV	ALQSKWEAFG	PPGSCRFPRC	FSEADEGVES	ASVSARVQML	ISTLQRDGAA	RGTSDERAAQ	RGHRAEGCHD
90	100	110	120	130	140	150	160
ARPAAKPTVH	KEPPALAVCG	LVADFDPMGE	EETTDFGPLV	LDSDSDDSVD	RDIEEAIQEY	LKAKSGAAQP	GAGGAQPGAA
170	180	190	200	210	220	230	240
QPSRAAGGGS	RCKPEPAHGS	APTALCPPKL	VPGSGGGPGS	QVGSSKDQGS	ASPVSVSSDD	SFEQSIRAEI	EQFLNEKRQH
250	260	270	280	290	300	310	320
ETQKCDGSVE	KKPDTNENSA	KSLLKSHQEP	PTKVVHRQGL	LGVQKEFAFR	KPPRLAKMNV	QPRSLRSKVT	TTQENEGSTK
330	340	350	360	370	380	390	400
PATPCRPSEA	AQNKGGIKRS	ASAARRGKRV	MSAAQASEAS	DSSSDDGIEE	AIQLYQLQKT	RKEADGDLPQ	RVQLREERAP
410	420	430	440	450	460	470	480
DPPAHSTSSA	TKSALPETHR	KTPSKKKLVA	TKTMDPGPGG	LDTDHAPKLL	KETKAPPPAS	PASRSEFVER	SSCRADTSAE
490	500	510	520	530	540	550	560
LMCAEAILDI	SKTILPAPVE	GSDGSLSASP	LFYSPNVPSR	SDGDSSSVDS	DDSIEQEIRT	FLALKAQSGS	LLARGESCPQ
570	580	590	600	610	620	630	640
AAQGPLLPPG	LNSQTGGHKT	PLSKTPDPLL	GCKRKRRGGG	HVRPSTPKKM	QEVVKDGSQD	ADHSQGRAEP	GHERRDLPIQ
650	660	670	680	690	700	710	720
GKASEALGGE	GTARGPGDTR	MSQGQGKTDE	ARRLDEKESS	EDKSSSLDSD	EDLDTAIKDL	LRSKRKLKKR	CREPRAACRK
730	740	750	760	770	780	790	800
KVRFSTAQTH	FLEQLGGLRR	DWKDRGPPVL	KSCLSKSKRD	SGEGPGKKPP	SVFGSTAERM	RQEGAASQDA	ALAFRVRRPA
810	820	830	840	850	860	870	880
SASASEGNPF	PRESQGPAPS	PGSLSDDSSS	VDSNDSIELE	IRKFLAEKAK	ESVSSSEVQA	EGPTALGTGG	PARPEVLCRK
890	900	910	920	930	940	950	960
EPAPPPGVCT	RSQRARGVPH	LAEGLRGTES	AGAQGTAGLF	SQGGKGLPAA	PARGDPVPPR	STSGGVSAKG	LSVSRRNVYV
970	980	990	1000	1010	1020	1030	1040
HKDQSPRGAE	PAAKSAFGQL	PSCATAGTEA	GGARGTFHMG	CGSPSFLTPS	PGAERDAGAQ	ADRTPPWSDF	AHQSRLPSPW
1050	1060	1070	1080	1090	1100	1110	1120
VLRSEGRDAV	WRGGVGSERD	KGSEGPARGL	PSLPLAGFSP	LLSTQLFHFG	KGVSWGGRQA	GLFSPHLGLP	LQGPSFSAFR
1130	1140	1150	1160	1170	1180	1190	1200
EAQAGPSPVF	GSPHLLAKKD	GGPWPTRKAQ	AGLSLHDRRS	SGSEESILDL	RYRRRVNRDD	QEQDALGSDA	SDFSDTSTED
1210							
SGGSSVVKV							

Cmpd.	No. of Cmpds.	m/z meas.	Δ m/z [ppm]	z	Rt [min]	Score	P	Range	Sequence	Modification
1424	1	627.5982	-62.69	3	46.2	13.9	0	475-492	R.ADTSAELMCAEAILDISK.T	

Detailed Protein Report

Protein 641: serine/threonine-protein kinase Nek4 isoform 2 [Homo sapiens]

**Accession:**	gi|302058308	**Score:**	13.8
**Database:**	refseq_human(refseq_human_20140103.fasta)	**MW [kDa]:**	84.3
**Database Date:**	2015-11-30	**pI:**	8.8
		**Sequence Coverage [%]:**	2.8
		**No. of unique Peptides:**	1

10	20	30	40	50	60	70	80
MPLAAYCYLR	VVGKGSYGEV	TLVKHRRDGK	QYLHEKHILH	RDLKTQNVFL	TRTNIIKVGD	LGIARVLENH	CDMASTLIGT
90	100	110	120	130	140	150	160
PYYMSPELFS	NKPYNYKSDV	WALGCCVYEM	ATLKHAFNAK	DMNSLVYRII	EGKLPPMPRD	YSPELAELIR	TMLSKRPEER
170	180	190	200	210	220	230	240
PSVRSILRQP	YIKRQISFFL	EATKIKTSKN	NIKNGDSQSK	PFATVVSGEA	ESNHEVIHPQ	PLSSEGSQTY	IMGEGKCLSQ
250	260	270	280	290	300	310	320
EKPRASGLLK	SPASLKAHTC	KQDLSNTTEL	ATISSVNIDI	LPAKGRDSVS	DGFVQENQPR	YLDASNELGG	ICSISQVEEE
330	340	350	360	370	380	390	400
MLQDNTKSSA	QPENLIPMWS	SDIVTGEKNE	PVKPLQPLIK	EQKPKDQSLA	LSPKLECSGT	ILAHSNLRLL	GSSDSPASAS
410	420	430	440	450	460	470	480
RVAGITGVCH	HAQDQVAGEC	IIEKQGRIHP	DLQPHNSGSE	PSLSRQRRQK	RREQTEHRGE	KRQVRRDLFA	FQESPPRFLP
490	500	510	520	530	540	550	560
SHPIVGKVDV	TSTQKEAENQ	RRVVTGSVSS	SRSSEMSSSK	DRPLSARERR	RLKQSQEEMS	SSGPSVRKAS	LSVAGPGKPQ
570	580	590	600	610	620	630	640
EEDQPLPARR	LSSDCSVTQE	RKQIHCLSED	ELSSSTSSTD	KSDGDYGEGK	GQTNEINALV	QLMTQTLKLD	SKESCEDVPV
650	660	670	680	690	700	710	720
ANPVSEFKLH	RKYRDTLILH	GKVAEEAEEI	HFKELPSAIM	PGSEKIRRLV	EVLRTDVIRG	LGVQLLEQVY	DLLEEEDEFD
730	740	750	760				
REVRLREHMG	EKYTTYSVKA	RQLKFFEENM	NF				

Cmpd.	No. of Cmpds.	m/z meas.	Δ m/z [ppm]	z	Rt [min]	Score	P	Range	Sequence	Modification
1866	1	716.4002	35.77	3	52.0	13.8	0	549-569	K.ASLSVAGPGKPQEEDQPLPAR.R	

Detailed Protein Report

Protein 642: **PREDICTED: apoptosis-resistant E3 ubiquitin protein ligase 1 isoform X2 [Homo sapiens]**

**Accession:**	gi|530404992	**Score:**	13.8
**Database:**	refseq_human(refseq_human_20140103.fasta)	**MW [kDa]:**	75.6
**Database Date:**	2015-11-30	**pI:**	6.3
		**Sequence Coverage [%]:**	1.4
		**No. of unique Peptides:**	1

10	20	30	40	50	60	70	80
MVVPSKTKIV	CHFSTLVLTC	GQPHTLQIVP	RDEYDNPTNN	SMSLRDEHNY	TLSIHELGPQ	EEESTGVSFE	KSVTSNRQTF
**90**	**100**	**110**	**120**	**130**	**140**	**150**	**160**
QVFLRLTLHS	RGCFHACISY	QNQPINNGEF	DIIVLSEDEK	NIVERNVSTS	GVSIYFEAYL	YNATNCSSTP	WHLPPMHMTS
**170**	**180**	**190**	**200**	**210**	**220**	**230**	**240**
SQRRPSTAVD	EEDEDSPSEC	HTPEKVKKPK	KVYCYVSPKQ	FSVKEFYLKI	IPWRLYTFRV	CPGTKFSYLG	PDPVHKLLTL
**250**	**260**	**270**	**280**	**290**	**300**	**310**	**320**
VVDDGIQPPV	ELSCKERNIL	AATFIRSLHK	NIGGSETFQD	KVNFFQRELR	QVHMKRPHSK	VTLKVSRHAL	LESSLKATRN
**330**	**340**	**350**	**360**	**370**	**380**	**390**	**400**
FSISDWSKNF	EVVFQDEEAL	DWGGPRREWF	ELICKALFDT	TNQLFTRFSD	NNQALVHPNP	NRPAHLRLKM	YEFAGRLVGK
**410**	**420**	**430**	**440**	**450**	**460**	**470**	**480**
CLYESSLGGA	YKQLVRARFT	RSFLAQIIGL	RMHYKYFETD	DPEFYKSKVC	FILNNDMSEM	ELVFAEEKYN	KSGQLDKVVE
**490**	**500**	**510**	**520**	**530**	**540**	**550**	**560**
LMTGGAQTPV	TNANKIFYLN	LLAQYRLASQ	VKEEVEHFLK	GLNELVPENL	LAIFDENELE	LLMCGTGDIS	VSDFKAHAVV
**570**	**580**	**590**	**600**	**610**	**620**	**630**	**640**
VGGSWHFREK	VMRWFWTVVS	SLTQEELARL	LQFTTGSSQL	PPGGFAALCP	SFQIIAAPTH	STLPTAHTCF	NQLCLPTYDS
**650**	**660**	**670**					
YEEVHRMLQL	AISEGCEGFG	ML					

Cmpd.	No. of Cmpds.	m/z meas.	Δ m/z [ppm]	z	Rt [min]	Score	P	Range	Sequence	Modification
2591	1	557.8482	105.51	2	60.7	13.8	1	388-396	R.LKMYEFAGR.L	

Detailed Protein Report

Protein 643: oxysterol-binding protein-related protein 11 [Homo sapiens]

Accession: gi|19923592
Database: refseq_human(refseq_human_20140103.fasta)
Database Date: 2015-11-30

Score: 13.8
MW [kDa]: 83.6
pI: 6.6
Sequence Coverage [%]: 2.4
No. of unique Peptides: 1

10	20	30	40	50	60	70	80
MQGGEPVSTM	KVSESEGKLE	GQATAVTPNK	NSSCGGGISS	SSSSRGGSAK	GWQYSDHMEN	VYGYLMKYTN	LVTGWQYRFF
90	100	110	120	130	140	150	160
VLNNEAGLLE	YFVNEQSRNQ	KPRGTLQLAG	AVISPSDEDS	HTFTVNAASG	EQYKLRATDA	KERQHWVSRL	QICTQHHTEA
170	180	190	200	210	220	230	240
IGKNNPPLKS	RSFSLASSSN	SPISQRRPSQ	NAISFFNVGH	SKLQSLSKRT	NLPPDHLVEV	REMMSHAEGQ	QRDLIRRIEC
250	260	270	280	290	300	310	320
LPTSGHLSSL	DQDLLMLKAT	SMATMNCLND	CFHILQLQHA	SHQKGSLPSG	TTIEWLEPKI	SLSNHYKNGA	DQPFATDQSK
330	340	350	360	370	380	390	400
PVAVPEEQPV	AESGLLAREP	EEINADDEIE	DTCDHKEDDL	GAVEEQRSVI	LHLLSQLKLG	MDLTRVVLPT	FILEKRSLLE
410	420	430	440	450	460	470	480
MYADFMSHPD	LFIAITNGAT	AEDRMIRFVE	YYLTSFHEGR	KGAIAKKPYN	PIIGETFHCS	WKMPKSEVAS	SVFSSSSTQG
490	500	510	520	530	540	550	560
VTNHAPLSGE	SLTQVGSDCY	TVRFVAEQVS	HHPPVSGFYA	ECTERKMCVN	AHVWTKSKFL	GMSIGVTMVG	EGILSLLEHG
570	580	590	600	610	620	630	640
EEYTFSLPCA	YARSILTVPW	VELGGKVSVN	CAKTGYSASI	TFHTKPFYGG	KLHRVTAEVK	HNITNTVVCR	VQGEWNSVLE
650	660	670	680	690	700	710	720
FTYSNGETKY	VDLTKLAVTK	KRVRPLEKQD	PFESRRLWKN	VTDSLRESEI	DKATEHKHTL	EERQRTEERH	RTETGTPWKT
730	740	750					
KYFIKEGDGW	VYHKPLWKII	PTTQPAE					

Cmpd.	No. of Cmpds.	m/z meas.	Δ m/z [ppm]	z	Rt [min]	Score	P	Range	Sequence	Modification
2097	1	701.2818	-9.24	3	54.8	13.8	0	339-356	R.EPEEINADDEIEDTCDHK.E	

Detailed Protein Report

Protein 644: PREDICTED: uroporphyrinogen decarboxylase isoform X2 [Homo sapiens]

Accession: gi|530363258
Database: refseq_human(refseq_human_20140103.fasta)
Database Date: 2015-11-30

Score: 13.8
MW [kDa]: 32.6
pI: 6.6
Sequence Coverage [%]: 3.7
No. of unique Peptides: 1

Alias proteins:

Accession	Name	Description
gi|530363260	refseq_human (refseq_human_20140103.fasta)	PREDICTED: uroporphyrinogen decarboxylase isoform X3 [Homo sapiens]

10	20	30	40	50	60	70	80
MLPSFSPTSL	LYPRCFLPLQ	ALGMEVTMVP	GKGPSFPEPL	REEQDLERLR	DPEVVASELG	YVFQAITLTR	QRLAGRVPLI
90	100	110	120	130	140	150	160
GFAGAPWTLM	TYMVEGGGSS	TMAQAKRWLY	QRPQASHQLL	RILTDALVPY	LVGQVVAGAQ	ALQLFESHAG	HLGPQLFNKF
170	180	190	200	210	220	230	240
ALPYIRDVAK	QVKARLREAG	LAPVPMIIFA	KDGHFALEEL	AQAGYEVVGL	DWTVAPKKAR	ECVGKTVTLQ	GNLDPCALYA
250	260	270	280	290	300		
SEEEIGQLVK	QMLDDFGPHR	YIANLGHGLY	PDMDPEHVGA	FVDAVHKHSR	LLRQN		

Cmpd.	No. of Cmpds.	m/z meas.	Δ m/z [ppm]	z	Rt [min]	Score	P	Range	Sequence	Modification
1090	1	646.7958	-117.34	2	42.3	13.8	1	160-170	K.FALPYIRDVAK.Q	

Detailed Protein Report

Protein 645: **phosphatidylinositol 3-kinase regulatory subunit beta [Homo sapiens]**

**Accession:**	gi|4826908	**Score:**	13.8
**Database:**	refseq_human(refseq_human_20140103.fasta)	**MW [kDa]:**	81.6
**Database Date:**	2015-11-30	**pI:**	6.1
**Modification(s):**	Oxidation	**Sequence Coverage [%]:**	1.9
		**No. of unique Peptides:**	1

10	20	30	40	50	60	70	80
MAGPEGFQYR	ALYPFRRERP	EDLELLPGDV	LVVSRAALQA	LGVAEGGERC	PQSVGWMPGL	NERTRQRGDF	PGTYVEFLGP
**90**	**100**	**110**	**120**	**130**	**140**	**150**	**160**
VALARPGPRP	RGPRPLPARP	RDGAPEPGLT	LPDLPEQFSP	PDVAPPLLVK	LVEAIERTGL	DSESHYRPEL	PAPRTDWSLS
**170**	**180**	**190**	**200**	**210**	**220**	**230**	**240**
DVDQWDTAAL	ADGIKSFLLA	LPAPLVTPEA	SAEARRALRE	AAGPVGPALE	PPTLPLHRAL	TLRFLLQHLG	RVARRAPALG
**250**	**260**	**270**	**280**	**290**	**300**	**310**	**320**
PAVRALGATF	GPLLLRAPPP	PSSPPPGGAP	DGSEPSPDFP	ALLVEKLLQE	HLEEQEVAPP	ALPPKPPKAK	PAPTVLANGG
**330**	**340**	**350**	**360**	**370**	**380**	**390**	**400**
SPPSLQDAEW	YWGDISREEV	NEKLRDTPDG	TFLVRDASSK	IQGEYTLTLR	KGGNNKLIKV	FHRDGHYGFS	EPLTFCSVVD
**410**	**420**	**430**	**440**	**450**	**460**	**470**	**480**
LINHYRHESL	AQYNAKLDTR	LLYPVSKYQQ	DQIVKEDSVE	AVGAQLKVYH	QQYQDKSREY	DQLYEEYTRT	SQELQMKRTA
**490**	**500**	**510**	**520**	**530**	**540**	**550**	**560**
IEAFNETIKI	FEEQGQTQEK	CSKEYLERFR	REGNEKEMQR	ILLNSERLKS	RIAEIHESRT	KLEQQLRAQA	SDNREIDKRM
**570**	**580**	**590**	**600**	**610**	**620**	**630**	**640**
NSLKPDLMQL	RKIRDQYLVW	LTQKGARQKK	INEWLGIKNE	TEDQYALMED	EDDLPHHEER	TWYVGKINRT	QAEEMLSGKR
**650**	**660**	**670**	**680**	**690**	**700**	**710**	**720**
DGTFLIRESS	QRGCYACSVV	VDGDTKHCVI	YRTATGFGFA	EPYNLYGSLK	ELVLHYQHAS	LVQHNDALTV	TLAHPVRAPG
**730**							
PGPPPAAR							

Cmpd.	No. of Cmpds.	m/z meas.	Δ m/z [ppm]	z	Rt [min]	Score	P	Range	Sequence	Modification
261	1	530.4911	-164.00	3	32.4	13.8	0	50-63	R.CPQSVGWMPGLNER.T	Oxidation: 8

Detailed Protein Report

Protein 646: metabotropic glutamate receptor 5 isoform b precursor [Homo sapiens]

**Accession:**	gi|4504143	**Score:**	13.8
**Database:**	refseq_human(refseq_human_20140103.fasta)	**MW [kDa]:**	128.8
**Database Date:**	2015-11-30	**pI:**	8.6
		**Sequence Coverage [%]:**	1.5
		**No. of unique Peptides:**	1

Quantitation

WD:WU	**Median:** 0.89	**CV:** 0.00 %	**No. of Peptides:** 1

Alias proteins:

Accession	Name	Description
gi|578822167	refseq_human (refseq_human_20140103.fasta)	PREDICTED: metabotropic glutamate receptor 5 isoform X3 [Homo sapiens]

10	20	30	40	50	60	70	80
MVLLLILSVL	LLKEDVRGSA	QSSERRVVAH	MPGDIIIGAL	FSVHHQPTVD	KVHERKCGAV	REQYGIQRVE	AMLHTLERIN
**90**	**100**	**110**	**120**	**130**	**140**	**150**	**160**
SDPTLLPNIT	LGCEIRDSCW	HSAVALEQSI	EFIRDSLISS	EEEEGLVRCV	DGSSSSFRSK	KPIVGVIGPG	SSSVAIQVQN
**170**	**180**	**190**	**200**	**210**	**220**	**230**	**240**
LLQLFNIPQI	AYSATSMDLS	DKTLFKYFMR	VVPSDAQQAR	AMVDIVKRYN	WTYVSAVHTE	GNYGESGMEA	FKDMSAKEGI
**250**	**260**	**270**	**280**	**290**	**300**	**310**	**320**
CIAHSYKIYS	NAGEQSFDKL	LKKLTSHLPK	ARVVACFCEG	MTVRGLLMAM	RRLGLAGEFL	LLGSDGWADR	YDVTDGYQRE
**330**	**340**	**350**	**360**	**370**	**380**	**390**	**400**
AVGGITIKLQ	SPDVKWFDDY	YLKLRPETNH	RNPWFQEFWQ	HRFQCRLEGF	PQENSKYNKT	CNSSLTLKTH	HVQDSKMGFV
**410**	**420**	**430**	**440**	**450**	**460**	**470**	**480**
INAIYSMAYG	LHNMQMSLCP	GYAGLCDAMK	PIDGRKLLES	LMKTNFTGVS	GDTILFDENG	DSPGRYEIMN	FKEMGKDYFD
**490**	**500**	**510**	**520**	**530**	**540**	**550**	**560**
YINVGSWDNG	ELKMDDDEVW	SKKSNIIRSV	CSEPCEKGQI	KVIRKGEVSC	CWTCTPCKEN	EYVFDEYTCK	ACQLGSWPTD
**570**	**580**	**590**	**600**	**610**	**620**	**630**	**640**
DLTGCDLIPV	QYLRWGDPEP	IAAVVFACLG	LLATLFVTVV	FIIYRDTPVV	KSSSRELCYI	ILAGICLGYL	CTFCLIAKPK
**650**	**660**	**670**	**680**	**690**	**700**	**710**	**720**
QIYCYLQRIG	IGLSPAMSYS	ALVTKTNRIA	RILAGSKKKI	CTKKPRFMSA	CAQLVIAFIL	ICIQLGIIVA	LFIMEPPDIM
**730**	**740**	**750**	**760**	**770**	**780**	**790**	**800**
HDYPSIREVY	LICNTTNLGV	VTPLGYNGLL	ILSCTFYAFK	TRNVPANFNE	AKYIAFTMYT	TCIIWLAFVP	IYFGSNYKII
**810**	**820**	**830**	**840**	**850**	**860**	**870**	**880**
TMCFSVSLSA	TVALGCMFVP	KVYIILAKPE	RNVRSAFTTS	TVVRMHVGDG	KSSSAASRSS	SLVNLWKRRG	SSGETLSSNG
**890**	**900**	**910**	**920**	**930**	**940**	**950**	**960**
KSVTWAQNEK	SSRGQHLWQR	LSIHINKKEN	PNQTAVIKPF	PKSTESRGLG	AGAGAGGSAG	GVGATGGAGC	AGAGPGGPES
**970**	**980**	**990**	**1000**	**1010**	**1020**	**1030**	**1040**
PDAGPKALYD	VAEAEEHFPA	PARPRSPSPI	STLSHRAGSA	SRTDDDVPSL	HSEPVARSSS	SQGSLMEQIS	SVVTRFTANI
**1050**	**1060**	**1070**	**1080**	**1090**	**1100**	**1110**	**1120**
SELNSMMLST	AAPSPGVGAP	LCSSYLIPKE	IQLPTTMTTF	AEIQPLPAIE	VTGGAQPAAG	AQAAGDAARE	SPAAGPEAAA
**1130**	**1140**	**1150**	**1160**	**1170**	**1180**	**1190**	
AKPDLEELVA	LTPPSPFRDS	VDSGSTTPNS	PVSESALCIP	SSPKYDTLII	RDYTQSSSSL		

Cmpd.	No. of Cmpds.	m/z meas.	Δ m/z [ppm]	z	Rt [min]	Score	P	Range	Sequence	Modification	Ratios
2722	1	941.9859	24.92	2	62.4	13.8	0	1018-1035	R.SSSSQGSLMEQISSVVTR.F		WD:WU 0.89

Detailed Protein Report

Protein 647: **PREDICTED: zinc finger protein 789 isoform X1 [Homo sapiens]**

**Accession:**	gi|530385765	**Score:**	13.8
**Database:**	refseq_human(refseq_human_20140103.fasta)	**MW [kDa]:**	49.2
**Database Date:**	2015-11-30	**pI:**	10.3
**Modification(s):**	Carbamidomethyl	**Sequence Coverage [%]:**	3.3
		**No. of unique Peptides:**	1

10	20	30	40	50	60	70	80
MELLSFEDVA	MYFTREEWGH	LNWGQKDLYR	DVMLENYRNM	VLLGFQFPKP	EMICQLENWD	EQWILDLPRT	GNRKASGSAC
**90**	**100**	**110**	**120**	**130**	**140**	**150**	**160**
PGSEARHKMK	KLTPKQKFSE	DLESYKISVV	MQESAEKLSE	KLHKCKEFVD	SCRLTFPTSG	DEYSRGFLQN	LNLIQDQNAQ
**170**	**180**	**190**	**200**	**210**	**220**	**230**	**240**
TRWKQGRYDE	DGKPFNQRSL	LLGHERILTR	AKSYECSECG	KVIRRKAWFD	QHQRIHFLEN	PFECKVCGQA	FRQRSALTVH
**250**	**260**	**270**	**280**	**290**	**300**	**310**	**320**
KQCHLQNKPY	RCHDCGKCFR	QLAYLVEHKR	IHTKEKPYKC	SKCEKTFSQN	STLIRHQVIH	SGEKRHKCLE	CGKAFGRHST
**330**	**340**	**350**	**360**	**370**	**380**	**390**	**400**
LLCHQQIHSK	PNTHKCSECG	QSFGRNVDLI	QHQRIHTKEE	FFQCGECGKT	FSFKRNLFRH	QVIHTGSQPY	QCVICGKSFK
**410**	**420**						
WHTSFIKHQG	THKGQIST						

Cmpd.	No. of Cmpds.	m/z meas.	Δ m/z [ppm]	z	Rt [min]	Score	P	Range	Sequence	Modification
2129	1	707.7420	-121.78	2	55.2	13.8	1	75-88	K.ASGSACPGSEARHK.M	Carbamidomethyl: 6

Detailed Protein Report

Protein 648: coiled-coil domain-containing protein 71L [Homo sapiens]

**Accession:**	gi|57242766	**Score:**	13.8
**Database:**	refseq_human(refseq_human_20140103.fasta)	**MW [kDa]:**	26.2
**Database Date:**	2015-11-30	**pI:**	12.4
**Modification(s):**	Oxidation	**Sequence Coverage [%]:**	6.0
		**No. of unique Peptides:**	1

Quantitation

WD:WU **Median:** 1.10 **CV:** 0.00 % **No. of Peptides:** 1

10	20	30	40	50	60	70	80
MRRSMKRRRR	RRPVAPATAA	RGGDFRAEDG	AGLEAREEKV	VYSRSQLSLA	DSTKALGDAF	KLFMPRSTEF	MSSDAELWSF
**90**	**100**	**110**	**120**	**130**	**140**	**150**	**160**
LCSLKHQFSP	HILRSKDVYG	YSSCRALVPD	PPGPPTARGQ	ARRPVPRAAA	RRRRRGARAA	AARRRKPRPP	PPPPPPPEES
**170**	**180**	**190**	**200**	**210**	**220**	**230**	**240**
CPAKPVAPGP	CFGGRTLEEI	WRAATPTLTT	FPTIRVGSDV	WGERSLAAAR	RRARQVLRVN	LEPMVRLRRF	PVPRA

Cmpd.	No. of Cmpds.	m/z meas.	Δ m/z [ppm]	z	Rt [min]	Score	P	Range	Sequence	Modification	Ratios
811	1	848.9801	-6.53	2	39.0	13.8	2	213-226	R.ARQVLRVNLEPMVR.L	Oxidation: 12	WD:WU 1.10

Detailed Protein Report

Protein 649: SH3 domain-containing protein 21 isoform 1 [Homo sapiens]

**Accession:**	gi|242117953	**Score:**	13.8
**Database:**	refseq_human(refseq_human_20140103.fasta)	**MW [kDa]:**	83.9
**Database Date:**	2015-11-30	**pI:**	5.9
		**Sequence Coverage [%]:**	1.6
		**No. of unique Peptides:**	1

10	20	30	40	50	60	70	80
MEVLVLAGYR	AQKEDELSLA	PGDVVRQVRW	VPARGWLRGE	FGGRYGLFPE	RLVQEIPETL	RGSGEARRPR	CARRRGHPAK
**90**	**100**	**110**	**120**	**130**	**140**	**150**	**160**
HPRPQRWCKV	NFSYSPEQAD	ELKLQAGEIV	EMIKEIEDGW	WLGKKNGQLG	AFPSNFVELL	DSGPPSLGNP	DMPSVSPGPQ
**170**	**180**	**190**	**200**	**210**	**220**	**230**	**240**
RPPKLSSLAY	DSPPDYLQTV	SHPEVYRVLF	DYQPEAPDEL	ALRRGDVVKV	LSKTTEDKGW	WEGECQGRRG	VFPDNFVLPP
**250**	**260**	**270**	**280**	**290**	**300**	**310**	**320**
PPIKKLVPRK	VVSRESAPIK	EPKKLMPKTS	LPTVKKLATA	TTGPSKAKTS	RTPSRDSQKL	TSRDSGPNGG	FQSGGSYHPG
**330**	**340**	**350**	**360**	**370**	**380**	**390**	**400**
RKRSKTQTPQ	QRSVSSQEEE	HSSPVKAPSV	KRTPMPDKTA	TPERPPAPEN	APSSKKIPAP	DKVPSPEKTL	TLGDKASIPG
**410**	**420**	**430**	**440**	**450**	**460**	**470**	**480**
NSTSGKIPAP	DKVPTPEKMV	TPEDKASIPE	NSIIPEETLT	VDKPSTPERV	FSVEESPALE	APPMDKVPNP	KMAPLGDEAP
**490**	**500**	**510**	**520**	**530**	**540**	**550**	**560**
TLEKVLTPEL	SEEEVSTRDD	IQFHHFSSEE	ALQKVKYFVA	KEDPSSQEEA	HTPEAPPPQP	PSSERCLGEM	KCTLVRGDSS
**570**	**580**	**590**	**600**	**610**	**620**	**630**	**640**
PRQAELKSGP	ASRPALEKPH	PHEEATTLPE	EAPSNDERTP	EEEAPPNEQR	PLREEVLPKE	GVASKEEVTL	KEELPPKEEV
**650**	**660**	**670**	**680**	**690**	**700**	**710**	**720**
APKEEVPPIE	RAFAQKTRPI	KPPPDSQETL	ALPSLVPQNY	TENKNEGVDV	TSLRGEVESL	RRALELMEVQ	LERKLTDIWE
**730**	**740**	**750**	**760**				
ELKSEKEQRR	RLEVQVMQGT	QKSQTPRVIH	TQTQTY				

Cmpd.	No. of Cmpds.	m/z meas.	Δ m/z [ppm]	z	Rt [min]	Score	P	Range	Sequence	Modification
328	1	658.3980	85.56	2	32.8	13.8	2	62-73	R.GSGEARRPRCAR.R	

Detailed Protein Report

Protein 650: rho GTPase-activating protein 12 isoform 5 [Homo sapiens]

**Accession:**	gi|398303814	**Score:**	13.8
**Database:**	refseq_human(refseq_human_20140103.fasta)	**MW [kDa]:**	87.7
**Database Date:**	2015-11-30	**pI:**	9.3
		**Sequence Coverage [%]:**	2.9
		**No. of unique Peptides:**	1

10	20	30	40	50	60	70	80
MKMADRSGKI	IPGQVYIEVE	YDYEYEAKDR	KIVIKQGERY	ILVKKTNDDW	WQVKPDENSK	AFYVPAQYVK	EVTRKALMPP
**90**	**100**	**110**	**120**	**130**	**140**	**150**	**160**
VKQVAGLPNN	STKIMQSLHL	QRSTENVNKL	PELSSFGKPS	SSVQGTGLIR	DANQNFGPSY	NQGQTVNLSL	DLTHNNGKFN
**170**	**180**	**190**	**200**	**210**	**220**	**230**	**240**
NDSHSPKVSS	QNRTRSFGHF	PGPEFLDVEK	TSFSQEQSCD	SAGEGSERIH	QDSESGDELS	SSSTEQIRAT	TPPNQGRPDS
**250**	**260**	**270**	**280**	**290**	**300**	**310**	**320**
PVYANLQELK	ISQSALPPLP	GSPAIQINGE	WETHKDSSGR	CYYYNRGTQE	RTWKPPRWTR	DASISKGDFQ	NPGDQEWLKH
**330**	**340**	**350**	**360**	**370**	**380**	**390**	**400**
VDDQGRQYYY	SADGSRSEWE	LPKYNASSQQ	QREIIKSRSL	DRRLQEPIVL	TKWRHSTIVL	DTNDKDQEKY	GLLNVTKIAE
**410**	**420**	**430**	**440**	**450**	**460**	**470**	**480**
NGKKVRKNWL	SSWAVLQGSS	LLFTKTQGSS	TSWFGSNQSK	PEFTVDLKGA	TIEMASKDKS	SKKNVFELKT	RQGTELLIQS
**490**	**500**	**510**	**520**	**530**	**540**	**550**	**560**
DNDTVINDWF	KVLSSTINNQ	AVETDEGIEE	EIPDSPGIEK	HDKEKEQKDP	KKLRSFKVSS	IDSSEQKKTK	KNLKKFLTRR
**570**	**580**	**590**	**600**	**610**	**620**	**630**	**640**
PTLQAVREKG	YIKDQVFGSN	LANLCQRENG	TVPKFVKLCI	EHVEEHGLDI	DGIYRVSGNL	AVIQKLRFAV	NHDEKLDLND
**650**	**660**	**670**	**680**	**690**	**700**	**710**	**720**
SKWEDIHVIT	GALKMFFREL	PEPLFTFNHF	NDFVNAIKQE	PRQRVAAVKD	LIRQLPKPNQ	DTMQILFRHL	RRVIENGEKN
**730**	**740**	**750**	**760**	**770**			
RMTYQSIAIV	FGPTLLKPEK	ETGNIAVHTV	YQNQIVELIL	LELSSIFGR			

Cmpd.	No. of Cmpds.	m/z meas.	Δ m/z [ppm]	z	Rt [min]	Score	P	Range	Sequence	Modification
1695	1	865.1582	65.82	3	49.5	13.8	1	470-491	K.TRQGTELLIQSDNDTVINDWFK.V	

Detailed Protein Report

Protein 651: **PREDICTED: ubiquitin carboxyl-terminal hydrolase isozyme L5 isoform X14 [Homo sapiens]**

**Accession:**	gi|578801073	**Score:**	13.7
**Database:**	refseq_human(refseq_human_20140103.fasta)	**MW [kDa]:**	23.7
**Database Date:**	2015-11-30	**pI:**	6.6
**Modification(s):**	Oxidation	**Sequence Coverage [%]:**	8.3
		**No. of unique Peptides:**	1

10	20	30	40	50	60	70	80
MKGLALSNSD	VIRQVHNSFA	RQQMFEFDTK	TSAKEEDAFH	FVSYVPVNGR	LYELDGLREG	PIDLGACNQD	DWISAVRPVI
**90**	**100**	**110**	**120**	**130**	**140**	**150**	**160**
EKRIQKYSEG	EIRFNLMAIV	SDRKMIYEQK	IAELQRQLAE	EPMDTDQGNS	MLSAIQSEVA	KNQMLIEEEV	QKLKRYKIEN
**170**	**180**	**190**	**200**	**210**			
IRRKHNYLPF	IMELLKTLAE	HQQLIPLVEK	AKEKQNAKKA	QETK			

Cmpd.	No. of Cmpds.	m/z meas.	Δ m/z [ppm]	z	Rt [min]	Score	P	Range	Sequence	Modification
2419	1	1051.5349	-7.05	2	58.6	13.7	2	94-110	R.FNLMAIVSDRKMIYEQK.I	Oxidation: 4

Detailed Protein Report

Protein 652: **amyloid beta A4 precursor protein-binding family B member 1 isoform b [Homo sapiens]**

Accession: gi|22035554
Database: refseq_human(refseq_human_20140103.fasta)
Database Date: 2015-11-30

Score: 13.7
MW [kDa]: 76.9
pI: 4.8
Sequence Coverage [%]: 3.7
No. of unique Peptides: 1

10	20	30	40	50	60	70	80
MSVPSSLSQS	AINANSHGGP	ALSLPLPLHA	AHNQLLNAKL	QATAVGPKDL	RSAMGEGGGP	EPGPANAKWL	KEGQNQLRRA
**90**	**100**	**110**	**120**	**130**	**140**	**150**	**160**
ATAHRDQNRN	VTLTLAEEAS	QEPEMAPLGP	KGLIHLYSEL	ELSAHNAANR	GLRGPGLIIS	TQEQGPDEGE	EKAAGEAEEE
**170**	**180**	**190**	**200**	**210**	**220**	**230**	**240**
EEDDDDEEEE	EDLSSPPGLP	EPLESVEAPP	RPQALTDGPR	EHSKSASLLF	GMRNSAASDE	DSSWATLSQG	SPSYGSPEDT
**250**	**260**	**270**	**280**	**290**	**300**	**310**	**320**
DSFWNPNAFE	TDSDLPAGWM	RVQDTSGTYY	WHIPTGTTQW	EPPGRASPSQ	GSSPQEESQL	TWTGFAHGEG	FEDGEFWKDE
**330**	**340**	**350**	**360**	**370**	**380**	**390**	**400**
PSDEAPMELG	LKEPEEGTLT	FPAQSLSPEP	LPQEEEKLPP	RNTNPGIKCF	AVRSLGWVEM	TEEELAPGRS	SVAVNNCIRQ
**410**	**420**	**430**	**440**	**450**	**460**	**470**	**480**
LSYHKNNLHD	PMSGGWGEGK	DLLLQLEDET	LKLVEPQSQA	LLHAQPIISI	RVWGVGRDSG	RDFAYVARDK	LTQMLKCHVF
**490**	**500**	**510**	**520**	**530**	**540**	**550**	**560**
RCEAPAKNIA	TSLHEICSKI	MAERRNARCL	VNGLSLDHSK	LVDVPFQVEF	PAPKNELVQK	FQVYYLGNVP	VAKPVGVDVI
**570**	**580**	**590**	**600**	**610**	**620**	**630**	**640**
NGALESVLSS	SSREQWTPSH	VSVAPATLTI	LHQQTEAVLG	ECRVRFLSFL	AVGRDVHTFA	FIMAAGPASF	CCHMFWCEPN
**650**	**660**	**670**	**680**	**690**	**700**	**710**	
AASLSEAVQA	ACMLRYQKCL	DARSQASTSC	LPAPPAESVA	RRVGWTVRRG	VQSLWGSLKP	KRLGAHTP	

Cmpd.	No. of Cmpds.	m/z meas.	Δ m/z [ppm]	z	Rt [min]	Score	P	Range	Sequence	Modification
2594	1	946.7123	-95.55	3	60.7	13.7	1	86-111	R.DQNRNVTLTLAEEASQEPEMAPLGPK.G	

Detailed Protein Report

Protein 653: **PREDICTED: nuclear receptor subfamily 2 group C member 2 isoform X1 [Homo sapiens]**

**Accession:**	gi|530373071	**Score:**	13.7
**Database:**	refseq_human(refseq_human_20140103.fasta)	**MW [kDa]:**	65.4
**Database Date:**	2015-11-30	**pI:**	5.9
		**Sequence Coverage [%]:**	2.0
		**No. of unique Peptides:**	1

10	20	30	40	50	60	70	80
MTSPSPRIQI	ISTDSAVASP	QRIQIVTDQQ	TGQKIQIVTA	VDASGSPKQQ	FILTSPDGAG	TGKVILASPE	TSSAKQLIFT
90	100	110	120	130	140	150	160
TSDNLVPGRI	QIVTDSASVE	RLLGKTDVQR	PQVVEYCVVC	GDKASGRHYG	AVSCEGCKGF	FKRSVRKNLT	YSCRSNQDCI
170	180	190	200	210	220	230	240
INKHHRNRCQ	FCRLKKCLEM	GMKMESVQSE	RKPFDVQREK	PSNCAASTEK	IYIRKDLRSP	LIATPTFVAD	KDGARQTGLL
250	260	270	280	290	300	310	320
DPGMLVNIQQ	PLIREDGTVL	LATDSKAETS	QGALGTLANV	VTSLANLSES	LNNGDTSEIQ	PEDQSASEIT	RAFDTLAKAL
330	340	350	360	370	380	390	400
NTTDSSSSPS	LADGIDTSGG	GSIHVISRDQ	STPIIEVEGP	LLSDTHVTFK	LTMPSPMPEY	LNVHYICESA	SRLLFLSMHW
410	420	430	440	450	460	470	480
ARSIPAFQAL	GQDCNTSLVR	ACWNELFTLG	LAQCAQVMSL	STILAAIVNH	LQNSIQEDKL	SGDRIKQVME	HIWKLQEFCN
490	500	510	520	530	540	550	560
SMAKLDIDGY	EYAYLKAIVL	FSPDHPGLTS	TSQIEKFQEK	AQMELQDYVQ	KTYSEDTYRL	ARILVRLPAL	RLMSSNITEE
570	580	590	600				
LFFTGLIGNV	SIDSIIPYIL	KMETAEYNGQ	ITGASL				

Cmpd.	No. of Cmpds.	m/z meas.	Δ m/z [ppm]	z	Rt [min]	Score	P	Range	Sequence	Modification
1400	1	695.9396	-46.71	2	46.3	13.7	2	540-551	R.LARILVRLPALR.L	

Detailed Protein Report

Protein 654: chromobox protein homolog 5 [Homo sapiens]

**Accession:**	gi|6912292	**Score:**	13.7
**Database:**	refseq_human(refseq_human_20140103.fasta)	**MW [kDa]:**	22.2
**Database Date:**	2015-11-30	**pI:**	5.6
		**Sequence Coverage [%]:**	4.7
		**No. of unique Peptides:**	1

Alias proteins:

Accession	Name	Description
gi|188035910	refseq_human (refseq_human_20140103.fasta)	chromobox protein homolog 5 [Homo sapiens]
gi|188035908	refseq_human (refseq_human_20140103.fasta)	chromobox protein homolog 5 [Homo sapiens]

```
        10         20         30         40         50         60         70         80
MGKKTKRTAD SSSSEDEEEY VVEKVLDRRV VKGQVEYLLK WKGFSEEHNT WEPEKNLDCP ELISEFMKKY KKMKEGENNK
        90        100        110        120        130        140        150        160
PREKSESNKR KSNFSNSADD IKSKKKREQS NDIARGFERG LEPEKIIGAT DSCGDLMFLM KWKDTDEADL VLAKEANVKC
       170        180        190        200
PQIVIAFYEE RLTWHAYPED AENKEKETAK S
```

Cmpd.	No. of Cmpds.	m/z meas.	Δ m/z [ppm]	z	Rt [min]	Score	P	Range	Sequence	Modification
403	1	544.7625	-24.48	2	34.1	13.7	1	107-115	K.REQSNDIAR.G	

Detailed Protein Report

Protein 655: **PREDICTED: STE20-related kinase adapter protein alpha isoform X6 [Homo sapiens]**

**Accession:**	gi|530413123	**Score:**	13.7
**Database:**	refseq_human(refseq_human_20140103.fasta)	**MW [kDa]:**	33.1
**Database Date:**	2015-11-30	**pI:**	6.4
		**Sequence Coverage [%]:**	5.1
		**No. of unique Peptides:**	1

10	20	30	40	50	60	70	80
MSFLVSKPER	IRRWVSEKFI	VEGLRDLELF	GEQPPGDTRR	KTNDASSESI	ASFSKQEVMS	SFLPEGGCYE	LLTVIGKGFE
**90**	**100**	**110**	**120**	**130**	**140**	**150**	**160**
DLMTVNLARY	KPTGEYVTVR	RINLEACSNE	MVTFLQGELH	VSKLFNHPNI	VPYRATFIAD	NELWVVTSFM	AYGSAKDLIC
**170**	**180**	**190**	**200**	**210**	**220**	**230**	**240**
THFMDGMNEL	AIAYILQGVL	KALDYIHHMG	YVHRSVKASH	ILISVDGKVY	LSGLRSNLSM	ISHGQRQRVV	HDFPKYSVKV
**250**	**260**	**270**	**280**	**290**	**300**		
LPWLSPEVLQ	QNLQGYDAKS	DIYSVGITAC	ELANGHVPFK	DMPATQGEDS	TVKGI		

Cmpd.	No. of Cmpds.	m/z meas.	Δ m/z [ppm]	z	Rt [min]	Score	P	Range	Sequence	Modification
58	1	516.8847	-52.61	3	29.7	13.7	1	281-295	K.DMPATQGEDSTVKGI.-	

Detailed Protein Report

Protein 656: zinc finger protein 624 [Homo sapiens]

Accession: gi|172072608
Database: refseq_human(refseq_human_20140103.fasta)
Database Date: 2015-11-30
Modification(s): Carbamidomethyl

Score: 13.7
MW [kDa]: 99.9
pI: 10.1
Sequence Coverage [%]: 1.3
No. of unique Peptides: 1

Alias proteins:

Accession	Name	Description
gi|578830001	refseq_human (refseq_human_20140103.fasta)	PREDICTED: zinc finger protein 624 isoform X1 [Homo sapiens]

10	20	30	40	50	60	70	80
MSLQDSTLSR	EGKPEGEIMA	AVFFSVGRLS	PEVTQPDEDL	HLQAEETQLV	KESVTFKDVA	IDFTLEEWRL	MDPTQRNLHK
**90**	**100**	**110**	**120**	**130**	**140**	**150**	**160**
DVMLENYRNL	VSLGLAVSKP	DMISHLENGK	GPWVTVREIS	RIPYPDMEPK	PATKKATRTK	AISEDLSQEA	ILEKLTENGL
**170**	**180**	**190**	**200**	**210**	**220**	**230**	**240**
WDSRMEGLWK	WNDRILRLQN	NQENHLSQRI	IPLKKTPTSQ	RGFRFESILI	PEPGIATEEL	HSRCQTQEEN	FTENLNLITD
**250**	**260**	**270**	**280**	**290**	**300**	**310**	**320**
THLGKIICKE	MKGSKAIRQT	SELTLGKKSN	NKEKPYKCST	CEKAFHYRSL	LIQHQRTHTK	EKPYECNECG	KTFSQPSYLS
**330**	**340**	**350**	**360**	**370**	**380**	**390**	**400**
QHKKIHTGEK	PYKCNECGKA	FIASSSLMVH	QRIHTKEKPY	QCNVCGKSFS	QCARLNQHQR	IQTGEKPYKC	SECGKAFSDK
**410**	**420**	**430**	**440**	**450**	**460**	**470**	**480**
SKLARHQETH	NGEKPYKCDD	CGKAFRNKSY	LSVHQKTHTE	EKPYQCNECG	KSFKNTTIFN	VHQRIHTGEK	PFRCNECGKA
**490**	**500**	**510**	**520**	**530**	**540**	**550**	**560**
YRSNSSLIVH	IRTHTGEKPY	ECNECGKAFN	RIANFTEHQR	IHTGEKPYKC	NECGKAFINY	SCLTVHHRMH	TGEKPYKCTE
**570**	**580**	**590**	**600**	**610**	**620**	**630**	**640**
CGKAFMRSSS	LIIHQRIHTE	EKPYLCNECG	ESFRIKSHLT	VHQRIHTGEK	PYKCTDCERA	FTKMVNLKEH	QKIHTGVKPY
**650**	**660**	**670**	**680**	**690**	**700**	**710**	**720**
KCYDCGKSFR	TKSYLIVHQR	THTGEKPYKC	NECEKAFTNT	SQLTVHQRRH	TGEKPYKCNE	CGKVFTSNSG	FNTHQRTHTG
**730**	**740**	**750**	**760**	**770**	**780**	**790**	**800**
EKPFKCNDCG	KAFSQMVHVT	EHQKIHSGEK	PYKCDVCGKA	FRRGSYLTVH	WRTHTGEKPY	TCKECGKGCI	TLSQLTLHQR
**810**	**820**	**830**	**840**	**850**	**860**	**870**	
IHTGERPYKC	EECGKAFRTN	SDFTVHLRMH	TGEKPYKCNE	CGKAFRSSSS	LTVHQRIHQR	ETQLI	

Cmpd.	No. of Cmpds.	m/z meas.	Δ m/z [ppm]	z	Rt [min]	Score	P	Range	Sequence	Modification
2758	1	701.4193	162.16	2	62.9	13.7	1	278-288	K.CSTCEKAFHYR.S	Carbamidomethyl: 1

Detailed Protein Report

Protein 657: **FERM domain-containing protein 6 isoform 1 [Homo sapiens]**

Accession: gi|22748721
Database: refseq_human(refseq_human_20140103.fasta)
Database Date: 2015-11-30

Score: 13.7
MW [kDa]: 70.9
pI: 7.4
Sequence Coverage [%]: 2.3
No. of unique Peptides: 1

Quantitation

WD:WU **Median:** 3.24 **CV:** 0.00 % **No. of Peptides:** 1

Alias proteins:

Accession	Name	Description
gi|578825482	refseq_human (refseq_human_20140103.fasta)	PREDICTED: FERM domain-containing protein 6 isoform X2 [Homo sapiens]
gi|109715866	refseq_human (refseq_human_20140103.fasta)	FERM domain-containing protein 6 isoform 1 [Homo sapiens]

10	20	30	40	50	60	70	80
MNKLNFHNNR	VMQDRRSVCI	FLPNDESLNI	IINVKILCHQ	LLVQVCDLLR	LKDCHLFGLS	VIQNNEHVYM	ELSQKLYKYC
**90**	**100**	**110**	**120**	**130**	**140**	**150**	**160**
PKEWKKEASK	GIDQFGPPMI	IHFRVQYYVE	NGRLISDRAA	RYYYYWHLRK	QVLHSQCVLR	EEAYFLLAAF	ALQADLGNFK
**170**	**180**	**190**	**200**	**210**	**220**	**230**	**240**
RNKHYGKYFE	PEAYFPSWVV	SKRGKDYILK	HIPNMHKDQF	ALTASEAHLK	YIKEAVRLDD	VAVHYYRLYK	DKREIEASLT
**250**	**260**	**270**	**280**	**290**	**300**	**310**	**320**
LGLTMRGIQI	FQNLDEEKQL	LYDFPWTNVG	KLVFVGKKFE	ILPDGLPSAR	KLIYYTGCPM	RSRHLLQLLS	NSHRLYMNLQ
**330**	**340**	**350**	**360**	**370**	**380**	**390**	**400**
PVLRHIRKLE	ENEEKKQYRE	SYISDNLDLD	MDQLEKRSRA	SGSSAGSMKH	KRLSRHSTAS	HSSSHTSGIE	ADTKPRDTGP
**410**	**420**	**430**	**440**	**450**	**460**	**470**	**480**
EDSYSSSAIH	RKLKTCSSMT	SHGSSHTSGV	ESGGKDRLEE	DLQDDEIEML	VDDPRDLEQM	NEESLEVSPD	MCIYITEDML
**490**	**500**	**510**	**520**	**530**	**540**	**550**	**560**
MSRKLNGHSG	LIVKEIGSST	SSSSETVVKL	RGQSTDSLPQ	TICRKPKTST	DRHSLSLDDI	RLYQKDFLRI	AGLCQDTAQS
**570**	**580**	**590**	**600**	**610**	**620**		
YTFGCGHELD	EEGLYCNSCL	AQQCINIQDA	FPVKRTSKYF	SLDLTHDEVP	EFVV		

Cmpd.	No. of Cmpds.	m/z meas.	Δ m/z [ppm]	z	Rt [min]	Score	P	Range	Sequence	Modification	Ratios
255	1	795.3881	-62.20	2	32.4	13.7	1	233-246	K.REIEASLTLGLTMR.G		WD:WU 3.24

Detailed Protein Report

Protein 658: schwannomin-interacting protein 1 isoform 4 [Homo sapiens]

**Accession:**	gi|308193348	**Score:**	13.7
**Database:**	refseq_human(refseq_human_20140103.fasta)	**MW [kDa]:**	27.5
**Database Date:**	2015-11-30	**pI:**	4.9
**Modification(s):**	Oxidation	**Sequence Coverage [%]:**	7.0
		**No. of unique Peptides:**	1

Quantitation

WD:WU **Median:** 1.13 **CV:** 0.00 % **No. of Peptides:** 1

10	20	30	40	50	60	70	80
MVHQDNCSYQ	AQKNERESIR	QKLALGSFFD	DGPGIYTSCS	KSGKPSLSSR	LQSGMNLQIC	FVNDSGSDKD	SDADDSKTET
**90**	**100**	**110**	**120**	**130**	**140**	**150**	**160**
SLDTPLSPMS	KQSSSYSDRD	TTEEESESLD	DMDFLTRQKK	LQAEAKMALA	MAKPMAKMQV	EVEKQNRKKS	PVADLLPHMP
**170**	**180**	**190**	**200**	**210**	**220**	**230**	**240**
HISECLMKRS	LKPTDLRDMT	IGQLQVIVND	LHSQIESLNE	ELVQLLLIRD	ELHTEQDAML	VDIEDLTRHA	ESQQKHMAEK
**250**							
MPAK							

Cmpd.	No. of Cmpds.	m/z meas.	Δ m/z [ppm]	z	Rt [min]	Score	P	Range	Sequence	Modification	Ratios
2028	2	909.8408	-159.06	2	53.7	13.7	1	121-137	K.LQAEAKMALAMAKPMAK.M	Oxidation: 15	WD:WU 1.13

Detailed Protein Report

Protein 659: electroneutral sodium bicarbonate exchanger 1 isoform c [Homo sapiens]

Accession: gi|385719213
Database: refseq_human(refseq_human_20140103.fasta)
Database Date: 2015-11-30
Modification(s): Oxidation

Score: 13.6
MW [kDa]: 117.1
pI: 6.5
Sequence Coverage [%]: 1.5
No. of unique Peptides: 1

10	20	30	40	50	60	70	80
MPLGRQSHRH	HRTHGQKHRR	RGRGKGASQG	EEGLEALAHD	TPSQRVQFIL	GTEEDEEHVP	HELFTELDEI	CMKEGEDAEW
90	100	110	120	130	140	150	160
KETARWLKFE	EDVEDGGERW	SKPYVATLSL	HSLFELRSCL	INGTVLLDMH	ANSIEEISDL	ILDQQELSSD	LNDSMRVKVR
170	180	190	200	210	220	230	240
EALLKKHHHQ	NEKKRNNLIP	IVRSFAEVGK	KQSDPHLMDK	HGQTVSPQSV	PTTNLEVKNG	VNCEHSPVDL	SKVDLHFMKK
250	260	270	280	290	300	310	320
IPTGAEASNV	LVGEVDILDR	PIVAFVRLSP	AVLLSGLTEV	PIPTRFLFIL	LGPVGKGQQY	HEIGRSMATI	MTDEIFHDVA
330	340	350	360	370	380	390	400
YKAKERDDLL	AGIDEFLDQV	TVLPPGEWDP	SIRIEPPKNV	PSQEKRKMPG	VPNGNVCHIE	QEPHGGHSGP	ELQRTGRLFG
410	420	430	440	450	460	470	480
GLVLDIKRKA	PWYWSDYRDA	LSLQCLASFL	FLYCACMSPV	ITFGGLLGEA	TEGRISAIES	LFGASMTGIA	YSLFAGQALT
490	500	510	520	530	540	550	560
ILGSTGPVLV	FEKILFKFCK	DYALSYLSLR	ACIGLWTAFL	CIVLVATDAS	SLVCYITRFT	EEAFASLICI	IFIYEAIEKL
570	580	590	600	610	620	630	640
IHLAETYPIH	MHSQLDHLSL	YYCRCTLPEN	PNNHTLQYWK	DHNIVTAEVH	WANLTVSECQ	EMHGEFMGSA	CGHHGPYTPD
650	660	670	680	690	700	710	720
VLFWSCILFF	TTFILSSTLK	TFKTSRYFPT	RVRSMVSDFA	VFLTIFTMVI	IDFLIGVPSP	KLQVPSVFKP	TRDDRGWIIN
730	740	750	760	770	780	790	800
PIGPNPWWTV	IAAIIPALLC	TILIFMDQQI	TAVIINRKEH	KLKKGCGYHL	DLLMVAIMLG	VCSIMGLPWF	VAATVLSITH
810	820	830	840	850	860	870	880
VNSLKLESEC	SAPGEQPKFL	GIREQRVTGL	MIFVLMGCSV	FMTAILKFIP	MPVLYGVFLY	MGVSSLQGIQ	FFDRLKLFGM
890	900	910	920	930	940	950	960
PAKHQPDFIY	LRHVPLRKVH	LFTLIQLTCL	VLLWVIKASP	AAIVFPMMVL	ALVFVRKVMD	LCFSKRELSW	LDDLMPESKK
970	980	990	1000	1010	1020	1030	1040
KKLDDAKKKA	KEEEEAEKML	EIGGDKFPLE	SRKLLSSPGK	NISCRCDPSE	INISDEMPKT	TVWKALSMNS	GNAKEKSLFN
1050							

Cmpd.	No. of Cmpds.	m/z meas.	Δ m/z [ppm]	z	Rt [min]	Score	P	Range	Sequence	Modification
2993	1	863.9668	48.28	2	66.2	13.6	2	1025-1040	K.ALSMNSGNAKEKSLFN.-	Oxidation: 4

Detailed Protein Report

Protein 660: **PREDICTED: putative uncharacterized protein LOC400499 [Homo sapiens]**

**Accession:**	gi|578846033	**Score:**	13.6
**Database:**	refseq_human(refseq_human_20140103.fasta)	**MW [kDa]:**	74.9
**Database Date:**	2015-11-30	**pI:**	10.1
		**Sequence Coverage [%]:**	2.3
		**No. of unique Peptides:**	1

10	20	30	40	50	60	70	80
MGWSDLQPAM	GGEAERFQAQ	LEVKLVTGGS	PVVFTGNLTR	QVGSKLAFSA	SLSHLLSDQA	NVTALLERKE	ENGRRVAALG
**90**	**100**	**110**	**120**	**130**	**140**	**150**	**160**
AELFVPGLVG	LRALGLLQQR	GQLWTNSLRI	QYSLLGQAKQ	AAHECSTSQK	LRADSGSDGA	YRLELRHELH	CTQILAFSHK
**170**	**180**	**190**	**200**	**210**	**220**	**230**	**240**
VQLWHEEDSR	HLHSQLEVSY	GKHWDKNSNK	RHLRVSQTFK	NDSGPALSNH	FMEFVLQVPE	RQVDCRVQLY	HLSLRLPYVE
**250**	**260**	**270**	**280**	**290**	**300**	**310**	**320**
SSSHLKVQYN	GRPLFVAGGQ	WKDTSRATLW	KWEGALNLDS	PWLMVSAAHR	LYWPHRAVFQ	AVLELTLGKA	WTLKDLVVSV
**330**	**340**	**350**	**360**	**370**	**380**	**390**	**400**
GCRSQGPNRE	GKIQVYTAAT	TYLRVSTVTV	LAQSLFHSWS	ELESAWNTAV	QGEIHAENSR	DRKILNCWLK	GPQQELNLTA
**410**	**420**	**430**	**440**	**450**	**460**	**470**	**480**
AYRHLEWPRK	TQVSLTAVWI	GAQGQPRGLQ	LEGELEELRQ	DRTLYRKRGA	LLLRHPLHLP	IPQSLLLQET	FTADRRHQRY
**490**	**500**	**510**	**520**	**530**	**540**	**550**	**560**
SLETRVVLNG	REETLQTMVL	GCQAGHPYVC	AGLMHPYDGK	VIPRNTEGCL	VTWNQHTAKN	REVEATLKVH	RKVVLHLKGL
**570**	**580**	**590**	**600**	**610**	**620**	**630**	**640**
HHDRSQHGEI	RHSLALDLAH	SYQLSFPQAL	SLDGGIIFRQ	SPQGTFNFGM	DARAAVNHNV	TSQASVQLNG	SDSDFAFFFQ
**650**	**660**	**670**					
LRHPHGPTFP	PNFQVWTLLP	WR					

Cmpd.	No. of Cmpds.	m/z meas.	Δ m/z [ppm]	z	Rt [min]	Score	P	Range	Sequence	Modification
170	1	793.8952	-14.93	2	31.4	13.6	1	315-329	K.DLVVSVGCRSQGPNR.E	

Detailed Protein Report

Protein 661: integral membrane protein 2B [Homo sapiens]

Accession: gi|11527402
Database: refseq_human(refseq_human_20140103.fasta)
Database Date: 2015-11-30

Score: 13.6
MW [kDa]: 30.3
pI: 4.9
Sequence Coverage [%]: 4.5
No. of unique Peptides: 1

10	20	30	40	50	60	70	80
MVKVTFNSAL	AQKEAKKDEP	KSGEEALIIP	PDAVAVDCKD	PDDVVPVGQR	RAWCWCMCFG	LAFMLAGVIL	GGAYLYKYFA
**90**	**100**	**110**	**120**	**130**	**140**	**150**	**160**
LQPDDVYYCG	IKYIKDDVIL	NEPSADAPAA	LYQTIEENIK	IFEEEEVEFI	SVPVPEFADS	DPANIVHDFN	KKLTAYLDLN
**170**	**180**	**190**	**200**	**210**	**220**	**230**	**240**
LDKCYVIPLN	TSIVMPPRNL	LELLINIKAG	TYLPQSYLIH	EHMVITDRIE	NIDHLGFFIY	RLCHDKETYK	LQRRETIKGI
**250**	**260**	**270**					
QKREASNCFA	IRHFENKFAV	ETLICS					

Cmpd.	No. of Cmpds.	m/z meas.	Δ m/z [ppm]	z	Rt [min]	Score	P	Range	Sequence	Modification
591	1	676.8296	-29.37	2	36.2	13.6	1	40-51	K.DPDDVVPVGQRR.A	

Detailed Protein Report

Protein 662: PREDICTED: helicase POLQ-like isoform X8 [Homo sapiens]

**Accession:**	gi|578808747	**Score:**	13.6
**Database:**	refseq_human(refseq_human_20140103.fasta)	**MW [kDa]:**	82.5
**Database Date:**	2015-11-30	**pI:**	9.0
**Modification(s):**	Oxidation	**Sequence Coverage [%]:**	1.4
		**No. of unique Peptides:**	1

10	20	30	40	50	60	70	80
MLQELLCCRK	DVLMILPYVA	IVQEKISGLS	SFGIELGFFV	EEYAGSKGRF	PPTKRREKKS	LYIATIEKGH	SLVNSLIETG
**90**	**100**	**110**	**120**	**130**	**140**	**150**	**160**
RIDSLGLVVV	DELHMIGEGS	RGATLEMTLA	KILYTSKTTQ	IIGMSATLNN	VEDLQKFLQA	EYYTSQFRPV	ELKEYLKIND
**170**	**180**	**190**	**200**	**210**	**220**	**230**	**240**
TIYEVDSKAE	NGMTFSRLLN	YKYSDTLKKM	DPDHLVALVT	EVIPNYSCLV	FCPSKKNCEN	VAEMICKFLS	KEYLKHKEKE
**250**	**260**	**270**	**280**	**290**	**300**	**310**	**320**
KCEVIKNLKN	IGNGNLCPVL	KRTIPFGVAY	HHSGLTSDER	KLLEEAYSTG	VLCLFTCTST	LAAGVNLPAR	RVILRAPYVA
**330**	**340**	**350**	**360**	**370**	**380**	**390**	**400**
KEFLKRNQYK	QMIGRAGRAG	IDTIGESILI	LQEKDKQQVL	ELITKPLENC	YSHLVQEFTK	GIQTLFLSLI	GLKIATNLDD
**410**	**420**	**430**	**440**	**450**	**460**	**470**	**480**
IYHFMNGTFF	GVQQKVLLKE	KSLWEITVES	LRYLTEKGLL	QKDTIYKSEE	EVQYNFHITK	LGRASFKGTI	DLAYCDILYR
**490**	**500**	**510**	**520**	**530**	**540**	**550**	**560**
DLKKGLEGLV	LESLLHLIYL	TTPYDLVSQC	NPDWMIYFRQ	FSQLSPAEQN	VAAILGVSES	FIGKKASGQA	IGKKVDKNVV
**570**	**580**	**590**	**600**	**610**	**620**	**630**	**640**
NRLYLSFVLY	TLLKETNIWT	VSEKFNMPRG	YIQNLLTGTA	SFSSCVLHFC	EELEEFWVYR	ALLVELTKKL	TYCVKAELIP
**650**	**660**	**670**	**680**	**690**	**700**	**710**	**720**
LMEVTGVLEG	RAKQLYSAGY	KSLMHLANAN	PEVLVRTIDH	LSRRQAKQIV	SSAKMLLHEK	AEALQEEVEE	LLRLPSDFPG
**730**							
AVASSTDKA							

Cmpd.	No. of Cmpds.	m/z meas.	Δ m/z [ppm]	z	Rt [min]	Score	P	Range	Sequence	Modification
1448	1	626.6972	-186.66	2	46.5	13.6	1	1-10	-.MLQELLCCRK.D	Oxidation: 1

Detailed Protein Report

Protein 663: poly [ADP-ribose] polymerase 10 [Homo sapiens]

**Accession:**	gi|157738665	**Score:**	13.6
**Database:**	refseq_human(refseq_human_20140103.fasta)	**MW [kDa]:**	109.9
**Database Date:**	2015-11-30	**pI:**	4.8
		**Sequence Coverage [%]:**	1.9
		**No. of unique Peptides:**	1

Quantitation

WD:WU **Median:** 0.92 **CV:** 0.00 % **No. of Peptides:** 1

10	20	30	40	50	60	70	80
MVAMAEAEAG	VAVEVRGLPP	AVPDELLTLY	FENRRRSGGG	PVLSWQRLGC	GGVLTFREPA	DAERVLAQAD	HELHGAQLSL
**90**	**100**	**110**	**120**	**130**	**140**	**150**	**160**
RPAPPRAPAR	LLLQGLPPGT	TPQRLEQHVQ	ALLRASGLPV	QPCCALASPR	PDRALVQLPK	PLSEADVRVL	EEQAQNLGLE
**170**	**180**	**190**	**200**	**210**	**220**	**230**	**240**
GTLVSLARVP	QARAVRVVGD	GASVDLLLLE	LYLENERRSG	GGPLEDLQRL	PGPLGTVASF	QQWQVAERVL	QQEHRLQGSE
**250**	**260**	**270**	**280**	**290**	**300**	**310**	**320**
LSLVPHYDIL	EPEELAENTS	GGDHPSTQGP	RATKHALLRT	GGLVTALQGA	GTVTMGSGEE	PGQSGASLRT	GPMVQGRGIM
**330**	**340**	**350**	**360**	**370**	**380**	**390**	**400**
TTGSGQEPGQ	SGTSLRTGPM	GSLGQAEQVS	SMPMGSLEHE	GLVSLRPVGL	QEQEGPMSLG	PVGSAGPVET	SKGLLGQEGL
**410**	**420**	**430**	**440**	**450**	**460**	**470**	**480**
VEIAMDSPEQ	EGLVGPMEIT	MGSLEKAGPV	SPGCVKLAGQ	EGLVEMVLLM	EPGAMRFLQL	YHEDLLAGLG	DVALLPLEGP
**490**	**500**	**510**	**520**	**530**	**540**	**550**	**560**
DMTGFRLCGA	QASCQAAEEF	LRSLLGSISC	HVLCLEHPGS	ARFLLGPEGQ	HLLQGLEAQF	QCVFGTERLA	TATLDTGLEE
**570**	**580**	**590**	**600**	**610**	**620**	**630**	**640**
VDPTEALPVL	PGNAHTLWTP	DSTGGDQEDV	SLEEVRELLA	TLEGLDLDGE	DWLPRELEEE	GPQEQPEEEV	TPGHEEEEPV
**650**	**660**	**670**	**680**	**690**	**700**	**710**	**720**
APSTVAPRWL	EEEAALQLAL	HRSLEPQGQV	AEQEEAAALR	QALTLSLLEQ	PPLEAEEPPD	GGTDGKAQLV	VHSAFEQDVE
**730**	**740**	**750**	**760**	**770**	**780**	**790**	**800**
ELDRALRAAL	EVHVQEETVG	PWRRTLPAEL	RARLERCHGV	SVALRGDCTI	LRGFGAHPAR	AARHLVALLA	GPWDQSLAFP
**810**	**820**	**830**	**840**	**850**	**860**	**870**	**880**
LAASGPTLAG	QTLKGPWNNL	ERLAENTGEF	QEVVRAFYDT	LDAARSSIRV	VRVERVSHPL	LQQQYELYRE	RLLQRCERRP
**890**	**900**	**910**	**920**	**930**	**940**	**950**	**960**
VEQVLYHGTT	APAVPDICAH	GFNRSFCGRN	ATVYGKGVYF	ARRASLSVQD	RYSPPNADGH	KAVFVARVLT	GDYGQGRRGL
**970**	**980**	**990**	**1000**	**1010**	**1020**	**1030**	
RAPPLRGPGH	VLLRYDSAVD	CICQPSIFVI	FHDTQALPTH	LITCEHVPRA	SPDDPSGLPG	RSPDT	

Cmpd.	No. of Cmpds.	m/z meas.	Δ m/z [ppm]	z	Rt [min]	Score	P	Range	Sequence	Modification	Ratios
1670	1	932.4465	-0.39	2	49.6	13.6	0	318-336	R.GIMTTGSGQEPGQSGTSLR.T		WD:WU 0.92

Detailed Protein Report

Protein 664: structural maintenance of chromosomes protein 1B [Homo sapiens]

**Accession:**	gi|71565160	**Score:**	13.6
**Database:**	refseq_human(refseq_human_20140103.fasta)	**MW [kDa]:**	143.7
**Database Date:**	2015-11-30	**pI:**	7.8
**Modification(s):**	Oxidation	**Sequence Coverage [%]:**	1.1
		**No. of unique Peptides:**	1

Quantitation

WD:WU **Median:** 0.95 **CV:** 0.00 % **No. of Peptides:** 1

10	20	30	40	50	60	70	80
MAHLELLLVE	NFKSWRGRQV	IGPFRRFTCI	IGPNGSGKSN	VMDALSFVMG	EKIANLRVKN	IQELIHGAHI	GKPISSSASV
90	100	110	120	130	140	150	160
KIIYVEESGE	EKTFARIIRG	GCSEFRFNDN	LVSRSVYIAE	LEKIGIIVKA	QNCLVFQGTV	ESISVKKPKE	RTQFFEEIST
170	180	190	200	210	220	230	240
SGELIGEYEE	KKRKLQKAEE	DAQFNFNKKK	NIAAERRQAK	LEKEEAERYQ	SLLEELKMNK	IQLQLFQLYH	NEKKIHLLNT
250	260	270	280	290	300	310	320
KLEHVNRDLS	VKRESLSHHE	NIVKARKKEH	GMLTRQLQQT	EKELKSVETL	LNQKRPQYIK	AKENTSHHLK	KLDVAKKSIK
330	340	350	360	370	380	390	400
DSEKQCSKQE	DDIKALETEL	ADLDAAWRSF	EKQIEEEILH	KKRDIELEAS	QLDRYKELKE	QVRKKVATMT	QQLEKLQWEQ
410	420	430	440	450	460	470	480
KTDEERLAFE	KRRHGEVQGN	LKQIKEQIED	HKKRIEKLEE	YTKTCMDCLK	EKKQQEETLV	DEIEKTKSRM	SEFNEELNLI
490	500	510	520	530	540	550	560
RSELQNAGID	THEGKRQQKR	AEVLEHLKRL	YPDSVFGRLF	DLCHPIHKKY	QLAVTKVFGR	FITAIVVASE	KVAKDCIRFL
570	580	590	600	610	620	630	640
KEERAEPETF	LALDYLDIKP	INERLRELKG	CKMVIDVIKT	QFPQLKKVIQ	FVCGNGLVCE	TMEEARHIAL	SGPERQKTVA
650	660	670	680	690	700	710	720
LDGTLFLKSG	VISGGSSDLK	YKARCWDEKE	LKNLRDRRSQ	KIQELKGLMK	TLRKETDLKQ	IQTLIQGTQT	RLKYSQNELE
730	740	750	760	770	780	790	800
MIKKKHLVAF	YQEQSQLQSE	LLNIESQCIM	LSEGIKERQR	RIKEFQEKID	KVEDDIFQHF	CEEIGVENIR	EFENKHVKRQ
810	820	830	840	850	860	870	880
QEIDQKRLEF	EKQKTRLNVQ	LEYSRSHLKK	KLNKINTLKE	TIQKGSEDID	HLKKAEENCL	QTVNELMAKQ	QQLKDIRVTQ
890	900	910	920	930	940	950	960
NSSAEKVQTQ	IEEERKKFLA	VDREVGKLQK	EVVSIQTSLE	QKRLEKHNLL	LDCKVQDIEI	ILLSGSLDDI	IEVEMGTEAE
970	980	990	1000	1010	1020	1030	1040
STQATIDIYE	KEEAFEIDYS	SLKEDLKALQ	SDQEIEAHLR	LLLQQVASQE	DILLKTAAPN	LRALENLKTV	RDKFQESTDA
1050	1060	1070	1080	1090	1100	1110	1120
FEASRKEARL	CRQEFEQVKK	RRYDLFTQCF	EHVSISIDQI	YKKLCRNNSA	QAFLSPENPE	EPYLEGISYN	CVAPGKRFMP
1130	1140	1150	1160	1170	1180	1190	1200
MDNLSGGEKC	VAALALLFAV	HSFRPAPFFV	LDEVDAALDN	TNIGKVSSYI	KEQTQDQFQM	IVISLKEEFY	SRADALIGIY
1210	1220	1230	1240				
PEYDDCMFSR	VLTLDLSQYP	DTEGQESSKR	HGESR				

Cmpd.	No. of Cmpds.	m/z meas.	Δ m/z [ppm]	z	Rt [min]	Score	P	Range	Sequence	Modification	Ratios	
2132	1	757.3003	-55.47	2	55.0	13.6	1	1117-1129	K.RFMPMDNLSGGEK.C	Oxidation: 3, 5	WD:WU	0.95

Detailed Protein Report

Protein 665: sodium/hydrogen exchanger 5 precursor [Homo sapiens]

**Accession:**	gi|4759144	**Score:**	13.6
**Database:**	refseq_human(refseq_human_20140103.fasta)	**MW [kDa]:**	98.9
**Database Date:**	2015-11-30	**pI:**	7.9
		**Sequence Coverage [%]:**	1.3
		**No. of unique Peptides:**	1

10	20	30	40	50	60	70	80
MLRAALSLLA	LPLAGAAEEP	TQKPESPGEP	PPGLELFRWQ	WHEVEAPYLV	ALWILVASLA	KIVFHLSRKV	TSLVPESCLL
**90**	**100**	**110**	**120**	**130**	**140**	**150**	**160**
ILLGLVLGGI	VLAVAKKAEY	QLEPGTFFLF	LLPPIVLDSG	YFMPSRLFFD	NLGAILTYAV	VGTLWNAFTT	GAALWGLQQA
**170**	**180**	**190**	**200**	**210**	**220**	**230**	**240**
GLVAPRVQAG	LLDFLLFGSL	ISAVDPVAVL	AVFEEVHVNE	TLFIIVFGES	LLNDAVTVVL	YKVCNSFVEM	GSANVQATDY
**250**	**260**	**270**	**280**	**290**	**300**	**310**	**320**
LKGVASLFVV	SLGGAAVGLV	FAFLLALTTR	FTKRVRIIEP	LLVFLLAYAA	YLTAEMASLS	AILAVTMCGL	GCKKYVEANI
**330**	**340**	**350**	**360**	**370**	**380**	**390**	**400**
SHKSRTTVKY	TMKTLASCAE	TVIFMLLGIS	AVDSSKWAWD	SGLVLGTLIF	ILFFRALGVV	LQTWVLNQFR	LVPLDKIDQV
**410**	**420**	**430**	**440**	**450**	**460**	**470**	**480**
VMSYGGLRGA	VAFALVILLD	RTKVPAKDYF	VATTIVVVFF	TVIVQGLTIK	PLVKWLKVKR	SEHHKPTLNQ	ELHEHTFDHI
**490**	**500**	**510**	**520**	**530**	**540**	**550**	**560**
LAAVEDVVGH	HGYHYWRDRW	EQFDKKYLSQ	LLMRRSAYRI	RDQIWDVYYR	LNIRDAISFV	DQGGHVLSST	GLTLPSMPSR
**570**	**580**	**590**	**600**	**610**	**620**	**630**	**640**
NSVAETSVTN	LLRESGSGAC	LDLQVIDTVR	SGRDREDAVM	HHLLCGGLYK	PRRRYKASCS	RHFISEDAQE	RQDKEVFQQN
**650**	**660**	**670**	**680**	**690**	**700**	**710**	**720**
MKRRLESFKS	TKHNICFTKS	KPRPRKTGRR	KKDGVANAEA	TNGKHRGLGF	QDTAAVILTV	ESEEEEEESD	SSETEKEDDE
**730**	**740**	**750**	**760**	**770**	**780**	**790**	**800**
GIIFVARATS	EVLQEGKVSG	SLEVCPSPRI	IPPSPTCAEK	ELPWKSGQGD	LAVYVSSETT	KIVPVDMQTG	WNQSISSLES
**810**	**820**	**830**	**840**	**850**	**860**	**870**	**880**
LASPPCNQAP	ILTCLPPHPR	GTEEPQVPLH	LPSDPRSSFA	FPPSLAKAGR	SRSESSADLP	QQQELQPLMG	HKDHTHLSPG
**890**	**900**						
TATSHWCIQF	NRGSRL						

Cmpd.	No. of Cmpds.	m/z meas.	Δ m/z [ppm]	z	Rt [min]	Score	P	Range	Sequence	Modification
1364	1	669.3350	-19.03	2	45.8	13.6	0	397-408	K.IDQVVMSYGGLR.G	

Detailed Protein Report

Protein 666: multiple epidermal growth factor-like domains protein 11 precursor [Homo sapiens]

**Accession:**	gi|181336739	**Score:**	13.5
**Database:**	refseq_human(refseq_human_20140103.fasta)	**MW [kDa]:**	110.8
**Database Date:**	2015-11-30	**pI:**	5.8
**Modification(s):**	Carbamidomethyl	**Sequence Coverage [%]:**	2.0
		**No. of unique Peptides:**	1

10	20	30	40	50	60	70	80
MVLSLTGLIA	FSFLQATLAL	NPEDPNVCSH	WESYAVTVQE	SYAHPFDQIY	YTRCTDILNW	FKCTRHRISY	KTAYRRGLRT
**90**	**100**	**110**	**120**	**130**	**140**	**150**	**160**
MYRRRSQCCP	GYYESGDFCI	PLCTEECVHG	RCVSPDTCHC	EPGWGGPDCS	SGCDSDHWGP	HCSNRCQCQN	GALCNPITGA
**170**	**180**	**190**	**200**	**210**	**220**	**230**	**240**
CVCAAGFRGW	RCEELCAPGT	HGKGCQLPCQ	CRHGASCDPR	AGECLCAPGY	TGVYCEELCP	PGSHGAHCEL	RCPCQNGGTC
**250**	**260**	**270**	**280**	**290**	**300**	**310**	**320**
HHITGECACP	PGWTGAVCAQ	PCPPGTFGQN	CSQDCPCHHG	GQCDHVTGQC	HCTAGYMGDR	CQEECPFGSF	GFQCSQHCDC
**330**	**340**	**350**	**360**	**370**	**380**	**390**	**400**
HNGGQCSPTT	GACECEPGYK	GPRCQERLCP	EGLHGPGCTL	PCPCDADNTI	SCHPVTGACT	CQPGWSGHHC	NESCPVGYYG
**410**	**420**	**430**	**440**	**450**	**460**	**470**	**480**
DGCQLPCTCQ	NGADCHSITG	GCTCAPGFMG	EVCAVSCAAG	TYGPNCSSIC	SCNNGGTCSP	VDGSCTCKEG	WQGLDCTLPC
**490**	**500**	**510**	**520**	**530**	**540**	**550**	**560**
PSGTWGLNCN	ESCTCANGAA	CSPIDGSCSC	TPGWLGDTCE	LPCPDGTFGL	NCSEHCDCSH	ADGCDPVTGH	CCCLAGWTGI
**570**	**580**	**590**	**600**	**610**	**620**	**630**	**640**
RCDSTCPPGR	WGPNCSVSCS	CENGGSCSPE	DGSCECAPGF	RGPLCQRICP	PGFYGHGCAQ	PCPLCVHSSR	PCHHISGICE
**650**	**660**	**670**	**680**	**690**	**700**	**710**	**720**
CLPGFSGALC	NQVCAGGYFG	QDCAQLCSCA	NNGTCSPIDG	SCQCFPGWIG	KDCSQACPPG	FWGPACFHAC	SCHNGASCSA
**730**	**740**	**750**	**760**	**770**	**780**	**790**	**800**
EDGACHCTPG	WTGLFCTQRC	PAAFFGKDCG	RVCQCQNGAS	CDHISGKCTC	RTGFTGQHCE	QRCAPGTFGY	GCQQLCECMN
**810**	**820**	**830**	**840**	**850**	**860**	**870**	**880**
NSTCDHVTGT	CYCSPGFKGI	RCDQAALMME	ELNPYTKISP	ALGAERHSVG	AVTGIMLLLF	LIVVLLGLFA	WHRRRQKEKG
**890**	**900**	**910**	**920**	**930**	**940**	**950**	**960**
RDLAPRVSYT	PAMRMTSTDY	SLSGACGMDR	RQNTYIMDKG	FKDYMKESVC	SSSTCSLNSS	ENPYATIKDP	PILTCKLPES
**970**	**980**	**990**	**1000**	**1010**	**1020**	**1030**	**1040**
SYVEMKSPVH	MGSPYTDVPS	LSTSNKNIYE	VEPTVSVVQE	GCGHNSSYIQ	NAYDLPRNSH	IPGHYDLLPV	RQSPANGPSQ
**1050**							
DKQS							

Cmpd.	No. of Cmpds.	m/z meas.	Δ m/z [ppm]	z	Rt [min]	Score	P	Range	Sequence	Modification
2524	1	821.0832	83.31	3	59.8	13.5	1	172-192	R.CEELCAPGTHGKGCQLPCQCR.H	Carbamidomethyl: 5, 14, 18, 20

Detailed Protein Report

Protein 667: uncharacterized protein C2orf54 isoform 2 [Homo sapiens]

**Accession:**	gi|544710097	**Score:**	13.5
**Database:**	refseq_human(refseq_human_20140103.fasta)	**MW [kDa]:**	30.9
**Database Date:**	2015-11-30	**pI:**	9.0
**Modification(s):**	Oxidation	**Sequence Coverage [%]:**	4.3
		**No. of unique Peptides:**	1

10	20	30	40	50	60	70	80
MDTGSLNAAS	LREEQLHLSL	LVSSGWRTIS	FHVVPVVRRK	LGAPALEGVQ	QMPGFPEGSL	RRILSQGVDL	VPASAQLWRT
**90**	**100**	**110**	**120**	**130**	**140**	**150**	**160**
STDYLLTRLL	GELGSLQGHR	LDSLSILDRV	NHESWRDSGQ	TDGLTFGHLK	MVLLWASVLF	PAPEDWAELQ	GAVYRLLVVL
**170**	**180**	**190**	**200**	**210**	**220**	**230**	**240**
LCCLATRKLP	HFLHPQRNLL	QGSGLDLGAI	YQRVEGFASQ	PEAALRIHAT	HLGRSPPPRI	GSGLKALLQL	PASDPTYWAT
**250**	**260**	**270**	**280**				
AYFDVLLDKF	QVFNIQDKDR	ISAMQSIFQK	TRTLGGEES				

Cmpd.	No. of Cmpds.	m/z meas.	Δ m/z [ppm]	z	Rt [min]	Score	P	Range	Sequence	Modification
914	1	626.3206	27.28	2	40.2	13.5	0	1-12	-.MDTGSLNAASLR.E	Oxidation: 1

Detailed Protein Report

Protein 668: PREDICTED: cyclin-L2 isoform X7 [Homo sapiens]

Accession: gi|578799847
Database: refseq_human(refseq_human_20140103.fasta)
Database Date: 2015-11-30

Score: 13.5
MW [kDa]: 33.8
pI: 11.5
Sequence Coverage [%]: 2.3
No. of unique Peptides: 1

Quantitation

WD:WU **Median:** 1.11 **CV:** 0.00 % **No. of Peptides:** 1

10	20	30	40	50	60	70	80
MNDSLRTDVF	VRFQPESIAC	ACIYLAARTL	EIPLPNRPHW	FLLFGATEEE	IQEICLKILQ	LYARKKVDLT	HLEGEVEKRK
**90**	**100**	**110**	**120**	**130**	**140**	**150**	**160**
HAIEEAKAQA	RGLLPGGTQV	LDGTSGFSPA	PKLVESPKEG	KGSKPSPLSV	KNTKRRLEGA	KKAKADSPVN	GLPKGRESRS
**170**	**180**	**190**	**200**	**210**	**220**	**230**	**240**
RSRSREQSYS	RSPSRSASPK	RRKSDSGSTS	GGSKSQSRSR	SRSDSPPRQA	PRSAPYKGSE	IRGSRKSKDC	KYPQKPHKSR
**250**	**260**	**270**	**280**	**290**	**300**		
SRSSSRSRSR	SRERADNPGK	YKKKSHYYRD	QRRERSRSYE	RTGRRYERDH	PGHSRHRR		

Cmpd.	No. of Cmpds.	m/z meas.	Δ m/z [ppm]	z	Rt [min]	Score	P	Range	Sequence	Modification	Ratios
510	1	469.1462	-224.93	2	34.8	13.5	2	282-288	R.TGRRYER.D		WD:WU 1.11

Detailed Protein Report

Protein 669: PREDICTED: cytochrome P450 2U1 isoform X4 [Homo sapiens]

Accession: gi|530377130
Database: refseq_human(refseq_human_20140103.fasta)
Database Date: 2015-11-30

Score: 13.5
MW [kDa]: 37.1
pI: 10.4
Sequence Coverage [%]: 6.6
No. of unique Peptides: 1

10	20	30	40	50	60	70	80
MSSPGPSQPP	AEDPPWPARL	LRAPLGLLRL	DPSGGALLLC	GLVALLGWSW	LRRRRARGIP	PGPTPWPLVG	NFGHVLLPPF
**90**	**100**	**110**	**120**	**130**	**140**	**150**	**160**
LRRRSWLSSR	TRAAGIDPSV	IGPQVLLAHL	ARVYGSIFSF	FIGHYLVVVL	SDFHSVREAL	VQQAEVFSDR	PRVPLISIVT
**170**	**180**	**190**	**200**	**210**	**220**	**230**	**240**
KEKEKVHEEI	ERVIGANRAP	SLTDKAQMPY	TEATIMEVQR	LTVVVPLAIP	HMTSENTVLQ	GYTIPKGTLI	LPNLWSVHRD
**250**	**260**	**270**	**280**	**290**	**300**	**310**	**320**
PAIWEKPEDF	YPNRFLDDQG	QLIKKETFIP	FGIGKRVCMG	EQLAKMELFL	MFVSLMQSFA	FALPEDSKKP	LLTGRFGLTL
**330**	**340**						
APHPFNITIS	RR						

Cmpd.	No. of Cmpds.	m/z meas.	Δ m/z [ppm]	z	Rt [min]	Score	P	Range	Sequence	Modification
332	1	752.4965	78.30	3	32.8	13.5	1	91-112	R.TRAAGIDPSVIGPQVLLAHLAR.V	

Detailed Protein Report

Protein 670: lysozyme C precursor [Homo sapiens]

**Accession:**	gi|4557894	**Score:**	13.5
**Database:**	refseq_human(refseq_human_20140103.fasta)	**MW [kDa]:**	16.5
**Database Date:**	2015-11-30	**pI:**	10.6
**Modification(s):**	Carbamidomethyl	**Sequence Coverage [%]:**	18.9
		**No. of unique Peptides:**	1

```
        10         20         30         40         50         60         70         80
MKALIVLGLV LLSVTVQGKV FERCELARTL KRLGMDGYRG ISLANWMCLA KWESGYNTRA TNYNAGDRST DYGIFQINSR
        90        100        110        120        130        140        150
YWCNDGKTPG AVNACHLSCS ALLQDNIADA VACAKRVVRD PQGIRAWVAW RNRCQNRDVR QYVQGCGV
```

Cmpd.	No. of Cmpds.	m/z meas.	Δ m/z [ppm]	z	Rt [min]	Score	P	Range	Sequence	Modification
2481	1	938.4534	2.74	3	59.3	13.5	0	88-115	K.TPGAVNACHLSCSALLQDNIADAVACAK.R	Carbamidomethyl: 26

Detailed Protein Report

Protein 671: neuronal pentraxin receptor [Homo sapiens]

**Accession:**	gi|17402888	**Score:**	13.5
**Database:**	refseq_human(refseq_human_20140103.fasta)	**MW [kDa]:**	52.8
**Database Date:**	2015-11-30	**pI:**	5.8
		**Sequence Coverage [%]:**	3.4
		**No. of unique Peptides:**	1

10	20	30	40	50	60	70	80
MKFLAVLLAA	GMLAFLGAVI	CIIASVPLAA	SPARALPGGA	DNASVASGAA	ASPGPQRSLS	ALHGAGGSAG	PPALPGAPAA
**90**	**100**	**110**	**120**	**130**	**140**	**150**	**160**
SAHPLPPGPL	FSRFLCTPLA	AACPSGAQQG	DAAGAAPGER	EELLLLQSTA	EQLRQTALQQ	EARIRADQDT	IRELTGKLGR
**170**	**180**	**190**	**200**	**210**	**220**	**230**	**240**
CESGLPRGLQ	GAGPRRDTMA	DGPWDSPALI	LELEDAVRAL	RDRIDRLEQE	LPARVNLSAA	PAPVSAVPTG	LHSKMDQLEG
**250**	**260**	**270**	**280**	**290**	**300**	**310**	**320**
QLLAQVLALE	KERVALSHSS	RRQRQEVEKE	LDVLQGRVAE	LEHGSSAYSP	PDAFKISIPI	RNNYMYARVR	KALPELYAFT
**330**	**340**	**350**	**360**	**370**	**380**	**390**	**400**
ACMWLRSRSS	GTGQGTPFSY	SVPGQANEIV	LLEAGHEPME	LLINDKVAQL	PLSLKDNGWH	HICIAWTTRD	GLWSAYQDGE
**410**	**420**	**430**	**440**	**450**	**460**	**470**	**480**
LQGSGENLAA	WHPIKPHGIL	ILGQEQDTLG	GRFDATQAFV	GDIAQFNLWD	HALTPAQVLG	IANCTAPLLG	NVLPWEDKLV
**490**	**500**	**510**					
EAFGGATKAA	FDVCKGRAKA						

Cmpd.	No. of Cmpds.	m/z meas.	Δ m/z [ppm]	z	Rt [min]	Score	P	Range	Sequence	Modification
2151	1	863.8612	-97.34	2	55.4	13.5	1	479-495	K.LVEAFGGATKAAFDVCK.G	

Detailed Protein Report

Protein 672: PREDICTED: gastrin/cholecystokinin type B receptor isoform X1 [Homo sapiens]

**Accession:**	gi|530395959	**Score:**	13.5
**Database:**	refseq_human(refseq_human_20140103.fasta)	**MW [kDa]:**	55.8
**Database Date:**	2015-11-30	**pI:**	11.5
**Modification(s):**	Oxidation	**Sequence Coverage [%]:**	3.3
		**No. of unique Peptides:**	1

10	20	30	40	50	60	70	80
MELLKLNRSV	QGTGPGPGAS	LCRPGAPLLN	SSSVGNLSCE	PPRIRGAGTR	ELELAIRITL	YAVIFLMSVG	GNMLIIVVLG
**90**	**100**	**110**	**120**	**130**	**140**	**150**	**160**
LSRRLRTVTN	AFLLSLAVSD	LLLAVACMPF	TLLPNLMGTF	IFGTVICKAV	SYLMGVSVSV	STLSLVAIAL	ERYSAICRPL
**170**	**180**	**190**	**200**	**210**	**220**	**230**	**240**
QARVWQTRSH	AARVIVATWL	LSGLLMVPYP	VYTVVQPVGP	RVLQCVHRWP	SARVRQTWSV	LLLLLLFFIP	GVVMAVAYGL
**250**	**260**	**270**	**280**	**290**	**300**	**310**	**320**
ISRELYLGLR	FDGDSDSDSQ	SRVRNQGGLP	GGAGPREQNL	GEAELWRATG	PAGVGGTEMK	VRVRRKLEME	LSWERRSGGD
**330**	**340**	**350**	**360**	**370**	**380**	**390**	**400**
WAGDWGDSPF	SLTAHPLCSG	AVHQNGRCRP	ETGAVGEDSD	GCYVQLPRSR	PALELTALTA	PGPGSGSRPT	QAKLLAKKRV
**410**	**420**	**430**	**440**	**450**	**460**	**470**	**480**
VRMLLVIVVL	FFLCWLPVYS	ANTWRAFDGP	GAHRALSGAP	ISFIHLLSYA	SACVNPLVYC	FMHRRFRQAC	LETCARCCPR
**490**	**500**	**510**	**520**				
PPRARPRALP	DEDPPTPSIA	SLSRLSYTTI	STLGPG				

Cmpd.	No. of Cmpds.	m/z meas.	Δ m/z [ppm]	z	Rt [min]	Score	P	Range	Sequence	Modification
1548	1	851.5588	119.65	2	47.7	13.5	2	288-304	R.ATGPAGVGGTEMKVRVR.R	Oxidation: 12

Detailed Protein Report

Protein 673: methyl-CpG-binding domain protein 4 isoform 4 [Homo sapiens]

Accession: gi|442796454
Database: refseq_human(refseq_human_20140103.fasta)
Database Date: 2015-11-30
Modification(s): Carbamidomethyl

Score: 13.5
MW [kDa]: 60.9
pI: 9.9
Sequence Coverage [%]: 2.2
No. of unique Peptides: 1

10	20	30	40	50	60	70	80
MGTTGLESLS	LGDRGAAPTV	TSSERLVPDP	PNDLRKEDVA	MELERVGEDE	EQMMIKRSSE	CNPLLQEPIA	SAQFGATAGT
**90**	**100**	**110**	**120**	**130**	**140**	**150**	**160**
ECRKSVPCGW	ERVVKQRLFG	KTAGRFDVYF	ISPQGLKFRS	KSSLANYLHK	NGETSLKPED	FDFTVLSKRG	IKSRYKDCSM
**170**	**180**	**190**	**200**	**210**	**220**	**230**	**240**
AALTSHLQNQ	SNNSNWNLRT	RSKCKKDVFM	PPSSSSELQE	SRGLSNFTST	HLLLKEDEGV	DDVNFRKVRK	PKGKVTILKG
**250**	**260**	**270**	**280**	**290**	**300**	**310**	**320**
IPIKKTKKGC	RKSCSGFVQS	DSKRESVCNK	ADAESEPVAQ	KSQLDRTVCI	SDAGACGETL	SVTSEENSLV	KKKERSLSSG
**330**	**340**	**350**	**360**	**370**	**380**	**390**	**400**
SNFCSEQKTS	GIINKFCSAK	DSEHNEKYED	TFLESEEIGT	KVEVVERKEH	LHTDILKRGS	EMDNNCSPTR	KDFTGEKIFQ
**410**	**420**	**430**	**440**	**450**	**460**	**470**	**480**
EDTIPRTQIE	RRKTSLYFSS	KYNKEALSPP	RRKAFKKWTP	PRSPFNLVQE	TLFHDPWKLL	IATIFLNRTS	GKMAIPVLWK
**490**	**500**	**510**	**520**	**530**	**540**	**550**	
FLEKYPSAEV	ARTADWRDVS	ELLKPLGLYD	LRAKTIVKFS	DEYLTKQWKY	PIELHGIGAP		

Cmpd.	No. of Cmpds.	m/z meas.	Δ m/z [ppm]	z	Rt [min]	Score	P	Range	Sequence	Modification
711	1	665.1654	-215.52	2	37.7	13.5	1	252-263	R.KSCSGFVQSDSK.R	Carbamidomethyl: 3

Detailed Protein Report

Protein 674: integral membrane protein GPR180 precursor [Homo sapiens]

**Accession:**	gi|30725877	**Score:**	13.5
**Database:**	refseq_human(refseq_human_20140103.fasta)	**MW [kDa]:**	49.4
**Database Date:**	2015-11-30	**pI:**	7.2
		**Sequence Coverage [%]:**	3.0
		**No. of unique Peptides:**	1

10	20	30	40	50	60	70	80
MGGLRLLAVA	LTCCWWPQGS	QGKTLRGSFS	STAAQDAQGQ	RIGHFEFHGD	HALLCVRINN	IAVAVGKEAK	LYLFQAQEWL
**90**	**100**	**110**	**120**	**130**	**140**	**150**	**160**
KLQQSSHGYS	CSEKLSKAQL	TMTMNQTEHN	LTVSQIPSPQ	TWHVFYADKY	TCQDDKENSQ	VEDIPFEMVL	LNPDAEGNPF
**170**	**180**	**190**	**200**	**210**	**220**	**230**	**240**
DHFSAGESGL	HEFFFLLVLV	YFVIACIYAQ	SLWQAIKKGG	PMHMILKVLT	TALLLQAGSA	LANYIHFSSY	SKDGIGVPFM
**250**	**260**	**270**	**280**	**290**	**300**	**310**	**320**
GSLAEFFDIA	SQIQMLYLLL	SLCMGWTIVR	MKKSQSRPLQ	WDSTPASTGI	AVFIVMTQSV	LLLWEQFEDI	SHHSYHSHHN
**330**	**340**	**350**	**360**	**370**	**380**	**390**	**400**
LAGILLIVLR	ICLALSLGCG	LYQIITVERS	TLKREFYITF	AKGCILWFLC	HPVLACISVI	FSDYQRDKVI	TIGVILCQSV
**410**	**420**	**430**	**440**	**450**			
SMVILYRLFL	SHSLYWEVSS	LSSVTLPLTI	SSGHKSRPHF				

Cmpd.	No. of Cmpds.	m/z meas.	Δ m/z [ppm]	z	Rt [min]	Score	P	Range	Sequence	Modification
1390	1	727.2022	-165.33	2	45.7	13.5	0	82-94	K.LQQSSHGYSCSEK.L	

Detailed Protein Report

Protein 675: **PREDICTED: retinal rod rhodopsin-sensitive cGMP 3',5'-cyclic phosphodiesterase subunit gamma isoform X1 [Homo sapiens]**

**Accession:**	gi|578831043	**Score:**	13.5
**Database:**	refseq_human(refseq_human_20140103.fasta)	**MW [kDa]:**	15.1
**Database Date:**	2015-11-30	**pI:**	10.8
		**Sequence Coverage [%]:**	20.4
		**No. of unique Peptides:**	1

10	20	30	40	50	60	70	80
MKQRDKGRGW	HPAEGGPALT	AQPPETRPAL	DRSRRESRSQ	GCRGVSVSLT	MNLEPPKAEF	RSATRVAGGP	VTPRKGPPKF
**90**	**100**	**110**	**120**	**130**	**140**		
KQRQTRQFKS	KPPKKGVQGF	GDDIPGMEGL	GTDITVICPW	EAFNHLELHE	LAQYGII		

Cmpd.	No. of Cmpds.	m/z meas.	Δ m/z [ppm]	z	Rt [min]	Score	P	Range	Sequence	Modification
1451	1	994.1693	-10.60	3	46.9	13.5	2	7-34	K.GRGWHPAEGGPALTAQPPETRPALDRSR.R	

Detailed Protein Report

Protein 676: **PREDICTED: hephaestin isoform X3 [Homo sapiens]**

**Accession:**	gi|578838557	**Score:**	13.5
**Database:**	refseq_human(refseq_human_20140103.fasta)	**MW [kDa]:**	97.3
**Database Date:**	2015-11-30	**pI:**	5.8
**Modification(s):**	Oxidation	**Sequence Coverage [%]:**	2.4
		**No. of unique Peptides:**	1

Quantitation

WD:WU **Median:** 0.29 **CV:** 0.00 % **No. of Peptides:** 1

10	20	30	40	50	60	70	80
MWAMESGHLL	WALLFMQSLW	PQLTDGATRV	YYLGIRDVQW	NYAPKGRNVI	TNQPLDSDIV	ASSFLKSDKN	RIGGTYKKTI
90	100	110	120	130	140	150	160
YKEYKDDSYT	DEVAQPAWLG	FLGPVLQAEV	GDVILIHLKN	FATRPYTIHP	HGVFYEKDSE	GSLYPDGSSG	PLKADDSVPP
170	180	190	200	210	220	230	240
GGSHIYNWTI	PEGHAPTDAD	PACLTWIYHS	HVDAPRDIAT	GLIGPLITCK	RGALDGNSPP	QRQDVDHDFF	LLFSVVDENL
250	260	270	280	290	300	310	320
SWHLNENIAT	YCSDPASVDK	EDETFQESNR	MHAINGFVFG	NLPELNMCAQ	KRVAWHLFGM	GNEIDVHTAF	FHGQMLTTRG
330	340	350	360	370	380	390	400
HHTDVANIFP	ATFVTAEMVP	WEPGTWLISC	QVNSHFRDGM	QALYKVKSCS	MAPPVDLLTG	KVRQYFIEAH	EIQWDYGPMG
410	420	430	440	450	460	470	480
HDGSTGKNLR	EPGSISDKFF	QKSSSRIGGT	YWKVRYEAFQ	DETFQEKMHL	EEDRHLGILG	PVIRAEVGDT	IQVVFYNRAS
490	500	510	520	530	540	550	560
QPFSMQPHGV	FYEKDYEGTV	YNDGTFEIYC	QAGSHREAGM	RAIYNVSQCP	GHQATPRQRY	QAARIYYIMA	EEVEWDYCPD
570	580	590	600	610	620	630	640
RSWEREWHNQ	SEKDSYGYIF	LSNKDGLLGS	RYKKAVFREY	TDGTFRIPRP	RTGPEEHLGI	LGPLIKGEVG	DILTVVFKNN
650	660	670	680	690	700	710	720
ASRPYSVHAH	GVLESTTVWP	LAAEPAINGK	LYANLRGLTM	YQGERVAWYM	LAMGQDVDLH	TIHFHAESFL	YRNGENYRAD
730	740	750	760	770	780	790	800
VVDLFPGTFE	VVEMVASNPG	TWLMHCHVTD	HVHAGMETLF	TVFSRTEHLS	PLTVITKETE	KAVPPRDIEE	GNVKMLGMQI
810	820	830	840	850	860		
PIKNVEMLAS	VLVAISVTLL	LVVLALGGVV	WYQHRQRKLR	RNRRSILDDS	FKLLSFKQ		

Cmpd.	No. of Cmpds.	m/z meas.	Δ m/z [ppm]	z	Rt [min]	Score	P	Range	Sequence	Modification	Ratios	
2643	1	784.0056	-47.59	3	61.3	13.5	0	271-291	R. MHAINGFVFGNLPELNMCAQK.R	Oxidation: 17	WD:WU	0.29

Detailed Protein Report

Protein 677: alpha-protein kinase 3 [Homo sapiens]

**Accession:**	gi|115511036	**Score:**	13.4
**Database:**	refseq_human(refseq_human_20140103.fasta)	**MW [kDa]:**	201.1
**Database Date:**	2015-11-30	**pI:**	8.8
		**Sequence Coverage [%]:**	0.7
		**No. of unique Peptides:**	1

10	20	30	40	50	60	70	80
MEVAWLVYVL	GQQPLARQGE	GQSRLVPGRG	LVLWLPGLPR	SSPSWPAVDL	APLAPARPRG	PLICHTGHEQ	AGREPGPGSS
90	100	110	120	130	140	150	160
TKGPVLHDQD	TRCAFLPRPP	GPLQTRRYCR	HQGRQGSGLG	AGPGAGTWAP	APPGVSKPRC	PGRARPGEGQ	QQVTTARPPA
170	180	190	200	210	220	230	240
INRGARQPRA	GAAAAGRGPG	AGAWRTGEAA	ASAGPAVGEG	GAMGSRRAPS	RGWGAGGRSG	AGGDGEDDGP	VWIPSPASRS
250	260	270	280	290	300	310	320
YLLSVRPETS	LSSNRLSHPS	SGRSTFCSII	AQLTEETQPL	FETTLKSRSV	SEDSDVRFTC	IVTGYPEPEV	TWYKDDTELD
330	340	350	360	370	380	390	400
RYCGLPKYEI	THQGNRHTLQ	LYRCREEDAA	IYQASAQNSK	GIVSCSGVLE	VGTMTEYKIH	QRWFAKLKRK	AAAKLREIEQ
410	420	430	440	450	460	470	480
SWKHEKAVPG	EVDTLRKLSP	DRFQRKRRLS	GAQAPGPSVP	TREPEGGTLA	AWQEGETETA	QHSGLGLINS	FASGEVTTNG
490	500	510	520	530	540	550	560
EAAPENGEDG	EHGLLTYICD	AMELGPQRAL	KEESGAKKKK	KDEESKQGLR	KPELEKAAQS	RRSSENCIPS	SDEPDSCGTQ
570	580	590	600	610	620	630	640
GPVGVEQVQT	QPRGRAARGP	GSSGTDSTRK	PASAVGTPDK	AQKAPGPGPG	QEVYFSLKDM	YLENTQAVRP	LGEEGPQTLS
650	660	670	680	690	700	710	720
VRAPGESPKG	KAPLRARSEG	VPGAPGQPTH	SLTPQPTRPF	NRKRFAPPKP	KGEATTDSKP	ISSLSQAPEC	GAQSLGKAPP
730	740	750	760	770	780	790	800
QASVQVPTPP	ARRRHGTRDS	TLQGQAGHRT	PGEVLECQTT	TAPTMSASSS	SDVASIGVST	SGSQGIIEPM	DMETQEDGRT
810	820	830	840	850	860	870	880
SANQRTGSKK	NVQADGKIQV	DGRTRGDGTQ	TAQRTRADRK	TQVDAGTQES	KRPQSDRSAQ	KGMMTQGRAE	TQLETTQAGE
890	900	910	920	930	940	950	960
KIQEDRKAQA	DKGTQEDRRM	QGEKGMQGEK	GTQSEGSAPT	AMEGQSEQEV	ATSLGPPSRT	PKLPPTAGPR	APLNIECFVQ
970	980	990	1000	1010	1020	1030	1040
TPEGSCFPKK	PGCLPRSEEA	VVTASRNHEQ	TVLGPLSGNL	MLPAQPPHEG	SVEQVGGERC	RGPQSSGPVE	AKQEDSPFQC
1050	1060	1070	1080	1090	1100	1110	1120
PKEERPGGVP	CMDQGGCPLA	GLSQEVPTMP	SLPGTGLTAS	PKAGPCSTPT	SQHGSTATFL	PSEDQVLMSS	APTLHLGLGT
1130	1140	1150	1160	1170	1180	1190	1200
PTQSHPPETM	ATSSEGACAQ	VPDVEGRTPG	PRSCDPGLID	SLKNYLLLLL	KLSSTETSGA	GGESQVGAAT	GGLVPSATLT
1210	1220	1230	1240	1250	1260	1270	1280
PTVEVAGLSP	RTSRRILERV	ENNHLVQSAQ	TLLLSPCTSR	RLTGLLDREV	QAGRQALAAA	RGSWGPGPSS	LTVPAIVVDE
1290	1300	1310	1320	1330	1340	1350	1360
EDPGLASEGA	SEGEGEVSPE	GPGLLGASQE	SSMAGRLGEA	GGQAAPGQGP	SAESIAQEPS	QEEKFPGEAL	TGLPAATPEE
1370	1380	1390	1400	1410	1420	1430	1440
LALGARRKRF	LPKVRAAGDG	EATTPEERES	PTVSPRGPRK	SLVPGSPGTP	GRERRSPTQG	RKASMLEVPR	AEEELAAGDL
1450	1460	1470	1480	1490	1500	1510	1520
GPSPKAGGLD	TEVALDEGKQ	ETLAKPRKAK	DLLKAPQVIR	KIRVEQFPDA	SGSLKLWCQF	FNILSDSVLT	WAKDQRPVGE
1530	1540	1550	1560	1570	1580	1590	1600
VGRSAGDEGP	AALAIVQASP	VDCGVYRCTI	HNEHGSASTD	FCLSPEVLSG	FISREEGEVG	EEIEMTPMVF	AKGLADSGCW
1610	1620	1630	1640	1650	1660	1670	1680
GDKLFGRLVS	EELRGGGYGC	GLRKASQAKV	IYGLEPIFES	GRTCIIKVSS	LLVFGPSSET	SLVGRNYDVT	IQGCKIQNMS
1690	1700	1710	1720	1730	1740	1750	1760
REYCKIFAAE	ARAAPGFGEV	PEIIPLYLIY	RPANNIPYAT	LEEDLGKPLE	SYCSREWGCA	EAPTASGSSE	AMQKCQTFQH
1770	1780	1790	1800	1810	1820	1830	1840
WLYQWTNGSF	LVTDLAGVDW	KMTDVQIATK	LRGYQGLKES	CFPALLDRFA	SSHQCNAYCE	LLGLTPLKGP	EAAHPQAKAK
1850	1860	1870	1880	1890	1900	1910	
GSKSPSAGRK	GSQLSPQPQK	KGLPSPQGTR	KSAPSSKATP	QASEPVTTQL	LGQPPTQEEG	SKAQGMR	

Detailed Protein Report

Cmpd.	No. of Cmpds.	m/z meas.	Δ m/z [ppm]	z	Rt [min]	Score	P	Range	Sequence	Modification
1359	1	669.3184	-65.41	2	45.3	13.4	0	429-442	R.LSGAQAPGPSVPTR.E	

Detailed Protein Report

Protein 678: photoreceptor-specific nuclear receptor isoform a [Homo sapiens]

**Accession:**	gi|7706515	**Score:**	13.4
**Database:**	refseq_human(refseq_human_20140103.fasta)	**MW [kDa]:**	39.6
**Database Date:**	2015-11-30	**pI:**	8.0
**Modification(s):**	Carbamidomethyl, Oxidation	**Sequence Coverage [%]:**	6.3
		**No. of unique Peptides:**	1

10	20	30	40	50	60	70	80
METRPTALMS	STVAAAAPAA	GAASRKESPG	RWGLGEDPTG	VSPSLQCRVC	GDSSSGKHYG	IYACNGCSGF	FKRSVRRRLI
**90**	**100**	**110**	**120**	**130**	**140**	**150**	**160**
YRCQVGAGMC	PVDKAHRNQC	QACRLKKCLQ	AGMNQDAVQN	ERQPRSTAQV	HLDSMESNTE	SRPESLVAPP	APAGRSPRGP
**170**	**180**	**190**	**200**	**210**	**220**	**230**	**240**
TPMSAARALG	HHFMASLITA	ETCAKLEPED	ADENIDVTSN	DPEFPSSPYS	SSSPCGLDSI	HETSARLLFM	AVKWAKNLPV
**250**	**260**	**270**	**280**	**290**	**300**	**310**	**320**
FSSLPFRDQV	ILLEEAWSEL	FLLGAIQWSL	PLDSCPLLAP	PEASAAGGAQ	GRLTLASMET	RVLQETISRF	RALAVDPTEF
**330**	**340**	**350**	**360**	**370**			
ACMKALVLFK	PETRGLKDPE	HVEALQDQSQ	VMLSQHSKAH	HPSQPVR			

Cmpd.	No. of Cmpds.	m/z meas.	Δ m/z [ppm]	z	Rt [min]	Score	P	Range	Sequence	Modification
2476	1	900.2164	98.29	3	59.2	13.4	2	302-324	R.VLQETISRFRALAVDPTEFACMK.A	Carbamidomethyl: 21; Oxidation: 22

Detailed Protein Report

Protein 679: erythroid membrane-associated protein precursor [Homo sapiens]

**Accession:**	gi|19923536	**Score:**	13.4
**Database:**	refseq_human(refseq_human_20140103.fasta)	**MW [kDa]:**	52.6
**Database Date:**	2015-11-30	**pI:**	9.7
		**Sequence Coverage [%]:**	4.0
		**No. of unique Peptides:**	1

Alias proteins:

Accession	Name	Description
gi|578798289	refseq_human (refseq_human_20140103.fasta)	PREDICTED: erythroid membrane-associated protein isoform X2 [Homo sapiens]
gi|63054855	refseq_human (refseq_human_20140103.fasta)	erythroid membrane-associated protein precursor [Homo sapiens]

10	20	30	40	50	60	70	80
MEMASSAGSW	LSGCLIPLVF	LRLSVHVSGH	AGDAGKFHVA	LLGGTAELLC	PLSLWPGTVP	KEVRWLRSPF	PQRSQAVHIF
**90**	**100**	**110**	**120**	**130**	**140**	**150**	**160**
RDGKDQDEDL	MPEYKGRTVL	VRDAQEGSVT	LQILDVRLED	QGSYRCLIQV	GNLSKEDTVI	LQVAAPSVGS	LSPSAVALAV
**170**	**180**	**190**	**200**	**210**	**220**	**230**	**240**
ILPVLVLLIM	VCLCLIWKQR	RAKEKLLYEH	VTEVDNLLSD	HAKEKGKLHK	AVKKLRSELK	LKRAAANSGW	RRARLHFVAV
**250**	**260**	**270**	**280**	**290**	**300**	**310**	**320**
TLDPDTAHPK	LILSEDQRCV	RLGDRRQPVP	DNPQRFDFVV	SILGSEYFTT	GCHYWEVYVG	DKTKWILGVC	SESVSRKGKV
**330**	**340**	**350**	**360**	**370**	**380**	**390**	**400**
TASPANGHWL	LRQSRGNEYE	ALTSPQTSFR	LKEPPRCVGI	FLDYEAGVIS	FYNVTNKSHI	FTFTHNFSGP	LRPFFEPCLH
**410**	**420**	**430**	**440**	**450**	**460**	**470**	**480**
DGGKNTAPLV	ICSELHKSEE	SIVPRPEGKG	HANGDVSLKV	NSSLLPPKAP	ELKDIILSLP	PDLGPALQEL	KAPSF

Cmpd.	No. of Cmpds.	m/z meas.	Δ m/z [ppm]	z	Rt [min]	Score	P	Range	Sequence	Modification
1582	2	715.4259	42.30	3	48.5	13.4	2	232-250	R.RARLHFVAVTLDPDTAHPK.L	

Detailed Protein Report

Protein 680: cerebral cavernous malformations 2 protein-like [Homo sapiens]

**Accession:**	gi|31542255	**Score:**	13.4
**Database:**	refseq_human(refseq_human_20140103.fasta)	**MW [kDa]:**	46.7
**Database Date:**	2015-11-30	**pI:**	10.4
		**Sequence Coverage [%]:**	4.4
		**No. of unique Peptides:**	1

Alias proteins:

Accession	Name	Description
gi|578835776	refseq_human (refseq_human_20140103.fasta)	PREDICTED: cerebral cavernous malformations 2 protein-like isoform X1 [Homo sapiens]

10	20	30	40	50	60	70	80
MEYEVKKGKK	GFVSPIRRLV	FPKAGRRAAC	RSSVSRRPLH	SMPLYPPDYL	IDPQILLCDY	LEKEVKFLGH	LTWVTSSLNP
**90**	**100**	**110**	**120**	**130**	**140**	**150**	**160**
SSRDELLQLL	DTARQLKELP	LKTTAEQDSI	LSLSARCLLL	TWRDNEELIL	RIPTHEIAAA	SYLQDDALHL	LVLKTGLGVD
**170**	**180**	**190**	**200**	**210**	**220**	**230**	**240**
PVPAGVDASP	GGAGRDPGPP	GGAPEKRRVG	TAERRHTICS	LDWRMGWGGG	AAEARAGGGG	GGSLERQRAG	ARASGSWERR
**250**	**260**	**270**	**280**	**290**	**300**	**310**	**320**
QTFSGSWERR	HGGGGGGGGA	GKPGGSWERR	QAGSGGGGSW	ERRHPGPNPL	DPQDPSPDAY	CNLVILAVAN	RDAAEESCAL
**330**	**340**	**350**	**360**	**370**	**380**	**390**	**400**
ICQVFQIIYG	DQSIECVDRA	GYHYTSTPER	PWLCSRIMAP	RTPLKHVTAA	RPHLLSMAPT	AAAATTAVWA	WSSYRITWSR
**410**	**420**	**430**	**440**				
CGVSWGPSRS	SSLRCCCGST	GWGCPSRTIA	QAC				

Cmpd.	No. of Cmpds.	m/z meas.	Δ m/z [ppm]	z	Rt [min]	Score	P	Range	Sequence	Modification
2317	1	819.4008	25.39	2	57.3	13.4	0	251-269	R.HGGGGGGGGAGKPGGSWER.R	

Detailed Protein Report

Protein 681: prickle-like protein 4 [Homo sapiens]

Accession: gi|118722347
Database: refseq_human(refseq_human_20140103.fasta)
Database Date: 2015-11-30
Modification(s): Carbamidomethyl

Score: 13.4
MW [kDa]: 41.7
pI: 5.0
Sequence Coverage [%]: 4.9
No. of unique Peptides: 1

10	20	30	40	50	60	70	80
MSVQNSGWPH	QEDSPKPQDP	GPPANSDSDS	GHLPGEDPED	THAQGPAVLS	LGSLCLDTNQ	APNWTGLQTL	LQQLPPQDID
**90**	**100**	**110**	**120**	**130**	**140**	**150**	**160**
ERYCLALGEE	ERAELQLFCA	RRKQEALGQG	VARLVLPKLE	GHTCEKCREL	LKPGEYGVFA	ARAGEQRCWH	QPCFACQACG
**170**	**180**	**190**	**200**	**210**	**220**	**230**	**240**
QALINLIYFY	HDGQLYCGRH	HAELLRPRCP	ACDQLIFSWR	CTEAEGQRWH	ENHFCCQDCA	GPLGGGRYAL	PGGSPCCPSC
**250**	**260**	**270**	**280**	**290**	**300**	**310**	**320**
FENRYSDAGS	SWAGALEGQA	FLGETGLDRT	EGRDQTSVNS	ATLSRTLLAA	AGGSSLQTQR	GLPGSSPQQE	NRPGDKAEAP
**330**	**340**	**350**	**360**	**370**	**380**	**390**	
KGQEQCRLET	IRDPKDTPFS	TCSSSSDSEP	EGFFLGERLP	QSWKTPGSLQ	AEDSNASKTH	CTMC	

Cmpd.	No. of Cmpds.	m/z meas.	Δ m/z [ppm]	z	Rt [min]	Score	P	Range	Sequence	Modification
1234	1	1136.1482	87.82	2	43.7	13.4	1	83-101	R.YCLALGEEERAELQLFCAR.R	Carbamidomethyl: 2

Detailed Protein Report

Protein 682: **PREDICTED: uveal autoantigen with coiled-coil domains and ankyrin repeats isoform X3 [Homo sapiens]**

**Accession:**	gi|530406156	**Score:**	13.4
**Database:**	refseq_human(refseq_human_20140103.fasta)	**MW [kDa]:**	160.2
**Database Date:**	2015-11-30	**pI:**	6.8
		**Sequence Coverage [%]:**	1.1
		**No. of unique Peptides:**	1

10	20	30	40	50	60	70	80
MMNCWFSCTP	KNRHAADWNK	YDDRLMKAAE	RGDVEKVTSI	LAKKGVNPGK	LDVEGRSVFH	VVTSKGNLEC	LNAILIHGVD
90	100	110	120	130	140	150	160
ITTSDTAGRN	ALHLAAKYGH	ALCLQKLLQY	NCPTEHADLQ	GRTALHDAAM	ADCPSSIQLL	CDHGASVNAK	DVDGRTPLVL
170	180	190	200	210	220	230	240
ATQMSRPTIC	QLLIDRGADV	NSRDKQNRTA	LMLGCEYGCR	DAVEVLIKNG	ADISLLDALG	HDSSYYARIG	DNLDILTLLK
250	260	270	280	290	300	310	320
TASENTNKGR	ELWKKGPSLQ	QRNLTHMQDE	VNVKSHQREH	QNIQDLEIEN	EDLKERLRKI	QQEQRILLDK	VNGLQLQLNE
330	340	350	360	370	380	390	400
REKLKSLLAA	KEKQHEESLR	TIEALKNRFK	YFESDHLGSG	SHFSNRKEDM	LLKQGQMYMA	DSQCTSPGIP	AHMQSRSMLR
410	420	430	440	450	460	470	480
PLELSLPSQT	SYSENEILKK	ELEAMRTFCE	SAKQDRLKLQ	NELAHKVAEC	KALALECERV	KEDSDEQIKQ	LEDALKDVQK
490	500	510	520	530	540	550	560
RMYESEGKVK	QMQTHFLALK	EHLTSEAASG	NHRLTEELKD	QLKDLKVKYE	GASAEVGKLR	NQIKQNEMIV	EEFKRDEGKL
570	580	590	600	610	620	630	640
IEENKRLQKE	LSMCEMEREK	KGRKVTEMEG	QAKELSAKLA	LSIPAEKFEN	MKSSLSNEVN	EKAKKLVEME	REHEKSLSEI
650	660	670	680	690	700	710	720
RQLKRELENV	KAKLAQHVKP	EEHEQVKSRL	EQKSGELGKK	ITELTLKNQT	LQKEIEKVYL	DNKLLKEQAH	NLTIEMKNHY
730	740	750	760	770	780	790	800
VPLKVSEDMK	KSHDAIIDDL	NRKLLDVTQK	YTEKKLEMEK	LLLENDSLSK	DVSRLETVFV	PPEKHEKEII	ALKSNIVELK
810	820	830	840	850	860	870	880
KQLSELKKKC	GEDQEKIHAL	TSENTNLKKM	MSNQYVPVKT	HEEVKMTLND	TLAKTNRELL	DVKKKFEDIN	QEFVKIKDKN
890	900	910	920	930	940	950	960
EILKRNLENT	QNQIKAEYIS	LAEHEAKMSS	LSQSMRKVQD	SNAEILANYR	KGQEEIVTLH	AEIKAQKKEL	DTIQECIKVK
970	980	990	1000	1010	1020	1030	1040
YAPIVSFEEC	ERKFKATEKE	LKDQLSEQTQ	KYSVSEEEVK	KNKQENDKLK	KEIFTLQKDL	RDKTVLIEKS	HEMERALSRK
1050	1060	1070	1080	1090	1100	1110	1120
TDELNKQLKD	LSQKYTEVKN	VKEKLVEENA	KQTSEILAVQ	NLLQKQHVPL	EQVEALKKSL	NGTIENLKEE	LKSMQRCYEK
1130	1140	1150	1160	1170	1180	1190	1200
EQQTVTKLHQ	LLENQKNSSV	PLAEHLQIKE	AFEKEVGIIK	ASLREKEEES	QNKMEEVSKL	QSEVQNTKQA	LKKLETREVV
1210	1220	1230	1240	1250	1260	1270	1280
DLSKYKATKS	DLETQISSLN	EKLANLNRKY	EEVCEEVLHA	KKKEISAKDE	KELLHFSIEQ	EIKDQKERCD	KSLTTITELQ
1290	1300	1310	1320	1330	1340	1350	1360
RRIQESAKQI	EAKDNKITEL	LNDVERLKQA	LNGLSQLTYT	SGNPTKRQSQ	LIDTLQHQVK	SLEQQLADAD	RQHQEVIAIY
1370	1380	1390	1400				
RTHLLSAAQG	HMDEDVQEAL	LQIIQMRQGL	VC				

Cmpd.	No. of Cmpds.	m/z meas.	Δ m/z [ppm]	z	Rt [min]	Score	P	Range	Sequence	Modification
1985	1	599.9122	-110.16	3	53.2	13.4	2	959-973	K.VKYAPIVSFEECERK.F	

Detailed Protein Report

Protein 683: **dolichyl-diphosphooligosaccharide--protein glycosyltransferase subunit STT3A isoform b [Homo sapiens]**

**Accession:**	gi|511630717	**Score:**	13.4
**Database:**	refseq_human(refseq_human_20140103.fasta)	**MW [kDa]:**	69.5
**Database Date:**	2015-11-30	**pI:**	8.7
		**Sequence Coverage [%]:**	1.5
		**No. of unique Peptides:**	1

10	20	30	40	50	60	70	80
MITSAAIYHV	LHFFHITIDI	RNVCVFLAPL	FSSFTTIVTY	HLTKELKDAG	AGLLAAAMIA	VVPGYISRSV	AGSYDNEGIA
**90**	**100**	**110**	**120**	**130**	**140**	**150**	**160**
IFCMLLTYYM	WIKAVKTGSI	CWAAKCALAY	FYMVSSWGGY	VFLINLIPLH	VLVLMLTGRF	SHRIYVAYCT	VYCLGTILSM
**170**	**180**	**190**	**200**	**210**	**220**	**230**	**240**
QISFVGFQPV	LSSEHMAAFG	VFGLCQIHAF	VDYLRSKLNP	QQFEVLFRSV	ISLVGFVLLT	VGALLMLTGK	ISPWTGRFYS
**250**	**260**	**270**	**280**	**290**	**300**	**310**	**320**
LLDPSYAKNN	IPIIASVSEH	QPTTWSSYYF	DLQLLVFMFP	VGLYYCFSNL	SDARIFIIMY	GVTSMYFSAV	MVRLMLVLAP
**330**	**340**	**350**	**360**	**370**	**380**	**390**	**400**
VMCILSGIGV	SQVLSTYMKN	LDISRPDKKS	KKQQDSTYPI	KNEVASGMIL	VMAFFLITYT	FHSTWVTSEA	YSSPSIVLSA
**410**	**420**	**430**	**440**	**450**	**460**	**470**	**480**
RGGDGSRIIF	DDFREAYYWL	RHNTPEDAKV	MSWWDYGYQI	TAMANRTILV	DNNTWNNTHI	SRVGQAMAST	EEKAYEIMRE
**490**	**500**	**510**	**520**	**530**	**540**	**550**	**560**
LDVSYVLVIF	GGLTGYSSDD	INKFLWMVRI	GGSTDTGKHI	KENDYYTPTG	EFRVDREGSP	VLLNCLMYKM	CYYRFGQVYT
**570**	**580**	**590**	**600**	**610**	**620**		
EAKRPPGFDR	VRNAEIGNKD	FELDVLEEAY	TTEHWLVRIY	KVKDLDNRGL	SRT		

Cmpd.	No. of Cmpds.	m/z meas.	Δ m/z [ppm]	z	Rt [min]	Score	P	Range	Sequence	Modification
1173	1	500.6606	-239.16	2	43.3	13.4	1	571-579	R.VRNAEIGNK.D	

Detailed Protein Report

Protein 684: malcavernin isoform 4 [Homo sapiens]

**Accession:**	gi|269308190	**Score:**	13.4
**Database:**	refseq_human(refseq_human_20140103.fasta)	**MW [kDa]:**	39.4
**Database Date:**	2015-11-30	**pI:**	5.6
		**Sequence Coverage [%]:**	4.0
		**No. of unique Peptides:**	1

Quantitation

WD:WU **Median:** 0.16 **CV:** 0.00 % **No. of Peptides:** 1

10	20	30	40	50	60	70	80
MEEEGKKGKK	PGIVSPFKRV	FLKGEKSRDK	KAHEKVTERR	PLHTVVLSLP	ERVEPDRLLS	DYIEKEVKYL	GQLTSIPGYL
**90**	**100**	**110**	**120**	**130**	**140**	**150**	**160**
NPSSRTEILH	FIDNAKRAHQ	LPGHLTQEHD	AVLSLSAYNV	KLAWRDGEDI	ILRVPIHDIA	AVSYVRDDAA	HLVVLKTDDS
**170**	**180**	**190**	**200**	**210**	**220**	**230**	**240**
STKVDIKETY	EVEASTFCFP	ESVDVGGASP	HSKTISESEL	SASATELLQD	YMLTLRTKLS	SQEIQQFAAL	LHEYRNGASI
**250**	**260**	**270**	**280**	**290**	**300**	**310**	**320**
HEFCINLRQL	YGDSRKFLLL	GLRPFIPEKD	SQHFENFLET	IGVKDGRGII	TDSFGRHRRA	LSTTSSSTTN	GNRATGSSDD
**330**	**340**	**350**	**360**				
RSAPSEGDEW	DRMISDISSD	IEALGCSMDQ	DSA				

Cmpd.	No. of Cmpds.	m/z meas.	Δ m/z [ppm]	z	Rt [min]	Score	P	Range	Sequence	Modification	Ratios
1332	1	698.9025	94.05	2	45.0	13.4	0	300-313	R.ALSTTSSSTTNGNR.A		WD:WU 0.16

Detailed Protein Report

Protein 685: pyridoxal phosphate phosphatase PHOSPHO2 [Homo sapiens]

**Accession:**	gi|56606064	**Score:**	13.4
**Database:**	refseq_human(refseq_human_20140103.fasta)	**MW [kDa]:**	27.8
**Database Date:**	2015-11-30	**pI:**	6.4
		**Sequence Coverage [%]:**	4.6
		**No. of unique Peptides:**	1

Alias proteins:

Accession	Name	Description
gi|313151197	refseq_huma (refseq_human_20140103.fasta)	pyridoxal phosphate phosphatase PHOSPHO2 [Homo sapiens]
gi|313151195	refseq_huma (refseq_human_20140103.fasta)	pyridoxal phosphate phosphatase PHOSPHO2 [Homo sapiens]
gi|313151193	refseq_huma (refseq_human_20140103.fasta)	pyridoxal phosphate phosphatase PHOSPHO2 [Homo sapiens]
gi|313151191	refseq_huma (refseq_human_20140103.fasta)	pyridoxal phosphate phosphatase PHOSPHO2 [Homo sapiens]

10	20	30	40	50	60	70	80
MKILLVFDFD	NTIIDDNSDT	WIVQCAPNKK	LPIELRDSYR	KGFWTEFMGR	VFKYLGDKGV	REHEMKRAVT	SLPFTPGMVE
**90**	**100**	**110**	**120**	**130**	**140**	**150**	**160**
LFNFIRKNKD	KFDCIIISDS	NSVFIDWVLE	AASFHDIFDK	VFTNPAAFNS	NGHLTVENYH	THSCNRCPKN	LCKKVVLIEF
**170**	**180**	**190**	**200**	**210**	**220**	**230**	**240**
VDKQLQQGVN	YTQIVYIGDG	GNDVCPVTFL	KNDDVAMPRK	GYTLQKTLSR	MSQNLEPMEY	SVVVWSSGVD	IISHLQFLIK
**250**							
D							

Cmpd.	No. of Cmpds.	m/z meas.	Δ m/z [ppm]	z	Rt [min]	Score	P	Range	Sequence	Modification
1387	1	695.3741	-31.67	2	46.1	13.4	2	30-40	K.KLPIELRDSYR.K	

Detailed Protein Report

Protein 686: sodium/hydrogen exchanger 11 [Homo sapiens]

**Accession:**	gi|224465231	**Score:**	13.4
**Database:**	refseq_human(refseq_human_20140103.fasta)	**MW [kDa]:**	129.0
**Database Date:**	2015-11-30	**pI:**	6.5
**Modification(s):**	Oxidation	**Sequence Coverage [%]:**	1.2
		**No. of unique Peptides:**	1

Quantitation

WD:WU **Median:** 1.14 **CV:** 0.00 % **No. of Peptides:** 1

10	20	30	40	50	60	70	80
MSSYFWAQNE	SNRPDLLCGQ	PADYLVEEKH	FTTLVCFIVV	LGGLLKMCLK	NCEVIVLTIL	SLSGFVIGHM	AYNSVEVHQI
**90**	**100**	**110**	**120**	**130**	**140**	**150**	**160**
VYPLLRTSSF	SLYSYFSPLI	IFMVALDVEF	YTLKKMFWQV	LLTGLISFST	ASIIIGYVVI	KFNKDSWDLQ	SCLLFSITLG
**170**	**180**	**190**	**200**	**210**	**220**	**230**	**240**
IIDPLRSVNS	LKTIGISKIY	IDLIRGESLI	ICSIASIFFG	NFRGNRIHFS	IFRDLHVGIE	LSYDILGSII	FGYWCAKIIQ
**250**	**260**	**270**	**280**	**290**	**300**	**310**	**320**
CILADVFSNM	LTNIILCFSM	VYMTFYIVEF	LGMSGTLALA	AVGLNLDSLT	FKPKIELVIT	KFLRIFSSVY	EHLIYAFFGI
**330**	**340**	**350**	**360**	**370**	**380**	**390**	**400**
VIGCGELSHY	EFHTIPFIFI	LFTTVNLVRL	LTILLVSPIL	MHSNYEYNWR	WGVVITWSGI	KGVFNLLWAP	DVYNLAERKV
**410**	**420**	**430**	**440**	**450**	**460**	**470**	**480**
EVPQMFILYV	QVISLLTMGI	NSYVMTQSAR	KLDLCVLSLP	RQMILQNATQ	HIQEIVQNTI	TLFKTEKILT	NVNWTLVEDK
**490**	**500**	**510**	**520**	**530**	**540**	**550**	**560**
TRIEYIPFSH	VSHNDMKTES	TTDEALMEEA	RLHVAAIQMS	SFEKQRNNGI	LEIEAARILI	GAAKCYYSIQ	GKFMSIYDVS
**570**	**580**	**590**	**600**	**610**	**620**	**630**	**640**
TYMRTRSWLI	KFKNVLTFLE	YCIEKIHFIP	PESNTFLTFI	FHIVFSEEFE	YTGQIINLIY	IYPMIIHLWP	MARGLNVSAL
**650**	**660**	**670**	**680**	**690**	**700**	**710**	**720**
ISINYYFMFL	YVLESTLKII	ILKRKYFQQC	WNTLEFFILV	IGIIDIFCVY	FVKLRPDNLA	LIQLTVIMGY	LRIIRFLPLF
**730**	**740**	**750**	**760**	**770**	**780**	**790**	**800**
KIIVPILIRI	ADVQIKKRLS	LMYSITKGYI	KSQEDAKLLI	KQIAVCESIY	QKLCEILETN	KQDAVKELVL	MEHEGRDVVI
**810**	**820**	**830**	**840**	**850**	**860**	**870**	**880**
ALKTKQAIRN	VIAKALKNLT	FLCSRGIIDK	HEVIEINKVL	LKKLKALNNF	PKAIPPPTPD	IYLHNIIWLE	GKDVLIDFFK
**890**	**900**	**910**	**920**	**930**	**940**	**950**	**960**
ERAKLACFDS	GDTICKGGEM	PQGIYLIISG	MAILHSLSPT	FGIESNQRCD	RGSRDMFTEF	CTTGDIIGEL	SCLLKREIEY
**970**	**980**	**990**	**1000**	**1010**	**1020**	**1030**	**1040**
TVICETSLQA	CFISLEDLYE	GFDAFWPSLE	YKIWLKLALS	TAYQYFESSL	IDEDLRFQNC	VMFNQAYVET	LSSYSDMIID
**1050**	**1060**	**1070**	**1080**	**1090**	**1100**	**1110**	**1120**
NMTMKFVIIV	YGSVIDTKTE	EPYFAPCIIP	TTCEQVQGTS	DLSKLLIIQA	SELTQRNSNT	NVMASVNTVF	EQPGKNINGR
**1130**							
QKMS							

Cmpd.	No. of Cmpds.	m/z meas.	Δ m/z [ppm]	z	Rt [min]	Score	P	Range	Sequence	Modification	Ratios
2425	1	893.4053	-14.86	2	58.6	13.4	1	553-566	K.FMSIYDVSTYMRTR.S	Oxidation: 2	WD:WU 1.14

Detailed Protein Report

Protein 687: uncharacterized protein ARIH2OS [Homo sapiens]

Accession: gi|176866318
Database: refseq_human(refseq_human_20140103.fasta)
Database Date: 2015-11-30
Modification(s): Carbamidomethyl

Score: 13.4
MW [kDa]: 30.4
pI: 12.4
Sequence Coverage [%]: 8.3
No. of unique Peptides: 1

10	20	30	40	50	60	70	80
MLGQRAGDGE	RPGLPGDGEG	GVPARPGRRA	ERPPQRPAKV	NKAVTCAAHL	PGAAASRPLS	PNKPDRVRPG	QRDRIGAKRQ
90	100	110	120	130	140	150	160
RRRRADAGQA	RAASSRRVVP	TAPEVLGAVA	SLPDRGRPTV	ARVATGSRLE	GLFSAASLKL	SALTQSLTRV	RQAPTASGAT
170	180	190	200	210	220	230	240
IRLPASPVEM	FLTSAFLTGF	SFHCLYSGIG	HGEDILASVE	QITIVSRPLS	GQRGAGPGNS	AYTPRRSQGG	PRAATTPGFR
250	260	270	280	290	300		
FPCRGLVRRA	VLRLTVTVQD	CILTALLAVS	FHSIGVVIMT	SSYLLGPVVK			

Cmpd.	No. of Cmpds.	m/z meas.	Δ m/z [ppm]	z	Rt [min]	Score	P	Range	Sequence	Modification
573	1	819.8424	95.39	3	35.6	13.4	0	43-66	K.AVTCAAHLPGAAASRPLSPNKPDR.V	Carbamidomethyl: 4

Detailed Protein Report

**Protein 688:**	**glucose-fructose oxidoreductase domain-containing protein 1 isoform 2 [Homo sapiens]**		
**Accession:**	gi|336285206	**Score:**	13.3
**Database:**	refseq_human(refseq_human_20140103.fasta)	**MW [kDa]:**	31.8
**Database Date:**	2015-11-30	**pI:**	5.7
**Modification(s):**	Oxidation	**Sequence Coverage [%]:**	6.6
		**No. of unique Peptides:**	1

Alias proteins:

Accession	Name	Description
gi|336285213	refseq_human (refseq_human_20140103.fasta)	glucose-fructose oxidoreductase domain-containing protein 1 isoform 2 [Homo sapiens]

10	20	30	40	50	60	70	80
MTSAAHYYPK	LMSIMGNVLR	FLPAFVRMKQ	LIEEGYVGEP	LVCEVQVHGG	SLLGKKYNWS	CDDLMGGGGL	HSVGTYIIDL
**90**	**100**	**110**	**120**	**130**	**140**	**150**	**160**
LTFLTGQKAV	KVHGLLKTFV	KQTDHIKGIR	QITSDDFCTF	QMVLEGGVCC	TVTLNFNVPG	EFKQDVTVVG	SAGRLLAVGT
**170**	**180**	**190**	**200**	**210**	**220**	**230**	**240**
DLYGQRNSAP	EQELLVQDAT	PVSNSLLPEK	AFSDIPSPYL	RGTIKMMQAV	RQAFQDQDDR	RTWDGRPLTM	AATFDDCLYA
**250**	**260**	**270**	**280**	**290**			
LCVVDTIKRS	SQTGEWQNIA	IMTEEPELSP	AYLISEAMRR	SRMSLYC			

Cmpd.	No. of Cmpds.	m/z meas.	Δ m/z [ppm]	z	Rt [min]	Score	P	Range	Sequence	Modification
348	1	752.5027	120.14	3	33.0	13.3	2	11-29	K.LMSIMGNVLRFLPAFVRMK.Q	Oxidation: 5, 18

Detailed Protein Report

Protein 689: PREDICTED: PEX5-related protein isoform X2 [Homo sapiens]

Accession: gi|530374762
Database: refseq_human(refseq_human_20140103.fasta)
Database Date: 2015-11-30

Score: 13.3
MW [kDa]: 72.3
pI: 5.1
Sequence Coverage [%]: 2.5
No. of unique Peptides: 1

10	20	30	40	50	60	70	80
MSDSEMDGRT	HIPSLLNALL	SRNRVMQMSY	LKSKEQGYGK	LSSDEDLEII	VDQKQGKGSR	AADKAVAMVM	KEIPREESAE
90	100	110	120	130	140	150	160
EKPLLTMTSQ	LVNEQQESRP	LLSPSIDDFL	CETKSEAIAR	PVTSNTAVLT	TGLDLLDLSE	PVSQTQTKAK	KSEPSSKTSS
170	180	190	200	210	220	230	240
LKKKADGSDL	ISTDAEQRGQ	PLRVPETSSL	DLDIQTQLEK	WDDVKFHGDR	NTKGHPMAER	KSSSSRTGSK	ELLWSSEHRS
250	260	270	280	290	300	310	320
QPELSGGKSA	LNSESASELE	LVAPTQARLT	KEHRWGSALL	SRNHSLEEEF	ERAKAAVESD	TEFWDKMQAE	WEEMARRNWI
330	340	350	360	370	380	390	400
SENQEAQNQV	TISASEKGYY	FHTENPFKDW	PGAFEEGLKR	LKEGDLPVTI	LFMEAAILQD	PGDAEAWQFL	GITQAENENE
410	420	430	440	450	460	470	480
QAAIVALQRC	LELQPNNLKA	LMALAVSYTN	TGHQQDACDA	LKNWIKQNPK	YKYLVKSKKG	SPGLTRRMSK	SPVDSSVLEG
490	500	510	520	530	540	550	560
VKELYLEAAH	QNGDMIDPDL	QTGLGVLFHL	SGEFNRAIDA	FNAALTVRPE	DYSLWNRLGA	TLANGDRSEE	AVEAYTRALE
570	580	590	600	610	620	630	640
IQPGFIRSRY	NLGISCINLG	AYREAVSNFL	TALSLQRKSR	NQQQVPHPAI	SGNIWAALRI	ALSLMDQPEL	FQAANLGDLD
650	660						
VLLRAFNLDP							

Cmpd.	No. of Cmpds.	m/z meas.	Δ m/z [ppm]	z	Rt [min]	Score	P	Range	Sequence	Modification
1782	2	938.9355	-35.07	2	50.6	13.3	2	25-40	R.VMQMSYLKSKEQGYGK.L	

Detailed Protein Report

Protein 690: **alcohol dehydrogenase 4 [Homo sapiens]**

**Accession:**	gi|71565152	**Score:**	13.3
**Database:**	refseq_human(refseq_human_20140103.fasta)	**MW [kDa]:**	40.2
**Database Date:**	2015-11-30	**pI:**	9.4
**Modification(s):**	Carbamidomethyl	**Sequence Coverage [%]:**	3.2
		**No. of unique Peptides:**	1

10	20	30	40	50	60	70	80
MGTKGKVIKC	KAAIAWEAGK	PLCIEEVEVA	PPKAHEVRIQ	IIATSLCHTD	ATVIDSKFEG	LAFPVIVGHE	AAGIVESIGP
**90**	**100**	**110**	**120**	**130**	**140**	**150**	**160**
GVTNVKPGDK	VIPLYAPLCR	KCKFCLSPLT	NLCGKISNLK	SPASDQQLME	DKTSRFTCKG	KPVYHFFGTS	TFSQYTVVSD
**170**	**180**	**190**	**200**	**210**	**220**	**230**	**240**
INLAKIDDDA	NLERVCLLGC	GFSTGYGAAI	NNAKVTPGST	CAVFGLGGVG	LSAVMGCKAA	GASRIIGIDI	NSEKFVKAKA
**250**	**260**	**270**	**280**	**290**	**300**	**310**	**320**
LGATDCLNPR	DLHKPIQEVI	IELTKGGVDF	ALDCAGGSET	MKAALDCTTA	GWGSCTFIGV	AAGSKGLTIF	PEELIIGRTI
**330**	**340**	**350**	**360**	**370**	**380**	**390**	
NGTFFGGWKS	VDSIPKLVTD	YKNKKFNLDA	LVTHTLPFDK	ISEAFDLMNQ	GKSVRTILIF		

Cmpd.	No. of Cmpds.	m/z meas.	Δ m/z [ppm]	z	Rt [min]	Score	P	Range	Sequence	Modification
1369	1	676.8982	87.95	2	45.4	13.3	0	104-115	K.FCLSPLTNLCGK.I	Carbamidomethyl: 10

Detailed Protein Report

Protein 691: **PREDICTED: probable ATP-dependent RNA helicase DDX60-like isoform X7 [Homo sapiens]**

**Accession:**	gi|578809624	**Score:**	13.3
**Database:**	refseq_human(refseq_human_20140103.fasta)	**MW [kDa]:**	152.1
**Database Date:**	2015-11-30	**pI:**	7.0
**Modification(s):**	Carbamidomethyl	**Sequence Coverage [%]:**	0.8
		**No. of unique Peptides:**	1

Alias proteins:

Accession	Name	Description
gi|578809626	refseq_human (refseq_human_20140103.fasta)	PREDICTED: probable ATP-dependent RNA helicase DDX60-like isoform X8 [Homo sapiens]

```
        10         20         30         40         50         60         70         80
MGSKDHAVFF REMTQLILNE MPKAGYSSIL NDFVESNFFV IDGDSLLVTC LGVKSFKWGQ NLHFFYLVEC YLVDLLSNGG
        90        100        110        120        130        140        150        160
QFTIVFFKDA EYAYFDFPEL LSLRTALILH LQHNTNIDVQ TEFSGCLSQD WKLFLEQHYP YFLIVSEEGL SDLQTYLFNF
       170        180        190        200        210        220        230        240
LIIHSWGMKV NVVLSSGHES DTLRFYAYTM ESTDRNQTFS KENETVIQSA YKSLIQHLEE IRVLVLATHF EHLKWNDMME
       250        260        270        280        290        300        310        320
EAYQTLFLLQ HLWSEGSDIQ RVLCVTSCSL SLRMYHRVLV HSNCLSLQEV EDFCRLRCLC VAFQLHLPLS QRACSRVITC
       330        340        350        360        370        380        390        400
SWIRNSDSFL KMNKWCEYFI LSNLNVFGCW NLNLNHVSDL YDEQLLKNIA FYYEFESTQE PHLNLGDSIR RDYEDLWNVV
       410        420        430        440        450        460        470        480
SHLVKEFNVG KSFPLRTTRR HFLRQEKSVI QEISLEKMPS VGFIPMTSAV IDEFVGDMMK DLPILKSDDP VVPSLFKQKT
       490        500        510        520        530        540        550        560
SDELLHWHAQ RLLSDDYDRI KCHVDEQSRD PHVLDFLKKI QDYQQFYGKS LESISTKVIV TQTTRPKEDS SGASGEILQN
       570        580        590        600        610        620        630        640
TKPHQITKKS KKKSFLKEDQ NKAQQNDDLL FSIEEEMKNN LHSGIRKLED YLTSCASNSV KFGVEMLGLI ACFKAWKKHC
       650        660        670        680        690        700        710        720
RGEGKISKDL SIAVQMMKRI HSLLERYPEI LEAEHHQYIA KCLKYLGFND LANSLDPTLI GDDKNKKKYS IDIGPARFQL
       730        740        750        760        770        780        790        800
QYMGHYLIRD ERKDRDPRVQ DFIPNAWQQE LLDVVDKNES AVIVAPTSSG KTYASYYCME KVLRESDVGV VVYVAPAKSL
       810        820        830        840        850        860        870        880
VGQVAATVEN RFTKTLPAGR TLCGAFTRDY CHNVLNCQVL ITVPECFEIL LLAPHRQKWV ERIRYVIFDE VHYLGREVGA
       890        900        910        920        930        940        950        960
KFWELLLVII RCPFLVLSAT INNPNLLTKW LQSVKQYWKQ ADKIMEEKCI SEKQADKCLN FLQDHSYKNQ SYEVRLVLCG
       970        980        990       1000       1010       1020       1030       1040
ERYNDLEKHI CSVKHDDVYF DHFHPCAALT TDIIEKYGFP PDLTLTPQES IQLYDTMAQV WETWPRAQEL CPEEFILFKN
      1050       1060       1070       1080       1090       1100       1110       1120
KIVIKKLDAR KYEENLKAEL TNWIKNGQVK KVKRVLKNLS PDSLSSSKDM VKMFPLLVEK LRQMDKLPAI FFLFKNDDVG
      1130       1140       1150       1160       1170       1180       1190       1200
KRAGSVCTFL EKTETKSHPH TECHSYVFAI DEVLEKVRKT QKRISTKKNP KKAEKLERKK VYRAEYINFL ENLKILEISE
      1210       1220       1230       1240       1250       1260       1270       1280
DCTYADVKAL HTEITRNKDS TLERVLPRVR FTRHGKELKA LAQRGIGYHH SSMYFKEKEF VEILFVKGLI RVVTATETLA
      1290       1300       1310
LGIHMPCKSV VFAQDSVYLD ALNYRQIM
```

Cmpd.	No. of Cmpds.	m/z meas.	Δ m/z [ppm]	z	Rt [min]	Score	P	Range	Sequence	Modification
1104	1	586.3508	80.45	2	42.5	13.3	2	639-648	K.HCRGEGKISK.D	Carbamidomethyl: 2

Detailed Protein Report

Protein 692: kanadaptin [Homo sapiens]

**Accession:**	gi|155722990	**Score:**	13.3
**Database:**	refseq_human(refseq_human_20140103.fasta)	**MW [kDa]:**	88.8
**Database Date:**	2015-11-30	**pI:**	5.0
**Modification(s):**	Carbamidomethyl	**Sequence Coverage [%]:**	2.0
		**No. of unique Peptides:**	1

10	20	30	40	50	60	70	80
MLAPLRNAPG	REGATSPSPP	TDATGSLGEW	DVDRNVKTEG	WVSKERISKL	HRLRMADILS	QSETLASQDL	SGDFKKPALP
90	100	110	120	130	140	150	160
VSPAARSKAP	ASSSSNPEEV	QKEGPTALQD	SNSGEPDIPP	PQPDCGDFRS	LQEEQSRPPT	AVSSPGGPAR	APPYQEPPWG
170	180	190	200	210	220	230	240
GPATAPYSLE	TLKGGTILGT	RSLKGTSYCL	FGRLSGCDVC	LEHPSVSRYH	AVLQHRASGP	DGECDSNGPG	FYLYDLGSTH
250	260	270	280	290	300	310	320
GTFLNKTRIP	PRTYCRVHVG	HVVRFGGSTR	LFILQGPEED	REAESELTVT	QLKELRKQQQ	ILLEKKMLGE	DSDEEEEMDT
330	340	350	360	370	380	390	400
SERKINAGSQ	DDEMGCTWGM	GEDAVEDDAE	ENPIVLEFQQ	EREAFYIKDP	KKALQGFFDR	EGEELEYEFD	EQGHSTWLCR
410	420	430	440	450	460	470	480
VRLPVDDSTG	KQLVAEAIHS	GKKKEAMIQC	SLEACRILDT	LGLLRQEAVS	RKRKAKNWED	EDFYDSDDDT	FLDRTGLIEK
490	500	510	520	530	540	550	560
KRLNRMKKAG	KIDEKPETFE	SLVAKLNDAE	RELSEISERL	KASSQVLSES	PSQDSLDAFM	SEMKSGSTLD	GVSRKKLHLR
570	580	590	600	610	620	630	640
TFELRKEQQR	LKGLIKIVKP	AEIPELKKTE	TQTTGAENKA	KKLTLPLFGA	MKGGSKFKLK	TGTVGKLPPK	RPELPPTLMR
650	660	670	680	690	700	710	720
MKDEPEVEEE	EEEEEEEEKE	KEEHEKKKLE	DGSLSRPQPE	IEPEAAVQEM	RPPTDLTHFK	ETQTHENMSQ	LSEEEQNKDY
730	740	750	760	770	780	790	800
QDCSKTTSLC	AGPSASKNEY	EKSRGELKKK	KTPGPGKLPP	TLSSKYPEDD	PDYCVWVPPE	GQSGDGRTHL	NDKYGY

Cmpd.	No. of Cmpds.	m/z meas.	Δ m/z [ppm]	z	Rt [min]	Score	P	Range	Sequence	Modification
2677	1	973.4892	-48.43	2	61.8	13.3	2	249-264	R.IPPRTYCRVHVGHVVR.F	Carbamidomethyl: 7

Detailed Protein Report

Protein 693: BH3-interacting domain death agonist isoform 1 [Homo sapiens]

**Accession:**	gi|37574726	**Score:**	13.3
**Database:**	refseq_human(refseq_human_20140103.fasta)	**MW [kDa]:**	26.8
**Database Date:**	2015-11-30	**pI:**	6.7
**Modification(s):**	Carbamidomethyl	**Sequence Coverage [%]:**	4.6
		**No. of unique Peptides:**	1

```
        10         20         30         40         50         60         70         80
MCSGAGVMMA RWAARGRAGW RSTVRILSPL GHCEPGVSRS CRAAQAMDCE VNNGSSLRDE CITNLLVFGF LQSCSDNSFR
        90        100        110        120        130        140        150        160
RELDALGHEL PVLAPQWEGY DELQTDGNRS SHSRLGRIEA DSESQEDIIR NIARHLAQVG DSMDRSIPPG LVNGLALQLR
       170        180        190        200        210        220        230        240
NTSRSEEDRN RDLATALEQL LQAYPRDMEK EKTMLVLALL LAKKVASHTP SLLRDVFHTT VNFINQNLRT YVRSLARNGM
       250
D
```

Cmpd.	No. of Cmpds.	m/z meas.	Δ m/z [ppm]	z	Rt [min]	Score	P	Range	Sequence	Modification
836	1	585.7806	55.65	2	39.3	13.3	0	1-11	-.MCSGAGVMMAR.W	Carbamidomethyl: 2

Detailed Protein Report

Protein 694: protein ECT2 isoform b [Homo sapiens]

Accession: gi|21735572
Database: refseq_human(refseq_human_20140103.fasta)
Database Date: 2015-11-30
Modification(s): Carbamidomethyl

Score: 13.3
MW [kDa]: 100.0
pI: 7.9
Sequence Coverage [%]: 1.9
No. of unique Peptides: 1

Alias proteins:

Accession	Name	Description
gi|385198081	refseq_human (refseq_human_20140103.fasta)	protein ECT2 isoform b [Homo sapiens]

10	20	30	40	50	60	70	80
MAENSVLTST	TGRTSLADSS	IFDSKVTEIS	KENLLIGSTS	YVEEEMPQIE	TRVILVQEAG	KQEELIKALK	DIKVGFVKME
**90**	**100**	**110**	**120**	**130**	**140**	**150**	**160**
SVEEFEGLDS	PEFENVFVVT	DFQDSVFNDL	YKADCRVIGP	PVVLNCSQKG	EPLPFSCRPL	YCTSMMNLVL	CFTGFRKKEE
**170**	**180**	**190**	**200**	**210**	**220**	**230**	**240**
LVRLVTLVHH	MGGVIRKDFN	SKVTHLVANC	TQGEKFRVAV	SLGTPIMKPE	WIYKAWERRN	EQDFYAAVDD	FRNEFKVPPF
**250**	**260**	**270**	**280**	**290**	**300**	**310**	**320**
QDCILSFLGF	SDEEKTNMEE	MTEMQGGKYL	PLGDERCTHL	VVEENIVKDL	PFEPSKKLYV	VKQEWFWGSI	QMDARAGETM
**330**	**340**	**350**	**360**	**370**	**380**	**390**	**400**
YLYEKANTPE	LKKSVSMLSL	NTPNSNRKRR	RLKETLAQLS	RETDVSPFPP	RKRPSAEHSL	SIGSLLDISN	TPESSINYGD
**410**	**420**	**430**	**440**	**450**	**460**	**470**	**480**
TPKSCTKSSK	SSTPVPSKQS	ARWQVAKELY	QTESNYVNIL	ATIIQLFQVP	LEEEGQRGGP	ILAPEEIKTI	FGSIPDIFDV
**490**	**500**	**510**	**520**	**530**	**540**	**550**	**560**
HTKIKDDLED	LIVNWDESKS	IGDIFLKYSK	DLVKTYPPFV	NFFEMSKETI	IKCEKQKPRF	HAFLKINQAK	PECGRQSLVE
**570**	**580**	**590**	**600**	**610**	**620**	**630**	**640**
LLIRPVQRLP	SVALLLNDLK	KHTADENPDK	STLEKAIGSL	KEVMTHINED	KRKTEAQKQI	FDVVYEVDGC	PANLLSSHRS
**650**	**660**	**670**	**680**	**690**	**700**	**710**	**720**
LVQRVETISL	GEHPCDRGEQ	VTLFLFNDCL	EIARKRHKVI	GTFRSPHGQT	RPPASLKHIH	LMPLSQIKKV	LDIRETEDCH
**730**	**740**	**750**	**760**	**770**	**780**	**790**	**800**
NAFALLVRPP	TEQANVLLSF	QMTSDELPKE	NWLKMLCRHV	ANTICKADAE	NLIYTADPES	FEVNTKDMDS	TLSRASRAIK
**810**	**820**	**830**	**840**	**850**	**860**	**870**	**880**
KTSKKVTRAF	SFSKTPKRAL	RRALMTSHGS	VEGRSPSSND	KHVMSRLSST	SSLAGIPSPS	LVSLPSFFER	RSHTLSRSTT
**890**							
HLI							

Cmpd.	No. of Cmpds.	m/z meas.	Δ m/z [ppm]	z	Rt [min]	Score	P	Range	Sequence	Modification
2201	1	928.4175	-66.81	2	55.9	13.3	1	113-129	K.ADCRVIGPPVVLNCSQK.G	Carbamidomethyl: 14

Detailed Protein Report

Protein 695: syntaxin-7 [Homo sapiens]

Accession: gi|170932494
Database: refseq_human(refseq_human_20140103.fasta)
Database Date: 2015-11-30

Score: 13.2
MW [kDa]: 29.8
pI: 5.3
Sequence Coverage [%]: 5.7
No. of unique Peptides: 1

10	20	30	40	50	60	70	80
MSYTPGVGGD	PAQLAQRISS	NIQKITQCSV	EIQRTLNQLG	TPQDSPELRQ	QLQQKQQYTN	QLAKETDKYI	KEFGSLPTTP
**90**	**100**	**110**	**120**	**130**	**140**	**150**	**160**
SEQRQRKIQK	DRLVAEFTTS	LTNFQKVQRQ	AAEREKEFVA	RVRASSRVSG	SFPEDSSKER	NLVSWESQTQ	PQVQVQDEEI
**170**	**180**	**190**	**200**	**210**	**220**	**230**	**240**
TEDDLRLIHE	RESSIRQLEA	DIMDINEIFK	DLGMMIHEQG	DVIDSIEANV	ENAEVHVQQA	NQQLSRAADY	QRKSRKTLCI
**250**	**260**	**270**					
IILILVIGVA	IISLIIWGLN	H					

Cmpd.	No. of Cmpds.	m/z meas.	Δ m/z [ppm]	z	Rt [min]	Score	P	Range	Sequence	Modification
2771	1	616.3212	-15.13	3	63.2	13.2	1	50-64	R.QQLQQKQQYTNQLAK.E	

Detailed Protein Report

Protein 696: PREDICTED: collagen alpha-1(XXII) chain isoform X2 [Homo sapiens]

**Accession:**	gi|578815852	**Score:**	13.2
**Database:**	refseq_human(refseq_human_20140103.fasta)	**MW [kDa]:**	129.6
**Database Date:**	2015-11-30	**pI:**	7.8
**Modification(s):**	Oxidation	**Sequence Coverage [%]:**	1.4
		**No. of unique Peptides:**	1

10	20	30	40	50	60	70	80
MAGLRGNAVA	GLLWMLLLWS	GGGGCQAQRA	GCKSVHYDLV	FLLDTSSSVG	KEDFEKVRQW	VANLVDTFEV	GPDRTRVGVV
90	100	110	120	130	140	150	160
RYSDRPTTAF	ELGLFGSQEE	VKAAARRLAY	HGGNTNTGDA	LRYITARSFS	PHAGGRPRDR	AYKQVAILLT	DGRSQDLVLD
170	180	190	200	210	220	230	240
AAAAAHRAGI	RIFAVGVGEA	LKEELEEIAS	EPKSAHVFHV	SDFNAIDKIR	GKLRRRLCEN	VLCPSVRVEG	DRFKHTNGGT
250	260	270	280	290	300	310	320
KEITGFDLMD	LFSVKEILGK	RENGAQSSYV	RMGSFPVVQS	TEDVFPQGLP	DEYAFVTTFR	FRKTSRKEDW	YIWQVIDQYS
330	340	350	360	370	380	390	400
IPQVSIRLDG	ENKAVEYNAV	AGPQGEKGDV	GPAGPPGVPG	SVVQQEGLKG	EQGAPGPRGH	QGAPGPPGAR	GPIGPEGRDG
410	420	430	440	450	460	470	480
PPGLQGLRGK	KGDMGPPGIP	GLLGLQGPPG	PPGVPGPPGP	GGSPGLPGEI	GFPGKPGPPG	PTGPPGKDGP	NGPPGPPGTK
490	500	510	520	530	540	550	560
GEPGERGEDG	LPGKPGLRGE	IGEQGLAGRP	GEKGEAGLPG	APGFPGVRGE	KGDQGEKGEL	GLPGLKGDRG	EKGEAGPAGP
570	580	590	600	610	620	630	640
PGLPGTTSLF	TPHPRMPGEQ	GPKGEKGDPG	LPGEPGLQGR	PGELGPQGPT	GPPGAKGQEG	AHGAPGAAGN	PGAPGHVGAP
650	660	670	680	690	700	710	720
GPSGPPGSVG	APGLRGTPGK	DGERGEKGAA	GEEGSPGPVG	PRGDPGAPGL	PGPPGKGKDG	EPGLRGSPGL	PGPLGTKGDR
730	740	750	760	770	780	790	800
GAPGIPGSPG	SRGDPGIGVA	GPPGPSGPPG	DKGSPGSRGL	PGFPGPQGPA	GRDGAPGNPG	ERGPPGKPGL	SSLLSPGDIN
810	820	830	840	850	860	870	880
LLAKDVCNDC	PPGPPGLPGL	PGFKGDKGVP	GKPGREGTEG	KKGEAGPPGL	PGPPGIAGPQ	GSQGERGADG	EVGQKGDQGH
890	900	910	920	930	940	950	960
PGVPGFMGPP	GNPGPPGADG	IAGAAGPPGI	QGSPGKEGPP	GPQGPSGLPG	IPGEEGKEGR	DGKPGPPGEP	GKAGEPGLPG
970	980	990	1000	1010	1020	1030	1040
PEGARGPPGF	KGHTGDSGAP	GPRGESGAMG	LPGQEGLPGK	DGDTGPTGPQ	GPQGPRGPPG	KNGSPGSPGE	PGPSGTPGQK
1050	1060	1070	1080	1090	1100	1110	1120
GSKGENGSPG	LPGFLGPRGP	PGEPGEKGVP	GKEGVPGKPG	EPGFKGERGD	PGIKGDKGPP	GGKGQPGDPG	IPGHKGHTGL
1130	1140	1150	1160	1170	1180	1190	1200
MGPQGLPGEN	GPVGPPGPPG	QPGFPGLRGE	SPSMETLRRL	IQEELGKQLE	TRLAYLLAQM	PPAYMKSSQG	RPGPPGPPGK
1210	1220	1230	1240	1250	1260	1270	1280
DGLPGRAGPM	GEPGRPGQGG	LEGPSGPIGP	KGERGAKGDP	GAPGVGLRGE	MGPPGIPGQP	GEPGYAKDGL	PGIPGPQGET
1290	1300	1310	1320				
GPAGHPGLPG	PPGPPGQCDP	SQCAYFASLA	ARPGNVKGP				

Cmpd.	No. of Cmpds.	m/z meas.	Δ m/z [ppm]	z	Rt [min]	Score	P	Range	Sequence	Modification
1945	1	927.8884	-53.65	2	53.0	13.2	0	1249-1267	R.GEMGPPGIPGQPGEPGYAK.D	Oxidation: 3

Detailed Protein Report

Protein 697: ras-GEF domain-containing family member 1C [Homo sapiens]

Accession: gi|40255197
Database: refseq_human(refseq_human_20140103.fasta)
Database Date: 2015-11-30

Score: 13.2
MW [kDa]: 52.8
pI: 8.6
Sequence Coverage [%]: 3.9
No. of unique Peptides: 1

Alias proteins:

Accession	Name	Description
gi|578811061	refseq_human (refseq_human_20140103.fasta)	PREDICTED: ras-GEF domain-containing family member 1C isoform X1 [Homo sapiens]

```
        10         20         30         40         50         60         70         80
MPQTLSASDM VTPGSLSPPP TEPTDGEQAG QPLLDGAPSS ASLETLIQHL VPTADYYPEK AYIFTFLLSS RLFIEPRELL
        90        100        110        120        130        140        150        160
ARVCHLCIEQ QQLDKPVLDK ARVRKFGPKL LQLLAEWTET FPRDFQEEST IGHLKDVVGR IAPCDEAYRK RMHQLLQALH
       170        180        190        200        210        220        230        240
QKLAALRQGP EGLVGADKPI SYRTKPPASI HRELLGVCSD PYTLAQQLTH VELERLRHIG PEEFVQAFVN KDPLASTKPC
       250        260        270        280        290        300        310        320
FSDKTSNLEA YVKWFNRLCY LVATEICMPA KKKQRAQVIE FFIDVARECF NIGNFNSLMA IISGMNMSPV SRLKKTWAKV
       330        340        350        360        370        380        390        400
RTAKFFILEH QMDPTGNFCN YRTALRGAAH RSLTAHSSRE KIVIPFFSLL IKDIYFLNEG CANRLPNGHV NFEKFLELAK
       410        420        430        440        450        460        470
QVGEFITWKQ VECPFEQDAS ITHYLYTAPI FSEDGLYLAS YESESPENQT EKERWKALRS SILGKT
```

Cmpd.	No. of Cmpds.	m/z meas.	Δ m/z [ppm]	z	Rt [min]	Score	P	Range	Sequence	Modification
1850	1	715.9797	-114.03	3	51.8	13.2	1	106-123	K.FGPKLLQLLAEWTETFPR.D	

Detailed Protein Report

Protein 698: P2Y purinoceptor 8 [Homo sapiens]

Accession: gi|30023826
Database: refseq_human(refseq_human_20140103.fasta)
Database Date: 2015-11-30

Score: 13.2
MW [kDa]: 40.6
pI: 10.7
Sequence Coverage [%]: 5.6
No. of unique Peptides: 1

Alias proteins:

Accession	Name	Description
gi|578838987	refseq_human (refseq_human_20140103.fasta)	PREDICTED: P2Y purinoceptor 8 isoform X6 [Homo sapiens]
gi|578837768	refseq_human (refseq_human_20140103.fasta)	PREDICTED: P2Y purinoceptor 8 isoform X5 [Homo sapiens]
gi|530423005	refseq_human (refseq_human_20140103.fasta)	PREDICTED: P2Y purinoceptor 8 isoform X4 [Homo sapiens]
gi|530423003	refseq_human (refseq_human_20140103.fasta)	PREDICTED: P2Y purinoceptor 8 isoform X3 [Homo sapiens]
gi|530420927	refseq_human (refseq_human_20140103.fasta)	PREDICTED: P2Y purinoceptor 8 isoform X2 [Homo sapiens]
gi|530420925	refseq_human (refseq_human_20140103.fasta)	PREDICTED: P2Y purinoceptor 8 isoform X1 [Homo sapiens]

10	20	30	40	50	60	70	80
MQVPNSTGPD	NATLQMLRNP	AIAVALPVVY	SLVAAVSIPG	NLFSLWVLCR	RMGPRSPSVI	FMINLSVTDL	MLASVLPFQI
**90**	**100**	**110**	**120**	**130**	**140**	**150**	**160**
YYHCNRHHWV	FGVLLCNVVT	VAFYANMYSS	ILTMTCISVE	RFLGVLYPLS	SKRWRRRRYA	VAACAGTWLL	LLTALSPLAR
**170**	**180**	**190**	**200**	**210**	**220**	**230**	**240**
TDLTYPVHAL	GIITCFDVLK	WTMLPSVAMW	AVFLFTIFIL	LFLIPFVITV	ACYTATILKL	LRTEEAHGRE	QRRRAVGLAA
**250**	**260**	**270**	**280**	**290**	**300**	**310**	**320**
VVLLAFVTCF	APNNFVLLAH	IVSRLFYGKS	YYHVYKLTLC	LSCLNNCLDP	FVYYFASREF	QLRLREYLGC	RRVPRDTLDT
**330**	**340**	**350**	**360**				
RRESLFSART	TSVRSEAGAH	PEGMEGATRP	GLQRQESVF				

Cmpd.	No. of Cmpds.	m/z meas.	Δ m/z [ppm]	z	Rt [min]	Score	P	Range	Sequence	Modification
961	1	1025.8925	-94.87	2	40.3	13.2	0	335-354	R.SEAGAHPEGMEGATRPGLQR.Q	

Detailed Protein Report

Protein 699: G-protein coupled receptor 35 isoform a [Homo sapiens]

Accession: gi|33695097
Database: refseq_human(refseq_human_20140103.fasta)
Database Date: 2015-11-30

Score: 13.2
MW [kDa]: 34.0
pI: 10.3
Sequence Coverage [%]: 5.8
No. of unique Peptides: 1

10	20	30	40	50	60	70	80
MNGTYNTCGS	SDLTWPPAIK	LGFYAYLGVL	LVLGLLLNSL	ALWVFCCRMQ	QWTETRIYMT	NLAVADLCLL	CTLPFVLHSL
**90**	**100**	**110**	**120**	**130**	**140**	**150**	**160**
RDTSDTPLCQ	LSQGIYLTNR	YMSISLVTAI	AVDRYVAVRH	PLRARGLRSP	RQAAAVCAVL	WVLVIGSLVA	RWLLGIQEGG
**170**	**180**	**190**	**200**	**210**	**220**	**230**	**240**
FCFRSTRHNF	NSMAFPLLGF	YLPLAVVVFC	SLKVVTALAQ	RPPTDVGQAE	ATRKAARMVW	ANLLVFVVCF	LPLHVGLTVR
**250**	**260**	**270**	**280**	**290**	**300**	**310**	
LAVGWNACAL	LETIRRALYI	TSKLSDANCC	LDAICYYYMA	KEFQEASALA	VAPSAKAHKS	QDSLCVTLA	

Cmpd.	No. of Cmpds.	m/z meas.	Δ m/z [ppm]	z	Rt [min]	Score	P	Range	Sequence	Modification
2988	1	619.0064	18.19	3	66.1	13.2	1	282-299	K.EFQEASALAVAPSAKAHK.S	

Detailed Protein Report

Protein 700: alpha-2-macroglobulin-like protein 1 isoform 2 [Homo sapiens]

Accession: gi|539846446
Database: refseq_human(refseq_human_20140103.fasta)
Database Date: 2015-11-30
Modification(s): Oxidation

Score: 13.2
MW [kDa]: 105.9
pI: 5.7
Sequence Coverage [%]: 1.8
No. of unique Peptides: 1

10	20	30	40	50	60	70	80
MLIGKGSLVM	EGQKHLNSKK	KGLKASFSLS	LTFTSRLAPD	PSLVIYAIFP	SGGVVADKIQ	FSVEMCFDNQ	VSLGFSPSQQ
**90**	**100**	**110**	**120**	**130**	**140**	**150**	**160**
LPGAEVELQL	QAAPGSLCAL	RAVDESVLLL	RPDRELSNRS	VYGMFPFWYG	HYPYQVAEYD	QCPVSGPWDF	PQPLIDPMPQ
**170**	**180**	**190**	**200**	**210**	**220**	**230**	**240**
GHSSQRSIIW	RPSFSEGTDL	FSFFRDVGLK	ILSNAKIKKP	VDCSHRSPEY	STAMGAGGGH	PEAFESSTPL	HQAEDSQVRQ
**250**	**260**	**270**	**280**	**290**	**300**	**310**	**320**
YFPETWLWDL	FPIGNSGKEA	VHVTVPDAIT	EWKAMSFCTS	QSRGFGLSPT	VGLTAFKPFF	VDLTLPYSVV	RGESFRLTAT
**330**	**340**	**350**	**360**	**370**	**380**	**390**	**400**
IFNYLKDCIR	VQTDLAKSHE	YQLESWADSQ	TSSCLCADEA	KTHHWNITAV	KLGHINFTIS	TKILDSNEPC	GGQKGFVPQK
**410**	**420**	**430**	**440**	**450**	**460**	**470**	**480**
GRSDTLIKPV	LVKPEGVLVE	KTHSSLLCPK	GKVASESVSL	ELPVDIVPDS	TKAYVTVLGD	IMGTALQNLD	GLVQMPSGCG
**490**	**500**	**510**	**520**	**530**	**540**	**550**	**560**
EQNMVLFAPI	IYVLQYLEKA	GLLTEEIRSR	AVGFLEIGYQ	KELMYKHSNG	SYSAFGERDG	NGNTWLTAFV	TKCFGQAQKF
**570**	**580**	**590**	**600**	**610**	**620**	**630**	**640**
IFIDPKNIQD	ALKWMAGNQL	PSGCYANVGN	LLHTAMKGGV	DDEVSLTAYV	TAALLEMGKD	VDDPMVSQGL	RCLKNSATST
**650**	**660**	**670**	**680**	**690**	**700**	**710**	**720**
TNLYTQALLA	YIFSLAGEMD	IRNILLKQLD	QQAIISGESI	YWSQKPTPSS	NASPWSEPAA	VDVELTAYAL	LAQLTKPSLT
**730**	**740**	**750**	**760**	**770**	**780**	**790**	**800**
QKEIAKATSI	VAWLAKQRNA	YGGFSSTQDT	VVALQALAKY	ATTAYMPSEE	INLVVKSTEN	FQRTFNIQSV	NRLVFQQDTL
**810**	**820**	**830**	**840**	**850**	**860**	**870**	**880**
PNVPGMYTLE	ASGQGCVYVQ	TVLRYNILPP	TNMKTFSLSV	EIGKARCEQP	TSPRSLTLTI	HTSYVGSRSS	SNMAIVEVKM
**890**	**900**	**910**	**920**	**930**	**940**	**950**	**960**
LSGFSPMEGT	NQLLLQQPLV	KKVEFGTDTL	NIYLDELIKN	TQTYTFTISQ	SVLVTNLKPA	TIKVYDYYLP	DEQATIQYSD
**970**							
PCE							

Cmpd.	No. of Cmpds.	m/z meas.	Δ m/z [ppm]	z	Rt [min]	Score	P	Range	Sequence	Modification
2228	1	972.9750	-10.10	2	56.2	13.2	0	760-776	K.YATTAYMPSEEINLVVK.S	Oxidation: 7

Detailed Protein Report

Protein 701: pentraxin-4 [Homo sapiens]

Accession: gi|61966763
Database: refseq_human(refseq_human_20140103.fasta)
Database Date: 2015-11-30

Score: 13.2
MW [kDa]: 51.3
pI: 10.8
Sequence Coverage [%]: 3.2
No. of unique Peptides: 1

Quantitation

WD:WU **Median:** 2.88 **CV:** 0.00 % **No. of Peptides:** 1

10	20	30	40	50	60	70	80
MGSGNWEVTG	PPCGSRCERP	PGLPRGVHGQ	FRSAVGLSGL	RWFRRFQEVT	WTHLQNIASN	YNVSYNVDVR	FRSLAEESQA
**90**	**100**	**110**	**120**	**130**	**140**	**150**	**160**
VAQAVNRSQA	SVQGELAQLK	AWVRKLQRRG	RKVDTRLRAL	DLTLGERSQQ	RARERKAHKA	QRDALQDSLA	RLEGLVHSQG
**170**	**180**	**190**	**200**	**210**	**220**	**230**	**240**
ARLAALEGRL	PVAHPGTAAL	GPALVPTPTQ	PEELGPTSLK	LQRDRQELRA	ASEHRGPPQD	SSAPLQGRRE	PPASGSHRVL
**250**	**260**	**270**	**280**	**290**	**300**	**310**	**320**
SGTAPKDPRQ	QAWSPQVPGE	ICGVGPTLVF	PNASTRNVVF	LSPGFVTALR	ALSFCSWVRT	ASGRLGTLLS	YATEDNDNKL
**330**	**340**	**350**	**360**	**370**	**380**	**390**	**400**
VLHGRDSLLP	GSIHFVIGDP	AFRELPLQLL	LDGQWHHICV	IWTSTQGRYW	LHVDRRLVAT	GSRFREGYEI	PPGGSLVLGQ
**410**	**420**	**430**	**440**	**450**	**460**	**470**	**480**
EQDSVGGGFD	SSEAFVGSMS	GLAIWDRALV	PGEVANLAIG	KEFPTGAILT	LANAALAGGF	VQGANCTCLE	RCP

Cmpd.	No. of Cmpds.	m/z meas.	Δ m/z [ppm]	z	Rt [min]	Score	P	Range	Sequence	Modification	Ratios
424	1	787.0347	168.04	2	33.8	13.2	0	73-87	R.SLAEESQAVAQAVNR.S		WD:WU 2.88

Detailed Protein Report

Protein 702: helicase SRCAP [Homo sapiens]

**Accession:**	gi|146219843	**Score:**	13.2
**Database:**	refseq_human(refseq_human_20140103.fasta)	**MW [kDa]:**	343.3
**Database Date:**	2015-11-30	**pI:**	5.6
		**Sequence Coverage [%]:**	0.6
		**No. of unique Peptides:**	1

Detailed Protein Report

10	20	30	40	50	60	70	80
MQSSPSPAHP	QLPVLQTQMV	SDGMTGSNPV	SPASSSSPAS	SGAGGISPQH	IAQDSSLDGP	PGPPDGATVP	LEGFSLSQAA
90	100	110	120	130	140	150	160
DLANKGPKWE	KSHAEIAEQA	KHEAEIETRI	AELRKEGFWS	LKRLPKVPEP	PRPKGHWDYL	CEEMQWLSAD	FAQERRWKRG
170	180	190	200	210	220	230	240
VARKVVRMVI	RHHEEQRQKE	ERARREEQAK	LRRIASTMAK	DVRQFWSNVE	KVVQFKQQSR	LEEKRKKALD	LHLDFIVGQT
250	260	270	280	290	300	310	320
EKYSDLLSQS	LNQPLTSSKA	GSSPCLGSSS	AASSPPPPAS	RLDDEDGDFQ	PQEDEEEDDE	ETIEVEEQQE	GNDAEAQRRE
330	340	350	360	370	380	390	400
IELLRREGEL	PLEELLRSLP	PQLLEGPSSP	SQTPSSHDSD	TRDGPEEGAE	EEPPQVLEIK	PPPSAVTQRN	KQPWHPDEDD
410	420	430	440	450	460	470	480
EEFTANEEEA	EDEEDTIAAE	EQLEGEVDHA	MELSELAREG	ELSMEELLQQ	YAGAYAPGSG	SSEDEDEDEV	DANSSDCEPE
490	500	510	520	530	540	550	560
GPVEAEEPPQ	EDSSSQSDSV	EDRSEDEEDE	HSEEEETSGS	SASEESESEE	SEDAQSQSQA	DEEEEDDDFG	VEYLLARDEE
570	580	590	600	610	620	630	640
QSEADAGSGP	PTPGPTTLGP	KKEITDIAAA	AESLQPKGYT	LATTQVKTPI	PLLLRGQLRE	YQHIGLDWLV	TMYEKKLNGI
650	660	670	680	690	700	710	720
LADEMGLGKT	IQTISLLAHL	ACEKGNWGPH	LIIVPTSVML	NWEMELKRWC	PSFKILTYYG	AQKERKLKRQ	GWTKPNAFHV
730	740	750	760	770	780	790	800
CITSYKLVLQ	DHQAFRRKNW	RYLILDEAQN	IKNFKSQRWQ	SLLNFNSQRR	LLLTGTPLQN	SLMELWSLMH	FLMPHVFQSH
810	820	830	840	850	860	870	880
REFKEWFSNP	LTGMIEGSQE	YNEGLVKRLH	KVLRPFLLRR	VKVDVEKQMP	KKYEHVIRCR	LSKRQRCLYD	DFMAQTTTKE
890	900	910	920	930	940	950	960
TLATGHFMSV	INILMQLRKV	CNHPNLFDPR	PVTSPFITPG	ICFSTASLVL	RATDVHPLQR	IDMGRFDLIG	LEGRVSRYEA
970	980	990	1000	1010	1020	1030	1040
DTFLPRHRLS	RRVLLEVATA	PDPPPRPKPV	KMKVNRMLQP	VPKQEGRTVV	VVNNPRAPLG	PVPVRPPPGP	ELSAQPTPGP
1050	1060	1070	1080	1090	1100	1110	1120
VPQVLPASLM	VSASPAGPPL	IPASRPPGPV	LLPPLQPNSG	SLPQVLPSPL	GVLSGTSRPP	TPTLSLKPTP	PAPVRLSPAP
1130	1140	1150	1160	1170	1180	1190	1200
PPGSSSLLKP	LTVPPGYTFP	PAAATTTSTT	TATATTTAVP	APTPAPQRLI	LSPDMQARLP	SGEVVSIGQL	ASLAQRPVAN
1210	1220	1230	1240	1250	1260	1270	1280
AGGSKPLTFQ	IQGNKLTLTG	AQVRQLAVGQ	PRPLQRNVVH	LVSAGGQHHL	ISQPAHVALI	QAVAPTPGPT	PVSVLPSSTP
1290	1300	1310	1320	1330	1340	1350	1360
STTPAPTGLS	LPLAANQVPP	TMVNNTGVVK	IVVRQAPRDG	LTPVPPLAPA	PRPPSSGLPA	VLNPRPTLTP	GRLPTPTLGT
1370	1380	1390	1400	1410	1420	1430	1440
ARAPMPTPTL	VRPLLKLVHS	PSPEVSASAP	GAAPLTISSP	LHVPSSLPGP	ASSPMPIPNS	SPLASPVSST	VSVPLSSSLP
1450	1460	1470	1480	1490	1500	1510	1520
ISVPTTLPAP	ASAPLTIPIS	APLTVSASGP	ALLTSVTPPL	APVVPAAPGP	PSLAPSGASP	SASALTLGLA	TAPSLSSSQT
1530	1540	1550	1560	1570	1580	1590	1600
PGHPLLLAPT	SSHVPGLNST	VAPACSPVLV	PASALASPFP	SAPNPAPAQA	SLLAPASSAS	QALATPLAPM	AAPQTAILAP
1610	1620	1630	1640	1650	1660	1670	1680
SPAPPLAPLP	VLAPSPGAAP	VLASSQTPVP	VMAPSSTPGT	SLASASPVPA	PTPVLAPSST	QTMLPAPVPS	PLPSPASTQT
1690	1700	1710	1720	1730	1740	1750	1760
LALAPALAPT	LGGSSPSQTL	SLGTGNPQGP	FPTQTLSLTP	ASSLVPTPAQ	TLSLAPGPPL	GPTQTLSLAP	APPLAPASPV
1770	1780	1790	1800	1810	1820	1830	1840
GPAPAHTLTL	APASSSASLL	APASVQTLTL	SPAPVPTLGP	AAAQTLALAP	ASTQSPASQA	SSLVVSASGA	APLPVTMVSR
1850	1860	1870	1880	1890	1900	1910	1920
LPVSKDEPDT	LTLRSGPPSP	PSTATSFGGP	RPRRQPPPPP	RSPFYLDSLE	EKRKRQRSER	LERIFQLSEA	HGALAPVYGT
1930	1940	1950	1960	1970	1980	1990	2000
EVLDFCTLPQ	PVASPIGPRS	PGPSHPTFWT	YTEAAHRAVL	FPQQRLDQLS	EIIERFIFVM	PPVEAPPPSL	HACHPPPWLA
2010	2020	2030	2040	2050	2060	2070	2080
PRQAAFQEQL	ASELWPRARP	LHRIVCNMRT	QFPDLRLIQY	DCGKLQTLAV	LLRQLKAEGH	RVLIFTQMTR	MLDVLEQFLT
2090	2100	2110	2120	2130	2140	2150	2160
YHGHLYLRLD	GSTRVEQRQA	LMERFNADKR	IFCFILSTRS	GGVGVNLTGA	DTVVFYDSDW	NPTMDAQAQD	RCHRIGQTRD
2170	2180	2190	2200	2210	2220	2230	2240

Detailed Protein Report

Cmpd.	No. of Cmpds.	m/z meas.	Δ m/z [ppm]	z	Rt [min]	Score	P	Range	Sequence	Modification
1437	1	1087.6372	55.64	2	46.7	13.2	0	880-898	K.ETLATGHFMSVINILMQLR.K	

Detailed Protein Report

Protein 703: **potassium voltage-gated channel subfamily KQT member 2 isoform e [Homo sapiens]**

**Accession:**	gi|26051268	**Score:**	13.2
**Database:**	refseq_human(refseq_human_20140103.fasta)	**MW [kDa]:**	44.2
**Database Date:**	2015-11-30	**pI:**	10.6
		**Sequence Coverage [%]:**	3.3
		**No. of unique Peptides:**	1

Quantitation

WD:WU **Median:** 2.72 **CV:** 0.00 % **No. of Peptides:** 1

10	20	30	40	50	60	70	80
MVQKSRNGGV	YPGPSGEKKL	KVGFVGLDPG	APDSTRDGAL	LIAGSEAPKR	GSILSKPRAG	GAGAGKPPKR	NAFYRKLQNF
**90**	**100**	**110**	**120**	**130**	**140**	**150**	**160**
LYNVLERPRG	WAFIYHAYVF	LLVFSCLVLS	VFSTIKEYEK	SSEGALYILE	IVTIVVFGVE	YFVRIWAAGC	CCRYRGWRGR
**170**	**180**	**190**	**200**	**210**	**220**	**230**	**240**
LKFARKPFCV	IDIMVLIASI	AVLAAGSQGN	VFATSALRSL	RFLQILRMIR	MDRRGGTWKL	LGSVVYAHSK	ELVTAWYIGF
**250**	**260**	**270**	**280**	**290**	**300**	**310**	**320**
LCLILASFLV	YLAEKGENDH	FDTYADALWW	GLITLTTIGY	GDKYPQTWNG	RLLAATFTLI	GVSFFALPAG	ILGSGFALKV
**330**	**340**	**350**	**360**	**370**	**380**	**390**	**400**
QEQHRQKHFE	KRRNPAAGLI	QSAWRFYATN	LSRTDLHSTW	QYYERTVTVP	MYRYRRRAPA	TKQLFHFLFS	ICS

Cmpd.	No. of Cmpds.	m/z meas.	Δ m/z [ppm]	z	Rt [min]	Score	P	Range	Sequence	Modification	Ratios
1607	1	720.3800	-23.52	2	48.4	13.2	1	333-345	R.RNPAAGLIQSAWR.F		WD:WU 2.72

Detailed Protein Report

Protein 704: metallothionein-1G [Homo sapiens]

**Accession:**	gi|10835230	**Score:**	13.2
**Database:**	refseq_human(refseq_human_20140103.fasta)	**MW [kDa]:**	6.1
**Database Date:**	2015-11-30	**pI:**	10.5
**Modification(s):**	Carbamidomethyl	**Sequence Coverage [%]:**	41.0
		**No. of unique Peptides:**	1

```
        10         20         30         40         50         60         70
MDPNCSCAAG VSCTCASSCK CKECKCTSCK KSCCSCCPVG CAKCAQGCIC KGASEKCSCC A
```

Cmpd.	No. of Cmpds.	m/z meas.	Δ m/z [ppm]	z	Rt [min]	Score	P	Range	Sequence	Modification
1987	2	862.6346	-31.70	3	53.2	13.2	2	1-25	-.MDPNCSCAAGVSCTCASSCKCKECK.C	Carbamidomethyl: 7

Detailed Protein Report

Protein 705: stathmin isoform a [Homo sapiens]

**Accession:**	gi|5031851	**Score:**	13.2
**Database:**	refseq_human(refseq_human_20140103.fasta)	**MW [kDa]:**	17.3
**Database Date:**	2015-11-30	**pI:**	5.7
		**Sequence Coverage [%]:**	12.1
		**No. of unique Peptides:**	1

Alias proteins:

Accession	Name	Description
gi|44890052	refseq_human (refseq_human_20140103.fasta)	stathmin isoform a [Homo sapiens]
gi|44890050	refseq_human (refseq_human_20140103.fasta)	stathmin isoform a [Homo sapiens]

```
        10         20         30         40         50         60         70         80
MASSDIQVKE LEKRASGQAF ELILSPRSKE SVPEFPLSPP KKKDLSLEEI QKKLEAAEER RKSHEAEVLK QLAEKREHEK
        90        100        110        120        130        140        150
EVLQKAIEEN NNFSKMAEEK LTHKMEANKE NREAQMAAKL ERLREKDKHI EEVRKNKESK DPADETEAD
```

Cmpd.	No. of Cmpds.	m/z meas.	Δ m/z [ppm]	z	Rt [min]	Score	P	Range	Sequence	Modification
2763	1	1022.5215	-40.47	2	63.0	13.2	2	10-27	K.ELEKRASGQAFELILSPR.S	

Detailed Protein Report

Protein 706: PREDICTED: protein PRR14L isoform X4 [Homo sapiens]

**Accession:**	gi|578837142	**Score:**	13.1
**Database:**	refseq_human(refseq_human_20140103.fasta)	**MW [kDa]:**	219.9
**Database Date:**	2015-11-30	**pI:**	6.2
		**Sequence Coverage [%]:**	0.8
		**No. of unique Peptides:**	1

Quantitation

WD:WU	**Median:** 0.75	**CV:** 0.00 %	**No. of Peptides:** 1

Detailed Protein Report

10	20	30	40	50	60	70	80
MQSSKELSHV	DLPEDFLRSK	EGNVQITAET	LLKSAEVQGM	KVNGTKTDNN	EGHKNGNVSK	DLSAGCGEFQ	EVDKIMTSDE
90	100	110	120	130	140	150	160
VSETSTLVTP	EPLTFVDPVL	TEATPKEKEC	EELKSCPWLS	LPGNSAISNV	DNGKEELCKP	NLVCEADDNH	QQLHGHHNEQ
170	180	190	200	210	220	230	240
PSSTHDSPTA	TSPLKENSEV	SCFTSDLSGP	ESRTISLENC	GFEGGGLLKR	SAEKTDSSYF	YRGDDQGKNL	ASREENEERL
250	260	270	280	290	300	310	320
LIPRSERGGP	FLFNAREPEK	EISGRCSGEK	EPVVSPKENI	HNNCIQDSLH	TGNSSSLMPN	SFTEATEVML	NKNDLKITVH
330	340	350	360	370	380	390	400
VQGNLTNPED	HKETFTNMSH	PGGHSEESSF	SSLMQIEEAG	QTTPVEPNIL	SKSFYTKDCN	SLVSIQRNLE	GNTQLNEASC
410	420	430	440	450	460	470	480
NDFLFERKSI	VSLMPEDQIS	PVSEVLKPKQ	GTALLLPSPE	FDYRPESEKV	IQTSHDDIPL	LDEQSIACEM	NELSCTNELV
490	500	510	520	530	540	550	560
VNKVESECVL	NQQVSLNSQE	HANLPTDSLL	HLNKEMPLAT	GRDAHQSHHP	PLEGRADVIA	DIQTIPIQTK	IKDISPPGNQ
570	580	590	600	610	620	630	640
TCGASSNCPT	LNIKPVSLER	KKEMADSGTK	ALHSRLRSNK	REAAGFPQVV	SVIECHSVQS	QDISSCHRVR	KNVSQENMCS
650	660	670	680	690	700	710	720
ASAAFKSSKI	SLQVDNSLIT	KYENAFQHRD	HCCQGTGHSV	EKSSCKVSYT	SQERELDGKE	TNGSLPGDKI	RNKMVAGLLN
730	740	750	760	770	780	790	800
SGISNKTIHT	SSSIKLSEEG	LEGKEQDVSK	ETVFCKYNIS	DHAIQELNQT	VNIPGPEKVL	DQSPTVMFSS	FKNVKSVETL
810	820	830	840	850	860	870	880
DQKADEVLDC	QSNQNRPDEC	KSEGQSAKEM	LSSDQRETVT	EPHGEVNHNQ	KDLLVSSGSN	NSLPCGSPKK	CNLKGAFVKM
890	900	910	920	930	940	950	960
SGCDESTEGM	VDIVYTDCSN	KLAEGVLDVK	ASNLLDCGAR	QEKLAFQEDS	RSTLSRRELD	AAHTGTTGQD	SDFPVTAAST
970	980	990	1000	1010	1020	1030	1040
VDFLKIKKSC	EENVCRSLKD	CEMEKCPDSC	AHEMESVADH	EPNKRILGRV	NLSLNDSHYG	QQDKGTSLRE	TQEMTEGSRL
1050	1060	1070	1080	1090	1100	1110	1120
EPNSEFGKES	TFGISSKESM	SCHDESSVSL	RSLKSIEIMP	SQENSETNVN	SEETDLKNLC	KPKDGEMLCE	NVKDCTVLPE
1130	1140	1150	1160	1170	1180	1190	1200
MKEIVSRDWS	NSSDRDSVCT	CVEKNACKAC	HPHENSSDRH	LPLTVKTDIK	VKGEETEEHQ	RGRLGYLTVG	EQSEELVTRE
1210	1220	1230	1240	1250	1260	1270	1280
TGDGDPVSNI	SQTHFKCRGI	LNHAEKQQSP	EVLDYMLQKE	EKYIRQQKAH	TISQQCISSS	LLLDDAQNQN	QPKADKDEST
1290	1300	1310	1320	1330	1340	1350	1360
MINEITLAKL	AKDSIVAQTQ	KLEDQKEERL	HHPLRKDTES	CTSPCLLGAP	RKAQDPSSAG	CDQIHGAFAK	KGVLPLKKQP
1370	1380	1390	1400	1410	1420	1430	1440
HRTCKKVSYQ	EQIIVGRKIG	KIRSSAFLKS	SSNPIPTKAH	RLLSLCTLSA	PTRLEPETAP	TKSLVSHIPK	QMSTPCHPLR
1450	1460	1470	1480	1490	1500	1510	1520
SLNFRKTTKE	SALLNKLSIL	ASKLAPAMKT	QKLRYRRCSS	ELLPMAKSYK	RLRYKRLLDG	FSSSTEQLNP	YLAASGWDKR
1530	1540	1550	1560	1570	1580	1590	1600
PNSKPMALYS	LESIKMTFID	LSNKMPSLLF	GSEIFPVSFH	VKSSSSDCTT	ESSRTFPEHC	APARLALGEA	LQCPSQPPKW
1610	1620	1630	1640	1650	1660	1670	1680
TFSFFLSHGC	PGMATFREDT	GVHSQTHTQA	PPQPPAPLQD	YGGTAIVQTR	ADCSVLGLHT	LLALCSPGCY	RIWTKKRSFS
1690	1700	1710	1720	1730	1740	1750	1760
SHMPTMQRLF	MTQFTQGLKG	LRSPASIADK	VFCSLPYSVG	RVLSIWSQHG	PSVCSFEISS	LHSPHCKRQP	SLGTTSSHTM
1770	1780	1790	1800	1810	1820	1830	1840
LPYVPLPGME	ATYNTSGSQT	RLEPPFPALV	PKSCLVAESA	VSKLLLSASE	FQVRGLDELD	GVKAACPCPQ	SSPPEQKEAE
1850	1860	1870	1880	1890	1900	1910	1920
PEKRPKKVSQ	IRIRKTIPRP	DPNLTPMGLP	RPKRLKKKEF	SLEEIYTNKN	YKSPPANRCL	ETIFEEPKER	NGTLISISQQ
1930	1940	1950	1960	1970	1980	1990	
KRKRVLEFQD	FTVPRKRRAR	GKVKVAGSFT	RAQKAAVQSR	ELDALLIQKL	MELETFFAKE	EEQEQSSGC	

Cmpd.	No. of Cmpds.	m/z meas.	Δ m/z [ppm]	z	Rt [min]	Score	P	Range	Sequence	Modification	Ratios
140	1	793.8794	36.37	2	31.0	13.1	0	632-646	K.NVSQENMCSASAAFK.S		WD:WU 0.75

Detailed Protein Report

Protein 707: ryanodine receptor 3 isoform 2 [Homo sapiens]

**Accession:**	gi|345842488	**Score:**	13.1
**Database:**	refseq_human(refseq_human_20140103.fasta)	**MW [kDa]:**	551.1
**Database Date:**	2015-11-30	**pI:**	5.4
**Modification(s):**	Carbamidomethyl	**Sequence Coverage [%]:**	0.4
		**No. of unique Peptides:**	1

BRUKER

Detailed Protein Report

10	20	30	40	50	60	70	80
MAEGGEGGED	EIQFLRTEDE	VVLQCIATIH	KEQRKFCLAA	EGLGNRLCFL	EPTSEAKYIP	PDLCVCNFVL	EQSLSVRALQ
90	100	110	120	130	140	150	160
EMLANTGENG	GEGAAQGGGH	RTLLYGHAVL	LRHSFSGMYL	TCLTTSRSQT	DKLAFDVGLR	EHATGEACWW	TIHPASKQRS
170	180	190	200	210	220	230	240
EGEKVRIGDD	LILVSVSSER	YLHLSVSNGN	IQVDASFMQT	LWNVHPTCSG	SSIEEGYLLG	GHVVRLFHGH	DECLTIPSTD
250	260	270	280	290	300	310	320
QNDSQHRRIF	YEAGGAGTRA	RSLWRVEPLR	ISWSGSNIRW	GQAFRLRHLT	TGHYLALTED	QGLILQDRAK	SDTKSTAFSF
330	340	350	360	370	380	390	400
RASKELKEKL	DSSHKRDIEG	MGVPEIKYGD	SVCFVQHIAS	GLWVTYKAQD	AKTSRLGPLK	RKVILHQEGH	MDDGLTLQRC
410	420	430	440	450	460	470	480
QREESQAARI	IRNTTALFSQ	FVSGNNRTAA	PITLPIEEVL	QTLQDLIAYF	QPPEEEMRHE	DKQNKLRSLK	NRQNLFKEEG
490	500	510	520	530	540	550	560
MLALVLNCID	RLNVYNSVAH	FAGIAREESG	MAWKEILNLL	YKLLAALIRG	NRNNCAQFSN	NLDWLISKLD	RLESSSGILE
570	580	590	600	610	620	630	640
VLHCILTESP	EALNLIAEGH	IKSIISLLDK	HGRNHKVLDI	LCSLCLCNGV	AVRANQNLIC	DNLLPRRNLL	LQTRLINDVT
650	660	670	680	690	700	710	720
SIRPNIFLGV	AEGSAQYKKW	YFELIIDQVD	PFLTAEPTHL	RVGWASSSGY	APYPGGGEGW	GGNGVGDDLY	SYGFDGLHLW
730	740	750	760	770	780	790	800
SGRIPRAVAS	INQHLLRSDD	VVSCCLDLGV	PSISFRINGQ	PVQGMFENFN	TDGLFFPVMS	FSAGVKVRFL	MGGRHGEFKF
810	820	830	840	850	860	870	880
LPPSGYAPCY	EALLPKEKMR	LEPVKEYKRD	ADGIRDLLGT	TQFLSQASFI	PCPVDTSQVI	LPPHLEKIRD	RLAENIHELW
890	900	910	920	930	940	950	960
GMNKIELGWT	FGKIRDDNKR	QHPCLVEFSK	LPETEKNYNL	QMSTETLKTL	LALGCHIAHV	NPAAEEDLKK	VKLPKNYMMS
970	980	990	1000	1010	1020	1030	1040
NGYKPAPLDL	SDVKLLPPQE	ILVDKLAENA	HNVWAKDRIK	QGWTYGIQQD	LKNKRNPRLV	PYALLDERTK	KSNRDSLREA
1050	1060	1070	1080	1090	1100	1110	1120
VRTFVGYGYN	IEPSDQELAD	SAVEKVSIDK	IRFFRVERSY	AVRSGKWYFE	FEVVTGGDMR	VGWARPGCRP	DVELGADDQA
1130	1140	1150	1160	1170	1180	1190	1200
FVFEGNRGQR	WHQGSGYFGR	TWQPGDVVGC	MINLDDASMI	FTLNGELLIT	NKGSELAFAD	YEIENGFVPI	CCLGLSQIGR
1210	1220	1230	1240	1250	1260	1270	1280
MNLGTDASTF	KFYTMCGLQE	GFEPFAVNMN	RDVAMWFSKR	LPTFVNVPKD	HPHIEVMRID	GTMDSPPCLK	VTHKTFGTQN
1290	1300	1310	1320	1330	1340	1350	1360
SNADMIYCRL	SMPVECHSSF	SHSPCLDSEA	FQKRKQMQEI	LSHTTTQCYY	AIRIFAGQDP	SCVWVGWVTP	DYHLYSEKFD
1370	1380	1390	1400	1410	1420	1430	1440
LNKNCTVTVT	LGDERGRVHE	SVKRSNCYMV	WGGDIVASSQ	RSNRSNVDLE	IGCLVDLAMG	MLSFSANGKE	LGTCYQVEPN
1450	1460	1470	1480	1490	1500	1510	1520
TKVFPAVFLQ	PTSTSLFQFE	LGKLKNAMPL	SAAIFRSEEK	NPVPQCPPRL	DVQTIQPVLW	SRMPNSFLKV	ETERVSERHG
1530	1540	1550	1560	1570	1580	1590	1600
WVVQCLEPLQ	MMALHIPEEN	RCVDILELCE	QEDLMRFHYH	TLRLYSAVCA	LGNSRVAYAL	CSHVDLSQLF	YAIDNKYLPG
1610	1620	1630	1640	1650	1660	1670	1680
LLRSGFYDLL	ISIHLASAKE	RKLMMKNEYI	IPITSTTRNI	RLFPDESKRH	GLPGVGLRTC	LKPGFRFSTP	CFVVTGEDHQ
1690	1700	1710	1720	1730	1740	1750	1760
KQSPEIPLES	LRTKALSMLT	EAVQCSGAHI	RDPVGGSVEF	QFVPVLKLIG	TLLVMGVFDD	DDVRQILLLI	DPSVFGEHSA
1770	1780	1790	1800	1810	1820	1830	1840
GTEEGAEKEE	VTQVEEKAVE	AGEKAGKEAP	VKGLLQTRLP	ESVKLQMCEL	LSYLCDCELQ	HRVEAIVAFG	DIYVSKLQAN
1850	1860	1870	1880	1890	1900	1910	1920
QKFRYNELMQ	ALNMSAALTA	RKTKEFRSPP	QEQINMLLNF	QLGENCPCPE	EIREELYDFH	EDLLLHCGVP	LEEEEEEEED
1930	1940	1950	1960	1970	1980	1990	2000
TSWTGKLCAL	VYKIKGPPKP	EKEQPTEEEE	RCPTTLKELI	SQTMICWAQE	DQIQDSELVR	MMFNLLRRQY	DSIGELLQAL
2010	2020	2030	2040	2050	2060	2070	2080
RKTYTISHTS	VSDTINLLAA	LGQIRSLLSV	RMGKEEELLM	INGLGDIMNN	KVFYQHPNLM	RVLGMHETVM	EVMVNVLGTE
2090	2100	2110	2120	2130	2140	2150	2160
KSQIAFPKMV	ASCCRFLCYF	CRISRQNQKA	MFEHLSYLLE	NSSVGLASPS	MRGSTPLDVA	ASSVMDNNEL	ALSLEEPDLE
2170	2180	2190	2200	2210	2220	2230	2240

Detailed Protein Report

Cmpd.	No. of Cmpds.	m/z meas.	Δ m/z [ppm]	z	Rt [min]	Score	P	Range	Sequence	Modification
58	1	845.3504	-92.64	3	29.9	13.1	2	382-402	R.KVILHQEGHMDDGLTLQRCQR.E	Carbamidomethyl: 19

Detailed Protein Report

Protein 708: protein ARV1 [Homo sapiens]

Accession: gi|12232479
Database: refseq_human(refseq_human_20140103.fasta)
Database Date: 2015-11-30

Score: 13.1
MW [kDa]: 31.0
pI: 9.5
Sequence Coverage [%]: 3.3
No. of unique Peptides: 1

10	20	30	40	50	60	70	80
MGNGGRSGLQ	QGKGNVDGVA	ATPTAASASC	QYRCIECNQE	AKELYRDYNH	GVLKITICKS	CQKPVDKYIE	YDPVIILINA
**90**	**100**	**110**	**120**	**130**	**140**	**150**	**160**
ILCKAQAYRH	ILFNTQINIH	GKLCIFCLLC	EAYLRWWQLQ	DSNQNTAPDD	LIRYAKEWDF	YRMFAIAALE	QTAYFIGIFT
**170**	**180**	**190**	**200**	**210**	**220**	**230**	**240**
FLWVERPMTA	KKKPNFILLL	KALLLSSYGK	LLLIPAVIWE	HDYTSVCLKL	IKVFVLTSNF	QAIRVTLNIN	RKLSFLAVLS
**250**	**260**	**270**	**280**				
GLLLESIMVY	FFQSMEWDVG	SDYAIFKSQD	F				

Cmpd.	No. of Cmpds.	m/z meas.	Δ m/z [ppm]	z	Rt [min]	Score	P	Range	Sequence	Modification
2796	1	639.3467	71.37	2	63.5	13.1	1	134-142	R.YAKEWDFYR.M	

Detailed Protein Report

Protein 709: uncharacterized protein LOC100132994 [Homo sapiens]

**Accession:**	gi|223468690	**Score:**	13.1
**Database:**	refseq_human(refseq_human_20140103.fasta)	**MW [kDa]:**	54.4
**Database Date:**	2015-11-30	**pI:**	10.1
**Modification(s):**	Carbamidomethyl	**Sequence Coverage [%]:**	3.1
		**No. of unique Peptides:**	1

Quantitation

WD:WU	**Median:** 0.78	**CV:** 0.00 %	**No. of Peptides:** 1

Alias proteins:

Accession	Name	Description
gi|223468693	refseq_human (refseq_human_20140103.fasta)	putative uncharacterized protein CXorf49 [Homo sapiens]

10	20	30	40	50	60	70	80
MSSPDKVSVC	GAGFDLEGGK	KAGSRTASPG	APGAHSHGLD	LGVPGSGDGK	SESGFTDPEG	FSFESESELI	EQGRVVLWGR
**90**	**100**	**110**	**120**	**130**	**140**	**150**	**160**
EGRPGTPVDD	QGDVVDYSFY	LADEPAAIVP	PPSVQGHPFP	EGAAAEGSAE	NWADAEVGPS	GRDVLGHSPG	KWQQASAGRL
**170**	**180**	**190**	**200**	**210**	**220**	**230**	**240**
HLCGPGPVRA	WKNPERGSKS	RWSLRVDPQQ	PSAKGPTRLP	THDSDSADES	SDLPLMKVGI	CRNEGSQAKP	GSPKKRADTS
**250**	**260**	**270**	**280**	**290**	**300**	**310**	**320**
RQASFHCKES	YLPVPGRFLT	SAPRGLTPVA	ERPAVGELED	SPQKKMQSRA	WGKVEVRPSC	SGAAAAGALP	QGLSRRKMAG
**330**	**340**	**350**	**360**	**370**	**380**	**390**	**400**
GKKSLGGASQ	LALGRGFPAC	GERLSAAPPE	PATFPPFSGV	RPQGMSKKPQ	KPKHSSPGKK	PAGRKTRESQ	AAAREDNDPN
**410**	**420**	**430**	**440**	**450**	**460**	**470**	**480**
RDEVPRAQLP	THRPGLPRLS	VRRGEFSSSD	PNIRAPQLPG	TSEPSAYSPG	GLVPRRHAPS	GNQQPPVHPP	RPERQQQPPG
**490**	**500**	**510**	**520**				
AQGCPRCIWL	QREIEDLTQQ	LAAMQFLTDK	FQDL				

Cmpd.	No. of Cmpds.	m/z meas.	Δ m/z [ppm]	z	Rt [min]	Score	P	Range	Sequence	Modification	Ratios
198	1	938.4465	-16.72	2	31.3	13.1	1	242-257	R.QASFHCKESYLPVPGR.F	Carbamidomethyl: 6	WD:WU 0.78

Detailed Protein Report

Protein 710: E3 ubiquitin-protein ligase MSL2 isoform 2 [Homo sapiens]

**Accession:**	gi|224028284	**Score:**	13.1
**Database:**	refseq_human(refseq_human_20140103.fasta)	**MW [kDa]:**	54.3
**Database Date:**	2015-11-30	**pI:**	8.7
**Modification(s):**	Carbamidomethyl	**Sequence Coverage [%]:**	3.2
		**No. of unique Peptides:**	1

Alias proteins:

Accession	Name	Description
gi|578807532	refseq_human (refseq_human_20140103.fasta)	PREDICTED: E3 ubiquitin-protein ligase MSL2 isoform X3 [Homo sapiens]
gi|530374862	refseq_human (refseq_human_20140103.fasta)	PREDICTED: E3 ubiquitin-protein ligase MSL2 isoform X2 [Homo sapiens]
gi|530374860	refseq_human (refseq_human_20140103.fasta)	PREDICTED: E3 ubiquitin-protein ligase MSL2 isoform X1 [Homo sapiens]

10	20	30	40	50	60	70	80
MMMKPSCSWC	KDYEQFEENK	QLSILVNCYK	KLCEYITQTT	LARDIIEAVD	CSSDILALLN	DGSLFCEETE	KPSDSSFTLC
**90**	**100**	**110**	**120**	**130**	**140**	**150**	**160**
LTHSPLPSTS	EPTTDPQASL	SPMSESTLSI	AIGSSVINGL	PTYNGLSIDR	FGINIPSPEH	SNTIDVCNTV	DIKTEDLSDS
**170**	**180**	**190**	**200**	**210**	**220**	**230**	**240**
LPPVCDTVAT	DLCSTGIDIC	SFSEDIKPGD	SLLLSVEEVL	RSLETVSNTE	VCCPNLQPNL	EATVSNGPFL	QLSSQSLSHN
**250**	**260**	**270**	**280**	**290**	**300**	**310**	**320**
VFMSTSPALH	GLSCTAATPK	IAKLNRKRSR	SESDSEKVQP	LPISTIIRGP	TLGASAPVTV	KRESKISLQP	IATVPNGGTT
**330**	**340**	**350**	**360**	**370**	**380**	**390**	**400**
PKISKTVLLS	TKSMKKSHEH	GSKKSHSKTK	PGILKKDKAV	KEKIPSHHFM	PGSPTKTVYK	KPQEKKGCKC	GRATQNPSVL
**410**	**420**	**430**	**440**	**450**	**460**	**470**	**480**
TCRGQRCPCY	SNRKACLDCI	CRGCQNSYMA	NGEKKLEAFA	VPEKALEQTR	LTLGINVTSI	AVRNASTSTS	VINVTGSPVT
**490**	**500**	**510**					
TFLAASTHDD	KSLDEAIDMR	FDC					

Cmpd.	No. of Cmpds.	m/z meas.	Δ m/z [ppm]	z	Rt [min]	Score	P	Range	Sequence	Modification
341	1	635.5460	-95.49	3	33.3	13.1	2	407-422	R.CPCYSNRKACLDCICR.G	Carbamidomethyl: 1

Detailed Protein Report

Protein 711: U11/U12 small nuclear ribonucleoprotein 25 kDa protein [Homo sapiens]

Accession: gi|13443018
Database: refseq_human(refseq_human_20140103.fasta)
Database Date: 2015-11-30

Score: 13.1
MW [kDa]: 15.3
pI: 9.0
Sequence Coverage [%]: 5.3
No. of unique Peptides: 1

10	20	30	40	50	60	70	80
MDVFQEGLAM	VVQDPLLCDL	PIQVTLEEVN	SQIALEYGQA	MTVRVCKMDG	EVMPVVVVQS	ATVLDLKKAI	QRYVQLKQER
90	100	110	120	130	140		
EGGIQHISWS	YVWRTYHLTS	AGEKLTEDRK	KLRDYGIRNR	DEVSFIKKLR	QK		

Cmpd.	No. of Cmpds.	m/z meas.	Δ m/z [ppm]	z	Rt [min]	Score	P	Range	Sequence	Modification
1247	1	419.0865	-321.45	2	43.9	13.1	0	121-127	R.DEVSFIK.K	

Detailed Protein Report

Protein 712: **ran GTPase-activating protein 1 [Homo sapiens]**

**Accession:**	gi|4506411	**Score:**	13.1
**Database:**	refseq_human(refseq_human_20140103.fasta)	**MW [kDa]:**	63.5
**Database Date:**	2015-11-30	**pI:**	4.5
**Modification(s):**	Carbamidomethyl	**Sequence Coverage [%]:**	2.0
		**No. of unique Peptides:**	1

Alias proteins:

Accession	Name	Description
gi|578837322	refseq_human (refseq_human_20140103.fasta)	PREDICTED: ran GTPase-activating protein 1 isoform X3 [Homo sapiens]
gi|520975387	refseq_human (refseq_human_20140103.fasta)	ran GTPase-activating protein 1 [Homo sapiens]

10	20	30	40	50	60	70	80
MASEDIAKLA	ETLAKTQVAG	GQLSFKGKSL	KLNTAEDAKD	VIKEIEDFDS	LEALRLEGNT	VGVEAARVIA	KALEKKSELK
90	100	110	120	130	140	150	160
RCHWSDMFTG	RLRTEIPPAL	ISLGEGLITA	GAQLVELDLS	DNAFGPDGVQ	GFEALLKSSA	CFTLQELKLN	NCGMGIGGGK
170	180	190	200	210	220	230	240
ILAAALTECH	RKSSAQGKPL	ALKVFVAGRN	RLENDGATAL	AEAFRVIGTL	EEVHMPQNGI	NHPGITALAQ	AFAVNPLLRV
250	260	270	280	290	300	310	320
INLNDNTFTE	KGAVAMAETL	KTLRQVEVIN	FGDCLVRSKG	AVAIADAIRG	GLPKLKELNL	SFCEIKRDAA	LAVAEAMADK
330	340	350	360	370	380	390	400
AELEKLDLNG	NTLGEEGCEQ	LQEVLEGFNM	AKVLASLSDD	EDEEEEEEGE	EEEEEAEEEE	EEDEEEEEEE	EEEEEEEPQQ
410	420	430	440	450	460	470	480
RGQGEKSATP	SRKILDPNTG	EPAPVLSSPP	PADVSTFLAF	PSPEKLLRLG	PKSSVLIAQQ	TDTSDPEKVV	SAFLKVSSVF
490	500	510	520	530	540	550	560
KDEATVRMAV	QDAVDALMQK	AFNSSSFNSN	TFLTRLLVHM	GLLKSEDKVK	AIANLYGPLM	ALNHMVQQDY	FPKALAPLLL
570	580	590					
AFVTKPNSAL	ESCSFARHSL	LQTLYKV					

Cmpd.	No. of Cmpds.	m/z meas.	Δ m/z [ppm]	z	Rt [min]	Score	P	Range	Sequence	Modification
2615	1	589.1847	-155.39	2	61.0	13.1	0	149-160	K.LNNCGMGIGGGK.I	Carbamidomethyl: 4

Detailed Protein Report

Protein 713: **matrix metalloproteinase-25 preproprotein [Homo sapiens]**

**Accession:**	gi|11968059	**Score:**	13.1
**Database:**	refseq_human(refseq_human_20140103.fasta)	**MW [kDa]:**	62.5
**Database Date:**	2015-11-30	**pI:**	9.5
		**Sequence Coverage [%]:**	2.0
		**No. of unique Peptides:**	1

10	20	30	40	50	60	70	80
MRLRLRLLAL	LLLLLAPPAR	APKPSAQDVS	LGVDWLTRYG	YLPPPHPAQA	QLQSPEKLRD	AIKVMQRFAG	LPETGRMDPG
**90**	**100**	**110**	**120**	**130**	**140**	**150**	**160**
TVATMRKPRC	SLPDVLGVAG	LVRRRRRYAL	SGSVWKKRTL	TWRVRSFPQS	SQLSQETVRV	LMSYALMAWG	MESGLTFHEV
**170**	**180**	**190**	**200**	**210**	**220**	**230**	**240**
DSPQGQEPDI	LIDFARAFHQ	DSYPFDGLGG	TLAHAFFPGE	HPISGDTHFD	DEETWTFGSK	DGEGTDLFAV	AVHEFGHALG
**250**	**260**	**270**	**280**	**290**	**300**	**310**	**320**
LGHSSAPNSI	MRPFYQGPVG	DPDKYRLSQD	DRDGLQQLYG	KAPQTPYDKP	TRKPLAPPPQ	PPASPTHSPS	FPIPDRCEGN
**330**	**340**	**350**	**360**	**370**	**380**	**390**	**400**
FDAIANIRGE	TFFFKGPWFW	RLQPSGQLVS	PRPARLHRFW	EGLPAQVRVV	QAAYARHRDG	RILLFSGPQF	WVFQDRQLEG
**410**	**420**	**430**	**440**	**450**	**460**	**470**	**480**
GARPLTELGL	PPGEEVDAVF	SWPQNGKTYL	VRGRQYWRYD	EAAARPDPGY	PRDLSLWEGA	PPSPDDVTVS	NAGDTYFFKG
**490**	**500**	**510**	**520**	**530**	**540**	**550**	**560**
AHYWRFPKNS	IKTEPDAPQP	MGPNWLDCPA	PSSGPRAPRP	PKATPVSETC	DCQCELNQAA	GRWPAPIPLL	LLPLLVGGVA
**570**							
SR							

Cmpd.	No. of Cmpds.	m/z meas.	Δ m/z [ppm]	z	Rt [min]	Score	P	Range	Sequence	Modification
869	1	647.8024	-99.59	2	39.7	13.1	2	108-118	R.YALSGSVWKKR.T	

Detailed Protein Report

Protein 714: UDP-GlcNAc:betaGal beta-1,3-N-acetylglucosaminyltransferase 7 [Homo sapiens]

**Accession:**	gi|21687139	**Score:**	13.1
**Database:**	refseq_human(refseq_human_20140103.fasta)	**MW [kDa]:**	46.0
**Database Date:**	2015-11-30	**pI:**	10.0
		**Sequence Coverage [%]:**	4.0
		**No. of unique Peptides:**	1

10	20	30	40	50	60	70	80
MSLWKKTVYR	SLCLALALLV	AVTVFQRSLT	PGQFLQEPPP	PTLEPQKAQK	PNGQLVNPNN	FWKNPKDVAA	PTPMASQGPQ
**90**	**100**	**110**	**120**	**130**	**140**	**150**	**160**
AWDVTTTNCS	ANINLTHQPW	FQVLEPQFRQ	FLFYRHCRYF	PMLLNHPEKC	RGDVYLLVVV	KSVITQHDRR	EAIRQTWGRE
**170**	**180**	**190**	**200**	**210**	**220**	**230**	**240**
RQSAGGGRGA	VRTLFLLGTA	SKQEERTHYQ	QLLAYEDRLY	GDILQWGFLD	TFFNLTLKEI	HFLKWLDIYC	PHVPFIFKGD
**250**	**260**	**270**	**280**	**290**	**300**	**310**	**320**
DDVFVNPTNL	LEFLADRQPQ	ENLFVGDVLQ	HARPIRRKDN	KYYIPGALYG	KASYPPYAGG	GGFLMAGSLA	RRLHHACDTL
**330**	**340**	**350**	**360**	**370**	**380**	**390**	**400**
ELYPIDDVFL	GMCLEVLGVQ	PTAHEGFKTF	GISRNRNSRM	NKEPCFFRAM	LVVHKLLPPE	LLAMWGLVHS	NLTCSRKLQV
**410**							
L							

Cmpd.	No. of Cmpds.	m/z meas.	Δ m/z [ppm]	z	Rt [min]	Score	P	Range	Sequence	Modification
2975	1	619.0047	20.87	3	66.0	13.1	0	48-63	K.AQKPNGQLVNPNNFWK.N	

Detailed Protein Report

Protein 715: **small conductance calcium-activated potassium channel protein 1 [Homo sapiens]**

**Accession:**	gi|25777643	**Score:**	13.1
**Database:**	refseq_human(refseq_human_20140103.fasta)	**MW [kDa]:**	59.9
**Database Date:**	2015-11-30	**pI:**	9.8
		**Sequence Coverage [%]:**	2.2
		**No. of unique Peptides:**	1

10	20	30	40	50	60	70	80
MNSHSYNGSV	GRPLGSGPGA	LGRDPPDPEA	GHPPQPPHSP	GLQVVVAKSE	PARPSPGSPR	GQPQDQDDDE	DDEEDEAGRQ
**90**	**100**	**110**	**120**	**130**	**140**	**150**	**160**
RASGKPSNVG	HRLGHRRALF	EKRKRLSDYA	LIFGMFGIVV	MVTETELSWG	VYTKESLYSF	ALKCLISLST	AILLGLVVLY
**170**	**180**	**190**	**200**	**210**	**220**	**230**	**240**
HAREIQLFMV	DNGADDWRIA	MTCERVFLIS	LELAVCAIHP	VPGHYRFTWT	ARLAFTYAPS	VAEADVDVLL	SIPMFLRLYL
**250**	**260**	**270**	**280**	**290**	**300**	**310**	**320**
LGRVMLLHSK	IFTDASSRSI	GALNKITFNT	RFVMKTLMTI	CPGTVLLVFS	ISSWIIAAWT	VRVCERYHDK	QEVTSNFLGA
**330**	**340**	**350**	**360**	**370**	**380**	**390**	**400**
MWLISITFLS	IGYGDMVPHT	YCGKGVCLLT	GIMGAGCTAL	VVAVVARKLE	LTKAEKHVHN	FMMDTQLTKR	VKNAAANVLR
**410**	**420**	**430**	**440**	**450**	**460**	**470**	**480**
ETWLIYKHTR	LVKKPDQARV	RKHQRKFLQA	IHQAQKLRSV	KIEQGKLNDQ	ANTLTDLAKT	QTVMYDLVSE	LHAQHEELEA
**490**	**500**	**510**	**520**	**530**	**540**	**550**	
RLATLESRLD	ALGASLQALP	GLIAQAIRPP	PPPLPPRPGP	GPQDQAARSS	PCRWTPVAPS	DCG	

Cmpd.	No. of Cmpds.	m/z meas.	Δ m/z [ppm]	z	Rt [min]	Score	P	Range	Sequence	Modification
2958	1	724.8727	-73.27	2	65.7	13.1	2	408-419	K.HTRLVKKPDQAR.V	

Detailed Protein Report

Protein 716: ICOS ligand isoform a precursor [Homo sapiens]

Accession: gi|27477039
Database: refseq_human(refseq_human_20140103.fasta)
Database Date: 2015-11-30
Modification(s): Oxidation

Score: 13.0
MW [kDa]: 33.3
pI: 5.0
Sequence Coverage [%]: 5.3
No. of unique Peptides: 1

Alias proteins:

Accession	Name	Description
gi|578836292	refseq_human (refseq_human_20140103.fasta)	PREDICTED: ICOS ligand-like isoform X2 [Homo sapiens]
gi|578797797	refseq_human (refseq_human_20140103.fasta)	PREDICTED: ICOS ligand-like isoform X2 [Homo sapiens]

```
        10         20         30         40         50         60         70         80
MRLGSPGLLF LLFSSLRADT QEKEVRAMVG SDVELSCACP EGSRFDLNDV YVYWQTSESK TVVTYHIPQN SSLENVDSRY
        90        100        110        120        130        140        150        160
RNRALMSPAG MLRGDFSLRL FNVTPQDEQK FHCLVLSQSL GFQEVLSVEV TLHVAANFSV PVVSAPHSPS QDELTFTCTS
       170        180        190        200        210        220        230        240
INGYPRPNVY WINKTDNSLL DQALQNDTVF LNMRGLYDVV SVLRIARTPS VNIGCCIENV LLQQNLTVGS QTGNDIGERD
       250        260        270        280        290        300        310
KITENPVSTG EKNAATWSIL AVLCLLVVVA VAIGWVCRDR CLQHSYAGAW AVSPETELTG HV
```

Cmpd.	No. of Cmpds.	m/z meas.	Δ m/z [ppm]	z	Rt [min]	Score	P	Range	Sequence	Modification
2987	2	869.4191	-26.75	2	66.1	13.0	1	84-99	R.ALMSPAGMLRGDFSLR.L	Oxidation: 3

Detailed Protein Report

Protein 717: PREDICTED: UPF0692 protein C19orf54 isoform X1 [Homo sapiens]

**Accession:**	gi|530416253	**Score:**	13.0
**Database:**	refseq_human(refseq_human_20140103.fasta)	**MW [kDa]:**	33.2
**Database Date:**	2015-11-30	**pI:**	11.1
**Modification(s):**	Carbamidomethyl	**Sequence Coverage [%]:**	4.2
		**No. of unique Peptides:**	1

Alias proteins:

Accession	Name	Description
gi|578834306	refseq_human (refseq_human_20140103.fasta)	PREDICTED: UPF0692 protein C19orf54 isoform X6 [Homo sapiens]
gi|578834304	refseq_human (refseq_human_20140103.fasta)	PREDICTED: UPF0692 protein C19orf54 isoform X5 [Homo sapiens]

10	20	30	40	50	60	70	80
MTSPCSPPLK	PPISPPKTPV	PQASSIPSPP	LPPSPLDFSA	LPSPPWSQQT	PVPPPLPLPP	PPAATGPAPR	HVFGLEKSQL
**90**	**100**	**110**	**120**	**130**	**140**	**150**	**160**
LKEAFDKAGP	VPKGREDVKR	LLKLHKDRFR	GDLRWILFCA	DLPSLIQEGP	QCGLVALWMA	GTLLSPPSGV	PLERLIRVAT
**170**	**180**	**190**	**200**	**210**	**220**	**230**	**240**
ERGYTAQGEM	FSVADMGRLA	QEVLGCQAKL	LSGGLGGPNR	DLVLQHLVTG	HPLLIPYDED	FNHEPCQRKG	HKAHWAGSCW
**250**	**260**	**270**	**280**	**290**	**300**	**310**	
VFGLCPVSAT	LRTLSCRACS	TQCWARPANH	HPCQRRAPRE	LSTCCPSRAR	VGTISCGTTT	RSGRATCS	

Cmpd.	No. of Cmpds.	m/z meas.	Δ m/z [ppm]	z	Rt [min]	Score	P	Range	Sequence	Modification
2691	1	690.3641	10.83	2	62.0	13.0	1	289-301	R.ARVGTISCGTTTR.S	Carbamidomethyl: 8

Detailed Protein Report

Protein 718: **PREDICTED: double zinc ribbon and ankyrin repeat-containing protein 1 isoform X7 [Homo sapiens]**

**Accession:**	gi|578835454	**Score:**	13.0
**Database:**	refseq_human(refseq_human_20140103.fasta)	**MW [kDa]:**	76.5
**Database Date:**	2015-11-30	**pI:**	9.7
**Modification(s):**	Oxidation	**Sequence Coverage [%]:**	2.0
		**No. of unique Peptides:**	1

10	20	30	40	50	60	70	80
MTAGSVCVPQ	IIPLRVPQPG	KANHEIDNNT	LLEMKSDTPD	VNIYYTLDGS	KPEFLKRIGY	GENNTFKYIK	PITLPDGKIQ
90	100	110	120	130	140	150	160
VKAIAVSKDC	RQSGIVTKVF	HVDYEPPNIV	SPEDNVENVL	KDSSRQEFKN	GFVGSKLKKK	YKNSENQRSW	NVNLRKFPVP
170	180	190	200	210	220	230	240
RFCTRKRSEV	FDKHGDNENS	KGDRLSQMGL	CAECRSLVPM	NTPICVVCEA	PLALQLQPQA	SLHLKEKVIC	RACGTGNPAH
250	260	270	280	290	300	310	320
LRYCVTCEGA	LPSSQESMCS	GDKAPPPPTQ	KGGTISCYRC	GRWNLWEASF	CGWCGAMLGI	PAGCSVCPKC	GASNHLSARF
330	340	350	360	370	380	390	400
CGSCGICVKS	LVKLSLDRSL	ALAAEEPRPF	SEPRCAWQSL	NIPLPRSDVG	TKRDIGTQTV	GLFYPSGKLL	AKKEQELASQ
410	420	430	440	450	460	470	480
KQRQEKMSDH	KPLLTAISPG	RGYWRRQLDH	ISAHLRCYAQ	NNPEFRALIA	EPRMGKLISA	TVHEDGCEVS	IRLNYSQVSN
490	500	510	520	530	540	550	560
KNLYLNKAVN	FSDHLLSSAA	EGDGGLCGSR	SSWVSDYSQS	TSDTIEKIKR	IKNFKTKTFQ	EKKEQLIPEN	RLLLKEVGPT
570	580	590	600	610	620	630	640
GEGRVSVIEQ	LLDEGADPNC	CDEDNRPVIT	VAVMNKHHEA	IPVLVQRGAD	IDQQWGPLRN	TALHEATLLG	LAGRESTATL
650	660	670	680	690	700		
LGCNASIQKK	NAGGQTAYDL	ALNTGDDLVT	SLFAAKFGQG	LEDQLAQTRS	LSLDDC		

Cmpd.	No. of Cmpds.	m/z meas.	Δ m/z [ppm]	z	Rt [min]	Score	P	Range	Sequence	Modification
1524	2	829.3909	-6.37	2	47.8	13.0	0	22-35	K.ANHEIDNNTLLEMK.S	Oxidation: 13

Detailed Protein Report

Protein 719: excitatory amino acid transporter 5 isoform 3 [Homo sapiens]

**Accession:**	gi|567316040	**Score:**	13.0
**Database:**	refseq_human(refseq_human_20140103.fasta)	**MW [kDa]:**	51.2
**Database Date:**	2015-11-30	**pI:**	9.5
**Modification(s):**	Oxidation	**Sequence Coverage [%]:**	4.2
		**No. of unique Peptides:**	1

10	20	30	40	50	60	70	80
MVPHAILARG	RDVCRRNGLL	ILSVLSVIVG	CLLGFFLRTR	RLSPQEISYF	QFPGELLMRM	LKMMILPLVV	SRNMFPANLV
**90**	**100**	**110**	**120**	**130**	**140**	**150**	**160**
EATFKQYRTK	TTPVVKSPKV	APEEAPPRRI	LIYGVQEENG	SHVQNFALDL	TPPPEVVYKS	EPGTSDGMNV	LGIVFFSATM
**170**	**180**	**190**	**200**	**210**	**220**	**230**	**240**
GIMLGRMGDS	GAPLVSFCQC	LNESVMKIVA	VAVWYFPFGI	VFLIAGKILE	MDDPRAVGKK	LGFYSVTVVC	GLVLHGLFIL
**250**	**260**	**270**	**280**	**290**	**300**	**310**	**320**
PLLYFFITKK	NPIVFIRGIL	QALLIALATS	SSSATLPITF	KCLLENNHID	RRIARFVLPV	GATINMDGTA	LYEAVAAIFI
**330**	**340**	**350**	**360**	**370**	**380**	**390**	**400**
AQVNNYELDF	GQIITISITA	TAASIGAAGI	PQAGLVTMVI	VLTSVGLPTD	DITLIIAVDW	ALDRFRTMIN	VLGDALAAGI
**410**	**420**	**430**	**440**	**450**	**460**	**470**	**480**
MAHICRKDFA	RDTGTETCFP	SPETAALRDQ	ASEPPGDRGS	PAEWLCEECS	RGLRAHPGPH	LPPPRPRSSG	AG

Cmpd.	No. of Cmpds.	m/z meas.	Δ m/z [ppm]	z	Rt [min]	Score	P	Range	Sequence	Modification
1964	1	701.2760	-115.08	3	53.2	13.0	0	387-406	R.TMINVLGDALAAGIMAHICR.K	Oxidation: 2, 15

Detailed Protein Report

Protein 720: transcription factor p65 isoform 4 [Homo sapiens]

Accession: gi|345842445
Database: refseq_human(refseq_human_20140103.fasta)
Database Date: 2015-11-30
Modification(s): Carbamidomethyl

Score: 13.0
MW [kDa]: 49.5
pI: 7.8
Sequence Coverage [%]: 6.0
No. of unique Peptides: 1

10	20	30	40	50	60	70	80
MDELFPLIFP	AEPAQASGPY	VEIIEQPKQR	GMRFRYKCEG	RSAGSIPGER	STDTTKTHPT	IKINGYTGPG	TVRISLVTKD
**90**	**100**	**110**	**120**	**130**	**140**	**150**	**160**
PPHRPHPHEL	VGKDCRDGFY	EAELCPDRCI	HSFQNLGIQC	VKKRDLEQAI	SQRIQTNNNP	FQVPIEEQRG	DYDLNAVRLC
**170**	**180**	**190**	**200**	**210**	**220**	**230**	**240**
FQVTVRDPSG	RPLRLPPVLS	HPIFDNRAPN	TAELKICRVN	RNSGSCLGGD	EIFLLCDKVQ	KEDIEVYFTG	PGWEARGSFS
**250**	**260**	**270**	**280**	**290**	**300**	**310**	**320**
QADVHRQVAI	VFRTPPYADP	SLQAPVRVSM	QLRRPSDREL	SEPMEFQYLP	DTDDRHRIEE	KRKRTYETFK	SIMKKSPFSG
**330**	**340**	**350**	**360**	**370**	**380**	**390**	**400**
PTDPRPPPRR	IAVPSRSSAS	VPKPAPQPYP	FTSSLSTINY	DEFPTMVFPS	GQISQASALA	PAPPQVLPQA	PAPAPAPAMV
**410**	**420**	**430**	**440**	**450**			
SALAQRPPDP	APAPLGAPGL	PNGLLSGDED	FSSIADMDFS	ALLSQISS			

Cmpd.	No. of Cmpds.	m/z meas.	Δ m/z [ppm]	z	Rt [min]	Score	P	Range	Sequence	Modification
1871	1	1057.5041	3.81	3	51.7	13.0	2	97-123	R.DGFYEAELCPDRCIHSFQNLGIQCVKK.R	Carbamidomethyl: 24

Detailed Protein Report

Protein 721: PREDICTED: coiled-coil domain-containing protein 9 isoform X4 [Homo sapiens]

**Accession:**	gi|578834259	**Score:**	13.0
**Database:**	refseq_human(refseq_human_20140103.fasta)	**MW [kDa]:**	56.8
**Database Date:**	2015-11-30	**pI:**	5.4
**Modification(s):**	Oxidation	**Sequence Coverage [%]:**	3.4
		**No. of unique Peptides:**	1

10	20	30	40	50	60	70	80
MAATLDLKSK	EEKDAELDKR	IEALRRKNEA	LIRRYQEIEE	DRKKAELEGV	AVTAPRKGRS	VEKENVAVES	EKNLGPSRRS
**90**	**100**	**110**	**120**	**130**	**140**	**150**	**160**
PGTPRPPGAS	KGGRTPPQQG	GRAGMGRASR	SWEGSPGEQP	RGGGAGGRGR	RGRGRGSPHL	SGAGDTSISD	RKSKEWEERR
**170**	**180**	**190**	**200**	**210**	**220**	**230**	**240**
RQNIEKMNEE	MEKIAEYERN	QREGVLEPNP	VRNFLDDPRR	RSGPLEESER	DRREESRRHG	RNWGGPDFER	VRCGLEHERQ
**250**	**260**	**270**	**280**	**290**	**300**	**310**	**320**
GRRAGLGSAG	DMTLSMTGRE	RSEYLRWKQE	REKIDQERLQ	RHRKPTGQWR	REWDAEKTDG	MFKDGPVPAH	EPSHRYDDQA
**330**	**340**	**350**	**360**	**370**	**380**	**390**	**400**
WARPPKPPTF	GEFLSQHKAE	ASSRRRRKSS	RPQAKAAPRA	YSDHDDRWET	KEGAASPAPE	TPQPTSPETS	PKETPMQPPE
**410**	**420**	**430**	**440**	**450**	**460**	**470**	**480**
IPAPAHRPPE	DEGEENEGEE	DEEWEDISED	EEEEEIEVEE	GGGQSAPAFP	ESGPSLRGTQ	EAEEEGSEAT	PEAGPEGQET
**490**	**500**	**510**					
AEITDFQRVR	FCKVVAAPPL	PGAAR					

Cmpd.	No. of Cmpds.	m/z meas.	Δ m/z [ppm]	z	Rt [min]	Score	P	Range	Sequence	Modification
774	1	848.7818	-149.75	2	38.5	13.0	1	243-259	R.RAGLGSAGDMTLSMTGR.E	Oxidation: 10

Detailed Protein Report

Protein 722: gamma-glutamylcyclotransferase isoform 3 [Homo sapiens]

**Accession:**	gi|315360620	**Score:**	13.0
**Database:**	refseq_human(refseq_human_20140103.fasta)	**MW [kDa]:**	12.9
**Database Date:**	2015-11-30	**pI:**	9.6
		**Sequence Coverage [%]:**	14.0
		**No. of unique Peptides:**	1

```
         10         20         30         40         50         60         70         80
MANSGCKDVT GPDEESFLYF AYGSNLLTER IHLRNPSAAF FCVARLQDFK LDFGNSQGKT SQTWHGGIAT IFQSPGDEVW
         90        100        110        120
GVVWKMNKSN LNSLDELFAW VQKKMVCRWS IKRS
```

Cmpd.	No. of Cmpds.	m/z meas.	Δ m/z [ppm]	z	Rt [min]	Score	P	Range	Sequence	Modification
2373	1	907.3649	-100.81	2	57.9	13.0	1	35-50	R.NPSAAFFCVARLQDFK.L	

Detailed Protein Report

Protein 723: **peptidyl-prolyl cis-trans isomerase FKBP5 isoform 1 [Homo sapiens]**

**Accession:**	gi|4758384	**Score:**	13.0
**Database:**	refseq_human(refseq_human_20140103.fasta)	**MW [kDa]:**	51.2
**Database Date:**	2015-11-30	**pI:**	5.6
**Modification(s):**	Carbamidomethyl	**Sequence Coverage [%]:**	3.7
		**No. of unique Peptides:**	1

Alias proteins:

Accession	Name	Description
gi|224809329	refseq_human (refseq_human_20140103.fasta)	peptidyl-prolyl cis-trans isomerase FKBP5 isoform 1 [Homo sapiens]
gi|224809327	refseq_human (refseq_human_20140103.fasta)	peptidyl-prolyl cis-trans isomerase FKBP5 isoform 1 [Homo sapiens]

10	20	30	40	50	60	70	80
MTTDEGAKNN	EESPTATVAE	QGEDITSKKD	RGVLKIVKRV	GNGEETPMIG	DKVYVHYKGK	LSNGKKFDSS	HDRNEPFVFS
90	100	110	120	130	140	150	160
LGKGQVIKAW	DIGVATMKKG	EICHLLCKPE	YAYGSAGSLP	KIPSNATLFF	EIELLDFKGE	DLFEDGGIIR	RTKRKGEGYS
170	180	190	200	210	220	230	240
NPNEGATVEI	HLEGRCGGRM	FDCRDVAFTV	GEGEDHDIPI	GIDKALEKMQ	REEQCILYLG	PRYGFGEAGK	PKFGIEPNAE
250	260	270	280	290	300	310	320
LIYEVTLKSF	EKAKESWEMD	TKEKLEQAAI	VKEKGTVYFK	GGKYMQAVIQ	YGKIVSWLEM	EYGLSEKESK	ASESFLLAAF
330	340	350	360	370	380	390	400
LNLAMCYLKL	REYTKAVECC	DKALGLDSAN	EKGLYRRGEA	QLLMNEFESA	KGDFEKVLEV	NPQNKAARLQ	ISMCQKKAKE
410	420	430	440	450	460		
HNERDRRIYA	NMFKKFAEQD	AKEEANKAMG	KKTSEGVTNE	KGTDSQAMEE	EKPEGHV		

Cmpd.	No. of Cmpds.	m/z meas.	Δ m/z [ppm]	z	Rt [min]	Score	P	Range	Sequence	Modification
2372	1	911.8056	-124.45	2	57.9	13.0	1	336-352	K.AVECCDKALGLDSANEK.G	Carbamidomethyl: 4

Detailed Protein Report

Protein 724: myelin transcription factor 1 [Homo sapiens]

**Accession:**	gi|17975763	**Score:**	13.0
**Database:**	refseq_human(refseq_human_20140103.fasta)	**MW [kDa]:**	122.3
**Database Date:**	2015-11-30	**pI:**	4.7
		**Sequence Coverage [%]:**	0.9
		**No. of unique Peptides:**	1

Quantitation

WD:WU **Median:** 1.14 **CV:** 0.00 % **No. of Peptides:** 1

10	20	30	40	50	60	70	80
MSLENEDKRA	RTRSKALRGP	PETTAADLSC	PTPGCTGSGH	VRGKYSRHRS	LQSCPLAKKR	KLEGAEAEHL	VSKRKSHPLK
90	100	110	120	130	140	150	160
LALDEGYGVD	SDGSEDTEVK	DASVSDESEG	TLEGAEAETS	GQDEIHRPET	AEGRSPVKSH	FGSNPIGSAT	ASSKGSYSSY
170	180	190	200	210	220	230	240
QGIIATSLLN	LGQIAEETLV	EEDLGQAAKP	GPGIVHLLQE	AAEGAASEEG	EKGLFIQPED	AEEVVEVTTE	RSQDLCPQSL
250	260	270	280	290	300	310	320
EDAASEESSK	QKGILSHEEE	DEEEEEEEEE	EEEDEEEEEE	EEEEEEEEEE	EEEEEEEEEE	EEEEEEAAPD	VIFQEDTSHT
330	340	350	360	370	380	390	400
SAQKAPELRG	PESPSPKPEY	SVIVEVRSDD	DKDEDTHSRK	STVTDESEMQ	DMMTRGNLGL	LEQAIALKAE	QVRTVCEPGC
410	420	430	440	450	460	470	480
PPAEQSQLGL	GEPGKAAKPL	DTVRKSYYSK	DPSRAEKREI	KCPTPGCDGT	GHVTGLYPHH	RSLSGCPHKD	RIPPEILAMH
490	500	510	520	530	540	550	560
ENVLKCPTPG	CTGQGHVNSN	RNTHRSLSGC	PIAAAEKLAK	SHEKQQPQTG	DPSKSSSNSD	RILRPMCFVK	QLEVPPYGSY
570	580	590	600	610	620	630	640
RPNVAPATPR	ANLAKELEKF	SKVTFDYASF	DAQVFGKRML	APKIQTSETS	PKAFQCFDYS	QDAEAAHMAA	TAILNLSTRC
650	660	670	680	690	700	710	720
WEMPENLSTK	PQDLPSKSVD	IEVDENGTLD	LSMHKHRKRE	NAFPSSSSCS	SSPGVKSPDA	SQRHSSTSAP	SSSMTSPQSS
730	740	750	760	770	780	790	800
QASRQDEWDR	PLDYTKPSRL	REEEPEESEP	AAHSFASSEA	DDQEVSEENF	EERKYPGEVT	LTNFKLKFLS	KDIKKELLTC
810	820	830	840	850	860	870	880
PTPGCDGSGH	ITGNYASHRS	LSGCPLADKS	LRNLMAAHSA	DLKCPTPGCD	GSGHITGNYA	SHRSLSGCPR	AKKSGVKVAP
890	900	910	920	930	940	950	960
TKDDKEDPEL	MKCPVPGCVG	LGHISGKYAS	HRSASGCPLA	ARRQKEGSLN	GSSFSWKSLK	NEGPTCPTPG	CDGSGHANGS
970	980	990	1000	1010	1020	1030	1040
FLTHRSLSGC	PRATFAGKKG	KLSGDEVLSP	KFKTSDVLEN	DEEIKQLNQE	IRDLNESNSE	MEAAMVQLQS	QISSMEKNLK
1050	1060	1070	1080	1090	1100	1110	1120
NIEEENKLIE	EQNEALFLEL	SGLSQALIQS	LANIRLPHME	PICEQNFDAY	VSTLTDMYSN	QDPENKDLLE	SIKQAVRGIQ
1130							
V							

Cmpd.	No. of Cmpds.	m/z meas.	Δ m/z [ppm]	z	Rt [min]	Score	P	Range	Sequence	Modification	Ratios	
234	1	1074.6988	94.18	1	31.7	13.0	1	50-59	R.SLQSCPLAKK.R		WD:WU	1.14

Detailed Protein Report

Protein 725: zinc finger protein 92 homolog [Homo sapiens]

**Accession:**	gi|210147475	**Score:**	13.0
**Database:**	refseq_human(refseq_human_20140103.fasta)	**MW [kDa]:**	45.8
**Database Date:**	2015-11-30	**pI:**	11.6
		**Sequence Coverage [%]:**	4.3
		**No. of unique Peptides:**	1

Alias proteins:

Accession	Name	Description
gi|530422737	refseq_human (refseq_human_20140103.fasta)	PREDICTED: zinc finger protein 92 homolog isoform X1 [Homo sapiens]

10	20	30	40	50	60	70	80
MAAILLTTRP	KVPVSFEDVS	VYFTKTEWKL	LDLRQKVLYK	RVMLENYSHL	VSLGFSFSKP	HLISQLERGE	GPWVADIPRT
**90**	**100**	**110**	**120**	**130**	**140**	**150**	**160**
WATAGLHIGD	RTQSKTSTST	QKHSGRQLPG	ADPQGGKEGQ	AARSSVLQRG	AQGLGQSSAA	GPQGPKGAEK	RYLCQQCGKA
**170**	**180**	**190**	**200**	**210**	**220**	**230**	**240**
FSRSSNLIKH	RIIHSGEKPY	ACPECGKLFR	RSFALLEHQR	IHSGEKPYAC	PECSKTFTRS	SNLIKHQVIH	SGERPFACGD
**250**	**260**	**270**	**280**	**290**	**300**	**310**	**320**
CGKLFRRSFA	LLEHARVHSG	ERPYACPECG	KAFSRSSNLI	EHQRTHRGEK	PYACGQCAKA	FKGVSQLIHH	QRSHSGERPF
**330**	**340**	**350**	**360**	**370**	**380**	**390**	**400**
ACRECGKAFR	GRSGLSQHRR	VHSGEKPYEC	SDCGKAFGRR	ANLFKHQAVH	GARRPAKAET	ARRLAGPGST	GPGSAVAATS
**410**	**420**						
PPRPSTAARP	SRPSRR						

Cmpd.	No. of Cmpds.	m/z meas.	Δ m/z [ppm]	z	Rt [min]	Score	P	Range	Sequence	Modification
1499	1	682.7065	38.52	3	47.5	13.0	2	152-169	R.YLCQQCGKAFSRSSNLIK.H	

Detailed Protein Report

Protein 726: PREDICTED: ran-binding protein 10 isoform X5 [Homo sapiens]

**Accession:**	gi|578829104	**Score:**	13.0
**Database:**	refseq_human(refseq_human_20140103.fasta)	**MW [kDa]:**	42.6
**Database Date:**	2015-11-30	**pI:**	6.1
**Modification(s):**	Carbamidomethyl, Oxidation	**Sequence Coverage [%]:**	4.1
		**No. of unique Peptides:**	1

10	20	30	40	50	60	70	80
MVSSYLVHHG	YCATATAFAR	MTETPIQEEQ	ASIKNRQKIQ	KLVLEGRVGE	AIETTQRFYP	GLLEHNPNLL	FMLKCRQFVE
**90**	**100**	**110**	**120**	**130**	**140**	**150**	**160**
MVNGTDSEVR	SLSSRSPKSQ	DSYPGSPSLS	PRHGPSSSHM	HNTGADSPSC	SNGVASTKSK	QNHSKYPAPS	SSSSSSSSSS
**170**	**180**	**190**	**200**	**210**	**220**	**230**	**240**
SSSPSSVNYS	ESNSTDSTKS	QHHSSTSNQE	TSNPWLQLER	RPNQAAPTTP	PGPTPTSTPP	HSDSEMEMEA	EHYPNGVLGS
**250**	**260**	**270**	**280**	**290**	**300**	**310**	**320**
MSTRIVNGAY	KHEDLQTDES	SMDDRHPRRQ	LCGGNQAATE	RIILFGRELQ	ALSEQLGREY	GKNLAHTEML	QDAFSLLAYS
**330**	**340**	**350**	**360**	**370**	**380**	**390**	**400**
DPWSCPVGQQ	LDPIQREPVC	AALNSAILES	QNLPKQPPLM	LALGQASECL	RLMARAGLGS	CSFARVDDYL	H

Cmpd.	No. of Cmpds.	m/z meas.	Δ m/z [ppm]	z	Rt [min]	Score	P	Range	Sequence	Modification
1769	1	900.4638	3.38	2	50.8	13.0	0	356-371	K.QPPLMLALGQASECLR.L	Carbamidomethyl: 14; Oxidation: 5

Detailed Protein Report

Protein 727: NADH dehydrogenase [ubiquinone] 1 beta subcomplex subunit 8, mitochondrial isoform 2 [Homo sapiens]

Accession: gi|548923801
Database: refseq_human(refseq_human_20140103.fasta)
Database Date: 2015-11-30

Score: 13.0
MW [kDa]: 20.0
pI: 7.8
Sequence Coverage [%]: 9.3
No. of unique Peptides: 1

10	20	30	40	50	60	70	80
MAVARAGVLG	VQWLQRASRN	VMPLGARTAS	HMTKDMFPGP	YPRTPEERAA	AAKKYNMRVE	DYEPYPDDGM	GYGDYPKLPD
**90**	**100**	**110**	**120**	**130**	**140**	**150**	**160**
RSQHERDPWY	SWDQPGLRLN	WGEPMHWHLD	MYNRNRVDTS	PTPVSWHVMC	MQLFGFLAFM	IFMCWVGDVY	PVYQPVCRHH
**170**	**180**						
FSYNLGFLSA	LG						

Cmpd.	No. of Cmpds.	m/z meas.	Δ m/z [ppm]	z	Rt [min]	Score	P	Range	Sequence	Modification
1939	1	918.3463	-93.31	2	52.9	13.0	1	28-43	R.TASHMTKDMFPGPYPR.T	

Detailed Protein Report

Protein 728: centromere protein J [Homo sapiens]

Accession: gi|130980075
Database: refseq_human(refseq_human_20140103.fasta)
Database Date: 2015-11-30

Score: 13.0
MW [kDa]: 152.9
pI: 6.2
Sequence Coverage [%]: 1.3
No. of unique Peptides: 1

10	20	30	40	50	60	70	80
MFLMPTSSEL	NSGQNFLTQW	MTNPSRAGVI	LNRGFPILEA	DKEKRAAVDI	STSFPIKGTH	FSDSFSFINE	EDSLLEEQKL
90	100	110	120	130	140	150	160
ESNNPYKPQS	DKSETHTAFP	CIKKGPQVAA	CHSAPGHQEE	NKNDFIPDLA	SEFKEGAYKD	PLFKKLEQLK	EVQQKKQEQL
170	180	190	200	210	220	230	240
KRQQLEQLQR	LMEEQEKLLT	MVSGQCTLPG	LSLLPDDQSQ	KHRSPGNTTT	GERATCCFPS	YVYPDPTQEE	TYPSNILSHE
250	260	270	280	290	300	310	320
QSNFCRTAHG	DFVLTSKRAS	PNLFSEAQYQ	EAPVEKNNLK	EENRNHPTGE	SILCWEKVTE	QIQEANDKNL	QKHDDSSEVA
330	340	350	360	370	380	390	400
NIEERPIKAA	IGERKQTFED	YLEEQIQLEE	QELKQKQLKE	AEGPLPIKAK	PKQPFLKRGE	GLARFTNAKS	KFQKGKESKL
410	420	430	440	450	460	470	480
VTNQSTSEDQ	PLFKMDRQQL	QRKTALKNKE	LCADNPILKK	DSKARTKSGS	VTLSQKPKML	KCSNRKSLSP	SGLKIQTGKK
490	500	510	520	530	540	550	560
CDGQFRDQIK	FENKVTSNNK	ENVTECPKPC	DTGCTGWNKT	QGKDRLPLST	GPASRLAAKS	PIRETMKESE	SSLDVSLQKK
570	580	590	600	610	620	630	640
LETWEREKEK	ENLELDEFLF	LEQAADEISF	SSNSSFVLKI	LERDQQICKG	HRMSSTPVKA	VPQKTNPADP	ISHCNRSEDL
650	660	670	680	690	700	710	720
DHTAREKESE	CEVAPKQLHS	LSSADELREQ	PCKIRKAVQK	STSENQTEWN	ARDDEGVPNS	DSSTDSEEQL	DVTIKPSTED
730	740	750	760	770	780	790	800
RERGISSRED	SPQVCDDKGP	FKDTRTQEDK	RRDVDLDLSD	KDYSSDESIM	ESIKHKVSEP	SRSSSLSLSK	MDFDDERTWT
810	820	830	840	850	860	870	880
DLEENLCNHD	VVLGNESTYG	TPQTCYPNNE	IGILDKTIKR	KIAPVKRGED	LSKSRRSRSP	PTSELMMKFF	PSLKPKPKSD
890	900	910	920	930	940	950	960
SHLGNELKLN	ISQDQPPGDN	ARSQVLREKI	IELETEIEKF	KAENASLAKL	RIERESALEK	LRKEIADFEQ	QKAKELARIE
970	980	990	1000	1010	1020	1030	1040
EFKKEEMRKL	QKERKVFEKY	TTAARTFPDK	KEREEIQTLK	QQIADLREDL	KRKETKWSST	HSRLRSQIQM	LVRENTDLRE
1050	1060	1070	1080	1090	1100	1110	1120
EIKVMERFRL	DAWKRAEAIE	SSLEVEKKDK	LANTSVRFQN	SQISSGTQVE	KYKKNYLPMQ	GNPPRRSKSA	PPRDLGNLDK
1130	1140	1150	1160	1170	1180	1190	1200
GQAASPREPL	EPLNFPDPEY	KEEEEDQDIQ	GEISHPDGKV	EKVYKNGCRV	ILFPNGTRKE	VSADGKTITV	TFFNGDVKQV
1210	1220	1230	1240	1250	1260	1270	1280
MPDQRVIYYY	AAAQTTHTTY	PEGLEVLHFS	SGQIEKHYPD	GRKEITFPDQ	TVKNLFPDGQ	EESIFPDGTI	VRVQRDGNKL
1290	1300	1310	1320	1330	1340		
IEFNNGQREL	HTAQFKRREY	PDGTVKTVYA	NGHQETKYRS	GRIRVKDKEG	NVLMDTEL		

Cmpd.	No. of Cmpds.	m/z meas.	Δ m/z [ppm]	z	Rt [min]	Score	P	Range	Sequence	Modification
1097	1	612.4614	154.39	3	42.4	13.0	2	526-543	R.LPLSTGPASRLAAKSPIR.E	

Detailed Protein Report

Protein 729: splicing factor, arginine/serine-rich 19 [Homo sapiens]

**Accession:**	gi|226509740	**Score:**	13.0
**Database:**	refseq_human(refseq_human_20140103.fasta)	**MW [kDa]:**	139.2
**Database Date:**	2015-11-30	**pI:**	9.9
		**Sequence Coverage [%]:**	1.4
		**No. of unique Peptides:**	1

Quantitation

WD:WU	**Median:** 0.73	**CV:** 0.00 %	**No. of Peptides:** 1

Alias proteins:

Accession	Name	Description
gi|530416968	refseq_human (refseq_human_20140103.fasta)	PREDICTED: splicing factor, arginine/serine-rich 19 isoform X1 [Homo sapiens]

10	20	30	40	50	60	70	80
MEEEDESRGK	TEESGEDRGD	GPPDRDPTLS	PSAFILRAIQ	QAVGSSLQGD	LPNDKDGSRC	HGLRWRRCRS	PRSEPRSQES
90	100	110	120	130	140	150	160
GGTDTATVLD	MATDSFLAGL	VSVLDPPDTW	VPSRLDLRPG	ESEDMLELVA	EVRIGDRDPI	PLPVPSLLPR	LRAWRTGKTV
170	180	190	200	210	220	230	240
SPQSNSSRPT	CARHLTLGTG	DGGPAPPPAP	SSASSSPSPS	PSSSSPSPPP	PPPPPAPPAP	PAPRFDIYDP	FHPTDEAYSP
250	260	270	280	290	300	310	320
PPAPEQKYDP	FEPTGSNPSS	SAGTPSPEEE	EEEEEEEEEE	EEDEEEEEGL	SQSISRISET	LAGIYDDNSL	SQDFPGDESP
330	340	350	360	370	380	390	400
RPDAQPTQPT	PAPGTPPQVD	STRADGAMRR	RVFVVGTEAE	ACREGKVSVE	VVTAGGAALP	PPLLPPGDSE	IEEGEIVQPE
410	420	430	440	450	460	470	480
EEPRLALSLF	RPGGRAARPT	PAASATPTAQ	PLPQPPAPRA	PEGDDFLSLH	AESDGEGALQ	VDLGEPAPAP	PAADSRWGGL
490	500	510	520	530	540	550	560
DLRRKILTQR	RERYRQRSPS	PAPAPAPAAA	AGPPTRKKSR	RERKRSGEAK	EAASSSSGTQ	PAPPAPASPW	DSKKHRSRDR
570	580	590	600	610	620	630	640
KPGSHASSSA	RRRSRSRSRS	RSTRRRSRST	DRRRGGSRRS	RSREKRRRRR	RSASPPPATS	SSSSSRRERH	RGKHRDGGGS
650	660	670	680	690	700	710	720
KKKKKRSRSR	GEKRSGDGSE	KAPAPAPPPS	GSTSCGDRDS	RRRGAVPPSI	QDLTDHDLFA	IKRTITVGRL	DKSDPRGPSP
730	740	750	760	770	780	790	800
APASSPKREV	LYDSEGLSGE	ERGGKSSQKD	RRRSGAASSS	SSSREKGSRR	KALDGGDRDR	DRDRDRDRDR	SSKKARPPKE
810	820	830	840	850	860	870	880
SAPSSGPPPK	PPVSSGSGSS	SSSSSCSSRK	VKLQSKVAVL	IREGVSSTTP	AKDAASAGLG	SIGVKFSRDR	ESRSPFLKPD
890	900	910	920	930	940	950	960
ERAPTEMAKA	APGSTKPKKT	KVKAKAGAKK	TKGTKGKTKP	SKTRKKVRSG	GGSGGSGGQV	SLKKSKADSC	SQAAGTKGAE
970	980	990	1000	1010	1020	1030	1040
ETSWSGEERA	AKVPSTPPPK	AAPPPPALTP	DSQTVDSSCK	TPEVSFLPEE	ATEEAGVRGG	AEEEEEEEEE	EEEEEEEEEQ
1050	1060	1070	1080	1090	1100	1110	1120
QPATTTATST	AAAAPSTAPS	AGSTAGDSGA	EDGPASRVSQ	LPTLPPPMPW	NLPAGVDCTT	SGVLALTALL	FKMEEANLAS
1130	1140	1150	1160	1170	1180	1190	1200
RAKAQELIQA	TNQILSHRKP	PSSLGMTPAP	VPTSLGLPPG	PSSYLLPGSL	PLGGCGSTPP	TPTGLAATSD	KREGSSSSEG
1210	1220	1230	1240	1250	1260	1270	1280
RGDTDKYLKK	LHTQERAVEE	VKLAIKPYYQ	KKDITKEEYK	DILRKAVHKI	CHSKSGEINP	VKVSNLVRAY	VQRYRYFRKH
1290	1300	1310	1320				
GRKPGDPPGP	PRPPKEPGPP	DKGGPGLPLP	PL				

Cmpd.	No. of Cmpds.	m/z meas.	Δ m/z [ppm]	z	Rt [min]	Score	P	Range	Sequence	Modification	Ratios
54	1	809.3848	-64.53	2	29.9	13.0	2	927-944	K.VRSGGGSGGSGGQVSLKK.S		WD:WU 0.73

Detailed Protein Report

Protein 730: methylcytosine dioxygenase TET1 [Homo sapiens]

**Accession:**	gi|156139122	**Score:**	13.0
**Database:**	refseq_human(refseq_human_20140103.fasta)	**MW [kDa]:**	235.2
**Database Date:**	2015-11-30	**pI:**	9.4
**Modification(s):**	Oxidation	**Sequence Coverage [%]:**	0.8
		**No. of unique Peptides:**	1

Quantitation

WD:WU	**Median:** 0.65	**CV:** 0.00 %	**No. of Peptides:** 1

Detailed Protein Report

10	20	30	40	50	60	70	80
MSRSRHARPS	RLVRKEDVNK	KKKNSQLRKT	TKGANKNVAS	VKTLSPGKLK	QLIQERDVKK	KTEPKPPVPV	RSLLTRAGAA
90	100	110	120	130	140	150	160
RMNLDRTEVL	FQNPESLTCN	GFTMALRSTS	LSRRLSQPPL	VVAKSKKVPL	SKGLEKQHDC	DYKILPALGV	KHSENDSVPM
170	180	190	200	210	220	230	240
QDTQVLPDIE	TLIGVQNPSL	LKGKSQETTQ	FWSQRVEDSK	INIPTHSGPA	AEILPGPLEG	TRCGEGLFSE	ETLNDTSGSP
250	260	270	280	290	300	310	320
KMFAQDTVCA	PFPQRATPKV	TSQGNPSIQL	EELGSRVESL	KLSDSYLDPI	KSEHDCYPTS	SLNKVIPDLN	LRNCLALGGS
330	340	350	360	370	380	390	400
TSPTSVIKFL	LAGSKQATLG	AKPDHQEAFE	ATANQQEVSD	TTSFLGQAFG	AIPHQWELPG	ADPVHGEALG	ETPDLPEIPG
410	420	430	440	450	460	470	480
AIPVQGEVFG	TILDQQETLG	MSGSVVPDLP	VFLPVPPNPI	ATFNAPSKWP	EPQSTVSYGL	AVQGAIQILP	LGSGHTPQSS
490	500	510	520	530	540	550	560
SNSEKNSLPP	VMAISNVENE	KQVHISFLPA	NTQGFPLAPE	RGLFHASLGI	AQLSQAGPSK	SDRGSSQVSV	TSTVHVVNTT
570	580	590	600	610	620	630	640
VVTMPVPMVS	TSSSSYTTLL	PTLEKKKRKR	CGVCEPCQQK	TNCGECTYCK	NRKNSHQICK	KRKCEELKKK	PSVVVPLEVI
650	660	670	680	690	700	710	720
KENKRPQREK	KPKVLKADFD	NKPVNGPKSE	SMDYSRCGHG	EEQKLELNPH	TVENVTKNED	SMTGIEVEKW	TQNKKSQLTD
730	740	750	760	770	780	790	800
HVKGDFSANV	PEAEKSKNSE	VDKKRTKSPK	LFVQTVRNGI	KHVHCLPAET	NVSFKKFNIE	EFGKTLENNS	YKFLKDTANH
810	820	830	840	850	860	870	880
KNAMSSVATD	MSCDHLKGRS	NVLVFQQPGF	NCSSIPHSSH	SIINHHASIH	NEGDQPKTPE	NIPSKEPKDG	SPVQPSLLSL
890	900	910	920	930	940	950	960
MKDRRLTLEQ	VVAIEALTQL	SEAPSENSSP	SKSEKDEESE	QRTASLLNSC	KAILYTVRKD	LQDPNLQGEP	PKLNHCPSLE
970	980	990	1000	1010	1020	1030	1040
KQSSCNTVVF	NGQTTTLSNS	HINSATNQAS	TKSHEYSKVT	NSLSLFIPKS	NSSKIDTNKS	IAQGIITLDN	CSNDLHQLPP
1050	1060	1070	1080	1090	1100	1110	1120
RNNEVEYCNQ	LLDSSKKLDS	DDLSCQDATH	TQIEEDVATQ	LTQLASIIKI	NYIKPEDKKV	ESTPTSLVTC	NVQQKYNQEK
1130	1140	1150	1160	1170	1180	1190	1200
GTIQQKPPSS	VHNNHGSSLT	KQKNPTQKKT	KSTPSRDRRK	KKPTVVSYQE	NDRQKWEKLS	YMYGTICDIW	IASKFQNFGQ
1210	1220	1230	1240	1250	1260	1270	1280
FCPHDFPTVF	GKISSSTKIW	KPLAQTRSIM	QPKTVFPPLT	QIKLQRYPES	AEEKVKVEPL	DSLSLFHLKT	ESNGKAFTDK
1290	1300	1310	1320	1330	1340	1350	1360
AYNSQVQLTV	NANQKAHPLT	QPSSPPNQCA	NVMAGDDQIR	FQQVVKEQLM	HQRLPTLPGI	SHETPLPESA	LTLRNVNVVC
1370	1380	1390	1400	1410	1420	1430	1440
SGGITVVSTK	SEEEVCSSSF	GTSEFSTVDS	AQKNFNDYAM	NFFTNPTKNL	VSITKDSELP	TCSCLDRVIQ	KDKGPYYTHL
1450	1460	1470	1480	1490	1500	1510	1520
GAGPSVAAVR	EIMENRYGQK	GNAIRIEIVV	YTGKEGKSSH	GCPIAKWVLR	RSSDEEKVLC	LVRQRTGHHC	PTAVMVVLIM
1530	1540	1550	1560	1570	1580	1590	1600
VWDGIPLPMA	DRLYTELTEN	LKSYNGHPTD	RRCTLNENRT	CTCQGIDPET	CGASFSFGCS	WSMYFNGCKF	GRSPSPRRFR
1610	1620	1630	1640	1650	1660	1670	1680
IDPSSPLHEK	NLEDNLQSLA	TRLAPIYKQY	APVAYQNQVE	YENVARECRL	GSKEGRPFSG	VTACLDFCAH	PHRDIHNMNN
1690	1700	1710	1720	1730	1740	1750	1760
GSTVVCTLTR	EDNRSLGVIP	QDEQLHVLPL	YKLSDTDEFG	SKEGMEAKIK	SGAIEVLAPR	RKKRTCFTQP	VPRSGKKRAA
1770	1780	1790	1800	1810	1820	1830	1840
MMTEVLAHKI	RAVEKKPIPR	IKRKNNSTTT	NNSKPSSLPT	LGSNTETVQP	EVKSETEPHF	ILKSSDNTKT	YSLMPSAPHP
1850	1860	1870	1880	1890	1900	1910	1920
VKEASPGFSW	SPKTASATPA	PLKNDATASC	GFSERSSTPH	CTMPSGRLSG	ANAAAADGPG	ISQLGEVAPL	PTLSAPVMEP
1930	1940	1950	1960	1970	1980	1990	2000
LINSEPSTGV	TEPLTPHQPN	HQPSFLTSPQ	DLASSPMEED	EQHSEADEPP	SDEPLSDDPL	SPAEEKLPHI	DEYWSDSEHI
2010	2020	2030	2040	2050	2060	2070	2080
FLDANIGGVA	IAPAHGSVLI	ECARRELHAT	TPVEHPNRNH	PTRLSLVFYQ	HKNLNKPQHG	FELNKIKFEA	KEAKNKKMKA
2090	2100	2110	2120	2130	2140		
SEQKDQAANE	GPEQSSEVNE	LNQIPSHKAL	TLTHDNVVTV	SPYALTHVAG	PYNHWV		

Detailed Protein Report

Cmpd.	No. of Cmpds.	m/z meas.	Δ m/z [ppm]	z	Rt [min]	Score	P	Range	Sequence	Modification	Ratios	
1153	1	945.9628	20.41	2	43.1	13.0	0	1674-1690	R.DIHNMNNGSTVVCTLTR.E	Oxidation: 5	WD:WU	0.65

Detailed Protein Report

Protein 731: PREDICTED: centrosomal protein of 57 kDa isoform X1 [Homo sapiens]

**Accession:**	gi|578822479	**Score:**	13.0
**Database:**	refseq_human(refseq_human_20140103.fasta)	**MW [kDa]:**	52.4
**Database Date:**	2015-11-30	**pI:**	10.1
		**Sequence Coverage [%]:**	2.6
		**No. of unique Peptides:**	1

10	20	30	40	50	60	70	80
MAAASVSAAS	GSHLSNSFAE	PSRSNGSMVR	HSSSPYVVYP	SDKPFLNSDL	RRSPSKPTLA	YPESNSRAIF	SALKNLQDKI
**90**	**100**	**110**	**120**	**130**	**140**	**150**	**160**
RRLELERIQA	EESVKTLSRE	TIEYKKVLDE	QIQERENSKN	EESKHNQELT	SQLLAAENKC	NLLEKQLEYM	RNMIKHAEME
**170**	**180**	**190**	**200**	**210**	**220**	**230**	**240**
RTSVLEKQKK	MQELEAKLHE	EEQERKRMQA	KAAELQTGLE	TNRLIFEDKA	TPCVPNARRI	KKKKSKPPEK	KSSRNYFGAQ
**250**	**260**	**270**	**280**	**290**	**300**	**310**	**320**
PHYRLCLGDM	PFVAGKSTSP	SHAVVANVQL	VLHLMKQHSK	ALCNDRVINS	IPLAKQVSSR	GGKSKKLSVT	PPSSNGINEE
**330**	**340**	**350**	**360**	**370**	**380**	**390**	**400**
LSEVLQTLQD	EFGQMSFDHQ	QLAKLIQESP	TVELKDKLEC	ELEALVGRME	AKANQITKVR	KYQAQLEKQK	LEKQKKELKA
**410**	**420**	**430**	**440**	**450**	**460**	**470**	
TKKTLDEERN	SSSRSGITGT	TNKKDFMKLR	PGEKRRKNLQ	LLKDMQSIQN	SLQSSSLCWD	Y	

Cmpd.	No. of Cmpds.	m/z meas.	Δ m/z [ppm]	z	Rt [min]	Score	P	Range	Sequence	Modification
2651	1	651.8176	-28.29	2	61.4	13.0	0	192-203	K.AAELQTGLETNR.L	

Detailed Protein Report

Protein 732: gamma-aminobutyric acid receptor subunit delta precursor [Homo sapiens]

Accession: gi|34734071
Database: refseq_human(refseq_human_20140103.fasta)
Database Date: 2015-11-30

Score: 13.0
MW [kDa]: 50.7
pI: 9.3
Sequence Coverage [%]: 2.0
No. of unique Peptides: 1

10	20	30	40	50	60	70	80
MDAPARLLAP	LLLLCAQQLR	GTRAMNDIGD	YVGSNLEISW	LPNLDGLIAG	YARNFRPGIG	GPPVNVALAL	EVASIDHISE
**90**	**100**	**110**	**120**	**130**	**140**	**150**	**160**
ANMEYTMTVF	LHQSWRDSRL	SYNHTNETLG	LDSRFVDKLW	LPDTFIVNAK	SAWFHDVTVE	NKLIRLQPDG	VILYSIRITS
**170**	**180**	**190**	**200**	**210**	**220**	**230**	**240**
TVACDMDLAK	YPMDEQECML	DLESYGYSSE	DIVYYWSESQ	EHIHGLDKLQ	LAQFTITSYR	FTTELMNFKS	AGQFPRLSLH
**250**	**260**	**270**	**280**	**290**	**300**	**310**	**320**
FHLRRNRGVY	IIQSYMPSVL	LVAMSWVSFW	ISQAAVPARV	SLGITTVLTM	TTLMVSARSS	LPRASAIKAL	DVYFWICYVF
**330**	**340**	**350**	**360**	**370**	**380**	**390**	**400**
VFAALVEYAF	AHFNADYRKK	QKAKVKVSRP	RAEMDVRNAI	VLFSLSAAGV	TQELAISRRQ	RRVPGNLMGS	YRSVGVETGE
**410**	**420**	**430**	**440**	**450**	**460**		
TKKEGAARSG	GQGGIRARLR	PIDADTIDIY	ARAVFPAAFA	AVNVIYWAAY	AM		

Cmpd.	No. of Cmpds.	m/z meas.	Δ m/z [ppm]	z	Rt [min]	Score	P	Range	Sequence	Modification
1088	2	565.6602	-213.89	2	41.9	13.0	0	221-229	R.FTTELMNFK.S	

Detailed Protein Report

Protein 733: protein dispatched homolog 2 [Homo sapiens]

Accession: gi|25121980
Database: refseq_human(refseq_human_20140103.fasta)
Database Date: 2015-11-30

Score: 12.9
MW [kDa]: 151.9
pI: 9.3
Sequence Coverage [%]: 0.6
No. of unique Peptides: 1

Quantitation

WD:WU **Median:** 0.64 **CV:** 0.00 % **No. of Peptides:** 1

10	20	30	40	50	60	70	80
MDGDSSSSSG	GSGPAPGPGP	EGEQRPEGEP	LAPDGGSPDS	TQTKAVPPEA	SPERSCSLHS	CPLEDPSSSS	GPPPTTSTLQ
90	100	110	120	130	140	150	160
PVGPSSPLAP	AHFTYPRALQ	EYQGGSSLPG	LGDRAALCSH	GSSLSPSPAP	SQRDGTWKPP	AVQHHVVSVR	QERAFQMPKS
170	180	190	200	210	220	230	240
YSQLIAEWPV	AVLMLCLAVI	FLCTLAGLLG	ARLPDFSKPL	LGFEPRDTDI	GSKLVVWRAL	QALTGPRKLL	FLSPDLELNS
250	260	270	280	290	300	310	320
SSSHNTLRPA	PRGSAQESAV	RPRRMVEPLE	DRRQENFFCG	PPEKSYAKLV	FMSTSSGSLW	NLHAIHSMCR	MEQDQIRSHT
330	340	350	360	370	380	390	400
SFGALCQRTA	ANQCCPSWSL	GNYLAVLSNR	SSCLDTTQAD	AARTLALLRT	CALYYHSGAL	VPSCLGPGQN	KSPRCAQVPT
410	420	430	440	450	460	470	480
KCSQSSAIYQ	LLHFLLDRDF	LSPQTTDYQV	PSLKYSLLFL	PTPKGASLMD	IYLDRLATPW	GLADNYTSVT	GMDLGLKQEL
490	500	510	520	530	540	550	560
LRHFLVQDTV	YPLLALVAIF	FGMALYLRSL	FLTLMVLLGV	LGSLLVAFFL	YQVAFRMAYF	PFVNLAALLL	LSSVCANHTL
570	580	590	600	610	620	630	640
IFFDLWRLSK	SQLPSGGLAQ	RVGRTMHHFG	YLLLVSGLTT	SAAFYASYLS	RLPAVRCLAL	FMGTAVLVHL	ALTLVWLPAS
650	660	670	680	690	700	710	720
AVLHERYLAR	GCARRARGRW	EGSAPRRLLL	ALHRRLRGLR	RAAAGTSRLL	FQRLLPCGVI	KFRYIWICWF	AALAAGGAYI
730	740	750	760	770	780	790	800
AGVSPRLRLP	TLPPPGGQVF	RPSHPFERFD	AEYRQLFLFE	QLPQGEGGHM	PVVLVWGVLP	VDTGDPLDPR	SNSSLVRDPA
810	820	830	840	850	860	870	880
FSASGPEAQR	WLLALCHRAR	NQSFFDTLQE	GWPTLCFVET	LQRWMESPSC	ARLGPDLCCG	HSDFPWAPQF	FLHCLKMMAL
890	900	910	920	930	940	950	960
EQGPDGTQDL	GLRFDAHGSL	AALVLQFQTN	FRNSPDYNQT	QLFYNEVSHW	LAAELGMAPP	GLRRGWFTSR	LELYSLQHSL
970	980	990	1000	1010	1020	1030	1040
STEPAVVLGL	ALALAFATLL	LGTWNVPLSL	FSVAAVAGTV	LLTVGLLVLL	EWQLNTAEAL	FLSASVGLSV	DFTVNYCISY
1050	1060	1070	1080	1090	1100	1110	1120
HLCPHPDRLS	RVAFSLRQTS	CATAVGAAAL	FAAGVLMLPA	TVLLYRKLGI	ILMMVKCVSC	GFASFFFQSL	CCFFGPEKNC
1130	1140	1150	1160	1170	1180	1190	1200
GQILWPCAHL	PWDAGTGDPG	GEKAGRPRPG	SVGGMPGSCS	EQYELQPLAR	RRSPSFDTST	ATSKLSHRPS	VLSEDLQLHD
1210	1220	1230	1240	1250	1260	1270	1280
GPCCSRPPPA	PASPRELLLD	HQAVFSQCPA	LQTSSPYKQA	GPSPKTRARQ	DSQGEEAEPL	PASPEAPAHS	PKAKAADPPD
1290	1300	1310	1320	1330	1340	1350	1360
GFCSSASTLE	GLSVSDETCL	STSEPSARVP	DSVGVSPDDL	DDTGQPVLER	GQLNGKRDTL	WLALRETVYD	PSLPASHHSS
1370	1380	1390	1400	1410			
LSWKGRGGPG	DGSPVVLPNS	QPDLPDVWLR	RPSTHTSGYS	S			

Cmpd.	No. of Cmpds.	m/z meas.	Δ m/z [ppm]	z	Rt [min]	Score	P	Range	Sequence	Modification	Ratios
1845	1	1011.8041	245.60	1	51.7	12.9	0	811-818	R.WLLALCHR.A		WD:WU 0.64

Detailed Protein Report

Protein 734: PREDICTED: arrestin domain-containing protein 5 isoform X1 [Homo sapiens]

**Accession:**	gi|530425368	**Score:**	12.9
**Database:**	refseq_human(refseq_human_20140103.fasta)	**MW [kDa]:**	23.0
**Database Date:**	2015-11-30	**pI:**	7.9
		**Sequence Coverage [%]:**	8.3
		**No. of unique Peptides:**	1

Alias proteins:

Accession	Name	Description
gi|578833485	refseq_human (refseq_human_20140103.fasta)	PREDICTED: arrestin domain-containing protein 5 isoform X2 [Homo sapiens]

```
        10         20         30         40         50         60         70         80
MGREHILAKK RMYLLVQGTS TFHKETPFQN PLFVEAEEKV SYNCCRQGTV CLQIQMERNT FTPGEKVVFT TEINNQTSKC
        90        100        110        120        130        140        150        160
IKTVVFALYA HIQYEGFTPS AERRSRLDSS ELLRQEANTP VTRFNTTKVV STFNLPLLLS VSSSTQDGEI MHTRYELVTT
       170        180        190        200        210
VHLPWSLTSL KAKVPIIITS ASVDSAICQL SEDGVLPVNP DHQN
```

Cmpd.	No. of Cmpds.	m/z meas.	Δ m/z [ppm]	z	Rt [min]	Score	P	Range	Sequence	Modification
2805	1	965.1168	115.45	2	63.5	12.9	1	107-123	R.LDSSELLRQEANTPVTR.F	

Detailed Protein Report

Protein 735: perforin-1 precursor [Homo sapiens]

**Accession:**	gi|40254808	**Score:**	12.9
**Database:**	refseq_human(refseq_human_20140103.fasta)	**MW [kDa]:**	61.3
**Database Date:**	2015-11-30	**pI:**	9.3
**Modification(s):**	Carbamidomethyl	**Sequence Coverage [%]:**	2.2
		**No. of unique Peptides:**	1

Alias proteins:

Accession	Name	Description
gi|133908621	refseq_human (refseq_human_20140103.fasta)	perforin-1 precursor [Homo sapiens]

10	20	30	40	50	60	70	80
MAARLLLLGI	LLLLLPLPVP	APCHTAARSE	CKRSHKFVPG	AWLAGEGVDV	TSLRRSGSFP	VDTQRFLRPD	GTCTLCENAL
**90**	**100**	**110**	**120**	**130**	**140**	**150**	**160**
QEGTLQRLPL	ALTNWRAQGS	GCQRHVTRAK	VSSTEAVARD	AARSIRNDWK	VGLDVTPKPT	SNVHVSVAGS	HSQAANFAAQ
**170**	**180**	**190**	**200**	**210**	**220**	**230**	**240**
KTHQDQYSFS	TDTVECRFYS	FHVVHTPPLH	PDFKRALGDL	PHHFNASTQP	AYLRLISNYG	THFIRAVELG	GRISALTALR
**250**	**260**	**270**	**280**	**290**	**300**	**310**	**320**
TCELALEGLT	DNEVEDCLTV	EAQVNIGIHG	SISAEAKACE	EKKKKHKMTA	SFHQTYRERH	SEVVGGHHTS	INDLLFGIQA
**330**	**340**	**350**	**360**	**370**	**380**	**390**	**400**
GPEQYSAWVN	SLPGSPGLVD	YTLEPLHVLL	DSQDPRREAL	RRALSQYLTD	RARWRDCSRP	CPPGRQKSPR	DPCQCVCHGS
**410**	**420**	**430**	**440**	**450**	**460**	**470**	**480**
[truncated: 327,062 more chars]
