# Supplementary material for: Exploration of key factors in Gingival Crevicular fluids from patients undergoing Periodontally Accelerated Osteogenic Orthodontics (PAOO) using proteome analysis
Source: BMC Oral Health. 2023 Nov 27;23:934. doi: 10.1186/s12903-023-03606-7 (PMC10683118; doi:10.1186/s12903-023-03606-7)
Supplement: Supplementary file 3 — Supplementary Material 3 [file 12903_2023_3606_MOESM3_ESM.pdf]

Detailed Protein Report

Project Info

Name: teeth-151208 **Date:** Dec 8, 2015

Note:

Sample Info & Protocols

Name: ID-M **Date:** Dec 8, 2015

Fraction:

Volume: **Concentration:**

Note:

Buffer: **Organism:**

Search Result Info

Search Result	Location	Search Engine	Database
ZT_human_PE_2015-12-08 22:06:28	/teeth-151208/ID-M/"Combined MS/MS spectra"	Mascot	refseq_human

Protein 1: serum albumin preproprotein [Homo sapiens]

Accession: gi|4502027 **Score:** 1200.0

Database: refseq_human(refseq_human_20140103.fasta) **MW [kDa]:** 69.3

Database Date: 2015-11-30 **pI:** 5.9

Modification(s): Carbamidomethyl, Oxidation **Sequence Coverage [%]:** 43.3

No. of unique Peptides: 26

Quantitation

MD:MU **Median:** 0.95 **CV:** 48.22 % **No. of Peptides:** 11

```
        10         20         30         40         50         60         70         80
MKWVTFISLL FLFSSAYSRG VFRRDAHKSE VAHRFKDLGE ENFKALVLIA FAQYLQQCPF EDHVKLVNEV TEFAKTCVAD
        90        100        110        120        130        140        150        160
ESAENCDKSL HTLFGDKLCT VATLRETYGE MADCCAKQEP ERNECFLQHK DDNPNLPRLV RPEVDVMCTA FHDNEETFLK
       170        180        190        200        210        220        230        240
KYLYEIARRH PYFYAPELLF FAKRYKAAFT ECCQAADKAA CLLPKLDELR DEGKASSAKQ RLKCASLQKF GERAFKAWAV
       250        260        270        280        290        300        310        320
ARLSQRFPKA EFAEVSKLVT DLTKVHTECC HGDLLECADD RADLAKYICE NQDSISSKLK ECCEKPLLEK SHCIAEVEND
       330        340        350        360        370        380        390        400
EMPADLPSLA ADFVESKDVC KNYAEAKDVF LGMFLYEYAR RHPDYSVVLL LRLAKTYETT LEKCCAAADP HECYAKVFDE
       410        420        430        440        450        460        470        480
FKPLVEEPQN LIKQNCELFE QLGEYKFQNA LLVRYTKKVP QVSTPTLVEV SRNLGKVGSK CCKHPEAKRM PCAEDYLSVV
       490        500        510        520        530        540        550        560
LNQLCVLHEK TPVSDRVTKC CTESLVNRRP CFSALEVDET YVPKEFNAET FTFHADICTL SEKERQIKKQ TALVELVKHK
       570        580        590        600        610
PKATKEQLKA VMDDFAAFVE KCCKADDKET CFAEEGKKLV AASQAALGL
```

Cmpd.	No. of Cmpds.	m/z meas.	Δ m/z [ppm]	z	Rt [min]	Score	P	Range	Sequence	Modification	Ratios
218	5	613.6618	-235.22	2	32.4	67.0	1	35-44	R.FKDLGEENFK.A		
581	11	575.1784	-230.65	2	36.9	63.0	0	66-75	K.LVNEVTEFAK.T		MD:MU 0.44
357	5	509.1363	-266.02	2	33.8	49.0	0	89-97	K.SLHTLFGDK.L		
166	2	879.4220	9.29	3	31.5	20.2	2	118-138	K.QEPERNECFLQHKDDNPNLPR.L	Carbamidomethyl: 8	MD:MU 1.14
328	1	509.7430	1.26	2	33.5	11.8	0	123-130	R.NECFLQHK.D		
213	1	666.3221	11.20	3	32.0	43.3	1	123-138	R.NECFLQHKDDNPNLPR.L	Carbamidomethyl: 3	MD:MU 0.65
258	1	528.1494	-281.00	2	32.9	20.3	1	161-168	K.KYLYEIAR.R		MD:MU 1.34
404	7	464.1276	-264.45	2	34.7	29.4	0	162-168	K.YLYEIAR.R		
41	2	686.2847	-3.37	2	30.2	57.0	0	187-198	K.AAFTECCQAADK.A	Carbamidomethyl: 6, 7	
											MD:MU 1.06

Detailed Protein Report

Cmpd.	No. of Cmpds.	m/z meas.	Δ m/z [ppm]	z	Rt [min]	Score	P	Range	Sequence	Modification	Ratios	
31	3	537.7767	3.24	2	30.1	45.4	1	206-214	K.LDELRDEGK.A			
143	2	696.2767	-10.48	3	31.5	53.1	0	265-281	K.VHTECCHGDLLECADDR.A	Carbamidomethyl: 5, 6, 13	MD:MU	0.47
43	6	722.3176	-9.77	2	29.9	70.8	0	287-298	K.YICENQDSISSK.L	Carbamidomethyl: 3	MD:MU	1.10
86	2	773.8871	-19.34	2	30.5	20.6	1	299-310	K.LKECCEKPLLEK.S	Carbamidomethyl: 4, 5		
2841	5	812.4044	8.52	2	65.9	42.2	0	348-360	K.DVFLGMFLYEYAR.R			
2327	7	820.2531	-172.78	2	59.0	52.7	0	348-360	K.DVFLGMFLYEYAR.R	Oxidation: 6		
4	7	776.8063	4.85	2	28.8	27.2	0	384-396	K.CCAAADPHECYAK.V	Carbamidomethyl: 1, 2, 10		
2151	19	1022.9578	-91.39	2	56.8	49.0	0	397-413	K.VFDEFKPLVEEPQNLIK.Q			
1272	10	829.3522	-33.47	2	45.5	60.5	0	414-426	K.QNCELFEQLGEYK.F	Carbamidomethyl: 3	MD:MU	0.70
645	6	480.6456	-289.76	2	37.6	42.7	0	427-434	K.FQNALLVR.Y			
78	3	569.7455	-12.45	2	30.7	62.5	0	500-508	K.CCTESLVNR.R	Carbamidomethyl: 1, 2		
1168	1	637.6405	-12.97	3	44.2	54.8	0	509-524	R.RPCFSALEVDETYVPK.E	Carbamidomethyl: 3		
2158	4	753.9220	-119.94	3	56.9	37.4	0	525-543	K.EFNAETFTFHADICTLSEK.E	Carbamidomethyl: 14	MD:MU	1.34
421	2	564.7074	-257.77	2	34.6	52.0	1	549-558	K.KQTALVELVK.H		MD:MU	1.95
804	9	500.6657	-279.19	2	39.5	47.2	0	550-558	K.QTALVELVK.H		MD:MU	1.38
1982	11	671.7238	-144.76	2	54.9	77.6	0	570-581	K.AVMDDFAAFVEK.C			
21	2	543.2367	-19.25	3	29.6	43.1	2	585-598	K.ADDKETCFAEEGKK.L	Carbamidomethyl: 7		

Detailed Protein Report

Protein 2: keratin, type I cytoskeletal 13 isoform a [Homo sapiens]

Accession: gi|131412225
Database: refseq_human(refseq_human_20140103.fasta)
Database Date: 2015-11-30
Modification(s): Carbamidomethyl

Score: 557.4
MW [kDa]: 49.5
pI: 4.8
Sequence Coverage [%]: 26.4
No. of unique Peptides: 11

Quantitation

MD:MU **Median:** 0.48 **CV:** 24.79 % **No. of Peptides:** 9

10	20	30	40	50	60	70	80
MSLRLQSSSA	SYGGGFGGGS	CQLGGGRGVS	TCSTRFVSGG	SAGGYGGGVS	CGFGGGAGSG	FGGGYGGGLG	GGYGGGLGGG
**90**	**100**	**110**	**120**	**130**	**140**	**150**	**160**
FGGGFAGGFV	DFGACDGGLL	TGNEKITMQN	LNDRLASYLE	KVRALEEANA	DLEVKIRDWH	LKQSPASPER	DYSPYYKTIE
**170**	**180**	**190**	**200**	**210**	**220**	**230**	**240**
ELRDKILTAT	IENNRVILEI	DNARLAADDF	RLKYENELAL	RQSVEADING	LRRVLDELTL	SKTDLEMQIE	SLNEELAYMK
**250**	**260**	**270**	**280**	**290**	**300**	**310**	**320**
KNHEEEMKEF	SNQVVGQVNV	EMDATPGIDL	TRVLAEMREQ	YEAMAERNRR	DAEEWFHAKS	AELNKEVSTN	TAMIQTSKTE
**330**	**340**	**350**	**360**	**370**	**380**	**390**	**400**
ITELRRTLQG	LEIELQSQLS	MKAGLENTVA	ETECRYALQL	QQIQGLISSI	EAQLSELRSE	MECQNQEYKM	LLDIKTRLEQ
**410**	**420**	**430**	**440**	**450**	**460**		
EIATYRSLLE	GQDAKMIGFP	SSAGSVSPRS	TSVTTTSSAS	VTTTSNASGR	RTSDVRRP		

Cmpd.	No. of Cmpds.	m/z meas.	Δ m/z [ppm]	z	Rt [min]	Score	P	Range	Sequence	Modification	Ratios
269	2	651.1939	-213.19	2	32.7	78.3	0	124-135	R.ALEEANADLEVK.I		MD:MU 0.38
211	1	572.8141	-9.69	2	32.3	55.6	0	166-175	K.ILTATIENNR.V		MD:MU 0.53
528	4	521.6763	-233.65	2	35.9	61.6	0	176-184	R.VILEIDNAR.L		MD:MU 0.37
395	1	624.8212	-47.61	2	34.3	26.0	1	192-201	R.LKYENELALR.Q		MD:MU 0.79
379	3	601.3190	11.24	2	34.1	54.4	0	202-212	R.QSVEADINGLR.R		MD:MU 0.36
235	1	679.3515	-16.64	2	32.3	26.4	1	202-213	R.QSVEADINGLRR.V		MD:MU 0.60
604	2	509.1500	-284.72	2	36.9	50.6	0	214-222	R.VLDELTLSK.T		
142	3	725.3217	-19.13	2	31.2	69.4	0	343-355	K.AGLENTVAETECR.Y	Carbamidomethyl: 12	MD:MU 0.44
269	3	690.3609	-9.58	2	33.1	55.3	1	396-406	K.TRLEQEIATYR.S		MD:MU 0.55
768	1	696.8079	-61.40	2	39.0	28.8	0	416-429	K.MIGFPSSAGSVSPR.S		
33	2	1001.9745	-5.27	2	29.8	50.9	0	430-450	R.STSVTTTSSASVTTTSNASGR.R		MD:MU 0.45

Detailed Protein Report

Protein 3: keratin, type II cytoskeletal 6A [Homo sapiens]

**Accession:**	gi|5031839	**Score:**	421.5
**Database:**	refseq_human(refseq_human_20140103.fasta)	**MW [kDa]:**	60.0
**Database Date:**	2015-11-30	**pI:**	8.9
		**Sequence Coverage [%]:**	18.1
		**No. of unique Peptides:**	9

Quantitation

MD:MU **Median:** 0.81 **CV:** 49.78 % **No. of Peptides:** 5

10	20	30	40	50	60	70	80
MASTSTTIRS	HSSSRRGFSA	NSARLPGVSR	SGFSSVSVSR	SRGSGGLGGA	CGGAGFGSRS	LYGLGGSKRI	SIGGGSCAIS
90	100	110	120	130	140	150	160
GGYGSRAGGS	YGFGGAGSGF	GFGGGAGIGF	GLGGGAGLAG	GFGGPGFPVC	PPGGIQEVTV	NQSLLTPLNL	QIDPTIQRVR
170	180	190	200	210	220	230	240
AEEREQIKTL	NNKFASFIDK	VRFLEQQNKV	LETKWTLLQE	QGTKTVRQNL	EPLFEQYINN	LRRQLDSIVG	ERGRLDSELR
250	260	270	280	290	300	310	320
GMQDLVEDFK	NKYEDEINKR	TAAENEFVTL	KKDVDAAYMN	KVELQAKADT	LTDEINFLRA	LYDAELSQMQ	THISDTSVVL
330	340	350	360	370	380	390	400
SMDNNRNLDL	DSIIAEVKAQ	YEEIAQRSRA	EAESWYQTKY	EELQVTAGRH	GDDLRNTKQE	IAEINRMIQR	LRSEIDHVKK
410	420	430	440	450	460	470	480
QCANLQAAIA	DAEQRGEMAL	KDAKNKLEGL	EDALQKAKQD	LARLLKEYQE	LMNVKLALDV	EIATYRKLLE	GEECRLNGEG
490	500	510	520	530	540	550	560
VGQVNISVVQ	STVSSGYGGA	SGVGSGLGLG	GGSSYSYGSG	LGVGGGFSSS	SGRAIGGGLS	SVGGGSSTIK	YTTTSSSSRK
570							
SYKH							

Cmpd.	No. of Cmpds.	m/z meas.	Δ m/z [ppm]	z	Rt [min]	Score	P	Range	Sequence	Modification	Ratios
646	5	602.1748	-244.40	2	37.4	36.8	0	195-204	K.WTLLQEQGTK.T		
2813	2	682.9825	-67.79	3	65.6	26.1	1	208-223	R.QNLEPLFEQYINNLRR.Q		
1215	2	591.1296	-252.65	2	44.9	47.4	0	241-250	R.GMQDLVEDFK.N		
212	1	675.8653	-2.24	2	32.0	57.5	1	261-272	R.TAAENEFVTLKK.D		MD:MU 0.49
1892	12	704.2620	-138.19	2	53.7	69.2	0	288-299	K.ADTLTDEINFLR.A		MD:MU 0.59
2394	1	665.3448	-32.87	2	59.8	62.6	0	327-338	R.NLDLDSIIAEVK.A		MD:MU 0.65
167	1	583.1671	-220.95	2	31.8	56.5	0	360-369	K.YEELQVTAGR.H		MD:MU 1.09
303	1	577.1338	-255.87	2	33.1	22.3	0	447-455	K.EYQELMNVK.L		
1633	5	632.2990	-81.98	2	50.2	43.1	0	456-466	K.LALDVEIATYR.K		MD:MU 1.76

BRUKER

Detailed Protein Report

Protein 4: apolipoprotein A-I preproprotein [Homo sapiens]

**Accession:**	gi|4557321	**Score:**	380.9
**Database:**	refseq_human(refseq_human_20140103.fasta)	**MW [kDa]:**	30.8
**Database Date:**	2015-11-30	**pI:**	5.5
		**Sequence Coverage [%]:**	41.2
		**No. of unique Peptides:**	11

Quantitation

MD:MU **Median:** 0.60 **CV:** 53.99 % **No. of Peptides:** 3

Alias proteins:

Accession	Name	Description
gi|530398069	refseq_human (refseq_human_20140103.fasta)	PREDICTED: apolipoprotein A-I isoform X2 [Homo sapiens]
gi|530398067	refseq_human (refseq_human_20140103.fasta)	PREDICTED: apolipoprotein A-I isoform X1 [Homo sapiens]

```
        10         20         30         40         50         60         70         80
MKAAVLTLAV LFLTGSQARH FWQQDEPPQS PWDRVKDLAT VYVDVLKDSG RDYVSQFEGS ALGKQLNLKL LDNWDSVTST
        90        100        110        120        130        140        150        160
FSKLREQLGP VTQEFWDNLE KETEGLRQEM SKDLEEVKAK VQPYLDDFQK KWQEEMELYR QKVEPLRAEL QEGARQKLHE
       170        180        190        200        210        220        230        240
LQEKLSPLGE EMRDRARAHV DALRTHLAPY SDELRQRLAA RLEALKENGG ARLAEYHAKA TEHLSTLSEK AKPALEDLRQ
       250        260        270
GLLPVLESFK VSFLSALEEY TKKLNTQ
```

Cmpd.	No. of Cmpds.	m/z meas.	Δ m/z [ppm]	z	Rt [min]	Score	P	Range	Sequence	Modification	Ratios	
1864	3	731.8056	-169.23	2	53.2	27.6	1	35-47	R.VKDLATVYVDVLK.D			
879	1	700.6959	-203.14	2	40.4	28.4	0	52-64	R.DYVSQFEGSALGK.Q		MD:MU	1.22
1776	8	806.8793	-21.03	2	52.0	89.5	0	70-83	K.LLDNWDSVTSTFSK.L			
2784	1	734.7111	-0.04	3	66.2	16.8	1	84-101	K.LREQLGPVTQEFWDNLEK.E			
2222	1	966.8539	-120.62	2	57.6	15.7	0	86-101	R.EQLGPVTQEFWDNLEK.E			
556	4	626.8185	7.06	2	36.5	29.2	0	121-130	K.VQPYLDDFQK.K		MD:MU	0.48
345	1	690.7121	-216.32	2	34.0	32.1	1	121-131	K.VQPYLDDFQKK.W			
1373	1	896.6035	133.69	1	46.9	10.1	0	158-164	K.LHELQEK.L			
207	1	651.3188	-13.88	2	32.3	46.5	0	185-195	R.THLAPYSDELR.Q		MD:MU	0.38
1846	7	615.7644	-152.39	2	52.9	27.1	0	240-250	R.QGLLPVLESFK.V			
2827	19	693.8609	-0.42	2	65.8	57.9	0	251-262	K.VSFLSALEEYTK.K			

Detailed Protein Report

Protein 5: keratin, type I cytoskeletal 15 [Homo sapiens]

Accession: gi|24430190
Database: refseq_human(refseq_human_20140103.fasta)
Database Date: 2015-11-30

Score: 319.5
MW [kDa]: 49.2
pI: 4.6
Sequence Coverage [%]: 14.0
No. of unique Peptides: 1

Quantitation

MD:MU **Median:** 0.55 **CV:** 0.00 % **No. of Peptides:** 1

10	20	30	40	50	60	70	80
MTTTFLQTSS	STFGGGSTRG	GSLLAGGGGF	GGGSLSGGGG	SRSISASSAR	FVSSGSGGGY	GGGMRVCGFG	GGAGSVFGGG
90	100	110	120	130	140	150	160
FGGGVGGGFG	GGFGGGDGGL	LSGNEKITMQ	NLNDRLASYL	DKVRALEEAN	ADLEVKIHDW	YQKQTPTSPE	CDYSQYFKTI
170	180	190	200	210	220	230	240
EELRDKIMAT	TIDNSRVILE	IDNARLAADD	FRLKYENELA	LRQGVEADIN	GLRRVLDELT	LARTDLEMQI	EGLNEELAYL
250	260	270	280	290	300	310	320
KKNHEEEMKE	FSSQLAGQVN	VEMDAAPGVD	LTRVLAEMRE	QYEAMAEKNR	RDVEAWFFSK	TEELNKEVAS	NTEMIQTSKT
330	340	350	360	370	380	390	400
EITDLRRTMQ	ELEIELQSQL	SMKAGLENSL	AETECRYATQ	LQQIQGLIGG	LEAQLSELRC	EMEAQNQEYK	MLLDIKTRLE
410	420	430	440	450	460		
QEIATYRSLL	EGQDAKMAGI	GIREASSGGG	GSSSNFHINV	EESVDGQVVS	SHKREI		

Cmpd.	No. of Cmpds.	m/z meas.	Δ m/z [ppm]	z	Rt [min]	Score	P	Range	Sequence	Modification	Ratios
811	4	515.1698	-253.80	2	39.5	44.9	0	215-223	R.VLDELTLAR.T		MD:MU 0.55

Detailed Protein Report

Protein 6: keratin, type II cytoskeletal 5 [Homo sapiens]

**Accession:**	gi|119395754	**Score:**	312.8
**Database:**	refseq_human(refseq_human_20140103.fasta)	**MW [kDa]:**	62.3
**Database Date:**	2015-11-30	**pI:**	8.6
**Modification(s):**	Carbamidomethyl	**Sequence Coverage [%]:**	15.8
		**No. of unique Peptides:**	3

Quantitation

MD:MU **Median:** 0.39 **CV:** 8.17 % **No. of Peptides:** 2

10	20	30	40	50	60	70	80
MSRQSSVSFR	SGGSRSFSTA	SAITPSVSRT	SFTSVSRSGG	GGGGGFGRVS	LAGACGVGGY	GSRSLYNLGG	SKRISISTSG
**90**	**100**	**110**	**120**	**130**	**140**	**150**	**160**
GSFRNRFGAG	AGGGYGFGGG	AGSGFGFGGG	AGGGFGLGGG	AGFGGGFGGP	GFPVCPPGGI	QEVTVNQSLL	TPLNLQIDPS
**170**	**180**	**190**	**200**	**210**	**220**	**230**	**240**
IQRVRTEERE	QIKTLNNKFA	SFIDKVRFLE	QQNKVLDTKW	TLLQEQGTKT	VRQNLEPLFE	QYINNLRRQL	DSIVGERGRL
**250**	**260**	**270**	**280**	**290**	**300**	**310**	**320**
DSELRNMQDL	VEDFKNKYED	EINKRTTAEN	EFVMLKKDVD	AAYMNKVELE	AKVDALMDEI	NFMKMFFDAE	LSQMQTHVSD
**330**	**340**	**350**	**360**	**370**	**380**	**390**	**400**
TSVVLSMDNN	RNLDLDSIIA	EVKAQYEEIA	NRSRTEAESW	YQTKYEELQQ	TAGRHGDDLR	NTKHEISEMN	RMIQRLRAEI
**410**	**420**	**430**	**440**	**450**	**460**	**470**	**480**
DNVKKQCANL	QNAIADAEQR	GELALKDARN	KLAELEEALQ	KAKQDMARLL	REYQELMNTK	LALDVEIATY	RKLLEGEECR
**490**	**500**	**510**	**520**	**530**	**540**	**550**	**560**
LSGEGVGPVN	ISVVTSSVSS	GYGSGSGYGG	GLGGGLGGGL	GGGLAGGSSG	SYYSSSSGGV	GLGGGLSVGG	SGFSASSGRG
**570**	**580**	**590**	**600**				
LGVGFGSGGG	SSSSVKFVST	TSSSRKSFKS					

Cmpd.	No. of Cmpds.	m/z meas.	Δ m/z [ppm]	z	Rt [min]	Score	P	Range	Sequence	Modification	Ratios	
419	1	705.9605	130.39	2	34.6	27.4	1	266-277	R.TTAENEFVMLKK.D		MD:MU	0.42
553	2	851.4187	19.30	2	36.2	57.4	0	406-420	K.QCANLQNAIADAEQR.G	Carbamidomethyl: 2	MD:MU	0.36
334	2	720.3453	-20.22	2	33.5	59.4	0	560-576	R.GLGVGFGSGGGSSSSVK.F			

Detailed Protein Report

Protein 7: hemoglobin subunit beta [Homo sapiens]

Accession: gi|4504349
Database: refseq_human(refseq_human_20140103.fasta)
Database Date: 2015-11-30

Score: 296.6
MW [kDa]: 16.0
pI: 6.9
Sequence Coverage [%]: 44.9
No. of unique Peptides: 7

Quantitation

MD:MU **Median:** 1.50 **CV:** 23.74 % **No. of Peptides:** 4

```
        10         20         30         40         50         60         70         80
MVHLTPEEKS AVTALWGKVN VDEVGGEALG RLLVVYPWTQ RFFESFGDLS TPDAVMGNPK VKAHGKKVLG AFSDGLAHLD
        90        100        110        120        130        140        150
NLKGTFATLS ELHCDKLHVD PENFRLLGNV LVCVLAHHFG KEFTPPVQAA YQKVVAGVAN ALAHKYH
```

Cmpd.	No. of Cmpds.	m/z meas.	Δ m/z [ppm]	z	Rt [min]	Score	P	Range	Sequence	Modification	Ratios	
63	1	476.7531	-11.35	2	30.5	38.7	0	2-9	M.VHLTPEEK.S			
718	2	466.6322	-281.50	2	38.5	27.6	0	10-18	K.SAVTALWGK.V		MD:MU	1.85
391	2	657.8281	-12.11	2	34.2	66.6	0	19-31	K.VNVDEVGGEALGR.L		MD:MU	1.10
2156	10	637.7714	-148.92	2	56.8	53.2	0	32-41	R.LLVVYPWTQR.F		MD:MU	1.30
2205	2	599.9528	-78.86	3	57.5	18.4	1	67-83	K.KVLGAFSDGLAHLDNLK.G			
2318	1	557.2358	-118.33	3	58.8	59.7	0	68-83	K.VLGAFSDGLAHLDNLK.G			
266	3	563.7687	-29.94	2	33.0	32.4	0	97-105	K.LHVDPENFR.L		MD:MU	1.93

Detailed Protein Report

Protein 8: PREDICTED: titin isoform X6 [Homo sapiens]

**Accession:**	gi|578804834	**Score:**	273.6
**Database:**	refseq_human(refseq_human_20140103.fasta)	**MW [kDa]:**	3773.1
**Database Date:**	2015-11-30	**pI:**	6.0
**Modification(s):**	Carbamidomethyl, Oxidation	**Sequence Coverage [%]:**	0.8
		**No. of unique Peptides:**	19

Quantitation

MD:MU	**Median:** 2.39	**CV:** 0.00 %	**No. of Peptides:** 1

Detailed Protein Report

10	20	30	40	50	60	70	80
MTTQAPTFTQ	PLQSVVVLEG	STATFEAHIS	GFPVPEVSWF	RDGQVISTST	LPGVQISFSD	GRAKLTIPAV	TKANSGRYSL
90	100	110	120	130	140	150	160
KATNGSGQAT	STAELLVKAE	TAPPNFVQRL	QSMTVRQGSQ	VRLQVRVTGI	PTPVVKFYRD	GAEIQSSLDF	QISQEGDLYS
170	180	190	200	210	220	230	240
LLIAEAYPED	SGTYSVNATN	SVGRATSTAE	LLVQGEEEVP	AKKTKTIVST	AQISESRQTR	IEKKIEAHFD	ARSIATVEMV
250	260	270	280	290	300	310	320
IDGAAGQQLP	HKTPPRIPPK	PKSRSPTPPS	IAAKAQLARQ	QSPSPIRHSP	SPVRHVRAPT	PSPVRSVSPA	ARISTSPIRS
330	340	350	360	370	380	390	400
VRSPLLMRKT	QASTVATGPE	VPPPWKQEGY	VASSSEAEMR	ETTLTTSTQI	RTEERWEGRY	GVQEQVTISG	AAGAAASVSA
410	420	430	440	450	460	470	480
SASYAAEAVA	TGAKEVKQDA	DKSAAVATVV	AAVDMARVRE	PVISAVEQTA	QRTTTTAVHI	QPAQEQQVRK	EAEKTAVTKV
490	500	510	520	530	540	550	560
VVAADKAKEQ	ELKSRTKEVI	TTKQEQMHVT	HEQIRKETEK	TFVPKVVISA	AKAKEQETRI	SEEITKKQKQ	VTQEAIRQET
570	580	590	600	610	620	630	640
EITAASMVVV	ATAKSTKLET	VPGAQEETTT	QQDQMHLSYE	KIMKETRKTV	VPKVIVATPK	VKEQDLVSRG	REGITTKREQ
650	660	670	680	690	700	710	720
VQITQEKMRK	EAEKTALSTI	AVATAKAKEQ	ETILRTRETM	ATRQEQIQVT	HGKVDVGKKA	EAVATVVAAV	DQARVREPRE
730	740	750	760	770	780	790	800
PGHLEESYAQ	QTTLEYGYKE	RISAAKVAEP	PQRPASEPHV	VPKAVKPRVI	QAPSETHIKT	TDQKGMHISS	QIKKTTDLTT
810	820	830	840	850	860	870	880
ERLVHVDKRP	RTASPHFTVS	KISVPKTEHG	YEASIAGSAI	ATLQKELSAT	SSAQKITKSV	KAPTVKPSET	RVRAEPTPLP
890	900	910	920	930	940	950	960
QFPFADTPDT	YKSEAGVEVK	KEVGVSITGT	TVREERFEVL	HGREAKVTET	ARVPAPVEIP	VTPPTLVSGL	KNVTVIEGES
970	980	990	1000	1010	1020	1030	1040
VTLECHISGY	PSPTVTWYRE	DYQIESSIDF	QITFQSGIAR	LMIREAFAED	SGRFTCSAVN	EAGTVSTSCY	LAVQVSEEFE
1050	1060	1070	1080	1090	1100	1110	1120
KETTAVTEKF	TTEEKRFVES	RDVVMTDTSL	TEEQAGPGEP	AAPYFITKPV	VQKLVEGGSV	VFGCQVGGNP	KPHVYWKKSG
1130	1140	1150	1160	1170	1180	1190	1200
VPLTTGYRYK	VSYNKQTGEC	KLVISMTFAD	DAGEYTIVVR	NKHGETSASA	SLLEEADYEL	LMKSQQEMLY	QTQVTAFVQE
1210	1220	1230	1240	1250	1260	1270	1280
PKVGETAPGF	VYSEYEKEYE	KEQALIRKKM	AKDTVVVRTY	VEDQEFHISS	FEERLIKEIE	YRIIKTTLEE	LLEEDGEEKM
1290	1300	1310	1320	1330	1340	1350	1360
AVDISESEAV	ESGFDSRIKN	YRILEGMGVT	FHCKMSGYPL	PKIAWYKDGK	RIKHGERYQM	DFLQDGRASL	RIPVVLPEDE
1370	1380	1390	1400	1410	1420	1430	1440
GIYTAFASNI	KGNAICSGKL	YVEPAAPLGA	PTYIPTLEPV	SRIRSLSPRS	VSRSPIRMSP	ARMSPARMSP	ARMSPARMSP
1450	1460	1470	1480	1490	1500	1510	1520
GRRLEETDES	QLERLYKPVF	VLKPVSFKCL	EGQTARFDLK	VVGRPMPETF	WFHDGQQIVN	DYTHKVVIKE	DGTQSLIIVP
1530	1540	1550	1560	1570	1580	1590	1600
ATPSDSGEWT	VVAQNRAGRS	SISVILTVEA	VEHQVKPMFV	EKLKNVNIKE	GSRLEMKVRA	TGNPNPDIVW	LKNSDIIVPH
1610	1620	1630	1640	1650	1660	1670	1680
KYPKIRIEGT	KGEAALKIDS	TVSQDSAWYT	ATAINKAGRD	TTRCKVNVEV	EFAEPEPERK	LIIPRGTYRA	KEIAAPELEP
1690	1700	1710	1720	1730	1740	1750	1760
LHLRYGQEQW	EEGDLYDKEK	QQKPFFKKKL	TSLRLKRFGP	AHFECRLTPI	GDPTMVVEWL	HDGKPLEAAN	RLRMINEFGY
1770	1780	1790	1800	1810	1820	1830	1840
CSLDYGVAYS	RDSGIITCRA	TNKYGTDHTS	ATLIVKDEKS	LVEESQLPEG	RKGLQRIEEL	ERMAHEGALT	GVTTDQKEKQ
1850	1860	1870	1880	1890	1900	1910	1920
KPDIVLYPEP	VRVLEGETAR	FRCRVTGYPQ	PKVNWYLNGQ	LIRKSKRFRV	RYDGIHYLDI	VDCKSYDTGE	VKVTAENPEG
1930	1940	1950	1960	1970	1980	1990	2000
VIEHKVKLEI	QQREDFRSVL	RRAPEPRPEF	HVHEPGKLQF	EVQKVDRPVD	TTETKEVVKL	KRAERITHEK	VPEESEELRS
2010	2020	2030	2040	2050	2060	2070	2080
KFKRRTEEGY	YEAITAVELK	SRKKDESYEE	LLRKTKDELL	HWTKELTEEE	KKALAEEGKI	TIPTFKPDKI	ELSPSMEAPK
2090	2100	2110	2120	2130	2140	2150	2160
IFERIQSQTV	GQGSDAHFRV	RVVGKPDPEC	EWYKNGVKIE	RSDRIYWYWP	EDNVCELVIR	DVTAEDSASI	MVKAINIAGE
2170	2180	2190	2200	2210	2220	2230	2240

Detailed Protein Report

Cmpd.	No. of Cmpds.	m/z meas.	Δ m/z [ppm]	z	Rt [min]	Score	P	Range	Sequence	Modification	Ratios
619	1	690.3860	77.00	2	37.3	10.5	0	5395-5407	R.VTNEVGSSICSAR.V	Carbamidomethyl: 10	
2868	1	805.9212	74.64	2	66.3	17.2	0	6519-6533	K.AQNEVGSDTCVCTVK.L	Carbamidomethyl: 10	
1459	1	851.5485	120.85	2	48.0	14.8	0	7111-7125	K.DIEQTVGLPVTLTCR.L	Carbamidomethyl: 14	
2735	1	1045.0652	51.04	2	64.6	11.1	1	7556-7574	K.NDAGMRECSAVLTVLEPAR.I	Carbamidomethyl: 8	
1761	1	785.8716	-131.54	2	51.8	13.2	2	10202-10215	K.IHVAISKRVEPPPK.V		
1296	1	640.2561	-89.77	2	45.9	12.9	1	10647-10656	K.EVTIMEEKER.A	Oxidation: 5	
1495	1	620.4184	75.49	3	48.4	11.8	2	11427-11443	K.GPEISEKIIPPKKPPTK.V		
857	4	555.3421	99.88	2	40.3	14.9	0	11876-11886	K.GTAIFACDIAK.D		
2424	1	822.3339	-55.35	2	60.7	10.9	2	12942-12954	R.EKEMARFECELSR.E	Oxidation: 4	
2695	1	805.4194	19.35	2	64.9	15.3	2	12948-12960	R.FECELSRENAKVK.W	Carbamidomethyl: 3	
958	4	481.1097	-309.72	2	41.5	15.0	0	13690-13697	K.TWVLATDR.A		
2512	2	813.3358	-60.53	2	61.3	13.8	0	17628-17640	R.VPDLLEGCQYEFR.V	Carbamidomethyl: 8	
2371	2	805.2633	-115.33	2	60.0	12.7	0	26736-26748	R.EEEPLFDIDSEMR.K		
1812	1	658.3022	-97.61	2	52.7	11.3	0	27840-27851	K.AGEDVQVLIPFK.G		
2614	2	701.8585	19.90	2	62.7	22.1	1	28261-28272	K.MVCSSVARTTFK.V	Carbamidomethyl: 3; Oxidation: 1	
2485	1	862.3731	-106.42	2	61.5	10.8	0	29616-29633	R.AGSDLVLDAAVGGKPEPK.I		
496	1	677.6900	21.58	3	35.5	11.2	2	31650-31667	K.DAGFYVVCAKNRFGIDQK.T		
2811	1	1052.5183	-55.98	2	65.6	16.6	0	32829-32846	R.SLSPTYIELMRPVSELIR.S		
1561	1	745.7829	-73.36	2	49.4	15.1	0	33469-33482	K.TDTSDSGLYTCTVK.N		MD:MU 2.39

Detailed Protein Report

Protein 9: keratin, type I cytoskeletal 14 [Homo sapiens]

**Accession:**	gi|15431310	**Score:**	194.1
**Database:**	refseq_human(refseq_human_20140103.fasta)	**MW [kDa]:**	51.6
**Database Date:**	2015-11-30	**pI:**	4.9
		**Sequence Coverage [%]:**	10.0
		**No. of unique Peptides:**	1

10	20	30	40	50	60	70	80
MTTCSRQFTS	SSSMKGSCGI	GGGIGGGSSR	ISSVLAGGSC	RAPSTYGGGL	SVSSSRFSSG	GAYGLGGGYG	GGFSSSSSSF
**90**	**100**	**110**	**120**	**130**	**140**	**150**	**160**
GSGFGGGYGG	GLGAGLGGGF	GGGFAGGDGL	LVGSEKVTMQ	NLNDRLASYL	DKVRALEEAN	ADLEVKIRDW	YQRQRPAEIK
**170**	**180**	**190**	**200**	**210**	**220**	**230**	**240**
DYSPYFKTIE	DLRNKILTAT	VDNANVLLQI	DNARLAADDF	RTKYETELNL	RMSVEADING	LRRVLDELTL	ARADLEMQIE
**250**	**260**	**270**	**280**	**290**	**300**	**310**	**320**
SLKEELAYLK	KNHEEEMNAL	RGQVGGDVNV	EMDAAPGVDL	SRILNEMRDQ	YEKMAEKNRK	DAEEWFFTKT	EELNREVATN
**330**	**340**	**350**	**360**	**370**	**380**	**390**	**400**
SELVQSGKSE	ISELRRTMQN	LEIELQSQLS	MKASLENSLE	ETKGRYCMQL	AQIQEMIGSV	EEQLAQLRCE	MEQQNQEYKI
**410**	**420**	**430**	**440**	**450**	**460**	**470**	**480**
LLDVKTRLEQ	EIATYRRLLE	GEDAHLSSSQ	FSSGSQSSRD	VTSSSRQIRT	KVMDVHDGKV	VSTHEQVLRT	KN

Cmpd.	No. of Cmpds.	m/z meas.	Δ m/z [ppm]	z	Rt [min]	Score	P	Range	Sequence	Modification
120	1	713.3140	-53.30	2	30.9	15.6	0	42-56	R.APSTYGGGLSVSSSR.F	

Detailed Protein Report

Protein 10: hemoglobin subunit alpha [Homo sapiens]

**Accession:**	gi|4504345	**Score:**	192.4
**Database:**	refseq_human(refseq_human_20140103.fasta)	**MW [kDa]:**	15.2
**Database Date:**	2015-11-30	**pI:**	9.4
		**Sequence Coverage [%]:**	25.4
		**No. of unique Peptides:**	3

Quantitation

MD:MU **Median:** 1.77 **CV:** 0.00 % **No. of Peptides:** 1

Alias proteins:

Accession	Name	Description
gi|4504347	refseq_human (refseq_human_20140103.fasta)	hemoglobin subunit alpha [Homo sapiens]

```
        10         20         30         40         50         60         70         80
MVLSPADKTN VKAAWGKVGA HAGEYGAEAL ERMFLSFPTT KTYFPHFDLS HGSAQVKGHG KKVADALTNA VAHVDDMPNA
        90        100        110        120        130        140        150
LSALSDLHAH KLRVDPVNFK LLSHCLLVTL AAHLPAEFTP AVHASLDKFL ASVSTVLTSK YR
```

Cmpd.	No. of Cmpds.	m/z meas.	Δ m/z [ppm]	z	Rt [min]	Score	P	Range	Sequence	Modification	Ratios
146	4	765.3477	-30.12	2	31.6	78.8	0	18-32	K.VGAHAGEYGAEALER.M		MD:MU 1.77
1666	2	536.1902	-168.98	2	50.7	47.8	0	33-41	R.MFLSFPTTK.T		
1467	1	626.8443	-26.58	2	48.0	65.8	0	129-140	K.FLASVSTVLTSK.Y		

Detailed Protein Report

Protein 11: keratin, type II cytoskeletal 2 epidermal [Homo sapiens]

Accession: gi|47132620
Database: refseq_human(refseq_human_20140103.fasta)
Database Date: 2015-11-30

Score: 151.0
MW [kDa]: 65.4
pI: 8.9
Sequence Coverage [%]: 5.5
No. of unique Peptides: 1

Quantitation

MD:MU **Median:** 0.69 **CV:** 0.00 % **No. of Peptides:** 1

10	20	30	40	50	60	70	80
MSCQISCKSR	GRGGGGGGFR	GFSSGSAVVS	GGSRRSTSSF	SCLSRHGGGG	GGFGGGGFGS	RSLVGLGGTK	SISISVAGGG
**90**	**100**	**110**	**120**	**130**	**140**	**150**	**160**
GGFGAAGGFG	GRGGGFGGGS	SFGGGSGFSG	GGFGGGGFGG	GRFGGFGGPG	GVGGLGGPGG	FGPGGYPGGI	HEVSVNQSLL
**170**	**180**	**190**	**200**	**210**	**220**	**230**	**240**
QPLNVKVDPE	IQNVKAQERE	QIKTLNNKFA	SFIDKVRFLE	QQNQVLQTKW	ELLQQMNVGT	RPINLEPIFQ	GYIDSLKRYL
**250**	**260**	**270**	**280**	**290**	**300**	**310**	**320**
DGLTAERTSQ	NSELNNMQDL	VEDYKKKYED	EINKRTAAEN	DFVTLKKDVD	NAYMIKVELQ	SKVDLLNQEI	EFLKVLYDAE
**330**	**340**	**350**	**360**	**370**	**380**	**390**	**400**
ISQIHQSVTD	TNVILSMDNS	RNLDLDSIIA	EVKAQYEEIA	QRSKEEAEAL	YHSKYEELQV	TVGRHGDSLK	EIKIEISELN
**410**	**420**	**430**	**440**	**450**	**460**	**470**	**480**
RVIQRLQGEI	AHVKKQCKNV	QDAIADAEQR	GEHALKDARN	KLNDLEEALQ	QAKEDLARLL	RDYQELMNVK	LALDVEIATY
**490**	**500**	**510**	**520**	**530**	**540**	**550**	**560**
RKLLEGEECR	MSGDLSSNVT	VSVTSSTISS	NVASKAAFGG	SGGRGSSSGG	GYSSGSSSYG	SGGRQSGSRG	GSGGGGSISG
**570**	**580**	**590**	**600**	**610**	**620**	**630**	**640**
GGYGSGGGSG	GRYGSGGGSK	GGSISGGGYG	SGGGKHSSGG	GSRGGSSSGG	GYGSGGGGSS	SVKGSSGEAF	GSSVTFSFR

Cmpd.	No. of Cmpds.	m/z meas.	Δ m/z [ppm]	z	Rt [min]	Score	P	Range	Sequence	Modification	Ratios
354	1	738.3735	-30.88	2	33.8	45.3	0	198-209	R.FLEQQNQVLQTK.W		MD:MU 0.69

Detailed Protein Report

Protein 12: annexin A1 [Homo sapiens]

Accession: gi|4502101
Database: refseq_human(refseq_human_20140103.fasta)
Database Date: 2015-11-30
Modification(s): Carbamidomethyl

Score: 148.6
MW [kDa]: 38.7
pI: 6.7
Sequence Coverage [%]: 21.4
No. of unique Peptides: 5

Quantitation

MD:MU **Median:** 0.80 **CV:** 13.75 % **No. of Peptides:** 3

10	20	30	40	50	60	70	80
MAMVSEFLKQ	AWFIENEEQE	YVQTVKSSKG	GPGSAVSPYP	TFNPSSDVAA	LHKAIMVKGV	DEATIIDILT	KRNNAQRQQI
**90**	**100**	**110**	**120**	**130**	**140**	**150**	**160**
KAAYLQETGK	PLDETLKKAL	TGHLEEVVLA	LLKTPAQFDA	DELRAAMKGL	GTDEDTLIEI	LASRTNKEIR	DINRVYREEL
**170**	**180**	**190**	**200**	**210**	**220**	**230**	**240**
KRDLAKDITS	DTSGDFRNAL	LSLAKGDRSE	DFGVNEDLAD	SDARALYEAG	ERRKGTDVNV	FNTILTTRSY	PQLRRVFQKY
**250**	**260**	**270**	**280**	**290**	**300**	**310**	**320**
TKYSKHDMNK	VLDLELKGDI	EKCLTAIVKC	ATSKPAFFAE	KLHQAMKGVG	TRHKALIRIM	VSRSEIDMND	IKAFYQKMYG
**330**	**340**	**350**					
ISLCQAILDE	TKGDYEKILV	ALCGGN					

Cmpd.	No. of Cmpds.	m/z meas.	Δ m/z [ppm]	z	Rt [min]	Score	P	Range	Sequence	Modification	Ratios	
1254	1	786.0316	-32.49	3	45.3	18.2	0	30-53	K.GGPGSAVSPYPTFNPSSDVAALHA		MD:MU	0.84
352	1	631.8010	-5.09	2	33.8	31.5	0	114-124	K.TPAQFDADELR.A			
2152	1	851.8249	-142.79	2	57.1	38.0	0	129-144	K.GLGTDEDTLIEILASR.T			
125	1	607.2634	-11.26	2	31.0	50.4	0	167-177	K.DITSDTSGDFR.N		MD:MU	0.91
2020	1	678.7710	-93.49	2	55.4	10.5	0	270-281	K.CATSKPAFFAEK.L	Carbamidomethyl: 1	MD:MU	0.66

Detailed Protein Report

Protein 13: serotransferrin precursor [Homo sapiens]

Accession: gi|4557871
Database: refseq_human(refseq_human_20140103.fasta)
Database Date: 2015-11-30
Modification(s): Carbamidomethyl

Score: 131.2
MW [kDa]: 77.0
pI: 7.0
Sequence Coverage [%]: 5.0
No. of unique Peptides: 3

Quantitation

MD:MU **Median:** 1.14 **CV:** 68.81 % **No. of Peptides:** 3

10	20	30	40	50	60	70	80
MRLAVGALLV	CAVLGLCLAV	PDKTVRWCAV	SEHEATKCQS	FRDHMKSVIP	SDGPSVACVK	KASYLDCIRA	IAANEADAVT
90	100	110	120	130	140	150	160
LDAGLVYDAY	LAPNNLKPVV	AEFYGSKEDP	QTFYYAVAVV	KKDSGFQMNQ	LRGKKSCHTG	LGRSAGWNIP	IGLLYCDLPE
170	180	190	200	210	220	230	240
PRKPLEKAVA	NFFSGSCAPC	ADGTDFPQLC	QLCPGCGCST	LNQYFGYSGA	FKCLKDGAGD	VAFVKHSTIF	ENLANKADRD
250	260	270	280	290	300	310	320
QYELLCLDNT	RKPVDEYKDC	HLAQVPSHTV	VARSMGGKED	LIWELLNQAQ	EHFGKDKSKE	FQLFSSPHGK	DLLFKDSAHG
330	340	350	360	370	380	390	400
FLKVPPRMDA	KMYLGYEYVT	AIRNLREGTC	PEAPTDECKP	VKWCALSHHE	RLKCDEWSVN	SVGKIECVSA	ETTEDCIAKI
410	420	430	440	450	460	470	480
MNGEADAMSL	DGGFVYIAGK	CGLVPVLAEN	YNKSDNCEDT	PEAGYFAVAV	VKKSASDLTW	DNLKGKKSCH	TAVGRTAGWN
490	500	510	520	530	540	550	560
IPMGLLYNKI	NHCRFDEFFS	EGCAPGSKKD	SSLCKLCMGS	GLNLCEPNNK	EGYYGYTGAF	RCLVEKGDVA	FVKHQTVPQN
570	580	590	600	610	620	630	640
TGGKNPDPWA	KNLNEKDYEL	LCLDGTRKPV	EEYANCHLAR	APNHAVVTRK	DKEACVHKIL	RQQQHLFGSN	VTDCSGNFCL
650	660	670	680	690	700		
FRSETKDLLF	RDDTVCLAKL	HDRNTYEKYL	GEEYVKAVGN	LRKCSTSSLL	EACTFRRP		

Cmpd.	No. of Cmpds.	m/z meas.	Δ m/z [ppm]	z	Rt [min]	Score	P	Range	Sequence	Modification	Ratios	
431	2	637.3141	-25.64	2	35.0	51.8	0	226-236	K.HSTIFENLANK.A		MD:MU	0.72
678	1	638.8051	-22.74	2	38.0	31.2	0	300-310	K.EFQLFSSPHGK.D		MD:MU	2.74
229	1	761.3746	3.44	2	32.3	48.1	1	372-384	R.LKCDEWSVNSVGK.I	Carbamidomethyl: 3	MD:MU	0.75

Detailed Protein Report

Protein 14: **PREDICTED: haptoglobin-related protein isoform X1 [Homo sapiens]**

**Accession:**	gi|578828965	**Score:**	110.1
**Database:**	refseq_human(refseq_human_20140103.fasta)	**MW [kDa]:**	35.3
**Database Date:**	2015-11-30	**pI:**	9.0
**Modification(s):**	Carbamidomethyl	**Sequence Coverage [%]:**	9.1
		**No. of unique Peptides:**	2

Quantitation

MD:MU **Median:** 1.36 **CV:** 0.00 % **No. of Peptides:** 1

10	20	30	40	50	60	70	80
MPCSLPHVNL	RVRFTSPADV	GKEGMLMMMS	PSPRVYTLND	KKQWINKAVG	DKLPECEAVC	GKPKNPANPV	QRILGGHLDA
**90**	**100**	**110**	**120**	**130**	**140**	**150**	**160**
KGSFPWQAKM	VSHHNLTTGA	TLINEQWLLT	TAKNLFLNHS	ENATAKDIAP	TLTLYVGKKQ	LVEIEKVVLH	PNYHQVDIGL
**170**	**180**	**190**	**200**	**210**	**220**	**230**	**240**
IKLKQKVLVN	ERVMPICLPS	KNYAEVGRVG	YVSGWGQSDN	FKLTDHLKYV	MLPVADQYDC	ITHYEGSTCP	KWKAPKSPVG
**250**	**260**	**270**	**280**	**290**	**300**	**310**	**320**
VQPILNEHTF	CVGMSKYQED	TCYGDAGSAF	AVHDLEEDTW	YAAGILSFDK	SCAVAEYGVY	VKVTSIQHWV	QKTIAEN

Cmpd.	No. of Cmpds.	m/z meas.	Δ m/z [ppm]	z	Rt [min]	Score	P	Range	Sequence	Modification	Ratios	
131	2	619.9592	-30.51	3	31.4	56.4	1	48-64	K.AVGDKLPECEAVCGKPK.N	Carbamidomethyl: 9, 13	MD:MU	1.36
1679	2	645.8477	-32.77	2	50.8	53.6	0	127-138	K.DIAPTLTLYVGK.K			

Detailed Protein Report

Protein 15: neutrophil defensin 1 preproprotein [Homo sapiens]

Accession: gi|4758146
Database: refseq_human(refseq_human_20140103.fasta)
Database Date: 2015-11-30
Modification(s): Carbamidomethyl

Score: 98.5
MW [kDa]: 10.2
pI: 7.6
Sequence Coverage [%]: 19.1
No. of unique Peptides: 2

Quantitation

MD:MU **Median:** 0.77 **CV:** 0.00 % **No. of Peptides:** 1

Alias proteins:

Accession	Name	Description
gi|124248516	refseq_huma (refseq_human_20140103.fasta)	neutrophil defensin 1 precursor [Homo sapiens]

```
        10         20         30         40         50         60         70         80
MRTLAILAAI LLVALQAQAE PLQARADEVA AAPEQIAADI PEVVVSLAWD ESLAPKHPGS RKNMACYCRI PACIAGERRY
        90        100
GTCIYQGRLW AFCC
```

Cmpd.	No. of Cmpds.	m/z meas.	Δ m/z [ppm]	z	Rt [min]	Score	P	Range	Sequence	Modification	Ratios
108	1	493.6493	-220.14	2	30.7	45.9	0	70-78	R.IPACIAGER.R	Carbamidomethyl: 4	MD:MU 0.77
91	1	559.2458	-22.58	2	30.8	52.6	0	80-88	R.YGTCIYQGR.L	Carbamidomethyl: 4	

Detailed Protein Report

Protein 16: keratin, type II cytoskeletal 4 [Homo sapiens]

**Accession:**	gi|331999954	**Score:**	87.8
**Database:**	refseq_human(refseq_human_20140103.fasta)	**MW [kDa]:**	56.1
**Database Date:**	2015-11-30	**pI:**	6.2
		**Sequence Coverage [%]:**	6.9
		**No. of unique Peptides:**	3

Quantitation

MD:MU **Median:** 0.27 **CV:** 0.00 % **No. of Peptides:** 1

10	20	30	40	50	60	70	80
MIARQQCVRG	GPRGFSCGSA	IVGGGKRGAF	SSVSMSGGAG	RCSSGGFGSR	SLYNLRGNKS	ISMSVAGSRQ	GACFGGAGGF
**90**	**100**	**110**	**120**	**130**	**140**	**150**	**160**
GTGGFGGGFG	GSFSGKGGPG	FPVCPAGGIQ	EVTINQSLLT	PLHVEIDPEI	QKVRTEEREQ	IKLLNNKFAS	FIDKVQFLEQ
**170**	**180**	**190**	**200**	**210**	**220**	**230**	**240**
QNKVLETKWN	LLQQQTTTTS	SKNLEPLFET	YLSVLRKQLD	TLGNDKGRLQ	SELKTMQDSV	EDFKTKYEEE	INKRTAAEND
**250**	**260**	**270**	**280**	**290**	**300**	**310**	**320**
FVVLKKDVDA	AYLNKVELEA	KVDSLNDEIN	FLKVLYDAEL	SQMQTHVSDT	SVVLSMDNNR	NLDLDSIIAE	VRAQYEEIAQ
**330**	**340**	**350**	**360**	**370**	**380**	**390**	**400**
RSKAEAEALY	QTKVQQLQIS	VDQHGDNLKN	TKSEIAELNR	MIQRLRAEIE	NIKKQCQTLQ	VSVADAEQRG	ENALKDAHSK
**410**	**420**	**430**	**440**	**450**	**460**	**470**	**480**
RVELEAALQQ	AKEELARMLR	EYQELMSVKL	ALDIEIATYR	KLLEGEEYRM	SGECQSAVSI	SVVSGSTSTG	GISGGLGSGS
**490**	**500**	**510**	**520**	**530**			
GFGLSSGFGS	GSGSGFGFGG	SVSGSSSSKI	ISTTTLNKRR				

Cmpd.	No. of Cmpds.	m/z meas.	Δ m/z [ppm]	z	Rt [min]	Score	P	Range	Sequence	Modification	Ratios
326	1	667.8409	-42.57	2	33.4	33.2	1	235-246	R.TAAENDFVVLKK.D		
1586	1	703.8014	-85.66	2	49.7	20.9	0	262-273	K.VDSLNDEINFLK.V		
2400	1	679.3689	-1.20	2	60.3	33.7	0	301-312	R.NLDLDSIIAEVR.A		MD:MU 0.27

Detailed Protein Report

Protein 17: desmoplakin isoform I [Homo sapiens]

**Accession:**	gi|58530840	**Score:**	86.9
**Database:**	refseq_human(refseq_human_20140103.fasta)	**MW [kDa]:**	331.6
**Database Date:**	2015-11-30	**pI:**	6.4
**Modification(s):**	Carbamidomethyl, Oxidation	**Sequence Coverage [%]:**	3.4
		**No. of unique Peptides:**	5

Quantitation

MD:MU	**Median:** 1.47	**CV:** 0.00 %	**No. of Peptides:** 1

Detailed Protein Report

10	20	30	40	50	60	70	80
MSCNGGSHPR	INTLGRMIRA	ESGPDLRYEV	TSGGGGTSRM	YYSRRGVITD	QNSDGYCQTG	TMSRHQNQNT	IQELLQNCSD
90	100	110	120	130	140	150	160
CLMRAELIVQ	PELKYGDGIQ	LTRSRELDEC	FAQANDQMEI	LDSLIREMRQ	MGQPCDAYQK	RLLQLQEQMR	ALYKAISVPR
170	180	190	200	210	220	230	240
VRRASSKGGG	GYTCQSGSGW	DEFTKHVTSE	CLGWMRQQRA	EMDMVAWGVD	LASVEQHINS	HRGIHNSIGD	YRWQLDKIKA
250	260	270	280	290	300	310	320
DLREKSAIYQ	LEEEYENLLK	ASFERMDHLR	QLQNIIQATS	REIMWINDCE	EEELLYDWSD	KNTNIAQKQE	AFSIRMSQLE
330	340	350	360	370	380	390	400
VKEKELNKLK	QESDQLVLNQ	HPASDKIEAY	MDTLQTQWSW	ILQITKCIDV	HLKENAAYFQ	FFEEAQSTEA	YLKGLQDSIR
410	420	430	440	450	460	470	480
KKYPCDKNMP	LQHLLEQIKE	LEKEREKILE	YKRQVQNLVN	KSKKIVQLKP	RNPDYRSNKP	IILRALCDYK	QDQKIVHKGD
490	500	510	520	530	540	550	560
ECILKDNNER	SKWYVTGPGG	VDMLVPSVGL	IIPPPNPLAV	DLSCKIEQYY	EAILALWNQL	YINMKSLVSW	HYCMIDIEKI
570	580	590	600	610	620	630	640
RAMTIAKLKT	MRQEDYMKTI	ADLELHYQEF	IRNSQGSEMF	GDDDKRKIQS	QFTDAQKHYQ	TLVIQLPGYP	QHQTVTTTEI
650	660	670	680	690	700	710	720
THHGTCQDVN	HNKVIETNRE	NDKQETWMLM	ELQKIRRQIE	HCEGRMTLKN	LPLADQGSSH	HITVKINELK	SVQNDSQAIA
730	740	750	760	770	780	790	800
EVLNQLKDML	ANFRGSEKYC	YLQNEVFGLF	QKLENINGVT	DGYLNSLCTV	RALLQAILQT	EDMLKVYEAR	LTEEETVCLD
810	820	830	840	850	860	870	880
LDKVEAYRCG	LKKIKNDLNL	KKSLLATMKT	ELQKAQQIHS	QTSQQYPLYD	LDLGKFGEKV	TQLTDRWQRI	DKQIDFRLWD
890	900	910	920	930	940	950	960
LEKQIKQLRN	YRDNYQAFCK	WLYDAKRRQD	SLESMKFGDS	NTVMRFLNEQ	KNLHSEISGK	RDKSEEVQKI	AELCANSIKD
970	980	990	1000	1010	1020	1030	1040
YELQLASYTS	GLETLLNIPI	KRTMIQSPSG	VILQEAADVH	ARYIELLTRS	GDYYRFLSEM	LKSLEDLKLK	NTKIEVLEEE
1050	1060	1070	1080	1090	1100	1110	1120
LRLARDANSE	NCNKNKFLDQ	NLQKYQAECS	QFKAKLASLE	ELKRQAELDG	KSAKQNLDKC	YGQIKELNEK	ITRLTYEIED
1130	1140	1150	1160	1170	1180	1190	1200
EKRRRKSVED	RFDQQKNDYD	QLQKARQCEK	ENLGWQKLES	EKAIKEKEYE	IERLRVLLQE	EGTRKREYEN	ELAKVRNHYN
1210	1220	1230	1240	1250	1260	1270	1280
EEMSNLRNKY	ETEINITKTT	IKEISMQKED	DSKNLRNQLD	RLSRENRDLK	DEIVRLNDSI	LQATEQRRRA	EENALQQKAC
1290	1300	1310	1320	1330	1340	1350	1360
GSEIMQKKQH	LEIELKQVMQ	QRSEDNARHK	QSLEEAAKTI	QDKNKEIERL	KAEFQEEAKR	RWEYENELSK	VRNNYDEEII
1370	1380	1390	1400	1410	1420	1430	1440
SLKNQFETEI	NITKTTIHQL	TMQKEEDTSG	YRAQIDNLTR	ENRSLSEEIK	RLKNTLTQTT	ENLRRVEEDI	QQQKATGSEV
1450	1460	1470	1480	1490	1500	1510	1520
SQRKQQLEVE	LRQVTQMRTE	ESVRYKQSLD	DAAKTIQDKN	KEIERLKQLI	DKETNDRKCL	EDENARLQRV	QYDLQKANSS
1530	1540	1550	1560	1570	1580	1590	1600
ATETINKLKV	QEQELTRLRI	DYERVSQERT	VKDQDITRFQ	NSLKELQLQK	QKVEEELNRL	KRTASEDSCK	RKKLEEELEG
1610	1620	1630	1640	1650	1660	1670	1680
MRRSLKEQAI	KITNLTQQLE	QASIVKKRSE	DDLRQRDVL	DGHLREKQRT	QEELRRLSSE	VEALRRQLLQ	EQESVKQAHL
1690	1700	1710	1720	1730	1740	1750	1760
RNEHFQKAIE	DKSRSLNESK	IEIERLQSLT	ENLTKEHLML	EEELRNLRLE	YDDLRRGRSE	ADSDKNATIL	ELRSQLQISN
1770	1780	1790	1800	1810	1820	1830	1840
NRTLELQGLI	NDLQRERENL	RQEIEKFQKQ	ALEASNRIQE	SKNQCTQVVQ	ERESLLVKIK	VLEQDKARLQ	RLEDELNRAK
1850	1860	1870	1880	1890	1900	1910	1920
STLEAETRVK	QRLECEKQQI	QNDLNQWKTQ	YSRKEEAIRK	IESEREKSER	EKNSLRSEIE	RLQAEIKRIE	ERCRRKLEDS
1930	1940	1950	1960	1970	1980	1990	2000
TRETQSQLET	ERSRYQREID	KLRQRPYGSH	RETQTECEWT	VDTSKLVFDG	LRKKVTAMQL	YECQLIDKTT	LDKLLKGKKS
2010	2020	2030	2040	2050	2060	2070	2080
VEEVASEIQP	FLRGAGSIAG	ASASPKEKYS	LVEAKRKKLI	SPESTVMLLE	AQAATGGIID	PHRNEKLTVD	SAIARDLIDF
2090	2100	2110	2120	2130	2140	2150	2160
DDRQQIYAAE	KAITGFDDPF	SGKTVSVSEA	IKKNLIDRET	GMRLLEAQIA	SGGVVDPVNS	VFLPKDVALA	RGLIDRDLYR
2170	2180	2190	2200	2210	2220	2230	2240

Detailed Protein Report

Cmpd.	No. of Cmpds.	m/z meas.	Δ m/z [ppm]	z	Rt [min]	Score	P	Range	Sequence	Modification	Ratios	
2092	1	1045.4569	7.22	2	56.0	12.2	0	46-64	R.GVITDQNSDGYCQTGTMSR.H	Carbamidomethyl: 12		
2088	1	596.7468	-112.69	2	56.0	10.2	1	1330-1339	R.LKAEFQEEAK.R		MD:MU	1.47
1858	1	616.2551	-101.28	2	53.1	10.2	1	1488-1497	K.QLIDKETNDR.K			
2423	1	837.8362	-126.41	2	60.6	16.4	2	1545-1558	R.VSQERTVKDQDITR.F			
2393	1	1176.1976	83.64	2	59.8	12.7	2	2465-2484	R.RVVIVDPETNKEMSVQEAYK.K	Oxidation: 13		

Detailed Protein Report

**Protein 18:**	**PREDICTED: trinucleotide repeat-containing gene 18 protein isoform X2 [Homo sapiens]**		
**Accession:**	gi|578813686	**Score:**	85.6
**Database:**	refseq_human(refseq_human_20140103.fasta)	**MW [kDa]:**	232.1
**Database Date:**	2015-11-30	**pI:**	9.7
**Modification(s):**	Carbamidomethyl, Oxidation	**Sequence Coverage [%]:**	3.7
		**No. of unique Peptides:**	5

Quantitation

MD:MU	**Median:** 2.72	**CV:** 127.82 %	**No. of Peptides:** 2

Detailed Protein Report

10	20	30	40	50	60	70	80
MDGRDFGPQR	SVHGPPPPLL	SGLAMDSHRV	GAATAGRLPA	SGLPGPLPPG	KYMAGLNLHP	HPGEAFLGSF	VASGMGPSAS
90	100	110	120	130	140	150	160
SHGSPVPLPS	DLSFRSPTPS	NLPMVQLWAA	HAHEGFSHLP	SGLYPSYLHL	NHLEPPSSGS	PLLSQLGQPS	IFDTQKGQGP
170	180	190	200	210	220	230	240
GGDGFYLPTA	GAPGSLHSHA	PSARTPGGGH	SSGAPAKGSS	SRDGPAKERA	GRGGEPPPLF	GKKDPRARGE	EASGPRGVVD
250	260	270	280	290	300	310	320
LTQEARAEGR	QDRGPPRLAE	RLSPFLAESK	TKNAALQPSV	LTMCNGGAGD	VGLPALVAEA	GRGGAKEAAR	QDEGARLLRR
330	340	350	360	370	380	390	400
TETLLPGPRP	CPSPLPPPPA	PPKGPPAPPA	ATPAGVYTVF	REQGREHRVV	APTFVPSVEA	FDERPGPIQI	ASQARDARAR
410	420	430	440	450	460	470	480
EREAGRPGVL	QAPPGSPRPL	DRPEGLREKN	SVIRSLKRPP	PADAPTVRAT	RASPDPRAYV	PAKELLKPEA	DPRPCERAPR
490	500	510	520	530	540	550	560
GPAGPAAQQA	AKLFGLEPGR	PPPTGPEHKW	KPFELGNFAA	TQMAVLAAQH	HHSRAEEEAA	VVAASSSKKA	YLDPGAVLPR
570	580	590	600	610	620	630	640
SAATCGRPVA	DMHSAAHGSG	EASAMQSLIK	YSGSFARDAV	AVRPGGCGKK	SPFGGLGTMK	PEPAPTSAGA	SRAQARLPHS
650	660	670	680	690	700	710	720
GGPAAGGGRQ	LKRDPERPES	AKAFGREGSG	AQGEAEVRHP	PVGIAVAVAR	QKDSGGSGRL	GPGLVDQERS	LSLSNVKGHG
730	740	750	760	770	780	790	800
RADEDCVDDR	ARHREERLLG	ARLDRDQEKL	LRESKELADL	ARLHPTSCAP	NGLNPNLMVT	GGPALAGSGR	WSADPAAHLA
810	820	830	840	850	860	870	880
THPWLPRSGN	ASMWLAGHPY	GLGPPSLHQG	MAPAFPPGLG	GSLPSAYQFV	RDPQSGQLVV	IPSDHLPHFA	ELMERATVPP
890	900	910	920	930	940	950	960
LWPALYPPGR	SPLHHAQQLQ	LFSQQHFLRQ	QEFLYLQQQA	AQALELQRSA	QLVQERLKAQ	EHRAEMEEKG	SKRGLEAAGK
970	980	990	1000	1010	1020	1030	1040
AGLATAGPGL	LPRKPPGLAA	GPAGTYGKAV	SPPPSPRASP	VAALKAKVIQ	KLEDVSKPPA	YAYPATPSSH	PTSPPPASPP
1050	1060	1070	1080	1090	1100	1110	1120
PTPGITRKEE	APENVVEKKD	LELEKEAPSP	FQALFSDIPP	RYPFQALPPH	YGRPYPFLLQ	PTAAADADGL	APDVPLPADG
1130	1140	1150	1160	1170	1180	1190	1200
PERLALSPED	KPIRLSPSKI	TEPLREGPEE	EPLAEREVKA	EVEDMDEGPT	ELPPLESPLP	LPAAEAMATP	SPAGGCGGGL
1210	1220	1230	1240	1250	1260	1270	1280
LEAQALSATG	QSCAEPSECP	DFVEGPEPRV	DSPGRTEPCT	AALDLGVQLT	PETLVEAKEE	PVEVPVAVPV	VEAVPEEGLA
1290	1300	1310	1320	1330	1340	1350	1360
QVAPSESQPT	LEMSDCDVPA	GEGQCPSLEP	QEAVPVLGST	CFLEEASSDQ	FLPSLEDPLA	GMNALAAAAE	LPQARPLPSP
1370	1380	1390	1400	1410	1420	1430	1440
GAAGAQALEK	LEAAESLVLE	QSFLHGITLL	SEIAELELER	RSQEMGGAER	ALVARPSLES	LLAAGSHMLR	EVLDGPVVDP
1450	1460	1470	1480	1490	1500	1510	1520
LKNLRLPREL	KPNKKYSWMR	KKEERMYAMK	SSLEDMDALE	LDFRMRLAEV	QRQYKEKQRE	LVKLQRRRDS	EDRREEPHRS
1530	1540	1550	1560	1570	1580	1590	1600
LARRGPGRPR	KRTHAPSALS	PPRKRGKSGH	SSGKLSSKSL	LTSDDYELGA	GIRKRHKGSE	EEHDALIGMG	KARGRNQTWD
1610	1620	1630	1640	1650	1660	1670	1680
EHEASSDFIS	QLKIKKKKMA	SDQEQLASKL	DKALSLTKQD	KLKSPFKFSD	SAGGKSKTSG	GCGRYLTPYD	SLLGKNRKAL
1690	1700	1710	1720	1730	1740	1750	1760
AKGLGLSLKS	SREGKHKRAA	KTRKMEVGFK	ARGQPKSAHS	PFASEVSSYS	YNTDSEEDEE	FLKDEWPAQG	PSSSKLTPSL
1770	1780	1790	1800	1810	1820	1830	1840
LCSMVAKNSK	AAGGPKLTKR	GLAAPRTLKP	KPATSRKQPF	CLLLREAEAR	SSFSDSSEES	FDQDESSEEE	DEEEELEEED
1850	1860	1870	1880	1890	1900	1910	1920
EASGGGYRLG	ARERALSPGL	EESGLGLLAR	FAASALPSPT	VGPSLSVVQL	EAKQKARKKE	ERQSLLGTEF	EYTDSESEVK
1930	1940	1950	1960	1970	1980	1990	2000
VRKRSPAGLL	RPKKGLGEPG	PSLAAPTPGA	RGPDPSSPDK	AKLAVEKGRK	ARKLRGPKEP	GFEAGPEASD	DDLWTRRRSE
2010	2020	2030	2040	2050	2060	2070	2080
RIFLHDASAA	APAPVSTAPA	TKTSRCAKGG	PLSPRKDAGR	AKDRKDPRKV	PATVSKGRHW	KKKGKEAGPG	AGLPPPRAPA
2090	2100	2110	2120	2130	2140	2150	2160
LPSEARAPHA	SSLTAAKRSK	AKAKGKEVKK	ENRGKGGAVS	KLMESMAAEE	DFEPNQDSSF	SEDEHLPRGG	AVERPLTPGL
2170	2180	2190					

Detailed Protein Report

Cmpd.	No. of Cmpds.	m/z meas.	Δ m/z [ppm]	z	Rt [min]	Score	P	Range	Sequence	Modification	Ratios
1629	1	1052.2731	65.37	3	50.1	21.1	1	271-302	K. TKNAALQPSVLTMCNGGAGDVGL G	Carbamidomethyl: 14; Oxidation: 13	
1053	1	500.6425	-250.20	2	42.8	13.1	2	1545-1554	K.RGKSGHSSGK.L		MD:MU 7.28
307	1	846.3647	-83.38	2	33.2	17.3	2	1618-1632	K.KMASDQEQLASKLDK.A		
2682	1	734.8888	-6.21	2	63.7	11.3	2	1642-1655	K.LKSPFKFSDSAGGK.S		
177	1	613.7776	-171.05	2	31.9	22.8	1	1787-1797	R.TLKPKPATSRK.Q		MD:MU 1.02

Detailed Protein Report

Protein 19: heat shock protein beta-1 [Homo sapiens]

**Accession:**	gi|4504517	**Score:**	84.1
**Database:**	refseq_human(refseq_human_20140103.fasta)	**MW [kDa]:**	22.8
**Database Date:**	2015-11-30	**pI:**	6.0
		**Sequence Coverage [%]:**	13.2
		**No. of unique Peptides:**	2

Quantitation

MD:MU **Median:** 0.85 **CV:** 32.52 % **No. of Peptides:** 2

```
        10         20         30         40         50         60         70         80
MTERRVPFSL LRGPSWDPFR DWYPHSRLFD QAFGLPRLPE EWSQWLGGSS WPGYVRPLPP AAIESPAVAA PAYSRALSRQ
        90        100        110        120        130        140        150        160
LSSGVSEIRH TADRWRVSLD VNHFAPDELT VKTKDGVVEI TGKHEERQDE HGYISRCFTR KYTLPPGVDP TQVSSSLSPE
       170        180        190        200        210
GTLTVEAPMP KLATQSNEIT IPVTFESRAQ LGGPEAAKSD ETAAK
```

Cmpd.	No. of Cmpds.	m/z meas.	Δ m/z [ppm]	z	Rt [min]	Score	P	Range	Sequence	Modification	Ratios	
1713	7	582.2695	-76.42	2	51.2	61.7	0	28-37	R.LFDQAFGLPR.L		MD:MU	0.62
1635	1	953.4046	-99.49	2	50.2	22.4	0	172-188	K.LATQSNEITIPVTFESR.A		MD:MU	1.16

Detailed Protein Report

Protein 20: **PREDICTED: DNA-directed RNA polymerase, mitochondrial isoform X1 [Homo sapiens]**

**Accession:**	gi|530425287	**Score:**	84.0
**Database:**	refseq_human(refseq_human_20140103.fasta)	**MW [kDa]:**	145.0
**Database Date:**	2015-11-30	**pI:**	10.3
**Modification(s):**	Carbamidomethyl, Oxidation	**Sequence Coverage [%]:**	5.0
		**No. of unique Peptides:**	4

10	20	30	40	50	60	70	80
MPSRLRCILG	VYFRLGFPFA	ASVDHETTTP	GRRRGRMRRR	GPVGWPGAAC	VMSALCWGRG	AAGLKRALRP	CGRPGLPGKE
90	100	110	120	130	140	150	160
GTAGGVCGPR	RSSSASPQEQ	DQDRRKDWGH	VELLEVLQAR	VRQLQAESVS	EVVVNRVDVA	RLPECGSGDG	SLQPPRKVQM
170	180	190	200	210	220	230	240
GAKDATPVPC	GRWAKILEKD	KRTQQMRMQR	LKAKLQMPFQ	SGEFKALTRR	LQVEPRLLSK	QMAGCLEDCT	RQAPESPWEE
250	260	270	280	290	300	310	320
QLARLLQEAP	GKLSLDVEQA	PSGQHSQAQL	SGQQQRLLAF	FKCCLLTDQL	PLAHHLLVVH	HGQRQKRKLL	TLDMYNAVML
330	340	350	360	370	380	390	400
GWARQGAFKE	LVYVLFMVKD	AGLTPDLLSY	AAALQCMGRQ	DQDAGTIERC	LEQMSQEGLK	LQALFTAVLL	SEEDRATVLK
410	420	430	440	450	460	470	480
AVHKVKPTFS	LPPQLPPPVN	TSKLLRDVYA	KDGRVSYPKL	HLPLKTLQCL	FEKQLHMELA	SRVCVVSVEK	PTLPSKEVKH
490	500	510	520	530	540	550	560
ARKTLKTLRD	QWEKALCRAL	RETKNRLERE	VYEGRFSLYP	FLCLLDEREV	VRMLLQVLQA	LPAQGESFTT	LARELSARTF
570	580	590	600	610	620	630	640
SRHVVQRQRV	SGQVQALQNH	YRKYLCLLAS	DAEVPEPCLP	RQYWEELGAP	EALREQPWPL	PVQMELGKLL	AEMLVQATQM
650	660	670	680	690	700	710	720
PCSLDKPHRS	SRLVPVLYHV	YSFRNVQQIG	ILKPHPAYVQ	LLEKAAEPTL	TFEAVDVPML	CPPLPWTSPH	SGAFLLSPTK
730	740	750	760	770	780	790	800
LMRTVEGATQ	HQELLETCPP	TALHGALDAL	TQLGNCAWRV	NGRVLDLVLQ	LFQAKGCPQL	GVPAPPSEAP	QPPEAHLPHS
810	820	830	840	850	860	870	880
AAPARKAELR	RELAHCQKVA	REMHSLRAEA	LYRLSLAQHL	RDRVFWLPHN	MDFRGRTYPC	PPHFNHLGSD	VARALLEFAQ
890	900	910	920	930	940	950	960
GRPLGPHGLD	WLKIHLVNLT	GLKKREPLRK	RLAFAEEVMD	DILDSADQPL	TGRKWWMGAE	EPWQTLACCM	EVANAVRASD
970	980	990	1000	1010	1020	1030	1040
PAAYVSHLPV	HQDGSCNGLQ	HYAALGRDSV	GAASVNLEPS	DVPQDVYSGV	AAQVEVFRRQ	DAQRGMRVAQ	VLEGFITRKV
1050	1060	1070	1080	1090	1100	1110	1120
VKQTVMTVVY	GVTRYGGRLQ	IEKRLRELSD	FPQEFVWEAS	HYLVRQVFKS	LQEMFSGTRA	IQHWLTESAR	LISHMGSVVE
1130	1140	1150	1160	1170	1180	1190	1200
WVTPLGVPVI	QPYRLDSKVK	QIGGGIQSIT	YTHNGDISRK	PNTRKQKNGF	PPNFIHSLDS	SHMMLTALHC	YRSPPLCHRK
1210	1220	1230	1240	1250	1260	1270	1280
GLTFVSVHDC	YWTHAADVSV	MNQVCREQFV	RLHSEPILQD	LSRFLVKRFC	SEPQKILEAS	QLKETLQAVP	KPGAFDLEQV
1290							
KRSTYFFS							

Cmpd.	No. of Cmpds.	m/z meas.	Δ m/z [ppm]	z	Rt [min]	Score	P	Range	Sequence	Modification
2442	1	857.6864	-95.07	3	60.9	18.7	2	40-65	R.RGPVGWPGAACVMSALCWGRGAAGLK.R	
3	1	1244.4602	-117.14	2	28.9	10.3	1	41-65	R.GPVGWPGAACVMSALCWGRGAAGLK.R	Carbamidomethyl: 10; Oxidation: 12
2861	1	682.6750	3.46	3	66.2	16.6	0	584-601	K.YLCLLASDAEVPEPCLPR.Q	Carbamidomethyl: 3
2603	1	678.4838	186.95	1	63.1	15.6	0	1227-1231	R.EQFVR.L	

Detailed Protein Report

Protein 21: PREDICTED: nebulin isoform X22 [Homo sapiens]

**Accession:**	gi|530370465	**Score:**	82.6
**Database:**	refseq_human(refseq_human_20140103.fasta)	**MW [kDa]:**	961.5
**Database Date:**	2015-11-30	**pI:**	9.5
**Modification(s):**	Oxidation	**Sequence Coverage [%]:**	1.0
		**No. of unique Peptides:**	5

Detailed Protein Report

10	20	30	40	50	60	70	80
MADDEDYEEV	VEYYTEEVVY	EEVPGETITK	IYETTTTRTS	DYEQSETSKP	ALAQPALAQP	ASAKPVERRK	VIRKKVDPSK
90	100	110	120	130	140	150	160
FMTPYIAHSQ	KMQDLFSPNK	YKEKFEKTKG	QPYASTTDTP	ELRRIKKVQD	QLSEVKYRMD	GDVAKTICHV	DEKAKDIEHA
170	180	190	200	210	220	230	240
KKVSQQVSKV	LYKQNWEDTK	DKYLLPPDAP	ELVQAVKNTA	MFSKKLYTED	WEADKSLFYP	YNDSPELRRV	AQAQKALSDV
250	260	270	280	290	300	310	320
AYKKGLAEQQ	AQFTPLADPP	DIEFAKKVTN	QVSKQKYKED	YENKIKGKWS	ETPCFEVANA	RMNADNISTR	KYQEDFENMK
330	340	350	360	370	380	390	400
DQIYFMQTET	PEYKMNKKAG	VAASKVKYKE	DYEKNKGKAD	YNVLPASENP	QLRQLKAAGD	ALSDKLYKEN	YEKTKAKSIN
410	420	430	440	450	460	470	480
YCETPKFKLD	TVLQNFSSDK	KYKDSYLKDI	LGHYVGSFED	PYHSHCMKVT	AQNSDKNYKA	EYEEDRGKGF	FPQTITQEYE
490	500	510	520	530	540	550	560
AIKKLDQCKD	HTYKVHPDKT	KFTQVTDSPV	LLQAQVNSKQ	LSDLNYKAKH	ESEKFKCHIP	PDTPAFIQHK	VNAYNLSDNL
570	580	590	600	610	620	630	640
YKQDWEKSKA	KKFDIKVDAI	PLLAAKANTK	NTSDVMYKKD	YEKNKGKMIG	VLSINDDPKM	LHSLKVAKNQ	SDRLYKENYE
650	660	670	680	690	700	710	720
KTKAKSMNYC	ETPKYQLDTQ	LKNFSEARYK	DLYVKDVLGH	YVGSMEDPYH	THCMKVAAQN	SDKSYKAEYE	EDKGKCYFPQ
730	740	750	760	770	780	790	800
TITQEYEAIK	KLDQCKDHTY	KVHPDKTKFT	AVTDSPVLLQ	AQLNTKQLSD	LNYKAKHEGE	KFKCHIPADA	PQFIQHRVNA
810	820	830	840	850	860	870	880
YNLSDNVYKQ	DWEKSKAKKF	DIKVDAIPLL	AAKANTKNTS	DVMYKKDYEK	SKGKMIGALS	INDDPKMLHS	LKTAKNQSDR
890	900	910	920	930	940	950	960
EYRKDYEKSK	TIYTAPLDML	QVTQAKKSQA	IASDVDYKHI	LHSYSYPPDS	INVDLAKKAY	ALQSDVEYKA	DYNSWMKGCG
970	980	990	1000	1010	1020	1030	1040
WVPFGSLEME	KAKRASDILN	EKKYRQHPDT	LKFTSIEDAP	ITVQSKINQA	QRSDIAYKAK	GEEIIHKYNL	PPDLPQFIQA
1050	1060	1070	1080	1090	1100	1110	1120
KVNAYNISEN	MYKADLKDLS	KKGYDLRTDA	IPIRAAKAAR	QAASDVQYKK	DYEKAKGKMV	GFQSLQDDPK	LVHYMNVAKI
1130	1140	1150	1160	1170	1180	1190	1200
QSDREYKKDY	EKTKSKYNTP	HDMFNVVAAK	KAQDVVSNVN	YKHSLHHYTY	LPDAMDLELS	KNMMQIQSDN	VYKEDYNNWM
1210	1220	1230	1240	1250	1260	1270	1280
KGIGWIPIGS	LDVEKVKKAG	DALNEKKYRQ	HPDTLKFTSI	VDSPVMVQAK	QNTKQVSDIL	YKAKGEDVKH	KYTMSPDLPQ
1290	1300	1310	1320	1330	1340	1350	1360
FLQAKCNAYN	ISDVCYKRDW	YDLIAKGNNV	LGDAIPITAA	KASRNIASDY	KYKEAYEKSK	GKHVGFRSLQ	DDPKLVHYMN
1370	1380	1390	1400	1410	1420	1430	1440
VAKLQSDREY	KKNYENTKTS	YHTPGDMVSI	TAAKMAQDVA	TNVNYKQPLH	HYTYLPDAMS	LEHTRNVNQI	QSDNVYKDEY
1450	1460	1470	1480	1490	1500	1510	1520
NSFLKGIGWI	PIGSLEVEKV	KKAGDALNER	KYRQHPDTVK	FTSVPDSMGM	VLAQHNTKQL	SDLNYKVEGE	KLKHKYTIDP
1530	1540	1550	1560	1570	1580	1590	1600
ELPQFIQAKV	NALNMSDAHY	KADWKKTIAK	GYDLRPDAIP	IVAAKSSRNI	ASDCKYKEAY	EKAKGKQVGF	LSLQDDPKLV
1610	1620	1630	1640	1650	1660	1670	1680
HYMNVAKIQS	DREYKKGYEA	SKTKYHTPLD	MVSVTAAKKS	QEVATNANYR	QSYHHYTLLP	DALNVEHSRN	AMQIQSDNLY
1690	1700	1710	1720	1730	1740	1750	1760
KSDFTNWMKG	IGWVPIESLE	VEKAKKAGEI	LSEKKYRQHP	EKLKFTYAMD	TMEQALNKSN	KLNMDKRLYT	EKWNKDKTTI
1770	1780	1790	1800	1810	1820	1830	1840
HVMPDTPDIL	LSRVNQITMS	DKLYKAGWEE	EKKKGYDLRP	DAIAIKAARA	SRDIASDYKY	KKAYEQAKGK	HIGFRSLEDD
1850	1860	1870	1880	1890	1900	1910	1920
PKLVHFMQVA	KMQSDREYKK	GYEKSKTSFH	TPVDMLSVVA	AKKSQEVATN	ANYRNVIHTY	NMLPDAMSFE	LAKNMMQIQS
1930	1940	1950	1960	1970	1980	1990	2000
DNQYKADYAD	FMKGIGWLPL	GSLEAEKNKK	AMEIISEKKY	RQHPDTLKYS	TLMDSMNMVL	AQNNAKIMNE	HLYKQAWEAD
2010	2020	2030	2040	2050	2060	2070	2080
KTKVHIMPDI	PQIILAKANA	INMSDKLYKL	SLEESKKKGY	DLRPDAIPIK	AAKASRDIAS	DYKYKYNYEK	GKGKMVGFRS
2090	2100	2110	2120	2130	2140	2150	2160
LEDDPKLVHS	MQVAKMQSDR	EYKKNYENTK	TSYHTPADML	SVTAAKDAQA	NITNTNYKHL	IHKYILLPDA	MNIELTRNMN
2170	2180	2190	2200	2210	2220	2230	2240

Detailed Protein Report

Cmpd.	No. of Cmpds.	m/z meas.	Δ m/z [ppm]	z	Rt [min]	Score	P	Range	Sequence	Modification
2522	1	885.4523	-25.95	2	62.0	17.0	2	407-421	K.FKLDTVLQNFSSDKK.Y	
457	1	544.7651	77.28	2	35.3	13.1	0	646-654	K.SMNYCETPK.Y	Oxidation: 2
1248	1	796.3930	45.30	2	45.3	13.7	1	2813-2825	R.DDPKMMWSMHVAK.I	Oxidation: 5
2640	1	701.8709	47.97	2	63.1	10.8	0	6260-6270	R.CQYILSDLEYR.H	
2657	1	994.4910	10.30	2	63.3	10.6	1	8000-8015	K.LNQENFSSVLYKENMR.K	Oxidation: 15

Detailed Protein Report

Protein 22: dynein heavy chain 1, axonemal [Homo sapiens]

**Accession:**	gi|197927452	**Score:**	82.0
**Database:**	refseq_human(refseq_human_20140103.fasta)	**MW [kDa]:**	487.2
**Database Date:**	2015-11-30	**pI:**	5.5
**Modification(s):**	Oxidation	**Sequence Coverage [%]:**	1.9
		**No. of unique Peptides:**	6

Quantitation

MD:MU	**Median:** 1.02	**CV:** 0.00 %	**No. of Peptides:** 1

Alias proteins:

Accession	Name	Description
gi|578805960	refseq_human (refseq_human_20140103.fasta)	PREDICTED: dynein heavy chain 1, axonemal isoform X1 [Homo sapiens]

BRUKER

Detailed Protein Report

10	20	30	40	50	60	70	80
MEQPNSKGYS	LGRTPQGPEC	SSAPAVQVGT	HRGLEYNPGK	ILPGSDYGLG	NPPALDPKLP	HLPLPPAPPT	LSDLGQPRKS
90	100	110	120	130	140	150	160
PLTGTDKKYP	LMKQRGFYSD	ILSPGTLDQL	GEVCRGPRMS	QNLLRQADLD	KFTPRVGSFE	VPEDFQERME	QQCIGSTTRL
170	180	190	200	210	220	230	240
LAQTDFPLQA	YEPKMQVPFQ	VLPGQHPRKI	EIERRKQQYL	SLDIEQLLFS	QGIDSNKLMP	RHLDHQHPQT	IEQGHDPIFP
250	260	270	280	290	300	310	320
IYLPLKVFDN	EDFDCRTPRE	WINMGLEPGS	LDRKPVPGKA	LLPTDDFLGH	EDPKSQKLKY	KWCEVGVLDY	DEEKKLYLVH
330	340	350	360	370	380	390	400
KTDEKGLVRD	EMGRPILNAG	VTTEGRPPLQ	VCQYWVPRIQ	LLFCAEDPCM	FAQRVVQANA	LRKNTEALLL	YNLYVDCMPS
410	420	430	440	450	460	470	480
DGQHVISEQS	LSKIKQWALS	TPRMRKGPSV	LEHLSSLARE	VSLDYERSMN	KINFDHVVSS	KPETFSYVTL	PKKEEEQVPE
490	500	510	520	530	540	550	560
RGLVSVPKYH	FWEQKEDFTF	VSLLTRPEVI	TALSKVRAEC	NKVTAMSLFH	SSLSKYSHLE	EFEQIQSQTF	SQVQMFLKDS
570	580	590	600	610	620	630	640
WISSLKVAMR	SSLRDMSKGW	YNLYETNWEV	YLMSKLRKLM	ELVKYMLQDT	LRFLVQDSLA	SFSQFISDTC	CSVLNCTDDM
650	660	670	680	690	700	710	720
VWGDDLINSP	YRPRKNPLFI	MDLVLDSSGV	HYSTPLEQFE	ASLLNLFDKG	ILATHAVPQL	EKLVMEDIFI	SGDPLLESVG
730	740	750	760	770	780	790	800
LHEPLVEELR	ATIASAVSKA	MIPLQAYAKE	YRKYLELNNN	DIASFLKTYQ	TQGLLAQEVR	EVVLTHLREK	EILDSSLPSS
810	820	830	840	850	860	870	880
IIIGPFYINT	DNVKQSLSKK	RKALATSVLD	ILAKNLHKEV	DSICEEFRSI	SRKIYEKPNS	IEELAELREW	MKGIPERLVG
890	900	910	920	930	940	950	960
LEERIVKVMD	DYQVMDEFLY	NLSSDDFNDK	WIASNWPSKI	LGQIELVQQQ	HVEDEEKFRK	IQIMDQNNFQ	EKLEGLQLVV
970	980	990	1000	1010	1020	1030	1040
AGFSIHVEIS	RAHEIANEVR	RVKKQLKDCQ	QLAMLYNNRE	RIFSLPITNY	DKLSRMVKEF	QPYLDLWTTA	SDWLRWSESW
1050	1060	1070	1080	1090	1100	1110	1120
MNDPLSAIDA	EQLEKNVVEA	FKTMHKCVKQ	FKDMPACQEV	ALDIRARIEE	FKPYIPLIQG	LRNPGMRIRH	WETLSNQINI
1130	1140	1150	1160	1170	1180	1190	1200
NVRPKANLTF	ARCLEMNLQD	HIESISKVAE	VAGKEYAIEQ	ALDKMEKEWS	TILFNVLPYK	ATDTYILKSP	DEASQLLDDH
1210	1220	1230	1240	1250	1260	1270	1280
IVMTQNMSFS	PYKKPFEQRI	NSWENKLKLT	QEVLEEWLNC	QRSWLYLEPI	FSSEDINQQL	PVESKRYQTM	ERIWKKIMKN
1290	1300	1310	1320	1330	1340	1350	1360
AYENREVINV	CSDLRMLDSL	RDCNKILDLV	QKGLSEYLET	KRSAFPRFYF	LSDDELLEIL	SQTKDPTAVQ	PHLRKCFENI
1370	1380	1390	1400	1410	1420	1430	1440
ARLLFQEDLE	ITHMYSAEGE	EVQLCFSIYP	SSNVEDWLRE	VERSMKASVH	DIIEKAIRAY	PTMPRTQWVL	NWPGQVTIAG
1450	1460	1470	1480	1490	1500	1510	1520
CQTYWTMEVA	EALEAGNLRS	QLFPQLCQQL	SDLVALVRGK	LSRMQRAVLS	ALIVIEVHAK	DVVSKLIQEN	VVSVNDFQWI
1530	1540	1550	1560	1570	1580	1590	1600
SQLRYYWTNN	DLYIRAVNAE	FIYGYEYLGN	SGRLVITPLT	DRCYLTLTGA	LHLKFGGAPA	GPAGTGKTET	TKDLGKALAI
1610	1620	1630	1640	1650	1660	1670	1680
QTVVFNCSDQ	LDFMAMGKFF	KGLASAGAWA	CFDEFNRIDI	EVLSVVAQQI	TTIQKAQQQR	VERFMFEGVE	IPLVPSCAVF
1690	1700	1710	1720	1730	1740	1750	1760
ITMNPGYAGR	TELPDNLKAL	FRPVAMMVPD	YAMITEISLY	SFGFNEASVL	AKKITTTFKL	SSEQLSSQDH	YDFGMRAVKT
1770	1780	1790	1800	1810	1820	1830	1840
VISAAGNLKR	ENPSMNEELI	CLRAIRDVNV	PKFLQEDLKL	FSGIVSDLFP	TIKEEDTDYG	ILDEAIREAC	RNSNLKDVEG
1850	1860	1870	1880	1890	1900	1910	1920
FLTKCIQLYE	TTVVRHGLML	VGPTGSGKST	CYRVLAAAMT	SLKGQPSISG	GMYEAVNYYV	LNPKSITMGQ	LYGEFDLLTH
1930	1940	1950	1960	1970	1980	1990	2000
EWTDGIFSSF	IRAGAITSDT	NKKWYMFDGP	VDAIWIENMN	TVLDDNKKLC	LSSGEIIKLT	EAMTMMFEVQ	DLAVASPATV
2010	2020	2030	2040	2050	2060	2070	2080
SRCGMVYLEP	SILGLMPFIE	CWLRKLPPLL	KPYEEHFKAL	FVSFLEESIS	FVRSSVKEVI	ASTNCNLTMS	LLKLLDCFFK
2090	2100	2110	2120	2130	2140	2150	2160
PFLPREGLKK	IPSEKLSRIV	ELIEPWFIFS	LIWSVGATGD	SSGRTSFSHW	LRLKMENEQL	TLLFPEEGLV	FDYRLEDAGI
2170	2180	2190	2200	2210	2220	2230	2240

Detailed Protein Report

Cmpd.	No. of Cmpds.	m/z meas.	Δ m/z [ppm]	z	Rt [min]	Score	P	Range	Sequence	Modification	Ratios
1891	1	918.3580	-147.46	2	53.5	15.4	2	559-574	K.DSWISSLKVAMRSSLR.D		
449	1	565.3223	-66.47	2	35.3	17.9	2	596-604	K.LRKLMELVK.Y		
870	1	573.1350	-254.46	2	40.3	10.7	1	2478-2486	R.DRLVNEEDR.S		
178	1	715.8622	76.35	2	31.9	12.3	0	2496-2506	R.CMEQWEVTFNK.V	Oxidation: 2	MD:MU 1.02
2816	1	788.8905	-16.60	2	65.6	10.2	1	3081-3094	R.LREVEDGIATMQAK.Y	Oxidation: 11	
51	2	706.3163	-75.95	3	30.3	15.4	2	3699-3718	K.KLSAISLGQGQGPRAEAMMR.S	Oxidation: 18	

Detailed Protein Report

Protein 23: PREDICTED: histone-lysine N-methyltransferase 2A isoform X1 [Homo sapiens]

**Accession:**	gi|578822195	**Score:**	78.6
**Database:**	refseq_human(refseq_human_20140103.fasta)	**MW [kDa]:**	323.9
**Database Date:**	2015-11-30	**pI:**	9.2
**Modification(s):**	Carbamidomethyl, Oxidation	**Sequence Coverage [%]:**	2.7
		**No. of unique Peptides:**	5

Detailed Protein Report

10	20	30	40	50	60	70	80
MLAQADKLPM	TDKRVASLLK	KAKAQLCKIE	KSKSLKQTDQ	PKAQGQESDS	SETSVRGPRI	KHVCRRAAVA	LGRKRAVFPD
90	100	110	120	130	140	150	160
DMPTLSALPW	EEREKILSSM	GNDDKSSIAG	SEDAEPLAPP	IKPIKPVTRN	KAPQEPPVKK	GRRSRRCGQC	PGCQVPEDCG
170	180	190	200	210	220	230	240
VCTNCLDKPK	FGGRNIKKQC	CKMRKCQNLQ	WMPSKAYLQK	QAKAVKKKEK	KSKTSEKKDS	KESSVVKNVV	DSSQKPTPSA
250	260	270	280	290	300	310	320
REDPAPKKSS	SEPPPRKPVE	EKSEEGNVSA	PGPESKQATT	PASRKSSKQV	SQPALVIPPQ	PPTTGPPRKE	VPKTTPSEPK
330	340	350	360	370	380	390	400
KKQPPPPESG	PEQSKQKKVA	PRPSIPVKQK	PKEKEKPPPV	NKQENAGTLN	ILSTLSNGNS	SKQKIPADGV	HRIRVDFKED
410	420	430	440	450	460	470	480
CEAENVWEMG	GLGILTSVPI	TPRVVCFLCA	SSGHVEFVYC	QVCCEPFHKF	CLEENERPLE	DQLENWCCRR	CKFCHVCGRQ
490	500	510	520	530	540	550	560
HQATKQLLEC	NKCRNSYHPE	CLGPNYPTKP	TKKKKVWICT	KCVRCKSCGS	TTPGKGWDAQ	WSHDFSLCHD	CAKLFAKGNF
570	580	590	600	610	620	630	640
CPLCDKCYDD	DDYESKMMQC	GKCDRWVHSK	CENLSGTEDE	MYEILSNLPE	SVAYTCVNCT	ERHPAEWRLA	LEKELQISLK
650	660	670	680	690	700	710	720
QVLTALLNSR	TTSHLLRYRQ	AAKPPDLNPE	TEESIPSRSS	PEGPDPPVLT	EVSKQDDQQP	LDLEGVKRKM	DQGNYTSVLE
730	740	750	760	770	780	790	800
FSDDIVKIIQ	AAINSDGGQP	EIKKANSMVK	SFFIRQMERV	FPWFSVKKSR	FWEPNKVSSN	SGMLPNAVLP	PSLDHNYAQW
810	820	830	840	850	860	870	880
QEREENSHTE	QPPLMKKIIP	APKPKGPGEP	DSPTPLHPPT	PPILSTDRSR	EDSPELNPPP	GIEDNRQCAL	CLTYGDDSAN
890	900	910	920	930	940	950	960
DAGRLLYIGQ	NEWTHVNCAL	WSAEVFEDDD	GSLKNVHMAV	IRGKQLRCEF	CQKPGATVGC	CLTSCTSNYH	FMCSRAKNCV
970	980	990	1000	1010	1020	1030	1040
FLDDKKVYCQ	RHRDLIKGEV	VPENGFEVFR	RVFVDFEGIS	LRRKFLNGLE	PENIHMMIGS	MTIDCLGILN	DLSDCEDKLF
1050	1060	1070	1080	1090	1100	1110	1120
PIGYQCSRVY	WSTTDARKRC	VYTCKIVECR	PPVVEPDINS	TVEHDENRTI	AHSPTSFTES	SSKESQNTAE	IISPPSPDRP
1130	1140	1150	1160	1170	1180	1190	1200
PHSQTSGSCY	YHVISKVPRI	RTPSYSPTQR	SPGCRPLPSA	GSPTPTTHEI	VTVGDPLLSS	GLRSIGSRRH	STSSLSPQRS
1210	1220	1230	1240	1250	1260	1270	1280
KLRIMSPMRT	GNTYSRNNVS	SVSTTGTATD	LESSAKVVDH	VLGPLNSSTS	LGQNTSTSSN	LQRTVVTVGN	KNSHLDGSSS
1290	1300	1310	1320	1330	1340	1350	1360
SEMKQSSASD	LVSKSSSLKG	EKTKVLSSKS	SEGSAHNVAY	PGIPKLAPQV	HNTTSRELNV	SKIGSFAEPS	SVSFSSKEAL
1370	1380	1390	1400	1410	1420	1430	1440
SFPHLHLRGQ	RNDRDQHTDS	TQSANSSPDE	DTEVKTLKLS	GMSNRSSIIN	EHMGSSSRDR	RQKGKKSCKE	TFKEKHSSKS
1450	1460	1470	1480	1490	1500	1510	1520
FLEPGQVTTG	EEGNLKPEFM	DEVLTPEYMG	QRPCNNVSSD	KIGDKGLSMP	GVPKAPPMQV	EGSAKELQAP	RKRTVKVTLT
1530	1540	1550	1560	1570	1580	1590	1600
PLKMENESQS	KNALKESSPA	SPLQIESTSP	TEPISASENP	GDGPVAQPSP	NNTSCQDSQS	NNYQNLPVQD	RNLMLPDGPK
1610	1620	1630	1640	1650	1660	1670	1680
PQEDGSFKRR	YPRRSARARS	NMFFGLTPLY	GVRSYGEEDI	PFYSSSTGKK	RGKRSAEGQV	DGADDLSTSD	EDDLYYYNFT
1690	1700	1710	1720	1730	1740	1750	1760
RTVISSGGEE	RLASHNLFRE	EEQCDLPKIS	QLDGVDDGTE	SDTSVTATTR	KSSQIPKRNG	KENGTENLKI	DRPEDAGEKE
1770	1780	1790	1800	1810	1820	1830	1840
HVTKSSVGHK	NEPKMDNCHS	VSRVKTQGQD	SLEAQLSSLE	SSRRVHTSTP	SDKNLLDTYN	TELLKSDSDN	NNSDDCGNIL
1850	1860	1870	1880	1890	1900	1910	1920
PSDIMDFVLK	NTPSMQALGE	SPESSSSELL	NLGEGLGLDS	NREKDMGLFE	VFSQQLPTTE	PVDSSVSSSI	SAEEQFELPL
1930	1940	1950	1960	1970	1980	1990	2000
ELPSDLSVLT	TRSPTVPSQN	PSRLAVISDS	GEKRVTITEK	SVASSESDPA	LLSPGVDPTP	EGHMTPDHFI	QGHMDADHIS
2010	2020	2030	2040	2050	2060	2070	2080
SPPCGSVEQG	HGNNQDLTRN	SSTPGLQVPV	SPTVPIQNQK	YVPNSTDSPG	PSQISNAAVQ	TTPPHLKPAT	EKLIVVNQNM
2090	2100	2110	2120	2130	2140	2150	2160
QPLYVLQTLP	NGVTQKIQLT	SSVSSTPSVM	ETNTSVLGPM	GGGLTLTTGL	NPSLPTSQSL	FPSASKGLLP	MSHHQHLHSF
2170	2180	2190	2200	2210	2220	2230	2240

Detailed Protein Report

Cmpd.	No. of Cmpds.	m/z meas.	Δ m/z [ppm]	z	Rt [min]	Score	P	Range	Sequence	Modification
1751	1	1061.1573	33.03	3	51.7	15.9	2	144-170	R.SRRCGQCPGCQVPEDCGVCTNCLDKPK.F	Carbamidomethyl: 4, 10, 16, 19, 22
2129	11	682.2818	44.75	3	56.8	21.4	1	567-582	K.CYDDDDYESKMMQCGK.C	Carbamidomethyl: 1, 14
53	1	1096.9888	-34.10	2	30.4	17.5	1	1264-1284	R.TVVTVGNKNSHLDGSSSSEMK.Q	Oxidation: 20
11	1	820.3475	-49.73	1	29.3	12.9	1	2734-2739	K.HCRNYK.F	
395	1	625.3909	142.58	2	34.6	10.9	2	2950-2960	K.LPCNCGAKKCR.K	Carbamidomethyl: 5

Detailed Protein Report

Protein 24: collagen alpha-1(XIV) chain precursor [Homo sapiens]

**Accession:**	gi|55743096	**Score:**	77.8
**Database:**	refseq_human(refseq_human_20140103.fasta)	**MW [kDa]:**	193.4
**Database Date:**	2015-11-30	**pI:**	5.0
		**Sequence Coverage [%]:**	4.0
		**No. of unique Peptides:**	4

Quantitation

MD:MU	**Median:** 0.83	**CV:** 0.00 %	**No. of Peptides:** 1

Alias proteins:

Accession	Name	Description
gi|578816162	refseq_human (refseq_human_20140103.fasta)	PREDICTED: collagen alpha-1(XIV) chain isoform X2 [Homo sapiens]

Detailed Protein Report

10	20	30	40	50	60	70	80
MKIFQRKMRY	WLLPPFLAIV	YFCTIVQGQV	APPTRLRYNV	ISHDSIQISW	KAPRGKFGGY	KLLVTPTSGG	KTNQLNLQNT
90	100	110	120	130	140	150	160
ATKAIIQGLM	PDQNYTVQII	AYNKDKESKP	AQGQFRIKDL	EKRKDPKPRV	KVVDRGNGSR	PSSPEEVKFV	CQTPAIADIV
170	180	190	200	210	220	230	240
ILVDGSWSIG	RFNFRLVRHF	LENLVTAFDV	GSEKTRIGLA	QYSGDPRIEW	HLNAFSTKDE	VIEAVRNLPY	KGGNTLTGLA
250	260	270	280	290	300	310	320
LNYIFENSFK	PEAGSRTGVS	KIGILITDGK	SQDDIIPPSR	NLRESGVELF	AIGVKNADVN	ELQEIASEPD	STHVYNVAEF
330	340	350	360	370	380	390	400
DLMHTVVESL	TRTLCSRVEE	QDREIKASAH	AITGPPTELI	TSEVTARSFM	VNWTHAPGNV	EKYRVVYYPT	RGGKPDEVVV
410	420	430	440	450	460	470	480
DGTVSSTVLK	NLMSLTEYQI	AVFAIYAHTA	SEGLRGTETT	LALPMASDLL	LYDVTENSMR	VKWDAVPGAS	GYLILYAPLT
490	500	510	520	530	540	550	560
EGLAGDEKEM	KIGETHTDIE	LSGLLPNTEY	TVTVYAMFGE	EASDPVTGQE	TTLALSPPRN	LRISNVGSNS	ARLTWDPTSR
570	580	590	600	610	620	630	640
QINGYRIVYN	NADGTEINEV	EVDPITTFPL	KGLTPLTEYT	IAIFSIYDEG	QSEPLTGVFT	TEEVPAQQYL	EIDEVTTDSF
650	660	670	680	690	700	710	720
RVTWHPLSAD	EGLHKLMWIP	VYGGKTEEVV	LKEEQDSHVI	EGLEPGTEYE	VSLLAVLDDG	SESEVVTAVG	TTLDSFWTEP
730	740	750	760	770	780	790	800
ATTIVPTTSV	TSVFQTGIRN	LVVGDETTSS	LRVKWDISDS	DVQQFRVTYM	TAQGDPEEEV	IGTVMVPGSQ	NNLLLKPLLP
810	820	830	840	850	860	870	880
DTEYKVTVTP	IYTDGEGVSV	SAPGKTLPSS	GPQNLRVSEE	WYNRLRITWD	PPSSPVKGYR	IVYKPVSVPG	PTLETFVGAD
890	900	910	920	930	940	950	960
INTILITNLL	SGMDYNVKIF	ASQASGFSDA	LTGMVKTLFL	GVTNLQAKHV	EMTSLCAHWQ	VHRHATAYRV	VIESLQDRQK
970	980	990	1000	1010	1020	1030	1040
QESTVGGGTT	RHCFYGLQPD	SEYKISVYTK	LQEIEGPSVS	IMEKTQSLPT	RPPTFPPTIP	PAKEVCKAAK	ADLVFMVDGS
1050	1060	1070	1080	1090	1100	1110	1120
WSIGDENFNK	IISFLYSTVG	ALNKIGTDGT	QVAMVQFTDD	PRTEFKLNAY	KTKETLLDAI	KHISYKGGNT	KTGKAIKYVR
1130	1140	1150	1160	1170	1180	1190	1200
DTLFTAESGT	RRGIPKVIVV	ITDGRSQDDV	NKISREMQLD	GYSIFAIGVA	DADYSELVSI	GSKPSARHVF	FVDDFDAFKK
1210	1220	1230	1240	1250	1260	1270	1280
IEDELITFVC	ETASATCPVV	HKDGIDLAGF	KMMEMFGLVE	KDFSSVEGVS	MEPGTFNVFP	CYQLHKDALV	SQPTRYLHPE
1290	1300	1310	1320	1330	1340	1350	1360
GLPSDYTISF	LFRILPDTPQ	EPFALWEILN	KNSDPLVGVI	LDNGGKTLTY	FNYDQSGDFQ	TVTFEGPEIR	KIFYGSFHKL
1370	1380	1390	1400	1410	1420	1430	1440
HIVVSETLVK	VVIDCKQVGE	KAMNASANIT	SDGVEVLGKM	VRSRGPGGNS	APFQLQMFDI	VCSTSWANTD	KCCELPGLRD
1450	1460	1470	1480	1490	1500	1510	1520
DESCPDLPHS	CSCSETNEVA	LGPAGPPGGP	GLRGPKGQQG	EPGPKGPDGP	RGEIGLPGPQ	GPPGPQGPSG	LSIQGMPGMP
1530	1540	1550	1560	1570	1580	1590	1600
GEKGEKGDTG	LPGPQGIPGG	VGSPGRDGSP	GQRGLPGKDG	SSGPPGPPGP	IGIPGTPGVP	GITGSMGPQG	ALGPPGVPGA
1610	1620	1630	1640	1650	1660	1670	1680
KGERGERGDL	QSQAMVRSVA	RQVCEQLIQS	HMARYTAILN	QIPSHSSSIR	TVQGPPGEPG	RPGSPGAPGE	QGPPGTPGFP
1690	1700	1710	1720	1730	1740	1750	1760
GNAGVPGTPG	ERGLTGIKGE	KGNPGVGTQG	PRGPPGPAGP	SGESRPGSPG	PPGSPGPRGP	PGHLGVPGPQ	GPSGQPGYCD
1770	1780	1790	1800				
PSSCSAYGVR	APHPDQPEFT	PVQDELEAME	LWGPGV				

Cmpd.	No. of Cmpds.	m/z meas.	Δ m/z [ppm]	z	Rt [min]	Score	P	Range	Sequence	Modification	Ratios
2684	12	673.3700	31.83	2	63.7	19.1	0	208-218	R.IEWHLNAFSTK.D		
74	1	809.3972	-57.78	2	30.6	10.2	1	740-754	R.NLVVGDETTSSLRVK.W		MD:MU 0.83
2197	1	585.1748	-243.43	2	57.7	10.6	0	826-836	K.TLPSSGPQNLR.V		
834	1	683.9031	-31.20	2	40.0	13.1	1	1133-1145	R.GIPKVIVVITDGR.S		

Detailed Protein Report

Protein 25: keratin, type II cytoskeletal 1 [Homo sapiens]

Accession: gi|119395750
Database: refseq_human(refseq_human_20140103.fasta)
Database Date: 2015-11-30

Score: 77.3
MW [kDa]: 66.0
pI: 8.8
Sequence Coverage [%]: 6.2
No. of unique Peptides: 1

10	20	30	40	50	60	70	80
MSRQFSSRSG	YRSGGGFSSG	SAGIINYQRR	TTSSSTRRSG	GGGGRFSSCG	GGGGSFGAGG	GFGSRSLVNL	GGSKSISISV
**90**	**100**	**110**	**120**	**130**	**140**	**150**	**160**
ARGGGRGSGF	GGGYGGGGFG	GGGFGGGGFG	GGGIGGGGFG	GFGSGGGGFG	GGGFGGGGYG	GGYGPVCPPG	GIQEVTINQS
**170**	**180**	**190**	**200**	**210**	**220**	**230**	**240**
LLQPLNVEID	PEIQKVKSRE	REQIKSLNNQ	FASFIDKVRF	LEQQNQVLQT	KWELLQQVDT	STRTHNLEPY	FESFINNLRR
**250**	**260**	**270**	**280**	**290**	**300**	**310**	**320**
RVDQLKSDQS	RLDSELKNMQ	DMVEDYRNKY	EDEINKRTNA	ENEFVTIKKD	VDGAYMTKVD	LQAKLDNLQQ	EIDFLTALYQ
**330**	**340**	**350**	**360**	**370**	**380**	**390**	**400**
AELSQMQTQI	SETNVILSMD	NNRSLDLDSI	IAEVKAQYED	IAQKSKAEAE	SLYQSKYEEL	QITAGRHGDS	VRNSKIEISE
**410**	**420**	**430**	**440**	**450**	**460**	**470**	**480**
LNRVIQRLRS	EIDNVKKQIS	NLQQSISDAE	QRGENALKDA	KNKLNDLEDA	LQQAKEDLAR	LLRDYQELMN	TKLALDLEIA
**490**	**500**	**510**	**520**	**530**	**540**	**550**	**560**
TYRTLLEGEE	SRMSGECAPN	VSVSVSTSHT	TISGGGSRGG	GGGGYGSGGS	SYGSGGGSYG	SGGGGGGGRG	SYGSGGSSYG
**570**	**580**	**590**	**600**	**610**	**620**	**630**	**640**
SGGGSYGSGG	GGGGHGSYGS	GSSSGGYRGG	SGGGGGGSSG	GRGSGGGSSG	GSIGGRGSSS	GGVKSSGGSS	SVKFVSTTYS
**650**							
GVTR							

Cmpd.	No. of Cmpds.	m/z meas.	Δ m/z [ppm]	z	Rt [min]	Score	P	Range	Sequence	Modification
1168	1	922.9918	16.42	2	44.2	12.8	1	417-432	K.KQISNLQQSISDAEQR.G	

Detailed Protein Report

Protein 26: actin, cytoplasmic 1 [Homo sapiens]

Accession: gi|4501885
Database: refseq_human(refseq_human_20140103.fasta)
Database Date: 2015-11-30

Score: 75.8
MW [kDa]: 41.7
pI: 5.2
Sequence Coverage [%]: 8.3
No. of unique Peptides: 2

Quantitation

MD:MU	**Median:** 1.16	**CV:** 0.00 %	**No. of Peptides:** 1

10	20	30	40	50	60	70	80
MDDDIAALVV	DNGSGMCKAG	FAGDDAPRAV	FPSIVGRPRH	QGVMVGMGQK	DSYVGDEAQS	KRGILTLKYP	IEHGIVTNWD
90	100	110	120	130	140	150	160
DMEKIWHHTF	YNELRVAPEE	HPVLLTEAPL	NPKANREKMT	QIMFETFNTP	AMYVAIQAVL	SLYASGRTTG	IVMDSGDGVT
170	180	190	200	210	220	230	240
HTVPIYEGYA	LPHAILRLDL	AGRDLTDYLM	KILTERGYSF	TTTAEREIVR	DIKEKLCYVA	LDFEQEMATA	ASSSSLEKSY
250	260	270	280	290	300	310	320
ELPDGQVITI	GNERFRCPEA	LFQPSFLGME	SCGIHETTFN	SIMKCDVDIR	KDLYANTVLS	GGTTMYPGIA	DRMQKEITAL
330	340	350	360	370	380		
APSTMKIKII	APPERKYSVW	IGGSILASLS	TFQQMWISKQ	EYDESGPSIV	HRKCF		

Cmpd.	No. of Cmpds.	m/z meas.	Δ m/z [ppm]	z	Rt [min]	Score	P	Range	Sequence	Modification	Ratios
1159	1	651.9980	-43.48	3	44.1	29.5	0	96-113	R.VAPEEHPVLLTEAPLNPK.A		
102	2	758.7219	-175.32	2	31.0	46.3	0	360-372	K.QEYDESGPSIVHR.K		MD:MU 1.16

Detailed Protein Report

Protein 27: alpha-enolase isoform 1 [Homo sapiens]

**Accession:**	gi|4503571	**Score:**	75.7
**Database:**	refseq_human(refseq_human_20140103.fasta)	**MW [kDa]:**	47.1
**Database Date:**	2015-11-30	**pI:**	7.7
		**Sequence Coverage [%]:**	10.1
		**No. of unique Peptides:**	2

Quantitation

MD:MU **Median:** 0.96 **CV:** 9.80 % **No. of Peptides:** 2

10	20	30	40	50	60	70	80
MSILKIHARE	IFDSRGNPTV	EVDLFTSKGL	FRAAVPSGAS	TGIYEALELR	DNDKTRYMGK	GVSKAVEHIN	KTIAPALVSK
90	100	110	120	130	140	150	160
KLNVTEQEKI	DKLMIEMDGT	ENKSKFGANA	ILGVSLAVCK	AGAVEKGVPL	YRHIADLAGN	SEVILPVPAF	NVINGGSHAG
170	180	190	200	210	220	230	240
NKLAMQEFMI	LPVGAANFRE	AMRIGAEVYH	NLKNVIKEKY	GKDATNVGDE	GGFAPNILEN	KEGLELLKTA	IGKAGYTDKV
250	260	270	280	290	300	310	320
VIGMDVAASE	FFRSGKYDLD	FKSPDDPSRY	ISPDQLADLY	KSFIKDYPVV	SIEDPFDQDD	WGAWQKFTAS	AGIQVVGDDL
330	340	350	360	370	380	390	400
TVTNPKRIAK	AVNEKSCNCL	LLKVNQIGSV	TESLQACKLA	QANGWGVMVS	HRSGETEDTF	IADLVVGLCT	GQIKTGAPCR
410	420	430	440				
SERLAKYNQL	LRIEEELGSK	AKFAGRNFRN	PLAK				

Cmpd.	No. of Cmpds.	m/z meas.	Δ m/z [ppm]	z	Rt [min]	Score	P	Range	Sequence	Modification	Ratios	
1942	2	902.8469	-142.55	2	54.2	18.2	0	33-50	R.AAVPSGASTGIYEALELR.D		MD:MU	1.06
1560	2	713.3678	1.67	2	49.2	40.1	0	270-281	R.YISPDQLADLYK.S		MD:MU	0.87

Detailed Protein Report

Protein 28: dynein heavy chain 2, axonemal [Homo sapiens]

**Accession:**	gi|75677365	**Score:**	75.0
**Database:**	refseq_human(refseq_human_20140103.fasta)	**MW [kDa]:**	507.4
**Database Date:**	2015-11-30	**pI:**	5.9
**Modification(s):**	Carbamidomethyl, Oxidation	**Sequence Coverage [%]:**	1.8
		**No. of unique Peptides:**	6

Quantitation

MD:MU	**Median:**	0.73	**CV:**	69.90 %	**No. of Peptides:**	3

Alias proteins:

Accession	Name	Description
gi|530409951	refseq_human (refseq_human_20140103.fasta)	PREDICTED: dynein heavy chain 2, axonemal isoform X1 [Homo sapiens]

Detailed Protein Report

10	20	30	40	50	60	70	80
MSSKAEKKQR	LSGRGSSQAS	WSGRATRAAV	ATQEQGNAPA	VSEPELQAEL	PKEEPEPRLE	GPQAQSEESV	EPEADVKPLF
90	100	110	120	130	140	150	160
LSRAALTGLA	DAVWTQEHDA	ILEHFAQDPT	ESILTIFIDP	CFGLKLELGM	PVQTQNQLVY	FIRQAPVPIT	WENFEATVQF
170	180	190	200	210	220	230	240
GTVRGPYIPA	LLRLLGGVFA	PQIFANTGWP	ESIRNHFASH	LHKFLACLTD	TRYKLEGHTV	LYIPAEAMNM	KPEMVIKDKE
250	260	270	280	290	300	310	320
LVQRLETSMI	HWTRQIKEML	SAQETVETGE	NLGPLEEIEF	WRNRCMDLSG	ISKQLVKKGV	KHVESILHLA	KSSYLAPFMK
330	340	350	360	370	380	390	400
LAQQIQDGSR	QAQSNLTFLS	ILKEPYQELA	FMKPKDISSK	LPKLISLIRI	IWVNSPHYNT	RERLTSLFRK	VCDCQYHFAR
410	420	430	440	450	460	470	480
WEDGKQGPLP	CFFGAQGPQI	TRNLLEIEDI	FHKNLHTLRA	VRGGILDVKN	TCWHEDYNKF	RAGIKDLEVM	TQNLITSAFE
490	500	510	520	530	540	550	560
LVRDVPHGVL	LLDTFHRLAS	REAIKRTYDK	KAVDLYMLFN	SELALVNRER	NKKWPDLEPY	VAQYSGKARW	VHILRRRIDR
570	580	590	600	610	620	630	640
VMTCLAGAHF	LPRIGTGKES	VHTYQQMVQA	IDELVRKTFQ	EWTSSLDKDC	IRRLDTPLLR	ISQEKAGMLD	VNFDKSLLIL
650	660	670	680	690	700	710	720
FAEIDYWERL	LFETPHYVVN	VAERAEDLRI	LRENLLLVAR	DYNRIIAMLS	PDEQALFKER	IRLLDKKIHP	GLKKLHWALK
730	740	750	760	770	780	790	800
GASAFFITEC	RIHASKVQMI	VNEFKASTLT	IGWRAQEMSE	KLLVRISGKR	VYRDLEFEED	QREHRAAVQQ	KLMNLHQDVV
810	820	830	840	850	860	870	880
TIMTNSYEVF	KNDGPEIQQQ	WMLYMIRLDR	MMEDALRLNV	KWSLLELSKA	INGDGKTSPN	PLFQVLVILK	NDLQGSVAQV
890	900	910	920	930	940	950	960
EFSPTLQTLA	GVVNDIGNHL	FSTISVFCHL	PDILTKRKLH	REPIQTVVEQ	DEDIKKIQTQ	ISSGMTNNAS	LLQNYLKTWD
970	980	990	1000	1010	1020	1030	1040
MYREIWEINK	DSFIHRYQRL	NPPVSSFVAD	IARYTEVANN	VQKEETVTNI	QFVLLDCSHL	KFSLVQHCNE	WQNKFATLLR
1050	1060	1070	1080	1090	1100	1110	1120
EMAAGRLLEL	HTYLKENAEK	ISRPPQTLEE	LGVSLQLVDA	LKHDLANVET	QIPPIHEQFA	ILEKYEVPVE	DSVLEMLDSL
1130	1140	1150	1160	1170	1180	1190	1200
NGEWVVFQQT	LLDSKQMLKK	HKEKFKTGLI	HSADDFKKKA	HTLLEDFEFK	GHFTSNVGYM	SALDQITQVR	AMLMAMREEE
1210	1220	1230	1240	1250	1260	1270	1280
NSLRANLGIF	KIEQPPSKDL	QNLEKELDAL	QQIWEIARDW	EENWNEWKTG	RFLILQTETM	ETTAHGLFRR	LTKLAKEYKD
1290	1300	1310	1320	1330	1340	1350	1360
RNWEIIETTR	SKIEQFKRTM	PLISDLRNPA	LRERHWDQVR	DEIQREFDQE	SESFTLEQIV	ELGMDQHVEK	IGEISASATK
1370	1380	1390	1400	1410	1420	1430	1440
ELAIEVALQN	IAKTWDVTQL	DIVPYKDKGH	HRLRGTEEVF	QALEDNQVAL	STMKASRFVK	AFEKDVDHWE	RCLSLILEVI
1450	1460	1470	1480	1490	1500	1510	1520
EMILTVQRQW	MYLENIFLGE	DIRKQLPNES	TLFDQVNSNW	KAIMDRMNKD	NNALRSTHHP	GLLDTLIEMN	TILEDIQKSL
1530	1540	1550	1560	1570	1580	1590	1600
DMYLETKRHI	FPRFYFLSND	DLLEILGQSR	NPEAVQPHLK	KCFDNIKLLR	IQKVGGPSSK	WEAVGMFSGD	GEYIDFLHSV
1610	1620	1630	1640	1650	1660	1670	1680
FLEGPVESWL	GDVEQTMRVT	LRDLLRNCHL	ALRKFLNKRD	KWVKEWAGQV	VITASQIQWT	ADVTKCLLTA	KERADKKILK
1690	1700	1710	1720	1730	1740	1750	1760
VMKKNQVSIL	NKYSEAIRGN	LTKIMRLKIV	ALVTIEIHAR	DVLEKLYKSG	LMDVNSFDWL	SQLRFYWEKD	LDDCVIRQTN
1770	1780	1790	1800	1810	1820	1830	1840
TQFQYNYEYL	GNSGRLVITP	LTDRCYMTLT	TALHLHRGGS	PKGPAGTGKT	ETVKDLGKAL	GIYVIVVNCS	EGLDYKSMGR
1850	1860	1870	1880	1890	1900	1910	1920
MYSGLAQTGA	WGCFDEFNRI	NIEVLSVVAH	QILCILSALA	AGLTHFHFDG	FEINLVWSCG	IFITMNPGYA	GRTELPENLK
1930	1940	1950	1960	1970	1980	1990	2000
SMFRPIAMVV	PDSTLIAEII	LFGEGFGNCK	ILAKKVYTLY	SLAVQQLSRQ	DHYDFGLRAL	TSLLRYAGKK	RRLQPDLTDE
2010	2020	2030	2040	2050	2060	2070	2080
EVLLLSMRDM	NIAKLTSVDA	PLFNAIVQDL	FPNIELPVID	YGKLRETVEQ	EIRDMGLQST	PFTLTKVFQL	YETKNSRHST
2090	2100	2110	2120	2130	2140	2150	2160
MIVGCTGSGK	TASWRILQAS	LSSLCRAGDP	NFNIVREFPL	NPKALSLGEL	YGEYDLSTNE	WTDGILSSVM	RTACADEKPD
2170	2180	2190	2200	2210	2220	2230	2240

Detailed Protein Report

Cmpd.	No. of Cmpds.	m/z meas.	Δ m/z [ppm]	z	Rt [min]	Score	P	Range	Sequence	Modification	Ratios	
58	1	827.0195	-82.92	3	30.1	13.2	1	1393-1414	R. LRGTEEVFQALEDNQVALSTMK. A			
271	1	701.3595	-80.46	2	33.1	11.1	2	1681-1692	K.VMKKNQVSILNK.Y		MD:MU	0.62
1377	1	976.4425	-17.17	2	46.8	10.3	1	2078-2095	R.HSTMIVGCTGSGKTASWR.I	Carbamidomethyl: 8; Oxidation: 4	MD:MU	1.69
312	1	712.8533	-32.33	2	33.2	10.2	2	3248-3258	K.LEMLKKQYDEK.L		MD:MU	0.37
2410	1	1023.8441	-100.67	2	60.0	14.9	2	4373-4389	K.KSAKGMYSCPCYYYPNR.A	Oxidation: 6		
2695	1	1044.5484	84.19	2	63.9	15.3	2	4373-4389	K.KSAKGMYSCPCYYYPNR.A	Carbamidomethyl: 9		

Detailed Protein Report

Protein 29: immunoglobulin lambda-like polypeptide 5 isoform 2 [Homo sapiens]

**Accession:**	gi|372466586	**Score:**	73.2
**Database:**	refseq_human(refseq_human_20140103.fasta)	**MW [kDa]:**	14.7
**Database Date:**	2015-11-30	**pI:**	7.7
		**Sequence Coverage [%]:**	24.5
		**No. of unique Peptides:**	2

```
         10         20         30         40         50         60         70         80
MACCAQWLHR KAGTQTLEPQ LEAADPACGA CGAGQPKANP TVTLFPPSSE ELQANKATLV CLISDFYPGA VTVAWKADGS
         90        100        110        120        130        140
PVKAGVETTK PSKQSNNKYA ASSYLSLTPE QWKSHRSYSC QVTHEGSTVE KTVAPTECS
```

Cmpd.	No. of Cmpds.	m/z meas.	Δ m/z [ppm]	z	Rt [min]	Score	P	Range	Sequence	Modification
1468	1	1022.0062	-16.69	2	48.0	16.1	0	38-56	K.ANPTVTLFPPSSEELQANK.A	
1686	4	872.3793	-61.61	2	50.9	57.0	0	99-113	K.YAASSYLSLTPEQWK.S	

Detailed Protein Report

Protein 30: **smoothelin isoform e [Homo sapiens]**

**Accession:**	gi|333360860	**Score:**	72.4
**Database:**	refseq_human(refseq_human_20140103.fasta)	**MW [kDa]:**	104.8
**Database Date:**	2015-11-30	**pI:**	9.9
**Modification(s):**	Carbamidomethyl, Oxidation	**Sequence Coverage [%]:**	4.0
		**No. of unique Peptides:**	2

10	20	30	40	50	60	70	80
MGGMGTPGDL	HAACPSTTRR	DALCLGPEFE	FDLRHSAGCP	GDLGFFPLDP	DTEAGGSGCL	PTWLPLRRLA	RGRLEVTADL
90	100	110	120	130	140	150	160
AERRRIRSAI	RELQRQELER	EEEALASKRF	RAERQDNKEN	WLHSQQREAE	QRAALARLAG	QLESMNDVEE	LTALLRSAGE
170	180	190	200	210	220	230	240
YEERKLIRAA	IRRVRAQEIE	AATLAGRLYS	GRPNSGSRED	SKGLAAHRLE	QCEVPEREEQ	EQQAEVSKPT	PTPEGTSQDV
250	260	270	280	290	300	310	320
TTVTLLLRAP	PGSTSSSPAS	PSSSPTPASP	EPPLEPAEAQ	CLTAEVPGSP	EPPPSPPKTT	SPEPQESPTL	PSTEGQVVNK
330	340	350	360	370	380	390	400
LLSGPKETPA	AQSPTRGPSD	TKRADVAGPR	PCQRSLSVLS	PRQPAQNRES	TPLASGPSSF	QRAGSVRDRV	HKFTSDSPMA
410	420	430	440	450	460	470	480
ARLQDGTPQA	ALSPLTPARL	LGPSLTSTTP	ASSSSGSSSR	GPSDTSSRFS	KEQRGVAQPL	AQLRSCPQEE	GPRGRGLAAR
490	500	510	520	530	540	550	560
PLENRAGGPV	ARSEEPGAPL	PVAVGTAEPG	GSMKTTFTIE	IKDGRGQAST	GRVLLPTGNQ	RAELTLGLRA	PPTLLSTSSG
570	580	590	600	610	620	630	640
GKSTITRVNS	PGTLARLGSV	THVTSFSHAP	PSSRGGCSIK	MEAEPAEPLA	AAVEAANGAE	QTRVNKAPEG	RSPLSAEELM
650	660	670	680	690	700	710	720
TIEDEGVLDK	MLDQSTDFEE	RKLIRAALRE	LRQRKRDQRD	KERERRLQEA	RGRPGEGRGN	TATETTTRHS	QRAADGSAVS
730	740	750	760	770	780	790	800
TVTKTERLVH	SNDGTRTART	TTVESSFVRR	SENGSGSTMM	QTKTFSSSSS	SKKMGSIFDR	EDQASPRAGS	LAALEKRQAE
810	820	830	840	850	860	870	880
KKKELMKAQS	LPKTSASQAR	KAMIEKLEKE	GAAGSPGGPR	AAVQRSTSFG	VPNANSIKQM	LLDWCRAKTR	GYEHVDIQNF
890	900	910	920	930	940	950	960
SSSWSDGMAF	CALVHNFFPE	AFDYGQLSPQ	NRRQNFEVAF	SSAETHADCP	QLLDTEDMVR	LREPDWKCVY	TYIQEFYRCL
970	980						
VQKGLVKTKK	S						

Cmpd.	No. of Cmpds.	m/z meas.	Δ m/z [ppm]	z	Rt [min]	Score	P	Range	Sequence	Modification
1663	1	1044.8761	-92.00	2	50.6	17.6	1	1-20	-.MGGMGTPGDLHAACPSTTRR.D	Carbamidomethyl: 14; Oxidation: 1
1621	1	502.6927	-108.16	2	50.1	15.3	1	523-532	K.DGRGQASTGR.V	

Detailed Protein Report

Protein 31: glyceraldehyde-3-phosphate dehydrogenase isoform 2 [Homo sapiens]

Accession: gi|378404908
Database: refseq_human(refseq_human_20140103.fasta)
Database Date: 2015-11-30
Modification(s): Carbamidomethyl

Score: 69.2
MW [kDa]: 31.5
pI: 7.9
Sequence Coverage [%]: 10.6
No. of unique Peptides: 2

Quantitation

MD:MU **Median:** 0.89 **CV:** 12.19 % **No. of Peptides:** 2

10	20	30	40	50	60	70	80
MVYMFQYDST	HGKFHGTVKA	ENGKLVINGN	PITIFQERDP	SKIKWGDAGA	EYVVESTGVF	TTMEKAGAHL	QGGAKRVIIS
**90**	**100**	**110**	**120**	**130**	**140**	**150**	**160**
APSADAPMFV	MGVNHEKYDN	SLKIISNASC	TTNCLAPLAK	VIHDNFGIVE	GLMTTVHAIT	ATQKTVDGPS	GKLWRDGRGA
**170**	**180**	**190**	**200**	**210**	**220**	**230**	**240**
LQNIIPASTG	AAKAVGKVIP	ELNGKLTGMA	FRVPTANVSV	VDLTCRLEKP	AKYDDIKKVV	KQASEGPLKG	ILGYTEHQVV
**250**	**260**	**270**	**280**	**290**	**300**		
SSDFNSDTHS	STFDAGAGIA	LNDHFVKLIS	WYDNEFGYSN	RVVDLMAHMA	SKE		

Cmpd.	No. of Cmpds.	m/z meas.	Δ m/z [ppm]	z	Rt [min]	Score	P	Range	Sequence	Modification	Ratios	
676	1	917.4657	2.44	2	38.0	53.0	0	104-120	K.IISNASCTTNCLAPLAK.V	Carbamidomethyl: 7, 11	MD:MU	0.79
1572	1	882.3573	-53.81	2	49.4	16.2	0	268-281	K.LISWYDNEFGYSNR.V		MD:MU	1.00

Detailed Protein Report

Protein 32: PREDICTED: ceruloplasmin isoform X3 [Homo sapiens]

**Accession:**	gi|578807065	**Score:**	67.3
**Database:**	refseq_human(refseq_human_20140103.fasta)	**MW [kDa]:**	122.1
**Database Date:**	2015-11-30	**pI:**	5.4
**Modification(s):**	Carbamidomethyl	**Sequence Coverage [%]:**	2.6
		**No. of unique Peptides:**	2

10	20	30	40	50	60	70	80
MKILILGIFL	FLCSTPAWAK	EKHYYIGIIE	TTWDYASDHG	EKKLISVDTE	HSNIYLQNGP	DRIGRLYKKA	LYLQYTDETF
90	100	110	120	130	140	150	160
RTTIEKPVWL	GFLGPIIKAE	TGDKVYVHLK	NLASRPYTFH	SHGITYYKEH	EGAIYPDNTT	DFQRADDKVY	PGEQYTYMLL
170	180	190	200	210	220	230	240
ATEEQSPGEG	DGNCVTRIYH	SHIDAPKDIA	SGLIGPLIIC	KKDSLDKEKE	KHIDREFVVM	FSVVDENFSW	YLEDNIKTYC
250	260	270	280	290	300	310	320
SEPEKVDKDN	EDFQESNRMY	SVNGYTFGSL	PGLSMCAEDR	VKWYLFGMGN	EVDVHAAFFH	GQALTNKNYR	IDTINLFPAT
330	340	350	360	370	380	390	400
LFDAYMVAQN	PGEWMLSCQN	LNHLKAGLQA	FFQVQECNKS	SSKDNIRGKH	VRHYYIAAEE	IIWNYAPSGI	DIFTKENLTA
410	420	430	440	450	460	470	480
PGSDSAVFFE	QGTTRIGGSY	KKLVYREYTD	ASFTNRKERG	PEEEHLGILG	PVIWAEVGDT	IRVTFHNKGA	YPLSIEPIGV
490	500	510	520	530	540	550	560
RFNKNNEGTY	YSPNYNPQSR	SVPPSASHVA	PTETFTYEWT	VPKEVGPTNA	DPVCLAKMYY	SAVEPTKDIF	TGLIGPMKIC
570	580	590	600	610	620	630	640
KKGSLHANGR	QKDVDKEFYL	FPTVFDENES	LLLEDNIRMF	TTAPDQVDKE	DEDFQESNKM	HSMNGFMYGN	QPGLTMCKGD
650	660	670	680	690	700	710	720
SVVWYLFSAG	NEADVHGIYF	SGNTYLWRGE	RRDTANLFPQ	TSLTLHMWPD	TEGTFNVECL	TTDHYTGGMK	QKYTVNQCRR
730	740	750	760	770	780	790	800
QSEDSTFYLG	ERTYYIAAVE	VEWDYSPQRE	WEKELHHLQE	QNVSNAFLDK	GEFYIGSKYK	KVVYRQYTDS	TFRVPVERKA
810	820	830	840	850	860	870	880
EEEHLGILGP	QLHADVGDKV	KIIFKNMATR	PYSIHAHGVQ	TESSTVTPTL	PGETLTYVWK	IPERSGAGTE	DSACIPWAYY
890	900	910	920	930	940	950	960
STVDQVKDLY	SGLIGPLIVC	RRPYLKVFNP	RRKLEFALLF	LVFDENESWY	LDDNIKTYSD	HPEKVNKDDE	EFIESNKMHA
970	980	990	1000	1010	1020	1030	1040
INGRMFGNLQ	GLTMHVGDEV	NWYLMGMGNE	IDLHTVHFHG	HSFQYKHRGV	YSSDVFDIFP	GTYQTLEMFP	RTPGIWLLHC
1050	1060	1070					
HVTDHIHAGM	ETTYTVLQNE	GGTSM					

Cmpd.	No. of Cmpds.	m/z meas.	Δ m/z [ppm]	z	Rt [min]	Score	P	Range	Sequence	Modification
2752	4	735.4129	-14.37	2	64.8	37.1	0	188-201	K.DIASGLIGPLIICK.K	Carbamidomethyl: 13
2789	2	788.4137	-22.96	2	65.3	30.2	0	888-901	K.DLYSGLIGPLIVCR.R	Carbamidomethyl: 13

Detailed Protein Report

Protein 33: hemoglobin subunit gamma-2 [Homo sapiens]

**Accession:**	gi|6715607	**Score:**	64.7
**Database:**	refseq_human(refseq_human_20140103.fasta)	**MW [kDa]:**	16.1
**Database Date:**	2015-11-30	**pI:**	6.8
		**Sequence Coverage [%]:**	15.6
		**No. of unique Peptides:**	1

10	20	30	40	50	60	70	80
MGHFTEEDKA	TITSLWGKVN	VEDAGGETLG	RLLVVYPWTQ	RFFDSFGNLS	SASAIMGNPK	VKAHGKKVLT	SLGDAIKHLD
**90**	**100**	**110**	**120**	**130**	**140**	**150**	
DLKGTFAQLS	ELHCDKLHVD	PENFKLLGNV	LVTVLAIHFG	KEFTPEVQAS	WQKMVTGVAS	ALSSRYH	

Cmpd.	No. of Cmpds.	m/z meas.	Δ m/z [ppm]	z	Rt [min]	Score	P	Range	Sequence	Modification
1800	1	658.7973	-43.08	2	52.5	11.5	0	19-31	K.VNVEDAGGETLGR.L	

Detailed Protein Report

Protein 34: lactotransferrin isoform 2 [Homo sapiens]

Accession: gi|312433998
Database: refseq_human(refseq_human_20140103.fasta)
Database Date: 2015-11-30
Modification(s): Carbamidomethyl

Score: 64.6
MW [kDa]: 73.1
pI: 9.3
Sequence Coverage [%]: 3.5
No. of unique Peptides: 2

Quantitation

MD:MU **Median:** 1.05 **CV:** 105.87 % **No. of Peptides:** 2

10	20	30	40	50	60	70	80
MRKVRGPPVS	CIKRDSPIQC	IQAIAENRAD	AVTLDGGFIY	EAGLAPYKLR	PVAAEVYGTE	RQPRTHYYAV	AVVKKGGSFQ
**90**	**100**	**110**	**120**	**130**	**140**	**150**	**160**
LNELQGLKSC	HTGLRRTAGW	NVPIGTLRPF	LNWTGPPEPI	EAAVARFFSA	SCVPGADKGQ	FPNLCRLCAG	TGENKCAFSS
**170**	**180**	**190**	**200**	**210**	**220**	**230**	**240**
QEPYFSYSGA	FKCLRDGAGD	VAFIRESTVF	EDLSDEAERD	EYELLCPDNT	RKPVDKFKDC	HLARVPSHAV	VARSVNGKED
**250**	**260**	**270**	**280**	**290**	**300**	**310**	**320**
AIWNLLRQAQ	EKFGKDKSPK	FQLFGSPSGQ	KDLLFKDSAI	GFSRVPPRID	SGLYLGSGYF	TAIQNLRKSE	EEVAARRARV
**330**	**340**	**350**	**360**	**370**	**380**	**390**	**400**
VWCAVGEQEL	RKCNQWSGLS	EGSVTCSSAS	TTEDCIALVL	KGEADAMSLD	GGYVYTAGKC	GLVPVLAENY	KSQQSSDPDP
**410**	**420**	**430**	**440**	**450**	**460**	**470**	**480**
NCVDRPVEGY	LAVAVVRRSD	TSLTWNSVKG	KKSCHTAVDR	TAGWNIPMGL	LFNQTGSCKF	DEYFSQSCAP	GSDPRSNLCA
**490**	**500**	**510**	**520**	**530**	**540**	**550**	**560**
LCIGDEQGEN	KCVPNSNERY	YGYTGAFRCL	AENAGDVAFV	KDVTVLQNTD	GNNNEAWAKD	LKLADFALLC	LDGKRKPVTE
**570**	**580**	**590**	**600**	**610**	**620**	**630**	**640**
ARSCHLAMAP	NHAVVSRMDK	VERLKQVLLH	QQAKFGRNGS	DCPDKFCLFQ	SETKNLLFND	NTECLARLHG	KTTYEKYLGP
**650**	**660**	**670**					
QYVAGITNLK	KCSTSPLLEA	CEFLRK					

Cmpd.	No. of Cmpds.	m/z meas.	Δ m/z [ppm]	z	Rt [min]	Score	P	Range	Sequence	Modification	Ratios	
852	1	598.3199	18.32	2	40.1	36.0	0	261-271	K.FQLFGSPSGQK.D		MD:MU	0.44
2333	1	668.3646	18.05	2	59.0	28.6	0	543-554	K.LADFALLCLDGK.R	Carbamidomethyl: 8	MD:MU	2.50

Detailed Protein Report

Protein 35: alpha-2-macroglobulin precursor [Homo sapiens]

**Accession:**	gi|66932947	**Score:**	64.3
**Database:**	refseq_human(refseq_human_20140103.fasta)	**MW [kDa]:**	163.2
**Database Date:**	2015-11-30	**pI:**	6.0
		**Sequence Coverage [%]:**	2.6
		**No. of unique Peptides:**	3

Quantitation

MD:MU **Median:** 2.19 **CV:** 0.00 % **No. of Peptides:** 1

10	20	30	40	50	60	70	80
MGKNKLLHPS	LVLLLLVLLP	TDASVSGKPQ	YMVLVPSLLH	TETTEKGCVL	LSYLNETVTV	SASLESVRGN	RSLFTDLEAE
90	100	110	120	130	140	150	160
NDVLHCVAFA	VPKSSSNEEV	MFLTVQVKGP	TQEFKKRTTV	MVKNEDSLVF	VQTDKSIYKP	GQTVKFRVVS	MDENFHPLNE
170	180	190	200	210	220	230	240
LIPLVYIQDP	KGNRIAQWQS	FQLEGGLKQF	SFPLSSEPFQ	GSYKVVVQKK	SGGRTEHPFT	VEEFVLPKFE	VQVTVPKIIT
250	260	270	280	290	300	310	320
ILEEEMNVSV	CGLYTYGKPV	PGHVTVSICR	KYSDASDCHG	EDSQAFCEKF	SGQLNSHGCF	YQQVKTKVFQ	LKRKEYEMKL
330	340	350	360	370	380	390	400
HTEAQIQEEG	TVVELTGRQS	SEITRTITKL	SFVKVDSHFR	QGIPFFGQVR	LVDGKGVPIP	NKVIFIRGNE	ANYYSNATTD
410	420	430	440	450	460	470	480
EHGLVQFSIN	TTNVMGTSLT	VRVNYKDRSP	CYGYQWVSEE	HEEAHHTAYL	VFSPSKSFVH	LEPMSHELPC	GHTQTVQAHY
490	500	510	520	530	540	550	560
ILNGGTLLGL	KKLSFYYLIM	AKGGIVRTGT	HGLLVKQEDM	KGHFSISIPV	KSDIAPVARL	LIYAVLPTGD	VIGDSAKYDV
570	580	590	600	610	620	630	640
ENCLANKVDL	SFSPSQSLPA	SHAHLRVTAA	PQSVCALRAV	DQSVLLMKPD	AELSASSVYN	LLPEKDLTGF	PGPLNDQDDE
650	660	670	680	690	700	710	720
DCINRHNVYI	NGITYTPVSS	TNEKDMYSFL	EDMGLKAFTN	SKIRKPKMCP	QLQQYEMHGP	EGLRVGFYES	DVMGRGHARL
730	740	750	760	770	780	790	800
VHVEEPHTET	VRKYFPETWI	WDLVVVNSAG	VAEVGVTVPD	TITEWKAGAF	CLSEDAGLGI	SSTASLRAFQ	PFFVELTMPY
810	820	830	840	850	860	870	880
SVIRGEAFTL	KATVLNYLPK	CIRVSVQLEA	SPAFLAVPVE	KEQAPHCICA	NGRQTVSWAV	TPKSLGNVNF	TVSAEALESQ
890	900	910	920	930	940	950	960
ELCGTEVPSV	PEHGRKDTVI	KPLLVEPEGL	EKETTFNSLL	CPSGGEVSEE	LSLKLPPNVV	EESARASVSV	LGDILGSAMQ
970	980	990	1000	1010	1020	1030	1040
NTQNLLQMPY	GCGEQNMVLF	APNIYVLDYL	NETQQLTPEI	KSKAIGYLNT	GYQRQLNYKH	YDGSYSTFGE	RYGRNQGNTW
1050	1060	1070	1080	1090	1100	1110	1120
LTAFVLKTFA	QARAYIFIDE	AHITQALIWL	SQRQKDNGCF	RSSGSLLNNA	IKGGVEDEVT	LSAYITIALL	EIPLTVTHPV
1130	1140	1150	1160	1170	1180	1190	1200
VRNALFCLES	AWKTAQEGDH	GSHVYTKALL	AYAFALAGNQ	DKRKEVLKSL	NEEAVKKDNS	VHWERPQKPK	APVGHFYEPQ
1210	1220	1230	1240	1250	1260	1270	1280
APSAEVEMTS	YVLLAYLTAQ	PAPTSEDLTS	ATNIVKWITK	QQNAQGGFSS	TQDTVVALHA	LSKYGAATFT	RTGKAAQVTI
1290	1300	1310	1320	1330	1340	1350	1360
QSSGTFSSKF	QVDNNNRLLL	QQVSLPELPG	EYSMKVTGEG	CVYLQTSLKY	NILPEKEEFP	FALGVQTLPQ	TCDEPKAHTS
1370	1380	1390	1400	1410	1420	1430	1440
FQISLSVSYT	GSRSASNMAI	VDVKMVSGFI	PLKPTVKMLE	RSNHVSRTEV	SSNHVLIYLD	KVSNQTLSLF	FTVLQDVPVR
1450	1460	1470	1480				
DLKPAIVKVY	DYYETDEFAI	AEYNAPCSKD	LGNA				

Cmpd.	No. of Cmpds.	m/z meas.	Δ m/z [ppm]	z	Rt [min]	Score	P	Range	Sequence	Modification	Ratios
1949	1	574.7312	-144.41	2	54.2	14.8	0	361-370	R.QGIPFFGQVR.L		
2267	1	942.4047	-130.24	2	58.2	14.1	0	824-841	R.VSVQLEASPAFLAVPVEK.E		
234	1	605.7801	-74.07	2	32.3	35.4	0	935-945	K.LPPNVVEESAR.A		MD:MU 2.19

Detailed Protein Report

Protein 36: PREDICTED: probable E3 ubiquitin-protein ligase MYCBP2 isoform X2 [Homo sapiens]

**Accession:**	gi|530402184	**Score:**	63.7
**Database:**	refseq_human(refseq_human_20140103.fasta)	**MW [kDa]:**	504.8
**Database Date:**	2015-11-30	**pI:**	6.6
**Modification(s):**	Carbamidomethyl, Oxidation	**Sequence Coverage [%]:**	2.0
		**No. of unique Peptides:**	3

Detailed Protein Report

10	20	30	40	50	60	70	80
MMMCAATASP	AAASSGLGGD	GFYPAATFSS	SPAPGALFMP	VPDGSVAAAG	LGLGLPAADS	RGHYQLLLSG	RALADRYRRI
90	100	110	120	130	140	150	160
YTAALNDRDQ	GGGSAGHPAS	RNKKILNKKK	LKRKQKSKSK	VKTRSKSENL	ENTVIIPDIK	LHSNPSAFNI	YCNVRHCVLE
170	180	190	200	210	220	230	240
WQKKEISLAA	ASKNSVQSGE	SDSDEEEESK	EPPIKLPKII	EVGLCEVFEL	IKETRFSHPS	LCLRSLQALL	NVLQGQQPEG
250	260	270	280	290	300	310	320
LQSEPPEVLE	SLFQLLLEIT	VRSTGMNDST	GQSLTALSCA	CLFSLVASWG	ETGRTLQAIS	AILTNNGSHA	CQTIQVPTIL
330	340	350	360	370	380	390	400
NSLQRSVQAV	LVGKIQIQDW	FSNGIKKAAL	MHKWPLKEIS	VDEDDQCLLQ	NDGFFLYLLC	KDGLYKIGSG	YSGTVRGHIY
410	420	430	440	450	460	470	480
NSTSRIRNRK	EKKSWLGYAQ	GYLLYRDVNN	HSMTAIRISP	ETLEQDGTVM	LPDCHTEGQN	ILFTDGEYIN	QIAASRDDGF
490	500	510	520	530	540	550	560
VVRIFATSTE	PVLQQELQLK	LARKCLHACG	ISLFDLEKDL	HIISTGFDEE	SAILGAGREF	ALMKTANGKI	YYTGKYQSLG
570	580	590	600	610	620	630	640
IKQGGPSAGK	WVELPITKSP	KIVHFSVGHD	GSHALLVAED	GSIFFTGSAS	KGEDGESTKS	RRQSKPYKPK	KIIKMEGKIV
650	660	670	680	690	700	710	720
VYTACNNGSS	SVISKDGELY	MFGKDAIYSD	SSSLVTDLKG	HFVTQVAMGK	AHTCVLMKNG	EVWTFGVNNK	GQCGRDTGAM
730	740	750	760	770	780	790	800
NQGGKGFGVE	NMATAMDEDL	EEELDEKDEK	SMMCPPGMHK	WKLEQCMVCT	VCGDCTGYGA	SCVSSGRPDR	VPGGICGCGS
810	820	830	840	850	860	870	880
GESGCAVCGC	CKACARELDG	QEARQRGILD	AVKEMIPLDL	LLAVPVPGVN	IEEHLQLRQE	EKRQRVIRRH	RLEEGRGPLV
890	900	910	920	930	940	950	960
FAGPIFMNHR	EQALARLRSH	PAQLKHKRDK	HKDGSGERGE	KDASKITTYP	PGSVRFDCEL	RAVQVSCGFH	HSVVLMENGD
970	980	990	1000	1010	1020	1030	1040
VYTFGYGQHG	QLGHGDVNSR	GCPTLVQALP	GPSTQVTAGS	NHTAVLLMDG	QVFTFGSFSK	GQLGRPILDV	PYWNAKPAPM
1050	1060	1070	1080	1090	1100	1110	1120
PNIGSKYGRK	ATWIGASGDQ	TFLRIDEALI	NSHVLATSEI	FASKHIIGLV	PASISEPPPF	KCLLINKVDG	SCKTFNDSEQ
1130	1140	1150	1160	1170	1180	1190	1200
EDLQGFGVCL	DPVYDVIWRF	RPNTRELWCY	NAVVADARLP	SAADMQSRCS	ILSPELALPT	GSRALTTRSH	AALHILGCLD
1210	1220	1230	1240	1250	1260	1270	1280
TLAAMQDLKM	GVASTEEETQ	AVMKVYSKED	YSVVNRFESH	GGGWGYSAHS	VEAIRFSADT	DILLGGLGLF	GGRGEYTAKI
1290	1300	1310	1320	1330	1340	1350	1360
KLFELGPDGG	DHETDGDLLA	ETDVLAYDCA	AREKYAMMFD	EPVLLQAGWW	YVAWARVSGP	SSDCGSHGQA	SITTDDGVVF
1370	1380	1390	1400	1410	1420	1430	1440
QFKSSKKSNN	GTDVNAGQIP	QLLYRLPTSD	GSASKGKQQT	SEPVHILKRS	FARTVSVECF	ESLLSILHWS	WTTLVLGVEE
1450	1460	1470	1480	1490	1500	1510	1520
LRGLKGFQFT	ATLLDLERLR	FVGTCCLRLL	RVYTCEIYPV	SATGKAVVEE	TSKLAECIGK	TRTLLRKILS	EGVDHCMVKL
1530	1540	1550	1560	1570	1580	1590	1600
DNDPQGYLSQ	PLSLLEAVLQ	ECHNTFTACF	HSFYPTPALQ	WACLCDLLNC	LDQDIQEANF	KTSSSRLLAA	VMSALCHTSV
1610	1620	1630	1640	1650	1660	1670	1680
KLTSIFPIAY	DGEVLLRSIV	KQVSTENDST	LVHRFPLLVA	HMEKLSQSEE	NISGMTSFRE	VLEKMLVIVV	LPVRNSLRRE
1690	1700	1710	1720	1730	1740	1750	1760
NELFSSHLVS	NTCGLLASIV	SELTASALGS	EVDGLNSLHS	VKASANRFTK	TSQGRSWNTG	NGSPDAICFS	VDKPGIVVVG
1770	1780	1790	1800	1810	1820	1830	1840
FSVYGGGGIH	EYELEVLVDD	SEHAGDSTHS	HRWTSLELVK	GTYTTDDSPS	DIAEIRLDKV	VPLKENVKYA	VRLRNYGSRT
1850	1860	1870	1880	1890	1900	1910	1920
ANGDGGMTTV	QCPDGVTFTF	STCSLSSNGT	NQTRGQIPQI	LYYRSEFDGD	LQSQLLSKAN	EEDKNCSRAL	SVVSTVVRAS
1930	1940	1950	1960	1970	1980	1990	2000
KDLLHRALAV	DADDIPELLS	SSSLFSMLLP	LIIAYIGPVA	AAIPKVAVEV	FGLVQQLLPS	VAILNQKYAP	PAFNPNQSTD
2010	2020	2030	2040	2050	2060	2070	2080
STTGNQPEQG	LSACTTSSHY	AVIESEHPYK	PACVMHYKVT	FPECVRWMTI	EFDPQCGTAQ	SEDVLRLLIP	VRTVQNSGYG
2090	2100	2110	2120	2130	2140	2150	2160
PKLTSVHENL	NSWIELKKFS	GSSGWPTMVL	VLPGNEALFS	LETASDYVKD	DKASFYGFKC	FAIGYEFSPG	PDEGVIQLEK
2170	2180	2190	2200	2210	2220	2230	2240

Detailed Protein Report

Cmpd.	No. of Cmpds.	m/z meas.	Δ m/z [ppm]	z	Rt [min]	Score	P	Range	Sequence	Modification
1222	1	927.2551	104.81	3	44.9	11.3	1	1184-1209	R.ALTTRSHAALHILGCLDTLAAMQDLK.M	Oxidation: 22
2398	1	1023.7079	112.56	2	59.9	13.0	2	2442-2460	K.VKDPPKGMIPPGTQLVKPK.S	Oxidation: 8
1461	1	799.6263	-94.96	3	48.0	13.1	1	4259-4279	K.AMVEFREHTGKPTTSSSEACR.F	Carbamidomethyl: 20; Oxidation: 2

Detailed Protein Report

Protein 37: keratin, type II cytoskeletal 1b [Homo sapiens]

**Accession:**	gi|194018511	**Score:**	62.2
**Database:**	refseq_human(refseq_human_20140103.fasta)	**MW [kDa]:**	61.9
**Database Date:**	2015-11-30	**pI:**	5.6
		**Sequence Coverage [%]:**	4.8
		**No. of unique Peptides:**	1

10	20	30	40	50	60	70	80
MSHQFSSQSA	FSSMSRRVYS	TSSSAGSGGG	SPAVGSVCYA	RGRCGGGGYG	IHGRGFGSRS	LYNLGGSRSI	SINLMGRSTS
90	100	110	120	130	140	150	160
GFCQGGGVGG	FGGGRGFGVG	STGAGGFGGG	GFGGAGFGTS	NFGLGGFGPY	CPPGGIQEVT	INQSLLEPLH	LEVDPEIQRI
170	180	190	200	210	220	230	240
KTQEREQIMV	LNNKFASFID	KVRFLEQQNQ	VLQTKWELLQ	QVNTSTGTNN	LEPLLENYIG	DLRRQVDLLS	AEQMRQNAEV
250	260	270	280	290	300	310	320
RSMQDVVEDY	KSKYEDEINK	RTGSENDFVV	LKKDVDAAYV	SKVDLESRVD	TLTGEVNFLK	YLFLTELSQV	QTHISDTNVI
330	340	350	360	370	380	390	400
LSMDNNRSLD	LDSIIDAVRT	QYELIAQRSK	DEAEALYQTK	YQELQITAGR	HGDDLKNSKM	EIAELNRTVQ	RLQAEISNVK
410	420	430	440	450	460	470	480
KQIEQMQSLI	SDAEERGEQA	LQDAWQKLQD	LEEALQQSKE	ELARLLRDYQ	AMLGVKLSLD	VEIATYRQLL	EGEESRMSGE
490	500	510	520	530	540	550	560
LQSHVSISVQ	NSQVSVNGGA	GGGGSYGSGG	YGGGSGGGYG	GGRSYRGGGA	RGRSGGGYGS	GCGGGGGSYG	GSGRSGRGSS
570	580						
RVQIIQTSTN	TSHRRILE						

Cmpd.	No. of Cmpds.	m/z meas.	Δ m/z [ppm]	z	Rt [min]	Score	P	Range	Sequence	Modification
2780	1	922.4553	-14.26	2	66.1	16.9	1	361-376	K.YQELQITAGRHGDDLK.N	

Detailed Protein Report

Protein 38: nucleolar transcription factor 1 isoform b [Homo sapiens]

Accession: gi|115529449
Database: refseq_human(refseq_human_20140103.fasta)
Database Date: 2015-11-30
Modification(s): Oxidation

Score: 61.6
MW [kDa]: 84.9
pI: 5.3
Sequence Coverage [%]: 5.2
No. of unique Peptides: 4

Quantitation

MD:MU **Median:** 0.84 **CV:** 21.21 % **No. of Peptides:** 2

Alias proteins:

Accession	Name	Description
gi|115529451	refseq_human (refseq_human_20140103.fasta)	nucleolar transcription factor 1 isoform b [Homo sapiens]

10	20	30	40	50	60	70	80
MNGEADCPTD	LEMAAPKGQD	RWSQEDMLTL	LECMKNNLPS	NDSSKFKTTE	SHMDWEKVAF	KDFSGDMCKL	KWVEISNEVR
90	100	110	120	130	140	150	160
KFRTLTELIL	DAQEHVKNPY	KGKKLKKHPD	FPKKPLTPYF	RFFMEKRAKY	AKLHPEMSNL	DLTKILSKKY	KELPEKKKMK
170	180	190	200	210	220	230	240
YIQDFQREKQ	EFERNLARFR	EDHPDLIQNA	KKSDIPEKPK	TPQQLWYTHE	KKVYLKVRPD	EIMRDYIQKH	PELNISEEGI
250	260	270	280	290	300	310	320
TKSTLTKAER	QLKDKFDGRP	TKPPPNSYSL	YCAELMANMK	DVPSTERMVL	CSQQWKLLSQ	KEKDAYHKKC	DQKKKDYEVE
330	340	350	360	370	380	390	400
LLRFLESLPE	EEQQRVLGEE	KMLNINKKQA	TSPASKKPAQ	EGGKGGSEKP	KRPVSAMFIF	SEEKRRQLQE	ERPELSESEL
410	420	430	440	450	460	470	480
TRLLARMWND	LSEKKKAKYK	AREAALKAQS	ERKPGGEREE	RGKLPESPKR	AEEIWQQSVI	GDYLARFKND	RVKALKAMEM
490	500	510	520	530	540	550	560
TWNNMEKKEK	LMWIKKAAED	QKRYERELSE	MRAPPAATNS	SKKMKFQGEP	KKPPMNGYQK	FSQELLSNGE	LNHLPLKERM
570	580	590	600	610	620	630	640
VEIGSRWQRI	SQSQKEHYKK	LAEEQQKQYK	VHLDLWVKSL	SPQDRAAYKE	YISNKRKSMT	KLRGPNPKSS	RTTLQSKSES
650	660	670	680	690	700	710	720
EEDDEEDEDD	EDEDEEEEDD	ENGDSSEDGG	DSSESSSEDE	SEDGDENEED	DEDEDDDEDD	DEDEDNESEG	SSSSSSSSGD
730							
SSDSDSN							

Cmpd.	No. of Cmpds.	m/z meas.	Δ m/z [ppm]	z	Rt [min]	Score	P	Range	Sequence	Modification	Ratios
1966	1	871.8504	-139.97	2	54.7	11.1	1	108-121	K.HPDFPKKPLTPYFR.F		MD:MU 0.68
2702	8	856.8935	-0.41	2	65.0	15.0	1	474-487	K.ALKAMEMTWNNMEK.K	Oxidation: 5	
2869	1	856.9447	59.29	2	66.4	17.0	1	474-487	K.ALKAMEMTWNNMEK.K	Oxidation: 7	
1591	1	472.2351	-22.65	2	49.6	18.5	0	513-522	R.APPAATNSSK.K		MD:MU 1.04

Detailed Protein Report

Protein 39: fibrillin-2 precursor [Homo sapiens]

**Accession:**	gi|66346695	**Score:**	60.6
**Database:**	refseq_human(refseq_human_20140103.fasta)	**MW [kDa]:**	314.6
**Database Date:**	2015-11-30	**pI:**	4.6
**Modification(s):**	Carbamidomethyl, Oxidation	**Sequence Coverage [%]:**	1.2
		**No. of unique Peptides:**	3

Quantitation

MD:MU	**Median:** 1.84	**CV:** 0.00 %	**No. of Peptides:** 1

BRUKER

Detailed Protein Report

10	20	30	40	50	60	70	80
MGRRRRLCLQ	LYFLWLGCVV	LWAQGTAGQP	QPPPPKPPRP	QPPPQQVRSA	TAGSEGGFLA	PEYREEGAAV	ASRVRRRGQQ
90	100	110	120	130	140	150	160
DVLRGPNVCG	SRFHSYCCPG	WKTLPGGNQC	IVPICRNSCG	DGFCSRPNMC	TCSSGQISST	CGSKSIQQCS	VRCMNGGTCA
170	180	190	200	210	220	230	240
DDHCQCQKGY	IGTYCGQPVC	ENGCQNGGRC	IGPNRCACVY	GFTGPQCERD	YRTGPCFTQV	NNQMCQGQLT	GIVCTKTLCC
250	260	270	280	290	300	310	320
ATIGRAWGHP	CEMCPAQPQP	CRRGFIPNIR	TGACQDVDEC	QAIPGICQGG	NCINTVGSFE	CRCPAGHKQS	ETTQKCEDID
330	340	350	360	370	380	390	400
ECSIIPGICE	TGECSNTVGS	YFCVCPRGYV	TSTDGSRCID	QRTGMCFSGL	VNGRCAQELP	GRMTKMQCCC	EPGRCWGIGT
410	420	430	440	450	460	470	480
IPEACPVRGS	EEYRRLCMDG	LPMGGIPGSA	GSRPGGTGGN	GFAPSGNGNG	YGPGGTGFIP	IPGGNGFSPG	VGGAGVGAGG
490	500	510	520	530	540	550	560
QGPIITGLTI	LNQTIDICKH	HANLCLNGRC	IPTVSSYRCE	CNMGYKQDAN	GDCIDVDECT	SNPCTNGDCV	NTPGSYYCKC
570	580	590	600	610	620	630	640
HAGFQRTPTK	QACIDIDECI	QNGVLCKNGR	CVNTDGSFQC	ICNAGFELTT	DGKNCVDHDE	CTTTNMCLNG	MCINEDGSFK
650	660	670	680	690	700	710	720
CICKPGFVLA	PNGRYCTDVD	ECQTPGICMN	GHCINSEGSF	RCDCPPGLAV	GMDGRVCVDT	HMRSTCYGGI	KKGVCVRPFP
730	740	750	760	770	780	790	800
GAVTKSECCC	ANPDYGFGEP	CQPCPAKNSA	EFHGLCSSGV	GITVDGRDIN	ECALDPDICA	NGICENLRGS	YRCNCNSGYE
810	820	830	840	850	860	870	880
PDASGRNCID	IDECLVNRLL	CDNGLCRNTP	GSYSCTCPPG	YVFRTETETC	EDINECESNP	CVNGACRNNL	GSFNCECSPG
890	900	910	920	930	940	950	960
SKLSSTGLIC	IDSLKGTCWL	NIQDSRCEVN	INGATLKSEC	CATLGAAWGS	PCERCELDTA	CPRGLARIKG	VTCEDVNECE
970	980	990	1000	1010	1020	1030	1040
VFPGVCPNGR	CVNSKGSFHC	ECPEGLTLDG	TGRVCLDIRM	EQCYLKWDED	ECIHPVPGKF	RMDACCCAVG	AAWGTECEEC
1050	1060	1070	1080	1090	1100	1110	1120
PKPGTKEYET	LCPRGAGFAN	RGDVLTGRPF	YKDINECKAF	PGMCTYGKCR	NTIGSFKCRC	NSGFALDMEE	RNCTDIDECR
1130	1140	1150	1160	1170	1180	1190	1200
ISPDLCGSGI	CVNTPGSFEC	ECFEGYESGF	MMMKNCMDID	ECERNPLLCR	GGTCVNTEGS	FQCDCPLGHE	LSPSREDCVD
1210	1220	1230	1240	1250	1260	1270	1280
INECSLSDNL	CRNGKCVNMI	GTYQCSCNPG	YQATPDRQGC	TDIDECMIMN	GGCDTQCTNS	EGSYECSCSE	GYALMPDGRS
1290	1300	1310	1320	1330	1340	1350	1360
CADIDECENN	PDICDGGQCT	NIPGEYRCLC	YDGFMASMDM	KTCIDVNECD	LNSNICMFGE	CENTKGSFIC	HCQLGYSVKK
1370	1380	1390	1400	1410	1420	1430	1440
GTTGCTDVDE	CEIGAHNCDM	HASCLNIPGS	FKCSCREGWI	GNGIKCIDLD	ECSNGTHQCS	INAQCVNTPG	SYRCACSEGF
1450	1460	1470	1480	1490	1500	1510	1520
TGDGFTCSDV	DECAENINLC	ENGQCLNVPG	AYRCECEMGF	TPASDSRSCQ	DIDECSFQNI	CVFGTCNNLP	GMFHCICDDG
1530	1540	1550	1560	1570	1580	1590	1600
YELDRTGGNC	TDIDECADPI	NCVNGLCVNT	PGRYECNCPP	DFQLNPTGVG	CVDNRVGNCY	LKFGPRGDGS	LSCNTEIGVG
1610	1620	1630	1640	1650	1660	1670	1680
VSRSSCCCSL	GKAWGNPCET	CPPVNSTEYY	TLCPGGEGFR	PNPITIILED	IDECQELPGL	CQGGNCINTF	GSFQCECPQG
1690	1700	1710	1720	1730	1740	1750	1760
YYLSEDTRIC	EDIDECFAHP	GVCGPGTCYN	TLGNYTCICP	PEYMQVNGGH	NCMDMRKSFC	YRSYNGTTCE	NELPFNVTKR
1770	1780	1790	1800	1810	1820	1830	1840
MCCCTYNVGK	AWNKPCEPCP	TPGTADFKTI	CGNIPGFTFD	IHTGKAVDID	ECKEIPGICA	NGVCINQIGS	FRCECPTGFS
1850	1860	1870	1880	1890	1900	1910	1920
YNDLLLVCED	IDECSNGDNL	CQRNADCINS	PGSYRCECAA	GFKLSPNGAC	VDRNECLEIP	NVCSHGLCVD	LQGSYQCICH
1930	1940	1950	1960	1970	1980	1990	2000
NGFKASQDQT	MCMDVDECER	HPCGNGTCKN	TVGSYNCLCY	PGFELTHNND	CLDIDECSSF	FGQVCRNGRC	FNEIGSFKCL
2010	2020	2030	2040	2050	2060	2070	2080
CNEGYELTPD	GKNCIDTNEC	VALPGSCSPG	TCQNLEGSFR	CICPPGYEVK	SENCIDINEC	DEDPNICLFG	SCTNTPGGFQ
2090	2100	2110	2120	2130	2140	2150	2160
CLCPPGFVLS	DNGRRCFDTR	QSFCFTNFEN	GKCSVPKAFN	TTKAKCCCSK	MPGEGWGDPC	ELCPKDDEVA	FQDLCPYGHG
2170	2180	2190	2200	2210	2220	2230	2240

Detailed Protein Report

Cmpd.	No. of Cmpds.	m/z meas.	Δ m/z [ppm]	z	Rt [min]	Score	P	Range	Sequence	Modification	Ratios
534	2	730.1886	-131.49	2	36.0	10.1	1	383-394	R.MTKMQCCCEPGR.C	Carbamidomethyl: 6; Oxidation: 4	
522	1	730.1851	-136.21	2	35.9	15.8	1	383-394	R.MTKMQCCCEPGR.C	Carbamidomethyl: 8; Oxidation: 4	
304	5	642.1738	-171.43	2	33.2	24.4	1	2529-2539	K.TCKDLDECQTK.Q		MD:MU 1.84

Detailed Protein Report

Protein 40: laminin subunit alpha-2 isoform a precursor [Homo sapiens]

**Accession:**	gi|28559088	**Score:**	59.5
**Database:**	refseq_human(refseq_human_20140103.fasta)	**MW [kDa]:**	343.7
**Database Date:**	2015-11-30	**pI:**	6.0
**Modification(s):**	Carbamidomethyl	**Sequence Coverage [%]:**	2.8
		**No. of unique Peptides:**	5

Quantitation

MD:MU	**Median:** 0.58	**CV:** 0.00 %	**No. of Peptides:** 1

Detailed Protein Report

10	20	30	40	50	60	70	80
MPGAAGVLLL	LLLSGGLGGV	QAQRPQQQRQ	SQAHQQRGLF	PAVLNLASNA	LITTNATCGE	KGPEMYCKLV	EHVPGQPVRN
90	100	110	120	130	140	150	160
PQCRICNQNS	SNPNQRHPIT	NAIDGKNTWW	QSPSIKNGIE	YHYVTITLDL	QQVFQIAYVI	VKAANSPRPG	NWILERSLDD
170	180	190	200	210	220	230	240
VEYKPWQYHA	VTDTECLTLY	NIYPRTGPPS	YAKDDEVICT	SFYSKIHPLE	NGEIHISLIN	GRPSADDPSP	ELLEFTSARY
250	260	270	280	290	300	310	320
IRLRFQRIRT	LNADLMMFAH	KDPREIDPIV	TRRYYYSVKD	ISVGGMCICY	GHARACPLDP	ATNKSRCECE	HNTCGDSCDQ
330	340	350	360	370	380	390	400
CCPGFHQKPW	RAGTFLTKTE	CEACNCHGKA	EECYYDENVA	RRNLSLNIRG	KYIGGGVCIN	CTQNTAGINC	ETCTDGFFRP
410	420	430	440	450	460	470	480
KGVSPNYPRP	CQPCHCDPIG	SLNEVCVKDE	KHARRGLAPG	SCHCKTGFGG	VSCDRCARGY	TGYPDCKACN	CSGLGSKNED
490	500	510	520	530	540	550	560
PCFGPCICKE	NVEGGDCSRC	KSGFFNLQED	NWKGCDECFC	SGVSNRCQSS	YWTYGKIQDM	SGWYLTDLPG	RIRVAPQQDD
570	580	590	600	610	620	630	640
LDSPQQISIS	NAEARQALPH	SYYWSAPAPY	LGNKLPAVGG	QLTFTISYDL	EEEEEDTERV	LQLMIILEGN	DLSISTAQDE
650	660	670	680	690	700	710	720
VYLHPSEEHT	NVLLLKEESF	TIHGTHFPVR	RKEFMTVLAN	LKRVLLQITY	SFGMDAIFRL	SSVNLESAVS	YPTDGSIAAA
730	740	750	760	770	780	790	800
VEVCQCPPGY	TGSSCESCWP	RHRRVNGTIF	GGICEPCQCF	GHAESCDDVT	GECLNCKDHT	GGPYCDKCLP	GFYGEPTKGT
810	820	830	840	850	860	870	880
SEDCQPCACP	LNIPSNNFSP	TCHLDRSLGL	ICDGCPVGYT	GPRCERCAEG	YFGQPSVPGG	SCQPCQCNDN	LDFSIPGSCD
890	900	910	920	930	940	950	960
SLSGSCLICK	PGTTGRYCEL	CADGYFGDAV	DAKNCQPCRC	NAGGSFSEVC	HSQTGQCECR	ANVQGQRCDK	CKAGTFGLQS
970	980	990	1000	1010	1020	1030	1040
ARGCVPCNCN	SFGSKSFDCE	ESGQCWCQPG	VTGKKCDRCA	HGYFNFQEGG	CTACECSHLG	NNCDPKTGRC	ICPPNTIGEK
1050	1060	1070	1080	1090	1100	1110	1120
CSKCAPNTWG	HSITTGCKAC	NCSTVGSLDF	QCNVNTGQCN	CHPKFSGAKC	TECSRGHWNY	PRCNLCDCFL	PGTDATTCDS
1130	1140	1150	1160	1170	1180	1190	1200
ETKKCSCSDQ	TGQCTCKVNV	EGIHCDRCRP	GKFGLDAKNP	LGCSSCYCFG	TTTQCSEAKG	LIRTWVTLKA	EQTILPLVDE
1210	1220	1230	1240	1250	1260	1270	1280
ALQHTTTKGI	VFQHPEIVAH	MDLMREDLHL	EPFYWKLPEQ	FEGKKLMAYG	GKLKYAIYFE	AREETGFSTY	NPQVIIRGGT
1290	1300	1310	1320	1330	1340	1350	1360
PTHARIIVRH	MAAPLIGQLT	RHEIEMTEKE	WKYYGDDPRV	HRTVTREDFL	DILYDIHYIL	IKATYGNFMR	QSRISEISME
1370	1380	1390	1400	1410	1420	1430	1440
VAEQGRGTTM	TPPADLIEKC	DCPLGYSGLS	CEACLPGFYR	LRSQPGGRTP	GPTLGTCVPC	QCNGHSSLCD	PETSICQNCQ
1450	1460	1470	1480	1490	1500	1510	1520
HHTAGDFCER	CALGYYGIVK	GLPNDCQQCA	CPLISSSNNF	SPSCVAEGLD	DYRCTACPRG	YEGQYCERCA	PGYTGSPGNP
1530	1540	1550	1560	1570	1580	1590	1600
GGSCQECECD	PYGSLPVPCD	PVTGFCTCRP	GATGRKCDGC	KHWHAREGWE	CVFCGDECTG	LLLGDLARLE	QMVMSINLTG
1610	1620	1630	1640	1650	1660	1670	1680
PLPAPYKMLY	GLENMTQELK	HLLSPQRAPE	RLIQLAEGNL	NTLVTEMNEL	LTRATKVTAD	GEQTGQDAER	TNTRAKSLGE
1690	1700	1710	1720	1730	1740	1750	1760
FIKELARDAE	AVNEKAIKLN	ETLGTRDEAF	ERNLEGLQKE	IDQMIKELRR	KNLETQKEIA	EDELVAAEAL	LKKVKKLFGE
1770	1780	1790	1800	1810	1820	1830	1840
SRGENEEMEK	DLREKLADYK	NKVDDAWDLL	REATDKIREA	NRLFAVNQKN	MTALEKKKEA	VESGKRQIEN	TLKEGNDILD
1850	1860	1870	1880	1890	1900	1910	1920
EANRLADEIN	SIIDYVEDIQ	TKLPPMSEEL	NDKIDDLSQE	IKDRKLAEKV	SQAESHAAQL	NDSSAVLDGI	LDEAKNISFN
1930	1940	1950	1960	1970	1980	1990	2000
ATAAFKAYSN	IKDYIDEAEK	VAKEAKDLAH	EATKLATGPR	GLLKEDAKGC	LQKSFRILNE	AKKLANDVKE	NEDHLNGLKT
2010	2020	2030	2040	2050	2060	2070	2080
RIENADARNG	DLLRTLNDTL	GKLSAIPNDT	AAKLQAVKDK	ARQANDTAKD	VLAQITELHQ	NLDGLKKNYN	KLADSVAKTN
2090	2100	2110	2120	2130	2140	2150	2160
AVVKDPSKNK	IIADADATVK	NLEQEADRLI	DKLKPIKELE	DNLKKNISEI	KELINQARKQ	ANSIKVSVSS	GGDCIRTYKP
2170	2180	2190	2200	2210	2220	2230	2240

Detailed Protein Report

Cmpd.	No. of Cmpds.	m/z meas.	Δ m/z [ppm]	z	Rt [min]	Score	P	Range	Sequence	Modification	Ratios
2021	1	665.3031	-44.41	2	55.2	14.0	1	295-306	R.ACPLDPATNKSR.C	Carbamidomethyl: 2	MD:MU 0.58
1220	1	1018.6209	-90.98	3	44.9	11.4	0	307-331	R. CECEHNTCGDSCDQCCPGFHQK A	Carbamidomethyl: 12, 15, 16	
2360	1	1073.7380	-80.56	3	59.8	11.8	2	1380-1408	K. CDCPLGYSGLSCEACLPGFYRLR T	Carbamidomethyl: 1, 12	
1065	1	836.1966	-317.81	1	42.9	11.8	1	1756-1762	K.KLFGESR.G		
1656	1	803.8745	-81.91	2	50.5	10.4	2	1969-1982	K.GCLQKSFRILNEAK.K		

Detailed Protein Report

Protein 41: macrophage mannose receptor 1 precursor [Homo sapiens]

**Accession:**	gi|4505245	**Score:**	59.5
**Database:**	refseq_human(refseq_human_20140103.fasta)	**MW [kDa]:**	165.9
**Database Date:**	2015-11-30	**pI:**	6.1
**Modification(s):**	Carbamidomethyl, Oxidation	**Sequence Coverage [%]:**	3.7
		**No. of unique Peptides:**	5

10	20	30	40	50	60	70	80
MRLPLLLVFA	SVIPGAVLLL	DTRQFLIYNE	DHKRCVDAVS	PSAVQTAACN	QDAESQKFRW	VSESQIMSVA	FKLCLGVPSK
90	100	110	120	130	140	150	160
TDWVAITLYA	CDSKSEFQKW	ECKNDTLLGI	KGEDLFFNYG	NRQEKNIMLY	KGSGLWSRWK	IYGTTDNLCS	RGYEAMYTLL
170	180	190	200	210	220	230	240
GNANGATCAF	PFKFENKWYA	DCTSAGRSDG	WLWCGTTTDY	DTDKLFGYCP	LKFEGSESLW	NKDPLTSVSY	QINSKSALTW
250	260	270	280	290	300	310	320
HQARKSCQQQ	NAELLSITEI	HEQTYLTGLT	SSLTSGLWIG	LNSLSFNSGW	QWSDRSPFRY	LNWLPGSPSA	EPGKSCVSLN
330	340	350	360	370	380	390	400
PGKNAKWENL	ECVQKLGYIC	KKGNTTLNSF	VIPSESDVPT	HCPSQWWPYA	GHCYKIHRDE	KKIQRDALTT	CRKEGGDLTS
410	420	430	440	450	460	470	480
IHTIEELDFI	ISQLGYEPND	ELWIGLNDIK	IQMYFEWSDG	TPVTFTKWLR	GEPSHENNRQ	EDCVVMKGKD	GYWADRGCEW
490	500	510	520	530	540	550	560
PLGYICKMKS	RSQGPEIVEV	EKGCRKGWKK	HHFYCYMIGH	TLSTFAEANQ	TCNNENAYLT	TIEDRYEQAF	LTSFVGLRPE
570	580	590	600	610	620	630	640
KYFWTGLSDI	QTKGTFQWTI	EEEVRFTHWN	SDMPGRKPGC	VAMRTGIAGG	LWDVLKCDEK	AKFVCKHWAE	GVTHPPKPTT
650	660	670	680	690	700	710	720
TPEPKCPEDW	GASSRTSLCF	KLYAKGKHEK	KTWFESRDFC	RALGGDLASI	NNKEEQQTIW	RLITASGSYH	KLFWLGLTYG
730	740	750	760	770	780	790	800
SPSEGFTWSD	GSPVSYENWA	YGEPNNYQNV	EYCGELKGDP	TMSWNDINCE	HLNNWICQIQ	KGQTPKPEPT	PAPQDNPPVT
810	820	830	840	850	860	870	880
EDGWVIYKDY	QYYFSKEKET	MDNARAFCKR	NFGDLVSIQS	ESEKKFLWKY	VNRNDAQSAY	FIGLLISLDK	KFAWMDGSKV
890	900	910	920	930	940	950	960
DYVSWATGEP	NFANEDENCV	TMYSNSGFWN	DINCGYPNAF	ICQRHNSSIN	ATTVMPTMPS	VPSGCKEGWN	FYSNKCFKIF
970	980	990	1000	1010	1020	1030	1040
GFMEEERKNW	QEARKACIGF	GGNLVSIQNE	KEQAFLTYHM	KDSTFSAWTG	LNDVNSEHTF	LWTDGRGVHY	TNWGKGYPGG
1050	1060	1070	1080	1090	1100	1110	1120
RRSSLSYEDA	DCVVIIGGAS	NEAGKWMDDT	CDSKRGYICQ	TRSDPSLTNP	PATIQTDGFV	KYGKSSYSLM	RQKFQWHEAE
1130	1140	1150	1160	1170	1180	1190	1200
TYCKLHNSLI	ASILDPYSNA	FAWLQMETSN	ERVWIALNSN	LTDNQYTWTD	KWRVRYTNWA	ADEPKLKSAC	VYLDLDGYWK
1210	1220	1230	1240	1250	1260	1270	1280
TAHCNESFYF	LCKRSDEIPA	TEPPQLPGRC	PESDHTAWIP	FHGHCYYIES	SYTRNWGQAS	LECLRMGSSL	VSIESAAESS
1290	1300	1310	1320	1330	1340	1350	1360
FLSYRVEPLK	SKTNFWIGLF	RNVEGTWLWI	NNSPVSFVNW	NTGDPSGERN	DCVALHASSG	FWSNIHCSSY	KGYICKRPKI
1370	1380	1390	1400	1410	1420	1430	1440
IDAKPTHELL	TTKADTRKMD	PSKPSSNVAG	VVIIVILLIL	TGAGLAAYFF	YKKRRVHLPQ	EGAFENTLYF	NSQSSPGTSD
1450	1460						
MKDLVGNIEQ	NEHSVI						

Cmpd.	No. of Cmpds.	m/z meas.	Δ m/z [ppm]	z	Rt [min]	Score	P	Range	Sequence	Modification
1884	1	621.2922	12.19	2	53.6	15.7	1	95-103	K.SEFQKWECK.N	Carbamidomethyl: 8
1876	1	914.3217	-127.99	2	53.5	10.7	2	477-491	R.GCEWPLGYICKMKSR.S	Carbamidomethyl: 10
2392	1	736.0727	87.47	3	60.2	10.6	1	586-604	R.FTHWNSDMPGRKPGCVAMR.T	Oxidation: 8
671	1	459.6231	-245.68	2	38.0	11.7	0	597-604	R.KPGCVAMR.T	Carbamidomethyl: 4
196	1	651.1714	-173.27	2	31.8	10.9	1	819-829	K.ETMDNARAFCK.R	Oxidation: 3

Detailed Protein Report

Protein 42: androglobin [Homo sapiens]

Accession: gi|168823435
Database: refseq_human(refseq_human_20140103.fasta)
Database Date: 2015-11-30

Score: 58.9
MW [kDa]: 189.6
pI: 9.1
Sequence Coverage [%]: 3.2
No. of unique Peptides: 3

10	20	30	40	50	60	70	80
MASKQTKKKE	VHRINSAHGS	DKSKDFYPFG	SNVQSGSTEQ	KKGKFPLWPE	WSEADINSEK	WDAGKGAKEK	DKTGKSPVFH
90	100	110	120	130	140	150	160
FFEDPEGKIE	LPPSLKIYSW	KRPQDILFSQ	TPVVVKNEIT	FDLFSANEHL	LCSELMRWII	SEIYAVWKIF	NGGILSNYFK
170	180	190	200	210	220	230	240
GTSGEPPLLP	WKPWEHIYSL	CKAVKGHMPL	FNSYGKYVVK	LYWMGCWRKI	TIDDFLPFDE	DNNLLLPATT	YEFELWPMLL
250	260	270	280	290	300	310	320
SKAIIKLANI	DIHVADRREL	GEFTVIHALT	GWLPEVISLH	PGYMDKVWEL	LKEILPEFKL	SDEASSESKI	AVLDSKLKEP
330	340	350	360	370	380	390	400
GKEGKEGKEI	KDGKEVKDVK	EFKPESSLTT	LKAPEKSDKV	PKEKADARDI	GKKRSKDGEK	EKFKFSLHGS	RPSSEVQYSV
410	420	430	440	450	460	470	480
QSLSDCSSAI	QTSHMVVYAT	FTPLYLFENK	IFSLEKMADS	AEKLREYGLS	HICSHPVLVT	RSRSCPLVAP	PKPPPLPPWK
490	500	510	520	530	540	550	560
LIRQKKETVI	TDEAQELIVK	KPERFLEISS	PFLNYRMTPF	TIPTEMHFVR	SLIKKGIPPG	SDLPSVSETD	ETATHSQTDL
570	580	590	600	610	620	630	640
SQITKATSQG	NTASQVILGK	GTDEQTDFGL	GDAHQSDGLN	LEREIVSQTT	ATQEKSQEEL	PTTNNSVSKE	IWLDFEDFCV
650	660	670	680	690	700	710	720
CFQNIYIFHK	PSSYCLNFQK	SEFKFSEERV	SYYLFVDSLK	PIELLVCFSA	LVRWGEYGAL	TKDSPPIEPG	LLTAETFSWK
730	740	750	760	770	780	790	800
SLKPGSLVLK	IHTYATKATV	VRLPVGRHML	LFNAYSPVGH	SIHICSMVSF	VIGDEHVVLP	NFEPESCRFT	EQSLLIMKAI
810	820	830	840	850	860	870	880
GNVIANFKDK	GKLSAALKDL	QTAHYPVPFH	DKELTAQHFR	VFHLSLWRLM	KKVQITKPPP	NFKFAFRAMV	LDLELLNSSL
890	900	910	920	930	940	950	960
EEVSLVEWLD	VKYCMPTSDK	EYSAEEVAAA	IKIQAMWRGT	YVRLLMKARI	PDTKENISVA	DTLQKVWAVL	EMNLEQYAVS
970	980	990	1000	1010	1020	1030	1040
LLRLMFKSKC	KSLESYPCYQ	DEETKIAFAD	YTVTYQEQPP	NSWFIVFRET	FLVHQDMILV	PKVYTTLPIC	ILHIVNNDTM
1050	1060	1070	1080	1090	1100	1110	1120
EQVPKVFQKV	VPYLYTKNKK	GYTFVAEAFT	GDTYVAASRW	KLRLIGSSAP	LPCLSRDSPC	NSFAIKEIRD	YYIPNDKKIL
1130	1140	1150	1160	1170	1180	1190	1200
FRYSVKVLTP	QPATIQVRTS	KPDAFIKLQV	LENEETMVSS	TGKGQAIIPA	FHFLKSEKGL	SSQSSKHILS	FHSASKKEQE
1210	1220	1230	1240	1250	1260	1270	1280
VYVKKKAAQG	IQKSPKGRAV	SAIQDIGLPL	VEEETTSTPT	REDSSSTPLQ	NYKYIIQCSV	LYNSWPLTES	QLTFVQALKD
1290	1300	1310	1320	1330	1340	1350	1360
LKKSNTKAYG	ERHEELINLG	SPDSHTISEG	QKSSVTSKTT	RKGKEKSSEK	EKTAKEKQAP	RFEPQISTVH	PQQEDPNKPY
1370	1380	1390	1400	1410	1420	1430	1440
WILRLVTEHN	ESELFEVKKD	TERADEIRAM	KQAWETTEPG	RAIKASQARL	HYLSGFIKKT	SDAESPPISE	SQTKPKEEVE
1450	1460	1470	1480	1490	1500	1510	1520
TAARGVKEPN	SKNSAGSESK	EMTQTGSGSA	VWKKWQLTKG	LRDVAKSTSS	ESGGVSSPGK	EEREQSTRKE	NIQTGPRTRS
1530	1540	1550	1560	1570	1580	1590	1600
PTILETSPRL	IRKALEFMDL	SQYVRKTDTD	PLLQTDELNQ	QQAMQKAEEI	HQFRQHRTRV	LSIRNIDQEE	RLKLKDEVLD
1610	1620	1630	1640	1650	1660	1670	
MYKEMQDSLD	EARQKIFDIR	EEYRNKLLEA	EHLKLETLAA	QEAAMKLETE	KMTPAPDTQK	KKKGKKK	

Cmpd.	No. of Cmpds.	m/z meas.	Δ m/z [ppm]	z	Rt [min]	Score	P	Range	Sequence	Modification
2723	1	921.1582	-5.50	3	65.3	17.8	1	138-160	R.WIISEIYAVWKIFNGGILSNYFK.G	
2431	1	590.4218	113.13	3	60.3	14.9	2	849-863	R.LMKKVQITKPPPNFK.F	
2707	2	933.4993	-17.55	2	65.1	26.2	2	913-927	K.IQAMWRGTYVRLLMK.A	

Detailed Protein Report

Protein 43: Alstrom syndrome protein 1 [Homo sapiens]

**Accession:**	gi|110349786	**Score:**	58.4
**Database:**	refseq_human(refseq_human_20140103.fasta)	**MW [kDa]:**	460.9
**Database Date:**	2015-11-30	**pI:**	5.8
**Modification(s):**	Carbamidomethyl	**Sequence Coverage [%]:**	1.1
		**No. of unique Peptides:**	2

Quantitation

MD:MU	**Median:** 3.08	**CV:** 0.00 %	**No. of Peptides:** 1

Detailed Protein Report

10	20	30	40	50	60	70	80
MEPEDLPWPG	ELEEEEEEEE	EEEEEEEEEA	AAAAAAANVDD	VVVVEEVEEE	AGRELDSDSH	YGPQHLESID	DEEDEEAKAW
90	100	110	120	130	140	150	160
LQAHPGRILP	PLSPPQHRYS	EGERTSLEKI	VPLTCHVWQQ	IVYQGNSRTQ	ISDTNVVCLE	TTAQRGSGDD	QKTESWHCLP
170	180	190	200	210	220	230	240
QEMDSSQTLD	TSQTRFNVRT	EDTEVTDFPS	LEEGILTQSE	NQVKEPNRDL	FCSPLLVIQD	SFASPDLPLL	TCLTQDQEFA
250	260	270	280	290	300	310	320
PDSLFHQSEL	SFAPLRGIPD	KSEDTEWSSR	PSEVSEALFQ	ATAEVASDLA	SSRFSVSQHP	LIGSTAVGSQ	CPFLPSEQGN
330	340	350	360	370	380	390	400
NEETISSVDE	LKIPKDCDRY	DDLCSYMSWK	TRKDTQWPEN	NLADKDQVSV	ATSFDITDEN	IATKRSDHFD	AARSYGGQYWT
410	420	430	440	450	460	470	480
QEDSSKQAET	YLTKGLQGKV	ESDVITLDGL	NENAVVCSER	VAELQRKPTR	ESEYHSSDLR	MLRMSPDTVP	KAPKHLKAGD
490	500	510	520	530	540	550	560
TSKGGIAKVT	QSNLKSGITT	TPVDSDIGSH	LSLSLEDLSQ	LAVSSPLETT	TGQHTDTLNQ	KTLADTHLTE	ETLKVTAIPE
570	580	590	600	610	620	630	640
PADQKTATPT	VLSSSHSHRG	KPSIFYQQGL	PDSHLTEEAL	KVSAAPGLAD	QTTGMSTLTS	TSYSHREKPG	TFYQQELPES
650	660	670	680	690	700	710	720
NLTEEPLEVS	AAPGPVEQKT	GIPTVSSTSH	SHVEDLLFFY	RQTLPDGHLT	DQALKVSAVS	GPADQKTGTA	TVLSTPHSHR
730	740	750	760	770	780	790	800
EKPGIFYQQE	FADSHQTEET	LTKVSATPGP	ADQKTEIPAV	QSSSYSQREK	PSILYPQDLA	DSHLPEEGLK	VSAVAGPADQ
810	820	830	840	850	860	870	880
KTGLPTVPSS	AYSHREKLLV	FYQQALLDSH	LPEEALKVSA	VSGPADGKTG	TPAVTSTSSA	SSSLGEKPSA	FYQQTLPNSH
890	900	910	920	930	940	950	960
LTEEALKVSI	VPGPGDQKTG	IPSAPSSFYS	HREKPIIFSQ	QTLPDFLFPE	EALKVSAVSV	LAAQKTGTPT	VSSNSHSHSE
970	980	990	1000	1010	1020	1030	1040
KSSVFYQQEL	PDSDLPRESL	KMSAIPGLTD	QKTVPTPTVP	SGSFSHREKP	SIFYQQEWPD	SYATEKALKV	STGPGPADQK
1050	1060	1070	1080	1090	1100	1110	1120
TEIPAVQSSS	YPQREKPSVL	YPQVLSDSHL	PEESLKVSAF	PGPADQMTDT	PAVPSTFYSQ	REKPGIFYQQ	TLPESHLPKE
1130	1140	1150	1160	1170	1180	1190	1200
ALKISVAPGL	ADQKTGTPTV	TSTSYSQHRE	KPSIFHQQAL	PGTHIPEEAQ	KVSAVTGPGN	QKTWIPRVLS	TFYSQREKPG
1210	1220	1230	1240	1250	1260	1270	1280
IFYQQTLPGS	HIPEEAQKVS	PVLGPADQKT	GTPTPTSASY	SHTEKPGIFY	QQVLPDNHPT	EEALKISVAS	EPVDQTTGTP
1290	1300	1310	1320	1330	1340	1350	1360
AVTSTSYSQY	REKPSIFYQQ	SLPSSHLTEE	AKNVSAVPGP	ADQKTVIPIL	PSTFYSHTEK	PGVFYQQVLP	HSHPTEEALK
1370	1380	1390	1400	1410	1420	1430	1440
ISVASEPVDQ	TTGTPTVTST	SYSQHTEKPS	IFYQQSLPGS	HLTEEAKNVS	AVPGPGDRKT	GIPTLPSTFY	SHTEKPGSFY
1450	1460	1470	1480	1490	1500	1510	1520
QQVLPHSHLP	EEALEVSVAP	GPVDQTIGTP	TVTSPSSSFG	EKPIVIYKQA	FPEGHLPEES	LKVSVAPGPV	GQTTGAPTIT
1530	1540	1550	1560	1570	1580	1590	1600
SPSYSQHRAK	SGSFYQLALL	GSQIPEEALR	VSSAPGPADQ	TTGIPTITST	SYSFGEKPIV	NYKQAFPDGH	LPEEALKVSI
1610	1620	1630	1640	1650	1660	1670	1680
VSGPTEKKTD	IPAGPLGSSA	LGEKPITFYR	QALLDSPLNK	EVVKVSAAPG	PADQKTETLP	VHSTSYSNRG	KPVIFYQQTL
1690	1700	1710	1720	1730	1740	1750	1760
SDSHLPEEAL	KVPPVPGPDA	QKTETPSVSS	SLYSYREKPI	VFYQQALPDS	ELTQEALKVS	AVPQPADQKT	GLSTVTSSFY
1770	1780	1790	1800	1810	1820	1830	1840
SHTEKPNISY	QQELPDSHLT	EEALKVSNVP	GPADQKTGVS	TVTSTSYSHR	EKPIVSYQRE	LPHFTEAGLK	ILRVPGPADQ
1850	1860	1870	1880	1890	1900	1910	1920
KTGINILPSN	SYPQREHSVI	SYEQELPDLT	EVTLKAIGVP	GPADQKTGIQ	IASSSSYSNR	EKASIFHQQE	LPDVTEEALN
1930	1940	1950	1960	1970	1980	1990	2000
VFVVPGQGDR	KTEIPTVPLS	YYSRREKPSV	ISQQELPDSH	LTEEALKVSP	VSIPAEQKTG	IPIGLSSSYS	HSHKEKLKIS
2010	2020	2030	2040	2050	2060	2070	2080
TVHIPDDQKT	EFPAATLSSY	SQIEKPKIST	VIGPNDQKTP	SQTAFHSSYS	QTVKPNILFQ	QQLPDRDQSK	GILKISAVPE
2090	2100	2110	2120	2130	2140	2150	2160
LTDVNTGKPV	SLSSSYFHRE	KSNIFSPQEL	PGSHVTEDVL	KVSTIPGPAG	QKTVLPTALP	SSFSHREKPD	IFYQKDLPDR
2170	2180	2190	2200	2210	2220	2230	2240

Detailed Protein Report

Cmpd.	No. of Cmpds.	m/z meas.	Δ m/z [ppm]	z	Rt [min]	Score	P	Range	Sequence	Modification	Ratios
881	1	555.8212	17.82	2	40.6	26.2	0	1219-1229	K.VSPVLGPADQK.T		MD:MU 3.08
160	1	896.7503	-5.56	3	31.7	21.2	1	2566-2589	R.GMGCKPEAVCSHIIIESHEKGCFR.T	Carbamidomethyl: 4	

Detailed Protein Report

Protein 44: PREDICTED: rho GTPase-activating protein 26 isoform X6 [Homo sapiens]

Accession: gi|530380462
Database: refseq_human(refseq_human_20140103.fasta)
Database Date: 2015-11-30

Score: 58.2
MW [kDa]: 69.9
pI: 9.8
Sequence Coverage [%]: 5.7
No. of unique Peptides: 3

Quantitation

MD:MU **Median:** 0.47 **CV:** 0.00 % **No. of Peptides:** 1

10	20	30	40	50	60	70	80
MKRETRRSAF	CVQGWSGGDP	ARVVLWGSAG	GRGRARHAGV	PAVGPECRAR	RSQRADPIVS	TAPAPLGAEL	LSLECPTVRM
90	100	110	120	130	140	150	160
ERALPRGRCL	PLGKDLSSAK	RKFADSLNEF	KFQCIGDAET	DDEMCIARSL	QEFATVLRNL	EDERIRMIEN	ASEVLITPLE
170	180	190	200	210	220	230	240
KFRKEQIGAA	KEAKKKYDKE	TEKYCGILEK	HLNLSSKKKE	SQLQEADSQV	DLVRQHFYEV	SLEYVFKVQE	VQERKMFEFV
250	260	270	280	290	300	310	320
EPLLAFLQGL	FTFYHHGYEL	AKDFGDFKTQ	LTISIQNTRN	RFEGTRSEVE	SLMKKMKENP	LEHKTISPYT	MEGYLYVQEK
330	340	350	360	370	380	390	400
RHFGTSWVKH	YCTYQRDSKQ	ITMVPFDQKS	GGKGGEDESV	ILKSCTRRKT	DSIEKRFCFD	VEAVDRPGVI	TMQALSEEDR
410	420	430	440	450	460	470	480
RLWMEAMDGR	EPVYNSNKDS	QSEGTAQLDS	IGFSIIRKCI	HAVETRGINE	QGLYRIVGVN	SRVQKLLSVL	MDPKTASETE
490	500	510	520	530	540	550	560
TDICAEWEIK	TITSALKTYL	RMLPGPLMMY	QFQRSFIKAA	KLENQESRVS	EIHSLVHRLP	EKNRQMLQLL	MNHLAKLIVP
570	580	590	600	610	620		
GTLKVTGSVD	MGKSSISLLF	KEMKLLKVRK	QQMLERMWRN	RNDFTLLVGV			

Cmpd.	No. of Cmpds.	m/z meas.	Δ m/z [ppm]	z	Rt [min]	Score	P	Range	Sequence	Modification	Ratios
1954	10	710.2864	-113.94	2	54.5	21.4	1	35-48	R.ARHAGVPAVGPECR.A		MD:MU 0.47
286	1	716.6484	355.53	1	32.9	20.0	0	165-171	K.EQIGAAK.E		
42	2	768.2950	-133.93	2	29.9	16.8	1	340-353	K.QITMVPFDQKSGGK.G		

Detailed Protein Report

**Protein 45:**	**PREDICTED: neuroblast differentiation-associated protein AHNAK isoform X5 [Homo sapiens]**		
**Accession:**	gi|530397287	**Score:**	57.2
**Database:**	refseq_human(refseq_human_20140103.fasta)	**MW [kDa]:**	614.8
**Database Date:**	2015-11-30	**pI:**	5.8
**Modification(s):**	Oxidation	**Sequence Coverage [%]:**	0.9
		**No. of unique Peptides:**	4

Quantitation

MD:MU	**Median:**	1.04	**CV:**	0.00 %	**No. of Peptides:**	1	

BRUKER

Detailed Protein Report

10	20	30	40	50	60	70	80
MEKEETTREL	LLPNWQGSGS	HGLTIAQRDD	GVFVQEVTQN	SPAARTGVVK	EGDQIVGATI	YFDNLQSGEV	TQLLNTMGHH
90	100	110	120	130	140	150	160
TVGLKLHRKG	DRSPEPGQTW	TREVFSSCSS	EVVLSGDDEE	YQRIYTTKIK	PRLKSEDGVE	GDLGETQSRT	ITVTRRVTAY
170	180	190	200	210	220	230	240
TVDVTGREGA	KDIDISSPEF	KIKIPRHELT	EISNVDVETQ	SGKTVIRLPS	GSGAASPTGS	AVDIRAGAIS	ASGPELQGAG
250	260	270	280	290	300	310	320
HSKLQVTMPG	IKVGGSGVNV	NAKGLDLGGR	GGVQVPAVDI	SSSLGGRAVE	VQGPSLESGD	HGKIKFPTMK	VPKFGVSTGR
330	340	350	360	370	380	390	400
EGQTPKAGLR	VSAPEVSVGH	KGGKPGLTIQ	APQLEVSVPS	ANIEGLEGKL	KGPQITGPSL	EGDLGLKGAK	PQGHIGVDAS
410	420	430	440	450	460	470	480
APQIGGSITG	PSVEVQAPDI	DVQGPGSKLN	VPKMKVPKFS	VSGAKGEETG	IDVTLPTGEV	TVPGVSGDVS	LPEIATGGLE
490	500	510	520	530	540	550	560
GKMKGTKVKT	PEMIIQKPKI	SMQDVDLSLG	SPKLKGDIKV	SAPGVQGDVK	GPQVALKGSR	VDIETPNLEG	TLTGPRLGSP
570	580	590	600	610	620	630	640
SGKTGTCRIS	MSEVDLNVAA	PKVKGGVDVT	LPRVEGKVKV	PEVDVRGPKV	DVSAPDVEAH	GPEWNLKMPK	MKMPTFSTPG
650	660	670	680	690	700	710	720
AKGEGPDVHM	TLPKGDISIS	GPKVNVEAPD	VNLEGLGGKL	KGPDVKLPDM	SVKTPKISMP	DVDLHVKGTK	VKGEYDVTVP
730	740	750	760	770	780	790	800
KLEGELKGPK	VDIDAPDVDV	HGPDWHLKMP	KMKMPKFSVP	GFKAEGPEVD	VNLPKADVDI	SGPKIDVTAP	DVSIEEPEGK
810	820	830	840	850	860	870	880
LKGPKFKMPE	MNIKVPKISM	PDVDLHLKGP	NVKGEYDVTM	PKVESEIKVP	DVELKSAKMD	IDVPDVEVQG	PDWHLKMPKM
890	900	910	920	930	940	950	960
KMPKFSMPGF	KAEGPEVDVN	LPKADVDISG	PKVGVEVPDV	NIEGPEGKLK	GPKFKMPEMN	IKAPKISMPD	VDLHMKGPKV
970	980	990	1000	1010	1020	1030	1040
KGEYDMTVPK	LEGDLKGPKV	DVSAPDVEMQ	GPDWNLKMPK	IKMPKFSMPS	LKGEGPEFDV	NLSKANVDIS	APKVDTNAPD
1050	1060	1070	1080	1090	1100	1110	1120
LSLEGPEGKL	KGPKFKMPEM	HFRAPKMSLP	DVDLDLKGPK	MKGNVDISAP	KIEGEMQVPD	VDIRGPKVDI	KAPDVEGQGL
1130	1140	1150	1160	1170	1180	1190	1200
DWSLKIPKMK	MPKFSMPSLK	GEGPEVDVNL	PKADVVVSGP	KVDIEAPDVS	LEGPEGKLKG	PKFKMPEMHF	KTPKISMPDV
1210	1220	1230	1240	1250	1260	1270	1280
DLHLKGPKVK	GDVDVSVPKV	EGEMKVPDVE	IKGPKMDIDA	PDVEVQGPDW	HLKMPKMKMP	KFSMPGFKGE	GREVDVNLPK
1290	1300	1310	1320	1330	1340	1350	1360
ADIDVSGPKV	DVEVPDVSLE	GPEGKLKGPK	FKMPEMHFKA	PKISMPDVDL	NLKGPKLKGD	VDVSLPEVEG	EMKVPDVDIK
1370	1380	1390	1400	1410	1420	1430	1440
GPKVDISAPD	VDVHGPDWHL	KMPKVKMPKF	SMPGFKGEGP	EVDVKLPKAD	VDVSGPKMDA	EVPDVNIEGP	DAKLKGPKFK
1450	1460	1470	1480	1490	1500	1510	1520
MPEMSIKPQK	ISIPDVGLHL	KGPKMKGDYD	VTVPKVEGEI	KAPDVDIKGP	KVDINAPDVE	VHGPDWHLKM	PKVKMPKFSM
1530	1540	1550	1560	1570	1580	1590	1600
PGFKGEGPEV	DMNLPKADLG	VSGPKVDIDV	PDVNLEAPEG	KLKGPKFKMP	SMNIQTHKIS	MPDVGLNLKA	PKLKTDVDVS
1610	1620	1630	1640	1650	1660	1670	1680
LPKVEGDLKG	PEIDVKAPKM	DVNVGDIDIE	GPEGKLKGPK	FKMPEMHFKA	PKISMPDVDL	HLKGPKVKGD	MDVSVPKVEG
1690	1700	1710	1720	1730	1740	1750	1760
EMKVPDVDIK	GPKVDIDAPD	VEVHDPDWHL	KMPKMKMPKF	SMPGFKAEGP	EVDVNLPKAD	IDVSGPSVDT	DAPDLDIEGP
1770	1780	1790	1800	1810	1820	1830	1840
EGKLKGSKFK	MPKLNIKAPK	VSMPDVDLNL	KGPKLKGEID	ASVPELEGDL	RGPQVDVKGP	FVEAEVPDVD	LECPDAKLKG
1850	1860	1870	1880	1890	1900	1910	1920
PKFKMPEMHF	KAPKISMPDV	DLHLKGPKVK	GDADVSVPKL	EGDLTGPSVG	VEVPDVELEC	PDAKLKGPKF	KMPDMHFKAP
1930	1940	1950	1960	1970	1980	1990	2000
KISMPDVDLH	LKGPKVKGDV	DVSVPKLEGD	LTGPSVGVEV	PDVELECPDA	KLKGPKFKMP	EMHFKTPKIS	MPDVDLHLKG
2010	2020	2030	2040	2050	2060	2070	2080
PKVKGDMDVS	VPKVEGEMKV	PDVDIKGPKM	DIDAPDVDVH	GPDWHLKMPK	MKMPKFSMPG	FKAEGPEVDV	NLPKADVVVS
2090	2100	2110	2120	2130	2140	2150	2160
GPKVDVEVPD	VSLEGPEGKL	KGPKLKMPEM	HFKAPKISMP	DVDLHLKGPK	VKGDVDVSLP	KLEGDLTGPS	VDVEVPDVEL
2170	2180	2190	2200	2210	2220	2230	2240

Detailed Protein Report

Cmpd.	No. of Cmpds.	m/z meas.	Δ m/z [ppm]	z	Rt [min]	Score	P	Range	Sequence	Modification	Ratios
2716	1	849.3583	-170.01	2	65.2	15.5	2	485-499	K.GTKVKTPEMIIQKPK.I		
863	1	578.2806	-119.29	2	40.2	10.1	2	1771-1780	K.MPKLNIKAPK.V	Oxidation: 1	
1213	1	764.7682	-167.48	2	44.8	12.8	1	2851-2865	K.GDVDVTGPKVEGDLK.G		
1557	1	472.2329	-53.94	2	49.3	18.8	0	2979-2987	K.GDVDISLPK.V		MD:MU 1.04

Detailed Protein Report

Protein 46: **phosphatidylinositol 3,4,5-trisphosphate 5-phosphatase 1 isoform b [Homo sapiens]**

**Accession:**	gi|40254823	**Score:**	56.6
**Database:**	refseq_human(refseq_human_20140103.fasta)	**MW [kDa]:**	133.1
**Database Date:**	2015-11-30	**pI:**	7.9
**Modification(s):**	Oxidation	**Sequence Coverage [%]:**	3.6
		**No. of unique Peptides:**	3

10	20	30	40	50	60	70	80
MVPCWNHGNI	TRSKAEELLS	RTGKDGSFLV	RASESISRAY	ALCVLYRNCV	YTYRILPNED	DKFTVQASEG	VSMRFFTKLD
**90**	**100**	**110**	**120**	**130**	**140**	**150**	**160**
QLIEFYKKEN	MGLVTHLQYP	VPLEEEDTGD	DPEEDTESVV	SPPELPPRNI	PLTASSCEAK	EVPFSNENPR	ATETSRPSLS
**170**	**180**	**190**	**200**	**210**	**220**	**230**	**240**
ETLFQRLQSM	DTSGLPEEHL	KAIQDYLSTQ	LAQDSEFVKT	GSSSLPHLKK	LTTLLCKELY	GEVIRTLPSL	ESLQRLFDQQ
**250**	**260**	**270**	**280**	**290**	**300**	**310**	**320**
LSPGLRPRPQ	VPGEANPINM	VSKLSQLTSL	LSSIEDKVKA	LLHEGPESPH	RPSLIPPVTF	EVKAESLGIP	QKMQLKVDVE
**330**	**340**	**350**	**360**	**370**	**380**	**390**	**400**
SGKLIIKKSK	DGSEDKFYSH	KKILQLIKSQ	KFLNKLVILV	ETEKEKILRK	EYVFADSKKR	EGFCQLLQQM	KNKHSEQPEP
**410**	**420**	**430**	**440**	**450**	**460**	**470**	**480**
DMITIFIGTW	NMGNAPPPKK	ITSWFLSKGQ	GKTRDDSADY	IPHDIYVIGT	QEDPLSEKEW	LEILKHSLQE	ITSVTFKTVA
**490**	**500**	**510**	**520**	**530**	**540**	**550**	**560**
IHTLWNIRIV	VLAKPEHENR	ISHICTDNVK	TGIANTLGNK	GAVGVSFMFN	GTSLGFVNSH	LTSGSEKKLR	RNQNYMNILR
**570**	**580**	**590**	**600**	**610**	**620**	**630**	**640**
FLALGDKKLS	PFNITHRFTH	LFWFGDLNYR	VDLPTWEAET	IIQKIKQQQY	ADLLSHDQLL	TERREQKVFL	HFEEEEITFA
**650**	**660**	**670**	**680**	**690**	**700**	**710**	**720**
PTYRFERLTR	DKYAYTKQKA	TGMKYNLPSW	CDRVLWKSYP	LVHVVCQSYG	STSDIMTSDH	SPVFATFEAG	VTSQFVSKNG
**730**	**740**	**750**	**760**	**770**	**780**	**790**	**800**
PGTVDSQGQI	EFLRCYATLK	TKSQTKFYLE	FHSSCLESFV	KSQEGENEEG	SEGELVVKFG	ETLPKLKPII	SDPEYLLDQH
**810**	**820**	**830**	**840**	**850**	**860**	**870**	**880**
ILISIKSSDS	DESYGEGCIA	LRLEATETQL	PIYTPLTHHG	ELTGHFQGEI	KLQTSQGKTR	EKLYDFVKTE	RDESSGPKTL
**890**	**900**	**910**	**920**	**930**	**940**	**950**	**960**
KSLTSHDPMK	QWEVTSRAPP	CSGSSITEII	NPNYMGVGPF	GPPMPLHVKQ	TLSPDQQPTA	WSYDQPPKDS	PLGPCRGESP
**970**	**980**	**990**	**1000**	**1010**	**1020**	**1030**	**1040**
PTPPGQPPIS	PKKFLPSTAN	RGLPPRTQES	RPSDLGKNAG	DTLPQEDLPL	TKPEMFENPL	YGSLSSFPKP	APRKDQESPK
**1050**	**1060**	**1070**	**1080**	**1090**	**1100**	**1110**	**1120**
MPRKEPPPCP	EPGILSPSIV	LTKAQEADRG	EGPGKQVPAP	RLRSFTCSSS	AEGRAAGGDK	SQGKPKTPVS	SQAPVPAKRP
**1130**	**1140**	**1150**	**1160**	**1170**	**1180**	**1190**	
IKPSRSEINQ	QTPPTPTPRP	PLPVKSPAVL	HLQHSKGRDY	RDNTELPHHG	KHRPEEGPPG	PLGRTAMQ	

Cmpd.	No. of Cmpds.	m/z meas.	Δ m/z [ppm]	z	Rt [min]	Score	P	Range	Sequence	Modification
499	2	584.8345	31.59	2	35.6	27.2	0	79-87	K.LDQLIEFYK.K	
234	1	850.8764	-41.35	2	32.6	12.8	0	167-181	R.LQSMDTSGLPEEHLK.A	Oxidation: 4
1543	1	1008.6543	64.47	2	49.1	16.6	1	1107-1125	K.TPVSSQAPVPAKRPIKPSR.S	

Detailed Protein Report

Protein 47: protocadherin Fat 2 precursor [Homo sapiens]

**Accession:**	gi|13787217	**Score:**	56.5
**Database:**	refseq_human(refseq_human_20140103.fasta)	**MW [kDa]:**	479.0
**Database Date:**	2015-11-30	**pI:**	4.9
		**Sequence Coverage [%]:**	1.5
		**No. of unique Peptides:**	3

Alias proteins:

Accession	Name	Description
gi|578810809	refseq_human (refseq_human_20140103.fasta)	PREDICTED: protocadherin Fat 2 isoform X1 [Homo sapiens]

Detailed Protein Report

10	20	30	40	50	60	70	80
MTIALLGFAI	FLLHCATCEK	PLEGILSSSA	WHFTHSHYNA	TIYENSSPKT	YVESFEKMGI	YLAEPQWAVR	YRIISGDVAN
90	100	110	120	130	140	150	160
VFKTEEYVVG	NFCFLRIRTK	SSNTALLNRE	VRDSYTLIIQ	ATEKTLELEA	LTRVVVHILD	QNDLKPLFSP	PSYRVTISED
170	180	190	200	210	220	230	240
MPLKSPICKV	TATDADLGQN	AEFYYAFNTR	SEMFAIHPTS	GVVTVAGKLN	VTWRGKHELQ	VLAVDRMRKI	SEGNGFGSLA
250	260	270	280	290	300	310	320
ALVVHVEPAL	RKPPAIASVV	VTPPDSNDGT	TYATVLVDAN	SSGAEVESVE	VVGGDPGKHF	KAIKSYARSN	EFSLVSVKDI
330	340	350	360	370	380	390	400
NWMEYLHGFN	LSLQARSGSG	PYFYSQIRGF	HLPPSKLSSL	KFEKAVYRVQ	LSEFSPPGSR	VVMVRVTPAF	PNLQYVLKPS
410	420	430	440	450	460	470	480
SENVGFKLNA	RTGLITTTKL	MDFHDRAHYQ	LHIRTSPGQA	STVVVIDIVD	CNNHAPLFNR	SSYDGTLDEN	IPPGTSVLAV
490	500	510	520	530	540	550	560
TATDRDHGEN	GYVTYSIAGP	KALPFSIDPY	LGIISTSKPM	DYELMKRIYT	FRVRASDWGS	PFRREKEVSI	FLQLRNLNDN
570	580	590	600	610	620	630	640
QPMFEEVNCT	GSIRQDWPVG	KSIMTMSAID	VDELQNLKYE	IVSGNELEYF	DLNHFSGVIS	LKRPFINLTA	GQPTSYSLKI
650	660	670	680	690	700	710	720
TASDGKNYAS	PTTLNITVVK	DPHFEVPVTC	DKTGVLTQFT	KTILHFIGLQ	NQESSDEEFT	SLSTYQINHY	TPQFEDHFPQ
730	740	750	760	770	780	790	800
SIDVLESVPI	NTPLARLAAT	DPDAGFNGKL	VYVIADGNEE	GCFDIELETG	LLTVAAPLDY	EATNFYILNV	TVYDLGTPQK
810	820	830	840	850	860	870	880
SSWKLLTVNV	KDWNDNAPRF	PPGGYQLTIS	EDTEVGTTIA	ELTTKDADSE	DNGRVRYTLL	SPTEKFSLHP	LTGELVVTGH
890	900	910	920	930	940	950	960
LDRESEPRYI	LKVEARDQPS	KGHQLFSVTD	LIITLEDVND	NSPQCITEHN	RLKVPEDLPP	GTVLTFLDAS	DPDLGPAGEV
970	980	990	1000	1010	1020	1030	1040
RYVLMDGAHG	TFRVDLMTGA	LILERELDFE	RRAGYNLSLW	ASDGGRPLAR	RTLCHVEVIV	LDVNENLHPP	HFASFVHQGQ
1050	1060	1070	1080	1090	1100	1110	1120
VQENSPSGTQ	VIVVAAQDDD	SGLDGELQYF	LRAGTGLAAF	SINQDTGMIQ	TLAPLDREFA	SYYWLTVLAV	DRGSVPLSSV
1130	1140	1150	1160	1170	1180	1190	1200
TEVYIEVTDA	NDNPPQMSQA	VFYPSIQEDA	PVGTSVLQLD	AWDPDSSSKG	KLTFNITSGN	YMGFFMIHPV	TGLLSTAQQL
1210	1220	1230	1240	1250	1260	1270	1280
DRENKDEHIL	EVTVLDNGEP	SLKSTSRVVV	GILDVNDNPP	IFSHKLFNVR	LPERLSPVSP	GPVYRLVASD	LDEGLNGRVT
1290	1300	1310	1320	1330	1340	1350	1360
YSIEDSDEEA	FSIDLVTGVV	SSSSTFTAGE	YNILTIKATD	SGQPPLSASV	RLHIEWIPWP	RPSSIPLAFD	ETYYSFTVME
1370	1380	1390	1400	1410	1420	1430	1440
TDPVNHMVGV	ISVEGRPGLF	WFNISGGDKD	MDFDIEKTTG	SIVIARPLDT	RRRSNYNLTV	EVTDGSRTIA	TQVHIFMIAN
1450	1460	1470	1480	1490	1500	1510	1520
INHHRPQFLE	TRYEVRVPQD	TVPGVELLRV	QAIDQDKGKS	LIYTIHGSQD	PGSASLFQLD	PSSGVLVTVG	KLDLGSGPSQ
1530	1540	1550	1560	1570	1580	1590	1600
HTLTVMVRDQ	EIPIKRNFVW	VTIHVEDGNL	HPPRFTQLHY	EASVPDTIAP	GTELLQVRAM	DADRGVNAEV	HYSLLKGNSE
1610	1620	1630	1640	1650	1660	1670	1680
GFFNINALLG	IITLAQKLDQ	ANHAPHTLTV	KAEDQGSPQW	HDLATVIIHV	YPSDRSAPIF	SKSEYFVEIP	ESIPVGSPIL
1690	1700	1710	1720	1730	1740	1750	1760
LVSAMSPSEV	TYELREGNKD	GVFSMNSYSG	LISTQKKLDH	EKISSYQLKI	RGSNMAGAFT	DVMVVVDIID	ENDNAPMFLK
1770	1780	1790	1800	1810	1820	1830	1840
STFVGQISEA	APLYSMIMDK	NNNPFVIHAS	DSDKEANSLL	VYKILEPEAL	KFFKIDPSMG	TLTIVSEMDY	ESMPSFQFCV
1850	1860	1870	1880	1890	1900	1910	1920
YVHDQGSPVL	FAPRPAQVII	HVRDVNDSPP	RFSEQIYEVA	IVGPIHPGME	LLMVRASDED	SEVNYSIKTG	NADEAVTIHP
1930	1940	1950	1960	1970	1980	1990	2000
VTGSISVLNP	AFLGLSRKLT	IRASDGLYQD	TALVKISLTQ	VLDKSLQFDQ	DVYWAAVKEN	LQDRKALVIL	GAQGNHLNDT
2010	2020	2030	2040	2050	2060	2070	2080
LSYFLLNGTD	MFHMVQSAGV	LQTRGVAFDR	EQQDTHELAV	EVRDNRTPQR	VAQGLVRVSI	EDVNDNPPKF	KHLPYYTIIQ
2090	2100	2110	2120	2130	2140	2150	2160
DGTEPGDVLF	QVSATDEDLG	TNGAVTYEFA	EDYTYFRIDP	YLGDISLKKP	FDYQALNKYH	LKVIARDGGT	PSLQSEEEVL
2170	2180	2190	2200	2210	2220	2230	2240
VTVRNKSNPL	FQSPYYKVRV	PENITLYTPI	LHTQARSPEG	LRLIYNIVEE	EPLMLFTTDF	KTGVLTVTGP	LDYESKTKHV
2250	2260	2270	2280	2290	2300	2310	2320
FTVRATDTAL	GSFSEATVEV	LVEDVNDNPP	TFSQLVYTTS	ISEGLPAQTP	VIQLLASDQD	SGRNRDVSYQ	IVEDGSDVSK
2330	2340	2350	2360	2370	2380	2390	2400

Detailed Protein Report

Cmpd.	No. of Cmpds.	m/z meas.	Δ m/z [ppm]	z	Rt [min]	Score	P	Range	Sequence	Modification
2633	1	741.3552	-72.85	2	63.0	15.7	1	71-83	R.YRIISGDVANVFK.T	
1721	1	1030.6326	87.08	2	51.3	16.6	1	993-1011	R.AGYNLSLWASDGGRPLARR.T	
65	2	1096.9820	-88.55	2	30.5	12.9	2	3376-3395	K.GELQVAKALDREQASSYSLK.L	

Detailed Protein Report

Protein 48: pyruvate kinase PKM isoform a [Homo sapiens]

**Accession:**	gi|33286418	**Score:**	56.5
**Database:**	refseq_human(refseq_human_20140103.fasta)	**MW [kDa]:**	57.9
**Database Date:**	2015-11-30	**pI:**	9.0
**Modification(s):**	Carbamidomethyl	**Sequence Coverage [%]:**	5.5
		**No. of unique Peptides:**	2

Quantitation

MD:MU **Median:** 0.57 **CV:** 0.00 % **No. of Peptides:** 1

Alias proteins:

Accession	Name	Description
gi|530405979	refseq_human (refseq_human_20140103.fasta)	PREDICTED: pyruvate kinase PKM isoform X3 [Homo sapiens]

10	20	30	40	50	60	70	80
MSKPHSEAGT	AFIQTQQLHA	AMADTFLEHM	CRLDIDSPPI	TARNTGIICT	IGPASRSVET	LKEMIKSGMN	VARLNFSHGT
**90**	**100**	**110**	**120**	**130**	**140**	**150**	**160**
HEYHAETIKN	VRTATESFAS	DPILYRPVAV	ALDTKGPEIR	TGLIKGSGTA	EVELKKGATL	KITLDNAYME	KCDENILWLD
**170**	**180**	**190**	**200**	**210**	**220**	**230**	**240**
YKNICKVVEV	GSKIYVDDGL	ISLQVKQKGA	DFLVTEVENG	GSLGSKKGVN	LPGAAVDLPA	VSEKDIQDLK	FGVEQDVDMV
**250**	**260**	**270**	**280**	**290**	**300**	**310**	**320**
FASFIRKASD	VHEVRKVLGE	KGKNIKIISK	IENHEGVRRF	DEILEASDGI	MVARGDLGIE	IPAEKVFLAQ	KMMIGRCNRA
**330**	**340**	**350**	**360**	**370**	**380**	**390**	**400**
GKPVICATQM	LESMIKKPRP	TRAEGSDVAN	AVLDGADCIM	LSGETAKGDY	PLEAVRMQHL	IAREAEAAIY	HLQLFEELRR
**410**	**420**	**430**	**440**	**450**	**460**	**470**	**480**
LAPITSDPTE	ATAVGAVEAS	FKCCSGAIIV	LTKSGRSAHQ	VARYRPRAPI	IAVTRNPQTA	RQAHLYRGIF	PVLCKDPVQE
**490**	**500**	**510**	**520**	**530**	**540**		
AWAEDVDLRV	NFAMNVGKAR	GFFKKGDVVI	VLTGWRPGSG	FTNTMRVVPV	P		

Cmpd.	No. of Cmpds.	m/z meas.	Δ m/z [ppm]	z	Rt [min]	Score	P	Range	Sequence	Modification	Ratios
566	1	680.2094	-215.66	2	36.4	14.7	0	44-56	R.NTGIICTIGPASR.S	Carbamidomethyl: 6	
111	1	628.6466	11.60	3	30.8	41.8	0	74-89	R.LNFSHGTHEYHAETIK.N		MD:MU 0.57

Detailed Protein Report

Protein 49: PREDICTED: splicing factor, proline- and glutamine-rich isoform X5 [Homo sapiens]

Accession: gi|530363148
Database: refseq_human(refseq_human_20140103.fasta)
Database Date: 2015-11-30
Modification(s): Oxidation

Score: 55.1
MW [kDa]: 72.2
pI: 9.7
Sequence Coverage [%]: 5.1
No. of unique Peptides: 3

Quantitation

MD:MU **Median:** 0.23 **CV:** 0.00 % **No. of Peptides:** 1

Alias proteins:

Accession	Name	Description
gi|530363150	refseq_human (refseq_human_20140103.fasta)	PREDICTED: splicing factor, proline- and glutamine-rich isoform X6 [Homo sapiens]

10	20	30	40	50	60	70	80
MSRDRFRSRG	GGGGGFHRRG	GGGGRGGLHD	FRSPPPGMGL	NQNRGPMGPG	PGQSGPKPPI	PPPPPHQQQQ	QPPPQQPPPQ
**90**	**100**	**110**	**120**	**130**	**140**	**150**	**160**
QPPPHQPPPH	PQPHQQQQPP	PPPQDSSKPV	VAQGPGPAPG	VGSAPPASSS	APPATPPTSG	APPGSGPGPT	PTPPPAVTSA
**170**	**180**	**190**	**200**	**210**	**220**	**230**	**240**
PPGAPPPTPP	SSGVPTTPPQ	AGGPPPPPAA	VPGPGPGPKQ	GPGPGGPKGG	KMPGGPKPGG	GPGLSTPGGH	PKPPHRGGGE
**250**	**260**	**270**	**280**	**290**	**300**	**310**	**320**
PRGGRQHHPP	YHQQHHQGPP	PGGPGGRSEE	KISDSEGFKA	NLSLLRRPGE	KTYTQRCRLF	VGNLPADITE	DEFKRLFAKY
**330**	**340**	**350**	**360**	**370**	**380**	**390**	**400**
GEPGEVFINK	GKGFGFIKLE	SRALAEIAKA	ELDDTPMRGR	QLRVRFATHA	AALSVRNLSP	YVSNELLEEA	FSQFGPIERA
**410**	**420**	**430**	**440**	**450**	**460**	**470**	**480**
VVIVDDRGRS	TGKGIVEFAS	KPAARKAFER	CSEGVFLLTT	TPRPVIVEPL	EQLDDEDGLP	EKLAQKNPMY	QKERETPPRF
**490**	**500**	**510**	**520**	**530**	**540**	**550**	**560**
AQHGTFEYEY	SQRWKSLDEM	EKQQREQVEK	NMKDAKDKLE	SEMEDAYHEH	QANLLRQDLM	RRQEELRRME	ELHNQEMQKR
**570**	**580**	**590**	**600**	**610**	**620**	**630**	**640**
KEMQLRQEEE	RRRREEEMMI	RQREMEEQMR	RQREESYSRM	GYMDPRERDM	RMGGGGAMNM	GDPYGSGGQK	FPPLGGGGGI
**650**	**660**	**670**					
GYEANPGVPP	ATMSGSMMGS	DMVRMIDVG					

Cmpd.	No. of Cmpds.	m/z meas.	Δ m/z [ppm]	z	Rt [min]	Score	P	Range	Sequence	Modification	Ratios
376	4	678.1939	-185.67	2	34.0	29.1	1	268-279	R.SEEKISDSEGFK.A		MD:MU 0.23
14	1	1103.5206	70.68	2	29.5	14.3	1	609-630	R. DMRMGGGGAMNMGDPYGSGGQ F	Oxidation: 2, 12	
20	1	735.9950	41.81	3	29.6	11.7	1	609-630	R. DMRMGGGGAMNMGDPYGSGGQ F	Oxidation: 4, 10	

Detailed Protein Report

Protein 50: PREDICTED: MAX gene-associated protein isoform X6 [Homo sapiens]

**Accession:**	gi|530405566	**Score:**	55.0
**Database:**	refseq_human(refseq_human_20140103.fasta)	**MW [kDa]:**	341.2
**Database Date:**	2015-11-30	**pI:**	6.7
**Modification(s):**	Carbamidomethyl, Oxidation	**Sequence Coverage [%]:**	1.4
		**No. of unique Peptides:**	3

Detailed Protein Report

10	20	30	40	50	60	70	80
MEEKQQIILA	NQDGGTVAGA	APTFFVILKQ	PGNGKTDQGI	LVTNQDACAL	ASSVSSPVKS	KGKICLPADC	TVGGITVTLD
90	100	110	120	130	140	150	160
NNSMWNEFYH	RSTEMILTKQ	GRRMFPYCRY	WITGLDSNLK	YILVMDISPV	DNHRYKWNGR	WWEPSGKAEP	HVLGRVFIHP
170	180	190	200	210	220	230	240
ESPSTGHYWM	HQPVSFYKLK	LTNNTLDQEG	HIILHSMHRY	LPRLHLVPAE	KAVEVIQLNG	PGVHTFTFPQ	TEFFAVTAYQ
250	260	270	280	290	300	310	320
NIQITQLKID	YNPFAKGFRD	DGLNNKPQRD	GKQKNSSDQE	GNNISSSSGH	RVRLTEGQGS	EIQPGDLDPL	SRGHETSGKG
330	340	350	360	370	380	390	400
LEKTSLNIKR	DFLGFMDTDS	ALSEVPQLKQ	EISECLIASS	FEDDSRVASP	LDQNGSFNVV	IKEEPLDDYD	YELGECPEGV
410	420	430	440	450	460	470	480
TVKQEETDEE	TDVYSNSDDD	PILEKQLKRH	NKVDNPEADH	LSSKWLPSSP	SGVAKAKMFK	LDTGKMPVVY	LEPCAVTRST
490	500	510	520	530	540	550	560
VKISELPDNM	LSTSRKDKSS	MLAELEYLPT	YIENSNETAF	CLGKESENGL	RKHSPDLRVV	QKYPLLKEPQ	WKYPDISDSI
570	580	590	600	610	620	630	640
STERILDDSK	DSVGDSLSGK	EDLGRKRTTM	LKIATAAKVV	NANQNASPNV	PGKRGRPRKL	KLCKAGRPPK	NTGKSLISTK
650	660	670	680	690	700	710	720
NTPVSPGSTF	PDVKPDLEDV	DGVLFVSFES	KEALDIHAVD	GTTEESSSLQ	ASTTNDSGYR	ARISQLEKEL	IEDLKTLRHK
730	740	750	760	770	780	790	800
QVIHPGLQEV	GLKLNSVDPT	MSIDLKYLGV	QLPLAPATSF	PFWNLTGTNP	ASPDAGFPFV	SRTGKTNDFT	KIKGWRGKFH
810	820	830	840	850	860	870	880
SASASRNEGG	NSESSLKNRS	AFCSDKLDEY	LENEGKLMET	SMGFSSNAPT	SPVVYQLPTK	STSYVRTLDS	VLKKQSTISP
890	900	910	920	930	940	950	960
STSYSLKPHS	VPPVSRKAKS	QNRQATFSGR	TKSSYKSILP	YPVSPKQKYS	HVILGDKVTK	NSSGIISENQ	ANNFVVPTLD
970	980	990	1000	1010	1020	1030	1040
ENIFPKQISL	RQAQQQQQQQ	QGSRPPGLSK	SQVKLMDLED	CALWEGKPRT	YITEERADVS	LTTLLTAQAS	LKTKPIHTII
1050	1060	1070	1080	1090	1100	1110	1120
RKRAPPCNND	FCRLGCVCSS	LALEKRQPAH	CRRPDCMFGC	TCLKRKVVLV	KGGSKTKHFQ	RKAAHRDPVF	YDTLGEEARE
1130	1140	1150	1160	1170	1180	1190	1200
EEEGIREEEE	QLKEKKKRKK	LEYTICETEP	EQPVRHYPLW	VKVEGEVDPE	PVYIPTPSVI	EPMKPLLLPQ	PEVLSPTVKG
1210	1220	1230	1240	1250	1260	1270	1280
KLLTGIKSPR	SYTPKPNPVI	REEDKDPVYL	YFESMMTCAR	VRVYERKKED	QRQPSSSSSP	SPSFQQQTSC	HSSPENHNNA
1290	1300	1310	1320	1330	1340	1350	1360
KEPDSEQQPL	KQLTCDLEDD	SDKLQEKSWK	SSCNEGESSS	TSYMHQRSPG	GPTKLIEIIS	DCNWEEDRNK	ILSILSQHIN
1370	1380	1390	1400	1410	1420	1430	1440
SNMPQSLKVG	SFIIELASQR	KSRGEKNPPV	YSSRVKISMP	SCQDQDDMAE	KSGSETPDGP	LSPGKMEDIS	PVQTDALDSV
1450	1460	1470	1480	1490	1500	1510	1520
RERLHGGKGL	PFYAGLSPAG	KLVAYKRKPS	SSTSGLIQVN	GKSYPQAKLL	LGQMGALHPA	NRLAAYITGR	LRPSVLDLST
1530	1540	1550	1560	1570	1580	1590	1600
LSTVISKVAS	NAKVAASRKP	RTLLPSTSNS	KMASSSGTAT	NRPGKNLKAF	VPAKRPIAAR	PSPGGVFTQF	VMSKVGALQQ
1610	1620	1630	1640	1650	1660	1670	1680
KIPGVSTPQT	LAGTQKFSIR	PSPVMVVTPV	VSSEPVQVCS	PVTAAVTTTT	PQVFLENTTA	VTPMTAISDV	ETKETTYSSG
1690	1700	1710	1720	1730	1740	1750	1760
ATTTGVVEVS	ETNTSTSVTS	TQSTATVNLT	KTTGITTPVA	SVAFPKSLVA	SPSTITLPVA	STASTSLVVV	TAAASSSMVT
1770	1780	1790	1800	1810	1820	1830	1840
TPTSSLGSVP	IILSGINGSP	PVSQRPENAA	QIPVATPQVS	PNTVKRAGPR	LLLIPVQQGS	PTLRPVSNTQ	LQGHRMVLQP
1850	1860	1870	1880	1890	1900	1910	1920
VRSPSGMNLF	RHPNGQIVQL	LPLHQLRGSN	TQPNLQPVMF	RNPGSVMGIR	LPAPSKPSET	PPSSTSSSAF	SVMNPVIQAV
1930	1940	1950	1960	1970	1980	1990	2000
GSSSAVNVIT	QAPSLLSSGA	SFVSQAGTLT	LRISPPEPQS	FASKTGSETK	ITYSSGGQPV	GTASLIPLQS	GSFALLQLPG
2010	2020	2030	2040	2050	2060	2070	2080
QKPVPSSILQ	HVASLQMKRE	SQNPDQKDET	NSIKREQETK	KVLQSEGEAV	DPEANVIKQN	SGAATSEETL	NDSLEDRGDH
2090	2100	2110	2120	2130	2140	2150	2160
LDEECLPEEG	CATVKPSEHS	CITGSHTDQD	YKDVNEEYGA	RNRKSSKEKV	AVLEVRTISE	KASNKTVQNL	SKVQHQKLGD
2170	2180	2190	2200	2210	2220	2230	2240

Detailed Protein Report

Cmpd.	No. of Cmpds.	m/z meas.	Δ m/z [ppm]	z	Rt [min]	Score	P	Range	Sequence	Modification
1755	1	636.2889	-110.13	2	51.9	13.3	0	1211-1221	R.SYTPKPNPVIR.E	
2040	1	841.7905	46.48	3	55.6	10.9	2	2189-2210	K.FELSGSKVMEQQSNLQPEAKEK.E	Oxidation: 9
939	1	583.7035	-110.96	2	41.3	10.6	1	2234-2243	R.KCVGASQECK.K	Carbamidomethyl: 2, 9

Detailed Protein Report

Protein 51: small proline-rich protein 2A [Homo sapiens]

**Accession:**	gi|5174693	**Score:**	54.8
**Database:**	refseq_human(refseq_human_20140103.fasta)	**MW [kDa]:**	8.0
**Database Date:**	2015-11-30	**pI:**	10.3
**Modification(s):**	Carbamidomethyl	**Sequence Coverage [%]:**	36.1
		**No. of unique Peptides:**	2

Quantitation

MD:MU **Median:** 0.85 **CV:** 34.64 % **No. of Peptides:** 2

```
        10        20        30        40        50        60        70        80
MSYQQQQCKQ PCQPPPVCPT PKCPEPCPPP KCPEPCPPPK CPQPCPPQQC QQKYPPVTPS PPCQSKYPPK SK
```

Cmpd.	No. of Cmpds.	m/z meas.	Δ m/z [ppm]	z	Rt [min]	Score	P	Range	Sequence	Modification	Ratios	
85	4	753.3519	-18.26	2	30.5	41.0	0	10-22	K.QPCQPPPVCPTPK.C	Carbamidomethyl: 3, 9	MD:MU	1.19
171	1	729.5012	195.91	2	31.9	13.8	0	54-66	K.YPPVTPSPPCQSK.Y	Carbamidomethyl: 10	MD:MU	0.61

Detailed Protein Report

Protein 52: dynein heavy chain 11, axonemal [Homo sapiens]

**Accession:**	gi|459642365	**Score:**	54.3
**Database:**	refseq_human(refseq_human_20140103.fasta)	**MW [kDa]:**	520.0
**Database Date:**	2015-11-30	**pI:**	6.0
**Modification(s):**	Carbamidomethyl	**Sequence Coverage [%]:**	1.1
		**No. of unique Peptides:**	4

Detailed Protein Report

10	20	30	40	50	60	70	80
MAAQVAAREA	RDFREAPTLR	LTSGAGLEAV	GAVELEEEEE	NEEEAAARRA	RSFAQDARVR	FLGGRLAMML	GFTEEKWSQY
90	100	110	120	130	140	150	160
LESEDNRQVL	GEFLESTSPA	CLVFSFAASG	RLAASQEIPR	DANHKLVFIS	KKITESIGVN	DFSQVVLFGE	LPALSLGHVS
170	180	190	200	210	220	230	240
AFLDEILVPV	LSNKNNHKSW	SCFTSQDMEY	HIEVMKKKMY	IFRGKMSRRT	LLPIPTVAGK	MDLDQNCSEN	KPPSNERIIL
250	260	270	280	290	300	310	320
HAIESVVIEW	SHQIQEIIER	DSVQRLLNGL	HLSPQAELDF	WMMRRENLSC	IYDQLQAPVV	LKMVKILTTK	QSSYFPTLKD
330	340	350	360	370	380	390	400
IFLAVENALL	EAQDVELYLR	PLRRHIQCLQ	ETEFPQTRIL	IAPLFHTICL	IWSHSKFYNT	PARVIVLLQE	FCNLFINQAT
410	420	430	440	450	460	470	480
AYLSPEDLLR	GEIEESLEKV	QVAVNILKTF	KNSFFNYRKK	LASYFMGRKL	RPWDFQSHLV	FCRFDKFLDR	LIKIEDIFAT
490	500	510	520	530	540	550	560
TLEFEKLERL	EFGGTKGAIL	NGQVHEMSEE	LMELCKLFKQ	STYDPSDCTN	MEFESDYVAF	KSKTLEFDRR	LGTIICEAFF
570	580	590	600	610	620	630	640
NCNGLEAAFK	LLTIFGNFLE	KPVVMEIFSL	HYSTLVHMFN	TELDVCKQLY	NEHMKQIECG	HVVLNKNMPF	TSGNMKWAQQ
650	660	670	680	690	700	710	720
VLQRLQMFWS	NFASLRYLFL	GNPDHALVYQ	KYVEMTTLLD	QFESRIYNEW	KSNVDEICEF	NLNQPLVKFS	AINGLLCVNF
730	740	750	760	770	780	790	800
DPKLVAVLRE	VKYLLMLKKQ	DIPDSALAIF	KKRNTILKYI	GNLDLLVQGY	NKLKQTLLEV	EYPLIEDELR	AIDEQLTAAT
810	820	830	840	850	860	870	880
TWLTWQDDCW	GYIERVRAAT	SELEHRVERT	QKNVKVIQQT	MRGWARCVLP	PRREHRREAA	FTLEDKGDLF	TKKYKLIQGD
890	900	910	920	930	940	950	960
GCKIHNLVEE	NRKLFKANPS	LDTWKIYVEF	IDDIVVEGFF	QAIMHDLDFF	LKNTEKQLKP	APFFQAQMIL	LPPEIVFKPS
970	980	990	1000	1010	1020	1030	1040
LDREAGDGFY	DLVEEMLCNS	FRMSAQMNRI	ATHLEIKNYQ	NDMDNMLGLA	EVRQEIMNRV	VNVINKVLDF	RNTLETHTYL
1050	1060	1070	1080	1090	1100	1110	1120
WVDDRAEFMK	HFLLYGHAVS	SDEMDAHANE	EIPEQPPTLE	QFKEQIDIYE	ALYVQMSKFE	DFRVFDSWFK	VDMKPFKVSL
1130	1140	1150	1160	1170	1180	1190	1200
LTIIKKWSWM	FQEHLLRFVI	DSLNELQEFI	KETDSGLQRE	LNEGDHDGLV	DIMVHLLAVR	SRQRATDELF	EPLKETITLL
1210	1220	1230	1240	1250	1260	1270	1280
ESYGQKMPEQ	VYIQLEELPE	RWETTKKIAA	TVRHEVSPLH	NAEVTLIRKK	CILFDAKQAE	FRERFRHYAP	LGFNAENPYT
1290	1300	1310	1320	1330	1340	1350	1360
ALDKANEELE	ALEEEMLQMQ	ESTRLFEVAL	PEYKQMKQCR	KEIKLLKGLW	DVIIYVRRSI	DNWTKTQWRQ	IHVEQMDVEL
1370	1380	1390	1400	1410	1420	1430	1440
RRFAKEIWSL	NKEVRVWDAY	TGLEGTVKDM	TASLRAITEL	QSPALRDRHW	HQLMKAIGVK	FLINEATTLA	DLLALRLHRV
1450	1460	1470	1480	1490	1500	1510	1520
EDDVRRIVDK	AVKELGTEKV	ITEISQTWAT	MKFSYEVHYR	TGIPLLKSDE	QLFETLEHNQ	VQLQTLLQSK	YVEYFIEQVL
1530	1540	1550	1560	1570	1580	1590	1600
SWQNKLNIAD	LVIFTWMEVQ	RTWSHLESIF	VCSEDIRIQL	VKDARRFDGV	DAEFKELMFK	TAKVENVLEA	TCRPNLYEKL
1610	1620	1630	1640	1650	1660	1670	1680
KDLQSRLSLC	EKALAEYLET	KRIAFPRFYF	VSSADLLDIL	SKGAQPKQVT	CHLAKLFDSI	ADLQFEDNQD	VSAHRAVGMY
1690	1700	1710	1720	1730	1740	1750	1760
SKEKEYVPFQ	AECECVGHVE	TWLLQLEQTM	QETVRHSITE	AIVAYEEKPR	ELWIFDFPAQ	VALTSSQIWW	TTDVGIAFSR
1770	1780	1790	1800	1810	1820	1830	1840
LEEGYETALK	DFHKKQISQL	NTLITLLLGE	LPPGDRQKIM	TICTIDVHAR	DVVAKLISQK	VVSPQAFTWL	SQLRHRWEDT
1850	1860	1870	1880	1890	1900	1910	1920
QKHCFVNICD	AQFQYFYEYL	GNSPRLVITP	LTDRCYITLT	QSLHLTMSGA	PAGPAGTGKT	ETTKDLGRAL	GMMVYVFNCS
1930	1940	1950	1960	1970	1980	1990	2000
EQMDYKSIGN	IYKGLVQTGA	WGCFDEFNRI	SVEVLSVVAV	QVKMIHDAIR	NRKKRFVFLG	EAITLKPSVG	IFITMNPGYA
2010	2020	2030	2040	2050	2060	2070	2080
GRTELPENLK	ALFRPCAMVA	PDIELICEIL	LVAEGFVDAR	ALARKFITLY	TLCKELLSKQ	DHYDWGLRAI	KSVLVVAGSL
2090	2100	2110	2120	2130	2140	2150	2160
KRGDKNRPED	QVLMRALRDF	NMPKIVTDDI	PVFLGLVGDL	FPALDVPRRR	KLHFEQMVRQ	STLELRLQPE	ESFILKVVQL
2170	2180	2190	2200	2210	2220	2230	2240

Detailed Protein Report

Cmpd.	No. of Cmpds.	m/z meas.	Δ m/z [ppm]	z	Rt [min]	Score	P	Range	Sequence	Modification
1302	1	817.1657	-329.27	1	46.0	11.8	0	1-8	-.MAAQVAAR.E	
2700	1	920.8814	-102.30	2	65.0	16.0	2	857-872	R.REAAFTLEDKGDLFTK.K	
2478	1	589.2111	-228.33	2	60.9	14.1	0	2296-2304	R.LLFEIHHLR.S	
1863	1	965.8216	-153.66	2	53.3	12.4	2	4470-4484	R.QETKQTYECPVYRTK.L	Carbamidomethyl: 9

Detailed Protein Report

Protein 53: obscurin isoform a [Homo sapiens]

**Accession:**	gi|403501448	**Score:**	53.8
**Database:**	refseq_human(refseq_human_20140103.fasta)	**MW [kDa]:**	721.1
**Database Date:**	2015-11-30	**pI:**	5.4
		**Sequence Coverage [%]:**	0.5
		**No. of unique Peptides:**	3

Quantitation

MD:MU	**Median:**	0.66	**CV:**	0.00 %	**No. of Peptides:**	1	

BRUKER

Detailed Protein Report

10	20	30	40	50	60	70	80
MDQPQFSGAP	RFLTRPKAFV	VSVGKDATLS	CQIVGNPTPQ	VSWEKDQQPV	AAGARFRLAQ	DGDLYRLTIL	DLALGDSGQY
90	100	110	120	130	140	150	160
VCRARNAIGE	AFAAVGLQVD	AEAACAEQAP	HFLLRPTSIR	VREGSEATFR	CRVGGSPRPA	VSWSKDGRRL	GEPDGPRVRV
170	180	190	200	210	220	230	240
EELGEASALR	IRAARPRDGG	TYEVRAENPL	GAASAAAALV	VDSDAADTAS	RPGTSTAALL	AHLQRRREAM	RAEGAPASPP
250	260	270	280	290	300	310	320
STGTRTCTVT	EGKHARLSCY	VTGEPKPETV	WKKDGQLVTE	GRRHVVYEDA	QENFVLKILF	CKQSDRGLYT	CTASNLVGQT
330	340	350	360	370	380	390	400
YSSVLVVVRE	PAVPFKKRLQ	DLEVREKESA	TFLCEVPQPS	TEAAWFKEET	RLWASAKYGI	EEEGTERRLT	VRNVSADDDA
410	420	430	440	450	460	470	480
VYICETPEGS	RTVAELAVQG	NLLRKLPRKT	AVRVGDTAMF	CVELAVPVGP	VHWLRNQEEV	VAGGRVAISA	EGTRHTLTIS
490	500	510	520	530	540	550	560
QCCLEDVGQV	AFMAGDCQTS	TQFCVSAPRK	PPLQPPVDPV	VKARMESSVI	LSWSPPPHGE	RPVTIDGYLV	EKKKLGTYTW
570	580	590	600	610	620	630	640
IRCHEAEWVA	TPELTVADVA	EEGNFQFRVS	ALNSFGQSPY	LEFPGTVHLA	PKLAVRTPLK	AVQAVEGGEV	TFSVDLTVAS
650	660	670	680	690	700	710	720
AGEWFLDGQA	LKASSVYEIH	CDRTRHTLTI	REVPASLHGA	QLKFVANGIE	SSIRMEVRAA	PGLTANKPPA	AAAREVLARL
730	740	750	760	770	780	790	800
HEEAQLLAEL	SDQAAAVTWL	KDGRTLSPGP	KYEVQASAGR	RVLLVRDVAR	DDAGLYECVS	RGGRIAYQLS	VQGLARFLHK
810	820	830	840	850	860	870	880
DMAGSCVDAV	AGGPAQFECE	TSEAHVHVHW	YKDGMELGHS	GERFLQEDVG	TRHRLVAATV	TRQDEGTYSC	RVGEDSVDFR
890	900	910	920	930	940	950	960
LRVSEPKVVF	AKEQLARRKL	QAEAGASATL	SCEVAQAQTE	VTWYKDGKKL	SSSSKVCMEA	TGCTRRLVVQ	QAGQADAGEY
970	980	990	1000	1010	1020	1030	1040
SCEAGGQRLS	FHLDVKEPKV	VFAKDQVAHS	EVQAEAGASA	TLSCEVAQAQ	TEVMWYKDGK	KLSSSLKVHV	EAKGCRRRLV
1050	1060	1070	1080	1090	1100	1110	1120
VQQAGKTDAG	DYSCEARGQR	VSFRLHITEP	KMMFAKEQSV	HNEVQAEAGA	SAMLSCEVAQ	AQTEVTWYKD	GKKLSSSSKV
1130	1140	1150	1160	1170	1180	1190	1200
GMEVKGCTRR	LVLPQAGKAD	AGEYSCEAGG	QRVSFHLHIT	EPKGVFAKEQ	SVHNEVQAEA	GTTAMLSCEV	AQPQTEVTWY
1210	1220	1230	1240	1250	1260	1270	1280
KDGKKLSSSS	KVRMEVKGCT	RRLVVQQVGK	ADAGEYSCEA	GGQRVSFQLH	ITEPKAVFAK	EQLVHNEVRT	EAGASATLSC
1290	1300	1310	1320	1330	1340	1350	1360
EVAQAQTEVT	WYKDGKKLSS	SSKVRIEAAG	CMRQLVVQQA	GQADAGEYTC	EAGGQRLSFH	LDVSEPKAVF	AKEQLAHRKV
1370	1380	1390	1400	1410	1420	1430	1440
QAEAGAIATL	SCEVAQAQTE	VTWYKDGKKL	SSSSKVRMEA	VGCTRRLVVQ	QACQADTGEY	SCEAGGQRLS	FSLDVAEPKV
1450	1460	1470	1480	1490	1500	1510	1520
VFAKEQPVHR	EVQAQAGAST	TLSCEVAQAQ	TEVMWYKDGK	KLSFSSKVRM	EAVGCTRRLV	VQQAGQAVAG	EYSCEAGSQR
1530	1540	1550	1560	1570	1580	1590	1600
LSFHLHVAEP	KAVFAKEQPA	SREVQAEAGT	SATLSCEVAQ	AQTEVTWYKD	GKKLSSSSKV	RMEAVGCTRR	LVVQEAGQAD
1610	1620	1630	1640	1650	1660	1670	1680
AGEYSCKAGD	QRLSFHLHVA	EPKVVFAKEQ	PAHREVQAEA	GASATLSCEV	AQAQTEVTWY	KDGKKLSSSS	KVRVEAVGCT
1690	1700	1710	1720	1730	1740	1750	1760
RRLVVQQAGQ	AEAGEYSCEA	GGQQLSFRLQ	VAELEPQISE	RPCRREPLVV	KEHEDIILTA	TLATPSAATV	TWLKDGVEIR
1770	1780	1790	1800	1810	1820	1830	1840
RSKRHETASQ	GDTHTLTVHG	AQVLDSAIYS	CRVGAEGQDF	PVQVEEVAAK	FCRLLEPVCG	ELGGTVTLAC	ELSPACAEVV
1850	1860	1870	1880	1890	1900	1910	1920
WRCGNTQLRV	GKRFQMVAEG	PVRSLTVLGL	RAEDAGEYVC	ESRDDHTSAQ	LTVSVPRVVK	FMSGLSTVVA	EEGGEATFQC
1930	1940	1950	1960	1970	1980	1990	2000
VVSPSDVAVV	WFRDGALLQP	SEKFAISQSG	ASHSLTISDL	VLEDAGQITV	EAEGASSSAA	LRVREAPVLF	KKKLEPQTVE
2010	2020	2030	2040	2050	2060	2070	2080
ERSSVTLEVE	LTRPWPELRW	TRNATALAPG	KNVEIHAEGA	RHRLVLHNVG	FADRGFFGCE	TPDDKTQAKL	TVEMRQVRLV
2090	2100	2110	2120	2130	2140	2150	2160
RGLQAVEARE	QGTATMEVQL	SHADVDGSWT	RDGLRFQQGP	TCHLAVRGPM	HTLTLSGLRP	EDSGLMVFKA	EGVHTSARLV
2170	2180	2190	2200	2210	2220	2230	2240
VTELPVSFSR	PLQDVVTTEK	EKVTLECELS	RPNVDVRWLK	DGVELRAGKT	MAIAAQGACR	SLTIYRCEFA	DQGVYVCDAH
2250	2260	2270	2280	2290	2300	2310	2320
DAQSSASVKV	QGRTYTLIYR	RVLAEDAGEI	QFVAENAESR	AQLRVKELPV	TLVRPLRDKI	AMEKHRGVLE	CQVSRASAQV
2330	2340	2350	2360	2370	2380	2390	2400

Detailed Protein Report

Cmpd.	No. of Cmpds.	m/z meas.	Δ m/z [ppm]	z	Rt [min]	Score	P	Range	Sequence	Modification	Ratios
1991	2	643.3110	-83.65	2	54.8	19.9	2	338-347	K.RLQDLEVREK.E		MD:MU 0.66
2661	1	686.8980	-30.78	2	63.3	13.1	2	881-892	R.LRVSEPKVVFAK.E		
2091	1	709.3658	-116.08	2	56.0	20.8	2	5981-5992	R.KYLLQARTAIIK.S		

Detailed Protein Report

Protein 54: fibrillin-1 precursor [Homo sapiens]

**Accession:**	gi|281485550	**Score:**	53.6
**Database:**	refseq_human(refseq_human_20140103.fasta)	**MW [kDa]:**	312.1
**Database Date:**	2015-11-30	**pI:**	4.7
**Modification(s):**	Carbamidomethyl, Oxidation	**Sequence Coverage [%]:**	1.7
		**No. of unique Peptides:**	3

Quantitation

MD:MU	**Median:** 2.40	**CV:** 0.00 %	**No. of Peptides:** 1

Detailed Protein Report

10	20	30	40	50	60	70	80
MRRGRLLEIA	LGFTVLLASY	TSHGADANLE	AGNVKETRAS	RAKRRGGGGH	DALKGPNVCG	SRYNAYCCPG	WKTLPGGNQC
90	100	110	120	130	140	150	160
IVPICRHSCG	DGFCSRPNMC	TCPSGQIAPS	CGSRSIQHCN	IRCMNGGSCS	DDHCLCQKGY	IGTHCGQPVC	ESGCLNGGRC
170	180	190	200	210	220	230	240
VAPNRCACTY	GFTGPQCERD	YRTGPCFTVI	SNQMCQGQLS	GIVCTKTLCC	ATVGRAWGHP	CEMCPAQPHP	CRRGFIPNIR
250	260	270	280	290	300	310	320
TGACQDVDEC	QAIPGLCQGG	NCINTVGSFE	CKCPAGHKLN	EVSQKCEDID	ECSTIPGICE	GGECTNTVSS	YFCKCPPGFY
330	340	350	360	370	380	390	400
TSPDGTRCID	VRPGYCYTAL	TNGRCSNQLP	QSITKMQCCC	DAGRCWSPGV	TVAPEMCPIR	ATEDFNKLCS	VPMVIPGRPE
410	420	430	440	450	460	470	480
YPPPPLGPIP	PVLPVPPGFP	PGPQIPVPRP	PVEYLYPSRE	PPRVLPVNVT	DYCQLVRYLC	QNGRCIPTPG	SYRCECNKGF
490	500	510	520	530	540	550	560
QLDLRGECID	VDECEKNPCA	GGECINNQGS	YTCQCRAGYQ	STLTRTECRD	IDECLQNGRI	CNNGRCINTD	GSFHCVCNAG
570	580	590	600	610	620	630	640
FHVTRDGKNC	EDMDECSIRN	MCLNGMCINE	DGSFKCICKP	GFQLASDGRY	CKDINECETP	GICMNGRCVN	TDGSYRCECF
650	660	670	680	690	700	710	720
PGLAVGLDGR	VCVDTHMRST	CYGGYKRGQC	IKPLFGAVTK	SECCCASTEY	AFGEPCQPCP	AQNSAEYQAL	CSSGPGMTSA
730	740	750	760	770	780	790	800
GSDINECALD	PDICPNGICE	NLRGTYKCIC	NSGYEVDSTG	KNCVDINECV	LNSLLCDNGQ	CRNTPGSFVC	TCPKGFIYKP
810	820	830	840	850	860	870	880
DLKTCEDIDE	CESSPCINGV	CKNSPGSFIC	ECSSESTLDP	TKTICIETIK	GTCWQTVIDG	RCEININGAT	LKSQCCSSLG
890	900	910	920	930	940	950	960
AAWGSPCTLC	QVDPICGKGY	SRIKGTQCED	IDECEVFPGV	CKNGLCVNTR	GSFKCQCPSG	MTLDATGRIC	LDIRLETCFL
970	980	990	1000	1010	1020	1030	1040
RYEDEECTLP	IAGRHRMDAC	CCSVGAAWGT	EECEECPMRN	TPEYEELCPR	GPGFATKEIT	NGKPFFKDIN	ECKMIPSLCT
1050	1060	1070	1080	1090	1100	1110	1120
HGKCRNTIGS	FKCRCDSGFA	LDSEERNCTD	IDECRISPDL	CGRGQCVNTP	GDFECKCDEG	YESGFMMMKN	CMDIDECQRD
1130	1140	1150	1160	1170	1180	1190	1200
PLLCRGGVCH	NTEGSYRCEC	PPGHQLSPNI	SACIDINECE	LSAHLCPNGR	CVNLIGKYQC	ACNPGYHSTP	DRLFCVDIDE
1210	1220	1230	1240	1250	1260	1270	1280
CSIMNGGCET	FCTNSEGSYE	CSCQPGFALM	PDQRSCTDID	ECEDNPNICD	GGQCTNIPGE	YRCLCYDGFM	ASEDMKTCVD
1290	1300	1310	1320	1330	1340	1350	1360
VNECDLNPNI	CLSGTCENTK	GSFICHCDMG	YSGKKGKTGC	TDINECEIGA	HNCGKHAVCT	NTAGSFKCSC	SPGWIGDGIK
1370	1380	1390	1400	1410	1420	1430	1440
CTDLDECSNG	THMCSQHADC	KNTMGSYRCL	CKEGYTGDGF	TCTDLDECSE	NLNLCGNGQC	LNAPGGYRCE	CDMGFVPSAD
1450	1460	1470	1480	1490	1500	1510	1520
GKACEDIDEC	SLPNICVFGT	CHNLPGLFRC	ECEIGYELDR	SGGNCTDVNE	CLDPTTCISG	NCVNTPGSYI	CDCPPDFELN
1530	1540	1550	1560	1570	1580	1590	1600
PTRVGCVDTR	SGNCYLDIRP	RGDNGDTACS	NEIGVGVSKA	SCCCSLGKAW	GTPCEMCPAV	NTSEYKILCP	GGEGFRPNPI
1610	1620	1630	1640	1650	1660	1670	1680
TVILEDIDEC	QELPGLCQGG	KCINTFGSFQ	CRCPTGYYLN	EDTRVCDDVN	ECETPGICGP	GTCYNTVGNY	TCICPPDYMQ
1690	1700	1710	1720	1730	1740	1750	1760
VNGGNNCMDM	RRSLCYRNYY	ADNQTCDGEL	LFNMTKKMCC	CSYNIGRAWN	KPCEQCPIPS	TDEFATLCGS	QRPGFVIDIY
1770	1780	1790	1800	1810	1820	1830	1840
TGLPVDIDEC	REIPGVCENG	VCINMVGSFR	CECPVGFFYN	DKLLVCEDID	ECQNGPVCQR	NAECINTAGS	YRCDCKPGYR
1850	1860	1870	1880	1890	1900	1910	1920
FTSTGQCNDR	NECQEIPNIC	SHGQCIDTVG	SFYCLCHTGF	KTNDDQTMCL	DINECERDAC	GNGTCRNTIG	SFNCRCNHGF
1930	1940	1950	1960	1970	1980	1990	2000
ILSHNNDCID	VDECASGNGN	LCRNGQCINT	VGSFQCQCNE	GYEVAPDGRT	CVDINECLLE	PRKCAPGTCQ	NLDGSYRCIC
2010	2020	2030	2040	2050	2060	2070	2080
PPGYSLQNEK	CEDIDECVEE	PEICALGTCS	NTEGSFKCLC	PEGFSLSSSG	RRCQDLRMSY	CYAKFEGGKC	SSPKSRNHSK
2090	2100	2110	2120	2130	2140	2150	2160
QECCCALKGE	GWGDPCELCP	TEPDEAFRQI	CPYGSGIIVG	PDDSAVDMDE	CKEPDVCKHG	QCINTDGSYR	CECPFGYILA
2170	2180	2190	2200	2210	2220	2230	2240

Detailed Protein Report

Cmpd.	No. of Cmpds.	m/z meas.	Δ m/z [ppm]	z	Rt [min]	Score	P	Range	Sequence	Modification	Ratios
1152	1	991.7003	-40.24	3	44.0	14.6	1	356-380	K. MQCCCDAGRCWSPGVTVAPEMQ A	Carbamidomethyl: 3, 4, 5, 22; Oxidation: 1, 21	
821	5	635.1373	-218.44	2	39.8	23.3	1	2482-2492	R.SCKDLDECATK.Q	Carbamidomethyl: 8	MD:MU 2.40
2864	1	703.3686	82.89	2	66.3	15.8	0	2681-2694	R.IGQGHCVSGMGMGR.G	Oxidation: 12	

Detailed Protein Report

Protein 55: PREDICTED: uncharacterized protein LOC285556 isoform X2 [Homo sapiens]

**Accession:**	gi|578843586	**Score:**	53.6
**Database:**	refseq_human(refseq_human_20140103.fasta)	**MW [kDa]:**	190.0
**Database Date:**	2015-11-30	**pI:**	9.8
**Modification(s):**	Carbamidomethyl	**Sequence Coverage [%]:**	2.6
		**No. of unique Peptides:**	3

Alias proteins:

Accession	Name	Description
gi|578843588	refseq_human (refseq_human_20140103.fasta)	PREDICTED: uncharacterized protein LOC285556 isoform X3 [Homo sapiens]

Detailed Protein Report

10	20	30	40	50	60	70	80
MLSFHFWKSR	GQPTDAASSV	ADGIQTPRCC	RQCQANNWTG	QLSYRTLATV	SAGAAAPQPQ	TTSTASSRSL	PTSLRLAAAP
90	100	110	120	130	140	150	160
PQGLKNWEVV	AAVAAVPTAL	GPVQIRGTLL	RATLQPLRGQ	RRTQDFPSDH	HCLFLSLKPG	QGLIMEAAPP	ELNSKARQAE
170	180	190	200	210	220	230	240
VGDGVSSAQD	SQELKQQLWP	LPKPSASSQR	EAKYVDMCAS	AEVQRESPQT	MKLTLGHCPG	GQRASRSPKE	KAQDEPSSKT
250	260	270	280	290	300	310	320
PSPQNNPASS	QLSRSQHSAS	EEGGNFSSSS	SSSPMNKAEE	DGLSKMEDST	TSTGALATSS	SSLGFESESG	ESEGCQAVGG
330	340	350	360	370	380	390	400
EGEKISGGRG	GGKGGGGGGA	GDGTECRDII	AKSQGSRDPP	KVEEAHYITT	HEIQLSEVEQ	DMDFDVGLAS	RWDFEDNNVI
410	420	430	440	450	460	470	480
YSFVDYASFG	GSDETPGDIT	SLTEEDDDNS	CYLSTTPSTN	TTRTPSPTSS	DLARPNAGRS	GRDTSSTEVG	SGPSDSGPTP
490	500	510	520	530	540	550	560
PPTGPGTAPL	TEPLPETPEA	ASGAAAAAAS	SCGSAASQIL	LSIKPASRAI	NEPSNVRAKQ	NIIYAAKHEG	DMSLRVSTAA
570	580	590	600	610	620	630	640
EHNSSSLKQN	PAAAVAQDHA	KKFIAVPARL	QTRCGAIRAK	ELVDYSSGAS	SAVSELDDAD	KEVRNLTSRA	FRSLAYPYFE
650	660	670	680	690	700	710	720
ALNISSRESS	TTLSEVGFGR	WSTFLDLKCG	GVGARVEQSL	LRSSAASVAA	GLRKGSGARA	TADQLYIQSK	KSQTKALEFV
730	740	750	760	770	780	790	800
VSKVEGEIKH	VETPLCFQKQ	VQTGSRVVTL	LEPLNVRSES	KASSAPGPGR	ATKGPGKGPG	SAYTDDGSET	SEGSKPTSRA
810	820	830	840	850	860	870	880
DGPQKSKFAS	SLLKNVISKK	MQREHEFKME	RGEVMDTSHH	LSGTSKETEG	ARGSERQRER	GLQRQSSRHS	EAGSEYTVVS
890	900	910	920	930	940	950	960
MSDAGGEGSV	AGSKSPVFKA	STPRERNAGP	GRNFTDGHTE	VCEIKKSASE	TVKGIFLRSQ	NSAFRSWKEK	EAEKREEQAP
970	980	990	1000	1010	1020	1030	1040
IGKLKLPKGG	DWRADLGEIS	ASKNTIMSRL	FVPNIQQTPK	DKQPRKQATK	YPAAQATSTA	VIRPKAPEIK	IRLGSVQQPS
1050	1060	1070	1080	1090	1100	1110	1120
SDFNIAKLLT	PKLAGGSASN	LFKTIEDNSR	AQQKLFRGDN	LEKVPHFQVR	DIRDKSKAQG	PLHQVRDVRK	LIKGSGDSSD
1130	1140	1150	1160	1170	1180	1190	1200
KGSVTPEQGL	TGPKPRQLSA	AAGGSGSLSP	MVITCQAVVN	QREDSMDREP	RESMGKGGGS	RVLNSSSPEG	TVLVHRASGR
1210	1220	1230	1240	1250	1260	1270	1280
LPVATIAPNK	PEQGSYLPVL	KIVSKASTQK	TPEKLKEEEV	KEEGKATKPA	RNALEKLTAA	VRSMEELYSF	NRNEWKRKSD
1290	1300	1310	1320	1330	1340	1350	1360
PLPMMMDSHV	LSLIASEERE	GVVVADGDHD	KLSKRLGEVE	ERGTGNKAGV	VLRGAPIERL	QRRNSNPSAE	SVSARAAAFE
1370	1380	1390	1400	1410	1420	1430	1440
NLARERPRSL	YIPPVHKDVE	RTQPLQPLPP	LPSNRNVFTV	SASSIQKTGG	VAGKFPQGPS	PESPSAAKGI	KSQGLRSLKI
1450	1460	1470	1480	1490	1500	1510	1520
SPATRAPPDE	VTNRKSGSNL	EKSNSDCENY	LTIPLKGSSA	AGELLSRPGA	SREGPPNSSA	ATLCSLPPLS	ARSQVPSSSK
1530	1540	1550	1560	1570	1580	1590	1600
GSQVSGTSRP	AWRTKPDNPR	ETVAAPPGPQ	SPEHPPTTIY	HQPPLPFTLQ	GAQPQVLCFS	PPSMPAPAPA	ASAPVPTDPF
1610	1620	1630	1640	1650	1660	1670	1680
QQAQPQQTQR	KMLLDVTTGQ	YYLVDTPVQP	MTRRLFDPET	GQYVDVPMTS	QQQAVAPMSI	SVPPLALSPG	AYGPTYMIYP
1690	1700	1710	1720	1730	1740	1750	1760
GFLPTVLPTN	ALQPTPIARA	PRGSELSPLV	AEPSSKEAAA	TFTEAPYFMA	SGQSPASSTS	SAPAATSQLL	GAKAFAQLHG
1770	1780	1790	1800				
KPVISITSQP	LGPRIIAPPS	FDGTTMSFVV	EHR				

Cmpd.	No. of Cmpds.	m/z meas.	Δ m/z [ppm]	z	Rt [min]	Score	P	Range	Sequence	Modification
886	1	575.7576	46.75	2	40.6	12.5	0	334-347	K.GGGGGGAGDGTECR.D	
1275	1	874.2865	-135.51	2	45.6	10.1	1	334-352	K.GGGGGGAGDGTECRDIIAK.S	Carbamidomethyl: 13
1984	1	634.7974	-113.89	2	54.7	11.7	2	955-965	K.REEQAPIGKLK.L	

Detailed Protein Report

Protein 56: dystonin isoform 1eA precursor [Homo sapiens]

**Accession:**	gi|34577049	**Score:**	51.1
**Database:**	refseq_human(refseq_human_20140103.fasta)	**MW [kDa]:**	590.7
**Database Date:**	2015-11-30	**pI:**	5.4
		**Sequence Coverage [%]:**	0.9
		**No. of unique Peptides:**	3

Quantitation

MD:MU	**Median:** 1.56	**CV:** 11.67 %	**No. of Peptides:** 2

BRUKER

Detailed Protein Report

10	20	30	40	50	60	70	80
MHSSSYSYRS	SDSVFSNTTS	TRTSLDSNEN	LLLVHCGPTL	INSCISFGSE	SFDGHRLEML	QQIANRVQRD	SVICEDKLIL
90	100	110	120	130	140	150	160
AGNALQSDSK	RLESGVQFQN	EAEIAGYILE	CENLLRQHVI	DVQILIDGKY	YQADQLVQRV	AKLRDEIMAL	RNECSSVYSK
170	180	190	200	210	220	230	240
GRILTTEQTK	LMISGITQSL	NSGFAQTLHP	SLTSGLTQSL	TPSLTSSSMT	SGLSSGMTSR	LTPSVTPAYT	PGFPSGLVPN
250	260	270	280	290	300	310	320
FSSGVEPNSL	QTLKLMQIRK	PLLKSSLLDQ	NLTEEEINMK	FVQDLLNWVD	EMQVQLDRTE	WGSDLPSVES	HLENHKNVHR
330	340	350	360	370	380	390	400
AIEEFESSLK	EAKISEIQMT	APLKLTYAEK	LHRLESQYAK	LLNTSRNQER	HLDTLHNFVS	RATNELIWLN	EKEEEEVAYD
410	420	430	440	450	460	470	480
WSERNTNIAR	KKDYHAELMR	ELDQKEENIK	SVQEIAEQLL	LENHPARLTI	EAYRAAMQTQ	WSWILQLCQC	VEQHIKENTA
490	500	510	520	530	540	550	560
YFEFFNDAKE	ATDYLRNLKD	AIQRKYSCDR	SSSIHKLEDL	VQESMEEKEE	LLQYKSTIAN	LMGKAKTIIQ	LKPRNSDCPL
570	580	590	600	610	620	630	640
KTSIPIKAIC	DYRQIEITIY	KDDECVLANN	SHRAKWKVIS	PTGNEAMVPS	VCFTVPPPNK	EAVDLANRIE	QQYQNVLTLW
650	660	670	680	690	700	710	720
HESHINMKSV	VSWHYLINEI	DRIRASNVAS	IKTMLPGEHQ	QVLSNLQSRF	EDFLEDSQES	QVFSGSDITQ	LEKEVNVCKQ
730	740	750	760	770	780	790	800
YYQELLKSAE	REEQEESVYN	LYISEVRNIR	LRLENCEDRL	IRQIRTPLER	DDLHESVFRI	TEQEKLKKEL	ERLKDDLGTI
810	820	830	840	850	860	870	880
TNKCEEFFSQ	AAASSSVPTL	RSELNVVLQN	MNQVYSMSST	YIDKLKTVNL	VLKNTQAAEA	LVKLYETKLC	EEEAVIADKN
890	900	910	920	930	940	950	960
NIENLISTLK	QWRSEVDEKR	QVFHALEDEL	QKAKAISDEM	FKTYKERDLD	FDWHKEKADQ	LVERWQNVHV	QIDNRLRDLE
970	980	990	1000	1010	1020	1030	1040
GIGKSLKYYR	DTYHPLDDWI	QQVETTQRKI	QENQPENSKT	LATQLNQQKM	LVSEIEMKQS	KMDECQKYAE	QYSATVKDYE
1050	1060	1070	1080	1090	1100	1110	1120
LQTMTYRAMV	DSQQKSPVKR	RRMQSSADLI	IQEFMDLRTR	YTALVTLMTQ	YIKFAGDSLK	RLEEEEKSLE	EEKKEHVEKA
1130	1140	1150	1160	1170	1180	1190	1200
KELQKWVSNI	SKTLKDAEKA	GKPPFSKQKI	SSEEISTKKE	QLSEALQTIQ	LFLAKHGDKM	TDEERNELEK	QVKTLQESYN
1210	1220	1230	1240	1250	1260	1270	1280
LLFSESLKQL	QESQTSGDVK	VEEKIVAERQ	QEYKEKLQGI	CDLLTQTENR	LIGHQEAFMI	GDGTVELKKY	QSKQEELQKD
1290	1300	1310	1320	1330	1340	1350	1360
MQGSAQALAE	VVKNTENFLK	ENGEKLSQED	KALIEQKLNE	AKIKCEQLNL	KAEQSKKELD	KVVTTAIKEE	TEKVAAVKQL
1370	1380	1390	1400	1410	1420	1430	1440
EESKTKIENL	LDWLSNVDKD	SERAGTKHKQ	VIEQNGTHFQ	EGDGKSAIGE	EDEVNGNLLE	TDVDGQVGTT	QENLNQQYQK
1450	1460	1470	1480	1490	1500	1510	1520
VKAQHEKIIS	QHQAVIIATQ	SAQVLLEKQG	QYLSPEEKEK	LQKNMKELKV	HYETALAESE	KKMKLTHSLQ	EELEKFDADY
1530	1540	1550	1560	1570	1580	1590	1600
TEFEHWLQQS	EQELENLEAG	ADDINGLMTK	LKRQKSFSED	VISHKGDLRY	ITISGNRVLE	AAKSCSKRDG	GKVDTSATHR
1610	1620	1630	1640	1650	1660	1670	1680
EVQRKLDHAT	DRFRSLYSKC	NVLGNNLKDL	VDKYQHYEDA	SCGLLAGLQA	CEATASKHLS	EPIAVDPKNL	QRQLEETKAL
1690	1700	1710	1720	1730	1740	1750	1760
QGQISSQQVA	VEKLKKTAEV	LLDARGSLLP	AKNDIQKTLD	DIVGRYEDLS	KSVNERNEKL	QITLTRSLSV	QDGLDEMLDW
1770	1780	1790	1800	1810	1820	1830	1840
MGNVESSLKE	QGQVPLNSTA	LQDIISKNIM	LEQDIAGRQS	SINAMNEKVK	KFMETTDPST	ASSLQAKMKD	LSARFSEASH
1850	1860	1870	1880	1890	1900	1910	1920
KHKETLAKME	ELKTKVELFE	NLSEKLQTFL	ETKTQALTEV	DVPGKDVTEL	SQYMQESTSE	FLEHKKHLEV	LHSLLKEISS
1930	1940	1950	1960	1970	1980	1990	2000
HGLPSDKALV	LEKTNNLSKK	FKEMEDTIKE	KKEAVTSCQE	QLDAFQVLVK	SLKSWIKETT	KKVPIVQPSF	GAEDLGKSLE
2010	2020	2030	2040	2050	2060	2070	2080
DTKKLQEKWS	LKTPEIQKVN	NSGISLCNLI	SAVTTPAKAI	AAVKSGGAVL	NGEGTATNTE	EFWANKGLTS	IKKDMTDISH
2090	2100	2110	2120	2130	2140	2150	2160
GYEDLGLLLK	DKIAELNTKL	SKLQKAQEES	SAMMQWLQKM	NKTATKWQQT	PAPTDTEAVK	TQVEQNKSFE	AELKQNVNKV
2170	2180	2190	2200	2210	2220	2230	2240

Detailed Protein Report

Cmpd.	No. of Cmpds.	m/z meas.	Δ m/z [ppm]	z	Rt [min]	Score	P	Range	Sequence	Modification	Ratios
585	1	558.7340	-142.02	2	36.6	17.2	1	143-151	K.LRDEIMALR.N		MD:MU 1.75
2450	1	684.2042	-212.00	2	61.0	10.9	1	2455-2466	K.EEQNKSHPISAK.L		
1580	3	781.3390	-110.38	2	49.5	12.4	2	4358-4371	R.SLKEKTSLADDNLK.L		MD:MU 1.39

Detailed Protein Report

Protein 57: coagulation factor VIII isoform a precursor [Homo sapiens]

**Accession:**	gi|4503647	**Score:**	51.0
**Database:**	refseq_human(refseq_human_20140103.fasta)	**MW [kDa]:**	266.8
**Database Date:**	2015-11-30	**pI:**	7.0
**Modification(s):**	Oxidation	**Sequence Coverage [%]:**	3.2
		**No. of unique Peptides:**	4

Quantitation

MD:MU	**Median:**	1.95	**CV:**	0.00 %	**No. of Peptides:**	1	

Detailed Protein Report

10	20	30	40	50	60	70	80
MQIELSTCFF	LCLLRFCFSA	TRRYYLGAVE	LSWDYMQSDL	GELPVDARFP	PRVPKSFPFN	TSVVYKKTLF	VEFTDHLFNI
90	100	110	120	130	140	150	160
AKPRPPWMGL	LGPTIQAEVY	DTVVITLKNM	ASHPVSLHAV	GVSYWKASEG	AEYDDQTSQR	EKEDDKVFPG	GSHTYVWQVL
170	180	190	200	210	220	230	240
KENGPMASDP	LCLTYSYLSH	VDLVKDLNSG	LIGALLVCRE	GSLAKEKTQT	LHKFILLFAV	FDEGKSWHSE	TKNSLMQDRD
250	260	270	280	290	300	310	320
AASARAWPKM	HTVNGYVNRS	LPGLIGCHRK	SVYWHVIGMG	TTPEVHSIFL	EGHTFLVRNH	RQASLEISPI	TFLTAQTLLM
330	340	350	360	370	380	390	400
DLGQFLLFCH	ISSHQHDGME	AYVKVDSCPE	EPQLRMKNNE	EAEDYDDDLT	DSEMDVVRFD	DDNSPSFIQI	RSVAKKHPKT
410	420	430	440	450	460	470	480
WVHYIAAEEE	DWDYAPLVLA	PDDRSYKSQY	LNNGPQRIGR	KYKKVRFMAY	TDETFKTREA	IQHESGILGP	LLYGEVGDTL
490	500	510	520	530	540	550	560
LIIFKNQASR	PYNIYPHGIT	DVRPLYSRRL	PKGVKHLKDF	PILPGEIFKY	KWTVTVEDGP	TKSDPRCLTR	YYSSFVNMER
570	580	590	600	610	620	630	640
DLASGLIGPL	LICYKESVDQ	RGNQIMSDKR	NVILFSVFDE	NRSWYLTENI	QRFLPNPAGV	QLEDPEFQAS	NIMHSINGYV
650	660	670	680	690	700	710	720
FDSLQLSVCL	HEVAYWYILS	IGAQTDFLSV	FFSGYTFKHK	MVYEDTLTLF	PFSGETVFMS	MENPGLWILG	CHNSDFRNRG
730	740	750	760	770	780	790	800
MTALLKVSSC	DKNTGDYYED	SYEDISAYLL	SKNNAIEPRS	FSQNSRHPST	RQKQFNATTI	PENDIEKTDP	WFAHRTPMPK
810	820	830	840	850	860	870	880
IQNVSSSDLL	MLLRQSPTPH	GLSLSDLQEA	KYETFSDDPS	PGAIDSNNSL	SEMTHFRPQL	HHSGDMVFTP	ESGLQLRLNE
890	900	910	920	930	940	950	960
KLGTTAATEL	KKLDFKVSST	SNNLISTIPS	DNLAAGTDNT	SSLGPPSMPV	HYDSQLDTTL	FGKKSSPLTE	SGGPLSLSEE
970	980	990	1000	1010	1020	1030	1040
NNDSKLLESG	LMNSQESSWG	KNVSSTESGR	LFKGKRAHGP	ALLTKDNALF	KVSISLLKTN	KTSNNSATNR	KTHIDGPSLL
1050	1060	1070	1080	1090	1100	1110	1120
IENSPSVWQN	ILESDTEFKK	VTPLIHDRML	MDKNATALRL	NHMSNKTTSS	KNMEMVQQKK	EGPIPPDAQN	PDMSFFKMLF
1130	1140	1150	1160	1170	1180	1190	1200
LPESARWIQR	THGKNSLNSG	QGPSPKQLVS	LGPEKSVEGQ	NFLSEKNKVV	VGKGEFTKDV	GLKEMVFPSS	RNLFLTNLDN
1210	1220	1230	1240	1250	1260	1270	1280
LHENNTHNQE	KKIQEEIEKK	ETLIQENVVL	PQIHTVTGTK	NFMKNLFLLS	TRQNVEGSYD	GAYAPVLQDF	RSLNDSTNRT
1290	1300	1310	1320	1330	1340	1350	1360
KKHTAHFSKK	GEEENLEGLG	NQTKQIVEKY	ACTTRISPNT	SQQNFVTQRS	KRALKQFRLP	LEETELEKRI	IVDDTSTQWS
1370	1380	1390	1400	1410	1420	1430	1440
KNMKHLTPST	LTQIDYNEKE	KGAITQSPLS	DCLTRSHSIP	QANRSPLPIA	KVSSFPSIRP	IYLTRVLFQD	NSSHLPAASY
1450	1460	1470	1480	1490	1500	1510	1520
RKKDSGVQES	SHFLQGAKKN	NLSLAILTLE	MTGDQREVGS	LGTSATNSVT	YKKVENTVLP	KPDLPKTSGK	VELLPKVHIY
1530	1540	1550	1560	1570	1580	1590	1600
QKDLFPTETS	NGSPGHLDLV	EGSLLQGTEG	AIKWNEANRP	GKVPFLRVAT	ESSAKTPSKL	LDPLAWDNHY	GTQIPKEEWK
1610	1620	1630	1640	1650	1660	1670	1680
SQEKSPEKTA	FKKKDTILSL	NACESNHAIA	AINEGQNKPE	IEVTWAKQGR	TERLCSQNPP	VLKRHQREIT	RTTLQSDQEE
1690	1700	1710	1720	1730	1740	1750	1760
IDYDDTISVE	MKKEDFDIYD	EDENQSPRSF	QKKTRHYFIA	AVERLWDYGM	SSSPHVLRNR	AQSGSVPQFK	KVVFQEFTDG
1770	1780	1790	1800	1810	1820	1830	1840
SFTQPLYRGE	LNEHLGLLGP	YIRAEVEDNI	MVTFRNQASR	PYSFYSSLIS	YEEDQRQGAE	PRKNFVKPNE	TKTYFWKVQH
1850	1860	1870	1880	1890	1900	1910	1920
HMAPTKDEFD	CKAWAYFSDV	DLEKDVHSGL	IGPLLVCHTN	TLNPAHGRQV	TVQEFALFFT	IFDETKSWYF	TENMERNCRA
1930	1940	1950	1960	1970	1980	1990	2000
PCNIQMEDPT	FKENYRFHAI	NGYIMDTLPG	LVMAQDQRIR	WYLLSMGSNE	NIHSIHFSGH	VFTVRKKEEY	KMALYNLYPG
2010	2020	2030	2040	2050	2060	2070	2080
VFETVEMLPS	KAGIWRVECL	IGEHLHAGMS	TLFLVYSNKC	QTPLGMASGH	IRDFQITASG	QYGQWAPKLA	RLHYSGSINA
2090	2100	2110	2120	2130	2140	2150	2160
WSTKEPFSWI	KVDLLAPMII	HGIKTQGARQ	KFSSLYISQF	IIMYSLDGKK	WQTYRGNSTG	TLMVFFGNVD	SSGIKHNIFN
2170	2180	2190	2200	2210	2220	2230	2240

Detailed Protein Report

Cmpd.	No. of Cmpds.	m/z meas.	Δ m/z [ppm]	z	Rt [min]	Score	P	Range	Sequence	Modification	Ratios
2041	1	856.8157	-171.77	2	55.6	11.8	2	1060-1073	K.KVTPLIHDRMLMDK.N	Oxidation: 12	
2156	1	953.3232	-120.41	2	57.2	11.3	0	1101-1117	K.EGPIPPDAQNPDMSFFK.M	Oxidation: 13	
2184	1	1061.5230	-9.68	3	57.5	13.4	0	1523-1553	K. DLFPTETSNGSPGHLDLVEGSLL[illegible] W		
2720	1	710.3296	-131.82	2	65.3	14.5	0	2092-2104	K.VDLLAPMIIHGIK.T		MD:MU 1.95

Detailed Protein Report

Protein 58: PREDICTED: uncharacterized protein KIAA1551 isoform X1 [Homo sapiens]

**Accession:**	gi|530399223	**Score:**	51.0
**Database:**	refseq_human(refseq_human_20140103.fasta)	**MW [kDa]:**	194.7
**Database Date:**	2015-11-30	**pI:**	9.7
**Modification(s):**	Carbamidomethyl, Oxidation	**Sequence Coverage [%]:**	2.1
		**No. of unique Peptides:**	3

Quantitation

MD:MU	**Median:** 0.59	**CV:** 0.00 %	**No. of Peptides:** 1

10	20	30	40	50	60	70	80
MNWNEKPKSA	TLPPLYPKSQ	PPFLHQSLIN	QITTTSQSSF	SYPGSNQEAC	MYPGNSNPIS	QPLLNIQNYP	QQISVSDMHN
**90**	**100**	**110**	**120**	**130**	**140**	**150**	**160**
GTVVASHTSV	ERITYANVNG	PKQLTHNLQM	SSGVTQNVWL	NSPMRNPVHS	HIGATVSHQT	DFGANVPNMP	ALQSQLITSD
**170**	**180**	**190**	**200**	**210**	**220**	**230**	**240**
TYSMQMQMIP	SNSTRLPVAY	QGNQGLNQSF	SEQQVDWTQQ	CISKGLTYPD	YRPPPKLYRY	SPQSFLPDST	IQKQNFIPHT
**250**	**260**	**270**	**280**	**290**	**300**	**310**	**320**
SLQVKNSQLL	NSVLTLPSRQ	TSAVPSQQYA	TQTDKRPPPP	PYNCRYGSQP	LQSTQHITKH	LSMEVPQSRE	MLSSEIRTSF
**330**	**340**	**350**	**360**	**370**	**380**	**390**	**400**
QQQWQNPNEN	VSTIGNFTNL	KVNTNSKQPF	NSPIRSSVDG	VQTLAQTNEE	KIMDSCNPTS	NQVLDTSVAK	EKLVRDIKTL
**410**	**420**	**430**	**440**	**450**	**460**	**470**	**480**
VEIKQKFSEL	ARKIKINKDL	LMAAGCIKMT	NTSYSEPAQN	SKLSLKQTAK	IQSGPQITPV	MPENAERQTP	TVVESAETNK
**490**	**500**	**510**	**520**	**530**	**540**	**550**	**560**
TQCMLNSDIQ	EVNCRRFNQV	DSVLPNPVYS	EKRPMPDSSH	DVKVLTSKTS	AVEMTQAVLN	TQLSSENVTK	VEQNSPAVCE
**570**	**580**	**590**	**600**	**610**	**620**	**630**	**640**
TISVPKSMST	EEYKSKIQNE	NMLLLALLSQ	ARKTQKTVLK	DANQTIQDSK	PDSCEMNPNT	QMTGNQLNLK	NMETPSTSNV
**650**	**660**	**670**	**680**	**690**	**700**	**710**	**720**
SGRVLDNSFC	SGQESSTKGM	PAKSDSSCSM	EVLATCLSLW	KKQPSDTAKE	KECDKLRTNT	TAVGISKPAN	IHVKSPCSVV
**730**	**740**	**750**	**760**	**770**	**780**	**790**	**800**
GNSNSQNKIS	NPSQQTALSM	VMHNYESSGI	NITKGTELQI	AVVSPLVLSE	VKTLSVKGIT	PAVLPETVYP	VIKEGSVCSL
**810**	**820**	**830**	**840**	**850**	**860**	**870**	**880**
QNQLAENAKA	TAALKVDVSG	PVASTATSTK	IFPLTQKEKQ	NESTNGNSEV	TPNVNQGKHN	KLESAIHSPM	NDQQISQESR
**890**	**900**	**910**	**920**	**930**	**940**	**950**	**960**
NSTVVSSDTL	QIDNICSLVE	GDTSYNSQIA	KIFSSLPLKM	VEPQKPSLPN	QQGIGSREPE	KQLDNTTENK	DFGFQKDKPV
**970**	**980**	**990**	**1000**	**1010**	**1020**	**1030**	**1040**
QCTDVSHKIC	DQSKSEPPLE	SSFNNLETNR	VILEKSSLEH	ATEKSTANDT	CSSAAIQEDI	YPQEIDASSN	YTPQDPARNE
**1050**	**1060**	**1070**	**1080**	**1090**	**1100**	**1110**	**1120**
IHSDKAPVLY	LHDQLSELLK	EFPYGIEAVN	TREGSVGQQT	TYQTSEDQTA	DKTSSDSKDP	ADQIQITILS	SEQMKEIFPE
**1130**	**1140**	**1150**	**1160**	**1170**	**1180**	**1190**	**1200**
QDDQPYVVDK	LAEPQKEEPI	TEVVSQCDLQ	APAAGQSRDS	VILDSEKDDI	HCCALGWLSM	VYEGVPQCQC	NSIKNSSSEE
**1210**	**1220**	**1230**	**1240**	**1250**	**1260**	**1270**	**1280**
EKQKEQCSPL	DTNSCKQGER	TSDRDVTVVQ	FKSLVNNPKT	PPDGKSHFPE	LQDDSRKDTP	KTKHKSLPRT	EQELVAGQFS
**1290**	**1300**	**1310**	**1320**	**1330**	**1340**	**1350**	**1360**
SKCDKLNPLQ	NHKRKKLRFH	EVTFHSSNKM	TASYEQASQE	TRQKKHVTQN	SRPLKTKTAF	LPNKDVYKKH	SSLGQSLSPE
**1370**	**1380**	**1390**	**1400**	**1410**	**1420**	**1430**	**1440**
KIKLKLKSVS	FKQKRKLDQG	NVLDMEVKKK	KHDKQEQKGS	VGATFKLGDS	LSNPNERAIV	KEKMVSNTKS	VDTKASSSKF
**1450**	**1460**	**1470**	**1480**	**1490**	**1500**	**1510**	**1520**
SRILTPKEYL	QRQKHKEALS	NKASKKICVK	NVPCDSEHMR	PSKLAVQVES	CGKSNEKHSS	GVQTSKESLN	GLTSHGKNLK
**1530**	**1540**	**1550**	**1560**	**1570**	**1580**	**1590**	**1600**
IHHSQESKTY	NILRNVKEKV	GGKQPDKIWI	DKTKLDKLTN	ISNEAQFSQM	PPQVKDQKKL	YLNRVGFKCT	ERESISLTKL
**1610**	**1620**	**1630**	**1640**	**1650**	**1660**	**1670**	**1680**
ESSPRKLHKD	KRQENKHKTF	LPVKGNTEKS	NMLEFKLCPD	ILLKNTNSVE	ERKDVKPHPR	KEQAPLQVSG	IKSTKEDWLK
**1690**	**1700**	**1710**	**1720**	**1730**	**1740**	**1750**	
FVATKKRTQK	DSQERDNVNS	RLSKRSFSAD	GFEMLQNPVK	DSKEMFQTYK	QMYLEKRSRS	LGSSPVK	

Detailed Protein Report

Cmpd.	No. of Cmpds.	m/z meas.	Δ m/z [ppm]	z	Rt [min]	Score	P	Range	Sequence	Modification	Ratios
2628	1	644.4984	138.82	1	63.6	19.8	1	391-395	K.EKLVR.D		MD:MU 0.59
2689	1	731.8632	-38.63	2	64.8	14.6	1	416-428	K.INKDLLMAAGCIK.M	Carbamidomethyl: 11; Oxidation: 7	
2006	1	1044.4022	-89.89	2	55.0	16.6	1	957-974	K.DKPVQCTDVSHKICDQSK.S	Carbamidomethyl: 6	

Detailed Protein Report

Protein 59: PREDICTED: Golgin subfamily A member 4 isoform X7 [Homo sapiens]

**Accession:**	gi|530372330	**Score:**	50.7
**Database:**	refseq_human(refseq_human_20140103.fasta)	**MW [kDa]:**	259.0
**Database Date:**	2015-11-30	**pI:**	5.2
**Modification(s):**	Oxidation	**Sequence Coverage [%]:**	2.4
		**No. of unique Peptides:**	4

10	20	30	40	50	60	70	80
MFKKLKQKIS	EEQQQLQQAL	APAQASSNSS	TPTRMRSRTS	SFTEQLDEGT	PNRESGDTQS	FAQKLQLRVP	SVESLFRSPI
90	100	110	120	130	140	150	160
KESLFRSSSK	ESLVRTSSRE	SLNRLDLDSS	TASFDPPSDM	DSEAEDLVGN	SDSLNKEQLI	QRLRRMERSL	SSYRGKYSEG
170	180	190	200	210	220	230	240
ILSQSQDKSL	RRIAELREEL	QMDQQAKKHL	QEEFDASLEE	KDQYISVLQT	QVSLLKQRLR	NGPMNVDVLK	PLPQLEPQAE
250	260	270	280	290	300	310	320
VFTKEENPES	DGEPVVEDGT	SVKTLETLQQ	RVKRQENLLK	RCKETIQSHK	EQCTLLTSEK	EALQEQLDER	LQELEKIKDL
330	340	350	360	370	380	390	400
HMAEKTKLIT	QLRDAKNLIE	QLEQDKGMVI	AETKRQMHET	LEMKEEEIAQ	LRSRIKQMTT	QGEELREQKE	KSERAAFEEL
410	420	430	440	450	460	470	480
EKALSTAQKT	EEARRKLKAE	MDEQIKTIEK	TSEEERISLQ	QELSRVKQEV	VDVMKKSSEE	QIAKLQKLHE	KELARKEQEL
490	500	510	520	530	540	550	560
TKKLQTRERE	FQEQMKVALE	KSQSEYLKIS	QEKEQQESLA	LEELELQKKA	ILTESENKLR	DLQQEAETYR	TRILELESSL
570	580	590	600	610	620	630	640
EKSLQENKNQ	SKDLAVHLEA	EKNKHNKEIT	VMVEKHKTEL	ESLKHQQDAL	WTEKLQVLKQ	QYQTEMEKLR	EKCEQEKETL
650	660	670	680	690	700	710	720
LKDKEIIFQA	HIEEMNEKTL	EKLDVKQTEL	ESLSSELSEV	LKARHKLEEE	LSVLKDQTDK	MKQELEAKMD	EQKNHHQQQV
730	740	750	760	770	780	790	800
DSIIKEHEVS	IQRTEKALKD	QINQLELLLK	ERDKHLKEHQ	AHVENLEADI	KRSEGELQQA	SAKLDVFQSY	QSATHEQTKA
810	820	830	840	850	860	870	880
YEEQLAQLQQ	KLLDLETERI	LLTKQVAEVE	AQKKDVCTEL	DAHKIQVQDL	MQQLEKQNSE	MEQKVKSLTQ	VYESKLEDGN
890	900	910	920	930	940	950	960
KEQEQTKQIL	VEKENMILQM	REGQKKEIEI	LTQKLSAKED	SIHILNEEYE	TKFKNQEKKM	EKVKQKAKEM	QETLKKKLLD
970	980	990	1000	1010	1020	1030	1040
QEAKLKKELE	NTALELSQKE	KQFNAKMLEM	AQANSAGISD	AVSRLETNQK	EQIESLTEVH	RRELNDVISI	WEKKLNQQAE
1050	1060	1070	1080	1090	1100	1110	1120
ELQEIHEIQL	QEKEQEVAEL	KQKILLFGCE	KEEMNKEITW	LKEEGVKQDT	TLNELQEQLK	QKSAHVNSLA	QDETKLKAHL
1130	1140	1150	1160	1170	1180	1190	1200
EKLEVDLNKS	LKENTFLQEQ	LVELKMLAEE	DKRKVSELTS	KLKTTDEEFQ	SLKSSHEKSN	KSLEDKSLEF	KKLSEELAIQ
1210	1220	1230	1240	1250	1260	1270	1280
LDICCKKTEA	LLEAKTNELI	NISSSKTNAI	LSRISHCQHR	TTKVKEALLI	KTCTVSELEA	QLRQLTEEQN	TLNISFQQAT
1290	1300	1310	1320	1330	1340	1350	1360
HQLEEKENQI	KSMKADIESL	VTEKEALQKE	GGNQQQAASE	KESCITQLKK	ELSENINAVT	LMKEELKEKK	VEISSLSKQL
1370	1380	1390	1400	1410	1420	1430	1440
TDLNVQLQNS	ISLSEKEAAI	SSLRKQYDEE	KCELLDQVQD	LSFKVDTLSK	EKISALEQVD	DWSNKFSEWK	KKAQSRFTQH
1450	1460	1470	1480	1490	1500	1510	1520
QNTVKELQIQ	LELKSKEAYE	KDEQINLLKE	ELDQQNKRFD	CLKGEMEDDK	SKMEKKESNL	ETELKSQTAR	IMELEDHITQ
1530	1540	1550	1560	1570	1580	1590	1600
KTIEIESLNE	VLKNYNQQKD	IEHKELVQKL	QHFQELGEEK	DNRVKEAEEK	ILTLENQVYS	MKAELETKKK	ELEHVNLSVK
1610	1620	1630	1640	1650	1660	1670	1680
SKEEELKALE	DRLESESAAK	LAELKRKAEQ	KIAAIKKQLL	SQMEEKEEQY	KKGTESHLSE	LNTKLQERER	EVHILEEKLK
1690	1700	1710	1720	1730	1740	1750	1760
SVESSQSETL	IVPRSAKNVA	AYTEQEEADS	QGCVQKTYEE	KISVLQRNLT	EKEKLLQRVG	QEKEETVSSH	FEMRCQYQER
1770	1780	1790	1800	1810	1820	1830	1840
LIKLEHAEAK	QHEDQSMIGH	LQEELEEKNK	KYSLIVAQHV	EKEGGKNNIQ	AKQNLENVFD	DVQKTLQEKE	LTCQILEQKI
1850	1860	1870	1880	1890	1900	1910	1920
KELDSCLVRQ	KEVHRVEMEE	LTSKYEKLQA	LQQMDGRNKP	TELLEENTEE	KSKSHLVQPK	LLSNMEAQHN	DLEFKLAGAE
1930	1940	1950	1960	1970	1980	1990	2000
REKQKLGKEI	VRLQKDLRML	RKEHQQELEI	LKKEYDQERE	EKIKQEQEDL	ELKHNSTLKQ	LMREFNTQLA	QKEQELEMTI
2010	2020	2030	2040	2050	2060	2070	2080
KETINKAQEV	EAELLESHQE	ETNQLLKKIA	EKDDDLKRTA	KRYEEILDAR	EEEMTAKVRD	LQTQLEELQK	KYQQKLEQEE
2090	2100	2110	2120	2130	2140	2150	2160
NPGNDNVTIM	ELQTQLAQKT	TLISDSKLKE	QEFREQIHNL	EDRLKKYEKN	VYATTVGTPY	KGGNLYHTDV	SLFGEPTEFE
2170	2180	2190	2200	2210	2220		

Detailed Protein Report

Cmpd.	No. of Cmpds.	m/z meas.	Δ m/z [ppm]	z	Rt [min]	Score	P	Range	Sequence	Modification
2557	1	715.3634	85.34	2	61.9	10.2	1	1479-1490	R.FDCLKGEMEDDK.S	
2790	2	683.3645	24.15	3	65.3	12.8	1	1813-1829	K.QNLENVFDDVQKTLQEK.E	
190	1	716.5084	147.04	2	32.1	12.0	2	1840-1851	K.IKELDSCLVRQK.E	
1522	1	620.4115	154.27	3	48.7	15.7	2	1850-1864	R.QKEVHRVEMEELTSK.Y	Oxidation: 9

Detailed Protein Report

Protein 60: **PREDICTED: striated muscle preferentially expressed protein kinase isoform X3 [Homo sapiens]**

**Accession:**	gi|530369683	**Score:**	50.6
**Database:**	refseq_human(refseq_human_20140103.fasta)	**MW [kDa]:**	345.1
**Database Date:**	2015-11-30	**pI:**	9.8
**Modification(s):**	Carbamidomethyl	**Sequence Coverage [%]:**	1.2
		**No. of unique Peptides:**	3

Quantitation

MD:MU	**Median:** 0.39	**CV:** 0.00 %	**No. of Peptides:** 1

BRUKER

Detailed Protein Report

10	20	30	40	50	60	70	80
MKKLWVKKRF	QKTGHSRRAF	GRLTHVFRSC	RKQSYDSETA	EDDISDVQGT	QRLELRDDGA	FSTPTGGSDT	LVGTSLDTPP
90	100	110	120	130	140	150	160
TSVTGTSEEQ	VSWWGSGQTV	LEQEAGSGGG	TRRLPGSPRQ	AQATGAGPRH	LGVEPLVRAS	RANLVGASWG	SEDSLSVASD
170	180	190	200	210	220	230	240
LYGSAFSLYR	GRALSIHVSV	PQSGLRREEP	DLQPQLASEA	PRRPAQPPPS	KSALLPPPSP	RVGKRSPPGP	PAQPAATPTS
250	260	270	280	290	300	310	320
PHRRTQEPVL	PEDTTTEEKR	GKKSKSSGPS	LAGTAESRPQ	TPLSEASGRL	SALGRSPRLV	RAGSRILDKL	QFFEERRRSL
330	340	350	360	370	380	390	400
ERSDSPPAPL	RPWVPLRKAR	SLEQPKSERG	APWGTPGASQ	EELRAPGSVA	ERRRLFQQKA	ASLDERTRQR	SPASDLELRF
410	420	430	440	450	460	470	480
AQELGRIRRS	TSREELVRSH	ESLRATLQRA	PSPREPGEPP	LFSRPSTPKT	SRAVSPAAAQ	PPSPSSAEKP	GDEPGRPRSR
490	500	510	520	530	540	550	560
GPAGRTEPGE	GPQQEVRRRD	QFPLTRSRAI	QECRSPVPPP	AADPPEARTK	APPGRKREPP	AQAVRFLPWA	TPGLEGAAVP
570	580	590	600	610	620	630	640
QTLEKNRAGP	EAEKRLRRGP	EEDGPWGPWD	RRGARSQGKG	RRARPTSPEL	ESSDDSYVSA	GEEPLEAPVF	EIPLQNVVVA
650	660	670	680	690	700	710	720
PGADVLLKCI	ITANPPPQVS	WHKDGSALRS	EGRLLLRAEG	ERHTLLLREA	RAADAGSYMA	TATNELGQAT	CAASLTVRPG
730	740	750	760	770	780	790	800
GSTSPFSSPI	TSDEEYLSPP	EEFPEPGETW	PRTPTMKPSP	SQNRRSSDTG	SKAPPTFKVS	LMDQSVREGQ	DVIMSIRVQG
810	820	830	840	850	860	870	880
EPKPVVSWLR	NRQPVRPDQR	RFAEEAEGGL	CRLRILAAER	GDAGFYTCKA	VNEYGARQCE	ARLEVRAHPE	SRSLAVLAPL
890	900	910	920	930	940	950	960
QDVDVGAGEM	ALFECLVAGP	TDVEVDWLCR	GRLLQPALLK	CKMHFDGRKC	KLLLTSVHED	DSGVYTCKLS	TAKDELTCSA
970	980	990	1000	1010	1020	1030	1040
RLTVRPSLAP	LFTRLLEDVE	VLEGRAARFD	CKISGTPPPV	VTWTHFGCPM	EESENLRLRQ	DGGLHSLHIA	HVGSEDEGLY
1050	1060	1070	1080	1090	1100	1110	1120
AVSAVNTHGQ	AHCSAQLYVE	EPRTAASGPS	SKLEKMPSIP	EEPEQGELER	LSIPDFLRPL	QDLEVGLAKE	AMLECQVTGL
1130	1140	1150	1160	1170	1180	1190	1200
PYPTISWFHN	GHRIQSSDDR	RMTQYRDVHR	LVFPAVGPQH	AGVYKSVIAN	KLGKAACYAH	LYVTDVVPGP	PDGAPQVVAV
1210	1220	1230	1240	1250	1260	1270	1280
TGRMVTLTWN	PPRSLDMAID	PDSLTYTVQH	QVLGSDQWTA	LVTGLREPGW	AATGLRKGVQ	HIFRVLSTTV	KSSSKPSPPS
1290	1300	1310	1320	1330	1340	1350	1360
EPVQLLEHGP	TLEEAPAMLD	KPDIVYVVEG	QPASVTVTFN	HVEAQVVWRS	CRGALLEARA	GVYELSQPDD	DQYCLRICRV
1370	1380	1390	1400	1410	1420	1430	1440
SRRDMGALTC	TARNRHGTQT	CSVTLELAEA	PRFESIMEDV	EVGAGETARF	AVVVEGKPLP	DIMWYKDEVL	LTESSHVSFV
1450	1460	1470	1480	1490	1500	1510	1520
YEENECSLVV	LSTGAQDGGV	YTCTAQNLAG	EVSCKAELAV	HSAQTAMEVE	GVGEDEDHRG	RRLSDFYDIH	QEIGRGAFSY
1530	1540	1550	1560	1570	1580	1590	1600
LRRIVERSSG	LEFAAKFIPS	QAKPKASARR	EARLLARLQH	DCVLYFHEAF	ERRRGLVIVT	ELCTEELLER	IARKPTVCES
1610	1620	1630	1640	1650	1660	1670	1680
EIRAYMRQVL	EGIHYLHQSH	VLHLDVKPEN	LLVWDGAAGE	QQVRICDFGN	AQELTPGEPQ	YCQYGTPEFV	APEIVNQSPV
1690	1700	1710	1720	1730	1740	1750	1760
SGVTDIWPVG	VVAFLCLTGI	SPFVGENDRT	TLMNIRNYNV	AFEETTFLSL	SREARGFLIK	VLVQDRLRPT	AEETLEHPWF
1770	1780	1790	1800	1810	1820	1830	1840
KTQAKGAEVS	TDHLKLFLSR	RRWQRSQISY	KCHLVLRPIP	ELLRAPPERV	WVTMPRRPPP	SGGLSSSSDS	EEEELEELPS
1850	1860	1870	1880	1890	1900	1910	1920
VPRPLQPEFS	GSRVSLTDIP	TEDEALGTPE	TGAATPMDWQ	EQGRAPSQDQ	EAPSPEALPS	PGQEPAAGAS	PRRGELRRGS
1930	1940	1950	1960	1970	1980	1990	2000
SAESALPRAG	PRELGRGLHK	AASVELPQRR	SPSPGATRLA	RGGLGEGEYA	QRLQALRQRL	LRGGPEDGKV	SGLRGPLLES
2010	2020	2030	2040	2050	2060	2070	2080
LGGRARDPRM	ARAASSEAAP	HHQPPLENRG	LQKSSSFSQG	EAEPRGRHRR	AGAPLEIPVA	RLGARRLQES	PSLSALSEAQ
2090	2100	2110	2120	2130	2140	2150	2160
PSSPARPSAP	KPSTPKSAEP	SATTPSDAPQ	PPAPQPAQDK	APEPRPEPVR	ASKPAPPPQA	LQTLALPLTP	YAQIIQSLQL
2170	2180	2190	2200	2210	2220	2230	2240

Detailed Protein Report

Cmpd.	No. of Cmpds.	m/z meas.	Δ m/z [ppm]	z	Rt [min]	Score	P	Range	Sequence	Modification	Ratios	
661	1	480.6345	-248.82	2	37.8	10.5	1	10-17	R.FQKTGHSR.R			
92	1	531.9231	-79.62	3	30.5	25.4	2	1995-2009	R.GPLLESLGGRARDPR.M		MD:MU	0.39
43	1	796.8047	-128.81	2	30.2	14.7	2	2883-2896	R.FGVVRACRENATGR.T	Carbamidomethyl: 7		

Detailed Protein Report

Protein 61: semaphorin-5B isoform 3 precursor [Homo sapiens]

Accession: gi|373432662
Database: refseq_human(refseq_human_20140103.fasta)
Database Date: 2015-11-30
Modification(s): Carbamidomethyl

Score: 50.6
MW [kDa]: 116.0
pI: 9.0
Sequence Coverage [%]: 4.7
No. of unique Peptides: 3

Quantitation

MD:MU **Median:** 2.06 **CV:** 0.00 % **No. of Peptides:** 1

```
        10         20         30         40         50         60         70         80
MVLAGPLAVS LLLPSLTLLV SHLSSSQDVS SEPSSEQQLC ALSKHPTVAF EDLQPWVSNF TYPGARDFSQ LALDPSGNQL
        90        100        110        120        130        140        150        160
IVGARNYLFR LSLANVSLLQ ATEWASSEDT RRSCQSKGKT EEECQNYVRV LIVAGRKVFM CGTNAFSPMC TSRQVGNLSR
       170        180        190        200        210        220        230        240
TIEKINGVAR CPYDPRHNST AVISSQGELY AATVIDFSGR DPAIYRSLGS GPPLRTAQYN SKWLNEPNFV AAYDIGLFAY
       250        260        270        280        290        300        310        320
FFLRENAVEH DCGRTVYSRV ARVCKNDVGG RFLLEDTWTT FMKARLNCSR PGEVPFYYNE LQSAFHLPEQ DLIYGVFTTN
       330        340        350        360        370        380        390        400
VNSIAASAVC AFNLSAISQA FNGPFRYQEN PRAAWLPIAN PIPNFQCGTL PETGPNENLT ERSLQDAQRL FLMSEAVQPV
       410        420        430        440        450        460        470        480
TPEPCVTQDS VRFSHLVVDL VQAKDTLYHV LYIGTESGTI LKALSTASRS LHGCYLEELH VLPPGRREPL RSLRILHSAR
       490        500        510        520        530        540        550        560
ALFVGLRDGV LRVPLERCAA YRSQGACLGA RDPYCGWDGK QQRCSTLEDS SNMSLWTQNI TACPVRNVTR DGGFGPWSPW
       570        580        590        600        610        620        630        640
QPCEHLDGDN SGSCLCRARS CDSPRPRCGG LDCLGPAIHI ANCSRNGAWT PWSSWALCST SCGIGFQVRQ RSCSNPAPRH
       650        660        670        680        690        700        710        720
GGRICVGKSR EERFCNENTP CPVPIFWASW GSWSKCSSNC GGGMQSRRRA CENGNSCLGC GVEFKTCNPE GCPEVRRNTP
       730        740        750        760        770        780        790        800
WTPWLPVNVT QGGARQEQRF RFTCRAPLAD PHGLQFGRRR TETRTCPADG SGSCDTDALV EVLLRSGSTS PHTVSGGWAA
       810        820        830        840        850        860        870        880
WGPWSSCSRD CELGFRVRKR TCTNPEPRNG GLPCVGDAAE YQDCNPQACP GEDICLGLHT EEALCATQAC PEGWSPWSEW
       890        900        910        920        930        940        950        960
SKCTDDGAQS RSRHCEELLP GSSACAGNSS QSRPCPYSEI PVILPASSME EATDCAGFNL IHLVATGISC FLGSGLLTLA
       970        980        990       1000       1010       1020       1030       1040
VYLSCQHCQR QSQESTLVHP ATPNHLHYKG GGTPKNEKYT PMEFKTLNKN NLIPDDRANF YPLQQTNVYT TTYYPSPLNK
      1050       1060
HSFRPEASPG QRCFPNS
```

Cmpd.	No. of Cmpds.	m/z meas.	Δ m/z [ppm]	z	Rt [min]	Score	P	Range	Sequence	Modification	Ratios
2274	5	682.6007	-55.67	3	58.3	18.2	2	113-129	R.SCQSKGKTEEECQNYVR.V	Carbamidomethyl: 2	
1589	1	648.3135	13.53	2	49.6	11.6	1	632-643	R.SCSNPAPRHGGR.I	Carbamidomethyl: 2	MD:MU 2.06
1478	1	1061.4566	-34.44	2	48.1	20.8	0	765-785	R.TCPADGSGSCDTDALVEVLLR.S		

Detailed Protein Report

Protein 62: PREDICTED: dynein heavy chain 3, axonemal isoform X1 [Homo sapiens]

**Accession:**	gi|578828592	**Score:**	50.6
**Database:**	refseq_human(refseq_human_20140103.fasta)	**MW [kDa]:**	466.0
**Database Date:**	2015-11-30	**pI:**	6.0
**Modification(s):**	Carbamidomethyl, Oxidation	**Sequence Coverage [%]:**	1.2
		**No. of unique Peptides:**	2

Detailed Protein Report

10	20	30	40	50	60	70	80
MGDMDCSSQK	IAKSDSIHHM	SHSQGQPELP	PLPASANEEP	SGLYQTVMSH	SFYPPLMQRT	SWTLAAPFKE	QHHHRGPSDS
90	100	110	120	130	140	150	160
IANNYSLMAQ	DLKLKDLLKV	YQPATISVPR	DRTGQGLPSS	GNRSSSEPMR	KKTKFSSRNK	EDSTRIKLAF	KTSIFSPMKK
170	180	190	200	210	220	230	240
EVKTSLTFPG	SRPMSPEQQL	DVMLQQEMEM	ESKEKKPSES	DLERYYYYLT	NGIRKDMIAP	EEGEVMVRIS	KLISNTLLTS
250	260	270	280	290	300	310	320
PFLEPLMVVL	VQEKENDYYC	SLMKSIVDYI	LMDPMERKRL	FIESIPRLFP	QRVIRAPVPW	HSVYRSAKKW	NEEHLHTVNP
330	340	350	360	370	380	390	400
MMLRLKELWF	AEFRDLRFVR	TAEILAGKLP	LQPQEFWDVI	QKHCLEAHQT	LLNKWIPTCA	QLFTSRKEHW	IHFAPKSNYD
410	420	430	440	450	460	470	480
SSRNIEEYFA	SVASFMSLQL	RELVIKSLED	LVSLFMIHKD	GNDFKEPYQE	MKFFIPQLIM	IKLEVSEPII	VFNPSFDGCW
490	500	510	520	530	540	550	560
ELIRDSFLEI	IKNSNGIPKV	ESVLFPELKG	YNLLLGTVNA	EEKLVSDFLI	QTFKVFQKNQ	VGPCKYLNVY	KKYVDLLDNT
570	580	590	600	610	620	630	640
AEQNIAAFLK	ENHDIDDFVT	KINAIKKRRN	EIASMNITVP	LAMFCLDATA	LNHDLCERAQ	NLKDHLIQFQ	VDVNRDTNTS
650	660	670	680	690	700	710	720
ICNQYSHIAD	KVSEVPANTK	ELVSLIEFLK	KSSAVTVFKL	RRQLRDASER	LEFLMDYADL	PYEDIKLNST	LFLWPDQIED
730	740	750	760	770	780	790	800
IFDNSRNLLL	HKRDQAEMDL	IKRCSEFELR	LEGYHRELES	FRKREVMTTE	EMKHNVEKLN	ELSKNLNRAF	AEFELINKEE
810	820	830	840	850	860	870	880
ELLEKEKSTY	PLLQAMLKNK	VPYEQLWSTA	YEFSIKSEEW	MNGPLFLLNA	EQIAEEIGNM	WRTTYKLIKT	LSDVPAPRRL
890	900	910	920	930	940	950	960
AENVKIKIDK	FKQYIPILSI	SCNPGMKDRH	WQQISEIVGY	EIKPTETTCL	SNMLEFGFGK	FVEKLEPIGA	AASKEYSLEK
970	980	990	1000	1010	1020	1030	1040
NLDRMKLDWV	NVTFSFVKYR	DTDTNILCAI	DDIQMLLDDH	VIKTQTMCGS	PFIKPIEAEC	RKWEEKLIRI	QDNLDAWLKC
1050	1060	1070	1080	1090	1100	1110	1120
QATWLYLEPI	FSSEDIIAQM	PEEGRKFGIV	DSYWKSLMSQ	AVKDNRILVA	ADQPRMAEKL	QEANFLLEDI	QKGLNDYLEK
1130	1140	1150	1160	1170	1180	1190	1200
KRLFFPRFFF	LSNDELLEIL	SETKDPLRVQ	PHLKKCFEGI	AKLEFTDNLE	IVGMISSEKE	TVPFIQKIYP	ANAKGMVEKW
1210	1220	1230	1240	1250	1260	1270	1280
LQQVEQMMLA	SMREVIGLGI	EAYVKVPRNH	WVLQWPGQVV	ICVSSIFWTQ	EVSQALAENT	LLDFLKKSND	QIAQIVQLVR
1290	1300	1310	1320	1330	1340	1350	1360
GKLSSGARLT	LGALTVIDVH	ARDVVAKLSE	DRVSDLNDFQ	WISQLRYYWV	AKDVQVQIIT	TEALYGYEYL	GNSPRLVITP
1370	1380	1390	1400	1410	1420	1430	1440
LTDRCYRTLM	GALKLNLGGA	PEGPAGTGKT	ETTKDLAKAL	AKQCVVFNCS	DGLDYKAMGK	FFKGLAQAGA	WACFDEFNRI
1450	1460	1470	1480	1490	1500	1510	1520
EVEVLSVVAQ	QILSIQQAII	RKLKTFIFEG	TELSLNPTCA	VFITMNPGYA	GRAELPDNLK	ALFRTVAMMV	PDYALIGEIS
1530	1540	1550	1560	1570	1580	1590	1600
LYSMGFLDSR	SLAQKIVATY	RLCSEQLSSQ	HHYDYGMRAV	KSVLTAAGNL	KLKYPEENES	VLLLRALLDV	NLAKFLAQDV
1610	1620	1630	1640	1650	1660	1670	1680
PLFQGIISDL	FPGVVLPKPD	YEVFLKVLND	NIKKMKLQPV	PWFIGKIIQI	YEMMLVRHGY	MIVGDPMGGK	TSAYKVLAAA
1690	1700	1710	1720	1730	1740	1750	1760
LGDLHAANQM	EEFAVEYKII	NPKAITMGQL	YGCFDQVSHE	WMDGVLANAF	REQASSLSDD	RKWIIFDGPV	DAIWIENMNT
1770	1780	1790	1800	1810	1820	1830	1840
VLDDNKKLCL	MSGEIIQMNS	KMSLIFEPAD	LEQASPATVS	RCGMIYMEPH	QLGWKPLKDS	YMDTLPSSLT	KEHKELVNDM
1850	1860	1870	1880	1890	1900	1910	1920
FMWLVQPCLE	FGRLHCKFVV	QTSPIHLAFS	MMRLYSSLLD	EIRAVEEEEM	ELGEGLSSQQ	IFLWLQGLFL	FSLVWTVAGT
1930	1940	1950	1960	1970	1980	1990	2000
INADSRKKFD	VFFRNLIMGM	DDNHPRPKSV	KLTKNNIFPE	RGSIYDFYFI	KQASGHWETW	TQYITKEEEK	VPAGAKVSEL
2010	2020	2030	2040	2050	2060	2070	2080
IIPTMETARQ	SFFLKTYLDH	EIPMLFVGPT	GTGKSAITNN	FLLHLPKNTY	LPNCINFSAR	TSANQTQDII	MSKLDRRRKG
2090	2100	2110	2120	2130	2140	2150	2160
LFGPPIGKKA	VVFVDDLNMP	AKEVYGAQPP	IELLRQWIDH	GYWFDKKDTT	RLDIVDMLLV	TAMGPPGGGR	NDITGRFTRH
2170	2180	2190	2200	2210	2220	2230	2240

Detailed Protein Report

Cmpd.	No. of Cmpds.	m/z meas.	Δ m/z [ppm]	z	Rt [min]	Score	P	Range	Sequence	Modification
301	1	874.1753	76.12	3	33.1	22.7	0	463-484	K.LEVSEPIIVFNPSFDGCWELIR.D	Carbamidomethyl: 17
2330	1	557.1827	-146.58	3	59.0	16.5	0	1935-1948	R.NLIMGMDDNHPRPK.S	Oxidation: 4, 6

Detailed Protein Report

Protein 63: neurogenic locus notch homolog protein 1 preproprotein [Homo sapiens]

**Accession:**	gi|148833508	**Score:**	50.5
**Database:**	refseq_human(refseq_human_20140103.fasta)	**MW [kDa]:**	272.3
**Database Date:**	2015-11-30	**pI:**	4.8
		**Sequence Coverage [%]:**	2.2
		**No. of unique Peptides:**	2

Quantitation

MD:MU	**Median:** 9.77	**CV:** 0.00 %	**No. of Peptides:** 1

BRUKER

Detailed Protein Report

10	20	30	40	50	60	70	80
MPPLLAPLLC	LALLPALAAR	GPRCSQPGET	CLNGGKCEAA	NGTEACVCGG	AFVGPRCQDP	NPCLSTPCKN	AGTCHVVDRR
90	100	110	120	130	140	150	160
GVADYACSCA	LGFSGPLCLT	PLDNACLTNP	CRNGGTCDLL	TLTEYKCRCP	PGWSGKSCQQ	ADPCASNPCA	NGGQCLPFEA
170	180	190	200	210	220	230	240
SYICHCPPSF	HGPTCRQDVN	ECGQKPGLCR	HGGTCHNEVG	SYRCVCRATH	TGPNCERPYV	PCSPSPCQNG	GTCRPTGDVT
250	260	270	280	290	300	310	320
HECACLPGFT	GQNCEENIDD	CPGNNCKNGG	ACVDGVNTYN	CRCPPEWTGQ	YCTEDVDECQ	LMPNACQNGG	TCHNTHGGYN
330	340	350	360	370	380	390	400
CVCVNGWTGE	DCSENIDDCA	SAACFHGATC	HDRVASFYCE	CPHGRTGLLC	HLNDACISNP	CNEGSNCDTN	PVNGKAICTC
410	420	430	440	450	460	470	480
PSGYTGPACS	QDVDECSLGA	NPCEHAGKCI	NTLGSFECQC	LQGYTGPRCE	IDVNECVSNP	CQNDATCLDQ	IGEFQCICMP
490	500	510	520	530	540	550	560
GYEGVHCEVN	TDECASSPCL	HNGRCLDKIN	EFQCECPTGF	TGHLCQYDVD	ECASTPCKNG	AKCLDGPNTY	TCVCTEGYTG
570	580	590	600	610	620	630	640
THCEVDIDEC	DPDPCHYGSC	KDGVATFTCL	CRPGYTGHHC	ETNINECSSQ	PCRHGGTCQD	RDNAYLCFCL	KGTTGPNCEI
650	660	670	680	690	700	710	720
NLDDCASSPC	DSGTCLDKID	GYECACEPGY	TGSMCNINID	ECAGNPCHNG	GTCEDGINGF	TCRCPEGYHD	PTCLSEVNEC
730	740	750	760	770	780	790	800
NSNPCVHGAC	RDSLNGYKCD	CDPGWSGTNC	DINNNECESN	PCVNGGTCKD	MTSGYVCTCR	EGFSGPNCQT	NINECASNPC
810	820	830	840	850	860	870	880
LNQGTCIDDV	AGYKCNCLLP	YTGATCEVVL	APCAPSPCRN	GGECRQSEDY	ESFSCVCPTG	WQGQTCEVDI	NECVLSPCRH
890	900	910	920	930	940	950	960
GASCQNTHGG	YRCHCQAGYS	GRNCETDIDD	CRPNPCHNGG	SCTDGINTAF	CDCLPGFRGT	FCEEDINECA	SDPCRNGANC
970	980	990	1000	1010	1020	1030	1040
TDCVDSYTCT	CPAGFSGIHC	ENNTPDCTES	SCFNGGTCVD	GINSFTCLCP	PGFTGSYCQH	DVNECDSQPC	LHGGTCQDGC
1050	1060	1070	1080	1090	1100	1110	1120
GSYRCTCPQG	YTGPNCQNLV	HWCDSSPCKN	GGKCWQTHTQ	YRCECPSGWT	GLYCDVPSVS	CEVAAQRQGV	DVARLCQHGG
1130	1140	1150	1160	1170	1180	1190	1200
LCVDAGNTHH	CRCQAGYTGS	YCEDLVDECS	PSPCQNGATC	TDYLGGYSCK	CVAGYHGVNC	SEEIDECLSH	PCQNGGTCLD
1210	1220	1230	1240	1250	1260	1270	1280
LPNTYKCSCP	RGTQGVHCEI	NVDDCNPPVD	PVSRSPKCFN	NGTCVDQVGG	YSCTCPPGFV	GERCEGDVNE	CLSNPCDARG
1290	1300	1310	1320	1330	1340	1350	1360
TQNCVQRVND	FHCECRAGHT	GRRCESVING	CKGKPCKNGG	TCAVASNTAR	GFICKCPAGF	EGATCENDAR	TCGSLRCLNG
1370	1380	1390	1400	1410	1420	1430	1440
GTCISGPRSP	TCLCLGPFTG	PECQFPASSP	CLGGNPCYNQ	GTCEPTSESP	FYRCLCPAKF	NGLLCHILDY	SFGGGAGRDI
1450	1460	1470	1480	1490	1500	1510	1520
PPPLIEEACE	LPECQEDAGN	KVCSLQCNNH	ACGWDGGDCS	LNFNDPWKNC	TQSLQCWKYF	SDGHCDSQCN	SAGCLFDGFD
1530	1540	1550	1560	1570	1580	1590	1600
CQRAEGQCNP	LYDQYCKDHF	SDGHCDQGCN	SAECEWDGLD	CAEHVPERLA	AGTLVVVVLM	PPEQLRNSSF	HFLRELSRVL
1610	1620	1630	1640	1650	1660	1670	1680
HTNVVFKRDA	HGQQMIFPYY	GREEELRKHP	IKRAAEGWAA	PDALLGQVKA	SLLPGGSEGG	RRRRELDPMD	VRGSIVYLEI
1690	1700	1710	1720	1730	1740	1750	1760
DNRQCVQASS	QCFQSATDVA	AFLGALASLG	SLNIPYKIEA	VQSETVEPPP	PAQLHFMYVA	AAAFVLLFFV	GCGVLLSRKR
1770	1780	1790	1800	1810	1820	1830	1840
RRQHGQLWFP	EGFKVSEASK	KKRREPLGED	SVGLKPLKNA	SDGALMDDNQ	NEWGDEDLET	KKFRFEEPVV	LPDLDDQTDH
1850	1860	1870	1880	1890	1900	1910	1920
RQWTQQHLDA	ADLRMSAMAP	TPPQGEVDAD	CMDVNVRGPD	GFTPLMIASC	SGGGLETGNS	EEEEDAPAVI	SDFIYQGASL
1930	1940	1950	1960	1970	1980	1990	2000
HNQTDRTGET	ALHLAARYSR	SDAAKRLLEA	SADANIQDNM	GRTPLHAAVS	ADAQGVFQIL	IRNRATDLDA	RMHDGTTPLI
2010	2020	2030	2040	2050	2060	2070	2080
LAARLAVEGM	LEDLINSHAD	VNAVDDLGKS	ALHWAAAVNN	VDAAVVLLKN	GANKDMQNNR	EETPLFLAAR	EGSYETAKVL
2090	2100	2110	2120	2130	2140	2150	2160
LDHFANRDIT	DHMDRLPRDI	AQERMHHDIV	RLLDEYNLVR	SPQLHGAPLG	GTPTLSPPLC	SPNGYLGSLK	PGVQGKKVRK
2170	2180	2190	2200	2210	2220	2230	2240

Detailed Protein Report

Cmpd.	No. of Cmpds.	m/z meas.	Δ m/z [ppm]	z	Rt [min]	Score	P	Range	Sequence	Modification	Ratios
1668	1	697.0259	81.08	3	50.6	14.2	1	1331-1350	R.GFICKCPAGFEGATCENDAR.T		
2073	4	698.2445	-185.84	2	55.8	21.5	0	1992-2004	R.MHDGTTPLILAAR.L		MD:MU 9.77

Detailed Protein Report

Protein 64: **zinc finger protein 281 isoform 2 [Homo sapiens]**

**Accession:**	gi|526253066	**Score:**	50.2
**Database:**	refseq_human(refseq_human_20140103.fasta)	**MW [kDa]:**	93.2
**Database Date:**	2015-11-30	**pI:**	9.3
**Modification(s):**	Carbamidomethyl, Oxidation	**Sequence Coverage [%]:**	4.4
		**No. of unique Peptides:**	3

10	20	30	40	50	60	70	80
MKIGSGFLSG	GGGTGSSGGS	GSGGGGSGGG	GGGGSSGRRA	EMEPTFPQAP	AAEPPPPPAP	DMTFKKEPAA	SAAAFPSQRT
**90**	**100**	**110**	**120**	**130**	**140**	**150**	**160**
SWGFLQSLVS	IKQEKPADPE	EQQSHHHHHH	HHYGGLFAGA	EERSPGLGGG	EGGSHGVIQD	LSILHQHVQQ	QPAQHHRDVL
**170**	**180**	**190**	**200**	**210**	**220**	**230**	**240**
LSSSSRTDDH	HGTEEPKQDT	NVKKAKRPKP	ESQGIKAKRK	PSASSKPSLV	GDGEGAILSP	SQKPHICDHC	SAAFRSSYHL
**250**	**260**	**270**	**280**	**290**	**300**	**310**	**320**
RRHVLIHTGE	RPFQCSQCSM	GFIQKYLLQR	HEKIHSREKP	FGCDQCSMKF	IQKYHMERHK	RTHSGEKPYK	CDTCQQYFSR
**330**	**340**	**350**	**360**	**370**	**380**	**390**	**400**
TDRLLKHRRT	CGEVIVKGAT	SAEPGSSNHT	NMGNLAVLSQ	GNTSSSRRKT	KSKSIAIENK	EQKTGKTNES	QISNNINMQS
**410**	**420**	**430**	**440**	**450**	**460**	**470**	**480**
YSVEMPTVSS	SGGIIGTGID	ELQKRVPKLI	FKKGSRKNTD	KNYLNFVSPL	PDIVGQKSLS	GKPSGSLGIV	SNNSVETIGL
**490**	**500**	**510**	**520**	**530**	**540**	**550**	**560**
LQSTSGKQGQ	ISSNYDDAMQ	FSKKRRYLPT	ASSNSAFSIN	VGHMVSQQSV	IQSAGVSVLD	NEAPLSLIDS	SALNAEIKSC
**570**	**580**	**590**	**600**	**610**	**620**	**630**	**640**
HDKSGIPDEV	LQSILDQYSN	KSESQKEDPF	NIAEPRVDLH	TSGEHSELVQ	EENLSPGTQT	PSNDKASMLQ	EYSKYLQQAF
**650**	**660**	**670**	**680**	**690**	**700**	**710**	**720**
EKSTNASFTL	GHGFQFVSLS	SPLHNHTLFP	EKQIYTTSPL	ECGFGQSVTS	VLPSSLPKPP	FGMLFGSQPG	LYLSALDATH
**730**	**740**	**750**	**760**	**770**	**780**	**790**	**800**
QQLTPSQELD	DLIDSQKNLE	TSSAFQSSSQ	KLTSQKEQKN	LESSTGFQIP	SQELASQIDP	QKDIEPRTTY	QIENFAQAFG
**810**	**820**	**830**	**840**	**850**	**860**		
SQFKSGSRVP	MTFITNSNGE	VDHRVRTSVS	DFSGYTNMMS	DVSEPCSTRV	KTPTSQSYR		

Cmpd.	No. of Cmpds.	m/z meas.	Δ m/z [ppm]	z	Rt [min]	Score	P	Range	Sequence	Modification
2793	1	840.4111	74.23	2	65.3	14.1	1	311-323	K.CDTCQQYFSRTDR.L	Carbamidomethyl: 4
2653	1	811.8976	73.50	2	64.2	24.6	1	311-323	K.CDTCQQYFSRTDR.L	
2862	1	693.8668	91.67	4	66.2	11.6	1	825-849	R.VRTSVSDFSGYTNMMSDVSEPCSTR.V	Oxidation: 14

Detailed Protein Report

Protein 65: ubiquitin carboxyl-terminal hydrolase 43 isoform 2 [Homo sapiens]

**Accession:**	gi|389616163	**Score:**	50.1
**Database:**	refseq_human(refseq_human_20140103.fasta)	**MW [kDa]:**	122.2
**Database Date:**	2015-11-30	**pI:**	10.2
		**Sequence Coverage [%]:**	3.5
		**No. of unique Peptides:**	2

Quantitation

MD:MU **Median:** 1.49 **CV:** 0.00 % **No. of Peptides:** 1

10	20	30	40	50	60	70	80
MDLGPGDAAG	GGPLAPRPRR	RRSLRRLFSR	FLLALGSRSR	PGDSPPRPQP	GHCDGDGEGG	FACAPGPVPA	APGSPGEERP
90	100	110	120	130	140	150	160
PGPQPQLQLP	AGDGARPPGA	QGLKNHGNTC	FMNAVVQCLS	NTDLLAEFLA	LGRYRAAPGR	AEVTEQLAAL	VRALWTREYT
170	180	190	200	210	220	230	240
PQLSAEFKNA	VSKYGSQFQG	NSQHDALEFL	LWLLDRVHED	LEGSSRGPVS	EKLPPEATKT	SENCLSPSAQ	LPLGQSFVQS
250	260	270	280	290	300	310	320
HFQAQYRSSL	TCPHCLKQSN	TFDPFLCVSL	PIPLRQTRFL	SVTLVFPSKS	QRFLRVGLAV	PILSTVAALR	KMVAEEGGVP
330	340	350	360	370	380	390	400
ADEVILVELY	PSGFQRSFFD	EEDLNTIAEG	DNVYAFQVPP	SPSQGTLSAH	PLGLSASPRL	AAREGQRFSL	SLHSESKVLI
410	420	430	440	450	460	470	480
LFCNLVGSGQ	QASRFGPPFL	IREDRAVSWA	QLQQSILSKV	RHLMKSEAPV	QNLGSLFSIR	VVGLSVACSY	LSPKDSRPLC
490	500	510	520	530	540	550	560
HWAVDRVLHL	RRPGGPPHVK	LAVEWDSSVK	ERLFGSLQEE	RAQDADSVWQ	QQQAHQQHSC	TLDECFQFYT	KEEQLAQDDA
570	580	590	600	610	620	630	640
WKCPHCQVLQ	QGMVKLSLWT	LPDILIIHLK	RFCQVGERRN	KLSTLVKFPL	SGLNMAPHVA	QRSTSPEAGL	GPWPSWKQPD
650	660	670	680	690	700	710	720
CLPTSYPLDF	LYDLYAVCNH	HGNLQAYCRN	SLDGQWYSYD	DSTVEPLRED	EVNTRGAYIL	FYQKRNSIPP	WSASSSMRGS
730	740	750	760	770	780	790	800
TSSSLSDHWL	LRLGSHAGST	RGSLLSWSSA	PCPSLPQVPD	SPIFTNSLCN	QEKGGLEPRR	LVRGVKGRSI	SMKAPTTSRA
810	820	830	840	850	860	870	880
KQGPFKTMPL	RWSFGSKEKP	PGASVELVEY	LESRRRPRST	SQSIVSLLTG	TAGEDEKSAS	PRSNVALPAN	SEDGGRAIER
890	900	910	920	930	940	950	960
GPAGVPCPSA	QPNHCLAPGN	SDGPNTARKL	KENAGQDIKL	PRKFDLPLTV	MPSVEHEKPA	RPEGQKAMNW	KESFQMGSKS
970	980	990	1000	1010	1020	1030	1040
SPPSPYMGFS	GNSKDSRRGT	SELDRPLQGT	LTLLRSVFRK	KENRRNERAE	VSPQVPPVSL	VSGGLSPAMD	GQAPGSPPAL
1050	1060	1070	1080	1090	1100	1110	1120
RIPEGLARGL	GSRLERDVWS	APSSLRLPRK	ASRAPRGSAL	GMSQRTVPGE	QASYGTFQRV	KYHTLSLGRK	KTLPESSF

Cmpd.	No. of Cmpds.	m/z meas.	Δ m/z [ppm]	z	Rt [min]	Score	P	Range	Sequence	Modification	Ratios
2741	1	656.8579	50.64	2	64.6	11.9	0	158-168	R.EYTPQLSAEFK.N		
2554	5	812.4077	-74.32	2	62.4	18.3	2	1042-1056	R.IPEGLARGLGSRLER.D		MD:MU 1.49

Detailed Protein Report

Protein 66: **leucine zipper protein 1 [Homo sapiens]**

**Accession:**	gi|216548085	**Score:**	50.0
**Database:**	refseq_human(refseq_human_20140103.fasta)	**MW [kDa]:**	120.2
**Database Date:**	2015-11-30	**pI:**	9.4
		**Sequence Coverage [%]:**	5.4
		**No. of unique Peptides:**	2

Alias proteins:

Accession	Name	Description
gi|216548091	refseq_human (refseq_human_20140103.fasta)	leucine zipper protein 1 [Homo sapiens]

10	20	30	40	50	60	70	80
MAEFTSYKET	ASSRHLRFKL	QSLSRRLDEL	EEATKNLQKA	EDELLDLQDK	VIQAEGSNSS	MLAEIEVLRQ	RVLRIEGKDE
**90**	**100**	**110**	**120**	**130**	**140**	**150**	**160**
EIKRAEDLCR	LMKEKLEEEE	NLTRELKSEI	ERLQKRMAEL	EKLEEAFSRS	KNDCTQLCLS	LNEERNLTKK	ISSELEMLRV
**170**	**180**	**190**	**200**	**210**	**220**	**230**	**240**
KVKELESSED	RLDKTEQSLA	SELEKLKSLT	LSFVSERKYL	NEKEKENEKL	IKELTQKLEQ	NKKMNRDYTR	NASNLERNDL
**250**	**260**	**270**	**280**	**290**	**300**	**310**	**320**
RIEDGISSTL	PSKESRRKGG	LDYLKQVENE	TRNKSENEKN	RNQEDNKVKD	LNQEIEKLKT	QIKHFESLEE	ELKKMKSKNN
**330**	**340**	**350**	**360**	**370**	**380**	**390**	**400**
DLQDNYLSEQ	NKNKLLASQL	EEIKLQIKKQ	KELENGEVEG	EDAFLSSKGR	HERTKFRGHG	SEASVSKHTA	RELSPQHKRE
**410**	**420**	**430**	**440**	**450**	**460**	**470**	**480**
RLRNREFALN	NENYSLSNRQ	VSSPSFTNRR	AAKASHMGVS	TDSGTQETKK	TEDRFVPGSS	QSEGKKSREQ	PSVLSRYPPA
**490**	**500**	**510**	**520**	**530**	**540**	**550**	**560**
AQEHSKAWKG	TSKPGTESGL	KGKVEKTTRT	FSDTTHGSVP	SDPLGRADKA	SDTSSETVFG	KRGHVLGNGS	QVTQAANSGC
**570**	**580**	**590**	**600**	**610**	**620**	**630**	**640**
SKAIGALASS	RRSSSEGLSK	GKKAANGLEA	DNSCPNSKAP	VLSKYPYSCR	SQENILQGFS	TSHKEGVNQP	AAVVMEDSSP
**650**	**660**	**670**	**680**	**690**	**700**	**710**	**720**
HEALRCRVIK	SSGREKPDSD	DDLDIASLVT	AKLVNTTITP	EPEPKPQPNS	REKAKTRGAP	RTSLFENDKD	AGMENESVKS
**730**	**740**	**750**	**760**	**770**	**780**	**790**	**800**
VRASTNTMEL	PDTNGAGVKS	QRPFSPREAL	RSRAIIKPVI	VDKDVKKIMG	GSGTETTLEK	QKPVSKPGPN	KVTSSITIYP
**810**	**820**	**830**	**840**	**850**	**860**	**870**	**880**
SDSSSPRAAP	GEALRERHTS	TSNIQVGLAE	LTSVSNHVSS	PFELSIHKHD	ITLQLAEAER	MADGPLKDRP	ETVVSRSSII
**890**	**900**	**910**	**920**	**930**	**940**	**950**	**960**
IKPSDPVERN	SHAPPAETIR	WKSHSAPSEV	GFSDARHVTV	RNAWKSRRDL	KSLEDPPTRI	GKNVESTNSN	AYTQRSSTDF
**970**	**980**	**990**	**1000**	**1010**	**1020**	**1030**	**1040**
SELEQPRSCL	FEQGTRRVGP	SSGDAPEPSS	RRTQSSLTVS	EVLTRRNRVG	DTITVAAWNH	SASMEEEGED	CTLSVYRQLH
**1050**	**1060**	**1070**	**1080**				
NSLDPSELPG	KQGLPESGRV	RAEERLRPTR	PCAEEN				

Cmpd.	No. of Cmpds.	m/z meas.	Δ m/z [ppm]	z	Rt [min]	Score	P	Range	Sequence	Modification
2080	1	1044.4112	-88.13	2	55.9	13.0	1	510-529	R.TFSDTTHGSVPSDPLGRADK.A	
2108	1	698.2650	-143.11	2	56.2	11.7	0	849-860	K.HDITLQLAEAER.M	

Detailed Protein Report

Protein 67: PREDICTED: protein AHNAK2 isoform X1 [Homo sapiens]

**Accession:**	gi|530403029	**Score:**	49.8
**Database:**	refseq_human(refseq_human_20140103.fasta)	**MW [kDa]:**	605.3
**Database Date:**	2015-11-30	**pI:**	5.1
		**Sequence Coverage [%]:**	0.8
		**No. of unique Peptides:**	3

Detailed Protein Report

10	20	30	40	50	60	70	80
MSRPEAVQEA	TEVTLKTEVE	AGASGYSVTG	GGDQGIFVKQ	VLKDSSAAKL	FNLREGDQLL	STTVFFENIK	YEDALKILQY
90	100	110	120	130	140	150	160
SEPYKVQFKI	RRQLPAPQDE	EWASSDAQHG	PQGKEKEDTD	VADGCRETPT	KTLEGDGDQE	RLISKPRVGR	GRQSQRERLS
170	180	190	200	210	220	230	240
WPKFQSIKSK	RGPGPQRSHS	SSEAYEPRDA	HDVSPTSTDT	EAQLTVERQE	QKAGPGSQRR	RKFLNLRFRT	GSGQGPSSTG
250	260	270	280	290	300	310	320
QPGRGFQSGV	GRAGVLEELG	PWGDSLEETG	AATGSRREER	AEQDREVMPA	QSMPLPTELG	DPRLCEGTPQ	EGGLRAARLH
330	340	350	360	370	380	390	400
GKTLEGQAQE	TAVAQRKPRA	QPTPGMSREG	EGEGLQSLEI	GIARLSLRDT	TEGGTQIGPP	EIRVRVHDLK	TPKFAFSTEK
410	420	430	440	450	460	470	480
EPERERRLST	PQRGKRQDAS	SKAGTGLKGE	EVEGAGWMPG	REPTTHAEAQ	GDEGDGEEGL	QRTRITEEQD	KGREDTEGQI
490	500	510	520	530	540	550	560
RMPKFKIPSL	GWSPSKHTKT	GREKATEDTE	QGREGEATAT	ADRREQRRTE	EGLKDKEDSD	SMTNTTKIQL	IHDEKRLKKE
570	580	590	600	610	620	630	640
QILTEKEVAT	KDSKFKMPKF	KMPLFGASAP	GKSMEASVDV	SAPKVEADVS	LLSMQGDLKT	TDLSVQTPSA	DLEVQDGQVD
650	660	670	680	690	700	710	720
VKLPEGPLPE	GASLKGHLPK	VQRPSLKMPK	VDLKGPKLDL	KGPKAEVTAP	DVKMSLSSME	VDVQAPRAKL	DGARLEGDLS
730	740	750	760	770	780	790	800
LADKEVTAKD	SKFKMPKFKM	PSFGVSAPGK	SMEDSVDVSA	PKVEADVSLS	SMQGDLKATD	LSIQPPSADL	EVQAGQVDVK
810	820	830	840	850	860	870	880
LPEGPVPEGA	GPKVHLPKVE	MPSFKMPKVD	LKGPQIDVKG	PKLDLKGPKA	EVTAPDGEVS	LPSMEVDVQA	QKAKLDGAWL
890	900	910	920	930	940	950	960
EGDLSLADKD	VTAKDSKFKM	PKFKMPSFGV	SAPGKSIKAL	VDVSAPKVEA	DLSLPSMQGD	LKTTDLSIQP	ASTDLKVQAD
970	980	990	1000	1010	1020	1030	1040
QVDVKLPEGH	LPEGAGLKGH	LPKVEMPSFK	MPKVALKGPQ	VDVKGPKLDL	KSPKAEVTAP	DVEVSLPSVE	VDVEAPGAKL
1050	1060	1070	1080	1090	1100	1110	1120
DSARLEGELS	LADKDVTAKD	SRFKMPKFKM	PSFGASAPGK	SIEASVDVSA	PKVEADVSLP	SMQGDLKTTD	LSIQPPSADL
1130	1140	1150	1160	1170	1180	1190	1200
EVHAGQVDVK	LLEGHVPEGA	GFKGHLPKVQ	MPSLKMPKVD	LKGPQVEVRG	PKLDLKGHKA	EVTAHEVAVS	LPSVEVDMQA
1210	1220	1230	1240	1250	1260	1270	1280
PGAKLDGAQL	DGDLSLADKD	VTAKDSKFKM	PKFKMPSFGV	SAPGKSIEAS	VDLSAPKVEA	DMSLPSMQGD	LKTTDLSIQP
1290	1300	1310	1320	1330	1340	1350	1360
PSTDLELQAG	QLDVKLPEGP	VPEGAGLKGH	LPKLQMPSFK	VPKVDLKGPE	IDIKGPKLDL	KDPKVEVTAP	DVEVSLPSVE
1370	1380	1390	1400	1410	1420	1430	1440
VDVEAPGAKL	DGGRLEEDMS	LADKDLTTKD	SKFKMPKFKM	PSFGVSAPGK	SIEASVDVSA	PKVEADVSLP	SMQGDLKATD
1450	1460	1470	1480	1490	1500	1510	1520
LSIQPPSADL	EVQAGQVDVK	LPEGPVSEGA	GLKGHLPKVQ	MPSFKMPKVD	LKGPQIDVKG	PKLDLKGPKV	EVTAPDVKMS
1530	1540	1550	1560	1570	1580	1590	1600
LSSMEVDVQA	PRAKLDGAQL	EGDLSLADKA	VTAKDSKFKM	PKFKMPSFGV	SAPGKSIEAS	VDVSEPKVEA	DVSLPSMQGD
1610	1620	1630	1640	1650	1660	1670	1680
LKTTDLSIQS	PSADLEVQAG	QVNVKLPEGP	LPEGAGFKGH	LPKVQMPSLK	MPKVALKGPQ	MDVKGPKLDL	KGPKAEVMAP
1690	1700	1710	1720	1730	1740	1750	1760
DVEVSLPSVE	VDVEAPGAKL	DSVRLEGDLS	LADKDVTAKD	SKFKMPKFKM	PSFGVSAPGK	SIEASVDVSA	PKVEAEVSLP
1770	1780	1790	1800	1810	1820	1830	1840
SMQGDLKTTD	LCIPLPSADL	VVQAGQVDMK	LPEGQVPEGA	GLKGHLPKVD	MPSFKMPKVD	LKGPQTDVKG	AKLDLKGPKA
1850	1860	1870	1880	1890	1900	1910	1920
EVTAPDVEVS	LPSMEVDVQA	QKAKLDGARL	EGDLSLADKD	MTAKDSKFKM	PKFKMPSFGV	SAPGRSIEAS	VDVPAPKVEA
1930	1940	1950	1960	1970	1980	1990	2000
DVSLPSMQGD	LKTTDLSIQP	PSADLKVQTG	QVDVKLPEGH	VPEGAGLKGH	LPKVEMPSLK	MPKVDLKGPQ	VDIKGPKLDL
2010	2020	2030	2040	2050	2060	2070	2080
KDPKVEMRVP	DVEVSLPSME	VDVQAPRAKL	DSAHLQGDLT	LANKDLTTKD	SKFKMPKFKM	PSFGVSAPGK	SIEASVDVSP
2090	2100	2110	2120	2130	2140	2150	2160
PKVEADMSLP	SMQGDLKTTD	LSIQPLSADV	KVQAGQVDVK	LLEGPVPEEV	GLKGHLPKLQ	MPSFKVPKVD	LKGPEIDIKG
2170	2180	2190	2200	2210	2220	2230	2240

Detailed Protein Report

Cmpd.	No. of Cmpds.	m/z meas.	Δ m/z [ppm]	z	Rt [min]	Score	P	Range	Sequence	Modification
2566	1	913.4598	-15.26	2	62.5	16.5	1	369-385	R.DTTEGGTQIGPPEIRVR.V	
2037	1	596.7447	-195.99	2	55.6	14.7	2	384-393	R.VRVHDLKTPK.F	
597	1	1230.6275	14.93	2	36.8	18.6	1	685-707	K.AEVTAPDVKMSLSSMEVDVQAPR.A	

Detailed Protein Report

Protein 68: 14-3-3 protein zeta/delta [Homo sapiens]

**Accession:**	gi|4507953	**Score:**	49.3
**Database:**	refseq_human(refseq_human_20140103.fasta)	**MW [kDa]:**	27.7
**Database Date:**	2015-11-30	**pI:**	4.6
**Modification(s):**	Carbamidomethyl	**Sequence Coverage [%]:**	9.0
		**No. of unique Peptides:**	2

Alias proteins:

Accession	Name	Description
gi|530389321	refseq_human (refseq_human_20140103.fasta)	PREDICTED: 14-3-3 protein zeta/delta isoform X4 [Homo sapiens]
gi|530389319	refseq_human (refseq_human_20140103.fasta)	PREDICTED: 14-3-3 protein zeta/delta isoform X3 [Homo sapiens]
gi|530389317	refseq_human (refseq_human_20140103.fasta)	PREDICTED: 14-3-3 protein zeta/delta isoform X2 [Homo sapiens]
gi|208973244	refseq_human (refseq_human_20140103.fasta)	14-3-3 protein zeta/delta [Homo sapiens]
gi|208973242	refseq_human (refseq_human_20140103.fasta)	14-3-3 protein zeta/delta [Homo sapiens]
gi|208973240	refseq_human (refseq_human_20140103.fasta)	14-3-3 protein zeta/delta [Homo sapiens]
gi|208973238	refseq_human (refseq_human_20140103.fasta)	14-3-3 protein zeta/delta [Homo sapiens]
gi|21735625	refseq_human (refseq_human_20140103.fasta)	14-3-3 protein zeta/delta [Homo sapiens]

10	20	30	40	50	60	70	80
MDKNELVQKA	KLAEQAERYD	DMAACMKSVT	EQGAELSNEE	RNLLSVAYKN	VVGARRSSWR	VVSSIEQKTE	GAEKKQQMAR
**90**	**100**	**110**	**120**	**130**	**140**	**150**	**160**
EYREKIETEL	RDICNDVLSL	LEKFLIPNAS	QAESKVFYLK	MKGDYYRYLA	EVAAGDDKKG	IVDQSQQAYQ	EAFEISKKEM
**170**	**180**	**190**	**200**	**210**	**220**	**230**	**240**
QPTHPIRLGL	ALNFSVFYYE	ILNSPEKACS	LAKTAFDEAI	AELDTLSEES	YKDSTLIMQL	LRDNLTLWTS	DTQGDEAEAG
**250**							
EGGEN							

Cmpd.	No. of Cmpds.	m/z meas.	Δ m/z [ppm]	z	Rt [min]	Score	P	Range	Sequence	Modification
2739	2	709.8489	-20.41	2	64.6	22.3	0	92-103	R.DICNDVLSLLEK.F	Carbamidomethyl: 3
2531	5	595.3252	-14.86	2	62.1	27.0	0	213-222	K.DSTLIMQLLR.D	

Detailed Protein Report

Protein 69: usherin isoform B [Homo sapiens]

**Accession:**	gi|219842266	**Score:**	49.2
**Database:**	refseq_human(refseq_human_20140103.fasta)	**MW [kDa]:**	575.2
**Database Date:**	2015-11-30	**pI:**	6.4
**Modification(s):**	Carbamidomethyl	**Sequence Coverage [%]:**	1.2
		**No. of unique Peptides:**	2

Detailed Protein Report

10	20	30	40	50	60	70	80
MNCPVLSLGS	GFLFQVIEML	IFAYFASISL	TESRGLFPRL	ENVGAFKKVS	IVPTQAVCGL	PDRSTFCHSS	AAAESIQFCT
90	100	110	120	130	140	150	160
QRFCIQDCPY	RSSHPTYTAL	FSAGLSSCIT	PDKNDLHPNA	HSNSASFIFG	NHKSCFSSPP	SPKLMASFTL	AVWLKPEQQG
170	180	190	200	210	220	230	240
VMCVIEKTVD	GQIVFKLTIS	EKETMFYYRT	VNGLQPPIKV	MTLGRILVKK	WIHLSVQVHQ	TKISFFINGV	EKDHTPFNAR
250	260	270	280	290	300	310	320
TLSGSITDFA	SGTVQIGQSL	NGLEQFVGRM	QDFRLYQVAL	TNREILEVFS	GDLLRLHAQS	HCRCPGSHPR	VHPLAQRYCI
330	340	350	360	370	380	390	400
PNDAGDTADN	RVSRLNPEAH	PLSFVNDNDV	GTSWVSNVFT	NITQLNQGVT	ISVDLENGQY	QVFYIIIQFF	SPQPTEIRIQ
410	420	430	440	450	460	470	480
RKKENSLDWE	DWQYFARNCG	AFGMKNNGDL	EKPDSVNCLQ	LSNFTPYSRG	NVTFSILTPG	PNYRPGYNNF	YNTPSLQEFV
490	500	510	520	530	540	550	560
KATQIRFHFH	GQYYTTETAV	NLRHRYYAVD	EITISGRCQC	HGHADNCDTT	SQPYRCLCSQ	ESFTEGLHCD	RCLPLYNDKP
570	580	590	600	610	620	630	640
FRQGDQVYAF	NCKPCQCNSH	SKSCHYNISV	DPFPFEHFRG	GGGVCDDCEH	NTTGRNCELC	KDYFFRQVGA	DPSAIDVCKP
650	660	670	680	690	700	710	720
CDCDTVGTRN	GSILCDQIGG	QCNCKRHVSG	RQCNQCQNGF	YNLQELDPDG	CSPCNCNTSG	TVDGDITCHQ	NSGQCKCKAN
730	740	750	760	770	780	790	800
VIGLRCDHCN	FGFKFLRSFN	DVGCEPCQCN	LHGSVNKFCN	PHSGQCECKK	EAKGLQCDTC	RENFYGLDVT	NCKACDCDTA
810	820	830	840	850	860	870	880
GSLPGTVCNA	KTGQCICKPN	VEGRQCNKCL	EGNFYLRQNN	SFLCLPCNCD	KTGTINGSLL	CNKSTGQCPC	KLGVTGLRCN
890	900	910	920	930	940	950	960
QCEPHRYNLT	IDNFQHCQMC	ECDSLGTLPG	TICDPISGQC	LCVPNRQGRR	CNQCQPGFYI	SPGNATGCLP	CSCHTTGAVN
970	980	990	1000	1010	1020	1030	1040
HICNSLTGQC	VCQDASIAGQ	RCDQCKDHYF	GFDPQTGRCQ	PCNCHLSGAL	NETCHLVTGQ	CFCKQFVTGS	KCDACVPSAS
1050	1060	1070	1080	1090	1100	1110	1120
HLDVNNLLGC	SKTPFQQPPP	RGQVQSSSAI	NLSWSPPDSP	NAHWLTYSLL	RDGFEIYTTE	DQYPYSIQYF	LDTDLLPYTK
1130	1140	1150	1160	1170	1180	1190	1200
YSYYIETTNV	HGSTRSVAVT	YKTKPGVPEG	NLTLSYIIPI	GSDSVTLTWT	TLSNQSGPIE	KYILSCAPLA	GGQPCVSYEG
1210	1220	1230	1240	1250	1260	1270	1280
HETSATIWNL	VPFAKYDFSV	QACTSGGCLH	SLPITVTTAQ	APPQRLSPPK	MQKISSTELH	VEWSPPAELN	GIIIRYELYM
1290	1300	1310	1320	1330	1340	1350	1360
RRLRSTKETT	SEESRVFQSS	GWLSPHSFVE	SANENALKPP	QTMTTITGLE	PYTKYEFRVL	AVNMAGSVSS	AWVSERTGES
1370	1380	1390	1400	1410	1420	1430	1440
APVFMIPPSV	FPLSSYSLNI	SWEKPADNVT	RGKVVGYDIN	MLSEQSPQQS	IPMAFSQLLH	TAKSQELSYT	VEGLKPYRIY
1450	1460	1470	1480	1490	1500	1510	1520
EFTITLCNSV	GCVTSASGAG	QTLAAAPAQL	RPPLVKGINS	TTIHLKWFPP	EELNGPSPIY	QLERRESSLP	ALMTTMMKGI
1530	1540	1550	1560	1570	1580	1590	1600
RFIGNGYCKF	PSSTHPVNTD	FTGIKASFRT	KVPEGLIVFA	ASPGNQEEYF	ALQLKKGRLY	FLFDPQGSPV	EVTTTNDHGK
1610	1620	1630	1640	1650	1660	1670	1680
QYSDGKWHEI	IAIRHQAFGQ	ITLDGIYTGS	SAILNGSTVI	GDNTGVFLGG	LPRSYTILRK	DPEIIQKGFV	GCLKDVHFMK
1690	1700	1710	1720	1730	1740	1750	1760
NYNPSAIWEP	LDWQSSEEQI	NVYNSWEGCP	ASLNEGAQFL	GAGFLELHPY	MFHGGMNFEI	SFKFRTDQLN	GLLLFVYNKD
1770	1780	1790	1800	1810	1820	1830	1840
GPDFLAMELK	SGILTFRLNT	SLAFTQVDLL	LGLSYCNGKW	NKVIIKKEGS	FISASVNGLM	KHASESGDQP	LVVNSPVYVG
1850	1860	1870	1880	1890	1900	1910	1920
GIPQELLNSY	QHLCLEQGFG	GCMKDVKFTR	GAVVNLASVS	SGAVRVNLDG	CLSTDSAVNC	RGNDSILVYQ	GKEQSVYEGG
1930	1940	1950	1960	1970	1980	1990	2000
LQPFTEYLYR	VIASHEGGSV	YSDWSRGRTT	GAAPQSVPTP	SRVRSLNGYS	IEVTWDEPVV	RGVIEKYILK	AYSEDSTRPP
2010	2020	2030	2040	2050	2060	2070	2080
RMPSASAEFV	NTSNLTGILT	GLLPFKNYAV	TLTACTLAGC	TESSHALNIS	TPQEAPQEVQ	PPVAKSLPSS	LLLSWNPPKK
2090	2100	2110	2120	2130	2140	2150	2160
ANGIITQYCL	YMDGRLIYSG	SEENYTVTDL	AVFTPHQFLL	SACTHVGCTN	SSWVLLYTAQ	LPPEHVDSPV	LTVLDSRTIH
2170	2180	2190	2200	2210	2220	2230	2240

Detailed Protein Report

Cmpd.	No. of Cmpds.	m/z meas.	Δ m/z [ppm]	z	Rt [min]	Score	P	Range	Sequence	Modification
2688	2	885.4613	98.77	2	63.8	13.3	0	838-851	R.QNNSFLCLPCNCDK.T	Carbamidomethyl: 7, 10, 12
206	1	898.4111	-54.24	3	32.3	10.4	2	5085-5108	K.RMSPLNVYPPGENHMGLADTKIPR.S	

Detailed Protein Report

Protein 70: PREDICTED: microtubule-actin cross-linking factor 1 isoform X21 [Homo sapiens]

**Accession:**	gi|578798822	**Score:**	49.0
**Database:**	refseq_human(refseq_human_20140103.fasta)	**MW [kDa]:**	853.1
**Database Date:**	2015-11-30	**pI:**	5.2
**Modification(s):**	Carbamidomethyl	**Sequence Coverage [%]:**	0.6
		**No. of unique Peptides:**	4

Quantitation

MD:MU	**Median:** 0.26	**CV:** 0.00 %	**No. of Peptides:** 1

10	20	30	40	50	60	70	80
MPLLDSSYLP	PTIFILTHVL	GVAGVLYWKR	HARGRADERD	RVQKKTFTKW	VNKHLMKVRK	HINDLYEDLR	DGHNLISLLE
90	100	110	120	130	140	150	160
VLSGIKLPRE	KGRMRFHRLQ	NVQIALDFLK	QRQVKLVNIR	NDDITDGNPK	LTLGLIWTII	LHFQISDIYI	SGESGDMSAK
170	180	190	200	210	220	230	240
EKLLLWTQKV	TAGYTGIKCT	NFSSCWSDGK	MFNALIHRYR	PDLVDMERVQ	IQSNRENLEQ	AFEVAERLGV	TRLLDAEDVD
250	260	270	280	290	300	310	320
VPSPDEKSVI	TYVSSIYDAF	PKVPEGGEGI	SATEVDSRWQ	EYQSRVDSLI	PWIKQHTILM	SDKTFPQNPV	ELKALYNQYI
330	340	350	360	370	380	390	400
HFKETEILAK	EREKGRIEEL	YKLLEVWIEF	GRIKLPQGYH	PNDVEEEWGK	LIIEMLEREK	SLRPAVERLE	LLLQIANKIQ
410	420	430	440	450	460	470	480
NGALNCEEKL	TLAKNTLQAD	AAHLESGQPV	QCESDVIMYI	QECEGLIRQL	QVDLQILRDE	NYYQLEELAF	RVMRLQDELV
490	500	510	520	530	540	550	560
TLRLECTNLY	RKGHFTSLEL	VPPSTLTTTH	LKAEPLTKAT	HSSSTSWFRK	PMTRAELVAI	SSSEDEGNLR	FVYELLSWVE
570	580	590	600	610	620	630	640
EMQMKLERAE	WGNDLPSVEL	QLETQQHIHT	SVEELGSSVK	EARLYEGKMS	QNFHTSYAET	LGKLETQYCK	LKETSSFRMR
650	660	670	680	690	700	710	720
HLQSLHKFVS	RATAELIWLN	EKEEEELAYD	WSDNNSNISA	KRNYFSELTM	ELEEKQDVFR	SLQDTAELLS	LENHPAKQTV
730	740	750	760	770	780	790	800
EAYSAAVQSQ	LQWMKQLCLC	VEQHVKENTA	YFQFFSDARE	LESFLRNLQD	SIKRKYSCDH	NTSLSRLEDL	LQDSMDEKEQ
810	820	830	840	850	860	870	880
LIQSKSSVAS	LVGRSKTIVQ	LKPRSPDHVL	KNTISVKAVC	DYRQIEITIC	KNDECVLEDN	SQRTKWKVIS	PTGNEAMVPS
890	900	910	920	930	940	950	960
VCFLIPPPNK	DAIEMASRVE	QSYQKVMALW	HQLHVNTKSL	ISWNYLRKDL	DLVQTWNLEK	LRSSAPGECH	QIMKNLQAHY
970	980	990	1000	1010	1020	1030	1040
EDFLQDSRDS	VLFSVADRLR	LEEEVEACKA	RFQHLMKSME	NEDKEETVAK	MYISELKNIR	LRLEEYEQRV	VKRIQSLASS
1050	1060	1070	1080	1090	1100	1110	1120
RTDRDAWQDN	ALRIAEQEHT	QEDLQQLRSD	LDAVSMKCDS	FLHQSPSSSS	VPTLRSELNL	LVEKMDHVYG	LSTVYLNKLK
1130	1140	1150	1160	1170	1180	1190	1200
TVDVIVRSIQ	DAELLVKGYE	IKLSQEEVVL	ADLSALEAHW	STLRHWLSDV	KDKNSVFSVL	DEEIAKAKVV	AEQMSRLTPE
1210	1220	1230	1240	1250	1260	1270	1280
RNLDLERYQE	KGSQLQERWH	RVIAQLEIRQ	SELESIQEVL	GDYRACHGTL	IKWIEETTAQ	QEMMKPGQAE	DSRVLSEQLS
1290	1300	1310	1320	1330	1340	1350	1360
QQTALFAEIE	RNQTKLDQCQ	KFSQQYSTIV	KDYELQLMTY	KAFVESQQKS	PGKRRRMLSS	SDAITQEFMD	LRTRYTALVT
1370	1380	1390	1400	1410	1420	1430	1440
LTTQHVKYIS	DALRRLEEEE	KVVEEEKQEH	VEKVKELLGW	VSTLARNTQG	KATSSETKES	TDIEKAILEQ	QVLSEELTTK
1450	1460	1470	1480	1490	1500	1510	1520
KEQVSEAIKT	SQIFLAKHGH	KLSEKEKKQI	SEQLNALNKA	YHDLCDGSAN	QLQQLQSQLA	HQTEQKECRA	VAGVIDLGTV
1530	1540	1550	1560	1570	1580	1590	1600
EIFPIFKAMQ	KGLLDQDTGL	VLLESQVIMS	GLIAPETGEN	LSLEEGIARN	LINPQMYQQL	RELQDALALI	SRLTESRGPL
1610	1620	1630	1640	1650	1660	1670	1680
SVVEAIEKRI	ISETVGLKIL	EAHLATGGFS	LSPSENCINL	EEAFHQGLIS	AWLHSVLESY	LRTSKNLIDP	NTAEKIGLLD
1690	1700	1710	1720	1730	1740	1750	1760
LMQRCIVHQE	SGFKLLPVKQ	LAGGMVSLKS	GRKVSIFRAV	QEGLIDRQVT	VRLLEAQLFA	GGIVDPRTGH	RLTVEEAVRH
1770	1780	1790	1800	1810	1820	1830	1840
NLIDQDMACA	ILIRQLQTGG	IIDTVTGQRL	TIDEAVSNDL	VAAKIALVIL	ESLWSFMGLL	WPESGEILPI	TDALEQGIVS
1850	1860	1870	1880	1890	1900	1910	1920
TELAHKILSN	RQHIKALFLP	ATTEILSWKK	AIESGILDRD	LANNLKSICI	PDVMPHMQLA	DSAEQNINPG	AAVLPCSKSH
1930	1940	1950	1960	1970	1980	1990	2000
PKATASQSEN	LLFQLMTHSY	INVQNGQRLL	LLDKELMETL	TSRDEYQTSP	PKVVEIGHQR	QKTPEGLQES	ANVKISGTFS
2010	2020	2030	2040	2050	2060	2070	2080
SGWTVRLPEF	QFSSQNKEYP	DREDCTTEKG	KKTTVETEDS	SVENPEQDLF	VEQKERNPNI	DALKVINKVK	LEVQRQLIGT
2090	2100	2110	2120	2130	2140	2150	2160
QREDQTAVSV	RENASRGHLL	TIPPAEAEGV	PLVVDKDVFS	VETPKKEHQP	LRNTSFTCQN	EQAHTLETEY	IHDETGGSHI
2170	2180	2190	2200	2210	2220	2230	2240

Detailed Protein Report

Cmpd.	No. of Cmpds.	m/z meas.	Δ m/z [ppm]	z	Rt [min]	Score	P	Range	Sequence	Modification	Ratios
324	1	755.2309	-145.08	2	33.4	13.7	1	998-1010	K.SMENEDKEETVAK.M		
371	1	625.3885	104.40	2	34.3	11.3	2	4866-4875	R.LKDCMQKAQK.Y	Carbamidomethyl: 4	
1272	1	673.3365	1.16	2	45.6	11.4	1	5890-5899	R.QQQEEMRQLR.E		
11	1	650.7452	-159.65	2	29.7	12.6	2	6624-6633	K.RAKQFHEAWK.K		MD:MU 0.26

Detailed Protein Report

Protein 71:	PREDICTED: arf-GAP with Rho-GAP domain, ANK repeat and PH domain-containing protein 3 isoform X7 [Homo sapiens]		
**Accession:**	gi|578810904	**Score:**	48.8
**Database:**	refseq_human(refseq_human_20140103.fasta)	**MW [kDa]:**	149.1
**Database Date:**	2015-11-30	**pI:**	6.5
**Modification(s):**	Oxidation	**Sequence Coverage [%]:**	1.7
		**No. of unique Peptides:**	1

Quantitation

MD:MU **Median:** 1.02 **CV:** 0.00 % **No. of Peptides:** 1

10	20	30	40	50	60	70	80
MDPGCLYYGV	QPVGTPGAPD	RRESRGVCQG	RAEHRLSRQD	LEAREDAGYA	SLELPGDSTL	LSPTLETEET	SDDLISPYAS
90	100	110	120	130	140	150	160
FSFTADRLTP	LLSGWLDKLS	PQGNYVFQRR	FVQFNGRSLM	YFGSDKDPFP	KGVIPLTAIE	MTRSSKDNKF	QVITGQRVFV
170	180	190	200	210	220	230	240
FRTESEAQRD	MWCSTLQSCL	KEQRLLGHPR	PPQPPRPLRT	GMLELRGHKA	KVFAALSPGE	LALYKSEQAF	SLGIGICFIE
250	260	270	280	290	300	310	320
LQGCSVRETK	SRSFDLLTPH	RCFSFTAESG	GARQSWAAAL	QEAVTETLSD	YEVAEKIWSN	RANRQCADCG	SSRPDWAAVN
330	340	350	360	370	380	390	400
LGVVICKQCA	GQHRALGSGI	SKVQSLKLDT	SVWSNEIVQL	FIVLGNDRAN	RFWAGTLPPG	EGLHPDATPG	PRGEFISRKY
410	420	430	440	450	460	470	480
RLGLFRKPHP	QYPDHSQLLQ	ALCAAVARPN	LLKNMTQLLC	VEAFEGEEPW	FPPAPDGSCP	GLLPSDPSPG	VYNEVVVRAT
490	500	510	520	530	540	550	560
YSGFLYCSPV	SNKAGPSPPR	RGRDAPPRLW	CVLGAALEMF	ASENSPEPLS	LIQPQDIVCL	GVSPPPTDPG	DRFPFSFELI
570	580	590	600	610	620	630	640
LAGGRIQHFG	TDGADSLEAW	TSAVGKWFSP	LSCHQLLGPG	LLRLGRLWLR	SPSHTAPAPG	LWLSGFGLLR	GDHLFLCSAP
650	660	670	680	690	700	710	720
GPGPPAPEDM	VHLRRLQEIS	VVSAADTPDK	KEHLVLVETG	RTLYLQGEGR	LDFTAWNAAI	GGAAGGGGTG	LQEQQMSRGD
730	740	750	760	770	780	790	800
IPIIVDACIS	FVTQHGLRLE	GVYRKGGARA	RSLRLLAEFR	RDARSVKLRP	GEHFVEDVTD	TLKRFFRELD	DPVTSARLLP
810	820	830	840	850	860	870	880
RWREAAELPQ	KNQRLEKYKD	VIGCLPRVNR	RTLATLIGHL	YRVQKCAALN	QMCTRNLALL	FAPSVFQTDG	RGEHEVRVLQ
890	900	910	920	930	940	950	960
ELIDGYISVF	DIDSDQVAQI	DLEVSLITTW	KDVQLSQAGD	LIMEVYIEQQ	LPDNCVTLKV	SPTLTAEELT	NQVLEMRGTA
970	980	990	1000	1010	1020	1030	1040
AGMDLWVTFE	IREHGELERP	LHPKEKVLEQ	ALQWCQLPEP	CSASLLLKKV	PLAQAGCLFT	GIRRESPRVG	LLRCREEPPR
1050	1060	1070	1080	1090	1100	1110	1120
LLGSRFQERF	FLLRGRCLLL	LKEKKSSKPE	REWPLEGAKV	YLGIRKKLKP	PTPWGFTLIL	EKMHLYLSCT	DEDEMWDWTT
1130	1140	1150	1160	1170	1180	1190	1200
SILKAQHDDQ	QPVVLRRHSS	SDLARQKFGT	MPLLPIRGDD	SGATLLSANQ	TLRRLHNRRT	LSMFFPMKSS	QGSVEEQEEL
1210	1220	1230	1240	1250	1260	1270	1280
EEPVYEEPVY	EEVGAFPELI	QDTSTSFSTT	REWTVKPENP	LTSQKSLDQP	FLSKSSTLGQ	EERPPEPPPG	PPSKSSPQAR
1290	1300	1310	1320	1330	1340	1350	
GSLEEQLLQE	LSSLILRKGE	TTAGLGSPSQ	PSSPQSPSPT	GLPTQTPGFP	TQPPCTSSPP	SSQPLT	

Cmpd.	No. of Cmpds.	m/z meas.	Δ m/z [ppm]	z	Rt [min]	Score	P	Range	Sequence	Modification	Ratios	
164	1	715.8153	8.42	2	31.4	24.0	0	170-181	R.DMWCSTLQSCLK.E	Oxidation: 2	MD:MU	1.02

Detailed Protein Report

Protein 72: chromodomain-helicase-DNA-binding protein 2 isoform 1 [Homo sapiens]

Accession: gi|118421089
Database: refseq_human(refseq_human_20140103.fasta)
Database Date: 2015-11-30
Modification(s): Oxidation

Score: 48.5
MW [kDa]: 211.2
pI: 8.8
Sequence Coverage [%]: 3.4
No. of unique Peptides: 4

10	20	30	40	50	60	70	80
MMRNKDKSQE	EDSSLHSNAS	SHSASEEASG	SDSGSQSESE	QGSDPGSGHG	SESNSSSESS	ESQSESESES	AGSKSQPVLP
90	100	110	120	130	140	150	160
EAKEKPASKK	ERIADVKKMW	EEYPDVYGVR	RSNRSRQEPS	RFNIKEEASS	GSESGSPKRR	GQRQLKKQEK	WKQEPSEDEQ
170	180	190	200	210	220	230	240
EQGTSAESEP	EQKKVKARRP	VPRRTVPKPR	VKKQPKTQRG	KRKKQDSSDE	DDDDDEAPKR	QTRRRAAKNV	SYKEDDDFET
250	260	270	280	290	300	310	320
DSDDLIEMTG	EGVDEQQDNS	ETIEKVLDSR	LGKKGATGAS	TTVYAIEANG	DPSGDFDTEK	DEGEIQYLIK	WKGWSYIHST
330	340	350	360	370	380	390	400
WESEESLQQQ	KVKGLKKLEN	FKKKEDEIKQ	WLGKVSPEDV	EYFNCQQELA	SELNKQYQIV	ERVIAVKTSK	STLGQTDFPA
410	420	430	440	450	460	470	480
HSRKPAPSNE	PEYLCKWMGL	PYSECSWEDE	ALIGKKFQNC	IDSFHSRNNS	KTIPTRECKA	LKQRPRFVAL	KKQPAYLGGE
490	500	510	520	530	540	550	560
NLELRDYQLE	GLNWLAHSWC	KNNSVILADE	MGLGKTIQTI	SFLSYLFHQH	QLYGPFLIVV	PLSTLTSWQR	EFEIWAPEIN
570	580	590	600	610	620	630	640
VVVYIGDLMS	RNTIREYEWI	HSQTKRLKFN	ALITTYEILL	KDKTVLGSIN	WAFLGVDEAH	RLKNDDSLLY	KTLIDFKSNH
650	660	670	680	690	700	710	720
RLLITGTPLQ	NSLKELWSLL	HFIMPEKFEF	WEDFEEDHGK	GRENGYQSLH	KVLEPFLLRR	VKKDVEKSLP	AKVEQILRVE
730	740	750	760	770	780	790	800
MSALQKQYYK	WILTRNYKAL	AKGTRGSTSG	FLNIVMELKK	CCNHCYLIKP	PEENERENGQ	EILLSLIRSS	GKLILLDKLL
810	820	830	840	850	860	870	880
TRLRERGNRV	LIFSQMVRML	DILAEYLTIK	HYPFQRLDGS	IKGEIRKQAL	DHFNADGSED	FCFLLSTRAG	GLGINLASAD
890	900	910	920	930	940	950	960
TVVIFDSDWN	PQNDLQAQAR	AHRIGQKKQV	NIYRLVTKGT	VEEEIIERAK	KKMVLDHLVI	QRMDTTGRTI	LENNSGRSNS
970	980	990	1000	1010	1020	1030	1040
NPFNKEELTA	ILKFGAEDLF	KELEGEESEP	QEMDIDEILR	LAETRENEVS	TSATDELLSQ	FKVANFATME	DEEELEERPH
1050	1060	1070	1080	1090	1100	1110	1120
KDWDEIIPEE	QRKKVEEEER	QKELEEIYML	PRIRSSTKKA	QTNDSDSDTE	SKRQAQRSSA	SESETEDSDD	DKKPKRRGRP
1130	1140	1150	1160	1170	1180	1190	1200
RSVRKDLVEG	FTDAEIRRFI	KAYKKFGLPL	ERLECIARDA	ELVDKSVADL	KRLGELIHNS	CVSAMQEYEE	QLKENASEGK
1210	1220	1230	1240	1250	1260	1270	1280
GPGKRRGPTI	KISGVQVNVK	SIIQHEEEFE	MLHKSIPVDP	EEKKKYCLTC	RVKAAHFDVE	WGVEDDSRLL	LGIYEHGYGN
1290	1300	1310	1320	1330	1340	1350	1360
WELIKTDPEL	KLTDKILPVE	TDKKPQGKQL	QTRADYLLKL	LRKGLEKKGA	VTGGEEAKLK	KRKPRVKKEN	KVPRLKEEHG
1370	1380	1390	1400	1410	1420	1430	1440
IELSSPRHSD	NPSEEGEVKD	DGLEKSPMKK	KQKKKENKEN	KEKQMSSRKD	KEGDKERKKS	KDKKEKPKSG	DAKSSSKSKR
1450	1460	1470	1480	1490	1500	1510	1520
SQGPVHITAG	SEPVPIGEDE	DDDLDQETFS	ICKERMRPVK	KALKQLDKPD	KGLNVQEQLE	HTRNCLLKIG	DRIAECLKAY
1530	1540	1550	1560	1570	1580	1590	1600
SDQEHIKLWR	RNLWIFVSKF	TEFDARKLHK	LYKMAHKKRS	QEEEEQKKKD	DVTGGKKPFR	PEASGSSRDS	LISQSHTSHN
1610	1620	1630	1640	1650	1660	1670	1680
LHPQKPHLPA	SHGPQMHGHP	RDNYNHPNKR	HFSNADRGDW	QRERKFNYGG	GNNNPPWGSD	RHHQYEQHWY	KDHHYGDRRH
1690	1700	1710	1720	1730	1740	1750	1760
MDAHRSGSYR	PNNMSRKRPY	DQYSSDRDHR	GHRDYYDRHH	HDSKRRRSDE	FRPQNYHQQD	FRRMSDHRPA	MGYHGQGPSD
1770	1780	1790	1800	1810	1820	1830	
HYRSFHTDKL	GEYKQPLPPL	HPAVSDPRSP	PSQKSPHDSK	SPLDHRSPLE	RSLEQKNNPD	YNWNVRKT	

Cmpd.	No. of	m/z meas.	Δ m/z	z	Rt	Score	P	Range	Sequence	Modification

Detailed Protein Report

	Cmpds.		[ppm]		[min]					
1421	1	846.6046	143.39	1	47.5	10.1	2	147-152	K.KQEKWK.Q	
2644	1	913.4624	-16.46	2	63.1	14.1	1	743-759	K.GTRGSTSGFLNIVMELK.K	Oxidation: 14
1792	1	898.7912	-12.20	3	52.2	11.6	1	1212-1234	K.ISGVQVNVKSIIQHEEEFEMLHK.S	
1619	1	1015.0002	-35.61	2	50.0	12.7	2	1531-1546	R.RNLWIFVSKFTEFDAR.K	

Detailed Protein Report

Protein 73: ATP-binding cassette sub-family A member 3 [Homo sapiens]

**Accession:**	gi|116734710	**Score:**	48.1
**Database:**	refseq_human(refseq_human_20140103.fasta)	**MW [kDa]:**	191.2
**Database Date:**	2015-11-30	**pI:**	8.2
		**Sequence Coverage [%]:**	1.4
		**No. of unique Peptides:**	2

Quantitation

MD:MU	**Median:** 1.13	**CV:** 0.00 %	**No. of Peptides:** 1

10	20	30	40	50	60	70	80
MAVLRQLALL	LWKNYTLQKR	KVLVTVLELF	LPLLFSGILI	WLRLKIQSEN	VPNATIYPGQ	SIQELPLFFT	FPPPGDTWEL
90	100	110	120	130	140	150	160
AYIPSHSDAA	KTVTETVRRA	LVINMRVRGF	PSEKDFEDYI	RYDNCSSSVL	AAVVFEHPFN	HSKEPLPLAV	KYHLRFSYTR
170	180	190	200	210	220	230	240
RNYMWTQTGS	FFLKETEGWH	TTSLFPLFPN	PGPREPTSPD	GGEPGYIREG	FLAVQHAVDR	AIMEYHADAA	TRQLFQRLTV
250	260	270	280	290	300	310	320
TIKRFPYPPF	IADPFLVAIQ	YQLPLLLLLS	FTYTALTIAR	AVVQEKERRL	KEYMRMMGLS	SWLHWSAWFL	LFFLFLLIAA
330	340	350	360	370	380	390	400
SFMTLLFCVK	VKPNVAVLSR	SDPSLVLAFL	LCFAISTISF	SFMVSTFFSK	ANMAAAFGGF	LYFFTYIPYF	FVAPRYNWMT
410	420	430	440	450	460	470	480
LSQKLCSCLL	SNVAMAMGAQ	LIGKFEAKGM	GIQWRDLLSP	VNVDDDFCFG	QVLGMLLLDS	VLYGLVTWYM	EAVFPGQFGV
490	500	510	520	530	540	550	560
PQPWYFFIMP	SYWCGKPRAV	AGKEEEDSDP	EKALRNEYFE	AEPEDLVAGI	KIKHLSKVFR	VGNKDRAAVR	DLNLNLYEGQ
570	580	590	600	610	620	630	640
ITVLLGHNGA	GKTTTLSMLT	GLFPPTSGRA	YISGYEISQD	MVQIRKSLGL	CPQHDILFDN	LTVAEHLYFY	AQLKGLSRQK
650	660	670	680	690	700	710	720
CPEEVKQMLH	IIGLEDKWNS	RSRFLSGGMR	RKLSIGIALI	AGSKVLILDE	PTSGMDAISR	RAIWDLLQRQ	KSDRTIVLTT
730	740	750	760	770	780	790	800
HFMDEADLLG	DRIAIMAKGE	LQCCGSSLFL	KQKYGAGYHM	TLVKEPHCNP	EDISQLVHHH	VPNATLESSA	GAELSFILPR
810	820	830	840	850	860	870	880
ESTHRFEGLF	AKLEKKQKEL	GIASFGASIT	TMEEVFLRVG	KLVDSSMDIQ	AIQLPALQYQ	HERRASDWAV	DSNLCGAMDP
890	900	910	920	930	940	950	960
SDGIGALIEE	ERTAVKLNTG	LALHCQQFWA	MFLKKAAYSW	REWKMVAAQV	LVPLTCVTLA	LLAINYSSEL	FDDPMLRLTL
970	980	990	1000	1010	1020	1030	1040
GEYGRTVVPF	SVPGTSQLGQ	QLSEHLKDAL	QAEGQEPREV	LGDLEEFLIF	RASVEGGGFN	ERCLVAASFR	DVGERTVVNA
1050	1060	1070	1080	1090	1100	1110	1120
LFNNQAYHSP	ATALAVVDNL	LFKLLCGPHA	SIVVSNFPQP	RSALQAAKDQ	FNEGRKGFDI	ALNLLFAMAF	LASTFSILAV
1130	1140	1150	1160	1170	1180	1190	1200
SERAVQAKHV	QFVSGVHVAS	FWLSALLWDL	ISFLIPSLLL	LVVFKAFDVR	AFTRDGHMAD	TLLLLLLYGW	AIIPLMYLMN
1210	1220	1230	1240	1250	1260	1270	1280
FFFLGAATAY	TRLTIFNILS	GIATFLMVTI	MRIPAVKLEE	LSKTLDHVFL	VLPNHCLGMA	VSSFYENYET	RRYCTSSEVA
1290	1300	1310	1320	1330	1340	1350	1360
AHYCKKYNIQ	YQENFYAWSA	PGVGRFVASM	AASGCAYLIL	LFLIETNLLQ	RLRGILCALR	RRRTLTELYT	RMPVLPEDQD
1370	1380	1390	1400	1410	1420	1430	1440
VADERTRILA	PSPDSLLHTP	LIIKELSKVY	EQRVPLLAVD	RLSLAVQKGE	CFGLLGFNGA	GKTTTFKMLT	GEESLTSGDA
1450	1460	1470	1480	1490	1500	1510	1520
FVGGHRISSD	VGKVRQRIGY	CPQFDALLDH	MTGREMLVMY	ARLRGIPERH	IGACVENTLR	GLLLEPHANK	LVRTYSGGNK
1530	1540	1550	1560	1570	1580	1590	1600
RKLSTGIALI	GEPAVIFLDE	PSTGMDPVAR	RLLWDTVARA	RESGKAIIIT	SHSMEECEAL	CTRLAIMVQG	QFKCLGSPQH
1610	1620	1630	1640	1650	1660	1670	1680
LKSKFGSGYS	LRAKVQSEGQ	QEALEEFKAF	VDLTFPGSVL	EDEHQGMVHY	HLPGRDLSWA	KVFGILEKAK	EKYGVDDYSV
1690	1700	1710					
SQISLEQVFL	SFAHLQPPTA	EEGR					

Detailed Protein Report

Cmpd.	No. of Cmpds.	m/z meas.	Δ m/z [ppm]	z	Rt [min]	Score	P	Range	Sequence	Modification	Ratios
198	1	424.2139	-143.01	3	32.2	15.7	1	672-684	R.KLSIGIALIAGSK.V		
448	2	607.1621	-217.19	2	34.9	32.4	0	988-998	K.DALQAEGQEPR.E		MD:MU 1.13

Detailed Protein Report

Protein 74: dynein heavy chain 9, axonemal isoform 2 [Homo sapiens]

**Accession:**	gi|114155133	**Score:**	47.9
**Database:**	refseq_human(refseq_human_20140103.fasta)	**MW [kDa]:**	511.6
**Database Date:**	2015-11-30	**pI:**	5.6
		**Sequence Coverage [%]:**	0.8
		**No. of unique Peptides:**	2

Detailed Protein Report

10	20	30	40	50	60	70	80
MRLAEERAAL	AAENADGEPG	ADRRLRLLGT	YVAMSLRPAA	GAWERCAGSA	EAEQLLQAFL	GRDAAEGPRP	LLVVRPGPRG
90	100	110	120	130	140	150	160
LAIRPGLEVG	PESGLAGAKA	LFFLRTGPEP	PGPDSFRGAV	VCGDLPAAPL	EHLAALFSEV	VLPVLANEKN	RLNWPHMICE
170	180	190	200	210	220	230	240
DVRRHAHSLQ	CDLSVILEQV	KGKTLLPLPA	GSEKMEFADS	KSETVLDSID	KSVIYAIESA	VIKWSYQVQV	VLKRESSQPL
250	260	270	280	290	300	310	320
LQGENPTPKV	ELEFWKSRYE	DLKYIYNQLR	TITVRGMAKL	LDKLQSSYFP	AFKAMYRDVV	AALAEAQDIH	VHLIPLQRHL
330	340	350	360	370	380	390	400
EALENAEFPE	VKPQLRPLLH	VVCLIWATCK	SYRSPGRLTV	LLQEICNLLI	QQASNYLSPE	DLLRSEVEES	QRKLQVVSDT
410	420	430	440	450	460	470	480
LSFFKQEFQD	RRENLHTYFK	ENQEVKEWDF	QSSLVFVRLD	GFLGQLHVVE	GLLKTALDFH	KLGKVEFSGV	RGNALSQQVQ
490	500	510	520	530	540	550	560
QMHEEFQEMY	RLLSGSSSDC	LYLQSTDFEN	DVSEFNQKVE	DLDRRLGTIF	IQAFDDAPGL	EHAFKLLDIA	GNLLERPLVA
570	580	590	600	610	620	630	640
RDTSDKYLVL	IQMFNKDLDA	VRMIYSQHVQ	EEAELGFSPV	HKNMPTVAGG	LRWAQELRQR	IQGPFSNFGR	ITHPCMESAE
650	660	670	680	690	700	710	720
GKRMQQKYED	MLSLLEKYET	RLYEDWCRTV	SEKSQYNLSQ	PLLKRDPETK	EITINFNPQL	ISVLKEMSYL	EPREMKHMPE
730	740	750	760	770	780	790	800
TAAAMFSSRD	FYRQLVANLE	LMANWYNKVM	KTLLEVEFPL	VEEELQNIDL	RLRAAEETLN	WKTEGICDYV	TEITSSIHDL
810	820	830	840	850	860	870	880
EQRIQKTKDN	VEEIQNIMKT	WVTPIFKTKD	GKRESLLSLD	DRHDRMEKYY	NLIKESGLKI	HALVQENLGL	FSADPTSNIW
890	900	910	920	930	940	950	960
KTYVNSIDNL	LLNGFFLAIE	CSLKYLLENT	ECKAGLTPIF	EAQLSLAIPE	LVFYPSLESG	VKGGFCDIVE	GLITSIFRIP
970	980	990	1000	1010	1020	1030	1040
SLVPRLSPQN	GSPHYQVDLD	GIPDLANMRR	TLMERVQRMM	GLCCGYQSTF	SQYSYLYVED	RKEVLGQFLL	YGHILTPEEI
1050	1060	1070	1080	1090	1100	1110	1120
EDHVEDGIPE	NPPLLSQFKV	QIDSYETLYE	EVCRLEPIKV	FDGWMKIDIR	PFKASLLNII	KRWSLLFKQH	LVDHVTHSLA
1130	1140	1150	1160	1170	1180	1190	1200
NLDAFIKKSE	SGLLKKVEKG	DFQGLVEIMG	HLMAVKERQS	NTDEMFEPLK	QTIELLKTYE	QELPETVFKQ	LEELPEKWNN
1210	1220	1230	1240	1250	1260	1270	1280
IKKVAITVKQ	QVAPLQANEV	TLLRQRCTAF	DAEQQQFWEQ	FHKEAPFRFD	SIHPHQMLDA	RHIEIQQMES	TMASISESAS
1290	1300	1310	1320	1330	1340	1350	1360
LFEVNVPDYK	QLRQCRKEVC	QLKELWDTIG	MVTSSIHAWE	TTPWRNINVE	AMELECKQFA	RHIRNLDKEV	RAWDAFTGLE
1370	1380	1390	1400	1410	1420	1430	1440
STVWNTLSSL	RAVAELQNPA	IRERHWRQLM	QATGVSFTMD	QDTTLAHLLQ	LQLHHYEDEV	RGIVDKAAKE	MGMEKTLKEL
1450	1460	1470	1480	1490	1500	1510	1520
QTTWAGMEFQ	YEPHPRTNVP	LLCSDEDLIE	VLEDNQVQLQ	NLVMSKYVAF	FLEEVSGWQK	KLSTVDAVIS	IWFEVQRTWT
1530	1540	1550	1560	1570	1580	1590	1600
HLESIFTGSE	DIRAQLPQDS	KRFEGIDIDF	KELAYDAQKI	PNVVQTTNKP	GLYEKLEDIQ	GRLCLCEKAL	AEYLDTKRLA
1610	1620	1630	1640	1650	1660	1670	1680
FPRFYFLSSS	DLLDILSNGT	APQQVQRHLS	KLFDNMAKMR	FQLDASGEPT	KTSLGMYSKE	EEYVAFSEPC	DCSGQVEIWL
1690	1700	1710	1720	1730	1740	1750	1760
NHVLGHMKAT	VRHEMTEGVT	AYEEKPREQW	LFDHPAQVAL	TCTQIWWTTE	VGMAFARLEE	GYESAMKDYY	KKQVAQLKTL
1770	1780	1790	1800	1810	1820	1830	1840
ITMLIGQLSK	GDRQKIMTIC	TIDVHARDVV	AKMIAQKVDN	AQAFLWLSQL	RHRWDDEVKH	CFANICDAQF	LYSYEYLGNT
1850	1860	1870	1880	1890	1900	1910	1920
PRLVITPLTD	RCYITLTQSL	HLTMSGAPAG	PAGTGKTETT	KDLGRALGIL	VYVFNCSEQM	DYKSCGNIYK	GLAQTGAWGC
1930	1940	1950	1960	1970	1980	1990	2000
FDEFNRISVE	VLSVVAVQVK	SIQDAIRDKK	QWFSFLGEEI	SLNPSVGIFI	TMNPGYAGRT	ELPENLKSLF	RPCAMVVPDF
2010	2020	2030	2040	2050	2060	2070	2080
ELICEIMLVA	EGFIEAQSLA	RKFITLYQLC	KELLSKQDHY	DWGLRAIKSV	LVVAGSLKRG	DPDRPEDQVL	MRSLRDFNIP
2090	2100	2110	2120	2130	2140	2150	2160
KIVTDDMPIF	MGLIGDLFPA	LDVPRRRDPN	FEALVRKAIV	DLKLQAEDNF	VLKVVQLEEL	LAVRHSVFVV	GGAGTGKSQV
2170	2180	2190	2200	2210	2220	2230	2240

Cmpd.	No. of Cmpds.	m/z meas.	Δ m/z [ppm]	z	Rt [min]	Score	P	Range	Sequence	Modification
2873	2	731.8707	-28.75	2	66.4	25.7	1	1075-1086	R.LEPIKVFDGWMK.I	
54	1	796.7965	-120.13	2	30.4	11.9	0	3448-3461	R.MSVENATILINCER.W	

Detailed Protein Report

Protein 75: **PERQ amino acid-rich with GYF domain-containing protein 2 isoform c [Homo sapiens]**

**Accession:**	gi|156766047	**Score:**	47.1
**Database:**	refseq_human(refseq_human_20140103.fasta)	**MW [kDa]:**	149.4
**Database Date:**	2015-11-30	**pI:**	5.3
**Modification(s):**	Oxidation	**Sequence Coverage [%]:**	2.8
		**No. of unique Peptides:**	3

Quantitation

MD:MU **Median:** 0.58 **CV:** 0.00 % **No. of Peptides:** 1

10	20	30	40	50	60	70	80
MAAETQTLNF	GPEWLRALSS	GGSITSPPLS	PALPKYKLAD	YRYGREEMLA	LFLKDNKIPS	DLLDKEFLPI	LQEEPLPPLA
90	100	110	120	130	140	150	160
LVPFTEEEQR	NFSMSVNSAA	VLRLTGRGGG	GTVVGAPRGR	SSSRGRGRGR	GECGFYQRSF	DEVEGVFGRG	GGREMHRSQS
170	180	190	200	210	220	230	240
WEERGDRRFE	KPGRKDVGRP	NFEEGGPTSV	GRKHEFIRSE	SENWRIFREE	QNGEDEDGGW	RLAGSRRDGE	RWRPHSPGWR
250	260	270	280	290	300	310	320
EHMERRRRFE	FDFRDRDDER	GYRRVRSGSG	SIDDDRDSLP	EWCLEDAEEE	MGTFDSSGAF	LSLKKVQKEP	IPEEQEMDFR
330	340	350	360	370	380	390	400
PVDEGEECSD	SEGSHNEEAK	EPDKTNKKEG	EKTDRVGVEA	SEETPQTSSS	SARPGTPSDH	QSQEASQFER	KDEPKTEQTE
410	420	430	440	450	460	470	480
KAEEETRMEN	SLPAKVPSRG	DEMVADVQQP	LSQIPSDTAS	PLLILPPPVP	NPSPTLRPVE	TPVVGAPGMG	SVSTEPDDEE
490	500	510	520	530	540	550	560
GLKHLEQQAE	KMVAYLQDSA	LDDERLASKL	QEHRAKGVSI	PLMHEAMQKW	YYKDPQGEIQ	GPFNNQEMAE	WFQAGYFTMS
570	580	590	600	610	620	630	640
LLVKRACDES	FQPLGDIMKM	WGRVPFSPGP	APPPHMGELD	QERLTRQQEL	TALYQMQHLQ	YQQFLIQQQY	AQVLAQQQKA
650	660	670	680	690	700	710	720
ALSSQQQQQL	ALLLQQFQTL	KMRISDQNII	PSVTRSVSVP	DTGSIWELQP	TASQPTVWEG	GSVWDLPLDT	TTPGPALEQL
730	740	750	760	770	780	790	800
QQLEKAKAAK	LEQERREAEM	RAKREEEERK	RQEELRRQQE	EILRRQQEEE	RKRREEEELA	RRKQEEALRR	QREQEIALRR
810	820	830	840	850	860	870	880
QREEEERQQQ	EEALRRLEER	RREEEERRKQ	EELLRKQEEE	AAKWAREEEE	AQRRLEENRL	RMEEEAARLR	HEEEERKRKE
890	900	910	920	930	940	950	960
LEVQRQKELM	RQRQQQQEAL	RRLQQQQQQQ	QLAQMKLPSS	STWGQQSNTT	ACQSQATLSL	AEIQKLEEER	ERQLREEQRR
970	980	990	1000	1010	1020	1030	1040
QQRELMKALQ	QQQQQQQQKL	SGWGNVSKPS	GTTKSLLEIQ	QEEARQMQKQ	QQQQQQHQQP	NRARNNTHSN	LHTSIGNSVW
1050	1060	1070	1080	1090	1100	1110	1120
GSINTGPPNQ	WASDLVSSIW	SNADTKNSNM	GFWDDAVKEV	GPRNSTNKNK	NNASLSKSVG	VSNRQNKKVE	EEEKLLKLFQ
1130	1140	1150	1160	1170	1180	1190	1200
GVNKAQDGFT	QWCEQMLHAL	NTANNLDVPT	FVSFLKEVES	PYEVHDYIRA	YLGDTSEAKE	FAKQFLERRA	KQKANQQRQQ
1210	1220	1230	1240	1250	1260	1270	1280
QQLPQQQQQQ	PPQQPPQQPQ	QQDSVWGMNH	STLHSVFQTN	QSNNQQSNFE	AVQSGKKKKK	QKMVRADPSL	LGFSVNASSE
1290	1300						
RLNMGEIETL	DDY						

Cmpd.	No. of Cmpds.	m/z meas.	Δ m/z [ppm]	z	Rt [min]	Score	P	Range	Sequence	Modification	Ratios
1750	1	863.3936	-7.87	2	51.7	13.5	1	565-579	K.RACDESFQPLGDIMK.M	Oxidation: 14	
1628	1	1071.6266	33.70	1	50.2	10.0	1	758-765	R.QQEEILRR.Q		
196	2	838.3951	-10.67	2	32.2	23.6	0	1010-1022	K.QQQQQQQHQQPNR.A		MD:MU 0.58

Detailed Protein Report

Protein 76: PREDICTED: dynein heavy chain 10, axonemal isoform X1 [Homo sapiens]

**Accession:**	gi|530401632	**Score:**	46.5
**Database:**	refseq_human(refseq_human_20140103.fasta)	**MW [kDa]:**	528.4
**Database Date:**	2015-11-30	**pI:**	5.5
		**Sequence Coverage [%]:**	0.9
		**No. of unique Peptides:**	2

Quantitation

MD:MU	**Median:** 0.86	**CV:** 0.00 %	**No. of Peptides:** 1

Detailed Protein Report

10	20	30	40	50	60	70	80
MDDLRVLWMR	DRVYAAFGIT	DPQLFEDLLN	RDDGQGEDLI	LHFLNQASEE	EGPSALFIYR	TMVPEEVEVE	IDEIPVLSEE
90	100	110	120	130	140	150	160
GEEEEETYSQ	KVESVDKVRA	KRVSLRTESL	GQPLNREDEE	MDKEISEKLP	SKRTAKHIME	KMHLHMLCTP	LPEEFLDQNV
170	180	190	200	210	220	230	240
VFFLRNTKEA	ISEATDMKEA	MEIMPETLEY	GIINANVLHF	LKNIICQVFL	PALSFNQHRT	STTVGVTSGE	VSNSSEHESD
250	260	270	280	290	300	310	320
LPPMPGEAVE	YHSIQLIRDE	FLMNVQKFAS	NIQRTMQQLE	GEIKLEMPII	SVEGEVSDLA	ADPETVDILE	QCVINWLNQI
330	340	350	360	370	380	390	400
STAVEAQLKK	TPQGKGPLAE	IEFWRERNAT	LSALHEQTKL	PIVRKVLDVI	KESDSMLVAN	LQPVFTELFK	FHTEASDNVR
410	420	430	440	450	460	470	480
FLSTVERYFK	NITHGSGFHV	VLDTIPAMMS	ALRMVWIISR	HYNKDERMIP	LMERIAWEIA	ERVCRVVNLR	TLFKENRASA
490	500	510	520	530	540	550	560
QSKTLEARNT	LRLWKKAYFD	TRAKIEASGR	EDRWEFDRKR	LFERTDYMAT	ICQDLSDVLQ	ILEEFYNIFG	PELKAVTGDP
570	580	590	600	610	620	630	640
KRIDDVLCRV	DGLVTPMENL	TFDPFSIKSS	QFWKYVMDEF	KIEVLVIEKE	AKHFIDESFK	TLRSAEAAFD	MLLKFKHIRS
650	660	670	680	690	700	710	720
REAVNRQMMM	KFNDILAQYC	KEIDIINKIF	VQNLENPPLY	KNHPPVAGAI	YWERSLFFRI	KHTILRFQEV	QEILDSDRGQ
730	740	750	760	770	780	790	800
EVKQKYLEVG	RTMKEYEDRK	YEQWMEVTEQ	VLPALMKKSL	LTKSSIATEE	PSTLERGAVF	AINFSPALRE	IINETKYLEQ
810	820	830	840	850	860	870	880
LGFTVPELAR	NVALQEDKFL	RYTAGIQRML	DHYHMLIGTL	NDAESVLLKD	HSQELLRVFR	SGYKRLNWNS	LGIGDYITGC
890	900	910	920	930	940	950	960
KQAIGKFESL	VHQIHKNADD	ISSRLTLIEA	INLFKYPAAK	SEEELPGVKE	FFEHIERERA	SDVDHMVRWY	LAIGPLLTKV
970	980	990	1000	1010	1020	1030	1040
EGLVVHTNTG	KAPKLASYYK	YWEKKIYEVL	TKLILKNLQS	FNSLILGNVP	LFHTETILTA	PEIILHPNTN	EIDKMCFHCV
1050	1060	1070	1080	1090	1100	1110	1120
RNCVEITKHF	VRWMNGSCIE	CPPQKGEEEE	VVIINFYNDI	SLNPQIIEQA	VMIPQNVHRI	LINLMKYLQK	WKRYRPLWKL
1130	1140	1150	1160	1170	1180	1190	1200
DKAIVMEKFA	AKKPPCVAYD	EKLQFYSKIA	YEVMRHPLIK	DEHCIRLQLR	HLANTVQENA	KSWVISLGKL	LNESAKEELY
1210	1220	1230	1240	1250	1260	1270	1280
NLHEEMEHLA	KNLRKIPNTL	EDLKFVLATI	AEIRSKSLVM	ELRYRDVQER	YRTMAMYNLF	PPDAEKELVD	KIESIWSNLF
1290	1300	1310	1320	1330	1340	1350	1360
NDSVNVEHAL	GDIKRTFTEL	TRGEIMNYRV	QIEEFAKRFY	SEGPGSVGDD	LDKGVELLGV	YERELARHEK	SRQELANAEK
1370	1380	1390	1400	1410	1420	1430	1440
LFDLPITMYP	ELLKVQKEMS	GLRMIYELYE	GLKVAKEEWS	QTLWINLNVQ	ILQEGIEGFL	RALRKLPRPV	RGLSVTYYLE
1450	1460	1470	1480	1490	1500	1510	1520
AKMKAFKDSI	PLLLDLKNEA	LRDRHWKELM	EKTSVFFEMT	ETFTLENMFA	MELHKHTDVL	NEIVTAAIKE	VAIEKAVKEI
1530	1540	1550	1560	1570	1580	1590	1600
LDTWENMKFT	VVKYCKGTQE	RGYILGSVDE	IIQSLDDNTF	NLQSISGSRF	VGPFLQTVHK	WEKTLSLIGE	VIEIWMLVQR
1610	1620	1630	1640	1650	1660	1670	1680
KWMYLESIFI	GGDIRSQLPE	EAKKFDNIDK	VFKRIMGETL	KDPVIKRCCE	APNRLSDLQN	VSEGLEKCQK	SLNDYLDSKR
1690	1700	1710	1720	1730	1740	1750	1760
NAFPRFFFIS	DDELLSILGS	SDPLCVQEHM	IKMYDNIASL	RFNDGDSGEK	LVSAMISAEG	EVMEFRKILR	AEGRVEDWMT
1770	1780	1790	1800	1810	1820	1830	1840
AVLNEMRRTN	RLITKEAIFR	YCEDRSRVDW	MLLYQGMVVL	AASQVWWTWE	VEDVFHKAQK	GEKQAMKNYG	RKMHRQIDEL
1850	1860	1870	1880	1890	1900	1910	1920
VTRITMPLSK	NDRKKYNTVL	IIDVHARDIV	DSFIRGSILE	AREFDWESQL	RFYWDREPDE	LNIRQCTGTF	GYGYEYMGLN
1930	1940	1950	1960	1970	1980	1990	2000
GRLVITPLTD	RIYLTLTQAL	SMYLGGAPAG	PAGTGKTETT	KDLAKALGLL	CVVTNCGEGM	DYRAVGKIFS	GLAQCGAWGC
2010	2020	2030	2040	2050	2060	2070	2080
FDEFNRIDAS	VLSVISSQIQ	TIRNALIHQL	TTFQFEGQEI	SLDSRMGIFI	TMNPGYAGRT	ELPESVKALF	RPVVVIVPDL
2090	2100	2110	2120	2130	2140	2150	2160
QQICEIMLFS	EGFLEAKTLA	KKMTVLYKLA	REQLSKQYHY	DFGLRALKSV	LVMAGELKRG	SSDLREDVVL	MRALRDMNLP
2170	2180	2190	2200	2210	2220	2230	2240

Detailed Protein Report

Cmpd.	No. of Cmpds.	m/z meas.	Δ m/z [ppm]	z	Rt [min]	Score	P	Range	Sequence	Modification	Ratios
1643	1	938.9588	-53.24	2	50.3	11.9	2	624-639	R.SAEAAFDMLLKFKHIR.S		
115	2	679.8485	-38.99	2	30.8	24.0	0	2780-2791	R.VFNGLVLTNPER.F		MD:MU 0.86

Detailed Protein Report

Protein 77: PREDICTED: G-protein coupled receptor 124 isoform X3 [Homo sapiens]

**Accession:**	gi|578815277	**Score:**	46.5
**Database:**	refseq_human(refseq_human_20140103.fasta)	**MW [kDa]:**	140.0
**Database Date:**	2015-11-30	**pI:**	9.9
**Modification(s):**	Carbamidomethyl	**Sequence Coverage [%]:**	2.6
		**No. of unique Peptides:**	3

10	20	30	40	50	60	70	80
MGAGGRRMRG	APARLLLPLL	PWLLLLLAPE	ARGAPGCPLS	IRSCKCSGER	PKGLSGGVPG	PARRRVVCSG	GDLPEPPEPG
**90**	**100**	**110**	**120**	**130**	**140**	**150**	**160**
LLPNGTVTLD	LRNNIISTVQ	PGAFLGLGEL	KRLDLSNNRI	GCLTSETFQG	LPRLLRLNIS	GNIFSSLQPG	VFDELPALKV
**170**	**180**	**190**	**200**	**210**	**220**	**230**	**240**
VDLGTEFLTC	DCHLRWLLPW	AQNRSLQLSE	HTLCAYPSAL	HAQALGSLQE	AQLCCEGALE	LHTHHLIPSL	RQVVFQGDRL
**250**	**260**	**270**	**280**	**290**	**300**	**310**	**320**
PFQCSASYLG	NDTRIRWYHN	RAPVEGDEQA	GILLAESLIH	DCTFITSELT	LSHIGVWASG	EWECTVSMAQ	GNASKKVEIV
**330**	**340**	**350**	**360**	**370**	**380**	**390**	**400**
VLETSASYCP	AERVANNRGD	FRWPRTLAGI	TAYQSCLQYP	FTSVPLGGGA	PGTRASRRCD	RAGRWEPGDY	SHCLYTNDIT
**410**	**420**	**430**	**440**	**450**	**460**	**470**	**480**
RVLYTFVLMP	INASNALTLA	HQLRVYTAEA	ASFSDMMDVV	YVAQMIQKFL	GYVDQIKELV	EVMVDMASNL	MLVDEHLLWL
**490**	**500**	**510**	**520**	**530**	**540**	**550**	**560**
AQREDKACSR	IVGALERIGG	AALSPHAQHI	SVNARNVALE	AYLIKPHSYV	GLTCTAFQRR	EGGVPGTRPG	SPGQNPPPEP
**570**	**580**	**590**	**600**	**610**	**620**	**630**	**640**
EPPADQQLRF	RCTTGRPNVS	LSSFHIKNSV	ALASIQLPPS	LFSSLPAALA	PPVPPDCTLQ	LLVFRNGRLF	HSHSNTSRPG
**650**	**660**	**670**	**680**	**690**	**700**	**710**	**720**
AAGPGKRRGV	ATPVIFAGTS	GCGVGNLTEP	VAVSLRHWAE	GAEPVAAWWS	QEGPGEAGGW	TSEGCQLRSS	QPNVSALHCQ
**730**	**740**	**750**	**760**	**770**	**780**	**790**	**800**
HLGNVAVLME	LSAFPREVGG	AGAGLHPVVY	PCTALLLLCL	FATIITYILN	HSSIRVSRKG	WHMLLNLCFH	IAMTSAVFAG
**810**	**820**	**830**	**840**	**850**	**860**	**870**	**880**
GITLTNYQMV	CQAVGITLHY	SSLSTLLWMG	VKARVLHKEL	TWRAPPPQEG	DPALPTPSPM	LRFYLIAGGI	PLIICGITAA
**890**	**900**	**910**	**920**	**930**	**940**	**950**	**960**
VNIHNYRDHS	PYCWLVWRPS	LGAFYIPVAL	ILLITWIYFL	CAGLRLRGPL	AQNPKAGNSR	ASLEAGEELR	GSTRLRGSGP
**970**	**980**	**990**	**1000**	**1010**	**1020**	**1030**	**1040**
LLSDSGSLLA	TGSARVGTPG	PPEDGDSLYS	PGVQLGALVT	THFLYLAMWA	CGALAVSQRW	LPRVVCSCLY	GVAASALGLF
**1050**	**1060**	**1070**	**1080**	**1090**	**1100**	**1110**	**1120**
VFTHHCARRR	DVRASWRACC	PPASPAAPHA	PPRALPAAAE	DGSPVFGEGP	PSLKSSPSGS	SGHPLALGPC	KLTNLQLAQS
**1130**	**1140**	**1150**	**1160**	**1170**	**1180**	**1190**	**1200**
QVCEAGAAAG	GEGEPEPAGT	RGNLAHRHPN	NVHHGRRAHK	SRAKGHRAGE	ACGKNRLKAL	RGGAAGALEL	LSSESGSLHN
**1210**	**1220**	**1230**	**1240**	**1250**	**1260**	**1270**	**1280**
SPTDSYLGSS	RNSPGAGLQL	EGEPMLTPSE	GSDTSAAPLS	EAGRAGQRRS	ASRDSLKGGG	ALEKESHRRS	YPLNAASLNG
**1290**	**1300**	**1310**	**1320**				
APKGGKYDDV	TLMGAEVASG	GCMKTGLWKS	ETTV				

Cmpd.	No. of Cmpds.	m/z meas.	Δ m/z [ppm]	z	Rt [min]	Score	P	Range	Sequence	Modification
2732	2	673.3595	29.93	2	64.5	15.8	1	33-45	R.GAPGCPLSIRSCK.C	Carbamidomethyl: 5
2870	14	673.3611	32.18	2	66.4	16.5	1	33-45	R.GAPGCPLSIRSCK.C	Carbamidomethyl: 12
1660	1	716.0007	12.17	3	50.5	14.1	1	1284-1304	K.GGKYDDVTLMGAEVASGGCMK.T	Carbamidomethyl: 19

Detailed Protein Report

Protein 78: **insulin receptor-related protein precursor [Homo sapiens]**

Accession: gi|31657140
Database: refseq_human(refseq_human_20140103.fasta)
Database Date: 2015-11-30
Modification(s): Carbamidomethyl

Score: 46.5
MW [kDa]: 143.6
pI: 6.0
Sequence Coverage [%]: 3.0
No. of unique Peptides: 2

10	20	30	40	50	60	70	80
MAVPSLWPWG	ACLPVIFLSL	GFGLDTVEVC	PSLDIRSEVA	ELRQLENCSV	VEGHLQILLM	FTATGEDFRG	LSFPRLTQVT
90	100	110	120	130	140	150	160
DYLLLFRVYG	LESLRDLFPN	LAVIRGTRLF	LGYALVIFEM	PHLRDVALPA	LGAVLRGAVR	VEKNQELCHL	STIDWGLLQP
170	180	190	200	210	220	230	240
APGANHIVGN	KLGEECADVC	PGVLGAAGEP	CAKTTFSGHT	DYRCWTSSHC	QRVCPCPHGM	ACTARGECCH	TECLGGCSQP
250	260	270	280	290	300	310	320
EDPRACVACR	HLYFQGACLW	ACPPGTYQYE	SWRCVTAERC	ASLHSVPGRA	STFGIHQGSC	LAQCPSGFTR	NSSSIFCHKC
330	340	350	360	370	380	390	400
EGLCPKECKV	GTKTIDSIQA	AQDLVGCTHV	EGSLILNLRQ	GYNLEPQLQH	SLGLVETITG	FLKIKHSFAL	VSLGFFKNLK
410	420	430	440	450	460	470	480
LIRGDAMVDG	NYTLYVLDNQ	NLQQLGSWVA	AGLTIPVGKI	YFAFNPRLCL	EHIYRLEEVT	GTRGRQNKAE	INPRTNGDRA
490	500	510	520	530	540	550	560
ACQTRTLRFV	SNVTEADRIL	LRWERYEPLE	ARDLLSFIVY	YKESPFQNAT	EHVGPDACGT	QSWNLLDVEL	PLSRTQEPGV
570	580	590	600	610	620	630	640
TLASLKPWTQ	YAVFVRAITL	TTEEDSPHQG	AQSPIVYLRT	LPAAPTVPQD	VISTSNSSSH	LLVRWKPPTQ	RNGNLTYYLV
650	660	670	680	690	700	710	720
LWQRLAEDGD	LYLNDYCHRG	LRLPTSNNDP	RFDGEDGDPE	AEMESDCCPC	QHPPPGQVLP	PLEAQEASFQ	KKFENFLHNA
730	740	750	760	770	780	790	800
ITIPISPWKV	TSINKSPQRD	SGRHRRAAGP	LRLGGNSSDF	EIQEDKVPRE	RAVLSGLRHF	TEYRIDIHAC	NHAAHTVGCS
810	820	830	840	850	860	870	880
AATFVFARTM	PHREADGIPG	KVAWEASSKN	SVLLRWLEPP	DPNGLILKYE	IKYRRLGEEA	TVLCVSRLRY	AKFGGVHLAL
890	900	910	920	930	940	950	960
LPPGNYSARV	RATSLAGNGS	WTDSVAFYIL	GPEEEDAGGL	HVLLTATPVG	LTLLIVLAAL	GFFYGKKRNR	TLYASVNPEY
970	980	990	1000	1010	1020	1030	1040
FSASDMYVPD	EWEVPREQIS	IIRELGQGSF	GMVYEGLARG	LEAGEESTPV	ALKTVNELAS	PRECIEFLKE	ASVMKAFKCH
1050	1060	1070	1080	1090	1100	1110	1120
HVVRLLGVVS	QGQPTLVIME	LMTRGDLKSH	LRSLRPEAEN	NPGLPQPALG	EMIQMAGEIA	DGMAYLAANK	FVHRDLAARN
1130	1140	1150	1160	1170	1180	1190	1200
CMVSQDFTVK	IGDFGMTRDV	YETDYYRKGG	KGLLPVRWMA	PESLKDGIFT	THSDVWSFGV	VLWEIVTLAE	QPYQGLSNEQ
1210	1220	1230	1240	1250	1260	1270	1280
VLKFVMDGGV	LEELEGCPLQ	LQELMSRCWQ	PNPRLRPSFT	HILDSIQEEL	RPSFRLLSFY	YSPECRGARG	SLPTTDAEPD
1290	1300						
SSPTPRDCSP	QNGGPGH						

Cmpd.	No. of Cmpds.	m/z meas.	Δ m/z [ppm]	z	Rt [min]	Score	P	Range	Sequence	Modification
561	1	730.2206	-117.87	2	36.3	16.7	0	213-225	R.VCPCPHGMACTAR.G	Carbamidomethyl: 2, 4
2751	1	922.4677	-48.57	2	65.7	17.3	2	499-512	R.ILLRWERYEPLEAR.D	

Detailed Protein Report

Protein 79: plastin-2 [Homo sapiens]

**Accession:**	gi|167614506	**Score:**	46.4
**Database:**	refseq_human(refseq_human_20140103.fasta)	**MW [kDa]:**	70.2
**Database Date:**	2015-11-30	**pI:**	5.2
**Modification(s):**	Carbamidomethyl, Oxidation	**Sequence Coverage [%]:**	8.0
		**No. of unique Peptides:**	4

Alias proteins:

Accession	Name	Description
gi|530402335	refseq_human (refseq_human_20140103.fasta)	PREDICTED: plastin-2 isoform X2 [Homo sapiens]

10	20	30	40	50	60	70	80
MARGSVSDEE	MMELREAFAK	VDTDGNGYIS	FNELNDLFKA	ACLPLPGYRV	REITENLMAT	GDLDQDGRIS	FDEFIKIFHG
**90**	**100**	**110**	**120**	**130**	**140**	**150**	**160**
LKSTDVAKTF	RKAINKKEGI	CAIGGTSEQS	SVGTQHSYSE	EEKYAFVNWI	NKALENDPDC	RHVIPMNPNT	NDLFNAVGDG
**170**	**180**	**190**	**200**	**210**	**220**	**230**	**240**
IVLCKMINLS	VPDTIDERTI	NKKKLTPFTI	QENLNLALNS	ASAIGCHVVN	IGAEDLKEGK	PYLVLGLLWQ	VIKIGLFADI
**250**	**260**	**270**	**280**	**290**	**300**	**310**	**320**
ELSRNEALIA	LLREGESLED	LMKLSPEELL	LRWANYHLEN	AGCNKIGNFS	TDIKDSKAYY	HLLEQVAPKG	DEEGVPAVVI
**330**	**340**	**350**	**360**	**370**	**380**	**390**	**400**
DMSGLREKDD	IQRAECMLQQ	AERLGCRQFV	TATDVVRGNP	KLNLAFIANL	FNRYPALHKP	ENQDIDWGAL	EGETREERTF
**410**	**420**	**430**	**440**	**450**	**460**	**470**	**480**
RNWMNSLGVN	PRVNHLYSDL	SDALVIFQLY	EKIKVPVDWN	RVNKPPYPKL	GGNMKKLENC	NYAVELGKNQ	AKFSLVGIGG
**490**	**500**	**510**	**520**	**530**	**540**	**550**	**560**
QDLNEGNRTL	TLALIWQLMR	RYTLNILEEI	GGGQKVNDDI	IVNWVNETLR	EAKKSSSISS	FKDPKISTSL	PVLDLIDAIQ
**570**	**580**	**590**	**600**	**610**	**620**	**630**	
PGSINYDLLK	TENLNDDEKL	NNAKYAISMA	RKIGARVYAL	PEDLVEVNPK	MVMTVFACLM	GKGMKRV	

Cmpd.	No. of Cmpds.	m/z meas.	Δ m/z [ppm]	z	Rt [min]	Score	P	Range	Sequence	Modification
545	1	652.1661	-227.16	2	36.1	14.5	0	402-412	R.NWMNSLGVNPR.V	Oxidation: 3
2352	1	836.1191	173.33	2	59.3	10.6	2	442-456	R.VNKPPYPKLGGNMKK.L	
1806	1	649.7882	-96.92	2	52.6	11.2	2	531-542	R.EAKKSSSISSFK.D	
2744	1	710.3515	31.99	2	65.6	10.2	0	611-622	K.MVMTVFACLMGK.G	Carbamidomethyl: 8; Oxidation: 1, 3

Detailed Protein Report

**Protein 80:**	**collagen alpha-3(VI) chain isoform 4 precursor [Homo sapiens]**		
**Accession:**	gi|240255535	**Score:**	46.4
**Database:**	refseq_human(refseq_human_20140103.fasta)	**MW [kDa]:**	278.0
**Database Date:**	2015-11-30	**pI:**	9.1
		**Sequence Coverage [%]:**	2.0
		**No. of unique Peptides:**	2

Detailed Protein Report

10	20	30	40	50	60	70	80
MRKHRHLPLV	AVFCLFLSGF	PTTHAQQQQA	VIEVNKRDIV	FLVDGSSALG	LANFNAIRDF	IAKVIQRLEI	GQDLIQVAVA
90	100	110	120	130	140	150	160
QYADTVRPEF	YFNTHPTKRE	VITAVRKMKP	LDGSALYTGS	ALDFVRNNLF	TSSAGYRAAE	GIPKLLVLIT	GGKSLDEISQ
170	180	190	200	210	220	230	240
PAQELKRSSI	MAFAIGNKGA	DQAELEEIAF	DSSLVFIPAE	FRAAPLQGML	PGLLAPLRTL	SGTPEESKRD	ILFLFDGSAN
250	260	270	280	290	300	310	320
LVGQFPVVRD	FLYKIIDELN	VKPEGTRIAV	AQYSDDVKVE	SRFDEHQSKP	EILNLVKRMK	IKTGKALNLG	YALDYAQRYI
330	340	350	360	370	380	390	400
FVKSAGSRIE	DGVLQFLVLL	VAGRSSDRVD	GPASNLKQSG	VVPFIFQAKN	ADPAELEQIV	LSPAFILAAE	SLPKIGDLHP
410	420	430	440	450	460	470	480
QIVNLLKSVH	NGAPAPVSGE	KDVVFLLDGS	EGVRSGFPLL	KEFVQRVVES	LDVGQDRVRV	AVVQYSDRTR	PEFYLNSYMN
490	500	510	520	530	540	550	560
KQDVVNAVRQ	LTLLGGPTPN	TGAALEFVLR	NILVSSAGSR	ITEGVPQLLI	VLTADRSGDD	VRNPSVVVKR	GGAVPIGIGI
570	580	590	600	610	620	630	640
GNADITEMQT	ISFIPDFAVA	IPTFRQLGTV	QQVISERVTQ	LTREELSRLQ	PVLQPLPSPG	VGGKRDVVFL	IDGSQSAGPE
650	660	670	680	690	700	710	720
FQYVRTLIER	LVDYLDVGFD	TTRVAVIQFS	DDPKVEFLLN	AHSSKDEVQN	AVQRLRPKGG	RQINVGNALE	YVSRNIFKRP
730	740	750	760	770	780	790	800
LGSRIEEGVP	QFLVLISSGK	SDDEVDDPAV	ELKQFGVAPF	TIARNADQEE	LVKISLSPEY	VFSVSTFREL	PSLEQKLLTP
810	820	830	840	850	860	870	880
ITTLTSEQIQ	KLLASTRYPP	PAVESDAADI	VFLIDSSEGV	RPDGFAHIRD	FVSRIVRRLN	IGPSKVRVGV	VQFSNDVFPE
890	900	910	920	930	940	950	960
FYLKTYRSQA	PVLDAIRRLR	LRGGSPLNTG	KALEFVARNL	FVKSAGSRIE	DGVPQHLVLV	LGGKSQDDVS	RFAQVIRSSG
970	980	990	1000	1010	1020	1030	1040
IVSLGVGDRN	IDRTELQTIT	NDPRLVFTVR	EFRELPNIEE	RIMNSFGPSA	ATPAPPGVDT	PPPSRPEKKK	ADIVFLLDGS
1050	1060	1070	1080	1090	1100	1110	1120
INFRRDSFQE	VLRFVSEIVD	TVYEDGDSIQ	VGLVQYNSDP	TDEFFLKDFS	TKRQIIDAIN	KVVYKGGRHA	NTKVGLEHLR
1130	1140	1150	1160	1170	1180	1190	1200
VNHFVPEAGS	RLDQRVPQIA	FVITGGKSVE	DAQDVSLALT	QRGVKVFAVG	VRNIDSEEVG	KIASNSATAF	RVGNVQELSE
1210	1220	1230	1240	1250	1260	1270	1280
LSEQVLETLH	DAMHETLCPG	VTDAAKACNL	DVILGFDGSR	DQNVFVAQKG	FESKVDAILN	RISQMHRVSC	SGGRSPTVRV
1290	1300	1310	1320	1330	1340	1350	1360
SVVANTPSGP	VEAFDFDEYQ	PEMLEKFRNM	RSQHPYVLTE	DTLKVYLNKF	RQSSPDSVKV	VIHFTDGADG	DLADLHRASE
1370	1380	1390	1400	1410	1420	1430	1440
NLRQEGVRAL	ILVGLERVVN	LERLMHLEFG	RGFMYDRPLR	LNLLDLDYEL	AEQLDNIAEK	ACCGVPCKCS	GQRGDRGPIG
1450	1460	1470	1480	1490	1500	1510	1520
SIGPKGIPGE	DGYRGYPGDE	GGPGERGPPG	VNGTQGFQGC	PGQRGVKGSR	GFPGEKGEVG	EIGLDGLDGE	DGDKGLPGSS
1530	1540	1550	1560	1570	1580	1590	1600
GEKGNPGRRG	DKGPRGEKGE	RGDVGIRGDP	GNPGQDSQER	GPKGETGDLG	PMGVPGRDGV	PGGPGETGKN	GGFGRRGPPG
1610	1620	1630	1640	1650	1660	1670	1680
AKGNKGGPGQ	PGFEGEQGTR	GAQGPAGPAG	PPGLIGEQGI	SGPRGSGGAA	GAPGERGRTG	PLGRKGEPGE	PGPKGGIGNR
1690	1700	1710	1720	1730	1740	1750	1760
GPRGETGDDG	RDGVGSEGRR	GKKGERGFPG	YPGPKGNPGE	PGLNGTTGPK	GIRGRRGNSG	PPGIVGQKGD	PGYPGPAGPK
1770	1780	1790	1800	1810	1820	1830	1840
GNRGDSIDQC	ALIQSIKDKC	PCCYGPLECP	VFPTELAFAL	DTSEGVNQDT	FGRMRDVVLS	IVNDLTIAES	NCPRGARVAV
1850	1860	1870	1880	1890	1900	1910	1920
VTYNNEVTTE	IRFADSKRKS	VLLDKIKNLQ	VALTSKQQSL	ETAMSFVARN	TFKRVRNGFL	MRKVAVFFSN	TPTRASPQLR
1930	1940	1950	1960	1970	1980	1990	2000
EAVLKLSDAG	ITPLFLTRQE	DRQLINALQI	NNTAVGHALV	LPAGRDLTDF	LENVLTCHVC	LDICNIDPSC	GFGSWRPSFR
2010	2020	2030	2040	2050	2060	2070	2080
DRRAAGSDVD	IDMAFILDSA	ETTTLFQFNE	MKKYIAYLVR	QLDMSPDPKA	SQHFARVAVV	QHAPSESVDN	ASMPPVKVEF
2090	2100	2110	2120	2130	2140	2150	2160
SLTDYGSKEK	LVDFLSRGMT	QLQGTRALGS	AIEYTIENVF	ESAPNPRDLK	IVVLMLTGEV	PEQQLEEAQR	VILQAKCKGY
2170	2180	2190	2200	2210	2220	2230	2240

Detailed Protein Report

Cmpd.	No. of Cmpds.	m/z meas.	Δ m/z [ppm]	z	Rt [min]	Score	P	Range	Sequence	Modification
605	1	472.1588	-273.18	2	37.1	12.8	1	99-106	K.REVITAVR.K	
1827	1	1056.0066	84.56	3	52.9	23.4	2	2131-2158	K.IVVLMLTGEVPEQQLEEAQRVILQAKCK.G	

Detailed Protein Report

Protein 81: protein kinase C-binding protein NELL2 isoform c precursor [Homo sapiens]

**Accession:**	gi|223029474	**Score:**	46.3
**Database:**	refseq_human(refseq_human_20140103.fasta)	**MW [kDa]:**	91.1
**Database Date:**	2015-11-30	**pI:**	5.3
**Modification(s):**	Carbamidomethyl	**Sequence Coverage [%]:**	6.0
		**No. of unique Peptides:**	2

10	20	30	40	50	60	70	80
METGLGAPLF	KAWLLISVWG	LGVDPSLQID	VLTELELGES	TTGVRQVPGL	HNGTKAFLFQ	DTPRSIKAST	ATAEQFFQKL
90	100	110	120	130	140	150	160
RNKHEFTILV	TLKQTHLNSG	VILSIHHLDH	RYLELESSGH	RNEVRLHYRS	GSHRPHTEVF	PYILADDKWH	KLSLAISASH
170	180	190	200	210	220	230	240
LILHIDCNKI	YERVVEKPST	DLPLGTTFWL	GQRNNAHGYF	KGIMQDVQLL	VMPQGFIAQC	PDLNRTCPTC	NDFHGLVQKI
250	260	270	280	290	300	310	320
MELQDILAKT	SAKLSRAEQR	MNRLDQCYCE	RTCTMKGTTY	REFESWIDGC	KNCTCLNGTI	QCETLICPNP	DCPLKSALAY
330	340	350	360	370	380	390	400
VDGKCCKECK	SICQFQGRTY	FEGERNTVYS	SSGVCVLYEC	KDQTMKLVES	SGCPALDCPE	SHQITLSHSC	CKVCKGYDFC
410	420	430	440	450	460	470	480
SERHNCMENS	ICRNLNDRAV	CSCRDGFRAL	REDNAYCEDI	DECAEGRHYC	RENTMCVNTP	GSFMCICKTG	YIRIDDYSCT
490	500	510	520	530	540	550	560
EHDECITNQH	NCDENALCFN	TVGGHNCVCK	PGYTGNGTTC	KAFCKDGCRN	GGACIAANVC	ACPQGFTGPS	CETDIDECSD
570	580	590	600	610	620	630	640
GFVQCDSRAN	CINLPGWYHC	ECRDGYHDNG	MFSPSGESCE	DIDECGTGRH	SCANDTICFN	LDGGYDCRCP	HGKNCTGDCI
650	660	670	680	690	700	710	720
HDGKVKHNGQ	IWVLENDRCS	VCSCQNGFVM	CRRMVCDCEN	PTVDLFCCPE	CDPRLSSQCL	HQNGETLYNS	GDTWVQNCQQ
730	740	750	760	770	780	790	800
CRCLQGEVDC	WPLPCPDVEC	EFSILPENEC	CPRCVTDPCQ	ADTIRNDITK	TCLDEMNVVR	FTGSSWIKHG	TECTLCQCKN
810	820						
GHICCSVDPQ	CLQEL						

Cmpd.	No. of Cmpds.	m/z meas.	Δ m/z [ppm]	z	Rt [min]	Score	P	Range	Sequence	Modification
1492	1	450.0889	-239.47	2	48.4	12.4	1	522-529	K.AFCKDGCR.N	
1555	1	932.3749	-6.08	2	49.1	11.3	1	659-673	R.CSVCSCQNGFVMCRR.M	Carbamidomethyl: 1, 4, 6

Detailed Protein Report

Protein 82: PREDICTED: zinc finger protein 708 isoform X1 [Homo sapiens]

**Accession:**	gi|578833563	**Score:**	45.3
**Database:**	refseq_human(refseq_human_20140103.fasta)	**MW [kDa]:**	57.3
**Database Date:**	2015-11-30	**pI:**	10.4
**Modification(s):**	Carbamidomethyl	**Sequence Coverage [%]:**	6.0
		**No. of unique Peptides:**	3

10	20	30	40	50	60	70	80
MKRHEMAAKP	PAMCSHFAKD	LRPEQYIKNS	FQQVILRRYG	KCGYQKGCKS	VDEHKLHKGG	HKGLNRCVTT	TQSKIVQCDK
**90**	**100**	**110**	**120**	**130**	**140**	**150**	**160**
YVKVFHKYSN	AKRHKIRHTG	KNPFKCKECG	KSFCMLSQLT	QHEIIHTGEK	PYKCEECGKA	FKKSSNLTNH	KIIHTGEKPY
**170**	**180**	**190**	**200**	**210**	**220**	**230**	**240**
KCEECGKAFN	QSSTLTRHKI	IHTGEKLYKC	EECGKAFNRS	SNLTKHKIVH	TGEKPYKCEE	CGKAFKQSSN	LTNHKKIHTG
**250**	**260**	**270**	**280**	**290**	**300**	**310**	**320**
EKPYKCGECG	KAFTLSSHLT	THKRIHTGEK	PYKCEECGKA	FSVFSTLTKH	KIIHTEEKPY	KCEECGKAFN	RSSHLTNHKV
**330**	**340**	**350**	**360**	**370**	**380**	**390**	**400**
IHTGEKPYKC	EECGKAFTKS	STLTYHKVIH	TGKKPYKCEE	CGKAFSIFSI	LTKHKVIHTE	DKPYKCEECG	KTFNYSSNFT
**410**	**420**	**430**	**440**	**450**	**460**	**470**	**480**
NHKKIHTGEK	PYKCEECGKS	FILSSHLTTH	KIIHTGEKPY	KCKECGKAFN	QSSTLMKHKI	IHTGEKPYKC	EECGKAFNQS
**490**	**500**						
PNLTKHKRIH	TKEKPYKCK						

Cmpd.	No. of Cmpds.	m/z meas.	Δ m/z [ppm]	z	Rt [min]	Score	P	Range	Sequence	Modification
252	2	605.6643	-205.83	2	32.9	12.7	2	102-111	K.NPFKCKECGK.S	Carbamidomethyl: 8
1912	1	621.2738	-20.17	2	54.0	13.3	1	130-139	E.KPYKCEECGK.A	Carbamidomethyl: 5
1130	2	586.6095	-252.07	2	43.8	19.3	1	330-339	K.CEECGKAFTK.S	Carbamidomethyl: 4

Detailed Protein Report

Protein 83: profilin-1 [Homo sapiens]

**Accession:**	gi|4826898	**Score:**	45.1
**Database:**	refseq_human(refseq_human_20140103.fasta)	**MW [kDa]:**	15.0
**Database Date:**	2015-11-30	**pI:**	9.4
**Modification(s):**	Carbamidomethyl	**Sequence Coverage [%]:**	20.7
		**No. of unique Peptides:**	2

Quantitation

MD:MU **Median:** 1.60 **CV:** 84.69 % **No. of Peptides:** 2

```
        10         20         30         40         50         60         70         80
MAGWNAYIDN LMADGTCQDA AIVGYKDSPS VWAAVPGKTF VNITPAEVGV LVGKDRSSFY VNGLTLGGQK CSVIRDSLLQ
        90        100        110        120        130        140        150
DGEFSMDLRT KSTGGAPTFN VTVTKTDKTL VLLMGKEGVH GGLINKKCYE MASHLRRSQY
```

Cmpd.	No. of Cmpds.	m/z meas.	Δ m/z [ppm]	z	Rt [min]	Score	P	Range	Sequence	Modification	Ratios	
2399	1	822.4646	-8.92	2	60.3	29.2	0	39-54	K.TFVNITPAEVGVLVGK.D		MD:MU	0.76
1495	1	850.7379	-173.16	2	48.5	15.9	2	128-140	K.CYEMASHLRRSQY.-	Carbamidomethyl: 1	MD:MU	3.33

Detailed Protein Report

Protein 84: zinc finger protein 492 [Homo sapiens]

**Accession:**	gi|148806865	**Score:**	44.8
**Database:**	refseq_human(refseq_human_20140103.fasta)	**MW [kDa]:**	61.1
**Database Date:**	2015-11-30	**pI:**	10.3
**Modification(s):**	Carbamidomethyl, Oxidation	**Sequence Coverage [%]:**	8.3
		**No. of unique Peptides:**	2

Quantitation

MD:MU **Median:** 0.62 **CV:** 0.00 % **No. of Peptides:** 1

Alias proteins:

Accession	Name	Description
gi|578833408	refseq_human (refseq_human_20140103.fasta)	PREDICTED: zinc finger protein 492 isoform X1 [Homo sapiens]

10	20	30	40	50	60	70	80
MLENYRNLVF	VGIAASKPDL	ITCLEQGKEP	WNVKRHEMVA	EPPVVCSYFA	RDLWPKQGKK	NYFQKVILRR	YKKCGCENLQ
**90**	**100**	**110**	**120**	**130**	**140**	**150**	**160**
LRKYCKSMDE	CKVHKECYNG	LNQCLTTTQN	KIFQCDKYVK	VFHKFSNSNR	HTIRHTGKKS	FKCKECEKSF	CMLSHLAQHK
**170**	**180**	**190**	**200**	**210**	**220**	**230**	**240**
RIHSGEKPYK	CKECGKAYNE	TSNLSTHKRI	HTGKKPYKCE	ECGKAFNRLS	HLTTHKIIHT	GKKPYKCEEC	GKAFNQSANL
**250**	**260**	**270**	**280**	**290**	**300**	**310**	**320**
TTHKRIHTGE	KPYKCEECGR	AFSQSSTLTA	HKIIHAGEKP	YKCEECGKAF	SQSSTLTTHK	IIHTGEKFYK	CEECGKAFSQ
**330**	**340**	**350**	**360**	**370**	**380**	**390**	**400**
LSHLTTHKRI	HSGEKPYKCE	ECGKAFKQSS	TLTTHKRIHA	GEKFYKCEVC	SKAFSRFSHL	TTHKRIHTGE	KPYKCEECGK
**410**	**420**	**430**	**440**	**450**	**460**	**470**	**480**
AFNLSSQLTT	HKIIHTGEKP	YKCEECGKAF	NQSSTLSKHK	VIHTGEKPYK	YEECGKAFNQ	SSHLTTHKMI	HTGEKPYKCE
**490**	**500**	**510**	**520**	**530**	**540**		
ECGKAFNNSS	ILNRHKMIHT	GEKLYKPESC	NNACDNIAKI	SKYKRNCAGE	K		

Cmpd.	No. of Cmpds.	m/z meas.	Δ m/z [ppm]	z	Rt [min]	Score	P	Range	Sequence	Modification	Ratios
1839	1	582.2374	12.53	2	53.0	13.4	1	84-92	K.YCKSMDECK.V	Carbamidomethyl: 8	MD:MU 0.62
2688	1	992.0980	-39.40	3	64.8	18.1	2	495-519	R. HKMIHTGEKLYKPESCNNACDNIA I	Carbamidomethyl: 16, 20; Oxidation: 3	

Detailed Protein Report

Protein 85: PREDICTED: plectin isoform X9 [Homo sapiens]

**Accession:**	gi|530389159	**Score:**	44.7
**Database:**	refseq_human(refseq_human_20140103.fasta)	**MW [kDa]:**	513.0
**Database Date:**	2015-11-30	**pI:**	5.5
		**Sequence Coverage [%]:**	0.8
		**No. of unique Peptides:**	1

Detailed Protein Report

10	20	30	40	50	60	70	80
MKIVPDERDR	VQKKTFTKWV	NKHLIKHWRA	EAQRHISDLY	EDLRDGHNLI	SLLEVLSGDS	LPREKGRMRF	HKLQNVQIAL
90	100	110	120	130	140	150	160
DYLRHRQVKL	VNIRNDDIAD	GNPKLTLGLI	WTIILHFQIS	DIQVSGQSED	MTAKEKLLLW	SQRMVEGYQG	LRCDNFTSSW
170	180	190	200	210	220	230	240
RDGRLFNAII	HRHKPLLIDM	NKVYRQTNLE	NLDQAFSVAE	RDLGVTRLLD	PEDVDVPQPD	EKSIITYVSS	LYDAMPRVPD
250	260	270	280	290	300	310	320
VQDGVRANEL	QLRWQEYREL	VLLLLQWMRH	HTAAFEERRF	PSSFEEIEIL	WSQFLKFKEM	ELPAKEADKN	RSKGIYQSLE
330	340	350	360	370	380	390	400
GAVQAGQLKV	PPGYHPLDVE	KEWGKLHVAI	LEREKQLRSE	FERLECLQRI	VTKLQMEAGL	CEEQLNQADA	LLQSDVRLLA
410	420	430	440	450	460	470	480
AGKVPQRAGE	VERDLDKADS	MIRLLFNDVQ	TLKDGRHPQG	EQMYRRVYRL	HERLVAIRTE	YNLRLKAGVA	APATQVAQVT
490	500	510	520	530	540	550	560
LQSVQRRPEL	EDSTLRYLQD	LLAWVEENQH	RVDGAEWGVD	LPSVEAQLGS	HRGLHQSIEE	FRAKIERARS	DEGQLSPATR
570	580	590	600	610	620	630	640
GAYRDCLGRL	DLQYAKLLNS	SKARLRSLES	LHSFVAAATK	ELMWLNEKEE	EEVGFDWSDR	NTNMTAKKES	YSALMRELEL
650	660	670	680	690	700	710	720
KEKKIKELQN	AGDRLLREDH	PARPTVESFQ	AALQTQWSWM	LQLCCCIEAH	LKENAAYFQF	FSDVREAEGQ	LQKLQEALRR
730	740	750	760	770	780	790	800
KYSCDRSATV	TRLEDLLQDA	QDEKEQLNEY	KGHLSGLAKR	AKAVVQLKPR	HPAHPMRGRL	PLLAVCDYKQ	VEVTVHKGDE
810	820	830	840	850	860	870	880
CQLVGPAQPS	HWKVLSSSGS	EAAVPSVCFL	VPPPNQEAQE	AVTRLEAQHQ	ALVTLWHQLH	VDMKSLLAWQ	SLRRDVQLIR
890	900	910	920	930	940	950	960
SWSLATFRTL	KPEEQRQALH	SLELHYQAFL	RDSQDAGGFG	PEDRLMAERE	YGSCSHHYQQ	LLQSLEQGAQ	EESRCQRCIS
970	980	990	1000	1010	1020	1030	1040
ELKDIRLQLE	ACETRTVHRL	RLPLDKEPAR	ECAQRIAEQQ	KAQAEVEGLG	KGVARLSAEA	EKVLALPEPS	PAAPTLRSEL
1050	1060	1070	1080	1090	1100	1110	1120
ELTLGKLEQV	RSLSAIYLEK	LKTISLVIRG	TQGAEEVLRA	HEEQLKEAQA	VPATLPELEA	TKASLKKLRA	QAEAQQPTFD
1130	1140	1150	1160	1170	1180	1190	1200
ALRDELRGAQ	EVGERLQQRH	GERDVEVERW	RERVAQLLER	WQAVLAQTDV	RQRELEQLGR	QLRYYRESAD	PLGAWLQDAR
1210	1220	1230	1240	1250	1260	1270	1280
RRQEQIQAMP	LADSQAVREQ	LRQEQALLEE	IERHGEKVEE	CQRFAKQYIN	AIKDYELQLV	TYKAQLEPVA	SPAKKPKVQS
1290	1300	1310	1320	1330	1340	1350	1360
GSESVIQEYV	DLRTHYSELT	TLTSQYIKFI	SETLRRMEEE	ERLAEQQRAE	ERERLAEVEA	ALEKQRQLAE	AHAQAKAQAE
1370	1380	1390	1400	1410	1420	1430	1440
REAKELQQRM	QEEVVRREEA	AVDAQQQKRS	IQEELQQLRQ	SSEAEIQAKA	RQAEAAERSR	LRIEEEIRVV	RLQLEATERQ
1450	1460	1470	1480	1490	1500	1510	1520
RGGAEGELQA	LRARAEEAEA	QKRQAQEEAE	RLRRQVQDES	QRKRQAEVEL	ASRVKAEAEA	AREKQRALQA	LEELRLQAEE
1530	1540	1550	1560	1570	1580	1590	1600
AERRLRQAEV	ERARQVQVAL	ETAQRSAEAE	LQSKRASFAE	KTAQLERSLQ	EEHVAVAQLR	EEAERRAQQQ	AEAERAREEA
1610	1620	1630	1640	1650	1660	1670	1680
ERELERWQLK	ANEALRLRLQ	AEEVAQQKSL	AQAEAEKQKE	EAEREARRRG	KAEEQAVRQR	ELAEQELEKQ	RQLAEGTAQQ
1690	1700	1710	1720	1730	1740	1750	1760
RLAAEQELIR	LRAETEQGEQ	QRQLLEEELA	RLQREAAAAT	QKRQELEAEL	AKVRAEMEVL	LASKARAEEE	SRSTSEKSKQ
1770	1780	1790	1800	1810	1820	1830	1840
RLEAEAGRFR	ELAEEAARLR	ALAEEAKRQR	QLAEEDAARQ	RAEAERVLAE	KLAAIGEATR	LKTEAEIALK	EKEAENERLR
1850	1860	1870	1880	1890	1900	1910	1920
RLAEDEAFQR	RRLEEQAAQH	KADIEERLAQ	LRKASDSELE	RQKGLVEDTL	RQRRQVEEEI	LALKASFEKA	AAGKAELELE
1930	1940	1950	1960	1970	1980	1990	2000
LGRIRSNAED	TLRSKEQAEL	EAARQRQLAA	EEERRREAE	ERVQKSLAAE	EEAARQRKAA	LEEVERLKAK	VEEARRLRER
2010	2020	2030	2040	2050	2060	2070	2080
AEQESARQLQ	LAQEAAQKRL	QAEEKAHAFA	VQQKEQELQQ	TLQQEQSVLD	QLRGEAEAAR	RAAEEAEEAR	VQAERERAAQS
2090	2100	2110	2120	2130	2140	2150	2160
RRQVEEAERL	KQSAEEQAQA	RAQAQAAAEK	LRKEAEQEAA	RRAQAEQAAL	RQKQAADAEM	EKHKKFAEQT	LRQKAQVEQE
2170	2180	2190	2200	2210	2220	2230	2240

Detailed Protein Report

Cmpd.	No. of Cmpds.	m/z meas.	Δ m/z [ppm]	z	Rt [min]	Score	P	Range	Sequence	Modification
1692	1	582.1401	-225.95	2	51.1	12.2	0	2348-2357	R.QLEMSAEAER.L	

Detailed Protein Report

Protein 86: PREDICTED: integrin beta-8 isoform X2 [Homo sapiens]

**Accession:**	gi|530384769	**Score:**	44.6
**Database:**	refseq_human(refseq_human_20140103.fasta)	**MW [kDa]:**	71.2
**Database Date:**	2015-11-30	**pI:**	9.2
**Modification(s):**	Carbamidomethyl	**Sequence Coverage [%]:**	7.4
		**No. of unique Peptides:**	2

Alias proteins:

Accession	Name	Description
gi|530384771	refseq_human (refseq_human_20140103.fasta)	PREDICTED: integrin beta-8 isoform X3 [Homo sapiens]

10	20	30	40	50	60	70	80
MLKVHPLKKY	PVDLYYLVDV	SASMHNNIEK	LNSVGNDLSR	KMAFFSRDFR	LGFGSYVDKT	VSPYISIHPE	RIHNQCSDYN
**90**	**100**	**110**	**120**	**130**	**140**	**150**	**160**
LDCMPPHGYI	HVLSLTENIT	EFEKAVHRQK	ISGNIDTPEG	GFDAMLQAAV	CESHIGWRKE	AKRLLLVMTD	QTSHLALDSK
**170**	**180**	**190**	**200**	**210**	**220**	**230**	**240**
LAGIVVPNDG	NCHLKNNVYV	KSTTMEHPSL	GQLSEKLIDN	NINVIFAVQG	KQFHWYKDLL	PLLPGTIAGE	IESKAANLNN
**250**	**260**	**270**	**280**	**290**	**300**	**310**	**320**
LVVEAYQKLI	SEVKVQVENQ	VQGIYFNITA	ICPDGSRKPG	MEGCRNVTSN	DEVLFNVTVT	MKKCDVTGGK	NYAIIKPIGF
**330**	**340**	**350**	**360**	**370**	**380**	**390**	**400**
NETAKIHIHR	NCSCQCEDNR	GPKGKCVDET	FLDSKCFQCD	ENKCHFDEDQ	FSSESCKSHK	DQPVCSGRGV	CVCGKCSCHK
**410**	**420**	**430**	**440**	**450**	**460**	**470**	**480**
IKLGKVYGKY	CEKDDFSCPY	HHGNLCAGHG	ECEAGRCQCF	SGWEGDRCQC	PSAAAQHCVN	SKGQVCSGRG	TCVCGRCECT
**490**	**500**	**510**	**520**	**530**	**540**	**550**	**560**
DPRSIGRFCE	HCPTCYTACK	ENWNCMQCLH	PHNLSQAILD	QCKTSCALME	QQHYVDQTSE	CFSSPSYLRI	FFIIFIVTFL
**570**	**580**	**590**	**600**	**610**	**620**	**630**	**640**
IGLLKVLIIR	QVILQWNSNK	IKSSSDYRVS	ASKKDKLILQ	SVCTRAVTYR	REKPEEIKMD	ISKLNAHETF	RCNF

Cmpd.	No. of Cmpds.	m/z meas.	Δ m/z [ppm]	z	Rt [min]	Score	P	Range	Sequence	Modification
1358	1	955.9298	-52.51	2	46.6	10.6	0	286-302	R.NVTSNDEVLFNVTVTMK.K	
2775	1	746.3843	84.07	3	66.0	16.7	2	381-400	K.DQPVCSGRGVCVCGKCSCHK.I	Carbamidomethyl: 5, 11, 18

Detailed Protein Report

Protein 87: **PREDICTED: T-lymphoma invasion and metastasis-inducing protein 1 isoform X4 [Homo sapiens]**

**Accession:**	gi|530419050	**Score:**	44.2
**Database:**	refseq_human(refseq_human_20140103.fasta)	**MW [kDa]:**	161.7
**Database Date:**	2015-11-30	**pI:**	6.4
**Modification(s):**	Carbamidomethyl	**Sequence Coverage [%]:**	3.4
		**No. of unique Peptides:**	3

Quantitation

MD:MU **Median:** 3.23 **CV:** 0.00 % **No. of Peptides:** 1

10	20	30	40	50	60	70	80
MGNAESQHVE	HEFYGEKHAS	LGRKHTSRSL	RLSHKTRRTR	HASSGKVIHR	NSEVSTRSSS	TPSIPQSLAE	NGLEPFSQDG
**90**	**100**	**110**	**120**	**130**	**140**	**150**	**160**
TLEDFGSPIW	VDRVDMGLRP	VSYTDSSVTP	SVDSSIVLTA	ASVQSMPDTE	ESRLYGDDAT	YLAEGGRRQH	SYTSNGPTFM
**170**	**180**	**190**	**200**	**210**	**220**	**230**	**240**
ETASFKKKRS	KSADIWREDS	LEFSLSDLSQ	EHLTSNEEIL	GSAEEKDCEE	ARGMETRASP	RQLSTCQRAN	SLGDLYAQKN
**250**	**260**	**270**	**280**	**290**	**300**	**310**	**320**
SGVTANGGPG	SKFAGYCRNL	VSDIPNLANH	KMPPAAAEET	PPYSNYNTLP	CRKSHCLSEG	ATNPQISHSN	SMQGRRAKTT
**330**	**340**	**350**	**360**	**370**	**380**	**390**	**400**
QDVNAGEGSE	FADSGIEGAT	TDTDLLSRRS	NATNSSYSPT	TGRAFVGSDS	GSSSTGDAAR	QGVYENFRRE	LEMSTTNSES
**410**	**420**	**430**	**440**	**450**	**460**	**470**	**480**
LEEAGSAHSD	EQSSGTLSSP	GQSDILLTAA	QGTVRKAGAL	AVKNFLVHKK	NKKVESATRR	KWKHYWVSLK	GCTLFFYESD
**490**	**500**	**510**	**520**	**530**	**540**	**550**	**560**
GRSGIDHNSI	PKHAVWVENS	IVQAVPEHPK	KDFVFCLSNS	LGDAFLFQTT	SQTELENWIT	AIHSACATAV	ARHHHKEDTL
**570**	**580**	**590**	**600**	**610**	**620**	**630**	**640**
RLLKSEIKKL	EQKIDMDEKM	KKMGEMQLSS	VTDSKKKKTI	LDQIFVWEQN	LEQFQMDLFR	FRCYLASLQG	GELPNPKRLL
**650**	**660**	**670**	**680**	**690**	**700**	**710**	**720**
AFASRPTKVA	MGRLGIFSVS	SFHALVAART	GETGVRRRTQ	AMSRSASKRR	SRFSSLWGLD	TTSKKKQGRP	SINQVFGEGT
**730**	**740**	**750**	**760**	**770**	**780**	**790**	**800**
EAVKKSLEGI	FDDIVPDGKR	EKEVVLPNVH	QHNPDCDIWV	HEYFTPSWFC	LPNNQPALTV	VRPGDTARDT	LELICKTHQL
**810**	**820**	**830**	**840**	**850**	**860**	**870**	**880**
DHSAHYLRLK	FLIENKMQLY	VPQPEEDIYE	LLYKEIEICP	KVTQSIHIEK	SDTAADTYGF	SLSSVEEDGI	RRLYVNSVKE
**890**	**900**	**910**	**920**	**930**	**940**	**950**	**960**
TGLASKKGLK	AGDEILEINN	RAADALNSSM	LKDFLSQPSL	GLLVRTYPEL	EEGVELLESP	PHRVDGPADL	GESPLAFLTS
**970**	**980**	**990**	**1000**	**1010**	**1020**	**1030**	**1040**
NPGHSLCSEQ	GSSAETAPEE	TEGPDLESSD	ETDHSSKSTE	QVAAFCRSLH	EMNPSDQSPS	PQDSTGPQLA	TMRQLSDADK
**1050**	**1060**	**1070**	**1080**	**1090**	**1100**	**1110**	**1120**
LRKVICELLE	TERTYVKDLN	CLMERYLKPL	QKETFLTQDE	LDVLFGNLTE	MVEFQVEFLK	TLEDGVRLVP	DLEKLEKVDQ
**1130**	**1140**	**1150**	**1160**	**1170**	**1180**	**1190**	**1200**
FKKVLFSLGG	SFLYYADRFK	LYSAFCASHT	KVPKVLVKAK	TDTAFKAFLD	AQNPKQQHSS	TLESYLIKPI	QRILKYPLLL
**1210**	**1220**	**1230**	**1240**	**1250**	**1260**	**1270**	**1280**
RELFALTDAE	SEEHYHLDVA	IKTMNKVASH	INEMQKIHEE	FGAVFDQLIA	EQTGEKKEVA	DLSMGDLLLH	TTVIWLNPPA
**1290**	**1300**	**1310**	**1320**	**1330**	**1340**	**1350**	**1360**
SLGKWKKEPE	LAAFVFKTAV	VLVYKDGSKQ	KKKLVGSHRL	SIYEDWDPFR	FRHMIPTEAL	QVRALASADA	EANAVCEIVH
**1370**	**1380**	**1390**	**1400**	**1410**	**1420**	**1430**	**1440**
VKSESEGRPE	RVFHLCCSSP	ESRKDFLKAV	HSILRDKHRR	QLLKTESLPS	SQQYVPFGGK	RLCALKGARP	AMSRAGTVGI
**1450**	**1460**						
RTFKIPVTPT	PQ						

Cmpd.	No. of Cmpds.	m/z meas.	Δ m/z [ppm]	z	Rt [min]	Score	P	Range	Sequence	Modification	Ratios
44	2	750.8174	-45.94	2	30.3	15.2	0	134-147	R.LYGDDATYLAEGGR.R		MD:MU 3.23
1584	1	1022.9716	-69.17	2	49.7	16.6	1	707-725	K.QGRPSINQVFGEGTEAVKK.S		
1360	1	942.5032	8.18	2	46.6	12.4	2	1139-1154	R.FKLYSAFCASHTKVPK.V	Carbamidomethyl: 8	

Detailed Protein Report

Protein 88: **histone-lysine N-methyltransferase, H3 lysine-36 and H4 lysine-20 specific isoform a [Homo sapiens]**

**Accession:**	gi|27477095	**Score:**	44.2
**Database:**	refseq_human(refseq_human_20140103.fasta)	**MW [kDa]:**	267.2
**Database Date:**	2015-11-30	**pI:**	9.9
		**Sequence Coverage [%]:**	0.9
		**No. of unique Peptides:**	1

Quantitation

MD:MU	**Median:** 0.98	**CV:** 0.00 %	**No. of Peptides:** 1

Alias proteins:

Accession	Name	Description
gi|578811200	refseq_human (refseq_human_20140103.fasta)	PREDICTED: histone-lysine N-methyltransferase, H3 lysine-36 and H4 lysine-20 specific isoform X6 [Homo sapiens]
gi|530381148	refseq_human (refseq_human_20140103.fasta)	PREDICTED: histone-lysine N-methyltransferase, H3 lysine-36 and H4 lysine-20 specific isoform X3 [Homo sapiens]
gi|530381146	refseq_human (refseq_human_20140103.fasta)	PREDICTED: histone-lysine N-methyltransferase, H3 lysine-36 and H4 lysine-20 specific isoform X2 [Homo sapiens]

Detailed Protein Report

10	20	30	40	50	60	70	80
MPLKTRTALS	DDPDSSTSTL	GNMLELPGTS	SSSTSQELPF	CQPKKKSTPL	KYEVGDLIWA	KFKRRPWWPC	RICSDPLINT
90	100	110	120	130	140	150	160
HSKMKVSNRR	PYRQYYVEAF	GDPSERAWVA	GKAIVMFEGR	HQFEELPVLR	RRGKQKEKGY	RHKVPQKILS	KWEASVGLAE
170	180	190	200	210	220	230	240
QYDVPKGSKN	RKCIPGSIKL	DSEEDMPFED	CTNDPESEHD	LLLNGCLKSL	AFDSEHSADE	KEKPCAKSRA	RKSSDNPKRT
250	260	270	280	290	300	310	320
SVKKGHIQFE	AHKDERRGKI	PENLGLNFIS	GDISDTQASN	ELSRIANSLT	GSNTAPGSFL	FSSCGKNTAK	KEFETSNGDS
330	340	350	360	370	380	390	400
LLGLPEGALI	SKCSREKNKP	QRSLVCGSKV	KLCYIGAGDE	EKRSDSISIC	TTSDDGSSDL	DPIEHSSESD	NSVLEIPDAF
410	420	430	440	450	460	470	480
DRTENMLSMQ	KNEKIKYSRF	AATNTRVKAK	QKPLISNSHT	DHLMGCTKSA	EPGTETSQVN	LSDLKASTLV	HKPQSDFTND
490	500	510	520	530	540	550	560
ALSPKFNLSS	SISSENSLIK	GGAANQALLH	SKSKQPKFRS	IKCKHKENPV	MAEPPVINEE	CSLKCCSSDT	KGSPLASISK
570	580	590	600	610	620	630	640
SGKVDGLKLL	NNMHEKTRDS	SDIETAVVKH	VLSELKELSY	RSLGEDVSDS	GTSKPSKPLL	FSSASSQNHI	PIEPDYKFST
650	660	670	680	690	700	710	720
LLMMLKDMHD	SKTKEQRLMT	AQNLVSYRSP	GRGDCSTNSP	VGVSKVLVSG	GSTHNSEKKG	DGTQNSANPS	PSGGDSALSG
730	740	750	760	770	780	790	800
ELSASLPGLL	SDKRDLPASG	KSRSDCVTRR	NCGRSKPSSK	LRDAFSAQMV	KNTVNRKALK	TERKRKLNQL	PSVTLDAVLQ
810	820	830	840	850	860	870	880
GDRERGGSLR	GGAEDPSKED	PLQIMGHLTS	EDGDHFSDVH	FDSKVKQSDP	GKISEKGLSF	ENGKGPELDS	VMNSENDELN
890	900	910	920	930	940	950	960
GVNQVVPKKR	WQRLNQRRTK	PRKRMNRFKE	KENSECAFRV	LLPSDPVQEG	RDEFPEHRTP	SASILEEPLT	EQNHADCLDS
970	980	990	1000	1010	1020	1030	1040
AGPRLNVCDK	SSASIGDMEK	EPGIPSLTPQ	AELPEPAVRS	EKKRLRKPSK	WLLEYTEEYD	QIFAPKKKQK	KVQEQVHKVS
1050	1060	1070	1080	1090	1100	1110	1120
SRCEEESLLA	RGRSSAQNKQ	VDENSLISTK	EEPPVLEREA	PFLEGPLAQS	ELGGGHAELP	QLTLSVPVAP	EVSPRPALES
1130	1140	1150	1160	1170	1180	1190	1200
EELLVKTPGN	YESKRQRKPT	KKLLESNDLD	PGFMPKKGDL	GLSKKCYEAG	HLENGITESC	ATSYSKDFGG	GTTKIFDKPR
1210	1220	1230	1240	1250	1260	1270	1280
KRKRQRHAAA	KMQCKKVKND	DSSKEIPGSE	GELMPHRTAT	SPKETVEEGV	EHDPGMPASK	KMQGERGGGA	ALKENVCQNC
1290	1300	1310	1320	1330	1340	1350	1360
EKLGELLLCE	AQCCGAFHLE	CLGLTEMPRG	KFICNECRTG	IHTCFVCKQS	GEDVKRCLLP	LCGKFYHEEC	VQKYPPTVMQ
1370	1380	1390	1400	1410	1420	1430	1440
NKGFRCSLHI	CITCHAANPA	NVSASKGRLM	RCVRCPVAYH	ANDFCLAAGS	KILASNSIIC	PNHFTPRRGC	RNHEHVNVSW
1450	1460	1470	1480	1490	1500	1510	1520
CFVCSEGGSL	LCCDSCPAAF	HRECLNIDIP	EGNWYCNDCK	AGKKPHYREI	VWVKVGRYRW	WPAEICHPRA	VPSNIDKMRH
1530	1540	1550	1560	1570	1580	1590	1600
DVGEFPVLFF	GSNDYLWTHQ	ARVFPYMEGD	VSSKDKMGKG	VDGTYKKALQ	EAAARFEELK	AQKELRQLQE	DRKNDKKPPP
1610	1620	1630	1640	1650	1660	1670	1680
YKHIKVNRPI	GRVQIFTADL	SEIPRCNCKA	TDENPCGIDS	ECINRMLLYE	CHPTVCPAGG	RCQNQCFSKR	QYPEVEIFRT
1690	1700	1710	1720	1730	1740	1750	1760
LQRGWGLRTK	TDIKKGEFVN	EYVGELIDEE	ECRARIRYAQ	EHDITNFYML	TLDKDRIIDA	GPKGNYARFM	NHCCQPNCET
1770	1780	1790	1800	1810	1820	1830	1840
QKWSVNGDTR	VGLFALSDIK	AGTELTFNYN	LECLGNGKTV	CKCGAPNCSG	FLGVRPKNQP	IATEEKSKKF	KKKQQGKRRT
1850	1860	1870	1880	1890	1900	1910	1920
QGEITKERED	ECFSCGDAGQ	LVSCKKPGCP	KVYHADCLNL	TKRPAGKWEC	PWHQCDICGK	EAASFCEMCP	SSFCKQHREG
1930	1940	1950	1960	1970	1980	1990	2000
MLFISKLDGR	LSCTEHDPCG	PNPLEPGEIR	EYVPPPVPLP	PGPSTHLAEQ	STGMAAQAPK	MSDKPPADTN	QMLSLSKKAL
2010	2020	2030	2040	2050	2060	2070	2080
AGTCQRPLLP	ERPLERTDSR	PQPLDKVRDL	AGSGTKSQSL	VSSQRPLDRP	PAVAGPRPQL	SDKPSPVTSP	SSSPSVRSQP
2090	2100	2110	2120	2130	2140	2150	2160
LERPLGTADP	RLDKSIGAAS	PRPQSLEKTS	VPTGLRLPPP	DRLLITSSPK	PQTSDRPTDK	PHASLSQRLP	PPEKVLSAVV
2170	2180	2190	2200	2210	2220	2230	2240
QTLVAKEKAL	RPVDQNTQSK	NRAALVMDLI	DLTPRQKERA	ASPHQVTPQA	DEKMPVLESS	SWPASKGLGH	MPRAVEKGCV
2250	2260	2270	2280	2290	2300	2310	2320
SDPLQTSGKA	AAPSEDPWQA	VKSLTQARLL	SQPPAKAFLY	EPTTQASGRA	SAGAEQTPGP	LSQSPGLVKQ	AKQMVGGQQL
2330	2340	2350	2360	2370	2380	2390	2400

Detailed Protein Report

Cmpd.	No. of Cmpds.	m/z meas.	Δ m/z [ppm]	z	Rt [min]	Score	P	Range	Sequence	Modification	Ratios	
617	1	459.6161	-280.24	2	37.0	16.5	1	222-229	K.EKPCAKSR.A		MD:MU	0.98

Detailed Protein Report

**Protein 89:**	**PREDICTED: low-density lipoprotein receptor-related protein 2 isoform X1 [Homo sapiens]**		
**Accession:**	gi|578804333	**Score:**	44.1
**Database:**	refseq_human(refseq_human_20140103.fasta)	**MW [kDa]:**	522.2
**Database Date:**	2015-11-30	**pI:**	4.7
**Modification(s):**	Carbamidomethyl	**Sequence Coverage [%]:**	0.7
		**No. of unique Peptides:**	3

Detailed Protein Report

10	20	30	40	50	60	70	80
MDRGPAAVAC	TLLLALVACL	APASGQECDS	AHFRCGSGHC	IPADWRCDGT	KDCSDDADEI	GCAVVTCQQG	YFKCQSEGQC
90	100	110	120	130	140	150	160
IPNSWVCDQD	QDCDDGSDER	QDCSQSTCSS	HQITCSNGQC	IPSEYRCDHV	RDCPDGADEN	DCQYPTCEQL	TCDNGACYNT
170	180	190	200	210	220	230	240
SQKCDWKVDC	RDSSDEINCT	EICLHNEFSC	GNGECIPRAY	VCDHDNDCQD	GSDEHACNYP	TCGGYQFTCP	SGRCIYQNWV
250	260	270	280	290	300	310	320
CDGEDDCKDN	GDEDGCESGP	HDVHKCSPRE	WSCPESGRCI	SIYKVCDGIL	DCPGREDENN	TSTGKYCSMT	LCSALNCQYQ
330	340	350	360	370	380	390	400
CHETPYGGAC	FCPPGYIINH	NDSRTCVEFD	DCQIWGICDQ	KCESRPGRHL	CHCEEGYILE	RGQYCKANDS	FGEASIIFSN
410	420	430	440	450	460	470	480
GRDLLIGDIH	GRSFRILVES	QNRGVAVGVA	FHYHLQRVFW	TDTVQNKVFS	VDINGLNIQE	VLNVSVETPE	NLAVDWVNNK
490	500	510	520	530	540	550	560
IYLVETKVNR	IDMVNLDGSY	RVTLITENLG	HPRGIAVDPT	VGYLFFSDWE	SLSGEPKLER	AFMDGSNRKD	LVKTKLGWPA
570	580	590	600	610	620	630	640
GVTLDMISKR	VYWVDSRFDY	IETVTYDGIQ	RKTVVHGGSL	IPHPFGVSLF	EGQVFFTDWT	KMAVLKANKF	TETNPQVYYQ
650	660	670	680	690	700	710	720
ASLRPYGVTV	YHSLRQPYAT	NPCKDNNGGC	EQVCVLSHRT	DNDGLGFRCK	CTFGFQLDTD	ERHCIAVQNF	LIFSSQVAIR
730	740	750	760	770	780	790	800
GIPFTLSTQE	DVMVPVSGNP	SFFVGIDFDA	QDSTIFFSDM	SKHMIFKQKI	DGTGREILAA	NRVENVESLA	FDWISKNLYW
810	820	830	840	850	860	870	880
TDSHYKSISV	MRLADKTRRT	VVQYLNNPRS	VVVHPFAGYL	FFTDWFRPAK	IMRAWSDGSH	LLPVINTTLG	WPNGLAIDWA
890	900	910	920	930	940	950	960
ASRLYWVDAY	FDKIEHSTFD	GLDRRRLGHI	EQMTHPFGLA	IFGEHLFFTD	WRLGAIIRVR	KADGGEMTVI	RSGIAYILHL
970	980	990	1000	1010	1020	1030	1040
KSYDVNIQTG	SNACNQPTHP	NGDCSHFCFP	VPNFQRVCGC	PYGMRLASNH	LTCEGDPTNE	PPTEQCGLFS	FPCKNGRCVP
1050	1060	1070	1080	1090	1100	1110	1120
NYYLCDGVDD	CHDNSDEQLC	GTLNNTCSSS	AFTCGHGECI	PAHWRCDKRN	DCVDGSDEHN	CPTHAPASCL	DTQYTCDNHQ
1130	1140	1150	1160	1170	1180	1190	1200
CISKNWVCDT	DNDCGDGSDE	KNCNSTETCQ	PSQFNCPNHR	CIDLSFVCDG	DKDCVDGSDE	VGCVLNCTAS	QFKCASGDKC
1210	1220	1230	1240	1250	1260	1270	1280
IGVTNRCDGV	FDCSDNSDEA	GCPTRPPGMC	HSDEFQCQED	GICIPNFWEC	DGHPDCLYGS	DEHNACVPKT	CPSSYFHCDN
1290	1300	1310	1320	1330	1340	1350	1360
GNCIHRAWLC	DRDNDCGDMS	DEKDCPTQPF	RCPSWQWQCL	GHNICVNLSV	VCDGIFDCPN	GTDESPLCNE	PQPDGNSCSD
1370	1380	1390	1400	1410	1420	1430	1440
FNGGCTHECV	QEPFGAKCLC	PLGFLLANDS	KTCEDIDECD	ILGSCSQHCY	NMRGSFRCSC	DTGYMLESDG	RTCKVTASES
1450	1460	1470	1480	1490	1500	1510	1520
LLLLVASQNK	IIADSVTSQV	HNIYSLVENG	SYIVAVDFDS	ISGRIFWSDA	TQGKTWSAFQ	NGTDRRVVFD	SSIILTETIA
1530	1540	1550	1560	1570	1580	1590	1600
IDWVGRNLYW	TDYALETIEV	SKIDGSHRTV	LISKNLTNPR	GLALDPRMNE	HLLFWSDWGH	HPRIERASMD	GSMRTVIVQD
1610	1620	1630	1640	1650	1660	1670	1680
KIFWPCGLTI	DYPNRLLYFM	DSYLDYMDFC	DYNGHHRRQV	IASDLIIRHP	YALTLFEDSV	YWTDRATRRV	MRANKWHGGN
1690	1700	1710	1720	1730	1740	1750	1760
QSVVMYNIQW	PLGIVAVHPS	KQPNSVNPCA	FSRCSHLCLL	SSQGPHFYSC	VCPSGWSLSP	DLLNCLRDDQ	PFLITVRQHI
1770	1780	1790	1800	1810	1820	1830	1840
IFGISLNPEV	KSNDAMVPIA	GIQNGLDVEF	DDAEQYIYWV	ENPGEIHRVK	TDGTNRTVFA	SISMVGPSMN	LALDWISRNL
1850	1860	1870	1880	1890	1900	1910	1920
YSTNPRTQSI	EVLTLHGDIR	YRKTLIANDG	TALGVGFPIG	ITVDPARGKL	YWSDQGTDSG	VPAKIASANM	DGTSVKTLFT
1930	1940	1950	1960	1970	1980	1990	2000
GNLEHLECVT	LDIEEQKLYW	AVTGRGVIER	GNVDGTDRMI	LVHQLSHPWG	IAVHDSFLYY	TDEQYEVIER	VDKATGANKI
2010	2020	2030	2040	2050	2060	2070	2080
VLRDNVPNLR	GLQVYHRRNA	AESSNGCSNN	MNACQQICLP	VPGGLFSCAC	ATGFKLNPDN	RSCSPYNSFI	VVSMLSAIRG
2090	2100	2110	2120	2130	2140	2150	2160
FSLELSDHSE	TMVPVAGQGR	NALHVDVDVS	SGFIYWCDFS	SSVASDNAIR	RIKPDGSSLM	NIVTHGIGEN	GVRGIAVDWV
2170	2180	2190	2200	2210	2220	2230	2240

Detailed Protein Report

Cmpd.	No. of Cmpds.	m/z meas.	Δ m/z [ppm]	z	Rt [min]	Score	P	Range	Sequence	Modification
1173	1	786.4695	28.78	1	44.3	12.4	0	776-782	R.EILAANR.V	
23	1	788.3643	33.00	3	29.6	20.9	0	3802-3821	R.TCHPEYFQCTSGHCVHSELK.C	Carbamidomethyl: 2
1208	1	441.6040	-232.73	2	44.7	10.7	1	4264-4270	K.YDGTDRR.V	

Detailed Protein Report

Protein 90: **origin recognition complex subunit 6 [Homo sapiens]**

**Accession:**	gi|7657427	**Score:**	43.9
**Database:**	refseq_human(refseq_human_20140103.fasta)	**MW [kDa]:**	28.1
**Database Date:**	2015-11-30	**pI:**	9.8
**Modification(s):**	Carbamidomethyl	**Sequence Coverage [%]:**	20.6
		**No. of unique Peptides:**	3

10	20	30	40	50	60	70	80
MGSELIGRLA	PRLGLAEPDM	LRKAEEYLRL	SRVKCVGLSA	RTTETSSAVM	CLDLAASWMK	CPLDRAYLIK	LSGLNKETYQ
**90**	**100**	**110**	**120**	**130**	**140**	**150**	**160**
SCLKSFECLL	GLNSNIGIRD	LAVQFSCIEA	VNMASKILKS	YESSLPQTQQ	VDLDLSRPLF	TSAALLSACK	ILKLKVDKNK
**170**	**180**	**190**	**200**	**210**	**220**	**230**	**240**
MVATSGVKKA	IFDRLCKQLE	KIGQQVDREP	GDVATPPRKR	KKIVVEAPAK	EMEKVEEMPH	KPQKDEDLTQ	DYEEWKRKIL
**250**	**260**						
ENAASAQKAT	AE						

Cmpd.	No. of Cmpds.	m/z meas.	Δ m/z [ppm]	z	Rt [min]	Score	P	Range	Sequence	Modification
2133	2	701.2686	-80.31	3	56.8	17.2	0	42-60	R.TTETSSAVMCLDLAASWMK.C	Carbamidomethyl: 10
55	1	930.9009	-129.85	2	30.4	14.2	2	61-76	K.CPLDRAYLIKLSGLNK.E	Carbamidomethyl: 1
2540	1	941.9343	-22.57	2	61.7	12.6	0	100-116	R.DLAVQFSCIEAVNMASK.I	Carbamidomethyl: 8

Detailed Protein Report

Protein 91: coiled-coil domain-containing protein 168 [Homo sapiens]

**Accession:**	gi|226246554	**Score:**	43.9
**Database:**	refseq_human(refseq_human_20140103.fasta)	**MW [kDa]:**	801.4
**Database Date:**	2015-11-30	**pI:**	9.6
**Modification(s):**	Carbamidomethyl, Oxidation	**Sequence Coverage [%]:**	0.9
		**No. of unique Peptides:**	4

10	20	30	40	50	60	70	80
MSKQYYSFKK	GVGSGLEDNT	FMTLWDFLES	WIIQNDWVAI	FFIILLGIIF	EIILMKACAS	FWKKPTLPEK	GSSDVQETED
90	100	110	120	130	140	150	160
SCPKSRKLAP	ENWSVINSSS	GERVGTFLEK	RITSSLTSEE	KECNFEDRIL	FSREILWSGT	SESEDQVSPS	SESHVPSSNG
170	180	190	200	210	220	230	240
ISSSLPLFYS	EVEETCLSHT	EHPDREYETI	QFSSKKLFSM	MKTNKNKNSG	FSSDLSFSAS	RFTVENEDLD	VAPCPLAHLF
250	260	270	280	290	300	310	320
LSRDQVRLLE	ENVRNQIPSK	PKTKLGSRTT	YQCSRSQESL	NQNQPSVGMV	ISVQAQDSFP	GQNAFQNQGL	YEVQFTSQAQ
330	340	350	360	370	380	390	400
YINHNQESIK	SQPESKASNF	AQPEDVMKKP	FSSSTQDSFQ	SQDLDRNQHF	VEVPSIVEAK	YSVKGLESDE	HLGEDQHCVW
410	420	430	440	450	460	470	480
FIDSNKVKYS	IKGQDTIFKN	AEFLVLTLNP	NLVTEDMPQL	RSVKAQGQQQ	IVSSELNQDS	VYSSVPLLST	IKGQKNRRKT
490	500	510	520	530	540	550	560
PDSKSKLSLN	VPSLKAKKTP	TSQVFQITVC	HTLKNRNELG	CKNNTEKKEL	HERKDISDIA	LHLISVSKLI	LPYVKNYSRK
570	580	590	600	610	620	630	640
QLVKVMPGLI	KCGHFLQKQN	KSPDTEKINY	AGPLEETGIS	DITKKEKEYD	KENKRLKNIS	PKMLPQLEQS	FMVNTVQLKA
650	660	670	680	690	700	710	720
PCLLVETNGK	SKESLKDSIT	QAKGIGITEF	HVLNSKKPFD	LHIPKHKTSL	EEAISKPMQK	LVSSPEMESN	NRMKIQEDLQ
730	740	750	760	770	780	790	800
SSENSHLQLS	NGEELPTSTP	KTQRCFPREN	TQKQKDFLEL	VLELSNVGLL	ISPGSKMHKS	SEELEAIKIQ	VNTESVNLKE
810	820	830	840	850	860	870	880
SKPLILNVTE	DSDLRESEEL	ECNTGSNITN	MHQDKETSDA	FHSATYTTIS	QLPDTETHSI	SKAKADTLRI	IRLSHSASKQ
890	900	910	920	930	940	950	960
EKLPDEKETQ	NAEYIDKSCT	FKKPQQCDRK	EQEKEANSEL	TQGFRFSIHL	KQKPKYVKFQ	MEQISSGSSK	APNKEQEVQP
970	980	990	1000	1010	1020	1030	1040
QTLSTQTILE	NSPCPMMDPF	QVEKVKQSTD	RPTDRESAGD	PKNPLTMPEN	LPVGELLIET	TEYSVPFGGN	LQKTTDSHIA
1050	1060	1070	1080	1090	1100	1110	1120
EEKEDVKRYL	PAVALGSFNN	HLLTLPYFKR	QEIKKKLSET	KSVLSVKYVI	MKVKKPAISL	MPYINICGTS	NHRKKMGGNF
1130	1140	1150	1160	1170	1180	1190	1200
EIIIKQILQD	KIAAGMLLNV	IYPPMSILPN	TRMYSRLNAE	NHSHIKLVQE	ESQIEREEKY	PYFINEGNES	QNTLDAKLQD
1210	1220	1230	1240	1250	1260	1270	1280
EVKGVKETLP	KAVLHDSCNL	GLDAHLEKEI	KTEKEMHQPI	PFTETIIESV	VSPIMELSHA	ENVKSTQKTQ	TDCKCTADSE
1290	1300	1310	1320	1330	1340	1350	1360
TPSPISGKSL	IGDPLNQTRE	SYIPSNGSDT	REMGYCFAEE	KTEIPKDLPA	TSPETFNYCT	PVLSCSKVMK	KRVTFALTTS
1370	1380	1390	1400	1410	1420	1430	1440
TAKPKCVNTK	AVKPSISETV	SVTSHRKKSE	LDFKTKFKKI	NQTKGLVPEC	LNTLCSPMHS	RLQREFCLPA	SQLKQGETAD
1450	1460	1470	1480	1490	1500	1510	1520
KTYTDVFAKN	SISHDREEKL	QDGKEEEHKV	LLEAAPQLSQ	HLGSEAGQMK	EIHLESDPVL	NCLTLELHIN	GQRLQHQTGF
1530	1540	1550	1560	1570	1580	1590	1600
EQTTLETSLQ	MGPLEAEELQ	KANETENDIK	VLGGPKIPPP	KALQALENSD	GLILNAYQKD	NELVKSDEEL	NQPGSTNIQV
1610	1620	1630	1640	1650	1660	1670	1680
QPQTHFTQTI	LKSTSCPTLD	QFPFEKVESH	VRFSPLKSGE	AKVDEIIFYA	REGGISSDSS	HQKEQAGGTE	KKETAIFGSC
1690	1700	1710	1720	1730	1740	1750	1760
MPALSTPKTT	RNLKQFSDMK	TLVNPKCGII	KAKKPSISYM	LNIRAGAGPK	RRKELSCNLT	TKMKELHQGK	KGVDETYAFL
1770	1780	1790	1800	1810	1820	1830	1840
TMTPDINKYS	KVETEKDTLR	EKRLSSTQVK	QDTSPHEDSI	TSRDIKETLL	QDEEQEERKQ	EALLKVIPQH	LQHFMFRSGQ
1850	1860	1870	1880	1890	1900	1910	1920
GKDLDFHKLE	NQGSRKILFV	TKQDVPQQLQ	PAEPIQREET	KKCLQTQNGT	ICTVNSKLLP	LKSEDSVNGE	VLTGAIKRGV
1930	1940	1950	1960	1970	1980	1990	2000
PTDRKCMGEQ	HNSGKGEKAE	FNKDLQATVL	ELQKSPHGGE	AQKANLTDME	SGSSNAMNMN	VQHEREDKNI	QKMLTESVPC
2010	2020	2030	2040	2050	2060	2070	2080
YSQHLRFSTH	QMKDPDPCKS	GSEPKSPEGR	SWNLSHIVQK	TKQETHFRET	VLEPISGYMM	KQSPHMQEGI	KCMEGLKTSF
2090	2100	2110	2120	2130	2140	2150	2160
PKTGKSKIGS	IPRDTPWDEN	PRRKWDSSIS	EKTAWNQKNL	QTVLKPLDFS	SLMSSEYESR	SYTLEFIGKK	SMSPKCVTLK
2170	2180	2190	2200	2210	2220	2230	2240

Detailed Protein Report

Cmpd.	No. of Cmpds.	m/z meas.	Δ m/z [ppm]	z	Rt [min]	Score	P	Range	Sequence	Modification
2490	1	610.2540	-89.47	2	61.1	10.6	2	3706-3716	K.QEGKMQEGKGK.S	
1634	1	803.8641	-92.41	2	50.2	10.9	0	5427-5440	K.MCILFSKPLPSNLK.L	Oxidation: 1
2403	2	974.2328	75.63	3	59.9	11.7	2	5658-5682	R.DEDIYFTGFGTIRSGKRPEWLFTGK.K	
1685	1	916.4063	-73.78	2	50.8	10.7	2	6846-6860	R.ECRKETLIITPPSCK.S	Carbamidomethyl: 2, 14

Detailed Protein Report

Protein 92: **PREDICTED: RING finger protein 17 isoform X10 [Homo sapiens]**

**Accession:**	gi|578824967	**Score:**	43.6
**Database:**	refseq_human(refseq_human_20140103.fasta)	**MW [kDa]:**	168.9
**Database Date:**	2015-11-30	**pI:**	5.0
		**Sequence Coverage [%]:**	2.6
		**No. of unique Peptides:**	1

Quantitation

MD:MU	**Median:** 0.99	**CV:** 0.00 %	**No. of Peptides:** 1

10	20	30	40	50	60	70	80
MLDTNTAEEI	DEALNTAHHS	FEQLSIAGKA	LEHMQKQTIE	ERERVIEVVE	KQFDQLLAFF	DSRKKNLCEE	FARTTDDYLS
**90**	**100**	**110**	**120**	**130**	**140**	**150**	**160**
NLIKAKSYIE	EKKNNLNAAM	NIARALQLSP	SLRTYCDLNQ	IIRTLQLTSD	SELAQVSSPQ	LRNPPRLSVN	CSEIICMFNN
**170**	**180**	**190**	**200**	**210**	**220**	**230**	**240**
MGKIEFRDST	KCYPQENEIR	QNVQKKYNNK	KELSCYDTYP	PLEKKKVDMS	VLTSEAPPPP	LQPETNDVHL	EAKNFQPQKD
**250**	**260**	**270**	**280**	**290**	**300**	**310**	**320**
VATASPKTIA	VLPQMGSSPD	VIIEEIIEDN	VESSAELVFV	SHVIDPCHFY	IRKYSQIKDA	KVLEKKVNEF	CNRSSHLDPS
**330**	**340**	**350**	**360**	**370**	**380**	**390**	**400**
DILELGARIF	VSSIKNGMWC	RGTITELIPI	EGRNTRKPCS	PTRLFVHEVA	LIQIFMVDFG	NSEVLIVTGV	VDTHVRPEHS
**410**	**420**	**430**	**440**	**450**	**460**	**470**	**480**
AKQHIALNDL	CLVLRKSEPY	TEGLLKDIQP	LAQPCSLKDI	VPQNSNEGWE	EEAKVEFLKM	VNNKAVSMKV	FREEDGVLIV
**490**	**500**	**510**	**520**	**530**	**540**	**550**	**560**
DLQKPPPNKI	SSDMPVSLRD	ALVFMELAKF	KSQSLRSHFE	KNTTLHYHPP	ILPKEMTDVS	VTVCHINSPG	DFYLQLIEGL
**570**	**580**	**590**	**600**	**610**	**620**	**630**	**640**
DILFLLKTIE	EFYKSEDGEN	LEILCPVQDQ	ACVAKFEDGI	WYRAKVIGLP	GHQEVEVKYV	DFGNTAKITI	KDVRKIKDEF
**650**	**660**	**670**	**680**	**690**	**700**	**710**	**720**
LNAPEKAIKC	KLAYIEPYKR	TMQWSKEAKE	KFEEKAQDKF	MTCSVIKILE	DNVLLVELFD	SLGAPEMTTT	SINDQLVKEG
**730**	**740**	**750**	**760**	**770**	**780**	**790**	**800**
LASYEIGYIL	KDNSQKHIEV	WDPSPEEIIS	NEVHNLNPVS	AKSLPNENFQ	SLYNKELPVH	ICNVISPEKI	YVQWLLTENL
**810**	**820**	**830**	**840**	**850**	**860**	**870**	**880**
LNSLEEKMIA	AYENSKWEPV	KWENDMHCAV	KIQDKNQWRR	GQIIRMVTDT	LVEVLLYDVG	VELVVNVDCL	RKLEENLKTM
**890**	**900**	**910**	**920**	**930**	**940**	**950**	**960**
GRLSLECSLV	DIRPAGGSDK	WTATACDCLS	LYLTGAVATI	ILQVDSEENN	TTWPLPVKIF	CRDEKGERVD	VSKYLIKKGL
**970**	**980**	**990**	**1000**	**1010**	**1020**	**1030**	**1040**
ALRERRINNL	DNSHSLSEKS	LEVPLEQEDS	VVTNCIKTNF	DPDKKTADII	SEQKVSEFQE	KILEPRTTRG	YKPPAIPNMN
**1050**	**1060**	**1070**	**1080**	**1090**	**1100**	**1110**	**1120**
VFEATVSCVG	DDGTIFVVPK	LSEFELIKMT	NEIQSNLKCL	GLLEPYFWKK	GEACAVRGSD	TLWYRGKVME	VVGGAVRVQY
**1130**	**1140**	**1150**	**1160**	**1170**	**1180**	**1190**	**1200**
LDHGFTEKIP	QCHLYPILLY	PDIPQFCIPC	QLHNTTPVGN	VWQPDAIEVL	QQLLSKRQVD	IHIMELPKNP	WEKLSIHLYF
**1210**	**1220**	**1230**	**1240**	**1250**	**1260**	**1270**	**1280**
DGMSLSYFMA	YYKYCTSEHT	EEMLKEKPRS	DHDKKYEEEQ	WEIRFEELLS	AETDTPLLPP	YLSSSLPSPG	ELYAVQVKHV
**1290**	**1300**	**1310**	**1320**	**1330**	**1340**	**1350**	**1360**
VSPNEVYICL	DSIETSNQSN	QHSDTDDSGV	SGESESESLD	EALQRVNKKV	EALPPLTDFR	TEMPCLAEYD	DGLWYRAKIV
**1370**	**1380**	**1390**	**1400**	**1410**	**1420**	**1430**	**1440**
AIKEFNPLSI	LVQFVDYGST	AKLTLNRLCQ	IPSHLMRYPA	RAIKVLLAGF	KPPLRDLGET	RIPYCPKWSM	EALWAMIDCL
**1450**	**1460**	**1470**	**1480**	**1490**			
QGKQLYAVSM	APAPEQIVTL	YDDEQHPVHM	PLVEMGLADK	DE			

Cmpd.	No. of Cmpds.	m/z meas.	Δ m/z [ppm]	z	Rt [min]	Score	P	Range	Sequence	Modification	Ratios	
200	1	642.8816	92.45	2	32.2	16.1	1	596-605	K.FEDGIWYRAK.V		MD:MU	0.99

Detailed Protein Report

Protein 93: PREDICTED: centrosomal protein of 290 kDa isoform X4 [Homo sapiens]

**Accession:**	gi|530400856	**Score:**	43.3
**Database:**	refseq_human(refseq_human_20140103.fasta)	**MW [kDa]:**	234.7
**Database Date:**	2015-11-30	**pI:**	6.1
**Modification(s):**	Oxidation	**Sequence Coverage [%]:**	2.0
		**No. of unique Peptides:**	3

Quantitation

MD:MU	**Median:** 1.11	**CV:** 0.00 %	**No. of Peptides:** 1

Detailed Protein Report

10	20	30	40	50	60	70	80
MQKLSLRQKS	AIFCQQIHEN	RADMDKSQVA	TLEEEQVHSQ	VKYADINLKE	DIIKSEVPLQ	TEILKNKLKV	NLPDPVSITA
90	100	110	120	130	140	150	160
QSKLSQINSL	ENLIEQLRRE	LVFLRSQNEI	IAQEFLIKEA	ECRNADIELE	HHRSQAEQNE	FLSRELIEKE	RDLERSRTVI
170	180	190	200	210	220	230	240
AKFQNKLKEL	VEENKQLEEG	MKEILQAIKE	MQKDPDVKGG	ETSLIIPSLE	RLVNAIESKN	AEGIFDASLH	LKAQVDQLTG
250	260	270	280	290	300	310	320
RNEELRQELR	ESRKEAINYS	QQLAKANLKI	DHLEKETSLL	RQSEGSNVVF	KGIDLPDGIA	PSSASIINSQ	NEYLIHLLQE
330	340	350	360	370	380	390	400
LENKEKKLKN	LEDSLEDYNR	KFAVIRHQQS	LLYKEYLSEK	ETWKTESKTI	KEEKRKLEDQ	VQQDAIKVKE	YNNLLNALQM
410	420	430	440	450	460	470	480
DSDEMKKILA	ENSRKITVLQ	VNEKSLIRQY	TTLVELERQL	RKENEKQKNE	LLSMEAEVCE	KIGCLQRFKE	MAIFKIAALQ
490	500	510	520	530	540	550	560
KVVDNSVSLS	ELELANKQYN	ELTAKYRDIL	QKDNMLVQRT	SNLEHLECEN	ISLKEQVESI	NKELEITKEK	LHTIEQAWEQ
570	580	590	600	610	620	630	640
ETKLGNESSM	DKAKKSITNS	DIVSISKKIT	MLEMKELNER	QRAEHCQKMY	EHLRTSLKQM	EERNFELETK	FAELTKINLD
650	660	670	680	690	700	710	720
AQKVEQMLRD	ELADSVSKAV	SDADRQRILE	LEKNEMELKV	EVSKLREISD	IARRQVEILN	AQQQSRDKEV	ESLRMQLLDY
730	740	750	760	770	780	790	800
QAQSDEKSLI	AKLHQHNVSL	QLSEATALGK	LESITSKLQK	MEAYNLRLEQ	KLDEKEQALY	YARLEGRNRA	KHLRQTIQSL
810	820	830	840	850	860	870	880
RRQFSGALPL	AQQEKFSKTM	IQLQNDKLKI	MQEMKNSQQE	HRNMENKTLE	MELKLKGLEE	LISTLKDTKG	AQKVINWHMK
890	900	910	920	930	940	950	960
IEELRLQELK	LNRELVKDKE	EIKYLNNIIS	EYERTISSLE	EEIVQQNKFH	EERQMAWDQR	EVDLERQLDI	FDRQQNEILN
970	980	990	1000	1010	1020	1030	1040
AAQKFEEATG	SIPDPSLPLP	NQLEIALRKI	KENIRIILET	RATCKSLEEK	LKEKESALRL	AEQNILSRDK	VINELRLRLP
1050	1060	1070	1080	1090	1100	1110	1120
ATAEREKLIA	ELGRKEMEPK	SHHTLKIAHQ	TIANMQARLN	QKEEVLKKYQ	RLLEKAREEQ	REIVKKHEED	LHILHHRLEL
1130	1140	1150	1160	1170	1180	1190	1200
QADSSLNKFK	QTAWDLMKQS	PTPVPTNKHF	IRLAEMEQTV	AEQDDSLSSL	LVKLKKVSQD	LERQREITEL	KVKEFENIKL
1210	1220	1230	1240	1250	1260	1270	1280
QLQENHEDEV	KKVKAEVEDL	KYLLDQSQKE	SQCLKSELQA	QKEANSRAPT	TTMRNLVERL	KSQLALKEKQ	QKALSRALLE
1290	1300	1310	1320	1330	1340	1350	1360
LRAEMTAAAE	ERIISATSQK	EAHLNVQQIV	DRHTRELKTQ	VEDLNENLLK	LKEALKTSKN	RENSLTDNLN	DLNNELQKKQ
1370	1380	1390	1400	1410	1420	1430	1440
KAYNKILREK	EEIDQENDEL	KRQIKRLTSG	LQGKPLTDNK	QSLIEELQRK	VKKLENQLEG	KVEEVDLKPM	KEKNAKEELI
1450	1460	1470	1480	1490	1500	1510	1520
RWEEGKKWQA	KIEGIRNKLK	EKEGEVFTLT	KQLNTLKDLF	AKADKEKLTL	QRKLKTTGMT	VDQVLGIRAL	ESEKELEELK
1530	1540	1550	1560	1570	1580	1590	1600
KRNLDLENDI	LYMRAHQALP	RDSVVEDLHL	QNRYLQEKLH	ALEKQFSKDT	YSKPSISGIE	SDDHCQREQE	LQKENLKLSS
1610	1620	1630	1640	1650	1660	1670	1680
ENIELKFQLE	QANKDLPRLK	NQVRDLKEMC	EFLKKEKAEV	QRKLGHVRGS	GRSGKTIPEL	EKTIGLMKKV	VEKVQRENEQ
1690	1700	1710	1720	1730	1740	1750	1760
LKKASGILTS	EKMANIEQEN	EKLKAELEKL	KAHLGHQLSM	HYESKTKGTE	KIIAENERLR	KELKKETDAA	EKLRIAKNNL
1770	1780	1790	1800	1810	1820	1830	1840
EILNEKMTVQ	LEETGKRLQF	AESRGPQLEG	ADSKSWKSIV	VTRMYETKLK	ELETDIAKKN	QSITDLKQLV	KEATEREQKV
1850	1860	1870	1880	1890	1900	1910	1920
NKYNEDLEQQ	IKILKHVPEG	AETEQGLKRE	LQVLRLANHQ	LDKEKAELIH	QIEANKDQSG	AESTIPDADQ	LKEKIKDLET
1930	1940	1950	1960	1970	1980	1990	2000
QLKMSDLEKQ	HLKEEIKKLK	KELENFDPSF	FEEIEDLKYN	YKEEVKKNIL	LEEKVKKLSE	QLGVELTSPV	AASEEFEDEE
2010							
ESPVNFPIY							

Cmpd.	No. of Cmpds.	m/z meas.	Δ m/z [ppm]	z	Rt [min]	Score	P	Range	Sequence	Modification	Ratios

Detailed Protein Report

Cmpd.	No. of Cmpds.	m/z meas.	Δ m/z [ppm]	z	Rt [min]	Score	P	Range	Sequence	Modification	Ratios
1675	1	745.8585	-74.75	2	50.7	11.8	1	631-643	K.FAELTKINLDAQK.V		
1969	1	703.7594	-155.73	2	54.7	10.7	0	1496-1508	K.TTGMTVDQVLGIR.A	Oxidation: 4	
1615	1	494.1461	-276.33	2	50.1	10.7	2	1636-1643	K.EKAEVQRK.L		MD:MU 1.11

Detailed Protein Report

Protein 94: PREDICTED: tripartite motif-containing protein 2 isoform X2 [Homo sapiens]

**Accession:**	gi|578808962	**Score:**	43.2
**Database:**	refseq_human(refseq_human_20140103.fasta)	**MW [kDa]:**	84.7
**Database Date:**	2015-11-30	**pI:**	7.3
**Modification(s):**	Oxidation	**Sequence Coverage [%]:**	4.5
		**No. of unique Peptides:**	3

Quantitation

MD:MU **Median:** 9.77 **CV:** 0.00 % **No. of Peptides:** 1

```
        10         20         30         40         50         60         70         80
MHRSGRYGTQ QRAGSKTAGP PCQWSRMASE GTNIPSPVVR QIDKQFLICS ICLERYKNPK VLPCLHTFCE RCLQNYIPAH
        90        100        110        120        130        140        150        160
SLTLSCPVCR QTSILPEKGV AALQNNFFIT NLMDVLQRTP GSNAEESSIL ETVTAVAAGK PLSCPNHDGN VMEFYCQSCE
       170        180        190        200        210        220        230        240
TAMCRECTEG EHAEHPTVPL KDVVEQHKAS LQVQLDAVNK RLPEIDSALQ FISEIIHQLT NQKASIVDDI HSTFDELQKT
       250        260        270        280        290        300        310        320
LNVRKSVLLM ELEVNYGLKH KVLQSQLDTL LQGQESIKSC SNFTAQALNH GTETEVLLVK KQMSEKLNEL ADQDFPLHPR
       330        340        350        360        370        380        390        400
ENDQLDFIVE TEGLKKSIHN LGTILTTNAV ASETVATGEG LRQTIIGQPM SVTITTKDKD GELCKTGNAY LTAELSTPDG
       410        420        430        440        450        460        470        480
SVADGEILDN KNGTYEFLYT VQKEGDFTLS LRLYDQHIRG SPFKLKVIRS ADVSPTTEGV KRRVKSPGSG HVKQKAVKRP
       490        500        510        520        530        540        550        560
ASMYSTGKRK ENPIEDDLIF RVGTKGRNKG EFTNLQGVAA STNGKILIAD SNNQCVQIFS NDGQFKSRFG IRGRSPGQLQ
       570        580        590        600        610        620        630        640
RPTGVAVHPS GDIIIADYDN KWVSIFSSDG KFKTKIGSGK LMGPKGVSVD RNGHIIVVDN KACCVFIFQP NGKIVTRFGS
       650        660        670        680        690        700        710        720
RGNGDRQFAG TLDGPHFAAV NSNNEIIITD FHNHSVKVFN QEGEFMLKFG SNGEGNGQFN APTGVAVDSN GNIIVADWGN
       730        740        750        760        770        780
SRIQVFDGSG SFLSYINTSA DPLYGPQGLA LTSDGHVVVA DSGNHCFKVY RYLQ
```

Cmpd.	No. of Cmpds.	m/z meas.	Δ m/z [ppm]	z	Rt [min]	Score	P	Range	Sequence	Modification	Ratios
2668	1	673.3612	23.64	2	63.5	12.0	2	2-12	M.HRSGRYGTQQR.A		
2047	1	698.2586	-165.65	2	55.7	16.9	1	476-488	K.AVKRPASMYSTGK.R		MD:MU 9.77
428	2	587.8118	-7.20	2	34.7	14.2	1	601-611	K.LMGPKGVSVDR.N	Oxidation: 2	

Detailed Protein Report

Protein 95: RB1-inducible coiled-coil protein 1 isoform 2 [Homo sapiens]

**Accession:**	gi|134304846	**Score:**	43.2
**Database:**	refseq_human(refseq_human_20140103.fasta)	**MW [kDa]:**	182.7
**Database Date:**	2015-11-30	**pI:**	5.2
		**Sequence Coverage [%]:**	1.9
		**No. of unique Peptides:**	2

Alias proteins:

Accession	Name	Description
gi|578815756	refseq_human (refseq_human_20140103.fasta)	PREDICTED: RB1-inducible coiled-coil protein 1 isoform X1 [Homo sapiens]

10	20	30	40	50	60	70	80
MKLYVFLVNT	GTTLTFDTEL	TVQTVADLKH	AIQSKYKIAI	QHQVLVVNGG	ECMAADRRVC	TYSAGTDTNP	IFLFNKEMIL
90	100	110	120	130	140	150	160
CDRPPAIPKT	TFSTENDMEI	KVEESLMMPA	VFHTVASRTQ	LALEMYEVAK	KLCSFCEGLV	HDEHLQHQGW	AAIMANLEDC
170	180	190	200	210	220	230	240
SNSYQKLLFK	FESIYSNYLQ	SIEDIKLKLT	HLGTAVSVMA	KIPLLECLTR	HSYRECLGRL	DSLPEHEDSE	KAEMKRSTEL
250	260	270	280	290	300	310	320
VLSPDMPRTT	NESLLTSFPK	SVEHVSPDTA	DAESGKEIRE	SCQSTVHQQD	ETTIDTKDGD	LPFFNVSLLD	WINVQDRPND
330	340	350	360	370	380	390	400
VESLVRKCFD	SMSRLDPRII	RPFIAECRQT	IAKLDNQNMK	AIKGLEDRLY	ALDQMIASCG	RLVNEQKELA	QGFLANQKRA
410	420	430	440	450	460	470	480
ENLKDASVLP	DLCLSHANQL	MIMLQNHRKL	LDIKQKCTTA	KQELANNLHV	RLKWCCFVML	HADQDGEKLQ	ALLRLVIELL
490	500	510	520	530	540	550	560
ERVKIVEALS	TVPQMYCLAV	VEVVRRKMFI	KHYREWAGAL	VKDGKRLYEA	EKSKRESFGK	LFRKSFLRNR	LFRGLDSWPP
570	580	590	600	610	620	630	640
SFCTQKPRKF	DCELPDISLK	DLQFLQSFCP	SEVQPFLRVP	LLCDFEPLHQ	HVLALHNLVK	AAQSLDEMSQ	TITDLLSEQK
650	660	670	680	690	700	710	720
ASVSQTSPQS	ASSPRMESTA	GITTTTSPRT	PPPLTVQDPL	CPAVCPLEEL	SPDSIDAHTF	DFETIPHPNI	EQTIHQVSLD
730	740	750	760	770	780	790	800
LDSLAESPES	DFMSAVNEFV	IEENLSSPNP	ISDPQSPEMM	VESLYSSVIN	AIDSRRMQDT	NVCGKEDFGD	HTSLNVQLER
810	820	830	840	850	860	870	880
CRVVAQDSHF	SIQTIKEDLC	HFRTFVQKEQ	CDFSNSLKCT	AVEIRNIIEK	VKCSLEITLK	EKHQKELLSL	KNEYEGKLDG
890	900	910	920	930	940	950	960
LIKETEENEN	KIKKLKGELV	CLEEVLQNKD	NEFALVKHEK	EAVICLQNEK	DQKLLEMENI	MHSQNCEIKE	LKQSREIVLE
970	980	990	1000	1010	1020	1030	1040
DLKKLHVEND	EKLQLLRAEL	QSLEQSHLKE	LEDTLQVRHI	QEFEKVMTDH	RVSLEELKKE	NQQIINQIQE	SHAEIIQEKE
1050	1060	1070	1080	1090	1100	1110	1120
KQLQELKLKV	SDLSDTRCKL	EVELALKEAE	TDEIKILLEE	SRAQQKETLK	SLLEQETENL	RTEISKLNQK	IQDNNENYQV
1130	1140	1150	1160	1170	1180	1190	1200
GLAELRTLMT	IEKDQCISEL	ISRHEEESNI	LKAELNKVTS	LHNQAFEIEK	NLKEQIIELQ	SKLDSELSAL	ERQKDEKITQ
1210	1220	1230	1240	1250	1260	1270	1280
QEEKYEAIIQ	NLEKDRQKLV	SSQEQDREQL	IQKLNCEKDE	AIQTALKEFK	LEREVVEKEL	LEKVKHLENQ	IAKSPAIDST
1290	1300	1310	1320	1330	1340	1350	1360
RGDSSSLVAE	LQEKLQEEKA	KFLEQLEEQE	KRKNEEMQNV	RTSLIAEQQT	NFNTVLTREK	MRKENIINDL	SDKLKSTMQQ
1370	1380	1390	1400	1410	1420	1430	1440
QERDKDLIES	LSEDRARLLE	EKKKLEEEVS	KLRSSSFVPS	PYVATAPELY	GACAPELPGE	SDRSAVETAD	EGRVDSAMET
1450	1460	1470	1480	1490	1500	1510	1520
SMMSVQENIH	MLSEEKQRIM	LLERTLQLKE	EENKRLNQRL	MSQSMSSVSS	RHSEKIAIRD	FQVGDLVLII	LDERHDNYVL
1530	1540	1550	1560	1570	1580	1590	1600
FTVSPTLYFL	HSESLPALDL	KPASGASRRP	WVLGKVMEKE	YCQAKKAQNR	FKVPLGTKFY	RVKAVSWNKK	V

Cmpd.	No. of Cmpds.	m/z meas.	Δ m/z [ppm]	z	Rt [min]	Score	P	Range	Sequence	Modification
2032	1	687.8227	-80.45	2	55.5	10.2	1	388-399	K.ELAQGFLANQKR.A	
1335	1	497.7323	-72.04	2	46.4	14.6	1	508-514	K.MFIKHYR.E	

Detailed Protein Report

Protein 96: zinc finger protein 841 [Homo sapiens]

Accession: gi|211057416
Database: refseq_human(refseq_human_20140103.fasta)
Database Date: 2015-11-30
Modification(s): Carbamidomethyl

Score: 43.1
MW [kDa]: 106.0
pI: 10.4
Sequence Coverage [%]: 7.7
No. of unique Peptides: 3

10	20	30	40	50	60	70	80
MALPQGSLTF	RDVAVEFSQE	EWKCLDPVQK	ALYRDVMLEN	YRNLGFLGLC	LPDLNIISML	EQGKEPWTVV	SQVKIARNPN
**90**	**100**	**110**	**120**	**130**	**140**	**150**	**160**
CGECMKGVIT	GISPKCVIKE	LPPIQNSNTG	EKFQAVMLEG	HESYDTENFY	FREIRKNLQE	VDFQWKDGEI	NYKEGPMTHK
**170**	**180**	**190**	**200**	**210**	**220**	**230**	**240**
NNLTGQRVRH	SQGDVENKHM	ENQLILRFQS	GLGELQKFQT	AEKIYGCNQI	ERTVNNCFLA	SPLQRIFPGV	QTNISRKYGN
**250**	**260**	**270**	**280**	**290**	**300**	**310**	**320**
DFLQLSLPTQ	DEKTHIREKP	YIGNECGKAF	RVSSSLINHQ	MIHTTEKPYR	CNESGKAFHR	GSLLTVHQIV	HTRGKPYQCD
**330**	**340**	**350**	**360**	**370**	**380**	**390**	**400**
VCGRIFRQNS	DLVNHRRSHT	GDKPYICNEC	GKSFSKSSHL	AVHQRIHTGE	KPYKCNRCGK	CFSQSSSLAT	HQTVHTGDKP
**410**	**420**	**430**	**440**	**450**	**460**	**470**	**480**
YKCNECGKTF	KRNSSLTAHH	IIHAGKKPYT	CDVCGKVFYQ	NSQLVRHQII	HTGETPYKCN	ECGKVFFQRS	RLAGHRRIHT
**490**	**500**	**510**	**520**	**530**	**540**	**550**	**560**
GEKPYKCNEC	GKVFSQHSHL	AVHQRVHTGE	KPYKCNECGK	AFNWGSLLTV	HQRIHTGEKP	YKCNVCGKVF	NYGGYLSVHM
**570**	**580**	**590**	**600**	**610**	**620**	**630**	**640**
RCHTGEKPLH	CNKCGMVFTY	YSCLARHQRM	HTGEKPYKCN	VCGKVFIDSG	NLSIHRRSHT	GEKPFQCNEC	GKVFSYYSCL
**650**	**660**	**670**	**680**	**690**	**700**	**710**	**720**
ARHRKIHTGE	KPYKCNDCGK	AYTQRSSLTK	HLVIHTGENP	YHCNEFGEAF	IQSSKLARYH	RNPTGEKPHK	CSECGRTFSH
**730**	**740**	**750**	**760**	**770**	**780**	**790**	**800**
KTSLVYHQRR	HTGEMPYKCI	ECGKVFNSTT	TLARHRRIHT	GEKPYKCNEC	GKVFRYRSGL	ARHWSIHTGE	KPYKCNECGK
**810**	**820**	**830**	**840**	**850**	**860**	**870**	**880**
AFRVRSILLN	HQMMHTGEKP	YKCNECGKAF	IERSNLVYHQ	RNHTGEKPYK	CMECGKAFGR	RSCLTKHQRI	HSSEKPYKCN
**890**	**900**	**910**	**920**	**930**			
ECGKSYISRS	GLTKHQIKHA	GENLTTKLNV	ERPLDVVLTS	GIPK			

Cmpd.	No. of Cmpds.	m/z meas.	Δ m/z [ppm]	z	Rt [min]	Score	P	Range	Sequence	Modification
1460	1	1011.7530	-79.39	3	48.0	16.8	2	590-616	R.MHTGEKPYKCNVCGKVFIDSGNLSIHR.R	
1732	1	1061.1490	-2.43	3	51.5	11.3	2	617-642	R.RSHTGEKPFQCNECGKVFSYYSCLAR.H	Carbamidomethyl: 11, 14, 23
1286	1	738.4255	114.34	3	45.7	15.0	2	699-716	R.YHRNPTGEKPHKCSECGR.T	Carbamidomethyl: 13, 16

Detailed Protein Report

Protein 97: PREDICTED: 14-3-3 protein epsilon isoform X1 [Homo sapiens]

Accession: gi|530410617
Database: refseq_human(refseq_human_20140103.fasta)
Database Date: 2015-11-30
Modification(s): Oxidation

Score: 43.0
MW [kDa]: 27.4
pI: 4.6
Sequence Coverage [%]: 11.3
No. of unique Peptides: 1

Alias proteins:

Accession	Name	Description
gi|578840292	refseq_human (refseq_human_20140103.fasta)	PREDICTED: 14-3-3 protein epsilon isoform X1 [Homo sapiens]

10	20	30	40	50	60	70	80
MDDREDLVYQ	AKLAEQAERY	DEMVESMKKV	AGMDVELTVE	ERNLLSVAYK	NVIGARRASW	RIISSIEQKE	ENKGGEDKLK
**90**	**100**	**110**	**120**	**130**	**140**	**150**	**160**
MIREYRQMVE	TELKLICCDI	LDVLDKHLIP	AANTGESKVF	YYKMKGDYHR	YLAEFATGND	RKEAAENSLV	AYKAASDIAM
**170**	**180**	**190**	**200**	**210**	**220**	**230**	**240**
TELPPTHPIR	LGLALNFSVF	YYEILNSPDR	ACRLAKAAFD	DAIAELDTLS	EESYKDSTLI	MQLLRDNLTL	WTSDMQGDDS
**250**							

Cmpd.	No. of Cmpds.	m/z meas.	Δ m/z [ppm]	z	Rt [min]	Score	P	Range	Sequence	Modification
2589	1	1045.0814	94.73	2	62.4	16.1	2	13-29	K.LAEQAERYDEMVESMKK.V	Oxidation: 11, 15

Detailed Protein Report

Protein 98: PREDICTED: intraflagellar transport protein 88 homolog isoform X7 [Homo sapiens]

**Accession:**	gi|530402705	**Score:**	42.9
**Database:**	refseq_human(refseq_human_20140103.fasta)	**MW [kDa]:**	95.8
**Database Date:**	2015-11-30	**pI:**	9.3
**Modification(s):**	Oxidation	**Sequence Coverage [%]:**	4.4
		**No. of unique Peptides:**	2

10	20	30	40	50	60	70	80
MKVESGMKIK	VHNNAESETA	VGFLEVDQGC	PIFGFPGPHR	KKNCLGPHMK	FTNTKVQMMQ	NVHLAPETDE	DDLYSGYNDY
**90**	**100**	**110**	**120**	**130**	**140**	**150**	**160**
NPIYDIEELE	NDAAFQQAVR	TSHGRRPPIT	AKISSTAVTR	PIATGYGSKT	SLASSIGRPM	TGAIQDGVTR	PMTAVRAAGF
**170**	**180**	**190**	**200**	**210**	**220**	**230**	**240**
TKAALRGSAF	DPLSQSRGPA	SPLEAKKKDS	PEEKIKQLEK	EVNELVEESC	IANSCGDLKL	ALEKAKDAGR	KERVLVRQRE
**250**	**260**	**270**	**280**	**290**	**300**	**310**	**320**
QVTTPENINL	DLTYSVLFNL	ASQYSVNEMY	AEALNTYQVI	VKNKMFSNAG	ILKMNMGNIY	LKQRNYSKAI	KFYRMALDQV
**330**	**340**	**350**	**360**	**370**	**380**	**390**	**400**
PSVNKQMRIK	IMQNIGVTFI	QAGQYSDAIN	SYEHIMSMAP	NLKAGYNLTI	CYFAIGDREK	MKKAFQKLIT	VPLEIDEDKY
**410**	**420**	**430**	**440**	**450**	**460**	**470**	**480**
ISPSDDPHTN	LVTEAIKNDH	LRQMERERKA	MAEKYIMTSA	KLIAPVIETS	FAAGYDWCVE	VVKASQYVEL	ANDLEINKAV
**490**	**500**	**510**	**520**	**530**	**540**	**550**	**560**
TYLRQKDYNQ	AVEILKVLEK	KDSRVKSAAA	TNLSALYYMG	KDFAQASSYA	DIAVNSDRYN	PAALTNKGNT	VFANGDYEKA
**570**	**580**	**590**	**600**	**610**	**620**	**630**	**640**
AEFYKEALRN	DSSCTEALYN	IGLTYEKLNR	LDEALDCFLK	LHAILRNSAE	VLYQIANIYE	LMENPSQAIE	WLMQVVSVIP
**650**	**660**	**670**	**680**	**690**	**700**	**710**	**720**
TDPQVLSKLG	ELYDREGDKS	QAFQYYYESY	RYFPCNIEVI	EWLGAYYIDT	QFWEKAIQYF	ERASLIQPTQ	VKWQLMVASC
**730**	**740**	**750**	**760**	**770**	**780**	**790**	**800**
FRRSGNYQKA	LDTYKDTHRK	FPENVECSGS	VRTGHMERDP	LNLLPRKSVL	PGPEIAKCNY	FSGEKYYLSA	FLSSSLHRSW
**810**	**820**	**830**	**840**	**850**			
IKRCSRICQK	TEEVGKNERN	KGTAHKVRQR	WQWGLPWQKR	RKC			

Cmpd.	No. of Cmpds.	m/z meas.	Δ m/z [ppm]	z	Rt [min]	Score	P	Range	Sequence	Modification
2105	1	937.3619	-150.42	2	56.5	15.6	2	315-330	R.MALDQVPSVNKQMRIK.I	Oxidation: 13
2734	1	772.4219	29.93	2	65.4	13.8	0	786-798	K.YYLSAFLSSSLHR.S	

Detailed Protein Report

Protein 99: spectrin beta chain, non-erythrocytic 1 isoform 2 [Homo sapiens]

**Accession:**	gi|112382252	**Score:**	42.7
**Database:**	refseq_human(refseq_human_20140103.fasta)	**MW [kDa]:**	251.2
**Database Date:**	2015-11-30	**pI:**	5.3
		**Sequence Coverage [%]:**	1.8
		**No. of unique Peptides:**	2

Detailed Protein Report

10	20	30	40	50	60	70	80
MELQRTSSIS	GPLSPAYTGQ	VPYNYNQLEG	RFKQLQDERE	AVQKKTFTKW	VNSHLARVSC	RITDLYTDLR	DGRMLIKLLE
90	100	110	120	130	140	150	160
VLSGERLPKP	TKGRMRIHCL	ENVDKALQFL	KEQRVHLENM	GSHDIVDGNH	RLTLGLIWTI	ILRFQIQDIS	VETEDNKEKK
170	180	190	200	210	220	230	240
SAKDALLLWC	QMKTAGYPNV	NIHNFTTSWR	DGMAFNALIH	KHRPDLIDFD	KLKKSNAHYN	LQNAFNLAEQ	HLGLTKLLDP
250	260	270	280	290	300	310	320
EDISVDHPDE	KSIITYVVTY	YHYFSKMKAL	AVEGKRIGKV	LDNAIETEKM	IEKYESLASD	LLEWIEQTII	ILNNRKFANS
330	340	350	360	370	380	390	400
LVGVQQQLQA	FNTYRTVEKP	PKFTEKGNLE	VLLFTIQSKM	RANNQKVYMP	REGKLISDIN	KAWERLEKAE	HERELALRNE
410	420	430	440	450	460	470	480
LIRQEKLEQL	ARRFDRKAAM	RETWLSENQR	LVSQDNFGFD	LPAVEAATKK	HEAIETDIAA	YEERVQAVVA	VARELEAENY
490	500	510	520	530	540	550	560
HDIKRITARK	DNVIRLWEYL	LELLRARRQR	LEMNLGLQKI	FQEMLYIMDW	MDEMKVLVLS	QDYGKHLLGV	EDLLQKHTLV
570	580	590	600	610	620	630	640
EADIGIQAER	VRGVNASAQK	FATDGEGYKP	CDPQVIRDRV	AHMEFCYQEL	CQLAAERRAR	LEESRRLWKF	FWEMAEEEGW
650	660	670	680	690	700	710	720
IREKEKILSS	DDYGKDLTSV	MRLLSKHRAF	EDEMSGRSGH	FEQAIKEGED	MIAEEHFGSE	KIRERIIYIR	EQWANLEQLS
730	740	750	760	770	780	790	800
AIRKKRLEEA	SLLHQFQADA	DDIDAWMLDI	LKIVSSSDVG	HDEYSTQSLV	KKHKDVAEEI	ANYRPTLDTL	HEQASALPQE
810	820	830	840	850	860	870	880
HAESPDVRGR	LSGIEERYKE	VAELTRLRKQ	ALQDTLALYK	MFSEADACEL	WIDEKEQWLN	NMQIPEKLED	LEVIQHRFES
890	900	910	920	930	940	950	960
LEPEMNNQAS	RVAVVNQIAR	QLMHSGHPSE	KEIKAQQDKL	NTRWSQFREL	VDRKKDALLS	ALSIQNYHLE	CNETKSWIRE
970	980	990	1000	1010	1020	1030	1040
KTKVIESTQD	LGNDLAGVMA	LQRKLTGMER	DLVAIEAKLS	DLQKEAEKLE	SEHPDQAQAI	LSRLAEISDV	WEEMKTTLKN
1050	1060	1070	1080	1090	1100	1110	1120
REASLGEASK	LQQFLRDLDD	FQSWLSRTQT	AIASEDMPNT	LTEAEKLLTQ	HENIKNEIDN	YEEDYQKMRD	MGEMVTQGQT
1130	1140	1150	1160	1170	1180	1190	1200
DAQYMFLRQR	LQALDTGWNE	LHKMWENRQN	LLSQSHAYQQ	FLRDTKQAEA	FLNNQEYVLA	HTEMPTTLEG	AEAAIKKQED
1210	1220	1230	1240	1250	1260	1270	1280
FMTTMDANEE	KINAVVETGR	RLVSDGNINS	DRIQEKVDSI	DDRHRKNRET	ASELLMRLKD	NRDLQKFLQD	CQELSLWINE
1290	1300	1310	1320	1330	1340	1350	1360
KMLTAQDMSY	DEARNLHSKW	LKHQAFMAEL	ASNKEWLDKI	EKEGMQLISE	KPETEAVVKE	KLTGLHKMWE	VLESTTQTKA
1370	1380	1390	1400	1410	1420	1430	1440
QRLFDANKAE	LFTQSCADLD	KWLHGLESQI	QSDDYGKDLT	SVNILLKKQQ	MLENQMEVRK	KEIEELQSQA	QALSQEGKST
1450	1460	1470	1480	1490	1500	1510	1520
DEVDSKRLTV	QTKFMELLEP	LNERKHNLLA	SKEIHQFNRD	VEDEILWVGE	RMPLATSTDH	GHNLQTVQLL	IKKNQTLQKE
1530	1540	1550	1560	1570	1580	1590	1600
IQGHQPRIDD	IFERSQNIVT	DSSSLSAEAI	RQRLADLKQL	WGLLIEETEK	RHRRLEEAHR	AQQYYFDAAE	AEAWMSEQEL
1610	1620	1630	1640	1650	1660	1670	1680
YMMSEEKAKD	EQSAVSMLKK	HQILEQAVED	YAETVHQLSK	TSRALVADSH	PESERISMRQ	SKVDKLYAGL	KDLAEERRGK
1690	1700	1710	1720	1730	1740	1750	1760
LDERHRLFQL	NREVDDLEQW	IAEREVVAGS	HELGQDYEHV	TMLQERFREF	ARDTGNIGQE	RVDTVNHLAD	ELINSGHSDA
1770	1780	1790	1800	1810	1820	1830	1840
ATIAEWKDGL	NEAWADLLEL	IDTRTQILAA	SYELHKFYHD	AKEIFGRIQD	KHKKLPEELG	RDQNTVETLQ	RMHTTFEHDI
1850	1860	1870	1880	1890	1900	1910	1920
QALGTQVRQL	QEDAARLQAA	YAGDKADDIQ	KRENEVLEAW	KSLLDACESR	RVRLVDTGDK	FRFFSMVRDL	MLWMEDVIRQ
1930	1940	1950	1960	1970	1980	1990	2000
IEAQEKPRDV	SSVELLMNNH	QGIKAEIDAR	NDSFTTCIEL	GKSLLARKHY	ASEEIKEKLL	QLTEKRKEMI	DKWEDRWEWL
2010	2020	2030	2040	2050	2060	2070	2080
RLILEVHQFS	RDASVAEAWL	LGQEPYLSSR	EIGQSVDEVE	KLIKRHEAFE	KSAATWDERF	SALERLTTLE	LLEVRRQQEE
2090	2100	2110	2120	2130	2140	2150	2160
EERKRRPPSP	EPSTKVSEEA	ESQQQWDTSK	GEQVSQNGLP	AEQGSPRVSY	RSQTYQNYKN	FNSRRTASDQ	PWSGL

Detailed Protein Report

Cmpd.	No. of Cmpds.	m/z meas.	Δ m/z [ppm]	z	Rt [min]	Score	P	Range	Sequence	Modification
2681	1	1079.0243	7.01	2	64.7	12.9	1	627-642	R.LWKFFWEMAEEEGWIR.E	
452	4	607.8361	-46.62	2	35.3	12.6	2	820-829	K.EVAELTRLRK.Q	

Detailed Protein Report

Protein 100: dynein heavy chain 17, axonemal [Homo sapiens]

**Accession:**	gi|256542310	**Score:**	42.6
**Database:**	refseq_human(refseq_human_20140103.fasta)	**MW [kDa]:**	509.0
**Database Date:**	2015-11-30	**pI:**	5.4
**Modification(s):**	Carbamidomethyl, Oxidation	**Sequence Coverage [%]:**	0.9
		**No. of unique Peptides:**	2

Detailed Protein Report

10	20	30	40	50	60	70	80
MTMAPDVRLE	YLEEVASIVL	KFKPDKWSKL	IGAEENVALF	TEFFEKPDVQ	VLVLTLNAAG	MIIPCLGFPQ	SLKSKGVYFI
90	100	110	120	130	140	150	160
KTKSENINKD	NYRARLLYGD	ISPTPVDQLI	AVVEEVLSSL	LNQSENMAGW	PQVVSEDIVK	QVHRLKNEMF	VMSGKIKGKT
170	180	190	200	210	220	230	240
LLPIPEHLGS	LDGTLESMER	IPSSLDNLLL	HAIETTIIDW	SHQIRDVLSK	DSAQALLDGL	HPLPQVEFEF	WDTRLLNLKC
250	260	270	280	290	300	310	320
IHEQLNRPKV	NKIVEILEKA	KSCYWPALQN	VYTNVTEGLK	EANDIVLYLK	PLRILLEEME	QADFTMLPTF	IAKVLDTICF
330	340	350	360	370	380	390	400
IWATSEYYNT	PARIIVILQE	FCNQIIEMTR	TFLSPEEVLK	GLQGEIEEVL	SGISLAVNVL	KELYQTYDFC	CVNMKLFFKD
410	420	430	440	450	460	470	480
KEPVPWEFPS	SLAFSRINSF	FQRIQTIEEL	YKTAIEFLKL	EKIELGGVRG	NLLGSLVTRI	YDEVFELVKV	FADCKYDPLD
490	500	510	520	530	540	550	560
PGDSNFDRDY	ADFEIKIQDL	DRRLATIFCQ	GFDDCSCIKS	SAKLLYMCGG	LMERPLILAE	VAPRYSVMLE	LFDAELDNAK
570	580	590	600	610	620	630	640
ILYDAQMAAS	EEGNIPLIHK	NMPPVAGQLK	WSLELQERLE	VSMKHLKHVE	HPVMSGAEAK	LTYQKYDEMM	ELLRCHREKI
650	660	670	680	690	700	710	720
YQQWVAGVDQ	DCHFNLGQPL	ILRDAASNLI	HVNFSKALVA	VLREVKYLNF	QQQKEIPDSA	ESLFSENETF	RKFVGNLELI
730	740	750	760	770	780	790	800
VGWYNEIKTI	VKAVEFLLIK	SELEAIDVKL	LSAETTLFWN	GEGVFQYIQE	VREILHNLQN	RMQKAKQNIE	GISQAMKDWS
810	820	830	840	850	860	870	880
ANPLFERKDN	KKEALLDLDG	RIANLNKRYA	AVRDAGVKIQ	AMVAENAELF	RADTLSLPWK	DYVIYIDDMV	LDEFDQFIRK
890	900	910	920	930	940	950	960
SLSFLMDNMV	IDESIAPLFE	IRMELDEDGL	TFNPTLEVGS	DRGFLALIEG	LVNDIYNVAR	LIPRLAKDRM	NYKMDLEDNT
970	980	990	1000	1010	1020	1030	1040
DLIEMREEVS	SLVINAMKEA	EEYQDSFERY	SYLWTDNLQE	FMKNFLIYGC	AVTAEDLDTW	TDDTIPKTPP	TLAQFQEQID
1050	1060	1070	1080	1090	1100	1110	1120
SYEKLYEEVS	KCENTKVFHG	WLQCDCRPFK	QALLSTIRRW	GFMFKRHLSN	HVTNSLADLE	AFMKVARMGL	TKPLKEGDYD
1130	1140	1150	1160	1170	1180	1190	1200
GLVEVMGHLM	KVKERQAATD	NMFEPLKQTI	ELLKTYGEEM	PEEIHLKLQE	LPEHWANTKK	LAIQVKLTVA	PLQANEVSIL
1210	1220	1230	1240	1250	1260	1270	1280
RRKCQQFELK	QHEFRERFRR	EAPFSFSDPN	PYKSLNKQQK	SISAMEGIME	ALSKSGGLFE	VPVPDYKQLK	ACHREVRLLK
1290	1300	1310	1320	1330	1340	1350	1360
ELWDMVVVVN	TSIEDWKTTK	WKDINVEQMD	IDCKKFAKDM	RSLDKEMKTW	DAFVGLDNTV	KNVITSLRAV	SELQNPAIRE
1370	1380	1390	1400	1410	1420	1430	1440
RHWQQLMQAT	QVKFKMSEET	TLADLLQLNL	HSYEDEVRNI	VDKAVKESGM	EKVLKALDST	WSMMEFQHEP	HPRTGTMMLK
1450	1460	1470	1480	1490	1500	1510	1520
SSEVLVETLE	DNQVQLQNLM	MSKYLAHFLK	EVTSWQQKLS	TADSVISIWF	EVQRTWSHLE	SIFIGSEDIR	TQLPGDSQRF
1530	1540	1550	1560	1570	1580	1590	1600
DDINQEFKAL	MEDAVKTPNV	VEATSKPGLY	NKLEALKKSL	AICEKALAEY	LETKRLAFPR	FYFVSSADLL	DILSNGNDPV
1610	1620	1630	1640	1650	1660	1670	1680
EVSRHLSKLF	DSLCKLKFRL	DASDKPLKVG	LGMYSKEDEY	MVFDQECDLS	GQVEVWLNRV	LDRMCSTLRH	EIPEAVVTYE
1690	1700	1710	1720	1730	1740	1750	1760
EKPREQWILD	YPAQVALTCT	QIWWTTEVGL	AFARLEEGYE	NAIRDYNKKQ	ISQLNVLITL	LMGNLNAGDR	MKIMTICTID
1770	1780	1790	1800	1810	1820	1830	1840
VHARDVVAKM	IVAKVESSQA	FTWQAQLRHR	WDEEKRHCFA	NICDAQIQYS	YEYLGNTPRL	VITPLTDRCY	ITLTQSLHLI
1850	1860	1870	1880	1890	1900	1910	1920
MGGAPAGPAG	TGKTETTKDL	GRALGTMVYV	FNCSEQMDYK	SCGNIYKGLA	QTGAWGCFDE	FNRISVEVLS	VIAVQVKCVQ
1930	1940	1950	1960	1970	1980	1990	2000
DAIRAKKKAF	NFLGEIIGLI	PTVGIFITMN	PGYAGRAELP	ENLKALFRPC	AMVVPDFELI	CEIMLMAEGF	LEARLLARKF
2010	2020	2030	2040	2050	2060	2070	2080
ITLYTLCKEL	LSKQDHYDWG	LRAIKSVLVV	AGSLKRGDPS	RAEDQVLMRA	LRDFNIPKIV	TDDLPVFMGL	IGDLFPALDV
2090	2100	2110	2120	2130	2140	2150	2160
PRKRDLNFEK	IIKQSIVELK	LQAEDSFVLK	VVQLEELLQV	RHSVFIVGNA	GSGKSQVLKS	LNKTYQNLKR	KPVAVDLDPK
2170	2180	2190	2200	2210	2220	2230	2240
AVTCDELFGI	INPVTREWKD	GLFSTIMRDL	ANITHDGPKW	IILDGDIDPM	WIESLNTVMD	DNKVLTLASN	ERIPLNRTMR
2250	2260	2270	2280	2290	2300	2310	2320
LVFEISHLRT	ATPATVSRAG	ILYINPADLG	WNPVVSSWIE	RRKVQSEKAN	LMILFDKYLP	TCLDKLRFGF	KKITPVPEIT
2330	2340	2350	2360	2370	2380	2390	2400

Detailed Protein Report

Cmpd.	No. of Cmpds.	m/z meas.	Δ m/z [ppm]	z	Rt [min]	Score	P	Range	Sequence	Modification
1870	1	704.2582	-146.54	2	53.4	13.7	0	1255-1267	K.SGGLFEVPVPDYK.Q	
1689	1	1051.5326	21.27	2	50.9	16.9	1	4025-4041	K.CMLFALCYFHAVVAERR.K	Carbamidomethyl: 1; Oxidation: 2

Detailed Protein Report

Protein 101: PREDICTED: protein ELYS isoform X2 [Homo sapiens]

**Accession:**	gi|578802192	**Score:**	42.6
**Database:**	refseq_human(refseq_human_20140103.fasta)	**MW [kDa]:**	252.3
**Database Date:**	2015-11-30	**pI:**	6.2
**Modification(s):**	Carbamidomethyl	**Sequence Coverage [%]:**	1.9
		**No. of unique Peptides:**	3

Quantitation

MD:MU	**Median:** 0.47	**CV:** 0.00 %	**No. of Peptides:** 1

Detailed Protein Report

10	20	30	40	50	60	70	80
MRDLRAQVTS	GLLPFPEVTL	QALGEDEITL	ESVLRGKFAA	GKNGLACLAC	GPQLEVVNSI	TGERLSAYRF	SGVNEQPPVV
90	100	110	120	130	140	150	160
LAVKEFSWQK	RTGLLIGLEE	TEGSVLCLYD	LGISKVVKAV	VLPGRVTAIE	PIINHGGASA	STQHLHPSLR	WLFGVAAVVT
170	180	190	200	210	220	230	240
DVGQILLVDL	CLDDLSCNQN	EVEASDLEVL	TGIPAEVPHI	RESVMRQGRH	LCFQLVSPTG	TAVSTLSYIS	RTNQLAVGFS
250	260	270	280	290	300	310	320
DGYLALWNMK	SMKREYYIQL	ESGQVPVYAV	TFQEPENDPR	NCCYLWAVQS	TQDSEGDVLS	LHLLQLAFGN	RKCLASGQIL
330	340	350	360	370	380	390	400
YEGLEYCEER	YTLDLTGGMF	PLRGQTSNTK	LLGCQSIEKF	RSHGDREEGV	NEALSPDTSV	SVFTWQVNIY	GQGKPSVYLG
410	420	430	440	450	460	470	480
LFDINRWYHA	QMPDSLRSGE	YLHNCSYFAL	WSLESVVSRT	SPHGILDILV	HERSLNRGVP	PSYPPPEQFF	NPSTYNFDAT
490	500	510	520	530	540	550	560
CLLNSGVVHL	TCTGFQKETL	TFLKKSGPSL	NELIPDGYNR	CLVAGLLSPR	FVDVQPSSLS	QEEQLEAILS	AAIQTSSLGL
570	580	590	600	610	620	630	640
LTGYIRRWIT	EEQPNSATNL	RFVLEWTWNK	VVLTKEEFDR	LCVPLFDGSC	HFMDPQTIQS	IQQCYLLLSN	LNIVLSCFAS
650	660	670	680	690	700	710	720
EAREITERGL	IDLSNKFVVS	HLICQYAQVV	LWFSHSGLLP	EGIDDSVQLS	RLCYNYPVIQ	NYYTSRRQKF	ERLSRGKWNP
730	740	750	760	770	780	790	800
DCLMIDGLVS	QLGERIEKLW	KRDEGGTGKY	PPASLHAVLD	MYLLDGVTEA	AKHSITIYLL	LDIMYSFPNK	TDTPIESFPT
810	820	830	840	850	860	870	880
VFAISWGQVK	LIQGFWLIDH	NDYESGLDLL	FHPATAKPLS	WQHSKIIQAF	MSQGEHRQAL	RYIQTMKPTV	SSGNDVILHL
890	900	910	920	930	940	950	960
TVLLFNRCMV	EAWNFLRQHC	NRLNIEELLK	HMYEVCQEMG	LMEDLLKLPF	TDTEQECLVK	FLQSSASVQN	HEFLLVHHLQ
970	980	990	1000	1010	1020	1030	1040
RANYVPALKL	NQTLKINVMN	DRDPRLRERS	LARNSILDQY	GKILPRVHRK	LAIERAKPYH	LSTSSVFRLV	SRPKPLSAVP
1050	1060	1070	1080	1090	1100	1110	1120
KQVVTGTVLT	RSVFINNVLS	KIGEVWASKE	PINSTTPFNS	SKIEEPSPIV	YSLPAPELPE	AFFGTPISKA	SQKISRLLDL
1130	1140	1150	1160	1170	1180	1190	1200
VVQPVPRPSQ	CSEFIQQSSM	KSPLYLVSRS	LPSSSQLKGS	PQAISRASEL	HLLETPLVVK	KAKSLAMSVT	TSGFSEFTPQ
1210	1220	1230	1240	1250	1260	1270	1280
SILRSTLRST	PLASPSPSPG	RSPQRLKETR	ISFVEEDVHP	KWIPGAADDS	KLEVFTTPKK	CAVPVETEWL	KSKDRTTSFF
1290	1300	1310	1320	1330	1340	1350	1360
LNSPEKEHQE	MDEGSQSLEK	LDVSKGNSSV	SITSDETTLE	YQDAPSPEDL	EETVFTASKP	KSSSTALTTN	VTEQTEKDGD
1370	1380	1390	1400	1410	1420	1430	1440
KDVFASEVTP	SDLQKQMGNL	EDAETKDLLV	AAEAFSELNH	LSPVQGTEAS	LCAPSVYEGK	IFTQKSKVPV	LDEGLTSVET
1450	1460	1470	1480	1490	1500	1510	1520
YTPAIRANDN	KSMADVLGDG	GNSSLTISEG	PIVSERRLNQ	EVALNLKEDH	EVEVGVLKES	VDLPEEKLPI	SDSPPDTQEI
1530	1540	1550	1560	1570	1580	1590	1600
HVIEQEKLEA	QDSGEEARNL	SFNELYPSGT	LKLQYNFDTI	DQQFCDLADN	KDTAECDIAE	VDGELFVAQS	NFTLILEGEE
1610	1620	1630	1640	1650	1660	1670	1680
GEVEPGDFAS	SDVLPKAANT	ATEEKLVCSG	ENDNHGQIAN	LPSAVTSDQK	SQKVDTLPYV	PEPIKVAIAE	NLLDVIKDTR
1690	1700	1710	1720	1730	1740	1750	1760
SKEITSDTME	QSIHETIPLV	SQNIMCPTKL	VKSAFKTAQE	TSTMTMNVSQ	VDDVVSSKTR	TRGQRIQNVN	VKSAQQEASA
1770	1780	1790	1800	1810	1820	1830	1840
DVATPKMPGQ	SVRKKTRKAK	EISEASENIY	SDVRGLSQNQ	QIPQNSVTPR	RGRRKKEVNQ	DILENTSSVE	QELQITTGRE
1850	1860	1870	1880	1890	1900	1910	1920
SKRLKSSQLL	EPAVEETTKK	EVKVSSVTKR	TPRRIKRSVE	NQESVEIIND	LKVSTVTSPS	RMIRKLRSTN	LDASENTGNK
1930	1940	1950	1960	1970	1980	1990	2000
QDDKSSDKQL	RIKHVRRVRG	REVSPSDVRE	DSNLESSQLT	VQAEFDMSAI	PRKRGRPRKI	NPSEDVGSKA	VKEERSPKKK
2010	2020	2030	2040	2050	2060	2070	2080
EAPSIRRRST	RNTPAKSENV	DVGKPALGKS	ILVPNEELSM	VMSSKKKLTK	KTESQSQKRS	LHSVSEERTD	EMTHKETNEQ
2090	2100	2110	2120	2130	2140	2150	2160
EERLLATASF	TKSSRSSRTR	SSKAILLPDL	SEPNNEPLFS	PASEVPRKAK	AKKIEVPAQL	KELVSDLSSQ	FVISPPALRS
2170	2180	2190	2200	2210	2220	2230	2240
RQKNTSNKNK	LEDELKDDAQ	SVETLGKPKA	KRIRTSKTKQ	ASKNTEKESA	WSPPPIEIRL	ISPLASPADG	VKSKPRKTTE
2250	2260	2270					
VTGTGLGRNR	KKLSSYPKQI	LRRKML					

Detailed Protein Report

Cmpd.	No. of Cmpds.	m/z meas.	Δ m/z [ppm]	z	Rt [min]	Score	P	Range	Sequence	Modification	Ratios
2616	1	742.3664	-24.12	2	62.8	14.1	0	331-343	R.YTLDLTGGMFPLR.G		
2789	1	735.0657	-23.60	3	66.4	12.6	2	1010-1028	R.KLAIERAKPYHLSTSSVFR.L		
549	1	730.3654	-25.94	2	36.2	15.9	1	1260-1271	K.KCAVPVETEWLK.S	Carbamidomethyl: 2	MD:MU 0.47

Detailed Protein Report

Protein 102: PREDICTED: transcription termination factor 2 isoform X1 [Homo sapiens]

**Accession:**	gi|530363482	**Score:**	42.1
**Database:**	refseq_human(refseq_human_20140103.fasta)	**MW [kDa]:**	129.4
**Database Date:**	2015-11-30	**pI:**	9.5
**Modification(s):**	Oxidation	**Sequence Coverage [%]:**	3.4
		**No. of unique Peptides:**	2

10	20	30	40	50	60	70	80
MEEVRCPEHG	TFCFLKTGVR	DGPNKGKSFY	VCRADTCSFV	RATDIPVSHC	LLHEDFVVEL	QGLLLPQDKK	EYRLFFRCIR
90	100	110	120	130	140	150	160
SKAEGKRWCG	SIPWQDPDSK	EHSVSNKSQH	ASETFHHSSN	WLRNPFKVLD	KNQEPALWKQ	LIKGEGEEKK	ADKKQREKGD
170	180	190	200	210	220	230	240
QLFDQKKEQK	PEMMEKDLSS	GLVPKKKQSV	VQEKKQEEGA	EIQCEAETGG	THKRDFSEIK	SQQCQGNELT	RPSASSQEKS
250	260	270	280	290	300	310	320
SGKSQDVQRE	SEPLREKVTQ	LLPQNVHSHN	SISKPQKGGP	LNKEYTNWEA	KETKAKDGPS	IQATQKSLPQ	GHFQERPETH
330	340	350	360	370	380	390	400
SVPAPGGPAA	QAAPAAPGLS	LGEGREAATS	SDDEEEDDVV	FVSSKPGSPL	LFDSTLDLET	KENLQFPDRS	VQRKVSPASG
410	420	430	440	450	460	470	480
VSKKVEPSDP	VARRVYLTTQ	LKQKKSTLAS	VNIQALPDKG	QKLIKQIQEL	EEVLSGLTLS	PEQGTNEKSN	SQVPQQSHFT
490	500	510	520	530	540	550	560
KTTTGPPHLV	PPQPLPRRGT	QPVGSLELKS	ACQVTAGGSS	QCYRGHTNQD	HVHAVWKITS	EAIGQLHRSL	ESCPGETVVA
570	580	590	600	610	620	630	640
EDPAGLKVPL	LLHQKQALAW	LLWRESQKPQ	GGILDDMGLG	KTLTMIALIL	TQKNQEKKEE	KEKSTALTWL	SKDDSCDFTS
650	660	670	680	690	700	710	720
HGTLIICPAS	LIHHWKNEVE	KRVNSNKLRV	YLYHGPNRDS	RARVLSTYDI	VITTYSLVAK	EIPTNKQEAE	IPGANLNVEG
730	740	750	760	770	780	790	800
TSTPLLRIAW	ARIILDEAHN	VKNPRVQTSI	AVCKLQACAR	WAVTGTPIQN	NLLDMYSLLK	FLRCSPFDEF	NLWRSQVDNG
810	820	830	840	850	860	870	880
SKKGGERLSI	LTKSLLLRRT	KDQLDSTGRP	LVILPQRKFQ	LHHLKLSEDE	ETVYNVFFAR	SRSALQSYLK	RHESRGNQSG
890	900	910	920	930	940	950	960
RSPNNPFSRV	ALEFGSEEPR	HSEAADSPRS	STVHILSQLL	RLRQCCCHLS	LLKSALDPME	LKGEGLVLSL	EEQLSALTLS
970	980	990	1000	1010	1020	1030	1040
ELRDSEPSST	VSLNGTFFKM	ELFEGMREST	KISSLLAELE	AIQRNSASQK	SVIVSQWTNM	LKVVALHLKK	HGLTYATIDG
1050	1060	1070	1080	1090	1100	1110	1120
SVNPKQRMDL	VEAFNHSRGP	QVMLISLLAG	GVGLNLTGGN	HLFLLDMHWN	PSLEDQACDR	IYRVGQQKDV	VIHRFVCEGT
1130	1140	1150	1160	1170			
VEEKILQLQE	KKKDLAKQVL	SGSGESVTKL	TLADLRVLFG	I			

Cmpd.	No. of Cmpds.	m/z meas.	Δ m/z [ppm]	z	Rt [min]	Score	P	Range	Sequence	Modification
2664	1	701.8434	-43.89	2	63.4	13.3	2	71-80	K.EYRLFFRCIR.S	
2866	1	655.3693	90.53	2	66.3	14.3	1	167-176	K.KEQKPEMMEK.D	Oxidation: 7, 8

Detailed Protein Report

Protein 103: GRIP and coiled-coil domain-containing protein 1 [Homo sapiens]

**Accession:**	gi|22208850	**Score:**	42.1
**Database:**	refseq_human(refseq_human_20140103.fasta)	**MW [kDa]:**	87.8
**Database Date:**	2015-11-30	**pI:**	5.2
		**Sequence Coverage [%]:**	5.0
		**No. of unique Peptides:**	2

Quantitation

MD:MU **Median:** 0.29 **CV:** 0.00 % **No. of Peptides:** 1

10	20	30	40	50	60	70	80
MEKFGMNFGG	GPSKKDLLET	IETQKKQLLQ	YQARLKDVVR	AYKSLLKEKE	ALEASIKVLS	VSHEADVGLA	GVQLPGLTFP
**90**	**100**	**110**	**120**	**130**	**140**	**150**	**160**
DSVDDRCSTH	SEDSTGTATS	LDTAASLTST	KGEFGVEDDR	PARGPPPPKS	EEASWSESGV	SSSSGDGPFA	GGEVDKRLHQ
**170**	**180**	**190**	**200**	**210**	**220**	**230**	**240**
LKTQLATLTS	SLATVTQEKS	RMEASYLADK	KKMKQDLEDA	SNKAEEERAR	LEGELKGLQE	QIAETKARLI	TQQHDRAQEQ
**250**	**260**	**270**	**280**	**290**	**300**	**310**	**320**
SDHALMLREL	QKLLQEERTQ	RQDLELRLEE	TREALAGRAY	AAEQMEGFEL	QTKQLTREVE	ELKSELQAIR	DEKNQPDPRL
**330**	**340**	**350**	**360**	**370**	**380**	**390**	**400**
QELQEEAARL	KSHFQAQLQQ	EMRKTALAED	QLRQQSQVEE	QRVAALENQI	SEVSELLGTY	EKAKQKDQLA	IQKLKERILQ
**410**	**420**	**430**	**440**	**450**	**460**	**470**	**480**
LDLENKTLAL	AASSRSPLDS	HGEESSLDVN	VLKDKMEKLK	RLLQVAARKS	QVTLDVEKLC	DLEIMPSSEA	ADGEKATALY
**490**	**500**	**510**	**520**	**530**	**540**	**550**	**560**
YQQELKQLKE	EFERYKMRAQ	VVLKSKNTKD	GNLGKELEAA	QEQLAELKEK	YISLRLSCEE	LEHQHQQEAD	DWKQELARLQ
**570**	**580**	**590**	**600**	**610**	**620**	**630**	**640**
QLHRQELERC	QLDFRDRTLK	LEEELHKQRD	RALAVLTEKD	LELEQLRSVA	LASGLPGRRS	PVGGGGPGDP	ADTSSSDSLT
**650**	**660**	**670**	**680**	**690**	**700**	**710**	**720**
QALQLAAANE	PTFFLYAEQL	ARKEVEITSL	RKQKHRLEVE	VHQLQDRLLE	EGERHREEVA	ALQSHIEKNI	RDQSREGANL
**730**	**740**	**750**	**760**	**770**	**780**		
EYLKNIIYRF	LTLPDSLGRQ	QTLTAILTIL	HFSPEEKQVI	MRLPTSASWW	PSGKR		

Cmpd.	No. of Cmpds.	m/z meas.	Δ m/z [ppm]	z	Rt [min]	Score	P	Range	Sequence	Modification	Ratios
1918	2	572.7243	-166.46	2	54.0	17.8	1	344-353	R.KTALAEDQLR.Q		MD:MU 0.29
55	1	1096.9659	-93.00	2	30.1	11.7	0	363-382	R.VAALENQISEVSELLGTYEK.A		

Detailed Protein Report

Protein 104: collagen alpha-2(XI) chain isoform 3 preproprotein [Homo sapiens]

Accession: gi|111118968
Database: refseq_human(refseq_human_20140103.fasta)
Database Date: 2015-11-30

Score: 42.0
MW [kDa]: 159.8
pI: 9.5
Sequence Coverage [%]: 2.6
No. of unique Peptides: 3

```
        10         20         30         40         50         60         70         80
MERCSRCHRL LLLLPLVLGL SAAPGWAGAP PVDVLRALRF PSLPDGVRRA KGICPADVAY RVARPAQLSA PTRQLFPGGF
        90        100        110        120        130        140        150        160
PKDFSLLTVV RTRPGLQAPL LTLYSAQGVR QLGLELGRPV RFLYEDQTGR PQPPSQPVFR GLSLADGKWH RVAVAVKGQS
       170        180        190        200        210        220        230        240
VTLIVDCKKR VTRPLPRSAR PVLDTHGVII FGARILDEEV FEGDVQELAI VPGVQAAYES CEQKELECEG GQRERPQNQQ
       250        260        270        280        290        300        310        320
PHRAQRSPQQ QPSRLHRPQN QEPQSQAAHG PRGLKGEKGE PAVLEPGMLV EGPPGPEGPA GLIGPPGIQG NPGPVGDPGE
       330        340        350        360        370        380        390        400
RGPPGRAGLP GSDGAPGPPG TSLMLPFRFG SGGGDKGPVV AAQEAQAQAI LQQARLALRG PPGPMGYTGR PGPLGQPGSP
       410        420        430        440        450        460        470        480
GLKGESGDLG PQGPRGPQGL TGPPGKAGRR GRAGADGARG MPGDPGVKGD RGFDGLPGLP GEKGHRGDTG AQGLPGPPGE
       490        500        510        520        530        540        550        560
DGERGDDGEI GPRGLPGESG PRGLLGPKGP PGIPGPPGVR GMDGPQGPKG SLGPQGEPGP PGQQGTPGTQ GLPGPQGAIG
       570        580        590        600        610        620        630        640
PHGEKGPQGK PGLPGMPGSD GPPGHPGKEG PPGTKGNQGP SGPQGPLGYP GPRGVKGVDG IRGLKGHKGE KGEDGFPGFK
       650        660        670        680        690        700        710        720
GDIGVKGDRG EVGVPGSRGE DGPEGPKGRT GPTGDPGPPG LMGEKGKLGV PGLPGYPGRQ GPKGSLGFPG FPGASGEKGA
       730        740        750        760        770        780        790        800
RGLSGKSGPR GERGPTGPRG QRGPRGATGK SGAKGTSGGD GPHGPPGERG LPGPQGPNGF PGPKGPPGPP GKDGLPGHPG
       810        820        830        840        850        860        870        880
QRGEVGFQGK TGPPGPPGVV GPQGAAGETG PMGERGHPGP PGPPGEQGLP GTAGKEGTKG DPGPPGAPGK DGPAGLRGFP
       890        900        910        920        930        940        950        960
GERGLPGTAG GPGLKGNEGP SGPPGPAGSP GERGAAGSGG PIGPPGRPGP QGPPGAAGEK GVPGEKGPIG PTGRDGVQGP
       970        980        990       1000       1010       1020       1030       1040
VGLPGPAGPP GVAGEDGDKG EVGDPGQKGT KGNKGEHGPP GPPGPIGPVG QPGAAGADGE PGARGPQGHF GAKGDEGTRG
      1050       1060       1070       1080       1090       1100       1110       1120
FNGPPGPIGL QGLPGPSGEK GETGDVGPMG PPGPPGPRGP AGPNGADGPQ GPPGGVGNLG PPGEKGEPGE SGSPGIQGEP
      1130       1140       1150       1160       1170       1180       1190       1200
GVKGPRGERG EKGESGQPGE PGPPGPKGPT GDDGPKGNPG PVGFPGDPGP PGEGGPRGQD GAKGDRGEDG EPGQPGSPGP
      1210       1220       1230       1240       1250       1260       1270       1280
TGENGPPGPL GKRGPAGSPG SEGRQGGKGA KGDPGAIGAP GKTGPVGPAG PAGKPGPDGL RGLPGSVGQQ GRPGATGQAG
      1290       1300       1310       1320       1330       1340       1350       1360
PPGPVGPPGL PGLRGDAGAK GEKGHPGLIG LIGPPGEQGE KGDRGLPGPQ GSPGQKGEMG IPGASGPIGP GGPPGLPGPA
      1370       1380       1390       1400       1410       1420       1430       1440
GPKGAKGATG PGGPKGEKGV QGPPGHPGPP GEVIQPLPIQ MPKKTRRSVD GSRLMQEDEA IPTGGAPGSP GGLEEIFGSL
      1450       1460       1470       1480       1490       1500       1510       1520
DSLREEIEQM RRPTGTQDSP ARTCQDLKLC HPELPDGEYW VDPNQGCARD AFRVFCNFTA GGETCVTPRD DVTQFSYVDS
      1530       1540       1550       1560       1570       1580       1590       1600
EGSPVGVVQL TFLRLLSVSA HQDVSYPCSG AARDGPLRLR GANEDELSPE TSPYVKEFRD GCQTQQGRTV LEVRTPVLEQ
      1610       1620       1630
LPVLDASFSD LGAPPRRGGV LLGPVCFMG
```

Cmpd.	No. of Cmpds.	m/z meas.	Δ m/z [ppm]	z	Rt [min]	Score	P	Range	Sequence	Modification
2716	2	645.8279	-46.54	2	64.3	18.2	1	158-169	K.GQSVTLIVDCKK.R	
2503	1	862.3584	-77.51	2	61.7	12.3	1	668-685	K.GRTGPTGDPGPPGLMGEK.G	
230	2	575.1791	-198.73	2	32.6	11.4	0	1618-1629	R.GGVLLGPVCFMG.-	

Detailed Protein Report

Protein 105: **PREDICTED: chromodomain-helicase-DNA-binding protein 4 isoform X6 [Homo sapiens]**

**Accession:**	gi|578822562	**Score:**	41.9
**Database:**	refseq_human(refseq_human_20140103.fasta)	**MW [kDa]:**	216.7
**Database Date:**	2015-11-30	**pI:**	5.7
**Modification(s):**	Carbamidomethyl, Oxidation	**Sequence Coverage [%]:**	2.5
		**No. of unique Peptides:**	2

Detailed Protein Report

10	20	30	40	50	60	70	80
MASGLGSPSP	CSAGSEEEDM	DALLNNSLPP	PHPENEEDPE	EDLSETETPK	LKKKKKPKKP	RDPKIPKSKR	QKKERMLLCR
90	100	110	120	130	140	150	160
QLGDSSGEGP	EFVEEEEEVA	LRSDSEGSDY	TPGKKKKKKL	GPKKEKKSKS	KRKEEEEEED	DDDDSKEPKS	SAQLLEDWGM
170	180	190	200	210	220	230	240
EDIDHVFSEE	DYRTLTNYKA	FSQFVRPLIA	AKNPKIAVSK	MMMVLGAKWR	EFSTNNPFKG	SSGASVAAAA	AAAVAVVESM
250	260	270	280	290	300	310	320
VTATEVAPPP	PPVEVPIRKA	KTKEGKGPNA	RRKPKGSPRV	PDAKKPKPKK	VAPLKIKLGG	FGSKRKRSSS	EDDDLDVESD
330	340	350	360	370	380	390	400
FDDASINSYS	VSDGSTSRSS	RSRKKLRTTK	KKKKDHQDYC	EVCQQGGEII	LCDTCPRAYH	MVCLDPDMEK	APEGKWSCPH
410	420	430	440	450	460	470	480
CEKEGIQWEA	KEDNSEGEEI	LEEVGGDLEE	EDDHHMEFCR	VCKDGGELLC	CDTCPSSYHI	HCLNPPLPEI	PNGEWLCPRC
490	500	510	520	530	540	550	560
TCPALKGKVQ	KILIWKWGQP	PSPTPVPRPP	DADPNTPSPK	PLEGRPERQF	FVKWQGMSYW	HCSWVSELQL	ELHCQVMFRN
570	580	590	600	610	620	630	640
YQRKNDMDEP	PSGDFGGDEE	KSRKRKNKDP	KFAEMEERFY	RYGIKPEWMM	IHRILNHSVD	KKGHVHYLIK	WRDLPYDQAS
650	660	670	680	690	700	710	720
WESEDVEIQD	YDLFKQSYWN	HRELMRGEEG	RPGKKLKKVK	LRKLERPPET	PTVDPTVKYE	RQPEYLDATG	GTLHPYQMEG
730	740	750	760	770	780	790	800
LNWLRFSWAQ	GTDTILADEM	GLGKTVQTAV	FLYSLYKEGH	SKGPFLVSAP	LSTIINWERE	FEMWAPDMYV	VTYVGDKDSR
810	820	830	840	850	860	870	880
AIIRENEFSF	EDNAIRGGKK	ASRMKKEASV	KFHVLLTSYE	LITIDMAILG	SIDWACLIVD	EAHRLKNNQS	KFFRVLNGYS
890	900	910	920	930	940	950	960
LQHKLLLTGT	PLQNNLEELF	HLLNFLTPER	FHNLEGFLEE	FADIAKEDQI	KKLHDMLGPH	MLRRLKADVF	KNMPSKTELI
970	980	990	1000	1010	1020	1030	1040
VRVELSPMQK	KYYKYILTRN	FEALNARGGG	NQVSLLNVVM	DLKKCCNHPY	LFPVAAMEAP	KMPNGMYDGS	ALIRASGKLL
1050	1060	1070	1080	1090	1100	1110	1120
LLQKMLKNLK	EGGHRVLIFS	QMTKMLDLLE	DFLEHEGYKY	ERIDGGITGN	MRQEAIDRFN	APGAQQFCFL	LSTRAGGLGI
1130	1140	1150	1160	1170	1180	1190	1200
NLATADTVII	YDSDWNPHND	IQAFSRAHRI	GQNKKVMIYR	FVTRASVEER	ITQVAKKKMM	LTHLVVRPGL	GSKTGSMSKQ
1210	1220	1230	1240	1250	1260	1270	1280
ELDDILKFGT	EELFKDEATD	GGGDNKEGED	SSVIHYDDKA	IERLLDRNQD	ETEDTELQGM	NEYLSSFKVA	QYVVREEEMG
1290	1300	1310	1320	1330	1340	1350	1360
EEEEVEREII	KQEESVDPDY	WEKLLRHHYE	QQQEDLARNL	GKGKRIRKQV	NYNDGSQEDR	DWQDDQSDNQ	SDYSVASEEG
1370	1380	1390	1400	1410	1420	1430	1440
DEDFDERSEA	PRRPSRKGLR	NDKDKPLPPL	LARVGGNIEV	LGFNARQRKA	FLNAIMRYGM	PPQDAFTTQW	LVRDLRGKSE
1450	1460	1470	1480	1490	1500	1510	1520
KEFKAYVSLF	MRHLCEPGAD	GAETFADGVP	REGLSRQHVL	TRIGVMSLIR	KKVQEFEHVN	GRWSMPELAE	VEENKKMSQP
1530	1540	1550	1560	1570	1580	1590	1600
GSPSPKTPTP	STPGDTQPNT	PAPVPPAEDG	IKIEENSLKE	EESIEGEKEV	KSTAPETAIE	CTQAPAPASE	DEKVVVEPPE
1610	1620	1630	1640	1650	1660	1670	1680
GEEKVEKAEV	KERTEEPMET	EPKGKGAADV	EKVEEKSAID	LTPIVVEDKE	EKKEEEEKKE	VMLQNGETPK	DLNDEKQKKN
1690	1700	1710	1720	1730	1740	1750	1760
IKQRFMFNIA	DGGFTELHSL	WQNEERAATV	TKKTYEIWHR	RHDYWLLAGI	INHGYARWQD	IQNDPRYAIL	NEPFKGEMNR
1770	1780	1790	1800	1810	1820	1830	1840
GNFLEIKNKF	LARRFKLLEQ	ALVIEEQLRR	AAYLNMSEDP	SHPSMALNTR	FAEVECLAES	HQHLSKESMA	GNKPANAVLH
1850	1860	1870	1880	1890	1900	1910	
KGILKQLEEL	LSDMKADVTR	LPATIARIPP	VAVRLQMSER	NILSRLANRA	PEPTPQQVAQ	QQ	

Cmpd.	No. of Cmpds.	m/z meas.	Δ m/z [ppm]	z	Rt [min]	Score	P	Range	Sequence	Modification
2366	1	649.8737	-146.01	3	59.4	13.6	0	1005-1021	K.CCNHPYLFPVAAMEAPK.M	Carbamidomethyl: 1
505	1	699.1373	103.36	3	35.9	10.8	2	1868-1885	R.IPPVAVRLQMSERNILSR.L	Oxidation: 10

Detailed Protein Report

Protein 106: metallothionein-1G [Homo sapiens]

Accession: gi|10835230
Database: refseq_human(refseq_human_20140103.fasta)
Database Date: 2015-11-30
Modification(s): Carbamidomethyl

Score: 41.9
MW [kDa]: 6.1
pI: 10.5
Sequence Coverage [%]: 91.8
No. of unique Peptides: 3

```
        10         20         30         40         50         60         70
MDPNCSCAAG VSCTCASSCK CKECKCTSCK KSCCSCCPVG CAKCAQGCIC KGASEKCSCC A
```

Cmpd.	No. of Cmpds.	m/z meas.	Δ m/z [ppm]	z	Rt [min]	Score	P	Range	Sequence	Modification
834	1	703.6768	119.69	3	39.8	13.8	0	1-20	-.MDPNCSCAAGVSCTCASSCK.C	Carbamidomethyl: 5, 15, 19
2136	1	1019.7667	-122.67	2	56.9	14.5	2	26-43	K.CTSCKKSCCSCCPVGCAK.C	Carbamidomethyl: 1, 11, 12, 16
1283	1	882.7587	-88.50	2	45.6	13.5	2	44-61	K.CAQGCICKGASEKCSCCA.-	

Detailed Protein Report

Protein 107: ectonucleoside triphosphate diphosphohydrolase 4 isoform b [Homo sapiens]

**Accession:**	gi|193083168	**Score:**	41.8
**Database:**	refseq_human(refseq_human_20140103.fasta)	**MW [kDa]:**	69.4
**Database Date:**	2015-11-30	**pI:**	9.3
**Modification(s):**	Carbamidomethyl, Oxidation	**Sequence Coverage [%]:**	7.2
		**No. of unique Peptides:**	3

10	20	30	40	50	60	70	80
MGRIGISCLF	PASWHFSISP	VGCPRILNTN	LRQIMVISVL	AAAVSLLYFS	VVIIRNKYGR	LTRDKKFQRY	LARVTDIEAT
**90**	**100**	**110**	**120**	**130**	**140**	**150**	**160**
DTNNPNVNYG	IVVDCGSSGS	RVFVYCWPRH	NGNPHDLLDI	RQMRDKNRKP	VVMKIKPGIS	EFATSPEKVS	DYISPLLNFA
**170**	**180**	**190**	**200**	**210**	**220**	**230**	**240**
AEHVPRAKHK	ETPLYILCTA	GMRILPESQQ	KAILEDLLTD	IPVHFDFLFS	DSHAEVISGK	QEGVYAWIGI	NFVLGRFEHI
**250**	**260**	**270**	**280**	**290**	**300**	**310**	**320**
EDDDEAVVEV	NIPGSESSEA	IVRKRTAGIL	DMGGVSTQIA	YEVPKTEEVA	KNLLAEFNLG	CDVHQTEHVY	RVYVATFLGF
**330**	**340**	**350**	**360**	**370**	**380**	**390**	**400**
GGNAARQRYE	DRIFANTIQK	NRLLGKQTGL	TPDMPYLDPC	LPLDIKDEIQ	QNGQTIYLRG	TGDFDLCRET	IQPFMNKTNE
**410**	**420**	**430**	**440**	**450**	**460**	**470**	**480**
TQTSLNGVYQ	PPIHFQNSEF	YGFSEFYYCT	EDVLRMGGDY	NAAKFTKAAK	DYCATKWSIL	RERFDRGLYA	SHADLHRLKY
**490**	**500**	**510**	**520**	**530**	**540**	**550**	**560**
QCFKSAWMFE	VFHRGFSFPV	NYKSLKTALQ	VYDKEVQWTL	GAILYRTRFL	PLRDIQQEAF	RASHTHWRGV	SFVYNHYLFS
**570**	**580**	**590**	**600**	**610**			
GCFLVVLLAI	LLYLLRLRRI	HRRTPRSSSA	AALWMEEGLP	AQNAPGTL			

Cmpd.	No. of Cmpds.	m/z meas.	Δ m/z [ppm]	z	Rt [min]	Score	P	Range	Sequence	Modification
2648	1	994.9726	-51.73	2	63.2	14.2	2	167-183	R.AKHKETPLYILCTAGMR.I	Carbamidomethyl: 12
2772	1	696.6903	49.74	3	65.0	14.8	1	380-397	R.GTGDFDLCRETIQPFMNK.T	Oxidation: 16
1075	1	463.6184	-187.66	2	43.0	12.9	0	436-444	R.MGGDYNAAK.F	

Detailed Protein Report

Protein 108: dedicator of cytokinesis protein 6 [Homo sapiens]

**Accession:**	gi|157426887	**Score:**	41.5
**Database:**	refseq_human(refseq_human_20140103.fasta)	**MW [kDa]:**	229.4
**Database Date:**	2015-11-30	**pI:**	6.3
		**Sequence Coverage [%]:**	1.4
		**No. of unique Peptides:**	2

Quantitation

MD:MU	**Median:** 0.36	**CV:** 0.00 %	**No. of Peptides:** 1

Detailed Protein Report

10	20	30	40	50	60	70	80
MAASERRAFA	HKINRTVAAE	VRKQVSRERS	GSPHSSRRCS	SSLGVPLTEV	VEPLDFEDVL	LSRPPDAEPG	PLRDLVEFPA
90	100	110	120	130	140	150	160
DDLELLLQPR	ECRTTEPGIP	KDEKLDAQVR	AAVEMYIEDW	VIVHRRYQYL	SAAYSPVTTD	TQRERQKGLP	RQVFEQDASG
170	180	190	200	210	220	230	240
DERSGPEDSN	DSRRGSGSPE	DTPRSSGASS	IFDLRNLAAD	SLLPSLLERA	APEDVDRRNE	TLRRQHRPPA	LLTLYPAPDE
250	260	270	280	290	300	310	320
DEAVERCSRP	EPPREHFGQR	ILVKCLSLKF	EIEIEPIFGI	LALYDVREKK	KISENFYFDL	NSDSMKGLLR	AHGTHPAIST
330	340	350	360	370	380	390	400
LARSAIFSVT	YPSPDIFLVI	KLEKVLQQGD	ISECCEPYMV	LKEVDTAKNK	EKLEKLRLAA	EQFCTRLGRY	RMPFAWTAVH
410	420	430	440	450	460	470	480
LANIVSSAGQ	LDRDSDSEGE	RRPAWTDRRR	RGPQDRASSG	DDACSFSGFR	PATLTVTNFF	KQEAERLSDE	DLFKFLADMR
490	500	510	520	530	540	550	560
RPSSLLRRLR	PVTAQLKIDI	SPAPENPHFC	LSPELLHIKP	YPDPRGRPTK	EILEFPAREV	YAPHTSYRNL	LYVYPHSLNF
570	580	590	600	610	620	630	640
SSRQGSVRNL	AVRVQYMTGE	DPSQALPVIF	GKSSCSEFTR	EAFTPVVYHN	KSPEFYEEFK	LHLPACVTEN	HHLLFTFYHV
650	660	670	680	690	700	710	720
SCQPRPGTAL	ETPVGFTWIP	LLQHGRLRTG	PFCLPVSVDQ	PPPSYSVLTP	DVALPGMRWV	DGHKGVFSVE	LTAVSSVHPQ
730	740	750	760	770	780	790	800
DPYLDKFFTL	VHVLEEGAFP	FRLKDTVLSE	GNVEQELRAS	LAALRLASPE	PLVAFSHHVL	DKLVRLVIRP	PIISGQIVNL
810	820	830	840	850	860	870	880
GRGAFEAMAH	VVSLVHRSLE	AAQDARGHCP	QLAAYVHYAF	RLPGTEPSLP	DGAPPVTVQA	ATLARGSGRP	ASLYLARSKS
890	900	910	920	930	940	950	960
ISSSNPDLAV	APGSVDDEVS	RILASKLLHE	ELALQWVVSS	SAVREAILQH	AWFFFQLMVK	SMALHLLLGQ	RLDTPRKLRF
970	980	990	1000	1010	1020	1030	1040
PGRFLDDITA	LVGSVGLEVI	TRVHKDVELA	EHLNASLAFF	LSDLLSLVDR	GFVFSLVRAH	YKQVATRLQS	SPNPAALLTL
1050	1060	1070	1080	1090	1100	1110	1120
RMEFTRILCS	HEHYVTLNLP	CCPLSPPASP	SPSVSSTTSQ	SSTFSSQAPD	PKVTSMFELS	GPFRQQHFLA	GLLLTELALA
1130	1140	1150	1160	1170	1180	1190	1200
LEPEAEGAFL	LHKKAISAVH	SLLCGHDTDP	RYAEATVKAR	VAELYLPLLS	IARDTLPRLH	DFAEGPGQRS	RLASMLDSDT
1210	1220	1230	1240	1250	1260	1270	1280
EGEGDIAGTI	NPSVAMAIAG	GPLAPGSRAS	ISQGPPTASR	AGCALSAESS	RTLLACVLWV	LKNTEPALLQ	RWATDLTLPQ
1290	1300	1310	1320	1330	1340	1350	1360
LGRLLDLLYL	CLAAFEYKGK	KAFERINSLT	FKKSLDMKAR	LEEAILGTIG	ARQEMVRRSR	ERSPFGNPEN	VRWRKSVTHW
1370	1380	1390	1400	1410	1420	1430	1440
KQTSDRVDKT	KDEMEHEALV	EGNLATEASL	VVLDTLEIIV	QTVMLSEARE	SVLGAVLKVV	LYSLGSAQSA	LFLQHGLATQ
1450	1460	1470	1480	1490	1500	1510	1520
RALVSKFPEL	LFEEDTELCA	DLCLRLLRHC	GSRISTIRTH	ASASLYLLMR	QNFEIGHNFA	RVKMQVTMSL	SSLVGTTQNF
1530	1540	1550	1560	1570	1580	1590	1600
SEEHLRRSLK	TILTYAEEDM	GLRDSTFAEQ	VQDLMFNLHM	ILTDTVKMKE	HQEDPEMLID	LMYRIARGYQ	GSPDLRLTWL
1610	1620	1630	1640	1650	1660	1670	1680
QNMAGKHAEL	GNHAEAAQCM	VHAAALVAEY	LALLEDHRHL	PVGCVSFQNI	SSNVLEESAI	SDDILSPDEE	GFCSGKHFTE
1690	1700	1710	1720	1730	1740	1750	1760
LGLVGLLEQA	AGYFTMGGLY	EAVNEVYKNL	IPILEAHRDY	KKLAAVHGKL	QEAFTKIMHQ	SSGWERVFGT	YFRVGFYGAH
1770	1780	1790	1800	1810	1820	1830	1840
FGDLDEQEFV	YKEPSITKLA	EISHRLEEFY	TERFGDDVVE	IIKDSNPVDK	SKLDSQKAYI	QITYVEPYFD	TYELKDRVTY
1850	1860	1870	1880	1890	1900	1910	1920
FDRNYGLRTF	LFCTPFTPDG	RAHGELPEQH	KRKTLLSTDH	AFPYIKTRIR	VCHREETVLT	PVEVAIEDMQ	KKTRELAFAT
1930	1940	1950	1960	1970	1980	1990	2000
EQDPPDAKML	QMVLQGSVGP	TVNQGPLEVA	QVFLAEIPED	PKLFRHHNKL	RLCFKDFCKK	CEDALRKNKA	LIGPDQKEYH
2010	2020	2030	2040	2050			
RELERNYCRL	REALQPLLTQ	RLPQLMAPTP	PGLRNSLNRA	SFRKADL			

Cmpd.	No. of Cmpds.	m/z meas.	Δ m/z [ppm]	z	Rt [min]	Score	P	Range	Sequence	Modification	Ratios

Detailed Protein Report

Cmpd.	No. of Cmpds.	m/z meas.	Δ m/z [ppm]	z	Rt [min]	Score	P	Range	Sequence	Modification	Ratios
1750	1	628.2499	-105.50	2	51.8	11.8	2	28-38	R.ERSGSPHSSRR.C		
1355	1	695.8559	-59.65	2	46.5	14.1	0	2022-2034	R.LPQLMAPTPPGLR.N		MD:MU 0.36

Detailed Protein Report

Protein 109: **transcription initiation factor TFIID subunit 4 [Homo sapiens]**

**Accession:**	gi|110832843	**Score:**	41.5
**Database:**	refseq_human(refseq_human_20140103.fasta)	**MW [kDa]:**	110.0
**Database Date:**	2015-11-30	**pI:**	10.6
		**Sequence Coverage [%]:**	2.5
		**No. of unique Peptides:**	1

10	20	30	40	50	60	70	80
MAAGSDLLDE	VFFNSEVDEK	VVSDLVGSLE	SQLAASAAHH	HHLAPRTPEV	RAAAAGALGN	HVVSGSPAGA	AGAGPAAPAE
90	100	110	120	130	140	150	160
GAPGAAPEPP	PAGRARPGGG	GPQRPGPPSP	RRPLVPAGPA	PPAAKLRPPP	EGSAGSCAPV	PAAAAVAAGP	EPAPAGPAKP
170	180	190	200	210	220	230	240
AGPAALAARA	GPGPGPGPGP	GPGPGPGKPA	GPGAAQTLNG	SAALLNSHHA	AAPAVSLVNN	GPAALLPLPK	PAAPGTVIQT
250	260	270	280	290	300	310	320
PPFVGAAAPP	APAAPSPPAA	PAPAAPAAAP	PPPPPAPATL	ARPPGHPAGP	PTAAPAVPPP	AAAQNGGSAG	AAPAPAPAAG
330	340	350	360	370	380	390	400
GPAGVSGQPG	PGAAAAAPAP	GVKAESPKRV	VQAAPPAAQT	LAASGPASTA	ASMVIGPTMQ	GALPSPAAVP	PPAPGTPTGL
410	420	430	440	450	460	470	480
PKGAAGAVTQ	SLSRTPTATT	SGIRATLTPT	VLAPRLPQPP	QNPTNIQNFQ	LPPGMVLVRS	ENGQLLMIPQ	QALAQMQAQA
490	500	510	520	530	540	550	560
HAQPQTTMAP	RPATPTSAPP	VQISTVQAPG	TPIIARQVTP	TTIIKQVSQA	QTTVQPSATL	QRSPGVQPQL	VLGGAAQTAS
570	580	590	600	610	620	630	640
LGTATAVQTG	TPQRTVPGAT	TTSSAATETM	ENVKKCKNFL	STLIKLASSG	KQSTETAANV	KELVQNLLDG	KIEAEDFTSR
650	660	670	680	690	700	710	720
LYRELNSSPQ	PYLVPFLKRS	LPALRQLTPD	SAAFIQQSQQ	QPPPPTSQAT	TALTAVVLSS	SVQRTAGKTA	ATVTSALQPP
730	740	750	760	770	780	790	800
VLSLTQPTQV	GVGKQGQPTP	LVIQQPPKPG	ALIRPPQVTL	TQTPMVALRQ	PHNRIMLTTP	QQIQLNPLQP	VPVVKPAVLP
810	820	830	840	850	860	870	880
GTKALSAVSA	QAAAAQKNKL	KEPGGGSFRD	DDDINDVASM	AGVNLSEESA	RILATNSELV	GTLTRSCKDE	TFLLQAPLQR
890	900	910	920	930	940	950	960
RILEIGKKHG	ITELHPDVVS	YVSHATQQRL	QNLVEKISET	AQQKNFSYKD	DDRYEQASDV	RAQLKFFEQL	DQIEKQRKDE
970	980	990	1000	1010	1020	1030	1040
QEREILMRAA	KSRSRQEDPE	QLRLKQKAKE	MQQQELAQMR	QRDANLTALA	AIGPRKKRKV	DCPGPGSGAE	GSGPGSVVPG
1050	1060	1070	1080	1090			
SSGVGTPRQF	TRQRITRVNL	RDLIFCLENE	RETSHSLLLY	KAFLK			

Cmpd.	No. of Cmpds.	m/z meas.	Δ m/z [ppm]	z	Rt [min]	Score	P	Range	Sequence	Modification
1978	1	643.7875	-107.62	2	54.6	19.5	0	804-817	K.ALSAVSAQAAAAQK.N	

Detailed Protein Report

Protein 110: probable ATP-dependent RNA helicase DDX58 [Homo sapiens]

**Accession:**	gi|27881482	**Score:**	41.4
**Database:**	refseq_human(refseq_human_20140103.fasta)	**MW [kDa]:**	106.5
**Database Date:**	2015-11-30	**pI:**	6.0
**Modification(s):**	Oxidation	**Sequence Coverage [%]:**	3.8
		**No. of unique Peptides:**	1

10	20	30	40	50	60	70	80
MTTEQRRSLQ	AFQDYIRKTL	DPTYILSYMA	PWFREEEVQY	IQAEKNNKGP	MEAATLFLKF	LLELQEEGWF	RGFLDALDHA
90	100	110	120	130	140	150	160
GYSGLYEAIE	SWDFKKIEKL	EEYRLLLKRL	QPEFKTRIIP	TDIISDLSEC	LINQECEEIL	QICSTKGMMA	GAEKLVECLL
170	180	190	200	210	220	230	240
RSDKENWPKT	LKLALEKERN	KFSELWIVEK	GIKDVETEDL	EDKMETSDIQ	IFYQEDPECQ	NLSENSCPPS	EVSDTNLYSP
250	260	270	280	290	300	310	320
FKPRNYQLEL	ALPAMKGKNT	IICAPTGCGK	TFVSLLICEH	HLKKFPQGQK	GKVVFFANQI	PVYEQQKSVF	SKYFERHGYR
330	340	350	360	370	380	390	400
VTGISGATAE	NVPVEQIVEN	NDIIILTPQI	LVNNLKKGTI	PSLSIFTLMI	FDECHNTSKQ	HPYNMIMFNY	LDQKLGGSSG
410	420	430	440	450	460	470	480
PLPQVIGLTA	SVGVGDAKNT	DEALDYICKL	CASLDASVIA	TVKHNLEELE	QVVYKPQKFF	RKVESRISDK	FKYIIAQLMR
490	500	510	520	530	540	550	560
DTESLAKRIC	KDLENLSQIQ	NREFGTQKYE	QWIVTVQKAC	MVFQMPDKDE	ESRICKALFL	YTSHLRKYND	ALIISEHARM
570	580	590	600	610	620	630	640
KDALDYLKDF	FSNVRAAGFD	EIEQDLTQRF	EEKLQELESV	SRDPSNENPK	LEDLCFILQE	EYHLNPETIT	ILFVKTRALV
650	660	670	680	690	700	710	720
DALKNWIEGN	PKLSFLKPGI	LTGRGKTNQN	TGMTLPAQKC	ILDAFKASGD	HNILIATSVA	DEGIDIAQCN	LVILYEYVGN
730	740	750	760	770	780	790	800
VIKMIQTRGR	GRARGSKCFL	LTSNAGVIEK	EQINMYKEKM	MNDSILRLQT	WDEAVFREKI	LHIQTHEKFI	RDSQEKPKPV
810	820	830	840	850	860	870	880
PDKENKKLLC	RKCKALACYT	ADVRVIEECH	YTVLGDAFKE	CFVSRPHPKP	KQFSSFEKRA	KIFCARQNCS	HDWGIHVKYK
890	900	910	920	930			
TFEIPVIKIE	SFVVEDIATG	VQTLYSKWKD	FHFEKIPFDP	AEMSK			

Cmpd.	No. of Cmpds.	m/z meas.	Δ m/z [ppm]	z	Rt [min]	Score	P	Range	Sequence	Modification
1833	1	599.7809	-18.91	2	52.9	13.8	1	751-759	K.EQINMYKEK.M	Oxidation: 5

Detailed Protein Report

Protein 111: hemopexin precursor [Homo sapiens]

**Accession:**	gi|11321561	**Score:**	41.4
**Database:**	refseq_human(refseq_human_20140103.fasta)	**MW [kDa]:**	51.6
**Database Date:**	2015-11-30	**pI:**	6.6
		**Sequence Coverage [%]:**	9.5
		**No. of unique Peptides:**	1

Quantitation

MD:MU **Median:** 1.18 **CV:** 0.00 % **No. of Peptides:** 1

Alias proteins:

Accession	Name	Description
gi|530395269	refseq_human (refseq_human_20140103.fasta)	PREDICTED: hemopexin isoform X1 [Homo sapiens]

10	20	30	40	50	60	70	80
MARVLGAPVA	LGLWSLCWSL	AIATPLPPTS	AHGNVAEGET	KPDPDVTERC	SDGWSFDATT	LDDNGTMLFF	KGEFVWKSHK
**90**	**100**	**110**	**120**	**130**	**140**	**150**	**160**
WDRELISERW	KNFPSPVDAA	FRQGHNSVFL	IKGDKVWVYP	PEKKEKGYPK	LLQDEFPGIP	SPLDAAVECH	RGECQAEGVL
**170**	**180**	**190**	**200**	**210**	**220**	**230**	**240**
FFQGDREWFW	DLATGTMKER	SWPAVGNCSS	ALRWLGRYYC	FQGNQFLRFD	PVRGEVPPRY	PRDVRDYFMP	CPGRGHGHRN
**250**	**260**	**270**	**280**	**290**	**300**	**310**	**320**
GTGHGNSTHH	GPEYMRCSPH	LVLSALTSDN	HGATYAFSGT	HYWRLDTSRD	GWHSWPIAHQ	WPQGPSAVDA	AFSWEEKLYL
**330**	**340**	**350**	**360**	**370**	**380**	**390**	**400**
VQGTQVYVFL	TKGGYTLVSG	YPKRLEKEVG	TPHGIILDSV	DAAFICPGSS	RLHIMAGRRL	WWLDLKSGAQ	ATWTELPWPH
**410**	**420**	**430**	**440**	**450**	**460**	**470**	
EKVDGALCME	KSLGPNSCSA	NGPGLYLIHG	PNLYCYSDVE	KLNAAKALPQ	PQNVTSLLGC	TH	

Cmpd.	No. of Cmpds.	m/z meas.	Δ m/z [ppm]	z	Rt [min]	Score	P	Range	Sequence	Modification	Ratios
1637	2	919.3696	-84.22	2	50.2	24.4	0	387-402	K.SGAQATWTELPWPHEK.V		MD:MU 1.18

Detailed Protein Report

Protein 112: complement receptor type 1 isoform F precursor [Homo sapiens]

**Accession:**	gi|86793036	**Score:**	41.2
**Database:**	refseq_human(refseq_human_20140103.fasta)	**MW [kDa]:**	223.5
**Database Date:**	2015-11-30	**pI:**	6.6
**Modification(s):**	Carbamidomethyl	**Sequence Coverage [%]:**	1.8
		**No. of unique Peptides:**	1

Quantitation

MD:MU	**Median:** 0.35	**CV:** 0.00 %	**No. of Peptides:** 1

Detailed Protein Report

10	20	30	40	50	60	70	80
MGASSPRSPE	PVGPPAPGLP	FCCGGSLLAV	VVLLALPVAW	GQCNAPEWLP	FARPTNLTDE	FEFPIGTYLN	YECRPGYSGR
90	100	110	120	130	140	150	160
PFSIICLKNS	VWTGAKDRCR	RKSCRNPPDP	VNGMVHVIKG	IQFGSQIKYS	CTKGYRLIGS	SSATCIISGD	TVIWDNETPI
170	180	190	200	210	220	230	240
CDRIPCGLPP	TITNGDFIST	NRENFHYGSV	VTYRCNPGSG	GRKVFELVGE	PSIYCTSNDD	QVGIWSGPAP	QCIIPNKCTP
250	260	270	280	290	300	310	320
PNVENGILVS	DNRSLFSLNE	VVEFRCQPGF	VMKGPRRVKC	QALNKWEPEL	PSCSRVCQPP	PDVLHAERTQ	RDKDNFSPGQ
330	340	350	360	370	380	390	400
EVFYSCEPGY	DLRGAASMRC	TPQGDWSPAA	PTCEVKSCDD	FMGQLLNGRV	LFPVNLQLGA	KVDFVCDEGF	QLKGSSASYC
410	420	430	440	450	460	470	480
VLAGMESLWN	SSVPVCEQIF	CPSPPVIPNG	RHTGKPLEVF	PFGKTVNYTC	DPHPDRGTSF	DLIGESTIRC	TSDPQGNGVW
490	500	510	520	530	540	550	560
SSPAPRCGIL	GHCQAPDHFL	FAKLKTQTNA	SDFPIGTSLK	YECRPEYYGR	PFSITCLDNL	VWSSPKDVCK	RKSCKTPPDP
570	580	590	600	610	620	630	640
VNGMVHVITD	IQVGSRINYS	CTTGHRLIGH	SSAECILSGN	AAHWSTKPPI	CQRIPCGLPP	TIANGDFIST	NRENFHYGSV
650	660	670	680	690	700	710	720
VTYRCNPGSG	GRKVFELVGE	PSIYCTSNDD	QVGIWSGPAP	QCIIPNKCTP	PNVENGILVS	DNRSLFSLNE	VVEFRCQPGF
730	740	750	760	770	780	790	800
VMKGPRRVKC	QALNKWEPEL	PSCSRVCQPP	PDVLHAERTQ	RDKDNFSPGQ	EVFYSCEPGY	DLRGAASMRC	TPQGDWSPAA
810	820	830	840	850	860	870	880
PTCEVKSCDD	FMGQLLNGRV	LFPVNLQLGA	KVDFVCDEGF	QLKGSSASYC	VLAGMESLWN	SSVPVCEQIF	CPSPPVIPNG
890	900	910	920	930	940	950	960
RHTGKPLEVF	PFGKAVNYTC	DPHPDRGTSF	DLIGESTIRC	TSDPQGNGVW	SSPAPRCGIL	GHCQAPDHFL	FAKLKTQTNA
970	980	990	1000	1010	1020	1030	1040
SDFPIGTSLK	YECRPEYYGR	PFSITCLDNL	VWSSPKDVCK	RKSCKTPPDP	VNGMVHVITD	IQVGSRINYS	CTTGHRLIGH
1050	1060	1070	1080	1090	1100	1110	1120
SSAECILSGN	TAHWSTKPPI	CQRIPCGLPP	TIANGDFIST	NRENFHYGSV	VTYRCNLGSR	GRKVFELVGE	PSIYCTSNDD
1130	1140	1150	1160	1170	1180	1190	1200
QVGIWSGPAP	QCIIPNKCTP	PNVENGILVS	DNRSLFSLNE	VVEFRCQPGF	VMKGPRRVKC	QALNKWEPEL	PSCSRVCQPP
1210	1220	1230	1240	1250	1260	1270	1280
PEILHGEHTP	SHQDNFSPGQ	EVFYSCEPGY	DLRGAASLHC	TPQGDWSPEA	PRCAVKSCDD	FLGQLPHGRV	LFPLNLQLGA
1290	1300	1310	1320	1330	1340	1350	1360
KVSFVCDEGF	RLKGSSVSHC	VLVGMRSLWN	NSVPVCEHIF	CPNPPAILNG	RHTGTPSGDI	PYGKEISYTC	DPHPDRGMTF
1370	1380	1390	1400	1410	1420	1430	1440
NLIGESTIRC	TSDPHGNGVW	SSPAPRCELS	VRAGHCKTPE	QFPFASPTIP	INDFEFPVGT	SLNYECRPGY	FGKMFSISCL
1450	1460	1470	1480	1490	1500	1510	1520
ENLVWSSVED	NCRRKSCGPP	PEPFNGMVHI	NTDTQFGSTV	NYSCNEGFRL	IGSPSTTCLV	SGNNVTWDKK	APICEIISCE
1530	1540	1550	1560	1570	1580	1590	1600
PPPTISNGDF	YSNNRTSFHN	GTVVTYQCHT	GPDGEQLFEL	VGERSIYCTS	KDDQVGVWSS	PPPRCISTNK	CTAPEVENAI
1610	1620	1630	1640	1650	1660	1670	1680
RVPGNRSFFT	LTEIIRFRCQ	PGFVMVGSHT	VQCQTNGRWG	PKLPHCSRVC	QPPPEILHGE	HTLSHQDNFS	PGQEVFYSCE
1690	1700	1710	1720	1730	1740	1750	1760
PSYDLRGAAS	LHCTPQGDWS	PEAPRCTVKS	CDDFLGQLPH	GRVLLPLNLQ	LGAKVSFVCD	EGFRLKGRSA	SHCVLAGMKA
1770	1780	1790	1800	1810	1820	1830	1840
LWNSSVPVCE	QIFCPNPPAI	LNGRHTGTPF	GDIPYGKEIS	YACDTHPDRG	MTFNLIGESS	IRCTSDPQGN	GVWSSPAPRC
1850	1860	1870	1880	1890	1900	1910	1920
ELSVPAACPH	PPKIQNGHYI	GGHVSLYLPG	MTISYICDPG	YLLVGKGFIF	CTDQGIWSQL	DHYCKEVNCS	FPLFMNGISK
1930	1940	1950	1960	1970	1980	1990	2000
ELEMKKVYHY	GDYVTLKCED	GYTLEGSPWS	QCQADDRWDP	PLAKCTSRTH	DALIVGTLSG	TIFFILLIIF	LSWIILKHRK
2010	2020	2030	2040				
GNNAHENPKE	VAIHLHSQGG	SSVHPRTLQT	NEENSRVLP				

Cmpd.	No. of Cmpds.	m/z meas.	Δ m/z [ppm]	z	Rt [min]	Score	P	Range	Sequence	Modification	Ratios
73	1	826.3528	-22.94	3	30.6	30.9	1	334-356	R. GAASMRCTPQGDWSPAAPTCEV S	Carbamidomethyl: 7, 20	MD:MU 0.35

Detailed Protein Report

Protein 113: **PREDICTED: cleavage and polyadenylation specificity factor subunit 1 isoform X2 [Homo sapiens]**

**Accession:**	gi|578815945	**Score:**	41.1
**Database:**	refseq_human(refseq_human_20140103.fasta)	**MW [kDa]:**	151.9
**Database Date:**	2015-11-30	**pI:**	5.9
**Modification(s):**	Carbamidomethyl, Oxidation	**Sequence Coverage [%]:**	3.2
		**No. of unique Peptides:**	3

10	20	30	40	50	60	70	80
MSMASVQLAG	AKRDALLLSF	KDAKLSVVEY	DPGTHDLKTL	SLHYFEEPEL	RDGFVQNVHT	PRVRVDPDGR	CAAMLVYGTR
**90**	**100**	**110**	**120**	**130**	**140**	**150**	**160**
LVVLPFRRES	LAEEHEGLVG	EGQRSSFLPS	YIIDVRALDE	KLLNIIDLQF	LHGYYEPTLL	ILFEPNQTWP	GRVAVRQDTC
**170**	**180**	**190**	**200**	**210**	**220**	**230**	**240**
SIVAISLNIT	QKVHPVIWSL	TSLPFDCTQA	LAVPKPIGGV	VVFAVNSLLY	LNQSVPPYGV	ALNSLTTGTT	AFPLRTQEGV
**250**	**260**	**270**	**280**	**290**	**300**	**310**	**320**
RITLDCAQAT	FISYDKMVIS	LKGGEIYVLT	LITDGMRSVR	AFHFDKAAAS	VLTTSMVTME	PGYLFLGSRL	GNSLLLKYTE
**330**	**340**	**350**	**360**	**370**	**380**	**390**	**400**
KLQEPPASAV	REAADKEEPP	SKKKRVDATA	GWSAAGKSVP	QDEVDEIEVY	GSEAQSGTQL	ATYSFEVCDS	ILNIGPCANA
**410**	**420**	**430**	**440**	**450**	**460**	**470**	**480**
AVGEPAFLSE	EFQNSPEPDL	EIVVCSGHGK	NGALSVLQKS	IRPQVVTTFE	LPGCYDMWTV	IAPVRKEEED	NPKGEGTEQE
**490**	**500**	**510**	**520**	**530**	**540**	**550**	**560**
PSTTPEADDD	GRRHGFLILS	REDSTMILQT	GQEIMELDTS	GFATQGPTVF	AGNIGDNRYI	VQVSPLGIRL	LEGVNQLHFI
**570**	**580**	**590**	**600**	**610**	**620**	**630**	**640**
PVDLGAPIVQ	CAVADPYVVI	MSAEGHVTMF	LLKSDSYGGR	HHRLALHKPP	LHHQSKVITL	CLYRDLSGMF	TTESRLGGAR
**650**	**660**	**670**	**680**	**690**	**700**	**710**	**720**
DELGGRSGPE	AEGLGSETSP	TVDDEEEMLY	GDSGSLFSPS	KEEARRSSQP	PADRDPAPFR	AEPTHWCLLV	RENGTMEIYQ
**730**	**740**	**750**	**760**	**770**	**780**	**790**	**800**
LPDWRLVFLV	KNFPVGQRVL	VDSSFGQPTT	QGEARREEAT	RQGELPLVKE	VLLVALGSRQ	SRPYLLVHVD	QELLIYEAFP
**810**	**820**	**830**	**840**	**850**	**860**	**870**	**880**
HDSQLGQGNL	KVRFKKVPHN	INFREKKPKP	SKKKAEGGGA	EEGAGARGRV	ARFRYFEDIY	GYSGVFICGP	SPHWLLVTGR
**890**	**900**	**910**	**920**	**930**	**940**	**950**	**960**
GALRLHPMAI	DGPVDSFAPF	HNVNCPRGFL	YFNRQGELRI	SVLPAYLSYD	APWPVRKIPL	RCTAHYVAYH	VESKVYAVAT
**970**	**980**	**990**	**1000**	**1010**	**1020**	**1030**	**1040**
STNTPCARIP	RMTGEEKEFE	TIERDERYIH	PQQEAFSIQL	ISPVSWEAIP	NARIELQEWE	HVTCMKTVSL	RSEETVSGLK
**1050**	**1060**	**1070**	**1080**	**1090**	**1100**	**1110**	**1120**
GYVAAGTCLM	QGEEVTCRGR	ILIMDVIEVV	PEPGQPLTKN	KFKVLYEKEQ	KGPVTALCHC	NGHLVSAIGQ	KIFLWSLRAS
**1130**	**1140**	**1150**	**1160**	**1170**	**1180**	**1190**	**1200**
ELTGMAFIDT	QLYIHQMISV	KNFILAADVM	KSISLLRYQE	ESKTLSLVSR	DAKPLEVYSV	DFMVDNAQLG	FLVSDRDRNL
**1210**	**1220**	**1230**	**1240**	**1250**	**1260**	**1270**	**1280**
MVYMYLPEAK	ESFGGMRLLR	RADFHVGAHV	NTFWRTPCRG	ATEGLSKKSV	VWENKHITWF	ATLDGGIGLL	LPMQEKTYRR
**1290**	**1300**	**1310**	**1320**	**1330**	**1340**	**1350**	**1360**
LLMLQNALTT	MLPHHAGLNP	RAFRMLHVDR	RTLQNAVRNV	LDGELLNRYL	YLSTMERSEL	AKKIGTTPDI	ILDDLLETDR
**1370**							
VTAHF							

Cmpd.	No. of Cmpds.	m/z meas.	Δ m/z [ppm]	z	Rt [min]	Score	P	Range	Sequence	Modification
2669	1	948.3641	-105.24	2	64.6	16.7	1	88-104	R.RESLAEEHEGLVGEGQR.S	
297	1	626.2973	-49.17	3	33.4	14.0	1	955-971	K.VYAVATSTNTPCARIPR.M	Carbamidomethyl: 12
2457	1	596.2158	-123.37	2	61.1	10.4	0	1329-1337	R.YLYLSTMER.S	Oxidation: 7

Detailed Protein Report

Protein 114: histone H4 [Homo sapiens]

**Accession:**	gi|4504301	**Score:**	41.1
**Database:**	refseq_human(refseq_human_20140103.fasta)	**MW [kDa]:**	11.4
**Database Date:**	2015-11-30	**pI:**	11.8
		**Sequence Coverage [%]:**	7.8
		**No. of unique Peptides:**	1

Quantitation

MD:MU **Median:** 0.60 **CV:** 0.00 % **No. of Peptides:** 1

Alias proteins:

Accession	Name	Description
gi|77539758	refseq_human (refseq_human_20140103.fasta)	histone H4 [Homo sapiens]
gi|28173560	refseq_human (refseq_human_20140103.fasta)	histone H4 [Homo sapiens]
gi|11415030	refseq_human (refseq_human_20140103.fasta)	histone H4 [Homo sapiens]
gi|4504323	refseq_human (refseq_human_20140103.fasta)	histone H4 [Homo sapiens]
gi|4504321	refseq_human (refseq_human_20140103.fasta)	histone H4 [Homo sapiens]
gi|4504317	refseq_human (refseq_human_20140103.fasta)	histone H4 [Homo sapiens]
gi|4504315	refseq_human (refseq_human_20140103.fasta)	histone H4 [Homo sapiens]
gi|4504313	refseq_human (refseq_human_20140103.fasta)	histone H4 [Homo sapiens]
gi|4504311	refseq_human (refseq_human_20140103.fasta)	histone H4 [Homo sapiens]
gi|4504309	refseq_human (refseq_human_20140103.fasta)	histone H4 [Homo sapiens]
gi|4504307	refseq_human (refseq_human_20140103.fasta)	histone H4 [Homo sapiens]
gi|4504305	refseq_human (refseq_human_20140103.fasta)	histone H4 [Homo sapiens]
gi|4504303	refseq_human (refseq_human_20140103.fasta)	histone H4 [Homo sapiens]

```
        10         20         30         40         50         60         70         80
MSGRGKGGKG LGKGGAKRHR KVLRDNIQGI TKPAIRRLAR RGGVKRISGL IYEETRGVLK VFLENVIRDA VTYTEHAKRK
        90        100        110
TVTAMDVVYA LKRQGRTLYG FGG
```

Cmpd.	No. of Cmpds.	m/z meas.	Δ m/z [ppm]	z	Rt [min]	Score	P	Range	Sequence	Modification	Ratios	
1293	7	495.1457	-296.53	2	45.8	41.1	0	61-68	K.VFLENVIR.D		MD:MU	0.60

Detailed Protein Report

Protein 115: PREDICTED: muskelin isoform X4 [Homo sapiens]

Accession: gi|578814266
Database: refseq_human(refseq_human_20140103.fasta)
Database Date: 2015-11-30
Modification(s): Carbamidomethyl

Score: 40.9
MW [kDa]: 80.3
pI: 6.1
Sequence Coverage [%]: 3.7
No. of unique Peptides: 2

10	20	30	40	50	60	70	80
MAAGGAVAAA	PECRLLPYAL	HKWSSFSSTY	LPENILVDKP	NDQSSRWSSE	SNYPPQYLIL	KLERPAIVQN	ITFGKYEKTH
**90**	**100**	**110**	**120**	**130**	**140**	**150**	**160**
VCNLKKFKVF	GGMNEENMTE	LLSSGLKNDY	NKETFTLKHK	IDEQMFPCRF	IKIVPLLSWG	PSFNFSIWYV	ELSGIDDPDI
**170**	**180**	**190**	**200**	**210**	**220**	**230**	**240**
VQPCLNWYSK	YREQEAIRLC	LKHFRQHNYT	EAFESLQKKT	KIALEHPMLT	DIHDKLVLKG	DFDACEELIE	KAVNDGLFNQ
**250**	**260**	**270**	**280**	**290**	**300**	**310**	**320**
YISQQEYKPR	WSQIIPKSTK	GDGEDNRPGM	RGGHQMVIDV	QTETVYLFGG	WDGTQDLADF	WAYSVKENQW	TCISRDTEKE
**330**	**340**	**350**	**360**	**370**	**380**	**390**	**400**
NGPSARSCHK	MCIDIQRRQI	YTLGRYLDSS	VRNSKSLKSD	FYRYDIDTNT	WMLLSEDTAA	DGGPKLVFDH	QMCMDSEKHM
**410**	**420**	**430**	**440**	**450**	**460**	**470**	**480**
IYTFGGRILT	CNGSVDDSRA	SEPQFSGLFA	FNCQCQTWKL	LREDSCNAGP	EDIQSRIGHC	MLFHSKNRCL	YVFGGQRSKT
**490**	**500**	**510**	**520**	**530**	**540**	**550**	**560**
YLNDFFSYDV	DSDHVDIISD	GTKKDSGMVP	MTGFTQRATI	DPELNEIHVL	SGLSKDKEKR	EENVRNSFWI	YDIVRNSWSC
**570**	**580**	**590**	**600**	**610**	**620**	**630**	**640**
VYKNDQAAKD	NPTKSLQEEE	PCPRFAHQLV	YDELHKVHYL	FGGNPGKSCS	PKMRLDDFWS	LKLCRPSKDY	LLRHCKYLIR
**650**	**660**	**670**	**680**	**690**	**700**		
KHRFEEKAQV	DPLSALKYLQ	NDLYITVDHS	DPEETKEFQL	LASALFKSGS	DFTALA		

Cmpd.	No. of Cmpds.	m/z meas.	Δ m/z [ppm]	z	Rt [min]	Score	P	Range	Sequence	Modification
1158	1	477.6545	-288.11	2	44.1	17.4	0	15-22	R.LLPYALHK.W	
2161	2	1051.4053	-78.68	2	56.9	11.4	2	320-337	K.ENGPSARSCHKMCIDIQR.R	Carbamidomethyl: 9

Detailed Protein Report

Protein 116: **PREDICTED: tetratricopeptide repeat protein 13 isoform X7 [Homo sapiens]**

**Accession:**	gi|578802328	**Score:**	40.9
**Database:**	refseq_human(refseq_human_20140103.fasta)	**MW [kDa]:**	57.4
**Database Date:**	2015-11-30	**pI:**	9.3
		**Sequence Coverage [%]:**	7.0
		**No. of unique Peptides:**	1

10	20	30	40	50	60	70	80
MMLYHHGSLQ	EALKNFKRCL	QLEPYNEVCQ	YMKGLSHVAM	GQFYEGIKAQ	TKVMLNDPLP	GQKASPEYLK	VKYLREYSRY
**90**	**100**	**110**	**120**	**130**	**140**	**150**	**160**
LHAHLDTPLT	EYNIDVDLPG	SFKDHWAKNL	PFLIEDYEEQ	PGLQPHIKDV	LHQNFESYKP	EVQELICVAD	RLGSLMQYET
**170**	**180**	**190**	**200**	**210**	**220**	**230**	**240**
PGFLPNKRIH	RAMGLAALEV	MQAVQRTWTN	SKVRMNGKTR	LMQWRDMFDI	AVKWRRIADP	DQPVLWLDQM	PARSLSRGFN
**250**	**260**	**270**	**280**	**290**	**300**	**310**	**320**
NHINLIRGQV	INMRYLEYFE	KILHFIKDRI	LVYHGANNPK	GLLEVREALE	KVHKVEDLLP	IMKQFNTKTK	DGFTVNTKVP
**330**	**340**	**350**	**360**	**370**	**380**	**390**	**400**
SLKDQGKEYD	GFTITITGDK	VGNILFSVET	QTTEERTQLY	HAEIDALYKD	LTAKGKVLIL	SSEFGEADAV	CNLILSLVYY
**410**	**420**	**430**	**440**	**450**	**460**	**470**	**480**
FYNLMPLSRG	SSVIAYSVIV	GALMASGKEV	AGKIPKGKLV	DFEAMTAPGS	EAFSKVAKSW	MNLKSISPSY	KTLPSVSETF
**490**	**500**	**510**					
PTLRSMIEVL	NTDSSPRCLK	KL					

Cmpd.	No. of Cmpds.	m/z meas.	Δ m/z [ppm]	z	Rt [min]	Score	P	Range	Sequence	Modification
1611	1	820.8315	-147.56	2	49.9	12.3	1	234-247	R.SLSRGFNNHINLIR.G	

Detailed Protein Report

Protein 117: zinc finger protein 646 [Homo sapiens]

**Accession:**	gi|215820619	**Score:**	40.7
**Database:**	refseq_human(refseq_human_20140103.fasta)	**MW [kDa]:**	200.7
**Database Date:**	2015-11-30	**pI:**	6.8
**Modification(s):**	Carbamidomethyl, Oxidation	**Sequence Coverage [%]:**	2.1
		**No. of unique Peptides:**	3

Alias proteins:

Accession	Name	Description
gi|530409437	refseq_human (refseq_human_20140103.fasta)	PREDICTED: zinc finger protein 646 isoform X2 [Homo sapiens]
gi|530409435	refseq_human (refseq_human_20140103.fasta)	PREDICTED: zinc finger protein 646 isoform X1 [Homo sapiens]

Detailed Protein Report

10	20	30	40	50	60	70	80
MEDTPPSLSC	SDCQRHFPSL	PELSRHRELL	HPSPNQDSEE	ADSIPRPYRC	QQCGRGYRHP	GSLVNHRRTH	ETGLFPCTTC
90	100	110	120	130	140	150	160
GKDFSNPMAL	KSHMRTHAPE	GRRRHRPPRP	KEATPHLQGE	TVSTDSWGQR	LGSSEGWENQ	TKHTEETPDC	ESVPDPRAAS
170	180	190	200	210	220	230	240
GTWEDLPTRQ	REGLASHPGP	EDGADGWGPS	TNSARAPPLP	IPASSLLSNL	EQYLAESVVN	FTGGQEPTQS	PPAEEERRYK
250	260	270	280	290	300	310	320
CSQCGKTYKH	AGSLTNHRQS	HTLGIYPCAI	CFKEFSNLMA	LKNHSRLHAQ	YRPYHCPHCP	RVFRLPRELL	EHQQSHEGER
330	340	350	360	370	380	390	400
QEPRWEEKGM	PTTNGHTDES	SQDQLPSAQM	LNGSAELSTS	GELEDSGLEE	YRPFRCGDCG	RTYRHAGSLI	NHRKSHQTGV
410	420	430	440	450	460	470	480
YPCSLCSKQL	FNAAALKNHV	RAHHRPRQGV	GENGQPSVPP	APLLLAETTH	KEEEDPTTTL	DHRPYKCSEC	GRAYRHRGSL
490	500	510	520	530	540	550	560
VNHRHSHRTG	EYQCSLCPRK	YPNLMALRNH	VRVHCKAARR	SADIGAEGAP	SHLKVELPPD	PVEAEAAPHT	DQDHVCKHEE
570	580	590	600	610	620	630	640
EATDITPAAD	KTAAHICSIC	GLLFEDAESL	ERHGLTHGAG	EKENSRTETT	MSPPRAFACR	DCGKSYRHSG	SLINHRQTHQ
650	660	670	680	690	700	710	720
TGDFSCGACA	KHFHTMAAMK	NHLRRHSRRR	SRRHRKRAGG	ASGGREAKLL	AAESWTRELE	DNEGLESPQD	PSGESPHGAE
730	740	750	760	770	780	790	800
GNLESDGDCL	QAESEGDKCG	LERDETHFQG	DKESGGTGEG	LERKDASLLD	NLDIPGEEGG	GTHFCDSLTG	VDEDQKPATG
810	820	830	840	850	860	870	880
QPNSSSHSAN	AVTGWQAGAA	HTCSDCGHSF	PHATGLLSHR	PCHPPGIYQC	SLCPKEFDSL	PALRSHFQNH	RPGEATSAQP
890	900	910	920	930	940	950	960
FLCCLCGMIF	PGRAGYRLHR	RQAHSSSGMT	EGSEEEGEEE	GVAEAAPARS	PPLQLSEAEL	LNQLQREVEA	LDSAGYGHIC
970	980	990	1000	1010	1020	1030	1040
GCCGQTYDDL	GSLERHHQSQ	SSGTTADKAP	SPLGVAGDAM	EMVVDSVLED	IVNSVSGEGG	DAKSQEGAGT	PLGDSLCIQG
1050	1060	1070	1080	1090	1100	1110	1120
GESLLEAQPR	PFRCNQCGKT	YRHGGSLVNH	RKIHQTGDFL	CPVCSRCYPN	LAAYRNHLRN	HPRCKGSEPQ	VGPIPEAAGS
1130	1140	1150	1160	1170	1180	1190	1200
SELQVGPIPE	GGSNKPQHMA	EEGPGQAEVE	KLQEELKVEP	LEEVARVKEE	VWEETTVKGE	EIEPRLETAE	KGCQTEASSE
1210	1220	1230	1240	1250	1260	1270	1280
RPFSCEVCGR	SYKHAGSLIN	HRQSHQTGHF	GCQACSKGFS	NLMSLKNHRR	IHADPRRFRC	SECGKAFRLR	KQLASHQRVH
1290	1300	1310	1320	1330	1340	1350	1360
MERRGGGGTR	KATREDRPFR	CGQCGRTYRH	AGSLLNHRRS	HETGQYSCPT	CPKTYSNRMA	LKDHQRLHSE	NRRRRAGRSR
1370	1380	1390	1400	1410	1420	1430	1440
RTAVRCALCG	RSFPGRGSLE	RHLREHEETE	REPANGQGGL	DGTAASEANL	TGSQGLETQL	GGAEPVPHLE	DGVPRPGERS
1450	1460	1470	1480	1490	1500	1510	1520
QSPIRAASSE	APEPLSWGAG	KAGGWPVGGG	LGNHSGGWVP	QFLTRSEEPE	DSVHRSPCHA	GDCQLNGPTL	SHMDSWDNRD
1530	1540	1550	1560	1570	1580	1590	1600
NSSQLQPGSH	SSCSQCGKTY	CQSGSLLNHN	TNKTDRHYCL	LCSKEFLNPV	ATKSHSHNHI	DAQTFACPDC	GKAFESHQEL
1610	1620	1630	1640	1650	1660	1670	1680
ASHLQAHARG	HSQVPAQMEE	ARDPKAGTGE	DQVVLPGQGK	AQEAPSETPR	GPGESVERAR	GGQAVTSMAA	EDKERPFRCT
1690	1700	1710	1720	1730	1740	1750	1760
QCGRSYRHAG	SLLNHQKAHT	TGLYPCSLCP	KLLPNLLSLK	NHSRTHTDPK	RHCCSICGKA	FRTAARLEGH	GRVHAPREGP
1770	1780	1790	1800	1810	1820	1830	1840
FTCPHCPRHF	RRRISFVQHQ	QQHQEEWTVA	GSGAPVAPVT	GRGDLPLPPP	PTPTTPLLDP	SPQWPADLSF	SL

Cmpd.	No. of Cmpds.	m/z meas.	Δ m/z [ppm]	z	Rt [min]	Score	P	Range	Sequence	Modification
2169	1	701.2467	-130.95	3	57.3	10.5	1	308-324	R.ELLEHQQSHEGERQEPR.W	
1307	1	1023.7335	252.51	1	46.0	17.9	0	1557-1564	R.HYCLLCSK.E	Carbamidomethyl: 3
1325	1	640.6479	-226.44	2	46.2	12.3	0	1661-1673	R.GGQAVTSMAAEDK.E	Oxidation: 8

Detailed Protein Report

Protein 118: **PREDICTED: zinc finger protein 510 isoform X3 [Homo sapiens]**

**Accession:**	gi|530390743	**Score:**	40.6
**Database:**	refseq_human(refseq_human_20140103.fasta)	**MW [kDa]:**	72.1
**Database Date:**	2015-11-30	**pI:**	10.2
**Modification(s):**	Carbamidomethyl	**Sequence Coverage [%]:**	6.6
		**No. of unique Peptides:**	1

10	20	30	40	50	60	70	80
MAPVQKNLYR	DVMLENYSNL	VSVGYCCFKP	EVIFKLEQGE	EPWFSEEEFS	NQSHPKDYRG	DDLIKQNKKI	KDKHLEQAIC
**90**	**100**	**110**	**120**	**130**	**140**	**150**	**160**
INNKTLTTEE	EKVLGKPFTL	HVAAVASTKM	SCKCNSWEVN	LQSISEFIIN	NRNYSTKKIG	CGNVCENSPF	KINFEKTQTG
**170**	**180**	**190**	**200**	**210**	**220**	**230**	**240**
EKFYEHNKNM	KALNYNENLP	KHPKFQTLEQ	AFECNKIGKA	FNDKANCVKH	NSSHTGETSS	KDDEFRKNCD	KKTLFDHRRT
**250**	**260**	**270**	**280**	**290**	**300**	**310**	**320**
GTGKKHLHLN	QCGKSFEKST	VEEYNKLNMG	IKHYELNPSG	NNFNRKAHLT	DPQTAVIEEN	PLVSNDRTQT	WVKSSEYHEN
**330**	**340**	**350**	**360**	**370**	**380**	**390**	**400**
KKSYQTSVHR	VRRRSHSMMK	PYKCNECGKS	FCQKGHLIQH	QRTHTGEKPF	ECSECGKTFS	QKSHLSTHQR	IHTAEKPYKC
**410**	**420**	**430**	**440**	**450**	**460**	**470**	**480**
NECGKTFVQK	STLRGHQRIH	TGEKPYECSE	CGKTFVQKST	LRDHHRIHTG	EKSFQCNQCG	KTFGQKSNLR	IHQRTHTGEK
**490**	**500**	**510**	**520**	**530**	**540**	**550**	**560**
TYQCNECEKS	FWRKDHLIQH	QKTHTGEKPF	KCNECGKTFA	RTSTLRVHQR	IHTGEKPFKC	NECGKKFVRK	AILSDHQRIH
**570**	**580**	**590**	**600**	**610**	**620**	**630**	
TGEKPFQCNK	CGKTFGQKSN	LRIHQRTHSG	EKSYECNEYG	KLCKKSTLSL	YQKIQGEGNP	Y	

Cmpd.	No. of Cmpds.	m/z meas.	Δ m/z [ppm]	z	Rt [min]	Score	P	Range	Sequence	Modification
282	1	627.0085	98.28	3	33.2	10.2	2	481-494	K.TYQCNECEKSFWRK.D	Carbamidomethyl: 7

Detailed Protein Report

Protein 119: PREDICTED: diacylglycerol kinase alpha isoform X2 [Homo sapiens]

**Accession:**	gi|530399854	**Score:**	40.3
**Database:**	refseq_human(refseq_human_20140103.fasta)	**MW [kDa]:**	69.9
**Database Date:**	2015-11-30	**pI:**	9.3
**Modification(s):**	Carbamidomethyl, Oxidation	**Sequence Coverage [%]:**	3.9
		**No. of unique Peptides:**	2

Alias proteins:

Accession	Name	Description
gi|530399856	refseq_human (refseq_human_20140103.fasta)	PREDICTED: diacylglycerol kinase alpha isoform X3 [Homo sapiens]

10	20	30	40	50	60	70	80
MVLRNRRGWG	ERLSPLGLNL	KEVDKIILQM	MRVAEYLDWD	VSELRPILQE	MMKEIDYDGS	GSVSQAEWVR	AGATTVPLLV
**90**	**100**	**110**	**120**	**130**	**140**	**150**	**160**
LLGLEMTLKD	DGQHMWRPKR	FPRPVYCNLC	ESSIGLGKQG	LSCNLCKYTV	HDQCAMKALP	CEVSTYAKSR	KDIGVQSHVW
**170**	**180**	**190**	**200**	**210**	**220**	**230**	**240**
VRGGCESGRC	DRCQKKIRIY	HSLTGLHCVW	CHLEIHDDCL	QAVGHECDCG	LLRDHILPPS	SIYPSVLASG	PDRKNSKTSQ
**250**	**260**	**270**	**280**	**290**	**300**	**310**	**320**
KTMDDLNLST	SEALRIDPVP	NTHPLLVFVN	PKSGGKQGQR	VLWKFQYILN	PRQVFNLLKD	GPEIGLRLFK	DVPDSRILVC
**330**	**340**	**350**	**360**	**370**	**380**	**390**	**400**
GGDGTVGWIL	ETIDKANLPV	LPPVAVLPLG	TGNDLARCLR	WGGGYEGQNL	AKILKDLEMS	KVVHMDRWSV	EVIPQQTEEK
**410**	**420**	**430**	**440**	**450**	**460**	**470**	**480**
SDPVPFQIIN	NYFSIGVDAS	IAHRFHIMRE	KYPEKFNSRM	KNKLWYFEFA	TSESIFSTCK	KLEESLTVEI	CGKPLDLSNL
**490**	**500**	**510**	**520**	**530**	**540**	**550**	**560**
SLEGIAVLNI	PSMHGGSNLW	GDTRRPHGDI	YGINQALGAT	AKVITDPDIL	KTCVPDLSDK	RLEVVGLEGA	IEMGQIYTKL
**570**	**580**	**590**	**600**	**610**	**620**	**630**	
KNAGRRLAKC	SEITFHTTKT	LPMQIDGEPW	MQTPCTIKIT	HKNQMPMLMG	PPPRSTNFFG	FLS	

Cmpd.	No. of Cmpds.	m/z meas.	Δ m/z [ppm]	z	Rt [min]	Score	P	Range	Sequence	Modification
1596	1	480.7905	22.53	2	49.7	13.2	2	1-7	-.MVLRNRR.G	Oxidation: 1
1847	1	577.2815	93.89	2	52.9	16.5	1	163-172	R.GGCESGRCDR.C	Carbamidomethyl: 3, 8

Detailed Protein Report

Protein 120: PREDICTED: ubiquitin-protein ligase E3C isoform X3 [Homo sapiens]

**Accession:**	gi|530387311	**Score:**	40.3
**Database:**	refseq_human(refseq_human_20140103.fasta)	**MW [kDa]:**	116.7
**Database Date:**	2015-11-30	**pI:**	6.2
**Modification(s):**	Carbamidomethyl	**Sequence Coverage [%]:**	4.3
		**No. of unique Peptides:**	3

10	20	30	40	50	60	70	80
MFSFEGDFKT	RPKVSLGGAS	RKEEKASLLH	RTQEERRKRE	EERRRLKNAI	IIQSFIRGYR	DRKQQYSIQR	SAFDRCATLS
**90**	**100**	**110**	**120**	**130**	**140**	**150**	**160**
QSGGAFPIAN	GPNLTLLVRQ	LLFFYKQNED	SKRLIWLYQN	LIKHSSLFVK	QLDGSERLTC	LFQIKRLMSL	CCRLLQNCND
**170**	**180**	**190**	**200**	**210**	**220**	**230**	**240**
DSLNVALPMR	MLEVFSSENT	YLPVLQDASY	VVSVIEQILH	YMIHNGYYRS	LYLLINSKLP	SSIEYSDLSR	VPIAKILLEN
**250**	**260**	**270**	**280**	**290**	**300**	**310**	**320**
VLKPLHFTYN	SCPEGARQQV	FTAFTEEFLA	APFTDQIFHF	IIPALADAQT	VFPYEPFLNA	LLLIESRCSR	KSGGAPWLFY
**330**	**340**	**350**	**360**	**370**	**380**	**390**	**400**
FVLTVGENYL	GALSEEGLLV	YLRVLQTFLS	QLPVSPASAS	CHDSASDSEE	ESEEADKPSS	PEDGRLSVSY	ITEECLKKLD
**410**	**420**	**430**	**440**	**450**	**460**	**470**	**480**
TKQQTNTLLN	LVWRDSASEE	VFTTMASVCH	TLMVQHRMMV	PKVRLLYSLA	FNARFLRHLW	FLISSMSTRM	ITGSMVPLLQ
**490**	**500**	**510**	**520**	**530**	**540**	**550**	**560**
VISRGSPMSF	EDSSRIIPLF	YLFSSLFSHS	LISIHDNEFF	GDPIEVVGQR	QSSMMPFTLE	ELIMLSRCLR	DACLGIIKLA
**570**	**580**	**590**	**600**	**610**	**620**	**630**	**640**
YPETKPEVRE	EYITAFQSIG	VTTSSEMQQC	IQMEQKRWIQ	LFKVITNLVK	MLKSRDTRRN	FCPPNHWLSE	QEDIKADKIF
**650**	**660**	**670**	**680**	**690**	**700**	**710**	**720**
QRLIYADKQE	VQGDGPFLDG	INVTIRRNYI	YEDAYDKLSP	ENEPDLKKRI	RVHLLNAHGL	DEAGIDGGGI	FREFLNELLK
**730**	**740**	**750**	**760**	**770**	**780**	**790**	**800**
SGFNPNQGFF	KTTNEGLLYP	NPAAQMLVGD	SFARHYYFLG	RMLGKALYEN	MLVELPFAGF	FLSKLLGTSA	DVDIHHLASL
**810**	**820**	**830**	**840**	**850**	**860**	**870**	**880**
DPEVYKNLLF	LKSYEDDVEE	LGLNFTVVNN	DLGEAQVVEL	KFGGKDIPVT	SANRIAYIHL	VADYRLNRQI	RQHCLAFRQG
**890**	**900**	**910**	**920**	**930**	**940**	**950**	**960**
LANVVSLEWL	RMFDQQEIQV	LISGAQVPIS	LEDLKSFTNY	SGGYSADHPV	IKVFWRVVEG	FTDEEKRKLL	KFVTSCSRPP
**970**	**980**	**990**	**1000**	**1010**	**1020**	**1030**	
LLGFKELYPA	FCIHNGGSDL	ERLPTASTCM	NLLKLPEFYD	ETLLRSKLLY	AIECAAGFEL	S	

Cmpd.	No. of Cmpds.	m/z meas.	Δ m/z [ppm]	z	Rt [min]	Score	P	Range	Sequence	Modification
2476	4	817.2871	-207.41	1	61.3	12.6	0	124-130	K.HSSLFVK.Q	
259	1	862.8548	57.47	3	32.6	16.7	2	689-712	K.RIRVHLLNAHGLDEAGIDGGGIFR.E	
2651	1	856.4088	-69.02	2	64.2	10.9	2	866-878	R.LNRQIRQHCLAFR.Q	Carbamidomethyl: 9

Detailed Protein Report

Protein 121: PREDICTED: nipped-B-like protein isoform X4 [Homo sapiens]

**Accession:**	gi|530378766	**Score:**	40.1
**Database:**	refseq_human(refseq_human_20140103.fasta)	**MW [kDa]:**	257.1
**Database Date:**	2015-11-30	**pI:**	9.1
**Modification(s):**	Oxidation	**Sequence Coverage [%]:**	2.0
		**No. of unique Peptides:**	3

Detailed Protein Report

10	20	30	40	50	60	70	80
MVSIDLHQAG	RVDSQASITQ	DSDSIKKPEE	IKQCNDAPVS	VLQEDIVGSL	KSTPENHPET	PKKKSDPELS	KSEMKQSESR
90	100	110	120	130	140	150	160
LAESKPNENR	LVETKSSENK	LETKVETQTE	ELKQNESRTT	ECKQNESTIV	EPKQNENRLS	DTKPNDNKQN	NGRSETTKSR
170	180	190	200	210	220	230	240
PETPKQKGES	RPETPKQKSD	GHPETPKQKG	DGRPETPKQK	GESRPETPKQ	KNEGRPETPK	HRHDNRRDSG	KPSTEKKPEV
250	260	270	280	290	300	310	320
SKHKQDTKSD	SPRLKSERAE	ALKQRPDGRS	VSESLRRDHD	NKQKSDDRGE	SERHRGDQSR	VRRPETLRSS	SRNEHGIKSD
330	340	350	360	370	380	390	400
SSKTDKLERK	HRHESGDSRE	RPSSGEQKSR	PDSPRVKQGD	SNKSRSDKLG	FKSPTSKDDK	RTEGNKSKVD	TNKAHPDNKA
410	420	430	440	450	460	470	480
EFPSYLLGGR	SGALKNFVIP	KIKRDKDGNV	TQETKKMEMK	GEPKDKVEKI	GLVEDLNKGA	KPVVVLQKLS	LDDVQKLIKD
490	500	510	520	530	540	550	560
REDKSRSSLK	PIKNKPSKSN	KGSIDQSVLK	ELPPELLAEI	ESTMPLCERV	KMNKRKRSTV	NEKPKYAEIS	SDEDNDSDEA
570	580	590	600	610	620	630	640
FESSRKRHKK	DDDKAWEYEE	RDRRSSGDHR	RSGHSHEGRR	SSGGGRYRNR	SPSDSDMEDY	SPPPSLSEVA	RKMKKKEKQK
650	660	670	680	690	700	710	720
KRKAYEPKLT	PEEMMDSSTF	KRFTASIENI	LDNLEDMDFT	AFGDDDEIPQ	ELLLGKHQLN	ELGSESAKIK	AMGIMDKLST
730	740	750	760	770	780	790	800
DKTVKVLNIL	EKNIQDGSKL	STLLNHNNDT	EEEERLWRDL	IMERVTKSAD	ACLTTINIMT	SPNMPKAVYI	EDVIERVIQY
810	820	830	840	850	860	870	880
TKFHLQNTLY	PQYDPVYRLD	PHGGGLLSSK	AKRAKCSTHK	QRVIVMLYNK	VCDIVSSLSE	LLEIQLLTDT	TILQVSSMGI
890	900	910	920	930	940	950	960
TPFFVENVSE	LQLCAIKLVT	AVFSRYEKHR	QLILEEIFTS	LARLPTSKRS	LRNFRLNSSD	MDGEPMYIQM	VTALVLQLIQ
970	980	990	1000	1010	1020	1030	1040
CVVHLPSSEK	DSNAEEDSNK	KIDQDVVITN	SYETAMRTAQ	NFLSIFLKKC	GSKQGEEDYR	PLFENFVQDL	LSTVNKPEWP
1050	1060	1070	1080	1090	1100	1110	1120
AAELLLSLLG	RLLVHQFSNK	STEMALRVAS	LDYLGTVAAR	LRKDAVTSKM	DQGSIERILK	QVSGGEDEIQ	QLQKALLDYL
1130	1140	1150	1160	1170	1180	1190	1200
DENTETDPSL	VFSRKFYIAQ	WFRDTTLETE	KAMKSQKDEE	SSEGTHHAKE	IETTGQIMHR	AENRKKFLRS	IIKTTPSQFS
1210	1220	1230	1240	1250	1260	1270	1280
TLKMNSDTVD	YDDACLIVRY	LASMRPFAQS	FDIYLTQILR	VLGENAIAVR	TKAMKCLSEV	VAVDPSILAR	LDMQRGVHGR
1290	1300	1310	1320	1330	1340	1350	1360
LMDNSTSVRE	AAVELLGRFV	LCRPQLAEQY	YDMLIERILD	TGISVRKRVI	KILRDICIEQ	PTFPKITEMC	VKMIRRVNDE
1370	1380	1390	1400	1410	1420	1430	1440
EGIKKLVNET	FQKLWFTPTP	HNDKEAMTRK	ILNITDVVAA	CRDTGYDWFE	QLLQNLLKSE	EDSSYKPVKK	ACTQLVDNLV
1450	1460	1470	1480	1490	1500	1510	1520
EHILKYEESL	ADSDNKGVNS	GRLVACITTL	FLFSKIRPQL	MVKHAMTMQP	YLTTKCSTQN	DFMVICNVAK	ILELVVPLME
1530	1540	1550	1560	1570	1580	1590	1600
HPSETFLATI	EEDLMKLIIK	YGMTVVQHCV	SCLGAVVNKV	TQNFKFVWAC	FNRYYGAISK	LKSQHQEDPN	NTSLLTNKPA
1610	1620	1630	1640	1650	1660	1670	1680
LLRSLFTVGA	LCRHFDFDLE	DFKGNSKVNI	KDKVLELLMY	FTKHSDEEVQ	TKAIIGLGFA	FIQHPSLMFE	QEVKNLYNNI
1690	1700	1710	1720	1730	1740	1750	1760
LSDKNSSVNL	KIQVLKNLQT	YLQEEDTRMQ	QADRDWKKVA	KQEDLKEMGD	VSSGMSSSIM	QLYLKQVLEA	FFHTQSSVRH
1770	1780	1790	1800	1810	1820	1830	1840
FALNVIALTL	NQGLIHPVQC	VPYLIAMGTD	PEPAMRNKAD	QQLVEIDKKY	AGFIHMKAVA	GMKMSYQVQQ	AINTCLKDPV
1850	1860	1870	1880	1890	1900	1910	1920
RGFRQDESSS	ALCSHLYSMI	RGNRQHRRAF	LISLLNLFDD	TAKTDVTMLL	YIADNLACFP	YQTQEEPLFI	MHHIDITLSV
1930	1940	1950	1960	1970	1980	1990	2000
SGSNLLQSFK	ESMVKDKRKE	RKSSPSKENE	SSDSEEEVSR	PRKSRKRVDS	DSDSDSEDDI	NSVMKCLPEN	SAPLIEFANV
2010	2020	2030	2040	2050	2060	2070	2080
SQGILLLLML	KQHLKNLCGF	SDSKIQKYSP	SESAKVYDKA	INRKTGVHFH	PKQTLDFLRS	DMANSKITEE	VKRSIVKQYL
2090	2100	2110	2120	2130	2140	2150	2160
DFKLLMEHLD	PDEEEEEGEV	SASTNARNKA	ITSLLGGGSP	KNNTAAETED	DESDGEDRGG	GTSGSLRRSK	RNSDSTELAA
2170	2180	2190	2200	2210	2220	2230	2240
QMNESVDVMD	VIAICCPKYK	DRPQIARVVQ	KTSSGFSVQW	MAGSYSGSWT	EAKRRDGRKL	VPWVDTIKES	DIIYKKIALT
2250	2260	2270					
SANKLTNKVV	QTLRSLYAAK	DGTSS					

Detailed Protein Report

Cmpd.	No. of Cmpds.	m/z meas.	Δ m/z [ppm]	z	Rt [min]	Score	P	Range	Sequence	Modification
887	1	491.6754	-84.38	2	40.6	13.5	0	575-581	K.AWEYEER.D	
2607	1	913.4633	-59.64	2	62.6	12.0	1	909-923	K.HRQLILEEIFTSLAR.L	
425	1	946.2023	58.66	3	35.0	14.6	2	1692-1714	K.IQVLKNLQTYLQEEDTRMQQADR.D	Oxidation: 18

Detailed Protein Report

Protein 122: PDZ domain-containing protein MAGIX isoform d [Homo sapiens]

Accession: gi|532164745
Database: refseq_human(refseq_human_20140103.fasta)
Database Date: 2015-11-30

Score: 40.1
MW [kDa]: 26.7
pI: 11.6
Sequence Coverage [%]: 11.9
No. of unique Peptides: 2

10	20	30	40	50	60	70	80
MEPRTGDAAD	PRGSRGGRGP	SPLAGPSARQ	LLARLDARPL	AARAAVDVAA	LVRRAGATLR	LRRKEAVSVL	DSADIEVTDS
**90**	**100**	**110**	**120**	**130**	**140**	**150**	**160**
RLPHATIVDH	RPQVGDLVLH	INGESTQGLT	HAQAVERIRA	GGPQLHLVIR	RPLETHPGKP	RGVGEPRKGV	DRSPDPGGPE
**170**	**180**	**190**	**200**	**210**	**220**	**230**	**240**
VTGSRSSSTS	LVQHPPSRTT	LKKTRGSPEP	SPEAAADGPT	VSPPERRAED	PNDQIPGSPG	PWLVPSEERL	SRALGVRGAA
**250**	**260**						
QLAQEMAAGR	RRH						

Cmpd.	No. of Cmpds.	m/z meas.	Δ m/z [ppm]	z	Rt [min]	Score	P	Range	Sequence	Modification
1080	1	841.9573	58.27	2	43.1	19.2	1	149-165	K.GVDRSPDPGGPEVTGSR.S	
1710	2	1273.4767	-121.71	1	51.3	20.9	0	238-250	R.GAAQLAQEMAAGR.R	

Detailed Protein Report

Protein 123: ubiquitin carboxyl-terminal hydrolase 17 [Homo sapiens]

**Accession:**	gi|153792150	**Score:**	40.1
**Database:**	refseq_human(refseq_human_20140103.fasta)	**MW [kDa]:**	59.6
**Database Date:**	2015-11-30	**pI:**	9.5
**Modification(s):**	Carbamidomethyl	**Sequence Coverage [%]:**	8.5
		**No. of unique Peptides:**	2

```
        10         20         30         40         50         60         70         80
MEDDSLYLGG EWQFNHFSKL TSSRPDAAFA EIQRTSLPEK SPLSSEARVD LCDDLAPVAR QLAPRKKLPL SSRRPAAVGA
        90        100        110        120        130        140        150        160
GLQNMGNTCY ENASLQCLTY TPPLANYMLS REHSQTCQRP KCCMLCTMQA HITWALHSPG HVIQPSQALA AGFHRGKQED
       170        180        190        200        210        220        230        240
AHEFLMFTVD AMKKACLPGH KQVDHHSKDT TLIHQIFGGC WRSQIKCLHC HGISDTFDPY LDIALDIQAA QSVKQALEQL
       250        260        270        280        290        300        310        320
VKPEELNGEN AYHCGLCLQR APASKTLTLH TSAKVLILVL KRFSDVTGNK LAKNVQYPEC LDMQPYMSQQ NTGPLVYVLY
       330        340        350        360        370        380        390        400
AVLVHAGWSC HDGHYFSYVK AQEGQWYKMD DAKVTACSIT SVLSQQAYVL FYIQKSEWER HSESVSRGRE PRALGAEDTD
       410        420        430        440        450        460        470        480
RRATQGELKR DHPCLQAPEL DERLVERATQ ESTLDHWKFP QEQNKTKPEF NVRKVEGTLP PNVLVIHQSK YKCGMKNHHP
       490        500        510        520        530        540
EQQSSLLNLS STTRTDQESV NTGTLASLQG RTRRSKGKNK HSKRALLVCQ
```

Cmpd.	No. of Cmpds.	m/z meas.	Δ m/z [ppm]	z	Rt [min]	Score	P	Range	Sequence	Modification
1128	1	1019.1809	129.35	2	43.7	15.4	2	49-66	R.VDLCDDLAPVARQLAPRK.K	Carbamidomethyl: 4
184	1	424.2193	37.35	3	32.0	12.9	0	112-121	R.EHSQTCQRPK.C	Carbamidomethyl: 6

Detailed Protein Report

Protein 124: uncharacterized protein C9orf172 [Homo sapiens]

**Accession:**	gi|148762978	**Score:**	40.1
**Database:**	refseq_human(refseq_human_20140103.fasta)	**MW [kDa]:**	106.6
**Database Date:**	2015-11-30	**pI:**	10.2
**Modification(s):**	Carbamidomethyl	**Sequence Coverage [%]:**	4.9
		**No. of unique Peptides:**	3

10	20	30	40	50	60	70	80
MTRTDPPDLL	VSTVYQDIKV	ATPGPASKCS	PCERSVARPA	EPAPFNKRHC	RSFDFLEALD	GPAMETLPEP	PPPESAVPRA
**90**	**100**	**110**	**120**	**130**	**140**	**150**	**160**
RTREAEPRRR	ARSKSAPRAP	PGLTPAPASP	PVLPRRGREA	QRAARAEASP	RREPAYPALR	ALANELHPIK	LQPQRGGPGR
**170**	**180**	**190**	**200**	**210**	**220**	**230**	**240**
VAPLCAAAGR	CAPPEPPAGP	APHVRCRLDI	KPDDAVLQHA	TRGSRSCGPT	EAAHWARPAP	QFHGLTVPGP	RHMALSRTPT
**250**	**260**	**270**	**280**	**290**	**300**	**310**	**320**
PSDSYCADPR	AFYCDGPLPG	PRDYAERRSL	PFTTPPGPTQ	FFYTEEPQGF	RGSFAASPGP	TFDAYYPRPY	PSEELSGPSP
**330**	**340**	**350**	**360**	**370**	**380**	**390**	**400**
RRMGGYYAGE	VRTFPIQEPP	SRSYYGEAPR	AYGLPYGPRY	VPEEPRAHST	ARPFYTEDFG	RYRERDVLAR	TYPHPRSSPA
**410**	**420**	**430**	**440**	**450**	**460**	**470**	**480**
WADWGPRPYR	TLQVVPPSDP	DPLLASWHGG	TGTSPPRLAT	DSRHYSRSWD	NILAPGPRRE	DPLGRGRSYE	NLLGREVREP
**490**	**500**	**510**	**520**	**530**	**540**	**550**	**560**
RGVSPEGRRP	PVVVNLSTSP	RRYAALSLSE	TSLTEKGRAG	EGLGRNWYVT	PEITITDNDL	RATERPSARA	WELPGGRTRP
**570**	**580**	**590**	**600**	**610**	**620**	**630**	**640**
PPHAAPDGPT	SGRQRSLEQL	DELITDLVID	SRPTAGQASE	PAADCLGPQL	RRLLDSRPAG	SGAPALAPPR	SPPASAGSAE
**650**	**660**	**670**	**680**	**690**	**700**	**710**	**720**
EPAAPGEAAD	ASPEPSADED	DLMTCSNARC	RRTETMFNAC	LYFKSCHSCY	TYYCSRLCRR	EDWDAHKARC	VYGRVGSVCR
**730**	**740**	**750**	**760**	**770**	**780**	**790**	**800**
HVLQFCRDSG	PVHRAFSRIA	RVGFLSRGRG	VLFLGFPSPG	SADNFLRFGL	EGLLLSPTYL	SLRELATHAA	PLGSYARELA
**810**	**820**	**830**	**840**	**850**	**860**	**870**	**880**
AAGRLYEPAE	CFLLSVSVAV	GPGTAPPGTP	ALPAPAPRSH	GPTVRKFAKV	ALAAGSPARP	PPARSREPDM	ETLILTPPPG
**890**	**900**	**910**	**920**	**930**	**940**	**950**	**960**
TAGLDQDGEA	GRRAREVAFI	HIQRELRLRG	VFLRHEFPRV	YEQLCEFVEA	NRRFTPTTIY	PTDRRTGRPF	MCMIMAASEP
**970**	**980**						
RALDWVASAN	LLDDIM						

Cmpd.	No. of Cmpds.	m/z meas.	Δ m/z [ppm]	z	Rt [min]	Score	P	Range	Sequence	Modification
46	1	731.3139	-91.02	3	30.3	16.4	1	1-19	-.MTRTDPPDLLVSTVYQDIK.V	
2506	1	741.8376	-49.69	2	61.8	10.3	2	708-720	K.ARCVYGRVGSVCR.H	Carbamidomethyl: 3
1901	1	599.9186	-47.48	3	53.6	13.3	0	946-961	R.TGRPFMCMIMAASEPR.A	

Detailed Protein Report

Protein 125: **basement membrane-specific heparan sulfate proteoglycan core protein precursor [Homo sapiens]**

**Accession:**	gi|126012571	**Score:**	39.8
**Database:**	refseq_human(refseq_human_20140103.fasta)	**MW [kDa]:**	468.5
**Database Date:**	2015-11-30	**pI:**	6.1
		**Sequence Coverage [%]:**	0.9
		**No. of unique Peptides:**	2

BRUKER

Detailed Protein Report

10	20	30	40	50	60	70	80
MGWRAAGALL	LALLLHGRLL	AVTHGLRAYD	GLSLPEDIET	VTASQMRWTH	SYLSDDEDML	ADSISGDDLG	SGDLGSGDFQ
90	100	110	120	130	140	150	160
MVYFRALVNF	TRSIEYSPQL	EDAGSREFRE	VSEAVVDTLE	SEYLKIPGDQ	VVSVVFIKEL	DGWVFVELDV	GSEGNADGAQ
170	180	190	200	210	220	230	240
IQEMLLRVIS	SGSVASYVTS	PQGFQFRRLG	TVPQFPRACT	EAEFACHSYN	ECVALEYRCD	RRPDCRDMSD	ELNCEEPVLG
250	260	270	280	290	300	310	320
ISPTFSLLVE	TTSLPPRPET	TIMRQPPVTH	APQPLLPGSV	RPLPCGPQEA	ACRNGHCIPR	DYLCDGQEDC	EDGSDELDCG
330	340	350	360	370	380	390	400
PPPPCEPNEF	PCGNGHCALK	LWRCDGDFDC	EDRTDEANCP	TKRPEEVCGP	TQFRCVSTNM	CIPASFHCDE	ESDCPDRSDE
410	420	430	440	450	460	470	480
FGCMPPQVVT	PPRESIQASR	GQTVTFTCVA	IGVPTPIINW	RLNWGHIPSH	PRVTVTSEGG	RGTLIIRDVK	ESDQGAYTCE
490	500	510	520	530	540	550	560
AMNARGMVFG	IPDGVLELVP	QRGPCPDGHF	YLEHSAACLP	CFCFGITSVC	QSTRRFRDQI	RLRFDQPDDF	KGVNVTMPAQ
570	580	590	600	610	620	630	640
PGTPPLSSTQ	LQIDPSLHEF	QLVDLSRRFL	VHDSFWALPE	QFLGNKVDSY	GGSLRYNVRY	ELARGMLEPV	QRPDVVLMGA
650	660	670	680	690	700	710	720
GYRLLSRGHT	PTQPGALNQR	QVQFSEEHWV	HESGRPVQRA	ELLQVLQSLE	AVLIQTVYNT	KMASVGLSDI	AMDTTVTHAT
730	740	750	760	770	780	790	800
SHGRAHSVEE	CRCPIGYSGL	SCESCDAHFT	RVPGGPYLGT	CSGCNCNGHA	SSCDPVYGHC	LNCQHNTEGP	QCNKCKAGFF
810	820	830	840	850	860	870	880
GDAMKATATS	CRPCPCPYID	ASRRFSDTCF	LDTDGQATCD	ACAPGYTGRR	CESCAPGYEG	NPIQPGGKCR	PVNQEIVRCD
890	900	910	920	930	940	950	960
ERGSMGTSGE	ACRCKNNVVG	RLCNECADGS	FHLSTRNPDG	CLKCFCMGVS	RHCTSSSWSR	AQLHGASEEP	GHFSLTNAAS
970	980	990	1000	1010	1020	1030	1040
THTTNEGIFS	PTPGELGFSS	FHRLLSGPYF	WSLPSRFLGD	KVTSYGGELR	FTVTQRSQPG	STPLHGQPLV	VLQGNNIILE
1050	1060	1070	1080	1090	1100	1110	1120
HHVAQEPSPG	QPSTFIVPFR	EQAWQRPDGQ	PATREHLLMA	LAGIDTLLIR	ASYAQQPAES	RVSGISMDVA	VPEETGQDPA
1130	1140	1150	1160	1170	1180	1190	1200
LEVEQCSCPP	GYRGPSCQDC	DTGYTRTPSG	LYLGTCERCS	CHGHSEACEP	ETGACQGCQH	HTEGPRCEQC	QPGYYGDAQR
1210	1220	1230	1240	1250	1260	1270	1280
GTPQDCQLCP	CYGDPAAGQA	AHTCFLDTDG	HPTCDACSPG	HSGRHCERCA	PGYYGNPSQG	QPCQRDSQVP	GPIGCNCDPQ
1290	1300	1310	1320	1330	1340	1350	1360
GSVSSQCDAA	GQCQCKAQVE	GLTCSHCRPH	HFHLSASNPD	GCLPCFCMGI	TQQCASSAYT	RHLISTHFAP	GDFQGFALVN
1370	1380	1390	1400	1410	1420	1430	1440
PQRNSRLTGE	FTVEPVPEGA	QLSFGNFAQL	GHESFYWQLP	ETYQGDKVAA	YGGKLRYTLS	YTAGPQGSPL	SDPDVQITGN
1450	1460	1470	1480	1490	1500	1510	1520
NIMLVASQPA	LQGPERRSYE	IMFREEFWRR	PDGQPATREH	LLMALADLDE	LLIRATFSSV	PLAASISAVS	LEVAQPGPSN
1530	1540	1550	1560	1570	1580	1590	1600
RPRALEVEEC	RCPPGYIGLS	CQDCAPGYTR	TGSGLYLGHC	ELCECNGHSD	LCHPETGACS	QCQHNAAGEF	CELCAPGYYG
1610	1620	1630	1640	1650	1660	1670	1680
DATAGTPEDC	QPCACPLTNP	ENMFSRTCES	LGAGGYRCTA	CEPGYTGQYC	EQCGPGYVGN	PSVQGGQCLP	ETNQAPLVVE
1690	1700	1710	1720	1730	1740	1750	1760
VHPARSIVPQ	GGSHSLRCQV	SGSPPHYFYW	SREDGRPVPS	GTQQRHQGSE	LHFPSVQPSD	AGVYICTCRN	LHQSNTSRAE
1770	1780	1790	1800	1810	1820	1830	1840
LLVTEAPSKP	ITVTVEEQRS	QSVRPGADVT	FICTAKSKSP	AYTLVWTRLH	NGKLPTRAMD	FNGILTIRNV	QLSDAGTYVC
1850	1860	1870	1880	1890	1900	1910	1920
TGSNMFAMDQ	GTATLHVQAS	GTLSAPVVSI	HPPQLTVQPG	QLAEFRCSAT	GSPTPTLEWT	GGPGGQLPAK	AQIHGGILRL
1930	1940	1950	1960	1970	1980	1990	2000
PAVEPTDQAQ	YLCRAHSSAG	QQVARAVLHV	HGGGGPRVQV	SPERTQVHAG	RTVRLYCRAA	GVPSATITWR	KEGGSLPPQA
2010	2020	2030	2040	2050	2060	2070	2080
RSERTDIATL	LIPAITTADA	GFYLCVATSP	AGTAQARIQV	VVLSASDASP	PPVKIESSSP	SVTEGQTLDL	NCVVAGSAHA
2090	2100	2110	2120	2130	2140	2150	2160
QVTWYRRGGS	LPPHTQVHGS	RLRLPQVSPA	DSGEYVCRVE	NGSGPKEASI	TVSVLHGTHS	GPSYTPVPGS	TRPIRIEPSS
2170	2180	2190	2200	2210	2220	2230	2240

Detailed Protein Report

Cmpd.	No. of Cmpds.	m/z meas.	Δ m/z [ppm]	z	Rt [min]	Score	P	Range	Sequence	Modification
2417	1	1175.2100	-20.32	2	60.1	10.2	1	5-27	R.AAGALLLALLLHGRLLAVTHGLR.A	
362	1	457.1238	-295.03	2	33.9	17.6	0	4004-4012	R.SAEPLALGR.W	

Detailed Protein Report

Protein 126: PREDICTED: centlein isoform X2 [Homo sapiens]

**Accession:**	gi|578816553	**Score:**	39.7
**Database:**	refseq_human(refseq_human_20140103.fasta)	**MW [kDa]:**	155.6
**Database Date:**	2015-11-30	**pI:**	9.1
		**Sequence Coverage [%]:**	2.5
		**No. of unique Peptides:**	2

10	20	30	40	50	60	70	80
MAARSPPSPH	PSPPARQLGP	RSPRVGRGAE	VHAMRSEASG	FAGAAREVVA	DESDKIWADK	EFVWSLWKRL	QVTNPDLTQV
90	100	110	120	130	140	150	160
VSLVVEREKQ	KSEAKDRKVL	EILQVKDAKI	QEFEQRESVL	KQEINDLVKR	KIAVDEENAF	LRKEFSDLEK	KFKDKSQEIK
170	180	190	200	210	220	230	240
DTKECVQNKE	EQNRLVIKNL	EEENKKLSTR	CTDLLNDLEK	LRKQEAHLRK	EKYSTDAKIK	TFEDNLIEAR	KEVEVSQSKY
250	260	270	280	290	300	310	320
NALSLQLSNK	QTELIQKDMD	ITLVRKELQE	LQNLYKQNST	HTAQQAELIQ	QLQVLNMDTQ	KVLRNQEDVH	TAESISYQKL
330	340	350	360	370	380	390	400
YNELHICFET	TKSNEAMLRQ	SVTNLQDQLL	QKEQENAKLK	EKLQESQGAP	LPLPQESDPD	YSAQVPHRPS	LSSLETLMVS
410	420	430	440	450	460	470	480
QKSEIEYLQE	KLKIANEKLS	ENISANKGFS	RKSIMTSAEG	KHKEPPVKRS	RSLSPKSSFT	DSEELQKLRK	AERKIENLEK
490	500	510	520	530	540	550	560
ALQLKSQEND	ELRDAHEKRK	ERLQMLQTNY	RAVKEQLKQW	EEGSGMTEIR	KIKRADPQQL	RQEDSDAVWN	ELAYFKRENQ
570	580	590	600	610	620	630	640
ELMIQKMNLE	EELDELKVHI	SIDKAAIQEL	NRCVAERREE	QLFRSGEDDE	VKRSTPEKNG	KEMLEQTLQK	VTELENRLKS
650	660	670	680	690	700	710	720
FEKRSRKLKE	GNKKLMKEND	FLKSLLKQQQ	EDTETREKEL	EQIIKGSKDV	EKENTELQVK	ISELETEVTS	LRRQVAEANA
730	740	750	760	770	780	790	800
LRNENEELIN	PMEKSHQSAD	RAKSEMATMK	VRSGRYDCKT	TMTKVKFKAA	KKNCSVGRHH	TVLNHSIKVM	SNVFENLSKD
810	820	830	840	850	860	870	880
GWEDVSESSS	DSEAQTSQTL	GTIIVETSQK	ISPTEDGKDQ	KESDPTEDSQ	TQGKEIVQTY	LNIDGKTPKD	YFHDKNAKKP
890	900	910	920	930	940	950	960
TFQKKNCKMQ	KSSHTAVPTR	VNREKYKNIT	AQKSSSNIIL	LRERIISLQQ	QNSVLQNAKK	TAELSVKEYK	EVNEKLLHQQ
970	980	990	1000	1010	1020	1030	1040
QVSDQRFQTS	RQTIKKLNLD	LAGLRKEKED	LLKKLESSSE	ITSLAEENSQ	VTFPRIQVTS	LSPSRSMDLE	MKQLQYKLKN
1050	1060	1070	1080	1090	1100	1110	1120
ATNELTKQSS	NVKTLKFELL	AKEEHIKEMH	EKISRMERDI	TMKRHLIEDL	KFRQKVNLES	NKSFSEMLQN	LDKKVKTLTE
1130	1140	1150	1160	1170	1180	1190	1200
ECSNKKVSID	SLKQRLNVAV	KEKSQYEQMY	QKSKEELEKK	DLKLTLLVSR	ISETESAMAE	IETAASKQLQ	ELALQSEQVL
1210	1220	1230	1240	1250	1260	1270	1280
EGAQKTLLLA	NEKVEEFTTF	VKALAKELQN	DVHVVRRQIR	ELKKMKKNRD	ACKTSTHKAQ	TLAASILNIS	RSDLEEILDT
1290	1300	1310	1320	1330	1340	1350	
EDQVEIEKTK	IDAENDKEWM	LYIQKLLEGQ	LPFASYLLEA	VLEKINEKKK	LVEGYFTIMK	DIR	

Cmpd.	No. of Cmpds.	m/z meas.	Δ m/z [ppm]	z	Rt [min]	Score	P	Range	Sequence	Modification
1953	1	643.3047	-169.45	1	54.5	11.9	0	1136-1141	R.LNVAVK.E	
2736	1	683.3656	57.65	3	65.5	15.2	2	1144-1159	K.SQYEQMYQKSKEELEK.K	

Detailed Protein Report

Protein 127: protein piccolo isoform 2 [Homo sapiens]

**Accession:**	gi|150170670	**Score:**	39.7
**Database:**	refseq_human(refseq_human_20140103.fasta)	**MW [kDa]:**	537.6
**Database Date:**	2015-11-30	**pI:**	5.9
		**Sequence Coverage [%]:**	1.0
		**No. of unique Peptides:**	2

Quantitation

MD:MU	**Median:** 1.06	**CV:** 0.00 %	**No. of Peptides:** 1

Detailed Protein Report

10	20	30	40	50	60	70	80
MGNEASLEGE	GLPEGLAAAA	AAGGGASGAG	SPSHTAIPAG	MEADLSQLSE	EERRQIAAVM	SRAQGLPKGS	VPPAAAESPS
90	100	110	120	130	140	150	160
MHRKQELDSS	HPPKQSGRPP	DPGRPAQPGL	SKSRTTDTFR	SEQKLPGRSP	STISLKESKS	RTDLKEEHKS	SMMPGFLSEV
170	180	190	200	210	220	230	240
NALSAVSSVV	NKFNPFDLIS	DSEASQEETT	KKQKVVQKEQ	GKPEGIIKPP	LQQQPPKPIP	KQQGPGRDPL	QQDGTPKSIS
250	260	270	280	290	300	310	320
SQQPEKIKSQ	PPGTGKPIQG	PTQTPQTDHA	KLPLQRDASR	PQTKQADIVR	GESVKPSLPS	PSKPPIQQPT	PGKPPAQQPG
330	340	350	360	370	380	390	400
HEKSQPGPAK	PPAQPSGLTK	PLAQQPGTVK	PPVQPPGTTK	PPAQPLGPAK	PPAQQTGSEK	PSSEQPGPKA	LAQPPGVGKT
410	420	430	440	450	460	470	480
PAQQPGPAKP	PTQQVGTPKP	LAQQPGLQSP	AKAPGPTKTP	VQQPGPGKIP	AQQAGPGKTS	AQQTGPTKPP	SQLPGPAKPP
490	500	510	520	530	540	550	560
PQQPGPAKPP	PQQPGSAKPP	SQQPGSTKPP	PQQPGPAKPS	PQQPGSTKPP	SQQPGSAKPS	AQQPSPAKPS	AQQSTKPVSQ
570	580	590	600	610	620	630	640
TGSGKPLQPP	TVSPSAKQPP	SQGLPKTICP	LCNTTELLLH	VPEKANFNTC	TECQTTVCSL	CGFNPNPHLT	EVKEWLCLNC
650	660	670	680	690	700	710	720
QMKRALGGDL	APVPSSPQPK	LKTAPVTTTS	AVSKSSPQPQ	QTSPKKDAAP	KQDLSKAPEP	KKPPPLVKQP	TLHGSPSAKA
730	740	750	760	770	780	790	800
KQPPEADSLS	KPAPPKEPSV	PSEQDKAPVA	DDKPKQPKMV	KPTTDLVSSS	SATTKPDIPS	SKVQSQAEEK	TTPPLKTDSA
810	820	830	840	850	860	870	880
KPSQSFPPTG	EKVSPFDSKA	IPRPASDSKI	ISHPGPSSES	KGQKQVDPVQ	KKEEPKKAQT	KMSPKPDAKP	MPKGSPTPPG
890	900	910	920	930	940	950	960
PRPTAGQTVP	TPQQSPKPQE	QSRRFSLNLG	SITDAPKSQP	TTPQETVTGK	LFGFGASIFS	QASNLISTAG	QPGPHSQSGP
970	980	990	1000	1010	1020	1030	1040
GAPMKQAPAP	SQPPTSQGPP	KSTGQAPPAP	AKSIPVKKET	KAPAAEKLEP	KAEQAPTVKR	TETEKKPPPI	KDSKSLTAEP
1050	1060	1070	1080	1090	1100	1110	1120
QKAVLPTKLE	KSPKPESTCP	LCKTELNIGS	KDPPNFNTCT	ECKNQVCNLC	GFNPTPHLTE	IQEWLCLNCQ	TQRAISGQLG
1130	1140	1150	1160	1170	1180	1190	1200
DIRKMPPAPS	GPKASPMPVP	TESSSQKTAV	PPQVKLVKKQ	EQEVKTEAEK	VILEKVKETL	SMEKIPPMVT	TDQKQEESKL
1210	1220	1230	1240	1250	1260	1270	1280
EKDKASALQE	KKPLPEEKKL	IPEEEKIRSE	EKKPLLEEKK	PTPEDKKLLP	EAKTSAPEEQ	KHDLLKSQVQ	IAEEKLEGRV
1290	1300	1310	1320	1330	1340	1350	1360
APKTVQEGKQ	PQTKMEGLPS	GTPQSLPKED	DKTTKTIKEQ	PQPPCTAKPD	QVEPGKEKTE	KEDDKSDTSS	SQQPKSPQGL
1370	1380	1390	1400	1410	1420	1430	1440
SDTGYSSDGI	SSSLGEIPSL	IPTDEKDILK	GLKKDSFSQE	SSPSSPSDLA	KLESTVLSIL	EAQASTLADE	KSEKKTQPHE
1450	1460	1470	1480	1490	1500	1510	1520
VSPEQPKDQE	KTQSLSETLE	ITISEEEIKE	SQEERKDTFK	KDSQQDIPSS	KDHKEKSEFV	DDITTRREPY	DSVEESSESE
1530	1540	1550	1560	1570	1580	1590	1600
NSPVPQRKRR	TSVGSSSSDE	YKQEDSQGSG	EEEDFIRKQI	IEMSADEDAS	GSEDDEFIRN	QLKEISSSTE	SQKKEETKGK
1610	1620	1630	1640	1650	1660	1670	1680
GKITAGKHRR	LTRKSSTSID	EDAGRRHSWH	DEDDEAFDES	PELKYRETKS	QESEELVVTG	GGGLRRFKTI	ELNSTIADKY
1690	1700	1710	1720	1730	1740	1750	1760
SAESSQKKTS	LYFDEEPELE	MESLTDSPED	RSRGEGSSSL	HASSFTPGTS	PTSVSSLDED	SDSSPSHKKG	ESKQQRKARH
1770	1780	1790	1800	1810	1820	1830	1840
RPHGPLLPTI	EDSSEEEELR	EEEELLKEQE	KQREIEQQQR	KSSSKKSKKD	KDELRAQRRR	ERPKTPPSNL	SPIEDASPTE
1850	1860	1870	1880	1890	1900	1910	1920
ELRQAAEMEE	LHRSSCSEYS	PSIESDPEGF	EISPEKIIEV	QKVYKLPTAV	SLYSPTDEQS	IMQKEGSQKA	LKSAEEMYEE
1930	1940	1950	1960	1970	1980	1990	2000
MMHKTHKYKA	FPAANERDEV	FEKEPLYGGM	LIEDYIYESL	VEDTYNGSVD	GSLLTRQEEE	NGFMQQKGRE	QKIRLSEQIY
2010	2020	2030	2040	2050	2060	2070	2080
EDPMQKITDL	QKEFYELESL	HSVVPQEDIV	SSSFIIPESH	EIVDLGTMVT	STEEERKLLD	ADAAYEELMK	RQQMQLTPGS
2090	2100	2110	2120	2130	2140	2150	2160
SPTQAPIGED	MTESTMDFDR	MPDASLTSSV	LSGASLTDST	SSATLSIPDV	KITQHFSTEE	IEDEYVTDYT	REIQEIIAHE
2170	2180	2190	2200	2210	2220	2230	2240
SLILTYSEPS	ESATSVPPSD	TPSLTSSVSS	VCTTDSSSPI	TTLDSITTVY	TEPVDMITKF	EDSEEISSST	YFPGSIIDYP
2250	2260	2270	2280	2290	2300	2310	2320
EEISVSLDRT	APPDGRASAD	HIVISLSDMA	SSIIESVVPK	PEGPVADTVS	TDLLISEKDP	VKKAKKETGN	GIILEVLEAY
2330	2340	2350	2360	2370	2380	2390	2400

Detailed Protein Report

Cmpd.	No. of Cmpds.	m/z meas.	Δ m/z [ppm]	z	Rt [min]	Score	P	Range	Sequence	Modification	Ratios
2516	1	941.4560	-98.82	2	61.9	13.1	1	702-719	K.KPPPLVKQPTLHGSPSAK.A		
1917	1	902.8454	-85.60	2	54.0	12.9	0	3678-3694	R.SMSDPKPLSPTADESSR.A		MD:MU 1.06

Detailed Protein Report

Protein 128: PREDICTED: nesprin-2 isoform X8 [Homo sapiens]

**Accession:**	gi|578825626	**Score:**	39.6
**Database:**	refseq_human(refseq_human_20140103.fasta)	**MW [kDa]:**	553.3
**Database Date:**	2015-11-30	**pI:**	5.1
		**Sequence Coverage [%]:**	0.8
		**No. of unique Peptides:**	2

Quantitation

MD:MU	**Median:** 1.00	**CV:** 0.00 %	**No. of Peptides:** 1

Detailed Protein Report

10	20	30	40	50	60	70	80
MASSPELPTE	DEQGSWGIDD	LHISLQAEQE	DTQKKAFTCW	INSQLARHTS	PSVISDLFTD	IKKGHVLLDL	LEVLSGQQLP
90	100	110	120	130	140	150	160
RDKGSNTFQC	RINIEHALTF	LRNRSIKLIN	IHVTDIIDGN	PSIILGLIWT	IILHFHIEKL	AQTLSCNYNQ	PSLDDVSVVD
170	180	190	200	210	220	230	240
SSPASSPPAK	KCSKVQARWQ	MSARKALLLW	AQEQCATYES	VNVTDFKSSW	RNGMAFLAII	HALRPDLIDM	KSVKHRSNKD
250	260	270	280	290	300	310	320
NLREAFRIAE	QELKIPRLLE	PEDVDVVDPD	EKSIMTYVAQ	FLQYSKDAPG	TGEEAQGKVK	DAMGWLTLQK	EKLQKLLKDS
330	340	350	360	370	380	390	400
ENDTYFKKYN	SLLSFMESFN	EEKKSFLDVL	SIKRDLDELD	KDHLQLREAW	DGLDHQINAW	KIKLNYALPP	PLHQTEAWLQ
410	420	430	440	450	460	470	480
EVEELMDEDL	SASQDHSQAV	TLIQEKMTLF	KSLMDRFEHH	SNILLTFENK	DENHLPLVPP	NKLEEMKRRI	NNILEKKFIL
490	500	510	520	530	540	550	560
LLEFHYYKCL	VLGLVDEVKS	KLDIWNIKYG	SRESVELLLE	DWHKFIEEKE	FLARLDTSFQ	KCGEIYKNLA	GECQNINKQY
570	580	590	600	610	620	630	640
MMVKSDVCMY	RKNIYNVKST	LQKVLACWAT	YVENLRLLRA	CFEETKKEEI	KEVPFETLAQ	WNLEHATLNE	AGNFLVEVSN
650	660	670	680	690	700	710	720
DVVGSSISKE	LRRLNKRWRK	LVSKTQLEMN	LPLMIKKQDQ	PTFDNSGNIL	SKEEKATVEF	STDMSVELPE	NYNQNIKAGE
730	740	750	760	770	780	790	800
KHEKENEEFT	GQLKVAKDVE	KLIGQVEIWE	AEAKSVLDQD	DVDTSMEESL	KHLIAKGSMF	DELMARSEDM	LQMDIQNISS
810	820	830	840	850	860	870	880
QESFQHVLTT	GLQAKIQEAK	EKVQINVVKL	IAALKNLTDV	SPDLDIRLKM	EESQKELESY	MMRAQQLLGQ	RESPGELISK
890	900	910	920	930	940	950	960
HKEALIISNT	KSLAKYLKAV	EELKNNVTED	IKMSLEEKSR	DVCAKWESLH	HELSLYVQQL	KIDIEKGKLS	DNILKLEKQI
970	980	990	1000	1010	1020	1030	1040
NKEKKLIRRG	RTKGLIKEHE	ACFSEEGCLY	QLNHHMEVLR	ELCEELPSQK	SQQEVKRLLK	DYEQKIERLL	KCASEIHMTL
1050	1060	1070	1080	1090	1100	1110	1120
QPTAGGTSKN	EGTITTSENR	GGDPHSEAPF	AKSDNQPSTE	KAMEPTMKFS	LASVLRPLQE	ESIMEKDYSA	SINSLLERYD
1130	1140	1150	1160	1170	1180	1190	1200
TYRDILEHHL	QNNKFRITSD	FSSEEDRSSS	CLQAKLTDLQ	VIKNETDARW	KEFEIISLKL	ENHVNDIKKP	FVIKERDTLK
1210	1220	1230	1240	1250	1260	1270	1280
ERERELQMTL	NTRMESLETA	LRLVLPVEKA	SLLLCGSDLP	LHKMAIQGFH	LIDADRIYQH	LRNIQDSIAK	QIEICNRLEE
1290	1300	1310	1320	1330	1340	1350	1360
PGNFVLKELH	PFDLHAMQNI	ILKYKTQFEG	MNHRVQRSED	TLKALEDFLA	SLRTAKLSAE	PVTDLSASDT	QVAQENTLTV
1370	1380	1390	1400	1410	1420	1430	1440
KNKEGEIHLM	KDKAKHLDKC	LKMLDMSFKD	AERGDDTSCE	NLLDAFSIKL	SETHGYGVQE	EFTEENKLLE	ACIFKNNELL
1450	1460	1470	1480	1490	1500	1510	1520
KNIQDVQSQI	SKIGLKDPTV	PAVKHRKKSL	IRLDKVLDEY	EEEKRHLQEM	ANSLPHFKDG	REKTVNQQCQ	NTVVLWENTK
1530	1540	1550	1560	1570	1580	1590	1600
ALVTECLEQC	GRVLELLKQY	QNFKSILTTL	IQKEESVISL	QASYMGKENL	KKRIAEIEIV	KEEFNEHLEV	VDKINQVCKN
1610	1620	1630	1640	1650	1660	1670	1680
LQFYLNKMKT	FEEPPFEKEA	NIIVDRWLDI	NEKTEDYYEN	LGRALALWDK	LFNLKNVIDE	WTEKALQKME	LHQLTEEDRE
1690	1700	1710	1720	1730	1740	1750	1760
RLKEELQVHE	QKTSEFSRRV	AEIQFLLQSS	EIPLELQVME	SSILNKMEHV	QKCLTGESNC	HALSGSTAEL	REDLDQAKTQ
1770	1780	1790	1800	1810	1820	1830	1840
IGMTESLLKA	LSPSDSLEIF	TKLEEIQQQI	LQQKHSMILL	ENQIGCLTPE	LSELKKQYES	VSDLFNTKKS	VLQDHFSKLL
1850	1860	1870	1880	1890	1900	1910	1920
NDQCKNFNDW	FSNIKVNLKE	CFESSETKKS	VEQKLQKLSD	FLTLEGRNSK	IKQVDSVLKH	VKKHLPKAHV	KELISWLVGQ
1930	1940	1950	1960	1970	1980	1990	2000
EFELEKMESI	CQARAKELED	SLQQLLRLQD	DHRNLRKWLT	NQEEKWKGME	EPGEKTELFC	QALARKREQF	ESVAQLNNSL
2010	2020	2030	2040	2050	2060	2070	2080
KEYGFTEEEE	IIMEATCLMD	RYQTLLRQLS	EIEEEDKLLP	TEDQSFNDLA	HDVIHWIKEI	KESLMVLNSS	EGKMPLEERI
2090	2100	2110	2120	2130	2140	2150	2160
QKIKEIILLK	PEGDARIETI	MKQAESSEAP	LVQKTLTDIS	NQWDNTLHLA	STYLSHQEKL	LLEGEKYLQS	KEDLRLMLIE
2170	2180	2190	2200	2210	2220	2230	2240

Detailed Protein Report

Cmpd.	No. of Cmpds.	m/z meas.	Δ m/z [ppm]	z	Rt [min]	Score	P	Range	Sequence	Modification	Ratios
2639	1	686.8883	34.43	2	63.0	14.6	0	451-462	K.DENHLPLVPPNK.L		
1518	1	882.6077	144.13	2	48.8	11.3	2	3618-3632	K.SEQFEELQSILKKGK.L		MD:MU 1.00

Detailed Protein Report

Protein 129: **coiled-coil domain-containing protein 178 isoform 2 [Homo sapiens]**

**Accession:**	gi|157671917	**Score:**	39.5
**Database:**	refseq_human(refseq_human_20140103.fasta)	**MW [kDa]:**	97.5
**Database Date:**	2015-11-30	**pI:**	5.9
		**Sequence Coverage [%]:**	3.4
		**No. of unique Peptides:**	3

10	20	30	40	50	60	70	80
MTENKTVSSS	STRDDQTNIG	LTCQEVKALR	EKAWSRTNEG	NAMSQSLVLY	GASKENSEGF	HESKMTNTEG	VNKGIYFSYP
**90**	**100**	**110**	**120**	**130**	**140**	**150**	**160**
CRRHSCAVVN	IPAPCVNKMI	SHIQDVESKI	QEHLKRFETS	FEEWSRTSST	KDLKEDWSVT	TPVKEVKPGE	KRDEKCPELK
**170**	**180**	**190**	**200**	**210**	**220**	**230**	**240**
QEMETLLSEA	IRLIKSLETD	RADAEEALKQ	QRSRKNMINM	KIDSWSVWKL	QELPLAVQKE	HEAYLSDVIE	LQWHLEDKAN
**250**	**260**	**270**	**280**	**290**	**300**	**310**	**320**
QLQHFEKQKT	ELEEANAKIQ	ADIDYMNEHG	PLLDSKQNQE	LQDLKNHYKK	KMEVMDLHRK	VNEELEEALE	ACENARLKAQ
**330**	**340**	**350**	**360**	**370**	**380**	**390**	**400**
QIKEEIDKDI	YQDEKTIEAY	KREIYQLNSL	FDHYSSSVIN	VNTNIEEKEE	EVTEAIRETK	SSKNELHSLS	KMLEDLRRVY
**410**	**420**	**430**	**440**	**450**	**460**	**470**	**480**
DQLTWKQKSH	ENQYLEAVND	FYAAKKTWDI	ELSDVAKDFS	AISLACTKLT	EDNKKLEIDI	NKITVKTNES	IRKKSKYESE
**490**	**500**	**510**	**520**	**530**	**540**	**550**	**560**
IKYLTIMKLK	NDKHLKNIYK	EAYRIGTLFH	LTKHKTDEME	DKIAEVRRKF	KGREEFLKKL	TQGEVAAGMV	LQKKLYSIYE
**570**	**580**	**590**	**600**	**610**	**620**	**630**	**640**
VQALERKELI	KNRAICAMSL	AELQEPLLQL	EDEAERIRSL	DKEHSVSKRS	AIFKDLEATK	SKTMIFYAKI	NELNEELKAK
**650**	**660**	**670**	**680**	**690**	**700**	**710**	**720**
EEEKKSFDQT	LEILKNKFIT	MRFKREHAQT	VFDHYMQEKK	DCEERIFEED	QRFRVLLAVR	QKTLQDTQKI	IADSLEENLR
**730**	**740**	**750**	**760**	**770**	**780**	**790**	**800**
LAQEYQQLQI	TFLKEKDNYF	NIYDKQLSLD	TSIRDKKQLC	QLQRRMHTLW	QEHFKLVVLF	SQMRLANFQT	DSQESIQKIL
**810**	**820**	**830**					
AVQEESSNLM	QHILGFFQTL	TDGTCENDG					

Cmpd.	No. of Cmpds.	m/z meas.	Δ m/z [ppm]	z	Rt [min]	Score	P	Range	Sequence	Modification
1067	1	942.2514	-302.71	1	42.9	13.2	1	145-152	K.EVKPGEKR.D	
299	1	559.1629	-222.10	2	33.4	13.3	2	152-160	K.RDEKCPELK.Q	
2736	2	694.8753	-15.16	2	64.6	13.1	2	603-614	K.EHSVSKRSAIFK.D	

Detailed Protein Report

Protein 130: **replication factor C subunit 1 isoform 1 [Homo sapiens]**

Accession: gi|32528306
Database: refseq_human(refseq_human_20140103.fasta)
Database Date: 2015-11-30
Modification(s): Carbamidomethyl

Score: 39.5
MW [kDa]: 128.1
pI: 9.9
Sequence Coverage [%]: 4.1
No. of unique Peptides: 2

Quantitation

MD:MU **Median:** 1.04 **CV:** 0.00 % **No. of Peptides:** 1

10	20	30	40	50	60	70	80
MDIRKFFGVI	PSGKKLVSET	VKKNEKTKSD	EETLKAKKGI	KEIKVNSSRK	EDDFKQKQPS	KKKRIIYDSD	SESEETLQVK
90	100	110	120	130	140	150	160
NAKKPPEKLP	VSSKPGKISR	QDPVTYISET	DEEDDFMCKK	AASKSKENGR	STNSHLGTSN	MKKNEENTKT	KNKPLSPIKL
170	180	190	200	210	220	230	240
TPTSVLDYFG	TGSVQRSNKK	MVASKRKELS	QNTDESGLND	EAIAKQLQLD	EDAELERQLH	EDEEFARTLA	MLDEEPKTKK
250	260	270	280	290	300	310	320
ARKDTEAGET	FSSVQANLSK	AEKHKYPHKV	KTAQVSDERK	SYSPRKQSKY	ESSKESQQHS	KSSADKIGEV	SSPKASSKLA
330	340	350	360	370	380	390	400
IMKRKEESSY	KEIEPVASKR	KENAIKLKGE	TKTPKKTKSS	PAKKESVSPE	DSEKKRTNYQ	AYRSYLNREG	PKALGSKEIP
410	420	430	440	450	460	470	480
KGAENCLEGL	IFVITGVLES	IERDEAKSLI	ERYGGKVTGN	VSKKTNYLVM	GRDSGQSKSD	KAAALGTKII	DEDGLLNLIR
490	500	510	520	530	540	550	560
TMPGKKSKYE	IAVETEMKKE	SKLERTPQKN	VQGKRKISPS	KKESESKKSR	PTSKRDSLAK	TIKKETDVFW	KSLDFKEQVA
570	580	590	600	610	620	630	640
EETSGDSKAR	NLADDSSENK	VENLLWVDKY	KPTSLKTIIG	QQGDQSCANK	LLRWLRNWQK	SSSEDKKHAK	FGKFSGKDDG
650	660	670	680	690	700	710	720
SSFKAALLSG	PPGVGKTTTA	SLVCQELGYS	YVELNASDTR	SKSSLKAIVA	ESLNNTSIKG	FYSNGAASSV	STKHALIMDE
730	740	750	760	770	780	790	800
VDGMAGNEDR	GGIQELIGLI	KHTKIPIICM	CNDRNHPKIR	SLVHYCFDLR	FQRPRVEQIK	GAMMSIAFKE	GLKIPPPAMN
810	820	830	840	850	860	870	880
EIILGANQDI	RQVLHNLSMW	CARSKALTYD	QAKADSHRAK	KDIKMGPFDV	ARKVFAAGEE	TAHMSLVDKS	DLFFHDYSIA
890	900	910	920	930	940	950	960
PLFVQENYIH	VKPVAAGGDM	KKHLMLLSRA	ADSICDGDLV	DSQIRSKQNW	SLLPAQAIYA	SVLPGELMRG	YMTQFPTFPS
970	980	990	1000	1010	1020	1030	1040
WLGKHSSTGK	HDRIVQDLAL	HMSLRTYSSK	RTVNMDYLSL	LRDALVQPLT	SQGVDGVQDV	VALMDTYYLM	KEDFENIMEI
1050	1060	1070	1080	1090	1100	1110	1120
SSWGGKPSPF	SKLDPKVKAA	FTRAYNKEAH	LTPYSLQAIK	ASRHSTSPSL	DSEYNEELNE	DDSQSDEKDQ	DAIETDAMIK
1130	1140	1150					
KKTKSSKPSK	PEKDKEPRKG	KGKSSKK					

Cmpd.	No. of Cmpds.	m/z meas.	Δ m/z [ppm]	z	Rt [min]	Score	P	Range	Sequence	Modification	Ratios
1265	1	990.4490	-59.66	2	45.4	11.3	2	745-760	K.IPIICMCNDRNHPKIR.S	Carbamidomethyl: 5	
6	1	776.7890	-162.05	2	28.8	17.4	1	776-789	R.VEQIKGAMMSIAFK.E		MD:MU 1.04

Detailed Protein Report

Protein 131: protein unc-13 homolog A [Homo sapiens]

**Accession:**	gi|283837842	**Score:**	39.1
**Database:**	refseq_human(refseq_human_20140103.fasta)	**MW [kDa]:**	192.9
**Database Date:**	2015-11-30	**pI:**	5.1
**Modification(s):**	Carbamidomethyl, Oxidation	**Sequence Coverage [%]:**	2.5
		**No. of unique Peptides:**	3

10	20	30	40	50	60	70	80
MSLLCVGVKK	AKFDGAQEKF	NTYVTLKVQN	VKSTTIAVRG	SQPSWEQDFM	FEINRLDLGL	TVEVWNKGLI	WDTMVGTVWI
90	100	110	120	130	140	150	160
PLRTIRQSNE	EGPGEWLTLD	SQVIMADSEI	CGTKDPTFHR	ILLDTRFELP	LDIPEEEARY	WAKKLEQLNA	MRDQDEYSFQ
170	180	190	200	210	220	230	240
DEQDKPLPVP	SNQCCNWNYF	GWGEQHNDDP	DSAVDDRDSD	YRSETSNSIP	PPYYTTSQPN	ASVHQYSVRP	PPLGSRESYS
250	260	270	280	290	300	310	320
DSMHSYEEFS	EPQALSPTGS	SRYASSGELS	QGSSQLSEDF	DPDEHSLQGS	DMEDERDRDS	YHSCHSSVSY	HKDSPRWDQD
330	340	350	360	370	380	390	400
EEELEEDLED	FLEEEELPED	EEELEEEEEE	VPDDLGSYAQ	REDVAVAEPK	DFKRISLPPA	APGKEDKAPV	APTEAPDMAK
410	420	430	440	450	460	470	480
VAPKPATPDK	VPAAEQIPEA	EPPKDEESFR	PREDEEGQEG	QDSMSRAKAN	WLRAFNKVRM	QLQEARGEGE	MSKSLWFKGG
490	500	510	520	530	540	550	560
PGGGLIIIDS	MPDIRKRKPI	PLVSDLAMSL	VQSRKAGITS	ALASSTLNNE	ELKNHVYKKT	LQALIYPISC	TTPHNFEVWT
570	580	590	600	610	620	630	640
ATTPTYCYEC	EGLLWGIARQ	GMRCTECGVK	CHEKCQDLLN	ADCLQRAAEK	SSKHGAEDRT	QNIIMVLKDR	MKIRERNKPE
650	660	670	680	690	700	710	720
IFELIQEIFA	VTKTAHTQQM	KAVKQSVLDG	TSKWSAKISI	TVVCAQGLQA	KDKTGSSDPY	VTVQVGKTKK	RTKTIYGNLN
730	740	750	760	770	780	790	800
PVWEENFHFE	CHNSSDRIKV	RVWDEDDDIK	SRVKQRFKRE	SDDFLGQTII	EVRTLSGEMD	VWYNLDKRTD	KSAVSGAIRL
810	820	830	840	850	860	870	880
HISVEIKGEE	KVAPYHVQYT	CLHENLFHFV	TDVQNNGVVK	IPDAKGDDAW	KVYYDETAQE	IVDEFAMRYG	VESIYQAMTH
890	900	910	920	930	940	950	960
FACLSSKYMC	PGVPAVMSTL	LANINAYYAH	TTASTNVSAS	DRFAASNFGK	ERFVKLLDQL	HNSLRIDLSM	YRNNFPASSP
970	980	990	1000	1010	1020	1030	1040
ERLQDLKSTV	DLLTSITFFR	MKVQELQSPP	RASQVVKDCV	KACLNSTYEY	IFNNCHELYS	REYQTDPAKK	GEVLPEEQGP
1050	1060	1070	1080	1090	1100	1110	1120
SIKNLDFWSK	LITLIVSIIE	EDKNSYTPCL	NQFPQELNVG	KISAEVMWNL	FAQDMKYAME	EHDKHRLCKS	ADYMNLHFKV
1130	1140	1150	1160	1170	1180	1190	1200
KWLYNEYVTE	LPAFKDRVPE	YPAWFEPFVI	QWLDENEEVS	RDFLHGALER	DKKDGFQQTS	EHALFSCSVV	DVFSQLNQSF
1210	1220	1230	1240	1250	1260	1270	1280
EIIKKLECPD	PQIVGHYMRR	FAKTISNVLL	QYADIISKDF	ASYCSKEKEK	VPCILMNNTQ	QLRVQLEKMF	EAMGGKELDA
1290	1300	1310	1320	1330	1340	1350	1360
EASDILKELQ	VKLNNVLDEL	SRVFATSFQP	HIEECVKQMG	DILSQVKGTG	NVPASACSSV	AQDADNVLQP	IMDLLDSNLT
1370	1380	1390	1400	1410	1420	1430	1440
LFAKICEKTV	LKRVLKELWK	LVMNTMEKTI	VLPPLTDQTM	IGNLLRKHGK	GLEKGRVKLP	SHSDGTQMIF	NAAKELGQLS
1450	1460	1470	1480	1490	1500	1510	1520
KLKDHMVREE	AKSLTPKQCA	VVELALDTIK	QYFHAGGVGL	KKTFLEKSPD	LQSLRYALSL	YTQATDLLIK	TFVQTQSAQG
1530	1540	1550	1560	1570	1580	1590	1600
LGVEDPVGEV	SVHVELFTHP	GTGEHKVTVK	VVAANDLKWQ	TSGIFRPFIE	VNIIGPQLSD	KKRKFATKSK	NNSWAPKYNE
1610	1620	1630	1640	1650	1660	1670	1680
SFQFTLSADA	GPECYELQVC	VKDYCFARED	RTVGLAVLQL	RELAQRGSAA	CWLPLGRRIH	MDDTGLTVLR	ILSQRSNDEV
1690	1700	1710					
AKEFVKLKSD	TRSAEEGGAA	PAP					

Cmpd.	No. of Cmpds.	m/z meas.	Δ m/z [ppm]	z	Rt [min]	Score	P	Range	Sequence	Modification
2705	1	805.4372	84.57	2	65.1	14.8	1	460-473	R.MQLQEARGEGEMSK.S	Oxidation: 1
2064	1	753.2991	-61.50	2	55.9	14.2	0	595-606	K.CQDLLNADCLQR.A	Carbamidomethyl: 1, 9

Detailed Protein Report

Cmpd.	No. of Cmpds.	m/z meas.	Δ m/z [ppm]	z	Rt [min]	Score	P	Range	Sequence	Modification
2215	1	682.1038	136.60	3	57.9	10.1	1	1122-1137	K.WLYNEYVTELPAFKDR.V	

Detailed Protein Report

Protein 132: PREDICTED: RAS guanyl-releasing protein 2 isoform X1 [Homo sapiens]

**Accession:**	gi|578821076	**Score:**	39.0
**Database:**	refseq_human(refseq_human_20140103.fasta)	**MW [kDa]:**	75.4
**Database Date:**	2015-11-30	**pI:**	10.1
**Modification(s):**	Carbamidomethyl, Oxidation	**Sequence Coverage [%]:**	8.4
		**No. of unique Peptides:**	3

10	20	30	40	50	60	70	80
MGVCACVWEC	TCVERHCLAC	VHTCEDVSAC	VAAGLKAGLA	QVNSTSRRRP	RPRVCILGWT	SWGFGPSPVG	GSRSAAWAQP
**90**	**100**	**110**	**120**	**130**	**140**	**150**	**160**
TPRRRPWQAP	WTWTRAARWR	SCSAGASKPS	LAAKLLHIYQ	QSRKDNSNSL	QVKTCHLVRY	WISAFPAEFD	LNPELAEQIK
**170**	**180**	**190**	**200**	**210**	**220**	**230**	**240**
ELKALLDQEG	NRRHSSLIDI	DSVPTYKWKR	QVTQRNPVGQ	KKRKMSLLFD	HLEPMELAEH	LTYLEYRSFC	KILFQDYHSF
**250**	**260**	**270**	**280**	**290**	**300**	**310**	**320**
VTHGCTVDNP	VLERFISLFN	SVSQWVQLMI	LSKPTAPQRA	LVITHFVHVA	EKLLQLQNFN	TLMAVVGGLS	HSSISRLKET
**330**	**340**	**350**	**360**	**370**	**380**	**390**	**400**
HSHVSPETIK	LWEGLTELVT	ATGNYGNYRR	RLAACVGFRF	PILGVHLKDL	VALQLALPDW	LDPARTRLNG	AKMKQLFSIL
**410**	**420**	**430**	**440**	**450**	**460**	**470**	**480**
EELAMVTSLR	PPVQANPDLL	SLLTVSLDQY	QTEDELYQLS	LQREPRSKSS	PTSPTSCTPP	PRPPVLEEWT	SAAKPKLDQA
**490**	**500**	**510**	**520**	**530**	**540**	**550**	**560**
LVVEHIEKMV	ESVFRNFDVD	GDGHISQEEF	QIIRGNFPYL	SAFGDLDQNQ	DGCISREEMV	SYFLRSSSVL	GGRMGFVHNF
**570**	**580**	**590**	**600**	**610**	**620**	**630**	**640**
QESNSLRPVA	CRHCKALILG	IYKQGLKCRA	CGVNCHKQCK	DRLSVECRRR	AQSVSLEGSA	PSPSPMHSHH	HRAFSFSLPR
**650**	**660**	**670**					
PGRRGSRPPE	IREEEVQTVE	DGVFDIHL					

Cmpd.	No. of Cmpds.	m/z meas.	Δ m/z [ppm]	z	Rt [min]	Score	P	Range	Sequence	Modification
2165	1	938.2937	-80.66	2	56.9	12.3	0	1-15	-.MGVCACVWECTCVER.H	Carbamidomethyl: 4, 6, 10; Oxidation: 1
1804	1	1056.6673	56.24	3	52.5	10.2	2	360-387	R.FPILGVHLKDLVALQLALPDWLDPARTR.L	
2600	1	731.3793	68.68	2	63.1	16.6	2	590-602	R.ACGVNCHKQCKDR.L	

Detailed Protein Report

Protein 133: 14-3-3 protein sigma [Homo sapiens]

Accession: gi|5454052
Database: refseq_human(refseq_human_20140103.fasta)
Database Date: 2015-11-30

Score: 38.9
MW [kDa]: 27.8
pI: 4.5
Sequence Coverage [%]: 11.3
No. of unique Peptides: 1

10	20	30	40	50	60	70	80
MERASLIQKA	KLAEQAERYE	DMAAFMKGAV	EKGEELSCEE	RNLLSVAYKN	VVGGQRAAWR	VLSSIEQKSN	EEGSEEKGPE
90	100	110	120	130	140	150	160
VREYREKVET	ELQGVCDTVL	GLLDSHLIKE	AGDAESRVFY	LKMKGDYYRY	LAEVATGDDK	KRIIDSARSA	YQEAMDISKK
170	180	190	200	210	220	230	240
EMPPTNPIRL	GLALNFSVFH	YEIANSPEEA	ISLAKTTFDE	AMADLHTLSE	DSYKDSTLIM	QLLRDNLTLW	TADNAGEEGG
250							
EAPQEPQS							

Cmpd.	No. of Cmpds.	m/z meas.	Δ m/z [ppm]	z	Rt [min]	Score	P	Range	Sequence	Modification
2105	1	1051.3989	-101.77	2	56.2	11.9	2	10-27	K.AKLAEQAERYEDMAAFMK.G	

Detailed Protein Report

Protein 134: centromere protein F [Homo sapiens]

**Accession:**	gi|55770834	**Score:**	38.8
**Database:**	refseq_human(refseq_human_20140103.fasta)	**MW [kDa]:**	357.3
**Database Date:**	2015-11-30	**pI:**	4.9
**Modification(s):**	Oxidation	**Sequence Coverage [%]:**	0.9
		**No. of unique Peptides:**	1

Detailed Protein Report

10	20	30	40	50	60	70	80
MSWALEEWKE	GLPTRALQKI	QELEGQLDKL	KKEKQQRQFQ	LDSLEAALQK	QKQKVENEKT	EGTNLKRENQ	RLMEICESLE
90	100	110	120	130	140	150	160
KTKQKISHEL	QVKESQVNFQ	EGQLNSGKKQ	IEKLEQELKR	CKSELERSQQ	AAQSADVSLN	PCNTPQKIFT	TPLTPSQYYS
170	180	190	200	210	220	230	240
GSKYEDLKEK	YNKEVEERKR	LEAEVKALQA	KKASQTLPQA	TMNHRDIARH	QASSSVFSWQ	QEKTPSHLSS	NSQRTPIRRD
250	260	270	280	290	300	310	320
FSASYFSGEQ	EVTPSRSTLQ	IGKRDANSSF	FDNSSSPHLL	DQLKAQNQEL	RNKINELELR	LQGHEKEMKG	QVNKFQELQL
330	340	350	360	370	380	390	400
QLEKAKVELI	EKEKVLNKCR	DELVRTTAQY	DQASTKYTAL	EQKLKKLTED	LSCQRQNAES	ARCSLEQKIK	EKEKEFQEEL
410	420	430	440	450	460	470	480
SRQQRSFQTL	DQECIQMKAR	LTQELQQAKN	MHNVLQAELD	KLTSVKQQLE	NNLEEFKQKL	CRAEQAFQAS	QIKENELRRS
490	500	510	520	530	540	550	560
MEEMKKENNL	LKSHSEQKAR	EVCHLEAELK	NIKQCLNQSQ	NFAEEMKAKN	TSQETMLRDL	QEKINQQENS	LTLEKLKLAV
570	580	590	600	610	620	630	640
ADLEKQRDCS	QDLLKKREHH	IEQLNDKLSK	TEKESKALLS	ALELKKKEYE	ELKEEKTLFS	CWKSENEKLL	TQMESEKENL
650	660	670	680	690	700	710	720
QSKINHLETC	LKTQQIKSHE	YNERVRTLEM	DRENLSVEIR	NLHNVLDSKS	VEVETQKLAY	MELQQKAEFS	DQKHQKEIEN
730	740	750	760	770	780	790	800
MCLKTSQLTG	QVEDLEHKLQ	LLSNEIMDKD	RCYQDLHAEY	ESLRDLLKSK	DASLVTNEDH	QRSLLAFDQQ	PAMHHSFANI
810	820	830	840	850	860	870	880
IGEQGSMPSE	RSECRLEADQ	SPKNSAILQN	RVDSLEFSLE	SQKQMNSDLQ	KQCEELVQIK	GEIEENLMKA	EQMHQSFVAE
890	900	910	920	930	940	950	960
TSQRISKLQE	DTSAHQNVVA	ETLSALENKE	KELQLLNDKV	ETEQAEIQEL	KKSNHLLEDS	LKELQLLSET	LSLEKKEMSS
970	980	990	1000	1010	1020	1030	1040
IISLNKREIE	ELTQENGTLK	EINASLNQEK	MNLIQKSESF	ANYIDEREKS	ISELSDQYKQ	EKLILLQRCE	ETGNAYEDLS
1050	1060	1070	1080	1090	1100	1110	1120
QKYKAAQEKN	SKLECLLNEC	TSLCENRKNE	LEQLKEAFAK	EHQEFLTKLA	FAEERNQNLM	LELETVQQAL	RSEMTDNQNN
1130	1140	1150	1160	1170	1180	1190	1200
SKSEAGGLKQ	EIMTLKEEQN	KMQKEVNDLL	QENEQLMKVM	KTKHECQNLE	SEPIRNSVKE	RESERNQCNF	KPQMDLEVKE
1210	1220	1230	1240	1250	1260	1270	1280
ISLDSYNAQL	VQLEAMLRNK	ELKLQESEKE	KECLQHELQT	IRGDLETSNL	QDMQSQEISG	LKDCEIDAEE	KYISGPHELS
1290	1300	1310	1320	1330	1340	1350	1360
TSQNDNAHLQ	CSLQTTMNKL	NELEKICEIL	QAEKYELVTE	LNDSRSECIT	ATRKMAEEVG	KLLNEVKILN	DDSGLLHGEL
1370	1380	1390	1400	1410	1420	1430	1440
VEDIPGGEFG	EQPNEQHPVS	LAPLDESNSY	EHLTLSDKEV	QMHFAELQEK	FLSLQSEHKI	LHDQHCQMSS	KMSELQTYVD
1450	1460	1470	1480	1490	1500	1510	1520
SLKAENLVLS	TNLRNFQGDL	VKEMQLGLEE	GLVPSLSSSC	VPDSSSLSSL	GDSSFYRALL	EQTGDMSLLS	NLEGAVSANQ
1530	1540	1550	1560	1570	1580	1590	1600
CSVDEVFCSS	LQEENLTRKE	TPSAPAKGVE	ELESLCEVYR	QSLEKLEEKM	ESQGIMKNKE	IQELEQLLSS	ERQELDCLRK
1610	1620	1630	1640	1650	1660	1670	1680
QYLSENEQWQ	QKLTSVTLEM	ESKLAAEKKQ	TEQLSLELEV	ARLQLQGLDL	SSRSLLGIDT	EDAIQGRNES	CDISKEHTSE
1690	1700	1710	1720	1730	1740	1750	1760
TTERTPKHDV	HQICDKDAQQ	DLNLDIEKIT	ETGAVKPTGE	CSGEQSPDTN	YEPPGEDKTQ	GSSECISELS	FSGPNALVPM
1770	1780	1790	1800	1810	1820	1830	1840
DFLGNQEDIH	NLQLRVKETS	NENLRLLHVI	EDRDRKVESL	LNEMKELDSK	LHLQEVQLMT	KIEACIELEK	IVGELKKENS
1850	1860	1870	1880	1890	1900	1910	1920
DLSEKLEYFS	CDHQELLQRV	ETSEGLNSDL	EMHADKSSRE	DIGDNVAKVN	DSWKERFLDV	ENELSRIRSE	KASIEHEALY
1930	1940	1950	1960	1970	1980	1990	2000
LEADLEVVQT	EKLCLEKDNE	NKQKVIVCLE	EELSVVTSER	NQLRGELDTM	SKKTTALDQL	SEKMKEKTQE	LESHQSECLH
2010	2020	2030	2040	2050	2060	2070	2080
CIQVAEAEVK	EKTELLQTLS	SDVSELLKDK	THLQEKLQSL	EKDSQALSLT	KCELENQIAQ	LNKEKELLVK	ESESLQARLS
2090	2100	2110	2120	2130	2140	2150	2160
ESDYEKLNVS	KALEAALVEK	GEFALRLSST	QEEVHQLRRG	IEKLRVRIEA	DEKKQLHIAE	KLKEREREND	SLKDKVENLE
2170	2180	2190	2200	2210	2220	2230	2240

Detailed Protein Report

Cmpd.	No. of Cmpds.	m/z meas.	Δ m/z [ppm]	z	Rt [min]	Score	P	Range	Sequence	Modification
2707	1	821.4735	62.12	2	64.1	11.9	2	301-314	R.LQGHEKEMKGQVNK.F	Oxidation: 8

Detailed Protein Report

Protein 135: PREDICTED: zinc finger protein 737 isoform X4 [Homo sapiens]

Accession: gi|530414419
Database: refseq_human(refseq_human_20140103.fasta)
Database Date: 2015-11-30
Modification(s): Carbamidomethyl

Score: 38.7
MW [kDa]: 60.3
pI: 10.4
Sequence Coverage [%]: 6.5
No. of unique Peptides: 2

10	20	30	40	50	60	70	80
MGPLQFRDVA	IEFSLEEWHC	LDTAQRNLYR	NVMLENYRNL	VFLGIVVSKP	DLITCLEQGK	KPLTMKKHEM	VANPSVTCSH
**90**	**100**	**110**	**120**	**130**	**140**	**150**	**160**
FARDLWPEQS	IKDSFQKVTL	RRYENYGHDN	LQFKKGCESV	DECKVHKRGY	NGLNQYLTTT	QSKIFQCDKY	VKVIHKFSNS
**170**	**180**	**190**	**200**	**210**	**220**	**230**	**240**
NRHKIRHTGK	KPFKCIECGK	AFNQSSTLTT	HKKIHTGEKP	FKCEECGKAF	NWSSHLTTHK	RIHTGEKRYK	CEDCGKAFSR
**250**	**260**	**270**	**280**	**290**	**300**	**310**	**320**
FSYLTAHKII	HSGEKPYKCE	ECGKAFKRSS	NLTTHKIIHT	GEKPYKCEEC	GKAFKRSSIL	TAHKIIHSGE	KPYKCEECGK
**330**	**340**	**350**	**360**	**370**	**380**	**390**	**400**
AFKHPSVLTT	HKRIHTGEKP	YKCEECGKAF	NWSSHLTTHK	RIHTGEKPYK	CEECGEAFKY	SSSLTTHKII	HTGQQPFKCE
**410**	**420**	**430**	**440**	**450**	**460**	**470**	**480**
ECGKAFKCFS	ILTTHKRIHT	GEKPYKCEEC	GKAFNSSSHL	TAHKRIHTGE	KPYKCERCGK	AFKRSFILTR	HKRIHTGEKP
**490**	**500**	**510**	**520**	**530**			
YKCEECGKGF	KCPSTLTTHK	RSGCPTRKLL	LSSVSTLNNS	EWK			

Cmpd.	No. of Cmpds.	m/z meas.	Δ m/z [ppm]	z	Rt [min]	Score	P	Range	Sequence	Modification
1955	1	1022.8979	-86.37	2	54.5	10.5	2	399-416	K.CEECGKAFKCFSILTTHK.R	
2857	1	1051.5637	63.44	2	66.2	11.3	2	399-416	K.CEECGKAFKCFSILTTHK.R	Carbamidomethyl: 1

Detailed Protein Report

Protein 136: PREDICTED: caspase-10 isoform X3 [Homo sapiens]

Accession: gi|530371073
Database: refseq_human(refseq_human_20140103.fasta)
Database Date: 2015-11-30

Score: 38.6
MW [kDa]: 34.9
pI: 6.5
Sequence Coverage [%]: 10.1
No. of unique Peptides: 1

10	20	30	40	50	60	70	80
MKSQGQHWYS	SSDKNCKVSF	REKLLIIDSN	LGVQDVENLK	FLCIGLVPNK	KLEKSSSASD	VFEHLLAEDL	LSEEDPFFLA
**90**	**100**	**110**	**120**	**130**	**140**	**150**	**160**
ELLYIIRQKK	LLQHLNCTKE	EVERLLPTRQ	RVSLFRNLLY	ELSEGIDSEN	LKDMIFLLKD	SLPKTEMTSL	SFLAFLEKQG
**170**	**180**	**190**	**200**	**210**	**220**	**230**	**240**
KIDEDNLTCL	EDLCKTVVPK	LLRNIEKYKR	EKAIQIVTPP	VDKEAESYQG	EEELVSQTDV	KTFLEALPQE	SWQNKHAGSN
**250**	**260**	**270**	**280**	**290**	**300**	**310**	
GNRATNGAPS	LVSRGMQGAS	ANTLNSETST	KRAAVYRMNR	NHRGLCVIVN	NHSFTSLKDR	QGTHKDAG	

Cmpd.	No. of Cmpds.	m/z meas.	Δ m/z [ppm]	z	Rt [min]	Score	P	Range	Sequence	Modification
1677	1	933.3261	-154.07	2	50.9	26.9	1	236-254	K.HAGSNGNRATNGAPSLVSR.G	

Detailed Protein Report

Protein 137: zinc finger protein 610 isoform a [Homo sapiens]

**Accession:**	gi|239787098	**Score:**	38.5
**Database:**	refseq_human(refseq_human_20140103.fasta)	**MW [kDa]:**	53.5
**Database Date:**	2015-11-30	**pI:**	10.3
		**Sequence Coverage [%]:**	10.6
		**No. of unique Peptides:**	2

Alias proteins:

Accession	Name	Description
gi|239787104	refseq_human (refseq_human_20140103.fasta)	zinc finger protein 610 isoform a [Homo sapiens]
gi|239787101	refseq_human (refseq_human_20140103.fasta)	zinc finger protein 610 isoform a [Homo sapiens]

```
        10         20         30         40         50         60         70         80
MLCDEEAQKR KAKESGMALP QGRLTFMDVA IEFSQEEWKS LDPGQRALYR DVMLENYRNL VFLGICLPDL SIISMLKQRR
        90        100        110        120        130        140        150        160
EPLILQSQVK IVKNTDGREC VRSVNTGRSC VLGSNAENKP IKNQLGLTLE AHLSELQLFQ AGRKIYRSNQ VEKFTNHRSS
       170        180        190        200        210        220        230        240
VSPLQKISSS FTTHIFNKYR NDLIDFPLLP QEEKAYIRGK SYEYECSEDG EVFRVRASLT NHQVIHTAEK PYKCTECGKV
       250        260        270        280        290        300        310        320
FSRNSHLVEH WRIHTGQKPY KCSECDKVFN RNSNLARHQR IHTGEKPHKC NECGKAFREC SGLTTHLVIH TGEKPYKCNE
       330        340        350        360        370        380        390        400
CGKNFRHKFS LTNHQRSHTA EKPYKCNECG KVFSLLSYLA RHQIIHSTEK PYKCNECGRA FHKRPGLMAH LLIHTGEKPY
       410        420        430        440        450        460        470
KCNECDKVFG RKLYLTNHQR IHTGERPYKC NACGKVFNQN PHLSRHRKIH AGENSLRTLQ ME
```

Cmpd.	No. of Cmpds.	m/z meas.	Δ m/z [ppm]	z	Rt [min]	Score	P	Range	Sequence	Modification
2725	2	1045.0312	-59.71	2	64.4	11.5	0	59-77	R.NLVFLGICLPDLSIISMLK.Q	
1493	1	949.3248	-78.23	2	48.5	12.6	1	199-214	R.GKSYEYECSEDGEVFR.V	

Detailed Protein Report

Protein 138: syntaxin-binding protein 5 isoform b [Homo sapiens]

Accession: gi|189217915
Database: refseq_human(refseq_human_20140103.fasta)
Database Date: 2015-11-30
Modification(s): Oxidation

Score: 38.5
MW [kDa]: 127.5
pI: 7.1
Sequence Coverage [%]: 3.5
No. of unique Peptides: 2

10	20	30	40	50	60	70	80
MRKFNIRKVL	DGLTAGSSSA	SQQQQQQHPP	GNREPEIQET	LQSEHFQLCK	TVRHGFPYQP	SALAFDPVQK	ILAVGTQTGA
**90**	**100**	**110**	**120**	**130**	**140**	**150**	**160**
LRLFGRPGVE	CYCQHDSGAA	VIQLQFLINE	GALVSALADD	TLHLWNLRQK	RPAILHSLKF	CRERVTFCHL	PFQSKWLYVG
**170**	**180**	**190**	**200**	**210**	**220**	**230**	**240**
TERGNIHIVN	VESFTLSGYV	IMWNKAIELS	SKSHPGPVVH	ISDNPMDEGK	LLIGFESGTV	VLWDLKSKKA	DYRYTYDEAI
**250**	**260**	**270**	**280**	**290**	**300**	**310**	**320**
HSVAWHHEGK	QFICSHSDGT	LTIWNVRSPA	KPVQTITPHG	KQLKDGKKPE	PCKPILKVEF	KTTRSGEPFI	ILSGGLSYDT
**330**	**340**	**350**	**360**	**370**	**380**	**390**	**400**
VGRRPCLTVM	HGKSTAVLEM	DYSIVDFLTL	CETPYPNDFQ	EPYAVVVLLE	KDLVLIDLAQ	NGYPIFENPY	PLSIHESPVT
**410**	**420**	**430**	**440**	**450**	**460**	**470**	**480**
CCEYFADCPV	DLIPALYSVG	ARQKRQGYSK	KEWPINGGNW	GLGAQSYPEI	IITGHADGSV	KFWDASAITL	QVLYKLKTSK
**490**	**500**	**510**	**520**	**530**	**540**	**550**	**560**
VFEKSRNKDD	RPNTDIVDED	PYAIQIISWC	PESRMLCIAG	VSAHVIIYRF	SKQEVITEVI	PMLEVRLLYE	INDVETPEGE
**570**	**580**	**590**	**600**	**610**	**620**	**630**	**640**
QPPPLPTPVG	GSNPQPIPPQ	SHPSTSSSSS	DGLRDNVPCL	KVKNSPLKQS	PGYQTELVIQ	LVWVGGEPPQ	QITSLAVNSS
**650**	**660**	**670**	**680**	**690**	**700**	**710**	**720**
YGLVVFGNCN	GIAMVDYLQK	AVLLNLGTIE	LYGSNDPYRR	EPRSPRKSRQ	PSGAGLCDIS	EGTVVPEDRC	KSPTSGSSSP
**730**	**740**	**750**	**760**	**770**	**780**	**790**	**800**
HNSDDEQKMN	NFIEKVKTKS	RKFSKMVAND	IAKMSRKLSL	PTDLKPDLDV	KDNSFSRSRS	SSVTSIDKES	REAISALHFC
**810**	**820**	**830**	**840**	**850**	**860**	**870**	**880**
ETFTRKTDSS	PSPCLWVGTT	LGTVLVIALN	LPPGGEQRLL	QPVIVSPSGT	ILRLKGAILR	MAFLDTTGCL	IPPAYEPWRE
**890**	**900**	**910**	**920**	**930**	**940**	**950**	**960**
HNVPEEKDEK	EKLKKRRPVS	VSPSSSQEIS	ENQYAVICSE	KQAKVISLPT	QNCAYKQNIT	ETSFVLRGDI	VALSNSICLA
**970**	**980**	**990**	**1000**	**1010**	**1020**	**1030**	**1040**
CFCANGHIMT	FSLPSLRPLL	DVYYLPLTNM	RIARTFCFTN	NGQALYLVSP	TEIQRLTYSQ	ETCENLQEML	GELFTPVETP
**1050**	**1060**	**1070**	**1080**	**1090**	**1100**	**1110**	**1120**
EAPNRGFFKG	LFGGGAQSLD	REELFGESSS	GKASRSLAQH	IPGPGGIEGV	KGAASGVVGE	LARARLALDE	RGQKLGDLEE
**1130**	**1140**	**1150**	**1160**				
RTAAMLSSAE	SFSKHAHEIM	LKYKDKKWYQ	F				

Cmpd.	No. of Cmpds.	m/z meas.	Δ m/z [ppm]	z	Rt [min]	Score	P	Range	Sequence	Modification
1682	1	796.3351	-89.13	2	50.8	10.5	1	143-155	R.ERVTFCHLPFQSK.W	
2747	1	807.3929	-21.85	2	65.6	15.2	2	743-756	K.FSKMVANDIAKMSR.K	Oxidation: 4

Detailed Protein Report

Protein 139: **PREDICTED: UDP-glucose:glycoprotein glucosyltransferase 1 isoform X3 [Homo sapiens]**

**Accession:**	gi|578804605	**Score:**	38.5
**Database:**	refseq_human(refseq_human_20140103.fasta)	**MW [kDa]:**	176.2
**Database Date:**	2015-11-30	**pI:**	5.3
**Modification(s):**	Carbamidomethyl, Oxidation	**Sequence Coverage [%]:**	3.6
		**No. of unique Peptides:**	3

```
        10         20         30         40         50         60         70         80
MGCKGDASGV CYKMGVLVVL TVLWLFSSVK ADSKAITTSL TTKWFSTPLL LEASEFLAED SQEKFWNFVE ASQNIGSSDH
        90        100        110        120        130        140        150        160
DGTDYSYYHA ILEAAFQFLS PLQQNLFKFC LSLRSYSATI QAFQQIAADE PPPEGCNSFF SVHGKKTCES DTLEALLLTA
       170        180        190        200        210        220        230        240
SERPKPLLFK GDHRYPSSNP ESPVVIFYSE IGSEEFSNFH RQLISKSNAG KINYVFRHYI FNPRKEPVYL SGYGVELAIK
       250        260        270        280        290        300        310        320
STEYKAKDDT QVKGTEVNTT VIGENDPIDE VQGFLFGKLR DLHPDLEGQL KELRKHLVES TNEMAPLKVW QLQDLSFQTA
       330        340        350        360        370        380        390        400
ARILASPVEL ALVVMKDLSQ NFPTKARAIT KTAVSSELRT EVEENQKYFK GTLGLQPGDS ALFINGLHMD LDTQDIFSLF
       410        420        430        440        450        460        470        480
DVLRNEARVM EGLHRLGIEG LSLHNVLKLN IQPSEADYAV DIRSPAISWV NNLEVDSRYN SWPSSLQELL RPTFPGVIRQ
       490        500        510        520        530        540        550        560
IRKNLHNMVF IVDPAHETTA ELMNTAEMFL SNHIPLRIGF IFVVNDSEDV DGMQDAGVAV LRAYNYVAQE VDDYHAFQTL
       570        580        590        600        610        620        630        640
THIYNKVRTG EKVKVEHVVS VLEKKYPYVE VNSILGIDSA YDRNRKEARG YYEQTGVGPL PVVLFNGMPF EREQLDPDEL
       650        660        670        680        690        700        710        720
ETITMHKILE TTTFFQRAVY LGELPHDQDV VEYIMNQPNV VPRINSRILT AERDYLDLTA SNNFFVDDYA RFTILDSQGK
       730        740        750        760        770        780        790        800
TAAVANSMNY LTKKGMSSKE IYDDSFIRPV TFWIVGDFDS PSGRQLLYDA IKHQKSSNNV RISMINNPAK EISYENTQIS
       810        820        830        840        850        860        870        880
RAIWAALQTQ TSNAAKNFIT KMAKEGAAEA LAAGADIAEF SVGGMDFSLF KEVFESSKMD FILSHAVYCR DVLKLKKGQR
       890        900        910        920        930        940        950        960
AVISNGRIIG PLEDSELFNQ DDFHLLENII LKTSGQKIKS HIQQLRVEED VASDLVMKVD ALLSAQPKGD PRIEYQFFED
       970        980        990       1000       1010       1020       1030       1040
RHSAIKLRPK EGETYFDVVA VVDPVTREAQ RLAPLLLVLA QLINMNLRVF MNCQSKLSDM PLKSFYRYVL EPEISFTSDN
      1050       1060       1070       1080       1090       1100       1110       1120
SFAKGPIAKF LDMPQSPLFT LNLNTPESWM VESVRTPYDL DNIYLEEVDS VVAAEYELEY LLLEGHCYDI TTGQPPRGLQ
      1130       1140       1150       1160       1170       1180       1190       1200
FTLGTSANPV IVDTIVMANL GYFQLKANPG AWILRLRKGR SEDIYRIYSH DGTDSPPDAD EVVIVLNNFK SKIIKVKVQK
      1210       1220       1230       1240       1250       1260       1270       1280
KADMVNEDLL SDGTSENESG FWDSFKWGFT GQKTEEVKQD KDDIINIFSV ASGHLYERFL RIMMLSVLKN TKTPVKFWFL
      1290       1300       1310       1320       1330       1340       1350       1360
KNYLSPTFKE FIPYMANEYN FQYELVQYKW PRWLHQQTEK QRIIWGYKIL FLDVLFPLVV DKFLFVDADQ IVRTDLKELR
      1370       1380       1390       1400       1410       1420       1430       1440
DFNLDGAPYG YTPFCDSRRE MDGYRFWKSG YWASHLAGRK YHISALYVVD LKKFRKIAAG DRLRGQYQGL SQDPNSLSNL
      1450       1460       1470       1480       1490       1500       1510       1520
DQDLPNNMIH QVPIKSLPQE WLWCETWCDD ASKKRAKTID LCNNPMTKEP KLEAAVRIVP EWQDYDQEIK QLQIRFQKEK
      1530       1540       1550
ETGALYKEKT KEPSREGPQK REEL
```

Cmpd.	No. of Cmpds.	m/z meas.	Δ m/z [ppm]	z	Rt [min]	Score	P	Range	Sequence	Modification
1963	1	696.2530	-54.87	2	54.6	12.7	1	1-13	-.MGCKGDASGVCYK.M	Carbamidomethyl: 11; Oxidation: 1
1565	1	1018.8365	-18.21	3	49.3	12.6	2	606-632	R.KEARGYYEQTGVGPLPVVLFNGMPFER.E	
1888	1	600.2525	-61.03	3	53.5	13.2	0	633-647	R.EQLDPDELETITMHK.I	

Detailed Protein Report

Protein 140: numb-like protein [Homo sapiens]

Accession: gi|10863899
Database: refseq_human(refseq_human_20140103.fasta)
Database Date: 2015-11-30

Score: 38.3
MW [kDa]: 64.9
pI: 10.1
Sequence Coverage [%]: 8.7
No. of unique Peptides: 2

10	20	30	40	50	60	70	80
MSRSAAASGG	PRRPERHLPP	APCGAPGPPE	TCRTEPDGAG	TMNKLRQSLR	RRKPAYVPEA	SRPHQWQADE	DAVRKGTCSF
90	100	110	120	130	140	150	160
PVRYLGHVEV	EESRGMHVCE	DAVKKLKAMG	RKSVKSVLWV	SADGLRVVDD	KTKDLLVDQT	IEKVSFCAPD	RNLDKAFSYI
170	180	190	200	210	220	230	240
CRDGTTRRWI	CHCFLALKDS	GERLSHAVGC	AFAACLERKQ	RREKECGVTA	AFDASRTSFA	REGSFRLSGG	GRPAEREAPD
250	260	270	280	290	300	310	320
KKKAEAAAAP	TVAPGPAQPG	HVSPTPATTS	PGEKGEAGTP	VAAGTTAAAI	PRRHAPLEQL	VRQGSFRGFP	ALSQKNSPFK
330	340	350	360	370	380	390	400
RQLSLRLNEL	PSTLQRRTDF	QVKGTVPEME	PPGAGDSDSI	NALCTQISSS	FASAGAPAPG	PPPATTGTSA	WGEPSVPPAA
410	420	430	440	450	460	470	480
AFQPGHKRTP	SEAERWLEEV	SQVAKAQQQQ	QQQQQQQQQQ	QQQQQQAASV	APVPTMPPAL	QPFPAPVGPF	DAAPAQVAVF
490	500	510	520	530	540	550	560
LPPPHMQPPF	VPAYPGLGYP	PMPRVPVVGI	TPSQMVANAF	CSAAQLQPQP	ATLLGKAGAF	PPPAIPSAPG	SQARPRPNGA
570	580	590	600	610			
PWPPEPAPAP	APELDPFEAQ	WAALEGKATV	EKPSNPFSGD	LQKTFEIEL			

Cmpd.	No. of Cmpds.	m/z meas.	Δ m/z [ppm]	z	Rt [min]	Score	P	Range	Sequence	Modification
2693	1	1023.5273	37.29	2	64.9	10.3	2	203-221	R.EKECGVTAAFDASRTSFAR.E	
2525	1	834.4332	-13.93	2	62.0	11.3	2	227-242	R.LSGGGRPAEREAPDKK.K	

Detailed Protein Report

Protein 141: ubiquilin-like protein [Homo sapiens]

Accession: gi|145580580
Database: refseq_human(refseq_human_20140103.fasta)
Database Date: 2015-11-30
Modification(s): Oxidation

Score: 38.3
MW [kDa]: 52.9
pI: 5.6
Sequence Coverage [%]: 8.8
No. of unique Peptides: 2

Quantitation

MD:MU **Median:** 3.04 **CV:** 0.00 % **No. of Peptides:** 1

10	20	30	40	50	60	70	80
MWHAISRTSR	MSQSGCPSGL	LADKNISSSA	TRVIVKTAGN	QKDFMVADDI	SVRQFKEMLL	AHFQCQMDQL	VLVFMGCLLK
90	100	110	120	130	140	150	160
DHDTLSQRGI	MDGHTIYLVI	KSKQGSRSLA	HSFRDLPTND	PCHRDRNTKG	NSSRVHQPTG	MNQAPVELAH	FVGSDAPKVH
170	180	190	200	210	220	230	240
TQNLEVSHPE	CKAQMLENPS	IQRLLSNMEF	MWQFISEHLD	TQQLMQQNPE	VSRLLLDNSE	ILLQTLELAR	NLAMIQEIMQ
250	260	270	280	290	300	310	320
IQQPSQNLEY	PLNPQPYLGL	ETMPGGNNAL	GQNYADINDQ	MLNSMQDPFG	GNPFTALLAG	QVLEQVQSSP	PPPPPSQEQQ
330	340	350	360	370	380	390	400
DQLTQHPATR	VIYNSSGGFS	SNTSANDTLN	KVNHTSKANT	AMISTKGQSH	ICATRQPAWI	PALPSIELTQ	QLQEEYKDAT
410	420	430	440	450	460	470	480
VSLSSSRQTL	KGDLQLSDEQ	SSSQITGGMM	QLLMNNPYLA	AQIMLFTSMP	QLSEQWRQQL	PTFLQQTQIS	DLLSA	

Cmpd.	No. of Cmpds.	m/z meas.	Δ m/z [ppm]	z	Rt [min]	Score	P	Range	Sequence	Modification	Ratios
177	1	642.3095	14.02	2	31.6	11.3	0	43-53	K.DFMVADDISVR.Q	Oxidation: 3	MD:MU 3.04
2144	1	953.3984	-72.07	2	57.0	10.6	1	358-375	K.ANTAMISTKGQSHICATR.Q	Oxidation: 5	

Detailed Protein Report

Protein 142: PREDICTED: zinc finger protein 675 isoform X3 [Homo sapiens]

Accession: gi|530414622
Database: refseq_human(refseq_human_20140103.fasta)
Database Date: 2015-11-30
Modification(s): Carbamidomethyl

Score: 38.3
MW [kDa]: 58.2
pI: 10.2
Sequence Coverage [%]: 5.6
No. of unique Peptides: 2

10	20	30	40	50	60	70	80
MVNEPPVMCS	HFAQEFWPEQ	NIKDSFEKVT	LRRYEKCGND	NFQLKGCKSV	DECKLHKGGY	NGLNQCLPTM	QSKMFQCDKY
90	100	110	120	130	140	150	160
VKVFNKFSHS	DRHKIKHMEN	KPFKCKECGR	SFCMLSHLTR	HERNYTKVNF	CKCEECEKAV	NQSSKLTKHK	RIYTCEKLYK
170	180	190	200	210	220	230	240
CQECDRTFNQ	FSNLTEYKKD	YAREKPYKCE	ECGKAFNQSS	HLTTHKIIHT	GEKPYKCEEC	GKAFNQFSNL	TTHKKIHTGE
250	260	270	280	290	300	310	320
QPYICEECGK	AFTQSSTLTT	HKRIHTGEKP	YKCEECGKAF	NRSSKLTEHK	NIHTGEQPYK	CEECGKAFNR	SSNLTEHRKI
330	340	350	360	370	380	390	400
HTEEKPYKCK	ECGKAFKHSS	ALTTHKRIHT	GEKPYKCEEC	GKAFNRSSKL	TEHKKLHTGK	KPYKCEECGK	AFIQSSKLTE
410	420	430	440	450	460	470	480
HKKIHSGEIP	YKCEECGKAF	KHSSSLTTHK	RIHTGEKPYK	CEECGKAFSR	SSKLTEHKII	HTGEKPYKCE	RCDKAFNQSA
490	500						
NLTKHKKIHT	GEKLQNWNV						

Cmpd.	No. of Cmpds.	m/z meas.	Δ m/z [ppm]	z	Rt [min]	Score	P	Range	Sequence	Modification
1793	1	682.2364	-94.72	3	52.4	10.6	2	37-54	K.CGNDNFQLKGCKSVDECK.L	Carbamidomethyl: 17
2097	2	701.2282	-113.98	3	56.4	14.4	2	37-54	K.CGNDNFQLKGCKSVDECK.L	Carbamidomethyl: 1, 17

Detailed Protein Report

Protein 143: **PREDICTED: histone-lysine N-methyltransferase setd3 isoform X3 [Homo sapiens]**

**Accession:**	gi|530404751	**Score:**	38.2
**Database:**	refseq_human(refseq_human_20140103.fasta)	**MW [kDa]:**	56.3
**Database Date:**	2015-11-30	**pI:**	5.7
		**Sequence Coverage [%]:**	3.4
		**No. of unique Peptides:**	2

Quantitation

MD:MU **Median:** 1.36 **CV:** 0.00 % **No. of Peptides:** 1

```
        10         20         30         40         50         60         70         80
MCRSGLWLRK YGKSKKAEEL FLWVPRKLLM TVESAKNSVL GPLYSQDRIL QAMGNIALAF HLLCERASPN SFWQPYIQTL
        90        100        110        120        130        140        150        160
PSEYDTPLYF EEDEVRYLQS TQAIHDVFSQ YKNTARQYAY FYKVIQTHPH ANKLPLKDSF TYEDYRWAVS SVMTRQNQIP
       170        180        190        200        210        220        230        240
TEDGSRVTLA LIPLWDMCNH TNGLITTGYN LEDDRCECVA LQDFRAGEQI YIFYGTRSNA EFVIHSGFFF DNNSHDRVKI
       250        260        270        280        290        300        310        320
KLGVSKSDRL YAMKAEVLAR AGIPTSSVFA LHFTEPPISA QLLAFLRVFC MTEEELKEHL LGDSAIDRIF TLGNSEFPVS
       330        340        350        360        370        380        390        400
WDNEVKLWTF LEDRASLLLK TYKTTIEEDK SVLKNHDLSV RAKMAIKLRL GEKEILEKAV KSAAVNREYY RQQMEEKAPL
       410        420        430        440        450        460        470        480
PKYEESNLGL LESSVGDSRL PLVLRNLEEE AGVQDALNIR EAISKAKATE NGLVNGENSI PNGTRSENES LNQESKRAVE
       490        500
DAKGSSSDST AGVKE
```

Cmpd.	No. of Cmpds.	m/z meas.	Δ m/z [ppm]	z	Rt [min]	Score	P	Range	Sequence	Modification	Ratios
832	1	491.6524	-172.14	2	39.8	26.5	0	117-123	R.QYAYFYK.V		
263	1	592.1181	-248.62	2	33.0	11.7	0	196-205	R.CECVALQDFR.A		MD:MU 1.36

Detailed Protein Report

Protein 144: PREDICTED: protein unc-45 homolog A isoform X3 [Homo sapiens]

**Accession:**	gi|530407244	**Score:**	38.1
**Database:**	refseq_human(refseq_human_20140103.fasta)	**MW [kDa]:**	87.2
**Database Date:**	2015-11-30	**pI:**	5.6
**Modification(s):**	Carbamidomethyl, Oxidation	**Sequence Coverage [%]:**	3.4
		**No. of unique Peptides:**	3

10	20	30	40	50	60	70	80
MSSTDAKVEQ	MFQILLDPEE	KGTEKKQKAS	QNLVVLARED	AGAEKIFRSN	GVQLLQRLLD	MGETDLMLAA	LRTLVGICSE
**90**	**100**	**110**	**120**	**130**	**140**	**150**	**160**
HQSRTVATLS	ILGTRRVVSI	LGVESQAVSL	AACHLLQVMF	DALKEGVKKG	FRGKEGAIIV	DPARELKVLI	SNLLDLLTEV
**170**	**180**	**190**	**200**	**210**	**220**	**230**	**240**
GVSGQGRDNA	LTLLIKAVPR	KSLKDPNNSL	TLWVIDQGLK	KILEVGGSLQ	DPPGELAVTA	NSRMSASILL	SKLFDDLKCD
**250**	**260**	**270**	**280**	**290**	**300**	**310**	**320**
AERENFHRLC	ENYIKSWFEG	QGLAGKLRAI	QTVSCLLQGP	CDAGNRALEL	SGVMESVIAL	CASEQEEEQL	VAVEALIHAA
**330**	**340**	**350**	**360**	**370**	**380**	**390**	**400**
GKAKRASFIT	ANGVSLLKDL	YKCSEKDSIR	IRALVGLCKL	GSAGGTDFSM	KQFAEGSTLK	LAKQCRKWLC	NDQIDAGTRR
**410**	**420**	**430**	**440**	**450**	**460**	**470**	**480**
WAVEGLAYLT	FDADVKEEFV	EDAAALKALF	QLSRLEERSV	LFAVASALVN	CTNSYDYEEP	DPKMVELAKY	AKQHVPEQHP
**490**	**500**	**510**	**520**	**530**	**540**	**550**	**560**
KDKPSFVRAR	VKKLLAAGVV	SAMVCMVKTE	SPVLTSSCRE	LLSRVFLALV	EEVEDRGTVV	AQGGGRALIP	LALEGTDVGQ
**570**	**580**	**590**	**600**	**610**	**620**	**630**	**640**
TKAAQALAKL	TITSNPEMTF	PGERIYEVVR	PLVSLLHLNC	SGLQNFEALM	ALTNLAGISE	RLRQKILKEK	AVPMIEGYMF
**650**	**660**	**670**	**680**	**690**	**700**	**710**	**720**
EEHEMIRRAA	TECMCNLAMS	KEVQDLFEAQ	GNDRLKLLVL	YSGEDDELLQ	RAAAGGLAML	TSMRPTLCSR	IPQVTTHWLE
**730**	**740**	**750**	**760**	**770**	**780**	**790**	**800**
ILQALLLSSN	QELQHRGAVV	VLNMVEASRE	IASTLMESEM	MEILSVLAKG	DHSPVTRAAA	ACLDKAVEYG	LIQPNQDGE

Cmpd.	No. of Cmpds.	m/z meas.	Δ m/z [ppm]	z	Rt [min]	Score	P	Range	Sequence	Modification
1687	1	793.3789	34.81	2	51.0	14.1	1	648-661	R.RAATECMCNLAMSK.E	Carbamidomethyl: 6
2677	1	731.3736	106.62	2	64.7	11.8	0	649-661	R.AATECMCNLAMSK.E	Carbamidomethyl: 5; Oxidation: 6, 11
150	1	977.7995	37.53	3	31.3	12.2	1	649-674	R.AATECMCNLAMSKEVQDLFEAQGNDR.L	Carbamidomethyl: 5

Detailed Protein Report

Protein 145: **PREDICTED: nuclear receptor subfamily 1 group D member 2 isoform X2 [Homo sapiens]**

**Accession:**	gi|578806859	**Score:**	38.1
**Database:**	refseq_human(refseq_human_20140103.fasta)	**MW [kDa]:**	49.9
**Database Date:**	2015-11-30	**pI:**	9.5
**Modification(s):**	Oxidation	**Sequence Coverage [%]:**	12.1
		**No. of unique Peptides:**	2

10	20	30	40	50	60	70	80
MEVNAGGVIA	YISSSSSASS	PASCHSEGSE	NSFQSSSSSV	PSSPNSSNSD	TNGNPKNGDL	ANIEGILKND	RIDCSMKTSK
**90**	**100**	**110**	**120**	**130**	**140**	**150**	**160**
SSAPGMTKSH	SGVTKFSGMV	LLCKVCGDVA	SGFHYGVHAC	EGCKGFFRRS	IQQNIQYKKC	LKNENCSIMR	MNRNRCQQCR
**170**	**180**	**190**	**200**	**210**	**220**	**230**	**240**
FKKCLSVGMS	RDAVRFGRIP	KREKQRMLIE	MQSAMKTMMN	SQFSGHLQND	TLVEHHEQTA	LPAQEQLRPK	PQLEQENIKS
**250**	**260**	**270**	**280**	**290**	**300**	**310**	**320**
SSPPSSDFAK	EEVIGMVTRA	HKDTFMYNQE	QQENSAESMQ	PQRGERIPKN	MEQYNLNHDH	CGNGLSSHFP	CSESQQHLNG
**330**	**340**	**350**	**360**	**370**	**380**	**390**	**400**
QFKGRNIMHY	PNGHAICIAN	GHCMNFSNAY	TQRVCDRVPI	DGFSQNENKN	SYLCNTGGRM	HLVCPMSKSP	YVDPHKSGHE
**410**	**420**	**430**	**440**	**450**			
IWEEFSMSFT	PAVKEVVEFA	KRIPGFRDLS	QHDQVNLLKA	GTFEIDLE			

Cmpd.	No. of Cmpds.	m/z meas.	Δ m/z [ppm]	z	Rt [min]	Score	P	Range	Sequence	Modification
1576	1	1011.7169	-72.08	3	49.6	10.7	1	96-124	K.FSGMVLLCKVCGDVASGFHYGVHACEGCK.G	Oxidation: 4
80	1	877.8666	-76.26	2	30.7	12.1	2	183-196	R.EKQRMLIEMQSAMK.T	Oxidation: 9, 13

Detailed Protein Report

Protein 146: **PREDICTED: UPF0505 protein C16orf62 isoform X2 [Homo sapiens]**

**Accession:**	gi|530408446	**Score:**	38.1
**Database:**	refseq_human(refseq_human_20140103.fasta)	**MW [kDa]:**	107.9
**Database Date:**	2015-11-30	**pI:**	8.9
**Modification(s):**	Carbamidomethyl, Oxidation	**Sequence Coverage [%]:**	5.7
		**No. of unique Peptides:**	2

10	20	30	40	50	60	70	80
MAPAGVVTRA	VRSGELPLTS	MASAENEACA	VRSVACPSQA	WRLQKVLCGR	CGAASCPSQT	WRPRGAGSGG	VRMGSRADGP
90	100	110	120	130	140	150	160
RTSGHVTGKM	AVFPWHSRNR	NYKAEFASCR	LEAVPLEFGD	YHPLKPITVT	ESKTKKVNRK	GSTSSTSSSS	SSSVVDPLSS
170	180	190	200	210	220	230	240
VLDGTDPLSM	FAATADPAAL	AAAMDSSRRK	RDRDDNSVVG	SDFEPWTNKR	GEILARYTTT	EKLSINLFMG	SEKGKAGTAT
250	260	270	280	290	300	310	320
LAMSEKVRTR	LEELDDFEEG	SQKELLNLTQ	QDYVNRIEEL	NQSLKDAWAS	DQKVKALKIV	IQCSKLLSDT	SVIQFYPSKF
330	340	350	360	370	380	390	400
VLITDILDTF	GKLVYERIFS	MCVDSRSVLP	DHFSPENAND	TAKETCLNWF	FKIASIRELI	PRFYVEASIL	KCNKFLSKTG
410	420	430	440	450	460	470	480
ISECLPRLTC	MIRGIGDPLV	SVYARAYLCR	ALLTEMMERC	KKLGNNALLL	NSVMSAFRAE	FIATRSMDFI	GMIKECDESG
490	500	510	520	530	540	550	560
FPKHLLFRSL	GLNLALADPP	ESDRLQILNE	AWKVITKLKN	PQDYINCAEV	WVEYTCKHFT	KREVNTVLAD	VIKHMTPDRA
570	580	590	600	610	620	630	640
FEDSYPQLQL	IIKKVIAHFH	DFSVLFSVEK	FLPFLDMFQK	ESVRVEVCKC	IMDAFIKHQQ	EPTKDPVILN	ALLHVCKTMH
650	660	670	680	690	700	710	720
DSVNALTLED	EKRMLSYLIN	GFIKMVSFGR	DFEQQLSFYV	ESRSMFCNLE	PVLVQLIHAC	VAYCFITIPS	LAGIFTRLNL
730	740	750	760	770	780	790	800
YLHSGQVALA	NQCLSQADAF	FKAAISLVPE	VPKMINIDGK	MRPSESFLLE	FLCNFFSTLL	IVPDHPEHGV	LFLVRELLNV
810	820	830	840	850	860	870	880
IQDYTWEDNS	DEKIRIYTCV	LHLLSAMSQE	TYLYHIDKVD	SNDSLYGGDS	KFLAENNKLC	ETVMAQILEH	LKTLAKDEAL
890	900	910	920	930	940	950	960
KRQSSLGLSF	FNSILAHGDL	RNNKLNQLSV	NLWHLAQRHG	CADTRTMVKT	LEYIKKQSKQ	PDMTHLTELA	LRLPLQTRT

Cmpd.	No. of Cmpds.	m/z meas.	Δ m/z [ppm]	z	Rt [min]	Score	P	Range	Sequence	Modification
1659	1	1273.7346	60.72	2	50.7	10.5	2	554-574	K.HMTPDRAFEDSYPQLQLIIKK.V	Oxidation: 2
1481	2	949.1566	5.84	3	48.3	12.1	2	902-925	R.NNKLNQLSVNLWHLAQRHGCADTR.T	Carbamidomethyl: 20

Detailed Protein Report

Protein 147: PREDICTED: uncharacterized protein LOC102723569 isoform X1 [Homo sapiens]

Accession: gi|578835246
Database: refseq_human(refseq_human_20140103.fasta)
Database Date: 2015-11-30
Modification(s): Carbamidomethyl, Oxidation

Score: 38.0
MW [kDa]: 28.7
pI: 10.2
Sequence Coverage [%]: 15.3
No. of unique Peptides: 1

Quantitation

MD:MU **Median:** 1.33 **CV:** 0.00 % **No. of Peptides:** 1

10	20	30	40	50	60	70	80
MCMASNAVCS	APETLAGYRI	IKMLPGSLKR	EDTRKQVKII	LLLFCIQQAS	YYGTAVMGQA	YLPELGTQWG	PPWLSLTGVT
**90**	**100**	**110**	**120**	**130**	**140**	**150**	**160**
TGCRQTPVLS	ILLARPAESS	ESRVGYWMPR	REEGAREVSP	KVLQGPQQAW	TEEVWTAWPC	PEPLCIITAV	PNLHPTLSLP
**170**	**180**	**190**	**200**	**210**	**220**	**230**	**240**
IPGPGALLLC	SRDSWSSLRQ	HTVLLWRRPS	PTHAGMLQPV	PEQVVAGGCW	QRRDNGPAPG	GKCWLQGTES	SPCHSPCYPL
**250**	**260**	**270**					
GNLKGISKNS	GSATLLSFLW	NM					

Cmpd.	No. of Cmpds.	m/z meas.	Δ m/z [ppm]	z	Rt [min]	Score	P	Range	Sequence	Modification	Ratios
2778	2	958.9830	67.11	2	66.1	25.9	0	2-19	M.CMASNAVCSAPETLAGYR.I	Carbamidomethyl: 1; Oxidation: 2	MD:MU 1.33

Detailed Protein Report

Protein 148: **PREDICTED: vacuolar protein sorting-associated protein 8 homolog isoform X4 [Homo sapiens]**

**Accession:**	gi|530374200	**Score:**	37.9
**Database:**	refseq_human(refseq_human_20140103.fasta)	**MW [kDa]:**	117.1
**Database Date:**	2015-11-30	**pI:**	5.3
**Modification(s):**	Oxidation	**Sequence Coverage [%]:**	3.8
		**No. of unique Peptides:**	1

10	20	30	40	50	60	70	80
MENEPDHENV	EQSLCAKTSE	EELNKSFNLE	ASLSKFSYID	MDKELEFKND	LIDDKEFDIP	QVDTPPTLES	ILNETDDEDE
**90**	**100**	**110**	**120**	**130**	**140**	**150**	**160**
SFILEDPTLL	NIDTIDSHSY	DTSSVASSDS	GDRTNLKRKK	KLPDSFSLHG	SVMRHSLLKG	ISAQIVSAAD	KVDAGLPTAI
**170**	**180**	**190**	**200**	**210**	**220**	**230**	**240**
AVSSLIAVGT	SHGLALIFGK	DQNQALRLCL	GSTSVGGQYG	AISALSINND	CSRLLCGFAK	GQITMWDLAS	GKLLRSITDA
**250**	**260**	**270**	**280**	**290**	**300**	**310**	**320**
HPPGTAILHI	KFTDDPTLAI	CNDSGGSVFE	LTFKRVMGVR	TCESRCLFSG	SKGEVCCIEP	LHSKPELKDH	PITQFSLLAM
**330**	**340**	**350**	**360**	**370**	**380**	**390**	**400**
ASLTKILVIG	LKPSLKVWMT	FPYGRMDPSS	VPLLAWHFVA	VQNYVNPMLA	FCRGDVVHFL	LVKRDESGAI	HVTKQKHLHL
**410**	**420**	**430**	**440**	**450**	**460**	**470**	**480**
YYDLINFTWI	NSRTVVLLDS	VEKLHVIDRQ	TQEELETVEI	SEVQLVYNSS	HFKSLATGGN	VSQALALVGE	KACYQSISSY
**490**	**500**	**510**	**520**	**530**	**540**	**550**	**560**
GGQIFYLGTK	SVYVMMLRSW	RERVDHLLKQ	DCLTEALALA	WSFHEGKAKA	VVGLSGDASK	RKAIVADRMV	EILFHYADRA
**570**	**580**	**590**	**600**	**610**	**620**	**630**	**640**
LKKCPDQGKI	QVMEQHFQDM	VPVIVDYCLL	LQRKDLLFSQ	MYDKLSENSV	AKGVFLECLE	PYILSDKLVG	ITPQVMKDLI
**650**	**660**	**670**	**680**	**690**	**700**	**710**	**720**
VHFQDKKLME	NVEALIVHMD	ITSLDIQQVV	LMCWENRLYD	AMIYVYNRGM	NEFISPMEKL	FRVIAPPLNA	GKTLTDEQVV
**730**	**740**	**750**	**760**	**770**	**780**	**790**	**800**
MGNKLLVYIS	CCLAGRAYPL	GDIPEDLVPL	VKNQVFEFLI	RLHSAEASPE	EEIYPYIRTL	LHFDTREFLN	VLALTFEDFK
**810**	**820**	**830**	**840**	**850**	**860**	**870**	**880**
NDKQAVEYQQ	RIVDILLKVM	VENSDFTPSQ	VGCLFTFLAR	QLAKPDNTLF	VNRTLFDQVL	EFLCSPDDDS	RHSERQQVLL
**890**	**900**	**910**	**920**	**930**	**940**	**950**	**960**
ELLQAGGIVQ	FEESRLIRMA	EKAEFYQICE	FMYEREHQYD	KIIDCYLRDP	LREEEVFNYI	HNILSIPGHS	AEEKQSVWQK
**970**	**980**	**990**	**1000**	**1010**	**1020**	**1030**	**1040**
AMDHIEELVS	LKPCKAAELV	ATHFSGHIET	VIKKLQNQVL	LFKFLRSLLD	PSCRFLMQWG	CLKDNTKGHV	FFVRP

Cmpd.	No. of Cmpds.	m/z meas.	Δ m/z [ppm]	z	Rt [min]	Score	P	Range	Sequence	Modification
1784	1	840.7706	-147.85	2	52.3	10.4	1	36-48	K.FSYIDMDKELEFK.N	Oxidation: 6

Detailed Protein Report

Protein 149: mucin-2 precursor [Homo sapiens]

**Accession:**	gi|557129045	**Score:**	37.8
**Database:**	refseq_human(refseq_human_20140103.fasta)	**MW [kDa]:**	539.8
**Database Date:**	2015-11-30	**pI:**	5.4
**Modification(s):**	Carbamidomethyl, Oxidation	**Sequence Coverage [%]:**	1.1
		**No. of unique Peptides:**	2

Quantitation

MD:MU	**Median:** 1.17	**CV:** 0.00 %	**No. of Peptides:** 1

Detailed Protein Report

10	20	30	40	50	60	70	80
MGLPLARLAA	VCLALSLAGG	SELQTEGRTR	NHGHNVCSTW	GNFHYKTFDG	DVFRFPGPCD	YNFASDCRGS	YKEFAVHLKR
90	100	110	120	130	140	150	160
GPGQAEAPAG	VESILLTIKD	DTIYLTRHLA	VLNGAVVSTP	HYSPGLLIEK	SDAYTKVYSR	AGLTLMWNRE	DALMLELDTK
170	180	190	200	210	220	230	240
FRNHTCGLCG	DYNGLQSYSE	FLSDGVLFSP	LEFGNMQKIN	QPDVVCEDPE	EEVAPASCSE	HRAECERLLT	AEAFADCQDL
250	260	270	280	290	300	310	320
VPLEPYLRAC	QQDRCRCPGG	DTCVCSTVAE	FSRQCSHAGG	RPGNWRTATL	CPKTCPGNLV	YLESGSPCMD	TCSHLEVSSL
330	340	350	360	370	380	390	400
CEEHRMDGCF	CPEGTVYDDI	GDSGCVPVSQ	CHCRLHGHLY	TPGQEITNDC	EQCVCNAGRW	VCKDLPCPGT	CALEGGSHIT
410	420	430	440	450	460	470	480
TFDGKTYTFH	GDCYYVLAKG	DHNDSYALLG	ELAPCGSTDK	QTCLKTVVLL	ADKKKNVVVF	KSDGSVLLNE	LQVNLPHVTA
490	500	510	520	530	540	550	560
SFSVFRPSSY	HIMVSMAIGV	RLQVQLAPVM	QLFVTLDQAS	QGQVQGLCGN	FNGLEGDDFK	TASGLVEATG	AGFANTWKAQ
570	580	590	600	610	620	630	640
STCHDKLDWL	DDPCSLNIES	ANYAEHWCSL	LKKTETPFGR	CHSAVDPAEY	YKRCKYDTCN	CQNNEDCLCA	ALSSYARACT
650	660	670	680	690	700	710	720
AKGVMLWGWR	EHVCNKDVGS	CPNSQVFLYN	LTTCQQTCRS	LSEADSHCLE	GFAPVDGCGC	PDHTFLDEKG	RCVPLAKCSC
730	740	750	760	770	780	790	800
YHRGLYLEAG	DVVVRQEERC	VCRDGRLHCR	QIRLIGQSCT	APKIHMDCSN	LTALATSKPR	ALSCQTLAAG	YYHTECVSGC
810	820	830	840	850	860	870	880
VCPDGLMDDG	RGGCVVEKEC	PCVHNNDLYS	SGAKIKVDCN	TCTCKRGRWV	CTQAVCHGTC	SIYGSGHYIT	FDGKYYDFDG
890	900	910	920	930	940	950	960
HCSYVAVQDY	CGQNSSLGSF	SIITENVPCG	TTGVTCSKAI	KIFMGRTELK	LEDKHRVVIQ	RDEGHHVAYT	TREVGQYLVV
970	980	990	1000	1010	1020	1030	1040
ESSTGIIVIW	DKRTTVFIKL	APSYKGTVCG	LCGNFDHRSN	NDFTTRDHMV	VSSELDFGNS	WKEAPTCPDV	STNPEPCSLN
1050	1060	1070	1080	1090	1100	1110	1120
PHRRSWAEKQ	CSILKSSVFS	ICHSKVDPKP	FYEACVHDSC	SCDTGGDCEC	FCSAVASYAQ	ECTKEGACVF	WRTPDLCPIF
1130	1140	1150	1160	1170	1180	1190	1200
CDYYNPPHEC	EWHYEPCGNR	SFETCRTING	IHSNISVSYL	EGCYPRCPKD	RPIYEEDLKK	CVTADKCGCY	VEDTHYPPGA
1210	1220	1230	1240	1250	1260	1270	1280
SVPTEETCKS	CVCTNSSQVV	CRPEEGKILN	QTQDGAFCYW	EICGPNGTVE	KHFNICSITT	RPSTLTTFTT	ITLPTTPTTF
1290	1300	1310	1320	1330	1340	1350	1360
TTTTTTTTPT	SSTVLSTTPK	LCCLWSDWIN	EDHPSSGSDD	GDRETFDGVC	GAPEDIECRS	VKDPHLSLEQ	LGQKVQCDVS
1370	1380	1390	1400	1410	1420	1430	1440
VGFICKNEDQ	FGNGPFGLCY	DYKIRVNCCW	PMDKCITTPS	PPTTTPSPPP	TSTTTLPPTT	TPSPPTTTTT	TPPPTTTPSP
1450	1460	1470	1480	1490	1500	1510	1520
PITTTTTPPP	TTTPSPPIST	TTTPPPTTTP	SPPTTTPSPP	TTTPSPPTTT	TTTPPPTTTP	SPPTTTPITP	PASTTTLPPT
1530	1540	1550	1560	1570	1580	1590	1600
TTPSPPTTTT	TTPPPTTTPS	PPTTTPITPP	TSTTTLPPTT	TPSPPPTTTT	TPPPTTTPSP	PTTTTPSPPT	ITTTTPPPTT
1610	1620	1630	1640	1650	1660	1670	1680
TPSPPTTTTT	TPPPTTTPSP	PTTTPITPPT	STTTLPPTTT	PSPPPTTTTT	PPPTTTPSPP	TTTTPSPPIT	TTTTPPPTTT
1690	1700	1710	1720	1730	1740	1750	1760
PSSPITTTPS	PPTTTMTTPS	PTTTPSSPIT	TTTTPSSTTT	PSPPPTTMTT	PSPTTTPSPP	TTTMTTLPPT	TTSSPLTTTP
1770	1780	1790	1800	1810	1820	1830	1840
LPPSITPPTF	SPFSTTTPTT	PCVPLCNWTG	WLDSGKPNFH	KPGGDTELIG	DVCGPGWAAN	ISCRATMYPD	VPIGQLGQTV
1850	1860	1870	1880	1890	1900	1910	1920
VCDVSVGLIC	KNEDQKPGGV	IPMAFCLNYE	INVQCCECVT	QPTTMTTTTT	ENPTPPTTTP	ITTTTTVTPT	PTPTGTQTPT
1930	1940	1950	1960	1970	1980	1990	2000
TTPITTTTTV	TPTPTPTGTQ	TPTTTPITTT	TTVTPTPTPT	GTQTPTTTPI	TTTTTVTPTP	TPTGTQTPTT	TPITTTTTVT
2010	2020	2030	2040	2050	2060	2070	2080
PTPTPTGTQT	PTTTPITTTT	TVTPTPTPTG	TQTPTTTPIT	TTTTVTPTPT	PTGTQTPTTT	PITTTTTVTP	TPTPTGTQTP
2090	2100	2110	2120	2130	2140	2150	2160
TTTPITTTTT	VTPTPTPTGT	QTPTTTPITT	TTTVTPTPTP	TGTQTPTTTP	ITTTTTVTPT	PTPTGTQTPT	TTPITTTTTV
2170	2180	2190	2200	2210	2220	2230	2240
TPTPTPTGTQ	TPTTTPITTT	TTVTPTPTPT	GTQTPTTTPI	TTTTTVTPTP	TPTGTQTPTT	TPITTTTTVT	PTPTPTGTQT
2250	2260	2270	2280	2290	2300	2310	2320
PTTTPITTTT	TVTPTPTPTG	TQTPTTTPIT	TTTTVTPTPT	PTGTQTPTTT	PITTTTTVTP	TPTPTGTQTP	TTTPITTTTT
2330	2340	2350	2360	2370	2380	2390	2400

Detailed Protein Report

Cmpd.	No. of Cmpds.	m/z meas.	Δ m/z [ppm]	z	Rt [min]	Score	P	Range	Sequence	Modification	Ratios
2678	1	857.6944	26.58	3	64.7	10.2	0	616-637	K. YDTCNCQNNEDCLCAALSSYAR. A	Carbamidomethyl: 4, 12	
2127	2	937.3845	-87.61	2	56.5	16.1	0	764-780	K.IHMDCSNLTALATSKPR.A	Oxidation: 3	MD:MU 1.17

Detailed Protein Report

Protein 150: PREDICTED: uncharacterized protein C22orf15 isoform X12 [Homo sapiens]

**Accession:**	gi|578836949	**Score:**	37.8
**Database:**	refseq_human(refseq_human_20140103.fasta)	**MW [kDa]:**	22.9
**Database Date:**	2015-11-30	**pI:**	11.0
**Modification(s):**	Carbamidomethyl	**Sequence Coverage [%]:**	14.6
		**No. of unique Peptides:**	2

10	20	30	40	50	60	70	80
MFIKVMFGAG	CSVLVNTSCR	LVNLTAHLRQ	KAGLPPDATI	ALLAEDGNLV	SLEEDLKEGA	SRAQTMGNSL	LKERAIYVLV
**90**	**100**	**110**	**120**	**130**	**140**	**150**	**160**
RIIKGEDMAS	TRYESLLENL	DDHYPELAEE	LRRLSGLSSV	GHNWRKRMGT	RRGRHEQSPT	SRPRKVQTDN	NPAGLLLQNL
**170**	**180**	**190**	**200**	**210**			
ELLRSCISKR	GKVRQDLRPP	GAPKLWVQRM	GCRGRDKKGR	GGRYP			

Cmpd.	No. of Cmpds.	m/z meas.	Δ m/z [ppm]	z	Rt [min]	Score	P	Range	Sequence	Modification
1676	1	1045.0153	-7.55	2	50.7	23.1	1	2-20	M.FIKVMFGAGCSVLVNTSCR.L	Carbamidomethyl: 18
2567	1	709.8660	-0.61	2	62.5	14.7	2	185-195	K.LWVQRMGCRGR.D	Carbamidomethyl: 8

Detailed Protein Report

Protein 151: zinc finger CCCH domain-containing protein 13 [Homo sapiens]

**Accession:**	gi|116008442	**Score:**	37.8
**Database:**	refseq_human(refseq_human_20140103.fasta)	**MW [kDa]:**	184.8
**Database Date:**	2015-11-30	**pI:**	10.0
**Modification(s):**	Oxidation	**Sequence Coverage [%]:**	1.9
		**No. of unique Peptides:**	3

Alias proteins:

Accession	Name	Description
gi|530402208	refseq_human (refseq_human_20140103.fasta)	PREDICTED: zinc finger CCCH domain-containing protein 13 isoform X12 [Homo sapiens]

```
        10         20         30         40         50         60         70         80
MSKIRRKVTV ENTKTISDST SRRPSVFERL GPSTGSTAET QCRNWLKTGN CLYGNTCRFV HGPSPRGKGY SSNYRRSPER
        90        100        110        120        130        140        150        160
PTGDLRERMK NKRQDVDTEP QKRNTEESSS PVRKESSRGR HREKEDIKIT KERTPESEEE NVEWETNRDD SDNGDINYDY
       170        180        190        200        210        220        230        240
VHELSLEMKR QKIQRELMKL EQENMEKREE IIIKKEVSPE VVRSKLSPSP SLRKSSKSPK RKSSPKSSSA SKKDRKTSAV
       250        260        270        280        290        300        310        320
SSPLLDQQRN SKTNQSKKKG PRTPSPPPPI PEDIALGKKY KEKYKVKDRI EEKTRDGKDR GRDFERQREK RDKPRSTSPA
       330        340        350        360        370        380        390        400
GQHHSPISSR HHSSSSQSGS SIQRHSPSPR RKRTPSPSYQ RTLTPPLRRS ASPYPSHSLS SPQRKQSPPR HRSPMREKGR
       410        420        430        440        450        460        470        480
HDHERTSQSH DRRHERREDT RGKRDREKDS REEREYEQDQ SSSRDHRDDR EPRDGRDRRD ARDTRDREL RDSRDMRDSR
       490        500        510        520        530        540        550        560
EMRDYSRDTK ESRDPRDSRS TRDAHDYRDR EGRDTHRKED TYPEESRSYG RNHLREESSR TEIRNESRNE SRSEIRNDRM
       570        580        590        600        610        620        630        640
GRSRGRVPEL PEKGSRGSRG SQIDSHSSNS NYHDSWETRS SYPERDRYPE RDNRDQARDS SFERRHGERD RRDNRERDQR
       650        660        670        680        690        700        710        720
PSSPIRHQGR NDELERDERR EERRVDRVDD RRDERARERD RERERDRERE RERERERDRE REKERELERE RARERERERE
       730        740        750        760        770        780        790        800
KERDRERDRD RDHDRERERE RERDREKERE REREERERER ERERERERER ERERERARER DKERERQRDW EDKDKGRDDR
       810        820        830        840        850        860        870        880
REKREEIRED RNPRDGHDER KSKKRYRNEG SPSPRQSPKR RREHSPDSDA YNSGDDKNEK HRLLSQVVRP QESRSLSPSH
       890        900        910        920        930        940        950        960
LTEDRQGRWK EEDRKPERKE SSRRYEEQEL KEKVSSVDKQ REQTEILESS RMRAQDIIGH HQSEDRETSD RAHDENKKKA
       970        980        990       1000       1010       1020       1030       1040
KIQKKPIKKK KEDDVGIERG NIETTSEDGQ VFSPKKGQKK KSIEKKRKKS KGDSDISDEE AAQQSKKKRG PRTPPITTKE
      1050       1060       1070       1080       1090       1100       1110       1120
ELVEMCNGKN GILEDSQKKE DTAFSDWSDE DVPDRTEVTE AEHTATATTP GSTPSPLSSL LPPPPPVATA TATTVPATLA
      1130       1140       1150       1160       1170       1180       1190       1200
ATTAAAATSF STSAITISTS ATPTNTTNNT FANEDSHRKC HRTRVEKVET PHVTIEDAQH RKPMDQKRSS SLGSNRSNRS
      1210       1220       1230       1240       1250       1260       1270       1280
HTSGRLRSPS NDSAHRSGDD QSGRKRVLHS GSRDREKTKS LEITGERKSR IDQLKRGEPS RSTSSDRQDS RSHSSRRSSP
      1290       1300       1310       1320       1330       1340       1350       1360
ESDRQVHSRS GSFDSRDRLQ ERDRYEHDRE RERERRDTRQ REWDRDADKD WPRNRDRDRL RERERERERD KRRDLDRERE
      1370       1380       1390       1400       1410       1420       1430       1440
RLISDSVERD RDRDRDRTFE SSQIESVKRC EAKLEGEHER DLESTSRDSL ALDKERMDKD LGSVQGFEET NKSERTESLE
      1450       1460       1470       1480       1490       1500       1510       1520
AGDDESKLDD AHSLGSGAGE GYEPISDDEL DEILAGDAEK REDQQDEEKM PDPLDVIDVD WSGLMPKHPK EPREPGAALL
      1530       1540       1550       1560       1570
KFTPGAVMLR VGISKKLAGS ELFAKVKETC QRLLEKPKGS FILL
```

Cmpd.	No. of Cmpds.	m/z meas.	Δ m/z [ppm]	z	Rt [min]	Score	P	Range	Sequence	Modification
760	1	856.2336	-297.82	1	38.9	11.7	0	206-213	K.LSPSPSLR.K	
1864	1	606.2622	-6.93	2	53.3	12.3	2	475-483	R.DMRDSREMR.D	Oxidation: 8

Detailed Protein Report

Cmpd.	No. of Cmpds.	m/z meas.	Δ m/z [ppm]	z	Rt [min]	Score	P	Range	Sequence	Modification
1287	1	795.9839	117.71	2	45.8	13.8	2	528-540	R.SYGRNHLREESSR.T	

Detailed Protein Report

Protein 152: **PREDICTED: 1-acyl-sn-glycerol-3-phosphate acyltransferase gamma isoform X1 [Homo sapiens]**

**Accession:**	gi|530419317	**Score:**	37.7
**Database:**	refseq_human(refseq_human_20140103.fasta)	**MW [kDa]:**	52.2
**Database Date:**	2015-11-30	**pI:**	9.9
**Modification(s):**	Carbamidomethyl, Oxidation	**Sequence Coverage [%]:**	6.0
		**No. of unique Peptides:**	2

10	20	30	40	50	60	70	80
MCITALSVAP	KGWVPAACRQ	GAYHRGTSMP	WGSLSSHGCA	LGAVPGPSQK	HRAGPAGPDA	MGFCVCEPLE	KDGCPQRGAV
**90**	**100**	**110**	**120**	**130**	**140**	**150**	**160**
HPLLSSAMGL	LAFLKTQFVL	HLLVGFVFVV	SGLVINFVQL	CTLALWPVSK	QLYRRLNCRL	AYSLWSQLVM	LLEWWSCTEC
**170**	**180**	**190**	**200**	**210**	**220**	**230**	**240**
TLFTDQATVE	RFGKEHAVII	LNHNFEIDFL	CGWTMCERFG	VLGSSKVLAK	KELLYVPLIG	WTWYFLEIVF	CKRKWEEDRD
**250**	**260**	**270**	**280**	**290**	**300**	**310**	**320**
TVVEGLRRLS	DYPEYMWFLL	YCEGTRFTET	KHRVSMEVAA	AKGLPVLKYH	LLPRTKGFTT	AVKCLRGTVA	AVYDVTLNFR
**330**	**340**	**350**	**360**	**370**	**380**	**390**	**400**
GNKNPSLLGI	LYGKKYEADM	CVRRFPLEDI	PLDEKEAAQW	LHKLYQEKDA	LQEIYNQKGM	FPGEQFKPAR	RPWTLLNFLS
**410**	**420**	**430**	**440**	**450**	**460**	**470**	
WATILLSPLF	SFVLGVFASG	SPLLILTFLG	FVGAASFGVR	RLIGVTEIEK	GSSYGNQEFK	KKE	

Cmpd.	No. of Cmpds.	m/z meas.	Δ m/z [ppm]	z	Rt [min]	Score	P	Range	Sequence	Modification
2765	2	702.0547	47.21	3	65.9	20.2	1	1-19	-.MCITALSVAPKGWVPAACR.Q	Carbamidomethyl: 2, 18; Oxidation: 1
2735	1	905.4845	9.33	1	65.5	17.5	0	274-282	R.VSMEVAAAK.G	

BRUKER

Detailed Protein Report

Protein 153: PREDICTED: probable ATP-dependent RNA helicase DDX60-like isoform X6 [Homo sapiens]

Accession: gi|578809622
Database: refseq_human(refseq_human_20140103.fasta)
Database Date: 2015-11-30
Modification(s): Carbamidomethyl, Oxidation

Score: 37.7
MW [kDa]: 193.4
pI: 9.2
Sequence Coverage [%]: 2.2
No. of unique Peptides: 3

Quantitation

MD:MU **Median:** 1.16 **CV:** 0.00 % **No. of Peptides:** 1

10	20	30	40	50	60	70	80
MGSKDHAVFF	REMTQLILNE	MPKAGYSSIL	NDFVESNFFV	IDGDSLLVTC	LGVKSFKWGQ	NLHFFYLVEC	YLVDLLSNGG
90	100	110	120	130	140	150	160
QFTIVFFKDA	EYAYFDFPEL	LSLRTALILH	LQHNTNIDVQ	TEFSGCLSQD	WKLFLEQHYP	YFLIVSEEGL	SDLQTYLFNF
170	180	190	200	210	220	230	240
LIIHSWGMKV	NVVLSSGHES	DTLRFYAYTM	ESTDRNQTFS	KENETVIQSA	YKSLIQHLEE	IRVLVLATHF	EHLKWNDMME
250	260	270	280	290	300	310	320
EAYQTLFLLQ	HLWSEGSDIQ	RVLCVTSCSL	SLRMYHRVLV	HSNCLSLQEV	EDFCRLRCLC	VAFQLHLPLS	QRACSRVITC
330	340	350	360	370	380	390	400
SWIRNSDSFL	KMNKWCEYFI	LSNLNVFGCW	NLNLNHVSDL	YDEQLLKNIA	FYYEFESTQE	PHLNLGDSIR	RDYEDLWNVV
410	420	430	440	450	460	470	480
SHLVKEFNVG	KSFPLRTTRR	HFLRQEKSVI	QEISLEKMPS	VGFIPMTSAV	IDEFVGDMMK	DLPILKSDDP	VVPSLFKQKT
490	500	510	520	530	540	550	560
SDELLHWHAQ	RLLSDDYDRI	KCHVDEQSRD	PHVLDFLKKI	QDYQQFYGKS	LESISTKVIV	TQTTRPKEDS	SGASGEILQN
570	580	590	600	610	620	630	640
TKPHQITKKS	KKKSFLKEDQ	NKAQQNDDLL	FSIEEEMKNN	LHSGIRKLED	YLTSCASNSV	KFGVEMLGLI	ACFKAWKKHC
650	660	670	680	690	700	710	720
RGEGKISKDL	SIAVQMMKRI	HSLLERYPEI	LEAEHHQYIA	KCLKYLGFND	LANSLDPTLI	GDDKNKKKYS	IDIGPARFQL
730	740	750	760	770	780	790	800
QYMGHYLIRD	ERKDRDPRVQ	DFIPNAWQQE	LLDVVDKNES	AVIVAPTSSG	KTYASYYCME	KVLRESDVGV	VVYVAPAKSL
810	820	830	840	850	860	870	880
VGQVAATVEN	RFTKTLPAGR	TLCGAFTRDY	CHNVLNCQVL	ITVPECFEIL	LLAPHRQKWV	ERIRYVIFDE	VHYLGREVGA
890	900	910	920	930	940	950	960
KFWELLLVII	RCPFLVLSAT	INNPNLLTKW	LQSVKQYWKQ	ADKIMEEKCI	SEKQADKCLN	FLQDHSYKNQ	SYEVRLVLCG
970	980	990	1000	1010	1020	1030	1040
ERYNDLEKHI	CSVKHDDVYF	DHFHPCAALT	TDIIEKYGFP	PDLTLTPQES	IQLYDTMAQV	WETWPRAQEL	CPEEFILFKN
1050	1060	1070	1080	1090	1100	1110	1120
KIVIKKLDAR	KYEENLKAEL	TNWIKNGQVK	KVKRVLKNLS	PDSLSSSKDM	VKMFPLLVEK	LRQMDKLPAI	FFLFKNDDVG
1130	1140	1150	1160	1170	1180	1190	1200
KRAGSVCTFL	EKTETKSHPH	TECHSYVFAI	DEVLEKVRKT	QKRISTKKNP	KKAEKLERKK	VYRAEYINFL	ENLKILEISE
1210	1220	1230	1240	1250	1260	1270	1280
DCTYADVKAL	HTEITRNKDS	TLERVLPRVR	FTRHGKELKA	LAQRGIGYHH	SSMYFKEKEF	VEILFVKGLI	RVVTATETLA
1290	1300	1310	1320	1330	1340	1350	1360
LGIHMPCKSV	VFAQDSVYLD	ALNYRQMSGR	AGRRGQDLLG	NVYFFDIPLP	KIKRLLASSV	PELRGQFPLS	ITLVLRLMLL
1370	1380	1390	1400	1410	1420	1430	1440
ASKGDDPEDA	KAKDYLNKKG	NPKKFAGLAS	YLHGHEPSNL	VFVNFLKRGL	FHNLCKPAWK	GSQQFSQDVM	EKLVLVLANL
1450	1460	1470	1480	1490	1500	1510	1520
FGRKYIPAKF	QNANLSFSQS	KVILAELPED	FKAALYEYNL	AVMKDFASFL	LIASKSVNMK	KEHQLPLSRI	KFTGKECEDS
1530	1540	1550	1560	1570	1580	1590	1600
QLVSHLMSCK	KGRVAISPFV	CLSGNTDNDL	LRPETINQVI	LRTVGVSGTQ	APLLWPWKLD	NRGRRMPLNA	YVLNFYKHNC
1610	1620	1630	1640	1650	1660	1670	1680
LTRLDQKNGM	RMGQLLKCLK	DFAFNIQAIS	DSLSELCENK	RDNVVLAFKQ	LSQTFYEKLQ	EMQIQMSQNH	LE

Cmpd.	No. of Cmpds.	m/z meas.	Δ m/z	z	Rt [min]	Score	P	Range	Sequence	Modification	Ratios

Detailed Protein Report

			[ppm]								
1058	1	586.1731	-222.69	2	42.8	12.2	2	639-648	K.HCRGEGKISK.D	Carbamidomethyl: 2	
225	1	641.7812	-36.28	2	32.5	12.3	1	924-933	K.IMEEKCISEK.Q	Carbamidomethyl: 6; Oxidation: 2	
1639	1	926.8312	-90.99	2	50.2	13.2	1	1516-1531	K.ECEDSQLVSHLMSCKK.G	Oxidation: 12	MD:MU 1.16

Detailed Protein Report

Protein 154: **PREDICTED: cytoplasmic tyrosine-protein kinase BMX isoform X1 [Homo sapiens]**

**Accession:**	gi|578837945	**Score:**	37.5
**Database:**	refseq_human(refseq_human_20140103.fasta)	**MW [kDa]:**	77.9
**Database Date:**	2015-11-30	**pI:**	9.3
**Modification(s):**	Oxidation	**Sequence Coverage [%]:**	5.3
		**No. of unique Peptides:**	1

10	20	30	40	50	60	70	80
MDTKSILEEL	LLKRSQQKKK	MSPNNYKERL	FVLTKTNLSY	YEYDKMKRGS	RKGSIEIKKI	RCVEKVNLEE	QTPVERQYPF
90	100	110	120	130	140	150	160
QIVYKDGLLY	VYASNEESRS	QWLKALQKEI	RGNPHLLVKY	HSGFFVDGKF	LCCQQSCKAA	PGCTLWEAYA	NLHTAVNEEK
170	180	190	200	210	220	230	240
HRVPTFPDRV	LKIPRAVPVL	KMDAPSSSTT	LAQYDNESKK	NYGSQPPSSS	TSLAQYDSNS	KKIYGSQPNF	NMQYIPREDF
250	260	270	280	290	300	310	320
PDWWQVRKLK	SSSSEDVASS	NQKERNVNHT	TSKISWEFPE	SSSSEEEENL	DDYDWFAGNI	SRSQSEQLLR	QKGKEGAFMV
330	340	350	360	370	380	390	400
RNSSQVGMYT	VSLFSKAVND	KKGTVKHYHV	HTNAENKLYL	AENYCFDSIP	KLIHYHQHNS	AGMITRLRHP	VSTKANKVPD
410	420	430	440	450	460	470	480
SVSLGNGIWE	LKREEITLLK	ELGSGQFGVV	QLGKWKGQYD	VAVKMIKEGS	MSEDEFFQEA	QTMMKLSHPK	LVKFYGVCSK
490	500	510	520	530	540	550	560
EYPIYIVTEY	ISNGCLLNYL	RSHGKGLEPS	QLLEMCYDVC	EGMAFLESHQ	FIHRDLAARN	CLVDRDLCVK	VSDFGMTRYV
570	580	590	600	610	620	630	640
LDDQYVSSVG	TKFPVKWSAP	EVFHYFKYSS	KSDVWAFGIL	MWEVFSLGKQ	PYDLYDNSQV	VLKVSQGHRL	YRPHLASDTI
650	660	670	680				
YQIMYSCWHE	LPEKRPTFQQ	LLSSIEPLRE	KDKH				

Cmpd.	No. of Cmpds.	m/z meas.	Δ m/z [ppm]	z	Rt [min]	Score	P	Range	Sequence	Modification
2282	1	701.1125	131.58	3	58.4	20.9	0	592-609	K.SDVWAFGILMWEVFSLGK.Q	Oxidation: 10

Detailed Protein Report

Protein 155: **PREDICTED: tudor domain-containing protein 7 isoform X2 [Homo sapiens]**

Accession: gi|578817240
Database: refseq_human(refseq_human_20140103.fasta)
Database Date: 2015-11-30
Modification(s): Oxidation

Score: 37.4
MW [kDa]: 74.0
pI: 6.0
Sequence Coverage [%]: 6.6
No. of unique Peptides: 2

Quantitation

MD:MU **Median:** 0.75 **CV:** 0.00 % **No. of Peptides:** 1

10	20	30	40	50	60	70	80
MAFGYLSFHI	FTKSYIKKTL	IKEFYNSLNT	GLIFARYVGK	DYSAAQELME	DEMKEYYSKN	PKITPVQAVN	VGQLLAVNAE
**90**	**100**	**110**	**120**	**130**	**140**	**150**	**160**
EDAWLRAQVI	STEENKIKVC	YVDYGFSENV	EKSKAYKLNP	KFCSLSFQAT	KCKLAGLEVL	SDDPDLVKVV	ESLTCGKIFA
**170**	**180**	**190**	**200**	**210**	**220**	**230**	**240**
VEILDKADIP	LVVLYDTSGE	DDININATCL	KAICDKSLEV	HLQVDAMYTN	VKVTNICSDG	TLYCQVPCKG	LNKLSDLLRK
**250**	**260**	**270**	**280**	**290**	**300**	**310**	**320**
IEDYFHCKHM	TSECFVSLPF	CGKICLFHCK	GKWLRVEITN	VHSSRALDVQ	FLDSGTVTSV	KVSELREIPP	RFLQEMIAIP
**330**	**340**	**350**	**360**	**370**	**380**	**390**	**400**
PQAIKCCLAD	LPQSIGMWTP	DAVLWLRDSV	LNCSDCSIKV	TKVDETRGIA	HVYLFTPKNF	PDPHRSINRQ	ITNADLWKHQ
**410**	**420**	**430**	**440**	**450**	**460**	**470**	**480**
KDVFLSAISS	GADSPNSKNG	NMPMSGNTGE	NFRKNLTDVI	KKSMVDHTSA	FSTEELPPPV	HLSKPGEHMD	VYVPVACHPG
**490**	**500**	**510**	**520**	**530**	**540**	**550**	**560**
YFVIQPWQEI	HKLEVLMEEM	ILYYSVSEER	HIAVEKDQVY	AAKVENKWHR	VLLKGILTNG	LVSVYELDYG	KHELVNIRKV
**570**	**580**	**590**	**600**	**610**	**620**	**630**	**640**
QPLVDMFRKL	PFQAVTAQLA	GVKCNQWSEE	ASMVFRNHVE	KKPLVALVQT	VIENANPWDR	KVVVYLVDTS	LPDTDTWIHD
**650**	**660**						
FMSEYLIELS	KVN						

Cmpd.	No. of Cmpds.	m/z meas.	Δ m/z [ppm]	z	Rt [min]	Score	P	Range	Sequence	Modification	Ratios
1797	1	851.2615	-133.71	2	52.3	11.4	0	249-263	K.HMTSECFVSLPFCGK.I	Oxidation: 2	MD:MU 0.75
2544	1	885.4222	38.31	2	61.8	14.1	1	419-434	K.NGNMPMSGNTGENFRK.N	Oxidation: 4	

Detailed Protein Report

Protein 156: PREDICTED: utrophin isoform X8 [Homo sapiens]

**Accession:**	gi|578812959	**Score:**	37.4
**Database:**	refseq_human(refseq_human_20140103.fasta)	**MW [kDa]:**	287.1
**Database Date:**	2015-11-30	**pI:**	5.2
**Modification(s):**	Oxidation	**Sequence Coverage [%]:**	0.8
		**No. of unique Peptides:**	1

BRUKER

Detailed Protein Report

10	20	30	40	50	60	70	80
MSLMILPRWR	RPCKKKKTLD	EILENQKPAL	HKLAEETKAL	EKNVHPDVEK	LYKQEFDDVQ	GKWNKLKVLV	SKDLHLLEEI
90	100	110	120	130	140	150	160
ALTLRAFEAD	STVIEKWMDG	VKDFLMKQQA	AQGDDAGLQR	QLDQCSAFVN	EIETIESSLK	NMKEIETNLR	SGPVAGIKTW
170	180	190	200	210	220	230	240
VQTRLGDYQT	QLEKLSKEIA	TQKSRLSESQ	EKAANLKKDL	AEMQEWMTQA	EEEYLERDFE	YKSPEELESA	VEEMKRAKED
250	260	270	280	290	300	310	320
VLQKEVRVKI	LKDNIKLLAA	KVPSGGQELT	SELNVVLENY	QLLCNRIRGK	CHTLEEVWSC	WIELLHYLDL	ETTWLNTLEE
330	340	350	360	370	380	390	400
RMKSTEVLPE	KTDAVNEALE	SLESVLRHPA	DNRTQIRELG	QTLIDGGILD	DIISEKLEAF	NSRYEDLSHL	AESKQISLEK
410	420	430	440	450	460	470	480
QLQVLRETDQ	MLQVLQESLG	ELDKQLTTYL	TDRIDAFQVP	QEAQKIQAEI	SAHELTLEEL	RRNMRSQPLT	SPESRTARGG
490	500	510	520	530	540	550	560
SQMDVLQRKL	REVSTKFQLF	QKPANFEQRM	LDCKRVLDGV	KAELHVLDVK	DVDPDVIQTH	LDKCMKLYKT	LSEVKLEVET
570	580	590	600	610	620	630	640
VIKTGRHIVQ	KQQTDNPKGM	DEQLTSLKVL	YNDLGAQVTE	GKQDLERASQ	LARKMKKEAA	SLSEWLSATE	TELVQKSTSE
650	660	670	680	690	700	710	720
GLLGDLDTEI	SWAKNVLKDL	EKRKADLNTI	TESSAALQNL	IEGSEPILEE	RLCVLNAGWS	RVRTWTEDWC	NTLMNHQNQL
730	740	750	760	770	780	790	800
EIFDGNVAHI	STWLYQAEAL	LDEIEKKPTS	KQEEIVKRLV	SELDDANLQV	ENVRDQALIL	MNARGSSSRE	LVEPKLAELN
810	820	830	840	850	860	870	880
RNFEKVSQHI	KSAKLLIAQE	PLYQCLVTTE	TFETGVPFSD	LEKLENDIEN	MLKFVEKHLE	SSDEDEKMDE	ESAQIEEVLQ
890	900	910	920	930	940	950	960
RGEEMLHQPM	EDNKKEKIRL	QLLLLHTRYN	KIKAIPIQQR	KMGQLASGIR	SSLLPTDYLV	EINKILLCMD	DVELSLNVPE
970	980	990	1000	1010	1020	1030	1040
LNTAIYEDFS	FQEDSLKNIK	DQLDKLGEQI	AVIHEKQPDV	ILEASGPEAI	QIRDTLTQLN	AKWDRINRMY	SDRKGCFDRA
1050	1060	1070	1080	1090	1100	1110	1120
MEEWRQFHCD	LNDLTQWITE	AEELLVDTCA	PGGSLDLEKA	RIHQQELEVG	ISSHQPSFAA	LNRTGDGIVQ	KLSQADGSFL
1130	1140	1150	1160	1170	1180	1190	1200
KEKLAGLNQR	WDAIVAEVKD	RQPRLKGESK	QVMKYRHQLD	EIICWLTKAE	HAMQKRSTTE	LGENLQELRD	LTQEMEVHAE
1210	1220	1230	1240	1250	1260	1270	1280
KLKWLNRTEL	EMLSDKSLSL	PERDKISESL	RTVNMTWNKI	CREVPTTLKE	CIQEPSSVSQ	TRIAAHPNVQ	KVVLVSSASD
1290	1300	1310	1320	1330	1340	1350	1360
IPVQSHRTSE	ISIPADLDKT	ITELADWLVL	IDQMLKSNIV	TVGDVEEINK	TVSRMKITKA	DLEQRHPQLD	YVFTLAQNLK
1370	1380	1390	1400	1410	1420	1430	1440
NKASSSDMRT	AITEKLERVK	NQWDGTQHGV	ELRQQQLEDM	IIDSLQWDDH	REETEELMRK	YEARLYILQQ	ARRDPLTKQI
1450	1460	1470	1480	1490	1500	1510	1520
SDNQILLQEL	GPGDGIVMAF	DNVLQKLLEE	YGSDDTRNVK	ETTEYLKTSW	INLKQSIADR	QNALEAEWRT	VQASRRDLEN
1530	1540	1550	1560	1570	1580	1590	1600
FLKWIQEAET	TVNVLVDASH	RENALQDSIL	ARELKQQMQD	IQAEIDAHND	IFKSIDGNRQ	KMVKALGNSE	EATMLQHRLD
1610	1620	1630	1640	1650	1660	1670	1680
DMNQRWNDLK	AKSASIRAHL	EASAEKWNRL	LMSLEELIKW	LNMKDEELKK	QMPIGGDVPA	LQLQYDHCKA	LRRELKEKEY
1690	1700	1710	1720	1730	1740	1750	1760
SVLNAVDQAR	VFLADQPIEA	PEEPRRNLQS	KTELTPEERA	QKIAKAMRKQ	SSEVKEKWES	LNAVTSNWQK	QVDKALEKLR
1770	1780	1790	1800	1810	1820	1830	1840
DLQGAMDDLD	ADMKEAESVR	NGWKPVGDLL	IDSLQDHIEK	IMAFREEIAP	INFKVKTVND	LSSQLSPLDL	HPSLKMSRQL
1850	1860	1870	1880	1890	1900	1910	1920
DDLNMRWKLL	QVSVDDRLKQ	LQEAHRDFGP	SSQHFLSTSV	QLPWQRSISH	NKVPYYINHQ	TQTTCWDHPK	MTELFQSLAD
1930	1940	1950	1960	1970	1980	1990	2000
LNNVRFSAYR	TAIKIRRLQK	ALCLDLLELS	TTNEIFKQHK	LNQNDQLLSV	PDVINCLTTT	YDGLEQMHKD	LVNVPLCVDM
2010	2020	2030	2040	2050	2060	2070	2080
CLNWLLNVYD	TGRTGKIRVQ	SLKIGLMSLS	KGLLEEKYRY	LFKEVAGPTE	MCDQRQLGLL	LHDAIQIPRQ	LGEVAAFGGS
2090	2100	2110	2120	2130	2140	2150	2160
NIEPSVRSCF	QQNNNKPEIS	VKEFIDWMHL	EPQSMVWLPV	LHRVAAAETA	KHQAKCNICK	ECPIVGFRYR	SLKHFNYDVC
2170	2180	2190	2200	2210	2220	2230	2240
QSCFFSGRTA	KGHKLHYPMV	EYCIPTTSGE	DVRDFTKVLK	NKFRSKKYFA	KHPRLGYLPV	QTVLEGDNLE	TPITLISMWP
2250	2260	2270	2280	2290	2300	2310	2320
EHYDPSQSPQ	LFHDDTHSRI	EQYATRLAQM	ERTNGSFLTD	SSSTTGSVED	EHALIQQYCQ	TLGGESPVSQ	PQSPAQILKS
2330	2340	2350	2360	2370	2380	2390	2400

Detailed Protein Report

Cmpd.	No. of Cmpds.	m/z meas.	Δ m/z [ppm]	z	Rt [min]	Score	P	Range	Sequence	Modification
1249	1	764.3778	23.73	2	45.3	11.3	2	1023-1033	K.WDRINRMYSDR.K	Oxidation: 7

Detailed Protein Report

Protein 157: peptidyl-prolyl cis-trans isomerase A [Homo sapiens]

Accession: gi|10863927
Database: refseq_human(refseq_human_20140103.fasta)
Database Date: 2015-11-30

Score: 37.2
MW [kDa]: 18.0
pI: 9.0
Sequence Coverage [%]: 27.3
No. of unique Peptides: 2

10	20	30	40	50	60	70	80
MVNPTVFFDI	AVDGEPLGRV	SFELFADKVP	KTAENFRALS	TGEKGFGYKG	SCFHRIIPGF	MCQGGDFTRH	NGTGGKSIYG
**90**	**100**	**110**	**120**	**130**	**140**	**150**	**160**
EKFEDENFIL	KHTGPGILSM	ANAGPNTNGS	QFFICTAKTE	WLDGKHVVFG	KVKEGMNIVE	AMERFGSRNG	KTSKKITIAD
**170**							
CGQLE							

Cmpd.	No. of Cmpds.	m/z meas.	Δ m/z [ppm]	z	Rt [min]	Score	P	Range	Sequence	Modification
2768	1	973.5119	7.60	2	65.0	13.8	0	2-19	M.VNPTVFFDIAVDGEPLGR.V	
2048	2	753.7914	-137.80	2	55.7	13.1	2	152-165	K.TSKKITIADCGQLE.-	

Detailed Protein Report

Protein 158: **polycystic kidney disease and receptor for egg jelly-related protein precursor [Homo sapiens]**

**Accession:**	gi|5174633	**Score:**	37.0
**Database:**	refseq_human(refseq_human_20140103.fasta)	**MW [kDa]:**	255.3
**Database Date:**	2015-11-30	**pI:**	10.0
**Modification(s):**	Carbamidomethyl, Oxidation	**Sequence Coverage [%]:**	1.5
		**No. of unique Peptides:**	3

Detailed Protein Report

10	20	30	40	50	60	70	80
MRPGPALLLL	GVGLSLSVGR	LPLPPVPRGA	QAAVSGAPGG	LLRGAPGLGV	RGGRALLSLR	PSAVRAGGAV	LSGRGSLCFP
90	100	110	120	130	140	150	160
HGGTGRRWYC	LDLRVLLSAQ	RLPWPAAPAL	ALVDLQLSAR	GGRLSLTWSV	RLPRSPGRLA	WAFRLRLLGP	GAARPASPAA
170	180	190	200	210	220	230	240
RVSPRSAAPG	PRPQQGFVAR	TECPTDGPAR	VMLQAVNSSS	HRAVESSVSC	QINACVIQRV	RINTDQKGAP	VRLSMQAEAT
250	260	270	280	290	300	310	320
INASVQLDCP	AARAIAQYWQ	VFSVPAVGQA	PDWTQPLDLP	QLEIRNSPLF	IHIPNNSLQW	GVYVFNFTVS	ITTGNPKMPE
330	340	350	360	370	380	390	400
VKDSDAVYVW	IVRSSLQAVM	LGDANITANF	TEQLILDGST	SSDPDADSPL	QGLQFFWYCT	TDPRNYGGDR	IILGSKEVCH
410	420	430	440	450	460	470	480
PEQANLKWPW	ASGPVLTLLP	ETLKGDHVYF	FRMVIRKDSR	TAFSDKRVHV	LQGPKAIAHI	TCIENCERNF	IVSDRFSLFL
490	500	510	520	530	540	550	560
NCTNCASRDF	YKWSILSSSG	GEMLFDWMGE	TVTGRNGAYL	SIKAFAFRHF	LEAEFSISLY	LACWSGVTSV	FRHSFIINHG
570	580	590	600	610	620	630	640
PQIGECKINP	AKGIALITKF	VVQCSNFRDK	HVPLTYKIIV	SDLHSVGEIS	SVKENTLGTI	LYLGPQSTVP	PSFLPVGMLA
650	660	670	680	690	700	710	720
SQYGLKIYAQ	VYDSLGAFSQ	VTLHATAQAP	TDKNSSKTVL	NQLLSFTVGP	SSLLSTLIQK	KDFLPAGYLL	YIVASVLNNM
730	740	750	760	770	780	790	800
KTELPLRDDR	VNLRKHLIDQ	SFLLPVSTLV	EIGQVVMTIT	KLTQKPSEFT	WDAQKRATMR	VWQANQALQE	YQQKDKRFRS
810	820	830	840	850	860	870	880
EQIEIVSTGI	LMSLSNILKM	TSPHQVVKDP	FYVIESLSDT	ILANKVPGNK	TTSMRTPNFN	MYVKKVEKWG	INQLFRNEKH
890	900	910	920	930	940	950	960
CRNCFYPTLN	VSSVPGLSAN	GPISTMFCDF	TNDLFPWLND	QENTSVEVSG	FRMTGVADNG	SVLEITPDVA	EVYLVRKNLT
970	980	990	1000	1010	1020	1030	1040
FAAFNLTVGP	NSEVDGSLKK	TTGGFSFQVD	STVLREVLVH	IVTEVMVLFT	VLVYTGSQIT	PTALVATFLV	PHDIPPFASQ
1050	1060	1070	1080	1090	1100	1110	1120
SALFDPACTV	KKARVVCLPV	SLLQLIAQHS	HSPHCTVSIV	LQAPRFVMKL	NDKLVRISIF	SVQCLDMYGI	QSEWREGYCI
1130	1140	1150	1160	1170	1180	1190	1200
LGEKTSWYEV	HCICKNVVRA	RRQLGTIGLT	GIHLHTHYVM	AKVIVIPNPV	DLRLNIIKSL	HQNPVTLFTV	LFIILLYVGL
1210	1220	1230	1240	1250	1260	1270	1280
AFWALYRDEM	DQHLRGHVIV	LPDNDPYDNL	CYLVTIFTGS	RWGSGTRANV	FVQLRGTVST	SDVHCLSHPH	FTTLYRGSIN
1290	1300	1310	1320	1330	1340	1350	1360
TFLLTTKSDL	GDIHSIRVWH	NNEGRSPSWY	LSRIKVENLF	SRHIWLFICQ	KWLSVDTTLD	RTFHVTHPDE	RLTRKDFFFI
1370	1380	1390	1400	1410	1420	1430	1440
DVSSNLRKNH	MWFSIFASVV	AKTFNRLQRL	SCCLAMLLSS	LLCNIMFFNL	NRQEQTESRE	RKYMRSMMIG	IESVLITIPV
1450	1460	1470	1480	1490	1500	1510	1520
QLLITFLFTC	SQRKPQADLK	EVSPQKHPLM	SEASEHWEEY	LRKWHAYETA	KVHPREVAKP	ASKGKPRLPK	ASPKATSKPK
1530	1540	1550	1560	1570	1580	1590	1600
HRHRKAQIKT	PETLGPNTNS	NNNIEDDQDV	HSEQHPSQKD	LQQLKKKPRI	VLPWWCVYVA	WFLVFATSSI	SSFFIVFYGL
1610	1620	1630	1640	1650	1660	1670	1680
TYGYDKSIEW	LFASFCSFCQ	SVLLVQPSKI	ILLSGFRTNK	PKYCKNLSWS	TKYKYTEIRL	DGMRMHPEEM	QRIHDQIVRI
1690	1700	1710	1720	1730	1740	1750	1760
RGTRMYQPLT	EDEIRIFKRK	KRIKRRALLF	LSYILTHFIF	LALLLILIVL	LRHTDCFYYN	QFIRDRFSMD	LATVTKLEDI
1770	1780	1790	1800	1810	1820	1830	1840
YRWLNSVLLP	LLHNDLNPTF	LPESSSKILG	LPLMRQVRAK	SSEKMCLPAE	KFVQNSIRRE	IHCHPKYGID	PEDTKNYSGF
1850	1860	1870	1880	1890	1900	1910	1920
WNEVDKQAID	ESTNGFTYKP	QGTQWLYYSY	GLLHTYGSGG	YALYFFPEQQ	RFNSTLRLKE	LQESNWLDEK	TWAVVLELTT
1930	1940	1950	1960	1970	1980	1990	2000
FNPDINLFCS	ISVIFEVSQL	GVVNTSISLH	SFSLADFDRK	ASAEIYLYVA	ILIFFLAYVV	DEGCIIMQER	ASYVRSVYNL
2010	2020	2030	2040	2050	2060	2070	2080
LNFALKCIFT	VLIVLFLRKH	FLATGIIRFY	LSNPEDFIPF	HAVSQVDHIM	RIILGFLLFL	TILKTLRYSR	FFYDVRLAQR
2090	2100	2110	2120	2130	2140	2150	2160
AIQAALPGIC	HMAFVVSVYF	FVYMAFGYLV	FGQHEWNYSN	LIHSTQTVFS	YCVSAFQNTE	FSNNRILGVL	FLSSFMLVMI
2170	2180	2190	2200	2210	2220	2230	2240

Detailed Protein Report

Cmpd.	No. of Cmpds.	m/z meas.	Δ m/z [ppm]	z	Rt [min]	Score	P	Range	Sequence	Modification
2763	1	741.8475	25.09	2	64.9	12.5	0	1125-1135	K.TSWYEVHCICK.N	Carbamidomethyl: 8, 10
2608	1	648.3616	-63.29	2	62.6	12.8	1	1788-1798	K.ILGLPLMRQVR.A	
2593	3	648.3609	90.56	2	62.4	11.8	1	1801-1811	K.SSEKMCLPAEK.F	Carbamidomethyl: 6; Oxidation: 5

Detailed Protein Report

Protein 159: sacsin isoform 1 [Homo sapiens]

**Accession:**	gi|163659918	**Score:**	36.9
**Database:**	refseq_human(refseq_human_20140103.fasta)	**MW [kDa]:**	520.8
**Database Date:**	2015-11-30	**pI:**	6.7
**Modification(s):**	Carbamidomethyl	**Sequence Coverage [%]:**	1.1
		**No. of unique Peptides:**	3

Detailed Protein Report

10	20	30	40	50	60	70	80
METKENRWVP	VTVLPGCVGC	RTVAALASWT	VRDVKERIFA	ETGFPVSEQR	LWRGGRELSD	WIKIGDLTSK	NCHLFVNLQS
90	100	110	120	130	140	150	160
KGLKGGGRFG	QTTPPLVDFL	KDILRRYPEG	GQILKELIQN	AEDAGATEVK	FLYDETQYGT	ETLWSKDMAP	YQGPALYVYN
170	180	190	200	210	220	230	240
NAVFTPEDWH	GIQEIARSRK	KDDPLKVGRF	GIGFNSVYHI	TDVPCIFSGD	QIGMLDPHQT	LFGPHESGQC	WNLKDDSKEI
250	260	270	280	290	300	310	320
SELSDQFAPF	VGIFGSTKET	FINGNFPGTF	FRFPLRLQPS	QLSSNLYNKQ	KVLELFESFR	ADADTVLLFL	KSVQDVSLYV
330	340	350	360	370	380	390	400
READGTEKLV	FRVTSSESKA	LKHERPNSIK	ILGTAISNYC	KKTPSNNITC	VTYHVNIVLE	EESTKDAQKT	SWLVCNSVGG
410	420	430	440	450	460	470	480
RGISSKLDSL	ADELKFVPII	GIAMPLSSRD	DEAKGATSDF	SGKAFCFLPL	PPGEESSTGL	PVHISGFFGL	TDNRRSIKWR
490	500	510	520	530	540	550	560
ELDQWRDPAA	LWNEFLVMNV	VPKAYATLIL	DSIKRLEMEK	SSDFPLSVDV	IYKLWPEASK	VKVHWQPVLE	PLFSELLQNA
570	580	590	600	610	620	630	640
VIYSISCDWV	RLEQVYFSEL	DENLEYTKTV	LNYLQSSGKQ	IAKVPGNVDA	AVQLTAASGT	TPVRKVTPAW	VRQVLRKCAH
650	660	670	680	690	700	710	720
LGCAEEKLHL	LEFVLSDQAY	SELLGLELLP	LQNGNFVPFS	SSVSDQDVIY	ITSAEYPRSL	FPSLEGRFIL	DNLKPHLVAA
730	740	750	760	770	780	790	800
LKEAAQTRGR	PCTQLQLLNP	ERFARLIKEV	MNTFWPGREL	IVQWYPFDEN	RNHPSVSWLK	MVWKNLYIHF	SEDLTLFDEM
810	820	830	840	850	860	870	880
PLIPRTILEE	GQTCVELIRL	RIPSLVILDD	ESEAQLPEFL	ADIVQKLGGF	VLKKLDASIQ	HPLIKKYIHS	PLPSAVLQIM
890	900	910	920	930	940	950	960
EKMPLQKLCN	QITSLLPTHK	DALRKFLASL	TDSSEKEKRI	IQELAIFKRI	NHSSDQGISS	YTKLKGCKVL	HHTAKLPADL
970	980	990	1000	1010	1020	1030	1040
RLSISVIDSS	DEATIRLANM	LKIEQLKTTS	CLKLVLKDIE	NAFYSHEEVT	QLMLWVLENL	SSLKNENPNV	LEWLTPLKFI
1050	1060	1070	1080	1090	1100	1110	1120
QISQEQMVSA	GELFDPDIEV	LKDLFCNEEG	TYFPPSVFTS	PDILHSLRQI	GLKNEASLKE	KDVVQVAKKI	EALQVGACPD
1130	1140	1150	1160	1170	1180	1190	1200
QDVLLKKAKT	LLLVLNKNHT	LLQSSEGKMT	LKKIKWVPAC	KERPPNYPGS	LVWKGDLCNL	CAPPDMCDVG	HAILIGSSLP
1210	1220	1230	1240	1250	1260	1270	1280
LVESIHVNLE	KALGIFTKPS	LSAVLKHFKI	VVDWYSSKTF	SDEDYYQFQH	ILLEIYGFMH	DHLNEGKDSF	RALKFPWVWT
1290	1300	1310	1320	1330	1340	1350	1360
GKKFCPLAQA	VIKPIHDLDL	QPYLHNVPKT	MAKFHQLFKV	CGSIEELTSD	HISMVIQKIY	LKSDQDLSEQ	ESKQNLHLML
1370	1380	1390	1400	1410	1420	1430	1440
NIIRWLYSNQ	IPASPNTPVP	IHHSKNPSKL	IMKPIHECCY	CDIKVDDLND	LLEDSVEPII	LVHEDIPMKT	AEWLKVPCLS
1450	1460	1470	1480	1490	1500	1510	1520
TRLINPENMG	FEQSGQREPL	TVRIKNILEE	YPSVSDIFKE	LLQNADDANA	TECSFLIDMR	RNMDIRENLL	DPGMAACHGP
1530	1540	1550	1560	1570	1580	1590	1600
ALWSFNNSQF	SDSDFVNITR	LGESLKRGEV	DKVGKFGLGF	NSVYHITDIP	IIMSREFMIM	FDPNINHISK	HIKDKSNPGI
1610	1620	1630	1640	1650	1660	1670	1680
KINWSKQQKR	LRKFPNQFKP	FIDVFGCQLP	LTVEAPYSYN	GTLFRLSFRT	QQEAKVSEVS	STCYNTADIY	SLVDEFSLCG
1690	1700	1710	1720	1730	1740	1750	1760
HRLIIFTQSV	KSMYLKYLKI	EETNPSLAQD	TVIIKKKSCS	SKALNTPVLS	VLKEAAKLMK	TCSSSNKKLP	SDEPKSSCIL
1770	1780	1790	1800	1810	1820	1830	1840
QITVEEFHHV	FRRIADLQSP	LFRGPDDDPA	ALFEMAKSGQ	SKKPSDELSQ	KTVECTTWLL	CTCMDTGEAL	KFSLSESGRR
1850	1860	1870	1880	1890	1900	1910	1920
LGLVPCGAVG	VQLSEIQDQK	WTVKPHIGEV	FCYLPLRIKT	GLPVHINGCF	AVTSNRKEIW	KTDTKGRWNT	TFMRHVIVKA
1930	1940	1950	1960	1970	1980	1990	2000
YLQVLSVLRD	LATSGELMDY	TYYAVWPDPD	LVHDDFSVIC	QGFYEDIAHG	KGKELTKVFS	DGSTWVSMKN	VRFLDDSILK
2010	2020	2030	2040	2050	2060	2070	2080
RRDVGSAAFK	IFLKYLKKTG	SKNLCAVELP	SSVKLGFEEA	GCKQILLENT	FSEKQFFSEV	FFPNIQEIEA	ELRDPLMIFV
2090	2100	2110	2120	2130	2140	2150	2160
LNEKVDEFSG	VLRVTPCIPC	SLEGHPLVLP	SRLIHPEGRV	AKLFDIKDGR	FPYGSTQDYL	NPIILIKLVQ	LGMAKDDILW
2170	2180	2190	2200	2210	2220	2230	2240

Detailed Protein Report

Cmpd.	No. of Cmpds.	m/z meas.	Δ m/z [ppm]	z	Rt [min]	Score	P	Range	Sequence	Modification
2078	1	1029.6855	128.38	2	56.1	10.5	0	1861-1877	K.WTVKPHIGEVFCYLPLR.I	
71	1	845.3572	-104.63	2	30.3	12.1	1	2019-2034	K.TGSKNLCAVELPSSVK.L	Carbamidomethyl: 7
2171	1	1022.8841	-140.49	2	57.3	14.3	2	2478-2495	K.DTTVKYCHADIPREVAVK.L	

Detailed Protein Report

Protein 160: PREDICTED: mucin-5AC [Homo sapiens]

**Accession:**	gi|578821001	**Score:**	36.8
**Database:**	refseq_human(refseq_human_20140103.fasta)	**MW [kDa]:**	520.3
**Database Date:**	2015-11-30	**pI:**	6.6
**Modification(s):**	Carbamidomethyl, Oxidation	**Sequence Coverage [%]:**	0.9
		**No. of unique Peptides:**	3

Detailed Protein Report

10	20	30	40	50	60	70	80
MSVGRRKLAL	LWALALALAC	TRHTGHAQDG	SSESSYKHHP	ALSPIARGPS	GVPLRGATVF	PSLRTIPVVR	ASNPAHNGRV
90	100	110	120	130	140	150	160
CSTWGSFHYK	TFDGDVFRFP	GLCNYVFSEH	CGAAYEDFNI	QLRRSQESAA	PTLSRVLMKV	DGVVIQLTKG	SVLVNGHPVL
170	180	190	200	210	220	230	240
LPFSQSGVLI	QQSSSYTKVE	ARLGLVLMWN	HDDSLLLELD	TKYANKTCGL	CGDFNGMPVV	SELLSHNTKL	TPMEFGNLQK
250	260	270	280	290	300	310	320
MDDPTDQCQD	PVPEPPRNCS	TGFGICEELL	HGQLFSGCVA	LVDVGSYLEA	CRQDLCFCED	TDLLSCVCHT	LAEYSRQCTH
330	340	350	360	370	380	390	400
AGGLPQDWRG	PDFCPQKCPN	NMQYHECRSP	CADTCSNQEH	SRACEDHCVA	GCFCPEGTVL	DDIGQTGCVP	VSKCACVYNG
410	420	430	440	450	460	470	480
AAYAPGATYS	TDCTNCTCSG	GRWSCQEVPC	PGTCSVLGGA	HFSTFDGKQY	TVHGDCSYVL	TKPCDSSAFT	VLAELRRCGL
490	500	510	520	530	540	550	560
TDSETCLKSV	TLSLDGAQTV	VVIKASGEVF	LNQIYTQLPI	SAANVTIFRP	STFFIIAQTS	LGLQLNLQLV	PTMQLFMQLA
570	580	590	600	610	620	630	640
PKLRGQTCGL	CGNFNSIQAD	DFRTLSGVVE	ATAAAFFNTF	KTQAACPNIR	NSFEDPCSLS	VENEKYAQHW	CSQLTDADGP
650	660	670	680	690	700	710	720
FGRCHAAVKP	GTYYSNCMFD	TCNCERSEDC	LCAALSSYVH	ACAAKGVQLG	GWRDGVCTKP	MTTCPKSMTY	HYHVSTCQPT
730	740	750	760	770	780	790	800
CRSLSEGDIT	CSVGFIPVDG	CICPKGTFLD	DTGKCVQASN	CPCYHRGSMI	PNGESVHDSG	AICTCTHGKL	SCIGGQAPAP
810	820	830	840	850	860	870	880
VCAAPMVFFD	CRNATPGDTG	AGCQKSCHTL	DMTCYSPQCV	PGCVCPDGLV	ADGEGGCITA	EDCPCVHNEA	SYRAGQTIRV
890	900	910	920	930	940	950	960
GCNTCTCDSR	MWRCTDDPCL	ATCAVYGDGH	YLTFDGQSYS	FNGDCEYTLV	QNHCGGKDST	QDSFRVVTEN	VPCGTTGTTC
970	980	990	1000	1010	1020	1030	1040
SKAIKIFLGG	FELKLSHGKV	EVIGTDESQE	VPYTIRQMGI	YLVVDTDIGL	VLLWDKKTSI	FINLSPEFKG	RVCGLCGNFD
1050	1060	1070	1080	1090	1100	1110	1120
DIAVNDFATR	SRSVVGDVLE	FGNSWKLSPS	CPDALAPKDP	CTANPFRKSW	AQKQCSILHG	PTFAACHAHV	EPARYYEACV
1130	1140	1150	1160	1170	1180	1190	1200
NDACACDSGG	DCECFCTAVA	AYAQACHEVG	LCVSWRTPSI	CPLFCDYYNP	EGQCEWHYQP	CGVPCLRTCR	NPRGDCLRDV
1210	1220	1230	1240	1250	1260	1270	1280
RGLEGCYPKC	PPEAPIFDED	KMQCVATCPT	PPLPPRCHVH	GKSYRPGAVV	PSDKNCQSCL	CTERGVECTY	KAEACVCTYN
1290	1300	1310	1320	1330	1340	1350	1360
GQRFHPGDVI	YHTTDGTGGC	ISARCGANGT	IERRVYPCSP	TTPVPPTTFS	FSTPPLVVSS	THTPSNGPSS	AHTGPPSSAW
1370	1380	1390	1400	1410	1420	1430	1440
PTTAGTSPRT	RLPTASASLP	PVCGEKCLWS	PWMDVSRPGR	GTDSGDFDTL	ENLRAHGYRV	CESPRSVECR	AEDAPGVPLR
1450	1460	1470	1480	1490	1500	1510	1520
ALGQRVQCSP	DVGLTCRNRE	QASGLCYNYQ	IRVQCCTPLP	CSTSSSPAQT	TPPTTSKTTE	TRASGSSAPS	STPGTVSLST
1530	1540	1550	1560	1570	1580	1590	1600
ARTTPAPGTA	TSVKKTFSTP	SPPPVPATST	SSMSTTAPGT	SVVSSKPTPT	EPSTSSCLQE	LCTWTEWIDG	SYPAPGINGG
1610	1620	1630	1640	1650	1660	1670	1680
DFDTFQNLRD	EGYTFCESPR	SVQCRAESFP	NTPLADLGQD	VICSHTEGLI	CLNKNQLPPI	CYNYEIRIQC	CETVNVCRDI
1690	1700	1710	1720	1730	1740	1750	1760
TRLPKTVATT	RPTPHPTGAQ	TQTTFTTHMP	SASTEQPTAT	SRGGPTATSV	TQGTHTTLVT	RNCHPRCTWT	KWFDVDFPSP
1770	1780	1790	1800	1810	1820	1830	1840
GPHGGDKETY	NNIIRSGEKI	CRRPEEITRL	QCRAKSHPEV	SIEHLGQVVQ	CSREEGLVCR	NQDQQGPFKM	CLNYEVRVLC
1850	1860	1870	1880	1890	1900	1910	1920
CETPRGCHMT	STPGSTSSSP	AQTTPSTTSK	TTETQASGSS	APSSTPGTVS	LSTARTTPAP	GTATSVKKTF	STPSPPPVPA
1930	1940	1950	1960	1970	1980	1990	2000
TSTSSMSTTA	PGTSVVSSKP	TPTEPSTSSC	LQELCTWTEW	IDGSYPAPGI	NGGDFDTFQN	LRDEGYTFCE	SPRSVQCRAE
2010	2020	2030	2040	2050	2060	2070	2080
SFPNTPLADL	GQDVICSHTE	GLICLNKNQL	PPICYNYEIR	IQCCETVNVC	RDITRPPKTV	ATTRPTPHPT	GAQTQTTFTT
2090	2100	2110	2120	2130	2140	2150	2160
HMPSASTEQP	TATSRGGPTA	TSVTQGTHTT	PVTRNCHPRC	TWTTWFDVDF	PSPGPHGGDK	ETYNNIIRSG	EKICRRPEEI
2170	2180	2190	2200	2210	2220	2230	2240

Detailed Protein Report

Cmpd.	No. of Cmpds.	m/z meas.	Δ m/z [ppm]	z	Rt [min]	Score	P	Range	Sequence	Modification
1356	1	863.9173	-123.12	2	46.6	12.1	1	7-22	R.KLALLWALALALACTR.H	
2649	1	734.8562	20.51	2	63.2	12.1	0	317-329	R.QCTHAGGLPQDWR.G	
2548	1	1044.5297	54.57	2	61.8	12.6	1	2203-2218	K.MCLNYEVRVLCCETPK.G	Carbamidomethyl: 2, 11, 12; Oxidation: 1

Detailed Protein Report

Protein 161: podocan isoform 2 [Homo sapiens]

**Accession:**	gi|312283621	**Score:**	36.7
**Database:**	refseq_human(refseq_human_20140103.fasta)	**MW [kDa]:**	72.1
**Database Date:**	2015-11-30	**pI:**	6.3
		**Sequence Coverage [%]:**	6.2
		**No. of unique Peptides:**	3

Alias proteins:

Accession	Name	Description
gi|312283625	refseq_huma (refseq_human_20140103.fasta)	podocan isoform 2 [Homo sapiens]

10	20	30	40	50	60	70	80
MEGEEAEQPA	WFRQPWRPGA	SDSAPPAGTM	AQSRVLLLLL	LLPPQLHLGP	VLAVRAPGFG	RSGGHSLSPE	ENEFAEEEPV
**90**	**100**	**110**	**120**	**130**	**140**	**150**	**160**
LVLSPEEPGP	GPAAVSCPRD	CACSQEGVVD	CGGIDLREFP	GDLPEHTNHL	SLQNNQLEKI	YPEELSRLHR	LETLNLQNNR
**170**	**180**	**190**	**200**	**210**	**220**	**230**	**240**
LTSRGLPEKA	FEHLTNLNYL	YLANNKLTLA	PRFLPNALIS	VDFAANYLTK	IYGLTFGQKP	NLRSVYLHNN	KLADAGLPDN
**250**	**260**	**270**	**280**	**290**	**300**	**310**	**320**
MFNGSSNVEV	LILSSNFLRH	VPKHLPPALY	KLHLKNNKLE	KIPPGAFSEL	SSLRELYLQN	NYLTDEGLDN	ETFWKLSSLE
**330**	**340**	**350**	**360**	**370**	**380**	**390**	**400**
YLDLSSNNLS	RVPAGLPRSL	VLLHLEKNAI	RSVDANVLTP	IRSLEYLLLH	SNQLREQGIH	PLAFQGLKRL	HTVHLYNNAL
**410**	**420**	**430**	**440**	**450**	**460**	**470**	**480**
ERVPSGLPRR	VRTLMILHNQ	ITGIGREDFA	TTYFLEELNL	SYNRITSPQV	HRDAFRKLRL	LRSLDLSGNR	LHTLPPGLPR
**490**	**500**	**510**	**520**	**530**	**540**	**550**	**560**
NVHVLKVKRN	ELAALARGAL	VGMAQLRELY	LTSNRLRSRA	LGPRAWVDLA	HLQLLDIAGN	QLTEIPEGLP	ESLEYLYLQN
**570**	**580**	**590**	**600**	**610**	**620**	**630**	**640**
NKISAVPANA	FDSTPNLKGI	FLRFNKLAVG	SVVDSAFRRL	KHLQVLDIEG	NLEFGDISKD	RGRLGKEKEE	EEEEEEEEEE
**650**							
TR							

Cmpd.	No. of Cmpds.	m/z meas.	Δ m/z [ppm]	z	Rt [min]	Score	P	Range	Sequence	Modification
127	1	724.8357	13.78	2	31.3	12.7	0	2-13	M.EGEEAEQPAWFR.Q	
1952	1	753.7987	-167.47	2	54.5	11.4	0	211-223	K.IYGLTFGQKPNLR.S	
16	1	819.8519	-141.56	2	29.5	12.6	1	348-362	K.NAIRSVDANVLTPIR.S	

Detailed Protein Report

Protein 162: myeloperoxidase precursor [Homo sapiens]

Accession: gi|4557759
Database: refseq_human(refseq_human_20140103.fasta)
Database Date: 2015-11-30
Modification(s): Carbamidomethyl

Score: 36.7
MW [kDa]: 83.8
pI: 10.1
Sequence Coverage [%]: 3.0
No. of unique Peptides: 2

10	20	30	40	50	60	70	80
MGVPFFSSLR	CMVDLGPCWA	GGLTAEMKLL	LALAGLLAIL	ATPQPSEGAA	PAVLGEVDTS	LVLSSMEEAK	QLVDKAYKER
**90**	**100**	**110**	**120**	**130**	**140**	**150**	**160**
RESIKQRLRS	GSASPMELLS	YFKQPVAATR	TAVRAADYLH	VALDLLERKL	RSLWRRPFNV	TDVLTPAQLN	VLSKSSGCAY
**170**	**180**	**190**	**200**	**210**	**220**	**230**	**240**
QDVGVTCPEQ	DKYRTITGMC	NNRRSPTLGA	SNRAFVRWLP	AEYEDGFSLP	YGWTPGVKRN	GFPVALARAV	SNEIVRFPTD
**250**	**260**	**270**	**280**	**290**	**300**	**310**	**320**
QLTPDQERSL	MFMQWGQLLD	HDLDFTPEPA	ARASFVTGVN	CETSCVQQPP	CFPLKIPPND	PRIKNQADCI	PFFRSCPACP
**330**	**340**	**350**	**360**	**370**	**380**	**390**	**400**
GSNITIRNQI	NALTSFVDAS	MVYGSEEPLA	RNLRNMSNQL	GLLAVNQRFQ	DNGRALLPFD	NLHDDPCLLT	NRSARIPCFL
**410**	**420**	**430**	**440**	**450**	**460**	**470**	**480**
AGDTRSSEMP	ELTSMHTLLL	REHNRLATEL	KSLNPRWDGE	RLYQEARKIV	GAMVQIITYR	DYLPLVLGPT	AMRKYLPTYR
**490**	**500**	**510**	**520**	**530**	**540**	**550**	**560**
SYNDSVDPRI	ANVFTNAFRY	GHTLIQPFMF	RLDNRYQPME	PNPRVPLSRV	FFASWRVVLE	GGIDPILRGL	MATPAKLNRQ
**570**	**580**	**590**	**600**	**610**	**620**	**630**	**640**
NQIAVDEIRE	RLFEQVMRIG	LDLPALNMQR	SRDHGLPGYN	AWRRFCGLPQ	PETVGQLGTV	LRNLKLARKL	MEQYGTPNNI
**650**	**660**	**670**	**680**	**690**	**700**	**710**	**720**
DIWMGGVSEP	LKRKGRVGPL	LACIIGTQFR	KLRDGDRFWW	ENEGVFSMQQ	RQALAQISLP	RIICDNTGIT	TVSKNNIFMS
**730**	**740**	**750**					
NSYPRDFVNC	STLPALNLAS	WREAS					

Cmpd.	No. of Cmpds.	m/z meas.	Δ m/z [ppm]	z	Rt [min]	Score	P	Range	Sequence	Modification
873	1	575.3008	19.33	2	40.5	19.5	0	396-405	R.IPCFLAGDTR.S	Carbamidomethyl: 3
1741	1	640.7565	-196.31	2	51.7	17.2	0	537-548	R.VVLEGGIDPILR.G	

Detailed Protein Report

Protein 163: PREDICTED: guanine nucleotide exchange factor VAV3 isoform X4 [Homo sapiens]

Accession: gi|530361574
Database: refseq_human(refseq_human_20140103.fasta)
Database Date: 2015-11-30
Modification(s): Carbamidomethyl, Oxidation

Score: 36.7
MW [kDa]: 72.1
pI: 6.9
Sequence Coverage [%]: 4.8
No. of unique Peptides: 2

Quantitation

MD:MU **Median:** 1.43 **CV:** 0.00 % **No. of Peptides:** 1

10	20	30	40	50	60	70	80
MEPWKQCAQW	LIHCKVLPTN	HRVTWDSAQV	FDLAQTLRDG	VLLCQLLNNL	RAHSINLKEI	NLRPQMSQFL	CLKNIRTFLT
**90**	**100**	**110**	**120**	**130**	**140**	**150**	**160**
ACCETFGMRK	SELFEAFDLF	DVRDFGKVIE	TLSRLSRTPI	ALATGIRPFP	TEESINDEDI	YKGLPDLIDE	TLVEDEEDLY
**170**	**180**	**190**	**200**	**210**	**220**	**230**	**240**
DCVYGEDEGG	EVYEDLMKAE	EAHQPKCPEN	DIRSCCLAEI	KQTEEKYTET	LESIEKYFMA	PLKRFLTAAE	FDSVFINIPE
**250**	**260**	**270**	**280**	**290**	**300**	**310**	**320**
LVKLHRNLMQ	EIHDSIVNKN	DQNLYQVFIN	YKERLVIYGQ	YCSGVESAIS	SLDYISKTKE	DVKLKLEECS	KRANNGKFTL
**330**	**340**	**350**	**360**	**370**	**380**	**390**	**400**
RDLLVVPMQR	VLKYHLLLQE	LVKHTTDPTE	KANLKLALDA	MKDLAQYVNE	VKRDNETLRE	IKQFQLSIEN	LNQPVLLFGR
**410**	**420**	**430**	**440**	**450**	**460**	**470**	**480**
PQGDGEIRIT	TLDKHTKQER	HIFLFDLAVI	VCKRKGDNYE	MKEIIDLQQY	KIANNPTTDK	ENKKWSYGFY	LIHTQGQNGL
**490**	**500**	**510**	**520**	**530**	**540**	**550**	**560**
EFYCKTKDLK	KKWLEQFEMA	LSNIRPDYAD	SNFHDFKMHT	FTRVTSCKVC	QMLLRGTFYQ	GYLCFKCGAR	AHKECLGRVD
**570**	**580**	**590**	**600**	**610**	**620**	**630**	
NCGRVNSGEQ	GTLKLPEKRT	NGLRRTPKQV	DPDVPCLLHF	FISMAPATRS	IVKSQKKNKK	F	

Cmpd.	No. of Cmpds.	m/z meas.	Δ m/z [ppm]	z	Rt [min]	Score	P	Range	Sequence	Modification	Ratios
154	1	968.4666	19.86	2	31.6	10.4	1	74-89	K.NIRTFLTACCETFGMR.K	Carbamidomethyl: 10; Oxidation: 15	MD:MU 1.43
103	1	807.7358	-182.29	2	31.0	26.3	2	551-564	R.AHKECLGRVDNCGR.V	Carbamidomethyl: 12	

Detailed Protein Report

Protein 164: PREDICTED: testis-expressed sequence 2 protein isoform X4 [Homo sapiens]

Accession: gi|530412523
Database: refseq_human(refseq_human_20140103.fasta)
Database Date: 2015-11-30
Modification(s): Carbamidomethyl, Oxidation

Score: 36.5
MW [kDa]: 125.0
pI: 5.6
Sequence Coverage [%]: 3.0
No. of unique Peptides: 2

10	20	30	40	50	60	70	80
MTSLYGRHAE	KTTDMPKPSA	PKVHVQRSVS	RDTIAIHFSA	SGEEEEEEEE	EFREYFEEGL	DDQSIVTGLE	AKEDLYLEPQ
90	100	110	120	130	140	150	160
VGHDPAGPAA	SPVLADGLSV	SQAPAILPVS	KNTVKLLESP	VPAAQVLSTV	PLAVSPGSSS	SGPLASSPSV	SSLSEQKTSS
170	180	190	200	210	220	230	240
SSPLSSPSKS	PILSSSASTS	TLSSAKPFMS	LVKSLSTEVE	PKESPHPARH	RHLMKTLVKS	LSTDTSRQES	DTVSYKPPDS
250	260	270	280	290	300	310	320
KLNLHLFKQF	TQPRNTGGDS	KTAPSSPLTS	PSDTRSFFKV	PEMEAKIEDT	KRRLSEVIYE	PFQLLSKIIG	EESGSHRPKA
330	340	350	360	370	380	390	400
LSSSASELSN	LSSLNGHLES	NNNYSIKEEE	CDSEGDGYGS	DSNIPRSDHP	KSTGEPTREI	ELKSSQGSSL	KDLGLKTSSL
410	420	430	440	450	460	470	480
VLEKCSLSAL	VSKEDEEFCE	LYTEDFDLET	EGESKVDKLS	DIPLKPEVLA	EDGVVLDSED	EVDSAVQHPE	LPVKTLGFFI
490	500	510	520	530	540	550	560
MCVYVYLILP	LPHYVSGLFL	GIGLGFMTAV	CVIWFFTPPS	AHKYHKLHKN	LRHWNTRSLD	IKEPEILKGW	MNEIYNYDPE
570	580	590	600	610	620	630	640
TYHATLTHSV	FVRLEGGTLR	LSKPNKNISR	RASYNEPKPE	VTYISQKIYD	LSDSKIYLVP	KTLARKRIWN	KKYPICIELG
650	660	670	680	690	700	710	720
QQDDFMSKAQ	TDKETSEEKP	PAEGSEDPKK	PPRPQEGTRS	SQRDQILYLF	GRTGREKEEW	FRRFILASKL	KSEIKKSSGV
730	740	750	760	770	780	790	800
SGGKPGLLPA	HSRHNSPSGH	LTHSRSSSKG	SVEEIMSQPK	QKELAGSVRQ	KMLLDYSVYM	GRCVPQESRS	PQRSPLQSAE
810	820	830	840	850	860	870	880
SSPTAGKKLP	EVPPSEEEEQ	EAWVNALLGR	IFWDFLGEKY	WSDLVSKKIQ	MKLSKIKLPY	FMNELTLTEL	DMGVAVPKIL
890	900	910	920	930	940	950	960
QAFKPYVDHQ	GLWIDLEMSY	NGSFLMTLET	KMNLTKLGKE	PLVEALKVGE	IGKEGPRAFC	LADSDEESSS	AGSSEEDDAP
970	980	990	1000	1010	1020	1030	1040
EPSGGDKQLL	PGAEGYVGGH	RTSKIMRFVD	KITKSKYFQK	ATETEFIKKK	IEEVSNTPLL	LTVEVQECRG	TLAVNIPPPP
1050	1060	1070	1080	1090	1100	1110	1120
TDRVWYGFRK	PPHVELKARP	KLGEREVTLV	HVTDWIEKKL	EQEFQKVFVM	PNMDDVYITI	MHSAMDPRST	SCLLKDPPVE
1130							
AADQP							

Cmpd.	No. of Cmpds.	m/z meas.	Δ m/z [ppm]	z	Rt [min]	Score	P	Range	Sequence	Modification
2499	1	1044.5086	15.43	2	61.7	15.0	1	632-648	K.KYPICIELGQQDDFMSK.A	Carbamidomethyl: 5; Oxidation: 15
2481	1	449.6389	-282.20	2	61.0	10.9	0	920-927	K.EPLVEALK.V	

Detailed Protein Report

Protein 165: endothelin-2 preproprotein [Homo sapiens]

**Accession:**	gi|4503463	**Score:**	36.5
**Database:**	refseq_human(refseq_human_20140103.fasta)	**MW [kDa]:**	19.9
**Database Date:**	2015-11-30	**pI:**	12.1
**Modification(s):**	Carbamidomethyl	**Sequence Coverage [%]:**	14.0
		**No. of unique Peptides:**	2

```
        10         20         30         40         50         60         70         80
MVSVPTTWCS VALALLVALH EGKGQAAATL EQPASSSHAQ GTHLRLRRCS CSSWLDKECV YFCHLDIIWV NTPEQTAPYG
        90        100        110        120        130        140        150        160
LGNPPRRRRR SLPRRCQCSS ARDPACATFC LRRPWTEAGA VPSRKSPADV FQTGKTGATT GELLQRLRDI STVKSLFAKR
       170        180
QQEAMREPRS THSRWRKR
```

Cmpd.	No. of Cmpds.	m/z meas.	Δ m/z [ppm]	z	Rt [min]	Score	P	Range	Sequence	Modification
2021	1	682.2548	-70.07	3	55.4	12.1	2	95-112	R.RCQCSSARDPACATFCLR.R	Carbamidomethyl: 16
1575	1	682.3132	15.52	3	49.5	12.7	2	95-112	R.RCQCSSARDPACATFCLR.R	Carbamidomethyl: 2

Detailed Protein Report

Protein 166: centrosome-associated protein 350 [Homo sapiens]

**Accession:**	gi|171184451	**Score:**	36.5
**Database:**	refseq_human(refseq_human_20140103.fasta)	**MW [kDa]:**	350.7
**Database Date:**	2015-11-30	**pI:**	5.9
		**Sequence Coverage [%]:**	1.5
		**No. of unique Peptides:**	1

Quantitation

MD:MU	**Median:** 1.23	**CV:** 0.00 %	**No. of Peptides:** 1

Detailed Protein Report

10	20	30	40	50	60	70	80
MRSSKSKEVP	LPNPRNSQSK	DTVQADITTS	WDALSQTKAA	LRHIENKLEV	APTSTAVCDS	VMDTKKSSTS	ATRKISRKDG
90	100	110	120	130	140	150	160
RYLDDSWVNA	PISKSTKSRK	EKSRSPLRAT	TLESNVKKNN	RVEFREPLVS	YREIHGAPSN	FSSSHLESKH	VYCVDVNEEK
170	180	190	200	210	220	230	240
TESGNWMIGS	REERNIRSCD	FESSQSSVIN	DTVVRFLNDR	PAIDALQNSE	CLIRMGASMR	TEEEMPNRTK	GSENNLKLSV
250	260	270	280	290	300	310	320
NNMAHDTDPK	ALRLTDSSPS	STSTSNSQRL	DILKRRQHDV	KLEKLKERIR	KQWEHSEETN	GRGQKLGHID	HPVMVVNVDN
330	340	350	360	370	380	390	400
SVTAKVRKVA	TAPPAPAYKG	FNPSETKIRT	PDGKVWQEAE	FQNMSRELYR	DLALHFADDI	SIKEKPAEKS	KEKKVVKPVR
410	420	430	440	450	460	470	480
KVQKVAQLSS	TECRTGSSHL	ISTSSWRDGQ	KLVKKILGPA	PRMEPKEQRT	ASSDRGGRER	TAKSGGHIGR	AESDPRLDVL
490	500	510	520	530	540	550	560
HRHLQRNSER	SRSKSRSENN	IKKLASSLPD	NKQEENTALN	KDFLPIEIRG	ILDDLQLDST	AHTAKQDTVE	LQNQKSSAPV
570	580	590	600	610	620	630	640
HAPRSHSPVK	RKPDKITANE	DPPVISKRRH	YDTDEVRQYI	VRQQEERKRK	QNEEKKAQKE	ATEQKNKRLQ	ELYRKQKEAF
650	660	670	680	690	700	710	720
TKVKNVPPSE	PSATRRLQET	YSKLLLEKTL	LEEPSHQHVT	QETQAKPGYQ	PSGESDKENK	VQERPPSASS	SSDMSLSEPP
730	740	750	760	770	780	790	800
QPLARKDLME	STWMQPERLS	PQVHHSQPQP	FAGTAGSLLS	HLLSLEHVGI	LHKDFESILP	TRKNHNMASR	PLTFTPQPYV
810	820	830	840	850	860	870	880
TSPAAYTDAL	LKPSASQYKS	KLDRIEALKA	TAASLSSRIE	SEAKKLAGAS	INYGSAWNTE	YDVQQAPQED	GPWTKAVTPP
890	900	910	920	930	940	950	960
VKDDNEDVFS	ARIQKMLGSC	VSHATFDDDL	PGVGNLSEFK	KLPEMIRPQS	AISSFRVRSP	GPKPEGLLAQ	LCKRQTDSSS
970	980	990	1000	1010	1020	1030	1040
SDMQACSQDK	AKISLGSSID	SVSEGPLLSE	GSLSEEEGDQ	DGQPLLKVAE	ILKEKEFCPG	ERNSYEPIKE	FQKEAEKFLP
1050	1060	1070	1080	1090	1100	1110	1120
LFGHIGGTQS	KGPWEELAKG	SPHSVINIFT	KSYQLYGKGF	EDKLDRGTST	SRPLNATATP	LSGVSYEDDF	VSSPGTGTST
1130	1140	1150	1160	1170	1180	1190	1200
EKKSTLEPHS	TLSPQEDHSN	RKSAYDPSSV	DVTSQHSSGA	QSAASSRSST	SSKGKKGKKE	KTEWLDSFTG	NVQNSLLDEE
1210	1220	1230	1240	1250	1260	1270	1280
KAERGSHQGK	KSGTSSKLSV	KDFEQTLDTD	STLEDLSGHS	VSVSSDKGRS	QKTPTSPLSP	SSQKSLQFDV	AGTSSERSKS
1290	1300	1310	1320	1330	1340	1350	1360
SVMPPTITGF	KPNAPLTDLN	PAASRTTTEN	MAPIPGSKRF	SPAGLHHRMA	AELSYLNAIE	ESVRQLSDVE	RVRGISLAQQ
1370	1380	1390	1400	1410	1420	1430	1440
ESVSLAQIIK	AQQQRHERDL	ALLKLKAEQE	ALESQRQLEE	TRNKAAQVHA	ESLQQVVQSQ	REVTEVLQEA	TCKIAAQQSE
1450	1460	1470	1480	1490	1500	1510	1520
TARLTTDAAR	QICEMAELTR	THISDAVVAS	GAPLAILYDH	QRQHLPDFVK	QLRTRTETDR	KSPSVSLSQS	KEGTLDSKHQ
1530	1540	1550	1560	1570	1580	1590	1600
KYSASYDSYS	ESSGYKNHDR	RSSSGSSRQE	SPSVPSCKEN	EKKLNGEKIE	SSIDEQVQTA	ADDSLRSDSV	PSLPDEKDST
1610	1620	1630	1640	1650	1660	1670	1680
SIATEYSLKF	DESMTEDEIE	EQSFRSLLPS	ESHRRFNMEK	RRGHHDDSDE	EASPEKTTLS	TAKELNMPFS	GGQDSFSKFT
1690	1700	1710	1720	1730	1740	1750	1760
MEMVRQYMKE	EEMRAAHQSS	LLRLREKALK	EKTKAELAWL	EHQKKHLRDK	GEDDKMPPLR	KKQRGLLLRL	QQEKAEIKRL
1770	1780	1790	1800	1810	1820	1830	1840
QEANKAARKE	RQLILKQQEE	IEKIRQTTIK	LQEKLKSAGE	SKLDSHSDDD	TKDNKATSPG	PTDLETRSPS	PISISSSETS
1850	1860	1870	1880	1890	1900	1910	1920
SIMQKLKKMR	SRMDEKFLTK	REQKLMQRRQ	HAEELLEWKR	RLDAEEAEIR	QMEKQALAAW	DKELIKPKTP	KKELEDQRTE
1930	1940	1950	1960	1970	1980	1990	2000
QKEIASEEES	PVPLYSHLNS	ESSIPEELGS	PAVEYVPSES	IGQEQPGSPD	HSILTEEMIC	SQELESSTSP	SKHSLPKSCT
2010	2020	2030	2040	2050	2060	2070	2080
SVSKQESSKG	SHRTGGQCHL	PIKSHQHCYS	WSDESLSMTQ	SETTSDQSDI	EGRIRALKDE	LRKRKSVVNQ	LKKEQKKRQK
2090	2100	2110	2120	2130	2140	2150	2160
ERLKAQEASL	IKQLESYDEF	IKKTEAELSQ	DLETSPTAKP	QIKTLSSASE	KPKIKPLTPL	HRSETAKNWK	SLTESERSRG
2170	2180	2190	2200	2210	2220	2230	2240

Detailed Protein Report

Cmpd.	No. of Cmpds.	m/z meas.	Δ m/z [ppm]	z	Rt [min]	Score	P	Range	Sequence	Modification	Ratios	
316	1	966.9340	-27.85	2	33.3	11.1	2	1795-1812	K.LKSAGESKLDSHSDDDTK.D		MD:MU	1.23

Detailed Protein Report

Protein 167: staphylococcal nuclease domain-containing protein 1 [Homo sapiens]

**Accession:**	gi|77404397	**Score:**	36.4
**Database:**	refseq_human(refseq_human_20140103.fasta)	**MW [kDa]:**	101.9
**Database Date:**	2015-11-30	**pI:**	6.8
**Modification(s):**	Oxidation	**Sequence Coverage [%]:**	4.7
		**No. of unique Peptides:**	1

10	20	30	40	50	60	70	80
MASSAQSGGS	SGGPAVPTVQ	RGIIKMVLSG	CAIIVRGQPR	GGPPPERQIN	LSNIRAGNLA	RRAAATQPDA	KDTPDEPWAF
**90**	**100**	**110**	**120**	**130**	**140**	**150**	**160**
PAREFLRKKL	IGKEVCFTIE	NKTPQGREYG	MIYLGKDTNG	ENIAESLVAE	GLATRREGMR	ANNPEQNRLS	ECEEQAKAAK
**170**	**180**	**190**	**200**	**210**	**220**	**230**	**240**
KGMWSEGNGS	HTIRDLKYTI	ENPRHFVDSH	HQKPVNAIIE	HVRDGSVVRA	LLLPDYYLVT	VMLSGIKCPT	FRREADGSET
**250**	**260**	**270**	**280**	**290**	**300**	**310**	**320**
PEPFAAEAKF	FTESRLLQRD	VQIILESCHN	QNILGTILHP	NGNITELLLK	EGFARCVDWS	IAVYTRGAEK	LRAAERFAKE
**330**	**340**	**350**	**360**	**370**	**380**	**390**	**400**
RRLRIWRDYV	APTANLDQKD	KQFVAKVMQV	LNADAIVVKL	NSGDYKTIHL	SSIRPPRLEG	ENTQDKNKKL	RPLYDIPYMF
**410**	**420**	**430**	**440**	**450**	**460**	**470**	**480**
EAREFLRKKL	IGKKVNVTVD	YIRPASPATE	TVPAFSERTC	ATVTIGGINI	AEALVSKGLA	TVIRYRQDDD	QRSSHYDELL
**490**	**500**	**510**	**520**	**530**	**540**	**550**	**560**
AAEARAIKNG	KGLHSKKEVP	IHRVADISGD	TQKAKQFLPF	LQRAGRSEAV	VEYVFSGSRL	KLYLPKETCL	ITFLLAGIEC
**570**	**580**	**590**	**600**	**610**	**620**	**630**	**640**
PRGARNLPGL	VQEGEPFSEE	ATLFTKELVL	QREVEVEVES	MDKAGNFIGW	LHIDGANLSV	LLVEHALSKV	HFTAERSSYY
**650**	**660**	**670**	**680**	**690**	**700**	**710**	**720**
KSLLSAEEAA	KQKKEKVWAH	YEEQPVEEVM	PVLEEKERSA	SYKPVFVTEI	TDDLHFYVQD	VETGTQLEKL	MENMRNDIAS
**730**	**740**	**750**	**760**	**770**	**780**	**790**	**800**
HPPVEGSYAP	RRGEFCIAKF	VDGEWYRARV	EKVESPAKIH	VFYIDYGNRE	VLPSTRLGTL	SPAFSTRVLP	AQATEYAFAF
**810**	**820**	**830**	**840**	**850**	**860**	**870**	**880**
IQVPQDDDAR	TDAVDSVVRD	IQNTQCLLNV	EHLSAGCPHV	TLQFADSKGD	VGLGLVKEGL	VMVEVRKEKQ	FQKVITEYLN
**890**	**900**	**910**	**920**				
AQESAKSARL	NLWRYGDFRA	DDADEFGYSR					

Cmpd.	No. of Cmpds.	m/z meas.	Δ m/z [ppm]	z	Rt [min]	Score	P	Range	Sequence	Modification
2148	1	966.3470	-130.61	2	56.7	12.8	2	161-177	K.KGMWSEGNGSHTIRDLK.Y	Oxidation: 3

Detailed Protein Report

Protein 168: **ATP-binding cassette sub-family B member 5 isoform 2 [Homo sapiens]**

**Accession:**	gi|148612844	**Score:**	36.3
**Database:**	refseq_human(refseq_human_20140103.fasta)	**MW [kDa]:**	89.8
**Database Date:**	2015-11-30	**pI:**	8.7
		**Sequence Coverage [%]:**	6.8
		**No. of unique Peptides:**	3

10	20	30	40	50	60	70	80
MVDENDIRAL	NVRHYRDHIG	VVSQEPVLFG	TTISNNIKYG	RDDVTDEEME	RAAREANAYD	FIMEFPNKFN	TLVGEKGAQM
**90**	**100**	**110**	**120**	**130**	**140**	**150**	**160**
SGGQKQRIAI	ARALVRNPKI	LILDEATSAL	DSESKSAVQA	ALEKASKGRT	TIVVAHRLST	IRSADLIVTL	KDGMLAEKGA
**170**	**180**	**190**	**200**	**210**	**220**	**230**	**240**
HAELMAKRGL	YYSLVMSQDI	KKADEQMESM	TYSTERKTNS	LPLHSVKSIK	SDFIDKAEES	TQSKEISLPE	VSLLKILKLN
**250**	**260**	**270**	**280**	**290**	**300**	**310**	**320**
KPEWPFVVLG	TLASVLNGTV	HPVFSIIFAK	IITMFGNNDK	TTLKHDAEIY	SMIFVILGVI	CFVSYFMQGL	FYGRAGEILT
**330**	**340**	**350**	**360**	**370**	**380**	**390**	**400**
MRLRHLAFKA	MLYQDIAWFD	EKENSTGGLT	TILAIDIAQI	QGATGSRIGV	LTQNATNMGL	SVIISFIYGW	EMTFLILSIA
**410**	**420**	**430**	**440**	**450**	**460**	**470**	**480**
PVLAVTGMIE	TAAMTGFANK	DKQELKHAGK	IATEALENIR	TIVSLTREKA	FEQMYEEMLQ	TQHRNTSKKA	QIIGSCYAFS
**490**	**500**	**510**	**520**	**530**	**540**	**550**	**560**
HAFIYFAYAA	GFRFGAYLIQ	AGRMTPEGMF	IVFTAIAYGA	MAIGETLVLA	PEYSKAKSGA	AHLFALLEKK	PNIDSRSQEG
**570**	**580**	**590**	**600**	**610**	**620**	**630**	**640**
KKPDTCEGNL	EFREVSFFYP	CRPDVFILRG	LSLSIERGKT	VAFVGSSGCG	KSTSVQLLQR	LYDPVQGQVL	FDGVDAKELN
**650**	**660**	**670**	**680**	**690**	**700**	**710**	**720**
VQWLRSQIAI	VPQEPVLFNC	SIAENIAYGD	NSRVVPLDEI	KEAANAANIH	SFIEGLPEKY	NTQVGLKGAQ	LSGGQKQRLA
**730**	**740**	**750**	**760**	**770**	**780**	**790**	**800**
IARALLQKPK	ILLLDEATSA	LDNDSEKVVQ	HALDKARTGR	TCLVVTHRLS	AIQNADLIVV	LHNGKIKEQG	THQELLRNRD
**810**	**820**						
IYFKLVNAQS	VQ						

Cmpd.	No. of Cmpds.	m/z meas.	Δ m/z [ppm]	z	Rt [min]	Score	P	Range	Sequence	Modification
1505	1	1022.6536	107.11	2	48.5	10.6	1	97-115	R.NPKILILDEATSALDSESK.S	
439	1	565.3076	0.55	2	35.1	13.4	1	708-718	K.GAQLSGGQKQR.L	
2759	1	905.4805	-31.89	3	65.8	12.2	1	761-785	R.TCLVVTHRLSAIQNADLIVVLHNGK.I	

Detailed Protein Report

Protein 169: T-complex protein 11 homolog isoform 3 [Homo sapiens]

**Accession:**	gi|387849264	**Score:**	36.3
**Database:**	refseq_human(refseq_human_20140103.fasta)	**MW [kDa]:**	56.8
**Database Date:**	2015-11-30	**pI:**	5.0
**Modification(s):**	Oxidation	**Sequence Coverage [%]:**	5.7
		**No. of unique Peptides:**	1

10	20	30	40	50	60	70	80
MTRGGGGGDT	ISKMPDVKES	VPPKYPGDSE	GRSCKPETSG	PPQEDKSGSE	DPPPCLTETV	NEVSKLSNKI	GMNCDYYMEE
**90**	**100**	**110**	**120**	**130**	**140**	**150**	**160**
KVLPPSSLEG	KVKETVHNAF	WDHLKEQLSA	TPPDFSCALE	LLKEIKEILL	SLLLPRQNRL	RIEIEEALDM	DLLKQEAEHG
**170**	**180**	**190**	**200**	**210**	**220**	**230**	**240**
ALKVLYLSKY	VLNMMALLCA	PVRDEAVQKL	ENITDPVWLL	RGIFQVLGRM	KMDMVNYTIQ	SLQPHLQEHS	IQYERAKFQE
**250**	**260**	**270**	**280**	**290**	**300**	**310**	**320**
LLNKQPSLLN	HTTKWLTQAA	GDLTMSPPTC	PDTSDSSSVA	GPSPNEAANN	PEPLSPTMVL	CQGFLNLLLW	DLENEEFPET
**330**	**340**	**350**	**360**	**370**	**380**	**390**	**400**
LLMDRTRLQE	LKSQLHQLTV	MASVLLVASS	FSGSVLFGSP	QFVDKLKRIT	KSLLEDFHSR	PEEAILTVSE	QVSQEIHQSL
**410**	**420**	**430**	**440**	**450**	**460**	**470**	**480**
KNMGLVALSS	DNTASLMGQL	QNIAKKENCV	CSVIDQRIHL	FLKCCLVLGV	QRSLLDLPGG	LTLIEAELAE	LGQKFVNLTH
**490**	**500**	**510**	**520**				
HNQQVFGPYY	TEILKTLISP	AQALETKVES	V				

Cmpd.	No. of Cmpds.	m/z meas.	Δ m/z [ppm]	z	Rt [min]	Score	P	Range	Sequence	Modification
2154	1	1052.4435	-57.89	2	57.1	16.6	2	14-32	K.MPDVKESVPPKYPGDSEGR.S	Oxidation: 1

Detailed Protein Report

Protein 170: **exportin-5 [Homo sapiens]**

**Accession:**	gi|22748937	**Score:**	36.0
**Database:**	refseq_human(refseq_human_20140103.fasta)	**MW [kDa]:**	136.2
**Database Date:**	2015-11-30	**pI:**	5.5
**Modification(s):**	Oxidation	**Sequence Coverage [%]:**	2.7
		**No. of unique Peptides:**	2

10	20	30	40	50	60	70	80
MAMDQVNALC	EQLVKAVTVM	MDPNSTQRYR	LEALKFCEEF	KEKCPICVPC	GLRLAEKTQV	AIVRHFGLQI	LEHVVKFRWN
90	100	110	120	130	140	150	160
GMSRLEKVYL	KNSVMELIAN	GTLNILEEEN	HIKDALSRIV	VEMIKREWPQ	HWPDMLIELD	TLSKQGETQT	ELVMFILLRL
170	180	190	200	210	220	230	240
AEDVVTFQTL	PPQRRRDIQQ	TLTQNMERIF	SFLLNTLQEN	VNKYQQVKTD	TSQESKAQAN	CRVGVAALNT	LAGYIDWVSM
250	260	270	280	290	300	310	320
SHITAENCKL	LEILCLLLNE	QELQLGAAEC	LLIAVSRKGK	LEDRKPLMVL	FGDVAMHYIL	SAAQTADGGG	LVEKHYVFLK
330	340	350	360	370	380	390	400
RLCQVLCALG	NQLCALLGAD	SDVETPSNFG	KYLESFLAFT	THPSQFLRSS	TQMTWGALFR	HEILSRDPLL	LAIIPKYLRA
410	420	430	440	450	460	470	480
SMTNLVKMGF	PSKTDSPSCE	YSRFDFDSDE	DFNAFFNSSR	AQQGEVMRLA	CRLDPKTSFQ	MAGEWLKYQL	STFLDAGSVN
490	500	510	520	530	540	550	560
SCSAVGTGEG	SLCSVFSPSF	VQWEAMTLFL	ESVITQMFRT	LNREEIPVND	GIELLQMVLN	FDTKDPLILS	CVLTNVSALF
570	580	590	600	610	620	630	640
PFVTYRPEFL	PQVFSKLFSS	VTFETVEESK	APRTRAVRNV	RRHACSSIIK	MCRDYPQLVL	PNFDMLYNHV	KQLLSNELLL
650	660	670	680	690	700	710	720
TQMEKCALME	ALVLISNQFK	NYERQKVFLE	ELMAPVASIW	LSQDMHRVLS	DVDAFIAYVG	TDQKSCDPGL	EDPCGLNRAR
730	740	750	760	770	780	790	800
MSFCVYSILG	VVKRTCWPTD	LEEAKAGGFV	VGYTSSGNPI	FRNPCTEQIL	KLLDNLLALI	RTHNTLYAPE	MLAKMAEPFT
810	820	830	840	850	860	870	880
KALDMLDAEK	SAILGLPQPL	LELNDSPVFK	TVLERMQRFF	STLYENCFHI	LGKAGPSMQQ	DFYTVEDLAT	QLLSSAFVNL
890	900	910	920	930	940	950	960
NNIPDYRLRP	MLRVFVKPLV	LFCPPEHYEA	LVSPILGPLF	TYLHMRLSQK	WQVINQRSLL	CGEDEAADEN	PESQEMLEEQ
970	980	990	1000	1010	1020	1030	1040
LVRMLTREVM	DLITVCCVSK	KGADHSSAPP	ADGDDEEMMA	TEVTPSAMAE	LTDLGKCLMK	HEDVCTALLI	TAFNSLAWKD
1050	1060	1070	1080	1090	1100	1110	1120
TLSCQRTTSQ	LCWPLLKQVL	SGTLLADAVT	WLFTSVLKGL	QMHGQHDGCM	ASLVHLAFQI	YEALRPRYLE	IRAVMEQIPE
1130	1140	1150	1160	1170	1180	1190	1200
IQKDSLDQFD	CKLLNPSLQK	VADKRRKDQF	KRLIAGCIGK	PLGEQFRKEV	HIKNLPSLFK	KTKPMLETEV	LDNDGGGLAT
1210							
IFEP							

Cmpd.	No. of Cmpds.	m/z meas.	Δ m/z [ppm]	z	Rt [min]	Score	P	Range	Sequence	Modification
629	1	459.6075	-259.30	2	37.2	12.3	0	441-448	R.AQQGEVMR.L	
1960	1	731.2880	-122.56	2	54.6	12.4	0	721-733	R.MSFCVYSILGVVK.R	Oxidation: 1

Detailed Protein Report

Protein 171: **PREDICTED: Fanconi anemia-associated protein of 100 kDa isoform X2 [Homo sapiens]**

**Accession:**	gi|578831470	**Score:**	35.9
**Database:**	refseq_human(refseq_human_20140103.fasta)	**MW [kDa]:**	77.0
**Database Date:**	2015-11-30	**pI:**	5.0
		**Sequence Coverage [%]:**	3.3
		**No. of unique Peptides:**	1

10	20	30	40	50	60	70	80
MQLFEQPCPG	EDPRPGGQIG	EVELSSYTPP	AGVPGKPAAP	HFLPVLCSVS	PSGSRVPHDL	LGGSGGFTLE	DALFGLLFGA
90	100	110	120	130	140	150	160
DATLLQSPVV	LCGLPDGQLC	CVILKALVTS	RSAPGDPNAL	VKILHHLEEP	VIFIGALKTE	PQAAEAAENF	LPDEDVHCDC
170	180	190	200	210	220	230	240
LVAFGHHGRM	LAIKASWDES	GKLVPELREY	CLPGPVLCAA	CGGGGRVYHS	TPSDLCVVDL	SRGSTPLGPE	QPEEGPGGLP
250	260	270	280	290	300	310	320
PMLCPASLNI	CSVVSLSASP	RTHEGGTKLL	ALSAKGRLMT	CSLDLDSEMP	GPARMTTESA	GQKIKELLSG	IGNISERVSF
330	340	350	360	370	380	390	400
LKKAVDQRNK	ALTSLNEAMN	VSCALLSSGT	GPRPISCTTS	TTWSRLQTQD	VLMATCVLEN	SSSFSLDQGW	TLCIQVLTSS
410	420	430	440	450	460	470	480
CALDLDSACS	AITYTIPVDQ	LGPGARREVT	LPLGPGENGG	LDLPVTVSCT	LFYSLREVVG	GALAPSDSED	PFLDECPSDV
490	500	510	520	530	540	550	560
LPEQEGVCLP	LSRHTVDMLQ	CLRFPGLAPP	HTRAPSPLGP	TRDPVATFLE	TCREPGSQPA	GPASLRAEYL	PPSVASIKVS
570	580	590	600	610	620	630	640
AELLRAALKD	GHSGVPLCCA	TLQWLLAENA	AVDVVRARAL	SSIQGVAPDG	ANVHLIVREV	AMTDLCPAGP	IQAVEIQVES
650	660	670	680	690	700	710	720
SSLADICRAH	HAVVGRMQTM	VTEQATQGSS	APDLRVQYLR	QIHANHETLL	REVQTLRDRL	CTEDEASSCA	TAQRLLQVYR
730	740						
QLRHPSLILL							

Cmpd.	No. of Cmpds.	m/z meas.	Δ m/z [ppm]	z	Rt [min]	Score	P	Range	Sequence	Modification
1236	1	765.0123	96.72	2	45.1	17.1	1	304-317	K.IKELLSGIGNISER.V	

Detailed Protein Report

Protein 172: PREDICTED: diphthine synthase isoform X1 [Homo sapiens]

Accession: gi|530362777
Database: refseq_human(refseq_human_20140103.fasta)
Database Date: 2015-11-30
Modification(s): Carbamidomethyl

Score: 35.8
MW [kDa]: 27.1
pI: 9.2
Sequence Coverage [%]: 7.9
No. of unique Peptides: 1

10	20	30	40	50	60	70	80
MLYLIGLGLG	DAKDITVKGL	EVVRRCSRVY	LEAYTSVLTV	GKEALEEFYG	RKLVVADREE	VEQEADNILK	DADISDVAFL
**90**	**100**	**110**	**120**	**130**	**140**	**150**	**160**
VVGDPFGATT	HSDLVLRATK	LGIPYRVIHN	ASIMNAVGCC	GLQLYKFGET	VSIVFWTDTW	RPESFFDKVK	KNRQNGMHTL
**170**	**180**	**190**	**200**	**210**	**220**	**230**	**240**
CLLDIKVKEQ	SLENLINSYR	GDTLCWLSQG	WSRRPENCSR	HFKANVHCGL	GRTIAFLDHH	RRQHTSNGDG	DAKSVFHTRK
**250**							

Cmpd.	No. of Cmpds.	m/z meas.	Δ m/z [ppm]	z	Rt [min]	Score	P	Range	Sequence	Modification
1993	1	698.2622	-135.48	2	55.0	20.1	1	201-212	R.HFKANVHCGLGR.T	Carbamidomethyl: 8

Detailed Protein Report

Protein 173: **PREDICTED: coiled-coil domain-containing protein 146 isoform X3 [Homo sapiens]**

**Accession:**	gi|530386260	**Score:**	35.8
**Database:**	refseq_human(refseq_human_20140103.fasta)	**MW [kDa]:**	97.2
**Database Date:**	2015-11-30	**pI:**	9.8
**Modification(s):**	Oxidation	**Sequence Coverage [%]:**	3.2
		**No. of unique Peptides:**	3

Quantitation

MD:MU **Median:** 0.66 **CV:** 25.52 % **No. of Peptides:** 2

10	20	30	40	50	60	70	80
MQTSSPTTRL	FLRIKEKSLK	EEKIIIVKEF	EKITKPGEME	KKMKILREST	EELRKEIMQK	KLEIKNLRED	LASKQKQLLK
**90**	**100**	**110**	**120**	**130**	**140**	**150**	**160**
EQKELEELLG	HQVVLKDEVA	HHQTIPVQIG	KEIEKITRKK	VEMEKKKIVL	EQEVKTLNDS	LKKVENKVSA	IVDEKENVIK
**170**	**180**	**190**	**200**	**210**	**220**	**230**	**240**
EVEGKRALLE	IKEREHNQLV	KLLELARENE	ATSLTERGIL	DLNLRNSLID	KQNYHDELSR	KQREKERDFR	NLRKMELLLK
**250**	**260**	**270**	**280**	**290**	**300**	**310**	**320**
VSWDALRQTQ	ALHQRLLLEM	EAIPKDDSTL	SERRRELHKE	VEVAKRNLAQ	QKIISEMESK	LVEQQLAEEN	KLLKEQENMK
**330**	**340**	**350**	**360**	**370**	**380**	**390**	**400**
ELVVNLLRMT	QIKIDEKEQK	SKDFLKAQQK	YTNIVKEMKA	KDLEIRIHKK	KKCEIYRRLR	EFAKLYDTIR	NERNKFVNLL
**410**	**420**	**430**	**440**	**450**	**460**	**470**	**480**
HKAHQKVNEI	KERHKMSLNE	LEILRNSAVS	QERKLQNSML	KHANNVTIRE	SMQNDVRKIV	SKLQEMKEKK	EAQLNNIDRL
**490**	**500**	**510**	**520**	**530**	**540**	**550**	**560**
ANTITMIEEE	MVQLRKRYEK	AVQHRNESGV	QLIEREEEIC	IFYEKINIQE	KMKLNGEIEI	HLLEEKIQFL	KMKIAEKQRQ
**570**	**580**	**590**	**600**	**610**	**620**	**630**	**640**
ICVTQKLLPA	KRSLDADLAV	LQIQFSQCTD	RIKDLEKQFV	KPDGENRARF	LPGKDLTEKE	MIQKLDKLEL	QLAKKEEKLL
**650**	**660**	**670**	**680**	**690**	**700**	**710**	**720**
EKDFIYEQVS	RLTDRLCSKT	QGCKQDTLLL	AKKMNGYQRR	IKNATEKMMA	LVAELSMKQA	LTIELQKEVR	EKEDFIFTCN
**730**	**740**	**750**	**760**	**770**	**780**	**790**	**800**
SRIEKGLPLN	KEIEKEWLKV	LRDEEMHALA	IAEKSQEFLE	ADNRQLPNGV	YTTAEQRPNA	YIPEADATLP	LPKPYGALAP
**810**	**820**	**830**					
FKPSEPGANM	RHIRKPVIKP	VEI					

Cmpd.	No. of Cmpds.	m/z meas.	Δ m/z [ppm]	z	Rt [min]	Score	P	Range	Sequence	Modification	Ratios	
1670	1	524.8066	68.22	2	50.8	14.1	0	33-41	K.ITKPGEMEK.K	Oxidation: 7	MD:MU	0.85
1783	1	870.2900	-263.69	1	52.3	10.4	0	396-402	K.FVNLLHK.A			
595	1	572.7739	-48.16	2	36.8	11.3	0	506-515	R.NESGVQLIER.E		MD:MU	0.51

Detailed Protein Report

Protein 174: testis-specific serine/threonine-protein kinase 2 [Homo sapiens]

**Accession:**	gi|194294513	**Score:**	35.6
**Database:**	refseq_human(refseq_human_20140103.fasta)	**MW [kDa]:**	40.9
**Database Date:**	2015-11-30	**pI:**	9.8
**Modification(s):**	Carbamidomethyl	**Sequence Coverage [%]:**	2.8
		**No. of unique Peptides:**	1

10	20	30	40	50	60	70	80
MDDATVLRKK	GYIVGINLGK	GSYAKVKSAY	SERLKFNVAV	KIIDRKKTPT	DFVERFLPRE	MDILATVNHG	SIIKTYEIFE
**90**	**100**	**110**	**120**	**130**	**140**	**150**	**160**
TSDGRIYIIM	ELGVQGDLLE	FIKCQGALHE	DVARKMFRQL	SSAVKYCHDL	DIVHRDLKCE	NLLLDKDFNI	KLSDFGFSKR
**170**	**180**	**190**	**200**	**210**	**220**	**230**	**240**
CLRDSNGRII	LSKTFCGSAA	YAAPEVLQSI	PYQPKVYDIW	SLGVILYIMV	CGSMPYDDSD	IRKMLRIQKE	HRVDFPRSKN
**250**	**260**	**270**	**280**	**290**	**300**	**310**	**320**
LTCECKDLIY	RMLQPDVSQR	LHIDEILSHS	WLQPPKPKAT	SSASFKREGE	GKYRAECKLD	TKTGLRPDHR	PDHKLGAKTQ
**330**	**340**	**350**	**360**				
HRLLVVPENE	NRMEDRLAET	SRAKDHHISG	AEVGKAST				

Cmpd.	No. of Cmpds.	m/z meas.	Δ m/z [ppm]	z	Rt [min]	Score	P	Range	Sequence	Modification
185	1	642.3289	7.21	2	32.0	16.0	2	293-302	K.YRAECKLDTK.T	Carbamidomethyl: 5

Detailed Protein Report

Protein 175: **PREDICTED: LOW QUALITY PROTEIN: double homeobox protein 4-like protein 4 [Homo sapiens]**

**Accession:**	gi|578798033	**Score:**	35.5
**Database:**	refseq_human(refseq_human_20140103.fasta)	**MW [kDa]:**	45.9
**Database Date:**	2015-11-30	**pI:**	10.8
**Modification(s):**	Carbamidomethyl	**Sequence Coverage [%]:**	5.0
		**No. of unique Peptides:**	2

10	20	30	40	50	60	70	80
MTLPTPSEST	LPAEARGWGQ	RRRLVWTPRQ	REALXACFYR	NPYMGIATRV	QLAQSIGIPE	TRVXIYFQNE	RSRQLRQHRR
**90**	**100**	**110**	**120**	**130**	**140**	**150**	**160**
ESRPWPVRRG	PQEGRTAVIG	SQTALLLRAF	EKDRLPGIEA	REELARERGL	LESRIQIWFQ	IRRARHQGQA	GRAPAQAGGR
**170**	**180**	**190**	**200**	**210**	**220**	**230**	**240**
SNAAPAXCHP	ARSWVAFAHT	GRWGRVFPHP	XVPCAPGALP	QGAFVNQGAR	AFLVFQHSQA	APVEGISQPA	LGRGDFSYTA
**250**	**260**	**270**	**280**	**290**	**300**	**310**	**320**
PAPLEGALSH	PQSPPXPPHT	GKSRENRDPQ	RDGLLGTCVV	GQPGPAQAGP	QGQGVLAPPA	SQGIPWWGWA	WGPQASGAAW
**330**	**340**	**350**	**360**	**370**	**380**	**390**	**400**
EPQARAASPR	QPMPPEDSAR	QGQMQGMPAP	SQALQELGRS	SALPSGLLLD	KFLASLVFLQ	QVQSFLETET	PGELEALEEA
**410**	**420**						
VSLEEPLSEQ	EYRALLEEI						

Cmpd.	No. of Cmpds.	m/z meas.	Δ m/z [ppm]	z	Rt [min]	Score	P	Range	Sequence	Modification
2748	1	826.4475	66.34	3	65.6	12.9	2	0-0	.QREALCACFYRNPYMGIATR.	Carbamidomethyl: 6, 8
2114	1	1131.4444	-110.45	1	56.6	10.9	0	173-182	R.SWVAFAHTGR.W	

Detailed Protein Report

Protein 176: 60 kDa heat shock protein, mitochondrial [Homo sapiens]

**Accession:**	gi|31542947	**Score:**	35.1
**Database:**	refseq_human(refseq_human_20140103.fasta)	**MW [kDa]:**	61.0
**Database Date:**	2015-11-30	**pI:**	5.6
**Modification(s):**	Oxidation	**Sequence Coverage [%]:**	4.4
		**No. of unique Peptides:**	1

Alias proteins:

Accession	Name	Description
gi|530370277	refseq_human (refseq_human_20140103.fasta)	PREDICTED: 60 kDa heat shock protein, mitochondrial isoform X1 [Homo sapiens]
gi|41399285	refseq_human (refseq_human_20140103.fasta)	60 kDa heat shock protein, mitochondrial [Homo sapiens]

10	20	30	40	50	60	70	80
MLRLPTVFRQ	MRPVSRVLAP	HLTRAYAKDV	KFGADARALM	LQGVDLLADA	VAVTMGPKGR	TVIIEQSWGS	PKVTKDGVTV
**90**	**100**	**110**	**120**	**130**	**140**	**150**	**160**
AKSIDLKDKY	KNIGAKLVQD	VANNTNEEAG	DGTTTATVLA	RSIAKEGFEK	ISKGANPVEI	RRGVMLAVDA	VIAELKKQSK
**170**	**180**	**190**	**200**	**210**	**220**	**230**	**240**
PVTTPEEIAQ	VATISANGDK	EIGNIISDAM	KKVGRKGVIT	VKDGKTLNDE	LEIIEGMKFD	RGYISPYFIN	TSKGQKCEFQ
**250**	**260**	**270**	**280**	**290**	**300**	**310**	**320**
DAYVLLSEKK	ISSIQSIVPA	LEIANAHRKP	LVIIAEDVDG	EALSTLVLNR	LKVGLQVVAV	KAPGFGDNRK	NQLKDMAIAT
**330**	**340**	**350**	**360**	**370**	**380**	**390**	**400**
GGAVFGEEGL	TLNLEDVQPH	DLGKVGEVIV	TKDDAMLLKG	KGDKAQIEKR	IQEIIEQLDV	TTSEYEKEKL	NERLAKLSDG
**410**	**420**	**430**	**440**	**450**	**460**	**470**	**480**
VAVLKVGGTS	DVEVNEKKDR	VTDALNATRA	AVEEGIVLGG	GCALLRCIPA	LDSLTPANED	QKIGIEIIKR	TLKIPAMTIA
**490**	**500**	**510**	**520**	**530**	**540**	**550**	**560**
KNAGVEGSLI	VEKIMQSSSE	VGYDAMAGDF	VNMVEKGIID	PTKVVRTALL	DAAGVASLLT	TAEVVVTEIP	KEEKDPGMGA
**570**	**580**						
MGGMGGGMGG	GMF						

Cmpd.	No. of Cmpds.	m/z meas.	Δ m/z [ppm]	z	Rt [min]	Score	P	Range	Sequence	Modification
29	1	1001.9624	-98.76	2	30.0	16.6	2	1-16	-.MLRLPTVFRQMRPVSR.V	Oxidation: 11

Detailed Protein Report

Protein 177: **PREDICTED: RANBP2-like and GRIP domain-containing protein 5/6 isoform X5 [Homo sapiens]**

**Accession:**	gi|530369376	**Score:**	35.1
**Database:**	refseq_human(refseq_human_20140103.fasta)	**MW [kDa]:**	165.9
**Database Date:**	2015-11-30	**pI:**	6.1
		**Sequence Coverage [%]:**	2.9
		**No. of unique Peptides:**	1

Alias proteins:

Accession	Name	Description
gi|530369509	refseq_human (refseq_human_20140103.fasta)	PREDICTED: RANBP2-like and GRIP domain-containing protein 5/6 isoform X5 [Homo sapiens]

10	20	30	40	50	60	70	80
MKGHFYMYAG	SLLLKMGQHG	NNVQWRALSE	LAALCYLIAF	QVPRPKIKLR	EGKAGQNLLE	MMACDRLSQS	GHMLLSLSRG
90	100	110	120	130	140	150	160
KQDFLKEVVE	TFANKIGQSA	LYDALFSSQS	PKDTSFLGSD	DIGKIDVQEP	ELEDLARYDV	GAIRAHNGSL	QHLTWLGLQW
170	180	190	200	210	220	230	240
NSLPALPGIR	KWLKQLFHRL	PHETSRLETN	APESICILDL	EVFLLGVVYT	SHLQLKEKCN	SHHSSYQPLC	LPFPVCKQLC
250	260	270	280	290	300	310	320
TERQKSWWDA	VCTLIHRKAV	PGNLAKLRLL	VQHEINTLRA	QEKHGLQPAL	LVHWAKYLQK	TGSGLNSFYG	QLEYIGRSVH
330	340	350	360	370	380	390	400
YWKKVLPLLK	IIKKNSIPEP	IDPLFKHFHS	VDIQASEIVE	YEEDAHITFA	MLDAVNGNIE	DAVTAFESIK	SVVSYWNLAL
410	420	430	440	450	460	470	480
IFHRKAEDIE	NDALSPEEQE	ECRNYLTKTR	DYLIKIIDDG	DSNLSVVKKL	PVPLESVKQM	LNSVMQELED	YSEGGPLYKN
490	500	510	520	530	540	550	560
GSLRNADSEI	KHSTPSPTKY	SLSPSKSYKY	SPETPPRWTE	DRNSLLNMIC	QQVEAIKKEM	QELKLNSSKS	ASRHRWPTEN
570	580	590	600	610	620	630	640
YGPDSVPDGY	QGSQTFHGAP	LTVATTGPSV	YYSQSPAYNS	QYLLRPAANV	TPTKGSSNTE	FKSTKEGFSI	PVSADGFKFG
650	660	670	680	690	700	710	720
ISEPGNQEKK	REKPLENDTG	FQAQDISGRK	KGRGVIFGQT	SSTFTFADVA	KSTSGEGFQF	GKKDLNFKGF	SGAGEKLFSS
730	740	750	760	770	780	790	800
RYGKMANKAN	TSGDFEKDDD	AYKTEDSDDI	HFEPVVQMPE	KVELVTGEEG	EKVLYSQGVK	LFRFDAEVRQ	WKERGLGNLK
810	820	830	840	850	860	870	880
ILKNEVNGKL	RMLMRREQVL	KVCANHWITT	TMNLKPLSGS	DRAWMWSASD	FSDGDAKLER	LAAKFKTPEL	AEEFKQKFEE
890	900	910	920	930	940	950	960
CQRLLLDIPL	QTPHKLVDTG	RAAKLIQRAE	EMKSGLKDFK	TFLTNDQTKV	TEEENKGSGT	GAAGASDTTI	KPNAENTGPT
970	980	990	1000	1010	1020	1030	1040
LEWDNYDLRE	DALDDSVSSS	SVHASPLASS	PVRKNLFRFD	ESTTGSNFSF	KSALSLSKSP	AKLNQSGTSV	GTDEESVVTQ
1050	1060	1070	1080	1090	1100	1110	1120
EEERDGQYFE	PVVPLPDLVE	VSSGEENEQV	VFSHRAEIYR	YDKDVGQWKE	RGIGDIKILQ	NYDNKQVRIV	MRRDQVLKLC
1130	1140	1150	1160	1170	1180	1190	1200
ANHRITPDMS	LQNMKGTERV	WVWTACDFAD	GERKVEHLAV	RFKLQDVADS	FKKIFDEAKT	AQEKDSLITP	HVSRSSTPRE
1210	1220	1230	1240	1250	1260	1270	1280
SPCGKIAVAI	LEETTRERTD	VIQGDDVADA	ASEVEVSSTS	ETTTKAVVSP	PKFVFVSESV	KRIFSSEKSK	PFVFGNSSAT
1290	1300	1310	1320	1330	1340	1350	1360
GSLFGFSFNA	PLKSNNSETS	SVAQSGSESK	VEPKKCELSK	NSDIEQSSDS	KVKNLSASFP	TEESSINYTF	KTPEKEPPLW
1370	1380	1390	1400	1410	1420	1430	1440
HAEFTKEELV	QKLRSTTKSA	DHLNGLLREI	EATNAVLMEQ	IKLLKSEIRR	LERNQEREKS	AANLEYLKNV	LLQFIFLKPG
1450	1460	1470	1480	1490			
SERERLLPVI	NTMLQLSPEE	KGKLAAVAQD	EEENASRSSG				

Cmpd.	No. of Cmpds.	m/z meas.	Δ m/z [ppm]	z	Rt [min]	Score	P	Range	Sequence	Modification
1535	1	1023.6824	101.59	2	49.0	12.2	1	1429-1445	K.NVLLQFIFLKPGSERER.L	

Detailed Protein Report

Protein 178: PREDICTED: probable tubulin polyglutamylase TTLL2 isoform X1 [Homo sapiens]

Accession: gi|578812991
Database: refseq_human(refseq_human_20140103.fasta)
Database Date: 2015-11-30

Score: 35.0
MW [kDa]: 59.5
pI: 9.4
Sequence Coverage [%]: 5.2
No. of unique Peptides: 2

10	20	30	40	50	60	70	80
MAEDEPSGAL	LKPLVFRVDE	TTPAVVQSVL	LERGWNKFDK	QEQNAEDWNL	YWRTSSFRMT	EHNSVKPWQQ	LNHHPGTTKL
90	100	110	120	130	140	150	160
TRKDCLAKHL	KHMRRMYGTS	LYQFIPLTFV	MPNDYTKFVA	EYFQERQMLG	TKHSYWICKP	AELSRGRGIL	IFSDFKDFIF
170	180	190	200	210	220	230	240
DDMYIVQKYI	SNPLLIGRYK	CDLRIYVCVT	GFKPLTIYVY	QEGLVRFATE	KFDLSNLQNN	YAHLTNSSIN	KSGASYEKIK
250	260	270	280	290	300	310	320
EVIGHGCKWT	LSRFFSYLRS	WDVDDLLLWK	KIHRMVILTI	LAIAPSVPFA	ANCFELFGFD	ILIDDNLKPW	LLEVNYSPAL
330	340	350	360	370	380	390	400
TLDCSTDVLV	KRKLVHDIID	LIYLNGLRNE	GREASNATHG	NSNIDAAKSD	RGGLDAPDCL	PYDSLSFTSR	MYNEDDSVVE
410	420	430	440	450	460	470	480
KAVSVRPEAA	PASQLEGEMS	GQDFHLSTRE	MPQSKPKLRS	RHTPHKTLMP	YASLFQSHSC	KTKTSPCVLS	DRGKAPDPQA
490	500	510	520				
GNFVLVFPFN	EATLGASRNG	LNVKRIIQEL	QKLMNKQHS				

Cmpd.	No. of Cmpds.	m/z meas.	Δ m/z [ppm]	z	Rt [min]	Score	P	Range	Sequence	Modification
429	1	542.2853	-31.85	2	35.0	16.8	1	239-248	K.IKEVIGHGCK.W	
89	1	647.9192	-109.54	3	30.5	18.2	1	447-463	K.TLMPYASLFQSHSCKTK.T	

Detailed Protein Report

Protein 179: **leucine-rich repeat and calponin homology domain-containing protein 1 isoform 3 [Homo sapiens]**

Accession: gi|256017180
Database: refseq_human(refseq_human_20140103.fasta)
Database Date: 2015-11-30
Modification(s): Carbamidomethyl

Score: 35.0
MW [kDa]: 77.2
pI: 5.5
Sequence Coverage [%]: 4.2
No. of unique Peptides: 1

10	20	30	40	50	60	70	80
MATPGSEPQP	FVPALSVATL	HPLHHPHHHH	HHHQHHGGTG	APGGAGGGGG	GSGGFNLPLN	RGLERALEEA	ANSGGLNLSA
**90**	**100**	**110**	**120**	**130**	**140**	**150**	**160**
RKLKEFPRTA	APGHDLSDTV	QADLSKNRLV	EVPMELCHFV	SLEILNLYHN	CIRVIPEAIV	NLQMLTYLNL	SRNQLSALPA
**170**	**180**	**190**	**200**	**210**	**220**	**230**	**240**
CLCGLPLKVL	IASNNKLGSL	PEEIGQLKQL	MELDVSCNEI	TALPQQIGQL	KSLRELNVRR	NYLKVLPQEL	VDLPLVKFDF
**250**	**260**	**270**	**280**	**290**	**300**	**310**	**320**
SCNKVLVIPI	CFREMKQLQV	LLLENNPLQS	PPAQICTKGK	VHIFKYLSIQ	ACQIKTADSL	YLHTMERPHL	HQHVEDGKKD
**330**	**340**	**350**	**360**	**370**	**380**	**390**	**400**
SDSGVGSDNG	DKRLSATEPS	DEDTVSLNVP	MSNIMEEEQI	IKEDSCHRLS	PVKGEFHQEF	QPEPSLLGDS	TNSGEERDQF
**410**	**420**	**430**	**440**	**450**	**460**	**470**	**480**
TDRADGLHSE	FMNYKARAED	CEELLRIEED	VHWQTEGIIS	SSKDQDMDIA	MIEQLREAVD	LLQDPNGLST	DITERSVLNL
**490**	**500**	**510**	**520**	**530**	**540**	**550**	**560**
YPMGSAEALE	LQDSALNGQI	QLETSPVCEV	QSDLTLQSNG	SQYSPNEIRE	NSPAVSPTTN	STAPFGLKPR	SVFLRPQRNL
**570**	**580**	**590**	**600**	**610**	**620**	**630**	**640**
ESIDPQFTIR	RKMEQMREEK	ELVEQLRESI	EMRLKVSLHE	DLGAALMDGV	VLCHLVNHIR	PRSVASIHVP	SPAVPKLSMA
**650**	**660**	**670**	**680**	**690**	**700**		
KCRRNVENFL	EACRKLGVPE	EKLCLPHHIL	EEKGLVKVGI	TIQALLDITV	TKALFT		

Cmpd.	No. of Cmpds.	m/z meas.	Δ m/z [ppm]	z	Rt [min]	Score	P	Range	Sequence	Modification
599	1	690.3992	85.05	2	36.8	23.5	1	645-655	R.NVENFLEACRK.L	Carbamidomethyl: 9

Detailed Protein Report

Protein 180: G-protein coupled receptor 98 precursor [Homo sapiens]

**Accession:**	gi|113722120	**Score:**	34.9
**Database:**	refseq_human(refseq_human_20140103.fasta)	**MW [kDa]:**	692.6
**Database Date:**	2015-11-30	**pI:**	4.4
**Modification(s):**	Oxidation	**Sequence Coverage [%]:**	0.7
		**No. of unique Peptides:**	2

Detailed Protein Report

10	20	30	40	50	60	70	80
MSVFLGPGMP	SASLLVNLLS	ALLILFVFGE	TEIRFTGQTE	FVVNETSTTV	IRLIIERIGE	PANVTAIVSL	YGEDAGDFFD
90	100	110	120	130	140	150	160
TYAAAFIPAG	ETNRTVYIAV	CDDDLPEPDE	TFIFHLTLQK	PSANVKLGWP	RTVTVTILSN	DNAFGIISFN	MLPSIAVSEP
170	180	190	200	210	220	230	240
KGRNESMPLT	LIREKGTYGM	VMVTFEVEGG	PNPPDEDLSP	VKGNITFPPG	RATVIYNLTV	LDDEVPENDE	IFLIQLKSVE
250	260	270	280	290	300	310	320
GGAEINTSRN	SIEIIIKKND	SPVRFLQSIY	LVPEEDHILI	IPVVRGKDNN	GNLIGSDEYE	VSISYAVTTG	NSTAHAQQNL
330	340	350	360	370	380	390	400
DFIDLQPNTT	VVFPPFIHES	HLKFQIVDDT	IPEIAESFHI	MLLKDTLQGD	AVLISPSVVQ	VTIKPNDKPY	GVLSFNSVLF
410	420	430	440	450	460	470	480
ERTVIIDEDR	ISRYEEITVV	RNGGTHGNVS	ANWVLTRNST	DPSPVTADIR	PSSGVLHFAQ	GQMLATIPLT	VVDDDLPEEA
490	500	510	520	530	540	550	560
EAYLLQILPH	TIRGGAEVSE	PAELLFYIQD	SDDVYGLITF	FPMENQKIES	SPGERYLSLS	FTRLGGTKGD	VRLLYSVLYI
570	580	590	600	610	620	630	640
PAGAVDPLQA	KEGILNISRR	NDLIFPEQKT	QVTTKLPIRN	DAFLQNGAHF	LVQLETVELL	NIIPLIPPIS	PRFGEICNIS
650	660	670	680	690	700	710	720
LLVTPAIANG	EIGFLSNLPI	ILHEPEDFAA	EVVYIPLHRD	GTDGQATVYW	SLKPSGFNSK	AVTPDDIGPF	NGSVLFLSGQ
730	740	750	760	770	780	790	800
SDTTINITIK	GDDIPEMNET	VTLSLDRVNV	ENQVLKSGYT	SRDLIILEND	DPGGVFEFSP	ASRGPYVIKE	GESVELHIIR
810	820	830	840	850	860	870	880
SRGSLVKQFL	HYRVEPRDSN	EFYGNTGVLE	FKPGEREIVI	TLLARLDGIP	ELDEHYWVVL	SSHGERESKL	GSATIVNITI
890	900	910	920	930	940	950	960
LKNDDPHGII	EFVSDGLIVM	INESKGDAIY	SAVYDVVRNR	GNFGDVSVSW	VVSPDFTQDV	FPVQGTVVFG	DQEFSKNITI
970	980	990	1000	1010	1020	1030	1040
YSLPDEIPEE	MEEFTVILLN	GTGGAKVGNR	TTATLRIRRN	DDPIYFAEPR	VVRVQEGETA	NFTVLRNGSV	DVTCMVQYAT
1050	1060	1070	1080	1090	1100	1110	1120
KDGKATARER	DFIPVEKGET	LIFEVGSRQQ	SISIFVNEDG	IPETDEPFYI	ILLNSTGDTV	VYQYGVATVI	IEANDDPNGI
1130	1140	1150	1160	1170	1180	1190	1200
FSLEPIDKAV	EEGKTNAFWI	LRHRGYFGSV	SVSWQLFQND	SALQPGQEFY	ETSGTVNFMD	GEEAKPIILH	AFPDKIPEFN
1210	1220	1230	1240	1250	1260	1270	1280
EFYFLKLVNI	SGGSPGPGGQ	LAETNLQVTV	MVPFNDDPFG	VFILDPECLE	REVAEDVLSE	DDMSYITNFT	ILRQQGVFGD
1290	1300	1310	1320	1330	1340	1350	1360
VQLGWEILSS	EFPAGLPPMI	DFLLVGIFPT	TVHLQQHMRR	HHSGTDALYF	TGLEGAFGTV	NPKYHPSRNN	TIANFTFSAW
1370	1380	1390	1400	1410	1420	1430	1440
VMPNANTNGF	IIAKDDGNGS	IYYGVKIQTN	ESHVTLSLHY	KTLGSNATYI	AKTTVMKYLE	ESVWLHLLII	LEDGIIEFYL
1450	1460	1470	1480	1490	1500	1510	1520
DGNAMPRGIK	SLKGEAITDG	PGILRIGAGI	NGNDRFTGLM	QDVRSYERKL	TLEEIYELHA	MPAKSDLHPI	SGYLEFRQGE
1530	1540	1550	1560	1570	1580	1590	1600
TNKSFIISAR	DDNDEEGEEL	FILKLVSVYG	GARISEENTT	ARLTIQKSDN	ANGLFGFTGA	CIPEIAEEGS	TISCVVERTR
1610	1620	1630	1640	1650	1660	1670	1680
GALDYVHVFY	TISQIETDGI	NYLVDDFANA	SGTITFLPWQ	RSEVLNIYVL	DDDIPELNEY	FRVTLVSAIP	GDGKLGSTPT
1690	1700	1710	1720	1730	1740	1750	1760
SGASIDPEKE	TTDITIKASD	HPYGLLQFST	GLPPQPKDAM	TLPASSVPHI	TVEEEDGEIR	LLVIRAQGLL	GRVTAEFRTV
1770	1780	1790	1800	1810	1820	1830	1840
SLTAFSPEDY	QNVAGTLEFQ	PGERYKYIFI	NITDNSIPEL	EKSFKVELLN	LEGGVAELFR	VDGSGSGDGD	MEFFLPTIHK
1850	1860	1870	1880	1890	1900	1910	1920
RASLGVASQI	LVTIAASDHA	HGVFEFSPES	LFVSGTEPED	GYSTVTLNVI	RHHGTLSPVT	LHWNIDSDPD	GDLAFTSGNI
1930	1940	1950	1960	1970	1980	1990	2000
TFEIGQTSAN	ITVEILPDED	PELDKAFSVS	VLSVSSGSLG	AHINATLTVL	ASDDPYGIFI	FSEKNRPVKV	EEATQNITLS
2010	2020	2030	2040	2050	2060	2070	2080
IIRLKGLMGK	VLVSYATLDD	MEKPPYFPPN	LARATQGRDY	IPASGFALFG	ANQSEATIAI	SILDDDEPER	SESVFIELLN
2090	2100	2110	2120	2130	2140	2150	2160
STLVAKVQSR	SIPNSPRLGP	KVETIAQLII	IANDDAFGTL	QLSAPIVRVA	ENHVGPIINV	TRTGGAFADV	SVKFKAVPIT
2170	2180	2190	2200	2210	2220	2230	2240

Detailed Protein Report

Cmpd.	No. of Cmpds.	m/z meas.	Δ m/z [ppm]	z	Rt [min]	Score	P	Range	Sequence	Modification
137	1	793.8712	-87.93	2	31.4	10.7	0	1990-2003	K.VEEATQNITLSIIR.L	
780	1	696.6836	-200.93	2	39.1	12.4	0	5266-5277	R.CAQMEPNALPFR.G	Oxidation: 4

Detailed Protein Report

Protein 181: sodium-driven chloride bicarbonate exchanger isoform 1 [Homo sapiens]

Accession: gi|295821221
Database: refseq_human(refseq_human_20140103.fasta)
Database Date: 2015-11-30
Modification(s): Oxidation

Score: 34.9
MW [kDa]: 125.9
pI: 6.0
Sequence Coverage [%]: 3.5
No. of unique Peptides: 2

Quantitation

MD:MU **Median:** 1.37 **CV:** 0.00 % **No. of Peptides:** 1

10	20	30	40	50	60	70	80
MEIKDQGAQM	EPLLPTRNDE	EAVVDRGGTR	SILKTHFEKE	DLEGHRTLFI	GVHVPLGGRK	SHRRHRHRGH	KHRKRDRERD
90	100	110	120	130	140	150	160
SGLEDGRESP	SFDTPSQRVQ	FILGTEDDDE	EHIPHDLFTE	LDEICWREGE	DAEWRETARW	LKFEEDVEDG	GERWSKPYVA
170	180	190	200	210	220	230	240
TLSLHSLFEL	RSCILNGTVL	LDMHANTLEE	IADMVLDQQV	SSGQLNEDVR	HRVHEALMKQ	HHHQNQKKLT	NRIPIVRSFA
250	260	270	280	290	300	310	320
DIGKKQSEPN	SMDKNAGQVV	SPQSAPACVE	NKNDVSRENS	TVDFSKGLGG	QQKGHTSPCG	MKQRHEKGPP	HQQEREVDLH
330	340	350	360	370	380	390	400
FMKKIPPGAE	ASNILVGELE	FLDRTVVAFV	RLSPAVLLQG	LAEVPIPTRF	LFILLGPLGK	GQQYHEIGRS	IATLMTDEVF
410	420	430	440	450	460	470	480
HDVAYKAKDR	NDLVSGIDEF	LDQVTVLPPG	EWDPSIRIEP	PKNVPSQEKR	KIPAVPNGTA	AHGEAEPHGG	HSGPELQRTG
490	500	510	520	530	540	550	560
RIFGGLILDI	KRKAPYFWSD	FRDAFSLQCL	ASFLFLYCAC	MSPVITFGGL	LGEATEGRIS	AIESLFGASM	TGIAYSLFGG
570	580	590	600	610	620	630	640
QPLTILGSTG	PVLVFEKILF	KFCKEYGLSY	LSLRASIGLW	TATLCIILVA	TDASSLVCYI	TRFTEEAFAS	LICIIFIYEA
650	660	670	680	690	700	710	720
LEKLFELSEA	YPINMHNDLE	LLTQYSCNCV	EPHNPSNGTL	KEWRESNISA	SDIIWENLTV	SECKSLHGEY	VGRACGHDHP
730	740	750	760	770	780	790	800
YVPDVLFWSV	ILFFSTVTLS	ATLKQFKTSR	YFPTKVRSIV	SDFAVFLTIL	CMVLIDYAIG	IPSPKLQVPS	VFKPTRDDRG
810	820	830	840	850	860	870	880
WFVTPLGPNP	WWTVIAAIIP	ALLCTILIFM	DQQITAVIIN	RKEHKLKKGC	GYHLDLLMVA	VMLGVCSIMG	LPWFVAATVL
890	900	910	920	930	940	950	960
SITHVNSLKL	ESECSAPGEQ	PKFLGIREQR	VTGLMIFILM	GSSVFMTSIL	KFIPMPVLYG	VFLYMGASSL	KGIQFFDRIK
970	980	990	1000	1010	1020	1030	1040
LFWMPAKHQP	DFIYLRHVPL	RKVHLFTIIQ	MSCLGLLWII	KVSRAAIVFP	MMVLALVFVR	KLMDLLFTKR	ELSWLDDLMP
1050	1060	1070	1080	1090	1100	1110	1120
ESKKKKLEDA	EKEEEQSMLA	MEDEGTVQLP	LEGHYRDDPS	VINISDEMSK	TALWRNLLIT	ADNSKDKESS	FPSKSSPS

Cmpd.	No. of Cmpds.	m/z meas.	Δ m/z [ppm]	z	Rt [min]	Score	P	Range	Sequence	Modification	Ratios
1817	1	847.8355	-100.68	2	52.5	10.3	1	278-293	R.ENSTVDFSKGLGGQQK.G		
1399	1	783.2967	-72.60	2	47.2	11.8	0	1077-1090	R.DDPSVINISDEMSK.T	Oxidation: 12	MD:MU 1.37

Detailed Protein Report

Protein 182: transcription regulator protein BACH1 [Homo sapiens]

**Accession:**	gi|4502353	**Score:**	34.6
**Database:**	refseq_human(refseq_human_20140103.fasta)	**MW [kDa]:**	81.9
**Database Date:**	2015-11-30	**pI:**	4.8
**Modification(s):**	Carbamidomethyl	**Sequence Coverage [%]:**	3.7
		**No. of unique Peptides:**	2

Alias proteins:

Accession	Name	Description
gi|530418993	refseq_human (refseq_human_20140103.fasta)	PREDICTED: transcription regulator protein BACH1 isoform X3 [Homo sapiens]
gi|530418991	refseq_human (refseq_human_20140103.fasta)	PREDICTED: transcription regulator protein BACH1 isoform X2 [Homo sapiens]
gi|530418989	refseq_human (refseq_human_20140103.fasta)	PREDICTED: transcription regulator protein BACH1 isoform X1 [Homo sapiens]
gi|45827690	refseq_human (refseq_human_20140103.fasta)	transcription regulator protein BACH1 [Homo sapiens]

```
        10         20         30         40         50         60         70         80
MSLSENSVFA YESSVHSTNV LLSLNDQRKK DVLCDVTIFV EGQRFRAHRS VLAACSSYFH SRIVGQADGE LNITLPEEVT
        90        100        110        120        130        140        150        160
VKGFEPLIQF AYTAKLILSK ENVDEVCKCV EFLSVHNIEE SCFQFLKFKF LDSTADQQEC PRKKCFSSHC QKTDLKLSLL
       170        180        190        200        210        220        230        240
DQRDLETDEV EEFLENKNVQ TPQCKLRRYQ GNAKASPPLQ DSASQTYESM CLEKDAALAL PSLCPKYRKF QKAFGTDRVR
       250        260        270        280        290        300        310        320
TGESSVKDIH ASVQPNERSE NECLGGVPEC RDLQVMLKCD ESKLAMEPEE TKKDPASQCP TEKSEVTPFP HNSSIDPHGL
       330        340        350        360        370        380        390        400
YSLSLLHTYD QYGDLNFAGM QNTTVLTEKP LSGTDVQEKT FGESQDLPLK SDLGTREDSS VASSDRSSVE REVAEHLAKG
       410        420        430        440        450        460        470        480
FWSDICSTDT PCQMQLSPAV AKDGSEQISQ KRSECPWLGI RISESPEPGQ RTFTTLSSVN CPFISTLSTE GCSSNLEIGN
       490        500        510        520        530        540        550        560
DDYVSEPQQE PCPYACVISL GDDSETDTEG DSESCSAREQ ECEVKLPFNA QRIISLSRND FQSLLKMHKL TPEQLDCIHD
       570        580        590        600        610        620        630        640
IRRRSKNRIA AQRCRKRKLD CIQNLESEIE KLQSEKESLL KERDHILSTL GETKQNLTGL CQKVCKEAAL SQEQIQILAK
       650        660        670        680        690        700        710        720
YSAADCPLSF LISEKDKSTP DGELALPSIF SLSDRPPAVL PPCARGNSEP GYARGQESQQ MSTATSEQAG PAEQCRQSGG
       730        740
ISDFCQQMTD KCTTDE
```

Cmpd.	No. of Cmpds.	m/z meas.	Δ m/z [ppm]	z	Rt [min]	Score	P	Range	Sequence	Modification
504	1	667.7055	-215.81	2	35.6	22.1	1	615-626	K.QNLTGLCQKVCK.E	
1770	1	851.2192	-170.05	2	52.1	12.5	0	717-731	R.QSGGISDFCQQMTDK.C	Carbamidomethyl: 9

Detailed Protein Report

Protein 183: myosin-9 [Homo sapiens]

**Accession:**	gi|12667788	**Score:**	34.6
**Database:**	refseq_human(refseq_human_20140103.fasta)	**MW [kDa]:**	226.4
**Database Date:**	2015-11-30	**pI:**	5.4
**Modification(s):**	Oxidation	**Sequence Coverage [%]:**	1.2
		**No. of unique Peptides:**	2

Quantitation

MD:MU	**Median:** 1.09	**CV:** 0.00 %	**No. of Peptides:** 1

BRUKER

Detailed Protein Report

10	20	30	40	50	60	70	80
MAQQAADKYL	YVDKNFINNP	LAQADWAAKK	LVWVPSDKSG	FEPASLKEEV	GEEAIVELVE	NGKKVKVNKD	DIQKMNPPKF
90	100	110	120	130	140	150	160
SKVEDMAELT	CLNEASVLHN	LKERYYSGLI	YTYSGLFCVV	INPYKNLPIY	SEEIVEMYKG	KKRHEMPPHI	YAITDTAYRS
170	180	190	200	210	220	230	240
MMQDREDQSI	LCTGESGAGK	TENTKKVIQY	LAYVASSHKS	KKDQGELERQ	LLQANPILEA	FGNAKTVKND	NSSRFGKFIR
250	260	270	280	290	300	310	320
INFDVNGYIV	GANIETYLLE	KSRAIRQAKE	ERTFHIFYYL	LSGAGEHLKT	DLLLEPYNKY	RFLSNGHVTI	PGQQDKDMFQ
330	340	350	360	370	380	390	400
ETMEAMRIMG	IPEEEQMGLL	RVISGVLQLG	NIVFKKERNT	DQASMPDNTA	AQKVSHLLGI	NVTDFTRGIL	TPRIKVGRDY
410	420	430	440	450	460	470	480
VQKAQTKEQA	DFAIEALAKA	TYERMFRWLV	LRINKALDKT	KRQGASFIGI	LDIAGFEIFD	LNSFEQLCIN	YTNEKLQQLF
490	500	510	520	530	540	550	560
NHTMFILEQE	EYQREGIEWN	FIDFGLDLQP	CIDLIEKPAG	PPGILALLDE	ECWFPKATDK	SFVEKVMQEQ	GTHPKFQKPK
570	580	590	600	610	620	630	640
QLKDKADFCI	IHYAGKVDYK	ADEWLMKNMD	PLNDNIATLL	HQSSDKFVSE	LWKDVDRIIG	LDQVAGMSET	ALPGAFKTRK
650	660	670	680	690	700	710	720
GMFRTVGQLY	KEQLAKLMAT	LRNTNPNFVR	CIIPNHEKKA	GKLDPHLVLD	QLRCNGVLEG	IRICRQGFPN	RVVFQEFRQR
730	740	750	760	770	780	790	800
YEILTPNSIP	KGFMDGKQAC	VLMIKALELD	SNLYRIGQSK	VFFRAGVLAH	LEEERDLKIT	DVIIGFQACC	RGYLARKAFA
810	820	830	840	850	860	870	880
KRQQQLTAMK	VLQRNCAAYL	KLRNWQWWRL	FTKVKPLLQV	SRQEEEMMAK	EEELVKVREK	QLAAENRLTE	METLQSQLMA
890	900	910	920	930	940	950	960
EKLQLQEQLQ	AETELCAEAE	ELRARLTAKK	QELEEICHDL	EARVEEEEER	CQHLQAEKKK	MQQNIQELEE	QLEEEESARQ
970	980	990	1000	1010	1020	1030	1040
KLQLEKVTTE	AKLKKLEEEQ	IILEDQNCKL	AKEKKLLEDR	IAEFTTNLTE	EEEKSKSLAK	LKNKHEAMIT	DLEERLRREE
1050	1060	1070	1080	1090	1100	1110	1120
KQRQELEKTR	RKLEGDSTDL	SDQIAELQAQ	IAELKMQLAK	KEEELQAALA	RVEEEAAQKN	MALKKIRELE	SQISELQEDL
1130	1140	1150	1160	1170	1180	1190	1200
ESERASRNKA	EKQKRDLGEE	LEALKTELED	TLDSTAAQQE	LRSKREQEVN	ILKKTLEEEA	KTHEAQIQEM	RQKHSQAVEE
1210	1220	1230	1240	1250	1260	1270	1280
LAEQLEQTKR	VKANLEKAKQ	TLENERGELA	NEVKVLLQGK	GDSEHKRKKV	EAQLQELQVK	FNEGERVRTE	LADKVTKLQV
1290	1300	1310	1320	1330	1340	1350	1360
ELDNVTGLLS	QSDSKSSKLT	KDFSALESQL	QDTQELLQEE	NRQKLSLSTK	LKQVEDEKNS	FREQLEEEEE	AKHNLEKQIA
1370	1380	1390	1400	1410	1420	1430	1440
TLHAQVADMK	KKMEDSVGCL	ETAEEVKRKL	QKDLEGLSQR	HEEKVAAYDK	LEKTKTRLQQ	ELDDLLVDLD	HQRQSACNLE
1450	1460	1470	1480	1490	1500	1510	1520
KKQKKFDQLL	AEEKTISAKY	AEERDRAEAE	AREKETKALS	LARALEEAME	QKAELERLNK	QFRTEMEDLM	SSKDDVGKSV
1530	1540	1550	1560	1570	1580	1590	1600
HELEKSKRAL	EQQVEEMKTQ	LEELEDELQA	TEDAKLRLEV	NLQAMKAQFE	RDLQGRDEQS	EEKKKQLVRQ	VREMEAELED
1610	1620	1630	1640	1650	1660	1670	1680
ERKQRSMAVA	ARKKLEMDLK	DLEAHIDSAN	KNRDEAIKQL	RKLQAQMKDC	MRELDDTRAS	REEILAQAKE	NEKKLKSMEA
1690	1700	1710	1720	1730	1740	1750	1760
EMIQLQEELA	AAERAKRQAQ	QERDELADEI	ANSSGKGALA	LEEKRRLEAR	IAQLEEELEE	EQGNTELIND	RLKKANLQID
1770	1780	1790	1800	1810	1820	1830	1840
QINTDLNLER	SHAQKNENAR	QQLERQNKEL	KVKLQEMEGT	VKSKYKASIT	ALEAKIAQLE	EQLDNETKER	QAACKQVRRT
1850	1860	1870	1880	1890	1900	1910	1920
EKKLKDVLLQ	VDDERRNAEQ	YKDQADKAST	RLKQLKRQLE	EAEEEAQRAN	ASRRKLQREL	EDATETADAM	NREVSSLKNK
1930	1940	1950	1960	1970			
LRRGDLPFVV	PRRMARKGAG	DGSDEEVDGK	ADGAEAKPAE				

Cmpd.	No. of Cmpds.	m/z meas.	Δ m/z [ppm]	z	Rt [min]	Score	P	Range	Sequence	Modification	Ratios
1950	1	856.8003	-136.41	2	54.5	12.5	1	910-923	K.KQELEEICHDLEAR.V		
502	2	567.1670	-259.21	2	35.6	22.1	2	1604-1613	K.QRSMAVAARK.K	Oxidation: 4	MD:MU 1.09

Detailed Protein Report

Protein 184: neuron navigator 2 isoform 3 [Homo sapiens]

**Accession:**	gi|161169015	**Score:**	34.5
**Database:**	refseq_human(refseq_human_20140103.fasta)	**MW [kDa]:**	254.8
**Database Date:**	2015-11-30	**pI:**	9.7
**Modification(s):**	Carbamidomethyl, Oxidation	**Sequence Coverage [%]:**	1.3
		**No. of unique Peptides:**	2

Detailed Protein Report

10	20	30	40	50	60	70	80
MESVSESSQQ	QKRKPVIHGL	EDQKRIYTDW	ANHYLAKSGH	KRLIRDLQQD	VTDGVLLAQI	IQVVANEKIE	DINGCPKNRS
90	100	110	120	130	140	150	160
QMIENIDACL	NFLAAKGINI	QGLSAEEIRN	GNLKAILGLF	FSLSRYKQQQ	QQPQKQHLSS	PLPPAVSQVA	GAPSQCQAGT
170	180	190	200	210	220	230	240
PQQQVPVTPQ	APCQPHQPAP	HQQSKAQAEM	QSRLPGPTAR	VSAAGSEAKT	RGGSTTANNR	RSQSFNNYDK	SKPVTSPPPP
250	260	270	280	290	300	310	320
PSSHEKEPLA	SSASSHPGMS	DNAPASLESG	SSSTPTNCST	SSAIPQPGAA	TKPWRSKSLS	VKHSATVSML	SVKPPGPEAP
330	340	350	360	370	380	390	400
RPTPEAMKPA	PNNQKSMLEK	LKLFNSKGGS	KAGEGPGSRD	TSCERLETLP	SFEESEELEA	ASRMLTTVGP	ASSSPKIALK
410	420	430	440	450	460	470	480
GIAQRTFSRA	LTNKKSSLKG	NEKEKEKQQR	EKDKEKSKDL	AKRASVTERL	DLKEEPKEDP	SGAAVPEMPK	KSSKIASFIP
490	500	510	520	530	540	550	560
KGGKLNSAKK	EPMAPSHSGI	PKPGMKSMPG	KSPSAPAPSK	EGERSRSGKL	SSGLPQQKPQ	LDGRHSSSSS	SLASSEGKGP
570	580	590	600	610	620	630	640
GGTTLNHSIS	SQTVSGSVGT	TQTTGSNTVS	VQLPQPQQQY	NHPNTATVAP	FLYRSQTDTE	GNVTAESSST	GVSVEPSHFT
650	660	670	680	690	700	710	720
KTGQPALEEL	TGEDPEARRL	RTVKNIADLR	QNLEETMSSL	RGTQVTHSTL	ETTFDTNVTT	EMSGRSILSL	TGRPTPLSWR
730	740	750	760	770	780	790	800
LGQSSPRLQA	GDAPSMGNGY	PPRANASRFI	NTESGRYVYS	APLRRQLASR	GSSVCHVDVS	DKAGDEMDLE	GISMDAPGYM
810	820	830	840	850	860	870	880
SDGDVLSKNI	RTDDITSGYM	TDGGLGLYTR	RLNRLPDGMA	VVRETLQRNT	SLGLGDADSW	DDSSSVSSGI	SDTIDNLSTD
890	900	910	920	930	940	950	960
DINTSSSISS	YANTPASSRK	NLDVQTDAEK	HSQVERNSLW	SGDDVKKSDG	GSDSGIKMEP	GSKWRRNPSD	VSDESDKSTS
970	980	990	1000	1010	1020	1030	1040
GKKNPVISQT	GSWRRGMTAQ	VGITMPRTKP	SAPAGALKTP	GTGKTDDAKV	SEKGRLSPKA	SQVKRSPSDA	GRSSGDESKK
1050	1060	1070	1080	1090	1100	1110	1120
PLPSSSRTPT	ANANSFGFKK	QSGSAAGLAM	ITASGVTVTS	RSATLGKIPK	SSALVSRSAG	RKSSMDGAQN	QDDGYLALSS
1130	1140	1150	1160	1170	1180	1190	1200
RTNLQYRSLP	RPSKSNSRNG	AGNRSSTSSI	DSNISSKSAG	LPVPKLREPS	KTALGSSLPG	LVNQTDKEKG	ISSDNESVAS
1210	1220	1230	1240	1250	1260	1270	1280
CNSVKVNPAA	QPVSSPAQTS	LQPGAKYPDV	ASPTLRRLFG	GKPTKQVPIA	TAENMKNSVV	ISNPHATMTQ	QGNLDSPSGS
1290	1300	1310	1320	1330	1340	1350	1360
GVLSSGSSSP	LYSKNVDLNQ	SPLASSPSSA	HSAPSNSLTW	GTNASSSSAV	SKDGLGFQSV	SSLHTSCESI	DISLSSGGVP
1370	1380	1390	1400	1410	1420	1430	1440
SHNSSTGLIA	SSKDDSLTPF	VRTNSVKTTL	SESPLSSPAA	SPKFCRSTLP	RKQDSDPHLD	RNTLPKKGLR	YTPTSQLRTQ
1450	1460	1470	1480	1490	1500	1510	1520
EDAKEWLRSH	SAGGLQDTAA	NSPFSSGSSV	TSPSGTRFNF	SQLASPTTVT	QMSLSNPTML	RTHSLSNADG	QYDPYTDSRF
1530	1540	1550	1560	1570	1580	1590	1600
RNSSMSLDEK	SRTMSRSGSF	RDGFEEVHGS	SLSLVSSTSS	VYSTPEEKCQ	SEIRKLRREL	DASQEKVSAL	TTQLTANAHL
1610	1620	1630	1640	1650	1660	1670	1680
VAAFEQSLGN	MTIRLQSLTM	TAEQKDSELN	ELRKTIELLK	KQNAAAQAAI	NGVINTPELN	CKGNGTAQSA	DLRIRRQHSS
1690	1700	1710	1720	1730	1740	1750	1760
DSVSSINSAT	SHSSVGSNIE	SDSKKKKRKN	WLRSSFKQAF	GKKKSPKSAS	SHSDIEEMTD	SSLPSSPKLP	HNGSTGSTPL
1770	1780	1790	1800	1810	1820	1830	1840
LRNSHSNSLI	SECMDSEAET	VMQLRNELRD	KEMKLTDIRL	EALSSAHQLD	QLREAMNRMQ	SEIEKLKAEN	DRLKSESQGS
1850	1860	1870	1880	1890	1900	1910	1920
GCSRAPSQVS	ISASPRQSMG	LSQHSLNLTE	STSLDMLLDD	TGECSARKEG	GRHVKIVVSF	QEEMKWKEDS	RPHLFLIGCI
1930	1940	1950	1960	1970	1980	1990	2000
GVSGKTKWDV	LDGVVRRLFK	EYIIHVDPVS	QLGLNSDSVL	GYSIGEIKRS	NTSETPELLP	CGYLVGENTT	ISVTVKGLAE
2010	2020	2030	2040	2050	2060	2070	2080
NSLDSLVFES	LIPKPILQRY	VSLLIEHRRI	ILSGPSGTGK	TYLANRLSEY	IVLREGRELT	DGVIATFNVD	HKSSKELRQY
2090	2100	2110	2120	2130	2140	2150	2160
LSNLADQCNS	ENNAVDMPLV	IILDNLHHVS	SLGEIFNGLL	NCKYHKCPYI	IGTMNQATSS	TPNLQLHHNF	RWVLCANHTE
2170	2180	2190	2200	2210	2220	2230	2240

Detailed Protein Report

Cmpd.	No. of Cmpds.	m/z meas.	Δ m/z [ppm]	z	Rt [min]	Score	P	Range	Sequence	Modification
183	1	904.3719	-34.40	2	31.7	10.6	2	348-365	K.GGSKAGEGPGSRDTSCER.L	Carbamidomethyl: 16
2463	1	647.1849	-205.84	2	61.2	24.0	0	976-987	R.GMTAQVGITMPR.T	Oxidation: 2, 10

Detailed Protein Report

Protein 185: PREDICTED: DNA polymerase kappa isoform X3 [Homo sapiens]

**Accession:**	gi|530379363	**Score:**	34.5
**Database:**	refseq_human(refseq_human_20140103.fasta)	**MW [kDa]:**	57.6
**Database Date:**	2015-11-30	**pI:**	9.2
**Modification(s):**	Oxidation	**Sequence Coverage [%]:**	10.1
		**No. of unique Peptides:**	2

10	20	30	40	50	60	70	80
MDSTKEKCDS	YKDDLLLRMG	LNDNKAGMEG	LDKEKINKII	MEATKGSRFY	GNELKKEKQV	NQRIENMMQQ	KAQITSQQLR
**90**	**100**	**110**	**120**	**130**	**140**	**150**	**160**
KAQLQVDRFA	MELEQSRNLS	NTIVHIDMDA	FYAAVEMRDN	PELKDKPIAV	GSMSMLSTSN	YHARRFGVRA	AMPGFIAKRL
**170**	**180**	**190**	**200**	**210**	**220**	**230**	**240**
CPQLIIVPPN	FDKYRAVSKE	VKEILADYDP	NFMAMSLDEA	YLNITKHLEE	RQNWPEDKRR	YFIKMGSSVE	NDLQNLYIQR
**250**	**260**	**270**	**280**	**290**	**300**	**310**	**320**
SITFTDNPGK	EVNKLSEHER	SISPLLFEES	PSDVQPPGDP	FQVNFEEQNN	PQILQNSVVF	GTSAQEVVKE	IRFRIEQKTT
**330**	**340**	**350**	**360**	**370**	**380**	**390**	**400**
LTASAGIAPN	TMLAKVCSDK	NKPNGQYQIL	PNRQAVMDFI	KDLPIRKVSG	IGKVTEKMLK	ALGIITCTEL	YQQRALLSLL
**410**	**420**	**430**	**440**	**450**	**460**	**470**	**480**
FSETSWHYFL	HISLGLGSTH	LTRDGERKSM	SVERTFSEIN	KAEEQYSLCQ	ELCSELAQDL	QKERLKVFGY	LVFPMKRTGN
**490**	**500**	**510**					
TNKGALLAFY	RLETKPCQPL	SVH					

Cmpd.	No. of Cmpds.	m/z meas.	Δ m/z [ppm]	z	Rt [min]	Score	P	Range	Sequence	Modification
2396	1	1045.2249	47.66	3	60.3	12.4	1	180-206	K.EVKEILADYDPNFMAMSLDEAYLNITK.H	
1772	1	807.4927	33.69	2	52.1	10.9	2	465-477	R.LKVFGYLVFPMKR.T	Oxidation: 11

Detailed Protein Report

Protein 186: PREDICTED: methylcytosine dioxygenase TET3 isoform X1 [Homo sapiens]

**Accession:**	gi|530367326	**Score:**	34.5
**Database:**	refseq_human(refseq_human_20140103.fasta)	**MW [kDa]:**	193.6
**Database Date:**	2015-11-30	**pI:**	9.0
		**Sequence Coverage [%]:**	0.8
		**No. of unique Peptides:**	1

Alias proteins:

Accession	Name	Description
gi|566559863	refseq_human (refseq_human_20140103.fasta)	methylcytosine dioxygenase TET3 [Homo sapiens]

Detailed Protein Report

10	20	30	40	50	60	70	80
MSQFQVPLAV	QPDLPGLYDF	PQRQVMVGSF	PGSGLSMAGS	ESQLRGGGDG	RKKRKRCGTC	EPCRRLENCG	ACTSCTNRRT
90	100	110	120	130	140	150	160
HQICKLRKCE	VLKKKVGLLK	EVEIKAGEGA	GPWGQGAAVK	TGSELSPVDG	PVPGQMDSGP	VYHGDSRQLS	ASGVPVNGAR
170	180	190	200	210	220	230	240
EPAGPSLLGT	GGPWRVDQKP	DWEAAPGPAH	TARLEDAHDL	VAFSAVAEAV	SSYGALSTRL	YETFNREMSR	EAGNNSRGPR
250	260	270	280	290	300	310	320
PGPEGCSAGS	EDLDTLQTAL	ALARHGMKPP	NCNCDGPECP	DYLEWLEGKI	KSVVMEGGEE	RPRLPGPLPP	GEAGLPAPST
330	340	350	360	370	380	390	400
RPLLSSEVPQ	ISPQEGLPLS	QSALSIAKEK	NISLQTAIAI	EALTQLSSAL	PQPSHSTPQA	SCPLPEALSP	PAPFRSPQSY
410	420	430	440	450	460	470	480
LRAPSWPVVP	PEEHSSFAPD	SSAFPPATPR	TEFPEAWGTD	TPPATPRSSW	PMPRPSPDPM	AELEQLLGSA	SDYIQSVFKR
490	500	510	520	530	540	550	560
PEALPTKPKV	KVEAPSSSPA	PAPSPVLQRE	APTPSSEPDT	HQKAQTALQQ	HLHHKRSLFL	EQVHDTSFPA	PSEPSAPGWW
570	580	590	600	610	620	630	640
PPPSSPVPRL	PDRPPKEKKK	KLPTPAGGPV	GTEKAAPGIK	PSVRKPIQIK	KSRPREAQPL	FPPVRQIVLE	GLRSPASQEV
650	660	670	680	690	700	710	720
QAHPPAPLPA	SQGSAVPLPP	EPSLALFAPS	PSRDSLLPPT	QEMRSPSPMT	ALQPGSTGPL	PPADDKLEEL	IRQFEAEFGD
730	740	750	760	770	780	790	800
SFGLPGPPSV	PIQDPENQQT	CLPAPESPFA	TRSPKQIKIE	SSGAVTVLST	TCFHSEEGGQ	EATPTKAENP	LTPTLSGFLE
810	820	830	840	850	860	870	880
SPLKYLDTPT	KSLLDTPAKR	AQAEFPTCDC	VEQIVEKDEG	PYYTHLGSGP	TVASIRELME	ERYGEKGKAI	RIEKVIYTGK
890	900	910	920	930	940	950	960
EGKSSRGCPI	AKWVIRRHTL	EEKLLCLVRH	RAGHHCQNAV	IVILILAWEG	IPRSLGDTLY	QELTDTLRKY	GNPTSRRCGL
970	980	990	1000	1010	1020	1030	1040
NDDRTCACQG	KDPNTCGASF	SFGCSWSMYF	NGCKYARSKT	PRKFRLAGDN	PKEEEVLRKS	FQDLATEVAP	LYKRLAPQAY
1050	1060	1070	1080	1090	1100	1110	1120
QNQVTNEEIA	IDCRLGLKEG	RPFAGVTACM	DFCAHAHKDQ	HNLYNGCTVV	CTLTKEDNRC	VGKIPEDEQL	HVLPLYKMAN
1130	1140	1150	1160	1170	1180	1190	1200
TDEFGSEENQ	NAKVGSGAIQ	VLTAFPREVR	RLPEPAKSCR	QRQLEARKAA	AEKKKIQKEK	LSTPEKIKQE	ALELAGITSD
1210	1220	1230	1240	1250	1260	1270	1280
PGLSLKGGLS	QQGLKPSLKV	EPQNHFSSFK	YSGNAVVESY	SVLGNCRPSD	PYSMNSVYSY	HSYYAQPSLT	SVNGFHSKYA
1290	1300	1310	1320	1330	1340	1350	1360
LPSFSYYGFP	SSNPVFPSQF	LGPGAWGHSG	SSGSFEKKPD	LHALHNSLSP	AYGGAEFAEL	PSQAVPTDAH	HPTPHHQQPA
1370	1380	1390	1400	1410	1420	1430	1440
YPGPKEYLLP	KAPLLHSVSR	DPSPFAQSSN	CYNRSIKQEP	VDPLTQAEPV	PRDAGKMGKT	PLSEVSQNGG	PSHLWGQYSG
1450	1460	1470	1480	1490	1500	1510	1520
GPSMSPKRTN	GVGGSWGVFS	SGESPAIVPD	KLSSFGASCL	APSHFTDGQW	GLFPGEGQQA	ASHSGGRLRG	KPWSPCKFGN
1530	1540	1550	1560	1570	1580	1590	1600
STSALAGPSL	TEKPWALGAG	DFNSALKGSP	GFQDKLWNPM	KGEEGRIPAA	GASQLDRAWQ	SFGLPLGSSE	KLFGALKSEE
1610	1620	1630	1640	1650	1660	1670	1680
KLWDPFSLEE	GPAEEPPSKG	AVKEEKGGGG	AEEEEEELWS	DSEHNFLDEN	IGGVAVAPAH	GSILIECARR	ELHATTPLKK
1690	1700	1710	1720	1730	1740	1750	1760
PNRCHPTRIS	LVFYQHKNLN	QPNHGLALWE	AKMKQLAERA	RARQEEAARL	GLGQQEAKLY	GKKRKWGGTV	VAEPQQKEKK
1770	1780	1790	1800				
GVVPTRQALA	VPTDSAVTVS	SYAYTKVTGP	YSRWI				

Cmpd.	No. of Cmpds.	m/z meas.	Δ m/z [ppm]	z	Rt [min]	Score	P	Range	Sequence	Modification
806	1	546.9707	-505.05	1	39.6	11.3	0	1562-1566	K.GEEGR.I	

Detailed Protein Report

Protein 187: scm-like with four MBT domains protein 1 [Homo sapiens]

Accession: gi|53692193
Database: refseq_human(refseq_human_20140103.fasta)
Database Date: 2015-11-30
Modification(s): Oxidation

Score: 34.5
MW [kDa]: 98.1
pI: 5.8
Sequence Coverage [%]: 4.5
No. of unique Peptides: 2

Alias proteins:

Accession	Name	Description
gi|578806223	refseq_human (refseq_human_20140103.fasta)	PREDICTED: scm-like with four MBT domains protein 1 isoform X4 [Homo sapiens]
gi|578806221	refseq_human (refseq_human_20140103.fasta)	PREDICTED: scm-like with four MBT domains protein 1 isoform X3 [Homo sapiens]
gi|530372640	refseq_human (refseq_human_20140103.fasta)	PREDICTED: scm-like with four MBT domains protein 1 isoform X2 [Homo sapiens]

10	20	30	40	50	60	70	80
MNGEQQLDAD	AGSGMEEVEL	SWEDYLEETG	STAVPYGSFK	HVDTRLQNGF	APGMKLEVAV	RTDPETYWVA	TVITTCEQLL
**90**	**100**	**110**	**120**	**130**	**140**	**150**	**160**
LLRYDGYGED	RRADFWCDIR	KADLYPIGWC	EQNKKTLEAP	EGIRDKVSDW	DEFLRQTLIG	ACSPPVPLLE	GLRNGRNPLD
**170**	**180**	**190**	**200**	**210**	**220**	**230**	**240**
LIAPGSRLEC	QAFQDSLSTW	IVTVVENIGG	RLKLRYEGLE	SSDNYEHWLY	YLDPFLHHVG	WAAQQGYELQ	PPSAIRHLKN
**250**	**260**	**270**	**280**	**290**	**300**	**310**	**320**
EAEWQEILAK	VKEEEEEPLP	SYLFKDKQVI	GIHTFSVNMK	LEAVDPWSPF	GISPATVVKV	FDEKYFLVEM	DDLRPENHAR
**330**	**340**	**350**	**360**	**370**	**380**	**390**	**400**
RSFVCHADSP	GIFPVQWSLK	NGLHISPPPG	YPSQDFDWAD	YLKQCGAEAA	PQRCFPPLIS	EHEFKENMKL	EAVNPILPEE
**410**	**420**	**430**	**440**	**450**	**460**	**470**	**480**
VCVATITAVR	GSYLWLQLEG	SKKPIPECIV	SVESMDIFPL	GWCETNGHPL	STPRRARVYK	QRKIAVVQPE	KQVPSSRTVH
**490**	**500**	**510**	**520**	**530**	**540**	**550**	**560**
EGLRNQELNS	TESVMINGKY	CCPKIYFNHR	CFSGPYLNKG	RIAELPQCVG	PGNCVLVLRE	VLTLLINAAY	KPSRVLRELQ
**570**	**580**	**590**	**600**	**610**	**620**	**630**	**640**
LDKDSVWHGC	GEVLKAKYKG	KSYRATVEIV	KTADRVTEFC	RQTCIKLECC	PNLFGPRMVL	DKCSENCSVL	TKTKYTHYYG
**650**	**660**	**670**	**680**	**690**	**700**	**710**	**720**
KKKNKRIGRP	PGGHSNLACA	LKKASKRRKR	RKNVFVHKKK	RSSASVDNTP	AGSPQGSGGE	DEDDPDEGDD	DSLSEGSTSE
**730**	**740**	**750**	**760**	**770**	**780**	**790**	**800**
QQDELQEESE	MSEKKSCSSS	PTQSEISTSL	PPDRQRRKRE	LRTFSFSDDE	NKPPSPKEIR	IEVAERLHLD	SNPLKWSVAD
**810**	**820**	**830**	**840**	**850**	**860**	**870**	
VVRFIRSTDC	APLARIFLDQ	EIDGQALLLL	TLPTVQECMD	LKLGPAIKLC	HHIERIKFAF	YEQFAN	

Cmpd.	No. of Cmpds.	m/z meas.	Δ m/z [ppm]	z	Rt [min]	Score	P	Range	Sequence	Modification
2196	1	873.3355	-156.25	2	57.3	10.7	1	46-61	R.LQNGFAPGMKLEVAVR.T	Oxidation: 9
1978	2	656.8089	-9.85	2	54.8	12.3	1	623-634	K.CSENCSVLTKTK.Y	

Detailed Protein Report

Protein 188: proteoglycan 4 isoform D precursor [Homo sapiens]

**Accession:**	gi|189181724	**Score:**	34.4
**Database:**	refseq_human(refseq_human_20140103.fasta)	**MW [kDa]:**	136.5
**Database Date:**	2015-11-30	**pI:**	10.1
		**Sequence Coverage [%]:**	1.4
		**No. of unique Peptides:**	1

10	20	30	40	50	60	70	80
MAWKTLPIYL	LLLLSVFVIQ	QVSSQELSCK	GRCFESFERG	RECDCDAQCK	KYDKCCPDYE	SFCAEVKDNK	KNRTKKKPTP
**90**	**100**	**110**	**120**	**130**	**140**	**150**	**160**
KPPVVDEAGS	GLDNGDFKVT	TPDTSTTQHN	KVSTSPKITT	AKPINPRPSL	PPNSDTSKET	SLTVNKETTV	ETKETTTTNK
**170**	**180**	**190**	**200**	**210**	**220**	**230**	**240**
QTSTDGKEKT	TSAKETQSIE	KTSAKDLAPT	SKVLAKPTPK	AETTTKGPAL	TTPKEPTPTT	PKEPASTTPK	EPTPTTIKSA
**250**	**260**	**270**	**280**	**290**	**300**	**310**	**320**
PTTPKEPAPT	TTKSAPTTPK	EPAPTTTKEP	APTTPKEPAP	TTTKEPAPTT	TKSAPTTPKE	PAPTTPKKPA	PTTPKEPAPT
**330**	**340**	**350**	**360**	**370**	**380**	**390**	**400**
TPKEPTPTTP	KEPAPTTKEP	APTTPKEPAP	TAPKKPAPTT	PKEPAPTTPK	EPAPTTTKEP	SPTTPKEPAP	TTTKSAPTTT
**410**	**420**	**430**	**440**	**450**	**460**	**470**	**480**
KEPAPTTTKS	APTTPKEPSP	TTTKEPAPTT	PKEPAPTTPK	KPAPTTPKEP	APTTPKEPAP	TTTKKPAPTT	PKEPAPTTPK
**490**	**500**	**510**	**520**	**530**	**540**	**550**	**560**
ETAPTTPKKL	TPTTPEKLAP	TTPEKPAPTT	PEELAPTTPE	EPTPTTPEEP	APTTPKAAAP	NTPKEPAPTT	PKEPAPTTPK
**570**	**580**	**590**	**600**	**610**	**620**	**630**	**640**
EPAPTTPKET	APTTPKGTAP	TTLKEPAPTT	PKKPAPKELA	PTTTKEPTST	TCDKPAPTTP	KGTAPTTPKE	PAPTTPKEPA
**650**	**660**	**670**	**680**	**690**	**700**	**710**	**720**
PTTPKGTAPT	TLKEPAPTTP	KKPAPKELAP	TTTKGPTSTT	SDKPAPTTPK	ETAPTTPKEP	APTTPKKPAP	TTPETPPPTT
**730**	**740**	**750**	**760**	**770**	**780**	**790**	**800**
SEVSTPTTTK	EPTTIHKSPD	ESTPELSAEP	TPKALENSPK	EPGVPTTKTP	AATKPEMTTT	AKDKTTERDL	RTTPETTTAA
**810**	**820**	**830**	**840**	**850**	**860**	**870**	**880**
PKMTKETATT	TEKTTESKIT	ATTTQVTSTT	TQDTTPFKIT	TLKTTTLAPK	VTTTKKTITT	TEIMNKPEET	AKPKDRATNS
**890**	**900**	**910**	**920**	**930**	**940**	**950**	**960**
KATTPKPQKP	TKAPKKPTST	KKPKTMPRVR	KPKTTPTPRK	MTSTMPELNP	TSRIAEAMLQ	TTTRPNQTPN	SKLVEVNPKS
**970**	**980**	**990**	**1000**	**1010**	**1020**	**1030**	**1040**
EDAGGAEGET	PHMLLRPHVF	MPEVTPDMDY	LPRVPNQGII	INPMLSDETN	ICNGKPVDGL	TTLRNGTLVA	FRGHYFWMLS
**1050**	**1060**	**1070**	**1080**	**1090**	**1100**	**1110**	**1120**
PFSPPSPARR	ITEVWGIPSP	IDTVFTRCNC	EGKTFFFKDS	QYWRFTNDIK	DAGYPKPIFK	GFGGLTGQIV	AALSTAKYKN
**1130**	**1140**	**1150**	**1160**	**1170**	**1180**	**1190**	**1200**
WPESVYFFKR	GGSIQQYIYK	QEPVQKCPGR	RPALNYPVYG	ETTQVRRRRF	ERAIGPSQTH	TIRIQYSPAR	LAYQDKGVLH
**1210**	**1220**	**1230**	**1240**	**1250**	**1260**	**1270**	**1280**
NEVKVSILWR	GLPNVVTSAI	SLPNIRKPDG	YDYYAFSKDQ	YYNIDVPSRT	ARAITTRSGQ	TLSKVWYNCP	

Cmpd.	No. of Cmpds.	m/z meas.	Δ m/z [ppm]	z	Rt [min]	Score	P	Range	Sequence	Modification
2237	1	994.8741	-142.64	2	57.8	23.9	2	785-802	K.TTERDLRTTPETTTAAPK.M	

Detailed Protein Report

Protein 189: DNA-directed RNA polymerase II subunit RPB1 [Homo sapiens]

**Accession:**	gi|4505939	**Score:**	34.4
**Database:**	refseq_human(refseq_human_20140103.fasta)	**MW [kDa]:**	217.1
**Database Date:**	2015-11-30	**pI:**	7.1
		**Sequence Coverage [%]:**	2.2
		**No. of unique Peptides:**	1

Detailed Protein Report

10	20	30	40	50	60	70	80
MHGGGPPSGD	SACPLRTIKR	VQFGVLSPDE	LKRMSVTEGG	IKYPETTEGG	RPKLGGLMDP	RQGVIERTGR	CQTCAGNMTE
90	100	110	120	130	140	150	160
CPGHFGHIEL	AKPVFHVGFL	VKTMKVLRCV	CFFCSKLLVD	SNNPKIKDIL	AKSKGQPKKR	LTHVYDLCKG	KNICEGGEEM
170	180	190	200	210	220	230	240
DNKFGVEQPE	GDEDLTKEKG	HGGCGRYQPR	IRRSGLELYA	EWKHVNEDSQ	EKKILLSPER	VHEIFKRISD	EECFVLGMEP
250	260	270	280	290	300	310	320
RYARPEWMIV	TVLPVPPLSV	RPAVVMQGSA	RNQDDLTHKL	ADIVKINNQL	RRNEQNGAAA	HVIAEDVKLL	QFHVATMVDN
330	340	350	360	370	380	390	400
ELPGLPRAMQ	KSGRPLKSLK	QRLKGKEGRV	RGNLMGKRVD	FSARTVITPD	PNLSIDQVGV	PRSIAANMTF	AEIVTPFNID
410	420	430	440	450	460	470	480
RLQELVRRGN	SQYPGAKYII	RDNGDRIDLR	FHPKPSDLHL	QTGYKVERHM	CDGDIVIFNR	QPTLHKMSMM	GHRVRILPWS
490	500	510	520	530	540	550	560
TFRLNLSVTT	PYNADFDGDE	MNLHLPQSLE	TRAEIQELAM	VPRMIVTPQS	NRPVMGIVQD	TLTAVRKFTK	RDVFLERGEV
570	580	590	600	610	620	630	640
MNLLMFLSTW	DGKVPQPAIL	KPRPLWTGKQ	IFSLIIPGHI	NCIRTHSTHP	DDEDSGPYKH	ISPGDTKVVV	ENGELIMGIL
650	660	670	680	690	700	710	720
CKKSLGTSAG	SLVHISYLEM	GHDITRLFYS	NIQTVINNWL	LIEGHTIGIG	DSIADSKTYQ	DIQNTIKKAK	QDVIEVIEKA
730	740	750	760	770	780	790	800
HNNELEPTPG	NTLRQTFENQ	VNRILNDARD	KTGSSAQKSL	SEYNNFKSMV	VSGAKGSKIN	ISQVIAVVGQ	QNVEGKRIPF
810	820	830	840	850	860	870	880
GFKHRTLPHF	IKDDYGPESR	GFVENSYLAG	LTPTEFFFHA	MGGREGLIDT	AVKTAETGYI	QRRLIKSMES	VMVKYDATVR
890	900	910	920	930	940	950	960
NSINQVVQLR	YGEDGLAGES	VEFQNLATLK	PSNKAFEKKF	RFDYTNERAL	RRTLQEDLVK	DVLSNAHIQN	ELEREFERMR
970	980	990	1000	1010	1020	1030	1040
EDREVLRVIF	PTGDSKVVLP	CNLLRMIWNA	QKIFHINPRL	PSDLHPIKVV	EGVKELSKKL	VIVNGDDPLS	RQAQENATLL
1050	1060	1070	1080	1090	1100	1110	1120
FNIHLRSTLC	SRRMAEEFRL	SGEAFDWLLG	EIESKFNQAI	AHPGEMVGAL	AAQSLGEPAT	QMTLNTFHYA	GVSAKNVTLG
1130	1140	1150	1160	1170	1180	1190	1200
VPRLKELINI	SKKPKTPSLT	VFLLGQSARD	AERAKDILCR	LEHTTLRKVT	ANTAIYYDPN	PQSTVVAEDQ	EWVNVYYEMP
1210	1220	1230	1240	1250	1260	1270	1280
DFDVARISPW	LLRVELDRKH	MTDRKLTMEQ	IAEKINAGFG	DDLNCIFNDD	NAEKLVLRIR	IMNSDENKMQ	EEEEVVDKMD
1290	1300	1310	1320	1330	1340	1350	1360
DDVFLRCIES	NMLTDMTLQG	IEQISKVYMH	LPQTDNKKKI	IITEDGEFKA	LQEWILETDG	VSLMRVLSEK	DVDPVRTTSN
1370	1380	1390	1400	1410	1420	1430	1440
DIVEIFTVLG	IEAVRKALER	ELYHVISFDG	SYVNYRHLAL	LCDTMTCRGH	LMAITRHGVN	RQDTGPLMKC	SFEETVDVLM
1450	1460	1470	1480	1490	1500	1510	1520
EAAAHGESDP	MKGVSENIML	GQLAPAGTGC	FDLLLDAEKC	KYGMEIPTNI	PGLGAAGPTG	MFFGSAPSPM	GGISPAMTPW
1530	1540	1550	1560	1570	1580	1590	1600
NQGATPAYGA	WSPSVGSGMT	PGAAGFSPSA	ASDASGFSPG	YSPAWSPTPG	SPGSPGPSSP	YIPSPGGAMS	PSYSPTSPAY
1610	1620	1630	1640	1650	1660	1670	1680
EPRSPGGYTP	QSPSYSPTSP	SYSPTSPSYS	PTSPNYSPTS	PSYSPTSPSY	SPTSPSYSPT	SPSYSPTSPS	YSPTSPSYSP
1690	1700	1710	1720	1730	1740	1750	1760
TSPSYSPTSP	SYSPTSPSYS	PTSPSYSPTS	PSYSPTSPSY	SPTSPSYSPT	SPSYSPTSPS	YSPTSPNYSP	TSPNYTPTSP
1770	1780	1790	1800	1810	1820	1830	1840
SYSPTSPSYS	PTSPNYTPTS	PNYSPTSPSY	SPTSPSYSPT	SPSYSPSSPR	YTPQSPTYTP	SSPSYSPSSP	SYSPTSPKYT
1850	1860	1870	1880	1890	1900	1910	1920
PTSPSYSPSS	PEYTPTSPKY	SPTSPKYSPT	SPKYSPTSPT	YSPTTPKYSP	TSPTYSPTSP	VYTPTSPKYS	PTSPTYSPTS
1930	1940	1950	1960	1970	1980		
PKYSPTSPTY	SPTSPKGSTY	SPTSPGYSPT	SPTYSLTSPA	ISPDDSDEEN			

Cmpd.	No. of Cmpds.	m/z meas.	Δ m/z [ppm]	z	Rt [min]	Score	P	Range	Sequence	Modification
652	1	918.1693	7.00	3	37.5	17.7	2	358-382	K.RVDFSARTVITPDPNLSIDQVGVPR.S	

Detailed Protein Report

Protein 190: **monofunctional C1-tetrahydrofolate synthase, mitochondrial isoform 3 [Homo sapiens]**

**Accession:**	gi|337756507	**Score:**	34.3
**Database:**	refseq_human(refseq_human_20140103.fasta)	**MW [kDa]:**	99.2
**Database Date:**	2015-11-30	**pI:**	7.0
		**Sequence Coverage [%]:**	3.7
		**No. of unique Peptides:**	1

10	20	30	40	50	60	70	80
MQSRRARPRR	EVIQNSKEVL	SLLQEKNPAF	KPVLAIIQAG	DDNLMQEINQ	NLAEEAGLNI	THICLPPDSS	EAEIIDEILK
**90**	**100**	**110**	**120**	**130**	**140**	**150**	**160**
INEDTRVHGL	ALQISENLFS	NKVLNALKPE	KDVDGVTDIN	LGKLVRGDAH	ECFVSPVAKA	VIELLEKSVG	VNLDGKKILV
**170**	**180**	**190**	**200**	**210**	**220**	**230**	**240**
VGAHGSLEAA	LQCLFQRKGS	MTMSIQWKTR	QLQSKLHEAD	IVVLGSPKPE	EIPLTWIQPG	TTVLNCSHDF	LSGKVGCGSP
**250**	**260**	**270**	**280**	**290**	**300**	**310**	**320**
RIHFGGLIEE	DDVILLAAAL	RIQNMVSSGR	RWLREQQHRR	WRLHCLKLQP	LSPVPSDIEI	SRGQTPKAVD	VLAKEIGLLA
**330**	**340**	**350**	**360**	**370**	**380**	**390**	**400**
DEIEIYGKSK	AKVRLSVLER	LKDQADGKYV	LVAGITPTPL	GEGKSTVTIG	LVQALTAHLN	VNSFACLRQP	SQGPTFGVKG
**410**	**420**	**430**	**440**	**450**	**460**	**470**	**480**
GAAGGGYAQV	IPMEEFNLHL	TGDIHAITAA	NNLLAAAIDT	RILHENTQTD	KALYNRLVPL	VNGVREFSEI	QLARLKKLGI
**490**	**500**	**510**	**520**	**530**	**540**	**550**	**560**
NKTDPSTLTE	EEVSKFARLD	IDPSTITWQR	VLDTNDRFLR	KITIGQGNTE	KGHYRQAQFD	IAVASEIMAV	LALTDSLADM
**570**	**580**	**590**	**600**	**610**	**620**	**630**	**640**
KARLGRMVVA	SDKSGQPVTA	DDLGVTGALT	VLMKDAIKPN	LMQTLEGTPV	FVHAGPFANI	AHGNSSVLAD	KIALKLVGEE
**650**	**660**	**670**	**680**	**690**	**700**	**710**	**720**
GFVVTEAGFG	ADIGMEKFFN	IKCRASGLVP	NVVVLVATVR	ALKMHGGGPS	VTAGVPLKKE	YTEENIQLVA	DGCCNLQKQI
**730**	**740**	**750**	**760**	**770**	**780**	**790**	**800**
QITQLFGVPV	VVALNVFKTD	TRAEIDLVCE	LAKRAGAFDA	VPCYHWSVGG	KGSVDLARAV	REAASKRSRF	QFLYDVQVPI
**810**	**820**	**830**	**840**	**850**	**860**	**870**	**880**
VDKIRTIAQA	VYGAKDIELS	PEAQAKIDRY	TQQGFGNLPI	CMAKTHLSLS	HQPDKKGVPR	DFILPISDVR	ASIGAGFIYP
**890**	**900**	**910**	**920**				
LVGTMSTMPG	LPTRPCFYDI	DLDTETEQVK	GLF				

Cmpd.	No. of Cmpds.	m/z meas.	Δ m/z [ppm]	z	Rt [min]	Score	P	Range	Sequence	Modification
228	1	801.7963	-174.05	2	32.6	23.3	2	262-274	R.IQNMVSSGRRWLR.E	

Detailed Protein Report

Protein 191: phospholipase B1, membrane-associated isoform 2 precursor [Homo sapiens]

Accession: gi|283436114
Database: refseq_human(refseq_human_20140103.fasta)
Database Date: 2015-11-30
Modification(s): Carbamidomethyl

Score: 34.2
MW [kDa]: 161.6
pI: 5.5
Sequence Coverage [%]: 2.5
No. of unique Peptides: 2

10	20	30	40	50	60	70	80
MGLRPGIFLL	ELLLLLGQGT	PQIHTSPRKS	TLEGQLWPET	LKNSPFPCNP	NKLGVNMPSK	SVHSLKPSDI	KFVAAIGNLE
90	100	110	120	130	140	150	160
IPPDPGTGDL	EKQDWTERPQ	QVCMGVMTVL	SDIIRYFSPS	VPMPVCHTGK	RVIPHDGAED	LWIQAQELVR	NMKENLQLDF
170	180	190	200	210	220	230	240
QFDWKLINVF	FSNASQCYLC	PSAQQAPSLS	TVLLSQNGLA	AGGVDELMGV	LDYLQQEVPR	AFVNLVDLSE	VAEVSRQYHG
250	260	270	280	290	300	310	320
TWLSPAPEPC	NCSEETTRLA	KVVMQWSYQE	AWNSLLASSR	YSEQESFTVV	FQPFFYETTP	SLHSEDPRLQ	DSTTLAWHLW
330	340	350	360	370	380	390	400
NRMMEPAGEK	DEPLSVKHGR	PMKCPSQESP	YLFSYRNSNY	LTRLQKPQDK	LEVREGAEIR	CPDKDPSDTV	PTSVHRLKPA
410	420	430	440	450	460	470	480
DINVIGALGD	SLTAGNGAGS	TPGNVLDVLT	QYRGLSWSVG	GDENIGTVTT	LANILREFNP	SLKGFSVGTG	KETSPNAFLN
490	500	510	520	530	540	550	560
QAVAGGRAED	LPVQARRLVD	LMKNDTRIHF	QEDWKIITLF	IGGNDLCDFC	NDLVPRAFVN	LVTVLEIVNL	RELYQEKKVY
570	580	590	600	610	620	630	640
CPRMILRSLC	PCVLKFDDNS	TELATLIEFN	KKFQEKTHQL	IESGRYDTRE	DFTVVVQPFF	ENVDMPKTSE	GLPDNSFFAP
650	660	670	680	690	700	710	720
DCFHFSSKSH	SRAASALWNN	MLEPVGQKTT	RHKFENKINI	TCPNQVQPFL	RTYKNSMQGH	GTWLPCRDRA	PSALHPTSVH
730	740	750	760	770	780	790	800
ALRPADIQVV	AALGDSLTAG	NGIGSKPDDL	PDVTTQYRGL	SYSAGGDGSL	ENVTTLPNIL	REFNRNLTGY	AVGTGDANDT
810	820	830	840	850	860	870	880
NAFLNQAVPG	AKAEDLMSQV	QTLMQKMKDD	HRVNFHEDWK	VITVLIGGSD	LCDYCTDSNL	YSAANFVHHL	RNALDVLHRE
890	900	910	920	930	940	950	960
VPRVLVNLVD	FLNPTIMRQV	FLGNPDKCPV	QQASVLCNCV	LTLRENSQEL	ARLEAFSRAY	RSSMRELVGS	GRYDTQEDFS
970	980	990	1000	1010	1020	1030	1040
VVLQPFFQNI	QLPVLADGLP	DTSFFAPDCI	HPNQKFHSQL	ARALWTNMLE	PLGSKTETLD	LRAEMPITCP	TQNEPFLRTP
1050	1060	1070	1080	1090	1100	1110	1120
RNSNYTYPIK	PAIENWGSDF	LCTEWKASNS	VPTSVHQLRP	ADIKVVAALG	DSLTTAVGAR	PNNSSDLPTS	WRGLSWSIGG
1130	1140	1150	1160	1170	1180	1190	1200
DGNLETHTTL	PNILKKFNPY	LLGFSTSTWE	GTAGLNVAAE	GARARDMPAQ	AWDLVERMKN	SPDINLEKDW	KLVTLFIGVN
1210	1220	1230	1240	1250	1260	1270	1280
DLCHYCENPE	AHLATEYVQH	IQQALDILSE	ELPRAFVNVV	EVMELASLYQ	GQGGKCAMLA	AQNNCTCLRH	SQSSLEKQEL
1290	1300	1310	1320	1330	1340	1350	1360
KKVNWNLQHG	ISSFSYWHQY	TQREDFAVVV	QPFFQNTLTP	LNERGDTDLT	FFSEDCFHFS	DRGHAEMAIA	LWNNMLEPVG
1370	1380	1390	1400	1410	1420	1430	1440
RKTTSNNFTH	SRAKLKCPSP	ESPYLYTLRN	SRLLPDQAEE	APEVLYWAVP	VAAGVGLVVG	IIGTVVWRCR	RGGRREDPPM
1450							
SLRTVAL							

Cmpd.	No. of Cmpds.	m/z meas.	Δ m/z [ppm]	z	Rt [min]	Score	P	Range	Sequence	Modification
413	1	587.7939	24.16	2	34.5	11.6	1	375-384	R.EGAEIRCPDK.D	Carbamidomethyl: 7
2292	1	816.2731	-168.26	2	58.9	11.1	0	472-487	K.ETSPNAFLNQAVAGGR.A	

Detailed Protein Report

Protein 192: **PREDICTED: transforming acidic coiled-coil-containing protein 2 isoform X20 [Homo sapiens]**

**Accession:**	gi|578818751	**Score:**	34.0
**Database:**	refseq_human(refseq_human_20140103.fasta)	**MW [kDa]:**	296.6
**Database Date:**	2015-11-30	**pI:**	4.6
**Modification(s):**	Carbamidomethyl	**Sequence Coverage [%]:**	1.6
		**No. of unique Peptides:**	2

Detailed Protein Report

10	20	30	40	50	60	70	80
MGNENSTSDN	QRTLSAQTPR	SAQPPGNSQN	IKRKQQDTPG	SPDHRDASSI	GSVGLGGFCT	ASESSASLDP	CLVSPEVTEP
90	100	110	120	130	140	150	160
RKDPQGARGP	EGSLLPSPPP	SQEREHPSSS	MPFAECPPEG	CLASPAAAPE	DGPQTQSPRR	EPAPNAPGDI	AAAFPAERDS
170	180	190	200	210	220	230	240
STPYQEIAAV	PSAGRERQPK	EEGQKSSFSF	SSGIDQSPGM	SPVPLREPMK	APLCGEGDQP	GGFESQEKEA	AGGFPPAESR
250	260	270	280	290	300	310	320
QGVASVQVTP	EAPAAAQQGT	ESSAVLEKSP	LKPMAPIPQD	PAPRASDRER	GQGEAPPQYL	TDDLEFLRAC	HLPRSNSGAA
330	340	350	360	370	380	390	400
PEAEVNAASQ	ESCQQPVGAY	LPHAELPWGL	PSPALVPEAG	GSGKEALDTI	DVQGHPQTGM	RGTKPNQVVC	VAAGGQPEGG
410	420	430	440	450	460	470	480
LPVSPEPSLL	TPTEEAHPAS	SLASFPAAQI	PIAVEEPGSS	SRESVSKAGM	PVSADAAKEV	VDAGLVGLER	QVSDLGSKGE
490	500	510	520	530	540	550	560
HPEGDPGEVP	APSPQERGEH	LNTEQSHEVQ	PGVPPPPLPK	EQSHEVQPGA	PPPPLPKAPS	ESARGPPGPT	DGAKVHEDST
570	580	590	600	610	620	630	640
SPAVAKEGSR	SPGDSPGGKE	EAPEPPDGGD	PGNLQGEDSQ	AFSSKRDPEV	GKDELSKPSS	DAESRDHPSS	HSAQPPRKGG
650	660	670	680	690	700	710	720
AGHTDGPHSQ	TAEADASGLP	HKLGEEDPVL	PPVPDGAGEP	TVPEGAIWEG	SGLQPKCPDT	LQSREGLGRM	ESFLTLESEK
730	740	750	760	770	780	790	800
SDFPPTPVAE	VAPKAQEGES	TLEIRKMGSC	DGEGLLTSPD	QPRGPACDAS	RQEFHAGVPH	PPQGENLAAD	LGLTALILDQ
810	820	830	840	850	860	870	880
DQQGIPSCPG	EGWIRGAASE	WPLLSSEKHL	QPSQAQPETS	IFDVLKEQAQ	PPENGKETSP	SHPGFKDQGA	DSSQIHVPVE
890	900	910	920	930	940	950	960
PQEDNNLPTH	GGQEQALGSE	LQSQLPKGTL	SDTPTSSPTD	MVWESSLTEE	SELSAPTRQK	LPALGEKRPE	GACGDGQSSR
970	980	990	1000	1010	1020	1030	1040
VSPPAADVLK	DFSLAGNFSR	KETCCTGQGP	NKSQQALADA	LEEGSQHEEA	CQRHPGASEA	ADGCSPLWGL	SKREMASGNT
1050	1060	1070	1080	1090	1100	1110	1120
GEAPPCQPDS	VALLDAVPCL	PALAPASPGV	TPTQDAPETE	ACDETQEGRQ	QPVPAPQQKM	ECWATSDAES	PKLLASFPSA
1130	1140	1150	1160	1170	1180	1190	1200
GEQGGEAGAA	ETGGSAGAGD	PGKQQAPEKP	GEATLSCGLL	QTEHCLTSGE	EASTSALRES	CQAEHPMASC	QDALLPAREL
1210	1220	1230	1240	1250	1260	1270	1280
GGIPRSTMDF	STHQAVPDPK	ELLLSGPPEV	AAPDTPYLHV	DSAAQRGAED	SGVKAVSSAD	PRAPGESPCP	VGEPPLALEN
1290	1300	1310	1320	1330	1340	1350	1360
AASLKLFAGS	LAPLLQPGAA	GGEIPAVQAS	SGSPKARTTE	GPVDSMPCLD	RMPLLAKGKQ	ATGEEKAATA	PGAGAKASGE
1370	1380	1390	1400	1410	1420	1430	1440
GMAGDAAGET	EGSMERMGEP	SQDPKQGTSG	GVDTSSEQIA	TLTGFPDFRE	HIAKIFEKPV	LGALATPGEK	AGAGRSAVGK
1450	1460	1470	1480	1490	1500	1510	1520
DLTRPLGPEK	LLDGPPGVDV	TLLPAPPARL	QVEKKQQLAG	EAEISHLALQ	DPASDKLLGP	AGLTWERNLP	GAGVGKEMAG
1530	1540	1550	1560	1570	1580	1590	1600
VPPTLREDER	PEGPGAAWPG	LEGQAYSQLE	RSRQELASGL	PSPAATQELP	VERAAAFQVA	PHSHGEEAVA	QDRIPSGKQH
1610	1620	1630	1640	1650	1660	1670	1680
QETSACDSPH	GEDGPGDFAH	TGVPGHVPRS	TCAPSPQREV	LTVPEANSEP	WTLDTLGGER	RPGVTAGILE	MRNALGNQST
1690	1700	1710	1720	1730	1740	1750	1760
PAPPTGEVAD	TPLEPGKVAG	AAGEAEGDIT	LSTAETQACA	SGDLPEAGTT	RTFSVVAGDL	VLPGSCQDPA	CSDKAPGMEG
1770	1780	1790	1800	1810	1820	1830	1840
TAALHGDSPA	RPQQAKEQPG	PERPIPAGDG	KVCVSSPPEP	DETHDPKLQH	LAPEELHTDR	ESPRPGPSML	PSVPKKDAPR
1850	1860	1870	1880	1890	1900	1910	1920
VMDKVTSDET	RGAEGTESSP	VADDIIQPAA	PADLESPTLA	ASSYHGDVVG	QVSTDLIAQR	SSDSEEAFET	PESTTPVKAP
1930	1940	1950	1960	1970	1980	1990	2000
PAPPPPPPEV	IPEPEVSTQP	PPEEPGCGSE	TVPVPDGPRS	DSVEGSPFRP	PSHSFSAVFD	EDKPIASSGT	YNLDFDNIEL
2010	2020	2030	2040	2050	2060	2070	2080
VDTFQTLEPR	ASDAKNQEGK	VNTRRKSTDS	VPISKSTLSR	SLSLQASDFD	GASSSGNPEA	VALAPDAYST	GSSSASSTLK
2090	2100	2110	2120	2130	2140	2150	2160
RTKKPRPPSL	KKKQTTKKPT	ETPPVKETQQ	EPDEESLVPS	GENLASETKT	ESAKTEGPSP	ALLEETPLEP	AVGPKAACPL
2170	2180	2190	2200	2210	2220	2230	2240
DSESAEGVVP	PASGGGRVQN	SPPVGRKTLP	LTTAPEAGEV	TPSDSGGQED	SPAKGLSVRL	EFDYSEDKSS	WDNQQENPPP
2250	2260	2270	2280	2290	2300	2310	2320
TKKIGKKPVA	KMPLRRPKMK	KTPEKLDNTP	ASPPRSPAEP	NDIPIAKGTY	TFDIDKWDDP	NFNPFSSTSK	MQESPKLPQQ
2330	2340	2350	2360	2370	2380	2390	2400

Detailed Protein Report

Cmpd.	No. of Cmpds.	m/z meas.	Δ m/z [ppm]	z	Rt [min]	Score	P	Range	Sequence	Modification
858	1	474.1144	-271.75	2	40.2	10.8	1	1338-1346	K.GKQATGEEK.A	
1463	1	1023.6735	127.47	2	48.1	13.1	2	2806-2822	K.NKEIEELTKICDELIAK.M	Carbamidomethyl: 11

Detailed Protein Report

Protein 193: PREDICTED: uncharacterized protein C1orf159 isoform X8 [Homo sapiens]

**Accession:**	gi|578799328	**Score:**	33.9
**Database:**	refseq_human(refseq_human_20140103.fasta)	**MW [kDa]:**	30.9
**Database Date:**	2015-11-30	**pI:**	11.7
**Modification(s):**	Oxidation	**Sequence Coverage [%]:**	8.9
		**No. of unique Peptides:**	2

10	20	30	40	50	60	70	80
MSVVPILEGQ	PHSISLAPHA	PPCGRLCASA	PGMALRHLAL	LAGLLVGVAS	KSMENTAQLP	ECCVDVVGVN	ASCPGASLCG
**90**	**100**	**110**	**120**	**130**	**140**	**150**	**160**
PGCYRRWNAD	GSASCVRCGN	GTLPAYNGSE	CRSFAGPGAP	FPMNRSSGTP	GRPHPGAPRV	AASLFLGTFF	ISSGLILSVA
**170**	**180**	**190**	**200**	**210**	**220**	**230**	**240**
GFFYLKRSSK	LPRACYRRNK	APALQPGEAA	AMIPPPQSSG	PTLTSPVSDP	FQYGSRATSG	GSGPWTGPRI	PLPSRGRPVS
**250**	**260**	**270**	**280**	**290**	**300**	**310**	
AMSDLEAETT	PRTWRQGPGG	RPLGGNQHKV	LASPGARDSP	GHSLGSESLR	LPATDAKGSR	AA	

Cmpd.	No. of Cmpds.	m/z meas.	Δ m/z [ppm]	z	Rt [min]	Score	P	Range	Sequence	Modification
2311	2	682.9198	139.69	2	58.8	23.8	0	113-125	R.SFAGPGAPFPMNR.S	Oxidation: 11
2555	1	701.8644	-5.90	2	61.9	10.1	0	256-269	R.QGPGGRPLGGNQHK.V	

Detailed Protein Report

Protein 194: **voltage-dependent P/Q-type calcium channel subunit alpha-1A isoform 4 [Homo sapiens]**

**Accession:**	gi|187828892	**Score:**	33.7
**Database:**	refseq_human(refseq_human_20140103.fasta)	**MW [kDa]:**	282.4
**Database Date:**	2015-11-30	**pI:**	9.6
		**Sequence Coverage [%]:**	1.8
		**No. of unique Peptides:**	2

Detailed Protein Report

10	20	30	40	50	60	70	80
MARFGDEMPA	RYGGGGSGAA	AGVVVGSGGG	RGAGGSRQGG	QPGAQRMYKQ	SMAQRARTMA	LYNPIPVRQN	CLTVNRSLFL
90	100	110	120	130	140	150	160
FSEDNVVRKY	AKKITEWPPF	EYMILATIIA	NCIVLALEQH	LPDDDKTPMS	ERLDDTEPYF	IGIFCFEAGI	KIIALGFAFH
170	180	190	200	210	220	230	240
KGSYLRNGWN	VMDFVVVLTG	ILATVGTEFD	LRTLRAVRVL	RPLKLVSGIP	SLQVVLKSIM	KAMIPLLQIG	LLLFFAILIF
250	260	270	280	290	300	310	320
AIIGLEFYMG	KFHTTCFEEG	TDDIQGESPA	PCGTEEPART	CPNGTKCQPY	WEGPNNGITQ	FDNILFAVLT	VFQCITMEGW
330	340	350	360	370	380	390	400
TDLLYNSNDA	SGNTWNWLYF	IPLIIIGSFF	MLNLVLGVLS	GEFAKERERV	ENRRAFLKLR	RQQQIERELN	GYMEWISKAE
410	420	430	440	450	460	470	480
EVILAEDETD	GEQRHPFDAL	RRTTIKKSKT	DLLNPEEAED	QLADIASVGS	PFARASIKSA	KLENSTFFHK	KERRMRFYIR
490	500	510	520	530	540	550	560
RMVKTQAFYW	TVLSLVALNT	LCVAIVHYNQ	PEWLSDFLYY	AEFIFLGLFM	SEMFIKMYGL	GTRPYFHSSF	NCFDCGVIIG
570	580	590	600	610	620	630	640
SIFEVIWAVI	KPGTSFGISV	LRALRLLRIF	KVTKYWASLR	NLVVSLLNSM	KSIISLLFLL	FLFIVVFALL	GMQLFGGQFN
650	660	670	680	690	700	710	720
FDEGTPPTNF	DTFPAAIMTV	FQILTGEDWN	EVMYDGIKSQ	GGVQGGMVFS	IYFIVLTLFG	NYTLLNVFLA	IAVDNLANAQ
730	740	750	760	770	780	790	800
ELTKDEQEEE	EAANQKLALQ	KAKEVAEVSP	LSAANMSIAV	KEQQKNQKPA	KSVWEQRTSE	MRKQNLLASR	EALYNEMDPD
810	820	830	840	850	860	870	880
ERWKAAYTRH	LRPDMKTHLD	RPLVVDPQEN	RNNNTNKSRA	AEPTVDQRLG	QQRAEDFLRK	QARYHDRARD	PSGSAGLDAR
890	900	910	920	930	940	950	960
RPWAGSQEAE	LSREGPYGRE	SDHHAREGSL	EQPGFWEGEA	ERGKAGDPHR	RHVHRQGGSR	ESRSGSPRTG	ADGEHRRHRA
970	980	990	1000	1010	1020	1030	1040
HRRPGEEGPE	DKAERRARHR	EGSRPARGGE	GEGEGPDGGE	RRRRHRHGAP	ATYEGDARRE	DKERRHRRRK	ENQGSGVPVS
1050	1060	1070	1080	1090	1100	1110	1120
GPNLSTTRPI	QQDLGRQDPP	LAEDIDNMKN	NKLATAESAA	PHGSLGHAGL	PQSPAKMGNS	TDPGPMLAIP	AMATNPQNAA
1130	1140	1150	1160	1170	1180	1190	1200
SRRTPNNPGN	PSNPGPPKTP	ENSLIVTNPS	GTQTNSAKTA	RKPDHTTVDI	PPACPPPLNH	TVVQVNKNAN	PDPLPKKEEE
1210	1220	1230	1240	1250	1260	1270	1280
KKEEEEDDRG	EDGPKPMPPY	SSMFILSTTN	PLRRLCHYIL	NLRYFEMCIL	MVIAMSSIAL	AAEDPVQPNA	PRNNVLRYFD
1290	1300	1310	1320	1330	1340	1350	1360
YVFTGVFTFE	MVIKMIDLGL	VLHQGAYFRD	LWNILDFIVV	SGALVAFAFT	GNSKGKDINT	IKSLRVLRVL	RPLKTIKRLP
1370	1380	1390	1400	1410	1420	1430	1440
KLKAVFDCVV	NSLKNVFNIL	IVYMLFMFIF	AVVAVQLFKG	KFFHCTDESK	EFEKDCRGKY	LLYEKNEVKA	RDREWKKYEF
1450	1460	1470	1480	1490	1500	1510	1520
HYDNVLWALL	TLFTVSTGEG	WPQVLKHSVD	ATFENQGPSP	GYRMEMSIFY	VVYFVVFPFF	FVNIFVALII	ITFQEQGDKM
1530	1540	1550	1560	1570	1580	1590	1600
MEEYSLEKNE	RACIDFAISA	KPLTRHMPQN	KQSFQYRMWQ	FVVSPPFEYT	IMAMIALNTI	VLMMKFYGAS	VAYENALRVF
1610	1620	1630	1640	1650	1660	1670	1680
NIVFTSLFSL	ECVLKVMAFG	ILNYFRDAWN	IFDFVTVLGS	ITDILVTEFG	NNFINLSFLR	LFRAARLIKL	LRQGYTIRIL
1690	1700	1710	1720	1730	1740	1750	1760
LWTFVQSFKA	LPYVCLLIAM	LFFIYAIIGM	QVFGNIGIDV	EDEDSDEDEF	QITEHNNFRT	FFQALMLLFR	SATGEAWHNI
1770	1780	1790	1800	1810	1820	1830	1840
MLSCLSGKPC	DKNSGILTRE	CGNEFAYFYF	VSFIFLCSFL	MLNLFVAVIM	DNFEYLTRDS	SILGPHHLDE	YVRVWAEYDP
1850	1860	1870	1880	1890	1900	1910	1920
AAWGRMPYLD	MYQMLRHMSP	PLGLGKKCPA	RVAYKRLLRM	DLPVADDNTV	HFNSTLMALI	RTALDIKIAK	GGADKQQMDA
1930	1940	1950	1960	1970	1980	1990	2000
ELRKEMMAIW	PNLSQKTLDL	LVTPHKSTDL	TVGKIYAAMM	IMEYYRQSKA	KKLQAMREEQ	DRTPLMFQRM	EPPSPTQEGG
2010	2020	2030	2040	2050	2060	2070	2080
PGQNALPSTQ	LDPGGALMAH	ESGLKESPSW	VTQRAQEMFQ	KTGTWSPEQG	PPTDMPNSQP	NSQSVEMREM	GRDGYSDSEH
2090	2100	2110	2120	2130	2140	2150	2160
YLPMEGQGRA	ASMPRLPAEN	QRRRGRPRGN	NLSTISDTSP	MKRSASVLGP	KARRLDDYSL	ERVPPEENQR	HHQRRRDRSH
2170	2180	2190	2200	2210	2220	2230	2240
RASERSLGRY	TDVDTGLGTD	LSMTTQSGDL	PSKERDQERG	RPKDRKHRQH	HHHHHHHHHP	PPPDKDRYAQ	ERPDHGRARA
2250	2260	2270	2280	2290	2300	2310	2320
RDQRWSRSPS	EGREHMAHRQ	GSSSVSGSPA	PSTSGTSTPR	RGRRQLPQTP	STPRPHVSYS	PVIRKAGGSG	PPQQQQQQQQ
2330	2340	2350	2360	2370	2380	2390	2400

Detailed Protein Report

Cmpd.	No. of Cmpds.	m/z meas.	Δ m/z [ppm]	z	Rt [min]	Score	P	Range	Sequence	Modification
66	1	796.7909	-127.42	2	30.5	10.0	0	12-31	R.YGGGGSGAAAGVVVGSGGGR.G	
2706	1	741.8621	-28.64	2	64.1	11.6	2	2371-2384	R.VPGPARSESPRACR.H	

Detailed Protein Report

Protein 195: zinc finger protein 177 isoform b [Homo sapiens]

**Accession:**	gi|289547607	**Score:**	33.5
**Database:**	refseq_human(refseq_human_20140103.fasta)	**MW [kDa]:**	36.4
**Database Date:**	2015-11-30	**pI:**	9.8
**Modification(s):**	Carbamidomethyl	**Sequence Coverage [%]:**	8.1
		**No. of unique Peptides:**	2

Alias proteins:

Accession	Name	Description
gi|289547609	r e f s e q _ h u m a n (refseq_human_20140103.fasta)	zinc finger protein 177 isoform b [Homo sapiens]

10	20	30	40	50	60	70	80
MAAGWLTTWS	QNSVTFQEVA	VDFSQEEWAL	LDPAQKNLYK	DVMLENFRNL	ASVGYQLCRH	SLISKVDQEQ	LKTDERGILQ
**90**	**100**	**110**	**120**	**130**	**140**	**150**	**160**
GDCADWETQL	KPKDTIAMQN	IPGGKTSNGI	NTNCVRTHSG	EMPYECSDCG	KAFIFQSSLK	KHMRSHTGEK	PYECDHCGKS
**170**	**180**	**190**	**200**	**210**	**220**	**230**	**240**
FSQSSHLNVH	KRTHTGEKPY	DCKECGKAFT	VPSSLQKHVR	THTGEKPYEC	SDCGKAFIDQ	SSLKKHTRSH	TGEKPYECNQ
**250**	**260**	**270**	**280**	**290**	**300**	**310**	**320**
CGKSFSTGSY	LIVHKRTHTG	EKTYECKECG	KAFRNSSCLR	VHVRTHTGEK	PYKCIQCEKA	FSTSTNLIMH	KRIHNGQKLH
**330**							
E							

Cmpd.	No. of Cmpds.	m/z meas.	Δ m/z [ppm]	z	Rt [min]	Score	P	Range	Sequence	Modification
2666	3	973.5093	-8.38	2	63.4	16.7	1	49-65	R.NLASVGYQLCRHSLISK.V	Carbamidomethyl: 10
1517	1	1008.6274	90.62	1	48.8	16.8	0	216-224	K.AFIDQSSLK.K	

Detailed Protein Report

Protein 196: **PREDICTED: ras-specific guanine nucleotide-releasing factor 2 isoform X1 [Homo sapiens]**

**Accession:**	gi|530379425	**Score:**	33.5
**Database:**	refseq_human(refseq_human_20140103.fasta)	**MW [kDa]:**	133.5
**Database Date:**	2015-11-30	**pI:**	8.3
**Modification(s):**	Oxidation	**Sequence Coverage [%]:**	2.1
		**No. of unique Peptides:**	1

10	20	30	40	50	60	70	80
MQKSVRYNEG	HALYLAFLAR	KEGTKRGFLS	KKTAEASRWH	EKWFALYQNV	LFYFEGEQSC	RPAGMYLLEG	CSCERTPAPP
**90**	**100**	**110**	**120**	**130**	**140**	**150**	**160**
RAGAGQGGVR	DALDKQYYFT	VLFGHEGQKP	LELRCEEEQD	GKEWMEAIHQ	ASYADILIER	EVLMQKYIHL	VQIVETEKIA
**170**	**180**	**190**	**200**	**210**	**220**	**230**	**240**
ANQLRHQLED	QDTEIERLKS	EIIALNKTKE	RMRPYQSNQE	DEDPDIKKIK	KVQSFMRGWL	CRRKWKTIVQ	DYICSPHAES
**250**	**260**	**270**	**280**	**290**	**300**	**310**	**320**
MRKRNQIVFT	MVEAESEYVH	QLYILVNGFL	RPLRMAASSK	KPPISHDDVS	SIFLNSETIM	FLHEIFHQGL	KARIANWPTL
**330**	**340**	**350**	**360**	**370**	**380**	**390**	**400**
ILADLFDILL	PMLNIYQEFV	RNHQYSLQVL	ANCKQNRDFD	KLLKQYEANP	ACEGRMLETF	LTYPMFQIPR	YIITLHELLA
**410**	**420**	**430**	**440**	**450**	**460**	**470**	**480**
HTPHEHVERK	SLEFAKSKLE	ELSRVMHDEV	SDTENIRKNL	AIERMIVEGC	DILLDTSQTF	IRQGSLIQVP	SVERGKLSKV
**490**	**500**	**510**	**520**	**530**	**540**	**550**	**560**
RLGSLSLKKE	GERQCFLFTK	HFLICTRSSG	GKLHLLKTGG	VLSLIDCTLI	EEPDASDDDS	KGSGQVFGHL	DFKIVVEPPD
**570**	**580**	**590**	**600**	**610**	**620**	**630**	**640**
AAAFTVVLLA	PSRQEKAAWM	SDISQCVDNI	RCNGLMTIVF	EENSKVTVPH	MIKSDARLHK	DDTDICFSKT	LNSCKVPQIR
**650**	**660**	**670**	**680**	**690**	**700**	**710**	**720**
YASVERLLER	LTDLRFLSID	FLNTFLHTYR	IFTTAAVVLG	KLSDIYKRPF	TSIPVRSLEL	FFATSQNNRG	EHLVDGKSPR
**730**	**740**	**750**	**760**	**770**	**780**	**790**	**800**
LCRKFSSPPP	LAVSRTSSPV	RARKLSLTSP	LNSKIGALDL	TTSSSPTTTT	QSPAASPPPH	TGQIPLDLSR	GLSSPEQSPG
**810**	**820**	**830**	**840**	**850**	**860**	**870**	**880**
TVEENVDNPR	VDLCNKLKRS	IQKGFNNTER	TCDKEFIIRR	TATNRVLNVL	RHWVSKHAQD	FELNNELKMN	VLNLLEEVLR
**890**	**900**	**910**	**920**	**930**	**940**	**950**	**960**
DPDLLPQERK	AAANILRALS	QDDQDDIHLK	LEDIIQMTDC	MKAECFESLS	AMELAEQITL	LDHVIFRSIP	YEEFLGQGWM
**970**	**980**	**990**	**1000**	**1010**	**1020**	**1030**	**1040**
KLDKNERTPY	IMKTSQHFND	MSNLVASQIM	NYADVSSRAN	AIEKWVAVAD	ICRCLHNYNG	VLEITSALNR	SAIYRLKKTW
**1050**	**1060**	**1070**	**1080**	**1090**	**1100**	**1110**	**1120**
AKVSKQTKAL	MDKLQKTVSS	EGRFKNLRET	LKNCNPPAVP	YLGMYLTDLA	FIEEGTPNFT	EEGLVNFSKM	RMISHIIREI
**1130**	**1140**	**1150**	**1160**	**1170**			
RQFQQTSYRI	DHQPKVAQYL	LDKDLIIDED	TLYELSLKIE	PRLPA			

Cmpd.	No. of Cmpds.	m/z meas.	Δ m/z [ppm]	z	Rt [min]	Score	P	Range	Sequence	Modification
1027	1	984.1548	34.58	3	42.4	33.5	1	898-922	R.ALSQDDQDDIHLKLEDIIQMTDCMK.A	Oxidation: 20, 24

Detailed Protein Report

Protein 197: PREDICTED: probable E3 ubiquitin-protein ligase HERC1 isoform X6 [Homo sapiens]

**Accession:**	gi|530406610	**Score:**	33.3
**Database:**	refseq_human(refseq_human_20140103.fasta)	**MW [kDa]:**	524.9
**Database Date:**	2015-11-30	**pI:**	5.7
**Modification(s):**	Carbamidomethyl, Oxidation	**Sequence Coverage [%]:**	1.1
		**No. of unique Peptides:**	3

Detailed Protein Report

10	20	30	40	50	60	70	80
MATMIPPVKL	KWLEHLNSSW	ITEDSESIAT	REGVAVLYSK	LVSNKEVVPL	PQQVLCLKGP	QLPDFERESL	SSDEQDHYLD
90	100	110	120	130	140	150	160
ALLSSQLALA	KMVCSDSPFA	GALRKRLLVL	QRVFYALSNK	YHDKGKVKQQ	QHSPESSSGS	ADVHSVSERP	RSSTDALIEM
170	180	190	200	210	220	230	240
GVRTGLSLLF	ALLRQSWMMP	VSGPGLSLCN	DVIHTAIEVV	SSLPPLSLAN	ESKIPPMGLD	CLSQVTTFLK	GVTIPNSGAD
250	260	270	280	290	300	310	320
TLGRRLASEL	LLGLAAQRGS	LRYLLEWIEM	ALGASAVVHT	MEKGKLLSSQ	EGMISFDCFM	TILMQMRRSL	GSSADRSQWR
330	340	350	360	370	380	390	400
EPTRTSDGLC	SLYEAALCLF	EEIEAGQYCT	FVISTDGSVR	ACGKGSYGRL	GLGDSNNQST	LKKLTFEPHR	SIKKVSSSKG
410	420	430	440	450	460	470	480
SDGHTLAFTT	EGEVFSWGDG	DYGKLGHGNS	STQKYPKLIQ	GPLQGKVVVC	VSAGYRHSAA	VTEDGELYTW	GEGDFGRLGH
490	500	510	520	530	540	550	560
GDSNSRNIPT	LVKDISNVGE	VSCGSSHTIA	LSKDGRTVWS	FGGGDNGKLG	HGDTNRVYKP	KVIEALQGMF	IRKVCAGSQS
570	580	590	600	610	620	630	640
SLALTSTGQV	YAWGCGACLG	CGSSEATALR	PKLIEELAAT	RIVDVSIGDS	HCLALSHDNE	VYAWGNNSMG	QCGQGNSTGP
650	660	670	680	690	700	710	720
ITKPKKVSGL	DGIAIQQISA	GTSHSLAWTA	LPRDRQVVAW	HRPYCVDLEE	STFSHLRSFL	ERYCDKINSE	IPPLPFPSSR
730	740	750	760	770	780	790	800
EHHSFLKLCL	KLLSNHLALA	LAGGVATSIL	GRQAGPLRNL	LFRLMDSTVP	DEIQEVVIET	LSVGATMLLP	PLRERMELLH
810	820	830	840	850	860	870	880
SLLPQGPDRW	ESLSKGQRMQ	LDIILTSLQD	HTHVASLLGY	SSPSDAADLS	SVCTGYGNLS	DQPYGTQSCH	PDTHLAEILM
890	900	910	920	930	940	950	960
KTLLRNLGFY	TDQAFGELEK	NSDKFLLGTS	SSENSQPAHL	HELLCSLQKQ	LLAFCHINNI	SENSSSVALL	HKHLQLLLPH
970	980	990	1000	1010	1020	1030	1040
ATDIYSRSAN	LLKESPWNGS	VGEKLRDVIY	VSAAGSMLCQ	IVNSLLLLPV	SVARPLLSYL	LDLLPPLDCL	NRLLPAADLL
1050	1060	1070	1080	1090	1100	1110	1120
EDQELQWPLH	GGPELIDPAG	LPLPQPAQSW	VWLVDLERTI	ALLIGRCLGG	MLQGSPVSPE	EQDTAYWMKT	PLFSDGVEMD
1130	1140	1150	1160	1170	1180	1190	1200
TPQLDKCMSC	LLEVALSGNE	EQKPFDYKLR	PEIAVYVDLA	LGCSKEPARS	LWISMQDYAV	SKDWDSATLS	NESLLDTVSR
1210	1220	1230	1240	1250	1260	1270	1280
FVLAALLKHT	NLLSQACGES	RYQPGKHLSE	VYRCVYKVRS	RLLACKNLEL	IQTRSSSRDR	WISENQDSAD	VDPQEHSFTR
1290	1300	1310	1320	1330	1340	1350	1360
TIDEEAEMEE	QAERDREEGH	PEPEDEEEER	EHEVMTAGKI	FQCFLSAREV	ARSRDRDRMN	SGAGSGARAD	DPPPQSQQER
1370	1380	1390	1400	1410	1420	1430	1440
RVSTDLPEGQ	DVYTAACNSV	IHRCALLILG	VSPVIDELQK	RREEGQLQQP	STSASEGGGL	MTRSESLTAE	SRLVHTSPNY
1450	1460	1470	1480	1490	1500	1510	1520
RLIKSRSESD	LSQPESDEEG	YALSGRRNVD	LDLAASHRKR	GPMHSQLESL	SDSWARLKHS	RDWLCNSSYS	FESDFDLTKS
1530	1540	1550	1560	1570	1580	1590	1600
LGVHTLIENV	VSFVSGDVGN	APGFKEPEES	MSTSPQASII	AMEQQQLRAE	LRLEALHQIL	VLLSGMEEKG	SISLAGSRLS
1610	1620	1630	1640	1650	1660	1670	1680
SGFQSSTLLT	SVRLQFLAGC	FGLGTVGHTG	GKGESGRLHH	YQDGIRAAKR	NIQIEIQVAV	HKIYQQLSAT	LERALQANKH
1690	1700	1710	1720	1730	1740	1750	1760
HIEAQQRLLL	VTVFALSVHY	QPVDVSLAIS	TGLLNVLSQL	CGTDTMLGQP	LQLLPKTGVS	QLSTALKVAS	TRLLQILAIT
1770	1780	1790	1800	1810	1820	1830	1840
TGTYADKLSP	KVVQSLLDLL	CSQLKNLLSQ	TGVLHMASFG	EGEQEDGEEE	EKKVDSSGET	EKKDFRAALR	KQHAAELHLG
1850	1860	1870	1880	1890	1900	1910	1920
DFLVFLRRVV	SSKAIQSKMA	SPKWTEVLLN	IASQKCSSGI	PLVGNLRTRL	LALHVLEAVL	PACESGVEDD	QMAQIVERLF
1930	1940	1950	1960	1970	1980	1990	2000
SLLSDCMWET	PIAQAKHAIQ	IKEKEQEIKL	QKQGELEEED	ENLPIQEVSF	DPEKAQCCLV	ENGQILTHGS	GGKGYGLAST
2010	2020	2030	2040	2050	2060	2070	2080
GVTSGCYQWK	FYIVKENRGN	EGTCVGVSRW	PVHDFNHRTT	SDMWLYRAYS	GNLYHNGEQT	LTLSSFTQGD	FITCVLDMEA
2090	2100	2110	2120	2130	2140	2150	2160
RTISFGKNGE	EPKLAFEDVD	AAELYPCVMF	YSSNPGEKVK	ICDMQMRGTP	RDLLPGDPIC	SPVAAVLAEA	TIQLIRILHR
2170	2180	2190	2200	2210	2220	2230	2240

Detailed Protein Report

Cmpd.	No. of Cmpds.	m/z meas.	Δ m/z [ppm]	z	Rt [min]	Score	P	Range	Sequence	Modification
1613	1	954.4635	-82.64	2	49.9	11.5	1	537-552	R.VYKPKVIEALQGMFIR.K	Oxidation: 13
1890	1	1023.4178	-75.16	2	53.5	10.4	1	1209-1226	K.HTNLLSQACGESRYQPGK.H	Carbamidomethyl: 9
2149	1	701.2767	-121.55	3	57.0	11.4	1	4282-4301	R.QISAGRCHSAAWTAPPVPPR.A	

Detailed Protein Report

Protein 198: mitochondrial intermediate peptidase [Homo sapiens]

Accession: gi|156105687
Database: refseq_human(refseq_human_20140103.fasta)
Database Date: 2015-11-30

Score: 33.3
MW [kDa]: 80.6
pI: 6.6
Sequence Coverage [%]: 2.9
No. of unique Peptides: 1

10	20	30	40	50	60	70	80
MLCVGRLGGL	GARAAALPPR	RAGRGSLEAG	IRARRVSTSW	SPVGAAFNVK	PQGSRLDLFG	ERRGLFGVPE	LSAPEGFHIA
**90**	**100**	**110**	**120**	**130**	**140**	**150**	**160**
QEKALRKTEL	LVDRACSTPP	GPQTVLIFDE	LSDSLCRVAD	LADFVKIAHP	EPAFREAAEE	ACRSIGTMVE	KLNTNVDLYQ
**170**	**180**	**190**	**200**	**210**	**220**	**230**	**240**
SLQKLLADKK	LVDSLDPETR	RVAELFMFDF	EISGIHLDKE	KRKRAVDLNV	KILDLSSTFL	MGTNFPNKIE	KHLLPEHIRR
**250**	**260**	**270**	**280**	**290**	**300**	**310**	**320**
NFTSAGDHII	IDGLHAESPD	DLVREAAYKI	FLYPNAGQLK	CLEELLSSRD	LLAKLVGYST	FSHRALQGTI	AKNPETVMQF
**330**	**340**	**350**	**360**	**370**	**380**	**390**	**400**
LEKLSDKLSE	RTLKDFEMIR	GMKMKLNPQN	SEVMPWDPPY	YSGVIRAERY	NIEPSLYCPF	FSLGACMEGL	NILLNRLLGI
**410**	**420**	**430**	**440**	**450**	**460**	**470**	**480**
SLYAEQPAKG	EVWSEDVRKL	AVVHESEGLL	GYIYCDFFQR	ADKPHQDCHF	TIRGGRLKED	GDYQLPVVVL	MLNLPRSSRS
**490**	**500**	**510**	**520**	**530**	**540**	**550**	**560**
SPTLLTPGMM	ENLFHEMGHA	MHSMLGRTRY	QHVTGTRCPT	DFAEVPSILM	EYFANDYRVV	NQFARHYQTG	QPLPKNMVSR
**570**	**580**	**590**	**600**	**610**	**620**	**630**	**640**
LCESKKVCAA	ADMQLQVFYA	TLDQIYHGKH	PLRNSTTDIL	KETQEKFYGL	PYVPNTAWQL	RFSHLVGYGA	RYYSYLMSRA
**650**	**660**	**670**	**680**	**690**	**700**	**710**	**720**
VASMVWKECF	LQDPFNRAAG	ERYRREMLAH	GGGREPMLMV	EGMLQKCPSV	DDFVSALVSD	LDLDFETFLM	DSE

Cmpd.	No. of Cmpds.	m/z meas.	Δ m/z [ppm]	z	Rt [min]	Score	P	Range	Sequence	Modification
2877	3	703.3682	28.70	2	66.7	15.9	0	675-686	R.EPMLMVEGMLQK.C	

Detailed Protein Report

Protein 199: histone acetyltransferase KAT6B isoform 1 [Homo sapiens]

**Accession:**	gi|100816397	**Score:**	33.3
**Database:**	refseq_human(refseq_human_20140103.fasta)	**MW [kDa]:**	231.2
**Database Date:**	2015-11-30	**pI:**	5.6
		**Sequence Coverage [%]:**	1.9
		**No. of unique Peptides:**	1

Alias proteins:

Accession	Name	Description
gi|530393350	refseq_human (refseq_human_20140103.fasta)	PREDICTED: histone acetyltransferase KAT6B isoform X1 [Homo sapiens]

Detailed Protein Report

10	20	30	40	50	60	70	80
MVKLANPLYT	EWILEAIQKI	KKQKQRPSEE	RICHAVSTSH	GLDKKTVSEQ	LELSVQDGSV	LKVTNKGLAS	YKDPDNPGRF
90	100	110	120	130	140	150	160
SSVKPGTFPK	SAKGSRGSCN	DLRNVDWNKL	LRRAIEGLEE	PNGSSLKNIE	KYLRSQSDLT	STTNNPAFQQ	RLRLGAKRAV
170	180	190	200	210	220	230	240
NNGRLLKDGP	QYRVNYGSLD	GKGAPQYPSA	FPSSLPPVSL	LPHEKDQPRA	DPIPICSFCL	GTKESNREKK	PEELLSCADC
250	260	270	280	290	300	310	320
GSSGHPSCLK	FCPELTTNVK	ALRWQCIECK	TCSACRVQGR	NADNMLFCDS	CDRGFHMECC	DPPLSRMPKG	MWICQVCRPK
330	340	350	360	370	380	390	400
KKGRKLLHEK	AAQIKRRYAK	PIGRPKNKLK	QRLLSVTSDE	GSMNAFTGRG	SPGRGQKTKV	CTTPSSGHAA	SGKDSSSRLA
410	420	430	440	450	460	470	480
VTDPTRPGAT	TKITTTSTYI	SASTLKVNKK	TKGLIDGLTK	FFTPSPDGRR	SRGEIIDFSK	HYRPRKKVSQ	KQSCTSHVLA
490	500	510	520	530	540	550	560
TGTTQKLKPP	PSSLPPPTPI	SGQSPSSQKS	STATSSPSPQ	SSSSQCSVPS	LSSLTTNSQL	KALFDGLSHI	YTTQGQSRKK
570	580	590	600	610	620	630	640
GHPSYAPPKR	MRRKTELSST	AKSKAHFFGK	RDIRSRFISH	SSSSSWGMAR	GSIFKAIAHF	KRTTFLKKHR	MLGRLKYKVT
650	660	670	680	690	700	710	720
PQMGTPSPGK	GSLTDGRIKP	DQDDDTEIKI	NIKQESADVN	VIGNKDVVTE	EDLDVFKQAQ	ELSWEKIECE	SGVEDCGRYP
730	740	750	760	770	780	790	800
SVIEFGKYEI	QTWYSSPYPQ	EYARLPKLYL	CEFCLKYMKS	KNILLRHSKK	CGWFHPPANE	IYRRKDLSVF	EVDGNMSKIY
810	820	830	840	850	860	870	880
CQNLCLLAKL	FLDHKTLYYD	VEPFLFYVLT	KNDEKGCHLV	GYFSKEKLCQ	QKYNVSCIMI	MPQHQRQGFG	RFLIDFSYLL
890	900	910	920	930	940	950	960
SRREGQAGSP	EKPLSDLGRL	SYLAYWKSVI	LEYLYHHHER	HISIKAISRA	TGMCPHDIAT	TLQHLHMIDK	RDGRFVIIRR
970	980	990	1000	1010	1020	1030	1040
EKLILSHMEK	LKTCSRANEL	DPDSLRWTPI	LISNAAVSEE	EREAEKEAER	LMEQASCWEK	EEQEILSTRA	NSRQSPAKVQ
1050	1060	1070	1080	1090	1100	1110	1120
SKNKYLHSPE	SRPVTGERGQ	LLELSKESSE	EEEEEEDEEE	EEEEEEEEED	EEEEEEEEEE	EEEENIQSSP	PRLTKPQSVA
1130	1140	1150	1160	1170	1180	1190	1200
IKRKRPFVLK	KKRGRKRRRI	NSSVTTETIS	ETTEVLNEPF	DNSDEERPMP	QLEPTCEIEV	EEDGRKPVLR	KAFQHQPGKK
1210	1220	1230	1240	1250	1260	1270	1280
RQTEEEEGKD	NHCFKNADPC	RNNMNDDSSN	LKEGSKDNPE	PLKCKQVWPK	GTKRGLSKWR	QNKERKTGFK	LNLYTPPETP
1290	1300	1310	1320	1330	1340	1350	1360
MEPDEQVTVE	EQKETSEGKT	SPSPIRIEEE	VKETGEALLP	QEENRREETC	APVSPNTSPG	EKPEDDLIKP	EEEEEEEEEE
1370	1380	1390	1400	1410	1420	1430	1440
EEEEEEEEGE	EEEGGGNVEK	DPDGAKSQEK	EEPEISTEKE	DSARLDDHEE	EEEEDEEPSH	NEDHDADDED	DSHMESAEVE
1450	1460	1470	1480	1490	1500	1510	1520
KEELPRESFK	EVLENQETFL	DLNVQPGHSN	PEVLMDCGVD	LTASCNSEPK	ELAGDPEAVP	ESDEEPPPGE	QAQKQDQKNS
1530	1540	1550	1560	1570	1580	1590	1600
KEVDTEFKEG	NPATMEIDSE	TVQAVQSLTQ	ESSEQDDTFQ	DCAETQEACR	SLQNYTRADQ	SPQIATTLDD	CQQSDHSSPV
1610	1620	1630	1640	1650	1660	1670	1680
SSVHSHPGQS	VRSVNSPSVP	ALENSYAQIS	PDQSAISVPS	LQNMETSPMM	DVPSVSDHSQ	QVVDSGFSDL	GSIESTTENY
1690	1700	1710	1720	1730	1740	1750	1760
ENPSSYDSTM	GGSICGNGSS	QNSCSYSNLT	SSSLTQSSCA	VTQQMSNISG	SCSMLQQTSI	SSPPTCSVKS	PQGCVVERPP
1770	1780	1790	1800	1810	1820	1830	1840
SSSQQLAQCS	MAANFTPPMQ	LAEIPETSNA	NIGLYERMGQ	SDFGAGHYPQ	PSATFSLAKL	QQLTNTLIDH	SLPYSHSAAV
1850	1860	1870	1880	1890	1900	1910	1920
TSYANSASLS	TPLSNTGLVQ	LSQSPHSVPG	GPQAQATMTP	PPNLTPPPMN	LPPPLLQRNM	AASNIGISHS	QRLQTQIASK
1930	1940	1950	1960	1970	1980	1990	2000
GHISMRTKSA	SLSPAAATHQ	SQIYGRSQTV	AMQGPARTLT	MQRGMNMSVN	LMPAPAYNVN	SVNMNMNTLN	AMNGYSMSQP
2010	2020	2030	2040	2050	2060	2070	2080
MMNSGYHSNH	GYMNQTPQYP	MQMQMGMMGT	QPYAQQPMQT	PPHGNMMYTA	PGHHGYMNTG	MSKQSLNGSY	MRR

Cmpd.	No. of Cmpds.	m/z meas.	Δ m/z [ppm]	z	Rt [min]	Score	P	Range	Sequence	Modification

Detailed Protein Report

Cmpd.	No. of Cmpds.	m/z meas.	Δ m/z [ppm]	z	Rt [min]	Score	P	Range	Sequence	Modification
1354	1	684.3815	59.97	2	46.5	10.7	0	32-44	R.ICHAVSTSHGLDK.K	

Detailed Protein Report

Protein 200: **A disintegrin and metalloproteinase with thrombospondin motifs 19 preproprotein [Homo sapiens]**

**Accession:**	gi|112789555	**Score:**	33.3
**Database:**	refseq_human(refseq_human_20140103.fasta)	**MW [kDa]:**	134.0
**Database Date:**	2015-11-30	**pI:**	9.1
**Modification(s):**	Carbamidomethyl	**Sequence Coverage [%]:**	3.4
		**No. of unique Peptides:**	1

10	20	30	40	50	60	70	80
MRLTHICCCC	LLYQLGFLSN	GIVSELQFAP	DREEWEVVFP	ALWRREPVDP	AGGSGGSADP	GWVRGVGGGG	SARAQAAGSS
90	100	110	120	130	140	150	160
REVRSVAPVP	LEEPVEGRSE	SRLRPPPPSE	GEEDEELESQ	ELPRGSSGAA	ALSPGAPASW	QPPPPPQPPP	SPPPAQHAEP
170	180	190	200	210	220	230	240
DGDEVLLRIP	AFSRDLYLLL	RRDGRFLAPR	FAVEQRPNPG	PGPTGAASAP	QPPAPPDAGC	FYTGAVLRHP	GSLASFSTCG
250	260	270	280	290	300	310	320
GGLMGFIQLN	EDFIFIEPLN	DTMAITGHPH	RVYRQKRSME	EKVTEKSALH	SHYCGIISDK	GRPRSRKIAE	SGRGKRYSYK
330	340	350	360	370	380	390	400
LPQEYNIETV	VVADPAMVSY	HGADAARRFI	LTILNMVFNL	FQHKSLSVQV	NLRVIKLILL	HETPPELYIG	HHGEKMLESF
410	420	430	440	450	460	470	480
CKWQHEEFGK	KNDIHLEMST	NWGEDMTSVD	AAILITRKDF	CVHKDEPCDT	VGIAYLSGMC	SEKRKCIIAE	DNGLNLAFTI
490	500	510	520	530	540	550	560
AHEMGHNMGI	NHDNDHPSCA	DGLHIMSGEW	IKGQNLGDVS	WSRCSKEDLE	RFLRSKASNC	LLQTNPQSVN	SVMVPSKLPG
570	580	590	600	610	620	630	640
MTYTADEQCQ	ILFGPLASFC	QEMQHVICTG	LWCKVEGEKE	CRTKLDPPMD	GTDCDLGKWC	KAGECTSRTS	APEHLAGEWS
650	660	670	680	690	700	710	720
LWSPCSRTCS	AGISSRERKC	PGLDSEARDC	NGPRKQYRIC	ENPPCPAGLP	GFRDWQCQAY	SVRTSSPKHI	LQWQAVLDEE
730	740	750	760	770	780	790	800
KPCALFCSPV	GKEQPILLSE	KVMDGTSCGY	QGLDICANGR	CQKVGCDGLL	GSLAREDHCG	VCNGNGKSCK	IIKGDFNHTR
810	820	830	840	850	860	870	880
GAGYVEVLVI	PAGARRIKVV	EEKPAHSYLA	LRDAGKQSIN	SDWKIEHSGA	FNLAGTTVHY	VRRGLWEKIS	AKGPTTAPLH
890	900	910	920	930	940	950	960
LLVLLFQDQN	YGLHYEYTIP	SDPLPENQSS	KAPEPLFMWT	HTSWEDCDAT	CGGGERKTTV	SCTKIMSKNI	SIVDNEKCKY
970	980	990	1000	1010	1020	1030	1040
LTKPEPQIRK	CNEQPCQTRW	MMTEWTPCSR	TCGKGMQSRQ	VACTQQLSNG	TLIRARERDC	IGPKPASAQR	CEGQDCMTVW
1050	1060	1070	1080	1090	1100	1110	1120
EAGVWSECSV	KCGKGIRHRT	VRCTNPRKKC	VLSTRPREAE	DCEDYSKCYV	WRMGDWSKCS	ITCGKGMQSR	VIQCMHKITG
1130	1140	1150	1160	1170	1180	1190	1200
RHGNECFSSE	KPAAYRPCHL	QPCNEKINVN	TITSPRLAAL	TFKCLGDQWP	VYCRVIREKN	LCQDMRWYQR	CCETCRDFYA
1210							
QKLQQKS							

Cmpd.	No. of Cmpds.	m/z meas.	Δ m/z [ppm]	z	Rt [min]	Score	P	Range	Sequence	Modification
109	1	1098.0910	-64.88	3	31.1	12.4	2	619-647	K.WCKAGECTSRTSAPEHLAGEWSLWSPCSR.T	Carbamidomethyl: 2

Detailed Protein Report

Protein 201: PREDICTED: serine/threonine-protein kinase 31 isoform X3 [Homo sapiens]

**Accession:**	gi|578813577	**Score:**	33.3
**Database:**	refseq_human(refseq_human_20140103.fasta)	**MW [kDa]:**	95.7
**Database Date:**	2015-11-30	**pI:**	4.8
		**Sequence Coverage [%]:**	3.1
		**No. of unique Peptides:**	1

10	20	30	40	50	60	70	80
MWVQGHSSRA	SATESVSFSG	IVQMDEDTHY	DKVEDVVGSH	IEDAVTFWAQ	SINRNKDIMK	IGCSLSEVCP	QASSVLGNLD
**90**	**100**	**110**	**120**	**130**	**140**	**150**	**160**
PNKIYGGLFS	EDQCWYRCKV	LKIISVEKCL	VRYIDYGNTE	ILNRSDIVEI	PLELQFSSVA	KKYKLWGLHI	PSDQEVTQFD
**170**	**180**	**190**	**200**	**210**	**220**	**230**	**240**
QQEKAAAVDL	TNHLEYTLKT	YIDTRMKNLA	AKMEILKEMR	HVDISVRFGK	DLSDAIQVLD	EGCFTTPASL	NGLEIIWAEY
**250**	**260**	**270**	**280**	**290**	**300**	**310**	**320**
SLAQENIKTC	EYVSEGNILI	AQRNEMQQKL	YMSVEDFILE	VDESSLNKRL	KTLQDLSVSL	EAVYGQAKEG	ANSDEILKKF
**330**	**340**	**350**	**360**	**370**	**380**	**390**	**400**
YDWKCDKREE	FTSVRSETDA	SLHRLVAWFQ	RTLKVFDLSV	EGSLISEDAM	DNIDEILEKT	ESSVCKELEI	ALVDQGDADK
**410**	**420**	**430**	**440**	**450**	**460**	**470**	**480**
EIISNTYSQV	LQKIHSEERL	IATVQAKYKD	SIEFKKQLIE	YLNKSPSVDH	LLSIKKTLKS	LKALLRWKLV	EKSNLEESDD
**490**	**500**	**510**	**520**	**530**	**540**	**550**	**560**
PDGSQIEKIK	EEITQLRNNV	FQEIYHEREE	YEMLTSLAQK	WFPELPLLHP	EIGLLKYMNS	GGLLTMSLER	DLLDAEPMKE
**570**	**580**	**590**	**600**	**610**	**620**	**630**	**640**
LSSKRPLVRS	EVNGQIILLK	GYSVDVDTEA	KVIERAATYH	RAWREAEGDS	GLLPLIFLFL	CKSDPMAYLM	VPYYPRANLN
**650**	**660**	**670**	**680**	**690**	**700**	**710**	**720**
AVQANMPLNS	EETLKVMKGV	AQGLHTLHKA	DIIHGSLHQN	NVFALNREQG	IVGDFDFTKS	VSQRASVNMM	VGDLSLMSPE
**730**	**740**	**750**	**760**	**770**	**780**	**790**	**800**
LKMGKPASPG	SDLYAYGCLL	LWLSVQNQEF	EINKDGIPKV	DQFHLDDKVK	SLLCSLICYR	SSMTAEQVLN	AECFLMPKEQ
**810**	**820**	**830**	**840**	**850**			
SVPNPEKDTE	YTLYKKEEEI	KTENLDKCME	KTRNGEANFD	C			

Cmpd.	No. of Cmpds.	m/z meas.	Δ m/z [ppm]	z	Rt [min]	Score	P	Range	Sequence	Modification
277	3	592.1990	-132.59	2	33.2	18.2	0	581-591	K.GYSVDVDTEAK.V	

Detailed Protein Report

Protein 202: spectrin beta chain, non-erythrocytic 5 [Homo sapiens]

**Accession:**	gi|485837026	**Score:**	33.2
**Database:**	refseq_human(refseq_human_20140103.fasta)	**MW [kDa]:**	416.5
**Database Date:**	2015-11-30	**pI:**	6.2
		**Sequence Coverage [%]:**	0.9
		**No. of unique Peptides:**	2

Detailed Protein Report

10	20	30	40	50	60	70	80
MAGQPHSPRE	LLGAAGHRSR	RPSTELRVPP	SPSLTMDSQY	ETGHIRKLQA	RHMQMQEKTF	TKWINNVFQC	GQAGIKIRNL
90	100	110	120	130	140	150	160
YTELADGIHL	LRLLELISGE	ALPPPSRGRL	RVHFLENSSR	ALAFLRAKVP	VPLIGPENIV	DGDQTLILGL	IWVIILRFQI
170	180	190	200	210	220	230	240
SHISLDKEEF	GASAALLSTK	EALLVWCQRK	TASYTNVNIT	DFSRSWSDGL	GFNALIHAHR	PDLLDYGSLR	PDRPLHNLAF
250	260	270	280	290	300	310	320
AFLVAEQELG	IAQLLDPEDV	AAAQPDERSI	MTYVSLYYHY	CSRLHQGQTV	QRRLTKILLQ	LQETELLQTQ	YEQLVADLLR
330	340	350	360	370	380	390	400
WIAEKQMQLE	ARDFPDSLPA	MRQLLAAFTI	FRTQEKPPRL	QQRGAAEALL	FRLQTALQAQ	NRRPFLPHEG	LGLAELSQCW
410	420	430	440	450	460	470	480
AGLEWAEAAR	SQALQQRLLQ	LQRLETLARR	FQHKAALRES	FLKDAEQVLD	QARAPPASLA	TVEAAVQRLG	MLEAGILPQE
490	500	510	520	530	540	550	560
GRFQALAEIA	DILRQEQYHS	WADVARRQEE	VTVRWQRLLQ	HLQGQRKQVA	DMQAVLSLLQ	EVEAASHQLE	ELQEPARSTA
570	580	590	600	610	620	630	640
CGQQLAEVVE	LLQRHDLLEA	QVSAHGAHVS	HLAQQTAELD	SSLGTSVEVL	QAKARTLAQL	QQSLVALVRA	RRALLEQTLQ
650	660	670	680	690	700	710	720
RAEFLRNCEE	EEAWLKECGQ	RVGNAALGRD	LSQIAGALQK	HKALEAEVHR	HQAVCVDLVR	RGRDLSARRP	PTQPDPGERA
730	740	750	760	770	780	790	800
EAVQGGWQLL	QTRVVGRGAR	LQTALLVLQY	FADAAEAASW	LRERRSSLER	ASCGQDQAAA	ETLLRRHVRL	ERVLRAFAAE
810	820	830	840	850	860	870	880
LRRLEEQGRA	ASARASLFTV	NSALSPPGES	LRNPGPWSEA	SCHPGPGDAW	KMALPAEPDP	DFDPNTILQT	QDHLSQDYES
890	900	910	920	930	940	950	960
LRALAQLRRA	RLEEAMALFG	FCSSCGELQL	WLEKQTVLLQ	RVQPQADTLE	VMQLKYENFL	TALAVGKGLW	AEVSSSAEQL
970	980	990	1000	1010	1020	1030	1040
RQRYPGNSTQ	IQRQQEELSQ	RWGQLEALKR	EKAVQLAHSV	EVCSFLQECG	PTQVQLRDVL	LQLEALQPGS	SEDTCHALQL
1050	1060	1070	1080	1090	1100	1110	1120
AQKKTLVLER	RVHFLQSVVV	KVEEPGYAES	QPLQGQVETL	QGLLKQVQEQ	VAQRARRQAE	TQARQSFLQE	SQQLLLWAES
1130	1140	1150	1160	1170	1180	1190	1200
VQAQLRSKEV	SVDVASAQRL	LREHQDLLEE	IHLWQERLQQ	LDAQSQPMAA	LDCPDSQEVP	NTLRVLGQQG	QELKVLWEQR
1210	1220	1230	1240	1250	1260	1270	1280
QQWLQEGLEL	QKFGREVDGF	TATCANHQAW	LHLDNLGEDV	REALSLLQQH	REFGRLLSTL	GPRAEALRAH	GEKLVQSQHP
1290	1300	1310	1320	1330	1340	1350	1360
AAHTVREQLQ	SIQAQWTRLQ	GRSEQRRRQL	LASLQLQEWK	QDVAELMQWM	EEKGLMAAHE	PSGARRNILQ	TLKRHEAAES
1370	1380	1390	1400	1410	1420	1430	1440
ELLATRRHVE	ALQQVGRELL	SRRPCGQEDI	QTRLQGLRSK	WEALNRKMTE	RGDELQQAGQ	QEQLLRQLQD	AKEQLEQLEG
1450	1460	1470	1480	1490	1500	1510	1520
ALQSSETGQD	LRSSQRLQKR	HQQLESESRT	LAAKMAALAS	MAHGMAASPA	ILEETQKHLR	RLELLQGHLA	IRGLQLQASV
1530	1540	1550	1560	1570	1580	1590	1600
ELHQFCHLSN	MELSWVAEHM	PHGSPTSYTE	CLNGAQSLHR	KHKELQVEVK	AHQGQVQRVL	SSGRSLAASG	HPQAQHIVEQ
1610	1620	1630	1640	1650	1660	1670	1680
CQELEGHWAE	LERACEARAQ	CLQQAVTFQQ	YFLDVSELEG	WVEEKRPLVS	SRDYGRDEAA	TLRLINKHQA	LQEELAIYWS
1690	1700	1710	1720	1730	1740	1750	1760
SMEELDQTAQ	TLTGPEVPEQ	QRVVQERLRE	QLRALQELAA	TRDRELEGTL	RLHEFLREAE	DLQGWLASQK	QAAKGGESLG
1770	1780	1790	1800	1810	1820	1830	1840
EDPEHALHLC	TKFAKFQHQV	EMGSQRVAAC	RLLAESLLER	GHSAGPMVRQ	RQQDLQTAWS	ELWELTQARG	HALRDTETTL
1850	1860	1870	1880	1890	1900	1910	1920
RVHRDLLEVL	TQVQEKATSL	PNNVARDLCG	LEAQLRSHQG	LERELVGTER	QLQELLETAG	RVQKLCPGPQ	AHAVQQRQQA
1930	1940	1950	1960	1970	1980	1990	2000
VTQAWAVLQR	RMEQRRAQLE	RARLLARFRT	AVRDYASWAA	RVRQDLQVEE	SSQEPSSGPL	KLSAHQWLRA	ELEAREKLWQ
2010	2020	2030	2040	2050	2060	2070	2080
QATQLGQQAL	LAAGTPTKEV	QEELRALQDQ	RDQVYQTWAR	KQERLQAEQQ	EQLFLRECGR	LEEILAAQEV	SLKTSALGSS
2090	2100	2110	2120	2130	2140	2150	2160
VEEVEQLIRK	HEVFLKVLTA	QDKKEAALRE	RLKTLRRPRV	RDRLPILLQR	RMRVKELAES	RGHALHASLL	MASFTQAATQ
2170	2180	2190	2200	2210	2220	2230	2240

Detailed Protein Report

Cmpd.	No. of Cmpds.	m/z meas.	Δ m/z [ppm]	z	Rt [min]	Score	P	Range	Sequence	Modification
1932	1	724.3207	-73.16	2	54.2	11.4	1	962-973	R.QRYPGNSTQIQR.Q	
2799	2	665.3651	-30.72	2	65.4	11.8	0	3278-3291	R.LGQLHPAAPGGLAK.V	

Detailed Protein Report

Protein 203: **serine/threonine-protein kinase 3 isoform 2 [Homo sapiens]**

**Accession:**	gi|372622373	**Score:**	33.2
**Database:**	refseq_human(refseq_human_20140103.fasta)	**MW [kDa]:**	59.4
**Database Date:**	2015-11-30	**pI:**	5.0
		**Sequence Coverage [%]:**	5.6
		**No. of unique Peptides:**	1

10	20	30	40	50	60	70	80
MLQLMDSGIT	ICLRNGAASV	FKKKEWSTQG	EENKQDSKLK	KLSEDSLTKQ	PEEVFDVLEK	LGEGSYGSVF	KAIHKESGQV
**90**	**100**	**110**	**120**	**130**	**140**	**150**	**160**
VAIKQVPVES	DLQEIIKEIS	IMQQCDSPYV	VKYYGSYFKN	TDLWIVMEYC	GAGSVSDIIR	LRNKTLIEDE	IATILKSTLK
**170**	**180**	**190**	**200**	**210**	**220**	**230**	**240**
GLEYLHFMRK	IHRDIKAGNI	LLNTEGHAKL	ADFGVAGQLT	DTMAKRNTVI	GTPFWMAPEV	IQEIGYNCVA	DIWSLGITSI
**250**	**260**	**270**	**280**	**290**	**300**	**310**	**320**
EMAEGKPPYA	DIHPMRAIFM	IPTNPPPTFR	KPELWSDDFT	DFVKKCLVKN	PEQRATATQL	LQHPFIKNAK	PVSILRDLIT
**330**	**340**	**350**	**360**	**370**	**380**	**390**	**400**
EAMEIKAKRH	EEQQRELEEE	EENSDEDELD	SHTMVKTSVE	SVGTMRATST	MSEGAQTMIE	HNSTMLESDL	GTMVINSEDE
**410**	**420**	**430**	**440**	**450**	**460**	**470**	**480**
EEEDGTMKRN	ATSPQVQRPS	FMDYFDKQDF	KNKSHENCNQ	NMHEPFPMSK	NVFPDNWKVP	QDGDFDFLKN	LSLEELQMRL
**490**	**500**	**510**	**520**				
KALDPMMERE	IEELRQRYTA	KRQPILDAMD	AKKRRQQNF				

Cmpd.	No. of Cmpds.	m/z meas.	Δ m/z [ppm]	z	Rt [min]	Score	P	Range	Sequence	Modification
2001	2	731.8177	-100.79	2	55.1	18.2	0	2-14	M.LQLMDSGITICLR.N	

Detailed Protein Report

Protein 204: **PREDICTED: LIM and senescent cell antigen-like-containing domain protein 1 isoform X7 [Homo sapiens]**

**Accession:**	gi|578804328	**Score:**	33.0
**Database:**	refseq_human(refseq_human_20140103.fasta)	**MW [kDa]:**	39.4
**Database Date:**	2015-11-30	**pI:**	9.8
**Modification(s):**	Carbamidomethyl	**Sequence Coverage [%]:**	16.0
		**No. of unique Peptides:**	2

10	20	30	40	50	60	70	80
MPSQSSNMAN	ALASATCERC	KGGFAPAEKI	VNSNGELYHE	QCFVCAQCFQ	QFPEGLFYEF	EGRKYCEHDF	QMLFAPCCHQ
**90**	**100**	**110**	**120**	**130**	**140**	**150**	**160**
CGEFIIGRVI	KAMNNSWHPE	CFRCDLCQEV	LADIGFVKNA	GRHLCRPCHN	REKARGLGKY	ICQKCHAIID	EQPLIFKNDP
**170**	**180**	**190**	**200**	**210**	**220**	**230**	**240**
YHPDHFNCAN	CGKELTADAR	ELKGELYCLP	CHDKMGVPIC	GACRRPIEGR	VVNAMGKQWH	VEHFVCAKCE	KPFLGHRHYE
**250**	**260**	**270**	**280**	**290**	**300**	**310**	**320**
RKGLAYCETH	YNQLFGDVCF	HCNRVIEGDV	VSALNKAWCV	NCFACSTCNT	KLTLKDKFVE	IDLKPVCKHC	YEKMPEEFKR
**330**	**340**	**350**					
RLAKREREAK	DKDKQKKKKP	VCL					

Cmpd.	No. of Cmpds.	m/z meas.	Δ m/z [ppm]	z	Rt [min]	Score	P	Range	Sequence	Modification
1893	1	573.2288	-144.49	2	53.7	11.3	1	174-183	K.ELTADARELK.G	
1542	1	978.1217	1.51	3	49.0	11.6	1	265-291	R.VIEGDVVSALNKAWCVNCFACSTCNTK.L	Carbamidomethyl: 21

Detailed Protein Report

Protein 205: **rapamycin-insensitive companion of mTOR isoform 3 [Homo sapiens]**

**Accession:**	gi|550544215	**Score:**	33.0
**Database:**	refseq_human(refseq_human_20140103.fasta)	**MW [kDa]:**	155.8
**Database Date:**	2015-11-30	**pI:**	6.8
		**Sequence Coverage [%]:**	1.9
		**No. of unique Peptides:**	2

Quantitation

MD:MU **Median:** 1.85 **CV:** 0.00 % **No. of Peptides:** 1

10	20	30	40	50	60	70	80
MGIIATFRSW	AGIINLCKPG	NSGIQSLIGV	LCIPNMEIRR	GLLEVLYDIF	RLPLPVVTEE	FIEALLSVDP	GRFQDSWRLS
90	100	110	120	130	140	150	160
DGFVAAEAKT	ILPHRARSRP	DLMDNYLALI	LSAFIRNGLL	EGLVEVITNS	DDHISVRATI	LLGELLHMAN	TILPHSHSHH
170	180	190	200	210	220	230	240
LHCLPTLMNM	AASFDIPKEK	RLRASAALNC	LKRFHEMKKR	GPKPYSLHLD	HIIQKAIATH	QKRDQYLRVQ	KDIFILKDTE
250	260	270	280	290	300	310	320
EALLINLRDS	QVLQHKENLE	WNWNLIGTIL	KWPNVNLRNY	KDEQLHRFVR	RLLYFYKPSS	KLYANLDLDF	AKAKQLTVVG
330	340	350	360	370	380	390	400
CQFTEFLLES	EEDGQGYLED	LVKDIVQWLN	ASSGMKPERS	LQNNGLLTTL	SQHYFLFIGT	LSCHPHGVKM	LEKCSVFQCL
410	420	430	440	450	460	470	480
LNLCSLKNQD	HLLKLTVSSL	DYSRDGLARV	ILSKILTAAT	DACRLYATKH	LRVLLRANVE	FFNNWGIELL	VTQLHDKNKT
490	500	510	520	530	540	550	560
ISSEALDILD	EACEDKANLH	ALIQMKPALS	HLGDKGLLLL	LRFLSIPKGF	SYLNERGYVA	KQLEKWHREY	NSKYVDLIEE
570	580	590	600	610	620	630	640
QLNEALTTYR	KPVDGDNYVR	RSNQRLQRPH	VYLPIHLYGQ	LVHHKTGCHL	LEVQNIITEL	CRNVRTPDLD	KWEEIKKLKA
650	660	670	680	690	700	710	720
SLWALGNIGS	SNWGLNLLQE	ENVIPDILKL	AKQCEVLSIR	GTCVYVLGLI	AKTKQGCDIL	KCHNWDAVRH	SRKHLWPVVP
730	740	750	760	770	780	790	800
DDVEQLCNEL	SSIPSTLSLN	SESTSSRHNS	ESESVPSSMF	ILEDDRFGSS	STSTFFLDIN	EDTEPTFYDR	SGPIKDKNSF
810	820	830	840	850	860	870	880
PFFASSKLVK	NRILNSLTLP	NKKHRSSSDP	KGGKLSSESK	TSNRRIRTLT	EPSVDFNHSD	DFTPISTVQK	TLQLETSFMG
890	900	910	920	930	940	950	960
NKHIEDTGST	PSIGENDLKF	TKNFGTENHR	ENTSRERLVV	ESSTSSHMKI	RSQSFNTDTT	TSGISSMSSS	PSRETVGVDA
970	980	990	1000	1010	1020	1030	1040
TTMDTDCGSM	STVVSTKTIK	TSHYLTPQSN	HLSLSKSNSV	SLVPPGSSHT	LPRRAQSLKA	PSIATIKSLA	DCNFSYTSSR
1050	1060	1070	1080	1090	1100	1110	1120
DAFGYATLKR	LQQQRMHPSL	SHSEALASPA	KDVLFTDTIT	MKANSFESRL	TPSRFMKALS	YASLDKEDLL	SPINQNTLQR
1130	1140	1150	1160	1170	1180	1190	1200
SSSVRSMVSS	ATYGGSDDYI	GLALPVDIND	IFQVKDIPYF	QTKNIPPHDD	RGARAFAHDA	GESTEDTGLQ	EHTDDNCLYC
1210	1220	1230	1240	1250	1260	1270	1280
VCIEILGFQP	SNQLSAICSH	SDFQDIPYSD	WCEQTIHNPL	EVVPSKFSGI	SGCSDGVSQE	GSASSTKSTE	LLLGVKTIPD
1290	1300	1310	1320	1330	1340	1350	1360
DTPMCRILLR	KEVLRLVINL	SSSVSTKCHE	TGLLTIKEKY	PQTFDDICLY	SEVSHLLSHC	TFRLPCRRFI	QELFQDVQFL
1370	1380	1390					
QMHEEAEAVL	ATPPKQPIVD	TSAES					

Cmpd.	No. of Cmpds.	m/z meas.	Δ m/z [ppm]	z	Rt [min]	Score	P	Range	Sequence	Modification	Ratios
2600	6	741.8765	-155.82	2	62.5	20.5	1	516-528	K.GLLLLLRFLSIPK.G		
275	1	795.2750	-158.71	2	33.1	12.4	1	916-929	R.ERLVVESSTSSHMK.I		MD:MU 1.85

Detailed Protein Report

Protein 206: **PREDICTED: NEDD8 ultimate buster 1 isoform X3 [Homo sapiens]**

**Accession:**	gi|530387034	**Score:**	32.9
**Database:**	refseq_human(refseq_human_20140103.fasta)	**MW [kDa]:**	67.7
**Database Date:**	2015-11-30	**pI:**	5.7
		**Sequence Coverage [%]:**	5.3
		**No. of unique Peptides:**	1

10	20	30	40	50	60	70	80
MKTNGGRCRI	RALCWSRREW	RGAGMAQKKY	LQAKLTQFLR	EDRIQLWKPP	YTDENKKVGL	ALKDLAKQYS	DRLECCENEV
**90**	**100**	**110**	**120**	**130**	**140**	**150**	**160**
EKVIEEIRCK	AIERGTGNDN	YRTTGIATIE	VFLPPRLKKD	RKNLLETRLH	ITGRELRSKI	AETFGLQENY	IKIVINKKQL
**170**	**180**	**190**	**200**	**210**	**220**	**230**	**240**
QLGKTLEEQG	VAHNVKAMVL	ELKQSEEDAR	KNFQLEEEEQ	NEAKLKEKQI	QRTKRGLEIL	AKRAAETVVD	PEMTPYLDIA
**250**	**260**	**270**	**280**	**290**	**300**	**310**	**320**
NQTGRSIRIP	PSERKALMLA	MGYHEKGRAF	LKRKEYGIAL	PCLLDADKYF	CECCRELLDT	VDNYAVLQLD	IVWCYFRLEQ
**330**	**340**	**350**	**360**	**370**	**380**	**390**	**400**
LECLDDAEKK	LNLAQKCFKN	CYGENHQRLV	HIKGNCGKEK	VLFLRLYLLQ	GIRNYHSGND	VEAYEYLNKA	RQLFKELYID
**410**	**420**	**430**	**440**	**450**	**460**	**470**	**480**
PSKVDNLLQL	GFTAQEARLG	LRACDGNVDH	AATHITNRRE	ILLSNPQMWW	LNDSNPETDN	RQESPSQENI	DRLVYMGFDA
**490**	**500**	**510**	**520**	**530**	**540**	**550**	**560**
LVAEAALRVF	RGNVQLAAQT	LAHNGGSLPP	ELPLSPEDSL	SPPATSPSDS	AGTSSASTDE	DMETEAVNEI	LEDIPEHEED
**570**	**580**	**590**	**600**				
YLDSTLEDEE	IIIAEYLSYV	ENRKSATKKN					

Cmpd.	No. of Cmpds.	m/z meas.	Δ m/z [ppm]	z	Rt [min]	Score	P	Range	Sequence	Modification
2129	1	596.7885	-98.31	2	56.5	10.5	2	330-339	K.KLNLAQKCFK.N	

Detailed Protein Report

Protein 207: **E3 ubiquitin-protein ligase RNF213 isoform 3 [Homo sapiens]**

**Accession:**	gi|366039979	**Score:**	32.9
**Database:**	refseq_human(refseq_human_20140103.fasta)	**MW [kDa]:**	591.0
**Database Date:**	2015-11-30	**pI:**	6.0
**Modification(s):**	Oxidation	**Sequence Coverage [%]:**	0.5
		**No. of unique Peptides:**	2

Detailed Protein Report

10	20	30	40	50	60	70	80
MECPSCQHVS	KEETPKFCSQ	CGERLPPAAP	IADSENNNST	MASASEGEME	CGQELKEEGG	PCLFPGSDSW	QENPEEPCSK
90	100	110	120	130	140	150	160
ASWTVQESKK	KKRKKKKKGN	KSASSELASL	PLSPASPCHL	TLLSNPWPQD	TALPHSQAQQ	SGPTGQPSQP	PGTATTPLEG
170	180	190	200	210	220	230	240
DGLSAPTEVG	DSPLQAQALG	EAGVATGSEA	QSSPQFQDHT	EGEDQDASIP	SGGRGLSQEG	TGPPTSAGEG	HSRTEDAAQE
250	260	270	280	290	300	310	320
LLLPESKGGS	SEPGTELQTT	EQQAGASASM	AVDAVAEPAN	AVKGAGKEMK	EKTQRMKQPP	ATTPPFKTHC	QEAETKTKDE
330	340	350	360	370	380	390	400
MAAAEEKVGK	NEQGEPEDLK	KPEGKNRSAA	AVKNEKEQKN	QEADVQEVKA	STLSPGGGVT	VFFHAIISLH	FPFNPDLHKV
410	420	430	440	450	460	470	480
FIRGGEEFGE	SKWDSNICEL	HYTRDLGHDR	VLVEGIVCIS	KKHLDKYIPY	KYVIYNGESF	EYEFIYKHQQ	KKGEYVNRCL
490	500	510	520	530	540	550	560
FIKSSLLGSG	DWHQYYDIVY	MKPHGRLQKV	MNHITDGPRK	DLVKGKQIAA	ALMLDSTFSI	LQTWDTINLN	SFFTQFEQFC
570	580	590	600	610	620	630	640
FVLQQPMIYE	GQAQLWTDLQ	YREKEVKRYL	WQHLKKHVVP	LPDGKSTDFL	PVDCPVRSKL	KTGLIVLFVV	EKIELLLEGS
650	660	670	680	690	700	710	720
LDWLCHLLTS	DASSPDEFHR	DLSHILGIPQ	SWRLYLVNLC	QRCMDTRTYT	WLGALPVLHC	CMELAPRHKD	AWRQPEDTWA
730	740	750	760	770	780	790	800
ALEGLSFSPF	REQMLDTSSL	LQFMREKQHL	LSIDEPLFRS	WFSLLPLSHL	VMYMENFIEH	LGRFPAHILD	CLSGIYYRLP
810	820	830	840	850	860	870	880
GLEQVLNTQD	VQDVQNVQNI	LEMLLRLLDT	YRDKIPEEAL	SPSYLTVCLK	LHEAICSSTK	LLKFYELPAL	SAEIVCRMIR
890	900	910	920	930	940	950	960
LLSLVDSAGQ	RDETGNNSVQ	TVFQGTLAAT	KRWLREVFTK	NMLTSSGASF	TYVKEIEVWR	RLVEIQFPAE	HGWKESLLGD
970	980	990	1000	1010	1020	1030	1040
MEWRLTKEEP	LSQITAYCNS	CWDTKGLEDS	VAKTFEKCII	EAVSSACQSQ	TSILQGFSYS	DLRKFGIVLS	AVITKSWPRT
1050	1060	1070	1080	1090	1100	1110	1120
ADNFNDILKH	LLTLADVKHV	FRLCGTDEKI	LANVTEDAKR	LIAVADSVLT	KVVGDLLSGT	ILVGQLELII	KHKNQFLDIW
1130	1140	1150	1160	1170	1180	1190	1200
QLREKSLSPQ	DEQCAVEEAL	DWRREELLLL	KKEKRCVDSL	LKMCGNVKHL	IQVDFGVLAV	RHSQDLSSKR	LNDTVTVRLS
1210	1220	1230	1240	1250	1260	1270	1280
TSSNSQRATH	YHLSSQVQEM	AGKIDLLRDS	HIFQLFWREA	AEPLSEPKED	QEAAELLSEP	EEESERHILE	LEEVYDYLYQ
1290	1300	1310	1320	1330	1340	1350	1360
PSYRKFIKLH	QDLKSGEVTL	AEIDVIFKDF	VNKYTDLDSE	LKIMCTVDHQ	DQRDWIKDRV	EQIKEYHHLH	QAVHAAKVIL
1370	1380	1390	1400	1410	1420	1430	1440
QVKESLGLNG	DFSVLNTLLN	FTDNFDDFRR	ETLDQINQEL	IQAKKLLQDI	SEARCKGLQA	LSLRKEFICW	VREALGGINE
1450	1460	1470	1480	1490	1500	1510	1520
LKVFVDLASI	SAGENDIDVD	RVACFHDAVQ	GYASLLFKLD	PSVDFSAFMK	HLKKLWKALD	KDQYLPRKLC	DSARNLEWLK
1530	1540	1550	1560	1570	1580	1590	1600
TVNESHGSVE	RSSLTLATAI	NQRGIYVIQA	PKGGQKISPD	TVLHLILPES	PGSHEESREY	SLEEVKELLN	KLMLMSGKKD
1610	1620	1630	1640	1650	1660	1670	1680
RNNTEVERFS	EVFCSVQRLS	QAFIDLHSAG	NMLFRTWIAM	AYCSPKQGVS	LQMDFGLDLV	TELKEGGDVT	ELLAALCRQM
1690	1700	1710	1720	1730	1740	1750	1760
EHFLDSWKRF	VTQKRMEHFY	LNFYTAEQLV	YLSTELRKQP	PSDAALTMLS	FIKSNCTLRD	VLRASVGCGS	EAARYRMRRV
1770	1780	1790	1800	1810	1820	1830	1840
MEELPLMLLS	EFSLVDKLRI	IMEQSMRCLP	AFLPDCLDLE	TLGHCLAHLA	GMGGSPVERC	LPRGLQVGQP	NLVVCGHSEV
1850	1860	1870	1880	1890	1900	1910	1920
LPAALAVYMQ	TPSQPLPTYD	EVLLCTPATT	FEEVALLLRR	CLTLGSLGHK	VYSLLFADQL	SYEVARQAEE	LFHNLCTQQH
1930	1940	1950	1960	1970	1980	1990	2000
REDYQLVMVC	DGDWEHCYLP	SAFSQHKVFV	TPQAPLEAIQ	AYLAGHYRVP	KQTLSAAAVF	NDRLCVGIVA	SERAGVGKSL
2010	2020	2030	2040	2050	2060	2070	2080
YVKRLHDKMK	MQLNVKNVPL	KTIRLIDPQV	DESRVLGALL	PFLDAQYQKV	PVLFHLDVTS	SVQTGIWVFL	FKLLILQYLM
2090	2100	2110	2120	2130	2140	2150	2160
DINGKMWLRN	PCHLYIVEIL	ERRTSVPSRS	SSALRTRVPQ	FSFLDIFPKV	TCRPPKEVID	MELSALRSDT	EPGMDLWEFC
2170	2180	2190	2200	2210	2220	2230	2240

Detailed Protein Report

Cmpd.	No. of Cmpds.	m/z meas.	Δ m/z [ppm]	z	Rt [min]	Score	P	Range	Sequence	Modification
1737	1	863.0655	110.54	2	51.5	19.4	1	746-759	R.EKQHLLSIDEPLFR.S	
2481	1	856.9085	-24.89	2	61.4	13.6	1	4661-4674	R.LLNFDTELSTKEMR.N	Oxidation: 13

Detailed Protein Report

Protein 208: **PREDICTED: agrin isoform X5 [Homo sapiens]**

**Accession:**	gi|578799096	**Score:**	32.9
**Database:**	refseq_human(refseq_human_20140103.fasta)	**MW [kDa]:**	200.0
**Database Date:**	2015-11-30	**pI:**	6.0
**Modification(s):**	Carbamidomethyl, Oxidation	**Sequence Coverage [%]:**	3.5
		**No. of unique Peptides:**	2

10	20	30	40	50	60	70	80
MAGRSHPGPL	RPLLPLLVVA	ACVLPGAGGT	CPERALERRE	EEANVVLTGT	VEEILNVDPV	QHTYSCKVRV	WRYLKGKDLV
90	100	110	120	130	140	150	160
ARESLLDGGN	KVVISGFGDP	LICDNQVSTG	DTRIFFVNPA	PPYLWPAHKN	ELMLNSSLMR	ITLRNLEEVE	FCVEDKPGTH
170	180	190	200	210	220	230	240
FTPVPPTPPD	ACRGMLCGFG	AVCEPNAEGP	GRASCVCKKS	PCPSVVAPVC	GSDASTYSNE	CELQRAQCSQ	QRRIRLLSRG
250	260	270	280	290	300	310	320
PCGSRDPCSN	VTCSFGSTCA	RSADGLTASC	LCPATCRGAP	EGTVCGSDGA	DYPGECQLLR	RACARQENVF	KKFDGPCDPC
330	340	350	360	370	380	390	400
QGALPDPSRS	CRVNPRTRRP	EMLLRPESCP	ARQAPVCGDD	GVTYENDCVM	GRSGAARGLL	LQKVRSGQCQ	GRDQCPEPCR
410	420	430	440	450	460	470	480
FNAVCLSRRG	RPRCSCDRVT	CDGAYRPVCA	QDGRTYDSDC	WRQQAECRQQ	RAIPSKHQGP	CDQAPSPCLG	VQCAFGATCA
490	500	510	520	530	540	550	560
VKNGQAACEC	LQACSSLYDP	VCGSDGVTYG	SACELEATAC	TLGREIQVAR	KGPCDRCGQC	RFGALCEAET	GRCVCPSECV
570	580	590	600	610	620	630	640
ALAQPVCGSD	GHTYPSECML	HVHACTHQIS	LHVASAGPCE	TCGDAVCAFG	AVCSAGQCVC	PRCEHPPPGP	VCGSDGVTYG
650	660	670	680	690	700	710	720
SACELREAAC	LQQTQIEEAR	AGPCEQAECG	SGGSGSGEDG	DCEQELCRQR	GGIWDEDSED	GPCVCDFSCQ	SVPGSPVCGS
730	740	750	760	770	780	790	800
DGVTYSTECE	LKKARCESQR	GLYVAAQGAC	RGPTFAPLPP	VAPLHCAQTP	YGCCQDNITA	ARGVGLAGCP	SACQCNPHGS
810	820	830	840	850	860	870	880
YGGTCDPATG	QCSCRPGVGG	LRCDRCEPGF	WNFRGIVTDG	RSGCTPCSCD	PQGAVRDDCE	QMTGLCSCKP	GVAGPKCGQC
890	900	910	920	930	940	950	960
PDGRALGPAG	CEADASAPAT	CAEMRCEFGA	RCVEESGSAH	CVCPMLTCPE	ANATKVCGSD	GVTYGNECQL	KTIACRQGLQ
970	980	990	1000	1010	1020	1030	1040
ISIQSLGPCQ	EAVAPSTHPT	SASVTVTTPG	LLLSQALPAP	PGALPLAPSS	TAHSQTTPPP	SSRPRTTASV	PRTTVWPVLT
1050	1060	1070	1080	1090	1100	1110	1120
VPPTAPSPAP	SLVASAFGES	GSTDGSSDEE	LSGDQEASGG	GSGGLEPLEG	SSVATPGPPV	ERASCYNSAL	GCCSDGKTPS
1130	1140	1150	1160	1170	1180	1190	1200
LDAEGSNCPA	TKVFQGVLEL	EGVEGQELFY	TPEMADPKSE	LFGETARSIE	STLDDLFRNS	DVKKDFRSVR	LRDLGPGKSV
1210	1220	1230	1240	1250	1260	1270	1280
RAIVDVHFDP	TTAFRAPDVA	RALLRQIQVS	RRRSLGVRRP	LQEHVRFMDF	DWFPAFITGA	TSGAIAAGAT	ARATTASRLP
1290	1300	1310	1320	1330	1340	1350	1360
SSAVTPRAPH	PSHTSQPVAK	TTAAPTTRRP	PTTAPSRVPG	RRPPAPQQPP	KPCDSQPCFH	GGTCQDWALG	GGFTCSCPAG
1370	1380	1390	1400	1410	1420	1430	1440
RGGAVCEKVL	GAPVPAFEGR	SFLAFPTLRA	YHTLRLALEF	RALEPQGLLL	YNGNARGKDF	LALALLDGRV	QLRFDTGSGP
1450	1460	1470	1480	1490	1500	1510	1520
AVLTSAVPVE	PGQWHRLELS	RHWRRGTLSV	DGETPVLGES	PSGTDGLNLD	TDLFVGGVPE	DQAAVALERT	FVGAGLRGCI
1530	1540	1550	1560	1570	1580	1590	1600
RLLDVNNQRL	ELGIGPGAAT	RGSGVGECGD	HPCLPNPCHG	GAPCQNLEAG	RFHCQCPPGR	VGPTCADEKS	PCQPNPCHGA
1610	1620	1630	1640	1650	1660	1670	1680
APCRVLPEGG	AQCECPLGRE	GTFCQTASGQ	DGSGPFLADF	NGFSHLELRG	LHTFARDLGE	KMALEVVFLA	RGPSGLLLYN
1690	1700	1710	1720	1730	1740	1750	1760
GQKTDGKGDF	VSLALRDRRL	EFRYDLGKGA	AVIRSREPVT	LGAWTRVSLE	RNGRKGALRV	GDGPRVLGES	PKSRKVPHTV
1770	1780	1790	1800	1810	1820	1830	1840
LNLKEPLYVG	GAPDFSKLAR	AAAVSSGFDG	AIQLVSLGGR	QLLTPEHVLR	QVDVTSFAGH	PCTRASGHPC	LNGASCVPRE
1850	1860	1870	1880	1890	1900	1910	
AAYVCLCPGG	FSGPHCEKGL	VEKSAGDVDT	LAFDGRTFVE	YLNAVTESEL	ANEIPVETL	DSGALHR	

Detailed Protein Report

Cmpd.	No. of Cmpds.	m/z meas.	Δ m/z [ppm]	z	Rt [min]	Score	P	Range	Sequence	Modification
1808	1	774.2788	-97.08	2	52.4	10.1	0	1118-1132	K.TPSLDAEGSNCPATK.V	Carbamidomethyl: 11
2489	1	981.1592	15.45	3	61.0	10.5	0	1133-1158	K.VFQGVLELEGVEGQELFYTPEMADPK.S	Oxidation: 22

Detailed Protein Report

Protein 209: endonuclease 8-like 3 [Homo sapiens]

**Accession:**	gi|157388969	**Score:**	32.8
**Database:**	refseq_human(refseq_human_20140103.fasta)	**MW [kDa]:**	67.8
**Database Date:**	2015-11-30	**pI:**	10.3
**Modification(s):**	Carbamidomethyl	**Sequence Coverage [%]:**	5.6
		**No. of unique Peptides:**	1

10	20	30	40	50	60	70	80
MVEGPGCTLN	GEKIRARVLP	GQAVTGVRGS	ALRSLQGRAL	RLAASTVVVS	PQAAALNNDS	SQNVLSLFNG	YVYSGVETLG
**90**	**100**	**110**	**120**	**130**	**140**	**150**	**160**
KELFMYFGPK	ALRIHFGMKG	FIMINPLEYK	YKNGASPVLE	VQLTKDLICF	FDSSVELRNS	MESQQRIRMM	KELDVCSPEF
**170**	**180**	**190**	**200**	**210**	**220**	**230**	**240**
SFLRAESEVK	KQKGRMLGDV	LMDQNVLPGV	GNIIKNEALF	DSGLHPAVKV	CQLTDEQIHH	LMKMIRDFSI	LFYRCRKAGL
**250**	**260**	**270**	**280**	**290**	**300**	**310**	**320**
ALSKHYKVYK	RPNCGQCHCR	ITVCRFGDNN	RMTYFCPHCQ	KENPQHVDIC	KLPTRNTIIS	WTSSRVDHVM	DSVARKSEEH
**330**	**340**	**350**	**360**	**370**	**380**	**390**	**400**
WTCVVCTLIN	KPSSKACDAC	LTSRPIDSVL	KSEENSTVFS	HLMKYPCNTF	GKPHTEVKIN	RKTAFGTTTL	VLTDFSNKSS
**410**	**420**	**430**	**440**	**450**	**460**	**470**	**480**
TLERKTKQNQ	ILDEEFQNSP	PASVCLNDIQ	HPSKKTTNDI	TQPSSKVNIS	PTISSESKLF	SPAHKKPKTA	QYSSPELKSC
**490**	**500**	**510**	**520**	**530**	**540**	**550**	**560**
NPGYSNSELQ	INMTDGPRTL	NPDSPRCSKH	NRLCILRVVR	KDGENKGRQF	YACPLPREAQ	CGFFEWADLS	FPFCNHGKRS
**570**	**580**	**590**	**600**	**610**			
TMKTVLKIGP	NNGKNFFVCP	LGKEKQCNFF	QWAENGPGIK	IIPGC			

Cmpd.	No. of Cmpds.	m/z meas.	Δ m/z [ppm]	z	Rt [min]	Score	P	Range	Sequence	Modification
1708	1	735.0267	-63.51	3	51.1	14.2	2	564-583	K.TVLKIGPNNGKNFFVCPLGK.E	Carbamidomethyl: 16

Detailed Protein Report

Protein 210: **PREDICTED: latent-transforming growth factor beta-binding protein 1 isoform X5 [Homo sapiens]**

**Accession:**	gi|530367595	**Score:**	32.8
**Database:**	refseq_human(refseq_human_20140103.fasta)	**MW [kDa]:**	134.0
**Database Date:**	2015-11-30	**pI:**	9.1
**Modification(s):**	Carbamidomethyl, Oxidation	**Sequence Coverage [%]:**	2.8
		**No. of unique Peptides:**	1

10	20	30	40	50	60	70	80
MAGAWLRWGL	LLWAGLLASS	AHGRLRRITY	VVHPGPGLAA	GALPLSGPPR	SRTFNVALNA	RYSRSSAAAG	APSRASPGVP
90	100	110	120	130	140	150	160
SERTRRTSKP	GGAALQGLRP	PPPPPPEPAR	PAVPGGQLHP	NPGGHPAAAP	FTKQGRQVVR	SKVPQETQSG	GGSRLQVHQK
170	180	190	200	210	220	230	240
QQLQGVNVCG	GRCCHGWSKA	PGSQRCTKPS	CVPPCQNGGM	CLRPQLCVCK	PGTKGKACET	IAAQDTSSPV	FGGQSPGAAS
250	260	270	280	290	300	310	320
SWGPPEQAAK	HTSSKKADTL	PRVSPVAQMT	LTLKPKPSVG	LPQQIHSQVT	PLSSQSVVIH	HGQTQEYVLK	PKYFPAQKGI
330	340	350	360	370	380	390	400
SGEQSTEGSF	PLRYVQDQVA	APFQLSNHTG	RIKVVFTPSI	CKVTCTKGSC	QNSCEKGNTT	TLISENGHAA	DTLTATNFRV
410	420	430	440	450	460	470	480
VICHLPCMNG	GQCSSRDKCQ	CPPNFTGKLC	QIPVHGASVP	KLYQHSQQPG	KALGTHVIHS	THTLPLTVTS	QQGVKVKFPP
490	500	510	520	530	540	550	560
NIVNIHVKHP	PEASVQIHQV	SRIDGPTGQK	TKEAQPGQSQ	VSYQGLPVQK	TQTIHSTYSH	QQVIPHVYPV	AAKTQLGRCF
570	580	590	600	610	620	630	640
QETIGSQCGK	ALPGLSKQED	CCGTVGTSWG	FNKCQKCPKK	PSYHGYNQMM	ECLPGYKRVN	NTFCQDINEC	QLQGVCPNGE
650	660	670	680	690	700	710	720
CLNTMGSYRC	TCKIGFGPDP	TFSSCVPDPP	VISEEKGPCY	RLVSSGRQCM	HPLSVHLTKQ	LCCCSVGKAW	GPHCEKCPLP
730	740	750	760	770	780	790	800
GTAAFKEICP	GGMGYTVSGV	HRRRPIHHHV	GKGPVFVKPK	NTQPVAKSTH	PPPLPAKEEP	VEALTFSREH	GPGVAEPEVA
810	820	830	840	850	860	870	880
TAPPEKEIPS	LDQEKTKLEP	GQPQLSPGIS	TIHLHPQFPV	VIEKTSPPVP	VEVAPEASTS	SASQVIAPTQ	VTEINECTVN
890	900	910	920	930	940	950	960
PDICGAGHCI	NLPVRYTCIC	YEGYRFSEQQ	RKCVDIDECT	QVQHLCSQGR	CENTEGSFLC	ICPAGFMASE	EGTNCIDVDE
970	980	990	1000	1010	1020	1030	1040
CLRPDVCGEG	HCVNTVGAFR	CEYCDSGYRM	TQRGRCEDID	ECLNPSTCPD	EQCVNSPGSY	QCVPCTEGFR	GWNGQCLDVD
1050	1060	1070	1080	1090	1100	1110	1120
ECLEPNVCAN	GDCSNLEGSY	MCSCHKGYTR	TPDHKHCRDI	DECQQGNLCV	NGQCKNTEGS	FRCTCGQGYQ	LSAAKDQCED
1130	1140	1150	1160	1170	1180	1190	1200
IDECQHRHLC	AHGQCRNTEG	SFQCVCDQGY	RASGLGDHCE	DINECLEDKS	VCQRGDCINT	AGSYDCTCPD	GFQLDDNKTC
1210	1220	1230	1240	1250			
QDINECEHPG	LCGPQGECLN	TEGSFHCVCQ	QGFSISADGR	TCEDVNE			

Cmpd.	No. of Cmpds.	m/z meas.	Δ m/z [ppm]	z	Rt [min]	Score	P	Range	Sequence	Modification
65	1	1240.0095	73.75	3	30.2	22.4	1	180-214	K.APGSQRCTKPSCVPPCQNGGMCLRPQLCVCKPGTK.G	Carbamidomethyl: 7; Oxidation: 21

Detailed Protein Report

Protein 211: putative ribosomal RNA methyltransferase NOP2 isoform 4 [Homo sapiens]

**Accession:**	gi|385198063	**Score:**	32.6
**Database:**	refseq_human(refseq_human_20140103.fasta)	**MW [kDa]:**	69.6
**Database Date:**	2015-11-30	**pI:**	6.2
		**Sequence Coverage [%]:**	4.5
		**No. of unique Peptides:**	1

10	20	30	40	50	60	70	80
MGRKLDPTKE	KRGPGRKARK	QKGAETELVR	FLPAVSDENS	KRLSSRARKR	AAKRRLGSVE	APKTNKSPEA	KPLPGKLPKG
**90**	**100**	**110**	**120**	**130**	**140**	**150**	**160**
AVQTAGKKGP	QSLFNAPRGK	KRPAPGSDEE	EEEEDSEEDG	MVNHGDLWGS	EDDADTVDDY	GADSNSEDEE	EGEALLPIER
**170**	**180**	**190**	**200**	**210**	**220**	**230**	**240**
AARKQKAREA	AAGIQWSEEE	TEDEEEEKEV	TPESGPPKVE	EADGGLQINV	DEEPFVLPPA	GEMEQDAQAP	DLQRVHKRIQ
**250**	**260**	**270**	**280**	**290**	**300**	**310**	**320**
DIVGILRDFG	AQREEGRSRS	EYLNRLKKDL	AIYYSYGDFL	LGKLMDLFPL	SELVEFLEAN	EVPRPVTLRT	NTLKTRRRDL
**330**	**340**	**350**	**360**	**370**	**380**	**390**	**400**
AQALINRGVN	LDPLGKWSKT	GLVVYDSSVP	IGATPEYLAG	HYMLQGASSM	LPVMALAPQE	HERILDMCCA	PGGKTSYMAQ
**410**	**420**	**430**	**440**	**450**	**460**	**470**	**480**
LMKNTGVILA	NDANAERLKS	VVGNLHRLGV	TNTIISHYDG	RQFPKVVGGF	DRVLLDAPCS	GTGVISKDPA	VKTNKDEKDI
**490**	**500**	**510**	**520**	**530**	**540**	**550**	**560**
LRCAHLQKEL	LLSAIDSVNA	TSKTGGYLVY	CTCSITVEEN	EWVVDYALKK	RNVRLVPTGL	DFGQEGFTRF	RERRFHPSLR
**570**	**580**	**590**	**600**	**610**	**620**	**630**	
STRRFYPHTH	NMDGFFIAKF	KKFSNSIPQS	QTDGVLLCRS	GWTAVVQSQL	IATSTFQVQA	ILVPQTPK	

Cmpd.	No. of Cmpds.	m/z meas.	Δ m/z [ppm]	z	Rt [min]	Score	P	Range	Sequence	Modification
2674	1	933.1060	151.43	2	64.6	13.8	0	583-599	K.FSNSIPQSQTDGVLLCR.S	

Detailed Protein Report

Protein 212: BPI fold-containing family B member 6 precursor [Homo sapiens]

**Accession:**	gi|28372525	**Score:**	32.4
**Database:**	refseq_human(refseq_human_20140103.fasta)	**MW [kDa]:**	49.7
**Database Date:**	2015-11-30	**pI:**	9.6
		**Sequence Coverage [%]:**	9.9
		**No. of unique Peptides:**	2

10	20	30	40	50	60	70	80
MLRILCLALC	SLLTGTRADP	GALLRLGMDI	MNQVQSAMDE	SHILEKMAAE	AGKKQPGMKP	IKGITNLKVK	DVQLPVITLN
**90**	**100**	**110**	**120**	**130**	**140**	**150**	**160**
FVPGVGIFQC	VSTGMTVTGK	SFMGGNMEII	VALNITATNR	LLRDEETGLP	VFKSEGCEVI	LVNVKTNLPS	NMLPKMVNKF
**170**	**180**	**190**	**200**	**210**	**220**	**230**	**240**
LDSTLHKVLP	GLMCPAIDAV	LVYVNRKWTN	LSDPMPVGQM	GTVKYVLMSA	PATTASYIQL	DFSPVVQQQK	GKTIKLADAG
**250**	**260**	**270**	**280**	**290**	**300**	**310**	**320**
EALTFPEGYA	KGSSQLLLPA	TFLSAELALL	QKSFHVNIQD	TMIGELPPQT	TKTLARFIPE	VAVAYPKSKP	LTTQIKIKKP
**330**	**340**	**350**	**360**	**370**	**380**	**390**	**400**
PKVTMKTGKS	LLHLHSTLEM	FAARWRSKAP	MSLFLLEVHF	NLKVQYSVHE	NQLQMATSLD	RLLSLSRKSS	SIGNFNEREL
**410**	**420**	**430**	**440**	**450**	**460**		
TGFITSYLEE	AYIPVVNDVL	QVGLPLPDFL	AMNYNLAELD	IVENALMLDL	KLG		

Cmpd.	No. of Cmpds.	m/z meas.	Δ m/z [ppm]	z	Rt [min]	Score	P	Range	Sequence	Modification
1223	1	873.6526	163.19	2	45.0	15.9	1	2-17	M.LRILCLALCSLLTGTR.A	
1632	1	1061.4621	-60.44	3	50.1	16.5	1	18-46	R.ADPGALLRLGMDIMNQVQSAMDESHILEK.M	

Detailed Protein Report

Protein 213: **PREDICTED: serine/threonine-protein kinase B-raf isoform X2 [Homo sapiens]**

**Accession:**	gi|530387108	**Score:**	32.4
**Database:**	refseq_human(refseq_human_20140103.fasta)	**MW [kDa]:**	83.9
**Database Date:**	2015-11-30	**pI:**	7.9
**Modification(s):**	Oxidation	**Sequence Coverage [%]:**	2.1
		**No. of unique Peptides:**	2

Quantitation

MD:MU **Median:** 1.36 **CV:** 0.00 % **No. of Peptides:** 1

10	20	30	40	50	60	70	80
MAALSGGGGG	GAEPGQALFN	GDMEPEAGAG	AGAAASSAAD	PAIPEEVWNI	KQMIKLTQEH	IEALLDKFGG	EHNPPSIYLE
90	100	110	120	130	140	150	160
AYEEYTSKLD	ALQQREQQLL	ESLGNGTDFS	VSSSASMDTV	TSSSSSSLSV	LPSSLSVFQN	PTDVARSNPK	SPQKPIVRVF
170	180	190	200	210	220	230	240
LPNKQRTVVP	ARCGVTVRDS	LKKALMMRGL	IPECCAVYRI	QDGEKKPIGW	DTDISWLTGE	ELHVEVLENV	PLTTHNFVRK
250	260	270	280	290	300	310	320
TFFTLAFCDF	CRKLLFQGFR	CQTCGYKFHQ	RCSTEVPLMC	VNYDQLDLLF	VSKFFEHHPI	PQEEASLAET	ALTSGSSPSA
330	340	350	360	370	380	390	400
PASDSIGPQI	LTSPSPSKSI	PIPQPFRPAD	EDHRNQFGQR	DRSSSAPNVH	INTIEPVNID	DLIRDQGFRG	DGGSTTGLSA
410	420	430	440	450	460	470	480
TPPASLPGSL	TNVKALQKSP	GPQRERKSSS	SSEDRNRMKT	LGRRDSSDDW	EIPDGQITVG	QRIGSGSFGT	VYKGKWHGDV
490	500	510	520	530	540	550	560
AVKMLNVTAP	TPQQLQAFKN	EVGVLRKTRH	VNILLFMGYS	TKPQLAIVTQ	WCEGSSLYHH	LHIIETKFEM	IKLIDIARQT
570	580	590	600	610	620	630	640
AQGMDYLHAK	SIIHRDLKSN	NIFLHEDLTV	KIGDFGLATV	KSRWSGSHQF	EQLSGSILWM	APEVIRMQDK	NPYSFQSDVY
650	660	670	680	690	700	710	720
AFGIVLYELM	TGQLPYSNIN	NRDQIIFMVG	RGYLSPDLSK	VRSNCPKAMK	RLMAECLKKK	RDERPLFPQE	NLQPSSSHHH
730	740	750	760				
GSICSYFLSL	VFVQFVNIKT	QFCSSNLFLK	IQNFQCIS				

Cmpd.	No. of Cmpds.	m/z meas.	Δ m/z [ppm]	z	Rt [min]	Score	P	Range	Sequence	Modification	Ratios
116	1	929.4622	17.68	2	30.9	16.0	1	184-199	K.ALMMRGLIPECCAVYR.I	Oxidation: 3, 4	MD:MU 1.36
2507	1	913.4337	-18.76	2	61.8	16.4	1	184-199	K.ALMMRGLIPECCAVYR.I		

Detailed Protein Report

Protein 214: **PREDICTED: angiopoietin-1 receptor isoform X3 [Homo sapiens]**

Accession: gi|530390111
Database: refseq_human(refseq_human_20140103.fasta)
Database Date: 2015-11-30

Score: 32.3
MW [kDa]: 120.9
pI: 6.5
Sequence Coverage [%]: 2.4
No. of unique Peptides: 2

10	20	30	40	50	60	70	80
MDSLASLVLC	GVSLLLSGTV	EGAMDLILIN	SLPLVSDAET	SLTCIASGWR	PHEPITIGRD	FEALMNQHQD	PLEVTQDVTR
**90**	**100**	**110**	**120**	**130**	**140**	**150**	**160**
EWAKKVVWKR	EKASKINGAY	FCEGRVRGEA	IRIRTMKMRQ	QASFLPATLT	MTVDKGDNVN	ISFKKVLIKE	EDAVIYKNGS
**170**	**180**	**190**	**200**	**210**	**220**	**230**	**240**
FIHSVPRHEV	PDILEVHLPH	AQPQDAGVYS	ARYIGGNLFT	SAFTRLIVRR	CEAQKWGPEC	NHLCTACMNN	GVCHEDTGEC
**250**	**260**	**270**	**280**	**290**	**300**	**310**	**320**
ICPPGFMGRT	CEKACELHTF	GRTCKERCSG	QEGCKSYVFC	LPDPYGCSCA	TGWKGLQCNE	GIQRMTPKIV	DLPDHIEVNS
**330**	**340**	**350**	**360**	**370**	**380**	**390**	**400**
GKFNPICKAS	GWPLPTNEEM	TLVKPDGTVL	HPKDFNHTDH	FSVAIFTIHR	ILPPDSGVWV	CSVNTVAGMV	EKPFNISVKV
**410**	**420**	**430**	**440**	**450**	**460**	**470**	**480**
LPKPLNAPNV	IDTGHNFAVI	NISSEPYFGD	GPIKSKKLLY	KPVNHYEAWQ	HIQVTNEIVT	LNYLEPRTEY	ELCVQLVRRG
**490**	**500**	**510**	**520**	**530**	**540**	**550**	**560**
EGGEGHPGPV	RRFTTASIGL	PPPRGLNLLP	KSQTTLNLTW	QPIFPSSEDD	FYVEVERRSV	QKSDQQNIKV	PGNLTSVLLN
**570**	**580**	**590**	**600**	**610**	**620**	**630**	**640**
NLHPREQYVV	RARVNTKAQG	EWSEDLTAWT	LSDILPPQPE	NIKISNITHS	SAVISWTILD	GYSISSITIR	YKVQGKNEDQ
**650**	**660**	**670**	**680**	**690**	**700**	**710**	**720**
HVDVKIKNAT	ITQYQLKGLE	PETAYQVDIF	AENNIGSSNP	AFSHELVTLP	ESQAPADLGG	GKMLLIAILG	SAGMTCLTVL
**730**	**740**	**750**	**760**	**770**	**780**	**790**	**800**
LAFLIILQLK	RANVQRRMAQ	AFQNREEPAV	QFNSGTLALN	RKVKNNPDPT	IYPVLDWNDI	KFQDVIGEGN	FGQVLKARIK
**810**	**820**	**830**	**840**	**850**	**860**	**870**	**880**
KDGLRMDAAI	KRMKEYASKD	DHRDFAGELE	VLCKLGHHPN	IINLLGACEH	RGYLYLAIEY	APHGNLLDFL	RKSRVLETDP
**890**	**900**	**910**	**920**	**930**	**940**	**950**	**960**
AFAIANSTAS	TLSSQQLLHF	AADVARGMDY	LSQKQFIHRD	LAARNILVGE	NYVAKIADFG	LSRGQEVYVK	KTMGRLPVRW
**970**	**980**	**990**	**1000**	**1010**	**1020**	**1030**	**1040**
MAIESLNYSV	YTTNSDVWSY	GVLLWEIVSL	GGTPYCGMTC	AELYEKLPQG	YRLEKPLNCD	DEVYDLMRQC	WREKPYERPS
**1050**	**1060**	**1070**	**1080**	**1090**			
FAQILVSLNR	MLEERKTYVN	TTLYEKFTYA	GIDCSAEEAA				

Cmpd.	No. of Cmpds.	m/z meas.	Δ m/z [ppm]	z	Rt [min]	Score	P	Range	Sequence	Modification
710	1	676.9374	132.70	2	38.2	12.4	0	468-478	R.TEYELCVQLVR.R	
2675	1	841.3883	-72.13	2	63.6	20.0	1	936-950	K.IADFGLSRGQEVYVK.K	

Detailed Protein Report

Protein 215: stAR-related lipid transfer protein 8 isoform a [Homo sapiens]

Accession: gi|215820650
Database: refseq_human(refseq_human_20140103.fasta)
Database Date: 2015-11-30
Modification(s): Carbamidomethyl

Score: 32.3
MW [kDa]: 121.7
pI: 5.9
Sequence Coverage [%]: 2.4
No. of unique Peptides: 2

Quantitation

MD:MU **Median:** 0.67 **CV:** 0.00 % **No. of Peptides:** 1

10	20	30	40	50	60	70	80
MPLLDVFWSC	FRKVKCFPLL	QVKKNAEAEA	KRACEWLQAT	GFPQYVQLFE	EGSFPLDIGS	VKKNHGFLDE	DSLGALCRRL
**90**	**100**	**110**	**120**	**130**	**140**	**150**	**160**
MTLNNCASMK	LEVHFQSKQN	EDSEEEEQCT	ISSHWAFQQE	SKCWSPMGSS	DLLAPPSPGL	PATSSCESVL	TELSATSLPV
**170**	**180**	**190**	**200**	**210**	**220**	**230**	**240**
ITVSLPPEPA	DLPLPGRAPS	SSDRPLLSPT	QGQEGPQDKA	KKRHRNRSFL	KHLESLRRKE	KSGSQQAEPK	HSPATSEKVS
**250**	**260**	**270**	**280**	**290**	**300**	**310**	**320**
KASSFRSCRG	FLSAGFYRAK	NWAATSAGGS	GANTRKAWEA	WPVASFRHPQ	WTHRGDCLVH	VPGDHKPGTF	PRSLSIESLC
**330**	**340**	**350**	**360**	**370**	**380**	**390**	**400**
PEDGHRLADW	QPGRRWGCEG	RRGSCGSTGS	HASTYDNLPE	LYPAEPVMVG	AEAEDEDDEE	SGGSYAHLDD	ILQHVWGLQQ
**410**	**420**	**430**	**440**	**450**	**460**	**470**	**480**
RVELWSRAMY	PDLGPGDEEE	EEATSSVEIA	TVEVKCQAEA	LSQMEVPAHG	ESPAWAQAEV	QPAVLAPAQA	PAEAEPVAQE
**490**	**500**	**510**	**520**	**530**	**540**	**550**	**560**
EAEAPAPAPA	PAPAQDSEQE	AHSGGEPTFA	SSLSVEEGHS	ISDTVASSSE	LDSSGNSMNE	AEAAGPLAGL	QASMPRERRD
**570**	**580**	**590**	**600**	**610**	**620**	**630**	**640**
SGVGASLTRP	CRKLRWHSFQ	NSHRPSLNSE	SLEINRQFAG	QINLLHKGSL	LRLTAFMEKY	TVPHKQGWVW	SMPKFMRRNK
**650**	**660**	**670**	**680**	**690**	**700**	**710**	**720**
TPDYRGQHVF	GVPPLIHVQR	TGQPLPQSIQ	QAMRYLRSQC	LDQVGIFRKS	GVKSRIQNLR	QMNETSPDNV	CYEGQSAYDV
**730**	**740**	**750**	**760**	**770**	**780**	**790**	**800**
ADLLKQYFRD	LPEPIFTSKL	TTTFLQIYQL	LPKDQWLAAA	QAATLLLPDE	NREVLQTLLY	FLSDIASAEE	NQMTAGNLAV
**810**	**820**	**830**	**840**	**850**	**860**	**870**	**880**
CLAPSIFHLN	VSKKDSPSPR	IKSKRSLIGR	PGPRDLSDNM	AATQGLSHMI	SDCKKLFQVP	QDMVLQLCSS	YSAAELSPPG
**890**	**900**	**910**	**920**	**930**	**940**	**950**	**960**
PALAELRQAQ	AAGVSLSLYM	EENIQDLLRD	AAERFKGWMS	VPGPQHTELA	CRKAPDGHPL	RLWKASTEVA	APPAVVLHRV
**970**	**980**	**990**	**1000**	**1010**	**1020**	**1030**	**1040**
LRERALWDED	LLRAQVLEAL	MPGVELYHYV	TDSMAPHPCR	DFVVLRMWRS	DLPRGGCLLV	SQSLDPEQPV	PESGVRALML
**1050**	**1060**	**1070**	**1080**	**1090**	**1100**	**1110**	
TSQYLMEPCG	LGRSRLTHIC	RADLRGRSPD	WYNKVFGHLC	AMEVAKIRDS	FPTLQAAGPE	TKL	

Cmpd.	No. of Cmpds.	m/z meas.	Δ m/z [ppm]	z	Rt [min]	Score	P	Range	Sequence	Modification	Ratios
1731	1	916.4065	-38.11	2	51.4	19.9	1	63-78	K.KNHGFLDEDSLGALCR.R	Carbamidomethyl: 15	MD:MU 0.67
1810	1	641.7727	-44.44	2	52.6	12.4	0	80-90	R.LMTLNNCASMK.L	Carbamidomethyl: 7	

Detailed Protein Report

Protein 216: PREDICTED: tenomodulin isoform X1 [Homo sapiens]

**Accession:**	gi|530422140	**Score:**	32.3
**Database:**	refseq_human(refseq_human_20140103.fasta)	**MW [kDa]:**	30.0
**Database Date:**	2015-11-30	**pI:**	4.6
**Modification(s):**	Oxidation	**Sequence Coverage [%]:**	11.4
		**No. of unique Peptides:**	2

10	20	30	40	50	60	70	80
MEHTFYSNGE	KKKIYMEIDP	VTRTEIFRSG	NGTDETLEVH	DFKNGYTGIY	FVGLQKCFIK	TQIKVIPEFS	EPEEEIDENE
**90**	**100**	**110**	**120**	**130**	**140**	**150**	**160**
EITTTFFEQS	VIWVPAEKPI	ENRDFLKNSK	ILEICDNVTM	YWINPTLISV	SELQDFEEEG	EDLHFPANEK	KGIEQNEQWV
**170**	**180**	**190**	**200**	**210**	**220**	**230**	**240**
VPQVKVEKTR	HARQASEEEL	PINDYTENGI	EFDPMLDERG	YCCIYCRRGN	RYCRRVCEPL	LGYYPYPYCY	QGGRVICRVI
**250**	**260**						
MPCNWWVARM	LGRV						

Cmpd.	No. of Cmpds.	m/z meas.	Δ m/z [ppm]	z	Rt [min]	Score	P	Range	Sequence	Modification
1182	1	679.6416	-214.99	2	44.4	15.1	0	1-11	-.MEHTFYSNGEK.K	Oxidation: 1
72	1	1069.4912	-86.67	2	30.6	17.2	2	151-168	K.KGIEQNEQWVVPQVKVEK.T	

Detailed Protein Report

Protein 217: PHD and RING finger domain-containing protein 1 isoform 4 [Homo sapiens]

**Accession:**	gi|557440810	**Score:**	32.2
**Database:**	refseq_human(refseq_human_20140103.fasta)	**MW [kDa]:**	178.1
**Database Date:**	2015-11-30	**pI:**	10.1
**Modification(s):**	Oxidation	**Sequence Coverage [%]:**	1.3
		**No. of unique Peptides:**	2

10	20	30	40	50	60	70	80
MDDDSLDELV	ARSPGPDGHP	QVGPADPAES	SVGSSGDSGD	DSDSEHGDGT	DGEDEGASEE	EDLEDRSGSE	DSEDDGETLL
**90**	**100**	**110**	**120**	**130**	**140**	**150**	**160**
EVAGTQGKLE	AAGSFNSDDD	AESCPICLNA	FRDQAVGTPE	NCAHYFCLDC	IVEWSKNANS	CPVDRTLFKC	ICIRAQFGGK
**170**	**180**	**190**	**200**	**210**	**220**	**230**	**240**
ILRKIPVENT	KASEEEEDPT	FCEVCGRSDR	EDRLLLCDGC	DAGYHMECLD	PPLQEVPVDE	WFCPECAAPG	VVLAADAGPV
**250**	**260**	**270**	**280**	**290**	**300**	**310**	**320**
SEEEVSLLLA	DVVPTTSRLR	PRAGRTRAIA	RTRQSERVRA	TVNRNRISTA	RRVQHTPGRL	GSSLLDEAIE	AVATGLSTAV
**330**	**340**	**350**	**360**	**370**	**380**	**390**	**400**
YQRPLTPRTP	ARRKRKTRRR	KKVPGRKKTP	SGPSAKSKSS	ATRSKKRQHR	VKKRRGKKVK	SEATTRSRIA	RTLGLRRPVH
**410**	**420**	**430**	**440**	**450**	**460**	**470**	**480**
SSCIPSVLKP	VEPSLGLLRA	DIGAASLSLF	GDPYELDPFD	SSEELSANPL	SPLSAKRRAL	SRSALQSHQP	VARPVSVGLS
**490**	**500**	**510**	**520**	**530**	**540**	**550**	**560**
RRRLPAAVPE	PDLEEEPVPD	LLGSILSGQS	LLMLGSSDVI	IHRDGSLSAK	RAAPVSFQRN	SGSLSRGEEG	FKGCLQPRAL
**570**	**580**	**590**	**600**	**610**	**620**	**630**	**640**
PSGSPAQGPS	GNRPQSTGLS	CQGRSRTPAR	TAGAPVRLDL	PAAPGAVQAR	NLSNGSVPGF	RQSHSPWFNG	TNKHTLPLAS
**650**	**660**	**670**	**680**	**690**	**700**	**710**	**720**
AASKISSRDS	KPPCRSVVPG	PPLKPAPRRT	DISELPRIPK	IRRDDGGGRR	DAAPAHGQSI	EIPSACISRL	TGREGTGQPG
**730**	**740**	**750**	**760**	**770**	**780**	**790**	**800**
RGTRAESEAS	SRVPREPGVH	TGSSRPPAPS	SHGSLAPLGP	SRGKGVGSTF	ESFRINIPGN	MAHSSQLSSP	GFCNTFRPVD
**810**	**820**	**830**	**840**	**850**	**860**	**870**	**880**
DKEQRKENPS	PLFSIKKTKQ	LRSEVYDPSD	PTGSDSSAPG	SSPERSGPGL	LPSEITRTIS	INSPKAQTVQ	AVRCVTSYTV
**890**	**900**	**910**	**920**	**930**	**940**	**950**	**960**
ESIFGTEPEP	PLGPSSAMSK	LRGAVAAEGA	SDTEREEPTE	SQGLAARLRR	PSPPEPWDEE	DGASCSTFFG	SEERTVTCVT
**970**	**980**	**990**	**1000**	**1010**	**1020**	**1030**	**1040**
VVEPEAPPSP	DVLQAATHRV	VELRPPSRSR	STSSSRSRKK	AKRKRVSREH	GRTRSGTRSE	SRDRSSRSAS	PSVGEERPRR
**1050**	**1060**	**1070**	**1080**	**1090**	**1100**	**1110**	**1120**
QRSKAKSRRS	SSDRSSSRER	AKRKKAKDKS	REHRRGPWGH	SRRTSRSRSG	SPGSSSYEHY	ESRKKKKRRS	ASRPRGRECS
**1130**	**1140**	**1150**	**1160**	**1170**	**1180**	**1190**	**1200**
PTSSLERLCR	HKHQRERSHE	RPDRKESVAW	PRDRRKRRSR	SPSSEHRARE	HRRPRSREKW	PQTRSHSPER	KGAVREASPA
**1210**	**1220**	**1230**	**1240**	**1250**	**1260**	**1270**	**1280**
PLAQGEPGRE	DLPTRLPALG	EAHVSPEVAT	ADKAPLQAPP	VLEVAAECEP	DDLDLDYGDS	VEAGHVFDDF	SSDAVFIQLD
**1290**	**1300**	**1310**	**1320**	**1330**	**1340**	**1350**	**1360**
DMSSPPSPES	TDSSPERDFP	LKPALPPASL	AVAAIQREVS	LMHDEDPSQP	PPLPEGTQEP	HLLRPDAAEK	AEAPSSPDVA
**1370**	**1380**	**1390**	**1400**	**1410**	**1420**	**1430**	**1440**
PAGKEDSPSA	SGRVQEAARP	EEVVSQTPLL	RSRALVKRVT	WNLQESESSA	PAEDRAPRAP	LHRPQKPREG	AWDMEDVAPT
**1450**	**1460**	**1470**	**1480**	**1490**	**1500**	**1510**	**1520**
GVRQVFSELP	FPSHVLPEPG	FPDTDPSQVY	SPGLPPAPAQ	PSSIPPCALV	SQPTVQFILQ	GSLPLVGCGA	AQTLAPVPAA
**1530**	**1540**	**1550**	**1560**	**1570**	**1580**	**1590**	**1600**
LTPASEPASQ	ATAASNSEEK	TPAPRLAAEK	TKKEEYMKKL	HMQERAVEEV	KLAIKPFYQK	REVTKEEYKD	ILRKAVQKIC
**1610**	**1620**	**1630**	**1640**	**1650**			
HSKSGEINPV	KVANLVKAYV	DKYRHMRRHK	KPEAGEEPPT	QGAEG			

Cmpd.	No. of Cmpds.	m/z meas.	Δ m/z [ppm]	z	Rt [min]	Score	P	Range	Sequence	Modification
1686	1	697.6744	-192.66	2	51.0	20.4	0	1-12	-.MDDDSLDELVAR.S	Oxidation: 1
1064	1	500.6481	-289.29	2	42.9	11.8	2	347-356	R.KKTPSGPSAK.S	

Detailed Protein Report

Protein 218: **keratin, type II cuticular Hb4 [Homo sapiens]**

**Accession:**	gi|15431316	**Score:**	32.2
**Database:**	refseq_human(refseq_human_20140103.fasta)	**MW [kDa]:**	64.8
**Database Date:**	2015-11-30	**pI:**	9.0
**Modification(s):**	Carbamidomethyl, Oxidation	**Sequence Coverage [%]:**	4.0
		**No. of unique Peptides:**	1

10	20	30	40	50	60	70	80
MSCRSYRVSS	GHRVGNFSSC	SAMTPQNLNR	FRANSVSCWS	GPGFRGLGSF	GSRSVITFGS	YSPRIAAVGS	RPIHCGVRFG
**90**	**100**	**110**	**120**	**130**	**140**	**150**	**160**
AGCGMGFGDG	RGVGLGPRAD	SCVGLGFGAG	SGIGYGFGGP	GFGYRVGGVG	VPAAPSITAV	TVNKSLLTPL	NLEIDPNAQR
**170**	**180**	**190**	**200**	**210**	**220**	**230**	**240**
VKKDEKEQIK	TLNNKFASFI	DKVRFLEQQN	KLLETKWSFL	QEQKCIRSNL	EPLFESYITN	LRRQLEVLVS	DQARLQAERN
**250**	**260**	**270**	**280**	**290**	**300**	**310**	**320**
HLQDVLEGFK	KKYEEEVVCR	ANAENEFVAL	KKDVDAAFMN	KSDLEANVDT	LTQEIDFLKT	LYMEEIQLLQ	SHISETSVIV
**330**	**340**	**350**	**360**	**370**	**380**	**390**	**400**
KMDNSRDLNL	DGIIAEVKAQ	YEEVARRSRA	DAEAWYQTKY	EEMQVTAGQH	CDNLRNIRNE	INELTRLIQR	LKAEIEHAKA
**410**	**420**	**430**	**440**	**450**	**460**	**470**	**480**
QRAKLEAAVA	EAEQQGEATL	SDAKCKLADL	ECALQQAKQD	MARQLCEYQE	LMNAKLGLDI	EIATYRRLLE	GEESRLCEGV
**490**	**500**	**510**	**520**	**530**	**540**	**550**	**560**
GPVNISVSSS	RGGLVCGPEP	LVAGSTLSRG	GVTFSGSSSV	CATSGVLASC	GPSLGGARVA	PATGDLLSTG	TRSGSMLISE
**570**	**580**	**590**	**600**	**610**			
ACVPSVPCPL	PTQGGFSSCS	GGRSSSVRFV	STTTSCRTKY				

Cmpd.	No. of Cmpds.	m/z meas.	Δ m/z [ppm]	z	Rt [min]	Score	P	Range	Sequence	Modification
603	1	652.6252	-207.98	2	36.9	13.1	0	79-91	R.FGAGCGMGFGDGR.G	Carbamidomethyl: 5; Oxidation: 7

Detailed Protein Report

Protein 219: fibulin-1 isoform D precursor [Homo sapiens]

**Accession:**	gi|34734066	**Score:**	32.1
**Database:**	refseq_human(refseq_human_20140103.fasta)	**MW [kDa]:**	77.2
**Database Date:**	2015-11-30	**pI:**	5.0
**Modification(s):**	Oxidation	**Sequence Coverage [%]:**	3.6
		**No. of unique Peptides:**	1

10	20	30	40	50	60	70	80
MERAAPSRRV	PLPLLLLGGL	ALLAAGVDAD	VLLEACCADG	HRMATHQKDC	SLPYATESKE	CRMVQEQCCH	SQLEELHCAT
**90**	**100**	**110**	**120**	**130**	**140**	**150**	**160**
GISLANEQDR	CATPHGDNAS	LEATFVKRCC	HCCLLGRAAQ	AQGQSCEYSL	MVGYQCGQVF	RACCVKSQET	GDLDVGGLQE
**170**	**180**	**190**	**200**	**210**	**220**	**230**	**240**
TDKIIEVEEE	QEDPYLNDRC	RGGGPCKQQC	RDTGDEVVCS	CFVGYQLLSD	GVSCEDVNEC	ITGSHSCRLG	ESCINTVGSF
**250**	**260**	**270**	**280**	**290**	**300**	**310**	**320**
RCQRDSSCGT	GYELTEDNSC	KDIDECESGI	HNCLPDFICQ	NTLGSFRCRP	KLQCKSGFIQ	DALGNCIDIN	ECLSISAPCP
**330**	**340**	**350**	**360**	**370**	**380**	**390**	**400**
IGHTCINTEG	SYTCQKNVPN	CGRGYHLNEE	GTRCVDVDEC	APPAEPCGKG	HRCVNSPGSF	RCECKTGYYF	DGISRMCVDV
**410**	**420**	**430**	**440**	**450**	**460**	**470**	**480**
NECQRYPGRL	CGHKCENTLG	SYLCSCSVGF	RLSVDGRSCE	DINECSSSPC	SQECANVYGS	YQCYCRRGYQ	LSDVDGVTCE
**490**	**500**	**510**	**520**	**530**	**540**	**550**	**560**
DIDECALPTG	GHICSYRCIN	IPGSFQCSCP	SSGYRLAPNG	RNCQDIDECV	TGIHNCSINE	TCFNIQGGFR	CLAFECPENY
**570**	**580**	**590**	**600**	**610**	**620**	**630**	**640**
RRSAATLQQE	KTDTVRCIKS	CRPNDVTCVF	DPVHTISHTV	ISLPTFREFT	RPEEIIFLRA	ITPPHPASQA	NIIFDITEGN
**650**	**660**	**670**	**680**	**690**	**700**	**710**	
LRDSFDIIKR	YMDGMTVGVV	RQVRPIVGPF	HAVLKLEMNY	VVGGVVSHRN	VVNVHIFVSE	YWF	

Cmpd.	No. of Cmpds.	m/z meas.	Δ m/z [ppm]	z	Rt [min]	Score	P	Range	Sequence	Modification
2101	2	788.3311	-88.71	2	56.1	18.9	0	676-689	K.LEMNYVVGGVVSHR.N	Oxidation: 3

Detailed Protein Report

Protein 220: E3 ubiquitin-protein ligase RNF213 isoform 2 [Homo sapiens]

**Accession:**	gi|66529203	**Score:**	32.1
**Database:**	refseq_human(refseq_human_20140103.fasta)	**MW [kDa]:**	118.4
**Database Date:**	2015-11-30	**pI:**	5.4
		**Sequence Coverage [%]:**	3.0
		**No. of unique Peptides:**	1

10	20	30	40	50	60	70	80
MECPSCQHVS	KEETPKFCSQ	CGERLPPAAP	IADSENNNST	MASASEGEME	CGQELKEEGG	PCLFPGSDSW	QENPEEPCSK
**90**	**100**	**110**	**120**	**130**	**140**	**150**	**160**
ASWTVQESKK	KKRKKKKKGN	KSASSELASL	PLSPASPCHL	TLLSNPWPQD	TALPHSQAQQ	SGPTGQPSQP	PGTATTPLEG
**170**	**180**	**190**	**200**	**210**	**220**	**230**	**240**
DGLSAPTEVG	DSPLQAQALG	EAGVATGSEA	QSSPQFQDHT	EGEDQDASIP	SGGRGLSQEG	TGPPTSAGEG	HSRTEDAAQE
**250**	**260**	**270**	**280**	**290**	**300**	**310**	**320**
LLLPESKGGS	SEPGTELQTT	EQQAGASASM	AVDAVAEPAN	AVKGAGKEMK	EKTQRMKQPP	ATTPPFKTHC	QEAETKTKDE
**330**	**340**	**350**	**360**	**370**	**380**	**390**	**400**
MAAAEEKVGK	NEQGEPEDLK	KPEGKNRSAA	AVKNEKEQKN	QEADVQEVKA	STLSPGGGVT	VFFHAIISLH	FPFNPDLHKV
**410**	**420**	**430**	**440**	**450**	**460**	**470**	**480**
FIRGGEEFGE	SKWDSNICEL	HYTRDLGHDR	VLVEGIVCIS	KKHLDKYIPY	KYVIYNGESF	EYEFIYKHQQ	KKGEYVNRCL
**490**	**500**	**510**	**520**	**530**	**540**	**550**	**560**
FIKSSLLGSG	DWHQYYDIVY	MKPHGRLQKV	MNHITDGPRK	DLVKGKQIAA	ALMLDSTFSI	LQTWDTINLN	SFFTQFEQFC
**570**	**580**	**590**	**600**	**610**	**620**	**630**	**640**
FVLQQPMIYE	GQAQLWTDLQ	YREKEVKRYL	WQHLKKHVVP	LPDGKSTDFL	PVDCPVRSKL	KTGLIVLFVV	EKIELLLEGS
**650**	**660**	**670**	**680**	**690**	**700**	**710**	**720**
LDWLCHLLTS	DASSPDEFHR	DLSHILGIPQ	SWRLYLVNLC	QRCMDTRTYT	WLGALPVLHC	CMELAPRHKD	AWRQPEDTWA
**730**	**740**	**750**	**760**	**770**	**780**	**790**	**800**
ALEGLSFSPF	REQMLDTSSL	LQFMREKQHL	LSIDEPLFRS	WFSLLPLSHL	VMYMENFIEH	LGRFPAHILD	CLSGIYYRLP
**810**	**820**	**830**	**840**	**850**	**860**	**870**	**880**
GLEQVLNTQD	VQDVQNVQNI	LEMLLRLLDT	YRDKIPEEAL	SPSYLTVCLK	LHEAICSSTK	LLKFYELPAL	SAEIVCRMIR
**890**	**900**	**910**	**920**	**930**	**940**	**950**	**960**
LLSLVDSAGQ	RDETGNNSVQ	TVFQGTLAAT	KRWLREVFTK	NMLTSSGASF	TYVKEIEVWR	RLVEIQFPAE	HGWKESLLGD
**970**	**980**	**990**	**1000**	**1010**	**1020**	**1030**	**1040**
MEWRLTKEEP	LSQITAYCNS	CWDTKGLEDS	VAKTFEKCII	EAVSSACQVN	NLSSWETDSG	SQLCSAMTQL	RAMKHPLGLS
**1050**	**1060**	**1070**					
SSANSEIGKW	APSSLAKGNG	AEI					

Cmpd.	No. of Cmpds.	m/z meas.	Δ m/z [ppm]	z	Rt [min]	Score	P	Range	Sequence	Modification
2673	1	913.9847	10.52	2	63.5	12.7	1	1032-1049	R.AMKHPLGLSSSANSEIGK.W	

Detailed Protein Report

Protein 221: PREDICTED: histone-lysine N-methyltransferase ASH1L isoform X3 [Homo sapiens]

**Accession:**	gi|530365072	**Score:**	32.1
**Database:**	refseq_human(refseq_human_20140103.fasta)	**MW [kDa]:**	191.3
**Database Date:**	2015-11-30	**pI:**	10.4
		**Sequence Coverage [%]:**	1.7
		**No. of unique Peptides:**	1

10	20	30	40	50	60	70	80
MDPRNTAMLG	LGSDSEGFSR	KSPSAISTGT	LVSKREVELE	KNTKEEEDLR	KRNRERNIEA	GKDDGLTDAQ	QQFSVKETNF
**90**	**100**	**110**	**120**	**130**	**140**	**150**	**160**
SEGNLKLKIG	LQAKRTKKPP	KNLENYVCRP	AIKTTIKHPR	KALKSGKMTD	EKNEHCPSKR	DPSKLYKKAD	DVAAIECQSE
**170**	**180**	**190**	**200**	**210**	**220**	**230**	**240**
EVIRLHSQGE	NNPLSKKLSP	VHSEMADYIN	ATPSTLLGSR	DPDLKDRALL	NGGTSVTEKL	AQLIATCPPS	KSSKTKPKKL
**250**	**260**	**270**	**280**	**290**	**300**	**310**	**320**
GTGTTAGLVS	KDLIRKAGVG	SVAGIIHKDL	IKKPTISTAV	GLVTKDPGKK	PVFNAAVGLV	NKDSVKKLGT	GTTAVFINKN
**330**	**340**	**350**	**360**	**370**	**380**	**390**	**400**
LGKKPGTITT	VGLLSKDSGK	KLGIGIVPGL	VHKESGKKLG	LGTVVGLVNK	DLGKKLGSTV	GLVAKDCAKK	IVASSAMGLV
**410**	**420**	**430**	**440**	**450**	**460**	**470**	**480**
NKDIGKKLMS	CPLAGLISKD	AINLKAEALL	PTQEPLKASC	STNINNQESQ	ELSESLKDSA	TSKTFEKNVV	RQNKESILEK
**490**	**500**	**510**	**520**	**530**	**540**	**550**	**560**
FSVRKEIINL	EKEMFNEGTC	IQQDSFSSSE	KGSYETSKHE	KQPPVYCTSP	DFKMGGASDV	STAKSPFSAV	GESNLPSPSP
**570**	**580**	**590**	**600**	**610**	**620**	**630**	**640**
TVSVNPLTRS	PPETSSQLAP	NPLLLSSTTE	LIEEISESVG	KNQFTSESTH	LNVGHRSVGH	SISIECKGID	KEVNDSKTTH
**650**	**660**	**670**	**680**	**690**	**700**	**710**	**720**
IDIPRISSSL	GKKPSLTSES	SIHTITPSVV	NFTSLFSNKP	FLKLGAVSAS	DKHCQVAESL	STSLQSKPLK	KRKGRKPRWT
**730**	**740**	**750**	**760**	**770**	**780**	**790**	**800**
KVVARSTCRS	PKGLELERSE	LFKNVSCSSL	SNSNSEPAKF	MKNIGPPSFV	DHDFLKRRLP	KLSKSTAPSL	ALLADSEKPS
**810**	**820**	**830**	**840**	**850**	**860**	**870**	**880**
HKSFATHKLS	SSMCVSSDLL	SDIYKPKRGR	PKSKEMPQLE	GPPKRTLKIP	ASKVFSLQSK	EEQEPPILQP	EIEIPSFKQG
**890**	**900**	**910**	**920**	**930**	**940**	**950**	**960**
LSVSPFPKKR	GRPKRQMRSP	VKMKPPVLSV	APFVATESPS	KLESESDNHR	SSSDFFESED	QLQDPDDLDD	SHRPSVCSMS
**970**	**980**	**990**	**1000**	**1010**	**1020**	**1030**	**1040**
DLEMEPDKKI	TKRNNGQLMK	TIIRKINKMK	TLKRKKLLNQ	ILSSSVESSN	KGKVQSKLHN	TVSSLAATFG	SKLGQQINVS
**1050**	**1060**	**1070**	**1080**	**1090**	**1100**	**1110**	**1120**
KKGTIYIGKR	RGRKPKTVLN	GILSGSPTSL	AVLEQTAQQA	AGSALGQILP	PLLPSSASSS	EILPSPICSQ	SSGTSGGQSP
**1130**	**1140**	**1150**	**1160**	**1170**	**1180**	**1190**	**1200**
VSSDAGFVEP	SSVPYLHLHS	RQGSMIQTLA	MKKASKGRRR	LSPPTLLPNS	PSHLSELTSL	KEATPSPISE	SHSDETIPSD
**1210**	**1220**	**1230**	**1240**	**1250**	**1260**	**1270**	**1280**
SGIGTDNNST	SDRAEKFCGQ	KKRRHSFEHV	SLIPPETSTV	LSSLKEKHKH	KCKRRNHDYL	SYDKMKRQKR	KRKKKYPQLR
**1290**	**1300**	**1310**	**1320**	**1330**	**1340**	**1350**	**1360**
NRQDPDFIAE	LEELISRLSE	IRITHRSHHF	IPRDLLPTIF	RINFNSFYTH	PSFPLDPLHY	IRKPDLKKKR	GRPPKMREAM
**1370**	**1380**	**1390**	**1400**	**1410**	**1420**	**1430**	**1440**
AEMPFMHSLS	FPLSSTGFYP	SYGMPYSPSP	LTAAPIGLGY	YGRYPPTLYP	PPPSPSFTTP	LPPPSYMHAG	HLLLNPAKYH
**1450**	**1460**	**1470**	**1480**	**1490**	**1500**	**1510**	**1520**
KKKHKLLRQE	AFLTTSRTPL	LSMSTYPSVP	PEMAYGWMVE	HKHRHRHKHR	EHRSSEQPQV	SMDTGSSRSV	LESLKRYRFG
**1530**	**1540**	**1550**	**1560**	**1570**	**1580**	**1590**	**1600**
KDAVGERYKH	KEKHRCHMSC	PHLSPSKSLI	NREEQWVHRE	PSESSPLALG	LQTPLQIDCS	ESSPSLSLGG	FTPNSEPASS
**1610**	**1620**	**1630**	**1640**	**1650**	**1660**	**1670**	**1680**
DEHTNLFTSA	IGSCRVSNPN	SSGRKKLTDS	PGLFSAQDTS	LNRLHRKESL	PSNERAVQTL	AGSQPTSDKP	SQRPSESTNC
**1690**	**1700**	**1710**	**1720**	**1730**	**1740**	**1750**	
SPTRKRSSSE	STSSTDKTED	WYEYLEVTYI	LMIIETMRIN	MFPVGNKRSS	LSKSKISCFW	G	

Cmpd.	No. of Cmpds.	m/z meas.	Δ m/z [ppm]	z	Rt [min]	Score	P	Range	Sequence	Modification
2429	1	856.6272	147.06	2	60.7	15.4	1	273-289	K.KPTISTAVGLVTKDPGK.K	

Detailed Protein Report

Protein 222: fanconi-associated nuclease 1 isoform a [Homo sapiens]

Accession: gi|226246523
Database: refseq_human(refseq_human_20140103.fasta)
Database Date: 2015-11-30

Score: 32.1
MW [kDa]: 114.2
pI: 7.4
Sequence Coverage [%]: 3.7
No. of unique Peptides: 2

Alias proteins:

Accession	Name	Description
gi|578840975	refseq_human (refseq_human_20140103.fasta)	PREDICTED: fanconi-associated nuclease 1 isoform X4 [Homo sapiens]
gi|578840973	refseq_human (refseq_human_20140103.fasta)	PREDICTED: fanconi-associated nuclease 1 isoform X3 [Homo sapiens]
gi|578840971	refseq_human (refseq_human_20140103.fasta)	PREDICTED: fanconi-associated nuclease 1 isoform X2 [Homo sapiens]
gi|578840969	refseq_human (refseq_human_20140103.fasta)	PREDICTED: fanconi-associated nuclease 1 isoform X1 [Homo sapiens]
gi|530405539	refseq_human (refseq_human_20140103.fasta)	PREDICTED: fanconi-associated nuclease 1 isoform X4 [Homo sapiens]
gi|530405537	refseq_human (refseq_human_20140103.fasta)	PREDICTED: fanconi-associated nuclease 1 isoform X3 [Homo sapiens]
gi|530405535	refseq_human (refseq_human_20140103.fasta)	PREDICTED: fanconi-associated nuclease 1 isoform X2 [Homo sapiens]
gi|530405533	refseq_human (refseq_human_20140103.fasta)	PREDICTED: fanconi-associated nuclease 1 isoform X1 [Homo sapiens]

```
        10         20         30         40         50         60         70         80
MMSEGKPPDK KRPRRSLSIS KNKKKASNSI ISCFNNAPPA KLACPVCSKM VPRYDLNRHL DEMCANNDFV QVDPGQVGLI
        90        100        110        120        130        140        150        160
NSNVSMVDLT SVTLEDVTPK KSPPPKTNLT PGQSDSAKRE VKQKISPYFK SNDVVCKNQD ELRNRSVKVI CLGSLASKLS
       170        180        190        200        210        220        230        240
RKYVKAKKSI DKDEEFAGSS PQSSKSTVVK SLIDNSSEIE DEDQILENSS QKENVFKCDS LKEECIPEHM VRGSKIMEAE
       250        260        270        280        290        300        310        320
SQKATRECEK SALTPGFSDN AIMLFSPDFT LRNTLKSTSE DSLVKQECIK EVVEKREACH CEEVKMTVAS EAKIQLSDSE
       330        340        350        360        370        380        390        400
AKSHSSADDA SAWSNIQEAP LQDDSCLNND IPHSIPLEQG SSCNGPGQTT GHPYYLRSFL VVLKTVLENE DDMLLFDEQE
       410        420        430        440        450        460        470        480
KGIVTKFYQL SATGQKLYVR LFQRKLSWIK MTKLEYEEIA LDLTPVIEEL TNAGFLQTES ELQELSEVLE LLSAPELKSL
       490        500        510        520        530        540        550        560
AKTFHLVNPN GQKQQLVDAF LKLAKQRSVC TWGKNKPGIG AVILKRAKAL AGQSVRICKG PRAVFSRILL LFSLTDSMED
       570        580        590        600        610        620        630        640
EDAACGGQGQ LSTVLLVNLG RMEFPSYTIN RKTHIFQDRD DLIRYAAATH MLSDISSAMA NGNWEEAKEL AQCAKRDWNR
       650        660        670        680        690        700        710        720
LKNHPSLRCH EDLPLFLRCF TVGWIYTRIL SRFVEILQRL HMYEEAVREL ESLLSQRIYC PDSRGRWWDR LALNLHQHLK
       730        740        750        760        770        780        790        800
RLEPTIKCIT EGLADPEVRT GHRLSLYQRA VRLRESPSCK KFKHLFQQLP EMAVQDVKHV TITGRLCPQR GMCKSVFVME
       810        820        830        840        850        860        870        880
AGEAADPTTV LCSVEELALA HYRRSGFDQG IHGEGSTFST LYGLLLWDII FMDGIPDVFR NACQAFPLDL CTDSFFTSRR
       890        900        910        920        930        940        950        960
PALEARLQLI HDAPEESLRA WVAATWHEQE GRVASLVSWD RFTSLQQAQD LVSCLGGPVL SGVCRHLAAD FRHCRGGLPD
       970        980        990       1000       1010       1020
LVVWNSQSRH FKLVEVKGPN DRLSHKQMIW LAELQKLGAE VEVCHVVAVG AKSQSLS
```

Cmpd.	No. of Cmpds.	m/z meas.	Δ m/z [ppm]	z	Rt [min]	Score	P	Range	Sequence	Modification
60	3	768.2851	-144.27	2	30.4	19.5	2	233-246	R.GSKIMEAESQKATR.E	

Detailed Protein Report

Cmpd.	No. of Cmpds.	m/z meas.	Δ m/z [ppm]	z	Rt [min]	Score	P	Range	Sequence	Modification
83	1	826.6984	-67.49	3	30.8	12.5	0	922-945	R.FTSLQQAQDLVSCLGGPVLSGVCR.H	

Detailed Protein Report

Protein 223: zinc finger protein 518A isoform a [Homo sapiens]

**Accession:**	gi|57242807	**Score:**	32.0
**Database:**	refseq_human(refseq_human_20140103.fasta)	**MW [kDa]:**	166.7
**Database Date:**	2015-11-30	**pI:**	10.2
**Modification(s):**	Carbamidomethyl, Oxidation	**Sequence Coverage [%]:**	2.8
		**No. of unique Peptides:**	2

Alias proteins:

Accession	Name	Description
gi|512388675	refseq_human (refseq_human_20140103.fasta)	zinc finger protein 518A isoform a [Homo sapiens]
gi|512388673	refseq_human (refseq_human_20140103.fasta)	zinc finger protein 518A isoform a [Homo sapiens]

```
        10         20         30         40         50         60         70         80
MPSEQKQLFC DEKQTTLKKD YDVKNEIVDR SAPKPKISGS IHYALKNVKI DLPKINIPNE VLLKHEVDKY RKLFQSKQQT
        90        100        110        120        130        140        150        160
ARKSISIKTV SCVEECTLLH KSERAEEEGV KMSAKILNFS CLKCRDNTRY SPNDLQKHFQ MWHHGELPSY PCEMCNFSAN
       170        180        190        200        210        220        230        240
DFQVFKQHRR THRSTLVKCD ICNNESVYTL LNLTKHFTST HCVNGNFQCE KCKFSTQDVG TFVQHIHRHN EIHYKCGKCH
       250        260        270        280        290        300        310        320
HVCFTKGELQ KHLHIHSGTF PFTCQYCSYG ATRREHLVRH VITLHKEHLY AKEKLEKDKY EKRMAKTSAG LKLILKRYKI
       330        340        350        360        370        380        390        400
GASRKTFWKR KKINSGSDRS IEKNTQVLKK MNKTQTKSED QSHVVQEHLS EEKDERLHCE NNDKAPESES EKPTPLSTGQ
       410        420        430        440        450        460        470        480
GNRAEEGPNA SSGFMKTAVL GPTLKNVMMK NNKLAVSPNY NATFMGFKMM DGKQHIVLKL VPIKQNVCSP GSQSGAAKDG
       490        500        510        520        530        540        550        560
TANLQPQTLD TNGFLTGVTT ELNDTVYMKA ATPFSCSSSI LSGKASSEKE MTLISQRNNM LQTMDYEKSV SSLSATSELV
       570        580        590        600        610        620        630        640
TASVNLTTKF ETRDNVDFWG NHLTQSHPEV LGTTIKSPDK VNCVAKPNAY NSGDMHNYCI NYGNCELPVE SSNQGSLPFH
       650        660        670        680        690        700        710        720
NYSKVNNSNK RRRFSGTAVY ENPQRESSSS KTVVQQPISE SFLSLVRQES SKPDSLLASI SLLNDKDGTL KAKSEIEEQY
       730        740        750        760        770        780        790        800
VLEKGQNIDG QNLYSNENQN LECATEKSKW EDFSNVDSPM MPRITSVFSL QSQQASEFLP PEVNQLLQDV LKIKPDVKQD
       810        820        830        840        850        860        870        880
SSNTPNKGLP LHCDQSFQKH EREGKIVESS KDFKVQGIFP VPPGSVGINV PTNDLNLKFG KEKQVSSIPQ DVRDSEKMPR
       890        900        910        920        930        940        950        960
ISGFGTLLKT QSDAIITQQL VKDKLRATTQ NLGSFYMQSP LLNSEQKKTI IVQTSKGFLI PLNITNKPGL PVIPGNALPL
       970        980        990       1000       1010       1020       1030       1040
VNSQGIPASL FVNKKPGMVL TLNNGKLEGV SAVKTEGAPA RGTVTKEPCK TPILKVEPNN NCLTPGLCSS IGSCLSMKSS
      1050       1060       1070       1080       1090       1100       1110       1120
SENTLPLKGP YILKPTSSVK AVLIPNMLSE QQSTKLNISD SVKQQNEIFP KPPLYTFLPD GKQAVFLKCV MPNKTELLKP
      1130       1140       1150       1160       1170       1180       1190       1200
KLVQNSTYQN IQPKKPEGTP QRILLKIFNP VLNVTAANNL SVSNSASSLQ KDNVPSNQII GGEQKEPESR DALPFLLDDL
      1210       1220       1230       1240       1250       1260       1270       1280
MPANEIVITS TATCPESSEE PICVSDCSES RVLRCKTNCR IERNFNRKKT SKKIFSKTKT HGSKDSETAF VSRNRNCKRK
      1290       1300       1310       1320       1330       1340       1350       1360
CRDSYQEPPR RKATLHRKCK EKAKPEDVRE TFGFSRPRLS KDSIRTLRLF PFSSKQLVKC PRRNQPVVVL NHPDADAPEV
      1370       1380       1390       1400       1410       1420       1430       1440
VSVMKTIAKF NGHVLKVSLS KRTINALLKP VCYNPPKTTY DDFSKRHKTF KPVSSVKERF VLKLTLKKTS KNNYQIVKTT
      1450       1460       1470       1480       1490
SENILKAKFN CWFCGRVFDN QDTWAGHGQR HLMEATRDWN MLE
```

Cmpd.	No. of Cmpds.	m/z meas.	Δ m/z [ppm]	z	Rt [min]	Score	P	Range	Sequence	Modification
1117	1	1019.4619	-129.58	2	43.5	17.7	2	31-49	R.SAPKPKISGSIHYALKNVK.I	
63	1	814.0267	-13.11	3	30.1	14.3	0	1016-	K.VEPNNNCLTPGLCSSIGSCLSMK.S	Carbamidomethyl: 7;

Detailed Protein Report

Cmpd.	No. of Cmpds.	m/z meas.	Δ m/z [ppm]	z	Rt [min]	Score	P	Range	Sequence	Modification
								1038		Oxidation: 22

Detailed Protein Report

Protein 224: PREDICTED: SH3 domain-containing protein 21 isoform X4 [Homo sapiens]

**Accession:**	gi|578799774	**Score:**	32.0
**Database:**	refseq_human(refseq_human_20140103.fasta)	**MW [kDa]:**	84.7
**Database Date:**	2015-11-30	**pI:**	5.6
		**Sequence Coverage [%]:**	2.7
		**No. of unique Peptides:**	2

Quantitation

MD:MU **Median:** 0.32 **CV:** 0.00 % **No. of Peptides:** 1

10	20	30	40	50	60	70	80
MEVLVLAGYR	AQKEDELSLA	PGDVVRQVRW	VPARGWLRGE	FGGRYGLFPE	RLVQEIPETL	RGSGEARRPR	CARRRGHPAK
90	100	110	120	130	140	150	160
HPRPQRWCKV	NFSYSPEQAD	ELKLQAGEIV	EMIKEIEDGW	WLGKKNGQLG	AFPSNFVELL	DSGPPSLGNP	DMPSVSPGPQ
170	180	190	200	210	220	230	240
RPPKLSSLAY	DSPPDYLQTV	SHPEVYRVLF	DYQPEAPDEL	ALRRGDVVKV	LSKTTEDKGW	WEGECQGRRG	VFPDNFVLPP
250	260	270	280	290	300	310	320
PPIKKLVPRK	VVSRESAPIK	EPKKLMPKTS	LPTVKKLATA	TTGPSKAKTS	RTPSRDSQKL	TSRDSGPNGG	FQSGGSYHPG
330	340	350	360	370	380	390	400
RKRSKTQTPQ	QRSVSSQEEE	HSSPVKAPSV	KRTPMPDKTA	TPERPPAPEN	APSSKKIPAP	DKVPSPEKTL	TLGDKASIPG
410	420	430	440	450	460	470	480
NSTSGKIPAP	DKVPTPEKMV	TPEDKASIPE	NSIIPEETLT	VDKPSTPERV	FSVEESPALE	APPMDKVPNP	KMAPLGDEAP
490	500	510	520	530	540	550	560
TLEKVLTPEL	SEEEVSTRDD	IQFHHFSSEE	ALQKVKYFVA	KEDPSSQEEA	HTPEAPPPQP	PSSERCLGEM	KCTLVRGDSS
570	580	590	600	610	620	630	640
PRQAELKSGP	ASRPALEKPH	PHEEATTLPE	EAPSNDERTP	EEEAPPNEQR	PLREEVLPKE	GVASKEEVTL	KEELPPKEEV
650	660	670	680	690	700	710	720
APKEEVPPIE	RAFAQKTRPI	KPPPDSQETL	ALPSLVPQNY	TENKNEGVDV	TSLRGEVESL	RRALELMEEE	ADRHLGGAEE
730	740	750	760	770	780		
REGAAPAAGG	PVYEPRALWG	QGDVCPGVCR	GLPWPSQARW	EKRQPCPGGF			

Cmpd.	No. of Cmpds.	m/z meas.	Δ m/z [ppm]	z	Rt [min]	Score	P	Range	Sequence	Modification	Ratios
949	1	473.1120	-311.20	2	41.4	19.1	1	695-702	R.GEVESLRR.A		MD:MU 0.32
2858	1	805.9291	-2.54	2	66.2	12.9	2	751-763	R.GLPWPSQARWEKR.Q		

Detailed Protein Report

Protein 225: polycystic kidney disease protein 1-like 3 precursor [Homo sapiens]

**Accession:**	gi|31559825	**Score:**	32.0
**Database:**	refseq_human(refseq_human_20140103.fasta)	**MW [kDa]:**	195.8
**Database Date:**	2015-11-30	**pI:**	9.6
		**Sequence Coverage [%]:**	2.1
		**No. of unique Peptides:**	1

10	20	30	40	50	60	70	80
MFFKGGSWLW	LYIRTSIILG	SELNSPAPHG	QNNCYQLNRF	QCSFEEAQHY	CHVQRGFLAH	IWNKEVQDLI	RDYLEEGKKW
90	100	110	120	130	140	150	160
WIGQNVMPLK	KHQDNKYPAD	VAANGPPKPL	SCTYLSRNFI	RISSKGDKCL	LKYYFICQTG	DFLDGDAHYE	RNGNNSHLYQ
170	180	190	200	210	220	230	240
RHKKTKRGVA	IARDKMPPGP	GHLPTTCHYP	LPAHLSKTLC	HPISQFPSVL	SSITSQVTSA	ASEPSSQPLP	VITQLTMPVS
250	260	270	280	290	300	310	320
VTHAGQSLAE	TTSSPKEEGH	PNTFTSYLQV	SLQKASGQVI	DEIAGNFSRA	VHGLQALNKL	QEACEFLQKL	TALTPRFSKP
330	340	350	360	370	380	390	400
AQVNLINSLI	YLSEELLRIP	FQNNNSLGFK	VPPTVCPFHS	LNNVTKAGEG	SWLESKRHTE	PVEDILEMSL	VEFGNIGEAF
410	420	430	440	450	460	470	480
LEQNQSPESS	VTLTSANATL	LLSRQNISTL	PLSSYTLGHP	APVRLGFPSA	LALKELLNKH	PGVNVQITGL	AFNPFKDLDN
490	500	510	520	530	540	550	560
RNIVGSIGSV	LLSANRKLLQ	VHDLMEDIEI	MLWRNVSLET	HPTSLNMSTH	QLTITVNVTS	LEKSLIVSID	PDSPLLMTLY
570	580	590	600	610	620	630	640
LGFQYQPNCT	HFHLNITLPK	DKVWQKDEEY	TWVLNPEHLQ	HGIGTYYITA	VLSERQEGAQ	QTPSLVSVIT	AVTQCYYWEI
650	660	670	680	690	700	710	720
HNQTWSSAGC	QVGPQSTILR	TQCLCNHLTF	FASDFFVVPR	TVNVEDTIKL	FLRVTNNPVG	VSLLASLLGF	YVITVVWARK
730	740	750	760	770	780	790	800
KDQADMQKVK	VTVLADNDPS	AQFHYLIQVY	TGYRRSAATT	AKVVITLYGS	EGRSEPHHLC	DPQKTVFERG	GLDVFLLTTW
810	820	830	840	850	860	870	880
TSLGNLHSLR	LWHDNSGVSP	SWYVSQVIVC	DMAVKRKWHF	LCNCWLAVDL	GDCELDRVFI	PVSKRELFSF	RHLFSSMIVE
890	900	910	920	930	940	950	960
KFTQDYLWLS	IATRHPWNQF	TRVQRLSCCM	TLLLCNMVIN	VMFWKINSTT	AKRDEQMRPF	AVAWSELLVS	IHTAVILFPI
970	980	990	1000	1010	1020	1030	1040
NLVIGRLFPL	IEPQETLPLF	PPIQASCLSD	ASVEPLSATM	VVEELKETVR	FLLRRNTYLL	SKCEQPPWSS	WDITKLVKLL
1050	1060	1070	1080	1090	1100	1110	1120
SSLVSSHLEG	QGCHQQGERH	WARVVPENHH	HFCCYLHRVL	QRLKSHLGTL	GLTQGHQSCD	FLDAASQLQK	LQELLETHIL
1130	1140	1150	1160	1170	1180	1190	1200
PTEQEPSREV	TSFAILSSEE	GKKPISNGLS	KWLTSVCWLL	LGFTSLASAF	FTALYSLELS	KDQATSWMIS	IILSVLQNIF
1210	1220	1230	1240	1250	1260	1270	1280
ISQPVKVVFF	TFLYSLMMSR	MPRLNKENEQ	QTKRILALLA	KCSSSVPGSR	DKNNPVYVAP	AINSPTKHPE	RTLKKKKLFK
1290	1300	1310	1320	1330	1340	1350	1360
LTGDILVQIL	FLTLLMTAIY	SAKNSNRFYL	HQAIWKTFSH	QFSEIKLLQD	FYPWANHILL	PSLYGDYRGK	NAVLEPSHCK
1370	1380	1390	1400	1410	1420	1430	1440
CGVQLIFQIP	RTKTYEKVDE	GQLAFCDNGH	TCGRPKSLFP	GLHLRRFSYI	CSPRPMVLIP	TDELHERLTS	KNENGFSYIM
1450	1460	1470	1480	1490	1500	1510	1520
RGAFFTSLRL	ESFTSLQMSK	KGCVWSIISQ	VIYYLLVCYY	AFIQGCQLKQ	QKWRFFTGKR	NILDTSIILI	SFILLGLDMK
1530	1540	1550	1560	1570	1580	1590	1600
SISLHKKNMA	RYRDDQDRFI	SFYEAVKVNS	AATHLVGFPV	LLATVQLWNL	LRHSPRLRVI	SRTLSRAWDE	VVGFLLIILI
1610	1620	1630	1640	1650	1660	1670	1680
LLTGYAIAFN	LLFGCSISDY	RTFFSSAVTV	VGLLMGISHQ	EEVFALDPVL	GTFLILTSVI	LMVLVVINLF	VSAILMAFGK
1690	1700	1710	1720	1730	1740		
ERKSLKKEAA	LIDTLLQKLS	NLLGISWPQK	TSSEQAATTA	VGSDTEVLDE	LP		

Cmpd.	No. of Cmpds.	m/z meas.	Δ m/z [ppm]	z	Rt [min]	Score	P	Range	Sequence	Modification
146	1	896.8772	102.59	3	31.2	18.7	2	1242-1267	K.CSSSVPGSRDKNNPVYVAPAINSPTK.H	

Detailed Protein Report

Protein 226: sorting nexin-8 [Homo sapiens]

Accession: gi|23943858
Database: refseq_human(refseq_human_20140103.fasta)
Database Date: 2015-11-30
Modification(s): Oxidation

Score: 31.9
MW [kDa]: 52.5
pI: 7.2
Sequence Coverage [%]: 5.8
No. of unique Peptides: 1

10	20	30	40	50	60	70	80
MTGRAMDPLP	AAAVGAAAEA	EADEEADPPA	SDLPTPQAIE	PQAIVQQVPA	PSRMQMPQGN	PLLLSHTLQE	LLARDTVQVE
**90**	**100**	**110**	**120**	**130**	**140**	**150**	**160**
LIPEKKGLFL	KHVEYEVSSQ	RFKSSVYRRY	NDFVVFQEML	LHKFPYRMVP	ALPPKRMLGA	DREFIEARRR	ALKRFVNLVA
**170**	**180**	**190**	**200**	**210**	**220**	**230**	**240**
RHPLFSEDVV	LKLFLSFSGS	DVQNKLKESA	QCVGDEFLNC	KLATRAKDFL	PADIQAQFAI	SRELIRNIYN	SFHKLRDRAE
**250**	**260**	**270**	**280**	**290**	**300**	**310**	**320**
RIASRAIDNA	ADLLIFGKEL	SAIGSDTTPL	PSWAALNSST	WGSLKQALKG	LSVEFALLAD	KAAQQGKQEE	NDVVEKLNLF
**330**	**340**	**350**	**360**	**370**	**380**	**390**	**400**
LDLLQSYKDL	CERHEKGVLH	KHQRALHKYS	LMKRQMMSAT	AQNREPESVE	QLESRIVEQE	NAIQTMELRN	YFSLYCLHQE
**410**	**420**	**430**	**440**	**450**	**460**	**470**	
TQLIHVYLPL	TSHILRAFVN	SQIQGHKEMS	KVWNDLRPKL	SCLFAGPHST	LTPPCSPPED	GLCPH	

Cmpd.	No. of Cmpds.	m/z meas.	Δ m/z [ppm]	z	Rt [min]	Score	P	Range	Sequence	Modification
1502	1	978.1127	119.49	2	48.6	18.2	1	109-123	R.RYNDFVVFQEMLLHK.F	Oxidation: 11

Detailed Protein Report

Protein 227: **peroxisome biogenesis factor 1 isoform 3 [Homo sapiens]**

**Accession:**	gi|544186104	**Score:**	31.9
**Database:**	refseq_human(refseq_human_20140103.fasta)	**MW [kDa]:**	119.5
**Database Date:**	2015-11-30	**pI:**	5.7
		**Sequence Coverage [%]:**	0.9
		**No. of unique Peptides:**	1

10	20	30	40	50	60	70	80
MMKELQTKQL	QSNTVGITES	NENESEIPVD	SSSVASLWTM	IGSIFSFQSE	KKQETSWGLT	EINAFKNMQS	KVVPLDNIFR
**90**	**100**	**110**	**120**	**130**	**140**	**150**	**160**
VCKSQPPSIY	NASATSVFHK	HCAIHVFPWD	QEYFDVEPSF	TVTYGKLVKL	LSPKQQQSKT	KQNVLSPEKE	KQMSEPLDQK
**170**	**180**	**190**	**200**	**210**	**220**	**230**	**240**
KIRSDHNEED	EKACVLQVVW	NGLEELNNAI	KYTKNVEVLH	LGKVWIPDDL	RKRLNIEMHA	VVRITPVEVT	PKIPRSLKLQ
**250**	**260**	**270**	**280**	**290**	**300**	**310**	**320**
PRENLPKDIS	EEDIKTVFYS	WLQQSTTTML	PLVISEEEFI	KLETKDGLKE	FSLSIVHSWE	KEKDKNIFLL	SPNLLQKTTI
**330**	**340**	**350**	**360**	**370**	**380**	**390**	**400**
QVLLDPMVKE	ENSEEIDFIL	PFLKLSSLGG	VNSLGVSSLE	HITHSLLGRP	LSRQLMSLVA	GLRNGALLLT	GGKGSGKSTL
**410**	**420**	**430**	**440**	**450**	**460**	**470**	**480**
AKAICKEAFD	KLDAHVERVD	CKALRGKRLE	NIQKTLEVAF	SEAVWMQPSV	VLLDDLDLIA	GLPAVPEHEH	SPDAVQSQRL
**490**	**500**	**510**	**520**	**530**	**540**	**550**	**560**
AHALNDMIKE	FISMGSLVAL	IATSQSQQSL	HPLLVSAQGV	HIFQCVQHIQ	PPNQEQRCEI	LCNVIKNKLD	CDINKFTDLD
**570**	**580**	**590**	**600**	**610**	**620**	**630**	**640**
LQHVAKETGG	FVARDFTVLV	DRAIHSRLSR	QSISTREKLV	LTTLDFQKAL	RGFLPASLRS	VNLHKPRDLG	WDKIGGLHEV
**650**	**660**	**670**	**680**	**690**	**700**	**710**	**720**
RQILMDTIQL	PAKYPELFAN	LPIRQRTGIL	LYGPPGTGKT	LLAGVIARES	RMNFISVKGP	ELLSKYIGAS	EQAVRDIFIR
**730**	**740**	**750**	**760**	**770**	**780**	**790**	**800**
AQAAKPCILF	FDEFESIAPR	RGHDNTGVTD	RVVNQLLTQL	DGVEGLQGVY	VLAATSRPDL	IDPALLRPGR	LDKCVYCPPP
**810**	**820**	**830**	**840**	**850**	**860**	**870**	**880**
DQVSRLEILN	VLSDSLPLAD	DVDLQHVASV	TDSFTGADLK	ALLYNAQLEA	LHGMLLSSGL	QDGSSSSDSD	LSLSSMVFLN
**890**	**900**	**910**	**920**	**930**	**940**	**950**	**960**
HSSGSDDSAG	DGECGLDQSL	VSLEMSEILP	DESKFNMYRL	YFGSSYESEL	GNGTSSDLSS	QCLSAPSSMT	QDLPGVPGKD
**970**	**980**	**990**	**1000**	**1010**	**1020**	**1030**	**1040**
QLFSQPPVLR	TASQEGCQEL	TQEQRDQLRA	DISIIKGRYR	SQSGEDESMN	QPGPIKTRLA	ISQSHLMTAL	GHTRPSISED
**1050**	**1060**	**1070**	**1080**				
DWKNFAELYE	SFQNPKRRKN	QSGTMFRPGQ	KVTLA				

Cmpd.	No. of Cmpds.	m/z meas.	Δ m/z [ppm]	z	Rt [min]	Score	P	Range	Sequence	Modification
1073	5	1207.9210	130.55	1	43.0	31.9	2	233-242	K.IPRSLKLQPR.E	

Detailed Protein Report

Protein 228: fibronectin isoform 6 preproprotein [Homo sapiens]

**Accession:**	gi|47132549	**Score:**	31.9
**Database:**	refseq_human(refseq_human_20140103.fasta)	**MW [kDa]:**	239.5
**Database Date:**	2015-11-30	**pI:**	5.5
**Modification(s):**	Carbamidomethyl	**Sequence Coverage [%]:**	1.5
		**No. of unique Peptides:**	2

Detailed Protein Report

10	20	30	40	50	60	70	80
MLRGPGPGLL	LLAVQCLGTA	VPSTGASKSK	RQAQQMVQPQ	SPVAVSQSKP	GCYDNGKHYQ	INQQWERTYL	GNALVCTCYG
90	100	110	120	130	140	150	160
GSRGFNCESK	PEAEETCFDK	YTGNTYRVGD	TYERPKDSMI	WDCTCIGAGR	GRISCTIANR	CHEGGQSYKI	GDTWRRPHET
170	180	190	200	210	220	230	240
GGYMLECVCL	GNGKGEWTCK	PIAEKCFDHA	AGTSYVVGET	WEKPYQGWMM	VDCTCLGEGS	GRITCTSRNR	CNDQDTRTSY
250	260	270	280	290	300	310	320
RIGDTWSKKD	NRGNLLQCIC	TGNGRGEWKC	ERHTSVQTTS	SGSGPFTDVR	AAVYQPQPHP	QPPPYGHCVT	DSGVVYSVGM
330	340	350	360	370	380	390	400
QWLKTQGNKQ	MLCTCLGNGV	SCQETAVTQT	YGGNSNGEPC	VLPFTYNGRT	FYSCTTEGRQ	DGHLWCSTTS	NYEQDQKYSF
410	420	430	440	450	460	470	480
CTDHTVLVQT	RGGNSNGALC	HFPFLYNNHN	YTDCTSEGRR	DNMKWCGTTQ	NYDADQKFGF	CPMAAHEEIC	TTNEGVMYRI
490	500	510	520	530	540	550	560
GDQWDKQHDM	GHMMRCTCVG	NGRGEWTCIA	YSQLRDQCIV	DDITYNVNDT	FHKRHEEGHM	LNCTCFGQGR	GRWKCDPVDQ
570	580	590	600	610	620	630	640
CQDSETGTFY	QIGDSWEKYV	HGVRYQCYCY	GRGIGEWHCQ	PLQTYPSSSG	PVEVFITETP	SQPNSHPIQW	NAPQPSHISK
650	660	670	680	690	700	710	720
YILRWRPKNS	VGRWKEATIP	GHLNSYTIKG	LKPGVVYEGQ	LISIQQYGHQ	EVTRFDFTTT	STSTPVTSNT	VTGETTPFSP
730	740	750	760	770	780	790	800
LVATSESVTE	ITASSFVVSW	VSASDTVSGF	RVEYELSEEG	DEPQYLDLPS	TATSVNIPDL	LPGRKYIVNV	YQISEDGEQS
810	820	830	840	850	860	870	880
LILSTSQTTA	PDAPPDPTVD	QVDDTSIVVR	WSRPQAPITG	YRIVYSPSVE	GSSTELNLPE	TANSVTLSDL	QPGVQYNITI
890	900	910	920	930	940	950	960
YAVEENQEST	PVVIQQETTG	TPRSDTVPSP	RDLQFVEVTD	VKVTIMWTPP	ESAVTGYRVD	VIPVNLPGEH	GQRLPISRNT
970	980	990	1000	1010	1020	1030	1040
FAEVTGLSPG	VTYYFKVFAV	SHGRESKPLT	AQQTTKLDAP	TNLQFVNETD	STVLVRWTPP	RAQITGYRLT	VGLTRRGQPR
1050	1060	1070	1080	1090	1100	1110	1120
QYNVGPSVSK	YPLRNLQPAS	EYTVSLVAIK	GNQESPKATG	VFTTLQPGSS	IPPYNTEVTE	TTIVITWTPA	PRIGFKLGVR
1130	1140	1150	1160	1170	1180	1190	1200
PSQGGEAPRE	VTSDSGSIVV	SGLTPGVEYV	YTIQVLRDGQ	ERDAPIVNKV	VTPLSPPTNL	HLEANPDTGV	LTVSWERSTT
1210	1220	1230	1240	1250	1260	1270	1280
PDITGYRITT	TPTNGQQGNS	LEEVVHADQS	SCTFDNLSPG	LEYNVSVYTV	KDDKESVPIS	DTIIPAVPPP	TDLRFTNIGP
1290	1300	1310	1320	1330	1340	1350	1360
DTMRVTWAPP	PSIDLTNFLV	RYSPVKNEED	VAELSISPSD	NAVVLTNLLP	GTEYVVSVSS	VYEQHESTPL	RGRQKTGLDS
1370	1380	1390	1400	1410	1420	1430	1440
PTGIDFSDIT	ANSFTVHWIA	PRATITGYRI	RHHPEHFSGR	PREDRVPHSR	NSITLTNLTP	GTEYVVSIVA	LNGREESPLL
1450	1460	1470	1480	1490	1500	1510	1520
IGQQSTVSDV	PRDLEVVAAT	PTSLLISWDA	PAVTVRYYRI	TYGETGGNSP	VQEFTVPGSK	STATISGLKP	GVDYTITVYA
1530	1540	1550	1560	1570	1580	1590	1600
VTGRGDSPAS	SKPISINYRT	EIDKPSQMQV	TDVQDNSISV	KWLPSSSPVT	GYRVTTTPKN	GPGPTKTKTA	GPDQTEMTIE
1610	1620	1630	1640	1650	1660	1670	1680
GLQPTVEYVV	SVYAQNPSGE	SQPLVQTAVT	TIPAPTDLKF	TQVTPTSLSA	QWTPPNVQLT	GYRVRVTPKE	KTGPMKEINL
1690	1700	1710	1720	1730	1740	1750	1760
APDSSSVVVS	GLMVATKYEV	SVYALKDTLT	SRPAQGVVTT	LENVSPPRRA	RVTDATETTI	TISWRTKTET	ITGFQVDAVP
1770	1780	1790	1800	1810	1820	1830	1840
ANGQTPIQRT	IKPDVRSYTI	TGLQPGTDYK	IYLYTLNDNA	RSSPVVIDAS	TAIDAPSNLR	FLATTPNSLL	VSWQPPRARI
1850	1860	1870	1880	1890	1900	1910	1920
TGYIIKYEKP	GSPPREVVPR	PRPGVTEATI	TGLEPGTEYT	IYVIALKNNQ	KSEPLIGRKK	TGQEALSQTT	ISWAPFQDTS
1930	1940	1950	1960	1970	1980	1990	2000
EYIISCHPVG	TDEEPLQFRV	PGTSTSATLT	GLTRGATYNI	IVEALKDQQR	HKVREEVVTV	GNSVNEGLNQ	PTDDSCFDPY
2010	2020	2030	2040	2050	2060	2070	2080
TVSHYAVGDE	WERMSESGFK	LLCQCLGFGS	GHFRCDSSRW	CHDNGVNYKI	GEKWDRQGEN	GQMMSCTCLG	NGKGEFKCDP
2090	2100	2110	2120	2130	2140	2150	2160
HEATCYDDGK	TYHVGEQWQK	EYLGAICSCT	CFGGQRGWRC	DNCRRPGGEP	SPEGTTGQSY	NQYSQRYHQR	TNTNVNCPIE
2170	2180						

Detailed Protein Report

Cmpd.	No. of Cmpds.	m/z meas.	Δ m/z [ppm]	z	Rt [min]	Score	P	Range	Sequence	Modification
1763	1	1044.9953	44.20	2	51.9	16.6	1	534-550	K.RHEEGHMLNCTCFGQGR.G	Carbamidomethyl: 10, 12
1706	1	796.3662	-51.72	2	51.1	15.3	0	1525-1539	R.GDSPASSKPISINYR.T	

Detailed Protein Report

Protein 229: PREDICTED: zinc finger protein 554 isoform X2 [Homo sapiens]

Accession: gi|578832959
Database: refseq_human(refseq_human_20140103.fasta)
Database Date: 2015-11-30
Modification(s): Carbamidomethyl

Score: 31.9
MW [kDa]: 43.7
pI: 9.8
Sequence Coverage [%]: 7.0
No. of unique Peptides: 1

10	20	30	40	50	60	70	80
MTVLRNQDST	YKKVALQEEP	ASGINMIKLI	REDGGWKQLE	DSHEDPQGLL	SQKASLHVVA	VPQEKATAWH	GFGENGNLSP
**90**	**100**	**110**	**120**	**130**	**140**	**150**	**160**
ALVLSQGSSK	GNHLCGSELD	ITSLASDSVL	NHHQLGYADR	RPCESNECGN	AIRQNSHFIQ	HGGKMFVYLE	NGQSLNHGMA
**170**	**180**	**190**	**200**	**210**	**220**	**230**	**240**
LTIHNKINTA	EKPFECHQCG	KVFNRRHSLS	EHQRIHTGEK	PYECQECGRA	FTHSSTLTRH	LRTHTGEKPY	GCGECGKAFN
**250**	**260**	**270**	**280**	**290**	**300**	**310**	**320**
RISSLTQHQR	IHTGEKPYKC	EDCGKSFCQS	SYLILHKRTH	TGEKPYECSE	CGKAFSDRSS	LNQHERTHTG	ENPYECKQCG
**330**	**340**	**350**	**360**	**370**	**380**	**390**	
RAFSQRSSLV	RHERTHTGEK	PYRCQECGKA	FSQSSSLVTH	QKTHSSQKTY	KIIDCGKAFY	QNRHLIGY	

Cmpd.	No. of Cmpds.	m/z meas.	Δ m/z [ppm]	z	Rt [min]	Score	P	Range	Sequence	Modification
2623	1	812.4211	89.86	2	63.6	11.3	0	223-237	R.THTGEKPYGCGECGK.A	Carbamidomethyl: 13

Detailed Protein Report

Protein 230: **PREDICTED: unhealthy ribosome biogenesis protein 2 homolog isoform X1 [Homo sapiens]**

**Accession:**	gi|530366678	**Score:**	31.9
**Database:**	refseq_human(refseq_human_20140103.fasta)	**MW [kDa]:**	166.5
**Database Date:**	2015-11-30	**pI:**	6.9
		**Sequence Coverage [%]:**	1.4
		**No. of unique Peptides:**	1

10	20	30	40	50	60	70	80
MAAVYSGISL	KLKSKTTSWE	DKLKLAHFAW	ISHQCFLPNK	EQVLLDWARQ	SLVAFYKKKL	ELKEDIVERL	WIYIDNILHS
90	100	110	120	130	140	150	160
RKLQNLLKNG	KTINLQISLV	KIINERVAEF	SLSGSQRNIC	AVLRCCQGIL	STPALAVIYT	AKQELMVALL	SQLCWSACRQ
170	180	190	200	210	220	230	240
PEGAVVAQLF	EVIHLALGHY	LLILQQQVNP	RRAFGDVTAH	LLQPCLVLRH	LLSGGTWTQA	GQGQLRQVLS	RDIRSQIEAM
250	260	270	280	290	300	310	320
FRGGIFQPEL	LSSYKEGLLD	QQQGDVKTGA	MKNLLAPMDT	VLNRLVDAGY	CAASLHTSVV	ANSVALLYKL	FLDSYFKEGN
330	340	350	360	370	380	390	400
QLLCFQVLPR	LFGCLKISHL	QEEQSKALST	SDWTTELLVV	EQLLNSVANN	NIYNIAADRI	RHEEAQFRFY	RHVAELLINH
410	420	430	440	450	460	470	480
AQAPIPAWFR	CLKTLISLNH	LILEPDLDDL	LASAWIDAEV	TEFRTKKAQE	ALIRTVFQTY	AKLRQVPRLF	EEVLGVICRP
490	500	510	520	530	540	550	560
AAEALRQPVL	ASGPSTVLSA	CLLELPPSQI	LDTWSLVLEK	FQSLVLPYLQ	SDADMALKSL	SLSLLLHCIM	FNMRSLDSST
570	580	590	600	610	620	630	640
PLPIVRRTQC	MMERMMRELV	QPLLALLPDT	PGPEPELWLQ	KVSDSVLLLS	YTWAQVDAMF	SLNCSQYHSM	SGPLIGVALE
650	660	670	680	690	700	710	720
ISNLPSLLPG	VKTQHWKKIE	KFTAQFSSLG	TYCLEQLYLQ	KMKRTLMQTS	FRSEGAIQSL	RCDAAFIIGS	GRKSLNQRTT
730	740	750	760	770	780	790	800
ASWDGQVGMV	SGLTYPVAHW	HLIVSNLTIL	ISYLCPDDVG	YLASVLLRTL	PMGKAQEVSI	DEEAYITLEK	ISKAFLHSPL
810	820	830	840	850	860	870	880
FPEMQSLHSA	FLTCVTTSCS	SILCSGAQRD	SGLVSQQLPW	LFEKDHMVVG	HWENRFAKAG	PEGIEPRGEI	AQNLLSLVKS
890	900	910	920	930	940	950	960
DFPIQLEGEQ	LESILGLLEV	ISALQLDSLL	PPYHVHYFLV	LLSMAVTKLG	CSCSSSLALK	FLTTCYQLLG	YLQKGKSARS
970	980	990	1000	1010	1020	1030	1040
VFKIMYGSDI	FEVVLTSLFR	ASSRFLIEMD	DPAWLEFLQV	IGTFLEELMQ	MLIQMKLSLV	LNFRKITAFL	SSSKPYTEAA
1050	1060	1070	1080	1090	1100	1110	1120
SSKQLENQNP	QGRQLLLVSL	TRLCHVLGPF	LKEQKLGQEA	PAALSELLQQ	VVLQTGAVLQ	LCSVPGARGW	RLPSVLISSV
1130	1140	1150	1160	1170	1180	1190	1200
STLLEADLGQ	HCRDGGADIS	QGSDRTLLSH	VALYQGVYSQ	ILLELPALAG	HDQSFQAALQ	FLTLFFLAPE	LHPKKDSVFT
1210	1220	1230	1240	1250	1260	1270	1280
SMFHSVRRVL	ADPEIPVQVT	QDIEPHLGAL	FTQMLEVGTT	EDLRLVMQCI	LQGLDVSNMW	KADVQLLNRE	ASQEQPVSLT
1290	1300	1310	1320	1330	1340	1350	1360
VVGPVLDVLA	ALLRQGEEAI	GNPHHVSLAF	SILLTVPLDH	LKPLEYGSVF	PRLHNVLFSI	LQCHPKVMLK	AIPSFLNSFN
1370	1380	1390	1400	1410	1420	1430	1440
RLVFSVMREG	RQKDKGSIDD	LPTVLKCARL	VERMYSHIAA	RAEEFAVFSP	FMVAQYVLEV	QKVTLYPAVK	SLLQEGIYLI
1450	1460	1470	1480	1490			
LDLCIEPDVQ	FLRASLQPGM	RDIFKELYND	YLKYHKAKHE	GEKRYTA			

Cmpd.	No. of Cmpds.	m/z meas.	Δ m/z [ppm]	z	Rt [min]	Score	P	Range	Sequence	Modification
598	1	661.8686	26.39	2	36.8	13.9	2	12-22	K.LKSKTTSWEDK.L	

Detailed Protein Report

Protein 231: **PREDICTED: cortactin-binding protein 2 isoform X2 [Homo sapiens]**

**Accession:**	gi|530386510	**Score:**	31.8
**Database:**	refseq_human(refseq_human_20140103.fasta)	**MW [kDa]:**	106.1
**Database Date:**	2015-11-30	**pI:**	9.1
**Modification(s):**	Carbamidomethyl	**Sequence Coverage [%]:**	3.0
		**No. of unique Peptides:**	1

10	20	30	40	50	60	70	80
MSGGPAPLAG	RPTLLQQAAA	QGNVTLLSML	LNEEGLDINY	SCEDGHSALY	SAAKNGHTDC	VRLLLSAEAQ	VNAADKNGFT
**90**	**100**	**110**	**120**	**130**	**140**	**150**	**160**
PLCAAAAQGH	FECVELLISY	DANINHAADG	GQTPLYLACK	NGNKECIKLL	LEAGTNRSVK	TTDGWTPVHA	AVDTGNVDSL
**170**	**180**	**190**	**200**	**210**	**220**	**230**	**240**
KLLMYHRIPA	HGNSFNEEES	ESSVFDLDGG	EESPEGISKP	VVPADLINHA	NREGWTAAHI	AASKGFKNCL	EILCRHGGLE
**250**	**260**	**270**	**280**	**290**	**300**	**310**	**320**
PERRDKCNRT	VHDVATDDCK	HLLENLNALK	IPLRISVGEI	EPSNYGSDDL	ECENTICALN	IRKQTSWDDF	SKAVSQALTN
**330**	**340**	**350**	**360**	**370**	**380**	**390**	**400**
HFQAISSDGW	WSLEDVTCNN	TTDSNIGLSA	RSIRSITLGN	VPWSVGQSFA	QSPWDFMRKN	KAEHITVLLS	GPQEGCLSSV
**410**	**420**	**430**	**440**	**450**	**460**	**470**	**480**
TYASMIPLQM	MQNYLRLVEQ	YHNVIFHGPE	GSLQDYIVHQ	LALCLKHRQM	AAGFSCEIVR	AEVDAGFSKE	QLLDLFISSA
**490**	**500**	**510**	**520**	**530**	**540**	**550**	**560**
CLIPVKQSPS	KKKIIIILEN	LEKSSLSELL	RDFLAPLENR	STESPCTFQK	GNGLSECYYF	HENCFLMGTI	AKACLQGSDL
**570**	**580**	**590**	**600**	**610**	**620**	**630**	**640**
LVQQHFRWVQ	LRWDGEPMQG	LLQRFLRRKV	VNKFKGQAPS	PCDPVCKIVD	WALSVWRQLN	SCLARLGTPE	ALLGPKYFLS
**650**	**660**	**670**	**680**	**690**	**700**	**710**	**720**
CPVVPGHAQV	TVKWMSKLWN	GVIAPRVQEA	ILSRASVKRQ	PGFGQTTAKR	HPSQGQQAVV	KAALSILLNK	AVLHGCPLPR
**730**	**740**	**750**	**760**	**770**	**780**	**790**	**800**
AELDQHTADF	KGGSFPLSIV	SSYNTCNKKK	GESGAWRKVN	TSPRRKSGRF	SLPTWNKPDL	STEGMKNKTI	SQLNCNRNAS
**810**	**820**	**830**	**840**	**850**	**860**	**870**	**880**
LSKQKSLEND	LSLTLNLDQR	LSLGSDDEAD	LVKELQSMCS	SKSESDISKI	ADSRDDLRMF	DSSGNNPVLS	ATINNLRMPV
**890**	**900**	**910**	**920**	**930**	**940**	**950**	**960**
SQKEVSPLSS	HQTTECSNSK	SKTELGVSRV	KSFLPVPRSK	VTQCSQNTKR	SSSSSNTRQI	EINNNSKEVN	WNLHKNEHLE
**970**							
KPNK							

Cmpd.	No. of Cmpds.	m/z meas.	Δ m/z [ppm]	z	Rt [min]	Score	P	Range	Sequence	Modification
2703	2	973.4604	-78.66	2	65.0	13.9	0	470-486	K.EQLLDLFISSACLIPVK.Q	Carbamidomethyl: 12

Detailed Protein Report

Protein 232: **rho GTPase-activating protein 42 [Homo sapiens]**

**Accession:**	gi|221307575	**Score:**	31.8
**Database:**	refseq_human(refseq_human_20140103.fasta)	**MW [kDa]:**	98.5
**Database Date:**	2015-11-30	**pI:**	8.9
**Modification(s):**	Oxidation	**Sequence Coverage [%]:**	2.5
		**No. of unique Peptides:**	1

10	20	30	40	50	60	70	80
MGLPTLEFSD	SYLDSPDFRE	RLQCHEIELE	RTNKFIKELI	KDGSLLIGAL	RNLSMAVQKF	SQSLQDFQFE	CIGDAETDDE
**90**	**100**	**110**	**120**	**130**	**140**	**150**	**160**
ISIAQSLKEF	ARLLIAVEEE	RRRLIQNAND	VLIAPLEKFR	KEQIGAAKDG	KKKFDKESEK	YYSILEKHLN	LSAKKKESHL
**170**	**180**	**190**	**200**	**210**	**220**	**230**	**240**
QEADTQIDRE	HQNFYEASLE	YVFKIQEVQE	KKKFEFVEPL	LSFLQGLFTF	YHEGYELAQE	FAPYKQQLQF	NLQNTRNNFE
**250**	**260**	**270**	**280**	**290**	**300**	**310**	**320**
STRQEVERLM	QRMKSANQDY	RPPSQWTMEG	YLYVQEKRPL	GFTWIKHYCT	YDKGSKTFTM	SVSEMKSSGK	MNGLVTSSPE
**330**	**340**	**350**	**360**	**370**	**380**	**390**	**400**
MFKLKSCIRR	KTDSIDKRFC	FDIEVVERHG	IITLQAFSEA	NRKLWLEAMD	GKEPIYTLPA	IISKKEEMYL	NEAGFNFVRK
**410**	**420**	**430**	**440**	**450**	**460**	**470**	**480**
CIQAVETRGI	TILGLYRIGG	VNSKVQKLMN	TTFSPKSPPD	IDIDIELWDN	KTITSGLKNY	LRCLAEPLMT	YKLHKDFIIA
**490**	**500**	**510**	**520**	**530**	**540**	**550**	**560**
VKSDDQNYRV	EAVHALVHKL	PEKNREMLDI	LIKHLVKVSL	HSQQNLMTVS	NLGVIFGPTL	MRAQEETVAA	MMNIKFQNIV
**570**	**580**	**590**	**600**	**610**	**620**	**630**	**640**
VEILIEHYEK	IFHTAPDPSI	PLPQPQSRSG	SRRTRAICLS	TGSRKPRGRY	TPCLAEPDSD	SYSSSPDSTP	MGSIESLSSH
**650**	**660**	**670**	**680**	**690**	**700**	**710**	**720**
SSEQNSTTKS	ASCQPREKSG	GIPWIATPSS	SNGQKSLGLW	TTSPESSSRE	DATKTDAESD	CQSVASVTSP	GDVSPPIDLV
**730**	**740**	**750**	**760**	**770**	**780**	**790**	**800**
KKEPYGLSGL	KRASASSLRS	ISAAEGNKSY	SGSIQSLTSV	GSKETPKASP	NPDLPPKMCR	RLRLDTASSN	GYQRPGSVVA
**810**	**820**	**830**	**840**	**850**	**860**	**870**	**880**
AKAQLFENVG	SPKPVSSGRQ	AKAMYSCKAE	HSHELSFPQG	AIFSNVYPSV	EPGWLKATYE	GKTGLVPENY	VVFL

Cmpd.	No. of Cmpds.	m/z meas.	Δ m/z [ppm]	z	Rt [min]	Score	P	Range	Sequence	Modification
150	1	849.3532	-94.77	2	31.6	11.8	1	311-325	K.MNGLVTSSPEMFKLK.S	Oxidation: 11

Detailed Protein Report

Protein 233: serine/threonine-protein kinase Nek8 [Homo sapiens]

**Accession:**	gi|30039692	**Score:**	31.7
**Database:**	refseq_human(refseq_human_20140103.fasta)	**MW [kDa]:**	74.8
**Database Date:**	2015-11-30	**pI:**	9.2
**Modification(s):**	Carbamidomethyl	**Sequence Coverage [%]:**	4.9
		**No. of unique Peptides:**	1

10	20	30	40	50	60	70	80
MEKYERIRVV	GRGAFGIVHL	CLRKADQKLV	IIKQIPVEQM	TKEERQAAQN	ECQVLKLLNH	PNVIEYYENF	LEDKALMIAM
**90**	**100**	**110**	**120**	**130**	**140**	**150**	**160**
EYAPGGTLAE	FIQKRCNSLL	EEETILHFFV	QILLALHHVH	THLILHRDLK	TQNILLDKHR	MVVKIGDFGI	SKILSSKSKA
**170**	**180**	**190**	**200**	**210**	**220**	**230**	**240**
YTVVGTPCYI	SPELCEGKPY	NQKSDIWALG	CVLYELASLK	RAFEAANLPA	LVLKIMSGTF	APISDRYSPE	LRQLVLSLLS
**250**	**260**	**270**	**280**	**290**	**300**	**310**	**320**
LEPAQRPPLS	HIMAQPLCIR	ALLNLHTDVG	SVRMRRAEKS	VAPSNTGSRT	TSVRCRGIPR	GPVRPAIPPP	LSSVYAWGGG
**330**	**340**	**350**	**360**	**370**	**380**	**390**	**400**
LGTPLRLPML	NTEVVQVAAG	RTQKAGVTRS	GRLILWEAPP	LGAGGGSLLP	GAVEQPQPQF	ISRFLEGQSG	VTIKHVACGD
**410**	**420**	**430**	**440**	**450**	**460**	**470**	**480**
FFTACLTDRG	IIMTFGSGSN	GCLGHGSLTD	ISQPTIVEAL	LGYEMVQVAC	GASHVLALST	ERELFAWGRG	DSGRLGLGTR
**490**	**500**	**510**	**520**	**530**	**540**	**550**	**560**
ESHSCPQQVP	MPPGQEAQRV	VCGIDSSMIL	TVPGQALACG	SNRFNKLGLD	HLSLGEEPVP	HQQVEEALSF	TLLGSAPLDQ
**570**	**580**	**590**	**600**	**610**	**620**	**630**	**640**
EPLLSIDLGT	AHSAAVTASG	DCYTFGSNQH	GQLGTNTRRG	SRAPCKVQGL	EGIKMAMVAC	GDAFTVAIGA	ESEVYSWGKG
**650**	**660**	**670**	**680**	**690**	**700**		
ARGRLGRRDE	DAGLPRPVQL	DETHPYTVTS	VSCCHGNTLL	AVRSVTDEPV	PP		

Cmpd.	No. of Cmpds.	m/z meas.	Δ m/z [ppm]	z	Rt [min]	Score	P	Range	Sequence	Modification
1879	2	918.3405	-130.83	2	53.4	19.3	2	280-296	K.SVAPSNTGSRTTSVRCR.G	Carbamidomethyl: 16

Detailed Protein Report

Protein 234: transient receptor potential cation channel subfamily M member 8 [Homo sapiens]

**Accession:**	gi|109689695	**Score:**	31.7
**Database:**	refseq_human(refseq_human_20140103.fasta)	**MW [kDa]:**	127.6
**Database Date:**	2015-11-30	**pI:**	7.0
**Modification(s):**	Carbamidomethyl	**Sequence Coverage [%]:**	1.5
		**No. of unique Peptides:**	1

10	20	30	40	50	60	70	80
MSFRAARLSM	RNRRNDTLDS	TRTLYSSASR	STDLSYSESD	LVNFIQANFK	KRECVFFTKD	SKATENVCKC	GYAQSQHMEG
90	100	110	120	130	140	150	160
TQINQSEKWN	YKKHTKEFPT	DAFGDIQFET	LGKKGKYIRL	SCDTDAEILY	ELLTQHWHLK	TPNLVISVTG	GAKNFALKPR
170	180	190	200	210	220	230	240
MRKIFSRLIY	IAQSKGAWIL	TGGTHYGLMK	YIGEVVRDNT	ISRSSEENIV	AIGIAAWGMV	SNRDTLIRNC	DAEGYFLAQY
250	260	270	280	290	300	310	320
LMDDFTRDPL	YILDNNHTHL	LLVDNGCHGH	PTVEAKLRNQ	LEKYISERTI	QDSNYGGKIP	IVCFAQGGGK	ETLKAINTSI
330	340	350	360	370	380	390	400
KNKIPCVVVE	GSGQIADVIA	SLVEVEDALT	SSAVKEKLVR	FLPRTVSRLP	EEETESWIKW	LKEILECSHL	LTVIKMEEAG
410	420	430	440	450	460	470	480
DEIVSNAISY	ALYKAFSTSE	QDKDNWNGQL	KLLLEWNQLD	LANDEIFTND	RRWESADLQE	VMFTALIKDR	PKFVRLFLEN
490	500	510	520	530	540	550	560
GLNLRKFLTH	DVLTELFSNH	FSTLVYRNLQ	IAKNSYNDAL	LTFVWKLVAN	FRRGFRKEDR	NGRDEMDIEL	HDVSPITRHP
570	580	590	600	610	620	630	640
LQALFIWAIL	QNKKELSKVI	WEQTRGCTLA	ALGASKLLKT	LAKVKNDINA	AGESEELANE	YETRAVELFT	ECYSSDEDLA
650	660	670	680	690	700	710	720
EQLLVYSCEA	WGGSNCLELA	VEATDQHFIA	QPGVQNFLSK	QWYGEISRDT	KNWKIILCLF	IIPLVGCGFV	SFRKKPVDKH
730	740	750	760	770	780	790	800
KKLLWYYVAF	FTSPFVVFSW	NVVFYIAFLL	LFAYVLLMDF	HSVPHPPELV	LYSLVFVLFC	DEVRQWYVNG	VNYFTDLWNV
810	820	830	840	850	860	870	880
MDTLGLFYFI	AGIVFRLHSS	NKSSLYSGRV	IFCLDYIIFT	LRLIHIFTVS	RNLGPKIIML	QRMLIDVFFF	LFLFAVWMVA
890	900	910	920	930	940	950	960
FGVARQGILR	QNEQRWRWIF	RSVIYEPYLA	MFGQVPSDVD	GTTYDFAHCT	FTGNESKPLC	VELDEHNLPR	FPEWITIPLV
970	980	990	1000	1010	1020	1030	1040
CIYMLSTNIL	LVNLLVAMFG	YTVGTVQENN	DQVWKFQRYF	LVQEYCSRLN	IPFPFIVFAY	FYMVVKKCFK	CCCKEKNMES
1050	1060	1070	1080	1090	1100	1110	
SVCCFKNEDN	ETLAWEGVMK	ENYLVKINTK	ANDTSEEMRH	RFRQLDTKLN	DLKGLLKEIA	NKIK	

Cmpd.	No. of Cmpds.	m/z meas.	Δ m/z [ppm]	z	Rt [min]	Score	P	Range	Sequence	Modification
2072	1	731.2846	-37.73	2	56.1	12.0	1	1035-1046	K.EKNMESSVCCFK.N	Carbamidomethyl: 9

Detailed Protein Report

Protein 235: receptor-type tyrosine-protein phosphatase O isoform b precursor [Homo sapiens]

**Accession:**	gi|4506323	**Score:**	31.6
**Database:**	refseq_human(refseq_human_20140103.fasta)	**MW [kDa]:**	134.9
**Database Date:**	2015-11-30	**pI:**	5.6
		**Sequence Coverage [%]:**	2.4
		**No. of unique Peptides:**	1

10	20	30	40	50	60	70	80
MGHLPTGIHG	ARRLLPLLWL	FVLFKNATAF	HVTVQDDNNI	VVSLEASDVI	SPASVYVVKI	TGESKNYFFE	FEEFNSTLPP
90	100	110	120	130	140	150	160
PVIFKASYHG	LYYIITLVVV	NGNVVTKPSR	SITVLTKPLP	VTSVSIYDYK	PSPETGVLFE	IHYPEKYNVF	TRVNISYWEG
170	180	190	200	210	220	230	240
KDFRTMLYKD	FFKGKTVFNH	WLPGMCYSNI	TFQLVSEATF	NKSTLVEYSG	VSHEPKQHRT	APYPPQNISV	RIVNLNKNNW
250	260	270	280	290	300	310	320
EEQSGNFPEE	SFMRSQDTIG	KEKLFHFTEE	TPEIPSGNIS	SGWPDFNSSD	YETTSQPYWW	DSASAAPESE	DEFVSVLPME
330	340	350	360	370	380	390	400
YENNSTLSET	EKSTSGSFSF	FPVQMILTWL	PPKPPTAFDG	FHIHIEREEN	FTEYLMVDEE	AHEFVAELKE	PGKYKLSVTT
410	420	430	440	450	460	470	480
FSSSGSCETR	KSQSAKSLSF	YISPSGEWIE	ELTEKPQHVS	VHVLSSTTAL	MSWTSSQENY	NSTIVSVVSL	TCQKQKESQR
490	500	510	520	530	540	550	560
LEKQYCTQVN	SSKPIIENLV	PGAQYQVVIY	LRKGPLIGPP	SDPVTFAIVP	TGIKDLMLYP	LGPTAVVLSW	TRPYLGVFRK
570	580	590	600	610	620	630	640
YVVEMFYFNP	ATMTSEWTTY	YEIAATVSLT	ASVRIANLLP	AWYYNFRVTM	VTWGDPELSC	CDSSTISFIT	APVAPEITSV
650	660	670	680	690	700	710	720
EYFNSLLYIS	WTYGDDTTDL	SHSRMLHWMV	VAEGKKKIKK	SVTRNVMTAI	LSLPPGDIYN	LSVTACTERG	SNTSMLRLVK
730	740	750	760	770	780	790	800
LEPAPPKSLF	AVNKTQTSVT	LLWVEEGVAD	FFEVFCQQVG	SSQKTKLQEP	VAVSSHVVTI	SSLLPATAYN	CSVTSFSHDS
810	820	830	840	850	860	870	880
PSVPTFIAVS	TMVTEMNPNV	VVISVLAILS	TLLIGLLLVT	LIILRKKHLQ	MARECGAGTF	VNFASLERDG	KLPYNWSKNG
890	900	910	920	930	940	950	960
LKKRKLTNPV	QLDDFDAYIK	DMAKDSDYKF	SLQFEELKLI	GLDIPHFAAD	LPLNRCKNRY	TNILPYDFSR	VRLVSMNEEE
970	980	990	1000	1010	1020	1030	1040
GADYINANYI	PGYNSPQEYI	ATQGPLPETR	NDFWKMVLQQ	KSQIIVMLTQ	CNEKRRVKCD	HYWPFTEEPI	AYGDITVEMI
1050	1060	1070	1080	1090	1100	1110	1120
SEEEQDDWAC	RHFRINYADE	MQDVMHFNYT	AWPDHGVPTA	NAAESILQFV	HMVRQQATKS	KGPMIIHCSA	GVGRTGTFIA
1130	1140	1150	1160	1170	1180	1190	
LDRLLQHIRD	HEFVDILGLV	SEMRSYRMSM	VQTEEQYIFI	HQCVQLMWMK	KKQQFCISDV	IYENVSKS	

Cmpd.	No. of Cmpds.	m/z meas.	Δ m/z [ppm]	z	Rt [min]	Score	P	Range	Sequence	Modification
1528	1	977.3724	-122.90	2	48.9	18.9	1	203-219	K.STLVEYSGVSHEPKQHR.T	

Detailed Protein Report

Protein 236: zinc finger protein 585B [Homo sapiens]

**Accession:**	gi|289547716	**Score:**	31.6
**Database:**	refseq_human(refseq_human_20140103.fasta)	**MW [kDa]:**	88.0
**Database Date:**	2015-11-30	**pI:**	10.2
**Modification(s):**	Carbamidomethyl	**Sequence Coverage [%]:**	3.6
		**No. of unique Peptides:**	2

10	20	30	40	50	60	70	80
MPASWTSPQK	SSALAPEDHG	SSYEGSVSFR	DVAIDFSREE	WRHLDLSQRN	LYRDVMLETY	SHLLSVGYQV	PKPEVVMLEQ
**90**	**100**	**110**	**120**	**130**	**140**	**150**	**160**
GKEPWALQGE	RPRHSCPGEK	LWDHNQHRKI	IGYKPASSQD	QKIYSGEKSY	ECAEFGKSFT	WKSQFKVHLK	VPTGEKLYVC
**170**	**180**	**190**	**200**	**210**	**220**	**230**	**240**
IECGRAFVQK	PEFITHQKTH	MREKPYKCNE	CGKSFFQVSS	LFRHHRIHTG	EKLYECSECG	KGFPYNSDLS	IHEKIHTGER
**250**	**260**	**270**	**280**	**290**	**300**	**310**	**320**
HHECTDCGKA	FTQKSTLKIH	QKIHTGERSY	ICIECGQAFI	QKTQLIAHRR	IHSGEKPYEC	NNCGKSFISK	SQLQVHQRVH
**330**	**340**	**350**	**360**	**370**	**380**	**390**	**400**
TRVKPYICTE	YGKVFSNNSN	LITHEKIQSR	EKSSICTECG	KAFTYRSELI	IHQRIHTGEK	PYECSDCGRA	FTQKSALTVH
**410**	**420**	**430**	**440**	**450**	**460**	**470**	**480**
QRIHTGEKSY	ICMKCGLAFI	RKAHLITHQI	IHTGEKPYKC	GHCGKLFTSK	SQLHVHKRIH	TGEKPYVCNK	CGKAFTNRSN
**490**	**500**	**510**	**520**	**530**	**540**	**550**	**560**
LITHQKTHTG	EKSYICSKCG	KAFTQRSDLI	THQRIHTGEK	PYECNTCGKA	FTQKSNLNIH	QKIHTGERQY	ECHECGKAFN
**570**	**580**	**590**	**600**	**610**	**620**	**630**	**640**
QKSILIVHQK	IHTGEKPYVC	TECGRAFIRK	SNFITHQRIH	TGEKPYECSD	CGKSFTSKSQ	LLVHQPVHTG	EKPYVCAECG
**650**	**660**	**670**	**680**	**690**	**700**	**710**	**720**
KAFSGRSNLS	KHQKTHTGEK	PYICSECGKT	FRQKSELITH	HRIHTGEKPY	ECSDCGKSFT	KKSQLQVHQR	IHTGEKPYVC
**730**	**740**	**750**	**760**	**770**			
AECGKAFSNR	SNLNKHQTTH	TGDKPYKCGI	CGKGFVQKSV	FSVHQSSHA			

Cmpd.	No. of Cmpds.	m/z meas.	Δ m/z [ppm]	z	Rt [min]	Score	P	Range	Sequence	Modification
2053	1	575.2161	-125.57	3	55.6	15.5	1	151-165	K.VPTGEKLYVCIECGR.A	Carbamidomethyl: 10
1964	3	753.7894	-100.09	2	54.7	16.1	0	222-234	K.GFPYNSDLSIHEK.I	

Detailed Protein Report

Protein 237: centromere-associated protein E isoform 2 [Homo sapiens]

**Accession:**	gi|557878612	**Score:**	31.3
**Database:**	refseq_human(refseq_human_20140103.fasta)	**MW [kDa]:**	301.6
**Database Date:**	2015-11-30	**pI:**	5.3
**Modification(s):**	Carbamidomethyl	**Sequence Coverage [%]:**	1.0
		**No. of unique Peptides:**	1

10	20	30	40	50	60	70	80
MAEEGAVAVC	VRVRPLNSRE	ESLGETAQVY	WKTDNNVIYQ	VDGSKSFNFD	RVFHGNETTK	NVYEEIAAPI	IDSAIQGYNG
90	100	110	120	130	140	150	160
TIFAYGQTAS	GKTYTMMGSE	DHLGVIPRAI	HDIFQKIKKF	PDREFLLRVS	YMEIYNETIT	DLLCGTQKMK	PLIIREDVNR
170	180	190	200	210	220	230	240
NVYVADLTEE	VVYTSEMALK	WITKGEKSRH	YGETKMNQRS	SRSHTIFRMI	LESREKGEPS	NCEGSVKVSH	LNLVDLAGSE
250	260	270	280	290	300	310	320
RAAQTGAAGV	RLKEGCNINR	SLFILGQVIK	KLSDGQVGGF	INYRDSKLTR	ILQNSLGGNA	KTRIICTITP	VSFDETLTAL
330	340	350	360	370	380	390	400
QFASTAKYMK	NTPYVNEVST	DEALLKRYRK	EIMDLKKQLE	EVSLETRAQA	MEKDQLAQLL	EEKDLLQKVQ	NEKIENLTRM
410	420	430	440	450	460	470	480
LVTSSSLTLQ	QELKAKRKRR	VTWCLGKINK	MKNSNYADQF	NIPTNITTKT	HKLSINLLRE	IDESVCSESD	VFSNTLDTLS
490	500	510	520	530	540	550	560
EIEWNPATKL	LNQENIESEL	NSLRADYDNL	VLDYEQLRTE	KEEMELKLKE	KNDLDEFEAL	ERKTKKDQEN	ELSSKVELLR
570	580	590	600	610	620	630	640
EKEDQIKKLQ	EYIDSQKLEN	IKMDLSYSLE	SIEDPKQMKQ	TLFDAETVAL	DAKRESAFLR	SENLELKEKM	KELATTYKQM
650	660	670	680	690	700	710	720
ENDIQLYQSQ	LEAKKKMQVD	LEKELQSAFN	EITKLTSLID	GKVPKDLLCN	LELEGKITDL	QKELNKEVEE	NEALREEVIL
730	740	750	760	770	780	790	800
LSELKSLPSE	VERLRKEIQD	KSEELHIITS	EKDKLFSEVV	HKESRVQGLL	EEIGKTKDDL	ATTQSNYKST	DQEFQNFKTL
810	820	830	840	850	860	870	880
HMDFEQKYKM	VLEENERMNQ	EIVNLSKEAQ	KFDSSLGALK	TELSYKTQEL	QEKTREVQER	LNEMEQLKEQ	LENRDSTLQT
890	900	910	920	930	940	950	960
VEREKTLITE	KLQQTLEEVK	TLTQEKDDLK	QLQESLQIER	DQLKSDIHDT	VNMNIDTQEQ	LRNALESLKQ	HQETINTLKS
970	980	990	1000	1010	1020	1030	1040
KISEEVSRNL	HMEENTGETK	DEFQQKMVGI	DKKQDLEAKN	TQTLTADVKD	NEIIEQQRKI	FSLIQEKNEL	QQMLESVIAE
1050	1060	1070	1080	1090	1100	1110	1120
KEQLKTDLKE	NIEMTIENQE	ELRLLGDELK	KQQEIVAQEK	NHAIKKEGEL	SRTCDRLAEV	EEKLKEKSQQ	LQEKQQQLLN
1130	1140	1150	1160	1170	1180	1190	1200
VQEEMSEMQK	KINEIENLKN	ELKNKELTLE	HMETERLELA	QKLNENYEEV	KSITKERKVL	KELQKSFETE	RDHLRGYIRE
1210	1220	1230	1240	1250	1260	1270	1280
IEATGLQTKE	ELKIAHIHLK	EHQETIDELR	RSVSEKTAQI	INTQDLEKSH	TKLQEEIPVL	HEEQELLPNV	KEVSETQETM
1290	1300	1310	1320	1330	1340	1350	1360
NELELLTEQS	TTKDSTTLAR	IEMERLRLNE	KFQESQEEIK	SLTKERDNLK	TIKEALEVKH	DQLKEHIRET	LAKIQESQSK
1370	1380	1390	1400	1410	1420	1430	1440
QEQSLNMKEK	DNETTKIVSE	MEQFKPKDSA	LLRIEIEMLG	LSKRLQESHD	EMKSVAKEKD	DLQRLQEVLQ	SESDQLKENI
1450	1460	1470	1480	1490	1500	1510	1520
KEIVAKHLET	EEELKVAHCC	LKEQEETINE	LRVNLSEKET	EISTIQKQLE	AINDKLQNKI	QEIYEKEEQF	NIKQISEVQE
1530	1540	1550	1560	1570	1580	1590	1600
KVNELKQFKE	HRKAKDSALQ	SIESKMLELT	NRLQESQEEI	QIMIKEKEEM	KRVQEALQIE	RDQLKENTKE	IVAKMKESQE
1610	1620	1630	1640	1650	1660	1670	1680
KEYQFLKMTA	VNETQEKMCE	IEHLKEQFET	QKLNLENIET	ENIRLTQILH	ENLEEMRSVT	KERDDLRSVE	ETLKVERDQL
1690	1700	1710	1720	1730	1740	1750	1760
KENLRETITR	DLEKQEELKI	VHMHLKEHQE	TIDKLRGIVS	EKTNEISNMQ	KDLEHSNDAL	KAQDLKIQEE	LRIAHMHLKE
1770	1780	1790	1800	1810	1820	1830	1840
QQETIDKLRG	IVSEKTDKLS	NMQKDLENSN	AKLQEKIQEL	KANEHQLITL	KKDVNETQKK	VSEMEQLKKQ	IKDQSLTLSK
1850	1860	1870	1880	1890	1900	1910	1920
LEIENLNLAQ	KLHENLEEMK	SVMKERDNLR	RVEETLKLER	DQLKESLQET	KARDLEIQQE	LKTARMLSKE	HKETVDKLRE
1930	1940	1950	1960	1970	1980	1990	2000
KISEKTIQIS	DIQKDLDKSK	DELQKKQDRQ	NHQVKPEKRL	LSDGQQHLTE	SLREKCSRIK	ELLKRYSEMD	DHYECLNRLS
2010	2020	2030	2040	2050	2060	2070	2080
LDLEKEIEFQ	KELSMRVKAN	LSLPYLQTKH	IEKLFTANQR	CSMEFHRIMK	KLKYVLSYVT	KIKEEQHESI	NKFEMDFIDE
2090	2100	2110	2120	2130	2140	2150	2160
VEKQKELLIK	IQHLQQDCDV	PSRELRDLKL	NQNMDLHIEE	ILKDFSESEF	PSIKTEFQQV	LSNRKEMTQF	LEEWLNTRFD
2170	2180	2190	2200	2210	2220	2230	2240
IEKLKNGIQK	ENDRICQVNN	FFNNRIIAIM	NESTEFEERS	ATISKEWEQD	LKSLKEKNEK	LFKNYQTLKT	SLASGAQVNP
2250	2260	2270	2280	2290	2300	2310	2320
TTQDNKNPHV	TSRATQLTTE	KIRELENSLH	EAKESAMHKE	SKIIKMQKEL	EVTNDIIAKL	QAKVHESNKC	LEKTKETIQV
2330	2340	2350	2360	2370	2380	2390	2400

Detailed Protein Report

Cmpd.	No. of Cmpds.	m/z meas.	Δ m/z [ppm]	z	Rt [min]	Score	P	Range	Sequence	Modification
1821	1	920.3533	-110.00	2	52.8	20.0	2	2474-2489	R.EAHKQVTCENSPKSPK.V	Carbamidomethyl: 8

Detailed Protein Report

Protein 238: ubiquitin-conjugating enzyme E2 J2 isoform 3 [Homo sapiens]

**Accession:**	gi|37577130	**Score:**	31.1
**Database:**	refseq_human(refseq_human_20140103.fasta)	**MW [kDa]:**	22.9
**Database Date:**	2015-11-30	**pI:**	7.8
		**Sequence Coverage [%]:**	5.8
		**No. of unique Peptides:**	1

Alias proteins:

Accession	Name	Description
gi|37577132	refseq_human (refseq_human_20140103.fasta)	ubiquitin-conjugating enzyme E2 J2 isoform 3 [Homo sapiens]

```
         10         20         30         40         50         60         70         80
MTPYEGGYYH GKLIFPREFP FKPPSIYMIT PNGRFKCNTR LCLSITDFHP DTWNPAWSVS TILTGLLSFM VEKGPTLGSI
         90        100        110        120        130        140        150        160
ETSDFTKRQL AVQSLAFNLK DKVFCELFPE VVEEIKQKQK AQDELSSRPQ TLPLPDVVPD GETHLVQNGI QLLNGHAPGA
        170        180        190        200        210
VPNLAGLQQA NRHHGLLGGA LANLFVIVGF AAFAYTVKYV LRSIAQE
```

Cmpd.	No. of Cmpds.	m/z meas.	Δ m/z [ppm]	z	Rt [min]	Score	P	Range	Sequence	Modification
188	1	636.3948	167.51	2	32.1	12.9	0	2-12	M.TPYEGGYYHGK.L	

Detailed Protein Report

Protein 239: prosaposin receptor GPR37 precursor [Homo sapiens]

**Accession:**	gi|4885323	**Score:**	31.1
**Database:**	refseq_human(refseq_human_20140103.fasta)	**MW [kDa]:**	67.1
**Database Date:**	2015-11-30	**pI:**	9.9
**Modification(s):**	Oxidation	**Sequence Coverage [%]:**	4.2
		**No. of unique Peptides:**	1

10	20	30	40	50	60	70	80
MRAPGALLAR	MSRLLLLLLL	KVSASSALGV	APASRNETCL	GESCAPTVIQ	RRGRDAWGPG	NSARDVLRAR	APREEQGAAF
**90**	**100**	**110**	**120**	**130**	**140**	**150**	**160**
LAGPSWDLPA	APGRDPAAGR	GAEASAAGPP	GPPTRPPGPW	RWKGARGQEP	SETLGRGNPT	ALQLFLQISE	EEEKGPRGAG
**170**	**180**	**190**	**200**	**210**	**220**	**230**	**240**
ISGRSQEQSV	KTVPGASDLF	YWPRRAGKLQ	GSHHKPLSKT	ANGLAGHEGW	TIALPGRALA	QNGSLGEGIH	EPGGPRRGNS
**250**	**260**	**270**	**280**	**290**	**300**	**310**	**320**
TNRRVRLKNP	FYPLTQESYG	AYAVMCLSVV	IFGTGIIGNL	AVMCIVCHNY	YMRSISNSLL	ANLAFWDFLI	IFFCLPLVIF
**330**	**340**	**350**	**360**	**370**	**380**	**390**	**400**
HELTKKWLLE	DFSCKIVPYI	EVASLGVTTF	TLCALCIDRF	RAATNVQMYY	EMIENCSSTT	AKLAVIWVGA	LLLALPEVVL
**410**	**420**	**430**	**440**	**450**	**460**	**470**	**480**
RQLSKEDLGF	SGRAPAERCI	IKISPDLPDT	IYVLALTYDS	ARLWWYFGCY	FCLPTLFTIT	CSLVTARKIR	KAEKACTRGN
**490**	**500**	**510**	**520**	**530**	**540**	**550**	**560**
KRQIQLESQM	NCTVVALTIL	YGFCIIPENI	CNIVTAYMAT	GVSQQTMDLL	NIISQFLLFF	KSCVTPVLLF	CLCKPFSRAF
**570**	**580**	**590**	**600**	**610**	**620**		
MECCCCCCEE	CIQKSSTVTS	DDNDNEYTTE	LELSPFSTIR	REMSTFASVG	THC		

Cmpd.	No. of Cmpds.	m/z meas.	Δ m/z [ppm]	z	Rt [min]	Score	P	Range	Sequence	Modification
2128	2	721.2347	-109.30	2	56.8	13.5	1	601-613	R.REMSTFASVGTHC.-	Oxidation: 3

Detailed Protein Report

Protein 240: PREDICTED: thyroglobulin isoform X9 [Homo sapiens]

**Accession:**	gi|530389281	**Score:**	31.1
**Database:**	refseq_human(refseq_human_20140103.fasta)	**MW [kDa]:**	232.1
**Database Date:**	2015-11-30	**pI:**	5.1
**Modification(s):**	Carbamidomethyl	**Sequence Coverage [%]:**	1.2
		**No. of unique Peptides:**	1

Quantitation

MD:MU	**Median:** 0.53	**CV:** 0.00 %	**No. of Peptides:** 1

Detailed Protein Report

10	20	30	40	50	60	70	80
MALVLEIFTL	LASICWVSAN	IFEYQVDAQP	LRPCELQRET	AFLKQADYVP	QCAEDGSFQT	VQCQNDGRSC	WCVGANGSEV
90	100	110	120	130	140	150	160
LGSRQPGRPV	ACLSFCQLQK	QQILLSGYIN	STDTSYLPQC	QDSGDYAPVQ	CDVQQVQCWC	VDAEGMEVYG	TRQLGRPKRC
170	180	190	200	210	220	230	240
PRSCEIRNRR	LLHGVGDKSP	PQCSAEGEFM	PVQCKFVNTT	DMMIFDLVHS	YNRFPDAFVT	FSSFQRRFPE	VSGYCHCADS
250	260	270	280	290	300	310	320
QGRELAETGL	ELLLDEIYDT	IFAGLDLPST	FTETTLYRIL	QRRFLAVQSV	ISGRFRCPTK	CEVERFTATS	FGHPYVPSCR
330	340	350	360	370	380	390	400
RNGDYQAVQC	QTEGPCWCVD	AQGKEMHGTR	QQGEPPSCAE	GQSCASERQQ	ALSRLYFGTS	GYFSQHDLFS	SPEKRWASPR
410	420	430	440	450	460	470	480
VARFATSCPP	TIKELFVDSG	LLRPMVEGQS	QQFSVSENLL	KEAIRAIFPS	RGLARLALQF	TTNPKRLQQN	LFGGKFLVNV
490	500	510	520	530	540	550	560
GQFNLSGALG	TRGTFNFSQF	FQQLGLASFL	NGGRQEDLAK	PLSVGLDSNS	STGTPEAAKK	DGTMNKPTVG	SFGFEINLQE
570	580	590	600	610	620	630	640
NQNALKFLAS	LLELPEFLLF	LQHAISVPED	VARDLGDVME	TVLSSQTCEQ	TPERLFVPSC	TTEGSYEDVQ	CFSGECWCVN
650	660	670	680	690	700	710	720
SWGKELPGSR	VRGGQPRCPT	DCEKQRARMQ	SLMGSQPAGS	TLFVPACTSE	GHFLPVQCFN	SECYCVDAEG	QAIPGTRSAI
730	740	750	760	770	780	790	800
GKPKKCPTPC	QLQSEQAFLR	TVQALLSNSS	MLPTLSDTYI	PQCSTDGQWR	QVQCNGPPEQ	VFELYQRWEA	QNKGQDLTPA
810	820	830	840	850	860	870	880
KLLVKIMSYR	EAASGNFSLF	IQSLYEAGQQ	DVFPVLSQYP	SLQDVPLAAL	EGKRPQPREN	ILLEPYLFWQ	ILNGQLSQYP
890	900	910	920	930	940	950	960
GSYSDFSTPL	AHFDLRNCWC	VDEAGQELEG	MRSEPSKLPT	CPGSCEEAKL	RVLQFIRETE	EIVSASNSSR	FPLGESFLVA
970	980	990	1000	1010	1020	1030	1040
KGIRLRNEDL	GLPPLFPPRE	AFAEQFLRGS	DYAIRLAAQS	TLSFYQRRRF	SPDDSAGASA	LLRSGPYMPQ	CDAFGSWEPV
1050	1060	1070	1080	1090	1100	1110	1120
QCHAGTGHCW	CVDEKGGFIP	GSLTARSLQI	PQCPTTCEKS	RTSGLLSSWK	QARSQENPSP	KDLFVPACLE	TGEYARLQAS
1130	1140	1150	1160	1170	1180	1190	1200
GAGTWCVDPA	SGEELRPGSS	SSAQCPSLCN	VLKSGVLSRR	VSPGYVPACR	AEDGGFSPVQ	CDQAQGSCWC	VMDSGEEVPG
1210	1220	1230	1240	1250	1260	1270	1280
TRVTGGQPAC	ESPRCPLPFN	ASEVVGGTIL	CETISGPTGS	AMQQCQLLCR	QGSWSVFPPG	PLICSLESGR	WESQLPQPRA
1290	1300	1310	1320	1330	1340	1350	1360
CQRPQLWQTI	QTQGHFQLQL	PPGKMCSADY	ADLLQTFQVF	ILDELTARGF	CQIQVKTFGT	LVSIPVCNNS	SVQVGCLTRE
1370	1380	1390	1400	1410	1420	1430	1440
RLGVNVTWKS	RLEDIPVASL	PDLHDIERAL	VGKDLLGRFT	DLIQSGSFQL	HLDSKTFPAE	TIRFLQGDHF	GTSPRTWFGC
1450	1460	1470	1480	1490	1500	1510	1520
SEGFYQVLTS	EASQDGLGCV	KCPEGSYSQD	EECIPCPVGF	YQEQAGSLAC	VPCPVGRTTI	SAGAFSQTHC	VTDCQRNEAG
1530	1540	1550	1560	1570	1580	1590	1600
LQCDQNGQYR	ASQKDRGSGK	AFCVDGEGRR	LPWWETEAPL	EDSQCLMMQK	FEKVPESKVI	FDANAPVAVR	SKVPDSEFPV
1610	1620	1630	1640	1650	1660	1670	1680
MQCLTDCTED	EACSFFTVST	TEPEISCDFY	AWTSDNVACM	TSDQKRDALG	NSKATSFGSL	RCQVKVRSHG	QDSPAVYLKK
1690	1700	1710	1720	1730	1740	1750	1760
GQGSTTTLQK	RFEPTGFQNM	LSGLYNPIVF	SASGANLTDA	HLFCLLACDR	DLCCDGFVLT	QVQGGAIICG	LLSSPSVLLC
1770	1780	1790	1800	1810	1820	1830	1840
NVKDWMDPSE	AWANATCPGV	TYDQESHQVI	LRLGDQEFIK	SLTPLEGTQD	TFTNFQQVYL	WKDSDMGSRP	ESMGCRKDTV
1850	1860	1870	1880	1890	1900	1910	1920
PRPASPTEAG	LTTELFSPVD	LNQVIVNGNQ	SLSSQKHWLF	KHLFSAQQAN	LWCLSRCVQE	HSFCQLAEIT	ESASLYFTCT
1930	1940	1950	1960	1970	1980	1990	2000
LYPEAQVCDD	IMESNAQGCR	LILPQMPKAL	FRKKVILEDK	VKNFYTRLPF	QKLMGISIRN	KVPMSEKSIS	NGFFECERRC
2010	2020	2030	2040	2050	2060	2070	2080
DADPCCTGFG	FLNVSQLKGG	EVTCLTLNSL	GIQMCSEENG	GAWRILDCGS	PDIEVHTYPF	GWYQKPIAQN	NAPSFCPLVV
2090	2100	2110					
LPSLTEKVFK	VREVRGTQLK	RME					

Detailed Protein Report

Cmpd.	No. of Cmpds.	m/z meas.	Δ m/z [ppm]	z	Rt [min]	Score	P	Range	Sequence	Modification	Ratios	
865	1	623.9049	-34.22	3	40.3	10.8	0	228-243	R.FPEVSGYCHCADSQGR.E	Carbamidomethyl: 8, 10	MD:MU	0.53

Detailed Protein Report

Protein 241: complement C4-A isoform 2 preproprotein [Homo sapiens]

Accession: gi|356582273
Database: refseq_human(refseq_human_20140103.fasta)
Database Date: 2015-11-30

Score: 31.1
MW [kDa]: 187.6
pI: 6.7
Sequence Coverage [%]: 1.8
No. of unique Peptides: 1

10	20	30	40	50	60	70	80
MRLLWGLIWA	SSFFTLSLQK	PRLLLFSPSV	VHLGVPLSVG	VQLQDVPRGQ	VVKGSVFLRN	PSRNNVPCSP	KVDFTLSSER
90	100	110	120	130	140	150	160
DFALLSLQVP	LKDAKSCGLH	QLLRGPEVQL	VAHSPWLKDS	LSRTTNIQGI	NLLFSSRRGH	LFLQTDQPIY	NPGQRVRYRV
170	180	190	200	210	220	230	240
FALDQKMRPS	TDTITVMVEN	SHGLRVRKKE	VYMPSSIFQD	DFVIPDISEP	GTWKISARFS	DGLESNSSTQ	FEVKKYVLPN
250	260	270	280	290	300	310	320
FEVKITPGKP	YILTVPGHLD	EMQLDIQARY	IYGKPVQGVA	YVRFGLLDED	GKKTFFRGLE	SQTKLVNGQS	HISLSKAEFQ
330	340	350	360	370	380	390	400
DALEKLNMGI	TDLQGLRLYV	AAAIIESPGG	EMEEAELTSW	YFVSSPFSLD	LSKTKRHLVP	GAPFLLQALV	REMSGSPASG
410	420	430	440	450	460	470	480
IPVKVSATVS	SPGSVPEVQD	IQQNTDGSGQ	VSIPIIIPQT	ISELQLSVSA	GSPHPAIARL	TVAAPPSGGP	GFLSIERPDS
490	500	510	520	530	540	550	560
RPPRVGDTLN	LNLRAVGSGA	TFSHYYYMIL	SRGQIVFMNR	EPKRTLTSVS	VFVDHHLAPS	FYFVAFYYHG	DHPVANSLRV
570	580	590	600	610	620	630	640
DVQAGACEGK	LELSVDGAKQ	YRNGESVKLH	LETDSLALVA	LGALDTALYA	AGSKSHKPLN	MGKVFEAMNS	YDLGCGPGGG
650	660	670	680	690	700	710	720
DSALQVFQAA	GLAFSDGDQW	TLSRKRLSCP	KEKTTRKKRN	VNFQKAINEK	LGQYASPTAK	RCCQDGVTRL	PMMRSCEQRA
730	740	750	760	770	780	790	800
ARVQQPDCRE	PFLSCCQFAE	SLRKKSRDKG	QAGLQRALEI	LQEEDLIDED	DIPVRSFFPE	NWLWRVETVD	RFQILTLWLP
810	820	830	840	850	860	870	880
DSLTTWEIHG	LSLSKTKGLC	VATPVQLRVF	REFHLHLRLP	MSVRRFEQLE	LRPVLYNYLD	KNLTVSVHVS	PVEGLCLAGG
890	900	910	920	930	940	950	960
GGLAQQVLVP	AGSARPVAFS	VVPTAAAAVS	LKVVARGSFE	FPVGDAVSKV	LQIEKEGAIH	REELVYELNP	LDHRGRTLEI
970	980	990	1000	1010	1020	1030	1040
PGNSDPNMIP	DGDFNSYVRV	TASDPLDTLG	SEGALSPGGV	ASLLRLPRGC	GEQTMIYLAP	TLAASRYLDK	TEQWSTLPPE
1050	1060	1070	1080	1090	1100	1110	1120
TKDHAVDLIQ	KGYMRIQQFR	KADGSYAAWL	SRDSSTWLTA	FVLKVLSLAQ	EQVGGSPEKL	QETSNWLLSQ	QQADGSFQDP
1130	1140	1150	1160	1170	1180	1190	1200
CPVLDRSMQG	GLVGNDETVA	LTAFVTIALH	HGLAVFQDEG	AEPLKQRVEA	SISKANSFLG	EKASAGLLGA	HAAAITAYAL
1210	1220	1230	1240	1250	1260	1270	1280
TLTKAPVDLL	GVAHNNLMAM	AQETGDNLYW	GSVTGSQSNA	VSPTPAPRNP	SDPMPQAPAL	WIETTAYALL	HLLLHEGKAE
1290	1300	1310	1320	1330	1340	1350	1360
MADQASAWLT	RQGSFQGGFR	STQDTVIALD	ALSAYWIASH	TTEERGLNVT	LSSTGRNGFK	SHALQLNNRQ	IRGLEEELQF
1370	1380	1390	1400	1410	1420	1430	1440
SLGSKINVKV	GGNSKGTLKV	LRTYNVLDMK	NTTCQDLQIE	VTVKGHVEYT	MEANEDYEDY	EYDELPAKDD	PDAPLQPVTP
1450	1460	1470	1480	1490	1500	1510	1520
LQLFEGRRNR	RRREAPKLTS	LSDRYVSHFE	TEGPHVLLYF	DSVPTSRECV	GFEAVQEVPV	GLVQPASATL	YDYYNPERRC
1530	1540	1550	1560	1570	1580	1590	1600
SVFYGAPSKS	RLLATLCSAE	VCQCAEGKCP	RQRRALERGL	QDEDGYRMKF	ACYYPRVEYG	FQVKVLREDS	RAAFRLFETK
1610	1620	1630	1640	1650	1660	1670	1680
ITQVLHFTKD	VKAAANQMRN	FLVRASCRLR	LEPGKEYLIM	GLDGATYDLE	GHPQYLLDSN	SWIEEMPSER	LCRSTRQRAA
1690	1700						
CAQLNDFLQE	YGTQGCQV						

Cmpd.	No. of Cmpds.	m/z meas.	Δ m/z [ppm]	z	Rt [min]	Score	P	Range	Sequence	Modification
1442	2	1061.4940	-67.98	2	47.8	19.8	1	119-137	K.DSLSRTTNIQGINLLFSSR.R	

Detailed Protein Report

Protein 242: PREDICTED: zinc finger protein 468 isoform X2 [Homo sapiens]

Accession: gi|578835091
Database: refseq_human(refseq_human_20140103.fasta)
Database Date: 2015-11-30

Score: 31.0
MW [kDa]: 54.3
pI: 10.3
Sequence Coverage [%]: 4.5
No. of unique Peptides: 2

10	20	30	40	50	60	70	80
MLKTLSSTGQ	GNTEVIHTGT	LHRQASHHIG	EFCFHEIEKD	IHGFEFQWKE	DETNGHAAPM	TEIKELAGST	GQHDQRHAGN
**90**	**100**	**110**	**120**	**130**	**140**	**150**	**160**
KRIKDQLGSS	FHLHLPEPHI	FQSEGKIGNQ	VEKSINNASS	VSTSQRICCR	PKTHISNKYG	NNSLHSSLLT	QKWEVHMREK
**170**	**180**	**190**	**200**	**210**	**220**	**230**	**240**
SFECIQSFKS	FNCSSLLKKH	QIIHLEEKQC	KCDVCGKVFN	QKRYLACHRR	CHTGEKPYKC	NECGKTFGHN	SSLFIHKALH
**250**	**260**	**270**	**280**	**290**	**300**	**310**	**320**
TGEKPYECEE	CDKVFSRKSH	LERHKRIHTG	EKPYKCKVCD	EAFAYNSYLA	KHTILHTGEK	PYTCNECGKV	FNRLSTLARH
**330**	**340**	**350**	**360**	**370**	**380**	**390**	**400**
HRLHTGEKPY	KCEECDKVFS	RKSHLERHRR	IHSGEKPYKC	EECCKVFSRK	SNLERHRRIH	TGEKPYKCKV	CDKAFQRDSH
**410**	**420**	**430**	**440**	**450**	**460**	**470**	
LAQHQRVHTG	EKPYKCNECG	KTFGQTSSLI	IHRRLHTGEK	PYKCNECGKT	FSQMSSLVYH	HRLHSGEKP	

Cmpd.	No. of Cmpds.	m/z meas.	Δ m/z [ppm]	z	Rt [min]	Score	P	Range	Sequence	Modification
1828	7	673.2559	-104.81	2	52.9	19.8	1	159-169	R.EKSFECIQSFK.S	
232	1	563.7487	-90.74	2	32.3	11.2	1	170-179	K.SFNCSSLLKK.H	

Detailed Protein Report

Protein 243: PREDICTED: pericentrin isoform X7 [Homo sapiens]

**Accession:**	gi|530419256	**Score:**	31.0
**Database:**	refseq_human(refseq_human_20140103.fasta)	**MW [kDa]:**	305.6
**Database Date:**	2015-11-30	**pI:**	4.9
		**Sequence Coverage [%]:**	1.1
		**No. of unique Peptides:**	1

Detailed Protein Report

10	20	30	40	50	60	70	80
MEVEQEQRRR	KVEAGRTKLA	HFRQRKTKGD	SSHSEKKTAK	RKGSAVDASV	QEESPVTKED	SALCGGGDIC	KSTSCDDTPD
90	100	110	120	130	140	150	160
GAGGAFAAQP	EDCDGEKRED	LEQLQQKQVN	DHPPEQCGMF	TVSDHPPEQH	GMFTVGDHPP	EQRGMFTVSD	HPPEQHGMFT
170	180	190	200	210	220	230	240
VSDHPPEQRG	MFTISDHQPE	QRGMFTVSDH	TPEQRGIFTI	SDHPAEQRGM	FTKECEQECE	LAITDLESGR	EDEAGLHQSQ
250	260	270	280	290	300	310	320
AVHGLELEAL	RLSLSNMHTA	QLELTQANLQ	KEKETALTEL	REMLNSRRAQ	ELALLQSRQQ	HELELLREQH	AREKEEVVLR
330	340	350	360	370	380	390	400
CGQEAAELKE	KLQSEMEKNA	QIVKTLKEDW	ESEKDLCLEN	LRKELSAKHQ	SEMEDLQNQF	QKELAEQRAE	LEKIFQDKNQ
410	420	430	440	450	460	470	480
AERALRNLES	HHQAAIEKLR	EDLQSEHGRC	LEDLEFKFKE	SEKEKQLELE	NLQASYEDLK	AQSQEEIRRL	WSQLDSARTS
490	500	510	520	530	540	550	560
RQELSELHEQ	LLARTSRVED	LEQLKQREKT	QHESELEQLR	IYFEKKLRDA	EKTYQEDLTL	LQQRLQGARE	DALLDSVEVG
570	580	590	600	610	620	630	640
LSCVGLEEKP	EKGRKDHVDE	LEPERHKESL	PRFQAELEES	HRHQLEALES	PLCIQHEGHV	SDRCCVETSA	LGHEWRLEPS
650	660	670	680	690	700	710	720
EGHSQELPWV	HLQGVQDGDL	EADTERAARV	LGLETEHKVQ	LSLLQTELKE	EIELLKIENR	NLYGKLQHET	RLKDDLEKVK
730	740	750	760	770	780	790	800
HNLIEDHQKE	LNNAKQKTEL	MKQEFQRKET	DWKVMKEELQ	REAEEKLTLM	LLELREKAES	EKQTIINKFE	LREAEMRQLQ
810	820	830	840	850	860	870	880
DQQAAQILDL	ERSLTEQQGR	LQQLEQDLTS	DDALHCSQCG	REPPTAQDGE	LAALHVKEDC	ALQLMLARSR	FLEERKEITE
890	900	910	920	930	940	950	960
KFSAEQDAFL	QEAQEQHARE	LQLLQERHQQ	QLLSVTAELE	ARHQAALGEL	TASLESKQGA	LLAARVAELQ	TKHAADLGAL
970	980	990	1000	1010	1020	1030	1040
ETRHLSSLDS	LESCYLSEFQ	TIREEHRQAL	ELLRADFEEQ	LWKKDSLHQT	ILTQELEKLK	RKHEGELQSV	RDHLRTEVST
1050	1060	1070	1080	1090	1100	1110	1120
ELAGTVAHEL	QGVHQGEFGS	EKKTALHEKE	ETLRLQSAQA	QPFHQEEKES	LSLQLQKKNH	QVQQLKDQVL	SLSHEIEECR
1130	1140	1150	1160	1170	1180	1190	1200
SELEVLQQRR	ERENREGANL	LSMLKADVNL	SHSERGALQD	ALRRLLGLFG	ETLRAAVTLR	SRIGERVGLC	LDDAGAGLAL
1210	1220	1230	1240	1250	1260	1270	1280
STAPALEETW	SDVALPELDR	TLSECAEMSS	VAEISSHMRE	SFLMSPESVR	ECEQPIRRVF	QSLSLAVDGL	MEMALDSSRQ
1290	1300	1310	1320	1330	1340	1350	1360
LEEARQIHSR	FEKEFSFKNE	ETAQVVRKHQ	ELLECLKEES	AAKAELALEL	HKTQGTLEGF	KVETADLKEV	LAGKEDSEHR
1370	1380	1390	1400	1410	1420	1430	1440
LVLELESLRR	QLQQAAQEQA	ALREECTRLW	SRGEATATDA	EAREAGTAVT	AAHKDSALRK	EVEDLTKEQS	ETRKQAEKDR
1450	1460	1470	1480	1490	1500	1510	1520
SALLSQMKIL	ESELEEQLSQ	HRGCAKQAEA	VTALEQQVAS	LDKHLRNQRQ	FMDEQAAERE	HEREEFQQEI	QRLEGQLRQA
1530	1540	1550	1560	1570	1580	1590	1600
AKPQPWGPRD	SQQAPLDGEV	ELLQQKLREK	LDEFNELAIQ	KESADRQVLM	QEEEIKRLEE	MNINIRKKVA	QLQEEVEKQK
1610	1620	1630	1640	1650	1660	1670	1680
NIVKGLEQDK	EVLKKQQMSS	LLLASTLQST	LDAGRCPEPP	SGSPPEGPEI	QLEVTQRALL	RRESEVLDLK	EQLEKMKGDL
1690	1700	1710	1720	1730	1740	1750	1760
ESKNEEILHL	NLKLDMQNSQ	TAVSLRELEE	ENTSLKVIYT	RSSEIEELKA	TIENLQENQK	RLQKEKAEEI	EQLHEVIEKL
1770	1780	1790	1800	1810	1820	1830	1840
QHELSLMGPV	VHEVSDSQAG	SLQSELLCSQ	AGGPRGQALQ	GELEAALEAK	EALSRLLADQ	ERRHSQALEA	LQQRLQGAEE
1850	1860	1870	1880	1890	1900	1910	1920
AAELQLAELE	RNVALREAEV	EDMASRIQEF	EAALKAKEAT	IAERNLEIDA	LNQRKAAHSA	ELEAVLLALA	RIRRALEQQP
1930	1940	1950	1960	1970	1980	1990	2000
LAAGAAPPEL	QWLRAQCARL	SRQLQVLHQR	FLRCQVELDR	RQARRATAHT	RVPGAHPQPR	MDGGAKAQVT	GDVEASHDAA
2010	2020	2030	2040	2050	2060	2070	2080
LEPVVPDPQG	DLQPVLVTLK	DAPLCKQEGV	MSVLTVCQRQ	LQSELLLVKN	EMRLSLEDGG	KGKEKVLEDC	QLPKVDLVAQ
2090	2100	2110	2120	2130	2140	2150	2160
VKQLQEKLNR	LLYSMTFQNV	DAADTKSLWP	MASAHLLESS	WSDDSCDGEE	PDISPHIDTC	DANTATGGVT	DVIKNQAIDA
2170	2180	2190	2200	2210	2220	2230	2240

Detailed Protein Report

Cmpd.	No. of Cmpds.	m/z meas.	Δ m/z [ppm]	z	Rt [min]	Score	P	Range	Sequence	Modification
2759	2	1044.5699	44.47	2	64.9	15.2	2	1291-1307	R.FEKEFSFKNEETAQVVR.K	

Detailed Protein Report

Protein 244: PREDICTED: tubulin polyglutamylase TTLL4 isoform X5 [Homo sapiens]

**Accession:**	gi|578805262	**Score:**	31.0
**Database:**	refseq_human(refseq_human_20140103.fasta)	**MW [kDa]:**	127.2
**Database Date:**	2015-11-30	**pI:**	9.9
		**Sequence Coverage [%]:**	1.8
		**No. of unique Peptides:**	1

10	20	30	40	50	60	70	80
MASAGTQHYS	IGLRQKNSFK	QSGPSGTVPA	TPPEKPSEGR	VWPQAHQQVK	PIWKLEKKQV	ETLSAGLGPG	LLGVPPQPAY
**90**	**100**	**110**	**120**	**130**	**140**	**150**	**160**
FFCPSTLCSS	GTTAVIAGHS	SSCYLHSLPD	LFNSTLLYRR	SSYRQKPYQQ	LESFCLRSSP	SEKSPFSLPQ	KSLPVSLTAN
**170**	**180**	**190**	**200**	**210**	**220**	**230**	**240**
KATSSMVFSM	AQPMASSSTE	PYLCLAAAGE	NPSGKSLASA	ISGKIPSPLS	SSYKPMLNNN	SFMWPNSTPV	PLLQTTQGLK
**250**	**260**	**270**	**280**	**290**	**300**	**310**	**320**
PVSPPKIQPV	SWHHSGGTGD	CAPQPVDHKV	PKSIGTVPAD	ASAHIALSTA	SSHDTSTTSV	ASSWYNRNNL	AMRAEPLSCA
**330**	**340**	**350**	**360**	**370**	**380**	**390**	**400**
LDDSSDSQDP	TKEIRFTEAV	RKLTARGFEK	MPRQGCQLEQ	SSFLNPSFQW	NVLNRSRRWK	PPAVNQQFPQ	EDAGSVRRVL
**410**	**420**	**430**	**440**	**450**	**460**	**470**	**480**
PGASDTLGLD	NTVFCTKRIS	IHLLASHASG	LNHNPACESV	IDSSAFGEGK	APGPPFPQTL	GIANVATRLS	SIQLGQSEKE
**490**	**500**	**510**	**520**	**530**	**540**	**550**	**560**
RPEEARELDS	SDRDISSATD	LQPDQAETED	TEEELVDGLE	DCCSRDENEE	EEGDSECSSL	SAVSPSESVA	MISRSCMEIL
**570**	**580**	**590**	**600**	**610**	**620**	**630**	**640**
TKPLSNHEKV	VRPALIYSLF	PNVPPTIYFG	TRDERVEKLP	WEQRKLLRWK	MSTVTPNIVK	QTIGRSHFKI	SKRNDDWLGC
**650**	**660**	**670**	**680**	**690**	**700**	**710**	**720**
WGHHMKSPSF	RSIREHQKLN	HFPGSFQIGR	KDRLWRNLSR	MQSRFGKKEF	SFFPQSFILP	QDAKLLRKAW	ESSSRQKWIV
**730**	**740**	**750**	**760**	**770**	**780**	**790**	**800**
KPPASARGIG	IQVIHKWSQL	PKRRPLLVQR	YLHKPYLISG	SKFDLRIYVY	VTSYDPLRIY	LFSDGLVRFA	SCKYSPSMKS
**810**	**820**	**830**	**840**	**850**	**860**	**870**	**880**
LGNKFMHLTN	YSVNKKNAEY	QANADEMACQ	GHKWALKALW	NYLSQKGVNS	DAIWEKIKDV	VVKTIISSEP	YVTSLLKMYV
**890**	**900**	**910**	**920**	**930**	**940**	**950**	**960**
RRPYSCHELF	GFDIMLDENL	KPWVLEVNIS	PSLHSSSPLD	ISIKGQMIRD	LLNLAGFVLP	NAEDIISSPS	SCSSSTTSLP
**970**	**980**	**990**	**1000**	**1010**	**1020**	**1030**	**1040**
TSPGDKCRMA	PEHVTAQKMK	KAYYLTQKIP	DQDFYASVLD	VLTPDDVRIL	VEMEDEFSRR	GQFERIFPSH	ISSRYLRFFE
**1050**	**1060**	**1070**	**1080**	**1090**	**1100**	**1110**	**1120**
QPRYFNILTT	QWEQKYHGNK	LKGVDLLRSW	CYKGFHMGVV	SDSAPVWSLP	TSLLTISKDD	VILNAFSKSE	TSKLGNPVPQ
**1130**	**1140**						
RTVRTPAKSP	AFLPRRYL						

Cmpd.	No. of Cmpds.	m/z meas.	Δ m/z [ppm]	z	Rt [min]	Score	P	Range	Sequence	Modification
445	1	592.8266	3.11	2	34.9	13.4	1	596-604	R.VEKLPWEQR.K	

Detailed Protein Report

Protein 245: dystrophin Dp260-2 isoform [Homo sapiens]

**Accession:**	gi|5032291	**Score:**	30.9
**Database:**	refseq_human(refseq_human_20140103.fasta)	**MW [kDa]:**	270.9
**Database Date:**	2015-11-30	**pI:**	5.9
		**Sequence Coverage [%]:**	0.6
		**No. of unique Peptides:**	1

Detailed Protein Report

10	20	30	40	50	60	70	80
MSARKLRNLS	YKKAVRRQKL	LEQSIQSAQE	TEKSLHLIQE	SLTFIDKQLA	AYIADKVDAA	QMPQEAQKIQ	SDLTSHEISL
90	100	110	120	130	140	150	160
EEMKKHNQGK	EAAQRVLSQI	DVAQKKLQDV	SMKFRLFQKP	ANFEQRLQES	KMILDEVKMH	LPALETKSVE	QEVVQSQLNH
170	180	190	200	210	220	230	240
CVNLYKSLSE	VKSEVEMVIK	TGRQIVQKKQ	TENPKELDER	VTALKLHYNE	LGAKVTERKQ	QLEKCLKLSR	KMRKEMNVLT
250	260	270	280	290	300	310	320
EWLAATDMEL	TKRSAVEGMP	SNLDSEVAWG	KATQKEIEKQ	KVHLKSITEV	GEALKTVLGK	KETLVEDKLS	LLNSNWIAVT
330	340	350	360	370	380	390	400
SRAEEWLNLL	LEYQKHMETF	DQNVDHITKW	IIQADTLLDE	SEKKKPQQKE	DVLKRLKAEL	NDIRPKVDST	RDQAANLMAN
410	420	430	440	450	460	470	480
RGDHCRKLVE	PQISELNHRF	AAISHRIKTG	KASIPLKELE	QFNSDIQKLL	EPLEAEIQQG	VNLKEEDFNK	DMNEDNEGTV
490	500	510	520	530	540	550	560
KELLQRGDNL	QQRITDERKR	EEIKIKQQLL	QTKHNALKDL	RSQRRKKALE	ISHQWYQYKR	QADDLLKCLD	DIEKKLASLP
570	580	590	600	610	620	630	640
EPRDERKIKE	IDRELQKKKE	ELNAVRRQAE	GLSEDGAAMA	VEPTQIQLSK	RWREIESKFA	QFRRLNFAQI	HTVREETMMV
650	660	670	680	690	700	710	720
MTEDMPLEIS	YVPSTYLTEI	THVSQALLEV	EQLLNAPDLC	AKDFEDLFKQ	EESLKNIKDS	LQQSSGRIDI	IHSKKTAALQ
730	740	750	760	770	780	790	800
SATPVERVKL	QEALSQLDFQ	WEKVNKMYKD	RQGRFDRSVE	KWRRFHYDIK	IFNQWLTEAE	QFLRKTQIPE	NWEHAKYKWY
810	820	830	840	850	860	870	880
LKELQDGIGQ	RQTVVRTLNA	TGEEIIQQSS	KTDASILQEK	LGSLNLRWQE	VCKQLSDRKK	RLEEQKNILS	EFQRDLNEFV
890	900	910	920	930	940	950	960
LWLEEADNIA	SIPLEPGKEQ	QLKEKLEQVK	LLVEELPLRQ	GILKQLNETG	GPVLVSAPIS	PEEQDKLENK	LKQTNLQWIK
970	980	990	1000	1010	1020	1030	1040
VSRALPEKQG	EIEAQIKDLG	QLEKKLEDLE	EQLNHLLLWL	SPIRNQLEIY	NQPNQEGPFD	VQETEIAVQA	KQPDVEEILS
1050	1060	1070	1080	1090	1100	1110	1120
KGQHLYKEKP	ATQPVKRKLE	DLSSEWKAVN	RLLQELRAKQ	PDLAPGLTTI	GASPTQTVTL	VTQPVVTKET	AISKLEMPSS
1130	1140	1150	1160	1170	1180	1190	1200
LMLEVPALAD	FNRAWTELTD	WLSLLDQVIK	SQRVMVGDLE	DINEMIIKQK	ATMQDLEQRR	PQLEELITAA	QNLKNKTSNQ
1210	1220	1230	1240	1250	1260	1270	1280
EARTIITDRI	ERIQNQWDEV	QEHLQNRRQQ	LNEMLKDSTQ	WLEAKEEAEQ	VLGQARAKLE	SWKEGPYTVD	AIQKKITETK
1290	1300	1310	1320	1330	1340	1350	1360
QLAKDLRQWQ	TNVDVANDLA	LKLLRDYSAD	DTRKVHMITE	NINASWRSIH	KRVSEREAAL	EETHRLLQQF	PLDLEKFLAW
1370	1380	1390	1400	1410	1420	1430	1440
LTEAETTANV	LQDATRKERL	LEDSKGVKEL	MKQWQDLQGE	IEAHTDVYHN	LDENSQKILR	SLEGSDDAVL	LQRRLDNMNF
1450	1460	1470	1480	1490	1500	1510	1520
KWSELRKKSL	NIRSHLEASS	DQWKRLHLSL	QELLVWLQLK	DDELSRQAPI	GGDFPAVQKQ	NDVHRAFKRE	LKTKEPVIMS
1530	1540	1550	1560	1570	1580	1590	1600
TLETVRIFLT	EQPLEGLEKL	YQEPRELPPE	ERAQNVTRLL	RKQAEEVNTE	WEKLNLHSAD	WQRKIDETLE	RLQELQEATD
1610	1620	1630	1640	1650	1660	1670	1680
ELDLKLRQAE	VIKGSWQPVG	DLLIDSLQDH	LEKVKALRGE	IAPLKENVSH	VNDLARQLTT	LGIQLSPYNL	STLEDLNTRW
1690	1700	1710	1720	1730	1740	1750	1760
KLLQVAVEDR	VRQLHEAHRD	FGPASQHFLS	TSVQGPWERA	ISPNKVPYYI	NHETQTTCWD	HPKMTELYQS	LADLNNVRFS
1770	1780	1790	1800	1810	1820	1830	1840
AYRTAMKLRR	LQKALCLDLL	SLSAACDALD	QHNLKQNDQP	MDILQIINCL	TTIYDRLEQE	HNNLVNVPLC	VDMCLNWLLN
1850	1860	1870	1880	1890	1900	1910	1920
VYDTGRTGRI	RVLSFKTGII	SLCKAHLEDK	YRYLFKQVAS	STGFCDQRRL	GLLLHDSIQI	PRQLGEVASF	GGSNIEPSVR
1930	1940	1950	1960	1970	1980	1990	2000
SCFQFANNKP	EIEAALFLDW	MRLEPQSMVW	LPVLHRVAAA	ETAKHQAKCN	ICKECPIIGF	RYRSLKHFNY	DICQSCFFSG
2010	2020	2030	2040	2050	2060	2070	2080
RVAKGHKMHY	PMVEYCTPTT	SGEDVRDFAK	VLKNKFRTKR	YFAKHPRMGY	LPVQTVLEGD	NMETPVTLIN	FWPVDSAPAS
2090	2100	2110	2120	2130	2140	2150	2160
SPQLSHDDTH	SRIEHYASRL	AEMENSNGSY	LNDSISPNES	IDDEHLLIQH	YCQSLNQDSP	LSQPRSPAQI	LISLESEERG
2170	2180	2190	2200	2210	2220	2230	2240

Detailed Protein Report

Cmpd.	No. of Cmpds.	m/z meas.	Δ m/z [ppm]	z	Rt [min]	Score	P	Range	Sequence	Modification
982	1	712.1299	-301.49	1	41.8	17.8	1	747-751	K.MYKDR.Q	

Detailed Protein Report

Protein 246: **PREDICTED: SH2 domain-containing adapter protein E isoform X1 [Homo sapiens]**

**Accession:**	gi|530364158	**Score:**	30.9
**Database:**	refseq_human(refseq_human_20140103.fasta)	**MW [kDa]:**	47.7
**Database Date:**	2015-11-30	**pI:**	9.9
		**Sequence Coverage [%]:**	5.0
		**No. of unique Peptides:**	1

10	20	30	40	50	60	70	80
MQWSPTPGAS	ACLGWASSLA	CSTAPTLLGR	AGRGPLMAAK	WFKEFPLNLK	TVSERAKPGG	GGGKLRKNSE	AGGAGPGPGK
**90**	**100**	**110**	**120**	**130**	**140**	**150**	**160**
GRKNSAAELG	SGRAGVGPKD	SRLSRDSLQG	LIQAAAGKGR	KNSRATEEEP	HRGATKSSGC	STYINRLIKV	DTQEKNGKSN
**170**	**180**	**190**	**200**	**210**	**220**	**230**	**240**
YPSSSSSSSS	SSSSASSSPS	SLGPELDKGK	IIKQQETVII	LEDYADPYDA	KRTKGQRDAE	RVGENDGYME	PYDAQQMITE
**250**	**260**	**270**	**280**	**290**	**300**	**310**	**320**
IRRRGSKDPL	VKALQLLDSP	CEPADGGLKS	ETLAKRRSSK	DLLGKPPQLY	DTPYEPAEGG	PRAEGKARPP	DSRLPENDER
**330**	**340**	**350**	**360**	**370**	**380**	**390**	**400**
PAAEYEQPWE	WKKEQIVRAL	SVQFEGAERP	SFREETVRQH	HRQKSWTQKI	LKPALSDHSE	GEKVDPGLPL	EKQPWYHGAI
**410**	**420**	**430**	**440**	**450**			
SRAEAESRLQ	PCKEAGYLVR	NSESGNSRYS	IALKNHHLLS				

Cmpd.	No. of Cmpds.	m/z meas.	Δ m/z [ppm]	z	Rt [min]	Score	P	Range	Sequence	Modification
2769	1	863.9639	30.41	2	65.0	11.1	0	253-269	K.ALQLLDSPCEPADGGLK.S	

Detailed Protein Report

Protein 247: PREDICTED: short transient receptor potential channel 7 isoform X1 [Homo sapiens]

**Accession:**	gi|530380051	**Score:**	30.8
**Database:**	refseq_human(refseq_human_20140103.fasta)	**MW [kDa]:**	55.5
**Database Date:**	2015-11-30	**pI:**	9.0
		**Sequence Coverage [%]:**	4.8
		**No. of unique Peptides:**	2

10	20	30	40	50	60	70	80
MKFVAHAVSF	TIFLGLLVVN	ASDRFEGVKT	LPNETFTDYP	KQIFRVKTTQ	FSWTEMLIMK	WVLGMIWSEC	KEIWEEGPRE
**90**	**100**	**110**	**120**	**130**	**140**	**150**	**160**
YVLHLWNLLD	FGMLSIFVAS	FTARFMAFLK	ATEAQLYVDQ	HVQDDTLHNV	SLPPEVAYFT	YARDKWWPSD	PQIISEGLYA
**170**	**180**	**190**	**200**	**210**	**220**	**230**	**240**
IAVVLSFSRI	AYILPANESF	GPLQISLGRT	VKDIFKFMVI	FIMVFVAFMI	GMFNLYSYYR	GAKYNPAFTT	VEESFKTLFW
**250**	**260**	**270**	**280**	**290**	**300**	**310**	**320**
SIFGLSEVIS	VVLKYDHKFI	ENIGYVLYGV	YNVTMVVVLL	NMLIAMINNS	YQEIEEDADV	EWKFARAKLW	LSYFDEGRTL
**330**	**340**	**350**	**360**	**370**	**380**	**390**	**400**
PAPFNLVPSP	KSFYYLIMRI	KMCLIKLCKS	KAKSCENDLE	MGMLNSKFKK	TRYQAGMRNS	ENLTANNTLS	KPTRYQKIMK
**410**	**420**	**430**	**440**	**450**	**460**	**470**	**480**
RLIKRYVLKA	QVDRENDEVN	EGELKEIKQD	ISSLRYELLE	EKSQATGELA	DLIQQLSEKF	GKNLNKDHLR	VNKGKDI

Cmpd.	No. of Cmpds.	m/z meas.	Δ m/z [ppm]	z	Rt [min]	Score	P	Range	Sequence	Modification
965	1	607.1283	-333.12	1	41.6	14.2	0	342-346	K.MCLIK.L	
1931	1	1022.8987	-78.15	2	54.2	16.6	2	352-369	K.AKSCENDLEMGMLNSKFK.K	

Detailed Protein Report

Protein 248: PREDICTED: PR domain zinc finger protein 1 isoform X1 [Homo sapiens]

**Accession:**	gi|530383954	**Score:**	30.8
**Database:**	refseq_human(refseq_human_20140103.fasta)	**MW [kDa]:**	73.8
**Database Date:**	2015-11-30	**pI:**	9.9
**Modification(s):**	Oxidation	**Sequence Coverage [%]:**	3.6
		**No. of unique Peptides:**	1

10	20	30	40	50	60	70	80
MRYVNPAHSP	REQNLAACQN	GMNIYFYTIK	PIPANQELLV	WYCRDFAERL	HYPYPGELTM	MNLTQTQSSL	KQPSTEKNEL
**90**	**100**	**110**	**120**	**130**	**140**	**150**	**160**
CPKNVPKREY	SVKEILKLDS	NPSKGKDLYR	SNISPLTSEK	DLDDFRRRGS	PEMPFYPRVV	YPIRAPLPED	FLKASLAYGI
**170**	**180**	**190**	**200**	**210**	**220**	**230**	**240**
ERPTYITRSP	IPSSTTPSPS	ARSSPDQSLK	SSSPHSSPGN	TVSPVGPGSQ	EHRDSYAYLN	ASYGTEGLGS	YPGYAPLPHL
**250**	**260**	**270**	**280**	**290**	**300**	**310**	**320**
PPAFIPSYNA	HYPKFLLPPY	GMNCNGLSAV	SSMNGINNFG	LFPRLCPVYS	NLLGGGSLPH	PMLNPTSLPS	SLPSDGARRL
**330**	**340**	**350**	**360**	**370**	**380**	**390**	**400**
LQPEHPREVL	VPAPHSAFSF	TGAAASMKDK	ACSPTSGSPT	AGTAATAEHV	VQPKATSAAM	AAPSSDEAMN	LIKNKRNMTG
**410**	**420**	**430**	**440**	**450**	**460**	**470**	**480**
YKTLPYPLKK	QNGKIKYECN	VCAKTFGQLS	NLKVHLRVHS	GERPFKCQTC	NKGFTQLAHL	QKHYLVHTGE	KPHECQVCHK
**490**	**500**	**510**	**520**	**530**	**540**	**550**	**560**
RFSSTSNLKT	HLRLHSGEKP	YQCKVCPAKF	TQFVHLKLHK	RLHTRERPHK	CSQCHKNYIH	LCSLKVHLKG	NCAAAPAPGL
**570**	**580**	**590**	**600**	**610**	**620**	**630**	**640**
PLEDLTRINE	EIEKFDISDN	ADRLEDVEDD	ISVISVVEKE	ILAVVRKEKE	ETGLKVSLQR	NMGNGLLSSG	CSLYESSDLP
**650**	**660**	**670**					
LMKLPPSNPL	PLVPVKVKQE	TVEPMDP					

Cmpd.	No. of Cmpds.	m/z meas.	Δ m/z [ppm]	z	Rt [min]	Score	P	Range	Sequence	Modification
1164	3	598.6419	-220.44	2	44.1	12.0	0	129-138	R.GSPEMPFYPR.V	Oxidation: 5

Detailed Protein Report

Protein 249: MAP7 domain-containing protein 2 isoform 4 [Homo sapiens]

**Accession:**	gi|270483747	**Score:**	30.7
**Database:**	refseq_human(refseq_human_20140103.fasta)	**MW [kDa]:**	76.6
**Database Date:**	2015-11-30	**pI:**	9.4
		**Sequence Coverage [%]:**	3.5
		**No. of unique Peptides:**	1

10	20	30	40	50	60	70	80
MEGFLKSDER	QRLAKERREE	REKCLAAREQ	QILEKQKRAR	LQYEKQMEER	WRKLEEQRQR	EDQKRAAVEE	KRKQKLREEE
90	100	110	120	130	140	150	160
ERLEAMMRRS	LERTQQLELK	KKYSWGAPLA	IGPGGHDACD	KLSTSTMSLP	KPTEPPMNKR	LSSSTVAISY	SPDRAPLGPL
170	180	190	200	210	220	230	240
NPSYKSSPTR	NIEKKKATST	STSGAGDVGK	EALSGGEASL	VEKVKRGQRT	ATSLPVVNFG	SPLRRCEFSG	GIPKRPSSPV
250	260	270	280	290	300	310	320
ISKTATKAYP	QSPKTTKPPY	PGSPVKYRLP	ALSGQDMPKR	KAEKEKSNKE	REGTLAQQAA	GPQGEEALEK	HVVDKHASEK
330	340	350	360	370	380	390	400
HAAAAGGKAE	NSAALGKPTA	GTTDAGEAAK	ILAEKRRQAR	LQKEQEEQER	LEKEEQDRLE	REELKRKAEE	ERLRLEEEAR
410	420	430	440	450	460	470	480
KQEEERKRQE	EEKKKQEGEE	KRKAGEEAKR	KAEEELLLKE	KQEQEKQEKA	MIEKQKEAAE	TKAREVAEQM	RLEREQIMLQ
490	500	510	520	530	540	550	560
IEQERLERKK	RIDEIMKRTR	KSDVSPQVKK	EDPKVGVQPA	VCVEKKTKLV	VPNKMEINGL	NTCQEVNGVD	HAAPETYPQD
570	580	590	600	610	620	630	640
IFSNGLKPAG	GLIHLDALDG	KSNSLDDSTE	EVQSMDVSPV	SKEELISIPE	FSPVSEMIPG	VSLDQNGTGN	ARALQDLLDF
650	660	670	680	690			
TGPPTFPKRS	SENLSLDDCN	KNLIEGFNSP	GQETPLNTFC				

Cmpd.	No. of Cmpds.	m/z meas.	Δ m/z [ppm]	z	Rt [min]	Score	P	Range	Sequence	Modification
1648	1	575.1737	-226.87	2	50.5	13.4	1	311-320	K.HVVDKHASEK.H	

Detailed Protein Report

Protein 250: chromodomain-helicase-DNA-binding protein 8 isoform 2 [Homo sapiens]

**Accession:**	gi|114326455	**Score:**	30.7
**Database:**	refseq_human(refseq_human_20140103.fasta)	**MW [kDa]:**	262.2
**Database Date:**	2015-11-30	**pI:**	6.2
**Modification(s):**	Oxidation	**Sequence Coverage [%]:**	1.6
		**No. of unique Peptides:**	1

Detailed Protein Report

10	20	30	40	50	60	70	80
MKGESKRITL	VLQQPQSGGP	QGHRHVVLGS	LPGKIVLQGN	QLAALTQAKN	AQGQPAKVVT	IQLQVQQPQQ	KIQIVPQPPS
90	100	110	120	130	140	150	160
SQPQPQQPPS	TQPVTLSSVQ	QAQIMGPGQS	PGQRLSVPVK	VVLQPQAGSS	QGASSGLSVV	KVLSASEVAA	LSSPASSAPH
170	180	190	200	210	220	230	240
SGGKTGMEEN	RRLEHQKKQE	KANRIVAEAI	ARARARGEQN	IPRVLNEDEL	PSVRPEEEGE	KKRRKKSAGE	RLKEEKPKKS
250	260	270	280	290	300	310	320
KTSGASKTKG	KSKLNTITPV	VGKKRKRNTS	SDNSDVEVMP	AQSPREDEES	SIQKRRSNRQ	VKRKKYTEDL	DIKITDDEEE
330	340	350	360	370	380	390	400
EEVDVTGPIK	PEPILPEPVQ	EPDGETLPSM	QFFVENPSEE	DAAIVDKVLS	MRIVKKELPS	GQYTEAEEFF	VKYKNYSYLH
410	420	430	440	450	460	470	480
CEWATISQLE	KDKRIHQKLK	RFKTKMAQMR	HFFHEDEEPF	NPDYVEVDRI	LDESHSIDKD	NGEPVIYYLV	KWCSLPYEDS
490	500	510	520	530	540	550	560
TWELKEDVDE	GKIREFKRIQ	SRHPELKRVN	RPQASAWKKL	ELSHEYKNRN	QLREYQLEGV	NWLLFNWYNR	QNCILADEMG
570	580	590	600	610	620	630	640
LGKTIQSIAF	LQEVYNVGIH	GPFLVIAPLS	TITNWEREFN	TWTEMNTIVY	HGSLASRQMI	QQYEMYCKDS	RGRLIPGAYK
650	660	670	680	690	700	710	720
FDALITTFEM	ILSDCPELRE	IEWRCVIIDE	AHRLKNRNCK	LLDSLKHMDL	EHKVLLTGTP	LQNTVEELFS	LLHFLEPSQF
730	740	750	760	770	780	790	800
PSESEFLKDF	GDLKTEEQVQ	KLQAILKPMM	LRRLKEDVEK	NLAPKQETII	EVELTNIQKK	YYRAILEKNF	SFLSKGAGHT
810	820	830	840	850	860	870	880
NMPNLLNTMM	ELRKCCNHPY	LINGAEEKIL	TEFREACHII	PHDFHLQAMV	RSAGKLVLID	KLLPKLKAGG	HKVLIFSQMV
890	900	910	920	930	940	950	960
RCLDILEDYL	IQRRYLYERI	DGRVRGNLRQ	AAIDRFSKPD	SDRFVFLLCT	RAGGLGINLT	AADTCIIFDS	DWNPQNDLQA
970	980	990	1000	1010	1020	1030	1040
QARCHRIGQS	KAVKVYRLIT	RNSYEREMFD	KASLKLGLDK	AVLQSMSGRD	GNITGIQQFS	KKEIEDLLRK	GAYAAIMEED
1050	1060	1070	1080	1090	1100	1110	1120
DEGSKFCEED	IDQILLRRTT	TITIESEGKG	STFAKASFVA	SENRTDISLD	DPNFWQKWAK	KADLDMDLLN	SKNNLVIDTP
1130	1140	1150	1160	1170	1180	1190	1200
RVRKQTRHFS	TLKDDDLVEF	SDLESEDDER	PRSRRHDRHH	AYGRTDCFRV	EKHLLVYGWG	RWRDILSHGR	FKRRMTERDV
1210	1220	1230	1240	1250	1260	1270	1280
ETICRAILVY	CLLHYRGDEN	IKGFIWDLIS	PAENGKTKEL	QNHSGLSIPV	PRGRKGKKVK	SQSTFDIHKA	DWIRKYNPDT
1290	1300	1310	1320	1330	1340	1350	1360
LFQDESYKKH	LKHQCNKVLL	RVRMLYYLRQ	EVIGDQAEKV	LGGAIASEID	IWFPVVDQLE	VPTTWWDSEA	DKSLLIGVFK
1370	1380	1390	1400	1410	1420	1430	1440
HGYEKYNTMR	ADPALCFLEK	AGRPDDKAIA	AEHRVLDNFS	DIVEGVDFDK	DCEDPEYKPL	QGPPKDQDDE	GDPLMMMDEE
1450	1460	1470	1480	1490	1500	1510	1520
ISVIDGDEAQ	VTQQPGHLFW	PPGSALTARL	RRLVTAYQRS	YKREQMKIEA	AERGDRRRRR	CEAAFKLKEI	ARREKQQRWT
1530	1540	1550	1560	1570	1580	1590	1600
RREQTDFYRV	VSTFGVEYDP	DTMQFHWDRF	RTFARLDKKT	DESLTKYFHG	FVAMCRQVCR	LPPAAGDEPP	DPNLFIEPIT
1610	1620	1630	1640	1650	1660	1670	1680
EERASRTLYR	IELLRRLREQ	VLCHPLLEDR	LALCQPPGPE	LPKWWEPVRH	DGELLRGAAR	HGVSQTDCNI	MQDPDFSFLA
1690	1700	1710	1720	1730	1740	1750	1760
ARMNYMQNHQ	AGAPAPSLSR	CSTPLLHQQY	TSRTASPLPL	RPDAPVEKSP	EETATQVPSL	ESLTLKLEHE	VVARSRPTPQ
1770	1780	1790	1800	1810	1820	1830	1840
DYEMRVSPSD	TTPLVSRSVP	PVKLEDEDDS	DSELDLSKLS	PSSSSSSSSS	SSSSSTDESE	DEKEEKLTDQ	SRSKLYDEES
1850	1860	1870	1880	1890	1900	1910	1920
LLSLTMSQDG	FPNEDGEQMT	PELLLLQERQ	RASEWPKDRV	LINRIDLVCQ	AVLSGKWPSS	RRSQEMVTGG	ILGPGNHLLD
1930	1940	1950	1960	1970	1980	1990	2000
SPSLTPGEYG	DSPVPTPRSS	SAASMAEEEA	SAVSTAAAQF	TKLRRGMDEK	EFTVQIKDEE	GLKLTFQKHK	LMANGVMGDG
2010	2020	2030	2040	2050	2060	2070	2080
HPLFHKKKGN	RKKLVELEVE	CMEEPNHLDV	DLETRIPVIN	KVDGTLLVGE	DAPRRAELEM	WLQGHPEFAV	DPRFLAYMED
2090	2100	2110	2120	2130	2140	2150	2160
RRKQKWQRCK	KNNKAELNCL	GMEPVQTANS	RNGKKGHHTE	TVFNRVLPGP	IAPESSKKRA	RRMRPDLSKM	MALMQGGSTG
2170	2180	2190	2200	2210	2220	2230	2240

Detailed Protein Report

Cmpd.	No. of Cmpds.	m/z meas.	Δ m/z [ppm]	z	Rt [min]	Score	P	Range	Sequence	Modification
1309	1	863.9074	-143.00	2	46.0	10.4	2	742-755	K.LQAILKPMMLRRLK.E	Oxidation: 8

Detailed Protein Report

Protein 251: **PREDICTED: ras-GEF domain-containing family member 1B isoform X2 [Homo sapiens]**

**Accession:**	gi|530377246	**Score:**	30.7
**Database:**	refseq_human(refseq_human_20140103.fasta)	**MW [kDa]:**	50.3
**Database Date:**	2015-11-30	**pI:**	9.0
		**Sequence Coverage [%]:**	6.3
		**No. of unique Peptides:**	1

Quantitation

MD:MU **Median:** 3.28 **CV:** 0.00 % **No. of Peptides:** 1

10	20	30	40	50	60	70	80
MPQTPPFSAM	FDSSGYNRNL	YQSAEDSCGG	LYYHDNNLLS	GSLEALIQHL	VPNVDYYPDN	QMRKIAPKIL	QLLTEWTETF
90	100	110	120	130	140	150	160
PYDFRDERMM	RNLKDLAHRI	ASGEETYRKN	VQQMMQCLIR	KLAALSQYEE	VLAKISSTST	DRLTVLKTKP	QSIQRDIITV
170	180	190	200	210	220	230	240
CNDPYTLAQQ	LTHIELERLN	YIGPEEFVQA	FVQKDPLDND	KSCYSERKKT	RNLEAYVEWF	NRLSYLVATE	ICMPVKKKHR
250	260	270	280	290	300	310	320
ARMIEYFIDV	ARECFNIGNF	NSLMAIISGM	NMSPVSRLKK	TWAKVKTAKF	DILEHQMDPS	SNFYNYRTAL	RGAAQRSLTA
330	340	350	360	370	380	390	400
HSSREKIVIP	FFSLLIKDIY	FLNEGCANRL	PNGHVNFEKF	WELAKQVSEF	MTWKQVECPF	ERDRKILQYL	LTVPVFSEDA
410	420	430	440				
LYLASYESEG	PENHIEKDRW	KSLRSSLLGR	V				

Cmpd.	No. of Cmpds.	m/z meas.	Δ m/z [ppm]	z	Rt [min]	Score	P	Range	Sequence	Modification	Ratios
2529	1	913.4616	-10.11	2	62.1	13.5	2	209-222	K.KTRNLEAYVEWFNR.L		MD:MU 3.28

Detailed Protein Report

Protein 252: **PREDICTED: collagen alpha-2(I) chain-like [Homo sapiens]**

**Accession:**	gi|530375734	**Score:**	30.6
**Database:**	refseq_human(refseq_human_20140103.fasta)	**MW [kDa]:**	28.8
**Database Date:**	2015-11-30	**pI:**	12.9
		**Sequence Coverage [%]:**	5.1
		**No. of unique Peptides:**	1

10	20	30	40	50	60	70	80
MAPFHLSHAP	AGARKVKLRH	PRKWSPSGSP	QPPAQLQPLE	APRLPGEVRT	SGRLATWFCR	LLARRSAAQR	KSKSRKDLNR
**90**	**100**	**110**	**120**	**130**	**140**	**150**	**160**
RVQSPLTPHS	FHYFSSQGIG	GGGGGRDKFA	GRLAWSNPRG	WDRPRGSGGA	LPRPEGDCGG	ARPPAGGAGR	WASPGAAGRW
**170**	**180**	**190**	**200**	**210**	**220**	**230**	**240**
SLPGRVCGAF	AQPAPCPHLT	SSPPRPLGGP	LRCAAQARSV	AARAVRPRSL	GQGPGAQGVG	RAGRERGRPS	TASGSARAGA
**250**	**260**	**270**	**280**				
ALPAAVRPRE	GPRGPSACAL	GRRRLCVGRP	GGPRGTD				

Cmpd.	No. of Cmpds.	m/z meas.	Δ m/z [ppm]	z	Rt [min]	Score	P	Range	Sequence	Modification
2843	4	731.8776	7.32	2	66.0	30.6	0	1-14	-.MAPFHLSHAPAGAR.K	

Detailed Protein Report

Protein 253: **PREDICTED: serine/arginine repetitive matrix protein 1-like [Homo sapiens]**

Accession: gi|578794871
Database: refseq_human(refseq_human_20140103.fasta)
Database Date: 2015-11-30

Score: 30.5
MW [kDa]: 45.0
pI: 12.4
Sequence Coverage [%]: 7.5
No. of unique Peptides: 1

Quantitation

MD:MU **Median:** 3.28 **CV:** 0.00 % **No. of Peptides:** 1

10	20	30	40	50	60	70	80
MTVATKSQRS	LSARCGTELA	QQGKPGPRAL	SFSLAQPQCS	AGYGLRKPQL	PYVKNGGQEI	HGLLRARVHP	CMAVNAPPQV
90	100	110	120	130	140	150	160
MTKSTTSFPF	LAMENKSALL	QGSFWGAPPK	PREEAGEPSL	QPRAAQRRHR	TCESLCAPLR	APATRRRDSE	PAPAATPHPP
170	180	190	200	210	220	230	240
ARQPARPVRP	PAQASQPRVA	RSLVGPRPLV	LPLAYSPPAR	EKPGRSEARR	PGPGCYIGRR	PNPAWTRQRP	PAPPLGSPPP
250	260	270	280	290	300	310	320
RAAQHPGGPP	NCRRGSASFF	RVGKAVPRSW	EINHLQKAEG	ARPARTRVET	TEDQKGSRKD	GCSGSRCHQY	RPPPQNQKKN
330	340	350	360	370	380	390	400
LKGCPPHRKR	MATLARGYKG	SRAICRDVSL	NLWQSRRSTE	SCIGHRINYR	NPLQWSHAEG	VRRSSGPGVI	SKAVGAEMWR
410	420						
TGAHHFLGSS	AVL						

Cmpd.	No. of Cmpds.	m/z meas.	Δ m/z [ppm]	z	Rt [min]	Score	P	Range	Sequence	Modification	Ratios
2683	1	913.4777	3.59	2	63.7	10.3	2	146-162	R.RRDSEPAPAATPHPPAR.Q		MD:MU 3.28

Detailed Protein Report

Protein 254: F-box only protein 41 [Homo sapiens]

**Accession:**	gi|289803020	**Score:**	30.5
**Database:**	refseq_human(refseq_human_20140103.fasta)	**MW [kDa]:**	94.4
**Database Date:**	2015-11-30	**pI:**	9.8
**Modification(s):**	Carbamidomethyl	**Sequence Coverage [%]:**	2.4
		**No. of unique Peptides:**	1

Alias proteins:

Accession	Name	Description
gi|578802734	refseq_human (refseq_human_20140103.fasta)	PREDICTED: F-box only protein 41 isoform X2 [Homo sapiens]

```
        10         20         30         40         50         60         70         80
MASLDLPYRC PRCGEHKRFR SLSSLRAHLE YSHTYETLYI LSKTNSICDG AAAAAAAAAA ASGFPLAPEP AALLAVPGAR
        90        100        110        120        130        140        150        160
REVFESTSFQ GKEQAAGPSP AAPHLLHHHH HHAPLAHFPG DLVPASLPCE ELAEPGLVPA AAARYALREI EIPLGELFAR
       170        180        190        200        210        220        230        240
KSVASSACST PPPGPGPGPC PGPASASPAS PSPADVAYEE GLARLKIRAL EKLEVDRRLE RLSEEVEQKI AGQVGRLQAE
       250        260        270        280        290        300        310        320
LERKAAELET ARQESARLGR EKEELEERAS ELSRQVDVSV ELLASLKQDL VHKEQELSRK QQEVVQIDQF LKETAAREAS
       330        340        350        360        370        380        390        400
AKLRLQQFIE ELLERADRAE RQLQVISSSC GSTPSASLGR GGGGGGAGPN ARGPGRMREH HVGPAVPNTY AVSRHGSSPS
       410        420        430        440        450        460        470        480
TGASSRVPAA SQSSGCYDSD SLELPRPEEG APEDSGPGGL GTRAQAANGG SERSQPPRSS GLRRQAIQNW QRRPRRHSTE
       490        500        510        520        530        540        550        560
GEEGDVSDVG SRTTESEAEG PLDAPRPGPA MAGPLSSCRL SARPEGGSGR GRRAERVSPS RSNEVISPEI LKMRAALFCI
       570        580        590        600        610        620        630        640
FTYLDTRTLL HAAEVCRDWR FVARHPAVWT RVLLENARVC SKFLAMLAQW CTQAHSLTLQ NLKPRQRGKK ESKEEYARST
       650        660        670        680        690        700        710        720
RGCLEAGLES LLKAAGGNLL ILRISHCPNI LTDRSLWLAS CYCRALQAVT YRSATDPVGH EVIWALGAGC REIVSLQVAP
       730        740        750        760        770        780        790        800
LHPCQQPTRF SNRCLQMIGR CWPHLRALGV GGAGCGVQGL ASLARNCMRL QVLELDHVSE ITQEVAAEVC REGLKGLEML
       810        820        830        840        850        860        870        880
VLTATPVTPK ALLHFNSICR NLKSIVVQIG IADYFKEPSS PEAQKLFEDM VTKLQALRRR PGFSKILHIK VEGGC
```

Cmpd.	No. of Cmpds.	m/z meas.	Δ m/z [ppm]	z	Rt [min]	Score	P	Range	Sequence	Modification
2595	2	813.9315	23.86	2	62.4	14.8	1	568-580	R.TLLHAAEVCRDWR.F	Carbamidomethyl: 9

Detailed Protein Report

Protein 255: **solute carrier organic anion transporter family member 1B1 [Homo sapiens]**

**Accession:**	gi|225543525	**Score:**	30.4
**Database:**	refseq_human(refseq_human_20140103.fasta)	**MW [kDa]:**	76.4
**Database Date:**	2015-11-30	**pI:**	9.7
		**Sequence Coverage [%]:**	3.5
		**No. of unique Peptides:**	1

10	20	30	40	50	60	70	80
MDQNQHLNKT	AEAQPSENKK	TRYCNGLKMF	LAALSLSFIA	KTLGAIIMKS	SIIHIERRFE	ISSSLVGFID	GSFEIGNLLV
**90**	**100**	**110**	**120**	**130**	**140**	**150**	**160**
IVFVSYFGSK	LHRPKLIGIG	CFIMGIGGVL	TALPHFFMGY	YRYSKETNIN	SSENSTSTLS	TCLINQILSL	NRASPEIVGK
**170**	**180**	**190**	**200**	**210**	**220**	**230**	**240**
GCLKESGSYM	WIYVFMGNML	RGIGETPIVP	LGLSYIDDFA	KEGHSSLYLG	ILNAIAMIGP	IIGFTLGSLF	SKMYVDIGYV
**250**	**260**	**270**	**280**	**290**	**300**	**310**	**320**
DLSTIRITPT	DSRWVGAWWL	NFLVSGLFSI	ISSIPFFFLP	QTPNKPQKER	KASLSLHVLE	TNDEKDQTAN	LTNQGKNITK
**330**	**340**	**350**	**360**	**370**	**380**	**390**	**400**
NVTGFFQSFK	SILTNPLYVM	FVLLTLLQVS	SYIGAFTYVF	KYVEQQYGQP	SSKANILLGV	ITIPIFASGM	FLGGYIIKKF
**410**	**420**	**430**	**440**	**450**	**460**	**470**	**480**
KLNTVGIAKF	SCFTAVMSLS	FYLLYFFILC	ENKSVAGLTM	TYDGNNPVTS	HRDVPLSYCN	SDCNCDESQW	EPVCGNNGIT
**490**	**500**	**510**	**520**	**530**	**540**	**550**	**560**
YISPCLAGCK	SSSGNKKPIV	FYNCSCLEVT	GLQNRNYSAH	LGECPRDDAC	TRKFYFFVAI	QVLNLFFSAL	GGTSHVMLIV
**570**	**580**	**590**	**600**	**610**	**620**	**630**	**640**
KIVQPELKSL	ALGFHSMVIR	ALGGILAPIY	FGALIDTTCI	KWSTNNCGTR	GSCRTYNSTS	FSRVYLGLSS	MLRVSSLVLY
**650**	**660**	**670**	**680**	**690**	**700**		
IILIYAMKKK	YQEKDINASE	NGSVMDEANL	ESLNKNKHFV	PSAGADSETH	C		

Cmpd.	No. of Cmpds.	m/z meas.	Δ m/z [ppm]	z	Rt [min]	Score	P	Range	Sequence	Modification
216	1	487.1727	-168.54	3	32.4	17.4	2	10-22	K.TAEAQPSENKKTR.Y	

Detailed Protein Report

Protein 256: **PREDICTED: bifunctional heparan sulfate N-deacetylase/N-sulfotransferase 2 isoform X2 [Homo sapiens]**

Accession: gi|530394565
Database: refseq_human(refseq_human_20140103.fasta)
Database Date: 2015-11-30

Score: 30.4
MW [kDa]: 59.2
pI: 9.8
Sequence Coverage [%]: 6.3
No. of unique Peptides: 2

10	20	30	40	50	60	70	80
MLLKHRKEFW	WFPHMWSHMQ	PHLFHNRSVL	ADQMRLNKQF	ALEHGIPTDL	GYAVAPHHSG	VYPIHTQLYE	AWKSVWGIQV
90	100	110	120	130	140	150	160
TSTEEYPHLR	PARYRRGFIH	NGIMVLPRQT	CGLFTHTIFY	NEYPGGSREL	DRSIRGGELF	LTVLLNPISI	FMTHLSNYGN
170	180	190	200	210	220	230	240
DRLGLYTFES	LVRFLQCWTR	LRLQTLPPVP	LAQKYFELFP	QERSPLWQNP	CDDKRHKDIW	SKEKTCDRLP	KFLIVGPQKT
250	260	270	280	290	300	310	320
GTTAIHFFLS	LHPAVTSSFP	SPSTFEEIQF	FNSPNYHKGI	DWYMDFFPVP	SNASTDFLFE	KSATYFDSEV	VPRRGAALLP
330	340	350	360	370	380	390	400
RAKIITVLTN	PADRAYSWYQ	HQRAHGDPVA	LNYTFYQVIS	ASSQTPLALR	SLQNRCLVPG	YYSTHLQRWL	TYYPSGQLLI
410	420	430	440	450	460	470	480
VDGQELRTNP	AASMESIQKF	LGITPFLNYT	RTLRFDDDKG	FWCQGLEGGK	TRCLGRSKGR	RYPDMDTESR	LFLTDFFRNH
490	500	510					
NLELSKLLSR	LGQPVPSWLR	EELQHSSLG					

Cmpd.	No. of Cmpds.	m/z meas.	Δ m/z [ppm]	z	Rt [min]	Score	P	Range	Sequence	Modification
618	1	677.3937	25.26	2	37.3	10.3	0	97-108	R.GFIHNGIMVLPR.Q	
1699	1	1045.7064	77.05	2	51.0	20.0	2	315-334	R.GAALLPRAKIITVLTNPADR.A	

Detailed Protein Report

Protein 257: E3 ubiquitin-protein ligase listerin [Homo sapiens]

**Accession:**	gi|231573214	**Score:**	30.4
**Database:**	refseq_human(refseq_human_20140103.fasta)	**MW [kDa]:**	205.0
**Database Date:**	2015-11-30	**pI:**	6.1
		**Sequence Coverage [%]:**	1.0
		**No. of unique Peptides:**	1

10	20	30	40	50	60	70	80
MDRVGGGKGP	GGSYYRVSSS	AARSRKLCPG	RVNRGLSAQS	TAATATMGGK	NKQRTKGNLR	PSNSGRAAEL	LAKEQGTVPG
**90**	**100**	**110**	**120**	**130**	**140**	**150**	**160**
FIGFGTSQSD	LGYVPAIQGA	EEIDSLVDSD	FRMVLRKLSK	KDVTTKLKAM	QEFGTMCTER	DTETVKGVLP	YWPRIFCKIS
**170**	**180**	**190**	**200**	**210**	**220**	**230**	**240**
LDHDRRVREA	TQQAFEKLIL	KVKKQLAPYL	KSLMGYWLMA	QCDTYTPAAF	AAKDAFEAAF	PPSKQPEAIA	FCKDEITSVL
**250**	**260**	**270**	**280**	**290**	**300**	**310**	**320**
QDHLIKETPD	TLSDPQTVPE	EEREAKFYRV	VTCSLLALKR	LLCLLPDNEL	DSLEEKFKSL	LSQNKFWKYG	KHSVPQIRSA
**330**	**340**	**350**	**360**	**370**	**380**	**390**	**400**
YFELVSALCQ	RIPQLMKEEA	SKVSPSVLLS	IDDSDPIVCP	ALWEAVLYTL	TTIEDCWLHV	NAKKSVFPKL	STVIREGGRG
**410**	**420**	**430**	**440**	**450**	**460**	**470**	**480**
LATVIYPYLL	PFISKLPQSI	TNPKLDFFKN	FLTSLVAGLS	TERTKTSSLE	SSAVISAFFE	CLRFIMQQNL	GEEEIEQMLV
**490**	**500**	**510**	**520**	**530**	**540**	**550**	**560**
NDQLIPFIDA	VLKDPGLQHG	QLFNHLAETL	SSWEAKADTE	KDEKTAHNLE	NVLIHFWERL	SEICVAKISE	PEADVESVLG
**570**	**580**	**590**	**600**	**610**	**620**	**630**	**640**
VSNLLQVLQK	PKSSLKSSKK	KNGKVRFADE	ILESNKENEK	CVSSEGEKIE	GWELTTEPSL	THNSSGLLSP	LRKKPLEDLV
**650**	**660**	**670**	**680**	**690**	**700**	**710**	**720**
CKLADISINY	VNERKSEQHL	RFLSTLLDSF	SSSRVFKMLL	GDEKQSIVQA	KPLEIAKLVQ	KNPAVQFLYQ	KLIGWLNEDQ
**730**	**740**	**750**	**760**	**770**	**780**	**790**	**800**
RKDFGFLVDI	LYSALRCCDN	DMERKKVLDD	LTKVDLKWNS	LLKIIEKACP	SSDKHALVTP	WLKGDILGEK	LVNLADCLCN
**810**	**820**	**830**	**840**	**850**	**860**	**870**	**880**
EDLESRVSSE	SHFSERWTLL	SLVLSQHVKN	DYLIGDVYVE	RIIVRLHETL	FKTKKLSEAE	SSDSSVSFIC	DVAYNYFSSA
**890**	**900**	**910**	**920**	**930**	**940**	**950**	**960**
KGCLLMPSSE	DLLLTLFQLC	AQSKEKTHLP	DFLICKLKNT	WLSGVNLLVH	QTDSSYKEST	FLHLSALWLK	NQVQASSLDI
**970**	**980**	**990**	**1000**	**1010**	**1020**	**1030**	**1040**
NSLQVLLSAV	DDLLNTLLES	EDSYLMGVYI	GSVMPNDSEW	EKMRQSLPMQ	WLHRPLLEGR	LSLNYECFKT	DFKEQDIKTL
**1050**	**1060**	**1070**	**1080**	**1090**	**1100**	**1110**	**1120**
PSHLCTSALL	SKMVLIALRK	ETVLENNELE	KIIAELLYSL	QWCEELDNPP	IFLIGFCEIL	QKMNITYDNL	RVLGNTSGLL
**1130**	**1140**	**1150**	**1160**	**1170**	**1180**	**1190**	**1200**
QLLFNRSREH	GTLWSLIIAK	LILSRSISSD	EVKPHYKRKE	SFFPLTEGNL	HTIQSLCPFL	SKEEKKEFSA	QCIPALLGWT
**1210**	**1220**	**1230**	**1240**	**1250**	**1260**	**1270**	**1280**
KKDLCSTNGG	FGHLAIFNSC	LQTKSIDDGE	LLHGILKIII	SWKKEHEDIF	LFSCNLSEAS	PEVLGVNIEI	IRFLSLFLKY
**1290**	**1300**	**1310**	**1320**	**1330**	**1340**	**1350**	**1360**
CSSPLAESEW	DFIMCSMLAW	LETTSENQAL	YSIPLVQLFA	CVSCDLACDL	SAFFDSTTLD	TIGNLPVNLI	SEWKEFFSQG
**1370**	**1380**	**1390**	**1400**	**1410**	**1420**	**1430**	**1440**
IHSLLLPILV	TVTGENKDVS	ETSFQNAMLK	PMCETLTYIS	KEQLLSHKLP	ARLVADQKTN	LPEYLQTLLN	TLAPLLLFRA
**1450**	**1460**	**1470**	**1480**	**1490**	**1500**	**1510**	**1520**
RPVQIAVYHM	LYKLMPELPQ	YDQDNLKSYG	DEEEEPALSP	PAALMSLLSI	QEDLLENVLG	CIPVGQIVTI	KPLSEDFCYV
**1530**	**1540**	**1550**	**1560**	**1570**	**1580**	**1590**	**1600**
LGYLLTWKLI	LTFFKAASSQ	LRALYSMYLR	KTKSLNKLLY	HLFRLMPENP	TYAETAVEVP	NKDPKTFFTE	ELQLSIRETT
**1610**	**1620**	**1630**	**1640**	**1650**	**1660**	**1670**	**1680**
MLPYHIPHLA	CSVYHMTLKD	LPAMVRLWWN	SSEKRVFNIV	DRFTSKYVSS	VLSFQEISSV	QTSTQLFNGM	TVKARATTRE
**1690**	**1700**	**1710**	**1720**	**1730**	**1740**	**1750**	**1760**
VMATYTIEDI	VIELIIQLPS	NYPLGSIIVE	SGKRVGVAVQ	QWRNWMLQLS	TYLTHQNGSI	MEGLALWKNN	VDKRFEGVED
**1770**	**1780**	**1790**	**1800**	**1810**	**1820**		
CMICFSVIHG	FNYSLPKKAC	RTCKKKFHSA	CLYKWFTSSN	KSTCPLCRET	FF		

Cmpd.	No. of	m/z meas.	Δ m/z	z	Rt	Score	P	Range	Sequence	Modification

Detailed Protein Report

	Cmpds.		[ppm]		[min]					
57	1	731.6721	-129.70	3	30.4	30.4	2	1127-1145	R.SREHGTLWSLIIAKLILSR.S	

Detailed Protein Report

Protein 258: contactin-2 precursor [Homo sapiens]

**Accession:**	gi|4827022	**Score:**	30.3
**Database:**	refseq_human(refseq_human_20140103.fasta)	**MW [kDa]:**	113.3
**Database Date:**	2015-11-30	**pI:**	8.9
		**Sequence Coverage [%]:**	3.6
		**No. of unique Peptides:**	2

10	20	30	40	50	60	70	80
MGTATRRKPH	LLLVAAVALV	SSSAWSSALG	SQTTFGPVFE	DQPLSVLFPE	ESTEEQVLLA	CRARASPPAT	YRWKMNGTEM
**90**	**100**	**110**	**120**	**130**	**140**	**150**	**160**
KLEPGSRHQL	VGGNLVIMNP	TKAQDAGVYQ	CLASNPVGTV	VSREAILRFG	FLQEFSKEER	DPVKAHEGWG	VMLPCNPPAH
**170**	**180**	**190**	**200**	**210**	**220**	**230**	**240**
YPGLSYRWLL	NEFPNFIPTD	GRHFVSQTTG	NLYIARTNAS	DLGNYSCLAT	SHMDFSTKSV	FSKFAQLNLA	AEDTRLFAPS
**250**	**260**	**270**	**280**	**290**	**300**	**310**	**320**
IKARFPAETY	ALVGQQVTLE	CFAFGNPVPR	IKWRKVDGSL	SPQWTTAEPT	LQIPSVSFED	EGTYECEAEN	SKGRDTVQGR
**330**	**340**	**350**	**360**	**370**	**380**	**390**	**400**
IIVQAQPEWL	KVISDTEADI	GSNLRWGCAA	AGKPRPTVRW	LRNGEPLASQ	NRVEVLAGDL	RFSKLSLEDS	GMYQCVAENK
**410**	**420**	**430**	**440**	**450**	**460**	**470**	**480**
HGTIYASAEL	AVQALAPDFR	LNPVRRLIPA	ARGGEILIPC	QPRAAPKAVV	LWSKGTEILV	NSSRVTVTPD	GTLIIRNISR
**490**	**500**	**510**	**520**	**530**	**540**	**550**	**560**
SDEGKYTCFA	ENFMGKANST	GILSVRDATK	ITLAPSSADI	NLGDNLTLQC	HASHDPTMDL	TFTWTLDDFP	IDFDKPGGHY
**570**	**580**	**590**	**600**	**610**	**620**	**630**	**640**
RRTNVKETIG	DLTILNAQLR	HGGKYTCMAQ	TVVDSASKEA	TVLVRGPPGP	PGGVVVRDIG	DTTIQLSWSR	GFDNHSPIAK
**650**	**660**	**670**	**680**	**690**	**700**	**710**	**720**
YTLQARTPPA	GKWKQVRTNP	ANIEGNAETA	QVLGLTPWMD	YEFRVIASNI	LGTGEPSGPS	SKIRTREAAP	SVAPSGLSGG
**730**	**740**	**750**	**760**	**770**	**780**	**790**	**800**
GGAPGELIVN	WTPMSREYQN	GDGFGYLLSF	RRQGSTHWQT	ARVPGADAQY	FVYSNESVRP	YTPFEVKIRS	YNRRGDGPES
**810**	**820**	**830**	**840**	**850**	**860**	**870**	**880**
LTALVYSAEE	EPRVAPTKVW	AKGVSSSEMN	VTWEPVQQDM	NGILLGYEIR	YWKAGDKEAA	ADRVRTAGLD	TSARVSGLHP
**890**	**900**	**910**	**920**	**930**	**940**	**950**	**960**
NTKYHVTVRA	YNRAGTGPAS	PSANATTMKP	PPRRPPGNIS	WTFSSSSLSI	KWDPVVPFRN	ESAVTGYKML	YQNDLHLTPT
**970**	**980**	**990**	**1000**	**1010**	**1020**	**1030**	**1040**
LHLTGKNWIE	IPVPEDIGHA	LVQIRTTGPG	GDGIPAEVHI	VRNGGTSMMV	ENMAVRPAPH	PGTVISHSVA	MLILIGSLEL
**1050**							

Cmpd.	No. of Cmpds.	m/z meas.	Δ m/z [ppm]	z	Rt [min]	Score	P	Range	Sequence	Modification
159	1	619.6506	-71.74	3	31.7	14.0	1	599-617	K.EATVLVRGPPGPPGGVVVR.D	
1718	1	857.4054	-57.25	2	51.4	16.3	0	685-702	R.VIASNILGTGEPSGPSSK.I	

Detailed Protein Report

Protein 259: spindle assembly abnormal protein 6 homolog [Homo sapiens]

**Accession:**	gi|35038601	**Score:**	30.3
**Database:**	refseq_human(refseq_human_20140103.fasta)	**MW [kDa]:**	74.4
**Database Date:**	2015-11-30	**pI:**	7.9
		**Sequence Coverage [%]:**	4.4
		**No. of unique Peptides:**	2

10	20	30	40	50	60	70	80
MSQVLFHQLV	PLQVKCKDCE	ERRVSIRMSI	ELQSVSNPVH	RKDLVIRLTD	DTDPFFLYNL	VISEEDFQSL	KFQQGLLVDF
**90**	**100**	**110**	**120**	**130**	**140**	**150**	**160**
LAFPQKFIDL	LQQCTQEHAK	EIPRFLLQLV	SPAAILDNSP	AFLNVVETNP	FKHLTHLSLK	LLPGNDVEIK	KFLAGCLKCS
**170**	**180**	**190**	**200**	**210**	**220**	**230**	**240**
KEEKLSLMQS	LDDATKQLDF	TRKTLAEKKQ	ELDKLRNEWA	SHTAALTNKH	SQELTNEKEK	ALQAQVQYQQ	QHEQQKKDLE
**250**	**260**	**270**	**280**	**290**	**300**	**310**	**320**
ILHQQNIHQL	QNRLSELEAA	NKDLTERKYK	GDSTIRELKA	KLSGVEEELQ	RTKQEVLSLR	RENSTLDVEC	HEKEKHVNQL
**330**	**340**	**350**	**360**	**370**	**380**	**390**	**400**
QTKVAVLEQE	IKDKDQLVLR	TKEAFDTIQE	QKVVLEENGE	KNQVQLGKLE	ATIKSLSAEL	LKANEIIKKL	QGDLKTLMGK
**410**	**420**	**430**	**440**	**450**	**460**	**470**	**480**
LKLKNTVTIQ	QEKLLAEKEE	KLQKEQKELQ	DVGQSLRIKE	QEVCKLQEQL	EATVKKLEES	KQLLKNNEKL	ITWLNKELNE
**490**	**500**	**510**	**520**	**530**	**540**	**550**	**560**
NQLVRKQDVL	GPSTTPPAHS	SSNTIRSGIS	PNLNVVDGRL	TYPTCGIGYP	VSSAFAFQNT	FPHSISAKNT	SHPGSGTKVQ
**570**	**580**	**590**	**600**	**610**	**620**	**630**	**640**
FNLQFTKPNA	SLGDVQSGAT	ISMPCSTDKE	NGENVGLESK	YLKKREDSIP	LRGLSQNLFS	NSDHQRDGTL	GALHTSSKPT
**650**	**660**						
ALPSASSAYF	PGQLPNS						

Cmpd.	No. of Cmpds.	m/z meas.	Δ m/z [ppm]	z	Rt [min]	Score	P	Range	Sequence	Modification
1496	1	829.1351	164.97	2	48.5	18.4	2	375-389	K.SLSAELLKANEIIKK.L	
239	1	801.7845	-124.85	2	32.7	11.9	0	613-626	R.GLSQNLFSNSDHQR.D	

Detailed Protein Report

Protein 260: neuroblastoma-amplified sequence [Homo sapiens]

**Accession:**	gi|41393547	**Score:**	30.3
**Database:**	refseq_human(refseq_human_20140103.fasta)	**MW [kDa]:**	268.4
**Database Date:**	2015-11-30	**pI:**	5.6
**Modification(s):**	Carbamidomethyl	**Sequence Coverage [%]:**	1.4
		**No. of unique Peptides:**	2

Quantitation

MD:MU	**Median:** 1.95	**CV:** 0.00 %	**No. of Peptides:** 1

Detailed Protein Report

10	20	30	40	50	60	70	80
MAAPESGPAL	SPGTAEGEEE	TILYDLLVNT	EWPPETEVQP	RGNQKHGASF	IITKAIRDRL	LFLRQYIWYS	PAPFLLPDGL
90	100	110	120	130	140	150	160
VRLVNKQINW	HLVLASNGKL	LAAVQDQCVE	IRSAKDDFTS	IIGKCQVPKD	PKPQWRRVAW	SYDCTLLAYA	ESTGTVRVFD
170	180	190	200	210	220	230	240
LMGSELFVIS	PASSFIGDLS	YAIAGLIFLE	YKASAQWSAE	LLVINYRGEL	RSYLVSVGTN	QSYQESHCFS	FSSHYPHGIN
250	260	270	280	290	300	310	320
TAIYHPGHRL	LLVGGCETAE	VGMSKASSCG	LSAWRVLSGS	PYYKQVTNGG	DGVTAVPKTL	GLLRMLSVKF	YSRQGQEQDG
330	340	350	360	370	380	390	400
IFKMSLSPDG	MLLAAIHFSG	KLSIWAIPSL	KQQGEWGQNE	QPGYDDLNPD	WRLSTEKRKK	IKDKESFYPL	IDVNWWADSA
410	420	430	440	450	460	470	480
VTLARCSGAL	TVSSVKTLKN	LLGKSCEWFE	PSPQVTATHD	GGFLSLECEI	KLAPKRSRLE	TRAGEEDEGE	EDSDSDYEIS
490	500	510	520	530	540	550	560
AKARYFGYIK	QGLYLVTEME	RFAPPRKRPR	TITKNYRLVS	LRSTTPEELY	QRKIESEEYE	EALSLAHTYG	LDTDLVYQRQ
570	580	590	600	610	620	630	640
WRKSAVNVAS	IQNYLSKIKK	RSWVLHECLE	RVPENVDAAK	ELLQYGLKGT	DLEALLAIGK	GADDGRFTLP	GEIDIDSISY
650	660	670	680	690	700	710	720
EELSPPDEEP	AKNKKEKELK	KRQELLKLVN	FSKLTLEQKE	LCRCRRKLLT	YLDRLATYEE	ILGVPHASEQ	RYDAEFFKKF
730	740	750	760	770	780	790	800
RNQNIVLSAR	TYAQESNVQA	LEILFTYHGS	DLLPHRLAIL	SNFPETTSPH	EYSVLLPEAC	FNGDSLMIIP	WHEHKHRAKD
810	820	830	840	850	860	870	880
WCEELACRMV	VEPNLQDESE	FLYAAQPELL	RFRMTQLTVE	KVMDWYQTRA	EEIEHYARQV	DCALSLIRLG	MERNIPGLLV
890	900	910	920	930	940	950	960
LCDNLVTLET	LVYEARCDVT	LTLKELQQMK	DIEKLRLLMN	SCSEDKYVTS	AYQWMVPFLH	RCEKQSPGVA	NELLKEYLVT
970	980	990	1000	1010	1020	1030	1040
LAKGDLKFPL	KIFQHSKPDL	QQKIIPDQDQ	LMAIALECIY	TCERNDQLCL	CYDLLECLPE	RGYGDKTEAT	TKLHDMVDQL
1050	1060	1070	1080	1090	1100	1110	1120
EQILSVSELL	EKHGLEKPIS	FVKNTQSSSE	EARKLMVRLT	RHTGRKQPPV	SESHWRTLLQ	DMLTMQQNVY	TCLDSDACYE
1130	1140	1150	1160	1170	1180	1190	1200
IFTESLLCSS	RLENIHLAGQ	MMHCSACSEN	PPAGIAHKGK	PHYRVSYEKS	IDLVLAASRE	YFNSSTNLTD	SCMDLARCCL
1210	1220	1230	1240	1250	1260	1270	1280
QLITDRPPAI	QEELDLIQAV	GCLEEFGVKI	LPLQVRLCPD	RISLIKECIS	QSPTCYKQST	KLLGLAELLR	VAGENPEERR
1290	1300	1310	1320	1330	1340	1350	1360
GQVLILLVEQ	ALRFHDYKAA	SMHCQELMAT	GYPKSWDVCS	QLGQSEGYQD	LATRQELMAF	ALTHCPPSSI	ELLLAASSSL
1370	1380	1390	1400	1410	1420	1430	1440
QTEILYQRVN	FQIHHEGGEN	ISASPLTSKA	VQEDEVGVPG	SNSADLLRWT	TATTMKVLSN	TTTTTKAVLQ	AVSDGQWWKK
1450	1460	1470	1480	1490	1500	1510	1520
SLTYLRPLQG	QKCGGAYQIG	TTANEDLEKQ	GCHPFYESVI	SNPFVAESEG	TYDTYQHVPV	ESFAEVLLRT	GKLAEAKNKG
1530	1540	1550	1560	1570	1580	1590	1600
EVFPTTEVLL	QLASEALPND	MTLALAYLLA	LPQVLDANRC	FEKQSPSALS	LQLAAYYYSL	QIYARLAPCF	RDKCHPLYRA
1610	1620	1630	1640	1650	1660	1670	1680
DPKELIKMVT	RHVTRHEHEA	WPEDLISLTK	QLHCYNERLL	DFTQAQILQG	LRKGVDVQRF	TADDQYKRET	ILGLAETLEE
1690	1700	1710	1720	1730	1740	1750	1760
SVYSIAISLA	QRYSVSRWEV	FMTHLEFLFT	DSGLSTLEIE	NRAQDLHLFE	TLKTDPEAFH	QHMVKYIYPT	IGGFDHERLQ
1770	1780	1790	1800	1810	1820	1830	1840
YYFTLLENCG	CADLGNCAIK	PETHIRLLKK	FKVVASGLNY	KKLTDENMSP	LEALEPVLSS	QNILSISKLV	PKIPEKDGQM
1850	1860	1870	1880	1890	1900	1910	1920
LSPSSLYTIW	LQKLFWTGDP	HLIKQVPGSS	PEWLHAYDVC	MKYFDRLHPG	DLITVVDAVT	FSPKAVTKLS	VEARKEMTRK
1930	1940	1950	1960	1970	1980	1990	2000
AIKTVKHFIE	KPRKRNSEDE	AQEAKDSKVT	YADTLNHLEK	SLAHLETLSH	SFILSLKNSE	QETLQKYSHL	YDLSRSEKEK
2010	2020	2030	2040	2050	2060	2070	2080
LHDEAVAICL	DGQPLAMIQQ	LLEVAVGPLD	ISPKDIVQSA	IMKIISALSG	GSADLGGPRD	PLKVLEGVVA	AVHASVDKGE
2090	2100	2110	2120	2130	2140	2150	2160
ELVSPEDLLE	WLRPFCADDA	WPVRPRIHVL	QILGQSFHLT	EEDSKLLVFF	RTEAILKASW	PQRQVDIADI	ENEENRYCLF
2170	2180	2190	2200	2210	2220	2230	2240

Detailed Protein Report

Cmpd.	No. of Cmpds.	m/z meas.	Δ m/z [ppm]	z	Rt [min]	Score	P	Range	Sequence	Modification	Ratios	
2612	1	1045.0458	32.19	2	62.7	16.8	1	266-284	K.ASSCGLSAWRVLSGSPYYK.Q	Carbamidomethyl: 4		
70	1	877.8816	-65.42	2	30.6	13.5	2	1987-2000	K.YSHLYDLSRSEKEK.L		MD:MU	1.95

Detailed Protein Report

Protein 261: zinc finger protein 761 [Homo sapiens]

Accession: gi|209954795
Database: refseq_human(refseq_human_20140103.fasta)
Database Date: 2015-11-30
Modification(s): Carbamidomethyl

Score: 30.3
MW [kDa]: 87.7
pI: 10.3
Sequence Coverage [%]: 3.8
No. of unique Peptides: 1

10	20	30	40	50	60	70	80
MAFSQGLLTF	RDVAIEFSQE	EWKCLDPAQR	TLYRDVMLEN	YRNLVSLDIS	SKCTMKEFLS	TAQGNREVFH	AGTLQIHESH
90	100	110	120	130	140	150	160
HNGDFCYQDV	DKDIHDYEFQ	WQEDERNGHE	APMTKIKKLT	GITERYDQSH	ARNKPIKDQL	GSSFHSHLPE	MHIFQTEEKI
170	180	190	200	210	220	230	240
DNQVVKSVHD	ASLVSTAQRI	SCRPKTHISN	NHGNNFWNSS	LLTQKQEVHM	REKSFQCNES	GKAFNYSSLL	RKHQIIHLAD
250	260	270	280	290	300	310	320
KYKCDVCGKL	FNQKRNLACH	RRCHTGENPY	KCNECGKTFS	QTSSLTCHRR	LHTGEKPYKC	EECDKAFHFK	SILERHRIIH
330	340	350	360	370	380	390	400
TEEKPYKCNE	CGKTFRQKSI	LTRHHRLHTG	EKPYKCNECG	KTFSHKSSLT	CHHRLHTGEK	PYKCNECGKT	FSHKSSLTCH
410	420	430	440	450	460	470	480
RRLHTGEKPY	KCEECDKAYS	FRSNFEIHRK	IHTEDNAYKC	NECGKTFSRT	SSLTCHRRRH	TGEQPYKCEE	CDKAFRFKSN
490	500	510	520	530	540	550	560
LERHRRIHTG	EKPYKCNECG	KTFSRKSYLT	CHHRLHTGEK	AYKCNECGKT	FSWKSSLTCH	RRLHSGEKPY	KCKECGKTFN
570	580	590	600	610	620	630	640
QQLTLKRHRR	LHSGENPYKC	EDSDKAYSFK	SNLEIHQKIH	TEENPYKCNE	CGKTFSRTSS	LTCHRRLHTG	EKPYKCEECD
650	660	670	680	690	700	710	720
KAFRVKSNLE	GHRRIHTGEK	PYKCNECGKT	FSRKSYFICH	HRLHTGEKPY	KCNECGKNFS	QKSSLICHHR	LHTGEKPYKC
730	740	750					
NECGKTFSQK	SNLTCHRRLH	TGEKQV					

Cmpd.	No. of Cmpds.	m/z meas.	Δ m/z [ppm]	z	Rt [min]	Score	P	Range	Sequence	Modification
1972	1	665.2897	-23.77	2	54.8	11.9	2	496-506	K.CNECGKTFSRK.S	Carbamidomethyl: 4

Detailed Protein Report

Protein 262: PREDICTED: calcium-dependent secretion activator 2 isoform X14 [Homo sapiens]

Accession: gi|530386656
Database: refseq_human(refseq_human_20140103.fasta)
Database Date: 2015-11-30

Score: 30.2
MW [kDa]: 142.8
pI: 5.7
Sequence Coverage [%]: 2.6
No. of unique Peptides: 2

Quantitation

MD:MU **Median:** 3.60 **CV:** 0.00 % **No. of Peptides:** 1

10	20	30	40	50	60	70	80
MLDPSSSEEE	SDEGLEEESR	DVLVAAGSSQ	RAPPAPTREG	RRDAPGRAGG	GGAARSVSPS	PSVLSEGRDE	PQRQLDDEQE
**90**	**100**	**110**	**120**	**130**	**140**	**150**	**160**
RRIRLQLYVF	VVRCIAYPFN	AKQPTDMARR	QQKLNKQQLQ	LLKERFQAFL	NGETQIVADE	AFCNAVRSYY	EVFLKSDRVA
**170**	**180**	**190**	**200**	**210**	**220**	**230**	**240**
RMVQSGGCSA	NDFREVFKKN	IEKRVRSLPE	IDGLSKETVL	SSWIAKYDAI	YRGEEDLCKQ	PNRMALSAVS	ELILSKEQLY
**250**	**260**	**270**	**280**	**290**	**300**	**310**	**320**
EMFQQILGIK	KLEHQLLYNA	CQLDNADEQA	AQIRRELDGR	LQLADKMAKE	RKFPKFIAKD	MENMYIEELR	SSVNLLMANL
**330**	**340**	**350**	**360**	**370**	**380**	**390**	**400**
ESLPVSKGGP	EFKLQKLKRS	QNSAFLDIGD	ENEIQLSKSD	VVLSFTLEIV	IMEVQGLKSV	APNRIVYCTM	EVEGEKLQTD
**410**	**420**	**430**	**440**	**450**	**460**	**470**	**480**
QAEASRPQWG	TQGDFTTTHP	RPVVKVKLFT	ESTGVLALED	KELGRVILYP	TSNSSKSAEL	HRMVVPKNSQ	DSDLKIKLAV
**490**	**500**	**510**	**520**	**530**	**540**	**550**	**560**
RMDKPAHMKH	SGYLYALGQK	VWKRWKKRYF	VLVQVSQYTF	AMCSYREKKS	EPQELMQLEG	YTVDYTDPHP	GLQGGCMFFN
**570**	**580**	**590**	**600**	**610**	**620**	**630**	**640**
AVKEGDTVIF	ASDDEQDRIL	WVQAMYRATG	QSYKPVPAIQ	TQKLNPKGGT	LHADAQLYAD	RFQKHGMDEF	ISANPCKLDH
**650**	**660**	**670**	**680**	**690**	**700**	**710**	**720**
AFLFRILQRQ	TLDHRLNDSY	SCLGWFSPGQ	VFVLDEYCAR	YGVRGCHRHL	CYLAELMEHS	ENGAVIDPTL	LHYSFAFCAS
**730**	**740**	**750**	**760**	**770**	**780**	**790**	**800**
HVHGNRPDGI	GTVSVEEKER	FEEIKERLSS	LLENQISHFR	YCFPFGRPEG	ALKATLSLLE	RVLMKDIATP	IPAEEVKKVV
**810**	**820**	**830**	**840**	**850**	**860**	**870**	**880**
RKCLEKAALI	NYTRLTEYAK	IEETMNQASP	ARKLEEILHL	AELCIEVLQQ	NEEHHAEAFA	WWPDLLAEHA	EKFWALFTVD
**890**	**900**	**910**	**920**	**930**	**940**	**950**	**960**
MDTALEAQPQ	DSWDSFPLFQ	LLNNFLRNDT	LLCNGKFHKH	LQEIFVPLVV	RYVDLMESSI	AQSIHRGFEQ	ETWQPVNNGS
**970**	**980**	**990**	**1000**	**1010**	**1020**	**1030**	**1040**
ATSEDLFWKL	DALQMFVFDL	HWPEQEFAHH	LEQRLKLMAS	DMLEACVKRT	RTAFELKLQK	ASKTTDLRIP	ASVCTMFNVL
**1050**	**1060**	**1070**	**1080**	**1090**	**1100**	**1110**	**1120**
VDAKKQSTKL	CALDGGQEQQ	YHSKIDDLID	NSVKEIISLL	VSKFVSVLEG	VLSKLSRYDE	GTFFSSILSF	TVKAAAKYVD
**1130**	**1140**	**1150**	**1160**	**1170**	**1180**	**1190**	**1200**
VPKPGMDLAD	TYIMFVRQNQ	DILREKVNEE	MYIEKLFDQW	YSSSMKVICV	WLTDRLDLQL	HIYQLKTLIK	IVKKTYRDFR
**1210**	**1220**	**1230**	**1240**	**1250**	**1260**		
LQGVLEGTLN	SKTYDTVHRR	LTVEEATASV	SEGGGLQGIT	MKDSDEEEEG			

Cmpd.	No. of Cmpds.	m/z meas.	Δ m/z [ppm]	z	Rt [min]	Score	P	Range	Sequence	Modification	Ratios
2170	1	951.3588	-156.41	2	57.0	13.7	1	446-462	R.VILYPTSNSSKSAELHR.M		MD:MU 3.60
1840	1	920.8515	-111.10	2	53.0	16.5	0	1098-1113	R.YDEGTFFSSILSFTVK.A		

Detailed Protein Report

Protein 263: **PREDICTED: glutamine-rich protein 2 isoform X2 [Homo sapiens]**

**Accession:**	gi|578831523	**Score:**	30.1
**Database:**	refseq_human(refseq_human_20140103.fasta)	**MW [kDa]:**	72.2
**Database Date:**	2015-11-30	**pI:**	9.9
		**Sequence Coverage [%]:**	3.6
		**No. of unique Peptides:**	2

10	20	30	40	50	60	70	80
MRSRPAKPTW	RRSSSCWHRW	VERLQRILEG	EGNQEAGKEL	KAGELRLQLG	VLRVTVADIE	KELAELRESQ	DRGKAAMENS
**90**	**100**	**110**	**120**	**130**	**140**	**150**	**160**
VSEASLYLQD	QLDKLRMIIE	SMLTSSSTLL	SMSMAPHKAH	TLAPGQIDPE	ATCPACSLDV	SHQVSTLVRR	YEQLQDMVNS
**170**	**180**	**190**	**200**	**210**	**220**	**230**	**240**
LAVSRPSKKA	KLQRQDEELL	GRVQSAILQV	QGDCEKLNIT	TSNLIEDHRQ	KQKDIAMLYQ	GLEKLEKEKA	NREHLEMEID
**250**	**260**	**270**	**280**	**290**	**300**	**310**	**320**
VKADKSALAT	KVSRVQFDAT	TEQLNHMMQE	LVAKMSGQEQ	DWQKMLDRLL	TEMDNKLDRL	ELDPVKQLLE	DRWKSLRQQL
**330**	**340**	**350**	**360**	**370**	**380**	**390**	**400**
RERPPLYQAD	EAAAMRRQLL	AHFHCLSCDR	PLETPVTGHA	IPVTPAGPGL	PGHHSIRPYT	VFELEQVRQH	SRNLKLGSAF
**410**	**420**	**430**	**440**	**450**	**460**	**470**	**480**
PRGDLAQMEQ	SVGRLRSMHS	KMLMNIEKVQ	IHFGGSTKAS	SQIIRELLHA	QCLGSPCYKR	VTDMADYTYS	TVPRRCGGSH
**490**	**500**	**510**	**520**	**530**	**540**	**550**	**560**
TLTYPYHRSR	PQHLPRGLYP	TEEIQIAMKH	DEVDILGLDG	HIYKGRMDTR	LPGILRKDSS	GTSKRKSQQP	RPHVHRPPSL
**570**	**580**	**590**	**600**	**610**	**620**	**630**	**640**
SSNGQLPSRP	QSAQISAGNT	SVSSRQQKDR	PSSEGRLSQP	NTAHPPSSAA	VANRGLERHV	DMPPGEGLEE	PTRGPRSSTA
**650**							
Q							

Cmpd.	No. of Cmpds.	m/z meas.	Δ m/z [ppm]	z	Rt [min]	Score	P	Range	Sequence	Modification
2513	1	410.0863	-280.49	2	61.4	19.3	1	68-74	R.ESQDRGK.A	
2139	1	937.3370	-142.40	2	56.6	10.9	1	322-337	R.ERPPLYQADEAAAMRR.Q	

Detailed Protein Report

Protein 264: **PREDICTED: ubiquitin carboxyl-terminal hydrolase 40 isoform X4 [Homo sapiens]**

**Accession:**	gi|530371419	**Score:**	30.1
**Database:**	refseq_human(refseq_human_20140103.fasta)	**MW [kDa]:**	125.8
**Database Date:**	2015-11-30	**pI:**	5.5
		**Sequence Coverage [%]:**	1.3
		**No. of unique Peptides:**	1

10	20	30	40	50	60	70	80
MRQHDVQELN	RILFSALETS	LVGTSGHDLI	YRLYHGTIVN	QIVCKECKNV	SERQEDFLDL	TVAVKNVSGL	EDALWNMYVE
90	100	110	120	130	140	150	160
EEVFDCDNLY	HCGTCDRLVK	AAKSAKLRKL	PPFLTVSLLR	FNFDFVKCER	YKETSCYTFP	LRINLKPFCE	QSELDDLEYI
170	180	190	200	210	220	230	240
YDLFSVIIHK	GGCYGGHYHV	YIKDVDHLGN	WQFQEEKSKP	DVNLKDLQSE	EEIDHPLMIL	KAILLEEENN	LIPVDQLGQK
250	260	270	280	290	300	310	320
LLKKIGISWN	KKYRKQHGPL	RKFLQLHSQI	FLLSSDESTV	RLLKNSSLQA	ESDFQRNDQQ	IFKMLPPESP	GLNNSISCPH
330	340	350	360	370	380	390	400
WFDINDSKVQ	PIREKDIEQQ	FQGKESAYML	FYRKSQLQRP	PEARANPRYG	VPCHLLNEMD	AANIELQTKR	AECDSANNTF
410	420	430	440	450	460	470	480
ELHLHLGPQY	HFFNGALHPV	VSQTESVWDL	TFDKRKTLGD	LRQSIFQLLE	FWEGDMVLSV	AKLVPAGLHI	YQSLGGDELT
490	500	510	520	530	540	550	560
LCETEIADGE	DIFVWNGVEV	GGVHIQTGID	CEPLLLNVLH	LDTSSDGEKC	CQVIESPHVF	PANAEVGTVL	TALAIPAGVI
570	580	590	600	610	620	630	640
FINSAGCPGG	EGWTAIPKED	MRKTFREQGL	RNGSSILIQD	SHDDNSLLTK	EEKWVTSMNE	IDWLHVKNLC	QLESEEKQVK
650	660	670	680	690	700	710	720
ISATVNTMVF	DIRIKAIKEL	KLMKELADNS	CLRPIDRNGK	LLCPVPDSYT	LKEAELKMGS	SLGLCLGKAP	SSSQLFLFFA
730	740	750	760	770	780	790	800
MGSDVQPGTE	MEIVVEETIS	VRDCLKLMLK	KSGLQGDAWH	LRKMDWCYEA	GEPLCEEDAT	LKELLICSGD	TLLLIEGQLP
810	820	830	840	850	860	870	880
PLGFLKVPIW	WYQLQGPSGH	WESHQDQTNC	TSSWGRVWRA	TSSQGASGNE	PAQVSLLYLG	DIEISEDATL	AELKSQAMTL
890	900	910	920	930	940	950	960
PPFLEFGVPS	PAHLRAWTVE	RKRPGRLLRT	DRQPLREYKL	GRRIEICLEP	LQKGENLGPQ	DVLLRTQVRI	PGERTYAPAL
970	980	990	1000	1010	1020	1030	1040
DLVWNAAQGG	TAGSLRQRVA	DFYRLPVEKI	EIAKYFPEKF	EWLPISSWNQ	QITKRKKKKK	QDYLQGAPYY	LKDGDTIGVK
1050	1060	1070	1080	1090	1100	1110	
NLLIDDDDDF	STIRDDTGKE	KQKQRALGRR	KSQEALHEQS	SYILSSAETP	ARPRAPETSL	SIHVGSFR	

Cmpd.	No. of Cmpds.	m/z meas.	Δ m/z [ppm]	z	Rt [min]	Score	P	Range	Sequence	Modification
2823	1	826.4280	41.02	2	65.7	30.1	0	1041-1054	K.NLLIDDDDDFSTIR.D	

Detailed Protein Report

Protein 265: PREDICTED: pericentriolar material 1 protein isoform X28 [Homo sapiens]

**Accession:**	gi|578815347	**Score:**	30.1
**Database:**	refseq_human(refseq_human_20140103.fasta)	**MW [kDa]:**	180.4
**Database Date:**	2015-11-30	**pI:**	5.3
		**Sequence Coverage [%]:**	2.1
		**No. of unique Peptides:**	2

10	20	30	40	50	60	70	80
MATGGGPFED	GMNDQDLPNW	SNENVDDRLN	NMDWGAQQKK	ANRSSEKNKK	KFGVESDKRV	TNDISPESSP	GVGRRRTKTP
**90**	**100**	**110**	**120**	**130**	**140**	**150**	**160**
HTFPHSRYMS	QMSVPEQAEL	EKLKQRINFS	DLDQRSIGSD	SQGRATAANN	KRQLSENRKP	FNFLPMQINT	NKSKDASTNP
**170**	**180**	**190**	**200**	**210**	**220**	**230**	**240**
PNRETIGSAQ	CKELFASALS	NDLLQNCQVS	EEDGRGEPAM	ESSQIVSRLV	QIRDYITKAS	SMREDLVEKN	ERSANVERLT
**250**	**260**	**270**	**280**	**290**	**300**	**310**	**320**
HLIDHLKEQE	KSYMKFLKKI	LARENEEEDV	RTIDSAVGSG	SVAESTSLNI	DVQSEASDTT	EEASFSLRIR	PCIEDKLGNS
**330**	**340**	**350**	**360**	**370**	**380**	**390**	**400**
ASQEQVSDID	VTTSPKGKGD	RPQNDRELRP	NRKYSQKRGF	PSKARDPQQE	PMEEIENLKK	QHDLLKRMLQ	QQEQLRALQG
**410**	**420**	**430**	**440**	**450**	**460**	**470**	**480**
RQAALLALQH	KAEQAIAVMD	DSVVAETAGS	LSGVSITSEL	NEELNDLIQR	FHNQLRDSQP	PAVPDNRRQA	ESLSLTREVS
**490**	**500**	**510**	**520**	**530**	**540**	**550**	**560**
QSRKPSASER	LPDEKVELFS	KMRVLQEKKQ	KMDKLLGELH	TLRDQHLNNS	SSSPQRSVDQ	RSTSAPSASV	GLAPVVNGES
**570**	**580**	**590**	**600**	**610**	**620**	**630**	**640**
NSLTSSVPYP	TASLVSQNES	ENEGHLNPSE	KLQKLNEVRK	RLNELRELVH	YYEQTSDMMT	DAVNENRKDE	ETEESEYDSE
**650**	**660**	**670**	**680**	**690**	**700**	**710**	**720**
HENSEPVTNI	RNPQVASTWN	EVNSHSNAQC	VSNNRDGRTV	NSNCEINNRS	AANIRALNMP	PSLADCRYNR	EGEQEIHVAQ
**730**	**740**	**750**	**760**	**770**	**780**	**790**	**800**
GEDDEEEEEE	AEEEGVSGAS	LSSHRSSLVD	EHPEDAEFEQ	KINRLMAAKQ	KLRQLQDLVA	MVQDDDAAQG	VISASASNLD
**810**	**820**	**830**	**840**	**850**	**860**	**870**	**880**
DFYPAEEDTK	QNSNNTRGNA	NKTQKDTGVN	EKAREKFYEA	KLQQQQRELK	QLQEERKKLI	DIQEKIQALQ	TACPDLQLSA
**890**	**900**	**910**	**920**	**930**	**940**	**950**	**960**
ASVGNCPTKK	YMPAVTSTPT	VNQHETSTSK	SVFEPEDSSI	VDNELWSEMR	RHEMLREELR	QRRKQLEALM	AEHQRRQGLA
**970**	**980**	**990**	**1000**	**1010**	**1020**	**1030**	**1040**
ETASPVAVSL	RSDGSENLCT	PQQSRTEKTM	ATWGGSTQCA	LDEEGDEDGY	LSEGIVRTDE	EEEEEQDASS	NDNFSVCPSN
**1050**	**1060**	**1070**	**1080**	**1090**	**1100**	**1110**	**1120**
SVNHNSYNGK	ETKNRWKNNC	PFSADENYRP	LAKTRQQNIS	MQRQENLRWV	SELSYVEEKE	QWQEQINQLK	KQLDFSVSIC
**1130**	**1140**	**1150**	**1160**	**1170**	**1180**	**1190**	**1200**
QTLMQDQQTL	SCLLQTLLTG	PYSVMPSNVA	SPQVHFIMHQ	LNQCYTQLTW	QQNNVQRLKQ	MLNELMRQQN	QHPEKPGGKE
**1210**	**1220**	**1230**	**1240**	**1250**	**1260**	**1270**	**1280**
RGSSASHPPS	PSLFCPFSFP	TQPVNLFNIP	GFTNFSSFAP	GMNFSPLFPS	NFGDFSQNIS	TPSEQQQPLA	QNSSGKTEYM
**1290**	**1300**	**1310**	**1320**	**1330**	**1340**	**1350**	**1360**
AFPKPFESSS	SIGAEKPRNK	KLPEEEVESS	RTPWLYEQEG	EVEKPFIKTG	FSVSVEKSTS	SNRKNQLDTN	GRRRQFDEES
**1370**	**1380**	**1390**	**1400**	**1410**	**1420**	**1430**	**1440**
LESFSSMPDP	VDPTTVTKTF	KTRKASAQAS	LASKDKTPKS	KSKKRNSTQL	KSRVKNIRYE	SASMSSTCEP	CKSRNRHSAQ
**1450**	**1460**	**1470**	**1480**	**1490**	**1500**	**1510**	**1520**
TEEPVQAKVF	SRKNHEQLEK	IIKCNRSTEI	SSAHARRILQ	QSNRNACNEA	PETGSDFSMF	EALRDTIYSE	VATLISQNES
**1530**	**1540**	**1550**	**1560**	**1570**	**1580**	**1590**	**1600**
RPHFLIELFH	ELQLLNTDYL	RQRALYALQD	IVSRHISESH	EKGENVKSVN	SGTWIASNSE	LTPSESLATT	DDVFIQEK

Cmpd.	No. of Cmpds.	m/z meas.	Δ m/z [ppm]	z	Rt [min]	Score	P	Range	Sequence	Modification
86	2	809.3666	-141.33	2	30.8	12.3	1	397-411	R.ALQGRQAALLALQHK.A	
1791	1	732.8049	117.99	3	52.4	17.8	1	1544-1562	R.ALYALQDIVSRHISESHEK.G	

Detailed Protein Report

Protein 266: ral GTPase-activating protein subunit alpha-2 [Homo sapiens]

**Accession:**	gi|118600961	**Score:**	30.0
**Database:**	refseq_human(refseq_human_20140103.fasta)	**MW [kDa]:**	210.6
**Database Date:**	2015-11-30	**pI:**	5.7
**Modification(s):**	Oxidation	**Sequence Coverage [%]:**	2.1
		**No. of unique Peptides:**	2

10	20	30	40	50	60	70	80
MFSRRSHGDV	KKSTQKVLDP	KKDVLTRLKH	LRALLDNVDA	NDLKQFFETN	YSQIYFIFYE	NFIALENSLK	LKGNNKSQRE
**90**	**100**	**110**	**120**	**130**	**140**	**150**	**160**
ELDSILFLFE	KILQFLPERI	FFRWHYQSIG	STLKKLLHTG	NSIKIRCEGI	RLFLLWLQAL	QTNCAEEQVL	IFACLVPGFP
**170**	**180**	**190**	**200**	**210**	**220**	**230**	**240**
AVMSSRGPCT	LETLINPSPS	VADVKIYPEE	ITPLLPAISG	EKIAEDQTCF	FLQILLKYMV	IQAASLEWKN	KENQDTGFKF
**250**	**260**	**270**	**280**	**290**	**300**	**310**	**320**
LFTLFRKYYL	PHLFPSFTKL	TNIYKPVLDI	PHLRPKPVYI	TTTRDNENIY	STKIPYMAAR	VVFIKWIVTF	FLEKKYLTAT
**330**	**340**	**350**	**360**	**370**	**380**	**390**	**400**
QNTKNGVDVL	PKIIQTVGGG	AVQERAPELD	GGGPTEQDKS	HSNSSTLSDR	RLSNSSLCSI	EEEHRMVYEM	VQRILLSTRG
**410**	**420**	**430**	**440**	**450**	**460**	**470**	**480**
YVNFVNEVFH	QAFLLPSCEI	AVTRKVVQVY	RKWILQDKPV	FMEEPDRKDV	AQEDAEKLGF	SETDSKEASS	ESSGHKRSSS
**490**	**500**	**510**	**520**	**530**	**540**	**550**	**560**
WGRTYSFTSA	MSRGCVTEEE	NTNVKAGVQA	LLQVFLTNSA	NIFLLEPCAE	VPVLLKEQVD	ACKAVLIIFR	RMIMELTMNK
**570**	**580**	**590**	**600**	**610**	**620**	**630**	**640**
KTWEQMLQIL	LRITEAVMQK	PKDKQIKDLF	AQSLAGLLFR	TLMVAWIRAN	LCVYISRELW	DDFLGVLSSL	TEWEELINEW
**650**	**660**	**670**	**680**	**690**	**700**	**710**	**720**
ANIMDSLTAV	LARTVYGVEM	TNLPLDKLSE	QKEKKQRGKG	CVLDPQKGTT	VGRSFSLSWR	SHPDVTEPMR	FRSATTSGAP
**730**	**740**	**750**	**760**	**770**	**780**	**790**	**800**
GVEKARNIVR	QKATEVEECQ	QSENAPAAGS	GHLTVGQQQQ	VLRSSSTSDI	PEPLCSDSSQ	GQKAENTQNS	SSSEPQPIQE
**810**	**820**	**830**	**840**	**850**	**860**	**870**	**880**
NKGHVKREHE	GITILVRRSS	SPAELDLKDD	LQQTQGKCRE	RQKSESTNSD	TTLGCTNEAE	LSMGPWQTCE	EDPELNTPTD
**890**	**900**	**910**	**920**	**930**	**940**	**950**	**960**
VVADADARHW	LQLSPTDASN	LTDSSECLTD	DCSIIAGGSL	TGWHPDSAAV	LWRRVLGILG	DVNNIQSPKI	HARVFCYLYE
**970**	**980**	**990**	**1000**	**1010**	**1020**	**1030**	**1040**
LWYKLAKIRD	NLAISLDNQS	SPSPPVLIPP	LRMFASWLFK	AATLPNEYKE	GKLQAYRLIC	AMMTRRQDVL	PNSDFLVHFY
**1050**	**1060**	**1070**	**1080**	**1090**	**1100**	**1110**	**1120**
LVMHLGLTSE	DQDILNTIIR	HCPPRFFSLG	FPGFSMLVGD	FITAAARVLS	TDILTAPRSE	AVTVLGSLVC	FPNTYQEIPL
**1130**	**1140**	**1150**	**1160**	**1170**	**1180**	**1190**	**1200**
LQSVPEVNEA	ITGTEDVKHY	LINILLKNAT	EEPNEYARCI	AVCSLGVWIC	EELAQCTSHP	QVKEAINVIG	VTLKFPNKIV
**1210**	**1220**	**1230**	**1240**	**1250**	**1260**	**1270**	**1280**
AQVACDVLQL	LVSYWEKLQM	FETSLPRKMA	EILVATVAFL	LPSAEYSSVE	TDKKFIVSLL	LCLLDWCMAL	PVSVLLHPVS
**1290**	**1300**	**1310**	**1320**	**1330**	**1340**	**1350**	**1360**
TAVLEEQHSA	RAPLLDYIYR	VLHCCVCGSS	TYTQQSHYIL	TLADLSSTDY	DPFLPLANVK	SSEPVQYHSS	AELGNLLTVE
**1370**	**1380**	**1390**	**1400**	**1410**	**1420**	**1430**	**1440**
EEKKRRSLEL	IPLTARMVMA	HLVNHLGHYP	LSGGPAILHS	LVSENHDNAH	VEGSELSFEV	FRSPNLQLFV	FNDSTLISYL
**1450**	**1460**	**1470**	**1480**	**1490**	**1500**	**1510**	**1520**
QTPTEGPVGG	SPVGSLSDVR	VIVRDISGKY	SWDGKVLYGP	LEGCLAPNGR	NPSFLISSWH	RDTFGPQKDS	SQVEEGDDVL
**1530**	**1540**	**1550**	**1560**	**1570**	**1580**	**1590**	**1600**
DKLLENIGHT	SPECLLPSQL	NLNEPSLTPC	GMNYDQEKEI	IEVILRQNAQ	EDEYIQSHNF	DSAMKVTSQG	QPSPVEPRGP
**1610**	**1620**	**1630**	**1640**	**1650**	**1660**	**1670**	**1680**
FYFCRLLLDD	LGMNSWDRRK	NFHLLKKNSK	LLRELKNLDS	RQCRETHKIA	VFYIAEGQED	KCSILSNERG	SQAYEDFVAG
**1690**	**1700**	**1710**	**1720**	**1730**	**1740**	**1750**	**1760**
LGWEVDLSTH	CGFMGGLQRN	GSTGQTAPYY	ATSTVEVIFH	VSTRMPSDSD	DSLTKKLRHL	GNDEVHIVWS	EHSRDYRRGI
**1770**	**1780**	**1790**	**1800**	**1810**	**1820**	**1830**	**1840**
IPTAFGDVSI	IIYPMKNHMF	FIAITKKPEV	PFFGPLFDGA	IVSGKLLPSL	VCATCINASR	AVKCLIPLYQ	SFYEERALYL
**1850**	**1860**	**1870**	**1880**				
EAIIQNHREV	MTFEDFAAQV	FSPSPSYSLS	GTD				

Detailed Protein Report

Cmpd.	No. of Cmpds.	m/z meas.	Δ m/z [ppm]	z	Rt [min]	Score	P	Range	Sequence	Modification
1746	3	1023.9594	-58.70	2	51.6	16.7	1	433-448	K.WILQDKPVFMEEPDRK.D	Oxidation: 10
1736	2	1273.5367	-62.63	2	51.5	13.3	0	1341-1363	K.SSEPVQYHSSAELGNLLTVEEEK.K	

Detailed Protein Report

Protein 267: polypeptide N-acetylgalactosaminyltransferase 14 isoform 3 [Homo sapiens]

**Accession:**	gi|359465585	**Score:**	29.9
**Database:**	refseq_human(refseq_human_20140103.fasta)	**MW [kDa]:**	61.5
**Database Date:**	2015-11-30	**pI:**	6.4
**Modification(s):**	Carbamidomethyl	**Sequence Coverage [%]:**	4.5
		**No. of unique Peptides:**	1

10	20	30	40	50	60	70	80
MSFPMSLARS	SVPFADSGLS	SSQPSDADWD	DLWDQFDERR	YLNAKKWRVG	DDPYKLYAFN	QRESERISSN	RAIPDTRHLR
**90**	**100**	**110**	**120**	**130**	**140**	**150**	**160**
CTLLVYCTDL	PPTSIIITFH	NEARSTLLRT	IRSVLNRTPT	HLIREIILVD	DFSNDPDDCK	QLIKLPKVKC	LRNNERQGLV
**170**	**180**	**190**	**200**	**210**	**220**	**230**	**240**
RSRIRGADIA	QGTTLTFLDS	HCEVNRDWLQ	PLLHRVKEDY	TRVVCPVIDI	INLDTFTYIE	SASELRGGFD	WSLHFQWEQL
**250**	**260**	**270**	**280**	**290**	**300**	**310**	**320**
SPEQKARRLD	PTEPIRTPII	AGGLFVIDKA	WFDYLGKYDM	DMDIWGGENF	EISFRVWMCG	GSLEIVPCSR	VGHVFRKKHP
**330**	**340**	**350**	**360**	**370**	**380**	**390**	**400**
YVFPDGNANT	YIKNTKRTAE	VWMDEYKQYY	YAARPFALER	PFGNVESRLD	LRKNLRCQSF	KWYLENIYPE	LSIPKESSIQ
**410**	**420**	**430**	**440**	**450**	**460**	**470**	**480**
KGNIRQRQKC	LESQRQNNQE	TPNLKLSPCA	KVKGEDAKSQ	VWAFTYTQQI	LQEELCLSVI	TLFPGAPVVL	VLCKNGDDRQ
**490**	**500**	**510**	**520**	**530**	**540**		
QWTKTGSHIE	HIASHLCLDT	DMFGDGTENG	KEIVVNPCES	SLMSQHWDMV	SS		

Cmpd.	No. of Cmpds.	m/z meas.	Δ m/z [ppm]	z	Rt [min]	Score	P	Range	Sequence	Modification
14	1	847.2854	-131.18	2	29.8	15.5	0	296-310	R.VWMCGGSLEIVPCSR.V	Carbamidomethyl: 13

Detailed Protein Report

Protein 268: thrombospondin type-1 domain-containing protein 7B [Homo sapiens]

Accession: gi|122937257
Database: refseq_human(refseq_human_20140103.fasta)
Database Date: 2015-11-30
Modification(s): Carbamidomethyl

Score: 29.9
MW [kDa]: 175.5
pI: 9.1
Sequence Coverage [%]: 2.4
No. of unique Peptides: 1

10	20	30	40	50	60	70	80
MHNTADEVVL	YHKLAGPWGR	CTGDCGPGGV	QSRAVWCFHV	DGWTSHLSNC	GESNRPPKER	SCFRVCDWHS	DLFQWEVSDW
90	100	110	120	130	140	150	160
HHCVLVPYAR	GEVKPRTAEC	VTAQHGLQHR	MVRCIQKLNR	TVVANEICEH	FALQPPTEQA	CLIPCPRDCV	VSEFLPWSNC
170	180	190	200	210	220	230	240
SKGCGKKLQH	RTRAVIAPPL	FGGLQCPNLT	ESRACDAPIS	CPLGEEEYTF	SLKVGPWSKC	RLPHLKEINP	SGRTVLDFNS
250	260	270	280	290	300	310	320
DSNERVTFKH	QSYKAHHHSK	SWAIEIGYQT	RQVSCTRSDG	QNAMLSLCLQ	DSFPLTVQSC	IMPKDCETSQ	WSSWSPCSKT
330	340	350	360	370	380	390	400
CRSGSLLPGF	RSRSRNVKHM	AIGGGKECPE	LLEKEACIVE	GELLQQCPRY	SWRTSEWKEC	QVSLLLEQQD	PHWHVTGPVC
410	420	430	440	450	460	470	480
GGGIQTREVY	CAQSVPAAAA	LRAKEVSRPV	EKALCVGPAP	LPSQLCNIPC	STDCIVSSWS	AWGLCIHENC	HDPQGKKGFR
490	500	510	520	530	540	550	560
TRQRHVLMES	TGPAGHCPHL	VESVPCEDPM	CYRWLASEGI	CFPDHGKCGL	GHRILKAVCQ	NDRGEDVSGS	LCPVPPPPER
570	580	590	600	610	620	630	640
KSCEIPCRMD	CVLSEWTEWS	SCSQSCSNKN	SDGKQTRSRT	ILALAGEGGK	PCPPSQALQE	HRLCNDHSCM	QLHWETSPWG
650	660	670	680	690	700	710	720
PCSEDTLVTA	LNATIGWNGE	ATCGVGIQTR	RVFCVKSHVG	QVMTKRCPDS	TRPETVRPCF	LPCKKDCIVT	AFSEWTPCPR
730	740	750	760	770	780	790	800
MCQAGNATVK	QSRYRIIIQE	AANGGQECPD	TLYEERECED	VSLCPVYRWK	PQKWSPCILV	PESVWQGITG	SSEACGKGLQ
810	820	830	840	850	860	870	880
TRAVSCISDD	NRSAEMMECL	KQTNGMPLLV	QECTVPCRED	CTFTAWSKFT	PCSTNCEATK	SRRRQLTGKS	RKKEKCQDSD
890	900	910	920	930	940	950	960
LYPLVETELC	PCDEFISQPY	GNWSDCILPE	GRREPHRGLR	VQADSKECGE	GLRFRAVACS	DKNGRPVDPS	FCSSSGYIQE
970	980	990	1000	1010	1020	1030	1040
KCVIPCPFDC	KLSDWSSWGS	CSSSCGIGVR	IRSKWLKEKP	YNGGRPCPKL	DLKNQAQVHE	AVPCYSECNQ	YSWVVEHWSS
1050	1060	1070	1080	1090	1100	1110	1120
CKINNELRSL	RCGGGTQSRK	IRCVNTADGE	GGAVDSNLCN	QDEIPPETQS	CSLMCPNECV	MSEWGLWSKC	PQSCDPHTMQ
1130	1140	1150	1160	1170	1180	1190	1200
RRTRHLLRPS	LNSRTCAEDS	QVQPCLLNEN	CFQFQYNLTE	WSTCQLSENA	PCGQGVRTRL	LSCVCSDGKP	VSMDQCEQHN
1210	1220	1230	1240	1250	1260	1270	1280
LEKPQRMSIP	CLVECVVNCQ	LSGWTAWTEC	SQTCGHGGRM	SRTRFIIMPT	QGEGRPCPTE	LTQEKTCPVT	PCYSWVLGNW
1290	1300	1310	1320	1330	1340	1350	1360
SACKLEGGDC	GEGVQIRSLS	CMVHSGSISH	AAGRVEDALC	GEMPFQDSIL	KQLCSVPCPG	DCHLTEWSEW	STCELTCIDG
1370	1380	1390	1400	1410	1420	1430	1440
RSFETVGRQS	RSRTFIIQSF	ENQDSCPQQV	LETRPCTGGK	CYHYTWKASL	WNNNERTVWC	QRSDGVNVTG	GCSPQARPAA
1450	1460	1470	1480	1490	1500	1510	1520
IRQCIPACRK	PFSYCTQGGV	CGCEKGYTEI	MKSNGFLDYC	MKVPGSEDKK	ADVKNLSGKN	RPVNSKIHDI	FKGWSLQPLD
1530	1540	1550	1560	1570	1580		
PDGRVKIWVY	GVSGGAFLIM	IFLIFTSYLV	CKKPKPHQST	PPQQKPLTLA	YDGDLDM		

Cmpd.	No. of Cmpds.	m/z meas.	Δ m/z [ppm]	z	Rt [min]	Score	P	Range	Sequence	Modification
1180	1	883.7188	-3.03	3	44.4	19.1	1	1443-1465	R.QCIPACRKPFSYCTQGGVCGCEK.G	Carbamidomethyl: 2, 6, 13

Detailed Protein Report

Protein 269: FCH domain only protein 1 isoform a [Homo sapiens]

**Accession:**	gi|239049440	**Score:**	29.9
**Database:**	refseq_human(refseq_human_20140103.fasta)	**MW [kDa]:**	97.0
**Database Date:**	2015-11-30	**pI:**	6.5
**Modification(s):**	Carbamidomethyl	**Sequence Coverage [%]:**	2.7
		**No. of unique Peptides:**	2

10	20	30	40	50	60	70	80
MSYFGEHFWG	EKNHGFEVLY	HSVKQGPIST	KELADFIRER	ATIEETYSKA	MAKLSKLASN	GTPMGTFAPL	WEVFRVSSDK
90	100	110	120	130	140	150	160
LALCHLELTR	KLQDLIKDVL	RYGEEQLKTH	KKCKEEVVST	LDAVQVLSGV	SQLLPKSREN	YLNRCMDQER	LRRESTSQKE
170	180	190	200	210	220	230	240
MDKAETKTKK	AAESLRRSVE	KYNSARADFE	QKMLDSALRF	QAMEETHLRH	MKALLGSYAH	SVEDTHVQIG	QVHEEFKQNI
250	260	270	280	290	300	310	320
ENVSVEMLLR	KFAESKGTGR	EKPGPLDFEA	YSAAALQEAM	KRLRGAKAFR	LPGLSRRERE	PEPPAAVDFL	EPDSGTCPEV
330	340	350	360	370	380	390	400
DEEGFTVRPD	VTQNSTAEPS	RFSSSDSDFD	DEEPRKFYVH	IKPAPARAPA	CSPEAAAAQL	RATAGSLILP	PGPGGTMKRH
410	420	430	440	450	460	470	480
SSRDAAGKPQ	RPRSAPRTSS	CAERLQSEEQ	VSKNLFGPPL	ESAFDHEDFT	GSSSLGFTSS	PSPFSSSSPE	NVEDSGLDSP
490	500	510	520	530	540	550	560
SHAAPGPSPD	SWVPRPGTPQ	SPPSCRAPPP	EARGIRAPPL	PDSPQPLASS	PGPWGLEALA	GGDLMPAPAD	PTAREGLAAP
570	580	590	600	610	620	630	640
PRRLRSRKVS	CPLTRSNGDL	SRSLSPSPLG	SSAASTALER	PSFLSQTGHG	VSRGPSPVVL	GSQDALPIAT	AFTEYVHAYF
650	660	670	680	690	700	710	720
RGHSPSCLAR	VTGELTMTFP	AGIVRVFSGT	PPPPVLSFRL	VHTTAIEHFQ	PNADLLFSDP	SQSDPETKDF	WLNMAALTEA
730	740	750	760	770	780	790	800
LQRQAEQNPT	ASYYNVVLLR	YQFSRPGPQS	VPLQLSAHWQ	CGATLTQVSV	EYGYRPGATA	VPTPLTNVQI	LLPVGEPVTN
810	820	830	840	850	860	870	880
VRLQPAATWN	LEEKRLTWRL	PDVSEAGGSG	RLSASWEPLS	GPSTPSPVAA	QFTSEGTTLS	GVDLELVGSG	YRMSLVKRRF
890	900						
ATAAPPQGCT	W						

Cmpd.	No. of Cmpds.	m/z meas.	Δ m/z [ppm]	z	Rt [min]	Score	P	Range	Sequence	Modification
1900	1	693.7929	-23.70	2	53.8	12.5	0	2-12	M.SYFGEHFWGEK.N	
2590	1	731.8881	54.25	2	62.9	17.4	1	879-891	R.RFATAAPPQGCTW.-	Carbamidomethyl: 11

Detailed Protein Report

Protein 270: **probable G-protein coupled receptor 27 [Homo sapiens]**

Accession: gi|9506747
Database: refseq_human(refseq_human_20140103.fasta)
Database Date: 2015-11-30
Modification(s): Carbamidomethyl

Score: 29.9
MW [kDa]: 39.8
pI: 10.7
Sequence Coverage [%]: 12.0
No. of unique Peptides: 2

10	20	30	40	50	60	70	80
MANASEPGGS	GGGEAAALGL	KLATLSLLLC	VSLAGNVLFA	LLIVRERSLH	RAPYYLLLDL	CLADGLRALA	CLPAVMLAAR
**90**	**100**	**110**	**120**	**130**	**140**	**150**	**160**
RAAAAAGAPP	GALGCKLLAF	LAALFCFHAA	FLLLGVGVTR	YLAIAHHRFY	AERLAGWPCA	AMLVCAAWAL	ALAAAFPPVL
**170**	**180**	**190**	**200**	**210**	**220**	**230**	**240**
DGGGDDEDAP	CALEQRPDGA	PGALGFLLLL	AVVVGATHLV	YLRLLFFIHD	RRKMRPARLV	PAVSHDWTFH	GPGATGQAAA
**250**	**260**	**270**	**280**	**290**	**300**	**310**	**320**
NWTAGFGRGP	TPPALVGIRP	AGPGRGARRL	LVLEEFKTEK	RLCKMFYAVT	LLFLLLWGPY	VVASYLRVLV	RPGAVPQAYL
**330**	**340**	**350**	**360**	**370**	**380**		
TASVWLTFAQ	AGINPVVCFL	FNRELRDCFR	AQFPCCQSPR	TTQATHPCDL	KGIGL		

Cmpd.	No. of Cmpds.	m/z meas.	Δ m/z [ppm]	z	Rt [min]	Score	P	Range	Sequence	Modification
1926	1	857.3073	-135.85	2	54.2	12.0	0	2-21	M.ANASEPGGSGGGEAAALGLK.L	
250	1	910.4923	61.03	3	32.8	17.9	2	351-375	R.AQFPCCQSPRTTQATHPCDLKGIGL.-	Carbamidomethyl: 6

Detailed Protein Report

Protein 271: **PREDICTED: transforming acidic coiled-coil-containing protein 3 isoform X2 [Homo sapiens]**

**Accession:**	gi|530375987	**Score:**	29.9
**Database:**	refseq_human(refseq_human_20140103.fasta)	**MW [kDa]:**	53.9
**Database Date:**	2015-11-30	**pI:**	5.2
**Modification(s):**	Carbamidomethyl	**Sequence Coverage [%]:**	5.2
		**No. of unique Peptides:**	1

10	20	30	40	50	60	70	80
MSLQVLNDKN	VSNEKNTENC	DFLFSPPEVT	GRSSVLRVSQ	KENVPPKNLA	KAMKVTFQTP	LRDPQTHRIL	SPSMASKLEA
**90**	**100**	**110**	**120**	**130**	**140**	**150**	**160**
PFTQDDTLGL	ENSHPVWTQK	EKQLHSASAE	DTPVVQLAAE	TPTAESKERA	LNSASTSLPT	SCPGSEPVPT	HQQGQPALEL
**170**	**180**	**190**	**200**	**210**	**220**	**230**	**240**
KEESFRDPAE	VLGTGAEVDY	LEQFGTSSFK	ESALRKQSLY	LKFDPLLRDS	PGRPVPVATE	TSSMHGANET	PSGRPREAKL
**250**	**260**	**270**	**280**	**290**	**300**	**310**	**320**
VEFDFLGALD	IPVPGPPPGV	PAPGGPPLST	GPIVDLLQYS	QKDLDAVVRT	QVKATQEENR	ELRSRCEELH	GKNLELGKIM
**330**	**340**	**350**	**360**	**370**	**380**	**390**	**400**
DRFEEVVYQA	MEEVQKQKEL	SKAEIQKVLK	EKDQLTTDLN	SMEKSFSDLF	KRFEKQKEVI	EGYRKNEESL	KKCVEDYLAR
**410**	**420**	**430**	**440**	**450**	**460**	**470**	**480**
ITQEGQRYQA	LKAHAEEKLQ	LANEEIAQVR	SKAQAEALAL	QASLRKEQMR	IQSLEKTVEQ	KTKENEELTR	ICDDLISKME
**490**							
KI							

Cmpd.	No. of Cmpds.	m/z meas.	Δ m/z [ppm]	z	Rt [min]	Score	P	Range	Sequence	Modification
1875	1	917.8351	-120.99	2	53.5	12.8	1	464-478	K.ENEELTRICDDLISK.M	Carbamidomethyl: 9

Detailed Protein Report

Protein 272: ATP-binding cassette sub-family A member 12 isoform b [Homo sapiens]

**Accession:**	gi|27881501	**Score:**	29.9
**Database:**	refseq_human(refseq_human_20140103.fasta)	**MW [kDa]:**	256.8
**Database Date:**	2015-11-30	**pI:**	7.6
**Modification(s):**	Oxidation	**Sequence Coverage [%]:**	1.1
		**No. of unique Peptides:**	2

Detailed Protein Report

10	20	30	40	50	60	70	80
MFTYIKIITS	GDSDNITHVW	NEDDGQTLSP	SSLAAQLLIL	ENFEDALLNI	SANSPYIPYL	ACVRNVTDSL	ARGSPENLRL
90	100	110	120	130	140	150	160
LQSTIRFKKS	FLRNGSYEDY	FPPVPEVLKS	KLSQLRNLTE	LLCESETFSL	IEKSCQLSDM	SFGSLCEESE	FDLQLLEAAE
170	180	190	200	210	220	230	240
LGTEIAASLL	YHDNVISKKV	RDLLTGDPSK	INLNMDQFLE	QALQMNYLEN	ITQLIPIIEA	MLHVNNSADA	SEKPGQLLEM
250	260	270	280	290	300	310	320
FKNVEELKED	LRRTTGMSNR	TIDKLLAIPI	PDNRAEIISQ	VFWLHSCDTN	ITTPKLEDAM	KEFCNLSLSE	RSRQSYLIGL
330	340	350	360	370	380	390	400
TLLHYLNIYN	FTYKVFFPRK	DQKPVEKMME	LFIRLKEILN	QMASGTHPLL	DKMRSLKQMH	LPRSVPLTQA	MYRSNRMNTP
410	420	430	440	450	460	470	480
QGSFSTISQA	LCSQGITTEY	LTAMLPSSQR	PKGNHTKDFL	TYKLTKEQIA	SKYGIPINST	PFCFSLYKDI	INMPAGPVIW
490	500	510	520	530	540	550	560
AFLKPMLLGR	ILYAPYNPVT	KAIMEKSNVT	LRQLAELREK	SQEWMDKSPL	FMNSFHLLNQ	AIPMLQNTLR	NPFVQVFVKF
570	580	590	600	610	620	630	640
SVGLDAVELL	KQIDELDILR	LKLENNIDII	DQLNTLSSLT	VNISSCVLYD	RIQAAKTIDE	MEREAKRLYK	SNELFGSVIF
650	660	670	680	690	700	710	720
KLPSNRSWHR	GYDSGNVFLP	PVIKYTIRMS	LKTAQTTRSL	RTKIWAPGPH	NSPSHNQIYG	RAFIYLQDSI	ERAIIELQTG
730	740	750	760	770	780	790	800
RNSQEIAVQV	QAIPYPCFMK	DNFLTSVSYS	LPIVLMVAWV	VFIAAFVKKL	VYEKDLRLHE	YMKMMGVNSC	SHFFAWLIES
810	820	830	840	850	860	870	880
VGFLLVTIVI	LIIILKFGNI	LPKTNGFILF	LYFSDYSFSV	IAMSYLISVF	FNNTNIAALI	GSLIYIIAFF	PFIVLVTVEN
890	900	910	920	930	940	950	960
ELSYVLKVFM	SLLSPTAFSY	ASQYIARYEE	QGIGLQWENM	YTSPVQDDTT	SFGWLCCLIL	ADSFIYFLIA	WYVRNVFPGT
970	980	990	1000	1010	1020	1030	1040
YGMAAPWYFP	ILPSYWKERF	GCAEVKPEKS	NGLMFTNIMM	QNTNPSASPE	YMFSSNIEPE	PKDLTVGVAL	HGVTKIYGSK
1050	1060	1070	1080	1090	1100	1110	1120
VAVDNLNLNF	YEGHITSLLG	PNGAGKTTTI	SMLTGLFGAS	AGTIFVYGKD	IKTDLHTVRK	NMGVCMQHDV	LFSYLTTKEH
1130	1140	1150	1160	1170	1180	1190	1200
LLLYGSIKVP	HWTKKQLHEE	VKRTLKDTGL	YSHRHKRVGT	LSGGMKRKLS	ISIALIGGSR	VVILDEPSTG	VDPCSRRSIW
1210	1220	1230	1240	1250	1260	1270	1280
DVISKNKTAR	TIILSTHHLD	EAEVLSDRIA	FLEQGGLRCC	GSPFYLKEAF	GDGYHLTLTK	KKSPNLNANA	VCDTMAVTAM
1290	1300	1310	1320	1330	1340	1350	1360
IQSHLPEAYL	KEDIGGELVY	VLPPFSTKVS	GAYLSLLRAL	DNGMGDLNIG	CYGISDTTVE	EVFLNLTKES	QKNSAMSLEH
1370	1380	1390	1400	1410	1420	1430	1440
LTQKKIGNSN	ANGISTPDDL	SVSSSNFTDR	DDKILTRGER	LDGFGLLLKK	IMAILIKRFH	HTRRNWKGLI	AQVILPIVFV
1450	1460	1470	1480	1490	1500	1510	1520
TTAMGLGTLR	NSSNSYPEIQ	ISPSLYGTSE	QTAFYANYHP	STEALVSAMW	DFPGIDNMCL	NTSDLQCLNK	DSLEKWNTSG
1530	1540	1550	1560	1570	1580	1590	1600
EPITNFGVCS	CSENVQECPK	FNYSPPHRRT	YSSQVIYNLT	GQRVENYLIS	TANEFVQKRY	GGWSFGLPLT	KDLRFDITGV
1610	1620	1630	1640	1650	1660	1670	1680
PANRTLAKVW	YDPEGYHSLP	AYLNSLNNFL	LRVNMSKYDA	ARHGIIMYSH	PYPGVQDQEQ	ATISSLIDIL	VALSILMGYS
1690	1700	1710	1720	1730	1740	1750	1760
VTTASFVTYV	VREHQTKAKQ	LQHISGIGVT	CYWVTNFIYD	MVFYLVPVAF	SIGIIAIFKL	PAFYSENNLG	AVSLLLLLFG
1770	1780	1790	1800	1810	1820	1830	1840
YATFSWMYLL	AGLFHETGMA	FITYVCVNLF	FGINSIVSLS	VVYFLSKEKP	NDPTLELISE	TLKRIFLIFP	QFCFGYGLIE
1850	1860	1870	1880	1890	1900	1910	1920
LSQQQSVLDF	LKAYGVEYPN	ETFEMNKLGA	MFVALVSQGT	MFFSLRLLIN	ESLIKKLRLF	FRKFNSSHVR	ETIDEDEDVR
1930	1940	1950	1960	1970	1980	1990	2000
AERLRVESGA	AEFDLVQLYC	LTKTYQLIHK	KIIAVNNISI	GIPAGECFGL	LGVNGAGKTT	IFKMLTGDII	PSSGNILIRN
2010	2020	2030	2040	2050	2060	2070	2080
KTGSLGHVDS	HSSLVGYCPQ	EDALDDLVTV	EEHLYFYARV	HGIPEKDIKE	TVHKLLRRLH	LMPFKDRATS	MCSYGTKRKL
2090	2100	2110	2120	2130	2140	2150	2160
STALALIGKP	SILLLDEPSS	GMDPKSKRHL	WKIISEEVQN	KCSVILTSHS	MEECEALCTR	LAIMVNGKFQ	CIGSLQHIKS
2170	2180	2190	2200	2210	2220	2230	2240
RFGRGFTVKV	HLKNNKVTME	TLTKFMQLHF	PKTYLKDQHL	SMLEYHVPVT	AGGVANIFDL	LETNKTALNI	TNFLVSQTTL
2250	2260	2270	2280				
EEVFINFAKD	QKSYETADTS	SQGSTISVDS	QDDQMES				

Detailed Protein Report

Cmpd.	No. of Cmpds.	m/z meas.	Δ m/z [ppm]	z	Rt [min]	Score	P	Range	Sequence	Modification
1898	1	890.3676	-104.02	2	53.6	10.7	1	341-354	K.DQKPVEKMMELFIR.L	Oxidation: 8
2766	1	702.8107	-71.83	2	65.0	19.2	1	612-623	R.IQAAKTIDEMER.E	

Detailed Protein Report

Protein 273: zinc finger C4H2 domain-containing protein isoform 2 [Homo sapiens]

**Accession:**	gi|295842335	**Score:**	29.9
**Database:**	refseq_human(refseq_human_20140103.fasta)	**MW [kDa]:**	23.5
**Database Date:**	2015-11-30	**pI:**	7.9
		**Sequence Coverage [%]:**	13.9
		**No. of unique Peptides:**	1

Alias proteins:

Accession	Name	Description
gi|345091006	refseq_human (refseq_human_20140103.fasta)	zinc finger C4H2 domain-containing protein isoform 2 [Homo sapiens]

```
        10         20         30         40         50         60         70         80
MEKIKARLKA EFEALESEER HLKEYKQEMD LLLQEKMAHV EELRLIHADI NVMENTIKQS ENDLNKLLES TRRLHDEYKP
        90        100        110        120        130        140        150        160
LKEHVDALRM TLGLQRLPDL CEEEEKLSLD YFEKQKAEWQ TEPQEPPIPE SLAAAAAAAQ QLQVARKQDT RQTATFRQQP
       170        180        190        200        210
PPMKACLSCH QQIHRNAPIC PLCKAKSRSR NPKKPKRKQD E
```

Cmpd.	No. of Cmpds.	m/z meas.	Δ m/z [ppm]	z	Rt [min]	Score	P	Range	Sequence	Modification
2312	1	1051.3738	-133.19	2	58.8	13.3	1	158-175	R.QQPPPMKACLSCHQQIHR.N	

Detailed Protein Report

Protein 274: PREDICTED: uncharacterized protein LOC102724905 [Homo sapiens]

Accession: gi|578835678
Database: refseq_human(refseq_human_20140103.fasta)
Database Date: 2015-11-30
Modification(s): Carbamidomethyl

Score: 29.8
MW [kDa]: 22.0
pI: 10.3
Sequence Coverage [%]: 8.4
No. of unique Peptides: 1

10	20	30	40	50	60	70	80
MKCSTSNCII	SIKDYTSIRI	NVAEVDKVTS	RFNGQFKTYA	FGGSPGTGVT	GGKGREGGRS	FCAGEGNERN	GKGTAGCEAR
**90**	**100**	**110**	**120**	**130**	**140**	**150**	**160**
DVPGSAAGPA	RGRAAPLHPA	PGPPLRGAIL	QAAVAGTGGG	GCSGHCGAEA	RRQDGNHASC	SQLGLDSTNG	DFSYGFELGP
**170**	**180**	**190**	**200**	**210**	**220**		
WCISKCNSAN	CNGKHMDRST	PHHLGAKRCR	LQASSLLENR	GLQALEEPAD	PGLLA		

Cmpd.	No. of Cmpds.	m/z meas.	Δ m/z [ppm]	z	Rt [min]	Score	P	Range	Sequence	Modification
2384	1	1045.1618	131.45	2	60.1	16.4	2	2-19	M.KCSTSNCIISIKDYTSIR.I	Carbamidomethyl: 7

Detailed Protein Report

Protein 275: structural maintenance of chromosomes protein 4 isoform 2 [Homo sapiens]

**Accession:**	gi|570700827	**Score:**	29.7
**Database:**	refseq_human(refseq_human_20140103.fasta)	**MW [kDa]:**	144.4
**Database Date:**	2015-11-30	**pI:**	6.7
		**Sequence Coverage [%]:**	2.3
		**No. of unique Peptides:**	1

10	20	30	40	50	60	70	80
MPRKGTQPST	ARRREEGPPP	PSPDGASSDA	EPEPPSGRTE	SPATAAAMTN	EAGAPRLMIT	HIVNQNFKSY	AGEKILGPFH
**90**	**100**	**110**	**120**	**130**	**140**	**150**	**160**
KRFSCIIGPN	GSGKSNVIDS	MLFVFGYRAQ	KIRSKKLSVL	IHNSDEHKDI	QSCTVEVHFQ	KIIDKEGDDY	EVIPNSNFYV
**170**	**180**	**190**	**200**	**210**	**220**	**230**	**240**
SRTACRDNTS	VYHISGKKKT	FKDVGNLLRS	HGIDLDHNRF	LILQGEVEQI	AMMKPKGQTE	HDEGMLEYLE	DIIGCGRLNE
**250**	**260**	**270**	**280**	**290**	**300**	**310**	**320**
PIKVLCRRVE	ILNEHRGEKL	NRVKMVEKEK	DALEGEKNIA	IEFLTLENEI	FRKKNHVCQY	YIYELQKRIA	EMETQKEKIH
**330**	**340**	**350**	**360**	**370**	**380**	**390**	**400**
EDTKEINEKS	NILSNEMKAK	NKDVKDTEKK	LNKITKFIEE	NKEKFTQLDL	EDVQVREKLK	HATSKAKKLE	KQLQKDKEKV
**410**	**420**	**430**	**440**	**450**	**460**	**470**	**480**
EEFKSIPAKS	NNIINETTTR	NNALEKEKEK	EEKKLKEVMD	SLKQETQGLQ	KEKESREKEL	MGFSKSVNEA	RSKMDVAQSE
**490**	**500**	**510**	**520**	**530**	**540**	**550**	**560**
LDIYLSRHNT	AVSQLTKAKE	ALIAASETLK	ERKAAIRDIE	GKLPQTEQEL	KEKEKELQKL	TQEETNFKSL	VHDLFQKVEE
**570**	**580**	**590**	**600**	**610**	**620**	**630**	**640**
AKSSLAMNRS	RGKVLDAIIQ	EKKSGRIPGI	YGRLGDLGAI	DEKYDVAISS	CCHALDYIVV	DSIDIAQECV	NFLKRQNIGV
**650**	**660**	**670**	**680**	**690**	**700**	**710**	**720**
ATFIGLDKMA	VWAKKMTEIQ	TPENTPRLFD	LVKVKDEKIR	QAFYFALRDT	LVADNLDQAT	RVAYQKDRRW	RVVTLQGQII
**730**	**740**	**750**	**760**	**770**	**780**	**790**	**800**
EQSGTMTGGG	SKVMKGRMGS	SLVIEISEEE	VNKMESQLQN	DSKKAMQIQE	QKVQLEERVV	KLRHSEREMR	NTLEKFTASI
**810**	**820**	**830**	**840**	**850**	**860**	**870**	**880**
QRLIEQEEYL	NVQVKELEAN	VLATAPDKKK	QKLLEENVSA	FKTEYDAVAE	KAGKVEAEVK	RLHNTIVEIN	NHKLKAQQDK
**890**	**900**	**910**	**920**	**930**	**940**	**950**	**960**
LDKINKQLDE	CASAITKAQV	AIKTADRNLQ	KAQDSVLRTE	KEIKDTEKEV	DDLTAELKSL	EDKAAEVVKN	TNAAEESLPE
**970**	**980**	**990**	**1000**	**1010**	**1020**	**1030**	**1040**
IQKEHRNLLQ	ELKVIQENEH	ALQKDALSIK	LKLEQIDGHI	AEHNSKIKYW	HKEISKISLH	PIEDNPIEEI	SVLSPEDLEA
**1050**	**1060**	**1070**	**1080**	**1090**	**1100**	**1110**	**1120**
IKNPDSITNQ	IALLEARCHE	MKPNLGAIAE	YKKKEELYLQ	RVAELDKITY	ERDSFRQAYE	DLRKQRLNEF	MAGFYIITNK
**1130**	**1140**	**1150**	**1160**	**1170**	**1180**	**1190**	**1200**
LKENYQMLTL	GGDAELELVD	SLDPFSEGIM	FSVRPPKKSW	KKIFNLSGGE	KTLSSLALVF	ALHHYKPTPL	YFMDEIDAAL
**1210**	**1220**	**1230**	**1240**	**1250**	**1260**	**1270**	
DFKNVSIVAF	YIYEQTKNAQ	FIIISLRNNM	FEISDRLIGI	YKTYNITKSV	AVNPKEIASK	GLC	

Cmpd.	No. of Cmpds.	m/z meas.	Δ m/z [ppm]	z	Rt [min]	Score	P	Range	Sequence	Modification
289	4	862.8586	-89.06	2	33.0	18.8	2	457-471	R.EKELMGFSKSVNEAR.S	

Detailed Protein Report

Protein 276: PREDICTED: integrin beta-4 isoform X9 [Homo sapiens]

**Accession:**	gi|578830859	**Score:**	29.7
**Database:**	refseq_human(refseq_human_20140103.fasta)	**MW [kDa]:**	198.8
**Database Date:**	2015-11-30	**pI:**	5.8
**Modification(s):**	Carbamidomethyl	**Sequence Coverage [%]:**	1.2
		**No. of unique Peptides:**	1

10	20	30	40	50	60	70	80
MVVAVRNRAG	SEGLESPSWG	LREGQQEGQE	GRRKRMAGPR	PSPWARLLLA	ALISVSLSGT	LANRCKKAPV	KSCTECVRVD
90	100	110	120	130	140	150	160
KDCAYCTDEM	FRDRRCNTQA	ELLAAGCQRE	SIVVMESSFQ	ITEETQIDTT	LRRSQMSPQG	LRVRLRPGEE	RHFELEVFEP
170	180	190	200	210	220	230	240
LESPVDLYIL	MDFSNSMSDD	LDNLKKMGQN	LARVLSQLTS	DYTIGFGKFV	DKVSVPQTDM	RPEKLKEPWP	NSDPPFSFKN
250	260	270	280	290	300	310	320
VISLTEDVDE	FRNKLQGERI	SGNLDAPEGG	FDAILQTAVC	TRDIGWRPDS	THLLVFSTES	AFHYEADGAN	VLAGIMSRND
330	340	350	360	370	380	390	400
ERCHLDTTGT	YTQYRTQDYP	SVPTLVRLLA	KHNIIPIFAV	TNYSYSYYEK	LHTYFPVSSL	GVLQEDSSNI	VELLEEAFNR
410	420	430	440	450	460	470	480
IRSNLDIRAL	DSPRGLRTEV	TSKMFQKTRT	GSFHIRRGEV	GIYQVQLRAL	EHVDGTHVCQ	LPEDQKGNIH	LKPSFSDGLK
490	500	510	520	530	540	550	560
MDAGIICDVC	TCELQKEVRS	ARCSFNGDFV	CGQCVCSEGW	SGQTCNCSTG	SLSDIQPCLR	EGEDKPCSGR	GECQCGHCVC
570	580	590	600	610	620	630	640
YGEGRYEGQF	CEYDNFQCPR	TSGFLCNDRG	RCSMGQCVCE	PGWTGPSCDC	PLSNATCIDS	NGGICNGRGH	CECGRCHCHQ
650	660	670	680	690	700	710	720
QSLYTDTICE	INYSAIHPGL	CEDLRSCVQC	QAWGTGEKKG	RTCEECNFKV	KMVDELKRAE	EVVVRCSFRD	EDDDCTYSYT
730	740	750	760	770	780	790	800
MEGDGAPGPN	STVLVHKKKD	CPPGSFWWLI	PLLLLLLPLL	ALLLLLCWKY	CACCKACLAL	LPCCNRGHMV	GFKEDHYMLR
810	820	830	840	850	860	870	880
ENLMASDHLD	TPMLRSGNLK	GRDVVRWKVT	NNMQRPGFAT	HAASINPTEL	VPYGLSLRLA	RLCTENLLKP	DTRECAQLRQ
890	900	910	920	930	940	950	960
EVEENLNEVY	RQISGVHKLQ	QTKFRQQPNA	GKKQDHTIVD	TVLMAPRSAK	PALLKLTEKQ	VEQRAFHDLK	VAPGYYTLTA
970	980	990	1000	1010	1020	1030	1040
DQDARGMVEF	QEGVELVDVR	VPLFIRPEDD	DEKQLLVEAI	DVPAGTATLG	RRLVNITIIK	EQARDVVSFE	QPEFSVSRGD
1050	1060	1070	1080	1090	1100	1110	1120
QVARIPVIRR	VLDGGKSQVS	YRTQDGTAQG	NRDYIPVEGE	LLFQPGEAWK	ELQVKLLELQ	EVDSLLRGRQ	VRRFHVQLSN
1130	1140	1150	1160	1170	1180	1190	1200
PKFGAHLGQP	HSTTIIIRDP	DELDRSFTSQ	MLSSQPPPHG	DLGAPQNPNA	KAAGSRKIHF	NWLPPSGKPM	GYRVKYWIQG
1210	1220	1230	1240	1250	1260	1270	1280
DSESEAHLLD	SKVPSVELTN	LYPYCDYEMK	VCAYGAQGEG	PYSSLVSCRT	HQEVPSEPGR	LAFNVVSSTV	TQLSWAEPAE
1290	1300	1310	1320	1330	1340	1350	1360
TNGEITAYEV	CYGLVNDDNR	PIGPMKKVLV	DNPKNRMLLI	ENLRESQPYR	YTVKARNGAG	WGPEREAIIN	LATQPKRPMS
1370	1380	1390	1400	1410	1420	1430	1440
IPIIPDIPIV	DAQSGEDYDS	FLMYSDDVLR	SPSGSQRPSV	SDDTEHLVNG	RMDFAFPGST	NSLHRMTTTS	AAAYGTHLSP
1450	1460	1470	1480	1490	1500	1510	1520
HVPHRVLSTS	STLTRDYNSL	TRSEHSHSTT	LPRDYSTLTS	VSSHDSRLTA	GVPDTPTRLV	FSALGPTSLR	VSWQEPRCER
1530	1540	1550	1560	1570	1580	1590	1600
PLQGYSVEYQ	LLNGGELHRL	NIPNPAQTSV	VVEDLLPNHS	YVFRVRAQSQ	EGWGREREGV	ITIESQVHPQ	SPLCPLPGSA
1610	1620	1630	1640	1650	1660	1670	1680
FTLSTPSAPG	PLVFTALSPD	SLQLSWERPR	RPNGDIVGYL	VTCEMAQGGG	PATAFRVDGD	SPESRLTVPG	LSENVPYKFK
1690	1700	1710	1720	1730	1740	1750	1760
VQARTTEGFG	PEREGIITIE	SQDGGPFPQL	GSRAGLFQHP	LQSEYSSITT	THTSATEPFL	VDGLTLGAQH	LEAGGSLTRH
1770	1780	1790					
VTQEFVSRTL	TTSGTLSTHM	DQQFFQT					

Cmpd.	No. of	m/z meas.	Δ m/z	z	Rt	Score	P	Range	Sequence	Modification

Detailed Protein Report

	Cmpds.		[ppm]		[min]					
2157	1	841.3003	-27.93	2	57.2	16.5	1	82-94	K.DCAYCTDEMFRDR.R	Carbamidomethyl: 2

Detailed Protein Report

Protein 277: zinc-binding alcohol dehydrogenase domain-containing protein 2 [Homo sapiens]

**Accession:**	gi|28557745	**Score:**	29.7
**Database:**	refseq_human(refseq_human_20140103.fasta)	**MW [kDa]:**	40.1
**Database Date:**	2015-11-30	**pI:**	9.2
**Modification(s):**	Oxidation	**Sequence Coverage [%]:**	9.0
		**No. of unique Peptides:**	2

Quantitation

MD:MU **Median:** 0.80 **CV:** 0.00 % **No. of Peptides:** 1

```
        10         20         30         40         50         60         70         80
MLRLVPTGAR AIVDMSYARH FLDFQGSAIP QAMQKLVVTR LSPNFREAVT LSRDCPVPLP GDGDLLVRNR FVGVNASDIN
        90        100        110        120        130        140        150        160
YSAGRYDPSV KPPFDIGFEG IGEVVALGLS ASARYTVGQA VAYMAPGSFA EYTVVPASIA TPVPSVKPEY LTLLVSGTTA
       170        180        190        200        210        220        230        240
YISLKELGGL SEGKKVLVTA AAGGTGQFAM QLSKKAKCHV IGTCSSDEKS AFLKSLGCDR PINYKTEPVG TVLKQEYPEG
       250        260        270        280        290        300        310        320
VDVVYESVGG AMFDLAVDAL ATKGRLIVIG FISGYQTPTG LSPVKAGTLP AKLLKKSASV QGFFLNHYLS KYQAAMSHLL
       330        340        350        360        370        380
EMCVSGDLVC EVDLGDLSPE GRFTGLESIF RAVNYMYMGK NTGKIVVELP HSVNSKL
```

Cmpd.	No. of Cmpds.	m/z meas.	Δ m/z [ppm]	z	Rt [min]	Score	P	Range	Sequence	Modification	Ratios
40	4	1068.4865	-82.29	2	29.9	13.9	2	1-19	-.MLRLVPTGARAIVDMSYAR.H	Oxidation: 1	
117	1	849.3965	-46.47	2	30.9	15.8	0	297-311	K.SASVQGFFLNHYLSK.Y		MD:MU 0.80

Detailed Protein Report

Protein 278: cyclic nucleotide-gated cation channel beta-1 isoform c [Homo sapiens]

**Accession:**	gi|554790418	**Score:**	29.7
**Database:**	refseq_human(refseq_human_20140103.fasta)	**MW [kDa]:**	139.1
**Database Date:**	2015-11-30	**pI:**	4.6
		**Sequence Coverage [%]:**	1.9
		**No. of unique Peptides:**	1

10	20	30	40	50	60	70	80
MLGWVQRVLP	QPPGTPRKTK	MQEEEEVEPE	PEMEAAEVEPE	PNPEEAETES	ESMPPEESFK	EEEVAVADPS	PQETKEAALT
90	100	110	120	130	140	150	160
STISLRAQGA	EISEMNSPSR	RVLTWLMKGV	EKVIPQPVHS	ITEDPAQILG	HGSTGDTGCT	DEPNEALEAQ	DTRPGLRLLL
170	180	190	200	210	220	230	240
WLEQNLERVL	PQPPKSSEVW	RDEPAVATAP	PGRPQEMGPK	LQARETPSLP	TPIPLQPKEE	PKEAPAPEPQ	PGSQAQTSSL
250	260	270	280	290	300	310	320
PPTRDPARLV	AWVLHRLEMA	LPQPVLHGKI	GEQEPDSPGI	CDVQTISILP	GGQVEPDLVL	EEVEPPWEDA	HQDVSTSPQG
330	340	350	360	370	380	390	400
TEVVPAYEEE	NKAVEKMPRE	LSRIEEEKED	EEEEEEEEEE	EEEEEVTEVL	LDSCVVSQVG	VGQSEEDGTR	PQSTSDQKLW
410	420	430	440	450	460	470	480
EEVGEEAKKE	AEEKAKEEAE	EVAEEEAEKE	PQDWAETKEE	PEAEAEAASS	GVPATKQHPE	VQVEDTDADS	CPLMAEENPP
490	500	510	520	530	540	550	560
STVLPPPSPA	KSDTLIVPSS	ASGTHRKKLP	SEDDEAEELK	ALSPAESPVV	AWSDPTTPKD	TDGQDRAAST	ASTNSAIIND
570	580	590	600	610	620	630	640
RLQELVKLFK	ERTEKVKEKL	IDPDVTSDEE	SPKPSPAKKA	PEPAPDTKPA	EAEPVEEEHY	CDMLCCKFKH	RPWKKYQFPQ
650	660	670	680	690	700	710	720
SIDPLTNLMY	VLWLFFVVMA	WNWNCWLIPV	RWAFPYQTPD	NIHHWLLMDY	LCDLIYFLDI	TVFQTRLQFV	RGGDIITDKK
730	740	750	760	770	780	790	800
DMRNNYLKSR	RFKMDLLSLL	PLDFLYLKVG	VNPLLRLPRC	LKYMAFFEFN	SRLESILSKA	YVYRVIRTTA	YLLYSLHLNS
810	820	830	840	850	860	870	880
CLYYWASAYQ	GLGSTHWVYD	GVGNSYIRCY	YFAVKTLITI	GGLPDPKTLF	EIVFQLLNYF	TGVFAFSVMI	GQMRDVVGAA
890	900	910	920	930	940	950	960
TAGQTYYRSC	MDSTVKYMNF	YKIPKSVQNR	VKTWYEYTWH	SQGMLDESEL	MVQLPDKMRL	DLAIDVNYNI	VSKVALFQGC
970	980	990	1000	1010	1020	1030	1040
DRQMIFDMLK	RLRSVVYLPN	DYVCKKGEIG	REMYIIQAGQ	VQVLGGPDGK	SVLVTLKAGS	VFGEISLLAV	GGGNRRTANV
1050	1060	1070	1080	1090	1100	1110	1120
VAHGFTNLFI	LDKKDLNEIL	VHYPESQKLL	RKKARRMLRS	NNKPKEEKSV	LILPPRAGTP	KLFNAALAMT	GKMGGKGAKG
1130	1140	1150	1160	1170	1180	1190	1200
GKLAHLRARL	KELAALEAAA	KQQELVEQAK	SSQDVKGEEG	SAAPDQHTHP	KEAATDPPAP	RTPPEPPGSP	PSSPPPASLG
1210	1220	1230	1240	1250			
RPEGEEEGPA	EPEEHSVRIC	MSPGPEPGEQ	ILSVKMPEER	EEKAE			

Cmpd.	No. of Cmpds.	m/z meas.	Δ m/z [ppm]	z	Rt [min]	Score	P	Range	Sequence	Modification
1512	1	851.5801	69.44	1	48.6	11.1	1	1120-1127	K.GGKLAHLR.A	

Detailed Protein Report

Protein 279: transient receptor potential cation channel subfamily V member 5 [Homo sapiens]

**Accession:**	gi|17505200	**Score:**	29.6
**Database:**	refseq_human(refseq_human_20140103.fasta)	**MW [kDa]:**	82.5
**Database Date:**	2015-11-30	**pI:**	6.0
		**Sequence Coverage [%]:**	3.0
		**No. of unique Peptides:**	1

10	20	30	40	50	60	70	80
MGGFLPKAEG	PGSQLQKLLP	SFLVREQDWD	QHLDKLHMLQ	QKRILESPLL	RASKENDLSV	LRQLLLDCTC	DVRQRGALGE
90	100	110	120	130	140	150	160
TALHIAALYD	NLEAALVLME	AAPELVFEPT	TCEAFAGQTA	LHIAVVNQNV	NLVRALLTRR	ASVSARATGT	AFRRSPRNLI
170	180	190	200	210	220	230	240
YFGEHPLSFA	ACVNSEEIVR	LLIEHGADIR	AQDSLGNTVL	HILILQPNKT	FACQMYNLLL	SYDGHGDHLQ	PLDLVPNHQG
250	260	270	280	290	300	310	320
LTPFKLAGVE	GNTVMFQHLM	QKRRHIQWTY	GPLTSILYDL	TEIDSWGEEL	SFLELVVSSD	KREARQILEQ	TPVKELVSFK
330	340	350	360	370	380	390	400
WNKYGRPYFC	ILAALYLLYM	ICFTTCCVYR	PLKFRGGNRT	HSRDITILQQ	KLLQEAYETR	EDIIRLVGEL	VSIVGAVIIL
410	420	430	440	450	460	470	480
LLEIPDIFRV	GASRYFGKTI	LGGPFHVIII	TYASLVLVTM	VMRLTNTNGE	VVPMSFALVL	GWCSVMYFTR	GFQMLGPFTI
490	500	510	520	530	540	550	560
MIQKMIFGDL	MRFCWLMAVV	ILGFASAFYI	IFQTEDPTSL	GQFYDYPMAL	FTTFELFLTV	IDAPANYDVD	LPFMFSIVNF
570	580	590	600	610	620	630	640
AFAIIATLLM	LNLFIAMMGD	THWRVAQERD	ELWRAQVVAT	TVMLERKLPR	CLWPRSGICG	CEFGLGDRWF	LRVENHNDQN
650	660	670	680	690	700	710	720
PLRVLRYVEV	FKNSDKEDDQ	EHPSEKQPSG	AESGTLARAS	LALPTSSLSR	TASQSSSHRG	WEILRQNTLG	HLNLGLNLSE
730							
GDGEEVYHF							

Cmpd.	No. of Cmpds.	m/z meas.	Δ m/z [ppm]	z	Rt [min]	Score	P	Range	Sequence	Modification
87	1	829.3594	10.76	2	30.8	10.5	1	653-666	K.NSDKEDDQEHPSEK.Q	

Detailed Protein Report

Protein 280: spindle and kinetochore-associated protein 1 [Homo sapiens]

**Accession:**	gi|21450832	**Score:**	29.6
**Database:**	refseq_human(refseq_human_20140103.fasta)	**MW [kDa]:**	29.5
**Database Date:**	2015-11-30	**pI:**	6.8
**Modification(s):**	Carbamidomethyl	**Sequence Coverage [%]:**	12.5
		**No. of unique Peptides:**	1

Alias proteins:

Accession	Name	Description
gi|88014625	refseq_human (refseq_human_20140103.fasta)	spindle and kinetochore-associated protein 1 [Homo sapiens]

```
        10         20         30         40         50         60         70         80
MASSDLEQLC SHVNEKIGNI KKTLSLRNCG QEPTLKTVLN KIGDEIIVIN ELLNKLELEI QYQEQTNNSL KELCESLEED
        90        100        110        120        130        140        150        160
YKDIEHLKEN VPSHLPQVTV TQSCVKGSDL DPEEPIKVEE PEPVKKPPKE QRSIKEMPFI TCDEFNGVPS YMKSRLTYNQ
       170        180        190        200        210        220        230        240
INDVIKEINK AVISKYKILH QPKKSMNSVT RNLYHRFIDE ETKDTKGRYF IVEADIKEFT TLKADKKFHV LLNILRHCRR
       250        260
LSEVRGGGLT RYVIT
```

Cmpd.	No. of Cmpds.	m/z meas.	Δ m/z [ppm]	z	Rt [min]	Score	P	Range	Sequence	Modification
1627	1	853.3841	-128.05	2	50.1	16.5	2	227-239	K.KFHVLLNILRHCR.R	Carbamidomethyl: 12

Detailed Protein Report

Protein 281: probable ATP-dependent RNA helicase DDX46 [Homo sapiens]

**Accession:**	gi|41327773	**Score:**	29.6
**Database:**	refseq_human(refseq_human_20140103.fasta)	**MW [kDa]:**	117.3
**Database Date:**	2015-11-30	**pI:**	9.9
**Modification(s):**	Carbamidomethyl, Oxidation	**Sequence Coverage [%]:**	3.0
		**No. of unique Peptides:**	2

```
        10         20         30         40         50         60         70         80
MGRESRHYRK RSASRGRSGS RSRSRSPSDK RSKRGDDRRS RSRDRDRRRE RSRSRDKRRS RSRDRKRLRR SRSRERDRSR
        90        100        110        120        130        140        150        160
ERRRSRSRDR RRSRSRSRGR RSRSSSPGNK SKKTENRSRS KEKTDGGESS KEKKKDKDDK EDEKEKDAGN FDQNKLEEEM
       170        180        190        200        210        220        230        240
RKRKERVEKW REEQRKKAME NIGELKKEIE EMKQGKKWSL EDDDDDEDDP AEAEKEGNEM EGEELDPLDA YMEEVKEEVK
       250        260        270        280        290        300        310        320
KFNMRSVKGG GGNEKKSGPT VTKVVTVVTT KKAVVDSDKK KGELMENDQD AMEYSSEEEE VDLQTALTGY QTKQRKLLEP
       330        340        350        360        370        380        390        400
VDHGKIEYEP FRKNFYVEVP ELAKMSQEEV NVFRLEMEGI TVKGKGCPKP IKSWVQCGIS MKILNSLKKH GYEKPTPIQT
       410        420        430        440        450        460        470        480
QAIPAIMSGR DLIGIAKTGS GKTIAFLLPM FRHIMDQRSL EEGEGPIAVI MTPTRELALQ ITKECKKFSK TLGLRVVCVY
       490        500        510        520        530        540        550        560
GGTGISEQIA ELKRGAEIIV CTPGRMIDML AANSGRVTNL RRVTYVVLDE ADRMFDMGFE PQVMRIVDNV RPDRQTVMFS
       570        580        590        600        610        620        630        640
ATFPRAMEAL ARRILSKPIE VQVGGRSVVC SDVEQQVIVI EEEKKFLKLL ELLGHYQESG SVIIFVDKQE HADGLLKDLM
       650        660        670        680        690        700        710        720
RASYPCMSLH GGIDQYDRDS IINDFKNGTC KLLVATSVAA RGLDVKHLIL VVNYSCPNHY EDYVHRAGRT GRAGNKGYAY
       730        740        750        760        770        780        790        800
TFITEDQARY AGDIIKALEL SGTAVPPDLE KLWSDFKDQQ KAEGKIIKKS SGFSGKGFKF DETEQALANE RKKLQKAALG
       810        820        830        840        850        860        870        880
LQDSDDEDAA VDIDEQIESM FNSKKRVKDM AAPGTSSVPA PTAGNAEKLE IAKRLALRIN AQKNLGIESQ DVMQQATNAI
       890        900        910        920        930        940        950        960
LRGGTILAPT VSAKTIAEQL AEKINAKLNY VPLEKQEEER QDGGQNESFK RYEEELEIND FPQTARWKVT SKEALQRISE
       970        980        990       1000       1010       1020       1030       1040
YSEAAITIRG TYFPPGKEPK EGERKIYLAI ESANELAVQK AKAEITRLIK EELIRLQNSY QPTNKGRYKV L
```

Cmpd.	No. of Cmpds.	m/z meas.	Δ m/z [ppm]	z	Rt [min]	Score	P	Range	Sequence	Modification
1641	1	1052.5617	14.08	2	50.3	18.9	2	364-382	K.GKGCPKPIKSWVQCGISMK.I	Carbamidomethyl: 4
1054	1	752.4550	172.69	2	42.8	10.7	0	534-545	R.MFDMGFEPQVMR.I	Oxidation: 1

Detailed Protein Report

Protein 282: ATP-binding cassette sub-family A member 13 [Homo sapiens]

**Accession:**	gi|31657092	**Score:**	29.6
**Database:**	refseq_human(refseq_human_20140103.fasta)	**MW [kDa]:**	575.8
**Database Date:**	2015-11-30	**pI:**	6.0
		**Sequence Coverage [%]:**	0.5
		**No. of unique Peptides:**	1

Detailed Protein Report

10	20	30	40	50	60	70	80
MGHAGCQFKA	LLWKNWLCRL	RNPVLFLAEF	FWPCILFVIL	TVLRFQEPPR	YRDICYLQPR	DLPSCGVIPF	VQSLLCNTGS
90	100	110	120	130	140	150	160
RCRNFSYEGS	MEHHFRLSRF	QTAADPKKVN	NLAFLKEIQD	LAEEIHGMMD	KAKNLKRLWV	ERSNTPDSSY	GSSFFTMDLN
170	180	190	200	210	220	230	240
KTEEVILKLE	SLHQQPHIWD	FLLLLPRLHT	SHDHVEDGMD	VAVNLLQTIL	NSLISLEDLD	WLPLNQTFSQ	VSELVLNVTI
250	260	270	280	290	300	310	320
STLTFLQQHG	VAVTEPVYHL	SMQNIVWDPQ	KVQYDLKSQF	GFDDLHTEQI	LNSSAELKEI	PTDTSLEKMV	CSVLSSTSED
330	340	350	360	370	380	390	400
EAEKWGHVGG	CHPKWSEAKN	YLVHAVSWLR	VYQQVFVQWQ	QGSLLQKTLT	GMGHSLEALR	NQFEEESKPW	KVVEALHTAL
410	420	430	440	450	460	470	480
LLLNDSLSAD	GPKDNHTFPK	ILQHLWKLQS	LLQNLPQWPA	LKRFLQLDGA	LRNAIAQNLH	FVQEVLICLE	TSANDFKWFE
490	500	510	520	530	540	550	560
LNQLKLEKDV	FFWELKQMLA	KNAVCPNGRF	SEKEVFLPPG	NSSIWGGLQG	LLCYCNSSET	SVLNKLLGSV	EDADRILQEV
570	580	590	600	610	620	630	640
ITWHKNMSVL	IPEEYLDWQE	LEMQLSEASL	SCTRLFLLLG	ADPSPENDVF	SSDCKHQLVS	TVIFHTLEKT	QFFLEQAYYW
650	660	670	680	690	700	710	720
KAFKKFIRKT	CEVAQYVNMQ	ESFQNRLLAF	PEESPCFEEN	MDWKMISDNY	FQFLNNLLKS	PTASISRALN	FTKHLLMMEK
730	740	750	760	770	780	790	800
KLHTLEDEQM	NFLLSFVEFF	EKLLLPNLFD	SSIVPSFHSL	PSLTEDILNI	SSLWTNHLKS	LKRDPSATDA	QKLLEFGNEV
810	820	830	840	850	860	870	880
IWKMQTLGSH	WIRKEPKNLL	RFIELILFEI	NPKLLELWAY	GISKGKRAKL	ENFFTLLNFS	VPENEILSTS	FNFSQLFHSD
890	900	910	920	930	940	950	960
WPKSPAMNID	FVRLSEAIIT	SLHEFGFLEQ	EQISEALNTV	YAIRNASDLF	SALSEPQKQE	VDKILTHIHL	NVFQDKDSAL
970	980	990	1000	1010	1020	1030	1040
LLQIYSSFYR	YIYELLNIQS	RGSSLTFLTQ	ISKHILDIIK	QFNFQNISKA	FAFLFKTAEV	LGGISNVSYC	QQLLSIFNFL
1050	1060	1070	1080	1090	1100	1110	1120
ELQAQSFMST	EGQELEVIHT	TLTGLKQLLI	IDEDFRISLF	QYMSQFFNSS	VEDLLDNKCL	ISDNKHISSV	NYSTSEESSF
1130	1140	1150	1160	1170	1180	1190	1200
VFPLAQIFSN	LSANVSVFNK	FMSIHCTVSW	LQMWTEIWET	ISQLFKFDMN	VFTSLHHGFT	QLLDELEDDV	KVSKSCQGIL
1210	1220	1230	1240	1250	1260	1270	1280
PTHNVARLIL	NLFKNVTQAN	DFHNWEDFLD	LRDFLVALGN	ALVSVKKLNL	EQVEKSLFTM	EAALHQLKTF	PFNESTSREF
1290	1300	1310	1320	1330	1340	1350	1360
LNSLLEVFIE	FSSTSEYIVR	NLDSINDFLS	NNLTNYGEKF	ENIITELREA	IVFLRNVSHD	RDLFSCADIF	QNVTECILED
1370	1380	1390	1400	1410	1420	1430	1440
GFLYVNTSQR	MLRILDTLNS	TFSSENTISS	LKGCIVWLDV	INHLYLLSNS	SFSQGHLQNI	LGNFRDIENK	MNSILKIVTW
1450	1460	1470	1480	1490	1500	1510	1520
VLNIKKPLCS	SNGSHINCVN	IYLKDVTDFL	NIVLTTVFEK	EKKPKFEILL	ALLNDSTKQV	RMSINNLTTD	FDFASQSNWR
1530	1540	1550	1560	1570	1580	1590	1600
YFTELILRPI	EMSDEIPNQF	QNIWLHLITL	GKEFQKLVKG	IYFNILENNS	SSKTENLLNI	FATSPKEKDV	NSVGNSIYHL
1610	1620	1630	1640	1650	1660	1670	1680
ASYLAFSLSH	DLQNSPKIII	SPEIMKATGL	GIQLIRDVFN	SLMPVVHHTS	PQNAGYMQAL	KKVTSVMRTL	KKADIDLLVD
1690	1700	1710	1720	1730	1740	1750	1760
QLEQVSVNLM	DFFKNISSVG	TGNLVVNLLV	GLMEKFADSS	HSWNVNHLLQ	LSRLFPKDVV	DAVIDVYYVL	PHAVRLLQGV
1770	1780	1790	1800	1810	1820	1830	1840
PGKNITEGLK	DVYSFTLLHG	ITISNITKED	FAIVIKILLD	TIELVSDKPD	IISEALACFP	VVWCWNHTNS	GFRQNSKIDP
1850	1860	1870	1880	1890	1900	1910	1920
CNVHGLMSSS	FYGKVASILD	HFHLSPQGED	SPCSNESSRM	EITRKVVCII	HELVDWNSIL	LELSEVFHVN	ISLVKTVQKF
1930	1940	1950	1960	1970	1980	1990	2000
WHKILPFVPP	SINQTRDSIS	ELCPSGSIKQ	VALQIIEKLK	NVNFTKVTSG	ENILDKLSSL	NKILNINEDT	ETSVQNIISS
2010	2020	2030	2040	2050	2060	2070	2080
NLERTVQLIS	EDWSLEKSTH	NLLSLFMMLQ	NANVTGSSLE	ALSSFIEKSE	TPYNFEELWP	KFQQIMKDLT	QDFRIRHLLS
2090	2100	2110	2120	2130	2140	2150	2160
EMNKGIKSIN	SMALQKITLQ	FAHFLEILDS	PSLKTLEIIE	DFLLVTKNWL	QEYANEDYSR	MIETLFIPVT	NESSTEDIAL
2170	2180	2190	2200	2210	2220	2230	2240
LAKAIATFWG	SLKNISRAGN	FDVAFLTHLL	NQEQLTNFSV	VQLLFENILI	NLINNLAGNS	QEAAWNLNDT	DLQIMNFINL
2250	2260	2270	2280	2290	2300	2310	2320
ILNHMQSETS	RKTVLSLRSI	VDFTEQFLKT	FFSLFLKEDS	ENKISLLLKY	FHKDVIAEMS	FVPKDKILEI	LKLDQFLTLM
2330	2340	2350	2360	2370	2380	2390	2400

Detailed Protein Report

Cmpd.	No. of Cmpds.	m/z meas.	Δ m/z [ppm]	z	Rt [min]	Score	P	Range	Sequence	Modification
2092	1	1030.3822	-211.70	1	56.3	15.0	1	3972-3981	K.QTRALSGGLK.R	

Detailed Protein Report

Protein 283: PREDICTED: uncharacterized protein KIAA1109 isoform X13 [Homo sapiens]

**Accession:**	gi|578809469	**Score:**	29.6
**Database:**	refseq_human(refseq_human_20140103.fasta)	**MW [kDa]:**	409.1
**Database Date:**	2015-11-30	**pI:**	6.0
		**Sequence Coverage [%]:**	0.9
		**No. of unique Peptides:**	2

Detailed Protein Report

10	20	30	40	50	60	70	80
MDQRKNESIV	PSITQLEDFL	TEHNSNVVWL	LVATILSCGW	IIYLTYYNSR	NVGLILTLVL	NRLYKHGYIH	IGSFSFSVLS
90	100	110	120	130	140	150	160
GKVMVREIYY	ITEDMSIRIQ	DGFIIFRWWK	MYNPKQKQHD	PKAETRLYIT	VNDFEFHVYN	RSDLYGRLQE	LFGLEPTIIP
170	180	190	200	210	220	230	240
PKKDDDKTRE	IGRTRTQSKI	ERVKVKTESQ	DPTSSWRSLI	PVIKVNVSTG	RLAFGNHYQP	QTLCINFDDA	FLTYTTKPPS
250	260	270	280	290	300	310	320
SHLDQFMHIV	KGKLENVRVM	LVPSPRYVGL	QNDEPPRLMG	EGFVVMQSND	VDIYYYMDEP	GLVPEETEEN	IEGEMSSEDC
330	340	350	360	370	380	390	400
KLQDLPPCWG	LDIVCGKGTD	FNYGPWADRQ	RDCLWKFFFP	PDYQVLKVSE	IAQPGRPRQI	LAFELRMNII	ADATIDLLFT
410	420	430	440	450	460	470	480
KNRETNAVHV	NVGAGSYLEI	NIPMTVEENG	YTPAIKGQLL	HVDATTSMQY	RTLLEAEMLA	FHINASYPRI	WNMPQTWQCE
490	500	510	520	530	540	550	560
LEVYKATYHF	IFAQKNFFTD	LIQDWSSDSP	PDIFSFVPYT	WNFKIMFHQF	EMIWAANQHN	WIDCSTKQQE	NVYLAACGET
570	580	590	600	610	620	630	640
LNIDFSLPFT	DFVPATCNTK	FSLRGEDVDL	HLFLPDCHPS	KYSLFMLVKN	CHPNKMIHDT	GIPAECQSGQ	KTVKPKWRNV
650	660	670	680	690	700	710	720
TQEKSGWVEC	WTVPSVMLTI	DYTWHPIYPQ	KADEQLKQSL	SEMEETMLSV	LRPSQKTSDR	VVSSPSTSSR	PPIDPSELPP
730	740	750	760	770	780	790	800
DKLHVEMELS	PDSQITLYGP	LLNAFLCIKE	NYFGEDDMYM	DFEEVISSPV	LSLSTSSSSG	WTAVGMENDK	KENEGSAKSI
810	820	830	840	850	860	870	880
HPLALRPWDI	TVLVNLYKVH	GRLPVHGTTD	GPECPTAFLE	RLCFEMKKGF	RETMLQLILS	PLNVFVSDNY	QQRPPVDEVL
890	900	910	920	930	940	950	960
REGHINLSGL	QLRAHAMFSA	EGLPLGSDSL	EYAWLIDVQA	GSLTAKVTAP	QLACLLEWGQ	TFVFHVVCRE	YELERPKSVI
970	980	990	1000	1010	1020	1030	1040
ICQHGIDRRF	CESKLSCIPG	PCPTSDDLKY	TMIRLAVDGA	DIYIVEHGCA	TNIKMGAIRV	ANCNLHNQSV	GEGISAAIQD
1050	1060	1070	1080	1090	1100	1110	1120
FQVRQYIEQL	NNCRIGLQPA	VLRRAYWLEA	GSANLGLITV	DIALAADHHS	KHEAQRHFLE	THDARTKRLW	FLWPDDILKN
1130	1140	1150	1160	1170	1180	1190	1200
KRCRNKCGCL	GGCRFFGGTV	TGLDFFKLEE	LTPSSSSAFS	STSAESDMYY	GQSLLQPGEW	IITKEIPKII	DGNVNGMKRK
1210	1220	1230	1240	1250	1260	1270	1280
EWENKSVGIE	VERKTQHLSL	QVPLRSHSSS	SSSEENSSSS	AAQPLLAGEK	ESPSSVADDH	LVQKEFLHGT	KRDDGQASIP
1290	1300	1310	1320	1330	1340	1350	1360
TEISGNSPVS	PNTQDKSVGQ	SPLRSPLKRQ	ASVCSTRLGS	TKSLTAAFYG	DKQPVTVGVQ	FSSDVSRSDE	NVLDSPKQRR
1370	1380	1390	1400	1410	1420	1430	1440
SFGSFPYTPS	ADSNSFHQYR	SMDSSMSMAD	SEAYFSAAEE	FEPISSDEGP	GTYPGRKKKK	KQTQQIDYSR	GSIYHSVEGP
1450	1460	1470	1480	1490	1500	1510	1520
LTGHGESIQD	SRTLPFKTHP	SQASFVSALG	GEDDVIEHLY	IVEGEKTVES	EQITPQQPVM	NCYQTYLTQF	QVINWSVKHP
1530	1540	1550	1560	1570	1580	1590	1600
TNKRTSKSSL	HRPLDLDTPT	SEESSSSFEQ	LSVPTFKVIK	QGLTANSLLD	RGMQLSGSTS	NTPYTPLEKK	LADNTDDETL
1610	1620	1630	1640	1650	1660	1670	1680
TEEWTLDQPV	SQTRTTAIVE	VKGTVDIVLT	PLVAEALDRY	IEAMVHCAST	RHPAAIVDDL	HAKVLREAVQ	NSKTTFSENL
1690	1700	1710	1720	1730	1740	1750	1760
SSKQDIRGTK	TEQSTIGTTN	QGQAQTNLTM	KQDNVTIKGL	QTNVSIPKVN	LCLLQASVEE	SPTTAPSRSV	THVSLVALCF
1770	1780	1790	1800	1810	1820	1830	1840
DRIATQVRMN	RGVVEETSNN	AEPGRTSNFD	RYVHATKMQP	QSSGSLRSNA	GAEKGKEIAA	KLNIHRVHGQ	LRGLDTTDIG
1850	1860	1870	1880	1890	1900	1910	1920
TCAITAIPFE	KSKVLFTLEE	LDEFTFVDET	DQQAVPDVTR	IGPSQEKWGW	IMFECGLENL	TIKGGRQSGA	VLYNSFGIMG
1930	1940	1950	1960	1970	1980	1990	2000
KASDTERGGV	LTSNNSSDSP	TGSGYNTDVS	DDNLPCDRTS	PSSDLNGNSV	SDEQDEGVES	DDLKKDLPLM	PPPPDSCSMK
2010	2020	2030	2040	2050	2060	2070	2080
LTIKEIWFSF	AAPTNVRSHT	HAFSRQLNLL	STATPAVGAW	LVPIDQLKSS	LNKLETEGTL	RICAVMGCIM	TEALENKSVH
2090	2100	2110	2120	2130	2140	2150	2160
FPLRSKYNRL	TKVARFLQEN	PSCLLCNILH	HYLHQANYSI	IDDATMSDGL	PALVTLKKGL	VALARQWMKF	IVVTPAFKGV
2170	2180	2190	2200	2210	2220	2230	2240

Detailed Protein Report

Cmpd.	No. of Cmpds.	m/z meas.	Δ m/z [ppm]	z	Rt [min]	Score	P	Range	Sequence	Modification
117	1	774.3916	-26.77	3	31.2	15.7	1	66-86	K.HGYIHIGSFSFSVLSGKVMVR.E	
1348	1	669.3232	-39.50	2	46.5	13.8	2	116-126	K.QKQHDPKAETR.L	

Detailed Protein Report

Protein 284: caspase recruitment domain-containing protein 9 isoform 2 [Homo sapiens]

**Accession:**	gi|16554562	**Score:**	29.6
**Database:**	refseq_human(refseq_human_20140103.fasta)	**MW [kDa]:**	56.6
**Database Date:**	2015-11-30	**pI:**	5.5
		**Sequence Coverage [%]:**	3.3
		**No. of unique Peptides:**	1

10	20	30	40	50	60	70	80
MSDYENDDEC	WSVLEGFRVT	LTSVIDPSRI	TPYLRQCKVL	NPDDEEQVLS	DPNLVIRKRK	VGVLLDILQR	TGHKGYVAFL
**90**	**100**	**110**	**120**	**130**	**140**	**150**	**160**
ESLELYYPQL	YKKVTGKEPA	RVFSMIIDAS	GESGLTQLLM	TEVMKLQKKV	QDLTALLSSK	DDFIKELRVK	DSLLRKHQER
**170**	**180**	**190**	**200**	**210**	**220**	**230**	**240**
VQRLKEECEA	GSRELKRCKE	ENYDLAMRLA	HQSEEKGAAL	MRNRDLQLEI	DQLKHSLMKA	EDDCKVERKH	TLKLRHAMEQ
**250**	**260**	**270**	**280**	**290**	**300**	**310**	**320**
RPSQELLWEL	QQEKALLQAR	VQELEASVQE	GKLDRSSPYI	QVLEEDWRQA	LRDHQEQANT	IFSLRKDLRQ	GEARRLRCME
**330**	**340**	**350**	**360**	**370**	**380**	**390**	**400**
EKEMFELQCL	ALRKDSKMYK	DRIEAILLQM	EEVAIERDQA	IATREELHAQ	HARGLQEKDA	LRKQVRELGE	KADELQLQVF
**410**	**420**	**430**	**440**	**450**	**460**	**470**	**480**
QCEAQLLAVE	GRLRRQQLET	LVLSSDLEDG	SPRRSQELSL	PQDLEDTQLS	DKGCLAGGGS	PKQPFAALHQ	EQVLRNPHDA
**490**	**500**						
GPAGLPGIGA	VC						

Cmpd.	No. of Cmpds.	m/z meas.	Δ m/z [ppm]	z	Rt [min]	Score	P	Range	Sequence	Modification
1435	1	627.2993	-161.59	2	47.6	11.8	1	60-70	R.KVGVLLDILQR.T	

Detailed Protein Report

Protein 285: transformation/transcription domain-associated protein isoform 2 [Homo sapiens]

**Accession:**	gi|4507691	**Score:**	29.5
**Database:**	refseq_human(refseq_human_20140103.fasta)	**MW [kDa]:**	434.1
**Database Date:**	2015-11-30	**pI:**	9.3
**Modification(s):**	Oxidation	**Sequence Coverage [%]:**	1.1
		**No. of unique Peptides:**	1

Detailed Protein Report

10	20	30	40	50	60	70	80
MAFVATQGAT	VVDQTTLMKK	YLQFVAALTD	VNTPDETKLK	MMQEVSENFE	NVTSSPQYST	FLEHIIPRFL	TFLQDGEVQF
90	100	110	120	130	140	150	160
LQEKPAQQLR	KLVLEIIHRI	PTNEHLRPHT	KNVLSVMFRF	LETENEENVL	ICLRIIIELH	KQFRPPITQE	IHHFLDFVKQ
170	180	190	200	210	220	230	240
IYKELPKVVN	RYFENPQVIP	ENTVPPPEMV	GMITTIAVKV	NPEREDSETR	THSIIPRGSL	SLKVLAELPI	IVVLMYQLYK
250	260	270	280	290	300	310	320
LNIHNVVAEF	VPLIMNTIAI	QVSAQARQHK	LYNKELYADF	IAAQIKTLSF	LAYIIRIYQE	LVTKYSQQMV	KGMLQLLSNC
330	340	350	360	370	380	390	400
PAETAHLRKE	LLIAAKHILT	TELRNQFIPC	MDKLFDESIL	IGSGYTARET	LRPLAYSTLA	DLVHHVRQHL	PLSDLSLAVQ
410	420	430	440	450	460	470	480
LFAKNIDDES	LPSSIQTMSC	KLLLNLVDCI	RSKSEQESGN	GRDVLMRMLE	VFVLKFHTIA	RYQLSAIFKK	CKPQSELGAV
490	500	510	520	530	540	550	560
EAALPGVPTA	PAAPGPAPSP	APVPAPPPPP	PPPPPATPVT	PAPVPPFEKQ	GEKDKEDKQT	FQVTDCRSLV	KTLVCGVKTI
570	580	590	600	610	620	630	640
TWGITSCKAP	GEAQFIPNKQ	LQPKETQIYI	KLVKYAMQAL	DIYQVQIAGN	GQTYIRVANC	QTVRMKEEKE	VLEHFAGVFT
650	660	670	680	690	700	710	720
MMNPLTFKEI	FQTTVPYMVE	RISKNYALQI	VANSFLANPT	TSALFATILV	EYLLDRLPEM	GSNVELSNLY	LKLFKLVFGS
730	740	750	760	770	780	790	800
VSLFAAENEQ	MLKPHLHKIV	NSSMELAQTA	KEPYNYFLLL	RALFRSIGGG	SHDLLYQEFL	PLLPNLLQGL	NMLQSGLHKQ
810	820	830	840	850	860	870	880
HMKDLFVELC	LTVPVRLSSL	LPYLPMLMDP	LVSALNGSQT	LVSQGLRTLE	LCVDNLQPDF	LYDHIQPVRA	ELMQALWRTL
890	900	910	920	930	940	950	960
RNPADSISHV	AYRVLGKFGG	SNRKMLKESQ	KLHYVVTEVQ	GPSITVEFSD	CKASLQLPME	KAIETALDCL	KSANTEPYYR
970	980	990	1000	1010	1020	1030	1040
RQAWEVIKCF	LVAMMSLEDN	KHALYQLLAH	PNFTEKTIPN	VIISHRYKAQ	DTPARKTFEQ	ALTGAFMSAV	IKDLRPSALP
1050	1060	1070	1080	1090	1100	1110	1120
FVASLIRHYT	MVAVAQQCGP	FLLPCYQVGS	QPSTAMFHSE	ENGSKGMDPL	VLIDAIAICM	AYEEKELCKI	GEVALAVIFD
1130	1140	1150	1160	1170	1180	1190	1200
VASIILGSKE	RACQLPLFSY	IVERLCACCY	EQAWYAKLGG	VVSIKFLMER	LPLTWVLQNQ	QTFLKALLFV	MMDLTGEVSN
1210	1220	1230	1240	1250	1260	1270	1280
GAVAMAKTTL	EQLLMRCATP	LKDEERAEEI	VAAQEKSFHH	VTHDLVREVT	SPNSTVRKQA	MHSLQVLAQV	TGKSVTVIME
1290	1300	1310	1320	1330	1340	1350	1360
PHKEVLQDMV	PPKKHLLRHQ	PANAQIGLME	GNTFCTTLQP	RLFTMDLNVV	EHKVFYTELL	NLCEAEDSAL	TKLPCYKSLP
1370	1380	1390	1400	1410	1420	1430	1440
SLVPLRIAAL	NALAACNYLP	QSREKIIAAL	FKALNSTNSE	LQEAGEACMR	KFLEGATIEV	DQIHTHMRPL	LMMLGDYRSL
1450	1460	1470	1480	1490	1500	1510	1520
TLNVVNRLTS	VTRLFPNSFN	DKFCDQMMQH	LRKWMEVVVI	THKGGQRSDG	NEMKICSAII	NLFHLIPAAP	QTLVKPLLEV
1530	1540	1550	1560	1570	1580	1590	1600
VMKTERAMLI	EAGSPFREPL	IKFLTRHPSQ	TVELFMMEAT	LNDPQWSRMF	MSFLKHKDAR	PLRDVLAANP	NRFITLLLPG
1610	1620	1630	1640	1650	1660	1670	1680
GAQTAVRPGS	PSTSTMRLDL	QFQAIKIISI	IVKNDDSWLA	SQHSLVSQLR	RVWVSENFQE	RHRKENMAAT	NWKEPKLLAY
1690	1700	1710	1720	1730	1740	1750	1760
CLLNYCKRNY	GDIELLFQLL	RAFTGRFLCN	MTFLKEYMEE	EIPKNYSIAQ	KRALFFRFVD	FNDPNFGDEL	KAKVLQHILN
1770	1780	1790	1800	1810	1820	1830	1840
PAFLYSFEKG	EGEQLLGPPN	PEGDNPESIT	SVFITKVLDP	EKQADMLDSL	RIYLLQYATL	LVEHAPHHIH	DNNKNRNSKL
1850	1860	1870	1880	1890	1900	1910	1920
RRLMTFAWPC	LLSKACVDPA	CKYSGHLLLA	HIIAKFAIHK	KIVLQVFHSL	LKAHAMEARA	IVRQAMAILT	PAVPARMEDG
1930	1940	1950	1960	1970	1980	1990	2000
HQMLTHWTRK	IIVEEGHTVP	QLVHILHLIV	QHFKVYYPVR	HHLVQHMVSA	MQRLGFTPSV	TIEQRRLAVD	LSEVVIKWEL
2010	2020	2030	2040	2050	2060	2070	2080
QRIKDQQPDS	DMDPNSSGEG	VNSVSSSIKR	GLSVDSAQEV	KRFRTATGAI	SAVFGRSQSL	PGADSLLAKP	IDKQHTDTVV
2090	2100	2110	2120	2130	2140	2150	2160
NFLIRVACQV	NDNTNTAGSP	GEVLSRRCVN	LLKTALRPDM	WPKSELKLQW	FDKLLMTVEQ	PNQVNYGNIC	TGLEVLSFLL
2170	2180	2190	2200	2210	2220	2230	2240
TVLQSPAILS	SFKPLQRGIA	ACMTCGNTKV	LRAVHSLLSR	LMSIFPTEPS	TSSVASKYEE	LECLYAAVGK	VIYEGLTNYE
2250	2260	2270	2280	2290	2300	2310	2320
KATNANPSQL	FGTLMILKSA	CSNNPSYIDR	LISVFMRSLQ	KMVREHLNPQ	AASGSTEATS	GTSELVMLSL	ELVKTRLAVM
2330	2340	2350	2360	2370	2380	2390	2400

Detailed Protein Report

Cmpd.	No. of Cmpds.	m/z meas.	Δ m/z [ppm]	z	Rt [min]	Score	P	Range	Sequence	Modification
873	1	946.4119	-18.65	3	40.4	15.0	1	2003-2029	R.IKDQQPDSDMDPNSSGEGVNSVSSSIK.R	Oxidation: 10

Detailed Protein Report

Protein 286: **PREDICTED: MAP kinase-activating death domain protein isoform X20 [Homo sapiens]**

**Accession:**	gi|530395950	**Score:**	29.5
**Database:**	refseq_human(refseq_human_20140103.fasta)	**MW [kDa]:**	136.1
**Database Date:**	2015-11-30	**pI:**	5.7
**Modification(s):**	Carbamidomethyl	**Sequence Coverage [%]:**	1.3
		**No. of unique Peptides:**	1

10	20	30	40	50	60	70	80
MVQKKKFCPR	LLDYLVIVGA	RHPSSDSVAQ	TPELLRRYPL	EDHTEFPLPP	DVVFFCQPEG	CLSVRQRRMS	LRDDTSFVFT
90	100	110	120	130	140	150	160
LTDKDTGVTR	YGICVNFYRS	FQKRISKEKG	EGGAGSRGKE	GTHATCASEE	GGTESSESGS	SLQPLSADST	PDVNQSPRGK
170	180	190	200	210	220	230	240
RRAKAGSRSR	NSTLTSLCVL	SHYPFFSTFR	ECLYTLKRLV	DCCSERLLGK	KLGIPRGVQR	DTMWRIFTGS	LLVEEKSSAL
250	260	270	280	290	300	310	320
LHDLREIEAW	IYRLLRSPVP	VSGQKRVDIE	VLPQELQPAL	TFALPDPSRF	TLVDFPLHLP	LELLGVDACL	QVLTCILLEH
330	340	350	360	370	380	390	400
KVVLQSRDYN	ALSMSVMAFV	AMIYPLEYMF	PVIPLLPTCM	ASAEQLLLAP	TPYIIGVPAS	FFLYKLDFKM	PDDVWLVDLD
410	420	430	440	450	460	470	480
SNRVIAPTNA	EVLPILPEPE	SLELKKHLKQ	ALASMSLNTQ	PILNLEKFHE	GQEIPLLLGR	PSNDLQSTPS	TEFNPLIYGN
490	500	510	520	530	540	550	560
DVDSVDVATR	VAMVRFFNSA	NVLQGFQMHT	RTLRLFPRPV	VAFQAGSFLA	SRPRQTPFAE	KLARTQAVEY	FGEWILNPTN
570	580	590	600	610	620	630	640
YAFQRIHNNM	FDPALIGDKP	KWYAHQLQPI	HYRVYDSNSQ	LAEALSVPPE	RDSDSEPTDD	SGSDSMDYDD	SSSSYSSLGD
650	660	670	680	690	700	710	720
FVSEMMKCDI	NGDTPNVDPL	THAALGDASE	VEIDELQNQK	EAEEPGPDSE	NSQENPPLRS	SSSTTASSSP	STVIHGANSE
730	740	750	760	770	780	790	800
PADSTEMDDK	AAVGVSKPLP	SVPPSIGKSN	VDRRQAEIGE	GSVRRRIYDN	PYFEPQYGFP	PEEDEDEQGE	SYTPRFSQHV
810	820	830	840	850	860	870	880
SGNRAQKLLR	PNSLRLASDS	DAESDSRASS	PNSTVSNTST	EGFGGIMSFA	SSLYRNHSTS	FSLSNLTLPT	KGAREKATPF
890	900	910	920	930	940	950	960
PSLKVFGLNT	LMEIVTEAGP	GSGEGNRRAL	VDQKSSVIKH	SPTVKREPPS	PQGRSSNSSE	NQQFLKEVVH	SVLDGQGVGW
970	980	990	1000	1010	1020	1030	1040
LNMKKVRRLL	ESEQLRVFVL	SKLNRMVQSE	DDARQDIIPD	VEISRKVYKG	MLDLLKCTVL	SLEQSYAHAG	LGGMASIFGL
1050	1060	1070	1080	1090	1100	1110	1120
LEIAQTHYYS	KEPDKRKRSP	TESVNTPVGK	DPGLAGRGDP	KAMAQLRVPQ	LGPRAPSATG	KGPKELDTRS	LKEENFIASI
1130	1140	1150	1160	1170	1180	1190	1200
ELWNKHQEVK	KQKALEKQRP	EVIKPVFDLG	ETEEKKSQIS	ADSGVSLTSS	SQRTDQDSVI	GVSPAVMIRS	SSQDSEVSTV
1210	1220	1230	1240				
VGEHHTGILV	CVYEISPRAS	HLIKKVLPEA	I				

Cmpd.	No. of Cmpds.	m/z meas.	Δ m/z [ppm]	z	Rt [min]	Score	P	Range	Sequence	Modification
1913	1	682.2568	-100.43	3	54.0	15.5	2	191-206	R.ECLYTLKRLVDCCSER.L	Carbamidomethyl: 12, 13

Detailed Protein Report

Protein 287: **PREDICTED: telomerase-binding protein EST1A isoform X2 [Homo sapiens]**

**Accession:**	gi|530410168	**Score:**	29.4
**Database:**	refseq_human(refseq_human_20140103.fasta)	**MW [kDa]:**	101.9
**Database Date:**	2015-11-30	**pI:**	9.8
		**Sequence Coverage [%]:**	3.6
		**No. of unique Peptides:**	1

10	20	30	40	50	60	70	80
MAEGLERVRI	SASELRGILA	TLAPQAGSRE	NMKELKEARP	RKDNRRPDLE	IYKPGLSRLR	NKPKIKEPPG	SEEFKDEIVN
**90**	**100**	**110**	**120**	**130**	**140**	**150**	**160**
DRDCSAVENG	TQPVKDVCKE	LNNQEQNGPI	DPENNRGQES	FPRTAGQEDR	SLKIIKRTKK	PDLQIYQPGR	RLQTVSKESA
**170**	**180**	**190**	**200**	**210**	**220**	**230**	**240**
SRVEEEEVLN	QVEQLRVEED	ECRGNVAKEE	VANKPDRAEI	EKSPGGGRVG	AAKGEKGKRM	GKGEGVRETH	DDPARGRPGS
**250**	**260**	**270**	**280**	**290**	**300**	**310**	**320**
AKRYSRSDKR	RNRYRTRSTS	SAGSNNSAEG	AGLTDNGCRR	RRQDRTKERP	RLKKQVSVSS	TDSLDEDRID	EPDGLGPRRS
**330**	**340**	**350**	**360**	**370**	**380**	**390**	**400**
SERKRHLERN	WSGRGEGEQK	NSAKEYRGTL	RVTFDAEAMN	KESPMVRSAR	DDMDRGKPDK	GLSSGGKGSE	KQESKNPKQE
**410**	**420**	**430**	**440**	**450**	**460**	**470**	**480**
LRGRGRGILI	LPAHTTLSVN	SAGSPESAPL	GPRLLFGSGS	KGSRSWGRGG	TTRRLWDPNN	PDQKPALKTQ	TPQLHFLDTD
**490**	**500**	**510**	**520**	**530**	**540**	**550**	**560**
DEVSPTSWGD	SRQAQASYYK	FQNSDNPYYY	PRTPGPASQY	PYTGYNPLQY	PVGPTNGVYP	GPYYPGYPTP	SGQYVCSPLP
**570**	**580**	**590**	**600**	**610**	**620**	**630**	**640**
TSTMSPEEVE	QHMRNLQQQE	LHRLLRVADN	QELQLSNLLS	RDRISPEGLE	KMAQLRAELL	QLYERCILLD	IEFSDNQNVD
**650**	**660**	**670**	**680**	**690**	**700**	**710**	**720**
QILWKNAFYQ	VIEKFRQLVK	DPNVENPEQI	RNRLLELLDE	GSDFFDSLLQ	KLQVTYKFKL	EDYMDGLAIR	SKPLRKTVKY
**730**	**740**	**750**	**760**	**770**	**780**	**790**	**800**
ALISAQRCMI	CQGDIARYRE	QASDTANYGK	ARSWYLKAQH	IAPKNGRPYN	QLALLAVYTR	RKLDAVYYYM	RSLAASNPIL
**810**	**820**	**830**	**840**	**850**	**860**	**870**	**880**
TAKESLMSLF	EETKRKAEQM	EKKQHEEFDL	SPDQWRKGKK	STFRHVGDDT	TRLEIWIHPS	HPRSSQGTES	GKDSEQENGL
**890**	**900**						
GSLSPSDATS	PRRNLQFLA						

Cmpd.	No. of Cmpds.	m/z meas.	Δ m/z [ppm]	z	Rt [min]	Score	P	Range	Sequence	Modification
1729	1	1051.5453	20.59	2	51.4	14.5	2	65-82	K.IKEPPGSEEFKDEIVNDR.D	

Detailed Protein Report

Protein 288: DNA repair and recombination protein RAD54-like [Homo sapiens]

Accession: gi|216548186
Database: refseq_human(refseq_human_20140103.fasta)
Database Date: 2015-11-30
Modification(s): Carbamidomethyl

Score: 29.4
MW [kDa]: 84.3
pI: 9.8
Sequence Coverage [%]: 4.7
No. of unique Peptides: 2

Alias proteins:

Accession	Name	Description
gi|216548193	refseq_human (refseq_human_20140103.fasta)	DNA repair and recombination protein RAD54-like [Homo sapiens]

10	20	30	40	50	60	70	80
MRRSLAPSQL	AKRKPEGRSC	DDEDWQPGLV	TPRKRKSSSE	TQIQECFLSP	FRKPLSQLTN	QPPCLDSSQH	EAFIRSILSK
**90**	**100**	**110**	**120**	**130**	**140**	**150**	**160**
PFKVPIPNYQ	GPLGSRALGL	KRAGVRRALH	DPLEKDALVL	YEPPPLSAHD	QLKLDKEKLP	VHVVVDPILS	KVLRPHQREG
**170**	**180**	**190**	**200**	**210**	**220**	**230**	**240**
VKFLWECVTS	RRIPGSHGCI	MADEMGLGKT	LQCITLMWTL	LRQSPECKPE	IDKAVVVSPS	SLVKNWYNEV	GKWLGGRIQP
**250**	**260**	**270**	**280**	**290**	**300**	**310**	**320**
LAIDGGSKDE	IDQKLEGFMN	QRGARVSSPI	LIISYETFRL	HVGVLQKGSV	GLVICDEGHR	LKNSENQTYQ	ALDSLNTSRR
**330**	**340**	**350**	**360**	**370**	**380**	**390**	**400**
VLISGTPIQN	DLLEYFSLVH	FVNSGILGTA	HEFKKHFELP	ILKGRDAAAS	EADRQLGEER	LRELTSIVNR	CLIRRTSDIL
**410**	**420**	**430**	**440**	**450**	**460**	**470**	**480**
SKYLPVKIEQ	VVCCRLTPLQ	TELYKRFLRQ	AKPAEELLEG	KMSVSSLSSI	TSLKKLCNHP	ALIYDKCVEE	EDGFVGALDL
**490**	**500**	**510**	**520**	**530**	**540**	**550**	**560**
FPPGYSSKAL	EPQLSGKMLV	LDYILAVTRS	RSSDKVVLVS	NYTQTLDLFE	KLCRARRYLY	VRLDGTMSIK	KRAKVVERFN
**570**	**580**	**590**	**600**	**610**	**620**	**630**	**640**
SPSSPDFVFM	LSSKAGGCGL	NLIGANRLVM	FDPDWNPAND	EQAMARVWRD	GQKKTCYIYR	LLSAGTIEEK	IFQRQSHKKA
**650**	**660**	**670**	**680**	**690**	**700**	**710**	**720**
LSSCVVDEEQ	DVERHFSLGE	LKELFILDEA	SLSDTHDRLH	CRRCVNSRQI	RPPPDGSDCT	SDLAGWNHCT	DKWGLRDEVL
**730**	**740**	**750**					
QAAWDAASTA	ITFVFHQRSH	EEQRGLR					

Cmpd.	No. of Cmpds.	m/z meas.	Δ m/z [ppm]	z	Rt [min]	Score	P	Range	Sequence	Modification
656	1	677.1638	133.53	3	37.8	16.7	2	134-151	K.LDKEKLPVHVVVDPILSK.V	
1883	2	1022.8975	-139.69	2	53.6	12.7	2	614-630	K.KTCYIYRLLSAGTIEEK.I	Carbamidomethyl: 3

Detailed Protein Report

Protein 289: PREDICTED: integrin beta-7 isoform X3 [Homo sapiens]

**Accession:**	gi|578823673	**Score:**	29.4
**Database:**	refseq_human(refseq_human_20140103.fasta)	**MW [kDa]:**	85.0
**Database Date:**	2015-11-30	**pI:**	5.7
**Modification(s):**	Carbamidomethyl	**Sequence Coverage [%]:**	3.3
		**No. of unique Peptides:**	1

10	20	30	40	50	60	70	80
MVALPMVLVL	LLVLSRGESE	LDAKIPSTGD	ATEWRNPHLS	MLGSCQPAPS	CQKCILSHPS	CAWCKQLNFT	ASGEAEARRC
**90**	**100**	**110**	**120**	**130**	**140**	**150**	**160**
ARREELLARG	CPLEELEEPR	GQQEVLQDQP	LSQGARGEGA	TQLAPQRVRV	TLRPGEPQQL	QVRFLRAEGY	PVDLYYLMDL
**170**	**180**	**190**	**200**	**210**	**220**	**230**	**240**
SYSMKDDLER	VRQLGHALLV	RLQEVTHSVR	IGFGSFVDKT	VLPFVSTVPS	KLRHPCPTRL	ERCQSPFSFH	HVLSLTGDAQ
**250**	**260**	**270**	**280**	**290**	**300**	**310**	**320**
AFEREVGRQS	VSGNLDSPEG	GFDAILQAAL	CQEQIGWRNV	SRLLVFTSDD	TFHTAGDGKL	GGIFMPSDGH	CHLDSNGLYS
**330**	**340**	**350**	**360**	**370**	**380**	**390**	**400**
RSTEFDYPSV	GQVAQALSAA	NIQPIFAVTS	AALPVYQELS	KLIPKSAVGE	LSEDSSNVVQ	LIMDAYNSLS	STVTLEHSSL
**410**	**420**	**430**	**440**	**450**	**460**	**470**	**480**
PPGVHISYES	QCEGPEKREG	KAEDRGQCNH	VRINQTVTFW	VSLQATHCLP	EPHLLRLRAL	GFSEELIVEL	HTLCDCNCSD
**490**	**500**	**510**	**520**	**530**	**540**	**550**	**560**
TQPQAPHCSD	GQGHLQCGVC	SCAPGRLGRL	CECSVAELSS	PDLESGCRAP	NGTGPLCSGK	GHCQCGRCSC	SGQSSGHLCE
**570**	**580**	**590**	**600**	**610**	**620**	**630**	**640**
CDDASCERHE	GILCGGFGRC	QCGVCHCHAN	RTGRACECSG	DMDSCISPEG	GLCSGHGRCK	CNRCQCLDGY	YGALCDQCPG
**650**	**660**	**670**	**680**	**690**	**700**	**710**	**720**
CKTPCERHRD	CAECGAFRTG	PLATNCSTAC	AHTNVTLALA	PILDDGWCKE	RTLDNQLFFF	LVEDDARGTV	VLRVRPQEKG
**730**	**740**	**750**	**760**	**770**	**780**	**790**	
ADHTQAIVLG	CVGGIVAVGL	GLVLAYRLSV	EIYDRREYSR	FEKEQQQLNW	KQLLGFGSRL	AS	

Cmpd.	No. of Cmpds.	m/z meas.	Δ m/z [ppm]	z	Rt [min]	Score	P	Range	Sequence	Modification
2663	2	731.3721	63.85	2	64.5	13.4	0	54-65	K.CILSHPSCAWCK.Q	Carbamidomethyl: 1, 11

Detailed Protein Report

Protein 290: palladin isoform 2 [Homo sapiens]

Accession: gi|93102361
Database: refseq_human(refseq_human_20140103.fasta)
Database Date: 2015-11-30

Score: 29.4
MW [kDa]: 122.0
pI: 6.5
Sequence Coverage [%]: 2.9
No. of unique Peptides: 1

10	20	30	40	50	60	70	80
MSGTSSHESF	YDSLSDMQEE	SKNTDFFPGL	SAFLSQEEIN	KSLDLARRAI	ADSETEDFDS	EKEISQIFST	SPASLCEHPS
90	100	110	120	130	140	150	160
HKETKLGEHA	SRRPQDNRST	PVQPLAEKQT	KSISSPVSKR	KPAMSPLLTR	PSYIRSLRKA	EKRGAKTPST	NVKPKTPHQR
170	180	190	200	210	220	230	240
KGGPQSQLCD	KAANLIEELT	SIFKAAKPRN	RSPNGESSSP	DSGYLSPKNQ	PSALLSASAS	QSPMEDQGEM	EREVKSPGAR
250	260	270	280	290	300	310	320
HCYQDNQDLA	VPHNRKSHPQ	PHSALHFPAA	PRFIQKLRSQ	EVAEGSRVYL	ECRVTGNPTP	RVRWFCEGKE	LHNTPDIQIH
330	340	350	360	370	380	390	400
CEGGDLHTLI	IAEAFEDDTG	RYTCLATNPS	GSDTTSAEVF	IEGASSTDSD	SESLAFKSRA	GAMPQAQKKT	TSVSLTIGSS
410	420	430	440	450	460	470	480
SPKTGVTTAV	IQPLSVPVQQ	VHSPTSYLCR	PDGTTTAYFP	PVFTKELQNT	AVAEGQVVVL	ECRVRGAPPL	QVQWFRQGSE
490	500	510	520	530	540	550	560
IQDSPDFRIL	QKKPRSTAEP	EEICTLVIAE	TFPEDAGIFT	CSARNDYGSA	TSTAQLVVTS	ANTENCSYES	MGESNNDHFQ
570	580	590	600	610	620	630	640
HFPPPPPILE	TSSLELASKK	PSEIQQVNNP	ELGLSRAALQ	MQFNAAERET	NGVHPSRGVN	GLINGKANSN	KSLPTPAVLL
650	660	670	680	690	700	710	720
SPTKEPPPLL	AKPKLGFPKK	ASRTARIASD	EEIQGTKDAV	IQDLERKLRF	KEDLLNNGQP	RLTYEERMAR	RLLGADSATV
730	740	750	760	770	780	790	800
FNIQEPEEET	ANQEYKVSSC	EQRLISEIEY	RLERSPVDES	GDEVQYGDVP	VENGMAPFFE	MKLKHYKIFE	GMPVTFTCRV
810	820	830	840	850	860	870	880
AGNPKPKIYW	FKDGKQISPK	SDHYTIQRDL	DGTCSLHTTA	STLDDDGNYT	IMAANPQGRI	SCTGRLMVQA	VNQRGRSPRS
890	900	910	920	930	940	950	960
PSGHPHVRRP	RSRSRDSGDE	NEPIQERFFR	PHFLQAPGDL	TVQEGKLCRM	DCKVSGLPTP	DLSWQLDGKP	VRPDSAHKML
970	980	990	1000	1010	1020	1030	1040
VRENGVHSLI	IEPVTSRDAG	IYTCIATNRA	GQNSFSLELV	VAAKEAHKPP	VFIEKLQNTG	VADGYPVRLE	CRVLGVPPPQ
1050	1060	1070	1080	1090	1100	1110	
IFWKKENESL	THSTDRVSMH	QDNHGYICLL	IQGATKEDAG	WYTVSAKNEA	GIVSCTARLD	VYISRH	

Cmpd.	No. of Cmpds.	m/z meas.	Δ m/z [ppm]	z	Rt [min]	Score	P	Range	Sequence	Modification
2009	2	945.8529	-132.43	2	55.0	12.3	1	1016-1032	K.LQNTGVADGYPVRLECR.V	

Detailed Protein Report

Protein 291: **PREDICTED: myosin phosphatase Rho-interacting protein isoform X6 [Homo sapiens]**

**Accession:**	gi|578829807	**Score:**	29.4
**Database:**	refseq_human(refseq_human_20140103.fasta)	**MW [kDa]:**	258.7
**Database Date:**	2015-11-30	**pI:**	5.3
**Modification(s):**	Oxidation	**Sequence Coverage [%]:**	1.6
		**No. of unique Peptides:**	2

Detailed Protein Report

10	20	30	40	50	60	70	80
MRRGRAEPLA	RRERQAGQAC	AAAAAAVAAA	PTMSAAKENP	CRKFQANIFN	KSKCQNCFKP	RESHLLNDED	LTQAKPIYGG
90	100	110	120	130	140	150	160
WLLLAPDGTD	FDNPVHRSRK	WQRRFFILYE	HGLLRYALDE	MPTTLPQGTI	NMNQCTDVVD	GEGRTGQKFS	LCILTPEKEH
170	180	190	200	210	220	230	240
FIRAETKEIV	SGWLEMLMVY	PRTNKQNQKK	KRKVEPPTPQ	EPGPAKVAVT	SSSSSSSSSS	SIPSAEKVPT	TKSTLWQEEM
250	260	270	280	290	300	310	320
RTKDQPDGSS	LSPAQSPSQS	QPPAASSLRE	PGLESKEEES	AMSSDRMDCG	RKVRVESGYF	SLEKTKQDLK	AEEQQLPPPL
330	340	350	360	370	380	390	400
SPPSPSTPNH	RRSQVIEKFE	ALDIEKAEHM	ETNAVGPSPS	SDTRQGRSEK	RAFPRKRDFT	NEAPPAPLPD	ASASPLSPHR
410	420	430	440	450	460	470	480
RAKSLDRRST	EPSVTPDLLN	FKKGWLTKQY	EDGQWKKHWF	VLADQSLRYY	RDSVAEEAAD	LDGEIDLSAC	YDVTEYPVQR
490	500	510	520	530	540	550	560
NYGFQIHTKE	GEFTLSAMTS	GIRRNWIQTI	MKHVHPTTAP	DVTSSLPEEK	NKSSCSFETC	PRPTEKQEAE	LGEPDPEQKR
570	580	590	600	610	620	630	640
SRARERRREG	RSKTFDWAEF	RPIQQALAQE	RVGGVGPADT	HEPLRPEAEP	GELERERARR	REERRKRFGM	LDATDGPGTE
650	660	670	680	690	700	710	720
DAALRMEVDR	SPGLPMSDLK	THNVHVEIEQ	RWHQVETTPL	REEKQVPIAP	VHLSSEDGGD	RLSTHELTSL	LEKELEQSQK
730	740	750	760	770	780	790	800
EASDLLEQNR	LLQDQLRVAL	GREQSAREGY	VLQTEVAASP	SGAWQRLHRV	NQDLQSELEA	QCQRQELITH	QIQTLKRSYG
810	820	830	840	850	860	870	880
EAKDTIRHHE	AEIRSLQARL	SNAAAELAIK	EQALAKLKGD	LKREQGRVRE	QLEERQHSEA	ALSSQLRASE	QKLKSAEALL
890	900	910	920	930	940	950	960
LEKTQELRGL	ETQQALQRDR	QKEVQRLQER	IADLSQQLGA	SEQAQRLMEE	KLQRNYELLL	ESCEKEKQAL	LQNLKEVEDK
970	980	990	1000	1010	1020	1030	1040
ASAYEDQLQG	QAQQVETLQK	EKLSATFEGS	EQVHQLEEQL	EAREASVRRL	AEHVQSLCDE	RDLLRQRFQE	LTERVATSDE
1050	1060	1070	1080	1090	1100	1110	1120
DVAELREKLR	RREADNQSLE	HSYQRVSSQL	QSMHTLLREK	EEELERIKEA	HEKVLEKKEQ	DLNEALVKMV	ALGSSLEETE
1130	1140	1150	1160	1170	1180	1190	1200
IKLQAKEEIL	RKFASESPKD	MEEPRSTPEE	TERDGTLLPG	QPVQATRAPL	GLPHTRLEDE	DEDLGAPPGE	EYGDGSPSRE
1210	1220	1230	1240	1250	1260	1270	1280
DSMVPPKSVE	VLDREGHQQG	TAKLDQGAPG	VKRQRIRFST	IQCQRYIHPE	GSEKTWTSST	SSDTSQDRSP	SEESMSSEPA
1290	1300	1310	1320	1330	1340	1350	1360
PSVLPATGDS	DTYLSIIHSL	ETKLYVTEEK	LKDVTVRLES	QQGQSREALL	ALHHQWAGTE	AQLREQLRAS	LLQVGALASQ
1370	1380	1390	1400	1410	1420	1430	1440
LEQERQERAR	RVEGHVGELG	DFQVKNSQAL	MCLENCREQL	RSLPRASQED	EQDARAASLA	SVESALVSAI	QALQHWPAPA
1450	1460	1470	1480	1490	1500	1510	1520
HGGARAQLET	GGTEENGKPA	SLQQCSQSEL	TEQEQVRLLS	DQIALEASLI	SQIADSLKNT	TSDVSRMLHE	ISWSGQPPME
1530	1540	1550	1560	1570	1580	1590	1600
SAGAPVDTWA	RKVLVDGEFW	SQVESLRKHL	GTLGGEAVGA	SGDGQQSIPQ	GLAPILANAT	WVRAELSFAT	QSVRESFHRR
1610	1620	1630	1640	1650	1660	1670	1680
LQSIQETLRG	TQTALRQHKC	LLREILGAYQ	TPDFERVMQQ	VLEALRLPAG	HEDGVQLSWD	LSPLGEVLGR	DSDSSQEPFD
1690	1700	1710	1720	1730	1740	1750	1760
VSDQSPGAFV	AIQEELAQQL	KEKASLLEEI	AAALPSLPPV	ESLRDCQKLL	QVSQSLSYNT	CLGGLGQYSS	LLVQDAIIQA
1770	1780	1790	1800	1810	1820	1830	1840
QVCYASCRIR	LEYEKELQLC	KESWQTREPS	CSEQAQAARA	LREEYEELLR	KQKSEYLDVI	AIVERENAEL	KAKAAQLDHQ
1850	1860	1870	1880	1890	1900	1910	1920
QQCLEDAESK	HSMSMFTLRG	RYEEEIRCVV	EQLTRTESTL	QAERSRVLSQ	LDASVRDRQD	MERHHGEQIQ	TLEDRFQLKV
1930	1940	1950	1960	1970	1980	1990	2000
RELQTIHEEE	LRTLQEHYSQ	SLRCLQDTLC	LHQGPHPKAL	PAPAPNWQAT	QGEADSMTGL	RERIQELEAQ	MDVMREELGH
2010	2020	2030	2040	2050	2060	2070	2080
KDLEGDAATL	REKYQRDLES	LKATCERGFA	AMEETHQKKI	EDLQRQHQRE	LEKLREEKDR	LLAEETAATI	SAIEAMKNAH
2090	2100	2110	2120	2130	2140	2150	2160
REEMERELEK	SQRSQISSVN	SDVEALRRQY	LEELQSVQRE	LEVLSEQYSQ	KCLENAHLAQ	ALEAERQALR	QCQRENQELN
2170	2180	2190	2200	2210	2220	2230	2240

Detailed Protein Report

Cmpd.	No. of Cmpds.	m/z meas.	Δ m/z [ppm]	z	Rt [min]	Score	P	Range	Sequence	Modification
1449	1	1011.7606	-72.73	3	47.9	13.8	2	646-671	R.MEVDRSPGLPMSDLKTHNVHVEIEQR.W	Oxidation: 11
448	1	593.8428	14.41	2	35.2	15.6	0	1637-1646	R.VMQQVLEALR.L	

Detailed Protein Report

Protein 292: probable G-protein coupled receptor 179 precursor [Homo sapiens]

**Accession:**	gi|93352554	**Score:**	29.3
**Database:**	refseq_human(refseq_human_20140103.fasta)	**MW [kDa]:**	257.2
**Database Date:**	2015-11-30	**pI:**	5.4
**Modification(s):**	Carbamidomethyl, Oxidation	**Sequence Coverage [%]:**	0.7
		**No. of unique Peptides:**	2

Quantitation

MD:MU	**Median:** 0.63	**CV:** 0.00 %	**No. of Peptides:** 1

Detailed Protein Report

10	20	30	40	50	60	70	80
MGTRGAVMPP	PMWGLLGCCF	VCAWALGGPR	PIRSLPPLSS	QVKPGSVPMQ	VPLEGAEAAL	AYLYSGDAQQ	LSQVNCSERY
90	100	110	120	130	140	150	160
EARGAGAMPG	LPPSLQGAAG	TLAQAANFLN	MLLQANDIRE	SSVEEDVEWY	QALVRSVAEG	DPRVYRALLT	FNPPPGASHL
170	180	190	200	210	220	230	240
QLALQATRTG	EETILQDLSG	NWVQEENPPG	DLDTPALKKR	VLTNDLGSLG	SPKWPQADGY	VGDTQQVRLS	PPFLECQEGR
250	260	270	280	290	300	310	320
LRPGWLITLS	ATFYGLKPDL	SPEVRGQVQM	DVDLQSVDIN	QCASGPGWYS	NTHLCDLNST	QCVPLESQGF	VLGRYLCRCR
330	340	350	360	370	380	390	400
PGFYGASPSG	GLEESDFQTT	GQFGFPEGRS	GRLLQCLPCP	EGCTSCMDAT	PCLVEEAAVL	RAAVLACQAC	CMLAIFLSML
410	420	430	440	450	460	470	480
VSYRCRRNKR	IWASGVVLLE	TVLFGFLLLY	FPVFILYFKP	SVFRCIALRW	VRLLGFAIVY	GTIILKLYRV	LQLFLSRTAQ
490	500	510	520	530	540	550	560
RSALLSSGRL	LRRLGLLLLP	VLGFLAVWTV	GALERGIQHA	PLVIRGHTPS	GRHFYLCHHD	RWDYIMVVAE	LLLLCWGSFL
570	580	590	600	610	620	630	640
CYATRAVLSA	FHEPRYMGIA	LHNELLLSAA	FHTARFVLVP	SLHPDWTLLL	FFFHTHSTVT	TTLALIFIPK	FWKLGAPPRE
650	660	670	680	690	700	710	720
EMVDEVCEDE	LDLQHSGSYL	GSSIASAWSE	HSLDPGDIRD	ELKKLYAQLE	VHKTKEMAAN	NPHLPKKRGS	SCQGLGRSFM
730	740	750	760	770	780	790	800
RYLAEFPEAL	ARQHSRDSGS	PGHGSLPGSS	RRRLLSSSLQ	EPEGTPALHK	SRSTYDQRRE	QDPPLLDSLL	RRKLAKKASR
810	820	830	840	850	860	870	880
TESRESVEGP	PALGFRSASA	HNLTVGERLP	RARPASLQKS	LSVASSREKA	LLMASQAYLE	ETYRQAKERE	ERKKAKAAMA
890	900	910	920	930	940	950	960
SLVRRPSARR	LERPRGAPLS	APPSPAKSSS	VDSSHTSGRL	HEEARRRLPH	PPIRHQVSTP	ILALSGGLGE	PRMLSPTSTL
970	980	990	1000	1010	1020	1030	1040
APALLPALAP	TPAPALAPVP	VSPQSPNLLT	YICPWENAEL	PAKQENVPQE	GPSGPERGHH	SPAPARARLW	RALSVAVEKS
1050	1060	1070	1080	1090	1100	1110	1120
RAGENEMDAE	DAHHQREAND	VDEDRPKIFP	KSHSLKAPVQ	QGSMRSLGLA	IKALTRSRST	YREKESVEES	PEGQNSGTAG
1130	1140	1150	1160	1170	1180	1190	1200
ESMGAPSRSP	RLGRPKAVSK	QAALIPSDDK	ESLQNQQNAH	TSRMLQVCQR	EGSREQEDRG	RRMTQGLGER	KAERAGKTGL
1210	1220	1230	1240	1250	1260	1270	1280
AMLRQVSRDK	NIKQSKETPV	GWQELPKAGL	QSLGSADHRV	AEVCPWEVTE	SETRQPDSGN	KAEICPWETS	EGAPESRALR
1290	1300	1310	1320	1330	1340	1350	1360
QDPGDSQKKR	GEARGKSEPI	DVVPMMRKKP	ERLVREQEAV	CPWESADRGG	LSPGSAPQDP	GRIRDKSEAG	DSVEARKVEK
1370	1380	1390	1400	1410	1420	1430	1440
PGWEAAGPEA	HTPDITKAEP	CPWEASEGGE	DGKPAQEAVK	DLPQEKQKTR	KATFWKEQKP	GGDLESLCPW	ESTDFRGPSA
1450	1460	1470	1480	1490	1500	1510	1520
VSIQAPGSSE	CSGSLGSGIA	EVCLWEAGDA	PAIQKAEICP	WELDDNVMGQ	EMLSLGTGRE	SLQEKEKASR	KGSFGEMGEQ
1530	1540	1550	1560	1570	1580	1590	1600
TVKAVQKLSQ	QQESVCPRES	TVPGHSSPCL	DNSSSKAGSQ	FLCNGGSRAT	QVCPQEDLRP	EAQEATPAKT	EICPWEVNER
1610	1620	1630	1640	1650	1660	1670	1680
TREEWTSAQV	PRGGESQKDK	EKMPGKSEIE	DVTAWEKPEG	QIQKQEAVGP	WESVDPGSFS	PQPRPQDTER	PQTLLQMSGS
1690	1700	1710	1720	1730	1740	1750	1760
VGSKAADICP	LDVEENLTAG	KAEICPWEVG	AGAGEERALG	AEAIRKSPND	TGKVSADLGP	RERAVTAPEK	PQKPTPEWEV
1770	1780	1790	1800	1810	1820	1830	1840
ACPWGSVGPG	ACSQHPGTLD	ADGPKAGFQE	LDHMGCRPGE	VCPWEAQEAA	TSEKAKICPW	EVSEGTTGKG	LDQKAGSESA
1850	1860	1870	1880	1890	1900	1910	1920
EQREKALEKG	RLTSLGEDVS	KGMAKLCQQQ	ETICIWENKD	LRESPAQAPK	ISDLPSSMSS	EVAEGHSLEA	TEKGDLRQDP
1930	1940	1950	1960	1970	1980	1990	2000
KTGSFPEHIT	QEKAPAADTE	EFTTEDGEKT	SHELQSVCPW	ETTAPADSVS	HLDRQRPDQP	KASSQRLVST	GGRAADVCPW
2010	2020	2030	2040	2050	2060	2070	2080
DVPDAGVYKS	DSSAKAETCP	WEVTERIPVK	GVSRQDGKGD	SQEEKGRAPE	KSEPKGVPVQ	KKPEMADFRQ	QEAVCPWESQ
2090	2100	2110	2120	2130	2140	2150	2160
DGKGLSPQPA	PDASDRSRGS	SEAAGSVETR	VAEVCLWEVV	EAPSAKKAEI	CPWEAGGGAA	EEGEQERESQ	GQGEMFLQKA
2170	2180	2190	2200	2210	2220	2230	2240

Detailed Protein Report

Cmpd.	No. of Cmpds.	m/z meas.	Δ m/z [ppm]	z	Rt [min]	Score	P	Range	Sequence	Modification	Ratios
479	1	475.6002	-274.91	2	35.6	13.9	0	1164-1170	R.MLQVCQR.E	Carbamidomethyl: 5; Oxidation: 1	MD:MU 0.63
799	1	509.1480	-293.13	2	39.5	15.4	1	1195-1204	R.AGKTGLAMLR.Q		

Detailed Protein Report

Protein 293: **PREDICTED: receptor-type tyrosine-protein phosphatase F isoform X12 [Homo sapiens]**

**Accession:**	gi|578799535	**Score:**	29.2
**Database:**	refseq_human(refseq_human_20140103.fasta)	**MW [kDa]:**	192.7
**Database Date:**	2015-11-30	**pI:**	5.8
		**Sequence Coverage [%]:**	1.6
		**No. of unique Peptides:**	1

10	20	30	40	50	60	70	80
MAPEPAPGRT	MVPLVPALVM	LGLVAGAHGD	SKPVFIKVPE	DQTGLSGGVA	SFVCQATGEP	KPRITWMKKG	KKVSSQRFEV
90	100	110	120	130	140	150	160
IEFDDGAGSV	LRIQPLRVQR	DEAIYECTAT	NSLGEINTSA	KLSVLEEEQL	PPGFPSIDMG	PQLKVVEKAR	TATMLCAAGG
170	180	190	200	210	220	230	240
NPDPEISWFK	DFLPVDPATS	NGRIKQLRSG	ALQIESSEES	DQGKYECVAT	NSAGTRYSAP	ANLYVRDQRE	VRRVAPRFSI
250	260	270	280	290	300	310	320
PPSSQEVMPG	GSVNLTCVAV	GAPMPYVKWM	MGAEELTKED	EMPVGRNVLE	LSNVVRSANY	TCVAISSLGM	IEATAQVTVK
330	340	350	360	370	380	390	400
ALPKPPIDLV	VTETTATSVT	LTWDSGNSEP	VTYYGIQYRA	AGTEGPFQEV	DGVATTRYSI	GGLSPFSEYA	FRVLAVNSIG
410	420	430	440	450	460	470	480
RGPPSEAVRA	RTGEQAPSSP	PRRVQARMLS	ASTMLVQWEP	PEEPNGLVRG	YRVYYTPDSR	RPPNAWHKHN	TDAGLLTTVG
490	500	510	520	530	540	550	560
SLLPGITYSL	RVLAFTAVGD	GPPSPTIQVK	TQQGVPAQPA	DFQAEVESDT	RIQLSWLLPP	QERIIMYELV	YWAAEDEDQQ
570	580	590	600	610	620	630	640
HKVTFDPTSS	YTLEDLKPDT	LYRFQLAARS	DMGVGVFTPT	IEARTAQSTP	SAPPQKVMCV	SMGSTTVRVS	WVPPPADSRN
650	660	670	680	690	700	710	720
GVITQYSVAY	EAVDGEDRGR	HVVDGISREH	SSWDLVGLEK	WTEYRVWVRA	HTDVGPGPES	SPVLVRTDED	VPSGPPRKVE
730	740	750	760	770	780	790	800
VEPLNSTAVH	VYWKLPVPSK	QHGQIRGYQV	TYVRLENGEP	RGLPIIQDVM	LAEAQWRPEE	SEDYETTISG	LTPETTYSVT
810	820	830	840	850	860	870	880
VAAYTTKGDG	ARSKPKIVTT	TGAVFAKNFR	VAAAMKTSVL	LSWEVPDSYK	SAVPFKILYN	GQSVEVDGHS	MRKLIADLQP
890	900	910	920	930	940	950	960
NTEYSFVLMN	RGSSAGGLQH	LVSIRTAPDL	LPHKPLPASA	YIEDGRFDLS	MPHVQDPSLV	RWFYIVVVPI	DRVGGSMLTP
970	980	990	1000	1010	1020	1030	1040
RWSTPEELEL	DELLEAIEQG	GEEQRRRRRQ	AERLKPYVAA	QLDVLPETFT	LGDKKNYRGF	YNRPLSPDLS	YQCFVLASLK
1050	1060	1070	1080	1090	1100	1110	1120
EPMDQKRYAS	SPYSDEIVVQ	VTPAQQQEEP	EMLWVTGPVL	AVILIIILIVI	AILLFKRKRT	HSPSSKDEQS	IGLKDSLLAH
1130	1140	1150	1160	1170	1180	1190	1200
SSDPVEMRRL	NYQTPGSSVP	SCPNTSSMRD	HPPIPITDLA	DNIERLKAND	GLKFSQEYES	IDPGQQFTWE	NSNLEVNKPK
1210	1220	1230	1240	1250	1260	1270	1280
NRYANVIAYD	HSRVILTSID	GVPGSDYINA	NYIDGYRKQN	AYIATQGPLP	ETMGDFWRMV	WEQRTATVVM	MTRLEEKSRV
1290	1300	1310	1320	1330	1340	1350	1360
KCDQYWPARG	TETCGLIQVT	LLDTVELATY	TVRTFALHKS	GSSEKRELRQ	FQFMAWPDHG	VPEYPTPILA	FLRRVKACNP
1370	1380	1390	1400	1410	1420	1430	1440
LDAGPMVVHC	SAGVGRTGCF	IVIDAMLERM	KHEKTVDIYG	HVTCMRSQRN	YMVQTEDQYV	FIHEALLEAA	TCGHTEVPAR
1450	1460	1470	1480	1490	1500	1510	1520
NLYAHIQKLG	QVPPGESVTA	MELEFKLLAS	SKAHTSRFIS	ANLPCNKFKN	RLVNIMPYEL	TRVCLQPIRG	VEGSDYINAS
1530	1540	1550	1560	1570	1580	1590	1600
FLDGYRQQKA	YIATQGPLAE	STEDFWRMLW	EHNSTIIVML	TKLREMGREK	CHQYWPAERS	ARYQYFVVDP	MAEYNMPQYI
1610	1620	1630	1640	1650	1660	1670	1680
LREFKVTDAR	DGQSRTIRQF	QFTDWPEQGV	PKTGEGFIDF	IGQVHKTKEQ	FGQDGPITVH	CSAGVGRTGV	FITLSIVLER
1690	1700	1710	1720	1730			
MRYEGVVDMF	QTVKTLRTQR	PAMVQTEDQY	QLCYRAALEY	LGSFDHYAT			

Cmpd.	No. of Cmpds.	m/z meas.	Δ m/z [ppm]	z	Rt [min]	Score	P	Range	Sequence	Modification

Detailed Protein Report

Cmpd.	No. of Cmpds.	m/z meas.	Δ m/z [ppm]	z	Rt [min]	Score	P	Range	Sequence	Modification
2797	1	1024.5150	17.18	2	65.4	15.2	2	1390-1406	R.MKHEKTVDIYGHVTCMR.S	

Detailed Protein Report

Protein 294: **PREDICTED: probable JmjC domain-containing histone demethylation protein 2C isoform X6 [Homo sapiens]**

**Accession:**	gi|530393271	**Score:**	29.1
**Database:**	refseq_human(refseq_human_20140103.fasta)	**MW [kDa]:**	250.9
**Database Date:**	2015-11-30	**pI:**	9.0
**Modification(s):**	Oxidation	**Sequence Coverage [%]:**	0.7
		**No. of unique Peptides:**	2

Detailed Protein Report

10	20	30	40	50	60	70	80
MNSQAAVPKQ	NTHQQQQQRS	IRPNKRKGSD	SSIPDEEKMK	EEKYDYISRG	ENPKGKNKHL	MNKRRKPEED	EKKLNMKRLR
90	100	110	120	130	140	150	160
TDNVSDFSES	SDSENSNKRI	IDNSSEQKPE	NELKNKNTSK	INGEEGKPHN	NEKAGEETLK	NSQPPWDQIQ	EDKKHEEAEK
170	180	190	200	210	220	230	240
RKSVDTQLQE	DMIIHSSEQS	TVSDHNSNDL	LPQECNMDKT	HTMELLPKEK	FVSRPPTPKC	VIDITNDTNL	EKVAQENSST
250	260	270	280	290	300	310	320
FGLQTLQKMD	PNVSDSKHSI	ANAKFLETAK	KDSDQSWVSD	VVKVDLTQSS	VTNASSGNDH	LNMEKEKYVS	YISPLSAVSV
330	340	350	360	370	380	390	400
MEDKLHKRSP	PPETIKSKLN	TSVDTHKIKS	SPSPEVVKPK	ITHSPDSVKS	KATYVNSQAT	GERRLANKIE	HELSRCSFHP
410	420	430	440	450	460	470	480
IPTRSSTLET	TKSPLIIDKN	EHFTVYRDPA	LIGSETGANH	ISPFLSQHPF	PLHSSSHRTC	LNPGTHHPAL	TPAPHLLAGS
490	500	510	520	530	540	550	560
SSQTPLPTIN	THPLTSGPHH	AVHHPHLLPT	VLPGVPTASL	LGGHPRLESA	HASSLSHLAL	AHQQQQQLLQ	HQSPHLLGQA
570	580	590	600	610	620	630	640
HPSASYNQLG	LYPIIWQYPN	GTHAYSGLGL	PSSKWVHPEN	AVNAEASLRR	NSPSPWLHQP	TPVTSADGIG	LLSHIPVRPS
650	660	670	680	690	700	710	720
SAEPHRPLKI	TAHSSPPLTK	TLVDHHKEEL	ERKAFMEPLR	SVASTSAKND	LDLNRSQTGK	DCHLHRHFVD	PVLNQLQRPP
730	740	750	760	770	780	790	800
QETGERLNKY	KEEHRRILQE	SIDVAPFTTK	IKGLEGEREN	YSRVASSSSS	PKSHIIKQDM	DVERSVSDLY	KMKHSVPQSL
810	820	830	840	850	860	870	880
PQSNYFTTLS	NSVVNEPPRS	YPSKEVSNIY	GDKQSNALAA	AAANPQTLTS	FITSLSKPPP	LIKHQPESEG	LVGKIPEHLP
890	900	910	920	930	940	950	960
HQIASHSVTT	FRNDCRSPTH	LTVSSTNTLR	SMPALHRAPV	FHPPIHHSLE	RKEGSYSSLS	PPTLTPVMPV	NAGGKVQESQ
970	980	990	1000	1010	1020	1030	1040
KPPTLIPEPK	DSQANFKSSS	EQSLTEMWRP	NNNLSKEKTE	WHVEKSSGKL	QAAMASVIVR	PSSSTKTDSM	PAMQLASKDR
1050	1060	1070	1080	1090	1100	1110	1120
VSERSSAGAH	KTDCLKLAEA	GETGRIILPN	VNSDSVHTKS	EKNFQAVSQG	SVPSSVMSAV	NTMCNTKTDV	ITSAADTTSV
1130	1140	1150	1160	1170	1180	1190	1200
SSWGGSEVIS	SLSNTILAST	SSECVSSKSV	SQPVAQKQEC	KVSTTAPVTL	ASSKTGSVVQ	PSSGFSGTTD	FIHLKKHKAA
1210	1220	1230	1240	1250	1260	1270	1280
LAAAQYKSSN	ASETEPNAIK	NQTLSASLPL	DSTVICSTIN	KANSVGNGQA	SQTSQPNYHT	KLKKAWLTRH	SEEDKNTNKM
1290	1300	1310	1320	1330	1340	1350	1360
ENSGNSVSEI	IKPCSVNLIA	STSSDIQNSV	DSKIIVDKYV	KDDKVNRRKA	KRTYESGSES	GDSDESESKS	EQRTKRQPKP
1370	1380	1390	1400	1410	1420	1430	1440
TYKKKQNDLQ	KRKGEIEEDL	KPNGVLSRSA	KERSKLKLQS	NSNTGIPRSV	LKDWRKVKKL	KQTGESFLQD	DSCCEIGPNL
1450	1460	1470	1480	1490	1500	1510	1520
QKCRECRLIR	SKKGEEPAHS	PVFCRFYYFR	RLSFSKNGVV	RIDGFSSPDQ	YDDEAMSLWT	HENFEDDELD	IETSKYILDI
1530	1540	1550	1560	1570	1580	1590	1600
IGDKFCQLVT	SEKTALSWVK	KDAKIAWKRA	VRGVREMCDA	CEATLFNIHW	VCQKCGFVVC	LDCYKAKERK	SSRDKELYAW
1610	1620	1630	1640	1650	1660	1670	1680
MKCVKGQPHD	HKHLMPTQII	PGSVLTDLLD	AMHTLREKYG	IKSHCHCTNK	QNLQVGNFPT	MNGVSQVLQN	VLNHSNKISL
1690	1700	1710	1720	1730	1740	1750	1760
CMPESQQQNT	PPKSEKNGGS	SPESDVGTDN	KLTPPESQSP	LHWLADLAEQ	KAREEKKENK	ELTLENQIKE	EREQDNSESP
1770	1780	1790	1800	1810	1820	1830	1840
NGRTSPLVSQ	NNEQGSTLRD	LLTTTAGKLR	VGSTDAGIAF	APVYSMGAPS	SKSGRTMPNI	LDDIIASVVE	NKIPPSKTSK
1850	1860	1870	1880	1890	1900	1910	1920
INVKPELKEE	PEESIISAVD	ENNKLYSDIP	HSWICEKHIL	WLKDYKNSSN	WKLFKECWKQ	GQPAVVSGVH	KKMNISLWKA
1930	1940	1950	1960	1970	1980	1990	2000
ESISLDFGDH	QADLLNCKDS	IISNANVKEF	WDGFEEVSKR	QKNKSGETVV	LKLKDWPSGE	DFKTMMPARY	EDLLKSLPLP
2010	2020	2030	2040	2050	2060	2070	2080
EYCNPEGKFN	LASHLPGFFV	RPDLGPRLCS	AYGVVAAKDH	DIGTTNLHIE	VSDVVNILVY	VGIAKGNGIL	SKAGILKKFE
2090	2100	2110	2120	2130	2140	2150	2160
EEDLDDILRK	RLKDSSEIPG	ALWHIYAGKD	VDKIREFLQK	ISKEQGLEVL	PEHDPIRDQS	WYVNKKLRQR	LLEEYGVRTC
2170	2180	2190	2200	2210	2220	2230	2240

Detailed Protein Report

Cmpd.	No. of Cmpds.	m/z meas.	Δ m/z [ppm]	z	Rt [min]	Score	P	Range	Sequence	Modification
343	1	528.7540	-68.44	2	33.9	11.7	2	57-64	K.NKHLMNKR.R	Oxidation: 5
1263	1	1019.5782	7.40	1	45.5	17.4	1	1912-1919	K.KMNISLWK.A	

Detailed Protein Report

Protein 295: PREDICTED: contactin-associated protein-like 3 isoform X8 [Homo sapiens]

Accession: gi|578816738
Database: refseq_human(refseq_human_20140103.fasta)
Database Date: 2015-11-30

Score: 29.1
MW [kDa]: 126.5
pI: 9.6
Sequence Coverage [%]: 3.2
No. of unique Peptides: 1

Quantitation

MD:MU **Median:** 1.18 **CV:** 0.00 % **No. of Peptides:** 1

10	20	30	40	50	60	70	80
MASVAWAVLK	VLLLLPTQTW	SPVGAGNPPD	CDAPLASALP	RSSFSSSSEL	SSSHGPGFSR	LNRRDGAGGW	TPLVSNKYQW
90	100	110	120	130	140	150	160
LQIDLGERME	VTAVATQGGY	GSSDWVTSYL	LMFSDGGRNW	KQYRREESIW	GFPGNTNADS	VVHYRLQPPF	EARFLRFLPL
170	180	190	200	210	220	230	240
AWNPRGRIGM	RIEVYGCAYK	SEVVYFDGQS	ALLYRLDKKP	LKPIRDVISL	KFKAMQSNGI	LLHREGQHGN	HITLELIKGK
250	260	270	280	290	300	310	320
LVFFLNSGNA	KLPSTIAPVT	LTLGSLLDDQ	HWHSVLIELL	DTQVNFTVDK	HTHHFQAKGD	SSYLDLNFEI	SFGGIPTPGR
330	340	350	360	370	380	390	400
SRAFRRKSFH	GCLENLYYNG	VDVTELAKKH	KPQILMMGNV	SFSCPQPQTV	PVTFLSSRSY	LALPGNSGED	KVSVTFQFRT
410	420	430	440	450	460	470	480
WNRAGHLLFG	ELRRGSGSFV	LFLKDGKLKL	SLFQPGQSPR	NVTAGAGLND	GQWHSVSFSA	KWSHMNVVVD	DDTAVQPLVA
490	500	510	520	530	540	550	560
VLIDSGDTYY	FGDAAWTVVQ	HGGPDAVTLR	GAPSGHPRSA	VSFAYAAGAG	QLRSAVNLAE	RCEQRLALRC	GTARRPDSRD
570	580	590	600	610	620	630	640
GTPLSWWVGR	TNETHTSWGG	SLPDAQKCTC	GLEGNCIDSQ	YYCNCDAGRN	EWTSDTIVLS	QKEHLPVTQI	VMTDAGRPHS
650	660	670	680	690	700	710	720
EAAYTLGPLL	CRGDQSFWNS	ASFNTETSYL	HFPAFHGELT	ADVCFFFKTT	VSSGVFMENL	GITDFIRIEL	RAPTEVTFSF
730	740	750	760	770	780	790	800
DVGNGPCEVT	VQSPTPFNDN	QWHHVRAERN	VKGASLQVDQ	LPQKMQPAPA	DGHVRLQLNS	QLFIGGTATR	QRGFLGCIRS
810	820	830	840	850	860	870	880
LQLNGVALDL	EERATVTPGV	EPGCAGHCST	YGHLCRNGGR	CREKRRGVTC	DCAFSAYDGP	FCSNEISAYF	ATGSSMTYHF
890	900	910	920	930	940	950	960
QEHYTLSENS	SSLVSSLHRD	VTLTREMITL	SFRTTRTPSL	LLYVSSFYEE	YLSVILANNG	SLQIRYKLDR	HQNPDAFTFD
970	980	990	1000	1010	1020	1030	1040
FKNMADGQLH	QVKINREEAV	VMVEVNQSTK	KQVILSSGTE	FNAVKSLILG	KVLEAAGADP	DTRRAATSGF	TGCLSAVRFG
1050	1060	1070	1080	1090	1100	1110	1120
RAAPLKAALR	PSGPSRVTVR	GHVAPMARCA	AGAASGSPAR	ELAPRLAGGA	GRSGPADEGE	PLVNADRRDS	AVIGGVIAVV
1130	1140	1150	1160				
IFILLCITAI	AIRIYQQRKL	RKENESKVSK	KEEC				

Cmpd.	No. of Cmpds.	m/z meas.	Δ m/z [ppm]	z	Rt [min]	Score	P	Range	Sequence	Modification	Ratios
1567	1	919.5953	159.08	2	49.4	13.7	1	1061-1080	R.GHVAPMARCAAGAASGSPAR.E		MD:MU 1.18

Detailed Protein Report

Protein 296: interferon regulatory factor 2-binding protein 2 isoform B [Homo sapiens]

Accession: gi|116734706
Database: refseq_human(refseq_human_20140103.fasta)
Database Date: 2015-11-30
Modification(s): Carbamidomethyl

Score: 29.1
MW [kDa]: 59.4
pI: 10.1
Sequence Coverage [%]: 4.4
No. of unique Peptides: 2

10	20	30	40	50	60	70	80
MAAAVAVAAA	SRRQSCYLCD	LPRMPWAMIW	DFTEPVCRGC	VNYEGADRVE	FVIETARQLK	RAHGCFPEGR	SPPGAAASAA
**90**	**100**	**110**	**120**	**130**	**140**	**150**	**160**
AKPPPLSAKD	ILLQQQQQLG	HGGPEAAPRA	PQALERYPLA	AAAERPPRLG	SDFGSSRPAA	SLAQPPTPQP	PPVNGILVPN
**170**	**180**	**190**	**200**	**210**	**220**	**230**	**240**
GFSKLEEPPE	LNRQSPNPRR	GHAVPPTLVP	LMNGSATPLP	TALGLGGRAA	ASLAAVSGTA	AASLGSAQPT	DLGAHKRPAS
**250**	**260**	**270**	**280**	**290**	**300**	**310**	**320**
VSSSAAVEHE	QREAAAKEKQ	PPPPAHRGPA	DSLSTAAGAA	ELSAEGAGKS	RGSGEQDWVN	RPKTVRDTLL	ALHQHGHSGP
**330**	**340**	**350**	**360**	**370**	**380**	**390**	**400**
FESKFKKEPA	LTAVARTARK	RKPSPEPEGE	VGPPKINGEA	QPWLSTSTEG	LKIPMTPTSS	FVSPPPPTAS	PHSNRTTPPE
**410**	**420**	**430**	**440**	**450**	**460**	**470**	**480**
AAQNGQSPMA	ALILVADNAG	GSHASKDANQ	VHSTTRRNSN	SPPSPSSMNQ	RRLGPREVGG	QGAGNTGGLE	PVHPASLPDS
**490**	**500**	**510**	**520**	**530**	**540**	**550**	**560**
SLATSAPLCC	TLCHERLEDT	HFVQCPSVPS	HKFCFPCSRQ	SIKQQGASGE	VYCPSGEKCP	LVGSNVPWAF	MQGEIATILA
**570**	**580**						
GDVKVKKERD	S						

Cmpd.	No. of Cmpds.	m/z meas.	Δ m/z [ppm]	z	Rt [min]	Score	P	Range	Sequence	Modification
1850	1	724.2703	-144.49	2	53.0	18.8	0	342-355	R.KPSPEPEGEVGPPK.I	
2516	1	715.2357	-144.98	2	61.4	10.3	1	513-523	K.FCFPCSRQSIK.Q	Carbamidomethyl: 2, 5

Detailed Protein Report

Protein 297: **PREDICTED: TGF-beta-activated kinase 1 and MAP3K7-binding protein 3 isoform X3 [Homo sapiens]**

Accession: gi|578837866
Database: refseq_human(refseq_human_20140103.fasta)
Database Date: 2015-11-30
Modification(s): Carbamidomethyl

Score: 29.1
MW [kDa]: 75.0
pI: 9.6
Sequence Coverage [%]: 5.9
No. of unique Peptides: 2

10	20	30	40	50	60	70	80
MAQSSPQLDI	QVLHDLRQRF	PEIPEGVVSQ	CMLQNNNNLE	ACCRALSQES	SKYLYMEYHS	PDDNRMNRNR	LLHINLGIHS
90	100	110	120	130	140	150	160
PSSYHPGDGA	QLNGGRTLVH	SSSDGHIDPQ	HAAGKQLICL	VQEPHSAPAV	VAATPNYNPF	FMNEQNRSAA	TPPSQPPQQP
170	180	190	200	210	220	230	240
SSMQTGMNPS	AMQGPSPPPP	PPSYMHIPRY	STNPITVTVS	QNLPSGQTVP	RALQILPQIP	SNLYGSPGSI	YIRQTSQSSS
250	260	270	280	290	300	310	320
GRQTPQSTPW	QSSPQGPVPH	YSQRPLPVYP	HQQNYQPSQY	SPKQQQIPQS	AYHSPPPSQC	PSPFSSPQHQ	VQPSQLGHIF
330	340	350	360	370	380	390	400
MPPSPSTTPP	HPYQQGPPSY	QKQGSHSVAY	LPYTASSLSK	GSMKKIEITV	EPSQRPGTAI	NRSPSPISNQ	PSPRNQHSLY
410	420	430	440	450	460	470	480
TATTPPSSSP	SRGISSQPKP	PFSVNPVYIT	YTQPTGPSCT	PSPSPRVIPN	PTTVFKITVG	RATTENLLNL	VDQEERSAAP
490	500	510	520	530	540	550	560
EPIQPISVIP	GSGGEKGSHK	YQRSSSSGSD	DYAYTQALLL	HQRARMERLA	KQLKLEKEEL	ERLKSEVNGM	EHDLMQRRLR
570	580	590	600	610	620	630	640
RVSCTTAIPT	PEEMTRLRSM	NRQLQINVDC	TLKEVDLLQS	RGNFDPKAMN	NFYDNIEPGP	VVPPKPSKKE	HRTGSTQSPR
650	660	670	680				
TQPRDEDYEG	APWNCDSCTF	LNHPALNRCE	QCEMPRYT				

Cmpd.	No. of Cmpds.	m/z meas.	Δ m/z [ppm]	z	Rt [min]	Score	P	Range	Sequence	Modification
1041	1	955.6888	-79.15	3	42.6	18.2	0	20-44	R.FPEIPEGVVSQCMLQNNNNLEACCR.A	Carbamidomethyl: 23
2667	1	821.4359	-63.35	2	63.5	10.8	1	447-461	R.VIPNPTTVFKITVGR.A	

Detailed Protein Report

Protein 298: ADP/ATP translocase 1 [Homo sapiens]

**Accession:**	gi|55749577	**Score:**	29.1
**Database:**	refseq_human(refseq_human_20140103.fasta)	**MW [kDa]:**	33.0
**Database Date:**	2015-11-30	**pI:**	10.3
**Modification(s):**	Carbamidomethyl, Oxidation	**Sequence Coverage [%]:**	11.7
		**No. of unique Peptides:**	1

10	20	30	40	50	60	70	80
MGDHAWSFLK	DFLAGGVAAA	VSKTAVAPIE	RVKLLLQVQH	ASKQISAEKQ	YKGIIDCVVR	IPKEQGFLSF	WRGNLANVIR
**90**	**100**	**110**	**120**	**130**	**140**	**150**	**160**
YFPTQALNFA	FKDKYKQLFL	GGVDRHKQFW	RYFAGNLASG	GAAGATSLCF	VYPLDFARTR	LAADVGKGAA	QREFHGLGDC
**170**	**180**	**190**	**200**	**210**	**220**	**230**	**240**
IIKIFKSDGL	RGLYQGFNVS	VQGIIIYRAA	YFGVYDTAKG	MLPDPKNVHI	FVSWMIAQSV	TAVAGLVSYP	FDTVRRRMMM
**250**	**260**	**270**	**280**	**290**	**300**		
QSGRKGADIM	YTGTVDCWRK	IAKDEGAKAF	FKGAWSNVLR	GMGGAFVLVL	YDEIKKYV		

Cmpd.	No. of Cmpds.	m/z meas.	Δ m/z [ppm]	z	Rt [min]	Score	P	Range	Sequence	Modification
2202	1	1051.1584	137.54	2	57.7	12.3	2	246-263	K.GADIMYTGTVDCWRKIAK.D	Carbamidomethyl: 12; Oxidation: 5

Detailed Protein Report

Protein 299: xylosyltransferase 1 precursor [Homo sapiens]

Accession: gi|28269693
Database: refseq_human(refseq_human_20140103.fasta)
Database Date: 2015-11-30
Modification(s): Oxidation

Score: 29.0
MW [kDa]: 107.5
pI: 10.0
Sequence Coverage [%]: 2.8
No. of unique Peptides: 2

10	20	30	40	50	60	70	80
MVAAPCARRL	ARRSHSALLA	ALTVLLLQTL	VVWNFSSLDS	GAGERRGGAA	VGGGEQPPPA	PAPRRERRDL	PAEPAAARGG
90	100	110	120	130	140	150	160
GGGGGGGGGG	RGPQARARGG	GPGEPRGQQP	ASRGALPARA	LDPHPSPLIT	LETQDGYFSH	RPKEKVRTDS	NNENSVPKDF
170	180	190	200	210	220	230	240
ENVDNSNFAP	RTQKQKHQPE	LAKKPPSRQK	ELLKRKLEQQ	EKGKGHTFPG	KGPGEVLPPG	DRAAANSSHG	KDVSRPPHAR
250	260	270	280	290	300	310	320
KTGGSSPETK	YDQPPKCDIS	GKEAISALSR	AKSKHCRQEI	GETYCRHKLG	LLMPEKVTRF	CPLEGKANKN	VQWDEDSVEY
330	340	350	360	370	380	390	400
MPANPVRIAF	VLVVHGRASR	QLQRMFKAIY	HKDHFYYIHV	DKRSNYLHRQ	VLQVSRQYSN	VRVTPWRMAT	IWGGASLLST
410	420	430	440	450	460	470	480
YLQSMRDLLE	MTDWPWDFFI	NLSAADYPIR	TNDQLVAFLS	RYRDMNFLKS	HGRDNARFIR	KQGLDRLFLE	CDAHMWRLGD
490	500	510	520	530	540	550	560
RRIPEGIAVD	GGSDWFLLNR	RFVEYVTFST	DDLVTKMKQF	YSYTLLPAES	FFHTVLENSP	HCDTMVDNNL	RITNWNRKLG
570	580	590	600	610	620	630	640
CKCQYKHIVD	WCGCSPNDFK	PQDFHRFQQT	ARPTFFARKF	EAVVNQEIIG	QLDYYLYGNY	PAGTPGLRSY	WENVYDEPDG
650	660	670	680	690	700	710	720
IHSLSDVTLT	LYHSFARLGL	RRAETSLHTD	GENSCRYYPM	GHPASVHLYF	LADRFQGFLI	KHHATNLAVS	KLETLETWVM
730	740	750	760	770	780	790	800
PKKVFKIASP	PSDFGRLQFS	EVGTDWDAKE	RLFRNFGGLL	GPMDEPVGMQ	KWGKGPNVTV	TVIWVDPVNV	IAATYDILIE
810	820	830	840	850	860	870	880
STAEFTHYKP	PLNLPLRPGV	WTVKILHHWV	PVAETKFLVA	PLTFSNRQPI	KPEEALKLHN	GPLRNAYMEQ	SFQSLNPVLS
890	900	910	920	930	940	950	960
LPINPAQVEQ	ARRNAASTGT	ALEGWLDSLV	GGMWTAMDIC	ATGPTACPVM	QTCSQTAWSS	FSPDPKSELG	AVKPDGRLR

Cmpd.	No. of Cmpds.	m/z meas.	Δ m/z [ppm]	z	Rt [min]	Score	P	Range	Sequence	Modification
1789	1	916.3008	-236.11	1	52.4	13.7	0	289-296	K.LGLLMPEK.V	Oxidation: 5
2109	1	701.2880	-90.93	3	56.5	15.4	0	388-406	R.MATIWGGASLLSTYLQSMR.D	Oxidation: 18

Detailed Protein Report

Protein 300: **ATP-dependent DNA helicase Q1 [Homo sapiens]**

**Accession:**	gi|14591902	**Score:**	29.0
**Database:**	refseq_human(refseq_human_20140103.fasta)	**MW [kDa]:**	73.4
**Database Date:**	2015-11-30	**pI:**	9.0
		**Sequence Coverage [%]:**	4.3
		**No. of unique Peptides:**	1

Alias proteins:

Accession	Name	Description
gi|578823000	refseq_human (refseq_human_20140103.fasta)	PREDICTED: ATP-dependent DNA helicase Q1 isoform X5 [Homo sapiens]
gi|530399347	refseq_human (refseq_human_20140103.fasta)	PREDICTED: ATP-dependent DNA helicase Q1 isoform X4 [Homo sapiens]
gi|530399345	refseq_human (refseq_human_20140103.fasta)	PREDICTED: ATP-dependent DNA helicase Q1 isoform X3 [Homo sapiens]
gi|530399343	refseq_human (refseq_human_20140103.fasta)	PREDICTED: ATP-dependent DNA helicase Q1 isoform X2 [Homo sapiens]
gi|530399341	refseq_human (refseq_human_20140103.fasta)	PREDICTED: ATP-dependent DNA helicase Q1 isoform X1 [Homo sapiens]
gi|14591904	refseq_human (refseq_human_20140103.fasta)	ATP-dependent DNA helicase Q1 [Homo sapiens]

```
        10         20         30         40         50         60         70         80
MASVSALTEE LDSITSELHA VEIQIQELTE RQQELIQKKK VLTKKIKQCL EDSDAGASNE YDSSPAAWNK EDFPWSGKVK
        90        100        110        120        130        140        150        160
DILQNVFKLE KFRPLQLETI NVTMAGKEVF LVMPTGGGKS LCYQLPALCS DGFTLVICPL ISLMEDQLMV LKQLGISATM
       170        180        190        200        210        220        230        240
LNASSSKEHV KWVHAEMVNK NSELKLIYVT PEKIAKSKMF MSRLEKAYEA RRFTRIAVDE VHCCSQWGHD FRPDYKALGI
       250        260        270        280        290        300        310        320
LKRQFPNASL IGLTATATNH VLTDAQKILC IEKCFTFTAS FNRPNLYYEV RQKPSNTEDF IEDIVKLING RYKGQSGIIY
       330        340        350        360        370        380        390        400
CFSQKDSEQV TVSLQNLGIH AGAYHANLEP EDKTTVHRKW SANEIQVVVA TVAFGMGIDK PDVRFVIHHS MSKSMENYYQ
       410        420        430        440        450        460        470        480
ESGRAGRDDM KADCILYYGF GDIFRISSMV VMENVGQQKL YEMVSYCQNI SKCRRVLMAQ HFDEVWNSEA CNKMCDNCCK
       490        500        510        520        530        540        550        560
DSAFERKNIT EYCRDLIKIL KQAEELNEKL TPLKLIDSWM GKGAAKLRVA GVVAPTLPRE DLEKIIAHFL IQQYLKEDYS
       570        580        590        600        610        620        630        640
FTAYATISYL KIGPKANLLN NEAHAITMQV TKSTQNSFRA ESSQTCHSEQ GDKKMEEKNS GNFQKKAANM LQQSGSKNTG
       650
AKKRKIDDA
```

Cmpd.	No. of Cmpds.	m/z meas.	Δ m/z [ppm]	z	Rt [min]	Score	P	Range	Sequence	Modification
2269	1	701.1276	94.64	3	58.2	13.1	1	540-556	R.EDLEKIIAHFLIQQYLK.E	

Detailed Protein Report

Protein 301: ectodysplasin-A isoform 2 [Homo sapiens]

**Accession:**	gi|54112101	**Score:**	28.9
**Database:**	refseq_human(refseq_human_20140103.fasta)	**MW [kDa]:**	41.0
**Database Date:**	2015-11-30	**pI:**	9.6
		**Sequence Coverage [%]:**	8.7
		**No. of unique Peptides:**	1

10	20	30	40	50	60	70	80
MGYPEVERRE	LLPAAAPRER	GSQGCGCGGA	PARAGEGNSC	LLFLGFFGLS	LALHLLTLCC	YLELRSELRR	ERGAESRLGG
**90**	**100**	**110**	**120**	**130**	**140**	**150**	**160**
SGTPGTSGTL	SSLGGLDPDS	PITSHLGQPS	PKQQPLEPGE	AALHSDSQDG	HQMALLNFFF	PDEKPYSEEE	SRRVRRNKRS
**170**	**180**	**190**	**200**	**210**	**220**	**230**	**240**
KSNEGADGPV	KNKKKGKKAG	PPGPNGPPGP	PGPPGPQGPP	GIPGIPGIPG	TTVMGPPGPP	GPPGPQGPPG	LQGPSGAADK
**250**	**260**	**270**	**280**	**290**	**300**	**310**	**320**
AGTRENQPAV	VHLQGQGSAI	QVKNDLSGGV	LNDWSRITMN	PKVFKLHPRS	GELEVLVDGT	YFIYSQVYYI	NFTDFASYEV
**330**	**340**	**350**	**360**	**370**	**380**	**390**	
VVDEKPFLQC	TRSIETGKTN	YNTCYTAGVC	LLKARQKIAV	KMVHADISIN	MSKHTTFFGA	IRLGEAPAS	

Cmpd.	No. of Cmpds.	m/z meas.	Δ m/z [ppm]	z	Rt [min]	Score	P	Range	Sequence	Modification
1518	1	1059.0796	50.76	2	48.7	16.6	1	264-282	K.NDLSGGVLNDWSRITMNPK.V	

Detailed Protein Report

Protein 302: **PREDICTED: MDS1 and EVI1 complex locus protein EVI1 isoform X16 [Homo sapiens]**

Accession: gi|578807160
Database: refseq_human(refseq_human_20140103.fasta)
Database Date: 2015-11-30
Modification(s): Carbamidomethyl

Score: 28.9
MW [kDa]: 93.9
pI: 6.2
Sequence Coverage [%]: 3.2
No. of unique Peptides: 2

10	20	30	40	50	60	70	80
MRSKGRARKL	ATNNECVYGN	YPEIPLEEMP	DADGVASTPS	LNIQEPCSPA	TSSEAFTPKE	GSPYKAPIYI	PDDIPIPAEF
90	100	110	120	130	140	150	160
ELRESNMPGA	GLGIWTKRKI	EVGEKFGPYV	GEQRSNLKDP	SYGWEILDEF	YNVKFCIDAS	QPDVGSWLKY	IRFAGCYDQH
170	180	190	200	210	220	230	240
NLVACQINDQ	IFYRVVADIA	PGEELLLFMK	SEDYPHETMA	PDIHEERQYR	CEDCDQLFES	KAELADHQKF	PCSTPHSAFS
250	260	270	280	290	300	310	320
MVEEDFQQKL	ESENDLQEIH	TIQECKECDQ	VFPDLQSLEK	HMLSHTEERE	YKCDQCPKAF	NWKSNLIRHQ	MSHDSGKHYE
330	340	350	360	370	380	390	400
CENCAKQVFT	DPSNLQRHIR	SQHVGARAHA	CPECGKTFAT	SSGLKQHKHI	HSSVKPFICE	VCHKSYTQFS	NLCRHKRMHA
410	420	430	440	450	460	470	480
DCRTQIKCKD	CGQMFSTTSS	LNKHRRFCEG	KNHFAAGGFF	GQGISLPGTP	AMDKTSMVNM	SHANPGLADY	FGANRHPAGL
490	500	510	520	530	540	550	560
TFPTAPGFSF	SFPGLFPSGL	YHRPPLIPAS	SPVKGLSSTE	QTNKSQSPLM	THPQILPATQ	DILKALSKHP	SVGDNKPVEL
570	580	590	600	610	620	630	640
QPERSSEERP	FEKISDQSES	SDLDDVSTPS	GSDLETTSGS	DLESDIESDK	EKFKENGKMF	KDKVSPLQNL	ASINNKKEYS
650	660	670	680	690	700	710	720
NHSIFSPSLE	EQTAVSGAVN	DSIKAIASIA	EKYFGSTGLV	GLQDKKVGAL	PYPSMFPLPF	FPAFSQSMYP	FPDRDLRSLP
730	740	750	760	770	780	790	800
LKMEPQSPGE	VKKLQKGSSE	SPFDLTTKRK	DEKPLTPVPS	KPPVTPATSQ	DQPLDLSMGS	RSRASGTKLT	EPRKNHVFGG
810	820	830	840	850			
KKGSNVESRP	ASDGSLQHAR	PTPFFMDPIY	RCQLLKTWQK	S			

Cmpd.	No. of Cmpds.	m/z meas.	Δ m/z [ppm]	z	Rt [min]	Score	P	Range	Sequence	Modification
2185	2	1052.4145	-10.10	2	57.2	11.8	1	309-326	R.HQMSHDSGKHYECENCAK.Q	
668	1	602.8050	-39.80	2	37.9	17.1	1	832-840	R.CQLLKTWQK.S	Carbamidomethyl: 1

Detailed Protein Report

Protein 303: **PREDICTED: bromodomain adjacent to zinc finger domain protein 2A isoform X11 [Homo sapiens]**

**Accession:**	gi|578823227	**Score:**	28.8
**Database:**	refseq_human(refseq_human_20140103.fasta)	**MW [kDa]:**	170.8
**Database Date:**	2015-11-30	**pI:**	6.2
		**Sequence Coverage [%]:**	1.8
		**No. of unique Peptides:**	1

```
        10         20         30         40         50         60         70         80
MEVACILHTR GGVQKTGERR EMEANDHFNF TGLPPAPAAS GLKPSPSSGE GLYTNGSPMN FPQQGKSLNG DVNVNGLSTV
        90        100        110        120        130        140        150        160
SHTTTSGILN SAPHSSSTSH LHHPSVAYDC LWNYSQYPSA NPGSNLKDPP LLSQFSGGQY PLNGILGGSR QPSSPSHNTN
       170        180        190        200        210        220        230        240
LRAGSQEFWA NGTQSPMGLN FDSQELYDSF PDQNFEVMPN GPPSFFTSPQ TSPMLGSSIQ TFAPSQEVGS GIHPDEAAEK
       250        260        270        280        290        300        310        320
EMTSVVAENG TGLVGSLELE EEQPELKMCG YNGSVPSVES LHQEVSVLVP DPTVSCLDDP SHLPDQLEDT PILSEDSLEP
       330        340        350        360        370        380        390        400
FNSLAPEPVS GGLYGIDDTE LMGAEDKLPL EDSPVISALD CPSLNNATAF SLLADDSQTS TSIFASPTSP PVLGESVLQD
       410        420        430        440        450        460        470        480
NSFDLNNGSD AEQEEMETQS SDFPPSLTQP APDQSSTIQL HPATSPAVSP TTSPAVSLVV SPAASPEISP EVCPAASTVV
       490        500        510        520        530        540        550        560
SPAVFSVVSP ASSAVLPAVS LEVPLTASVT SPKASPVTSP AAAFPTASPA NKDVSSFLET TADVEEITGE GLTASGSGDV
       570        580        590        600        610        620        630        640
MRRRIATPEE VRLPLQHGWR REVRIKKGSH RWQGETWYYG PCGKRMKQFP EVIKYLSRNV VHSVRREHFS FSPRMPVGDF
       650        660        670        680        690        700        710        720
FEERDTPEGL QWVQLSAEEI PSRIQAITGK RGRPRNTEKA KTKEVPKVKR GRGRPPKVKI TELLNKTDNR PLKKLEAQET
       730        740        750        760        770        780        790        800
LNEEDKAKIA KSKKKMRQKV QRGECQTTIQ GQARNKRKQE TKSLKQKEAK KKSKAEKEKG KTKQEKLKEK VKREKKEKVK
       810        820        830        840        850        860        870        880
MKEKEEVTKA KPACKADKTL ATQRRLEERQ RQQMILEEMK KPTEDMCLTD HQPLPDFSRV PGLTLPSGAF SDCLTIVEFL
       890        900        910        920        930        940        950        960
HSFGKVLGFD PAKDVPSLGV LQEGLLCQGD SLGEVQDLLV RLLKAALHDP GFPSYCQSLK ILGEKVSEIP LTRDNVSEIL
       970        980        990       1000       1010       1020       1030       1040
RCFLMAYGVE PALCDRLRTQ PFQAQPPQQK AAVLAFLVHE LNGSTLIINE IDKTLESMSS YRKNKWIVEG RLRRLKTVLA
      1050       1060       1070       1080       1090       1100       1110       1120
KRTGRSEVEM EGPEECLGRR RSSRIMEETS GMEEEEEEES IAAVPGRRGR RDGEVDATAS SIPELERQIE KLSKRQLFFR
      1130       1140       1150       1160       1170       1180       1190       1200
KKLLHSSQML RAVSLGQDRY RRRYWVLPYL AGIFVEGTEG NLVPEEVIKK ETDSLKVAAH ASLNPALFSM KMELAGSNTT
      1210       1220       1230       1240       1250       1260       1270       1280
ASSPARARGR PRKTKPGSMQ PRHLKSPVRG QDSEQPQAQL QPEAQLHAPA QPQPQLQLQL QSHKGFLEQE GSPLSLGQSQ
      1290       1300       1310       1320       1330       1340       1350       1360
HDLSQSAFLS WLSQTQSHSS LLSSSVLTPD SSPGKLDPAP SQPPEEPEPD EAESSPDPQA LWFNISAQMP CNAAPTPPPA
      1370       1380       1390       1400       1410       1420       1430       1440
VSEDQPTPSP QQLASSKPMN RPSAANPCSP VQFSSTPLAG LAPKRRAGDP GEMPQSPTGL GQPKRRGRPP SKFFKQMEQR
      1450       1460       1470       1480       1490       1500       1510       1520
YLTQLTAQPV PPEMCSGWWW IRDPEMLDAM LKALHPRGIR EKALHKHLNK HRDFLQEVCL RPSADPIFEP RQLPAFQEGI
      1530       1540       1550       1560
MSWSPKEKTY ETDLAVLQWV EELEQRVIMS DLQIR
```

Cmpd.	No. of Cmpds.	m/z meas.	Δ m/z [ppm]	z	Rt [min]	Score	P	Range	Sequence	Modification
2577	1	941.5794	21.02	2	62.2	13.8	2	698-713	K.VKITELLNKTDNRPLK.K	

Detailed Protein Report

Protein 304: latent-transforming growth factor beta-binding protein 4 isoform c precursor [Homo sapiens]

**Accession:**	gi|110347437	**Score:**	28.8
**Database:**	refseq_human(refseq_human_20140103.fasta)	**MW [kDa]:**	165.6
**Database Date:**	2015-11-30	**pI:**	4.8
		**Sequence Coverage [%]:**	1.7
		**No. of unique Peptides:**	2

10	20	30	40	50	60	70	80
MAGGVRLLWV	SLLVLLAQLG	PQPGLGRLGE	RLRVRFTPVV	CGLRCVHGPT	GSRCTPTCAP	RNATSVDSGA	PGGAAPGGPG
**90**	**100**	**110**	**120**	**130**	**140**	**150**	**160**
FRAFLCPLIC	HNGGVCVKPD	RCLCPPDFAG	KFCQLHSSGA	RPPAPAVPGL	TRSVYTMPLA	NHRDDEHGVA	SMVSVHVEHP
**170**	**180**	**190**	**200**	**210**	**220**	**230**	**240**
QEASVVVHQV	ERVSGPWEEA	DAEAVARAEA	AARAEAAAPY	TVLAQSAPRE	DGYSDASGFG	YCFRELRGGE	CASPLPGLRT
**250**	**260**	**270**	**280**	**290**	**300**	**310**	**320**
QEVCCRGAGL	AWGVHDCQLC	SERLGNSERV	SAPDGPCPTG	FERVNGSCED	VDECATGGRC	QHGECANTRG	GYTCVCPDGF
**330**	**340**	**350**	**360**	**370**	**380**	**390**	**400**
LLDSSRSSCI	SQHVISEAKG	PCFRVLRDGG	CSLPILRNIT	KQICCCSRVG	KAWGRGCQLC	PPFGSEGFRE	ICPAGPGYHY
**410**	**420**	**430**	**440**	**450**	**460**	**470**	**480**
SASDLRYNTR	PLGQEPPRVS	LSQPRTLPAT	SRPSAGFLPT	HRLEPRPEPR	PDPRPGPELP	LPSIPAWTGP	EIPESGPSSG
**490**	**500**	**510**	**520**	**530**	**540**	**550**	**560**
MCQRNPQVCG	PGRCISRPSG	YTCACDSGFR	LSPQGTRCID	VDECRRVPPP	CAPGRCENSP	GSFRCVCGPG	FRAGPRAAEC
**570**	**580**	**590**	**600**	**610**	**620**	**630**	**640**
LDVDECHRVP	PPCDLGRCEN	TPGSFLCVCP	AGYQAAPHGA	SCQDVDECTQ	SPGLCGRGAC	KNLPGSFRCV	CPAGFRGSAC
**650**	**660**	**670**	**680**	**690**	**700**	**710**	**720**
EEDVDECAQE	PPPCGPGRCD	NTAGSFHCAC	PAGFRSRGPG	APCQDVDECA	RSPPPCTYGR	CENTEGSFQC	VCPMGFQPNT
**730**	**740**	**750**	**760**	**770**	**780**	**790**	**800**
AGSECEDVDE	CENHLACPGQ	ECVNSPGSFQ	CRTCPSGHHL	HRGRCTDVDE	CSSGAPPCGP	HGHCTNTEGS	FRCSCAPGYR
**810**	**820**	**830**	**840**	**850**	**860**	**870**	**880**
APSGRPGPCA	DVNECLEGDF	CFPHGECLNT	DGSFACTCAP	GYRPGPRGAS	CLDVDECSEE	DLCQSGICTN	TDGSFECICP
**890**	**900**	**910**	**920**	**930**	**940**	**950**	**960**
PGHRAGPDLA	SCLDVDECRE	RGPALCGSQR	CENSPGSYRC	VRDCDPGYHA	GPEGTCDDVD	ECQEYGPEIC	GAQRCENTPG
**970**	**980**	**990**	**1000**	**1010**	**1020**	**1030**	**1040**
SYRCTPACDP	GYQPTPGGGC	QDVDECRNRS	FCGAHAVCQN	LPGSFQCLCD	QGYEGARDGR	HCVDVNECET	LQGVCGAALC
**1050**	**1060**	**1070**	**1080**	**1090**	**1100**	**1110**	**1120**
ENVEGSFLCV	CPNSPEEFDP	MTGRCVPPRT	SAGTFPGSQP	QAPASPVLPA	RPPPPPLPRR	PSTPRQGPVG	SGRRECYFDT
**1130**	**1140**	**1150**	**1160**	**1170**	**1180**	**1190**	**1200**
AAPDACDNIL	ARNVTWQECC	CTVGEGWGSG	CRIQQCPGTE	TAEYQSLCPH	GRGYLAPSGD	LSLRRDVDEC	QLFRDQVCKS
**1210**	**1220**	**1230**	**1240**	**1250**	**1260**	**1270**	**1280**
GVCVNTAPGY	SCYCSNGYYY	HTQRLECIDN	DECADEEPAC	EGGRCVNTVG	SYHCTCEPPL	VLDGSQRRCV	SNESQSLDDN
**1290**	**1300**	**1310**	**1320**	**1330**	**1340**	**1350**	**1360**
LGVCWQEVGA	DLVCSHPRLD	RQATYTECCC	LYGEAWGMDC	ALCPAQDSDD	FEALCNVLRP	PAYSPPRPGG	FGLPYEYGPD
**1370**	**1380**	**1390**	**1400**	**1410**	**1420**	**1430**	**1440**
LGPPYQGLPY	GPELYPPPAL	PYDPYPPPPG	PFARREAPYG	APRFDMPDFE	DDGGPYGESE	APAPPGPGTR	WPYRSRDTRR
**1450**	**1460**	**1470**	**1480**	**1490**	**1500**	**1510**	**1520**
SFPEPEEPPE	GGSYAGSLAE	PYEELEAEEC	GILDGCTNGR	CVRVPEGFTC	RCFDGYRLDM	TRMACVDINE	CDEAEAASPL
**1530**	**1540**	**1550**	**1560**				
CVNARCLNTD	GSFRCICRPG	FAPTHQPHHC	APARPRA				

Cmpd.	No. of Cmpds.	m/z meas.	Δ m/z [ppm]	z	Rt [min]	Score	P	Range	Sequence	Modification
1055	1	941.2810	-147.80	2	42.8	15.8	1	902-919	R.GPALCGSQRCENSPGSYR.C	
373	1	457.1205	-287.06	2	34.0	13.0	1	1106-1114	R.QGPVGSGRR.E	

Detailed Protein Report

Protein 305: relA-associated inhibitor [Homo sapiens]

**Accession:**	gi|63003907	**Score:**	28.7
**Database:**	refseq_human(refseq_human_20140103.fasta)	**MW [kDa]:**	89.0
**Database Date:**	2015-11-30	**pI:**	6.4
**Modification(s):**	Oxidation	**Sequence Coverage [%]:**	4.1
		**No. of unique Peptides:**	1

Alias proteins:

Accession	Name	Description
gi|215820635	refseq_human (refseq_human_20140103.fasta)	relA-associated inhibitor [Homo sapiens]

10	20	30	40	50	60	70	80
MDSEAFQSAR	DFLDMNFQSL	AMKHMDLKQM	ELDTAAAKVD	ELTKQLESLW	SDSPAPPGPQ	AGPPSRPPRY	SSSSIPEPFG
90	100	110	120	130	140	150	160
SRGSPRKAAT	DGADTPFGRS	ESAPTLHPYS	PLSPKGRPSS	PRTPLYLQPD	AYGSLDRATS	PRPRAFDGAG	SSLGRAPSPR
170	180	190	200	210	220	230	240
PGPGPLRQQG	PPTPFDFLGR	AGSPRGSPLA	EGPQAFFPER	GPSPRPPATA	YDAPASAFGS	SLLGSGGSAF	APPLRAQDDL
250	260	270	280	290	300	310	320
TLRRRPPKAW	NESDLDVAYE	KKPSQTASYE	RLDVFARPAS	PSLQLLPWRE	SSLDGLGGTG	KDNLTSATLP	RNYKVSPLAS
330	340	350	360	370	380	390	400
DRRSDAGSYR	RSLGSAGPSG	TLPRSWQPVS	RIPMPPSSPQ	PRGAPRQRPI	PLSMIFKLQN	AFWEHGASRA	MLPGSPLFTR
410	420	430	440	450	460	470	480
APPPKLQPQP	QPQPQPQSQP	QPQLPPQPQT	QPQTPTPAPQ	HPQQTWPPVN	EGPPKPPTEL	EPEPEIEGLL	TPVLEAGDVD
490	500	510	520	530	540	550	560
EGPVARPLSP	TRLQPALPPE	AQSVPELEEV	ARVLAEIPRP	LKRRGSMEQA	PAVALPPTHK	KQYQQIISRL	FHRHGGPGPG
570	580	590	600	610	620	630	640
GPEPELSPIT	EGSEARAGPP	APAPPAPIPP	PAPSQSSPPE	QPQSMEMRSV	LRKAGSPRKA	RRARLNPLVL	LLDAALTGEL
650	660	670	680	690	700	710	720
EVVQQAVKEM	NDPSQPNEEG	ITALHNAICG	ANYSIVDFLI	TAGANVNSPD	SHGWTPLHCA	ASCNDTVICM	ALVQHGAAIF
730	740	750	760	770	780	790	800
ATTLSDGATA	FEKCDPYREG	YADCATYLAD	VEQSMGLMNS	GAVYALWDYS	AEFGDELSFR	EGESVTVLRR	DGPEETDWWW
810	820	830					
AALHGQEGYV	PRNYFGLFPR	VKPQRSKV					

Cmpd.	No. of Cmpds.	m/z meas.	Δ m/z [ppm]	z	Rt [min]	Score	P	Range	Sequence	Modification
100	1	1301.5284	-95.57	2	31.0	12.3	1	378-400	K.LQNAFWEHGASRAMLPGSPLFTR.A	Oxidation: 14

Detailed Protein Report

Protein 306: **zinc finger and SCAN domain-containing protein 1 [Homo sapiens]**

**Accession:**	gi|110431336	**Score:**	28.7
**Database:**	refseq_human(refseq_human_20140103.fasta)	**MW [kDa]:**	45.3
**Database Date:**	2015-11-30	**pI:**	10.0
		**Sequence Coverage [%]:**	10.0
		**No. of unique Peptides:**	1

10	20	30	40	50	60	70	80
MLPRPKAPAS	PRRPQTPTPS	EQDADPGPAS	PRDTEAQRLR	FRQFQYHVAS	GPHLALGQLW	TLCRQWLRPE	ARSKEQMLEL
90	100	110	120	130	140	150	160
LVLEQFLGAL	PSKMRTWVQS	QGPRSCREAA	SLVEDLTQMC	QQEVLVSLDS	VEPQDWSFGE	EEDGKSPRSQ	KEPSQASELI
170	180	190	200	210	220	230	240
LDAVAAAPAL	PEESEWLETT	QLQQSLHTRA	EAEAPRAPGL	LGSRARLPLK	PSIWDEPEDL	LAGPSSDLRA	EGTVISSPKG
250	260	270	280	290	300	310	320
PSAQRISPRR	RNRNTDQSGR	HQPSLKHTKG	GTQEAVAGIS	VVPRGPRGGR	PFQCADCGMV	FTWVTHFIEH	QKTHREEGPF
330	340	350	360	370	380	390	400
PCPECGKVFL	HNSVLTEHGK	IHLLEPPRKK	APRSKGPRES	VPPRDGAQGP	VAPRSPKRPF	QCSVCGKAFP	WMVHLIDHQK
410							
LHTAHGHM							

Cmpd.	No. of Cmpds.	m/z meas.	Δ m/z [ppm]	z	Rt [min]	Score	P	Range	Sequence	Modification
2130	2	1052.4295	-73.05	2	56.8	17.6	0	13-32	R.RPQTPTPSEQDADPGPASPR.D	

Detailed Protein Report

Protein 307: protein MROH8 isoform 1 [Homo sapiens]

**Accession:**	gi|194595501	**Score:**	28.6
**Database:**	refseq_human(refseq_human_20140103.fasta)	**MW [kDa]:**	119.2
**Database Date:**	2015-11-30	**pI:**	9.5
		**Sequence Coverage [%]:**	2.7
		**No. of unique Peptides:**	1

10	20	30	40	50	60	70	80
MPLSRRGRGS	RLAGSRAATS	NLPRATALSP	KSAGRGALSI	SHAQLLTRRR	HSSEQVPPES	EPRADFRSGK	WLQEPATGDA
**90**	**100**	**110**	**120**	**130**	**140**	**150**	**160**
RDSRQALRAR	MSSKHRICSQ	EEVVIPCAYD	SDSESVDLEL	SNLEIIKKGS	SSIELTDLDI	PDIPGLHCEP	LSHSPRHLTQ
**170**	**180**	**190**	**200**	**210**	**220**	**230**	**240**
QDPLSEAIVE	KLIQSIQKVF	NGELKGELEK	LKFLGDLSSL	SQALPYDETA	KSFIHSHIAD	IVHTLNVLVQ	EERPHSLSSS
**250**	**260**	**270**	**280**	**290**	**300**	**310**	**320**
MRQEVFVTIA	DLSYQDVHLL	LGSEDRAELF	SLTIKSIITL	PSVRTLTQIQ	EIMPNGTCNT	ECLYRQTFQA	FSEMLQSLVV
**330**	**340**	**350**	**360**	**370**	**380**	**390**	**400**
KDPHLENLDT	IIKHLVPWLQ	SVKDHERERA	TASMAQVLKC	LSKHLNLKLP	LRFQRLGHLV	ALMALLCGDP	QEKVAEEAAE
**410**	**420**	**430**	**440**	**450**	**460**	**470**	**480**
GIHSLLHITL	RLKYITHDKK	DQQNLKRALT	KCREFLELHS	SAAKCFYNCP	FRIAQVFEGF	LDSNELCQFI	MTTFDTLKTL
**490**	**500**	**510**	**520**	**530**	**540**	**550**	**560**
KHPCIQRSAG	ELLLTLAKNT	ESQFEKVPEI	MGVICAQLSI	ISQPRVRQQI	INTVSLFISR	PKYTDIVLSF	LLCHPVPYNR
**570**	**580**	**590**	**600**	**610**	**620**	**630**	**640**
HLAEVWRMLS	VELPSTTWIL	WRLLRKLQKC	HNEPAQEKMA	YVAVAATDAL	YEVFLGNRLR	AATFRLFPQL	LMTLLIQIHH
**650**	**660**	**670**	**680**	**690**	**700**	**710**	**720**
SIGLTMSDVD	IPSGLYTEQE	VPSEVTPLCF	AMQATKTLLL	RTCCLQEFNI	MEKNKGWALL	GGKDGHLQGL	FLLANALLER
**730**	**740**	**750**	**760**	**770**	**780**	**790**	**800**
NQLLAQKVMY	LLVPLLNRGN	DKHKLTSAGF	FVELLRSPVA	KRLPSIYSVA	RFKDWLQDGN	HLFRILGLRG	LYNLVGHQEM
**810**	**820**	**830**	**840**	**850**	**860**	**870**	**880**
REDIKSLLPY	IVDSLRETDE	KIVLSAIQIL	LQLVRTMDFT	TLAAMMRTLF	SLFGDVRSDV	HRFSVTLFGA	AIKSVKNPDK
**890**	**900**	**910**	**920**	**930**	**940**	**950**	**960**
KSIENQVLDS	LVPLLLYSQD	ENDAVAEESR	QVLTICAQFL	KWKLPQEVYS	KDPWHIKPTE	AGTICRFFEK	KCKGKINILE
**970**	**980**	**990**	**1000**	**1010**	**1020**	**1030**	**1040**
QTLMYSKNPK	LPIRRSAVLF	VGLLSKYMDH	NELRRMGTDW	IEDDLRDLLC	DPEPSLCIIA	SQTLLLVQMA	RAEPKPKQRV
**1050**	**1060**						
NWLQKLMGRS	SA						

Cmpd.	No. of Cmpds.	m/z meas.	Δ m/z [ppm]	z	Rt [min]	Score	P	Range	Sequence	Modification
1490	1	977.1102	85.91	2	48.4	10.9	1	774-789	K.DWLQDGNHLFRILGLR.G	

Detailed Protein Report

Protein 308: hydrocephalus-inducing protein homolog isoform a [Homo sapiens]

**Accession:**	gi|401664560	**Score:**	28.6
**Database:**	refseq_human(refseq_human_20140103.fasta)	**MW [kDa]:**	575.5
**Database Date:**	2015-11-30	**pI:**	5.7
**Modification(s):**	Oxidation	**Sequence Coverage [%]:**	0.8
		**No. of unique Peptides:**	2

Detailed Protein Report

10	20	30	40	50	60	70	80
MTSRRLEESM	GAVQMGLVNM	FKGFQSKVLP	PLSPKVVTEE	EVNRMLTPSE	FLKEMSLTTE	QRLAKTRLMC	RPQIIELLDM
90	100	110	120	130	140	150	160
GETTHQKFSG	IDLDQALFQP	FPSEIIFQNY	TPCEVYEVPL	ILRNNDKIPR	LVKVVEESSP	YFKVISPKDI	GHKVAPGVPS
170	180	190	200	210	220	230	240
IFRILFTPEE	NKDYAHTLTC	VTEREKFIVP	IKARGARAIL	DFPDKLNFST	CPVKYSTQKI	LLVRNIGNKN	AVFHIKTCRP
250	260	270	280	290	300	310	320
FSIEPAIGTL	NVGESMQLEV	EFEPQSVGDH	SGRLIVCYDT	GEKVFVSLYG	AAIDMNIRLD	KNSLTIEKTY	ISLANQRTIT
330	340	350	360	370	380	390	400
IHNRSNIIAH	FLWKVFATQQ	EEDREKYRAC	DDLIKEEKDE	TDEFFEECIT	DPLLREHLSV	LSRTFANQRR	LVQGDSKLFF
410	420	430	440	450	460	470	480
NNVFTVEPLE	GDVWPNSSAE	ITVYFNPLEA	KLYQQTIYCD	ILGREIRLPL	RIKGEGMGPK	IHFNFELLDI	GKVFTGSAHC
490	500	510	520	530	540	550	560
YEAILYNKGS	IDALFNMTPP	TSALGACFVF	SPKEGIIEPS	GVQAIQISFS	STILGNFEEE	FLVNVNGSPE	PVKLTIRGCV
570	580	590	600	610	620	630	640
IGPTFHFNVP	ALHFGDVSFG	FPHTLICSLN	NTSLIPMTYK	LRIPGDGLGH	KSISYCEQHV	DYKRPSWTKE	EISSMKPKEF
650	660	670	680	690	700	710	720
TISPDCGTIR	PQGFAAIRVT	LCSNTVQKYE	LALVVDVEGI	GEEVLALLIT	ARCVVPALHL	VNTEVDFGHC	FLKYPYEKTL
730	740	750	760	770	780	790	800
QLANQDDLPG	FYEVQPQVCE	EVPTVLFSSP	TPSGVISPSS	TIHIPLVLET	QVTGEHRSTV	YISIFGSQDP	PLVCHLKSAG
810	820	830	840	850	860	870	880
EGPVIYVHPN	QVDFGNIYVL	KDSSRILNLC	NQSFIPAFFQ	AHMAHKKSLW	TIEPNEGMVP	PETDVQLALT	ANLNDTLTFK
890	900	910	920	930	940	950	960
DCVILDIENS	STYRIPVQAS	GTGSTIVSDK	PFAPELNLGA	HFSLDTHYYH	FKLINKGRRI	QQLFWMNDSF	RPQAKLSKKG
970	980	990	1000	1010	1020	1030	1040
RVKKGHAHVQ	PQPSGSQEPR	DPQSPVFHLH	PASMELYPGQ	AIDVILEGYS	ATPRIVKEKL	VCHAIIGAQK	GKSLVMAVNI
1050	1060	1070	1080	1090	1100	1110	1120
TCEFVAPLIQ	LSTKQLIYRL	EKKPNSILKP	DYQPLAIKNI	STLPVNLLLS	TSGPFFICET	DKSLLPATPE	PIKLEIDEEK
1130	1140	1150	1160	1170	1180	1190	1200
NLLIKFDPSY	RNDLNNWVAE	EILAIKYVEH	PQIDSLDLRG	EVHYPNLSFE	TKELDFGCIL	NDTELIRYVT	ITNCSPLVVK
1210	1220	1230	1240	1250	1260	1270	1280
FRWFFLVNDE	ENQIRFVTLP	KKPYSAPVSQ	MESIPATSEA	ASPPAILVTV	ESPEMDLNDF	VKTVLVDEDA	RPEEKELRKT
1290	1300	1310	1320	1330	1340	1350	1360
KASSVISDEI	KISSTEIERI	YSSQSQVEDQ	ESLQTCEQNE	MLSIGIEEVF	DILPLFGVLQ	PHSSHQISFT	FYGHANIIAQ
1370	1380	1390	1400	1410	1420	1430	1440
AKALCEVEEG	PTYEITLKGE	ASLVNYSFDT	KDIHYGLQLF	DHVTEREITL	TNMGKVGFEF	KVLTDHQSSP	DNLLPGVPLI
1450	1460	1470	1480	1490	1500	1510	1520
LPVSGFISSH	QEQVLKVYYL	PGVPEVFKRS	FQIQIAHLDP	ENITLSGEGI	FPQICLDLPR	NLTANEKYEM	FLNQARKNTD
1530	1540	1550	1560	1570	1580	1590	1600
KEYNKCEMLD	HFDIITEEVP	EDEPAEVSAH	LQMEVERLIV	QSYVLEHQKT	TTPDPMDDPC	FSHRSRRKLA	KIQLPEYILD
1610	1620	1630	1640	1650	1660	1670	1680
FGYIILGEVR	THIIKIINTS	HFPVSFHADK	RVLHETGFST	ELDRVKNLPH	CETEIFEVRF	DPQGANLPVG	SKEVILPIKV
1690	1700	1710	1720	1730	1740	1750	1760
VGGPTVHICL	QAKVTIPTMT	LSRGKVDFAT	IQCGQCLVET	IQLSNHLQVP	CEWFVQSQKP	VDKLEKHMPK	YLRQKLRAEL
1770	1780	1790	1800	1810	1820	1830	1840
KPKTRIFEIQ	PISGVLDPGE	KSNVQVKFMP	KEEKFYSQTL	VFQIAQSAQK	LTLLARGQGL	EPRLEFSPSV	LDLGPLLLCA
1850	1860	1870	1880	1890	1900	1910	1920
PGDEAEVIVK	NPCNFPIEFY	SLEFDQQYLI	EEKILRKLKG	YDSYNTLLLP	PRNPGEKLPP	ELYEYFKEIK	KSKEEQMRAK
1930	1940	1950	1960	1970	1980	1990	2000
YLENLAQENE	EEDITSSDQG	TSNSTKRTSL	SRGISVTSNL	EEWHALLVES	KTYLEEEEDE	ESLEKIIFQT	DKLQSIDSHS
2010	2020	2030	2040	2050	2060	2070	2080
MEEVGEVENN	PVSKAIARHL	GIDISAEGRL	AKNRKGIAII	IHGTPLSGKS	ANAVSVAKYY	NAACLSIDSI	VLEAVANSNN
2090	2100	2110	2120	2130	2140	2150	2160
IPGIRARELC	IRAAIEQSVK	EGEEAAQEAA	VGQNVIGQGR	LSTDTLGKLA	SEMTLVAPEI	KPGKSVRGSV	VITKSKADSH
2170	2180	2190	2200	2210	2220	2230	2240
GSGSQKQHHS	HQSETPQISS	SPLPPGPIHR	WLSVSPSVGG	ETGLMSCVLP	DELLVQILAE	RIQLSDCYRG	VVFDGLDTLF
2250	2260	2270	2280	2290	2300	2310	2320
AQNAAAALLC	LLKAIGSREH	IYILNMAQDY	AAMKAQEKAK	KEQEERKHKG	ALEKEKERLQ	NMDEEEYDAL	TEEEKLTFDR
2330	2340	2350	2360	2370	2380	2390	2400

Detailed Protein Report

Cmpd.	No. of Cmpds.	m/z meas.	Δ m/z [ppm]	z	Rt [min]	Score	P	Range	Sequence	Modification
1684	1	1273.9976	-90.58	2	50.8	13.8	2	1-22	-.MTSRRLEESMGAVQMGLVNMFK.G	Oxidation: 1, 10
1422	1	982.5936	82.29	2	47.5	14.9	1	1282-1299	K.ASSVISDEIKISSTEIER.I	

Detailed Protein Report

Protein 309: WASH complex subunit FAM21B [Homo sapiens]

**Accession:**	gi|154090959	**Score:**	28.6
**Database:**	refseq_human(refseq_human_20140103.fasta)	**MW [kDa]:**	136.9
**Database Date:**	2015-11-30	**pI:**	4.5
		**Sequence Coverage [%]:**	1.8
		**No. of unique Peptides:**	2

Quantitation

MD:MU **Median:** 3.67 **CV:** 0.00 % **No. of Peptides:** 1

10	20	30	40	50	60	70	80
MPRLSGKSVR	VYDEEVEEPV	LKAEAEKTEQ	EKTREQKEVD	LIPKVQEAVN	YGLQVLDSAF	EQLDIKAGNS	DSEEDDANGR
90	100	110	120	130	140	150	160
VELILEPKDL	YIDRPLPYLI	GSKLFMEQED	VGLGELSSEE	GSVGSDRGSI	VDTEEEKEEE	ESDEDFAHHS	DNEQNRHTTQ
170	180	190	200	210	220	230	240
MSDEEEDDDG	CDLFADSEKE	EEDIEDIEEN	TRPKRSRPTS	FADELAARIK	GDAVGRVDEE	PTTLPSGEAK	PRKTLKEKKE
250	260	270	280	290	300	310	320
RRTPSDDEED	NLFTPPKLTD	EDFSPFGSGG	GLFSGGKGLF	DDEDEESDLF	TEAPQDRQAG	ASVKEESSSS	KPGKKIPAGA
330	340	350	360	370	380	390	400
VSVFLGDTDV	FGAASVPSMK	EPQKPEQPTP	RKSPYGPPPT	GLFDDDDGDD	DDDFFSAPHS	KPSKTGKVQS	TADIFGDEEG
410	420	430	440	450	460	470	480
DLFKEKAVAS	PEATVSQTDE	NKARAEKKVT	LSSSKNLKPS	SETKTQKGLF	SDEEDSEDLF	SSQSASKLKG	ASLLPGKLPT
490	500	510	520	530	540	550	560
SVSLFDDEDE	EDNLFGGTAA	KKQTLCLQAQ	REEKAKASEL	SKKKASALLF	SSDEEDQWNI	PASQTHLASD	SRSKGEPRDS
570	580	590	600	610	620	630	640
GTLQSQEAKA	VKKTSLFEED	EEDDLFAIAK	DSQKKTQRVS	LLFEDDVDSR	GSLFGSPPTS	VPPATKKKET	VSEAPPLLFS
650	660	670	680	690	700	710	720
DEEEKEAQLG	VKSVDKKVES	AKESLKFGRT	DVAESEKEGL	LTRSAQETVK	HSDLFSSSSP	WDKGTKPRTK	TVLSLFDEEE
730	740	750	760	770	780	790	800
DKMEDQNIIQ	APQKEVGKGR	DPDAHPKSTG	VFQDEELLFS	HKLQKDNDPD	VDLFAGTKKT	KLLEPSVGSL	FGDDEDDDLF
810	820	830	840	850	860	870	880
SSAKSQPLVQ	EKKRVVKKDH	SVDSFKNQKH	PESIQGSKEK	GIWKPETPQD	SSGLAPFKTK	EPSTRIGKIQ	ANLAINPAAL
890	900	910	920	930	940	950	960
LPTAASQISE	VKPVLPELAF	PSSEHRRSHG	LESVPVLPGS	GEAGVSFDLP	AQADTLHSAN	KSRVKMRGKR	RPQTRAARRL
970	980	990	1000	1010	1020	1030	1040
AAQESSETED	MSVPRGPIAQ	WADGAISPNG	HRPQLRAASG	EDSTEEALAA	AAAPWEGGPV	PGVDRSPFAK	SLGHSRGEAD
1050	1060	1070	1080	1090	1100	1110	1120
LFDSGDIFST	GTGSQSVERT	KPKAKIAENP	ANPPVGGKAK	SPMFPALGEA	SSDDDLFQSA	KPKPAKKTNT	FPLLEDEDDL
1130	1140	1150	1160	1170	1180	1190	1200
FTDQKVKKNE	TKSNSQQDVI	LTTQDIFEDD	IFATEAIKPS	QKTREKEKTL	ESNLFDDNID	IFADLTVKPK	EKSKKKVEAK
1210	1220	1230	1240	1250	1260		
SIFDDDMDDI	FSSGIQAKTT	KPKSRSAQAA	PEPRFEHKVS	NIFDDPLNAF	GGQ		

Cmpd.	No. of Cmpds.	m/z meas.	Δ m/z [ppm]	z	Rt [min]	Score	P	Range	Sequence	Modification	Ratios
500	1	599.8540	27.43	2	35.6	16.5	1	35-44	R.EQKEVDLIPK.V		
2876	2	703.3637	1.39	2	66.6	12.1	2	735-747	K.EVGKGRDPDAHPK.S		MD:MU 3.67

Detailed Protein Report

Protein 310: probable exonuclease mut-7 homolog isoform a [Homo sapiens]

**Accession:**	gi|145279204	**Score:**	28.6
**Database:**	refseq_human(refseq_human_20140103.fasta)	**MW [kDa]:**	96.5
**Database Date:**	2015-11-30	**pI:**	9.9
**Modification(s):**	Carbamidomethyl	**Sequence Coverage [%]:**	3.0
		**No. of unique Peptides:**	1

10	20	30	40	50	60	70	80
MDPGDPAGDP	AAGERHRMGR	DPLLLLQALQ	TLWSTRERKQ	LREEAWRGFA	ALDDPLAGLL	DMLESCRGQR	GEGPSLAAWI
90	100	110	120	130	140	150	160
SHQLQCWLQA	QPCPSLAQHS	LRLKQLQARA	VKVLTESPPS	LAAPLASIFQ	LQDADRSCLL	AHVHRLHHEG	RFREAATLGA
170	180	190	200	210	220	230	240
TLKLQSELGV	EKMSIPLLLQ	DKVALVERYV	AGFPDLQRRL	LVLMDSWCQP	GFDIKDVARR	YPEVTSLSLE	KLSPKALSRQ
250	260	270	280	290	300	310	320
VLRLQERYGV	APALCPNAAI	QQRLAALRHL	CHKRFVEKSL	SQENWTDHVQ	GLVGQSPWLQ	EQLSQLLVSH	SDPVTAAQCA
330	340	350	360	370	380	390	400
MELLLPEERL	PAAVAVELRR	FRLQGRATEA	DSRLEVKDMK	DRYYQLPIPR	ENVHLLASWE	DLTRHEGALL	QCHQVVGVDV
410	420	430	440	450	460	470	480
EWTPVFVAGG	RPRPSLLQVA	VEGHVFLLDV	LALSQPPTGQ	GAQAFSRLVA	QLLSDPSITK	LGYGMVGDLQ	KLGTSCPALA
490	500	510	520	530	540	550	560
HVEKQILGGM	DLLLVHRQMR	VASVPAPAVD	RARELRGLSL	LVQQVLGTAL	DKTQQLSNWD	RRPLCEEQVI	YAAADAYCLL
570	580	590	600	610	620	630	640
EVHQALCREP	ARFHLSEDLA	GSRRPRHRER	PGARKPPGLQ	KASAPAAPRQ	VPVAVAVSEG	AAPQIPARAF	RVVCDNMLQG
650	660	670	680	690	700	710	720
LARSLRCLGV	DARMLGNGED	HRRAAEVARQ	EGRIILTSGQ	PFHKLRAQVG	AGRCLSVDCS	LKAQQQAKAV	LKHFNVRVTH
730	740	750	760	770	780	790	800
ADIFSRCQAC	NCDQYLKVSR	DMMKQLMWLS	SHQEGPRSSG	DEATQSQAVQ	EPGPAPDAAP	EGCTYDRPCR	WLQMADLRAE
810	820	830	840	850	860	870	880
TPDMLADGTR	LQLAGVPVGV	LRTPGLRCFY	CCTGCGKVFW	DGSHLGRVAT	HFRDMLESAP	SPCEPSPAPS	PASSPF

Cmpd.	No. of Cmpds.	m/z meas.	Δ m/z [ppm]	z	Rt [min]	Score	P	Range	Sequence	Modification
1319	1	571.1608	-69.20	2	46.1	14.7	0	828-837	R.CFYCCTGCGK.V	Carbamidomethyl: 5

Detailed Protein Report

BRUKER

Protein 311: WD repeat-containing protein 89 [Homo sapiens]

Accession: gi|18087841
Database: refseq_human(refseq_human_20140103.fasta)
Database Date: 2015-11-30

Score: 28.6
MW [kDa]: 43.2
pI: 5.7
Sequence Coverage [%]: 7.5
No. of unique Peptides: 2

Alias proteins:

Accession	Name	Description
gi|384871679	refseq_human (refseq_human_20140103.fasta)	WD repeat-containing protein 89 [Homo sapiens]
gi|57165359	refseq_human (refseq_human_20140103.fasta)	WD repeat-containing protein 89 [Homo sapiens]

```
        10         20         30         40         50         60         70         80
MEKIEEQFAN LHIVKCSLGT KEPTYLLGID TSKTVQAGKE NLVAVLCSNG SIRIYDKERL NVLREFSGYP GLLNGVRFAN
        90        100        110        120        130        140        150        160
SCDSVYSACT DGTVKCWDAR VAREKPVQLF KGYPSNIFIS FDINCNDHII CAGTEKVDDD ALLVFWDARM NSQNLSTTKD
       170        180        190        200        210        220        230        240
SLGAYSETHS DDVTQVRFHP SNPNMVVSGS SDGLVNVFDI NIDNEEDALV TTCNSISSVS CIGWSGKGYK QIYCMTHDEG
       250        260        270        280        290        300        310        320
FYWWDLNHLD TDEPVTRLNI QDVREVVNMK EDALDYLIGG LYHEKTDTLH VIGGTNKGRI HLMNCSMSGL THVTSLQGGH
       330        340        350        360        370        380        390
AATVRSFCWN VQDDSLLTGG EDAQLLLWKP GAIEKTFTKK ESMKIASSVH QRVRVHSNDS YKRRKKQ
```

Cmpd.	No. of Cmpds.	m/z meas.	Δ m/z [ppm]	z	Rt [min]	Score	P	Range	Sequence	Modification
2497	1	709.8401	-97.59	2	61.6	14.1	2	54-64	R.IYDKERLNVLR.E	
633	1	1042.1848	135.88	2	37.2	14.5	2	365-382	K.IASSVHQRVRVHSNDSYK.R	

Detailed Protein Report

Protein 312: PREDICTED: coiled-coil domain-containing protein 38 isoform X2 [Homo sapiens]

**Accession:**	gi|578823287	**Score:**	28.6
**Database:**	refseq_human(refseq_human_20140103.fasta)	**MW [kDa]:**	42.7
**Database Date:**	2015-11-30	**pI:**	9.1
		**Sequence Coverage [%]:**	9.2
		**No. of unique Peptides:**	1

10	20	30	40	50	60	70	80
MSSNLLPTLN	SGGKVKDGST	KEDRPYKIFF	RDLFLVKENE	MAAKETEKFM	NRNMKVYQKT	TFSSRMKSHS	YLSQLAFYPK
**90**	**100**	**110**	**120**	**130**	**140**	**150**	**160**
RSGRSFEKFG	PGPAPIPRLI	EGSDTKRTVH	EFINDQRDRF	LLEYALSTKR	NTIKKFEKDI	AMRERQLKKA	EKKLQDDALA
**170**	**180**	**190**	**200**	**210**	**220**	**230**	**240**
FEEFLRENDQ	RSVDALKMAA	QETINKLQMT	AELKKASMEV	QAVKSEIAKT	EFLLREYMKY	GFFLLQMSPK	HWQIQQALKR
**250**	**260**	**270**	**280**	**290**	**300**	**310**	**320**
AQASKSKANI	ILPKILAKLS	LHSSNKEGIL	EESGRTAVLS	EDASQGRDSQ	GKPSRSLTRT	PEKKKSNLAE	SFGSEDSLEF
**330**	**340**	**350**	**360**	**370**	**380**		
LLDDEMDVDL	EPALYFKEPE	ELLQVLRELE	EQNLTLFQYS	QDVDENLEEK			

Cmpd.	No. of Cmpds.	m/z meas.	Δ m/z [ppm]	z	Rt [min]	Score	P	Range	Sequence	Modification
39	1	750.3346	-92.35	2	29.8	15.7	1	85-98	R.SFEKFGPGPAPIPR.L	

Detailed Protein Report

Protein 313: laminin subunit gamma-1 precursor [Homo sapiens]

Accession: gi|145309326
Database: refseq_human(refseq_human_20140103.fasta)
Database Date: 2015-11-30
Modification(s): Carbamidomethyl

Score: 28.5
MW [kDa]: 177.5
pI: 4.9
Sequence Coverage [%]: 1.6
No. of unique Peptides: 2

Quantitation

MD:MU **Median:** 1.91 **CV:** 0.00 % **No. of Peptides:** 1

10	20	30	40	50	60	70	80
MRGSHRAAPA	LRPRGRLWPV	LAVLAAAAAA	GCAQAAMDEC	TDEGGRPQRC	MPEFVNAAFN	VTVVATNTCG	TPPEEYCVQT
90	100	110	120	130	140	150	160
GVTGVTKSCH	LCDAGQPHLQ	HGAAFLTDYN	NQADTTWWQS	QTMLAGVQYP	SSINLTLHLG	KAFDITYVRL	KFHTSRPESF
170	180	190	200	210	220	230	240
AIYKRTREDG	PWIPYQYYSG	SCENTYSKAN	RGFIRTGGDE	QQALCTDEFS	DISPLTGGNV	AFSTLEGRPS	AYNFDNSPVL
250	260	270	280	290	300	310	320
QEWVTATDIR	VTLNRLNTFG	DEVFNDPKVL	KSYYYAISDF	AVGGRCKCNG	HASECMKNEF	DKLVCNCKHN	TYGVDCEKCL
330	340	350	360	370	380	390	400
PFFNDRPWRR	ATAESASECL	PCDCNGRSQE	CYFDPELYRS	TGHGGHCTNC	QDNTDGAHCE	RCRENFFRLG	NNEACSSCHC
410	420	430	440	450	460	470	480
SPVGSLSTQC	DSYGRCSCKP	GVMGDKCDRC	QPGFHSLTEA	GCRPCSCDPS	GSIDECNIET	GRCVCKDNVE	GFNCERCKPG
490	500	510	520	530	540	550	560
FFNLESSNPR	GCTPCFCFGH	SSVCTNAVGY	SVYSISSTFQ	IDEDGWRAEQ	RDGSEASLEW	SSERQDIAVI	SDSYFPRYFI
570	580	590	600	610	620	630	640
APAKFLGKQV	LSYGQNLSFS	FRVDRRDTRL	SAEDLVLEGA	GLRVSVPLIA	QGNSYPSETT	VKYVFRLHEA	TDYPWRPALT
650	660	670	680	690	700	710	720
PFEFQKLLNN	LTSIKIRGTY	SERSAGYLDD	VTLASARPGP	GVPATWVESC	TCPVGYGGQF	CEMCLSGYRR	ETPNLGPYSP
730	740	750	760	770	780	790	800
CVLCACNGHS	ETCDPETGVC	NCRDNTAGPH	CEKCSDGYYG	DSTAGTSSDC	QPCPCPGGSS	CAVVPKTKEV	VCTNCPTGTT
810	820	830	840	850	860	870	880
GKRCELCDDG	YFGDPLGRNG	PVRLCRLCQC	SDNIDPNAVG	NCNRLTGECL	KCIYNTAGFY	CDRCKDGFFG	NPLAPNPADK
890	900	910	920	930	940	950	960
CKACNCNLYG	TMKQQSSCNP	VTGQCECLPH	VTGQDCGACD	PGFYNLQSGQ	GCERCDCHAL	GSTNGQCDIR	TGQCECQPGI
970	980	990	1000	1010	1020	1030	1040
TGQHCERCEV	NHFGFGPEGC	KPCDCHPEGS	LSLQCKDDGR	CECREGFVGN	RCDQCEENYF	YNRSWPGCQE	CPACYRLVKD
1050	1060	1070	1080	1090	1100	1110	1120
KVADHRVKLQ	ELESLIANLG	TGDEMVTDQA	FEDRLKEAER	EVMDLLREAQ	DVKDVDQNLM	DRLQRVNNTL	SSQISRLQNI
1130	1140	1150	1160	1170	1180	1190	1200
RNTIEETGNL	AEQARAHVEN	TERLIEIASR	ELEKAKVAAA	NVSVTQPEST	GDPNNMTLLA	EEARKLAERH	KQEADDIVRV
1210	1220	1230	1240	1250	1260	1270	1280
AKTANDTSTE	AYNLLLRTLA	GENQTAFEIE	ELNRKYEQAK	NISQDLEKQA	ARVHEEAKRA	GDKAVEIYAS	VAQLSPLDSE
1290	1300	1310	1320	1330	1340	1350	1360
TLENEANNIK	MEAENLEQLI	DQKLKDYEDL	REDMRGKELE	VKNLLEKGKT	EQQTADQLLA	RADAAKALAE	EAAKKGRDTL
1370	1380	1390	1400	1410	1420	1430	1440
QEANDILNNL	KDFDRRVNDN	KTAAEEALRK	IPAINQTITE	ANEKTREAQQ	ALGSAAADAT	EAKNKAHEAE	RIASAVQKNA
1450	1460	1470	1480	1490	1500	1510	1520
TSTKAEAERT	FAEVTDLDNE	VNNMLKQLQE	AEKELKRKQD	DADQDMMMAG	MASQAAQEAE	INARKAKNSV	TSLLSIINDL
1530	1540	1550	1560	1570	1580	1590	1600
LEQLGQLDTV	DLNKLNEIEG	TLNKAKDEMK	VSDLDRKVSD	LENEAKKQEA	AIMDYNRDIE	EIMKDIRNLE	DIRKTLPSGC
1610							
FNTPSIEKP							

Cmpd.	No. of Cmpds.	m/z meas.	Δ m/z [ppm]	z	Rt [min]	Score	P	Range	Sequence	Modification	Ratios
2564	2	941.9632	50.20	2	62.0	15.2	1	303-318	K.LVCNCKHNTYGVDCEK.C	Carbamidomethyl: 14	

Detailed Protein Report

Cmpd.	No. of Cmpds.	m/z meas.	Δ m/z [ppm]	z	Rt [min]	Score	P	Range	Sequence	Modification	Ratios
1858	1	590.7710	-122.60	2	53.3	13.3	2	1037-1046	R.LVKDKVADHR.V		MD:MU 1.91

Detailed Protein Report

Protein 314: testis-specific serine kinase substrate [Homo sapiens]

**Accession:**	gi|11119430	**Score:**	28.5
**Database:**	refseq_human(refseq_human_20140103.fasta)	**MW [kDa]:**	65.0
**Database Date:**	2015-11-30	**pI:**	5.6
		**Sequence Coverage [%]:**	5.2
		**No. of unique Peptides:**	1

10	20	30	40	50	60	70	80
MASVVVKTIW	QSKEIHEAGD	TPTGVESCSQ	LVPEAPRRVT	SRAKGIPKKK	KAVSFHGVEP	QMSHQPMHWC	LNLKRSSACT
**90**	**100**	**110**	**120**	**130**	**140**	**150**	**160**
NVSLLNLAAM	EPTDSTGTDS	TVEDLSGQLT	LAGPPASPTL	PWDPDDADIT	EILSGVNSGL	VRAKDSITSL	KEKTNRVNQH
**170**	**180**	**190**	**200**	**210**	**220**	**230**	**240**
VQSLQSECSV	LSENLERRRQ	EAEELEGYCI	QLKENCWKVT	RSVEDAEIKT	NVLKQNSALL	EEKLRYLQQQ	LQDETPRRQE
**250**	**260**	**270**	**280**	**290**	**300**	**310**	**320**
AELQEPEEKQ	EPEEKQEPEE	KQKPEAGLSW	NSLGPAATSQ	GCPGPPGSPD	KPSRPHGLVP	AGWGMGPRAG	EGPYVSEQEL
**330**	**340**	**350**	**360**	**370**	**380**	**390**	**400**
QKLFTGIEEL	RREVSSLTAR	WHQEEGAVQE	ALRLLGGLGG	RVDGFLGQWE	RAQREQAQTA	RDLQELRGRA	DELCTMVERS
**410**	**420**	**430**	**440**	**450**	**460**	**470**	**480**
AVSVASLRSE	LEGLGPLKPI	LEEFGRQFQN	SRRGPDLSMN	LDRSHQGNCA	RCASQGSQLS	TESLQQLLDR	ALTSLVDEVK
**490**	**500**	**510**	**520**	**530**	**540**	**550**	**560**
QRGLTPACPS	CQRLHKKILE	LERQALAKHV	RAEALSSTLR	LAQDEALRAK	NLLLTDKMKP	EEKMATLDHL	HLKMCSLHDH
**570**	**580**	**590**	**600**				
LSNLPLEGST	GTMGGGSSAG	TPPKQGGSAP	EQ				

Cmpd.	No. of Cmpds.	m/z meas.	Δ m/z [ppm]	z	Rt [min]	Score	P	Range	Sequence	Modification
24	1	819.8334	-120.26	2	29.6	15.2	2	483-497	R.GLTPACPSCQRLHKK.I	

Detailed Protein Report

Protein 315: caseinolytic peptidase B protein homolog isoform 3 [Homo sapiens]

Accession: gi|385719196
Database: refseq_human(refseq_human_20140103.fasta)
Database Date: 2015-11-30

Score: 28.4
MW [kDa]: 72.2
pI: 9.7
Sequence Coverage [%]: 3.4
No. of unique Peptides: 1

10	20	30	40	50	60	70	80
MLGSLVLRRK	ALAPRLLLRL	LRSPTLRGHG	GASGRNVTTG	SLGEPQWLRV	ATGGRPGTSP	ALFSGRGAAT	GGRQGGRFDT
90	100	110	120	130	140	150	160
KCLAAATWGR	LPGPEETLPG	QDSWNGVPSR	AGLGMCALAA	ALVVHCYSKS	PSNKDAALLE	AARANNMQEV	SSVVQVLLAA
170	180	190	200	210	220	230	240
GADPNLGDDF	SSVYKTAKEQ	GIHSLEVLIT	REDDFNNRLN	NRASFKGCTA	LHYAVLADDY	RTVKELLDGG	ANPLQRNEMG
250	260	270	280	290	300	310	320
HTPLDYAREG	EVMKLLRTSE	AKYQEKQRKR	EAEERRRFPL	EQRLKEHIIG	QESAIATVGA	AIRRKENGWY	DEEHPLVFLF
330	340	350	360	370	380	390	400
LGSSGIGKTE	LAKQTAKYMH	KDAKKGFIRL	DMSEFQERHE	VAKFIGSPPG	YVGHEEGGQL	TKKLKQCPNA	VVLFDEVDKA
410	420	430	440	450	460	470	480
HPDVLTIMLQ	LFDEGRLTDG	KGKTIDCKDA	IFIMTSNVAS	DEIAQHALQL	RQEALEMSRN	RIAENLGDVQ	ISDKITISKN
490	500	510	520	530	540	550	560
FKENVIRPIL	KAHFRRDEFL	GRINEIVYFL	PFCHSELIQL	VNKELNFWAK	RAKQRHNITL	LWDREVADVL	VDGYNVHYGA
570	580	590	600	610	620	630	640
RSIKHEVERR	VVNQLAAAYE	QDLLPGGCTL	RITVEDSDKQ	LLKSPELPSP	QAEKRLPKLR	LEIIDKDSKT	RRLDIRAPLH
650							
PEKVCNTI							

Cmpd.	No. of Cmpds.	m/z meas.	Δ m/z [ppm]	z	Rt [min]	Score	P	Range	Sequence	Modification
1569	1	788.7801	-136.01	2	49.5	16.8	0	386-399	K.QCPNAVVLFDEVDK.A	

Detailed Protein Report

Protein 316: sortilin-related receptor preproprotein [Homo sapiens]

**Accession:**	gi|4507157	**Score:**	28.4
**Database:**	refseq_human(refseq_human_20140103.fasta)	**MW [kDa]:**	248.3
**Database Date:**	2015-11-30	**pI:**	5.2
**Modification(s):**	Carbamidomethyl	**Sequence Coverage [%]:**	0.7
		**No. of unique Peptides:**	2

Quantitation

MD:MU	**Median:** 1.16	**CV:** 0.00 %	**No. of Peptides:** 1

Detailed Protein Report

10	20	30	40	50	60	70	80
MATRSSRRES	RLPFLFTLVA	LLPPGALCEV	WTQRLHGGSA	PLPQDRGFLV	VQGDPRELRL	WARGDARGAS	RADEKPLRRK
90	100	110	120	130	140	150	160
RSAALQPEPI	KVYGQVSLND	SHNQMVVHWA	GEKSNVIVAL	ARDSLALARP	KSSDVYVSYD	YGKSFKKISD	KLNFGLGNRS
170	180	190	200	210	220	230	240
EAVIAQFYHS	PADNKRYIFA	DAYAQYLWIT	FDFCNTLQGF	SIPFRAADLL	LHSKASNLLL	GFDRSHPNKQ	LWKSDDFGQT
250	260	270	280	290	300	310	320
WIMIQEHVKS	FSWGIDPYDK	PNTIYIERHE	PSGYSTVFRS	TDFFQSRENQ	EVILEEVRDF	QLRDKYMFAT	KVVHLLGSEQ
330	340	350	360	370	380	390	400
QSSVQLWVSF	GRKPMRAAQF	VTRHPINEYY	IADASEDQVF	VCVSHSNNRT	NLYISEAEGL	KFSLSLENVL	YYSPGGAGSD
410	420	430	440	450	460	470	480
TLVRYFANEP	FADFHRVEGL	QGVYIATLIN	GSMNEENMRS	VITFDKGGTW	EFLQAPAFTG	YGEKINCELS	QGCSLHLAQR
490	500	510	520	530	540	550	560
LSQLLNLQLR	RMPILSKESA	PGLIIATGSV	GKNLASKTNV	YISSSAGARW	REALPGPHYY	TWGDHGGIIT	AIAQGMETNE
570	580	590	600	610	620	630	640
LKYSTNEGET	WKTFIFSEKP	VFVYGLLTEP	GEKSTVFTIF	GSNKENVHSW	LILQVNATDA	LGVPCTENDY	KLWSPSDERG
650	660	670	680	690	700	710	720
NECLLGHKTV	FKRRTPHATC	FNGEDFDRPV	VVSNCSCTRE	DYECDFGFKM	SEDLSLEVCV	PDPEFSGKSY	SPPVPCPVGS
730	740	750	760	770	780	790	800
TYRRTRGYRK	ISGDTCSGGD	VEARLEGELV	PCPLAEENEF	ILYAVRKSIY	RYDLASGATE	QLPLTGLRAA	VALDFDYEHN
810	820	830	840	850	860	870	880
CLYWSDLALD	VIQRLCLNGS	TGQEVIINSG	LETVEALAFE	PLSQLLYWVD	AGFKKIEVAN	PDGDFRLTIV	NSSVLDRPRA
890	900	910	920	930	940	950	960
LVLVPQEGVM	FWTDWGDLKP	GIYRSNMDGS	AAYHLVSEDV	KWPNGISVDD	QWIYWTDAYL	ECIERITFSG	QQRSVILDNL
970	980	990	1000	1010	1020	1030	1040
PHPYAIAVFK	NEIYWDDWSQ	LSIFRASKYS	GSQMEILANQ	LTGLMDMKIF	YKGKNTGSNA	CVPRPCSLLC	LPKANNSRSC
1050	1060	1070	1080	1090	1100	1110	1120
RCPEDVSSSV	LPSGDLMCDC	PQGYQLKNNT	CVKEENTCLR	NQYRCSNGNC	INSIWWCDFD	NDCGDMSDER	NCPTTICDLD
1130	1140	1150	1160	1170	1180	1190	1200
TQFRCQESGT	CIPLSYKCDL	EDDCGDNSDE	SHCEMHQCRS	DEYNCSSGMC	IRSSWVCDGD	NDCRDWSDEA	NCTAIYHTCE
1210	1220	1230	1240	1250	1260	1270	1280
ASNFQCRNGH	CIPQRWACDG	DTDCQDGSDE	DPVNCEKKCN	GFRCPNGTCI	PSSKHCDGLR	DCSDGSDEQH	CEPLCTHFMD
1290	1300	1310	1320	1330	1340	1350	1360
FVCKNRQQCL	FHSMVCDGII	QCRDGSDEDA	AFAGCSQDPE	FHKVCDEFGF	QCQNGVCISL	IWKCDGMDDC	GDYSDEANCE
1370	1380	1390	1400	1410	1420	1430	1440
NPTEAPNCSR	YFQFRCENGH	CIPNRWKCDR	ENDCGDWSDE	KDCGDSHILP	FSTPGPSTCL	PNYYRCSSGT	CVMDTWVCDG
1450	1460	1470	1480	1490	1500	1510	1520
YRDCADGSDE	EACPLLANVT	AASTPTQLGR	CDRFEFECHQ	PKTCIPNWKR	CDGHQDCQDG	RDEANCPTHS	TLTCMSREFQ
1530	1540	1550	1560	1570	1580	1590	1600
CEDGEACIVL	SERCDGFLDC	SDESDEKACS	DELTVYKVQN	LQWTADFSGD	VTLTWMRPKK	MPSASCVYNV	YYRVVGESIW
1610	1620	1630	1640	1650	1660	1670	1680
KTLETHSNKT	NTVLKVLKPD	TTYQVKVQVQ	CLSKAHNTND	FVTLRTPEGL	PDAPRNLQLS	LPREAEGVIV	GHWAPPIHTH
1690	1700	1710	1720	1730	1740	1750	1760
GLIREYIVEY	SRSGSKMWAS	QRAASNFTEI	KNLLVNTLYT	VRVAAVTSRG	IGNWSDSKSI	TTIKGKVIPP	PDIHIDSYGE
1770	1780	1790	1800	1810	1820	1830	1840
NYLSFTLTME	SDIKVNGYVV	NLFWAFDTHK	QERRTLNFRG	SILSHKVGNL	TAHTSYEISA	WAKTDLGDSP	LAFEHVMTRG
1850	1860	1870	1880	1890	1900	1910	1920
VRPPAPSLKA	KAINQTAVEC	TWTGPRNVVY	GIFYATSFLD	LYRNPKSLTT	SLHNKTVIVS	KDEQYLFLVR	VVVPYQGPSS
1930	1940	1950	1960	1970	1980	1990	2000
DYVVVKMIPD	SRLPPRHLHV	VHTGKTSVVI	KWESPYDSPD	QDLLYAIAVK	DLIRKTDRSY	KVKSRNSTVE	YTLNKLEPGG
2010	2020	2030	2040	2050	2060	2070	2080
KYHIIVQLGN	MSKDSSIKIT	TVSLSAPDAL	KIITENDHVL	LFWKSLALKE	KHFNESRGYE	IHMFDSAMNI	TAYLGNTTDN
2090	2100	2110	2120	2130	2140	2150	2160
FFKISNLKMG	HNYTFTVQAR	CLFGNQICGE	PAILLYDELG	SGADASATQA	ARSTDVAAVV	VPILFLILLS	LGVGFAILYT
2170	2180	2190	2200	2210	2220		

Detailed Protein Report

Cmpd.	No. of Cmpds.	m/z meas.	Δ m/z [ppm]	z	Rt [min]	Score	P	Range	Sequence	Modification	Ratios
2231	1	599.9323	-0.25	3	57.8	14.4	1	1239-1254	K.CNGFRCPNGTCIPSSK.H	Carbamidomethyl: 1, 11	
1162	1	610.6492	-203.57	2	44.2	14.0	0	1244-1254	R.CPNGTCIPSSK.H	Carbamidomethyl: 1, 6	MD:MU 1.16

Detailed Protein Report

Protein 317: 60 kDa SS-A/Ro ribonucleoprotein isoform 4 [Homo sapiens]

Accession: gi|291084635
Database: refseq_human(refseq_human_20140103.fasta)
Database Date: 2015-11-30
Modification(s): Carbamidomethyl

Score: 28.3
MW [kDa]: 58.4
pI: 9.5
Sequence Coverage [%]: 3.9
No. of unique Peptides: 2

10	20	30	40	50	60	70	80
MEESVNQMQP	LNEKQIANSQ	DGYVWQVTDM	NRLHRFLCFG	SEGGTYYIKE	QKLGLENAEA	LIRLIEDGRG	CEVIQEIKSF
**90**	**100**	**110**	**120**	**130**	**140**	**150**	**160**
SQEGRTTKQE	PMLFALAICS	QCSDISTKQA	AFKAVSEVCR	IPTHLFTFIQ	FKKDLKESMK	CGMWGRALRK	AIADWYNEKG
**170**	**180**	**190**	**200**	**210**	**220**	**230**	**240**
GMALALAVTK	YKQRNGWSHK	DLLRLSHLKP	SSEGLAIVTK	YITKGWKEVH	ELYKEKALSV	ETEKLLKYLE	AVEKVKRTRD
**250**	**260**	**270**	**280**	**290**	**300**	**310**	**320**
ELEVIHLIEE	HRLVREHLLT	NHLKSKEVWK	ALLQEMPLTA	LLRNLGKMTA	NSVLEPGNSE	VSLVCEKLCN	EKLLKKARIH
**330**	**340**	**350**	**360**	**370**	**380**	**390**	**400**
PFHILIALET	YKTGHGLRGK	LKWRPDEEIL	KALDAAFYKT	FKTVEPTGKR	FLLAVDVSAS	MNQRVLGSIL	NASTVAAAMC
**410**	**420**	**430**	**440**	**450**	**460**	**470**	**480**
MVVTRTEKDS	YVVAFSDEMV	PCPVTTDMTL	QQVLMAMSQI	PAGGTDCSLP	MIWAQKTNTP	ADVFIVFTDN	ETFAGGVHPA
**490**	**500**	**510**	**520**				
IALREYRKKM	DIPAKLIVCG	MTSNGFTIAD	PDDRDTVK				

Cmpd.	No. of Cmpds.	m/z meas.	Δ m/z [ppm]	z	Rt [min]	Score	P	Range	Sequence	Modification
1633	1	821.2438	-171.33	2	50.3	12.3	0	36-49	R.FLCFGSEGGTYYIK.E	Carbamidomethyl: 3
2854	2	702.3710	-10.04	1	66.1	16.1	0	64-69	R.LIEDGR.G	

Detailed Protein Report

Protein 318: PREDICTED: latrophilin-1 isoform X5 [Homo sapiens]

**Accession:**	gi|578833089	**Score:**	28.2
**Database:**	refseq_human(refseq_human_20140103.fasta)	**MW [kDa]:**	96.9
**Database Date:**	2015-11-30	**pI:**	6.5
**Modification(s):**	Carbamidomethyl	**Sequence Coverage [%]:**	4.3
		**No. of unique Peptides:**	1

10	20	30	40	50	60	70	80
MARLAAVLWN	LCVTAVLVTS	ATQGLSRAGL	PFGLMRRELA	CEGYPIELRC	PGSDVIMVEN	ANYGRTDDKI	CDADPFQMEN
**90**	**100**	**110**	**120**	**130**	**140**	**150**	**160**
VQCYLPDAFK	IMSQRCNNRT	QCVVVAGSDA	FPDPCPGTYK	YLEVQYDCVP	YKVEQKVFVC	PGTLQKVLEP	TSTHESEHQS
**170**	**180**	**190**	**200**	**210**	**220**	**230**	**240**
GAWCKDPLQA	GDRIYVMPWI	PYRTDTLTEY	ASWEDYVAAR	HTTTYRLPNR	VDGTGFVVYD	GAVFYNKERT	RNIVKYDLRT
**250**	**260**	**270**	**280**	**290**	**300**	**310**	**320**
RIKSGETVIN	TANYHDTSPY	RWGGKTDIDL	AVDENGLWVI	YATEGNNGRL	VVSQLNPYTL	RFEGTWETGY	DKRSASNAFM
**330**	**340**	**350**	**360**	**370**	**380**	**390**	**400**
VCGVLYVLRS	VYVDDDSEAA	GNRVDYAFNT	NANREEPVSL	TFPNPYQFIS	SVDYNPRDNQ	LYVWNNYFVV	RYSLEFGPPD
**410**	**420**	**430**	**440**	**450**	**460**	**470**	**480**
PSAGPATSPP	LSTTTTARPT	PLTSTASPAA	TTPLRRAPLT	THPVGAINQL	GPDLPPATAP	VPSTRRPPAP	NLHVSPELFC
**490**	**500**	**510**	**520**	**530**	**540**	**550**	**560**
EPREVRRVQW	PATQQGMLVE	RPCPKGTRGI	ASFQCLPALG	LWNPRGPDLS	NCTSPWVNQV	AQKIKSGENA	ANIASELARH
**570**	**580**	**590**	**600**	**610**	**620**	**630**	**640**
TRGSIYAGDV	SSSVKLMEQL	LDILDAQLQA	LRPIERESAG	KNYNKMHKRE	RTCKDYIKAV	VETVDNLLRP	EALESWKDMN
**650**	**660**	**670**	**680**	**690**	**700**	**710**	**720**
ATEQVHTATM	LLDVLEEGAF	LLADNVREPA	RFLAAKENVV	LEVTVLNTEG	QVQELVFPQE	EYPRKNSIQL	SAKTIKQNSR
**730**	**740**	**750**	**760**	**770**	**780**	**790**	**800**
NGVVKVVFIL	YNNLGLFLST	ENATVKLAGE	AGPGGPGGAS	LVVNSQVIAA	SINKESSRVF	LMDPVIFTVA	HLEDKNHFNA
**810**	**820**	**830**	**840**	**850**	**860**	**870**	**880**
NCSFWNYSER	SMLGYWSTQG	CRLVESNKTH	TTCACSHLTN	FAVLMAHREI	ALWRSKLVLR	WSSLHKPSRA	

Cmpd.	No. of Cmpds.	m/z meas.	Δ m/z [ppm]	z	Rt [min]	Score	P	Range	Sequence	Modification
2050	1	900.3480	-63.50	3	55.8	14.3	1	96-120	R.CNNRTQCVVVAGSDAFPDPCPGTYK.Y	Carbamidomethyl: 7

Detailed Protein Report

Protein 319: RNA polymerase II subunit A C-terminal domain phosphatase isoform 3 [Homo sapiens]

**Accession:**	gi|321267522	**Score:**	28.2
**Database:**	refseq_human(refseq_human_20140103.fasta)	**MW [kDa]:**	92.3
**Database Date:**	2015-11-30	**pI:**	4.9
		**Sequence Coverage [%]:**	4.5
		**No. of unique Peptides:**	1

10	20	30	40	50	60	70	80
MKGLCAECGQ	DLTQLQSKNG	KQQVPLSTAT	VSMVHSVPEL	MVSSEQAEQL	GREDQQRLHR	NRKLVLMVDL	DQTLIHTTEQ
**90**	**100**	**110**	**120**	**130**	**140**	**150**	**160**
HCQQMSNKGI	FHFQLGRGEP	MLHTRLRPHC	KDFLEKIAKL	YELHVFTFGS	RLYAHTIAGF	LDPEKKLFSH	RILSRDECID
**170**	**180**	**190**	**200**	**210**	**220**	**230**	**240**
PFSKTGNLRN	LFPCGDSMVC	IIDDREDVWK	FAPNLITVKK	YVYFQGTGDM	NAPPGSRESQ	TRKKVNHSRG	TEVSEPSPPV
**250**	**260**	**270**	**280**	**290**	**300**	**310**	**320**
RDPEGVTQAP	GVEPSNGLEK	PARELNGSEA	ATPRDSPRPG	KPDERDIWPP	AQAPTSSQEL	AGAPEPQGSC	AQGGRVAPGQ
**330**	**340**	**350**	**360**	**370**	**380**	**390**	**400**
RPAQGATGTD	LDFDLSSDSE	SSSESEGTKS	SSSASDGESE	GKRGRQKPKA	APEGAGALAQ	GSSLEPGRPA	APSLPGEAEP
**410**	**420**	**430**	**440**	**450**	**460**	**470**	**480**
GAHAPDKEPE	LGGQEEGERD	GLCGLGNGCA	DRKEAETESQ	NSELSGVTAG	ESLDQSMEEE	EEEDTDEDDH	LIYLEEILVR
**490**	**500**	**510**	**520**	**530**	**540**	**550**	**560**
VHTDYYAKYD	RYLNKEIEEA	PDIRKIVPEL	KSKVLADVAI	IFSGLHPTNF	PIEKTREHYH	ATALGAKILT	RLVLSPDAPD
**570**	**580**	**590**	**600**	**610**	**620**	**630**	**640**
RATHLIAARA	GTEKVLQAQE	CGHLHVVNPD	WLWSCLERWD	KVEEQLFPLR	DDHTKAQREN	SPAAFPDREG	VPPTALFHPM
**650**	**660**	**670**	**680**	**690**	**700**	**710**	**720**
PVLPKAQPGP	EVRIYDSNTG	KLIRTGARGP	PAPSSSLPIR	QEPSSFRAVP	PPQPQMFGEE	LPDAQDGEQP	GPSRRKRQPS
**730**	**740**	**750**	**760**	**770**	**780**	**790**	**800**
MSETMPLYTL	CKEDLESMDK	EVDDILGEGS	DDSDSEKRRP	EEQEEEPQPR	KPGTRRERTL	GAPASSERSA	AGGRGPRGHK
**810**	**820**	**830**	**840**	**850**			
RKLNEEDAAS	ESSRESSNED	EGSSSEADEM	AKALEAELND	LM			

Cmpd.	No. of Cmpds.	m/z meas.	Δ m/z [ppm]	z	Rt [min]	Score	P	Range	Sequence	Modification
1973	1	899.3577	-124.16	2	54.6	12.7	2	779-797	R.TLGAPASSERSAAGGRGPR.G	

Detailed Protein Report

Protein 320: **PREDICTED: RING finger protein 112 isoform X3 [Homo sapiens]**

**Accession:**	gi|578830030	**Score:**	28.2
**Database:**	refseq_human(refseq_human_20140103.fasta)	**MW [kDa]:**	66.2
**Database Date:**	2015-11-30	**pI:**	9.8
**Modification(s):**	Carbamidomethyl	**Sequence Coverage [%]:**	3.4
		**No. of unique Peptides:**	1

10	20	30	40	50	60	70	80
MGNSGNSWSH	TPFPKLELGL	GPQPMAPREL	PTCSICLERL	RDPISLDCGH	DFCIRCFSTH	RLPGCEPPCC	PECRKICKQK
**90**	**100**	**110**	**120**	**130**	**140**	**150**	**160**
RGLRSLGEKM	KLLPQRPLPP	ALQETCPVRA	EPLLLVRINA	SGGLILRMGA	INRCLKHPLA	RDTPVCLLAV	LGEQHSGKSF
**170**	**180**	**190**	**200**	**210**	**220**	**230**	**240**
LLNHLLQGLP	GLESGEGGRP	RGGEASLQGC	RWGANGLARG	IWMWSHPFLL	GKEGKKVAVF	LVDTGDAMSP	ELSRETRIKL
**250**	**260**	**270**	**280**	**290**	**300**	**310**	**320**
CALTTMLSSY	QILSTSQELK	DTDLDYLEMF	VHVAEVMGKH	YGMVPIQHLD	LLVRDSSHPN	KAGQGHVGNI	FQSTPLAASQ
**330**	**340**	**350**	**360**	**370**	**380**	**390**	**400**
RLSGRYPKVQ	ELLQGKRARC	CLLPAPGRRR	MNQGHASPGD	TDDDFRHLLG	AYVSDVLSAA	PQHAKSRCQG	YWNEGRAVAR
**410**	**420**	**430**	**440**	**450**	**460**	**470**	**480**
GDRRLLTGQQ	LAQEIKNLSG	WMGRTGPGFT	SPDEMAAQLH	DLRKVEAAKR	EFEEYVRQQD	VATKRIFSAL	RVLPDTMRNL
**490**	**500**	**510**	**520**	**530**	**540**	**550**	**560**
LSTQKDAILA	RHGVALLCKG	RDQTLEALEA	ELQATAKAFM	DSYTMRFCGH	LAAVGGAVGA	GLMGLAGGVV	GAGMAAAALA
**570**	**580**	**590**	**600**	**610**	**620**		
AEAGMVAAGA	AVGATGAAVV	GGGVGAGLAA	TVGCMEKEED	ERLLEGDREP	LLQEE		

Cmpd.	No. of Cmpds.	m/z meas.	Δ m/z [ppm]	z	Rt [min]	Score	P	Range	Sequence	Modification
2148	1	1062.3966	-148.73	1	57.0	17.9	1	128-136	R.MGAINRCLK.H	Carbamidomethyl: 7

Detailed Protein Report

Protein 321: prolactin-inducible protein precursor [Homo sapiens]

**Accession:**	gi|4505821	**Score:**	28.1
**Database:**	refseq_human(refseq_human_20140103.fasta)	**MW [kDa]:**	16.6
**Database Date:**	2015-11-30	**pI:**	9.3
		**Sequence Coverage [%]:**	8.2
		**No. of unique Peptides:**	1

Quantitation

MD:MU **Median:** 0.27 **CV:** 0.00 % **No. of Peptides:** 1

```
         10         20         30         40         50         60         70         80
MRLLQLLFRA SPATLLLVLC LQLGANKAQD NTRKIIIKNF DIPKSVRPND EVTAVLAVQT ELKECMVVKT YLISSIPLQG
         90        100        110        120        130        140        150
AFNYKYTACL CDDNPKTFYW DFYTNRTVQI AAVVDVIREL GICPDDAAVI PIKNNRFYTI EILKVE
```

Cmpd.	No. of Cmpds.	m/z meas.	Δ m/z [ppm]	z	Rt [min]	Score	P	Range	Sequence	Modification	Ratios	
2176	2	642.3028	-132.15	2	57.4	28.1	0	107-118	R.TVQIAAVVDVIR.E		MD:MU	0.27

Detailed Protein Report

Protein 322: **PREDICTED: uncharacterized protein LOC101928409 [Homo sapiens]**

**Accession:**	gi|530358441	**Score:**	28.1
**Database:**	refseq_human(refseq_human_20140103.fasta)	**MW [kDa]:**	60.4
**Database Date:**	2015-11-30	**pI:**	11.3
		**Sequence Coverage [%]:**	3.8
		**No. of unique Peptides:**	1

10	20	30	40	50	60	70	80
MLGTVASPRP	GSLFSPPLAV	IKPGYEQGFK	SSLPHPQVYR	AAPELSDMLR	CGGQRRGGGI	ALCSLGVKSN	MNVKYCGKTE
**90**	**100**	**110**	**120**	**130**	**140**	**150**	**160**
SFQSRQRGPS	AGGPGRPESL	LPRVRLPEAL	LLAEDCRGNV	GEGEMDGGSR	AYKSDKYAHT	LTVTASHHAP	PPPTHMEGFE
**170**	**180**	**190**	**200**	**210**	**220**	**230**	**240**
LFHLPELCSP	SQDAQTTGRT	QMKPDHSPRP	SHRGRWVPKR	LLRLHPSSRV	VVRLVAEGAD	GARFGNTPHC	ILFHFTSTKE
**250**	**260**	**270**	**280**	**290**	**300**	**310**	**320**
ESRIGGRPPS	CFLMQITQHS	NRKSKLQGLL	LRGALEKEER	VVRARAGASE	AKSHSRGGWD	AWGLKANARG	RDGVGSGGRS
**330**	**340**	**350**	**360**	**370**	**380**	**390**	**400**
ALQSRPLPPA	SLEGGSGTCA	RSLSPGLCAS	AWRLPGKNLI	YFLPSRVSSL	PPRAAPFRRP	EPAPGPRGCS	GERGLGMSGG
**410**	**420**	**430**	**440**	**450**	**460**	**470**	**480**
VRAPPHPRAL	LGGDIQELKE	IPQEEVETSE	RRGNRLFCKK	TERRLEGLRK	AADGSQDNSV	RPVLQGNFNP	TRTQAKTIYI
**490**	**500**	**510**	**520**	**530**	**540**	**550**	**560**
YIYELTQNRA	TLVPTPPQVG	RHIQVALSED	GTWRLAGVAL	WRSRSPVSSY	EVSLLLQSSL	QRGDLATSLP	LGFPVNPL

Cmpd.	No. of Cmpds.	m/z meas.	Δ m/z [ppm]	z	Rt [min]	Score	P	Range	Sequence	Modification
2625	1	885.4475	-93.73	1	62.9	13.6	0	515-522	R.LAGVALWR.S	

Detailed Protein Report

Protein 323: contactin-associated protein-like 4 isoform 2 [Homo sapiens]

**Accession:**	gi|148664242	**Score:**	28.0
**Database:**	refseq_human(refseq_human_20140103.fasta)	**MW [kDa]:**	137.4
**Database Date:**	2015-11-30	**pI:**	6.1
**Modification(s):**	Oxidation	**Sequence Coverage [%]:**	3.1
		**No. of unique Peptides:**	2

10	20	30	40	50	60	70	80
MWNYDCDDPL	VSALPQASFS	SSSELSSSHG	PGFARLNRRD	GAGGWSPLVS	NKYQWLQIDL	GERMEVTAVA	TQGGYGSSNW
90	100	110	120	130	140	150	160
VTSYLLMFSD	SGWNWKQYRQ	EDSIWGFSGN	ANADSVVYYR	LQPSIKARFL	RFIPLEWNPK	GRIGMRIEVF	GCAYRSEVVD
170	180	190	200	210	220	230	240
LDGKSSLLYR	FDQKSLSPIK	DIISLKFKTM	QSDGILLHRE	GPNGDHITLQ	LRRARLFLLI	NSGEAKLPST	STLVNLTLGS
250	260	270	280	290	300	310	320
LLDDQHWHSV	LIQRLGKQVN	FTVDEHRHHF	HARGEFNLMN	LDYEGNVSFS	CSQPQSMPVT	FLSSRSYLAL	PDFSGEEEVS
330	340	350	360	370	380	390	400
ATFQFRTWNK	AGLLLFSELQ	LISGGILLFL	SDGKLKSNLY	QPGKLPSDIT	AGVELNDGQW	HSVSLSAKKN	HLSVAVDGQM
410	420	430	440	450	460	470	480
ASAAPLLGPE	QIYSGGTYYF	GGCPDKSFGS	KCKSPLGGFQ	GCMRLISISG	KVVDLISVQQ	GSLGNFSDLQ	IDSCGISDRC
490	500	510	520	530	540	550	560
LPNYCEHGGE	CSQSWSTFHC	NCTNTGYRGA	TCHNSIYEQS	CEAYKHRGNT	SGFYYIDSDG	SGPLEPFLLY	CNMTETAWTI
570	580	590	600	610	620	630	640
IQHNGSDLTR	VRNTNPENPY	AGFFEYVASM	EQLQATINRA	EHCEQEFTYY	CKKSRLVNKQ	DGTPLSWWVG	RTNETQTYWG
650	660	670	680	690	700	710	720
GSSPDLQKCT	CGLEGNCIDS	QYYCNCDADR	NEWTNDTGLL	AYKEHLPVTK	IVITDTGRLH	SEAAYKLGPL	LCQGDRSFWN
730	740	750	760	770	780	790	800
SASFDTEASY	LHFPTFHGEL	SADVSFFFKT	TASSGVFLEN	LGIADFIRIE	LRSPTVVTFS	FDVGNGPFEI	SVQSPTHFND
810	820	830	840	850	860	870	880
NQWHHVRVER	NMKEASLQVD	QLTPKTQPAP	ADGHVLLQLN	SQLFVGGTAT	RQRGFLGCIR	SLQLNGMTLD	LEERAQVTPE
890	900	910	920	930	940	950	960
VQPGCRGHCS	SYGKLCRNGG	KCRERPIGFF	CDCTFSAYTG	PFCSNEISAY	FGSGSSVIYN	FQENYLLSKN	SSSHAASFHG
970	980	990	1000	1010	1020	1030	1040
DMKLSREMIK	FSFRTTRTPS	LLLFVSSFYK	EYLSVIIAKN	GSLQIRYKLN	KYQEPDVVNF	DFKNMADGQL	HHIMINREEG
1050	1060	1070	1080	1090	1100	1110	1120
VVFIEIDDNR	RRQVHLSSGT	EFSAVKSLVL	GRILEHSDVD	QDTALAGAQG	FTGCLSAVQL	SHVAPLKAAL	HPSHPDPVTV
1130	1140	1150	1160	1170	1180	1190	1200
TGHVTESSCM	AQPGTDATSR	ERTHSFADHS	GTIDDREPLA	NAIKSDSAVI	GGLIAVVIFI	LLCITAIAVR	IYQQKRLYKR
1210	1220	1230	1240				
SEAKRSENVD	SAEAVLKSEL	NIQNAVNENQ	KEYFF				

Cmpd.	No. of Cmpds.	m/z meas.	Δ m/z [ppm]	z	Rt [min]	Score	P	Range	Sequence	Modification
2542	1	715.3361	-67.84	2	61.7	15.1	2	964-974	K.LSREMIKFSFR.T	Oxidation: 5
1571	1	1061.1650	-10.98	3	49.3	12.9	1	1024-1050	K.NMADGQLHHIMINREEGVVFIEIDDNR.R	Oxidation: 2

Detailed Protein Report

Protein 324: **PREDICTED: probable ATP-dependent RNA helicase DDX11 isoform X15 [Homo sapiens]**

**Accession:**	gi|578822806	**Score:**	28.0
**Database:**	refseq_human(refseq_human_20140103.fasta)	**MW [kDa]:**	94.9
**Database Date:**	2015-11-30	**pI:**	6.8
**Modification(s):**	Oxidation	**Sequence Coverage [%]:**	4.1
		**No. of unique Peptides:**	2

10	20	30	40	50	60	70	80
MPLDGVRDSG	MASRGPAAKD	GENMVSASQK	KGEIWSMANE	TQKVGAIHFP	FPFTPYSIQE	DFMAELYRVL	EAGKIGIFES
90	100	110	120	130	140	150	160
PTGTGKSLSL	ICGALSWLRD	FEQKKREEEA	RLLETGTGPL	HDEKDESLCL	SSSCEGAAGT	PRPAGEPAWV	TQFVQKKEER
170	180	190	200	210	220	230	240
DLVDRLKAEQ	ARRKQREERL	QQLQHRVQLK	YAAKRLRQEE	EERENLLRLS	REMLETGPEA	ERLEQLESGE	EELVLAEYES
250	260	270	280	290	300	310	320
DEEKKVASRV	DEDEDDLEEE	HITKIYYCSR	THSQLAQFVH	EVKKSPFGKD	VRLVSLGSRQ	NLCVNEDVKS	LGSVQLINDR
330	340	350	360	370	380	390	400
CVDMQRSRHE	KKKGAEEEKP	KRRRQEKQAA	CPFYNHEQMG	LLRDEALAEV	KDMEQLLALG	KEARACPYYG	SRLAIPAAQL
410	420	430	440	450	460	470	480
VVLPYQMLLH	AATRQAAGIR	LQDQVVIIDE	AHNLIDTITG	MHSVEVSGSQ	LCQAHSQLLQ	YVERYGKRLK	AKNLMYLKQI
490	500	510	520	530	540	550	560
LYLLEKFVAV	LGGNIKQNPN	TQSLSQTGTE	LKTINDFLFQ	SQIDNINLFK	VQRYCEKSMI	SRKLFGFTER	YGAVFSSREQ
570	580	590	600	610	620	630	640
PKLAGFQQFL	QSLQPRTTEA	LAAPADESQA	STLRPASPLM	HIQGFLAALT	TANQDGRVIL	SRQGSLSQST	LKFLLLNPAV
650	660	670	680	690	700	710	720
HFAQVVKECR	AVVIAGGTMQ	PVSDFRQQLL	ACAGVEAERV	VEFSCGHVIP	PDNILPLVIC	SGISNQPLEF	TFQKRELPQM
730	740	750	760	770	780	790	800
MDEVGRILCN	LCGVVPGGVV	CFFPSYEYLR	QVHAHWEKGG	LLGRLAARKK	IFQEPKSAHQ	VEQVLLAYSR	CIQACGQERG
810	820	830	840	850			
QVTGALLLSV	VGGKMSEGIN	FSDNLGRPEP	PARHPQGRLW	WRTCA			

Cmpd.	No. of Cmpds.	m/z meas.	Δ m/z [ppm]	z	Rt [min]	Score	P	Range	Sequence	Modification
2190	1	966.3871	-90.93	2	57.6	10.3	2	1-19	-.MPLDGVRDSGMASRGPAAK.D	Oxidation: 11
1689	1	932.9135	-80.83	2	51.0	17.7	2	543-558	R.KLFGFTERYGAVFSSR.E	

Detailed Protein Report

Protein 325: PREDICTED: inactive ribonuclease-like protein 10 isoform X1 [Homo sapiens]

Accession: gi|530403621
Database: refseq_human(refseq_human_20140103.fasta)
Database Date: 2015-11-30

Score: 27.9
MW [kDa]: 27.0
pI: 5.0
Sequence Coverage [%]: 3.3
No. of unique Peptides: 1

10	20	30	40	50	60	70	80
MWGAPLPRRP	VWDVRSASAG	PQPCLGGKMK	LNLVQIFFML	LMLLLGLGMG	LGLGLHMATA	VLEESDQPLN	EFWSSDSQDK
**90**	**100**	**110**	**120**	**130**	**140**	**150**	**160**
AEATEEGDGT	QTTETLVLSN	KEVVQPGWPE	DPILGEDEVG	GNKMLRASAL	FQSNKDYLRL	DQTDRECNDM	MAHKMKEPSQ
**170**	**180**	**190**	**200**	**210**	**220**	**230**	**240**
SCIAQYAFIH	EDLNTVKAVC	NSPVIACELK	GGKCHKSSRP	FDLTLCELSQ	PDQVTPNCNY	LTSVIKKHII	ITCNDMKRQL
**250**							
PTGQ							

Cmpd.	No. of Cmpds.	m/z meas.	Δ m/z [ppm]	z	Rt [min]	Score	P	Range	Sequence	Modification
1593	1	927.7563	290.39	1	49.8	27.9	0	1-8	-.MWGAPLPR.R	

Detailed Protein Report

Protein 326: lysine-specific demethylase 3A [Homo sapiens]

**Accession:**	gi|20357522	**Score:**	27.9
**Database:**	refseq_human(refseq_human_20140103.fasta)	**MW [kDa]:**	147.2
**Database Date:**	2015-11-30	**pI:**	9.4
**Modification(s):**	Carbamidomethyl, Oxidation	**Sequence Coverage [%]:**	2.4
		**No. of unique Peptides:**	2

Alias proteins:

Accession	Name	Description
gi|226442779	refseq_human (refseq_human_20140103.fasta)	lysine-specific demethylase 3A [Homo sapiens]

10	20	30	40	50	60	70	80
MVLTLGESWP	VLVGRRFLSL	SAADGSDGSH	DSWDVERVAE	WPWLSGTIRA	VSHTDVTKKD	LKVCVEFDGE	SWRKRRWIEV
**90**	**100**	**110**	**120**	**130**	**140**	**150**	**160**
YSLLRRAFLV	EHNLVLAERK	SPEISERIVQ	WPAITYKPLL	DKAGLGSITS	VRFLGDQQRV	FLSKDLLKPI	QDVNSLRLSL
**170**	**180**	**190**	**200**	**210**	**220**	**230**	**240**
TDNQIVSKEF	QALIVKHLDE	SHLLKGDKNL	VGSEVKIYSL	DPSTQWFSAT	VINGNPASKT	LQVNCEEIPA	LKIVDPSLIH
**250**	**260**	**270**	**280**	**290**	**300**	**310**	**320**
VEVVHDNLVT	CGNSARIGAV	KRKSSENNGT	LVSKQAKSCS	EASPSMCPVQ	SVPTTVFKEI	LLGCTAATPP	SKDPRQQSTP
**330**	**340**	**350**	**360**	**370**	**380**	**390**	**400**
QAANSPPNLG	AKIPQGCHKQ	SLPEEISSCL	NTKSEALRTK	PDVCKAGLLS	KSSQIGTGDL	KILTEPKGSC	TQPKTNTDQE
**410**	**420**	**430**	**440**	**450**	**460**	**470**	**480**
NRLESVPQAL	TGLPKECLPT	KASSKAELEI	ANPPELQKHL	EHAPSPSDVS	NAPEVKAGVN	SDSPNNCSGK	KVEPSALACR
**490**	**500**	**510**	**520**	**530**	**540**	**550**	**560**
SQNLKESSVK	VDNESCCSRS	NNKIQNAPSR	KSVLTDPAKL	KKLQQSGEAF	VQDDSCVNIV	AQLPKCRECR	LDSLRKDKEQ
**570**	**580**	**590**	**600**	**610**	**620**	**630**	**640**
QKDSPVFCRF	FHFRRLQFNK	HGVLRVEGFL	TPNKYDNEAI	GLWLPLTKNV	VGIDLDTAKY	ILANIGDHFC	QMVISEKEAM
**650**	**660**	**670**	**680**	**690**	**700**	**710**	**720**
STIEPHRQVA	WKRAVKGVRE	MCDVCDTTIF	NLHWVCPRCG	FGVCVDCYRM	KRKNCQQGAA	YKTFSWLKCV	KSQIHEPENL
**730**	**740**	**750**	**760**	**770**	**780**	**790**	**800**
MPTQIIPGKA	LYDVGDIVHS	VRAKWGIKAN	CPCSNRQFKL	FSKPASKEDL	KQTSLAGEKP	TLGAVLQQNP	SVLEPAAVGG
**810**	**820**	**830**	**840**	**850**	**860**	**870**	**880**
EAASKPAGSM	KPACPASTSP	LNWLADLTSG	NVNKENKEKQ	PTMPILKNEI	KCLPPLPPLS	KSSTVLHTFN	STILTPVSNN
**890**	**900**	**910**	**920**	**930**	**940**	**950**	**960**
NSGFLRNLLN	SSTGKTENGL	KNTPKILDDI	FASLVQNKTT	SDLSKRPQGL	TIKPSILGFD	TPHYWLCDNR	LLCLQDPNNK
**970**	**980**	**990**	**1000**	**1010**	**1020**	**1030**	**1040**
SNWNVFRECW	KQGQPVMVSG	VHHKLNSELW	KPESFRKEFG	EQEVDLVNCR	TNEIITGATV	GDFWDGFEDV	PNRLKNEKEP
**1050**	**1060**	**1070**	**1080**	**1090**	**1100**	**1110**	**1120**
MVLKLKDWPP	GEDFRDMMPS	RFDDLMANIP	LPEYTRRDGK	LNLASRLPNY	FVRPDLGPKM	YNAYGLITPE	DRKYGTTNLH
**1130**	**1140**	**1150**	**1160**	**1170**	**1180**	**1190**	**1200**
LDVSDAANVM	VYVGIPKGQC	EQEEEVLKTI	QDGDSDELTI	KRFIEGKEKP	GALWHIYAAK	DTEKIREFLK	KVSEEQGQEN
**1210**	**1220**	**1230**	**1240**	**1250**	**1260**	**1270**	**1280**
PADHDPIHDQ	SWYLDRSLRK	RLHQEYGVQG	WAIVQFLGDV	VFIPAGAPHQ	VHNLYSCIKV	AEDFVSPEHV	KHCFWLTQEF
**1290**	**1300**	**1310**	**1320**	**1330**			
RYLSQTHTNH	EDKLQVKNVI	YHAVKDAVAM	LKASESSFGK	P			

Cmpd.	No. of Cmpds.	m/z meas.	Δ m/z [ppm]	z	Rt [min]	Score	P	Range	Sequence	Modification
81	1	821.0339	-27.87	3	30.4	17.2	2	457-480	K.AGVNSDSPNNCSGKKVEPSALACR.S	Carbamidomethyl: 23
624	1	472.1185	-315.94	2	37.4	10.7	0	840-847	K.QPTMPILK.N	Oxidation: 4

Detailed Protein Report

Protein 327: PREDICTED: regulator of G-protein signaling 22 isoform X8 [Homo sapiens]

**Accession:**	gi|578815920	**Score:**	27.8
**Database:**	refseq_human(refseq_human_20140103.fasta)	**MW [kDa]:**	121.2
**Database Date:**	2015-11-30	**pI:**	6.5
**Modification(s):**	Carbamidomethyl	**Sequence Coverage [%]:**	2.1
		**No. of unique Peptides:**	1

10	20	30	40	50	60	70	80
MPEKRLTAEP	PTITEEEFED	SLATDDFLVD	YFNEFLSLPT	FSEAIRFNAD	YGVFEVANDA	PQFLEKQLKK	ILQNQQPRNP
90	100	110	120	130	140	150	160
IYDVVRKGKN	EVKPVQMNAP	DEDETINVNY	NIMCLSREEG	IKWIKKERLP	AFLESDCYFE	YRLAKLVSQV	RWSKSGMNFT
170	180	190	200	210	220	230	240
VGSNFSPWIV	KKPPSLPPPA	TEEDNLVIMK	KFYVSLGEAS	YTQTKDWFAL	AKQSQQTVST	FSLPCCVPYN	KLKSPAISSV
250	260	270	280	290	300	310	320
SENFIFDDGV	HPRTKKDPSK	TNKLISEFEE	EEGEEEEVSV	SLQDTPSQAL	LRVYLEKKQD	VDESLTMHFS	TCEEFLSSYI
330	340	350	360	370	380	390	400
YFILRGAIQQ	IVGKPVGETP	DYINFNNITK	VSFDDCFESI	HGKNFLSELV	QTTKERSEEI	EQTSLSSKNE	SAGPESRADW
410	420	430	440	450	460	470	480
CISHRTYDIG	NRKEFERFKK	FIKGTLGERY	WWLWMDIERL	KVLKDPGRHQ	RHLEKMKKCY	LVSNGDYYLS	AEILSKFKLL
490	500	510	520	530	540	550	560
DGSQWNEEHL	RNIQSEVLKP	LLLYWAPRFC	VTHSASTKYA	SAELKFWHLR	QAKPRKDIDP	FPQMATLLPL	RPKSCIPQIP
570	580	590	600	610	620	630	640
EIQKEEFSLS	QPPKSPNKSP	EVKTATQKPW	KRELLYPGSS	KDDVIEKGSK	YMSESSKVIH	LTSFTDISEC	LKPQLDRRYA
650	660	670	680	690	700	710	720
YTEEPRVKTV	SDVGALGGSD	MENLLQSLYV	ENRAGFFFTK	FCEHSGNKLW	KNSVYFWFDL	QAYHQLFYQE	TLQPFKVCKQ
730	740	750	760	770	780	790	800
AQYLFATYVA	PSATLDIGLQ	QEKKKEIYMK	IQPPFEDLFD	TAEEYILLLL	LEPWTKMVKS	DQIAYKKVEL	VEETRQLDST
810	820	830	840	850	860	870	880
YFRKLQALHK	ETFSKKAEVQ	MKDIAEELLL	QKAEKKIGVW	KDLCHSHCDE	SVIQKKITTI	INCFINSSIP	PALQIDIPVE
890	900	910	920	930	940	950	960
QAQKIIEHRK	ELGPYVFREA	QMTIFGVLFK	FWPQFCEFRK	NLTDENIMSV	LERRQEYNKQ	KKKLAVLEDE	KSGKDGIKQY
970	980	990	1000	1010	1020	1030	1040
ANTSVPAIKT	ALLSDSFLGL	QPYGRQPTWC	YSKYIEALEQ	ERILLKIQEE	LEKKLFAGLQ	PLTNFKASSS	TMSLKKNMSA
1050							
HSSQK							

Cmpd.	No. of Cmpds.	m/z meas.	Δ m/z [ppm]	z	Rt [min]	Score	P	Range	Sequence	Modification
2278	1	557.5521	-50.47	3	58.7	12.0	0	842-855	K.DLCHSHCDESVIQK.K	Carbamidomethyl: 7

Detailed Protein Report

Protein 328: **PREDICTED: leukocyte immunoglobulin-like receptor subfamily B member 3-like isoform X4 [Homo sapiens]**

**Accession:**	gi|578842508	**Score:**	27.8
**Database:**	refseq_human(refseq_human_20140103.fasta)	**MW [kDa]:**	69.3
**Database Date:**	2015-11-30	**pI:**	6.2
		**Sequence Coverage [%]:**	5.4
		**No. of unique Peptides:**	1

10	20	30	40	50	60	70	80
MTPALTALLC	LGLSLGPRTR	VQAGPFPKPT	LWAEPGSVIS	WGSPVTIWCQ	GSLEAQEYRL	DKEGSPEPLD	RNNPLEPKNK
**90**	**100**	**110**	**120**	**130**	**140**	**150**	**160**
ARFSIPSMTE	HHAGRYRCHY	YSSAGWSEPS	DPLELVMTGF	YNKPTLSALP	SPVVASGGNM	TLRCGSQKGY	HHFVLMKEGE
**170**	**180**	**190**	**200**	**210**	**220**	**230**	**240**
HQLPRTLDSQ	QLHSGGFQAL	FPVGPVNPSH	RWRFTCYYYY	MNTPQVWSHP	SDPLEILPSG	VSRKPSLLTL	QGPVLAPGQS
**250**	**260**	**270**	**280**	**290**	**300**	**310**	**320**
LTLQCGSDVG	YDRFVLYKEG	ERDFLQRPGQ	QPQAGLSQAN	FTLGPVSPSH	GGQYRCYGAH	NLSSEWSAPS	DPLNILMAGQ
**330**	**340**	**350**	**360**	**370**	**380**	**390**	**400**
IYDTVSLSAQ	PGPTVASGEN	VTLLCQSWWQ	FDTFLLTKEG	AAHPPLRLRS	MYGAHKYQAE	FPMSPVTSAH	AGTYRCYGSY
**410**	**420**	**430**	**440**	**450**	**460**	**470**	**480**
SSNPHLLSFP	SEPLELMVSG	HSGGSSLPPT	GPPSTPGLGR	YLEVLIGVSV	AFVLLLFLLL	FLLLRRQRHS	KHRTSDQRKT
**490**	**500**	**510**	**520**	**530**	**540**	**550**	**560**
DFQRPAGAAE	TEPKDRGLLR	RSSPAADVQE	ENLYAAVKDT	QSEDRVELDS	QSPHDEDPQA	VTYAPVKHSS	PRREMASPPS
**570**	**580**	**590**	**600**	**610**	**620**	**630**	**640**
SLSGEFLDTK	DRQVEEDRQM	DTEAAASEAS	QDVTYAQLHS	LTLRRKATEP	PPSQEGEPPA	EPSIYATLAI	H

Cmpd.	No. of Cmpds.	m/z meas.	Δ m/z [ppm]	z	Rt [min]	Score	P	Range	Sequence	Modification
1409	1	927.1324	25.05	3	47.2	16.3	2	501-525	R.RSSPAADVQEENLYAAVKDTQSEDR.V	

Detailed Protein Report

Protein 329: **leucine-rich repeats and immunoglobulin-like domains protein 1 precursor [Homo sapiens]**

Accession: gi|54607118
Database: refseq_human(refseq_human_20140103.fasta)
Database Date: 2015-11-30

Score: 27.8
MW [kDa]: 119.0
pI: 6.7
Sequence Coverage [%]: 2.9
No. of unique Peptides: 1

Quantitation

MD:MU **Median:** 1.61 **CV:** 0.00 % **No. of Peptides:** 1

10	20	30	40	50	60	70	80
MARPVRGGLG	APRRSPCLLL	LWLLLLRLEP	VTAAAGPRAP	CAAACTCAGD	SLDCGGRGLA	ALPGDLPSWT	RSLNLSYNKL
**90**	**100**	**110**	**120**	**130**	**140**	**150**	**160**
SEIDPAGFED	LPNLQEVYLN	NNELTAVPSL	GAASSHVVSL	FLQHNKIRSV	EGSQLKAYLS	LEVLDLSLNN	ITEVRNTCFP
**170**	**180**	**190**	**200**	**210**	**220**	**230**	**240**
HGPPIKELNL	AGNRIGTLEL	GAFDGLSRSL	LTLRLSKNRI	TQLPVRAFKL	PRLTQLDLNR	NRIRLIEGLT	FQGLNSLEVL
**250**	**260**	**270**	**280**	**290**	**300**	**310**	**320**
KLQRNNISKL	TDGAFWGLSK	MHVLHLEYNS	LVEVNSGSLY	GLTALHQLHL	SNNSIARIHR	KGWSFCQKLH	ELVLSFNNLT
**330**	**340**	**350**	**360**	**370**	**380**	**390**	**400**
RLDEESLAEL	SSLSVLRLSH	NSISHIAEGA	FKGLRSLRVL	DLDHNEISGT	IEDTSGAFSG	LDSLSKLTLF	GNKIKSVAKR
**410**	**420**	**430**	**440**	**450**	**460**	**470**	**480**
AFSGLEGLEH	LNLGGNAIRS	VQFDAFVKMK	NLKELHISSD	SFLCDCQLKW	LPPWLIGRML	QAFVTATCAH	PESLKGQSIF
**490**	**500**	**510**	**520**	**530**	**540**	**550**	**560**
SVPPESFVCD	DFLKPQIITQ	PETTMAMVGK	DIRFTCSAAS	SSSSPMTFAW	KKDNEVLTNA	DMENFVHVHA	QDGEVMEYTT
**570**	**580**	**590**	**600**	**610**	**620**	**630**	**640**
ILHLRQVTFG	HEGRYQCVIT	NHFGSTYSHK	ARLTVNVLPS	FTKTPHDITI	RTTTMARLEC	AATGHPNPQI	AWQKDGGTDF
**650**	**660**	**670**	**680**	**690**	**700**	**710**	**720**
PAARERRMHV	MPDDDVFFIT	DVKIDDAGVY	SCTAQNSAGS	ISANATLTVL	ETPSLVVPLE	DRVVSVGETV	ALQCKATGNP
**730**	**740**	**750**	**760**	**770**	**780**	**790**	**800**
PPRITWFKGD	RPLSLTERHH	LTPDNQLLVV	QNVVAEDAGR	YTCEMSNTLG	TERAHSQLSV	LPAAGCRKDG	TTVGIFTIAV
**810**	**820**	**830**	**840**	**850**	**860**	**870**	**880**
VSSIVLTSLV	WVCIIYQTRK	KSEEYSVTNT	DETVVPPDVP	SYLSSQGTLS	DRQETVVRTE	GGPQANGHIE	SNGVCPRDAS
**890**	**900**	**910**	**920**	**930**	**940**	**950**	**960**
HFPEPDTHSV	ACRQPKLCAG	SAYHKEPWKA	MEKAEGTPGP	HKMEHGGRVV	CSDCNTEVDC	YSRGQAFHPQ	PVSRDSAQPS
**970**	**980**	**990**	**1000**	**1010**	**1020**	**1030**	**1040**
APNGPEPGGS	DQEHSPHHQC	SRTAAGSCPE	CQGSLYPSNH	DRMLTAVKKK	PMASLDGKGD	SSWTLARLYH	PDSTELQPAS
**1050**	**1060**	**1070**	**1080**	**1090**	**1100**		
SLTSGSPERA	EAQYLLVSNG	HLPKACDASP	ESTPLTGQLP	GKQRVPLLLA	PKS		

Cmpd.	No. of Cmpds.	m/z meas.	Δ m/z [ppm]	z	Rt [min]	Score	P	Range	Sequence	Modification	Ratios
45	1	1096.9782	-54.15	2	29.9	16.3	1	431-449	K.NLKELHISSDSFLCDCQLK.W		MD:MU 1.61

Detailed Protein Report

Protein 330: coiled-coil domain-containing protein 80 precursor [Homo sapiens]

**Accession:**	gi|41152074	**Score:**	27.8
**Database:**	refseq_human(refseq_human_20140103.fasta)	**MW [kDa]:**	108.1
**Database Date:**	2015-11-30	**pI:**	10.2
**Modification(s):**	Oxidation	**Sequence Coverage [%]:**	4.2
		**No. of unique Peptides:**	2

Quantitation

MD:MU **Median:** 3.85 **CV:** 0.00 % **No. of Peptides:** 1

Alias proteins:

Accession	Name	Description
gi|41152076	refseq_human (refseq_human_20140103.fasta)	coiled-coil domain-containing protein 80 precursor [Homo sapiens]

10	20	30	40	50	60	70	80
MTWRMGPRFT	MLLAMWLVCG	SEPHPHATIR	GSHGGRKVPL	VSPDSSRPAR	FLRHTGRSRG	IERSTLEEPN	LQPLQRRRSV
**90**	**100**	**110**	**120**	**130**	**140**	**150**	**160**
PVLRLARPTE	PPARSDINGA	AVRPEQRPAA	RGSPREMIRD	EGSSARSRML	RFPSGSSSPN	ILASFAGKNR	VWVISAPHAS
**170**	**180**	**190**	**200**	**210**	**220**	**230**	**240**
EGYYRLMMSL	LKDDVYCELA	ERHIQQIVLF	HQAGEEGGKV	RRITSEGQIL	EQPLDPSLIP	KLMSFLKLEK	GKFGMVLLKK
**250**	**260**	**270**	**280**	**290**	**300**	**310**	**320**
TLQVEERYPY	PVRLEAMYEV	IDQGPIRRIE	KIRQKGFVQK	CKASGVEGQV	VAEGNDGGGG	AGRPSLGSEK	KKEDPRRAQV
**330**	**340**	**350**	**360**	**370**	**380**	**390**	**400**
PPTRESRVKV	LRKLAATAPA	LPQPPSTPRA	TTLPPAPATT	VTRSTSRAVT	VAARPMTTTA	FPTTQRPWTP	SPSHRPPTTT
**410**	**420**	**430**	**440**	**450**	**460**	**470**	**480**
EVITARRPSV	SENLYPPSRK	DQHRERPQTT	RRPSKATSLE	SFTNAPPTTI	SEPSTRAAGP	GRFRDNRMDR	REHGHRDPNV
**490**	**500**	**510**	**520**	**530**	**540**	**550**	**560**
VPGPPKPAKE	KPPKKKAQDK	ILSNEYEEKY	DLSRPTASQL	EDELQVGNVP	LKKAKESKKH	EKLEKPEKEK	KKKMKNENAD
**570**	**580**	**590**	**600**	**610**	**620**	**630**	**640**
KLLKSEKQMK	KSEKKSKQEK	EKSKKKKGGK	TEQDGYQKPT	NKHFTQSPKK	SVADLLGSFE	GKRRLLLITA	PKAENNMYVQ
**650**	**660**	**670**	**680**	**690**	**700**	**710**	**720**
QRDEYLESFC	KMATRKISVI	TIFGPVNNST	MKIDHFQLDN	EKPMRVVDDE	DLVDQRLISE	LRKEYGMTYN	DFFMVLTDVD
**730**	**740**	**750**	**760**	**770**	**780**	**790**	**800**
LRVKQYYEVP	ITMKSVFDLI	DTFQSRIKDM	EKQKKEGIVC	KEDKKQSLEN	FLSRFRWRRR	LLVISAPNDE	DWAYSQQLSA
**810**	**820**	**830**	**840**	**850**	**860**	**870**	**880**
LSGQACNFGL	RHITILKLLG	VGEEVGGVLE	LFPINGSSVV	EREDVPAHLV	KDIRNYFQVS	PEYFSMLLVG	KDGNVKSWYP
**890**	**900**	**910**	**920**	**930**	**940**	**950**	**960**
SPMWSMVIVY	DLIDSMQLRR	QEMAIQQSLG	MRCPEDEYAG	YGYHSYHQGY	QDGYQDDYRH	HESYHHGYPY	

Cmpd.	No. of Cmpds.	m/z meas.	Δ m/z [ppm]	z	Rt [min]	Score	P	Range	Sequence	Modification	Ratios	
1256	5	698.8457	-68.14	2	45.4	14.9	0	350-363	R.ATTLPPAPATTVTR.S		MD:MU	3.85
1645	1	1062.1080	-81.53	3	50.3	12.9	2	697-722	R.LISELRKEYGMTYNDFFMVLTDVDV	Oxidation: 18		

Detailed Protein Report

Protein 331: PREDICTED: E3 ubiquitin-protein ligase HERC2 isoform X5 [Homo sapiens]

**Accession:**	gi|578827509	**Score:**	27.8
**Database:**	refseq_human(refseq_human_20140103.fasta)	**MW [kDa]:**	517.6
**Database Date:**	2015-11-30	**pI:**	5.9
		**Sequence Coverage [%]:**	0.4
		**No. of unique Peptides:**	1

Detailed Protein Report

10	20	30	40	50	60	70	80
MPSESFCLAA	QARLDSKWLK	TDIQLAFTRD	GLCGLWNEMV	KDGEIVYTGT	ESTQNGELPP	RKDDSVEPSG	TKKEDLNDKE
90	100	110	120	130	140	150	160
KKDEEETPAP	IYRAKSILDS	WVWGKQPDVN	ELKECLSVLV	KEQQALAVQS	ATTTLSALRL	KQRLVILERY	FIALNRTVFQ
170	180	190	200	210	220	230	240
ENVKVKWKSS	GISLPPVDKK	RDVHGTPATK	GPGSIPLQDQ	HLALAILLEL	AVQRGTLSQM	LSAILLLLQL	WDSGAQETDN
250	260	270	280	290	300	310	320
ERSAQGTSAP	LLPLLQRFQS	IICRKDAPHS	EGDMHLLSGP	LSPNESFLRY	LTLPQDNELA	IDLRQTAVVV	MAHLDRLATP
330	340	350	360	370	380	390	400
CMPPLCSSPT	SHKGSLQEVI	GWGLIGWKYY	ANVIGPIQCE	GLANLGVTQI	ACAEKRFLIL	SRNGRVYTQA	YNSDTLAPQL
410	420	430	440	450	460	470	480
VQGLASRNIV	KIAAHSDGHH	YLALAATGEV	YSWGCGDGGR	LGHGDTVPLE	EPKVISAFSG	KQAGKHVVHI	ACGSTYSAAI
490	500	510	520	530	540	550	560
TAEGELYTWG	RGNYGRLGHG	SSEDEAIPML	VAGLKGLKVI	DVACGSGDAQ	TLAVTENGQV	WSWGDGDYGK	LGRGGSDGCK
570	580	590	600	610	620	630	640
TPKLIEKLQD	LDVVKVRCGS	QFSIALTKDG	QVYSWGKGDN	QRLGHGTEEH	VRYPKLLEGL	QGKKVIDVAA	GSTHCLALTE
650	660	670	680	690	700	710	720
DSEVHSWGSN	DQCQHFDTLR	VTKPEPAALP	GLDTKHIVGI	ACGPAQSFAW	SSCSEWSIGL	RVPFVVDICS	MTFEQLDLLL
730	740	750	760	770	780	790	800
RQVSEGMDGS	ADWPPPQEKE	CVAVATLNLL	RLQLHAAISH	QVDPEFLGLG	LGSILLNSLK	QTVVTLASSA	GVLSTVQSAA
810	820	830	840	850	860	870	880
QAVLQSGWSV	LLPTAEERAR	ALSALLPCAV	SGNEVNISPG	RRFMIDLLVG	SLMADGGLES	ALHAAITAEI	QDIEAKKEAQ
890	900	910	920	930	940	950	960
KEKEIDEQEA	NASTFHRSRT	PLDKDLINTG	ICESSGKQCL	PLVQLIQQLL	RNIASQTVAR	LKDVARRISS	CLDFEQHSRE
970	980	990	1000	1010	1020	1030	1040
RSASLDLLLR	FQRLLISKLY	PGESIGQTSD	ISSPELMGVG	SLLKKYTALL	CTHIGDILPV	AASIASTSWR	HFAEVAYIVE
1050	1060	1070	1080	1090	1100	1110	1120
GDFTGVLLPE	LVVSIVLLLS	KNAGLMQEAG	AVPLLGGLLE	HLDRFNHLAP	GKERDDHEEL	AWPGIMESFF	TGQNCRNNEE
1130	1140	1150	1160	1170	1180	1190	1200
VTLIRKADLE	NHNKDGGFWT	VIDGKVYDIK	DFQTQSLTGN	SILAQFAGED	PVVALEAALQ	FEDTRESMHA	FCVGQYLEPD
1210	1220	1230	1240	1250	1260	1270	1280
QEIVTIPDLG	SLSSPLIDTE	RNLGLLLGLH	ASYLAMSTPL	SPVEIECAKW	LQSSIFSGGL	QTSQIHYSYN	EEKDEDHCSS
1290	1300	1310	1320	1330	1340	1350	1360
PGGTPASKSR	LCSHRRALGD	HSQAFLQAIA	DNNIQDHNVK	DFLCQIERYC	RQCHLTTPIM	FPPEHPVEEV	GRLLLCCLLK
1370	1380	1390	1400	1410	1420	1430	1440
HEDLGHVALS	LVHAGALGIE	QVKHRTLPKS	VVDVCRVVYQ	AKCSLIKTHQ	EQGRSYKEVC	APVIERLRFL	FNELRPAVCN
1450	1460	1470	1480	1490	1500	1510	1520
DLSIMSKFKL	LSSLPRWRRI	AQKIIERRK	KRVPKKPEST	DDEEKIGNEE	SDLEEACILP	HSPINVDKRP	IAIKSPKDKW
1530	1540	1550	1560	1570	1580	1590	1600
QPLLSTVTGV	HKYKWLKQNV	QGLYPQSPLL	STIAEFALKE	EPVDVEKMRK	CLLKQLERAE	VRLEGIDTIL	KLASKNFLLP
1610	1620	1630	1640	1650	1660	1670	1680
SVQYAMFCGW	QRLIPEGIDI	GEPLTDCLKD	VDLIPPFNRM	LLEVTFGKLY	AWAVQNIRNV	LMDASAKFKE	LGIQPVPLQT
1690	1700	1710	1720	1730	1740	1750	1760
ITNENPSGPS	LGTIPQARFL	LVMLSMLTLQ	HGANNLDLLL	NSGMLALTQT	ALRLIGPSCD	NVEEDMNASA	QGASATVLEE
1770	1780	1790	1800	1810	1820	1830	1840
TRKETAPVQL	PVSGPELAAM	MKIGTRVMRG	VDWKWGDQDG	PPPGLGRVIG	ELGEDGWIRV	QWDTGSTNSY	RMGKEGKYDL
1850	1860	1870	1880	1890	1900	1910	1920
KLAELPAAAQ	PSAEDSDTED	DSEAEQTERN	IHPTAMMFTS	TINLLQTLCL	SAGVHAEIMQ	SEATKTLCGL	LRMLVESGTT
1930	1940	1950	1960	1970	1980	1990	2000
DKTSSPNRLV	YREQHRSWCT	LGFVRSIALT	PQVCGALSSP	QWITLLMKVV	EGHAPFTATS	LQRQILAVHL	LQAVLPSWDK
2010	2020	2030	2040	2050	2060	2070	2080
TERARDMKCL	VEKLFDFLGS	LLTTCSSDVP	LLRESTLRRR	RVRPQASLTA	THSSTLAEEV	VALLRTLHSL	TQWNGLINKY
2090	2100	2110	2120	2130	2140	2150	2160
INSQLRSITH	SFVGRPSEGA	QLEDYFPDSE	NPEVGGLMAV	LAVIGGIDGR	LRLGGQVMHD	EFGEGTVTRI	TPKGKITVQF
2170	2180	2190	2200	2210	2220	2230	2240

Detailed Protein Report

Cmpd.	No. of Cmpds.	m/z meas.	Δ m/z [ppm]	z	Rt [min]	Score	P	Range	Sequence	Modification
360	1	543.1311	-259.95	2	33.8	14.2	0	3920-3930	K.LYATGYGAGGR.L	

Detailed Protein Report

Protein 332: **PREDICTED: coiled-coil domain-containing protein 91 isoform X6 [Homo sapiens]**

**Accession:**	gi|578822930	**Score:**	27.8
**Database:**	refseq_human(refseq_human_20140103.fasta)	**MW [kDa]:**	51.6
**Database Date:**	2015-11-30	**pI:**	5.4
**Modification(s):**	Oxidation	**Sequence Coverage [%]:**	5.8
		**No. of unique Peptides:**	1

Quantitation

MD:MU **Median:** 0.58 **CV:** 0.00 % **No. of Peptides:** 1

10	20	30	40	50	60	70	80
MWVQQHRPEN	TFGDQRETCN	FHCELPSRVY	GRTSLKLLMP	MWNKVSGVHL	SPSSPEIVLD	RDHSSSIGCL	SSDAIISSPE
90	100	110	120	130	140	150	160
NTHAANSIVS	QTIPKAQIQQ	STHTHLDISL	FPLGLTDEKS	NGTIALVDDS	EDPGANVSNI	QLQQKISSLE	IKLKVSEEEK
170	180	190	200	210	220	230	240
QRIKQDVESL	MEKHNVLEKG	FLKEKEQEAI	SFQDRYKELQ	EKHKQELEDM	RKAGHEALSI	IVDEYKALLQ	SSVKQQVEAI
250	260	270	280	290	300	310	320
EKQYISAIEK	QAHKCEELLN	AQHQRLLEML	DTEKELLKEK	IKEALIQQSQ	EQKEILEKCL	EEERQRNKEA	LVSAAKLEKE
330	340	350	360	370	380	390	400
AVKDAVLKVV	EEERKNLEKA	HAEERELWKT	EHAKDQEKVS	QEIQKAIQEQ	RKISQETVKA	AIIEEQKRSE	KAVEEAVKRT
410	420	430	440	450			
RDELIEYIKE	QKRLDQVIRQ	RSLSSLELFL	SCAQKQLSAL	IATEPVDIE			

Cmpd.	No. of Cmpds.	m/z meas.	Δ m/z [ppm]	z	Rt [min]	Score	P	Range	Sequence	Modification	Ratios
1996	1	665.3129	-29.71	2	55.1	11.2	2	203-212	K.HKQELEDMRK.A	Oxidation: 8	MD:MU 0.58

Detailed Protein Report

Protein 333: engulfment and cell motility protein 1 isoform 2 [Homo sapiens]

**Accession:**	gi|18765702	**Score:**	27.7
**Database:**	refseq_human(refseq_human_20140103.fasta)	**MW [kDa]:**	28.7
**Database Date:**	2015-11-30	**pI:**	6.0
		**Sequence Coverage [%]:**	10.1
		**No. of unique Peptides:**	1

Alias proteins:

Accession	Name	Description
gi|86788140	refseq_human (refseq_human_20140103.fasta)	engulfment and cell motility protein 1 isoform 2 [Homo sapiens]

10	20	30	40	50	60	70	80
MQVVKEQVMR	ALTTKPSSLD	QFKSKLQNLS	YTEILKIRQS	ERMNQEDFQS	RPILELKEKI	QPEILELIKQ	QRLNRLVEGT
**90**	**100**	**110**	**120**	**130**	**140**	**150**	**160**
CFRKLNARRR	QDKFWYCRLS	PNHKVLHYGD	LEESPQGEVP	HDSLQDKLPV	ADIKAVVTGK	DCPHMKEKGA	LKQNKEVLEL
**170**	**180**	**190**	**200**	**210**	**220**	**230**	**240**
AFSILYDSNC	QLNFIAPDKH	EYCIWTDGLN	ALLGKDMMSD	LTRNDLDTLL	SMEIKLRLLD	LENIQIPDAP	PPIPKEPSNY
**250**							
DFVYDCN							

Cmpd.	No. of Cmpds.	m/z meas.	Δ m/z [ppm]	z	Rt [min]	Score	P	Range	Sequence	Modification
1550	1	1011.5446	-2.78	2	49.2	15.4	2	128-146	K.LPVADIKAVVTGKDCPHMK.E	

Detailed Protein Report

Protein 334: glutathione S-transferase P [Homo sapiens]

Accession: gi|4504183
Database: refseq_human(refseq_human_20140103.fasta)
Database Date: 2015-11-30

Score: 27.7
MW [kDa]: 23.3
pI: 5.3
Sequence Coverage [%]: 7.6
No. of unique Peptides: 1

10	20	30	40	50	60	70	80
MPPYTVVYFP	VRGRCAALRM	LLADQGQSWK	EEVVTVETWQ	EGSLKASCLY	GQLPKFQDGD	LTLYQSNTIL	RHLGRTLGLY
**90**	**100**	**110**	**120**	**130**	**140**	**150**	**160**
GKDQQEAALV	DMVNDGVEDL	RCKYISLIYT	NYEAGKDDYV	KALPGQLKPF	ETLLSQNQGG	KTFIVGDQIS	FADYNLLDLL
**170**	**180**	**190**	**200**	**210**	**220**		
LIHEVLAPGC	LDAFPLLSAY	VGRLSARPKL	KAFLASPEYV	NLPINGNGKQ			

Cmpd.	No. of Cmpds.	m/z meas.	Δ m/z [ppm]	z	Rt [min]	Score	P	Range	Sequence	Modification
1594	1	942.4301	-51.36	2	49.6	27.7	0	56-71	K.FQDGDLTLYQSNTILR.H	

Detailed Protein Report

Protein 335: **pleckstrin homology domain-containing family A member 7 [Homo sapiens]**

**Accession:**	gi|239047795	**Score:**	27.6
**Database:**	refseq_human(refseq_human_20140103.fasta)	**MW [kDa]:**	127.1
**Database Date:**	2015-11-30	**pI:**	9.9
		**Sequence Coverage [%]:**	2.7
		**No. of unique Peptides:**	1

10	20	30	40	50	60	70	80
MAAATVGRDT	LPEHWSYGVC	RDGRVFFIND	QLRCTTWLHP	RTGEPVNSGH	MIRSDLPRGW	EEGFTEEGAS	YFIDHNQQTT
**90**	**100**	**110**	**120**	**130**	**140**	**150**	**160**
AFRHPVTGQF	SPENSEFILQ	EEPNPHMSKQ	DRNQRPSSMV	SETSTAGTAS	TLEAKPGPKI	IKSSSKVHSF	GKRDQAIRRN
**170**	**180**	**190**	**200**	**210**	**220**	**230**	**240**
PNVPVVVRGW	LHKQDSSGMR	LWKRRWFVLA	DYCLFYYKDS	REEAVLGSIP	LPSYVISPVA	PEDRISRKYS	FKAVHTGMRA
**250**	**260**	**270**	**280**	**290**	**300**	**310**	**320**
LIYNSSTAGS	QAEQSGMRTY	YFSADTQEDM	NAWVRAMNQA	AQVLSRSSLK	RDMEKVERQA	VPQANHTESC	HECGRVGPGH
**330**	**340**	**350**	**360**	**370**	**380**	**390**	**400**
TRDCPHRGHD	DIVNFERQEQ	EGEQYRSQRD	PLEGKRDRSK	ARSPYSPAEE	DALFMDLPTG	PRGQQAQPQR	AEKNGMLPAS
**410**	**420**	**430**	**440**	**450**	**460**	**470**	**480**
YGPGEQNGTG	GYQRAFPPRT	NPEKHSQRKS	NLAQVEHWAR	AQKGDSRSLP	LDQTLPRQGP	GQSLSFPENY	QTLPKSTRHP
**490**	**500**	**510**	**520**	**530**	**540**	**550**	**560**
SGGSSPPPRN	LPSDYKYAQD	RASHLKMSSE	ERRAHRDGTV	WQLYEWQQRQ	QFRHGSPTAP	ICLGSPEFTD	QGRSRSMLEV
**570**	**580**	**590**	**600**	**610**	**620**	**630**	**640**
PRSISVPPSP	SDIPPPGPPR	VFPPRRPHTP	AERVTVKPPD	QRRSVDISLG	DSPRRARGHA	VKNSSHVDRR	SMPSMGYMTH
**650**	**660**	**670**	**680**	**690**	**700**	**710**	**720**
TVSAPSLHGK	SADDTYLQLK	KDLEYLDLKM	TGRDLLKDRS	LKPVKIAESD	TDVKLSIFCE	QDRVLQDLED	KIRALKENKD
**730**	**740**	**750**	**760**	**770**	**780**	**790**	**800**
QLESVLEVLH	RQMEQYRDQP	QHLEKIAYQQ	KLLQEDLVHI	RAELSRESTE	MENAWNEYLK	LENDVEQLKQ	TLQEQHRRAF
**810**	**820**	**830**	**840**	**850**	**860**	**870**	**880**
FFQEKSQIQK	DLWRIEDVTA	GLSANKENFR	ILVESVKNPE	RKTVPLFPHP	PVPSLSTSES	KPPPQPSPPT	SPVRTPLEVR
**890**	**900**	**910**	**920**	**930**	**940**	**950**	**960**
LFPQLQTYVP	YRPHPPQLRK	VTSPLQSPTK	AKPKVEDEAP	PRPPLPELYS	PEDQPPAVPP	LPREATIIRH	TSVRGLKRQS
**970**	**980**	**990**	**1000**	**1010**	**1020**	**1030**	**1040**
DERKRDRELG	QCVNGDSRVE	LRSYVSEPEL	ATLSGDMAQP	SLGLVGPESR	YQTLPGRGLS	GSTSRLQQSS	TIAPYVTLRR
**1050**	**1060**	**1070**	**1080**	**1090**	**1100**	**1110**	**1120**
GLNAESSKAT	FPRPKSALER	LYSGDHQRGK	MSAEEQLERM	KRHQKALVRE	RKRTLGQGER	TGLPSSRYLS	RPLPGDLGSV
**1130**							
C							

Cmpd.	No. of Cmpds.	m/z meas.	Δ m/z [ppm]	z	Rt [min]	Score	P	Range	Sequence	Modification
2724	1	973.5090	30.70	2	64.4	12.7	2	966-982	R.DRELGQCVNGDSRVELR.S	

Detailed Protein Report

Protein 336: PREDICTED: rap guanine nucleotide exchange factor 3 isoform X4 [Homo sapiens]

**Accession:**	gi|530399604	**Score:**	27.6
**Database:**	refseq_human(refseq_human_20140103.fasta)	**MW [kDa]:**	55.9
**Database Date:**	2015-11-30	**pI:**	9.9
**Modification(s):**	Carbamidomethyl, Oxidation	**Sequence Coverage [%]:**	5.1
		**No. of unique Peptides:**	1

10	20	30	40	50	60	70	80
MPSAQLCAAL	LHHFHVEPAG	GSEQERSTYV	CNKRQQILRL	VSQWVALYGS	MLHTDPVATS	FLQKLSDLVG	RDTRLSNLLR
**90**	**100**	**110**	**120**	**130**	**140**	**150**	**160**
EQWPERRRCH	RLENGCGNAS	PQMKARNLPV	WLPNQDEPLP	GSSCAIQVGD	KVPYDICRPD	HSVLTLQLPV	TASVREVMAA
**170**	**180**	**190**	**200**	**210**	**220**	**230**	**240**
LAQEDGWTKG	QVLVKVNSAG	DAIGLQPDAR	GVATSLGLNE	RLFVVNPQEV	HELIPHPDQL	GPTVGSAEGL	DLVSAKDLAG
**250**	**260**	**270**	**280**	**290**	**300**	**310**	**320**
QLTDHDWSLF	NSIHQVELIH	YVLGPQHLRD	VTTANLERFM	RRFNELQYWV	ATELCLCPVP	GPRAQLLRKF	IKLAAHLKEQ
**330**	**340**	**350**	**360**	**370**	**380**	**390**	**400**
KNLNSFFAVM	FGLSNSAISR	LAHTWERLPH	KVRKLYSALE	RLLDPSWNHR	VYRLALAKLS	PPVIPFMPLL	LKDMTFIHEG
**410**	**420**	**430**	**440**	**450**	**460**	**470**	**480**
NHTLVENLIN	FEKMRMMARA	ARMLHHCRSH	NPVPLSPLRS	RVSHLHEDSQ	VARISTCSEQ	SLSTRSPAST	WAYVQQLKVI
**490**	**500**						
DNQRELSRLS	RELEP						

Cmpd.	No. of Cmpds.	m/z meas.	Δ m/z [ppm]	z	Rt [min]	Score	P	Range	Sequence	Modification
1805	1	937.7928	-121.06	2	52.4	13.0	1	89-104	R.CHRLENGCGNASPQMK.A	Carbamidomethyl: 1, 8; Oxidation: 15

Detailed Protein Report

Protein 337: PREDICTED: DNA (cytosine-5)-methyltransferase 1 isoform X1 [Homo sapiens]

**Accession:**	gi|578833065	**Score:**	27.5
**Database:**	refseq_human(refseq_human_20140103.fasta)	**MW [kDa]:**	169.3
**Database Date:**	2015-11-30	**pI:**	8.8
		**Sequence Coverage [%]:**	1.9
		**No. of unique Peptides:**	1

10	20	30	40	50	60	70	80
MADANSPPKP	LSKPRTPRRS	KSDGEAKPEP	SPSPRITRKS	TRQTTITSHF	AKGPAKRKPQ	EESERAKSDE	SIKEEDKDQD
90	100	110	120	130	140	150	160
EKRRRVTSRE	RVARPLPAEE	PERAKSGTRT	EKEEERDEKE	EKRLRSQTKE	PTPKQKLKEE	PDREARAGVQ	ADEDEDGDEK
170	180	190	200	210	220	230	240
DEKKHRSQPK	DLAAKRRPEE	KEPEKVNPQI	SDEKDEDEKE	EKRRKTTPKE	PTEKKMARAK	TVMNSKTHPP	KCIQCGQYLD
250	260	270	280	290	300	310	320
DPDLKYGQHP	PDAVDEPQML	TNEKLSIFDA	NESGFESYEA	LPQHKLTCFS	VYCKHGHLCP	IDTGLIEKNI	ELFFSGSAKP
330	340	350	360	370	380	390	400
IYDDDPSLEG	GVNGKNLGPI	NEWWITGFDG	GEKALIGFST	SFAEYILMDP	SPEYAPIFGL	MQEKIYISKI	VVEFLQSNSD
410	420	430	440	450	460	470	480
STYEDLINKI	ETTVPPSGLN	LNRFTEDSLL	RHAQFVVEQV	ESYDEAGDSD	EQPIFLTPCM	RDLIKLAGVT	LGQRRAQARR
490	500	510	520	530	540	550	560
QTIRHSTREK	DRGPTKATTT	KLVYQIFDTF	FAEQIEKDDR	EDKENAFKRR	RCGVCEVCQQ	PECGKCKACK	DMVKFGGSGR
570	580	590	600	610	620	630	640
SKQACQERRC	PNMAMKEADD	DEEVDDNIPE	MPSPKKMHQG	KKKKQNKNRI	SWVGEAVKTD	GKKSYYKKVC	IDAETLEVGD
650	660	670	680	690	700	710	720
CVSVIPDDSS	KPLYLARVTA	LWEDSSNGQM	FHAHWFCAGT	DTVLGATSDP	LELFLVDECE	DMQLSYIHSK	VKVIYKAPSE
730	740	750	760	770	780	790	800
NWAMEGGMDP	ESLLEGDDGK	TYFYQLWYDQ	DYARFESPPK	TQPTEDNKFK	FCVSCARLAE	MRQKEIPRVL	EQLEDLDSRV
810	820	830	840	850	860	870	880
LYYSATKNGI	LYRVGDGVYL	PPEAFTFNIK	LSSPVKRPRK	EPVDEDLYPE	HYRKYSDYIK	GSNLDAPEPY	RIGRIKEIFC
890	900	910	920	930	940	950	960
PKKSNGRPNE	TDIKIRVNKF	YRPENTHKST	PASYHADINL	LYWSDEEAVV	DFKAVQGRCT	VEYGEDLPEC	VQVYSMGGPN
970	980	990	1000	1010	1020	1030	1040
RFYFLEAYNA	KSKSFEDPPN	HARSPGNKGK	GKGKGKGKPK	SQACEPSEPE	IEIKLPKLRT	LDVFSGCGGL	SEGFHQAGIS
1050	1060	1070	1080	1090	1100	1110	1120
DTLWAIEMWD	PAAQAFRLNN	PGSTVFTEDC	NILLKLVMAG	ETTNSRGQRL	PQKGDVEMLC	GGPPCQGFSG	MNRFNSRTYS
1130	1140	1150	1160	1170	1180	1190	1200
KFKNSLVVSF	LSYCDYYRPR	FFLLENVRNF	VSFKRSMVLK	LTLRCLVRMG	YQCTFGVLQA	GQYGVAQTRR	RAIILAAAPG
1210	1220	1230	1240	1250	1260	1270	1280
EKLPLFPEPL	HVFAPRACQL	SVVVDDKKFV	SNITRLSSGP	FRTITVRDTM	SDLPEVRNGA	SALEISYNGE	PQSWFQRQLR
1290	1300	1310	1320	1330	1340	1350	1360
GAQYQPILRD	HICKDMSALV	AARMRHIPLA	PGSDWRDLPN	IEVRLSDGTM	ARKLRYTHHD	RKNGRSSSGA	LRGVCSCVEA
1370	1380	1390	1400	1410	1420	1430	1440
GKACDPAARQ	FNTLIPWCLP	HTGNRHNHWA	GLYGRLEWDG	FFSTTVTNPE	PMGKQGRVLH	PEQHRVVSVR	ECARSQGFPD
1450	1460	1470	1480	1490	1500		
TYRLFGNILD	KHRQVGNAVP	PPLAKAIGLE	IKLCMLAKAR	ESASAKIKEE	EAAKD		

Cmpd.	No. of Cmpds.	m/z meas.	Δ m/z [ppm]	z	Rt [min]	Score	P	Range	Sequence	Modification
273	1	685.7423	-204.14	2	32.8	12.1	2	778-788	R.LAEMRQKEIPR.V	

Detailed Protein Report

Protein 338: partitioning defective 3 homolog isoform 8 [Homo sapiens]

Accession: gi|296278200
Database: refseq_human(refseq_human_20140103.fasta)
Database Date: 2015-11-30
Modification(s): Oxidation

Score: 27.5
MW [kDa]: 138.2
pI: 8.4
Sequence Coverage [%]: 2.7
No. of unique Peptides: 1

10	20	30	40	50	60	70	80
MKVTVCFGRT	RVVVPCGDGH	MKVFSLIQQA	VTRYRKAIAK	DPNYWIQVHR	LEHGDGGILD	LDDILCDVAD	DKDRLVAVFD
**90**	**100**	**110**	**120**	**130**	**140**	**150**	**160**
EQDPHHGGDG	TSASSTGTQS	PEIFGSELGT	NNVSAFQPYQ	ATSEIEVTPS	VLRANMPLHV	RRSSDPALIG	LSTSVSDSNF
**170**	**180**	**190**	**200**	**210**	**220**	**230**	**240**
SSEEPSRKNP	TRWSTTAGFL	KQNTAGSPKT	CDRKDEDGTE	EDNSRVEPVG	HADTGLEHIP	NFSLDDMVKL	VEVPNDGGPL
**250**	**260**	**270**	**280**	**290**	**300**	**310**	**320**
GIHVVPFSAR	GGRTLGLLVK	RLEKGGKAEH	ENLFRENDCI	VRINDGDLRN	RRFEQAQHMF	RQAMRTPIIW	FHVVPAANKE
**330**	**340**	**350**	**360**	**370**	**380**	**390**	**400**
QYEQLSQSEK	NNYYSSRFSP	DSQYIDNRSV	NSAGLHTVQR	APRLNHPPEQ	IDSHSRLPHS	AHPSGKPPSA	PASAPQNVFS
**410**	**420**	**430**	**440**	**450**	**460**	**470**	**480**
TTVSSGYNTK	KIGKRLNIQL	KKGTEGLGFS	ITSRDVTIGG	SAPIYVKNIL	PRGAAIQDGR	LKAGDRLIEV	NGVDLVGKSQ
**490**	**500**	**510**	**520**	**530**	**540**	**550**	**560**
EEVVSLLRST	KMEGTVSLLV	FRQEDAFHPR	ELKAEDEDIV	LTPDGTREFL	TFEVPLNDSG	SAGLGVSVKG	NRSKENHADL
**570**	**580**	**590**	**600**	**610**	**620**	**630**	**640**
GIFVKSIING	GAASKDGRLR	VNDQLIAVNG	ESLLGKTNQD	AMETLRRSMS	TEGNKRGMIQ	LIVARRISKC	NELKSPGSPP
**650**	**660**	**670**	**680**	**690**	**700**	**710**	**720**
GPELPIETAL	DDRERRISHS	LYSGIEGLDE	SPSRNAALSR	IMGKYQLSPT	VNMPQDDTVI	IEDDRLPVLP	PHLSDQSSSS
**730**	**740**	**750**	**760**	**770**	**780**	**790**	**800**
SHDDVGFVTA	DAGTWAKAAI	SDSADCSLSP	DVDPVLAFQR	EGFGRQSMSE	KRTKQFSDAS	QLDFVKTRKS	KSMDLGSSPS
**810**	**820**	**830**	**840**	**850**	**860**	**870**	**880**
RDVGPSLGLK	KSSSLESLQT	AVAEVTLNGD	IPFHRPRPRI	IRGRGCNESF	RAAIDKSYDK	PAVDDDDEGM	ETLEEDTEES
**890**	**900**	**910**	**920**	**930**	**940**	**950**	**960**
SRSGRESVST	ASDQPSHSLE	RQMNGNQEKG	DKTDRKKDKT	GKEKKKDRDK	EKDKMKAKKG	MLKGLGDMFR	IQAKTREFRE
**970**	**980**	**990**	**1000**	**1010**	**1020**	**1030**	**1040**
RQARERDYAE	IQDFHRTFGC	DDELMYGGVS	SYEGSMALNA	RPQSPREGHM	MDALYAQVKK	PRNSKPSPVD	SNRSTPSNHD
**1050**	**1060**	**1070**	**1080**	**1090**	**1100**	**1110**	**1120**
RIQRLRQEFQ	QAKQDEDVED	RRRTYSFEQP	WPNARPATQS	GRHSVSVEVQ	MQRQRQEERE	SSQQAQRQYS	SLPRQSRKNA
**1130**	**1140**	**1150**	**1160**	**1170**	**1180**	**1190**	**1200**
SSVSQDSWEQ	NYSPGEGFQS	AKENPRYSSY	QGSRNGYLGG	HGFNARVMLE	TQELLRQEQR	RKEQQMKKQP	PSEGPSNYDS
**1210**	**1220**	**1230**	**1240**	**1250**			
YKKVQDPSYA	PPKGPFRQDV	PPSPSQVARL	NRLQTPEKGR	PFYS			

Cmpd.	No. of Cmpds.	m/z meas.	Δ m/z [ppm]	z	Rt [min]	Score	P	Range	Sequence	Modification
2354	1	711.9476	-118.31	3	59.3	10.7	2	790-810	K.SKSMDLGSSPSRDVGPSLGLK.K	Oxidation: 4

Detailed Protein Report

Protein 339: metastasis-associated in colon cancer protein 1 [Homo sapiens]

**Accession:**	gi|157502191	**Score:**	27.5
**Database:**	refseq_human(refseq_human_20140103.fasta)	**MW [kDa]:**	96.6
**Database Date:**	2015-11-30	**pI:**	6.5
		**Sequence Coverage [%]:**	3.2
		**No. of unique Peptides:**	1

10	20	30	40	50	60	70	80
MLITERKHFR	SGRIAQSMSE	ANLIDMEAGK	LSKSCNITEC	QDPDLLHNWP	DAFTLRGNNA	SKVANPFWNQ	LSASNPFLDD
**90**	**100**	**110**	**120**	**130**	**140**	**150**	**160**
ITQLRNNRKR	NNISILKEDP	FLFCREIENG	NSFDSSGDEL	DVHQLLRQTS	SRNSGRSKSV	SELLDILDDT	AHAHQSIHNS
**170**	**180**	**190**	**200**	**210**	**220**	**230**	**240**
DQILLHDLEW	LKNDREAYKM	AWLSQRQLAR	SCLDLNTISQ	SPGWAQTQLA	EVTIACKVNH	QGGSVQLPES	DITVHVPQGH
**250**	**260**	**270**	**280**	**290**	**300**	**310**	**320**
VAVGEFQEVS	LRAFLDPPHM	LNHDLSCTVS	PLLEIMLGNL	NTMEALLLEM	KIGAEVRKDP	FSQVMTEMVC	LHSLGKEGPF
**330**	**340**	**350**	**360**	**370**	**380**	**390**	**400**
KVLSNCYIYK	DTIQVKLIDL	SQVMYLVVAA	QAKALPSPAA	TIWDYIHKTT	SIGIYGPKYI	HPSFTVVLTV	CGHNYMPGQL
**410**	**420**	**430**	**440**	**450**	**460**	**470**	**480**
TISDIKKGGK	NISPVVFQLW	GKQSFLLDKP	QDLSISIFSC	DPDFEVKTEG	ERKEIKQKQL	EAGEVVHQQF	LFSLVEHREM
**490**	**500**	**510**	**520**	**530**	**540**	**550**	**560**
HLFDFCVQVE	PPNGEPVAQF	SITTPDPTPN	LKRLSNLPGY	LQKKEEIKSA	PLSPKILVKY	PTFQDKTLNF	SNYGVTLKAV
**570**	**580**	**590**	**600**	**610**	**620**	**630**	**640**
LRQSKIDYFL	EYFKGDTIAL	LGEGKVKAIG	QSKVKEWYVG	VLRGKIGLVH	CKNVKVISKE	QVMFMSDSVF	TTRNLLEQIV
**650**	**660**	**670**	**680**	**690**	**700**	**710**	**720**
LPLKKLTYIY	SVVLTLVSEK	VYDWKVLADV	LGYSHLSLED	FDQIQADKES	EKVSYVIKKL	KEDCHTERNT	RKFLYELIVA
**730**	**740**	**750**	**760**	**770**	**780**	**790**	**800**
LLKMDCQELV	ARLIQEAAVL	TSAVKLGKGW	RELAEKLVRL	TKQQMEAYEI	PHRGNTGDVA	VEMMWKPAYD	FLYTWSAHYG
**810**	**820**	**830**	**840**	**850**	**860**		
NNYRDVLQDL	QSALDRMKNP	VTKHWRELTG	VLILVNSLEV	LRVTAFSTSE	EV		

Cmpd.	No. of Cmpds.	m/z meas.	Δ m/z [ppm]	z	Rt [min]	Score	P	Range	Sequence	Modification
2511	1	841.8162	-157.28	2	61.3	16.7	0	354-368	K.ALPSPAATIWDYIHK.T	

Detailed Protein Report

Protein 340: kinesin-like protein KIF20B isoform 2 [Homo sapiens]

**Accession:**	gi|46049114	**Score:**	27.5
**Database:**	refseq_human(refseq_human_20140103.fasta)	**MW [kDa]:**	206.0
**Database Date:**	2015-11-30	**pI:**	5.5
**Modification(s):**	Oxidation	**Sequence Coverage [%]:**	1.7
		**No. of unique Peptides:**	1

10	20	30	40	50	60	70	80
MESNFNQEGV	PRPSYVFSAD	PIARPSEINF	DGIKLDLSHE	FSLVAPNTEA	NSFESKDYLQ	VCLRIRPFTQ	SEKELESEGC
**90**	**100**	**110**	**120**	**130**	**140**	**150**	**160**
VHILDSQTVV	LKEPQCILGR	LSEKSSGQMA	QKFSFSKVFG	PATTQKEFFQ	GCIMQPVKDL	LKGQSRLIFT	YGLTNSGKTY
**170**	**180**	**190**	**200**	**210**	**220**	**230**	**240**
TFQGTEENIG	ILPRTLNVLF	DSLQERLYTK	MNLKPHRSRE	YLRLSSEQEK	EEIASKSALL	RQIKEVTVHN	DSDDTLYGSL
**250**	**260**	**270**	**280**	**290**	**300**	**310**	**320**
TNSLNISEFE	ESIKDYEQAN	LNMANSIKFS	VWVSFFEIYN	EYIYDLFVPV	SSKFQKRKML	RLSQDVKGYS	FIKDLQWIQV
**330**	**340**	**350**	**360**	**370**	**380**	**390**	**400**
SDSKEAYRLL	KLGIKHQSVA	FTKLNNASSR	SHSIFTVKIL	QIEDSEMSRV	IRVSELSLCD	LAGSERTMKT	QNEGERLRET
**410**	**420**	**430**	**440**	**450**	**460**	**470**	**480**
GNINTSLLTL	GKCINVLKNS	EKSKFQQHVP	FRESKLTHYF	QSFFNGKGKI	CMIVNISQCY	LAYDETLNVL	KFSAIAQKVC
**490**	**500**	**510**	**520**	**530**	**540**	**550**	**560**
VPDTLNSSQE	KLFGPVKSSQ	DVSLDSNSNS	KILNVKRATI	SWENSLEDLM	EDEDLVEELE	NAEETQNVET	KLLDEDLDKT
**570**	**580**	**590**	**600**	**610**	**620**	**630**	**640**
LEENKAFISH	EEKRKLLDLI	EDLKKKLINE	KKEKLTLEFK	IREEVTQEFT	QYWAQREADF	KETLLQEREI	LEENAERRLA
**650**	**660**	**670**	**680**	**690**	**700**	**710**	**720**
IFKDLVGKCD	TREEAAKDIC	ATKVETEEAT	ACLELKFNQI	KAELAKTKGE	LIKTKEELKK	RENESDSLIQ	ELETSNKKII
**730**	**740**	**750**	**760**	**770**	**780**	**790**	**800**
TQNQRIKELI	NIIDQKEDTI	NEFQNLKSHM	ENTFKCNDKA	DTSSLIINNK	LICNETVEVP	KDSKSKICSE	RKRVNENELQ
**810**	**820**	**830**	**840**	**850**	**860**	**870**	**880**
QDEPPAKKGS	IHVSSAITED	QKKSEEVRPN	IAEIEDIRVL	QENNEGLRAF	LLTIENELKN	EKEEKAELNK	QIVHFQQELS
**890**	**900**	**910**	**920**	**930**	**940**	**950**	**960**
LSEKKNLTLS	KEVQQIQSNY	DIAIAELHVQ	KSKNQEQEEK	IMKLSNEIET	ATRSITNNVS	QIKLMHTKID	ELRTLDSVSQ
**970**	**980**	**990**	**1000**	**1010**	**1020**	**1030**	**1040**
ISNIDLLNLR	DLSNGSEEDN	LPNTQLDLLG	NDYLVSKQVK	EYRIQEPNRE	NSFHSSIEAI	WEECKEIVKA	SSKKSHQIEE
**1050**	**1060**	**1070**	**1080**	**1090**	**1100**	**1110**	**1120**
LEQQIEKLQA	EVKGYKDENN	RLKEKEHKNQ	DDLLKEKETL	IQQLKEELQE	KNVTLDVQIQ	HVVEGKRALS	ELTQGVTCYK
**1130**	**1140**	**1150**	**1160**	**1170**	**1180**	**1190**	**1200**
AKIKELETIL	ETQKVECSHS	AKLEQDILEK	ESIILKLERN	LKEFQEHLQD	SVKNTKDLNV	KELKLKEEIT	QLTNNLQDMK
**1210**	**1220**	**1230**	**1240**	**1250**	**1260**	**1270**	**1280**
HLLQLKEEEE	ETNRQETEKL	KEELSASSAR	TQNLKADLQR	KEEDYADLKE	KLTDAKKQIK	QVQKEVSVMR	DEDKLLRIKI
**1290**	**1300**	**1310**	**1320**	**1330**	**1340**	**1350**	**1360**
NELEKKKNQC	SQELDMKQRT	IQQLKEQLNN	QKVEEAIQQY	ERACKDLNVK	EKIIEDMRMT	LEEQEQTQVE	QDQVLEAKLE
**1370**	**1380**	**1390**	**1400**	**1410**	**1420**	**1430**	**1440**
EVERLATELE	KWKEKCNDLE	TKNNQRSNKE	HENNTDVLGK	LTNLQDELQE	SEQKYNADRK	KWLEEKMMLI	TQAKEAENIR
**1450**	**1460**	**1470**	**1480**	**1490**	**1500**	**1510**	**1520**
NKEMKKYAED	RERFFKQQNE	MEILTAQLTE	KDSDLQKWRE	ERDQLVAALE	IQLKALISSN	VQKDNEIEQL	KRIISETSKI
**1530**	**1540**	**1550**	**1560**	**1570**	**1580**	**1590**	**1600**
ETQIMDIKPK	RISSADPDKL	QTEPLSTSFE	ISRNKIEDGS	VVLDSCEVST	ENDQSTRFPK	PELEIQFTPL	QPNKMAVKHP
**1610**	**1620**	**1630**	**1640**	**1650**	**1660**	**1670**	**1680**
GCTTPVTVKI	PKARKRKSNE	MEEDLVKCEN	KKNATPRTNL	KFPISDDRNS	SVKKEQKVAI	RPSSKKTYSL	RSQASIIGVN
**1690**	**1700**	**1710**	**1720**	**1730**	**1740**	**1750**	**1760**
LATKKKEGTL	QKFGDFLQHS	PSILQSKAKK	IIETMSSSKL	SNVEASKENV	SQPKRAKRKL	YTSEISSPID	ISGQVILMDQ
**1770**	**1780**	**1790**					
KMKESDHQII	KRRLRTKTAK						

Cmpd.	No. of	m/z meas.	Δ m/z	z	Rt	Score	P	Range	Sequence	Modification

Detailed Protein Report

	Cmpds.		[ppm]		[min]					
20	1	1103.5042	-42.73	2	29.9	14.1	2	93-112	K.EPQCILGRLSEKSSGQMAQK.F	Oxidation: 17

Detailed Protein Report

Protein 341: unconventional myosin-XV [Homo sapiens]

**Accession:**	gi|118402590	**Score:**	27.4
**Database:**	refseq_human(refseq_human_20140103.fasta)	**MW [kDa]:**	395.0
**Database Date:**	2015-11-30	**pI:**	9.8
		**Sequence Coverage [%]:**	0.7
		**No. of unique Peptides:**	1

Detailed Protein Report

10	20	30	40	50	60	70	80
MAKEEDEEKK	AKKGKKGKKA	PEPEKPKRSL	KGTSRLFMGF	RDRTPKISKK	GQFRSASAFF	WGLHTGPQKT	KRKRKARTVL
90	100	110	120	130	140	150	160
KSTSKLMTQM	RMGKKKRAMK	GKKPSFMVIR	FPGRRGYGRL	RPRARSLSKA	STAINWLTKK	FLLKKAEESG	SEQATVDAWL
170	180	190	200	210	220	230	240
QRSSSRMGSR	KLPFPSGAEI	LRPGGRLRRF	PRSRSIYASG	EPLGFLPFED	EAPFHHSGSR	KSLYGLEGFQ	DLGEYYDYHR
250	260	270	280	290	300	310	320
DGDDYYDRQS	LHRYEEQEPY	LAGLGPYSPA	WPPYGDHYYG	YPPEDPYDYY	HPDYYGGPFD	PGYTYGYGYD	DYEPPYAPPS
330	340	350	360	370	380	390	400
GYSSPYSYHD	GYEGEAHPYG	YYLDPYAPYD	APYPPYDLPY	HTPYDVPYFD	PYGVHYTVPY	AEGVYGGGDE	AIYPPEVPYF
410	420	430	440	450	460	470	480
YPEESASAFV	YPWVPPPIPS	PHNPYAHAMD	DIAELEEPED	AGVERQGTSF	RLPSAAFFEQ	QGMDKPARSK	LSLIRKFRLF
490	500	510	520	530	540	550	560
PRPQVKLFGK	EKLEVPLPPS	LDIPLPLGDA	DEEEDEEELP	PVSAVPYGHP	FWGFLTPRQR	NLQRALSAFG	AHRGLGFGPE
570	580	590	600	610	620	630	640
FGRPVPRPAT	SLARFLKKTL	SEKKPIARLR	GSQKARAGGP	AVREAAYKRF	GYKLAGMDPE	KPGTPIVLRR	AQPRARSSND
650	660	670	680	690	700	710	720
ARRPPAPQPA	PRTLSHWSAL	LSPPVPPRPP	SSGPPPAPPL	SPALSGLPRP	ASPYGSLRRH	PPPWAAPAHV	PPAPQASWWA
730	740	750	760	770	780	790	800
FVEPPAVSPE	VPPDLLAFPG	PRPSFRGSRR	RGAAFGFPGA	SPRASRRRAW	SPLASPQPSL	RSSPGLGYCS	PLAPPSPQLS
810	820	830	840	850	860	870	880
LRTGPFQPPF	LPPARRPRSL	QESPAPRRAA	GRLGPPGSPL	PGSPRPPSPP	LGLCHSPRRS	SLNLPSRLPH	TWRRLSEPPT
890	900	910	920	930	940	950	960
RAVKPQVRLP	FHRPPRAGAW	RAPLEHRESP	REPEDSETPW	TVPPLAPSWD	VDMPPTQRPP	SPWPGGAGSR	RGFSRPPPVP
970	980	990	1000	1010	1020	1030	1040
ENPFLQLLGP	VPSPTLQPED	PAADMTRVFL	GRHHEPGPGQ	LTKSAGPTPE	KPEEEATLGD	PQLPAETKPP	TPAPPKDVTP
1050	1060	1070	1080	1090	1100	1110	1120
PKDITPPKDV	LPEQKTLRPS	LSYPLAACDQ	TRATWPPWHR	WGTLPQAAAP	LAPIRAPEPL	PKGGERRQAA	PGRFAVVMPR
1130	1140	1150	1160	1170	1180	1190	1200
VQKLSSFQRV	GPATLKPQVQ	PIQDPKPRAC	SLRWSCLWLR	ADAYGPWPRV	HTHPQSCHLG	PGAACLSLRG	SWEEVGPPSW
1210	1220	1230	1240	1250	1260	1270	1280
RNKMHSIRNL	PSMRFREQHG	EDGVEDMTQL	EDLQETTVLS	NLKIRFERNL	IYTYIGSILV	SVNPYQMFGI	YGPEQVQQYN
1290	1300	1310	1320	1330	1340	1350	1360
GRALGENPPH	LFAVANLAFA	KMLDAKQNQC	IIISGESGSG	KTEATKLILR	YLAAMNQKRE	VMQQIKILEA	TPLLESFGNA
1370	1380	1390	1400	1410	1420	1430	1440
KTVRNDNSSR	FGKFVEIFLE	GGVISGAITS	QYLLEKSRIV	FQAKNERNYH	IFYELLAGLP	AQLRQAFSLQ	EAETYYYLNQ
1450	1460	1470	1480	1490	1500	1510	1520
GGNCEIAGKS	DADDFRRLLA	AMEVLGFSSE	DQDSIFRILA	SILHLGNVYF	EKYETDAQEV	ASVVSAREIQ	AVAELLQISP
1530	1540	1550	1560	1570	1580	1590	1600
EGLQKAITFK	VTETMREKIF	TPLTVESAVD	ARDAIAKVLY	ALLFSWLITR	VNALVSPRQD	TLSIAILDIY	GFEDLSFNSF
1610	1620	1630	1640	1650	1660	1670	1680
EQLCINYANE	NLQYLFNKIV	FQEEQEEYIR	EQIDWQEITF	ADNQPCINLI	SLKPYGILRI	LDDQCCFPQA	TDHTFLQKCH
1690	1700	1710	1720	1730	1740	1750	1760
YHHGANPLYS	KPKMPLPEFT	IKHYAGKVTY	QVHKFLDKNH	DQVRQDVLDL	FVRSRTRVVA	HLFSSHAPQA	APQRLGKSSS
1770	1780	1790	1800	1810	1820	1830	1840
VTRLYKAHTV	AAKFQQSLLD	LVEKMERCNP	LFMRCLKPNH	KKEPGLFEPD	VVMAQLRYSG	VLETVRIRKE	GFPVRLPFQG
1850	1860	1870	1880	1890	1900	1910	1920
FIDRYCCLVA	LKHDLPANGD	MCVSVLSRLC	KVMPNMYRVG	VSKLFLKEHL	YQLLESMREH	VLNLAALTLQ	RCLRGFFIKR
1930	1940	1950	1960	1970	1980	1990	2000
RFRSLRHKII	LLQSRARGYL	ARQRYQQMRR	SLVKFRSLVH	AYVSRRRYLK	LRAEWRCQVE	GALLWEQEEL	SKREVVAVGH
2010	2020	2030	2040	2050	2060	2070	2080
LEVPAELAGL	LQAVAGLGLA	QVPQVAPVRT	PRLQAEPRVT	LPLDINNYPM	AKFVQCHFKE	PAFGMLTVPL	RTPLTQLPAE
2090	2100	2110	2120	2130	2140	2150	2160
HHAEAVSIFK	LILRFMGDPH	LHGARENIFG	NYIVQKGLAV	PELRDEILAQ	LANQVWHNHN	AHNAERGWLL	LAACLSGFAP
2170	2180	2190	2200	2210	2220	2230	2240

Detailed Protein Report

Cmpd.	No. of Cmpds.	m/z meas.	Δ m/z [ppm]	z	Rt [min]	Score	P	Range	Sequence	Modification
2655	1	673.3551	-22.70	2	64.3	13.5	2	597-609	R.AGGPAVREAAYKR.F	

Detailed Protein Report

Protein 342: **kinesin heavy chain isoform 5A [Homo sapiens]**

**Accession:**	gi|45446749	**Score:**	27.4
**Database:**	refseq_human(refseq_human_20140103.fasta)	**MW [kDa]:**	117.3
**Database Date:**	2015-11-30	**pI:**	5.6
**Modification(s):**	Carbamidomethyl	**Sequence Coverage [%]:**	2.6
		**No. of unique Peptides:**	2

Quantitation

MD:MU **Median:** 0.75 **CV:** 0.00 % **No. of Peptides:** 1

10	20	30	40	50	60	70	80
MAETNNECSI	KVLCRFRPLN	QAEILRGDKF	IPIFQGDDSV	VIGGKPYVFD	RVFPPNTTQE	QVYHACAMQI	VKDVLAGYNG
**90**	**100**	**110**	**120**	**130**	**140**	**150**	**160**
TIFAYGQTSS	GKTHTMEGKL	HDPQLMGIIP	RIARDIFNHI	YSMDENLEFH	IKVSYFEIYL	DKIRDLLDVT	KTNLSVHEDK
**170**	**180**	**190**	**200**	**210**	**220**	**230**	**240**
NRVPFVKGCT	ERFVSSPEEI	LDVIDEGKSN	RHVAVTNMNE	HSSRSHSIFL	INIKQENMET	EQKLSGKLYL	VDLAGSEKVS
**250**	**260**	**270**	**280**	**290**	**300**	**310**	**320**
KTGAEGAVLD	EAKNINKSLS	ALGNVISALA	EGTKSYVPYR	DSKMTRILQD	SLGGNCRTTM	FICCSPSSYN	DAETKSTLMF
**330**	**340**	**350**	**360**	**370**	**380**	**390**	**400**
GQRAKTIKNT	ASVNLELTAE	QWKKKYEKEK	EKTKAQKETI	AKLEAELSRW	RNGENVPETE	RLAGEEAALG	AELCEETPVN
**410**	**420**	**430**	**440**	**450**	**460**	**470**	**480**
DNSSIVVRIA	PEERQKYEEE	IRRLYKQLDD	KDDEINQQSQ	LIEKLKQQML	DQEELLVSTR	GDNEKVQREL	SHLQSENDAA
**490**	**500**	**510**	**520**	**530**	**540**	**550**	**560**
KDEVKEVLQA	LEELAVNYDQ	KSQEVEEKSQ	QNQLLVDELS	QKVATMLSLE	SELQRLQEVS	GHQRKRIAEV	LNGLMKDLSE
**570**	**580**	**590**	**600**	**610**	**620**	**630**	**640**
FSVIVGNGEI	KLPVEISGAI	EEEFTVARLY	ISKIKSEVKS	VVKRCRQLEN	LQVECHRKME	VTGRELSSCQ	LLISQHEAKI
**650**	**660**	**670**	**680**	**690**	**700**	**710**	**720**
RSLTEYMQSV	ELKKRHLEES	YDSLSDELAK	LQAQETVHEV	ALKDKEPDTQ	DADEVKKALE	LQMESHREAH	HRQLARLRDE
**730**	**740**	**750**	**760**	**770**	**780**	**790**	**800**
INEKQKTIDE	LKDLNQKLQL	ELEKLQADYE	KLKSEEHEKS	TKLQELTFLY	ERHEQSKQDL	KGLEETVARE	LQTLHNLRKL
**810**	**820**	**830**	**840**	**850**	**860**	**870**	**880**
FVQDVTTRVK	KSAEMEPEDS	GGIHSQKQKI	SFLENNLEQL	TKVHKQLVRD	NADLRCELPK	LEKRLRATAE	RVKALEGALK
**890**	**900**	**910**	**920**	**930**	**940**	**950**	**960**
EAKEGAMKDK	RRYQQEVDRI	KEAVRYKSSG	KRGHSAQIAK	PVRPGHYPAS	SPTNPYGTRS	PECISYTNSL	FQNYQNLYLQ
**970**	**980**	**990**	**1000**	**1010**	**1020**	**1030**	**1040**
ATPSSTSDMY	FANSCTSSGA	TSSGGPLASY	QKANMDNGNA	TDINDNRSDL	PCGYEAEDQA	KLFPLHQETA	AS

Cmpd.	No. of Cmpds.	m/z meas.	Δ m/z [ppm]	z	Rt [min]	Score	P	Range	Sequence	Modification	Ratios
2155	1	713.2696	-111.00	2	57.1	15.2	0	607-617	R.QLENLQVECHR.K	Carbamidomethyl: 9	
1747	1	851.2332	-164.00	2	51.8	12.2	0	812-827	K.SAEMEPEDSGGIHSQK.Q		MD:MU 0.75

Detailed Protein Report

Protein 343: transmembrane protease serine 12 precursor [Homo sapiens]

Accession: gi|32698940
Database: refseq_human(refseq_human_20140103.fasta)
Database Date: 2015-11-30

Score: 27.4
MW [kDa]: 38.6
pI: 10.0
Sequence Coverage [%]: 8.6
No. of unique Peptides: 2

10	20	30	40	50	60	70	80
MRLGLLSVAL	LFVGSSHLYS	DHYSPSGRHR	LGPSPEPAAS	SQQAEAVRKR	LRRRREGGAH	AKDCGTAPLK	DVLQGSRIIG
**90**	**100**	**110**	**120**	**130**	**140**	**150**	**160**
GTEAQAGAWP	WVVSLQIKYG	RVLVHVCGGT	LVRERWVLTA	AHCTKDASDP	LMWTAVIGTN	NIHGRYPHTK	KIKIKAIIIH
**170**	**180**	**190**	**200**	**210**	**220**	**230**	**240**
PNFILESYVN	DIALFHLKKA	VRYNDYIQPI	CLPFDVFQIL	DGNTKCFISG	WGRTKEEGNA	TNILQDAEVH	YISREMCNSE
**250**	**260**	**270**	**280**	**290**	**300**	**310**	**320**
RSYGGIIPNT	SFCAGDEDGA	FDTCRGDSGG	PLMCYLPEYK	RFFVMGITSY	GHGCGRRGFP	GVYIGPSFYQ	KWLTEHFFHA
**330**	**340**	**350**					
STQGILTINI	LRGQILIALC	FVILLATT					

Cmpd.	No. of Cmpds.	m/z meas.	Δ m/z [ppm]	z	Rt [min]	Score	P	Range	Sequence	Modification
1840	3	897.8543	-109.51	2	52.9	14.9	0	31-48	R.LGPSPEPAASSQQAEAVR.K	
2601	1	626.8465	-26.97	2	63.1	12.5	0	102-113	R.VLVHVCGGTLVR.E	

Detailed Protein Report

Protein 344: PREDICTED: rab11 family-interacting protein 4 isoform X1 [Homo sapiens]

**Accession:**	gi|530411171	**Score:**	27.4
**Database:**	refseq_human(refseq_human_20140103.fasta)	**MW [kDa]:**	67.7
**Database Date:**	2015-11-30	**pI:**	4.8
**Modification(s):**	Carbamidomethyl	**Sequence Coverage [%]:**	5.1
		**No. of unique Peptides:**	1

10	20	30	40	50	60	70	80
MSPQLDTKSP	SAHAGLPATG	GGGAAPSTGA	QKCERQRRGR	DCSCASGLPA	ASPRAADPRP	LASAPLPGSG	PRMRTPPALG
**90**	**100**	**110**	**120**	**130**	**140**	**150**	**160**
SQGSEVTGPT	FADGELIPRE	PGFFPEDEEE	AMTLAPPEGP	QELYTDSPME	STQSLEGSVG	SPAEKDGGLG	GLFLPEDKSL
**170**	**180**	**190**	**200**	**210**	**220**	**230**	**240**
VHTPSMTTSD	LSTHSTTSLI	SNEEQFEDYG	EGDDVDCAPS	SPCPDDETRT	NVYSDLGSSV	SSSAGQTPRK	MRHVYNSELL
**250**	**260**	**270**	**280**	**290**	**300**	**310**	**320**
DVYCSQCCKK	INLLNDLEAR	LKNLKANSPN	RKISSTAFGR	QLMHSSNFSS	SNGSTEDLFR	DSIDSCDNDI	TEKVSFLEKK
**330**	**340**	**350**	**360**	**370**	**380**	**390**	**400**
VTELENDSLT	NGDLKSKLKQ	ENTQLVHRVH	ELEEMVKDQE	TTAEQALEEE	ARRHREAYGK	LEREKATEVE	LLNARVQQLE
**410**	**420**	**430**	**440**	**450**	**460**	**470**	**480**
EENTELRTTV	TRLKSQTEKL	DEERQRMSDR	LEDTSLRLKD	EMDLYKRMMD	KLRQNRLEFQ	KEREATQELI	EDLRKELEHL
**490**	**500**	**510**	**520**	**530**	**540**	**550**	**560**
QMYKLDCERP	GRGRSASSGL	GEFNARAREV	ELEHEVKRLK	QENYKLRDQN	DDLNGQILSL	SLYEAKNLFA	AQTKAQSLAA
**570**	**580**	**590**	**600**	**610**			
EIDTASRDEL	MEALKEQEEI	NFRLRQYMDK	IILAILDHNP	SILEIKH			

Cmpd.	No. of Cmpds.	m/z meas.	Δ m/z [ppm]	z	Rt [min]	Score	P	Range	Sequence	Modification
1981	1	696.2626	-62.02	2	54.7	16.3	0	41-54	R.DCSCASGLPAASPR.A	Carbamidomethyl: 2

Detailed Protein Report

Protein 345: PREDICTED: suppressor of tumorigenicity 7 protein-like isoform X10 [Homo sapiens]

**Accession:**	gi|530362849	**Score:**	27.3
**Database:**	refseq_human(refseq_human_20140103.fasta)	**MW [kDa]:**	35.9
**Database Date:**	2015-11-30	**pI:**	9.9
**Modification(s):**	Oxidation	**Sequence Coverage [%]:**	5.6
		**No. of unique Peptides:**	2

10	20	30	40	50	60	70	80
MADRGGVGEA	AAVGASPASV	PGLNPTLGWR	ERLRAGLAGT	GASLWFVAGL	GLLYALRIPL	RLCENLAAVT	VFLNSLTPKF
**90**	**100**	**110**	**120**	**130**	**140**	**150**	**160**
YVALTGTSSL	ISGLIFIFEW	WYFHKHGTSF	IEQVSVSHLQ	PLMGGTESSI	SEPGSPSRNR	ENETSRQNLS	ECKVWRNPLN
**170**	**180**	**190**	**200**	**210**	**220**	**230**	**240**
LFRGAEYRRY	TWVTGKEPLT	YYDMNLSAQD	HQTFFTCDTD	FLRPSDTVMQ	KAWRERNPPA	RIKAAYQALE	LNNDCATAYV
**250**	**260**	**270**	**280**	**290**	**300**	**310**	**320**
LLAEEEATTI	VDAERLFKQA	LKAGETIYRQ	SQQCQHQSPQ	HEAQLRRDTN	VLVYIKRRLA	MCARKLGRIR	EAVKIMRDI

Cmpd.	No. of Cmpds.	m/z meas.	Δ m/z [ppm]	z	Rt [min]	Score	P	Range	Sequence	Modification
2033	1	625.2626	-137.48	2	55.3	14.2	1	259-269	K.QALKAGETIYR.Q	
2273	2	836.1691	-303.52	1	58.7	13.2	1	298-304	R.RLAMCAR.K	Oxidation: 4

Detailed Protein Report

Protein 346: PREDICTED: homeobox protein Hox-A6 isoform X1 [Homo sapiens]

**Accession:**	gi|578813472	**Score:**	27.3
**Database:**	refseq_human(refseq_human_20140103.fasta)	**MW [kDa]:**	23.6
**Database Date:**	2015-11-30	**pI:**	12.3
**Modification(s):**	Carbamidomethyl	**Sequence Coverage [%]:**	13.7
		**No. of unique Peptides:**	2

10	20	30	40	50	60	70	80
MWMRTVNMNF	FIISLARIEP	HSFAPLRPAC	FGALPSSRAR	LCFVRRWRAK	GSPGQARPGG	SRWRLGALRS	RRRGSSFATR
**90**	**100**	**110**	**120**	**130**	**140**	**150**	**160**
NLANFLRPLA	PGKRTILLSG	CPSDSCQRGR	SFWEPPSEGA	VYGSHGRRGR	QTYTRYQTLE	LEKEFHFNRY	LTRRRRIEIA
**170**	**180**	**190**	**200**	**210**			
NALCLTERQI	KIWFQNRRMK	WKKENKLINS	TQPSGEDSEA	KAGE			

Cmpd.	No. of Cmpds.	m/z meas.	Δ m/z [ppm]	z	Rt [min]	Score	P	Range	Sequence	Modification
2501	1	834.4079	-45.05	2	61.7	10.3	2	47-62	R.WRAKGSPGQARPGGSR.W	
2519	1	701.8340	-53.57	2	61.5	17.0	0	157-168	R.IEIANALCLTER.Q	Carbamidomethyl: 8

Detailed Protein Report

Protein 347: **PREDICTED: phosphoenolpyruvate carboxykinase [GTP], mitochondrial isoform X2 [Homo sapiens]**

Accession: gi|578825848
Database: refseq_human(refseq_human_20140103.fasta)
Database Date: 2015-11-30
Modification(s): Oxidation

Score: 27.3
MW [kDa]: 55.9
pI: 6.7
Sequence Coverage [%]: 7.5
No. of unique Peptides: 2

10	20	30	40	50	60	70	80
MSPADFQRAV	DERFPGCMQG	RTMYVLPFSM	GPVGSPLSRI	GVQLTDSAYV	VASMRIMTRL	GTPVLQALGD	GDFVKCLHSV
**90**	**100**	**110**	**120**	**130**	**140**	**150**	**160**
GQPLTGQGEP	VSQWPCNPEK	TLIGHVPDQR	EIISFGSGYG	GNSLLGKKCF	ALRIASRLAR	DEGWLAEHML	ILGITSPAGK
**170**	**180**	**190**	**200**	**210**	**220**	**230**	**240**
KRYVAAAFPS	ACGKTNLAMM	RPALPGWKVE	CVGDDIAWMR	FDSEGRLRAI	NPENGFFGVA	PGTSATTNPN	AMATIQSNTI
**250**	**260**	**270**	**280**	**290**	**300**	**310**	**320**
FTNVAETSDG	GVYWEGIDQP	LPPGVTVTSW	LGKPWKPGDK	EPCAHPNSRF	CAPARQCPIM	DPAWEAPEGV	PIDAIIFGGR
**330**	**340**	**350**	**360**	**370**	**380**	**390**	**400**
RPKGVPLVYE	AFNWRHGVFV	GSAMRSESTA	AAEHKGKIIM	HDPFAMRPFF	GYNFGHYLEH	WLSMEGRKGA	QLPRIFHVNW
**410**	**420**	**430**	**440**	**450**	**460**	**470**	**480**
FRRDEAGHFL	WPGFGENARV	LDWICRRLEG	EDSARETPIG	LVPKEGALDL	SGLRAIDTTQ	LFSLPKDFWE	QEVRDIRSYL
**490**	**500**	**510**					
TEQVNQDLPK	EVLAELEALE	RRVHKM					

Cmpd.	No. of Cmpds.	m/z meas.	Δ m/z [ppm]	z	Rt [min]	Score	P	Range	Sequence	Modification
2104	1	1044.4007	-95.08	2	56.2	12.0	1	336-355	R.HGVFVGSAMRSESTAAAEHK.G	Oxidation: 9
972	1	984.1646	135.28	2	41.7	15.3	2	427-444	R.RLEGEDSARETPIGLVPK.E	

Detailed Protein Report

Protein 348: DNA damage-inducible transcript 4-like protein [Homo sapiens]

Accession: gi|21687001
Database: refseq_human(refseq_human_20140103.fasta)
Database Date: 2015-11-30
Modification(s): Carbamidomethyl, Oxidation

Score: 27.3
MW [kDa]: 21.7
pI: 9.0
Sequence Coverage [%]: 13.0
No. of unique Peptides: 2

Alias proteins:

Accession	Name	Description
gi|530377132	refseq_human (refseq_human_20140103.fasta)	PREDICTED: DNA damage-inducible transcript 4-like protein isoform X1 [Homo sapiens]

10	20	30	40	50	60	70	80
MVATGSLSSK	NPASISELLD	CGYHPESLLS	DFDYWDYVVP	EPNLNEVIFE	ESTCQNLVKM	LENCLSKSKQ	TKLGCSKVLV
**90**	**100**	**110**	**120**	**130**	**140**	**150**	**160**
PEKLTQRIAQ	DVLRLSSTEP	CGLRGCVMHV	NLEIENVCKK	LDRIVCDSSV	VPTFELTLVF	KQENCSWTSF	RDFFFSRGRF
**170**	**180**	**190**	**200**				
SSGFRRTLIL	SSGFRLVKKK	LYSLIGTTVI	EGS				

Cmpd.	No. of Cmpds.	m/z meas.	Δ m/z [ppm]	z	Rt [min]	Score	P	Range	Sequence	Modification
1701	1	606.5258	-149.20	3	51.2	15.7	0	105-119	R.GCVMHVNLEIENVCK.K	Carbamidomethyl: 2, 14; Oxidation: 4
2065	1	575.2075	-231.33	2	55.7	11.6	1	166-175	R.RTLILSSGFR.L	

Detailed Protein Report

Protein 349: **A disintegrin and metalloproteinase with thrombospondin motifs 20 preproprotein [Homo sapiens]**

**Accession:**	gi|124430557	**Score:**	27.3
**Database:**	refseq_human(refseq_human_20140103.fasta)	**MW [kDa]:**	214.6
**Database Date:**	2015-11-30	**pI:**	7.2
**Modification(s):**	Carbamidomethyl	**Sequence Coverage [%]:**	1.2
		**No. of unique Peptides:**	1

Quantitation

MD:MU	**Median:** 0.59	**CV:** 0.00 %	**No. of Peptides:** 1

Detailed Protein Report

10	20	30	40	50	60	70	80
MWVAKWLTGL	LYHLSLFITR	SWEVDFHPRQ	EALVRTLTSY	EVVIPERVNE	FGEVFPQSHH	FSRQKRSSEA	LEPMPFRTHY
90	100	110	120	130	140	150	160
RFTAYGQLFQ	LNLTADASFL	AAGYTEVHLG	TPERGAWESD	AGPSDLRHCF	YRGQVNSQED	YKAVVSLCGG	LTGTFKGQNG
170	180	190	200	210	220	230	240
EYFLEPIMKA	DGNEYEDGHN	KPHLIYRQDL	NNSFLQTLKY	CSVSESQIKE	TSLPFHTYSN	MNEDLNVMKE	RVLGHTSKNV
250	260	270	280	290	300	310	320
PLKDERRHSR	KKRLISYPRY	IEIMVTADAK	VVSAHGSNLQ	NYILTLMSIV	ATIYKDPSIG	NLIHIVVVKL	VMIHREEEGP
330	340	350	360	370	380	390	400
VINFDGATTL	KNFCSWQQTQ	NDLDDVHPSH	HDTAVLITRE	DICSSKEKCN	MLGLSYLGTI	CDPLQSCFIN	EEKGLISAFT
410	420	430	440	450	460	470	480
IAHELGHTLG	VQHDDNPRCK	EMKVTKYHVM	APALSFHMSP	WSWSNCSRKY	VTEFLDTGYG	ECLLDKPDEE	IYNLPSELPG
490	500	510	520	530	540	550	560
SRYDGNKQCE	LAFGPGSQMC	PHINICMHLW	CTSTEKLHKG	CFTQHVPPAD	GTDCGPGMHC	RHGLCVNKET	ETRPVNGEWG
570	580	590	600	610	620	630	640
PWEPYSSCSR	TCGGGIESAT	RRCNRPEPRN	GGNYCVGRRM	KFRSCNTDSC	PKGTQDFREK	QCSDFNGKHL	DISGIPSNVR
650	660	670	680	690	700	710	720
WLPRYSGIGT	KDRCKLYCQV	AGTNYFYLLK	DMVEDGTPCG	TETHDICVQG	QCMAAGCDHV	LNSSAKIDKC	GVCGGDNSSC
730	740	750	760	770	780	790	800
KTITGVFNSS	HYGYNVVVKI	PAGATNVDIR	QYSYSGQPDD	SYLALSDAEG	NFLFNGNFLL	STSKKEINVQ	GTRTVIEYSG
810	820	830	840	850	860	870	880
SNNAVERINS	TNRQEKELIL	QVLCVGNLYN	PDVHYSFNIP	LEERSDMFTW	DPYGPWEGCT	KMCQGLQRRN	ITCIHKSDHS
890	900	910	920	930	940	950	960
VVSDKECDHL	PLPSFVTQSC	NTDCELRWHV	IGKSECSSQC	GQGYRTLDIH	CMKYSIHEGQ	TVQVDDHYCG	DQLKPPTQEL
970	980	990	1000	1010	1020	1030	1040
CHGNCVFTRW	HYSEWSQCSR	SCGGGERSRE	SYCMNNFGHR	LADNECQELS	RVTRENCNEF	SCPSWAASEW	SECLVTCGKG
1050	1060	1070	1080	1090	1100	1110	1120
TKQRQVWCQL	NVDHLSDGFC	NSSTKPESLS	PCELHTCASW	QVGPWGPCTT	TCGHGYQMRD	VKCVNELASA	VLEDTECHEA
1130	1140	1150	1160	1170	1180	1190	1200
SRPSDRQSCV	LTPCSFISKL	ETALLPTVLI	KKMAQWRHGS	WTPCSVSCGR	GTQARYVSCR	DALDRIADES	YCAHLPRPAE
1210	1220	1230	1240	1250	1260	1270	1280
IWDCFTPCGE	WQAGDWSPCS	ASCGHGKTTR	QVLCMNYHQP	IDENYCDPEV	RPLMEQECSL	AACPPAHSHF	PSSPVQPSYY
1290	1300	1310	1320	1330	1340	1350	1360
LSTNLPLTQK	LEDNENQVVH	PSVRGNQWRT	GPWGSCSSSC	SGGLQHRAVV	CQDENGQSAS	YCDAASKPPE	LQQCGPGPCP
1370	1380	1390	1400	1410	1420	1430	1440
QWNYGNWGEC	SQTCGGGIKS	RLVICQFPNG	QILEDHNCEI	VNKPPSVIQC	HMHACPADVS	WHQEPWTSCS	ASCGKGRKYR
1450	1460	1470	1480	1490	1500	1510	1520
EVFCIDQFQR	KLEDTNCSQV	QKPPTHKACR	SVRCPSWKAN	SWNECSVTCG	SGVQQRDVYC	RLKGVGQVVE	EMCDQSTRPC
1530	1540	1550	1560	1570	1580	1590	1600
SQRRCWSQDC	VQHKGMERGR	LNCSTSCERK	DSHQRMECTD	NQIRQVNEIV	YNSSTISLTS	KNCRNPPCNY	IVVTADSSQC
1610	1620	1630	1640	1650	1660	1670	1680
ANNCGFSYRQ	RITYCTEIPS	TKKHKLHRLR	PIVYQECPVV	PSSQVYQCIN	SCLHLATWKV	GKWSKCSVTC	GIGIMKRQVK
1690	1700	1710	1720	1730	1740	1750	1760
CITKHGLSSD	LCLNHLKPGA	QKKCYANDCK	SFTTCKEIQV	KNHIRKDGDY	YLNIKGRIIK	IYCADMYLEN	PKEYLTLVQG
1770	1780	1790	1800	1810	1820	1830	1840
EENFSEVYGF	RLKNPYQCPF	NGSRREDCEC	DNGHLAAGYT	VFSKIRIDLT	SMQIKTTDLL	FSKTIFGNAV	PFATAGDCYS
1850	1860	1870	1880	1890	1900	1910	1920
AFRCPQGQFS	INLSGTGMKI	SSTAKWLTQG	SYTSVSIRRS	EDGTRFFGKC	GGYCGKCLPH	MTTGLPIQVI	

Cmpd.	No. of Cmpds.	m/z meas.	Δ m/z [ppm]	z	Rt [min]	Score	P	Range	Sequence	Modification	Ratios	
1189	1	599.1520	-198.95	2	44.5	12.7	1	1541-1550	R.LNCSTSCERK.D	Carbamidomethyl: 7	MD:MU	0.59

Detailed Protein Report

Protein 350: PREDICTED: uncharacterized protein LOC101930265 [Homo sapiens]

Accession: gi|530355909
Database: refseq_human(refseq_human_20140103.fasta)
Database Date: 2015-11-30
Modification(s): Carbamidomethyl, Oxidation

Score: 27.1
MW [kDa]: 38.6
pI: 10.5
Sequence Coverage [%]: 7.8
No. of unique Peptides: 1

10	20	30	40	50	60	70	80
MGAQVRTWKP	RCLHGNPGAC	METRVRAWKP	GCVHGNPGVC	METQECAWKL	RCVHGSPGAW	METQMCAWEP	RCVDGNPGVC
90	100	110	120	130	140	150	160
METQVCAWEP	RCVRGNSGAC	MEPRCVDGNP	GVCMGAQVCG	RKPRCVHGSP	GVCMETRVCA	WEPGCVHGSP	GVCIKTQVCA
170	180	190	200	210	220	230	240
WEPRCVHGSP	GVCMETQVCA	WEPRCVHGSP	GVCMETQVCA	WEPRCVHGSP	GVCMETQVCA	WEPRCVHGNP	GVCMGAQVCV
250	260	270	280	290	300	310	320
WKTSCVHGNP	GVCMGAQVCV	WKTSCVHGNP	GVCMEAQVCA	WKTRCVHGSP	VCAWEPRRVH	GKPGVCIGAQ	VCAWEPRCVD
330	340	350	360	370			
GNPGVCTGAQ	VCAWEPGCVH	ESLGVCMGTR	VCAWKPVAAL	L			

Cmpd.	No. of Cmpds.	m/z meas.	Δ m/z [ppm]	z	Rt [min]	Score	P	Range	Sequence	Modification
883	1	759.6775	-182.21	2	40.5	10.1	0	12-24	R.CLHGNPGACMETR.V	Carbamidomethyl: 1, 9; Oxidation: 10

Detailed Protein Report

Protein 351: PREDICTED: coiled-coil domain-containing protein 108 isoform X2 [Homo sapiens]

**Accession:**	gi|578804012	**Score:**	27.0
**Database:**	refseq_human(refseq_human_20140103.fasta)	**MW [kDa]:**	87.3
**Database Date:**	2015-11-30	**pI:**	9.5
**Modification(s):**	Carbamidomethyl	**Sequence Coverage [%]:**	3.4
		**No. of unique Peptides:**	1

10	20	30	40	50	60	70	80
MDSLCVSSAY	ISLISSERPV	HNWRGKSVQK	KQAESKSQIK	LHTQSAPFGL	CPKDMMLTQA	PSSVVRSRNS	RNHTVNSGGS
90	100	110	120	130	140	150	160
CLSASTVAIP	AINDSSAAMS	ACSTISAQPA	SSMDTQMHSP	KKQERVNKRV	IWGIEVAEEL	HWKGWELGKE	TTRNLVLKNR
170	180	190	200	210	220	230	240
SLKLQKMKYR	PPKTKFFFTV	IPQPIFLSPG	ITLTLPIVFR	PLEAKEYMDQ	LWFEKAEGMF	CVGLRATLPC	HRLICRPPSL
250	260	270	280	290	300	310	320
QLPMCAVGDT	TEAFFCLDNV	GDLPTFFTWE	FSSPFQMLPA	TGLLEPGQAS	QIKVTFQPLT	AVIYEVQATC	WYGAGSRQRS
330	340	350	360	370	380	390	400
SIQLQAVAKC	AQLLVSIKHK	CPEDQDAEGF	QKLLYFGSVA	VGCTSERQIR	LHNPSAVNAP	FRIEISPDEL	AEDQAFSCPT
410	420	430	440	450	460	470	480
AHGIVLPGEK	KCVSVFFHPK	TLDTRTVDYC	SIMPSGCASK	TLLKVVGFCR	GPAVSLQHYC	VNFSWVNLGE	RSEQPLWIEN
490	500	510	520	530	540	550	560
QSDCTAHFQF	AIDCLESVFT	IRPAFGTLVG	KARMTLHCAF	QPTHPIICFR	RVACLIHHQD	PLFLDLMGTC	HSDSTKPAIL
570	580	590	600	610	620	630	640
KPQHLTWYRT	HLARGLTLYP	PDILDAMLKE	KKLAQDQNGA	LMIPIQDLED	MPAPQYPYIP	PMTEFFFDGT	SDITIFPPPI
650	660	670	680	690	700	710	720
SVEPVEVDFG	ACPGPEAPNP	VPLCLMNHTK	GKIMVVWTRR	SDCPFWVTPE	SCDVPPLKSM	AMRLHFQPPH	PNCLYTVELE
730	740	750	760	770	780	790	
AFAIYKVCAR	NEREECGVSA	RSLSGLVGWQ	EVTEGSFRLH	PLRARLSLGW	TVTPMSLSPP	KLLA	

Cmpd.	No. of Cmpds.	m/z meas.	Δ m/z [ppm]	z	Rt [min]	Score	P	Range	Sequence	Modification
640	1	712.1825	-155.63	2	37.3	10.0	0	341-352	K.CPEDQDAEGFQK.L	Carbamidomethyl: 1

Detailed Protein Report

Protein 352: **ankyrin repeat and BTB/POZ domain-containing protein BTBD11 isoform a [Homo sapiens]**

**Accession:**	gi|65786661	**Score:**	27.0
**Database:**	refseq_human(refseq_human_20140103.fasta)	**MW [kDa]:**	120.8
**Database Date:**	2015-11-30	**pI:**	6.4
		**Sequence Coverage [%]:**	0.9
		**No. of unique Peptides:**	1

10	20	30	40	50	60	70	80
MARRGKKPVV	RTLEDLTLDS	GYGGAADSVR	SSNLSLCCSD	SHPASPYGGS	CWPPLADSMH	SRHNSFDTVN	TALVEDSEGL
**90**	**100**	**110**	**120**	**130**	**140**	**150**	**160**
DCAGQHCSRL	LPDLDEVPWT	LQELEALLLR	SRDPRAGPAV	PGGLPKDALA	KLSTLVSRAL	VRIAKEAQRL	SLRFAKCTKY
**170**	**180**	**190**	**200**	**210**	**220**	**230**	**240**
EIQSAMEIVL	SWGLAAHCTA	AALAALSLYN	MSSAGGDRLG	RGKSARCGLT	FSVGRVYRWM	VDSRVALRIH	EHAAIYLTAC
**250**	**260**	**270**	**280**	**290**	**300**	**310**	**320**
MESLFRDIYS	RVVASGVPRS	CSGPGSGSGS	GPGPSSGPGA	APAADKEREA	PGGGAASGGA	CSAASSASGG	SSCCAPPAAA
**330**	**340**	**350**	**360**	**370**	**380**	**390**	**400**
AAAVPPAAAA	NHHHHHHHAL	HEAPKFTVET	LEHTVNNDSE	IWGLLQPYQH	LICGKNASGV	LCLPDSLNLH	RDPQRSNKPG
**410**	**420**	**430**	**440**	**450**	**460**	**470**	**480**
ELPMFSQSEL	RTIEQSLLAT	RVGSIAELSD	LVSRAMHHLQ	PLNAKHHGNG	TPLHHKQGAL	YWEPEALYTL	CYFMHCPQME
**490**	**500**	**510**	**520**	**530**	**540**	**550**	**560**
WENPNVEPSK	VNLQVERPFL	VLPPLMEWIR	VAVAHAGHRR	SFSMDSDDVR	QAARLLLPGV	DCEPRQLRAD	DCFCASRKLD
**570**	**580**	**590**	**600**	**610**	**620**	**630**	**640**
AVAIEAKFKQ	DLGFRMLNCG	RTDLVKQAVS	LLGPDGINTM	SEQGMTPLMY	ACVRGDEAMV	QMLLDAGADL	NVEVVSTPHK
**650**	**660**	**670**	**680**	**690**	**700**	**710**	**720**
YPSVHPETRH	WTALTFAVLH	GHIPVVQLLL	DAGAKVEGSV	EHGEENYSET	PLQLAAAVGN	FELVSLLLER	GADPLIGTMY
**730**	**740**	**750**	**760**	**770**	**780**	**790**	**800**
RNGISTTPQG	DMNSFSQAAA	HGHRNVFRKL	LAQPEKEKSD	ILSLEEILAE	GTDLAETAPP	PLCASRNSKA	KLRALREAMY
**810**	**820**	**830**	**840**	**850**	**860**	**870**	**880**
HSAEHGYVDV	TIDIRSIGVP	WTLHTWLESL	RIAFQQHRRP	LIQCLLKEFK	TIQEEEYTEE	LVTQGLPLMF	EILKASKNEV
**890**	**900**	**910**	**920**	**930**	**940**	**950**	**960**
ISQQLCVIFT	HCYGPYPIPK	LTEIKRKQTS	RLDPHFLNNK	EMSDVTFLVE	GRPFYAHKVL	LFTASPRFKA	LLSSKPTNDG
**970**	**980**	**990**	**1000**	**1010**	**1020**	**1030**	**1040**
TCIEIGYVKY	SIFQLVMQYL	YYGGPESLLI	KNNEIMELLS	AAKFFQLEAL	QRHCEIICAK	SINTDNCVDI	YNHAKFLGVT
**1050**	**1060**	**1070**	**1080**	**1090**	**1100**	**1110**	
ELSAYCEGYF	LKNMMVLIEN	EAFKQLLYDK	NGEGTGQDVL	QDLQRTLAIR	IQSIHLSSSK	GSVV	

Cmpd.	No. of Cmpds.	m/z meas.	Δ m/z [ppm]	z	Rt [min]	Score	P	Range	Sequence	Modification
1645	1	583.7942	-77.28	2	50.5	27.0	2	150-159	R.LSLRFAKCTK.Y	

Detailed Protein Report

Protein 353: ankyrin repeat and SOCS box protein 16 [Homo sapiens]

Accession: gi|24586688
Database: refseq_human(refseq_human_20140103.fasta)
Database Date: 2015-11-30
Modification(s): Carbamidomethyl

Score: 27.0
MW [kDa]: 49.6
pI: 9.9
Sequence Coverage [%]: 4.6
No. of unique Peptides: 2

Quantitation

MD:MU **Median:** 0.98 **CV:** 0.00 % **No. of Peptides:** 1

10	20	30	40	50	60	70	80
MARETFPFTS	SMLRSLRLQQ	EWLEWEDRRR	AAAQQCRSRR	CPSSPRARLT	RPHRSCRDPA	VHQALFSGNL	QQVQALFQDE
**90**	**100**	**110**	**120**	**130**	**140**	**150**	**160**
EAANMIVETV	SNQLAWSAEQ	GFWVLTPKTK	QTAPLAIATA	RGYTDCARHL	IRQGAELDAR	VGGRAALHEA	CARAQFDCVR
**170**	**180**	**190**	**200**	**210**	**220**	**230**	**240**
LLLTFGAKAN	VLTEEGTTPL	HLCTIPESLQ	CAKLLLEAGA	TVNLAAGESQ	ETPLHVAAAR	GLEQHVALYL	EHGADVGLRT
**250**	**260**	**270**	**280**	**290**	**300**	**310**	**320**
SQGETALNTA	CAGAEGPGSC	RRHQAAARRL	LEAGADARAA	GRKRHTPLHN	ACANGCGGLA	ELLLRYGARA	EVPNGAGHTP
**330**	**340**	**350**	**360**	**370**	**380**	**390**	**400**
MDCALQAVQD	SPNWEPEVLF	AALLDYGAQP	VRPEMLKHCA	NFPRALEVLL	NAYPCVPSCE	TWVEAVLPEL	WKEHEAFYSS
**410**	**420**	**430**	**440**	**450**	**460**		
ALCMVNQPRQ	LQHLARLAVR	ARLGSRCRQG	ATRLPLPPLL	RDYLLLRVEG	CIQ		

Cmpd.	No. of Cmpds.	m/z meas.	Δ m/z [ppm]	z	Rt [min]	Score	P	Range	Sequence	Modification	Ratios
660	1	459.6081	-321.96	2	37.8	16.3	2	421-428	R.ARLGSRCR.Q		MD:MU 0.98
1199	1	874.6540	165.48	2	44.7	10.7	2	427-441	R.CRQGATRLPLPPLLR.D	Carbamidomethyl: 1	

Detailed Protein Report

Protein 354: **PREDICTED: ranBP2-like and GRIP domain-containing protein 4 isoform X1 [Homo sapiens]**

**Accession:**	gi|578804143	**Score:**	27.0
**Database:**	refseq_human(refseq_human_20140103.fasta)	**MW [kDa]:**	197.0
**Database Date:**	2015-11-30	**pI:**	5.9
		**Sequence Coverage [%]:**	1.8
		**No. of unique Peptides:**	1

10	20	30	40	50	60	70	80
MSCSKAYGER	YVASVQGSAP	SPRKKSTRGF	YFAKLYYEAK	EYDLAKKYIC	TYINVREMDP	RAHRFLGLLY	ELEENTEKAV
90	100	110	120	130	140	150	160
ECYRRSVELN	PTQKDLVLKI	AELLCKNDVT	DGRAKYWVER	AAKLFPGSPA	IYKLKEQLLD	CEGEDGWNKL	FDLIQSELYV
170	180	190	200	210	220	230	240
RPDDVHVNIR	LVELYRSTKR	LKDAVARCHE	AERNIALRSS	LEWNSCVVQT	LKEYLESLQC	LESDKSDWRA	TNTDLLLAYA
250	260	270	280	290	300	310	320
NLMLLTLSTR	DVQESRELLE	SFDSALQSAK	SSLGGNDELS	ATFLEMKGHF	YMHAGSLLLK	MGQHGNNVQW	RALSELAALC
330	340	350	360	370	380	390	400
YLIAFQVPRP	KIKLIKGEAG	QNLLEMMACD	RLSQSGHMLL	NLSRGKQDFL	KVVVETFANK	SGQSALYDAL	FSSQSPKDTS
410	420	430	440	450	460	470	480
FLGSDDIGNI	DVQEPELEDL	ARYDVGAIRA	HNGSLQHLTW	LGLQWNSLPA	LPGIRKWLKQ	LFHHLPQETS	RLETNAPESI
490	500	510	520	530	540	550	560
CILDLEVFLL	GVVYTSHLQL	KEKCNSHHSS	YQPLCLPLPV	CKQLCTERQK	SWWDAVCTLI	HRKAVPGNSA	KLRLLVQHEI
570	580	590	600	610	620	630	640
NTLRAQEKHG	LQPALLVHWA	KCLQKMGSGL	NSFYDQREYI	GRSVHYWKKV	LPLLKIIKKK	NSIPEPIDPL	FKHFHSVDIQ
650	660	670	680	690	700	710	720
ASEIVEYEED	AHVTFAILDA	VNGNIEDAMT	AFESIKSVVS	YWNLALIFHR	KAEDIANDAL	SPEEQEECKN	YLRKTRGYLI
730	740	750	760	770	780	790	800
KILDDSDSNL	SVVKKLPVPL	ESVKEMLKSV	MQELENYSEG	DPLYKNGSLR	NADSEIKHST	PSPTKYSLSP	SKSYKYSPKT
810	820	830	840	850	860	870	880
PPRWAEDQNS	LLKMIRQEVK	AIKEEMQELK	LNSSKSASHH	RWPTENYGPD	SVPDGYQGSQ	TFHGAPLTVA	TTGPSVYYSQ
890	900	910	920	930	940	950	960
SPAYNSQYLL	RPAANVTPTK	GSSNTEFKST	KEGFSIPVSA	DGFKFGISEP	GNQEKESEKP	LENDTGFQAQ	DISGQKNGRG
970	980	990	1000	1010	1020	1030	1040
VIFGQTSSTF	TFADVAKSTS	GEGFQFGKKD	PNFKGFSGAG	EKLFSSQCGK	MANKANTSGD	FEKDDDAYKT	EDSDDIHFEP
1050	1060	1070	1080	1090	1100	1110	1120
VVQMPEKVEL	VIGEEGEKVL	YSQGVKLFRF	DAEVRQWKER	GLGNLKILKN	EVNGKPRMLM	RREQVLKVCA	NHWITTTMNL
1130	1140	1150	1160	1170	1180	1190	1200
KPLSGSDRAW	MWSASDFSDG	DAKLERLAAK	FKTPELAEEF	KQKFEECQQL	LLDIPLQTPH	KLVDTGRAAK	LIQRAEEMKS
1210	1220	1230	1240	1250	1260	1270	1280
GLKDFKTFLT	NDQTKVTEEE	NKGSGTGAAG	ASDTTIKPNP	ENTGPTLEWD	NCDLREDALD	DSVSSSSVHA	SPLASSPVRK
1290	1300	1310	1320	1330	1340	1350	1360
NLFHFGESTT	GSNFSFKSAL	SPSKSPAKLN	QSGTSVGTDE	ESDVTQEEER	DGQYFEPVVP	LPDLVEVSSG	EENEKVVFSH
1370	1380	1390	1400	1410	1420	1430	1440
RAELYRYDKD	VGQWKERGIG	DIKILQNYDN	KQVRIVMRRD	QVLKLCANHT	ITPDMSLQNM	KGTERVWVWT	ACDFADGERK
1450	1460	1470	1480	1490	1500	1510	1520
VEHLAVRFKL	QDVADSFKKI	FDEAKTAQEK	DSLITPHVSR	SSTPRESPCG	KIAVAVLEET	TRERTDVIQG	DDVADAASEV
1530	1540	1550	1560	1570	1580	1590	1600
EVSSTSETTT	KAVVSPPKFV	FGSESVKRIF	SSEKSKPFAF	GNSSATGSLF	GFSFNASLKS	NNSETSSVAQ	SGSESKVEPK
1610	1620	1630	1640	1650	1660	1670	1680
KCELSKNSDI	EQSSDSKVKN	LSASFPMEES	SINYTFKTPE	KEPPLWHAEF	TKEELVQKLS	STTKSADHLN	GLLREAEATS
1690	1700	1710	1720	1730	1740	1750	1760
AVLMEQIKLL	KSEIRRLERN	QEQEESAANV	EHLKNVLLQF	IFLKPGSERE	RLLPVINTML	QLSPEEKGKL	AAVAQGG

Cmpd.	No. of Cmpds.	m/z meas.	Δ m/z [ppm]	z	Rt [min]	Score	P	Range	Sequence	Modification

Detailed Protein Report

Cmpd.	No. of Cmpds.	m/z meas.	Δ m/z [ppm]	z	Rt [min]	Score	P	Range	Sequence	Modification
2527	1	863.4144	10.20	2	62.0	14.8	0	1700-1714	R.NQEQEESAANVEHLK.N	

Detailed Protein Report

Protein 355: PREDICTED: ryanodine receptor 2 isoform X7 [Homo sapiens]

**Accession:**	gi|578802309	**Score:**	27.0
**Database:**	refseq_human(refseq_human_20140103.fasta)	**MW [kDa]:**	557.1
**Database Date:**	2015-11-30	**pI:**	5.6
		**Sequence Coverage [%]:**	0.5
		**No. of unique Peptides:**	1

10	20	30	40	50	60	70	80
MADGGEGEDE	IQFLRTDDEV	VLQCTATIHK	EQQKLCLAAE	GFGNRLCFLE	STSNSKNVPP	DLSICTFVLE	QSLSVRALQE
90	100	110	120	130	140	150	160
MLANTVEKSE	GQVDVEKWKF	MMKTAQGGGH	RTLLYGHAIL	LRHSYSGMYL	CCLSTSRSST	DKLAFDVGLQ	EDTTGEACWW
170	180	190	200	210	220	230	240
TIHPASKQRS	EGEKVRVGDD	LILVSVSSER	YLHLSYGNGS	LHVDAAFQQT	LWSVAPISSG	SEAAQGYLIG	GDVLRLLHGH
250	260	270	280	290	300	310	320
MDECLTVPSG	EHGEEQRRTV	HYEGGAVSVH	ARSLWRLETL	RVAWSGSHIR	WGQPFRLRHV	TTGKYLSLME	DKNLLLMDKE
330	340	350	360	370	380	390	400
KADVKSTAFT	FRSSKEKLDV	GVRKEVDGMG	TSEIKYGDSV	CYIQHVDTGL	WLTYQSVDVK	SVRMGSIQRK	AIMHHEGHMD
410	420	430	440	450	460	470	480
DGISLSRSQH	EESRTARVIR	STVFLFNRFI	RGLDALSKKA	KASTVDLPIE	SVSLSLQDLI	GYFHPPDEHL	EHEDKQNRLR
490	500	510	520	530	540	550	560
ALKNRQNLFQ	EEGMINLVLE	CIDRLHVYSS	AAHFADVAGR	EAGESWKSIL	NSLYEELLAAL	IRGNRKNCAQ	FSGSLDWLIS
570	580	590	600	610	620	630	640
RLERLEASSG	ILEVLHCVLV	ESPEALNIIK	EGHIKSIISL	LDKHGRNHKV	LDVLCSLCVC	HGVAVRSNQH	LICDNLLPGR
650	660	670	680	690	700	710	720
DLLLQTRLVN	HVSSMRPNIF	LGVSEGSAQY	KKWYYELMVD	HTEPFVTAEA	THLRVGWAST	EGYSPYPGGG	EEWGGNGVGD
730	740	750	760	770	780	790	800
DLFSYGFDGL	HLWSGCIART	VSSPNQHLLR	TDDVISCCLD	LSAPSISFRI	NGQPVQGMFE	NFNIDGLFFP	VVSFSAGIKV
810	820	830	840	850	860	870	880
RFLLGGRHGE	FKFLPPPGYA	PCYEAVLPKE	KLKVEHSREY	KQERTYTRDL	LGPTVSLTQA	AFTPIPVDTS	QIVLPPHLER
890	900	910	920	930	940	950	960
IREKLAENIH	ELWVMNKIEL	GWQYGPVRDD	NKRQHPCLVE	FSKLPEQERN	YNLQMSLETL	KTLLALGCHV	GISDEHAEDK
970	980	990	1000	1010	1020	1030	1040
VKKMKLPKNY	QLTSGYKPAP	MDLSFIKLTP	SQEAMVDKLA	ENAHNVWARD	RIRQGWTYGI	QQDVKNRRNP	RLVPYTLLDD
1050	1060	1070	1080	1090	1100	1110	1120
RTKKSNKDSL	REAVRTLLGY	GYNLEAPDQD	HAARAEVCSG	TGERFRIFRA	EKTYAVKAGR	WYFEFETVTA	GDMRVGWSRP
1130	1140	1150	1160	1170	1180	1190	1200
GCQPDQELGS	DERAFAFDGF	KAQRWHQGNE	HYGRSWQAGD	VVGCMVDMNE	HTMMFTLNGE	ILLDDSGSEL	AFKDFDVGDG
1210	1220	1230	1240	1250	1260	1270	1280
FIPVCSLGVA	QVGRMNFGKD	VSTLKYFTIC	GLQEGYEPFA	VNTNRDITMW	LSKRLPQFLQ	VPSNHEHIEV	TRIDGTIDSS
1290	1300	1310	1320	1330	1340	1350	1360
PCLKVTQKSF	GSQNSNTDIM	FYRLSMPIEC	AEVFSKTVAG	GLPGAGLFGP	KNDLEDYDAD	SDFEVLMKTA	HGHLVPDRVD
1370	1380	1390	1400	1410	1420	1430	1440
KDKEATKPEF	NNHKDYAQEK	PSRLKQRFLL	RRTKPDYSTS	HSARLTEDVL	ADDRDDYDFL	MQTSTYYYSV	RIFPGQEPAN
1450	1460	1470	1480	1490	1500	1510	1520
VWVGWITSDF	HQYDTGFDLD	RVRTVTVTLG	DEKGKVHESF	AIDSLCGFGI	KRSNCYMVCA	GESMSPGQGR	NNNGLEIGCV
1530	1540	1550	1560	1570	1580	1590	1600
VDAASGLLTF	IANGKELSTY	YQVEPSTKLF	PAVFAQATSP	NVFQFELGRI	KNVMPLSAGL	FKSEHKNPVP	QCPPRLHVQF
1610	1620	1630	1640	1650	1660	1670	1680
LSHVLWSRMP	NQFLKVDVSR	ISERQGWLVQ	CLDPLQFMSL	HIPEENRSVD	ILELTEQEEL	LKFHYHTLRL	YSAVCALGNH
1690	1700	1710	1720	1730	1740	1750	1760
RVAHALCSHV	DEPQLLYAIE	NKYMPGLLRA	GYYDLLIDIH	LSSYATARLM	MNNEYIVPMT	EETKSITLFP	DENKKHGLPG
1770	1780	1790	1800	1810	1820	1830	1840
IGLSTSLRPR	MQFSSPSFVS	ISNECYQYSP	EFPLDILKSK	TIQMLTEAVK	EGSLHARDPV	GGTTEFLFVP	LIKLFYTLLI
1850	1860	1870	1880	1890	1900	1910	1920
MGIFHNEDLK	HILQLIEPSV	FKEAATPEEE	SDTLEKELSV	DDAKLQGAGE	EEAKGGKRPK	EGLLQMKLPE	PVKLQMCLLL
1930	1940	1950	1960	1970	1980	1990	2000
QYLCDCQVRH	RIEAIVAFSD	DFVAKLQDNQ	RFRYNEVMQA	LNMSAALTAR	KTKEFRSPPQ	EQINMLLNFK	DDKSECPCPE
2010	2020	2030	2040	2050	2060	2070	2080
EIRDQLLDFH	EDLMTHCGIE	LDEDGSLDGN	SDLTIRGRLL	SLVEKVTYLK	KKQAEKPVES	DSKKSSTLQQ	LISETMVRWA
2090	2100	2110	2120	2130	2140	2150	2160
QESVIEDPEL	VRAMFVLLHR	QYDGIGGLVR	ALPKTYTING	VSVEDTINLL	ASLGQIRSLL	SVRMGKEEEK	LMIRGLGDIM
2170	2180	2190	2200	2210	2220	2230	2240

Detailed Protein Report

Cmpd.	No. of Cmpds.	m/z meas.	Δ m/z [ppm]	z	Rt [min]	Score	P	Range	Sequence	Modification
2416	1	447.0974	-322.64	2	60.5	12.0	1	3605-3612	R.TALTEKCK.L	

Detailed Protein Report

Protein 356: LIM/homeobox protein Lhx5 [Homo sapiens]

Accession: gi|11641283
Database: refseq_human(refseq_human_20140103.fasta)
Database Date: 2015-11-30

Score: 27.0
MW [kDa]: 44.4
pI: 9.1
Sequence Coverage [%]: 8.5
No. of unique Peptides: 1

Quantitation

MD:MU **Median:** 0.86 **CV:** 0.00 % **No. of Peptides:** 1

10	20	30	40	50	60	70	80
MMVHCAGCER	PILDRFLLNV	LDRAWHIKCV	QCCECKTNLS	EKCFSREGKL	YCKNDFFRRF	GTKCAGCAQG	ISPSDLVRKA
90	100	110	120	130	140	150	160
RSKVFHLNCF	TCMVCNKQLS	TGEELYVIDE	NKFVCKDDYL	SSSSLKEGSL	NSVSSCTDRS	LSPDLQDALQ	DDPKETDNST
170	180	190	200	210	220	230	240
SSDKETANNE	NEEQNSGTKR	RGPRTTIKAK	QLETLKAAFA	ATPKPTRHIR	EQLAQETGLN	MRVIQVWFQN	RRSKERRMKQ
250	260	270	280	290	300	310	320
LSALGARRHA	FFRSPRRMRP	LGGRLDESEM	LGSTPYTYYG	DYQGDYYAPG	SNYDFFAHGP	PSQAQSPADS	SFLAASGPGS
330	340	350	360	370	380	390	400
TPLGALEPPL	AGPHAADNPR	FTDMISHPDT	PSPEPGLPGT	LHPMPGEVFS	GGPSPPFPMS	GTSGYSGPLS	HPNPELNEAA
410							
VW							

Cmpd.	No. of Cmpds.	m/z meas.	Δ m/z [ppm]	z	Rt [min]	Score	P	Range	Sequence	Modification	Ratios
1688	1	916.3971	-76.21	2	51.0	10.9	2	64-81	K.CAGCAQGISPSDLVRKAR.S		MD:MU 0.86

Detailed Protein Report

Protein 357: chordin-like protein 1 isoform 4 precursor [Homo sapiens]

Accession: gi|291084494
Database: refseq_human(refseq_human_20140103.fasta)
Database Date: 2015-11-30

Score: 26.9
MW [kDa]: 43.5
pI: 9.9
Sequence Coverage [%]: 8.7
No. of unique Peptides: 1

10	20	30	40	50	60	70	80
MRKKWKMGGM	KYIFSLLFFL	LLEGGKTEQV	KHSETYCMFQ	DKKYRVGERW	HPYLEPYGLV	YCVNCICSEN	GNVLCSRVRC
90	100	110	120	130	140	150	160
PNVHCLSPVH	IPHLCCPRCP	GDGELSWEHS	DGDIFRQPAN	REARHSYHRS	HYDPPPSRQA	GGLSRFPGAR	SHRGALMDSQ
170	180	190	200	210	220	230	240
QASGTIVQIV	INNKHKHGQV	CVSNGKTYSH	GESWHPNLRA	FGIVECVLCT	CNVTKQECKK	IHCPNRYPCK	YPQKIDGKCC
250	260	270	280	290	300	310	320
KVCPGKKAKE	ELPGQSFDNK	GYFCGEETMP	VYESVFMEDG	ETTRKIALET	ERPPQVEVHV	WTIRKGILQH	FHIEKISKRM
330	340	350	360	370	380		
FEELPHFKLV	TRTTLSQWKI	FTEGEAQISQ	MCSSRVCRTE	LEDLVKVLYL	ERSEKGHC		

Cmpd.	No. of Cmpds.	m/z meas.	Δ m/z [ppm]	z	Rt [min]	Score	P	Range	Sequence	Modification
1642	1	701.3735	65.53	3	50.3	10.1	2	27-43	K.TEQVKHSETYCMFQDKK.Y	

Detailed Protein Report

Protein 358: **polyadenylate-binding protein 4 isoform 3 [Homo sapiens]**

**Accession:**	gi|208431836	**Score:**	26.9
**Database:**	refseq_human(refseq_human_20140103.fasta)	**MW [kDa]:**	69.5
**Database Date:**	2015-11-30	**pI:**	10.0
**Modification(s):**	Oxidation	**Sequence Coverage [%]:**	3.0
		**No. of unique Peptides:**	1

10	20	30	40	50	60	70	80
MNAAASSYPM	ASLYVGDLHS	DVTEAMLYEK	FSPAGPVLSI	RVCRDMITRR	SLGYAYVNFQ	QPADAERALD	TMNFDVIKGK
**90**	**100**	**110**	**120**	**130**	**140**	**150**	**160**
PIRIMWSQRD	PSLRKSGVGN	VFIKNLDKSI	DNKALYDTFS	AFGNILSCKV	VCDENGSKGY	AFVHFETQEA	ADKAIEKMNG
**170**	**180**	**190**	**200**	**210**	**220**	**230**	**240**
MLLNDRKVFV	GRFKSRKERE	AELGAKAKEF	TNVYIKNFGE	EVDDESLKEL	FSQFGKTLSV	KVMRDPNGKS	KGFGFVSYEK
**250**	**260**	**270**	**280**	**290**	**300**	**310**	**320**
HEDANKAVEE	MNGKEISGKI	IFVGRAQKKV	ERQAELKRKF	EQLKQERISR	YQGVNLYIKN	LDDTIDDEKL	RKEFSPFGSI
**330**	**340**	**350**	**360**	**370**	**380**	**390**	**400**
TSAKVMLEDG	RSKGFGFVCF	SSPEEATKAV	TEMNGRIVGS	KPLYVALAQR	KEERKAHLTN	QYMQRVAGMR	ALPANAILNQ
**410**	**420**	**430**	**440**	**450**	**460**	**470**	**480**
FQPAAGGYFV	PAVPQAQGRP	PYYTPNQLAQ	MRPNPRWQQG	GRPQGFQGMP	SAIRQSGPRP	TLRHLAPTGN	APASRGLPTT
**490**	**500**	**510**	**520**	**530**	**540**	**550**	**560**
TQRVGVPTAV	QNLAPRAAVA	AAAPRAVAPY	KYASSVRSPH	PAIQPLQAPQ	PAVHVQGQEP	LTASMLAAAP	PQEQKQMLGE
**570**	**580**	**590**	**600**	**610**	**620**	**630**	**640**
RLFPLIQTMH	SNLAGKITGM	LLEIDNSELL	HMLESPESLR	SKVDEAVAVL	QAHHAKKEAA	QKVGAVAAAT	S

Cmpd.	No. of Cmpds.	m/z meas.	Δ m/z [ppm]	z	Rt [min]	Score	P	Range	Sequence	Modification
2821	1	1051.5459	48.78	2	65.7	12.8	2	241-259	K.HEDANKAVEEMNGKEISGK.I	Oxidation: 11

Detailed Protein Report

Protein 359: PREDICTED: core histone macro-H2A.1 isoform X3 [Homo sapiens]

**Accession:**	gi|530380257	**Score:**	26.9
**Database:**	refseq_human(refseq_human_20140103.fasta)	**MW [kDa]:**	39.0
**Database Date:**	2015-11-30	**pI:**	10.3
		**Sequence Coverage [%]:**	9.0
		**No. of unique Peptides:**	2

10	20	30	40	50	60	70	80
MSSRGGKKKS	TKTSRSAKAG	VIFPVGRMLR	YIKKGHPKYR	IGVGAPVYMA	AVLEYLTAEI	LELAGNAARD	NKKGRVTPRH
**90**	**100**	**110**	**120**	**130**	**140**	**150**	**160**
ILLAVANDEE	LNQLLKGVTI	ASGGVLPNIH	PELLAKKRGS	KGKLEAIITP	PPAKKAKSPS	QKKPVSKKAG	GKKGARKSKK
**170**	**180**	**190**	**200**	**210**	**220**	**230**	**240**
QGEVSKAASA	DSTTEGTPAD	GFTVLSTKSL	FLGQKLQVVQ	ADIASIDSDA	VVHPTNTDFY	IGGEVGNTLE	KKGGKEFVEA
**250**	**260**	**270**	**280**	**290**	**300**	**310**	**320**
VLELRKKNGP	LEVAGAAVSA	GHGLPAKFVI	HCNSPVWGAD	KCEELLEKTV	KNCLALADDK	KLKSIAFPSI	GSGRNGFPKQ
**330**	**340**	**350**	**360**	**370**			
TAAQLILKAI	SSYFVSTMSS	SIKTVYFVLF	DSESIGIYVQ	EMAKLDAN			

Cmpd.	No. of Cmpds.	m/z meas.	Δ m/z [ppm]	z	Rt [min]	Score	P	Range	Sequence	Modification
2553	1	639.3415	-83.77	2	61.9	15.2	1	124-135	K.LEAIITPPPAKK.A	
2834	1	806.4289	45.18	3	65.9	11.7	1	268-288	K.FVIHCNSPVWGADKCEELLEK.T	

Detailed Protein Report

Protein 360: transmembrane protein 151A [Homo sapiens]

**Accession:**	gi|23397566	**Score:**	26.8
**Database:**	refseq_human(refseq_human_20140103.fasta)	**MW [kDa]:**	51.2
**Database Date:**	2015-11-30	**pI:**	9.3
**Modification(s):**	Carbamidomethyl, Oxidation	**Sequence Coverage [%]:**	9.4
		**No. of unique Peptides:**	1

10	20	30	40	50	60	70	80
MPEDGAGDGG	EVPALIPDGE	PLREEQRPLK	QSLGSSLCRE	SHWKCLLLTL	LIHACGAVVA	WCRLATVPRL	VLGPEAALAR
**90**	**100**	**110**	**120**	**130**	**140**	**150**	**160**
GAGGPPPTYP	ASPCSDGYLY	IPLAFVSLLY	LLYLAECWHC	HVRSCQAPRT	DAHTVLALIR	RLQQAPPCVW	WKATSYHYVR
**170**	**180**	**190**	**200**	**210**	**220**	**230**	**240**
RTRQITRYRN	GDAYTTTQVY	HERADSRTAR	GEFDYSAHGV	RDVSKELVGL	AEHAATRLRF	TKCFSFGSAE	AEASYLTQRA
**250**	**260**	**270**	**280**	**290**	**300**	**310**	**320**
RFFSANEGLD	DYLEAREGMH	LKDVDFRESL	MVFADPRSPP	WYARAWVFWL	VSAATLSWPL	RVVAAYGTAH	VHYQVEKLFG
**330**	**340**	**350**	**360**	**370**	**380**	**390**	**400**
ASSPPPGAVP	SGPPLSRVAT	VDFTELEWHI	CSNRQLVPSY	SEAVVMGAGS	GAYLRGCQRC	RRSVSSNSLP	PARPSGPRLP
**410**	**420**	**430**	**440**	**450**	**460**	**470**	
FSRSRLSLGA	GGRATPGVFR	SLSGGPLGRR	GEDTEPLESP	PCYEDALYFP	VLIVHGDSGC	QGDGQGAL	

Cmpd.	No. of Cmpds.	m/z meas.	Δ m/z [ppm]	z	Rt [min]	Score	P	Range	Sequence	Modification
136	1	977.7569	-49.80	3	31.1	10.2	2	355-381	R.QLVPSYSEAVVMGAGSGAYLRGCQRCR.R	Carbamidomethyl: 23; Oxidation: 12

Detailed Protein Report

Protein 361: PREDICTED: RAD51-associated protein 2 isoform X1 [Homo sapiens]

**Accession:**	gi|530367071	**Score:**	26.8
**Database:**	refseq_human(refseq_human_20140103.fasta)	**MW [kDa]:**	132.8
**Database Date:**	2015-11-30	**pI:**	7.1
		**Sequence Coverage [%]:**	2.7
		**No. of unique Peptides:**	1

Quantitation

MD:MU **Median:** 1.32 **CV:** 0.00 % **No. of Peptides:** 1

```
        10         20         30         40         50         60         70         80
MAELRKPTSS LTPPEDPDSQ PPSSKRLCLE EPGGVFKAGW RLPLVPRLSE AEKVWELSPR PFKGLLVSTN AIFDNSTDSC
        90        100        110        120        130        140        150        160
VEKSVSGKQI CNLKCSNLKF QMSSCLQSPP SQSPDSDLRA SGRSEAGLHD REAFSVHRSN SSKAGVSQLL PSTSIHDIHG
       170        180        190        200        210        220        230        240
IRNENRKQQF VQGRDNVHKE NPFLDVTFYK ETKSPFHEIK NRCKANSVVP SNKRENNISS SVLKISKSQN QPSLEIAKPS
       250        260        270        280        290        300        310        320
YFRDSGTISV PQFPMDLNSK MSSVYLKEIA KKKNDKKEAY VRDFTNIYWS QNRPDVKKQK LQNDKKTVEA ENIFSKCYEN
       330        340        350        360        370        380        390        400
DYPSLSSQNT CKRKDLISSN YCNCSSIQCN VRDSRKNFAI LENANWEEAE CLDSYVLTRL EKSQNWDCNV RHILRRNRGN
       410        420        430        440        450        460        470        480
CWIINNCKTK CENMKKTEEK WNWLLLLEID LLSKEDYHCA KVINAYEEQS KLLVREILGS QTALITTVWL NGKGENDNTL
       490        500        510        520        530        540        550        560
QLRYNTTQKV FHVNNPFESF IIEIFYFHKS ISGNKKDNSI LTCCNILKCK KQIGIIGIQN LITRNMNTNI KNGILSIYLQ
       570        580        590        600        610        620        630        640
DSVSEPLDIL LKTNIAFLLN NFDSLTRIEN DFELEEECIF KCMLYLKYPK NIVENHTAYL VKILTSSRLL EDNMKPMLKK
       650        660        670        680        690        700        710        720
RKLFRTEQVF EKSKKKLINS FSMTTQNTGF PIFETYEKIP LLMDFDDMDE ISLIREITCQ NMSCPQQVVN VENWAHYNSS
       730        740        750        760        770        780        790        800
TVKAHGNSCP QFIQNNRGYI NENFYEVNMH SQDLNMERKQ GHNKISNFDC EHIFEDLCNV RQQAIPASHN IIHNEETHTT
       810        820        830        840        850        860        870        880
SITQVLNFWN LLSEIEEKKY DLILKEEVKV TAESLTNSCQ VHKDTKIEKE EKDSFFPMDD MFSVQSVSLI SKEVNVEENK
       890        900        910        920        930        940        950        960
YVNQNYVTNT NEYESILPER EIANSKDFHR KNDSALYINH QFETGLSEGN DECFQDLAAK YLSTEALTIV KDFEMKRKFD
       970        980        990       1000       1010       1020       1030       1040
LVLEELRMFH EISRENELLS TVETNNGQEN YFGENDAEKV KMEIEKDLKM VVVNKIRASS SFHDTIAGPN MGKSHQSLFK
      1050       1060       1070       1080       1090       1100       1110       1120
WKTVPNNGEQ EVPNESCYPS RSEEELLYST SEKDCETPLP KRPAFLPDEC KEEFNYLLRG GSHFPHGISR VRPLKTCSRP
      1130       1140       1150       1160
IRIGLSRKAR IKQLHPYLKQ MCYGNLKENF
```

Cmpd.	No. of Cmpds.	m/z meas.	Δ m/z [ppm]	z	Rt [min]	Score	P	Range	Sequence	Modification	Ratios
1446	1	698.8871	-14.31	2	47.7	15.2	2	261-272	K.MSSVYLKEIAKK.K		MD:MU 1.32

Detailed Protein Report

Protein 362: PREDICTED: uncharacterized protein LOC100996701 [Homo sapiens]

Accession: gi|397139476
Database: refseq_human(refseq_human_20140103.fasta)
Database Date: 2015-11-30
Modification(s): Carbamidomethyl

Score: 26.8
MW [kDa]: 22.6
pI: 12.1
Sequence Coverage [%]: 15.0
No. of unique Peptides: 1

Quantitation

MD:MU **Median:** 0.67 **CV:** 0.00 % **No. of Peptides:** 1

10	20	30	40	50	60	70	80
MAALCLRKGP	LPLCPRPSHG	LKAMGGEGSC	QDDTESGALI	FLPSDDAQPG	KALRPQRSGP	GGSERRGRGW	GRAGALEEQV
**90**	**100**	**110**	**120**	**130**	**140**	**150**	**160**
RQGPSAQRHP	RTQACSRPCS	PSPHCSCGKG	KHGALPQGQC	SAWLELTMVT	VPCCHHCSHC	PGGQPGPQLH	CAWTVWSWAV
**170**	**180**	**190**	**200**	**210**	**220**		
PSSASRACGD	GHRRSTCQAQ	GSCTGLPPLR	GCLSRLVPGC	PCPHLRQQDK	GKWN		

Cmpd.	No. of Cmpds.	m/z meas.	Δ m/z [ppm]	z	Rt [min]	Score	P	Range	Sequence	Modification	Ratios	
1676	1	916.3092	-152.23	2	50.9	15.0	1	174-190	R.RSTCQAQGSCTGLPPLR.G	Carbamidomethyl: 10	MD:MU	0.67

Detailed Protein Report

Protein 363: **PREDICTED: double zinc ribbon and ankyrin repeat-containing protein 1 isoform X3 [Homo sapiens]**

**Accession:**	gi|530425777	**Score:**	26.8
**Database:**	refseq_human(refseq_human_20140103.fasta)	**MW [kDa]:**	63.6
**Database Date:**	2015-11-30	**pI:**	9.4
**Modification(s):**	Carbamidomethyl	**Sequence Coverage [%]:**	6.5
		**No. of unique Peptides:**	2

10	20	30	40	50	60	70	80
MLTLESFQSP	GFAHVSGQKC	LTSTEIMRIQ	RETDFLKCAH	CLAPRPSDPF	ARFCQECGSP	VPPIFGCRLP	PPEGAQMGLC
**90**	**100**	**110**	**120**	**130**	**140**	**150**	**160**
AECRSLVPMN	TPICVVCEAP	LALQLQPQAS	LHLKEKVICR	ACGTGNPAHL	RYCVTCEGAL	PSSQESMCSG	DKAPPPPTQK
**170**	**180**	**190**	**200**	**210**	**220**	**230**	**240**
GGTISCYRCG	RWNLWEASFC	GWCGAMLGIP	AGCSVCPKCG	ASNHLSARFC	GSCGICVKSL	VKLSLDRSLA	LAAEEPRPFS
**250**	**260**	**270**	**280**	**290**	**300**	**310**	**320**
EPRCAWQSLN	IPLPRSDVGT	KRDIGTQTVG	LFYPSGKLLA	KKEQELASQK	QRQEKMSDHK	PLLTAISPGR	GYWRRQLDHI
**330**	**340**	**350**	**360**	**370**	**380**	**390**	**400**
SAHLRCYAQN	NPEFRALIAE	PRMGKLISAT	VHEDGCEVSI	RLNYSQVSNK	NLYLNKAVNF	SDHLLSSAAE	GDGGLCGSRS
**410**	**420**	**430**	**440**	**450**	**460**	**470**	**480**
SWVSDYSQST	SDTIEKIKRI	KNFKTKTFQE	KKEQLIPENR	LLLKEVGPTG	EGRVSVIEQL	LDEGADPNCC	DEDNRPVITV
**490**	**500**	**510**	**520**	**530**	**540**	**550**	**560**
AVMNKHHEAI	PVLVQRGADI	DQQWGPLRNT	ALHEATLLGL	AGRESTATLL	GCNASIQKKN	AGGQTAYDLA	LNTGDDLVTS
**570**	**580**	**590**					
LFAAKFGQGL	EDQLAQTRSL	SLDDC					

Cmpd.	No. of Cmpds.	m/z meas.	Δ m/z [ppm]	z	Rt [min]	Score	P	Range	Sequence	Modification
2106	1	1052.4447	-67.35	3	56.5	13.1	1	1-28	-.MLTLESFQSPGFAHVSGQKCLTSTEIMR.I	Carbamidomethyl: 20
1819	1	649.7840	1.98	2	52.7	13.7	0	326-335	R.CYAQNNPEFR.A	Carbamidomethyl: 1

Detailed Protein Report

Protein 364: PREDICTED: phenylalanine--tRNA ligase beta subunit isoform X1 [Homo sapiens]

**Accession:**	gi|578803432	**Score:**	26.8
**Database:**	refseq_human(refseq_human_20140103.fasta)	**MW [kDa]:**	54.8
**Database Date:**	2015-11-30	**pI:**	7.8
		**Sequence Coverage [%]:**	3.5
		**No. of unique Peptides:**	1

Alias proteins:

Accession	Name	Description
gi|578803436	refseq_human (refseq_human_20140103.fasta)	PREDICTED: phenylalanine--tRNA ligase beta subunit isoform X3 [Homo sapiens]
gi|578803434	refseq_human (refseq_human_20140103.fasta)	PREDICTED: phenylalanine--tRNA ligase beta subunit isoform X2 [Homo sapiens]

10	20	30	40	50	60	70	80
MPDGKIQKLI	ITEETAKIRP	FAVAAVLRNI	KFTKDRYDSF	IELQEKLHQN	ICRKRALVAI	GTHDLDTLSG	PFTYTAKRPS
**90**	**100**	**110**	**120**	**130**	**140**	**150**	**160**
DIKFKPLNKT	KEYTACELMN	IYKTDNHLKH	YLHIIENKPL	YPVIYDSNGV	VLSMPPIING	DHSRITVNTR	NIFIECTGTD
**170**	**180**	**190**	**200**	**210**	**220**	**230**	**240**
FTKAKIVLDI	IVTMFSEYCE	NQFTVEAAEV	VFPNGKSHTF	PELAYRKEMV	RADLINKKVG	IRETPENLAK	LLTRMYLKSE
**250**	**260**	**270**	**280**	**290**	**300**	**310**	**320**
VIGDGNQIEI	EIPPTRADII	HACDIVEDAA	IAYGYNNIQM	TLPKTYTIAN	QFPLNKLTEL	LRHDMAAAGF	TEALTFALCS
**330**	**340**	**350**	**360**	**370**	**380**	**390**	**400**
QEDIADKLGV	DISATKAVHI	SNPKTAEFQV	ARTTLLPGLL	KTIAANRKMP	LPLKLFEISD	IVIKDSNTDV	GAKNYRHLCA
**410**	**420**	**430**	**440**	**450**	**460**	**470**	**480**
VYYNKNPGFE	IIHGLLDRIM	QLLDVPPGED	KGGYVIKASE	GPAFFPGRCA	EIFARGQSVG	KLGVLHPDVI	TKFELTMPCS
**490**	**500**						
SLEINVGPFL							

Cmpd.	No. of Cmpds.	m/z meas.	Δ m/z [ppm]	z	Rt [min]	Score	P	Range	Sequence	Modification
1331	1	721.8048	-169.98	2	46.2	11.2	1	78-89	K.RPSDIKFKPLNK.T	

Detailed Protein Report

Protein 365: **complexin-3 precursor [Homo sapiens]**

Accession: gi|71725345
Database: refseq_human(refseq_human_20140103.fasta)
Database Date: 2015-11-30
Modification(s): Oxidation

Score: 26.7
MW [kDa]: 17.5
pI: 4.7
Sequence Coverage [%]: 19.6
No. of unique Peptides: 1

10	20	30	40	50	60	70	80
MAFMVKTMVG	GQLKNLTGSL	GGGEDKGDGD	KSAAEAQGMS	REEYEEYQKQ	LVEEKMERDA	QFTQRKAERA	TLRSHFRDKY
**90**	**100**	**110**	**120**	**130**	**140**	**150**	**160**
RLPKNETDES	QIQMAGGDVE	LPRELAKMIE	EDTEEEEEKA	SVLGQLASLP	GLNLGSLKDK	AQATLGDLKQ	SAEKCHVM

Cmpd.	No. of Cmpds.	m/z meas.	Δ m/z [ppm]	z	Rt [min]	Score	P	Range	Sequence	Modification
1306	1	721.2739	-148.07	2	45.9	11.1	1	2-14	M.AFMVKTMVGGQLK.N	Oxidation: 3, 7

Detailed Protein Report

Protein 366: partitioning defective 3 homolog B isoform a [Homo sapiens]

Accession: gi|119120907
Database: refseq_human(refseq_human_20140103.fasta)
Database Date: 2015-11-30

Score: 26.7
MW [kDa]: 121.1
pI: 9.4
Sequence Coverage [%]: 2.8
No. of unique Peptides: 1

Quantitation

MD:MU **Median:** 2.39 **CV:** 0.00 % **No. of Peptides:** 1

10	20	30	40	50	60	70	80
MKVTVCFGRT	GIVVPCKEGQ	LRVGELTQQA	LQRYLKTREK	GPGYWVKIHH	LEYTDGGILD	PDDVLADVVE	DKDKLIAVFE
90	100	110	120	130	140	150	160
EQEPLHKIES	PSGNPADRQS	PDAFETEVAA	QLAAFKPIGG	EIEVTPSALK	LGTPLLVRRS	SDPVPGPPAD	TQPSASHPGG
170	180	190	200	210	220	230	240
QSLKLVVPDS	TQNLEDREVL	NGVQTELLTS	PRTKDTLSDM	TRTVEISGEG	GPLGIHVVPF	FSSLSGRILG	LFIRGIEDNS
250	260	270	280	290	300	310	320
RSKREGLFHE	NECIVKINNV	DLVDKTFAQA	QDVFRQAMKS	PSVLLHVLPP	QNREQYEKSV	IGSLNIFGNN	DGVLKTKVPP
330	340	350	360	370	380	390	400
PVHGKSGLKT	ANLTGTDSPE	TDASASLQQN	KSPRVPRLGG	KPSSPSLSPL	MGFGSNKNAK	KIKIDLKKGP	EGLGFTVVTR
410	420	430	440	450	460	470	480
DSSIHGPGPI	FVKNILPKGA	AIKDGRLQSG	DRILEVNGRD	VTGRTQEELV	AMLRSTKQGE	TASLVIARQE	GHFLPRELKG
490	500	510	520	530	540	550	560
EPDCCALSLE	TSEQLTFEIP	LNDSGSAGLG	VSLKGNKSRE	TGTDLGIFIK	SIIHGGAAFK	DGRLRMNDQL	IAVNGESLLG
570	580	590	600	610	620	630	640
KSNHEAMETL	RRSMSMEGNI	RGMIQLVILR	RPERPMEDPA	ECGAFSKPCF	ENCQNAVTTS	RRNDNSILHP	LGTCSPQDKQ
650	660	670	680	690	700	710	720
KGLLLPNDGW	AESEVPPSPT	PHSALGLGLE	DYSHSSGVDS	AVYFPDQHIN	FRSVTPARQP	ESINLKASKS	MDLVPDESKV
730	740	750	760	770	780	790	800
HSLAGQKSES	PSKDFGPTLG	LKKSSSLESL	QTAVAEVRKN	DLPFHRPRPH	MVRGRGCNES	FRAAIDKSYD	GPEEIEADGL
810	820	830	840	850	860	870	880
SDKSSHSGQG	ALNCESAPQG	NSELEDMENK	ARKVKKTKEK	EKKKEKGKLK	VKEKKRKEEN	EDPERKIKKK	GFGAMLRFGK
890	900	910	920	930	940	950	960
KKEDKGGKAE	QKGTLKHGGL	REEELEKMKE	ERESGRPTGG	STDRIQKLRK	EYYQARREGF	PLYEDDEGRA	RPSEYDLLWV
970	980	990	1000	1010	1020	1030	1040
PGRGPDGNAH	NLRFEGMERQ	YASLPRGGPA	DPVDYLPAAP	RGLYKERELP	YYPGAHPMHP	PKGSYPRPTE	LRVADLRYPQ
1050	1060	1070	1080	1090	1100	1110	
HYPPPPAPQH	KGPFRQDVPP	SPPQHQRMPA	YQETGRPGPR	GGSPDQYPYR	TQDSRQKNPM	TAAV	

Cmpd.	No. of Cmpds.	m/z meas.	Δ m/z [ppm]	z	Rt [min]	Score	P	Range	Sequence	Modification	Ratios	
268	1	549.5949	-92.30	3	33.0	10.2	1	23-36	R.VGELTQQALQRYLK.T		MD:MU	2.39

Detailed Protein Report

Protein 367: **PREDICTED: neuroligin-2 isoform X1 [Homo sapiens]**

Accession: gi|530410532
Database: refseq_human(refseq_human_20140103.fasta)
Database Date: 2015-11-30

Score: 26.7
MW [kDa]: 88.9
pI: 5.7
Sequence Coverage [%]: 4.5
No. of unique Peptides: 2

Quantitation

MD:MU **Median:** 2.27 **CV:** 59.28 % **No. of Peptides:** 2

10	20	30	40	50	60	70	80
MWLLALCLVG	LAGAQRGGGG	PGGGAPGGPG	LGLGSLGEER	FPVVNTAYGR	VRGVRRELNN	EILGPVVQFL	GVPYATPPLG
90	100	110	120	130	140	150	160
ARRFQPPEAP	ASWPGVRNAT	TLPPACPQNL	HGALPAIMLP	VWFTDNLEAA	ATYVQNQSED	CLYLNLYVPT	EDDIRDPGKK
170	180	190	200	210	220	230	240
PVMLFLHGGS	YMEGTGNMFD	GSVLAAYGNV	IVATLNYRLG	VLGFLSTGDQ	AAKGNYGLLD	QIQALRWLSE	NIAHFGGDPE
250	260	270	280	290	300	310	320
RITIFGSGAG	ASCVNLLILS	HHSEGLFQKA	IAQSGTAISS	WSVNYQPLKY	TRLLAAKVGC	DREDSAEAVE	CLRRKPSREL
330	340	350	360	370	380	390	400
VDQDVQPARY	HIAFGPVVDG	DVVPDDPEIL	MQQGEFLNYD	MLIGVNQGEG	LKFVEDSAES	EDGVSASAFD	FTVSNFVDNL
410	420	430	440	450	460	470	480
YGYPEGKDVL	RETIKFMYTD	WADRDNGEMR	RKTLLALFTD	HQWVAPAVAT	AKLHADYQSP	VYFYTFYHHC	QAEGRPEWAD
490	500	510	520	530	540	550	560
AAHGDELPYV	FGVPMVGATD	LFPCNFSKND	VMLSAVVMTY	WTNFAKTGDP	NQPVPQDTKF	IHTKPNRFEE	VVWSKFNSKE
570	580	590	600	610	620	630	640
KQYLHIGLKP	RVRDNYRANK	VAFWLELVPH	LHNLHTELFT	TTTRLPPYAT	RWPPRPPAGA	PGTRRPPPPA	TLPPEPEPEP
650	660	670	680	690	700	710	720
GPRAYDRFPG	DSRDYSTELS	VTVAVGASLL	FLNILAFAAL	YYKRDRRQEL	RCRRLSPPGG	SGSGVPGGGP	LLPAAGRELP
730	740	750	760	770	780	790	800
PEEELVSLQL	KRGGGVGADP	AEALRPACPP	DYTLALRRAP	DDVPLLAPGA	LTLLPSGLGP	PPPPPPPSLH	PFGPFPPPPP
810	820						
TATSHNNTLP	HPHSTTRV						

Cmpd.	No. of Cmpds.	m/z meas.	Δ m/z [ppm]	z	Rt [min]	Score	P	Range	Sequence	Modification	Ratios	
1419	1	981.9479	-37.80	2	47.5	11.8	0	17-40	R. GGGGPGGGAPGGPGLGLGSLGE F		MD:MU	3.94
1556	1	698.7932	-65.19	2	49.1	14.9	0	527-539	K.TGDPNQPVPQDTK.F		MD:MU	1.31

Detailed Protein Report

Protein 368: prolow-density lipoprotein receptor-related protein 1 precursor [Homo sapiens]

**Accession:**	gi|126012562	**Score:**	26.7
**Database:**	refseq_human(refseq_human_20140103.fasta)	**MW [kDa]:**	504.3
**Database Date:**	2015-11-30	**pI:**	5.0
**Modification(s):**	Carbamidomethyl, Oxidation	**Sequence Coverage [%]:**	0.6
		**No. of unique Peptides:**	2

Quantitation

MD:MU	**Median:** 0.80	**CV:** 0.00 %	**No. of Peptides:** 1

Detailed Protein Report

10	20	30	40	50	60	70	80
MLTPPLLLLL	PLLSALVAAA	IDAPKTCSPK	QFACRDQITC	ISKGWRCDGE	RDCPDGSDEA	PEICPQSKAQ	RCQPNEHNCL
90	100	110	120	130	140	150	160
GTELCVPMSR	LCNGVQDCMD	GSDEGPHCRE	LQGNCSRLGC	QHHCVPTLDG	PTCYCNSSFQ	LQADGKTCKD	FDECSVYGTC
170	180	190	200	210	220	230	240
SQLCTNTDGS	FICGCVEGYL	LQPDNRSCKA	KNEPVDRPPV	LLIANSQNIL	ATYLSGAQVS	TITPTSTRQT	TAMDFSYANE
250	260	270	280	290	300	310	320
TVCWVHVGDS	AAQTQLKCAR	MPGLKGFVDE	HTINISLSLH	HVEQMAIDWL	TGNFYFVDDI	DDRIFVCNRN	GDTCVTLLDL
330	340	350	360	370	380	390	400
ELYNPKGIAL	DPAMGKVFFT	DYGQIPKVER	CDMDGQNRTK	LVDSKIVFPH	GITLDLVSRL	VYWADAYLDY	IEVVDYEGKG
410	420	430	440	450	460	470	480
RQTIIQGILI	EHLYGLTVFE	NYLYATNSDN	ANAQQKTSVI	RVNRFNSTEY	QVVTRVDKGG	ALHIYHQRRQ	PRVRSHACEN
490	500	510	520	530	540	550	560
DQYGKPGGCS	DICLLANSHK	ARTCRCRSGF	SLGSDGKSCK	KPEHELFLVY	GKGRPGIIRG	MDMGAKVPDE	HMIPIENLMN
570	580	590	600	610	620	630	640
PRALDFHAET	GFIYFADTTS	YLIGRQKIDG	TERETILKDG	IHNVEGVAVD	WMGDNLYWTD	DGPKKTISVA	RLEKAAQTRK
650	660	670	680	690	700	710	720
TLIEGKMTHP	RAIVVDPLNG	WMYWTDWEED	PKDSRRGRLE	RAWMDGSHRD	IFVTSKTVLW	PNGLSLDIPA	GRLYWVDAFY
730	740	750	760	770	780	790	800
DRIETILLNG	TDRKIVYEGP	ELNHAFGLCH	HGNYLFWTEY	RSGSVYRLER	GVGGAPPTVT	LLRSERPPIF	EIRMYDAQQQ
810	820	830	840	850	860	870	880
QVGTNKCRVN	NGGCSSLCLA	TPGSRQCACA	EDQVLDADGV	TCLANPSYVP	PPQCQPGEFA	CANSRCIQER	WKCDGDNDCL
890	900	910	920	930	940	950	960
DNSDEAPALC	HQHTCPSDRF	KCENNRCIPN	RWLCDGDNDC	GNSEDESNAT	CSARTCPPNQ	FSCASGRCIP	ISWTCDLDDD
970	980	990	1000	1010	1020	1030	1040
CGDRSDESAS	CAYPTCFPLT	QFTCNNGRCI	NINWRCDNDN	DCGDNSDEAG	CSHSCSSTQF	KCNSGRCIPE	HWTCDGDNDC
1050	1060	1070	1080	1090	1100	1110	1120
GDYSDETHAN	CTNQATRPPG	GCHTDEFQCR	LDGLCIPLRW	RCDGDTDCMD	SSDEKSCEGV	THVCDPSVKF	GCKDSARCIS
1130	1140	1150	1160	1170	1180	1190	1200
KAWVCDGDND	CEDNSDEENC	ESLACRPPSH	PCANNTSVCL	PPDKLCDGND	DCGDGSDEGE	LCDQCSLNNG	GCSHNCSVAP
1210	1220	1230	1240	1250	1260	1270	1280
GEGIVCSCPL	GMELGPDNHT	CQIQSYCAKH	LKCSQKCDQN	KFSVKCSCYE	GWVLEPDGES	CRSLDPFKPF	IIFSNRHEIR
1290	1300	1310	1320	1330	1340	1350	1360
RIDLHKGDYS	VLVPGLRNTI	ALDFHLSQSA	LYWTDVVEDK	IYRGKLLDNG	ALTSFEVVIQ	YGLATPEGLA	VDWIAGNIYW
1370	1380	1390	1400	1410	1420	1430	1440
VESNLDQIEV	AKLDGTLRTT	LLAGDIEHPR	AIALDPRDGI	LFWTDWDASL	PRIEAASMSG	AGRRTVHRET	GSGGWPNGLT
1450	1460	1470	1480	1490	1500	1510	1520
VDYLEKRILW	IDARSDAIYS	ARYDGSGHME	VLRGHEFLSH	PFAVTLYGGE	VYWTDWRTNT	LAKANKWTGH	NVTVVQRTNT
1530	1540	1550	1560	1570	1580	1590	1600
QPFDLQVYHP	SRQPMAPNPC	EANGGQGPCS	HLCLINYNRT	VSCACPHLMK	LHKDNTTCYE	FKKFLLYARQ	MEIRGVDLDA
1610	1620	1630	1640	1650	1660	1670	1680
PYYNYIISFT	VPDIDNVTVL	DYDAREQRVY	WSDVRTQAIK	RAFINGTGVE	TVVSADLPNA	HGLAVDWVSR	NLFWTSYDTN
1690	1700	1710	1720	1730	1740	1750	1760
KKQINVARLD	GSFKNAVVQG	LEQPHGLVVH	PLRGKLYWTD	GDNISMANMD	GSNRTLLFSG	QKGPVGLAID	FPESKLYWIS
1770	1780	1790	1800	1810	1820	1830	1840
SGNHTINRCN	LDGSGLEVID	AMRSQLGKAT	ALAIMGDKLW	WADQVSEKMG	TCSKADGSGS	VVLRNSTTLV	MHMKVYDESI
1850	1860	1870	1880	1890	1900	1910	1920
QLDHKGTNPC	SVNNGDCSQL	CLPTSETTRS	CMCTAGYSLR	SGQQACEGVG	SFLLYSVHEG	IRGIPLDPND	KSDALVPVSG
1930	1940	1950	1960	1970	1980	1990	2000
TSLAVGIDFH	AENDTIYWVD	MGLSTISRAK	RDQTWREDVV	TNGIGRVEGI	AVDWIAGNIY	WTDQGFDVIE	VARLNGSFRY
2010	2020	2030	2040	2050	2060	2070	2080
VVISQGLDKP	RAITVHPEKG	YLFWTEWGQY	PRIERSRLDG	TERVVLVNVS	ISWPNGISVD	YQDGKLYWCD	ARTDKIERID
2090	2100	2110	2120	2130	2140	2150	2160
LETGENREVV	LSSNNMDMFS	VSVFEDFIYW	SDRTHANGSI	KRGSKDNATD	SVPLRTGIGV	QLKDIKVFNR	DRQKGTNVCA
2170	2180	2190	2200	2210	2220	2230	2240

Detailed Protein Report

Cmpd.	No. of Cmpds.	m/z meas.	Δ m/z [ppm]	z	Rt [min]	Score	P	Range	Sequence	Modification	Ratios	
138	1	849.3804	-44.02	2	31.5	11.0	1	1560-1573	R.TVSCACPHLMKLHK.D	Carbamidomethyl: 4, 6; Oxidation: 10	MD:MU	0.80
2236	2	873.3305	-116.11	2	57.8	15.6	1	1952-1966	R.DQTWREDVVTNGIGR.V			

Detailed Protein Report

Protein 369: PRAME family member 1 [Homo sapiens]

Accession: gi|12738831
Database: refseq_human(refseq_human_20140103.fasta)
Database Date: 2015-11-30

Score: 26.6
MW [kDa]: 55.1
pI: 9.9
Sequence Coverage [%]: 3.8
No. of unique Peptides: 1

10	20	30	40	50	60	70	80
MSIQAPPRLL	ELAGQSLLRD	QALSISAMEE	LPRVLYLPLF	MEAFSRRHFQ	TLTVMVQAWP	FTCLPLGSLM	KTLHLETLKA
**90**	**100**	**110**	**120**	**130**	**140**	**150**	**160**
LLEGLHMLLT	QKDRPRRWKL	QVLDLRDVDE	NFWARWPGAW	ALSCFPETTS	KRQTAEDCPR	MGEHQPLKVF	IDICLKEIPQ
**170**	**180**	**190**	**200**	**210**	**220**	**230**	**240**
DECLRYLFQW	VYQRRGLVHL	CCSKLVNYLT	PIKYLRKSLK	IIYLNSIQEL	EIRNMSWPRL	IRKLRCYLKE	MKNLRKLVFS
**250**	**260**	**270**	**280**	**290**	**300**	**310**	**320**
RCHHYTSDNE	LQGRLVAKFS	SVFLRLEHLQ	LLKIKLITFF	SGHLEQLIRC	LQNPLENLEL	TYGYLLEEDM	KCLSQYPSLG
**330**	**340**	**350**	**360**	**370**	**380**	**390**	**400**
YLKHLNLSYV	LLFRISLEPL	GALLEKIAAS	LKTLILEGCQ	IHYSQLSAIL	PALSRCSQLT	TFYFGRNCMS	IDALKDLLRH
**410**	**420**	**430**	**440**	**450**	**460**	**470**	**480**
TSGLSKLSLE	TYPAPEESLN	SLVRVNWEIF	TPLRAELMCT	LREVRQPKRI	FIGPTPCPSC	GSSPSEELEL	HLCC

Cmpd.	No. of Cmpds.	m/z meas.	Δ m/z [ppm]	z	Rt [min]	Score	P	Range	Sequence	Modification
1448	1	428.2129	-107.14	3	47.7	10.6	0	335-346	R.ISLEPLGALLEK.I	

Detailed Protein Report

Protein 370: PREDICTED: protein CASP isoform X6 [Homo sapiens]

Accession: gi|578813926
Database: refseq_human(refseq_human_20140103.fasta)
Database Date: 2015-11-30
Modification(s): Carbamidomethyl, Oxidation

Score: 26.6
MW [kDa]: 172.0
pI: 5.9
Sequence Coverage [%]: 1.5
No. of unique Peptides: 2

Quantitation

MD:MU **Median:** 9.99 **CV:** 0.00 % **No. of Peptides:** 1

10	20	30	40	50	60	70	80
MERAAGALGD	AFSGRVLESR	APGARWDRGM	PGGGPRAESP	APRGDRAGAP	RPAPESPPCP	RGECLPATPR	PEERRTLRVR
90	100	110	120	130	140	150	160
PARASSAPAA	PGQPRDSARW	MLCVAGARLK	RELDATATVL	ANRQDESEQS	RKRLIEQSRE	FKKNTPEDLR	KQVAPLLKSF
170	180	190	200	210	220	230	240
QGEIDALSKR	SKEAEAAFLN	VYKRLIDVPD	PVPALDLGQQ	LQLKVQRLHD	IETENQKLRE	TLEEYNKEFA	EVKNQEVTIK
250	260	270	280	290	300	310	320
ALKEKIREYE	QTLKNQAETI	ALEKEQKLQN	DFAEKERKLQ	ETQMSTTSKL	EEAEHKVQSL	QTALEKTRTE	LFDLKTKYDE
330	340	350	360	370	380	390	400
ETTAKADEIE	MIMTDLERAN	QRAEVAQREA	ETLREQLSSA	NHSLQLASQI	QKAPDVEQAI	EVLTRSSLEV	ELAAKEREIA
410	420	430	440	450	460	470	480
QLVEDVQRLQ	ASLTKLRENS	ASQISQLEQQ	LSAKNSTLKQ	LEEKLKGQAD	YEEVKKELNI	LKSMEFAPSE	GAGTQDAAKP
490	500	510	520	530	540	550	560
LEVLLLEKNR	SLQSENAALR	ISNSDLSGSA	RRKGKDQPES	RRPGSLPAPP	PSQLPRNPGE	QASNTNGTHQ	FSPAGLSQDF
570	580	590	600	610	620	630	640
FSSSLASPSL	PLASTGKFAL	NSLLQRQLMQ	SFYSKAMQEA	GSTSMIFSTG	PYSTNSISSQ	SPLQQSPDVN	GMAPSPSQSE
650	660	670	680	690	700	710	720
SAGSVSEGEE	MDTAEIARQV	KEQLIKHNIG	QRIFGHYVLG	LSQGSVSEIL	ARPKPWNKLT	VRGKEPFHKM	KQFLSDEQNI
730	740	750	760	770	780	790	800
LALRSIQGRQ	RGNITTRIRA	SETGSDEAIK	SILEQAKREL	QVQKTAEPAQ	PSSASGSGNS	DDAIRSILQQ	ARREMEAQQA
810	820	830	840	850	860	870	880
ALDPALKQAP	LSQSDITILT	PKLLSTSPMP	TVSSYPPLAI	SLKKPSAAPE	AGASALPNPP	ALKKEAQDAP	GLDPQGAADC
890	900	910	920	930	940	950	960
AQGVLRQVKN	EVGRSGAWKD	HWWSAVQPER	RNAASSEEAK	AEETGGGKEK	GSGGSGGGSQ	PRAERSQLQG	PSSSEYWKEW
970	980	990	1000	1010	1020	1030	1040
PSAESPYSQS	SELSLTGASR	SETPQNSPLP	SSPIVPMSKP	TKPSVPPLTP	EQYEVYMYQE	VDTIELTRQV	KEKLAKNGIC
1050	1060	1070	1080	1090	1100	1110	1120
QRIFGEKVLG	LSQGSVSDML	SRPKPWSKLT	QKGREPFIRM	QLWLNGELGQ	GVLPVQGQQQ	GPVLHSVTSL	QDPLQQGCVS
1130	1140	1150	1160	1170	1180	1190	1200
SESTPKTSAS	CSPAPESPMS	SSESVKSLTE	LVQQPCPPIE	ASKDSKPPEP	SDPPASDSQP	TTPLPLSGHS	ALSIQELVAM
1210	1220	1230	1240	1250	1260	1270	1280
SPELDTYGIT	KRVKEVLTDN	NLGQRLFGET	ILGLTQGSVS	DLLARPKPWH	KLSLKGREPF	VRMQLWLNDP	NNVEKLMDMK
1290	1300	1310	1320	1330	1340	1350	1360
RMEKKAYMKR	RHSSVSDSQP	CEPPSVGTEY	SQGASPQPQH	QLKKPRVVLA	PEEKEALKRA	YQQKPYPSPK	TIEDLATQLN
1370	1380	1390	1400	1410	1420	1430	1440
LKTSTVINWF	HNYRSRIRRE	LFIEEIQAGS	QGQAGASDSP	SARSGRAAPS	SEGDSCDGVE	ATEGPGSADT	EEPKSQGEAE
1450	1460	1470	1480	1490	1500	1510	1520
REEVPRPAEQ	TEPPPSGTPG	PDDARDDDHE	GGPVEGPGPL	PSPASATATA	APAAPEDAAT	SAAAAPGEGP	AAPSSAPPPS
1530	1540	1550	1560	1570	1580	1590	
NSSSSSAPRR	PSSLQSLFGL	PEAAGARDSR	DNPLRKKKAA	NLNSIIHRLE	KAASREEPIE	WEF	

Cmpd.	No. of Cmpds.	m/z meas.	Δ m/z [ppm]	z	Rt [min]	Score	P	Range	Sequence	Modification	Ratios
1855	1	738.7765	-167.06	2	53.2	12.2	2	100-111	R.WMLCVAGARLKR.E	Carbamidomethyl: 4; Oxidation: 2	
597	3	661.1918	-220.59	2	37.0	14.5	1	1037-1047	K.NGICQRIFGEK.V	Carbamidomethyl: 4	MD:MU 9.99

Detailed Protein Report

Protein 371: PREDICTED: coiled-coil domain-containing protein 111 isoform X3 [Homo sapiens]

**Accession:**	gi|578808887	**Score:**	26.6
**Database:**	refseq_human(refseq_human_20140103.fasta)	**MW [kDa]:**	58.1
**Database Date:**	2015-11-30	**pI:**	8.8
		**Sequence Coverage [%]:**	2.2
		**No. of unique Peptides:**	1

Quantitation

MD:MU **Median:** 5.03 **CV:** 0.00 % **No. of Peptides:** 1

10	20	30	40	50	60	70	80
MNRKWEAKLK	QIEERASHYE	RKPLSSVYRP	RLSKPEEPPS	IWRLFHRQAQ	AFNFVKSCKE	DVHVFALECK	VGDGQRIYLV
**90**	**100**	**110**	**120**	**130**	**140**	**150**	**160**
TTYAEFWFYY	KSRKNLLHCY	EVIPENAVCK	LYFDLEFNKP	ANPGADGKKM	VALLIEYVCK	ALQELYGVNC	SAEDVLNLDS
**170**	**180**	**190**	**200**	**210**	**220**	**230**	**240**
STDEKFSRHL	IFQLHDVAFK	DNIHVGNFLR	KILQPALDLL	GSEDDDSAPE	TTGHGFPHFS	EAPARQGFSF	NKMFTEKATE
**250**	**260**	**270**	**280**	**290**	**300**	**310**	**320**
ESWTSNSKKL	ERLGSAEQSS	PDLSFLVVKN	NMGEKHLFVD	LGVYTRNRNF	RLYKSSKIGK	RVALEVTEDN	KFFPIQSKDV
**330**	**340**	**350**	**360**	**370**	**380**	**390**	**400**
SDEYQYFLSS	LVSNVRFSDT	LRILTCEPSQ	NKQKGVGYFN	SIGTSVETIE	GFQCSPYPEV	DHFVLSLVNK	DGIKGGIRRW
**410**	**420**	**430**	**440**	**450**	**460**	**470**	**480**
NYFFPEELLV	YDICKYRWCE	NIGRAHKSNN	IMILVDLKNE	VWYQKCHDPV	CKAENFKSDC	FPLPAEVCLL	FLFKELDCPL
**490**	**500**	**510**					
VKKLNPPLLF	RRGVLKSMCT	EL					

Cmpd.	No. of Cmpds.	m/z meas.	Δ m/z [ppm]	z	Rt [min]	Score	P	Range	Sequence	Modification	Ratios
1088	1	540.6458	-238.97	2	43.2	12.3	1	493-502	R.GVLKSMCTEL.-		MD:MU 5.03

Detailed Protein Report

Protein 372: **spectrin beta chain, non-erythrocytic 4 isoform sigma1 [Homo sapiens]**

**Accession:**	gi|115430237	**Score:**	26.6
**Database:**	refseq_human(refseq_human_20140103.fasta)	**MW [kDa]:**	288.8
**Database Date:**	2015-11-30	**pI:**	5.7
		**Sequence Coverage [%]:**	1.1
		**No. of unique Peptides:**	1

Detailed Protein Report

10	20	30	40	50	60	70	80
MAQVPGEVDN	MEGLPAPNNN	PAARWESPDR	GWEREQPAAS	TAAASLFECS	RIKALADERE	AVQKKTFTKW	VNSHLARVGC
90	100	110	120	130	140	150	160
HIGDLYVDLR	DGFVLTRLLE	VLSGEQLPRP	TRGRMRIHSL	ENVDKALQFL	KEQRVHLENV	GSHDIVDGNH	RLTLGLVWTI
170	180	190	200	210	220	230	240
ILRFQIQVIK	IETEDNRETR	SAKDALLLWC	QMKTAGYPEV	NIQNFTTSWR	DGLAFNALIH	RHRPDLVDFS	KLTKSNANYN
250	260	270	280	290	300	310	320
LQRAFRTAEQ	HLGLARLLDP	EDVNMEAPDE	KSIITYVVSF	YHYFSKMKAL	AVEGKRIGKV	LDQVLEVGKI	IERYEELAAE
330	340	350	360	370	380	390	400
LLAWIHRTVG	LISNQKFANS	LSGVQQQLQA	FTAYCTLEKP	VKFQEKGNLE	VLLFSIQSKL	RACNRRLFVP	REGCGIWDID
410	420	430	440	450	460	470	480
KAWGELEKAE	HEREAALRAE	LIRQEKLELL	AQRFDHKVAM	RESWLNENQR	LVSQDNFGYE	LPAVEAAMKK	HEAIEADIAA
490	500	510	520	530	540	550	560
YEERVQGVAE	LAQALAAEGY	YDIRRVAAQR	DSVLRQWALL	TGLVGARRTR	LEQNLALQKV	FQEMVYMVDW	MEEMQAQLLS
570	580	590	600	610	620	630	640
RECGQHLVEA	DDLLQKHGLL	EGDIAAQSER	VEALNAAALR	FSQLQGYQPC	DPQVICNRVN	HVHGCLAELQ	EQAARRRAEL
650	660	670	680	690	700	710	720
EASRSLWALL	QELEEAESWA	RDKERLLEAA	GGGGAAGAAG	AAGTAGGAHD	LSSTARLLAQ	HKILQGELGG	RRALLQQALR
730	740	750	760	770	780	790	800
CGEELVAAGG	AVGPGADTVH	LVGLAERAAS	ARRRWQRLEE	AAARRERRLQ	EARALHQFGA	DLDGLLDWLR	DAYRLAAAGD
810	820	830	840	850	860	870	880
FGHDEASSRR	LARQHRALTG	EVEAHRGPVS	GLRRQLATLG	GASGAGPLVV	ALQVRVVEAE	QLFAEVTEVA	ALRRQWLRDA
890	900	910	920	930	940	950	960
LAVYRMFGEV	HACELWIGEK	EQWLLSMRVP	DSLDDVEVVQ	HRFESLDQEM	NSLMGRVLDV	NHTVQELVEG	GHPSSDEVRS
970	980	990	1000	1010	1020	1030	1040
CQDHLNSRWN	RIVELVEQRK	EEMSAVLLVE	NHVLEVAEVR	AQVREKRRAV	ESAPRAGGAL	QWRLSGLEAA	LQALEPRQAA
1050	1060	1070	1080	1090	1100	1110	1120
LLEEAALLAE	RFPAQAARLH	QGAEELGAEW	GALASAAQAC	GEAVAAAGRL	QRFLHDLDAF	LDWLVRAQEA	AGGSEGPLPN
1130	1140	1150	1160	1170	1180	1190	1200
SLEEADALLA	RHAALKEEVD	QREEDYARIV	AASEALLAAD	GAELGPGLAL	DEWLPHLELG	WHKLLGLWEA	RREALVQAHI
1210	1220	1230	1240	1250	1260	1270	1280
YQLFLRDLRQ	ALVVLRNQEM	ALSGAELPGT	VESVEEALKQ	HRDFLTTMEL	SQQKMQVAVQ	AAEGLLRQGN	IYGEQAQEAV
1290	1300	1310	1320	1330	1340	1350	1360
TRLLEKNQEN	QLRAQQWMQK	LHDQLELQHF	LRDCHELDGW	IHEKMLMARD	GTREDNHKLH	KRWLRHQAFM	AELAQNKEWL
1370	1380	1390	1400	1410	1420	1430	1440
EKIEREGQQL	MQEKPELAAS	VRKKLGEIRQ	CWAELESTTQ	AKARQLFEAS	KADQLVQSFA	ELDKKLLHME	SQLQDVDPGG
1450	1460	1470	1480	1490	1500	1510	1520
DLATVNSQLK	KLQSMESQVE	EWYREVGELQ	AQTAALPLEP	ASKELVGERQ	NAVGERLVRL	LEPLQERRRL	LLASKELHQV
1530	1540	1550	1560	1570	1580	1590	1600
AHDLDDELAW	VQERLPLAMQ	TERGNGLQAV	QQHIKKNQGL	RREIQAHGPR	LEEVLERAGA	LASLRSPEAE	AVRRGLEQLQ
1610	1620	1630	1640	1650	1660	1670	1680
SAWAGLREAA	ERRQQVLDAA	FQVEQYYFDV	AEVEAWLGEQ	ELLMMSEDKG	KDEQSTLQLL	KKHLQLEQGV	ENYEESIAQL
1690	1700	1710	1720	1730	1740	1750	1760
SRQCRALLEM	GHPDSEQISR	RQSQVDRLYV	ALKELGEERR	VALEQQYWLY	QLSRQVSELE	HWIAEKEVVA	GSPELGQDFE
1770	1780	1790	1800	1810	1820	1830	1840
HVSVLQEKFS	EFASETGMAG	RERLAAVNQM	VDELIECGHT	AAATMAEWKD	GLNEAWAELL	ELMGTRAQLL	AASRELHKFF
1850	1860	1870	1880	1890	1900	1910	1920
SDARELQGQI	EEKRRRLPRL	TTPPEPRPSA	SSMQRTLRAF	EHDLQLLVSQ	VRQLQEGAAQ	LRTVYAGEHA	EAIASREQEV
1930	1940	1950	1960	1970	1980	1990	2000
LQGWKELLSA	CEDARLHVSS	TADALRFHSQ	VRDLLSWMDG	IASQIGAADK	PRDVSSVEVL	MNYHQGLKTE	LEARVPELTT
2010	2020	2030	2040	2050	2060	2070	2080
CQELGRSLLL	NKSAMADEIQ	AQLDKLGTRK	EEVSEKWDRH	WEWLQQMLEV	HQFAQEAVVA	DAWLTAQEPL	LQSRELGSSV
2090	2100	2110	2120	2130	2140	2150	2160
DEVEQLIRRH	EAFRKAAAAW	EERFSSLRRL	TTIEKIKAEQ	SKQPPTPLLG	RKFFGDPTEL	AAKAAPLLRP	GGYERGLEPL
2170	2180	2190	2200	2210	2220	2230	2240

Detailed Protein Report

Cmpd.	No. of Cmpds.	m/z meas.	Δ m/z [ppm]	z	Rt [min]	Score	P	Range	Sequence	Modification
1079	4	530.6190	-276.49	2	43.1	12.2	2	2553-2562	R.EGGDRRASGR.R	

Detailed Protein Report

Protein 373: **spectrin beta chain, erythrocytic isoform b [Homo sapiens]**

**Accession:**	gi|67782319	**Score:**	26.5
**Database:**	refseq_human(refseq_human_20140103.fasta)	**MW [kDa]:**	246.3
**Database Date:**	2015-11-30	**pI:**	5.0
**Modification(s):**	Oxidation	**Sequence Coverage [%]:**	1.0
		**No. of unique Peptides:**	1

Detailed Protein Report

10	20	30	40	50	60	70	80
MTSATEFENV	GNQPPYSRIN	ARWDAPDDEL	DNDNSSARLF	ERSRIKALAD	EREVVQKKTF	TKWVNSHLAR	VSCRITDLYK
90	100	110	120	130	140	150	160
DLRDGRMLIK	LLEVLSGEML	PKPTKGKMRI	HCLENVDKAL	QFLKEQRVHL	ENMGSHDIVD	GNHRLVLGLI	WTIILRFQIQ
170	180	190	200	210	220	230	240
DIVVQTQEGR	ETRSAKDALL	LWCQMKTAGY	PHVNVTNFTS	SWKDGLAFNA	LIHKHRPDLI	DFDKLKDSNA	RHNLEHAFNV
250	260	270	280	290	300	310	320
AERQLGIIPL	LDPEDVFTEN	PDEKSIITYV	VAFYHYFSKM	KVLAVEGKRV	GKVIDHAIET	EKMIEKYSGL	ASDLLTWIEQ
330	340	350	360	370	380	390	400
TITVLNSRKF	ANSLTGVQQQ	LQAFSTYRTV	EKPPKFQEKG	NLEVLLFTIQ	SRMRANNQKV	YTPHDGKLVS	DINRAWESLE
410	420	430	440	450	460	470	480
EAEYRRELAL	RNELIRQEKL	EQLARRFDRK	AAMRETWLSE	NQRLVAQDNF	GYDLAAVEAA	KKKHEAIETD	TAAYEERVRA
490	500	510	520	530	540	550	560
LEDLAQELEK	ENYHDQKRIT	ARKDNILRLW	SYLQELLQSR	RQRLETTLAL	QKLFQDMLHS	IDWMDEIKAH	LLSAEFGKHL
570	580	590	600	610	620	630	640
LEVEDLLQKH	KLMEADIAIQ	GDKVKAITAA	TLKFTEGKGY	QPCDPQVIQD	RISHLEQCFE	ELSNMAAGRK	AQLEQSKRLW
650	660	670	680	690	700	710	720
KFFWEMDEAE	SWIKEKEQIY	SSLDYGKDLT	SVLILQRKHK	AFEDELRGLD	AHLEQIFQEA	HGMVARKQFG	HPQIEARIKE
730	740	750	760	770	780	790	800
VSAQWDQLKD	LAAFCKKNLQ	DAENFFQFQG	DADDLKAWLQ	DAHRLLSGED	VGQDEGATRA	LGKKHKDFLE	ELEESRGVME
810	820	830	840	850	860	870	880
HLEQQAQGFP	EEFRDSPDVT	HRLQALRELY	QQVVAQADLR	QQRLQEALDL	YTVFGETDAC	ELWMGEKEKW	LAEMEMPDTL
890	900	910	920	930	940	950	960
EDLEVVQHRF	DILDQEMKTL	MTQIDGVNLA	ANSLVESGHP	RSREVKQYQD	HLNTRWQAFQ	TLVSERREAV	DSALRVHNYC
970	980	990	1000	1010	1020	1030	1040
VDCEETSKWI	TDKTKVVEST	KDLGRDLAGI	IAIQRKLSGL	ERDVAAIQAR	VDALERESQQ	LMDSHPEQKE	DIGQRQKHLE
1050	1060	1070	1080	1090	1100	1110	1120
ELWQGLQQSL	QGQEDLLGEV	SQLQAFLQDL	DDFQAWLSIT	QKAVASEDMP	ESLPEAEQLL	QQHAGIKDEI	DGHQDSYQRV
1130	1140	1150	1160	1170	1180	1190	1200
KESGEKVIQG	QTDPEYLLLG	QRLEGLDTGW	NALGRMWESR	SHTLAQCLGF	QEFQKDAKQA	EAILSNQEYT	LAHLEPPDSL
1210	1220	1230	1240	1250	1260	1270	1280
EAAEAGIRKF	EDFLGSMENN	RDKVLSPVDS	GNKLVAEGNL	YSDKIKEKVQ	LIEDRHRKNN	EKAQEASVLL	RDNLELQNFL
1290	1300	1310	1320	1330	1340	1350	1360
QNCQELTLWI	NDKLLTSQDV	SYDEARNLHN	KWLKHQAFVA	ELASHEGWLE	NIDAEGKQLM	DEKPQFTALV	SQKLEALHRL
1370	1380	1390	1400	1410	1420	1430	1440
WDELQATTKE	KTQHLSAARS	SDLRLQTHAD	LNKWISAMED	QLRSDDPGKD	LTSVNRMLAK	LKRVEDQVNV	RKEELGELFA
1450	1460	1470	1480	1490	1500	1510	1520
QVPSMGEEGG	DADLSIEKRF	LDLLEPLGRR	KKQLESSRAK	LQISRDLEDE	TLWVEERLPL	AQSADYGTNL	QTVQLFMKKN
1530	1540	1550	1560	1570	1580	1590	1600
QTLQNEILGH	TPRVEDVLQR	GQQLVEAAEI	DCQDLEERLG	HLQSSWDRLR	EAAAGRLQRL	RDANEAQQYY	LDADEAEAWI
1610	1620	1630	1640	1650	1660	1670	1680
GEQELYVISD	EIPKDEEGAI	VMLKRHLRQQ	RAVEDYGRNI	KQLASRAQGL	LSAGHPEGEQ	IIRLQGQVDK	HYAGLKDVAE
1690	1700	1710	1720	1730	1740	1750	1760
ERKRKLENMY	HLFQLKRETD	DLEQWISEKE	LVASSPEMGQ	DFDHVTLLRD	KFRDFARETG	AIGQERVDNV	NAFIERLIDA
1770	1780	1790	1800	1810	1820	1830	1840
GHSEAATIAE	WKDGLNEMWA	DLLELIDTRM	QLLAASYDLH	RYFYTGAEIL	GLIDEKHREL	PEDVGLDAST	AESFHRVHTA
1850	1860	1870	1880	1890	1900	1910	1920
FERELHLLGV	QVQQFQDVAT	RLQTAYAGEK	AEAIQNKEQE	VSAAWQALLD	ACAGRRTQLV	DTADKFRFFS	MARDLLSWME
1930	1940	1950	1960	1970	1980	1990	2000
SIIRQIETQE	RPRDVSSVEL	LMKYHQGINA	EIETRSKNFS	ACLELGESLL	QRQHQASEEI	REKLQQVMSR	RKEMNEKWEA
2010	2020	2030	2040	2050	2060	2070	2080
RWERLRMLLE	VCQFSRDASV	AEAWLIAQEP	YLASGDFGHT	VDSVEKLIKR	HEAFEKSTAS	WAERFAALEK	PTTLELKERQ
2090	2100	2110	2120	2130	2140		
IAERPAEETG	PQEEEGETAG	EAPVSHHAAT	ERTSPVSLWS	RLSSSWESLQ	PEPSHPY		

Cmpd.	No. of Cmpds.	m/z meas.	Δ m/z [ppm]	z	Rt [min]	Score	P	Range	Sequence	Modification
2770	1	905.4785	51.90	3	66.0	26.5	1	868-889	K.EKWLAEMEMPDTLEDLEVVQHR.F	Oxidation: 9

Detailed Protein Report

Protein 374: tumor necrosis factor alpha-induced protein 3 [Homo sapiens]

**Accession:**	gi|5454132	**Score:**	26.5
**Database:**	refseq_human(refseq_human_20140103.fasta)	**MW [kDa]:**	89.6
**Database Date:**	2015-11-30	**pI:**	9.8
**Modification(s):**	Carbamidomethyl	**Sequence Coverage [%]:**	3.7
		**No. of unique Peptides:**	1

Quantitation

MD:MU **Median:** 1.10 **CV:** 0.00 % **No. of Peptides:** 1

Alias proteins:

Accession	Name	Description
gi|530384006	refseq_human (refseq_human_20140103.fasta)	PREDICTED: tumor necrosis factor alpha-induced protein 3 isoform X1 [Homo sapiens]
gi|395393997	refseq_human (refseq_human_20140103.fasta)	tumor necrosis factor alpha-induced protein 3 [Homo sapiens]
gi|395393995	refseq_human (refseq_human_20140103.fasta)	tumor necrosis factor alpha-induced protein 3 [Homo sapiens]

10	20	30	40	50	60	70	80
MAEQVLPQAL	YLSNMRKAVK	IRERTPEDIF	KPTNGIIHHF	KTMHRYTLEM	FRTCQFCPQF	REIIHKALID	RNIQATLESQ
90	100	110	120	130	140	150	160
KKLNWCREVR	KLVALKTNGD	GNCLMHATSQ	YMWGVQDTDL	VLRKALFSTL	KETDTRNFKF	RWQLESLKSQ	EFVETGLCYD
170	180	190	200	210	220	230	240
TRNWNDEWDN	LIKMASTDTP	MARSGLQYNS	LEEIHIFVLC	NILRRPIIVI	SDKMLRSLES	GSNFAPLKVG	GIYLPLHWPA
250	260	270	280	290	300	310	320
QECYRYPIVL	GYDSHHFVPL	VTLKDSGPEI	RAVPLVNRDR	GRFEDLKVHF	LTDPENEMKE	KLLKEYLMVI	EIPVQGWDHG
330	340	350	360	370	380	390	400
TTHLINAAKL	DEANLPKEIN	LVDDYFELVQ	HEYKKWQENS	EQGRREGHAQ	NPMEPSVPQL	SLMDVKCETP	NCPFFMSVNT
410	420	430	440	450	460	470	480
QPLCHECSER	RQKNQNKLPK	LNSKPGPEGL	PGMALGASRG	EAYEPLAWNP	EESTGGPHSA	PPTAPSPFLF	SETTAMKCRS
490	500	510	520	530	540	550	560
PGCPFTLNVQ	HNGFCERCHN	ARQLHASHAP	DHTRHLDPGK	CQACLQDVTR	TFNGICSTCF	KRTTAEASSS	LSTSLPPSCH
570	580	590	600	610	620	630	640
QRSKSDPSRL	VRSPSPHSCH	RAGNDAPAGC	LSQAARTPGD	RTGTSKCRKA	GCVYFGTPEN	KGFCTLCFIE	YRENKHFAAA
650	660	670	680	690	700	710	720
SGKVSPTASR	FQNTIPCLGR	ECGTLGSTMF	EGYCQKCFIE	AQNQRFHEAK	RTEEQLRSSQ	RRDVPRTTQS	TSRPKCARAS
730	740	750	760	770	780	790	800
CKNILACRSE	ELCMECQHPN	QRMGPGAHRG	EPAPEDPPKQ	RCRAPACDHF	GNAKCNGYCN	ECFQFKQMYG	

Cmpd.	No. of Cmpds.	m/z meas.	Δ m/z [ppm]	z	Rt [min]	Score	P	Range	Sequence	Modification	Ratios	
1527	1	932.4083	-48.26	2	48.9	13.5	1	53-66	R.TCQFCPQFREIIHK.A	Carbamidomethyl: 2, 5	MD:MU	1.10

Detailed Protein Report

Protein 375: **PREDICTED: serine/threonine-protein phosphatase with EF-hands 2 isoform X1 [Homo sapiens]**

Accession: gi|578809184
Database: refseq_human(refseq_human_20140103.fasta)
Database Date: 2015-11-30
Modification(s): Carbamidomethyl

Score: 26.5
MW [kDa]: 69.0
pI: 9.4
Sequence Coverage [%]: 3.8
No. of unique Peptides: 1

10	20	30	40	50	60	70	80
MGSGTSTQHH	FAFQNAERAF	KAAALIQRWY	RRYVARLEMR	RRCTWSIFQS	IEYAGQQDQV	KLHDFFSYLM	DHFIPSSHND
**90**	**100**	**110**	**120**	**130**	**140**	**150**	**160**
RDFLTRIFTE	DRFAQDSEMK	KCSDYESIEV	PDSYTGPRLS	FPLLPDHATA	LVEAFRLKQQ	LHARYVLNLL	YETKKHLVQL
**170**	**180**	**190**	**200**	**210**	**220**	**230**	**240**
PNINRVSTCY	SEEITVCGDL	HGQLDDLIFI	FYKNGLPSPE	RSYVFNGDFV	DRGKDSVEIL	MILFAFMLVY	PKEFHLNRGN
**250**	**260**	**270**	**280**	**290**	**300**	**310**	**320**
HEDHMVNLRY	GFTKEVMNKY	KVHGKEILRT	LQDVFCWLPL	ATLIDEKVLI	LHGGVSDITD	LELLDKIERS	KIVSTMRCKT
**330**	**340**	**350**	**360**	**370**	**380**	**390**	**400**
RQKSEKQMEE	KRRANQKSSA	QGPIPWFLPE	SRSLPSSPLR	LGSYKAQKTS	RSSSIPCSGS	LDGRELSRQV	RSSVELELER
**410**	**420**	**430**	**440**	**450**	**460**	**470**	**480**
CRQQAGLLVT	GEKEEPSRSA	SEADSEAGEL	RKPTQEEWRQ	VVDILWSDPM	AQEGCKANTI	RGGGCYFGPD	VTQQLLQKYN
**490**	**500**	**510**	**520**	**530**	**540**	**550**	**560**
MQFLIRSHEC	KPEGYEFCHN	RKVLTIFSAS	NYYEVGSNRG	AYVKLGPALT	PHIVQYQANK	VTHTLTMRQR	ISRVEESALR
**570**	**580**	**590**	**600**				
ALREKLFAHS	SDLLSEFKKH	DADKVGLASS	QLCYYQQK				

Cmpd.	No. of Cmpds.	m/z meas.	Δ m/z [ppm]	z	Rt [min]	Score	P	Range	Sequence	Modification
2068	1	946.8198	-78.17	2	55.7	14.8	0	487-501	R.SHECKPEGYEFCHNR.K	Carbamidomethyl: 4

Detailed Protein Report

Protein 376: zinc finger protein 483 isoform a [Homo sapiens]

Accession: gi|190014620
Database: refseq_human(refseq_human_20140103.fasta)
Database Date: 2015-11-30
Modification(s): Carbamidomethyl

Score: 26.5
MW [kDa]: 85.0
pI: 9.9
Sequence Coverage [%]: 4.6
No. of unique Peptides: 1

10	20	30	40	50	60	70	80
MQAVVPLNKM	TAISPEPQTL	ASTEQNEVPR	VVTSGEQEAI	LRGNAADAES	FRQRFRWFCY	SEVAGPRKAL	SQLWELCNQW
**90**	**100**	**110**	**120**	**130**	**140**	**150**	**160**
LRPDIHTKEQ	ILELLVFEQF	LTILPGEIRI	WVKSQHPESS	EEVVTLIEDL	TQMLEEKDPV	SQDSTVSQEE	NSKEDKMVTV
**170**	**180**	**190**	**200**	**210**	**220**	**230**	**240**
CPNTESCESI	TLKDVAVNFS	RGEWKKLEPF	QKELYKEVLL	ENLRNLEFLD	FPVSKLELIS	QLKWVELPWL	LEEVSKSSRL
**250**	**260**	**270**	**280**	**290**	**300**	**310**	**320**
DESALDKIIE	RCLRDDDHGL	MEESQQYCGS	SEEDHGNQGN	SKGRVAQNKT	LGSGSRGKKF	DPDKSPFGHN	FKETSDLIKH
**330**	**340**	**350**	**360**	**370**	**380**	**390**	**400**
LRVYLRKKSR	RYNESKKPFS	FHSDLVLNRK	EKTAGEKSRK	SNDGGKVLSH	SSALTEHQKR	QKIHLGDRSQ	KCSKCGIIFI
**410**	**420**	**430**	**440**	**450**	**460**	**470**	**480**
RRSTLSRRKT	PMCEKCRKDS	CQEAALNKDE	GNESGEKTHK	CSKCGKAFGY	SASLTKHRRI	HTGEKPYMCN	ECGKAFSDSS
**490**	**500**	**510**	**520**	**530**	**540**	**550**	**560**
SLTPHHRTHS	GEKPFKCDDC	GKGFTLSAHL	IKHQRIHTGE	KPYKCKDCGR	PFSDSSSLIQ	HQRIHTGEKP	YTCSNCGKSF
**570**	**580**	**590**	**600**	**610**	**620**	**630**	**640**
SHSSSLSKHQ	RIHTGEKPYK	CGECGKAFRQ	NSCLTRHQRI	HTGEKPYLCN	DCGMTFSHFT	SVIYHQRLHS	GEKPYKCNQC
**650**	**660**	**670**	**680**	**690**	**700**	**710**	**720**
EKAFPTHSLL	SRHQRIHTGV	KPYKCKECGK	SFSQSSSLNE	HHRIHTGEKP	YECNYCGATF	SRSSILVEHL	KIHTGRREYE
**730**	**740**	**750**					
CNECEKTFKS	NSGLIRHRGF	HSAE					

Cmpd.	No. of Cmpds.	m/z meas.	Δ m/z [ppm]	z	Rt [min]	Score	P	Range	Sequence	Modification
173	1	1318.5770	-87.18	2	31.6	12.9	1	68-88	R.KALSQLWELCNQWLRPDIHTK.E	Carbamidomethyl: 10

Detailed Protein Report

Protein 377: PREDICTED: zinc finger protein 41 isoform X5 [Homo sapiens]

**Accession:**	gi|530421580	**Score:**	26.4
**Database:**	refseq_human(refseq_human_20140103.fasta)	**MW [kDa]:**	79.5
**Database Date:**	2015-11-30	**pI:**	10.2
**Modification(s):**	Carbamidomethyl	**Sequence Coverage [%]:**	4.5
		**No. of unique Peptides:**	2

Alias proteins:

Accession	Name	Description
gi|578838101	refseq_human (refseq_human_20140103.fasta)	PREDICTED: zinc finger protein 41 isoform X15 [Homo sapiens]
gi|530421582	refseq_human (refseq_human_20140103.fasta)	PREDICTED: zinc finger protein 41 isoform X6 [Homo sapiens]

10	20	30	40	50	60	70	80
MLEGEAPHQS	CSGEAIGKMQ	QQGIPGGIFF	HCERFDQPIG	EDSLCSILEE	LWQDNDQLEQ	RQENQNNLLS	HVKVLIKERG
**90**	**100**	**110**	**120**	**130**	**140**	**150**	**160**
YEHKNIEKII	HVTTKLVPSI	KRLHNCDTIL	KHTLNSHNHN	RNSATKNLGK	IFGNGNNFPH	SPSSTKNENA	KTGANSCEHD
**170**	**180**	**190**	**200**	**210**	**220**	**230**	**240**
HYEKHLSHKQ	APTHHQKIHP	EEKLYVCTEC	VMGFTQKSHL	FEHQRIHAGE	KSRECDKSNK	VFPQKPQVDV	HPSVYTGEKP
**250**	**260**	**270**	**280**	**290**	**300**	**310**	**320**
YLCTQCGKVF	TLKSNLITHQ	KIHTGQKPYK	CSECGKAFFQ	RSDLFRHLRI	HTGEKPYECS	ECGKGFSQNS	DLSIHQKTHT
**330**	**340**	**350**	**360**	**370**	**380**	**390**	**400**
GEKHYECNEC	GKAFTRKSAL	RMHQRIHTGE	KPYVCADCGK	AFIQKSHFNT	HQRIHTGEKP	YECSDCGKSF	TKKSQLHVHQ
**410**	**420**	**430**	**440**	**450**	**460**	**470**	**480**
RIHTGEKPYI	CTECGKVFTH	RTNLTTHQKT	HTGEKPYMCA	ECGKAFTDQS	NLIKHQKTHT	GEKPYKCNGC	GKAFIWKSRL
**490**	**500**	**510**	**520**	**530**	**540**	**550**	**560**
KIHQKSHIGE	RHYECKDCGK	AFIQKSTLSV	HQRIHTGEKP	YVCPECGKAF	IQKSHFIAHH	RIHTGEKPYE	CSDCGKCFTK
**570**	**580**	**590**	**600**	**610**	**620**	**630**	**640**
KSQLRVHQKI	HTGEKPNICA	ECGKAFTDRS	NLITHQKIHT	REKPYECGDC	GKTFTWKSRL	NIHQKSHTGE	RHYECSKCGK
**650**	**660**	**670**	**680**	**690**	**700**		
AFIQKATLSM	HQIIHTGKKP	YACTECQKAF	TDRSNLIKHQ	KMHSGEKRYK	ASD		

Cmpd.	No. of Cmpds.	m/z meas.	Δ m/z [ppm]	z	Rt [min]	Score	P	Range	Sequence	Modification
1222	1	955.6757	154.26	2	44.9	10.3	2	80-95	R.GYEHKNIEKIIHVTTK.L	
2726	1	884.9432	75.73	2	64.4	16.1	0	430-444	K.THTGEKPYMCAECGK.A	Carbamidomethyl: 10, 13

Detailed Protein Report

Protein 378: PREDICTED: LIM/homeobox protein Lhx9 isoform X2 [Homo sapiens]

**Accession:**	gi|530365098	**Score:**	26.4
**Database:**	refseq_human(refseq_human_20140103.fasta)	**MW [kDa]:**	37.1
**Database Date:**	2015-11-30	**pI:**	10.2
**Modification(s):**	Carbamidomethyl, Oxidation	**Sequence Coverage [%]:**	8.2
		**No. of unique Peptides:**	2

```
        10         20         30         40         50         60         70         80
MEIVGCRAED NSCPFRPPAM LFHGISGGHI QGIMEEMERR SKTEARLAKG AQLNGRDAGM PPLSPEKPAL CAGCGGKISD
        90        100        110        120        130        140        150        160
RYYLLAVDKQ WHLRCLKCCE CKLALESELT CFAKDGSIYC KEDYYRRFSV QRCARCHLGI SASEMVMRAR DSVYHLSCFT
       170        180        190        200        210        220        230        240
CSTCNKTLTT GDHFGMKDSL VYCRAHFETL LQGEYPPQLS YTELAAKSGG LALPYFNGTG TVQKGRPRKR KSPALGVDIV
       250        260        270        280        290        300        310        320
NYNSGCNENE ADHLDRDQQP YPPSQKTKRM RTSFKHHQLR TMKSYFAINH NPDAKDLKQL AQKTGLTKRV LQGEQILGHY
       330        340
SQTSRRLKIP
```

Cmpd.	No. of Cmpds.	m/z meas.	Δ m/z [ppm]	z	Rt [min]	Score	P	Range	Sequence	Modification
2658	1	932.9537	71.73	2	64.4	15.8	0	151-166	R.DSVYHLSCFTCSTCNK.T	Carbamidomethyl: 11
929	1	612.4347	236.48	2	41.1	10.6	0	167-177	K.TLTTGDHFGMK.D	Oxidation: 10

Detailed Protein Report

Protein 379: proline-rich protein 18 [Homo sapiens]

**Accession:**	gi|58331144	**Score:**	26.4
**Database:**	refseq_human(refseq_human_20140103.fasta)	**MW [kDa]:**	30.9
**Database Date:**	2015-11-30	**pI:**	12.1
		**Sequence Coverage [%]:**	5.1
		**No. of unique Peptides:**	2

10	20	30	40	50	60	70	80
MPFPPMPPPP	APAPGAQAAR	QLPRRPCAAG	DKKKRPPQRP	EGLLSSSWPS	ATLKRPPARR	GPGLDRTQPP	APPGVSPQAL
**90**	**100**	**110**	**120**	**130**	**140**	**150**	**160**
PSRARAPATC	APPRPAGSGH	SPARTTYAAT	SAGTGTTAAG	TSSGAGPCPD	SAARFCLNLT	PEAVLVIQKR	HLEKQLLARP
**170**	**180**	**190**	**200**	**210**	**220**	**230**	**240**
RRPFPSPSAE	PRRLLAPCLP	ARAAGPRRGG	PASDPDAPPT	AGQGRRAPPP	GAQLLHGGLQ	VPQLSPRPGA	LRPMLKVSLL
**250**	**260**	**270**	**280**	**290**	**300**		
NERHRYDDVE	YEEEPEAVDE	GLVRKCTEWL	RGVESAAAAR	GRAGALDSRR	HLSTL		

Cmpd.	No. of Cmpds.	m/z meas.	Δ m/z [ppm]	z	Rt [min]	Score	P	Range	Sequence	Modification
1740	1	555.2376	-174.09	2	51.7	12.3	1	173-182	R.RLLAPCLPAR.A	
2875	2	703.3735	-49.65	2	66.6	14.1	1	174-187	R.LLAPCLPARAAGPR.R	

Detailed Protein Report

Protein 380: **spermatogenesis-associated protein 31E1 [Homo sapiens]**

**Accession:**	gi|155029550	**Score:**	26.4
**Database:**	refseq_human(refseq_human_20140103.fasta)	**MW [kDa]:**	157.0
**Database Date:**	2015-11-30	**pI:**	10.3
		**Sequence Coverage [%]:**	1.8
		**No. of unique Peptides:**	1

10	20	30	40	50	60	70	80
MGNLVIPLGK	GRAGRVESGQ	RIPPPAPRPS	VECTGDDIAL	QMEKMLFPLK	SPSATWLSPS	STPWMMDFIL	TSVCGLVLLF
90	100	110	120	130	140	150	160
LLLLYVHSDP	PSPPPGRKRS	SREPQRERSG	RSRSRKISAL	KACRILLREL	EETRDLNYLL	ESHLRKLAGE	GSSHLPLGGD
170	180	190	200	210	220	230	240
PLGDVCKPVP	AKAHQPHGKC	MQDPSPASLS	PPAPPAPLAS	TLSPGPMTFS	EPFGPHSTLS	ASGPPEPLLP	LKCPATQPHV
250	260	270	280	290	300	310	320
VFPPSPQPHG	PLASSPPPPD	SSLAGLQCGS	TTCPVPQSSP	LHNQVLPPPT	RVISGLGCSS	DPIWDLYCWR	EAATTWGLST
330	340	350	360	370	380	390	400
YSHGKSQPRH	LPDHTSEASF	WGDPTPKHME	VGGCTFIHPD	VQKLLETLIA	KRALMKMWQE	KERKRADHPH	MTSLGKEWDI
410	420	430	440	450	460	470	480
TTLNPFWNVS	TQPQQLPRPQ	QVSDATTVGN	HLQQKRSQLF	WDLPSLNSES	LATTVWVSRN	PSSQNAHSVP	LDKASTSLPG
490	500	510	520	530	540	550	560
EPEVEASSQL	SQAPPQPHHM	AQPQHFTPAW	PQSQPPPLAE	IQTQAHLSPP	VPSLGCSSPP	QIRGCGASYP	TSQERTQSVI
570	580	590	600	610	620	630	640
PTGKEYLEWP	LKKRPKWKRV	LPSLLKKSQA	VLSQPTAHLP	QERPASWSPK	SAPILPGVVT	SPELPEHWWQ	GRNAIHQEQS
650	660	670	680	690	700	710	720
CGPPSRLQAS	GDLLQPDGEF	PGRPQSQAED	TQQALLPSQP	SDFAGKGRKD	VQKTGFRSSG	RFSDKGCLGS	KLGPDPSRDQ
730	740	750	760	770	780	790	800
GSGRTSVKAL	DEDKEAEGDL	RRSWKYQSVS	STPRDPDKEH	LENKLQIHLA	RKVGEIKEGW	IPMPVRRSWL	MAKCAVPKSD
810	820	830	840	850	860	870	880
THRKPGKLAS	WRGGKAHVNT	SQELSFLHPC	TQQILEVHLV	RFCVRHSWGT	DLQSLEPINV	WSGEAQAPPF	PQSTFTPWAS
890	900	910	920	930	940	950	960
WVSRVESVPK	VPIFLGKRPQ	NGPGDNRTTS	KSVPTVSGPL	AAPPPEQEGV	QRPPRGSQSA	DTHGRSEAFP	TGHKGRGCSQ
970	980	990	1000	1010	1020	1030	1040
PPTCSLVGRT	WQSRTVLESG	KPKPRLEGSM	GSEMAGNEAW	LESESMSPGD	PCSSRALQVL	SIGSQWARAE	DALQALKVGE
1050	1060	1070	1080	1090	1100	1110	1120
KPPTWEVTLG	ASVRASSGSV	QEDLRSTGAL	GTTGNPSASS	VCVAQDPEQL	HLKAQVVSEI	ALIVQVDSEE	QLPGRAPGIL
1130	1140	1150	1160	1170	1180	1190	1200
LQDGATGLCL	PGRHMDMLTA	ADRLPTQAPL	STSQSVSGKN	MTASQGPCAL	LWKGGDSPGQ	QEPGSPKAKA	PQKSQKTLGC
1210	1220	1230	1240	1250	1260	1270	1280
ADKGEAHRRP	RTGEQGHRSK	GPRTSEASGR	SHPAQAREIG	DKQERKYNQL	QLEKGQTPPE	SHFQRKISHH	PQGLHPRKGG
1290	1300	1310	1320	1330	1340	1350	1360
TRWEDVLQKG	KPGADAFQSW	GSGPPRQFMD	CMADKAWTIS	RVVGQILVDK	LGLQWGRGPS	EVNRHKGDFR	AQENVPSCCH
1370	1380	1390	1400	1410	1420	1430	1440
RGHCHQERSR	EMRALACSPK	ATPKGHHCPV	KNRGIRDRDS	SWAPPPREPV	SPAGPHHHRP	RMASTSGGPH	PQLQELMSAQ
1450							
RCLAS							

Cmpd.	No. of Cmpds.	m/z meas.	Δ m/z [ppm]	z	Rt [min]	Score	P	Range	Sequence	Modification
1848	1	805.3101	-128.16	2	53.1	14.3	2	1394-1407	R.GIRDRDSSWAPPPR.E	

Detailed Protein Report

Protein 381: probable global transcription activator SNF2L2 isoform c [Homo sapiens]

**Accession:**	gi|574957245	**Score:**	26.4
**Database:**	refseq_human(refseq_human_20140103.fasta)	**MW [kDa]:**	172.5
**Database Date:**	2015-11-30	**pI:**	6.7
		**Sequence Coverage [%]:**	1.8
		**No. of unique Peptides:**	1

10	20	30	40	50	60	70	80
MSTPTDPGAM	PHPGPSPGPG	PSPGPILGPS	PGPGPSPGSV	HSMMGPSPGP	PSVSHPMPTM	GSTDFPQEGM	HQMHKPIDGI
**90**	**100**	**110**	**120**	**130**	**140**	**150**	**160**
HDKGIVEDIH	CGSMKGTGMR	PPHPGMGPPQ	SPMDQHSQGY	MSPHPSPLGA	PEHVSSPMSG	GGPTPPQMPP	SQPGALIPGD
**170**	**180**	**190**	**200**	**210**	**220**	**230**	**240**
PQAMSQPNRG	PSPFSPVQLH	QLRAQILAYK	MLARGQPLPE	TLQLAVQGKR	TLPGLQQQQQ	QQQQQQQQQQ	QQQQQQQQPQ
**250**	**260**	**270**	**280**	**290**	**300**	**310**	**320**
QQPPQPQTQQ	QQQPALVNYN	RPSGPGPELS	GPSTPQKLPV	PAPGGRPSPA	PPAAAQPPAA	AVPGPSVPQP	APGQPSPVLQ
**330**	**340**	**350**	**360**	**370**	**380**	**390**	**400**
LQQKQSRISP	IQKPQGLDPV	EILQEREYRL	QARIAHRIQE	LENLPGSLPP	DLRTKATVEL	KALRLLNFQR	QLRQEVVACM
**410**	**420**	**430**	**440**	**450**	**460**	**470**	**480**
RRDTTLETAL	NSKAYKRSKR	QTLREARMTE	KLEKQQKIEQ	ERKRRQKHQE	YLNSILQHAK	DFKEYHRSVA	GKIQKLSKAV
**490**	**500**	**510**	**520**	**530**	**540**	**550**	**560**
ATWHANTERE	QKKETERIEK	ERMRRLMAED	EEGYRKLIDQ	KKDRRLAYLL	QQTDEYVANL	TNLVWEHKQA	QAAKEKKKRR
**570**	**580**	**590**	**600**	**610**	**620**	**630**	**640**
RRKKKAEENA	EGGESALGPD	GEPIDESSQM	SDLPVKVTHT	ETGKVLFGPE	APKASQLDAW	LEMNPGYEVA	PRSDSEESDS
**650**	**660**	**670**	**680**	**690**	**700**	**710**	**720**
DYEEEDEEEE	SSRQETEEKI	LLDPNSEEVS	EKDAKQIIET	AKQDVDDEYS	MQYSARGSQS	YYTVAHAISE	RVEKQSALLI
**730**	**740**	**750**	**760**	**770**	**780**	**790**	**800**
NGTLKHYQLQ	GLEWMVSLYN	NNLNGILADE	MGLGKTIQTI	ALITYLMEHK	RLNGPYLIIV	PLSTLSNWTY	EFDKWAPSVV
**810**	**820**	**830**	**840**	**850**	**860**	**870**	**880**
KISYKGTPAM	RRSLVPQLRS	GKFNVLLTTY	EYIIKDKHIL	AKIRWKYMIV	DEGHRMKNHH	CKLTQVDLNE	EETILIIRRL
**890**	**900**	**910**	**920**	**930**	**940**	**950**	**960**
HKVLRPFLLR	RLKKEVESQL	PEKVEYVIKC	DMSALQKILY	RHMQAKGILL	TDGSEKDKKG	KGGAKTLMNT	IMQLRKICNH
**970**	**980**	**990**	**1000**	**1010**	**1020**	**1030**	**1040**
PYMFQHIEES	FAEHLGYSNG	VINGAELYRA	SGKFELLDRI	LPKLRATNHR	VLLFCQMTSL	MTIMEDYFAF	RNFLYLRLDG
**1050**	**1060**	**1070**	**1080**	**1090**	**1100**	**1110**	**1120**
TTKSEDRAAL	LKKFNEPGSQ	YFIFLLSTRA	GGLGLNLQAA	DTVVIFDSDW	NPHQDLQAQD	RAHRIGQQNE	VRVLRLCTVN
**1130**	**1140**	**1150**	**1160**	**1170**	**1180**	**1190**	**1200**
SVEEKILAAA	KYKLNVDQKV	IQAGMFDQKS	SSHERRAFLQ	AILEHEEENE	EEDEVPDDET	LNQMIARREE	EFDLFMRMDM
**1210**	**1220**	**1230**	**1240**	**1250**	**1260**	**1270**	**1280**
DRRREDARNP	KRKPRLMEED	ELPSWIIKDD	AEVERLTCEE	EEEKIFGRGS	RQRRDVDYSD	ALTEKQWLRA	IEDGNLEEME
**1290**	**1300**	**1310**	**1320**	**1330**	**1340**	**1350**	**1360**
EEVRLKKRKR	RRNVDKDPAK	EDVEKAKKRR	GRPPAEKLSP	NPPKLTKQMN	AIIDTVINYK	DSSGRQLSEV	FIQLPSRKEL
**1370**	**1380**	**1390**	**1400**	**1410**	**1420**	**1430**	**1440**
PEYYELIRKP	VDFKKIKERI	RNHKYRSLGD	LEKDVMLLCH	NAQTFNLEGS	QIYEDSIVLQ	SVFKSARQKI	AKEEESEDES
**1450**	**1460**	**1470**	**1480**	**1490**	**1500**	**1510**	**1520**
NEEEEEEDEE	ESESEAKSVK	VKIKLNKKDD	KGRDKGKGKK	RPNRGKAKPV	VSDFDSDEEQ	DEREQSEGSG	TDDE

Cmpd.	No. of Cmpds.	m/z meas.	Δ m/z [ppm]	z	Rt [min]	Score	P	Range	Sequence	Modification
2825	2	1024.0347	-1.30	2	65.7	15.7	1	1053-1069	K.KFNEPGSQYFIFLLSTR.A	

Detailed Protein Report

Protein 382: zinc finger protein 275 [Homo sapiens]

Accession: gi|298231195
Database: refseq_human(refseq_human_20140103.fasta)
Database Date: 2015-11-30
Modification(s): Carbamidomethyl

Score: 26.3
MW [kDa]: 37.0
pI: 10.0
Sequence Coverage [%]: 6.7
No. of unique Peptides: 2

10	20	30	40	50	60	70	80
MMSHPCVSLL	GVPVLNPALV	PHLAQGQVLL	VSDPSPNTDP	AKYSESTSAT	RHQMKGEDAQ	PQEMASTSFP	RASGPSPEFR
**90**	**100**	**110**	**120**	**130**	**140**	**150**	**160**
QHGDSDGKRG	SPQNLPIEHH	FACKECGDTF	RLKVLLVQHQ	RVHSEEKGWE	CGDCGKVFRG	VAEFNEHRKS	HVAAEPQPGP
**170**	**180**	**190**	**200**	**210**	**220**	**230**	**240**
SRALENAAEK	REQMEREAKP	FECEECGKRF	KKNAGLSQHL	RVHSREKPFD	CEECGRSFKV	NTHLFRHQKL	HTSEKPFACK
**250**	**260**	**270**	**280**	**290**	**300**	**310**	**320**
ACSRDFLDRQ	ELLKHQRMHT	GHLPFDCDDC	GKSFRGVNGL	AEHQRIHSGA	KPYGCPHCGK	LFRRSSELTK	HRRIHTGEKP
**330**							
YACGLPWAL							

Cmpd.	No. of Cmpds.	m/z meas.	Δ m/z [ppm]	z	Rt [min]	Score	P	Range	Sequence	Modification
2741	1	806.4367	70.79	2	65.5	13.9	0	286-300	R.IHSGAKPYGCPHCGK.L	Carbamidomethyl: 13
803	1	820.2164	-287.56	1	39.4	12.4	1	304-310	R.RSSELTK.H	

Detailed Protein Report

Protein 383: SH3 and multiple ankyrin repeat domains protein 3 [Homo sapiens]

**Accession:**	gi|380748963	**Score:**	26.3
**Database:**	refseq_human(refseq_human_20140103.fasta)	**MW [kDa]:**	184.6
**Database Date:**	2015-11-30	**pI:**	9.6
**Modification(s):**	Oxidation	**Sequence Coverage [%]:**	1.8
		**No. of unique Peptides:**	1

10	20	30	40	50	60	70	80
MDGPGASAVV	VRVGIPDLQQ	TKCLRLDPAA	PVWAAKQRVL	CALNHSLQDA	LNYGLFQPPS	RGRAGKFLDE	ERLLQEYPPN
**90**	**100**	**110**	**120**	**130**	**140**	**150**	**160**
LDTPLPYLEF	RYKRRVYAQN	LIDDKQFAKL	HTKANLKKFM	DYVQLHSTDK	VARLLDKGLD	PNFHDPDSGE	CPLSLAAQLD
**170**	**180**	**190**	**200**	**210**	**220**	**230**	**240**
NATDLLKVLK	NGGAHLDFRT	RDGLTAVHCA	TRQRNAAALT	TLLDLGASPD	YKDSRGLTPL	YHSALGGGDA	LCCELLLHDH
**250**	**260**	**270**	**280**	**290**	**300**	**310**	**320**
AQLGITDENG	WQEIHQACRF	GHVQHLEHLL	FYGADMGAQN	ASGNTALHIC	ALYNQESCAR	VLLFRGANRD	VRNYNSQTAF
**330**	**340**	**350**	**360**	**370**	**380**	**390**	**400**
QVAIIAGNFE	LAEVIKTHKD	SDVVPFRETP	SYAKRRRLAG	PSGLASPRPL	QRSASDINLK	GEAQPAASPG	PSLRSLPHQL
**410**	**420**	**430**	**440**	**450**	**460**	**470**	**480**
LLQRLQEEKD	RDRDADQESN	ISGPLAGRAG	QSKISPSGPG	GPGPAPGPGP	APPAPPAPPP	RGPKRKLYSA	VPGRKFIAVK
**490**	**500**	**510**	**520**	**530**	**540**	**550**	**560**
AHSPQGEGEI	PLHRGEAVKV	LSIGEGGFWE	GTVKGRTGWF	PADCVEEVQM	RQHDTRPETR	EDRTKRLFRH	YTVGSYDSLT
**570**	**580**	**590**	**600**	**610**	**620**	**630**	**640**
SHSDYVIDDK	VAVLQKRDHE	GFGFVLRGAK	AETPIEEFTP	TPAFPALQYL	ESVDVEGVAW	RAGLRTGDFL	IEVNGVNVVK
**650**	**660**	**670**	**680**	**690**	**700**	**710**	**720**
VGHKQVVALI	RQGGNRLVMK	VVSVTRKPEE	DGARRRAPPP	PKRAPSTTLT	LRSKSMTAEL	EELASIRRRK	GEKLDEMLAA
**730**	**740**	**750**	**760**	**770**	**780**	**790**	**800**
AAEPTLRPDI	ADADSRAATV	KQRPTSRRIT	PAEISSLFER	QGLPGPEKLP	GSLRKGIPRT	KSVGEDEKLA	SLLEGRFPRS
**810**	**820**	**830**	**840**	**850**	**860**	**870**	**880**
TSMQDPVREG	RGIPPPPQTA	PPPPPAPYYF	DSGPPPAFSP	PPPPGRAYDT	VRSSFKPGLE	ARLGAGAAGL	YEPGAALGPL
**890**	**900**	**910**	**920**	**930**	**940**	**950**	**960**
PYPERQKRAR	SMIILQDSAP	ESGDAPRPPP	AATPPERPKR	RPRPPGPDSP	YANLGAFSAS	LFAPSKPQRR	KSPLVKQLQV
**970**	**980**	**990**	**1000**	**1010**	**1020**	**1030**	**1040**
EDAQERAALA	VGSPGPGGGS	FAREPSPTHR	GPRPGGLDYG	AGDGPGLAFG	GPGPAKDRRL	EERRRSTVFL	SVGAIEGSAP
**1050**	**1060**	**1070**	**1080**	**1090**	**1100**	**1110**	**1120**
GADLPSLQPS	RSIDERLLGT	GPTAGRDLLL	PSPVSALKPL	VSGPSLGPSG	STFIHPLTGK	PLDPSSPLAL	ALAARERALA
**1130**	**1140**	**1150**	**1160**	**1170**	**1180**	**1190**	**1200**
SQAPSRSPTP	VHSPDADRPG	PLFVDVQARD	PERGSLASPA	FSPRSPAWIP	VPARREAEKV	PREERKSPED	KKSMILSVLD
**1210**	**1220**	**1230**	**1240**	**1250**	**1260**	**1270**	**1280**
TSLQRPAGLI	VVHATSNGQE	PSRLGGAEEE	RPGTPELAPA	PMQSAAVAEP	LPSPRAQPPG	GTPADAGPGQ	GSSEEEPELV
**1290**	**1300**	**1310**	**1320**	**1330**	**1340**	**1350**	**1360**
FAVNLPPAQL	SSSDEETREE	LARIGLVPPP	EEFANGVLLA	TPLAGPGPSP	TTVPSPASGK	PSSEPPPAPE	SAADSGVEEA
**1370**	**1380**	**1390**	**1400**	**1410**	**1420**	**1430**	**1440**
DTRSSSDPHL	ETTSTISTVS	SMSTLSSESG	ELTDTHTSFA	DGHTFLLEKP	PVPPKPKLKS	PLGKGPVTFR	DPLLKQSSDS
**1450**	**1460**	**1470**	**1480**	**1490**	**1500**	**1510**	**1520**
ELMAQQHHAA	SAGLASAAGP	ARPRYLFQRR	SKLWGDPVES	RGLPGPEDDK	PTVISELSSR	LQQLNKDTRS	LGEEPVGGLG
**1530**	**1540**	**1550**	**1560**	**1570**	**1580**	**1590**	**1600**
SLLDPAKKSP	IAAARLFSSL	GELSSISAQR	SPGGPGGGAS	YSVRPSGRYP	VARRAPSPVK	PASLERVEGL	GAGAGGAGRP
**1610**	**1620**	**1630**	**1640**	**1650**	**1660**	**1670**	**1680**
FGLTPPTILK	SSSLSIPHEP	KEVRFVVRSV	SARSRSPSPS	PLPSPASGPG	PGAPGPRRPF	QQKPLQLWSK	FDVGDWLESI
**1690**	**1700**	**1710**	**1720**	**1730**	**1740**		
HLGEHRDRFE	DHEIEGAHLP	ALTKDDFVEL	GVTRVGHRMN	IERALRQLDG	S		

Cmpd.	No. of Cmpds.	m/z meas.	Δ m/z [ppm]	z	Rt [min]	Score	P	Range	Sequence	Modification
1868	1	840.7873	-166.49	2	53.4	13.8	1	693-707	R.SKSMTAELEELASIR.R	Oxidation: 4

Detailed Protein Report

Protein 384: **PREDICTED: polyribonucleotide nucleotidyltransferase 1, mitochondrial isoform X1 [Homo sapiens]**

Accession: gi|530368250
Database: refseq_human(refseq_human_20140103.fasta)
Database Date: 2015-11-30

Score: 26.3
MW [kDa]: 77.3
pI: 6.2
Sequence Coverage [%]: 4.7
No. of unique Peptides: 2

10	20	30	40	50	60	70	80
MVTAVSKTKP	SPSQFMPLVV	DYRQKAAAAG	RIPTNYLRRE	IGTSDKEILT	SRIIDRSIRP	LFPAGYFYDT	QVLCNLLAVD
90	100	110	120	130	140	150	160
GVNEPDVLAI	NGASVALSLS	DIPWNGPVGA	VRIGIIDGEY	VVNPTRKEMS	SSTLNLVVAG	APKSQIVMLE	ASAENILQQD
170	180	190	200	210	220	230	240
FCHAIKVGVK	YTQQIIQGIQ	QLVKETGVTK	RTPQKLFTPS	PEIVKYTHKL	AMERLYAVFT	DYEHDKVSRD	EAVNKIRLDT
250	260	270	280	290	300	310	320
EEQLKEKFPE	ADPYEIIESF	NVVAKEVFRS	IVLNEYKRCD	GRDLTSLRNV	SCEVDMFKTL	HGSALFQRGQ	TQVLCTVTFD
330	340	350	360	370	380	390	400
SLESGIKSDQ	VITAINGIKD	KNFMLHYEFP	PYATNEIGKV	TGLNRRELGH	GALAEKALYP	VIPRDFPFTI	RVTSEVLESN
410	420	430	440	450	460	470	480
GSSSMASACG	GSLALMDSGV	PISSAVAGVA	IGLVTKTDPE	KGEIEDYRLL	TDILGIEDYN	GDMDFKIAGT	NKGITALQAD
490	500	510	520	530	540	550	560
IKLPGIPIKI	VMEAIQQASV	AKKEILQIMN	KTISKPRASR	KENGPVVETV	QVPLSKRAKF	VGPGGYNLKK	LQAETGVTIS
570	580	590	600	610	620	630	640
QVDEETFSVF	APTPSAMHEA	RDFITEICKD	DQEQQLEFGA	VYTATITEIR	DTGVMVKLYP	NMTAVLLHNT	QLDQRKIKHP
650	660	670	680	690	700	710	
TALGLEVGQE	IQVKYFGRDP	ADGRMRLSRK	VLQSPATTVV	RTLNDRSSIV	MGEPISQSSS	NSQ	

Cmpd.	No. of Cmpds.	m/z meas.	Δ m/z [ppm]	z	Rt [min]	Score	P	Range	Sequence	Modification
2331	1	973.5348	3.64	2	59.0	11.2	2	40-56	R.EIGTSDKEILTSRIIDR.S	
2737	1	885.4690	-31.70	2	64.6	15.2	1	671-686	K.VLQSPATTVVRTLNDR.S	

Detailed Protein Report

Protein 385: mitochondrial inner membrane protein OXA1L [Homo sapiens]

Accession: gi|223718097
Database: refseq_human(refseq_human_20140103.fasta)
Database Date: 2015-11-30

Score: 26.3
MW [kDa]: 55.2
pI: 10.6
Sequence Coverage [%]: 3.2
No. of unique Peptides: 1

Quantitation

MD:MU **Median:** 1.09 **CV:** 0.00 % **No. of Peptides:** 1

10	20	30	40	50	60	70	80
MVTWLYRFLP	TSNMAAKLRS	LLPPDLRLQF	WLHARLQKCF	LSRGCGSYCA	GAKASPLPGK	MAMGLMCGRR	ELLRLLQSGR
90	100	110	120	130	140	150	160
RVHSVAGPSQ	WLGKPLTTRL	LFPAAPCCCR	PHYLFLAASG	PRSLSTSAIS	FAEVQVQAPP	VVAATPSPTA	VPEVASGETA
170	180	190	200	210	220	230	240
DVVQTAAEQS	FAELGLGSYT	PVGLIQNLLE	FMHVDLGLPW	WGAIAACTVF	ARCLIFPLIV	TGQREAARIH	NHLPEIQKFS
250	260	270	280	290	300	310	320
SRIREAKLAG	DHIEYYKASS	EMALYQKKHG	IKLYKPLILP	VTQAPIFISF	FIALREMANL	PVPSLQTGGL	WWFQDLTVSD
330	340	350	360	370	380	390	400
PIYILPLAVT	ATMWAVLELG	AETGVQSSDL	QWMRNVIRMM	PLITLPITMH	FPTAVFMYWL	SSNLFSLVQV	SCLRIPAVRT
410	420	430	440	450	460	470	480
VLKIPQRVVH	DLDKLPPREG	FLESFKKGWK	NAEMTRQLRE	REQRMRNQLE	LAARGPLRQT	FTHNPLLQPG	KDNPPNIPSS
490	500						
SSKPKSKYPW	HDTLG						

Cmpd.	No. of Cmpds.	m/z meas.	Δ m/z [ppm]	z	Rt [min]	Score	P	Range	Sequence	Modification	Ratios	
1726	1	546.7057	-111.00	2	51.5	15.2	1	428-436	K.GWKNAEMTR.Q		MD:MU	1.09

Detailed Protein Report

Protein 386: PREDICTED: E3 ubiquitin-protein ligase UBR4 isoform X13 [Homo sapiens]

**Accession:**	gi|578798776	**Score:**	26.2
**Database:**	refseq_human(refseq_human_20140103.fasta)	**MW [kDa]:**	573.4
**Database Date:**	2015-11-30	**pI:**	5.7
**Modification(s):**	Carbamidomethyl	**Sequence Coverage [%]:**	0.6
		**No. of unique Peptides:**	2

Detailed Protein Report

10	20	30	40	50	60	70	80
MATSGGEEAA	AAAPAPGTPA	TGADTTPGWE	VAVRPLLSAS	YSAFEMKELP	QLVASVIESE	SEILHHEKQY	EPFYSSFVAL
90	100	110	120	130	140	150	160
STHYITTVCS	LIPRNQLQSV	AAACKVLIEF	SLLRLENPDE	ACAVSQKHLI	LLIKGLCTGC	SRLDRTEIIT	FTAMMKSAKL
170	180	190	200	210	220	230	240
PQTVKTLSDV	EDQKELASPV	SPELRQKEVQ	MNFLNQLTSV	FNPRTVASQP	ISTQTLVEGE	NDEQSSTDQA	SAIKTKNVFI
250	260	270	280	290	300	310	320
AQNVASLQEL	GGSEKLLRVC	LNLPYFLRYI	NRFQDAVLAN	SFFIMPATVA	DATAVRNGFH	SLVIDVTMAL	DTLSLPVLEP
330	340	350	360	370	380	390	400
LNPSRLQDVT	VLSLSCLYAG	VSVATCMAIL	HVGSAQQVRT	GSTSSKEDDY	ESDAATIVQK	CLEIYDMIGQ	AISSSRRAGG
410	420	430	440	450	460	470	480
EHYQNFQLLG	AWCLLNSLFL	ILNLSPTALA	DKGKEKDPLA	ALRVRDILSR	TKEGVGSPKL	GPGKGHQGFG	VLSVILANHA
490	500	510	520	530	540	550	560
IKLLTSLFQD	LQVEALHKGW	ETDGPPAALS	IMAQSTSIQR	IQRLIDSVPL	MNLLLTLLST	SYRKACVLQR	QRKGSMSSDA
570	580	590	600	610	620	630	640
SASTDSNTYY	EDDFSSTEED	SSQDDDSEPI	LGQWFEETIS	PSKEKAAPPP	PPPPPPLESS	PRVKSPSKQA	PGEKGNILAS
650	660	670	680	690	700	710	720
RKDPELFLGL	ASNILNFITS	SMLNSRNNFI	RNYLSVSLSE	HHMATLASII	KEVDKDGLKG	SSDEEFAAAL	YHFNHSLVTS
730	740	750	760	770	780	790	800
DLQSPNLQNT	LLQQLGVAPF	SEGPWPLYIH	PQSLSVLSRL	LLIWQHKASA	QGDPDVPECL	KVWDRFLSTM	KQNALQGVVP
810	820	830	840	850	860	870	880
SETEDLNVEH	LQMLLLIFHN	FTETGRRAIL	SLFVQIIQEL	SVNMDAQMRF	VPLILARLLL	IFDYLLHQYS	KAPVYLFEQV
890	900	910	920	930	940	950	960
QHNLLSPPFG	WASGSQDSNS	RRATTPLYHG	FKEVEENWSK	HFSSDAVPHP	RFYCVLSPEA	SEDDLNRLDS	VACDVLFSKL
970	980	990	1000	1010	1020	1030	1040
VKYDELYAAL	TALLAAGSQL	DTVRRKENKN	VTALEACALQ	YYFLILWRIL	GILPPSKTYI	NQLSMNSPEM	SECDILHTLR
1050	1060	1070	1080	1090	1100	1110	1120
WSSRLRISSY	VNWIKDHLIK	QGMKAEHASS	LLELASTTKC	SSVKYDVEIV	EEYFARQISS	FCSIDCTTIL	QLHEIPSLQS
1130	1140	1150	1160	1170	1180	1190	1200
IYTLDAAISK	VQVSLDEHFS	KMAAETDPHK	SSEITKNLLP	ATLQLIDTYA	SFTRAYLLQN	FNEEGTTEKP	SKEKLQGFAA
1210	1220	1230	1240	1250	1260	1270	1280
VLAIGSSRCK	ANTLGPTLVQ	NLPSSVQTVC	ESWNNINTNE	FPNIGSWRNA	FANDTIPSES	YISAVQAAHL	GTLCSQSLPL
1290	1300	1310	1320	1330	1340	1350	1360
AASLKHTLLS	LVRLTGDLIV	WSDEMNPPQV	IRTLLPLLLE	SSTESVAEIS	SNSLERILGP	AESDEFLARV	YEKLITGCYN
1370	1380	1390	1400	1410	1420	1430	1440
ILANHADPNS	GLDESILEEC	LQYLEKQLES	SQARKAMEEF	FSDSGELVQI	MMATANENLS	AKFCNRVLKF	FTKLFQLTEK
1450	1460	1470	1480	1490	1500	1510	1520
SPNPSLLHLC	GSLAQLACVE	PVRLQAWLTR	MTTSPPKDSD	QLDVIQENRQ	LLQLLTTYIV	RENSQVGEGV	CAVLLGTLTP
1530	1540	1550	1560	1570	1580	1590	1600
MATEMLANGD	GTGFPELMVV	MATLASAGQG	AGHLQLHNAA	VDWLSRCKKY	LSQKNVVEKL	NANVMHGKHV	MILECTCHIM
1610	1620	1630	1640	1650	1660	1670	1680
SYLADVTNAL	SQSNGQGPSH	LSVDGEERAI	EVDSDWVEEL	AVEEEDSQAE	DSDEDSLCNK	LCTFTITQKE	FMNQHWYHCH
1690	1700	1710	1720	1730	1740	1750	1760
TCKMVDGVGV	CTVCAKVCHK	DHEISYAKYG	SFFCDCGAKE	DGSCLALVKR	TPSSGMSSTM	KESAFQSEPR	ISESLVRHAS
1770	1780	1790	1800	1810	1820	1830	1840
TSSPADKAKV	TISDGKVADE	EKPKKSSLCR	TVEGCREELQ	NQANFSFAPL	VLDMLNFLMD	AIQTNFQQAS	AVGSSSRAQQ
1850	1860	1870	1880	1890	1900	1910	1920
ALSELHTVEK	AVEMTDQLMV	PTLGSQEGAF	ENVRMNYSGD	QGQTIRQLIS	AHVLRRVAMC	VLSSPHGRRQ	HLAVSHEKGK
1930	1940	1950	1960	1970	1980	1990	2000
ITVLQLSALL	KQADSSKRKL	TLTRLASAPV	PFTVLSLTGN	PCKEDYLAVC	GLKDCHVLTF	SSSGSVSDHL	VLHPQLATGN
2010	2020	2030	2040	2050	2060	2070	2080
FIIKAVWLPG	SQTELAIVTA	DFVKIYDLCV	DALSPTFYFL	LPSSKIRDVT	FLFNEEGKNI	IVIMSSAGYI	YTQLMEEASS
2090	2100	2110	2120	2130	2140	2150	2160
AQQGPFYVTN	VLEINHEDLK	QDSNSQVAGG	GVSVYYSHVL	QMLFFSYCQG	KSFAATISRT	TLEVLQLFPI	NIKSSNGGSK
2170	2180	2190	2200	2210	2220	2230	2240

Detailed Protein Report

Cmpd.	No. of Cmpds.	m/z meas.	Δ m/z [ppm]	z	Rt [min]	Score	P	Range	Sequence	Modification
270	1	530.5999	-29.12	3	33.1	13.6	2	1777-1790	K.VADEEKPKKSSLCR.T	
1759	2	920.8290	-80.75	2	52.0	12.7	0	4469-4485	K.MAGVMAQCGGLECMLNR.L	Carbamidomethyl: 13

Detailed Protein Report

Protein 387: eukaryotic translation initiation factor 3 subunit B [Homo sapiens]

**Accession:**	gi|33239445	**Score:**	26.2
**Database:**	refseq_human(refseq_human_20140103.fasta)	**MW [kDa]:**	92.4
**Database Date:**	2015-11-30	**pI:**	4.7
		**Sequence Coverage [%]:**	2.8
		**No. of unique Peptides:**	1

Quantitation

MD:MU	**Median:** 0.46	**CV:** 0.00 %	**No. of Peptides:** 1

Alias proteins:

Accession	Name	Description
gi|83367072	refseq_human (refseq_human_20140103.fasta)	eukaryotic translation initiation factor 3 subunit B [Homo sapiens]

10	20	30	40	50	60	70	80
MQDAENVAVP	EAAEERAEPG	QQQPAAEPPP	AEGLLRPAGP	GAPEAAGTEA	SSEEVGIAEA	GPESEVRTEP	AAEAEAASGP
**90**	**100**	**110**	**120**	**130**	**140**	**150**	**160**
SESPSPPAAE	ELPGSHAEPP	VPAQGEAPGE	QARDERSDSR	AQAVSEDAGG	NEGRAAEAEP	RALENGDADE	PSFSDPEDFV
**170**	**180**	**190**	**200**	**210**	**220**	**230**	**240**
DDVSEEELLG	DVLKDRPQEA	DGIDSVIVVD	NVPQVGPDRL	EKLKNVIHKI	FSKFGKITND	FYPEEDGKTK	GYIFLEYASP
**250**	**260**	**270**	**280**	**290**	**300**	**310**	**320**
AHAVDAVKNA	DGYKLDKQHT	FRVNLFTDFD	KYMTISDEWD	IPEKQPFKDL	GNLRYWLEEA	ECRDQYSVIF	ESGDRTSIFW
**330**	**340**	**350**	**360**	**370**	**380**	**390**	**400**
NDVKDPVSIE	ERARWTETYV	RWSPKGTYLA	TFHQRGIALW	GGEKFKQIQR	FSHQGVQLID	FSPCERYLVT	FSPLMDTQDD
**410**	**420**	**430**	**440**	**450**	**460**	**470**	**480**
PQAIIIWDIL	TGHKKRGFHC	ESSAHWPIFK	WSHDGKFFAR	MTLDTLSIYE	TPSMGLLDKK	SLKISGIKDF	SWSPGGNIIA
**490**	**500**	**510**	**520**	**530**	**540**	**550**	**560**
FWVPEDKDIP	ARVTLMQLPT	RQEIRVRNLF	NVVDCKLHWQ	KNGDYLCVKV	DRTPKGTQGV	VTNFEIFRMR	EKQVPVDVVE
**570**	**580**	**590**	**600**	**610**	**620**	**630**	**640**
MKETIIAFAW	EPNGSKFAVL	HGEAPRISVS	FYHVKNNGKI	ELIKMFDKQQ	ANTIFWSPQG	QFVVLAGLRS	MNGALAFVDT
**650**	**660**	**670**	**680**	**690**	**700**	**710**	**720**
SDCTVMNIAE	HYMASDVEWD	PTGRYVVTSV	SWWSHKVDNA	YWLWTFQGRL	LQKNNKDRFC	QLLWRPRPPT	LLSQEQIKQI
**730**	**740**	**750**	**760**	**770**	**780**	**790**	**800**
KKDLKKYSKI	FEQKDRLSQS	KASKELVERR	RTMMEDFRKY	RKMAQELYME	QKNERLELRG	GVDTDELDSN	VDDWEEETIE
**810**	**820**						
FFVTEEIIPL	GNQE						

Cmpd.	No. of Cmpds.	m/z meas.	Δ m/z [ppm]	z	Rt [min]	Score	P	Range	Sequence	Modification	Ratios
450	1	680.6717	-200.25	2	34.9	12.9	0	121-134	R.AQAVSEDAGGNEGR.A		MD:MU 0.46

Detailed Protein Report

Protein 388: metabotropic glutamate receptor 4 isoform 5 [Homo sapiens]

Accession: gi|378548216
Database: refseq_human(refseq_human_20140103.fasta)
Database Date: 2015-11-30

Score: 26.2
MW [kDa]: 83.5
pI: 9.9
Sequence Coverage [%]: 3.2
No. of unique Peptides: 2

10	20	30	40	50	60	70	80
MSCKIPQISY	ASTAPDLSDN	SRYDFFSRVV	PSDTYQAQAM	VDIVRALKWN	YVSTVASEGS	YGESGVEAFI	QKSREDGGVC
**90**	**100**	**110**	**120**	**130**	**140**	**150**	**160**
IAQSVKIPRE	PKAGEFDKII	RRLLETSNAR	AVIIFANEDD	IRRVLEAARR	ANQTGHFFWM	GSDSWGSKIA	PVLHLEEVAE
**170**	**180**	**190**	**200**	**210**	**220**	**230**	**240**
GAVTILPKRM	SVRGFDRYFS	SRTLDNNRRN	IWFAEFWEDN	FHCKLSRHAL	KKGSHVKKCT	NRERIGQDSA	YEQEGKVQFV
**250**	**260**	**270**	**280**	**290**	**300**	**310**	**320**
IDAVYAMGHA	LHAMHRDLCP	GRVGLCPRMD	PVDGTQLLKY	IRNVNFSGIA	GNPVTFNENG	DAPGRYDIYQ	YQLRNDSAEY
**330**	**340**	**350**	**360**	**370**	**380**	**390**	**400**
KVIGSWTDHL	HLRIERMHWP	GSGQQLPRSI	CSLPCQPGER	KKTVKGMPCC	WHCEPCTGYQ	YQVDRYTCKT	CPYDMRPTEN
**410**	**420**	**430**	**440**	**450**	**460**	**470**	**480**
RTGCRPIPII	KLEWGSPWAV	LPLFLAVVGI	AATLFVVITF	VRYNDTPIVK	ASGRELSYVL	LAGIFLCYAT	TFLMIAEPDL
**490**	**500**	**510**	**520**	**530**	**540**	**550**	**560**
GTCSLRRIFL	GLGMSISYAA	LLTKTNRIYR	IFEQGKRSVS	APRFISPASQ	LAITFSLISL	QLLGICVWFV	VDPSHSVVDF
**570**	**580**	**590**	**600**	**610**	**620**	**630**	**640**
QDQRTLDPRF	ARGVLKCDIS	DLSLICLLGY	SMLLMVTCTV	YAIKTRGVPE	TFNEAKPIGF	TMYTTCIVWL	AFIPIFFGTS
**650**	**660**	**670**	**680**	**690**	**700**	**710**	**720**
QSADKLYIQT	TTLTVSVSLS	ASVSLGMLYM	PKVYIILFHP	EQNVPKRKRS	LKAVVTAATM	SNKFTQKGNF	RPNGEAKSEL
**730**	**740**	**750**					
CENLEAPALA	TKQTYVTYTN	HAI					

Cmpd.	No. of Cmpds.	m/z meas.	Δ m/z [ppm]	z	Rt [min]	Score	P	Range	Sequence	Modification
2591	1	701.8584	-42.25	2	62.4	10.3	2	90-101	R.EPKAGEFDKIIR.R	
2594	1	741.8530	35.29	2	63.0	15.9	0	390-401	K.TCPYDMRPTENR.T	

Detailed Protein Report

Protein 389: NF-X1-type zinc finger protein NFXL1 [Homo sapiens]

**Accession:**	gi|89363020	**Score:**	26.2
**Database:**	refseq_human(refseq_human_20140103.fasta)	**MW [kDa]:**	101.3
**Database Date:**	2015-11-30	**pI:**	10.5
		**Sequence Coverage [%]:**	2.9
		**No. of unique Peptides:**	1

Alias proteins:

Accession	Name	Description
gi|520261838	refseq_human (refseq_human_20140103.fasta)	NF-X1-type zinc finger protein NFXL1 [Homo sapiens]
gi|520261712	refseq_human (refseq_human_20140103.fasta)	NF-X1-type zinc finger protein NFXL1 [Homo sapiens]

10	20	30	40	50	60	70	80
MEASWRQVAG	GRGRSRGRAT	AAPSGNGVHL	RGAGGGREKG	SVGAVPSGTS	PGGVATTAAA	GSRHSPAGSQ	ALQTTAASEL
90	100	110	120	130	140	150	160
MSQKKFEEIK	KANQAAARKL	VEEQFSSSSE	EGDEDFEGKQ	GKILANTFIT	YTTQTDGDTR	ELERTKQYVN	EAFQAGAMTC
170	180	190	200	210	220	230	240
LICIASVKRN	QAVWSCSGCF	CIFHMPCIQK	WAKDSQFLVS	SVTDDDFGKK	DCPWPCPKCR	FEYKRSETPS	RYYCYCGKVE
250	260	270	280	290	300	310	320
DPPLDPWLVP	HSCGQVCERE	FKPPCGHKCL	LLCHPGPCPP	CPKMVTTTCY	CKKAKPIPRR	CSAKEWSCQL	PCGQKLLCGQ
330	340	350	360	370	380	390	400
HKCENPCHAG	SCQPCPRVSR	QKCVCGKKVA	ERSCASPLWH	CDQVCGKTLP	CGNHTCEQVC	HVGACGECPR	SGKRFCPCQK
410	420	430	440	450	460	470	480
SKFSLPCTED	VPTCGDSCDK	VLECGIHRCS	QRCHRGPCET	CRQEVEKHCR	CGKHTKRMPC	HKPYLCETKC	VKMRDCQKHQ
490	500	510	520	530	540	550	560
CRRKCCPGNC	PPCDQNCGRT	LGCRNHKCPS	VCHRGSCYPC	PETVDVKCNC	GNTKVTVPCG	RERTTRPPKC	KEQCSRPPTC
570	580	590	600	610	620	630	640
HHTSQEKHRC	HFGSCPPCHQ	PCQKVLEKCG	HLCPAPCHDQ	ALIKQTGRHQ	PTGPWEQPSE	PAFIQTALPC	PPCQVPIPME
650	660	670	680	690	700	710	720
CLGKHEVSPL	PCHAVGPYSC	KRVCGRILDC	QNHTCMKECH	KVTKTDGCTG	KNKAGPECLH	CEEGCSKSRP	LGCLHPCILR
730	740	750	760	770	780	790	800
CHPGECPPCV	QMLRIKCHCK	ITSLYVECRK	ITTADVNEKN	LLSCCKNQCP	KELPCGHRCK	EMCHPGECPF	NCNQKVKLRC
810	820	830	840	850	860	870	880
PCKRIKKELQ	CNKVRENQVS	IECDTTCKEM	KRKASEIKEA	EAKAALEEEK	RRQQAELEAF	ENRLKGRRKK	NRKRDEVAVE
890	900	910	920				
LSLWQKHKYY	LISVCGVVVV	VFAWYITHDV	N				

Cmpd.	No. of Cmpds.	m/z meas.	Δ m/z [ppm]	z	Rt [min]	Score	P	Range	Sequence	Modification
490	1	587.1540	-200.57	2	35.5	10.2	2	475-483	R.DCQKHQCRR.K	

Detailed Protein Report

Protein 390: **tubulin--tyrosine ligase-like protein 12 [Homo sapiens]**

Accession: gi|11056036
Database: refseq_human(refseq_human_20140103.fasta)
Database Date: 2015-11-30
Modification(s): Carbamidomethyl

Score: 26.2
MW [kDa]: 74.4
pI: 5.2
Sequence Coverage [%]: 4.7
No. of unique Peptides: 1

10	20	30	40	50	60	70	80
MEAERGPERR	PAERSSPGQT	PEEGAQALAE	FAALHGPALR	ASGVPERYWG	RLLHKLEHEV	FDAGEVFGIM	QVEEVEEEED
**90**	**100**	**110**	**120**	**130**	**140**	**150**	**160**
EAAREVRKQQ	PNPGNELCYK	VIVTRESGLQ	AAHPNSIFLI	DHAWTCRVEH	ARQQLQQVPG	LLHRMANLMG	IEFHGELPST
**170**	**180**	**190**	**200**	**210**	**220**	**230**	**240**
EAVALVLEEM	WKFNQTYQLA	HGTAEEKMPV	WYIMDEFGSR	IQHADVPSFA	TAPFFYMPQQ	VAYTLLWPLR	DLDTGEEVTR
**250**	**260**	**270**	**280**	**290**	**300**	**310**	**320**
DFAYGETDPL	IRKCMLLPWA	PTDMLDLSSC	TPEPPAEHYQ	AILEENKEKL	PLDINPVVHP	HGHIFKVYTD	VQQVASSLTH
**330**	**340**	**350**	**360**	**370**	**380**	**390**	**400**
PRFTLTQSEA	DADILFNFSH	FKDYRKLSQE	RPGVLLNQFP	CENLLTVKDC	LASIARRAGG	PEGPPWLPRT	FNLRTELPQF
**410**	**420**	**430**	**440**	**450**	**460**	**470**	**480**
VSYFQQRERW	GEDNHWICKP	WNLARSLDTH	VTKSLHSIIR	HRESTPKVVS	KYIESPVLFL	REDVGKVKFD	IRYIVLLRSV
**490**	**500**	**510**	**520**	**530**	**540**	**550**	**560**
RPLRLFVYDV	FWLRFSNRAF	ALNDLDDYEK	HFTVMNYDPD	VVLKQVHCEE	FIPEFEKQYP	EFPWTDVQAE	IFRAFTELFQ
**570**	**580**	**590**	**600**	**610**	**620**	**630**	**640**
VACAKPPPLG	LCDYPSSRAM	YAVDLMLKWD	NGPDGRRVMQ	PQILEVNFNP	DCERACRYHP	TFFNDVFSTL	FLDQPGGCHV
**650**							
TCLV							

Cmpd.	No. of Cmpds.	m/z meas.	Δ m/z [ppm]	z	Rt [min]	Score	P	Range	Sequence	Modification
1692	1	696.9748	-35.52	3	50.9	10.5	0	598-614	R.VMQPQILEVNFNPDCER.A	Carbamidomethyl: 15

Detailed Protein Report

Protein 391: **multiple epidermal growth factor-like domains protein 8 isoform 2 precursor [Homo sapiens]**

**Accession:**	gi|145701025	**Score:**	26.1
**Database:**	refseq_human(refseq_human_20140103.fasta)	**MW [kDa]:**	295.7
**Database Date:**	2015-11-30	**pI:**	6.5
**Modification(s):**	Carbamidomethyl, Oxidation	**Sequence Coverage [%]:**	1.8
		**No. of unique Peptides:**	2

Detailed Protein Report

10	20	30	40	50	60	70	80
MALGKVLAMA	LVLALAVLGS	LSPGARAGDC	KGQRQVLREA	PGFVTDGAGN	YSVNGNCEWL	IEAPSPQHRI	LLDFLFLDTE
90	100	110	120	130	140	150	160
CTYDYLFVYD	GDSPRGPLLA	SLSGSTRPPP	IEASSGKMLL	HLFSDANYNL	LGFNASFRFS	LCPGGCQSHG	QCQPPGVCAC
170	180	190	200	210	220	230	240
EPGWGGPDCG	LQECSAYCGS	HGTCASPLGP	CRCEPGFLGR	ACDLHLWENQ	GAGWWHNVSA	RDPAFSARIG	AAGAFLSPPG
250	260	270	280	290	300	310	320
LLAVFGGQDL	NNALGDLVLY	NFSANTWESW	DLSPAPAARH	SHVAVAWAGS	LVLMGGELAD	GSLTNDVWAF	SPLGRGHWEL
330	340	350	360	370	380	390	400
LAPPASSSSG	PPGLAGHAAA	LVDDVWLYVS	GGRTPHDLFS	SGLFRFRLDS	TSGGYWEQVI	PAGGRPPAAT	GHSMVFHAPS
410	420	430	440	450	460	470	480
RALLVHGGHR	PSTARFSVRV	NSTELFHVDR	HVWTTLKGRD	GLQGPRERAF	HTASVLGNYM	VVYGGNVHTH	YQEEKCYEDG
490	500	510	520	530	540	550	560
IFFYHLGCHQ	WVSGAELAPP	GTPEGRAAPP	SGRYSHVAAV	LGGSVLLVAG	GYSGRPRGDL	MAYKVPPFVF	QAPAPDYHLD
570	580	590	600	610	620	630	640
YCSMYTDHSV	CSRDPECSWC	QGACQAAPPP	GTPLGACPAA	SCLGLGRLLG	DCQACLAFSS	PTAPPRGPGT	LGWCVHNESC
650	660	670	680	690	700	710	720
LPRPEQARCR	GEQISGTVGW	WGPAPVFVTS	LEACVTQSFL	PGLHLLTFQQ	PPNTSQPDKE	EVGRWVAHQE	KETRRLQRPG
730	740	750	760	770	780	790	800
SARLFPLPGR	DHKYAVEIQG	QLNGSAGPGH	SELTLLWDRT	GVPGGSEISF	FFLEPYRSSS	CTSYSSCLGC	LADQGCGWCL
810	820	830	840	850	860	870	880
TSATCHLRQG	GAHCGDDGAG	GSLLVLVPTL	CPLCEEHRDC	HACTQDPFCE	WHQSTSRKGD	AACSRRGRGR	GALKSPEECP
890	900	910	920	930	940	950	960
PLCSQRLTCE	DCLANSSQCA	WCQSTHTCFL	FAAYLARYPH	GGCRGWDDSV	HSEPRCRSCD	GFLTCHECLQ	SHECGWCGNE
970	980	990	1000	1010	1020	1030	1040
DNPTLGRCLQ	GDFSGPLGGG	NCSLWVGEGL	GLPVALPARW	AYARCPDVDE	CRLGLARCHP	RATCLNTPLS	YECHCQRGYQ
1050	1060	1070	1080	1090	1100	1110	1120
GDGISHCNRT	CLEDCGHGVC	SGPPDFTCVC	DLGWTSDLPP	PTPAPGPPAP	RCSRDCGCSF	HSHCRKRGPG	FCDECQDWTW
1130	1140	1150	1160	1170	1180	1190	1200
GEHCERCRPG	SFGNATGSRG	CRPCQCNGHG	DPRRGHCDNL	SGLCFCQDHT	EGAHCQLCSP	GYYGDPRAGG	SCFRECGGRA
1210	1220	1230	1240	1250	1260	1270	1280
LLTNVSSVAL	GSRRVGGLLP	PGGGAARAGP	GLSYCVWVVS	ATEELQPCAP	GTLCPPLTLT	FSPDSSTPCT	LSYVLAFDGF
1290	1300	1310	1320	1330	1340	1350	1360
PRFLDTGVVQ	SDRSLIAAFC	GQRRDRPLTV	QALSGLLVLH	WEANGSSSWG	FNASVGSARC	GSGGPGSCPV	PQECVPQDGA
1370	1380	1390	1400	1410	1420	1430	1440
AGAGLCRCPQ	GWAGPHCRMA	LCPENCNAHT	GAGTCNQSLG	VCICAEGFGG	PDCATKLDGG	QLVWETLMDS	RLSADTASRF
1450	1460	1470	1480	1490	1500	1510	1520
LHRLGHTMVD	GPDATLWMFG	GLGLPQGLLG	NLYRYSVSER	RWTQMLAGAE	DGGPGPSPRS	FHAAAYVPAG	RGAMYLLGGL
1530	1540	1550	1560	1570	1580	1590	1600
TAGGVTRDFW	VLNLTTLQWR	QEKAPQTVEL	PAVAGHTLTA	RRGLSLLLVG	GYSPENGFNQ	QLLEYQLATG	TWVSGAQSGT
1610	1620	1630	1640	1650	1660	1670	1680
PPTGLYGHSA	VYHEATDSLY	VFGGFRFHVE	LAAPSPELYS	LHCPDRTWSL	LAPSQGAKRD	RMRNVRGSSR	GLGQVPGEQP
1690	1700	1710	1720	1730	1740	1750	1760
GSWGFREVRK	KMALWAALAG	TGGFLEEISP	HLKEPRPRLF	HASALLGDTM	VVLGGRSDPD	EFSSDVLLYQ	VNCNAWLLPD
1770	1780	1790	1800	1810	1820	1830	1840
LTRSASVGPP	MEESVAHAVA	AVGSRLYISG	GFGGVALGRL	LALTLPPDPC	RLLSSPEACN	QSGACTWCHG	ACLSGDQAHR
1850	1860	1870	1880	1890	1900	1910	1920
LGCGGSPCSP	MPRSPEECRR	LRTCSECLAR	HPRTLQPGDG	EASTPRCKWC	TNCPEGACIG	RNGSCTSEND	CRINQREVFW
1930	1940	1950	1960	1970	1980	1990	2000
AGNCSEAACG	AADCEQCTRE	GKCMWTRQFK	RTGETRRILS	VQPTYDWTCF	SHSLLNVSPM	PVESSPPLPC	PTPCHLLPNC
2010	2020	2030	2040	2050	2060	2070	2080
TSCLDSKGAD	GGWQHCVWSS	SLQQCLSPSY	LPLRCMAGGC	GRLLRGPESC	SLGCAQATQC	ALCLRRPHCG	WCAWGGQDGG
2090	2100	2110	2120	2130	2140	2150	2160
GRCMEGGLSG	PRDGLTCGRP	GASWAFLSCP	PEDECANGHH	DCNETQNCHD	QPHGYECSCK	TGYTMDNMTG	LCRPVCAQGC
2170	2180	2190	2200	2210	2220	2230	2240

Detailed Protein Report

Cmpd.	No. of Cmpds.	m/z meas.	Δ m/z [ppm]	z	Rt [min]	Score	P	Range	Sequence	Modification
1619	1	1023.8271	-75.44	3	50.1	15.1	2	1-31	-.MALGKVLAMALVLALAVLGSLSPGARAGDCK.G	Carbamidomethyl: 30; Oxidation: 1
112	1	668.6652	6.60	3	31.1	11.1	0	608-626	R.LLGDCQACLAFSSPTAPPR.G	Carbamidomethyl: 8

Detailed Protein Report

Protein 392: PREDICTED: dynamin-binding protein isoform X1 [Homo sapiens]

**Accession:**	gi|578819186	**Score:**	26.1
**Database:**	refseq_human(refseq_human_20140103.fasta)	**MW [kDa]:**	172.2
**Database Date:**	2015-11-30	**pI:**	5.1
		**Sequence Coverage [%]:**	2.0
		**No. of unique Peptides:**	1

10	20	30	40	50	60	70	80
MEAGSVVRAI	FDFCPSVSEE	LPLFVGDIIE	VLAVVDEFWL	LGKKEDVTGQ	FPSSFVEIVT	IPSLKEGERL	FVCICEFTSQ
90	100	110	120	130	140	150	160
ELDNLPLHRG	DLVILDGIPT	AGWLQGRSCW	GARGFFPSSC	VRELCLSSQS	RQWHSQSALF	QIPEYSMGQA	RALMGLSAQL
170	180	190	200	210	220	230	240
DEELDFREGD	VITIIGVPEP	GWFEGELEGR	RGIFPEGFVE	LLGPLRTVDE	SVSSGNQDDC	IVNGEVDTPV	GEEEIGPDED
250	260	270	280	290	300	310	320
EEEPGTYGVA	LYRFQALEPN	ELDFEVGDKI	RILATLEDGW	LEGSLKGRTG	IFPYRFVKLC	PDTRVEETMA	LPQEGSLARI
330	340	350	360	370	380	390	400
PETSLDCLEN	TLGVEEQRHE	TSDHEAEEPD	CIISEAPTSP	LGHLTSEYDT	DRNSYQDEDT	AGGPPRSPGV	EWEMPLATDS
410	420	430	440	450	460	470	480
PTSDPTEVVN	GISSQPQVPF	HPNLQKSQYY	STVGGSHPHS	EQYPDLLPLE	ARTRDYASLP	PKRMYSQLKT	LQKPVLPLYR
490	500	510	520	530	540	550	560
GSSVSASRVV	KPRQSSPQLH	NLASYTKKHH	TSSVYSISER	LEMKPGPQAQ	GLVMEAATHS	QGDGSTDLDS	KLTQQLIEFE
570	580	590	600	610	620	630	640
KSLAGPGTEP	DKILRHFSIM	DFNSEKDIVR	GSSKLITEQE	LPERRKALRP	PPPRPCTPVS	TSPHLLVDQN	LKPAPPLVVR
650	660	670	680	690	700	710	720
PSRPAPLPPS	AQQRTNAVSP	KLLSRHRPTC	ETLEKEGPGH	MGRSLDQTSP	CPLVLVRIEE	MERDLDMYSR	AQEELNLMLE
730	740	750	760	770	780	790	800
EKQDESSRAE	TLEDLKFCES	NIESLNMELQ	QLREMTLLSS	QSSSLVAPSG	SVSAENPEQR	MLEKRAKVIE	ELLQTERDYI
810	820	830	840	850	860	870	880
RDLEMCIERI	MVPMQQAQVP	NIDFEGLFGN	MQMVIKVSKQ	LLAALEISDA	VGPVFLGHRD	ELEGTYKIYC	QNHDEAIALL
890	900	910	920	930	940	950	960
EIYEKDEKIQ	KHLQDSLADL	KSLYNEWGCT	NYINLGSFLI	KPVQRVMRYP	LLLMELLNST	PESHPDKVPL	TNAVLAVKEI
970	980	990	1000	1010	1020	1030	1040
NVNINEYKRR	KDLVLKYRKG	DEDSLMEKIS	KLNIHSIIKK	SNRVSSHLKH	LTGFAPQIKD	EVFEETEKNF	RMQERLIKSF
1050	1060	1070	1080	1090	1100	1110	1120
IRDLSLYLQH	IRKERTERLV	ISPLNQLLSM	FTGPHKLVQK	RFDKLLDFYN	CTERAEKLKD	KKTLEELQSA	RNNYEALNAQ
1130	1140	1150	1160	1170	1180	1190	1200
LLDELPKFHQ	YAQGLFTNCV	HGYAEAHCDF	VHQALEQLKP	LLSLLKVAGR	EGNLIAIFHE	EHSRVLQQLQ	VFTFFPESLP
1210	1220	1230	1240	1250	1260	1270	1280
ATKKPFERKT	IDRQSARKPL	LGLPSYMLQS	EELRASLLAR	YPPEKLFQAE	RNFNAAQDLD	VSLLEGDLVG	VIKKKDPMGS
1290	1300	1310	1320	1330	1340	1350	1360
QNRWLIDNGV	TKGFVYSSFL	KPYNPRRSHS	DASVGSHSST	ESEHGSSSPR	FPRQNSGSTL	TFNPSSMAVS	FTSGSCQKQP
1370	1380	1390	1400	1410	1420	1430	1440
QDASPPPKEC	DQGTLSASLN	PSNSESSPSR	CPSDPDSTSQ	PRSGDSADVA	RDVKQPTATP	RSYRNFRHPE	IVGYSVPGRN
1450	1460	1470	1480	1490	1500	1510	1520
GQSQDLVKGC	ARTAQAPEDR	STEPDGSEAE	GNQVYFAVYT	FKARNPNELS	VSANQKLKIL	EFKDVTGNTE	WWLAEVNGKK
1530	1540						
GYVPSNYIRK	TEYT						

Cmpd.	No. of Cmpds.	m/z meas.	Δ m/z [ppm]	z	Rt [min]	Score	P	Range	Sequence	Modification
2669	1	995.0095	-40.18	2	63.5	15.5	2	1235-1251	R.ASLLARYPPEKLFQAER.N	

Detailed Protein Report

Protein 393: PREDICTED: rootletin isoform X5 [Homo sapiens]

**Accession:**	gi|578800146	**Score:**	26.1
**Database:**	refseq_human(refseq_human_20140103.fasta)	**MW [kDa]:**	196.6
**Database Date:**	2015-11-30	**pI:**	5.4
		**Sequence Coverage [%]:**	1.3
		**No. of unique Peptides:**	2

10	20	30	40	50	60	70	80
MVFLPSLLGG	GCPTGPRSQP	CAHCPLPVLL	PATEMASLLS	LQEENQLLQQ	ELSRVEDLLA	QSRAERDELA	IKYNAVSERL
90	100	110	120	130	140	150	160
EQALRLEPGE	LETQEPRGLV	RQSVELRRQL	QEEQASYRRK	LQAYQEGQQR	QAQLVQRLQG	KILQYKKRCS	ELEQQLLERS
170	180	190	200	210	220	230	240
GELEQQRLRD	TEHSQDLESA	LIRLEEEQQR	SASLAQVNAM	LREQLDQAGS	ANQALSEDIR	KVTNDWTRCR	KELEHREAAW
250	260	270	280	290	300	310	320
RREEESFNAY	FSNEHSRLLL	LWRQVVGFRR	LVSEVKMFTE	RDLLQLGGEL	ARTSRAVQEA	GLGLSTGLRL	AESRAEAALE
330	340	350	360	370	380	390	400
KQALLQAQLE	EQLRDKVLRE	KDLAQQQMQS	DLDKADLSAR	VTELGLAVKR	LEKQNLEKDQ	VNKDLTEKLE	ALESLRLQEQ
410	420	430	440	450	460	470	480
AALETEDGEG	LQQTLRDLAQ	AVLSDSESGV	QLSGSERTAD	ASNGSLRGLS	GQRTPSPPRR	SSPGRGRSPR	RGPSPACSDS
490	500	510	520	530	540	550	560
STLALIHSAL	HKRQLQVQDM	RGRYEASQDL	LGTLRKQLSD	SESERRALEE	QLQRLRDKTD	GAMQAHEDAQ	REVQRLRSAN
570	580	590	600	610	620	630	640
ELLSREKSNL	AHSLQVAQQQ	AEELRQEREK	LQAAQEELRR	QRDRLEEEQE	DAVQDGARVR	RELERSHRQL	EQLEGKRSVL
650	660	670	680	690	700	710	720
AKELVEVREA	LSRATLQRDM	LQAEKAEVAE	ALTKAEAGRV	ELELSMTKLR	AEEASLQDSL	SKLSALNESL	AQDKLDLNRL
730	740	750	760	770	780	790	800
VAQLEEEKSA	LQGRQRQAEQ	EATVAREEQE	RLEELRLEQE	VARQGLEGSL	RVAEQAQEAL	EQQLPTLRHE	RSQLQEQLAQ
810	820	830	840	850	860	870	880
LSRQLSGREQ	ELEQARREAQ	RQVEALERAA	REKEALAKEH	AGLAVQLVAA	EREGRTLSEE	ATRLRLEKEA	LEGSLFEVQR
890	900	910	920	930	940	950	960
QLAQLEARRE	QLEAEGQALL	LAKETLTGEL	AGLRQQIIAT	QEKASLDKEL	MAQKLVQAER	EAQASLREQR	AAHEEDLQRL
970	980	990	1000	1010	1020	1030	1040
QREKEAAWRE	LEAERAQLQS	QLQREQEELL	ARLEAEKEEL	SEEIAALQQE	RDEGLLLAES	EKQQALSLKE	SEKTALSEKL
1050	1060	1070	1080	1090	1100	1110	1120
MGTRHSLATI	SLEMERQKRD	AQSRQEQDRS	TVNALTSELR	DLRAQREEAA	AAHAQEVRRL	QEQARDLGKQ	RDSCLREAEE
1130	1140	1150	1160	1170	1180	1190	1200
LRTQLRLLED	ARDGLRRELL	EAQRKLRESQ	EGREVQRQEA	GELRRSLGEG	AKEREALRRS	NEELRSAVKK	AESERISLKL
1210	1220	1230	1240	1250	1260	1270	1280
ANEDKEQKLA	LLEEARTAVG	KEAGELRTGL	QEVERSRLEA	RRELQELRRQ	MKMLDSENTR	LGRELAELQG	RLALGERAEK
1290	1300	1310	1320	1330	1340	1350	1360
ESRRETLGLR	QRLLKGEASL	EVMRQELQVA	QRKLQEQEGE	FRTRERRLLG	SLEEARGTEK	QQLDHARGLE	LKLEAARAEA
1370	1380	1390	1400	1410	1420	1430	1440
AELGLRLSAA	EGRAQGLEAE	LARVEVQRRA	AEAQLGGLRS	ALRRGLGLGR	APSPAPRPVP	GSPARDAPAE	GSGEGLNSPS
1450	1460	1470	1480	1490	1500	1510	1520
TLECSPGSQP	PSPGPATSPA	SPDLDPEAVR	GALREFLQEL	RSAQRERDEL	RTQTSALNRQ	LAEMEAERDS	ATSRARQLQK
1530	1540	1550	1560	1570	1580	1590	1600
AVAESEEARR	SVDGRLSGVQ	AELALQEESV	RRSERERRAT	LDQVATLERS	LQATESELRA	SQEKISKMKA	NETKLEGDKR
1610	1620	1630	1640	1650	1660	1670	1680
RLKEVLDASE	SRTVKLELQR	RSLEGELQRS	RLGLSDREAQ	AQALQDRVDS	LQRQVADSEV	KAGTLQLTVE	RLNGALAKVE
1690	1700	1710	1720	1730	1740		
ESEGALRDKV	RGLTEALAQS	SASLNSTRDK	NLHLQKALTA	CEHDRQVLQR	YRIERTP		

Cmpd.	No. of Cmpds.	m/z meas.	Δ m/z [ppm]	z	Rt [min]	Score	P	Range	Sequence	Modification
427	1	457.6259	-312.42	2	34.7	12.4	0	1209-1216	K.LALLEEAR.T	

Detailed Protein Report

Cmpd.	No. of Cmpds.	m/z meas.	Δ m/z [ppm]	z	Rt [min]	Score	P	Range	Sequence	Modification
2749	1	863.9191	-72.24	2	64.7	13.7	2	1616-1629	K.LELQRRSLEGELQR.S	

Detailed Protein Report

Protein 394: nitric oxide synthase, inducible [Homo sapiens]

**Accession:**	gi|24041029	**Score:**	26.1
**Database:**	refseq_human(refseq_human_20140103.fasta)	**MW [kDa]:**	131.0
**Database Date:**	2015-11-30	**pI:**	9.1
**Modification(s):**	Carbamidomethyl, Oxidation	**Sequence Coverage [%]:**	2.3
		**No. of unique Peptides:**	2

10	20	30	40	50	60	70	80
MACPWKFLFK	TKFHQYAMNG	EKDINNNVEK	APCATSSPVT	QDDLQYHNLS	KQQNESPQPL	VETGKKSPES	LVKLDATPLS
90	100	110	120	130	140	150	160
SPRHVRIKNW	GSGMTFQDTL	HHKAKGILTC	RSKSCLGSIM	TPKSLTRGPR	DKPTPPDELL	PQAIEFVNQY	YGSFKEAKIE
170	180	190	200	210	220	230	240
EHLARVEAVT	KEIETTGTYQ	LTGDELIFAT	KQAWRNAPRC	IGRIQWSNLQ	VFDARSCSTA	REMFEHICRH	VRYSTNNGNI
250	260	270	280	290	300	310	320
RSAITVFPQR	SDGKHDFRVW	NAQLIRYAGY	QMPDGSIRGD	PANVEFTQLC	IDLGWKPKYG	RFDVVPLVLQ	ANGRDPELFE
330	340	350	360	370	380	390	400
IPPDLVLEVA	MEHPKYEWFR	ELELKWYALP	AVANMLLEVG	GLEFPGCPFN	GWYMGTEIGV	RDFCDVQRYN	ILEEVGRRMG
410	420	430	440	450	460	470	480
LETHKLASLW	KDQAVVEINI	AVLHSFQKQN	VTIMDHHSAA	ESFMKYMQNE	YRSRGGCPAD	WIWLVPPMSG	SITPVFHQEM
490	500	510	520	530	540	550	560
LNYVLSPFYY	YQVEAWKTHV	WQDEKRRPKR	REIPLKVLVK	AVLFACMLMR	KTMASRVRVT	ILFATETGKS	EALAWDLGAL
570	580	590	600	610	620	630	640
FSCAFNPKVV	CMDKYRLSCL	EEERLLLVVT	STFGNGDCPG	NGEKLKKSLF	MLKELNNKFR	YAVFGLGSSM	YPRFCAFAHD
650	660	670	680	690	700	710	720
IDQKLSHLGA	SQLTPMGEGD	ELSGQEDAFR	SWAVQTFKAA	CETFDVRGKQ	HIQIPKLYTS	NVTWDPHHYR	LVQDSQPLDL
730	740	750	760	770	780	790	800
SKALSSMHAK	NVFTMRLKSR	QNLQSPTSSR	ATILVELSCE	DGQGLNYLPG	EHLGVCPGNQ	PALVQGILER	VVDGPTPHQT
810	820	830	840	850	860	870	880
VRLEALDESG	SYWVSDKRLP	PCSLSQALTY	FLDITTPPTQ	LLLQKLAQVA	TEEPERQRLE	ALCQPSEYSK	WKFTNSPTFL
890	900	910	920	930	940	950	960
EVLEEFPSLR	VSAGFLLSQL	PILKPRFYSI	SSSRDHTPTE	IHLTVAVVTY	HTRDGQGPLH	HGVCSTWLNS	LKPQDPVPCF
970	980	990	1000	1010	1020	1030	1040
VRNASGFHLP	EDPSHPCILI	GPGTGIAPFR	SFWQQRLHDS	QHKGVRGGRM	TLVFGCRRPD	EDHIYQEEML	EMAQKGVLHA
1050	1060	1070	1080	1090	1100	1110	1120
VHTAYSRLPG	KPKVYVQDIL	RQQLASEVLR	VLHKEPGHLY	VCGDVRMARD	VAHTLKQLVA	AKLKLNEEQV	EDYFFQLKSQ
1130	1140	1150	1160				
KRYHEDIFGA	VFPYEAKKDR	VAVQPSSLEM	SAL				

Cmpd.	No. of Cmpds.	m/z meas.	Δ m/z [ppm]	z	Rt [min]	Score	P	Range	Sequence	Modification
2024	1	687.3070	-6.35	2	55.4	12.0	0	267-278	R.YAGYQMPDGSIR.G	Oxidation: 6
2767	1	841.9071	-64.44	2	65.0	14.1	1	517-530	K.VLVKAVLFACMLMR.K	Carbamidomethyl: 10; Oxidation: 11, 13

Detailed Protein Report

Protein 395: PREDICTED: ankyrin repeat domain-containing protein 11 isoform X4 [Homo sapiens]

**Accession:**	gi|578828959	**Score:**	26.1
**Database:**	refseq_human(refseq_human_20140103.fasta)	**MW [kDa]:**	286.8
**Database Date:**	2015-11-30	**pI:**	6.4
**Modification(s):**	Oxidation	**Sequence Coverage [%]:**	1.1
		**No. of unique Peptides:**	1

10	20	30	40	50	60	70	80
MGLSGIRAGY	PLSERQQVAL	LMQMTAEESA	NSPVDTTPKH	PSQSTVCQKG	TPNSASKTKD	KVNKRNERGE	TRLHRAAIRG
90	100	110	120	130	140	150	160
DARRIKELIS	EGADVNVKDF	AGWTALHEAC	NRGYYDVAKQ	LLAAGAEVNT	KGLDDDTPLH	DAANNGHYKV	VKLLLRYGGN
170	180	190	200	210	220	230	240
PQQSNRKGET	PLKVANSPTM	VNLLLGKGTY	TSSEESSTES	SEEEDAPSFA	PSSSVDGNNT	DSEFEKGLKH	KAKNPEPQKA
250	260	270	280	290	300	310	320
TAPVKDEYEF	DEDDEQDRVP	PVDDKHLLKK	DYRKETKSNS	FISIPKMEVK	SYTKNNTIAP	KKASHRILSD	TSDEEDASVT
330	340	350	360	370	380	390	400
VGTGEKLRLS	AHTILPGSKT	REPSNAKQQK	EKNKVKKKRK	KETKGREVRF	GKRSDKFCSS	ESESESSESG	EDDRDSLGSS
410	420	430	440	450	460	470	480
GCLKGSPLVL	KDPSLFSSLS	ASSTSSHGSS	AAQKQNPSHT	DQHTKHWRTD	NWKTISSPAW	SEVSSLSDST	RTRLTSESDY
490	500	510	520	530	540	550	560
SSEGSSVESL	KPVRKRQEHR	KRASLSEKKS	PFLSSAEGAV	PKLDKEGKVV	KKHKTKHKHK	NKEKGQCSIS	QELKLKSFTY
570	580	590	600	610	620	630	640
EYEDSKQKSD	KAILLENDLS	TENKLKVLKH	DRDHFKKEEK	LSKMKLEEKE	WLFKDEKSLK	RIKDTNKDIS	RSFREEKDRS
650	660	670	680	690	700	710	720
NKAEKERSLK	EKSPKEEKLR	LYKEERKKKS	KDRPSKLEKK	NDLKEDKISK	EKEKIFKEDK	EKLKKEKVYR	EDSAFDEYCN
730	740	750	760	770	780	790	800
KNQFLENEDT	KFSLSDDQRD	RWFSDLSDSS	FDFKGEDSWD	SPVTDYRDMK	SDSVAKLILE	TVKEDSKERR	RDSRAREKRD
810	820	830	840	850	860	870	880
YREPFFRKKD	RDYLDKNSEK	RKEQTEKHKS	VPGYLSEKDK	KRRESAEAGR	DRKDALESCK	ERRDGRAKPE	EAHREELKEC
890	900	910	920	930	940	950	960
GCESGFKDKS	DGDFGKGLEP	WERHHPAREK	EKKDGPDKER	KEKTKPERYK	EKSSDKDKSE	KSILEKCQKD	KEFDKCFKEK
970	980	990	1000	1010	1020	1030	1040
KDTKEKHKDT	HGKDKERKAS	LDQGKEKKEK	AFPGIISEDF	SEKKDDKKGK	EKSWYIADIF	TDESEDDRDS	CMGSGFKMGE
1050	1060	1070	1080	1090	1100	1110	1120
ASDLPRTDGL	QEKEEGREAY	ASDRHRKSSD	KQHPERQKDK	EPRDRRKDRG	AADAGRDKKE	KVFEKHKEKK	DKESTEKYKD
1130	1140	1150	1160	1170	1180	1190	1200
RKDRASVDST	QDKKNKQKLP	EKAEKKHAAE	DKAKSKHKEK	SDKEHSKERK	SSRSADAEKS	LLEKLEEEAL	HEYREDSNDK
1210	1220	1230	1240	1250	1260	1270	1280
ISEVSSDSFT	DRGQEPGLTA	FLEVSFTEPP	GDDKPRESAC	LPEKLKEKER	HRHSSSSSKK	SHDRERAKKE	KAEKKEKGED
1290	1300	1310	1320	1330	1340	1350	1360
YKEGGSRKDS	GQYEKDFLEA	DAYGVSYNMK	ADIEDELDKT	IELFSTEKKD	KNDSEREPSK	KIEKELKPYG	SSAINILKEK
1370	1380	1390	1400	1410	1420	1430	1440
KKREKHREKW	RDEKERHRDR	HADGLLRHHR	DELLRHHRDE	QKPATRDKDS	PPRVLKDKSR	DEGPRLGDAK	LKEKFKDGAE
1450	1460	1470	1480	1490	1500	1510	1520
KEKGDPVKMS	NGNDKVAPSK	DPGKKDARPR	EKLLGDGDLM	MTSFERMLSQ	KDLEIEERHK	RHKERMKQME	KLRHRSGDPK
1530	1540	1550	1560	1570	1580	1590	1600
LKEKAKPADD	GRKKGLDIPA	KKPPGLDPPF	KDKKLKESTP	IPPAAENKLH	PASGADSKDW	LAGPHMKEVL	PASPRPDQSR
1610	1620	1630	1640	1650	1660	1670	1680
PTGVPTPTSV	LSCPSYEEVM	HTPRTPSCSA	DDYADLVFDC	ADSQHSTPVP	TAPTSACSPS	FFDRFSVASS	GLSENASQAP
1690	1700	1710	1720	1730	1740	1750	1760
ARPLSTNLYR	SVSVDIRRTP	EEEFSVGDKL	FRQQSVPAAS	SYDSPMPPSM	EDRAPLPPVP	AEKFACLSPG	YYSPDYGLPS
1770	1780	1790	1800	1810	1820	1830	1840
PKVDALHCPP	AAVVTVTPSP	EGVFSSLQAK	PSPSPRAELL	VPSLEGALPP	DLDTSEDQQA	TAAIIPPEPS	YLEPLDEGPF
1850	1860	1870	1880	1890	1900	1910	1920
SAVITEEPVE	WAHPSEQALA	SSLIGGTSEN	PVSWPVGSDL	LLKSPQRFPE	SPKRFCPADP	LHSAAPGPFS	ASEAPYPAPP
1930	1940	1950	1960	1970	1980	1990	2000
ASPAPYALPV	AEPGLEDVKD	GVDAVPAAIS	TSEAAPYAPP	SGLESFFSNC	KSLPEAPLDV	APEPACVAAV	AQVEALGPLE
2010	2020	2030	2040	2050	2060	2070	2080
NSFLDGSRGL	SHLGQVEPVP	WADAFAGPED	DLDLGPFSLP	ELPLQTKDAA	DGEAEPVEES	LAPPEEMPPG	APGVINGGDV
2090	2100	2110	2120	2130	2140	2150	2160
STVVAEEPPA	LPPDQASTRL	PAELEPEPSG	EPKLDVALEA	AVEAETVPEE	RARGDPDSSV	EPAPVPPEQR	PLGSGDQGAE
2170	2180	2190	2200	2210	2220	2230	2240

Detailed Protein Report

Cmpd.	No. of Cmpds.	m/z meas.	Δ m/z [ppm]	z	Rt [min]	Score	P	Range	Sequence	Modification
1507	1	619.9684	9.93	3	48.5	11.3	1	1471-1486	R.EKLLGDGDLMMTSFER.M	Oxidation: 11

Detailed Protein Report

Protein 396: glutamate decarboxylase 2 [Homo sapiens]

**Accession:**	gi|4503875	**Score:**	26.1
**Database:**	refseq_human(refseq_human_20140103.fasta)	**MW [kDa]:**	65.4
**Database Date:**	2015-11-30	**pI:**	6.5
**Modification(s):**	Oxidation	**Sequence Coverage [%]:**	4.1
		**No. of unique Peptides:**	1

Alias proteins:

Accession	Name	Description
gi|197276620	refseq_human (refseq_human_20140103.fasta)	glutamate decarboxylase 2 [Homo sapiens]

10	20	30	40	50	60	70	80
MASPGSGFWS	FGSEDGSGDS	ENPGTARAWC	QVAQKFTGGI	GNKLCALLYG	DAEKPAESGG	SQPPRAAARK	AACACDQKPC
90	100	110	120	130	140	150	160
SCSKVDVNYA	FLHATDLLPA	CDGERPTLAF	LQDVMNILLQ	YVVKSFDRST	KVIDFHYPNE	LLQEYNWELA	DQPQNLEEIL
170	180	190	200	210	220	230	240
MHCQTTLKYA	IKTGHPRYFN	QLSTGLDMVG	LAADWLTSTA	NTNMFTYEIA	PVFVLLEYVT	LKKMREIIGW	PGGSGDGIFS
250	260	270	280	290	300	310	320
PGGAISNMYA	MMIARFKMFP	EVKEKGMAAL	PRLIAFTSEH	SHFSLKKGAA	ALGIGTDSVI	LIKCDERGKM	IPSDLERRIL
330	340	350	360	370	380	390	400
EAKQKGFVPF	LVSATAGTTV	YGAFDPLLAV	ADICKKYKIW	MHVDAAWGGG	LLMSRKHKWK	LSGVERANSV	TWNPHKMMGV
410	420	430	440	450	460	470	480
PLQCSALLVR	EEGLMQNCNQ	MHASYLFQQD	KHYDLSYDTG	DKALQCGRHV	DVFKLWLMWR	AKGTTGFEAH	VDKCLELAEY
490	500	510	520	530	540	550	560
LYNIIKNREG	YEMVFDGKPQ	HTNVCFWYIP	PSLRTLEDNE	ERMSRLSKVA	PVIKARMMEY	GTTMVSYQPL	GDKVNFFRMV
570	580	590					
ISNPAATHQD	IDFLIEEIER	LGQDL					

Cmpd.	No. of Cmpds.	m/z meas.	Δ m/z [ppm]	z	Rt [min]	Score	P	Range	Sequence	Modification
2204	1	767.2614	-182.11	2	57.4	12.9	0	397-410	K.MMGVPLQCSALLVR.E	Oxidation: 2

Detailed Protein Report

Protein 397: PREDICTED: L-fucose kinase isoform X3 [Homo sapiens]

**Accession:**	gi|578828897	**Score:**	26.1
**Database:**	refseq_human(refseq_human_20140103.fasta)	**MW [kDa]:**	81.7
**Database Date:**	2015-11-30	**pI:**	6.4
**Modification(s):**	Carbamidomethyl	**Sequence Coverage [%]:**	1.7
		**No. of unique Peptides:**	1

10	20	30	40	50	60	70	80
MTSSASEFLL	SLTLPGAPGA	QIVHSQVEEQ	QLLAAGSSVV	SCLLEGPVQL	GPGSVLQHCH	LQGPIHIGAG	CLVTGLDTAH
**90**	**100**	**110**	**120**	**130**	**140**	**150**	**160**
SKALHGRELR	DLVLQGHHTR	LHGSPGHAFT	LVGRLDSWER	QGAGTYLNVP	WSEFFKRTGV	RAWDLWDPET	LPAEYCLPSA
**170**	**180**	**190**	**200**	**210**	**220**	**230**	**240**
RLFPVLHPSR	ELGPQDLLWM	LDHQEDGGEA	LRAWRASWRL	SWEQLQPCLD	RAATLASRRD	LFFRQALHKA	RHVLEARQDL
**250**	**260**	**270**	**280**	**290**	**300**	**310**	**320**
SLRPLIWAAV	REGCPGPLLA	TLDQVAAGAG	DPGVAARALA	CVADVLGCMA	EGRGGLRSGP	AANPEWMRPF	SYLECGDLAA
**330**	**340**	**350**	**360**	**370**	**380**	**390**	**400**
GVEALAQERD	KWLSRPALLV	RAARHYEGAG	QILIRQAVMS	AQHFVSTEQV	ELPGPGQWVV	AECPARVDFS	GGWSDTPPLA
**410**	**420**	**430**	**440**	**450**	**460**	**470**	**480**
YELGGAVLGL	AVRVDGRRPI	GARARRIPEP	ELWLAVGPRQ	DEMTVKIVCR	CLADLRDYCQ	PHAPGALLKA	AFICAGIVHV
**490**	**500**	**510**	**520**	**530**	**540**	**550**	**560**
HSELQLSEQL	LRTFGGGFEL	HTWSELPHGS	GLGTSSILAG	TALAALQRAA	GRVVGTEALI	HAVLHLEQVL	TTGGGWQDQV
**570**	**580**	**590**	**600**	**610**	**620**	**630**	**640**
GGLMPGIKVG	RSRAQLPLKV	EVEEVTVPEG	FVQKLNDHLL	LVYTGKTRLA	RNLLQDVLRS	WYARLPAVVQ	NAHSLVRQTE
**650**	**660**	**670**	**680**	**690**	**700**	**710**	**720**
ECAEGFRQGS	LPLLGQCLTS	YWEQKKLMAP	GCEPLTVRRM	MDVLAPHVHG	QSLAGAGGGG	FLYLLTKEPQ	QKEALEAVLA
**730**	**740**	**750**	**760**				
KTEGLGNYSI	HLVEVDTQGL	SLKLLGTEAS	TCCPFP				

Cmpd.	No. of Cmpds.	m/z meas.	Δ m/z [ppm]	z	Rt [min]	Score	P	Range	Sequence	Modification
2085	1	698.2938	-34.34	2	56.0	13.4	0	744-756	K.LLGTEASTCCPFP.-	Carbamidomethyl: 9

Detailed Protein Report

Protein 398: nuclear receptor corepressor 1 isoform 1 [Homo sapiens]

**Accession:**	gi|22538461	**Score:**	26.0
**Database:**	refseq_human(refseq_human_20140103.fasta)	**MW [kDa]:**	270.0
**Database Date:**	2015-11-30	**pI:**	6.7
		**Sequence Coverage [%]:**	1.2
		**No. of unique Peptides:**	1

BRUKER

Detailed Protein Report

10	20	30	40	50	60	70	80
MSSSGYPPNQ	GAFSTEQSRY	PPHSVQYTFP	NTRHQQEFAV	PDYRSSHLEV	SQASQLLQQQ	QQQQLRRRPS	LLSEFHPGSD
90	100	110	120	130	140	150	160
RPQERRTSYE	PFHPGPSPVD	HDSLESKRPR	LEQVSDSHFQ	RVSAAVLPLV	HPLPEGLRAS	ADAKKDPAFG	GKHEAPSSPI
170	180	190	200	210	220	230	240
SGQPCGDDQN	ASPSKLSKEE	LIQSMDRVDR	EIAKVEQQIL	KLKKKQQQLE	EEAAKPPEPE	KPVSPPPVEQ	KHRSIVQIIY
250	260	270	280	290	300	310	320
DENRKKAEEA	HKIFEGLGPK	VELPLYNQPS	DTKVYHENIK	TNQVMRKKLI	LFFKRRNHAR	KQREQKICQR	YDQLMEAWEK
330	340	350	360	370	380	390	400
KVDRIENNPR	RKAKESKTRE	YYEKQFPEIR	KQREQQERFQ	RVGQRGAGLS	ATIARSEHEI	SEIIDGLSEQ	ENNEKQMRQL
410	420	430	440	450	460	470	480
SVIPPMMFDA	EQRRVKFINM	NGLMEDPMKV	YKDRQFMNVW	TDHEKEIFKD	KFIQHPKNFG	LIASYLERKS	VPDCVLYYYL
490	500	510	520	530	540	550	560
TKKNENYKAL	VRRNYGKRRG	RNQQIARPSQ	EEKVEEKEED	KAEKTEKKEE	EKKDEEEKDE	KEDSKENTKE	KDKIDGTAEE
570	580	590	600	610	620	630	640
TEEREQATPR	GRKTANSQGR	RKGRITRSMT	NEAAAASAAA	AAATEEPPPP	LPPPPEPIST	EPVETSRWTE	EEMEVAKKGL
650	660	670	680	690	700	710	720
VEHGRNWAAI	AKMVGTKSEA	QCKNFYFNYK	RRHNLDNLLQ	QHKQKTSRKP	REERDVSQCE	SVASTVSAQE	DEDIEASNEE
730	740	750	760	770	780	790	800
ENPEDSEVEA	VKPSEDSPEN	ATSRGNTEPA	VELEPTTETA	PSTSPSLAVP	STKPAEDESV	ETQVNDSISA	ETAEQMDVDQ
810	820	830	840	850	860	870	880
QEHSAEEGSV	CDPPPATKAD	SVDVEVRVPE	NHASKVEGDN	TKERDLDRAS	EKVEPRDEDL	VVAQQINAQR	PEPQSDNDSS
890	900	910	920	930	940	950	960
ATCSADEDVD	GEPERQRMFP	MDSKPSLLNP	TGSILVSSPL	KPNPLDLPQL	QHRAAVIPPM	VSCTPCNIPI	GTPVSGYALY
970	980	990	1000	1010	1020	1030	1040
QRHIKAMHES	ALLEEQRQRQ	EQIDLECRSS	TSPCGTSKSP	NREWEVLQPA	PHQVITNLPE	GVRLPTTRPT	RPPPPLIPSS
1050	1060	1070	1080	1090	1100	1110	1120
KTTVASEKPS	FIMGGSISQG	TPGTYLTSHN	QASYTQETPK	PSVGSISLGL	PRQQESAKSA	TLPYIKQEEF	SPRSQNSQPE
1130	1140	1150	1160	1170	1180	1190	1200
GLLVRAQHEG	VVRGTAGAIQ	EGSITRGTPT	SKISVESIPS	LRGSITQGTP	ALPQTGIPTE	ALVKGSISRM	PIEDSSPEKG
1210	1220	1230	1240	1250	1260	1270	1280
REEAASKGHV	IYEGKSGHIL	SYDNIKNARE	GTRSPRTAHE	ISLKRSYESV	EGNIKQGMSM	RESPVSAPLE	GLICRALPRG
1290	1300	1310	1320	1330	1340	1350	1360
SPHSDLKERT	VLSGSIMQGT	PRATTESFED	GLKYPKQIKR	ESPPIRAFEG	AITKGKPYDG	ITTIKEMGRS	IHEIPRQDIL
1370	1380	1390	1400	1410	1420	1430	1440
TQESRKTPEV	VQSTRPIIEG	SISQGTPIKF	DNNSGQSAIK	HNVKSLITGP	SKLSRGMPPL	EIVPENIKVV	ERGKYEDVKA
1450	1460	1470	1480	1490	1500	1510	1520
GETVRSRHTS	VVSSGPSVLR	STLHEAPKAQ	LSPGIYDDTS	ARRTPVSYQN	TMSRGSPMMN	RTSDVTISSN	KSTNHERKST
1530	1540	1550	1560	1570	1580	1590	1600
LTPTQRESIP	AKSPVPGVDP	VVSHSPFDPH	HRGSTAGEVY	RSHLPTHLDP	AMPFHRALDP	AAAAYLFQRQ	LSPTPGYPSQ
1610	1620	1630	1640	1650	1660	1670	1680
YQLYAMENTR	QTILNDYITS	QQMQVNLRPD	VARGLSPREQ	PLGLPYPATR	GIIDLTNMPP	TILVPHPGGT	STPPMDRITY
1690	1700	1710	1720	1730	1740	1750	1760
IPGTQITFPP	RPYNSASMSP	GHPTHLAAAA	SAERERERER	EKERERERIA	AASSDLYLRP	GSEQPGRPGS	HGYVRSPSPS
1770	1780	1790	1800	1810	1820	1830	1840
VRTQETMLQQ	RPSVFQGTNG	TSVITPLDPT	AQLRIMPLPA	GGPSISQGLP	ASRYNTAADA	LAALVDAAAS	APQMDVSKTK
1850	1860	1870	1880	1890	1900	1910	1920
ESKHEAARLE	ENLRSRSAAV	SEQQQLEQKT	LEVEKRSVQC	LYTSSAFPSG	KPQPHSSVVY	SEAGKDKGPP	PKSRYEEELR
1930	1940	1950	1960	1970	1980	1990	2000
TRGKTTITAA	NFIDVIITRQ	IASDKDARER	GSQSSDSSSS	LSSHRYETPS	DAIEVISPAS	SPAPPQEKLQ	TYQPEVVKAN
2010	2020	2030	2040	2050	2060	2070	2080
QAENDPTRQY	EGPLHHYRPQ	QESPSPQQQL	PPSSQAEGMG	QVPRTHRLIT	LADHICQIIT	QDFARNQVSS	QTPQQPPTST
2090	2100	2110	2120	2130	2140	2150	2160
FQNSPSALVS	TPVRTKTSNR	YSPESQAQSV	HHQRPGSRVS	PENLVDKSRG	SRPGKSPERS	HVSSEPYEPI	SPPQVPVVHE
2170	2180	2190	2200	2210	2220	2230	2240

Detailed Protein Report

Cmpd.	No. of Cmpds.	m/z meas.	Δ m/z [ppm]	z	Rt [min]	Score	P	Range	Sequence	Modification
911	1	492.2027	-52.98	2	40.9	10.7	0	2270-2278	K.ALMGSFDDK.V	

Detailed Protein Report

Protein 399: **PREDICTED: phosphatidylinositol 4,5-bisphosphate 3-kinase catalytic subunit beta isoform isoform X3 [Homo sapiens]**

**Accession:**	gi|530374778	**Score:**	26.0
**Database:**	refseq_human(refseq_human_20140103.fasta)	**MW [kDa]:**	121.0
**Database Date:**	2015-11-30	**pI:**	7.1
**Modification(s):**	Oxidation	**Sequence Coverage [%]:**	2.1
		**No. of unique Peptides:**	1

10	20	30	40	50	60	70	80
MCFSFIMPPA	MADILDIWAV	DSQIASDGSI	PVDFLLPTGI	YIQLEVPREA	TISYIKQMLW	KQVHNYPMFN	LLMDIDSYMF
**90**	**100**	**110**	**120**	**130**	**140**	**150**	**160**
ACVNQTAVYE	ELEDETRRLC	DVRPFLPVLK	LVTRSCDPGE	KLDSKIGVLI	GKGLHEFDSL	KDPEVNEFRR	KMRKFSEEKI
**170**	**180**	**190**	**200**	**210**	**220**	**230**	**240**
LSLVGLSWMD	WLKQTYPPEH	EPSIPENLED	KLYGGKLIVA	VHFENCQDVF	SFQVSPNMNP	IKVNELAIQK	RLTIHGKEDE
**250**	**260**	**270**	**280**	**290**	**300**	**310**	**320**
VSPYDYVLQV	SGRVEYVFGD	HPLIQFQYIR	NCVMNRALPH	FILVECCKIK	KMYEQEMIAI	EAAINRNSSN	LPLPLPPKKT
**330**	**340**	**350**	**360**	**370**	**380**	**390**	**400**
RIISHVWENN	NPFQIVLVKG	NKLNTEETVK	VHVRAGLFHG	TELLCKTIVS	SEVSGKNDHI	WNEPLEFDIN	ICDLPRMARL
**410**	**420**	**430**	**440**	**450**	**460**	**470**	**480**
CFAVYAVLDK	VKTKKSTKTI	NPSKYQTIRK	AGKVHYPVAW	VNTMVFDFKG	QLRTGDIILH	SWSSFPDELE	EMLNPMGTVQ
**490**	**500**	**510**	**520**	**530**	**540**	**550**	**560**
TNPYTENATA	LHVKFPENKK	QPYYYPPFDK	SRGGKKFLPV	LKEILDRDPL	SQLCENEMDL	IWTLRQDCRE	IFPQSLPKLL
**570**	**580**	**590**	**600**	**610**	**620**	**630**	**640**
LSIKWNKLED	VAQLQALLQI	WPKLPPREAL	ELLDFNYPDQ	YVREYAVGCL	RQMSDEELSQ	YLLQLVQVLK	YEPFLDCALS
**650**	**660**	**670**	**680**	**690**	**700**	**710**	**720**
RFLLERALGN	RRIGQFLFWH	LRSEVHIPAV	SVQFGVILEA	YCRGSVGHMK	VLSKQVEALN	KLKTLNSLIK	LNAVKLNRAK
**730**	**740**	**750**	**760**	**770**	**780**	**790**	**800**
GKEAMHTCLK	QSAYREALSD	LQSPLNPCVI	LSELYVEKCK	YMDSKMKPLW	LVYNNKVFGE	DSVGVIFKNG	DDLRQDMLTL
**810**	**820**	**830**	**840**	**850**	**860**	**870**	**880**
QMLRLMDLLW	KEAGLDLRML	PYGCLATGDR	SGLIEVVSTS	ETIADIQLNS	SNVAAAAAFN	KDALLNWLKE	YNSGDDLDRA
**890**	**900**	**910**	**920**	**930**	**940**	**950**	**960**
IEEFTLSCAG	YCVASYVLGI	GDRHSDNIMV	KKTGQLFHID	FGHILGNFKS	KFGIKRERVP	FILTYDFIHV	IQQGKTGNTE
**970**	**980**	**990**	**1000**	**1010**	**1020**	**1030**	**1040**
KFGRFRQCCE	DAYLILRRHG	NLFITLFALM	LTAGLPELTS	VKDIQYLKDS	LALGKSEEEA	LKQFKQKFDE	ALRESWTTKV
**1050**	**1060**						
NWMAHTVRKD	YRS						

Cmpd.	No. of Cmpds.	m/z meas.	Δ m/z [ppm]	z	Rt [min]	Score	P	Range	Sequence	Modification
2763	1	959.4928	32.66	1	65.8	10.7	0	904-911	R.HSDNIMVK.K	Oxidation: 6

Detailed Protein Report

Protein 400: FERM domain-containing protein 6 isoform 1 [Homo sapiens]

**Accession:**	gi|22748721	**Score:**	25.9
**Database:**	refseq_human(refseq_human_20140103.fasta)	**MW [kDa]:**	70.9
**Database Date:**	2015-11-30	**pI:**	7.4
**Modification(s):**	Carbamidomethyl	**Sequence Coverage [%]:**	4.4
		**No. of unique Peptides:**	2

Alias proteins:

Accession	Name	Description
gi|578825482	refseq_human (refseq_human_20140103.fasta)	PREDICTED: FERM domain-containing protein 6 isoform X2 [Homo sapiens]
gi|109715866	refseq_human (refseq_human_20140103.fasta)	FERM domain-containing protein 6 isoform 1 [Homo sapiens]

10	20	30	40	50	60	70	80
MNKLNFHNNR	VMQDRRSVCI	FLPNDESLNI	IINVKILCHQ	LLVQVCDLLR	LKDCHLFGLS	VIQNNEHVYM	ELSQKLYKYC
**90**	**100**	**110**	**120**	**130**	**140**	**150**	**160**
PKEWKKEASK	GIDQFGPPMI	IHFRVQYYVE	NGRLISDRAA	RYYYYWHLRK	QVLHSQCVLR	EEAYFLLAAF	ALQADLGNFK
**170**	**180**	**190**	**200**	**210**	**220**	**230**	**240**
RNKHYGKYFE	PEAYFPSWVV	SKRGKDYILK	HIPNMHKDQF	ALTASEAHLK	YIKEAVRLDD	VAVHYYRLYK	DKREIEASLT
**250**	**260**	**270**	**280**	**290**	**300**	**310**	**320**
LGLTMRGIQI	FQNLDEEKQL	LYDFPWTNVG	KLVFVGKKFE	ILPDGLPSAR	KLIYYTGCPM	RSRHLLQLLS	NSHRLYMNLQ
**330**	**340**	**350**	**360**	**370**	**380**	**390**	**400**
PVLRHIRKLE	ENEEKKQYRE	SYISDNLDLD	MDQLEKRSRA	SGSSAGSMKH	KRLSRHSTAS	HSSSHTSGIE	ADTKPRDTGP
**410**	**420**	**430**	**440**	**450**	**460**	**470**	**480**
EDSYSSSAIH	RKLKTCSSMT	SHGSSHTSGV	ESGGKDRLEE	DLQDDEIEML	VDDPRDLEQM	NEESLEVSPD	MCIYITEDML
**490**	**500**	**510**	**520**	**530**	**540**	**550**	**560**
MSRKLNGHSG	LIVKEIGSST	SSSSETVVKL	RGQSTDSLPQ	TICRKPKTST	DRHSLSLDDI	RLYQKDFLRI	AGLCQDTAQS
**570**	**580**	**590**	**600**	**610**	**620**		
YTFGCGHELD	EEGLYCNSCL	AQQCINIQDA	FPVKRTSKYF	SLDLTHDEVP	EFVV		

Cmpd.	No. of Cmpds.	m/z meas.	Δ m/z [ppm]	z	Rt [min]	Score	P	Range	Sequence	Modification
237	1	795.3910	-58.65	2	32.3	12.8	1	233-246	K.REIEASLTLGLTMR.G	
1818	1	731.8030	-66.05	2	52.7	13.2	0	512-524	R.GQSTDSLPQTICR.K	Carbamidomethyl: 12

Detailed Protein Report

Protein 401: **PREDICTED: chromodomain-helicase-DNA-binding protein 7 isoform X4 [Homo sapiens]**

**Accession:**	gi|578815680	**Score:**	25.9
**Database:**	refseq_human(refseq_human_20140103.fasta)	**MW [kDa]:**	214.1
**Database Date:**	2015-11-30	**pI:**	5.2
		**Sequence Coverage [%]:**	1.6
		**No. of unique Peptides:**	1

10	20	30	40	50	60	70	80
MILTDCPELR	NIPWRCVVID	EAHRLKNRNC	KLLEGLKMMD	LEHKVLLTGT	PLQNTVEELF	SLLHFLEPSR	FPSETTFMQE
90	100	110	120	130	140	150	160
FGDLKTEEQV	QKLQAILKPM	MLRRLKEDVE	KNLAPKEETI	IEVELTNIQK	KYYRAILEKN	FTFLSKGGGQ	ANVPNLLNTM
170	180	190	200	210	220	230	240
MELRKCCNHP	YLINGAEEKI	LEEFKETHNA	ESPDFQLQAM	IQAAGKLVLI	DKLLPKLKAG	GHRVLIFSQM	VRCLDILEDY
250	260	270	280	290	300	310	320
LIQRRYPYER	IDGRVRGNLR	QAAIDRFSKP	DSDRFVFLLC	TRAGGLGINL	TAADTCIIFD	SDWNPQNDLQ	AQARCHRIGQ
330	340	350	360	370	380	390	400
SKSVKIYRLI	TRNSYEREMF	DKASLKLGLD	KAVLQSMSGR	ENATNGVQQL	SKKEIEDLLR	KGAYGALMDE	EDEGSKFCEE
410	420	430	440	450	460	470	480
DIDQILLRRT	HTITIESEGK	GSTFAKASFV	ASGNRTDISL	DDPNFWQKWA	KKAELDIDAL	NGRNNLVIDT	PRVRKQTRLY
490	500	510	520	530	540	550	560
SAVKEDELME	FSDLESDSEE	KPCAKPRRPQ	DKSQGYARSE	CFRVEKNLLV	YGWGRWTDIL	SHGRYKRQLT	EQDVETICRT
570	580	590	600	610	620	630	640
ILVYCLNHYK	GDENIKSFIW	DLITPTADGQ	TRALVNHSGL	SAPVPRGRKG	KKVKAQSTQP	VVQDADWLAS	CNPDALFQED
650	660	670	680	690	700	710	720
SYKKHLKHHC	NKVLLRVRML	YYLRQEVIGD	QADKILEGAD	SSEADVWIPE	PFHAEVPADW	WDKEADKSLL	IGVFKHGYEK
730	740	750	760	770	780	790	800
YNSMRADPAL	CFLERVGMPD	AKAIAAEQRG	TDMLADGGDG	GEFDREDEDP	EYKPTRTPFK	DEIDEFANSP	SEDKEESMEI
810	820	830	840	850	860	870	880
HATGKHSESN	AELGQLYWPN	TSTLTTRLRR	LITAYQRSYK	RQQMRQEALM	KTDRRRRRPR	EEVRALEAER	EAIISEKRQK
890	900	910	920	930	940	950	960
WTRREEADFY	RVVSTFGVIF	DPVKQQFDWN	QFRAFARLDK	KSDESLEKYF	SCFVAMCRRV	CRMPVKPDDE	PPDLSSIIEP
970	980	990	1000	1010	1020	1030	1040
ITEERASRTL	YRIELLRKIR	EQVLHHPQLG	ERLKLCQPSL	DLPEWWECGR	HDRDLLVGAA	KHGVSRTDYH	ILNDPELSFL
1050	1060	1070	1080	1090	1100	1110	1120
DAHKNFAQNR	GAGNTSSLNP	LAVGFVQTPP	VISSAHIQDE	RVLEQAEGKV	EEPENPAAKE	KCEGKEEEEE	TDGSGKESKQ
1130	1140	1150	1160	1170	1180	1190	1200
ECEAEASSVK	NELKGVEVGA	DTGSKSISEK	GSEEDEEEKL	EDDDKSEESS	QPEAGAVSRG	KNFDEESNAS	MSTARDETRD
1210	1220	1230	1240	1250	1260	1270	1280
GFYMEDGDPS	VAQLLHERTF	AFSFWPKDRV	MINRLDNICE	AVLKGKWPVN	RRQMFDFQGL	IPGYTPTTVD	SPLQKRSFAE
1290	1300	1310	1320	1330	1340	1350	1360
LSMVGQASIS	GSEDITTSPQ	LSKEDALNLS	VPRQRRRRRR	KIEIEAERAA	KRRNLMEMVA	QLRESQVVSE	NGQEKVVDLS
1370	1380	1390	1400	1410	1420	1430	1440
KASREATSST	SNFSSLSSKF	ILPNVSTPVS	DAFKTQMELL	QAGLSRTPTR	HLLNGSLVDG	EPPMKRRRGR	RKNVEGLDLL
1450	1460	1470	1480	1490	1500	1510	1520
FMSHKRTSLS	AEDAEVTKAF	EEDIETPPTR	NIPSPGQLDP	DTRIPVINLE	DGTRLVGEDA	PKNKDLVEWL	KLHPTYTVDM
1530	1540	1550	1560	1570	1580	1590	1600
PSYVPKNADV	LFSSFQKPKQ	KRHRCRNPNK	LDINTLTGEE	RVPVVNKRNG	KKMGGAMAPP	MKDLPRWLEE	NPEFAVAPDW
1610	1620	1630	1640	1650	1660	1670	1680
TDIVKQSGFV	PESMFDRLLT	GPVVRGEGAS	RRGRRPKSEI	ARAAAAAAAV	ASTSGINPLL	VNSLFAGMDL	TSLQNLQNLQ
1690	1700	1710	1720	1730	1740	1750	1760
SLQLAGLMGF	PPGLATAATA	GGDAKNPAAV	LPLMLPGMAG	LPNVFGLGGL	LNNPLSAATG	NTTTASSQGE	PEDSTSKGEE
1770	1780	1790	1800	1810	1820	1830	1840
KGNENEDENK	DSEKSTDAVS	AADSANGSVG	AATAPAGLPS	NPLAFNPFLL	STMAPGLFYP	SMFLPPGLGG	LTLPGFPALA
1850	1860	1870	1880	1890	1900	1910	1920
GLQNAVGSSE	EKAADKAEGG	PFKDGETLEG	SDAEESLDKT	AESSLLEDEI	AQGEELDSLD	GGDEIENNEN	DE

Cmpd.	No. of	m/z meas.		z	Rt	Score	P	Range	Sequence	Modification

Detailed Protein Report

	Cmpds.		Δ m/z [ppm]		[min]					
1713	1	806.6994	-162.85	2	51.4	15.5	0	750-765	R.GTDMLADGGDGGEFDR.E	

Detailed Protein Report

Protein 402: **PREDICTED: U3 small nucleolar RNA-associated protein 6 homolog isoform X1 [Homo sapiens]**

**Accession:**	gi|530411102	**Score:**	25.9
**Database:**	refseq_human(refseq_human_20140103.fasta)	**MW [kDa]:**	55.6
**Database Date:**	2015-11-30	**pI:**	6.6
**Modification(s):**	Oxidation	**Sequence Coverage [%]:**	8.4
		**No. of unique Peptides:**	2

10	20	30	40	50	60	70	80
MRLRILLYTG	YKVFSSVPQQ	NGKTMFNFGS	PMWLFVRSGL	LKLDLARYSL	PCWRFIPTNQ	YFRMELMHAE	KLRKEKEEFE
**90**	**100**	**110**	**120**	**130**	**140**	**150**	**160**
KASMDVENPD	YSEEILKGEL	AWIIYKNSVS	IIKGAEFHVS	LLSIAQLFDF	AKDLQKEIYD	DLQALHTDDP	LTWDYVARRE
**170**	**180**	**190**	**200**	**210**	**220**	**230**	**240**
LEIESQTEEQ	PTTKQAKAVE	VGRKEERCCA	VYEEAVKTLP	TEAMWKCYIT	FCLERFTKKS	NSGFLRGKRL	ERTMTVFRKA
**250**	**260**	**270**	**280**	**290**	**300**	**310**	**320**
HELKLLSECQ	YKQLSVSLLC	YNFLREALEV	AVAGTELFRD	SGTMWQLKLQ	VLIESKSPDI	AMLFEEAFVH	LKPQVCLPLW
**330**	**340**	**350**	**360**	**370**	**380**	**390**	**400**
ISWAEWSEGA	KSQEDTEAVF	KKALLAVIGA	DSVTLKNKYL	DWAYRSGGYK	KARAVFKSLQ	ESRPFSVDFF	RKMIQFEKEQ
**410**	**420**	**430**	**440**	**450**	**460**	**470**	**480**
ESCNMANIRE	YYERALREFG	SADSDLWMDY	MKEELNHPLG	RPENCGQIYW	RAMKMLQGES	AEAFVAKHAM	HQTGHL

Cmpd.	No. of Cmpds.	m/z meas.	Δ m/z [ppm]	z	Rt [min]	Score	P	Range	Sequence	Modification
2485	1	1045.1567	132.37	2	61.0	12.6	1	160-177	R.ELEIESQTEEQPTTKQAK.A	
15	1	803.7134	-7.51	3	29.5	13.3	1	455-476	K.MLQGESAEAFVAKHAMHQTGHL.-	Oxidation: 1

Detailed Protein Report

Protein 403: toll-like receptor 9 precursor [Homo sapiens]

Accession: gi|8394456
Database: refseq_human(refseq_human_20140103.fasta)
Database Date: 2015-11-30

Score: 25.9
MW [kDa]: 115.8
pI: 9.7
Sequence Coverage [%]: 1.9
No. of unique Peptides: 2

10	20	30	40	50	60	70	80
MGFCRSALHP	LSLLVQAIML	AMTLALGTLP	AFLPCELQPH	GLVNCNWLFL	KSVPHFSMAA	PRGNVTSLSL	SSNRIHHLHD
90	100	110	120	130	140	150	160
SDFAHLPSLR	HLNLKWNCPP	VGLSPMHFPC	HMTIEPSTFL	AVPTLEELNL	SYNNIMTVPA	LPKSLISLSL	SHTNILMLDS
170	180	190	200	210	220	230	240
ASLAGLHALR	FLFMDGNCYY	KNPCRQALEV	APGALLGLGN	LTHLSLKYNN	LTVVPRNLPS	SLEYLLLSYN	RIVKLAPEDL
250	260	270	280	290	300	310	320
ANLTALRVLD	VGGNCRRCDH	APNPCMECPR	HFPQLHPDTF	SHLSRLEGLV	LKDSSLSWLN	ASWFRGLGNL	RVLDLSENFL
330	340	350	360	370	380	390	400
YKCITKTKAF	QGLTQLRKLN	LSFNYQKRVS	FAHLSLAPSF	GSLVALKELD	MHGIFFRSLD	ETTLRPLARL	PMLQTLRLQM
410	420	430	440	450	460	470	480
NFINQAQLGI	FRAFPGLRYV	DLSDNRISGA	SELTATMGEA	DGGEKVWLQP	GDLAPAPVDT	PSSEDFRPNC	STLNFTLDLS
490	500	510	520	530	540	550	560
RNNLVTVQPE	MFAQLSHLQC	LRLSHNCISQ	AVNGSQFLPL	TGLQVLDLSH	NKLDLYHEHS	FTELPRLEAL	DLSYNSQPFG
570	580	590	600	610	620	630	640
MQGVGHNFSF	VAHLRTLRHL	SLAHNNIHSQ	VSQQLCSTSL	RALDFSGNAL	GHMWAEGDLY	LHFFQGLSGL	IWLDLSQNRL
650	660	670	680	690	700	710	720
HTLLPQTLRN	LPKSLQVLRL	RDNYLAFFKW	WSLHFLPKLE	VLDLAGNQLK	ALTNGSLPAG	TRLRRLDVSC	NSISFVAPGF
730	740	750	760	770	780	790	800
FSKAKELREL	NLSANALKTV	DHSWFGPLAS	ALQILDVSAN	PLHCACGAAF	MDFLLEVQAA	VPGLPSRVKC	GSPGQLQGLS
810	820	830	840	850	860	870	880
IFAQDLRLCL	DEALSWDCFA	LSLLAVALGL	GVPMLHHLCG	WDLWYCFHLC	LAWLPWRGRQ	SGRDEDALPY	DAFVVFDKTQ
890	900	910	920	930	940	950	960
SAVADWVYNE	LRGQLEECRG	RWALRLCLEE	RDWLPGKTLF	ENLWASVYGS	RKTLFVLAHT	DRVSGLLRAS	FLLAQQRLLE
970	980	990	1000	1010	1020	1030	1040
DRKDVVVLVI	LSPDGRRSRY	VRLRQRLCRQ	SVLLWPHQPS	GQRSFWAQLG	MALTRDNHHF	YNRNFCQGPT	AE

Cmpd.	No. of Cmpds.	m/z meas.	Δ m/z [ppm]	z	Rt [min]	Score	P	Range	Sequence	Modification
1454	1	1059.0764	13.42	2	48.0	15.1	1	788-807	R.VKCGSPGQLQGLSIFAQDLR.L	
399	1	945.4882	8.09	2	34.3	10.8	0	790-807	K.CGSPGQLQGLSIFAQDLR.L	

Detailed Protein Report

Protein 404: **endoplasmic reticulum-Golgi intermediate compartment protein 2 [Homo sapiens]**

**Accession:**	gi|50959176	**Score:**	25.9
**Database:**	refseq_human(refseq_human_20140103.fasta)	**MW [kDa]:**	42.5
**Database Date:**	2015-11-30	**pI:**	6.3
**Modification(s):**	Oxidation	**Sequence Coverage [%]:**	5.6
		**No. of unique Peptides:**	2

10	20	30	40	50	60	70	80
MRRLNRKKTL	SLVKELDAFP	KVPESYVETS	ASGGTVSLIA	FTTMALLTIM	EFSVYQDTWM	KYEYEVDKDF	SSKLRINIDI
**90**	**100**	**110**	**120**	**130**	**140**	**150**	**160**
TVAMKCQYVG	ADVLDLAETM	VASADGLVYE	PTVFDLSPQQ	KEWQRMLQLI	QSRLQEEHSL	QDVIFKSAFK	STSTALPPRE
**170**	**180**	**190**	**200**	**210**	**220**	**230**	**240**
DDSSQSPNAC	RIHGHLYVNK	VAGNFHITVG	KAIPHPRGHA	HLAALVNHES	YNFSHRIDHL	SFGELVPAII	NPLDGTEKIA
**250**	**260**	**270**	**280**	**290**	**300**	**310**	**320**
IDHNQMFQYF	ITVVPTKLHT	YKISADTHQF	SVTERERIIN	HAAGSHGVSG	IFMKYDLSSL	MVTVTEEHMP	FWQFFVRLCG
**330**	**340**	**350**	**360**	**370**	**380**		
IVGGIFSTTG	MLHGIGKFIV	EIICCRFRLG	SYKPVNSVPF	EDGHTDNHLP	LLENNTH		

Cmpd.	No. of Cmpds.	m/z meas.	Δ m/z [ppm]	z	Rt [min]	Score	P	Range	Sequence	Modification
2603	1	701.8580	-71.21	2	62.6	14.2	1	74-85	K.LRINIDITVAMK.C	Oxidation: 11
1411	1	1095.6964	116.34	1	47.3	11.8	0	338-346	K.FIVEIICCR.F	

Detailed Protein Report

Protein 405: protein TALPID3 isoform 3 [Homo sapiens]

Accession: gi|346644665
Database: refseq_human(refseq_human_20140103.fasta)
Database Date: 2015-11-30
Modification(s): Oxidation

Score: 25.9
MW [kDa]: 161.7
pI: 5.2
Sequence Coverage [%]: 2.1
No. of unique Peptides: 2

Quantitation

MD:MU **Median:** 1.29 **CV:** 0.00 % **No. of Peptides:** 1

10	20	30	40	50	60	70	80
MVSESDFSKD	VAVQVLPLDK	IEENNKQKAN	DIFISQYTMG	QKDALRTVLK	QKAQSMPVFK	EVKVHLLEDA	GIEKDAVTQE
90	100	110	120	130	140	150	160
TRISPSGIDS	ATTVAAATAA	AIATAAPLIK	VQSDLEAKVN	SVTELLSKLQ	ETDKHLQRVT	EQQTSIQRKQ	EKLHCHDHEK
170	180	190	200	210	220	230	240
QMNVFMEQHI	RHLEKLQQQQ	IDIQTHFISA	ALKTSSFQPV	SMPSSRAVEK	YSVKPEHPNL	GSCNPSLYNT	FASKQAPLKE
250	260	270	280	290	300	310	320
VEDTSFDKQK	SPLETPAPRR	FAPVPVSRDD	ELSKRENLLE	EKENMEVSCH	RGNVRLLEQI	LNNNDSLTRK	SESSNTTSLT
330	340	350	360	370	380	390	400
RSKIGWTPEK	TNRFPSCEEL	ETTKVTMQKS	DDVLHDLGQK	EKETNSMVQP	KESLSMLKLP	DLPQNSVKLQ	TTNTTRSVLK
410	420	430	440	450	460	470	480
DAEKILRGVQ	NNKKVLEENL	EAIIRAKDGA	AMYSLINALS	TNREMSEKIR	IRKTVDEWIK	TISAEIQDEL	SRTDYEQKRF
490	500	510	520	530	540	550	560
DQKNQRTKKG	QNMTKDIRTN	TQDKTVNKSV	IPRKHSQKQI	EEHFRNLPMR	GMPASSLQKE	RKEGLLKATT	VIQDEDYMLQ
570	580	590	600	610	620	630	640
VYGKPVYQGH	RSTLKKGPYL	RFNSPSPKSR	PQRPKVIERV	KGTKVKSIRT	QTDFYATKPK	KMDSKMKHSV	PVLPHGDQQY
650	660	670	680	690	700	710	720
LFSPSREMPT	FSGTLEGHLI	PMAILLGQTQ	SNSDTMPPAG	VIVSKPHPVT	VTTSIPPSSR	KVETGVKKPN	IAIVEMKSEK
730	740	750	760	770	780	790	800
KDPPQLTVQV	LPSVDIDSIS	NSSADVLSPL	SSPKEASLPP	VQTWIKTPEI	MKVDEEEVKF	PGTNFDEIID	VIQEEEKCDE
810	820	830	840	850	860	870	880
IPDSEPILEF	NRSVKADSTK	YNGPPFPPVA	STFQPTADIL	DKVIERKETL	ENSLIQWVEQ	EIMSRIISGL	FPVQQQIAPS
890	900	910	920	930	940	950	960
ISVSVSETSE	PLTSDIVEGT	SSGALQLFVD	AGVPVNSNVI	KHFVNEALAE	TIAVMLGDRE	AKKQGPVATG	VSGDASTNET
970	980	990	1000	1010	1020	1030	1040
YLPARVCTPL	PTPQPTPPCS	PSSPAKECVL	VKTPDSSPCD	SDHDMAFPVK	EICAEKGDDM	PAIMLVNTPT	VTPTTTPPPA
1050	1060	1070	1080	1090	1100	1110	1120
AAVFTPTLSD	ISIDKLKVSS	PELPKPWGDG	DLPLEEENPN	SPQEELHPRA	IVMSVAKDEE	PESMDFPAQP	PPPEPVPFMP
1130	1140	1150	1160	1170	1180	1190	1200
FPAGTKAPSP	SQMPGSDSST	LESTLSVTVT	ETETLDKPIS	EGEILFSCGQ	KLAPKILEDI	GLYLTNLNDS	LSSTLHDAVE
1210	1220	1230	1240	1250	1260	1270	1280
MEDDPPSEGQ	VIRMSHKKFH	ADAILSFAKQ	NQESAVSQQA	VYHSEDLENS	VGELSEGQRP	QLTAAAENIL	MGHSLYMQPP
1290	1300	1310	1320	1330	1340	1350	1360
VTNTQSLDQQ	CDPKPLSRQF	DTVSGSIYED	SCASHGPMSL	GELELEPNSK	LVLPTTLLTA	QENDVNLPVA	AEDFSQYQLK
1370	1380	1390	1400	1410	1420	1430	1440
QNQDVKQVEH	KPSQSYLRVR	NKSDIAPSQQ	QVSPGDMDRT	QIELNPYLTC	VFSGGKAVPL	SASQMPPAKM	SVMLPSVNLE
1450	1460	1470					
DCSQSLSLST	MQEDMESSGA	DTF					

Cmpd.	No. of Cmpds.	m/z meas.	Δ m/z [ppm]	z	Rt [min]	Score	P	Range	Sequence	Modification	Ratios
2296	1	653.8345	23.68	2	58.6	11.0	1	361-371	K.EKETNSMVQPK.E	Oxidation: 7	MD:MU 1.29
1681	1	1044.8573	-121.61	2	50.9	14.9	1	1381-1399	R.NKSDIAPSQQQVSPGDMDR.T	Oxidation: 17	

Detailed Protein Report

Protein 406: **PREDICTED: serine/arginine repetitive matrix protein 1 isoform X6 [Homo sapiens]**

**Accession:**	gi|530360793	**Score:**	25.9
**Database:**	refseq_human(refseq_human_20140103.fasta)	**MW [kDa]:**	100.2
**Database Date:**	2015-11-30	**pI:**	12.3
**Modification(s):**	Oxidation	**Sequence Coverage [%]:**	2.6
		**No. of unique Peptides:**	1

10	20	30	40	50	60	70	80
MDAGFFRGTS	AEQDNRFSNK	QKKLLKQLKF	AECLEKKVDM	SKVNLEVIKP	WITKRVTEIL	GFEDDVVIEF	IFNQLEVKNP
**90**	**100**	**110**	**120**	**130**	**140**	**150**	**160**
DSKMMQINLT	GFLNGKNARE	FMGELWPLLL	SAQENIAGIP	SAFLELKKEE	IKQRQIEQEK	LASMKKQDED	KDKRDKEEKE
**170**	**180**	**190**	**200**	**210**	**220**	**230**	**240**
SSREKRERSR	SPRRTKSRSP	SPAPEKKEKT	PELPEPSVKV	KEPSVQEATS	TSDILKVPKP	EPIPEPKEPS	PEKNSKKEKE
**250**	**260**	**270**	**280**	**290**	**300**	**310**	**320**
KEKTRPRSRS	RSKSRSRTRS	RSPSHTRPRR	RHRSRSRSYS	PRRRPSPRRR	PSPRRRTPPR	RMPPPPRHRR	SRSPVRRRRR
**330**	**340**	**350**	**360**	**370**	**380**	**390**	**400**
SSASLSGSSS	SSSSSRSRSP	PKKPPKRTSS	PPRKTRRLSP	SASPPRRRHR	PSPPATPPPK	TRHSPTPQQS	NRTRKSRVSV
**410**	**420**	**430**	**440**	**450**	**460**	**470**	**480**
SPGRTSGKVT	KHKGTEKRES	PSPAPKPRKV	ELSESEEDKG	GKMAAADSVQ	QRRQYRRQNQ	QSSSDSGSSS	SSEDERPKRS
**490**	**500**	**510**	**520**	**530**	**540**	**550**	**560**
HVKNGEVGRR	RRHSPSRSAS	PSPRKRQKET	SPRMQMGKRW	QSPVTKSGRR	RRSPSPPPTR	RRRSPSPAPP	PRRRRTPTPP
**570**	**580**	**590**	**600**	**610**	**620**	**630**	**640**
PRRRTPSPPP	RRRSPSPRRY	SPPIQRRYSP	SPPPKRRTAS	PPPPPKRRAS	PSPPPKRRVS	HSPPPKQRSS	PVTKRRSPSL
**650**	**660**	**670**	**680**	**690**	**700**	**710**	**720**
SSKHRKGSSP	SRSTREARSP	QPNKRHSPSP	RPRAPQTSSS	PPPVRRGASS	SPQRRQSPSP	STRPIRRVSR	TPEPKKIKKA
**730**	**740**	**750**	**760**	**770**	**780**	**790**	**800**
ASPSPQSVRR	VSSSRSVSGS	PEPAAKKPPA	PPSPVQSQSP	STNWSPAVPV	KKAKSPTPSP	SPPRNSDQEG	GGKKKKKKKD
**810**	**820**	**830**	**840**	**850**	**860**	**870**	**880**
KKHKKDKKHK	KHKKHKKEKA	VAAAAAAAVT	PAAIAAATTT	LAQEEPVAAP	EPKKETESEA	EDNLDDLEKH	LREKALRSMR
**890**							
KAQVSPQS							

Cmpd.	No. of Cmpds.	m/z meas.	Δ m/z [ppm]	z	Rt [min]	Score	P	Range	Sequence	Modification
1844	1	590.7772	1.91	2	53.1	11.4	1	509-518	K.ETSPRMQMGK.R	Oxidation: 6

Detailed Protein Report

Protein 407: condensin complex subunit 1 [Homo sapiens]

**Accession:**	gi|178056552	**Score:**	25.8
**Database:**	refseq_human(refseq_human_20140103.fasta)	**MW [kDa]:**	157.1
**Database Date:**	2015-11-30	**pI:**	6.2
		**Sequence Coverage [%]:**	1.9
		**No. of unique Peptides:**	1

10	20	30	40	50	60	70	80
MAPQMYEFHL	PLSPEELLKS	GGVNQYVVQE	VLSIKHLPPQ	LRAFQAAFRA	QGPLAMLQHF	DTIYSILHHF	RSIDPGLKED
**90**	**100**	**110**	**120**	**130**	**140**	**150**	**160**
TLQFLIKVVS	RHSQELPAIL	DDTTLSGSDR	NAHLNALKMN	CYALIRLLES	FETMASQTNL	VDLDLGGKGK	KARTKAAHGF
**170**	**180**	**190**	**200**	**210**	**220**	**230**	**240**
DWEEERQPIL	QLLTQLLQLD	IRHLWNHSII	EEEFVSLVTG	CCYRLLENPT	INHQKNRPTR	EAITHLLGVA	LTRYNHMLSA
**250**	**260**	**270**	**280**	**290**	**300**	**310**	**320**
TVKIIQMLQH	FEHLAPVLVA	AVSLWATDYG	MKSIVGEIVR	EIGQKCPQEL	SRDPSGTKGF	AAFLTELAER	VPAILMSSMC
**330**	**340**	**350**	**360**	**370**	**380**	**390**	**400**
ILLDHLDGEN	YMMRNAVLAA	MAEMVLQVLS	GDQLEAAARD	TRDQFLDTLQ	AHGHDVNSFV	RSRVLQLFTR	IVQQKALPLT
**410**	**420**	**430**	**440**	**450**	**460**	**470**	**480**
RFQAVVALAV	GRLADKSVLV	CKNAIQLLAS	FLANNPFSCK	LSDADLAGPL	QKETQKLQEM	RAQRRTAAAS	AVLDPEEEWE
**490**	**500**	**510**	**520**	**530**	**540**	**550**	**560**
AMLPELKSTL	QQLLQLPQGE	EEIPEQIANT	ETTEDVKGRI	YQLLAKASYK	KAIILTREAT	GHFQESEPFS	HIDPEESEET
**570**	**580**	**590**	**600**	**610**	**620**	**630**	**640**
RLLNILGLIF	KGPAASTQEK	NPRESTGNMV	TGQTVCKNKP	NMSDPEESRG	NDELVKQEML	VQYLQDAYSF	SRKITEAIGI
**650**	**660**	**670**	**680**	**690**	**700**	**710**	**720**
ISKMMYENTT	TVVQEVIEFF	VMVFQFGVPQ	ALFGVRRMLP	LIWSKEPGVR	EAVLNAYRQL	YLNPKGDSAR	AKAQALIQNL
**730**	**740**	**750**	**760**	**770**	**780**	**790**	**800**
SLLLVDASVG	TIQCLEEILC	EFVQKDELKP	AVTQLLWERA	TEKVACCPLE	RCSSVMLLGM	MARGKPEIVG	SNLDTLVSIG
**810**	**820**	**830**	**840**	**850**	**860**	**870**	**880**
LDEKFPQDYR	LAQQVCHAIA	NISDRRKPSL	GKRHPPFRLP	QEHRLFERLR	ETVTKGFVHP	DPLWIPFKEV	AVTLIYQLAE
**890**	**900**	**910**	**920**	**930**	**940**	**950**	**960**
GPEVICAQIL	QGCAKQALEK	LEEKRTSQED	PKESPAMLPT	FLLMNLLSLA	GDVALQQLVH	LEQAVSGELC	RRRVLREEQE
**970**	**980**	**990**	**1000**	**1010**	**1020**	**1030**	**1040**
HKTKDPKEKN	TSSETTMEEE	LGLVGATADD	TEAELIRGIC	EMELLDGKQT	LAAFVPLLLK	VCNNPGLYSN	PDLSAAASLA
**1050**	**1060**	**1070**	**1080**	**1090**	**1100**	**1110**	**1120**
LGKFCMISAT	FCDSQLRLLF	TMLEKSPLPI	VRSNLMVATG	DLAIRFPNLV	DPWTPHLYAR	LRDPAQQVRK	TAGLVMTHLI
**1130**	**1140**	**1150**	**1160**	**1170**	**1180**	**1190**	**1200**
LKDMVKVKGQ	VSEMAVLLID	PEPQIAALAK	NFFNELSHKG	NAIYNLLPDI	ISRLSDPELG	VEEEPFHTIM	KQLLSYITKD
**1210**	**1220**	**1230**	**1240**	**1250**	**1260**	**1270**	**1280**
KQTESLVEKL	CQRFRTSRTE	RQQRDLAYCV	SQLPLTERGL	RKMLDNFDCF	GDKLSDESIF	SAFLSVVGKL	RRGAKPEGKA
**1290**	**1300**	**1310**	**1320**	**1330**	**1340**	**1350**	**1360**
IIDEFEQKLR	ACHTRGLDGI	KELEIGQAGS	QRAPSAKKPS	TGSRYQPLAS	TASDNDFVTP	EPRRTTRRHP	NTQQRASKKK
**1370**	**1380**	**1390**	**1400**	**1410**			
PKVVFSSDES	SEEDLSAEMT	EDETPKKTTP	ILRASARRHR	S			

Cmpd.	No. of Cmpds.	m/z meas.	Δ m/z [ppm]	z	Rt [min]	Score	P	Range	Sequence	Modification
1765	1	788.2766	-109.58	2	52.0	10.1	1	154-166	R.TKAAHGFDWEEER.Q	

Detailed Protein Report

Protein 408: PREDICTED: LIM/homeobox protein Lhx3 isoform X1 [Homo sapiens]

Accession: gi|530426634
Database: refseq_human(refseq_human_20140103.fasta)
Database Date: 2015-11-30
Modification(s): Carbamidomethyl

Score: 25.8
MW [kDa]: 42.3
pI: 9.5
Sequence Coverage [%]: 8.5
No. of unique Peptides: 2

10	20	30	40	50	60	70	80
MTLNMKETRW	GAPAPEIPLC	AGCDQHILDR	FILKALDRHW	HSKCLKCSDC	HTPLAERCFS	RGESVYCKDD	FFKRFGTKCA
**90**	**100**	**110**	**120**	**130**	**140**	**150**	**160**
ACQLGIPPTQ	VVRRAQDFVY	HLHCFACVVC	KRQLATGDEF	YLMEDSRLVC	KADYETAKQR	EAEATAKRPR	TTITAKQLET
**170**	**180**	**190**	**200**	**210**	**220**	**230**	**240**
LKSAYNTSPK	PARHVREQLS	SETGLDMRVV	QVWFQNRRAK	EKRLKKDAGR	QRWGQYFRNM	KRSRGGSKSD	KDSVQEGQDS
**250**	**260**	**270**	**280**	**290**	**300**	**310**	**320**
DAEVSFPDEP	SLAEMGPANG	LYGSLGEPTQ	ALGRPSGALG	NFSLEHGGLA	GPEQYRELRP	GSPYGVPPSP	AAPQSLPGPQ
**330**	**340**	**350**	**360**	**370**	**380**	**390**	
PLLSSLVYPD	TSLGLVPSGA	PGGPPPMRVL	AGNGPSSDLS	TGSSGGYPDF	PASPASWLDE	VDHAQF	

Cmpd.	No. of Cmpds.	m/z meas.	Δ m/z [ppm]	z	Rt [min]	Score	P	Range	Sequence	Modification
2005	1	788.2933	-81.32	2	55.0	10.3	1	44-57	K.CLKCSDCHTPLAER.C	
2172	1	1051.9909	-51.91	2	57.0	15.5	1	75-93	R.FGTKCAACQLGIPPTQVVR.R	Carbamidomethyl: 5, 8

Detailed Protein Report

Protein 409: protein-glutamine gamma-glutamyltransferase 5 isoform 2 [Homo sapiens]

**Accession:**	gi|4759230	**Score:**	25.8
**Database:**	refseq_human(refseq_human_20140103.fasta)	**MW [kDa]:**	71.9
**Database Date:**	2015-11-30	**pI:**	5.9
		**Sequence Coverage [%]:**	6.0
		**No. of unique Peptides:**	1

10	20	30	40	50	60	70	80
MAQGLEVALT	DLQSSRNNVR	HHTEEITVDH	LLVRRGQAFN	LTLYFRNRSF	QPGLDNIIFV	VETEDAVYLD	SEPQRQEYVM
**90**	**100**	**110**	**120**	**130**	**140**	**150**	**160**
NDYGFIYQGS	KNWIRPCPWN	YGQFEDKIID	ICLKLLDKSL	HFQTDPATDC	ALRGSPVYVS	RVVCAMINSN	DDNGVLNGNW
**170**	**180**	**190**	**200**	**210**	**220**	**230**	**240**
SENYTDGANP	AEWTGSVAIL	KQWNATGCQP	VRYGQCWVFA	AVMCTVMRCL	GIPTRVITNF	DSGHDTDGNL	IIDEYYDNTG
**250**	**260**	**270**	**280**	**290**	**300**	**310**	**320**
RILGNKKKDT	IWNFHVWNEC	WMARKDLPPA	YGGWQVLDAT	PQEMSNGVYC	CGPASVRAIK	EGEVDLNYDT	PFVFSMVNAD
**330**	**340**	**350**	**360**	**370**	**380**	**390**	**400**
CMSWLVQGGK	EQKLHQDTSS	VGNFISTKSI	QSDERDDITE	NYKYEEGSLQ	ERQVFLKALQ	KLKARSFHGS	QRGAELQPSR
**410**	**420**	**430**	**440**	**450**	**460**	**470**	**480**
PTSLSQDSPR	SLHTPSLRPS	DVVQVSLKFK	LLDPPNMGQD	ICFVLLALNM	SSQFKDLKVN	LSAQSLLHDG	SPLSPFWQDT
**490**	**500**	**510**	**520**	**530**	**540**	**550**	**560**
AFITLSPKEA	KTYPCKISYS	QYSQYLSTDK	LIRISALGEE	KSSPEKILVN	KIITLSYPSI	TINVLGAAVV	NQPLSIQVIF
**570**	**580**	**590**	**600**	**610**	**620**	**630**	**640**
SNPLSEQVED	CVLTVEGSGL	FKKQQKVFLG	VLKPQHQASI	ILETVPFKSG	QRQIQANMRS	NKFKDIKGYR	NVYVDFAL

Cmpd.	No. of Cmpds.	m/z meas.	Δ m/z [ppm]	z	Rt [min]	Score	P	Range	Sequence	Modification
1787	1	1023.8570	-1.46	3	52.3	10.0	1	429-455	K.FKLLDPPNMGQDICFVLLALNMSSQFK.D	

Detailed Protein Report

Protein 410: cytochrome P450 3A43 isoform 4 [Homo sapiens]

**Accession:**	gi|525458843	**Score:**	25.8
**Database:**	refseq_human(refseq_human_20140103.fasta)	**MW [kDa]:**	44.8
**Database Date:**	2015-11-30	**pI:**	9.8
		**Sequence Coverage [%]:**	4.3
		**No. of unique Peptides:**	1

10	20	30	40	50	60	70	80
MDLIPNFAME	TWVLVATSLV	LLYIYGTHSH	KLFKKLGIPG	PTPLPFLGTI	LFYLRRSLNK	IPSWAWWLTP	VIPALWEAEA
**90**	**100**	**110**	**120**	**130**	**140**	**150**	**160**
GGSPKVRSSR	PALPTWVFGI	LTENVMKNTE	KCGALFPFLT	PVFEALNIGL	FPKDVTHFLK	NSIERMKESR	LKDKQKHRVD
**170**	**180**	**190**	**200**	**210**	**220**	**230**	**240**
FFQQMIDSQN	SKETKSHKAL	SDLELVAQSI	IIIFAAYDTT	STTLPFIMYE	LATHPDVQQK	LQEEIDAVLP	NKAPVTYDAL
**250**	**260**	**270**	**280**	**290**	**300**	**310**	**320**
VQMEYLDMVV	NETLRLFPVV	SRVTRVCKKD	IEINGVFIPK	GLAVMVPIYA	LHHDPKYWTE	PEKFCPERFS	KKNKDSIDLY
**330**	**340**	**350**	**360**	**370**	**380**	**390**	**400**
RYIPFGAGPR	NCIGMRFALT	NIKLAVIRAL	QNFSFKPCKE	TQIPLKLDNL	PILQPEKPIV	LKVHLRDGIT	SGP

Cmpd.	No. of Cmpds.	m/z meas.	Δ m/z [ppm]	z	Rt [min]	Score	P	Range	Sequence	Modification
398	1	587.3194	-67.09	2	34.3	11.8	1	256-265	R.LFPVVSRVTR.V	

Detailed Protein Report

Protein 411: NMDA receptor-regulated protein 2 isoform c [Homo sapiens]

**Accession:**	gi|446713799	**Score:**	25.8
**Database:**	refseq_human(refseq_human_20140103.fasta)	**MW [kDa]:**	43.8
**Database Date:**	2015-11-30	**pI:**	9.7
		**Sequence Coverage [%]:**	5.7
		**No. of unique Peptides:**	1

10	20	30	40	50	60	70	80
MSSKMVISEP	GLNWDISPKN	GLKTFFSREN	YKDHSMAPSL	KELRVLSNRR	IGENLNASAS	SVENEPAVSS	ATQAKEKVKT
**90**	**100**	**110**	**120**	**130**	**140**	**150**	**160**
TIGMVLLPKP	RVPYPRFSRF	SQREQRSYVD	LLVKYAKIPA	NSKAVGINKN	DYLQYLDMKK	HVNEEVTEFL	KFLQNSAKKC
**170**	**180**	**190**	**200**	**210**	**220**	**230**	**240**
AQDYNMLSDD	ARLFTEKILR	ACIEQVKKYS	EFYTLHEVTS	LMGFFPFRVE	MGLKLEKTLL	ALGSVKYVKT	VFPSMPIKLQ
**250**	**260**	**270**	**280**	**290**	**300**	**310**	**320**
LSKDDIATIE	TSEQTAEAMH	YDISKDPNAE	KLVSRYHPQI	ALTSQSLFTL	LNNHGPTYKE	QWEIPVCIQV	IPVAGSKPVK
**330**	**340**	**350**	**360**	**370**	**380**	**390**	
VIYINSPLPQ	KKMTMRERNQ	IFHEVPLKFM	MSKNTSVPVS	AVFMDKPEEF	ISEMDVCNAF	FFP	

Cmpd.	No. of Cmpds.	m/z meas.	Δ m/z [ppm]	z	Rt [min]	Score	P	Range	Sequence	Modification
9	1	650.7714	-185.46	2	29.3	12.0	2	178-188	K.ILRACIEQVKK.Y	

Detailed Protein Report

Protein 412: **PREDICTED: diphosphomevalonate decarboxylase isoform X2 [Homo sapiens]**

**Accession:**	gi|578828983	**Score:**	25.7
**Database:**	refseq_human(refseq_human_20140103.fasta)	**MW [kDa]:**	24.8
**Database Date:**	2015-11-30	**pI:**	6.1
		**Sequence Coverage [%]:**	16.2
		**No. of unique Peptides:**	1

10	20	30	40	50	60	70	80
MGEQADGKDS	IARQVAPESH	WPELRVLILV	VSAEKKLTGS	TVGMRASVET	SPLLRFRAES	VVPARMAEMA	RCIRERDFPS
**90**	**100**	**110**	**120**	**130**	**140**	**150**	**160**
FAQLTMKDSN	QFHATCLDTF	PPISYLNAIS	WRIIHLVHRF	NAHHGDTKVA	YTFDAGPNAV	IFTLDDTVAE	FVAAVWHGFP
**170**	**180**	**190**	**200**	**210**	**220**	**230**	
PGSNGDTFLK	GLQVRPAPLS	AELQAALAME	PTPGGVKYII	VTQVGPGPQI	LDDPCAHLLG	PDGLPKPAA	

Cmpd.	No. of Cmpds.	m/z meas.	Δ m/z [ppm]	z	Rt [min]	Score	P	Range	Sequence	Modification
1514	1	1052.1817	93.63	2	48.6	11.8	2	36-55	K.KLTGSTVGMRASVETSPLLR.F	

Detailed Protein Report

Protein 413: PREDICTED: protein unc-13 homolog B isoform X1 [Homo sapiens]

**Accession:**	gi|578816361	**Score:**	25.7
**Database:**	refseq_human(refseq_human_20140103.fasta)	**MW [kDa]:**	268.9
**Database Date:**	2015-11-30	**pI:**	5.4
		**Sequence Coverage [%]:**	1.1
		**No. of unique Peptides:**	1

Detailed Protein Report

10	20	30	40	50	60	70	80
MSLLCVRVKR	AKFQGSPDKF	NTYVTLKVQN	VKSTTVAVRG	DQPSWEQDFM	FEISRLDLGL	SVEVWNKGLI	WDTMVGTVWI
90	100	110	120	130	140	150	160
ALKTIRQSDE	EGPGEWSTLE	AETLMKDDEI	CGTRNPTPHK	ILLDTRFELP	FDIPEEEARY	WTYKWEQINA	LGADNEYSSQ
170	180	190	200	210	220	230	240
EESQRKPLPT	AAAQCCHWTY	LGWGEQQTFE	DPDSAVDDRD	SDYRSETSNS	FPPPYHTASQ	PNASVHQFPV	PVRSPQQLLL
250	260	270	280	290	300	310	320
QGSSRDSCND	SMQSYDLDYP	ERRAISPTSS	SRYGSSCNVS	QGSSQLSELD	QYHEQDDDHR	ETDSIHSCHS	SHSLSRDGQA
330	340	350	360	370	380	390	400
GFGEQEKPLE	VTGQAEKEAA	CEPKEMKEDA	TTHPPPDLVL	QKDHFLGPQE	SFPEENASSP	FTQARAHWIR	AVTKVRLQLQ
410	420	430	440	450	460	470	480
EVGNLFYILT	RNKRKKQRTP	QTLREKMKRL	LQESEEEIMV	TLGPSSRLSP	DKAKAETMCG	TKSKSSGPAG	SLPEDNSLPP
490	500	510	520	530	540	550	560
CCGSAALAIG	GDRDGDLAQL	CSFGQQNNSQ	LPLGSTAYVS	GSGSQDPSSV	SITTSCQEPS	ERNQTKTLLS	RGHGQGCSRE
570	580	590	600	610	620	630	640
QREPLGDVVE	YIIRELQGIS	RLQTEIAELQ	QHLSQVQGSV	DEVSSCVDSV	LSEIEGLQVS	TCSLAKVCEG	EKAQEPHVDR
650	660	670	680	690	700	710	720
PSEEAILYLY	GLPEQDGENT	MELVESFLAK	HLCVNGMQCN	RYIKKAYRAG	TSPSPRPTVV	KLAHLEHRDF	ILQKSILLQN
730	740	750	760	770	780	790	800
VGVRIATREE	PSCPQGNKNP	QKESISFFQQ	QHQDYSQTSL	NQDEPVLQME	TGDRGPITGT	YQMKAQDQQR	EHQAPEQQGP
810	820	830	840	850	860	870	880
CFLLKNNLSK	ESDVPKLGDE	IKGASRTSQV	ISGSCDELSE	KKASLSTPHQ	FEEPALVLIS	KEEASGKSQF	FKQYSQKHEA
890	900	910	920	930	940	950	960
CKVGKPENDC	HDKSEASSCL	SLSGLLKTER	INAEDKLLGC	EAGLDILSSK	ELEDLLADKS	RRLAALSCDS	MMEEIIIGPE
970	980	990	1000	1010	1020	1030	1040
TFSDMVHIDL	NEEEECAAHV	LKDVFDKSSC	VLGGSQEDED	VEIKFYTSKL	GRAIHHFRSA	LQGVFQKLEN	SGSISPEDLE
1050	1060	1070	1080	1090	1100	1110	1120
SNESGSQSEN	SDRLLGTVSS	GGAQDCSLES	PGSQGSESLL	SVVSGGVGVS	TQGEQTPQDP	STFSLASNNS	LPSVALAPCL
1130	1140	1150	1160	1170	1180	1190	1200
GSETCSRPGS	PKQGRLSLEQ	VCTETVYLNK	CINNFKNVLR	EKRLRQKKLL	HELVQKANRL	SVEDIHSEEK	RGALQIPDDG
1210	1220	1230	1240	1250	1260	1270	1280
DPSLPQWLPE	GPAGGLYGID	SMPDLRRKKP	LPLVSDLAMS	LVQSRKAGIT	SAMATRTSLK	DEELKSHVYK	KTLQALIYPI
1290	1300	1310	1320	1330	1340	1350	1360
SCTTPHNFEV	WTATTPTYCY	ECEGLLWGIA	RQGMRCSECG	VKCHEKCQDL	LNADCLQRAA	EKSCKHGAED	RTQNIIMAMK
1370	1380	1390	1400	1410	1420	1430	1440
DRMKIRERNK	PEIFEVIRDV	FTVNKAAHVQ	QMKTVKQSVL	DGTSKWSAKI	TITVVCAQGL	QAKDKTGSSD	PYVTVQVSKT
1450	1460	1470	1480	1490	1500	1510	1520
KKRTKTIFGN	LNPVWEEKFH	FECHNSSDRI	KVRVWDEDDD	IKSRVKQRLK	RESDDFLGQT	IIEVRTLSGE	MDVWYNLEKR
1530	1540	1550	1560	1570	1580	1590	1600
TDKSAVSGAI	RLQISVEIKG	EEKVAPYHVQ	YTCLHENLFH	YLTDIQGSGG	VRIPEARGDD	AWKVYFDETA	QEIVDEFAMR
1610	1620	1630	1640	1650	1660	1670	1680
YGIESIYQAM	THFACLSSKY	MCPGVPAVMS	TLLANINAYY	AHTTASTNVS	ASDRFAASNF	GKERFVKLLD	QLHNSLRIDL
1690	1700	1710	1720	1730	1740	1750	1760
STYRNNFPAG	SPERLQDLKS	TVDLLTSITF	FRMKVQELQS	PPRASQVVKD	CVKACLNSTY	EYIFNNCHDL	YSRQYQLKQE
1770	1780	1790	1800	1810	1820	1830	1840
LPPEEQGPSI	RNLDFWPKLI	TLIVSIIEED	KNSYTPVLNQ	FPQELNVGKV	SAEVMWHLFA	QDMKYALEEH	EKDHLCKSAD
1850	1860	1870	1880	1890	1900	1910	1920
YMNLHFKVKW	LHNEYVRDLP	VLQGQVPEYP	AWFEQFVLQW	LDENEDVSLE	FLRGALERDK	KDGFQQTSEH	ALFSCSVVDV
1930	1940	1950	1960	1970	1980	1990	2000
FTQLNQSFEI	IRKLECPDPS	ILAHYMRRFA	KTIGKVLMQY	ADILSKDFPA	YCTKEKLPCI	LMNNVQQLRV	QLEKMFEAMG
2010	2020	2030	2040	2050	2060	2070	2080
GKELDLEAAD	SLKELQVKLN	TVLDELSMVF	GNSFQVRIDE	CVRQMADILG	QVRGTGNASP	DARASAAQDA	DSVLRPLMDF
2090	2100	2110	2120	2130	2140	2150	2160
LDGNLTLFAT	VCEKTVLKRV	LKELWRVVMN	TMERMIVLPP	LTDQTGTQLI	FTAAKELSHL	SKLKDHMVRE	ETRNLTPKQC
2170	2180	2190	2200	2210	2220	2230	2240
AVLDLALDTI	KQYFHAGGNG	LKKTFLEKSP	DLQSLRYALS	LYTQTTDTLI	KTFVRSQTTQ	GSGVDDPVGE	VSIQVDLFTH
2250	2260	2270	2280	2290	2300	2310	2320
PGTGEHKVTV	KVVAANDLKW	QTAGMFRPFV	EVTMVGPHQS	DKKRKFTTKS	KSNNWAPKYN	ETFHFLLGNE	EGPESYELQI
2330	2340	2350	2360	2370	2380	2390	2400

Detailed Protein Report

Cmpd.	No. of Cmpds.	m/z meas.	Δ m/z [ppm]	z	Rt [min]	Score	P	Range	Sequence	Modification
2611	1	709.8596	-31.02	2	63.3	11.5	0	917-930	K.LLGCEAGLDILSSK.E	

Detailed Protein Report

Protein 414: integrin beta-like protein 1 isoform 3 [Homo sapiens]

**Accession:**	gi|418203907	**Score:**	25.7
**Database:**	refseq_human(refseq_human_20140103.fasta)	**MW [kDa]:**	43.9
**Database Date:**	2015-11-30	**pI:**	5.0
**Modification(s):**	Carbamidomethyl	**Sequence Coverage [%]:**	10.0
		**No. of unique Peptides:**	1

10	20	30	40	50	60	70	80
MNLVAVMGAF	NKGHGKCDCG	KCKCDQGWYG	DACQYPTNCD	LTKKKSNQMC	KNSQDIICSN	AGTCHCGRCK	CDNSDGSGLV
**90**	**100**	**110**	**120**	**130**	**140**	**150**	**160**
YGKFCECDDR	ECIDDETEEI	CGGHGKCYCG	NCYCKAGWHG	DKCEFQCDIT	PWESKRRCTS	PDGKICSNRG	TCVCGECTCH
**170**	**180**	**190**	**200**	**210**	**220**	**230**	**240**
DVDPTGDWGD	IHGDTCECDE	RDCRAVYDRY	SDDFCSGHGQ	CNCGRCDCKA	GWYGKKCEHP	QSCTLSAEES	IRKCQGSSDL
**250**	**260**	**270**	**280**	**290**	**300**	**310**	**320**
PCSGRGKCEC	GKCTCYPPGD	RRVYGKTCEC	DDRRCEDLDG	VVCGGHGTCS	CGRCVCERGW	FGKLCQHPRK	CNMTEEQSKN
**330**	**340**	**350**	**360**	**370**	**380**	**390**	**400**
LCESADGILC	SGKGSCHCGK	CICSAEEWYI	SGEFCDCDDR	DCDKHDGLIC	TGNGICSCGN	CECWDGWNGN	ACEIWLGSEY
**410**							
P							

Cmpd.	No. of Cmpds.	m/z meas.	Δ m/z [ppm]	z	Rt [min]	Score	P	Range	Sequence	Modification
2134	1	1062.3814	-60.33	2	56.8	10.7	1	52-70	K.NSQDIICSNAGTCHCGRCK.C	Carbamidomethyl: 13, 15

Detailed Protein Report

Protein 415: PREDICTED: GRAM domain-containing protein 1C isoform X2 [Homo sapiens]

**Accession:**	gi|530374810	**Score:**	25.7
**Database:**	refseq_human(refseq_human_20140103.fasta)	**MW [kDa]:**	69.6
**Database Date:**	2015-11-30	**pI:**	6.9
		**Sequence Coverage [%]:**	3.8
		**No. of unique Peptides:**	2

10	20	30	40	50	60	70	80
MEGAPTVRQV	MNEGDSSLAT	DLQEDVEENP	SPTVEENNVV	VKKQGPNLHN	WSGDWSFWIS	SSTYKDRNEE	YRRQFTHLPD
**90**	**100**	**110**	**120**	**130**	**140**	**150**	**160**
TERLIADYAC	ALQRDILLQG	RLYLSENWLC	FYSNIFRWET	TISIALKNIT	FMTKEKTARL	IPNAIQIVTE	SEKFFFTSFG
**170**	**180**	**190**	**200**	**210**	**220**	**230**	**240**
ARDRSYLSIF	RLWQNVLLDK	SLTRQEFWQL	LQQNYGTELG	LNAEEMENLS	LSIEDVQPRS	PGRSSLDDSG	ERDEKLSKSI
**250**	**260**	**270**	**280**	**290**	**300**	**310**	**320**
SFTSESISRV	SETESFDGNS	SKGGLGKEES	QNEKQTKKSL	LPTLEKKLTR	VPSKSLDLNK	NEYLSLDKSS	TSDSVDEENV
**330**	**340**	**350**	**360**	**370**	**380**	**390**	**400**
PEKDLHGRLF	INRIFHISAD	RMFELLFTSS	RFMQKFASSR	NIIDVVSTPW	TAELGGDQLR	TMTYTIVLNS	PLTGKCTAAT
**410**	**420**	**430**	**440**	**450**	**460**	**470**	**480**
EKQTLYKESR	EARFYLVDSE	VLTHDVPYHD	YFYTVNRYCI	IRSSKQKCRL	RVSTDLKYRK	QPWGLVKSLI	EKNSWSSLED
**490**	**500**	**510**	**520**	**530**	**540**	**550**	**560**
YFKQLGKKKE	MENYNVTLIV	VMSIFVLLLV	LLNVTLFLKL	SKIEHAAQSF	YRLRLQEEKS	LNLASDMVSR	AETIQKNKDQ
**570**	**580**	**590**	**600**	**610**			
AHRLKGVLRD	SIVMLEQLKS	SLIMLQKTFD	LLNKNKTGMA	VES			

Cmpd.	No. of Cmpds.	m/z meas.	Δ m/z [ppm]	z	Rt [min]	Score	P	Range	Sequence	Modification
1154	1	981.6192	184.16	1	44.1	11.5	1	66-72	K.DRNEEYR.R	
2537	1	884.5766	142.07	2	62.2	14.2	2	588-603	K.TFDLLNKNKTGMAVES.-	

Detailed Protein Report

Protein 416: PREDICTED: acetyl-CoA acetyltransferase, cytosolic isoform X1 [Homo sapiens]

**Accession:**	gi|530383698	**Score:**	25.7
**Database:**	refseq_human(refseq_human_20140103.fasta)	**MW [kDa]:**	44.6
**Database Date:**	2015-11-30	**pI:**	9.9
**Modification(s):**	Carbamidomethyl	**Sequence Coverage [%]:**	9.4
		**No. of unique Peptides:**	2

Quantitation

MD:MU **Median:** 1.10 **CV:** 0.00 % **No. of Peptides:** 1

```
        10         20         30         40         50         60         70         80
MGSHPVLRIW GNRRATAASL GRSGGRLSSP RLLRVVAPTL TFAQTSRCSF NGALAAVPVQ DLGSTVIKEV LKRATVAPED
        90        100        110        120        130        140        150        160
VSEVIFGHVL AAGCGQNPVR QASVGAGIPY SVPAWSCQMI CGSGLKAVCL AVQSIGIGDS SIVVAGGMEN MSKAPHLAYL
       170        180        190        200        210        220        230        240
RTGVKIGEMP LTDSILCDGL TDAFHNCHMG ITAENVAKKW QVSREDQDKV AVLSQNRTEN AQKAGHFDKE IVPVLVSTRK
       250        260        270        280        290        300        310        320
GLIEVKTDEF PRHGSNIEAM SKLKPYFLTD GTGTVTPANA SGINDGAAAV VLMKKSEADK RGLTPLARIV SWSQVGVEPS
       330        340        350        360        370        380        390        400
IMGIGPIPAI KQAVTKAGWS LEDVDIFEIN EAFAAVSAAI VKELGLNPEK VNIEGGAIAL GHPLGASGCR ILVTLLHTLE
       410        420        430
RMGRSRGVAA LCIGGGMGIA MCVQRE
```

Cmpd.	No. of Cmpds.	m/z meas.	Δ m/z [ppm]	z	Rt [min]	Score	P	Range	Sequence	Modification	Ratios
2173	1	1045.4885	-59.90	2	57.0	15.6	0	48-68	R.CSFNGALAAVPVQDLGSTVIK.E		
1603	1	932.4687	12.96	2	49.8	10.1	0	407-425	R.GVAALCIGGGMGIAMCVQR.E	Carbamidomethyl: 16	MD:MU 1.10

Detailed Protein Report

Protein 417: tubulin beta-4B chain [Homo sapiens]

**Accession:**	gi|5174735	**Score:**	25.7
**Database:**	refseq_human(refseq_human_20140103.fasta)	**MW [kDa]:**	49.8
**Database Date:**	2015-11-30	**pI:**	4.6
**Modification(s):**	Carbamidomethyl, Oxidation	**Sequence Coverage [%]:**	9.2
		**No. of unique Peptides:**	1

10	20	30	40	50	60	70	80
MREIVHLQAG	QCGNQIGAKF	WEVISDEHGI	DPTGTYHGDS	DLQLERINVY	YNEATGGKYV	PRAVLVDLEP	GTMDSVRSGP
**90**	**100**	**110**	**120**	**130**	**140**	**150**	**160**
FGQIFRPDNF	VFGQSGAGNN	WAKGHYTEGA	ELVDSVLDVV	RKEAESCDCL	QGFQLTHSLG	GGTGSGMGTL	LISKIREEYP
**170**	**180**	**190**	**200**	**210**	**220**	**230**	**240**
DRIMNTFSVV	PSPKVSDTVV	EPYNATLSVH	QLVENTDETY	CIDNEALYDI	CFRTLKLTTP	TYGDLNHLVS	ATMSGVTTCL
**250**	**260**	**270**	**280**	**290**	**300**	**310**	**320**
RFPGQLNADL	RKLAVNMVPF	PRLHFFMPGF	APLTSRGSQQ	YRALTVPELT	QQMFDAKNMM	AACDPRHGRY	LTVAAVFRGR
**330**	**340**	**350**	**360**	**370**	**380**	**390**	**400**
MSMKEVDEQM	LNVQNKNSSY	FVEWIPNNVK	TAVCDIPPRG	LKMSATFIGN	STAIQELFKR	ISEQFTAMFR	RKAFLHWYTG
**410**	**420**	**430**	**440**	**450**			
EGMDEMEFTE	AESNMNDLVS	EYQQYQDATA	EEEGEFEEEA	EEVA			

Cmpd.	No. of Cmpds.	m/z meas.	Δ m/z [ppm]	z	Rt [min]	Score	P	Range	Sequence	Modification
1621	1	1061.1775	-37.83	3	50.0	12.9	2	351-379	K.TAVCDIPPRGLKMSATFIGNSTAIQELFK.R	Carbamidomethyl: 4; Oxidation: 13

Detailed Protein Report

BRUKER

Protein 418: methyl-CpG-binding domain protein 5 [Homo sapiens]

**Accession:**	gi|38202209	**Score:**	25.6
**Database:**	refseq_human(refseq_human_20140103.fasta)	**MW [kDa]:**	159.8
**Database Date:**	2015-11-30	**pI:**	10.0
		**Sequence Coverage [%]:**	1.7
		**No. of unique Peptides:**	1

10	20	30	40	50	60	70	80
MNGGKECDGG	DKEGGLPAIQ	VPVGWQRRVD	QNGVLYVSPS	GSLLSCLEQV	KTYLLTDGTC	KCGLECPLIL	PKVFNFDPGA
90	100	110	120	130	140	150	160
AVKQRTAEDV	KADEDVTKLC	IHKRKIIAVA	TLHKSMEAPH	PSLVLTSPGG	GTNATPVVPS	RAATPRSVRN	KSHEGITNSV
170	180	190	200	210	220	230	240
MPECKNPFKL	MIGSSNAMGR	LYVQELPGSQ	QQELHPVYPR	QRLGSSEHGQ	KSPFRGSHGG	LPSPASSGSQ	IYGDGSISPR
250	260	270	280	290	300	310	320
TDPLGSPDVF	TRSNPGFHGA	PNSSPIHLNR	TPLSPPSVML	HGSPVQSSCA	MAGRTNIPLS	PTLTTKSPVM	KKPMCNFSTN
330	340	350	360	370	380	390	400
MEIPRAMFHH	KPPQGPPPPP	PPSCALQKKP	LTSEKDPLGI	LDPIPSKPVN	QNPVIINPTS	FHSNVHSQVP	MMNVSMPPAV
410	420	430	440	450	460	470	480
VPLPSNLPLP	TVKPGHMNHG	SHVQRVQHSA	STSLSPSPVT	SPVHMMGTGI	GRIEASPQRS	RSSSTSSDHG	NFMMPPVGPQ
490	500	510	520	530	540	550	560
ATSSGIKVPP	RSPRSTIGSP	RPSMPSSPST	KSDGHHQYKD	IPNPLIAGIS	NVLNTPSSAA	FPTASAGSSS	VKSQPGLLGM
570	580	590	600	610	620	630	640
PLNQILNQHN	AASFPASSLL	SAAAKAQLAN	QNKLAGNNSS	SSSNSGAVAG	SGNTEGHSTL	NTMFPPTANM	LLPTGEGQSG
650	660	670	680	690	700	710	720
RAALRDKLMS	QQKDALRKRK	QPPTTVLSLL	RQSQMDSSAV	PKPGPDLLRK	QGQGSFPISS	MSQLLQSMSC	QSSHLSSNST
730	740	750	760	770	780	790	800
PGCGASNTAL	PCSANQLHFT	DPSMNSSVLQ	NIPLRGEAVH	CHNANTNFVH	SNSPVPNHHL	AGLINQIQAS	GNCGMLSQSG
810	820	830	840	850	860	870	880
MALGNSLHPN	PPQSRISTSS	TPVIPNSIVS	SYNQTSSEAG	GSGPSSSIAI	AGTNHPAITK	TTSVLQDGVI	VTTAAGNPLQ
890	900	910	920	930	940	950	960
SQLPIGSDFP	FVGQEHALHF	PSNSTSNNHL	PHPLNPSLLS	SLPISLPVNQ	QHLLNQNLLN	ILQPSAGEGD	MSSINNTLSN
970	980	990	1000	1010	1020	1030	1040
HQLTHLQSLL	NNNQMFPPNQ	QQQQLLQGYQ	NLQAFQGQST	IPCPANNNPM	ACLFQNFQVR	MQEDAALLNK	RISTQPGLTA
1050	1060	1070	1080	1090	1100	1110	1120
LPENPNTTLP	PFQDTPCELQ	PRIDPSLGQQ	VKDGLVVGGP	GDASVDAIYK	AVVDAASKGM	QVVITTAVNS	TTQISPIPAL
1130	1140	1150	1160	1170	1180	1190	1200
SAMSAFTASI	GDPLNLSSAV	SAVIHGRNMG	GVDHDGRLRN	SRGARLPKNL	DHGKNVNEGD	GFEYFKSASC	HTSKKQWDGE
1210	1220	1230	1240	1250	1260	1270	1280
QSPRGERNRW	KYEEFLDHPG	HIHSSPCHER	PNNVSTLPFL	PGEQHPILLP	PRNCPGDKIL	EENFRYNNYK	RTMMSFKERL
1290	1300	1310	1320	1330	1340	1350	1360
ENTVERCAHI	NGNRPRQSRG	FGELLSTAKQ	DLVLEEQSPS	SSNSLENSLV	KDYIHYNGDF	NAKSVNGCVP	SPSDAKSISS
1370	1380	1390	1400	1410	1420	1430	1440
EDDLRNPDSP	SSNELIHYRP	RTFNVGDLVW	GQIKGLTSWP	GKLVREDDVH	NSCQQSPEEG	KVEPEKLKTL	TEGLEAYSRV
1450	1460	1470	1480	1490	1500		
RKRNRKSGKL	NNHLEAAIHE	AMSELDKMSG	TVHQIPQGDR	QMRPPKPKRR	KISR		

Cmpd.	No. of Cmpds.	m/z meas.	Δ m/z [ppm]	z	Rt [min]	Score	P	Range	Sequence	Modification
2584	1	834.4230	41.03	2	62.8	12.5	0	312-325	K.KPMCNFSTNMEIPR.A	

Detailed Protein Report

Protein 419: PREDICTED: sterol regulatory element-binding protein 1 isoform X2 [Homo sapiens]

**Accession:**	gi|578830023	**Score:**	25.6
**Database:**	refseq_human(refseq_human_20140103.fasta)	**MW [kDa]:**	119.1
**Database Date:**	2015-11-30	**pI:**	9.9
**Modification(s):**	Oxidation	**Sequence Coverage [%]:**	2.4
		**No. of unique Peptides:**	1

10	20	30	40	50	60	70	80
MDCTFEDMLQ	LINNQDSDFP	GLFDPPYAGS	GAGGTDPASP	DTSSPGSLSP	PPATLSSSLE	AFLSGPQAAP	SPLSPPQPAP
**90**	**100**	**110**	**120**	**130**	**140**	**150**	**160**
TPLKMYPSMP	AFSPGPGIKE	ESVPLSILQT	PTPQPLPGAL	LPQSFPAPAP	PQFSSTPVLG	YPSPPGGFST	GSPPGNTQQP
**170**	**180**	**190**	**200**	**210**	**220**	**230**	**240**
LPGLPLASPP	GVPPVSLHTQ	VQSVVPQQLL	TVTAAPTAAP	VTTTVTSQIQ	QVPVLLQPHF	IKADSLLLTA	MKTDGATVKA
**250**	**260**	**270**	**280**	**290**	**300**	**310**	**320**
AGLSPLVSGT	TVQTGPLPTL	VSGGTILATV	PLVVDAEKLP	INRLAAGSKA	PASAQSRGEK	RTAHNAIEKR	YRSSINDKII
**330**	**340**	**350**	**360**	**370**	**380**	**390**	**400**
ELKDLVVGTE	AKLNKSAVLR	KAIDYIRFLQ	HSNQKLKQEN	LSLRTAVHKS	KSLKDLVSAC	GSGGNTDVLM	EGVKTEVEDT
**410**	**420**	**430**	**440**	**450**	**460**	**470**	**480**
LTPPPSDAGS	PFQSSPLSLG	SRGSGSGGSG	SDSEPDSPVF	EDSKAKPEQR	PSLHSRGMLD	RSRLALCTLV	FLCLSCNPLA
**490**	**500**	**510**	**520**	**530**	**540**	**550**	**560**
SLLGARGLPS	PSDTTSVYHS	PGRNVLGTES	RDGPGWAQWL	LPPVVWLLNG	LLVLVSLVLL	FVYGEPVTRP	HSGPAVYFWR
**570**	**580**	**590**	**600**	**610**	**620**	**630**	**640**
HRKQADLDLA	RGDFAQAAQQ	LWLALRALGR	PLPTSHLDLA	CSLLWNLIRH	LLQRLWVGRW	LAGRAGGLQQ	DCALRVDASA
**650**	**660**	**670**	**680**	**690**	**700**	**710**	**720**
SARDAALVYH	KLHQLHTMGK	HTGGHLTATN	LALSALNLAE	CAGDAVSVAT	LAEIYVAAAL	RVKTSLPRAL	HFLTRFFLSS
**730**	**740**	**750**	**760**	**770**	**780**	**790**	**800**
ARQACLAQSG	SVPPAMQWLC	HPVGHRFFVD	GDWSVLSTPW	ESLYSLAGNP	VDPLAQVTQL	FREHLLERAL	NCVTQPNPSP
**810**	**820**	**830**	**840**	**850**	**860**	**870**	**880**
GSADGDKEFS	DALGYLQLLN	SCSDAAGAPA	YSFSISSSMA	TTTGVDPVAK	WWASLTAVVI	HWLRRDEEAA	ERLCPLVEHL
**890**	**900**	**910**	**920**	**930**	**940**	**950**	**960**
PRVLQESERP	LPRAALHSFK	AARALLGCAK	AESGPASLTI	CEKASGYLQD	SLATTPASSS	IDKAVQLFLC	DLLLVVRTSL
**970**	**980**	**990**	**1000**	**1010**	**1020**	**1030**	**1040**
WRQQQPPAPA	PAAQGTSSRP	QASALELRGF	QRDLSSLRRL	AQSFRPAMRR	VFLHEATARL	MAGASPTRTH	QLLDRSLRRR
**1050**	**1060**	**1070**	**1080**	**1090**	**1100**	**1110**	**1120**
AGPGGKGGAV	AELEPRPTRR	EHAEALLLAS	CYLPPGFLSA	PGQRVGMLAE	AARTLEKLGD	RRLLHDCQQM	LMRLGGGTTV
**1130**							
TSS							

Cmpd.	No. of Cmpds.	m/z meas.	Δ m/z [ppm]	z	Rt [min]	Score	P	Range	Sequence	Modification
1578	1	798.3570	-33.20	2	49.4	11.7	0	85-99	K.MYPSMPAFSPGPGIK.E	Oxidation: 5

Detailed Protein Report

Protein 420: insulin-like peptide INSL6 precursor [Homo sapiens]

Accession: gi|38569396
Database: refseq_human(refseq_human_20140103.fasta)
Database Date: 2015-11-30
Modification(s): Carbamidomethyl

Score: 25.6
MW [kDa]: 24.8
pI: 10.4
Sequence Coverage [%]: 12.7
No. of unique Peptides: 2

```
        10         20         30         40         50         60         70         80
MPRLLRLSLL WLGLLLVRFS RELSDISSAR KLCGRYLVKE IEKLCGHANW SQFRFEEETP FSRLIAQASE KVEAYSPYQF
        90        100        110        120        130        140        150        160
ESPQTASPAR GRGTNPVSTS WEEAVNSWEM QSLPEYKDKK GYSPLGKTRE FSSSHNINVY IHENAKFQKK RRNKIKTLSN
       170        180        190        200        210        220
LFWGHHPQRK RRGYSEKCCL TGCTKEELSI ACLPYIDFKR LKEKRSSLVT KIY
```

Cmpd.	No. of Cmpds.	m/z meas.	Δ m/z [ppm]	z	Rt [min]	Score	P	Range	Sequence	Modification
1976	1	753.7938	-38.59	2	54.8	12.2	1	173-185	R.GYSEKCCLTGCTK.E	Carbamidomethyl: 7, 11
2810	1	1024.4996	19.87	3	65.5	13.4	2	173-199	R.GYSEKCCLTGCTKEELSIACLPYIDFK.R	Carbamidomethyl: 11

Detailed Protein Report

Protein 421: PREDICTED: kinesin-like protein KIF13A isoform X8 [Homo sapiens]

**Accession:**	gi|578811856	**Score:**	25.6
**Database:**	refseq_human(refseq_human_20140103.fasta)	**MW [kDa]:**	194.6
**Database Date:**	2015-11-30	**pI:**	5.3
		**Sequence Coverage [%]:**	1.2
		**No. of unique Peptides:**	2

10	20	30	40	50	60	70	80
MMGHAEQLGL	IPRLCCALFK	RISLEQNESQ	TFKVEVSYME	IYNEKVRDLL	DPKGSRQSLK	VREHKVLGPY	VDGLSQLAVT
90	100	110	120	130	140	150	160
SFEDIESLMS	EGNKSRTVAA	TNMNEESSRS	HAVFNIIITQ	TLYDLQSGNS	GEKVSKVSLV	DLAGSERVSK	TGAAGERLKE
170	180	190	200	210	220	230	240
GSNINKSLTT	LGLVISSLAD	QAAGKGKSKF	VPYRDSVLTW	LLKDNLGGNS	QTSMIATISP	AADNYEETLS	TLRYADRAKR
250	260	270	280	290	300	310	320
IVNHAVVNED	PNAKVIRELR	EEVEKLREQL	SQAEAMKAPE	LKEKLEESEK	LIKELTVTWE	EKLRKTEEIA	QERQRQLESM
330	340	350	360	370	380	390	400
GISLEMSGIK	VGDDKCYLVN	LNADPALNEL	LVYYLKDHTR	VGADTSQDIQ	LFGIGIQPQH	CEIDIASDGD	VTLTPKENAR
410	420	430	440	450	460	470	480
SCVNGTLVCS	TTQLWHGDRI	LWGNNHFFRI	NLPKRKRRDW	LKDFEKETGP	PEHDLDAASE	ASSEPDYNYE	FAQMEVIMKT
490	500	510	520	530	540	550	560
LNSNGGFPII	SLSAWWSSPW	WSYLKILFPL	MWFQTKLLHK	HWDKDPVQNV	VQVLEKQYLE	EKRSALEEQR	LMYERELEQL
570	580	590	600	610	620	630	640
RQQLSPDRQP	QSSGPDRLAY	SSQTAQQKVT	QWAEERDELF	RQSLAKLREQ	LVKANTLVRE	ANFLAEEMSK	LTDYQVTLQI
650	660	670	680	690	700	710	720
PAANLSANRK	RGAIVSEPAI	QVRRKGKSTQ	VWTIEKLENK	LIDMRDLYQE	WKEKVPEAKR	LYGKRGDPFY	EAQENHNLIG
730	740	750	760	770	780	790	800
VANVFLECLF	CDVKLQYAVP	IISQQGEVAG	RLHVEVMRVT	GAVPERVVED	DSSENSSESG	SLEVVDSSGE	IIHRVKKLTC
810	820	830	840	850	860	870	880
RVKIKEATGL	PLNLSNFVFC	QYTFWDQCES	TVAAPVVDPE	VPSPQSKDAQ	YTVTFSHCKD	YVVNVTEEFL	EFISDGALAI
890	900	910	920	930	940	950	960
EVWGHRCAGN	GSSIWEVDSL	HAKTRTLHDR	WNEVTRRIEM	WISILELNEL	GEYAAVELHQ	AKDVNTGGIF	QLRQGHSRRV
970	980	990	1000	1010	1020	1030	1040
QVTVKPVQHS	GTLPLMVEAI	LSVSIGCVTA	RSTKLQRGLD	SYQRDDEDGD	DMDSYQEEDL	NCVRERWSDA	LIKRREYLDE
1050	1060	1070	1080	1090	1100	1110	1120
QIKKVSNKTE	KTEDDVEREA	QLVEQWVGLT	EERNAVLVPA	PGSGIPGAPA	DWIPPPGMET	HIPVLFLDLN	ADDLSANEQL
1130	1140	1150	1160	1170	1180	1190	1200
VGPHASGVNS	ILPKEHGSQF	FYLPIIKHSD	DEVSATASWD	SSVHDSVHLN	RVTPQNERIY	LIVKTTVQLS	HPAAMELVLR
1210	1220	1230	1240	1250	1260	1270	1280
KRIAANIYNK	QSFTQSLKRR	ISLKNIFYSC	GVTYEIVSNI	PKATEEIEDR	ETLALLAARS	ENEGTSDGET	YIEKYTRGVL
1290	1300	1310	1320	1330	1340	1350	1360
QVENILSLER	LRQAVTVKEA	LSTKARHIRR	SLSTPNVHNV	SSSRPDLSGF	DEDDKGWPEN	QLDMSDYSSS	YQDVACYGTL
1370	1380	1390	1400	1410	1420	1430	1440
PRDSPRRNKE	GCTSETPHAL	TVSPFKAFSP	QPPKFFKPLM	PVKEEHKKRI	ALEARPLLSQ	ESMPPPQAHN	PGCIVPSGSN
1450	1460	1470	1480	1490	1500	1510	1520
GSSMPVEHNS	KREKKIDSEE	EENELEAINR	KLISSQPYVP	VEFADFSVYN	ASLENREWFS	SKVDLSNSRV	LEKEVSRSPT
1530	1540	1550	1560	1570	1580	1590	1600
TSSITSGYFS	HSASNATLSD	MVVPSSDSSD	QLAIQTKDAD	STEHSTPSLV	HDFRPSSNKE	LTEVEKGLVK	DKIIVVPLKE
1610	1620	1630	1640	1650	1660	1670	1680
NSALAKGSPS	SQSIPEKNSK	SLCRTGSCSE	LDACPSKISQ	PARGFCPREV	TVEHTTNILE	DHSFTEFMGV	SEGKDFDGLT
1690	1700	1710	1720	1730	1740		
DSSAGELSSR	RSLPNKTGGK	TVSDGLHHPS	QLHSKLENDQ	VIIPEAAFWV	LCCQ		

Cmpd.	No. of Cmpds.	m/z meas.	Δ m/z [ppm]	z	Rt [min]	Score	P	Range	Sequence	Modification
978	1	955.6278	74.00	1	41.8	12.8	1	46-53	K.VRDLLDPK.G	
2115	1	840.8591	-81.82	2	56.6	12.8	2	543-555	K.RSALEEQRLMYER.E	

Detailed Protein Report

Protein 422: abnormal spindle-like microcephaly-associated protein isoform 1 [Homo sapiens]

**Accession:**	gi|126116596	**Score:**	25.6
**Database:**	refseq_human(refseq_human_20140103.fasta)	**MW [kDa]:**	409.5
**Database Date:**	2015-11-30	**pI:**	11.2
		**Sequence Coverage [%]:**	0.7
		**No. of unique Peptides:**	1

Detailed Protein Report

10	20	30	40	50	60	70	80
MANRRVGRGC	WEVSPTERRP	PAGLRGPAAE	EEASSPPVLS	LSHFCRSPFL	CFGDVLLGAS	RTLSLALDNP	NEEVAEVKIS
90	100	110	120	130	140	150	160
HFPAADLGFS	VSQRCFVLQP	KEKIVISVNW	TPLKEGRVRE	IMTFLVNDVL	KHQAILLGNA	EEQKKKKRSL	WDTIKKKKIS
170	180	190	200	210	220	230	240
ASTSHNRRVS	NIQNVNKTFS	VSQKVDRVRS	PLQACENLAM	NEGGPPTENN	SLILEENKIP	ISPISPAFNE	CHGATCLPLS
250	260	270	280	290	300	310	320
VRRSTTYSSL	HASENRELLN	VHSANVSKVS	FNEKAVTETS	FNSVNVNGQR	GENSKLSLTP	NCSSTLNITQ	SQIHFLSPDS
330	340	350	360	370	380	390	400
FVNNSHGANN	ELELVTCLSS	DMFMKDNSQP	VHLESTIAHE	IYQKILSPDS	FIKDNYGLNQ	DLESESVNPI	LSPNQFLKDN
410	420	430	440	450	460	470	480
MAYMCTSQQT	CKVPLSNENS	QVPQSPEDWR	KSEVSPRIPE	CQGSKSPKAI	FEELVEMKSN	YYSFIKQNNP	KFSAVQDISS
490	500	510	520	530	540	550	560
HSHNKQPKRR	PILSATVTKR	KATCTRENQT	EINKPKAKRC	LNSAVGEHEK	VINNQKEKED	FHSYLPIIDP	ILSKSKSYKN
570	580	590	600	610	620	630	640
EVTPSSTTAS	VARKRKSDGS	MEDANVRVAI	TEHTEVREIK	RIHFSPSEPK	TSAVKKTKNV	TTPISKRISN	REKLNLKKKT
650	660	670	680	690	700	710	720
DLSIFRTPIS	KTNKRTKPII	AVAQSSLTFI	KPLKTDIPRH	PMPFAAKNMF	YDERWKEKQE	QGFTWWLNFI	LTPDDFTVKT
730	740	750	760	770	780	790	800
NISEVNAATL	LLGIENQHKI	SVPRAPTKEE	MSLRAYTARC	RLNRLRRAAC	RLFTSEKMVK	AIKKLEIEIE	ARRLIVRKDR
810	820	830	840	850	860	870	880
HLWKDVGERQ	KVLNWLLSYN	PLWLRIGLET	TYGELISLED	NSDVTGLAMF	ILNRLLWNPD	IAAEYRHPTV	PHLYRDGHEE
890	900	910	920	930	940	950	960
ALSKFTLKKL	LLLVCFLDYA	KISRLIDHDP	CLFCKDAEFK	ASKEILLAFS	RDFLSGEGDL	SRHLGLLGLP	VNHVQTPFDE
970	980	990	1000	1010	1020	1030	1040
FDFAVTNLAV	DLQCGVRLVR	TMELLTQNWD	LSKKLRIPAI	SRLQKMHNVD	IVLQVLKSRG	IELSDEHGNT	ILSKDIVDRH
1050	1060	1070	1080	1090	1100	1110	1120
REKTLRLLWK	IAFAFQVDIS	LNLDQLKEEI	AFLKHTKSIK	KTISLLSCHS	DDLINKKKGK	RDSGSFEQYS	ENIKLLMDWV
1130	1140	1150	1160	1170	1180	1190	1200
NAVCAFYNKK	VENFTVSFSD	GRVLCYLIHH	YHPCYVPFDA	ICQRTTQTVE	CTQTGSVVLN	SSSESDDSSL	DMSLKAFDHE
1210	1220	1230	1240	1250	1260	1270	1280
NTSELYKELL	ENEKKNFHLV	RSAVRDLGGI	PAMINHSDMS	NTIPDEKVVI	TYLSFLCARL	LDLRKEIRAA	RLIQTTWRKY
1290	1300	1310	1320	1330	1340	1350	1360
KLKTDLKRHQ	EREKAARIIQ	LAVINFLAKQ	RLRKRVNAAL	VIQKYWRRVL	AQRKLLMLKK	EKLEKVQNKA	ASLIQGYWRR
1370	1380	1390	1400	1410	1420	1430	1440
YSTRQRFLKL	KYYSIILQSR	IRMIIAVTSY	KRYLWATVTI	QRHWRAYLRR	KQDQQRYEML	KSSTLIIQSM	FRKWKQRKMQ
1450	1460	1470	1480	1490	1500	1510	1520
SQVKATVILQ	RAFREWHLRK	QAKEENSAII	IQSWYRMHKE	LRKYIYIRSC	VVIIQKRFRC	FQAQKLYKRR	KESILTIQKY
1530	1540	1550	1560	1570	1580	1590	1600
YKAYLKGKIE	RTNYLQKRAA	AIQLQAAFRR	LKAHNLCRQI	RAACVIQSYW	RMRQDRVRFL	NLKKTIIKFQ	AHVRKHQQRQ
1610	1620	1630	1640	1650	1660	1670	1680
KYKKMKKAAV	IIQTHFRAYI	FAMKVLASYQ	KTRSAVIVLQ	SAYRGMQARK	MYIHILTSVI	KIQSYYRAYV	SKKEFLSLKN
1690	1700	1710	1720	1730	1740	1750	1760
ATIKLQSTVK	MKQTRKQYLH	LRAAALFIQQ	CYRSKKIAAQ	KREEYMQMRE	SCIKLQAFVR	GYLVRKQMRL	QRKAVISLQS
1770	1780	1790	1800	1810	1820	1830	1840
YFRMRKARQY	YLKMYKAIIV	IQNYYHAYKA	QVNQRKNFLQ	VKKAATCLQA	AYRGYKVRQL	IKQQSIAALK	IQSAFRGYNK
1850	1860	1870	1880	1890	1900	1910	1920
RVKYQSVLQS	IIKIQRWYRA	YKTLHDTRTH	FLKTKAAVIS	LQSAYRGWKV	RKQIRREHQA	ALKIQSAFRM	AKAQKQFRLF
1930	1940	1950	1960	1970	1980	1990	2000
KTAALVIQQN	FRAWTAGRKQ	CMEYIELRHA	VLVLQSMWKG	KTLRRQLQRQ	HKCAIIIQSY	YRMHVQQKKW	KIMKKAALLI
2010	2020	2030	2040	2050	2060	2070	2080
QKYYRAYSIG	REQNHLYLKT	KAAVVTLQSA	YRGMKVRKRI	KDCNKAAVTI	QSKYRAYKTK	KKYATYRASA	IIIQRWYRGI
2090	2100	2110	2120	2130	2140	2150	2160
KITNHQHKEY	LNLKKTAIKI	QSVYRGIRVR	RHIQHMHRAA	TFIKAMFKMH	QSRISYHTMR	KAAIVIQVRC	RAYYQGKMQR
2170	2180	2190	2200	2210	2220	2230	2240
EKYLTILKAV	KVLQASFRGV	RVRRTLRKMQ	TAATLIQSNY	RRYRQQTYFN	KLKKITKTVQ	QRYWAMKERN	IQFQRYNKLR
2250	2260	2270	2280	2290	2300	2310	2320
HSVIYIQAIF	RGKKARRHLK	MMHIAATLIQ	RRFRTLMMRR	RFLSLKKTAI	LIQRKYRAHL	CTKHHLQFLQ	VQNAVIKIQS
2330	2340	2350	2360	2370	2380	2390	2400

Detailed Protein Report

Cmpd.	No. of Cmpds.	m/z meas.	Δ m/z [ppm]	z	Rt [min]	Score	P	Range	Sequence	Modification
402	1	711.3339	-88.13	2	34.3	14.6	2	178-189	K.TFSVSQKVDRVR.S	

Detailed Protein Report

Protein 423: **PREDICTED: leucine-rich repeat and coiled-coil domain-containing protein 1 isoform X6 [Homo sapiens]**

**Accession:**	gi|530388606	**Score:**	25.6
**Database:**	refseq_human(refseq_human_20140103.fasta)	**MW [kDa]:**	77.0
**Database Date:**	2015-11-30	**pI:**	6.0
		**Sequence Coverage [%]:**	3.2
		**No. of unique Peptides:**	1

10	20	30	40	50	60	70	80
MKPPYLKELY	VSSSLANCPM	LQESEKPKTE	IIKVDQSHSE	DNTYQSLVEQ	LDQEREKRWR	AEQAENKLMD	YIDELHKHAN
**90**	**100**	**110**	**120**	**130**	**140**	**150**	**160**
EKEDIHSLAL	LTTDRLKEII	FRERNSKGQL	EVMVHKLQNE	IKKLTVELMK	AKDQQEDHLK	HLRTLEKTLE	KMERQKRQQQ
**170**	**180**	**190**	**200**	**210**	**220**	**230**	**240**
AAQIRLIQEV	ELKASAADRE	IYLLRTSLHR	EREQAQQLHQ	LLALKEQEHR	KELETREFFT	DADFQDALAK	EIAKEEKKHE
**250**	**260**	**270**	**280**	**290**	**300**	**310**	**320**
QMIKEYQEKI	DVLSQQYMDL	ENEFRIALTV	EARRFQDVKD	GFENVATELA	KSKHALIWAQ	RKENESSSLI	KDLTCMVKEQ
**330**	**340**	**350**	**360**	**370**	**380**	**390**	**400**
KTKLAEVSKL	KQETAANLQN	QINTLEILIE	DDKQKSIQIE	LLKHEKVQLI	SELAAKESLI	FGLRTERKVW	GHELAQQGSS
**410**	**420**	**430**	**440**	**450**	**460**	**470**	**480**
LAQNRGKLEA	QIESLSRENE	CLRKTNESDS	DALRIKCKII	DDQTETIRKL	KDCLQEKDEH	IKRLQEKITE	IEKCTQEQLD
**490**	**500**	**510**	**520**	**530**	**540**	**550**	**560**
EKSSQLDEVL	EKLERHNERK	EKLKQQLKGK	EVELEEIRKA	YSTLNRKWHD	KGELLCHLET	QVKEVKEKFE	NKEKKLKAER
**570**	**580**	**590**	**600**	**610**	**620**	**630**	**640**
DKSIELQKNA	MEKLHSMDDA	FKRQVDAIVE	AHQAEIAQLA	NEKQKCIDSA	NLKVHQIEKE	MRELLEETCK	NKKTMEAKIK
**650**	**660**						
QLAFALNEIQ	QDM						

Cmpd.	No. of Cmpds.	m/z meas.	Δ m/z [ppm]	z	Rt [min]	Score	P	Range	Sequence	Modification
2425	1	679.2638	-89.31	2	60.7	11.3	1	452-462	K.DCLQEKDEHIK.R	

Detailed Protein Report

Protein 424: pro-MCH preproprotein [Homo sapiens]

Accession: gi|71361684
Database: refseq_human(refseq_human_20140103.fasta)
Database Date: 2015-11-30
Modification(s): Oxidation

Score: 25.6
MW [kDa]: 18.7
pI: 7.6
Sequence Coverage [%]: 15.8
No. of unique Peptides: 1

10	20	30	40	50	60	70	80
MAKMNLSSYI	LILTFSLFSQ	GILLSASKSI	RNLDDDMVFN	TFRLGKGFQK	EDTAEKSVIA	PSLEQYKNDE	SSFMNEEENK
**90**	**100**	**110**	**120**	**130**	**140**	**150**	**160**
VSKNTGSKHN	FLNHGLPLNL	AIKPYLALKG	SVAFPAENGV	QNTESTQEKR	EIGDEENSAK	FPIGRRDFDM	LRCMLGRVYR
**170**							
PCWQV							

Cmpd.	No. of Cmpds.	m/z meas.	Δ m/z [ppm]	z	Rt [min]	Score	P	Range	Sequence	Modification
118	1	929.9543	13.99	2	31.2	15.3	1	29-43	K.SIRNLDDDMVFNTFR.L	Oxidation: 9

Detailed Protein Report

Protein 425: **phosphatidylinositol 3,4,5-trisphosphate-dependent Rac exchanger 1 protein [Homo sapiens]**

**Accession:**	gi|34452732	**Score:**	25.6
**Database:**	refseq_human(refseq_human_20140103.fasta)	**MW [kDa]:**	186.1
**Database Date:**	2015-11-30	**pI:**	6.0
		**Sequence Coverage [%]:**	2.0
		**No. of unique Peptides:**	2

10	20	30	40	50	60	70	80
MEAPSGSEPG	GDGAGDCAHP	DPRAPGAAAP	SSGPGPCAAA	RESERQLRLR	LCVLNEILGT	ERDYVGTLRF	LQSAFLHRIR
90	100	110	120	130	140	150	160
QNVADSVEKG	LTEENVKVLF	SNIEDILEVH	KDFLAALEYC	LHPEPQSQHE	LGNVFLKFKD	KFCVYEEYCS	NHEKALRLLV
170	180	190	200	210	220	230	240
ELNKIPTVRA	FLLSCMLLGG	RKTTDIPLEG	YLLSPIQRIC	KYPLLLKELA	KRTPGKHPDH	PAVQSALQAM	KTVCSNINET
250	260	270	280	290	300	310	320
KRQMEKLEAL	EQLQSHIEGW	EGSNLTDICT	QLLLQGTLLK	ISAGNIQERA	FFLFDNLLVY	CKRKSRVTGS	KKSTKRTKSI
330	340	350	360	370	380	390	400
NGSLYIFRGR	INTEVMEVEN	VEDGTADYHS	NGYTVTNGWK	IHNTAKNKWF	VCMAKTAEEK	QKWLDAIIRE	REQRESLKLG
410	420	430	440	450	460	470	480
MERDAYVMIA	EKGEKLYHMM	MNKKVNLIKD	RRRKLSTVPK	CFLGNEFVAW	LLEIGEISKT	EEGVNLGQAL	LENGIIHHVS
490	500	510	520	530	540	550	560
DKHQFKNEQV	MYRFRYDDGT	YKARSELEDI	MSKGVRLYCR	LHSLYTPVIK	DRDYHLKTYK	SVLPGSKLVD	WLLAQGDCQT
570	580	590	600	610	620	630	640
REEAVALGVG	LCNNGFMHHV	LEKSEFRDES	QYFRFHADEE	MEGTSSKNKQ	LRNDFKLVEN	ILAKRLLILP	QEEDYGFDIE
650	660	670	680	690	700	710	720
EKNKAVVVKS	VQRGSLAEVA	GLQVGRKIYS	INEDLVFLRP	FSEVESILNQ	SFCSRRPLRL	LVATKAKEII	KIPDQPDTLC
730	740	750	760	770	780	790	800
FQIRGAAPPY	VYAVGRGSEA	MAAGLCAGQC	ILKVNGSNVM	NDGAPEVLEH	FQAFRSRREE	ALGLYQWIYH	THEDAQEARA
810	820	830	840	850	860	870	880
SQEASTEDPS	GEQAQEEDQA	DSAFPLLSLG	PRLSLCEDSP	MVTLTVDNVH	LEHGVVYEYV	STAGVRCHVL	EKIVEPRGCF
890	900	910	920	930	940	950	960
GLTAKILEAF	AANDSVFVEN	CRRLMALSSA	IVTMPHFEFR	NICDTKLESI	GQRIACYQEF	AAQLKSRVSP	PFKQAPLEPH
970	980	990	1000	1010	1020	1030	1040
PLCGLDFCPT	NCHINLMEVS	YPKTTPSVGR	SFSIRFGRKP	SLIGLDPEQG	HLNPMSYTQH	CITTMAAPSW	KCLPAAEGDP
1050	1060	1070	1080	1090	1100	1110	1120
QGQGLHDGSF	GPASGTLGQE	DRGLSFLLKQ	EDREIQDAYL	QLFTKLDVAL	KEMKQYVTQI	NRLLSTITEP	TSGGSCDASL
1130	1140	1150	1160	1170	1180	1190	1200
AEEASSLPLV	SEESEMDRSD	HGGIKKVCFK	VAEEDQEDSG	HDTMSYRDSY	SECNSNRDSV	LSYTSVRSNS	SYLGSDEMGS
1210	1220	1230	1240	1250	1260	1270	1280
GDELPCDMRI	PSDKQDKLHG	CLEHLFNQVD	SINALLKGPV	MSRAFEETKH	FPMNHSLQEF	KQKEECTIRG	RSLIQISIQE
1290	1300	1310	1320	1330	1340	1350	1360
DPWNLPNSIK	TLVDNIQRYV	EDGKNQLLLA	LLKCTDTELQ	LRRDAIFCQA	LVAAVCTFSE	QLLAALGYRY	NNNGEYEESS
1370	1380	1390	1400	1410	1420	1430	1440
RDASRKWLEQ	VAATGVLLHC	QSLLSPATVK	EERTMLEDIW	VTLSELDNVT	FSFKQLDENY	VANTNVFYHI	EGSRQALKVI
1450	1460	1470	1480	1490	1500	1510	1520
FYLDSYHFSK	LPSRLEGGAS	LRLHTALFTK	VLENVEGLPS	PGSQAAEDLQ	QDINAQSLEK	VQQYYRKLRA	FYLERSNLPT
1530	1540	1550	1560	1570	1580	1590	1600
DASTTAVKID	QLIRPINALD	ELCRLMKSFV	HPKPGAAGSV	GAGLIPISSE	LCYRLGACQM	VMCGTGMQRS	TLSVSLEQAA
1610	1620	1630	1640	1650	1660		
ILARSHGLLP	KCIMQATDIM	RKQGPRVEIL	AKNLRVKDQM	PQGAPRLYRL	CQPPVDGDL		

Cmpd.	No. of Cmpds.	m/z meas.	Δ m/z [ppm]	z	Rt [min]	Score	P	Range	Sequence	Modification
1541	1	789.6495	-94.76	3	49.1	12.7	0	562-583	R.EEAVALGVGLCNNGFMHHVLEK.S	
2660	1	734.8652	-30.72	2	63.3	12.9	0	1074-1085	R.EIQDAYLQLFTK.L	

Detailed Protein Report

Protein 426: tRNA selenocysteine 1-associated protein 1 [Homo sapiens]

**Accession:**	gi|8923460	**Score:**	25.6
**Database:**	refseq_human(refseq_human_20140103.fasta)	**MW [kDa]:**	32.5
**Database Date:**	2015-11-30	**pI:**	4.5
		**Sequence Coverage [%]:**	9.1
		**No. of unique Peptides:**	1

10	20	30	40	50	60	70	80
MAASLWMGDL	EPYMDENFIS	RAFATMGETV	MSVKIIRNRL	TGIPAGYCFV	EFADLATAEK	CLHKINGKPL	PGATPAKRFK
**90**	**100**	**110**	**120**	**130**	**140**	**150**	**160**
LNYATYGKQP	DNSPEYSLFV	GDLTPDVDDG	MLYEFFVKVY	PSCRGGKVVL	DQTGVSKGYG	FVKFTDELEQ	KRALTECQGA
**170**	**180**	**190**	**200**	**210**	**220**	**230**	**240**
VGLGSKPVRL	SVAIPKASRV	KPVEYSQMYS	YSYNQYYQQY	QNYYAQWGYD	QNTGSYSYSY	PQYGYTQSTM	QTYEEVGDDA
**250**	**260**	**270**	**280**	**290**			
LEDPMPQLDV	TEANKEFMEQ	SEELYDALMD	CHWQPLDTVS	SEIPAMM			

Cmpd.	No. of Cmpds.	m/z meas.	Δ m/z [ppm]	z	Rt [min]	Score	P	Range	Sequence	Modification
1961	1	686.2209	-167.75	2	54.6	10.7	0	22-34	R.AFATMGETVMSVK.I	

Detailed Protein Report

Protein 427: plexin-A1 precursor [Homo sapiens]

**Accession:**	gi|262118282	**Score:**	25.5
**Database:**	refseq_human(refseq_human_20140103.fasta)	**MW [kDa]:**	210.9
**Database Date:**	2015-11-30	**pI:**	6.5
**Modification(s):**	Oxidation	**Sequence Coverage [%]:**	1.7
		**No. of unique Peptides:**	2

10	20	30	40	50	60	70	80
MPLPPRSLQV	LLLLLLLLLL	LPGMWAEAGL	PRAGGGSQPP	FRTFSASDWG	LTHLVVHEQT	GEVYVGAVNR	IYKLSGNLTL
90	100	110	120	130	140	150	160
LRAHVTGPVE	DNEKCYPPPS	VQSCPHGLGS	TDNVNKLLLL	DYAANRLLAC	GSASQGICQF	LRLDDLFKLG	EPHHRKEHYL
170	180	190	200	210	220	230	240
SSVQEAGSMA	GVLIAGPPGQ	GQAKLFVGTP	IDGKSEYFPT	LSSRRLMANE	EDADMFGFVY	QDEFVSSQLK	IPSDTLSKFP
250	260	270	280	290	300	310	320
AFDIYYVYSF	RSEQFVYYLT	LQLDTQLTSP	DAAGEHFFTS	KIVRLCVDDP	KFYSYVEFPI	GCEQAGVEYR	LVQDAYLSRP
330	340	350	360	370	380	390	400
GRALAHQLGL	AEDEDVLFTV	FAQGQKNRVK	PPKESALCLF	TLRAIKEKIK	ERIQSCYRGE	GKLSLPWLLN	KELGCINSPL
410	420	430	440	450	460	470	480
QIDDDFCGQD	FNQPLGGTVT	IEGTPLFVDK	DDGLTAVAAY	DYRGRTVVFA	GTRSGRIRKI	LVDLSNPGGR	PALAYESVVA
490	500	510	520	530	540	550	560
QEGSPILRDL	VLSPNHQYLY	AMTEKQVTRV	PVESCVQYTS	CELCLGSRDP	HCGWCVLHSI	CSRRDACERA	DEPQRFAADL
570	580	590	600	610	620	630	640
LQCVQLTVQP	RNVSVTMSQV	PLVLQAWNVP	DLSAGVNCSF	EDFTESESVL	EDGRIHCRSP	SAREVAPITR	GQGDQRVVKL
650	660	670	680	690	700	710	720
YLKSKETGKK	FASVDFVFYN	CSVHQSCLSC	VNGSFPCHWC	KYRHVCTHNV	ADCAFLEGRV	NVSEDCPQIL	PSTQIYVPVG
730	740	750	760	770	780	790	800
VVKPITLAAR	NLPQPQSGQR	GYECLFHIPG	SPARVTALRF	NSSSLQCQNS	SYSYEGNDVS	DLPVNLSVVW	NGNFVIDNPQ
810	820	830	840	850	860	870	880
NIQAHLYKCP	ALRESCGLCL	KADPRFECGW	CVAERRCSLR	HHCAADTPAS	WMHARHGSSR	CTDPKILKLS	PETGPRQGGT
890	900	910	920	930	940	950	960
RLTITGENLG	LRFEDVRLGV	RVGKVLCSPV	ESEYISAEQI	VCEIGDASSV	RAHDALVEVC	VRDCSPHYRA	LSPKRFTFVT
970	980	990	1000	1010	1020	1030	1040
PTFYRVSPSR	GPLSGGTWIG	IEGSHLNAGS	DVAVSVGGRP	CSFSWRNSRE	IRCLTPPGQS	PGSAPIIINI	NRAQLTNPEV
1050	1060	1070	1080	1090	1100	1110	1120
KYNYTEDPTI	LRIDPEWSIN	SGGTLLTVTG	TNLATVREPR	IRAKYGGIER	ENGCLVYNDT	TMVCRAPSVA	NPVRSPPELG
1130	1140	1150	1160	1170	1180	1190	1200
ERPDELGFVM	DNVRSLLVLN	STSFLYYPDP	VLEPLSPTGL	LELKPSSPLI	LKGRNLLPPA	PGNSRLNYTV	LIGSTPCTLT
1210	1220	1230	1240	1250	1260	1270	1280
VSETQLLCEA	PNLTGQHKVT	VRAGGFEFSP	GTLQVYSDSL	LTLPAIVGIG	GGGGLLLLVI	VAVLIAYKRK	SRDADRTLKR
1290	1300	1310	1320	1330	1340	1350	1360
LQLQMDNLES	RVALECKEAF	AELQTDIHEL	TNDLDGAGIP	FLDYRTYAMR	VLFPGIEDHP	VLKEMEVQAN	VEKSLTLFGQ
1370	1380	1390	1400	1410	1420	1430	1440
LLTKKHFLLT	FIRTLEAQRS	FSMRDRGNVA	SLIMTALQGE	MEYATGVLKQ	LLSDLIEKNL	ESKNHPKLLL	RRTESVAEKM
1450	1460	1470	1480	1490	1500	1510	1520
LTNWFTFLLY	KFLKECAGEP	LFMLYCAIKQ	QMEKGPIDAI	TGEARYSLSE	DKLIRQQIDY	KTLTLNCVNP	ENENAPEVPV
1530	1540	1550	1560	1570	1580	1590	1600
KGLDCDTVTQ	AKEKLLDAAY	KGVPYSQRPK	AADMDLEWRQ	GRMARIILQD	EDVTTKIDND	WKRLNTLAHY	QVTDGSSVAL
1610	1620	1630	1640	1650	1660	1670	1680
VPKQTSAYNI	SNSSTFTKSL	SRYESMLRTA	SSPDSLRSRT	PMITPDLESG	TKLWHLVKNH	DHLDQREGDR	GSKMVSEIYL
1690	1700	1710	1720	1730	1740	1750	1760
TRLLATKGTL	QKFVDDLFET	IFSTAHRGSA	LPLAIKYMFD	FLDEQADKHQ	IHDADVRHTW	KSNCLPLRFW	VNVIKNPQFV
1770	1780	1790	1800	1810	1820	1830	1840
FDIHKNSITD	ACLSVVAQTF	MDSCSTSEHK	LGKDSPSNKL	LYAKDIPNYK	SWVERYYADI	AKMPAISDQD	MSAYLAEQSR
1850	1860	1870	1880	1890	1900		
LHLSQFNSMS	ALHEIYSYIT	KYKDEILAAL	EKDEQARRQR	LRSKLEQVVD	TMALSS		

Cmpd.	No. of Cmpds.	m/z meas.	Δ m/z [ppm]	z	Rt [min]	Score	P	Range	Sequence	Modification

Detailed Protein Report

Cmpd.	No. of Cmpds.	m/z meas.	Δ m/z [ppm]	z	Rt [min]	Score	P	Range	Sequence	Modification
2833	1	1051.5514	-6.99	2	65.8	13.1	1	1326-1343	R.TYAMRVLFPGIEDHPVLK.E	Oxidation: 4
1277	1	990.9708	-66.25	2	45.6	12.4	1	1440-1454	K.MLTNWFTFLLYKFLK.E	Oxidation: 1

Detailed Protein Report

Protein 428: chromosome-associated kinesin KIF4A [Homo sapiens]

Accession: gi|116686122
Database: refseq_human(refseq_human_20140103.fasta)
Database Date: 2015-11-30
Modification(s): Carbamidomethyl

Score: 25.5
MW [kDa]: 139.8
pI: 5.9
Sequence Coverage [%]: 2.9
No. of unique Peptides: 2

10	20	30	40	50	60	70	80
MKEEVKGIPV	RVALRCRPLV	PKEISEGCQM	CLSFVPGEPQ	VVVGTDKSFT	YDFVFDPSTE	QEEVFNTAVA	PLIKGVFKGY
90	100	110	120	130	140	150	160
NATVLAYGQT	GSGKTYSMGG	AYTAEQENEP	TVGVIPRVIQ	LLFKEIDKKS	DFEFTLKVSY	LEIYNEEILD	LLCPSREKAQ
170	180	190	200	210	220	230	240
INIREDPKEG	IKIVGLTEKT	VLVALDTVSC	LEQGNNSRTV	ASTAMNSQSS	RSHAIFTISL	EQRKKSDKNS	SFRSKLHLVD
250	260	270	280	290	300	310	320
LAGSERQKKT	KAEGDRLKEG	ININRGLLCL	GNVISALGDD	KKGGFVPYRD	SKLTRLLQDS	LGGNSHTLMI	ACVSPADSNL
330	340	350	360	370	380	390	400
EETLNTLRYA	DRARKIKNKP	IVNIDPQTAE	LNHLKQQVQQ	LQVLLLQAHG	GTLPGSITVE	PSENLQSLME	KNQSLVEENE
410	420	430	440	450	460	470	480
KLSRGLSEAA	GQTAQMLERI	ILTEQANEKM	NAKLEELRQH	AACKLDLQKL	VETLEDQELK	ENVEIICNLQ	QLITQLSDET
490	500	510	520	530	540	550	560
VACMAAAIDT	AVEQEAQVET	SPETSRSSDA	FTTQHALRQA	QMSKELVELN	KALALKEALA	RKMTQNDSQL	QPIQYQYQDN
570	580	590	600	610	620	630	640
IKELELEVIN	LQKEKEELVL	ELQTAKKDAN	QAKLSERRRK	RLQELEGQIA	DLKKKLNEQS	KLLKLKESTE	RTVSKLNQEI
650	660	670	680	690	700	710	720
RMMKNQRVQL	MRQMKEDAEK	FRQWKQKKDK	EVIQLKERDR	KRQYELLKLE	RNFQKQSNVL	RRKTEEAAAA	NKRLKDALQK
730	740	750	760	770	780	790	800
QREVADKRKE	TQSRGMEGTA	ARVKNWLGNE	IEVMVSTEEA	KRHLNDLLED	RKILAQDVAQ	LKEKKESGEN	PPPKLRRRTF
810	820	830	840	850	860	870	880
SLTEVRGQVS	ESEDSITKQI	ESLETEMEFR	SAQIADLQQK	LLDAESEDRP	KQRWENIATI	LEAKCALKYL	IGELVSSKIQ
890	900	910	920	930	940	950	960
VSKLESSLKQ	SKTSCADMQK	MLFEERNHFA	EIETELQAEL	VRMEQQHQEK	VLYLLSQLQQ	SQMAEKQLEE	SVSEKEQQLL
970	980	990	1000	1010	1020	1030	1040
STLKCQDEEL	EKMREVCEQN	QQLLRENEII	KQKLTLLQVA	SRQKHLPKDT	LLSPDSSFEY	VPPKPKPSRV	KEKFLEQSMD
1050	1060	1070	1080	1090	1100	1110	1120
IEDLKYCSEH	SVNEHEDGDG	DDDEGDDEEW	KPTKLVKVSR	KNIQGCSCKG	WCGNKQCGCR	KQKSDCGVDC	CCDPTKCRNR
1130	1140	1150	1160	1170	1180	1190	1200
QQGKDSLGTV	ERTQDSEGSF	KLEDPTEVTP	GLSFFNPVCA	TPNSKILKEM	CDVEQVLSKK	TPPAPSPFDL	PELKHVATEY
1210	1220	1230	1240				
QENKAPGKKK	KRALASNTSF	FSGCSPIEEE	AH				

Cmpd.	No. of Cmpds.	m/z meas.	Δ m/z [ppm]	z	Rt [min]	Score	P	Range	Sequence	Modification
2074	1	701.2275	-91.47	3	56.1	10.6	2	1082-1100	K.NIQGCSCKGWCGNKQCGCR.K	Carbamidomethyl: 18
1950	2	1023.4041	14.44	2	54.3	14.9	2	1104-1120	K.SDCGVDCCCDPTKCRNR.Q	Carbamidomethyl: 3, 7, 8

Detailed Protein Report

Protein 429: vacuolar protein sorting-associated protein 13B isoform 1 [Homo sapiens]

**Accession:**	gi|35493701	**Score:**	25.5
**Database:**	refseq_human(refseq_human_20140103.fasta)	**MW [kDa]:**	445.7
**Database Date:**	2015-11-30	**pI:**	6.0
**Modification(s):**	Carbamidomethyl	**Sequence Coverage [%]:**	0.9
		**No. of unique Peptides:**	2

10	20	30	40	50	60	70	80
MLESYVTPIL	MSYVNRYIKN	LKPSDLQLSL	WGGDVVLSKL	ELKLDVLEQE	LKLPFTFLSG	HIHELRIHVP	WTKLGSEPVV
90	100	110	120	130	140	150	160
ITINTMECIL	KLKDGIQDDH	ESCGSNSTNR	STAESTKSSI	KPRRMQQAAP	TDPDLPPGYV	QSLIRRVVNN	VNIVINNLIL
170	180	190	200	210	220	230	240
KYVEDDIVLS	VNITSAECYT	VGELWDRAFM	DISATDLVLR	KVINFSDCTV	CLDKRNASGK	IEFYQDPLLY	KCSFRTRLHF
250	260	270	280	290	300	310	320
TYENLNSKMP	SVIKIHTLVE	SLKLSITDQQ	LPMFIRIMQL	GIALYYGEIG	NFKEGEIEDL	TCHNKDMLGN	ITGSEDETRI
330	340	350	360	370	380	390	400
DMQYPAQHKG	QELYSQQDEE	QPQGWVSWAW	SFVPAIVSYD	DGEEDFVGND	PASTMHQQKA	QTLKDPIVSI	GFYCTKATVT
410	420	430	440	450	460	470	480
FKLTEMQVES	SYYSPQKVKS	KEVLCWEQEG	TTVEALMMGE	PFFDCQIGFV	GCRAMCLKGI	MGVKDFEENM	NRSETEACFF
490	500	510	520	530	540	550	560
ICGDNLSTKG	FTYLTNSLFD	YRSPENNGTR	AEFILDSTHH	KETYTEIAGM	QRFGAFYMDY	LYTMENTSGK	GSTNQQDFSS
570	580	590	600	610	620	630	640
GKSEDLGTVQ	EKSTKSLVIG	PLDFRLDSSA	VHRILKMIVC	ALEHEYEPYS	RLKSDIKDEN	ETILNPEEVA	LLEEYIPTRH
650	660	670	680	690	700	710	720
TSVTLLKCTC	TISMAEFNLL	DHLLPVIMGE	KNSSNFMNTT	NFQSLRPLPS	IRILVDKINL	EHSVPMYAEQ	LVHVVSSLTQ
730	740	750	760	770	780	790	800
PSDNLLHYCY	VHCYLKIFGF	QAGLTSLDCS	GSYCLPVPVI	PSFSTALYGK	LLKLPTCWTK	RSQIAITEGI	FELPNLTIQA
810	820	830	840	850	860	870	880
TRAQTLLLQA	IYQSWSHLGN	VSSSAVIEAL	INEIFLSIGV	KSKNPLPTLE	GSIQNVELKY	CSTSLVKCAS	GTMGSIKICA
890	900	910	920	930	940	950	960
KAPVDSGKEK	LIPLLQGPSD	TKDLHSTKWL	NESRKPESLL	APDLMAFTIQ	VPQYIDYCHN	SGAVLLCSIQ	GLAVNIDPIL
970	980	990	1000	1010	1020	1030	1040
YTWLIYQPQK	RTSRHMQQQP	VVAVPLVMPV	CRRKEDEVSI	GSAPLAKQQS	YQASEYASSP	VKTKTVTESR	PLSVPVKAML
1050	1060	1070	1080	1090	1100	1110	1120
NISESCRSPE	ERMKEFIGIV	WNAVKHLTLQ	LEVQSCCVFI	PNDSLPSPST	IVSGDIPGTV	RSWYHGQTSM	PGTLVLCLPQ
1130	1140	1150	1160	1170	1180	1190	1200
IKIISAGHKY	MEPLQEIPFV	IPRPILEEGD	AFPWTISLHN	FSIYTLLGKQ	VTLCLVEPMG	CTSTLAVTSQ	KLLATGPDTR
1210	1220	1230	1240	1250	1260	1270	1280
HSFVVCLHVD	LESLEIKCSN	PQVQLFYELT	DIMNKVWNKI	QKRGNLNLSP	TSPETMAGPV	PTSPVRSSIG	TAPPDTSTCS
1290	1300	1310	1320	1330	1340	1350	1360
PSADIGTTTE	GDSIQAGEES	PFSDSVTLEQ	TTSNIGGTSG	RVSLWMQWVL	PKITIKLFAP	DPENKGTEVC	MVSELEDLSA
1370	1380	1390	1400	1410	1420	1430	1440
SIDVQDVYTK	VKCKIESFNI	DHYRSRPGEG	WQSGHFEGVF	LQCKEKSVTT	TKLLDGTHQQ	HGFLSLTYTK	AVTKNVRHKL
1450	1460	1470	1480	1490	1500	1510	1520
TSRNERRSFH	KLSEGLMDGS	PHFLHEILLS	AQAFDIVLYF	PLLNAIASIF	QAKLPKTQKE	KRKSPGQPMR	THTLTSRNLP
1530	1540	1550	1560	1570	1580	1590	1600
LIYVNTSVIR	IFIPKTEEMQ	PTVEANQAAK	EDTVVLKIGS	VAMAPQADNP	LGRSVLRKDI	YQRALNLGIL	RDPGSEIEDR
1610	1620	1630	1640	1650	1660	1670	1680
QYQIDLQSIN	IGTAQWHQLK	PEKESVSGGV	VTETERNSQN	PALEWNMASS	IRRHQERRAI	LTPVLTDFSV	RITGAPAVIF
1690	1700	1710	1720	1730	1740	1750	1760
TKVVSPENLH	TEEILVCGHS	LEVNITTNLD	FFLSVAQVQL	LHQLIVANMT	GLEPSNKAAE	ISKQEQKKVD	IFDGGMAETS
1770	1780	1790	1800	1810	1820	1830	1840
SRYSGAQDSG	IGSDSVKIRI	VQIEQHSGAS	QHRIARPSRQ	SSIVKNLNFI	PFDIFITASR	ISLMTYSCMA	LSKSKSQEQK
1850	1860	1870	1880	1890	1900	1910	1920
NNEKTDKSSL	NLPEVDSDVA	KPNQACISTV	TAEDLLRSSI	SFPSGKKIGV	LSLESLHAST	RSSARQALGI	TIVRQPGRRG
1930	1940	1950	1960	1970	1980	1990	2000
TGDLQLEPFL	YFIVSQPSLL	LSCHHRKQRV	EVSIFDAVLK	GVASDYKCID	PGKTLPEALD	YCTVWLQTVP	GEIDSKSGIP
2010	2020	2030	2040	2050	2060	2070	2080
PSFITLQIKD	FLNGPADVNL	DISKPLKANL	SFTKLDQINL	FLKKIKNAHS	LAHSEETSAM	SNTMVNKDDL	PVSKYYRGKL
2090	2100	2110	2120	2130	2140	2150	2160
SKPKIHGDGV	QKISAQENMW	RAVSCFQKIS	VQTTQIVISM	ETVPHTSKPC	LLASLSNLNG	SLSVKATQKV	PGIILGSSFL
2170	2180	2190	2200	2210	2220	2230	2240
LSINDFLLKT	SLKERSRILI	GPCCATANLE	AKWCKHSGNP	GPEQSIPKIS	IDLRGGLLQV	FWGQEHLNCL	VLLHELLNGY
2250	2260	2270	2280	2290	2300	2310	2320
LNEEGNFEVQ	VSEPVPQMSS	PVEKNQTFKS	EQSSDDLRTG	LFQYVQDAES	LKLPGVYEVL	FYNETEDCPG	MMLWRYPEPR
2330	2340	2350	2360	2370	2380	2390	2400

Detailed Protein Report

Cmpd.	No. of Cmpds.	m/z meas.	Δ m/z [ppm]	z	Rt [min]	Score	P	Range	Sequence	Modification
1794	2	1023.9088	-120.39	2	52.3	12.4	1	385-402	K.DPIVSIGFYCTKATVTFK.L	Carbamidomethyl: 10
1721	1	682.3288	-51.59	3	51.5	13.2	1	2196-2214	K.HSGNPGPEQSIPKISIDLR.G	

Detailed Protein Report

Protein 430: vascular endothelial growth factor receptor 3 isoform 2 precursor [Homo sapiens]

Accession: gi|103472027
Database: refseq_human(refseq_human_20140103.fasta)
Database Date: 2015-11-30
Modification(s): Carbamidomethyl

Score: 25.4
MW [kDa]: 145.5
pI: 5.9
Sequence Coverage [%]: 2.1
No. of unique Peptides: 2

10	20	30	40	50	60	70	80
MQRGAALCLR	LWLCLGLLDG	LVSGYSMTPP	TLNITEESHV	IDTGDSLSIS	CRGQHPLEWA	WPGAQEAPAT	GDKDSEDTGV
**90**	**100**	**110**	**120**	**130**	**140**	**150**	**160**
VRDCEGTDAR	PYCKVLLLHE	VHANDTGSYV	CYYKYIKARI	EGTTAASSYV	FVRDFEQPFI	NKPDTLLVNR	KDAMWVPCLV
**170**	**180**	**190**	**200**	**210**	**220**	**230**	**240**
SIPGLNVTLR	SQSSVLWPDG	QEVVWDDRRG	MLVSTPLLHD	ALYLQCETTW	GDQDFLSNPF	LVHITGNELY	DIQLLPRKSL
**250**	**260**	**270**	**280**	**290**	**300**	**310**	**320**
ELLVGEKLVL	NCTVWAEFNS	GVTFDWDYPG	KQAERGKWVP	ERRSQQTHTE	LSSILTIHNV	SQHDLGSYVC	KANNGIQRFR
**330**	**340**	**350**	**360**	**370**	**380**	**390**	**400**
ESTEVIVHEN	PFISVEWLKG	PILEATAGDE	LVKLPVKLAA	YPPPEFQWYK	DGKALSGRHS	PHALVLKEVT	EASTGTYTLA
**410**	**420**	**430**	**440**	**450**	**460**	**470**	**480**
LWNSAAGLRR	NISLELVVNV	PPQIHEKEAS	SPSIYSRHSR	QALTCTAYGV	PLPLSIQWHW	RPWTPCKMFA	QRSLRRRQQQ
**490**	**500**	**510**	**520**	**530**	**540**	**550**	**560**
DLMPQCRDWR	AVTTQDAVNP	IESLDTWTEF	VEGKNKTVSK	LVIQNANVSA	MYKCVVSNKV	GQDERLIYFY	VTTIPDGFTI
**570**	**580**	**590**	**600**	**610**	**620**	**630**	**640**
ESKPSEELLE	GQPVLLSCQA	DSYKYEHLRW	YRLNLSTLHD	AHGNPLLLDC	KNVHLFATPL	AASLEEVAPG	ARHATLSLSI
**650**	**660**	**670**	**680**	**690**	**700**	**710**	**720**
PRVAPEHEGH	YVCEVQDRRS	HDKHCHKKYL	SVQALEAPRL	TQNLTDLLVN	VSDSLEMQCL	VAGAHAPSIV	WYKDERLLEE
**730**	**740**	**750**	**760**	**770**	**780**	**790**	**800**
KSGVDLADSN	QKLSIQRVRE	EDAGRYLCSV	CNAKGCVNSS	ASVAVEGSED	KGSMEIVILV	GTGVIAVFFW	VLLLLIFCNM
**810**	**820**	**830**	**840**	**850**	**860**	**870**	**880**
RRPAHADIKT	GYLSIIMDPG	EVPLEEQCEY	LSYDASQWEF	PRERLHLGRV	LGYGAFGKVV	EASAFGIHKG	SSCDTVAVKM
**890**	**900**	**910**	**920**	**930**	**940**	**950**	**960**
LKEGATASEH	RALMSELKIL	IHIGNHLNVV	NLLGACTKPQ	GPLMVIVEFC	KYGNLSNFLR	AKRDAFSPCA	EKSPEQRGRF
**970**	**980**	**990**	**1000**	**1010**	**1020**	**1030**	**1040**
RAMVELARLD	RRRPGSSDRV	LFARFSKTEG	GARRASPDQE	AEDLWLSPLT	MEDLVCYSFQ	VARGMEFLAS	RKCIHRDLAA
**1050**	**1060**	**1070**	**1080**	**1090**	**1100**	**1110**	**1120**
RNILLSESDV	VKICDFGLAR	DIYKDPDYVR	KGSARLPLKW	MAPESIFDKV	YTTQSDVWSF	GVLLWEIFSL	GASPYPGVQI
**1130**	**1140**	**1150**	**1160**	**1170**	**1180**	**1190**	**1200**
NEEFCQRLRD	GTRMRAPELA	TPAIRRIMLN	CWSGDPKARP	AFSELVEILG	DLLQGRGLQE	EEEVCMAPRS	SQSSEEGSFS
**1210**	**1220**	**1230**	**1240**	**1250**	**1260**	**1270**	**1280**
QVSTMALHIA	QADAEDSPPS	LQRHSLAARY	YNWVSFPGCL	ARGAETRGSS	RMKTFEEFPM	TPTTYKGSVD	NQTDSGMVLA
**1290**	**1300**						
SEEFEQIESR	HRQESGFR						

Cmpd.	No. of Cmpds.	m/z meas.	Δ m/z [ppm]	z	Rt [min]	Score	P	Range	Sequence	Modification
2767	1	683.0222	-25.88	3	65.9	12.6	1	354-370	K.LPVKLAAYPPPEFQWYK.D	
1787	2	620.2385	-164.56	2	52.2	12.8	2	1032-1041	R.KCIHRDLAAR.N	Carbamidomethyl: 2

Detailed Protein Report

Protein 431: nuclear receptor coactivator 4 isoform 3 [Homo sapiens]

Accession: gi|14149617
Database: refseq_human(refseq_human_20140103.fasta)
Database Date: 2015-11-30
Modification(s): Carbamidomethyl

Score: 25.4
MW [kDa]: 69.7
pI: 5.7
Sequence Coverage [%]: 3.1
No. of unique Peptides: 1

Alias proteins:

Accession	Name	Description
gi|223890287	refseq_human (refseq_human_20140103.fasta)	nuclear receptor coactivator 4 isoform 3 [Homo sapiens]
gi|223890284	refseq_human (refseq_human_20140103.fasta)	nuclear receptor coactivator 4 isoform 3 [Homo sapiens]

10	20	30	40	50	60	70	80
MNTFQDQSGS	SSNREPLLRC	SDARRDLELA	IGGVLRAEQQ	IKDNLREVKA	QIHSCISRHL	ECLRSREVWL	YEQVDLIYQL
**90**	**100**	**110**	**120**	**130**	**140**	**150**	**160**
KEETLQQQAQ	QLYSLLGQFN	CLTHQLECTQ	NKDLANQVSV	CLERLGSLTL	KPEDSTVLLF	EADTITLRQT	ITTFGSLKTI
**170**	**180**	**190**	**200**	**210**	**220**	**230**	**240**
QIPEHLMAHA	SSANIGPFLE	KRGCISMPEQ	KSASGIVAVP	FSEWLLGSKP	ASGYQAPYIP	STDPQDWLTQ	KQTLENSQTS
**250**	**260**	**270**	**280**	**290**	**300**	**310**	**320**
SRACNFFNNV	GGNLKGLENW	LLKSEKSSYQ	KCNSHSTTSS	FSIEMEKVGD	QELPDQDEMD	LSDWLVTPQE	SHKLRKPENG
**330**	**340**	**350**	**360**	**370**	**380**	**390**	**400**
SRETSEKFKL	LFQSYNVNDW	LVKTDSCTNC	QGNQPKGVEI	ENLGNLKCLN	DHLEAKKPLS	TPSMVTEDWL	VQNHQDPCKV
**410**	**420**	**430**	**440**	**450**	**460**	**470**	**480**
EEVCRANEPC	TSFAECVCDE	NCEKEALYKW	LLKKEGKDKN	GMPVEPKPEP	EKHKDSLNMW	LCPRKEVIEQ	TKAPKAMTPS
**490**	**500**	**510**	**520**	**530**	**540**	**550**	**560**
RIADSFQVIK	NSPLSEWLIR	PPYKEGSPKE	VPGTEDRAGK	QKFKSPMNTS	WCSFNTADWV	LPGKKMGNLS	QLSSGEDKWL
**570**	**580**	**590**	**600**	**610**	**620**		
LRKKAQEVLL	NSPLQEEHNF	PPDHYGLPAV	CDLFACMQLK	VDKEKWLYRT	PLQM		

Cmpd.	No. of Cmpds.	m/z meas.	Δ m/z [ppm]	z	Rt [min]	Score	P	Range	Sequence	Modification
39	1	1103.4999	78.53	2	30.2	25.4	0	406-424	R.ANEPCTSFAECVCDENCEK.E	Carbamidomethyl: 5, 17

Detailed Protein Report

Protein 432: WD repeat and FYVE domain-containing protein 1 [Homo sapiens]

Accession: gi|18482373
Database: refseq_human(refseq_human_20140103.fasta)
Database Date: 2015-11-30

Score: 25.3
MW [kDa]: 46.3
pI: 7.2
Sequence Coverage [%]: 6.8
No. of unique Peptides: 1

10	20	30	40	50	60	70	80
MAAEIHSRPQ	SSRPVLLSKI	EGHQDAVTAA	LLIPKEDGVI	TASEDRTIRV	WLKRDSGQYW	PSIYHTMASP	CSAMAYHHDS
90	100	110	120	130	140	150	160
RRIFVGQDNG	AVMEFHVSED	FNKMNFIKTY	PAHQNRVSAI	IFSLATEWVI	STGHDKCVSW	MCTRSGNMLG	RHFFTSWASC
170	180	190	200	210	220	230	240
LQYDFDTQYA	FVGDYSGQIT	LLKLEQNTCS	VITTLKGHEG	SVACLWWDPI	QRLLFSGASD	NSIIMWDIGG	RKGRTLLLQG
250	260	270	280	290	300	310	320
HHDKVQSLCY	LQLTRQLVSC	SSDGGIAVWN	MDVSREEAPQ	WLESDSCQKC	EQPFFWNIKQ	MWDTKTLGLR	QHHCRKCGQA
330	340	350	360	370	380	390	400
VCGKCSSKRS	SYPVMGFEFQ	VRVCDSCYDS	IKDEDRTSLA	TFHEGKHNIS	HMSMDIARGL	MVTCGTDRIV	KIWDMTPVVG
410	420						
CSLATGFSPH							

Cmpd.	No. of Cmpds.	m/z meas.	Δ m/z [ppm]	z	Rt [min]	Score	P	Range	Sequence	Modification
2193	1	1009.4117	-66.86	2	57.6	13.1	0	392-410	K.IWDMTPVVGCSLATGFSPH.-	

Detailed Protein Report

**Protein 433:**	**PREDICTED: leucine-rich repeat serine/threonine-protein kinase 2 isoform X1 [Homo sapiens]**		
**Accession:**	gi|530399725	**Score:**	25.3
**Database:**	refseq_human(refseq_human_20140103.fasta)	**MW [kDa]:**	285.2
**Database Date:**	2015-11-30	**pI:**	6.3
**Modification(s):**	Oxidation	**Sequence Coverage [%]:**	1.5
		**No. of unique Peptides:**	1

Detailed Protein Report

10	20	30	40	50	60	70	80
MASGSCQGCE	EDEETLKKLI	VRLNNVQEGK	QIETLVQILE	DLLVFTYSER	ASKLFQGKNI	HVPLLIVLDS	YMRVASVQQV
90	100	110	120	130	140	150	160
GWSLLCKLIE	VCPGTMQSLM	GPQDVGNDWE	VLGVHQLILK	MLTVHNASVN	LSVIGLKTLD	LLLTSGKITL	LILDEESDIF
170	180	190	200	210	220	230	240
MLIFDAMHSF	PANDEVQKLG	CKALHVLFER	VSEEQLTEFV	ENKDYMILLS	ALTNFKDEEE	IVLHVLHCLH	SLAIPCNNVE
250	260	270	280	290	300	310	320
VLMSGNVRCY	NIVVEAMKAF	PMSERIQEVS	CCLLHRLTLG	NFFNILVLNE	VHEFVVKAVQ	QYPENAALQI	SALSCLALLT
330	340	350	360	370	380	390	400
ETIFLNQDLE	EKNENQENDD	EGEEDKLFWL	EACYKALTWH	RKNKHVQEAA	CWALNNLLMY	QNSLHEKIGD	EDGHFPAHRE
410	420	430	440	450	460	470	480
VMLSMLMHSS	SKEVFQASAN	ALSTLLEQNV	NFRKILLSKG	IHLNVLELMQ	KHIHSPEVAE	SGCKMLNHLF	EGSNTSLDIM
490	500	510	520	530	540	550	560
AAVVPKILTV	MKRHETSLPV	QLEALRAILH	FIVPGMPEES	REDTEFHHKL	NMVKKQCFKN	DIHKLVLAAL	NRFIGNPGIQ
570	580	590	600	610	620	630	640
KCGLKVISSI	VHFPDALEML	SLEGAMDSVL	HTLQMYPDDQ	EIQCLGLSLI	GYLITKKNVF	IGTGHLLAKI	LVSSLYRFKD
650	660	670	680	690	700	710	720
VAEIQTKGFQ	TILAILKLSA	SFSKLLVHHS	FDLVIFHQMS	SNIMEQKDQQ	FLNLCCKCFA	KVAMDDYLKN	VMLERACDQN
730	740	750	760	770	780	790	800
NSIMVECLLL	LGADANQAKE	GSSLICQVCE	KESSPKLVEL	LLNSGSREQD	VRKALTISIG	KGDSQIISLL	LRRLALDVAN
810	820	830	840	850	860	870	880
NSICLGGFCI	GKVEPSWLGP	LFPDKTSNLR	KQTNIASTLA	RMVIRYQMKS	AVEEGTASGS	DGNFSEDVLS	KFDEWTFIPD
890	900	910	920	930	940	950	960
SSMDSVFAQS	DDLDSEGSEG	SFLVKKKSNS	ISVGEFYRDA	VLQRCSPNLQ	RHSNSLGPIF	DHEDLLKRKR	KILSSDDSLR
970	980	990	1000	1010	1020	1030	1040
SSKLQSHMRH	SDSISSLASE	REYITSLDLS	ANELRDIDAL	SQKCCISVHL	EHLEKLELHQ	NALTSFPQQL	CETLKSLTHL
1050	1060	1070	1080	1090	1100	1110	1120
DLHSNKFTSF	PSYLLKMSCI	ANLDVSRNDI	GPSVVLDPTV	KCPTLKQFNL	SYNQLSFVPE	NLTDVVEKLE	QLILEGNKIS
1130	1140	1150	1160	1170	1180	1190	1200
GICSPLRLKE	LKILNLSKNH	ISSLSENFLE	ACPKVESFSA	RMNFLAAMPF	LPPSMTILKL	SQNKFSCIPE	AILNLPHLRS
1210	1220	1230	1240	1250	1260	1270	1280
LDMSSNDIQY	LPGPAHWKSL	NLRELLFSHN	QISILDLSEK	AYLWSRVEKL	HLSHNKLKEI	PPEIGCLENL	TSLDVSYNLE
1290	1300	1310	1320	1330	1340	1350	1360
LRSFPNEMGK	LSKIWDLPLD	ELHLNFDFKH	IGCKAKDIIR	FLQQRLKKAV	PYNRMKLMIV	GNTGSGKTTL	LQQLMKTKKS
1370	1380	1390	1400	1410	1420	1430	1440
DLGMQSATVG	IDVKDWPIQI	RDKRKRDLVL	NVWDFAGREE	FYSTHPHFMT	QRALYLAVYD	LSKGQAEVDA	MKPWLFNIKA
1450	1460	1470	1480	1490	1500	1510	1520
RASSSPVILV	GTHLDVSDEK	QRKACMSKIT	KELLNKRGFP	AIRDYHFVNA	TEESDALAKL	RKTIINESLN	FKIRDQLVVG
1530	1540	1550	1560	1570	1580	1590	1600
QLIPDCYVEL	EKIILSERKN	VPIEFPVIDR	KRLLQLVREN	QLQLDENELP	HAVHFLNESG	VLLHFQDPAL	QLSDLYFVEP
1610	1620	1630	1640	1650	1660	1670	1680
KWLCKIMAQI	LTVKVEGCPK	HPKGIISRRD	VEKFLSKKRK	FPKNYMSQYF	KLLEKFQIAL	PIGEEYLLVP	SSLSDHRPVI
1690	1700	1710	1720	1730	1740	1750	1760
ELPHCENSEI	IIRLYEMPYF	PMGFWSRLIN	RLLEISPYML	SGRERALRPN	RMYWRQGIYL	NWSPEAYCLV	GSEVLDNHPE
1770	1780	1790	1800	1810	1820	1830	1840
SFLKITVPSC	RKGCILLGQV	VDHIDSLMEE	WFPGLLEIDI	CGEGETLLKK	WALYSFNDGE	EHQKILLDDL	MKKAEEGDLL
1850	1860	1870	1880	1890	1900	1910	1920
VNPDQPRLTI	PISQIAPDLI	LADLPRNIML	NNDELEFEQA	PEFLLGDGSF	GSVYRAAYEG	EEVAVKIFNK	HTSLRLLRQE
1930	1940	1950	1960	1970	1980	1990	2000
LVVLCHLHHP	SLISLLAAGI	RPRMLVMELA	SKGSLDRLLQ	QDKASLTRTL	QHRIALHVAD	GLRYLHSAMI	IYRDLKPHNV
2010	2020	2030	2040	2050	2060	2070	2080
LLFTLYPNAA	IIAKIADYGI	AQYCCRMGIK	TSEGTPGFRA	PEVARGNVIY	NQQADVYSFG	LLLYDILTTG	GRIVEGLKFP
2090	2100	2110	2120	2130	2140	2150	2160
NEFDELEIQG	KLPDPVKEYG	CAPWPMVEKL	IKQCLKENPQ	ERPTSAQVFD	ILNSAELVCL	TRRILLPKNV	IVECMVATHH
2170	2180	2190	2200	2210	2220	2230	2240
NSRNASIWLG	CGHTDRGQLS	FLDLNTEGYT	SEEVADSRIL	CLALVHLPVE	KESWIVSGTQ	SGTLLVINTE	DGKKRHTLEK
2250	2260	2270	2280	2290	2300	2310	2320
MTDSVTCLYC	NSFSKQSKQK	NFLLVGTADG	KLAIFEDKTV	KLKGAAPLKI	LNIGNVSTPL	MCLSESTNST	ERNVMWGGCG
2330	2340	2350	2360	2370	2380	2390	2400

Detailed Protein Report

Cmpd.	No. of Cmpds.	m/z meas.	Δ m/z [ppm]	z	Rt [min]	Score	P	Range	Sequence	Modification
1730	1	796.0646	-20.88	3	51.4	11.0	1	2457-2477	R.VMMTAQLGSLKNVMLVLGYNR.K	Oxidation: 2, 3, 14

Detailed Protein Report

Protein 434: **PREDICTED: zinc finger protein 33B isoform X3 [Homo sapiens]**

**Accession:**	gi|578819760	**Score:**	25.3
**Database:**	refseq_human(refseq_human_20140103.fasta)	**MW [kDa]:**	92.3
**Database Date:**	2015-11-30	**pI:**	9.9
		**Sequence Coverage [%]:**	4.4
		**No. of unique Peptides:**	1

10	20	30	40	50	60	70	80
MPHPLATSAF	LRFRLSFCLS	HVLRGRRSRD	FRGLPALLWR	MQPEERVSCI	FRVVSVFPRT	EQNEQGYCAH	KPEVIFRLEQ
90	100	110	120	130	140	150	160
GEEPWRLEEE	FPSQSFPVWT	ADHLKERSQE	NQSKHLWEVV	FINNEMLTKE	QGNVIGIPFN	MDVSSFPSRK	MFCQYDSRGM
170	180	190	200	210	220	230	240
SFNTVSELVI	SKINYLGKKS	DEFNACGKLL	LNIKHDETHT	REKNEVLKNR	NTLSHRENTL	QHEKIQTLDH	NFEYSICQET
250	260	270	280	290	300	310	320
LLEKAVFNTR	KRENAEENNC	DYNEFGRTFC	DSSSLLFHQI	PPSKDSHYEF	SDCEKFLCVK	STLSKHDGVP	VKHYDCGESG
330	340	350	360	370	380	390	400
NNFRRKLCLS	QLQKGDKGEK	HFECNECGKA	FWEKSHLTRH	QRVHTGEKHF	QCNQCGKTFW	EKSNLTKHQR	SHTGEKPFEC
410	420	430	440	450	460	470	480
NECGKAFSHK	SALTLHQRTH	TGEKPYQCNA	CGKTFYQKSD	LTKHQRTHTG	QKPYECYECG	KSFCMNSHLT	VHQRTHTGEK
490	500	510	520	530	540	550	560
PFECLECGKS	FCQKSHLTQH	QRTHIGDKPY	ECNACGKTFY	HKSVLTRHQI	IHTGLKPYEC	YECGKTFCLK	SDLTIHQRTH
570	580	590	600	610	620	630	640
TGEKPFACPE	CGKFFSHKST	LSQHYRTHTG	EKPYECHECG	KIFYNKSYLT	KHNRTHTGEK	PYECNECGKT	FCQKSQLTQH
650	660	670	680	690	700	710	720
QRIHIGEKPY	ECNECGKAFC	HKSALIVHQR	THTQEKPYKC	NECGKSFCVK	SGLILHERKH	TGEKPYECNE	CGKSFSHKSS
730	740	750	760	770	780	790	800
LTVHHRAHTG	EKSCQCNECG	KIFYRKSDLA	KHQRSHTGEK	PYECNTCRKT	FSQKSNLIVH	QRTHIGEKPY	E

Cmpd.	No. of Cmpds.	m/z meas.	Δ m/z [ppm]	z	Rt [min]	Score	P	Range	Sequence	Modification
2070	1	682.5545	-123.95	3	56.0	12.2	1	752-768	K.HQRSHTGEKPYECNTCR.K	

Detailed Protein Report

Protein 435: tristetraprolin [Homo sapiens]

**Accession:**	gi|393539038	**Score:**	25.3
**Database:**	refseq_human(refseq_human_20140103.fasta)	**MW [kDa]:**	34.7
**Database Date:**	2015-11-30	**pI:**	10.2
**Modification(s):**	Carbamidomethyl	**Sequence Coverage [%]:**	6.9
		**No. of unique Peptides:**	1

10	20	30	40	50	60	70	80
MANRYTMDLT	AIYESLLSLS	PDVPVPSDHG	GTESSPGWGS	SGPWSLSPSD	SSPSGVTSRL	PGRSTSLVEG	RSCGWVPPPP
**90**	**100**	**110**	**120**	**130**	**140**	**150**	**160**
GFAPLAPRLG	PELSPSPTSP	TATSTTPSRY	KTELCRTFSE	SGRCRYGAKC	QFAHGLGELR	QANRHPKYKT	ELCHKFYLQG
**170**	**180**	**190**	**200**	**210**	**220**	**230**	**240**
RCPYGSRCHF	IHNPSEDLAA	PGHPPVLRQS	ISFSGLPSGR	RTSPPPPGLA	GPSLSSSSFS	PSSSPPPPGD	LPLSPSAFSA
**250**	**260**	**270**	**280**	**290**	**300**	**310**	**320**
APGTPLARRD	PTPVCCPSCR	RATPISVWGP	LGGLVRTPSV	QSLGSDPDEY	ASSGSSLGGS	DSPVFEAGVF	APPQPVAAPR
**330**	**340**						
RLPIFNRISV	SE						

Cmpd.	No. of Cmpds.	m/z meas.	Δ m/z [ppm]	z	Rt [min]	Score	P	Range	Sequence	Modification
373	1	644.3883	111.01	2	34.3	10.7	0	130-140	K.CQFAHGLGELR.Q	Carbamidomethyl: 1

Detailed Protein Report

Protein 436: PREDICTED: adenomatous polyposis coli protein 2 isoform X5 [Homo sapiens]

**Accession:**	gi|578832904	**Score:**	25.3
**Database:**	refseq_human(refseq_human_20140103.fasta)	**MW [kDa]:**	243.7
**Database Date:**	2015-11-30	**pI:**	10.0
		**Sequence Coverage [%]:**	1.4
		**No. of unique Peptides:**	1

Quantitation

MD:MU	**Median:** 0.58	**CV:** 0.00 %	**No. of Peptides:** 1

Detailed Protein Report

10	20	30	40	50	60	70	80
MASSVAPYEQ	LVRQVEALKA	ENSHLRQELR	DNSSHLSKLE	TETSGMKEVL	KHLQGKLEQE	ARVLVSSGQT	EVLEQLKALQ
90	100	110	120	130	140	150	160
MDITSLYNLK	FQPPTLGPEP	AARTPEGSPV	HGSGPSKDSF	GELSRATIRL	LEELDRERCF	LLNEIEKEEK	EKLWYYSQLQ
170	180	190	200	210	220	230	240
GLSKRLDELP	HVETFSMQMD	LIRQQLEFEA	QHIRSLMEER	FGTSDEMVQR	AQIRASRLEQ	IDKELLEAQD	RVQQTEPQAL
250	260	270	280	290	300	310	320
LAVKSVPVDE	DPETEVPTHP	EDGTPQPGNS	KVEVVFWLLS	MLATRDQEDT	ARTLLAMSSS	PESCVAMRRS	GCLPLLLQIL
330	340	350	360	370	380	390	400
HGTEAAAGGR	AGAPGAPGAK	DARMRANAAL	HNIVFSQPDQ	GLARKEMRVL	HVLEQIRAYC	ETCWDWLQAR	DGGPEGGGAG
410	420	430	440	450	460	470	480
SAPIPIEPQI	CQATCAVMKL	SFDEEYRRAM	NELGGLQAVA	ELLQVDYEMH	KMTRDPLNLA	LRRYAGMTLT	NLTFGDVANK
490	500	510	520	530	540	550	560
ATLCARRGCM	EAIVAQLASD	SEELHQVVSS	ILRNLSWRAD	INSKKVLREA	GSVTALVQCV	LRATKESTLK	SVLSALWNLS
570	580	590	600	610	620	630	640
AHSTENKAAI	CQVDGALGFL	VSTLTYKCQS	NSLAIIESGG	GILRNVSSLV	ATREDYRQVL	RDHNCLQTLL	QHLTSHSLTI
650	660	670	680	690	700	710	720
VSNACGTLWN	LSARSARDQE	LLWDLGAVGM	LRNLVHSKHK	MIAMGSAAAL	RNLLAHRPAK	HQAAATAVSP	GSCVPSLYVR
730	740	750	760	770	780	790	800
KQRALEAELD	ARHLAQALEH	LEKQGPPAAE	AATKKPLPPL	RHLDGLAQDY	ASDSGCFDDD	DAPSSLAAAA	ATGEPASPAA
810	820	830	840	850	860	870	880
LSLFLGSPFL	QGQALARTPP	TRRGGKEAEK	DTSGEAAVAA	KAKAKLALAV	ARIDQLVEDI	SALHTSSDDS	FSLSSGDPGQ
890	900	910	920	930	940	950	960
EAPREGRAQS	CSPCRGPEGG	RREAGSRAHP	LLRLKAAHAS	LSNDSLNSGS	ASDGYCPREH	MLPCPLAALA	SRREDPRCGQ
970	980	990	1000	1010	1020	1030	1040
PRPSRLDLDL	PGCQAEPPAR	EATSADARVR	TIKLSPTYQH	VPLLEGASRA	GAEPLAGPGI	SPGARKQAWL	PADHLSKVPE
1050	1060	1070	1080	1090	1100	1110	1120
KLAAAPLSVA	SKALQKLAAQ	EGPLSLSRCS	SLSSLSSAGR	PGPSEGGDLD	DSDSSLEGLE	EAGPSEAELD	STWRAPGATS
1130	1140	1150	1160	1170	1180	1190	1200
LPVAIPAPRR	NRGRGLGVED	ATPSSSSENY	VQETPLVLSR	CSSVSSLGSF	ESPSIASSIP	SEPCSGQGSG	TISPSELPDS
1210	1220	1230	1240	1250	1260	1270	1280
PGQTMPPSRS	KTPPLAPAPQ	GPPEATQFSL	QWESYVKRFL	DIADCRERCR	LPSELDAGSV	RFTVEKPDEN	FSCASSLSAL
1290	1300	1310	1320	1330	1340	1350	1360
ALHEHYVQQD	VELRLLPSAC	PERGGGAGGA	GLHFAGHRRR	EEGPAPTGSR	PRGAADQELE	LLRECLGAAV	PARLRKVASA
1370	1380	1390	1400	1410	1420	1430	1440
LVPGRRALPV	PVYMLVPAPA	PAQEDDSCTD	SAEGTPVNFS	SAASLSDETL	QGPPRDQPGG	PAGRQRPTGR	PTSARQAMGH
1450	1460	1470	1480	1490	1500	1510	1520
RHKAGGAGRS	AEQSRGAGKN	RAGLELPLGR	PPSAPADKDG	SKPGRTRGDG	ALQSLCLTTP	TEEAVYCFYG	NDSDEEPPAA
1530	1540	1550	1560	1570	1580	1590	1600
APTPTHRRTS	AIPRAFTRER	PQGRKEAPAP	SKAAPAAPPP	ARTQPSLIAD	ETPPCYSLSS	SASSLSEPEP	SEPPAVHPRG
1610	1620	1630	1640	1650	1660	1670	1680
REPAVTKDPG	PGGGRDSSPS	PRAAEELLQR	CISSALPRRR	PPVSGLRRRK	PRATRLDERP	AEGSRERGEE	AAGSDRASDL
1690	1700	1710	1720	1730	1740	1750	1760
DSVEWRAIQE	GANSIVTWLH	QAAAATREAS	SESDSILSFV	SGLSVGSTLQ	PPKHRKGRQA	EGEMGSARRP	EKRGAASVKT
1770	1780	1790	1800	1810	1820	1830	1840
SGSPRSPAGP	EKPRGTQKTT	PGVPAVLRGR	TVIYVPSPAP	RAQPKGTPGP	RATPRKVAPP	CLAQPAAPAK	VPSPGQQRSR
1850	1860	1870	1880	1890	1900	1910	1920
SLHRPAKTSE	LATLSQPPRS	ATPPARLAKT	PSSSSSQTSP	ASQPLPRKRP	PVTQAAGALP	GPGASPVPKT	PARTLLAKQH
1930	1940	1950	1960	1970	1980	1990	2000
KTQRSPVRIP	FMQRPARRGP	PPLARAVPEP	GPRGRAGTEA	GPGARGGRLG	LVRVASALSS	GSESSDRSGF	RRQLTFIKES
2010	2020	2030	2040	2050	2060	2070	2080
PGLRRRRSEL	SSAESAASAP	QGASPRRGRP	ALPAVFLCSS	RCEELRAAPR	QGPAPARQRP	PAARPSPGER	PARRTTSESP
2090	2100	2110	2120	2130	2140	2150	2160
SRLPVRAPAA	RPETVKRYAS	LPHISVARRP	DGAVPAAPAS	ADAARRSSDG	EPRPLPRVAA	PGTTWRRIRD	EDVPHILRST
2170	2180	2190	2200	2210	2220	2230	2240

Detailed Protein Report

Cmpd.	No. of Cmpds.	m/z meas.	Δ m/z [ppm]	z	Rt [min]	Score	P	Range	Sequence	Modification	Ratios
1997	1	731.3112	-143.18	2	54.9	10.6	1	1816-1830	R.KVAPPCLAQPAAPAK.V		MD:MU 0.58

Detailed Protein Report

Protein 437: **chitotriosidase-1 isoform 2 precursor [Homo sapiens]**

Accession: gi|388890228
Database: refseq_human(refseq_human_20140103.fasta)
Database Date: 2015-11-30
Modification(s): Oxidation

Score: 25.3
MW [kDa]: 49.6
pI: 5.8
Sequence Coverage [%]: 5.8
No. of unique Peptides: 2

10	20	30	40	50	60	70	80
MVRSVAWAGF	MVLLMIPWGS	AAKLVCYFTN	WAQYRQGEAR	FLPKDLDPSL	CTHLIYAFAG	MTNHQLSTTE	WNDETLYQEF
**90**	**100**	**110**	**120**	**130**	**140**	**150**	**160**
NGLKKMFTDM	VATANNRQTF	VNSAIRFLRK	YSFDGLDLDW	EYPGSQGSPA	VDKERFTTLV	QDLANAFQQE	AQTSGKERLL
**170**	**180**	**190**	**200**	**210**	**220**	**230**	**240**
LSAAVPAGQT	YVDAGYEVDK	IAQNLDFVNL	MAYDFHGSWE	KVTGHNSPLY	KRQEESGAAA	SLNVDAAVQQ	WLQKGTPASK
**250**	**260**	**270**	**280**	**290**	**300**	**310**	**320**
LILGMPTYGR	SFTLASSSDT	RVGAPATGSG	TPGPFTKEGG	MLAYYEVCSW	KGATKQRIQD	QKVPYIFRDN	QWVGFDDVES
**330**	**340**	**350**	**360**	**370**	**380**	**390**	**400**
FKTKVSYLKQ	KGLGGAMVWA	LDLDDFAGFS	CNQGRYPLIQ	TLRQELSLPY	LPSGTPELEV	PKPGQPSEPE	HGPSPGQDTF
**410**	**420**	**430**	**440**	**450**			
CQGKADGLYP	NPRERSSFYS	CAAGRLFQQS	CPTGLVFSNS	CKCCTWN			

Cmpd.	No. of Cmpds.	m/z meas.	Δ m/z [ppm]	z	Rt [min]	Score	P	Range	Sequence	Modification
2531	1	701.8609	75.18	2	61.6	11.3	0	86-97	K.MFTDMVATANNR.Q	Oxidation: 1, 5
2659	1	826.4139	65.06	2	64.4	13.9	0	278-291	K.EGGMLAYYEVCSWK.G	Oxidation: 4

Detailed Protein Report

Protein 438: leucine-rich repeat-containing protein 14B [Homo sapiens]

Accession: gi|122937351
Database: refseq_human(refseq_human_20140103.fasta)
Database Date: 2015-11-30
Modification(s): Carbamidomethyl

Score: 25.2
MW [kDa]: 56.7
pI: 6.8
Sequence Coverage [%]: 5.4
No. of unique Peptides: 1

10	20	30	40	50	60	70	80
MDTMRSLRFI	SAEALVSHPQ	VARQSLDSVA	HNLYPLLFKA	SYLLEQAEVT	RAVLGRWPLE	EFRLGALLGP	GADHPQDLRD
**90**	**100**	**110**	**120**	**130**	**140**	**150**	**160**
RTCRACLEAL	VRGLADHVLQ	DRSRRRLRVA	DLTGIRDVQV	QRCPCGRALG	RWGRTQLLAR	TCCELQAEPL	AAGRPVEVLA
**170**	**180**	**190**	**200**	**210**	**220**	**230**	**240**
DLFVTEGNFE	AVVQALRPAG	PAPLRVHCPS	FRADSLSPSQ	LLHVLRLAGP	GALRKLEVVH	NVRLHAGHVQ	QLLAQVGFPR
**250**	**260**	**270**	**280**	**290**	**300**	**310**	**320**
LASLTLPTKA	FDAPPTYAST	PDGEDPLLAS	IARELSKMAQ	LTELSVAFST	LTGKIPTLLG	PLQTPLRVLD	LANCALNHTD
**330**	**340**	**350**	**360**	**370**	**380**	**390**	**400**
MAFLADCAHA	AHLEVLDLSG	HNLVSLYPST	FFRLLSQASR	TLRILTLEEC	GIVDSHVGML	ILGLSPCHRL	RQLKFLGNPL
**410**	**420**	**430**	**440**	**450**	**460**	**470**	**480**
SARALRRLFT	ALCELPELRC	IEFPVPKDCY	PEGAAYPQDE	LAMSKFNQQK	YDEIAEELRA	VLLRADREDI	QVSTPLFGSF
**490**	**500**	**510**	**520**				
DPDIQETSNE	LGAFLLQAFK	TALENFSRAL	KQIE				

Cmpd.	No. of Cmpds.	m/z meas.	Δ m/z [ppm]	z	Rt [min]	Score	P	Range	Sequence	Modification
1949	1	682.2553	-54.37	3	54.5	10.3	0	428-445	K.DCYPEGAAYPQDELAMSK.F	Carbamidomethyl: 2

Detailed Protein Report

Protein 439: C-type mannose receptor 2 precursor [Homo sapiens]

Accession: gi|110624774
Database: refseq_human(refseq_human_20140103.fasta)
Database Date: 2015-11-30
Modification(s): Carbamidomethyl

Score: 25.2
MW [kDa]: 166.6
pI: 5.5
Sequence Coverage [%]: 1.4
No. of unique Peptides: 2

Quantitation

MD:MU **Median:** 2.82 **CV:** 120.49 % **No. of Peptides:** 2

10	20	30	40	50	60	70	80
MGPGRPAPAP	WPRHLLRCVL	LLGCLHLGRP	GAPGDAALPE	PNVFLIFSHG	LQGCLEAQGG	QVRVTPACNT	SLPAQRWKWV
90	100	110	120	130	140	150	160
SRNRLFNLGT	MQCLGTGWPG	TNTTASLGMY	ECDREALNLR	WHCRTLGDQL	SLLLGARTSN	ISKPGTLERG	DQTRSGQWRI
170	180	190	200	210	220	230	240
YGSEEDLCAL	PYHEVYTIQG	NSHGKPCTIP	FKYDNQWFHG	CTSTGREDGH	LWCATTQDYG	KDERWGFCPI	KSNDCETFWD
250	260	270	280	290	300	310	320
KDQLTDSCYQ	FNFQSTLSWR	EAWASCEQQG	ADLLSITEIH	EQTYINGLLT	GYSSTLWIGL	NDLDTSGGWQ	WSDNSPLKYL
330	340	350	360	370	380	390	400
NWESDQPDNP	SEENCGVIRT	ESSGGWQNRD	CSIALPYVCK	KKPNATAEPT	PPDRWANVKV	ECEPSWQPFQ	GHCYRLQAEK
410	420	430	440	450	460	470	480
RSWQESKKAC	LRGGGDLVSI	HSMAELEFIT	KQIKQEVEEL	WIGLNDLKLQ	MNFEWSDGSL	VSFTHWHPFE	PNNFRDSLED
490	500	510	520	530	540	550	560
CVTIWGPEGR	WNDSPCNQSL	PSICKKAGQL	SQGAAEEDHG	CRKGWTWHSP	SCYWLGEDQV	TYSEARRLCT	DHGSQLVTIT
570	580	590	600	610	620	630	640
NRFEQAFVSS	LIYNWEGEYF	WTALQDLNST	GSFFWLSGDE	VMYTHWNRDQ	PGYSRGGCVA	LATGSAMGLW	EVKNCTSFRA
650	660	670	680	690	700	710	720
RYICRQSLGT	PVTPELPGPD	PTPSLTGSCP	QGWASDTKLR	YCYKVFSSER	LQDKKSWVQA	QGACQELGAQ	LLSLASYEEE
730	740	750	760	770	780	790	800
HFVANMLNKI	FGESEPEIHE	QHWFWIGLNR	RDPRGGQSWR	WSDGVGFSYH	NFDRSRHDDD	DIRGCAVLDL	ASLQWVAMQC
810	820	830	840	850	860	870	880
DTQLDWICKI	PRGTDVREPD	DSPQGRREWL	RFQEAEYKFF	EHHSTWAQAQ	RICTWFQAEL	TSVHSQAELD	FLSHNLQKFS
890	900	910	920	930	940	950	960
RAQEQHWWIG	LHTSESDGRF	RWTDGSIINF	ISWAPGKPRP	VGKDKKCVYM	TASREDWGDQ	RCLTALPYIC	KRSNVTKETQ
970	980	990	1000	1010	1020	1030	1040
PPDLPTTALG	GCPSDWIQFL	NKCFQVQGQE	PQSRVKWSEA	QFSCEQQEAQ	LVTITNPLEQ	AFITASLPNV	TFDLWIGLHA
1050	1060	1070	1080	1090	1100	1110	1120
SQRDFQWVEQ	EPLMYANWAP	GEPSGPSPAP	SGNKPTSCAV	VLHSPSAHFT	GRWDDRSCTE	ETHGFICQKG	TDPSLSPSPA
1130	1140	1150	1160	1170	1180	1190	1200
ALPPAPGTEL	SYLNGTFRLL	QKPLRWHDAL	LLCESRNASL	AYVPDPYTQA	FLTQAARGLR	TPLWIGLAGE	EGSRRYSWVS
1210	1220	1230	1240	1250	1260	1270	1280
EEPLNYVGWQ	DGEPQQPGGC	TYVDVDGAWR	TTSCDTKLQG	AVCGVSSGPP	PPRRISYHGS	CPQGLADSAW	IPFREHCYSF
1290	1300	1310	1320	1330	1340	1350	1360
HMELLLGHKE	ARQRCQRAGG	AVLSILDEME	NVFVWEHLQS	YEGQSRGAWL	GMNFNPKGGT	LVWQDNTAVN	YSNWGPPGLG
1370	1380	1390	1400	1410	1420	1430	1440
PSMLSHNSCY	WIQSNSGLWR	PGACTNITMG	VVCKLPRAEQ	SSFSPSALPE	NPAALVVVLM	AVLLLLALLT	AALILYRRRQ
1450	1460	1470	1480				
SIERGAFEGA	RYSRSSSSPT	EATEKNILVS	DMEMNEQQE				

Cmpd.	No. of Cmpds.	m/z meas.	Δ m/z [ppm]	z	Rt [min]	Score	P	Range	Sequence	Modification	Ratios	
1293	1	727.3351	-37.75	2	45.8	10.7	1	350-361	R.DCSIALPYVCKK.K	Carbamidomethyl: 2, 10	MD:MU	1.10
1150	3	500.6507	-138.75	2	44.0	14.5	0	818-826	R.EPDDSPQGR.R		MD:MU	7.28

Detailed Protein Report

Protein 440: PREDICTED: PDZ domain-containing protein 2 isoform X9 [Homo sapiens]

**Accession:**	gi|578809883	**Score:**	25.2
**Database:**	refseq_human(refseq_human_20140103.fasta)	**MW [kDa]:**	269.5
**Database Date:**	2015-11-30	**pI:**	9.0
		**Sequence Coverage [%]:**	1.4
		**No. of unique Peptides:**	1

Detailed Protein Report

10	20	30	40	50	60	70	80
MTNAASQKVG	RRTSNRVTAW	HGRKLAEYGR	WSCSKNRMGW	EFRDGRLSLG	DELLVINGHL	LVGLSHEEAV	AILRSATGMV
90	100	110	120	130	140	150	160
QLVVASKENS	AEDLLRLTSK	SLPDLTSSVE	DVSSWTDNED	QEADGEEDEG	TSSSVQRAMP	GTDEPQDVCG	AEESKGNLES
170	180	190	200	210	220	230	240
PKQGSNKIKL	KSRLSGGVHR	LESVEEYNEL	MVRNGDPRIR	MLEVSRDGRK	HSLPQLLDSS	SASQEYHIVK	KSTRSLSTTQ
250	260	270	280	290	300	310	320
VESPWRLIRP	SVISIIGLYK	EKGKGLGFSI	AGGRDCIRGQ	MGIFVKTIFP	NGSAAEDGRL	KEGDEILDVN	GIPIKGLTFQ
330	340	350	360	370	380	390	400
EAIHTFKQIR	SGLFVLTVRT	KLVSPSLTPC	STPTHMSRSA	SPNFNTSGGA	SAGGSDEGSS	SSLGRKTPGP	KDRIVMEVTL
410	420	430	440	450	460	470	480
NKEPRVGLGI	GACCLALENS	PPGIYIHSLA	PGSVAKMESN	LSRGDQILEV	NSVNVRHAAL	SKVHAILSKC	PPGPVRLVIG
490	500	510	520	530	540	550	560
RHPNPKVSEQ	EMDEVIARST	YQESKEANSS	PGLGTPLKSP	SLAKKDSLIS	ESELSQYFAH	DVPGPLSDFM	VAGSEDEDHP
570	580	590	600	610	620	630	640
GSGCSTSEEG	SLPPSTSTHK	EPGKPRANSL	VTLGSHRASG	LFHKQVTVAR	QASLPGSPQA	LRNPLLRQRK	VGCYDANDAS
650	660	670	680	690	700	710	720
DEEEFDREGD	CISLPGALPG	PIRPLSEDDP	RRVSISSSKG	MDVHNQEERP	RKTLVSKAIS	APLLGSSVDL	EESIPEGMVD
730	740	750	760	770	780	790	800
AASYAANLTD	SAEAPKGSPG	SWWKKELSGS	SSAPKLEYTV	RTDTQSPTNT	GSPSSPQQKS	EGLGSRHRPV	ARVSPHCKRS
810	820	830	840	850	860	870	880
EAEAKPSGSQ	TVNLTGRAND	PCDLDSRVQA	TSVKVTVAGF	QPGGAVEKES	LGKLTTGDAC	VSTSCELASA	LSHLDASHLT
890	900	910	920	930	940	950	960
ENLPKAASEL	GQQPMTELDS	SSDLISSPGK	KGAAHPDPSK	TSVDTGQVSR	PENPSQPASP	RVTKCKARSP	VRLPHEGSPS
970	980	990	1000	1010	1020	1030	1040
PGEKAAAPPD	YSKTRSASET	STPHNTRRVA	ALRGAGPGAE	GMTPAGAVLP	GDPLTSQEQR	QGAPGNHSKA	LEMTGIHAPE
1050	1060	1070	1080	1090	1100	1110	1120
SSQEPSLLEG	ADSVSSRAPQ	ASLSMLPSTD	NTKEACGHVS	GHCCPGGSRE	SPVTDIDSFI	KELDASAARS	PSSQTGDSGS
1130	1140	1150	1160	1170	1180	1190	1200
QEGSAQGHPP	AGAGGGSSCR	AEPVPGGQTS	SPRRAWAAGA	PAYPQWASQP	SVLDSINPDK	HFTVNKNFLS	NYSRNFSSFH
1210	1220	1230	1240	1250	1260	1270	1280
EDSTSLSGLG	DSTEPSLSSM	YGDAEDSSSD	PESLTEAPRA	SARDGWSPPR	SRVSLHKEDP	SESEEEQIEI	CSTRGCPNPP
1290	1300	1310	1320	1330	1340	1350	1360
SSPAHLPTQA	AICPASAKVL	SLKYSTPRES	VASPREKAAC	LPGSYTSGPD	SSQPSSLLEM	SSQEHETHAD	ISTSQNHRPS
1370	1380	1390	1400	1410	1420	1430	1440
CAEETTEVTS	ASSAMENSPL	SKVARHFHSP	PIILSSPNMV	NGLEHDLLDD	ETLNQYETSI	NAAASLSSFS	VDVPKNGESV
1450	1460	1470	1480	1490	1500	1510	1520
LENLHISESQ	DLDDLLQKPK	MIARRPIMAW	FKEINKHNQG	THLRSKTEKE	QPLMPARSPD	SKIQMVSSSQ	KKGVTVPHSP
1530	1540	1550	1560	1570	1580	1590	1600
PQPKTNLENK	DLSKKSPAEM	LLTNGQKAKC	GPKLKRLSLK	GKAKVNSEAP	AANAVKAGGT	DHRKPLISPQ	TSHKTLSKAV
1610	1620	1630	1640	1650	1660	1670	1680
SQRLHVADHE	DPDRNTTAAP	RSPQCVLESK	PPLATSGPLK	PSVSDTSIRT	FVSPLTSPKP	VPEQGMWSRF	HMAVLSEPDR
1690	1700	1710	1720	1730	1740	1750	1760
GCPTTPKSPK	CRAEGRAPRA	DSGPVSPAAS	RNGMSVAGNR	QSEPRLASHV	AADTAQPRPT	GEKGGNIMAS	DRLERTNQLK
1770	1780	1790	1800	1810	1820	1830	1840
IVEISAEAVS	ETVCGNKPAE	SDRRGGCLAQ	GNCQEKSEIR	LYRQVAESST	SHPSSLPSHA	SQAEQEMSRS	FSMAKLASSS
1850	1860	1870	1880	1890	1900	1910	1920
SSLQTAIRKA	EYSQGKSSLM	SDSRGVPRNS	IPGGPSGEDH	LYFTPRPATR	TYSMPAQFSS	HFGREGHPPH	SLGRSRDSQV
1930	1940	1950	1960	1970	1980	1990	2000
PVTSSVVPEA	KASRGGLPSL	ANGQGIYSVK	PLLDTSRNLP	ATDEGDIISV	QETSCLVTDK	IKVTRRHYCY	EQNWPHESTS
2010	2020	2030	2040	2050	2060	2070	2080
FFSVKQRIKS	FENLANADRP	VAKSGASPFL	SVSSKPPIGR	RSSGSIVSGS	LGHPGDAAAR	LLRRSLSSCS	ENQSEAGTLL
2090	2100	2110	2120	2130	2140	2150	2160
PQMAKSPSIM	TLTISRQNPP	ETSSKGSDSE	LKKSLGPLGI	PTPTMTLASP	VKRNKSSVRH	TQPSPVSRSK	LQELRALSMP
2170	2180	2190	2200	2210	2220	2230	2240

Detailed Protein Report

Cmpd.	No. of Cmpds.	m/z meas.	Δ m/z [ppm]	z	Rt [min]	Score	P	Range	Sequence	Modification
444	1	848.1390	136.74	2	35.2	10.0	1	2429-2444	R.IGRPTVLLGSSAVIRR.S	

Detailed Protein Report

Protein 441: PREDICTED: laminin subunit alpha-5 isoform X3 [Homo sapiens]

**Accession:**	gi|578836003	**Score:**	25.2
**Database:**	refseq_human(refseq_human_20140103.fasta)	**MW [kDa]:**	248.1
**Database Date:**	2015-11-30	**pI:**	6.2
**Modification(s):**	Carbamidomethyl	**Sequence Coverage [%]:**	1.8
		**No. of unique Peptides:**	1

Detailed Protein Report

10	20	30	40	50	60	70	80
MAKRLCAGSA	LCVRGPRGPA	PLLLVGLALL	GAARAREEAG	GGFSLHPPYF	NLAEGARIAA	SATCGEEAPA	RGSPRPTEDL
90	100	110	120	130	140	150	160
YCKLVGGPVA	GGDPNQTIRG	QYCDICTAAN	SNKAHPASNA	IDGTERWWQS	PPLSRGLEYN	EVNVTLDLGQ	VFHVAYVLIK
170	180	190	200	210	220	230	240
FANSPRPDLW	VLERSMDFGR	TYQPWQFFAS	SKRDCLERFG	PQTLERITRD	DAAICTTEYS	RIVPLENGEI	VVSLVNGRPG
250	260	270	280	290	300	310	320
AMNFSYSPLL	REFTKATNVR	LRFLRTNTLL	GHLMGKALRD	PTVTRRYYYS	IKDISIGGRC	VCHGHADACD	AKDPTDPFRL
330	340	350	360	370	380	390	400
QCTCQHNTCG	GTCDRCCPGF	NQQPWKPATA	NSANECQSCN	CYGHATDCYY	DPEVDRRRAS	QSLDGTYQGG	GVCIDCQHHT
410	420	430	440	450	460	470	480
TGVNCERCLP	GFYRSPNHPL	DSPHVCRRCN	CESDFTDGTC	EDLTGRCYCR	PNFSGERCDV	CAEGFTGFPS	CYPTPSSSND
490	500	510	520	530	540	550	560
TREQVLPAGQ	IVNCDCSAAG	TQGNACRKDP	RVGRCLCKPN	FQGTHCELCA	PGFYGPGCQP	CQCSSPGVAD	DRCDPDTGQC
570	580	590	600	610	620	630	640
RCRVGFEGAT	CDRCAPGYFH	FPLCQLCGCS	PAGTLPEGCD	EAGRCLCQPE	FAGPHCDRCR	PGYHGFPNCQ	ACTCDPRGAL
650	660	670	680	690	700	710	720
DQLCGAGGLC	RCRPGYTGTA	CQECSPGFHG	FPSCVPCHCS	AEGSLHAACD	PRSGQCSCRP	RVTGLRCDTC	VPGAYNFPYC
730	740	750	760	770	780	790	800
EAGSCHPAGL	APVDPALPEA	QVPCMCRAHV	EGPSCDRCKP	GFWGLSPSNP	EGCTRCSCDL	RGTLGGVAEC	QPGTGQCFCK
810	820	830	840	850	860	870	880
PHVCGQACAS	CKDGFFGLDQ	ADYFGCRSCR	CDIGGALGQS	CEPRTGVCRC	RPNTQGPTCS	EPARDHYLPD	LHHLRLELEE
890	900	910	920	930	940	950	960
AATPEGHAVR	FGFNPLEFEN	FSWRGYAQMA	PVQPRIVARL	NLTSPDLFWL	VFRYVNRGAM	SVSGRVSVRE	EGRSATCANC
970	980	990	1000	1010	1020	1030	1040
TAQSQPVAFP	PSTEPAFITV	PQRGFGEPFV	LNPGTWALRV	EAEGVLLDYV	VLLPSAYYEA	ALLQLRVTEA	CTYRPSAQQS
1050	1060	1070	1080	1090	1100	1110	1120
GDNCLLYTHL	PLDGFPSAAG	LEALCRQDNS	LPRPCPTEQL	SPSHPPLITC	TGSDVDVQLQ	VAVPQPGRYA	LVVEYANEDA
1130	1140	1150	1160	1170	1180	1190	1200
RQEVGVAVHT	PQRAPQQGLL	SLHPCLYSTL	CRGTARDTQD	HLAVFHLDSE	ASVRLTAEQA	RFFLHGVTLV	PIEEFSPEFV
1210	1220	1230	1240	1250	1260	1270	1280
EPRVSCISSH	GAFGPNSAAC	LPSRFPKPPQ	PIILRDCQVI	PLPPGLPLTH	AQDLTPAMSP	AGPRPRPPTA	VDPDAEPTLL
1290	1300	1310	1320	1330	1340	1350	1360
REPQATVVFT	THVPTLGRYA	FLLHGYQPAH	PTFPVEVLIN	AGRVWQGHAN	ASFCPHGYGC	RTLVVCEGQA	LLDVTHSELT
1370	1380	1390	1400	1410	1420	1430	1440
VTVRVPKGRW	LWLDYVLVVP	ENVYSFGYLR	EEPLDKSYDF	ISHCAAQGYH	ISPSSSSLFC	RNAAASLSLF	YNNGARPCGC
1450	1460	1470	1480	1490	1500	1510	1520
HEVGATGPTC	EPFGGQCPCH	AHVIGRDCSR	CATGYWGFPN	CRPCDCGARL	CDELTGQCIC	PPRTIPPDCL	LCQPQTFGCH
1530	1540	1550	1560	1570	1580	1590	1600
PLVGCEECNC	SGPGIQELTD	PTCDTDSGQC	KCRPNVTGRR	CDTCSPGFHG	YPRCRPCDCH	EAGTAPGVCD	PLTGQCYCKE
1610	1620	1630	1640	1650	1660	1670	1680
NVQGPKCDQC	SLGTFSLDAA	NPKGCTRCFC	FGATERCRSS	SYTRQEFVDM	EGWVLLSTDR	QVVPHERQPG	TEMLRADLRH
1690	1700	1710	1720	1730	1740	1750	1760
VPEAVPEAFP	ELYWQAPPSY	LGDRVSSYGG	TLRYELHSET	QRGDVFVPME	SRPDVVLQGN	QMSITFLEPA	YPTPGHVHRG
1770	1780	1790	1800	1810	1820	1830	1840
QLQLVEGNFR	HTETRNTVSR	EELMMVLASL	EQLQIRALFS	QISSAVFLRR	VALEVASPAG	QGALASNVEL	CLCPASYRGD
1850	1860	1870	1880	1890	1900	1910	1920
SCQECAPGFY	RDVKGLFLGR	CVPCQCHGHS	DRCLPGSGVC	VDCQHNTEGA	HCERCQAGFV	SSRDDPSAPC	VSCPCPLSVP
1930	1940	1950	1960	1970	1980	1990	2000
SNNFAEGCVL	RGGRTQCLCK	PGYAGASCER	CAPGFFGNPL	VLGSSCQPCD	CSGNGDPNLL	FSDCDPLTGA	CRGCLRHTTG
2010	2020	2030	2040	2050	2060	2070	2080
PRCEICAPGF	YGNALLPGNC	TRCDCTPCGT	EACDPHSGHC	LCKAGVTGRR	CDRCQEGHFG	FDGCGGCRPC	ACGPAAEGSE
2090	2100	2110	2120	2130	2140	2150	2160
CHPQSGQCHC	RPGTMGPQCR	ECAPGYWGLP	EQGCRRCQCP	GGRCDPHTGR	CNCPPGLSGE	RCDTCSQQHQ	VPVPGGPVGH
2170	2180	2190	2200	2210	2220	2230	2240

Detailed Protein Report

Cmpd.	No. of Cmpds.	m/z meas.	Δ m/z [ppm]	z	Rt [min]	Score	P	Range	Sequence	Modification
1502	1	1052.1977	73.86	3	48.4	12.0	0	782-812	R.GTLGGVAECQPGTGQCFCKPHVCGQACASCK.D	Carbamidomethyl: 16, 18

Detailed Protein Report

Protein 442: vitamin D3 receptor isoform VDRA [Homo sapiens]

**Accession:**	gi|4507883	**Score:**	25.2
**Database:**	refseq_human(refseq_human_20140103.fasta)	**MW [kDa]:**	48.3
**Database Date:**	2015-11-30	**pI:**	6.1
**Modification(s):**	Carbamidomethyl, Oxidation	**Sequence Coverage [%]:**	2.6
		**No. of unique Peptides:**	1

Alias proteins:

Accession	Name	Description
gi|578824200	refseq_human (refseq_human_20140103.fasta)	PREDICTED: vitamin D3 receptor isoform X1 [Homo sapiens]
gi|63054845	refseq_human (refseq_human_20140103.fasta)	vitamin D3 receptor isoform VDRA [Homo sapiens]

10	20	30	40	50	60	70	80
MEAMAASTSL	PDPGDFDRNV	PRICGVCGDR	ATGFHFNAMT	CEGCKGFFRR	SMKRKALFTC	PFNGDCRITK	DNRRHCQACR
**90**	**100**	**110**	**120**	**130**	**140**	**150**	**160**
LKRCVDIGMM	KEFILTDEEV	QRKREMILKR	KEEEALKDSL	RPKLSEEQQR	IIAILLDAHH	KTYDPTYSDF	CQFRPPVRVN
**170**	**180**	**190**	**200**	**210**	**220**	**230**	**240**
DGGGSHPSRP	NSRHTPSFSG	DSSSSCSDHC	ITSSDMMDSS	SFSNLDLSEE	DSDDPSVTLE	LSQLSMLPHL	ADLVSYSIQK
**250**	**260**	**270**	**280**	**290**	**300**	**310**	**320**
VIGFAKMIPG	FRDLTSEDQI	VLLKSSAIEV	IMLRSNESFT	MDDMSWTCGN	QDYKYRVSDV	TKAGHSLELI	EPLIKFQVGL
**330**	**340**	**350**	**360**	**370**	**380**	**390**	**400**
KKLNLHEEEH	VLLMAICIVS	PDRPGVQDAA	LIEAIQDRLS	NTLQTYIRCR	HPPPGSHLLY	AKMIQKLADL	RSLNEEHSKQ
**410**	**420**	**430**					
YRCLSFQPEC	SMKLTPLVLE	VFGNEIS					

Cmpd.	No. of Cmpds.	m/z meas.	Δ m/z [ppm]	z	Rt [min]	Score	P	Range	Sequence	Modification
2538	1	673.3328	74.17	2	61.7	14.1	0	403-413	R.CLSFQPECSMK.L	Carbamidomethyl: 8; Oxidation: 10

Detailed Protein Report

Protein 443: zinc finger protein 567 [Homo sapiens]

Accession: gi|34303941
Database: refseq_human(refseq_human_20140103.fasta)
Database Date: 2015-11-30
Modification(s): Carbamidomethyl, Oxidation

Score: 25.1
MW [kDa]: 71.5
pI: 10.3
Sequence Coverage [%]: 4.5
No. of unique Peptides: 2

10	20	30	40	50	60	70	80
MDVMLENYCH	LISVGCHMTK	PDVILKLERG	EEPWTSFAGH	TCLEENWKAE	DFLVKFKEHQ	EKYSRSVVSI	NHKKLVKEKS
**90**	**100**	**110**	**120**	**130**	**140**	**150**	**160**
KIYEKTFTLG	KNPVNSKNLP	PEYDTHGRIL	KNVSELIISN	LNPARKRLSE	YNGYGKSLLS	TKQETTHPEV	KSHNQSARAF
**170**	**180**	**190**	**200**	**210**	**220**	**230**	**240**
SHNEVLMQYQ	KTETPAQSFG	YNDCEKSFLQ	RGGLITHSRP	YKGENPSVYN	KKRRATNIEK	KHTCNECGKS	FCRKSVLILH
**250**	**260**	**270**	**280**	**290**	**300**	**310**	**320**
QGIHSEEKPY	QCHQCGNAFR	RKSYLIDHQR	THTGEKPFVC	NECGKSFRLK	TALTDHQRTH	TGEKSYECLQ	CRNAFRLKSH
**330**	**340**	**350**	**360**	**370**	**380**	**390**	**400**
LIRHQRTHTG	EKPYECNDCG	KSFRQKTTLS	LHQRIHTGEK	PYICKECGKS	FHQKANLTVH	QRTHTGEKPY	ICNECGKSFS
**410**	**420**	**430**	**440**	**450**	**460**	**470**	**480**
QKTTLALHEK	THNEEKPYIC	SECGKSFRQK	TTLVAHQRTH	TGEKSYECPH	CGKAFRMKSY	LIDHHRTHTG	EKPYECNECG
**490**	**500**	**510**	**520**	**530**	**540**	**550**	**560**
KSFSQKTNLN	LHQRIHTGEK	PYVCNECGKS	FRQKATLTVH	QKIHTGQKSY	ECPQCGKAFS	RKSYLIHHQR	THTGEKPYKC
**570**	**580**	**590**	**600**	**610**	**620**		
SECGKCFRQK	TNLIVHQRTH	TGEKPYVCNE	CGKSFSYKRN	LIVHQRTHKG	ENIEMQ		

Cmpd.	No. of Cmpds.	m/z meas.	Δ m/z [ppm]	z	Rt [min]	Score	P	Range	Sequence	Modification
1824	1	805.8049	-99.60	2	52.8	12.3	0	159-171	R.AFSHNEVLMQYQK.T	Oxidation: 9
1247	1	873.9372	79.81	2	45.3	12.8	0	172-186	K.TETPAQSFGYNDCEK.S	Carbamidomethyl: 13

Detailed Protein Report

Protein 444: non-receptor tyrosine-protein kinase TYK2 [Homo sapiens]

Accession: gi|187608615
Database: refseq_human(refseq_human_20140103.fasta)
Database Date: 2015-11-30
Modification(s): Oxidation

Score: 25.1
MW [kDa]: 133.6
pI: 6.8
Sequence Coverage [%]: 2.9
No. of unique Peptides: 2

10	20	30	40	50	60	70	80
MPLRHWGMAR	GSKPVGDGAQ	PMAAMGGLKV	LLHWAGPGGG	EPWVTFSESS	LTAEEVCIHI	AHKVGITPPC	FNLFALFDAQ
90	100	110	120	130	140	150	160
AQVWLPPNHI	LEIPRDASLM	LYFRIRFYFR	NWHGMNPREP	AVYRCGPPGT	EASSDQTAQG	MQLLDPASFE	YLFEQGKHEF
170	180	190	200	210	220	230	240
VNDVASLWEL	STEEEIHHFK	NESLGMAFLH	LCHLALRHGI	PLEEVAKKTS	FKDCIPRSFR	RHIRQHSALT	RLRLRNVFRR
250	260	270	280	290	300	310	320
FLRDFQPGRL	SQQMVMVKYL	ATLERLAPRF	GTERVPVCHL	RLLAQAEGEP	CYIRDSGVAP	TDPGPESAAG	PPTHEVLVTG
330	340	350	360	370	380	390	400
TGGIQWWPVE	EEVNKEEGSS	GSSGRNPQAS	LFGKKAKAHK	AVGQPADRPR	EPLWAYFCDF	RDITHVVLKE	HCVSIHRQDN
410	420	430	440	450	460	470	480
KCLELSLPSR	AAALSFVSLV	DGYFRLTADS	SHYLCHEVAP	PRLVMSIRDG	IHGPLLEPFV	QAKLRPEDGL	YLIHWSTSHP
490	500	510	520	530	540	550	560
YRLILTVAQR	SQAPDGMQSL	RLRKFPIEQQ	DGAFVLEGWG	RSFPSVRELG	AALQGCLLRA	GDDCFSLRRC	CLPQPGETSN
570	580	590	600	610	620	630	640
LIIMRGARAS	PRTLNLSQLS	FHRVDQKEIT	QLSHLGQGTR	TNVYEGRLRV	EGSGDPEEGK	MDDEDPLVPG	RDRGQELRVV
650	660	670	680	690	700	710	720
LKVLDPSHHD	IALAFYETAS	LMSQVSHTHL	AFVHGVCVRG	PENIMVTEYV	EHGPLDVWLR	RERGHVPMAW	KMVVAQQLAS
730	740	750	760	770	780	790	800
ALSYLENKNL	VHGNVCGRNI	LLARLGLAEG	TSPFIKLSDP	GVGLGALSRE	ERVERIPWLA	PECLPGGANS	LSTAMDKWGF
810	820	830	840	850	860	870	880
GATLLEICFD	GEAPLQSRSP	SEKEHFYQRQ	HRLPEPSCPQ	LATLTSQCLT	YEPTQRPSFR	TILRDLTRLQ	PHNLADVLTV
890	900	910	920	930	940	950	960
NPDSPASDPT	VFHKRYLKKI	RDLGEGHFGK	VSLYCYDPTN	DGTGEMVAVK	ALKADCGPQH	RSGWKQEIDI	LRTLYHEHII
970	980	990	1000	1010	1020	1030	1040
KYKGCCEDQG	EKSLQLVMEY	VPLGSLRDYL	PRHSIGLAQL	LLFAQQICEG	MAYLHAQHYI	HRDLAARNVL	LDNDRLVKIG
1050	1060	1070	1080	1090	1100	1110	1120
DFGLAKAVPE	GHEYYRVRED	GDSPVFWYAP	ECLKEYKFYY	ASDVWSFGVT	LYELLTHCDS	SQSPPTKFLE	LIGIAQGQMT
1130	1140	1150	1160	1170	1180	1190	
VLRLTELLER	GERLPRPDKC	PCEVYHLMKN	CWETEASFRP	TFENLIPILK	TVHEKYQGQA	PSVFSVC	

Cmpd.	No. of Cmpds.	m/z meas.	Δ m/z [ppm]	z	Rt [min]	Score	P	Range	Sequence	Modification
2015	1	731.8181	-73.05	2	55.1	13.5	0	282-294	R.LLAQAEGEPCYIR.D	
623	1	824.1088	42.34	3	37.4	11.6	0	680-700	R.GPENIMVTEYVEHGPLDVWLR.R	Oxidation: 6

Detailed Protein Report

Protein 445: PREDICTED: tubulin-specific chaperone D isoform X12 [Homo sapiens]

Accession: gi|578832042
Database: refseq_human(refseq_human_20140103.fasta)
Database Date: 2015-11-30

Score: 25.1
MW [kDa]: 129.5
pI: 5.8
Sequence Coverage [%]: 2.3
No. of unique Peptides: 1

10	20	30	40	50	60	70	80
MALSDEPAAG	GPEEEAEDET	LAFGAALEAF	GESAETRALL	GRLREVHGGG	AEREVALERF	RVIMDKYQEQ	PHLLDPHLEW
**90**	**100**	**110**	**120**	**130**	**140**	**150**	**160**
MMNLLLDIVQ	DQTSPASLVH	LAFKFLYIIT	KVRGYKTFLR	LFPHEVADVE	PVLDLVTIQN	PKDHEAWETR	YMLLLWLSVT
**170**	**180**	**190**	**200**	**210**	**220**	**230**	**240**
CLIPFDFSRL	DGNLLTQPGQ	ARMSIMDRIL	QIAESYLIVS	DKARDAAAVL	VSRFITRPDV	KQSKMAEFLD	WSLCNLARSS
**250**	**260**	**270**	**280**	**290**	**300**	**310**	**320**
FQTMQGVITM	DGTLQALAQI	FKHGKREDCL	PYAATVLRCL	DGCRLPESNQ	TLLRKLGVKL	VQRLGLTFLK	PKVAAWRYQR
**330**	**340**	**350**	**360**	**370**	**380**	**390**	**400**
GCRSLAANLQ	LLTQGQSEQK	PLILTEDDDE	DDDVPEGVER	VIEQLLVGLK	DKDTVVRWSA	AKGIGRMAGR	LPRALADDVV
**410**	**420**	**430**	**440**	**450**	**460**	**470**	**480**
GSVLDCFSFQ	ETDKAWHGGC	LALAELGRRG	LLLPSRLVDV	VAVILKALTY	DEKRGACSVG	TNVRDAACYV	CWAFARAYEP
**490**	**500**	**510**	**520**	**530**	**540**	**550**	**560**
QELKPFVTAI	SSALVIAAVF	DRDINCRRAA	SAAFQENVGR	QGTFPHGIDI	LTTADYFAVG	NRSNCFLVIR	VIRELAARAL
**570**	**580**	**590**	**600**	**610**	**620**	**630**	**640**
HNLAQQAPEF	SATQVFPRLL	SMTLSPDLHM	RHGSILACAE	VAYALYKLAA	QENRPVTDHL	DEQAVQGLKQ	IHQQLYDRQL
**650**	**660**	**670**	**680**	**690**	**700**	**710**	**720**
YRGLGGQLMR	QAVCVLIEKL	SLSKMPFRGD	TVIDGWQWLI	NDTLRHLHLI	SSHSRQQMKD	AAVSALAALC	SEYYMKEPGE
**730**	**740**	**750**	**760**	**770**	**780**	**790**	**800**
ADPAIQEELI	TQYLAELRNP	EEMTRCGFSL	ALGALPGFLL	KGRLQQVLTG	LRAVTHTSPE	DVSFAESRRD	GLKAIARICQ
**810**	**820**	**830**	**840**	**850**	**860**	**870**	**880**
TVGVKAGAPD	EAVCGENVSQ	IYCALLGCMD	DYTTDSRGDV	GTWVRKAAMT	SLMDLTLLLA	RSQPELIEAH	TCERIMCCVA
**890**	**900**	**910**	**920**	**930**	**940**	**950**	**960**
QQASEKIDRF	RAHAASVFLT	LLHFDSPPIP	HVPHRGELEK	LFPRSDVASV	NWSAPSQAFP	RITQLLGLPT	YRYHVLLGLV
**970**	**980**	**990**	**1000**	**1010**	**1020**	**1030**	**1040**
VSLGGLTEST	IRHSTQSLFE	YMKGIQSDPQ	ALGSFSGTLL	QIFEDNLLNE	RVSVPLLKTL	DHVLTHGCFD	IFTTEEDHPF
**1050**	**1060**	**1070**	**1080**	**1090**	**1100**	**1110**	**1120**
AVKLLALCKK	EIKNSKDIQK	LLSGIAVFCE	MVQFPGDVRR	QALLQLCLLL	CHRFPLIRKT	TASQVYETLL	TYSDVVGADV
**1130**	**1140**	**1150**	**1160**	**1170**			
LDEVVTVLSD	TAWDAELAVV	REQRNRLCDL	LGVPRPQLVP	QPGAC			

Cmpd.	No. of Cmpds.	m/z meas.	Δ m/z [ppm]	z	Rt [min]	Score	P	Range	Sequence	Modification
1258	2	727.3443	-38.23	2	45.4	13.9	0	415-428	K.AWHGGCLALAELGR.R	

Detailed Protein Report

Protein 446: structural maintenance of chromosomes protein 2 [Homo sapiens]

**Accession:**	gi|110347418	**Score:**	25.1
**Database:**	refseq_human(refseq_human_20140103.fasta)	**MW [kDa]:**	135.6
**Database Date:**	2015-11-30	**pI:**	9.1
		**Sequence Coverage [%]:**	2.6
		**No. of unique Peptides:**	1

Alias proteins:

Accession	Name	Description
gi|578816998	refseq_human (refseq_human_20140103.fasta)	PREDICTED: structural maintenance of chromosomes protein 2 isoform X1 [Homo sapiens]
gi|388240808	refseq_human (refseq_human_20140103.fasta)	structural maintenance of chromosomes protein 2 [Homo sapiens]
gi|110347425	refseq_human (refseq_human_20140103.fasta)	structural maintenance of chromosomes protein 2 [Homo sapiens]
gi|110347420	refseq_human (refseq_human_20140103.fasta)	structural maintenance of chromosomes protein 2 [Homo sapiens]

10	20	30	40	50	60	70	80
MHIKSIILEG	FKSYAQRTEV	NGFDPLFNAI	TGLNGSGKSN	ILDSICFLLG	ISNLSQVRAS	NLQDLVYKNG	QAGITKASVS
90	100	110	120	130	140	150	160
ITFDNSDKKQ	SPLGFEVHDE	ITVTRQVVIG	GRNKYLINGV	NANNTRVQDL	FCSVGLNVNN	PHFLIMQGRI	TKVLNMKPPE
170	180	190	200	210	220	230	240
ILSMIEEAAG	TRMYEYKKIA	AQKTIEKKEA	KLKEIKTILE	EEITPTIQKL	KEERSSYLEY	QKVMREIEHL	SRLYIAYQFL
250	260	270	280	290	300	310	320
LAEDTKVRSA	EELKEMQDKV	IKLQEELSEN	DKKIKALNHE	IEELEKRKDK	ETGGILRSLE	DALAEAQRVN	TKSQSAFDLK
330	340	350	360	370	380	390	400
KKNLACEESK	RKELEKNMVE	DSKTLAAKEK	EVKKITDGLH	ALQEASNKDA	EALAAAQQHF	NAVSAGLSSN	EDGAEATLAG
410	420	430	440	450	460	470	480
QMMACKNDIS	KAQTEAKQAQ	MKLKHAQQEL	KNKQAEVKKM	DSGYRKDQEA	LEAVKRLKEK	LEAEMKKLNY	EENKEESLLE
490	500	510	520	530	540	550	560
KRRQLSRDIG	RLKETYEALL	ARFPNLRFAY	KDPEKNWNRN	CVKGLVASLI	SVKDTSATTA	LELVAGERLY	NVVVDTEVTG
570	580	590	600	610	620	630	640
KKLLERGELK	RRYTIIPLNK	ISARCIAPET	LRVAQNLVGP	DNVHVALSLV	EYKPELQKAM	EFVFGTTFVC	DNMDNAKKVA
650	660	670	680	690	700	710	720
FDKRIMTRTV	TLGGDVFDPH	GTLSGGARSQ	AASILTKFQE	LKDVQDELRI	KENELRALEE	ELAGLKNTAE	KYRQLKQQWE
730	740	750	760	770	780	790	800
MKTEEADLLQ	TKLQQSSYHK	QQEELDALKK	TIEESEETLK	NTKEIQRKAE	EKYEVLENKM	KNAEAERERE	LKDAQKKLDC
810	820	830	840	850	860	870	880
AKTKADASSK	KMKEKQQEVE	AITLELEELK	REHTSYKQQL	EAVNEAIKSY	ESQIEVMAAE	VAKNKESVNK	AQEEVTKQKE
890	900	910	920	930	940	950	960
VITAQDTVIK	AKYAEVAKHK	EQNNDSQLKI	KELDHNISKH	KREAEDGAAK	VSKMLKDYDW	INAERHLFGQ	PNSAYDFKTN
970	980	990	1000	1010	1020	1030	1040
NPKEAGQRLQ	KLQEMKEKLG	RNVNMRAMNV	LTEAEERYND	LMKKKRIVEN	DKSKILTTIE	DLDQKKNQAL	NIAWQKVNKD
1050	1060	1070	1080	1090	1100	1110	1120
FGSIFSTLLP	GANAMLAPPE	GQTVLDGLEF	KVALGNTWKE	NLTELSGGQR	SLVALSLILS	MLLFKPAPIY	ILDEVDAALD
1130	1140	1150	1160	1170	1180	1190	1200
LSHTQNIGQM	LRTHFTHSQF	IVVSLKEGMF	NNANVLFKTK	FVDGVSTVAR	FTQCQNGKIS	KEAKSKAKPP	KGAHVEV

Cmpd.	No. of Cmpds.	m/z meas.	Δ m/z [ppm]	z	Rt [min]	Score	P	Range	Sequence	Modification
2304	1	1022.8910	-124.62	2	59.1	14.3	1	733-749	K.LQQSSYHKQQEELDALK.K	

Detailed Protein Report

Protein 447: **putative pre-mRNA-splicing factor ATP-dependent RNA helicase DHX16 isoform 2 [Homo sapiens]**

Accession: gi|256000749
Database: refseq_human(refseq_human_20140103.fasta)
Database Date: 2015-11-30
Modification(s): Oxidation

Score: 25.1
MW [kDa]: 112.4
pI: 6.5
Sequence Coverage [%]: 1.8
No. of unique Peptides: 1

10	20	30	40	50	60	70	80
MSEDPRGPKV	PRKAVVEKPA	RAAEREARAL	LEKNRSYRLL	EDSEESSEET	VSRAGSSLQK	KRKKRKHLRK	KREEEEEEEA
90	100	110	120	130	140	150	160
SEKGKKKTGG	SKQQTEKPES	EDEWERTERE	RLQDLEERDA	FAERVRQRDK	DRTRNVLERS	DKKAYEEAQK	RLKMAEEDRK
170	180	190	200	210	220	230	240
AMVPELRKKS	RREYLAKRER	EKLEDLEAEL	ADEEFLFGDV	ELSRHERQEL	KYKRRVRDLA	REYRAAGEQE	KLEATNRYHM
250	260	270	280	290	300	310	320
PKETRGQPAR	AVDLVEEESG	APGEEQRRWE	EARLGAASLK	FGARDAASQE	PKYQLVLEEE	ETIEFVRATQ	LQGDEEPSAP
330	340	350	360	370	380	390	400
PTSTQAQQKE	SIQAVRRSLP	VFPFREELLA	AIANHQVLII	EGETGSGKTT	QIPQYLFEEG	YTNKGMKIAC	TQPRRVAAMS
410	420	430	440	450	460	470	480
VAARVAREMG	VKLGNEVGYS	IRFEDCTSER	TVLRYMTDGM	LLREFLSEPD	LASYSVVMVD	EAHERTLHTD	ILFGLIKDVA
490	500	510	520	530	540	550	560
RFRPELKVLV	ASATMDTARF	STFFDDAPVF	RIPGRRFPVD	IFYTKAPEAD	YLEACVVSVL	QIHVTQPPGD	ILVFLTGQEE
570	580	590	600	610	620	630	640
IEAACEMLQD	RCRRLGSKIR	ELLVLPIYAN	LPSDMQARIF	QPTPPGARKV	VVATNIAETS	LTIEGIIYVL	DPGFCKQKSY
650	660	670	680	690	700	710	720
NPRTGMESLT	VTPCSKASAN	QRAGRAGRVA	AGKCFRLYTA	WAYQHELEET	TVPEIQRTSL	GNVVLLLKSL	GIHDLMHFDF
730	740	750	760	770	780	790	800
LDPPPYETLL	LALEQLYALG	ALNHLGELTT	SGRKMAELPV	DPMLSKMILA	SEKYSCSEEI	LTVAAMLSVN	NSIFYRPKDK
810	820	830	840	850	860	870	880
VVHADNARVN	FFLPGGDHLV	LLNVYTQWAE	SGYSSQWCYE	NFVQFRSMRR	ARDVREQLEG	LLERVEVGLS	SCQGDYIRVR
890	900	910	920	930	940	950	960
KAITAGYFYH	TARLTRSGYR	TVKQQQTVFI	HPNSSLFEQQ	PRWLLYHELV	LTTKEFMRQV	LEIESSWLLE	VAPHYYKAKE
970	980	990					
LEDPHAKKMP	KKIGKTREEL	G					

Cmpd.	No. of Cmpds.	m/z meas.	Δ m/z [ppm]	z	Rt [min]	Score	P	Range	Sequence	Modification
1748	1	1044.9652	-43.04	2	51.7	25.1	2	225-242	R.AAGEQEKLEATNRYHMPK.E	Oxidation: 16

Detailed Protein Report

Protein 448: neuroblastoma breakpoint family member 3 isoform 3 [Homo sapiens]

Accession: gi|374088176
Database: refseq_human(refseq_human_20140103.fasta)
Database Date: 2015-11-30
Modification(s): Oxidation

Score: 25.1
MW [kDa]: 65.1
pI: 4.2
Sequence Coverage [%]: 4.1
No. of unique Peptides: 1

Quantitation

MD:MU **Median:** 1.18 **CV:** 0.00 % **No. of Peptides:** 1

10	20	30	40	50	60	70	80
MPLTPTVQGF	QWTLRGPDVE	TSPFGAPRAA	SHGVGRHQEL	RDPTDYEDCK	DLIKSMLRDE	RLLTEEKLAE	ELGQAEELRQ
90	100	110	120	130	140	150	160
YKVLVHSQER	ELTQLREKLQ	EGRDASRSLN	QHLQALLTPD	EPDNSQGRDL	REQLAEGCRL	AQHLVQKLSP	ENDDDEDEDV
170	180	190	200	210	220	230	240
KVEEAEKVQE	LYAPREVQKA	EEKEVPEDSL	EECAITCSNS	HHPCESNQPY	GNTRITFEED	QVDSTLIDSS	SHDEWLDAVC
250	260	270	280	290	300	310	320
IIPENESDHE	QEEEKGPVSP	RNLQESEEEE	APQESWDEGD	WTLSIPPDMS	ASYQSDRSTF	HSVEEQQVGL	ALDIGRHWCD
330	340	350	360	370	380	390	400
QVKKEDQEAT	SPRLSRELLD	EKEPEVLQDS	LDRFYSTPFE	YLELPDLCQP	YRSDFYSLQE	QHLGLALDLD	RMKKDQEEEE
410	420	430	440	450	460	470	480
DQGPPCPRLS	RELPEVVEPE	DLQDSLDRWY	STPFSYPELP	DSCQPYGSCF	YSLEEEHVGF	SLDVDEIEKY	QEGEEDQKPP
490	500	510	520	530	540	550	560
CPRLNEVLME	AEEPEVLQDS	LDRCYSTTST	YFQLHASFQQ	YRSAFYSFEE	QDVSLALDVD	NRFFTLTVIR	HHLAFQMGVI
570							
FPH							

Cmpd.	No. of Cmpds.	m/z meas.	Δ m/z [ppm]	z	Rt [min]	Score	P	Range	Sequence	Modification	Ratios	
1974	2	696.2762	-133.49	2	54.8	13.9	2	51-61	K.DLIKSMLRDER.L	Oxidation: 6	MD:MU	1.18

Detailed Protein Report

Protein 449: zinc finger protein 30 isoform b [Homo sapiens]

Accession: gi|152963633
Database: refseq_human(refseq_human_20140103.fasta)
Database Date: 2015-11-30

Score: 25.0
MW [kDa]: 71.4
pI: 10.2
Sequence Coverage [%]: 4.2
No. of unique Peptides: 1

10	20	30	40	50	60	70	80
MAHKYVGLQY	HGSVTFEDVA	IAFSQQEWES	LDSSQRGLYR	DVMLENYRNL	VSMGHSRSKP	HVIALLEQWK	EPEVTVRKDG
90	100	110	120	130	140	150	160
RRWCTDLQLE	DDTIGCKEMP	TSENCPSFAL	HQKISRQKPR	ECQEYGKTLC	QDSKPVQHER	IHSSEKPNRC	KECGKNFSNG
170	180	190	200	210	220	230	240
HQLTIHQRLH	VGEKPYKYEK	CGKAFISGSA	FVKHGRIHTG	EKPLKCKQCG	KTISGSYQLT	VHKSIHTGKK	PYECGECGKA
250	260	270	280	290	300	310	320
FLVYGKLTRH	QSTHTGEKPF	GCEECGKAFS	TFSYLVQHQR	IHTSEKPYEC	KECGKAFSTS	SPLAKHQRIH	TGEKPYECKE
330	340	350	360	370	380	390	400
CGKSFTVYGQ	LTRHQSIHTG	EKPFECKECG	KAFRLSSFLH	AHQRIHAEIK	PYGCKECGRT	FSRASYLVQH	GRLHTGEKPY
410	420	430	440	450	460	470	480
ECKECGKAFS	TGSYLVQHQR	IHTGEKPYEC	KECGKAFISR	HQLTVHQRVH	TGEKPYECKE	CGKAFRVHVH	LTQHRKIHTD
490	500	510	520	530	540	550	560
VKPYECKECG	KTFSRASYLV	QHSRIHTGKK	PYECKECGKA	FSSGSYLVQH	QRIHTGEKPY	ECNKCGKAFT	VYGQLIGHQS
570	580	590	600	610	620	630	
VHTGEKPFEC	KECGKAFRLN	SFLTEHQRVH	TGEKPFKCKK	CGKTFRYSSA	LKVHLRKHMS	VIP	

Cmpd.	No. of Cmpds.	m/z meas.	Δ m/z [ppm]	z	Rt [min]	Score	P	Range	Sequence	Modification
1857	1	939.3363	-138.12	2	53.1	11.7	2	476-491	R.KIHTDVKPYECKECGK.T	

Detailed Protein Report

Protein 450: transcription elongation factor A protein-like 4 [Homo sapiens]

**Accession:**	gi|55749442	**Score:**	25.0
**Database:**	refseq_human(refseq_human_20140103.fasta)	**MW [kDa]:**	24.6
**Database Date:**	2015-11-30	**pI:**	5.0
		**Sequence Coverage [%]:**	10.2
		**No. of unique Peptides:**	1

Alias proteins:

Accession	Name	Description
gi|530422178	refseq_human (refseq_human_20140103.fasta)	PREDICTED: transcription elongation factor A protein-like 4 isoform X3 [Homo sapiens]
gi|530422176	refseq_human (refseq_human_20140103.fasta)	PREDICTED: transcription elongation factor A protein-like 4 isoform X2 [Homo sapiens]
gi|65505873	refseq_human (refseq_human_20140103.fasta)	transcription elongation factor A protein-like 4 [Homo sapiens]
gi|55749459	refseq_human (refseq_human_20140103.fasta)	transcription elongation factor A protein-like 4 [Homo sapiens]

10	20	30	40	50	60	70	80
MEKLYSENEG	MASNQGKMEN	EEQPQDERKP	EVTCTLEDKK	LENEGKTENK	GKTGDEEMLK	DKGKPESEGE	AKEGKSEREG
**90**	**100**	**110**	**120**	**130**	**140**	**150**	**160**
ESEMEGGSER	EGKPEIEGKP	ESEGEPGSET	RAAGKRPAED	DVPRKAKRKT	NKGLAHYLKE	YKEAIHDMNF	SNEDMIREFD
**170**	**180**	**190**	**200**	**210**	**220**		
NMAKVQDEKR	KSKQKLGAFL	WMQRNLQDPF	YPRGPREFRG	GCRAPRRDIE	DIPYV		

Cmpd.	No. of Cmpds.	m/z meas.	Δ m/z [ppm]	z	Rt [min]	Score	P	Range	Sequence	Modification
1816	1	673.2645	-102.29	2	52.7	13.7	1	63-75	K.GKPESEGEAKEGK.S	

Detailed Protein Report

Protein 451: PREDICTED: cytosolic carboxypeptidase 2 isoform X4 [Homo sapiens]

**Accession:**	gi|530395811	**Score:**	25.0
**Database:**	refseq_human(refseq_human_20140103.fasta)	**MW [kDa]:**	90.6
**Database Date:**	2015-11-30	**pI:**	9.4
**Modification(s):**	Oxidation	**Sequence Coverage [%]:**	3.7
		**No. of unique Peptides:**	2

10	20	30	40	50	60	70	80
MFPALETHLK	QTIPDPYEDF	MYRHLQYYGY	FKAQRGSLPN	SATHQHVRKN	NPQCLLNGSL	GEKDDLIPDT	LQKEKLLWPI
90	100	110	120	130	140	150	160
SLSSAVHRQI	EAINRDSHML	SLPHLRSRQL	LYDELDEVNP	RLREPQELFS	ILSTKRPLQA	PRWPIECEVI	KENIHHIEWA
170	180	190	200	210	220	230	240
PPQPEYFYQP	KGNEKVPEIV	GEKKGTVVYQ	LDSVPIEGSY	FTSSRVGGKR	GIVKELAVTL	QGPEDNTLLF	ESRFESGNLQ
250	260	270	280	290	300	310	320
KAVRVDTYEY	ELTLRTDLYT	NKHTQWFYFR	VQNTRKDATY	RFTIVNLLKP	KSLYTVGMKP	LLYSQLDANT	RNIGWRREGN
330	340	350	360	370	380	390	400
EIKYYKNNTD	DGQQPFYCLT	WTIQFPYDQD	TCFFAHFYPY	TYTDLQCYLL	SVANNPIQSQ	FCKLQTLCRS	LAGNTVYLLT
410	420	430	440	450	460	470	480
ITNPSQTPQE	AAAKKAVVLS	ARVHPGESNG	SWVMKGFLDF	ILSNSPDAQL	LRDIFVFKVL	PMLNPDGVIV	GNYRCSLAGR
490	500	510	520	530	540	550	560
DLNRHYKTIL	KESFPCIWYT	RNMIKRLLEE	REVLLYCDFH	GHSRKNNIFL	YGCNNNNRKY	WLHERVFPLM	LCKNAPDKFS
570	580	590	600	610	620	630	640
FHSCNFKVQK	CKEGTGRVVM	WRMGILNSYT	MESTFGGSTL	GNKRDTHFTI	EDLKSLGYHV	CDTLLDFCDP	DQMKFTQCLA
650	660	670	680	690	700	710	720
ELKELLRQEI	HKKFHELGQD	VDLEGSWSDI	SLSDIESSTS	GSDSSLSDGL	PVHLANIADE	TNLNRRDKDT	PLDPSMATLI
730	740	750	760	770	780	790	
LPKNKGRMQN	KKPGFTVSCS	PKRTINSSQE	PAPGMKPNWP	RSRYPATKRG	CAAMAAYPSL	HIYTYP	

Cmpd.	No. of Cmpds.	m/z meas.	Δ m/z [ppm]	z	Rt [min]	Score	P	Range	Sequence	Modification
179	1	879.8295	-147.14	2	31.6	13.6	1	33-48	K.AQRGSLPNSATHQHVR.K	
1977	1	746.3572	-41.97	2	54.8	11.3	1	546-558	R.VFPLMLCKNAPDK.F	Oxidation: 5

Detailed Protein Report

Protein 452: ran GTPase-activating protein 1 [Homo sapiens]

**Accession:**	gi|4506411	**Score:**	24.9
**Database:**	refseq_human(refseq_human_20140103.fasta)	**MW [kDa]:**	63.5
**Database Date:**	2015-11-30	**pI:**	4.5
**Modification(s):**	Oxidation	**Sequence Coverage [%]:**	4.3
		**No. of unique Peptides:**	1

Alias proteins:

Accession	Name	Description
gi|578837322	refseq_human (refseq_human_20140103.fasta)	PREDICTED: ran GTPase-activating protein 1 isoform X3 [Homo sapiens]
gi|520975387	refseq_human (refseq_human_20140103.fasta)	ran GTPase-activating protein 1 [Homo sapiens]

10	20	30	40	50	60	70	80
MASEDIAKLA	ETLAKTQVAG	GQLSFKGKSL	KLNTAEDAKD	VIKEIEDFDS	LEALRLEGNT	VGVEAARVIA	KALEKKSELK
**90**	**100**	**110**	**120**	**130**	**140**	**150**	**160**
RCHWSDMFTG	RLRTEIPPAL	ISLGEGLITA	GAQLVELDLS	DNAFGPDGVQ	GFEALLKSSA	CFTLQELKLN	NCGMGIGGGK
**170**	**180**	**190**	**200**	**210**	**220**	**230**	**240**
ILAAALTECH	RKSSAQGKPL	ALKVFVAGRN	RLENDGATAL	AEAFRVIGTL	EEVHMPQNGI	NHPGITALAQ	AFAVNPLLRV
**250**	**260**	**270**	**280**	**290**	**300**	**310**	**320**
INLNDNTFTE	KGAVAMAETL	KTLRQVEVIN	FGDCLVRSKG	AVAIADAIRG	GLPKLKELNL	SFCEIKRDAA	LAVAEAMADK
**330**	**340**	**350**	**360**	**370**	**380**	**390**	**400**
AELEKLDLNG	NTLGEEGCEQ	LQEVLEGFNM	AKVLASLSDD	EDEEEEEEGE	EEEEEAEEEE	EEDEEEEEEE	EEEEEEEPQQ
**410**	**420**	**430**	**440**	**450**	**460**	**470**	**480**
RGQGEKSATP	SRKILDPNTG	EPAPVLSSPP	PADVSTFLAF	PSPEKLLRLG	PKSSVLIAQQ	TDTSDPEKVV	SAFLKVSSVF
**490**	**500**	**510**	**520**	**530**	**540**	**550**	**560**
KDEATVRMAV	QDAVDALMQK	AFNSSSFNSN	TFLTRLLVHM	GLLKSEDKVK	AIANLYGPLM	ALNHMVQQDY	FPKALAPLLL
**570**	**580**	**590**					
AFVTKPNSAL	ESCSFARHSL	LQTLYKV					

Cmpd.	No. of Cmpds.	m/z meas.	Δ m/z [ppm]	z	Rt [min]	Score	P	Range	Sequence	Modification
1782	1	568.7446	-32.25	2	52.3	12.8	0	149-160	K.LNNCGMGIGGGK.I	Oxidation: 6

Detailed Protein Report

Protein 453: centrosomal protein of 63 kDa isoform c [Homo sapiens]

**Accession:**	gi|109255226	**Score:**	24.8
**Database:**	refseq_human(refseq_human_20140103.fasta)	**MW [kDa]:**	57.9
**Database Date:**	2015-11-30	**pI:**	5.9
		**Sequence Coverage [%]:**	8.7
		**No. of unique Peptides:**	1

10	20	30	40	50	60	70	80
MEALLEGIQN	RGHGGGFLTS	CEAELQELMK	QIDIMVAHKK	SEWEGRTHAL	ETCLKIREQE	LKSLRSQLDV	THKEVGMLHQ
**90**	**100**	**110**	**120**	**130**	**140**	**150**	**160**
QVEEHEKIKQ	EMTMEYKQEL	KKLHEELCIL	KRSYEKLQKK	QMREFRGNTK	NHREDRSEIE	RLTAKIEEFR	QKSLDWEKQR
**170**	**180**	**190**	**200**	**210**	**220**	**230**	**240**
LIYQQQVSSL	EAQRKALAEQ	SEIIQAQLVN	RKQKLESVEL	SSQSEIQHLS	SKLERANDTI	CANELEIERL	TMRVNDLVGT
**250**	**260**	**270**	**280**	**290**	**300**	**310**	**320**
SMTVLQEQQQ	KEEKLRESEK	LLEALQEEKR	ELKAALQSQE	NLIHEARIQK	EKLQEKVKAT	NTQHAVEAIS	LESVSATCKQ
**330**	**340**	**350**	**360**	**370**	**380**	**390**	**400**
LSQELMEKYE	ELKRMEAHNN	EYKAEIKKLK	EQILQGEQSY	SSALEGMKME	ISHLTQELHQ	RDITIASTKG	SSSDMEKRLR
**410**	**420**	**430**	**440**	**450**	**460**	**470**	**480**
AEMQKAEDKA	VEHKEILDQL	ESLKLENRHL	SEMVMKLELG	LHECSLPVSP	LGSIATRFLE	EEELRSHHIL	ERLDAHIEEL
**490**	**500**						
KRESEKTVRQ	FTALK						

Cmpd.	No. of Cmpds.	m/z meas.	Δ m/z [ppm]	z	Rt [min]	Score	P	Range	Sequence	Modification
2671	1	1079.8373	-57.12	3	64.6	12.7	1	437-465	K.LELGLHECSLPVSPLGSIATRFLEEEELR.S	

Detailed Protein Report

Protein 454: myomesin-1 isoform b [Homo sapiens]

Accession: gi|140560917
Database: refseq_human(refseq_human_20140103.fasta)
Database Date: 2015-11-30

Score: 24.8
MW [kDa]: 177.6
pI: 6.4
Sequence Coverage [%]: 1.5
No. of unique Peptides: 1

Quantitation

MD:MU **Median:** 3.08 **CV:** 0.00 % **No. of Peptides:** 1

10	20	30	40	50	60	70	80
MSLPFYQRCH	QHYDLSYRNK	DVRSTVSHYQ	REKKRSAVYT	QGSTAYSSRS	SAAHRRESEA	FRRASASSSQ	QQASQHALSS
90	100	110	120	130	140	150	160
EVSRKAASAY	DYGSSHGLTD	SSLLLDDYSS	KLSPKPKRAK	HSLLSGEEKE	NLPSDYMVPI	FSGRQKHVSG	ITDTEEERIK
170	180	190	200	210	220	230	240
EAAAYIAQRN	LLASEEGITT	SKQSTASKQT	TASKQSTASK	QSTASKQSTA	SRQSTASRQS	VVSKQATSAL	QQEETSEKKS
250	260	270	280	290	300	310	320
RKVVIREKAE	RLSLRKTLEE	TETYHAKLNE	DHLLHAPEFI	IKPRSHTVWE	KENVKLHCSI	AGWPEPRVTW	YKNQVPINVH
330	340	350	360	370	380	390	400
ANPGKYIIES	RYGMHTLEIN	GCDFEDTAQY	RASAMNVKGE	LSAYASVVVK	RYKGEFDETR	FHAGASTMPL	SFGVTPYGYA
410	420	430	440	450	460	470	480
SRFEIHFDDK	FDVSFGREGE	TMSLGCRVVI	TPEIKHFQPE	IQWYRNGVPL	SPSKWVQTLW	SGERATLTFS	HLNKEDEGLY
490	500	510	520	530	540	550	560
TIRVRMGEYY	EQYSAYVFVR	DADAEIEGAP	AAPLDVKCLE	ANKDYIIISW	KQPAVDGGSP	ILGYFIDKCE	VGTDSWSQCN
570	580	590	600	610	620	630	640
DTPVKFARFP	VTGLIEGRSY	IFRVRAVNKM	GIGFPSRVSE	PVAALDPAEK	ARLKSRPSAP	WTGQIIVTEE	EPSEGIVPGP
650	660	670	680	690	700	710	720
PTDLSVTEAT	RSYVVLSWKP	PGQRGHEGIM	YFVEKCEAGT	ENWQRVNTEL	PVKSPRFALF	DLAEGKSYCF	RVRCSNSAGV
730	740	750	760	770	780	790	800
GEPSEATEVT	VVGDKLDIPK	APGKIIPSRN	TDTSVVVSWE	ESKDAKELVG	YYIEASVAGS	GKWEPCNNNP	VKGSRFTCHG
810	820	830	840	850	860	870	880
LVTGQSYIFR	VRAVNAAGLS	EYSQDSEAIE	VKAAIAPPSP	PCDITCLESF	RDSMVLGWKQ	PDKIGGAEIT	GYYVNYREVI
890	900	910	920	930	940	950	960
DGVPGKWREA	NVKAVSEEAY	KISNLKENMV	YQFQVAAMNM	AGLGAPSAVS	ECFKCEEWTI	AVPGPPHSLK	CSEVRKDSLV
970	980	990	1000	1010	1020	1030	1040
LQWKPPVHSG	RTPVTGYFVD	LKEAKAKEDQ	WRGLNEAAIK	NVYLKVRGLK	EGVSYVFRVR	AINQAGVGKP	SDLAGPVVAE
1050	1060	1070	1080	1090	1100	1110	1120
TRPGTKEVVV	NVDDDGVISL	NFECDKMTPK	SEFSWSKDYV	STEDSPRLEV	ESKGNKTKMT	FKDLGMDDLG	IYSCDVTDTD
1130	1140	1150	1160	1170	1180	1190	1200
GIASSYLIDE	EELKRLLALS	HEHKFPTVPV	KSELAVEILE	KGQVRFWMQA	EKLSGNAKVN	YIFNEKEIFE	GPKYKMHIDR
1210	1220	1230	1240	1250	1260	1270	1280
NTGIIEMFME	KLQDEDEGTY	TFQLQDGKAT	NHSTVVLVGD	VFKKLQKEAE	FQRQEWIRKQ	GPHFVEYLSW	EVTGECNVLL
1290	1300	1310	1320	1330	1340	1350	1360
KCKVANIKKE	THIVWYKDER	EISVDEKHDF	KDGICTLLIT	EFSKKDAGIY	EVILKDDRGK	DKSRLKLVDE	AFKELMMEVC
1370	1380	1390	1400	1410	1420	1430	1440
KKIALSATDL	KIQSTAEGIQ	LYSFVTYYVE	DLKVNWSHNG	SAIRYSDRVK	TGVTGEQIWL	QINEPTPNDK	GKYVMELFDG
1450	1460	1470	1480	1490	1500	1510	1520
KTGHQKTVDL	SGQAYDEAYA	EFQRLKQAAI	AEKNRARVLG	GLPDVVTIQE	GKALNLTCNV	WGDPPPEVSW	LKNEKALASD
1530	1540	1550	1560	1570	1580	1590	
DHCNLKFEAG	RTAYFTINGV	STADSGKYGL	VVKNKYGSET	SDFTVSVFIP	EEEARMAALE	SLKGGKKAK	

Cmpd.	No. of Cmpds.	m/z meas.	Δ m/z [ppm]	z	Rt [min]	Score	P	Range	Sequence	Modification	Ratios
764	1	555.8059	19.32	2	38.9	10.8	0	697-706	R.FALFDLAEGK.S		MD:MU 3.08

Detailed Protein Report

Protein 455: NTPase KAP family P-loop domain-containing protein 1 [Homo sapiens]

**Accession:**	gi|254553319	**Score:**	24.8
**Database:**	refseq_human(refseq_human_20140103.fasta)	**MW [kDa]:**	91.8
**Database Date:**	2015-11-30	**pI:**	10.3
		**Sequence Coverage [%]:**	2.8
		**No. of unique Peptides:**	1

10	20	30	40	50	60	70	80
MHKHYKVHFA	KDAQSPNGHY	FWDPELGHRK	GCCHQWRQDS	AALRAHGPCR	PSPQSHWQLA	YHSHQVGGSG	WRRGLLPSVL
90	100	110	120	130	140	150	160
QQQRQPQSQP	SPPSPLRQRL	CPIHEAQKGL	PATSTVPKEP	ASAPQAPTLP	TTAPAMARSG	PALPSAAGVL	LKPSEPTDAR
170	180	190	200	210	220	230	240
PLPAPAACGS	FTAYSSDILT	EDDVYCSCLA	KTLCHVPVPV	TVGFYAPFGC	RLHMMLDKIT	ALMQQEAAQR	ESEELQHVQW
250	260	270	280	290	300	310	320
RPRAVSGWGV	PQLLWYLVFL	QPIITEVHLR	RRNVQFLFIR	FSAWQYAGTD	KLWAGLVTTL	CEGIRRHYGA	LPFSVYSVLG
330	340	350	360	370	380	390	400
NKPATRQDCC	QSEWHCRRRV	CLGLLALLAA	LGLGVGLLYL	SLGGHALGHG	SPSGSLLKVF	GGAATTLSGS	GLLMAVYSVG
410	420	430	440	450	460	470	480
KHLFVSQRKK	IERLVSREKF	GSQLGFMCEV	KKEVELLTDF	LCFLEIYQRR	RLRVVLEVTG	LDTCYPERVV	GVLNAINTLL
490	500	510	520	530	540	550	560
SDSHAPFIFI	LVVDPSILAA	CLESAGNMKG	TADNGYLFLN	RTVTLPFSVP	IMGRRTKLQF	LHDAVQSRDD	LLYREMTRKP
570	580	590	600	610	620	630	640
WLPGDAGGES	AQLLAVQAQA	GTERGQGRID	DEAARRIQEA	LFCLHDERDC	LYEYVPDNVV	SMRRIVNTVP	ITVRLLQQQQ
650	660	670	680	690	700	710	720
QQGDFGGPTP	RQAVAWVVLA	NQWPCRLSWA	LQCLEDRQQT	GGAPEGRARL	WDVFRDNSRE	LHTMTKALQN	VLDLDGDPEL
730	740	750	760	770	780	790	800
FERFLGADFP	FTVAEAQSLL	RCTVNLDHSI	RRRMGLIRAV	SALKPPSPPK	SPTRDTPHAA	HRANSASRAP	PSGRASGQAG
810	820	830	840				
EGHHTGDLAH	RGKLWPVACA	LFRPGQSSPG	GP				

Cmpd.	No. of Cmpds.	m/z meas.	Δ m/z [ppm]	z	Rt [min]	Score	P	Range	Sequence	Modification
1838	1	673.2555	-124.96	2	53.0	13.9	1	771-782	K.SPTRDTPHAAHR.A	

Detailed Protein Report

Protein 456: **PREDICTED: myotubularin-related protein 5 isoform X5 [Homo sapiens]**

**Accession:**	gi|530420851	**Score:**	24.8
**Database:**	refseq_human(refseq_human_20140103.fasta)	**MW [kDa]:**	208.2
**Database Date:**	2015-11-30	**pI:**	6.5
**Modification(s):**	Carbamidomethyl	**Sequence Coverage [%]:**	1.2
		**No. of unique Peptides:**	2

10	20	30	40	50	60	70	80
MARLADYFVL	VAFGPHPRGS	GEGQGQILQR	FPEKDWEDNP	FPQGIELFCQ	PSGWQLCPER	NPPTFFVAVL	TDINSERHYC
90	100	110	120	130	140	150	160
ACLTFWEPAE	PSQETTRVED	ATEREEEGDE	GGQTHLSPTA	PAPSAQLFAP	KTLVLVSRLD	HTEVFRNSLG	LIYAIHVEGL
170	180	190	200	210	220	230	240
NVCLENVIGN	LLTCTVPLAG	GSQRTISLGA	GDRQVIQTPL	ADSLPVSRCS	VALLFRQLGI	TNVLSLFCAA	LTEHKVLFLS
250	260	270	280	290	300	310	320
RSYQRLADAC	RGLLALLFPL	RYSFTYVPIL	PAQLLEVLST	PTPFIIGVNA	AFQAETQELL	DVIVADLDGG	TVTIPECVHI
330	340	350	360	370	380	390	400
PPLPEPLQSQ	THSVLSMVLD	PELELADLAF	PPPTTSTSSL	KMQDKELRAV	FLRLFAQLLQ	GYRWCLHVVR	IHPEPVIRFH
410	420	430	440	450	460	470	480
KAAFLGQRGL	VEDDFLMKVL	EGMAFAGFVS	ERGVPYRPTD	LFDELVAHEV	ARMRADENHP	QRVLRHVQEL	AEQLYKNENP
490	500	510	520	530	540	550	560
YPAVAMHKVQ	RPGESSHLRR	VPRPFPRLDE	GTVQWIVDQA	AAKMQGAPPA	VKAERRTTVP	SGPPMTAILE	RCSGLHVNSA
570	580	590	600	610	620	630	640
RRLEVVRNCI	SYVFEGKMLE	AKKLLPAVLR	ALKGRAARRC	LAQELHLHVQ	QNRAVLDHQQ	FDFVVRMMNC	CLQDCTSLDE
650	660	670	680	690	700	710	720
HGIAAALLPL	VTAFCRKLSP	GVTQFAYSCV	QEHVVWSTPQ	FWEAMFYGDV	QTHIRALYLE	PTEDLAPAQE	VGEAPSQEDE
730	740	750	760	770	780	790	800
RSALDVASEQ	RRLWPTLSRE	KQQELVQKEE	STVFSQAIHY	ANRMSYLLLP	LDSSKSRLLR	ERAGLGDLES	ASNSLVTNSM
810	820	830	840	850	860	870	880
AGSVAESYDT	ESGFEDAETC	DVAGAVVRFI	NRFVDKVCTE	SGVTSDHLKG	LHVMVPDIVQ	MHIETLEAVQ	RESRRLPPIQ
890	900	910	920	930	940	950	960
KPKLLRPRLL	PGEECVLDGL	RVYLLPDGRE	EGAGGSAGGP	ALLPAEGAVF	LTTYRVIFTG	MPTDPLVGEQ	VVVRSFPVAA
970	980	990	1000	1010	1020	1030	1040
LTKEKRISVQ	TPVDQLLQDG	LQLRSCTFQL	LKMAFDEEVG	SDSAELFRKQ	LHKLRYPPDI	RATFAFTLGS	AHTPGRPPRV
1050	1060	1070	1080	1090	1100	1110	1120
TKDKGPSLRT	LSRNLVKNAK	KTIGRQHVTR	KKYNPPSWEH	RGQPPPEDQE	DEISVSEELE	PSTLTPSSAL	KPSDRMTMSS
1130	1140	1150	1160	1170	1180	1190	1200
LVERACCRDY	QRLGLGTLSS	SLSRAKSEPF	RISPVNRMYA	ICRSYPGLLI	VPQSVQDNAL	QRVSRCYRQN	RFPVVCWRSG
1210	1220	1230	1240	1250	1260	1270	1280
RSKAVLLRSG	GLHGKGVVGL	FKAQNAPSPG	QSQADSSSLE	QEKYLQAVVS	SMPRYADASG	RNTLSGFSSA	HMGSHGKWGS
1290	1300	1310	1320	1330	1340	1350	1360
VRTSGRSSGL	GTDVGSRLAG	RDALAPPQAN	GGPPDPGFLR	PQRAALYILG	DKAQLKGVRS	DPLQQWELVP	IEVFEARQVK
1370	1380	1390	1400	1410	1420	1430	1440
ASFKKLLKAC	VPGCPAAEPS	PASFLRSLED	SEWLIQIHKL	LQVSVLVVEL	LDSGSSVLVG	LEDGWDITTQ	VVSLVQLLSD
1450	1460	1470	1480	1490	1500	1510	1520
PFYRTLEGFR	LLVEKEWLSF	GHRFSHRGAH	TLAGQSSGFT	PVFLQFLDCV	HQVHLQFPME	FEFSQFYLKF	LGYHHVSRRF
1530	1540	1550	1560	1570	1580	1590	1600
RTFLLDSDYE	RIELGLLYEE	KGERRGQVPC	RSVWEYVDRL	SKRTPVFHNY	MYAPEDAEVL	RPYSNVSNLK	VWDFYTEETL
1610	1620	1630	1640	1650	1660	1670	1680
AEGPPYDWEL	AQGPPEPPEE	ERSDGGAPQS	RRRVVWPCYD	SCPRAQPDAI	SRLLEELQRL	ETELGQPAER	WKDTWDRVKA
1690	1700	1710	1720	1730	1740	1750	1760
AQRLEGRPDG	RGTPSSLLVS	TAPHHRRSLG	VYLQEGPVGS	TLSLSLDSDQ	SSGSTTSGSR	QAARRSTSTL	YSQFQTAESE
1770	1780	1790	1800	1810	1820	1830	1840
NRSYEGTLYK	KGAFMKPWKA	RWFVLDKTKH	QLRYYDHRVD	TECKGVIDLA	EVEAVAPGTP	TMGAPKTVDE	KAFFDVKTTR
1850	1860	1870					
RVYNFCAQDV	PSAQQWVDRI	QSCLSDA					

Detailed Protein Report

Cmpd.	No. of Cmpds.	m/z meas.	Δ m/z [ppm]	z	Rt [min]	Score	P	Range	Sequence	Modification
292	1	591.6596	-216.66	2	33.3	11.6	1	242-251	R.SYQRLADACR.G	
348	1	800.7371	-149.37	2	33.7	13.1	1	1116-1128	R.MTMSSLVERACCR.D	Carbamidomethyl: 11, 12

Detailed Protein Report

Protein 457: **ankyrin repeat domain-containing protein 62 [Homo sapiens]**

**Accession:**	gi|472339087	**Score:**	24.7
**Database:**	refseq_human(refseq_human_20140103.fasta)	**MW [kDa]:**	106.4
**Database Date:**	2015-11-30	**pI:**	6.2
		**Sequence Coverage [%]:**	2.7
		**No. of unique Peptides:**	2

10	20	30	40	50	60	70	80
MEVRGSFLAA	CRRRMATWRK	NRDKDGFSNP	GYRVRQKDLG	MIHKAAIAGD	VNKVMESILL	RLNDLNDRDK	KNRTALLLAC
**90**	**100**	**110**	**120**	**130**	**140**	**150**	**160**
AHGRPGVVAD	LVARKCQLNL	TDSENRTALI	KAVQCQEEVC	ASILLEHGAN	PNVRDMYGNT	ALHYAIDNEN	ISMARKLLAY
**170**	**180**	**190**	**200**	**210**	**220**	**230**	**240**
GADIEARSQD	GHTSLLLAVN	RKKEQMVAFL	LKKKPDLTAI	DNFGRTALIL	AARNGSTSVV	YQLLQHNIDV	FCQDISGWTA
**250**	**260**	**270**	**280**	**290**	**300**	**310**	**320**
EDYAVASKFQ	AIRGMISEYK	ANKRCKSLQN	SNSEQDLEMT	SEGEQERLEG	CESSQPQVEE	KMKKCRNKKM	EVSRNVHADD
**330**	**340**	**350**	**360**	**370**	**380**	**390**	**400**
SDNYNDDVDE	LIHKIKNRKP	DNHQSPGKEN	GEFDRLARKT	SNEKSKVKSQ	IYFTDDLNDI	SGSSEKTSED	DELPYSDDEN
**410**	**420**	**430**	**440**	**450**	**460**	**470**	**480**
FMLLIEQSGM	ECKDFVSLSK	SKNATAACGR	SIEDQKCYCE	RLKVKFQKMK	NNISVLQKVL	SETDKTKSQS	EHQNLQGKKK
**490**	**500**	**510**	**520**	**530**	**540**	**550**	**560**
LCNLRFILQQ	QEEERIKAEE	LYEKDIEELK	IMEEQYRTQT	EVKKQSKLTL	KSLEVELKTV	RSNSNQNFHT	HERERDLWQE
**570**	**580**	**590**	**600**	**610**	**620**	**630**	**640**
NHLMRDEIAR	LRLEIDTIKH	QNQETENKYF	KDIEIIKENN	EDLEKTLKRN	EEALTKTITR	YSKELNVLMD	ENTMLNSELQ
**650**	**660**	**670**	**680**	**690**	**700**	**710**	**720**
KEKQSMSRLE	TEMESYRCRL	AAALCDHDQR	QSSKRDLQLA	FQSTVNEWCH	LQEDTNSHIQ	ILSQQLSKAE	STSSGLETEL
**730**	**740**	**750**	**760**	**770**	**780**	**790**	**800**
HYEREALKEK	TLHIEHMQGV	LSRTQRRLED	IEHMYQNDQP	ILEKYVRKQQ	SVEDGLFQLQ	SQNLLYQQQC	NDARKKADNQ
**810**	**820**	**830**	**840**	**850**	**860**	**870**	**880**
EKTIINIQVK	CEDTVEKLQA	ECRKLEENNK	GLMKECTLLK	ERQCQYEKEK	EEREVVRRQL	QREVDDALNK	QLLLEAMLEI
**890**	**900**	**910**	**920**				
SSERRINLED	EAQSLKKKLG	QMRSQVCMKL	SMSTVTL				

Cmpd.	No. of Cmpds.	m/z meas.	Δ m/z [ppm]	z	Rt [min]	Score	P	Range	Sequence	Modification
2195	1	900.3616	-160.41	2	57.3	12.4	1	45-61	K.AAIAGDVNKVMESILLR.L	
1860	1	915.3255	-218.76	1	53.3	12.3	0	451-458	K.NNISVLQK.V	

Detailed Protein Report

Protein 458: protein FAM117B [Homo sapiens]

Accession: gi|254910983
Database: refseq_human(refseq_human_20140103.fasta)
Database Date: 2015-11-30
Modification(s): Carbamidomethyl

Score: 24.7
MW [kDa]: 61.9
pI: 10.8
Sequence Coverage [%]: 5.1
No. of unique Peptides: 2

Quantitation

MD:MU **Median:** 1.70 **CV:** 0.00 % **No. of Peptides:** 1

10	20	30	40	50	60	70	80
MSQRVRRNGS	PTPAGSLGGG	AVATAGGPGS	RLQPMRATVP	FQLKQQQQQQ	HGSPTRSGGG	GGGNNNGGCC	GGASGPAGGG
**90**	**100**	**110**	**120**	**130**	**140**	**150**	**160**
GGGGPRTASR	STSPTRGGGN	AAARTSPTVA	TQTGASATST	RGTSPTRSAA	PGARGSPPRP	PPPPPLLGTV	SSPSSSPTHL
**170**	**180**	**190**	**200**	**210**	**220**	**230**	**240**
WTGEVSAAPP	PARVRHRRRS	PEQSRSSPEK	RSPSAPVCKA	GDKTRQPSSS	PSSIIRRTSS	LDTLAAPYLA	GHWPRDSHGQ
**250**	**260**	**270**	**280**	**290**	**300**	**310**	**320**
AAPCMRDKAT	QTESAWAEEY	SEKKKGSHKR	SASWGSTDQL	KEIAKLRQQL	QRSKHSSRHH	RDKERQSPFH	GNHAAINQCQ
**330**	**340**	**350**	**360**	**370**	**380**	**390**	**400**
APVPKSALIP	VIPITKSTGS	RFRNSVEGLN	QEIEIIIKET	GEKEEQLIPQ	DIPDGHRAPP	PLVQRSSSTR	SIDTQTPGGA
**410**	**420**	**430**	**440**	**450**	**460**	**470**	**480**
DRGSNNSSRS	QSVSPTSFLT	ISNEGSEESP	CSADDLLVDP	RDKENGNNSP	LPKYATSPKP	NNSYMFKREP	PEGCERVKVF
**490**	**500**	**510**	**520**	**530**	**540**	**550**	**560**
EECSPKQLHE	IPAFYCPDKN	KVNFIPKSGS	AFCLVSILKP	LLPTPDLTLK	GSGHSLTVTT	GMTTTLLQPI	AVASLSTNTE
**570**	**580**	**590**					
QDRVSRGTST	VMPSASLLPP	PEPIEEAEG					

Cmpd.	No. of Cmpds.	m/z meas.	Δ m/z [ppm]	z	Rt [min]	Score	P	Range	Sequence	Modification	Ratios
2120	1	753.8961	-98.38	3	56.7	11.3	0	57-86	R. SGGGGGGNNNGGCCGGASGPA(T		MD:MU 1.70
2079	1	772.9176	-77.41	3	55.9	13.4	0	57-86	R. SGGGGGGNNNGGCCGGASGPA(T	Carbamidomethyl: 14	

Detailed Protein Report

Protein 459: PREDICTED: beclin-1 isoform X2 [Homo sapiens]

Accession: gi|530413037
Database: refseq_human(refseq_human_20140103.fasta)
Database Date: 2015-11-30

Score: 24.7
MW [kDa]: 43.8
pI: 4.6
Sequence Coverage [%]: 6.3
No. of unique Peptides: 1

10	20	30	40	50	60	70	80
MEGSKTSNNS	TMQVSFVCQR	CSQPLKLDTS	FKILDRVTIQ	ELTAPLLTTA	QAKPGETQEE	ETNSGEEPFI	ETPRQDGVSR
90	100	110	120	130	140	150	160
RFIPPARMMS	TESANSFTLI	GEASDGGTME	NLSRRLKVTG	DLFDIMSGQT	DVDHPLCEEC	TDTLLDQLDT	QLNVTENECQ
170	180	190	200	210	220	230	240
NYKRCLEILE	QMNEDDSEQL	QMELKELALE	EERLIQELED	VEKNRKIVAE	NLEKVQAEAE	RLDQEEAQYQ	REYSEFKRQQ
250	260	270	280	290	300	310	320
LELDDELKSV	ENQMRYAQTQ	LDKLKKTNVF	NATFHIWHSG	QFGTINNFRL	GRLPSVPVEW	NEINAAWGQT	VLLLHALANK
330	340	350	360	370	380	390	
MGLKFQRMDV	EKGKIEDTGG	SGGSYSIKTQ	FNSEEQWTKA	LKFMLTNLKW	GLAWVSSQFY	NK	

Cmpd.	No. of Cmpds.	m/z meas.	Δ m/z [ppm]	z	Rt [min]	Score	P	Range	Sequence	Modification
1558	1	685.6866	-202.29	2	49.2	11.8	0	335-348	K.IEDTGGSGGSYSIK.T	

Detailed Protein Report

Protein 460: serine/threonine-protein kinase mTOR [Homo sapiens]

**Accession:**	gi|4826730	**Score:**	24.7
**Database:**	refseq_human(refseq_human_20140103.fasta)	**MW [kDa]:**	288.7
**Database Date:**	2015-11-30	**pI:**	6.8
		**Sequence Coverage [%]:**	0.8
		**No. of unique Peptides:**	1

Alias proteins:

Accession	Name	Description
gi|530360517	refseq_human (refseq_human_20140103.fasta)	PREDICTED: serine/threonine-protein kinase mTOR isoform X1 [Homo sapiens]

Detailed Protein Report

10	20	30	40	50	60	70	80
MLGTGPAAAT	TAATTSSNVS	VLQQFASGLK	SRNEETRAKA	AKELQHYVTM	ELREMSQEES	TRFYDQLNHH	IFELVSSSDA
90	100	110	120	130	140	150	160
NERKGGILAI	ASLIGVEGGN	ATRIGRFANY	LRNLLPSNDP	VVMEMASKAI	GRLAMAGDTF	TAEYVEFEVK	RALEWLGADR
170	180	190	200	210	220	230	240
NEGRRHAAVL	VLRELAISVP	TFFFQQVQPF	FDNIFVAVWD	PKQAIREGAV	AALRACLILT	TQREPKEMQK	PQWYRHTFEE
250	260	270	280	290	300	310	320
AEKGFDETLA	KEKGMNRDDR	IHGALLILNE	LVRISSMEGE	RLREEMEEIT	QQQLVHDKYC	KDLMGFGTKP	RHITPFTSFQ
330	340	350	360	370	380	390	400
AVQPQQSNAL	VGLLGYSSHQ	GLMGFGTSPS	PAKSTLVESR	CCRDLMEEKF	DQVCQWVLKC	RNSKNSLIQM	TILNLLPRLA
410	420	430	440	450	460	470	480
AFRPSAFTDT	QYLQDTMNHV	LSCVKKEKER	TAAFQALGLL	SVAVRSEFKV	YLPRVLDIIR	AALPPKDFAH	KRQKAMQVDA
490	500	510	520	530	540	550	560
TVFTCISMLA	RAMGPGIQQD	IKELLEPMLA	VGLSPALTAV	LYDLSRQIPQ	LKKDIQDGLL	KMLSLVLMHK	PLRHPGMPKG
570	580	590	600	610	620	630	640
LAHQLASPGL	TTLPEASDVG	SITLALRTLG	SFEFEGHSLT	QFVRHCADHF	LNSEHKEIRM	EAARTCSRLL	TPSIHLISGH
650	660	670	680	690	700	710	720
AHVVSQTAVQ	VVADVLSKLL	VVGITDPDPD	IRYCVLASLD	ERFDAHLAQA	ENLQALFVAL	NDQVFEIREL	AICTVGRLSS
730	740	750	760	770	780	790	800
MNPAFVMPFL	RKMLIQILTE	LEHSGIGRIK	EQSARMLGHL	VSNAPRLIRP	YMEPILKALI	LKLKDPDPDP	NPGVINNVLA
810	820	830	840	850	860	870	880
TIGELAQVSG	LEMRKWVDEL	FIIIMDMLQD	SSLLAKRQVA	LWTLGQLVAS	TGYVVEPYRK	YPTLLEVLLN	FLKTEQNQGT
890	900	910	920	930	940	950	960
RREAIRVLGL	LGALDPYKHK	VNIGMIDQSR	DASAVSLSES	KSSQDSSDYS	TSEMLVNMGN	LPLDEFYPAV	SMVALMRIFR
970	980	990	1000	1010	1020	1030	1040
DQSLSHHHTM	VVQAITFIFK	SLGLKCVQFL	PQVMPTFLNV	IRVCDGAIRE	FLFQQLGMLV	SFVKSHIRPY	MDEIVTLMRE
1050	1060	1070	1080	1090	1100	1110	1120
FWVMNTSIQS	TIILLIEQIV	VALGGEFKLY	LPQLIPHMLR	VFMHDNSPGR	IVSIKLLAAI	QLFGANLDDY	LHLLLPPIVK
1130	1140	1150	1160	1170	1180	1190	1200
LFDAPEAPLP	SRKAALETVD	RLTESLDFTD	YASRIIHPIV	RTLDQSPELR	STAMDTLSSL	VFQLGKKYQI	FIPMVNKVLV
1210	1220	1230	1240	1250	1260	1270	1280
RHRINHQRYD	VLICRIVKGY	TLADEEEDPL	IYQHRMLRSG	QGDALASGPV	ETGPMKKLHV	STINLQKAWG	AARRVSKDDW
1290	1300	1310	1320	1330	1340	1350	1360
LEWLRRLSLE	LLKDSSSPSL	RSCWALAQAY	NPMARDLFNA	AFVSCWSELN	EDQQDELIRS	IELALTSQDI	AEVTQTLLNL
1370	1380	1390	1400	1410	1420	1430	1440
AEFMEHSDKG	PLPLRDDNGI	VLLGERAAKC	RAYAKALHYK	ELEFQKGPTP	AILESLISIN	NKLQQPEAAA	GVLEYAMKHF
1450	1460	1470	1480	1490	1500	1510	1520
GELEIQATWY	EKLHEWEDAL	VAYDKKMDTN	KDDPELMLGR	MRCLEALGEW	GQLHQQCCEK	WTLVNDETQA	KMARMAAAAA
1530	1540	1550	1560	1570	1580	1590	1600
WGLGQWDSME	EYTCMIPRDT	HDGAFYRAVL	ALHQDLFSLA	QQCIDKARDL	LDAELTAMAG	ESYSRAYGAM	VSCHMLSELE
1610	1620	1630	1640	1650	1660	1670	1680
EVIQYKLVPE	RREIIRQIWW	ERLQGCQRIV	EDWQKILMVR	SLVVSPHEDM	RTWLKYASLC	GKSGRLALAH	KTLVLLLGVD
1690	1700	1710	1720	1730	1740	1750	1760
PSRQLDHPLP	TVHPQVTYAY	MKNMWKSARK	IDAFQHMQHF	VQTMQQQAQH	AIATEDQQHK	QELHKLMARC	FLKLGEWQLN
1770	1780	1790	1800	1810	1820	1830	1840
LQGINESTIP	KVLQYYSAAT	EHDRSWYKAW	HAWAVMNFEA	VLHYKHQNQA	RDEKKKLRHA	SGANITNATT	AATTAATATT
1850	1860	1870	1880	1890	1900	1910	1920
TASTEGSNSE	SEAESTENSP	TPSPLQKKVT	EDLSKTLLMY	TVPAVQGFFR	SISLSRGNNL	QDTLRVLTLW	FDYGHWPDVN
1930	1940	1950	1960	1970	1980	1990	2000
EALVEGVKAI	QIDTWLQVIP	QLIARIDTPR	PLVGRLIHQL	LTDIGRYHPQ	ALIYPLTVAS	KSTTTARHNA	ANKILKNMCE
2010	2020	2030	2040	2050	2060	2070	2080
HSNTLVQQAM	MVSEELIRVA	ILWHEMWHEG	LEEASRLYFG	ERNVKGMFEV	LEPLHAMMER	GPQTLKETSF	NQAYGRDLME
2090	2100	2110	2120	2130	2140	2150	2160
AQEWCRKYMK	SGNVKDLTQA	WDLYYHVFRR	ISKQLPQLTS	LELQYVSPKL	LMCRDLELAV	PGTYDPNQPI	IRIQSIAPSL
2170	2180	2190	2200	2210	2220	2230	2240

Detailed Protein Report

Cmpd.	No. of Cmpds.	m/z meas.	Δ m/z [ppm]	z	Rt [min]	Score	P	Range	Sequence	Modification
1142	1	586.6322	-235.21	2	43.9	12.9	0	2067-2076	K.ETSFNQAYGR.D	

Detailed Protein Report

Protein 461: **PREDICTED: leucine-rich repeat and IQ domain-containing protein 3 isoform X3 [Homo sapiens]**

Accession: gi|530361769
Database: refseq_human(refseq_human_20140103.fasta)
Database Date: 2015-11-30
Modification(s): Carbamidomethyl, Oxidation

Score: 24.7
MW [kDa]: 39.2
pI: 10.2
Sequence Coverage [%]: 9.3
No. of unique Peptides: 1

10	20	30	40	50	60	70	80
MFHGTVTEEL	TSHEEWSHYN	ENIREGQKDF	VFVKFNGLHL	KSMENLQSCI	SLRVCIFSNN	FITDIHPLQS	CIKLIKLDLH
90	100	110	120	130	140	150	160
GNQIKSLPNT	KFWNGLKNLK	LLYLHDNGFA	KLKNICVLSA	CPTLIALTMF	DCPVSLKKGY	RHVLVNSIWP	LKALDHHVIS
170	180	190	200	210	220	230	240
DEEIIQNWHL	PERFKACNHR	LFFNFCPALR	KGTTYEEEIN	NIKHITSKIN	AILAHNSPVL	IVQRWIRGFL	VRKNLSPVFF
250	260	270	280	290	300	310	320
HKKKQQEKII	RGYEAKWIYI	TKGYEDKLLK	DLFFKPETNI	KGKLAYWKHN	IYYPVDLKNS	SEHRKHVSSI	LCELKPKDLG
330	340						
MKSKTSRHLI	QKDGS						

Cmpd.	No. of Cmpds.	m/z meas.	Δ m/z [ppm]	z	Rt [min]	Score	P	Range	Sequence	Modification
1395	1	727.3741	43.73	2	47.1	12.0	0	42-53	K.SMENLQSCISLR.V	Carbamidomethyl: 8; Oxidation: 2

Detailed Protein Report

Protein 462: zinc finger protein 622 [Homo sapiens]

Accession: gi|15529978
Database: refseq_human(refseq_human_20140103.fasta)
Database Date: 2015-11-30
Modification(s): Carbamidomethyl

Score: 24.7
MW [kDa]: 54.2
pI: 5.8
Sequence Coverage [%]: 5.7
No. of unique Peptides: 2

Quantitation

MD:MU **Median:** 0.79 **CV:** 0.00 % **No. of Peptides:** 1

10	20	30	40	50	60	70	80
MATYTCITCR	VAFRDADMQR	AHYKTDWHRY	NLRRKVASMA	PVTAEGFQER	VRAQRAVAEE	ESKGSATYCT	VCSKKFASFN
90	100	110	120	130	140	150	160
AYENHLKSRR	HVELEKKAVQ	AVNRKVEMMN	EKNLEKGLGV	DSVDKDAMNA	AIQQAIKAQP	SMSPKKAPPA	PAKEARNVVA
170	180	190	200	210	220	230	240
VGTGGRGTHD	RDPSEKPPRL	QWFEQQAKKL	AKQQEEDSEE	EEEDLDGDDW	EDIDSDEELE	CEDTEAMDDV	VEQDAEEEEA
250	260	270	280	290	300	310	320
EEGPPLGAIP	ITDCLFCSHH	SSSLMKNVAH	MTKDHSFFIP	DIEYLSDIKG	LIKYLGEKVG	VGKICLWCNE	KGKSFYSTEA
330	340	350	360	370	380	390	400
VQAHMNDKSH	CKLFTDGDAA	LEFADFYDFR	SSYPDHKEGE	DPNKAEELPS	EKNLEYDDET	MELILPSGAR	VGHRSLMRYY
410	420	430	440	450	460	470	480
KQRFGLSRAV	AVAKNRKAVG	RVLQQYRALG	WTGSTGAALM	RERDMQYVQR	MKSKWMLKTG	MKNNATKQMH	FRVQVRF

Cmpd.	No. of Cmpds.	m/z meas.	Δ m/z [ppm]	z	Rt [min]	Score	P	Range	Sequence	Modification	Ratios
2592	1	822.7237	16.97	3	62.4	10.6	2	1-20	-.MATYTCITCRVAFRDADMQR.A	Carbamidomethyl: 6, 9	
1586	1	480.6461	-220.76	2	49.5	14.0	1	395-401	R.SLMRYYK.Q		MD:MU 0.79

Detailed Protein Report

Protein 463: ras GTPase-activating protein 1 isoform 2 [Homo sapiens]

Accession: gi|12545406
Database: refseq_human(refseq_human_20140103.fasta)
Database Date: 2015-11-30
Modification(s): Oxidation

Score: 24.6
MW [kDa]: 100.3
pI: 7.9
Sequence Coverage [%]: 4.5
No. of unique Peptides: 2

Quantitation

MD:MU **Median:** 0.27 **CV:** 0.00 % **No. of Peptides:** 1

10	20	30	40	50	60	70	80
MKGWYHGKLD	RTIAEERLRQ	AGKSGSYLIR	ESDRRPGSFV	LSFLSQMNVV	NHFRIIAMCG	DYYIGGRRFS	SLSDLIGYYS
90	100	110	120	130	140	150	160
HVSCLLKGEK	LLYPVAPPEP	VEDRRRVRAI	LPYTKVPDTD	EISFLKGDMF	IVHNELEDGW	MWVTNLRTDE	QGLIVEDLVE
170	180	190	200	210	220	230	240
EVGREEDPHE	GKIWFHGKIS	KQEAYNLLMT	VGQVCSFLVR	PSDNTPGDYS	LYFRTNENIQ	RFKICPTPNN	QFMMGGRYYN
250	260	270	280	290	300	310	320
SIGDIIDHYR	KEQIVEGYYL	KEPVPMQDQE	QVLNDTVDGK	EIYNTIRRKT	KDAFYKNIVK	KGYLLKKGKG	KRWKNLYFIL
330	340	350	360	370	380	390	400
EGSDAQLIYF	ESEKRATKPK	GLIDLSVCSV	YVVHDSLFGR	PNCFQIVVQH	FSEEHYIFYF	AGETPEQAED	WMKGLQAFCN
410	420	430	440	450	460	470	480
LRKSSPGTSN	KRLRQVSSLV	LHIEEAHKLP	VKHFTNPYCN	IYLNSVQVAK	THAREGQNPV	WSEEFVFDDL	PPDINRFEIT
490	500	510	520	530	540	550	560
LSNKTKKSKD	PDILFMRCQL	SRLQKGHATD	EWFLLSSHIP	LKGIEPGSLR	VRARYSMEKI	MPEEEYSEFK	ELILQKELHV
570	580	590	600	610	620	630	640
VYALSHVCGQ	DRTLLASILL	RIFLHEKLES	LLLCTLNDRE	ISMEDEATTL	FRATTLASTL	MEQYMKATAT	QFVHHALKDS
650	660	670	680	690	700	710	720
ILKIMESKQS	CELSPSKLEK	NEDVNTNLTH	LLNILSELVE	KIFMASEILP	PTLRYIYGCL	QKSVQHKWPT	NTTMRTRVVS
730	740	750	760	770	780	790	800
GFVFLRLICP	AILNPRMFNI	ISDSPSPIAA	RTLILVAKSV	QNLANLVEFG	AKEPYMEGVN	PFIKSNKHRM	IMFLDELGNV
810	820	830	840	850	860	870	880
PELPDTTEHS	RTDLSRDLAA	LHEICVAHSD	ELRTLSNERG	AQQHVLKKLL	AITELLQQKQ	NQYTKTNDVR	

Cmpd.	No. of Cmpds.	m/z meas.	Δ m/z [ppm]	z	Rt [min]	Score	P	Range	Sequence	Modification	Ratios
1295	1	1023.3898	-122.23	2	45.9	11.7	2	773-789	K.EPYMEGVNPFIKSNKHR.M		
82	1	951.7644	-30.51	3	30.7	12.9	1	788-811	K. HRMIMFLDELGNVPELPDTTEHSF T	Oxidation: 5	MD:MU 0.27

Detailed Protein Report

Protein 464: protein shisa-9 isoform 2 precursor [Homo sapiens]

**Accession:**	gi|291463301	**Score:**	24.6
**Database:**	refseq_human(refseq_human_20140103.fasta)	**MW [kDa]:**	24.4
**Database Date:**	2015-11-30	**pI:**	9.1
**Modification(s):**	Carbamidomethyl	**Sequence Coverage [%]:**	19.9
		**No. of unique Peptides:**	1

10	20	30	40	50	60	70	80
MRRVLRLLLG	CFLTELCARV	CRAQERAGHG	QLAQLGGVLL	LAGGNRSGAA	SGEASEGAEA	SDAPPTRAPT	PDFCRGYFDV
**90**	**100**	**110**	**120**	**130**	**140**	**150**	**160**
MGQWDPPFNC	SSGDFIFCCG	TCGFRFCCTF	KKRRLNQSTC	TNYDTPLWLN	TGKPPARKDD	PLHDPTKDKT	NLIVYIICGV
**170**	**180**	**190**	**200**	**210**	**220**	**230**	
VAVMVLVGIF	TKLGLEKAHR	PQREHMSRLY	DNLLFMEAQI	SFQEDEPAPG	EWSVGLQTWT	V	

Cmpd.	No. of Cmpds.	m/z meas.	Δ m/z [ppm]	z	Rt [min]	Score	P	Range	Sequence	Modification
2323	1	1175.1820	36.25	2	59.3	14.4	2	4-22	R.VLRLLLGCFLTELCARVCR.A	Carbamidomethyl: 8, 14, 18

Detailed Protein Report

Protein 465: sarcoplasmic/endoplasmic reticulum calcium ATPase 2 isoform a [Homo sapiens]

**Accession:**	gi|4502285	**Score:**	24.6
**Database:**	refseq_human(refseq_human_20140103.fasta)	**MW [kDa]:**	109.6
**Database Date:**	2015-11-30	**pI:**	5.1
**Modification(s):**	Carbamidomethyl, Oxidation	**Sequence Coverage [%]:**	1.8
		**No. of unique Peptides:**	1

10	20	30	40	50	60	70	80
MENAHTKTVE	EVLGHFGVNE	STGLSLEQVK	KLKERWGSNE	LPAEEGKTLL	ELVIEQFEDL	LVRILLLAAC	ISFVLAWFEE
90	100	110	120	130	140	150	160
GEETITAFVE	PFVILLILVA	NAIVGVWQER	NAENAIEALK	EYEPEMGKVY	RQDRKSVQRI	KAKDIVPGDI	VEIAVGDKVP
170	180	190	200	210	220	230	240
ADIRLTSIKS	TTLRVDQSIL	TGESVSVIKH	TDPVPDPRAV	NQDKKNMLFS	GTNIAAGKAM	GVVVATGVNT	EIGKIRDEMV
250	260	270	280	290	300	310	320
ATEQERTPLQ	QKLDEFGEQL	SKVISLICIA	VWIINIGHFN	DPVHGGSWIR	GAIYYFKIAV	ALAVAAIPEG	LPAVITTCLA
330	340	350	360	370	380	390	400
LGTRRMAKKN	AIVRSLPSVE	TLGCTSVICS	DKTGTLTTNQ	MSVCRMFILD	RVEGDTCSLN	EFTITGSTYA	PIGEVHKDDK
410	420	430	440	450	460	470	480
PVNCHQYDGL	VELATICALC	NDSALDYNEA	KGVYEKVGEA	TETALTCLVE	KMNVFDTELK	GLSKIERANA	CNSVIKQLMK
490	500	510	520	530	540	550	560
KEFTLEFSRD	RKSMSVYCTP	NKPSRTSMSK	MFVKGAPEGV	IDRCTHIRVG	STKVPMTSGV	KQKIMSVIRE	WGSGSDTLRC
570	580	590	600	610	620	630	640
LALATHDNPL	RREEMHLEDS	ANFIKYETNL	TFVGCVGMLD	PPRIEVASSV	KLCRQAGIRV	IMITGDNKGT	AVAICRRIGI
650	660	670	680	690	700	710	720
FGQDEDVTSK	AFTGREFDEL	NPSAQRDACL	NARCFARVEP	SHKSKIVEFL	QSFDEITAMT	GDGVNDAPAL	KKAEIGIAMG
730	740	750	760	770	780	790	800
SGTAVAKTAS	EMVLADDNFS	TIVAAVEEGR	AIYNNMKQFI	RYLISSNVGE	VVCIFLTAAL	GFPEALIPVQ	LLWVNLVTDG
810	820	830	840	850	860	870	880
LPATALGFNP	PDLDIMNKPP	RNPKEPLISG	WLFFRYLAIG	CYVGAATVGA	AAWWFIAADG	GPRVSFYQLS	HFLQCKEDNP
890	900	910	920	930	940	950	960
DFEGVDCAIF	ESPYPMTMAL	SVLVTIEMCN	ALNSLSENQS	LLRMPPWENI	WLVGSICLSM	SLHFLILYVE	PLPLIFQITP
970	980	990	1000				
LNVTQWLMVL	KISLPVILMD	ETLKFVARNY	LEPAILE				

Cmpd.	No. of Cmpds.	m/z meas.	Δ m/z [ppm]	z	Rt [min]	Score	P	Range	Sequence	Modification
2121	1	701.2646	-134.99	3	56.7	14.0	2	511-528	K.MFVKGAPEGVIDRCTHIR.V	Carbamidomethyl: 14; Oxidation: 1

Detailed Protein Report

Protein 466: CCR4-NOT transcription complex subunit 1 isoform c [Homo sapiens]

**Accession:**	gi|388454220	**Score:**	24.6
**Database:**	refseq_human(refseq_human_20140103.fasta)	**MW [kDa]:**	266.2
**Database Date:**	2015-11-30	**pI:**	6.6
**Modification(s):**	Oxidation	**Sequence Coverage [%]:**	1.1
		**No. of unique Peptides:**	1

Detailed Protein Report

10	20	30	40	50	60	70	80
MNLDSLSLAL	SQISYLVDNL	TKKNYRASQQ	EIQHIVNRHG	PEADRHLLRC	LFSHVDFSGD	GKSSGKDFHQ	TQFLIQECAL
90	100	110	120	130	140	150	160
LITKPNFIST	LSYAIDNPLH	YQKSLKPAPH	LFAQLSKVLK	LSKVQEVIFG	LALLNSSSSD	LRGFAAQFIK	QKLPDLLRSY
170	180	190	200	210	220	230	240
IDADVSGNQE	GGFQDIAIEV	LHLLLSHLLF	GQKGAFGVGQ	EQIDAFLKTL	RRDFPQERCP	VVLAPLLYPE	KRDILMDRIL
250	260	270	280	290	300	310	320
PDSGGVAKTM	MESSLADFMQ	EVGYGFCASI	EECRNIIVQF	GVREVTAAQV	ARVLGMMART	HSGLTDGIPL	QSISAPGSGI
330	340	350	360	370	380	390	400
WSDGKDKSDG	AQAHTWNVEV	LIDVLKELNP	SLNFKEVTYE	LDHPGFQIRD	SKGLHNVVYG	IQRGLGMEVF	PVDLIYRPWK
410	420	430	440	450	460	470	480
HAEGQLSFIQ	HSLINPEIFC	FADYPCHTVA	TDILKAPPED	DNREIATWKS	LDLIESLLRL	AEVGQYEQVK	QLFSFPIKHC
490	500	510	520	530	540	550	560
PDMLVLALLQ	INTSWHTLRH	ELISTLMPIF	LGNHPNSAII	LHYAWHGQGQ	SPSIRQLIMH	AMAEWYMRGE	QYDQAKLSRI
570	580	590	600	610	620	630	640
LDVAQDLKAL	SMLLNGTPFA	FVIDLAALAS	RREYLKLDKW	LTDKIREHGE	PFIQACMTFL	KRRCPSILGG	LAPEKDQPKS
650	660	670	680	690	700	710	720
AQLPPETLAT	MLACLQACAG	SVSQELSETI	LTMVANCSNV	MNKARQPPPG	VMPKGRPPSA	SSLDAISPVQ	IDPLAGMTSL
730	740	750	760	770	780	790	800
SIGGSAAPHT	QSMQGFPPNL	GSAFSTPQSP	AKAFPPLSTP	NQTTAFSGIG	GLSSQLPVGG	LGTGSLTGIG	TGALGLPAVN
810	820	830	840	850	860	870	880
NDPFVQRKLG	TSGLNQPTFQ	QTDLSQVWPE	ANQHFSKEID	DEANSYFQRI	YNHPPHPTMS	VDEVLEMLQR	FKDSTIKRER
890	900	910	920	930	940	950	960
EVFNCMLRNL	FEEYRFFPQY	PDKELHITAC	LFGGIIEKGL	VTYMALGLAL	RYVLEALRKP	FGSKMYYFGI	AALDRFKNRL
970	980	990	1000	1010	1020	1030	1040
KDYPQYCQHL	ASISHFMQFP	HHLQEYIEYG	QQSRDPPVKM	QGSITTPGSI	ALAQAQAQAQ	VPAKAPLAGQ	VSTMVTTSTT
1050	1060	1070	1080	1090	1100	1110	1120
TTVAKTVTVT	RPTGVSFKKD	VPPSINTTNI	DTLLVATDQT	ERIVEPPENI	QEKIAFIFNN	LSQSNMTQKV	EELKETVKEE
1130	1140	1150	1160	1170	1180	1190	1200
FMPWVSQYLV	MKRVSIEPNF	HSLYSNFLDT	LKNPEFNKMV	LNETYRNIKV	LLTSDKAAAN	FSDRSLLKNL	GHWLGMITLA
1210	1220	1230	1240	1250	1260	1270	1280
KNKPILHTDL	DVKSLLLEAY	VKGQQELLYV	VPFVAKVLES	SIRSVVFRPP	NPWTMAIMNV	LAELHQEHDL	KLNLKFEIEV
1290	1300	1310	1320	1330	1340	1350	1360
LCKNLALDIN	ELKPGNLLKD	KDRLKNLDEQ	LSAPKKDVKQ	PEELPPITTT	TTSTTPATNT	TCTATVPPQP	QYSYHDINVY
1370	1380	1390	1400	1410	1420	1430	1440
SLAGLAPHIT	LNPTIPLFQA	HPQLKQCVRQ	AIERAVQELV	HPVVDRSIKI	AMTTCEQIVR	KDFALDSEES	RMRIAAHHMM
1450	1460	1470	1480	1490	1500	1510	1520
RNLTAGMAMI	TCREPLLMSI	STNLKNSFAS	ALRTASPQQR	EMMDQAAAQL	AQDNCELACC	FIQKTAVEKA	GPEMDKRLAT
1530	1540	1550	1560	1570	1580	1590	1600
EFELRKHARQ	EGRRYCDPVV	LTYQAERMPE	QIRLKVGGVD	PKQLAVYEEF	ARNVPGFLPT	NDLSQPTGFL	AQPMKQAWAT
1610	1620	1630	1640	1650	1660	1670	1680
DDVAQIYDKC	ITELEQHLHA	IPPTLAMNPQ	AQALRSLLEV	VVLSRNSRDA	IAALGLLQKA	VEGLLDATSG	ADADLLLRYR
1690	1700	1710	1720	1730	1740	1750	1760
ECHLLVLKAL	QDGRAYGSPW	CNKQITRCLI	ECRDEYKYNV	EAVELLIRNH	LVNMQQYDLH	LAQSMENGLN	YMAVAFAMQL
1770	1780	1790	1800	1810	1820	1830	1840
VKILLVDERS	VAHVTEADLF	HTIETLMRIN	AHSRGNAPEG	LPQLMEVVRS	NYEAMIDRAH	GGPNFMMHSG	ISQASEYDDP
1850	1860	1870	1880	1890	1900	1910	1920
PGLREKAEYL	LREWVNLYHS	AAAGRDSTKA	FSAFVGQMHQ	QGILKTDDLI	TRFFRLCTEM	CVEISYRAQA	EQQHNPAANP
1930	1940	1950	1960	1970	1980	1990	2000
TMIRAKCYHN	LDAFVRLIAL	LVKHSGEATN	TVTKINLLNK	VLGIVVGVLL	QDHDVRQSEF	QQLPYHRIFI	MLLLELNAPE
2010	2020	2030	2040	2050	2060	2070	2080
HVLETINFQT	LTAFCNTFHI	LRPTKAPGFV	YAWLELISHR	IFIARMLAHT	PQQKGWPMYA	QLLIDLFKYL	APFLRNVELT
2090	2100	2110	2120	2130	2140	2150	2160
KPMQILYKGT	LRVLLVLLHD	FPEFLCDYHY	GFCDVIPPNC	IQLRNLILSA	FPRNMRLPDP	FTPNLKVDML	SEINIAPRIL
2170	2180	2190	2200	2210	2220	2230	2240

Detailed Protein Report

Cmpd.	No. of Cmpds.	m/z meas.	Δ m/z [ppm]	z	Rt [min]	Score	P	Range	Sequence	Modification
1883	1	946.8565	-104.22	2	53.4	12.0	0	1908-1924	R.AQAEQQHNPAANPTMIR.A	Oxidation: 15

Detailed Protein Report

Protein 467: keratin, type I cuticular Ha2 [Homo sapiens]

**Accession:**	gi|116488398	**Score:**	24.6
**Database:**	refseq_human(refseq_human_20140103.fasta)	**MW [kDa]:**	50.3
**Database Date:**	2015-11-30	**pI:**	4.6
**Modification(s):**	Carbamidomethyl	**Sequence Coverage [%]:**	4.2
		**No. of unique Peptides:**	2

10	20	30	40	50	60	70	80
MTSSCCVTNN	LQASLKSCPR	PASVCSSGVN	CRPELCLGYV	CQPMACLPSV	CLPTTFRPAS	CLSKTYLSSS	CQAASGISGS
**90**	**100**	**110**	**120**	**130**	**140**	**150**	**160**
MGPGSWYSEG	AFNGNEKETM	QFLNDRLASY	LTRVRQLEQE	NAELESRIQE	ASHSQVLTMT	PDYQSHFRTI	EELQQKILCT
**170**	**180**	**190**	**200**	**210**	**220**	**230**	**240**
KAENARMVVN	IDNAKLAADD	FRAKYEAELA	MRQLVEADIN	GLRRILDDLT	LCKADLEAQV	ESLKEELMCL	KKNHEEEVGS
**250**	**260**	**270**	**280**	**290**	**300**	**310**	**320**
LRCQLGDRLN	IEVDAAPPVD	LTRVLEEMRC	QYEAMVEANR	RDVEEWFNMQ	MEELNQQVAT	SSEQLQNYQS	DIIDLRRTVN
**330**	**340**	**350**	**360**	**370**	**380**	**390**	**400**
TLEIELQAQH	SLRDSLENTL	TESEARYSSQ	LAQMQCMITN	VEAQLAEIRA	DLERQNQEYQ	VLLDVRARLE	GEINTYRSLL
**410**	**420**	**430**	**440**	**450**			
ENEDCKLPCN	PCSTPSCTTC	VPSPCVPRTV	CVPRTVGMPC	SPCPQGRY			

Cmpd.	No. of Cmpds.	m/z meas.	Δ m/z [ppm]	z	Rt [min]	Score	P	Range	Sequence	Modification
2706	1	1051.5703	74.71	2	65.1	11.7	1	429-447	R.TVCVPRTVGMPCSPCPQGR.Y	Carbamidomethyl: 12, 15
2221	1	1051.4304	-58.38	2	57.6	12.8	1	429-447	R.TVCVPRTVGMPCSPCPQGR.Y	Carbamidomethyl: 3, 15

Detailed Protein Report

Protein 468: EPM2A-interacting protein 1 [Homo sapiens]

**Accession:**	gi|7662294	**Score:**	24.5
**Database:**	refseq_human(refseq_human_20140103.fasta)	**MW [kDa]:**	70.3
**Database Date:**	2015-11-30	**pI:**	5.7
		**Sequence Coverage [%]:**	6.4
		**No. of unique Peptides:**	1

10	20	30	40	50	60	70	80
MWMTPKRSKM	EVDEALVFRP	EWTQRYLVVE	PPEGDGALCL	VCRRLIVATR	ERDVRRHYEA	EHEYYERYVA	DGERAALVER
**90**	**100**	**110**	**120**	**130**	**140**	**150**	**160**
LRQGDLPVAS	FTPEERAARA	GLGLCRLLAL	KGRGWGEGDF	VYQCMEVLLR	EVLPEHVSVL	QGVDLSPDIT	RQRILSIDRN
**170**	**180**	**190**	**200**	**210**	**220**	**230**	**240**
LRNQLFNRAR	DFKAYSLALD	DQAFVAYENY	LLVFIRGVGP	ELEVQEDLLT	IINLTHHFSV	GALMSAILES	LQTAGLSLQR
**250**	**260**	**270**	**280**	**290**	**300**	**310**	**320**
MVGLTTTHTL	RMIGENSGLV	SYMREKAVSP	NCWNVIHYSG	FLHLELLSSY	DVDVNQIINT	ISEWIVLIKT	RGVRRPEFQT
**330**	**340**	**350**	**360**	**370**	**380**	**390**	**400**
LLTESESEHG	ERVNGRCLNN	WLRRGKTLKL	IFSLRKEMEA	FLVSVGATTV	HFSDKQWLCD	FGFLVDIMEH	LRELSEELRV
**410**	**420**	**430**	**440**	**450**	**460**	**470**	**480**
SKVFAAAAFD	HICTFEVKLN	LFQRHIEEKN	LTDFPALREV	VDELKQQNKE	DEKIFDPDRY	QMVICRLQKE	FERHFKDLRF
**490**	**500**	**510**	**520**	**530**	**540**	**550**	**560**
IKKDLELFSN	PFNFKPEYAP	ISVRVELTKL	QANTNLWNEY	RIKDLGQFYA	GLSAESYPII	KGVACKVASL	FDSNQICEKA
**570**	**580**	**590**	**600**	**610**			
FSYLTRNQHT	LSQPLTDEHL	QALFRVATTE	MEPGWDDLVR	ERNESNP			

Cmpd.	No. of Cmpds.	m/z meas.	Δ m/z [ppm]	z	Rt [min]	Score	P	Range	Sequence	Modification
829	1	859.6189	172.24	2	39.9	10.2	2	68-82	R.YVADGERAALVERLR.Q	

Detailed Protein Report

Protein 469: **PREDICTED: tubulin polymerization-promoting protein family member 3 isoform X1 [Homo sapiens]**

**Accession:**	gi|530423965	**Score:**	24.5
**Database:**	refseq_human(refseq_human_20140103.fasta)	**MW [kDa]:**	28.3
**Database Date:**	2015-11-30	**pI:**	10.1
**Modification(s):**	Carbamidomethyl	**Sequence Coverage [%]:**	10.6
		**No. of unique Peptides:**	2

10	20	30	40	50	60	70	80
METEKQGIIR	RSRLGCSPTR	EPAGSGAAEP	PGLPRPSVHS	CVVLSFRTHT	SGAAARSEHC	TQDPPSAWAK	HPVASKLPWT
**90**	**100**	**110**	**120**	**130**	**140**	**150**	**160**
LPLVNQGGMA	ASTDMAGLEE	SFRKFAIHGD	PKASGQEMNG	KNWAKLCKDC	KVADGKSVTG	TDVDIVFSKV	KGKSARVINY
**170**	**180**	**190**	**200**	**210**	**220**	**230**	**240**
EEFKKALEEL	ATKRFKGKSK	EEAFDAICQL	VAGKEPANVG	VTKAKTGGAV	DRLTDTSRYT	GSHKERFDES	GKGKGIAGRQ
**250**	**260**	**270**					
DILDDSGYVS	AYKNAGTYDA	KVKK					

Cmpd.	No. of Cmpds.	m/z meas.	Δ m/z [ppm]	z	Rt [min]	Score	P	Range	Sequence	Modification
396	1	595.2630	-86.70	2	34.3	12.8	2	11-20	R.RSRLGCSPTR.E	Carbamidomethyl: 6
2670	1	885.4398	-44.05	2	63.5	11.8	2	195-212	K.EPANVGVTKAKTGGAVDR.L	

Detailed Protein Report

Protein 470: PREDICTED: WD repeat-containing protein 87 isoform X3 [Homo sapiens]

**Accession:**	gi|578834971	**Score:**	24.5
**Database:**	refseq_human(refseq_human_20140103.fasta)	**MW [kDa]:**	316.9
**Database Date:**	2015-11-30	**pI:**	6.9
		**Sequence Coverage [%]:**	0.8
		**No. of unique Peptides:**	1

Detailed Protein Report

10	20	30	40	50	60	70	80
MSSPRLIPLW	KDLKLLLNDT	INKSKQPSED	PKNCLIVLSD	RSQVLFKESR	YPQNMPCVCY	YFSDAHFFAS	LSWVTSNTKE
90	100	110	120	130	140	150	160
IQAVAWMKSK	TEDMVEKRTF	SMTERLPPIQ	SMVHAGSFHI	LVVYCGDLIL	RLFGDHFRAF	KPLGKVPCRF	NISCLCYDPE
170	180	190	200	210	220	230	240
MKMLLSGILG	AVVTWVIELG	GTGLQIAHMV	SMPGDELVQD	IVLNGPSGSL	LALCETVVRV	LMHQGKGQLG	EVKRFTSTSS
250	260	270	280	290	300	310	320
GSSITCCFTC	FDQGFLYAGN	QAGEIQVWSL	QQGHPLHSFQ	AHQSGVICIR	SRPEAHTLLT	AGSDSLIKEW	NLTSGSLLRR
330	340	350	360	370	380	390	400
LELGEELYRL	QFIDSITFFC	QTAHSFSLHR	LPCFYSLFNV	CGSAPQQLRR	VCCGNNWFRI	LCTTEDGLLR	FVSPVTGDLL
410	420	430	440	450	460	470	480
VITWPFSILD	QAVDWAYDPG	KEELFVATGS	SEVLVFDTTR	CPCPAKYLLG	TSPNSQDFVQ	CLAYGHFNLG	RGLEGLIFSG
490	500	510	520	530	540	550	560
HQSGVIRVLS	QHSCARLEKF	MHFGAVLALS	TLSGGIFGGQ	GNSLLCSYGM	DDYVHLSEAV	LDGVKVQLRP	LASILSSCHL
570	580	590	600	610	620	630	640
THLILLPKSV	GAITETNCLR	LWKFHDFLSS	GSQNGLKFIE	TLPLHLCAIT	SFDVCLSLSL	FVTGSADGSV	RIWDFHGRLI
650	660	670	680	690	700	710	720
GILDSSLHFG	PVCFANDRGD	LLVTFNQSLY	LVSCLKLLPP	ALLTRLSFMS	ISDEVLEVPK	PFIPSFFFSF	ETMFVPKYIY
730	740	750	760	770	780	790	800
PGQAQQKLVG	LEKLVNNRAI	AFDHSVPHVI	EEDEEGSPVL	LRSSMHYSLQ	DMEDWMQVSP	ALRSETARRL	LNDTTNSNPL
810	820	830	840	850	860	870	880
IRELAWEGLK	RLGMITHLFA	MPLAQGLMDK	DERVRIKTLS	LMAEIGIHSR	TSLLQLTQKQ	ETFREMQQQM	IGEEPLDHLL
890	900	910	920	930	940	950	960
GMRATDLQIL	STQVEQRLNE	NLTLSHRDEK	PAFSLDVSMP	SELKSSLKPP	TVSEESEVAI	KPSKGQRRGQ	AGVKKHSQKW
970	980	990	1000	1010	1020	1030	1040
LRGLKKTKER	DSKQMSTEPG	LLEDESGTEA	APIEMEEASV	YSQWSSSTSV	IKLSKDVDSQ	EKDISKDHIA	LTLKRLQKIR
1050	1060	1070	1080	1090	1100	1110	1120
DKRDKKATAQ	KLKKKHKKKG	KEAKVINEET	TPPVMEQPVT	KKVKIQGRGA	SGISGRRSTA	GDGSSWRDDL	CRLMALRISG
1130	1140	1150	1160	1170	1180	1190	1200
SQTKMSENLN	AELVTFAQEM	LVDRHPSWEL	FQEICPLLKK	ESKVLLEDLD	WDVVPPEKKP	IFIQEGAIRE	DMIQGVTQEV
1210	1220	1230	1240	1250	1260	1270	1280
IRHKEVMPRE	EEQAQKKARD	MLGLEETQVI	LKKGKKVIFL	EPGNVTMGKE	ISKKEEKKTF	QKSPKQGRKA	VQKERKVGKI
1290	1300	1310	1320	1330	1340	1350	1360
KREMTKEERD	MSEEVEEMAT	LEEKVVKQEG	KLVMIERTPS	WQDWKKAWDE	WKQVHGETRK	SWKAWKEEWE	KRLLQEEEKL
1370	1380	1390	1400	1410	1420	1430	1440
HQAGEKLSPE	EEMLQEDKKL	KWEEWKQVWE	NMLSSKSKEQ	QYKDEEEVTL	EEEVSREGEE	KEQQVTEEQR	HIQEEHKWAR
1450	1460	1470	1480	1490	1500	1510	1520
IHRKRARAEK	KRAQEERKLA	QEEEKLAQEE	RQLAQEERKL	AQAYVKITQD	DREMAQAEGK	FAQKEETLAQ	RGEKLSQEAE
1530	1540	1550	1560	1570	1580	1590	1600
KLAQKRKKLA	KKWEKVAREE	EKLAKKGGKL	AEVKNILAQK	VEELPQREQN	LDWQEKELAQ	ELEELEWDME	ELSWKEEELN
1610	1620	1630	1640	1650	1660	1670	1680
QEEGKLVEEK	KKLAEEEEAL	AWQREKLSEE	ETKLAQEEEL	LIQEKEKLAQ	HKEKMPEEEE	RLGRKREQLI	EKKMKLAQKR
1690	1700	1710	1720	1730	1740	1750	1760
ERWINSMEEL	TKNKMILYQK	KNLAQEKKNL	AQEKEKLAQR	KENLLYNKER	LTHSKKQLVQ	VKNKLGMFNK	ILAQVEEKLT
1770	1780	1790	1800	1810	1820	1830	1840
QEKETVIKKK	EKLAETEKKL	VQVEDSLAKK	QEKLAQEKMK	LALEKAMVQG	KKRLRGELDI	AKEEKALNLE	MKRLAEEKMR
1850	1860	1870	1880	1890	1900	1910	1920
LVEGKETLSK	GETPETSRQR	KMTQVEQELF	ERKLSLEEKI	LLHEDRILAM	EESEIAKGKL	EFTRGQRIFV	QGQRKLAKAS
1930	1940	1950	1960	1970	1980	1990	2000
RKLIKKRESL	SKEPAKLNKI	LKALQKLTRD	ERKLTQEEIK	MTKMKRALFV	KERRLSIEQS	KLDIKEWDFS	EKRSELTKDE
2010	2020	2030	2040	2050	2060	2070	2080
KKLARKQRKL	ANKMRRMINK	EEKMTEEESK	LARKHSEVIL	DDEEEGGIEE	EEVIPFLKRR	WRKRKEAKRG	DKPKEKFSSQ
2090	2100	2110	2120	2130	2140	2150	2160
VDEVESEEHF	SEEMESLLDE	LEKQESLSSE	EEEEREEEEE	REEEEEREEE	EERKEEEEGE	EKQVEKEEEE	KKKKKKEKKK
2170	2180	2190	2200	2210	2220	2230	2240
EEVQEKEEVF	EEKEEIMSEE	ETESLSDEEE	EEESCSLEEE	VDREKEILKK	EKQFKLQEQR	RKSLRGRERV	LSILRGVPHG
2250	2260	2270	2280	2290	2300	2310	2320
KGRAIRLGVL	KSPLKKLMST	ALEMKEKTPV	PVPEKQISWE	DKKATVVEIP	RKFLGTMDKE	REVMGKYEPI	PPHVLGTVLE
2330	2340	2350	2360	2370	2380	2390	2400

Detailed Protein Report

Cmpd.	No. of Cmpds.	m/z meas.	Δ m/z [ppm]	z	Rt [min]	Score	P	Range	Sequence	Modification
698	1	512.1244	-202.48	2	38.3	13.5	0	1098-1107	R.STAGDGSSWR.D	

Detailed Protein Report

Protein 471: PREDICTED: E3 ubiquitin-protein ligase SHPRH isoform X8 [Homo sapiens]

**Accession:**	gi|578812645	**Score:**	24.5
**Database:**	refseq_human(refseq_human_20140103.fasta)	**MW [kDa]:**	173.4
**Database Date:**	2015-11-30	**pI:**	7.9
**Modification(s):**	Carbamidomethyl	**Sequence Coverage [%]:**	2.1
		**No. of unique Peptides:**	1

10	20	30	40	50	60	70	80
MSSRRKRAPP	VRVDEEKRQQ	LHWNMHEDRR	NEPIIISDDD	EQPCPGSDTS	SAHYIILSDS	LKEEVAHRDK	KRCSKVVSFS
90	100	110	120	130	140	150	160
KPIEKEETVG	IFSPLSVKLN	IVISPYHFDN	SWKAFLGELT	LQLLPAQSLI	ENFSERSITL	MSSESSNQFL	IYVHSKGEDV
170	180	190	200	210	220	230	240
EKQKKEPMSI	CDKGILVESS	FSGEMLEDLG	WLQKKRRIKL	YQKPEGNHII	KVGIYLLEAG	LAKLDFLSDA	NSRMKKFNQL
250	260	270	280	290	300	310	320
MKKVMEKLHN	SIIPDVLEED	EDDPESEPEG	QDIDELYHFV	KQTHQQETQS	IQVDVQHPAL	IPVLRPYQRE	AVNWMLQQEC
330	340	350	360	370	380	390	400
FRSSPATESA	LHFLWREIVT	SEGLKLYYNP	YTGCIIREYP	NSGPQLLGGI	LADEMGLGKT	VEVLALILTH	TRQDVKQDAL
410	420	430	440	450	460	470	480
TLPEGKVVNY	FIPSHYFGGK	LKKTEIQNIE	FEPKEKVQCP	PTRVMILTAV	KEMNGKKGVS	ILSIYKYVSS	IYRYDVQRNR
490	500	510	520	530	540	550	560
SLLKRMLKCL	IFEGLVKQIK	GHGFSGTFTL	GKNYKEEDIC	DKTKKQAVGS	PRKIQKETRK	SGNKDTDSEY	LPSDTSDDDD
570	580	590	600	610	620	630	640
DPYYYYYKSR	RNRSKLRKKL	VPSTKKGKSQ	PFINPDSQGH	CPATSDSGIT	DVAMSKSTCI	SEFNQEHETE	DCAESLNHAD
650	660	670	680	690	700	710	720
SDVPPSNTMS	PFNTSDYRFE	CICGELDQID	RKPRVQCLKC	HLWQHAKCVN	YDEKNLKIKP	FYCPHCLVAM	EPVSTRATLI
730	740	750	760	770	780	790	800
ISPSSICHQW	VDEINRHVRS	SSLRVLVYQG	VKKDGFLQPH	FLAEQDIVII	TYDVLRSELN	YVDIPHSNSE	DGRRLRNQKR
810	820	830	840	850	860	870	880
YMAIPSPLVA	VEWWRICLDE	AQMVECPTVK	AAEMAQRLSG	INRWCISGTP	VQRGLEDLFG	LVVFLGIEPY	CVKHWWVRLL
890	900	910	920	930	940	950	960
YRPYCKKNPQ	HLYSFIAKIL	WRSAKKDVID	QIQIPPQTEE	IHWLHFSPVE	RHFYHRQHEV	CCQDVVVKLR	KISDWALKLS
970	980	990	1000	1010	1020	1030	1040
SLDRRTVTSI	LYPLLRLRQA	CCHPQAVRGE	FLPLQKSTMT	MEELLTSLQK	KCGTECEEAH	RQLVCALNGL	AGIHIIKGEY
1050	1060	1070	1080	1090	1100	1110	1120
ALAAELYREV	LRSSEEHKGK	LKTDSLQRLH	ATHNLMELLI	ARHPGIPPTL	RDGRLEEEAK	QLREHYMSKC	NTEVAEAQQA
1130	1140	1150	1160	1170	1180	1190	1200
LYPVQQTIHE	LQRKIHSNSP	WWLNVIHRAI	EFTIDEELVQ	RVRNEITSNY	KQQTGKLSMS	EKFRDCRGLQ	FLLTTQMEEL
1210	1220	1230	1240	1250	1260	1270	1280
NKCQKLVREA	VKNLEGPPSR	NVIESATVCH	LRPARLPLNC	CVFCKADELF	TEYESKLFSN	TVKGQTAIFE	EMIEDEEGLV
1290	1300	1310	1320	1330	1340	1350	1360
DDRAPTTTRG	LWAISETERS	MKAILSFAKS	HRFDVEFVDE	GSTSMDLFEA	WKKEYKLLHE	YWMALRNRVS	AVDELAMATE
1370	1380	1390	1400	1410	1420	1430	1440
RLRVRDPREP	KPNPPVLHII	EPHEVEQNRI	KLLNDKAVAT	SQLQKKLGQL	LYLTNLEKSQ	DKTSGGVNPE	PCPICARQLG
1450	1460	1470	1480	1490	1500	1510	
KQWAVLTCGH	CFCNECISII	IEQYSVGSHR	SSIKCAICRQ	TTSHKEISYV	FTSEKANQEE	DIPVKVF	

Cmpd.	No. of Cmpds.	m/z meas.	Δ m/z [ppm]	z	Rt [min]	Score	P	Range	Sequence	Modification
1767	1	856.7921	-163.45	2	52.1	10.0	1	937-950	R.QHEVCCQDVVVKLR.K	Carbamidomethyl: 5

Detailed Protein Report

Protein 472: uncharacterized protein KIAA0513 isoform b [Homo sapiens]

**Accession:**	gi|557440789	**Score:**	24.4
**Database:**	refseq_human(refseq_human_20140103.fasta)	**MW [kDa]:**	45.5
**Database Date:**	2015-11-30	**pI:**	4.9
		**Sequence Coverage [%]:**	7.0
		**No. of unique Peptides:**	1

10	20	30	40	50	60	70	80
METPEVPVGS	LIDFGPEAPT	SSPLEAPPPV	LQDGDGSLGD	GASESETTES	ADSENDMGES	PSHPSWDQDR	RSSSNESFSS
**90**	**100**	**110**	**120**	**130**	**140**	**150**	**160**
NQSTESTQDE	ETLALRDFMR	GYVEKIFSGG	EDLDQEEKAK	FGEYCSSENG	KGREWFARYV	SAQRCNSKCV	SEATFYRLVQ
**170**	**180**	**190**	**200**	**210**	**220**	**230**	**240**
SFAVVLFECH	QMDDFGPAKN	LMTMCFTYYH	IGKPQLLPPE	SREKPAGSID	SYLKSANSWL	AEKKDIAERL	LKNTSARTEN
**250**	**260**	**270**	**280**	**290**	**300**	**310**	**320**
VKGFFGGLET	KLKGPLARRN	EEDENKPQEK	RPRAVTAYSP	EDEKKGEKIY	LYTHLKQQPI	WHTLRFWNAA	FFDAVHCERT
**330**	**340**	**350**	**360**	**370**	**380**	**390**	**400**
KRSPTTREKW	CHMTQEERDD	SLRFNENITF	GQLGTFTHNM	LAFGLNKKLC	NDFLKKQAVI	GNLDEEQYKL	LSDHIEQMAT
**410**							
E							

Cmpd.	No. of Cmpds.	m/z meas.	Δ m/z [ppm]	z	Rt [min]	Score	P	Range	Sequence	Modification
470	1	564.2533	-129.11	3	35.5	14.4	2	243-258	K.GFFGGLETKLKGPLAR.R	

Detailed Protein Report

Protein 473: serine/threonine-protein kinase PINK1, mitochondrial precursor [Homo sapiens]

**Accession:**	gi|14165272	**Score:**	24.4
**Database:**	refseq_human(refseq_human_20140103.fasta)	**MW [kDa]:**	62.7
**Database Date:**	2015-11-30	**pI:**	10.4
**Modification(s):**	Carbamidomethyl	**Sequence Coverage [%]:**	4.6
		**No. of unique Peptides:**	2

10	20	30	40	50	60	70	80
MAVRQALGRG	LQLGRALLLR	FTGKPGRAYG	LGRPGPAAGC	VRGERPGWAA	GPGAEPRRVG	LGLPNRLRFF	RQSVAGLAAR
**90**	**100**	**110**	**120**	**130**	**140**	**150**	**160**
LQRQFVVRAW	GCAGPCGRAV	FLAFGLGLGL	IEEKQAESRR	AVSACQEIQA	IFTQKSKPGP	DPLDTRRLQG	FRLEEYLIGQ
**170**	**180**	**190**	**200**	**210**	**220**	**230**	**240**
SIGKGCSAAV	YEATMPTLPQ	NLEVTKSTGL	LPGRGPGTSA	PGEGQERAPG	APAFPLAIKM	MWNISAGSSS	EAILNTMSQE
**250**	**260**	**270**	**280**	**290**	**300**	**310**	**320**
LVPASRVALA	GEYGAVTYRK	SKRGPKQLAP	HPNIIRVLRA	FTSSVPLLPG	ALVDYPDVLP	SRLHPEGLGH	GRTLFLVMKN
**330**	**340**	**350**	**360**	**370**	**380**	**390**	**400**
YPCTLRQYLC	VNTPSPRLAA	MMLLQLLEGV	DHLVQQGIAH	RDLKSDNILV	ELDPDGCPWL	VIADFGCCLA	DESIGLQLPF
**410**	**420**	**430**	**440**	**450**	**460**	**470**	**480**
SSWYVDRGGN	GCLMAPEVST	ARPGPRAVID	YSKADAWAVG	AIAYEIFGLV	NPFYGQGKAH	LESRSYQEAQ	LPALPESVPP
**490**	**500**	**510**	**520**	**530**	**540**	**550**	**560**
DVRQLVRALL	QREASKRPSA	RVAANVLHLS	LWGEHILALK	NLKLDKMVGW	LLQQSAATLL	ANRLTEKCCV	ETKMKMLFLA
**570**	**580**	**590**					
NLECETLCQA	ALLLCSWRAA	L					

Cmpd.	No. of Cmpds.	m/z meas.	Δ m/z [ppm]	z	Rt [min]	Score	P	Range	Sequence	Modification
2329	1	1002.8159	-41.27	3	59.0	13.9	2	527-553	K.MVGWLLQQSAATLLANRLTEKCCVETK.M	
274	1	605.6515	-235.18	2	33.1	10.5	1	544-553	R.LTEKCCVETK.M	Carbamidomethyl: 6

Detailed Protein Report

Protein 474: mitogen-activated protein kinase kinase kinase kinase 5 [Homo sapiens]

**Accession:**	gi|14589909	**Score:**	24.4
**Database:**	refseq_human(refseq_human_20140103.fasta)	**MW [kDa]:**	95.0
**Database Date:**	2015-11-30	**pI:**	8.7
**Modification(s):**	Carbamidomethyl	**Sequence Coverage [%]:**	3.5
		**No. of unique Peptides:**	1

Alias proteins:

Accession	Name	Description
gi|578825438	refseq_human (refseq_human_20140103.fasta)	PREDICTED: mitogen-activated protein kinase kinase kinase kinase 5 isoform X1 [Homo sapiens]
gi|38570135	refseq_human (refseq_human_20140103.fasta)	mitogen-activated protein kinase kinase kinase kinase 5 [Homo sapiens]

10	20	30	40	50	60	70	80
MEAPLRPAAD	ILRRNPQQDY	ELVQRVGSGT	YGDVYKARNV	HTGELAAVKI	IKLEPGDDFS	LIQQEIFMVK	ECKHCNIVAY
90	100	110	120	130	140	150	160
FGSYLSREKL	WICMEYCGGG	SLQDIYHVTG	PLSELQIAYV	CRETLQGLAY	LHTKGKMHRD	IKGANILLTD	HGDVKLADFG
170	180	190	200	210	220	230	240
VAAKITATIA	KRKSFIGTPY	WMAPEVAAVE	KNGGYNQLCD	IWAVGITAIE	LGELQPPMFD	LHPMRALFLM	SKSNFQPPKL
250	260	270	280	290	300	310	320
KDKTKWSSTF	HNFVKIALTK	NPKKRPTAER	LLTHTFVAQP	GLSRALAVEL	LDKVNNPDNH	AHYTEADDDD	FEPHAIIRHT
330	340	350	360	370	380	390	400
IRSTNRNARA	ERTASEINFD	KLQFEPPLRK	ETEARDEMGL	SSDPNFMLQW	NPFVDGANTG	KSTSKRAIPP	PLPPKPRISS
410	420	430	440	450	460	470	480
YPEDNFPDEE	KASTIKHCPD	SESRAPQILR	RQSSPSCGPV	AETSSIGNGD	GISKLMSENT	EGSAQAPQLP	RKKDKRDFPK
490	500	510	520	530	540	550	560
PAINGLPPTP	KVLMGACFSK	VFDGCPLKIN	CATSWIHPDT	KDQYIIFGTE	DGIYTLNLNE	LHEATMEQLF	PRKCTWLYVI
570	580	590	600	610	620	630	640
NNTLMSLSEG	KTFQLYSHNL	IALFEHAKKP	GLAAHIQTHR	FPDRILPRKF	ALTTKIPDTK	GCHKCCIVRN	PYTGHKYLCG
650	660	670	680	690	700	710	720
ALQSGIVLLQ	WYEPMQKFML	IKHFDFPLPS	PLNVFEMLVI	PEQEYPMVCV	AISKGTESNQ	VVQFETINLN	SASSWFTEIG
730	740	750	760	770	780	790	800
AGSQQLDSIH	VTQLERDTVL	VCLDKFVKIV	NLQGKLKSSK	KLASELSFDF	RIESVVCLQD	SVLAFWKHGM	QGKSFKSDEV
810	820	830	840	850			
TQEISDETRV	FRLLGSDRVV	VLESRPTENP	TAHSNLYILA	GHENSY			

Cmpd.	No. of Cmpds.	m/z meas.	Δ m/z [ppm]	z	Rt [min]	Score	P	Range	Sequence	Modification
1739	2	1023.8681	-109.14	2	51.7	12.0	1	71-87	K.ECKHCNIVAYFGSYLSR.E	Carbamidomethyl: 2

Detailed Protein Report

Protein 475: actin-binding Rho-activating protein [Homo sapiens]

Accession: gi|21040251
Database: refseq_human(refseq_human_20140103.fasta)
Database Date: 2015-11-30
Modification(s): Carbamidomethyl

Score: 24.4
MW [kDa]: 43.1
pI: 9.4
Sequence Coverage [%]: 7.9
No. of unique Peptides: 2

10	20	30	40	50	60	70	80
MAPGEKESGE	GPAKSALRKI	RTATLVISLA	RGWQQWANEN	SIRQAQEPTG	WLPGGTQDSP	QAPKPITPPT	SHQKAQSAPK
**90**	**100**	**110**	**120**	**130**	**140**	**150**	**160**
SPPRLPEGHG	DGQSSEKAPE	VSHIKKKEVS	KTVVSKTYER	GGDVSHLSHR	YERDAGVLEP	GQPENDIDRI	LHSHGSPTRR
**170**	**180**	**190**	**200**	**210**	**220**	**230**	**240**
RKCANLVSEL	TKGWRVMEQE	EPTWRSDSVD	TEDSGYGGEA	EERPEQDGVQ	VAVVRIKRPL	PSQVNRFTEK	LNCKAQQKYS
**250**	**260**	**270**	**280**	**290**	**300**	**310**	**320**
PVGNLKGRWQ	QWADEHIQSQ	KLNPFSEEFD	YELAMSTRLH	KGDEGYGRPK	EGTKTAERAK	RAEEHIYREM	MDMCFIICTM
**330**	**340**	**350**	**360**	**370**	**380**	**390**	
ARHRRDGKIQ	VTFGDLFDRY	VRISDKVVGI	LMRARKHGLV	DFEGEMLWQG	RDDHVVITLL	K	

Cmpd.	No. of Cmpds.	m/z meas.	Δ m/z [ppm]	z	Rt [min]	Score	P	Range	Sequence	Modification
1991	1	1022.8793	-58.83	2	55.0	10.7	1	309-324	R.EMMDMCFIICTMARHR.R	Carbamidomethyl: 6
1836	1	805.7966	-143.60	2	53.0	13.7	1	326-339	R.DGKIQVTFGDLFDR.Y	

Detailed Protein Report

Protein 476: alpha-1-antitrypsin precursor [Homo sapiens]

**Accession:**	gi|50363217	**Score:**	24.3
**Database:**	refseq_human(refseq_human_20140103.fasta)	**MW [kDa]:**	46.7
**Database Date:**	2015-11-30	**pI:**	5.3
		**Sequence Coverage [%]:**	3.3
		**No. of unique Peptides:**	1

Alias proteins:

Accession	Name	Description
gi|189163542	refseq_human (refseq_human_20140103.fasta)	alpha-1-antitrypsin precursor [Homo sapiens]
gi|189163540	refseq_human (refseq_human_20140103.fasta)	alpha-1-antitrypsin precursor [Homo sapiens]
gi|189163538	refseq_human (refseq_human_20140103.fasta)	alpha-1-antitrypsin precursor [Homo sapiens]
gi|189163536	refseq_human (refseq_human_20140103.fasta)	alpha-1-antitrypsin precursor [Homo sapiens]
gi|189163534	refseq_human (refseq_human_20140103.fasta)	alpha-1-antitrypsin precursor [Homo sapiens]
gi|189163532	refseq_human (refseq_human_20140103.fasta)	alpha-1-antitrypsin precursor [Homo sapiens]
gi|189163530	refseq_human (refseq_human_20140103.fasta)	alpha-1-antitrypsin precursor [Homo sapiens]
gi|189163528	refseq_human (refseq_human_20140103.fasta)	alpha-1-antitrypsin precursor [Homo sapiens]
gi|50363221	refseq_human (refseq_human_20140103.fasta)	alpha-1-antitrypsin precursor [Homo sapiens]
gi|50363219	refseq_human (refseq_human_20140103.fasta)	alpha-1-antitrypsin precursor [Homo sapiens]

10	20	30	40	50	60	70	80
MPSSVSWGIL	LLAGLCCLVP	VSLAEDPQGD	AAQKTDTSHH	DQDHPTFNKI	TPNLAEFAFS	LYRQLAHQSN	STNIFFSPVS
**90**	**100**	**110**	**120**	**130**	**140**	**150**	**160**
IATAFAMLSL	GTKADTHDEI	LEGLNFNLTE	IPEAQIHEGF	QELLRTLNQP	DSQLQLTTGN	GLFLSEGLKL	VDKFLEDVKK
**170**	**180**	**190**	**200**	**210**	**220**	**230**	**240**
LYHSEAFTVN	FGDTEEAKKQ	INDYVEKGTQ	GKIVDLVKEL	DRDTVFALVN	YIFFKGKWER	PFEVKDTEEE	DFHVDQVTTV
**250**	**260**	**270**	**280**	**290**	**300**	**310**	**320**
KVPMMKRLGM	FNIQHCKKLS	SWVLLMKYLG	NATAIFFLPD	EGKLQHLENE	LTHDIITKFL	ENEDRRSASL	HLPKLSITGT
**330**	**340**	**350**	**360**	**370**	**380**	**390**	**400**
YDLKSVLGQL	GITKVFSNGA	DLSGVTEEAP	LKLSKAVHKA	VLTIDEKGTE	AAGAMFLEAI	PMSIPPEVKF	NKPFVFLMIE
**410**	**420**						
QNTKSPLFMG	KVVNPTQK						

Cmpd.	No. of Cmpds.	m/z meas.	Δ m/z [ppm]	z	Rt [min]	Score	P	Range	Sequence	Modification
2521	1	821.3964	-47.50	2	61.5	24.3	0	50-63	K.ITPNLAEFAFSLYR.Q	

Detailed Protein Report

Protein 477: DIS3-like exonuclease 1 isoform 2 [Homo sapiens]

**Accession:**	gi|19115966	**Score:**	24.3
**Database:**	refseq_human(refseq_human_20140103.fasta)	**MW [kDa]:**	111.0
**Database Date:**	2015-11-30	**pI:**	5.9
		**Sequence Coverage [%]:**	2.5
		**No. of unique Peptides:**	1

Alias proteins:

Accession	Name	Description
gi|530405359	refseq_human (refseq_human_20140103.fasta)	PREDICTED: DIS3-like exonuclease 1 isoform X4 [Homo sapiens]
gi|530405355	refseq_human (refseq_human_20140103.fasta)	PREDICTED: DIS3-like exonuclease 1 isoform X2 [Homo sapiens]

10	20	30	40	50	60	70	80
MQTACQAVQH	QRGRRQYNKL	RNLLKDARHD	CILFANEFQQ	CCYLPRERGE	SMEKWQTRSI	YNAAVWYYHH	CQDRMPIVMV
90	100	110	120	130	140	150	160
TEDEEAIQQY	GSETEGVFVI	TFKNYLDNFW	PDLKAAHELC	DSILQSRRER	ENESQESHGK	EYPEHLPLEV	LEAGIKSGRY
170	180	190	200	210	220	230	240
IQGILNVNKH	RAQIEAFVRL	QGASSKDSDL	VSDILIHGMK	ARNRSIHGDV	VVVELLPKNE	WKGRTVALCE	NDCDDKASGE
250	260	270	280	290	300	310	320
SPSEPMPTGR	VVGILQKNWR	DYVVTFPSKE	EVQSQGKNAQ	KILVTPWDYR	IPKIRISTQQ	AETLQDFRVV	VRIDSWESTS
330	340	350	360	370	380	390	400
VYPNGHFVRV	LGRIGDLEGE	IATILVENSI	SVIPFSEAQM	CEMPVNTPES	PWKVSPEEEQ	KRKDLRKSHL	VFSIDPKGCE
410	420	430	440	450	460	470	480
DVDDTLSVRT	LNNGNLELGV	HIADVTHFVA	PNSYIDIEAR	TRATTYYLAD	RRYDMLPSVL	SADLCSLLGG	VDRYAVSIMW
490	500	510	520	530	540	550	560
ELDKASYEIK	KVWYGRTIIR	SAYKLFYEAA	QELLDGNLSV	VDDIPEFKDL	DEKSRQAKLE	ELVWAIGKLT	DIARHVRAKR
570	580	590	600	610	620	630	640
DGCGALELEG	VEVCVQLDDK	KNIHDLIPKQ	PLEVHETVAE	CMILANHWVA	KKIWESFPHQ	ALLRQHPPPH	QEFFSELREC
650	660	670	680	690	700	710	720
AKAKGFFIDT	RSNKTLADSL	DNANDPHDPI	VNRLLRSMAT	QAMSNALYFS	TGSCAEEEFH	HYGLALDKYT	HFTSPIRRYS
730	740	750	760	770	780	790	800
DIVVHRLLMA	AISKDKKMEI	KGNLFSNKDL	EELCRHINNR	NQAAQHSQKQ	STELFQCMYF	KDKDPATEER	CISDGVIYSI
810	820	830	840	850	860	870	880
RTNGVLLFIP	RFGIKGAAYL	KNKDGLVISC	GPDSCSEWKP	GSLQRFQNKI	TSTTTDGESV	TFHLFDHVTV	RISIQASRCH
890	900	910	920	930	940	950	960
SDTIRLEIIS	NKPYKIPNTE	LIHQSSPLLK	SELVKEVTKS	VEEAQLAQEV	KVNIIQEEYQ	EYRQTKGRSL	YTLLEEIRDL
970	980						
ALLDVSNNYG	I						

Cmpd.	No. of Cmpds.	m/z meas.	Δ m/z [ppm]	z	Rt [min]	Score	P	Range	Sequence	Modification
1844	1	655.2608	-189.42	2	52.9	10.1	2	812-823	R.FGIKGAAYLKNK.D	

Detailed Protein Report

Protein 478: gametogenetin-binding protein 2 [Homo sapiens]

**Accession:**	gi|13376245	**Score:**	24.3
**Database:**	refseq_human(refseq_human_20140103.fasta)	**MW [kDa]:**	79.0
**Database Date:**	2015-11-30	**pI:**	6.0
**Modification(s):**	Carbamidomethyl	**Sequence Coverage [%]:**	4.4
		**No. of unique Peptides:**	1

10	20	30	40	50	60	70	80
MARLVAVCRD	GEEEFPFERR	QIPLYIDDTL	TMVMEFPDNV	LNLDGHQNNG	AQLKQFIQRH	GMLKQQDLSI	AMVVTSREVL
**90**	**100**	**110**	**120**	**130**	**140**	**150**	**160**
SALSQLVPCV	GCRRSVERLF	SQLVESGNPA	LEPLTVGPKG	VLSVTRSCMT	DAKKLYTLFY	VHGSKLNDMI	DAIPKSKKNK
**170**	**180**	**190**	**200**	**210**	**220**	**230**	**240**
RCQLHSLDTH	KPKPLGGCWM	DVWELMSQEC	RDEVVLIDSS	CLLETLETYL	RKHRFCTDCK	NKVLRAYNIL	IGELDCSKEK
**250**	**260**	**270**	**280**	**290**	**300**	**310**	**320**
GYCAALYEGL	RCCPHERHIH	VCCETDFIAH	LLGRAEPEFA	GGRRERHAKT	IDIAQEEVLT	CLGIHLYERL	HRIWQKLRAE
**330**	**340**	**350**	**360**	**370**	**380**	**390**	**400**
EQTWQMLFYL	GVDALRKSFE	MTVEKVQGIS	RLEQLCEEFS	EEERVRELKQ	EKKRQKRKNR	RKNKCVCDIP	TPLQTADEKE
**410**	**420**	**430**	**440**	**450**	**460**	**470**	**480**
VSQEKETDFI	ENSSCKACGS	TEDGNTCVEV	IVTNENTSCT	CPSSGNLLGS	PKIKKGLSPH	CNGSDCGYSS	SMEGSETGSR
**490**	**500**	**510**	**520**	**530**	**540**	**550**	**560**
EGSDVACTEG	ICNHDEHGDD	SCVHHCEDKE	DDGDSCVECW	ANSEENDTKG	KNKKKKKKSK	ILKCDEHIQK	LGSCITDPGN
**570**	**580**	**590**	**600**	**610**	**620**	**630**	**640**
RETSGNTMHT	VFHRDKTKDT	HPESCCSSEK	GGQPLPWFEH	RKNVPQFAEP	TETLFGPDSG	KGAKSLVELL	DESECTSDEE
**650**	**660**	**670**	**680**	**690**	**700**		
IFISQDEIQS	FMANNQSFYS	NREQYRQHLK	EKFNKYCRLN	DHKRPICSGW	LTTAGAN		

Cmpd.	No. of Cmpds.	m/z meas.	Δ m/z [ppm]	z	Rt [min]	Score	P	Range	Sequence	Modification
124	1	896.3913	-30.48	3	30.9	13.9	1	551-574	K.LGSCITDPGNRETSGNTMHTVFHR.D	Carbamidomethyl: 4

Detailed Protein Report

Protein 479: PREDICTED: nucleolar GTP-binding protein 1-like [Homo sapiens]

Accession: gi|578839277
Database: refseq_human(refseq_human_20140103.fasta)
Database Date: 2015-11-30
Modification(s): Oxidation

Score: 24.3
MW [kDa]: 66.4
pI: 9.5
Sequence Coverage [%]: 4.9
No. of unique Peptides: 1

Quantitation

MD:MU **Median:** 6.95 **CV:** 0.00 % **No. of Peptides:** 1

10	20	30	40	50	60	70	80
MRKVKFTQQN	YHDRLSQILT	DFPKLDDIHP	FYADLMNILY	DKDHYKLALG	QINIAKNLVD	NVAKDYVRLM	KYGDSLYRCK
**90**	**100**	**110**	**120**	**130**	**140**	**150**	**160**
QLKRAALGRM	CTIIKRQKQS	LEYLEQVRQH	LSRLPTIDPN	TRTLLLCGYP	NVGKSSFINK	VTRADVDVQP	YAFTTKSLFV
**170**	**180**	**190**	**200**	**210**	**220**	**230**	**240**
GHMDYKYLRW	QVVDTPGILD	HPLEDRNTIE	MQAITALAHL	RAAVLYVMDL	SEQCGHGLKE	QLELFQNIRP	LFINKPLIVV
**250**	**260**	**270**	**280**	**290**	**300**	**310**	**320**
ANKCDVKRIT	ELSEEDQKIF	TDLQAEGFPV	IETSTLTEEG	VIQVKTEACD	RLLAHRVETK	MKGNKVNEVL	NRLHLAVPNK
**330**	**340**	**350**	**360**	**370**	**380**	**390**	**400**
RDDKERPPFI	PEGVVARRKR	MEIVEPRKKR	ERDLELEMGD	DYILDLQKYW	DLMNSSEKYD	KIPEIWEGHN	VADYIDPAIM
**410**	**420**	**430**	**440**	**450**	**460**	**470**	**480**
KKLEELEKEE	ELRTAAGEYD	SDSESEDEEM	MEIRQLAKQI	REKKKLKILQ	SKEKNTQGPR	MPRTAKKVQR	ADLENEMRSL
**490**	**500**	**510**	**520**	**530**	**540**	**550**	**560**
GVDMDDKDNA	HYAVQARRSR	SVTRKRKREE	SVPPSSIARS	RSCSRTPRDV	SGLRDVKVSF	LDREQKKGAF	RVFIVQNLQQ
**570**	**580**						
TVVQRSTVVE							

Cmpd.	No. of Cmpds.	m/z meas.	Δ m/z [ppm]	z	Rt [min]	Score	P	Range	Sequence	Modification	Ratios	
2112	1	596.7627	-113.88	2	56.3	12.1	1	85-95	R.AALGRMCTIIK.R	Oxidation: 6	MD:MU	6.95

Detailed Protein Report

Protein 480: PREDICTED: multiple PDZ domain protein isoform X3 [Homo sapiens]

**Accession:**	gi|530390235	**Score:**	24.3
**Database:**	refseq_human(refseq_human_20140103.fasta)	**MW [kDa]:**	213.7
**Database Date:**	2015-11-30	**pI:**	4.8
		**Sequence Coverage [%]:**	1.4
		**No. of unique Peptides:**	1

Detailed Protein Report

10	20	30	40	50	60	70	80
MLEAIDKNRA	LHAAERLQTK	LRERGDVANE	DKLSLLKSVL	QSPLFSQILS	LQTSVQQLKD	QVNIATSATS	NIEYAHVPHL
90	100	110	120	130	140	150	160
SPAVIPTLQN	ESFLLSPNNG	NLEALTGPGI	PHINGKPACD	EFDQLIKNMA	QGRHVEVFEL	LKPPSGGLGF	SVVGLRSENR
170	180	190	200	210	220	230	240
GELGIFVQEI	QEGSVAHRDG	RLKETDQILA	INGQALDQTI	THQQAISILQ	KAKDTVQLVI	ARGSLPQLVS	PIVSRSPSAA
250	260	270	280	290	300	310	320
STISAHSNPV	HWQHMETIEL	VNDGSGLGFG	IIGGKATGVI	VKTILPGGVA	DQHGRLCSGD	HILKIGDTDL	AGMSSEQVAQ
330	340	350	360	370	380	390	400
VLRQCGNRVK	LMIARGAIEE	RTAPTALGIT	LSSSPTSTPE	LRVDASTQKG	EESETFDVEL	TKNVQGLGIT	IAGYIGDKKL
410	420	430	440	450	460	470	480
EPSGIFVKSI	TKSSAVEHDG	RIQIGDQIIA	VDGTNLQGFT	NQQAVEVLRH	TGQTVLLTLM	RRGMKQEAEL	MSREDVTKDA
490	500	510	520	530	540	550	560
DLSPVNASII	KENYEKDEDF	LSSTRNTNIL	PTEEEGYPLL	SAEIEEIEDA	QKQEAALLTK	WQRIMGINYE	IVVAHVSKFS
570	580	590	600	610	620	630	640
ENSGLGISLE	ATVGHHFIRS	VLPEGPVGHS	GKLFSGDELL	EVNGITLLGE	NHQDVVNILK	ELPIEVTMVC	CRRTVPPTTQ
650	660	670	680	690	700	710	720
SELDSLDLCD	IELTEKPHVD	LGEFIGSSET	EDPVLAMTDA	GQSTEEVQAP	LAMWEAGIQH	IELEKGSKGL	GFSILDYQDP
730	740	750	760	770	780	790	800
IDPASTVIII	RSLVPGGIAE	KDGRLLPGDR	LMFVNDVNLE	NSSLEEAVEA	LKGAPSGTVR	IGVAKPLPLS	PEEGYVSAKE
810	820	830	840	850	860	870	880
DSFLYPPHSC	EEAGLADKPL	FRADLALVGT	NDADLVDEST	FESPYSPEND	SIYSTQASIL	SLHGSSCGDG	LNYGSSLPSS
890	900	910	920	930	940	950	960
PPKDVIENSC	DPVLDLHMSL	EELYTQNLLQ	RQDENTPSVD	ISMGPASGFT	INDYTPANAI	EQQYECENTI	VWTESHLPSE
970	980	990	1000	1010	1020	1030	1040
VISSAELPSV	LPDSAGKGSE	YLLEQSSLAC	NAECVMLQNV	SKESFERTIN	IAKGNSSLGM	TVSANKDGLG	MIVRSIIHGG
1050	1060	1070	1080	1090	1100	1110	1120
AISRDGRIAI	GDCILSINEE	STISVTNAQA	RAMLRRHSLI	GPDIKITYVP	AEHLEEFKIS	LGQQSGRVMA	LDIFSSYTGR
1130	1140	1150	1160	1170	1180	1190	1200
DIPELPEREE	GEGEESELQN	TAYSNWNQPR	RVELWREPSK	SLGISIVGGR	GMGSRLSNGE	VMRGIFIKHV	LEDSPAGKNG
1210	1220	1230	1240	1250	1260	1270	1280
TLKPGDRIVE	APSQSESEPE	KAPLCSVPPP	PPSAFAEMGS	DHTQSSASKI	SQDVDKEDEF	GYSWKNIRER	YGTLTGELHM
1290	1300	1310	1320	1330	1340	1350	1360
IELEKGHSGL	GLSLAGNKDR	SRMSVFIVGI	DPNGAAGKDG	RLQIADELLE	INGQILYGRS	HQNASSIIKC	APSKVKIIFI
1370	1380	1390	1400	1410	1420	1430	1440
RNKDAVNQMA	VCPGNAVEPL	PSNSENLQNK	ETEPTVTTSD	AAVDLSSFKN	VQHLELPKDQ	GGLGIAISEE	DTLSGVIIKS
1450	1460	1470	1480	1490	1500	1510	1520
LTEHGVAATD	GRLKVGDQIL	AVDDEIVVGY	PIEKFISLLK	TAKMTVKLTI	HAENPDSQAV	PSAAGAASGE	KKNSSQSLMV
1530	1540	1550	1560	1570	1580	1590	1600
PQSGSPEPES	IRNTSRSSTP	AIFASDPATC	PIIPGCETTI	EISKGRTGLG	LSIVGGSDTL	LGAIIIHEVY	EEGAACKDGR
1610	1620	1630	1640	1650	1660	1670	1680
LWAGDQILEV	NGIDLRKATH	DEAINVLRQT	PQRVRLTLYR	DEAPYKEEEV	CDTLTIELQK	KPGKGLGLSI	VGKRNDTGVF
1690	1700	1710	1720	1730	1740	1750	1760
VSDIVKGGIA	DADGRLMQGD	QILMVNGEDV	RNATQEAVAA	LLKCSLGTVT	LEVGRIKAGP	FHSERRPSQS	SQVSEGSLSS
1770	1780	1790	1800	1810	1820	1830	1840
FTFPLSGSST	SESLESSSKK	NALASEIQGL	RTVEMKKGPT	DSLGISIAGG	VGSPLGDVPI	FIAMMHPTGV	AAQTQKLRVG
1850	1860	1870	1880	1890	1900	1910	1920
DRIVTICGTS	TEGMTHTQAV	NLLKNASGSI	EMQVVAGGDV	SVVTGHQQEP	ASSSLSFTGL	TSSSIFQDDL	GPPQCKSITL
1930	1940	1950	1960	1970	1980	1990	2000
ERGPDGLGFS	IVGGYGSPHG	DLPIYVKTVF	AKGAASEDGR	LKRGDQIIAV	NGQSLEGVTH	EEAVAILKRT	KGTVTLMVLS
2010							

Cmpd.	No. of Cmpds.	m/z meas.	Δ m/z [ppm]	z	Rt [min]	Score	P	Range	Sequence	Modification

Detailed Protein Report

Cmpd.	No. of Cmpds.	m/z meas.	Δ m/z [ppm]	z	Rt [min]	Score	P	Range	Sequence	Modification
2017	1	788.3019	-141.73	2	55.1	14.1	0	1303-1318	R.MSVFIVGIDPNGAAGK.D	

Detailed Protein Report

Protein 481: **serine/threonine-protein kinase TNNI3K [Homo sapiens]**

**Accession:**	gi|7705748	**Score:**	24.3
**Database:**	refseq_human(refseq_human_20140103.fasta)	**MW [kDa]:**	92.8
**Database Date:**	2015-11-30	**pI:**	6.3
**Modification(s):**	Carbamidomethyl	**Sequence Coverage [%]:**	2.0
		**No. of unique Peptides:**	1

10	20	30	40	50	60	70	80
MGNYKSRPTQ	TCTDEWKKKV	SESYVITIER	LEDDLQIKEK	ELTELRNIFG	SDEAFSKVNL	NYRTENGLSL	LHLCCICGGK
**90**	**100**	**110**	**120**	**130**	**140**	**150**	**160**
KSHIRTLMLK	GLRPSRLTRN	GFTALHLAVY	KDNAELITSL	LHSGADIQQV	GYGGLTALHI	ATIAGHLEAA	DVLLQHGANV
**170**	**180**	**190**	**200**	**210**	**220**	**230**	**240**
NIQDAVFFTP	LHIAAYYGHE	QVTRLLLKFG	ADVNVSGEVG	DRPLHLASAK	GFLNIAKLLM	EEGSKADVNA	QDNEDHVPLH
**250**	**260**	**270**	**280**	**290**	**300**	**310**	**320**
FCSRFGHHDI	VKYLLQSDLE	VQPHVVNIYG	DTPLHLACYN	GKFEVAKEII	QISGTESLTK	ENIFSETAFH	SACTYGKSID
**330**	**340**	**350**	**360**	**370**	**380**	**390**	**400**
LVKFLLDQNV	ININHQGRDG	HTGLHSACYH	GHIRLVQFLL	DNGADMNLVA	CDPSRSSGEK	DEQTCLMWAY	EKGHDAIVTL
**410**	**420**	**430**	**440**	**450**	**460**	**470**	**480**
LKHYKRPQDE	LPCNEYSQPG	GDGSYVSVPS	PLGKIKSMTK	EKADILLLRA	GLPSHFHLQL	SEIEFHEIIG	SGSFGKVYKG
**490**	**500**	**510**	**520**	**530**	**540**	**550**	**560**
RCRNKIVAIK	RYRANTYCSK	SDVDMFCREV	SILCQLNHPC	VIQFVGACLN	DPSQFAIVTQ	YISGGSLFSL	LHEQKRILDL
**570**	**580**	**590**	**600**	**610**	**620**	**630**	**640**
QSKLIIAVDV	AKGMEYLHNL	TQPIIHRDLN	SHNILLYEDG	HAVVADFGES	RFLQSLDEDN	MTKQPGNLRW	MAPEVFTQCT
**650**	**660**	**670**	**680**	**690**	**700**	**710**	**720**
RYTIKADVFS	YALCLWEILT	GEIPFAHLKP	AAAAADMAYH	HIRPPIGYSI	PKPISSLLIR	GWNACPEGRP	EFSEVVMKLE
**730**	**740**	**750**	**760**	**770**	**780**	**790**	**800**
ECLCNIELMS	PASSNSSGSL	SPSSSSDCLV	NRGGPGRSHV	AALRSRFELE	YALNARSYAA	LSQSAGQYSS	QGLSLEEMKR
**810**	**820**	**830**	**840**				
SLQYTPIDKY	GYVSDPMSSM	HFHSCRNSSS	FEDSS				

Cmpd.	No. of Cmpds.	m/z meas.	Δ m/z [ppm]	z	Rt [min]	Score	P	Range	Sequence	Modification
2206	1	701.3221	4.21	3	57.5	10.6	1	1-17	-.MGNYKSRPTQTCTDEWK.K	Carbamidomethyl: 12

Detailed Protein Report

Protein 482: PREDICTED: protein sel-1 homolog 2 isoform X5 [Homo sapiens]

Accession: gi|578835602
Database: refseq_human(refseq_human_20140103.fasta)
Database Date: 2015-11-30

Score: 24.3
MW [kDa]: 66.4
pI: 9.7
Sequence Coverage [%]: 4.4
No. of unique Peptides: 1

Alias proteins:

Accession	Name	Description
gi|578835604	refseq_human (refseq_human_20140103.fasta)	PREDICTED: protein sel-1 homolog 2 isoform X6 [Homo sapiens]

10	20	30	40	50	60	70	80
MKPLSLLIEI	LIILGVTIKT	IKAEEHNKRQ	KERNVTTQVS	VNEIKQYLSH	ILEQRTSSNV	INKRENLLEK	KKNQRKIRIK
90	100	110	120	130	140	150	160
GIQNKDILKR	NKNHLQKQAE	KNFTDEGDQL	FKMGIKVLQQ	SKSQKQKEEA	YLLFAKAADM	GNLKAMEKMA	DALLFGNFGV
170	180	190	200	210	220	230	240
QNITAAIQLY	ESLAKEGSCK	AQNALGFLSS	YGIGMEYDQA	KALIYYTFGS	AGGNMMSQMI	LGYRYLSGIN	VLQNCEVALS
250	260	270	280	290	300	310	320
YYKKVADYIA	DTFEKSEGVP	VEKVRLTERP	ENLSSNSEIL	DWDIYQYYKF	LAERGDVQIQ	VSLGQLHLIG	RKGLDQDYYK
330	340	350	360	370	380	390	400
ALHYFLKAAK	AGSANAMAFI	GKMYLEGNAA	VPQNNATAFK	YFSMAASKGN	AIGLHGLGLL	YFHGKGVPLN	YAEALKYFQK
410	420	430	440	450	460	470	480
AAEKGWPDAQ	FQLGFMYYSG	SGIWKDYKLA	FKYFYLASQS	GQPLAIYYLA	KMYATGTGVV	RSCRTAVELY	KGVCELGHWA
490	500	510	520	530	540	550	560
EKFLTAYFAY	KDGDIDSSLV	QYALLAEMGY	EVAQSNSAFI	LESKKANILE	KEKMYPMALL	LWNRAAIQGH	SLGQKIVRHG
570	580	590	600				
CSNESRCPHT	CALCRHETGN	YAFAPGYPVF					

Cmpd.	No. of Cmpds.	m/z meas.	Δ m/z [ppm]	z	Rt [min]	Score	P	Range	Sequence	Modification
629	1	509.6880	-191.47	2	37.4	10.0	1	56-64	R.TSSNVINKR.E	

Detailed Protein Report

Protein 483: serine/threonine-protein kinase Nek11 isoform 2 [Homo sapiens]

**Accession:**	gi|41281753	**Score:**	24.3
**Database:**	refseq_human(refseq_human_20140103.fasta)	**MW [kDa]:**	54.0
**Database Date:**	2015-11-30	**pI:**	5.6
**Modification(s):**	Carbamidomethyl	**Sequence Coverage [%]:**	4.0
		**No. of unique Peptides:**	1

10	20	30	40	50	60	70	80
MLKFQEAAKC	VSGSTAISTY	PKTLIARRYV	LQQKLGSGSF	GTVYLVSDKK	AKRGEELKVL	KEISVGELNP	NETVQANLEA
**90**	**100**	**110**	**120**	**130**	**140**	**150**	**160**
QLLSKLDHPA	IVKFHASFVE	QDNFCIITEY	CEGRDLDDKI	QEYKQAGKIF	PENQIIEWFI	QLLLGVDYMH	ERRILHRDLK
**170**	**180**	**190**	**200**	**210**	**220**	**230**	**240**
SKNVFLKNNL	LKIGDFGVSR	LLMGSCDLAT	TLTGTPHYMS	PEALKHQGYD	TKSDIWSLAC	ILYEMCCMNH	AFAGSNFLSI
**250**	**260**	**270**	**280**	**290**	**300**	**310**	**320**
VLKIVEGDTP	SLPERYPKEL	NAIMESMLNK	NPSLRPSAIE	ILKIPYLDEQ	LQNLMCRYSE	MTLEDKNLDC	QKEAAHIINA
**330**	**340**	**350**	**360**	**370**	**380**	**390**	**400**
MQKRIHLQTL	RALSEVQKMT	PRERMRLRKL	QAADEKARKL	KKIVEEKYEE	NSKRMQELRS	RNFQQLSVDV	LHEKTHLKGM
**410**	**420**	**430**	**440**	**450**	**460**	**470**	**480**
EEKEEQPEGR	LSCSPQDEDE	ERWQGREEES	DEPTLENLPE	SQPIPSMDLH	ELESIVEDAT	SDLGYHATHS	

Cmpd.	No. of Cmpds.	m/z meas.	Δ m/z [ppm]	z	Rt [min]	Score	P	Range	Sequence	Modification
1841	1	682.2595	-115.74	3	53.0	24.3	1	4-22	K.FQEAAKCVSGSTAISTYPK.T	Carbamidomethyl: 7

Detailed Protein Report

Protein 484: cartilage intermediate layer protein 1 preproprotein [Homo sapiens]

**Accession:**	gi|192449445	**Score:**	24.3
**Database:**	refseq_human(refseq_human_20140103.fasta)	**MW [kDa]:**	132.5
**Database Date:**	2015-11-30	**pI:**	9.7
		**Sequence Coverage [%]:**	1.8
		**No. of unique Peptides:**	1

10	20	30	40	50	60	70	80
MVGTKAWVFS	FLVLEVTSVL	GRQTMLTQSV	RRVQPGKKNP	SIFAKPADTL	ESPGEWTTWF	NIDYPGGKGD	YERLDAIRFY
**90**	**100**	**110**	**120**	**130**	**140**	**150**	**160**
YGDRVCARPL	RLEARTTDWT	PAGSTGQVVH	GSPREGFWCL	NREQRPGQNC	SNYTVRFLCP	PGSLRRDTER	IWSPWSPWSK
**170**	**180**	**190**	**200**	**210**	**220**	**230**	**240**
CSAACGQTGV	QTRTRICLAE	MVSLCSEASE	EGQHCMGQDC	TACDLTCPMG	QVNADCDACM	CQDFMLHGAV	SLPGGAPASG
**250**	**260**	**270**	**280**	**290**	**300**	**310**	**320**
AAIYLLTKTP	KLLTQTDSDG	RFRIPGLCPD	GKSILKITKV	KFAPIVLTMP	KTSLKAATIK	AEFVRAETPY	MVMNPETKAR
**330**	**340**	**350**	**360**	**370**	**380**	**390**	**400**
RAGQSVSLCC	KATGKPRPDK	YFWYHNDTLL	DPSLYKHESK	LVLRKLQQHQ	AGEYFCKAQS	DAGAVKSKVA	QLIVIASDET
**410**	**420**	**430**	**440**	**450**	**460**	**470**	**480**
PCNPVPESYL	IRLPHDCFQN	ATNSFYYDVG	RCPVKTCAGQ	QDNGIRCRDA	VQNCCGISKT	EEREIQCSGY	TLPTKVAKEC
**490**	**500**	**510**	**520**	**530**	**540**	**550**	**560**
SCQRCTETRS	IVRGRVSAAD	NGEPMRFGHV	YMGNSRVSMT	GYKGTFTLHV	PQDTERLVLT	FVDRLQKFVN	TTKVLPFNKK
**570**	**580**	**590**	**600**	**610**	**620**	**630**	**640**
GSAVFHEIKM	LRRKEPITLE	AMETNIIPLG	EVVGEDPMAE	LEIPSRSFYR	QNGEPYIGKV	KASVTFLDPR	NISTATAAQT
**650**	**660**	**670**	**680**	**690**	**700**	**710**	**720**
DLNFINDEGD	TFPLRTYGMF	SVDFRDEVTS	EPLNAGKVKV	HLDSTQVKMP	EHISTVKLWS	LNPDTGLWEE	EGDFKFENQR
**730**	**740**	**750**	**760**	**770**	**780**	**790**	**800**
RNKREDRTFL	VGNLEIRERR	LFNLDVPESR	RCFVKVRAYR	SERFLPSEQI	QGVVISVINL	EPRTGFLSNP	RAWGRFDSVI
**810**	**820**	**830**	**840**	**850**	**860**	**870**	**880**
TGPNGACVPA	FCDDQSPDAY	SAYVLASLAG	EELQAVESSP	KFNPNAIGVP	QPYLNKLNYR	RTDHEDPRVK	KTAFQISMAK
**890**	**900**	**910**	**920**	**930**	**940**	**950**	**960**
PRPNSAEESN	GPIYAFENLR	ACEEAPPSAA	HFRFYQIEGD	RYDYNTVPFN	EDDPMSWTED	YLAWWPKPME	FRACYIKVKI
**970**	**980**	**990**	**1000**	**1010**	**1020**	**1030**	**1040**
VGPLEVNVRS	RNMGGTHRQT	VGKLYGIRDV	RSTRDRDQPN	VSAACLEFKC	SGMLYDQDRV	DRTLVKVIPQ	GSCRRASVNP
**1050**	**1060**	**1070**	**1080**	**1090**	**1100**	**1110**	**1120**
MLHEYLVNHL	PLAVNNDTSE	YTMLAPLDPL	GHNYGIYTVT	DQDPRTAKEI	ALGRCFDGTS	DGSSRIMKSN	VGVALTFNCV
**1130**	**1140**	**1150**	**1160**	**1170**	**1180**	**1190**	
ERQVGRQSAF	QYLQSTPAQS	PAAGTVQGRV	PSRRQQRASR	GGQRQGGVVA	SLRFPRVAQQ	PLIN	

Cmpd.	No. of Cmpds.	m/z meas.	Δ m/z [ppm]	z	Rt [min]	Score	P	Range	Sequence	Modification
1333	1	1061.5792	19.46	1	46.4	13.3	1	378-388	K.AQSDAGAVKSK.V	

Detailed Protein Report

Protein 485: **PREDICTED: serine/threonine-protein phosphatase 4 regulatory subunit 4 isoform X1 [Homo sapiens]**

**Accession:**	gi|530404339	**Score:**	24.2
**Database:**	refseq_human(refseq_human_20140103.fasta)	**MW [kDa]:**	87.3
**Database Date:**	2015-11-30	**pI:**	9.4
		**Sequence Coverage [%]:**	4.7
		**No. of unique Peptides:**	1

Alias proteins:

Accession	Name	Description
gi|578839938	refseq_human (refseq_human_20140103.fasta)	PREDICTED: serine/threonine-protein phosphatase 4 regulatory subunit 4 isoform X3 [Homo sapiens]
gi|578839936	refseq_human (refseq_human_20140103.fasta)	PREDICTED: serine/threonine-protein phosphatase 4 regulatory subunit 4 isoform X2 [Homo sapiens]
gi|578839934	refseq_human (refseq_human_20140103.fasta)	PREDICTED: serine/threonine-protein phosphatase 4 regulatory subunit 4 isoform X1 [Homo sapiens]
gi|578826014	refseq_human (refseq_human_20140103.fasta)	PREDICTED: serine/threonine-protein phosphatase 4 regulatory subunit 4 isoform X3 [Homo sapiens]
gi|530404341	refseq_human (refseq_human_20140103.fasta)	PREDICTED: serine/threonine-protein phosphatase 4 regulatory subunit 4 isoform X2 [Homo sapiens]

```
        10         20         30         40         50         60         70         80
MQLTAAMSFL TILQDESVSI HAYTHSFLQV ILLHLEHRDT GVSNAWLETL LSVIEVLPKE TLRHEILNPL VSKAQLSQTV
        90        100        110        120        130        140        150        160
QSRLVSCKIL GKLTNKFDAH TIKREILPLV KSLCQDVEYE VRSCMCRQLE NIAQGIGTEL TKSVVLPELI ELSRDEGSSV
       170        180        190        200        210        220        230        240
RLAAFETLVN LLDIFDTDDR SQTILPLVKS FCEKSFKADE SILISLSFHL GKLCHGLYGI FTPDQHLRFL EFYKKLCTLG
       250        260        270        280        290        300        310        320
LQQENGHNEN QIPPQILEQE KKYISVRKNC AYNFPAMIVF VDPKNFHMEL YSTFFCLCHD PEVPVRYTIA ICFYEVSKLL
       330        340        350        360        370        380        390        400
NSGVYLIHKE LITLLQDESL EVLDALIDHL PEILELMSTG GESSVQENKL SSLPDLIPAL TAAEQRAAAS LKWRTHEKLL
       410        420        430        440        450        460        470        480
QKYACLPHVI SSDQIYYRFL QRMFTIMMTN NVLPVQKAAS RTLCIFLRYN RKQEQRHEVI QKLIEQLGQG KSYWNRLRFL
       490        500        510        520        530        540        550        560
DTCEFIIEIF SKSFFCKYFF LPAIELTHDP VANVRMKLCY LLPKVKSTLK IPADKHLLQQ LEMCVRKLLC QEKDKDVLAI
       570        580        590        600        610        620        630        640
VKRTVLELDR MEMSMDAFQK KFYEKDLLDQ EKEREELLLL EMEQLEKEKQ QNDGRPMSDK MFEKKRRDTK TPTQSLPKNI
       650        660        670        680        690        700        710        720
PISVPGPSSV TPSTSKEIKK SKLIRSQSFN NQAFHAKYGN LEKCASKSST TGYTTSVSGL GKTSVLSLAD DSFRTRNASS
       730        740        750        760        770
VPSSFSPNTP LPSTSRGTGN SVDPKSSGSK DTQPRKATLK SRKSNP
```

Cmpd.	No. of Cmpds.	m/z meas.	Δ m/z [ppm]	z	Rt [min]	Score	P	Range	Sequence	Modification
119	1	566.9137	-154.93	3	30.9	13.4	2	89-103	K.ILGKLTNKFDAHTIK.R	

Detailed Protein Report

Protein 486: protein SMG8 [Homo sapiens]

**Accession:**	gi|47777319	**Score:**	24.2
**Database:**	refseq_human(refseq_human_20140103.fasta)	**MW [kDa]:**	109.6
**Database Date:**	2015-11-30	**pI:**	8.7
		**Sequence Coverage [%]:**	3.1
		**No. of unique Peptides:**	1

10	20	30	40	50	60	70	80
MAGPVSLRDL	LMGASAWMGS	ESPGGSPTEG	GGSAAGGPEP	PWREDEICVV	GIFGKTALRL	NSEKFSLVNT	VCDRQVFPLF
**90**	**100**	**110**	**120**	**130**	**140**	**150**	**160**
RHQDPGDPGP	GIRTEAGAVG	EAGGAEDPGA	AAGGSVRGSG	AVAEGNRTEA	GSQDYSLLQA	YYSQESKVLY	LLLTSICDNS
**170**	**180**	**190**	**200**	**210**	**220**	**230**	**240**
QLLRACRALQ	SGEAGGGLSL	PHAEAHEFWK	HQEKLQCLSL	LYLFSVCHIL	LLVHPTCSFD	ITYDRVFRAL	DGLRQKVLPL
**250**	**260**	**270**	**280**	**290**	**300**	**310**	**320**
LKTAIKDCPV	GKDWKLNCRP	CPPRLLFLFQ	LNGALKVEPP	RNQDPAHPDK	PKKHSPKRRL	QHALEDQIYR	IFRKSRVLTN
**330**	**340**	**350**	**360**	**370**	**380**	**390**	**400**
QSINCLFTVP	ANQAFVYIVP	GSQEEDPVGM	LLDQLRSHCT	VKDPESLLVP	APLSGPRRYQ	VMRQHSRQQL	SFHIDSSSSS
**410**	**420**	**430**	**440**	**450**	**460**	**470**	**480**
SSGQLVDFTL	REFLWQHVEL	VLSKKGFDDS	VGRNPQPSHF	ELPTYQKWIS	AASKLYEVAI	DGKEEDLGSP	TGELTSKILS
**490**	**500**	**510**	**520**	**530**	**540**	**550**	**560**
SIKVLEGFLD	IDTKFSENRC	QKALPMAHSA	YQSNLPHNYT	MTVHKNQLAQ	ALRVYSQHAR	GPAFHKYAMQ	LHEDCYKFWS
**570**	**580**	**590**	**600**	**610**	**620**	**630**	**640**
NGHQLCEERS	LTDQHCVHKF	HSLPKSGEKP	EADRNPPVLY	HNSRARSTGA	CNCGRKQAPR	DDPFDIKAAN	YDFYQLLEEK
**650**	**660**	**670**	**680**	**690**	**700**	**710**	**720**
CCGKLDHINF	PVFEPSTPDP	APAKNESSPA	PPDSDADKLK	EKEPQTQGES	TSLSLALSLG	QSTDSLGTYP	ADPQAGGDNP
**730**	**740**	**750**	**760**	**770**	**780**	**790**	**800**
EVHGQVEVKT	EKRPNFVDRQ	ASTVEYLPGM	LHSNCPKGLL	PKFSSWSLVK	LGPAKSYNFH	TGLDQQGFIP	GTNYLMPWDI
**810**	**820**	**830**	**840**	**850**	**860**	**870**	**880**
VIRTRAEDEG	DLDTNSWPAP	NKAIPGKRSA	VVMGRGRRRD	DIARAFVGFE	YEDSRGRRFM	CSGPDKVMKV	MGSGPKESAL
**890**	**900**	**910**	**920**	**930**	**940**	**950**	**960**
KALNSDMPLY	ILSSSQGRGL	KPHYAQLMRL	FVVVPDAPLQ	IILMPQVQPG	PPPCPVFYPE	KQEITLPPDG	LWVLRFPYAY
**970**	**980**	**990**	**1000**				
VTERGPCFPP	KENVQLMSYK	VLRGVLKAVT	Q				

Cmpd.	No. of Cmpds.	m/z meas.	Δ m/z [ppm]	z	Rt [min]	Score	P	Range	Sequence	Modification
2766	1	735.0575	1.78	3	65.9	14.0	1	357-377	R.SHCTVKDPESLLVPAPLSGPR.R	

Detailed Protein Report

Protein 487: mastermind-like protein 2 [Homo sapiens]

**Accession:**	gi|33286444	**Score:**	24.2
**Database:**	refseq_human(refseq_human_20140103.fasta)	**MW [kDa]:**	125.1
**Database Date:**	2015-11-30	**pI:**	10.0
**Modification(s):**	Carbamidomethyl	**Sequence Coverage [%]:**	1.5
		**No. of unique Peptides:**	1

10	20	30	40	50	60	70	80
MGDTAPPQAP	AGGLGGASGA	GLLGGGSVTP	RVHSAIVERL	RARIAVCRQH	HLSCEGRYER	GRAESSDRER	ESTLQLLSLV
**90**	**100**	**110**	**120**	**130**	**140**	**150**	**160**
QHGQGARKAG	KHTKATATAA	TTTAPPPPPA	APPAASQAAA	TAAPPPPPDY	HHHHQQHLLN	SSNNGGSGGI	NGEQQPPAST
**170**	**180**	**190**	**200**	**210**	**220**	**230**	**240**
PGDQRNSALI	ALQGSLKRKQ	VVNLSPANSK	RPNGFVDNSF	LDIKRIRVGE	NLSAGQGGLQ	INNGQSQIMS	GTLPMSQAPL
**250**	**260**	**270**	**280**	**290**	**300**	**310**	**320**
RKTNTLPSHT	HSPGNGLFNM	GLKEVKKEPG	ETLSCSKHMD	GQMTQENIFP	NRYGDDPGEQ	LMDPELQELF	NELTNISVPP
**330**	**340**	**350**	**360**	**370**	**380**	**390**	**400**
MSDLELENMI	NATIKQDDPF	NIDLGQQSQR	STPRPSLPME	KIVIKSEYSP	GLTQGPSGSP	QLRPPSAGPA	FSMANSALST
**410**	**420**	**430**	**440**	**450**	**460**	**470**	**480**
SSPIPSVPQS	QAQPQTGSGA	SRALPSWQEV	SHAQQLKQIA	ANRQQHARMQ	QHQQQHQPTN	WSALPSSAGP	SPGPFGQEKI
**490**	**500**	**510**	**520**	**530**	**540**	**550**	**560**
PSPSFGQQTF	SPQSSPMPGV	AGGSGQSKVM	ANYMYKAGPS	AQGGHLDVLM	QQKPQDLSRS	FINNPHPAME	PRQGNTKPLF
**570**	**580**	**590**	**600**	**610**	**620**	**630**	**640**
HFNSDQANQQ	MPSVLPSQNK	PSLLHYTQQQ	QQQQQQQQQQ	QQQQQQQQQQ	QQQQQQQQQQ	QSSISAQQQQ	QQQSSISAQQ
**650**	**660**	**670**	**680**	**690**	**700**	**710**	**720**
QQQQQQQQQQ	QQQQQQQQQQ	QQQQQPSSQP	AQSLPSQPLL	RSPLPLQQKL	LLQQMQNQPI	AGMGYQVSQQ	QRQDQHSVVG
**730**	**740**	**750**	**760**	**770**	**780**	**790**	**800**
QNTGPSPSPN	PCSNPNTGSG	YMNSQQSLLN	QQLMGKKQTL	QRQIMEQKQQ	LLLQQQMLAD	AEKIAPQDQI	NRHLSRPPPD
**810**	**820**	**830**	**840**	**850**	**860**	**870**	**880**
YKDQRRNVGN	MQPTAQYSGG	SSTISLNSNQ	ALANPVSTHT	ILTPNSSLLS	TSHGTRMPSL	STAVQNMGMY	GNLPCNQPNT
**890**	**900**	**910**	**920**	**930**	**940**	**950**	**960**
YSVTSGMNQL	TQQRNPKQLL	ANQNNPMMPR	PPTLGPSNNN	NVATFGAGSV	GNSQQLRPNL	THSMASMPPQ	RTSNVMITSN
**970**	**980**	**990**	**1000**	**1010**	**1020**	**1030**	**1040**
TTAPNWASQE	GTSKQQEALT	SAGVRFPTGT	PAAYTPNQSL	QQAVGSQQFS	QRAVAPPNQL	TPAVQMRPMN	QMSQTLNGQT
**1050**	**1060**	**1070**	**1080**	**1090**	**1100**	**1110**	**1120**
MGPLRGLNLR	PNQLSTQILP	NLNQSGTGLN	QSRTGINQPP	SLTPSNFPSP	NQSSRAFQGT	DHSSDLAFDF	LSQQNDNMGP
**1130**	**1140**	**1150**	**1160**				
ALNSDADFID	SLLKTEPGND	DWMKDINLDE	ILGNNS				

Cmpd.	No. of Cmpds.	m/z meas.	Δ m/z [ppm]	z	Rt [min]	Score	P	Range	Sequence	Modification
1771	1	1057.5151	4.71	2	52.0	24.2	2	44-60	R.IAVCRQHHLSCEGRYER.G	Carbamidomethyl: 11

Detailed Protein Report

Protein 488: connective tissue growth factor precursor [Homo sapiens]

**Accession:**	gi|4503123	**Score:**	24.2
**Database:**	refseq_human(refseq_human_20140103.fasta)	**MW [kDa]:**	38.0
**Database Date:**	2015-11-30	**pI:**	9.9
**Modification(s):**	Carbamidomethyl, Oxidation	**Sequence Coverage [%]:**	9.2
		**No. of unique Peptides:**	1

10	20	30	40	50	60	70	80
MTAASMGPVR	VAFVVLLALC	SRPAVGQNCS	GPCRCPDEPA	PRCPAGVSLV	LDGCGCCRVC	AKQLGELCTE	RDPCDPHKGL
**90**	**100**	**110**	**120**	**130**	**140**	**150**	**160**
FCDFGSPANR	KIGVCTAKDG	APCIFGGTVY	RSGESFQSSC	KYQCTCLDGA	VGCMPLCSMD	VRLPSPDCPF	PRRVKLPGKC
**170**	**180**	**190**	**200**	**210**	**220**	**230**	**240**
CEEWVCDEPK	DQTVVGPALA	AYRLEDTFGP	DPTMIRANCL	VQTTEWSACS	KTCGMGISTR	VTNDNASCRL	EKQSRLCMVR
**250**	**260**	**270**	**280**	**290**	**300**	**310**	**320**
PCEADLEENI	KKGKKCIRTP	KISKPIKFEL	SGCTSMKTYR	AKFCGVCTDG	RCCTPHRTTT	LPVEFKCPDG	EVMKKNMMFI
**330**	**340**	**350**					
KTCACHYNCP	GDNDIFESLY	YRKMYGDMA					

Cmpd.	No. of Cmpds.	m/z meas.	Δ m/z [ppm]	z	Rt [min]	Score	P	Range	Sequence	Modification
1888	1	921.3555	-133.61	2	53.7	12.6	1	262-277	K.ISKPIKFELSGCTSMK.T	Carbamidomethyl: 12; Oxidation: 15

Detailed Protein Report

Protein 489: secreted frizzled-related protein 4 precursor [Homo sapiens]

**Accession:**	gi|170784838	**Score:**	24.2
**Database:**	refseq_human(refseq_human_20140103.fasta)	**MW [kDa]:**	39.8
**Database Date:**	2015-11-30	**pI:**	10.3
**Modification(s):**	Carbamidomethyl, Oxidation	**Sequence Coverage [%]:**	11.3
		**No. of unique Peptides:**	1

10	20	30	40	50	60	70	80
MFLSILVALC	LWLHLALGVR	GAPCEAVRIP	MCRHMPWNIT	RMPNHLHHST	QENAILAIEQ	YEELVDVNCS	AVLRFFLCAM
**90**	**100**	**110**	**120**	**130**	**140**	**150**	**160**
YAPICTLEFL	HDPIKPCKSV	CQRARDDCEP	LMKMYNHSWP	ESLACDELPV	YDRGVCISPE	AIVTDLPEDV	KWIDITPDMM
**170**	**180**	**190**	**200**	**210**	**220**	**230**	**240**
VQERPLDVDC	KRLSPDRCKC	KKVKPTLATY	LSKNYSYVIH	AKIKAVQRSG	CNEVTTVVDV	KEIFKSSSPI	PRTQVPLITN
**250**	**260**	**270**	**280**	**290**	**300**	**310**	**320**
SSCQCPHILP	HQDVLIMCYE	WRSRMMLLEN	CLVEKWRDQL	SKRSIQWEER	LQEQRRTVQD	KKKTAGRTSR	SNPPKPKGKP
**330**	**340**	**350**					
PAPKPASPKK	NIKTRSAQKR	TNPKRV					

Cmpd.	No. of Cmpds.	m/z meas.	Δ m/z [ppm]	z	Rt [min]	Score	P	Range	Sequence	Modification
768	1	698.4267	127.49	2	39.2	13.0	0	265-275	R.MMLLENCLVEK.W	Carbamidomethyl: 7; Oxidation: 1

Detailed Protein Report

Protein 490: PREDICTED: SCL-interrupting locus protein isoform X2 [Homo sapiens]

**Accession:**	gi|578799618	**Score:**	24.2
**Database:**	refseq_human(refseq_human_20140103.fasta)	**MW [kDa]:**	92.6
**Database Date:**	2015-11-30	**pI:**	5.9
**Modification(s):**	Carbamidomethyl	**Sequence Coverage [%]:**	2.1
		**No. of unique Peptides:**	1

10	20	30	40	50	60	70	80
MVNNENPPLI	NHLEHLKPLQ	PQLYDEKHSP	EVEAGEPSLR	GIPNQLNQDK	PALLRHCKVR	QPPAYKKGNP	HTRNSIKPSS
**90**	**100**	**110**	**120**	**130**	**140**	**150**	**160**
HNGPSHDIFE	KLQTVSAGNV	QNEEYPIRPS	TLNSRQSSLA	PQSQPHDFVF	SPHNSGRPME	LQIPTPPLPS	YCSTNVCRCC
**170**	**180**	**190**	**200**	**210**	**220**	**230**	**240**
QHHSHIQYSP	LNSWQGANTV	GSIQDVQSEA	LQKHSLFHPS	GCPALYCNAF	CSSSSPIALR	PQGDMGSCSP	HSNIEPSPVA
**250**	**260**	**270**	**280**	**290**	**300**	**310**	**320**
RPPSHMDLCN	PQPCTVCMHT	PKTESDNGMM	GLSPDAYRFL	TEQDRQLRLL	QAQIQRLLEA	QSLMPCSPKT	TAVEDTVQAG
**330**	**340**	**350**	**360**	**370**	**380**	**390**	**400**
RQMELVSVEA	QSSPGLHMRK	GVSIAVSTGA	SLFWNAAGED	QEPDSQMKQD	DTKISSEDMN	FSVDINNEVT	SLPGSASSLK
**410**	**420**	**430**	**440**	**450**	**460**	**470**	**480**
AVDIPSFEES	NIAVEEEFNQ	PLSVSNSSSL	VVRKEPDVPV	FFPSGQLAES	VSMCLQTGPT	GGASNNSETS	EEPKIEHVMQ
**490**	**500**	**510**	**520**	**530**	**540**	**550**	**560**
PLLHQPSDNQ	KIYQDLLGQV	NHLLNSSSKE	TEQPSTKAVI	ISHECTRTQN	VYHTKKKTHH	SRLVDKDCVL	NATLKQLRSL
**570**	**580**	**590**	**600**	**610**	**620**	**630**	**640**
GVKIDSPTKV	KKNAHNVDHA	SVLACISPEA	VISGLNCMSF	ANVGMSGLSP	NGVDLSMEAN	AIALKYLNEN	QLSQLSVTRS
**650**	**660**	**670**	**680**	**690**	**700**	**710**	**720**
NQNNCDPFSL	LHINTDRSTV	GLSLISPNNM	SFATKKYMKR	YGLLQSSDNS	EDEEEPPDNA	DSKSEYLLNQ	NLRSIPEQLG
**730**	**740**	**750**	**760**	**770**	**780**	**790**	**800**
GQKEPSKNDH	EIINCSNCES	VGTNADTPVL	RNITNEVLQT	KAKQQLTEKP	AFLVKNLKPS	PAVNLRTGKA	EFTQHPEKEN
**810**	**820**	**830**	**840**	**850**			
EGDITIFPES	LQPSETLKQM	NSMNSVGTFL	DVKRLRQLPK	LF			

Cmpd.	No. of Cmpds.	m/z meas.	Δ m/z [ppm]	z	Rt [min]	Score	P	Range	Sequence	Modification
2867	3	1051.5491	-17.97	2	66.3	24.2	1	41-58	R.GIPNQLNQDKPALLRHCK.V	Carbamidomethyl: 17

Detailed Protein Report

Protein 491: PREDICTED: BRCA1-A complex subunit Abraxas isoform X1 [Homo sapiens]

**Accession:**	gi|530378285	**Score:**	24.2
**Database:**	refseq_human(refseq_human_20140103.fasta)	**MW [kDa]:**	41.6
**Database Date:**	2015-11-30	**pI:**	9.2
**Modification(s):**	Oxidation	**Sequence Coverage [%]:**	3.3
		**No. of unique Peptides:**	1

10	20	30	40	50	60	70	80
MDDVEVVYTI	DIQKYIPCYQ	LFSFYNSSGE	VNEQALKKIL	SNVKKNVVGW	YKFRRHSDQI	MTFRERLLHK	NLQEHFSNQD
**90**	**100**	**110**	**120**	**130**	**140**	**150**	**160**
LVFLLLTPSI	ITESCSTHRL	EHSLYKPQKG	LFHRVPLVVA	NLGMSEQLGY	KTVSGSCMST	GFSRAVQTHS	SKFFEEDGSL
**170**	**180**	**190**	**200**	**210**	**220**	**230**	**240**
KEVHKINEMY	ASLQEELKSI	CKKVEDSEQA	VDKLVKDVNR	LKREIEKRRG	AQIQAAREKN	IQKDPQENIF	LCQALRTFFP
**250**	**260**	**270**	**280**	**290**	**300**	**310**	**320**
NSEFLHSCVM	SLKNRHVSKS	SCNYNHHLDV	VDNLTLMVEH	TDIPEASPAS	TPQIIKHKAL	DLDDRWQFKR	SRLLDTQDKR
**330**	**340**	**350**	**360**	**370**			
SKADTGSSNQ	DKASKMSSPE	TDEEIEKMKG	FGEYSRSPTF				

Cmpd.	No. of Cmpds.	m/z meas.	Δ m/z [ppm]	z	Rt [min]	Score	P	Range	Sequence	Modification
1271	1	796.3744	-21.47	2	45.6	24.2	2	55-66	R.RHSDQIMTFRER.L	Oxidation: 7

Detailed Protein Report

Protein 492: PREDICTED: unconventional myosin-X isoform X3 [Homo sapiens]

**Accession:**	gi|578809923	**Score:**	24.2
**Database:**	refseq_human(refseq_human_20140103.fasta)	**MW [kDa]:**	234.6
**Database Date:**	2015-11-30	**pI:**	5.7
**Modification(s):**	Carbamidomethyl	**Sequence Coverage [%]:**	2.1
		**No. of unique Peptides:**	1

Detailed Protein Report

10	20	30	40	50	60	70	80
MDNFFTEGTR	VWLRENGQHF	PSTVNSCAEG	IVVFRTDYGQ	VFTYKQSTIT	HQKVTAMHPT	NEEGVDDMAS	LTELHGGSIM
90	100	110	120	130	140	150	160
YNLFQRYKRN	QIYTYIGSIL	ASVNPYQPIA	GLYEPATMEQ	YSRRHLGELP	PHIFAIANEC	YRCLWKRHDN	QCILISGESG
170	180	190	200	210	220	230	240
AGKTESTKLI	LKFLSVISQQ	SLELSLKEKT	SCVERAILES	SPIMEAFGNA	KTVYNNNSSR	FGKFVQLNIC	QKGNIQGGRI
250	260	270	280	290	300	310	320
VDYLLEKNRV	VRQNPGERNY	HIFYALLAGL	EHEEREEFYL	STPENYHYLN	QSGCVEDKTI	SDQESFREVI	TAMDVMQFSK
330	340	350	360	370	380	390	400
EEVREVSRLL	AGILHLGNIE	FITAGGAQVS	FKTALGRSAE	LLGLDPTQLT	DALTQRSMFL	RGEEILTPLN	VQQAVDSRDS
410	420	430	440	450	460	470	480
LAMALYACCF	EWVIKKINSR	IKGNEDFKSI	GILDIFGFEN	FEVNHFEQFN	INYANEKLQE	YFNKHIFSLE	QLEYSREGLV
490	500	510	520	530	540	550	560
WEDIDWIDNG	ECLDLIEKKL	GLLALINEES	HFPQATDSTL	LEKLHSQHAV	QYDVRGILEK	NRDTFRDDLL	NLLRESRFDF
570	580	590	600	610	620	630	640
IYDLFEHVSS	RNNQDTLKCG	SKHRRPTVSS	QFKDSLHSLM	ATLSSSNPFF	VRCIKPNMQK	MPDQFDQAVV	LNQLRYSGML
650	660	670	680	690	700	710	720
ETVRIRKAGY	AVRRPFQDFY	KRYKVLMRNL	ALPEDVRGKC	TSLLQLYDAS	NSEWQLGKTK	VFLRESLEQK	LEKRREEEVS
730	740	750	760	770	780	790	800
HAAMVIRAHV	LGFLARKQYR	KVLYCVVIIQ	KNYRAFLLRR	RFLHLKKAAI	VFQKQLRGQI	ARRVYRQLLA	EKREQEEKKK
810	820	830	840	850	860	870	880
QEEEEKKKRE	EEERERERER	REAELRAQQE	EETRKQQELE	ALQKSQKEAE	LTRELEKQKE	NKQVEEILRL	EKEIEDLQRM
890	900	910	920	930	940	950	960
KEQQELSLTE	ASLQKLQERR	DQELRRLEEE	ACRAAQEFLE	SLNFDEIDEC	VRNIERSLSV	GSEFSSELAE	SACEEKPNFN
970	980	990	1000	1010	1020	1030	1040
FSQPYPEEEV	DEGFEADDDA	FKDSPNPSEH	GHSDQRTSGI	RTSDDSSEED	PYMNDTVVPT	SPSADSTVLL	APSVQDSGSL
1050	1060	1070	1080	1090	1100	1110	1120
HNSSSGESTY	CMPQNAGDLP	SPDGDYDYDQ	DDYEDGAITS	GSSVTFSNSY	GSQWSPDYRC	SVGTYNSSGA	YRFSSEGAQS
1130	1140	1150	1160	1170	1180	1190	1200
SFEDSEEDFD	SRFDTDDELS	YRRDSVYSCV	TLPYFHSFLY	MKGGLMNSWK	RRWCVLKDET	FLWFRSKQEA	LKQGWLHKKG
1210	1220	1230	1240	1250	1260	1270	1280
GGSSTLSRRN	WKKRWFVLRQ	SKLMYFENDS	EEKLKGTVEV	RTAKEIIDNT	TKENGIDIIM	ADRTFHLIAE	SPEDASQWFS
1290	1300	1310	1320	1330	1340	1350	1360
VLSQVHASTD	QEIQEMHDEQ	ANPQNAVGTL	DVGLIDSVCA	SDSPDRPNSF	VIITANRVLH	CNADTPEEMH	HWITLLQRSK
1370	1380	1390	1400	1410	1420	1430	1440
GDTRVEGQEF	IVRGWLHKEV	KNSPKMSSLK	LKKRWFVLTH	NSLDYYKSSE	KNALKLGTLV	LNSLCSVVPP	DEKIFKETGY
1450	1460	1470	1480	1490	1500	1510	1520
WNVTVYGRKH	CYRLYTKLLN	EATRWSSAIQ	NVTDTKAPID	TPTQQLIQDI	KENCLNSDVV	EQIYKRNPIL	RYTHHPLHSP
1530	1540	1550	1560	1570	1580	1590	1600
LLPLPYGDIN	LNLLKDKGYT	TLQDEAIKIF	NSLQQLESMS	DPIPIIQGIL	QTGHDLRPLR	DELYCQLIKQ	TNKVPHPGSV
1610	1620	1630	1640	1650	1660	1670	1680
GNLYSWQILT	CLSCTFLPSR	GILKYLKFHL	KRIREQFPGS	EMEKYALFTY	ESLKKTKCRE	FVPSRDEIEA	LIHRQEMTST
1690	1700	1710	1720	1730	1740	1750	1760
VYCHGGGSCK	ITINSHTTAG	EVVEKLIRGL	AMEDSRNMFA	LFEYNGHVDK	AIESRTVVAD	VLAKFEKLAA	TSEVGDLPWK
1770	1780	1790	1800	1810	1820	1830	1840
FYFKLYCFLD	TDNVPKDSVE	FAFMFEQAHE	AVIHGHHPAP	EENLQVLAAL	RLQYLQGDYT	LHAAIPPLEE	VYSLQRLKAR
1850	1860	1870	1880	1890	1900	1910	1920
ISQSTKTFTP	CERLEKRRTS	FLEGTLRRSF	RTGSVVRQKV	EEEQMLDMWI	KEEVSSARAS	IIDKWRKFQG	MNQEQAMAKY
1930	1940	1950	1960	1970	1980	1990	2000
MALIKEWPGY	GSTLFDVECK	EGGFPQELWL	GVSADAVSVY	KRGEGRPLEV	FQYEHILSFG	APLANTYKIV	VDERELLFET
2010	2020	2030	2040				
SEVVDVAKLM	KAYISMIVKK	RYSTTRSASS	QGSSR				

Cmpd.	No. of Cmpds.	m/z meas.	Δ m/z [ppm]	z	Rt [min]	Score	P	Range	Sequence	Modification

Detailed Protein Report

Cmpd.	No. of Cmpds.	m/z meas.	Δ m/z [ppm]	z	Rt [min]	Score	P	Range	Sequence	Modification
1694	1	1108.8433	-4.24	3	51.1	11.2	1	1675-1705	R.QEMTSTVYCHGGGSCKITINSHTTAGEVVEK.L	Carbamidomethyl: 15

Detailed Protein Report

Protein 493: PREDICTED: uncharacterized protein LOC102724413 [Homo sapiens]

**Accession:**	gi|578838161	**Score:**	24.1
**Database:**	refseq_human(refseq_human_20140103.fasta)	**MW [kDa]:**	27.4
**Database Date:**	2015-11-30	**pI:**	12.6
**Modification(s):**	Carbamidomethyl	**Sequence Coverage [%]:**	13.1
		**No. of unique Peptides:**	2

Quantitation

MD:MU	**Median:** 1.98	**CV:** 0.00 %	**No. of Peptides:**	1

Alias proteins:

Accession	Name	Description
gi|578847088	refseq_human (refseq_human_20140103.fasta)	PREDICTED: uncharacterized protein LOC102724413 [Homo sapiens]

```
        10         20         30         40         50         60         70         80
MWPASGPGTR PAIRQPCTSF TPVPWLPTRP RASPAELARC PAAAPKLRAP RRLSRQPEAW SPTAQDWRAQ FVSPGWDCWF
        90        100        110        120        130        140        150        160
RVAHDHSPLN TRPARARAQT HKHTRLPSAQ PASRASAFPS SSPLGRFGSP WRLCAAPGKS PGRGDRAALF PPKVCAHASI
       170        180        190        200        210        220        230        240
LRKHSQACGF PSSREMTAAG EDLFAPCFAM ARGSEERTGR WGLRVDEGGA RETPWIRGWA GQRRRPAALA NPRPRGGQAG
       250        260
LGAGRPGCTC RR
```

Cmpd.	No. of Cmpds.	m/z meas.	Δ m/z [ppm]	z	Rt [min]	Score	P	Range	Sequence	Modification	Ratios
2132	1	899.3904	-102.48	2	56.5	14.0	1	82-97	R.VAHDHSPLNTRPARAR.A		MD:MU 1.98
2739	1	922.4796	7.26	2	65.5	10.1	2	127-143	R.FGSPWRLCAAPGKSPGR.G	Carbamidomethyl: 8	

Detailed Protein Report

Protein 494: PREDICTED: zinc finger protein 813 isoform X1 [Homo sapiens]

Accession: gi|578833930
Database: refseq_human(refseq_human_20140103.fasta)
Database Date: 2015-11-30

Score: 24.1
MW [kDa]: 62.1
pI: 10.5
Sequence Coverage [%]: 3.9
No. of unique Peptides: 1

10	20	30	40	50	60	70	80
MALPQGLLTF	RDVAIEFSQE	EWKCLDPAQR	TLYRDVMLEN	YRNLVSLDIS	SKCMMKEFSS	TAQGNREVIH	TGTLQRHESH
**90**	**100**	**110**	**120**	**130**	**140**	**150**	**160**
HTGDFRFQEI	DKDIHNLEFQ	WQEDERNSHE	APMTEIKKLT	GSADRYDQRH	AGNKPIKDQL	GSSFHSHLPE	LHMFQTQGKI
**170**	**180**	**190**	**200**	**210**	**220**	**230**	**240**
GNQVEKSIND	ASSISTSQRI	SCRPKTHISN	NYGNNFRNSS	LLTQKQEVHM	REKSFQCNES	GKAFNYSSLL	RKHQIIHLGE
**250**	**260**	**270**	**280**	**290**	**300**	**310**	**320**
KQYKCDVCGK	VFNRKRNLVC	HRRCHTGEKP	YRCNECGKTF	SQTYSLTCHR	RLHTGEKPYK	CEECDKAFSF	KSNLKRHRRI
**330**	**340**	**350**	**360**	**370**	**380**	**390**	**400**
HAGEKPYKCN	ECGKTFSQTS	SLTCHRRLHT	GEKPFKCNEC	GKTFSRKSSL	TCHHRLHTGE	KPYKCNECGK	TFSQELTLKC
**410**	**420**	**430**	**440**	**450**	**460**	**470**	**480**
HRRLHTGEKP	YKCNECGKGF	NRKTHLACHH	RLHTGEKPYK	CNECGKVFNR	KTHLAHHHRL	HTGDKPYKCN	ECGKVFNQKA
**490**	**500**	**510**	**520**	**530**	**540**		
HLARHHRLHT	GEKPYKCNEC	GKVFNQKANL	ARHHRLHTGE	KPYKFNECGK	AFN		

Cmpd.	No. of Cmpds.	m/z meas.	Δ m/z [ppm]	z	Rt [min]	Score	P	Range	Sequence	Modification
2454	1	611.6737	-159.08	2	61.0	12.2	0	77-86	R.HESHHTGDFR.F	

Detailed Protein Report

Protein 495: uncharacterized protein LOC100144595 [Homo sapiens]

Accession: gi|373838702
Database: refseq_human(refseq_human_20140103.fasta)
Database Date: 2015-11-30

Score: 24.1
MW [kDa]: 11.6
pI: 11.4
Sequence Coverage [%]: 13.7
No. of unique Peptides: 1

10	20	30	40	50	60	70	80
MATYILKLRA	CPWETLNGVL	LCRQAGVHWR	NLGLLQPPPP	GFKRFSCLSL	PSGWDYRHAP	PCRPNVLYFS	RDGVSLCWPG
90	100	110					
WSQSPDLMIH	LPRPPKVLGL	QV					

Cmpd.	No. of Cmpds.	m/z meas.	Δ m/z [ppm]	z	Rt [min]	Score	P	Range	Sequence	Modification
2266	1	787.7912	-131.21	2	58.2	24.1	0	10-23	R.ACPWETLNGVLLCR.Q	

Detailed Protein Report

Protein 496: exocyst complex component 2 [Homo sapiens]

Accession: gi|21361625
Database: refseq_human(refseq_human_20140103.fasta)
Database Date: 2015-11-30

Score: 24.0
MW [kDa]: 104.0
pI: 6.5
Sequence Coverage [%]: 4.0
No. of unique Peptides: 2

Alias proteins:

Accession	Name	Description
gi|578811797	refseq_human (refseq_human_20140103.fasta)	PREDICTED: exocyst complex component 2 isoform X2 [Homo sapiens]
gi|578811795	refseq_human (refseq_human_20140103.fasta)	PREDICTED: exocyst complex component 2 isoform X1 [Homo sapiens]

10	20	30	40	50	60	70	80
MSRSRQPPLV	TGISPNEGIP	WTKVTIRGEN	LGTGPTDLIG	LTICGHNCLL	TAEWMSASKI	VCRVGQAKND	KGDIIVTTKS
**90**	**100**	**110**	**120**	**130**	**140**	**150**	**160**
GGRGTSTVSF	KLLKPEKIGI	LDQSAVWVDE	MNYYDMRTDR	NKGIPPLSLR	PANPLGIEIE	KSKFSQKDLE	MLFHGMSADF
**170**	**180**	**190**	**200**	**210**	**220**	**230**	**240**
TSENFSAAWY	LIENHSNTSF	EQLKMAVTNL	KRQANKKSEG	SLAYVKGGLS	TFFEAQDALS	AIHQKLEADG	TEKVEGSMTQ
**250**	**260**	**270**	**280**	**290**	**300**	**310**	**320**
KLENVLNRAS	NTADTLFQEV	LGRKDKADST	RNALNVLQRF	KFLFNLPLNI	ERNIQKGDYD	VVINDYEKAK	SLFGKTEVQV
**330**	**340**	**350**	**360**	**370**	**380**	**390**	**400**
FKKYYAEVET	RIEALRELLL	DKLLETPSTL	HDQKRYIRYL	SDLHASGDPA	WQCIGAQHKW	ILQLMHSCKE	GYVKDLKGNP
**410**	**420**	**430**	**440**	**450**	**460**	**470**	**480**
GLHSPMLDLD	NDTRPSVLGH	LSQTASLKRG	SSFQSGRDDT	WRYKTPHRVA	FVEKLTKLVL	SQLPNFWKLW	ISYVNGSLFS
**490**	**500**	**510**	**520**	**530**	**540**	**550**	**560**
ETAEKSGQIE	RSKNVRQRQN	DFKKMIQEVM	HSLVKLTRGA	LLPLSIRDGE	AKQYGGWEVK	CELSGQWLAH	AIQTVRLTHE
**570**	**580**	**590**	**600**	**610**	**620**	**630**	**640**
SLTALEIPND	LLQTIQDLIL	DLRVRCVMAT	LQHTAEEIKR	LAEKEDWIVD	NEGLTSLPCQ	FEQCIVCSLQ	SLKGVLECKP
**650**	**660**	**670**	**680**	**690**	**700**	**710**	**720**
GEASVFQQPK	TQEEVCQLSI	NIMQVFIYCL	EQLSTKPDAD	IDTTHLSVDV	SSPDLFGSIH	EDFSLTSEQR	LLIVLSNCCY
**730**	**740**	**750**	**760**	**770**	**780**	**790**	**800**
LERHTFLNIA	EHFEKHNFQG	IEKITQVSMA	SLKELDQRLF	ENYIELKADP	IVGSLEPGIY	AGYFDWKDCL	PPTGVRNYLK
**810**	**820**	**830**	**840**	**850**	**860**	**870**	**880**
EALVNIIAVH	AEVFTISKEL	VPRVLSKVIE	AVSEELSRLM	QCVSSFSKNG	ALQARLEICA	LRDTVAVYLT	PESKSSFKQA
**890**	**900**	**910**	**920**	**930**			
LEALPQLSSG	ADKKLLEELL	NKFKSSMHLQ	LTCFQAASST	MMKT			

Cmpd.	No. of Cmpds.	m/z meas.	Δ m/z [ppm]	z	Rt [min]	Score	P	Range	Sequence	Modification
1723	1	1011.4144	-64.38	3	51.5	12.9	2	98-122	K.IGILDQSAVWVDEMNYYDMRTDRNK.G	
2856	1	655.3750	-67.75	2	66.1	11.1	1	516-527	K.LTRGALLPLSIR.D	

Detailed Protein Report

Protein 497: biorientation of chromosomes in cell division protein 1-like 1 [Homo sapiens]

**Accession:**	gi|87299628	**Score:**	23.9
**Database:**	refseq_human(refseq_human_20140103.fasta)	**MW [kDa]:**	330.3
**Database Date:**	2015-11-30	**pI:**	4.9
		**Sequence Coverage [%]:**	0.9
		**No. of unique Peptides:**	1

Detailed Protein Report

10	20	30	40	50	60	70	80
MATNPQPQPP	PPAPPPPPPQ	PQPQPPPPPP	GPGAGPGAGG	AGGAGAGAGD	PQLVAMIVNH	LKSQGLFDQF	RRDCLADVDT
90	100	110	120	130	140	150	160
KPAYQNLRQR	VDNFVANHLA	THTWSPHLNK	NQLRNNIRQQ	VLKSGMLESG	IDRIISQVVD	PKINHTFRPQ	VEKAVHEFLA
170	180	190	200	210	220	230	240
TLNHKEEGSG	NTAPDDEKPD	TSLITQGVPT	PGPSANVAND	AMSILETITS	LNQEASAARA	STETSNAKTS	ERASKKLPSQ
250	260	270	280	290	300	310	320
PTTDTSTDKE	RTSEDMADKE	KSTADSGGEG	LETAPKSEEF	SDLPCPVEEI	KNYTKEHNNL	ILLNKDVQQE	SSEQKNKSTD
330	340	350	360	370	380	390	400
KGEKKPDSNE	KGERKKEKKE	KTEKKFDHSK	KSEDTQKVKD	EKQAKEKEVE	SLKLPSEKNS	NKAKTVEGTK	EDFSLIDSDV
410	420	430	440	450	460	470	480
DGLTDITVSS	VHTSDLSSFE	EDTEEEVVTS	DSMEEGEITS	DDEEKNKQNK	TKTQTSDSSE	GKTKSVRHAY	VHKPYLYSKY
490	500	510	520	530	540	550	560
YSDSDDELTV	EQRRQSIAKE	KEERLLRRQI	NREKLEEKRK	QKAEKTKSSK	TKGQGRSSVD	LEESSTKSLE	PKAARIKEVL
570	580	590	600	610	620	630	640
KERKVLEKKV	ALSKKRKKDS	RNVEENSKKK	QQYEEDSKET	LKTSEHCEKE	KISSSKELKH	VHAKSEPSKP	ARRLSESLHV
650	660	670	680	690	700	710	720
VDENKNESKL	EREHKRRTST	PVIMEGVQEE	TDTRDVKRQV	ERSEICTEEP	QKQKSTLKNE	KHLKKDDSET	PHLKSLLKKE
730	740	750	760	770	780	790	800
VKSSKEKPER	EKTPSEDKLS	VKHKYKGDCM	HKTGDETELH	SSEKGLKVEE	NIQKQSQQTK	LSSDDKTERK	SKHRNERKLS
810	820	830	840	850	860	870	880
VLGKDGKPVS	EYIIKTDENV	RKENNKKERR	LSAEKTKAEH	KSRRSSDSKI	QKDSLGSKQH	GITLQRRSES	YSEDKCDMDS
890	900	910	920	930	940	950	960
TNMDSNLKPE	EVVHKEKRRT	KSLLEEKLVL	KSKSKTQGKQ	VKVVETELQE	GATKQATTPK	PDKEKNTEEN	DSEKQRKSKV
970	980	990	1000	1010	1020	1030	1040
EDKPFEETGV	EPVLETASSS	AHSTQKDSSH	RAKLPLAKEK	YKSDKDSTST	RLERKLSDGH	KSRSLKHSSK	DIKKKDENKS
1050	1060	1070	1080	1090	1100	1110	1120
DDKDGKEVDS	SHEKARGNSS	LMEKKLSRRL	CENRRGSLSQ	EMAKGEEKLA	ANTLSTPSGS	SLQRPKKSGD	MTLIPEQEPM
1130	1140	1150	1160	1170	1180	1190	1200
EIDSEPGVEN	VFEVSKTQDN	RNNNSQQDID	SENMKQKTSA	TVQKDELRTC	TADSKATAPA	YKPGRGTGVN	SNSEKHADHR
1210	1220	1230	1240	1250	1260	1270	1280
STLTKKMHIQ	SAVSKMNPGE	KEPIHRGTTE	VNIDSETVHR	MLLSAPSEND	RVQKNLKNTA	AEEHVAQGDA	TLEHSTNLDS
1290	1300	1310	1320	1330	1340	1350	1360
SPSLSSVTVV	PLRESYDPDV	IPLFDKRTVL	EGSTASTSPA	DHSALPNQSL	TVRESEVLKT	SDSKEGGEGF	TVDTPAKASI
1370	1380	1390	1400	1410	1420	1430	1440
TSKRHIPEAH	QATLLDGKQG	KVIMPLGSKL	TGVIVENENI	TKEGGLVDMA	KKENDLNAEP	NLKQTIKATV	ENGKKDGIAV
1450	1460	1470	1480	1490	1500	1510	1520
DHVVGLNTEK	YAETVKLKHK	RSPGKVKDIS	IDVERRNENS	EVDTSAGSGS	APSVLHQRNG	QTEDVATGPR	RAEKTSVATS
1530	1540	1550	1560	1570	1580	1590	1600
TEGKDKDVTL	SPVKAGPATT	TSSETRQSEV	ALPCTSIEAD	EGLIIGTHSR	NNPLHVGAEA	SECTVFAAAE	EGGAVVTEGF
1610	1620	1630	1640	1650	1660	1670	1680
AESETFLTST	KEGESGECAV	AESEDRAADL	LAVHAVKIEA	NVNSVVTEEK	DDAVTSAGSE	EKCDGSLSRD	SEIVEGTITF
1690	1700	1710	1720	1730	1740	1750	1760
ISEVESDGAV	TSAGTEIRAG	SISSEEVDGS	QGNMMRMGPK	KETEGTVTCT	GAEGRSDNFV	ICSVTGAGPR	EERMVTGAGV
1770	1780	1790	1800	1810	1820	1830	1840
VLGDNDAPPG	TSASQEGDGS	VNDGTEGESA	VTSTGITEDG	EGPASCTGSE	DSSEGFAISS	ESEENGESAM	DSTVAKEGTN
1850	1860	1870	1880	1890	1900	1910	1920
VPLVAAGPCD	DEGIVTSTGA	KEEDEEGEDV	VTSTGRGNEI	GHASTCTGLG	EESEGVLICE	SAEGDSQIGT	VVEHVEAEAG
1930	1940	1950	1960	1970	1980	1990	2000
AAIMNANENN	VDSMSGTEKG	SKDTDICSSA	KGIVESSVTS	AVSGKDEVTP	VPGGCEGPMT	SAASDQSDSQ	LEKVEDTTIS
2010	2020	2030	2040	2050	2060	2070	2080
TGLVGGSYDV	LVSGEVPECE	VAHTSPSEKE	DEDIITSVEN	EECDGLMATT	ASGDITNQNS	LAGGKNQGKV	LIISTSTTND
2090	2100	2110	2120	2130	2140	2150	2160
YTPQVSAITD	VEGGLSDALR	TEENMEGTRV	TTEEFEAPMP	SAVSGDDSQL	TASRSEEKDE	CAMISTSIGE	EFELPISSAT
2170	2180	2190	2200	2210	2220	2230	2240

Detailed Protein Report

Cmpd.	No. of Cmpds.	m/z meas.	Δ m/z [ppm]	z	Rt [min]	Score	P	Range	Sequence	Modification
582	1	848.5905	146.58	2	36.6	10.8	2	853-867	K.DSLGSKQHGITLQRR.S	

Detailed Protein Report

Protein 498: PREDICTED: protein SZT2 isoform X3 [Homo sapiens]

**Accession:**	gi|578798759	**Score:**	23.9
**Database:**	refseq_human(refseq_human_20140103.fasta)	**MW [kDa]:**	345.4
**Database Date:**	2015-11-30	**pI:**	6.0
**Modification(s):**	Carbamidomethyl, Oxidation	**Sequence Coverage [%]:**	1.0
		**No. of unique Peptides:**	2

Detailed Protein Report

10	20	30	40	50	60	70	80
MVGGVYSYDC	SFGHVPNVEL	MKFIAMATFG	SYLSTCPEPE	PGNLGLTVYH	RAFLLYSFLR	SGEALNPEYY	CGSQHRLFNE
90	100	110	120	130	140	150	160
HLVSASSNPA	LALRRKKHTE	KEVPADLVST	VSVRLREGYS	VREVTLAKGG	SQLEVKLVLL	WKHNMRIEYV	AMAPWPLEPE
170	180	190	200	210	220	230	240
GPRVTRVEVT	MEGGYDILHD	VSCALRQPIR	SLYRTHVIRR	FWNTLQSINQ	TDQMLAHLQS	FSSVPEHFTL	PDSTKSGVPL
250	260	270	280	290	300	310	320
FYIPPGSTTP	VLSLQPSGSD	SSHAQFAAYW	KPVLSMDANS	WQRWLHMHRL	VLILEHDTPI	PKHLHTPGSN	GRYSTIQCRI
330	340	350	360	370	380	390	400
SHSSLTSLLR	DWSSFVLVEG	YSYVKLLSSA	PDQPPNSFYM	VRIISKAPCM	VLRLGFPIGT	PAPARHKIVS	GLREEILRLR
410	420	430	440	450	460	470	480
FPHRVQSKEP	TPKVKRKGLG	GAGGGSSPSK	SPPVLGPQQA	LSDRPCLVVL	HKPLDKLLIR	YEKLPLDYRA	PFLLTLEPPG
490	500	510	520	530	540	550	560
PLPLVSGRSA	SSSLASLSRY	LYHQRWLWSV	PSGLAPALPL	SAIAQLLSIL	TEVRLSEGFH	FACSGEGIIN	MVLELPIQNE
570	580	590	600	610	620	630	640
PPGQAAAEEK	HTCVVQYILF	PPHSTSTKDS	FSTDDDNDVE	VEALEGDSEL	NLVTEVWVEP	QYGRVGPGPG	IWKHLQDLTY
650	660	670	680	690	700	710	720
SEIPQALHPR	DAACIGSMLS	FEYLIQLCQS	KEWGPLPPEP	RVSDGLDQGG	DTCVHEIPFH	FDLMGLLPQC	QQLQMFFLLL
730	740	750	760	770	780	790	800
AREPEGVPFA	EGSCPANDMV	LCLLHSCLGQ	ELSDREIPLT	PVDQAAFLSE	VLRRTCHVPG	AEGPLLGVHG	IPKEQAVGST
810	820	830	840	850	860	870	880
QATGDSAFTS	LSVGLPETLK	PLISAQPPQW	RCYARLVNPQ	HVFLTFLPAT	FSDVQRLAAC	GLEGPPQEET	KPKFGDWSGA
890	900	910	920	930	940	950	960
PSLKDLGGTG	IKATKSHVPV	LSVTLASDNA	QNQGELSPPF	RRDLQAYAGR	QASQTESADG	PRTRCPVYIY	SCSLEALREQ
970	980	990	1000	1010	1020	1030	1040
MVGMQPPQAP	RDLIFRTQFL	DHPSPSSAWM	EPRYKEAANH	CALLQEHAQR	CYVRGLFRSL	QQAQSVTSQD	LLTAVDACEE
1050	1060	1070	1080	1090	1100	1110	1120
LLQEIDITPF	LLALCGHTWG	LPHAPPSPGP	LSPGPFSSSM	EEGAEPRERA	ILASESSIET	EDLSEPEFQS	TRVPGIPDPG
1130	1140	1150	1160	1170	1180	1190	1200
PEISLTDVCQ	LRGEAHGALH	SVIQEKFLEI	SRLHFRTVPS	NPHYFFYCPP	SSRREDEGPR	DTVDRKISDL	EFSEAELMGE
1210	1220	1230	1240	1250	1260	1270	1280
EGDTSACCVV	TESDPELEVE	YRESRESDLG	PAGLDSASLS	DVDTVNPDED	SFSILGGDSP	TGPESFLHDL	PPLFLHLTCS
1290	1300	1310	1320	1330	1340	1350	1360
VRLRGQHSSV	PVCSLPTCLG	QVLSSLEGPP	VGGRVPLRDL	SVTLDVFMLT	LPLEVELPTA	SDPQHHRSTS	ESSASFPRSP
1370	1380	1390	1400	1410	1420	1430	1440
GQPSSLRSDD	GLGPPLPPPE	EERHPGLSNL	ATPHRLAIET	TMNEIRWLLE	DEMVGALRRG	GIPQSPALHR	AAAHIHSSPG
1450	1460	1470	1480	1490	1500	1510	1520
RSTCLRQTLP	LSFVFGPERS	LTQFKEEFRR	LHLPGHVLLE	DPDSGFFFVA	AGQQPGGSHG	EPSSAAWAWH	SHEDRAEGIE
1530	1540	1550	1560	1570	1580	1590	1600
GETLTASPQA	PGSPEDSEGV	PLISLPRVPQ	GGSQPGPSRG	LSLMSSQGSV	DSDHLGYDGG	SSGSDSEGPN	DTLGEKAPFT
1610	1620	1630	1640	1650	1660	1670	1680
LRTPPGPAPP	QPSLSGLPGP	CLPDFWLIVR	VLQDRVEVYA	HARSLIREDG	GPGTECRHLQ	QLLVRRVGEI	CREVNQRLLL
1690	1700	1710	1720	1730	1740	1750	1760
QDLHDSHVCN	SLLVAESEED	LWRSETPFHS	RQRAPLPSDD	YAADESCAPR	GYLAATMQFV	PGHFSCDVVW	GTVIRVHSRL
1770	1780	1790	1800	1810	1820	1830	1840
KMGPSMGVSR	AIQALRSVLN	AFSVVNRKNM	FVYQERATKA	VYYLRLLETS	CSDRPWKGDA	LPPSLALSRS	QEPIYSEEAS
1850	1860	1870	1880	1890	1900	1910	1920
GPRSPLDMVS	SRSSDAARPV	GQVDRHIQLL	VHGVGQAGPE	ITDELVRVLC	RRLDEATLDV	ITVMLVRNCK	LTPADVEFIQ
1930	1940	1950	1960	1970	1980	1990	2000
PPGSLPSEVL	HLALPTSCRP	WLPALAWYLR	QNLLIFLHSP	KYTDSNSRNH	FQHPLPPQGG	LPDLDIYLYN	KPGGQGTGGK
2010	2020	2030	2040	2050	2060	2070	2080
GVACITLAFV	DEGGAPLSLA	LWPPSSPGPP	DPLREEEFEQ	LTQVIRCPVV	VDSSSAQNGA	PRLRLDVWEK	GNISIVQLEE
2090	2100	2110	2120	2130	2140	2150	2160
KLRGAARQAL	ADAIIELQLL	PASLCTEDTP	TGSLRNGSLE	TKSSAGRAST	FPPAPVPGEP	VTPPSKAGRR	SFWDMLSKTE
2170	2180	2190	2200	2210	2220	2230	2240

Detailed Protein Report

Cmpd.	No. of Cmpds.	m/z meas.	Δ m/z [ppm]	z	Rt [min]	Score	P	Range	Sequence	Modification
2101	1	599.9147	-73.79	3	56.4	12.3	1	2656-2672	R.AMAILGTEGRGSFSCPK.T	Carbamidomethyl: 15; Oxidation: 2
2181	1	952.3242	-131.11	2	57.1	11.6	0	2746-2760	R.DLMHVHSFSYDFHLR.L	

Detailed Protein Report

Protein 499: PREDICTED: terminal uridylyltransferase 4 isoform X5 [Homo sapiens]

**Accession:**	gi|530362240	**Score:**	23.9
**Database:**	refseq_human(refseq_human_20140103.fasta)	**MW [kDa]:**	184.6
**Database Date:**	2015-11-30	**pI:**	9.3
**Modification(s):**	Oxidation	**Sequence Coverage [%]:**	1.8
		**No. of unique Peptides:**	1

10	20	30	40	50	60	70	80
MEESKTLKSE	NHEPKKNVIC	EESKAVQVIG	NQTLKARNDK	SVKEIENSSP	NRNSSKKNKQ	NDICIEKTEV	KSCKVNAANL
**90**	**100**	**110**	**120**	**130**	**140**	**150**	**160**
PGPKDLGLVL	RDQSHCKAKK	FPNSPVKAEK	ATISQAKSEK	ATSLQAKAEK	SPKSPNSVKA	EKASSYQMKS	EKVPSSPAEA
**170**	**180**	**190**	**200**	**210**	**220**	**230**	**240**
EKGPSLLLKD	MRQKTELQQI	GKKIPSSFTS	VDKVNIEAVG	GEKCALQNSP	RSQKQQTCTD	NTGDSDDSAS	GIEDVSDDLS
**250**	**260**	**270**	**280**	**290**	**300**	**310**	**320**
KMKNDESNKE	NSSEMDYLEN	ATVIDESALT	PEQRLGLKQA	EERLERDHIF	RLEKRSPEYT	NCRYLCKLCL	IHIENIQGAH
**330**	**340**	**350**	**360**	**370**	**380**	**390**	**400**
KHIKEKRHKK	NILEKQEESE	LRSLPPPSPA	HLAALSVAVI	ELAKEHGITD	DDLRVRQEIV	EEMSKVITTF	LPECSLRLYG
**410**	**420**	**430**	**440**	**450**	**460**	**470**	**480**
SSLTRFALKS	SDVNIDIKFP	PKMNHPDLLI	KVLGILKKNV	LYVDVESDFH	AKVPVVVCRD	RKSGLLCRVS	AGNDMACLTT
**490**	**500**	**510**	**520**	**530**	**540**	**550**	**560**
DLLTALGKIE	PVFIPLVLAF	RYWAKLCYID	SQTDGGIPSY	CFALMVMFFL	QQRKPPLLPC	LLGSWIEGFD	PKRMDDFQLK
**570**	**580**	**590**	**600**	**610**	**620**	**630**	**640**
GIVEEKFVKW	ECNSSSATEK	NSIAEENKAK	ADQPKDDTKK	TETDNQSNAM	KEKHGKSPLA	LETPNRVSLG	QLWLELLKFY
**650**	**660**	**670**	**680**	**690**	**700**	**710**	**720**
TLDFALEEYV	ICVRIQDILT	RENKNWPKRR	IAIEDPFSVK	RNVARSLNSQ	LVYEYVVERF	RAAYRYFACP	QTKGGNKSTV
**730**	**740**	**750**	**760**	**770**	**780**	**790**	**800**
DFKKREKGKI	SNKKPVKSNN	MATNGCILLG	ETTEKINAER	EQPVQCDEMD	CTSQRCIIDN	NNLLVNELDF	ADHGQDSSSL
**810**	**820**	**830**	**840**	**850**	**860**	**870**	**880**
STSKSSEIEP	KLDKKQDDLA	PSETCLKKEL	SQCNCIDLSK	SPDPDKSTGT	DCRSNLETES	SHQSVCTDTS	ATSCNCKATE
**890**	**900**	**910**	**920**	**930**	**940**	**950**	**960**
DASDLNDDDN	LPTQELYYVF	DKFILTSGKP	PTIVCSICKK	DGHSKNDCPE	DFRKIDLKPL	PPMTNRFREI	LDLVCKRCFD
**970**	**980**	**990**	**1000**	**1010**	**1020**	**1030**	**1040**
ELSPPCSEQH	NREQILIGLE	KFIQKEYDEK	ARLCLFGSSK	NGFGFRDSDL	DICMTLEGHE	NAEKLNCKEI	IENLAKILKR
**1050**	**1060**	**1070**	**1080**	**1090**	**1100**	**1110**	**1120**
HPGLRNILPI	TTAKVPIVKF	EHRRSGLEGD	ISLYNTLAQH	NTRMLATYAA	IDPRVQYLGY	TMKVFAKRCD	IGDASRGSLS
**1130**	**1140**	**1150**	**1160**	**1170**	**1180**	**1190**	**1200**
SYAYILMVLY	FLQQRKPPVI	PVLQEIFDGK	QIPQRMVDGW	NAFFFDKTEE	LKKRLPSLGK	NTESLGELWL	GLLRFYTEEF
**1210**	**1220**	**1230**	**1240**	**1250**	**1260**	**1270**	**1280**
DFKEYVISIR	QKKLLTTFEK	QWTSKCIAIE	DPFDLNHNLG	AGVSRKMTNF	IMKAFINGRK	LFGTPFYPLI	GREAEYFFDS
**1290**	**1300**	**1310**	**1320**	**1330**	**1340**	**1350**	**1360**
RVLTDGELAP	NDRCCRVCGK	IGHYMKDCPK	RKRLKKKDSE	EEKEGNEEEK	DSRDVLDPRD	LHDTRDFRDP	RDLRCFICGD
**1370**	**1380**	**1390**	**1400**	**1410**	**1420**	**1430**	**1440**
AGHVRRECPE	VKLARQRNSS	VAAAQLVRNL	VNAQQVAGSA	QQQGDQSIRT	RQSSECSESP	SYSPQPQPFP	QNSSQSAAIT
**1450**	**1460**	**1470**	**1480**	**1490**	**1500**	**1510**	**1520**
QPSSQPGSQP	KLGPPQQGAQ	PPHQVQMPLY	NFPQSPPAQY	SPMHNMGLLP	MHPLQIPAPS	WPIHGPVIHS	APGSAPSNIG
**1530**	**1540**	**1550**	**1560**	**1570**	**1580**	**1590**	**1600**
LNDPSIIFAQ	PAARPVAIPN	TSHDGHWPRT	VAPNSLVNSG	AVGNSEPGFR	GLTPPIPWEH	APRPHFPLVP	ASWPYGLHQN
**1610**	**1620**	**1630**	**1640**	**1650**			
FMHQGNARFQ	PNKPFYTQDR	CATRRCRERC	PHPPRGNVSE				

Cmpd.	No. of Cmpds.	m/z meas.	Δ m/z [ppm]	z	Rt [min]	Score	P	Range	Sequence	Modification
1985	4	862.2984	-161.67	2	54.7	12.6	2	553-566	K.RMDDFQLKGIVEEK.F	Oxidation: 2

Detailed Protein Report

Protein 500: **PREDICTED: uncharacterized protein C3orf17 isoform X4 [Homo sapiens]**

Accession: gi|530374250
Database: refseq_human(refseq_human_20140103.fasta)
Database Date: 2015-11-30
Modification(s): Carbamidomethyl

Score: 23.9
MW [kDa]: 49.6
pI: 10.7
Sequence Coverage [%]: 4.6
No. of unique Peptides: 1

10	20	30	40	50	60	70	80
MKVLGACKLL	LRLLDCCCKT	FLLTVKHLGL	QEFIILNLVM	VGLVSRLWVL	YKGVLKRLIL	LYEPLFGLLQ	EVARIQPMPY
**90**	**100**	**110**	**120**	**130**	**140**	**150**	**160**
FKDFTFPSDI	TEFLGQPYFE	AFKKKMPIAF	AAKGINKLLN	KLFLINEQSP	RASEETLLGI	SKKAKQMKIN	VQNNVDLGQP
**170**	**180**	**190**	**200**	**210**	**220**	**230**	**240**
VKNKRVFKEE	SSEFDVRAFC	NQLKHKATQE	TSFDFKCSQS	RLKTTKYSSQ	KVIGTPHAKS	FVQRFREAES	FTQLSEEIQM
**250**	**260**	**270**	**280**	**290**	**300**	**310**	**320**
AVVWCRSKKL	KAQAIFLGNK	LLKSNRLKHL	EAQGTSLPKK	LECIKTSICN	HLLRGSGIKT	SKHHLRQRRS	QNKFLRRQRK
**330**	**340**	**350**	**360**	**370**	**380**	**390**	**400**
PQRKLQSTLL	REIQQFSQGT	RKSATDTSAK	WRLSHCTVHR	TDLYPNSKQL	LNSGVSMPVI	QTKEKMIHEN	LRGIHENETD
**410**	**420**	**430**	**440**				
SWTVMQINKN	STSGTIKETD	DIDDIFALMG	V				

Cmpd.	No. of Cmpds.	m/z meas.	Δ m/z [ppm]	z	Rt [min]	Score	P	Range	Sequence	Modification
2818	1	732.4805	137.02	2	65.7	10.2	1	9-19	K.LLLRLLDCCCK.T	Carbamidomethyl: 8, 9, 10

Detailed Protein Report

Protein 501: cullin-7 isoform 2 [Homo sapiens]

**Accession:**	gi|41872646	**Score:**	23.9
**Database:**	refseq_human(refseq_human_20140103.fasta)	**MW [kDa]:**	191.0
**Database Date:**	2015-11-30	**pI:**	5.5
**Modification(s):**	Carbamidomethyl	**Sequence Coverage [%]:**	1.6
		**No. of unique Peptides:**	1

10	20	30	40	50	60	70	80
MVGELRYREF	RVPLGPGLHA	YPDELIRQRV	GHDGHPEYQI	RWLILRRGDE	GDGGSGQVDC	KAEHILLWMS	KDEIYANCHK
90	100	110	120	130	140	150	160
MLGEDGQVIG	PSQESAGEVG	ALDKSVLEEM	ETDVKSLIQR	ALRQLEECVG	TIPPAPLLHT	VHVLSAYASI	EPLTGVFKDP
170	180	190	200	210	220	230	240
RVLDLLMHML	SSPDYQIRWS	AGRMIQALSS	HDAGTRTQIL	LSLSQQEAIE	KHLDFDSRCA	LLALFAQATL	SEHPMSFEGI
250	260	270	280	290	300	310	320
QLPQVPGRVL	FSLVKRYLHV	TSLLDQLNDS	AAEPGAQNTS	APEELSGERG	QLELEFSMAM	GTLISELVQA	MRWDQASDRP
330	340	350	360	370	380	390	400
RSSARSPGSI	FQPQLADVSP	GLPAAQAQPS	FRRSRRFRPR	SEFASGNTYA	LYVRDTLQPG	MRVRMLDDYE	EISAGDEGEF
410	420	430	440	450	460	470	480
RQSNNGVPPV	QVFWESTGRT	YWVHWHMLEI	LGFEEDIEDM	VEADEYQGAV	ASRVLGRALP	AWRWRPMTEL	YAVPYVLPED
490	500	510	520	530	540	550	560
EDTEECEHLT	LAEWWELLFF	IKKLDGPDHQ	EVLQILQENL	DGEILDDEIL	AELAVPIELA	QDLLLTLPQR	LNDSALRDLI
570	580	590	600	610	620	630	640
NCHVYKKYGP	EALAGNQAYP	SLLEAQEDVL	LLDAQAQAKD	SEDAAKVEAK	EPPSQSPNTP	LQRLVEGYGP	AGKILLDLEQ
650	660	670	680	690	700	710	720
ALSSEGTQEN	KVKPLLLQLQ	RQPQPFLALM	QSLDTPETNR	TLHLTVLRIL	KQLVDFPEAL	LLPWHEAVDA	CMACLRSPNT
730	740	750	760	770	780	790	800
DREVLQELIF	FLHRLTSVSR	DYAVVLNQLG	ARDAISKALE	KHLGKLELAQ	ELRDMVFKCE	KHAHLYRKLI	TNILGGCIQM
810	820	830	840	850	860	870	880
VLGQIEDHRR	THQPINIPFF	DVFLRYLCQG	SSVEVKEDKC	WEKVEVSSNP	HRASKLTDHN	PKTYWESNGS	AGSHYITLHM
890	900	910	920	930	940	950	960
RRGILIRQLT	LLVASEDSSY	MPARVVVCGG	DSTSSLHTEL	NSVNVMPSAS	RVILLENLTR	FWPIIQIRIK	RCQQGGIDTR
970	980	990	1000	1010	1020	1030	1040
IRGLEILGPK	PTFWPVFREQ	LCRHTRLFYM	VRAQAWSQDM	AEDRRSLLHL	SSRLNGALRQ	EQNFADRFLP	DDEAAQALGK
1050	1060	1070	1080	1090	1100	1110	1120
TCWEALVSPV	VQNITSPDED	GISPLGWLLD	QYLECQEAVF	NPQSRGPAFF	SRVRRLTHLL	VHVEPCEAPP	PVVATPRPKG
1130	1140	1150	1160	1170	1180	1190	1200
RNRSHDWSSL	ATRGLPSSIM	RNLTRCWRAV	VEKQVNNFLT	SSWRDDDFVP	RYCEHFNILQ	NSSSELFGPR	AAFLLALQNG
1210	1220	1230	1240	1250	1260	1270	1280
CAGALLKLPF	LKAAHVSEQF	ARHIDQQIQG	SRIGGAQEME	RLAQLQQCLQ	AVLIFSGLEI	ATTFEHYYQH	YMADRLLGVV
1290	1300	1310	1320	1330	1340	1350	1360
SSWLEGAVLE	QIGPCFPNRL	PQQMLQSLST	SKELQRQFHV	YQLQQLDQEL	LKLEDTEKKI	QVGLGASGKE	HKSEKEEEAG
1370	1380	1390	1400	1410	1420	1430	1440
AAAVVDVAEG	EEEEEENEDL	YYEGAMPEVS	VLVLSRHSWP	VASICHTLNP	RTCLPSYLRG	TLNRYSNFYN	KSQSHPALER
1450	1460	1470	1480	1490	1500	1510	1520
GSQRRLQWTW	LGWAELQFGN	QTLHVSTVQM	WLLLYLNDLK	AVSVESLLAF	SGLSADMLNQ	AIGPLTSSRG	PLDLHEQKDI
1530	1540	1550	1560	1570	1580	1590	1600
PGGVLKIRDG	SKEPRSRWDI	VRLIPPQTYL	QAEGEDGQNL	EKRRNLLNCL	IVRILKAHGD	EGLHIDQLVC	LVLEAWQKGP
1610	1620	1630	1640	1650	1660	1670	1680
CPPRGLVSSL	GKGSACSSTD	VLSCILHLLG	KGTLRRHDDR	PQVLSYAVPV	TVMEPHTESL	NPGSSGPNPP	LTFHTLQIRS
1690	1700						
RGVPYASCTA	TQSFSTFR						

Cmpd.	No. of Cmpds.	m/z meas.	Δ m/z [ppm]	z	Rt [min]	Score	P	Range	Sequence	Modification
1825	1	768.7766	-57.45	2	52.8	12.4	1	47-61	R.RGDEGDGGSGQVDCK.A	Carbamidomethyl: 14

Detailed Protein Report

Protein 502: pleckstrin homology domain-containing family A member 4 isoform 2 [Homo sapiens]

**Accession:**	gi|238859653	**Score:**	23.8
**Database:**	refseq_human(refseq_human_20140103.fasta)	**MW [kDa]:**	64.2
**Database Date:**	2015-11-30	**pI:**	10.7
		**Sequence Coverage [%]:**	3.1
		**No. of unique Peptides:**	1

10	20	30	40	50	60	70	80
MEGSRPRSSL	SLASSASTIS	SLSSLSPKKP	TRAVNKIHAF	GKRGNALRRD	PNLPVHIRGW	LHKQDSSGLR	LWKRRWFVLS
**90**	**100**	**110**	**120**	**130**	**140**	**150**	**160**
GHCLFYYKDS	REESVLGSVL	LPSYNIRPDG	PGAPRGRRFT	FTAEHPGMRT	YVLAADTLED	LRGWLRALGR	ASRAEGDDYG
**170**	**180**	**190**	**200**	**210**	**220**	**230**	**240**
QPRSPARPQP	GEGPGGPGGP	PEVSRGEEGR	ISESPEVTRL	SRGRGRPRLL	TPSPTTDLHS	GLQMRRARSP	DLFTPLSRPP
**250**	**260**	**270**	**280**	**290**	**300**	**310**	**320**
SPLSLPRPRS	APARRPPAPS	GDTAPPARPH	TPLSRIDVRP	PLDWGPQRQT	LSRPPTPRRG	PPSEAGGGKP	PRSPQHWSQE
**330**	**340**	**350**	**360**	**370**	**380**	**390**	**400**
PRTQPGPPLE	STFHQSLETD	TLLTKLCGQD	RLLRRLQEEI	DQKQEEKEQL	EAALELTRQQ	LGQATREAGA	PGRAWGRQRL
**410**	**420**	**430**	**440**	**450**	**460**	**470**	**480**
LQDRLVSVRA	TLCHLTQERE	RVWDTYSGLE	QELGTLRETL	EYLLHLGSPQ	DRVSAQQQLW	MVEDTLAGLG	GPQKPPPHTE
**490**	**500**	**510**	**520**	**530**	**540**	**550**	**560**
PDSPSPVLQG	EESSERESLP	ESLELSSPRS	PETDWGRPPG	GDKDLASPHL	GLGSPRVSRA	SSPEGRHLPS	PQLGTKSKEH
**570**	**580**	**590**					
HPLLADFRRS	PGAGSQPLPS	PGY					

Cmpd.	No. of Cmpds.	m/z meas.	Δ m/z [ppm]	z	Rt [min]	Score	P	Range	Sequence	Modification
411	1	603.6782	-226.42	2	34.8	12.5	0	300-312	R.GPPSEAGGGKPPR.S	

Detailed Protein Report

Protein 503: **FYVE, RhoGEF and PH domain-containing protein 4 [Homo sapiens]**

**Accession:**	gi|198041928	**Score:**	23.8
**Database:**	refseq_human(refseq_human_20140103.fasta)	**MW [kDa]:**	86.6
**Database Date:**	2015-11-30	**pI:**	5.7
		**Sequence Coverage [%]:**	2.7
		**No. of unique Peptides:**	1

10	20	30	40	50	60	70	80
MEEIKPASAS	CVSKEKPSKV	SDLISRFEGG	SSLSNYSDLK	KESAVNLNAP	RTPGRHGLTT	TPQQKLLSQH	LPQRQGNDTD
**90**	**100**	**110**	**120**	**130**	**140**	**150**	**160**
KTQGAQTCVA	NGVMAAQNQM	ECEEEKAATL	SSDTSIQASE	PLLDTHIVNG	ERDETATAPA	SPTTDSCDGN	ASDSSYRTPG
**170**	**180**	**190**	**200**	**210**	**220**	**230**	**240**
IGPVLPLEER	GAETETKVQE	RENGESPLEL	EQLDQHHEMK	ETNEQKLHKI	ANELLLTERA	YVNRLDLLDQ	VFYCKLLEEA
**250**	**260**	**270**	**280**	**290**	**300**	**310**	**320**
NRGSFPAEMV	NKIFSNISSI	NAFHSKFLLP	ELEKRMQEWE	TTPRIGDILQ	KLAPFLKMYG	EYVKGFDNAM	ELVKNMTERI
**330**	**340**	**350**	**360**	**370**	**380**	**390**	**400**
PQFKSVVEEI	QKQKICGSLT	LQHHMLEPVQ	RIPRYEMLLK	DYLRKLPPDS	LDWNDAKKSL	EIISTAASHS	NSAIRKMENL
**410**	**420**	**430**	**440**	**450**	**460**	**470**	**480**
KKLLEIYEML	GEEEDIVNPS	NELIKEGQIL	KLAARNTSAQ	ERYLFLFNNM	LLYCVPKFSL	VGSKFTVRTR	VGIDGMKIVE
**490**	**500**	**510**	**520**	**530**	**540**	**550**	**560**
TQNEEYPHTF	QVSGKERTLE	LQASSAQDKE	EWIKALQETI	DAFHQRHETF	RNAIAKDNDI	HSEVSTAELG	KRAPRWIRDN
**570**	**580**	**590**	**600**	**610**	**620**	**630**	**640**
EVTMCMKCKE	PFNALTRRRH	HCRACGYVVC	WKCSDYKAQL	EYDGGKLSKV	CKDCYQIISG	FTDSEEKKRK	GILEIESAEV
**650**	**660**	**670**	**680**	**690**	**700**	**710**	**720**
SGNSVVCSFL	QYMEKSKPWQ	KAWCVIPKQD	PLVLYMYGAP	QDVRAQATIP	LLGYVVDEMP	RSADLPHSFK	LTQSKSVHSF
**730**	**740**	**750**	**760**	**770**			
AADSEELKQK	WLKVILLAVT	GETPGGPNEH	PATLDDHPEP	KKKSEC			

Cmpd.	No. of Cmpds.	m/z meas.	Δ m/z [ppm]	z	Rt [min]	Score	P	Range	Sequence	Modification
889	1	540.2259	-68.97	2	40.6	13.0	0	243-252	R.GSFPAEMVNK.I	

Detailed Protein Report

Protein 504: alpha-amylase 1 precursor [Homo sapiens]

Accession: gi|40254482
Database: refseq_human(refseq_human_20140103.fasta)
Database Date: 2015-11-30

Score: 23.8
MW [kDa]: 57.7
pI: 6.5
Sequence Coverage [%]: 6.8
No. of unique Peptides: 1

Alias proteins:

Accession	Name	Description
gi|578798954	refseq_human (refseq_human_20140103.fasta)	PREDICTED: alpha-amylase 1 isoform X3 [Homo sapiens]
gi|578798952	refseq_human (refseq_human_20140103.fasta)	PREDICTED: alpha-amylase 1 isoform X3 [Homo sapiens]
gi|578798950	refseq_human (refseq_human_20140103.fasta)	PREDICTED: alpha-amylase 1 isoform X2 [Homo sapiens]
gi|530362403	refseq_human (refseq_human_20140103.fasta)	PREDICTED: alpha-amylase 1 isoform X2 [Homo sapiens]
gi|530362397	refseq_human (refseq_human_20140103.fasta)	PREDICTED: alpha-amylase 1 isoform X2 [Homo sapiens]
gi|530362391	refseq_human (refseq_human_20140103.fasta)	PREDICTED: alpha-amylase 1 isoform X1 [Homo sapiens]
gi|56549664	refseq_human (refseq_human_20140103.fasta)	alpha-amylase 1 precursor [Homo sapiens]
gi|56549662	refseq_human (refseq_human_20140103.fasta)	alpha-amylase 1 precursor [Homo sapiens]
gi|56549660	refseq_human (refseq_human_20140103.fasta)	alpha-amylase 1 precursor [Homo sapiens]

10	20	30	40	50	60	70	80
MKLFWLLFTI	GFCWAQYSSN	TQQGRTSIVH	LFEWRWVDIA	LECERYLAPK	GFGGVQVSPP	NENVAIHNPF	RPWWERYQPV
**90**	**100**	**110**	**120**	**130**	**140**	**150**	**160**
SYKLCTRSGN	EDEFRNMVTR	CNNVGVRIYV	DAVINHMCGN	AVSAGTSSTC	GSYFNPGSRD	FPAVPYSGWD	FNDGKCKTGS
**170**	**180**	**190**	**200**	**210**	**220**	**230**	**240**
GDIENYNDAT	QVRDCRLSGL	LDLALGKDYV	RSKIAEYMNH	LIDIGVAGFR	IDASKHMWPG	DIKAILDKLH	NLNSNWFPEG
**250**	**260**	**270**	**280**	**290**	**300**	**310**	**320**
SKPFIYQEVI	DLGGEPIKSS	DYFGNGRVTE	FKYGAKLGTV	IRKWNGEKMS	YLKNWGEGWG	FMPSDRALVF	VDNHDNQRGH
**330**	**340**	**350**	**360**	**370**	**380**	**390**	**400**
GAGGASILTF	WDARLYKMAV	GFMLAHPYGF	TRVMSSYRWP	RYFENGKDVN	DWVGPPNDNG	VTKEVTINPD	TTCGNDWVCE
**410**	**420**	**430**	**440**	**450**	**460**	**470**	**480**
HRWRQIRNMV	NFRNVVDGQP	FTNWYDNGSN	QVAFGRGNRG	FIVFNNDDWT	FSLTLQTGLP	AGTYCDVISG	DKINGNCTGI
**490**	**500**	**510**	**520**				
KIYVSDDGKA	HFSISNSAED	PFIAIHAESK	L				

Cmpd.	No. of Cmpds.	m/z meas.	Δ m/z [ppm]	z	Rt [min]	Score	P	Range	Sequence	Modification
1297	1	863.8565	-56.34	2	45.8	11.6	0	368-383	K.DVNDWVGPPNDNGVTK.E	

Detailed Protein Report

Protein 505: ataxin-1 [Homo sapiens]

Accession: gi|51479158
Database: refseq_human(refseq_human_20140103.fasta)
Database Date: 2015-11-30

Score: 23.7
MW [kDa]: 86.9
pI: 9.2
Sequence Coverage [%]: 3.1
No. of unique Peptides: 2

Quantitation

MD:MU **Median:** 1.18 **CV:** 0.00 % **No. of Peptides:** 1

Alias proteins:

Accession	Name	Description
gi|189491748	refseq_human (refseq_human_20140103.fasta)	ataxin-1 [Homo sapiens]

10	20	30	40	50	60	70	80
MKSNQERSNE	CLPPKKREIP	ATSRSSEEKA	PTLPSDNHRV	EGTAWLPGNP	GGRGHGGGRH	GPAGTSVELG	LQQGIGLHKA
**90**	**100**	**110**	**120**	**130**	**140**	**150**	**160**
LSTGLDYSPP	SAPRSVPVAT	TLPAAYATPQ	PGTPVSPVQY	AHLPHTFQFI	GSSQYSGTYA	SFIPSQLIPP	TANPVTSAVA
**170**	**180**	**190**	**200**	**210**	**220**	**230**	**240**
SAAGATTPSQ	RSQLEAYSTL	LANMGSLSQT	PGHKAEQQQQ	QQQQQQQQHQ	HQQQQQQQQQ	QQQQQHLSRA	PGLITPGSPP
**250**	**260**	**270**	**280**	**290**	**300**	**310**	**320**
PAQQNQYVHI	SSSPQNTGRT	ASPPAIPVHL	HPHQTMIPHT	LTLGPPSQVV	MQYADSGSHF	VPREATKKAE	SSRLQQAIQA
**330**	**340**	**350**	**360**	**370**	**380**	**390**	**400**
KEVLNGEMEK	SRRYGAPSSA	DLGLGKAGGK	SVPHPYESRH	VVVHPSPSDY	SSRDPSGVRA	SVMVLPNSNT	PAADLEVQQA
**410**	**420**	**430**	**440**	**450**	**460**	**470**	**480**
THREASPSTL	NDKSGLHLGK	PGHRSYALSP	HTVIQTTHSA	SEPLPVGLPA	TAFYAGTQPP	VIGYLSGQQQ	AITYAGSLPQ
**490**	**500**	**510**	**520**	**530**	**540**	**550**	**560**
HLVIPGTQPL	LIPVGSTDME	ASGAAPAIVT	SSPQFAAVPH	TFVTTALPKS	ENFNPEALVT	QAAYPAMVQA	QIHLPVVQSV
**570**	**580**	**590**	**600**	**610**	**620**	**630**	**640**
ASPAAAPPTL	PPYFMKGSII	QLANGELKKV	EDLKTEDFIQ	SAEISNDLKI	DSSTVERIED	SHSPGVAVIQ	FAVGEHRAQV
**650**	**660**	**670**	**680**	**690**	**700**	**710**	**720**
SVEVLVEYPF	FVFGQGWSSC	CPERTSQLFD	LPCSKLSVGD	VCISLTLKNL	KNGSVKKGQP	VDPASVLLKH	SKADGLAGSR
**730**	**740**	**750**	**760**	**770**	**780**	**790**	**800**
HRYAEQENGI	NQGSAQMLSE	NGELKFPEKM	GLPAAPFLTK	IEPSKPAATR	KRRWSAPESR	KLEKSEDEPP	LTLPKPSLIP
**810**	**820**						
QEVKICIEGR	SNVGK						

Cmpd.	No. of Cmpds.	m/z meas.	Δ m/z [ppm]	z	Rt [min]	Score	P	Range	Sequence	Modification	Ratios
1037	3	612.4575	170.02	2	42.5	13.3	0	698-709	K.GQPVDPASVLLK.H		
1970	1	696.2657	-144.31	2	54.5	10.5	2	710-722	K.HSKADGLAGSRHR.Y		MD:MU 1.18

Detailed Protein Report

Protein 506: **PREDICTED: histone acetyltransferase KAT6A isoform X1 [Homo sapiens]**

**Accession:**	gi|530388073	**Score:**	23.7
**Database:**	refseq_human(refseq_human_20140103.fasta)	**MW [kDa]:**	224.1
**Database Date:**	2015-11-30	**pI:**	5.4
**Modification(s):**	Carbamidomethyl	**Sequence Coverage [%]:**	0.9
		**No. of unique Peptides:**	1

10	20	30	40	50	60	70	80
MVKLANPLYT	EWILEAIKKV	KKQKQRPSEE	RICNAVSSSH	GLDRKTVLEQ	LELSVKDGTI	LKVSNKGLNS	YKDPDNPGRI
90	100	110	120	130	140	150	160
ALPKPRNHGK	LDNKQNVDWN	KLIKRAVEGL	AESGGSTLKS	IERFLKGQKD	VSALFGGSAA	SGFHQQLRLA	IKRAIGHGRL
170	180	190	200	210	220	230	240
LKDGPLYRLN	TKATNVDGKE	SCESLSCLPP	VSLLPHEKDK	PVAEPIPICS	FCLGTKEQNR	EKKPEELISC	ADCGNSGHPS
250	260	270	280	290	300	310	320
CLKFSPELTV	RVKALRWQCI	ECKTCSSCRD	QGKNADNMLF	CDSCDRGFHM	ECCDPPLTRM	PKGMWICQIC	RPRKKGRKLL
330	340	350	360	370	380	390	400
QKKAAQIKRR	YTNPIGRPKN	RSKGPFSKVR	TGPGRGRKRK	ITLSSQSASS	SSEEGYLERI	DGLDFCRDSN	VSLKFNKKTK
410	420	430	440	450	460	470	480
GLIDGLTKFF	TPSPDGRKAR	GEVVDYSEQY	RIRKRGNRKS	STSDWPTDNQ	DGWDGKQENE	ERLFGSQEIM	TEKDMELFRD
490	500	510	520	530	540	550	560
IQEQALQKVG	VTGPPDPQVR	CPSVIEFGKY	EIHTWYSSPY	PQEYSRLPKL	YLCEFCLKYM	KSRTILQQHM	KKCGWFHPPA
570	580	590	600	610	620	630	640
NEIYRKNNIS	VFEVDGNVST	IYCQNLCLLA	KLFLDHKTLY	YDVEPFLFYV	LTQNDVKGCH	LVGYFSKEKH	CQQKYNVSCI
650	660	670	680	690	700	710	720
MILPQYQRKG	YGRFLIDFSY	LLSKREGQAG	SPEKPLSDLG	RLSYMAYWKS	VILECLYHQN	DKQISIKKLS	KLTGICPQDI
730	740	750	760	770	780	790	800
TSTLHHLRML	DFRSDQFVII	RREKLIQDHM	AKLQLNLRPV	DVDPECLRWT	PVIVSNSVVS	EEEEEEAEEG	ENEEPQCQER
810	820	830	840	850	860	870	880
ELEISVGKSV	SHENKEQDSY	SVESEKKPEV	MAPVSSTRLS	KQVLPHDSLP	ANSQPSRRGR	WGRKNRKTQE	RFGDKDSKLL
890	900	910	920	930	940	950	960
LEETSSAPQE	QYGECGEKSE	ATQEQYTESE	EQLVASEEQP	SQDGKPDLPK	RRLSEGVEPW	RGQLKKSPEA	LKCRLTEGSE
970	980	990	1000	1010	1020	1030	1040
RLPRRYSEGD	RAVLRGFSES	SEEEEEPESP	RSSSPPILTK	PTLKRKKPFL	HRRRRVRKRK	HHNSSVVTET	ISETTEVLDE
1050	1060	1070	1080	1090	1100	1110	1120
PFEDSDSERP	MPRLEPTFEI	DEEEEEEDEN	ELFPREYFRR	LSSQDVLRCQ	SSSKRKSKDE	EEDEESDDAD	DTPILKPVSL
1130	1140	1150	1160	1170	1180	1190	1200
LRKRDVKNSP	LEPDTSTPLK	KKKGWPKGKS	RKPIHWKKRP	GRKPGFKLSR	EIMPVSTQAC	VIEPIVSIPK	AGRKPKIQES
1210	1220	1230	1240	1250	1260	1270	1280
EETVEPKEDM	PLPEERKEEE	EMQAEAEEAE	EGEEEDAASS	EVPAASPADS	SNSPETETKE	PEVEEEEEKP	RVSEEQRQSE
1290	1300	1310	1320	1330	1340	1350	1360
EEQQELEEPE	PEEEEDAAAE	TAQNDDHDAD	DEDDGHLEST	KKKELEEQPT	REDVKEEPGV	QESFLDANMQ	KSREKIKDKE
1370	1380	1390	1400	1410	1420	1430	1440
ETELDSEEEQ	PSHDTSVVSE	QMAGSEDDHE	EDSHTKEELI	ELKEEEEIPH	SELDLETVQA	VQSLTQEESS	EHEGAYQDCE
1450	1460	1470	1480	1490	1500	1510	1520
ETLAACQTLQ	SYTQADEDPQ	MSMVEDCHAS	EHNSPISSVQ	SHPSQSVRSV	SSPNVPALES	GYTQISPEQG	SLSAPSMQNM
1530	1540	1550	1560	1570	1580	1590	1600
ETSPMMDVPS	VSDHSQQVVD	SGFSDLGSIE	STTENYENPS	SYDSTMGGSI	CGNSSSQSSC	SYGGLSSSSS	LTQSSCVVTQ
1610	1620	1630	1640	1650	1660	1670	1680
QMASMGSSCS	MMQQSSVQPA	ANCSIKSPQS	CVVERPPSNQ	QQQPPPPPPQ	QPQPPPPQPQ	PAPQPPPPQQ	QPQQQPQPQP
1690	1700	1710	1720	1730	1740	1750	1760
QQPPPPPPPQ	QQPPLSQCSM	NNSFTPAPMI	MEIPESGSTG	NISIYERIPG	DFGAGSYSQP	SATFSLAKLQ	QLTNTIMDPH
1770	1780	1790	1800	1810	1820	1830	1840
AMPYSHSPAV	TSYATSVSLS	NTGLAQLAPS	HPLAGTPQAQ	ATMTPPPNLA	STTMNLTSPL	LQCNMSATNI	GIPHTQRLQG
1850	1860	1870	1880	1890	1900	1910	1920
QMPVKGHISI	RSKSAPLPSA	AAHQQQLYGR	SPSAVAMQAG	PRALAVQRGM	NMGVNLMPTP	AYNVNSMNMN	TLNAMNSYRM
1930	1940	1950	1960	1970	1980	1990	2000
TQPMMNSSYH	SNPAYMNQTA	QYPMQMQMGM	MGSQAYTQQP	MQPNPHGNMM	YTGPSHHSYM	NAAGVPKQSL	NGPYMRR

Detailed Protein Report

Cmpd.	No. of Cmpds.	m/z meas.	Δ m/z [ppm]	z	Rt [min]	Score	P	Range	Sequence	Modification
1722	1	1044.9651	24.46	2	51.3	23.7	2	257-273	R.WQCIECKTCSSCRDQGK.N	Carbamidomethyl: 3, 6

Detailed Protein Report

Protein 507: PREDICTED: sperm-associated antigen 17 isoform X5 [Homo sapiens]

**Accession:**	gi|578798575	**Score:**	23.7
**Database:**	refseq_human(refseq_human_20140103.fasta)	**MW [kDa]:**	149.1
**Database Date:**	2015-11-30	**pI:**	5.6
**Modification(s):**	Oxidation	**Sequence Coverage [%]:**	2.8
		**No. of unique Peptides:**	1

10	20	30	40	50	60	70	80
MAPKKEKGGT	VNTSSKIWEP	SLIAAQFNQN	DWQASIAFVV	GNQIEDDLLI	QALTVAVQVP	QRKLFSMVSW	QDILQQINEI
90	100	110	120	130	140	150	160
NTLVGSASSK	KAKKPVGGNA	PLYYEVLTAA	KAIMDSGEKL	TLPLIGKLLK	FQLLQIKFKD	QQRRENEKKV	IEDKPKLEKD
170	180	190	200	210	220	230	240
KGKAKSPKEK	KAPSAKPAKG	KGKDQPEANA	PVKKTTQLKR	RGEDDHTNRY	IDDEPDDGAQ	HYIIVVGFNN	PQLLAIMAEL
250	260	270	280	290	300	310	320
GIPITSVIKI	SSENYEPLQT	HLAAVNQQQE	VLLQSEDLEA	EKLKKENAIK	ELKTFWKYLE	PVLNNEKPET	NLFDVARLEY
330	340	350	360	370	380	390	400
MVKAADFPSD	WSDGEMMLKL	GTDIFENIAC	LMYDILDWKR	QHQHYLESMQ	LINVPQVVNE	KPVLEAMPTS	EAPQPAVPAP
410	420	430	440	450	460	470	480
GKKKAQYEEP	QAPPPVTSVI	TTEVDMRYYN	YLLNPIREEF	ISVPLILHCM	LEQVVATEED	LVPPSLREPS	PRADGLDHRI
490	500	510	520	530	540	550	560
AAHIVSLLPS	LCLSEREKKN	LHDIFLSEEE	NESKAVPKGP	LLLNYHDAHA	HKKYALQDQK	NFDPVQIEQE	MQSKLPLWEF
570	580	590	600	610	620	630	640
LQFPLPPPWN	NTKRLATIHE	LMHFCTSDVL	SWNEVERAFK	VFTFESLKLS	EVDEKGKLKP	SGMMCGSDSE	MFNIPWDNPA
650	660	670	680	690	700	710	720
RFAKQIRQQY	VMKMNTQEAK	QKADIKIKDR	TLFVDQNLSM	SVQDNESNRE	PSDPSQCDAN	NMKHSDLNNL	KLSVPDNRQL
730	740	750	760	770	780	790	800
LEQESIMKAQ	PQHESLEQTT	NNEIKDDAVT	KADSHEKKPK	KMMVEADLED	IKKTQQRSLM	DWSFTEHFKP	KVLLQVLQEA
810	820	830	840	850	860	870	880
HKQYRCVDSY	YHTQDNSLLL	VFHNPMNRQR	LHCEYWNIAL	HSNVGFRNYL	ELVAKSIQDW	ITKEEAIYQE	SKMNEKIIRT
890	900	910	920	930	940	950	960
RAELELKSSA	NAKLTSASKI	FSIKESKSNK	GISKTEISDQ	EKEKEKEKIP	FILEGSLKAW	KEEQHRLAEE	ERLREEKKAE
970	980	990	1000	1010	1020	1030	1040
KKGKEAGKKK	GKDNAEKEDS	RSLKKKSPYK	EKSKEEQVKI	QEVTEESPHQ	PEPKITYPFH	GYNMGNIPTQ	ISGSNYYLYP
1050	1060	1070	1080	1090	1100	1110	1120
SDGGQIEVEK	TMFEKGPTFI	KVRVVKDNHN	FMIHLNDPKE	IVKKEEKGDY	YLEEEEEGDE	EQSLETEVSD	AKNKAFSKFG
1130	1140	1150	1160	1170	1180	1190	1200
SFSATLENGI	CLSISYYGSN	GMAPEDKDPD	LETILNIPSA	LTPTVVPVIV	TVPQSKAKGK	IKGKEKPKES	LKEEEHPKEE
1210	1220	1230	1240	1250	1260	1270	1280
EKKEEEVEPE	PVLQETLDVP	TFQSLNVSCP	SGLLLTFIGQ	ESTGQYVIDE	EPTWDIMVRQ	SYPQRVKHYE	FYKTVMPPAE
1290	1300	1310					
QEASRVITSQ	GTVVKYMLDG	STQIR					

Cmpd.	No. of Cmpds.	m/z meas.	Δ m/z [ppm]	z	Rt [min]	Score	P	Range	Sequence	Modification
2219	1	900.3315	-71.18	3	57.6	13.7	0	618-641	K.LKPSGMMCGSDSEMFNIPWDNPAR.F	Oxidation: 6

Detailed Protein Report

Protein 508: protein FAM160A1 [Homo sapiens]

**Accession:**	gi|158341646	**Score:**	23.7
**Database:**	refseq_human(refseq_human_20140103.fasta)	**MW [kDa]:**	116.5
**Database Date:**	2015-11-30	**pI:**	4.6
		**Sequence Coverage [%]:**	2.2
		**No. of unique Peptides:**	1

Alias proteins:

Accession	Name	Description
gi|530378111	refseq_human (refseq_human_20140103.fasta)	PREDICTED: protein FAM160A1 isoform X1 [Homo sapiens]

10	20	30	40	50	60	70	80
MMSSVSTESK	LQQAVSLQGV	DPETCMIVFK	NHWAQVVKIL	EKHDPLKNTQ	AKYGSIPPDE	ASAVQNYVEH	MLFLLIEEQA
**90**	**100**	**110**	**120**	**130**	**140**	**150**	**160**
KDAAMGPILE	FVVSENIMEK	LFLWSLRREF	TDETKIEQLK	MYEMLVTQSH	QPLLHHKPIL	KPLMMLLSSC	SGTTTPTVEE
**170**	**180**	**190**	**200**	**210**	**220**	**230**	**240**
KLVVLLNQLC	SILAKDPSIL	ELFFHTSEDQ	GAANFLIFSL	LIPFIHREGS	VGQQARDALL	FIMSLSAENT	MVAHHIVENT
**250**	**260**	**270**	**280**	**290**	**300**	**310**	**320**
YFCPVLATGL	SGLYSSLPTK	LEEKGEEWHC	LLKDDWLLLP	SLVQFMNSLE	FCNAVIQVAH	PLIRNQLVNY	IYNGFLVPVL
**330**	**340**	**350**	**360**	**370**	**380**	**390**	**400**
APALHKVTVE	EVMTTTAYLD	LFLRSISEPA	LLEIFLRFIL	LHQHENVHIL	DTLTSRINTP	FRLCVVSLAL	FRTLIGLHCE
**410**	**420**	**430**	**440**	**450**	**460**	**470**	**480**
DVMLQLVLRY	LIPCNHMMLS	QRWAVKERDC	YSVSAAKLLA	LTPVCCSSGI	TLTLGNQERD	YILWSKCMHD	TSGPVERPFP
**490**	**500**	**510**	**520**	**530**	**540**	**550**	**560**
EAFSESACIV	EYGKALDISY	LQYLWEAHTN	ILRCMRDCRV	WSALYDGDSP	DPEMFLQSLT	EEGSVSSACP	VFGLPQQLPR
**570**	**580**	**590**	**600**	**610**	**620**	**630**	**640**
KTGPQLAPRK	DKSQTELEWD	DSYDTGISSG	ADVGSPGPYD	DLEVSGPPAP	IDPPKHIQEM	KKNALLLFKG	SYIEESDFQD
**650**	**660**	**670**	**680**	**690**	**700**	**710**	**720**
DVMVYRLCAE	KDSEDMKDSQ	EEAARPPAEA	QAEVQSVPIN	NGPLLSTQPE	TDSEEEWNRD	NSDPFHSEPK	EPKQEREPEA
**730**	**740**	**750**	**760**	**770**	**780**	**790**	**800**
APESNSELAS	PAPEAEHSSN	LTAAHPESEE	LIAQYDQIIK	ELDSGAEGLM	EQNYPTPDPL	LLTKEEEGKE	ESKGEKEKEG
**810**	**820**	**830**	**840**	**850**	**860**	**870**	**880**
KKELEDEEDD	FDSFIAEMPA	VETVPSPFVG	RDEAAFASRH	PVRTQSTPFT	GPFISVVLSK	LENMLENSLH	VNLLLIGIIT
**890**	**900**	**910**	**920**	**930**	**940**	**950**	**960**
QLASYPQPLL	RSFLLNTNMV	FQPSVRSLYQ	VLASVKNKIE	QFASVERDFP	GLLIQAQQYL	LFRVDMSDMT	PAALTKDPIQ
**970**	**980**	**990**	**1000**	**1010**	**1020**	**1030**	**1040**
EASRTGSGKN	LLDGPPRVLQ	PFLTHRTKVA	EAPPNLPLPV	RNPMLAAALF	PEFLKELAAL	AQEHSILCYK	ILGDFEDSCC
**1050**							

Cmpd.	No. of Cmpds.	m/z meas.	Δ m/z [ppm]	z	Rt [min]	Score	P	Range	Sequence	Modification
468	1	686.9223	31.03	2	35.5	11.6	0	989-1001	K.VAEAPPNLPLPVR.N	

Detailed Protein Report

Protein 509: brain-derived neurotrophic factor isoform a preproprotein [Homo sapiens]

**Accession:**	gi|25306253	**Score:**	23.7
**Database:**	refseq_human(refseq_human_20140103.fasta)	**MW [kDa]:**	27.8
**Database Date:**	2015-11-30	**pI:**	9.8
		**Sequence Coverage [%]:**	7.7
		**No. of unique Peptides:**	1

Alias proteins:

Accession	Name	Description
gi|219842314	refseq_human (refseq_human_20140103.fasta)	brain-derived neurotrophic factor isoform a preproprotein [Homo sapiens]
gi|219842309	refseq_human (refseq_human_20140103.fasta)	brain-derived neurotrophic factor isoform a preproprotein [Homo sapiens]
gi|219842306	refseq_human (refseq_human_20140103.fasta)	brain-derived neurotrophic factor isoform a preproprotein [Homo sapiens]
gi|219842303	refseq_human (refseq_human_20140103.fasta)	brain-derived neurotrophic factor isoform a preproprotein [Homo sapiens]
gi|219842300	refseq_human (refseq_human_20140103.fasta)	brain-derived neurotrophic factor isoform a preproprotein [Homo sapiens]
gi|219842294	refseq_human (refseq_human_20140103.fasta)	brain-derived neurotrophic factor isoform a preproprotein [Homo sapiens]
gi|219842292	refseq_human (refseq_human_20140103.fasta)	brain-derived neurotrophic factor isoform a preproprotein [Homo sapiens]
gi|219842290	refseq_human (refseq_human_20140103.fasta)	brain-derived neurotrophic factor isoform a preproprotein [Homo sapiens]
gi|219842288	refseq_human (refseq_human_20140103.fasta)	brain-derived neurotrophic factor isoform a preproprotein [Homo sapiens]
gi|25306267	refseq_human (refseq_human_20140103.fasta)	brain-derived neurotrophic factor isoform a preproprotein [Homo sapiens]
gi|25306264	refseq_human (refseq_human_20140103.fasta)	brain-derived neurotrophic factor isoform a preproprotein [Homo sapiens]
gi|25306257	refseq_human (refseq_human_20140103.fasta)	brain-derived neurotrophic factor isoform a preproprotein [Homo sapiens]

```
        10         20         30         40         50         60         70         80
MTILFLTMVI SYFGCMKAAP MKEANIRGQG GLAYPGVRTH GTLESVNGPK AGSRGLTSLA DTFEHVIEEL LDEDQKVRPN
        90        100        110        120        130        140        150        160
EENNKDADLY TSRVMLSSQV PLEPPLLFLL EEYKNYLDAA NMSMRVRRHS DPARRGELSV CDSISEWVTA ADKKTAVDMS
       170        180        190        200        210        220        230        240
GGTVTVLEKV PVSKGQLKQY FYETKCNPMG YTKEGCRGID KRHWNSQCRT TQSYVRALTM DSKKRIGWRF IRIDTSCVCT
       250
LTIKRGR
```

Cmpd.	No. of Cmpds.	m/z meas.	Δ m/z [ppm]	z	Rt [min]	Score	P	Range	Sequence	Modification
2135	1	1149.9035	-125.59	2	56.6	23.7	2	175-193	K.GQLKQYFYETKCNPMGYTK.E	

Detailed Protein Report

Protein 510: PREDICTED: protein dopey-1 isoform X9 [Homo sapiens]

**Accession:**	gi|578812549	**Score:**	23.7
**Database:**	refseq_human(refseq_human_20140103.fasta)	**MW [kDa]:**	226.8
**Database Date:**	2015-11-30	**pI:**	5.7
		**Sequence Coverage [%]:**	1.7
		**No. of unique Peptides:**	1

Detailed Protein Report

10	20	30	40	50	60	70	80
MNTEELELLS	DSKYRNYVAA	IDKALKNFEY	SSEWADLISA	LGKLNKVLQN	NAKYQVVPKK	LTIGKRLAQC	LHPALPGGVH
90	100	110	120	130	140	150	160
RKALETYEII	FKIIGPKRLA	KDLFLYSSGL	FPLLANAAMS	VKPTLLSLYE	IYYLPLGKTL	KPGLQGLLTG	ILPGLEEGSE
170	180	190	200	210	220	230	240
YYERTNMLLE	KVAAAVDQSA	FYSALWGSLL	TSPAVRLPGI	TYVLAHLNRK	LSMEDQLYII	GSDIELMVEA	VSTSVQDSSV
250	260	270	280	290	300	310	320
LVQRSTLDLI	LFCFPFHMSQ	ATRPDMIRIL	SAALHVVLRR	DMSLNRRLYA	WLLGFDNNGA	IIGPRSTRHS	NPEEHATYYF
330	340	350	360	370	380	390	400
TTFSKELLVQ	AMVGILQVNG	FGEENTLMQD	LKPFRILISL	LDKPELGPVI	LEDVLIEVFR	TLYSQCKAEL	DLQTEPPFSK
410	420	430	440	450	460	470	480
DHAQLSSKLR	ENKKTAELIK	TANLLFNSFE	PYYMWDYVAR	WFEECCRRTL	HVRLQIGPGD	SNDSSELQLT	NFCLLVDFLL
490	500	510	520	530	540	550	560
DIVSLPTRSM	RVLCQETYIE	IQTEHLPQLL	LRMISALTSH	LQTLHLSELT	DSLRLCSKIL	SKVQPPLLSA	STGGVLQFPS
570	580	590	600	610	620	630	640
GQNNSVKEWE	DKKVSSVSHE	NPTEVFEDGE	NPPSSRSSES	GFTEFIQYQA	DRTDDIDREL	SEGQGAAAIP	IGSTSSETET
650	660	670	680	690	700	710	720
ASTVGSEETI	IQTPSVVTQG	TATRSRKTAQ	KTAMQCCLEY	VQQFLTRLIN	LYIIQNNSFS	QSLATEHQGD	LGREQGETSK
730	740	750	760	770	780	790	800
WDRNSQGDVK	EKNISKQKTS	KEYLSAFLAA	CQLFLECSSF	PVYIAEGNHT	SELRSEKLET	DCEHVQPPQW	LQTLMNACSQ
810	820	830	840	850	860	870	880
ASDFSVQSVA	ISLVMDLVGL	TQSVAMVTGE	NINSVEPAQP	LSPNQGRVAV	VIRPPLTQGN	LRYIAEKTEF	FKHVALTLWD
890	900	910	920	930	940	950	960
QLGDGTPQHH	QKSVELFYQL	HNLVPSSSIC	EDVISQQLTH	KDKKIRMEAH	AKFAVLWHLT	RDLHINKSSS	FVRSFDRSLF
970	980	990	1000	1010	1020	1030	1040
IMLDSLNSLD	GSTSSVGQAW	LNQVLQRHDI	ARVLEPLLLL	LLHPKTQRVS	VQRVQAERYW	NKSPCYPGEE	SDKHFMQNFA
1050	1060	1070	1080	1090	1100	1110	1120
CSNVSQVQLI	TSKGNGEKPL	TMDEIENFSL	TVNPLSDRLS	LLSTSSETIP	MVVSDFDLPD	QQIEILQSSD	SGCSQSSAGD
1130	1140	1150	1160	1170	1180	1190	1200
NLSYEVDPET	VNAQEDSQMP	KESSPDDDVQ	QVVFDLICKV	VSGLEVESAS	VTSQLEIEAM	PPKCSDIDPD	EETIKIEDDS
1210	1220	1230	1240	1250	1260	1270	1280
IQQSQNALLS	NESSQFLSVS	AEGGHECVAN	GISRNSSSPC	ISGTTHTLHD	SSVASIETKS	RQRSHSSIQF	SFKEKLSEKV
1290	1300	1310	1320	1330	1340	1350	1360
SEKETIVKES	GKQPGAKPKV	KLARKKDDDK	KKSSNEKLKQ	TSVFFSDGLD	LENWYSCGEG	DISEIESDMG	SPGSRKSPNF
1370	1380	1390	1400	1410	1420	1430	1440
NIHPLYQHVL	LYLQLYDSSR	TLYAFSAIKA	ILKTNPIAFV	NAISTTSVNN	AYTPQLSLLQ	NLLARHRISV	MGKDFYSHIP
1450	1460	1470	1480	1490	1500	1510	1520
VDSNHNFRSS	MYIEILISLC	LYYMRSHYPT	HVKVTAQDLI	GNRNMQMMSI	EILTLLFTEL	AKVIESSAKG	FPSFISDMLS
1530	1540	1550	1560	1570	1580	1590	1600
KCKVQKVILH	CLLSSIFSAQ	KWHSEKMAGK	NLVAVEEGFS	EDSLINFSED	EFDNGSTLQS	QLLKVLQRLI	VLEHRVMTIP
1610	1620	1630	1640	1650	1660	1670	1680
EENETGFDFV	VSDLEHISPH	QPMTSLQYLH	AQPITCQGMF	LCAVIRALHQ	HCACKMHPQW	IGLITSTLPY	MGKVLQRVVV
1690	1700	1710	1720	1730	1740	1750	1760
SVTLQLCRNL	DNLIQQYKYE	TGLSDSRPLW	MASIIPPDMI	LTLLEGITAI	IHYCLLDPTT	QYHQLLVSVD	QKHLFEARSG
1770	1780	1790	1800	1810	1820	1830	1840
ILSILHMIMS	SVTLLWSILH	QADSSEKMTI	AASASLTTIN	LGATKNLRQQ	ILELLGPISM	NHGVHFMAAI	AFVWNERRQN
1850	1860	1870	1880	1890	1900	1910	1920
KTTTRTKVIP	AASEEQLLLV	ELVRSISVMR	AETVIQTVKE	VLKQPPAIAK	DKKHLSLEVC	MLQFFYAYIQ	RIPVPNLVDS
1930	1940	1950	1960	1970	1980	1990	2000
WASLLILLKD	SIQLSLPAPG	QFLILGVLNE	FIMKNPSLEN	KKDQRDLQDV	THKIVDAIGA	IAGSSLEQTT	WLRRNLEVKP
2010	2020	2030					
SPKIMVDGTN	LESDVEGFGS	SFGYGFL					

Cmpd.	No. of Cmpds.	m/z meas.	Δ m/z [ppm]	z	Rt [min]	Score	P	Range	Sequence	Modification

Detailed Protein Report

Cmpd.	No. of Cmpds.	m/z meas.	Δ m/z [ppm]	z	Rt [min]	Score	P	Range	Sequence	Modification
2760	1	848.9242	-69.58	2	64.9	12.9	2	1284-1299	K.ETIVKESGKQPGAKPK.V	

Detailed Protein Report

Protein 511: CUB and sushi domain-containing protein 1 precursor [Homo sapiens]

**Accession:**	gi|259013213	**Score:**	23.6
**Database:**	refseq_human(refseq_human_20140103.fasta)	**MW [kDa]:**	388.5
**Database Date:**	2015-11-30	**pI:**	5.6
**Modification(s):**	Carbamidomethyl	**Sequence Coverage [%]:**	1.0
		**No. of unique Peptides:**	2

BRUKER

Detailed Protein Report

10	20	30	40	50	60	70	80
MTAWRRFQSL	LLLLGLLVLC	ARLLTAAKGQ	NCGGLVQGPN	GTIESPGFPH	GYPNYANCTW	IIITGERNRI	QLSFHTFALE
90	100	110	120	130	140	150	160
EDFDILSVYD	GQPQQGNLKV	RLSGFQLPSS	IVSTGSILTL	WFTTDFAVSA	QGFKALYEVL	PSHTCGNPGE	ILKGVLHGTR
170	180	190	200	210	220	230	240
FNIGDKIRYS	CLPGYILEGH	AILTCIVSPG	NGASWDFPAP	FCRAEGACGG	TLRGTSSSIS	SPHFPSEYEN	NADCTWTILA
250	260	270	280	290	300	310	320
EPGDTIALVF	TDFQLEEGYD	FLEISGTEAP	SIWLTGMNLP	SPVISSKNWL	RLHFTSDSNH	RRKGFNAQFQ	VKKAIELKSR
330	340	350	360	370	380	390	400
GVKMLPSKDG	SHKNSVLSQG	GVALVSDMCP	DPGIPENGRR	AGSDFRVGAN	VQFSCEDNYV	LQGSKSITCQ	RVTETLAAWS
410	420	430	440	450	460	470	480
DHRPICRART	CGSNLRGPSG	VITSPNYPVQ	YEDNAHCVWV	ITTTDPDKVI	KLAFEEFELE	RGYDTLTVGD	AGKVGDTRSV
490	500	510	520	530	540	550	560
LYVLTGSSVP	DLIVSMSNQM	WLHLQSDDSI	GSPGFKAVYQ	EIEKGGCGDP	GIPAYGKRTG	SSFLHGDTLT	FECPAAFELV
570	580	590	600	610	620	630	640
GERVITCQQN	NQWSGNKPSC	VFSCFFNFTA	SSGIILSPNY	PEEYGNNMNC	VWLIISEPGS	RIHLIFNDFD	VEPQFDFLAV
650	660	670	680	690	700	710	720
KDDGISDITV	LGTFSGNEVP	SQLASSGHIV	RLEFQSDHST	TGRGFNITYT	TFGQNECHDP	GIPINGRRFG	DRFLLGSSVS
730	740	750	760	770	780	790	800
FHCDDGFVKT	QGSESITCIL	QDGNVVWSST	VPRCEAPCGG	HLTASSGVIL	PPGWPGYYKD	SLHCEWIIEA	KPGHSIKITF
810	820	830	840	850	860	870	880
DRFQTEVNYD	TLEVRDGPAS	SSPLIGEYHG	TQAPQFLIST	GNFMYLLFTT	DNSRSSIGFL	IHYESVTLES	DSCLDPGIPV
890	900	910	920	930	940	950	960
NGHRHGGDFG	IRSTVTFSCD	PGYTLSDDEP	LVCERNHQWN	HALPSCDALC	GGYIQGKSGT	VLSPGFPDFY	PNSLNCTWTI
970	980	990	1000	1010	1020	1030	1040
EVSHGKGVQM	IFHTFHLESS	HDYLLITEDG	SFSEPVARLT	GSVLPHTIKA	GLFGNFTAQL	RFISDFSISY	EGFNITFSEY
1050	1060	1070	1080	1090	1100	1110	1120
DLEPCDDPGV	PAFSRRIGFH	FGVGDSLTFS	CFLGYRLEGA	TKLTCLGGGR	RVWSAPLPRC	VAECGASVKG	NEGTLLSPNF
1130	1140	1150	1160	1170	1180	1190	1200
PSNYDNNHEC	IYKIETEAGK	GIHLRTRSFQ	LFEGDTLKVY	DGKDSSSRPL	GTFTKNELLG	LILNSTSNHL	WLEFNTNGSD
1210	1220	1230	1240	1250	1260	1270	1280
TDQGFQLTYT	SFDLVKCEDP	GIPNYGYRIR	DEGHFTDTVV	LYSCNPGYAM	HGSNTLTCLS	GDRRVWDKPL	PSCIAECGGQ
1290	1300	1310	1320	1330	1340	1350	1360
IHAATSGRIL	SPGYPAPYDN	NLHCTWIIEA	DPGKTISLHF	IVFDTEMAHD	ILKVWDGPVD	SDILLKEWSG	SALPEDIHST
1370	1380	1390	1400	1410	1420	1430	1440
FNSLTLQFDS	DFFISKSGFS	IQFSTSIAAT	CNDPGMPQNG	TRYGDSREAG	DTVTFQCDPG	YQLQGQAKIT	CVQLNNRFFW
1450	1460	1470	1480	1490	1500	1510	1520
QPDPPTCIAA	CGGNLTGPAG	VILSPNYPQP	YPPGKECDWR	VKVNPDFVIA	LIFKSFNMEP	SYDFLHIYEG	EDSNSPLIGS
1530	1540	1550	1560	1570	1580	1590	1600
YQGSQAPERI	ESSGNSLFLA	FRSDASVGLS	GFAIEFKEKP	REACFDPGNI	MNGTRVGTDF	KLGSTITYQC	DSGYKILDPS
1610	1620	1630	1640	1650	1660	1670	1680
SITCVIGADG	KPSWDQVLPS	CNAPCGGQYT	GSEGVVLSPN	YPHNYTAGQI	CLYSITVPKE	FVVFGQFAYF	QTALNDLAEL
1690	1700	1710	1720	1730	1740	1750	1760
FDGTHAQARL	LSSLSGSHSG	ETLPLATSNQ	ILLRFSAKSG	ASARGFHFVY	QAVPRTSDTQ	CSSVPEPRYG	RRIGSEFSAG
1770	1780	1790	1800	1810	1820	1830	1840
SIVRFECNPG	YLLQGSTALH	CQSVPNALAQ	WNDTIPSCVV	PCSGNFTQRR	GTILSPGYPE	PYGNNLNCIW	KIIVTEGSGI
1850	1860	1870	1880	1890	1900	1910	1920
QIQVISFATE	QNWDSLEIHD	GGDVTAPRLG	SFSGTTVPAL	LNSTSNQLYL	HFQSDISVAA	AGFHLEYKTV	GLAACQEPAL
1930	1940	1950	1960	1970	1980	1990	2000
PSNSIKIGDR	YMVNDVLSFQ	CEPGYTLQGR	SHISCMPGTV	RRWNYPSPLC	IATCGGTLST	LGGVILSPGF	PGSYPNNLDC
2010	2020	2030	2040	2050	2060	2070	2080
TWRISLPIGY	GAHIQFLNFS	TEANHDFLEI	QNGPYHTSPM	IGQFSGTDLP	AALLSTTHET	LIHFYSDHSQ	NRQGFKLAYQ
2090	2100	2110	2120	2130	2140	2150	2160
AYELQNCPDP	PPFQNGYMIN	SDYSVGQSVS	FECYPGYILI	GHPVLTCQHG	INRNWNYPFP	RCDAPCGYNV	TSQNGTIYSP
2170	2180	2190	2200	2210	2220	2230	2240

Detailed Protein Report

Cmpd.	No. of Cmpds.	m/z meas.	Δ m/z [ppm]	z	Rt [min]	Score	P	Range	Sequence	Modification
77	1	845.3458	-32.83	3	30.7	11.7	0	893-915	R.STVTFSCDPGYTLSDDEPLVCER.N	
570	1	720.8066	-10.56	2	36.7	12.0	0	1217-1228	K.CEDPGIPNYGYR.I	Carbamidomethyl: 1

Detailed Protein Report

Protein 512: ubiquitin carboxyl-terminal hydrolase 34 [Homo sapiens]

**Accession:**	gi|110347427	**Score:**	23.6
**Database:**	refseq_human(refseq_human_20140103.fasta)	**MW [kDa]:**	404.0
**Database Date:**	2015-11-30	**pI:**	5.4
		**Sequence Coverage [%]:**	1.0
		**No. of unique Peptides:**	1

BRUKER

Detailed Protein Report

10	20	30	40	50	60	70	80
MCENCADLVE	VLNEISDVEG	GDGLQLRKEH	TLKIFTYINS	WTQRQCLCCF	KEYKHLEIFN	QVVCALINLV	IAQVQVLRDQ
90	100	110	120	130	140	150	160
LCKHCTTINI	DSTWQDESNQ	AEEPLNIDRE	CNEGSTERQK	SIEKKSNSTR	ICNLTEEESS	KSSDPFSLWS	TDEKEKLLLC
170	180	190	200	210	220	230	240
VAKIFQIQFP	LYTAYKHNTH	PTIEDISTQE	SNILGAFCDM	NDVEVPLHLL	RYVCLFCGKN	GLSLMKDCFE	YGTPETLPFL
250	260	270	280	290	300	310	320
IAHAFITVVS	NIRIWLHIPA	VMQHIIPFRT	YVIRYLCKLS	DQELRQSAAR	NMADLMWSTV	KEPLDTTLCF	DKESLDLAFK
330	340	350	360	370	380	390	400
YFMSPTLTMR	LAGLSQITNQ	LHTFNDVCNN	ESLVSDTETS	IAKELADWLI	SNNVVEHIFG	PNLHIEIIKQ	CQVILNFLAA
410	420	430	440	450	460	470	480
EGRLSTQHID	CIWAAAQLKH	CSRYIHDLFP	SLIKNLDPVP	LRHLLNLVSA	LEPSVHTEQT	LYLASMLIKA	LWNNALAAKA
490	500	510	520	530	540	550	560
QLSKQSSFAS	LLNTNIPIGN	KKEEEELRRT	APSPWSPAAS	PQSSDNSDTH	QSGGSDIEMD	EQLINRTKHV	QQRLSDTEES
570	580	590	600	610	620	630	640
MQGSSDETAN	SGEDGSSGPG	SSSGHSDGSS	NEVNSSHASQ	SAGSPGSEVQ	SEDIADIEAL	KEEDEDDDHG	HNPPKSSCGT
650	660	670	680	690	700	710	720
DLRNRKLESQ	AGICLGDSQG	MSERNGTSSG	TGKDLVFNTE	SLPSVDNRMR	MLDACSHSED	PEHDISGEMN	ATHIAQGSQE
730	740	750	760	770	780	790	800
SCITRTGDFL	GETIGNELFN	CRQFIGPQHH	HHHHHHHHHH	DGHMVDDMLS	ADDVSCSSSQ	VSAKSEKNMA	DFDGEESGCE
810	820	830	840	850	860	870	880
EELVQINSHA	ELTSHLQQHL	PNLASIYHEH	LSQGPVVHKH	QFNSNAVTDI	NLDNVCKKGN	TLLWDIVQDE	DAVNLSEGLI
890	900	910	920	930	940	950	960
NEAEKLLCSL	VCWFTDRQIR	MRFIEGCLEN	LGNNRSVVIS	LRLLPKLFGT	FQQFGSSYDT	HWITMWAEKE	LNMMKLFFDN
970	980	990	1000	1010	1020	1030	1040
LVYYIQTVRE	GRQKHALYSH	SAEVQVRLQF	LTCVFSTLGS	PDHFRLSLEQ	VDILWHCLVE	DSECYDDALH	WFLNQVRSKD
1050	1060	1070	1080	1090	1100	1110	1120
QHAMGMETYK	HLFLEKMPQL	KPETISMTGL	NLFQHLCNLA	RLATSAYDGC	SNSELCGMDQ	FWGIALRAQS	GDVSRAAIQY
1130	1140	1150	1160	1170	1180	1190	1200
INSYYINGKT	GLEKEQEFIS	KCMESLMIAS	SSLEQESHSS	LMVIERGLLM	LKTHLEAFRR	RFAYHLRQWQ	IEGTGISSHL
1210	1220	1230	1240	1250	1260	1270	1280
KALSDKQSLP	LRVVCQPAGL	PDKMTIEMYP	SDQVADLRAE	VTHWYENLQK	EQINQQAQLQ	EFGQSNRKGE	FPGGLMGPVR
1290	1300	1310	1320	1330	1340	1350	1360
MISSGHELTT	DYDEKALHEL	GFKDMQMVFV	SLGAPRRERK	GEGVQLPASC	LPPPQKDNIP	MLLLLQEPHL	TTLFDLLEML
1370	1380	1390	1400	1410	1420	1430	1440
ASFKPPSGKV	AVDDSESLRC	EELHLHAENL	SRRVWELLML	LPTCPNMLMA	FQNISDEQSN	DGFNWKELLK	IKSAHKLLYA
1450	1460	1470	1480	1490	1500	1510	1520
LEIIEALGKP	NRRIRRESTG	SYSDLYPDSD	DSSEDQVENS	KNSWSCKFVA	AGGLQQLLEI	FNSGILEPKE	QESWTVWQLD
1530	1540	1550	1560	1570	1580	1590	1600
CLACLLKLIC	QFAVDPSDLD	LAYHDVFAWS	GIAESHRKRT	WPGKSRKAAG	DHAKGLHIPR	LTEVFLVLVQ	GTSLIQRLMS
1610	1620	1630	1640	1650	1660	1670	1680
VAYTYDNLAP	RVLKAQSDHR	SRHEVSHYSM	WLLVSWAHCC	SLVKSSLADS	DHLQDWLKKL	TLLIPETAVR	HESCSGLYKL
1690	1700	1710	1720	1730	1740	1750	1760
SLSGLDGGDS	INRSFLLLAA	STLLKFLPDA	QALKPIRIDD	YEEEPILKPG	CKEYFWLLCK	LVDNIHIKDA	SQTTLLDLDA
1770	1780	1790	1800	1810	1820	1830	1840
LARHLADCIR	SREILDHQDG	NVEDDGLTGL	LRLATSVVKH	KPPFKFSREG	QEFLRDIFNL	LFLLPSLKDR	QQPKCKSHSS
1850	1860	1870	1880	1890	1900	1910	1920
RAAAYDLLVE	MVKGSVENYR	LIHNWVMAQH	MQSHAPYKWD	YWPHEDVRAE	CRFVGLTNLG	ATCYLASTIQ	QLYMIPEARQ
1930	1940	1950	1960	1970	1980	1990	2000
AVFTAKYSED	MKHKTTLLEL	QKMFTYLMES	ECKAYNPRPF	CKTYTMDKQP	LNTGEQKDMT	EFFTDLITKI	EEMSPELKNT
2010	2020	2030	2040	2050	2060	2070	2080
VKSLFGGVIT	NNVVSLDCEH	VSQTAEEFYT	VRCQVADMKN	IYESLDEVTI	KDTLEGDNMY	TCSHCGKKVR	AEKRACFKKL
2090	2100	2110	2120	2130	2140	2150	2160
PRILSFNTMR	YTFNMVTMMK	EKVNTHFSFP	LRLDMTPYTE	DFLMGKSERK	EGFKEVSDHS	KDSESYEYDL	IGVTVHTGTA
2170	2180	2190	2200	2210	2220	2230	2240
DGGHYYSFIR	DIVNPHAYKN	NKWYLFNDAE	VKPFDSAQLA	SECFGGEMTT	KTYDSVTDKF	MDFSFEKTHS	AYMLFYKRME
2250	2260	2270	2280	2290	2300	2310	2320
PEEENGREYK	FDVSSELLEW	IWHDNMQFLQ	DKNIFEHTYF	GFMWQLCSCI	PSTLPDPKAV	SLMTAKLSTS	FVLETFIHSK
2330	2340	2350	2360	2370	2380	2390	2400

Detailed Protein Report

Cmpd.	No. of Cmpds.	m/z meas.	Δ m/z [ppm]	z	Rt [min]	Score	P	Range	Sequence	Modification
2144	1	899.3487	-134.27	2	56.7	10.7	0	404-419	R.LSTQHIDCIWAAAQLK.H	

Detailed Protein Report

Protein 513: ryanodine receptor 3 isoform 2 [Homo sapiens]

**Accession:**	gi|345842488	**Score:**	23.6
**Database:**	refseq_human(refseq_human_20140103.fasta)	**MW [kDa]:**	551.1
**Database Date:**	2015-11-30	**pI:**	5.4
**Modification(s):**	Carbamidomethyl, Oxidation	**Sequence Coverage [%]:**	0.6
		**No. of unique Peptides:**	2

Detailed Protein Report

10	20	30	40	50	60	70	80
MAEGGEGGED	EIQFLRTEDE	VVLQCIATIH	KEQRKFCLAA	EGLGNRLCFL	EPTSEAKYIP	PDLCVCNFVL	EQSLSVRALQ
90	100	110	120	130	140	150	160
EMLANTGENG	GEGAAQGGGH	RTLLYGHAVL	LRHSFSGMYL	TCLTTSRSQT	DKLAFDVGLR	EHATGEACWW	TIHPASKQRS
170	180	190	200	210	220	230	240
EGEKVRIGDD	LILVSVSSER	YLHLSVSNGN	IQVDASFMQT	LWNVHPTCSG	SSIEEGYLLG	GHVVRLFHGH	DECLTIPSTD
250	260	270	280	290	300	310	320
QNDSQHRRIF	YEAGGAGTRA	RSLWRVEPLR	ISWSGSNIRW	GQAFRLRHLT	TGHYLALTED	QGLILQDRAK	SDTKSTAFSF
330	340	350	360	370	380	390	400
RASKELKEKL	DSSHKRDIEG	MGVPEIKYGD	SVCFVQHIAS	GLWVTYKAQD	AKTSRLGPLK	RKVILHQEGH	MDDGLTLQRC
410	420	430	440	450	460	470	480
QREESQAARI	IRNTTALFSQ	FVSGNNRTAA	PITLPIEEVL	QTLQDLIAYF	QPPEEEMRHE	DKQNKLRSLK	NRQNLFKEEG
490	500	510	520	530	540	550	560
MLALVLNCID	RLNVYNSVAH	FAGIAREESG	MAWKEILNLL	YKLLAALIRG	NRNNCAQFSN	NLDWLISKLD	RLESSSGILE
570	580	590	600	610	620	630	640
VLHCILTESP	EALNLIAEGH	IKSIISLLDK	HGRNHKVLDI	LCSLCLCNGV	AVRANQNLIC	DNLLPRRNLL	LQTRLINDVT
650	660	670	680	690	700	710	720
SIRPNIFLGV	AEGSAQYKKW	YFELIIDQVD	PFLTAEPTHL	RVGWASSSGY	APYPGGGEGW	GGNGVGDDLY	SYGFDGLHLW
730	740	750	760	770	780	790	800
SGRIPRAVAS	INQHLLRSDD	VVSCCLDLGV	PSISFRINGQ	PVQGMFENFN	TDGLFFPVMS	FSAGVKVRFL	MGGRHGEFKF
810	820	830	840	850	860	870	880
LPPSGYAPCY	EALLPKEKMR	LEPVKEYKRD	ADGIRDLLGT	TQFLSQASFI	PCPVDTSQVI	LPPHLEKIRD	RLAENIHELW
890	900	910	920	930	940	950	960
GMNKIELGWT	FGKIRDDNKR	QHPCLVEFSK	LPETEKNYNL	QMSTETLKTL	LALGCHIAHV	NPAAEEDLKK	VKLPKNYMMS
970	980	990	1000	1010	1020	1030	1040
NGYKPAPLDL	SDVKLLPPQE	ILVDKLAENA	HNVWAKDRIK	QGWTYGIQQD	LKNKRNPRLV	PYALLDERTK	KSNRDSLREA
1050	1060	1070	1080	1090	1100	1110	1120
VRTFVGYGYN	IEPSDQELAD	SAVEKVSIDK	IRFFRVERSY	AVRSGKWYFE	FEVVTGGDMR	VGWARPGCRP	DVELGADDQA
1130	1140	1150	1160	1170	1180	1190	1200
FVFEGNRGQR	WHQGSGYFGR	TWQPGDVVGC	MINLDDASMI	FTLNGELLIT	NKGSELAFAD	YEIENGFVPI	CCLGLSQIGR
1210	1220	1230	1240	1250	1260	1270	1280
MNLGTDASTF	KFYTMCGLQE	GFEPFAVNMN	RDVAMWFSKR	LPTFVNVPKD	HPHIEVMRID	GTMDSPPCLK	VTHKTFGTQN
1290	1300	1310	1320	1330	1340	1350	1360
SNADMIYCRL	SMPVECHSSF	SHSPCLDSEA	FQKRKQMQEI	LSHTTTQCYY	AIRIFAGQDP	SCVWVGWVTP	DYHLYSEKFD
1370	1380	1390	1400	1410	1420	1430	1440
LNKNCTVTVT	LGDERGRVHE	SVKRSNCYMV	WGGDIVASSQ	RSNRSNVDLE	IGCLVDLAMG	MLSFSANGKE	LGTCYQVEPN
1450	1460	1470	1480	1490	1500	1510	1520
TKVFPAVFLQ	PTSTSLFQFE	LGKLKNAMPL	SAAIFRSEEK	NPVPQCPPRL	DVQTIQPVLW	SRMPNSFLKV	ETERVSERHG
1530	1540	1550	1560	1570	1580	1590	1600
WVVQCLEPLQ	MMALHIPEEN	RCVDILELCE	QEDLMRFHYH	TLRLYSAVCA	LGNSRVAYAL	CSHVDLSQLF	YAIDNKYLPG
1610	1620	1630	1640	1650	1660	1670	1680
LLRSGFYDLL	ISIHLASAKE	RKLMMKNEYI	IPITSTTRNI	RLFPDESKRH	GLPGVGLRTC	LKPGFRFSTP	CFVVTGEDHQ
1690	1700	1710	1720	1730	1740	1750	1760
KQSPEIPLES	LRTKALSMLT	EAVQCSGAHI	RDPVGGSVEF	QFVPVLKLIG	TLLVMGVFDD	DDVRQILLLI	DPSVFGEHSA
1770	1780	1790	1800	1810	1820	1830	1840
GTEEGAEKEE	VTQVEEKAVE	AGEKAGKEAP	VKGLLQTRLP	ESVKLQMCEL	LSYLCDCELQ	HRVEAIVAFG	DIYVSKLQAN
1850	1860	1870	1880	1890	1900	1910	1920
QKFRYNELMQ	ALNMSAALTA	RKTKEFRSPP	QEQINMLLNF	QLGENCPCPE	EIREELYDFH	EDLLLHCGVP	LEEEEEEEED
1930	1940	1950	1960	1970	1980	1990	2000
TSWTGKLCAL	VYKIKGPPKP	EKEQPTEEEE	RCPTTLKELI	SQTMICWAQE	DQIQDSELVR	MMFNLLRRQY	DSIGELLQAL
2010	2020	2030	2040	2050	2060	2070	2080
RKTYTISHTS	VSDTINLLAA	LGQIRSLLSV	RMGKEEELLM	INGLGDIMNN	KVFYQHPNLM	RVLGMHETVM	EVMVNVLGTE
2090	2100	2110	2120	2130	2140	2150	2160
KSQIAFPKMV	ASCCRFLCYF	CRISRQNQKA	MFEHLSYLLE	NSSVGLASPS	MRGSTPLDVA	ASSVMDNNEL	ALSLEEPDLE
2170	2180	2190	2200	2210	2220	2230	2240

Detailed Protein Report

Cmpd.	No. of Cmpds.	m/z meas.	Δ m/z [ppm]	z	Rt [min]	Score	P	Range	Sequence	Modification
833	1	575.1451	-229.53	2	39.8	12.5	0	1250-1258	K.DHPHIEVMR.I	Oxidation: 8
2682	1	1051.5508	66.61	2	64.7	11.2	1	1384-1401	K.RSNCYMVWGGDIVASSQR.S	Carbamidomethyl: 4; Oxidation: 6

Detailed Protein Report

Protein 514: **PREDICTED: receptor-type tyrosine-protein phosphatase delta isoform X16 [Homo sapiens]**

**Accession:**	gi|578816637	**Score:**	23.6
**Database:**	refseq_human(refseq_human_20140103.fasta)	**MW [kDa]:**	211.2
**Database Date:**	2015-11-30	**pI:**	6.2
**Modification(s):**	Oxidation	**Sequence Coverage [%]:**	0.9
		**No. of unique Peptides:**	1

Detailed Protein Report

10	20	30	40	50	60	70	80
MVHVARLLLL	LLTFFLRTDA	ETPPRFTRTP	VDQTGVSGGV	ASFICQATGD	PRPKIVWNKK	GKKVSNQRFE	VIEFDDGSGS
**90**	**100**	**110**	**120**	**130**	**140**	**150**	**160**
VLRIQPLRTP	RDEAIYECVA	SNNVGEISVS	TRLTVLREDQ	IPRGFPTIDM	GPQLKVVERT	RTATMLCAAS	GNPDPEITWF
**170**	**180**	**190**	**200**	**210**	**220**	**230**	**240**
KDFLPVDTSN	NNGRIKQLRS	VRRVPPRFSI	PPTNHEIMPG	GSVNITCVAV	GSPMPYVKWM	LGAEDLTPED	DMPIGRNVLE
**250**	**260**	**270**	**280**	**290**	**300**	**310**	**320**
LNDVRQSANY	TCVAMSTLGV	IEAIAQITVK	ALPKPPGTPV	VTESTATSIT	LTWDSGNPEP	VSYYIIQHKP	KNSEELYKEI
**330**	**340**	**350**	**360**	**370**	**380**	**390**	**400**
DGVATTRYSV	AGLSPYSDYE	FRVVAVNNIG	RGPPSEPVLT	QTSEQAPSSA	PRDVQARMLS	STTILVQWKE	PEEPNGQIQG
**410**	**420**	**430**	**440**	**450**	**460**	**470**	**480**
YRVYYTMDPT	QHVNNWMKHN	VADSQITTIG	NLVPQKTYSV	KVLAFTSIGD	GPLSSDIQVI	TQTGVPGQPL	NFKAEPESET
**490**	**500**	**510**	**520**	**530**	**540**	**550**	**560**
SILLSWTPPR	SDTIANYELV	YKDGEHGEEQ	RITIEPGTSY	RLQGLKPNSL	YYFRLAARSP	QGLGASTAEI	SARTMQSKPS
**570**	**580**	**590**	**600**	**610**	**620**	**630**	**640**
APPQDISCTS	PSSTSILVSW	QPPPVEKQNG	IITEYSIKYT	AVDGEDDKPH	EILGIPSDTT	KYLLEQLEKW	TEYRITVTAH
**650**	**660**	**670**	**680**	**690**	**700**	**710**	**720**
TDVGPGPESL	SVLIRTNEDV	PSGPPRKVEV	EAVNSTSVKV	SWRSPVPNKQ	HGQIRGYQVH	YVRMENGEPK	GQPMLKDVML
**730**	**740**	**750**	**760**	**770**	**780**	**790**	**800**
ADAQWEFDDT	TEHDMIISGL	QPETSYSLTV	TAYTTKGDGA	RSKPKLVSTT	GAVPGKPRLV	INHTQMNTAL	IQWHPPVDTF
**810**	**820**	**830**	**840**	**850**	**860**	**870**	**880**
GPLQGYRLKF	GRKDMEPLTT	LEFSEKEDHF	TATDIHKGAS	YVFRLSARNK	VGFGEEMVKE	ISIPEEVPTG	FPQNLHSEGT
**890**	**900**	**910**	**920**	**930**	**940**	**950**	**960**
TSTSVQLSWQ	PPVLAERNGI	ITKYTLLYRD	INIPLLPMEQ	LIVPADTTMT	LTGLKPDTTY	DVKVRAHTSK	GPGPYSPSVQ
**970**	**980**	**990**	**1000**	**1010**	**1020**	**1030**	**1040**
FRTLPVDQAV	FAKNFHVKAV	MKTSVLLSWE	IPENYNSAMP	FKILYDDGKM	VEEVDGRATQ	KLIVNLKPEK	SYSFVLTNRG
**1050**	**1060**	**1070**	**1080**	**1090**	**1100**	**1110**	**1120**
NSAGGLQHRV	TAKTAPDVLR	TKPAFIGKTN	LDGMITVQLP	EVPANENIKG	YYIIIVPLKK	SRGKFIKPWE	SPDEMELDEL
**1130**	**1140**	**1150**	**1160**	**1170**	**1180**	**1190**	**1200**
LKEISRKRRS	IRYGREVELK	PYIAAHFDVL	PTEFTLGDDK	HYGGFTNKQL	QSGQEYVFFV	LAVMEHAESK	MYATSPYSDP
**1210**	**1220**	**1230**	**1240**	**1250**	**1260**	**1270**	**1280**
VVSMDLDPQP	ITDEEEGLIW	VVGPVLAVVF	IICIVIAILL	YKSSKPDRKR	AESDSRKSSI	PNNKEIPSHH	PTDPVELRRL
**1290**	**1300**	**1310**	**1320**	**1330**	**1340**	**1350**	**1360**
NFQTPGSDDS	GYPGNLHSSS	MASHPPIPIL	ELADHIERLK	ANDNLKFSQE	YESIDPGQQF	TWEHSNLEVN	KPKNRYANVI
**1370**	**1380**	**1390**	**1400**	**1410**	**1420**	**1430**	**1440**
AYDHSRVLLS	AIEGIPGSDY	VNANYIDGYR	KQNAYIATQG	SLPETFGDFW	RMIWEQRSAT	VVMMTKLEER	SRVKCDQYWP
**1450**	**1460**	**1470**	**1480**	**1490**	**1500**	**1510**	**1520**
SRGTETHGLV	QVTLLDTVEL	ATYCVRTFAL	YKNGSSEKRE	VRQFQFTAWP	DHGVPEHPTP	FLAFLRRVKT	CNPPDAGPMV
**1530**	**1540**	**1550**	**1560**	**1570**	**1580**	**1590**	**1600**
VHCSAGVGRT	GCFIVIDAML	ERIKHEKTVD	IYGHVTLMRA	QRNYMVQTED	QYIFIHDALL	EAVTCGNTEV	PARNLYAYIQ
**1610**	**1620**	**1630**	**1640**	**1650**	**1660**	**1670**	**1680**
KLTQIETGEN	VTGMELEFKR	LASSKAHTSR	FISANLPCNK	FKNRLVNIMP	YESTRVCLQP	IRGVEGSDYI	NASFIDGYRQ
**1690**	**1700**	**1710**	**1720**	**1730**	**1740**	**1750**	**1760**
QKAYIATQGP	LAETTEDFWR	MLWEHNSTIV	VMLTKLREMG	REKCHQYWPA	ERSARYQYFV	VDPMAEYNMP	QYILREFKVT
**1770**	**1780**	**1790**	**1800**	**1810**	**1820**	**1830**	**1840**
DARDGQSRTV	RQFQFTDWPE	QGVPKSGEGF	IDFIGQVHKT	KEQFGQDGPI	SVHCSAGVGR	TGVFITLSIV	LERMRYEGVV
**1850**	**1860**	**1870**	**1880**	**1890**			
DIFQTVKMLR	TQRPAMVQTE	DQYQFSYRAA	LEYLGSFDHY	AT			

Cmpd.	No. of Cmpds.	m/z meas.	Δ m/z [ppm]	z	Rt [min]	Score	P	Range	Sequence	Modification
1512	1	682.1037	142.22	3	48.7	23.6	2	810-826	K.FGRKDMEPLTTLEFSEK.E	Oxidation: 6

Detailed Protein Report

Protein 515: leukocyte receptor cluster member 1 [Homo sapiens]

**Accession:**	gi|24308289	**Score:**	23.6
**Database:**	refseq_human(refseq_human_20140103.fasta)	**MW [kDa]:**	30.5
**Database Date:**	2015-11-30	**pI:**	10.1
		**Sequence Coverage [%]:**	4.2
		**No. of unique Peptides:**	1

10	20	30	40	50	60	70	80
MNILPKKSWH	VRNKDNVARV	RRDEAQAREE	EKERERRVLL	AQQEARTEFL	RKKARHQNSL	PELEAAEAGA	PGSGPVDLFR
**90**	**100**	**110**	**120**	**130**	**140**	**150**	**160**
ELLEEGKGVI	RGNKEYEEEK	RQEKERQEKA	LGILTYLGQS	AAEAQTQPPW	YQLPPGRGGP	PPGPAPDEKI	KSRLDPLREM
**170**	**180**	**190**	**200**	**210**	**220**	**230**	**240**
QKHLGKKRQH	GGDEGSRSRK	EKEGSEKQRP	KEPPSLDQLR	AERLRREAAE	RSRAEALLAR	VQGRALQEGQ	PEEDETDDRR
**250**	**260**	**270**					
RRYNSQFNPQ	LARRPRQQDP	HLTH					

Cmpd.	No. of Cmpds.	m/z meas.	Δ m/z [ppm]	z	Rt [min]	Score	P	Range	Sequence	Modification
575	1	565.3015	-6.83	2	36.5	11.1	1	154-162	R.LDPLREMQK.H	

Detailed Protein Report

Protein 516: PREDICTED: calcyphosin-like protein isoform X2 [Homo sapiens]

Accession: gi|578809847
Database: refseq_human(refseq_human_20140103.fasta)
Database Date: 2015-11-30
Modification(s): Oxidation

Score: 23.5
MW [kDa]: 23.1
pI: 8.9
Sequence Coverage [%]: 10.6
No. of unique Peptides: 1

10	20	30	40	50	60	70	80
MQETASIVSV	RSNWWQKMAG	TARHDREMAI	QAKKKLTTAT	DPIERLRLQC	LARGSAGIKG	LGRVFRIMDD	DNNRTLDFKE
90	100	110	120	130	140	150	160
FMKGLNDYAV	VMEKEEVEEL	FRRFDKDGNG	TIDFNEFLLT	LRPPMSRARK	EVIMQAFRKL	DKTGDGVITI	EDLREVYNAK
170	180	190	200				
HHPKYQNGEW	SEEQVFRKFL	DNFDSPYDKD	GLVHKVGAH				

Cmpd.	No. of Cmpds.	m/z meas.	Δ m/z [ppm]	z	Rt [min]	Score	P	Range	Sequence	Modification
19	3	1205.0501	-38.96	2	29.8	23.5	0	107-127	K.DGNGTIDFNEFLLTLRPPMSR.A	Oxidation: 19

Detailed Protein Report

Protein 517: **E3 ubiquitin-protein ligase BRE1A [Homo sapiens]**

**Accession:**	gi|34878777	**Score:**	23.5
**Database:**	refseq_human(refseq_human_20140103.fasta)	**MW [kDa]:**	113.6
**Database Date:**	2015-11-30	**pI:**	5.6
**Modification(s):**	Oxidation	**Sequence Coverage [%]:**	2.5
		**No. of unique Peptides:**	1

Quantitation

MD:MU **Median:** 0.63 **CV:** 0.00 % **No. of Peptides:** 1

10	20	30	40	50	60	70	80
MSGIGNKRAA	GEPGTSMPPE	KKAAVEDSGT	TVETIKLGGV	SSTEELDIRT	LQTKNRKLAE	MLDQRQAIED	ELREHIEKLE
90	100	110	120	130	140	150	160
RRQATDDASL	LIVNRYWSQF	DENIRIILKR	YDLEQGLGDL	LTERKALVVP	EPEPDSDSNQ	ERKDDRERGE	GQEPAFSFLA
170	180	190	200	210	220	230	240
TLASSSSEEM	ESQLQERVES	SRRAVSQIVT	VYDKLQEKVE	LLSRKLNSGD	NLIVEEAVQE	LNSFLAQENM	RLQELTDLLQ
250	260	270	280	290	300	310	320
EKHRTMSQEF	SKLQSKVETA	ESRVSVLESM	IDDLQWDIDK	IRKREQRLNR	HLAEVLERVN	SKGYKVYGAG	SSLYGGTITI
330	340	350	360	370	380	390	400
NARKFEEMNA	ELEENKELAQ	NRLCELEKLR	QDFEEVTTQN	EKLKVELRSA	VEQVVKETPE	YRCMQSQFSV	LYNESLQLKA
410	420	430	440	450	460	470	480
HLDEARTLLH	GTRGTHQHQV	ELIERDEVSL	HKKLRTEVIQ	LEDTLAQVRK	EYEMLRIEFE	QTLAANEQAG	PINREMRHLI
490	500	510	520	530	540	550	560
SSLQNHNHQL	KGEVLRYKRK	LREAQSDLNK	TRLRSGSALL	QSQSSTEDPK	DEPAELKPDS	EDLSSQSSAS	KASQEDANEI
570	580	590	600	610	620	630	640
KSKRDEEERE	RERREKERER	EREREKEKER	EREKQKLKES	EKERDSAKDK	EKGKHDDGRK	KEAEIIKQLK	IELKKAQESQ
650	660	670	680	690	700	710	720
KEMKLLLDMY	RSAPKEQRDK	VQLMAAEKKS	KAELEDLRQR	LKDLEDKEKK	ENKKMADEDA	LRKIRAVEEQ	IEYLQKKLAM
730	740	750	760	770	780	790	800
AKQEEEALLS	EMDVTGQAFE	DMQEQNIRLM	QQLREKDDAN	FKLMSERIKS	NQIHKLLKEE	KEELADQVLT	LKTQVDAQLQ
810	820	830	840	850	860	870	880
VVRKLEEKEH	LLQSNIGTGE	KELGLRTQAL	EMNKRKAMEA	AQLADDLKAQ	LELAQKKLHD	FQDEIVENSV	TKEKDMFNFK
890	900	910	920	930	940	950	960
RAQEDISRLR	RKLETTKKPD	NVPKCDEILM	EEIKDYKARL	TCPCCNMRKK	DAVLTKCFHV	FCFECVKTRY	DTRQRKCPKC
970	980						
NAAFGANDFH	RIYIG						

Cmpd.	No. of Cmpds.	m/z meas.	Δ m/z [ppm]	z	Rt [min]	Score	P	Range	Sequence	Modification	Ratios	
463	1	475.6020	-277.84	2	35.1	11.6	0	827-834	R.TQALEMNK.R	Oxidation: 6	MD:MU	0.63

Detailed Protein Report

Protein 518: **PREDICTED: complement C1q tumor necrosis factor-related protein 1 isoform X4 [Homo sapiens]**

**Accession:**	gi|578830336	**Score:**	23.5
**Database:**	refseq_human(refseq_human_20140103.fasta)	**MW [kDa]:**	32.7
**Database Date:**	2015-11-30	**pI:**	7.0
**Modification(s):**	Oxidation	**Sequence Coverage [%]:**	4.8
		**No. of unique Peptides:**	1

10	20	30	40	50	60	70	80
MMSATGPGRK	MGSRGQGLLL	AYCLLLAFAS	GLVLSRVPHV	QGEQQEWEGT	EELPSPPDHA	ERAEEQHEKY	RPSQDQGLPA
**90**	**100**	**110**	**120**	**130**	**140**	**150**	**160**
SRCLRCCDPG	TSMYPATAVP	QINITILKGE	KGDRGDRGLQ	GKYGKTGSAG	ARGHTGPKGQ	KGSMGAPGER	CKSHYAAFSV
**170**	**180**	**190**	**200**	**210**	**220**	**230**	**240**
GRKKPMHSNH	YYQTVIFDTE	FVNLYDHFNM	FTGKFYCYVP	GLYFFSLNVH	TWNQKETYLH	IMKNEEEVVI	LFAQVGDRSI
**250**	**260**	**270**	**280**	**290**	**300**		
MQSQSLMLEL	REQDQVWVRL	YKGERENAIF	SEELDTYITF	SGYLVKHATE	P		

Cmpd.	No. of Cmpds.	m/z meas.	Δ m/z [ppm]	z	Rt [min]	Score	P	Range	Sequence	Modification
2717	1	741.8557	4.52	2	64.3	11.4	2	1-14	-.MMSATGPGRKMGSR.G	Oxidation: 11

Detailed Protein Report

Protein 519: actin-related protein 8 [Homo sapiens]

**Accession:**	gi|39812115	**Score:**	23.4
**Database:**	refseq_human(refseq_human_20140103.fasta)	**MW [kDa]:**	70.4
**Database Date:**	2015-11-30	**pI:**	8.8
**Modification(s):**	Oxidation	**Sequence Coverage [%]:**	2.6
		**No. of unique Peptides:**	1

Alias proteins:

Accession	Name	Description
gi|578806774	refseq_human (refseq_human_20140103.fasta)	PREDICTED: actin-related protein 8 isoform X2 [Homo sapiens]
gi|530373398	refseq_human (refseq_human_20140103.fasta)	PREDICTED: actin-related protein 8 isoform X1 [Homo sapiens]

10	20	30	40	50	60	70	80
MTQAEKGDTE	NGKEKGGEKE	KEQRGVKRPI	VPALVPESLQ	EQIQSNFIIV	IHPGSTTLRI	GRATDTLPAS	IPHVIARRHK
**90**	**100**	**110**	**120**	**130**	**140**	**150**	**160**
QQGQPLYKDS	WLLREGLNKP	ESNEQRQNGL	KMVDQAIWSK	KMSNGTRRIP	VSPEQARSYN	KQMRPAILDH	CSGNKWTNTS
**170**	**180**	**190**	**200**	**210**	**220**	**230**	**240**
HHPEYLVGEE	ALYVNPLDCY	NIHWPIRRGQ	LNIHPGPGGS	LTAVLADIEV	IWSHAIQKYL	EIPLKDLKYY	RCILLIPDIY
**250**	**260**	**270**	**280**	**290**	**300**	**310**	**320**
NKQHVKELVN	MILMKMGFSG	IVVHQESVCA	TYGSGLSSTC	IVDVGDQKTS	VCCVEDGVSH	RNTRLCLAYG	GSDVSRCFYW
**330**	**340**	**350**	**360**	**370**	**380**	**390**	**400**
LMQRAGFPYR	ECQLTNKMDC	LLLQHLKETF	CHLDQDISGL	QDHEFQIRHP	DSPALLYQFR	LGDEKLQAPM	ALFYPATFGI
**410**	**420**	**430**	**440**	**450**	**460**	**470**	**480**
VGQKMTTLQH	RSQGDPEDPH	DEHYLLATQS	KQEQSAKATA	DRKSASKPIG	FEGDLRGQSS	DLPERLHSQE	VDLGSAQGDG
**490**	**500**	**510**	**520**	**530**	**540**	**550**	**560**
LMAGNDSEEA	LTALMSRKTA	ISLFEGKALG	LDKAILHSID	CCSSDDTKKK	MYSSILVVGG	GLMFHKAQEF	LQHRILNKMP
**570**	**580**	**590**	**600**	**610**	**620**	**630**	
PSFRRIIENV	DVITRPKDMD	PRLIAWKGGA	VLACLDTTQE	LWIYQREWQR	FGVRMLRERA	AFVW	

Cmpd.	No. of Cmpds.	m/z meas.	Δ m/z [ppm]	z	Rt [min]	Score	P	Range	Sequence	Modification
775	1	585.7012	109.37	3	39.1	11.6	0	531-546	K.MYSSILVVGGGLMFHK.A	Oxidation: 1

Detailed Protein Report

Protein 520: myocyte-specific enhancer factor 2C isoform 3 [Homo sapiens]

Accession: gi|301069380
Database: refseq_human(refseq_human_20140103.fasta)
Database Date: 2015-11-30

Score: 23.4
MW [kDa]: 52.3
pI: 9.5
Sequence Coverage [%]: 6.4
No. of unique Peptides: 1

Quantitation

MD:MU **Median:** 0.93 **CV:** 0.00 % **No. of Peptides:** 1

Alias proteins:

Accession	Name	Description
gi|578810376	refseq_human (refseq_human_20140103.fasta)	PREDICTED: myocyte-specific enhancer factor 2C isoform X12 [Homo sapiens]

```
        10         20         30         40         50         60         70         80
MGRKKIQITR IMDERNRQVT FTKRKFGLMK KAYELSVLCD CEIALIIFNS TNKLFQYAST DMDKVLLKYT EYNEPHESRT
        90        100        110        120        130        140        150        160
NSDIVETLRK KGLNGCDSPD PDADDSALNK KENKGCESPD PDSSYALTPR TEEKYKKINE EFDNMIKSHK IPAVPPPNFE
       170        180        190        200        210        220        230        240
MPVSIPVSSH NSLVYSNPVS SLGNPNLLPL AHPSLQRNSM SPGVTHRPPS AGNTGGLMGG DLTSGAGTSA GNGYGNPRNS
       250        260        270        280        290        300        310        320
PGLLVSPGNL NKNMQAKSPP PMNLGMNNRK PDLRVLIPPG SKNTMPSVNQ RINNSQSAQS LATPVVSVAT PTLPGQGMGG
       330        340        350        360        370        380        390        400
YPSAISTTYG TEYSLSSADL SSLSGFNTAS ALHLGSVTGW QQQHLHNMPP SALSQLGACT STHLSQSSNL SLPSTQSLNI
       410        420        430        440        450        460        470        480
KSEPVSPPRD RTTTPSRYPQ HTRHEAGRSP VDSLSSCSSS YDGSDREDHR NEFHSPIGLT RPSPDERESP SVKRMRLSEG
       490
WAT
```

Cmpd.	No. of Cmpds.	m/z meas.	Δ m/z [ppm]	z	Rt [min]	Score	P	Range	Sequence	Modification	Ratios	
672	1	676.1898	-213.65	2	37.7	11.3	1	418-428	R.YPQHTRHEAGR.S		MD:MU	0.93

Detailed Protein Report

Protein 521: melanoma-associated antigen 8 [Homo sapiens]

Accession: gi|19923308
Database: refseq_human(refseq_human_20140103.fasta)
Database Date: 2015-11-30
Modification(s): Carbamidomethyl, Oxidation

Score: 23.4
MW [kDa]: 35.2
pI: 4.6
Sequence Coverage [%]: 4.4
No. of unique Peptides: 1

Quantitation

MD:MU **Median:** 1.68 **CV:** 0.00 % **No. of Peptides:** 1

Alias proteins:

Accession	Name	Description
gi|261878490	refseq_huma (refseq_human_20140103.fasta)	melanoma-associated antigen 8 [Homo sapiens]
gi|261878488	refseq_huma (refseq_human_20140103.fasta)	melanoma-associated antigen 8 [Homo sapiens]

10	20	30	40	50	60	70	80
MLLGQKSQRY	KAEEGLQAQG	EAPGLMDVQI	PTAEEQKAAS	SSSTLIMGTL	EEVTDSGSPS	PPQSPEGASS	SLTVTDSTLW
**90**	**100**	**110**	**120**	**130**	**140**	**150**	**160**
SQSDEGSSSN	EEEGPSTSPD	PAHLESLFRE	ALDEKVAELV	RFLLRKYQIK	EPVTKAEMLE	SVIKNYKNHF	PDIFSKASEC
**170**	**180**	**190**	**200**	**210**	**220**	**230**	**240**
MQVIFGIDVK	EVDPAGHSYI	LVTCLGLSYD	GLLGDDQSTP	KTGLLIIVLG	MILMEGSRAP	EEAIWEALSV	MGLYDGREHS
**250**	**260**	**270**	**280**	**290**	**300**	**310**	**320**
VYWKLRKLLT	QEWVQENYLE	YRQAPGSDPV	RYEFLWGPRA	LAETSYVKVL	EHVVRVNARV	RISYPSLHEE	ALGEEKGV

Cmpd.	No. of Cmpds.	m/z meas.	Δ m/z [ppm]	z	Rt [min]	Score	P	Range	Sequence	Modification	Ratios	
1776	1	806.7498	-172.60	2	52.2	23.4	0	157-170	K.ASECMQVIFGIDVK.E	Carbamidomethyl: 4; Oxidation: 5	MD:MU	1.68

Detailed Protein Report

Protein 522: gastric inhibitory polypeptide receptor precursor [Homo sapiens]

**Accession:**	gi|4503999	**Score:**	23.4
**Database:**	refseq_human(refseq_human_20140103.fasta)	**MW [kDa]:**	53.1
**Database Date:**	2015-11-30	**pI:**	10.1
**Modification(s):**	Carbamidomethyl, Oxidation	**Sequence Coverage [%]:**	1.5
		**No. of unique Peptides:**	1

10	20	30	40	50	60	70	80
MTTSPILQLL	LRLSLCGLLL	QRAETGSKGQ	TAGELYQRWE	RYRRECQETL	AAAEPPSGLA	CNGSFDMYVC	WDYAAPNATA
**90**	**100**	**110**	**120**	**130**	**140**	**150**	**160**
RASCPWYLPW	HHHVAAGFVL	RQCGSDGQWG	LWRDHTQCEN	PEKNEAFLDQ	RLILERLQVM	YTVGYSLSLA	TLLLALLILS
**170**	**180**	**190**	**200**	**210**	**220**	**230**	**240**
LFRRLHCTRN	YIHINLFTSF	MLRAAAILSR	DRLLPRPGPY	LGDQALALWN	QALAACRTAQ	IVTQYCVGAN	YTWLLVEGVY
**250**	**260**	**270**	**280**	**290**	**300**	**310**	**320**
LHSLLVLVGG	SEEGHFRYYL	LLGWGAPALF	VIPWVIVRYL	YENTQCWERN	EVKAIWWIIR	TPILMTILIN	FLIFIRILGI
**330**	**340**	**350**	**360**	**370**	**380**	**390**	**400**
LLSKLRTRQM	RCRDYRLRLA	RSTLTLVPLL	GVHEVVFAPV	TEEQARGALR	FAKLGFEIFL	SSFQGFLVSV	LYCFINKEVQ
**410**	**420**	**430**	**440**	**450**	**460**	**470**	
SEIRRGWHHC	RLRRSLGEEQ	RQLPERAFRA	LPSGSGPGEV	PTSRGLSSGT	LPGPGNEASR	ELESYC	

Cmpd.	No. of Cmpds.	m/z meas.	Δ m/z [ppm]	z	Rt [min]	Score	P	Range	Sequence	Modification
1715	6	1023.7648	265.10	1	51.4	23.4	2	327-333	R.TRQMRCR.D	Carbamidomethyl: 6; Oxidation: 4

Detailed Protein Report

Protein 523: **PREDICTED: collagen alpha-1(III) chain-like [Homo sapiens]**

Accession: gi|530433770
Database: refseq_human(refseq_human_20140103.fasta)
Database Date: 2015-11-30

Score: 23.4
MW [kDa]: 26.9
pI: 12.5
Sequence Coverage [%]: 14.5
No. of unique Peptides: 1

10	20	30	40	50	60	70	80
MIPAVSSAER	PGPVFVFSPP	PTPPRGREGA	GGGGPGTGGL	EGARQQPGRR	EPESERGPSP	APCLPSPAAA	WVEAAARQRA
**90**	**100**	**110**	**120**	**130**	**140**	**150**	**160**
GEQAVGRTER	LERPTLLRVP	LTPTRGSALP	FYPAPSRPPG	KPGVESGAGA	GRGTDSLPSL	HKGGGERTSR	GGGRPSAAFA
**170**	**180**	**190**	**200**	**210**	**220**	**230**	**240**
RQRRRRRLRR	KPGPEPAHLW	SLRRLPPGVP	IQVCPLPAVP	RGAGSVNFTR	GCKIGQSLRP	RPPRPSPDPL	AAGPVRWLLL
**250**	**260**						
STSTALFVFK	AFYGPR						

Cmpd.	No. of Cmpds.	m/z meas.	Δ m/z [ppm]	z	Rt [min]	Score	P	Range	Sequence	Modification
953	1	637.0944	77.56	3	41.4	11.8	1	184-201	R.RLPPGVPIQVCPLPAVPR.G	

Detailed Protein Report

Protein 524: **1-phosphatidylinositol 4,5-bisphosphate phosphodiesterase epsilon-1 isoform 2 [Homo sapiens]**

**Accession:**	gi|260166694	**Score:**	23.4
**Database:**	refseq_human(refseq_human_20140103.fasta)	**MW [kDa]:**	223.7
**Database Date:**	2015-11-30	**pI:**	6.2
**Modification(s):**	Oxidation	**Sequence Coverage [%]:**	1.1
		**No. of unique Peptides:**	1

BRUKER

Detailed Protein Report

10	20	30	40	50	60	70	80
MVSEGSAAGR	DFAGMEEVRQ	LHVRFCKGIK	IWHQAWFLCS	LLGREPQERE	AGCQLWLCTL	SAVLKVGWLF	PLSEVPNFTL
90	100	110	120	130	140	150	160
LKDGCGCWRL	KEDQIYNAVR	REETENTVGS	LLHFLTKLPA	SETAHGRISV	GPCLKQCVRD	TVCEYRATLQ	RTSISQYITG
170	180	190	200	210	220	230	240
SLLEATTSLG	ARSGLLSTFG	GSTGRMMLKE	RQPGPSVANS	NALPSSSAGI	SKELIDLQPL	IQFPEEVASI	LMEQEQTIYR
250	260	270	280	290	300	310	320
RVLPVDYLCF	LTRDLGTPEC	QSSLPCLKAS	ISASILTTQN	GEHNALEDLV	MRFNEVSSWV	TWLILTAGSM	EEKREVFSYL
330	340	350	360	370	380	390	400
VHVAKCCWNM	GNYNAVMEFL	AGLRSRKVLK	MWQFMDQSDI	ETMRSLKDAM	AQHESSCEYR	KVVTRALHIP	GCKVVPFCGV
410	420	430	440	450	460	470	480
FLKELCEVLD	GASGLMKLCP	RYNSQEETLE	FVADYSGQDN	FLQRVGQNGL	KNSEKESTVN	SIFQVIRSCN	RSLETDEEDS
490	500	510	520	530	540	550	560
PSEGNSSRKS	SLKDKSRWQF	IIGDLLDSDN	DIFEQSKEYD	SHGSEDSQKA	FDHGTELIPW	YVLSIQADVH	QFLLQGATVI
570	580	590	600	610	620	630	640
HYDQDTHLSA	RCFLQLQPDN	STLTWVKPTT	ASPASSKAKL	GVLNNTAEPG	KFPLLGNAGL	SSLTEGVLDL	FAVKAVYMGH
650	660	670	680	690	700	710	720
PGIDIHTVCV	QNKLGSMFLS	ETGVTLLYGL	QTTDNRLLHF	VAPKHTAKML	FSGLLELTRA	VRKMRKFPDQ	RQQWLRKQYV
730	740	750	760	770	780	790	800
SLYQEDGRYE	GPTLAHAVEL	FGGRRWSARN	PSPGTSAKNA	EKPNMQRNNT	LGISTTKKKK	KILMRGESGE	VTDDEMATRK
810	820	830	840	850	860	870	880
AKMHKECRSR	SGSDPQDINE	QEESEVNAIA	NPPNPLPSRR	AHSLTTAGSP	NLAAGTSSPI	RPVSSPVLSS	SNKSPSSAWS
890	900	910	920	930	940	950	960
SSSWHGRIKG	GMKGFQSFMV	SDSNMSFVEF	VELFKSFSVR	SRKDLKDLFD	VYAVPCNRSG	SESAPLYTNL	TIDENTSDLQ
970	980	990	1000	1010	1020	1030	1040
PDLDLLTRNV	SDLGLFIKSK	QQLSDNQRQI	SDAIAAASIV	TNGTGIESTS	LGIFGVGILQ	LNDFLVNCQG	EHCTYDEILS
1050	1060	1070	1080	1090	1100	1110	1120
IIQKFEPSIS	MCHQGLMSFE	GFARFLMDKE	NFASKNDESQ	ENIKELQLPL	SYYYIESSHN	TYLTGHQLKG	ESSVELYSQV
1130	1140	1150	1160	1170	1180	1190	1200
LLQGCRSVEL	DCWDGDDGMP	IIYHGHTLTT	KIPFKEVVEA	IDRSAFINSD	LPIIISIENH	CSLPQQRKMA	EIFKTVFGEK
1210	1220	1230	1240	1250	1260	1270	1280
LVTKFLFETD	FSDDPMLPSP	DQLRKKVLLK	NKKLKAHQTP	VDILKQKAHQ	LASMQVQAYN	GGNANPRPAN	NEEEEDEEDE
1290	1300	1310	1320	1330	1340	1350	1360
YDYDYESLSD	DNILEDRPEN	KSCNDKLQFE	YNEEIPKRIK	KADNSACNKG	KVYDMELGEE	FYLDQNKKES	RQIAPELSDL
1370	1380	1390	1400	1410	1420	1430	1440
VIYCQAVKFP	GLSTLNASGS	SRGKERKSRK	SIFGNNPGRM	SPGETASFNK	TSGKSSCEGI	RQTWEESSSP	LNPTTSLSAI
1450	1460	1470	1480	1490	1500	1510	1520
IRTPKCYHIS	SLNENAAKRL	CRRYSQKLTQ	HTACQLLRTY	PAATRIDSSN	PNPLMFWLHG	IQLVALNYQT	DDLPLHLNAA
1530	1540	1550	1560	1570	1580	1590	1600
MFEANGGCGY	VLKPPVLWDK	NCPMYQKFSP	LERDLDSMDP	AVYSLTIVSG	QNVCPSNSMG	SPCIEVDVLG	MPLDSCHFRT
1610	1620	1630	1640	1650	1660	1670	1680
KPIHRNTLNP	MWNEQFLFHV	HFEDLVFLRF	AVVENNSSAV	TAQRIIPLKA	LKRGYRHLQL	RNLHNEVLEI	SSLFINSRRM
1690	1700	1710	1720	1730	1740	1750	1760
EENSSGNTMS	ASSMFNTEER	KCLQTHRVTV	HGVPGPEPFT	VFTINGGTKA	KQLLQQILTN	EQDIKPVTTD	YFLMEEKYFI
1770	1780	1790	1800	1810	1820	1830	1840
SKEKNECRKQ	PFQRAIGPEE	EIMQILSSWF	PEEGYMGRIV	LKTQQENLEE	KNIVQDDKEV	ILSSEEESFF	VQVHDVSPEQ
1850	1860	1870	1880	1890	1900	1910	1920
PRTVIKAPRV	STAQDVIQQT	LCKAKYSYSI	LSNPNPSDYV	LLEEVVKDTT	NKKTTTPKSS	QRVLLDQECV	FQAQSKWKGA
1930	1940	1950	1960	1970	1980	1990	2000
GKFILKLKEQ	VQASREDKKK	GISFASELKK	LTKSTKQPRG	LTSPSQLLTS	ESIQTKEEKP	VGGLSSSDTM	DYRQ

Cmpd.	No. of Cmpds.	m/z meas.	Δ m/z [ppm]	z	Rt [min]	Score	P	Range	Sequence	Modification
2703	1	673.3636	55.13	2	64.1	13.1	1	1065-1075	R.FLMDKENFASK.N	Oxidation: 3

Detailed Protein Report

Protein 525: methyltransferase-like protein 17, mitochondrial isoform 1 precursor [Homo sapiens]

**Accession:**	gi|71999135	**Score:**	23.4
**Database:**	refseq_human(refseq_human_20140103.fasta)	**MW [kDa]:**	53.0
**Database Date:**	2015-11-30	**pI:**	10.4
**Modification(s):**	Carbamidomethyl	**Sequence Coverage [%]:**	6.7
		**No. of unique Peptides:**	1

10	20	30	40	50	60	70	80
MAAALKCLLT	LGRWCPGLGV	APQARALAAL	VPGVTQVDNK	SGFLQKRPHR	QHPGILKLPH	VRLPQALANG	AQLLLLGSAG
**90**	**100**	**110**	**120**	**130**	**140**	**150**	**160**
PTMENQVQTL	TSYLWSRHLP	VEPEELQRRA	RHLEKKFLEN	PDLSQTEEKL	RGAVLHALRK	TTYHWQELSY	TEGLSLVYMA
**170**	**180**	**190**	**200**	**210**	**220**	**230**	**240**
ARLDGGFAAV	SRAFHEIRAR	NPAFQPQTLM	DFGSGTGSVT	WAAHSIWGQS	LREYMCVDRS	AAMLVLAEKL	LKGGSESGEP
**250**	**260**	**270**	**280**	**290**	**300**	**310**	**320**
YIPGVFFRQF	LPVSPKVQFD	VVVSAFSLSE	LPSKADRTEV	VQTLWRKTGH	FLVLVENGTK	AGHSLLMDAR	DLVLKGKEKS
**330**	**340**	**350**	**360**	**370**	**380**	**390**	**400**
PLDPRPGFVF	APCPHELPCP	QLTNLACSFS	QAYHPIPFSW	NKKPKEEKFS	MVILARGSPE	EAHRWPRITQ	PVLKRPRHVH
**410**	**420**	**430**	**440**	**450**	**460**	**470**	**480**
CHLCCPDGHM	QHAVLTARRH	GRYGGCDQNQ	WDVAGSCSPR	QHLFPQGFVS	LCPCQLLGRS	FTCAYSVCVS	SIYGSGSL

Cmpd.	No. of Cmpds.	m/z meas.	Δ m/z [ppm]	z	Rt [min]	Score	P	Range	Sequence	Modification
2043	1	709.3053	-139.87	2	55.7	12.2	1	1-13	-.MAAALKCLLTLGR.W	Carbamidomethyl: 7

Detailed Protein Report

Protein 526: PREDICTED: ankyrin-2 isoform X16 [Homo sapiens]

**Accession:**	gi|578809070	**Score:**	23.4
**Database:**	refseq_human(refseq_human_20140103.fasta)	**MW [kDa]:**	454.5
**Database Date:**	2015-11-30	**pI:**	4.9
		**Sequence Coverage [%]:**	0.5
		**No. of unique Peptides:**	2

Quantitation

MD:MU	**Median:**	1.07	**CV:**	0.00 %	**No. of Peptides:**	1	

Detailed Protein Report

10	20	30	40	50	60	70	80
MGNAALCSSC	HADKSIRAQG	NMQELDKTPD	YYGCNSEDVK	EPGVWVTCPQ	MKPEAANIEK	SDSNASFLRA	ARAGNLDKVV
90	100	110	120	130	140	150	160
EYLKGGIDIN	TCNQNGLNAL	HLAAKEGHVG	LVQELLGRGS	SVDSATKKGN	TALHIASLAG	QAEVVKVLVK	EGANINAQSQ
170	180	190	200	210	220	230	240
NGFTPLYMAA	QENHIDVVKY	LLENGANQST	ATEDGFTPLA	VALQQGHNQA	VAILLENDTK	GKVRLPALHI	AARKDDTKSA
250	260	270	280	290	300	310	320
ALLLQNDHNA	DVQSKSGFTP	LHIAAHYGNV	NVATLLLNRG	AAVDFTARNG	ITPLHVASKR	GNTNMVKLLL	DRGGQIDAKT
330	340	350	360	370	380	390	400
RDGLTPLHCA	ARSGHDQVVE	LLLERGAPLL	ARTKNGLSPL	HMAAQGDHVE	CVKHLLQHKA	PVDDVTLDYL	TALHVAAHCG
410	420	430	440	450	460	470	480
HYRVTKLLLD	KRANPNARAL	NGFTPLHIAC	KKNRIKVMEL	LVKYGASIQA	ITESGLTPIH	VAAFMGHLNI	VLLLLQNGAS
490	500	510	520	530	540	550	560
PDVTNIRGET	ALHMAARAGQ	VEVVRCLLRN	GALVDARARE	EQTPLHIASR	LGKTEIVQLL	LQHMAHPDAA	TTNGYTPLHI
570	580	590	600	610	620	630	640
SAREGQVDVA	SVLLEAGAAH	SLATKKGFTP	LHVAAKYGSL	DVAKLLLQRR	AAADSAGKNG	LTPLHVAAHY	DNQKVALLLL
650	660	670	680	690	700	710	720
EKGASPHATA	KNGYTPLHIA	AKKNQMQIAS	TLLNYGAETN	IVTKQGVTPL	HLASQEGHTD	MVTLLLDKGA	NIHMSTKSGL
730	740	750	760	770	780	790	800
TSLHLAAQED	KVNVADILTK	HGADQDAHTK	LGYTPLIVAC	HYGNVKMVNF	LLKQGANVNA	KTKNGYTPLH	QAAQQGHTHI
810	820	830	840	850	860	870	880
INVLLQHGAK	PNATTANGNT	ALAIAKRLGY	ISVVDTLKVV	TEEVTTTTTT	ITEKHKLNVP	ETMTEVLDVS	DEEALKQFGD
890	900	910	920	930	940	950	960
HFIDGEALSD	SGDDTMTGDG	GEYLRPEDLK	ELGDDSLPSS	QFLDGMNYLR	YSLEGGRSDS	LRSFSSDRSH	TLSHASYLRD
970	980	990	1000	1010	1020	1030	1040
SAVMDDSVVI	PSHQVSTLAK	EAERNSYRLS	WGTENLDNVA	LSSSPIHSGF	LVSFMVDARG	GAMRGCRHNG	LRIIIPPRKC
1050	1060	1070	1080	1090	1100	1110	1120
TAPTRVTCRL	VKRHRLATMP	PMVEGEGLAS	RLIEVGPSGA	QFLGPVIVEI	PHFAALRGKE	RELVVLRSEN	GDSWKEHFCD
1130	1140	1150	1160	1170	1180	1190	1200
YTEDELNEIL	NGMDEVLDSP	EDLEKKRICR	IITRDFPQYF	AVVSRIKQDS	NLIGPEGGVL	SSTVVPQVQA	VFPEGALTKR
1210	1220	1230	1240	1250	1260	1270	1280
IRVGLQAQPM	HSELVKKILG	NKATFSPIVT	LEPRRRKFHK	PITMTIPVPK	ASSDVMLNGF	GGDAPTLRLL	CSITGGTTPA
1290	1300	1310	1320	1330	1340	1350	1360
QWEDITGTTP	LTFVNECVSF	TTNVSARFWL	IDCRQIQESV	TFASQVYREI	ICVPYMAKFV	VFAKSHDPIE	ARLRCFCMTD
1370	1380	1390	1400	1410	1420	1430	1440
DKVDKTLEQQ	ENFAEVARSR	DVEVLEGKPI	YVDCFGNLVP	LTKSGQHHIF	SFFAFKENRL	PLFVKVRDTT	QEPCGRLSFM
1450	1460	1470	1480	1490	1500	1510	1520
KEPKSTRGLV	HQAICNLNIT	LPIYTKESES	DQEQEEEIDM	TSEKNDETES	TETSVLKSHL	VNEVPVLASP	DLLSEVSEMK
1530	1540	1550	1560	1570	1580	1590	1600
QDLIKMTAIL	TTDVSDKAGS	IKVKELVKAA	EEEPGEPFEI	VERVKEDLEK	VNEILRSGTC	TRDESSVQSS	RSERGLVEEE
1610	1620	1630	1640	1650	1660	1670	1680
WVIVSDEEIE	EARQKAPLEI	TEYPCVEVRI	DKEIKGKVEK	DSTGLVNYLT	DDLNTCVPLP	KEQLQTVQDK	AGKKCEALAV
1690	1700	1710	1720	1730	1740	1750	1760
GRSSEKEGKD	IPPDETQSTQ	KQHKPSLGIK	KPVRRKLKEK	QKQKEEGLQA	SAEKAELKKG	SSEESLGEDP	GLAPEPLPTV
1770	1780	1790	1800	1810	1820	1830	1840
KATSPLIEET	PIGSIKDKVK	ALQKRVEDEQ	KGRSKLPIRV	KGKEDVPKKT	THRPHPAASP	SLKSERHAPG	SPSPKTERHS
1850	1860	1870	1880	1890	1900	1910	1920
TLSSSAKTER	HPPVSPSSKT	EKHSPVSPSA	KTERHSPASS	SSKTEKHSPV	SPSTKTERHS	PVSSTKTERH	PPVSPSGKTD
1930	1940	1950	1960	1970	1980	1990	2000
KRPPVSPSGR	TEKHPPVSPG	RTEKRLPVSP	SGRTDKHQPV	STAGKTEKHL	PVSPSGKTEK	QPPVSPTSKT	ERIEETMSVR
2010	2020	2030	2040	2050	2060	2070	2080
ELMKAFQSGQ	DPSKHKTGLF	EHKSAKQKQP	QEKGKVRVEK	EKGPILTQRE	AQKTENQTIK	RGQRLPVTGT	AESKRGVRVS
2090	2100	2110	2120	2130	2140	2150	2160
SIGVKKEDAA	GGKEKVLSHK	IPEPVQSVPE	EESHRESEVP	KEKMADEQGD	MDLQISPDRK	TSTDFSEVIK	QELEDNDKYQ
2170	2180	2190	2200	2210	2220	2230	2240

Detailed Protein Report

Cmpd.	No. of Cmpds.	m/z meas.	Δ m/z [ppm]	z	Rt [min]	Score	P	Range	Sequence	Modification	Ratios
182	1	745.2293	-195.45	2	31.7	10.9	1	15-27	K.SIRAQGNMQELDK.T		MD:MU 1.07
298	1	535.7922	-16.95	2	33.1	12.4	2	2027-2035	K.QKQPQEKGK.V		

Detailed Protein Report

Protein 527: transcription factor HIVEP3 isoform b [Homo sapiens]

**Accession:**	gi|189181750	**Score:**	23.3
**Database:**	refseq_human(refseq_human_20140103.fasta)	**MW [kDa]:**	259.2
**Database Date:**	2015-11-30	**pI:**	8.6
		**Sequence Coverage [%]:**	1.4
		**No. of unique Peptides:**	1

Detailed Protein Report

10	20	30	40	50	60	70	80
MDPEQSVKGT	KKAEGSPRKR	LTKGEAIQTS	VSSSVPYPGS	GTAATQESPA	QELLAPQFPF	GPSSVLREGS	QEKTGQQQKP
90	100	110	120	130	140	150	160
PKRPPIEASV	HISQLPQHPL	TPAFMSPGKP	EHLLEGSTWQ	LVDPMRPGPS	GSFVAPGLHP	QSQLLPSHAS	IIPPEDLPGV
170	180	190	200	210	220	230	240
PKVFVPRPSQ	VSLKPTEEAH	KKERKPQKPG	KYICQYCSRP	CAKPSVLQKH	IRSHTGERPY	PCGPCGFSFK	TKSNLYKHRK
250	260	270	280	290	300	310	320
SHAHRIKAGL	ASGMGGEMYP	HGLEMERIPG	EEFEEPTEGE	STDSEEETSA	TSGHPAELSP	RPKQPLLSSG	LYSSGSHSSS
330	340	350	360	370	380	390	400
HERCSLSQSS	TAQSLEDPPP	FVEPSSEHPL	SHKPEDTHTI	KQKLALRLSE	RKKVIDEQAF	LSPGSKGSTE	SGYFSRSESA
410	420	430	440	450	460	470	480
EQQVSPPNTN	AKSYAEIIFG	KCGRIGQRTA	MLTATSTQPL	LPLSTEDKPS	LVPLSVPRTQ	VIEHITKLIT	INEAVVDTSE
490	500	510	520	530	540	550	560
IDSVKPRRSS	LSRRSSMESP	KSSLYREPLS	SHSEKTKPEQ	SLLSLQHPPS	TAPPVPLLRS	HSMPSAACTI	STPHHPFRGS
570	580	590	600	610	620	630	640
YSFDDHITDS	EALSHSSHVF	TSHPRMLKRQ	PAIELPLGGE	YSSEEPGPSS	KDTASKPSDE	VEPKESELTK	KTKKGLKTKG
650	660	670	680	690	700	710	720
VIYECNICGA	RYKKRDNYEA	HKKYYCSELQ	IAKPISAGTH	TSPEAEKSQI	EHEPWSQMMH	YKLGTTLELT	PLRKRRKEKS
730	740	750	760	770	780	790	800
LGDEEEPPAF	ESTKSQFGSP	GPSDAARNLP	LESTKSPAEP	SKSVPSLEGP	TGFQPRTPKP	GSGSESGKER	RTTSKEISVI
810	820	830	840	850	860	870	880
QHTSSFEKSD	SLEQPSGLEG	EDKPLAQFPS	PPPAPHGRSA	HSLQPKLVRQ	PNIQVPEILV	TEEPDRPDTE	PEPPPKEPEK
890	900	910	920	930	940	950	960
TEEFQWPQRS	QTLAQLPAEK	LPPKKKRLRL	AEMAQSSGES	SFESSVPLSR	SPSQESNVSL	SGSSRSASFE	RDDHGKAEAP
970	980	990	1000	1010	1020	1030	1040
SPSSDMRPKP	LGTHMLTVPS	HHPHAREMRR	SASEQSPNVS	HSAHMTETRS	KSFDYGSLSL	TGPSAPAPVA	PPARVAPPER
1050	1060	1070	1080	1090	1100	1110	1120
RKCFLVRQAS	LSRPPESELE	VAPKGRQESE	EPQPSSSKPS	AKSSLSQISS	AATSHGGPPG	GKGPGQDRPP	LGPTVPYTEA
1130	1140	1150	1160	1170	1180	1190	1200
LQVFHHPVAQ	TPLHEKPYLP	PPVSLFSFQH	LVQHEPGQSP	EFFSTQAMSS	LLSSPYSMPP	LPPSLFQAPP	LPLQPTVLHP
1210	1220	1230	1240	1250	1260	1270	1280
GQLHLPQLMP	HPANIPFRQP	PSFLPMPYPT	SSALSSGFFL	PLQSQFALQL	PGDVESHLPQ	IKTSLAPLAT	GSAGLSPSTE
1290	1300	1310	1320	1330	1340	1350	1360
YSSDIRLPPV	APPASSSAPT	SAPPLALPAC	PDTMVSLVVP	VRVQTNMPSY	GSAMYTTLSQ	ILVTQSQGSS	ATVALPKFEE
1370	1380	1390	1400	1410	1420	1430	1440
PPSKGTTVCG	ADVHEVGPGP	SGLSEEQSRA	FPTPYLRVPV	TLPERKGTSL	SSESILSLEG	SSSTAGGSKR	VLSPAGSLEL
1450	1460	1470	1480	1490	1500	1510	1520
TMETQQQKRV	KEEEASKADE	KLELVKPCSV	VLTSTEDGKR	PEKSHLGNQG	QGRRELEMLS	SLSSDPSDTK	EIPPLPHPAL
1530	1540	1550	1560	1570	1580	1590	1600
SHGTAPGSEA	LKEYPQPSGK	PHRRGLTPLS	VKKEDSKEQP	DLPSLAPPSS	LPLSETSSRP	AKSQEGTDSK	KVLQFPSLHT
1610	1620	1630	1640	1650	1660	1670	1680
TTNVSWCYLN	YIKPNHIQHA	DRRSSVYAGW	CISLYNPNLP	GVSTKAALSL	LRSKQKVSKE	TYTMATAPHP	EAGRLVPSSS
1690	1700	1710	1720	1730	1740	1750	1760
RKPRMTEVHL	PSLVSPEGQK	DLARVEKEEE	RRGEPEEDAP	ASQRGEPARI	KIFEGGYKSN	EEYVYVRGRG	RGKYVCEECG
1770	1780	1790	1800	1810	1820	1830	1840
IRCKKPSMLK	KHIRTHTDVR	PYVCKHCHFA	FKTKGNLTKH	MKSKAHSKKC	QETGVLEELE	AEEGTSDDLF	QDSEGREGSE
1850	1860	1870	1880	1890	1900	1910	1920
AVEEHQFSDL	EDSDSDSDLD	EDEDEDEEES	QDELSRPSSE	APPPGPPHAL	RADSSPILGP	QPPDAPASGT	EATRGSSVSE
1930	1940	1950	1960	1970	1980	1990	2000
AERLTASSCS	MSSQSMPGLP	WLGPAPLGSV	EKDTGSALSY	KPVSPRRPWS	PSKEAGSRPP	LARKHSLTKN	DSSPQRCSPA
2010	2020	2030	2040	2050	2060	2070	2080
REPQASAPSP	PGLHVDPGRG	MGALPCGSPR	LQLSPLTLCP	LGRELAPRAH	VLSKLEGTTD	PGLPRYSPTR	RWSPGQAESP
2090	2100	2110	2120	2130	2140	2150	2160
PRSAPPGKWA	LAGPGSPSAG	EHGPGLGLDP	RVLFPPAPLP	HKLLSRSPET	CASPWKAESR	SPSCSPGPAH	PLSSRPFSAL
2170	2180	2190	2200	2210	2220	2230	2240
HDFHGHILAR	TEENIFSHLP	LHSQHLTRAP	CPLIPIGGIQ	MVQARPGAHP	TLLPGPTAAW	VSGFSGGGSD	LTGAREAQER
2250	2260	2270	2280	2290	2300	2310	2320
GRWSPTESSS	ASVSPVAKVS	KFTLSSELEG	GDYPKERERT	GGGPGRPPDW	TPHGTGAPAE	PTPTHSPCTP	PDTLPRPPQG
2330	2340	2350	2360	2370	2380	2390	2400

Detailed Protein Report

Cmpd.	No. of Cmpds.	m/z meas.	Δ m/z [ppm]	z	Rt [min]	Score	P	Range	Sequence	Modification
1349	1	942.4987	40.33	2	46.5	10.1	2	1985-2001	K.HSLTKNDSSPQRCSPAR.E	

Detailed Protein Report

Protein 528: **PREDICTED: zinc finger protein 823 isoform X1 [Homo sapiens]**

**Accession:**	gi|530415003	**Score:**	23.3
**Database:**	refseq_human(refseq_human_20140103.fasta)	**MW [kDa]:**	49.6
**Database Date:**	2015-11-30	**pI:**	10.4
**Modification(s):**	Carbamidomethyl	**Sequence Coverage [%]:**	6.5
		**No. of unique Peptides:**	1

10	20	30	40	50	60	70	80
MAAHHGDGPY	KCKLCGKAFV	WPSLFHLHER	THTGEKPYEC	KQCSKAFPFY	SSYLRHERIH	TGEKAYECKQ	CSKAFPDYST
**90**	**100**	**110**	**120**	**130**	**140**	**150**	**160**
YLRHERTHTG	EKPYKCTQCG	KAFSCYYYTR	LHERTHTGEQ	PYACKQCGKT	FYHHTSFRRH	MIRHTGDGPH	KCKICGKGFD
**170**	**180**	**190**	**200**	**210**	**220**	**230**	**240**
CPSSVRNHET	THTGEKPYEC	KQCGKVLSHS	SSFRSHMITH	TGDGPQKCKI	CGKAFGCPSL	FQRHERTHTG	EKPYQCKQCG
**250**	**260**	**270**	**280**	**290**	**300**	**310**	**320**
KAFSLAGSLR	RHEATHTGVK	PYKCQCGKAF	SDLSSFQNHE	TTHTGEKPYE	CKECGKAFSC	FKYLSQHKRT	HTVEKPYECK
**330**	**340**	**350**	**360**	**370**	**380**	**390**	**400**
TCRKAFSHFS	NLKVHERIHS	GEKPYECKEC	GKAFSWLTCL	LRHERIHTGE	KPYECLQCGK	AFTRSRFLRG	HEKTHTGEKL
**410**	**420**	**430**					
YECKECGKAL	SSLRSLHRHK	RTHWKDTL					

Cmpd.	No. of Cmpds.	m/z meas.	Δ m/z [ppm]	z	Rt [min]	Score	P	Range	Sequence	Modification
739	1	512.6267	-200.37	2	38.8	10.6	0	158-166	K.GFDCPSSVR.N	Carbamidomethyl: 4

Detailed Protein Report

Protein 529: dynein heavy chain 12, axonemal isoform 1 [Homo sapiens]

**Accession:**	gi|194440727	**Score:**	23.3
**Database:**	refseq_human(refseq_human_20140103.fasta)	**MW [kDa]:**	356.7
**Database Date:**	2015-11-30	**pI:**	5.8
**Modification(s):**	Oxidation	**Sequence Coverage [%]:**	1.6
		**No. of unique Peptides:**	2

Detailed Protein Report

10	20	30	40	50	60	70	80
MSDANKAAIA	AEKEALNLKL	PPIVHLPENI	GVDTPTQSKL	LKYRRSKEQQ	QKINQLVIDG	AKRNLDRTLG	KRTPLLPPPD
90	100	110	120	130	140	150	160
YPQTMTSEMK	KKGFNYIYMK	QCVESSPLVP	IQQEWLDHML	RLIPESLKEG	KEREELLESL	INEVSSDFEN	SMKRYLVQSV
170	180	190	200	210	220	230	240
LVKPPVKSLE	DEGGPLPESP	VGLDYSNPWH	SSYVQARNQI	FSNLHIIHPT	MKMLLDLGYT	TFADTVLLDF	TGIRAKGPID
250	260	270	280	290	300	310	320
CESLKTDLSI	QTRNAEEKIM	NTWYPKVINL	FTKKEALEGV	KPEKLDAFYS	CVSTLMSNQL	KDLLRRTVEG	FVKLFDPKDQ
330	340	350	360	370	380	390	400
QRLPIFKIEL	TFDDDKMEFY	PTFQDLEDNV	LSLVERIAEA	LQNVQTIPSW	LSGTSTPVNL	DTELPEHVLH	WAVDTLKAAV
410	420	430	440	450	460	470	480
HRNLEGARKH	YETYVEKYNW	LLDGTAVENI	ETFQTEDHTF	DEYTEFIEKF	LSLASEIMLL	PQWIHYTMVR	LDCEDLKTGL
490	500	510	520	530	540	550	560
TNKAKAFANI	LLNDIASKYR	KENECICSEF	EAIKEHALKV	PETTEEMMDL	ISYVEKARTV	GIEELILRIQ	ESKRQMSYFL
570	580	590	600	610	620	630	640
DVFLFPQEDL	ALNATVLMWP	RKINPIFDEN	DELIENAKHK	KENELMAKRE	KLILEIEKES	RRMEEFTEFA	ELERMQQYVT
650	660	670	680	690	700	710	720
DVRQLQKRIQ	ESEEAVQFIN	KEEELFKWEL	TKYPELDKLK	VNIEPYQKFF	NFVLKWQRSE	KRWMDGGFLD	LNGESMEADV
730	740	750	760	770	780	790	800
EEFSREIFKT	LKFFQTKLKK	ELQEKRKAAR	KRSLEEEKIE	EEPKDNATIT	MCRMRARHWK	QISEIVGYDL	TPDSGTTLRK
810	820	830	840	850	860	870	880
VLKLNLTPYL	EQFEVISAGA	SKEFSLEKAM	NTMIGTWEDI	AFHISLYRDT	GVCILSSVDE	IQAILDDQII	KTQTMRGSPF
890	900	910	920	930	940	950	960
IKPFEHEIKA	WEDRLIRIQE	TIDEWLKVQA	QWLYLEPIFC	SEDIMQQMPE	EGRQFQTVDR	HWRDIMKFCA	KDPKVLAATS
970	980	990	1000	1010	1020	1030	1040
LTGLLEKLQN	CNELLEKIMK	GLNAYLEKKR	LFFPRFFFLS	NDEMLEILSE	TKDPLRVQPH	LKKCFEGIAK	LEFLPNLDIK
1050	1060	1070	1080	1090	1100	1110	1120
AMYSSEGERV	ELIALISTSA	ARGAVEKWLI	QVEDLMLRSV	HDVIAAARLA	YPESARRDWV	REWPGQVVLC	ISQMFWTSET
1130	1140	1150	1160	1170	1180	1190	1200
QEVISGGTEG	LKKYYKELQN	QLNEIVELVR	GKLSKQTRTT	LGALVTIDVH	ARDVVMDMIK	MGVSHDTDFL	WLAQLRYYWE
1210	1220	1230	1240	1250	1260	1270	1280
NENARVRIIN	CNVKYAYEYL	GNSPRLVITP	LTDRCYRTLI	GAFYLNLGGA	PEGPAGTGKT	ETTKDLAKAL	AVQCVVFNCS
1290	1300	1310	1320	1330	1340	1350	1360
DGLDYLAMGK	FFKGLASSGA	WACFDEFNRI	ELEVLSVVAQ	QILCIQRAIQ	QKLVVFVFEG	TELKLNPNCF	VAITMNPGYA
1370	1380	1390	1400	1410	1420	1430	1440
GRSELPDNLK	VLFRTVAMMV	PNYALIAEIS	LYSYGFLNAR	PLSVKIVMTY	RLCSEQLSSQ	FHYDYGMRAV	KAVLVAAGNL
1450	1460	1470	1480	1490	1500	1510	1520
KLKYPNENED	ILLLRSIKDV	NEPKFLSHDI	PLFNGITSDL	FPGIKLPEAD	YHEFLECAHE	ACNVHNLQPV	KFFLEKIIQT
1530	1540	1550	1560	1570	1580	1590	1600
YEMMIVRHGF	MLVGEPFAAK	TKVLHVLADT	LTLMNEHGYG	EEEKVIYRTV	NPKSITMGQL	FGQFDPVSHE	WTDGIVANTF
1610	1620	1630	1640	1650	1660	1670	1680
REFALSETPD	RKWVVFDGPI	DTLWIESMNT	VLDDNKKLCL	MSGEIIQMSP	QMSLIFETMD	LSQASPATVS	RCGMIYLEPS
1690	1700	1710	1720	1730	1740	1750	1760
QLGWEPLVSS	WLNSLKGPLC	EPEYQALLRG	LFAWLIPPSL	NQRVELFQLN	YLYTTIVSKI	LKILITFRIS	NYFKYVPLKT
1770	1780	1790	1800	1810	1820	1830	1840
QCTFIKFFLH	QQACFIFSLI	WSIGGSCDTD	GRRVFDTFIR	LIILGKDDEN	PVPDSVGKWE	CPFDEKGLVY	DYMYELKNKG
1850	1860	1870	1880	1890	1900	1910	1920
RWVHWNELIK	NTNLGDKQIK	IQDIIVPTMD	TIRYTFLMDL	SITYAKPLLF	VGPTGTGKSV	YVKDKLMNHL	EKDQYFPFYI
1930	1940	1950	1960	1970	1980	1990	2000
NLSARTSANQ	VQNIIMARLD	KRRKGVFGPP	MGKKCIIFID	DMNMPALEKY	GAQPPIELLR	QFFDCGHWYD	LKDTSKITLV
2010	2020	2030	2040	2050	2060	2070	2080
DIELIAAMGP	PGGGRNPVTP	RCIRHFNICS	INSFSDETMV	RIFSSIVAFY	LRTHEFPPEY	FVIGNQIVNG	TMEIYKQSVE
2090	2100	2110	2120	2130	2140	2150	2160
NLLPTPTKSH	YTFNLRDFSR	VIRGCLLIER	DAVANKHTMI	RLFVHEVLRV	FYDRLINDDD	RRWLFQLTKT	VIKDHFKESF
2170	2180	2190	2200	2210	2220	2230	2240
HSIFSHLRKQ	NAPVTEEDLR	NLMFGDYMNP	DLEGDDRVYI	EIPNIHHFSD	VVDQCLDEYN	QTHKTRMNLV	IFRYVLEHLS
2250	2260	2270	2280	2290	2300	2310	2320
RICRVLKQSG	GNALLVGLGG	SGRQSLTRLA	TSMAKMHIFQ	PEISKSYGMN	EWREDMKSFI	AVPVTNRIVD	NKSKILEKRL
2330	2340	2350	2360	2370	2380	2390	2400

Detailed Protein Report

Cmpd.	No. of Cmpds.	m/z meas.	Δ m/z [ppm]	z	Rt [min]	Score	P	Range	Sequence	Modification
47	1	1103.4984	-39.62	2	29.9	12.9	1	73-91	R.TPLLPPPDYPQTMTSEMKK.K	Oxidation: 13, 17
2090	1	1159.8674	-54.47	3	56.0	10.4	2	1512-1540	K.FFLEKIIQTYEMMIVRHGFMLVGEPFAAK.T	Oxidation: 12, 20

Detailed Protein Report

Protein 530: granulysin isoform 519 [Homo sapiens]

Accession: gi|157502222
Database: refseq_human(refseq_human_20140103.fasta)
Database Date: 2015-11-30
Modification(s): Carbamidomethyl

Score: 23.3
MW [kDa]: 14.8
pI: 10.5
Sequence Coverage [%]: 10.8
No. of unique Peptides: 1

10	20	30	40	50	60	70	80
MEGLVFSRLS	PEYYDLARAH	LRDEEKSCPC	LAQEGPQGDL	LTKTQELGRD	YRTCLTIVQK	LKKMVDKPTQ	RSVSNAATRV
**90**	**100**	**110**	**120**	**130**	**140**		
CRTGRSRWRD	VCRNFMRRYQ	SRVTQGLVAG	ETAQQICEDL	RLCIPSTGPL			

Cmpd.	No. of Cmpds.	m/z meas.	Δ m/z [ppm]	z	Rt [min]	Score	P	Range	Sequence	Modification
74	1	767.7586	-180.19	2	30.3	23.3	2	72-85	R.SVSNAATRVCRTGR.S	Carbamidomethyl: 10

Detailed Protein Report

Protein 531: PREDICTED: zinc finger protein 728 isoform X1 [Homo sapiens]

**Accession:**	gi|578833255	**Score:**	23.2
**Database:**	refseq_human(refseq_human_20140103.fasta)	**MW [kDa]:**	68.5
**Database Date:**	2015-11-30	**pI:**	10.3
**Modification(s):**	Carbamidomethyl	**Sequence Coverage [%]:**	4.7
		**No. of unique Peptides:**	1

10	20	30	40	50	60	70	80
MGSLTFRDVA	IQFSLEEWQC	LDTAQQNLYR	NVMLENYRNL	VFLGIAAPKP	DLIIFLEQGK	EPWNMKRHEL	VKEPPVICSH
**90**	**100**	**110**	**120**	**130**	**140**	**150**	**160**
FAQDLWPEQG	REDSFQKVIL	RRYEKCGHEN	LQLKIGCTNV	DECKVHKKGY	NKLNQSLTTT	QSKVFQCGKY	ANIFHKCSNS
**170**	**180**	**190**	**200**	**210**	**220**	**230**	**240**
KRHKIRHTGK	KLLKCKEYVR	SFCMLSHLSQ	HKRIYTRENS	YKSEEHGKAF	NWSSALTYKR	IHTGEKPCKC	EECGKAFSKF
**250**	**260**	**270**	**280**	**290**	**300**	**310**	**320**
SILTKHKVIH	TGEKHYKCEE	CGKAFTRSSS	LIEHKRSHAG	EKPYKCEECG	KAFSKASTLT	AHKTIHAGEK	PYKCEECGKA
**330**	**340**	**350**	**360**	**370**	**380**	**390**	**400**
FNRSSNLMEH	KRIHTGEKPC	KCEECGKAFG	NFSTLTKHKV	IHTGEKPYKC	EECGKAFSWP	SSLTEHKRIH	AGDKPYKCEE
**410**	**420**	**430**	**440**	**450**	**460**	**470**	**480**
CGKTFKWSST	LTKHKIIHTG	EKPYKCEECG	KAFTTFSSLT	KHKVIHTGEK	HYKCEECGKV	FSWSSSLTTH	KRIHTGEKPY
**490**	**500**	**510**	**520**	**530**	**540**	**550**	**560**
KCEECGKAFS	KVANLTKHKV	IHTGEKQYKC	EECGKAFIWS	SRLSEHKRIH	TGEKPYKCEE	CGKAFSWVSV	LNKHKKIHAG
**570**	**580**	**590**	**600**				
KKFYKCEECG	KDFNQSSHLT	THKRIHTGGK	TLQM				

Cmpd.	No. of Cmpds.	m/z meas.	Δ m/z [ppm]	z	Rt [min]	Score	P	Range	Sequence	Modification
2120	1	924.3321	-133.13	2	56.4	11.9	2	230-245	K.CEECGKAFSKFSILTK.H	Carbamidomethyl: 1

Detailed Protein Report

Protein 532: mucin-19 precursor [Homo sapiens]

**Accession:**	gi|533112494	**Score:**	23.2
**Database:**	refseq_human(refseq_human_20140103.fasta)	**MW [kDa]:**	804.8
**Database Date:**	2015-11-30	**pI:**	4.8
**Modification(s):**	Carbamidomethyl	**Sequence Coverage [%]:**	0.5
		**No. of unique Peptides:**	1

Detailed Protein Report

10	20	30	40	50	60	70	80
MKLILWYLVV	ALWCFFKDVE	ALLYRQKSDG	KIAASRSGGF	SYGSSSSGDL	DRKKPLFSLE	FGSPGETEDK	SRQRQDAGSP
90	100	110	120	130	140	150	160
KSEDTPAGGF	FNSSSSSGDS	DRTKPFFSLG	LGAPGKAEDK	SGDSQDAGGS	KSEDTPPGGF	FYGSSSSGDS	DKKKPLFSFE
170	180	190	200	210	220	230	240
FGATGEDEDK	SRERWDAGNS	RSEDSPADST	NTRYGAGFSS	SGASLDVGFG	WGISDEKGLE	VSKADGRETR	GSGSAGGETI
250	260	270	280	290	300	310	320
VFGPDAGSSV	GTGSSGLKLG	AGKGDAAFGF	EVSDSNSFGD	TGISSKTVEG	NQTSSSGGSV	SIDLGDTSFR	SENQFVGGGS
330	340	350	360	370	380	390	400
LNSISNLWDS	GQEGFGINEI	GGNGMSGSVS	AEAGFKGFGS	DSSSSGDSSA	RNGFENSSGI	SEDSGVILGS	SDQHEVELSR
410	420	430	440	450	460	470	480
TGGNRKRSSD	PDEAGNLSPG	SDVSDSGGNT	WSSDSGSGGG	GVTSSSEYST	SGPLNTPEKG	SHIPEATPKY	SETNAIIGEI
490	500	510	520	530	540	550	560
STWSKGAYKS	FNGRIFFFES	SCPYTFCRHC	IESGGDFNIE	IKRNNDSEIE	KITVLIDNND	VSIFGDTILV	NGESVQIPYN
570	580	590	600	610	620	630	640
NKLIHIKKYG	EHNVLNSRRG	ILTLMWDKNN	KLSLTLHKQY	PTCGLCGNFN	STPGQDINEH	IANSKIPGDC	PNAVGKSYEV
650	660	670	680	690	700	710	720
CEDGIQHCNK	IIGTYFEKCG	KVAALSNDYK	MICIDEYCQT	RDKTSTCDTY	SELSRLCASD	GPGTFESWRS	DSDVVCGTQR
730	740	750	760	770	780	790	800
CPEQHIYKEC	GPSNPATCSN	VAPFQDSECV	SGCTCPEGYL	LDDIGEKGKC	VLKAECPCES	SGTVYQPGEV	REGPCGSQCT
810	820	830	840	850	860	870	880
CQDAKWSCTE	ALCPGRCKVE	GSSLTTFDGV	KYNFPGNCHF	LAVHNEDWSI	SVELRPCPSG	QTGTCLNSVT	LLLNSSVPVD
890	900	910	920	930	940	950	960
KYVFNSDGTV	TNDKIRNQGY	YYSDKIQIFN	ASSSYLQVET	YFHVKLQIQI	VPVMQLYVSM	PPNQFTDTVG	LCGSYNNKAE
970	980	990	1000	1010	1020	1030	1040
DDFMSSQNIL	EKTSQAFANS	WEMMSCPKGN	PSSCISIEKE	KFAERHCGIL	LDSSGPLASC	HPIVNPKPYH	EECKKYTCTC
1050	1060	1070	1080	1090	1100	1110	1120
ENSQDCLCTI	LGNYVKACAE	KETYIVGWRT	GLCEHSCPSG	LVFKYNVKAC	NSSCRSLSER	DRSCDVEDVP	VDGCTCPDAM
1130	1140	1150	1160	1170	1180	1190	1200
YQNNEGNCVL	KSQCDCYIND	EVMQPGKLIH	IDDNKCVCRD	GILLCQIPID	LTLQNCSGGA	EYVDCSDPKA	QRRTNRTCST
1210	1220	1230	1240	1250	1260	1270	1280
RNIPVFDENL	PCKRGCFCPE	GMVRNSKGIC	VFPNDCPCSF	GGREYDEGSV	TSVGCNECTC	IKGSWSCTQN	ECQTICHIYG
1290	1300	1310	1320	1330	1340	1350	1360
EGHVRTFDGK	SYSFDGLCQY	SFLEDYCGHE	NGTFRILTES	VPCCEDGLTC	SRKIIVAFQD	QNIVLQDGKV	TAVKSTESKK
1370	1380	1390	1400	1410	1420	1430	1440
CELNANAYSI	HTVGLYLILK	FQNGIIVIWD	KNTRLSVILD	PNWNGKVCGL	CGNNNGDLKD	DFTTRYSSVA	SGALEFGNSW
1450	1460	1470	1480	1490	1500	1510	1520
KTSQECSDTV	AQTFPCDSNP	YCKAWAVRKC	EILRDSTFRD	CHNKVDPSAY	HDACIEEACA	CDMEGKYLGF	CTAVAMYAEA
1530	1540	1550	1560	1570	1580	1590	1600
CSAVGVCVSW	RKPNLCPVYC	DYYNAPGECR	WHYEPCGTVT	AKTCKDQLVG	QKFSSLLEGC	YAKCPDSAPY	LDENTMKCVS
1610	1620	1630	1640	1650	1660	1670	1680
LSECSCFYND	VIPAGGVIED	NCGRTCYCIA	GQLECSETAP	TNSTFAVSTT	TATTILSTGA	AITLVTGGPS	TAASIPAITT
1690	1700	1710	1720	1730	1740	1750	1760
SSSETTGTTL	GPLTEPFTTG	ITETSVPIIS	TSGNAGMTGV	VSPTVTGASG	MAGTTGGVDA	ATTGAASENT	SERAGTPRVS
1770	1780	1790	1800	1810	1820	1830	1840
GETPAVGGGS	TPGEAGPGAT	VSGSTGVSAG	SITASPGASA	TSSESSKSGT	TGPSVGGKTG	ATSSEATSSE	GMSGVTGQSL
1850	1860	1870	1880	1890	1900	1910	1920
GSTAGSDSEI	TAKTSFTGSS	PPGKLTRPSP	GSPGHFSGGT	TEWGNVATTG	AAGENTSGAL	GSTEGSVEAT	TSAGSGNTAG
1930	1940	1950	1960	1970	1980	1990	2000
TSGTGDTGPG	NTAVSGTPVV	SPGATPGAPG	SSTPGEADIG	NTSFGKSGTP	TVSAASTTSS	PVSKHTDAAS	ATAVTISGSK
2010	2020	2030	2040	2050	2060	2070	2080
PGTPGTPGGA	TSGGKITSGW	SSSGTSTGAS	NTPGATGSST	GQTDTSGPSA	KVTGNYGQSS	EIPGTIKSSS	DVSGTMGQSD
2090	2100	2110	2120	2130	2140	2150	2160
TTSGPSVAVT	RTSEQSSGVT	VASEPSVGVS	GTTGPLAEIS	GTTRPLVSGL	RTTGSSAEGS	GTTGPSSRES	VTTRPLAEGS
2170	2180	2190	2200	2210	2220	2230	2240
GTSGQSVTGS	RATGLSATEL	GTTVSFTGGL	GTSRSSARET	RTTGPSADGS	GTTGPSVVRS	GTTRLSVGVT	RATESSPGVT
2250	2260	2270	2280	2290	2300	2310	2320
GTTTPSAEES	RTTGPSVLVT	GTTGQSGQGS	GTTGKSFIES	GPSVVGSGTT	GPTSAGLGTT	APSTRRSSTT	KPSVGRTGTT
2330	2340	2350	2360	2370	2380	2390	2400

Detailed Protein Report

Cmpd.	No. of Cmpds.	m/z meas.	Δ m/z [ppm]	z	Rt [min]	Score	P	Range	Sequence	Modification
2090	1	1131.3695	-118.05	2	56.3	11.2	1	8209-8227	K.LVKFQSNDTCCEIGYCEPR.T	Carbamidomethyl: 10

Detailed Protein Report

Protein 533: PREDICTED: radial spoke head 10 homolog B isoform X5 [Homo sapiens]

**Accession:**	gi|530384952	**Score:**	23.2
**Database:**	refseq_human(refseq_human_20140103.fasta)	**MW [kDa]:**	59.8
**Database Date:**	2015-11-30	**pI:**	6.8
**Modification(s):**	Oxidation	**Sequence Coverage [%]:**	2.7
		**No. of unique Peptides:**	1

10	20	30	40	50	60	70	80
MVKEKKKADK	KGEKSARSPS	SLSDNLDFSK	QDGNTTRQEM	SPAGVPLLGM	QLNEVKPKKD	RQNVQQNEDA	SQYEESILTK
90	100	110	120	130	140	150	160
LIVESYEGEK	VRGLYEGEGF	AAFQGGCTYR	GMFSEGLMHG	QGTYIWADGL	KYEGDFVKNV	PMNHGVYTWP	DGSMYEGEVV
170	180	190	200	210	220	230	240
NGMRNGFGMF	KCSTQPVSYI	GHWCNGKRHG	KGSIYYNQEG	TCWYEGDWVQ	NIKKGWGIRC	YKSGNIYEGQ	WEDNMRHGEG
250	260	270	280	290	300	310	320
RMRWLTTNEE	YTGRWERGIQ	NGFGTHTWFL	KRIRSSQYPL	RNEYIGEFVN	GYRHGRGKFY	YASGAMYDGE	WVSNKKHGMF
330	340	350	360	370	380	390	400
FCLQGRLTFK	NGRVYEGAFS	NDHIAGFPDL	EVEFISCLDL	SSGVAPRLSR	SAELIRKLDG	SESHSVLGSS	IELDLNLLLD
410	420	430	440	450	460	470	480
MYPETVQPEE	KKQVEYAVLR	NITELRRIYS	FYSSLGCGHS	LDNTFLMTKL	HFWRFLKDCK	FHHHKLTLAD	MDRILSANND
490	500	510	520				
IPVEEIHSPF	TTILLRTFLN	YLLHLAYHIY	HEEFQQFIP				

Cmpd.	No. of Cmpds.	m/z meas.	Δ m/z [ppm]	z	Rt [min]	Score	P	Range	Sequence	Modification
2029	1	857.8121	-55.71	2	55.5	23.2	0	223-236	K.SGNIYEGQWEDNMR.H	Oxidation: 13

Detailed Protein Report

Protein 534: **PREDICTED: F-box/LRR-repeat protein 17 isoform X3 [Homo sapiens]**

**Accession:**	gi|530380083	**Score:**	23.2
**Database:**	refseq_human(refseq_human_20140103.fasta)	**MW [kDa]:**	53.6
**Database Date:**	2015-11-30	**pI:**	10.2
		**Sequence Coverage [%]:**	7.1
		**No. of unique Peptides:**	1

10	20	30	40	50	60	70	80
MGHLLSKEPR	NRPSQKRPRC	CSWCRRRRPL	LRLPRRTPAK	VPPQPAAPRS	RDCFFRGPCM	LCFIVHSPGA	PAPAGPEEEP
**90**	**100**	**110**	**120**	**130**	**140**	**150**	**160**
PLSPPPRDGA	YAAASSSQHL	ARRYAALAAE	DCAAAARRFL	LSSAAAAAAA	AASASSPASC	CKELGLAAAA	AWEQQGRSLF
**170**	**180**	**190**	**200**	**210**	**220**	**230**	**240**
LASLGPVRFL	GPPAAVQLFR	GPTPSPAELP	TPPEMVCKRK	GAGVPACTPC	KQPRCGGGGC	GGGGGGGGGG	GPAGGGASPP
**250**	**260**	**270**	**280**	**290**	**300**	**310**	**320**
RPPDAGCCQA	PEQPPQPLCP	PPSSPTSEGA	PTEAGGDAVR	AGGTAPLSAQ	QQHECGDADC	RESPENPCDC	HREPPPETPD
**330**	**340**	**350**	**360**	**370**	**380**	**390**	**400**
INQLPPSILL	KIFSNLSLDE	RCLSASLVCK	YWRDLCLDFQ	FWKQLDLSSR	QQVTDELLEK	IASRSQNIIE	INISDCRSMS
**410**	**420**	**430**	**440**	**450**	**460**	**470**	**480**
DNGVCVLAFK	CPGLLRYTAY	RCKQLSDTSI	IAVASHCPLL	QKVHVGNQDK	LTDEGLKQLG	SKCRELKDIH	FGQCYKISDE
**490**	**500**	**510**					
GMIVIAKGCL	KLQRIYMQEN	KLILD					

Cmpd.	No. of Cmpds.	m/z meas.	Δ m/z [ppm]	z	Rt [min]	Score	P	Range	Sequence	Modification
191	1	879.7026	-94.05	3	32.1	12.5	2	465-487	R.ELKDIHFGQCYKISDEGMIVIAK.G	

Detailed Protein Report

Protein 535: nitric oxide-inducible gene protein [Homo sapiens]

**Accession:**	gi|194239694	**Score:**	23.1
**Database:**	refseq_human(refseq_human_20140103.fasta)	**MW [kDa]:**	111.5
**Database Date:**	2015-11-30	**pI:**	6.9
**Modification(s):**	Carbamidomethyl	**Sequence Coverage [%]:**	2.9
		**No. of unique Peptides:**	1

10	20	30	40	50	60	70	80
MNRRRKFLLA	SVLALQNSSF	IYPSCQKCFS	RIILVSKRSN	CPKCGSTGES	GNANYRYKLS	LKVAESNKLF	VITVFGSCLD
**90**	**100**	**110**	**120**	**130**	**140**	**150**	**160**
TFFGLTATGL	HRYIQDPNKI	PETLDNDTTQ	NLLTKAVETC	FVGQSFIFGV	TNFENQPGQG	SDASNFLQQC	SDHKRKAKAL
**170**	**180**	**190**	**200**	**210**	**220**	**230**	**240**
VACQIVLPDP	GIAGFTVIDY	FHQLLQTFNF	RKLQCDSQAP	NNHLLALDHS	NSDLSSIYTS	DSTSDFFKSC	SKDTFSKFWQ
**250**	**260**	**270**	**280**	**290**	**300**	**310**	**320**
PSLEFTCIVS	QLTDNDDFSA	SEQSKAFGTL	QQNRKSISIA	EATGSSSCHD	PIQDSWSLVS	YMDKKSTAEK	LGKELGLQAK
**330**	**340**	**350**	**360**	**370**	**380**	**390**	**400**
ELSAVHSSHH	EIGVNDSNLF	SLEMREPLES	SNTKSFHSAV	EIKNRSQHEL	PCFQHHGIDT	PTSLQKRSAC	CPPSLLRLEE
**410**	**420**	**430**	**440**	**450**	**460**	**470**	**480**
TASSSQDGDP	QIWDDLPFSE	SLNKFLAVLE	SEIAVTQADV	SSRKHHVDND	IDKFHADHSR	LSVTPQRTTG	ALHTPPIALR
**490**	**500**	**510**	**520**	**530**	**540**	**550**	**560**
SSQVIVKANC	SKDDFLFNCK	GNLSPSVEKE	SQPDNKVEAV	SVNHNGRDMS	EYFLPNPYLS	ALSSSSKDLE	TIVTLKKTIR
**570**	**580**	**590**	**600**	**610**	**620**	**630**	**640**
ISPHRESDHS	SLNNKYLNGC	GEISVSEMNE	KLTTLCYRKY	NDVSDLCKLE	NKQYCRWSKN	QDDSFTICRK	LTYPLETLCN
**650**	**660**	**670**	**680**	**690**	**700**	**710**	**720**
SPNRSTNTLK	EMPWGHINNN	VTQSYSIGYE	GSYDASADLF	DDIAKEMDIA	TEITKKSQDI	LLKWGTSLAE	SHPSESDFSL
**730**	**740**	**750**	**760**	**770**	**780**	**790**	**800**
RSLSEDFIQP	SQKLSLQSLS	DSRHSRTCSP	TPHFQSDSEY	NFENSQDFVP	CSQSTPISGF	HQTRIHGINR	AFKKPVFYSD
**810**	**820**	**830**	**840**	**850**	**860**	**870**	**880**
LDGNYEKIRI	FPENDKQQAS	PSCPKNIKTP	SQKIRSPIVS	GVSQPDVFNH	YPFAECHETD	SDEWVPPTTQ	KIFPSDMLGF
**890**	**900**	**910**	**920**	**930**	**940**	**950**	**960**
QGIGLGKCLA	AYHFPDQQEL	PRKKLKHIRQ	GTNKGLIKKK	LKNMLAAVVT	KKKTHKYNCK	SSGWISKCPD	IQVLAAPQLH
**970**	**980**	**990**	**1000**				
PILGPDSCSE	VKCCLPFSEK	GPPSVCETRS	AWSPELFS				

Cmpd.	No. of Cmpds.	m/z meas.	Δ m/z [ppm]	z	Rt [min]	Score	P	Range	Sequence	Modification
2056	1	1029.5727	121.71	2	55.8	10.9	2	38-56	K.RSNCPKCGSTGESGNANYR.Y	Carbamidomethyl: 4

Detailed Protein Report

Protein 536: DENN domain-containing protein 1B isoform 4 [Homo sapiens]

Accession: gi|304571967
Database: refseq_human(refseq_human_20140103.fasta)
Database Date: 2015-11-30
Modification(s): Carbamidomethyl

Score: 23.1
MW [kDa]: 8.3
pI: 9.3
Sequence Coverage [%]: 21.1
No. of unique Peptides: 1

10	20	30	40	50	60	70	80
MDCRTKANPD	RTFDLVLKVK	CHASENEEDS	PAYLPRIPPG	KVRRFAFCIK	KLENFPVGPG	VAPPVSGVDL	VPAETA

Cmpd.	No. of Cmpds.	m/z meas.	Δ m/z [ppm]	z	Rt [min]	Score	P	Range	Sequence	Modification
2093	2	937.8811	-22.59	2	56.3	23.1	0	21-36	K.CHASENEEDSPAYLPR.I	Carbamidomethyl: 1

Detailed Protein Report

Protein 537: PREDICTED: diacylglycerol kinase iota isoform X2 [Homo sapiens]

**Accession:**	gi|578814735	**Score:**	23.1
**Database:**	refseq_human(refseq_human_20140103.fasta)	**MW [kDa]:**	113.9
**Database Date:**	2015-11-30	**pI:**	9.1
**Modification(s):**	Carbamidomethyl	**Sequence Coverage [%]:**	4.9
		**No. of unique Peptides:**	2

10	20	30	40	50	60	70	80
MNPSSSAGEE	KGATGGSSSS	GSGAGSCCLG	AEGGADPRGA	GSAAAAGAAA	LDEPAAAGQK	EKDEALEEKL	RNLTFRKQVS
**90**	**100**	**110**	**120**	**130**	**140**	**150**	**160**
YRKAISRAGL	QHLAPAHPLS	LPVANGPAKE	PRATLDWSEN	AVNGEHLWLE	TNVSGDLCYL	GEENCQVRFA	KSALRRKCAV
**170**	**180**	**190**	**200**	**210**	**220**	**230**	**240**
CKIVVHTACI	EQLEKINFRC	KPTFREGGSR	SPRENFVRHH	WVHRRRQEGK	CKQCGKGFQQ	KFSFHSKEIV	AISCSWCKQA
**250**	**260**	**270**	**280**	**290**	**300**	**310**	**320**
FHNKVTCFML	HHIEEPCSLG	AHAAVIVPPT	WIIKVKKPQN	SLKASNRKKK	RTSFKRKASK	RGMEQENKGR	PFVIKPISSP
**330**	**340**	**350**	**360**	**370**	**380**	**390**	**400**
LMKPLLVFVN	PKSGGNQGTK	VLQMFMWYLN	PRQVFDLSQE	GPKDALELYR	KVPNLRILAC	GGDGTVGWIL	SILDELQLSP
**410**	**420**	**430**	**440**	**450**	**460**	**470**	**480**
QPPVGVLPLG	TGNDLARTLN	WGGGYTDEPV	SKILCQVEDG	TVVQLDRWNL	HVERNPDLPP	EELEDGVCKK	LDRKQLKYKE
**490**	**500**	**510**	**520**	**530**	**540**	**550**	**560**
EYHMDILPLN	VFNNYFSLGF	DAHVTLEFHE	SREANPEKFN	SRFRNKMFYA	GAAFSDFLQR	SSRDLSKHVK	VVCDGTDLTP
**570**	**580**	**590**	**600**	**610**	**620**	**630**	**640**
KIQELKFQCI	VFLNIPRYCA	GTMPWGNPGD	HHDFEPQRHD	DGYIEVIGFT	MASLAALQVG	GHGERLHQCR	EVMLLTYKSI
**650**	**660**	**670**	**680**	**690**	**700**	**710**	**720**
PMQVDGEPCR	LAPAMIRISL	RNQANMVQKS	KRRTSMPLLN	DPQSVPDRLR	IRVNKISLQD	YEGFHYDKEK	LREASIPLGI
**730**	**740**	**750**	**760**	**770**	**780**	**790**	**800**
LVVRGDCDLE	TCRMYIDRLQ	EDLQSVSSGS	QRVHYQDHET	SFPRALSAQR	LSPRWCFLDA	TSADRFYRID	RSQEHLHFVM
**810**	**820**	**830**	**840**	**850**	**860**	**870**	**880**
EISQDEIFIL	DPDMVVSQPA	GTPPGMPDLV	VEQASGISDW	WNPALRKRML	SDSGLGMIAP	YYEDSDLKDL	SHSRVLQSPV
**890**	**900**	**910**	**920**	**930**	**940**	**950**	**960**
SSEDHAILQA	VIAGDLMKLI	ESYKNGGSLL	IQGPDHCSLL	HYAAKTGNGE	IVKYILDHGP	SELLDMADSE	TGETALHKAA
**970**	**980**	**990**	**1000**	**1010**	**1020**	**1030**	
CQRNRAVCQL	LVDAGASLRK	TDSKGKTPQE	RAQQAGDPDL	AAYLESRQNY	KVIGHEDLET	AV	

Cmpd.	No. of Cmpds.	m/z meas.	Δ m/z [ppm]	z	Rt [min]	Score	P	Range	Sequence	Modification
1635	1	1100.4411	-20.69	3	50.3	12.6	1	2-38	M.NPSSSAGEEKGATGGSSSSGSGAGSCCLGAEGGADPR.G	Carbamidomethyl: 26
2495	1	735.4148	-22.06	2	61.6	10.5	2	275-287	K.VKKPQNSLKASNR.K	

Detailed Protein Report

Protein 538: UPF0598 protein C8orf82 [Homo sapiens]

Accession: gi|49169841
Database: refseq_human(refseq_human_20140103.fasta)
Database Date: 2015-11-30

Score: 23.1
MW [kDa]: 23.9
pI: 10.4
Sequence Coverage [%]: 14.8
No. of unique Peptides: 1

10	20	30	40	50	60	70	80
MWPPCGTLRT	LALARSRGAR	ACSGDGGVSY	TQGQSPEPRT	REYFYYVDHQ	GQLFLDDSKM	KNFITCFKDP	QFLVTFFSRL
**90**	**100**	**110**	**120**	**130**	**140**	**150**	**160**
RPNRSGRYEA	AFPFLSPCGR	ERNFLRCEDR	PVVFTHLLTA	DHGPPRLSYC	GGGEALAVPF	EPARLLPLAA	NGRLYHPAPE
**170**	**180**	**190**	**200**	**210**	**220**		
RAGGVGLVRS	ALAFELSACF	EYGPGAPALP	SHVRWQGRRL	ALTMDLAPLL	LAARSP		

Cmpd.	No. of Cmpds.	m/z meas.	Δ m/z [ppm]	z	Rt [min]	Score	P	Range	Sequence	Modification
367	1	846.3873	-98.49	2	34.2	12.6	1	154-169	R.LYHPAPERAGGVGLVR.S	

Detailed Protein Report

Protein 539: PREDICTED: zinc finger protein 236 isoform X1 [Homo sapiens]

**Accession:**	gi|530414335	**Score:**	23.1
**Database:**	refseq_human(refseq_human_20140103.fasta)	**MW [kDa]:**	197.2
**Database Date:**	2015-11-30	**pI:**	9.7
**Modification(s):**	Carbamidomethyl	**Sequence Coverage [%]:**	1.7
		**No. of unique Peptides:**	2

10	20	30	40	50	60	70	80
MRDHERNDKP	HRCDQCPQTF	NVEFNLTLHK	CTHSGEDPTC	PVCNKKFSRV	ASLKAHIMLH	EKEENLICSE	CGDEFTLQSQ
90	100	110	120	130	140	150	160
LAVHMEEHRQ	ELAGTRQHAC	KACKKEFETS	SELKEHMKTH	YKIRVSSTRS	YNRNIDRSGF	TYSCPHCGKT	FQKPSQLTRH
170	180	190	200	210	220	230	240
IRIHTGERPF	KCSECGKAFN	QKGALQTHMI	KHTGEKPHAC	AFCPAAFSQK	GNLQSHVQRV	HSEVKNGPTY	NCTECSCVFK
250	260	270	280	290	300	310	320
SLGSLNTHIS	KMHMGGPQNS	TSSTETAHVL	TATLFQTLPL	QQTEAQATSA	SSQPSSQAVS	DVIQQLLELS	EPAPVESGQS
330	340	350	360	370	380	390	400
PQPGQQLSIT	VGINQDILQQ	ALENSGLSSI	PAAAHPNDSC	HAKTSAPHAQ	NPDVSSVSNE	QTDPTDAEQE	KEQESPEKLD
410	420	430	440	450	460	470	480
KKEKKMIKKK	SPFLPGSIRE	ENGVRWHVCP	YCAKEFRKPS	DLVRHIRIHT	HEKPFKCPQC	FRAFAVKSTL	TAHIKTHTGI
490	500	510	520	530	540	550	560
KAFKCQYCMK	SFSTSGSLKV	HIRLHTGVRP	FACPHCDKKF	RTSGHRKTHI	ASHFKHTELR	KMRHQRKPAK	VRVGKTNIPV
570	580	590	600	610	620	630	640
PDIPLQEPIL	ITDLGLIQPI	PKNQFFQSYF	NNNFVNEADR	PYKCFYCHRA	YKKSCHLKQH	IRSHTGEKPF	KCSQCGRGFV
650	660	670	680	690	700	710	720
SAGVLKAHIR	THTGLKSFKC	LICNGAFTTG	GSLRRHMGIH	NDLRPYMCPY	CQKTFKTSLN	CKKHMKTHRY	ELAQQLQQHQ
730	740	750	760	770	780	790	800
QAASIDDSTV	DQQSMQASTQ	MQVEIESDEL	PQTAEVVAAN	PEAMLDLEPQ	HVVGTEEAGL	GQQLADQPLE	ADEDGFVAPQ
810	820	830	840	850	860	870	880
DPLRGHVDQF	EEQSPAQQSF	EPAGLPQGFT	VTDTYHQQPQ	FPPVQQLQDS	STLESQALST	SFHQQSLLQA	PSSDGMNVTT
890	900	910	920	930	940	950	960
RLIQESSQEE	LDLQAQGSQF	LEDNEDQSRR	SYRCDYCNKG	FKKSSHLKQH	VRSHTGEKPY	KCKLCGRGFV	SSGVLKSHEK
970	980	990	1000	1010	1020	1030	1040
THTGVKAFSC	SVCNASFTTN	GSLTRHMATH	MSMKPYKCPF	CEEGFRTTVH	CKKHMKRHQT	VPSAVSATGE	TEGGDICMEE
1050	1060	1070	1080	1090	1100	1110	1120
EEEHSDRNAS	RKSRPEVITF	TEEETAQLAK	IRPQESATVS	EKVLVQSAAE	KDRISELRDK	QAELQDEPKH	ANCCTYCPKS
1130	1140	1150	1160	1170	1180	1190	1200
FKKPSDLVRH	VRIHTGEKPY	KCDECGKSFT	VKSTLDCHVK	THTGQKLFSC	HVCSNAFSTK	GSLKVHMRLH	TGAKPFKCPH
1210	1220	1230	1240	1250	1260	1270	1280
CELRFRTSGR	RKTHMQFHYK	PDPKKARKPM	TRSSSEGLQP	VNLLNSSSTD	PNVFIMNNSV	LTGQFDQNLL	QPGLVGQAIL
1290	1300	1310	1320	1330	1340	1350	1360
PASVSAGGDL	TVSLTDGSLA	TLEGIQLQLA	ANLVGPNVQI	SGIDAASINN	ITLQIDPSIL	QQTLQQGNLL	AQQLTGEPGL
1370	1380	1390	1400	1410	1420	1430	1440
APQNSSLQTS	DSTVPASVVI	QPISGLSLQP	TVTSANLTIG	PLSEQDSVLT	TNSSGTQDLT	QVMTSQGLVS	PSGGPHEITL
1450	1460	1470	1480	1490	1500	1510	1520
TINNSSLSQV	LAQAAGPTAT	SSSGSPQEIT	LTISELNTTS	GSLPSTTPMS	PSAISTQNLV	MSSSGVGGDA	SVTLTLADTQ
1530	1540	1550	1560	1570	1580	1590	1600
GMLSGGLDTV	TLNITSQGQQ	FPALLTDPSL	SGQGGAGSPQ	VILVSHTPQS	ASAACEEIAY	QVAGVSGNLA	PGNQPEKEGR
1610	1620	1630	1640	1650	1660	1670	1680
AHQCLECDRA	FSSAAVLMHH	SKEVHGRERI	HGCPVCRKAF	KRATHLKEHM	QTHQAGPSLS	SQKPRVFKCD	TCEKAFAKPS
1690	1700	1710	1720	1730	1740	1750	1760
QLERHSRIHT	GERPFHCTLC	EKAFNQKSAL	QVHMKKHTGE	RPYKCAYCVM	GFTQKSNMKL	HMKRAHSYAG	ALQESAGHPE
1770	1780	1790	1800				
QDGEELSRTL	HLEEVVQEAA	GEWQALTHVF					

Cmpd.	No. of	m/z meas.	Δ m/z	z	Rt	Score	P	Range	Sequence	Modification

Detailed Protein Report

	Cmpds.		[ppm]		[min]					
2391	1	862.0720	174.01	2	60.2	10.8	2	105-118	K.KEFETSSELKEHMK.T	
1447	1	947.6235	148.95	2	47.9	12.3	1	1189-1204	R.LHTGAKPFKCPHCELR.F	Carbamidomethyl: 10

Detailed Protein Report

Protein 540: PREDICTED: midasin isoform X3 [Homo sapiens]

**Accession:**	gi|578812559	**Score:**	23.1
**Database:**	refseq_human(refseq_human_20140103.fasta)	**MW [kDa]:**	627.0
**Database Date:**	2015-11-30	**pI:**	5.4
		**Sequence Coverage [%]:**	0.6
		**No. of unique Peptides:**	1

Detailed Protein Report

10	20	30	40	50	60	70	80
MEHFLLEVAA	APLRLIAAKN	EKSRSELGRF	LAKQVWTPQD	RQCVLSTLAQ	LLLDKDCTVL	VGRQLRPLLL	DLLERNAEAI
90	100	110	120	130	140	150	160
KAGGQINHDL	HERLCVSMSK	LIGNHPDVLP	FALRYFKDTS	PVFQRLFLES	SDANPVRYGR	RRMKLRDLME	AAFKFLQQEQ
170	180	190	200	210	220	230	240
SVFRELWDWS	VCVPLLRSHD	TLVRWYTANC	LALVTCMNEE	HKLSFLKKIF	NSDELIHFRL	RLLEEAQLQD	LEKALVLANP
250	260	270	280	290	300	310	320
EVSLWRKQKE	LQYLQGHLVS	SDLSPRVTAV	CGVVLPGQLP	APGELGGNRS	SSREQELALR	SYVLVESVCK	SLQTLAMAVA
330	340	350	360	370	380	390	400
SQNAVLLEGP	IGCGKTSLVE	YLAAVTGRTK	PPQLLKVQLG	DQTDSKMLLG	MYRCTDVPGE	FVWQPGTLTQ	AATMGHWILL
410	420	430	440	450	460	470	480
EDIDYAPLDV	VSVLIPLLEN	GELLIPGRGD	CLKVAPGFQF	FATRRLLSCG	GNWYRPLNSH	ATLLDKYWTK	IHLDNLDKRE
490	500	510	520	530	540	550	560
LNEVLQSRYP	SLLAVVDHLL	DIYIQLTGEK	HHSWSDSSVG	CEQAPEEVSE	ARRENKRPTL	EGRELSLRDL	LNWCNRIAHS
570	580	590	600	610	620	630	640
FDSSSLSASL	NIFQEALDCF	TAMLSEHTSK	LKMAEVIGSK	LNISRKKAEF	FCQLYKPEIV	INELDLQVGR	VRLLRKQSEA
650	660	670	680	690	700	710	720
VHLQREKFTF	AATRPSSVLI	EQLAVCVSKG	EPVLLVGETG	TGKTSTIQYL	AHITGHRLRV	VNMNQQSDTA	DLLGGYKPVD
730	740	750	760	770	780	790	800
HKLIWLPLRE	AFEELFAQTF	SKKQNFTFLG	HIQTCYRQKR	WHDLLRLMQH	VHKSAVNKDG	KDSETGLLIK	EKWEAFGLRL
810	820	830	840	850	860	870	880
NHAQQQMKMT	ENTLLFAFVE	GTLAQAVKKG	EWILLDEINL	AAPEILECLS	GLLEGSSGSL	VLLDRGDTEP	LVRHPDFRLF
890	900	910	920	930	940	950	960
ACMNPATDVG	KRNLPPGIRN	RFTELYVEEL	ESKEDLQVLI	VDYLKGLSVN	KNTVQGIINF	YTALRKESGT	KLVDGTGHRP
970	980	990	1000	1010	1020	1030	1040
HYSLRTLCRA	LRFAASNPCG	NIQRSLYEGF	CLGFLTQLDR	ASHPIVQKLI	CQHIVPGNVK	SLLKQPIPEP	KGGRLIQVEG
1050	1060	1070	1080	1090	1100	1110	1120
YWIAVGDKEP	TIDETYILTS	SVKLNLRDIV	RVVSAGTYPV	LIQGETSVGK	TSLIQWLAAA	TGNHCVRINN	HEHTDIQEYI
1130	1140	1150	1160	1170	1180	1190	1200
GCYTSDSSGK	LVFKEGVLID	AMRKGYWIIL	DELNLAPTDV	LEALNRLLDD	NRELLVTETQ	EVVKAHPRFM	LFATQNPPGL
1210	1220	1230	1240	1250	1260	1270	1280
YGGRKVLSRA	FRNRFVELHF	DELPSSELET	ILHKRCSLPP	SYCSKLVKVM	LDLQSYRRSS	SVFAGKQGFI	TLRDLFRWAE
1290	1300	1310	1320	1330	1340	1350	1360
RYRLAEPTEK	EYDWLQHLAN	DGYMLLAGRV	RKQEEIDVIQ	EVLEKHFKKK	LCPQSLFSKE	NVLKLLGKLS	TQISTLECNF
1370	1380	1390	1400	1410	1420	1430	1440
GHIVWTEGMR	RLAMLVGRAL	EFGEPVLLVG	DTGCGKTTIC	QVFAALANQK	LYSVSCHLHM	ETSDFLGGLR	PVRQKPNDKE
1450	1460	1470	1480	1490	1500	1510	1520
EIDTSRLFEW	HDGPLVQAMK	EDGFFLLDEI	SLADDSVLER	LNSVLEVEKS	LVLAEKGSPE	DKDSEIELLT	AGKKFRILAT
1530	1540	1550	1560	1570	1580	1590	1600
MNPGGDFGKK	ELSPALRNRF	TEIWCPQSTS	REDLIQIISH	NLRPGLCLGR	IDPKGSDIPE	VMLDFIDWLT	HQEFGRKCVV
1610	1620	1630	1640	1650	1660	1670	1680
SIRDILSWVN	FMNKMGEEAA	LKRPEIISTV	TSFVHAACLV	YIDGIGSGVT	SSGFGTALLA	RKECLKFLIK	RLAKIVRLTE
1690	1700	1710	1720	1730	1740	1750	1760
YQKNELKIYD	RMKAKEFTGI	DNLWGIHPFF	IPRGPVLHRN	NIADYALSAG	TTAMNAQRLL	RATKLKKPIL	LEGSPGVGKT
1770	1780	1790	1800	1810	1820	1830	1840
SLVGALAKAS	GNTLVRINLS	EQTDITDLFG	ADLPVEGGKG	GEFAWRDGPL	LAALKAGHWV	VLDELNLASQ	SVLEGLNACF
1850	1860	1870	1880	1890	1900	1910	1920
DHRGEIYVPE	LGMSFQVQHE	KTKIFGCQNP	FRQGGGRKGL	PRSFLNRFTQ	VFVDPLTVID	MEFIASTLFP	AIEKNIVKKM
1930	1940	1950	1960	1970	1980	1990	2000
VAFNNQIDHE	VTVEKKWGQK	GGPWEFNLRD	LFRWCQLMLV	DQSPGCYDPG	QHVFLVYGER	MRTEEDKKKV	IAVFKDVFGS
2010	2020	2030	2040	2050	2060	2070	2080
NSNPYMGTRL	FRITPYDVQL	GYSVLSRGSC	VPHPSRHPLL	LLHQSFQPLE	SIMKCVQMSW	MVILVGPASV	GKTSLVQLLA
2090	2100	2110	2120	2130	2140	2150	2160
HLTGHTLKIM	AMNSAMDTTE	LLGGFEQVDL	IRPWRRLLEK	VEGTVRALLR	DSLLISADDA	EVVLRAWSHF	LLTYKPKCLG
2170	2180	2190	2200	2210	2220	2230	2240

Detailed Protein Report

Cmpd.	No. of Cmpds.	m/z meas.	Δ m/z [ppm]	z	Rt [min]	Score	P	Range	Sequence	Modification
1915	1	918.3456	-31.31	2	53.8	12.2	1	4832-4848	K.EEKEEAEADDGGQGEDK.I	

Detailed Protein Report

Protein 541: integrin alpha-IIb preproprotein [Homo sapiens]

Accession: gi|88758615
Database: refseq_human(refseq_human_20140103.fasta)
Database Date: 2015-11-30

Score: 23.0
MW [kDa]: 113.3
pI: 5.1
Sequence Coverage [%]: 1.4
No. of unique Peptides: 1

10	20	30	40	50	60	70	80
MARALCPLQA	LWLLEWVLLL	LGPCAAPPAW	ALNLDPVQLT	FYAGPNGSQF	GFSLDFHKDS	HGRVAIVVGA	PRTLGPSQEE
90	100	110	120	130	140	150	160
TGGVFLCPWR	AEGGQCPSLL	FDLRDETRNV	GSQTLQTFKA	RQGLGASVVS	WSDVIVACAP	WQHWNVLEKT	EEAEKTPVGS
170	180	190	200	210	220	230	240
CFLAQPESGR	RAEYSPCRGN	TLSRIYVEND	FSWDKRYCEA	GFSSVVTQAG	ELVLGAPGGY	YFLGLLAQAP	VADIFSSYRP
250	260	270	280	290	300	310	320
GILLWHVSSQ	SLSFDSSNPE	YFDGYWGYSV	AVGEFDGDLN	TTEYVVGAPT	WSWTLGAVEI	LDSYYQRLHR	LRGEQMASYF
330	340	350	360	370	380	390	400
GHSVAVTDVN	GDGRHDLLVG	APLYMESRAD	RKLAEVGRVY	LFLQPRGPHA	LGAPSLLLTG	TQLYGRFGSA	IAPLGDLDRD
410	420	430	440	450	460	470	480
GYNDIAVAAP	YGGPSGRGQV	LVFLGQSEGL	RSRPSQVLDS	PFPTGSAFGF	SLRGAVDIDD	NGYPDLIVGA	YGANQVAVYR
490	500	510	520	530	540	550	560
AQPVVKASVQ	LLVQDSLNPA	VKSCVLPQTK	TPVSCFNIQM	CVGATGHNIP	QKLSLNAELQ	LDRQKPRQGR	RVLLLGSQQA
570	580	590	600	610	620	630	640
GTTLNLDLGG	KHSPICHTTM	AFLRDEADFR	DKLSPIVLSL	NVSLPPTEAG	MAPAVVLHGD	THVQEQTRIV	LDCGEDDVCV
650	660	670	680	690	700	710	720
PQLQLTASVT	GSPLLVGADN	VLELQMDAAN	EGEGAYEAEL	AVHLPQGAHY	MRALSNVEGF	ERLICNQKKE	NETRVVLCEL
730	740	750	760	770	780	790	800
GNPMKKNAQI	GIAMLVSVGN	LEEAGESVSF	QLQIRSKNSQ	NPNSKIVLLD	VPVRAEAQVE	LRGNSFPASL	VVAAEEGERE
810	820	830	840	850	860	870	880
QNSLDSWGPK	VEHTYELHNN	GPGTVNGLHL	SIHLPGQSQP	SDLLYILDIQ	PQGGLQCFPQ	PPVNPLKVDW	GLPIPSPSPI
890	900	910	920	930	940	950	960
HPAHHKRDRR	QIFLPEPEQP	SRLQDPVLVS	CDSAPCTVVQ	CDLQEMARGQ	RAMVTVLAFL	WLPSLYQRPL	DQFVLQSHAW
970	980	990	1000	1010	1020	1030	1040
FNVSSLPYAV	PPLSLPRGEA	QVWTQLLRAL	EERAIPIWWV	LVGVLGGLLL	LTILVLAMWK	VGFFKRNRPP	LEEDDEEGE

Cmpd.	No. of Cmpds.	m/z meas.	Δ m/z [ppm]	z	Rt [min]	Score	P	Range	Sequence	Modification
183	1	862.4693	40.75	2	32.0	23.0	1	105-119	R.DETRNVGSQTLQTFK.A	

Detailed Protein Report

Protein 542: PREDICTED: PR domain zinc finger protein 15 isoform X1 [Homo sapiens]

Accession: gi|578836643
Database: refseq_human(refseq_human_20140103.fasta)
Database Date: 2015-11-30
Modification(s): Carbamidomethyl

Score: 23.0
MW [kDa]: 130.3
pI: 9.2
Sequence Coverage [%]: 4.1
No. of unique Peptides: 2

10	20	30	40	50	60	70	80
MPRRRPPASG	AAQFPERIAT	RSPDPIPLCT	FQRQVSEMAE	DGSEEIMFIW	CEDCSQYHDS	ECPELGPVVM	VKDSFVLSRA
**90**	**100**	**110**	**120**	**130**	**140**	**150**	**160**
RSSLPPNLEI	RRLEDGAEGV	FAITQLVKRT	QFGPFESRRV	AKWEKESAFP	LKVFQKDGHP	VCFDTSNEDD	CNWMMLVRPA
**170**	**180**	**190**	**200**	**210**	**220**	**230**	**240**
AEAEHQNLTA	YQHGSDVYFT	TSRDIPPGTE	LRVWYAAFYA	KKMDKPMLKQ	AGSGVHAAGT	PENSAPVESE	PSQWACKVCS
**250**	**260**	**270**	**280**	**290**	**300**	**310**	**320**
ATFLELQLLN	EHLLGHLEQA	KSLPPGSQSE	AAAPEKEQDT	PRGEPPAVPE	SENVATKEQK	KKPRRGRKPK	VSKAEQPLVI
**330**	**340**	**350**	**360**	**370**	**380**	**390**	**400**
VEDKEPTEQV	AEIITEVPPD	EPVSATPDER	IMELVLGKLA	TTTTDTSSVP	KFTHHQNNTI	TLKRSLILSS	RHGIRRKLIK
**410**	**420**	**430**	**440**	**450**	**460**	**470**	**480**
QLGEHKRVYQ	CNICSKIFQN	SSNLSRHVRS	HGDKLFKCEE	CAKLFSRKES	LKQHVSYKHS	RNEVDGEYRY	RCGTCEKTFR
**490**	**500**	**510**	**520**	**530**	**540**	**550**	**560**
IESALEFHNC	RTGLIAHPGE	GGPGGSRLRD	LPDDKTFQCE	MCFRFFSTNS	NLSKHKKKHG	DKKFACEVCS	KMFYRKDVML
**570**	**580**	**590**	**600**	**610**	**620**	**630**	**640**
DHQRRHLEGV	RRVKREDLEA	GGENLVRYKK	EPSGCPVCGK	DIALMDDHQR	EEFIGKIGIS	SEENDDNSDE	SADSEPHKYS
**650**	**660**	**670**	**680**	**690**	**700**	**710**	**720**
CKRCQLTFGR	GKEYLKHIME	VHKEKGYGCS	ICNRRFALKA	TYHAHMVIHR	ENLPDPNVQK	YIHPCEICGR	IFNSIGNLER
**730**	**740**	**750**	**760**	**770**	**780**	**790**	**800**
HKLIHTGVKS	HACEQCGKSF	ARKDMLKEHM	RVHDNVREYL	CAECGKGMKT	KHALRHHMKL	HKGIKEYECK	ECHRRFAQKV
**810**	**820**	**830**	**840**	**850**	**860**	**870**	**880**
NMLKHCKRHT	GIKDFMCELC	GKTFSERNTM	ETHKLIHTVG	KQWTCSVCDK	KYVTEYMLQK	HVQLTHDKVE	AQSCQLCGTK
**890**	**900**	**910**	**920**	**930**	**940**	**950**	**960**
VSTRASMSRH	MRRKHPEVLA	VRIDDLDHLP	ETTTIDASSI	GIVQPELTLE	QEDLAEGKHG	KAAKRSHKRK	QKPEEEAGAP
**970**	**980**	**990**	**1000**	**1010**	**1020**	**1030**	**1040**
VPEDATFSEY	SEKETEFTGS	VGDETNSAVQ	SIQQVVVTLG	DPNVTTPSSS	VGLTNITVTP	ITTAAATQFT	NLQPVAVGHL
**1050**	**1060**	**1070**	**1080**	**1090**	**1100**	**1110**	**1120**
TTPERQLQLD	NSILTVTFDT	VSGSAMLHNR	QNDVQIHPQP	EASNPQSVAH	FINLTTLVNS	ITPLGSQLSD	QHPLTWRAVP
**1130**	**1140**	**1150**	**1160**				
QTDVLPPSQP	QAPPQQAAQP	QVQAEQQQQQ	MYSY				

Cmpd.	No. of Cmpds.	m/z meas.	Δ m/z [ppm]	z	Rt [min]	Score	P	Range	Sequence	Modification
1448	1	699.6392	-84.48	3	47.9	11.3	1	184-201	R.DIPPGTELRVWYAAFYAK.K	
1744	1	1030.2489	59.15	3	51.6	11.7	2	481-509	R.IESALEFHNCRTGLIAHPGEGGPGGSRLR.D	Carbamidomethyl: 10

Detailed Protein Report

Protein 543: PREDICTED: insulin-like growth factor 1 receptor isoform X2 [Homo sapiens]

**Accession:**	gi|530407102	**Score:**	23.0
**Database:**	refseq_human(refseq_human_20140103.fasta)	**MW [kDa]:**	141.1
**Database Date:**	2015-11-30	**pI:**	5.4
**Modification(s):**	Carbamidomethyl, Oxidation	**Sequence Coverage [%]:**	1.4
		**No. of unique Peptides:**	1

10	20	30	40	50	60	70	80
MTNLKDIGLY	NLRNITRGAI	RIEKNADLCY	LSTVDWSLIL	DAVSNNYIVG	NKPPKECGDL	CPGTMEEKPM	CEKTTINNEY
**90**	**100**	**110**	**120**	**130**	**140**	**150**	**160**
NYRCWTTNRC	QKMCPSTCGK	RACTENNECC	HPECLGSCSA	PDNDTACVAC	RHYYYAGVCV	PACPPNTYRF	EGWRCVDRDF
**170**	**180**	**190**	**200**	**210**	**220**	**230**	**240**
CANILSAESS	DSEGFVIHDG	ECMQECPSGF	IRNGSQSMYC	IPCEGPCPKV	CEEEKKTKTI	DSVTSAQMLQ	GCTIFKGNLL
**250**	**260**	**270**	**280**	**290**	**300**	**310**	**320**
INIRRGNNIA	SELENFMGLI	EVVTGYVKIR	HSHALVSLSF	LKNLRLILGE	EQLEGNYSFY	VLDNQNLQQL	WDWDHRNLTI
**330**	**340**	**350**	**360**	**370**	**380**	**390**	**400**
KAGKMYFAFN	PKLCVSEIYR	MEEVTGTKGR	QSKGDINTRN	NGERASCESD	VLHFTSTTTS	KNRIIITWHR	YRPPDYRDLI
**410**	**420**	**430**	**440**	**450**	**460**	**470**	**480**
SFTVYYKEAP	FKNVTEYDGQ	DACGSNSWNM	VDVDLPPNKD	VEPGILLHGL	KPWTQYAVYV	KAVTLTMVEN	DHIRGAKSEI
**490**	**500**	**510**	**520**	**530**	**540**	**550**	**560**
LYIRTNASVP	SIPLDVLSAS	NSSSQLIVKW	NPPSLPNGNL	SYYIVRWQRQ	PQDGYLYRHN	YCSKDKIPIR	KYADGTIDIE
**570**	**580**	**590**	**600**	**610**	**620**	**630**	**640**
EVTENPKTEV	CGGEKGPCCA	CPKTEAEKQA	EKEEAEYRKV	FENFLHNSIF	VPRPERKRRD	VMQVANTTMS	SRSRNTTAAD
**650**	**660**	**670**	**680**	**690**	**700**	**710**	**720**
TYNITDPEEL	ETEYPFFESR	VDNKERTVIS	NLRPFTLYRI	DIHSCNHEAE	KLGCSASNFV	FARTMPAEGA	DDIPGPVTWE
**730**	**740**	**750**	**760**	**770**	**780**	**790**	**800**
PRPENSIFLK	WPEPENPNGL	ILMYEIKYGS	QVEDQRECVS	RQEYRKYGGA	KLNRLNPGNY	TARIQATSLS	GNGSWTDPVF
**810**	**820**	**830**	**840**	**850**	**860**	**870**	**880**
FYVQAKTGYE	NFIHLIIALP	VAVLLIVGGL	VIMLYVFHRK	RNNSRLGNGV	LYASVNPEYF	SAADVYVPDE	WEVAREKITM
**890**	**900**	**910**	**920**	**930**	**940**	**950**	**960**
SRELGQGSFG	MVYEGVAKGV	VKDEPETRVA	IKTVNEAASM	RERIEFLNEA	SVMKEFNCHH	VVRLLGVVSQ	GQPTLVIMEL
**970**	**980**	**990**	**1000**	**1010**	**1020**	**1030**	**1040**
MTRGDLKSYL	RSLRPEMENN	PVLAPPSLSK	MIQMAGEIAD	GMAYLNANKF	VHRDLAARNC	MVAEDFTVKI	GDFGMTRDIY
**1050**	**1060**	**1070**	**1080**	**1090**	**1100**	**1110**	**1120**
ETDYYRKGGK	GLLPVRWMSP	ESLKDGVFTT	YSDVWSFGVV	LWEIATLAEQ	PYQGLSNEQV	LRFVMEGGLL	DKPDNCPDML
**1130**	**1140**	**1150**	**1160**	**1170**	**1180**	**1190**	**1200**
FELMRMCWQY	NPKMRPSFLE	IISSIKEEME	PGFREVSFYY	SEENKLPEPE	ELDLEPENME	SVPLDPSASS	SSLPLPDRHS
**1210**	**1220**	**1230**	**1240**	**1250**			
GHKAENGPGP	GVLVLRASFD	ERQPYAHMNG	GRKNERALPL	PQSSTC			

Cmpd.	No. of Cmpds.	m/z meas.	Δ m/z [ppm]	z	Rt [min]	Score	P	Range	Sequence	Modification
1628	1	716.0279	112.96	3	50.1	12.0	0	56-73	K.ECGDLCPGTMEEKPMCEK.T	Carbamidomethyl: 2, 16; Oxidation: 10, 15

Detailed Protein Report

Protein 544: PREDICTED: rho guanine nucleotide exchange factor 12 isoform X3 [Homo sapiens]

**Accession:**	gi|530397943	**Score:**	22.9
**Database:**	refseq_human(refseq_human_20140103.fasta)	**MW [kDa]:**	161.8
**Database Date:**	2015-11-30	**pI:**	5.3
		**Sequence Coverage [%]:**	1.5
		**No. of unique Peptides:**	2

10	20	30	40	50	60	70	80
MRAGVQTGDR	IIKVNGTLVT	HSNHLEVVKL	IKSGSYVALT	VQGRPPGSPQ	IPLADSEVEP	SVIGHMSPIM	TSPHSPGASG
**90**	**100**	**110**	**120**	**130**	**140**	**150**	**160**
NMERITSPVL	MGEENNVVHN	QKVEILRKML	QKEQERLQLL	QEDYNRTPAQ	RLLKEIQEAK	KHIPQLQEQL	SKATGSAQDG
**170**	**180**	**190**	**200**	**210**	**220**	**230**	**240**
AVVTPSRPLG	DTLTVSEAET	DPGDVLGRTD	CSSGDASRPS	SDNADSPKSG	PKERIYLEEN	PEKSETIQDT	DTQSLVGSPS
**250**	**260**	**270**	**280**	**290**	**300**	**310**	**320**
TRIAPHIIGA	EDDDFGTEHE	QINGQCSCFQ	SIELLKSRPA	HLAVFLHHVV	SQFDPATLLC	YLYSDLYKHT	NSKETRRIFL
**330**	**340**	**350**	**360**	**370**	**380**	**390**	**400**
EFHQFFLDRS	AHLKVSVPDE	MSADLEKRRP	ELIPEDLHRH	YIQTMQERVH	PEVQRHLEDF	RQKRSMGLTL	AESELTKLDA
**410**	**420**	**430**	**440**	**450**	**460**	**470**	**480**
ERDKDRLTLE	KERTCAEQIV	AKIEEVLMTA	QAVEEDKSST	MQYVILMYMK	HLGVKVKEPR	NLEHKRGRIG	FLPKIKQSMK
**490**	**500**	**510**	**520**	**530**	**540**	**550**	**560**
KDKEGEEKGK	RRGFPSILGP	PRRPSRHDNS	AIGRAMELQK	ARHPKHLSTP	SSVSPEPQDS	AKLRQSGLAN	EGTDAGYLPA
**570**	**580**	**590**	**600**	**610**	**620**	**630**	**640**
NSMSSVASGA	SFSQEGGKEN	DTGSKQVGET	SAPGDTLDGT	PRTLNTVFDF	PPPPLDQVQE	EECEVERVTE	HGTPKPFRKF
**650**	**660**	**670**	**680**	**690**	**700**	**710**	**720**
DSVAFGESQS	EDEQFENDLE	TDPPNWQQLV	SREVLLGLKP	CEIKRQEVIN	ELFYTERAHV	RTLKVLDQVF	YQRVSREGIL
**730**	**740**	**750**	**760**	**770**	**780**	**790**	**800**
SPSELRKIFS	NLEDILQLHI	GLNEQMKAVR	KRNETSVIDQ	IGEDLLTWFS	GPGEEKLKHA	AATFCSNQPF	ALEMIKSRQK
**810**	**820**	**830**	**840**	**850**	**860**	**870**	**880**
KDSRFQTFVQ	DAESNPLCRR	LQLKDIIPTQ	MQRLTKYPLL	LDNIAKYTEW	PTEREKVKKA	ADHCRQILNY	VNQAVKEAEN
**890**	**900**	**910**	**920**	**930**	**940**	**950**	**960**
KQRLEDYQRR	LDTSSLKLSE	YPNVEELRNL	DLTKRKMIHE	GPLVWKVNRD	KTIDLYTLLL	EDILVLLQKQ	DDRLVLRCHS
**970**	**980**	**990**	**1000**	**1010**	**1020**	**1030**	**1040**
KILASTADSK	HTFSPVIKLS	TVLVRQVATD	NKALFVISMS	DNGAQIYELV	AQTVSEKTVW	QDLICRMAAS	VKEQSTKPIP
**1050**	**1060**	**1070**	**1080**	**1090**	**1100**	**1110**	**1120**
LPQSTPGEGD	NDEEDPSKLK	EEQHGISVTG	LQSPDRDLGL	ESTLISSKPQ	SHSLSTSGKS	EVRDLFVAER	QFAKEQHTDG
**1130**	**1140**	**1150**	**1160**	**1170**	**1180**	**1190**	**1200**
TLKEVGEDYQ	IAIPDSHLPV	SEERWALDAL	RNLGLLKQLL	VQQLGLTEKS	VQEDWQHFPR	YRTASQGPQT	DSVIQNSENI
**1210**	**1220**	**1230**	**1240**	**1250**	**1260**	**1270**	**1280**
KAYHSGEGHM	PFRTGTGDIA	TCYSPRTSTE	SFAPRDSVGL	APQDSQASNI	LVMDHMIMTP	EMPTMEPEGG	LDDSGEHFFD
**1290**	**1300**	**1310**	**1320**	**1330**	**1340**	**1350**	**1360**
AREAHSDENP	SEGDGAVNKE	EKDVNLRISG	NYLILDGYDP	VQESSTDEEV	ASSLTLQPMT	GIPAVESTHQ	QQHSPQNTHS
**1370**	**1380**	**1390**	**1400**	**1410**	**1420**	**1430**	**1440**
DGAISPFTPE	FLVQQRWGAM	EYSCFEIQSP	SSCADSQSQI	MEYIHKIEAD	LEHLKKVEES	YTILCQRLAG	SALTDKHSDK
**1450**							
S							

Cmpd.	No. of Cmpds.	m/z meas.	Δ m/z [ppm]	z	Rt [min]	Score	P	Range	Sequence	Modification
85	1	710.2749	-87.79	2	30.8	12.0	0	335-347	K.VSVPDEMSADLEK.R	
1443	1	1022.6499	109.61	1	47.7	10.9	1	458-465	K.EPRNLEHK.R	

Detailed Protein Report

Protein 545: **complement component 1 Q subcomponent-binding protein, mitochondrial precursor [Homo sapiens]**

**Accession:**	gi|4502491	**Score:**	22.9
**Database:**	refseq_human(refseq_human_20140103.fasta)	**MW [kDa]:**	31.3
**Database Date:**	2015-11-30	**pI:**	4.6
		**Sequence Coverage [%]:**	10.3
		**No. of unique Peptides:**	1

10	20	30	40	50	60	70	80
MLPLLRCVPR	VLGSSVAGLR	AAAPASPFRQ	LLQPAPRLCT	RPFGLLSVRA	GSERRPGLLR	PRGPCACGCG	CGSLHTDGDK
**90**	**100**	**110**	**120**	**130**	**140**	**150**	**160**
AFVDFLSDEI	KEERKIQKHK	TLPKMSGGWE	LELNGTEAKL	VRKVAGEKIT	VTFNINNSIP	PTFDGEEEPS	QGQKVEEQEP
**170**	**180**	**190**	**200**	**210**	**220**	**230**	**240**
ELTSTPNFVV	EVIKNDDGKK	ALVLDCHYPE	DEVGQEDEAE	SDIFSIREVS	FQSTGESEWK	DTNYTLNTDS	LDWALYDHLM
**250**	**260**	**270**	**280**	**290**			
DFLADRGVDN	TFADELVELS	TALEHQEYIT	FLEDLKSFVK	SQ			

Cmpd.	No. of Cmpds.	m/z meas.	Δ m/z [ppm]	z	Rt [min]	Score	P	Range	Sequence	Modification
1934	1	840.8059	-11.17	2	54.3	12.6	0	63-80	R.GPCACGCGCGSLHTDGDK.A	

Detailed Protein Report

Protein 546: histone-lysine N-methyltransferase SETD1B [Homo sapiens]

**Accession:**	gi|210032580	**Score:**	22.9
**Database:**	refseq_human(refseq_human_20140103.fasta)	**MW [kDa]:**	208.6
**Database Date:**	2015-11-30	**pI:**	4.7
		**Sequence Coverage [%]:**	0.8
		**No. of unique Peptides:**	1

Detailed Protein Report

10	20	30	40	50	60	70	80
MENSHPPHHH	HQQPPPQPGP	SGERRNHHWR	SYKLMIDPAL	KKGHHKLYRY	DGQHFSLAMS	SNRPVEIVED	PRVVGIWTKN
90	100	110	120	130	140	150	160
KELELSVPKF	KIDEFYVGPV	PPKQVTFAKL	NDNIRENFLR	DMCKKYGEVE	EVEILYNPKT	KKHLGIAKVV	FATVRGAKDA
170	180	190	200	210	220	230	240
VQHLHSTSVM	GNIIHVELDT	KGETRMRFYE	LLVTGRYTPQ	TLPVGELDAV	SPIVNETLQL	SDALKRLKDG	GLSAGCGSGS
250	260	270	280	290	300	310	320
SSVTPNSGGT	PFSQDTAYSS	CRLDTPNSYG	QGTPLTPRLG	TPFSQDSSYS	SRQPTPSYLF	SQDPAVTFKA	RRHESKFTDA
330	340	350	360	370	380	390	400
YNRRHEHHYV	HNSPAVTAVA	GATAAFRGSS	DLPFGAVGGT	GGSSGPPFKA	QPQDSATFAH	TPPPAQATPA	PGFKSAFSPY
410	420	430	440	450	460	470	480
QTPVAHFPPP	PEEPTATAAF	GARDSGEFRR	APAPPPLPPA	EPLAKEKPGT	PPGPPPPDTN	SMELGGRPTF	GWSPEPCDSP
490	500	510	520	530	540	550	560
GTPTLESSPA	GPEKPHDSLD	SRIEMLLKEQ	RTKLLFLREP	DSDTELQMEG	SPISSSSSQL	SPLAPFGTNS	QPGFRGPTPP
570	580	590	600	610	620	630	640
SSRPSSTGLE	DISPTPLPDS	DEDEELDLGL	GPRPPPEPGP	PDPAGLLSQT	AEVALDLVGD	RTPTSEKMDE	GQQSSGEDME
650	660	670	680	690	700	710	720
ISDDEMPSAP	ITSADCPKPM	VVTPGAAAVA	APSVLAPTLP	LPPPPGFPPL	PPPPPPPPPQ	PGFPMPPPLP	PPPPPPPPAH
730	740	750	760	770	780	790	800
PAVTVPPPPL	PAPPGVPPPP	ILPPLPPFPP	GLFPVMQVDM	SHVLGGQWGG	MPMSFQMQTQ	VLSRLMTGQG	ACPYPPFMAA
810	820	830	840	850	860	870	880
AAAAASAGLQ	FVNLPPYRGP	FSLSNSGPGR	GQHWPPLPKF	DPSVPPPGYM	PRQEDPHKAT	VDGVLLVVLK	ELKAIMKRDL
890	900	910	920	930	940	950	960
NRKMVEVVAF	RAFDEWWDKK	ERMAKASLTP	VKSGEHKDED	RPKPKDRIAS	CLLESWGKGE	GLGYEGLGLG	IGLRGAIRLP
970	980	990	1000	1010	1020	1030	1040
SFKVKRKEPP	DTTSSGDQKR	LRPSTSVDEE	DEESERERDR	DMADTPCELA	KRDPKGVGVR	RRPARPLELD	SGGEEDEKES
1050	1060	1070	1080	1090	1100	1110	1120
LSEEQESTEE	EEEAEEEEEE	EDDDDDDSDD	RDESENDDED	TALSEASEKD	EGDSDEEETV	SIVTSKAEAT	SSSESSESSE
1130	1140	1150	1160	1170	1180	1190	1200
FESSSESSPS	SSEDEEEVVA	REEEEEEEEE	EMVAEESMAS	AGPEDFEQDG	EEAALAPGAP	AVDSLGMEEE	VDIETEAVAP
1210	1220	1230	1240	1250	1260	1270	1280
EERPSMLDEP	PLPVGVEEPA	DSREPPEEPG	LSQEGAMLLS	PEPPAKEVEA	RPPLSPERAP	EHDLEVEPEP	PMMLPLPLQP
1290	1300	1310	1320	1330	1340	1350	1360
PLPPPRPPRP	PSPPPEPETT	DASHPSVPPE	PLAEDHPPHT	PGLCGSLAKS	QSTETVPATP	GGEPPLSGGS	SGLSLSSPQV
1370	1380	1390	1400	1410	1420	1430	1440
PGSPFSYPAP	SPSLSSGGLP	RTPGRDFSFT	PTFSEPSGPL	LLPVCPLPTG	RRDERSGPLA	SPVLLETGLP	LPLPLPLPLP
1450	1460	1470	1480	1490	1500	1510	1520
LALPAVLRAQ	ARAPTPLPPL	LPAPLASCPP	PMKRKPGRPR	RSPPSMLSLD	GPLVRPPAGA	ALGRELLLLP	GQPQTPVFPS
1530	1540	1550	1560	1570	1580	1590	1600
THDPRTVTLD	FRNAGIPAPP	PPLPPQPPPP	PPPPPVEPTK	LPFKELDNQW	PSEAIPPGPR	GRDEVTEEYM	ELAKSRGPWR
1610	1620	1630	1640	1650	1660	1670	1680
RPPKKRHEDL	VPPAGSPELS	PPQPLFRPRS	EFEEMTILYD	IWNGGIDEED	IRFLCVTYER	LLQQDNGMDW	LNDTLWVYHP
1690	1700	1710	1720	1730	1740	1750	1760
STSLSSAKKK	KRDDGIREHV	TGCARSEGFY	TIDKKDKLRY	LNSSRASTDE	PPADTQGMSI	PAQPHASTRA	GSERRSEQRR
1770	1780	1790	1800	1810	1820	1830	1840
LLSSFTGSCD	SDLLKFNQLK	FRKKKLKFCK	SHIHDWGLFA	MEPIAADEMV	IEYVGQNIRQ	VIADMREKRY	EDEGIGSSYM
1850	1860	1870	1880	1890	1900	1910	1920
FRVDHDTIID	ATKCGNFARF	INHSCNPNCY	AKVITVESQK	KIVIYSKQHI	NVNEEITYDY	KFPIEDVKIP	CLCGSENCRG
1930							
TLN							

Cmpd.	No. of Cmpds.	m/z meas.	Δ m/z [ppm]	z	Rt [min]	Score	P	Range	Sequence	Modification
837	1	801.1846	-354.77	1	40.0	10.6	2	878-883	K.RDLNRK.M	

Detailed Protein Report

Protein 547: hepatoma-derived growth factor isoform a [Homo sapiens]

**Accession:**	gi|4758516	**Score:**	22.9
**Database:**	refseq_human(refseq_human_20140103.fasta)	**MW [kDa]:**	26.8
**Database Date:**	2015-11-30	**pI:**	4.6
		**Sequence Coverage [%]:**	12.9
		**No. of unique Peptides:**	1

10	20	30	40	50	60	70	80
MSRSNRQKEY	KCGDLVFAKM	KGYPHWPARI	DEMPEAAVKS	TANKYQVFFF	GTHETAFLGP	KDLFPYEESK	EKFGKPNKRK
**90**	**100**	**110**	**120**	**130**	**140**	**150**	**160**
GFSEGLWEIE	NNPTVKASGY	QSSQKKSCVE	EPEPEPEAAE	GDGDKKGNAE	GSSDEEGKLV	IDEPAKEKNE	KGALKRRAGD
**170**	**180**	**190**	**200**	**210**	**220**	**230**	**240**
LLEDSPKRPK	EAENPEGEEK	EAATLEVERP	LPMEVEKNST	PSEPGSGRGP	PQEEEEEEDE	EEEATKEDAE	APGIRDHESL
**250**							

Cmpd.	No. of Cmpds.	m/z meas.	Δ m/z [ppm]	z	Rt [min]	Score	P	Range	Sequence	Modification
1973	1	682.2866	-59.46	3	54.8	11.7	1	127-146	K.GNAEGSSDEEGKLVIDEPAK.E	

Detailed Protein Report

Protein 548: **leucine-rich repeat and transmembrane domain-containing protein 2 precursor [Homo sapiens]**

**Accession:**	gi|84781739	**Score:**	22.8
**Database:**	refseq_human(refseq_human_20140103.fasta)	**MW [kDa]:**	41.1
**Database Date:**	2015-11-30	**pI:**	9.5
		**Sequence Coverage [%]:**	6.5
		**No. of unique Peptides:**	1

Alias proteins:

Accession	Name	Description
gi|578822678	refseq_human (refseq_human_20140103.fasta)	PREDICTED: leucine-rich repeat and transmembrane domain-containing protein 2 isoform X1 [Homo sapiens]
gi|255652964	refseq_human (refseq_human_20140103.fasta)	leucine-rich repeat and transmembrane domain-containing protein 2 precursor [Homo sapiens]
gi|255652962	refseq_human (refseq_human_20140103.fasta)	leucine-rich repeat and transmembrane domain-containing protein 2 precursor [Homo sapiens]

10	20	30	40	50	60	70	80
MLAPGSSPGQ	RGRLALQWRQ	VSWITCWIAL	YAVEALPTCP	FSCKCDSRSL	EVDCSGLGLT	TVPPDVPAAT	RTLLLLNNKL
**90**	**100**	**110**	**120**	**130**	**140**	**150**	**160**
SALPSWAFAN	LSSLQRLDLS	NNFLDRLPRS	IFGDLTNLTE	LQLRNNSIRT	LDRDLLRHSP	LLRHLDLSIN	GLAQLPPGLF
**170**	**180**	**190**	**200**	**210**	**220**	**230**	**240**
DGLLALRSLS	LRSNRLQNLD	RLTFEPLANL	QLLQVGDNPW	ECDCNLREFK	HWMEWFSYRG	GRLDQLACTL	PKELRGKDMR
**250**	**260**	**270**	**280**	**290**	**300**	**310**	**320**
MVPMEMFNYC	SQLEDENSSA	GLDIPGPPCT	KASPEPAKPK	PGAEPEPEPS	TACPQKQRHR	PASVRRAMGT	VIIAGVVCGV
**330**	**340**	**350**	**360**	**370**	**380**		
VCIMMVVAAA	YGCIYASLMA	KYHRELKKRQ	PLMGDPEGEH	EDQKQISSVA			

Cmpd.	No. of Cmpds.	m/z meas.	Δ m/z [ppm]	z	Rt [min]	Score	P	Range	Sequence	Modification
1746	1	1272.3522	103.26	2	51.8	22.8	0	144-167	R.HLDLSINGLAQLPPGLFDGLLALR.S	

Detailed Protein Report

Protein 549: zinc finger protein 674 isoform 2 [Homo sapiens]

**Accession:**	gi|226371655	**Score:**	22.8
**Database:**	refseq_human(refseq_human_20140103.fasta)	**MW [kDa]:**	66.5
**Database Date:**	2015-11-30	**pI:**	10.3
**Modification(s):**	Carbamidomethyl	**Sequence Coverage [%]:**	6.1
		**No. of unique Peptides:**	1

10	20	30	40	50	60	70	80
MAMSQESLTF	KDVFVDFTLE	EWQQLDSAQK	NLYRDVMLEN	YSHLVSVGHL	VGKPDVIFRL	GPGDESWMAD	GGTPVRTCAV
90	100	110	120	130	140	150	160
WEVDEQIDHY	KESQDKFLWQ	AAFIGKETLK	DESGQECKIC	RKIIYLNTDF	VSVKQRLPKY	YSWERCSKHH	LNFLGQNRSY
170	180	190	200	210	220	230	240
VRKKDDGCKA	YWKVCLHYNL	HKAQPAERFF	DPNQRGKALH	QKQALRKSQR	SQTGEKLYKC	TECGKVFIQK	ANLVVHQRTH
250	260	270	280	290	300	310	320
TGEKPYECCE	CAKAFSQKST	LIAHQRTHTG	EKPYECSECG	KTFIQKSTLI	KHQRTHTGEK	PFVCDKCPKA	FKSSYHLIRH
330	340	350	360	370	380	390	400
EKTHIRQAFY	KGIKCTTSSL	IYQRIHTSEK	PQCSEHGKAS	DEKPSPTKHW	RTHTKENIYE	CSKCGKSFRG	KSHLSVHQRI
410	420	430	440	450	460	470	480
HTGEKPYECS	ICGKTFSGKS	HLSVHHRTHT	GEKPYECRRC	GKAFGEKSTL	IVHQRMHTGE	KPYKCNECGK	AFSEKSPLIK
490	500	510	520	530	540	550	560
HQRIHTGERP	YECTDCKKAF	SRKSTLIKHQ	RIHTGEKPYK	CSECGKAFSV	KSTLIVHHRT	HTGEKPYECR	DCGKAFSGKS
570	580						
TLIKHQRSHT	GDKNL						

Cmpd.	No. of Cmpds.	m/z meas.	Δ m/z [ppm]	z	Rt [min]	Score	P	Range	Sequence	Modification
1639	1	1136.1099	81.18	2	50.4	11.8	2	540-559	R.THTGEKPYECRDCGKAFSGK.S	Carbamidomethyl: 13

Detailed Protein Report

Protein 550: fibrinogen-like protein 1 precursor [Homo sapiens]

**Accession:**	gi|42544189	**Score:**	22.8
**Database:**	refseq_human(refseq_human_20140103.fasta)	**MW [kDa]:**	36.4
**Database Date:**	2015-11-30	**pI:**	5.5
		**Sequence Coverage [%]:**	2.9
		**No. of unique Peptides:**	1

Alias proteins:

Accession	Name	Description
gi|42544202	refseq_huma (refseq_human_20140103.fasta)	fibrinogen-like protein 1 precursor [Homo sapiens]
gi|42544200	refseq_huma (refseq_human_20140103.fasta)	fibrinogen-like protein 1 precursor [Homo sapiens]
gi|42544198	refseq_huma (refseq_human_20140103.fasta)	fibrinogen-like protein 1 precursor [Homo sapiens]

10	20	30	40	50	60	70	80
MAKVFSFILV	TTALTMGREI	SALEDCAQEQ	MRLRAQVRLL	ETRVKQQQVK	IKQLLQENEV	QFLDKGDENT	VIDLGSKRQY
**90**	**100**	**110**	**120**	**130**	**140**	**150**	**160**
ADCSEIFNDG	YKLSGFYKIK	PLQSPAEFSV	YCDMSDGGGW	TVIQRRSDGS	ENFNRGWKDY	ENGFGNFVQK	HGEYWLGNKN
**170**	**180**	**190**	**200**	**210**	**220**	**230**	**240**
LHFLTTQEDY	TLKIDLADFE	KNSRYAQYKN	FKVGDEKNFY	ELNIGEYSGT	AGDSLAGNFH	PEVQWWASHQ	RMKFSTWDRD
**250**	**260**	**270**	**280**	**290**	**300**	**310**	**320**
HDNYEGNCAE	EDQSGWWFNR	CHSANLNGVY	YSGPYTAKTD	NGIVWYTWHG	WWYSLKSVVM	KIRPNDFIPN	VI

Cmpd.	No. of Cmpds.	m/z meas.	Δ m/z [ppm]	z	Rt [min]	Score	P	Range	Sequence	Modification
1516	1	513.1182	-194.06	2	48.6	22.8	0	127-135	R.SDGSENFNR.G	

Detailed Protein Report

Protein 551: **nucleolar protein 11 [Homo sapiens]**

Accession: gi|21361468
Database: refseq_human(refseq_human_20140103.fasta)
Database Date: 2015-11-30
Modification(s): Carbamidomethyl

Score: 22.7
MW [kDa]: 81.1
pI: 5.7
Sequence Coverage [%]: 3.1
No. of unique Peptides: 1

10	20	30	40	50	60	70	80
MAALEEEFTL	SSVVLSAGPE	GLLGVEQSDK	TDQFLVTDSG	RTVILYKVSD	QKPLGSWSVK	QGQIITCPAV	CNFQTGEYVV
**90**	**100**	**110**	**120**	**130**	**140**	**150**	**160**
VHDNKVLRIW	NNEDVNLDKV	FKATLSAEVY	RILSVQGTEP	LVLFKEGAVR	GLEALLADPQ	QKIETVISDE	EVIKWTKFFV
**170**	**180**	**190**	**200**	**210**	**220**	**230**	**240**
VFRHPVLIFI	TEKHGNYFAY	VQMFNSRILT	KYTLLLGQDE	NSVIKSFTAS	VDRKFISLMS	LSSDGCIYET	LIPIRPADPE
**250**	**260**	**270**	**280**	**290**	**300**	**310**	**320**
KNQSLVKSLL	LKAVVSGNAR	NGVALTALDQ	DHVAVLGSPL	AASKECLSVW	NIKFQTLQTS	KELPQGTSGQ	LWYYGEHLFM
**330**	**340**	**350**	**360**	**370**	**380**	**390**	**400**
LHGKSLTVIP	YKCEVSSLAG	ALGKLKHSQD	PGTHVVSHFV	NWETPQGCGL	GFQNSEQSRR	ILRRRKIEVS	LQPEVPPSKQ
**410**	**420**	**430**	**440**	**450**	**460**	**470**	**480**
LLSTIMKDSE	KHIEVEVRKF	LALKQTPDFH	TVIGDTVTGL	LERCKAEPSF	YPRNCLMQLI	QTHVLSYSLC	PDLMEIALKK
**490**	**500**	**510**	**520**	**530**	**540**	**550**	**560**
KDVQLLQLCL	QQFPDIPESV	TCACLKIFLS	IGDDSLQETD	VNMESVFDYS	INSVHDEKME	EQTEILQNGF	NPEEDKCNNC
**570**	**580**	**590**	**600**	**610**	**620**	**630**	**640**
DQELNKKPQD	ETKESTSCPV	VQKRAALLNA	ILHSAYSETF	LLPHLKDIPA	QHITLFLKYL	YFLYLKCSEN	ATMTLPGIHP
**650**	**660**	**670**	**680**	**690**	**700**	**710**	**720**
PTLNQIMDWI	CLLLDANFTV	VVMMPEAKRL	LINLYKLVKS	QISVYSELNK	IEVSFRELQK	LNQEKNNRGL	YSIEVLELF

Cmpd.	No. of Cmpds.	m/z meas.	Δ m/z [ppm]	z	Rt [min]	Score	P	Range	Sequence	Modification
913	1	645.6782	-230.66	2	40.9	11.4	1	574-584	K.ESTSCPVVQKR.A	Carbamidomethyl: 5

Detailed Protein Report

Protein 552: PREDICTED: mitochondrial Rho GTPase 2 isoform X9 [Homo sapiens]

**Accession:**	gi|578828338	**Score:**	22.7
**Database:**	refseq_human(refseq_human_20140103.fasta)	**MW [kDa]:**	52.3
**Database Date:**	2015-11-30	**pI:**	6.7
		**Sequence Coverage [%]:**	5.4
		**No. of unique Peptides:**	1

10	20	30	40	50	60	70	80
MGGPRRGPVG	NKSDLRSGSS	MEAVLPIMSQ	FPEIETCVEC	SAKNLRNISE	LFYYAQKAVL	HPTAPLYDPE	AKQKSCFGHP
**90**	**100**	**110**	**120**	**130**	**140**	**150**	**160**
LAPQALEDVK	TVVCRNVAGG	VREDRLTLDG	FLFLNTLFIQ	RGRHETTWTI	LRRFGYSDAL	ELTADYLSPL	IHVPPGCSTE
**170**	**180**	**190**	**200**	**210**	**220**	**230**	**240**
LNHLGYQFVQ	RVFEKHDQDR	DGALSPVELQ	SLFSVFPAAP	WGPELPRTVR	TEAGRLPLHG	YLCQWTLVTY	LDVRSCLGHL
**250**	**260**	**270**	**280**	**290**	**300**	**310**	**320**
GYLGYPTLCE	QDQAHAITVT	REKRLDQEKG	QTQRSVLLCK	VVGARGVGKS	AFLQAFLGRG	LGHQDTREQP	PGYAIDTVQV
**330**	**340**	**350**	**360**	**370**	**380**	**390**	**400**
NGQEKYLILC	EVGTDGLLAT	SLDATCDVAC	LMFDGSDPKS	FAHCASVYKH	HYMDGQTPCL	FVSSKADLPE	GVAVSGPSPA
**410**	**420**	**430**	**440**	**450**	**460**	**470**	**480**
EFCRKHRLPA	PVPFSCAGPA	EPSTTIFTQL	ATMAAFPHLV	HAELHPSSFW	LRGLLGVVGA	AVAAVLSFSL	YRVLVKSQ

Cmpd.	No. of Cmpds.	m/z meas.	Δ m/z [ppm]	z	Rt [min]	Score	P	Range	Sequence	Modification
2627	2	727.3858	-48.33	1	63.6	12.4	1	6-12	R.RGPVGNK.S	

Detailed Protein Report

Protein 553: gamma-tubulin complex component 6 [Homo sapiens]

**Accession:**	gi|56788364	**Score:**	22.7
**Database:**	refseq_human(refseq_human_20140103.fasta)	**MW [kDa]:**	200.4
**Database Date:**	2015-11-30	**pI:**	5.9
**Modification(s):**	Carbamidomethyl	**Sequence Coverage [%]:**	1.8
		**No. of unique Peptides:**	1

10	20	30	40	50	60	70	80
MASITQLFDD	LCEALLPAAK	THLGQRSVNR	KRAKRSLKKV	AYNALFTNLF	QDETQQLQPD	MSKLPARNKI	LMLSFDLRVG
90	100	110	120	130	140	150	160
GLGPKADRLE	ELVEELEAAP	CCPLLEVGSV	LDLLVQLAGS	GPPQVLPRKR	DYFLNNKHVG	RNVPYSGYDC	DDLSVFEMDV
170	180	190	200	210	220	230	240
QSLISREECL	CHSMIQETLQ	VMEAAPGTGL	PTVGLFSFGD	PCGDRFERDT	RVSLFGALVH	SRTYDMDVRL	GLPPVPDNAD
250	260	270	280	290	300	310	320
LSGLAIKVPP	SVDQWEDEGF	QSASNLTPDS	QSEPSVTPDV	DLWEAALTYE	ASKRRCWERV	GCPPGHREEP	YLTEAGRDAF
330	340	350	360	370	380	390	400
DKFCRLHQGE	LQLLAGGVLQ	APQPVLVKEC	ELVKDVLNVL	IGVVSATFSL	CQPAQAFVVK	RGVHVSGASP	ESISSLLSEV
410	420	430	440	450	460	470	480
AEYGTCYTRL	SHFSLQPVLD	SLYSKGLVFQ	AFTSGLRRYL	QYYRACVLST	PPTLSLLTIG	FLFKKLGRQL	RYLAELCGVG
490	500	510	520	530	540	550	560
AVLPGTCGGG	PRAAFPTGVK	LLSYLYQEAL	HNCSNEHYPV	LLSLLKTSCE	PYTRFIHDWV	YSGVFRDAYG	EFMIQVNHEY
570	580	590	600	610	620	630	640
LSFRDKLYWT	HGYVLISKEV	EDCVPVFLKH	IAHDIYVCGK	TINLLKLCCP	RHYLCWSDVP	VPRISVIFSL	EELKEIEKDC
650	660	670	680	690	700	710	720
AVYVGRMERV	ARHSSVSKEE	KELRMEIAKQ	ELIAHAREAA	SRVLSALSDR	QMSERMALDA	RKREQFQRLK	EQFVKDQERR
730	740	750	760	770	780	790	800
QAARQEELDD	DFSYARELRD	RERRLKSLEE	ELERKARQAL	VDHYSKLSAE	AARREQKALW	RIQRHRLESA	RLRFLLEDEK
810	820	830	840	850	860	870	880
HIQEMLKAVS	EAHQPQEPPD	VLLSVHPQVT	SPGPEHPEGG	QGCDSGSAEQ	HSPAWDGWNR	PGLLTPQPLK	PLAVGAGGRG
890	900	910	920	930	940	950	960
LQQAEGARPF	SDSLSIGDFL	PVGPGAEPSV	QTGMVPLLEV	ALQTINLDLP	PSAPGEAPAA	ASTQPSRPQE	YDFSTVLRPA
970	980	990	1000	1010	1020	1030	1040
VATSPAPGPL	QAAECSLGSS	GLQLWEDSCG	KMDACGSASR	ETLLPSHPPR	RAALEEGSSQ	PTERLFGQVS	GGGLPTGDYA
1050	1060	1070	1080	1090	1100	1110	1120
SEIAPTRPRW	NTHGHVSDAS	IRVGENVSDV	APTQPRWNTH	GHVSNASISL	GESVSDVAPT	RPRWNIHGHV	SNASIRVGEN
1130	1140	1150	1160	1170	1180	1190	1200
VSDVAPTRPR	WNTHGHVSNA	SIRVGENVSD	VAPTRPRWNT	HGHVSDASIS	LGESVSDMAP	ARPRWNTHGH	VSDASISLGE
1210	1220	1230	1240	1250	1260	1270	1280
SVSDMAPTRP	RWNTHGHVSD	TSIRVGENVS	DVAPIRSRCN	THGHVSDASI	SLGEPVSDVV	STRPRWNTHV	PIPPPHMVLG
1290	1300	1310	1320	1330	1340	1350	1360
ALSPEAEPNT	PRPQQSPPGH	TSQSALSLGA	QSTVLDCGPR	LPVEVGPSLS	SPSSGCGEGS	ISVGENVSDV	APTQPWWPNT
1370	1380	1390	1400	1410	1420	1430	1440
PGDSVSEELG	PGRSGDTEDL	SPNWPLNSQE	DTAAQSSPGR	GEEAEASAAE	AQGGEQAYLA	GLAGQYHLER	YPDSYESMSE
1450	1460	1470	1480	1490	1500	1510	1520
PPIAHLLRPV	LPRAFAFPVD	PQVQSAADET	AVQLSELLTL	PVLMKRSITA	PLAAHISLVN	KAAVDYFFVE	LHLEAHYEAL
1530	1540	1550	1560	1570	1580	1590	1600
RHFLLMEDGE	FAQSLSDLLF	EKLGAGQTPG	ELLNPLVLNS	VLSKALQCSL	HGDTPHASNL	SLALKYLPEV	FAPNAPDVLS
1610	1620	1630	1640	1650	1660	1670	1680
CLELRYKVDW	PLNIVITEGC	LSKYSGVFSF	LLQLKLMMWA	LKDVCFHLKR	TALLSHMAGS	VQFRQLQLFK	HEMQHFVKVI
1690	1700	1710	1720	1730	1740	1750	1760
QGYIANQILH	VTWCEFRARL	ATVGDLEEIQ	RAHAEYLHKA	VFRGLLTEKA	APVMNVIHSI	FSLVLKFRSQ	LISQAWGPPG
1770	1780	1790	1800	1810	1820		
GPRGAEHPNF	ALMQQSYNTF	KYYSHFLFKV	VTKLVNRGYQ	PHLEDFLLRI	NFNNYYQDA		

Cmpd.	No. of Cmpds.	m/z meas.	Δ m/z [ppm]	z	Rt [min]	Score	P	Range	Sequence	Modification
2334	1	1023.8623	-142.84	2	59.1	12.7	0	472-492	R.YLAELCGVGAVLPGTCGGGPR.A	Carbamidomethyl: 6

Detailed Protein Report

Protein 554: **PREDICTED: receptor-type tyrosine-protein phosphatase mu isoform X4 [Homo sapiens]**

**Accession:**	gi|578832170	**Score:**	22.6
**Database:**	refseq_human(refseq_human_20140103.fasta)	**MW [kDa]:**	161.1
**Database Date:**	2015-11-30	**pI:**	6.4
**Modification(s):**	Carbamidomethyl	**Sequence Coverage [%]:**	1.8
		**No. of unique Peptides:**	1

10	20	30	40	50	60	70	80
MPSGSFMLVN	ASGRPEGQRA	HLLLPQLKEN	DTHCIDFHYF	VSSKSNSPPG	LLNVYVKVNN	GPLGNPIWNI	SGDPTRTWNR
90	100	110	120	130	140	150	160
AELAISTFWP	NFYQVIFEVI	TSGHQGYLAI	DEVKVLGHPC	TRTPHFLRIQ	NVEVNAGQFA	TFQCSAIGRT	VAGDRLWLQG
170	180	190	200	210	220	230	240
IDVRDAPLKE	IKVTSSRRFI	ASFNVVNTTK	RDAGKYRCMI	RTEGGVGISN	YAELVVKEPP	VPIAPPQLAS	VGATYLWIQL
250	260	270	280	290	300	310	320
NANSINGDGP	IVAREVEYCT	ASGSWNDRQP	VDSTSYKIGH	LDPDTEYEIS	VLLTRPGEGG	TGSPGPALRT	RTKCADPMRG
330	340	350	360	370	380	390	400
PRKLEVVEVK	SRQITIRWEP	FGYNVTRCHS	YNLTVHYCYQ	VGGQEQVREE	VSWDTENSHP	QHTITNLSPY	TNVSVKLILM
410	420	430	440	450	460	470	480
NPEGRKESQE	LIVQTDEDLP	GAVPTESIQG	STFEEKIFLQ	WREPTQTYGV	ITLYEITYKA	VSSFDPEIDL	SNQSGRVSKL
490	500	510	520	530	540	550	560
GNETHFLFFG	LYPGTTYSFT	IRASTAKGFG	PPATNQFTTK	ISAPSMPAYE	LETPLNQTDN	TVTVMLKPAH	SRGAPVSVYQ
570	580	590	600	610	620	630	640
IVVEEERPRR	TKKTTEILKC	YPVPIHFQNA	SLLNSQYYFA	AEFPADSLQA	AQPFTIGDNK	TYNGYWNTPL	LPYKSYRIYF
650	660	670	680	690	700	710	720
QAASRANGET	KIDCVQVATK	AAIIVTQLTT	PYIRIAPAAG	DGQLTGAATP	KPVPEPEKQT	DHTVKIAGVI	AGILLFVIIF
730	740	750	760	770	780	790	800
LGVVLVMKKR	KLAKKRKETM	SSTRQEMTVM	VNSMDKSYAE	QGTNCDEAFS	FMDTHNLNGR	SVSSPSSFTM	KTNTLSTSVP
810	820	830	840	850	860	870	880
NSYYPDETHT	MASDTSSLVQ	SHTYKKREPA	DVPYQTGQLH	PAIRVADLLQ	HITQMKCAEG	YGFKEEYESF	FEGQSAPWDS
890	900	910	920	930	940	950	960
AKKDENRMKN	RYGNIIAYDH	SRVRLQTIEG	DTNSDYINGN	YIDGYHRPNH	YIATQGPMQE	TIYDFWRMVW	HENTASIIMV
970	980	990	1000	1010	1020	1030	1040
TNLVEVGRVK	CCKYWPDDTE	IYKDIKVTLI	ETELLAEYVI	RTFAVEKGGA	GGEANCSPSR	EVSQRGVHEI	REIRQFHFTG
1050	1060	1070	1080	1090	1100	1110	1120
WPDHGVPYHA	TGLLGFVRQV	KSKSPPSAGP	LVVHCSAGAG	RTGCFIVIDI	MLDMAEREGV	VDIYNCVREL	RSRRVNMVQT
1130	1140	1150	1160	1170	1180	1190	1200
EEQYVFIHDA	ILEACLCGDT	SVPASQVRSL	YYDMNKLDPQ	TNSSQIKEEF	RTLNMVTPTL	RVEDCSIALL	PRNHEKNRCM
1210	1220	1230	1240	1250	1260	1270	1280
DILPPDRCLP	FLITIDGESS	NYINAALMDS	YKQPSAFIVT	QHPLPNTVKD	FWRLVLDYHC	TSVVMLNDVD	PAQLCPQYWP
1290	1300	1310	1320	1330	1340	1350	1360
ENGVHRHGPI	QVEFVSADLE	EDIISRIFRI	YNAARPQDGY	RMVQQFQFLG	WPMYRDTPVS	KRSFLKLIRQ	VDKWQEEYNG
1370	1380	1390	1400	1410	1420	1430	1440
GEGRTVVHCL	NGGGRSGTFC	AISIVCEMLR	HQRTVDVFHA	VKTLRNNKPN	MVDLLDQYKF	CYEVALEYLN	SG

Cmpd.	No. of Cmpds.	m/z meas.	Δ m/z [ppm]	z	Rt [min]	Score	P	Range	Sequence	Modification
1436	1	927.4440	-10.35	3	47.6	22.6	1	1365-1390	R.TVVHCLNGGGRSGTFCAISIVCEMLR.H	Carbamidomethyl: 22

Detailed Protein Report

Protein 555: PREDICTED: exophilin-5 isoform X3 [Homo sapiens]

**Accession:**	gi|578822067	**Score:**	22.6
**Database:**	refseq_human(refseq_human_20140103.fasta)	**MW [kDa]:**	201.0
**Database Date:**	2015-11-30	**pI:**	6.8
**Modification(s):**	Carbamidomethyl	**Sequence Coverage [%]:**	2.0
		**No. of unique Peptides:**	2

10	20	30	40	50	60	70	80
MREESGMPPP	WDASLLENEF	FQVLDDLDSK	LAQEQSASSV	NTRTPLNYGS	RTQFGHFYSS	GNRHGNITER	HKKHYNETSN
**90**	**100**	**110**	**120**	**130**	**140**	**150**	**160**
MSIYDILRPG	TPREGFKTFS	PRTSTIYDMY	RTREPRVFKE	DYVQKNTFGS	TSLCFDSRQR	SALPATGHFT	ARSLHFPATT
**170**	**180**	**190**	**200**	**210**	**220**	**230**	**240**
QSKSGFIPPR	HQQSPKRTPL	SSIIWNRSDS	SRDRENQEEF	LRAPSPMEID	PADKYVYPRG	FQENKRYESY	HSQNVYQRVS
**250**	**260**	**270**	**280**	**290**	**300**	**310**	**320**
LNAPMENAMS	PDTFENSENM	PFYHQSNTFT	RSFFSNTFGR	SGEQRRFGQG	PFWGQEKGHS	FWSDFHRSRK	SFSSSDRDFE
**330**	**340**	**350**	**360**	**370**	**380**	**390**	**400**
MISMEANSVS	AIHGHNVSSE	HWESFSSGYG	TDVSRGQEEP	HPWQFDFQRS	TLDSMVVSHG	NETQLTPHFG	TPNVCSMTGS
**410**	**420**	**430**	**440**	**450**	**460**	**470**	**480**
SYHVKSSELV	SQQDSSPVEV	HINKEASSFG	IAQTLASSFK	TSFSQISDDR	RNPQSPNLQN	PTVTLQKIFP	NKPASHPMRS
**490**	**500**	**510**	**520**	**530**	**540**	**550**	**560**
HTEVTVTSSN	SVDSLPLAKS	QPNILVTEVN	NEKDLNESIS	EEDKQLSKMD	QTNKAGEIPQ	PVSQTGISNS	LPDFQNPLSQ
**570**	**580**	**590**	**600**	**610**	**620**	**630**	**640**
DSAKSNGFGF	NASTIISSKK	SPRVFSRKDT	SKMYIPHTDK	SNDIKQDKRF	TENRKLGSTA	SLPFIQEHRT	PPSFPRTDQG
**650**	**660**	**670**	**680**	**690**	**700**	**710**	**720**
CHQELTVNNE	DISRIITNNH	WSSALTDTQN	AQYSKCKLTP	GHKTSCDSLD	LSSAALPDSS	PSKNSSLDAP	VVPSTTVFSR
**730**	**740**	**750**	**760**	**770**	**780**	**790**	**800**
RSPSDKDPSL	GEREEKDNAG	KNQKNQFIVS	HSENQERNDS	PVPTHDEVVD	VKCHSHSPFR	NERGKGKIRH	HISCIEKLSK
**810**	**820**	**830**	**840**	**850**	**860**	**870**	**880**
TESISVPTSD	HRSLIEANQS	NSKVSELDTI	YCTLPRKSSS	FLIHGRQSGS	KIMAASLRNG	PPPFQIKNNV	EDAMGNYMLN
**890**	**900**	**910**	**920**	**930**	**940**	**950**	**960**
KFSPSSPESA	NECSKVLSDS	ALEAPEATER	MTNVKSSGST	SVRKGPLPFL	INRAMSCPSG	EPHASTGREG	RKKPLTSGMD
**970**	**980**	**990**	**1000**	**1010**	**1020**	**1030**	**1040**
ASELTPRAWE	RIISPVESDS	SVRDCSLTKR	QHQKENFQEY	TEKEGKMAAS	RRSVFALSNE	DPLPFCSDLS	GKERGKTLHK
**1050**	**1060**	**1070**	**1080**	**1090**	**1100**	**1110**	**1120**
VKTTSTFSVS	GDEDNVKCLE	VVSIYYTLPR	KPSKKFCNLL	QQYTQNTNLL	IESPQVETET	FPNALEKDKQ	NYSTREQSGT
**1130**	**1140**	**1150**	**1160**	**1170**	**1180**	**1190**	**1200**
PSCENLKMSV	NSDQTLTTEN	MTAFRLSNRG	PLAPTLQEMA	SVEAAVSLPE	EESKAREIFS	DNLAKTPLGD	SENKKERGKK
**1210**	**1220**	**1230**	**1240**	**1250**	**1260**	**1270**	**1280**
LQSETLHTSL	MLQRKNVSEE	KSENCQQSIN	SSNSGPSSLP	ALSEVNIGNS	QTRRSSWECT	GSGRAIPFTG	SGKCPQKDHT
**1290**	**1300**	**1310**	**1320**	**1330**	**1340**	**1350**	**1360**
STAVGDGSSG	SQPREGRGDI	GTNCQKMTNK	TLSHSESQVF	ALTPALHKLQ	LGEETQSDEP	NLESLQSEPR	ELPQRSQEAN
**1370**	**1380**	**1390**	**1400**	**1410**	**1420**	**1430**	**1440**
MTESRKAEDE	MQKSAWDQPS	LPEGNKNKTN	LDDLVKGENR	SSVKHRLAAM	SKASRKFPAK	DVSPRRHVAT	IFPQSGSRSG
**1450**	**1460**	**1470**	**1480**	**1490**	**1500**	**1510**	**1520**
FDHLSLGTVE	CNPLFPEPTP	KSAESIGESR	LSENGKHVKK	SENLLPITVL	PNREPSTHVS	NQKSNSISQR	HQNEFKNVSE
**1530**	**1540**	**1550**	**1560**	**1570**	**1580**	**1590**	**1600**
SPSKHENSKD	VTAAQNLVRE	SGAPSPITFT	SLREAEFSDN	QRRLSPPFPL	EPAQKSRVSS	PLASFLQQQR	SASSLEWEPE
**1610**	**1620**	**1630**	**1640**	**1650**	**1660**	**1670**	**1680**
PHLYRSKSLK	SINVHGDLLR	KSHPPKVRER	HFSESTSIDN	ALSRLTLGNE	FSVNNGYSRR	FRSFSELPSC	DGNESWAYRS
**1690**	**1700**	**1710**	**1720**	**1730**	**1740**	**1750**	**1760**
GTKTGPRSAI	SIYRPIDYGI	FGKEQQLAFL	ENVKRSLTQG	RLWKPSFLKN	PGFLKDDLRN	PPNPSESLSS	NSPSSQVPED
**1770**	**1780**	**1790**	**1800**	**1810**			
GLSPSEPLNI	YEDDPVDSDC	DTDTTTDDEY	YLDENDKESE	L			

Cmpd.	No. of	m/z meas.	Δ m/z	z	Rt	Score	P	Range	Sequence	Modification

Detailed Protein Report

	Cmpds.		[ppm]		[min]					
309	1	558.5767	-48.96	3	33.2	12.3	2	722-736	R.SPSDKDPSLGEREEK.D	
2720	1	1086.5521	56.79	2	64.3	10.3	1	1274-1294	K.CPQKDHTSTAVGDGSSGSQPR.E	Carbamidomethyl: 1

Detailed Protein Report

Protein 556: **uncharacterized protein KIAA0408 [Homo sapiens]**

**Accession:**	gi|59806363	**Score:**	22.5
**Database:**	refseq_human(refseq_human_20140103.fasta)	**MW [kDa]:**	79.1
**Database Date:**	2015-11-30	**pI:**	9.6
**Modification(s):**	Carbamidomethyl, Oxidation	**Sequence Coverage [%]:**	3.7
		**No. of unique Peptides:**	1

10	20	30	40	50	60	70	80
MDLHKQWENT	ETNWHKEKME	LLDQFDNERK	EWESQWKIMQ	KKIEELCREV	KLWRKININE	SAKIIDLYHE	KTIPEKVIES
90	100	110	120	130	140	150	160
SPNYPDLGQS	EFIRTNHKDG	LRKENKREQS	LVSGGNQMCK	EQKATKKSKV	GFLDPLATDN	QKECEAWPDL	RTSEEDSKSC
170	180	190	200	210	220	230	240
SGALSTALEE	LAKVSEELCS	FQEEIRKRSN	HRRMKSDSFL	QEMPNVTNIP	HGDPMINNDQ	CILPISLEKE	KQKNRKNLSC
250	260	270	280	290	300	310	320
TNVLQSNSTK	KCGIDTIDLK	RNETPPVPPP	RSTSRNFPSS	DSEQAYERWK	ERLDHNSWVP	HEGRSKRNYN	PHFPLRQQEM
330	340	350	360	370	380	390	400
SMLYPNEGKT	SKDGIIFSSL	VPEVKIDSKP	PSNEDVGLSM	WSCDIGIGAK	RSPSTSWFQK	TCSTPSNPKY	EMVIPDHPAK
410	420	430	440	450	460	470	480
SHPDLHVSND	CSSSVAESSS	PLRNFSCGFE	RTTRNEKLAA	KTDEFNRTVF	RTDRNCQAIQ	QNHSCSKSSE	DLKPCDTSST
490	500	510	520	530	540	550	560
HTGSISQSND	VSGIWKTNAH	MPVPMENVPD	NPTKKSTTGL	VRQMQGHLSP	RSYRNMLHEH	DWRPSNLSGR	PRSADPRSNY
570	580	590	600	610	620	630	640
GVVEKLLKTY	ETATESALQN	SKCFQDNWTK	CNSDVSGGAT	LSQHLEMLQM	EQQFQQKTAV	WGGQEVKQGI	DPKKITEESM
650	660	670	680	690	700		
SVNASHGKGF	SRPARPANRR	LPSRWASRSP	SAPPALRRTT	HNYTISLRSE	ALMV		

Cmpd.	No. of Cmpds.	m/z meas.	Δ m/z [ppm]	z	Rt [min]	Score	P	Range	Sequence	Modification
1324	1	727.1757	-204.08	2	46.2	10.1	0	108-120	R.EQSLVSGGNQMCK.E	Carbamidomethyl: 12; Oxidation: 11

Detailed Protein Report

Protein 557: regulator of G-protein signaling 3 isoform 3 [Homo sapiens]

**Accession:**	gi|62865654	**Score:**	22.5
**Database:**	refseq_human(refseq_human_20140103.fasta)	**MW [kDa]:**	67.2
**Database Date:**	2015-11-30	**pI:**	9.9
**Modification(s):**	Carbamidomethyl, Oxidation	**Sequence Coverage [%]:**	4.3
		**No. of unique Peptides:**	1

```
        10         20         30         40         50         60         70         80
MERSLHRVSL GSRRAHPDLS FYLTTFGQLR LSIDAQDRVL LLHIIEGKGL ISKQPGTCDP YVKISLIPED SRLRHQKTQT
        90        100        110        120        130        140        150        160
VPDCRDPAFH EHFFFPVQEE DDQKRLLVTV WNRASQSRQS GLIGCMSFGV KSLLTPDKEI SGWYYLLGEH LGRTKHLKVA
       170        180        190        200        210        220        230        240
RRRLRPLRDP LLRMPGGGDT ENGKKLKITI PRGKDGFGFT ICCDSPVRVQ AVDSGGPAER AGLQQLDTVL QLNERPVEHW
       250        260        270        280        290        300        310        320
KCVELAHEIR SCPSEIILLV WRMVPQVKPG PDGGVLRRAS CKSTHDLQSP PNKREKNCTH GVQARPEQRH SCHLVCDSSD
       330        340        350        360        370        380        390        400
GLLLGGWERY TEVAKRGGQH TLPALSRATA PTDPNYIILA PLNPGSQLLR PVYQEDTIPE ESGSPSKGKS YTGLGKKSRL
       410        420        430        440        450        460        470        480
MKTVQTMKGH GNYQNCPVVR PHATHSSYGT YVTLAPKVLV FPVFVQPLDL CNPARTLLLS EELLLYEGRN KAAEVTLFAY
       490        500        510        520        530        540        550        560
SDLLLFTKED EPGRCDVLRN PLYLQSVKLQ EGSSEDLKFC VLYLAEKAEC LFTLEAHSQE QKKRVCWCLS ENIAKQQQLA
       570        580        590        600        610
ASPPDSKKLH PFGSLQQEMG PVNSTNATQD RSFTSPGQTL IG
```

Cmpd.	No. of Cmpds.	m/z meas.	Δ m/z [ppm]	z	Rt [min]	Score	P	Range	Sequence	Modification
1945	1	700.2356	-147.65	2	54.4	10.8	0	119-131	R.QSGLIGCMSFGVK.S	Carbamidomethyl: 7; Oxidation: 8

Detailed Protein Report

Protein 558: PREDICTED: PAX3- and PAX7-binding protein 1 isoform X2 [Homo sapiens]

Accession: gi|578836733
Database: refseq_human(refseq_human_20140103.fasta)
Database Date: 2015-11-30

Score: 22.5
MW [kDa]: 86.2
pI: 5.1
Sequence Coverage [%]: 4.0
No. of unique Peptides: 2

10	20	30	40	50	60	70	80
MFRKARRVNV	RKRNDSEEEE	RERDEEQEPP	PLLPPPGTGE	EAGPGGGDRA	PGGESLLGPG	PSPPSALTPG	LGAEAGGGFP
90	100	110	120	130	140	150	160
GGAEPGNGLK	PRKRPRENKE	VPRASLLSFQ	DEEEENEEVF	KVKKSSYSKK	IVKLLKKEYK	EDLEKSKIKT	ELNSSAESEQ
170	180	190	200	210	220	230	240
PLDKTGHVKD	TNQEDGVIIS	EHGEDEMDME	SEKEEEKPKT	GGAFSNALSS	LNVLRPGEIP	DAAFIHAARK	KRQMARELGD
250	260	270	280	290	300	310	320
FTPHDNEPGK	GRLVREDEND	ASDDEDDDEK	RRIVFSVKEK	SQRQKIAEEI	GIEGSDDDAL	VTGEQDEELS	RWEQEQIRKG
330	340	350	360	370	380	390	400
INIPQVQASQ	PAEVNMYYQN	TYQTMPYGSS	YGIPYSYTAY	GSSDAKSQKT	DNTVPFKTPS	NEMTPVTIDL	VKKQLKDRLD
410	420	430	440	450	460	470	480
SMKELHKTNR	QQHEKHLQSR	VDSTRAIERL	EGSSGGIGER	YKFLQEMRGY	VQDLLECFSE	KVPLINELES	AIHQLYKQRA
490	500	510	520	530	540	550	560
SRLVQRRQDD	IKDESSEFSS	HSNKALMAPN	LDSFGRDRAL	YQEHAKRRIA	EREARRTRRR	QAREQTGKMA	DHLEGLSSDD
570	580	590	600	610	620	630	640
EETSTDITNF	NLEKDRISKE	SGKVFEDVLE	SFYSIDCIKS	QFEAWRSKYY	TSYKDAYIGL	CLPKLFNPLI	RLQLLTWTPL
650	660	670	680	690	700	710	720
EAKCRDFENM	LWFESLLFYG	CEEREQEKDD	VDVALLPTIV	EKVILPKLTV	IAENMWDPFS	TTQTSRMVGI	TLKLINGYPS
730	740	750	760				
VVNAENKNTQ	VYLKALLLRM	RRTLDDDVFM	PLYPKNC				

Cmpd.	No. of Cmpds.	m/z meas.	Δ m/z [ppm]	z	Rt [min]	Score	P	Range	Sequence	Modification
584	1	824.2497	-169.01	2	36.9	10.9	0	150-164	K.TELNSSAESEQPLDK.T	
156	1	822.7809	-180.37	2	31.7	11.6	0	378-392	K.TPSNEMTPVTIDLVK.K	

Detailed Protein Report

Protein 559: tyrosine-protein kinase Lck precursor [Homo sapiens]

**Accession:**	gi|112789546	**Score:**	22.4
**Database:**	refseq_human(refseq_human_20140103.fasta)	**MW [kDa]:**	58.0
**Database Date:**	2015-11-30	**pI:**	5.1
		**Sequence Coverage [%]:**	2.9
		**No. of unique Peptides:**	1

Alias proteins:

Accession	Name	Description
gi|112789548	refseq_human (refseq_human_20140103.fasta)	tyrosine-protein kinase Lck precursor [Homo sapiens]

10	20	30	40	50	60	70	80
MGCGCSSHPE	DDWMENIDVC	ENCHYPIVPL	DGKGTLLIRN	GSEVRDPLVT	YEGSNPPASP	LQDNLVIALH	SYEPSHDGDL
**90**	**100**	**110**	**120**	**130**	**140**	**150**	**160**
GFEKGEQLRI	LEQSGEWWKA	QSLTTGQEGF	IPFNFVAKAN	SLEPEPWFFK	NLSRKDAERQ	LLAPGNTHGS	FLIRESESTA
**170**	**180**	**190**	**200**	**210**	**220**	**230**	**240**
GSFSLSVRDF	DQNQGEVVKH	YKIRNLDNGG	FYISPRITFP	GLHELVRHYT	NASDGLCTRL	SRPCQTQKPQ	KPWWEDEWEV
**250**	**260**	**270**	**280**	**290**	**300**	**310**	**320**
PRETLKLVER	LGAGQFGEVW	MGYYNGHTKV	AVKSLKQGSM	SPDAFLAEAN	LMKQLQHQRL	VRLYAVVTQE	PIYIITEYME
**330**	**340**	**350**	**360**	**370**	**380**	**390**	**400**
NGSLVDFLKT	PSGIKLTINK	LLDMAAQIAE	GMAFIEERNY	IHRDLRAANI	LVSDTLSCKI	ADFGLARLIE	DNEYTAREGA
**410**	**420**	**430**	**440**	**450**	**460**	**470**	**480**
KFPIKWTAPE	AINYGTFTIK	SDVWSFGILL	TEIVTHGRIP	YPGMTNPEVI	QNLERGYRMV	RPDNCPEELY	QLMRLCWKER
**490**	**500**	**510**					
PEDRPTFDYL	RSVLEDFFTA	TEGQYQPQP					

Cmpd.	No. of Cmpds.	m/z meas.	Δ m/z [ppm]	z	Rt [min]	Score	P	Range	Sequence	Modification
2661	1	812.3984	-65.88	2	64.5	22.4	0	140-154	R.QLLAPGNTHGSFLIR.E	

Detailed Protein Report

Protein 560: PREDICTED: formin-binding protein 1 isoform X20 [Homo sapiens]

**Accession:**	gi|530390795	**Score:**	22.4
**Database:**	refseq_human(refseq_human_20140103.fasta)	**MW [kDa]:**	64.0
**Database Date:**	2015-11-30	**pI:**	5.0
		**Sequence Coverage [%]:**	3.4
		**No. of unique Peptides:**	1

Quantitation

MD:MU **Median:** 0.61 **CV:** 0.00 % **No. of Peptides:** 1

10	20	30	40	50	60	70	80
MSWGTELWDQ	FDNLEKHTQW	GIDILEKYIK	FVKERTEIEL	SYAKQLRNLS	KKYQPKKNSK	EEEEYKYTSC	KAFISNLNEM
**90**	**100**	**110**	**120**	**130**	**140**	**150**	**160**
NDYAGQHEVI	SENMASQIIV	DLARYVQELK	QERKSNFHDG	RKAQQHIETC	WKQLESSKRR	FERDCKEADR	AQQYFEKMDA
**170**	**180**	**190**	**200**	**210**	**220**	**230**	**240**
DINVTKADVE	KARQQAQIRH	QMAEDSKADY	SSILQKFNHE	QHEYYHTHIP	NIFQKIQEME	ERRIVRMGES	MKTYAEVDRQ
**250**	**260**	**270**	**280**	**290**	**300**	**310**	**320**
VIPIIGKCLD	GIVKAAESID	QKNDSQLVIE	AYKSGFEPPG	DIEFEDYTQP	MKRTVSDNSL	SNSRGEGKPD	LKFGGKSKGK
**330**	**340**	**350**	**360**	**370**	**380**	**390**	**400**
LWPFIKKNKG	ATPEDFSNLP	PEQRRKKLQQ	KVDELNKEIQ	KEMDQRDAIT	KMKDVYLKNP	QMGDPASLDH	KLAEVSQNIE
**410**	**420**	**430**	**440**	**450**	**460**	**470**	**480**
KLRVETQKFE	AWLAEVEGRL	PARSEQARRQ	SGLYDSQNPP	TVNNCAQDRE	SPDGSYTEEQ	SQESEMKVLA	TDFDDEFDDE
**490**	**500**	**510**	**520**	**530**	**540**	**550**	**560**
EPLPAIGTCK	ALYTFEGQNE	GTISVVEGET	LYVIEEDKGD	GWTRIRRNED	EEGYVPTSYV	EVCLDKNAKD	S

Cmpd.	No. of Cmpds.	m/z meas.	Δ m/z [ppm]	z	Rt [min]	Score	P	Range	Sequence	Modification	Ratios
812	1	603.7427	-137.75	2	39.7	11.4	2	223-232	R.RIVRMGESMK.T		MD:MU 0.61

Detailed Protein Report

Protein 561: PREDICTED: cyclin-dependent kinase-like 5 isoform X2 [Homo sapiens]

Accession: gi|530421243
Database: refseq_human(refseq_human_20140103.fasta)
Database Date: 2015-11-30

Score: 22.4
MW [kDa]: 110.0
pI: 10.1
Sequence Coverage [%]: 3.0
No. of unique Peptides: 1

Quantitation

MD:MU **Median:** 0.94 **CV:** 0.00 % **No. of Peptides:** 1

10	20	30	40	50	60	70	80
MKIPNIGNVM	NKFEILGVVG	EGAYGVVLKC	RHKETHEIVA	IKKFKDSEEN	EEVKETTLRE	LKMLRTLKQE	NIVELKEAFR
90	100	110	120	130	140	150	160
RRGKLYLVFE	YVEKNMLELL	EEMPNGVPPE	KVKSYIYQLI	KAIHWCHKND	IVHRDIKPEN	LLISHNDVLK	LCDFGFARNL
170	180	190	200	210	220	230	240
SEGNNANYTE	YVATRWYRSP	ELLLGAPYGK	SVDMWSVGCI	LGELSDGQPL	FPGESEIDQL	FTIQKVLGPL	PSEQMKLFYS
250	260	270	280	290	300	310	320
NPRFHGLRFP	AVNHPQSLER	RYLGILNSVL	LDLMKNLLKL	DPADRYLTEQ	CLNHPTFQTQ	RLLDRSPSRN	QAGKSTALQS
330	340	350	360	370	380	390	400
HHRSNSKDIQ	NLSVGLPRAD	EGLPANESFL	NGNLAGASLS	PLHTKTYQAS	SQPGSTSKDL	TNNNIPHLLS	PKEAKSKTEF
410	420	430	440	450	460	470	480
DFNIDPKPSE	GPGTKYLKSN	SRSQQNRHSF	MESSQSKAGT	LQPNEKQSRH	SYIDTIPQSS	RSPSYRTKAK	SHGALSDSKS
490	500	510	520	530	540	550	560
VSNLSEARAQ	IAEPSTSRYF	PSSCLDLNSP	TSPTPTRHSD	TRTLLSPSGR	NNRNEGTLDS	RRTTTRHSKT	MEELKLPEHM
570	580	590	600	610	620	630	640
DSSHSHSLSA	PHESFSYGLG	YTSPFSSQQR	PHRHSMYVTR	DKVRAKGLDG	SLSIGQGMAA	RANSLQLLSP	QPGEQLPPEM
650	660	670	680	690	700	710	720
TVARSSVKET	SREGTSSFHT	RQKSEGGVYH	DPHSDDGTAP	KENRHLYNDP	VPRRVGSFYR	VPSPRPDNSF	HENNVSTRVS
730	740	750	760	770	780	790	800
SLPSESSSGT	NHSKRQPAFD	PWKSPENISH	SEQLKEKEKQ	GFFRSMKKKK	KKSQTTDSTN	GENPSIKKSL	FPLFNSKNHL
810	820	830	840	850	860	870	880
KHSSSLKKLP	VVTPPMVPNS	DSPDLLTLQK	SIHSASTPSS	RPKEWRPEKI	SDLQTQSQPL	KSLRKLLHLS	SASNHPASSD
890	900	910	920	930	940	950	960
PRFQPLTAQQ	TKNSFSEIRI	HPLSQASGGS	SNIRQEPAPK	GRPALQLPGQ	MDPGWHVSSV	TRSATEGPSY	SEQLGAKSGP
970	980	990					
NGHPYNRTNR	SRMPNLNDLK	ETAL					

Cmpd.	No. of Cmpds.	m/z meas.	Δ m/z [ppm]	z	Rt [min]	Score	P	Range	Sequence	Modification	Ratios
198	1	671.1826	-201.27	2	31.9	11.2	0	366-378	K.TYQASSQPGSTSK.D		MD:MU 0.94

Detailed Protein Report

Protein 562: disrupted in schizophrenia 1 protein isoform f [Homo sapiens]

Accession: gi|257153469
Database: refseq_human(refseq_human_20140103.fasta)
Database Date: 2015-11-30

Score: 22.4
MW [kDa]: 74.3
pI: 6.9
Sequence Coverage [%]: 4.7
No. of unique Peptides: 1

10	20	30	40	50	60	70	80
MPGGGPQGAP	AAAGGGGVSH	RAGSRDCLPP	AACFRRRRLA	RRPGYMRSST	GPGIGFLSPA	VGTLFRFPGG	VSGEESHHSE
90	100	110	120	130	140	150	160
SRARQCGLDS	RGLLVRSPVS	KSAAAPTVTS	VRGTSAHFGI	QLRGGTRLPD	RLSWPCGPGS	AGWQQEFAAM	DSSETLDASW
170	180	190	200	210	220	230	240
EAACSDGARR	VRAAGSLPSA	ELSSNSCSPG	CGPEVPPTPP	GSHSAFTSSF	SFIRLSLGSA	GERGEAEGCP	PSREAESHCQ
250	260	270	280	290	300	310	320
SPQEMGAKAA	SLDGPHEDPR	CLSRPFSLLA	TRVSADLAQA	ARNSSRPERD	MHSLPDMDPG	SSSSLDPSLA	GCGGDGSSGS
330	340	350	360	370	380	390	400
GDAHSWDTLL	RKWEPVLRDC	LLRNRRQMEV	ISLRLKLQKL	QEDAVENDDY	DKAETLQQRL	EDLEQEKISL	HFQLPSRQPA
410	420	430	440	450	460	470	480
LSSFLGHLAA	QVQAALRRGA	TQQASGDDTH	TPLRMEPRLL	EPTAQDSLHV	SITRRDWLLQ	EKQQLQKEIE	ALQARMFVLE
490	500	510	520	530	540	550	560
AKDQQLRREI	EEQEQQLQWQ	GCDLTPLVGQ	LSLGQLQEVS	KALQDTLASA	GQIPFHAEPP	ETIRSLQERI	KSLNLSLKEI
570	580	590	600	610	620	630	640
TTKVCMSEKF	CSTLRKKVND	IETQLPALLE	AKMHAISGNH	FWTAKDLTEE	IRSLTSEREG	LEGLLSKLLV	LSSRNVKKLG
650	660	670	680	690			
SVKEDYNRLR	REVEHQETAY	ETSVKENTMK	YMETLKNKLC	R			

Cmpd.	No. of Cmpds.	m/z meas.	Δ m/z [ppm]	z	Rt [min]	Score	P	Range	Sequence	Modification
12	1	803.7126	-4.89	3	29.3	12.4	2	651-670	R.REVEHQETAYETSVKENTMK.Y	

Detailed Protein Report

Protein 563: tigger transposable element-derived protein 7 [Homo sapiens]

Accession: gi|22325392
Database: refseq_human(refseq_human_20140103.fasta)
Database Date: 2015-11-30

Score: 22.4
MW [kDa]: 63.2
pI: 9.6
Sequence Coverage [%]: 4.2
No. of unique Peptides: 1

Quantitation

MD:MU **Median:** 3.93 **CV:** 0.00 % **No. of Peptides:** 1

10	20	30	40	50	60	70	80
MNKRGKYTTL	NLEEKMKVLS	RIEAGRSLKS	VMDEFGISKS	TFYDIKKNKK	LILDFVLKQD	MPLVGAEKRK	RTTGAKYGDV
90	100	110	120	130	140	150	160
DDAVYMWYQQ	KRSAGVPVRG	VELQAAAERF	ARCFGRTDFK	ASTGWLFRFR	NRHAIGNRKG	CGEQVLSSVS	ENVEPFRQKL
170	180	190	200	210	220	230	240
SMIIKEEKLC	LAQLYSGDET	DLFWKSMPEN	SQASRKDICL	PGKKINKERL	SAFLCANADG	THKLKSIIIG	KSKLPKSVKE
250	260	270	280	290	300	310	320
DTSTLPVIYK	PSKDVWFTRE	LFSEWFFQNF	VPEVRHFQLN	VLRFHDEDVR	ALLLLDSCPA	HPSSESLTSE	DGRIKCMFFP
330	340	350	360	370	380	390	400
HNTSTLIQPM	NQGVILSCKR	LYRWKQLEES	LVIFEESDDE	QEKGDKGVSK	IKIYNIKSAI	FNWAKSWEEV	KQITIANAWE
410	420	430	440	450	460	470	480
NLLYKKEPEY	DFQGLEHGDY	REILEKCGEL	ETKLDDDRVW	LNGDEEKGCL	LKTKGGITKE	VVQKGGEAEK	QTAEFKLSAV
490	500	510	520	530	540	550	
RESLDYLLDF	VDATPEFQRF	HFTLKEMQQE	IVKKQFQSKI	HSRIGSFLKP	RPHNIKDSFS	GPSTSGSNH	

Cmpd.	No. of Cmpds.	m/z meas.	Δ m/z [ppm]	z	Rt [min]	Score	P	Range	Sequence	Modification	Ratios
2435	1	648.2013	-222.78	2	60.8	10.6	1	5-15	R.GKYTTLNLEEK.M		MD:MU 3.93

Detailed Protein Report

Protein 564: PREDICTED: G patch domain-containing protein 8 isoform X1 [Homo sapiens]

**Accession:**	gi|578830624	**Score:**	22.3
**Database:**	refseq_human(refseq_human_20140103.fasta)	**MW [kDa]:**	155.1
**Database Date:**	2015-11-30	**pI:**	9.6
		**Sequence Coverage [%]:**	1.8
		**No. of unique Peptides:**	1

Alias proteins:

Accession	Name	Description
gi|578830628	refseq_human (refseq_human_20140103.fasta)	PREDICTED: G patch domain-containing protein 8 isoform X3 [Homo sapiens]
gi|578830626	refseq_human (refseq_human_20140103.fasta)	PREDICTED: G patch domain-containing protein 8 isoform X2 [Homo sapiens]

10	20	30	40	50	60	70	80
MGMGRMEMEL	DYAEDATERR	RVLEVEKEDT	EELRQKYKDY	VDKEKAIAKA	LEDLRANFYC	ELCDKQYQKH	QEFDNHINSY
**90**	**100**	**110**	**120**	**130**	**140**	**150**	**160**
DHAHKQRLKD	LKQREFARNV	SSRSRKDEKK	QEKALRRLHE	LAEQRKQAEC	APGSGPMFKP	TTVAVDEEGG	EDDKDESATN
**170**	**180**	**190**	**200**	**210**	**220**	**230**	**240**
SGTGATASCG	LGSEFSTDKG	GPFTAVQITN	TTGLAQAPGL	ASQGISFGIK	NNLGTPLQKL	GVSFSFAKKA	PVKLESIASV
**250**	**260**	**270**	**280**	**290**	**300**	**310**	**320**
FKDHAEEGTS	EDGTKPDEKS	SDQGLQKVGD	SDGSSNLDGK	KEDEDPQDGG	SLASTLSKLK	RMKREEGAGA	TEPEYYHYIP
**330**	**340**	**350**	**360**	**370**	**380**	**390**	**400**
PAHCKVKPNF	PFLLFMRASE	QMDGDNTTHP	KNAPESKKGS	SPKPKSCIKA	AASQGAEKTV	SEVSEQPKET	SMTEPSEPGS
**410**	**420**	**430**	**440**	**450**	**460**	**470**	**480**
KAEAKKALGG	DVSDQSLESH	SQKVSETQMC	ESNSSKETSL	ATPAGKESQE	GPKHPTGPFF	PVLSKDESTA	LQWPSELLIF
**490**	**500**	**510**	**520**	**530**	**540**	**550**	**560**
TKAEPSISYS	CNPLYFDFKL	SRNKDARTKG	TEKPKDIGSS	SKDHLQGLDP	GEPNKSKEVG	GEKIVRSSGG	RMDAPASGSA
**570**	**580**	**590**	**600**	**610**	**620**	**630**	**640**
CSGLNKQEPG	GSHGSETEDT	GRSLPSKKER	SGKSHRHKKK	KKHKKSSKHK	RKHKADTEEK	SSKAESGEKS	KKRKKRKRKK
**650**	**660**	**670**	**680**	**690**	**700**	**710**	**720**
NKSSAPADSE	RGPKPEPPGS	GSPAPPRRRR	RAQDDSQRRS	LPAEEGSSGK	KDEGGGGSSS	QDHGGRKHKG	ELPPSSCQRR
**730**	**740**	**750**	**760**	**770**	**780**	**790**	**800**
AGTKRSSRSS	HRSQPSSGDE	DSDDASSHRL	HQKSPSQYSE	EEEEEDSGSE	HSRSRSRSGR	RHSSHRSSRR	SYSSSSDASS
**810**	**820**	**830**	**840**	**850**	**860**	**870**	**880**
DQSCYSRQRS	YSDDSYSDYS	DRSRRHSKRS	HDSDDSDYAS	SKHRSKRHKY	SSSDDDYSLS	CSQSRSRSRS	HTRERSRSRG
**890**	**900**	**910**	**920**	**930**	**940**	**950**	**960**
RSRSSSCSRS	RSKRRSRSTT	AHSWQRSRSY	SRDRSRSTRS	PSQRSGSRKR	SWGHESPEER	HSGRRDFIRS	KIYRSQSPHY
**970**	**980**	**990**	**1000**	**1010**	**1020**	**1030**	**1040**
FRSGRGEGPG	KKDDGRGDDS	KATGPPSQNS	NIGTGRGSEG	DCSPEDKNSV	TAKLLLEKIQ	SRKVERKPSV	SEEVQATPNK
**1050**	**1060**	**1070**	**1080**	**1090**	**1100**	**1110**	**1120**
AGPKLKDPPQ	GYFGPKLPPS	LGNKPVLPLI	GKLPATRKPN	KKCEESGLER	GEEQEQSETE	EGPPGSSDAL	FGHQFPSEET
**1130**	**1140**	**1150**	**1160**	**1170**	**1180**	**1190**	**1200**
TGPLLDPPPE	ESKSGEATAD	HPVAPLGTPA	HSDCYPGDPT	ISHNYLPDPS	DGDTLESLDS	SSQPGPVESS	LLPIAPDLEH
**1210**	**1220**	**1230**	**1240**	**1250**	**1260**	**1270**	**1280**
FPSYAPPSGD	PSIESTDGAE	DASLAPLESQ	PITFTPEEME	KYSKLQQAAQ	QHIQQQLLAK	QVKAFPASAA	LAPATPALQP
**1290**	**1300**	**1310**	**1320**	**1330**	**1340**	**1350**	**1360**
IHIQQPATAS	ATSITTVQHA	ILQHHAAAAA	AAIGIHPHPH	PQPLAQVHHI	PQPHLTPISL	SHLTHSIIPG	HPATFLASHP
**1370**	**1380**	**1390**	**1400**	**1410**	**1420**	**1430**	
IHIIPASAIH	PGPFTFHPVP	HAALYPTLLA	PRPAAAAATA	LHLHPLLHPI	FSGQDLQHPP	SHGT	

Cmpd.	No. of Cmpds.	m/z meas.	Δ m/z [ppm]	z	Rt [min]	Score	P	Range	Sequence	Modification
2163	1	1131.2917	-238.27	1	57.2	12.1	2	671-679	R.RAQDDSQRR.S	

Detailed Protein Report

Protein 565: RNA-binding protein MEX3A [Homo sapiens]

**Accession:**	gi|147902746	**Score:**	22.3
**Database:**	refseq_human(refseq_human_20140103.fasta)	**MW [kDa]:**	54.1
**Database Date:**	2015-11-30	**pI:**	7.8
**Modification(s):**	Carbamidomethyl, Oxidation	**Sequence Coverage [%]:**	6.7
		**No. of unique Peptides:**	1

10	20	30	40	50	60	70	80
MPSLVVSGIM	ERNGGFGELG	CFGGSAKDRG	LLEDERALQL	ALDQLCLLGL	GEPPAPTAGE	DGGGGGGGAP	AQPAAPPQPA
**90**	**100**	**110**	**120**	**130**	**140**	**150**	**160**
PPPPPAAPPA	APTAAPAAQT	PQPPTAPKGA	SDAKLCALYK	EAELRLKGSS	NTTECVPVPT	SEHVAEIVGR	QGCKIKALRA
**170**	**180**	**190**	**200**	**210**	**220**	**230**	**240**
KTNTYIKTPV	RGEEPVFMVT	GRREDVATAR	REIISAAEHF	SMIRASRNKS	GAAFGVAPAL	PGQVTIRVRV	PYRVVGLVVG
**250**	**260**	**270**	**280**	**290**	**300**	**310**	**320**
PKGATIKRIQ	QQTNTYIITP	SRDRDPVFEI	TGAPGNVERA	REEIETHIAV	RTGKILEYNN	ENDFLAGSPD	AAIDSRYSDA
**330**	**340**	**350**	**360**	**370**	**380**	**390**	**400**
WRVHQPGCKP	LSTFRQNSLG	CIGECGVDSG	FEAPRLGEQG	GDFGYGGYLF	PGYGVGKQDV	YYGVAETSPP	LWAGQENATP
**410**	**420**	**430**	**440**	**450**	**460**	**470**	**480**
TSVLFSSASS	SSSSSAKARA	GPPGAHRSPA	TSAGPELAGL	PRRPPGEPLQ	GFSKLGGGGL	RSPGGGRDCM	VCFESEVTAA
**490**	**500**	**510**	**520**	**530**			
LVPCGHNLFC	MECAVRICER	TDPECPVCHI	TATQAIRIFS				

Cmpd.	No. of Cmpds.	m/z meas.	Δ m/z [ppm]	z	Rt [min]	Score	P	Range	Sequence	Modification
1760	1	1273.5603	8.38	3	51.8	22.3	1	462-496	R.SPGGGRDCMVCFESEVTAALVPCGHNLFCMECAVR.I	Carbamidomethyl: 8, 11; Oxidation: 9

Detailed Protein Report

Protein 566: PREDICTED: pecanex-like protein 3 isoform X4 [Homo sapiens]

Accession: gi|530396772
Database: refseq_human(refseq_human_20140103.fasta)
Database Date: 2015-11-30

Score: 22.3
MW [kDa]: 115.4
pI: 6.0
Sequence Coverage [%]: 3.8
No. of unique Peptides: 1

10	20	30	40	50	60	70	80
MGSQVLQILR	QGVWASLTGG	WFFDPHQSTF	SNCFHLYVWI	FLLIFPFLLY	MVLPPSLMVA	GVYCLVVAVI	FATIKTVNYR
90	100	110	120	130	140	150	160
LHAMFDQGEI	VEKRSSTMGE	LEEEPAQGDS	NPPRDPGVEM	TVFRKVSSTP	PVRCSSQHSV	FGFNQVSELL	PRMEDSGPLR
170	180	190	200	210	220	230	240
DIKELVREQG	SNNVIVTSAD	REMLKLSSQE	KLIGDLPQTP	PGAVPDPSLA	STDSSEPSPL	AGDGAPWSGS	SMADTPMSPL
250	260	270	280	290	300	310	320
LKGSLSQELS	KSFLTLTQPD	RALVRTSSRR	EQRRGAGGYQ	PLDRRGSGEP	TPQKAGSSDS	CFSGTDRETL	SSFKSEKTNS
330	340	350	360	370	380	390	400
THLDSPPGGP	APEGSDTDPP	SEAELPASPD	AGVPSDDTLR	SFDTVIGAGT	PPGLAEPLLV	VRPKDLALLR	PSKRQPPLRR
410	420	430	440	450	460	470	480
HSPPGRAPRR	PLLEGGGFFE	DEDTSEGSEL	SPASSLRSQR	RYSTDSSSST	SCYSPESSRG	AAGGPRKRRA	PHGAEEGTAV
490	500	510	520	530	540	550	560
PPKRPYGTQR	TPSTASAKTH	ARVLSMDGAG	GDVLRPPLAG	CKAELEAQVG	VEQAASEPVV	LPAEARRGPA	ANQPGWRGEL
570	580	590	600	610	620	630	640
QEEGAVGGAA	EETGRRDRSS	SVRRTQAIRR	RHNAGSNPTP	PASVMGSPPS	SLQEAQRGRA	ASHSRALTLP	SALHFASSLL
650	660	670	680	690	700	710	720
LTRAGANVHE	ACTFDDTSEG	AVHYFYDESG	VRRSYTFGLA	GGGYENPVGQ	QGEQTANGAW	DRHSHSSSFH	SADVPEATGG
730	740	750	760	770	780	790	800
LNLLQPRPVV	LQGMQVRRVP	LEIPEFDLLD	QDSLHESQEQ	TLMEEAPPRA	QHSYKYWLLP	GRWTSVRYER	LALLALLDRT
810	820	830	840	850	860	870	880
RGVLENIFGV	GLSSLVAFLG	YLLLLKGFFT	DIWVFQFCLV	IASCQYSLLK	SVQPDAASPM	HGHNWVIAYS	RPVYFCICCL
890	900	910	920	930	940	950	960
LIWLLDALGS	AQPFPPVSLY	GLTLFSASFF	FCARDVATVF	TLCFPFVFLL	GLLPQVNTCL	MYLLEQIDMH	GFGGTAATSP
970	980	990	1000	1010	1020	1030	1040
LTAVFSLSRS	LLAAALLYGF	CLGAIKTPWP	EQHVPVLFSV	FCGLLVALSY	HLSRQSSDPT	VLWSLIRSKL	FPELEERSLE
1050	1060	1070					
TARAEPPDPL	PDKMRQSVLN	VSPGPAA					

Cmpd.	No. of Cmpds.	m/z meas.	Δ m/z [ppm]	z	Rt [min]	Score	P	Range	Sequence	Modification
2754	1	1086.0034	31.79	2	64.8	11.5	1	286-307	R.GSGEPTPQKAGSSDSCFSGTDR.E	

Detailed Protein Report

Protein 567: **PREDICTED: uncharacterized protein LOC101929845 [Homo sapiens]**

**Accession:**	gi|578845877	**Score:**	22.3
**Database:**	refseq_human(refseq_human_20140103.fasta)	**MW [kDa]:**	90.2
**Database Date:**	2015-11-30	**pI:**	12.2
**Modification(s):**	Carbamidomethyl	**Sequence Coverage [%]:**	4.9
		**No. of unique Peptides:**	1

10	20	30	40	50	60	70	80
MEESAEQAPR	CDETRVGRRR	HSWRNTCGGV	GRAGTAVRRD	AASQSCCPTC	TGVATRRHSW	RRRQSRQTKE	FIFSELLSNL
**90**	**100**	**110**	**120**	**130**	**140**	**150**	**160**
YSRGNEQTLV	EALAEQADQG	VHLLRAAVQP	ALAWEREDTR	GGVGRAGRPR	SSSSQSCCPN	CTRVGTRRHS	WRSRQSRHRG
**170**	**180**	**190**	**200**	**210**	**220**	**230**	**240**
VTRLAWEEED	TPGGVGRAGR	PRSSSSRSCC	PTCTRVGTCR	HSWRRRQSRQ	TKEFIFSELL	SNLYSRGNEK	TLVEASAEQA
**250**	**260**	**270**	**280**	**290**	**300**	**310**	**320**
DQGVHLLRAA	VQPVLAWERE	DTRGGVGRAG	RPRSSSSQSC	CPTCTRVGTR	RHSWRSRPSR	HSGVTRLAWE	EEDTPGGVGR
**330**	**340**	**350**	**360**	**370**	**380**	**390**	**400**
AGRPRSSSSR	SCCPTCTRVG	TCRHLWRSRQ	SRHSGATRCC	FSELLSNLHW	RGNEKTLVEA	LAEQADQGVH	LLRAAVQPVL
**410**	**420**	**430**	**440**	**450**	**460**	**470**	**480**
AWERADTRGG	VGRAGRPRSS	SSQSCCPTCT	RVGTRRHSWR	RRQSRQTKEF	IFSELLSKLH	SRGNEKTLME	ESAEQAQRCD
**490**	**500**	**510**	**520**	**530**	**540**	**550**	**560**
ETRVGRRRHS	WRSRQSRQTK	KFIFSELLSN	LYSRRNLQTL	VEASAEQADQ	GVHLLRAAVQ	PVLAWEREDT	RGGVGRAGRP
**570**	**580**	**590**	**600**	**610**	**620**	**630**	**640**
RSSSSQSCCP	TCTRVGTRRH	SWRRRQSRQT	KEFIFSELLS	NLHSRGNEKT	LMEESAEQAQ	RRDETRVGRR	RHSWRSRQSR
**650**	**660**	**670**	**680**	**690**	**700**	**710**	**720**
QTKEFIFSEL	LSNLYSRRNL	QTLVEESAEQ	AQRCDKMLLL	RAAVQPALAW	EREDTRGGVG	RAGRPRSSSS	QSCCPTCPRV
**730**	**740**	**750**	**760**	**770**	**780**	**790**	**800**
GTRRHSWRRR	QSRQTKEFIF	SELLSNLHSR	GNEKTLVEAS	AEQADQGVHL	LRAAVQTALA	WEREDTHGGV	GRAGTEV

Cmpd.	No. of Cmpds.	m/z meas.	Δ m/z [ppm]	z	Rt [min]	Score	P	Range	Sequence	Modification
48	1	1220.0219	-37.76	2	30.0	11.4	2	33-56	R.AGTAVRRDAASQSCCPTCTGVATR.R	Carbamidomethyl: 14

Detailed Protein Report

Protein 568: PREDICTED: protein FAM188A isoform X3 [Homo sapiens]

**Accession:**	gi|530392461	**Score:**	22.2
**Database:**	refseq_human(refseq_human_20140103.fasta)	**MW [kDa]:**	30.5
**Database Date:**	2015-11-30	**pI:**	4.5
**Modification(s):**	Oxidation	**Sequence Coverage [%]:**	14.3
		**No. of unique Peptides:**	1

Alias proteins:

Accession	Name	Description
gi|530392465	refseq_human (refseq_human_20140103.fasta)	PREDICTED: protein FAM188A isoform X5 [Homo sapiens]
gi|530392463	refseq_human (refseq_human_20140103.fasta)	PREDICTED: protein FAM188A isoform X4 [Homo sapiens]

10	20	30	40	50	60	70	80
MWGNKFGVLL	FLYSVLLTKG	IENIKNEIED	ASEPLIDPVY	GHGSQSLINL	LLTGHAVSNV	WDGDRECSGM	KLLGIHEQAA
**90**	**100**	**110**	**120**	**130**	**140**	**150**	**160**
VGFLTLMEAL	RYCKVGSYLK	SPKFPIWIVG	SETHLTVFFA	KDMALVAPEA	PSEQARRVFQ	TYDPEDNGFI	PDSLLEDVMK
**170**	**180**	**190**	**200**	**210**	**220**	**230**	**240**
ALDLVSDPEY	INLMKNKLDP	EGLGIILLGP	FLQEFFPDQG	SSGPESFTVY	HYNGLKQSNY	NEKVMYVEGT	AVVMGFEDPM
**250**	**260**	**270**	**280**				
LQTDDTPIKR	CLQTKWPYIE	LLWTTDRSPS	LN				

Cmpd.	No. of Cmpds.	m/z meas.	Δ m/z [ppm]	z	Rt [min]	Score	P	Range	Sequence	Modification
1733	1	1030.7459	-72.31	3	51.5	11.5	1	224-250	K.VMYVEGTAVVMGFEDPMLQTDDTPIKR.C	Oxidation: 2, 11, 17

Detailed Protein Report

Protein 569: PREDICTED: disks large-associated protein 1 isoform X7 [Homo sapiens]

**Accession:**	gi|578832238	**Score:**	22.2
**Database:**	refseq_human(refseq_human_20140103.fasta)	**MW [kDa]:**	81.4
**Database Date:**	2015-11-30	**pI:**	6.6
		**Sequence Coverage [%]:**	2.6
		**No. of unique Peptides:**	1

10	20	30	40	50	60	70	80
MIDLFKAEWV	SSVCVQVSRN	GRTDQSKLKR	LKGHLSQVAF	VKVKAVWSRK	AVLPAAKKMQ	RNRVPQDEWT	GYTPRGKDDE
**90**	**100**	**110**	**120**	**130**	**140**	**150**	**160**
IPCRRMRSGS	YIKAMGDEDS	GDSDTSPKPS	PKVAARRESY	LKATQPSLTE	LTTLKISNEH	SPKLQIRSHS	YLRAVSEVSI
**170**	**180**	**190**	**200**	**210**	**220**	**230**	**240**
NRSLDSLDPA	GLLTSPKFRS	RNESYMRAMS	TISQVSEMEV	NGQFESVCES	VFSELESQAV	EALDLPMPGC	FRMRSHSYVR
**250**	**260**	**270**	**280**	**290**	**300**	**310**	**320**
AIEKGCSQDD	ECVSLRSSSP	PRTTTTVRTI	QSSTGVIKLS	SAVEVSSCIT	TYKKTPPPVP	PRTTTKPFIS	ITAQSSTESA
**330**	**340**	**350**	**360**	**370**	**380**	**390**	**400**
QDAYMDGQGQ	RGDIISQSGL	SNSTESLDSM	KALTAAIEAA	NAQIHGPASQ	HMGNNTATVT	TTTTIATVTT	EDRKKDHFKK
**410**	**420**	**430**	**440**	**450**	**460**	**470**	**480**
NRCLSIGIQV	DDAEEPDKTG	ENKAPSKFQS	VGVQVEEEKC	FRRFTRSNSV	TTAVQADLDF	HDNLENSLES	IEDNSCPGPM
**490**	**500**	**510**	**520**	**530**	**540**	**550**	**560**
ARQFSRDAST	STVSIQGSGN	HYHACAADDD	FDTDFDPSIL	PPPDPWIDSI	TEDPLEAVQR	SVCHRDGHWF	LKLLQAERDR
**570**	**580**	**590**	**600**	**610**	**620**	**630**	**640**
MEGWCQQMER	EERENNLPED	ILGKIRTAVG	SAQLLMAQKF	YQFRELCEEN	LNPNAHPRPT	SQDLAGFWDM	LQLSIENISM
**650**	**660**	**670**	**680**	**690**	**700**	**710**	**720**
KFDELHQLKA	NNWKQMDPLD	KKERRAPPPV	PKKPAKGPAP	LIRERSLESS	QRQEARKRLM	AAKRAASVRQ	NSATESAESI
**730**	**740**						
EIYIPEAQTR	L						

Cmpd.	No. of Cmpds.	m/z meas.	Δ m/z [ppm]	z	Rt [min]	Score	P	Range	Sequence	Modification
1338	1	845.6519	163.33	1	46.4	12.0	2	698-704	K.RLMAAKR.A	

Detailed Protein Report

Protein 570: PREDICTED: tau-tubulin kinase 1 isoform X1 [Homo sapiens]

**Accession:**	gi|578812010	**Score:**	22.1
**Database:**	refseq_human(refseq_human_20140103.fasta)	**MW [kDa]:**	84.4
**Database Date:**	2015-11-30	**pI:**	8.9
		**Sequence Coverage [%]:**	4.3
		**No. of unique Peptides:**	1

10	20	30	40	50	60	70	80
MSGGGEQADI	LPANYVVKDR	WKVLKKIGGG	GFGEIYEAMD	LLTRENVALK	VESAQQPKQV	LKMEVAVLKK	LQGKDHVCRF
**90**	**100**	**110**	**120**	**130**	**140**	**150**	**160**
IGCGRNEKFN	YVVMQLQGRN	LADLRRSQPR	GTFTLSTTLR	LGKQILESIE	AIHSVGFLHR	DIKPSNFAMG	RLPSTYRKCY
**170**	**180**	**190**	**200**	**210**	**220**	**230**	**240**
MLDFGLARQY	TNTTGDVRPP	RNVAGFRGTV	RYASVNAHKN	REMGRHDDLW	SLFYMLVEFA	VGQLPWRKIK	DKEQVGMIKE
**250**	**260**	**270**	**280**	**290**	**300**	**310**	**320**
KYEHRMLLKH	MPSEFHLFLD	HIASLDYFTK	PDYQLIMSVF	ENSMKERGIA	ENEAFDWEKA	GTDALLSTST	STPPQQNTRQ
**330**	**340**	**350**	**360**	**370**	**380**	**390**	**400**
TAAMFGVVNV	TPVPGDLLRE	NTEDVLQGEH	LSDQENAPPI	LPGRPSEGLG	PSPHLVPHPG	GPEAEVWEET	DVNRNKLRIN
**410**	**420**	**430**	**440**	**450**	**460**	**470**	**480**
IGKVTAARAK	GSPCVEEEQS	RGMGVPSSPV	RAPPDSPTTP	VRSLRYRRVN	SPESERLSTA	DGRVELPERR	SRMDLPGSPS
**490**	**500**	**510**	**520**	**530**	**540**	**550**	**560**
RQACSSQPAQ	MLSVDTGHAD	RQASGRMDVS	ASVEQEALSN	AFRSVPLAEE	EDFDSKEWVI	IDKETELKDF	PPGAEPSTSG
**570**	**580**	**590**	**600**	**610**	**620**	**630**	**640**
TTDEEPEELR	PLPEEGEERR	RLGAEPTVRP	RGRSMQALAE	EDLQHLPPQP	LPPQLSQGDG	RSETSQPPTP	GSPSHSPLHS
**650**	**660**	**670**	**680**	**690**	**700**	**710**	**720**
GPRPRRRESD	PTGPQRQLEE	DRLSGHSLPR	YSPLRRLASS	VFSSSTLETE	HYPHPGGGGS	SGSSGSLIQR	SRSAESSPVR
**730**	**740**	**750**	**760**				
APHRRHAPLA	AGNHRLMPSV	LRISRSQLQQ	VWARFTHKT				

Cmpd.	No. of Cmpds.	m/z meas.	Δ m/z [ppm]	z	Rt [min]	Score	P	Range	Sequence	Modification
1658	1	697.8038	-143.18	2	50.5	11.5	1	111-123	R.GTFTLSTTLRLGK.Q	

Detailed Protein Report

Protein 571: conserved oligomeric Golgi complex subunit 5 isoform 2 [Homo sapiens]

Accession: gi|240849537
Database: refseq_human(refseq_human_20140103.fasta)
Database Date: 2015-11-30

Score: 22.1
MW [kDa]: 92.7
pI: 6.1
Sequence Coverage [%]: 4.3
No. of unique Peptides: 1

10	20	30	40	50	60	70	80
MGWVGGRRRD	SASPPGRSRS	AADDINPAPA	NMEGGGGSVA	VAGLGARGSG	AAAATVRELL	QDGCYSDFLN	EDFDVKTYTS
90	100	110	120	130	140	150	160
QSIHQAVIAE	QLAKLAQGIS	QLDRELHLQV	VARHEDLLAQ	ATGIESLEGV	LQMMQTRIGA	LQGAVDRIKA	KIVEPYNKIV
170	180	190	200	210	220	230	240
ARTAQLARLQ	VACDLLRRII	RILNLSKRLQ	GQLQGGSREI	TKAAQSLNEL	DYLSQGIDLS	GIEVIENDLL	FIARARLEVE
250	260	270	280	290	300	310	320
NQAKRLLEQG	LETQNPTQVG	TALQVFYNLG	TLKDTITSVV	DGYCATLEEN	INSALDIKVL	TQPSQSAVRG	GPGRSTMPTP
330	340	350	360	370	380	390	400
GNTAALRASF	WTNMEKLMDH	IYAVCGQVQH	LQKVLAKKRD	PVSHICFIEE	IVKDGQPEIF	YTFWNSVTQA	LSSQFHMATN
410	420	430	440	450	460	470	480
SSMFLKQAFE	GEYPKLLRLY	NDLWKRLQQY	SQHIQGNFNA	SGTTDLYVDL	QHMEDDAQDI	FIPKKPDYDP	EKALKDSLQP
490	500	510	520	530	540	550	560
YEAAYLSKSL	SRLFDPINLV	FPPGGRNPPS	SDELDGIIKT	IASELNVAAV	DTNLTLAVSK	NVAKTIQLYS	VKSEQLLSTQ
570	580	590	600	610	620	630	640
GDASQVIGPL	TEGQRRNVAV	VNSLYKLHQS	VTKAIHALME	NAVQPLLTSV	GDAIEAIIIT	MHQEDFSGSL	SSSGKPDVPC
650	660	670	680	690	700	710	720
SLYMKELQGF	IARVMSDYFK	HFECLDFVFD	NTEAIAQRAV	ELFIRHASLI	RPLGEGGKMR	LAADFAQMEL	AVGPFCRRVS
730	740	750	760	770	780	790	800
DLGKSYRMLR	SFRPLLFQAS	EHVASSPALG	DVIPFSIIIQ	FLFTRAPAEL	KSPFQRAEWS	HTRFSQWLDD	HPSEKDRLLL
810	820	830	840				
IRGALEAYVQ	SVRSREGKEF	APVYPIMVQL	LQKAMSALQ				

Cmpd.	No. of Cmpds.	m/z meas.	Δ m/z [ppm]	z	Rt [min]	Score	P	Range	Sequence	Modification
2238	1	682.1284	82.08	3	58.2	10.8	2	798-815	R.LLLIRGALEAYVQSVRSR.E	

Detailed Protein Report

Protein 572: guanine nucleotide-binding protein-like 3 isoform 2 [Homo sapiens]

**Accession:**	gi|45643127	**Score:**	22.1
**Database:**	refseq_human(refseq_human_20140103.fasta)	**MW [kDa]:**	60.5
**Database Date:**	2015-11-30	**pI:**	9.6
		**Sequence Coverage [%]:**	5.0
		**No. of unique Peptides:**	1

Quantitation

MD:MU **Median:** 1.31 **CV:** 0.00 % **No. of Peptides:** 1

Alias proteins:

Accession	Name	Description
gi|45643129	refseq_human (refseq_human_20140103.fasta)	guanine nucleotide-binding protein-like 3 isoform 2 [Homo sapiens]

10	20	30	40	50	60	70	80
MTCHKRYKIQ	KKVREHHRKL	RKEAKKRGHK	KPRKDPGVPN	SAPFKEALLR	EAELRKQRLE	ELKQQQKLDR	QKELEKKRKL
**90**	**100**	**110**	**120**	**130**	**140**	**150**	**160**
ETNPDIKPSN	VEPMEKEFGL	CKTENKAKSG	KQNSKKLYCQ	ELKKVIEASD	VVLEVLDARD	PLGCRCPQVE	EAIVQSGQKK
**170**	**180**	**190**	**200**	**210**	**220**	**230**	**240**
LVLILNKSDL	VPKENLESWL	NYLKKELPTV	VFRASTKPKD	KGKITKRVKA	KKNAAPFRSE	VCFGKEGLWK	LLGGFQETCS
**250**	**260**	**270**	**280**	**290**	**300**	**310**	**320**
KAIRVGVIGF	PNVGKSSIIN	SLKQEQMCNV	GVSMGLTRSM	QVVPLDKQIT	IIDSPSFIVS	PLNSSSALAL	RSPASIEVVK
**330**	**340**	**350**	**360**	**370**	**380**	**390**	**400**
PMEAASAILS	QADARQVVLK	YTVPGYRNSL	EFFTVLAQRR	GMHQKGGIPN	VEGAAKLLWS	EWTGASLAYY	CHPPTSWTPP
**410**	**420**	**430**	**440**	**450**	**460**	**470**	**480**
PYFNESIVVD	MKSGFNLEEL	EKNNAQSIRA	IKGPHLANSI	LFQSSGLTNG	IIEEKDIHEE	LPKRKERKQE	EREDDKDSDQ
**490**	**500**	**510**	**520**	**530**	**540**		
ETVDEEVDEN	SSGMFAAEET	GEALSEETTA	GEQSTRSFIL	DKIIEEDDAY	DFSTDYV		

Cmpd.	No. of Cmpds.	m/z meas.	Δ m/z [ppm]	z	Rt [min]	Score	P	Range	Sequence	Modification	Ratios
107	1	591.7687	-44.15	2	31.1	10.4	0	231-241	K.LLGGFQETCSK.A		MD:MU 1.31

Detailed Protein Report

Protein 573: **TFIIH basal transcription factor complex helicase XPD subunit isoform 2 [Homo sapiens]**

**Accession:**	gi|195947407	**Score:**	22.1
**Database:**	refseq_human(refseq_human_20140103.fasta)	**MW [kDa]:**	46.2
**Database Date:**	2015-11-30	**pI:**	9.7
		**Sequence Coverage [%]:**	2.5
		**No. of unique Peptides:**	1

10	20	30	40	50	60	70	80
MRELKRTLDA	KGHGVLEMPS	GTGKTVSLLA	LIMAYQRAYP	LEVTKLIYCS	RTVPEIEKVI	EELRKLLNFY	EKQEGEKLPF
**90**	**100**	**110**	**120**	**130**	**140**	**150**	**160**
LGLALSSRKN	LCIHPEVTPL	RFGKDVDGKC	HSLTASYVRA	QYQHDTSLPH	CRFYEEFDAH	GREVPLPAGI	YNLDDLKALG
**170**	**180**	**190**	**200**	**210**	**220**	**230**	**240**
RRQGWCPYFL	ARYSILHANV	VVYSYHYLLD	PKIADLVSKE	LARKAVVVFD	EAHNIDNVCI	DSMSVNLTRR	TLDRCQGNLE
**250**	**260**	**270**	**280**	**290**	**300**	**310**	**320**
TLQKTVLRIK	ETDEQRLRDE	YRRLVEGLRE	ASAARETDAH	LANPVLPDEV	LQEAVPGSIR	TAEHFLGFLR	RLLEYVKWRL
**330**	**340**	**350**	**360**	**370**	**380**	**390**	**400**
RVQHVVQESP	PAFLSGLAQR	VCIQRKPLRF	CAERLRSLLH	TLEITDLADF	SPLTLLANFA	TLVSTYAKGQ	AQHCGSSRNQ
**410**							
KRSHP							

Cmpd.	No. of Cmpds.	m/z meas.	Δ m/z [ppm]	z	Rt [min]	Score	P	Range	Sequence	Modification
1794	1	568.7414	-67.06	2	52.4	22.1	0	110-119	K.CHSLTASYVR.A	

Detailed Protein Report

Protein 574: **breast cancer anti-estrogen resistance protein 3 isoform 2 [Homo sapiens]**

**Accession:**	gi|387157911	**Score:**	22.1
**Database:**	refseq_human(refseq_human_20140103.fasta)	**MW [kDa]:**	82.3
**Database Date:**	2015-11-30	**pI:**	9.4
		**Sequence Coverage [%]:**	4.0
		**No. of unique Peptides:**	1

10	20	30	40	50	60	70	80
MPKECSAFHA	LSAALCCFYH	RKSFIGVKFS	KERHIMDRTP	EKLKKELEEE	LLLSSEDLRS	HAWYHGRIPR	QVSENLVQRD
**90**	**100**	**110**	**120**	**130**	**140**	**150**	**160**
GDFLVRDSLS	SPGNFVLTCQ	WKNLAQHFKI	NRTVLRLSEA	YSRVQYQFEM	ESFDSIPGLV	RCYVGNRRPI	SQQSGAIIFQ
**170**	**180**	**190**	**200**	**210**	**220**	**230**	**240**
PINRTVPLRC	LEEHYGTSPG	QAREGSLTKG	RPDVAKRLSL	TMGGVQAREQ	NLPRGNLLRN	KEKSGSQPAC	LDHMQDRRAL
**250**	**260**	**270**	**280**	**290**	**300**	**310**	**320**
SLKAHQSESY	LPIGCKLPPQ	SSGVDTSPCP	NSPVFRTGSE	PALSPAVVRR	VSSDARAGEA	LRGSDSQLCP	KPPPKPCKVP
**330**	**340**	**350**	**360**	**370**	**380**	**390**	**400**
FLKVPSSPSA	WLNSEANYCE	LNPAFATGCG	RGAKLPSCAQ	GSHTELLTAK	QNEAPGPRNS	GVNYLILDDD	DRERPWEPAA
**410**	**420**	**430**	**440**	**450**	**460**	**470**	**480**
AQMEKGQWDK	GEFVTPLLET	VSSFRPNEFE	SKFLPPENKP	LETAMLKRAK	ELFTNNDPKV	IAQHVLSMDC	RVARILGVSE
**490**	**500**	**510**	**520**	**530**	**540**	**550**	**560**
EMRRNMGVSS	GLELITLPHG	HQLRLDIIER	HNTMAIGIAV	DILGCTGTLE	DRAATLSKII	QVAVELKDSM	GDLYSFSALM
**570**	**580**	**590**	**600**	**610**	**620**	**630**	**640**
KALEMPQITR	LEKTWTALRH	QYTQTAILYE	KQLKPFSKLL	HEGRESTCVP	PNNVSVPLLM	PLVTLMERQA	VTFEGTDMWE
**650**	**660**	**670**	**680**	**690**	**700**	**710**	**720**
KNDQSCEIML	NHLATARFMA	EAADSYRMNA	ERILAGFQPD	EEMNEICKTE	FQMRLLWGSK	GAQVNQTERY	EKFNQILTAL
**730**	**740**						
SRKLEPPPVK	QAEL						

Cmpd.	No. of Cmpds.	m/z meas.	Δ m/z [ppm]	z	Rt [min]	Score	P	Range	Sequence	Modification
127	1	849.3900	-71.21	2	31.0	11.9	1	460-474	K.VIAQHVLSMDCRVAR.I	

Detailed Protein Report

Protein 575: PREDICTED: diacylglycerol kinase iota isoform X1 [Homo sapiens]

**Accession:**	gi|578814733	**Score:**	22.0
**Database:**	refseq_human(refseq_human_20140103.fasta)	**MW [kDa]:**	116.2
**Database Date:**	2015-11-30	**pI:**	9.2
**Modification(s):**	Carbamidomethyl, Oxidation	**Sequence Coverage [%]:**	3.2
		**No. of unique Peptides:**	1

10	20	30	40	50	60	70	80
MDAAGRGCHL	LPLPAARGPA	RAPAAAAAAA	ASPPGPCSGA	ACAPSAAAGA	GAMNPSSSAG	EEKGATGGSS	SSGSGAGSCC
90	100	110	120	130	140	150	160
LGAEGGADPR	GAGSAAAAGA	AALDEPAAAG	QKEKDEALEE	KLRNLTFRKQ	VSYRKAISRA	GLQHLAPAHP	LSLPVANGPA
170	180	190	200	210	220	230	240
KEPRATLDWS	ENAVNGEHLW	LETNVSGDLC	YLGEENCQVR	FAKSALRRKC	AVCKIVVHTA	CIEQLEKINF	RCKPTFREGG
250	260	270	280	290	300	310	320
SRSPRENFVR	HHWVHRRRQE	GKCKQCGKGF	QQKFSFHSKE	IVAISCSWCK	QAFHNKVTCF	MLHHIEEPCS	LGAHAAVIVP
330	340	350	360	370	380	390	400
PTWIIKVKKP	QNSLKASNRK	KKRTSFKRKA	SKRGMEQENK	GRPFVIKPIS	SPLMKPLLVF	VNPKSGGNQG	TKVLQMFMWY
410	420	430	440	450	460	470	480
LNPRQVFDLS	QEGPKDALEL	YRKVPNLRIL	ACGGDGTVGW	ILSILDELQL	SPQPPVGVLP	LGTGNDLART	LNWGGGYTDE
490	500	510	520	530	540	550	560
PVSKILCQVE	DGTVVQLDRW	NLHVERNPDL	PPEELEDGVC	KLPLNVFNNY	FSLGFDAHVT	LEFHESREAN	PEKFNSRFRN
570	580	590	600	610	620	630	640
KMFYAGAAFS	DFLQRSSRDL	SKHVKVVCDG	TDLTPKIQEL	KFQCIVFLNI	PRYCAGTMPW	GNPGDHHDFE	PQRHDDGYIE
650	660	670	680	690	700	710	720
VIGFTMASLA	ALQVGGHGER	LHQCREVMLL	TYKSIPMQVD	GEPCRLAPAM	IRISLRNQAN	MVQKSKRRTS	MPLLNDPQSV
730	740	750	760	770	780	790	800
PDRLRIRVNK	ISLQDYEGFH	YDKEKLREAS	IPLGILVVRG	DCDLETCRMY	IDRLQEDLQS	VSSGSQRVHY	QDHETSFPRA
810	820	830	840	850	860	870	880
LSAQRLSPRW	CFLDATSADR	FYRIDRSQEH	LHFVMEISQD	EIFILDPDMV	VSQPAGTPPG	MPDLVVEQAS	GISDWWNPAL
890	900	910	920	930	940	950	960
RKRMLSDSGL	GMIAPYYEDS	DLKDLSHSRV	LQSPVSSEDH	AILQAVIAGD	LMKLIESYKN	GGSLLIQGPD	HCSLLHYAAK
970	980	990	1000	1010	1020	1030	1040
TGNGEIVKYI	LDHGPSELLD	MADSETGETA	LHKAACQRNR	AVCQLLVDAG	ASLRKTDSKG	KTPQERAQQA	GDPDLAAYLE
1050	1060						
SRQNYKVIGH	EDLETAV						

Cmpd.	No. of Cmpds.	m/z meas.	Δ m/z [ppm]	z	Rt [min]	Score	P	Range	Sequence	Modification
1696	1	1102.1837	102.80	2	51.0	11.5	2	1-21	-.MDAAGRGCHLLPLPAARGPAR.A	Carbamidomethyl: 8; Oxidation: 1

Detailed Protein Report

Protein 576: **PREDICTED: zinc finger MYM-type protein 2 isoform X7 [Homo sapiens]**

**Accession:**	gi|578824999	**Score:**	22.0
**Database:**	refseq_human(refseq_human_20140103.fasta)	**MW [kDa]:**	129.3
**Database Date:**	2015-11-30	**pI:**	6.2
**Modification(s):**	Carbamidomethyl	**Sequence Coverage [%]:**	2.8
		**No. of unique Peptides:**	2

Quantitation

MD:MU **Median:** 0.48 **CV:** 27.16 % **No. of Peptides:** 2

10	20	30	40	50	60	70	80
MDTSSVGGLE	LTDQTPVLLG	STAMATSLTN	VGNSFSGPAN	PLVSRSNKFQ	NSSVEDDDDV	VFIEPVQPPP	PSVPVVADQR
**90**	**100**	**110**	**120**	**130**	**140**	**150**	**160**
TITFTSSKNE	ELQGNDSKIT	PSSKELASQK	GSVSETIVID	DEEDMETNQG	QEKNSSNFIE	RRPPETKNRT	NDVDFSTSSF
**170**	**180**	**190**	**200**	**210**	**220**	**230**	**240**
SRSKVNAGMG	NSGITTEPDS	EIQIANVTTL	ETGVSSVNDG	QLENTDGRDM	NLMITHVTSL	QNTNLGDVSN	GLQSSNFGVN
**250**	**260**	**270**	**280**	**290**	**300**	**310**	**320**
IQTYTPSLTS	QTKTGVGPFN	PGRMNVAGDV	FQNGESATHH	NPDSWISQSA	SFPRNQKQPG	VDSLSPVASL	PKQIFQPSVQ
**330**	**340**	**350**	**360**	**370**	**380**	**390**	**400**
QQPTKPVKVT	CANCKKPLQK	GQTAYQRKGS	AHLFCSTTCL	SSFSHKPAPK	KLCVMCKKDI	TTMKGTIVAQ	VDSSESFQEF
**410**	**420**	**430**	**440**	**450**	**460**	**470**	**480**
CSTSCLSLYE	DKQNPTKGAL	NKSRCTICGK	LTEIRHEVSF	KNMTHKLCSD	HCFNRYRMAN	GLIMNCCEQC	GEYLPSKGAG
**490**	**500**	**510**	**520**	**530**	**540**	**550**	**560**
NNVLVIDGQQ	KRFCCQSCVS	EYKQVGSHPS	FLKEVRDHMQ	DSFLMQPEKY	GKLTTCTGCR	TQCRFFDMTQ	CIGPNGYMEP
**570**	**580**	**590**	**600**	**610**	**620**	**630**	**640**
YCSTACMNSH	KTKYAKSQSL	GIICHFCKRN	SLPQYQATMP	DGKLYNFCNS	SCVAKFQALS	MQSSPNGQFV	APSDIQLKCN
**650**	**660**	**670**	**680**	**690**	**700**	**710**	**720**
YCKNSFCSKP	EILEWENKVH	QFCSKTCSDD	YKKLHCIVTY	CEYCQEEKTL	HETVNFSGVK	RPFCSEGCKL	LYKQDFARRL
**730**	**740**	**750**	**760**	**770**	**780**	**790**	**800**
GLRCVTCNYC	SQLCKKGATK	ELDGVVRDFC	SEDCCKKFQD	WYYKAARCDC	CKSQGTLKER	VQWRGEMKHF	CDQHCLLRFY
**810**	**820**	**830**	**840**	**850**	**860**	**870**	**880**
CQQNEPNMTT	QKGPENLHYD	QGCQTSRTKM	TGSAPPPSPT	PNKEMKNKAV	LCKPLTMTKA	TYCKPHMQTK	SCQTDDTWRT
**890**	**900**	**910**	**920**	**930**	**940**	**950**	**960**
EYVPVPIPVP	VYIPVPMHMY	SQNIPVPTTV	PVPVPVPVFL	PAPLDSSEKI	PAAIEELKSK	VSSDALDTEL	LTMTDMMSED
**970**	**980**	**990**	**1000**	**1010**	**1020**	**1030**	**1040**
EGKTETTNIN	SVIIETDIIG	SDLLKNSDPE	TQSSMPDVPY	EPDLDIEIDF	PRAAEELDME	NEFLLPPVFG	EEYEEQPRPR
**1050**	**1060**	**1070**	**1080**	**1090**	**1100**	**1110**	**1120**
SKKKGAKRKA	VSGYQSHDDS	SDNSECSFPF	KYTYGVNAWK	HWVKTRQLDE	DLLVLDELKS	SKSVKLKEDL	LSHTTAELNY
**1130**	**1140**	**1150**	**1160**				
GLAHFVNEIR	RPNGENYAPD	SIYYLCLGIQ	EDRNLLKIL				

Cmpd.	No. of Cmpds.	m/z meas.	Δ m/z [ppm]	z	Rt [min]	Score	P	Range	Sequence	Modification	Ratios	
1160	1	713.6536	1.59	3	44.1	11.9	2	442-457	K.NMTHKLCSDHCFNRYR.M	Carbamidomethyl: 7, 11	MD:MU	0.37
2179	1	966.3391	-135.18	2	57.4	10.1	2	720-736	R.LGLRCVTCNYCSQLCKK.G		MD:MU	0.63

Detailed Protein Report

Protein 577: PREDICTED: microtubule-associated protein 1B isoform X1 [Homo sapiens]

**Accession:**	gi|530379305	**Score:**	22.0
**Database:**	refseq_human(refseq_human_20140103.fasta)	**MW [kDa]:**	256.6
**Database Date:**	2015-11-30	**pI:**	4.6
**Modification(s):**	Oxidation	**Sequence Coverage [%]:**	0.8
		**No. of unique Peptides:**	1

Detailed Protein Report

10	20	30	40	50	60	70	80
MITDAARHKL	LVLTGQCFEN	TGELILQSGS	FSFQNFIEIF	TDQEIGELLS	TTHPANKASL	TLFCPEEGDW	KNSNLDRHNL
90	100	110	120	130	140	150	160
QDFINIKLNS	ASILPEMEGL	SEFTEYLSES	VEVPSPFDIL	EPPTSGGFLK	LSKPCCYIFP	GGRGDSALFA	VNGFNMLING
170	180	190	200	210	220	230	240
GSERKSCFWK	LIRHLDRVDS	ILLTHIGDDN	LPGINSMLQR	KIAELEEEQS	QGSTTNSDWM	KNLISPDLGV	VFLNVPENLK
250	260	270	280	290	300	310	320
NPEPNIKMKR	SIEEACFTLQ	YLNKLSMKPE	PLFRSVGNTI	DPVILFQKMG	VGKLEMYVLN	PVKSSKEMQY	FMQQWTGTNK
330	340	350	360	370	380	390	400
DKAEFILPNG	QEVDLPISYL	TSVSSLIVWH	PANPAEKIIR	VLFPGNSTQY	NILEGLEKLK	HLDFLKQPLA	TQKDLTGQVP
410	420	430	440	450	460	470	480
TPVVKQTKLK	QRADSRESLK	PAAKPLPSKS	VRKESKEETP	EVTKVNHVEK	PPKVESKEKV	MVKKDKPIKT	ETKPSVTEKE
490	500	510	520	530	540	550	560
VPSKEEPSPV	KAEVAEKQAT	DVKPKAAKEK	TVKKETKVKP	EDKKEEKEKP	KKEVAKKEDK	TPIKKEEKPK	KEEVKKEVKK
570	580	590	600	610	620	630	640
EIKKEEKKEP	KKEVKKETPP	KEVKKEVKKE	EKKEVKKEEK	EPKKEIKKLP	KDAKKSSTPL	SEAKKPAALK	PKVPKKEESV
650	660	670	680	690	700	710	720
KKDSVAAGKP	KEKGKIKVIK	KEGKAAEAVA	AAVGTGATTA	AVMAAAGIAA	IGPAKELEAE	RSLMSSPEDL	TKDFEELKAE
730	740	750	760	770	780	790	800
EVDVTKDIKP	QLELIEDEEK	LKETEPVEAY	VIQKEREVTK	GPAESPDEGI	TTTEGEGECE	QTPEELEPVE	KQGVDDIEKF
810	820	830	840	850	860	870	880
EDEGAGFEES	SETGDYEEKA	ETEEAEEPEE	DGEEHVCVSA	SKHSPTEDEE	SAKAEADAYI	REKRESVASG	DDRAEEDMDE
890	900	910	920	930	940	950	960
AIEKGEAEQS	EEEADEEDKA	EDAREEEYEP	EKMEAEDYVM	AVVDKAAEAG	GAEEQYGFLT	TPTKQLGAQS	PGREPASSIH
970	980	990	1000	1010	1020	1030	1040
DETLPGGSES	EATASDEENR	EDQPEEFTAT	SGYTQSTIEI	SSEPTPMDEM	STPRDVMSDE	TNNEETESPS	QEFVNITKYE
1050	1060	1070	1080	1090	1100	1110	1120
SSLYSQEYSK	PADVTPLNGF	SEGSKTDATD	GKDYNASAST	ISPPSSMEED	KFSRSALRDA	YCSEVKASTT	LDIKDSISAV
1130	1140	1150	1160	1170	1180	1190	1200
SSEKVSPSKS	PSLSPSPPSP	LEKTPLGERS	VNFSLTPNEI	KVSAEAEVAP	VSPEVTQEVV	EEHCASPEDK	TLEVVSPSQS
1210	1220	1230	1240	1250	1260	1270	1280
VTGSAGHTPY	YQSPTDEKSS	HLPTEVIEKP	PAVPVSFEFS	DAKDENERAS	VSPMDEPVPD	SESPIEKVLS	PLRSPPLIGS
1290	1300	1310	1320	1330	1340	1350	1360
ESAYESFLSA	DDKASGRGAE	SPFEEKSGKQ	GSPDQVSPVS	EMTSTSLYQD	KQEGKSTDFA	PIKEDFGQEK	KTDDVEAMSS
1370	1380	1390	1400	1410	1420	1430	1440
QPALALDERK	LGDVSPTQID	VSQFGSFKED	TKMSISEGTV	SDKSATPVDE	GVAEDTYSHM	EGVASVSTAS	VATSSFPEPT
1450	1460	1470	1480	1490	1500	1510	1520
TDDVSPSLHA	EVGSPHSTEV	DDSLSVSVVQ	TPTTFQETEM	SPSKEECPRP	MSISPPDFSP	KTAKSRTPVQ	DHRSEQSSMS
1530	1540	1550	1560	1570	1580	1590	1600
IEFGQESPEQ	SLAMDFSRQS	PDHPTVGAGV	LHITENGPTE	VDYSPSDMQD	SSLSHKIPPM	EEPSYTQDND	LSELISVSQV
1610	1620	1630	1640	1650	1660	1670	1680
EASPSTSSAH	TPSQIASPLQ	EDTLSDVAPP	RDMSLYASLT	SEKVQSLEGE	KLSPKSDISP	LTPRESSPLY	SPTFSDSTSA
1690	1700	1710	1720	1730	1740	1750	1760
VKEKTATCHS	SSSPPIDAAS	AEPYGFRASV	LFDTMQHHLA	LNRDLSTPGL	EKDSGGKTPG	DFSYAYQKPE	ETTRSPDEED
1770	1780	1790	1800	1810	1820	1830	1840
YDYESYEKTT	RTSDVGGYYY	EKIERTTKSP	SDSGYSYETI	GKTTKTPEDG	DYSYEIIEKT	TRTPEEGGYS	YDISEKTTSP
1850	1860	1870	1880	1890	1900	1910	1920
PEVSGYSYEK	TERSRRLLDD	ISNGYDDSED	GGHTLGDPSY	SYETTEKITS	FPESEGYSYE	TSTKTTRTPD	TSTYCYETAE
1930	1940	1950	1960	1970	1980	1990	2000
KITRTPQAST	YSYETSDLCY	TAEKKSPSEA	RQDVDLCLVS	SCEYKHPKTE	LSPSFINPNP	LEWFASEEPT	EESEKPLTQS
2010	2020	2030	2040	2050	2060	2070	2080
GGAPPPPGGK	QQGRQCDETP	PTSVSESAPS	QTDSDVPPET	EECPSITADA	NIDSEDESET	IPTDKTVTYK	HMDPPPAPVQ
2090	2100	2110	2120	2130	2140	2150	2160
DRSPSPRHPD	VSMVDPEALA	IEQNLGKALK	KDLKEKTKTK	KPGTKTKSSS	PVKKSDGKSK	PLAASPKPAG	LKESSDKVSR
2170	2180	2190	2200	2210	2220	2230	2240
VASPKKKESV	EKAAKPTTTP	EVKAARGEEK	DKETKNAANA	SASKSAKTAT	AGPGTTKTTK	SSAVPPGLPV	YLDLCYIPNH
2250	2260	2270	2280	2290	2300	2310	2320
SNSKNVDVEF	FKRVRSSYYV	VSGNDPAAEE	PSRAVLDALL	EGKAQWGSNM	QVTLIPTHDS	EVMREWYQET	HEKQQDLNIM
2330	2340	2350					

Detailed Protein Report

Cmpd.	No. of Cmpds.	m/z meas.	Δ m/z [ppm]	z	Rt [min]	Score	P	Range	Sequence	Modification
1859	1	1022.9020	-33.49	2	53.3	22.0	0	1073-1091	K.DYNASASTISPPSSMEEDK.F	Oxidation: 15

Detailed Protein Report

Protein 578: leucine-rich repeat-containing protein 8B [Homo sapiens]

**Accession:**	gi|21245134	**Score:**	22.0
**Database:**	refseq_human(refseq_human_20140103.fasta)	**MW [kDa]:**	92.3
**Database Date:**	2015-11-30	**pI:**	6.4
**Modification(s):**	Carbamidomethyl	**Sequence Coverage [%]:**	3.5
		**No. of unique Peptides:**	1

Alias proteins:

Accession	Name	Description
gi|578798841	refseq_human (refseq_human_20140103.fasta)	PREDICTED: leucine-rich repeat-containing protein 8B isoform X5 [Homo sapiens]
gi|530362288	refseq_human (refseq_human_20140103.fasta)	PREDICTED: leucine-rich repeat-containing protein 8B isoform X4 [Homo sapiens]
gi|530362286	refseq_human (refseq_human_20140103.fasta)	PREDICTED: leucine-rich repeat-containing protein 8B isoform X3 [Homo sapiens]
gi|530362284	refseq_human (refseq_human_20140103.fasta)	PREDICTED: leucine-rich repeat-containing protein 8B isoform X2 [Homo sapiens]
gi|530362282	refseq_human (refseq_human_20140103.fasta)	PREDICTED: leucine-rich repeat-containing protein 8B isoform X1 [Homo sapiens]
gi|197333692	refseq_human (refseq_human_20140103.fasta)	leucine-rich repeat-containing protein 8B [Homo sapiens]

```
        10         20         30         40         50         60         70         80
MITLTELKCL ADAQSSYHIL KPWWDVFWYY ITLIMLLVAV LAGALQLTQS RVLCCLPCKV EFDNHCAVPW DILKASMNTS
        90        100        110        120        130        140        150        160
SNPGTPLPLP LRIQNDLHRQ QYSYIDAVCY EKQLHWFAKF FPYLVLLHTL IFAACSNFWL HYPSTSSRLE HFVAILHKCF
       170        180        190        200        210        220        230        240
DSPWTTRALS ETVAEQSVRP LKLSKSKILL SSSGCSADID SGKQSLPYPQ PGLESAGIES PTSSVLDKKE GEQAKAIFEK
       250        260        270        280        290        300        310        320
VKRFRMHVEQ KDIIYRVYLK QIIVKVILFV LIITYVPYFL THITLEIDCS VDVQAFTGYK RYQCVYSLAE IFKVLASFYV
       330        340        350        360        370        380        390        400
ILVILYGLTS SYSLWWMLRS SLKQYSFEAL REKSNYSDIP DVKNDFAFIL HLADQYDPLY SKRFSIFLSE VSENKLKQIN
       410        420        430        440        450        460        470        480
LNNEWTVEKL KSKLVKNAQD KIELHLFMLN GLPDNVFELT EMEVLSLELI PEVKLPSAVS QLVNLKELRV YHSSLVVDHP
       490        500        510        520        530        540        550        560
ALAFLEENLK ILRLKFTEMG KIPRWVFHLK NLKELYLSGC VLPEQLSTMQ LEGFQDLKNL RTLYLKSSLS RIPQVVTDLL
       570        580        590        600        610        620        630        640
PSLQKLSLDN EGSKLVVLNN LKKMVNLKSL ELISCDLERI PHSIFSLNNL HELDLRENNL KTVEEIISFQ HLQNLSCLKL
       650        660        670        680        690        700        710        720
WHNNIAYIPA QIGALSNLEQ LSLDHNNIEN LPLQLFLCTK LHYLDLSYNH LTFIPEEIQY LSNLQYFAVT NNNIEMLPDG
       730        740        750        760        770        780        790        800
LFQCKKLQCL LLGKNSLMNL SPHVGELSNL THLELIGNYL ETLPPELEGC QSLKRNCLIV EENLLNTLPL PVTERLQTCL
       810
DKC
```

Cmpd.	No. of Cmpds.	m/z meas.	Δ m/z [ppm]	z	Rt [min]	Score	P	Range	Sequence	Modification
1622	1	1100.2202	-11.62	3	50.2	22.0	2	776-803	R.NCLIVEENLLNTLPLPVTERLQTCLDKC.-	Carbamidomethyl: 24, 28

Detailed Protein Report

Protein 579: PREDICTED: collagen alpha-4(IV) chain isoform X9 [Homo sapiens]

**Accession:**	gi|578803629	**Score:**	22.0
**Database:**	refseq_human(refseq_human_20140103.fasta)	**MW [kDa]:**	135.0
**Database Date:**	2015-11-30	**pI:**	10.1
**Modification(s):**	Oxidation	**Sequence Coverage [%]:**	2.5
		**No. of unique Peptides:**	2

Quantitation

MD:MU **Median:** 3.74 **CV:** 0.00 % **No. of Peptides:** 1

10	20	30	40	50	60	70	80
MWSLHIVLMR	CSFRLTKSLA	TGPWSLILIL	FSVQYVYGSG	KKYIGPCGGR	DCSVCHCVPE	KGSRGPPGPP	GPQGPIGPLG
90	100	110	120	130	140	150	160
APGPIGLSGE	KGMRGDRGPP	GAAGDKGDKG	PTGVPGFPGL	DGIPGHPGPP	GPRGKPGMSG	HNGSRGDPGF	PGGRGALGPG
170	180	190	200	210	220	230	240
GPLGHPGEKG	EKGNSVFILG	AVKGIQGDRG	DPGLPGLPGS	WGAGGPAGPT	GYPGEPGLVG	PPGQPGRPGL	KGNPGVGVKG
250	260	270	280	290	300	310	320
QMGDPGEVGQ	QGSPGPTLLV	EPPDFCLYKG	EKGIKGIPGM	VGLPGPPGRK	GESGIGAKGE	KGIPGFPGPR	GDPGSYGSPG
330	340	350	360	370	380	390	400
FPGLKGELGL	VGDPGLFGLI	GPKGDPGNRG	HPGPPGVLVT	PPLPLKGPPG	DPGFPGRYGE	TGDVGPPGPP	GLLGRPGEAC
410	420	430	440	450	460	470	480
AGMIGPPGPQ	GFPGLPGLPG	EAGIPGRPDS	APGKPGKPGS	PGLPGAPGLQ	GLPGSSVIYC	SVGNPGPQGI	KGKVGPPGGR
490	500	510	520	530	540	550	560
GPKGEKGNEG	LCACEPGPMG	PPGPPGLPGR	QGSKGDLGLP	GWLGTKGDPG	PPGAEGPPGL	PGKHGASGPP	GNKGAKGDMV
570	580	590	600	610	620	630	640
VSRVKGHKGE	RGPDGPPGFP	GQPGSHGRDG	HAGEKGDPGP	PGDHEDATPG	GKGFPGPLGP	PGKAGPVGPP	GLGFPGPPGE
650	660	670	680	690	700	710	720
RGHPGVPGHP	GVRGPDGLKG	QKGDTISCNV	TYPGRHGPPG	FDGPPGPKGF	PGPQGAPGLS	GSDGHKGRPG	TPGTAEIPGP
730	740	750	760	770	780	790	800
PGFRGDMGDP	GFGGEKGSSP	VGPPGPPGSP	GVNGQKGIPG	DPAFGHLGPP	GKRGLSGVPG	IKGPRGDPGC	PGAEGPAGIP
810	820	830	840	850	860	870	880
GFLGLKGPKG	REGHAGFPGV	PGPPGHSCER	GAPGIPGQPG	LPGYPGSPGA	PGGKGQPGDV	GPPGPAGMKG	LPGLPGRPGA
890	900	910	920	930	940	950	960
HGPPGLPGIP	GPFGDDGLPG	PPGPKGPRGL	PGFPGFPGER	GKPGAEGCPG	AKGEPGEKGM	SGLPGDRGLR	GAKGAIGPPG
970	980	990	1000	1010	1020	1030	1040
DEGEMAIISQ	KGTPGEPGPP	GDDGFPGERG	DKGTPGMQGR	RGEPGRYGPP	GFHRGEPGEK	GQPGPPGPPG	PPGSTGLRGF
1050	1060	1070	1080	1090	1100	1110	1120
IGFPGLPGDQ	GEPGSPGPPG	FSGIDGARGP	KGNKGDPASH	FGPPGPKGEP	GSPGCPGHFG	ASGEQGLPGI	QGPRGSPGRP
1130	1140	1150	1160	1170	1180	1190	1200
GPPGSSGPPG	CPGDHGMPGL	RGQPGEMGDP	GPRGLQGDPG	IPGPPGIKGP	SGSPGLNGLH	GLKGQKGTKG	ASGLHDVGPP
1210	1220	1230	1240	1250	1260	1270	1280
GPVGIPGLKG	ERGDPGSPGI	SPPGPRGKKG	PPGPPGSSGP	PGPAGATGRA	PKDIPDPGPP	GDQGPPGPDG	PRGAPGPPGL
1290	1300	1310	1320	1330	1340	1350	1360
PGSVDLLRGE	PGDCGLPGPP	GPPGPPGPPG	YKGFPGCDGK	DGQKGPVGFP	GPQGPHGFPG	PPGEKGLPGP	PGRKGPTGLP
1370	1380	1390	1400	1410	1420	1430	
GPRGEPGPPA	DVDDCPRIPG	LPGAPGMRGP	EGAMGLPGMR	GPSGPGGTRG	RQTALRKTCN		

Cmpd.	No. of Cmpds.	m/z meas.	Δ m/z [ppm]	z	Rt [min]	Score	P	Range	Sequence	Modification	Ratios
474	1	607.1354	-216.53	2	35.2	11.4	0	1142-1153	R.GQPGEMGDPGPR.G	Oxidation: 6	MD:MU 3.74
950	1	984.1390	132.77	2	41.4	10.5	1	1230-1252	K.GPPGPPGSSGPPGPAGATGRAPI D		

Detailed Protein Report

Protein 580: PREDICTED: A-kinase anchor protein 10, mitochondrial isoform X4 [Homo sapiens]

**Accession:**	gi|578829668	**Score:**	22.0
**Database:**	refseq_human(refseq_human_20140103.fasta)	**MW [kDa]:**	65.4
**Database Date:**	2015-11-30	**pI:**	5.2
**Modification(s):**	Oxidation	**Sequence Coverage [%]:**	2.1
		**No. of unique Peptides:**	1

10	20	30	40	50	60	70	80
MDSFSSSRTA	TLKKQPSHME	AAHFGDLGRS	CLDYQTQETK	SSLSKTLEQV	LHDTIVLPYF	IQFMELRRME	HLVKFWLEAE
**90**	**100**	**110**	**120**	**130**	**140**	**150**	**160**
SFHSTTWSRI	RAHSLNTVKQ	SSLAEPVSPS	KKHETTASFL	TDSLDKRLED	SGSAQLFMTH	SEGIDLNNRT	NSTQNHLLLS
**170**	**180**	**190**	**200**	**210**	**220**	**230**	**240**
QECDSAHSLR	LEMARAGTHQ	VSMETQESSS	TLTVASRNSP	ASPLKELSGK	LMKSIEQDAV	NTFTKYISPD	AAKPIPITEA
**250**	**260**	**270**	**280**	**290**	**300**	**310**	**320**
MRNDIIARIC	GEDGQVDPNC	FVLAQSIVFS	AMEQEHFSEF	LRSHHFCKYQ	IEVLTSGTVY	LADILFCESA	LFYFSEYMEK
**330**	**340**	**350**	**360**	**370**	**380**	**390**	**400**
EDAVNILQFW	LAADNFQSQL	AAKKGQYDGQ	EAQNDAMILY	DKYFSLQATH	PLGFDDVVRL	EIESNICREG	GPLPNCFTTP
**410**	**420**	**430**	**440**	**450**	**460**	**470**	**480**
LRQAWTTMEK	VFLPGFLSSN	LYYKYLNDLI	HSVRGDEFLG	GNVSLTAPGS	VGPPDESHPG	SSDSSASQSS	VKKASIKILK
**490**	**500**	**510**	**520**	**530**	**540**	**550**	**560**
NFDEAIIVDA	ASLDPESLYQ	RTYAGKMTFG	RVSDLGQFIR	ESEPEPDVRK	SKGSMFSQAM	KKWVQGNTDE	AQEELAWKIA
**570**	**580**	**590**					
KMIVSDIMQQ	AQYDQPLEKS	TKL					

Cmpd.	No. of Cmpds.	m/z meas.	Δ m/z [ppm]	z	Rt [min]	Score	P	Range	Sequence	Modification
1254	1	673.3228	-16.24	2	45.3	22.0	2	531-542	K.SKGSMFSQAMKK.W	Oxidation: 10

Detailed Protein Report

Protein 581: PREDICTED: myomegalin isoform X1 [Homo sapiens]

**Accession:**	gi|530432185	**Score:**	22.0
**Database:**	refseq_human(refseq_human_20140103.fasta)	**MW [kDa]:**	287.2
**Database Date:**	2015-11-30	**pI:**	5.4
		**Sequence Coverage [%]:**	1.2
		**No. of unique Peptides:**	1

Detailed Protein Report

10	20	30	40	50	60	70	80
MKEICRICAR	ELCGNQRRWI	FHTASKLNLQ	VLLSHVLGKD	VPRDGKAEFA	CSKCAFMLDR	IYRFDTVIAR	IEALSIERLQ
90	100	110	120	130	140	150	160
KLLLEKDRLK	FCIASMYRKN	NDDSGAEIKA	GNGTVDMSVL	PDARYSALLQ	EDFAYSGFEC	WVENEDQIQE	PHSCHGSEGP
170	180	190	200	210	220	230	240
GNRPRRCRGC	AALRVADSDY	EAICKVPRKV	ARSISCGPSS	RWSTSICTEE	PALSEVGPPD	LASTKVPPDG	ESMEEETPGS
250	260	270	280	290	300	310	320
SVESLDASVQ	ASPPQQKDEE	TERSAKELGK	CDCCSDDQAP	QHGCNHKLEL	ALSMIKGLDY	KPIQSPRGSR	LPIPVKSSLP
330	340	350	360	370	380	390	400
GAKPGPSMTD	GVSSGFLNRS	LKPLYKTPVS	YPLELSDLQE	LWDDLCEDYL	PLRVQPMTEE	LLKQQKLNSH	ETTITQQSVS
410	420	430	440	450	460	470	480
DSHLAELQEK	IQQTEATNKI	LQEKLNEMSY	ELKCAQESSQ	KQDGTIQNLK	ETLKSRERET	EELYQVIEGQ	NDTMAKLREM
490	500	510	520	530	540	550	560
LHQSQLGQLH	SSEGTSPAQQ	QVALLDLQSA	LFCSQLEIQK	LQRVVRQKER	QLADAKQCVQ	FVEAAAHESE	QQKEASWKHN
570	580	590	600	610	620	630	640
QELRKALQQL	QEELQNKSQQ	LRAWEAEKYN	EIRTQEQNIQ	HLNHSLSHKE	QLLQEFRELL	QYRDNSDKTL	EANEMLLEKL
650	660	670	680	690	700	710	720
RQRIHDKAVA	LERAIDEKFS	ALEEKEKELR	QLRLAVRERD	HDLERLRDVL	SSNEATMQSM	ESLLRAKGLE	VEQLSTTCQN
730	740	750	760	770	780	790	800
LQWLKEEMET	KFSRWQKEQE	SIIQQLQTSL	HDRNKEVEDL	SATLLCKLGP	GQSEIAEELC	QRLQRKERML	QDLLSDRNKQ
810	820	830	840	850	860	870	880
VLEHEMEIQG	LLQSVSTREQ	ESQAAAEKLV	QALMERNSEL	QALRQYLGGR	DSLMSQAPIS	NQQAEVTPTG	RLGKQTDQGS
890	900	910	920	930	940	950	960
MQIPSRDDST	SLTAKEDVSI	PRSTLGDLDT	VAGLEKELSN	AKEELELMAK	KERESQMELS	ALQSMMAVQE	EELQVQAADM
970	980	990	1000	1010	1020	1030	1040
ESLTRNIQIK	EDLIKDLQMQ	LVDPEDIPAM	ERLTQEVLLL	REKVASVESQ	GQEISGNRRQ	QLLLMLEGLV	DERSRLNEAL
1050	1060	1070	1080	1090	1100	1110	1120
QAERQLYSSL	VKFHAHPESS	ERDRTLQVEL	EGAQVLRSRL	EEVLGRSLER	LNRLETLAAI	GGAAAGDDTE	DTSTEFTDSI
1130	1140	1150	1160	1170	1180	1190	1200
EEEAAHHSHQ	QLVKVALEKS	LATVETQNPS	FSPPSPMGGD	SNRCLQEEML	HLRAEFHQHL	EEKRKAEEEL	KELKAQIEEA
1210	1220	1230	1240	1250	1260	1270	1280
GFSSVSHIRN	TMLSLCLENA	ELKEQMGEAM	SDGWEIEEDK	EKGEVMVETV	VTKEGLSESS	LQAEFRKLQG	KLKNAHNIIN
1290	1300	1310	1320	1330	1340	1350	1360
LLKEQLVLSS	KEGNSKLTPE	LLVHLTSTIE	RINTELVGSP	GKHQHQEEGN	VTVRPFPRPQ	SLDLGATFTV	DAHQLDNQSQ
1370	1380	1390	1400	1410	1420	1430	1440
PRDPGPQSAF	SLPGSTQHLR	SQLSQCKQRY	QDLQEKLLLS	EATVFAQANE	LEKYRVMLTG	ESLVKQDSKQ	IQVDLQDLGY
1450	1460	1470	1480	1490	1500	1510	1520
ETCGRSENEA	EREETTSPEC	EEHNSLKEMV	LMEGLCSEQG	RRGSTLASSS	ERKPLENQLG	KQEEFRVYGK	SENILVLRKD
1530	1540	1550	1560	1570	1580	1590	1600
IKDLKAQLQN	ANKVIQNLKS	RVRSLSVTSD	YSSSLERPRK	LRAVGTLEGS	SPHSVPDEDE	GWLSDGTGAF	YSPGLQAKKD
1610	1620	1630	1640	1650	1660	1670	1680
LESLIQRVSQ	LEAQLPKNGL	EEKLAEELRS	ASWPGKYDSL	IQDQARELSY	LRQKIREGRG	ICYLITRHAK	DTVKSFEDLL
1690	1700	1710	1720	1730	1740	1750	1760
RSNDIDYYLG	QSFREQLAQG	SQLTERLTSK	LSTKDHKSEK	DQAGLEPLAL	RLSRELQEKE	KVIEVLQAKL	DARSLTPSSS
1770	1780	1790	1800	1810	1820	1830	1840
HALSDSHRSP	SSTSFLSDEL	EACSDMDIVS	EYTHYEEKKA	SPSHSDSIHH	SSHSAVLSSK	PSSTSASQGA	KAESNSNPIS
1850	1860	1870	1880	1890	1900	1910	1920
LPTPQNTPKE	ANQAHSGFHF	HSIPKLASLP	QAPLPSAPSS	FLPFSPTGPL	LLGCCETPVV	SLAEAQQELQ	MLQKQLGESA
1930	1940	1950	1960	1970	1980	1990	2000
STVPPASTAT	LLSNDLEADS	SYYLNSAQPH	SPPRGTIELG	RILEPGYLGS	SGKWDVMRPQ	KGSVSGDLSS	GSSVYQLNSK
2010	2020	2030	2040	2050	2060	2070	2080
PTGADLLEEH	LGEIRNLRQR	LEESICINDR	LREQLEHRLT	STARGRGSTS	NFYSQGLESI	PQLCNENRVL	REDNRRLQAQ
2090	2100	2110	2120	2130	2140	2150	2160
LSHVSREHSQ	ETESLREALL	SSRSHLQELE	KELEHQKVER	QQLLEDLREK	QQEVLHFREE	RLSLQENDSR	LQHKLVLLQQ
2170	2180	2190	2200	2210	2220	2230	2240
QCEEKQQLFE	SLQSELQIYE	ALYGNSKKGL	KAYSLDACHQ	IPLSSDLSHL	VAEVRALRGQ	LEQSIQGNNC	LRLQLQQQLE
2250	2260	2270	2280	2290	2300	2310	2320
SGAGKASLSP	SSINQNFPAS	TDPGNKQLLL	QDSAVSPPVR	DVGMNSPALV	FPSSASSTPG	SETPIINRAN	GLGLDTSPVM
2330	2340	2350	2360	2370	2380	2390	2400

Detailed Protein Report

Cmpd.	No. of Cmpds.	m/z meas.	Δ m/z [ppm]	z	Rt [min]	Score	P	Range	Sequence	Modification
569	1	671.4345	38.57	2	36.4	10.8	1	993-1003	R.LTQEVLLLREK.V	

Detailed Protein Report

Protein 582: NACHT, LRR and PYD domains-containing protein 2 isoform 3 [Homo sapiens]

**Accession:**	gi|291463280	**Score:**	21.9
**Database:**	refseq_human(refseq_human_20140103.fasta)	**MW [kDa]:**	117.8
**Database Date:**	2015-11-30	**pI:**	5.8
**Modification(s):**	Carbamidomethyl	**Sequence Coverage [%]:**	2.6
		**No. of unique Peptides:**	2

10	20	30	40	50	60	70	80
MVSSAQMGFN	LQALLEQLSQ	DELSKFKYLI	TTFSLAHELQ	KIPHKEMASL	QVFEKMHRMD	LSERAKDEVR	EAALKSFNKR
90	100	110	120	130	140	150	160
KPLSLGITRK	ERPPLDVDEM	LERFKTEAQA	FTETKGNVIC	LGKEVFKGKK	PDKDNRCRYI	LKTKFREMWK	SWPGDSKEVQ
170	180	190	200	210	220	230	240
VMAERYKMLI	PFSNPRVLPG	PFSYTVVLYG	PAGLGKTTLA	QKLMLDWAED	NLIHKFKYAF	YLSCRELSRL	GPCSFAELVF
250	260	270	280	290	300	310	320
RDWPELQDDI	PHILAQARKI	LFVIDGFDEL	GAAPGALIED	ICGDWEKKKP	VPVLLGSLLN	RVMLPKAALL	VTTRPRALRD
330	340	350	360	370	380	390	400
LRILAEEPIY	IRVEGFLEED	RRAYFLRHFG	DEDQAMRAFE	LMRSNAALFQ	LGSAPAVCWI	VCTTLKLQME	KGEDPVPTCL
410	420	430	440	450	460	470	480
TRTGLFLRFL	CSRFPQGAQL	RGALRTLSLL	AAQGLWAQTS	VLHREDLERL	GVQESDLRLF	LDGDILRQDR	VSKGCYSFIH
490	500	510	520	530	540	550	560
LSFQQFLTAL	FYTLEKEEEE	DRDGHTWDIG	DVQKLLSGVE	RLRNPDLIQA	GYYSFGLANE	KRAKELEATF	GCRMSPDIKQ
570	580	590	600	610	620	630	640
ELLRCDISCK	GGHSTVTDLQ	ELLGCLYESQ	EEELVKEVMA	QFKEISLHLN	AVDVVPSSFC	VKHCRNLQKM	SLQVIKENLP
650	660	670	680	690	700	710	720
ENVTASESDA	EVERSQDDQH	MLPFWTDLCS	IFGSNKDLMG	LAINDSFLSA	SLVRILCEQI	ASDTCHLQRV	VFKNISPADA
730	740	750	760	770	780	790	800
HRNLCLALRG	HKTVTYLTLQ	GNDQDDMFPA	LCEVLRHPEC	NLRYLGLVSC	SATTQQWADL	SLALEVNQSL	TCVNLSDNEL
810	820	830	840	850	860	870	880
LDEGAKLLYT	TLRHPKCFLQ	RLSLENCHLT	EANCKDLAAV	LVVSRELTHL	CLAKNPIGNT	GVKFLCEGLR	YPECKLQTLV
890	900	910	920	930	940	950	960
LWNCDITSDG	CCDLTKLLQE	KSSLLCLDLG	LNHIGVKGMK	FLCEALRKPL	CNLRCLWLWG	CSIPPFSCED	LCSALSCNQS
970	980	990	1000	1010	1020	1030	1040
LVTLDLGQNP	LGSSGVKMLF	ETLTCSSGTL	RTLRLKIDDF	NDELNKLLEE	IEEKNPQLII	DTEKHHPWAE	RPSSHDFMI

Cmpd.	No. of Cmpds.	m/z meas.	Δ m/z [ppm]	z	Rt [min]	Score	P	Range	Sequence	Modification
1922	1	893.8122	-134.43	2	53.9	11.2	0	695-709	R.ILCEQIASDTCHLQR.V	Carbamidomethyl: 3
1197	1	786.4909	152.63	2	44.6	10.8	1	864-875	K.FLCEGLRYPECK.L	Carbamidomethyl: 3, 11

Detailed Protein Report

Protein 583: E3 ubiquitin-protein ligase HECW1 isoform b [Homo sapiens]

Accession: gi|559098413
Database: refseq_human(refseq_human_20140103.fasta)
Database Date: 2015-11-30

Score: 21.9
MW [kDa]: 175.6
pI: 5.2
Sequence Coverage [%]: 2.0
No. of unique Peptides: 2

10	20	30	40	50	60	70	80
MLLHLCSVKN	LYQNRFLGLA	AMASPSRNSQ	SRRRCKEPLR	YSYNPDQFHN	MDLRGGPHDG	VTIPRSTSDT	DLVTSDSRST
90	100	110	120	130	140	150	160
LMVSSSYYSI	GHSQDLVIHW	DIKEEVDAGD	WIGMYLIDEV	LSENFLDYKN	RGVNGSHRGQ	IIWKIDASSY	FVEPETKICF
170	180	190	200	210	220	230	240
KYYHGVSGAL	RATTPSVTVK	NSAAPIFKSI	GADETVQGQG	SRRLISFSLS	DFQAMGLKKG	MFFNPDPYLK	ISIQPGKHSI
250	260	270	280	290	300	310	320
FPALPHHGQE	RRSKIIGNTV	NPIWQAEQFS	FVSLPTDVLE	IEVKDKFAKS	RPIIKRFLGK	LSMPVQRLLE	RHAIGDRVVS
330	340	350	360	370	380	390	400
YTLGRRLPTD	HVSGQLQFRF	EITSSIHPDD	EEISLSTEPE	SAQIQDSPMN	NLMESGSGEP	RSEAPESSES	WKPEQLGEGS
410	420	430	440	450	460	470	480
VPDGPGNQSI	ELSRPAEEAA	VITEAGDQGM	VSVGPEGAGE	LLAQVQKDIQ	PAPSAEELAE	QLDLGEEASA	LLLEDGEAPA
490	500	510	520	530	540	550	560
STKEEPLEEE	ATTQSRAGRE	EEEKEQEEEG	DVSTLEQGEG	RLQLRASVKR	KSRPCSLPVS	ELETVIASAC	GDPETPRTHY
570	580	590	600	610	620	630	640
IRIHTLLHSM	PSAQGGSAAE	EEDGAEEEST	LKDSSEKDGL	SEVDTVAADP	SALEEDREEP	EGATPGTAHP	GHSGGHFPSL
650	660	670	680	690	700	710	720
ANGAAQDGDT	HPSTGSESDS	SPRQGGDHSC	EGCDASCCSP	SCYSSSCYST	SCYSSSCYSA	SCYSPSCYNG	NRFASHTRFS
730	740	750	760	770	780	790	800
SVDSAKISES	TVFSSQDDEE	EENSAFESVP	DSMQSPELDP	ESTNGAGPWQ	DELAAPSGHV	ERSPEGLESP	VAGPSNRRED
810	820	830	840	850	860	870	880
WEARIDSHGR	VFYVDHVNRT	TTWQRPTAAA	TPDGMRRSGS	IQQMEQLNRR	YQNIQRTIAT	ERSEEDSGSQ	SCEQAPAGGG
890	900	910	920	930	940	950	960
GGGGSDSEAE	SSQSSLDLRR	EGSLSPVNSQ	KITLLLQSPA	VKFITNPEFF	TVLHANYSAY	RVFTSSTCLK	HMILKVRRDA
970	980	990	1000	1010	1020	1030	1040
RNFERYQHNR	DLVNFINMFA	DTRLELPRGW	EIKTDQQGKS	FFVDHNSRAT	TFIDPRIPLQ	NGRLPNHLTH	RQHLQRLRSY
1050	1060	1070	1080	1090	1100	1110	1120
SAGEASEVSR	NRGASLLARP	GHSLVAAIRS	QHQHESLPLA	YNDKIVAFLR	QPNIFEMLQE	RQPSLARNHT	LREKIHYIRT
1130	1140	1150	1160	1170	1180	1190	1200
EGNHGLEKLS	CDADLVILLS	LFEEEIMSYV	PLQAAFHPGY	SFSPRCSPCS	SPQNSPGLQR	ASARAPSPYR	RDFEAKLRNF
1210	1220	1230	1240	1250	1260	1270	1280
YRKLEAKGFG	QGPGKIKLII	RRDHLLEGTF	NQVMAYSRKE	LQRNKLYVTF	VGEEGLDYSG	PSREFFFLLS	QELFNPYYGL
1290	1300	1310	1320	1330	1340	1350	1360
FEYSANDTYT	VQISPMSAFV	ENHLEWFRFS	GRILGLALIH	QYLLDAFFTR	PFYKALLRLP	CDLSDLEYLD	EEFHQSLQWM
1370	1380	1390	1400	1410	1420	1430	1440
KDNNITDILD	LTFTVNEEVF	GQVTERELKS	GGANTQVTEK	NKKEYIERMV	KWRVERGVVQ	QTEALVRGFY	EVVDSRLVSV
1450	1460	1470	1480	1490	1500	1510	1520
FDARELELVI	AGTAEIDLND	WRNNTEYRGG	YHDGHLVIRW	FWAAVERFNN	EQRLRLLQFV	TGTSSVPYEG	FAALRGSNGL
1530	1540	1550	1560	1570	1580		
RRFCIEKWGK	ITSLPRAHTC	FNRLDLPPYP	SYSMLYEKLL	TAVEETSTFG	LE		

Cmpd.	No. of Cmpds.	m/z meas.	Δ m/z [ppm]	z	Rt [min]	Score	P	Range	Sequence	Modification
405	1	631.2089	-107.18	3	34.4	10.9	0	505-521	K.EQEEEGDVSTLEQGEGR.L	
1838	1	731.2905	-123.49	2	52.8	11.1	1	1387-1400	R.ELKSGGANTQVTEK.N	

Detailed Protein Report

Protein 584: rho guanine nucleotide exchange factor 33 [Homo sapiens]

**Accession:**	gi|290463441	**Score:**	21.9
**Database:**	refseq_human(refseq_human_20140103.fasta)	**MW [kDa]:**	97.5
**Database Date:**	2015-11-30	**pI:**	6.4
		**Sequence Coverage [%]:**	3.9
		**No. of unique Peptides:**	1

10	20	30	40	50	60	70	80
MEKTKTKQGE	NEHMPVNNPS	TQIYQLQALA	SELKTGFTEA	MQELSRIQHG	EYALEEKVKS	CRCSMEEKVT	EMKNSLNYFK
90	100	110	120	130	140	150	160
EELSNAMSMI	QAITSKQEEM	QQKIEQLQQE	KRRESRKVKA	KKTQKEEHSS	QAGPAQAQGS	PFRSINIPEP	VLPSEDFTNL
170	180	190	200	210	220	230	240
LPSQAYEKAQ	ESRSVHVGDS	NVKGMMGPGV	NPTTPEAEEN	LKSCLSADIQ	SKGHLPSGMW	RQPKDGKEWG	EEYVTKDHPD
250	260	270	280	290	300	310	320
KLKEAGQGRH	SSLENVLCET	SLAAKRQTVA	LELLESERKY	VINISLILKI	KATFQGSDGK	RNSKERSLFP	GSLRYLVQQH
330	340	350	360	370	380	390	400
LDLLHALQER	VLKWPRQGVL	GDLFLKLTND	ENNFLDYYVA	YLRDLPECIS	LVHVVVLKEG	DEEIKSDIYT	LFFHIVQRIP
410	420	430	440	450	460	470	480
EYLIHLQNVL	KFTEQEHPDY	YLLLVCVQRL	RVFISHYTLL	FQCNEDLLIQ	KRKKLKKSSM	AKLYKGLASQ	CANAGQDASP
490	500	510	520	530	540	550	560
TAGPEAVRDT	GIHSEELLQP	YPSAPSSGPA	ITHLMPPVKK	SQQQQSLMES	MQPGKPSDWE	LEGRKHERPE	SLLAPTQFCA
570	580	590	600	610	620	630	640
AEQDVKALAG	PLQAIPEMDF	ESSPAEPLGN	VERSLRAPAE	LLPDARGFVP	AAYEEFEYGG	EIFALPAPYD	EEPFQAPALF
650	660	670	680	690	700	710	720
ENCSPASSES	SLDICFLRPV	SFAMEAERPE	HPLQPLPKSA	TSPAGSSSAY	KLEAAAQAHG	KAKPLSRSLK	EFPRAPPADG
730	740	750	760	770	780	790	800
VAPRLYSTRS	SSGGRAPIKA	ERAAQAHGPA	AAAVAARGAS	RTFFPQQRSQ	SEKQTYLEVR	REMHLEDTTR	FCPKEERESE
810	820	830	840	850	860	870	880
QTSFSDQNPR	QDQKGGFRSS	FRKLFKKKSS	GSEYREKTNE	NPSMDPSPTK	QDFFRNRLAL	ANDLDQGTAV	

Cmpd.	No. of Cmpds.	m/z meas.	Δ m/z [ppm]	z	Rt [min]	Score	P	Range	Sequence	Modification
1626	2	745.8661	-55.26	2	50.1	11.4	2	302-314	R.NSKERSLFPGSLR.Y	

Detailed Protein Report

Protein 585: **PREDICTED: nuclear body protein SP140 isoform X7 [Homo sapiens]**

**Accession:**	gi|530369725	**Score:**	21.9
**Database:**	refseq_human(refseq_human_20140103.fasta)	**MW [kDa]:**	77.3
**Database Date:**	2015-11-30	**pI:**	7.8
**Modification(s):**	Carbamidomethyl	**Sequence Coverage [%]:**	5.2
		**No. of unique Peptides:**	1

10	20	30	40	50	60	70	80
MAQQGQQGQM	ASGDSNLNFR	MVAEIQNVEG	QNLQEQVCPE	PIFRFFRENK	VEIASAITRP	FPFLMGLRDR	SFISEQMYEH
90	100	110	120	130	140	150	160
FQEAFRNLVP	VTRVMYCVLS	ELEKTFGWSH	LEALFSRINL	MAYPDLNEIY	RSFQNVCYEH	SPLQMNNVND	LEDRPRLLPY
170	180	190	200	210	220	230	240
GKQENSNACH	EMDDIAVPQE	ALSSSPRCEP	GFSSESCEQL	ALPKAGGGDA	EDAPSLLPGG	GVSSELENHP	MNEEGESEEL
250	260	270	280	290	300	310	320
ASSLLYDNVP	GAEQSAYENE	KCSCVMCFSE	EVPGSPEART	ESDQACGTMD	TVDIANNSTL	GKPKRKRRKK	RGHGWSRMRM
330	340	350	360	370	380	390	400
RRQENSQQND	NSKADGQVVS	SEKKANVNLK	DLSKIRGRKR	GKPGTRFTQS	DRAAQKRVRS	RASRKHKDET	VDFKAPLLPV
410	420	430	440	450	460	470	480
TCGGVKGILH	KKKLQQGILV	KCIQTEDGKW	FTPTEFEIKG	GHARSKNWRL	SVRCGGWPLR	WLMENGFLPD	PPRIRYRKKK
490	500	510	520	530	540	550	560
RILKSQNNSS	VDPCMRNLDE	CEVCRDGGEL	FCCDTCSRVF	HEDCHIPPVE	AERTPWNCIF	CRMKESPGSQ	QCCQESEVLE
570	580	590	600	610	620	630	640
RQMCPEEQLK	CEFLLLKVYC	CSESSFFAKI	PYYYYIREAC	QGLKEPMWLD	KIKKRLNEHG	YPQVEGFVQD	MRLIFQNHRA
650	660	670	680				
SYKYKDFGQM	GFRLEAEFEK	NFKEVFAIQE	TNGNN				

Cmpd.	No. of Cmpds.	m/z meas.	Δ m/z [ppm]	z	Rt [min]	Score	P	Range	Sequence	Modification
2587	1	941.5681	155.71	2	62.4	10.3	0	188-204	R.CEPGFSSESCEQLALPK.A	Carbamidomethyl: 1

Detailed Protein Report

Protein 586: inactive serine protease 35 precursor [Homo sapiens]

**Accession:**	gi|281427284	**Score:**	21.9
**Database:**	refseq_human(refseq_human_20140103.fasta)	**MW [kDa]:**	47.1
**Database Date:**	2015-11-30	**pI:**	10.3
**Modification(s):**	Oxidation	**Sequence Coverage [%]:**	10.2
		**No. of unique Peptides:**	2

Alias proteins:

Accession	Name	Description
gi|281427286	refseq_human (refseq_human_20140103.fasta)	inactive serine protease 35 precursor [Homo sapiens]

10	20	30	40	50	60	70	80
MENMLLWLIF	FTPGWTLIDG	SEMEWDFMWH	LRKVPRIVSE	RTFHLTSPAF	EADAKMMVNT	VCGIECQKEL	PTPSLSELED
**90**	**100**	**110**	**120**	**130**	**140**	**150**	**160**
YLSYETVFEN	GTRTLTRVKV	QDLVLEPTQN	ITTKGVSVRR	KRQVYGTDSR	FSILDKRFLT	NFPFSTAVKL	STGCSGILIS
**170**	**180**	**190**	**200**	**210**	**220**	**230**	**240**
PQHVLTAAHC	VHDGKDYVKG	SKKLRVGLLK	MRNKSGGKKR	RGSKRSRREA	SGGDQREGTR	EHLRERAKGG	RRRKKSGRGQ
**250**	**260**	**270**	**280**	**290**	**300**	**310**	**320**
RIAEGRPSFQ	WTRVKNTHIP	KGWARGGMGD	ATLDYDYALL	ELKRAHKKKY	MELGISPTIK	KMPGGMIHFS	GFDNDRADQL
**330**	**340**	**350**	**360**	**370**	**380**	**390**	**400**
VYRFCSVSDE	SNDLLYQYCD	AESGSTGSGV	YLRLKDPDKK	NWKRKIIAVY	SGHQWVDVHG	VQKDYNVAVR	ITPLKYAQIC
**410**	**420**						
LWIHGNDANC	AYG						

Cmpd.	No. of Cmpds.	m/z meas.	Δ m/z [ppm]	z	Rt [min]	Score	P	Range	Sequence	Modification
2001	1	698.2933	-125.88	2	54.9	10.1	1	289-300	K.KYMELGISPTIK.K	Oxidation: 3
1626	1	1135.9599	6.52	3	50.2	11.8	2	366-395	K.IIAVYSGHQWVDVHGVQKDYNVAVRITPLK.Y	

Detailed Protein Report

Protein 587: cleavage and polyadenylation specificity factor subunit 2 [Homo sapiens]

**Accession:**	gi|34101288	**Score:**	21.9
**Database:**	refseq_human(refseq_human_20140103.fasta)	**MW [kDa]:**	88.4
**Database Date:**	2015-11-30	**pI:**	4.8
**Modification(s):**	Oxidation	**Sequence Coverage [%]:**	1.4
		**No. of unique Peptides:**	1

Alias proteins:

Accession	Name	Description
gi|530403998	refseq_human (refseq_human_20140103.fasta)	PREDICTED: cleavage and polyadenylation specificity factor subunit 2 isoform X1 [Homo sapiens]

10	20	30	40	50	60	70	80
MTSIIKLTTL	SGVQEESALC	YLLQVDEFRF	LLDCGWDEHF	SMDIIDSLRK	HVHQIDAVLL	SHPDPLHLGA	LPYAVGKLGL
**90**	**100**	**110**	**120**	**130**	**140**	**150**	**160**
NCAIYATIPV	YKMGQMFMYD	LYQSRHNTED	FTLFTLDDVD	AAFDKIQQLK	FSQIVNLKGK	GHGLSITPLP	AGHMIGGTIW
**170**	**180**	**190**	**200**	**210**	**220**	**230**	**240**
KIVKDGEEEI	VYAVDFNHKR	EIHLNGCSLE	MLSRPSLLIT	DSFNATYVQP	RRKQRDEQLL	TNVLETLRGD	GNVLIAVDTA
**250**	**260**	**270**	**280**	**290**	**300**	**310**	**320**
GRVLELAQLL	DQIWRTKDAG	LGVYSLALLN	NVSYNVVEFS	KSQVEWMSDK	LMRCFEDKRN	NPFQFRHLSL	CHGLSDLARV
**330**	**340**	**350**	**360**	**370**	**380**	**390**	**400**
PSPKVVLASQ	PDLECGFSRD	LFIQWCQDPK	NSIILTYRTT	PGTLARFLID	NPSEKITEIE	LRKRVKLEGK	ELEEYLEKEK
**410**	**420**	**430**	**440**	**450**	**460**	**470**	**480**
LKKEAAKKLE	QSKEADIDSS	DESDIEEDID	QPSAHKTKHD	LMMKGEGSRK	GSFFKQAKKS	YPMFPAPEER	IKWDEYGEII
**490**	**500**	**510**	**520**	**530**	**540**	**550**	**560**
KPEDFLVPEL	QATEEEKSKL	ESGLTNGDEP	MDQDLSDVPT	KCISTTESIE	IKARVTYIDY	EGRSDGDSIK	KIINQMKPRQ
**570**	**580**	**590**	**600**	**610**	**620**	**630**	**640**
LIIVHGPPEA	SQDLAECCRA	FGGKDIKVYM	PKLHETVDAT	SETHIYQVRL	KDSLVSSLQF	CKAKDAELAW	IDGVLDMRVS
**650**	**660**	**670**	**680**	**690**	**700**	**710**	**720**
KVDTGVILEE	GELKDDGEDS	EMQVEAPSDS	SVIAQQKAMK	SLFGDDEKET	GEESEIIPTL	EPLPPHEVPG	HQSVFMNEPR
**730**	**740**	**750**	**760**	**770**	**780**	**790**	
LSDFKQVLLR	EGIQAEFVGG	VLVCNNQVAV	RRTETGRIGL	EGCLCQDFYR	IRDLLYEQYA	IV	

Cmpd.	No. of Cmpds.	m/z meas.	Δ m/z [ppm]	z	Rt [min]	Score	P	Range	Sequence	Modification
303	1	1292.7280	120.56	1	33.5	21.9	1	439-449	K.HDLMMKGEGSR.K	Oxidation: 4, 5

Detailed Protein Report

Protein 588: **52 kDa repressor of the inhibitor of the protein kinase [Homo sapiens]**

**Accession:**	gi|19923268	**Score:**	21.8
**Database:**	refseq_human(refseq_human_20140103.fasta)	**MW [kDa]:**	87.6
**Database Date:**	2015-11-30	**pI:**	5.5
**Modification(s):**	Oxidation	**Sequence Coverage [%]:**	2.5
		**No. of unique Peptides:**	1

Quantitation

MD:MU **Median:** 0.58 **CV:** 0.00 % **No. of Peptides:** 1

10	20	30	40	50	60	70	80
MPNFCAAPNC	TRKSTQSDLA	FFRFPRDPAR	CQKWVENCRR	ADLEDKTPDQ	LNKHYRLCAK	HFETSMICRT	SPYRTVLRDN
90	100	110	120	130	140	150	160
AIPTIFDLTS	HLNNPHSRHR	KRIKELSEDE	IRTLKQKKID	ETSEQEQKHK	ETNNSNAQNP	SEEEGEGQDE	DILPLTLEEK
170	180	190	200	210	220	230	240
ENKEYLKSLF	EILILMGKQN	IPLDGHEADE	IPEGLFTPDN	FQALLECRIN	SGEEVLRKRF	ETTAVNTLFC	SKTQQRQMLE
250	260	270	280	290	300	310	320
ICESCIREET	LREVRDSHFF	SIITDDVVDI	AGEEHLPVLV	RFVDESHNLR	EEFIGFLPYE	ADAEILAVKF	HTMITEKWGL
330	340	350	360	370	380	390	400
NMEYCRGQAY	IVSSGFSSKM	KVVASRLLEK	YPQAIYTLCS	SCALNMWLAK	SVPVMGVSVA	LGTIEEVCSF	FHRSPQLLLE
410	420	430	440	450	460	470	480
LDNVISVLFQ	NSKERGKELK	EICHSQWTGR	HDAFEILVEL	LQALVLCLDG	INSDTNIRWN	NYIAGRAFVL	CSAVSDFDFI
490	500	510	520	530	540	550	560
VTIVVLKNVL	SFTRAFGKNL	QGQTSDVFFA	AGSLTAVLHS	LNEVMENIEV	YHEFWFEEAT	NLATKLDIQM	KLPGKFRRAH
570	580	590	600	610	620	630	640
QGNLESQLTS	ESYYKETLSV	PTVEHIIQEL	KDIFSEQHLK	ALKCLSLVPS	VMGQLKFNTS	EEHHADMYRS	DLPNPDTLSA
650	660	670	680	690	700	710	720
ELHCWRIKWK	HRGKDIELPS	TIYEALHLPD	IKFFPNVYAL	LKVLCILPVM	KVENERYENG	RKRLKAYLRN	TLTDQRSSNL
730	740	750	760	770			
ALLNINFDIK	HDLDLMVDTY	IKLYTSKSEL	PTDNSETVEN	T			

Cmpd.	No. of Cmpds.	m/z meas.	Δ m/z [ppm]	z	Rt [min]	Score	P	Range	Sequence	Modification	Ratios
2085	1	731.3255	-123.74	2	56.2	10.1	2	546-557	K.LDIQMKLPGKFR.R	Oxidation: 5	MD:MU 0.58

Detailed Protein Report

Protein 589: **PREDICTED: glycylpeptide N-tetradecanoyltransferase 1 isoform X1 [Homo sapiens]**

**Accession:**	gi|530412338	**Score:**	21.8
**Database:**	refseq_human(refseq_human_20140103.fasta)	**MW [kDa]:**	61.3
**Database Date:**	2015-11-30	**pI:**	9.8
**Modification(s):**	Oxidation	**Sequence Coverage [%]:**	3.5
		**No. of unique Peptides:**	2

10	20	30	40	50	60	70	80
MPGGRPVPKG	TPHVVGRGAL	LSQLKMADES	ETAVKPPAPP	LPQMMEGNGN	GHEHCSDCEN	EEDNSYNRGG	LSPANDTGAK
**90**	**100**	**110**	**120**	**130**	**140**	**150**	**160**
KKKKKQKKKK	EKGSETDSAQ	DQPVKMNSLP	AERIQEIQKA	IELFSVGQGP	AKTMEEASKR	SYQFWDTQPV	PKLGEVVNTH
**170**	**180**	**190**	**200**	**210**	**220**	**230**	**240**
GPVEPDKDNI	RQEPYTLPQG	FTWDALDLGD	RGVLKELYTL	LNENYVEDDD	NMFRFDYSPE	FLLWALRPPG	WLPQWHCGVR
**250**	**260**	**270**	**280**	**290**	**300**	**310**	**320**
VVSSRKLVGF	ISAIPANIHI	YDTEKKMVEI	NFLCVHKKLR	SKRVAPVLIR	EITRRVHLEG	IFQAVYTAGV	VLPKPVGTCR
**330**	**340**	**350**	**360**	**370**	**380**	**390**	**400**
YWHRSLNPRK	LIEVKFSHLS	RNMTMQRTMK	LYRLPETPKT	AGLRPMETKD	IPVVHQLLTR	YLKQFHLTPV	MSQEEVEHWF
**410**	**420**	**430**	**440**	**450**	**460**	**470**	**480**
YPQENIIDTF	VVENANGEVT	DFLSFYTLPS	TIMNHPTHKS	LKAAYSFYNV	HTQTPLLDLM	SDALVLAKMV	RSRRGGLWRC
**490**	**500**	**510**	**520**	**530**	**540**	**550**	
AGKRQWSHGE	HSSRDAASHG	LEALSPPHLF	CLGRVPAVFR	QKLKTWREQG	ASEVKALNSS		

Cmpd.	No. of Cmpds.	m/z meas.	Δ m/z [ppm]	z	Rt [min]	Score	P	Range	Sequence	Modification
653	1	820.5245	82.19	1	37.7	10.3	2	469-474	K.MVRSRR.G	Oxidation: 1
2042	1	766.2799	-176.23	2	55.4	11.5	2	523-535	K.LKTWREQGASEVK.A	

Detailed Protein Report

Protein 590: PREDICTED: PR domain zinc finger protein 15 isoform X2 [Homo sapiens]

**Accession:**	gi|578836645	**Score:**	21.8
**Database:**	refseq_human(refseq_human_20140103.fasta)	**MW [kDa]:**	128.4
**Database Date:**	2015-11-30	**pI:**	9.2
**Modification(s):**	Carbamidomethyl, Oxidation	**Sequence Coverage [%]:**	3.7
		**No. of unique Peptides:**	1

10	20	30	40	50	60	70	80
MPRRRPPASG	AAQFPERIAT	RSPDPIPLCT	FQRQVSEMAE	DGSEEIMFIW	CEDCSQYHDS	ECPELGPVVM	VKDSFVLSRA
90	100	110	120	130	140	150	160
RSSLPPNLEI	RRLEDGAEGV	FAITQLVKRT	QFGPFESRRV	AKWEKESAFP	LKVFQKDGHP	VCFDTSNEDD	CNWMMLVRPA
170	180	190	200	210	220	230	240
AEAEHQNLTA	YQHGSDVYFT	TSRDIPPGTE	LRVWYAAFYA	KKMDKPMLKQ	AGSGVHAAGT	PENSAPVESE	PSQWACKVCS
250	260	270	280	290	300	310	320
ATFLELQLLN	EHLLGHLEQA	KSLPPGSQSE	AAAPEKEQDT	PRGEPPAVPE	SENVATKEQK	KKPRRGRKPK	VSKAEQPLVI
330	340	350	360	370	380	390	400
VEDKEPTEQV	AEIITEVPPD	EPVSATPDER	IMELVLGKLA	TTTTDTSSVP	KFTHHQNNTI	TLKRSLILSS	RHGIRRKLIK
410	420	430	440	450	460	470	480
QLGEHKRVYQ	CNICSKIFQN	SSNLSRHVRS	HGDKLFKCEE	CAKLFSRKES	LKQHVSYKHS	RNEVDGEYRY	RCGTCEKTFR
490	500	510	520	530	540	550	560
IESALEFHNC	RTDDKTFQCE	MCFRFFSTNS	NLSKHKKKHG	DKKFACEVCS	KMFYRKDVML	DHQRRHLEGV	RRVKREDLEA
570	580	590	600	610	620	630	640
GGENLVRYKK	EPSGCPVCGK	DIALMDDHQR	EEFIGKIGIS	SEENDDNSDE	SADSEPHKYS	CKRCQLTFGR	GKEYLKHIME
650	660	670	680	690	700	710	720
VHKEKGYGCS	ICNRRFALKA	TYHAHMVIHR	ENLPDPNVQK	YIHPCEICGR	IFNSIGNLER	HKLIHTGVKS	HACEQCGKSF
730	740	750	760	770	780	790	800
ARKDMLKEHM	RVHDNVREYL	CAECGKGMKT	KHALRHHMKL	HKGIKEYECK	ECHRRFAQKV	NMLKHCKRHT	GIKDFMCELC
810	820	830	840	850	860	870	880
GKTFSERNTM	ETHKLIHTVG	KQWTCSVCDK	KYVTEYMLQK	HVQLTHDKVE	AQSCQLCGTK	VSTRASMSRH	MRRKHPEVLA
890	900	910	920	930	940	950	960
VRIDDLDHLP	ETTTIDASSI	GIVQPELTLE	QEDLAEGKHG	KAAKRSHKRK	QKPEEEAGAP	VPEDATFSEY	SEKETEFTGS
970	980	990	1000	1010	1020	1030	1040
VGDETNSAVQ	SIQQVVVTLG	DPNVTTPSSS	VGLTNITVTP	ITTAAATQFT	NLQPVAVGHL	TTPERQLQLD	NSILTVTFDT
1050	1060	1070	1080	1090	1100	1110	1120
VSGSAMLHNR	QNDVQIHPQP	EASNPQSVAH	FINLTTLVNS	ITPLGSQLSD	QHPLTWRAVP	QTDVLPPSQP	QAPPQQAAQP
1130	1140						
QVQAEQQQQQ	MYSY						

Cmpd.	No. of Cmpds.	m/z meas.	Δ m/z [ppm]	z	Rt [min]	Score	P	Range	Sequence	Modification
1553	1	1018.4978	58.27	3	49.1	10.5	2	481-504	R.IESALEFHNCRTDDKTFQCEMCFR.F	Carbamidomethyl: 19, 22; Oxidation: 21

Detailed Protein Report

Protein 591: PREDICTED: growth/differentiation factor 9 isoform X1 [Homo sapiens]

**Accession:**	gi|530379837	**Score:**	21.7
**Database:**	refseq_human(refseq_human_20140103.fasta)	**MW [kDa]:**	41.8
**Database Date:**	2015-11-30	**pI:**	10.0
**Modification(s):**	Carbamidomethyl	**Sequence Coverage [%]:**	4.1
		**No. of unique Peptides:**	1

Alias proteins:

Accession	Name	Description
gi|578810283	refseq_human (refseq_human_20140103.fasta)	PREDICTED: growth/differentiation factor 9 isoform X2 [Homo sapiens]
gi|572153064	refseq_human (refseq_human_20140103.fasta)	growth/differentiation factor 9 isoform 2 [Homo sapiens]
gi|572153044	refseq_human (refseq_human_20140103.fasta)	growth/differentiation factor 9 isoform 2 [Homo sapiens]
gi|572152931	refseq_human (refseq_human_20140103.fasta)	growth/differentiation factor 9 isoform 2 [Homo sapiens]
gi|572152923	refseq_human (refseq_human_20140103.fasta)	growth/differentiation factor 9 isoform 2 [Homo sapiens]
gi|572152852	refseq_human (refseq_human_20140103.fasta)	growth/differentiation factor 9 isoform 2 [Homo sapiens]

10	20	30	40	50	60	70	80
MKKLYKTYAT	KEGIPKSNRS	HLYNTVRLFT	PCTRHKQAPG	DQVTGILPSV	ELLFNLDRIT	TVEHLLKSVL	LYNINNSVSF
**90**	**100**	**110**	**120**	**130**	**140**	**150**	**160**
SSAVKCVCNL	MIKEPKSSSR	TLGRAPYSFT	FNSQFEFGKK	HKWIQIDVTS	LLQPLVASNK	RSIHMSINFT	CMKDQLEHPS
**170**	**180**	**190**	**200**	**210**	**220**	**230**	**240**
AQNGLFNMTL	VSPSLILYLN	DTSAQAYHSW	YSLHYKRRPS	QGPDQERSLS	AYPVGEEAAE	DGRSSHHRHR	RGQETVSSEL
**250**	**260**	**270**	**280**	**290**	**300**	**310**	**320**
KKPLGPASFN	LSEYFRQFLL	PQNECELHDF	RLSFSQLKWD	NWIVAPHRYN	PRYCKGDCPR	AVGHRYGSPV	HTMVQNIIYE
**330**	**340**	**350**	**360**	**370**			
KLDSSVPRPS	CVPAKYSPLS	VLTIEPDGSI	AYKEYEDMIA	TKCTCR			

Cmpd.	No. of Cmpds.	m/z meas.	Δ m/z [ppm]	z	Rt [min]	Score	P	Range	Sequence	Modification
1385	1	905.0891	159.82	2	46.9	10.8	2	86-100	K.CVCNLMIKEPKSSSR.T	Carbamidomethyl: 1, 3

Detailed Protein Report

Protein 592: kinesin-like protein KIF1B isoform alpha [Homo sapiens]

**Accession:**	gi|41393559	**Score:**	21.7
**Database:**	refseq_human(refseq_human_20140103.fasta)	**MW [kDa]:**	130.3
**Database Date:**	2015-11-30	**pI:**	9.4
		**Sequence Coverage [%]:**	2.4
		**No. of unique Peptides:**	1

10	20	30	40	50	60	70	80
MSGASVKVAV	RVRPFNSRET	SKESKCIIQM	QGNSTSIINP	KNPKEAPKSF	SFDYSYWSHT	SPEDPCFASQ	NRVYNDIGKE
**90**	**100**	**110**	**120**	**130**	**140**	**150**	**160**
MLLHAFEGYN	VCIFAYGQTG	AGKSYTMMGK	QEESQAGIIP	QLCEELFEKI	NDNCNEEMSY	SVEVSYMEIY	CERVRDLLNP
**170**	**180**	**190**	**200**	**210**	**220**	**230**	**240**
KNKGNLRVRE	HPLLGPYVED	LSKLAVTSYT	DIADLMDAGN	KARTVAATNM	NETSSRSHAV	FTIVFTQKKH	DNETNLSTEK
**250**	**260**	**270**	**280**	**290**	**300**	**310**	**320**
VSKISLVDLA	GSERADSTGA	KGTRLKEGAN	INKSLTTLGK	VISALAEVSK	KKKKTDFIPY	RDSVLTWLLR	ENLGGNSRTA
**330**	**340**	**350**	**360**	**370**	**380**	**390**	**400**
MVAALSPADI	NYDETLSTLR	YADRAKQIKC	NAVINEDPNA	KLVRELKEEV	TRLKDLLRAQ	GLGDIIDTSM	GSLTSSPSSC
**410**	**420**	**430**	**440**	**450**	**460**	**470**	**480**
SLSSQVGLTS	VTSIQERIMS	TPGGEEAIER	LKESEKIIAE	LNETWEEKLR	KTEAIRMERE	ALLAEMGVAI	REDGGTLGVF
**490**	**500**	**510**	**520**	**530**	**540**	**550**	**560**
SPKKTPHLVN	LNEDPLMSEC	LLYYIKDGIT	RVGQADAERR	QDIVLSGAHI	KEEHCIFRSE	RSNSGEVIVT	LEPCERSETY
**570**	**580**	**590**	**600**	**610**	**620**	**630**	**640**
VNGKRVSQPV	QLRSGNRIIM	GKNHVFRFNH	PEQARAEREK	TPSAETPSEP	VDWTFAQREL	LEKQGIDMKQ	EMEKRLQEME
**650**	**660**	**670**	**680**	**690**	**700**	**710**	**720**
ILYKKEKEEA	DLLLEQQRLD	ADSDSGDDSD	KRSCEESWKL	ITSLREKLPP	SKLQTIVKKC	GLPSSGKKRE	PIKMYQIPQR
**730**	**740**	**750**	**760**	**770**	**780**	**790**	**800**
RRLSKDSKWV	TISDLKIQAV	KEICYEVALN	DFRHSRQEIE	ALAIVKMKEL	CAMYGKKDPN	ERDSWRAVAR	DVWDTVGVGD
**810**	**820**	**830**	**840**	**850**	**860**	**870**	**880**
EKIEDVMATG	KGSTDVDDLK	VHIDKLEDIL	QEVKKQNNMK	DEEIKVLRNK	MLKMEKVLPL	IGSQEQKSPG	SHKAKEPVGA
**890**	**900**	**910**	**920**	**930**	**940**	**950**	**960**
GVSSTSENNV	SKGDNGELAK	EERVSQLMNG	DPAFRRGRLR	WMRQEQIRFK	NLQQQEITKQ	LRRQNVPHRF	IPPENRKPRF
**970**	**980**	**990**	**1000**	**1010**	**1020**	**1030**	**1040**
PFKSNPKHRN	SWSPGTHIII	TEDEVIELRI	PKDDEARKGN	KEESQEKGGK	GAFKDPQFPW	GSQGMRSQDH	IQVSKQHINN
**1050**	**1060**	**1070**	**1080**	**1090**	**1100**	**1110**	**1120**
QQQPPQLRWR	SNSLNNGQPK	STRCQASASA	ESLNSHSGHP	TADVQTFQAK	RHIHQHRQSY	CNYNTGGQLE	GNAATSYQKQ
**1130**	**1140**	**1150**	**1160**				
TDKPSHCSQF	VTPPRMRRQF	SAPNLKAGRE	TTV				

Cmpd.	No. of Cmpds.	m/z meas.	Δ m/z [ppm]	z	Rt [min]	Score	P	Range	Sequence	Modification
1501	1	671.0963	120.59	3	48.4	10.5	1	737-753	K.IQAVKEICYEVALNDFR.H	

Detailed Protein Report

Protein 593: pantetheinase precursor [Homo sapiens]

**Accession:**	gi|223633991	**Score:**	21.6
**Database:**	refseq_human(refseq_human_20140103.fasta)	**MW [kDa]:**	57.0
**Database Date:**	2015-11-30	**pI:**	5.2
		**Sequence Coverage [%]:**	3.5
		**No. of unique Peptides:**	1

10	20	30	40	50	60	70	80
MTTQLPAYVA	ILLFYVSRAS	CQDTFTAAVY	EHAAILPNAT	LTPVSREEAL	ALMNRNLDIL	EGAITSAADQ	GAHIIVTPED
**90**	**100**	**110**	**120**	**130**	**140**	**150**	**160**
AIYGWNFNRD	SLYPYLEDIP	DPEVNWIPCN	NRNRFGQTPV	QERLSCLAKN	NSIYVVANIG	DKKPCDTSDP	QCPPDGRYQY
**170**	**180**	**190**	**200**	**210**	**220**	**230**	**240**
NTDVVFDSQG	KLVARYHKQN	LFMGENQFNV	PKEPEIVTFN	TTFGSFGIFT	CFDILFHDPA	VTLVKDFHVD	TIVFPTAWMN
**250**	**260**	**270**	**280**	**290**	**300**	**310**	**320**
VLPHLSAVEF	HSAWAMGMRV	NFLASNIHYP	SKKMTGSGIY	APNSSRAFHY	DMKTEEGKLL	LSQLDSHPSH	SAVVNWTSYA
**330**	**340**	**350**	**360**	**370**	**380**	**390**	**400**
SSIEALSSGN	KEFKGTVFFD	EFTFVKLTGV	AGNYTVCQKD	LCCHLSYKMS	ENIPNEVYAL	GAFDGLHTVE	GRYYLQICTL
**410**	**420**	**430**	**440**	**450**	**460**	**470**	**480**
LKCKTTNLNT	CGDSAETAST	RFEMFSLSGT	FGTQYVFPEV	LLSENQLAPG	EFQVSTDGRL	FSLKPTSGPV	LTVTLFGRLY
**490**	**500**	**510**	**520**				
EKDWASNASS	GLTAQARIIM	LIVIAPIVCS	LSW				

Cmpd.	No. of Cmpds.	m/z meas.	Δ m/z [ppm]	z	Rt [min]	Score	P	Range	Sequence	Modification
2233	1	1043.7218	141.72	2	57.8	21.6	0	1-18	-.MTTQLPAYVAILLFYVSR.A	

Detailed Protein Report

Protein 594: **complement C3 precursor [Homo sapiens]**

**Accession:**	gi|115298678	**Score:**	21.6
**Database:**	refseq_human(refseq_human_20140103.fasta)	**MW [kDa]:**	187.0
**Database Date:**	2015-11-30	**pI:**	6.0
		**Sequence Coverage [%]:**	0.8
		**No. of unique Peptides:**	1

10	20	30	40	50	60	70	80
MGPTSGPSLL	LLLLTHLPLA	LGSPMYSIIT	PNILRLESEE	TMVLEAHDAQ	GDVPVTVTVH	DFPGKKLVLS	SEKTVLTPAT
90	100	110	120	130	140	150	160
NHMGNVTFTI	PANREFKSEK	GRNKFVTVQA	TFGTQVVEKV	VLVSLQSGYL	FIQTDKTIYT	PGSTVLYRIF	TVNHKLLPVG
170	180	190	200	210	220	230	240
RTVMVNIENP	EGIPVKQDSL	SSQNQLGVLP	LSWDIPELVN	MGQWKIRAYY	ENSPQQVFST	EFEVKEYVLP	SFEVIVEPTE
250	260	270	280	290	300	310	320
KFYYIYNEKG	LEVTITARFL	YGKKVEGTAF	VIFGIQDGEQ	RISLPESLKR	IPIEDGSGEV	VLSRKVLLDG	VQNPRAEDLV
330	340	350	360	370	380	390	400
GKSLYVSATV	ILHSGSDMVQ	AERSGIPIVT	SPYQIHFTKT	PKYFKPGMPF	DLMVFVTNPD	GSPAYRVPVA	VQGEDTVQSL
410	420	430	440	450	460	470	480
TQGDGVAKLS	INTHPSQKPL	SITVRTKKQE	LSEAEQATRT	MQALPYSTVG	NSNNYLHLSV	LRTELRPGET	LNVNFLLRMD
490	500	510	520	530	540	550	560
RAHEAKIRYY	TYLIMNKGRL	LKAGRQVREP	GQDLVVLPLS	ITTDFIPSFR	LVAYYTLIGA	SGQREVVADS	VWVDVKDSCV
570	580	590	600	610	620	630	640
GSLVVKSGQS	EDRQPVPGQQ	MTLKIEGDHG	ARVVLVAVDK	GVFVLNKKNK	LTQSKIWDVV	EKADIGCTPG	SGKDYAGVFS
650	660	670	680	690	700	710	720
DAGLTFTSSS	GQQTAQRAEL	QCPQPAARRR	RSVQLTEKRM	DKVGKYPKEL	RKCCEDGMRE	NPMRFSCQRR	TRFISLGEAC
730	740	750	760	770	780	790	800
KKVFLDCCNY	ITELRRQHAR	ASHLGLARSN	LDEDIIAEEN	IVSRSEFPES	WLWNVEDLKE	PPKNGISTKL	MNIFLKDSIT
810	820	830	840	850	860	870	880
TWEILAVSMS	DKKGICVADP	FEVTVMQDFF	IDLRLPYSVV	RNEQVEIRAV	LYNYRQNQEL	KVRVELLHNP	AFCSLATTKR
890	900	910	920	930	940	950	960
RHQQTVTIPP	KSSLSVPYVI	VPLKTGLQEV	EVKAAVYHHF	ISDGVRKSLK	VVPEGIRMNK	TVAVRTLDPE	RLGREGVQKE
970	980	990	1000	1010	1020	1030	1040
DIPPADLSDQ	VPDTESETRI	LLQGTPVAQM	TEDAVDAERL	KHLIVTPSGC	GEQNMIGMTP	TVIAVHYLDE	TEQWEKFGLE
1050	1060	1070	1080	1090	1100	1110	1120
KRQGALELIK	KGYTQQLAFR	QPSSAFAAFV	KRAPSTWLTA	YVVKVFSLAV	NLIAIDSQVL	CGAVKWLILE	KQKPDGVFQE
1130	1140	1150	1160	1170	1180	1190	1200
DAPVIHQEMI	GGLRNNNEKD	MALTAFVLIS	LQEAKDICEE	QVNSLPGSIT	KAGDFLEANY	MNLQRSYTVA	IAGYALAQMG
1210	1220	1230	1240	1250	1260	1270	1280
RLKGPLLNKF	LTTAKDKNRW	EDPGKQLYNV	EATSYALLAL	LQLKDFDFVP	PVVRWLNEQR	YYGGGYGSTQ	ATFMVFQALA
1290	1300	1310	1320	1330	1340	1350	1360
QYQKDAPDHQ	ELNLDVSLQL	PSRSSKITHR	IHWESASLLR	SEETKENEGF	TVTAEGKGQG	TLSVVTMYHA	KAKDQLTCNK
1370	1380	1390	1400	1410	1420	1430	1440
FDLKVTIKPA	PETEKRPQDA	KNTMILEICT	RYRGDQDATM	SILDISMMTG	FAPDTDDLKQ	LANGVDRYIS	KYELDKAFSD
1450	1460	1470	1480	1490	1500	1510	1520
RNTLIIYLDK	VSHSEDDCLA	FKVHQYFNVE	LIQPGAVKVY	AYYNLEESCT	RFYHPEKEDG	KLNKLCRDEL	CRCAEENCFI
1530	1540	1550	1560	1570	1580	1590	1600
QKSDDKVTLE	ERLDKACEPG	VDYVYKTRLV	KVQLSNDFDE	YIMAIEQTIK	SGSDEVQVGQ	QRTFISPIKC	REALKLEEKK
1610	1620	1630	1640	1650	1660	1670	
HYLMWGLSSD	FWGEKPNLSY	IIGKDTWVEH	WPEEDECQDE	ENQKQCQDLG	AFTESMVVFG	CPN	

Cmpd.	No. of Cmpds.	m/z meas.	Δ m/z [ppm]	z	Rt [min]	Score	P	Range	Sequence	Modification
2396	1	701.3860	-50.24	2	59.8	21.6	0	892-904	K.SSLSVPYVIVPLK.T	

Detailed Protein Report

Protein 595: **PREDICTED: neurobeachin-like protein 1 isoform X8 [Homo sapiens]**

**Accession:**	gi|578804777	**Score:**	21.6
**Database:**	refseq_human(refseq_human_20140103.fasta)	**MW [kDa]:**	305.4
**Database Date:**	2015-11-30	**pI:**	6.0
**Modification(s):**	Carbamidomethyl	**Sequence Coverage [%]:**	0.7
		**No. of unique Peptides:**	2

Detailed Protein Report

10	20	30	40	50	60	70	80
MASRERLFEL	WMLYCTKKDP	DYLKLWLDTF	VSSYEQFLDV	DFEKLPTRVD	DMPPGISLLP	DNILQVLRIQ	LLQCVQKMAD
90	100	110	120	130	140	150	160
GLEEQQQALS	ILLVKFFIIL	CRNLSNVEEI	GTCSYINYVI	TMTTLYIQQL	KSKKKEKEMA	DQTCIEEFVI	HALAFCESLY
170	180	190	200	210	220	230	240
DPYRNWRHRI	SGRILSTVEK	SRQKYKPASL	TVEFVPFFYQ	CFQESEHLKE	SLKCCLLHLF	GAIVAGGQRN	ALQAISPATM
250	260	270	280	290	300	310	320
EVLMRVLADC	DSWEDGDPEE	VGRKAELTLK	CLTEVVHILL	SSNSDQRQVE	TSTILENYFK	LLNSDHSALP	NQRRSRQWEN
330	340	350	360	370	380	390	400
RFIALQIKML	NTITAMLDCT	DRPVLQAIFL	NSNCFEHLIR	LLQNCKEVFK	ERIGYTHMLE	VLKSLGQPPL	ELLKELMNMA
410	420	430	440	450	460	470	480
VEGDHTSVGI	LGISNVQPLL	LLIQWLPELQ	SHDLQIFISD	WLKRICCINR	QSRTTCVNAN	MGIRIIETLD	LHSSLHQTCA
490	500	510	520	530	540	550	560
ENLIAIHGSL	GSQSVSSEEI	RRLLRLLRVD	ESESVHPYVT	PVTRAILTMA	RKLSLESALQ	YFNLSHSMAG	ISVPPIQKWP
570	580	590	600	610	620	630	640
GSAFSFSAWF	CLDQDQLTLG	IANKGGKRKQ	LYSFFTGSGM	GFEAFITHSG	MLVVAVCTKR	EYATVMLPDH	SFCDSLWHNI
650	660	670	680	690	700	710	720
TVVHMPGKRP	FGQSFVYIYD	NGQQKVSAPL	RFPAMNEPFT	SCCIGSAGQR	TTTPPPSQIP	DPPFSSPITP	HRTSFGGILS
730	740	750	760	770	780	790	800
SASWGGTIEK	SKLITKLISA	GTQDSEWGCP	TSLEGQLGSV	IIFYEPLQPP	QVKALYLAGP	NCLSPWKCQE	SDMADLPGNI
810	820	830	840	850	860	870	880
LLYYTAKACK	NSICLDLSTN	CLHGRLTGNK	VVNWDIKDII	NCIGGLNVLF	PLLEQISHFS	EGQIPEEKNE	STVPESVTPV
890	900	910	920	930	940	950	960
EGDWLVWTST	KASESRLERN	LVATFILIVK	HFIQRHPINQ	GNLIHSHGVA	TLGALLQKVP	STLMDVNVLM	AVQLLIEQVS
970	980	990	1000	1010	1020	1030	1040
LEKNMQLLQQ	MYQYLLFDFR	IWNRGDFPFR	IGHIQYLSTI	IKDSRRVFRK	KYGVQFLLDT	LRIYYGNGCK	YNELSLDDIR
1050	1060	1070	1080	1090	1100	1110	1120
TIRTSLYGLI	KYFLCKGGSH	EEIQSIMGYI	AATNEEEQLF	GILDVLFSLL	RTSPTRGQLF	LLLFEPGNAD	ILYALLLNQK
1130	1140	1150	1160	1170	1180	1190	1200
YSDRLREIIF	KIMEQMLKCT	NVYERSKQHI	RLREVGYSGL	GLLLNEALVN	TSLIKNLTHQ	IINTDPVINF	KDLLSVVYIS
1210	1220	1230	1240	1250	1260	1270	1280
HRAHINVRVA	ICRKVLQILQ	FQPDAAHQIS	QQVGWQDTLV	RLFLKAKFEN	GNTLHKHSRA	VLMKDNDKNM	STEDTKKNSD
1290	1300	1310	1320	1330	1340	1350	1360
EKTDEEKITS	FASANVSSDQ	WSLEDRHSLD	SNTPLFPEDS	SVGELSFKSE	NQEEFWHSNP	SHLSLDLSGI	DSCEMSDSGS
1370	1380	1390	1400	1410	1420	1430	1440
QVPDSLPSTP	SPVESTKSFS	VHSDRESSIT	NDMGFSDDFS	LLESQERCEE	ELLQLLTHIL	NYVMCKGLEK	SDDDTWIERG
1450	1460	1470	1480	1490	1500	1510	1520
QVFSALSKPG	ISSELLRPSD	EIKLTLLQKM	LEWAISENRE	AKTNPVTAEN	AFRLVLIIQD	FLQSEGLVNS	NMWTEKLLED
1530	1540	1550	1560	1570	1580	1590	1600
MMLLFDCLSV	CYSESPVWVK	LSQIQIQLLL	GFIGRGNLQV	CAMASAKLNT	LLQTKVIENQ	DEACYILGKL	EHVLSQSIKE
1610	1620	1630	1640	1650	1660	1670	1680
QTEIYSFLIP	LVRTLVSKIY	ELLFMNLHLP	SLPFTNGSSS	FFEDFQEYCN	SNEWQVYIEK	YIVPYMKQYE	AHTFYDGHEN
1690	1700	1710	1720	1730	1740	1750	1760
MALYWKDCYE	ALMVNMHKRD	REGGESKLKF	QELFVEPFNR	KARQENLRYN	NMLKQLSSQQ	LATLRRWKAI	QLYLTCERGP
1770	1780	1790	1800	1810	1820	1830	1840
WAKRKQNPIH	WKLANVENYS	RMRLKLVPNY	NFKTHEEASA	LRDNLGIQHS	QPSSDTLLLE	VVKQVKVSDM	VEDKLDLPEE
1850	1860	1870	1880	1890	1900	1910	1920
DITARVNVDE	KEEQDQKEKL	VLMEDCELIT	IIDVIPGRLE	ITTQHIYFYD	GSIEKEDGVG	FDFKWPHSQI	REIHLRRYNL
1930	1940	1950	1960	1970	1980	1990	2000
RRSALEIFHV	DQSNYFLNFK	KEVRNKIYSR	LLSLHSPNSY	YGSRSPQELF	KASGLTQKWV	NREISNFDYL	IQINTMAGRT
2010	2020	2030	2040	2050	2060	2070	2080
YNDLAQYPVF	PWILQDYTSE	ELDLNNPAVF	RDLSKPIGVV	NEKNAKAMRE	KYENFEDPMG	TIDKFHYGTH	YSNSAGVMHY
2090	2100	2110	2120	2130	2140	2150	2160
LIRVEPFTTL	HIQLQSGRFD	CADRQFHSIP	ATWQALMDNP	YDVKELIPEF	FYFPEFLENQ	NQFNLGRLQI	SKELVNDVIL
2170	2180	2190	2200	2210	2220	2230	2240

Detailed Protein Report

Cmpd.	No. of Cmpds.	m/z meas.	Δ m/z [ppm]	z	Rt [min]	Score	P	Range	Sequence	Modification
2277	1	816.2926	-155.86	2	58.7	11.5	1	7-18	R.LFELWMLYCTKK.D	Carbamidomethyl: 9
2089	1	925.3261	-72.47	1	56.3	10.1	0	1269-1276	K.NMSTEDTK.K	

Detailed Protein Report

Protein 596: nuclear RNA export factor 5 [Homo sapiens]

**Accession:**	gi|254039592	**Score:**	21.5
**Database:**	refseq_human(refseq_human_20140103.fasta)	**MW [kDa]:**	42.1
**Database Date:**	2015-11-30	**pI:**	9.9
**Modification(s):**	Carbamidomethyl, Oxidation	**Sequence Coverage [%]:**	7.4
		**No. of unique Peptides:**	1

10	20	30	40	50	60	70	80
MRRNTQDENM	RKWFKVTIPY	GIKYDKAWLM	NSIQSNCSVP	FTPVDFHYIR	NRACFFVQVA	SAASALKDVS	YKIYDDENQK
**90**	**100**	**110**	**120**	**130**	**140**	**150**	**160**
ICIFVSHFTA	PYSVKNKLKP	GQMEMLKLTM	NKRYNVSQQA	LDLQNLRFDP	DLMGRDIDII	LNRRNCMAAT	LKITERNFPE
**170**	**180**	**190**	**200**	**210**	**220**	**230**	**240**
LLSLNLCNNK	LYQLDGLSDI	TEKAPKVKTL	NLSKNKLESA	WELGKVKGLK	LEELWLEGNP	LCSTFSDQSA	YVSAIRDCFP
**250**	**260**	**270**	**280**	**290**	**300**	**310**	**320**
KLLRLDGREL	SAPVIVDIDS	SETMKPCKEN	FTGSETLKHL	VLQFLQQSNL	CKYFKDSRNI	KILKDPYLQR	KLLKHTKCPR
**330**	**340**	**350**	**360**	**370**			
NVDSLSALPE	TQHDFTSILV	DMWYQTVNTC	FLPRAGPESQ	SLRPL			

Cmpd.	No. of Cmpds.	m/z meas.	Δ m/z [ppm]	z	Rt [min]	Score	P	Range	Sequence	Modification
2208	1	1061.5011	-52.58	3	57.8	21.5	2	81-107	K.ICIFVSHFTAPYSVKNKLKPGQMEMLK.L	Carbamidomethyl: 2; Oxidation: 23

Detailed Protein Report

Protein 597: forkhead box protein D3 [Homo sapiens]

Accession: gi|6912372
Database: refseq_human(refseq_human_20140103.fasta)
Database Date: 2015-11-30
Modification(s): Carbamidomethyl

Score: 21.5
MW [kDa]: 47.6
pI: 6.0
Sequence Coverage [%]: 7.5
No. of unique Peptides: 1

10	20	30	40	50	60	70	80
MTLSGGGSAS	DMSGQTVLTA	EDVDIDVVGE	GDDGLEEKDS	DAGCDSPAGP	PELRLDEADE	VPPAAPHHGQ	PQPPHQQPLT
**90**	**100**	**110**	**120**	**130**	**140**	**150**	**160**
LPKEAAGAGA	GPGGDVGAPE	ADGCKGGVGG	EEGGASGGGP	GAGSGSAGGL	APSKPKNSLV	KPPYSYIALI	TMAILQSPQK
**170**	**180**	**190**	**200**	**210**	**220**	**230**	**240**
KLTLSGICEF	ISNRFPYYRE	KFPAWQNSIR	HNLSLNDCFV	KIPREPGNPG	KGNYWTLDPQ	SEDMFDNGSF	LRRRKRFKRH
**250**	**260**	**270**	**280**	**290**	**300**	**310**	**320**
QQEHLREQTA	LMMQSFGAYS	LAAAAGAAGP	YGRPYGLHPA	AAAGAYSHPA	AAAAAAAAAA	LQYPYALPPV	APVLPPAVPL
**330**	**340**	**350**	**360**	**370**	**380**	**390**	**400**
LPSGELGRKA	AAFGSQLGPG	LQLQLNSLGA	AAAAAGTAGA	AGTTASLIKS	EPSARPSFSI	ENIIGGGPAA	PGGSAVGAGV
**410**	**420**	**430**	**440**	**450**	**460**	**470**	**480**
AGGTGGSGGG	STAQSFLRPP	GTVQSAALMA	THQPLSLSRT	TATIAPILSV	PLSGQFLQPA	ASAAAAAAAA	AQAKWPAQ

Cmpd.	No. of Cmpds.	m/z meas.	Δ m/z [ppm]	z	Rt [min]	Score	P	Range	Sequence	Modification
1915	1	856.8032	-169.79	2	54.0	10.8	1	191-204	R.HNLSLNDCFVKIPR.E	Carbamidomethyl: 8

Detailed Protein Report

Protein 598: **MAM and LDL-receptor class A domain-containing protein C10orf112 precursor [Homo sapiens]**

**Accession:**	gi|565671710	**Score:**	21.4
**Database:**	refseq_human(refseq_human_20140103.fasta)	**MW [kDa]:**	240.9
**Database Date:**	2015-11-30	**pI:**	4.9
		**Sequence Coverage [%]:**	1.4
		**No. of unique Peptides:**	1

Detailed Protein Report

10	20	30	40	50	60	70	80
MLFFLDRMLA	FPMNETFCCL	WIACVFNSTL	AQQGTESFQC	DNGVSLPPDS	ICDFTDQCGD	SSDERHCLNY	ERCDFEDGLC
90	100	110	120	130	140	150	160
HMTQDQSLQP	SWTKRSGMIG	LSPPFYDHNG	DVSAHFLSLV	SRVDSISSSL	RSRVFLPTND	QHDCQITFYY	FSCQVSGKLM
170	180	190	200	210	220	230	240
VGLQTACGGP	IQHLWQNTAA	LPNQWERNVI	KIQSSQRFQV	VFEGQMASTY	EQDEVIAIDD	ISFSSGCLPA	NDGILLCQEA
250	260	270	280	290	300	310	320
LNAERELCHP	DTDLCRFDAT	DEELRLCQAC	GFEFDMCEWT	SEASAGQISW	MRTKAREIPA	FESTPQQDQG	GDDEGYYVWV
330	340	350	360	370	380	390	400
GAKHGFTLNH	LDSRAYLNSS	VCHCLGKSCH	LQFYYAMESS	VLRVRLYNNK	EEEIFWTYNI	STHSQWVKAD	VLIPEDLKTF
410	420	430	440	450	460	470	480
KIIFEGTLLS	QRSFIALDHL	WVYACGQTQS	RKLCSADEFP	CTSGQCIAKE	SVCDSRQDCS	DESDEDPATC	SKHLTCDFES
490	500	510	520	530	540	550	560
GFCGWEPFLT	EDSHWKLMKG	LNNGEHHFPA	ADHTANINHG	SFIYLEAQRS	PGVAKLGSPV	LTKLLTASTP	CQVQFWYHLS
570	580	590	600	610	620	630	640
QHSNLSVFTR	TSLDGNLQKQ	GKIIRFSESQ	WSHAKIDLIA	EAGESTLPFQ	LILEATVLSS	NATVALDDIS	VSQECEISYK
650	660	670	680	690	700	710	720
SLPRTSTQSK	FSKCDFEANS	CDWFEAISGD	HFDWIRSSQS	ELSADFEHQA	PPRDHSLNAS	QGHFMFILKK	SSSLWQVAKL
730	740	750	760	770	780	790	800
QSPTFSQTGP	GCILSFWFYN	YGLSVGAAEL	QLHMENSHDS	TVIWRVLYNQ	GKQWLEATIQ	LGRLSQPFHL	SLDKVSLGIY
810	820	830	840	850	860	870	880
DGVSAIDDIR	FENCTLPLPA	ESCEGLDHFW	CRHTRACIEK	LRLCDLVDDC	GDRTDEVNCA	PELQCNFETG	ICNWEQDAKD
890	900	910	920	930	940	950	960
DFDWTRSQGP	TPTLNTGPMK	DNTLGTAKGH	YLYIESSEPQ	AFQDSAALLS	PILNATDTKG	CTFRFYYHMF	GKRIYRLAIY
970	980	990	1000	1010	1020	1030	1040
QRIWSDSRGQ	LLWQIFGNQG	NRWIRKHLNI	SSRQPFQILV	EASVGDGFTG	DIAIDDLSFM	DCTLYPGNLP	ADLPTPPETS
1050	1060	1070	1080	1090	1100	1110	1120
VPVTLPPHNC	TDNEFICRSD	GHCIEKMQKC	DFKYDCPDKS	DEASCVMEVC	SFEKRSLCKW	YQPIPVHLLQ	DSNTFRWGLG
1130	1140	1150	1160	1170	1180	1190	1200
NGISIHHGEE	NHRPSVDHTQ	NTTDGWYLYA	DSSNGKFGDT	ADILTPIISL	TGPKCTLVFW	THMNGATVGS	LQVLIKKDNV
1210	1220	1230	1240	1250	1260	1270	1280
TSKLWAQTGQ	QGAQWKRAEV	FLGIRSHTQI	VFRAKRGISY	IGDVAVDDIS	FQDCSPLLSP	ERKCTDHEFM	CANKHCIAKD
1290	1300	1310	1320	1330	1340	1350	1360
KLCDFVNDCA	DNSDETTFIC	RTSSGRCDFE	FDLCSWKQEK	DEDFDWNLKA	SSIPAAGTEP	AADHTLGNSS	GHYIFIKSLF
1370	1380	1390	1400	1410	1420	1430	1440
PQQPMRAARI	SSPVISKRSK	NCKIIFHYHM	YGNGIGALTL	MQVSVTNQTK	VLLNLTVEQG	NFWRREELSL	FGDEDFQLKF
1450	1460	1470	1480	1490	1500	1510	1520
EGRVGKGQRG	DIALDDIVLT	ENCLSLHDSV	QEELAVPLPT	GFCPLGYREC	HNGKCYRLEQ	SCNFVDNCGD	NTDENECGSS
1530	1540	1550	1560	1570	1580	1590	1600
CTFEKGWCGW	QNSQADNFDW	VLGVGSHQSL	RPPKDHTLGN	ENGHFMYLEA	TAVGLRGDKA	HFRSTMWRES	SAACTMSFWY
1610	1620	1630	1640	1650	1660	1670	1680
FVSAKATGSI	QILIKTEKGL	SKVWQESKQN	PGNHWQKADI	LLGKLRNFEV	IFQGIRTRDL	GGGAAIDDIE	FKNCTTVGEI
1690	1700	1710	1720	1730	1740	1750	1760
SELCPEITDF	LCRDKKCIAS	HLLCDYKPDC	SDRSDEAHCA	HYTSTTGSCN	FETSSGNWTT	ACSLTQDSED	DLDWAIGSRI
1770	1780	1790	1800	1810	1820	1830	1840
PAKALIPDSD	HTPGSGQHFL	YVNSSGSKEG	SVARITTSKS	FPASLGMCTV	RFWFYMIDPR	SMGILKVYTI	EESGLNILVW
1850	1860	1870	1880	1890	1900	1910	1920
SVIGNKRTGW	TYGSVPLSSN	SPFKVAFEAD	LDGNEDIFIA	LDDISFTPEC	VTGGPVPVQP	SPCEADQFSC	IYTLQCVPLS
1930	1940	1950	1960	1970	1980	1990	2000
GKCDGHEDCI	DGSDEMDCPL	SPTPPLCSNM	EFPCSTDECI	PSLLLCDGVP	DCHFNEDELI	CSNKSCSNGA	LVCASSNSCI
2010	2020	2030	2040	2050	2060	2070	2080
PAHQRCDGFA	DCMDFQLDES	SCSECPLNYC	RNGGTCVVEK	NGPMCRCRQG	WKGNRCHIKF	NPPATDFTYA	QNNTWTLLGI
2090	2100	2110	2120	2130	2140	2150	2160
GLAFLMTHIT	VAVLCFLANR	KVPIRKTEGS	GNCAFVNPVY	GNWSNPEKTE	SSVYSFSNPL	YGTTSGSLET	LSHHLK

Cmpd.	No. of Cmpds.	m/z meas.	Δ m/z [ppm]	z	Rt [min]	Score	P	Range	Sequence	Modification
2517	1	913.4615	10.45	2	61.9	10.8	1	1425-1439	R.REELSLFGDEDFQLK.F	

Detailed Protein Report

Protein 599: astrotactin-1 isoform 2 precursor [Homo sapiens]

Accession: gi|46488921
Database: refseq_human(refseq_human_20140103.fasta)
Database Date: 2015-11-30

Score: 21.4
MW [kDa]: 135.0
pI: 4.9
Sequence Coverage [%]: 3.2
No. of unique Peptides: 1

10	20	30	40	50	60	70	80
MALAGLCALL	ACCWGPAAVL	ATAAGDVDPS	KELECKLKSI	TVSALPFLRE	NDLSIMHSPS	ASEPKLLFSV	RNDFPGEMVV
90	100	110	120	130	140	150	160
VDDLENTELP	YFVLEISGNT	EDIPLVRWRQ	QWLENGTLLF	HIHHQDGAPS	LPGQDPTEEP	QHESAEEELR	ILHISVMGGM
170	180	190	200	210	220	230	240
IALLLSILCL	VMILYTRRRW	CKRRRVPQPQ	KSASAEAANE	IHYIPSVLIG	GHGRESLRNA	RVQGHNSSGT	LSIRETPILD
250	260	270	280	290	300	310	320
GYEYDITDLR	HHLQRECMNG	GEDFASQVTR	TLDSLQGCNE	KSGMDLTPGS	DNAKLSLMNK	YKDNIIATSP	VDSNHQQATL
330	340	350	360	370	380	390	400
LSHTSSSQRK	RINNKARAGS	AFLNPEGDSG	TEAENDPQLT	FYTDPSRSRR	RSRVGSPRSP	VNKTTLTLIS	ITSCVIGLVC
410	420	430	440	450	460	470	480
SSHVNCPLVV	KITLHVPEHL	IADGSRFILL	EGSQLDASDW	LNPAQVVLFS	QQNSSGPWAM	DLCARRLLDP	CEHQCDPETG
490	500	510	520	530	540	550	560
ECLCYEGYMK	DPVHKHLCIR	NEWGTNQGPW	PYTIFQRGFD	LVLGEQPSDK	IFRFTYTLGE	GMWLPLSKSF	VIPPAELAIN
570	580	590	600	610	620	630	640
PSAKCKTDMT	VMEDAVEVRE	ELMTSSSFDS	LEVLLDSFGP	VRDCSKDNGG	CSKNFRCISD	RKLDSTGCVC	PSGLSPMKDS
650	660	670	680	690	700	710	720
SGCYDRHIGV	DCSDGFNGGC	EQLCLQQMAP	FPDDPTLYNI	LMFCGCIEDY	KLGVDGRSCQ	LITETCPEGS	DCGESRELPM
730	740	750	760	770	780	790	800
NQTLFGEMFF	GYNNHSKEVA	AGQVLKGTFR	QNNFARGLDQ	QLPDGLVVAT	VPLENQCLEE	ISEPTPDPDF	LTGMVNFSEV
810	820	830	840	850	860	870	880
SGYPVLQHWK	VRSVMYHIKL	NQVAISQALS	NALHSLDGAT	SRADFVALLD	QFGNHYIQEA	IYGFEESCSI	WYPNKQVQRR
890	900	910	920	930	940	950	960
LWLEYEDISK	GNSPSDESEE	RERDPKVLTF	PEYITSLSDS	GTKHMAAGVR	MECHSKGRCP	SSCPLCHVTS	SPDTPAEPVL
970	980	990	1000	1010	1020	1030	1040
LEVTKAAPIY	ELVTNNQTQR	LLQEATMSSL	WCSGTGDVIE	DWCRCDSTAF	GADGLPTCAP	LPQPVLRLST	VHEPSSTLVV
1050	1060	1070	1080	1090	1100	1110	1120
LEWEHSEPPI	GVQIVDYLLR	QEKVTDRMDH	SKVETETVLS	FVDDIISGAK	SPCAMPSQVP	DKQLTTISLI	IRCLEPDTIY
1130	1140	1150	1160	1170	1180	1190	1200
MFTLWGVDNT	GRRSRPSDVI	VKTPCPVVDD	VKAQEIADKI	YNLFNGYTSG	KEQQTAYNTL	LDLGSPTLHR	VLYHYNQHYE
1210	1220						
SFGEFTWRCE	DELGPR						

Cmpd.	No. of Cmpds.	m/z meas.	Δ m/z [ppm]	z	Rt [min]	Score	P	Range	Sequence	Modification
2260	1	1061.4977	-63.82	3	58.5	11.2	2	185-214	R.RVPQPQKSASAEAANEIHYIPSVLIGGHGR.E	

Detailed Protein Report

Protein 600: PREDICTED: CAS1 domain-containing protein 1 isoform X2 [Homo sapiens]

Accession: gi|578814529
Database: refseq_human(refseq_human_20140103.fasta)
Database Date: 2015-11-30
Modification(s): Carbamidomethyl

Score: 21.4
MW [kDa]: 72.0
pI: 9.6
Sequence Coverage [%]: 2.2
No. of unique Peptides: 2

Quantitation

MD:MU **Median:** 1.76 **CV:** 0.00 % **No. of Peptides:** 1

10	20	30	40	50	60	70	80
MGRCVLFSRN	CSSIAPTWAQ	VAASKLGDSP	KARVRARRVP	LVLSPHTPMA	LPQPPPPAGA	RDPVTPEHWA	SGPPSQAQPL
**90**	**100**	**110**	**120**	**130**	**140**	**150**	**160**
LRQEAKEEEE	GEETGVQGAW	GTGTAEQRRR	GWGEAAESAA	AEEGQAEVGG	AAAAGSGSPA	GGAGGGLGSW	RPLLAWLQRR
**170**	**180**	**190**	**200**	**210**	**220**	**230**	**240**
QPQCCPCAAP	LSRSAAHCCH	GGTKMAALAY	NLGKREINHY	FSVRSAKVLA	LVAVLLLAAC	HLASRRYRGN	DSCEYLLSSG
**250**	**260**	**270**	**280**	**290**	**300**	**310**	**320**
RFLGEKVWQP	HSCMMHKYKI	SEAKNCLVDK	HIAFIGDSRI	RQLFYSFVKI	INPQFKEEGN	KHENIPFEDK	TASVKVDFLW
**330**	**340**	**350**	**360**	**370**	**380**	**390**	**400**
HPEVNGSMKQ	CIKVWTEDSI	AKPHVIVAGA	ATWSIKIHNG	SSEALSQYKM	NITSIAPLLE	KLAKTSDVYW	VLQDPVYEDL
**410**	**420**	**430**	**440**	**450**	**460**	**470**	**480**
LSENRKMITN	EKIDAYNEAA	VSILNSSTRN	SKSNVKMFSV	SKLIAQETIM	ESLDGLHLPE	SSRETTAMIL	MNVYCNKILK
**490**	**500**	**510**	**520**	**530**	**540**	**550**	**560**
PVDGSCCQPR	PPVTLIQKLA	ACFFTLSIIG	YLIFYIIHRN	AHRKNKPCTD	LESGEEKKNI	INTPVSSLEI	LLQSFCKLGL
**570**	**580**	**590**	**600**	**610**	**620**	**630**	**640**
IMAYFYMCDR	ANLFMKENKF	YTHSSFFIPI	IYILVLGVFY	NENTKETKVL	NREQTDEWKG	WMQLVILIYH	ISGASTIVGA
**650**	**660**						
IGTQEGEMTF	I						

Cmpd.	No. of Cmpds.	m/z meas.	Δ m/z [ppm]	z	Rt [min]	Score	P	Range	Sequence	Modification	Ratios
161	1	793.8859	18.63	2	31.7	10.1	1	160-173	R.RQPQCCPCAAPLSR.S	Carbamidomethyl: 8	
149	1	793.8480	-29.08	2	31.6	11.2	1	160-173	R.RQPQCCPCAAPLSR.S	Carbamidomethyl: 6	MD:MU 1.76

Detailed Protein Report

Protein 601: BRCA1-associated protein [Homo sapiens]

**Accession:**	gi|188497705	**Score:**	21.3
**Database:**	refseq_human(refseq_human_20140103.fasta)	**MW [kDa]:**	67.3
**Database Date:**	2015-11-30	**pI:**	5.6
**Modification(s):**	Carbamidomethyl	**Sequence Coverage [%]:**	2.5
		**No. of unique Peptides:**	1

Quantitation

MD:MU **Median:** 0.20 **CV:** 0.00 % **No. of Peptides:** 1

10	20	30	40	50	60	70	80
MSVSLVVIRL	ELAEHSPVPA	GFGFSAAAGE	MSDEEIKKTT	LASAVACLEG	KSPGEKVAII	HQHLGRREMT	DVIIETMKSN
**90**	**100**	**110**	**120**	**130**	**140**	**150**	**160**
PDELKTTVEE	RKSSEASPTA	QRSKDHSKEC	INAAPDSPSK	QLPDQISFFS	GNPSVEIVHG	IMHLYKTNKM	TSLKEDVRRS
**170**	**180**	**190**	**200**	**210**	**220**	**230**	**240**
AMLCILTVPA	AMTSHDLMKF	VAPFNEVIEQ	MKIIRDSTPN	QYMVLIKFRA	QADADSFYMT	CNGRQFNSIE	DDVCQLVYVE
**250**	**260**	**270**	**280**	**290**	**300**	**310**	**320**
RAEVLKSEDG	ASLPVMDLTE	LPKCTVCLER	MDESVNGILT	TLCNHSFHSQ	CLQRWDDTTC	PVCRYCQTPE	PVEENKCFEC
**330**	**340**	**350**	**360**	**370**	**380**	**390**	**400**
GVQENLWICL	ICGHIGCGRY	VSRHAYKHFE	ETQHTYAMQL	TNHRVWDYAG	DNYVHRLVAS	KTDGKIVQYE	CEGDTCQEEK
**410**	**420**	**430**	**440**	**450**	**460**	**470**	**480**
IDALQLEYSY	LLTSQLESQR	IYWENKIVRI	EKDTAEEINN	MKTKFKETIE	KCDNLEHKLN	DLLKEKQSVE	RKCTQLNTKV
**490**	**500**	**510**	**520**	**530**	**540**	**550**	**560**
AKLTNELKEE	QEMNKCLRAN	QVLLQNKLKE	EERVLKETCD	QKDLQITEIQ	EQLRDVMFYL	ETQQKINHLP	AETRQEIQEG
**570**	**580**	**590**	**600**				
QINIAMASAS	SPASSGGSGK	LPSRKGRSKR	GK				

Cmpd.	No. of Cmpds.	m/z meas.	Δ m/z [ppm]	z	Rt [min]	Score	P	Range	Sequence	Modification	Ratios
257	1	569.4703	-171.75	3	32.6	21.3	0	210-224	R.AQADADSFYMTCNGR.Q	Carbamidomethyl: 12	MD:MU 0.20

Detailed Protein Report

Protein 602: spermatogenesis-associated serine-rich protein 2 [Homo sapiens]

**Accession:**	gi|148746220	**Score:**	21.3
**Database:**	refseq_human(refseq_human_20140103.fasta)	**MW [kDa]:**	59.5
**Database Date:**	2015-11-30	**pI:**	9.7
		**Sequence Coverage [%]:**	4.0
		**No. of unique Peptides:**	1

10	20	30	40	50	60	70	80
MSRKQNQKDS	SGFIFDLQSN	TVLAQGGAFE	NMKEKINAVR	AIVPNKSNNE	IILVLQHFDN	CVDKTVQAFM	EGSASEVLKE
**90**	**100**	**110**	**120**	**130**	**140**	**150**	**160**
WTVTGKKKNK	KKKNKPKPAA	EPSNGIPDSS	KSVSIQEEQS	APSSEKGGMN	GYHVNGAIND	TESVDSLSEG	LETLSIDARE
**170**	**180**	**190**	**200**	**210**	**220**	**230**	**240**
LEDPESAMLD	TLDRTGSMLQ	NGVSDFETKS	LTMHSIHNSQ	QPRNAAKSLS	RPTTETQFSN	MGMEDVPLAT	SKKLSSNIEK
**250**	**260**	**270**	**280**	**290**	**300**	**310**	**320**
SVKDLQRCTV	SLARYRVVVK	EEMDASIKKM	KQAFAELESC	LMDREVALLA	EMDKVKAEAM	EILLSRQKKA	ELLKKMTHVA
**330**	**340**	**350**	**360**	**370**	**380**	**390**	**400**
VQMSEQQLVE	LRADIKHFVS	ERKYDEDLGR	VARFTCDVET	LKKSIDSFGQ	VSHPKNSYST	RSRCSSVTSV	SLSSPSDASA
**410**	**420**	**430**	**440**	**450**	**460**	**470**	**480**
ASSSTCASPP	SLTSANKKNF	APGETPAAIA	NSSGQPYQPL	REVLPGNRRG	GQGYRPQGQK	SNDPMNQGRH	DSMGRYRNSS
**490**	**500**	**510**	**520**	**530**	**540**	**550**	
WYSSGSRYQS	APSQAPGNTI	ERGQTHSAGT	NGTGVSMEPS	PPTPSFKKGL	PQRKPRTSQT	EAVNS	

Cmpd.	No. of Cmpds.	m/z meas.	Δ m/z [ppm]	z	Rt [min]	Score	P	Range	Sequence	Modification
1227	1	565.6011	-257.29	2	45.0	10.2	0	478-487	R.NSSWYSSGSR.Y	

Detailed Protein Report

Protein 603: disintegrin and metalloproteinase domain-containing protein 20 preproprotein [Homo sapiens]

**Accession:**	gi|50845382	**Score:**	21.3
**Database:**	refseq_human(refseq_human_20140103.fasta)	**MW [kDa]:**	87.0
**Database Date:**	2015-11-30	**pI:**	6.0
**Modification(s):**	Carbamidomethyl	**Sequence Coverage [%]:**	2.8
		**No. of unique Peptides:**	1

Alias proteins:

Accession	Name	Description
gi|530404802	refseq_human (refseq_human_20140103.fasta)	PREDICTED: disintegrin and metalloproteinase domain-containing protein 20 isoform X2 [Homo sapiens]
gi|530404800	refseq_human (refseq_human_20140103.fasta)	PREDICTED: disintegrin and metalloproteinase domain-containing protein 20 isoform X1 [Homo sapiens]

10	20	30	40	50	60	70	80
MVQLHQDTDP	QIPKGQPCTL	NSSEGGARPA	VPHTLFSSAL	DRWLHNDSFI	MAVGEPLVHI	RVTLLLLWFG	MFLSISGHSQ
**90**	**100**	**110**	**120**	**130**	**140**	**150**	**160**
ARPSQYFTSP	EVVIPLKVIS	RGRGAKAPGW	LSYSLRFGGQ	RYIVHMRVNK	LLFAAHLPVF	TYTEQHALLQ	DQPFIQDDCY
**170**	**180**	**190**	**200**	**210**	**220**	**230**	**240**
YHGYVEGVPE	SLVALSTCSG	GFLGMLQIND	LVYEIKPISV	SATFEHLVYK	IDSDDTQFPP	MRCGLTEEKI	AHQMELQLSY
**250**	**260**	**270**	**280**	**290**	**300**	**310**	**320**
NFTLKQSSFV	GWWTHQRFVE	LVVVVDNIRY	LFSQSNATTV	QHEVFNVVNI	VDSFYHPLEV	DVILTGIDIW	TASNPLPTSG
**330**	**340**	**350**	**360**	**370**	**380**	**390**	**400**
DLDNVLEDFS	IWKNYNLNNR	LQHDVAHLFI	KDTQGMKLGV	AYVKGICQNP	FNTGVDVFED	NRLVVFAITL	GHELGHNLGM
**410**	**420**	**430**	**440**	**450**	**460**	**470**	**480**
QHDTQWCVCE	LQWCIMHAYR	KVTTKFSNCS	YAQYWDSTIS	SGLCIQPPPY	PGNIFRLKYC	GNLVVEEGEE	CDCGTIRQCA
**490**	**500**	**510**	**520**	**530**	**540**	**550**	**560**
KDPCCLLNCT	LHPGAACAFG	ICCKDCKFLP	SGTLCRQQVG	ECDLPEWCNG	TSHQCPDDVY	VQDGISCNVN	AFCYEKTCNN
**570**	**580**	**590**	**600**	**610**	**620**	**630**	**640**
HDIQCKEIFG	QDARSASQSC	YQEINTQGNR	FGHCGIVGTT	YVKCWTPDIM	CGRVQCENVG	VIPNLIEHST	VQQFHLNDTT
**650**	**660**	**670**	**680**	**690**	**700**	**710**	**720**
CWGTDYHLGM	AIPDIGEVKD	GTVCGPEKIC	IRKKCASMVH	LSQACQPKTC	NMRGICNNKQ	HCHCNHEWAP	PYCKDKGYGG
**730**	**740**	**750**	**760**	**770**	**780**		
SADSGPPPKN	NMEGLNVMGK	LRYLSLLCLL	PLVAFLLFCL	HVLFKKRTKS	KEDEEG		

Cmpd.	No. of Cmpds.	m/z meas.	Δ m/z [ppm]	z	Rt [min]	Score	P	Range	Sequence	Modification
1299	1	727.3560	8.30	2	45.9	10.7	1	505-516	K.DCKFLPSGTLCR.Q	Carbamidomethyl: 2, 11

Detailed Protein Report

Protein 604: PREDICTED: probable cation-transporting ATPase 13A3 isoform X4 [Homo sapiens]

**Accession:**	gi|530375932	**Score:**	21.2
**Database:**	refseq_human(refseq_human_20140103.fasta)	**MW [kDa]:**	123.9
**Database Date:**	2015-11-30	**pI:**	6.1
		**Sequence Coverage [%]:**	3.0
		**No. of unique Peptides:**	1

10	20	30	40	50	60	70	80
MDREERKTIN	QGQEDEMEIY	GYNLSRWKLA	IVSLGVICSG	GFLLLLLYWM	PEWRVKATCV	RAAIKDCEVV	LLRTTDEFKM
**90**	**100**	**110**	**120**	**130**	**140**	**150**	**160**
WFCAKIRVLS	LETYPVSSPK	SMSNKLSNGH	AVCLIENPTE	ENRHRISKYS	QTESQQIRYF	THHSVKYFWN	DTIHNFDFLK
**170**	**180**	**190**	**200**	**210**	**220**	**230**	**240**
GLDEGVSCTS	IYEKHSAGLT	KGMHAYRKLL	YGVNEIAVKV	PSVFKLLIKE	VLNPFYIFQL	FSVILWSTDE	YYYYALAIVV
**250**	**260**	**270**	**280**	**290**	**300**	**310**	**320**
MSIVSIVSSL	YSIRKQYVML	HDMVATHSTV	RVSVCRVNEE	IEEIFSTDLV	PGDVMVIPLN	GTIMPCDAVL	INGTCIVNES
**330**	**340**	**350**	**360**	**370**	**380**	**390**	**400**
MLTGESVPVT	KTNLPNPSVD	VKGIGDELYN	PETHKRHTLF	CGTTVIQTRF	YTGELVKAIV	VRTGFSTSKG	QLVRSILYPK
**410**	**420**	**430**	**440**	**450**	**460**	**470**	**480**
PTDFKLYRDA	YLFLLCLVAV	AGIGFIYTII	NSILNEVQVG	VIIIESLDII	TITVPPALPA	AMTAGIVYAQ	RRLKKIGIFC
**490**	**500**	**510**	**520**	**530**	**540**	**550**	**560**
ISPQRINICG	QLNLVCFDKT	GTLTEDGLDL	WGIQRVENAR	FLSPEENVCN	EMLVKSQFVA	CMATCHSLTK	IEGVLSGDPL
**570**	**580**	**590**	**600**	**610**	**620**	**630**	**640**
DLKMFEAIGW	ILEEATEEET	ALHNRIMPTV	VRPPKQLLPE	STPAGNQEME	LFELPATYEI	GIVRQFPFSS	ALQRMSVVAR
**650**	**660**	**670**	**680**	**690**	**700**	**710**	**720**
VLGDRKMDAY	MKGAPEAIAG	LCKPETVPVD	FQNVLEDFTK	QGFRVIALAH	RKLESKLTWH	KVQNISRDAI	ENNMDFMGLI
**730**	**740**	**750**	**760**	**770**	**780**	**790**	**800**
IMQNKLKQET	PAVLEDLHKA	NIRTVMVTGD	SMLTAVSVAR	DCGMILPQDK	VIIAEALPPK	DGKVAKINWH	YADSLTQCSH
**810**	**820**	**830**	**840**	**850**	**860**	**870**	**880**
PSAIDPEAIP	VKLVHDSLED	LQMTRYHFAM	NGKSFSVILE	HFQDLVPKLM	LHGTVFARMA	PDQKTQLIEA	LQNVDYFVGM
**890**	**900**	**910**	**920**	**930**	**940**	**950**	**960**
CGDGANDCGA	LKRAHGGISL	SELEASVASP	FTSKTPSISC	VPNLIREGRA	ALITSFCVFK	FMALYSIIQY	FSVTLLYSIL
**970**	**980**	**990**	**1000**	**1010**	**1020**	**1030**	**1040**
SNLGDFQFLF	IDLAIILVVV	FTMSLNPAWK	ELVAQRPPSG	LISGALLFSV	LSQIIICIGF	QSLGFFWVKQ	QPWYEVWHPK
**1050**	**1060**	**1070**	**1080**	**1090**	**1100**	**1110**	
SDACNTTGSG	FWNSSHVDNE	TELDEHNIQN	YENTTVFFIS	SFQYLIVAIA	FSKGKPFRQP	CYKN	

Cmpd.	No. of Cmpds.	m/z meas.	Δ m/z [ppm]	z	Rt [min]	Score	P	Range	Sequence	Modification
2025	1	1044.3877	-140.05	2	55.2	10.4	0	894-914	R.AHGGISLSELEASVASPFTSK.T	

Detailed Protein Report

Protein 605: membrane-bound transcription factor site-1 protease preproprotein [Homo sapiens]

**Accession:**	gi|4506775	**Score:**	21.1
**Database:**	refseq_human(refseq_human_20140103.fasta)	**MW [kDa]:**	117.7
**Database Date:**	2015-11-30	**pI:**	9.5
**Modification(s):**	Oxidation	**Sequence Coverage [%]:**	1.7
		**No. of unique Peptides:**	1

10	20	30	40	50	60	70	80
MKLVNIWLLL	LVVLLCGKKH	LGDRLEKKSF	EKAPCPGCSH	LTLKVEFSST	VVEYEYIVAF	NGYFTAKARN	SFISSALKSS
**90**	**100**	**110**	**120**	**130**	**140**	**150**	**160**
EVDNWRIIPR	NNPSSDYPSD	FEVIQIKEKQ	KAGLLTLEDH	PNIKRVTPQR	KVFRSLKYAE	SDPTVPCNET	RWSQKWQSSR
**170**	**180**	**190**	**200**	**210**	**220**	**230**	**240**
PLRRASLSLG	SGFWHATGRH	SSRRLLRAIP	RQVAQTLQAD	VLWQMGYTGA	NVRVAVFDTG	LSEKHPHFKN	VKERTNWTNE
**250**	**260**	**270**	**280**	**290**	**300**	**310**	**320**
RTLDDGLGHG	TFVAGVIASM	RECQGFAPDA	ELHIFRVFTN	NQVSYTSWFL	DAFNYAILKK	IDVLNLSIGG	PDFMDHPFVD
**330**	**340**	**350**	**360**	**370**	**380**	**390**	**400**
KVWELTANNV	IMVSAIGNDG	PLYGTLNNPA	DQMDVIGVGG	IDFEDNIARF	SSRGMTTWEL	PGGYGRMKPD	IVTYGAGVRG
**410**	**420**	**430**	**440**	**450**	**460**	**470**	**480**
SGVKGGCRAL	SGTSVASPVV	AGAVTLLVST	VQKRELVNPA	SMKQALIASA	RRLPGVNMFE	QGHGKLDLLR	AYQILNSYKP
**490**	**500**	**510**	**520**	**530**	**540**	**550**	**560**
QASLSPSYID	LTECPYMWPY	CSQPIYYGGM	PTVVNVTILN	GMGVTGRIVD	KPDWQPYLPQ	NGDNIEVAFS	YSSVLWPWSG
**570**	**580**	**590**	**600**	**610**	**620**	**630**	**640**
YLAISISVTK	KAASWEGIAQ	GHVMITVASP	AETESKNGAE	QTSTVKLPIK	VKIIPTPPRS	KRVLWDQYHN	LRYPPGYFPR
**650**	**660**	**670**	**680**	**690**	**700**	**710**	**720**
DNLRMKNDPL	DWNGDHIHTN	FRDMYQHLRS	MGYFVEVLGA	PFTCFDASQY	GTLLMVDSEE	EYFPEEIAKL	RRDVDNGLSL
**730**	**740**	**750**	**760**	**770**	**780**	**790**	**800**
VIFSDWYNTS	VMRKVKFYDE	NTRQWWMPDT	GGANIPALNE	LLSVWNMGFS	DGLYEGEFTL	ANHDMYYASG	CSIAKFPEDG
**810**	**820**	**830**	**840**	**850**	**860**	**870**	**880**
VVITQTFKDQ	GLEVLKQETA	VVENVPILGL	YQIPAEGGGR	IVLYGDSNCL	DDSHRQKDCF	WLLDALLQYT	SYGVTPPSLS
**890**	**900**	**910**	**920**	**930**	**940**	**950**	**960**
HSGNRQRPPS	GAGSVTPERM	EGNHLHRYSK	VLEAHLGDPK	PRPLPACPRL	SWAKPQPLNE	TAPSNLWKHQ	KLLSIDLDKV
**970**	**980**	**990**	**1000**	**1010**	**1020**	**1030**	**1040**
VLPNFRSNRP	QVRPLSPGES	GAWDIPGGIM	PGRYNQEVGQ	TIPVFAFLGA	MVVLAFFVVQ	INKAKSRPKR	RKPRVKRPQL
**1050**	**1060**						
MQQVHPPKTP	SV						

Cmpd.	No. of Cmpds.	m/z meas.	Δ m/z [ppm]	z	Rt [min]	Score	P	Range	Sequence	Modification
1464	1	742.6304	-61.81	3	48.1	21.1	1	645-662	R.MKNDPLDWNGDHIHTNFR.D	Oxidation: 1

Detailed Protein Report

Protein 606: suppressor of IKBKE 1 isoform 2 [Homo sapiens]

**Accession:**	gi|156151377	**Score:**	21.1
**Database:**	refseq_human(refseq_human_20140103.fasta)	**MW [kDa]:**	23.7
**Database Date:**	2015-11-30	**pI:**	5.0
		**Sequence Coverage [%]:**	11.6
		**No. of unique Peptides:**	1

10	20	30	40	50	60	70	80
MSCTIEKILT	DAKTLLERLR	EHDAAAESLV	DQSAALHRRV	AAMREAGTAL	PDQYQEDASD	MKDMSKYKPH	ILLSQENTQI
**90**	**100**	**110**	**120**	**130**	**140**	**150**	**160**
RDLQQENREL	WISLEEHQDA	LELIMSKYRK	QMLQLMVAKK	AVDAEPVLKA	HQSHSAEIES	QIDRICEMGE	VMRKAVQVDD
**170**	**180**	**190**	**200**	**210**			
DQFCKIQEKL	AQLELENKEL	RELLSISSES	LQARKENSMD	TASQAIK			

Cmpd.	No. of Cmpds.	m/z meas.	Δ m/z [ppm]	z	Rt [min]	Score	P	Range	Sequence	Modification
1321	1	781.0677	172.15	2	46.2	10.9	1	182-195	R.ELLSISSESLQARK.E	

Detailed Protein Report

Protein 607: late cornified envelope protein 3E [Homo sapiens]

Accession: gi|30410033
Database: refseq_human(refseq_human_20140103.fasta)
Database Date: 2015-11-30
Modification(s): Carbamidomethyl

Score: 21.1
MW [kDa]: 9.5
pI: 12.6
Sequence Coverage [%]: 30.4
No. of unique Peptides: 2

```
        10         20         30         40         50         60         70         80
MSCQQNQKQC QPPPKCPSPK CPPKNPVQCL PPASSGCAPS SGGCGPSSEG GCFLNHHRRH HRCRRQRSNS CDRGSGQQGG
        90        100
GSGCCHGSGG CC
```

Cmpd.	No. of Cmpds.	m/z meas.	Δ m/z [ppm]	z	Rt [min]	Score	P	Range	Sequence	Modification
1406	1	956.6395	178.07	1	47.2	10.6	1	16-24	K.CPSPKCPPK.N	
2462	1	885.2390	-64.11	2	60.7	10.5	0	74-92	R.GSGQQGGGSGCCHGSGGCC.-	Carbamidomethyl: 12, 18, 19

Detailed Protein Report

Protein 608: PREDICTED: xylulose kinase isoform X4 [Homo sapiens]

Accession: gi|578806855
Database: refseq_human(refseq_human_20140103.fasta)
Database Date: 2015-11-30
Modification(s): Carbamidomethyl

Score: 21.1
MW [kDa]: 51.1
pI: 5.6
Sequence Coverage [%]: 7.2
No. of unique Peptides: 1

10	20	30	40	50	60	70	80
MAEHAPRRCC	LGWDFSTQQV	KVVAVDAELN	VFYEESVHFD	RDLPEFGTQG	GVHVHKDGLT	VTSPVLMWVQ	ALDIILEKMK
**90**	**100**	**110**	**120**	**130**	**140**	**150**	**160**
ASGFDFSQVL	ALSGAGQQHG	SIYWKAGAQQ	ALTSLSPDLR	LHQQLQDCFS	ISDCPVWMDS	STTAQCRQLE	AAVGGAQALS
**170**	**180**	**190**	**200**	**210**	**220**	**230**	**240**
CLTGSRAYER	FTGNQIAKIY	QQNPEAYSHT	ERISLVSSFA	ASLFLGSYSP	IDYSDGSGMN	LLQIQDKVWS	QACLGACAPH
**250**	**260**	**270**	**280**	**290**	**300**	**310**	**320**
LEEKLSPPVP	SCSVVGAISS	YYVQRYGFPP	GCKVVAFTGD	NPASLAGMRL	EEGDIAVSLG	TSDTLFLWLQ	EPMPALEGHI
**330**	**340**	**350**	**360**	**370**	**380**	**390**	**400**
FCNPVDSQHY	MALLCFKNGS	LMREKIRNES	VSRSWSDFSK	ALQSTEMGNG	GNLGFYFDVM	EITPEIIGRH	RFNTENHKVA
**410**	**420**	**430**	**440**	**450**	**460**	**470**	
AFPGDVEVRA	LIEGQFMAKR	IHAEGLGYRC	PRQRFWPQEE	HLTIEKSYRC	LQMCLMPRCM	L	

Cmpd.	No. of Cmpds.	m/z meas.	Δ m/z [ppm]	z	Rt [min]	Score	P	Range	Sequence	Modification
766	1	859.6992	-41.80	3	39.0	10.2	2	1-21	-.MAEHAPRRCCLGWDFSTQQVK.V	Carbamidomethyl: 9, 10

Detailed Protein Report

Protein 609: PREDICTED: 5'-3' exoribonuclease 1 isoform X6 [Homo sapiens]

**Accession:**	gi|578807507	**Score:**	20.9
**Database:**	refseq_human(refseq_human_20140103.fasta)	**MW [kDa]:**	108.1
**Database Date:**	2015-11-30	**pI:**	6.1
		**Sequence Coverage [%]:**	2.6
		**No. of unique Peptides:**	1

10	20	30	40	50	60	70	80
MGVPKFYRWI	SERYPCLSEV	VKEHQIPEFD	NLYLDMNGII	HQCSHPNDDD	VHFRISDDKI	FTDIFHYLEV	LFRIIKPRKV
90	100	110	120	130	140	150	160
FFMAVDGVAP	RAKMNQQRGR	RFRSAKEAED	KIKKAIEKGE	TLPTEARFDS	NCITPGTEFM	ARLHEHLKYF	VNMKISTDKS
170	180	190	200	210	220	230	240
WQGVTIYFSG	HETPGEGEHK	IMEFIRSEKA	KPDHDPNTRH	CLYGLDADLI	MLGLTSHEAH	FSLLREEVRF	GGKKTQRVCA
250	260	270	280	290	300	310	320
PEETTFHLLH	LSLMREYIDY	EFSVLKEKIT	FKYDIERIID	DWILMGFLVG	NDFIPHLPHL	HINHDALPLL	YGTYVTILPE
330	340	350	360	370	380	390	400
LGGYINESGH	LNLPRFEKYL	VKLSDFDREH	FSEVFVDLKW	FESKVGNKYL	NEAAGVAAEE	ARNYKEKKKL	KGQENSLCWT
410	420	430	440	450	460	470	480
ALDKNEGEMI	TSKDNLEDET	EDDDLFETEF	RQYKRTYYMT	KMGVDVVSDD	FLADQAACYV	QAIQWILHYY	YHGVQSWSWY
490	500	510	520	530	540	550	560
YPYHYAPFLS	DIHNISTLKI	HFELGKPFKP	FEQLLAVLPA	ASKNLLPACY	QHLMTNEDSP	IIEYYPPDFK	TDLNGKQQEW
570	580	590	600	610	620	630	640
EAVVLIPFID	EKRLLEAMET	CNHSLKKEER	KRNQHSECLM	CWYDRDTEFI	YPSPWPEKFP	AIERCCTRYK	IISLDAWRVD
650	660	670	680	690	700	710	720
INKNKITRID	QKALYFCGFP	TLKHIRHKFF	LKKSGVQVFQ	QSSRGENMML	EILVDAESDE	LTVENVASSV	LGKSVFVNWP
730	740	750	760	770	780	790	800
HLEEARVVAV	SDGETKFYLE	EPPGTQKLYS	GRTAPPSKVV	HLGDKEQSNW	AKEVQGISEH	YLRRKGIIIN	ETSAVVYAQL
810	820	830	840	850	860	870	880
LTGRKYQINQ	NGEVRLEKQW	SKQVVPFVYQ	TIVKDIRAFD	SRFSNIKTLD	DLFPLRSMVF	MLGTPYYGCT	GEVQDSGDVI
890	900	910	920	930			
TEGRIRVIFS	IPCEPNLDAL	IQNQHEKERG	AEKFCLQILS	KKRGNGSK			

Cmpd.	No. of Cmpds.	m/z meas.	Δ m/z [ppm]	z	Rt [min]	Score	P	Range	Sequence	Modification
263	1	795.8251	-113.37	2	32.7	10.2	1	806-818	K.YQINQNGEVRLEK.Q	

Detailed Protein Report

Protein 610: zinc finger protein 467 [Homo sapiens]

**Accession:**	gi|46409310	**Score:**	20.8
**Database:**	refseq_human(refseq_human_20140103.fasta)	**MW [kDa]:**	65.1
**Database Date:**	2015-11-30	**pI:**	11.0
**Modification(s):**	Carbamidomethyl	**Sequence Coverage [%]:**	5.4
		**No. of unique Peptides:**	1

Alias proteins:

Accession	Name	Description
gi|578813959	refseq_human (refseq_human_20140103.fasta)	PREDICTED: zinc finger protein 467 isoform X6 [Homo sapiens]
gi|530386928	refseq_human (refseq_human_20140103.fasta)	PREDICTED: zinc finger protein 467 isoform X3 [Homo sapiens]
gi|530386926	refseq_human (refseq_human_20140103.fasta)	PREDICTED: zinc finger protein 467 isoform X2 [Homo sapiens]
gi|530386924	refseq_human (refseq_human_20140103.fasta)	PREDICTED: zinc finger protein 467 isoform X1 [Homo sapiens]

10	20	30	40	50	60	70	80
MRETLEALSS	LGFSVGQPEM	APQSEPREGS	HNAQEQMSSS	REERALGVCS	GHEAPTPEEG	AHTEQAEAPC	RGQACSAQKA
90	100	110	120	130	140	150	160
QPVGTCPGEE	WMIRKVKVED	EDQEAEEEVE	WPQHLSLLPS	PFPAPDLGHL	AAAYKLEPGA	PGALSGLALS	GWGPMPEKPY
170	180	190	200	210	220	230	240
GCGECERRFR	DQLTLRLHQR	LHRGEGPCAC	PDCGRSFTQR	AHMLLHQRSH	RGERPFPCSE	CDKRFSKKAH	LTRHLRTHTG
250	260	270	280	290	300	310	320
ERPYPCAECG	KRFSQKIHLG	SHQKTHTGER	PFPCTECEKR	FRKKTHLIRH	QRIHTGERPY	QCAQCARSFT	HKQHLVRHQR
330	340	350	360	370	380	390	400
VHQTAGPARP	SPDSSASPHS	TAPSPTPSFP	GPKPFACSDC	GLSFGWKKNL	ATHQCLHRSE	GRPFGCDECA	LGATVDAPAA
410	420	430	440	450	460	470	480
KPLASAPGGP	GCGPGSDPVV	PQRAPSGERS	FFCPDCGRGF	SHGQHLARHP	RVHTGERPFA	CTQCDRRFGS	RPNLVAHSRA
490	500	510	520	530	540	550	560
HSGARPFACA	QCGRRFSRKS	HLGRHQAVHT	GSRPHACAVC	ARSFSSKTNL	VRHQAIHTGS	RPFSCPQCGK	SFSRKTHLVR
570	580	590	600				
HQLIHGEAAH	AAPDAALAAP	AWSAPPEVAP	PPLFF				

Cmpd.	No. of Cmpds.	m/z meas.	Δ m/z [ppm]	z	Rt [min]	Score	P	Range	Sequence	Modification
2174	1	849.0086	-71.63	3	57.0	10.8	1	533-554	R.HQAIHTGSRPFSCPQCGKSFSR.K	Carbamidomethyl: 13, 16

Detailed Protein Report

Protein 611: **PREDICTED: EGF-containing fibulin-like extracellular matrix protein 1 isoform X2 [Homo sapiens]**

**Accession:**	gi|530367362	**Score:**	20.7
**Database:**	refseq_human(refseq_human_20140103.fasta)	**MW [kDa]:**	59.3
**Database Date:**	2015-11-30	**pI:**	9.5
		**Sequence Coverage [%]:**	5.5
		**No. of unique Peptides:**	1

Quantitation

MD:MU **Median:** 4.40 **CV:** 0.00 % **No. of Peptides:** 1

10	20	30	40	50	60	70	80
MSTCPLWRGV	PASLGNEELA	REGKRGRQRG	PLEQGARTSL	RCALYRRARN	AGLSARASLS	SLLRPPSPSP	FPSLSPPPPA
90	100	110	120	130	140	150	160
AAAAAGLRQI	RPTGLPSPAH	SPRCPGPERS	AAAQVFLLCC	ARNSASSRFT	MLKALFLTML	TLALVKSQDT	EETITYTQCT
170	180	190	200	210	220	230	240
DGYEWDPVRQ	QCKDIDECDI	VPDACKGGMK	CVNHYGGYLC	LPKTAQIIVN	NEQPQQETQP	AEGTSGATTG	VVAASSMATS
250	260	270	280	290	300	310	320
GVLPGGGFVA	SAAAVAGPEM	QTGRNNFVIR	RNPADPQRIP	SNPSHRIQCA	AGYEQSEHNV	CQDIDECTAG	THNCRADQVC
330	340	350	360	370	380	390	400
INLRGSFACQ	CPPGYQKRGE	QCVDIDECRT	SSYLCQYQCV	NEPGKFSCMC	PQGYQVVRSR	TCQDINECET	TNECREDEMC
410	420	430	440	450	460	470	480
WNYHGGFRCY	PRNPCQDPYI	LTPENRCVCP	VSNAMCRELP	QSIVYKYMSI	RSDRSVPSDI	FQIQATTIYA	NTINTFRIKS
490	500	510	520	530	540	550	
GNENGEFYLR	QTSPVSAMLV	LVKSLSGPRE	HIVDLEMLTV	SSIGTFRTSS	VLRLTIIVGP	FSF	

Cmpd.	No. of Cmpds.	m/z meas.	Δ m/z [ppm]	z	Rt [min]	Score	P	Range	Sequence	Modification	Ratios	
555	1	676.8735	43.07	2	36.3	10.5	0	110-122	R.SAAAQVFLLCCAR.N		MD:MU	4.40

Detailed Protein Report

Protein 612: protein-methionine sulfoxide oxidase MICAL3 isoform 1 [Homo sapiens]

**Accession:**	gi|209862789	**Score:**	20.6
**Database:**	refseq_human(refseq_human_20140103.fasta)	**MW [kDa]:**	224.2
**Database Date:**	2015-11-30	**pI:**	5.3
		**Sequence Coverage [%]:**	1.3
		**No. of unique Peptides:**	1

Alias proteins:

Accession	Name	Description
gi|530426255	refseq_human (refseq_human_20140103.fasta)	PREDICTED: protein-methionine sulfoxide oxidase MICAL3 isoform X6 [Homo sapiens]

Detailed Protein Report

10	20	30	40	50	60	70	80
MEERKHETMN	PAHVLFDRFV	QATTCKGTLK	AFQELCDHLE	LKPKDYRSFY	HKLKSKLNYW	KAKALWAKLD	KRGSHKDYKK
90	100	110	120	130	140	150	160
GKACTNTKCL	IIGAGPCGLR	TAIDLSLLGA	KVVVIEKRDA	FSRNNVLHLW	PFTIHDLRGL	GAKKFYGKFC	AGAIDHISIR
170	180	190	200	210	220	230	240
QLQLILLKVA	LILGIEIHVN	VEFQGLIQPP	EDQENERIGW	RALVHPKTHP	VSEYEFEVII	GGDGRRNTLE	GFRRKEFRGK
250	260	270	280	290	300	310	320
LAIAITANFI	NRNTTAEAKV	EEISGVAFIF	NQKFFQELRE	ATGIDLENIV	YYKDDTHYFV	MTAKKQSLLD	KGVILHDYAD
330	340	350	360	370	380	390	400
TELLLSRENV	DQEALLSYAR	EAADFSTQQQ	LPSLDFAINH	YGQPDVAMFD	FTCMYASENA	ALVREQNGHQ	LLVALVGDSL
410	420	430	440	450	460	470	480
LEPFWPMGTG	IARGFLAAMD	SAWMVRSWSL	GTSPLEVLAE	RESIYRLLPQ	TTPENVSKNF	SQYSIDPVTR	YPNINVNFLR
490	500	510	520	530	540	550	560
PSQVRHLYDT	GETKDIHLEM	ESLVNSRTTP	KLTRNESVAR	SSKLLGWCQR	QTDGYAGVNV	TDLTMSWKSG	LALCAIIHRY
570	580	590	600	610	620	630	640
RPDLIDFDSL	DEQNVEKNNQ	LAFDIAEKEL	GISPIMTGKE	MASVGEPDKL	SMVMYLTQFY	EMFKDSLPSS	DTLDLNAEEK
650	660	670	680	690	700	710	720
AVLIASTRSP	ISFLSKLGQT	ISRKRSPKDK	KEKDLDGAGK	RRKTSQSEEE	EAPRGHRGER	PTLVSTLTDR	RMDVAVGNQN
730	740	750	760	770	780	790	800
KVKYMATQLL	AKFEENAPAQ	SIGIRRQGSM	KKEFPQNLGG	SDTCYFCQKR	VYVMERLSAE	GKFFHRSCFK	CEYCATTLRL
810	820	830	840	850	860	870	880
SAYAYDIEDG	KFYCKPHYCY	RLSGYAQRKR	PAVAPLSGKE	AKGPLQDGAT	TDANGRANAV	ASSTERTPGS	GVNGLEEPSI
890	900	910	920	930	940	950	960
AKRLRGTPER	IELENYRLSL	RQAEALQEVP	EETQAEHNLS	SVLDTGAEED	VASSSSESEM	EEEGEEEEEE	PRLPPSDLGG
970	980	990	1000	1010	1020	1030	1040
VPWKEAVRIH	ALLKGKSEEE	LEASKSFGPG	NEEEEEEEEE	YEEEEEEDYD	EEEEESSEAG	NQRLQQVMHA	ADPLEIQADV
1050	1060	1070	1080	1090	1100	1110	1120
HWTHIREREE	EERMAPASES	SASGAPLDEN	DLEEDVDSEP	AEIEGEAAED	GDPGDTGAEL	DDDQHWSDSP	SDADRELRLP
1130	1140	1150	1160	1170	1180	1190	1200
CPAEGEAELE	LRVSEDEEKL	PASPKHQERG	PSQATSPIRS	PQESALLFIP	VHSPSTEGPQ	LPPVPAATQE	KSPEERLFPE
1210	1220	1230	1240	1250	1260	1270	1280
PLLPKEKPKA	DAPSDLKAVH	SPIRSQPVTL	PEARTPVSPG	SPQPQPPVAA	STPPPSPLPI	CSQPQPSTEA	TVPSPTQSPI
1290	1300	1310	1320	1330	1340	1350	1360
RFQPAPAKTS	TPLAPLPVQS	QSDTKDRLGS	PLAVDEALRR	SDLVEEFWMK	SAEIRRSLGL	TPVDRSKGPE	PSFPTPAFRP
1370	1380	1390	1400	1410	1420	1430	1440
VSLKSYSVEK	SPQDEGLHLL	KPLSIPKRLG	LPKPEGEPLS	LPTPRSPSDR	ELRSAQEERR	ELSSSSGLGL	HGSSSNMKTL
1450	1460	1470	1480	1490	1500	1510	1520
GSQSFNTSDS	AMLTPPSSPP	PPPPPGEEPA	TLRRKLREAE	PNASVVPPPL	PATWMRPPRE	PAQPPREEVR	KSFVESVEEI
1530	1540	1550	1560	1570	1580	1590	1600
PFADDVEDTY	DDKTEDSSLQ	EKFFTPPSCW	PRPEKPRHPP	LAKENGRLPA	LEGTLQPQKR	GLPLVSAEAK	ELAEERMRAR
1610	1620	1630	1640	1650	1660	1670	1680
EKSVKSQALR	DAMARQLSRM	QQMELASGAP	RPRKASSAPS	QGKERRPDSP	TRPTLRGSEE	PTLKHEATSE	EVLSPPSDSG
1690	1700	1710	1720	1730	1740	1750	1760
GPDGSFTSSE	GSSGKSKKRS	SLFSPRRNKK	EKKSKGEGRP	PEKPSSNLLE	EAAAKPKSLW	KSVFSGYKKD	KKKKADDKSC
1770	1780	1790	1800	1810	1820	1830	1840
PSTPSSGATV	DSGKHRVLPV	VRAELQLRRQ	LSFSEDSDLS	SDDVLEKSSQ	KSRREPRTYT	EEELNAKLTR	RVQKAARRQA
1850	1860	1870	1880	1890	1900	1910	1920
KQEELKRLHR	AQIIQRQLQQ	VEERQRRLEE	RGVAVEKALR	GEAGMGKKDD	PKLMQEWFKL	VQEKNAMVRY	ESELMIFARE
1930	1940	1950	1960	1970	1980	1990	2000
LELEDRQSRL	QQELRERMAV	EDHLKTEEEL	SEEKQILNEM	LEVVEQRDSL	VALLEEQRLR	EREEDKDLEA	AMLSKGFSLN
2010							
WS							

Cmpd.	No. of Cmpds.	m/z meas.	Δ m/z [ppm]	z	Rt [min]	Score	P	Range	Sequence	Modification
2512	1	834.4289	-40.88	2	61.8	10.6	2	1306-1320	K.DRLGSPLAVDEALRR.S	

Detailed Protein Report

Protein 613: mitogen-activated protein kinase kinase kinase 15 [Homo sapiens]

Accession: gi|282847398
Database: refseq_human(refseq_human_20140103.fasta)
Database Date: 2015-11-30

Score: 20.6
MW [kDa]: 147.3
pI: 5.3
Sequence Coverage [%]: 1.4
No. of unique Peptides: 2

10	20	30	40	50	60	70	80
MESGGGNAPA	GALGAASESP	QCPPPPGVEG	AAGPAEPDGA	AEGAAGGSGE	GESGGGPRRA	LRAVYVRSES	SQGGAAGGPE
90	100	110	120	130	140	150	160
AGARQCLLRA	CEAEGAHLTS	VPFGELDFGE	TAVLDAFYDA	DVAVVDMSDV	SRQPSLFYHL	GVRESFDMAN	NVILYHDTDA
170	180	190	200	210	220	230	240
DTALSLKDMV	TQKNTASSGN	YYFIPYIVTP	CADYFCCESD	AQRRASEYMQ	PNWDNILGPL	CMPLVDRFIS	LLKDIHVTSC
250	260	270	280	290	300	310	320
VYYKETLLND	IRKAREKYQG	EELAKELARI	KLRMDNTEVL	TSDIIINLLL	SYRDIQDYDA	MVKLVETLEM	LPTCDLADQH
330	340	350	360	370	380	390	400
NIKFHYAFAL	NRRNSTGDRE	KALQIMLQVL	QSCDHPGPDM	FCLCGRIYKD	IFLDSDCKDD	TSRDSAIEWY	RKGFELQSSL
410	420	430	440	450	460	470	480
YSGINLAVLL	IVAGQQFETS	LELRKIGVRL	NSLLGRKGSL	EKMNNYWDVG	QFFSVSMLAH	DVGKAVQAAE	RLFKLKPPVW
490	500	510	520	530	540	550	560
YLRSLVQNLL	LIRRFKKTII	EHSPRQERLN	FWLDIIFEAT	NEVTNGLRFP	VLVIEPTKVY	QPSYVSINNE	AEERTVSLWH
570	580	590	600	610	620	630	640
VSPTEMKQMH	EWNFTASSIK	GISLSKFDER	CCFLYVHDNS	DDFQIYFSTE	EQCSRFFSLV	KEMITNTAGS	TVELEGETDG
650	660	670	680	690	700	710	720
DTLEYEYDHD	ANGERVVLGK	GTYGIVYAGR	DLSNQVRIAI	KEIPERDSRY	SQPLHEEIAL	HKYLKHRNIV	QYLGSVSENG
730	740	750	760	770	780	790	800
YIKIFMEQVP	GGSLSALLRS	KWGPMKEPTI	KFYTKQILEG	LKYLHENQIV	HRDIKGDNVL	VNTYSGVVKI	SDFGTSKRLA
810	820	830	840	850	860	870	880
GVNPCTETFT	GTLQYMAPEI	IDQGPRGYGA	PADIWSLGCT	IIEMATSKPP	FHELGEPQAA	MFKVGMFKIH	PEIPEALSAE
890	900	910	920	930	940	950	960
ARAFILSCFE	PDPHKRATTA	ELLREGFLRQ	VNKGKKNRIA	FKPSEGPRGV	VLALPTQGEP	MATSSSEHGS	VSPDSDAQPD
970	980	990	1000	1010	1020	1030	1040
ALFERTRAPR	HHLGHLLSVP	DESSALEDRG	LASSPEDRDQ	GLFLLRKDSE	RRAILYKILW	EEQNQVASNL	QECVAQSSEE
1050	1060	1070	1080	1090	1100	1110	1120
LHLSVGHIKQ	IIGILRDFIR	SPEHRVMATT	ISKLKVDLDF	DSSSISQIHL	VLFGFQDAVN	KILRNHLIRP	HWMFAMDNII
1130	1140	1150	1160	1170	1180	1190	1200
RRAVQAAVTI	LIPELRAHFE	PTCETEGVDK	DMDEAEEGYP	PATGPGQEAQ	PHQQHLSLQL	GELRQETNRL	LEHLVEKERE
1210	1220	1230	1240	1250	1260	1270	1280
YQNLLRQTLE	QKTQELYHLQ	LKLKSNCITE	NPAGPYGQRT	DKELIDWLRL	QGADAKTIEK	IVEEGYTLSD	ILNEITKEDL
1290	1300	1310	1320				
RYLRLRGGLL	CRLWSAVSQY	RRAQEASETK	DKA				

Cmpd.	No. of Cmpds.	m/z meas.	Δ m/z [ppm]	z	Rt [min]	Score	P	Range	Sequence	Modification
1857	1	599.2758	-1.14	2	53.3	10.5	0	294-303	R.DIQDYDAMVK.L	
1128	1	874.6829	210.03	1	43.7	10.1	0	897-904	R.ATTAELLR.E	

Detailed Protein Report

Protein 614: **PREDICTED: arf-GAP domain and FG repeat-containing protein 2 isoform X2 [Homo sapiens]**

**Accession:**	gi|578814185	**Score:**	20.5
**Database:**	refseq_human(refseq_human_20140103.fasta)	**MW [kDa]:**	47.4
**Database Date:**	2015-11-30	**pI:**	10.2
		**Sequence Coverage [%]:**	3.7
		**No. of unique Peptides:**	1

10	20	30	40	50	60	70	80
MVMAAKKGPG	PGGGVSGGKA	EAEAASEVWC	RRVRELGGCS	QAGNRHCFEC	AQRGVTYVDI	TVGSFVCTTC	SGLLRGLNPP
**90**	**100**	**110**	**120**	**130**	**140**	**150**	**160**
HRVKSISMTT	FTEPEVVFLQ	SRGNEVCRKI	WLGLFDARTS	LVPDSRDPQK	VKEFLQEKYE	KKRWYVPPDQ	VKGPTYTKGS
**170**	**180**	**190**	**200**	**210**	**220**	**230**	**240**
ASTPVQGSIP	EGKPLRTLLG	DPAPSLSVAA	STSSQPVSQS	HARTSQARST	QPPPHSSVKK	ASTDLLADIG	GDPFAAPQMA
**250**	**260**	**270**	**280**	**290**	**300**	**310**	**320**
PAFAAFPAFG	GQTPSQGGFA	NFDAFSSGPS	SSVFGSLPPA	GQASFQAQPT	PAASRMLTES	YSFGSSQGTP	FGATPLAPAS
**330**	**340**	**350**	**360**	**370**	**380**	**390**	**400**
QPNSLADVGS	FLGPGVPAAG	VPSSLFGMAG	QVPPLQSVTM	GGGGGSSTGL	AFGGPGFGMS	SAGPGFPQAV	PPTGAFASSF
**410**	**420**	**430**	**440**	**450**	**460**	**470**	
PAPLFPPQTP	LVQQQNGSSF	GDLGSAKLGQ	RPLSQPAGIS	TNPFMTGPSS	SPFASKPPTT	NPFL	

Cmpd.	No. of Cmpds.	m/z meas.	Δ m/z [ppm]	z	Rt [min]	Score	P	Range	Sequence	Modification
2745	1	973.4995	-29.64	2	64.7	20.5	1	110-126	K.IWLGLFDARTSLVPDSR.D	

Detailed Protein Report

Protein 615: PREDICTED: ankyrin repeat and protein kinase domain-containing protein 1 isoform X1 [Homo sapiens]

**Accession:**	gi|578822107	**Score:**	20.5
**Database:**	refseq_human(refseq_human_20140103.fasta)	**MW [kDa]:**	79.1
**Database Date:**	2015-11-30	**pI:**	6.7
		**Sequence Coverage [%]:**	1.7
		**No. of unique Peptides:**	1

Quantitation

MD:MU **Median:** 1.26 **CV:** 0.00 % **No. of Peptides:** 1

10	20	30	40	50	60	70	80
MGHPLPYLAP	PLHSSDVNYL	IEEAAKMKKI	KFQHIVSIYG	VCKQPLGIVM	EFMANGSLEK	VLSTHSLCWK	LRFRIIHETS
90	100	110	120	130	140	150	160
LAMNFLHSIK	PPLLHLDLKP	GNILLDSNMH	VKISDFGLSK	WMEQSTRMQY	IERSALRGML	SYIPPEMFLE	SNKAPGPKYD
170	180	190	200	210	220	230	240
VYSFAIVIWE	LLTQKKPYSG	FNMMMIIIRV	AAGMRPSLQP	VSDQWPSEAQ	QMVDLMKRCW	DQDPKKRPCF	LDITIETDIL
250	260	270	280	290	300	310	320
LSLLQSRVAV	PESKALARKV	SCKLSLRQPG	EVNEDISQEL	MDSDSGNYLK	RALQLSDRKN	LVPRDEELCI	YENKVTPLHF
330	340	350	360	370	380	390	400
LVAQGSVEQV	RLLLAHEVDV	DCQTASGYTP	LLIAAQDQQP	DLCALLLAHG	ADANRVDEDG	WAPLHFAAQN	GDDGTARLLL
410	420	430	440	450	460	470	480
DHGACVDAQE	REGWTPLHLA	AQNNFENVAR	LLVSRQADPN	LHEAEGKTPL	HVAAYFGHVS	LVKLLTSQGA	ELDAQQRNLR
490	500	510	520	530	540	550	560
TPLHLAVERG	KVRAIQHLLK	SGAVPDALDQ	SGYGPLHTAA	ARGKYLICKM	LLRYGASLEL	PTHQGWTPLH	LAAYKGHLEI
570	580	590	600	610	620	630	640
IHLLAESHAN	MGALGAVNWT	PLHLAARHGE	EAVVSALLQC	GADPNAAEQS	GWTPLHLAVQ	RSTFLSVINL	LEHHANVHAR
650	660	670	680	690	700	710	720
NKVGWTPAHL	AALKGNTAIL	KVLVEAGAQL	DVQDGVSCTP	LQLALRSRKQ	GIMSFLEGKE	PSVATLGGSK	PGAEMEI

Cmpd.	No. of Cmpds.	m/z meas.	Δ m/z [ppm]	z	Rt [min]	Score	P	Range	Sequence	Modification	Ratios
2290	1	697.2835	-121.83	2	58.5	20.5	0	32-43	K.FQHIVSIYGVCK.Q		MD:MU 1.26

Detailed Protein Report

Protein 616: coiled-coil domain-containing protein 181 [Homo sapiens]

**Accession:**	gi|10880975	**Score:**	20.4
**Database:**	refseq_human(refseq_human_20140103.fasta)	**MW [kDa]:**	59.9
**Database Date:**	2015-11-30	**pI:**	5.6
		**Sequence Coverage [%]:**	3.9
		**No. of unique Peptides:**	1

Alias proteins:

Accession	Name	Description
gi|530365168	refseq_human (refseq_human_20140103.fasta)	PREDICTED: coiled-coil domain-containing protein 181 isoform X4 [Homo sapiens]

10	20	30	40	50	60	70	80
MNENKDTDSK	KSEEYEDDFE	KDLEWLINEN	EKSDASIIEM	ACEKEENINQ	DLKENETVME	HTKRHSDPDK	SLQDEVSPRR
**90**	**100**	**110**	**120**	**130**	**140**	**150**	**160**
NDIISVPGIQ	PLDPISDSDS	ENSFQESKLE	SQKDLEEEED	EEVRRYIMEK	IVQANKLLQN	QEPVNDKRER	KLKFKDQLVD
**170**	**180**	**190**	**200**	**210**	**220**	**230**	**240**
LEVPPLEDTT	TFKNYFENER	NMFGKLSQLC	ISNDFGQEDV	LLSLTNGSCE	ENKDRTILVE	RDGKFELLNL	QDIASQGFLP
**250**	**260**	**270**	**280**	**290**	**300**	**310**	**320**
PINNANSTEN	DPQQLLPRSS	NSSVSGTKKE	DSTAKIHAVT	HSSTGEPLAY	IAQPPLNRKT	CPSSAVNSDR	SKGNGKSNHR
**330**	**340**	**350**	**360**	**370**	**380**	**390**	**400**
TQSAHISPVT	STYCLSPRQK	ELQKQLEEKR	EKLKREEERR	KIEEEKEKKR	ENDIVFKAWL	QKKREQVLEM	RRIQRAKEIE
**410**	**420**	**430**	**440**	**450**	**460**	**470**	**480**
DMNSRENRDP	QQAFRLWLKK	KHEEQMKERQ	TEELRKQEEC	LFFLKGTEGR	ERAFKQWLRR	KRMEKMAEQQ	AVRERTRQLR
**490**	**500**	**510**					
LEAKRSKQLQ	HHLYMSEAKP	FRFTDHYN					

Cmpd.	No. of Cmpds.	m/z meas.	Δ m/z [ppm]	z	Rt [min]	Score	P	Range	Sequence	Modification
2777	1	735.3766	1.06	3	66.0	20.4	1	321-340	R.TQSAHISPVTSTYCLSPRQK.E	

Detailed Protein Report

Protein 617: notch homolog 2 N-terminal-like protein [Homo sapiens]

**Accession:**	gi|46397353	**Score:**	20.4
**Database:**	refseq_human(refseq_human_20140103.fasta)	**MW [kDa]:**	25.8
**Database Date:**	2015-11-30	**pI:**	5.4
**Modification(s):**	Carbamidomethyl	**Sequence Coverage [%]:**	11.0
		**No. of unique Peptides:**	1

10	20	30	40	50	60	70	80
MCVTYHNGTG	YCKCPEGFLG	EYCQHRDPCE	KNRCQNGGTC	VAQAMLGKAT	CRCASGFTGE	DCQYSTSHPC	FVSRPCLNGG
**90**	**100**	**110**	**120**	**130**	**140**	**150**	**160**
TCHMLSRDTY	ECTCQVGFTG	KECQWTDACL	SHPCANGSTC	TTVANQFSCK	CLTGFTGQKC	ETDVNECDIP	GHCQHGGTCL
**170**	**180**	**190**	**200**	**210**	**220**	**230**	**240**
NLPGSYQCQC	LQGFTGQYCD	SLYVPCAPSP	CVNGGTCRQT	GDFTFECNCL	PETVRRGTEL	WERDREVWNG	KEHDEN

Cmpd.	No. of Cmpds.	m/z meas.	Δ m/z [ppm]	z	Rt [min]	Score	P	Range	Sequence	Modification
1871	2	1037.3636	-66.59	3	53.2	20.4	1	1-26	-.MCVTYHNGTGYCKCPEGFLGEYCQHR.D	Carbamidomethyl: 2, 23

Detailed Protein Report

Protein 618: sodium channel protein type 3 subunit alpha isoform 3 [Homo sapiens]

Accession: gi|126362955
Database: refseq_human(refseq_human_20140103.fasta)
Database Date: 2015-11-30
Modification(s): Carbamidomethyl

Score: 20.3
MW [kDa]: 221.3
pI: 5.5
Sequence Coverage [%]: 2.3
No. of unique Peptides: 2

10	20	30	40	50	60	70	80
MAQALLVPPG	PESFRLFTRE	SLAAIEKRAA	EEKAKKPKKE	QDNDDENKPK	PNSDLEAGKN	LPFIYGDIPP	EMVSEPLEDL
90	100	110	120	130	140	150	160
DPYYINKKTF	IVMNKGKAIF	RFSATSALYI	LTPLNPVRKI	AIKILVHSLF	SMLIMCTILT	NCVFMTLSNP	PDWTKNVEYT
170	180	190	200	210	220	230	240
FTGIYTFESL	IKILARGFCL	EDFTFLRDPW	NWLDFSVIVM	AYVTEFVSLG	NVSALRTFRV	LRALKTISVI	PGLKTIVGAL
250	260	270	280	290	300	310	320
IQSVKKLSDV	MILTVFCLSV	FALIGLQLFM	GNLRNKCLQW	PPSDSAFETN	TTSYFNGTMD	SNGTFVNVTM	STFNWKDYIG
330	340	350	360	370	380	390	400
DDSHFYVLDG	QKDPLLCGNG	SDAGQCPEGY	ICVKAGRNPN	YGYTSFDTFS	WAFLSLFRLM	TQDYWENLYQ	LTLRAAGKTY
410	420	430	440	450	460	470	480
MIFFVLVIFL	GSFYLVNLIL	AVVAMAYEEQ	NQATLEEAEQ	KEAEFQQMLE	QLKKQQEEAQ	AVAAASAASR	DFSGIGGLGE
490	500	510	520	530	540	550	560
LLESSSEASK	LSSKSAKEWR	NRRKKRRQRE	HLEGNNKGER	DSFPKSESED	SVKRSSFLFS	MDGNRLTSDK	KFCSPHQSLL
570	580	590	600	610	620	630	640
SIRGSLFSPR	RNSKTSIFSF	RGRAKDVGSE	NDFADDEHST	FEDSESRRDS	LFVPHRHGER	RNSNGTTTET	EVRKRRLSSY
650	660	670	680	690	700	710	720
QISMEMLEDS	SGRQRAVSIA	SILTNTMEEL	EESRQKCPPC	WYRFANVFLI	WDCCDAWLKV	KHLVNLIVMD	PFVDLAITIC
730	740	750	760	770	780	790	800
IVLNTLFMAM	EHYPMTEQFS	SVLTVGNLVF	TGIFTAEMVL	KIIAMDPYYY	FQEGWNIFDG	IIVSLSLMEL	GLSNVEGLSV
810	820	830	840	850	860	870	880
LRSFRLLRVF	KLAKSWPTLN	MLIKIIGNSV	GALGNLTLVL	AIIVFIFAVV	GMQLFGKSYK	ECVCKINDDC	TLPRWHMNDF
890	900	910	920	930	940	950	960
FHSFLIVFRV	LCGEWIETMW	DCMEVAGQTM	CLIVFMLVMV	IGNLVVLNLF	LALLLSSFSS	DNLAATDDDN	EMNNLQIAVG
970	980	990	1000	1010	1020	1030	1040
RMQKGIDYVK	NKMRECFQKA	FFRKPKVIEI	HEGNKIDSCM	SNNTGIEISK	ELNYLRDGNG	TTSGVGTGSS	VEKYVIDEND
1050	1060	1070	1080	1090	1100	1110	1120
YMSFINNPSL	TVTVPIAVGE	SDFENLNTEE	FSSESELEES	KEKLNATSSS	EGSTVDVVLP	REGEQAETEP	EEDLKPEACF
1130	1140	1150	1160	1170	1180	1190	1200
TEGCIKKFPF	CQVSTEEGKG	KIWWNLRKTC	YSIVEHNWFE	TFIVFMILLS	SGALAFEDIY	IEQRKTIKTM	LEYADKVFTY
1210	1220	1230	1240	1250	1260	1270	1280
IFILEMLLKW	VAYGFQTYFT	NAWCWLDFLI	VDVSLVSLVA	NALGYSELGA	IKSLRTLRAL	RPLRALSRFE	GMRVVVNALV
1290	1300	1310	1320	1330	1340	1350	1360
GAIPSIMNVL	LVCLIFWLIF	SIMGVNLFAG	KFYHCVNMTT	GNMFDISDVN	NLSDCQALGK	QARWKNVKVN	FDNVGAGYLA
1370	1380	1390	1400	1410	1420	1430	1440
LLQVATFKGW	MDIMYAAVDS	RDVKLQPVYE	ENLYMYLYFV	IFIIFGSFFT	LNLFIGVIID	NFNQQKKKFG	GQDIFMTEEQ
1450	1460	1470	1480	1490	1500	1510	1520
KKYYNAMKKL	GSKKPQKPIP	RPANKFQGMV	FDFVTRQVFD	ISIMILICLN	MVTMMVETDD	QGKYMTLVLS	RINLVFIVLF
1530	1540	1550	1560	1570	1580	1590	1600
TGEFVLKLVS	LRHYYFTIGW	NIFDFVVVIL	SIVGMFLAEM	IEKYFVSPTL	FRVIRLARIG	RILRLIKGAK	GIRTLLFALM
1610	1620	1630	1640	1650	1660	1670	1680
MSLPALFNIG	LLLFLVMFIY	AIFGMSNFAY	VKKEAGIDDM	FNFETFGNSM	ICLFQITTSA	GWDGLLAPIL	NSAPPDCDPD
1690	1700	1710	1720	1730	1740	1750	1760
TIHPGSSVKG	DCGNPSVGIF	FFVSYIIISF	LVVVNMYIAV	ILENFSVATE	ESAEPLSEDD	FEMFYEVWEK	FDPDATQFIE
1770	1780	1790	1800	1810	1820	1830	1840
FSKLSDFAAA	LDPPLLIAKP	NKVQLIAMDL	PMVSGDRIHC	LDILFAFTKR	VLGESGEMDA	LRIQMEDRFM	ASNPSKVSYE
1850	1860	1870	1880	1890	1900	1910	1920
PITTTLKRKQ	EEVSAAIIQR	NFRCYLLKQR	LKNISSNYNK	EAIKGRIDLP	IKQDMIIDKL	NGNSTPEKTD	GSSSTTSPPS
1930	1940	1950	1960				
YDSVTKPDKE	KFEKDKPEKE	SKGKEVRENQ	K				

Detailed Protein Report

Cmpd.	No. of Cmpds.	m/z meas.	Δ m/z [ppm]	z	Rt [min]	Score	P	Range	Sequence	Modification
2349	1	1073.7513	-75.47	3	59.7	10.2	2	675-699	R.QKCPPCWYRFANVFLIWDCCDAWLK.V	Carbamidomethyl: 6, 19
2533	1	1045.0400	-2.11	2	61.6	10.0	1	1082-1101	K.EKLNATSSSEGSTVDVVLPR.E	

Detailed Protein Report

Protein 619: mothers against decapentaplegic homolog 3 isoform 1 [Homo sapiens]

**Accession:**	gi|5174513	**Score:**	20.3
**Database:**	refseq_human(refseq_human_20140103.fasta)	**MW [kDa]:**	48.0
**Database Date:**	2015-11-30	**pI:**	6.8
**Modification(s):**	Oxidation	**Sequence Coverage [%]:**	4.5
		**No. of unique Peptides:**	1

10	20	30	40	50	60	70	80
MSSILPFTPP	IVKRLLGWKK	GEQNGQEEKW	CEKAVKSLVK	KLKKTGQLDE	LEKAITTQNV	NTKCITIPRS	LDGRLQVSHR
**90**	**100**	**110**	**120**	**130**	**140**	**150**	**160**
KGLPHVIYCR	LWRWPDLHSH	HELRAMELCE	FAFNMKKDEV	CVNPYHYQRV	ETPVLPPVLV	PRHTEIPAEF	PPLDDYSHSI
**170**	**180**	**190**	**200**	**210**	**220**	**230**	**240**
PENTNFPAGI	EPQSNIPETP	PPGYLSEDGE	TSDHQMNHSM	DAGSPNLSPN	PMSPAHNNLD	LQPVTYCEPA	FWCSISYYEL
**250**	**260**	**270**	**280**	**290**	**300**	**310**	**320**
NQRVGETFHA	SQPSMTVDGF	TDPSNSERFC	LGLLSNVNRN	AAVELTRRHI	GRGVRLYYIG	GEVFAECLSD	SAIFVQSPNC
**330**	**340**	**350**	**360**	**370**	**380**	**390**	**400**
NQRYGWHPAT	VCKIPPGCNL	KIFNNQEFAA	LLAQSVNQGF	EAVYQLTRMC	TIRMSFVKGW	GAEYRRQTVT	STPCWIELHL
**410**	**420**	**430**					
NGPLQWLDKV	LTQMGSPSIR	CSSVS					

Cmpd.	No. of Cmpds.	m/z meas.	Δ m/z [ppm]	z	Rt [min]	Score	P	Range	Sequence	Modification
1610	1	1100.1365	-2.93	2	50.0	20.3	2	1-19	-.MSSILPFTPPIVKRLLGWK.K	Oxidation: 1

Detailed Protein Report

Protein 620: uncharacterized protein C6orf203 isoform a [Homo sapiens]

**Accession:**	gi|7706029	**Score:**	20.1
**Database:**	refseq_human(refseq_human_20140103.fasta)	**MW [kDa]:**	27.9
**Database Date:**	2015-11-30	**pI:**	9.8
**Modification(s):**	Oxidation	**Sequence Coverage [%]:**	10.8
		**No. of unique Peptides:**	1

Alias proteins:

Accession	Name	Description
gi|215599508	refseq_human (refseq_human_20140103.fasta)	uncharacterized protein C6orf203 isoform a [Homo sapiens]

10	20	30	40	50	60	70	80
MAMASVKLLA	GVLRKPDAWI	GLWGVLRGTP	SSYKLCTSWN	RYLYFSSTKL	RAPNYKTLFY	NIFSLRLPGL	LLSPECIFPF
**90**	**100**	**110**	**120**	**130**	**140**	**150**	**160**
SVRLKSNIRS	TKSTKKSLQK	VDEEDSDEES	HHDEMSEQEE	ELEDDPTVVK	NYKDLEKAVQ	SFRYDVVLKT	GLDIGRNKVE
**170**	**180**	**190**	**200**	**210**	**220**	**230**	**240**
DAFYKGELRL	NEEKLWKKSR	TVKVGDTLDL	LIGEDKEAGT	ETVMRILLKK	VFEEKTESEK	YRVVLRRWKS	LKLPKKRMSK
**250**							

Cmpd.	No. of Cmpds.	m/z meas.	Δ m/z [ppm]	z	Rt [min]	Score	P	Range	Sequence	Modification
1123	1	946.1417	-80.24	3	43.7	20.1	2	2-27	M.AMASVKLLAGVLRKPDAWIGLWGVLR.G	Oxidation: 2

Detailed Protein Report

Protein 621: glycine receptor subunit alpha-4 isoform 2 precursor [Homo sapiens]

**Accession:**	gi|288683407	**Score:**	19.8
**Database:**	refseq_human(refseq_human_20140103.fasta)	**MW [kDa]:**	39.0
**Database Date:**	2015-11-30	**pI:**	9.3
**Modification(s):**	Oxidation	**Sequence Coverage [%]:**	5.0
		**No. of unique Peptides:**	1

Quantitation

MD:MU **Median:** 1.98 **CV:** 0.00 % **No. of Peptides:** 1

10	20	30	40	50	60	70	80
MTTLVPATLS	FLLLWTLPGQ	VLLRVALAKE	EVKSGTKGSQ	PMSPSDFLDK	LMGRTSGYDA	RIRPNFKGPP	VNVTCNIFIN
**90**	**100**	**110**	**120**	**130**	**140**	**150**	**160**
SFSSITKTTM	DYRVNVFLRQ	QWNDPRLSYR	EYPDDSLDLD	PSMLDSIWKP	DLFFANEKGA	NFHEVTTDNK	LLRIFKNGNV
**170**	**180**	**190**	**200**	**210**	**220**	**230**	**240**
LYSIRLTLIL	SCLMDLKNFP	MDIQTCTMQL	ESFGYTMKDL	VFEWLEDAPA	VQVAEGLTLP	QFILRDEKDL	GCCTKHYNTG
**250**	**260**	**270**	**280**	**290**	**300**	**310**	**320**
KFTCIEVKFH	LERQMGYYLI	QMYIPSLLIV	ILSWVSFWIN	MDAAPARVGL	GITTVLTMTT	QSSGSRASLP	KVKRHARELA
**330**	**340**	**350**					
CHRVPHFPFI	PMCPPAHHLP	WI					

Cmpd.	No. of Cmpds.	m/z meas.	Δ m/z [ppm]	z	Rt [min]	Score	P	Range	Sequence	Modification	Ratios
2077	1	599.9042	-74.64	3	56.1	19.8	1	34-50	K.SGTKGSQPMSPSDFLDK.L	Oxidation: 9	MD:MU 1.98

Detailed Protein Report

Protein 622: tubulin polyglutamylase TTLL6 isoform 2 [Homo sapiens]

Accession: gi|195963416
Database: refseq_human(refseq_human_20140103.fasta)
Database Date: 2015-11-30

Score: 19.7
MW [kDa]: 65.9
pI: 9.7
Sequence Coverage [%]: 3.6
No. of unique Peptides: 1

10	20	30	40	50	60	70	80
MRIGTPTAGP	GPSAVMEGCL	GVAELRKLST	FSAYLEDHSY	NVEQIWRDIE	DVIIKTLISA	HPIIRHNYHT	CFPNHTLNSA
90	100	110	120	130	140	150	160
CFEILGFDIL	LDHKLKPWLL	EVNHSPSFST	DSRLDKEVKD	GLLYDTLVLI	NLESCDKKKV	LEEERQRGQF	LQQCCSREMR
170	180	190	200	210	220	230	240
IEEAKGFRAV	QLKKTETYEK	ENCGGFRLIY	PSLNSEKYEK	FFQDNNSLFQ	NTVASRAREE	YARQLIQELR	LKREKKPFQM
250	260	270	280	290	300	310	320
KKKVEMQGES	AGEQVRKKGM	RGWQQKQQQK	DKAATQASKQ	YIQPLTLVSY	TPDLLLSVRG	ERKNETDSSL	NQEAPTEEAS
330	340	350	360	370	380	390	400
SVFPKLTSAK	PFSSLPDLRN	INLSSSKLEP	SKPNFSIKEA	KSASAVNVFT	GTVHLTSVET	TPESTTQLSI	SPKSPPTLAV
410	420	430	440	450	460	470	480
TASSEYSGPE	TDRVVSFKCK	KQQTPPHLTQ	KKMLKSFLPT	KSKSFWESPN	TNWTLLKSDM	NKPHLISELL	TKLQLSGKLS
490	500	510	520	530	540	550	560
FFPAHYNPKL	GMNNLSQNPS	LPGECHSRSD	SSGEKRQLDV	SSLLLQSPQS	YNVTLRDLLV	IATPAQLDPR	PCRSHASAMR
570	580	590					
DPCMQDQEAY	SHCLISGQKG	CERS					

Cmpd.	No. of Cmpds.	m/z meas.	Δ m/z [ppm]	z	Rt [min]	Score	P	Range	Sequence	Modification
271	1	1293.7773	110.12	2	32.8	19.7	1	27-47	R.KLSTFSAYLEDHSYNVEQIWR.D	

Detailed Protein Report

Protein 623: CMP-N-acetylneuraminate-beta-1,4-galactoside alpha-2,3-sialyltransferase isoform n [Homo sapiens]

**Accession:**	gi|394953753	**Score:**	19.7
**Database:**	refseq_human(refseq_human_20140103.fasta)	**MW [kDa]:**	26.7
**Database Date:**	2015-11-30	**pI:**	10.2
		**Sequence Coverage [%]:**	4.8
		**No. of unique Peptides:**	1

```
        10         20         30         40         50         60         70         80
MGLLVFVRNL LLALCLFLVL GFLYYSAWKL HLLQWEEDSK YDRLGFLLNL DSKLFSKPAP MFLDDSFRKW ARIREFVPPF
        90        100        110        120        130        140        150        160
GIKGQDNLIK AILSVTKEYR LTPALDSLRC RRCIIVGNGG VLANKSLGSR IDDYDIVVRL NSAPVKGFEK DVGSKTTLRI
       170        180        190        200        210        220        230        240
TYPEGAMQRP EQYERDSLFV LAGFKWQDFK WLKYIVYKER VSWTHNIQRE KEFLRKLVKA RVITDLSSGI
```

Cmpd.	No. of Cmpds.	m/z meas.	Δ m/z [ppm]	z	Rt [min]	Score	P	Range	Sequence	Modification
2153	1	1131.4149	-196.06	1	56.8	19.7	1	220-230	K.ARVITDLSSGI.-	

Detailed Protein Report

Protein 624: eukaryotic translation initiation factor 4 gamma 3 isoform 1 [Homo sapiens]

Accession: gi|311771714
Database: refseq_human(refseq_human_20140103.fasta)
Database Date: 2015-11-30
Modification(s): Carbamidomethyl, Oxidation

Score: 19.4
MW [kDa]: 180.4
pI: 5.2
Sequence Coverage [%]: 0.9
No. of unique Peptides: 1

10	20	30	40	50	60	70	80
MNSQPQTRSP	FFQRPQIQPP	RATIPNSSPS	IRPGAQTPTA	VYQANQHIMM	VNHLPMPYPV	PQGPQYCIPQ	YRHSGPPYVG
90	100	110	120	130	140	150	160
PPQQYPVQPP	GPGPFYPGPG	PGDFPNAYGT	PFYPSQPVYQ	SAPIIVPTQQ	QPPPAKREKK	TIRIRDPNQG	GKDITEEIMS
170	180	190	200	210	220	230	240
GGGSRNPTPP	IGRPTSTPTP	PQLPSQVPEH	SPVVYGTVES	AHLAASTPVT	AASDQKQEEK	PKPDPVLKSP	SPVLRLVLSG
250	260	270	280	290	300	310	320
EKKEQEGQTS	ETTAIVSIAE	LPLPPSPTTV	SSVARSTIAA	PTSSALSSQP	IFTTAIDDRC	ELSSPREDTI	PIPSLTSCTE
330	340	350	360	370	380	390	400
TSDPLPTNEN	DDDICKKPCS	VAPNDIPLVS	STNLINEING	VSEKLSATES	IVEIVKQEVL	PLTLELEILE	NPPEEMKLEC
410	420	430	440	450	460	470	480
IPAPITPSTV	PSFPPTPPTP	PASPPHTPVI	VPAAATTVSS	PSAAITVQRV	LEEDESIRTC	LSEDAKEIQN	KIEVEADGQT
490	500	510	520	530	540	550	560
EEILDSQNLN	SRRSPVPAQI	AITVPKTWKK	PKDRTRTTEE	MLEAELELKA	EEELSIDKVL	ESEQDKMSQG	FHPERDPSDL
570	580	590	600	610	620	630	640
KKVKAVEENG	EEAEPVRNGA	ESVSEGEGID	ANSGSTDSSG	DGVTFPFKPE	SWKPTDTEGK	KQYDREFLLD	FQFMPACIQK
650	660	670	680	690	700	710	720
PEGLPPISDV	VLDKINQPKL	PMRTLDPRIL	PRGPDFTPAF	ADFGRQTPGG	RGVPICKVQS	RHGLPILEQS	KAPTCPPLVM
730	740	750	760	770	780	790	800
SHPPMKSLPL	GLLNVGSRRS	QPGQRREPRK	IITVSVKEDV	HLKKAENAWK	PSQKRDSQAD	DPENIKTQEL	FRKVRSILNK
810	820	830	840	850	860	870	880
LTPQMFNQLM	KQVSGLTVDT	EERLKGVIDL	VFEKAIDEPS	FSVAYANMCR	CLVTLKVPMA	DKPGNTVNFR	KLLLNRCQKE
890	900	910	920	930	940	950	960
FEKDKADDDV	FEKKQKELEA	ASAPEERTRL	HDELEEAKDK	ARRRSIGNIK	FIGELFKLKM	LTEAIMHDCV	VKLLKNHDEE
970	980	990	1000	1010	1020	1030	1040
SLECLCRLLT	TIGKDLDFEK	AKPRMDQYFN	QMEKIVKERK	TSSRIRFMLQ	DVIDLRLCNW	VSRRADQGPK	TIEQIHKEAK
1050	1060	1070	1080	1090	1100	1110	1120
IEEQEEQRKV	QQLMTKEKRR	PGVQRVDEGG	WNTVQGAKNS	RVLDPSKFLK	ITKPTIDEKI	QLVPKAQLGS	WGKGSSGGAK
1130	1140	1150	1160	1170	1180	1190	1200
ASETDALRSS	ASSLNRFSAL	QPPAPSGSTP	STPVEFDSRR	TLTSRGSMGR	EKNDKPLPSA	TARPNTFMRG	GSSKDLLDNQ
1210	1220	1230	1240	1250	1260	1270	1280
SQEEQRREML	ETVKQLTGGV	DVERNSTEAE	RNKTRESAKP	EISAMSAHDK	AALSEEELER	KSKSIIDEFL	HINDFKEAMQ
1290	1300	1310	1320	1330	1340	1350	1360
CVEELNAQGL	LHVFVRVGVE	STLERSQITR	DHMGQLLYQL	VQSEKLSKQD	FFKGFSETLE	LADDMAIDIP	HIWLYLAELV
1370	1380	1390	1400	1410	1420	1430	1440
TPMLKEGGIS	MRELTIEFSK	PLLPVGRAGV	LLSEILHLLC	KQMSHKKVGA	LWREADLSWK	DFLPEGEDVH	NFLLEQKLDF
1450	1460	1470	1480	1490	1500	1510	1520
IESDSPCSSE	ALSKKELSAE	ELYKRLEKLI	IEDKANDEQI	FDWVEANLDE	IQMSSPTFLR	ALMTAVCKAA	IIADSSTFRV
1530	1540	1550	1560	1570	1580	1590	1600
DTAVIKQRVP	ILLKYLDSDT	EKELQALYAL	QASIVKLDQP	ANLLRMFFDC	LYDEEVISED	AFYKWESSKD	PAEQNGKGVA
1610	1620	1630					
LKSVTAFFTW	LREAEEESED	N					

Cmpd.	No. of Cmpds.	m/z meas.	Δ m/z [ppm]	z	Rt [min]	Score	P	Range	Sequence	Modification
2704	1	847.8687	-44.85	2	65.0	19.4	0	712-726	K.APTCPPLVMSHPPMK.S	Carbamidomethyl: 4; Oxidation: 9, 14

Detailed Protein Report

Protein 625: PREDICTED: protein spire homolog 1 isoform X1 [Homo sapiens]

**Accession:**	gi|530424894	**Score:**	19.4
**Database:**	refseq_human(refseq_human_20140103.fasta)	**MW [kDa]:**	79.4
**Database Date:**	2015-11-30	**pI:**	9.8
**Modification(s):**	Carbamidomethyl, Oxidation	**Sequence Coverage [%]:**	3.9
		**No. of unique Peptides:**	1

10	20	30	40	50	60	70	80
MMDDGKLGYS	QCMETEVIES	LGIIIYKALD	YGLKENEERE	LSPPLEQLID	HMANTVEADG	SNDEGYEAAE	EGLGDEDEKR
**90**	**100**	**110**	**120**	**130**	**140**	**150**	**160**
KISAIRSYRD	VMKLCAAHLP	TESDAPNHYQ	AVCRALFAET	MELHTFLTKI	KSAKENLKKI	QEMEKSDESS	TDLEELKNAD
**170**	**180**	**190**	**200**	**210**	**220**	**230**	**240**
WARFWVQVMR	DLRNGVKLKK	VQERQYNPLP	IEYQLTPYEM	LMDDIRCKRY	TLRKVMVNGD	IPPRLKKSAH	EIILDFIRSR
**250**	**260**	**270**	**280**	**290**	**300**	**310**	**320**
PPLNPVSARK	LKPTPPRPRS	LHERILEEIK	AERKLRPVSP	EEIRRSRLDV	TTPESTKNLV	ESSMVNGGLT	SQTKENGLST
**330**	**340**	**350**	**360**	**370**	**380**	**390**	**400**
SQQVPAQRKK	LLRAPTLAEL	DSSESEEETL	HKSTSSSSVS	PSFPEEPVLE	AVSTRKKPPK	FLPISSTPQP	ERRQPPQRRH
**410**	**420**	**430**	**440**	**450**	**460**	**470**	**480**
SIEKETPTNV	RQFLPPSRQS	SRSLVPRITS	VWPRTPFRPL	FSTIQTASLL	SSHPFEAAMF	GVAGAMYYLC	ERAFTSRWKS
**490**	**500**	**510**	**520**	**530**	**540**	**550**	**560**
SKEEFCYPVE	CLALTVEEVM	HIRQVLVKAE	LEKYQQYKDI	YTALKKGKLC	FCCRTRRFSF	FTWSYTCQFC	KRPVCSQCCK
**570**	**580**	**590**	**600**	**610**	**620**	**630**	**640**
KMRLPSKPYS	TLPIFSLGPS	ALQRGESSMR	SEKPSTAHHR	PLRSIARFSS	KSKSMDKSDE	ELQFPKELME	DWSTMEVCVD
**650**	**660**	**670**	**680**	**690**	**700**		
CKKFISEIIS	SSRRSLVLAN	KRARLKRKTQ	SFYMSSPGPS	EYCPSERTIS	EI		

Cmpd.	No. of Cmpds.	m/z meas.	Δ m/z [ppm]	z	Rt [min]	Score	P	Range	Sequence	Modification
2094	1	1052.4646	-27.32	3	56.3	19.4	1	1-27	-.MMDDGKLGYSQCMETEVIESLGIIIYK.A	Carbamidomethyl: 12; Oxidation: 1, 2

Detailed Protein Report

Protein 626: prostaglandin E2 receptor EP3 subtype isoform 8 [Homo sapiens]

**Accession:**	gi|38505186	**Score:**	19.4
**Database:**	refseq_human(refseq_human_20140103.fasta)	**MW [kDa]:**	46.7
**Database Date:**	2015-11-30	**pI:**	11.4
		**Sequence Coverage [%]:**	6.2
		**No. of unique Peptides:**	1

10	20	30	40	50	60	70	80
MKETRGYGGD	APFCTRLNHS	YTGMWAPERS	AEARGNLTRP	PGSGEDCGSV	SVAFPITMLL	TGFVGNALAM	LLVSRSYRRR
**90**	**100**	**110**	**120**	**130**	**140**	**150**	**160**
ESKRKKSFLL	CIGWLALTDL	VGQLLTTPVV	IVVYLSKQRW	EHIDPSGRLC	TFFGLTMTVF	GLSSLFIASA	MAVERALAIR
**170**	**180**	**190**	**200**	**210**	**220**	**230**	**240**
APHWYASHMK	TRATRAVLLG	VWLAVLAFAL	LPVLGVGQYT	VQWPGTWCFI	STGRGGNGTS	SSHNWGNLFF	ASAFAFLGLL
**250**	**260**	**270**	**280**	**290**	**300**	**310**	**320**
ALTVTFSCNL	ATIKALVSRC	RAKATASQSS	AQWGRITTET	AIQLMGIMCV	LSVCWSPLLI	MMLKMIFNQT	SVEHCKTHTE
**330**	**340**	**350**	**360**	**370**	**380**	**390**	**400**
KQKECNFFLI	AVRLASLNQI	LDPWVYLLLR	KILLRKFCQM	RKRRLREQLI	CSLQNSQIQR	ATAHCGQVQT	YRVLNREEME
**410**	**420**						
VLVSSINVYT	RISTVKTE						

Cmpd.	No. of Cmpds.	m/z meas.	Δ m/z [ppm]	z	Rt [min]	Score	P	Range	Sequence	Modification
2724	1	992.4655	-25.39	3	65.3	19.4	1	367-392	R.EQLICSLQNSQIQRATAHCGQVQTYR.V	

Detailed Protein Report

Protein 627: PREDICTED: mothers against decapentaplegic homolog 9 isoform X5 [Homo sapiens]

Accession: gi|530402395
Database: refseq_human(refseq_human_20140103.fasta)
Database Date: 2015-11-30

Score: 19.3
MW [kDa]: 40.6
pI: 10.0
Sequence Coverage [%]: 5.1
No. of unique Peptides: 1

10	20	30	40	50	60	70	80
MHSTTPISSL	FSFTSPAVKR	LLGWKQGDEE	EKWAEKAVDS	LVKKLKKKKG	AMDELERALS	CPGQPSKCVT	IPRSLDGRLQ
**90**	**100**	**110**	**120**	**130**	**140**	**150**	**160**
VSHRKGLPHV	IYCRVWRWPD	LQSHHELKPL	ECCEFPFGSK	QKEVCINPYH	YRRVETPDFR	PVCYEEPQHW	CSVAYYELNN
**170**	**180**	**190**	**200**	**210**	**220**	**230**	**240**
RVGETFQASS	RSVLIDGFTD	PSNNRNRFCL	GLLSNVNRNS	TIENTRRHIG	KGVHLYYVGG	EVYAECVSDS	SIFVQSRNCN
**250**	**260**	**270**	**280**	**290**	**300**	**310**	**320**
YQHGFHPATV	CKIPSGCSLK	VFNNQLFAQL	LAQSVHHGFE	VVYELTKMCT	IRMSFVKVAM	KAGEPRKFLS	GKERQLGLYM
**330**	**340**	**350**	**360**				
EVKAFSFITC	YFVCCKVLAN	ESSISKYGLI	KVSGYV				

Cmpd.	No. of Cmpds.	m/z meas.	Δ m/z [ppm]	z	Rt [min]	Score	P	Range	Sequence	Modification
1665	1	944.8090	-147.29	2	50.7	19.3	1	50-67	K.GAMDELERALSCPGQPSK.C	

Detailed Protein Report

Protein 628: **PREDICTED: cleavage and polyadenylation specificity factor subunit 7 isoform X5 [Homo sapiens]**

**Accession:**	gi|530397403	**Score:**	19.3
**Database:**	refseq_human(refseq_human_20140103.fasta)	**MW [kDa]:**	41.9
**Database Date:**	2015-11-30	**pI:**	10.1
		**Sequence Coverage [%]:**	3.9
		**No. of unique Peptides:**	1

Alias proteins:

Accession	Name	Description
gi|530397405	refseq_human (refseq_human_20140103.fasta)	PREDICTED: cleavage and polyadenylation specificity factor subunit 7 isoform X6 [Homo sapiens]

10	20	30	40	50	60	70	80
MWTTDQQLIQ	VIRSIGVYDV	VELKFAENRA	NGQSKGYAEV	VVASENSVHK	LLELLPGKVL	NGEKVDVRPA	TRQNLSQFEA
**90**	**100**	**110**	**120**	**130**	**140**	**150**	**160**
QARKRECVRV	PRGGIPPRAH	SRDSSDSADG	RATPSENLVP	SSARVDKPPS	VLPYFNRPPS	ALPLMGLPPP	PIPPPPPLSS
**170**	**180**	**190**	**200**	**210**	**220**	**230**	**240**
SFGVPPPPPG	IHYQHLMPPP	PRLPPHLAVP	PPGAIPPALH	LNPAFFPPPN	ATVGPPPDTY	MKASAPYNHH	GSRDSGPPPS
**250**	**260**	**270**	**280**	**290**	**300**	**310**	**320**
TVSEAEFEDI	MKRNRAISSS	AISKAVSGAS	AGDYSDAIET	LLTAIAVIKQ	SRVANDERCR	VLISSLKDCL	HGIEAKSYSV
**330**	**340**	**350**	**360**	**370**	**380**	**390**	
GASGSSSRKR	HRSRERSPSR	SRESSRRHRD	LLHNEDRHDD	YFQERNREHE	RHRDRERDRH	H	

Cmpd.	No. of Cmpds.	m/z meas.	Δ m/z [ppm]	z	Rt [min]	Score	P	Range	Sequence	Modification
2002	2	794.7799	-153.57	2	54.9	19.3	0	36-50	K.GYAEVVVASENSVHK.L	

Detailed Protein Report

Protein 629: **PREDICTED: exosome complex exonuclease RRP44 isoform X1 [Homo sapiens]**

**Accession:**	gi|530402172	**Score:**	19.3
**Database:**	refseq_human(refseq_human_20140103.fasta)	**MW [kDa]:**	90.5
**Database Date:**	2015-11-30	**pI:**	6.5
		**Sequence Coverage [%]:**	2.0
		**No. of unique Peptides:**	1

10	20	30	40	50	60	70	80
MSADNQLQVI	FITNDRRNKE	KAIEEGIPAF	TCEEYVKSLT	ANPELIDRLA	CLSEEGNEIE	SGKIIFSEHL	PLSKLQQGIK
**90**	**100**	**110**	**120**	**130**	**140**	**150**	**160**
SGTYLQGTFR	ASRENYLEAT	VWIHGDNEEN	KEIILQGLKH	LNRAVHEDIV	AVELLPKSQW	VAPSSVVLHD	EGQNEEDVEK
**170**	**180**	**190**	**200**	**210**	**220**	**230**	**240**
EEETERMLKT	AVSEKMLKPT	GRVVGIIKRN	WRPYCGMLSK	SDIKESRRHL	FTPADKRIPR	IRIETRQAST	LEGRRIIVAI
**250**	**260**	**270**	**280**	**290**	**300**	**310**	**320**
DGWPRNSRYP	NGHFVRNLGD	VGEKETETEV	LLLEHDVPHQ	PFSQAVLSFL	PKMPWSITEK	DMKNREDLRH	LCICSVDPPG
**330**	**340**	**350**	**360**	**370**	**380**	**390**	**400**
CTDIDDALHC	RELENGNLEV	GVHIADVSHF	IRPGNALDQE	SARRGTTVYL	CEKRIDMVPE	LLSSNLCSLK	CDVDRLAFSC
**410**	**420**	**430**	**440**	**450**	**460**	**470**	**480**
IWEMNHNAEI	LKTKFTKSVI	NSKASLTYAE	AQLRIDSANM	NDDITTSLRG	LNKLAKILKK	RRIEKGALTL	SSPEVRFHMD
**490**	**500**	**510**	**520**	**530**	**540**	**550**	**560**
SETHDPIDLQ	TKELRETNSM	VEEFMLLANI	SVAKKIHEEF	SEHALLRKHP	APPPSNYEIL	VKAARSRNLE	IKTDTAKSLA
**570**	**580**	**590**	**600**	**610**	**620**	**630**	**640**
ESLDQAESPT	FPYLNTLLRI	LATRCMMQAV	YFCSGMDNDF	HHYGLASPIY	THFTSPIRRY	ADVIVHRLLA	VAIGADCTYP
**650**	**660**	**670**	**680**	**690**	**700**	**710**	**720**
ELTDKHKLAD	ICKNLNFRHK	MAQYAQRASV	AFHTQLFFKS	KGIVSEEAYI	LFVRKNAIVV	LIPKYGLEGT	VFFEEKDKPN
**730**	**740**	**750**	**760**	**770**	**780**	**790**	**800**
PQLIYDDEIP	SLKIEDTVFH	VFDKVKVKIM	LDSSNLQHQK	IRMSLVEPQI	PGISIPTDTS	NMDLNGPKKK	KMKLGK

Cmpd.	No. of Cmpds.	m/z meas.	Δ m/z [ppm]	z	Rt [min]	Score	P	Range	Sequence	Modification
1167	1	977.5217	29.45	2	44.2	19.3	2	189-204	K.RNWRPYCGMLSKSDIK.E	

Detailed Protein Report

Protein 630: **protein Tob2 [Homo sapiens]**

Accession: gi|7706739
Database: refseq_human(refseq_human_20140103.fasta)
Database Date: 2015-11-30

Score: 19.2
MW [kDa]: 36.6
pI: 6.6
Sequence Coverage [%]: 8.1
No. of unique Peptides: 1

Alias proteins:

Accession	Name	Description
gi|578836864	refseq_human (refseq_human_20140103.fasta)	PREDICTED: protein Tob2 isoform X2 [Homo sapiens]
gi|530419484	refseq_human (refseq_human_20140103.fasta)	PREDICTED: protein Tob2 isoform X1 [Homo sapiens]

10	20	30	40	50	60	70	80
MQLEIKVALN	FIISYLYNKL	PRRRADLFGE	ELERLLKKKY	EGHWYPEKPL	KGSGFRCVHI	GEMVDPVVEL	AAKRSGLAVE
**90**	**100**	**110**	**120**	**130**	**140**	**150**	**160**
DVRANVPEEL	SVWIDPFEVS	YQIGEKGAVK	VLYLDDSEGC	GAPELDKEIK	SSFNPDAQVF	VPIGSQDSSL	SNSPSPSFGQ
**170**	**180**	**190**	**200**	**210**	**220**	**230**	**240**
SPSPTFIPRS	AQPITFTTAS	FAATKFGSTK	MKKGGGAASG	GGVASSGAGG	QQPPQQPRMA	RSPTNSLLKH	KSLSLSMHSL
**250**	**260**	**270**	**280**	**290**	**300**	**310**	**320**
NFITANPAPQ	SQLSPNAKEF	VYNGGGSPSL	FFDAADGQGS	GTPGPFGGSG	AGTCNSSSFD	MAQVFGGGAN	SLFLEKTPFV
**330**	**340**	**350**					
EGLSYNLNTM	QYPSQQFQPV	VLAN					

Cmpd.	No. of Cmpds.	m/z meas.	Δ m/z [ppm]	z	Rt [min]	Score	P	Range	Sequence	Modification
2780	1	841.6618	-109.74	3	65.2	19.2	2	191-218	K.MKKGGGAASGGGVASSGAGGQQPPQQPR.M	

Detailed Protein Report

Protein 631: PREDICTED: mucin-6 isoform X1 [Homo sapiens]

**Accession:**	gi|578840955	**Score:**	19.2
**Database:**	refseq_human(refseq_human_20140103.fasta)	**MW [kDa]:**	141.7
**Database Date:**	2015-11-30	**pI:**	5.7
**Modification(s):**	Carbamidomethyl	**Sequence Coverage [%]:**	1.1
		**No. of unique Peptides:**	1

10	20	30	40	50	60	70	80
MVQRWLLLSC	CGALLSAGLA	NTSYTSPGLQ	RLKDSPQTAP	DKGQCSTWGA	GHFSTFDHHV	YDFSGTCNYI	FAATCKDAFP
90	100	110	120	130	140	150	160
TFSVQLRRGP	DGSISRIIVE	LGASVVTVSE	AIISVKDIGV	ISLPYTSNGL	QITPFGQSVR	LVAKQLELEL	EVVWGPDSHL
170	180	190	200	210	220	230	240
MVLVERKYMG	QMCGLCGNFD	GKVTNEFVSE	EGKFLEPHKF	AALQKLDDPG	EICTFQDIPS	THVRQAQHAR	ICTQLLTLVA
250	260	270	280	290	300	310	320
PECSVSKEPF	VLSCQADVAA	APQPGPQNSS	CATLSEYSRQ	CSMVGQPVRR	WRSPGLCSVG	QCPANQVYQE	CGSACVKTCS
330	340	350	360	370	380	390	400
NPQHSCSSSC	TFGCFCPEGT	VLNDLSNNHT	CVPVTQCPCV	LHGAMYAPGE	VTIAACQTCR	CTLGRWVCTE	RPCPGHCSLE
410	420	430	440	450	460	470	480
GGSFVTTFDA	RPYRFHGTCT	YILLQSPQLP	EDGALMAVYD	KSGVSHSETS	LVAVVYLSRQ	DKIVISQDEV	VTNNGEAKWL
490	500	510	520	530	540	550	560
PYKTRNITVF	RQTSTHLQMA	TSFGLELVVQ	LRPIFQAYVT	VGPQFRGQTR	GLCGNFNGDT	TDDFTTSMGI	AEGTASLFVD
570	580	590	600	610	620	630	640
SWRAGNCPAA	LERETDPCSM	SQLNKVCAET	HCSMLLRTGT	VFERCHATVN	PAPFYKRCMY	QACNYEETFP	HICAALGDYV
650	660	670	680	690	700	710	720
HACSLRGVLL	WGWRSSVDNC	TIPCTGNTTF	SYNSQACERT	CLSLSDRATE	CHHSAVPVDG	CNCPDGTYLN	QKGECVRKAQ
730	740	750	760	770	780	790	800
CPCILEGYKF	ILAEQSTVIN	GITCHCINGR	LSCPQRPQMF	LASCQAPKTF	KSCSQSSENK	FGAACAPTCQ	MLATGVACVP
810	820	830	840	850	860	870	880
TKCEPGCVCA	EGLYENADGQ	CVPPEECPCE	FSGVSYPGGA	ELHTDCRTCS	CSRGRWACQQ	GTHCPSTCTL	YGEGHVITFD
890	900	910	920	930	940	950	960
GQRFVFDGNC	EYILATDVCG	VNDSQPTFKI	LTENVICGNS	GVTCSRAIKI	FLGGLSVVLA	DRNYTVTGEE	PHVQLGVTPG
970	980	990	1000	1010	1020	1030	1040
ALSLVVDISI	PGRYNLTLIW	NRHMTILIRI	ARASQDPLCG	LCGNFNGNMK	DDFETRSRYV	ASSELELVNS	WKESPLCGDV
1050	1060	1070	1080	1090	1100	1110	1120
SFVTDPCSLN	AFRRSWAERK	CSVINSQTFA	TCHSKVYHLP	YYEACVRDAC	GCDSGGDCEC	LCDAVAAYAQ	ACLDKGVCVD
1130	1140	1150	1160	1170	1180	1190	1200
WRTPAFCPIY	CGFYNTHTQD	GHGEYQYTQE	ANCTWHYQPC	LCPSQPQSVP	GSNIEGCYNC	SQDEYFDHEE	GVCVPCMPPT
1210	1220	1230	1240	1250	1260	1270	1280
TPQPPTTPQL	PTTGSRPTQV	WPMTGTSTTI	GLLSSTGPSP	SSNHTPASPT	QTPLLPATLT	SSKPTASSGG	KEPPAEPMER
1290	1300	1310	1320				
AAAGGPRYTS	GVGLGMALLP	GSHLPLSRFP	A				

Cmpd.	No. of Cmpds.	m/z meas.	Δ m/z [ppm]	z	Rt [min]	Score	P	Range	Sequence	Modification
1896	1	840.7895	-54.74	2	53.8	19.2	0	168-182	K.YMGQMCGLCGNFDGK.V	Carbamidomethyl: 9

Detailed Protein Report

Protein 632: probable G-protein coupled receptor 141 [Homo sapiens]

Accession: gi|32401435
Database: refseq_human(refseq_human_20140103.fasta)
Database Date: 2015-11-30

Score: 19.1
MW [kDa]: 35.4
pI: 10.1
Sequence Coverage [%]: 5.6
No. of unique Peptides: 1

10	20	30	40	50	60	70	80
MPGHNTSRNS	SCDPIVTPHL	ISLYFIVLIG	GLVGVISILF	LLVKMNTRSV	TTMAVINLVV	VHSVFLLTVP	FRLTYLIKKT
90	100	110	120	130	140	150	160
WMFGLPFCKF	VSAMLHIHMY	LTFLFYVVIL	VTRYLIFFKC	KDKVEFYRKL	HAVAASAGMW	TLVIVIVVPL	VVSRYGIHEE
170	180	190	200	210	220	230	240
YNEEHCFKFH	KELAYTYVKI	INYMIVIFVI	AVAVILLVFQ	VFIIMLMVQK	LRHSLLSHQE	FWAQLKNLFF	IGVILVCFLP
250	260	270	280	290	300	310	
YQFFRIYYLN	VVTHSNACNS	KVAFYNEIFL	SVTAISCYDL	LLFVFGGSHW	FKQKIIGLWN	CVLCR	

Cmpd.	No. of Cmpds.	m/z meas.	Δ m/z [ppm]	z	Rt [min]	Score	P	Range	Sequence	Modification
1711	1	1044.9814	-87.18	2	51.2	19.1	2	73-89	R.LTYLIKKTWMFGLPFCK.F	

Detailed Protein Report

Protein 633: protein FAM83G [Homo sapiens]

Accession: gi|115392150
Database: refseq_human(refseq_human_20140103.fasta)
Database Date: 2015-11-30

Score: 19.0
MW [kDa]: 90.8
pI: 5.9
Sequence Coverage [%]: 1.0
No. of unique Peptides: 1

Quantitation

MD:MU **Median:** 0.74 **CV:** 0.00 % **No. of Peptides:** 1

10	20	30	40	50	60	70	80
MAFSQVQCLD	DNHVNWRSSE	SKPEFFYSEE	QRLALEALVA	RGRDAFYEVL	KRENIRDFLS	ELELKRILET	IEVYDPGSED
**90**	**100**	**110**	**120**	**130**	**140**	**150**	**160**
PRGTGPSQGP	EDNGVGDGEE	ASGADGVPIE	AEPLPSLEYW	PQKSDRSIPQ	LDLGWPDTIA	YRGVTRASVY	MQPPIDGQAH
**170**	**180**	**190**	**200**	**210**	**220**	**230**	**240**
IKEVVRKMIS	QAQKVIAVVM	DMFTDVDIFK	DLLDAGFKRK	VAVYIIVDES	NVKYFLHMCE	RACMHLGHLK	NLRVRSSGGT
**250**	**260**	**270**	**280**	**290**	**300**	**310**	**320**
EFFTRSATKF	KGALAQKFMF	VDGDRAVCGS	YSFTWSAART	DRNVISVLSG	QVVEMFDRQF	QELYLMSHSV	SLKGIPMEKE
**330**	**340**	**350**	**360**	**370**	**380**	**390**	**400**
PEPEPIVLPS	VVPLVPAGTV	AKKLVNPKYA	LVKAKSVDEI	AKISSEKQEA	KKPLGLKGPA	LAEHPGELPE	LLPPIHPGLL
**410**	**420**	**430**	**440**	**450**	**460**	**470**	**480**
HLERANMFEY	LPTWVEPDPE	PGSDILGYIN	IIDPNIWNPQ	PSQMNRIKIR	DTSQASAQHQ	LWKQSQDSRP	RPEPCPPPEP
**490**	**500**	**510**	**520**	**530**	**540**	**550**	**560**
SAPQDGVPAE	NGLPQGDPEP	LPPVPKPRTV	PVADVLARDS	SDIGWVLELP	KEEAPQNGTD	HRLPRMAGPG	HAPLQRQLSV
**570**	**580**	**590**	**600**	**610**	**620**	**630**	**640**
TQDDPESLGV	GLPNGLDGVE	EEDDDDYVTL	SDQDSHSGSS	GRGPGPRRPS	VASSVSEEYF	EVREHSVPLR	RRHSEQVANG
**650**	**660**	**670**	**680**	**690**	**700**	**710**	**720**
PTPPPRRQLS	APHITRGTFV	GPQGGSPWAQ	SRGREEADAL	KRMQAQRSTD	KEAQGQQFHH	HRVPASGTRD	KDGFPGPPRY
**730**	**740**	**750**	**760**	**770**	**780**	**790**	**800**
RSAADSVQSS	TRNAGPAMAG	PHHWQAKGGQ	VPRLLPDPGS	PRLAQNARPM	TDGRATEEHP	SPFGIPYSKL	SQSKHLKART
**810**	**820**	**830**					
GGSQWASSDS	KRRAQAPRDR	KDP					

Cmpd.	No. of Cmpds.	m/z meas.	Δ m/z [ppm]	z	Rt [min]	Score	P	Range	Sequence	Modification	Ratios
546	4	492.6414	-231.48	2	36.4	19.0	0	44-51	R.DAFYEVLK.R		MD:MU 0.74

Detailed Protein Report

Protein 634: PREDICTED: zinc finger protein 606 isoform X2 [Homo sapiens]

**Accession:**	gi|530417293	**Score:**	18.9
**Database:**	refseq_human(refseq_human_20140103.fasta)	**MW [kDa]:**	81.7
**Database Date:**	2015-11-30	**pI:**	9.6
**Modification(s):**	Carbamidomethyl	**Sequence Coverage [%]:**	2.6
		**No. of unique Peptides:**	1

10	20	30	40	50	60	70	80
MLETYGHLLS	VGNQIAKPEV	ISLLEQGEEP	WSVEQACPQR	TCPEWVRNLE	SKALIPAQSI	FEEEQSHGMK	LERYIWDDPW
**90**	**100**	**110**	**120**	**130**	**140**	**150**	**160**
FSRLEVLGCK	DQLEMYHMNQ	STAMRQMVFM	QKQVLSQRSS	EFCGLGAEFS	QNLNFVPSQR	VSQIEHFYKP	DTHAQSWRCD
**170**	**180**	**190**	**200**	**210**	**220**	**230**	**240**
SAIMYADKVT	CENNDYDKTV	YQSIQPIYPA	RIQTGDNLFK	CTDAVKSFNH	IIHFGDHKGI	HTGEKLYEYK	ECHQIFNQSP
**250**	**260**	**270**	**280**	**290**	**300**	**310**	**320**
SFNEHPRLHV	GENQYNYKEY	ENIFYFSSFM	EHQKIGTVEK	AYKYNEWEKV	FGYDSFLTQH	TSTYTAEKPY	DYNECGTSFI
**330**	**340**	**350**	**360**	**370**	**380**	**390**	**400**
WSSYLIQHKK	THTGEKPYEC	DKCGKVFRNR	SALTKHERTH	TGIKPYECNK	CGKAFSWNSH	LIVHKRIHTG	EKPYVCNECG
**410**	**420**	**430**	**440**	**450**	**460**	**470**	**480**
KSFNWNSHLI	GHQRTHTGEK	PFECTECGKS	FSWSSHLIAH	MRMHTGEKPF	KCDECEKAFR	DYSALSKHER	THSGAKPYKC
**490**	**500**	**510**	**520**	**530**	**540**	**550**	**560**
TECGKSFSWS	SHLIAHQRTH	TGEKPYNCQE	CGKAFRERSA	LTKHEIIHSG	IKPYECNKCG	KSCSQMAHLV	RHQRTHTGEK
**570**	**580**	**590**	**600**	**610**	**620**	**630**	**640**
PYECNKCGKS	FSQSCHLVAH	RRIHTGEKPY	KCNQCERSFN	CSSHLIAHRR	THTGEKPYRC	NECGKAFNES	SSLIVHLRNH
**650**	**660**	**670**	**680**	**690**	**700**	**710**	
TGEKPYKCNH	CEKAFCKNSS	LIIHQRMHSG	EKRFICSECG	KAFSGHSALL	QHQRNHSEEK	LN	

Cmpd.	No. of Cmpds.	m/z meas.	Δ m/z [ppm]	z	Rt [min]	Score	P	Range	Sequence	Modification
1657	1	1044.8456	-136.96	2	50.6	18.9	2	468-485	K.HERTHSGAKPYKCTECGK.S	Carbamidomethyl: 13

Detailed Protein Report

Protein 635: something about silencing protein 10 [Homo sapiens]

Accession: gi|9966799
Database: refseq_human(refseq_human_20140103.fasta)
Database Date: 2015-11-30

Score: 18.9
MW [kDa]: 54.5
pI: 5.4
Sequence Coverage [%]: 4.0
No. of unique Peptides: 1

Quantitation

MD:MU **Median:** 1.07 **CV:** 0.00 % **No. of Peptides:** 1

10	20	30	40	50	60	70	80
MVGRSRRRGA	AKWAAVRAKA	GPTLTDENGD	DLGLPPSPGD	TSYYQDQVDD	FHEARSRAAL	AKGWNEVQSG	DEEDGEEEEE
90	100	110	120	130	140	150	160
EVLALDMDDE	DDEDGGNAGE	EEEEENADDD	GGSSVQSEAE	ASVDPSLSWG	QRKKLYYDTD	YGSKSRGRQS	QQEAEEEERE
170	180	190	200	210	220	230	240
EEEEAQIIQR	RLAQALQEDD	FGVAWVEAFA	KPVPQVDEAE	TRVVKDLAKV	SVKEKLKMLR	KESPELLELI	EDLKVKLTEV
250	260	270	280	290	300	310	320
KDELEPLLEL	VEQGIIPPGK	GSQYLRTKYN	LYLNYCSNIS	FYLILKARRV	PAHGHPVIER	LVTYRNLINK	LSVVDQKLSS
330	340	350	360	370	380	390	400
EIRHLLTLKD	DAVKKELIPK	AKSTKPKPKS	VSKTSAAACA	VTDLSDDSDF	DEKAKLKYYK	EIEDRQKLKR	KKEENSTEEQ
410	420	430	440	450	460	470	480
ALEDQNAKRA	ITYQIAKNRG	LTPRRKKIDR	NPRVKHREKF	RRAKIRRRGQ	VREVRKEEQR	YSGELSGIRA	GVKKSIKLK

Cmpd.	No. of Cmpds.	m/z meas.	Δ m/z [ppm]	z	Rt [min]	Score	P	Range	Sequence	Modification	Ratios
1704	1	697.0043	-68.98	3	51.1	18.9	0	242-260	K.DELEPLLELVEQGIIPPGK.G		MD:MU 1.07

Detailed Protein Report

Protein 636: PREDICTED: rho GTPase-activating protein 18 isoform X2 [Homo sapiens]

**Accession:**	gi|530384204	**Score:**	18.8
**Database:**	refseq_human(refseq_human_20140103.fasta)	**MW [kDa]:**	66.4
**Database Date:**	2015-11-30	**pI:**	6.5
		**Sequence Coverage [%]:**	1.5
		**No. of unique Peptides:**	1

10	20	30	40	50	60	70	80
MSWLSSSQGV	VLTAYHPSGK	DQTVGNSHAK	AGEEATSSRR	YGQYTMNQES	TTIKVMEKPP	FDRSISQDSL	DELSMEDYWI
**90**	**100**	**110**	**120**	**130**	**140**	**150**	**160**
ELENIKKSSE	NSQEDQEVVV	VKEPDEGELE	EEWLKEAGLS	NLFGESAGDP	QESIVFLSTL	TRTQAAAVQK	RVETVSQTLR
**170**	**180**	**190**	**200**	**210**	**220**	**230**	**240**
KKNKQYQIPD	VRDIFAQQRE	SKETAPGGTE	SQSLRTNENK	YQGRDDEASN	LVGEEKLIPP	EETPAPETDI	NLEVSFAEQA
**250**	**260**	**270**	**280**	**290**	**300**	**310**	**320**
LNQKESSKEK	IQKSKGDDAT	LPSFRLPKDK	TGTTRIGDLA	PQDMKKVCHL	ALIELTALYD	VLGIELKQQK	AVKIKTKDSG
**330**	**340**	**350**	**360**	**370**	**380**	**390**	**400**
LFCVPLTALL	EQDQRKVPGM	RIPLIFQKLI	SRIEERGLET	EGLLRIPGAA	IRIKNLCQEL	EAKFYEGTFN	WESVKQHDAA
**410**	**420**	**430**	**440**	**450**	**460**	**470**	**480**
SLLKLFIREL	PQPLLSVEYL	KAFQAVQNLP	TKKQQLQALN	LLVILLPDAN	RDTLKALLEF	LQRVIDNKEK	NKMTVMNVAM
**490**	**500**	**510**	**520**	**530**	**540**	**550**	**560**
VMAPNLFMCH	ALGLKSSEQR	EFVMAAGTAN	TMHLLIKYQK	LLWTIPKFIV	NQVRKQNTEN	HKKDKRAMKK	LLKKMAYDRE
**570**	**580**	**590**					
KYEKQDKSTN	DWGCPDSQER	RSFFV					

Cmpd.	No. of Cmpds.	m/z meas.	Δ m/z [ppm]	z	Rt [min]	Score	P	Range	Sequence	Modification
875	1	491.6668	-208.81	2	40.5	18.8	0	396-404	K.QHDAASLLK.L	

Detailed Protein Report

Protein 637: N-acylglucosamine 2-epimerase [Homo sapiens]

Accession: gi|213417820
Database: refseq_human(refseq_human_20140103.fasta)
Database Date: 2015-11-30
Modification(s): Oxidation

Score: 18.8
MW [kDa]: 48.8
pI: 5.9
Sequence Coverage [%]: 3.0
No. of unique Peptides: 1

10	20	30	40	50	60	70	80
MSKGLPARQD	MEKERETLQA	WKERVGQELD	RVVAFWMEHS	HDQEHGGFFT	CLGREGRVYD	DLKYVWLQGR	QVWMYCRLYR
**90**	**100**	**110**	**120**	**130**	**140**	**150**	**160**
TFERFRHAQL	LDAAKAGGEF	LLRYARVAPP	GKKCAFVLTR	DGRPVKVQRT	IFSECFYTMA	MNELWRATGE	VRYQTEAVEM
**170**	**180**	**190**	**200**	**210**	**220**	**230**	**240**
MDQIVHWVQE	DASGLGRPQL	QGAPAAEPMA	VPMMLLNLVE	QLGEADEELA	GKYAELGDWC	ARRILQHVQR	DGQAVLENVS
**250**	**260**	**270**	**280**	**290**	**300**	**310**	**320**
EGGKELPGCL	GRQQNPGHTL	EAGWFLLRHC	IRKGDPELRA	HVIDKFLLLP	FHSGWDPDHG	GLFYFQDADN	FCPTQLEWAM
**330**	**340**	**350**	**360**	**370**	**380**	**390**	**400**
KLWWPHSEAM	IAFLMGYSDS	GDPVLLRLFY	QVAEYTFRQF	RDPEYGEWFG	YLSREGKVAL	SIKGGPFKGC	FHVPRCLAMC
**410**	**420**	**430**					
EEMLGALLSR	PAPAPSPAPT	PACRGAE					

Cmpd.	No. of Cmpds.	m/z meas.	Δ m/z [ppm]	z	Rt [min]	Score	P	Range	Sequence	Modification
2036	1	753.8094	-85.39	2	55.6	18.8	2	1-13	-.MSKGLPARQDMEK.E	Oxidation: 11

Detailed Protein Report

Protein 638: **protein dispatched homolog 2 [Homo sapiens]**

**Accession:**	gi|25121980	**Score:**	18.7
**Database:**	refseq_human(refseq_human_20140103.fasta)	**MW [kDa]:**	151.9
**Database Date:**	2015-11-30	**pI:**	9.3
		**Sequence Coverage [%]:**	0.6
		**No. of unique Peptides:**	1

10	20	30	40	50	60	70	80
MDGDSSSSSG	GSGPAPGPGP	EGEQRPEGEP	LAPDGGSPDS	TQTKAVPPEA	SPERSCSLHS	CPLEDPSSSS	GPPPTTSTLQ
90	100	110	120	130	140	150	160
PVGPSSPLAP	AHFTYPRALQ	EYQGGSSLPG	LGDRAALCSH	GSSLSPSPAP	SQRDGTWKPP	AVQHHVVSVR	QERAFQMPKS
170	180	190	200	210	220	230	240
YSQLIAEWPV	AVLMLCLAVI	FLCTLAGLLG	ARLPDFSKPL	LGFEPRDTDI	GSKLVVWRAL	QALTGPRKLL	FLSPDLELNS
250	260	270	280	290	300	310	320
SSSHNTLRPA	PRGSAQESAV	RPRRMVEPLE	DRRQENFFCG	PPEKSYAKLV	FMSTSSGSLW	NLHAIHSMCR	MEQDQIRSHT
330	340	350	360	370	380	390	400
SFGALCQRTA	ANQCCPSWSL	GNYLAVLSNR	SSCLDTTQAD	AARTLALLRT	CALYYHSGAL	VPSCLGPGQN	KSPRCAQVPT
410	420	430	440	450	460	470	480
KCSQSSAIYQ	LLHFLLDRDF	LSPQTTDYQV	PSLKYSLLFL	PTPKGASLMD	IYLDRLATPW	GLADNYTSVT	GMDLGLKQEL
490	500	510	520	530	540	550	560
LRHFLVQDTV	YPLLALVAIF	FGMALYLRSL	FLTLMVLLGV	LGSLLVAFFL	YQVAFRMAYF	PFVNLAALLL	LSSVCANHTL
570	580	590	600	610	620	630	640
IFFDLWRLSK	SQLPSGGLAQ	RVGRTMHHFG	YLLLVSGLTT	SAAFYASYLS	RLPAVRCLAL	FMGTAVLVHL	ALTLVWLPAS
650	660	670	680	690	700	710	720
AVLHERYLAR	GCARRARGRW	EGSAPRRLLL	ALHRRLRGLR	RAAAGTSRLL	FQRLLPCGVI	KFRYIWICWF	AALAAGGAYI
730	740	750	760	770	780	790	800
AGVSPRLRLP	TLPPPGGQVF	RPSHPFERFD	AEYRQLFLFE	QLPQGEGGHM	PVVLVWGVLP	VDTGDPLDPR	SNSSLVRDPA
810	820	830	840	850	860	870	880
FSASGPEAQR	WLLALCHRAR	NQSFFDTLQE	GWPTLCFVET	LQRWMESPSC	ARLGPDLCCG	HSDFPWAPQF	FLHCLKMMAL
890	900	910	920	930	940	950	960
EQGPDGTQDL	GLRFDAHGSL	AALVLQFQTN	FRNSPDYNQT	QLFYNEVSHW	LAAELGMAPP	GLRRGWFTSR	LELYSLQHSL
970	980	990	1000	1010	1020	1030	1040
STEPAVVLGL	ALALAFATLL	LGTWNVPLSL	FSVAAVAGTV	LLTVGLLVLL	EWQLNTAEAL	FLSASVGLSV	DFTVNYCISY
1050	1060	1070	1080	1090	1100	1110	1120
HLCPHPDRLS	RVAFSLRQTS	CATAVGAAAL	FAAGVLMLPA	TVLLYRKLGI	ILMMVKCVSC	GFASFFFQSL	CCFFGPEKNC
1130	1140	1150	1160	1170	1180	1190	1200
GQILWPCAHL	PWDAGTGDPG	GEKAGRPRPG	SVGGMPGSCS	EQYELQPLAR	RRSPSFDTST	ATSKLSHRPS	VLSEDLQLHD
1210	1220	1230	1240	1250	1260	1270	1280
GPCCSRPPPA	PASPRELLLD	HQAVFSQCPA	LQTSSPYKQA	GPSPKTRARQ	DSQGEEAEPL	PASPEAPAHS	PKAKAADPPD
1290	1300	1310	1320	1330	1340	1350	1360
GFCSSASTLE	GLSVSDETCL	STSEPSARVP	DSVGVSPDDL	DDTGQPVLER	GQLNGKRDTL	WLALRETVYD	PSLPASHHSS
1370	1380	1390	1400	1410			
LSWKGRGGPG	DGSPVVLPNS	QPDLPDVWLR	RPSTHTSGYS	S			

Cmpd.	No. of Cmpds.	m/z meas.	Δ m/z [ppm]	z	Rt [min]	Score	P	Range	Sequence	Modification
1767	2	1011.7857	227.39	1	51.9	18.7	0	811-818	R.WLLALCHR.A	

Detailed Protein Report

Protein 639: PREDICTED: leucine-rich repeat-containing protein 16A isoform X6 [Homo sapiens]

**Accession:**	gi|578811792	**Score:**	18.7
**Database:**	refseq_human(refseq_human_20140103.fasta)	**MW [kDa]:**	105.2
**Database Date:**	2015-11-30	**pI:**	6.6
**Modification(s):**	Oxidation	**Sequence Coverage [%]:**	2.7
		**No. of unique Peptides:**	1

10	20	30	40	50	60	70	80
MTEESSDVPR	ELIESIKDVI	GRKIKISVKK	KVKLEVKGDK	VENKVLVLTS	CRAFLVTARI	PTKLELTFSY	LEIHGVVCSK
90	100	110	120	130	140	150	160
SAQMIVETEK	CSISMKMASP	EDVSEVLAHI	GTCLRKIFPG	LSPVRIMKKV	SMEPSERLAS	LQALWDSQTV	AEQGPCGGFS
170	180	190	200	210	220	230	240
QMYACVCDWL	GFSYREEVQW	DVDTIYLTQD	TRELNLQDFS	HLDHRDLIPI	IAALEYNQWF	TKLSSKDLKL	STDVCEQILR
250	260	270	280	290	300	310	320
VVSRSNRLEE	LVLENAGLRT	DFAQKLASAL	AHNPNSGLHT	INLAGNPLED	RGVSSLSIQF	AKLPKGLKHL	NLSKTSLSPK
330	340	350	360	370	380	390	400
GVNSLSQSLS	ANPLTASTLV	HLDLSGNVLR	GDDLSHMYNF	LAQPNAIVHL	DLSNTECSLD	MVCGALLRGC	LQYLAVLNLS
410	420	430	440	450	460	470	480
RTVFSHRKGK	EVPPSFKQFF	SSSLALMHIN	LSGTKLSPEP	LKALLLGLAC	NHNLKGVSLD	LSNCELGHCL	RSGGAQVLEG
490	500	510	520	530	540	550	560
CIAEIHNITS	LDISDNGLES	DLSTLIVWLS	KNRSIQHLAL	GKNFNNMKSK	NLTPVLDNLV	QMIQDEESPL	QSLSLADSKL
570	580	590	600	610	620	630	640
KTEVTIIINA	LGSNTSLTKV	DISGNGMGDM	GAKMLAKALQ	INTKLRTVIW	DKNNITAQGF	QDIAVAMEKN	YTLRFMPIPM
650	660	670	680	690	700	710	720
YDASQALKTN	PEKTEDALQK	IENYLLRNHE	TRKYLQEQAY	RLQQGIVTST	TQQMIDRICV	KVQDHLNSLR	NCGGDAIQED
730	740	750	760	770	780	790	800
LKSAERLMRD	AKNSKTLLPN	LYHVGGASWA	GASGLLSSPI	QETLESMAGE	VTRVVDEQLK	ALLESMVDAA	ENLCPNVMKK
810	820	830	840	850	860	870	880
AHIRQDLIHA	STEKISIPRT	FVKNVLLEQS	GIDILNKISE	VKLTVASFLS	DRIVDEILDA	LSHCHHKLAD	HFSRRGKTLP
890	900	910	920	930	940	950	
QQESLEIELA	EEKPVKRSII	TVEELTEIER	LEDLDTCMKP	HAFQHAMMTF	ASTTFRLCQK	SSKLSLLSS	

Cmpd.	No. of Cmpds.	m/z meas.	Δ m/z [ppm]	z	Rt [min]	Score	P	Range	Sequence	Modification
843	1	946.5224	65.61	3	40.1	18.7	2	91-116	K.CSISMKMASPEDVSEVLAHIGTCLRK.I	Oxidation: 5, 7

Detailed Protein Report

Protein 640: myosin-binding protein C, slow-type isoform 9 [Homo sapiens]

**Accession:**	gi|360039225	**Score:**	18.6
**Database:**	refseq_human(refseq_human_20140103.fasta)	**MW [kDa]:**	126.3
**Database Date:**	2015-11-30	**pI:**	6.0
		**Sequence Coverage [%]:**	1.4
		**No. of unique Peptides:**	1

10	20	30	40	50	60	70	80
MPEPTKKEDW	TLVETPPGEE	QAKQNANSQL	SILFIEKPQG	GTVKVGEDIT	FIAKVKAEDL	LRKPTIKWFK	GKWMDLASKA
**90**	**100**	**110**	**120**	**130**	**140**	**150**	**160**
GKHLQLKETF	ERHSRVYTFE	MQIIKAKDNF	AGNYRCEVTY	KDKFDSCSFD	LEVHESTGTT	PNIDIRSAFK	RSGEGQEDAG
**170**	**180**	**190**	**200**	**210**	**220**	**230**	**240**
ELDFSGLLKR	REVKQQEEEP	QVDVWELLKN	AKPSEYEKIA	FQYGITDLRG	MLKRLKRMRR	EEKKSAAFAK	ILDPAYQVDK
**250**	**260**	**270**	**280**	**290**	**300**	**310**	**320**
GGRVRFVVEL	ADPKLEVKWY	KNGQEIRPST	KYIFEHKGCQ	RILFINNCQM	TDDSEYYVTA	GDEKCSTELF	VREPPIMVTK
**330**	**340**	**350**	**360**	**370**	**380**	**390**	**400**
QLEDTTAYCG	ERVELECEVS	EDDANVKWFK	NGEEIIPGPK	SRYRIRVEGK	KHILIIEGAT	KADAAEYSVM	TTGGQSSAKL
**410**	**420**	**430**	**440**	**450**	**460**	**470**	**480**
SVDLKPLKIL	TPLTDQTVNL	GKEICLKCEI	SENIPGKWTK	NGLPVQESDR	LKVVHKGRIH	KLVIANALTE	DEGDYVFAPD
**490**	**500**	**510**	**520**	**530**	**540**	**550**	**560**
AYNVTLPAKV	HVIDPPKIIL	DGLDADNTVT	VIAGNKLRLE	IPISGEPPPK	AMWSRGDKAI	MEGSGRIRTE	SYPDSSTLVI
**570**	**580**	**590**	**600**	**610**	**620**	**630**	**640**
DIAERDDSGV	YHINLKNEAG	EAHASIKVKV	VDFPDPPVAP	TVTEVGDDWC	IMNWEPPAYD	GGSPILGYFI	ERKKKQSSRW
**650**	**660**	**670**	**680**	**690**	**700**	**710**	**720**
MRLNFDLCKE	TTFEPKKMIE	GVAYEVRIFA	VNAIGISKPS	MPSRPFVPLA	VTSPPTLLTV	DSVTDTTVTM	RWRPPDHIGA
**730**	**740**	**750**	**760**	**770**	**780**	**790**	**800**
AGLDGYVLEY	CFEGTEDWIV	ANKDLIDKTK	FTITGLPTDA	KIFVRVKAVN	AAGASEPKYY	SQPILVKEII	EPPKIRIPRH
**810**	**820**	**830**	**840**	**850**	**860**	**870**	**880**
LKQTYIRRVG	EAVNLVIPFQ	GKPRPELTWK	KDGAEIDKNQ	INIRNSETDT	IIFIRKAERS	HSGKYDLQVK	VDKFVETASI
**890**	**900**	**910**	**920**	**930**	**940**	**950**	**960**
DIQIIDRPGP	PQIVKIEDVW	GENVALTWTP	PKDDGNAAIT	GYTIQKADKK	SMEWFTVIEH	YHRTSATITE	LVIGNEYYFR
**970**	**980**	**990**	**1000**	**1010**	**1020**	**1030**	**1040**
VFSENMCGLS	EDATMTKESA	VIARDGKIYK	NPVYEDFDFS	EAPMFTQPLV	NTYAIAGYNA	TLNCSVRGNP	KPKITWMKNK
**1050**	**1060**	**1070**	**1080**	**1090**	**1100**	**1110**	**1120**
VAIVDDPRYR	MFSNQGVCTL	EIRKPSPYDG	GTYCCKAVND	LGTVEIECKL	EVKVIYQGVN	TPGQPVFLEG	QQQSLHNKDF
**1130**							

Cmpd.	No. of Cmpds.	m/z meas.	Δ m/z [ppm]	z	Rt [min]	Score	P	Range	Sequence	Modification
1458	1	982.5602	12.16	2	48.0	18.6	2	246-261	R.FVVELADPKLEVKWYK.N	

Detailed Protein Report

Protein 641: **DAN domain family member 5 precursor [Homo sapiens]**

**Accession:**	gi|22749329	**Score:**	18.6
**Database:**	refseq_human(refseq_human_20140103.fasta)	**MW [kDa]:**	20.2
**Database Date:**	2015-11-30	**pI:**	12.3
		**Sequence Coverage [%]:**	9.5
		**No. of unique Peptides:**	1

10	20	30	40	50	60	70	80
MLLGQLSTLL	CLLSGALPTG	SGRPEPQSPR	PQSWAAANQT	WALGPGALPP	LVPASALGSW	KAFLGLQKAR	QLGMGRLQRG
**90**	**100**	**110**	**120**	**130**	**140**	**150**	**160**
QDEVAAVTLP	LNPQEVIQGM	CKAVPFVQVF	SRPGCSAIRL	RNHLCFGHCS	SLYIPGSDPT	PLVLCNSCMP	ARKRWAPVVL
**170**	**180**	**190**					
WCLTGSSASR	RRVKISTMLI	EGCHCSPKA					

Cmpd.	No. of Cmpds.	m/z meas.	Δ m/z [ppm]	z	Rt [min]	Score	P	Range	Sequence	Modification
1379	1	1023.1562	107.05	2	47.0	18.6	2	155-172	R.WAPVVLWCLTGSSASRRR.V	

Detailed Protein Report

Protein 642: **PREDICTED: dyslexia-associated protein KIAA0319-like protein isoform X6 [Homo sapiens]**

**Accession:**	gi|578799795	**Score:**	18.5
**Database:**	refseq_human(refseq_human_20140103.fasta)	**MW [kDa]:**	76.2
**Database Date:**	2015-11-30	**pI:**	5.4
		**Sequence Coverage [%]:**	2.2
		**No. of unique Peptides:**	1

Alias proteins:

Accession	Name	Description
gi|578799797	refseq_human (refseq_human_20140103.fasta)	PREDICTED: dyslexia-associated protein KIAA0319-like protein isoform X7 [Homo sapiens]

10	20	30	40	50	60	70	80
MEGKHSQILK	LSKLTPGLYE	FKVIVEGQNA	HGEGYVNVTV	KPEPRKNRPP	IAIVSPQFQE	ISLPTTSTVI	DGSQSTDDDK
**90**	**100**	**110**	**120**	**130**	**140**	**150**	**160**
IVQYHWEELK	GPLREEKISE	DTAILKLSKL	VPGNYTFSLT	VVDSDGATNS	TTANLTVNKA	VDYPPVANAG	PNQVITLPQN
**170**	**180**	**190**	**200**	**210**	**220**	**230**	**240**
SITLFGNQST	DDHGITSYEW	SLSPSSKGKV	VEMQGVRTPT	LQLSAMQEGD	YTYQLTVTDT	IGQQATAQVT	VIVQPENNKP
**250**	**260**	**270**	**280**	**290**	**300**	**310**	**320**
PQADAGPDKE	LTLPVDSTTL	DGSKSSDDQK	IISYLWEKTQ	GPDGVQLENA	NSSVATVTGL	QVGTYVFTLT	VKDERNLQSQ
**330**	**340**	**350**	**360**	**370**	**380**	**390**	**400**
SSVNVIVKEE	INKPPIAKIT	GNVVITLPTS	TAELDGSKSS	DDKGIVSYLW	TRDEGSPAAG	EVLNHSDHHP	ILFLSNLVEG
**410**	**420**	**430**	**440**	**450**	**460**	**470**	**480**
TYTFHLKVTD	AKGESDTDRT	TVEVKPDPRK	NNLVEIILDI	NVSQLTERLK	GMFIRQIGVL	LGVLDSDIIV	QKIQPYTEQS
**490**	**500**	**510**	**520**	**530**	**540**	**550**	**560**
TKMVFFVQNE	PPHQIFKGHE	VAAMLKSELR	KQKADFLIFR	ALEVNTVTCQ	LNCSDHGHCD	SFTKRCICDP	FWMENFIKVQ
**570**	**580**	**590**	**600**	**610**	**620**	**630**	**640**
LRDGDSNCEW	SVLYVIIATF	VIVVALGILS	WTVICCCKRQ	KGKPKRKSKY	KILDATDQES	LELKPTSRAG	IKQKGLLLSS
**650**	**660**	**670**	**680**	**690**	**700**		
SLMHSESELD	SDDAIFTWPD	REKGKLLHGQ	NGSVPNGQTP	LKARSPREEI	L		

Cmpd.	No. of Cmpds.	m/z meas.	Δ m/z [ppm]	z	Rt [min]	Score	P	Range	Sequence	Modification
2117	1	788.3020	-135.99	2	56.3	18.5	0	250-264	K.ELTLPVDSTTLDGSK.S	

Detailed Protein Report

Protein 643: PREDICTED: E3 ubiquitin-protein ligase pellino homolog 3 isoform X2 [Homo sapiens]

**Accession:**	gi|578821247	**Score:**	18.5
**Database:**	refseq_human(refseq_human_20140103.fasta)	**MW [kDa]:**	27.9
**Database Date:**	2015-11-30	**pI:**	9.1
		**Sequence Coverage [%]:**	11.7
		**No. of unique Peptides:**	1

10	20	30	40	50	60	70	80
MADPRWPDGW	TDHQWSPGDA	PGRRLLRGLS	PGCLAGDLGL	WECVHIAGQP	LSPAAGQAVR	PFYCLWQVEN	ESNVLQDGSL
**90**	**100**	**110**	**120**	**130**	**140**	**150**	**160**
IDLCGATLLW	RTPAGLLRAP	TLKQLEAQRQ	EANAARPQCP	VGLSTLAFPS	PARGRTAPDK	QQPWVYVRCG	HVHGYHGWGC
**170**	**180**	**190**	**200**	**210**	**220**	**230**	**240**
RRERGPQERE	CPLCRLVGPY	VPLWLGQEAG	LCLDPGPPSH	AFAPCGHVCS	EKTARYWAQT	PLPHGTHAFH	AACPFCGAWL
**250**	**260**						
TGEHGCVRLI	FQGPLD						

Cmpd.	No. of Cmpds.	m/z meas.	Δ m/z [ppm]	z	Rt [min]	Score	P	Range	Sequence	Modification
36	1	1069.4752	-74.05	3	30.1	18.5	1	104-133	K.QLEAQRQEANAARPQCPVGLSTLAFPSPAR.G	

Detailed Protein Report

Protein 644: alpha-tocopherol transfer protein-like isoform 1 [Homo sapiens]

**Accession:**	gi|85861243	**Score:**	18.5
**Database:**	refseq_human(refseq_human_20140103.fasta)	**MW [kDa]:**	38.5
**Database Date:**	2015-11-30	**pI:**	6.0
		**Sequence Coverage [%]:**	2.9
		**No. of unique Peptides:**	1

Alias proteins:

Accession	Name	Description
gi|530418391	refseq_human (refseq_human_20140103.fasta)	PREDICTED: alpha-tocopherol transfer protein-like isoform X1 [Homo sapiens]
gi|85861250	refseq_human (refseq_human_20140103.fasta)	alpha-tocopherol transfer protein-like isoform 1 [Homo sapiens]

10	20	30	40	50	60	70	80
MSEESDSLRT	SPSVASLSEN	ELPPPPEPPG	YVCSLTEDLV	TKAREELQEK	PEWRLRDVQA	LRDMVRKEYP	NLSTSLDDAF
**90**	**100**	**110**	**120**	**130**	**140**	**150**	**160**
LLRFLRARKF	DYDRALQLLV	NYHSCRRSWP	EVFNNLKPSA	LKDVLASGFL	TVLPHTDPRG	CHVVCIRPDR	WIPSNYPITE
**170**	**180**	**190**	**200**	**210**	**220**	**230**	**240**
NIRAIYLTLE	KLIQSEETQV	NGIVILADYK	GVSLSKASHF	GPFIAKKVIG	ILQDGFPIRI	KAVHVVNEPR	IFKGIFAIIK
**250**	**260**	**270**	**280**	**290**	**300**	**310**	**320**
PFLKEKIANR	FFLHGSDLNS	LHTNLPRSIL	PKEYGGTAGE	LDTATWNAVL	LASEDDFVKE	FCQPVPACDS	ILGQTLLPEG
**330**	**340**	**350**					
LTSDAQCDDS	LRAVKSQLYS	CY					

Cmpd.	No. of Cmpds.	m/z meas.	Δ m/z [ppm]	z	Rt [min]	Score	P	Range	Sequence	Modification
22	1	537.7431	-87.57	2	29.9	18.5	0	197-206	K.ASHFGPFIAK.K	

Detailed Protein Report

Protein 645: putative ATP-dependent RNA helicase DHX57 [Homo sapiens]

**Accession:**	gi|39777586	**Score:**	18.4
**Database:**	refseq_human(refseq_human_20140103.fasta)	**MW [kDa]:**	155.5
**Database Date:**	2015-11-30	**pI:**	8.7
		**Sequence Coverage [%]:**	1.4
		**No. of unique Peptides:**	1

10	20	30	40	50	60	70	80
MSSSVRRKGK	PGKGGGKGSS	RGGRGGRSHA	SKSHGSGGGG	GGGGGGGGGN	RKASSRIWDD	GDDFCIFSES	RRPSRPSNSN
**90**	**100**	**110**	**120**	**130**	**140**	**150**	**160**
ISKGESRPKW	KPKAKVPLQT	LHMTSENQEK	VKALLRDLQE	QDADAGSERG	LSGEEEDDEP	DCCNDERYWP	AGQEPSLVPD
**170**	**180**	**190**	**200**	**210**	**220**	**230**	**240**
LDPLEYAGLA	SVEPYVPEFT	VSPFAVQKLS	RYGFNTERCQ	AVLRMCDGDV	GASLEHLLTQ	CFSETFGERM	KISEAVNQIS
**250**	**260**	**270**	**280**	**290**	**300**	**310**	**320**
LDECMEQRQE	EAFALKSICG	EKFIERIQNR	VWTIGLELEY	LTSRFRKSKP	KESTKNVQEN	SLEICKFYLK	GNCKFGSKCR
**330**	**340**	**350**	**360**	**370**	**380**	**390**	**400**
FKHEVPPNQI	VGRIERSVDD	SHLNAIEDAS	FLYELEIRFS	KDHKYPYQAP	LVAFYSTNEN	LPLACRLHIS	EFLYDKALTF
**410**	**420**	**430**	**440**	**450**	**460**	**470**	**480**
AETSEPVVYS	LITLLEEESE	IVKLLTNTHH	KYSDPPVNFL	PVPSRTRINN	PACHKTVIPN	NSFVSNQIPE	VEKASESEES
**490**	**500**	**510**	**520**	**530**	**540**	**550**	**560**
DEDDGPAPVI	VENESYVNLK	KKISKRYDWQ	AKSVHAENGK	ICKQFRMKQA	SRQFQSILQE	RQSLPAWEER	ETILNLLRKH
**570**	**580**	**590**	**600**	**610**	**620**	**630**	**640**
QVVVISGMTG	CGKTTQIPQF	ILDDSLNGPP	EKVANIICTQ	PRRISAISVA	ERVAKERAER	VGLTVGYQIR	LESVKSSATR
**650**	**660**	**670**	**680**	**690**	**700**	**710**	**720**
LLYCTTGVLL	RRLEGDTALQ	GVSHIIVDEV	HERTEESDFL	LLVLKDIVSQ	RPGLQVILMS	ATLNAELFSD	YFNSCPVITI
**730**	**740**	**750**	**760**	**770**	**780**	**790**	**800**
PGRTFPVDQF	FLEDAIAVTR	YVLQDGSPYM	RSMKQISKEK	LKARRNRTAF	EEVEEDLRLS	LHLQDQDSVK	DAVPDQQLDF
**810**	**820**	**830**	**840**	**850**	**860**	**870**	**880**
KQLLARYKGV	SKSVIKTMSI	MDFEKVNLEL	IEALLEWIVD	GKHSYPPGAI	LVFLPGLAEI	KMLYEQLQSN	SLFNNRRSNR
**890**	**900**	**910**	**920**	**930**	**940**	**950**	**960**
CVIHPLHSSL	SSEEQQAVFV	KPPAGVTKII	ISTNIAETSI	TIDDVVYVID	SGKMKEKRYD	ASKGMESLED	TFVSQANALQ
**970**	**980**	**990**	**1000**	**1010**	**1020**	**1030**	**1040**
RKGRAGRVAS	GVCFHLFTSH	HYNHQLLKQQ	LPEIQRVPLE	QLCLRIKILE	MFSAHNLQSV	FSRLIEPPHT	DSLRASKIRL
**1050**	**1060**	**1070**	**1080**	**1090**	**1100**	**1110**	**1120**
RDLGALTPDE	RLTPLGYHLA	SLPVDVRIGK	LMLFGSIFRC	LDPALTIAAS	LAFKSPFVSP	WDKKEEANQK	KLEFAFANSD
**1130**	**1140**	**1150**	**1160**	**1170**	**1180**	**1190**	**1200**
YLALLQAYKG	WQLSTKEGVR	ASYNYCRQNF	LSGRVLQEMA	SLKRQFTELL	SDIGFAREGL	RAREIEKRAQ	GGDGVLDATG
**1210**	**1220**	**1230**	**1240**	**1250**	**1260**	**1270**	**1280**
EEANSNAENP	KLISAMLCAA	LYPNVVQVKS	PEGKFQKTST	GAVRMQPKSA	ELKFVTKNDG	YVHIHPSSVN	YQVRHFDSPY
**1290**	**1300**	**1310**	**1320**	**1330**	**1340**	**1350**	**1360**
LLYHEKIKTS	RVFIRDCSMV	SVYPLVLFGG	GQVNVQLQRG	EFVVSLDDGW	IRFVAASHQV	AELVKELRCE	LDQLLQDKIK
**1370**	**1380**	**1390**					
NPSIDLCTCP	RGSRIISTIV	KLVTTQ					

Cmpd.	No. of Cmpds.	m/z meas.	Δ m/z [ppm]	z	Rt [min]	Score	P	Range	Sequence	Modification
1483	2	699.6611	-193.23	2	48.2	18.4	0	33-51	K.SHGSGGGGGGGGGGGGNR.K	

Detailed Protein Report

Protein 646: storkhead-box protein 1 isoform c [Homo sapiens]

Accession: gi|194328689
Database: refseq_human(refseq_human_20140103.fasta)
Database Date: 2015-11-30
Modification(s): Carbamidomethyl

Score: 18.4
MW [kDa]: 19.0
pI: 12.4
Sequence Coverage [%]: 9.5
No. of unique Peptides: 1

10	20	30	40	50	60	70	80
MARPVQLAPG	SLALVLCRLE	AQKAAGAAEE	PGGRAVFRAF	RRANARCFWN	ARLARAASRL	AFQGWLRRGV	LLVRAPPACL
**90**	**100**	**110**	**120**	**130**	**140**	**150**	**160**
QVLRDAWRRR	ALRPPRGFRI	RAVGDVFPVQ	MNPITQSQFV	PLGEVLCCAI	SDMNTAQIVV	TQESLLERLM	KHYPGHRVWD
**170**							
LIIQSFWMD							

Cmpd.	No. of Cmpds.	m/z meas.	Δ m/z [ppm]	z	Rt [min]	Score	P	Range	Sequence	Modification
1506	1	881.6342	118.58	2	48.6	18.4	1	69-84	R.GVLLVRAPPACLQVLR.D	Carbamidomethyl: 11

Detailed Protein Report

Protein 647: homeobox protein Nkx-2.4 [Homo sapiens]

**Accession:**	gi|157426823	**Score:**	18.4
**Database:**	refseq_human(refseq_human_20140103.fasta)	**MW [kDa]:**	36.2
**Database Date:**	2015-11-30	**pI:**	10.0
**Modification(s):**	Oxidation	**Sequence Coverage [%]:**	6.8
		**No. of unique Peptides:**	1

10	20	30	40	50	60	70	80
MSLSPKHTTP	FSVSDILSPI	EETYKKFSGA	MDGAPPGLGA	PLGAAAAYRA	PPPGPSSQAA	TVAGMQPSHA	MAGHNAAAAA
**90**	**100**	**110**	**120**	**130**	**140**	**150**	**160**
AAAAAAAAAA	ATYHMPPGVS	QFPHGAMGSY	CNGGLGNMGE	LPAYTDGMRG	GAATGWYGAN	PDPRYSSISR	FMGPSAGVNV
**170**	**180**	**190**	**200**	**210**	**220**	**230**	**240**
AGMGSLTGIA	DAAKSLGPLH	AAAAAAAPRR	KRRVLFSQAQ	VYELERRFKQ	QKYLSAPERE	HLASMIHLTP	TQVKIWFQNH
**250**	**260**	**270**	**280**	**290**	**300**	**310**	**320**
RYKMKRQAKD	KAAQQLQQEG	GLGPPPPPPP	SPRRVAVPVL	VKDGKPCQNG	ASTPTPGQAG	PQPPAPTPAP	ELEELSPSPP
**330**	**340**	**350**	**360**				
ALHGPGGGLA	ALDAAAGEYS	GGVLGANLLY	GRTW				

Cmpd.	No. of Cmpds.	m/z meas.	Δ m/z [ppm]	z	Rt [min]	Score	P	Range	Sequence	Modification
2128	1	1131.4206	-132.38	2	56.5	18.4	1	26-49	K.KFSGAMDGAPPGLGAPLGAAAAYR.A	Oxidation: 6

Detailed Protein Report

Protein 648: **PREDICTED: janus kinase and microtubule-interacting protein 3 isoform X5 [Homo sapiens]**

Accession: gi|530394820
Database: refseq_human(refseq_human_20140103.fasta)
Database Date: 2015-11-30
Modification(s): Carbamidomethyl, Oxidation

Score: 18.3
MW [kDa]: 105.7
pI: 6.1
Sequence Coverage [%]: 2.2
No. of unique Peptides: 1

Quantitation

MD:MU **Median:** 1.07 **CV:** 0.00 % **No. of Peptides:** 1

10	20	30	40	50	60	70	80
MSKRGMSSRA	KGDKAEALAA	LQAANEDLRA	KLTDIQIELQ	QEKSKVSKVE	REKNQELRQV	REHEQHKTAV	LLTELKTKLH
90	100	110	120	130	140	150	160
EEKMKELQAV	RETLLRQHEA	ELLRVIKIKD	NENQRLQALL	SALRDGGPEK	VKTVLLSEAK	EEAKKGFEVE	KVKMQQEISE
170	180	190	200	210	220	230	240
LKGAKRQVEE	ALTLVIQADK	IKAAEIRSVY	HLHQEEITRI	KKECEREIRR	LQLDEKDARR	FQLKIAELSA	IIRKLEDRNA
250	260	270	280	290	300	310	320
LLSEERNELL	KRVREAESQY	KPLLDKNKRL	SRKNEDLSHA	LRRMENKLKF	VTQENIEMRQ	RAGIIRRPSS	LNDLDQSQDE
330	340	350	360	370	380	390	400
REVDFLKLQI	VEQQNLIDEL	SKTLETAGYV	KSVLERDKLL	RFRKQRKKMA	KLPKPVVVET	FFGYDEEASL	ESDGSSVSYQ
410	420	430	440	450	460	470	480
TDRTDQTPCT	PDDDLEEGMA	KEETELRFRQ	LTMEYQALQR	AYALLQEQVG	GTLDAEREVK	TREQLQAEVQ	RAQARIEDLE
490	500	510	520	530	540	550	560
KALAEQGQDM	KWIEEKQALY	RRNQELVEKI	KQMETEEARL	RHEVQDARDQ	NELLEFRILE	LEERERKSPA	ISFHHTPFVD
570	580	590	600	610	620	630	640
GKSPLQVYCE	AEGVTDIVVA	ELMKKLDILG	DNAVSNLTNE	EQVVVIQART	VLTLAEKWLQ	QIEETEAALQ	RKMVDLESEK
650	660	670	680	690	700	710	720
ELFSKQKGYL	DEELDYRKQA	LDQANKHILE	LEAMLYDALQ	QEAGAKVAEL	LSEEEREKLK	VAVEQWKRQV	MSELRERDAQ
730	740	750	760	770	780	790	800
ILRERMELLQ	LAQQRIKELE	ERIEAQKRQI	KELEEKLSFS	GHSPSWHPDV	PHIESDPFPP	VGPESRDKMG	RRVSILKTQG
810	820	830	840	850	860	870	880
DLVSARPSRG	GRVRRGSVWG	VELPSTWDVP	ELELALPTSR	GLLRDGPGLG	CWTLGPFSRT	AAPIPFPGMC	SIPCQAGVRT
890	900	910	920				
ASVQKHQPRV	PSSLLVLTVA	ATGVWRKIFR	NNRGLA				

Cmpd.	No. of Cmpds.	m/z meas.	Δ m/z [ppm]	z	Rt [min]	Score	P	Range	Sequence	Modification	Ratios	
1669	1	1045.0250	19.09	2	50.8	18.3	0	860-879	R.TAAPIPFPGMCSIPCQAGVR.T	Carbamidomethyl: 15; Oxidation: 10	MD:MU	1.07

Detailed Protein Report

Protein 649: PREDICTED: zinc finger protein 831 isoform X4 [Homo sapiens]

**Accession:**	gi|530417824	**Score:**	18.3
**Database:**	refseq_human(refseq_human_20140103.fasta)	**MW [kDa]:**	132.1
**Database Date:**	2015-11-30	**pI:**	9.2
**Modification(s):**	Carbamidomethyl	**Sequence Coverage [%]:**	1.5
		**No. of unique Peptides:**	1

10	20	30	40	50	60	70	80
MEVPEPTCPA	PPARDQPAPT	PGPPGAPGGQ	ASPHLTLGPV	LLPPEQGLAP	PTVFLKALPI	PLYHTVPPGG	LQPRAPLVTG
90	100	110	120	130	140	150	160
SLDGGNVPFI	LSPVLQPEGP	GPTQVGKPAA	PTLTVNIVGT	LPVLSPGLGP	TLGSPGKVRN	AGKYLCPHCG	RDCLKPSVLE
170	180	190	200	210	220	230	240
KHIRSHTGER	PFPCATCGIA	FKTQSNLYKH	RRTQTHLNNS	RLSSESEGAG	GGLLEEGDKA	GEPPRPEGRG	ESRCQGMHEG
250	260	270	280	290	300	310	320
ASERPLSPGA	HVPLLAKNLD	VRTEAAPCPG	SAFADREAPW	DSAPMASPGL	PAASTQPWRK	LPEQKSPTAG	KPCALQRQQA
330	340	350	360	370	380	390	400
TAAEKPWDAK	APEGRLRKCE	STDSGYLSRS	DSAEQPHAPC	SPLHSLSEHS	AESEGEGGPG	PGPGVAGAEP	GAREAGLELE
410	420	430	440	450	460	470	480
KKRLEERIAQ	LISHNQAVVD	DAQLDNVRPR	KTGLSKQGSI	DLPTPYTYKD	SFHFDIRALE	PGRRRAPGPV	RSTWTPPDKS
490	500	510	520	530	540	550	560
RPLFFHSVPT	QLSTTVECVP	VTRSNSLPFV	EGSRTWLEPR	EPRDPWSRTQ	KPLSPRPGPA	RLGCRSGLSS	TDVPSGHPRA
570	580	590	600	610	620	630	640
LVRQAAVEDL	PGTPIGDALV	PAEDTDAKRT	AAREAMAGKG	RAGGRKCGQR	RLKMFSQEKW	QVYGDETFKR	IYQKMKASPH
650	660	670	680	690	700	710	720
GGKKAREVGM	GSGAELGFPL	QKEAAGSSGT	VPTQDRRTPV	HEDISAGATP	EPWGNPPALE	ASLVTEPTKH	GETVARRGDS
730	740	750	760	770	780	790	800
DRPRVEEAVS	SPALGGRDSP	CSGSRSPLVS	PNGRLELGWQ	MPPAPGPLKG	GDVEAPRPVW	PDPKLEGGAR	GVGDVQETCL
810	820	830	840	850	860	870	880
WAQTVLRWPS	RGSGEDKLPS	ERKKLKVEDL	HSWKQPEPVS	AETPGGPTQP	ASLSSQKQDA	DPGEVPGGSK	ESARQVGEPL
890	900	910	920	930	940	950	960
ESSGASLAAA	SVALKRVGPR	DKATPLHPAA	PAPAEHPSLA	TPPQAPRVLS	ALADNAFSPK	YLLRLPQAET	PLPLPIPWGP
970	980	990	1000	1010	1020	1030	1040
RHSQDSLCSS	GWPEERASFV	GSGLGTPLSP	SPASGPSPGE	ADSILEDPSC	SRPQDGRKGA	QLGGDKGDRM	ATSRPAAREL
1050	1060	1070	1080	1090	1100	1110	1120
PISAPGAPRE	ATSSPPTPTC	EAHLVQDMEG	DSHRIHRLCM	GSTLARARLS	GDVLNPWVPN	WELGEPPGNA	PEDPSSGPLV
1130	1140	1150	1160	1170	1180	1190	1200
GPDPCSPLQP	GSFLTALTRP	QGVPPGWPEL	ALSSHSGTSR	SHSTRSPHST	QNPFPSLKAE	PRLTWCCLSR	SVPLPAEQKA
1210	1220	1230	1240	1250	1260		
KAASVYLAVH	FPGSSLRDEG	PNGPPGSNGG	WTWTSPGEGG	PAQMSKSENP	LEFSSS		

Cmpd.	No. of Cmpds.	m/z meas.	Δ m/z [ppm]	z	Rt [min]	Score	P	Range	Sequence	Modification
2187	6	1023.8998	-84.84	2	57.2	18.3	1	258-276	K.NLDVRTEAAPCPGSAFADR.E	Carbamidomethyl: 11

Detailed Protein Report

Protein 650: prominin-2 precursor [Homo sapiens]

**Accession:**	gi|260764009	**Score:**	18.2
**Database:**	refseq_human(refseq_human_20140103.fasta)	**MW [kDa]:**	91.8
**Database Date:**	2015-11-30	**pI:**	5.8
		**Sequence Coverage [%]:**	1.8
		**No. of unique Peptides:**	1

Alias proteins:

Accession	Name	Description
gi|530368507	refseq_human (refseq_human_20140103.fasta)	PREDICTED: prominin-2 isoform X1 [Homo sapiens]
gi|260764013	refseq_human (refseq_human_20140103.fasta)	prominin-2 precursor [Homo sapiens]
gi|260764011	refseq_human (refseq_human_20140103.fasta)	prominin-2 precursor [Homo sapiens]

10	20	30	40	50	60	70	80
MKHTLALLAP	LLGLGLGLAL	SQLAAGATDC	KFLGPAEHLT	FTPAARARWL	APRVRAPGLL	DSLYGTVRRF	LSVVQLNPFP
90	100	110	120	130	140	150	160
SELVKALLNE	LASVKVNEVV	RYEAGYVVCA	VIAGLYLLLV	PTAGLCFCCC	RCHRRCGGRV	KTEHKALACE	RAALMVFLLL
170	180	190	200	210	220	230	240
TTLLLLIGVV	CAFVTNQRTH	EQMGPSIEAM	PETLLSLWGL	VSDVPQELQA	VAQQFSLPQE	QVSEELDGVG	VSIGSAIHTQ
250	260	270	280	290	300	310	320
LRSSVYPLLA	AVGSLGQVLQ	VSVHHLQTLN	ATVVELQAGQ	QDLEPAIREH	RDRLLELLQE	ARCQGDCAGA	LSWARTLELG
330	340	350	360	370	380	390	400
ADFSQVPSVD	HVLHQLKGVP	EANFSSMVQE	ENSTFNALPA	LAAMQTSSVV	QELKKAVAQQ	PEGVRTLAEG	FPGLEAASRW
410	420	430	440	450	460	470	480
AQALQEVEES	SRPYLQEVQR	YETYRWIVGC	VLCSVVLFVV	LCNLLGLNLG	IWGLSARDDP	SHPEAKGEAG	ARFLMAGVGL
490	500	510	520	530	540	550	560
SFLFAAPLIL	LVFATFLVGG	NVQTLVCQSW	ENGELFEFAD	TPGNLPPSMN	LSQLLGLRKN	ISIHQAYQQC	KEGAALWTVL
570	580	590	600	610	620	630	640
QLNDSYDLEE	HLDINQYTNK	LRQELQSLKV	DTQSLDLLSS	AARRDLEALQ	SSGLQRIHYP	DFLVQIQRPV	VKTSMEQLAQ
650	660	670	680	690	700	710	720
ELQGLAQAQD	NSVLGQRLQE	EAQGLRNLHQ	EKVVPQQSLV	AKLNLSVRAL	ESSAPNLQLE	TSDVLANVTY	LKGELPAWAA
730	740	750	760	770	780	790	800
RILRNVSECF	LAREMGYFSQ	YVAWVREEVT	QRIATCQPLS	GALDNSRVIL	CDMMADPWNA	FWFCLAWCTF	FLIPSIIFAV
810	820	830	840				
KTSKYFRPIR	KRLSSTSSEE	TQLFHIPRVT	SLKL				

Cmpd.	No. of Cmpds.	m/z meas.	Δ m/z [ppm]	z	Rt [min]	Score	P	Range	Sequence	Modification
17	1	814.3561	-94.62	2	29.5	18.2	0	32-46	K.FLGPAEHLTFTPAAR.A	

Detailed Protein Report

Protein 651: coiled-coil domain-containing protein 120 isoform 4 [Homo sapiens]

**Accession:**	gi|254039614	**Score:**	18.2
**Database:**	refseq_human(refseq_human_20140103.fasta)	**MW [kDa]:**	66.3
**Database Date:**	2015-11-30	**pI:**	10.5
		**Sequence Coverage [%]:**	2.6
		**No. of unique Peptides:**	1

10	20	30	40	50	60	70	80
MEVKAALFGE	AAPQVKSERL	RGLLDRQRTL	QEALSLKLQE	LRKVCLQEAE	LTGQLPPECP	LEPGERPQLV	RRRPPTARAY
90	100	110	120	130	140	150	160
PPPHPNQAHH	SLCPAEELAL	EALEREVSVQ	QQIAAAARRL	ALAPDLSTEQ	RRRRRQVQAD	ALRRLHELEE	QLRDVRARLG
170	180	190	200	210	220	230	240
LPVLPLPQPL	PLSTGSVITT	QGVCLGMRLA	QLSQEDVVLH	SESSSLSESG	ASHDNEEPHG	CFSLAERPSP	PKAWDQLRAV
250	260	270	280	290	300	310	320
SGGSPERRTP	WKPPPSDLYG	DLKSRRNSVA	SPTSPTRSLP	RSASSFEGRS	VPATPVLTRG	AGPQLCKPEG	LHSRQWSGSQ
330	340	350	360	370	380	390	400
DSQMGFPRAD	PASDRASLFV	ARTRRSNSSE	ALLVDRAAGG	GAGSPPAPLA	PSASGPPVCK	SSEVLYERPQ	PTPAFSSRTA
410	420	430	440	450	460	470	480
GPPDPPRAAR	PSSAAPASRG	APRLPPVCGD	FLLDYSLDRG	LPRSGGGTGW	GELPPAAEVP	GPLSRRDGLL	TMLPGPPPVY
490	500	510	520	530	540	550	560
AADSNSPLLR	TKDPHTRATR	TKPCGLPPEA	AEGPEVHPNP	LLWMPPPTRI	PSAGERSGHK	NLALEGLRDW	YIRNSGLAAG
570	580	590	600	610	620		
PQRRPVLPSV	GPPHPPFLHA	RCYEVGQALY	GAPSQAPLPH	SRSFTAPPVS	GRYGGCFY		

Cmpd.	No. of Cmpds.	m/z meas.	Δ m/z [ppm]	z	Rt [min]	Score	P	Range	Sequence	Modification
500	1	642.6194	-139.68	3	35.9	18.2	2	27-42	R.QRTLQEALSLKLQELR.K	

Detailed Protein Report

Protein 652: PREDICTED: retinoid isomerohydrolase isoform X1 [Homo sapiens]

**Accession:**	gi|578799557	**Score:**	18.1
**Database:**	refseq_human(refseq_human_20140103.fasta)	**MW [kDa]:**	65.6
**Database Date:**	2015-11-30	**pI:**	8.4
		**Sequence Coverage [%]:**	1.9
		**No. of unique Peptides:**	1

Quantitation

MD:MU **Median:** 0.85 **CV:** 0.00 % **No. of Peptides:** 1

10	20	30	40	50	60	70	80
MQKVLKPVAV	LKQCGYSPRT	PKAAQGYLRK	ALGADTGTSK	TGFSSPRRVE	HPAGGYKKLF	ETVEELSSPL	TAHVTGRIPL
90	100	110	120	130	140	150	160
WLTGSLLRCG	PGLFEVGSEP	FYHLFDGQAL	LHKFDFKEGH	VTYHRRFIRT	DAYVRAMTEK	RIVITEFGTC	AFPDPCKNIF
170	180	190	200	210	220	230	240
SRFFSYFRGV	EVTDNALVNV	YPVGEDYYAC	TETNFITKIN	PETLETIKQV	DLCNYVSVNG	ATAHPHIEND	GTVYNIGNCF
250	260	270	280	290	300	310	320
GKNFSIAYNI	VKIPPLQADK	EDPISKSEIV	VQFPCSDRFK	PSYVHSFGLT	PNYIVFVETP	VKINLFKFLS	SWSLWGANYM
330	340	350	360	370	380	390	400
DCFESNETMG	VWLHIADKKR	KKYLNNKYRT	SPFNLFHHIN	TYEDNGFLIV	DLCCWKGFEF	VYNYLYLANL	RENWEEVKKN
410	420	430	440	450	460	470	480
ARKAPQPEVR	RYVLPLNIDK	ADTGKNLVTL	PNTTATAILC	SDETIWLEPE	VLFSGPRQAF	EFPQINYQKY	CGKPYTYAYG
490	500	510	520	530	540	550	560
LGLNHFVPDR	LCKLNVKTKE	TWVWQEPDSY	PSEPIFVSHP	DALEEDDGVV	LSVVVSPGAG	QKPAYLLILN	AKDLSEVARA
570	580						
EVEINIPVTF	HGLFKKS						

Cmpd.	No. of Cmpds.	m/z meas.	Δ m/z [ppm]	z	Rt [min]	Score	P	Range	Sequence	Modification	Ratios
1724	1	524.7781	-13.64	2	51.3	18.1	1	30-40	R.KALGADTGTSK.T		MD:MU 0.85

Detailed Protein Report

Protein 653: **PREDICTED: parkin coregulated gene protein isoform X3 [Homo sapiens]**

**Accession:**	gi|530383379	**Score:**	18.1
**Database:**	refseq_human(refseq_human_20140103.fasta)	**MW [kDa]:**	24.0
**Database Date:**	2015-11-30	**pI:**	10.0
		**Sequence Coverage [%]:**	3.3
		**No. of unique Peptides:**	1

```
        10         20         30         40         50         60         70         80
MVAEKETLSL NKCPDKMPKR TKLLAQQPLP VHQPHSLVSE GFTVKAMMKN SVVRGPPAAG AFKERPTKPT AFRKFYERGD
        90        100        110        120        130        140        150        160
FPIALEHDSK GNKIAWKVEI EKLDYHHYLP LFFDGLCEMT FPYEFFARQG IHDMLEHGGN KILPVLPQLI IPIKNALNLR
       170        180        190        200        210        220
NRQVICVTLK VLQHLVVSAE MVGKALVPYY RQILPVLNIF KNMNGDTVPN Y
```

Cmpd.	No. of Cmpds.	m/z meas.	Δ m/z [ppm]	z	Rt [min]	Score	P	Range	Sequence	Modification
1618	1	804.1891	-319.55	1	50.1	18.1	0	6-12	K.ETLSLNK.C	

Detailed Protein Report

Protein 654: PREDICTED: RING finger protein 10 isoform X5 [Homo sapiens]

**Accession:**	gi|530401582	**Score:**	18.0
**Database:**	refseq_human(refseq_human_20140103.fasta)	**MW [kDa]:**	85.1
**Database Date:**	2015-11-30	**pI:**	7.0
		**Sequence Coverage [%]:**	2.1
		**No. of unique Peptides:**	1

10	20	30	40	50	60	70	80
MPLSSPNAAA	TASDMDKNSG	SNSSSASSGS	SKGQQPPRSA	SAGPAGESKP	KSDGKNSSGS	KRYNRKRELS	YPKNESFNNQ
90	100	110	120	130	140	150	160
SRRSSSQKSK	TFNKMPPQRG	GGSSKLFSSS	FNGGRRDEVA	EAQRAEFSPA	QFSGPKKINL	NHLLNFTFEP	RGQTGHFEGS
170	180	190	200	210	220	230	240
[truncated: 327,638 more chars]
