# Supplementary material for: Exploration of key factors in Gingival Crevicular fluids from patients undergoing Periodontally Accelerated Osteogenic Orthodontics (PAOO) using proteome analysis
Source: BMC Oral Health. 2023 Nov 27;23:934. doi: 10.1186/s12903-023-03606-7 (PMC10683118; doi:10.1186/s12903-023-03606-7)
Supplement: Supplementary file 4 — Supplementary Material 4 [file 12903_2023_3606_MOESM4_ESM.pdf]

Detailed Protein Report

Project Info

Name: teeth-151208 **Date:** Dec 8, 2015
Note:

Sample Info & Protocols

Name: id-DOWN **Date:** Dec 10, 2015
Fraction:
Volume: **Concentration:**
Note:
Buffer: **Organism:**

Search Result Info

Search Result	Location	Search Engine	Database
ZT_human_PE_2015-12-10 16:24:32	/teeth-151208/id-DOWN/"Combined MS/MS spectra"	Mascot	refseq_human

Protein 1: serum albumin preproprotein [Homo sapiens]

Accession: gi|4502027
Database: refseq_human(refseq_human_20140103.fasta)
Database Date: 2015-11-30
Modification(s): Carbamidomethyl, Oxidation

Score: 1455.3
MW [kDa]: 69.3
pI: 5.9
Sequence Coverage [%]: 55.5
No. of unique Peptides: 30

Quantitation

mdown:qdown **Median:** 1.04 **CV:** 57.29 % **No. of Peptides:** 18
Wdown:Qdown **Median:** 0.59 **CV:** 92.33 % **No. of Peptides:** 18

```
        10         20         30         40         50         60         70         80
MKWVTFISLL FLFSSAYSRG VFRRDAHKSE VAHRFKDLGE ENFKALVLIA FAQYLQQCPF EDHVKLVNEV TEFAKTCVAD
        90        100        110        120        130        140        150        160
ESAENCDKSL HTLFGDKLCT VATLRETYGE MADCCAKQEP ERNECFLQHK DDNPNLPRLV RPEVDVMCTA FHDNEETFLK
       170        180        190        200        210        220        230        240
KYLYEIARRH PYFYAPELLF FAKRYKAAFT ECCQAADKAA CLLPKLDELR DEGKASSAKQ RLKCASLQKF GERAFKAWAV
       250        260        270        280        290        300        310        320
ARLSQRFPKA EFAEVSKLVT DLTKVHTECC HGDLLECADD RADLAKYICE NQDSISSKLK ECCEKPLLEK SHCIAEVEND
       330        340        350        360        370        380        390        400
EMPADLPSLA ADFVESKDVC KNYAEAKDVF LGMFLYEYAR RHPDYSVVLL LRLAKTYETT LEKCCAAADP HECYAKVFDE
       410        420        430        440        450        460        470        480
FKPLVEEPQN LIKQNCELFE QLGEYKFQNA LLVRYTKKVP QVSTPTLVEV SRNLGKVGSK CCKHPEAKRM PCAEDYLSVV
       490        500        510        520        530        540        550        560
LNQLCVLHEK TPVSDRVTKC CTESLVNRRP CFSALEVDET YVPKEFNAET FTFHADICTL SEKERQIKKQ TALVELVKHK
       570        580        590        600        610
PKATKEQLKA VMDDFAAFVE KCCKADDKET CFAEEGKKLV AASQAALGL
```

Cmpd.	No. of Cmpds.	m/z meas.	Δ m/z [ppm]	z	Rt [min]	Score	P	Range	Sequence	Modification	Ratios
298	12	613.8004	-9.46	2	32.4	71.1	1	35-44	R.FKDLGEENFK.A		Wdown:Qdown 1.02<br>mdown:qdown 1.23
600	16	575.1620	-259.21	2	36.4	63.2	0	66-75	K.LVNEVTEFAK.T		Wdown:Qdown 0.99<br>mdown:qdown 3.00
357	7	509.1363	-266.02	2	33.8	49.0	0	89-97	K.SLHTLFGDK.L		Wdown:Qdown 1.33<br>mdown:qdown 0.62
171	1	659.8150	4.22	4	31.4	26.5	2	118-138	K.<br>QEPERNECFLQHKDDNPNLPR.L	Carbamidomethyl: 8	
328	1	509.7430	1.26	2	33.5	11.8	0	123-130	R.NECFLQHK.D		
213	1	666.3221	11.20	3	32.0	43.3	1	123-138	R.NECFLQHKDDNPNLPR.L	Carbamidomethyl: 3	Wdown:Qdown 0.83<br>mdown:qdown 0.76

Detailed Protein Report

Cmpd.	No. of Cmpds.	m/z meas.	Δ m/z [ppm]	z	Rt [min]	Score	P	Range	Sequence	Modification	Ratios
1425	1	695.3249	-29.21	4	46.6	14.9	1	139-161	R. LVRPEVDVMCTAFHDNEETFLKK. Y	Carbamidomethyl: 10	
532	9	464.1198	-281.27	2	35.3	30.2	0	162-168	K.YLYEIAR.R		Wdown:Qdown 0.79<br>mdown:qdown 1.28
33	3	686.2794	-11.06	2	29.6	70.0	0	187-198	K.AAFTECCQAADK.A	Carbamidomethyl: 6, 7	
20	5	537.7465	-52.90	2	29.5	44.4	1	206-214	K.LDELRDEGK.A		Wdown:Qdown 0.46<br>mdown:qdown 0.80
235	5	696.2743	-13.94	3	31.7	57.7	0	265-281	K.VHTECCHGDLLECADDR.A	Carbamidomethyl: 5, 6, 13	Wdown:Qdown 1.57<br>mdown:qdown 0.83
43	6	722.3176	-9.77	2	29.9	70.8	0	287-298	K.YICENQDSISSK.L	Carbamidomethyl: 3	mdown:qdown 0.86<br>Wdown:Qdown 0.45
75	2	773.8989	-4.03	2	30.2	28.9	1	299-310	K.LKECCEKPLLEK.S	Carbamidomethyl: 4, 5	Wdown:Qdown 0.73<br>mdown:qdown 0.81
2988	1	992.1219	2.21	3	66.1	32.9	0	311-337	K. SHCIAEVENDEMPADLPSLAADFV D	Carbamidomethyl: 3	
2549	3	820.4166	26.51	2	60.1	54.2	0	348-360	K.DVFLGMFLYEYAR.R	Oxidation: 6	
42	1	492.7365	-23.11	2	29.7	28.1	0	376-383	K.TYETTLEK.C		Wdown:Qdown 0.55
3	5	776.7905	-15.51	2	28.9	31.9	0	384-396	K.CCAAADPHECYAK.V	Carbamidomethyl: 1, 2, 10	mdown:qdown 1.78
2246	52	682.3456	-35.78	3	56.6	62.3	0	397-413	K.VFDEFKPLVEEPQNLIK.Q		
1582	31	829.3539	-31.44	2	48.3	77.6	0	414-426	K.QNCELFEQLGEYK.F	Carbamidomethyl: 3	Wdown:Qdown 0.15
855	16	480.6439	-293.27	2	39.2	53.0	0	427-434	K.FQNALLVR.Y		Wdown:Qdown 0.17<br>mdown:qdown 2.77
783	8	820.4638	-10.68	2	38.6	67.6	1	438-452	K.KVPQVSTPTLVEVSR.N		mdown:qdown 0.74<br>Wdown:Qdown 0.22
1019	2	756.4151	-13.09	2	41.5	37.1	0	439-452	K.VPQVSTPTLVEVSR.N		mdown:qdown 1.44<br>Wdown:Qdown 0.34
57	4	569.7652	22.14	2	29.9	51.3	0	500-508	K.CCTESLVNR.R	Carbamidomethyl: 1, 2	
1753	6	637.6399	-13.90	3	50.5	67.0	0	509-524	R.RPCFSALEVDETYVPK.E	Carbamidomethyl: 3	Wdown:Qdown 0.17<br>mdown:qdown 0.31
2255	7	753.9716	-54.07	3	56.7	52.9	0	525-543	K.EFNAETFTFHADICTLSEK.E	Carbamidomethyl: 14	
2154	1	848.9827	-91.45	3	55.4	11.2	1	525-545	K.EFNAETFTFHADICTLSEKER. Q	Carbamidomethyl: 14	mdown:qdown 0.86
421	2	564.7074	-257.77	2	34.6	52.0	1	549-558	K.KQTALVELVK.H		Wdown:Qdown 0.71<br>mdown:qdown 0.91
860	15	500.8124	13.72	2	39.6	52.8	0	550-558	K.QTALVELVK.H		mdown:qdown 0.90
2105	55	671.7288	-137.21	2	54.9	98.5	0	570-581	K.AVMDDFAAFVEK.C		Wdown:Qdown 1.72
21	2	543.2367	-19.25	3	29.6	43.1	2	585-598	K.ADDKETCFAEEGKK.L	Carbamidomethyl: 7	Wdown:Qdown 1.67<br>mdown:qdown 1.85

Detailed Protein Report

Protein 2: hemoglobin subunit beta [Homo sapiens]

**Accession:**	gi|4504349	**Score:**	732.7
**Database:**	refseq_human(refseq_human_20140103.fasta)	**MW [kDa]:**	16.0
**Database Date:**	2015-11-30	**pI:**	6.9
**Modification(s):**	Carbamidomethyl, Oxidation	**Sequence Coverage [%]:**	83.0
		**No. of unique Peptides:**	12

Quantitation

mdown:qdown	**Median:**	0.66	**CV:**	88.03 %	**No. of Peptides:**	6
Wdown:Qdown	**Median:**	0.10	**CV:**	57.84 %	**No. of Peptides:**	5

```
        10         20         30         40         50         60         70         80
MVHLTPEEKS AVTALWGKVN VDEVGGEALG RLLVVYPWTQ RFFESFGDLS TPDAVMGNPK VKAHGKKVLG AFSDGLAHLD
        90        100        110        120        130        140        150
NLKGTFATLS ELHCDKLHVD PENFRLLGNV LVCVLAHHFG KEFTPPVQAA YQKVVAGVAN ALAHKYH
```

Cmpd.	No. of Cmpds.	m/z meas.	Δ m/z [ppm]	z	Rt [min]	Score	P	Range	Sequence	Modification	Ratios
47	2	476.7443	-29.87	2	29.8	25.8	0	2-9	M.VHLTPEEK.S		
701	5	466.7367	-57.79	2	37.6	58.3	0	10-18	K.SAVTALWGK.V		mdown:qdown 0.34<br>Wdown:Qdown 0.07
391	7	657.8281	-12.11	2	34.2	66.6	0	19-31	K.VNVDEVGGEALGR.L		Wdown:Qdown 0.17<br>mdown:qdown 0.64
2103	16	637.8031	-99.32	2	56.4	50.0	0	32-41	R.LLVVYPWTQR.F		
2217	12	1029.8793	-95.37	2	56.2	51.5	0	42-60	R.FFESFGDLSTPDAVMGNPK.V		mdown:qdown 3.03
1677	1	1037.9101	-62.48	2	49.6	60.1	0	42-60	R.FFESFGDLSTPDAVMGNPK.V	Oxidation: 15	
2072	17	599.9066	-155.81	3	54.5	102.5	1	67-83	K.KVLGAFSDGLAHLDNLK.G		
2939	17	557.3045	4.87	3	65.5	94.6	0	68-83	K.VLGAFSDGLAHLDNLK.G		
595	2	739.7263	-168.37	2	36.3	53.2	0	84-96	K.GTFATLSELHCDK.L	Carbamidomethyl: 11	Wdown:Qdown 0.06<br>mdown:qdown 0.48
237	11	563.6399	-258.57	2	32.1	37.3	0	97-105	K.LHVDPENFR.L		mdown:qdown 0.81<br>Wdown:Qdown 0.19
563	3	689.7251	-186.44	2	35.9	45.0	0	122-133	K.EFTPPVQAAYQK.V		Wdown:Qdown 0.06<br>mdown:qdown 0.31
213	3	575.2065	-233.10	2	31.9	88.0	0	134-145	K.VVAGVANALAHK.Y		

Detailed Protein Report

Protein 3: keratin, type I cytoskeletal 13 isoform a [Homo sapiens]

**Accession:**	gi|131412225	**Score:**	661.4
**Database:**	refseq_human(refseq_human_20140103.fasta)	**MW [kDa]:**	49.5
**Database Date:**	2015-11-30	**pI:**	4.8
**Modification(s):**	Carbamidomethyl	**Sequence Coverage [%]:**	34.7
		**No. of unique Peptides:**	15

Quantitation

mdown:qdown	**Median:**	0.69	**CV:**	42.42 %	**No. of Peptides:**	12
Wdown:Qdown	**Median:**	2.00	**CV:**	67.65 %	**No. of Peptides:**	11

10	20	30	40	50	60	70	80
MSLRLQSSSA	SYGGGFGGGS	CQLGGGRGVS	TCSTRFVSGG	SAGGYGGGVS	CGFGGGAGSG	FGGGYGGGLG	GGYGGGLGGG
**90**	**100**	**110**	**120**	**130**	**140**	**150**	**160**
FGGGFAGGFV	DFGACDGGLL	TGNEKITMQN	LNDRLASYLE	KVRALEEANA	DLEVKIRDWH	LKQSPASPER	DYSPYYKTIE
**170**	**180**	**190**	**200**	**210**	**220**	**230**	**240**
ELRDKILTAT	IENNRVILEI	DNARLAADDF	RLKYENELAL	RQSVEADING	LRRVLDELTL	SKTDLEMQIE	SLNEELAYMK
**250**	**260**	**270**	**280**	**290**	**300**	**310**	**320**
KNHEEEMKEF	SNQVVGQVNV	EMDATPGIDL	TRVLAEMREQ	YEAMAERNRR	DAEEWFHAKS	AELNKEVSTN	TAMIQTSKTE
**330**	**340**	**350**	**360**	**370**	**380**	**390**	**400**
ITELRRTLQG	LEIELQSQLS	MKAGLENTVA	ETECRYALQL	QQIQGLISSI	EAQLSELRSE	MECQNQEYKM	LLDIKTRLEQ
**410**	**420**	**430**	**440**	**450**	**460**		
EIATYRSLLE	GQDAKMIGFP	SSAGSVSPRS	TSVTTTSSAS	VTTTSNASGR	RTSDVRRP		

Cmpd.	No. of Cmpds.	m/z meas.	Δ m/z [ppm]	z	Rt [min]	Score	P	Range	Sequence	Modification	Ratios
269	3	651.1939	-213.19	2	32.7	78.3	0	124-135	R.ALEEANADLEVK.I		mdown:qdown 1.21
288	1	572.8039	-27.55	2	32.3	45.4	0	166-175	K.ILTATIENNR.V		Wdown:Qdown 0.81<br>mdown:qdown 1.32
528	7	521.6763	-233.65	2	35.9	61.6	0	176-184	R.VILEIDNAR.L		Wdown:Qdown 2.79<br>mdown:qdown 0.61
342	4	624.7261	-199.84	2	33.4	46.8	1	192-201	R.LKYENELALR.Q		mdown:qdown 0.51<br>Wdown:Qdown 1.80
379	5	601.3190	11.24	2	34.1	54.4	0	202-212	R.QSVEADINGLR.R		mdown:qdown 0.76
235	1	679.3515	-16.64	2	32.3	26.4	1	202-213	R.QSVEADINGLRR.V		Wdown:Qdown 2.65<br>mdown:qdown 0.51
686	1	587.3207	-42.26	2	37.1	41.5	1	213-222	R.RVLDELTLSK.T		mdown:qdown 0.46
809	4	509.1457	-293.16	2	38.6	50.8	0	214-222	R.VLDELTLSK.T		Wdown:Qdown 0.84<br>mdown:qdown 0.64
467	1	566.6136	-252.15	2	34.5	12.6	0	291-299	R.DAEEWFHAK.S		Wdown:Qdown 3.86<br>mdown:qdown 1.18
173	1	705.3260	-34.92	2	30.9	13.5	0	306-318	K.EVSTNTAMIQTSK.T		Wdown:Qdown 4.32
2903	1	909.4716	-17.49	2	65.4	28.3	0	327-342	R.TLQGLEIELQSQLSMK.A		Wdown:Qdown 2.40
142	2	725.3217	-19.13	2	31.2	69.4	0	343-355	K.AGLENTVAETECR.Y	Carbamidomethyl: 12	Wdown:Qdown 3.00<br>mdown:qdown 0.72
258	3	690.3402	-39.52	2	32.4	52.8	1	396-406	K.TRLEQEIATYR.S		Wdown:Qdown 2.61<br>mdown:qdown 0.76
768	1	696.8079	-61.40	2	39.0	28.8	0	416-429	K.MIGFPSSAGSVSPR.S		
33	2	1001.9745	-5.27	2	29.8	50.9	0	430-450	R.STSVTTTSSASVTTTSNASGR.R		Wdown:Qdown 0.71<br>mdown:qdown 0.32

Detailed Protein Report

Protein 4: apolipoprotein A-I preproprotein [Homo sapiens]

**Accession:**	gi|4557321	**Score:**	494.8
**Database:**	refseq_human(refseq_human_20140103.fasta)	**MW [kDa]:**	30.8
**Database Date:**	2015-11-30	**pI:**	5.5
		**Sequence Coverage [%]:**	45.7
		**No. of unique Peptides:**	12

Quantitation

mdown:qdown	**Median:**	1.63	**CV:**	30.66 %	**No. of Peptides:**	2
Wdown:Qdown	**Median:**	0.86	**CV:**	124.09 %	**No. of Peptides:**	4

Alias proteins:

Accession	Name	Description
gi|530398069	r e f s e q _ h u m a n (refseq_human_20140103.fasta)	PREDICTED: apolipoprotein A-I isoform X2 [Homo sapiens]
gi|530398067	r e f s e q _ h u m a n (refseq_human_20140103.fasta)	PREDICTED: apolipoprotein A-I isoform X1 [Homo sapiens]

10	20	30	40	50	60	70	80
MKAAVLTLAV	LFLTGSQARH	FWQQDEPPQS	PWDRVKDLAT	VYVDVLKDSG	RDYVSQFEGS	ALGKQLNLKL	LDNWDSVTST
**90**	**100**	**110**	**120**	**130**	**140**	**150**	**160**
FSKLREQLGP	VTQEFWDNLE	KETEGLRQEM	SKDLEEVKAK	VQPYLDDFQK	KWQEEMELYR	QKVEPLRAEL	QEGARQKLHE
**170**	**180**	**190**	**200**	**210**	**220**	**230**	**240**
LQEKLSPLGE	EMRDRARAHV	DALRTHLAPY	SDELRQRLAA	RLEALKENGG	ARLAEYHAKA	TEHLSTLSEK	AKPALEDLRQ
**250**	**260**	**270**					
GLLPVLESFK	VSFLSALEEY	TKKLNTQ					

Cmpd.	No. of Cmpds.	m/z meas.	Δ m/z [ppm]	z	Rt [min]	Score	P	Range	Sequence	Modification	Ratios
1873	4	731.8942	-48.16	2	52.1	53.6	1	35-47	R.VKDLATVYVDVLK.D		
2093	3	618.2734	-120.22	2	54.7	58.3	0	37-47	K.DLATVYVDVLK.D		
805	1	908.4284	-0.73	2	38.9	11.7	1	48-64	K.DSGRDYVSQFEGSALGK.Q		Wdown:Qdown 0.52
943	3	700.8438	7.87	2	40.6	38.3	0	52-64	R.DYVSQFEGSALGK.Q		Wdown:Qdown 0.25
1807	8	806.8737	-27.99	2	51.3	94.1	0	70-83	K.LLDNWDSVTSTFSK.L		
2784	1	734.7111	-0.04	3	66.2	16.8	1	84-101	K.LREQLGPVTQEFWDNLEK.E		
681	5	626.6736	-224.11	2	37.1	28.6	0	121-130	K.VQPYLDDFQK.K		
860	1	642.2972	11.37	2	39.2	28.3	0	132-140	K.WQEEMELYR.Q		Wdown:Qdown 3.28<br>mdown:qdown 2.21
1373	1	896.6035	133.69	1	46.9	10.1	0	158-164	K.LHELQEK.L		
311	3	651.3061	-33.42	2	32.6	52.7	0	185-195	R.THLAPYSDELR.Q		Wdown:Qdown 1.30<br>mdown:qdown 1.21
2427	8	615.8382	-32.66	2	58.7	41.3	0	240-250	R.QGLLPVLESFK.V		
2300	6	693.8302	-44.61	2	57.2	61.0	0	251-262	K.VSFLSALEEYTK.K		

Detailed Protein Report

Protein 5: keratin, type II cytoskeletal 6A [Homo sapiens]

Accession: gi|5031839
Database: refseq_human(refseq_human_20140103.fasta)
Database Date: 2015-11-30
Modification(s): Carbamidomethyl

Score: 479.5
MW [kDa]: 60.0
pI: 8.9
Sequence Coverage [%]: 23.9
No. of unique Peptides: 11

Quantitation

mdown:qdown	**Median:** 0.60	**CV:** 168.38 %	**No. of Peptides:** 6
Wdown:Qdown	**Median:** 1.09	**CV:** 87.16 %	**No. of Peptides:** 5

10	20	30	40	50	60	70	80
MASTSTTIRS	HSSSRRGFSA	NSARLPGVSR	SGFSSVSVSR	SRGSGGLGGA	CGGAGFGSRS	LYGLGGSKRI	SIGGGSCAIS
90	100	110	120	130	140	150	160
GGYGSRAGGS	YGFGGAGSGF	GFGGGAGIGF	GLGGGAGLAG	GFGGPGFPVC	PPGGIQEVTV	NQSLLTPLNL	QIDPTIQRVR
170	180	190	200	210	220	230	240
AEEREQIKTL	NNKFASFIDK	VRFLEQQNKV	LETKWTLLQE	QGTKTVRQNL	EPLFEQYINN	LRRQLDSIVG	ERGRLDSELR
250	260	270	280	290	300	310	320
GMQDLVEDFK	NKYEDEINKR	TAAENEFVTL	KKDVDAAYMN	KVELQAKADT	LTDEINFLRA	LYDAELSQMQ	THISDTSVVL
330	340	350	360	370	380	390	400
SMDNNRNLDL	DSIIAEVKAQ	YEEIAQRSRA	EAESWYQTKY	EELQVTAGRH	GDDLRNTKQE	IAEINRMIQR	LRSEIDHVKK
410	420	430	440	450	460	470	480
QCANLQAAIA	DAEQRGEMAL	KDAKNKLEGL	EDALQKAKQD	LARLLKEYQE	LMNVKLALDV	EIATYRKLLE	GEECRLNGEG
490	500	510	520	530	540	550	560
VGQVNISVVQ	STVSSGYGGA	SGVGSGLGLG	GGSSYSYGSG	LGVGGGFSSS	SGRAIGGGLS	SVGGGSSTIK	YTTTSSSSRK
570							
SYKH							

Cmpd.	No. of Cmpds.	m/z meas.	Δ m/z [ppm]	z	Rt [min]	Score	P	Range	Sequence	Modification	Ratios
902	1	712.9682	207.39	2	39.8	10.1	0	43-59	R.GSGGLGGACGGAGFGSR.S	Carbamidomethyl: 9	
910	11	602.3097	-20.53	2	39.9	44.2	0	195-204	K.WTLLQEQGTK.T		Wdown:Qdown 0.86<br>mdown:qdown 0.45
2397	1	945.9922	3.32	2	58.3	17.5	0	208-222	R.QNLEPLFEQYINNLR.R		
1215	2	591.1296	-252.65	2	44.9	47.4	0	241-250	R.GMQDLVEDFK.N		mdown:qdown 2.16
212	1	675.8653	-2.24	2	32.0	57.5	1	261-272	R.TAAENEFVTLKK.D		Wdown:Qdown 2.26<br>mdown:qdown 0.75
1892	10	704.2620	-138.19	2	53.7	69.2	0	288-299	K.ADTLTDEINFLR.A		
2868	18	665.3564	-15.34	2	65.0	82.6	0	327-338	R.NLDLDSIIAEVK.A		Wdown:Qdown 1.64
145	3	583.2816	-24.68	2	31.1	63.8	0	360-369	K.YEELQVTAGR.H		mdown:qdown 1.13<br>Wdown:Qdown 1.72
303	1	577.1338	-255.87	2	33.1	22.3	0	447-455	K.EYQELMNVK.L		mdown:qdown 1.02
2191	11	632.2117	-219.91	2	56.0	48.4	0	456-466	K.LALDVEIATYR.K		
359	1	724.3637	-37.90	2	33.6	16.6	0	534-550	R.AIGGGLSSVGGGSSTIK.Y		Wdown:Qdown 0.28<br>mdown:qdown 0.06

Detailed Protein Report

Protein 6: hemoglobin subunit delta [Homo sapiens]

**Accession:**	gi|4504351	**Score:**	462.8
**Database:**	refseq_human(refseq_human_20140103.fasta)	**MW [kDa]:**	16.0
**Database Date:**	2015-11-30	**pI:**	9.1
**Modification(s):**	Carbamidomethyl, Oxidation	**Sequence Coverage [%]:**	59.9
		**No. of unique Peptides:**	2

10	20	30	40	50	60	70	80
MVHLTPEEKT	AVNALWGKVN	VDAVGGEALG	RLLVVYPWTQ	RFFESFGDLS	SPDAVMGNPK	VKAHGKKVLG	AFSDGLAHLD
**90**	**100**	**110**	**120**	**130**	**140**	**150**	
NLKGTFSQLS	ELHCDKLHVD	PENFRLLGNV	LVCVLARNFG	KEFTPQMQAA	YQKVVAGVAN	ALAHKYH	

Cmpd.	No. of Cmpds.	m/z meas.	Δ m/z [ppm]	z	Rt [min]	Score	P	Range	Sequence	Modification
2232	1	1030.8450	-118.44	2	56.4	33.3	0	42-60	R.FFESFGDLSSPDAVMGNPK.V	Oxidation: 15
1086	2	761.3593	7.32	2	42.3	31.4	0	84-96	K.GTFSQLSELHCDK.L	Carbamidomethyl: 11

BRUKER

Detailed Protein Report

Protein 7: keratin, type II cytoskeletal 5 [Homo sapiens]

**Accession:**	gi|119395754	**Score:**	385.2
**Database:**	refseq_human(refseq_human_20140103.fasta)	**MW [kDa]:**	62.3
**Database Date:**	2015-11-30	**pI:**	8.6
**Modification(s):**	Carbamidomethyl	**Sequence Coverage [%]:**	17.3
		**No. of unique Peptides:**	4

Quantitation

mdown:qdown	**Median:**	0.46	**CV:**	12.37 %	**No. of Peptides:**	4
Wdown:Qdown	**Median:**	3.63	**CV:**	71.17 %	**No. of Peptides:**	2

10	20	30	40	50	60	70	80
MSRQSSVSFR	SGGSRSFSTA	SAITPSVSRT	SFTSVSRSGG	GGGGGFGRVS	LAGACGVGGY	GSRSLYNLGG	SKRISISTSG
90	100	110	120	130	140	150	160
GSFRNRFGAG	AGGGYGFGGG	AGSGFGFGGG	AGGGFGLGGG	AGFGGGFGGP	GFPVCPPGGI	QEVTVNQSLL	TPLNLQIDPS
170	180	190	200	210	220	230	240
IQRVRTEERE	QIKTLNNKFA	SFIDKVRFLE	QQNKVLDTKW	TLLQEQGTKT	VRQNLEPLFE	QYINNLRRQL	DSIVGERGRL
250	260	270	280	290	300	310	320
DSELRNMQDL	VEDFKNKYED	EINKRTTAEN	EFVMLKKDVD	AAYMNKVELE	AKVDALMDEI	NFMKMFFDAE	LSQMQTHVSD
330	340	350	360	370	380	390	400
TSVVLSMDNN	RNLDLDSIIA	EVKAQYEEIA	NRSRTEAESW	YQTKYEELQQ	TAGRHGDDLR	NTKHEISEMN	RMIQRLRAEI
410	420	430	440	450	460	470	480
DNVKKQCANL	QNAIADAEQR	GELALKDARN	KLAELEEALQ	KAKQDMARLL	REYQELMNTK	LALDVEIATY	RKLLEGEECR
490	500	510	520	530	540	550	560
LSGEGVGPVN	ISVVTSSVSS	GYGSGSGYGG	GLGGGLGGGL	GGGLAGGSSG	SYYSSSSGGV	GLGGGLSVGG	SGFSASSGRG
570	580	590	600				
LGVGFGSGGG	SSSSVKFVST	TSSSRKSFKS					

Cmpd.	No. of Cmpds.	m/z meas.	Δ m/z [ppm]	z	Rt [min]	Score	P	Range	Sequence	Modification	Ratios
419	1	705.9605	130.39	2	34.6	27.4	1	266-277	R.TTAENEFVMLKK.D		mdown:qdown 0.38
553	1	851.4187	19.30	2	36.2	57.4	0	406-420	K.QCANLQNAIADAEQR.G	Carbamidomethyl: 2	mdown:qdown 0.48
804	1	572.1668	-261.37	2	38.5	48.3	0	432-441	K.LAELEEALQK.A		Wdown:Qdown 6.89<br>mdown:qdown 0.53
334	3	720.3453	-20.22	2	33.5	59.4	0	560-576	R.GLGVGFGSGGGSSSSVK.F		mdown:qdown 0.48<br>Wdown:Qdown 1.92

Detailed Protein Report

Protein 8: hemoglobin subunit alpha [Homo sapiens]

Accession: gi|4504345
Database: refseq_human(refseq_human_20140103.fasta)
Database Date: 2015-11-30
Modification(s): Oxidation

Score: 376.6
MW [kDa]: 15.2
pI: 9.4
Sequence Coverage [%]: 44.4
No. of unique Peptides: 6

Quantitation

mdown:qdown	**Median:** 0.87	**CV:** 366.85 %	**No. of Peptides:**	2
Wdown:Qdown	**Median:** 0.13	**CV:** 64.98 %	**No. of Peptides:**	2

Alias proteins:

Accession	Name	Description
gi|4504347	refseq_human (refseq_human_20140103.fasta)	hemoglobin subunit alpha [Homo sapiens]

```
        10         20         30         40         50         60         70         80
MVLSPADKTN VKAAWGKVGA HAGEYGAEAL ERMFLSFPTT KTYFPHFDLS HGSAQVKGHG KKVADALTNA VAHVDDMPNA
        90        100        110        120        130        140        150
LSALSDLHAH KLRVDPVNFK LLSHCLLVTL AAHLPAEFTP AVHASLDKFL ASVSTVLTSK YR
```

Cmpd.	No. of Cmpds.	m/z meas.	Δ m/z [ppm]	z	Rt [min]	Score	P	Range	Sequence	Modification	Ratios
39	1	586.3399	3.78	2	29.7	55.0	1	2-12	M.VLSPADKTNVK.A		
111	9	765.3757	6.39	2	30.7	80.1	0	18-32	K.VGAHAGEYGAEALER.M		Wdown:Qdown 0.24
1149	2	544.2847	11.90	2	43.1	35.6	0	33-41	R.MFLSFPTTK.T	Oxidation: 1	
1779	24	536.2595	-39.76	2	50.9	56.7	0	33-41	R.MFLSFPTTK.T		mdown:qdown 4.44
1032	12	917.4300	-21.34	2	41.7	79.5	0	42-57	K.TYFPHFDLSHGSAQVK.G		Wdown:Qdown 0.07<br>mdown:qdown 0.17
1623	13	626.8522	-14.06	2	49.0	69.7	0	129-140	K.FLASVSTVLTSK.Y		

Detailed Protein Report

Protein 9: serotransferrin precursor [Homo sapiens]

**Accession:**	gi|4557871	**Score:**	375.5
**Database:**	refseq_human(refseq_human_20140103.fasta)	**MW [kDa]:**	77.0
**Database Date:**	2015-11-30	**pI:**	7.0
**Modification(s):**	Carbamidomethyl	**Sequence Coverage [%]:**	17.5
		**No. of unique Peptides:**	10

Quantitation

mdown:qdown	**Median:**	0.71	**CV:**	25.98 %	**No. of Peptides:**	7
Wdown:Qdown	**Median:**	0.68	**CV:**	50.84 %	**No. of Peptides:**	7

```
        10         20         30         40         50         60         70         80
MRLAVGALLV CAVLGLCLAV PDKTVRWCAV SEHEATKCQS FRDHMKSVIP SDGPSVACVK KASYLDCIRA IAANEADAVT
        90        100        110        120        130        140        150        160
LDAGLVYDAY LAPNNLKPVV AEFYGSKEDP QTFYYAVAVV KKDSGFQMNQ LRGKKSCHTG LGRSAGWNIP IGLLYCDLPE
       170        180        190        200        210        220        230        240
PRKPLEKAVA NFFSGSCAPC ADGTDFPQLC QLCPGCGCST LNQYFGYSGA FKCLKDGAGD VAFVKHSTIF ENLANKADRD
       250        260        270        280        290        300        310        320
QYELLCLDNT RKPVDEYKDC HLAQVPSHTV VARSMGGKED LIWELLNQAQ EHFGKDKSKE FQLFSSPHGK DLLFKDSAHG
       330        340        350        360        370        380        390        400
FLKVPPRMDA KMYLGYEYVT AIRNLREGTC PEAPTDECKP VKWCALSHHE RLKCDEWSVN SVGKIECVSA ETTEDCIAKI
       410        420        430        440        450        460        470        480
MNGEADAMSL DGGFVYIAGK CGLVPVLAEN YNKSDNCEDT PEAGYFAVAV VKKSASDLTW DNLKGKKSCH TAVGRTAGWN
       490        500        510        520        530        540        550        560
IPMGLLYNKI NHCRFDEFFS EGCAPGSKKD SSLCKLCMGS GLNLCEPNNK EGYYGYTGAF RCLVEKGDVA FVKHQTVPQN
       570        580        590        600        610        620        630        640
TGGKNPDPWA KNLNEKDYEL LCLDGTRKPV EEYANCHLAR APNHAVVTRK DKEACVHKIL RQQQHLFGSN VTDCSGNFCL
       650        660        670        680        690        700
FRSETKDLLF RDDTVCLAKL HDRNTYEKYL GEEYVKAVGN LRKCSTSSLL EACTFRRP
```

Cmpd.	No. of Cmpds.	m/z meas.	Δ m/z [ppm]	z	Rt [min]	Score	P	Range	Sequence	Modification	Ratios
610	1	708.3500	-19.16	2	36.2	66.2	0	47-60	K.SVIPSDGPSVACVK.K	Carbamidomethyl: 12	mdown:qdown 0.73<br>Wdown:Qdown 0.87
1759	1	815.3781	-41.02	2	50.7	25.1	0	108-121	K.EDPQTFYYAVAVVK.K		
400	1	637.3157	-23.07	2	34.3	35.7	0	226-236	K.HSTIFENLANK.A		Wdown:Qdown 1.19<br>mdown:qdown 1.01
1032	2	638.7248	-148.43	2	41.4	30.2	0	300-310	K.EFQLFSSPHGK.D		
229	1	761.3746	3.44	2	32.3	48.1	1	372-384	R.LKCDEWSVNSVGK.I	Carbamidomethyl: 3	mdown:qdown 0.55<br>Wdown:Qdown 0.81
708	2	625.2611	-72.75	2	37.7	48.9	0	454-464	K.SASDLTWDNLK.G		mdown:qdown 0.46<br>Wdown:Qdown 0.33
817	2	789.3225	-12.58	2	39.0	49.5	0	495-508	R.FDEFFSEGCAPGSK.K	Carbamidomethyl: 9	mdown:qdown 0.87<br>Wdown:Qdown 1.21
1439	1	783.3662	-47.33	2	46.8	10.9	1	647-659	K.DLLFRDDTVCLAK.L	Carbamidomethyl: 10	Wdown:Qdown 0.41<br>mdown:qdown 0.82
232	3	500.6172	-271.08	2	32.1	36.9	0	669-676	K.YLGEEYVK.A		mdown:qdown 0.69<br>Wdown:Qdown 0.49
915	1	766.3408	-8.88	2	40.2	24.0	0	684-696	K.CSTSSLLEACTFR.R	Carbamidomethyl: 1, 10	

Detailed Protein Report

Protein 10: keratin, type I cytoskeletal 15 [Homo sapiens]

**Accession:**	gi|24430190	**Score:**	337.7
**Database:**	refseq_human(refseq_human_20140103.fasta)	**MW [kDa]:**	49.2
**Database Date:**	2015-11-30	**pI:**	4.6
		**Sequence Coverage [%]:**	14.0
		**No. of unique Peptides:**	1

Quantitation

mdown:qdown	**Median:**	1.55	**CV:**	0.00 %	**No. of Peptides:**	1
Wdown:Qdown	**Median:**	4.68	**CV:**	0.00 %	**No. of Peptides:**	1

10	20	30	40	50	60	70	80
MTTTFLQTSS	STFGGGSTRG	GSLLAGGGGF	GGGSLSGGGG	SRSISASSAR	FVSSGSGGGY	GGGMRVCGFG	GGAGSVFGGG
**90**	**100**	**110**	**120**	**130**	**140**	**150**	**160**
FGGGVGGGFG	GGFGGGDGGL	LSGNEKITMQ	NLNDRLASYL	DKVRALEEAN	ADLEVKIHDW	YQKQTPTSPE	CDYSQYFKTI
**170**	**180**	**190**	**200**	**210**	**220**	**230**	**240**
EELRDKIMAT	TIDNSRVILE	IDNARLAADD	FRLKYENELA	LRQGVEADIN	GLRRVLDELT	LARTDLEMQI	EGLNEELAYL
**250**	**260**	**270**	**280**	**290**	**300**	**310**	**320**
KKNHEEEMKE	FSSQLAGQVN	VEMDAAPGVD	LTRVLAEMRE	QYEAMAEKNR	RDVEAWFFSK	TEELNKEVAS	NTEMIQTSKT
**330**	**340**	**350**	**360**	**370**	**380**	**390**	**400**
EITDLRRTMQ	ELEIELQSQL	SMKAGLENSL	AETECRYATQ	LQQIQGLIGG	LEAQLSELRC	EMEAQNQEYK	MLLDIKTRLE
**410**	**420**	**430**	**440**	**450**	**460**		
QEIATYRSLL	EGQDAKMAGI	GIREASSGGG	GSSSNFHINV	EESVDGQVVS	SHKREI		

Cmpd.	No. of Cmpds.	m/z meas.	Δ m/z [ppm]	z	Rt [min]	Score	P	Range	Sequence	Modification	Ratios
811	5	515.1698	-253.80	2	39.5	44.9	0	215-223	R.VLDELTLAR.T		Wdown:Qdown 4.68<br>mdown:qdown 1.55

Detailed Protein Report

Protein 11: titin isoform IC [Homo sapiens]

**Accession:**	gi|388998877	**Score:**	303.6
**Database:**	refseq_human(refseq_human_20140103.fasta)	**MW [kDa]:**	3992.1
**Database Date:**	2015-11-30	**pI:**	6.0
**Modification(s):**	Carbamidomethyl, Oxidation	**Sequence Coverage [%]:**	0.9
		**No. of unique Peptides:**	23

Quantitation

mdown:qdown	**Median:**	0.58	**CV:**	49.87 %	**No. of Peptides:**	3
Wdown:Qdown	**Median:**	1.11	**CV:**	127.57 %	**No. of Peptides:**	4

Detailed Protein Report

10	20	30	40	50	60	70	80
MTTQAPTFTQ	PLQSVVVLEG	STATFEAHIS	GFPVPEVSWF	RDGQVISTST	LPGVQISFSD	GRAKLTIPAV	TKANSGRYSL
90	100	110	120	130	140	150	160
KATNGSGQAT	STAELLVKAE	TAPPNFVQRL	QSMTVRQGSQ	VRLQVRVTGI	PTPVVKFYRD	GAEIQSSLDF	QISQEGDLYS
170	180	190	200	210	220	230	240
LLIAEAYPED	SGTYSVNATN	SVGRATSTAE	LLVQGEEEVP	AKKTKTIVST	AQISESRQTR	IEKKIEAHFD	ARSIATVEMV
250	260	270	280	290	300	310	320
IDGAAGQQLP	HKTPPRIPPK	PKSRSPTPPS	IAAKAQLARQ	QSPSPIRHSP	SPVRHVRAPT	PSPVRSVSPA	ARISTSPIRS
330	340	350	360	370	380	390	400
VRSPLLMRKT	QASTVATGPE	VPPPWKQEGY	VASSSEAEMR	ETTLTTSTQI	RTEERWEGRY	GVQEQVTISG	AAGAAASVSA
410	420	430	440	450	460	470	480
SASYAAEAVA	TGAKEVKQDA	DKSAAVATVV	AAVDMARVRE	PVISAVEQTA	QRTTTTAVHI	QPAQEQVRKE	AEKTAVTKVV
490	500	510	520	530	540	550	560
VAADKAKEQE	LKSRTKEVIT	TKQEQMHVTH	EQIRKETEKT	FVPKVVISAA	KAKEQETRIS	EEITKKQKQV	TQEAIRQETE
570	580	590	600	610	620	630	640
ITAASMVVVA	TAKSTKLETV	PGAQEETTTQ	QDQMHLSYEK	IMKETRKTVV	PKVIVATPKV	KEQDLVSRGR	EGITTKREQV
650	660	670	680	690	700	710	720
QITQEKMRKE	AEKTALSTIA	VATAKAKEQE	TILRTRETMA	TRQEQIQVTH	GKVDVGKKAE	AVATVVAAVD	QARVREPREP
730	740	750	760	770	780	790	800
GHLEESYAQQ	TTLEYGYKER	ISAAKVAEPP	QRPASEPHVV	PKAVKPRVIQ	APSETHIKTT	DQKGMHISSQ	IKKTTDLTTE
810	820	830	840	850	860	870	880
RLVHVDKRPR	TASPHFTVSK	ISVPKTEHGY	EASIAGSAIA	TLQKELSATS	SAQKITKSVK	APTVKPSETR	VRAEPTPLPQ
890	900	910	920	930	940	950	960
FPFADTPDTY	KSEAGVEVKK	EVGVSITGTT	VREERFEVLH	GREAKVTETA	RVPAPVEIPV	TPPTLVSGLK	NVTVIEGESV
970	980	990	1000	1010	1020	1030	1040
TLECHISGYP	SPTVTWYRED	YQIESSIDFQ	ITFQSGIARL	MIREAFAEDS	GRFTCSAVNE	AGTVSTSCYL	AVQVSEEFEK
1050	1060	1070	1080	1090	1100	1110	1120
ETTAVTEKFT	TEEKRFVESR	DVVMTDTSLT	EEQAGPGEPA	APYFITKPVV	QKLVEGGSVV	FGCQVGGNPK	PHVYWKKSGV
1130	1140	1150	1160	1170	1180	1190	1200
PLTTGYRYKV	SYNKQTGECK	LVISMTFADD	AGEYTIVVRN	KHGETSASAS	LLEEADYELL	MKSQQEMLYQ	TQVTAFVQEP
1210	1220	1230	1240	1250	1260	1270	1280
KVGETAPGFV	YSEYEKEYEK	EQALIRKKMA	KDTVVVRTYV	EDQEFHISSF	EERLIKEIEY	RIIKTTLEEL	LEEDGEEKMA
1290	1300	1310	1320	1330	1340	1350	1360
VDISESEAVE	SGFDSRIKNY	RILEGMGVTF	HCKMSGYPLP	KIAWYKDGKR	IKHGERYQMD	FLQDGRASLR	IPVVLPEDEG
1370	1380	1390	1400	1410	1420	1430	1440
IYTAFASNIK	GNAICSGKLY	VEPAAPLGAP	TYIPTLEPVS	RIRSLSPRSV	SRSPIRMSPA	RMSPARMSPA	RMSPARMSPG
1450	1460	1470	1480	1490	1500	1510	1520
RRLEETDESQ	LERLYKPVFV	LKPVSFKCLE	GQTARFDLKV	VGRPMPETFW	FHDGQQIVND	YTHKVVIKED	GTQSLIIVPA
1530	1540	1550	1560	1570	1580	1590	1600
TPSDSGEWTV	VAQNRAGRSS	ISVILTVEAV	EHQVKPMFVE	KLKNVNIKEG	SRLEMKVRAT	GNPNPDIVWL	KNSDIIVPHK
1610	1620	1630	1640	1650	1660	1670	1680
YPKIRIEGTK	GEAALKIDST	VSQDSAWYTA	TAINKAGRDT	TRCKVNVEVE	FAEPEPERKL	IIPRGTYRAK	EIAAPELEPL
1690	1700	1710	1720	1730	1740	1750	1760
HLRYGQEQWE	EGDLYDKEKQ	QKPFFKKKLT	SLRLKRFGPA	HFECRLTPIG	DPTMVVEWLH	DGKPLEAANR	LRMINEFGYC
1770	1780	1790	1800	1810	1820	1830	1840
SLDYGVAYSR	DSGIITCRAT	NKYGTDHTSA	TLIVKDEKSL	VEESQLPEGR	KGLQRIEELE	RMAHEGALTG	VTTDQKEKQK
1850	1860	1870	1880	1890	1900	1910	1920
PDIVLYPEPV	RVLEGETARF	RCRVTGYPQP	KVNWYLNGQL	IRKSKRFRVR	YDGIHYLDIV	DCKSYDTGEV	KVTAENPEGV
1930	1940	1950	1960	1970	1980	1990	2000
IEHKVKLEIQ	QREDFRSVLR	RAPEPRPEFH	VHEPGKLQFE	VQKVDRPVDT	TETKEVVKLK	RAERITHEKV	PEESEELRSK
2010	2020	2030	2040	2050	2060	2070	2080
FKRRTEEGYY	EAITAVELKS	RKKDESYEEL	LRKTKDELLH	WTKELTEEEK	KALAEEGKIT	IPTFKPDKIE	LSPSMEAPKI
2090	2100	2110	2120	2130	2140	2150	2160
FERIQSQTVG	QGSDAHFRVR	VVGKPDPECE	WYKNGVKIER	SDRIYWYWPE	DNVCELVIRD	VTAEDSASIM	VKAINIAGET
2170	2180	2190	2200	2210	2220	2230	2240

Detailed Protein Report

Cmpd.	No. of Cmpds.	m/z meas.	Δ m/z [ppm]	z	Rt [min]	Score	P	Range	Sequence	Modification	Ratios
1676	1	839.3937	-10.73	2	49.5	10.3	2	1417-1431	R.MSPARMSPARMSPAR.M	Oxidation: 6, 11	Wdown:Qdown 0.30<br>mdown:qdown 0.35
1459	1	851.5485	120.85	2	48.0	14.8	0	7427-7441	K.DIEQTVGLPVTLTCR.L	Carbamidomethyl: 14	
2713	1	698.3756	114.21	2	62.8	14.9	0	8326-8337	K.VDHSDVGEYSCK.A	Carbamidomethyl: 11	
967	1	752.8772	-83.84	2	40.6	12.6	2	10852-10864	K.VPEEPKKPVPEKK.V		
1296	1	640.2561	-89.77	2	45.9	12.9	1	10963-10972	K.EVTIMEEKER.A	Oxidation: 5	
2926	1	713.8984	-28.68	2	65.7	11.1	1	11452-11464	K.VEPPPPPKVPEIK.K		
1036	8	555.3376	91.87	2	41.5	15.1	0	13889-13899	K.GTAIFACDIAK.D		Wdown:Qdown 2.51
2424	1	822.3339	-55.35	2	60.7	10.9	2	14955-14967	R.EKEMARFECELSR.E	Oxidation: 4	
2695	1	805.4194	19.35	2	64.9	15.3	2	14961-14973	R.FECELSRENAKVK.W	Carbamidomethyl: 3	
798	5	481.1628	-199.39	2	38.5	12.9	0	15703-15710	K.TWVLATDR.A		Wdown:Qdown 3.26
1108	1	540.7821	22.96	2	42.5	15.6	1	17633-17641	K.EIREGADYK.L		mdown:qdown 1.09
2888	1	900.4707	-22.65	2	64.8	13.7	0	18003-18018	K.NETVIEKPTDALQITK.E		
2673	3	813.4051	24.69	2	61.8	13.6	0	19641-19653	R.VPDLLEGCQYEFR.V	Carbamidomethyl: 8	
2371	2	805.2633	-115.33	2	60.0	12.7	0	28749-28761	R.EEEPLFDIDSEMR.K		
1812	1	658.3022	-97.61	2	52.7	11.3	0	29853-29864	K.AGEDVQVLIPFK.G		
2869	1	710.3695	-29.92	2	65.0	10.9	0	30145-30156	R.IGHNVHLELPYK.G		
2745	1	656.8669	67.42	2	62.7	10.9	0	30994-31005	K.YTLTVENNSGSK.S		
2485	1	862.3731	-106.42	2	61.5	10.8	0	31629-31646	R.AGSDLVLDAAVGGKPEPK.I		
98	1	529.8207	-183.86	3	30.5	11.1	1	32923-32936	K.DDGGSRVTGYYIER.K		
2686	1	937.4503	-21.14	2	62.5	12.9	0	33057-33072	K.QFTIGGLLEATEYEFR.V		
496	1	677.6900	21.58	3	35.5	11.2	2	33663-33680	K.DAGFYVVCAKNRFGIDQK.T		
37	1	731.6663	-74.70	3	29.7	10.4	2	35370-35388	K.TNLQFMGQAFKSIHEKVSK.I		Wdown:Qdown 0.61<br>mdown:qdown 0.52
1561	1	745.7829	-73.36	2	49.4	15.1	0	35482-35495	K.TDTSDSGLYTCTVK.N		

Detailed Protein Report

Protein 12: keratin, type I cytoskeletal 14 [Homo sapiens]

Accession: gi|15431310
Database: refseq_human(refseq_human_20140103.fasta)
Database Date: 2015-11-30
Modification(s): Oxidation

Score: 300.7
MW [kDa]: 51.6
pI: 4.9
Sequence Coverage [%]: 17.8
No. of unique Peptides: 4

Quantitation

mdown:qdown	**Median:**	1.20	**CV:**	147.60 %	**No. of Peptides:**	3
Wdown:Qdown	**Median:**	0.24	**CV:**	67.67 %	**No. of Peptides:**	2

10	20	30	40	50	60	70	80
MTTCSRQFTS	SSSMKGSCGI	GGGIGGGSSR	ISSVLAGGSC	RAPSTYGGGL	SVSSSRFSSG	GAYGLGGGYG	GGFSSSSSSF
**90**	**100**	**110**	**120**	**130**	**140**	**150**	**160**
GSGFGGGYGG	GLGAGLGGGF	GGGFAGGDGL	LVGSEKVTMQ	NLNDRLASYL	DKVRALEEAN	ADLEVKIRDW	YQRQRPAEIK
**170**	**180**	**190**	**200**	**210**	**220**	**230**	**240**
DYSPYFKTIE	DLRNKILTAT	VDNANVLLQI	DNARLAADDF	RTKYETELNL	RMSVEADING	LRRVLDELTL	ARADLEMQIE
**250**	**260**	**270**	**280**	**290**	**300**	**310**	**320**
SLKEELAYLK	KNHEEEMNAL	RGQVGGDVNV	EMDAAPGVDL	SRILNEMRDQ	YEKMAEKNRK	DAEEWFFTKT	EELNREVATN
**330**	**340**	**350**	**360**	**370**	**380**	**390**	**400**
SELVQSGKSE	ISELRRTMQN	LEIELQSQLS	MKASLENSLE	ETKGRYCMQL	AQIQEMIGSV	EEQLAQLRCE	MEQQNQEYKI
**410**	**420**	**430**	**440**	**450**	**460**	**470**	**480**
LLDVKTRLEQ	EIATYRRLLE	GEDAHLSSSQ	FSSGSQSSRD	VTSSSRQIRT	KVMDVHDGKV	VSTHEQVLRT	KN

Cmpd.	No. of Cmpds.	m/z meas.	Δ m/z [ppm]	z	Rt [min]	Score	P	Range	Sequence	Modification	Ratios
121	2	713.3373	-20.71	2	30.8	66.0	0	42-56	R.APSTYGGGLSVSSSR.F		
639	1	680.8203	-49.04	2	36.6	15.5	1	212-223	R.MSVEADINGLRR.V		mdown:qdown 0.43<br>Wdown:Qdown 0.44
1899	3	586.6929	-125.55	2	52.2	33.2	0	301-309	K.DAEEWFFTK.T		mdown:qdown 5.28
1027	2	636.9803	-7.37	3	41.6	10.2	0	337-352	R.TMQNLEIELQSQLSMK.A	Oxidation: 15	mdown:qdown 0.77<br>Wdown:Qdown 0.13

Detailed Protein Report

Protein 13: keratin, type II cytoskeletal 75 [Homo sapiens]

Accession: gi|153791158
Database: refseq_human(refseq_human_20140103.fasta)
Database Date: 2015-11-30

Score: 247.8
MW [kDa]: 59.5
pI: 8.7
Sequence Coverage [%]: 8.2
No. of unique Peptides: 1

10	20	30	40	50	60	70	80
MSRQSSITFQ	SGSRRGFSTT	SAITPAAGRS	RFSSVSVARS	AAGSGGLGRI	SSAGASFGSR	SLYNLGGAKR	VSINGCGSSC
**90**	**100**	**110**	**120**	**130**	**140**	**150**	**160**
RSGFGGRASN	RFGVNSGFGY	GGGVGGGFSG	PSFPVCPPGG	IQEVTVNQSL	LTPLHLQIDP	TIQRVRAEER	EQIKTLNNKF
**170**	**180**	**190**	**200**	**210**	**220**	**230**	**240**
ASFIDKVRFL	EQQNKVLETK	WALLQEQGSR	TVRQNLEPLF	DSYTSELRRQ	LESITTERGR	LEAELRNMQD	VVEDFKVRYE
**250**	**260**	**270**	**280**	**290**	**300**	**310**	**320**
DEINKRTAAE	NEFVALKKDV	DAAYMNKVEL	EAKVKSLPEE	INFIHSVFDA	ELSQLQTQVG	DTSVVLSMDN	NRNLDLDSII
**330**	**340**	**350**	**360**	**370**	**380**	**390**	**400**
AEVKAQYEDI	ANRSRAEAES	WYQTKYEELQ	VTAGRHGDDL	RNTKQEISEM	NRMIQRLRAE	IDSVKKQCSS	LQTAIADAEQ
**410**	**420**	**430**	**440**	**450**	**460**	**470**	**480**
RGELALKDAR	AKLVDLEEAL	QKAKQDMARL	LREYQELMNI	KLALDVEIAT	YRKLLEGEEC	RLSGEGVSPV	NISVVTSTLS
**490**	**500**	**510**	**520**	**530**	**540**	**550**	**560**
SGYGSGSSIG	GGNLGLGGGS	GYSFTTSGGH	SLGAGLGGSG	FSATSNRGLG	GSGSSVKFVS	TTSSSQKSYT	H

Cmpd.	No. of Cmpds.	m/z meas.	Δ m/z [ppm]	z	Rt [min]	Score	P	Range	Sequence	Modification
566	2	678.9555	96.02	2	35.7	53.0	1	413-424	K.LVDLEEALQKAK.Q	

Detailed Protein Report

Protein 14: alpha-1-antitrypsin precursor [Homo sapiens]

**Accession:**	gi|50363217	**Score:**	244.4
**Database:**	refseq_human(refseq_human_20140103.fasta)	**MW [kDa]:**	46.7
**Database Date:**	2015-11-30	**pI:**	5.3
		**Sequence Coverage [%]:**	23.0
		**No. of unique Peptides:**	5

Quantitation

mdown:qdown	**Median:** 1.75	**CV:** 13.54 %	**No. of Peptides:**	3
Wdown:Qdown	**Median:** 0.63	**CV:** 72.71 %	**No. of Peptides:**	3

Alias proteins:

Accession	Name	Description
gi|189163542	refseq_human (refseq_human_20140103.fasta)	alpha-1-antitrypsin precursor [Homo sapiens]
gi|189163540	refseq_human (refseq_human_20140103.fasta)	alpha-1-antitrypsin precursor [Homo sapiens]
gi|189163538	refseq_human (refseq_human_20140103.fasta)	alpha-1-antitrypsin precursor [Homo sapiens]
gi|189163536	refseq_human (refseq_human_20140103.fasta)	alpha-1-antitrypsin precursor [Homo sapiens]
gi|189163534	refseq_human (refseq_human_20140103.fasta)	alpha-1-antitrypsin precursor [Homo sapiens]
gi|189163532	refseq_human (refseq_human_20140103.fasta)	alpha-1-antitrypsin precursor [Homo sapiens]
gi|189163530	refseq_human (refseq_human_20140103.fasta)	alpha-1-antitrypsin precursor [Homo sapiens]
gi|189163528	refseq_human (refseq_human_20140103.fasta)	alpha-1-antitrypsin precursor [Homo sapiens]
gi|50363221	refseq_human (refseq_human_20140103.fasta)	alpha-1-antitrypsin precursor [Homo sapiens]
gi|50363219	refseq_human (refseq_human_20140103.fasta)	alpha-1-antitrypsin precursor [Homo sapiens]

```
        10         20         30         40         50         60         70         80
MPSSVSWGIL LLAGLCCLVP VSLAEDPQGD AAQKTDTSHH DQDHPTFNKI TPNLAEFAFS LYRQLAHQSN STNIFFSPVS
        90        100        110        120        130        140        150        160
IATAFAMLSL GTKADTHDEI LEGLNFNLTE IPEAQIHEGF QELLRTLNQP DSQLQLTTGN GLFLSEGLKL VDKFLEDVKK
       170        180        190        200        210        220        230        240
LYHSEAFTVN FGDTEEAKKQ INDYVEKGTQ GKIVDLVKEL DRDTVFALVN YIFFKGKWER PFEVKDTEEE DFHVDQVTTV
       250        260        270        280        290        300        310        320
KVPMMKRLGM FNIQHCKKLS SWVLLMKYLG NATAIFFLPD EGKLQHLENE LTHDIITKFL ENEDRRSASL HLPKLSITGT
       330        340        350        360        370        380        390        400
YDLKSVLGQL GITKVFSNGA DLSGVTEEAP LKLSKAVHKA VLTIDEKGTE AAGAMFLEAI PMSIPPEVKF NKPFVFLMIE
       410        420
QNTKSPLFMG KVVNPTQK
```

Cmpd.	No. of Cmpds.	m/z meas.	Δ m/z [ppm]	z	Rt [min]	Score	P	Range	Sequence	Modification	Ratios
2910	3	858.7649	-23.64	3	65.6	35.3	0	126-149	R. TLNQPDSQLQLTTGNGLFLSEGLK L		
722	1	729.3434	-11.09	3	37.9	16.3	1	161-179	K.LYHSEAFTVNFGDTEEAKK.Q		Wdown:Qdown 0.32
762	1	601.9916	0.24	3	38.4	25.3	0	284-298	K.LQHLENELTHDIITK.F		mdown:qdown 1.97
904	4	555.7989	-12.32	2	39.8	70.1	0	315-324	K.LSITGTYDLK.S		Wdown:Qdown 0.51<br>mdown:qdown 1.88
1068	4	508.1796	-258.32	2	42.1	41.2	0	325-334	K.SVLGQLGITK.V		mdown:qdown 1.45<br>Wdown:Qdown 1.52

Detailed Protein Report

Protein 15: annexin A1 [Homo sapiens]

Accession: gi|4502101
Database: refseq_human(refseq_human_20140103.fasta)
Database Date: 2015-11-30
Modification(s): Carbamidomethyl

Score: 234.0
MW [kDa]: 38.7
pI: 6.7
Sequence Coverage [%]: 27.2
No. of unique Peptides: 7

Quantitation

mdown:qdown	**Median:**	2.22	**CV:**	104.73 %	**No. of Peptides:**	3
Wdown:Qdown	**Median:**	2.63	**CV:**	93.07 %	**No. of Peptides:**	3

```
        10         20         30         40         50         60         70         80
MAMVSEFLKQ AWFIENEEQE YVQTVKSSKG GPGSAVSPYP TFNPSSDVAA LHKAIMVKGV DEATIIDILT KRNNAQRQQI
        90        100        110        120        130        140        150        160
KAAYLQETGK PLDETLKKAL TGHLEEVVLA LLKTPAQFDA DELRAAMKGL GTDEDTLIEI LASRTNKEIR DINRVYREEL
       170        180        190        200        210        220        230        240
KRDLAKDITS DTSGDFRNAL LSLAKGDRSE DFGVNEDLAD SDARALYEAG ERRKGTDVNV FNTILTTRSY PQLRRVFQKY
       250        260        270        280        290        300        310        320
TKYSKHDMNK VLDLELKGDI EKCLTAIVKC ATSKPAFFAE KLHQAMKGVG TRHKALIRIM VSRSEIDMND IKAFYQKMYG
       330        340        350
ISLCQAILDE TKGDYEKILV ALCGGN
```

Cmpd.	No. of Cmpds.	m/z meas.	Δ m/z [ppm]	z	Rt [min]	Score	P	Range	Sequence	Modification	Ratios
2228	1	694.3075	-115.27	2	56.3	17.4	0	59-71	K.GVDEATIIDILTK.R		
537	1	635.6517	-48.42	3	35.3	22.1	1	82-98	K.AAYLQETGKPLDETLKK.A		
352	1	631.8010	-5.09	2	33.8	31.5	0	114-124	K.TPAQFDADELR.A		Wdown:Qdown 2.57<br>mdown:qdown 1.16
2453	2	851.9230	-27.64	2	59.0	48.1	0	129-144	K.GLGTDEDTLIEILASR.T		
174	2	607.2652	-8.28	2	30.9	68.2	0	167-177	K.DITSDTSGDFR.N		Wdown:Qdown 6.99<br>mdown:qdown 7.48
2388	1	775.8834	-37.22	2	58.2	36.2	0	215-228	K.GTDVNVFNTILTTR.S		Wdown:Qdown 1.01<br>mdown:qdown 1.26
2020	1	678.7710	-93.49	2	55.4	10.5	0	270-281	K.CATSKPAFFAEK.L	Carbamidomethyl: 1	

Detailed Protein Report

Protein 16: alpha-2-macroglobulin precursor [Homo sapiens]

**Accession:**	gi|66932947	**Score:**	220.1
**Database:**	refseq_human(refseq_human_20140103.fasta)	**MW [kDa]:**	163.2
**Database Date:**	2015-11-30	**pI:**	6.0
		**Sequence Coverage [%]:**	7.9
		**No. of unique Peptides:**	8

Quantitation

mdown:qdown	**Median:**	1.56	**CV:**	27.61 %	**No. of Peptides:**	2
Wdown:Qdown	**Median:**	1.15	**CV:**	0.00 %	**No. of Peptides:**	1

10	20	30	40	50	60	70	80
MGKNKLLHPS	LVLLLLVLLP	TDASVSGKPQ	YMVLVPSLLH	TETTEKGCVL	LSYLNETVTV	SASLESVRGN	RSLFTDLEAE
90	100	110	120	130	140	150	160
NDVLHCVAFA	VPKSSSNEEV	MFLTVQVKGP	TQEFKKRTTV	MVKNEDSLVF	VQTDKSIYKP	GQTVKFRVVS	MDENFHPLNE
170	180	190	200	210	220	230	240
LIPLVYIQDP	KGNRIAQWQS	FQLEGGLKQF	SFPLSSEPFQ	GSYKVVVQKK	SGGRTEHPFT	VEEFVLPKFE	VQVTVPKIIT
250	260	270	280	290	300	310	320
ILEEEMNVSV	CGLYTYGKPV	PGHVTVSICR	KYSDASDCHG	EDSQAFCEKF	SGQLNSHGCF	YQQVKTKVFQ	LKRKEYEMKL
330	340	350	360	370	380	390	400
HTEAQIQEEG	TVVELTGRQS	SEITRTITKL	SFVKVDSHFR	QGIPFFGQVR	LVDGKGVPIP	NKVIFIRGNE	ANYYSNATTD
410	420	430	440	450	460	470	480
EHGLVQFSIN	TTNVMGTSLT	VRVNYKDRSP	CYGYQWVSEE	HEEAHHTAYL	VFSPSKSFVH	LEPMSHELPC	GHTQTVQAHY
490	500	510	520	530	540	550	560
ILNGGTLLGL	KKLSFYYLIM	AKGGIVRTGT	HGLLVKQEDM	KGHFSISIPV	KSDIAPVARL	LIYAVLPTGD	VIGDSAKYDV
570	580	590	600	610	620	630	640
ENCLANKVDL	SFSPSQSLPA	SHAHLRVTAA	PQSVCALRAV	DQSVLLMKPD	AELSASSVYN	LLPEKDLTGF	PGPLNDQDDE
650	660	670	680	690	700	710	720
DCINRHNVYI	NGITYTPVSS	TNEKDMYSFL	EDMGLKAFTN	SKIRKPKMCP	QLQQYEMHGP	EGLRVGFYES	DVMGRGHARL
730	740	750	760	770	780	790	800
VHVEEPHTET	VRKYFPETWI	WDLVVVNSAG	VAEVGVTVPD	TITEWKAGAF	CLSEDAGLGI	SSTASLRAFQ	PFFVELTMPY
810	820	830	840	850	860	870	880
SVIRGEAFTL	KATVLNYLPK	CIRVSVQLEA	SPAFLAVPVE	KEQAPHCICA	NGRQTVSWAV	TPKSLGNVNF	TVSAEALESQ
890	900	910	920	930	940	950	960
ELCGTEVPSV	PEHGRKDTVI	KPLLVEPEGL	EKETTFNSLL	CPSGGEVSEE	LSLKLPPNVV	EESARASVSV	LGDILGSAMQ
970	980	990	1000	1010	1020	1030	1040
NTQNLLQMPY	GCGEQNMVLF	APNIYVLDYL	NETQQLTPEI	KSKAIGYLNT	GYQRQLNYKH	YDGSYSTFGE	RYGRNQGNTW
1050	1060	1070	1080	1090	1100	1110	1120
LTAFVLKTFA	QARAYIFIDE	AHITQALIWL	SQRQKDNGCF	RSSGSLLNNA	IKGGVEDEVT	LSAYITIALL	EIPLTVTHPV
1130	1140	1150	1160	1170	1180	1190	1200
VRNALFCLES	AWKTAQEGDH	GSHVYTKALL	AYAFALAGNQ	DKRKEVLKSL	NEEAVKKDNS	VHWERPQKPK	APVGHFYEPQ
1210	1220	1230	1240	1250	1260	1270	1280
APSAEVEMTS	YVLLAYLTAQ	PAPTSEDLTS	ATNIVKWITK	QQNAQGGFSS	TQDTVVALHA	LSKYGAATFT	RTGKAAQVTI
1290	1300	1310	1320	1330	1340	1350	1360
QSSGTFSSKF	QVDNNNRLLL	QQVSLPELPG	EYSMKVTGEG	CVYLQTSLKY	NILPEKEEFP	FALGVQTLPQ	TCDEPKAHTS
1370	1380	1390	1400	1410	1420	1430	1440
FQISLSVSYT	GSRSASNMAI	VDVKMVSGFI	PLKPTVKMLE	RSNHVSRTEV	SSNHVLIYLD	KVSNQTLSLF	FTVLQDVPVR
1450	1460	1470	1480				
DLKPAIVKVY	DYYETDEFAI	AEYNAPCSKD	LGNA				

Cmpd.	No. of Cmpds.	m/z meas.	Δ m/z [ppm]	z	Rt [min]	Score	P	Range	Sequence	Modification	Ratios
1683	5	802.8311	-117.18	2	49.7	41.2	0	175-188	R.IAQWQSFQLEGGLK.Q		
980	1	542.6642	-270.62	2	41.0	12.6	0	522-531	K.GHFSISIPVK.S		
2454	1	922.9898	-34.93	2	59.0	33.4	0	540-557	R.LLIYAVLPTGDVIGDSAK.Y		
1177	1	509.7828	-34.15	2	43.4	37.2	0	812-820	K.ATVLNYLPK.C		
2405	1	942.4446	-87.99	2	58.4	18.6	0	824-841	R.VSVQLEASPAFLAVPVEK.E		

Detailed Protein Report

Cmpd.	No. of Cmpds.	m/z meas.	Δ m/z [ppm]	z	Rt [min]	Score	P	Range	Sequence	Modification	Ratios
234	1	605.7801	-74.07	2	32.3	35.4	0	935-945	K.LPPNVVEESAR.A		Wdown:Qdown 1.15<br>mdown:qdown 2.04
1151	1	796.4102	8.78	3	43.1	23.4	0	1241-1263	K.QQNAQGGFSSTQDTVVALHALSKY		
891	2	809.4242	-4.39	2	39.9	18.3	0	1408-1421	R.TEVSSNHVLIYLDK.V		mdown:qdown 1.19

Detailed Protein Report

Protein 17: actin, cytoplasmic 1 [Homo sapiens]

**Accession:**	gi|4501885	**Score:**	215.9
**Database:**	refseq_human(refseq_human_20140103.fasta)	**MW [kDa]:**	41.7
**Database Date:**	2015-11-30	**pI:**	5.2
		**Sequence Coverage [%]:**	21.1
		**No. of unique Peptides:**	5

Quantitation

mdown:qdown	**Median:**	1.44	**CV:**	61.08 %	**No. of Peptides:**	5
Wdown:Qdown	**Median:**	1.30	**CV:**	21.84 %	**No. of Peptides:**	2

```
        10         20         30         40         50         60         70         80
MDDDIAALVV DNGSGMCKAG FAGDDAPRAV FPSIVGRPRH QGVMVGMGQK DSYVGDEAQS KRGILTLKYP IEHGIVTNWD
        90        100        110        120        130        140        150        160
DMEKIWHHTF YNELRVAPEE HPVLLTEAPL NPKANREKMT QIMFETFNTP AMYVAIQAVL SLYASGRTTG IVMDSGDGVT
       170        180        190        200        210        220        230        240
HTVPIYEGYA LPHAILRLDL AGRDLTDYLM KILTERGYSF TTTAERЕIVR DIKEKLCYVA LDFEQEMATA ASSSSLEKSY
       250        260        270        280        290        300        310        320
ELPDGQVITI GNERFRCPEA LFQPSFLGME SCGIHETTFN SIMKCDVDIR KDLYANTVLS GGTTMYPGIA DRMQKEITAL
       330        340        350        360        370        380
APSTMKIKII APPERKYSVW IGGSILASLS TFQQMWISKQ EYDESGPSIV HRKCF
```

Cmpd.	No. of Cmpds.	m/z meas.	Δ m/z [ppm]	z	Rt [min]	Score	P	Range	Sequence	Modification	Ratios
659	3	599.7070	-249.11	2	36.8	51.4	0	29-39	R.AVFPSIVGRPR.H		mdown:qdown 1.29
2136	3	651.9448	-125.01	3	55.3	28.9	0	96-113	R.VAPEEHPVLLTEAPLNPK.A		mdown:qdown 3.26
1678	5	895.8924	-63.80	2	49.5	48.9	0	239-254	K.SYELPDGQVITIGNER.F		mdown:qdown 0.69<br>Wdown:Qdown 1.05
2043	1	1107.8969	-127.87	2	54.1	33.7	0	292-312	K.DLYANTVLSGGTTMYPGIADR.M		mdown:qdown 0.95
74	2	758.8545	-0.57	2	30.1	53.0	0	360-372	K.QEYDESGPSIVHR.K		Wdown:Qdown 1.61<br>mdown:qdown 2.23

BRUKER Detailed Protein Report

Protein 18: keratin, type II cytoskeletal 4 [Homo sapiens]

Accession: gi|331999954
Database: refseq_human(refseq_human_20140103.fasta)
Database Date: 2015-11-30

Score: 179.5
MW [kDa]: 56.1
pI: 6.2
Sequence Coverage [%]: 10.2
No. of unique Peptides: 6

Quantitation

mdown:qdown	**Median:**	0.69	**CV:**	18.18 %	**No. of Peptides:**	3
Wdown:Qdown	**Median:**	1.59	**CV:**	54.27 %	**No. of Peptides:**	3

10	20	30	40	50	60	70	80
MIARQQCVRG	GPRGFSCGSA	IVGGGKRGAF	SSVSMSGGAG	RCSSGGFGSR	SLYNLRGNKS	ISMSVAGSRQ	GACFGGAGGF
**90**	**100**	**110**	**120**	**130**	**140**	**150**	**160**
GTGGFGGGFG	GSFSGKGGPG	FPVCPAGGIQ	EVTINQSLLT	PLHVEIDPEI	QKVRTEEREQ	IKLLNNKFAS	FIDKVQFLEQ
**170**	**180**	**190**	**200**	**210**	**220**	**230**	**240**
QNKVLETKWN	LLQQQTTTTS	SKNLEPLFET	YLSVLRKQLD	TLGNDKGRLQ	SELKTMQDSV	EDFKTKYEEE	INKRTAAEND
**250**	**260**	**270**	**280**	**290**	**300**	**310**	**320**
FVVLKKDVDA	AYLNKVELEA	KVDSLNDEIN	FLKVLYDAEL	SQMQTHVSDT	SVVLSMDNNR	NLDLDSIIAE	VRAQYEEIAQ
**330**	**340**	**350**	**360**	**370**	**380**	**390**	**400**
RSKAEAEALY	QTKVQQLQIS	VDQHGDNLKN	TKSEIAELNR	MIQRLRAEIE	NIKKQCQTLQ	VSVADAEQRG	ENALKDAHSK
**410**	**420**	**430**	**440**	**450**	**460**	**470**	**480**
RVELEAALQQ	AKEELARMLR	EYQELMSVKL	ALDIEIATYR	KLLEGEEYRM	SGECQSAVSI	SVVSGSTSTG	GISGGLGSGS
**490**	**500**	**510**	**520**	**530**			
GFGLSSGFGS	GSGSGFGFGG	SVSGSSSSKI	ISTTTLNKRR				

Cmpd.	No. of Cmpds.	m/z meas.	Δ m/z [ppm]	z	Rt [min]	Score	P	Range	Sequence	Modification	Ratios
950	1	603.8017	-33.37	2	40.4	11.5	0	235-245	R.TAAENDFVVLK.K		Wdown:Qdown 0.78<br>mdown:qdown 0.88
530	2	667.8552	-21.26	2	35.2	56.2	1	235-246	R.TAAENDFVVLKK.D		mdown:qdown 0.64<br>Wdown:Qdown 2.40
1586	2	703.8014	-85.66	2	49.7	20.9	0	262-273	K.VDSLNDEINFLK.V		
2400	2	679.3689	-1.20	2	60.3	33.7	0	301-312	R.NLDLDSIIAEVR.A		
365	2	678.3483	-55.17	2	33.6	39.9	1	401-412	K.RVELEAALQQAK.E		mdown:qdown 0.58<br>Wdown:Qdown 2.17
1796	1	599.9815	-22.51	3	51.1	17.3	1	402-417	R.VELEAALQQAKEELAR.M		

Detailed Protein Report

Protein 19: keratin, type II cytoskeletal 2 epidermal [Homo sapiens]

**Accession:**	gi|47132620	**Score:**	176.3
**Database:**	refseq_human(refseq_human_20140103.fasta)	**MW [kDa]:**	65.4
**Database Date:**	2015-11-30	**pI:**	8.9
		**Sequence Coverage [%]:**	5.5
		**No. of unique Peptides:**	1

Quantitation

mdown:qdown	**Median:**	1.08	**CV:**	0.00 %	**No. of Peptides:**	1
Wdown:Qdown	**Median:**	0.55	**CV:**	0.00 %	**No. of Peptides:**	1

10	20	30	40	50	60	70	80
MSCQISCKSR	GRGGGGGGFR	GFSSGSAVVS	GGSRRSTSSF	SCLSRHGGGG	GGFGGGGFGS	RSLVGLGGTK	SISISVAGGG
90	100	110	120	130	140	150	160
GGFGAAGGFG	GRGGGFGGGS	SFGGGSGFSG	GGFGGGGFGG	GRFGGFGGPG	GVGGLGGPGG	FGPGGYPGGI	HEVSVNQSLL
170	180	190	200	210	220	230	240
QPLNVKVDPE	IQNVKAQERE	QIKTLNNKFA	SFIDKVRFLE	QQNQVLQTKW	ELLQQMNVGT	RPINLEPIFQ	GYIDSLKRYL
250	260	270	280	290	300	310	320
DGLTAERTSQ	NSELNNMQDL	VEDYKKKYED	EINKRTAAEN	DFVTLKKDVD	NAYMIKVELQ	SKVDLLNQEI	EFLKVLYDAE
330	340	350	360	370	380	390	400
ISQIHQSVTD	TNVILSMDNS	RNLDLDSIIA	EVKAQYEEIA	QRSKEEAEAL	YHSKYEELQV	TVGRHGDSLK	EIKIEISELN
410	420	430	440	450	460	470	480
RVIQRLQGEI	AHVKKQCKNV	QDAIADAEQR	GEHALKDARN	KLNDLEEALQ	QAKEDLARLL	RDYQELMNVK	LALDVEIATY
490	500	510	520	530	540	550	560
RKLLEGEECR	MSGDLSSNVT	VSVTSSTISS	NVASKAAFGG	SGGRGSSSGG	GYSSGSSSYG	SGGRQSGSRG	GSGGGGSISG
570	580	590	600	610	620	630	640
GGYGSGGGSG	GRYGSGGGSK	GGSISGGGYG	SGGGKHSSGG	GSRGGSSSGG	GYGSGGGGSS	SVKGSSGEAF	GSSVTFSFR

Cmpd.	No. of Cmpds.	m/z meas.	Δ m/z [ppm]	z	Rt [min]	Score	P	Range	Sequence	Modification	Ratios
354	1	738.3735	-30.88	2	33.8	45.3	0	198-209	R.FLEQQNQVLQTK.W		Wdown:Qdown 0.55<br>mdown:qdown 1.08

Detailed Protein Report

BRUKER

Protein 20: **PREDICTED: POTE ankyrin domain family member E isoform X1 [Homo sapiens]**

**Accession:**	gi|578804361	**Score:**	164.6
**Database:**	refseq_human(refseq_human_20140103.fasta)	**MW [kDa]:**	76.6
**Database Date:**	2015-11-30	**pI:**	4.9
		**Sequence Coverage [%]:**	8.6
		**No. of unique Peptides:**	1

10	20	30	40	50	60	70	80
MSQELEINKD	GDREVEEEMK	KHESNNVGLL	ENLTNGVTAG	NGDNGLIPQR	KSRTPENQQF	PDNESEEYHR	ICELLSDYKE
**90**	**100**	**110**	**120**	**130**	**140**	**150**	**160**
KQMPKYSSEN	SNPEQDLKLT	SEEESQRLKG	SENGQPEKRS	QEPEINKDGD	RELENFMAIE	EMKKHGSTHV	GFPENLTNGA
**170**	**180**	**190**	**200**	**210**	**220**	**230**	**240**
TAGNGDDGLI	PPRKSRTPES	QQFPDTENEE	YHSDEQNDTQ	KQFCEEQNTG	ILHDEILIHE	EKQIEVVEKM	NSELSLSCKK
**250**	**260**	**270**	**280**	**290**	**300**	**310**	**320**
EKDVLHENST	LREEIAMLRL	ELDTMKHQSQ	LREKKYLEDI	ESVKKKNDNL	LKALQLNELT	MDDDTAVLVI	DNGSGMCKAG
**330**	**340**	**350**	**360**	**370**	**380**	**390**	**400**
FAGDDAPRAV	FPSIVGRPRQ	QGMMGGMHQK	ESYVGKEAQS	KRGILTLKYP	MEHGIITNWD	DMEKIWHHTF	YNELRVAPEE
**410**	**420**	**430**	**440**	**450**	**460**	**470**	**480**
HPILLTEAPL	NPKANREKMT	QIMFETFNTP	AMYVAIQAVP	SLYTSGRTTG	IVMDSGDGVT	HTVPIYEGNA	LPHATLRLDL
**490**	**500**	**510**	**520**	**530**	**540**	**550**	**560**
AGRELPDYLM	KILTERGYRF	TTMAEREIVR	DIKEKLCYVA	LDFEQEMATA	ASSSSLEKSY	ELPDGQVITI	GNERFRCPEA
**570**	**580**	**590**	**600**	**610**	**620**	**630**	**640**
LFQPCFLGME	SCGIHETTFN	SIMKSDVDIR	KDLYTNTVLS	GGTTMYPGMA	HRMQKEIAAL	APSMMKIRII	APPKRKYSVW
**650**	**660**	**670**	**680**				
VGGSILASLS	TFQQMWISKQ	EYDESGPSIV	HRKCF				

Cmpd.	No. of Cmpds.	m/z meas.	Δ m/z [ppm]	z	Rt [min]	Score	P	Range	Sequence	Modification
1146	1	984.4853	-59.25	2	43.0	11.3	0	396-413	R.VAPEEHPILLTEAPLNPK.A	

Detailed Protein Report

Protein 21: keratin, type II cytoskeletal 1 [Homo sapiens]

**Accession:**	gi|119395750	**Score:**	154.8
**Database:**	refseq_human(refseq_human_20140103.fasta)	**MW [kDa]:**	66.0
**Database Date:**	2015-11-30	**pI:**	8.8
		**Sequence Coverage [%]:**	9.9
		**No. of unique Peptides:**	3

10	20	30	40	50	60	70	80
MSRQFSSRSG	YRSGGGFSSG	SAGIINYQRR	TTSSSTRRSG	GGGGRFSSCG	GGGGSFGAGG	GFGSRSLVNL	GGSKSISISV
**90**	**100**	**110**	**120**	**130**	**140**	**150**	**160**
ARGGGRGSGF	GGGYGGGGFG	GGGFGGGGFG	GGGIGGGGFG	GFGSGGGGFG	GGGFGGGGYG	GGYGPVCPPG	GIQEVTINQS
**170**	**180**	**190**	**200**	**210**	**220**	**230**	**240**
LLQPLNVEID	PEIQKVKSRE	REQIKSLNNQ	FASFIDKVRF	LEQQNQVLQT	KWELLQQVDT	STRTHNLEPY	FESFINNLRR
**250**	**260**	**270**	**280**	**290**	**300**	**310**	**320**
RVDQLKSDQS	RLDSELKNMQ	DMVEDYRNKY	EDEINKRTNA	ENEFVTIKKD	VDGAYMTKVD	LQAKLDNLQQ	EIDFLTALYQ
**330**	**340**	**350**	**360**	**370**	**380**	**390**	**400**
AELSQMQTQI	SETNVILSMD	NNRSLDLDSI	IAEVKAQYED	IAQKSKAEAE	SLYQSKYEEL	QITAGRHGDS	VRNSKIEISE
**410**	**420**	**430**	**440**	**450**	**460**	**470**	**480**
LNRVIQRLRS	EIDNVKKQIS	NLQQSISDAE	QRGENALKDA	KNKLNDLEDA	LQQAKEDLAR	LLRDYQELMN	TKLALDLEIA
**490**	**500**	**510**	**520**	**530**	**540**	**550**	**560**
TYRTLLEGEE	SRMSGECAPN	VSVSVSTSHT	TISGGGSRGG	GGGGYGSGGS	SYGSGGGSYG	SGGGGGGGRG	SYGSGGSSYG
**570**	**580**	**590**	**600**	**610**	**620**	**630**	**640**
SGGGSYGSGG	GGGGHGSYGS	GSSSGGYRGG	SGGGGGGSSG	GRGSGGGSSG	GSIGGRGSSS	GGVKSSGGSS	SVKFVSTTYS
**650**							
GVTR							

Cmpd.	No. of Cmpds.	m/z meas.	Δ m/z [ppm]	z	Rt [min]	Score	P	Range	Sequence	Modification
1307	1	738.3615	-22.47	2	45.1	53.5	0	212-223	K.WELLQQVDTSTR.T	
2843	2	651.8509	-15.86	2	64.1	16.0	0	344-355	R.SLDLDSIIAEVK.A	
1168	1	922.9918	16.42	2	44.2	12.8	1	417-432	K.KQISNLQQSISDAEQR.G	

Detailed Protein Report

Protein 22: alpha-enolase isoform 1 [Homo sapiens]

Accession: gi|4503571
Database: refseq_human(refseq_human_20140103.fasta)
Database Date: 2015-11-30
Modification(s): Carbamidomethyl

Score: 146.5
MW [kDa]: 47.1
pI: 7.7
Sequence Coverage [%]: 16.6
No. of unique Peptides: 5

Quantitation

mdown:qdown **Median:** 0.67 **CV:** 33.45 % **No. of Peptides:** 2
Wdown:Qdown **Median:** 1.72 **CV:** 51.45 % **No. of Peptides:** 2

10	20	30	40	50	60	70	80
MSILKIHARE	IFDSRGNPTV	EVDLFTSKGL	FRAAVPSGAS	TGIYEALELR	DNDKTRYMGK	GVSKAVEHIN	KTIAPALVSK
90	100	110	120	130	140	150	160
KLNVTEQEKI	DKLMIEMDGT	ENKSKFGANA	ILGVSLAVCK	AGAVEKGVPL	YRHIADLAGN	SEVILPVPAF	NVINGGSHAG
170	180	190	200	210	220	230	240
NKLAMQEFMI	LPVGAANFRE	AMRIGAEVYH	NLKNVIKEKY	GKDATNVGDE	GGFAPNILEN	KEGLELLKTA	IGKAGYTDKV
250	260	270	280	290	300	310	320
VIGMDVAASE	FFRSGKYDLD	FKSPDDPSRY	ISPDQLADLY	KSFIKDYPVV	SIEDPFDQDD	WGAWQKFTAS	AGIQVVGDDL
330	340	350	360	370	380	390	400
TVTNPKRIAK	AVNEKSCNCL	LLKVNQIGSV	TESLQACKLA	QANGWGVMVS	HRSGETEDTF	IADLVVGLCT	GQIKTGAPCR
410	420	430	440				
SERLAKYNQL	LRIEEELGSK	AKFAGRNFRN	PLAK				

Cmpd.	No. of Cmpds.	m/z meas.	Δ m/z [ppm]	z	Rt [min]	Score	P	Range	Sequence	Modification	Ratios
1541	1	703.8713	13.64	2	47.8	10.1	0	16-28	R.GNPTVEVDLFTSK.G		
2401	2	902.9549	-22.96	2	58.7	34.1	0	33-50	R.AAVPSGASTGIYEALELR.D		
1993	5	713.2706	-134.70	2	53.4	51.9	0	270-281	R.YISPDQLADLYK.S		
584	1	817.3947	-24.01	2	36.2	33.0	0	344-358	K.VNQIGSVTESLQACK.L	Carbamidomethyl: 14	Wdown:Qdown 1.06<br>mdown:qdown 0.48
307	1	846.3647	-102.88	2	33.2	17.4	1	407-420	K.YNQLLRIEEELGSK.A		mdown:qdown 0.92<br>Wdown:Qdown 2.78

Detailed Protein Report

Protein 23: actin, aortic smooth muscle [Homo sapiens]

**Accession:**	gi|4501883	**Score:**	130.9
**Database:**	refseq_human(refseq_human_20140103.fasta)	**MW [kDa]:**	42.0
**Database Date:**	2015-11-30	**pI:**	5.1
		**Sequence Coverage [%]:**	15.4
		**No. of unique Peptides:**	2

Quantitation

mdown:qdown	**Median:**	2.74	**CV:**	0.00 %	**No. of Peptides:**	1
Wdown:Qdown	**Median:**	0.67	**CV:**	0.00 %	**No. of Peptides:**	1

Alias proteins:

Accession	Name	Description
gi|213688375	refseq_human (refseq_human_20140103.fasta)	actin, aortic smooth muscle [Homo sapiens]

```
        10         20         30         40         50         60         70         80
MCEEEDSTAL VCDNGSGLCK AGFAGDDAPR AVFPSIVGRP RHQGVMVGMG QKDSYVGDEA QSKRGILTLK YPIEHGIITN
        90        100        110        120        130        140        150        160
WDDMEKIWHH SFYNELRVAP EEHPTLLTEA PLNPKANREK MTQIMFETFN VPAMYVAIQA VLSLYASGRT TGIVLDSGDG
       170        180        190        200        210        220        230        240
VTHNVPIYEG YALPHAIMRL DLAGRDLTDY LMKILTERGY SFVTTAEREI VRDIKEKLCY VALDFENEMA TAASSSSLEK
       250        260        270        280        290        300        310        320
SYELPDGQVI TIGNERFRCP ETLFQPSFIG MESAGIHETT YNSIMKCDID IRKDLYANNV LSGGTTMYPG IADRMQKEIT
       330        340        350        360        370        380
ALAPSTMKIK IIAPPERKYS VWIGGSILAS LSTFQQMWIS KQEYDEAGPS IVHRKCF
```

Cmpd.	No. of Cmpds.	m/z meas.	Δ m/z [ppm]	z	Rt [min]	Score	P	Range	Sequence	Modification	Ratios
2088	1	652.6205	-100.50	3	54.7	15.4	0	98-115	R.VAPEEHPTLLTEAPLNPK.A		
267	1	750.8467	-14.36	2	32.1	15.2	0	362-374	K.QEYDEAGPSIVHR.K		Wdown:Qdown 0.67<br>mdown:qdown 2.74

Detailed Protein Report

Protein 24: beta-actin-like protein 2 [Homo sapiens]

**Accession:**	gi|63055057	**Score:**	122.9
**Database:**	refseq_human(refseq_human_20140103.fasta)	**MW [kDa]:**	42.0
**Database Date:**	2015-11-30	**pI:**	5.3
**Modification(s):**	Oxidation	**Sequence Coverage [%]:**	14.6
		**No. of unique Peptides:**	1

10	20	30	40	50	60	70	80
MTDNELSALV	VDNGSGMCKA	GFGGDDAPRA	VFPSMIGRPR	HQGVMVGMGQ	KDCYVGDEAQ	SKRGVLTLKY	PIEHGVVTNW
**90**	**100**	**110**	**120**	**130**	**140**	**150**	**160**
DDMEKIWYHT	FYNELRVAPD	EHPILLTEAP	LNPKINREKM	TQIMFEAFNT	PAMYVAIQAV	LSLYASGRTT	GIVMDSGDGV
**170**	**180**	**190**	**200**	**210**	**220**	**230**	**240**
THIVPIYEGY	ALPHAILRLD	LAGRDLTDYL	MKILTERGYN	FTTTAEREIV	RDVKEKLCYV	ALDFEQEMVR	AAASSSPERS
**250**	**260**	**270**	**280**	**290**	**300**	**310**	**320**
YELPDGQVIT	IGNERFRCPE	AIFQPSFLGI	ESSGIHETTF	NSIMKCDVDI	RKDLYANTVL	SGGSTMYPGI	ADRMQKEIIT
**330**	**340**	**350**	**360**	**370**	**380**		
LAPSTMKIKI	IAPPERKYSV	WIGGSILASL	STFQQMWISK	QEYDEAGPPI	VHRKCF		

Cmpd.	No. of Cmpds.	m/z meas.	Δ m/z [ppm]	z	Rt [min]	Score	P	Range	Sequence	Modification
2065	1	1108.9201	-97.52	2	54.4	45.1	0	293-313	K.DLYANTVLSGGSTMYPGIADR.M	Oxidation: 14

Detailed Protein Report

Protein 25: pyruvate kinase PKM isoform f [Homo sapiens]

Accession: gi|332164781
Database: refseq_human(refseq_human_20140103.fasta)
Database Date: 2015-11-30
Modification(s): Carbamidomethyl

Score: 119.1
MW [kDa]: 58.5
pI: 8.7
Sequence Coverage [%]: 9.9
No. of unique Peptides: 4

Quantitation

mdown:qdown	**Median:**	0.45	**CV:**	16.33 %	**No. of Peptides:**	2
Wdown:Qdown	**Median:**	2.15	**CV:**	39.88 %	**No. of Peptides:**	2

```
        10         20         30         40         50         60         70         80
MTSAAMSKPH SEAGTAFIQT QQLHAAMADT FLEHMCRLDI DSPPITARNT GIICTIGPAS RSVETLKEMI KSGMNVARLN
        90        100        110        120        130        140        150        160
FSHGTHEYHA ETIKNVRTAT ESFASDPILY RPVAVALDTK GPEIRTGLIK GSGTAEVELK KGATLKITLD NAYMEKCDEN
       170        180        190        200        210        220        230        240
ILWLDYKNIC KVVEVGSKIY VDDGLISLQV KQKGADFLVT EVENGGSLGS KKGVNLPGAA VDLPAVSEKD IQDLKFGVEQ
       250        260        270        280        290        300        310        320
DVDMVFASFI RKASDVHEVR KVLGEKGKNI KIISKIENHE GVRRFDEILE ASDGIMVARG DLGIEIPAEK VFLAQKMMIG
       330        340        350        360        370        380        390        400
RCNRAGKPVI CATQMLESMI KKPRPTRAEG SDVANAVLDG ADCIMLSGET AKGDYPLEAV RMQHLIAREA EAAMFHRKLF
       410        420        430        440        450        460        470        480
EELVRASSHS TDLMEAMAMG SVEASYKCLA AALIVLTESG RSAHQVARYR PRAPIIAVTR NPQTARQAHL YRGIFPVLCK
       490        500        510        520        530        540
DPVQEAWAED VDLRVNFAMN VGKARGFFKK GDVVIVLTGW RPGSGFTNTM RVVPVP
```

Cmpd.	No. of Cmpds.	m/z meas.	Δ m/z [ppm]	z	Rt [min]	Score	P	Range	Sequence	Modification	Ratios
859	2	599.3167	-17.70	2	39.2	51.2	0	38-48	R.LDIDSPPITAR.N		Wdown:Qdown 3.15<br>mdown:qdown 0.39
566	1	680.2094	-215.66	2	36.4	14.7	0	49-61	R.NTGIICTIGPASR.S	Carbamidomethyl: 6	
111	1	628.6466	11.60	3	30.8	41.8	0	79-94	R.LNFSHGTHEYHAETIK.N		Wdown:Qdown 1.46<br>mdown:qdown 0.53
2063	1	731.7933	-161.16	2	54.4	11.5	0	179-191	K.IYVDDGLISLQVK.Q		

Detailed Protein Report

Protein 26: **haptoglobin isoform 2 preproprotein [Homo sapiens]**

**Accession:**	gi|186910296	**Score:**	114.6
**Database:**	refseq_human(refseq_human_20140103.fasta)	**MW [kDa]:**	38.4
**Database Date:**	2015-11-30	**pI:**	6.1
**Modification(s):**	Carbamidomethyl	**Sequence Coverage [%]:**	12.1
		**No. of unique Peptides:**	3

Quantitation

mdown:qdown	**Median:**	0.85	**CV:**	28.22 %	**No. of Peptides:**	2
Wdown:Qdown	**Median:**	0.57	**CV:**	18.62 %	**No. of Peptides:**	2

10	20	30	40	50	60	70	80
MSALGAVIAL	LLWGQLFAVD	SGNDVTDIAD	DGCPKPPEIA	HGYVEHSVRY	QCKNYYKLRT	EGDGVYTLNN	EKQWINKAVG
**90**	**100**	**110**	**120**	**130**	**140**	**150**	**160**
DKLPECEAVC	GKPKNPANPV	QRILGGHLDA	KGSFPWQAKM	VSHHNLTTGA	TLINEQWLLT	TAKNLFLNHS	ENATAKDIAP
**170**	**180**	**190**	**200**	**210**	**220**	**230**	**240**
TLTLYVGKKQ	LVEIEKVVLH	PNYSQVDIGL	IKLKQKVSVN	ERVMPICLPS	KDYAEVGRVG	YVSGWGRNAN	FKFTDHLKYV
**250**	**260**	**270**	**280**	**290**	**300**	**310**	**320**
MLPVADQDQC	IRHYEGSTVP	EKKTPKSPVG	VQPILNEHTF	CAGMSKYQED	TCYGDAGSAF	AVHDLEEDTW	YATGILSFDK
**330**	**340**	**350**					
SCAVAEYGVY	VKVTSIQDWV	QKTIAEN					

Cmpd.	No. of Cmpds.	m/z meas.	Δ m/z [ppm]	z	Rt [min]	Score	P	Range	Sequence	Modification	Ratios
136	2	854.8916	-43.33	2	31.0	42.7	1	58-72	K.LRTEGDGVYTLNNEK.Q		mdown:qdown 1.12<br>Wdown:Qdown 0.69
83	2	619.9584	-31.73	3	30.3	42.3	1	78-94	K.AVGDKLPECEAVCGKPK.N	Carbamidomethyl: 9, 13	Wdown:Qdown 0.48<br>mdown:qdown 0.64
819	1	602.3169	-8.58	2	38.7	29.6	0	333-342	K.VTSIQDWVQK.T		

Detailed Protein Report

Protein 27: PREDICTED: nebulin isoform X22 [Homo sapiens]

**Accession:**	gi|530370465	**Score:**	114.1
**Database:**	refseq_human(refseq_human_20140103.fasta)	**MW [kDa]:**	961.5
**Database Date:**	2015-11-30	**pI:**	9.5
**Modification(s):**	Oxidation	**Sequence Coverage [%]:**	1.2
		**No. of unique Peptides:**	7

Quantitation

mdown:qdown	**Median:**	1.64	**CV:**	0.00 %	**No. of Peptides:**	1
Wdown:Qdown	**Median:**	0.66	**CV:**	0.00 %	**No. of Peptides:**	1

Detailed Protein Report

10	20	30	40	50	60	70	80
MADDEDYEEV	VEYYTEEVVY	EEVPGETITK	IYETTTTRTS	DYEQSETSKP	ALAQPALAQP	ASAKPVERRK	VIRKKVDPSK
90	100	110	120	130	140	150	160
FMTPYIAHSQ	KMQDLFSPNK	YKEKFEKTKG	QPYASTTDTP	ELRRIKKVQD	QLSEVKYRMD	GDVAKTICHV	DEKAKDIEHA
170	180	190	200	210	220	230	240
KKVSQQVSKV	LYKQNWEDTK	DKYLLPPDAP	ELVQAVKNTA	MFSKKLYTED	WEADKSLFYP	YNDSPELRRV	AQAQKALSDV
250	260	270	280	290	300	310	320
AYKKGLAEQQ	AQFTPLADPP	DIEFAKKVTN	QVSKQKYKED	YENKIKGKWS	ETPCFEVANA	RMNADNISTR	KYQEDFENMK
330	340	350	360	370	380	390	400
DQIYFMQTET	PEYKMNKKAG	VAASKVKYKE	DYEKNKGKAD	YNVLPASENP	QLRQLKAAGD	ALSDKLYKEN	YEKTKAKSIN
410	420	430	440	450	460	470	480
YCETPKFKLD	TVLQNFSSDK	KYKDSYLKDI	LGHYVGSFED	PYHSHCMKVT	AQNSDKNYKA	EYEEDRGKGF	FPQTITQEYE
490	500	510	520	530	540	550	560
AIKKLDQCKD	HTYKVHPDKT	KFTQVTDSPV	LLQAQVNSKQ	LSDLNYKAKH	ESEKFKCHIP	PDTPAFIQHK	VNAYNLSDNL
570	580	590	600	610	620	630	640
YKQDWEKSKA	KKFDIKVDAI	PLLAAKANTK	NTSDVMYKKD	YEKNKGKMIG	VLSINDDPKM	LHSLKVAKNQ	SDRLYKENYE
650	660	670	680	690	700	710	720
KTKAKSMNYC	ETPKYQLDTQ	LKNFSEARYK	DLYVKDVLGH	YVGSMEDPYH	THCMKVAAQN	SDKSYKAEYE	EDKGKCYFPQ
730	740	750	760	770	780	790	800
TITQEYEAIK	KLDQCKDHTY	KVHPDKTKFT	AVTDSPVLLQ	AQLNTKQLSD	LNYKAKHEGE	KFKCHIPADA	PQFIQHRVNA
810	820	830	840	850	860	870	880
YNLSDNVYKQ	DWEKSKAKKF	DIKVDAIPLL	AAKANTKNTS	DVMYKKDYEK	SKGKMIGALS	INDDPKMLHS	LKTAKNQSDR
890	900	910	920	930	940	950	960
EYRKDYEKSK	TIYTAPLDML	QVTQAKKSQA	IASDVDYKHI	LHSYSYPPDS	INVDLAKKAY	ALQSDVEYKA	DYNSWMKGCG
970	980	990	1000	1010	1020	1030	1040
WVPFGSLEME	KAKRASDILN	EKKYRQHPDT	LKFTSIEDAP	ITVQSKINQA	QRSDIAYKAK	GEEIIHKYNL	PPDLPQFIQA
1050	1060	1070	1080	1090	1100	1110	1120
KVNAYNISEN	MYKADLKDLS	KKGYDLRTDA	IPIRAAKAAR	QAASDVQYKK	DYEKAKGKMV	GFQSLQDDPK	LVHYMNVAKI
1130	1140	1150	1160	1170	1180	1190	1200
QSDREYKKDY	EKTKSKYNTP	HDMFNVVAAK	KAQDVVSNVN	YKHSLHHYTY	LPDAMDLELS	KNMMQIQSDN	VYKEDYNNWM
1210	1220	1230	1240	1250	1260	1270	1280
KGIGWIPIGS	LDVEKVKKAG	DALNEKKYRQ	HPDTLKFTSI	VDSPVMVQAK	QNTKQVSDIL	YKAKGEDVKH	KYTMSPDLPQ
1290	1300	1310	1320	1330	1340	1350	1360
FLQAKCNAYN	ISDVCYKRDW	YDLIAKGNNV	LGDAIPITAA	KASRNIASDY	KYKEAYEKSK	GKHVGFRSLQ	DDPKLVHYMN
1370	1380	1390	1400	1410	1420	1430	1440
VAKLQSDREY	KKNYENTKTS	YHTPGDMVSI	TAAKMAQDVA	TNVNYKQPLH	HYTYLPDAMS	LEHTRNVNQI	QSDNVYKDEY
1450	1460	1470	1480	1490	1500	1510	1520
NSFLKGIGWI	PIGSLEVEKV	KKAGDALNER	KYRQHPDTVK	FTSVPDSMGM	VLAQHNTKQL	SDLNYKVEGE	KLKHKYTIDP
1530	1540	1550	1560	1570	1580	1590	1600
ELPQFIQAKV	NALNMSDAHY	KADWKKTIAK	GYDLRPDAIP	IVAAKSSRNI	ASDCKYKEAY	EKAKGKQVGF	LSLQDDPKLV
1610	1620	1630	1640	1650	1660	1670	1680
HYMNVAKIQS	DREYKKGYEA	SKTKYHTPLD	MVSVTAAKKS	QEVATNANYR	QSYHHYTLLP	DALNVEHSRN	AMQIQSDNLY
1690	1700	1710	1720	1730	1740	1750	1760
KSDFTNWMKG	IGWVPIESLE	VEKAKKAGEI	LSEKKYRQHP	EKLKFTYAMD	TMEQALNKSN	KLNMDKRLYT	EKWNKDKTTI
1770	1780	1790	1800	1810	1820	1830	1840
HVMPDTPDIL	LSRVNQITMS	DKLYKAGWEE	EKKKGYDLRP	DAIAIKAARA	SRDIASDYKY	KKAYEQAKGK	HIGFRSLEDD
1850	1860	1870	1880	1890	1900	1910	1920
PKLVHFMQVA	KMQSDREYKK	GYEKSKTSFH	TPVDMLSVVA	AKKSQEVATN	ANYRNVIHTY	NMLPDAMSFE	LAKNMMQIQS
1930	1940	1950	1960	1970	1980	1990	2000
DNQYKADYAD	FMKGIGWLPL	GSLEAEKNKK	AMEIISEKKY	RQHPDTLKYS	TLMDSMNMVL	AQNNAKIMNE	HLYKQAWEAD
2010	2020	2030	2040	2050	2060	2070	2080
KTKVHIMPDI	PQIILAKANA	INMSDKLYKL	SLEESKKKGY	DLRPDAIPIK	AAKASRDIAS	DYKYKYNYEK	GKGKMVGFRS
2090	2100	2110	2120	2130	2140	2150	2160
LEDDPKLVHS	MQVAKMQSDR	EYKKNYENTK	TSYHTPADML	SVTAAKDAQA	NITNTNYKHL	IHKYILLPDA	MNIELTRNMN
2170	2180	2190	2200	2210	2220	2230	2240

Detailed Protein Report

Cmpd.	No. of Cmpds.	m/z meas.	Δ m/z [ppm]	z	Rt [min]	Score	P	Range	Sequence	Modification	Ratios
2522	1	885.4523	-25.95	2	62.0	17.0	2	407-421	K.FKLDTVLQNFSSDKK.Y		
415	3	544.7701	86.52	2	34.2	12.0	0	646-654	K.SMNYCETPK.Y	Oxidation: 2	
1248	1	796.3930	45.30	2	45.3	13.7	1	2813-2825	R.DDPKMMWSMHVAK.I	Oxidation: 5	
1176	2	729.3599	-9.93	2	43.4	18.3	1	3617-3628	R.KAYDLQSDNLYK.S		Wdown:Qdown 0.66<br>mdown:qdown 1.64
993	1	662.3199	-62.62	2	41.2	12.9	2	7204-7214	K.VKDEVSDLKYK.E		
624	1	607.8333	-21.30	2	36.4	10.4	2	7714-7723	K.RDLELEVKGR.G		
2801	1	994.5013	20.67	2	63.5	13.2	1	8000-8015	K.LNQENFSSVLYKENMR.K	Oxidation: 15	

Detailed Protein Report

Protein 28: heat shock protein beta-1 [Homo sapiens]

Accession: gi|4504517
Database: refseq_human(refseq_human_20140103.fasta)
Database Date: 2015-11-30

Score: 107.1
MW [kDa]: 22.8
pI: 6.0
Sequence Coverage [%]: 21.5
No. of unique Peptides: 3

Quantitation

mdown:qdown **Median:** 1.04 **CV:** 0.00 % **No. of Peptides:** 1

10	20	30	40	50	60	70	80
MTERRVPFSL	LRGPSWDPFR	DWYPHSRLFD	QAFGLPRLPE	EWSQWLGGSS	WPGYVRPLPP	AAIESPAVAA	PAYSRALSRQ
**90**	**100**	**110**	**120**	**130**	**140**	**150**	**160**
LSSGVSEIRH	TADRWRVSLD	VNHFAPDELT	VKTKDGVVEI	TGKHEERQDE	HGYISRCFTR	KYTLPPGVDP	TQVSSSLSPE
**170**	**180**	**190**	**200**	**210**			
GTLTVEAPMP	KLATQSNEIT	IPVTFESRAQ	LGGPEAAKSD	ETAAK			

Cmpd.	No. of Cmpds.	m/z meas.	Δ m/z [ppm]	z	Rt [min]	Score	P	Range	Sequence	Modification	Ratios
1668	7	582.3006	-23.03	2	50.8	56.0	0	28-37	R.LFDQAFGLPR.L		
1666	3	953.4317	-71.06	2	49.5	35.6	0	172-188	K.LATQSNEITIPVTFESR.A		mdown:qdown 1.04
2886	2	822.4678	63.76	2	64.8	15.6	1	189-205	R.AQLGGPEAAKSDETAAK.-		

Detailed Protein Report

Protein 29: cadherin EGF LAG seven-pass G-type receptor 3 precursor [Homo sapiens]

**Accession:**	gi|145309304	**Score:**	103.8
**Database:**	refseq_human(refseq_human_20140103.fasta)	**MW [kDa]:**	358.0
**Database Date:**	2015-11-30	**pI:**	6.2
**Modification(s):**	Carbamidomethyl, Oxidation	**Sequence Coverage [%]:**	3.0
		**No. of unique Peptides:**	7

Quantitation

Wdown:Qdown	**Median:**	0.31	**CV:**	0.00 %	**No. of Peptides:**	1	

Detailed Protein Report

10	20	30	40	50	60	70	80
MMARRPPWRG	LGGRSTPILL	LLLLSLFPLS	QEELGGGGHQ	GWDPGLAATT	GPRAHIGGGA	LALCPESSGV	REDGGPGLGV
90	100	110	120	130	140	150	160
REPIFVGLRG	RRQSARNSRG	PPEQPNEELG	IEHGVQPLGS	RERETGQGPG	SVLYWRPEVS	SCGRTGPLQR	GSLSPGALSS
170	180	190	200	210	220	230	240
GVPGSGNSSP	LPSDFLIRHH	GPKPVSSQRN	AGTGSRKRVG	TARCCGELWA	TGSKGQGERA	TTSGAERTAP	RRNCLPGASG
250	260	270	280	290	300	310	320
SGPELDSAPR	TARTAPASGS	APRESRTAPE	PAPKRMRSRG	LFRCRFLPQR	PGPRPPGLPA	RPEARKVTSA	NRARFRRAAN
330	340	350	360	370	380	390	400
RHPQFPQYNY	QTLVPENEAA	GTAVLRVVAQ	DPDAGEAGRL	VYSLAALMNS	RSLELFSIDP	QSGLIRTAAA	LDRESMERHY
410	420	430	440	450	460	470	480
LRVTAQDHGS	PRLSATTMVA	VTVADRNDHS	PVFEQAQYRE	TLRENVEEGY	PILQLRATDG	DAPPNANLRY	RFVGPPAARA
490	500	510	520	530	540	550	560
AAAAAFEIDP	RSGLISTSGR	VDREHMESYE	LVVEASDQGQ	EPGPRSATVR	VHITVLDEND	NAPQFSEKRY	VAQVREDVRP
570	580	590	600	610	620	630	640
HTVVLRVTAT	DRDKDANGLV	HYNIISGNSR	GHFAIDSLTG	EIQVVAPLDF	EAEREYALRI	RAQDAGRPPL	SNNTGLASIQ
650	660	670	680	690	700	710	720
VVDINDHIPI	FVSTPFQVSV	LENAPLGHSV	IHIQAVDADH	GENARLEYSL	TGVAPDTPFV	INSATGWVSV	SGPLDRESVE
730	740	750	760	770	780	790	800
HYFFGVEARD	HGSPPLSASA	SVTVTVLDVN	DNRPEFTMKE	YHLRLNEDAA	VGTSVVSVTA	VDRDANSAIS	YQITGGNTRN
810	820	830	840	850	860	870	880
RFAISTQGGV	GLVTLALPLD	YKQERYFKLV	LTASDRALHD	HCYVHINITD	ANTHRPVFQS	AHYSVSVNED	RPMGSTIVVI
890	900	910	920	930	940	950	960
SASDDDVGEN	ARITYLLEDN	LPQFRIDADS	GAITLQAPLD	YEDQVTYTLA	ITARDNGIPQ	KADTTYVEVM	VNDVNDNAPQ
970	980	990	1000	1010	1020	1030	1040
FVASHYTGLV	SEDAPPFTSV	LQISATDRDA	HANGRVQYTF	QNGEDGDGDF	TIEPTSGIVR	TVRRLDREAV	SVYELTAYAV
1050	1060	1070	1080	1090	1100	1110	1120
DRGVPPLRTP	VSIQVMVQDV	NDNAPVFPAE	EFEVRVKENS	IVGSVVAQIT	AVDPDEGPNA	HIMYQIVEGN	IPELFQMDIF
1130	1140	1150	1160	1170	1180	1190	1200
SGELTALIDL	DYEARQEYVI	VVQATSAPLV	SRATVHVRLV	DQNDNSPVLN	NFQILFNNYV	SNRSDTFPSG	IIGRIPAYDP
1210	1220	1230	1240	1250	1260	1270	1280
DVSDHLFYSF	ERGNELQLLV	VNQTSGELRL	SRKLDNNRPL	VASMLVTVTD	GLHSVTAQCV	LRVVIITEEL	LANSLTVRLE
1290	1300	1310	1320	1330	1340	1350	1360
NMWQERFLSP	LLGRFLEGVA	AVLATPAEDV	FIFNIQNDTD	VGGTVLNVSF	SALAPRGAGA	GAAGPWFSSE	ELQEQLYVRR
1370	1380	1390	1400	1410	1420	1430	1440
AALAARSLLD	VLPFDDNVCL	REPCENYMKC	VSVLRFDSSA	PFLASASTLF	RPIQPIAGLR	CRCPPGFTGD	FCETELDLCY
1450	1460	1470	1480	1490	1500	1510	1520
SNPCRNGGAC	ARREGGYTCV	CRPRFTGEDC	ELDTEAGRCV	PGVCRNGGTC	TDAPNGGFRC	QCPAGGAFEG	PRCEVAARSF
1530	1540	1550	1560	1570	1580	1590	1600
PPSSFVMFRG	LRQRFHLTLS	LSFATVQQSG	LLFYNGRLNE	KHDFLALELV	AGQVRLTYST	GESNTVVSPT	VPGGLSDGQW
1610	1620	1630	1640	1650	1660	1670	1680
HTVHLRYYNK	PRTDALGGAQ	GPSKDKVAVL	SVDDCDVAVA	LQFGAEIGNY	SCAAAGVQTS	SKKSLDLTGP	LLLGGVPNLP
1690	1700	1710	1720	1730	1740	1750	1760
ENFPVSHKDF	IGCMRDLHID	GRRVDMAAFV	ANNGTMAGCQ	AKLHFCDSGP	CKNSGFCSER	WGSFSCDCPV	GFGGKDCQLT
1770	1780	1790	1800	1810	1820	1830	1840
MAHPHHFRGN	GTLSWNFGSD	MAVSVPWYLG	LAFRTRATQG	VLMQVQAGPH	STLLCQLDRG	LLSVTVTRGS	GRASHLLLDQ
1850	1860	1870	1880	1890	1900	1910	1920
VTVSDGRWHD	LRLELQEEPG	GRRGHHVLMV	SLDFSLFQDT	MAVGSELQGL	KVKQLHVGGL	PPGSAEEAPQ	GLVGCIQGVW
1930	1940	1950	1960	1970	1980	1990	2000
LGSTPSGSPA	LLPPSHRVNA	EPGCVVTNAC	ASGPCPPHAD	CRDLWQTFSC	TCQPGYYGPG	CVDACLLNPC	QNQGSCRHLP
2010	2020	2030	2040	2050	2060	2070	2080
GAPHGYTCDC	VGGYFGHHCE	HRMDQQCPRG	WWGSPTCGPC	NCDVHKGFDP	NCNKTNGQCH	CKEFHYRPRG	SDSCLPCDCY
2090	2100	2110	2120	2130	2140	2150	2160
PVGSTSRSCA	PHSGQCPCRP	GALGRQCNSC	DSPFAEVTAS	GCRVLYDACP	KSLRSGVWWP	QTKFGVLATV	PCPRGALGAA
2170	2180	2190	2200	2210	2220	2230	2240

Detailed Protein Report

Cmpd.	No. of Cmpds.	m/z meas.	Δ m/z [ppm]	z	Rt [min]	Score	P	Range	Sequence	Modification	Ratios
1647	5	942.4685	14.29	2	49.1	22.8	1	232-250	R.RNCLPGASGSGPELDSAPR.T		
2841	1	648.3701	87.56	1	64.1	13.5	0	1513-1518	R.CEVAAR.S		
2719	1	964.4996	-2.09	2	62.9	13.4	2	1519-1534	R.SFPPSSFVMFRGLRQR.F	Oxidation: 9	
2229	1	956.4132	-125.14	2	56.5	12.5	1	2144-2162	K.FGVLATVPCPRGALGAAVR.L	Carbamidomethyl: 9	
942	1	470.6329	-182.64	2	40.6	10.3	0	2331-2338	R.MEHPSSPR.G		Wdown:Qdown 0.31
2129	1	698.8200	-38.23	2	55.2	13.9	2	2331-2342	R.MEHPSSPRGARR.Y	Oxidation: 1	
118	1	1069.1641	-33.12	3	30.2	17.4	1	3083-3111	R.ERLEEAPAPVLRPLSRPGSQECML	Carbamidomethyl: 22; Oxidation: 23	

Detailed Protein Report

Protein 30: PREDICTED: dystonin isoform X9 [Homo sapiens]

**Accession:**	gi|530382434	**Score:**	101.7
**Database:**	refseq_human(refseq_human_20140103.fasta)	**MW [kDa]:**	872.0
**Database Date:**	2015-11-30	**pI:**	5.0
**Modification(s):**	Carbamidomethyl, Oxidation	**Sequence Coverage [%]:**	1.3
		**No. of unique Peptides:**	6

Quantitation

mdown:qdown	**Median:**	0.81	**CV:**	0.00 %	**No. of Peptides:**	1
Wdown:Qdown	**Median:**	0.70	**CV:**	0.00 %	**No. of Peptides:**	1

Detailed Protein Report

10	20	30	40	50	60	70	80
MSSGNASYRC	SMSSSADFSD	EDDFSQKSGS	ASPAPGDTLP	WNLPKHERSK	RKIQGGSVLD	PAERAVLRIA	DERDKVQKKT
90	100	110	120	130	140	150	160
FTKWINQHLM	KVRKHVNDLY	EDLRDGHNLI	SLLEVLSGDT	LPRERDFLKT	LRLVSATEAC	EYEQHEDVED	EDKGPREKGR
170	180	190	200	210	220	230	240
MRFHRLQNVQ	IALDYLKRRQ	VKLVNIRNDD	ITDGNPKLTL	GLIWTIILHF	QISDIHVTGE	SEDMSAKERL	LLWTQQATEG
250	260	270	280	290	300	310	320
YAGIRCENFT	TCWRDGKLFN	AIIHKYRPDL	IDMNTVAVQS	NLANLEHAFY	VAEKIGVIRL	LDPEDVDVSS	PDEKSVITYV
330	340	350	360	370	380	390	400
SSLYDAFPKV	PEGGEGIGAN	DVEVKWIEYQ	NMVNYLIQWI	RHHVTTMSER	TFPNNPVELK	ALYNQYLQFK	ETEIPPKETE
410	420	430	440	450	460	470	480
KSKIKRLYKL	LEIWIEFGRI	KLLQGYHPND	IEKEWGKLII	AMLEREKALR	PEVERLEMLQ	QIANRVQRDS	VICEDKLILA
490	500	510	520	530	540	550	560
GNALQSDSKR	LESGVQFQNE	AEIAGYILEC	ENLLRQHVID	VQILIDGKYY	QADQLVQRVA	KLRDEIMALR	NECSSVYSKG
570	580	590	600	610	620	630	640
RILTTEQTKL	MISGITQSLN	SGFAQTLHPS	LTSGLTQSLT	PSLTSSSMTS	GLSSGMTSRL	TPSVTPAYTP	GFPSGLVPNF
650	660	670	680	690	700	710	720
SSGVEPNSLQ	TLKLMQIRKP	LLKSSLLDQN	LTEEEINMKF	VQDLLNWVDE	MQVQLDRTEW	GSDLPSVESH	LENHKNVHRA
730	740	750	760	770	780	790	800
IEEFESSLKE	AKISEIQMTA	PLKLTYAEKL	HRLESQYAKL	LNTSRNQERH	LDTLHNFVSR	ATNELIWLNE	KEEEEVAYDW
810	820	830	840	850	860	870	880
SERNTNIARK	KDYHAELMRE	LDQKEENIKS	VQEIAEQLLL	ENHPARLTIE	AYRAAMQTQW	SWILQLCQCV	EQHIKENTAY
890	900	910	920	930	940	950	960
FEFFNDAKEA	TDYLRNLKDA	IQRKYSCDRS	SSIHKLEDLV	QESMEEKEEL	LQYKSTIANL	MGKAKTIIQL	KPRNSDCPLK
970	980	990	1000	1010	1020	1030	1040
TSIPIKAICD	YRQIEITIYK	DDECVLANNS	HRAKWKVISP	TGNEAMVPSV	CFTVPPPNKE	AVDLANRIEQ	QYQNVLTLWH
1050	1060	1070	1080	1090	1100	1110	1120
ESHINMKSVV	SWHYLINEID	RIRASNVASI	KTMLPGEHQQ	VLSNLQSRFE	DFLEDSQESQ	VFSGSDITQL	EKEVNVCKQY
1130	1140	1150	1160	1170	1180	1190	1200
YQELLKSAER	EEQEESVYNL	YISEVRNIRL	RLENCEDRLI	RQIRTPLERD	DLHESVFRIT	EQEKLKKELE	RLKDDLGTIT
1210	1220	1230	1240	1250	1260	1270	1280
NKCEEFFSQA	AASSSVPTLR	SELNVVLQNM	NQVYSMSSTY	IDKLKTVNLV	LKNTQAAEAL	VKLYETKLCE	EEAVIADKNN
1290	1300	1310	1320	1330	1340	1350	1360
IENLISTLKQ	WRSEVDEKRQ	VFHALEDELQ	KAKAISDEMF	KTYKERDLDF	DWHKEKADQL	VERWQNVHVQ	IDNRLRDLEG
1370	1380	1390	1400	1410	1420	1430	1440
IGKSLKYYRD	TYHPLDDWIQ	QVETTQRKIQ	ENQPENSKTL	ATQLNQQKML	VSEIEMKQSK	MDECQKYAEQ	YSATVKDYEL
1450	1460	1470	1480	1490	1500	1510	1520
QTMTYRAMVD	SQQKSPVKRR	RMQSSADLII	QEFMDLRTRY	TALVTLMTQY	IKFAGDSLKR	LEEEEKSLEE	EKKEHVEKAK
1530	1540	1550	1560	1570	1580	1590	1600
ELQKWVSNIS	KTLKDAEKAG	KPPFSKQKIS	SEEISTKKEQ	LSEALQTIQL	FLAKHGDKMT	DEERNELEKQ	VKTLQESYNL
1610	1620	1630	1640	1650	1660	1670	1680
LFSESLKQLQ	ESQTSGDVKV	EEKLDKVIAG	TIDQTTGEVL	SVFQAVLRGL	IDYDTGIRLL	ETQLMISGLI	SPELRKCFDL
1690	1700	1710	1720	1730	1740	1750	1760
KDAKSHGLID	EQILCQLKEL	SKAKEIISAA	SPTTIPVLDA	LAQSMITESM	AIKVLEILLS	TGSLVIPATG	EQLTLQKAFQ
1770	1780	1790	1800	1810	1820	1830	1840
QNLVSSALFS	KVLERQNMCK	DLIDPCTSEK	VSLIDMVQRS	TLQENTGMWL	LPVRPQEGGR	ITLKCGRNIS	ILRAAHEGLI
1850	1860	1870	1880	1890	1900	1910	1920
DRETMFRLLS	AQLLSGGLIN	SNSGQRMTVE	EAVREGVIDR	DTASSILTYQ	VQTGGIIQSN	PAKRLTVDEA	VQCDLITSSS
1930	1940	1950	1960	1970	1980	1990	2000
ALLVLEAQRG	YVGLIWPHSG	EIFPTSSSLQ	QELITNELAY	KILNGRQKIA	ALYIPESSQV	IGLDAAKQLG	IIDNNTASIL
2010	2020	2030	2040	2050	2060	2070	2080
KNITLPDKMP	DLGDLEACKN	ARRWLSFCKF	QPSTVHDYRQ	EEDVFDGEEP	VTTQTSEETK	KLFLSYLMIN	SYMDANTGQR
2090	2100	2110	2120	2130	2140	2150	2160
LLLYDGDLDE	AVGMLLEGCH	AEFDGNTAIK	ECLDVLSSSG	VFLNNASGRE	KDECTATPSS	FNKCHCGEPE	HEETPENRKC
2170	2180	2190	2200	2210	2220	2230	2240

Detailed Protein Report

Cmpd.	No. of Cmpds.	m/z meas.	Δ m/z [ppm]	z	Rt [min]	Score	P	Range	Sequence	Modification	Ratios
585	1	558.7340	-142.02	2	36.6	17.2	1	542-550	K.LRDEIMALR.N		Wdown:Qdown 0.70<br>mdown:qdown 0.81
1982	1	927.8655	-42.70	2	53.4	10.5	1	1776-1790	R.QNMCKDLIDPCTSEK.V	Carbamidomethyl: 4, 11; Oxidation: 3	
103	1	822.3979	-76.79	2	30.5	11.5	2	3803-3816	K.LNEAKIKCEQLNLK.A		
779	1	1105.2011	129.15	2	38.2	12.7	2	4203-4221	K.TLDDIVGRYEDLSKSVNER.N		
2450	1	684.2042	-212.00	2	61.0	10.9	1	4940-4951	K.EEQNKSHPISAK.L		
2575	1	588.6984	-194.01	2	60.9	10.5	0	6226-6235	K.LLDVMELAEK.F	Oxidation: 5	

Detailed Protein Report

Protein 31: PREDICTED: plectin isoform X7 [Homo sapiens]

**Accession:**	gi|530389155	**Score:**	101.6
**Database:**	refseq_human(refseq_human_20140103.fasta)	**MW [kDa]:**	514.1
**Database Date:**	2015-11-30	**pI:**	5.5
**Modification(s):**	Carbamidomethyl	**Sequence Coverage [%]:**	2.3
		**No. of unique Peptides:**	4

Quantitation

mdown:qdown	**Median:**	0.40	**CV:**	0.00 %	**No. of Peptides:**	1
Wdown:Qdown	**Median:**	0.34	**CV:**	0.00 %	**No. of Peptides:**	1

Detailed Protein Report

10	20	30	40	50	60	70	80
MDPSRAIQNE	ISSLKDERDR	VQKKTFTKWV	NKHLIKHWRA	EAQRHISDLY	EDLRDGHNLI	SLLEVLSGDS	LPREKGRMRF
90	100	110	120	130	140	150	160
HKLQNVQIAL	DYLRHRQVKL	VNIRNDDIAD	GNPKLTLGLI	WTIILHFQIS	DIQVSGQSED	MTAKEKLLLW	SQRMVEGYQG
170	180	190	200	210	220	230	240
LRCDNFTSSW	RDGRLFNAII	HRHKPLLIDM	NKVYRQTNLE	NLDQAFSVAE	RDLGVTRLLD	PEDVDVPQPD	EKSIITYVSS
250	260	270	280	290	300	310	320
LYDAMPRVPD	VQDGVRANEL	QLRWQEYREL	VLLLLQWMRH	HTAAFEERRF	PSSFEEIEIL	WSQFLKFKEM	ELPAKEADKN
330	340	350	360	370	380	390	400
RSKGIYQSLE	GAVQAGQLKV	PPGYHPLDVE	KEWGKLHVAI	LEREKQLRSE	FERLECLQRI	VTKLQMEAGL	CEEQLNQADA
410	420	430	440	450	460	470	480
LLQSDVRLLA	AGKVPQRAGE	VERDLDKADS	MIRLLFNDVQ	TLKDGRHPQG	EQMYRRVYRL	HERLVAIRTE	YNLRLKAGVA
490	500	510	520	530	540	550	560
APATQVAQVT	LQSVQRRPEL	EDSTLRYLQD	LLAWVEENQH	RVDGAEWGVD	LPSVEAQLGS	HRGLHQSIEE	FRAKIERARS
570	580	590	600	610	620	630	640
DEGQLSPATR	GAYRDCLGRL	DLQYAKLLNS	SKARLRSLES	LHSFVAAATK	ELMWLNEKEE	EEVGFDWSDR	NTNMTAKKES
650	660	670	680	690	700	710	720
YSALMRELEL	KEKKIKELQN	AGDRLLREDH	PARPTVESFQ	AALQTQWSWM	LQLCCCIEAH	LKENAAYFQF	FSDVREAEGQ
730	740	750	760	770	780	790	800
LQKLQEALRR	KYSCDRSATV	TRLEDLLQDA	QDEKEQLNEY	KGHLSGLAKR	AKAVVQLKPR	HPAHPMRGRL	PLLAVCDYKQ
810	820	830	840	850	860	870	880
VEVTVHKGDE	CQLVGPAQPS	HWKVLSSSGS	EAAVPSVCFL	VPPPNQEAQE	AVTRLEAQHQ	ALVTLWHQLH	VDMKSLLAWQ
890	900	910	920	930	940	950	960
SLRRDVQLIR	SWSLATFRTL	KPEEQRQALH	SLELHYQAFL	RDSQDAGGFG	PEDRLMAERE	YGSCSHHYQQ	LLQSLEQGAQ
970	980	990	1000	1010	1020	1030	1040
EESRCQRCIS	ELKDIRLQLE	ACETRTVHRL	RLPLDKEPAR	ECAQRIAEQQ	KAQAEVEGLG	KGVARLSAEA	EKVLALPEPS
1050	1060	1070	1080	1090	1100	1110	1120
PAAPTLRSEL	ELTLGKLEQV	RSLSAIYLEK	LKTISLVIRG	TQGAEEVLRA	HEEQLKEAQA	VPATLPELEA	TKASLKKLRA
1130	1140	1150	1160	1170	1180	1190	1200
QAEAQQPTFD	ALRDELRGAQ	EVGERLQQRH	GERDVEVERW	RERVAQLLER	WQAVLAQTDV	RQRELEQLGR	QLRYYRESAD
1210	1220	1230	1240	1250	1260	1270	1280
PLGAWLQDAR	RRQEQIQAMP	LADSQAVREQ	LRQEQALLEE	IERHGEKVEE	CQRFAKQYIN	AIKDYELQLV	TYKAQLEPVA
1290	1300	1310	1320	1330	1340	1350	1360
SPAKKPKVQS	GSESVIQEYV	DLRTHYSELT	TLTSQYIKFI	SETLRRMEEE	ERLAEQQRAE	ERERLAEVEA	ALEKQRQLAE
1370	1380	1390	1400	1410	1420	1430	1440
AHAQAKAQAE	REAKELQQRM	QEEVVRREEA	AVDAQQQKRS	IQEELQQLRQ	SSEAEIQAKA	RQAEAAERSR	LRIEEEIRVV
1450	1460	1470	1480	1490	1500	1510	1520
RLQLEATERQ	RGGAEGELQA	LRARAEEAEA	QKRQAQEEAE	RLRRQVQDES	QRKRQAEVEL	ASRVKAEAEA	AREKQRALQA
1530	1540	1550	1560	1570	1580	1590	1600
LEELRLQAEE	AERRLRQAEV	ERARQVQVAL	ETAQRSAEAE	LQSKRASFAE	KTAQLERSLQ	EEHVAVAQLR	EEAERRAQQQ
1610	1620	1630	1640	1650	1660	1670	1680
AEAERAREEA	ERELERWQLK	ANEALRLRLQ	AEEVAQQKSL	AQAEAEKQKE	EAEREARRRG	KAEEQAVRQR	ELAEQELEKQ
1690	1700	1710	1720	1730	1740	1750	1760
RQLAEGTAQQ	RLAAEQELIR	LRAETEQGEQ	QRQLLEEELA	RLQREAAAAT	QKRQELEAEL	AKVRAEMEVL	LASKARAEEE
1770	1780	1790	1800	1810	1820	1830	1840
SRSTSEKSKQ	RLEAEAGRFR	ELAEEAARLR	ALAEEAKRQR	QLAEEDAARQ	RAEAERVLAE	KLAAIGEATR	LKTEAEIALK
1850	1860	1870	1880	1890	1900	1910	1920
EKEAENERLR	RLAEDEAFQR	RRLEEQAAQH	KADIEERLAQ	LRKASDSELE	RQKGLVEDTL	RQRRQVEEEI	LALKASFEKA
1930	1940	1950	1960	1970	1980	1990	2000
AAGKAELELE	LGRIRSNAED	TLRSKEQAEL	EAARQRQLAA	EEERRRREAE	ERVQKSLAAE	EEAARQRKAA	LEEVERLKAK
2010	2020	2030	2040	2050	2060	2070	2080
VEEARRLRER	AEQESARQLQ	LAQEAAQKRL	QAEEKAHAFA	VQQKEQELQQ	TLQQEQSVLD	QLRGEAEAAR	RAAEEAEEAR
2090	2100	2110	2120	2130	2140	2150	2160
VQAEREAAQS	RRQVEEAERL	KQSAEEQAQA	RAQAQAAAEK	LRKEAEQEAA	RRAQAEQAAL	RQKQAADAEM	EKHKKFAEQT
2170	2180	2190	2200	2210	2220	2230	2240

Detailed Protein Report

Cmpd.	No. of Cmpds.	m/z meas.	Δ m/z [ppm]	z	Rt [min]	Score	P	Range	Sequence	Modification	Ratios
1213	1	713.6809	-62.89	3	43.8	10.9	2	2258-2277	K.QVAEEAARLSVAAQEAARLR.Q		
1692	1	582.1401	-225.95	2	51.1	12.2	0	2358-2367	R.QLEMSAEAER.L		
42	1	768.2950	-141.23	2	29.9	18.7	1	3585-3598	R.AQLMADFQAGRVTK.E		mdown:qdown 0.40<br>Wdown:Qdown 0.34
1932	1	1021.8810	-139.60	2	52.6	12.9	2	3847-3864	R.CRRDDGTGQLLLPLSDAR.K	Carbamidomethyl: 1	

Detailed Protein Report

Protein 32: lactotransferrin isoform 2 [Homo sapiens]

Accession: gi|312433998
Database: refseq_human(refseq_human_20140103.fasta)
Database Date: 2015-11-30
Modification(s): Carbamidomethyl

Score: 100.4
MW [kDa]: 73.1
pI: 9.3
Sequence Coverage [%]: 5.4
No. of unique Peptides: 3

Quantitation

mdown:qdown	**Median:**	0.43	**CV:**	0.00 %	**No. of Peptides:**	1
Wdown:Qdown	**Median:**	2.01	**CV:**	0.00 %	**No. of Peptides:**	1

10	20	30	40	50	60	70	80
MRKVRGPPVS	CIKRDSPIQC	IQAIAENRAD	AVTLDGGFIY	EAGLAPYKLR	PVAAEVYGTE	RQPRTHYYAV	AVVKKGGSFQ
90	100	110	120	130	140	150	160
LNELQGLKSC	HTGLRRTAGW	NVPIGTLRPF	LNWTGPPEPI	EAAVARFFSA	SCVPGADKGQ	FPNLCRLCAG	TGENKCAFSS
170	180	190	200	210	220	230	240
QEPYFSYSGA	FKCLRDGAGD	VAFIRESTVF	EDLSDEAERD	EYELLCPDNT	RKPVDKFKDC	HLARVPSHAV	VARSVNGKED
250	260	270	280	290	300	310	320
AIWNLLRQAQ	EKFGKDKSPK	FQLFGSPSGQ	KDLLFKDSAI	GFSRVPPRID	SGLYLGSGYF	TAIQNLRKSE	EEVAARRARV
330	340	350	360	370	380	390	400
VWCAVGEQEL	RKCNQWSGLS	EGSVTCSSAS	TTEDCIALVL	KGEADAMSLD	GGYVYTAGKC	GLVPVLAENY	KSQQSSDPDP
410	420	430	440	450	460	470	480
NCVDRPVEGY	LAVAVVRRSD	TSLTWNSVKG	KKSCHTAVDR	TAGWNIPMGL	LFNQTGSCKF	DEYFSQSCAP	GSDPRSNLCA
490	500	510	520	530	540	550	560
LCIGDEQGEN	KCVPNSNERY	YGYTGAFRCL	AENAGDVAFV	KDVTVLQNTD	GNNNEAWAKD	LKLADFALLC	LDGKRKPVTE
570	580	590	600	610	620	630	640
ARSCHLAMAP	NHAVVSRMDK	VERLKQVLLH	QQAKFGRNGS	DCPDKFCLFQ	SETKNLLFND	NTECLARLHG	KTTYEKYLGP
650	660	670					
QYVAGITNLK	KCSTSPLLEA	CEFLRK					

Cmpd.	No. of Cmpds.	m/z meas.	Δ m/z [ppm]	z	Rt [min]	Score	P	Range	Sequence	Modification	Ratios
1629	2	695.8567	-18.94	2	48.9	36.9	0	76-88	K.GGSFQLNELQGLK.S		
852	1	598.3199	18.32	2	40.1	36.0	0	261-271	K.FQLFGSPSGQK.D		mdown:qdown 0.43
2950	4	668.3368	-23.49	2	66.0	27.5	0	543-554	K.LADFALLCLDGK.R	Carbamidomethyl: 8	Wdown:Qdown 2.01

Detailed Protein Report

Protein 33: keratin, type I cytoskeletal 19 [Homo sapiens]

**Accession:**	gi|24234699	**Score:**	100.0
**Database:**	refseq_human(refseq_human_20140103.fasta)	**MW [kDa]:**	44.1
**Database Date:**	2015-11-30	**pI:**	4.9
**Modification(s):**	Oxidation	**Sequence Coverage [%]:**	13.5
		**No. of unique Peptides:**	1

10	20	30	40	50	60	70	80
MTSYSYRQSS	ATSSFGGLGG	GSVRFGPGVA	FRAPSIHGGS	GGRGVSVSSA	RFVSSSSSGA	YGGGYGGVLT	ASDGLLAGNE
**90**	**100**	**110**	**120**	**130**	**140**	**150**	**160**
KLTMQNLNDR	LASYLDKVRA	LEAANGELEV	KIRDWYQKQG	PGPSRDYSHY	YTTIQDLRDK	ILGATIENSR	IVLQIDNARL
**170**	**180**	**190**	**200**	**210**	**220**	**230**	**240**
AADDFRTKFE	TEQALRMSVE	ADINGLRRVL	DELTLARTDL	EMQIEGLKEE	LAYLKKNHEE	EISTLRGQVG	GQVSVEVDSA
**250**	**260**	**270**	**280**	**290**	**300**	**310**	**320**
PGTDLAKILS	DMRSQYEVMA	EQNRKDAEAW	FTSRTEELNR	EVAGHTEQLQ	MSRSEVTDLR	RTLQGLEIEL	QSQLSMKAAL
**330**	**340**	**350**	**360**	**370**	**380**	**390**	**400**
EDTLAETEAR	FGAQLAHIQA	LISGIEAQLG	DVRADSERQN	QEYQRLMDIK	SRLEQEIATY	RSLLEGQEDH	YNNLSASKVL
**410**							

Cmpd.	No. of Cmpds.	m/z meas.	Δ m/z [ppm]	z	Rt [min]	Score	P	Range	Sequence	Modification
1981	1	1051.4041	-77.63	2	53.4	11.4	1	248-264	K.ILSDMRSQYEVMAEQNR.K	Oxidation: 5, 12

Detailed Protein Report

Protein 34: dynein heavy chain 1, axonemal [Homo sapiens]

**Accession:**	gi|197927452	**Score:**	87.6
**Database:**	refseq_human(refseq_human_20140103.fasta)	**MW [kDa]:**	487.2
**Database Date:**	2015-11-30	**pI:**	5.5
**Modification(s):**	Carbamidomethyl, Oxidation	**Sequence Coverage [%]:**	2.0
		**No. of unique Peptides:**	6

Quantitation

mdown:qdown	**Median:** 1.42	**CV:** 70.92 %	**No. of Peptides:** 2

Alias proteins:

Accession	Name	Description
gi|578805960	refseq_human (refseq_human_20140103.fasta)	PREDICTED: dynein heavy chain 1, axonemal isoform X1 [Homo sapiens]

Detailed Protein Report

10	20	30	40	50	60	70	80
MEQPNSKGYS	LGRTPQGPEC	SSAPAVQVGT	HRGLEYNPGK	ILPGSDYGLG	NPPALDPKLP	HLPLPPAPPT	LSDLGQPRKS
90	100	110	120	130	140	150	160
PLTGTDKKYP	LMKQRGFYSD	ILSPGTLDQL	GEVCRGPRMS	QNLLRQADLD	KFTPRVGSFE	VPEDFQERME	QQCIGSTTRL
170	180	190	200	210	220	230	240
LAQTDFPLQA	YEPKMQVPFQ	VLPGQHPRKI	EIERRKQQYL	SLDIEQLLFS	QGIDSNKLMP	RHLDHQHPQT	IEQGHDPIFP
250	260	270	280	290	300	310	320
IYLPLKVFDN	EDFDCRTPRE	WINMGLEPGS	LDRKPVPGKA	LLPTDDFLGH	EDPKSQKLKY	KWCEVGVLDY	DEEKKLYLVH
330	340	350	360	370	380	390	400
KTDEKGLVRD	EMGRPILNAG	VTTEGRPPLQ	VCQYWVPRIQ	LLFCAEDPCM	FAQRVVQANA	LRKNTEALLL	YNLYVDCMPS
410	420	430	440	450	460	470	480
DGQHVISEQS	LSKIKQWALS	TPRMRKGPSV	LEHLSSLARE	VSLDYERSMN	KINFDHVVSS	KPETFSYVTL	PKKEEEQVPE
490	500	510	520	530	540	550	560
RGLVSVPKYH	FWEQKEDFTF	VSLLTRPEVI	TALSKVRAEC	NKVTAMSLFH	SSLSKYSHLE	EFEQIQSQTF	SQVQMFLKDS
570	580	590	600	610	620	630	640
WISSLKVAMR	SSLRDMSKGW	YNLYETNWEV	YLMSKLRKLM	ELVKYMLQDT	LRFLVQDSLA	SFSQFISDTC	CSVLNCTDDM
650	660	670	680	690	700	710	720
VWGDDLINSP	YRPRKNPLFI	MDLVLDSSGV	HYSTPLEQFE	ASLLNLFDKG	ILATHAVPQL	EKLVMEDIFI	SGDPLLESVG
730	740	750	760	770	780	790	800
LHEPLVEELR	ATIASAVSKA	MIPLQAYAKE	YRKYLELNNN	DIASFLKTYQ	TQGLLAQEVR	EVVLTHLREK	EILDSSLPSS
810	820	830	840	850	860	870	880
IIIGPFYINT	DNVKQSLSKK	RKALATSVLD	ILAKNLHKEV	DSICEEFRSI	SRKIYEKPNS	IEELAELREW	MKGIPERLVG
890	900	910	920	930	940	950	960
LEERIVKVMD	DYQVMDEFLY	NLSSDDFNDK	WIASNWPSKI	LGQIELVQQQ	HVEDEEKFRK	IQIMDQNNFQ	EKLEGLQLVV
970	980	990	1000	1010	1020	1030	1040
AGFSIHVEIS	RAHEIANEVR	RVKKQLKDCQ	QLAMLYNNRE	RIFSLPITNY	DKLSRMVKEF	QPYLDLWTTA	SDWLRWSESW
1050	1060	1070	1080	1090	1100	1110	1120
MNDPLSAIDA	EQLEKNVVEA	FKTMHKCVKQ	FKDMPACQEV	ALDIRARIEE	FKPYIPLIQG	LRNPGMRIRH	WETLSNQINI
1130	1140	1150	1160	1170	1180	1190	1200
NVRPKANLTF	ARCLEMNLQD	HIESISKVAE	VAGKEYAIEQ	ALDKMEKEWS	TILFNVLPYK	ATDTYILKSP	DEASQLLDDH
1210	1220	1230	1240	1250	1260	1270	1280
IVMTQNMSFS	PYKKPFEQRI	NSWENKLKLT	QEVLEEWLNC	QRSWLYLEPI	FSSEDINQQL	PVESKRYQTM	ERIWKKIMKN
1290	1300	1310	1320	1330	1340	1350	1360
AYENREVINV	CSDLRMLDSL	RDCNKILDLV	QKGLSEYLET	KRSAFPRFYF	LSDDELLEIL	SQTKDPTAVQ	PHLRKCFENI
1370	1380	1390	1400	1410	1420	1430	1440
ARLLFQEDLE	ITHMYSAEGE	EVQLCFSIYP	SSNVEDWLRE	VERSMKASVH	DIIEKAIRAY	PTMPRTQWVL	NWPGQVTIAG
1450	1460	1470	1480	1490	1500	1510	1520
CQTYWTMEVA	EALEAGNLRS	QLFPQLCQQL	SDLVALVRGK	LSRMQRAVLS	ALIVIEVHAK	DVVSKLIQEN	VVSVNDFQWI
1530	1540	1550	1560	1570	1580	1590	1600
SQLRYYWTNN	DLYIRAVNAE	FIYGYEYLGN	SGRLVITPLT	DRCYLTLTGA	LHLKFGGAPA	GPAGTGKTET	TKDLGKALAI
1610	1620	1630	1640	1650	1660	1670	1680
QTVVFNCSDQ	LDFMAMGKFF	KGLASAGAWA	CFDEFNRIDI	EVLSVVAQQI	TTIQKAQQQR	VERFMFEGVE	IPLVPSCAVF
1690	1700	1710	1720	1730	1740	1750	1760
ITMNPGYAGR	TELPDNLKAL	FRPVAMMVPD	YAMITEISLY	SFGFNEASVL	AKKITTTFKL	SSEQLSSQDH	YDFGMRAVKT
1770	1780	1790	1800	1810	1820	1830	1840
VISAAGNLKR	ENPSMNEELI	CLRAIRDVNV	PKFLQEDLKL	FSGIVSDLFP	TIKEEDTDYG	ILDEAIREAC	RNSNLKDVEG
1850	1860	1870	1880	1890	1900	1910	1920
FLTKCIQLYE	TTVVRHGLML	VGPTGSGKST	CYRVLAAAMT	SLKGQPSISG	GMYEAVNYYV	LNPKSITMGQ	LYGEFDLLTH
1930	1940	1950	1960	1970	1980	1990	2000
EWTDGIFSSF	IRAGAITSDT	NKKWYMFDGP	VDAIWIENMN	TVLDDNKKLC	LSSGEIIKLT	EAMTMMFEVQ	DLAVASPATV
2010	2020	2030	2040	2050	2060	2070	2080
SRCGMVYLEP	SILGLMPFIE	CWLRKLPPLL	KPYEEHFKAL	FVSFLEESIS	FVRSSVKEVI	ASTNCNLTMS	LLKLLDCFFK
2090	2100	2110	2120	2130	2140	2150	2160
PFLPREGLKK	IPSEKLSRIV	ELIEPWFIFS	LIWSVGATGD	SSGRTSFSHW	LRLKMENEQL	TLLFPEEGLV	FDYRLEDAGI
2170	2180	2190	2200	2210	2220	2230	2240

Detailed Protein Report

Cmpd.	No. of Cmpds.	m/z meas.	Δ m/z [ppm]	z	Rt [min]	Score	P	Range	Sequence	Modification	Ratios
405	3	565.3234	-64.51	2	34.1	17.4	2	596-604	K.LRKLMELVK.Y		
2181	1	990.9291	-65.49	2	55.9	12.3	2	1828-1844	R.EACRNSNLKDVEGFLTK.C	Carbamidomethyl: 3	
870	1	573.1350	-254.46	2	40.3	10.7	1	2478-2486	R.DRLVNEEDR.S		mdown:qdown 2.69
318	1	715.6717	-189.89	2	32.7	11.8	0	2496-2506	R.CMEQWEVTFNK.V	Oxidation: 2	
972	1	730.6935	389.04	1	40.6	12.0	0	3422-3428	K.AAEIQAK.V		mdown:qdown 0.75
41	1	706.3121	-81.89	3	29.9	12.9	2	3699-3718	K.KLSAISLGQGQGPRAEAMMR.S	Oxidation: 18	

Detailed Protein Report

Protein 35: fibrillin-2 precursor [Homo sapiens]

**Accession:**	gi|66346695	**Score:**	83.7
**Database:**	refseq_human(refseq_human_20140103.fasta)	**MW [kDa]:**	314.6
**Database Date:**	2015-11-30	**pI:**	4.6
**Modification(s):**	Carbamidomethyl, Oxidation	**Sequence Coverage [%]:**	2.0
		**No. of unique Peptides:**	5

Quantitation

mdown:qdown	**Median:**	1.53	**CV:**	38.07 %	**No. of Peptides:**	2
Wdown:Qdown	**Median:**	3.53	**CV:**	7.35 %	**No. of Peptides:**	2

Detailed Protein Report

10	20	30	40	50	60	70	80
MGRRRRLCLQ	LYFLWLGCVV	LWAQGTAGQP	QPPPPKPPRP	QPPPQQVRSA	TAGSEGGFLA	PEYREEGAAV	ASRVRRRGQQ
90	100	110	120	130	140	150	160
DVLRGPNVCG	SRFHSYCCPG	WKTLPGGNQC	IVPICRNSCG	DGFCSRPNMC	TCSSGQISST	CGSKSIQQCS	VRCMNGGTCA
170	180	190	200	210	220	230	240
DDHCQCQKGY	IGTYCGQPVC	ENGCQNGGRC	IGPNRCACVY	GFTGPQCERD	YRTGPCFTQV	NNQMCQGQLT	GIVCTKTLCC
250	260	270	280	290	300	310	320
ATIGRAWGHP	CEMCPAQPQP	CRRGFIPNIR	TGACQDVDEC	QAIPGICQGG	NCINTVGSFE	CRCPAGHKQS	ETTQKCEDID
330	340	350	360	370	380	390	400
ECSIIPGICE	TGECSNTVGS	YFCVCPRGYV	TSTDGSRCID	QRTGMCFSGL	VNGRCAQELP	GRMTKMQCCC	EPGRCWGIGT
410	420	430	440	450	460	470	480
IPEACPVRGS	EEYRRLCMDG	LPMGGIPGSA	GSRPGGTGGN	GFAPSGNGNG	YGPGGTGFIP	IPGGNGFSPG	VGGAGVGAGG
490	500	510	520	530	540	550	560
QGPIITGLTI	LNQTIDICKH	HANLCLNGRC	IPTVSSYRCE	CNMGYKQDAN	GDCIDVDECT	SNPCTNGDCV	NTPGSYYCKC
570	580	590	600	610	620	630	640
HAGFQRTPTK	QACIDIDECI	QNGVLCKNGR	CVNTDGSFQC	ICNAGFELTT	DGKNCVDHDE	CTTTNMCLNG	MCINEDGSFK
650	660	670	680	690	700	710	720
CICKPGFVLA	PNGRYCTDVD	ECQTPGICMN	GHCINSEGSF	RCDCPPGLAV	GMDGRVCVDT	HMRSTCYGGI	KKGVCVRPFP
730	740	750	760	770	780	790	800
GAVTKSECCC	ANPDYGFGEP	CQPCPAKNSA	EFHGLCSSGV	GITVDGRDIN	ECALDPDICA	NGICENLRGS	YRCNCNSGYE
810	820	830	840	850	860	870	880
PDASGRNCID	IDECLVNRLL	CDNGLCRNTP	GSYSCTCPPG	YVFRTETETC	EDINECESNP	CVNGACRNNL	GSFNCECSPG
890	900	910	920	930	940	950	960
SKLSSTGLIC	IDSLKGTCWL	NIQDSRCEVN	INGATLKSEC	CATLGAAWGS	PCERCELDTA	CPRGLARIKG	VTCEDVNECE
970	980	990	1000	1010	1020	1030	1040
VFPGVCPNGR	CVNSKGSFHC	ECPEGLTLDG	TGRVCLDIRM	EQCYLKWDED	ECIHPVPGKF	RMDACCCAVG	AAWGTECEEC
1050	1060	1070	1080	1090	1100	1110	1120
PKPGTKEYET	LCPRGAGFAN	RGDVLTGRPF	YKDINECKAF	PGMCTYGKCR	NTIGSFKCRC	NSGFALDMEE	RNCTDIDECR
1130	1140	1150	1160	1170	1180	1190	1200
ISPDLCGSGI	CVNTPGSFEC	ECFEGYESGF	MMMKNCMDID	ECERNPLLCR	GGTCVNTEGS	FQCDCPLGHE	LSPSREDCVD
1210	1220	1230	1240	1250	1260	1270	1280
INECSLSDNL	CRNGKCVNMI	GTYQCSCNPG	YQATPDRQGC	TDIDECMIMN	GGCDTQCTNS	EGSYECSCSE	GYALMPDGRS
1290	1300	1310	1320	1330	1340	1350	1360
CADIDECENN	PDICDGGQCT	NIPGEYRCLC	YDGFMASMDM	KTCIDVNECD	LNSNICMFGE	CENTKGSFIC	HCQLGYSVKK
1370	1380	1390	1400	1410	1420	1430	1440
GTTGCTDVDE	CEIGAHNCDM	HASCLNIPGS	FKCSCREGWI	GNGIKCIDLD	ECSNGTHQCS	INAQCVNTPG	SYRCACSEGF
1450	1460	1470	1480	1490	1500	1510	1520
TGDGFTCSDV	DECAENINLC	ENGQCLNVPG	AYRCECEMGF	TPASDSRSCQ	DIDECSFQNI	CVFGTCNNLP	GMFHCICDDG
1530	1540	1550	1560	1570	1580	1590	1600
YELDRTGGNC	TDIDECADPI	NCVNGLCVNT	PGRYECNCPP	DFQLNPTGVG	CVDNRVGNCY	LKFGPRGDGS	LSCNTEIGVG
1610	1620	1630	1640	1650	1660	1670	1680
VSRSSCCCSL	GKAWGNPCET	CPPVNSTEYY	TLCPGGEGFR	PNPITIILED	IDECQELPGL	CQGGNCINTF	GSFQCECPQG
1690	1700	1710	1720	1730	1740	1750	1760
YYLSEDTRIC	EDIDECFAHP	GVCGPGTCYN	TLGNYTCICP	PEYMQVNGGH	NCMDMRKSFC	YRSYNGTTCE	NELPFNVTKR
1770	1780	1790	1800	1810	1820	1830	1840
MCCCTYNVGK	AWNKPCEPCP	TPGTADFKTI	CGNIPGFTFD	IHTGKAVDID	ECKEIPGICA	NGVCINQIGS	FRCECPTGFS
1850	1860	1870	1880	1890	1900	1910	1920
YNDLLLVCED	IDECSNGDNL	CQRNADCINS	PGSYRCECAA	GFKLSPNGAC	VDRNECLEIP	NVCSHGLCVD	LQGSYQCICH
1930	1940	1950	1960	1970	1980	1990	2000
NGFKASQDQT	MCMDVDECER	HPCGNGTCKN	TVGSYNCLCY	PGFELTHNND	CLDIDECSSF	FGQVCRNGRC	FNEIGSFKCL
2010	2020	2030	2040	2050	2060	2070	2080
CNEGYELTPD	GKNCIDTNEC	VALPGSCSPG	TCQNLEGSFR	CICPPGYEVK	SENCIDINEC	DEDPNICLFG	SCTNTPGGFQ
2090	2100	2110	2120	2130	2140	2150	2160
CLCPPGFVLS	DNGRRCFDTR	QSFCFTNFEN	GKCSVPKAFN	TTKAKCCCSK	MPGEGWGDPC	ELCPKDDEVA	FQDLCPYGHG
2170	2180	2190	2200	2210	2220	2230	2240

Detailed Protein Report

Cmpd.	No. of Cmpds.	m/z meas.	Δ m/z [ppm]	z	Rt [min]	Score	P	Range	Sequence	Modification	Ratios
534	2	730.1886	-131.49	2	36.0	10.1	1	383-394	R.MTKMQCCCEPGR.C	Carbamidomethyl: 6; Oxidation: 4	
522	1	730.1851	-136.21	2	35.9	15.8	1	383-394	R.MTKMQCCCEPGR.C	Carbamidomethyl: 8; Oxidation: 4	
472	1	516.1120	-268.37	2	34.5	12.2	0	1884-1893	K.LSPNGACVDR.N		mdown:qdown 1.06<br>Wdown:Qdown 3.80
281	1	477.4617	-193.67	3	32.2	10.8	0	2436-2448	K.ICPHGPGYTTDGR.D	Carbamidomethyl: 2	
304	4	642.1738	-171.43	2	33.2	24.4	1	2529-2539	K.TCKDLDECQTK.Q		Wdown:Qdown 3.28<br>mdown:qdown 2.21

Detailed Protein Report

Protein 36: plastin-2 [Homo sapiens]

**Accession:**	gi|167614506	**Score:**	83.3
**Database:**	refseq_human(refseq_human_20140103.fasta)	**MW [kDa]:**	70.2
**Database Date:**	2015-11-30	**pI:**	5.2
**Modification(s):**	Carbamidomethyl, Oxidation	**Sequence Coverage [%]:**	12.1
		**No. of unique Peptides:**	5

Quantitation

Wdown:Qdown **Median:** 1.14 **CV:** 0.00 % **No. of Peptides:** 1

Alias proteins:

Accession	Name	Description
gi|530402335	refseq_human (refseq_human_20140103.fasta)	PREDICTED: plastin-2 isoform X2 [Homo sapiens]

10	20	30	40	50	60	70	80
MARGSVSDEE	MMELREAFAK	VDTDGNGYIS	FNELNDLFKA	ACLPLPGYRV	REITENLMAT	GDLDQDGRIS	FDEFIKIFHG
90	100	110	120	130	140	150	160
LKSTDVAKTF	RKAINKKEGI	CAIGGTSEQS	SVGTQHSYSE	EEKYAFVNWI	NKALENDPDC	RHVIPMNPNT	NDLFNAVGDG
170	180	190	200	210	220	230	240
IVLCKMINLS	VPDTIDERTI	NKKKLTPFTI	QENLNLALNS	ASAIGCHVVN	IGAEDLKEGK	PYLVLGLLWQ	VIKIGLFADI
250	260	270	280	290	300	310	320
ELSRNEALIA	LLREGESLED	LMKLSPEELL	LRWANYHLEN	AGCNKIGNFS	TDIKDSKAYY	HLLEQVAPKG	DEEGVPAVVI
330	340	350	360	370	380	390	400
DMSGLREKDD	IQRAECMLQQ	AERLGCRQFV	TATDVVRGNP	KLNLAFIANL	FNRYPALHKP	ENQDIDWGAL	EGETREERTF
410	420	430	440	450	460	470	480
RNWMNSLGVN	PRVNHLYSDL	SDALVIFQLY	EKIKVPVDWN	RVNKPPYPKL	GGNMKKLENC	NYAVELGKNQ	AKFSLVGIGG
490	500	510	520	530	540	550	560
QDLNEGNRTL	TLALIWQLMR	RYTLNILEEI	GGGQKVNDDI	IVNWVNETLR	EAKKSSSISS	FKDPKISTSL	PVLDLIDAIQ
570	580	590	600	610	620	630	
PGSINYDLLK	TENLNDDEKL	NNAKYAISMA	RKIGARVYAL	PEDLVEVNPK	MVMTVFACLM	GKGMKRV	

Cmpd.	No. of Cmpds.	m/z meas.	Δ m/z [ppm]	z	Rt [min]	Score	P	Range	Sequence	Modification	Ratios
2609	1	658.3945	34.02	2	61.3	14.5	1	77-88	K.IFHGLKSTDVAK.T		
545	1	652.1661	-227.16	2	36.1	14.5	0	402-412	R.NWMNSLGVNPR.V	Oxidation: 3	Wdown:Qdown 1.14
1806	1	649.7882	-96.92	2	52.6	11.2	2	531-542	R.EAKKSSSISSFK.D		
2231	1	793.3805	-58.93	2	56.5	14.2	0	597-610	R.VYALPEDLVEVNPK.M		
2744	1	710.3515	31.99	2	65.6	10.2	0	611-622	K.MVMTVFACLMGK.G	Carbamidomethyl: 8; Oxidation: 1, 3	

Detailed Protein Report

Protein 37: splicing factor, proline- and glutamine-rich [Homo sapiens]

**Accession:**	gi|4826998	**Score:**	81.7
**Database:**	refseq_human(refseq_human_20140103.fasta)	**MW [kDa]:**	76.1
**Database Date:**	2015-11-30	**pI:**	9.9
**Modification(s):**	Oxidation	**Sequence Coverage [%]:**	4.8
		**No. of unique Peptides:**	5

Quantitation

mdown:qdown	**Median:**	0.44	**CV:**	28.48 %	**No. of Peptides:**	2
Wdown:Qdown	**Median:**	1.11	**CV:**	74.66 %	**No. of Peptides:**	2

Alias proteins:

Accession	Name	Description
gi|530363144	refseq_human (refseq_human_20140103.fasta)	PREDICTED: splicing factor, proline- and glutamine-rich isoform X3 [Homo sapiens]
gi|530363142	refseq_human (refseq_human_20140103.fasta)	PREDICTED: splicing factor, proline- and glutamine-rich isoform X2 [Homo sapiens]
gi|530363140	refseq_human (refseq_human_20140103.fasta)	PREDICTED: splicing factor, proline- and glutamine-rich isoform X1 [Homo sapiens]

10	20	30	40	50	60	70	80
MSRDRFRSRG	GGGGGFHRRG	GGGGRGGLHD	FRSPPPGMGL	NQNRGPMGPG	PGQSGPKPPI	PPPPPHQQQQ	QPPPQQPPPQ
**90**	**100**	**110**	**120**	**130**	**140**	**150**	**160**
QPPPHQPPPH	PQPHQQQQPP	PPPQDSSKPV	VAQGPGPAPG	VGSAPPASSS	APPATPPTSG	APPGSGPGPT	PTPPPAVTSA
**170**	**180**	**190**	**200**	**210**	**220**	**230**	**240**
PPGAPPPTPP	SSGVPTTPPQ	AGGPPPPPAA	VPGPGPGPKQ	GPGPGGPKGG	KMPGGPKPGG	GPGLSTPGGH	PKPPHRGGGE
**250**	**260**	**270**	**280**	**290**	**300**	**310**	**320**
PRGGRQHHPP	YHQQHHQGPP	PGGPGGRSEE	KISDSEGFKA	NLSLLRRPGE	KTYTQRCRLF	VGNLPADITE	DEFKRLFAKY
**330**	**340**	**350**	**360**	**370**	**380**	**390**	**400**
GEPGEVFINK	GKGFGFIKLE	SRALAEIAKA	ELDDTPMRGR	QLRVRFATHA	AALSVRNLSP	YVSNELLEEA	FSQFGPIERA
**410**	**420**	**430**	**440**	**450**	**460**	**470**	**480**
VVIVDDRGRS	TGKGIVEFAS	KPAARKAFER	CSEGVFLLTT	TPRPVIVEPL	EQLDDEDGLP	EKLAQKNPMY	QKERETPPRF
**490**	**500**	**510**	**520**	**530**	**540**	**550**	**560**
AQHGTFEYEY	SQRWKSLDEM	EKQQREQVEK	NMKDAKDKLE	SEMEDAYHEH	QANLLRQDLM	RRQEELRRME	ELHNQEMQKR
**570**	**580**	**590**	**600**	**610**	**620**	**630**	**640**
KEMQLRQEEE	RRRREEEMMI	RQREMEEQMR	RQREESYSRM	GYMDPRERDM	RMGGGGAMNM	GDPYGSGGQK	FPPLGGGGGI
**650**	**660**	**670**	**680**	**690**	**700**	**710**	
GYEANPGVPP	ATMSGSMMGS	DMRTERFGQG	GAGPVGGQGP	RGMGPGTPAG	YGRGREEYEG	PNKKPRF	

Cmpd.	No. of Cmpds.	m/z meas.	Δ m/z [ppm]	z	Rt [min]	Score	P	Range	Sequence	Modification	Ratios
376	6	678.1939	-185.67	2	34.0	29.1	1	268-279	R.SEEKISDSEGFK.A		mdown:qdown 0.58<br>Wdown:Qdown 2.17
14	1	1103.5206	70.68	2	29.5	14.3	1	609-630	R.<br>DMRMGGGGAMNMGDPYGSGGQ<br>F	Oxidation: 2, 12	
18	2	736.0042	54.34	3	29.4	11.7	1	609-630	R.<br>DMRMGGGGAMNMGDPYGSGGQ<br>F	Oxidation: 2, 10	Wdown:Qdown 0.57<br>mdown:qdown 0.33
20	2	735.9950	41.81	3	29.6	11.7	1	609-630	R.<br>DMRMGGGGAMNMGDPYGSGGQ<br>F	Oxidation: 4, 10	
8	2	736.0117	64.51	3	29.3	14.9	1	609-630	R.<br>DMRMGGGGAMNMGDPYGSGGQ<br>F	Oxidation: 4, 12	

Detailed Protein Report

Protein 38: **PREDICTED: dynein heavy chain 3, axonemal isoform X1 [Homo sapiens]**

**Accession:**	gi|578828592	**Score:**	81.1
**Database:**	refseq_human(refseq_human_20140103.fasta)	**MW [kDa]:**	466.0
**Database Date:**	2015-11-30	**pI:**	6.0
**Modification(s):**	Carbamidomethyl, Oxidation	**Sequence Coverage [%]:**	2.0
		**No. of unique Peptides:**	3

Detailed Protein Report

10	20	30	40	50	60	70	80
MGDMDCSSQK	IAKSDSIHHM	SHSQGQPELP	PLPASANEEP	SGLYQTVMSH	SFYPPLMQRT	SWTLAAPFKE	QHHHRGPSDS
90	100	110	120	130	140	150	160
IANNYSLMAQ	DLKLKDLLKV	YQPATISVPR	DRTGQGLPSS	GNRSSSEPMR	KKTKFSSRNK	EDSTRIKLAF	KTSIFSPMKK
170	180	190	200	210	220	230	240
EVKTSLTFPG	SRPMSPEQQL	DVMLQQEMEM	ESKEKKPSES	DLERYYYYLT	NGIRKDMIAP	EEGEVMVRIS	KLISNTLLTS
250	260	270	280	290	300	310	320
PFLEPLMVVL	VQEKENDYYC	SLMKSIVDYI	LMDPMERKRL	FIESIPRLFP	QRVIRAPVPW	HSVYRSAKKW	NEEHLHTVNP
330	340	350	360	370	380	390	400
MMLRLKELWF	AEFRDLRFVR	TAEILAGKLP	LQPQEFWDVI	QKHCLEAHQT	LLNKWIPTCA	QLFTSRKEHW	IHFAPKSNYD
410	420	430	440	450	460	470	480
SSRNIEEYFA	SVASFMSLQL	RELVIKSLED	LVSLFMIHKD	GNDFKEPYQE	MKFFIPQLIM	IKLEVSEPII	VFNPSFDGCW
490	500	510	520	530	540	550	560
ELIRDSFLEI	IKNSNGIPKV	ESVLFPELKG	YNLLLGTVNA	EEKLVSDFLI	QTFKVFQKNQ	VGPCKYLNVY	KKYVDLLDNT
570	580	590	600	610	620	630	640
AEQNIAAFLK	ENHDIDDFVT	KINAIKKRRN	EIASMNITVP	LAMFCLDATA	LNHDLCERAQ	NLKDHLIQFQ	VDVNRDTNTS
650	660	670	680	690	700	710	720
ICNQYSHIAD	KVSEVPANTK	ELVSLIEFLK	KSSAVTVFKL	RRQLRDASER	LEFLMDYADL	PYEDIKLNST	LFLWPDQIED
730	740	750	760	770	780	790	800
IFDNSRNLLL	HKRDQAEMDL	IKRCSEFELR	LEGYHRELES	FRKREVMTTE	EMKHNVEKLN	ELSKNLNRAF	AEFELINKEE
810	820	830	840	850	860	870	880
ELLEKEKSTY	PLLQAMLKNK	VPYEQLWSTA	YEFSIKSEEW	MNGPLFLLNA	EQIAEEIGNM	WRTTYKLIKT	LSDVPAPRRL
890	900	910	920	930	940	950	960
AENVKIKIDK	FKQYIPILSI	SCNPGMKDRH	WQQISEIVGY	EIKPTETTCL	SNMLEFGFGK	FVEKLEPIGA	AASKEYSLEK
970	980	990	1000	1010	1020	1030	1040
NLDRMKLDWV	NVTFSFVKYR	DTDTNILCAI	DDIQMLLDDH	VIKTQTMCGS	PFIKPIEAEC	RKWEEKLIRI	QDNLDAWLKC
1050	1060	1070	1080	1090	1100	1110	1120
QATWLYLEPI	FSSEDIIAQM	PEEGRKFGIV	DSYWKSLMSQ	AVKDNRILVA	ADQPRMAEKL	QEANFLLEDI	QKGLNDYLEK
1130	1140	1150	1160	1170	1180	1190	1200
KRLFFPRFFF	LSNDELLEIL	SETKDPLRVQ	PHLKKCFEGI	AKLEFTDNLE	IVGMISSEKE	TVPFIQKIYP	ANAKGMVEKW
1210	1220	1230	1240	1250	1260	1270	1280
LQQVEQMMLA	SMREVIGLGI	EAYVKVPRNH	WVLQWPGQVV	ICVSSIFWTQ	EVSQALAENT	LLDFLKKSND	QIAQIVQLVR
1290	1300	1310	1320	1330	1340	1350	1360
GKLSSGARLT	LGALTVIDVH	ARDVVAKLSE	DRVSDLNDFQ	WISQLRYYWV	AKDVQVQIIT	TEALYGYEYL	GNSPRLVITP
1370	1380	1390	1400	1410	1420	1430	1440
LTDRCYRTLM	GALKLNLGGA	PEGPAGTGKT	ETTKDLAKAL	AKQCVVFNCS	DGLDYKAMGK	FFKGLAQAGA	WACFDEFNRI
1450	1460	1470	1480	1490	1500	1510	1520
EVEVLSVVAQ	QILSIQQAII	RKLKTFIFEG	TELSLNPTCA	VFITMNPGYA	GRAELPDNLK	ALFRTVAMMV	PDYALIGEIS
1530	1540	1550	1560	1570	1580	1590	1600
LYSMGFLDSR	SLAQKIVATY	RLCSEQLSSQ	HHYDYGMRAV	KSVLTAAGNL	KLKYPEENES	VLLLRALLDV	NLAKFLAQDV
1610	1620	1630	1640	1650	1660	1670	1680
PLFQGIISDL	FPGVVLPKPD	YEVFLKVLND	NIKKMKLQPV	PWFIGKIIQI	YEMMLVRHGY	MIVGDPMGGK	TSAYKVLAAA
1690	1700	1710	1720	1730	1740	1750	1760
LGDLHAANQM	EEFAVEYKII	NPKAITMGQL	YGCFDQVSHE	WMDGVLANAF	REQASSLSDD	RKWIIFDGPV	DAIWIENMNT
1770	1780	1790	1800	1810	1820	1830	1840
VLDDNKKLCL	MSGEIIQMNS	KMSLIFEPAD	LEQASPATVS	RCGMIYMEPH	QLGWKPLKDS	YMDTLPSSLT	KEHKELVNDM
1850	1860	1870	1880	1890	1900	1910	1920
FMWLVQPCLE	FGRLHCKFVV	QTSPIHLAFS	MMRLYSSLLD	EIRAVEEEEM	ELGEGLSSQQ	IFLWLQGLFL	FSLVWTVAGT
1930	1940	1950	1960	1970	1980	1990	2000
INADSRKKFD	VFFRNLIMGM	DDNHPRPKSV	KLTKNNIFPE	RGSIYDFYFI	KQASGHWETW	TQYITKEEEK	VPAGAKVSEL
2010	2020	2030	2040	2050	2060	2070	2080
IIPTMETARQ	SFFLKTYLDH	EIPMLFVGPT	GTGKSAITNN	FLLHLPKNTY	LPNCINFSAR	TSANQTQDII	MSKLDRRRKG
2090	2100	2110	2120	2130	2140	2150	2160
LFGPPIGKKA	VVFVDDLNMP	AKEVYGAQPP	IELLRQWIDH	GYWFDKKDTT	RLDIVDMLLV	TAMGPPGGGR	NDITGRFTRH
2170	2180	2190	2200	2210	2220	2230	2240

Detailed Protein Report

Cmpd.	No. of Cmpds.	m/z meas.	Δ m/z [ppm]	z	Rt [min]	Score	P	Range	Sequence	Modification
301	1	874.1753	76.12	3	33.1	22.7	0	463-484	K.LEVSEPIIVFNPSFDGCWELIR.D	Carbamidomethyl: 17
565	1	607.8337	-71.29	2	35.7	10.9	1	1562-1573	K.SVLTAAGNLKLK.Y	
315	1	1201.1883	61.60	2	32.6	16.3	1	2128-2150	K.DTTRLDIVDMLLVTAMGPPGGGR.N	Oxidation: 10

Detailed Protein Report

Protein 39: glyceraldehyde-3-phosphate dehydrogenase isoform 1 [Homo sapiens]

**Accession:**	gi|7669492	**Score:**	80.6
**Database:**	refseq_human(refseq_human_20140103.fasta)	**MW [kDa]:**	36.0
**Database Date:**	2015-11-30	**pI:**	9.3
**Modification(s):**	Carbamidomethyl	**Sequence Coverage [%]:**	13.7
		**No. of unique Peptides:**	3

Quantitation

mdown:qdown	**Median:**	0.50	**CV:**	0.00 %	**No. of Peptides:**	1
Wdown:Qdown	**Median:**	0.36	**CV:**	136.48 %	**No. of Peptides:**	2

```
        10         20         30         40         50         60         70         80
MGKVKVGVNG FGRIGRLVTR AAFNSGKVDI VAINDPFIDL NYMVYMFQYD STHGKFHGTV KAENGKLVIN GNPITIFQER
        90        100        110        120        130        140        150        160
DPSKIKWGDA GAEYVVESTG VFTTMEKAGA HLQGGAKRVI ISAPSADAPM FVMGVNHEKY DNSLKIISNA SCTTNCLAPL
       170        180        190        200        210        220        230        240
AKVIHDNFGI VEGLMTTVHA ITATQKTVDG PSGKLWRDGR GALQNIIPAS TGAAKAVGKV IPELNGKLTG MAFRVPTANV
       250        260        270        280        290        300        310        320
SVVDLTCRLE KPAKYDDIKK VVKQASEGPL KGILGYTEHQ VVSSDFNSDT HSSTFDAGAG IALNDHFVKL ISWYDNEFGY
       330        340
SNRVVDLMAH MASKE
```

Cmpd.	No. of Cmpds.	m/z meas.	Δ m/z [ppm]	z	Rt [min]	Score	P	Range	Sequence	Modification	Ratios
703	1	917.4101	-58.22	2	37.6	39.4	0	146-162	K.IISNASCTTNCLAPLAK.V	Carbamidomethyl: 7, 11	mdown:qdown 0.50<br>Wdown:Qdown 1.00
904	2	706.3818	-24.09	2	40.1	26.3	0	201-215	R.GALQNIIPASTGAAK.A		Wdown:Qdown 0.13
2067	1	882.3181	-98.33	2	54.4	15.0	0	310-323	K.LISWYDNEFGYSNR.V		

Detailed Protein Report

Protein 40: zinc finger protein 737 [Homo sapiens]

**Accession:**	gi|226530355	**Score:**	78.7
**Database:**	refseq_human(refseq_human_20140103.fasta)	**MW [kDa]:**	61.9
**Database Date:**	2015-11-30	**pI:**	10.3
**Modification(s):**	Carbamidomethyl	**Sequence Coverage [%]:**	9.3
		**No. of unique Peptides:**	6

Quantitation

Wdown:Qdown **Median:** 3.24 **CV:** 0.00 % **No. of Peptides:** 1

```
        10         20         30         40         50         60         70         80
MGPLQFRDVA IEFSLEEWHC LDTAQRNLYR NVMLENYRNL VFLGIVVSKP DLITCLEQGK KPLTMKKHEM VANPSVTCSH
        90        100        110        120        130        140        150        160
FARDLWPEQS IKDSFQKVTL RRYENYGHDN LQFKKGCESV DECKVHKRGY NGLNQYLTTT QSKIFQCDKY VKVIHKFSNS
       170        180        190        200        210        220        230        240
NRHKIRHTGK KPFKCIECGK AFNQSSTLTT HKKIHTGEKP FKCEECGKAF NWSSHLTTHK RIHTGEKRYK CEDCGKAFSR
       250        260        270        280        290        300        310        320
FSYLTAHKII HSGEKPYKCE ECGKAFKRSS NLTTHKIIHT GEKPYKCEEC GKAFKRSSIL TAHKIIHSGE KPYKCEECGK
       330        340        350        360        370        380        390        400
AFKHPSVLTT HKRIHTGEKP YKCEECGRAF KYFSSLTTHK IIHSGEKPYK CEECGKAFNW SSHLTTHKRI HTGEKPYKCE
       410        420        430        440        450        460        470        480
ECGEAFKYSS SLTTHKIIHT GQQPFKCEEC GKAFKCFSIL TTHKRIHTGE KPYKCEECGK AFNSSSHLTA HKRIHTGEKP
       490        500        510        520        530        540
YKCERCGKAF KRSFILTRHK RIHTGEKPYK CEECGKGFKC PSTLTTHKVI HTGEKL
```

Cmpd.	No. of Cmpds.	m/z meas.	Δ m/z [ppm]	z	Rt [min]	Score	P	Range	Sequence	Modification	Ratios
2780	1	922.4553	-14.26	2	66.1	16.9	1	128-143	K.RGYNGLNQYLTTTQSK.I		
2919	1	981.9597	-7.66	2	65.7	10.8	2	333-348	K.RIHTGEKPYKCEECGR.A	Carbamidomethyl: 11	
2966	1	953.4397	-17.72	2	66.3	13.9	2	333-348	K.RIHTGEKPYKCEECGR.A		Wdown:Qdown 3.24
1955	1	1022.8979	-86.37	2	54.5	10.5	2	427-444	K.CEECGKAFKCFSILTTHK.R		
2022	1	1051.4074	-85.21	2	53.9	10.6	2	427-444	K.CEECGKAFKCFSILTTHK.R	Carbamidomethyl: 10	
2081	3	1051.4297	-64.07	2	54.6	15.9	2	427-444	K.CEECGKAFKCFSILTTHK.R	Carbamidomethyl: 1	

Detailed Protein Report

Protein 41: **TNF receptor-associated factor 2 [Homo sapiens]**

**Accession:**	gi|22027612	**Score:**	77.4
**Database:**	refseq_human(refseq_human_20140103.fasta)	**MW [kDa]:**	55.8
**Database Date:**	2015-11-30	**pI:**	9.0
**Modification(s):**	Carbamidomethyl	**Sequence Coverage [%]:**	9.2
		**No. of unique Peptides:**	2

10	20	30	40	50	60	70	80
MAAASVTPPG	SLELLQPGFS	KTLLGTKLEA	KYLCSACRNV	LRRPFQAQCG	HRYCSFCLAS	ILSSGPQNCA	ACVHEGIYEE
**90**	**100**	**110**	**120**	**130**	**140**	**150**	**160**
GISILESSSA	FPDNAARREV	ESLPAVCPSD	GCTWKGTLKE	YESCHEGRCP	LMLTECPACK	GLVRLGEKER	HLEHECPERS
**170**	**180**	**190**	**200**	**210**	**220**	**230**	**240**
LSCRHCRAPC	CGADVKAHHE	VCPKFPLTCD	GCGKKKIPRE	KFQDHVKTCG	KCRVPCRFHA	IGCLETVEGE	KQQEHEVQWL
**250**	**260**	**270**	**280**	**290**	**300**	**310**	**320**
REHLAMLLSS	VLEAKPLLGD	QSHAGSELLQ	RCESLEKKTA	TFENIVCVLN	REVERVAMTA	EACSRQHRLD	QDKIEALSSK
**330**	**340**	**350**	**360**	**370**	**380**	**390**	**400**
VQQLERSIGL	KDLAMADLEQ	KVLEMEASTY	DGVFIWKISD	FARKRQEAVA	GRIPAIFSPA	FYTSRYGYKM	CLRIYLNGDG
**410**	**420**	**430**	**440**	**450**	**460**	**470**	**480**
TGRGTHLSLF	FVVMKGPNDA	LLRWPFNQKV	TLMLLDQNNR	EHVIDAFRPD	VTSSSFQRPV	NDMNIASGCP	LFCPVSKMEA
**490**	**500**	**510**					
KNSYVRDDAI	FIKAIVDLTG	L					

Cmpd.	No. of Cmpds.	m/z meas.	Δ m/z [ppm]	z	Rt [min]	Score	P	Range	Sequence	Modification
2037	1	1051.4158	-90.99	2	54.1	10.8	1	214-231	R.VPCRFHAIGCLETVEGEK.Q	Carbamidomethyl: 3, 10
2147	1	1022.9076	-91.14	2	57.0	15.3	1	214-231	R.VPCRFHAIGCLETVEGEK.Q	Carbamidomethyl: 10

Detailed Protein Report

Protein 42: **immunoglobulin lambda-like polypeptide 5 isoform 2 [Homo sapiens]**

**Accession:**	gi|372466586	**Score:**	77.0
**Database:**	refseq_human(refseq_human_20140103.fasta)	**MW [kDa]:**	14.7
**Database Date:**	2015-11-30	**pI:**	7.7
		**Sequence Coverage [%]:**	24.5
		**No. of unique Peptides:**	2

```
        10         20         30         40         50         60         70         80
MACCAQWLHR KAGTQTLEPQ LEAADPACGA CGAGQPKANP TVTLFPPSSE ELQANKATLV CLISDFYPGA VTVAWKADGS
        90        100        110        120        130        140
PVKAGVETTK PSKQSNNKYA ASSYLSLTPE QWKSHRSYSC QVTHEGSTVE KTVAPTECS
```

Cmpd.	No. of Cmpds.	m/z meas.	Δ m/z [ppm]	z	Rt [min]	Score	P	Range	Sequence	Modification
1891	3	1021.8855	-134.81	2	52.1	22.0	0	38-56	K.ANPTVTLFPPSSEELQANK.A	
1753	11	872.4147	-21.04	2	50.6	55.0	0	99-113	K.YAASSYLSLTPEQWK.S	

Detailed Protein Report

Protein 43: **PREDICTED: DNA-directed RNA polymerase, mitochondrial isoform X1 [Homo sapiens]**

**Accession:**	gi|530425287	**Score:**	75.6
**Database:**	refseq_human(refseq_human_20140103.fasta)	**MW [kDa]:**	145.0
**Database Date:**	2015-11-30	**pI:**	10.3
**Modification(s):**	Carbamidomethyl	**Sequence Coverage [%]:**	3.6
		**No. of unique Peptides:**	3

```
        10         20         30         40         50         60         70         80
MPSRLRCILG VYFRLGFPFA ASVDHETTTP GRRRGRMRRR GPVGWPGAAC VMSALCWGRG AAGLKRALRP CGRPGLPGKE
        90        100        110        120        130        140        150        160
GTAGGVCGPR RSSSASPQEQ DQDRRKDWGH VELLEVLQAR VRQLQAESVS EVVVNRVDVA RLPECGSGDG SLQPPRKVQM
       170        180        190        200        210        220        230        240
GAKDATPVPC GRWAKILEKD KRTQQMRMQR LKAKLQMPFQ SGEFKALTRR LQVEPRLLSK QMAGCLEDCT RQAPESPWEE
       250        260        270        280        290        300        310        320
QLARLLQEAP GKLSLDVEQA PSGQHSQAQL SGQQQRLLAF FKCCLLTDQL PLAHHLLVVH HGQRQKRKLL TLDMYNAVML
       330        340        350        360        370        380        390        400
GWARQGAFKE LVYVLFMVKD AGLTPDLLSY AAALQCMGRQ DQDAGTIERC LEQMSQEGLK LQALFTAVLL SEEDRATVLK
       410        420        430        440        450        460        470        480
AVHKVKPTFS LPPQLPPPVN TSKLLRDVYA KDGRVSYPKL HLPLKTLQCL FEKQLHMELA SRVCVVSVEK PTLPSKEVKH
       490        500        510        520        530        540        550        560
ARKTLKTLRD QWEKALCRAL RETKNRLERE VYEGRFSLYP FLCLLDEREV VRMLLQVLQA LPAQGESFTT LARELSARTF
       570        580        590        600        610        620        630        640
SRHVVQRQRV SGQVQALQNH YRKYLCLLAS DAEVPEPCLP RQYWEELGAP EALREQPWPL PVQMELGKLL AEMLVQATQM
       650        660        670        680        690        700        710        720
PCSLDKPHRS SRLVPVLYHV YSFRNVQQIG ILKPHPAYVQ LLEKAAEPTL TFEAVDVPML CPPLPWTSPH SGAFLLSPTK
       730        740        750        760        770        780        790        800
LMRTVEGATQ HQELLETCPP TALHGALDAL TQLGNCAWRV NGRVLDLVLQ LFQAKGCPQL GVPAPPSEAP QPPEAHLPHS
       810        820        830        840        850        860        870        880
AAPARKAELR RELAHCQKVA REMHSLRAEA LYRLSLAQHL RDRVFWLPHN MDFRGRTYPC PPHFNHLGSD VARALLEFAQ
       890        900        910        920        930        940        950        960
GRPLGPHGLD WLKIHLVNLT GLKKREPLRK RLAFAEEVMD DILDSADQPL TGRKWWMGAE EPWQTLACCM EVANAVRASD
       970        980        990       1000       1010       1020       1030       1040
PAAYVSHLPV HQDGSCNGLQ HYAALGRDSV GAASVNLEPS DVPQDVYSGV AAQVEVFRRQ DAQRGMRVAQ VLEGFITRKV
      1050       1060       1070       1080       1090       1100       1110       1120
VKQTVMTVVY GVTRYGGRLQ IEKRLRELSD FPQEFVWEAS HYLVRQVFKS LQEMFSGTRA IQHWLTESAR LISHMGSVVE
      1130       1140       1150       1160       1170       1180       1190       1200
WVTPLGVPVI QPYRLDSKVK QIGGGIQSIT YTHNGDISRK PNTRKQKNGF PPNFIHSLDS SHMMLTALHC YRSPPLCHRK
      1210       1220       1230       1240       1250       1260       1270       1280
GLTFVSVHDC YWTHAADVSV MNQVCREQFV RLHSEPILQD LSRFLVKRFC SEPQKILEAS QLKETLQAVP KPGAFDLEQV
      1290
KRSTYFFS
```

Cmpd.	No. of Cmpds.	m/z meas.	Δ m/z [ppm]	z	Rt [min]	Score	P	Range	Sequence	Modification
2442	1	857.6864	-95.07	3	60.9	18.7	2	40-65	R.RGPVGWPGAACVMSALCWGRGAAGLK.R	
125	1	793.8591	-42.14	2	30.8	18.8	1	158-172	K.VQMGAKDATPVPCGR.W	Carbamidomethyl: 13
2603	1	678.4838	186.95	1	63.1	15.6	0	1227-1231	R.EQFVR.L	

Detailed Protein Report

Protein 44: keratin, type II cytoskeletal 1b [Homo sapiens]

**Accession:**	gi|194018511	**Score:**	75.2
**Database:**	refseq_human(refseq_human_20140103.fasta)	**MW [kDa]:**	61.9
**Database Date:**	2015-11-30	**pI:**	5.6
		**Sequence Coverage [%]:**	9.0
		**No. of unique Peptides:**	1

10	20	30	40	50	60	70	80
MSHQFSSQSA	FSSMSRRVYS	TSSSAGSGGG	SPAVGSVCYA	RGRCGGGGYG	IHGRGFGSRS	LYNLGGSRSI	SINLMGRSTS
**90**	**100**	**110**	**120**	**130**	**140**	**150**	**160**
GFCQGGGVGG	FGGGRGFGVG	STGAGGFGGG	GFGGAGFGTS	NFGLGGFGPY	CPPGGIQEVT	INQSLLEPLH	LEVDPEIQRI
**170**	**180**	**190**	**200**	**210**	**220**	**230**	**240**
KTQEREQIMV	LNNKFASFID	KVRFLEQQNQ	VLQTKWELLQ	QVNTSTGTNN	LEPLLENYIG	DLRRQVDLLS	AEQMRQNAEV
**250**	**260**	**270**	**280**	**290**	**300**	**310**	**320**
RSMQDVVEDY	KSKYEDEINK	RTGSENDFVV	LKKDVDAAYV	SKVDLESRVD	TLTGEVNFLK	YLFLTELSQV	QTHISDTNVI
**330**	**340**	**350**	**360**	**370**	**380**	**390**	**400**
LSMDNNRSLD	LDSIIDAVRT	QYELIAQRSK	DEAEALYQTK	YQELQITAGR	HGDDLKNSKM	EIAELNRTVQ	RLQAEISNVK
**410**	**420**	**430**	**440**	**450**	**460**	**470**	**480**
KQIEQMQSLI	SDAEERGEQA	LQDAWQKLQD	LEEALQQSKE	ELARLLRDYQ	AMLGVKLSLD	VEIATYRQLL	EGEESRMSGE
**490**	**500**	**510**	**520**	**530**	**540**	**550**	**560**
LQSHVSISVQ	NSQVSVNGGA	GGGGSYGSGG	YGGGSGGGYG	GGRSYRGGGA	RGRSGGGYGS	GCGGGGGSYG	GSGRSGRGSS
**570**	**580**						
RVQIIQTSTN	TSHRRILE						

Cmpd.	No. of Cmpds.	m/z meas.	Δ m/z [ppm]	z	Rt [min]	Score	P	Range	Sequence	Modification
2213	1	1103.9435	-56.00	2	56.2	13.0	0	18-41	R.VYSTSSSAGSGGGSPAVGSVCYAR.G	

Detailed Protein Report

Protein 45: PREDICTED: zinc finger protein 665 isoform X5 [Homo sapiens]

Accession: gi|578834915
Database: refseq_human(refseq_human_20140103.fasta)
Database Date: 2015-11-30
Modification(s): Carbamidomethyl, Oxidation

Score: 72.6
MW [kDa]: 76.3
pI: 10.3
Sequence Coverage [%]: 7.5
No. of unique Peptides: 5

Quantitation

Wdown:Qdown **Median:** 0.88 **CV:** 0.00 % **No. of Peptides:** 1

```
        10         20         30         40         50         60         70         80
MVCPCSGLLL AVGGMPPRCQ GRTLEEEEEE GKGVRDGSSS DISCKCVNTD LPPKGKNNMG EAFYTVKLER LESCDTVGLS
        90        100        110        120        130        140        150        160
FQEVQKNTYD FECQWKDDEG NYKTVLMLQK ENLPGRRAQR DRRAAGNRHI ENQLGVSFQS HLPELQQFQH EGKIYEYNQV
       170        180        190        200        210        220        230        240
EKSPNNRGKH YKCDECGKVF SQNSRLTSHK RIHTGEKPYQ CNKCGKAFTV RSNLTIHQVI HTGEKPYKCN ECGKVFSQPS
       250        260        270        280        290        300        310        320
NLAGHQRIHT GEKPYKCNEC GKAFRAHSKL TTHQVIHTGE KPYKCKECGK CFTQNSHLAS HRRIHTGEKP YKCNECGKAF
       330        340        350        360        370        380        390        400
SVRSSLTTHQ TIHTGEKPYK CNECGKVFRH NSYLAKHRRI HTGEKPYKCN ECGKAFSMHS NLTKHQIIHT GEKPFKCNEC
       410        420        430        440        450        460        470        480
VKVFTQYSHL ANHRRIHTGE KPYRCDECGK AFSVRSSLTT HQAIHTGEKP YKCNDCGKVF TQNSHLASHR GIHSGEKPYK
       490        500        510        520        530        540        550        560
CDECGKAFSQ TSQLARHWRV HTGEKPYKCN ECGKAFSVHS SLTIHQTIHT GQKPYKCNDC GKVFRHNSYL AIHQRIHTGE
       570        580        590        600        610        620        630        640
KPYKCNECGK AFSVHSNLAT HQVIHTGEKP YKCNECGKVF TQNSHLANHR RIHTGEKPYR CNECGKAFSV RSTLTTHMAV
       650        660        670        680
HTGDKPYKCN QCGKVFTQNS NLAKHRRIHS G
```

Cmpd.	No. of Cmpds.	m/z meas.	Δ m/z [ppm]	z	Rt [min]	Score	P	Range	Sequence	Modification	Ratios
2105	6	937.3619	-106.09	2	56.5	15.4	0	1-18	-.MVCPCSGLLLAVGGMPPR.C	Carbamidomethyl: 5; Oxidation: 15	Wdown:Qdown 0.88
2957	4	937.4644	3.23	2	66.1	16.2	0	1-18	-.MVCPCSGLLLAVGGMPPR.C	Carbamidomethyl: 3; Oxidation: 15	
2976	1	754.4009	45.80	3	66.4	15.0	1	1-22	-.MVCPCSGLLLAVGGMPPRCQGR.T	Oxidation: 1	
1877	2	1057.5274	21.10	3	52.1	10.3	2	453-480	K.CNDCGKVFTQNSHLASHRGIHSGC	Carbamidomethyl: 4	
1830	2	1057.5034	-1.56	3	51.5	15.7	2	453-480	K.CNDCGKVFTQNSHLASHRGIHSGC	Carbamidomethyl: 1	

Detailed Protein Report

Protein 46: PREDICTED: protein AHNAK2 isoform X1 [Homo sapiens]

**Accession:**	gi|530403029	**Score:**	72.4
**Database:**	refseq_human(refseq_human_20140103.fasta)	**MW [kDa]:**	605.3
**Database Date:**	2015-11-30	**pI:**	5.1
**Modification(s):**	Oxidation	**Sequence Coverage [%]:**	1.4
		**No. of unique Peptides:**	5

Quantitation

mdown:qdown	**Median:**	7.41	**CV:**	0.00 %	**No. of Peptides:**	1
Wdown:Qdown	**Median:**	0.50	**CV:**	0.00 %	**No. of Peptides:**	1

Detailed Protein Report

10	20	30	40	50	60	70	80
MSRPEAVQEA	TEVTLKTEVE	AGASGYSVTG	GGDQGIFVKQ	VLKDSSAAKL	FNLREGDQLL	STTVFFENIK	YEDALKILQY
90	100	110	120	130	140	150	160
SEPYKVQFKI	RRQLPAPQDE	EWASSDAQHG	PQGKEKEDTD	VADGCRETPT	KTLEGDGDQE	RLISKPRVGR	GRQSQRERLS
170	180	190	200	210	220	230	240
WPKFQSIKSK	RGPGPQRSHS	SSEAYEPRDA	HDVSPTSTDT	EAQLTVERQE	QKAGPGSQRR	RKFLNLRFRT	GSGQGPSSTG
250	260	270	280	290	300	310	320
QPGRGFQSGV	GRAGVLEELG	PWGDSLEETG	AATGSRREER	AEQDREVMPA	QSMPLPTELG	DPRLCEGTPQ	EGGLRAARLH
330	340	350	360	370	380	390	400
GKTLEGQAQE	TAVAQRKPRA	QPTPGMSREG	EGEGLQSLEI	GIARLSLRDT	TEGGTQIGPP	EIRVRVHDLK	TPKFAFSTEK
410	420	430	440	450	460	470	480
EPERERRLST	PQRGKRQDAS	SKAGTGLKGE	EVEGAGWMPG	REPTTHAEAQ	GDEGDGEEGL	QRTRITEEQD	KGREDTEGQI
490	500	510	520	530	540	550	560
RMPKFKIPSL	GWSPSKHTKT	GREKATEDTE	QGREGEATAT	ADRREQRRTE	EGLKDKEDSD	SMTNTTKIQL	IHDEKRLKKE
570	580	590	600	610	620	630	640
QILTEKEVAT	KDSKFKMPKF	KMPLFGASAP	GKSMEASVDV	SAPKVEADVS	LLSMQGDLKT	TDLSVQTPSA	DLEVQDGQVD
650	660	670	680	690	700	710	720
VKLPEGPLPE	GASLKGHLPK	VQRPSLKMPK	VDLKGPKLDL	KGPKAEVTAP	DVKMSLSSME	VDVQAPRAKL	DGARLEGDLS
730	740	750	760	770	780	790	800
LADKEVTAKD	SKFKMPKFKM	PSFGVSAPGK	SMEDSVDVSA	PKVEADVSLS	SMQGDLKATD	LSIQPPSADL	EVQAGQVDVK
810	820	830	840	850	860	870	880
LPEGPVPEGA	GPKVHLPKVE	MPSFKMPKVD	LKGPQIDVKG	PKLDLKGPKA	EVTAPDGEVS	LPSMEVDVQA	QKAKLDGAWL
890	900	910	920	930	940	950	960
EGDLSLADKD	VTAKDSKFKM	PKFKMPSFGV	SAPGKSIKAL	VDVSAPKVEA	DLSLPSMQGD	LKTTDLSIQP	ASTDLKVQAD
970	980	990	1000	1010	1020	1030	1040
QVDVKLPEGH	LPEGAGLKGH	LPKVEMPSFK	MPKVALKGPQ	VDVKGPKLDL	KSPKAEVTAP	DVEVSLPSVE	VDVEAPGAKL
1050	1060	1070	1080	1090	1100	1110	1120
DSARLEGELS	LADKDVTAKD	SRFKMPKFKM	PSFGASAPGK	SIEASVDVSA	PKVEADVSLP	SMQGDLKTTD	LSIQPPSADL
1130	1140	1150	1160	1170	1180	1190	1200
EVHAGQVDVK	LLEGHVPEGA	GFKGHLPKVQ	MPSLKMPKVD	LKGPQVEVRG	PKLDLKGHKA	EVTAHEVAVS	LPSVEVDMQA
1210	1220	1230	1240	1250	1260	1270	1280
PGAKLDGAQL	DGDLSLADKD	VTAKDSKFKM	PKFKMPSFGV	SAPGKSIEAS	VDLSAPKVEA	DMSLPSMQGD	LKTTDLSIQP
1290	1300	1310	1320	1330	1340	1350	1360
PSTDLELQAG	QLDVKLPEGP	VPEGAGLKGH	LPKLQMPSFK	VPKVDLKGPE	IDIKGPKLDL	KDPKVEVTAP	DVEVSLPSVE
1370	1380	1390	1400	1410	1420	1430	1440
VDVEAPGAKL	DGGRLEEDMS	LADKDLTTKD	SKFKMPKFKM	PSFGVSAPGK	SIEASVDVSA	PKVEADVSLP	SMQGDLKATD
1450	1460	1470	1480	1490	1500	1510	1520
LSIQPPSADL	EVQAGQVDVK	LPEGPVSEGA	GLKGHLPKVQ	MPSFKMPKVD	LKGPQIDVKG	PKLDLKGPKV	EVTAPDVKMS
1530	1540	1550	1560	1570	1580	1590	1600
LSSMEVDVQA	PRAKLDGAQL	EGDLSLADKA	VTAKDSKFKM	PKFKMPSFGV	SAPGKSIEAS	VDVSEPKVEA	DVSLPSMQGD
1610	1620	1630	1640	1650	1660	1670	1680
LKTTDLSIQS	PSADLEVQAG	QVNVKLPEGP	LPEGAGFKGH	LPKVQMPSLK	MPKVALKGPQ	MDVKGPKLDL	KGPKAEVMAP
1690	1700	1710	1720	1730	1740	1750	1760
DVEVSLPSVE	VDVEAPGAKL	DSVRLEGDLS	LADKDVTAKD	SKFKMPKFKM	PSFGVSAPGK	SIEASVDVSA	PKVEAEVSLP
1770	1780	1790	1800	1810	1820	1830	1840
SMQGDLKTTD	LCIPLPSADL	VVQAGQVDMK	LPEGQVPEGA	GLKGHLPKVD	MPSFKMPKVD	LKGPQTDVKG	AKLDLKGPKA
1850	1860	1870	1880	1890	1900	1910	1920
EVTAPDVEVS	LPSMEVDVQA	QKAKLDGARL	EGDLSLADKD	MTAKDSKFKM	PKFKMPSFGV	SAPGRSIEAS	VDVPAPKVEA
1930	1940	1950	1960	1970	1980	1990	2000
DVSLPSMQGD	LKTTDLSIQP	PSADLKVQTG	QVDVKLPEGH	VPEGAGLKGH	LPKVEMPSLK	MPKVDLKGPQ	VDIKGPKLDL
2010	2020	2030	2040	2050	2060	2070	2080
KDPKVEMRVP	DVEVSLPSME	VDVQAPRAKL	DSAHLQGDLT	LANKDLTTKD	SKFKMPKFKM	PSFGVSAPGK	SIEASVDVSP
2090	2100	2110	2120	2130	2140	2150	2160
PKVEADMSLP	SMQGDLKTTD	LSIQPLSADV	KVQAGQVDVK	LLEGPVPEEV	GLKGHLPKLQ	MPSFKVPKVD	LKGPEIDIKG
2170	2180	2190	2200	2210	2220	2230	2240

Detailed Protein Report

Cmpd.	No. of Cmpds.	m/z meas.	Δ m/z [ppm]	z	Rt [min]	Score	P	Range	Sequence	Modification	Ratios
2566	1	913.4598	-15.26	2	62.5	16.5	1	369-385	R.DTTEGGTQIGPPEIRVR.V		
2037	1	596.7447	-195.99	2	55.6	14.7	2	384-393	R.VRVHDLKTPK.F		Wdown:Qdown 0.50<br>mdown:qdown 7.41
597	1	1230.6275	14.93	2	36.8	18.6	1	685-707	K.<br>AEVTAPDVKMSLSSMEVDVQAPR<br>A		
2190	3	648.2929	-93.26	2	56.0	10.4	1	5599-5610	R.FPKLGFSSSPTK.K		
1331	1	1023.0328	-5.50	2	45.4	12.2	1	5662-5679	R.VMVTSAARTELILPEQDR.K	Oxidation: 2	

Detailed Protein Report

Protein 47: centromere protein F [Homo sapiens]

**Accession:**	gi|55770834	**Score:**	71.9
**Database:**	refseq_human(refseq_human_20140103.fasta)	**MW [kDa]:**	357.3
**Database Date:**	2015-11-30	**pI:**	4.9
		**Sequence Coverage [%]:**	1.9
		**No. of unique Peptides:**	3

Quantitation

mdown:qdown	**Median:**	1.50	**CV:**	63.88 %	**No. of Peptides:**	2
Wdown:Qdown	**Median:**	1.57	**CV:**	0.00 %	**No. of Peptides:**	1

Detailed Protein Report

10	20	30	40	50	60	70	80
MSWALEEWKE	GLPTRALQKI	QELEGQLDKL	KKEKQQRQFQ	LDSLEAALQK	QKQKVENEKT	EGTNLKRENQ	RLMEICESLE
90	100	110	120	130	140	150	160
KTKQKISHEL	QVKESQVNFQ	EGQLNSGKKQ	IEKLEQELKR	CKSELERSQQ	AAQSADVSLN	PCNTPQKIFT	TPLTPSQYYS
170	180	190	200	210	220	230	240
GSKYEDLKEK	YNKEVEERKR	LEAEVKALQA	KKASQTLPQA	TMNHRDIARH	QASSSVFSWQ	QEKTPSHLSS	NSQRTPIRRD
250	260	270	280	290	300	310	320
FSASYFSGEQ	EVTPSRSTLQ	IGKRDANSSF	FDNSSSPHLL	DQLKAQNQEL	RNKINELELR	LQGHEKEMKG	QVNKFQELQL
330	340	350	360	370	380	390	400
QLEKAKVELI	EKEKVLNKCR	DELVRTTAQY	DQASTKYTAL	EQKLKKLTED	LSCQRQNAES	ARCSLEQKIK	EKEKEFQEEL
410	420	430	440	450	460	470	480
SRQQRSFQTL	DQECIQMKAR	LTQELQQAKN	MHNVLQAELD	KLTSVKQQLE	NNLEEFKQKL	CRAEQAFQAS	QIKENELRRS
490	500	510	520	530	540	550	560
MEEMKKENNL	LKSHSEQKAR	EVCHLEAELK	NIKQCLNQSQ	NFAEEMKAKN	TSQETMLRDL	QEKINQQENS	LTLEKLKLAV
570	580	590	600	610	620	630	640
ADLEKQRDCS	QDLLKKREHH	IEQLNDKLSK	TEKESKALLS	ALELKKKEYE	ELKEEKTLFS	CWKSENEKLL	TQMESEKENL
650	660	670	680	690	700	710	720
QSKINHLETC	LKTQQIKSHE	YNERVRTLEM	DRENLSVEIR	NLHNVLDSKS	VEVETQKLAY	MELQQKAEFS	DQKHQKEIEN
730	740	750	760	770	780	790	800
MCLKTSQLTG	QVEDLEHKLQ	LLSNEIMDKD	RCYQDLHAEY	ESLRDLLKSK	DASLVTNEDH	QRSLLAFDQQ	PAMHHSFANI
810	820	830	840	850	860	870	880
IGEQGSMPSE	RSECRLEADQ	SPKNSAILQN	RVDSLEFSLE	SQKQMNSDLQ	KQCEELVQIK	GEIEENLMKA	EQMHQSFVAE
890	900	910	920	930	940	950	960
TSQRISKLQE	DTSAHQNVVA	ETLSALENKE	KELQLLNDKV	ETEQAEIQEL	KKSNHLLEDS	LKELQLLSET	LSLEKKEMSS
970	980	990	1000	1010	1020	1030	1040
IISLNKREIE	ELTQENGTLK	EINASLNQEK	MNLIQKSESF	ANYIDEREKS	ISELSDQYKQ	EKLILLQRCE	ETGNAYEDLS
1050	1060	1070	1080	1090	1100	1110	1120
QKYKAAQEKN	SKLECLLNEC	TSLCENRKNE	LEQLKEAFAK	EHQEFLTKLA	FAEERNQNLM	LELETVQQAL	RSEMTDNQNN
1130	1140	1150	1160	1170	1180	1190	1200
SKSEAGGLKQ	EIMTLKEEQN	KMQKEVNDLL	QENEQLMKVM	KTKHECQNLE	SEPIRNSVKE	RESERNQCNF	KPQMDLEVKE
1210	1220	1230	1240	1250	1260	1270	1280
ISLDSYNAQL	VQLEAMLRNK	ELKLQESEKE	KECLQHELQT	IRGDLETSNL	QDMQSQEISG	LKDCEIDAEE	KYISGPHELS
1290	1300	1310	1320	1330	1340	1350	1360
TSQNDNAHLQ	CSLQTTMNKL	NELEKICEIL	QAEKYELVTE	LNDSRSECIT	ATRKMAEEVG	KLLNEVKILN	DDSGLLHGEL
1370	1380	1390	1400	1410	1420	1430	1440
VEDIPGGEFG	EQPNEQHPVS	LAPLDESNSY	EHLTLSDKEV	QMHFAELQEK	FLSLQSEHKI	LHDQHCQMSS	KMSELQTYVD
1450	1460	1470	1480	1490	1500	1510	1520
SLKAENLVLS	TNLRNFQGDL	VKEMQLGLEE	GLVPSLSSSC	VPDSSSLSSL	GDSSFYRALL	EQTGDMSLLS	NLEGAVSANQ
1530	1540	1550	1560	1570	1580	1590	1600
CSVDEVFCSS	LQEENLTRKE	TPSAPAKGVE	ELESLCEVYR	QSLEKLEEKM	ESQGIMKNKE	IQELEQLLSS	ERQELDCLRK
1610	1620	1630	1640	1650	1660	1670	1680
QYLSENEQWQ	QKLTSVTLEM	ESKLAAEKKQ	TEQLSLELEV	ARLQLQGLDL	SSRSLLGIDT	EDAIQGRNES	CDISKEHTSE
1690	1700	1710	1720	1730	1740	1750	1760
TTERTPKHDV	HQICDKDAQQ	DLNLDIEKIT	ETGAVKPTGE	CSGEQSPDTN	YEPPGEDKTQ	GSSECISELS	FSGPNALVPM
1770	1780	1790	1800	1810	1820	1830	1840
DFLGNQEDIH	NLQLRVKETS	NENLRLLHVI	EDRDRKVESL	LNEMKELDSK	LHLQEVQLMT	KIEACIELEK	IVGELKKENS
1850	1860	1870	1880	1890	1900	1910	1920
DLSEKLEYFS	CDHQELLQRV	ETSEGLNSDL	EMHADKSSRE	DIGDNVAKVN	DSWKERFLDV	ENELSRIRSE	KASIEHEALY
1930	1940	1950	1960	1970	1980	1990	2000
LEADLEVVQT	EKLCLEKDNE	NKQKVIVCLE	EELSVVTSER	NQLRGELDTM	SKKTTALDQL	SEKMKEKTQE	LESHQSECLH
2010	2020	2030	2040	2050	2060	2070	2080
CIQVAEAEVK	EKTELLQTLS	SDVSELLKDK	THLQEKLQSL	EKDSQALSLT	KCELENQIAQ	LNKEKELLVK	ESESLQARLS
2090	2100	2110	2120	2130	2140	2150	2160
ESDYEKLNVS	KALEAALVEK	GEFALRLSST	QEEVHQLRRG	IEKLRVRIEA	DEKKQLHIAE	KLKEREREND	SLKDKVENLE
2170	2180	2190	2200	2210	2220	2230	2240

Detailed Protein Report

Cmpd.	No. of Cmpds.	m/z meas.	Δ m/z [ppm]	z	Rt [min]	Score	P	Range	Sequence	Modification	Ratios
104	2	1043.8924	-98.05	2	30.5	15.3	0	128-147	R.SQQAAQSADVSLNPCNTPQK.I		Wdown:Qdown 1.57<br>mdown:qdown 0.83
1209	1	573.1437	-261.24	2	43.6	14.6	0	981-990	K.EINASLNQEK.M		mdown:qdown 2.69
2721	2	974.5198	32.44	2	63.0	14.2	1	2466-2482	K.AKEQNLSSQVECLELEK.A		

Detailed Protein Report

Protein 48: neurogenic locus notch homolog protein 1 preproprotein [Homo sapiens]

**Accession:**	gi|148833508	**Score:**	70.7
**Database:**	refseq_human(refseq_human_20140103.fasta)	**MW [kDa]:**	272.3
**Database Date:**	2015-11-30	**pI:**	4.8
**Modification(s):**	Carbamidomethyl, Oxidation	**Sequence Coverage [%]:**	2.2
		**No. of unique Peptides:**	5

Detailed Protein Report

10	20	30	40	50	60	70	80
MPPLLAPLLC	LALLPALAAR	GPRCSQPGET	CLNGGKCEAA	NGTEACVCGG	AFVGPRCQDP	NPCLSTPCKN	AGTCHVVDRR
90	100	110	120	130	140	150	160
GVADYACSCA	LGFSGPLCLT	PLDNACLTNP	CRNGGTCDLL	TLTEYKCRCP	PGWSGKSCQQ	ADPCASNPCA	NGGQCLPFEA
170	180	190	200	210	220	230	240
SYICHCPPSF	HGPTCRQDVN	ECGQKPGLCR	HGGTCHNEVG	SYRCVCRATH	TGPNCERPYV	PCSPSPCQNG	GTCRPTGDVT
250	260	270	280	290	300	310	320
HECACLPGFT	GQNCEENIDD	CPGNNCKNGG	ACVDGVNTYN	CRCPPEWTGQ	YCTEDVDECQ	LMPNACQNGG	TCHNTHGGYN
330	340	350	360	370	380	390	400
CVCVNGWTGE	DCSENIDDCA	SAACFHGATC	HDRVASFYCE	CPHGRTGLLC	HLNDACISNP	CNEGSNCDTN	PVNGKAICTC
410	420	430	440	450	460	470	480
PSGYTGPACS	QDVDECSLGA	NPCEHAGKCI	NTLGSFECQC	LQGYTGPRCE	IDVNECVSNP	CQNDATCLDQ	IGEFQCICMP
490	500	510	520	530	540	550	560
GYEGVHCEVN	TDECASSPCL	HNGRCLDKIN	EFQCECPTGF	TGHLCQYDVD	ECASTPCKNG	AKCLDGPNTY	TCVCTEGYTG
570	580	590	600	610	620	630	640
THCEVDIDEC	DPDPCHYGSC	KDGVATFTCL	CRPGYTGHHC	ETNINECSSQ	PCRHGGTCQD	RDNAYLCFCL	KGTTGPNCEI
650	660	670	680	690	700	710	720
NLDDCASSPC	DSGTCLDKID	GYECACEPGY	TGSMCNINID	ECAGNPCHNG	GTCEDGINGF	TCRCPEGYHD	PTCLSEVNEC
730	740	750	760	770	780	790	800
NSNPCVHGAC	RDSLNGYKCD	CDPGWSGTNC	DINNNECESN	PCVNGGTCKD	MTSGYVCTCR	EGFSGPNCQT	NINECASNPC
810	820	830	840	850	860	870	880
LNQGTCIDDV	AGYKCNCLLP	YTGATCEVVL	APCAPSPCRN	GGECRQSEDY	ESFSCVCPTG	WQGQTCEVDI	NECVLSPCRH
890	900	910	920	930	940	950	960
GASCQNTHGG	YRCHCQAGYS	GRNCETDIDD	CRPNPCHNGG	SCTDGINTAF	CDCLPGFRGT	FCEEDINECA	SDPCRNGANC
970	980	990	1000	1010	1020	1030	1040
TDCVDSYTCT	CPAGFSGIHC	ENNTPDCTES	SCFNGGTCVD	GINSFTCLCP	PGFTGSYCQH	DVNECDSQPC	LHGGTCQDGC
1050	1060	1070	1080	1090	1100	1110	1120
GSYRCTCPQG	YTGPNCQNLV	HWCDSSPCKN	GGKCWQTHTQ	YRCECPSGWT	GLYCDVPSVS	CEVAAQRQGV	DVARLCQHGG
1130	1140	1150	1160	1170	1180	1190	1200
LCVDAGNTHH	CRCQAGYTGS	YCEDLVDECS	PSPCQNGATC	TDYLGGYSCK	CVAGYHGVNC	SEEIDECLSH	PCQNGGTCLD
1210	1220	1230	1240	1250	1260	1270	1280
LPNTYKCSCP	RGTQGVHCEI	NVDDCNPPVD	PVSRSPKCFN	NGTCVDQVGG	YSCTCPPGFV	GERCEGDVNE	CLSNPCDARG
1290	1300	1310	1320	1330	1340	1350	1360
TQNCVQRVND	FHCECRAGHT	GRRCESVING	CKGKPCKNGG	TCAVASNTAR	GFICKCPAGF	EGATCENDAR	TCGSLRCLNG
1370	1380	1390	1400	1410	1420	1430	1440
GTCISGPRSP	TCLCLGPFTG	PECQFPASSP	CLGGNPCYNQ	GTCEPTSESP	FYRCLCPAKF	NGLLCHILDY	SFGGGAGRDI
1450	1460	1470	1480	1490	1500	1510	1520
PPPLIEEACE	LPECQEDAGN	KVCSLQCNNH	ACGWDGGDCS	LNFNDPWKNC	TQSLQCWKYF	SDGHCDSQCN	SAGCLFDGFD
1530	1540	1550	1560	1570	1580	1590	1600
CQRAEGQCNP	LYDQYCKDHF	SDGHCDQGCN	SAECEWDGLD	CAEHVPERLA	AGTLVVVVLM	PPEQLRNSSF	HFLRELSRVL
1610	1620	1630	1640	1650	1660	1670	1680
HTNVVFKRDA	HGQQMIFPYY	GREEELRKHP	IKRAAEGWAA	PDALLGQVKA	SLLPGGSEGG	RRRRELDPMD	VRGSIVYLEI
1690	1700	1710	1720	1730	1740	1750	1760
DNRQCVQASS	QCFQSATDVA	AFLGALASLG	SLNIPYKIEA	VQSETVEPPP	PAQLHFMYVA	AAAFVLLFFV	GCGVLLSRKR
1770	1780	1790	1800	1810	1820	1830	1840
RRQHGQLWFP	EGFKVSEASK	KKRREPLGED	SVGLKPLKNA	SDGALMDDNQ	NEWGDEDLET	KKFRFEEPVV	LPDLDDQTDH
1850	1860	1870	1880	1890	1900	1910	1920
RQWTQQHLDA	ADLRMSAMAP	TPPQGEVDAD	CMDVNVRGPD	GFTPLMIASC	SGGGLETGNS	EEEEDAPAVI	SDFIYQGASL
1930	1940	1950	1960	1970	1980	1990	2000
HNQTDRTGET	ALHLAARYSR	SDAAKRLLEA	SADANIQDNM	GRTPLHAAVS	ADAQGVFQIL	IRNRATDLDA	RMHDGTTPLI
2010	2020	2030	2040	2050	2060	2070	2080
LAARLAVEGM	LEDLINSHAD	VNAVDDLGKS	ALHWAAAVNN	VDAAVVLLKN	GANKDMQNNR	EETPLFLAAR	EGSYETAKVL
2090	2100	2110	2120	2130	2140	2150	2160
LDHFANRDIT	DHMDRLPRDI	AQERMHHDIV	RLLDEYNLVR	SPQLHGAPLG	GTPTLSPPLC	SPNGYLGSLK	PGVQGKKVRK
[illegible]	[illegible]	[illegible]	[illegible]	[illegible]	[illegible]	[illegible]	[illegible]

Detailed Protein Report

Cmpd.	No. of Cmpds.	m/z meas.	Δ m/z [ppm]	z	Rt [min]	Score	P	Range	Sequence	Modification
1878	2	716.0566	111.85	3	52.1	10.3	1	1331-1350	R.GFICKCPAGFEGATCENDAR.T	Carbamidomethyl: 6
1825	2	715.9982	30.20	3	51.5	11.6	1	1331-1350	R.GFICKCPAGFEGATCENDAR.T	Carbamidomethyl: 4
1618	4	697.0380	98.52	3	48.9	15.1	1	1331-1350	R.GFICKCPAGFEGATCENDAR.T	
2040	1	841.7905	116.85	3	55.6	14.8	0	1855-1877	R.MSAMAPTPPQGEVDADCMDVNVR.G	Carbamidomethyl: 17; Oxidation: 1, 4
2914	9	698.3691	-7.48	2	65.6	18.9	0	1992-2004	R.MHDGTTPLILAAR.L	

Detailed Protein Report

Protein 49: dynein heavy chain 17, axonemal [Homo sapiens]

**Accession:**	gi|256542310	**Score:**	70.2
**Database:**	refseq_human(refseq_human_20140103.fasta)	**MW [kDa]:**	509.0
**Database Date:**	2015-11-30	**pI:**	5.4
		**Sequence Coverage [%]:**	1.6
		**No. of unique Peptides:**	3

Detailed Protein Report

10	20	30	40	50	60	70	80
MTMAPDVRLE	YLEEVASIVL	KFKPDKWSKL	IGAEENVALF	TEFFEKPDVQ	VLVLTLNAAG	MIIPCLGFPQ	SLKSKGVYFI
90	100	110	120	130	140	150	160
KTKSENINKD	NYRARLLYGD	ISPTPVDQLI	AVVEEVLSSL	LNQSENMAGW	PQVVSEDIVK	QVHRLKNEMF	VMSGKIKGKT
170	180	190	200	210	220	230	240
LLPIPEHLGS	LDGTLESMER	IPSSLDNLLL	HAIETTIIDW	SHQIRDVLSK	DSAQALLDGL	HPLPQVEFEF	WDTRLLNLKC
250	260	270	280	290	300	310	320
IHEQLNRPKV	NKIVEILEKA	KSCYWPALQN	VYTNVTEGLK	EANDIVLYLK	PLRILLEEME	QADFTMLPTF	IAKVLDTICF
330	340	350	360	370	380	390	400
IWATSEYYNT	PARIIVILQE	FCNQIIEMTR	TFLSPEEVLK	GLQGEIEEVL	SGISLAVNVL	KELYQTYDFC	CVNMKLFFKD
410	420	430	440	450	460	470	480
KEPVPWEFPS	SLAFSRINSF	FQRIQTIEEL	YKTAIEFLKL	EKIELGGVRG	NLLGSLVTRI	YDEVFELVKV	FADCKYDPLD
490	500	510	520	530	540	550	560
PGDSNFDRDY	ADFEIKIQDL	DRRLATIFCQ	GFDDCSCIKS	SAKLLYMCGG	LMERPLILAE	VAPRYSVMLE	LFDAELDNAK
570	580	590	600	610	620	630	640
ILYDAQMAAS	EEGNIPLIHK	NMPPVAGQLK	WSLELQERLE	VSMKHLKHVE	HPVMSGAEAK	LTYQKYDEMM	ELLRCHREKI
650	660	670	680	690	700	710	720
YQQWVAGVDQ	DCHFNLGQPL	ILRDAASNLI	HVNFSKALVA	VLREVKYLNF	QQQKEIPDSA	ESLFSENETF	RKFVGNLELI
730	740	750	760	770	780	790	800
VGWYNEIKTI	VKAVEFLLIK	SELEAIDVKL	LSAETTLFWN	GEGVFQYIQE	VREILHNLQN	RMQKAKQNIE	GISQAMKDWS
810	820	830	840	850	860	870	880
ANPLFERKDN	KKEALLDLDG	RIANLNKRYA	AVRDAGVKIQ	AMVAENAELF	RADTLSLPWK	DYVIYIDDMV	LDEFDQFIRK
890	900	910	920	930	940	950	960
SLSFLMDNMV	IDESIAPLFE	IRMELDEDGL	TFNPTLEVGS	DRGFLALIEG	LVNDIYNVAR	LIPRLAKDRM	NYKMDLEDNT
970	980	990	1000	1010	1020	1030	1040
DLIEMREEVS	SLVINAMKEA	EEYQDSFERY	SYLWTDNLQE	FMKNFLIYGC	AVTAEDLDTW	TDDTIPKTPP	TLAQFQEQID
1050	1060	1070	1080	1090	1100	1110	1120
SYEKLYEEVS	KCENTKVFHG	WLQCDCRPFK	QALLSTIRRW	GFMFKRHLSN	HVTNSLADLE	AFMKVARMGL	TKPLKEGDYD
1130	1140	1150	1160	1170	1180	1190	1200
GLVEVMGHLM	KVKERQAATD	NMFEPLKQTI	ELLKTYGEEM	PEEIHLKLQE	LPEHWANTKK	LAIQVKLTVA	PLQANEVSIL
1210	1220	1230	1240	1250	1260	1270	1280
RRKCQQFELK	QHEFRERFRR	EAPFSFSDPN	PYKSLNKQQK	SISAMEGIME	ALSKSGGLFE	VPVPDYKQLK	ACHREVRLLK
1290	1300	1310	1320	1330	1340	1350	1360
ELWDMVVVVN	TSIEDWKTTK	WKDINVEQMD	IDCKKFAKDM	RSLDKEMKTW	DAFVGLDNTV	KNVITSLRAV	SELQNPAIRE
1370	1380	1390	1400	1410	1420	1430	1440
RHWQQLMQAT	QVKFKMSEET	TLADLLQLNL	HSYEDEVRNI	VDKAVKESGM	EKVLKALDST	WSMMEFQHEP	HPRTGTMMLK
1450	1460	1470	1480	1490	1500	1510	1520
SSEVLVETLE	DNQVQLQNLM	MSKYLAHFLK	EVTSWQQKLS	TADSVISIWF	EVQRTWSHLE	SIFIGSEDIR	TQLPGDSQRF
1530	1540	1550	1560	1570	1580	1590	1600
DDINQEFKAL	MEDAVKTPNV	VEATSKPGLY	NKLEALKKSL	AICEKALAEY	LETKRLAFPR	FYFVSSADLL	DILSNGNDPV
1610	1620	1630	1640	1650	1660	1670	1680
EVSRHLSKLF	DSLCKLKFRL	DASDKPLKVG	LGMYSKEDEY	MVFDQECDLS	GQVEVWLNRV	LDRMCSTLRH	EIPEAVVTYE
1690	1700	1710	1720	1730	1740	1750	1760
EKPREQWILD	YPAQVALTCT	QIWWTTEVGL	AFARLEEGYE	NAIRDYNKKQ	ISQLNVLITL	LMGNLNAGDR	MKIMTICTID
1770	1780	1790	1800	1810	1820	1830	1840
VHARDVVAKM	IVAKVESSQA	FTWQAQLRHR	WDEEKRHCFA	NICDAQIQYS	YEYLGNTPRL	VITPLTDRCY	ITLTQSLHLI
1850	1860	1870	1880	1890	1900	1910	1920
MGGAPAGPAG	TGKTETTKDL	GRALGTMVYV	FNCSEQMDYK	SCGNIYKGLA	QTGAWGCFDE	FNRISVEVLS	VIAVQVKCVQ
1930	1940	1950	1960	1970	1980	1990	2000
DAIRAKKKAF	NFLGEIIGLI	PTVGIFITMN	PGYAGRAELP	ENLKALFRPC	AMVVPDFELI	CEIMLMAEGF	LEARLLARKF
2010	2020	2030	2040	2050	2060	2070	2080
ITLYTLCKEL	LSKQDHYDWG	LRAIKSVLVV	AGSLKRGDPS	RAEDQVLMRA	LRDFNIPKIV	TDDLPVFMGL	IGDLFPALDV
2090	2100	2110	2120	2130	2140	2150	2160
PRKRDLNFEK	IIKQSIVELK	LQAEDSFVLK	VVQLEELLQV	RHSVFIVGNA	GSGKSQVLKS	LNKTYQNLKR	KPVAVDLDPK
2170	2180	2190	2200	2210	2220	2230	2240

Detailed Protein Report

Cmpd.	No. of Cmpds.	m/z meas.	Δ m/z [ppm]	z	Rt [min]	Score	P	Range	Sequence	Modification
1870	1	704.2582	-146.54	2	53.4	13.7	0	1255-1267	K.SGGLFEVPVPDYK.Q	
2850	1	869.4224	-91.80	2	64.2	14.1	0	3728-3742	K.LIFLAQVTFQVLSMK.K	
637	1	635.8011	-148.78	2	36.5	10.8	2	4247-4257	R.RSLKELNLGLK.G	

Detailed Protein Report

Protein 50: zinc finger protein 492 [Homo sapiens]

Accession: gi|148806865
Database: refseq_human(refseq_human_20140103.fasta)
Database Date: 2015-11-30
Modification(s): Carbamidomethyl, Oxidation

Score: 69.5
MW [kDa]: 61.1
pI: 10.3
Sequence Coverage [%]: 11.3
No. of unique Peptides: 3

Alias proteins:

Accession	Name	Description
gi|578833408	refseq_human (refseq_human_20140103.fasta)	PREDICTED: zinc finger protein 492 isoform X1 [Homo sapiens]

10	20	30	40	50	60	70	80
MLENYRNLVF	VGIAASKPDL	ITCLEQGKEP	WNVKRHEMVA	EPPVVCSYFA	RDLWPKQGKK	NYFQKVILRR	YKKCGCENLQ
**90**	**100**	**110**	**120**	**130**	**140**	**150**	**160**
LRKYCKSMDE	CKVHKECYNG	LNQCLTTTQN	KIFQCDKYVK	VFHKFSNSNR	HTIRHTGKKS	FKCKECEKSF	CMLSHLAQHK
**170**	**180**	**190**	**200**	**210**	**220**	**230**	**240**
RIHSGEKPYK	CKECGKAYNE	TSNLSTHKRI	HTGKKPYKCE	ECGKAFNRLS	HLTTHKIIHT	GKKPYKCEEC	GKAFNQSANL
**250**	**260**	**270**	**280**	**290**	**300**	**310**	**320**
TTHKRIHTGE	KPYKCEECGR	AFSQSSTLTA	HKIIHAGEKP	YKCEECGKAF	SQSSTLTTHK	IIHTGEKFYK	CEECGKAFSQ
**330**	**340**	**350**	**360**	**370**	**380**	**390**	**400**
LSHLTTHKRI	HSGEKPYKCE	ECGKAFKQSS	TLTTHKRIHA	GEKFYKCEVC	SKAFSRFSHL	TTHKRIHTGE	KPYKCEECGK
**410**	**420**	**430**	**440**	**450**	**460**	**470**	**480**
AFNLSSQLTT	HKIIHTGEKP	YKCEECGKAF	NQSSTLSKHK	VIHTGEKPYK	YEECGKAFNQ	SSHLTTHKMI	HTGEKPYKCE
**490**	**500**	**510**	**520**	**530**	**540**		
ECGKAFNNSS	ILNRHKMIHT	GEKLYKPESC	NNACDNIAKI	SKYKRNCAGE	K		

Cmpd.	No. of Cmpds.	m/z meas.	Δ m/z [ppm]	z	Rt [min]	Score	P	Range	Sequence	Modification
1839	1	582.2374	12.53	2	53.0	13.4	1	84-92	K.YCKSMDECK.V	Carbamidomethyl: 8
1912	1	621.2738	-20.17	2	54.0	13.3	1	195-204	K.KPYKCEECGK.A	Carbamidomethyl: 5
2688	1	992.0980	-39.40	3	64.8	18.1	2	495-519	R.HKMIHTGEKLYKPESCNNACDNIAK.I	Carbamidomethyl: 16, 20; Oxidation: 3

Detailed Protein Report

Protein 51: PREDICTED: neuroblast differentiation-associated protein AHNAK isoform X6 [Homo sapiens]

**Accession:**	gi|530397289	**Score:**	68.9
**Database:**	refseq_human(refseq_human_20140103.fasta)	**MW [kDa]:**	580.3
**Database Date:**	2015-11-30	**pI:**	5.7
**Modification(s):**	Oxidation	**Sequence Coverage [%]:**	1.2
		**No. of unique Peptides:**	4

Quantitation

mdown:qdown	**Median:**	0.54	**CV:**	0.00 %	**No. of Peptides:**	1
Wdown:Qdown	**Median:**	1.31	**CV:**	0.00 %	**No. of Peptides:**	1

Detailed Protein Report

10	20	30	40	50	60	70	80
MEKEETTREL	LLPNWQGSGS	HGLTIAQRDD	GVFVQEVTQN	SPAARTGVVK	EGDQIVGATI	YFDNLQSGEV	TQLLNTMGHH
90	100	110	120	130	140	150	160
TVGLKLHRKG	DRSPEPGQTW	TREVFSSCSS	EVVLSGDDEE	YQRIYTTKIK	PRLKSEDGVE	GDLGETQSRT	ITVTRRVTAY
170	180	190	200	210	220	230	240
TVDVTGREGA	KDIDISSPEF	KIKIPRHELT	EISNVDVETQ	SGKTVIRLPS	GSGAASPTGS	AVDIRAGAIS	ASGPELQGAG
250	260	270	280	290	300	310	320
HSKLQVTMPG	IKVGGSGVNV	NAKGLDLGGR	GGVQVPAVDI	SSSLGGRAVE	VQGPSLESGD	HGKIKFPTMK	VPKFGVSTGR
330	340	350	360	370	380	390	400
EGQTPKAGLR	VSAPEVSVGH	KGGKPGLTIQ	APQLEVSVPS	ANIEGLEGKL	KGPQITGPSL	EGDLGLKGAK	PQGHIGVDAS
410	420	430	440	450	460	470	480
APQIGGSITG	PSVEVQAPDI	DVQGPGSKLN	VPKMKVPKFS	VSGAKGEETG	IDVTLPTGEV	TVPGVSGDVS	LPEIATGGLE
490	500	510	520	530	540	550	560
GKMKGTKVKT	PEMIIQKPKI	SMQDVDLSLG	SPKLKGDIKV	SAPGVQGDVK	GPQVALKGSR	VDIETPNLEG	TLTGPRLGSP
570	580	590	600	610	620	630	640
SGKTGTCRIS	MSEVDLNVAA	PKVKGGVDVT	LPRVEGKVKV	PEVDVRGPKV	DVSAPDVEAH	GPEWNLKMPK	MKMPTFSTPG
650	660	670	680	690	700	710	720
AKGEGPDVHM	TLPKGDISIS	GPKVNVEAPD	VNLEGLGGKL	KGPDVKLPDM	SVKTPKISMP	DVDLHVKGTK	VKGEYDVTVP
730	740	750	760	770	780	790	800
KLEGELKGPK	VDIDAPDVDV	HGPDWHLKMP	KMKMPKFSVP	GFKAEGPEVD	VNLPKADVDI	SGPKIDVTAP	DVSIEEPEGK
810	820	830	840	850	860	870	880
LKGPKFKMPE	MNIKVPKISM	PDVDLHLKGP	NVKGEYDVTM	PKVESEIKVP	DVELKSAKMD	IDVPDVEVQG	PDWHLKMPKM
890	900	910	920	930	940	950	960
KMPKFSMPGF	KAEGPEVDVN	LPKADVDISG	PKVGVEVPDV	NIEGPEGKLK	GPKFKMPEMN	IKAPKISMPD	VDLHMKGPKV
970	980	990	1000	1010	1020	1030	1040
KGEYDMTVPK	LEGDLKGPKV	DVSAPDVEMQ	GPDWNLKMPK	IKMPKFSMPS	LKGEGPEFDV	NLSKANVDIS	APKVDTNAPD
1050	1060	1070	1080	1090	1100	1110	1120
LSLEGPEGKL	KGPKFKMPEM	HFRAPKMSLP	DVDLDLKGPK	MKGNVDISAP	KIEGEMQVPD	VDIRGPKVDI	KAPDVEGQGL
1130	1140	1150	1160	1170	1180	1190	1200
DWSLKIPKMK	MPKFSMPSLK	GEGPEVDVNL	PKADVVVSGP	KVDIEAPDVS	LEGPEGKLKG	PKFKMPEMHF	KTPKISMPDV
1210	1220	1230	1240	1250	1260	1270	1280
DLHLKGPKVK	GDVDVSVPKV	EGEMKVPDVE	IKGPKMDIDA	PDVEVQGPDW	HLKMPKMKMP	KFSMPGFKGE	GREVDVNLPK
1290	1300	1310	1320	1330	1340	1350	1360
ADIDVSGPKV	DVEVPDVSLE	GPEGKLKGPK	FKMPEMHFKA	PKISMPDVDL	NLKGPKLKGD	VDVSLPEVEG	EMKVPDVDIK
1370	1380	1390	1400	1410	1420	1430	1440
GPKVDISAPD	VDVHGPDWHL	KMPKVKMPKF	SMPGFKGEGP	EVDVKLPKAD	VDVSGPKMDA	EVPDVNIEGP	DAKLKGPKFK
1450	1460	1470	1480	1490	1500	1510	1520
MPEMSIKPQK	ISIPDVGLHL	KGPKMKGDYD	VTVPKVEGEI	KAPDVDIKGP	KVDINAPDVE	VHGPDWHLKM	PKVKMPKFSM
1530	1540	1550	1560	1570	1580	1590	1600
PGFKGEGPEV	DMNLPKADLG	VSGPKVDIDV	PDVNLEAPEG	KLKGPKFKMP	SMNIQTHKIS	MPDVGLNLKA	PKLKTDVDVS
1610	1620	1630	1640	1650	1660	1670	1680
LPKVEGDLKG	PEIDVKAPKM	DVNVGDIDIE	GPEGKLKGPK	FKMPEMHFKA	PKISMPDVDL	HLKGPKVKGD	MDVSVPKVEG
1690	1700	1710	1720	1730	1740	1750	1760
EMKVPDVDIK	GPKVDIDAPD	VEVHDPDWHL	KMPKMKMPKF	SMPGFKAEGP	EVDVNLPKAD	IDVSGPSVDT	DAPDLDIEGP
1770	1780	1790	1800	1810	1820	1830	1840
EGKLKGSKFK	MPKLNIKAPK	VSMPDVDLNL	KGPKLKGEID	ASVPELEGDL	RGPQVDVKGP	FVEAEVPDVD	LECPDAKLKG
1850	1860	1870	1880	1890	1900	1910	1920
PKFKMPEMHF	KAPKISMPDV	DLHLKGPKVK	GDADVSVPKL	EGDLTGPSVG	VEVPDVELEC	PDAKLKGPKF	KMPDMHFKAP
1930	1940	1950	1960	1970	1980	1990	2000
KISMPDVDLH	LKGPKVKGDV	DVSVPKLEGD	LTGPSVGVEV	PDVELECPDA	KLKGPKFKMP	EMHFKTPKIS	MPDVDLHLKG
2010	2020	2030	2040	2050	2060	2070	2080
PKVKGDMDVS	VPKVEGEMKV	PDVDIKGPKM	DIDAPDVDVH	GPDWHLKMPK	MKMPKFSMPG	FKAEGPEVDV	NLPKADVVVS
2090	2100	2110	2120	2130	2140	2150	2160
GPKVDVEVPD	VSLEGPEGKL	KGPKLKMPEM	HFKAPKISMP	DVDLHLKGPK	VKGDVDVSLP	KLEGDLTGPS	VDVEVPDVEL
2170	2180	2190	2200	2210	2220	2230	2240

Detailed Protein Report

Cmpd.	No. of Cmpds.	m/z meas.	Δ m/z [ppm]	z	Rt [min]	Score	P	Range	Sequence	Modification	Ratios
2716	1	849.3583	-170.01	2	65.2	15.5	2	485-499	K.GTKVKTPEMIIQKPK.I		
863	1	578.2806	-119.29	2	40.2	10.1	2	1771-1780	K.MPKLNIKAPK.V	Oxidation: 1	
1213	1	764.7682	-167.48	2	44.8	12.8	1	2851-2865	K.GDVDVTGPKVEGDLK.G		
1557	1	472.2329	-53.94	2	49.3	18.8	0	2979-2987	K.GDVDISLPK.V		Wdown:Qdown 1.31<br>mdown:qdown 0.54

Detailed Protein Report

Protein 52: PREDICTED: rho GTPase-activating protein 26 isoform X13 [Homo sapiens]

**Accession:**	gi|578810840	**Score:**	68.5
**Database:**	refseq_human(refseq_human_20140103.fasta)	**MW [kDa]:**	71.6
**Database Date:**	2015-11-30	**pI:**	9.8
		**Sequence Coverage [%]:**	8.4
		**No. of unique Peptides:**	2

10	20	30	40	50	60	70	80
MKRETRRSAF	CVQGWSGGDP	ARVVLWGSAG	GRGRARHAGV	PAVGPECRAR	RSQRADPIVS	TAPAPLGAEL	LSLECPTVRM
**90**	**100**	**110**	**120**	**130**	**140**	**150**	**160**
ERALPRGRCL	PLGKDLSSAK	RKFADSLNEF	KFQCIGDAET	DDEMCIARSL	QEFATVLRNL	EDERIRMIEN	ASEVLITPLE
**170**	**180**	**190**	**200**	**210**	**220**	**230**	**240**
KFRKEQIGAA	KEAKKKYDKE	TEKYCGILEK	HLNLSSKKKE	SQLQEADSQV	DLVRQHFYEV	SLEYVFKVQE	VQERKMFEFV
**250**	**260**	**270**	**280**	**290**	**300**	**310**	**320**
EPLLAFLQGL	FTFYHHGYEL	AKDFGDFKTQ	LTISIQNTRN	RFEGTRSEVE	SLMKKMKENP	LEHKTISPYT	MEGYLYVQEK
**330**	**340**	**350**	**360**	**370**	**380**	**390**	**400**
RHFGTSWVKH	YCTYQRDSKQ	ITMVPFDQKS	GGKGGEDESV	ILKSCTRRKT	DSIEKRFCFD	VEAVDRPGVI	TMQALSEEDR
**410**	**420**	**430**	**440**	**450**	**460**	**470**	**480**
RLWMEAMDGR	EPVYNSNKDS	QSEGTAQLDS	IGFSIIRKCI	HAVETRGINE	QGLYRIVGVN	SRVQKLLSVL	MDPKTASETE
**490**	**500**	**510**	**520**	**530**	**540**	**550**	**560**
TDICAEWEIK	TITSALKTYL	RMLPGPLMMY	QFQRSFIKAA	KLENQESRVS	EIHSLVHRLP	EKNRQMLQLL	MNHLANSAFV
**570**	**580**	**590**	**600**	**610**	**620**	**630**	
SLSHYCIRYA	GGLGKKCVKC	SLQWRTAANA	RWPSQRMLGK	CSCNQATKQS	FQGDFTYGDQ	GQIGVCCP	

Cmpd.	No. of Cmpds.	m/z meas.	Δ m/z [ppm]	z	Rt [min]	Score	P	Range	Sequence	Modification
1954	9	710.2864	-113.94	2	54.5	21.4	1	35-48	R.ARHAGVPAVGPECR.A	
286	1	716.6484	355.53	1	32.9	20.0	0	165-171	K.EQIGAAK.E	

Detailed Protein Report

Protein 53: zymogen granule protein 16 homolog B precursor [Homo sapiens]

Accession: gi|94536866
Database: refseq_human(refseq_human_20140103.fasta)
Database Date: 2015-11-30

Score: 68.1
MW [kDa]: 22.7
pI: 7.6
Sequence Coverage [%]: 14.4
No. of unique Peptides: 2

Quantitation

mdown:qdown	**Median:**	2.33	**CV:**	0.00 %	**No. of Peptides:**	1
Wdown:Qdown	**Median:**	2.74	**CV:**	0.00 %	**No. of Peptides:**	1

```
        10         20         30         40         50         60         70         80
MGAQGAQESI KAMWRVPGTT RRPVTGESPG MHRPEAMLLL LTLALLGGPT WAGKMYGPGG GKYFSTTEDY DHEITGLRVS
        90        100        110        120        130        140        150        160
VGLLLVKSVQ VKLGDSWDVK LGALGGNTQE VTLQPGEYIT KVFVAFQAFL RGMVMYTSKD RYFYFGKLDG QISSAYPSQE
       170        180        190        200        210
GQVLVGIYGQ YQLLGIKSIG FEWNYPLEEP TTEPPVNLTY SANSPVGR
```

Cmpd.	No. of Cmpds.	m/z meas.	Δ m/z [ppm]	z	Rt [min]	Score	P	Range	Sequence	Modification	Ratios
2510	2	464.1779	-296.31	2	60.1	35.2	0	79-87	R.VSVGLLLVK.S		
1743	6	1095.0646	-10.46	2	50.3	32.9	0	101-121	K.LGALGGNTQEVTLQPGEYITK.V		Wdown:Qdown 2.74<br>mdown:qdown 2.33

Detailed Protein Report

Protein 54: **PREDICTED: keratin, type I cytoskeletal 24 isoform X1 [Homo sapiens]**

**Accession:**	gi|578830527	**Score:**	67.2
**Database:**	refseq_human(refseq_human_20140103.fasta)	**MW [kDa]:**	46.1
**Database Date:**	2015-11-30	**pI:**	4.7
**Modification(s):**	Oxidation	**Sequence Coverage [%]:**	4.9
		**No. of unique Peptides:**	1

Quantitation

Wdown:Qdown **Median:** 0.97 **CV:** 0.00 % **No. of Peptides:** 1

10	20	30	40	50	60	70	80
MSCSSRASSS	RAGGSSSARV	SAGGSSFSSG	SRCGLGGSSA	QGFRGGASSC	SLSGGSSDKV	RALEEANTDL	ENKIKEWYDK
90	100	110	120	130	140	150	160
YGPGSGDGGS	GRDYSKYYSI	IEDLRNQIIA	ATVENAGIIL	HIDNARLAAD	DFRLKYENEL	CLRQSVEADI	NGLRKVLDDL
170	180	190	200	210	220	230	240
TMTRSDLEMQ	IESFTEELAY	LRKNHEEEMK	NMQGSSGGEV	TVEMNAAPGT	DLTKLLNDMR	AQYEELAEQN	RREAEERFNK
250	260	270	280	290	300	310	320
QSASLQAQIS	TDAGAATSAK	NEITELKRTL	QALEIELQSQ	LAMKSSLEGT	LADTEAGYVA	QLSEIQTQIS	ALEEEICQIW
330	340	350	360	370	380	390	400
GETKCQNAEY	KQLLDIKTRL	EVEIETYRRL	LDGEGGGSSF	AEFGGRNSGS	VNMGSRDLVS	GDSRSGSCSG	QGRDSSKTRV
410	420	430					
TKTIVEELVD	GKVVSSQVSS	ISEVKVK					

Cmpd.	No. of Cmpds.	m/z meas.	Δ m/z [ppm]	z	Rt [min]	Score	P	Range	Sequence	Modification	Ratios
587	1	512.7149	-24.47	2	35.9	12.9	0	367-376	R.NSGSVNMGSR.D	Oxidation: 7	Wdown:Qdown 0.97

Detailed Protein Report

Protein 55: macrophage mannose receptor 1 precursor [Homo sapiens]

**Accession:**	gi|4505245	**Score:**	66.0
**Database:**	refseq_human(refseq_human_20140103.fasta)	**MW [kDa]:**	165.9
**Database Date:**	2015-11-30	**pI:**	6.1
**Modification(s):**	Carbamidomethyl, Oxidation	**Sequence Coverage [%]:**	4.9
		**No. of unique Peptides:**	5

Quantitation

mdown:qdown	**Median:** 0.16	**CV:** 0.00 %	**No. of Peptides:**	1
Wdown:Qdown	**Median:** 0.51	**CV:** 117.59 %	**No. of Peptides:**	2

```
        10         20         30         40         50         60         70         80
MRLPLLLVFA SVIPGAVLLL DTRQFLIYNE DHKRCVDAVS PSAVQTAACN QDAESQKFRW VSESQIMSVA FKLCLGVPSK
        90        100        110        120        130        140        150        160
TDWVAITLYA CDSKSEFQKW ECKNDTLLGI KGEDLFFNYG NRQEKNIMLY KGSGLWSRWK IYGTTDNLCS RGYEAMYTLL
       170        180        190        200        210        220        230        240
GNANGATCAF PFKFENKWYA DCTSAGRSDG WLWCGTTTDY DTDKLFGYCP LKFEGSESLW NKDPLTSVSY QINSKSALTW
       250        260        270        280        290        300        310        320
HQARKSCQQQ NAELLSITEI HEQTYLTGLT SSLTSGLWIG LNSLSFNSGW QWSDRSPFRY LNWLPGSPSA EPGKSCVSLN
       330        340        350        360        370        380        390        400
PGKNAKWENL ECVQKLGYIC KKGNTTLNSF VIPSESDVPT HCPSQWWPYA GHCYKIHRDE KKIQRDALTT CRKEGGDLTS
       410        420        430        440        450        460        470        480
IHTIEELDFI ISQLGYEPND ELWIGLNDIK IQMYFEWSDG TPVTFTKWLR GEPSHENNRQ EDCVVMKGKD GYWADRGCEW
       490        500        510        520        530        540        550        560
PLGYICKMKS RSQGPEIVEV EKGCRKGWKK HHFYCYMIGH TLSTFAEANQ TCNNENAYLT TIEDRYEQAF LTSFVGLRPE
       570        580        590        600        610        620        630        640
KYFWTGLSDI QTKGTFQWTI EEEVRFTHWN SDMPGRKPGC VAMRTGIAGG LWDVLKCDEK AKFVCKHWAE GVTHPPKPTT
       650        660        670        680        690        700        710        720
TPEPKCPEDW GASSRTSLCF KLYAKGKHEK KTWFESRDFC RALGGDLASI NNKEEQQTIW RLITASGSYH KLFWLGLTYG
       730        740        750        760        770        780        790        800
SPSEGFTWSD GSPVSYENWA YGEPNNYQNV EYCGELKGDP TMSWNDINCE HLNNWICQIQ KGQTPKPEPT PAPQDNPPVT
       810        820        830        840        850        860        870        880
EDGWVIYKDY QYYFSKEKET MDNARAFCKR NFGDLVSIQS ESEKKFLWKY VNRNDAQSAY FIGLLISLDK KFAWMDGSKV
       890        900        910        920        930        940        950        960
DYVSWATGEP NFANEDENCV TMYSNSGFWN DINCGYPNAF ICQRHNSSIN ATTVMPTMPS VPSGCKEGWN FYSNKCFKIF
       970        980        990       1000       1010       1020       1030       1040
GFMEEERKNW QEARKACIGF GGNLVSIQNE KEQAFLTYHM KDSTFSAWTG LNDVNSEHTF LWTDGRGVHY TNWGKGYPGG
      1050       1060       1070       1080       1090       1100       1110       1120
RRSSLSYEDA DCVVIIGGAS NEAGKWMDDT CDSKRGYICQ TRSDPSLTNP PATIQTDGFV KYGKSSYSLM RQKFQWHEAE
      1130       1140       1150       1160       1170       1180       1190       1200
TYCKLHNSLI ASILDPYSNA FAWLQMETSN ERVWIALNSN LTDNQYTWTD KWRVRYTNWA ADEPKLKSAC VYLDLDGYWK
      1210       1220       1230       1240       1250       1260       1270       1280
TAHCNESFYF LCKRSDEIPA TEPPQLPGRC PESDHTAWIP FHGHCYYIES SYTRNWGQAS LECLRMGSSL VSIESAAESS
      1290       1300       1310       1320       1330       1340       1350       1360
FLSYRVEPLK SKTNFWIGLF RNVEGTWLWI NNSPVSFVNW NTGDPSGERN DCVALHASSG FWSNIHCSSY KGYICKRPKI
      1370       1380       1390       1400       1410       1420       1430       1440
IDAKPTHELL TTKADTRKMD PSKPSSNVAG VVIIVILLIL TGAGLAAYFF YKKRRVHLPQ EGAFENTLYF NSQSSPGTSD
      1450       1460
MKDLVGNIEQ NEHSVI
```

Cmpd.	No. of Cmpds.	m/z meas.	Δ m/z [ppm]	z	Rt [min]	Score	P	Range	Sequence	Modification	Ratios
1884	1	621.2922	12.19	2	53.6	15.7	1	95-103	K.SEFQKWECK.N	Carbamidomethyl: 8	
1638	1	1069.9824	-53.73	2	49.0	18.2	2	324-341	K.NAKWENLECVQKLGYICK.K		Wdown:Qdown 0.20<br>mdown:qdown 0.16
1876	1	914.3217	-127.99	2	53.5	10.7	2	477-491	R.GCEWPLGYICKMKSR.S	Carbamidomethyl: 10	
2392	1	736.0727	87.47	3	60.2	10.6	1	586-604	R.FTHWNSDMPGRKPGCVAMR.T	Oxidation: 8	

Detailed Protein Report

Cmpd.	No. of Cmpds.	m/z meas.	Δ m/z [ppm]	z	Rt [min]	Score	P	Range	Sequence	Modification	Ratios
196	1	651.1714	-173.27	2	31.8	10.9	1	819-829	K.ETMDNARAFCK.R	Oxidation: 3	Wdown:Qdown 1.30

Detailed Protein Report

Protein 56: dystrophin Dp260-2 isoform [Homo sapiens]

**Accession:**	gi|5032291	**Score:**	65.7
**Database:**	refseq_human(refseq_human_20140103.fasta)	**MW [kDa]:**	270.9
**Database Date:**	2015-11-30	**pI:**	5.9
**Modification(s):**	Carbamidomethyl	**Sequence Coverage [%]:**	2.4
		**No. of unique Peptides:**	5

Quantitation

Wdown:Qdown	**Median:** 0.50	**CV:** 3.56 %	**No. of Peptides:** 2

Detailed Protein Report

10	20	30	40	50	60	70	80
MSARKLRNLS	YKKAVRRQKL	LEQSIQSAQE	TEKSLHLIQE	SLTFIDKQLA	AYIADKVDAA	QMPQEAQKIQ	SDLTSHEISL
90	100	110	120	130	140	150	160
EEMKKHNQGK	EAAQRVLSQI	DVAQKKLQDV	SMKFRLFQKP	ANFEQRLQES	KMILDEVKMH	LPALETKSVE	QEVVQSQLNH
170	180	190	200	210	220	230	240
CVNLYKSLSE	VKSEVEMVIK	TGRQIVQKKQ	TENPKELDER	VTALKLHYNE	LGAKVTERKQ	QLEKCLKLSR	KMRKEMNVLT
250	260	270	280	290	300	310	320
EWLAATDMEL	TKRSAVEGMP	SNLDSEVAWG	KATQKEIEKQ	KVHLKSITEV	GEALKTVLGK	KETLVEDKLS	LLNSNWIAVT
330	340	350	360	370	380	390	400
SRAEEWLNLL	LEYQKHMETF	DQNVDHITKW	IIQADTLLDE	SEKKKPQQKE	DVLKRLKAEL	NDIRPKVDST	RDQAANLMAN
410	420	430	440	450	460	470	480
RGDHCRKLVE	PQISELNHRF	AAISHRIKTG	KASIPLKELE	QFNSDIQKLL	EPLEAEIQQG	VNLKEEDFNK	DMNEDNEGTV
490	500	510	520	530	540	550	560
KELLQRGDNL	QQRITDERKR	EEIKIKQQLL	QTKHNALKDL	RSQRRKKALE	ISHQWYQYKR	QADDLLKCLD	DIEKKLASLP
570	580	590	600	610	620	630	640
EPRDERKIKE	IDRELQKKKE	ELNAVRRQAE	GLSEDGAAMA	VEPTQIQLSK	RWREIESKFA	QFRRLNFAQI	HTVREETMMV
650	660	670	680	690	700	710	720
MTEDMPLEIS	YVPSTYLTEI	THVSQALLEV	EQLLNAPDLC	AKDFEDLFKQ	EESLKNIKDS	LQQSSGRIDI	IHSKKTAALQ
730	740	750	760	770	780	790	800
SATPVERVKL	QEALSQLDFQ	WEKVNKMYKD	RQGRFDRSVE	KWRRFHYDIK	IFNQWLTEAE	QFLRKTQIPE	NWEHAKYKWY
810	820	830	840	850	860	870	880
LKELQDGIGQ	RQTVVRTLNA	TGEEIIQQSS	KTDASILQEK	LGSLNLRWQE	VCKQLSDRKK	RLEEQKNILS	EFQRDLNEFV
890	900	910	920	930	940	950	960
LWLEEADNIA	SIPLEPGKEQ	QLKEKLEQVK	LLVEELPLRQ	GILKQLNETG	GPVLVSAPIS	PEEQDKLENK	LKQTNLQWIK
970	980	990	1000	1010	1020	1030	1040
VSRALPEKQG	EIEAQIKDLG	QLEKKLEDLE	EQLNHLLLWL	SPIRNQLEIY	NQPNQEGPFD	VQETEIAVQA	KQPDVEEILS
1050	1060	1070	1080	1090	1100	1110	1120
KGQHLYKEKP	ATQPVKRKLE	DLSSEWKAVN	RLLQELRAKQ	PDLAPGLTTI	GASPTQTVTL	VTQPVVTKET	AISKLEMPSS
1130	1140	1150	1160	1170	1180	1190	1200
LMLEVPALAD	FNRAWTELTD	WLSLLDQVIK	SQRVMVGDLE	DINEMIIKQK	ATMQDLEQRR	PQLEELITAA	QNLKNKTSNQ
1210	1220	1230	1240	1250	1260	1270	1280
EARTIITDRI	ERIQNQWDEV	QEHLQNRRQQ	LNEMLKDSTQ	WLEAKEEAEQ	VLGQARAKLE	SWKEGPYTVD	AIQKKITETK
1290	1300	1310	1320	1330	1340	1350	1360
QLAKDLRQWQ	TNVDVANDLA	LKLLRDYSAD	DTRKVHMITE	NINASWRSIH	KRVSEREAAL	EETHRLLQQF	PLDLEKFLAW
1370	1380	1390	1400	1410	1420	1430	1440
LTEAETTANV	LQDATRKERL	LEDSKGVKEL	MKQWQDLQGE	IEAHTDVYHN	LDENSQKILR	SLEGSDDAVL	LQRRLDNMNF
1450	1460	1470	1480	1490	1500	1510	1520
KWSELRKKSL	NIRSHLEASS	DQWKRLHLSL	QELLVWLQLK	DDELSRQAPI	GGDFPAVQKQ	NDVHRAFKRE	LKTKEPVIMS
1530	1540	1550	1560	1570	1580	1590	1600
TLETVRIFLT	EQPLEGLEKL	YQEPRELPPE	ERAQNVTRLL	RKQAEEVNTE	WEKLNLHSAD	WQRKIDETLE	RLQELQEATD
1610	1620	1630	1640	1650	1660	1670	1680
ELDLKLRQAE	VIKGSWQPVG	DLLIDSLQDH	LEKVKALRGE	IAPLKENVSH	VNDLARQLTT	LGIQLSPYNL	STLEDLNTRW
1690	1700	1710	1720	1730	1740	1750	1760
KLLQVAVEDR	VRQLHEAHRD	FGPASQHFLS	TSVQGPWERA	ISPNKVPYYI	NHETQTTCWD	HPKMTELYQS	LADLNNVRFS
1770	1780	1790	1800	1810	1820	1830	1840
AYRTAMKLRR	LQKALCLDLL	SLSAACDALD	QHNLKQNDQP	MDILQIINCL	TTIYDRLEQE	HNNLVNVPLC	VDMCLNWLLN
1850	1860	1870	1880	1890	1900	1910	1920
VYDTGRTGRI	RVLSFKTGII	SLCKAHLEDK	YRYLFKQVAS	STGFCDQRRL	GLLLHDSIQI	PRQLGEVASF	GGSNIEPSVR
1930	1940	1950	1960	1970	1980	1990	2000
SCFQFANNKP	EIEAALFLDW	MRLEPQSMVW	LPVLHRVAAA	ETAKHQAKCN	ICKECPIIGF	RYRSLKHFNY	DICQSCFFSG
2010	2020	2030	2040	2050	2060	2070	2080
RVAKGHKMHY	PMVEYCTPTT	SGEDVRDFAK	VLKNKFRTKR	YFAKHPRMGY	LPVQTVLEGD	NMETPVTLIN	FWPVDSAPAS
2090	2100	2110	2120	2130	2140	2150	2160
SPQLSHDDTH	SRIEHYASRL	AEMENSNGSY	LNDSISPNES	IDDEHLLIQH	YCQSLNQDSP	LSQPRSPAQI	LISLESEERG
2170	2180	2190	2200	2210	2220	2230	2240

Detailed Protein Report

Cmpd.	No. of Cmpds.	m/z meas.	Δ m/z [ppm]	z	Rt [min]	Score	P	Range	Sequence	Modification	Ratios
129	1	619.6434	29.86	3	30.9	11.2	2	392-407	R.DQAANLMANRGDHCRK.L	Carbamidomethyl: 14	Wdown:Qdown 0.48
2949	1	735.4039	12.58	2	65.6	12.3	1	482-493	K.ELLQRGDNLQQR.I		
982	1	712.1299	-301.49	1	41.8	17.8	1	747-751	K.MYKDR.Q		Wdown:Qdown 0.52
1755	1	636.2889	-138.71	2	51.9	13.1	1	951-960	K.LKQTNLQWIK.V		
1813	1	785.8684	-31.80	2	51.3	11.2	0	1315-1327	K.VHMITENINASWR.S		

Detailed Protein Report

Protein 57: PREDICTED: uncharacterized protein KIAA1551 isoform X1 [Homo sapiens]

**Accession:**	gi|530399223	**Score:**	65.1
**Database:**	refseq_human(refseq_human_20140103.fasta)	**MW [kDa]:**	194.7
**Database Date:**	2015-11-30	**pI:**	9.7
**Modification(s):**	Carbamidomethyl, Oxidation	**Sequence Coverage [%]:**	2.5
		**No. of unique Peptides:**	4

10	20	30	40	50	60	70	80
MNWNEKPKSA	TLPPLYPKSQ	PPFLHQSLIN	QITTTSQSSF	SYPGSNQEAC	MYPGNSNPIS	QPLLNIQNYP	QQISVSDMHN
90	100	110	120	130	140	150	160
GTVVASHTSV	ERITYANVNG	PKQLTHNLQM	SSGVTQNVWL	NSPMRNPVHS	HIGATVSHQT	DFGANVPNMP	ALQSQLITSD
170	180	190	200	210	220	230	240
TYSMQMQMIP	SNSTRLPVAY	QGNQGLNQSF	SEQQVDWTQQ	CISKGLTYPD	YRPPPKLYRY	SPQSFLPDST	IQKQNFIPHT
250	260	270	280	290	300	310	320
SLQVKNSQLL	NSVLTLPSRQ	TSAVPSQQYA	TQTDKRPPPP	PYNCRYGSQP	LQSTQHITKH	LSMEVPQSRE	MLSSEIRTSF
330	340	350	360	370	380	390	400
QQQWQNPNEN	VSTIGNFTNL	KVNTNSKQPF	NSPIRSSVDG	VQTLAQTNEE	KIMDSCNPTS	NQVLDTSVAK	EKLVRDIKTL
410	420	430	440	450	460	470	480
VEIKQKFSEL	ARKIKINKDL	LMAAGCIKMT	NTSYSEPAQN	SKLSLKQTAK	IQSGPQITPV	MPENAERQTP	TVVESAETNK
490	500	510	520	530	540	550	560
TQCMLNSDIQ	EVNCRRFNQV	DSVLPNPVYS	EKRPMPDSSH	DVKVLTSKTS	AVEMTQAVLN	TQLSSENVTK	VEQNSPAVCE
570	580	590	600	610	620	630	640
TISVPKSMST	EEYKSKIQNE	NMLLLALLSQ	ARKTQKTVLK	DANQTIQDSK	PDSCEMNPNT	QMTGNQLNLK	NMETPSTSNV
650	660	670	680	690	700	710	720
SGRVLDNSFC	SGQESSTKGM	PAKSDSSCSM	EVLATCLSLW	KKQPSDTAKE	KECDKLRTNT	TAVGISKPAN	IHVKSPCSVV
730	740	750	760	770	780	790	800
GNSNSQNKIS	NPSQQTALSM	VMHNYESSGI	NITKGTELQI	AVVSPLVLSE	VKTLSVKGIT	PAVLPETVYP	VIKEGSVCSL
810	820	830	840	850	860	870	880
QNQLAENAKA	TAALKVDVSG	PVASTATSTK	IFPLTQKEKQ	NESTNGNSEV	TPNVNQGKHN	KLESAIHSPM	NDQQISQESR
890	900	910	920	930	940	950	960
NSTVVSSDTL	QIDNICSLVE	GDTSYNSQIA	KIFSSLPLKM	VEPQKPSLPN	QQGIGSREPE	KQLDNTTENK	DFGFQKDKPV
970	980	990	1000	1010	1020	1030	1040
QCTDVSHKIC	DQSKSEPPLE	SSFNNLETNR	VILEKSSLEH	ATEKSTANDT	CSSAAIQEDI	YPQEIDASSN	YTPQDPARNE
1050	1060	1070	1080	1090	1100	1110	1120
IHSDKAPVLY	LHDQLSELLK	EFPYGIEAVN	TREGSVGQQT	TYQTSEDQTA	DKTSSDSKDP	ADQIQITILS	SEQMKEIFPE
1130	1140	1150	1160	1170	1180	1190	1200
QDDQPYVVDK	LAEPQKEEPI	TEVVSQCDLQ	APAAGQSRDS	VILDSEKDDI	HCCALGWLSM	VYEGVPQCQC	NSIKNSSSEE
1210	1220	1230	1240	1250	1260	1270	1280
EKQKEQCSPL	DTNSCKQGER	TSDRDVTVVQ	FKSLVNNPKT	PPDGKSHFPE	LQDDSRKDTP	KTKHKSLPRT	EQELVAGQFS
1290	1300	1310	1320	1330	1340	1350	1360
SKCDKLNPLQ	NHKRKKLRFH	EVTFHSSNKM	TASYEQASQE	TRQKKHVTQN	SRPLKTKTAF	LPNKDVYKKH	SSLGQSLSPE
1370	1380	1390	1400	1410	1420	1430	1440
KIKLKLKSVS	FKQKRKLDQG	NVLDMEVKKK	KHDKQEQKGS	VGATFKLGDS	LSNPNERAIV	KEKMVSNTKS	VDTKASSSKF
1450	1460	1470	1480	1490	1500	1510	1520
SRILTPKEYL	QRQKHKEALS	NKASKKICVK	NVPCDSEHMR	PSKLAVQVES	CGKSNEKHSS	GVQTSKESLN	GLTSHGKNLK
1530	1540	1550	1560	1570	1580	1590	1600
IHHSQESKTY	NILRNVKEKV	GGKQPDKIWI	DKTKLDKLTN	ISNEAQFSQM	PPQVKDQKKL	YLNRVGFKCT	ERESISLTKL
1610	1620	1630	1640	1650	1660	1670	1680
ESSPRKLHKD	KRQENKHKTF	LPVKGNTEKS	NMLEFKLCPD	ILLKNTNSVE	ERKDVKPHPR	KEQAPLQVSG	IKSTKEDWLK
1690	1700	1710	1720	1730	1740	1750	
FVATKKRTQK	DSQERDNVNS	RLSKRSFSAD	GFEMLQNPVK	DSKEMFQTYK	QMYLEKRSRS	LGSSPVK	

Cmpd.	No. of Cmpds.	m/z meas.	Δ m/z [ppm]	z	Rt [min]	Score	P	Range	Sequence	Modification
2628	1	644.4984	138.82	1	63.6	19.8	1	391-395	K.EKLVR.D	
2689	1	731.8632	-38.63	2	64.8	14.6	1	416-428	K.INKDLLMAAGCIK.M	Carbamidomethyl: 11;

Detailed Protein Report

Cmpd.	No. of Cmpds.	m/z meas.	Δ m/z [ppm]	z	Rt [min]	Score	P	Range	Sequence	Modification
										Oxidation: 7
2776	1	710.3755	7.84	2	63.8	11.0	0	816-830	K.VDVSGPVASTATSTK.I	
258	1	585.2801	-84.98	2	32.0	19.7	0	1662-1672	K.EQAPLQVSGIK.S	

Detailed Protein Report

**Protein 58:**	**ubiquitin carboxyl-terminal hydrolase 34 [Homo sapiens]**		
**Accession:**	gi|110347427	**Score:**	64.9
**Database:**	refseq_human(refseq_human_20140103.fasta)	**MW [kDa]:**	404.0
**Database Date:**	2015-11-30	**pI:**	5.4
		**Sequence Coverage [%]:**	1.7
		**No. of unique Peptides:**	2

Detailed Protein Report

10	20	30	40	50	60	70	80
MCENCADLVE	VLNEISDVEG	GDGLQLRKEH	TLKIFTYINS	WTQRQCLCCF	KEYKHLEIFN	QVVCALINLV	IAQVQVLRDQ
90	100	110	120	130	140	150	160
LCKHCTTINI	DSTWQDESNQ	AEEPLNIDRE	CNEGSTERQK	SIEKKSNSTR	ICNLTEEESS	KSSDPFSLWS	TDEKEKLLLC
170	180	190	200	210	220	230	240
VAKIFQIQFP	LYTAYKHNTH	PTIEDISTQE	SNILGAFCDM	NDVEVPLHLL	RYVCLFCGKN	GLSLMKDCFE	YGTPETLPFL
250	260	270	280	290	300	310	320
IAHAFITVVS	NIRIWLHIPA	VMQHIIPFRT	YVIRYLCKLS	DQELRQSAAR	NMADLMWSTV	KEPLDTTLCF	DKESLDLAFK
330	340	350	360	370	380	390	400
YFMSPTLTMR	LAGLSQITNQ	LHTFNDVCNN	ESLVSDTETS	IAKELADWLI	SNNVVEHIFG	PNLHIEIIKQ	CQVILNFLAA
410	420	430	440	450	460	470	480
EGRLSTQHID	CIWAAAQLKH	CSRYIHDLFP	SLIKNLDPVP	LRHLLNLVSA	LEPSVHTEQT	LYLASMLIKA	LWNNALAAKA
490	500	510	520	530	540	550	560
QLSKQSSFAS	LLNTNIPIGN	KKEEEELRRT	APSPWSPAAS	PQSSDNSDTH	QSGGSDIEMD	EQLINRTKHV	QQRLSDTEES
570	580	590	600	610	620	630	640
MQGSSDETAN	SGEDGSSGPG	SSSGHSDGSS	NEVNSSHASQ	SAGSPGSEVQ	SEDIADIEAL	KEEDEDDDHG	HNPPKSSCGT
650	660	670	680	690	700	710	720
DLRNRKLESQ	AGICLGDSQG	MSERNGTSSG	TGKDLVFNTE	SLPSVDNRMR	MLDACSHSED	PEHDISGEMN	ATHIAQGSQE
730	740	750	760	770	780	790	800
SCITRTGDFL	GETIGNELFN	CRQFIGPQHH	HHHHHHHHHH	DGHMVDDMLS	ADDVSCSSSQ	VSAKSEKNMA	DFDGEESGCE
810	820	830	840	850	860	870	880
EELVQINSHA	ELTSHLQQHL	PNLASIYHEH	LSQGPVVHKH	QFNSNAVTDI	NLDNVCKKGN	TLLWDIVQDE	DAVNLSEGLI
890	900	910	920	930	940	950	960
NEAEKLLCSL	VCWFTDRQIR	MRFIEGCLEN	LGNNRSVVIS	LRLLPKLFGT	FQQFGSSYDT	HWITMWAEKE	LNMMKLFFDN
970	980	990	1000	1010	1020	1030	1040
LVYYIQTVRE	GRQKHALYSH	SAEVQVRLQF	LTCVFSTLGS	PDHFRLSLEQ	VDILWHCLVE	DSECYDDALH	WFLNQVRSKD
1050	1060	1070	1080	1090	1100	1110	1120
QHAMGMETYK	HLFLEKMPQL	KPETISMTGL	NLFQHLCNLA	RLATSAYDGC	SNSELCGMDQ	FWGIALRAQS	GDVSRAAIQY
1130	1140	1150	1160	1170	1180	1190	1200
INSYYINGKT	GLEKEQEFIS	KCMESLMIAS	SSLEQESHSS	LMVIERGLLM	LKTHLEAFRR	RFAYHLRQWQ	IEGTGISSHL
1210	1220	1230	1240	1250	1260	1270	1280
KALSDKQSLP	LRVVCQPAGL	PDKMTIEMYP	SDQVADLRAE	VTHWYENLQK	EQINQQAQLQ	EFGQSNRKGE	FPGGLMGPVR
1290	1300	1310	1320	1330	1340	1350	1360
MISSGHELTT	DYDEKALHEL	GFKDMQMVFV	SLGAPRRERK	GEGVQLPASC	LPPPQKDNIP	MLLLLQEPHL	TTLFDLLEML
1370	1380	1390	1400	1410	1420	1430	1440
ASFKPPSGKV	AVDDSESLRC	EELHLHAENL	SRRVWELLML	LPTCPNMLMA	FQNISDEQSN	DGFNWKELLK	IKSAHKLLYA
1450	1460	1470	1480	1490	1500	1510	1520
LEIIEALGKP	NRRIRRESTG	SYSDLYPDSD	DSSEDQVENS	KNSWSCKFVA	AGGLQQLLEI	FNSGILEPKE	QESWTVWQLD
1530	1540	1550	1560	1570	1580	1590	1600
CLACLLKLIC	QFAVDPSDLD	LAYHDVFAWS	GIAESHRKRT	WPGKSRKAAG	DHAKGLHIPR	LTEVFLVLVQ	GTSLIQRLMS
1610	1620	1630	1640	1650	1660	1670	1680
VAYTYDNLAP	RVLKAQSDHR	SRHEVSHYSM	WLLVSWAHCC	SLVKSSLADS	DHLQDWLKKL	TLLIPETAVR	HESCSGLYKL
1690	1700	1710	1720	1730	1740	1750	1760
SLSGLDGGDS	INRSFLLLAA	STLLKFLPDA	QALKPIRIDD	YEEEPILKPG	CKEYFWLLCK	LVDNIHIKDA	SQTTLLDLDA
1770	1780	1790	1800	1810	1820	1830	1840
LARHLADCIR	SREILDHQDG	NVEDDGLTGL	LRLATSVVKH	KPPFKFSREG	QEFLRDIFNL	LFLLPSLKDR	QQPKCKSHSS
1850	1860	1870	1880	1890	1900	1910	1920
RAAAYDLLVE	MVKGSVENYR	LIHNWVMAQH	MQSHAPYKWD	YWPHEDVRAE	CRFVGLTNLG	ATCYLASTIQ	QLYMIPEARQ
1930	1940	1950	1960	1970	1980	1990	2000
AVFTAKYSED	MKHKTTLLEL	QKMFTYLMES	ECKAYNPRPF	CKTYTMDKQP	LNTGEQKDMT	EFFTDLITKI	EEMSPELKNT
2010	2020	2030	2040	2050	2060	2070	2080
VKSLFGGVIT	NNVVSLDCEH	VSQTAEEFYT	VRCQVADMKN	IYESLDEVTI	KDTLEGDNMY	TCSHCGKKVR	AEKRACFKKL
2090	2100	2110	2120	2130	2140	2150	2160
PRILSFNTMR	YTFNMVTMMK	EKVNTHFSFP	LRLDMTPYTE	DFLMGKSERK	EGFKEVSDHS	KDSESYEYDL	IGVTVHTGTA
2170	2180	2190	2200	2210	2220	2230	2240

Detailed Protein Report

Cmpd.	No. of Cmpds.	m/z meas.	Δ m/z [ppm]	z	Rt [min]	Score	P	Range	Sequence	Modification
2681	1	1079.0243	41.33	2	64.7	12.9	2	2052-2070	K.DTLEGDNMYTCSHCGKKVR.A	
597	2	642.8360	18.78	2	36.1	18.4	0	2940-2950	R.SCWTTLISAFR.I	

Detailed Protein Report

Protein 59: **PREDICTED: uncharacterized protein LOC101927628 [Homo sapiens]**

**Accession:**	gi|578827891	**Score:**	63.5
**Database:**	refseq_human(refseq_human_20140103.fasta)	**MW [kDa]:**	109.0
**Database Date:**	2015-11-30	**pI:**	12.3
**Modification(s):**	Carbamidomethyl	**Sequence Coverage [%]:**	7.8
		**No. of unique Peptides:**	5

10	20	30	40	50	60	70	80
MEESAEQAQR	RDETRVGRRR	HSWRSRQSRQ	TKEFIFSELL	SNLYSRRNLQ	TLVEESAEQA	QRCDEMLLLR	AAVQPALAWE
**90**	**100**	**110**	**120**	**130**	**140**	**150**	**160**
REDTRGGVGR	AGRPRSSSSQ	SCCPTCTHVG	TSRHSWRRRQ	SRQTKEFIFS	ELLSNLHSRG	NEKTLVKASA	EKADQGVHLL
**170**	**180**	**190**	**200**	**210**	**220**	**230**	**240**
RAAVQPALAW	EREDTRGGVG	RAGRPRSSSS	QSCCPNCTRV	GTRRHSWRSR	QSRQTKEFIF	SELLSNLYSR	RNLQTLVEES
**250**	**260**	**270**	**280**	**290**	**300**	**310**	**320**
AEQAQRCDEM	LLLRAAVQPA	LAWEREDTRG	GVGRAGRPRS	SSSQSCCPTC	TRVGTRRHSW	RRRQSRQTKE	FIFSELLSNL
**330**	**340**	**350**	**360**	**370**	**380**	**390**	**400**
HSRGNEKTLM	EESAEQAQRR	DETRVGRRRH	SWRSRQSRQT	KEFIFSELLS	NLYSHRNLQT	LVEESAEQAQ	RCDEMLLLRA
**410**	**420**	**430**	**440**	**450**	**460**	**470**	**480**
AVQPALAWER	EDTRGGVGRA	GRPRSSSSQS	CCPTCTHVGT	SRHSWRRRQS	RQTKEFIFSE	LLSNLHSRGN	EKTLVKASAE
**490**	**500**	**510**	**520**	**530**	**540**	**550**	**560**
KADQGVHLLR	AAVQPALAWE	REDTRGGVGR	AGRPRSSSSQ	SCCPNCTRVG	TRRHSWRSRQ	SRHRGVTRLA	WEEEDTPGGV
**570**	**580**	**590**	**600**	**610**	**620**	**630**	**640**
GRAGRPRSSS	SRSCCPTCTR	VGTCRHLWRS	RQSRHSGATR	CCFSELLSNL	HSRGNKKTLV	EALAEQADQG	VHLLRAAVQP
**650**	**660**	**670**	**680**	**690**	**700**	**710**	**720**
VLAWEREDTR	GGVGRAGRPR	SSSSQSCCPT	CTRVGTRRHS	CRSRQSRHSG	VTRLAWEEED	IPGGVGRAGR	ARSSSSRSCC
**730**	**740**	**750**	**760**	**770**	**780**	**790**	**800**
PTCTRVGTCR	HLWRSRQSRH	SGATRCCFSE	LLSNLHSRGN	QKTLVEASAE	QADQGVHLLR	AAVQPVLAWE	REDTRGGVGR
**810**	**820**	**830**	**840**	**850**	**860**	**870**	**880**
AGRPRSSSSQ	SCCPTCTRVG	TRRHSWRSRP	SRHSGVTRLA	WEEEDTPGGV	GRAGRPRSSS	SRSCCPTCTR	VGTCRHLWRS
**890**	**900**	**910**	**920**	**930**	**940**	**950**	**960**
RQSRHSGATR	CCFSELLSNL	HWRGNEKTLV	EASAEQADQG	VHLLRAAVQP	VLAWERADTR	GGVGRAGRPR	NSSSQSCCPT
**970**	**980**						
CTRVGMRRHS							

Cmpd.	No. of Cmpds.	m/z meas.	Δ m/z [ppm]	z	Rt [min]	Score	P	Range	Sequence	Modification
2702	1	856.8935	-53.50	2	65.0	10.9	1	71-85	R.AAVQPALAWEREDTR.G	
254	1	977.7698	-10.85	3	31.9	11.3	2	91-117	R.AGRPRSSSSQSCCPTCTHVGTSRHSWR.R	
2075	3	937.3867	-15.36	2	54.5	13.9	1	280-296	R.SSSSQSCCPTCTRVGTR.R	Carbamidomethyl: 7, 8
2276	2	937.4372	47.94	2	56.9	12.7	1	951-967	R.NSSSQSCCPTCTRVGMR.R	Carbamidomethyl: 8
2316	1	937.2798	-119.93	2	57.4	14.7	1	951-967	R.NSSSQSCCPTCTRVGMR.R	Carbamidomethyl: 7

Detailed Protein Report

Protein 60: hemoglobin subunit epsilon [Homo sapiens]

**Accession:**	gi|4885393	**Score:**	63.5
**Database:**	refseq_human(refseq_human_20140103.fasta)	**MW [kDa]:**	16.2
**Database Date:**	2015-11-30	**pI:**	9.4
		**Sequence Coverage [%]:**	15.0
		**No. of unique Peptides:**	1

Quantitation

mdown:qdown	**Median:** 7.41	**CV:** 0.00 %	**No. of Peptides:** 1
Wdown:Qdown	**Median:** 0.50	**CV:** 0.00 %	**No. of Peptides:** 1

```
        10         20         30         40         50         60         70         80
MVHFTAEEKA AVTSLWSKMN VEEAGGEALG RLLVVYPWTQ RFFDSFGNLS SPSAILGNPK VKAHGKKVLT SFGDAIKNMD
        90        100        110        120        130        140        150
NLKPAFAKLS ELHCDKLHVD PENFKLLGNV MVIILATHFG KEFTPEVQAA WQKLVSAVAI ALAHKYH
```

Cmpd.	No. of Cmpds.	m/z meas.	Δ m/z [ppm]	z	Rt [min]	Score	P	Range	Sequence	Modification	Ratios
2140	3	596.7831	-152.64	2	55.3	13.5	0	134-145	K.LVSAVAIALAHK.Y		Wdown:Qdown 0.50<br>mdown:qdown 7.41

Detailed Protein Report

Protein 61: dynein heavy chain 11, axonemal [Homo sapiens]

**Accession:**	gi|459642365	**Score:**	63.2
**Database:**	refseq_human(refseq_human_20140103.fasta)	**MW [kDa]:**	520.0
**Database Date:**	2015-11-30	**pI:**	6.0
**Modification(s):**	Carbamidomethyl	**Sequence Coverage [%]:**	1.4
		**No. of unique Peptides:**	5

Detailed Protein Report

10	20	30	40	50	60	70	80
MAAQVAAREA	RDFREAPTLR	LTSGAGLEAV	GAVELEEEEE	NEEEAAARRA	RSFAQDARVR	FLGGRLAMML	GFTEEKWSQY
90	100	110	120	130	140	150	160
LESEDNRQVL	GEFLESTSPA	CLVFSFAASG	RLAASQEIPR	DANHKLVFIS	KKITESIGVN	DFSQVVLFGE	LPALSLGHVS
170	180	190	200	210	220	230	240
AFLDEILVPV	LSNKNNHKSW	SCFTSQDMEY	HIEVMKKKMY	IFRGKMSRRT	LLPIPTVAGK	MDLDQNCSEN	KPPSNERIIL
250	260	270	280	290	300	310	320
HAIESVVIEW	SHQIQEIIER	DSVQRLLNGL	HLSPQAELDF	WMMRRENLSC	IYDQLQAPVV	LKMVKILTTK	QSSYFPTLKD
330	340	350	360	370	380	390	400
IFLAVENALL	EAQDVELYLR	PLRRHIQCLQ	ETEFPQTRIL	IAPLFHTICL	IWSHSKFYNT	PARVIVLLQE	FCNLFINQAT
410	420	430	440	450	460	470	480
AYLSPEDLLR	GEIEESLEKV	QVAVNILKTF	KNSFFNYRKK	LASYFMGRKL	RPWDFQSHLV	FCRFDKFLDR	LIKIEDIFAT
490	500	510	520	530	540	550	560
TLEFEKLERL	EFGGTKGAIL	NGQVHEMSEE	LMELCKLFKQ	STYDPSDCTN	MEFESDYVAF	KSKTLEFDRR	LGTIICEAFF
570	580	590	600	610	620	630	640
NCNGLEAAFK	LLTIFGNFLE	KPVVMEIFSL	HYSTLVHMFN	TELDVCKQLY	NEHMKQIECG	HVVLNKNMPF	TSGNMKWAQQ
650	660	670	680	690	700	710	720
VLQRLQMFWS	NFASLRYLFL	GNPDHALVYQ	KYVEMTTLLD	QFESRIYNEW	KSNVDEICEF	NLNQPLVKFS	AINGLLCVNF
730	740	750	760	770	780	790	800
DPKLVAVLRE	VKYLLMLKKQ	DIPDSALAIF	KKRNTILKYI	GNLDLLVQGY	NKLKQTLLEV	EYPLIEDELR	AIDEQLTAAT
810	820	830	840	850	860	870	880
TWLTWQDDCW	GYIERVRAAT	SELEHRVERT	QKNVKVIQQT	MRGWARCVLP	PRREHRREAA	FTLEDKGDLF	TKKYKLIQGD
890	900	910	920	930	940	950	960
GCKIHNLVEE	NRKLFKANPS	LDTWKIYVEF	IDDIVVEGFF	QAIMHDLDFF	LKNTEKQLKP	APFFQAQMIL	LPPEIVFKPS
970	980	990	1000	1010	1020	1030	1040
LDREAGDGFY	DLVEEMLCNS	FRMSAQMNRI	ATHLEIKNYQ	NDMDNMLGLA	EVRQEIMNRV	VNVINKVLDF	RNTLETHTYL
1050	1060	1070	1080	1090	1100	1110	1120
WVDDRAEFMK	HFLLYGHAVS	SDEMDAHANE	EIPEQPPTLE	QFKEQIDIYE	ALYVQMSKFE	DFRVFDSWFK	VDMKPFKVSL
1130	1140	1150	1160	1170	1180	1190	1200
LTIIKKWSWM	FQEHLLRFVI	DSLNELQEFI	KETDSGLQRE	LNEGDHDGLV	DIMVHLLAVR	SRQRATDELF	EPLKETITLL
1210	1220	1230	1240	1250	1260	1270	1280
ESYGQKMPEQ	VYIQLEELPE	RWETTKKIAA	TVRHEVSPLH	NAEVTLIRKK	CILFDAKQAE	FRERFRHYAP	LGFNAENPYT
1290	1300	1310	1320	1330	1340	1350	1360
ALDKANEELE	ALEEEMLQMQ	ESTRLFEVAL	PEYKQMKQCR	KEIKLLKGLW	DVIIYVRRSI	DNWTKTQWRQ	IHVEQMDVEL
1370	1380	1390	1400	1410	1420	1430	1440
RRFAKEIWSL	NKEVRVWDAY	TGLEGTVKDM	TASLRAITEL	QSPALRDRHW	HQLMKAIGVK	FLINEATTLA	DLLALRLHRV
1450	1460	1470	1480	1490	1500	1510	1520
EDDVRRIVDK	AVKELGTEKV	ITEISQTWAT	MKFSYEVHYR	TGIPLLKSDE	QLFETLEHNQ	VQLQTLLQSK	YVEYFIEQVL
1530	1540	1550	1560	1570	1580	1590	1600
SWQNKLNIAD	LVIFTWMEVQ	RTWSHLESIF	VCSEDIRIQL	VKDARRFDGV	DAEFKELMFK	TAKVENVLEA	TCRPNLYEKL
1610	1620	1630	1640	1650	1660	1670	1680
KDLQSRLSLC	EKALAEYLET	KRIAFPRFYF	VSSADLLDIL	SKGAQPKQVT	CHLAKLFDSI	ADLQFEDNQD	VSAHRAVGMY
1690	1700	1710	1720	1730	1740	1750	1760
SKEKEYVPFQ	AECECVGHVE	TWLLQLEQTM	QETVRHSITE	AIVAYEEKPR	ELWIFDFPAQ	VALTSSQIWW	TTDVGIAFSR
1770	1780	1790	1800	1810	1820	1830	1840
LEEGYETALK	DFHKKQISQL	NTLITLLLGE	LPPGDRQKIM	TICTIDVHAR	DVVAKLISQK	VVSPQAFTWL	SQLRHRWEDT
1850	1860	1870	1880	1890	1900	1910	1920
QKHCFVNICD	AQFQYFYEYL	GNSPRLVITP	LTDRCYITLT	QSLHLTMSGA	PAGPAGTGKT	ETTKDLGRAL	GMMVYVFNCS
1930	1940	1950	1960	1970	1980	1990	2000
EQMDYKSIGN	IYKGLVQTGA	WGCFDEFNRI	SVEVLSVVAV	QVKMIHDAIR	NRKKRFVFLG	EAITLKPSVG	IFITMNPGYA
2010	2020	2030	2040	2050	2060	2070	2080
GRTELPENLK	ALFRPCAMVA	PDIELICEIL	LVAEGFVDAR	ALARKFITLY	TLCKELLSKQ	DHYDWGLRAI	KSVLVVAGSL
2090	2100	2110	2120	2130	2140	2150	2160
KRGDKNRPED	QVLMRALRDF	NMPKIVTDDI	PVFLGLVGDL	FPALDVPRRR	KLHFEQMVRQ	STLELRLQPE	ESFILKVVQL
2170	2180	2190	2200	2210	2220	2230	2240

Detailed Protein Report

Cmpd.	No. of Cmpds.	m/z meas.	Δ m/z [ppm]	z	Rt [min]	Score	P	Range	Sequence	Modification
1302	1	817.1657	-329.27	1	46.0	11.8	0	1-8	-.MAAQVAAR.E	
2700	1	920.8814	-102.30	2	65.0	16.0	2	857-872	R.REAAFTLEDKGDLFTK.K	
882	1	647.8007	-79.74	2	39.8	12.8	2	1437-1446	R.LHRVEDDVRR.I	
36	1	779.3158	-100.01	2	29.7	10.1	1	2083-2095	R.GDKNRPEDQVLMR.A	
1863	1	965.8216	-153.66	2	53.3	12.4	2	4470-4484	R.QETKQTYECPVYRTK.L	Carbamidomethyl: 9

Detailed Protein Report

Protein 62: fibrillin-1 precursor [Homo sapiens]

**Accession:**	gi|281485550	**Score:**	63.1
**Database:**	refseq_human(refseq_human_20140103.fasta)	**MW [kDa]:**	312.1
**Database Date:**	2015-11-30	**pI:**	4.7
**Modification(s):**	Carbamidomethyl, Oxidation	**Sequence Coverage [%]:**	2.3
		**No. of unique Peptides:**	4

Quantitation

mdown:qdown	**Median:**	0.87	**CV:**	0.00 %	**No. of Peptides:**	1
Wdown:Qdown	**Median:**	0.57	**CV:**	87.02 %	**No. of Peptides:**	2

Detailed Protein Report

10	20	30	40	50	60	70	80
MRRGRLLEIA	LGFTVLLASY	TSHGADANLE	AGNVKETRAS	RAKRRGGGGH	DALKGPNVCG	SRYNAYCCPG	WKTLPGGNQC
90	100	110	120	130	140	150	160
IVPICRHSCG	DGFCSRPNMC	TCPSGQIAPS	CGSRSIQHCN	IRCMNGGSCS	DDHCLCQKGY	IGTHCGQPVC	ESGCLNGGRC
170	180	190	200	210	220	230	240
VAPNRCACTY	GFTGPQCERD	YRTGPCFTVI	SNQMCQGQLS	GIVCTKTLCC	ATVGRAWGHP	CEMCPAQPHP	CRRGFIPNIR
250	260	270	280	290	300	310	320
TGACQDVDEC	QAIPGLCQGG	NCINTVGSFE	CKCPAGHKLN	EVSQKCEDID	ECSTIPGICE	GGECTNTVSS	YFCKCPPGFY
330	340	350	360	370	380	390	400
TSPDGTRCID	VRPGYCYTAL	TNGRCSNQLP	QSITKMQCCC	DAGRCWSPGV	TVAPEMCPIR	ATEDFNKLCS	VPMVIPGRPE
410	420	430	440	450	460	470	480
YPPPPLGPIP	PVLPVPPGFP	PGPQIPVPRP	PVEYLYPSRE	PPRVLPVNVT	DYCQLVRYLC	QNGRCIPTPG	SYRCECNKGF
490	500	510	520	530	540	550	560
QLDLRGECID	VDECEKNPCA	GGECINNQGS	YTCQCRAGYQ	STLTRTECRD	IDECLQNGRI	CNNGRCINTD	GSFHCVCNAG
570	580	590	600	610	620	630	640
FHVTRDGKNC	EDMDECSIRN	MCLNGMCINE	DGSFKCICKP	GFQLASDGRY	CKDINECETP	GICMNGRCVN	TDGSYRCECF
650	660	670	680	690	700	710	720
PGLAVGLDGR	VCVDTHMRST	CYGGYKRGQC	IKPLFGAVTK	SECCCASTEY	AFGEPCQPCP	AQNSAEYQAL	CSSGPGMTSA
730	740	750	760	770	780	790	800
GSDINECALD	PDICPNGICE	NLRGTYKCIC	NSGYEVDSTG	KNCVDINECV	LNSLLCDNGQ	CRNTPGSFVC	TCPKGFIYKP
810	820	830	840	850	860	870	880
DLKTCEDIDE	CESSPCINGV	CKNSPGSFIC	ECSSESTLDP	TKTICIETIK	GTCWQTVIDG	RCEININGAT	LKSQCCSSLG
890	900	910	920	930	940	950	960
AAWGSPCTLC	QVDPICGKGY	SRIKGTQCED	IDECEVFPGV	CKNGLCVNTR	GSFKCQCPSG	MTLDATGRIC	LDIRLETCFL
970	980	990	1000	1010	1020	1030	1040
RYEDEECTLP	IAGRHRMDAC	CCSVGAAWGT	EECEECPMRN	TPEYEELCPR	GPGFATKEIT	NGKPFFKDIN	ECKMIPSLCT
1050	1060	1070	1080	1090	1100	1110	1120
HGKCRNTIGS	FKCRCDSGFA	LDSEERNCTD	IDECRISPDL	CGRGQCVNTP	GDFECKCDEG	YESGFMMMKN	CMDIDECQRD
1130	1140	1150	1160	1170	1180	1190	1200
PLLCRGGVCH	NTEGSYRCEC	PPGHQLSPNI	SACIDINECE	LSAHLCPNGR	CVNLIGKYQC	ACNPGYHSTP	DRLFCVDIDE
1210	1220	1230	1240	1250	1260	1270	1280
CSIMNGGCET	FCTNSEGSYE	CSCQPGFALM	PDQRSCTDID	ECEDNPNICD	GGQCTNIPGE	YRCLCYDGFM	ASEDMKTCVD
1290	1300	1310	1320	1330	1340	1350	1360
VNECDLNPNI	CLSGTCENTK	GSFICHCDMG	YSGKKGKTGC	TDINECEIGA	HNCGKHAVCT	NTAGSFKCSC	SPGWIGDGIK
1370	1380	1390	1400	1410	1420	1430	1440
CTDLDECSNG	THMCSQHADC	KNTMGSYRCL	CKEGYTGDGF	TCTDLDECSE	NLNLCGNGQC	LNAPGGYRCE	CDMGFVPSAD
1450	1460	1470	1480	1490	1500	1510	1520
GKACEDIDEC	SLPNICVFGT	CHNLPGLFRC	ECEIGYELDR	SGGNCTDVNE	CLDPTTCISG	NCVNTPGSYI	CDCPPDFELN
1530	1540	1550	1560	1570	1580	1590	1600
PTRVGCVDTR	SGNCYLDIRP	RGDNGDTACS	NEIGVGVSKA	SCCCSLGKAW	GTPCEMCPAV	NTSEYKILCP	GGEGFRPNPI
1610	1620	1630	1640	1650	1660	1670	1680
TVILEDIDEC	QELPGLCQGG	KCINTFGSFQ	CRCPTGYYLN	EDTRVCDDVN	ECETPGICGP	GTCYNTVGNY	TCICPPDYMQ
1690	1700	1710	1720	1730	1740	1750	1760
VNGGNNCMDM	RRSLCYRNYY	ADNQTCDGEL	LFNMTKKMCC	CSYNIGRAWN	KPCEQCPIPS	TDEFATLCGS	QRPGFVIDIY
1770	1780	1790	1800	1810	1820	1830	1840
TGLPVDIDEC	REIPGVCENG	VCINMVGSFR	CECPVGFFYN	DKLLVCEDID	ECQNGPVCQR	NAECINTAGS	YRCDCKPGYR
1850	1860	1870	1880	1890	1900	1910	1920
FTSTGQCNDR	NECQEIPNIC	SHGQCIDTVG	SFYCLCHTGF	KTNDDQTMCL	DINECERDAC	GNGTCRNTIG	SFNCRCNHGF
1930	1940	1950	1960	1970	1980	1990	2000
ILSHNNDCID	VDECASGNGN	LCRNGQCINT	VGSFQCQCNE	GYEVAPDGRT	CVDINECLLE	PRKCAPGTCQ	NLDGSYRCIC
2010	2020	2030	2040	2050	2060	2070	2080
PPGYSLQNEK	CEDIDECVEE	PEICALGTCS	NTEGSFKCLC	PEGFSLSSSG	RRCQDLRMSY	CYAKFEGGKC	SSPKSRNHSK
2090	2100	2110	2120	2130	2140	2150	2160
QECCCALKGE	GWGDPCELCP	TEPDEAFRQI	CPYGSGIIVG	PDDSAVDMDE	CKEPDVCKHG	QCINTDGSYR	CECPFGYILA
2170	2180	2190	2200	2210	2220	2230	2240

Detailed Protein Report

Cmpd.	No. of Cmpds.	m/z meas.	Δ m/z [ppm]	z	Rt [min]	Score	P	Range	Sequence	Modification	Ratios
25	1	796.8073	-13.58	2	29.5	12.2	0	166-179	R.CACTYGFTGPQCER.D	Carbamidomethyl: 1	Wdown:Qdown 0.27
1152	1	991.7003	-40.24	3	44.0	14.6	1	356-380	K. MQCCCDAGRCWSPGVTVAPEMC A	Carbamidomethyl: 3, 4, 5, 22; Oxidation: 1, 21	
2800	1	913.4636	99.50	2	63.5	13.3	0	1178-1192	K.YQCACNPGYHSTPDR.L	Carbamidomethyl: 3, 5	
976	9	635.1635	-177.36	2	40.7	23.1	1	2482-2492	R.SCKDLDECATK.Q	Carbamidomethyl: 8	Wdown:Qdown 1.21<br>mdown:qdown 0.87

BRUKER

Detailed Protein Report

Protein 63: coagulation factor VIII isoform a precursor [Homo sapiens]

**Accession:**	gi|4503647	**Score:**	63.0
**Database:**	refseq_human(refseq_human_20140103.fasta)	**MW [kDa]:**	266.8
**Database Date:**	2015-11-30	**pI:**	7.0
**Modification(s):**	Carbamidomethyl, Oxidation	**Sequence Coverage [%]:**	3.8
		**No. of unique Peptides:**	4

Quantitation

mdown:qdown	**Median:** 0.79	**CV:** 0.00 %	**No. of Peptides:** 1

Detailed Protein Report

10	20	30	40	50	60	70	80
MQIELSTCFF	LCLLRFCFSA	TRRYYLGAVE	LSWDYMQSDL	GELPVDARFP	PRVPKSFPFN	TSVVYKKTLF	VEFTDHLFNI
90	100	110	120	130	140	150	160
AKPRPPWMGL	LGPTIQAEVY	DTVVITLKNM	ASHPVSLHAV	GVSYWKASEG	AEYDDQTSQR	EKEDDKVFPG	GSHTYVWQVL
170	180	190	200	210	220	230	240
KENGPMASDP	LCLTYSYLSH	VDLVKDLNSG	LIGALLVCRE	GSLAKEKTQT	LHKFILLFAV	FDEGKSWHSE	TKNSLMQDRD
250	260	270	280	290	300	310	320
AASARAWPKM	HTVNGYVNRS	LPGLIGCHRK	SVYWHVIGMG	TTPEVHSIFL	EGHTFLVRNH	RQASLEISPI	TFLTAQTLLM
330	340	350	360	370	380	390	400
DLGQFLLFCH	ISSHQHDGME	AYVKVDSCPE	EPQLRMKNNE	EAEDYDDDLT	DSEMDVVRFD	DDNSPSFIQI	RSVAKKHPKT
410	420	430	440	450	460	470	480
WVHYIAAEEE	DWDYAPLVLA	PDDRSYKSQY	LNNGPQRIGR	KYKKVRFMAY	TDETFKTREA	IQHESGILGP	LLYGEVGDTL
490	500	510	520	530	540	550	560
LIIFKNQASR	PYNIYPHGIT	DVRPLYSRRL	PKGVKHLKDF	PILPGEIFKY	KWTVTVEDGP	TKSDPRCLTR	YYSSFVNMER
570	580	590	600	610	620	630	640
DLASGLIGPL	LICYKESVDQ	RGNQIMSDKR	NVILFSVFDE	NRSWYLTENI	QRFLPNPAGV	QLEDPEFQAS	NIMHSINGYV
650	660	670	680	690	700	710	720
FDSLQLSVCL	HEVAYWYILS	IGAQTDFLSV	FFSGYTFKHK	MVYEDTLTLF	PFSGETVFMS	MENPGLWILG	CHNSDFRNRG
730	740	750	760	770	780	790	800
MTALLKVSSC	DKNTGDYYED	SYEDISAYLL	SKNNAIEPRS	FSQNSRHPST	RQKQFNATTI	PENDIEKTDP	WFAHRTPMPK
810	820	830	840	850	860	870	880
IQNVSSSDLL	MLLRQSPTPH	GLSLSDLQEA	KYETFSDDPS	PGAIDSNNSL	SEMTHFRPQL	HHSGDMVFTP	ESGLQLRLNE
890	900	910	920	930	940	950	960
KLGTTAATEL	KKLDFKVSST	SNNLISTIPS	DNLAAGTDNT	SSLGPPSMPV	HYDSQLDTTL	FGKKSSPLTE	SGGPLSLSEE
970	980	990	1000	1010	1020	1030	1040
NNDSKLLESG	LMNSQESSWG	KNVSSTESGR	LFKGKRAHGP	ALLTKDNALF	KVSISLLKTN	KTSNNSATNR	KTHIDGPSLL
1050	1060	1070	1080	1090	1100	1110	1120
IENSPSVWQN	ILESDTEFKK	VTPLIHDRML	MDKNATALRL	NHMSNKTTSS	KNMEMVQQKK	EGPIPPDAQN	PDMSFFKMLF
1130	1140	1150	1160	1170	1180	1190	1200
LPESARWIQR	THGKNSLNSG	QGPSPKQLVS	LGPEKSVEGQ	NFLSEKNKVV	VGKGEFTKDV	GLKEMVFPSS	RNLFLTNLDN
1210	1220	1230	1240	1250	1260	1270	1280
LHENNTHNQE	KKIQEEIEKK	ETLIQENVVL	PQIHTVTGTK	NFMKNLFLLS	TRQNVEGSYD	GAYAPVLQDF	RSLNDSTNRT
1290	1300	1310	1320	1330	1340	1350	1360
KKHTAHFSKK	GEEENLEGLG	NQTKQIVEKY	ACTTRISPNT	SQQNFVTQRS	KRALKQFRLP	LEETELEKRI	IVDDTSTQWS
1370	1380	1390	1400	1410	1420	1430	1440
KNMKHLTPST	LTQIDYNEKE	KGAITQSPLS	DCLTRSHSIP	QANRSPLPIA	KVSSFPSIRP	IYLTRVLFQD	NSSHLPAASY
1450	1460	1470	1480	1490	1500	1510	1520
RKKDSGVQES	SHFLQGAKKN	NLSLAILTLE	MTGDQREVGS	LGTSATNSVT	YKKVENTVLP	KPDLPKTSGK	VELLPKVHIY
1530	1540	1550	1560	1570	1580	1590	1600
QKDLFPTETS	NGSPGHLDLV	EGSLLQGTEG	AIKWNEANRP	GKVPFLRVAT	ESSAKTPSKL	LDPLAWDNHY	GTQIPKEEWK
1610	1620	1630	1640	1650	1660	1670	1680
SQEKSPEKTA	FKKKDTILSL	NACESNHAIA	AINEGQNKPE	IEVTWAKQGR	TERLCSQNPP	VLKRHQREIT	RTTLQSDQEE
1690	1700	1710	1720	1730	1740	1750	1760
IDYDDTISVE	MKKEDFDIYD	EDENQSPRSF	QKKTRHYFIA	AVERLWDYGM	SSSPHVLRNR	AQSGSVPQFK	KVVFQEFTDG
1770	1780	1790	1800	1810	1820	1830	1840
SFTQPLYRGE	LNEHLGLLGP	YIRAEVEDNI	MVTFRNQASR	PYSFYSSLIS	YEEDQRQGAE	PRKNFVKPNE	TKTYFWKVQH
1850	1860	1870	1880	1890	1900	1910	1920
HMAPTKDEFD	CKAWAYFSDV	DLEKDVHSGL	IGPLLVCHTN	TLNPAHGRQV	TVQEFALFFT	IFDETKSWYF	TENMERNCRA
1930	1940	1950	1960	1970	1980	1990	2000
PCNIQMEDPT	FKENYRFHAI	NGYIMDTLPG	LVMAQDQRIR	WYLLSMGSNE	NIHSIHFSGH	VFTVRKKEEY	KMALYNLYPG
2010	2020	2030	2040	2050	2060	2070	2080
VFETVEMLPS	KAGIWRVECL	IGEHLHAGMS	TLFLVYSNKC	QTPLGMASGH	IRDFQITASG	QYGQWAPKLA	RLHYSGSINA
2090	2100	2110	2120	2130	2140	2150	2160
WSTKEPFSWI	KVDLLAPMII	HGIKTQGARQ	KFSSLYISQF	IIMYSLDGKK	WQTYRGNSTG	TLMVFFGNVD	SSGIKHNIFN
2170	2180	2190	2200	2210	2220	2230	2240

Detailed Protein Report

Cmpd.	No. of Cmpds.	m/z meas.	Δ m/z [ppm]	z	Rt [min]	Score	P	Range	Sequence	Modification	Ratios
2170	1	937.3499	-130.54	2	55.7	11.5	0	1-15	-.MQIELSTCFFLCLLR.F	Carbamidomethyl: 12	mdown:qdown 0.79
2041	1	856.8157	-171.77	2	55.6	11.8	2	1060-1073	K.KVTPLIHDRMLMDK.N	Oxidation: 12	
2184	1	1061.5230	-9.68	3	57.5	13.4	0	1523-1553	K. DLFPTETSNGSPGHLDLVEGSLLQ W		
2720	1	710.3296	-131.82	2	65.3	14.5	0	2092-2104	K.VDLLAPMIIHGIK.T		

Detailed Protein Report

Protein 64: profilin-1 [Homo sapiens]

Accession: gi|4826898
Database: refseq_human(refseq_human_20140103.fasta)
Database Date: 2015-11-30
Modification(s): Carbamidomethyl

Score: 62.6
MW [kDa]: 15.0
pI: 9.4
Sequence Coverage [%]: 20.7
No. of unique Peptides: 2

Quantitation

mdown:qdown	**Median:** 1.19	**CV:** 0.00 %	**No. of Peptides:**	1
Wdown:Qdown	**Median:** 1.07	**CV:** 0.00 %	**No. of Peptides:**	1

```
        10         20         30         40         50         60         70         80
MAGWNAYIDN LMADGTCQDA AIVGYKDSPS VWAAVPGKTF VNITPAEVGV LVGKDRSSFY VNGLTLGGQK CSVIRDSLLQ
        90        100        110        120        130        140        150
DGEFSMDLRT KSTGGAPTFN VTVTKTDKTL VLLMGKEGVH GGLINKKCYE MASHLRRSQY
```

Cmpd.	No. of Cmpds.	m/z meas.	Δ m/z [ppm]	z	Rt [min]	Score	P	Range	Sequence	Modification	Ratios
2934	9	822.4733	1.65	2	65.5	46.8	0	39-54	K.TFVNITPAEVGVLVGK.D		
1495	1	850.7379	-173.16	2	48.5	15.9	2	128-140	K.CYEMASHLRRSQY.-	Carbamidomethyl: 1	mdown:qdown 1.19<br>Wdown:Qdown 1.07

Detailed Protein Report

Protein 65:	PREDICTED: probable E3 ubiquitin-protein ligase MYCBP2 isoform X2 [Homo sapiens]		
**Accession:**	gi|530402184	**Score:**	62.5
**Database:**	refseq_human(refseq_human_20140103.fasta)	**MW [kDa]:**	504.8
**Database Date:**	2015-11-30	**pI:**	6.6
**Modification(s):**	Carbamidomethyl, Oxidation	**Sequence Coverage [%]:**	1.9
		**No. of unique Peptides:**	3

BRUKER

Detailed Protein Report

10	20	30	40	50	60	70	80
MMMCAATASP	AAASSGLGGD	GFYPAATFSS	SPAPGALFMP	VPDGSVAAAG	LGLGLPAADS	RGHYQLLLSG	RALADRYRRI
90	100	110	120	130	140	150	160
YTAALNDRDQ	GGGSAGHPAS	RNKKILNKKK	LKRKQKSKSK	VKTRSKSENL	ENTVIIPDIK	LHSNPSAFNI	YCNVRHCVLE
170	180	190	200	210	220	230	240
WQKKEISLAA	ASKNSVQSGE	SDSDEEEESK	EPPIKLPKII	EVGLCEVFEL	IKETRFSHPS	LCLRSLQALL	NVLQGQQPEG
250	260	270	280	290	300	310	320
LQSEPPEVLE	SLFQLLLEIT	VRSTGMNDST	GQSLTALSCA	CLFSLVASWG	ETGRTLQAIS	AILTNNGSHA	CQTIQVPTIL
330	340	350	360	370	380	390	400
NSLQRSVQAV	LVGKIQIQDW	FSNGIKKAAL	MHKWPLKEIS	VDEDDQCLLQ	NDGFFLYLLC	KDGLYKIGSG	YSGTVRGHIY
410	420	430	440	450	460	470	480
NSTSRIRNRK	EKKSWLGYAQ	GYLLYRDVNN	HSMTAIRISP	ETLEQDGTVM	LPDCHTEGQN	ILFTDGEYIN	QIAASRDDGF
490	500	510	520	530	540	550	560
VVRIFATSTE	PVLQQELQLK	LARKCLHACG	ISLFDLEKDL	HIISTGFDEE	SAILGAGREF	ALMKTANGKI	YYTGKYQSLG
570	580	590	600	610	620	630	640
IKQGGPSAGK	WVELPITKSP	KIVHFSVGHD	GSHALLVAED	GSIFFTGSAS	KGEDGESTKS	RRQSKPYKPK	KIIKMEGKIV
650	660	670	680	690	700	710	720
VYTACNNGSS	SVISKDGELY	MFGKDAIYSD	SSSLVTDLKG	HFVTQVAMGK	AHTCVLMKNG	EVWTFGVNNK	GQCGRDTGAM
730	740	750	760	770	780	790	800
NQGGKGFGVE	NMATAMDEDL	EEELDEKDEK	SMMCPPGMHK	WKLEQCMVCT	VCGDCTGYGA	SCVSSGRPDR	VPGGICGCGS
810	820	830	840	850	860	870	880
GESGCAVCGC	CKACARELDG	QEARQRGILD	AVKEMIPLDL	LLAVPVPGVN	IEEHLQLRQE	EKRQRVIRRH	RLEEGRGPLV
890	900	910	920	930	940	950	960
FAGPIFMNHR	EQALARLRSH	PAQLKHKRDK	HKDGSGERGE	KDASKITTYP	PGSVRFDCEL	RAVQVSCGFH	HSVVLMENGD
970	980	990	1000	1010	1020	1030	1040
VYTFGYGQHG	QLGHGDVNSR	GCPTLVQALP	GPSTQVTAGS	NHTAVLLMDG	QVFTFGSFSK	GQLGRPILDV	PYWNAKPAPM
1050	1060	1070	1080	1090	1100	1110	1120
PNIGSKYGRK	ATWIGASGDQ	TFLRIDEALI	NSHVLATSEI	FASKHIIGLV	PASISEPPPF	KCLLINKVDG	SCKTFNDSEQ
1130	1140	1150	1160	1170	1180	1190	1200
EDLQGFGVCL	DPVYDVIWRF	RPNTRELWCY	NAVVADARLP	SAADMQSRCS	ILSPELALPT	GSRALTTRSH	AALHILGCLD
1210	1220	1230	1240	1250	1260	1270	1280
TLAAMQDLKM	GVASTEEETQ	AVMKVYSKED	YSVVNRFESH	GGGWGYSAHS	VEAIRFSADT	DILLGGLGLF	GGRGEYTAKI
1290	1300	1310	1320	1330	1340	1350	1360
KLFELGPDGG	DHETDGDLLA	ETDVLAYDCA	AREKYAMMFD	EPVLLQAGWW	YVAWARVSGP	SSDCGSHGQA	SITTDDGVVF
1370	1380	1390	1400	1410	1420	1430	1440
QFKSSKKSNN	GTDVNAGQIP	QLLYRLPTSD	GSASKGKQQT	SEPVHILKRS	FARTVSVECF	ESLLSILHWS	WTTLVLGVEE
1450	1460	1470	1480	1490	1500	1510	1520
LRGLKGFQFT	ATLLDLERLR	FVGTCCLRLL	RVYTCEIYPV	SATGKAVVEE	TSKLAECIGK	TRTLLRKILS	EGVDHCMVKL
1530	1540	1550	1560	1570	1580	1590	1600
DNDPQGYLSQ	PLSLLEAVLQ	ECHNTFTACF	HSFYPTPALQ	WACLCDLLNC	LDQDIQEANF	KTSSSRLLAA	VMSALCHTSV
1610	1620	1630	1640	1650	1660	1670	1680
KLTSIFPIAY	DGEVLLRSIV	KQVSTENDST	LVHRFPLLVA	HMEKLSQSEE	NISGMTSFRE	VLEKMLVIVV	LPVRNSLRRE
1690	1700	1710	1720	1730	1740	1750	1760
NELFSSHLVS	NTCGLLASIV	SELTASALGS	EVDGLNSLHS	VKASANRFTK	TSQGRSWNTG	NGSPDAICFS	VDKPGIVVVG
1770	1780	1790	1800	1810	1820	1830	1840
FSVYGGGGIH	EYELEVLVDD	SEHAGDSTHS	HRWTSLELVK	GTYTTDDSPS	DIAEIRLDKV	VPLKENVKYA	VRLRNYGSRT
1850	1860	1870	1880	1890	1900	1910	1920
ANGDGGMTTV	QCPDGVTFTF	STCSLSSNGT	NQTRGQIPQI	LYYRSEFDGD	LQSQLLSKAN	EEDKNCSRAL	SVVSTVVRAS
1930	1940	1950	1960	1970	1980	1990	2000
KDLLHRALAV	DADDIPELLS	SSSLFSMLLP	LIIAYIGPVA	AAIPKVAVEV	FGLVQQLLPS	VAILNQKYAP	PAFNPNQSTD
2010	2020	2030	2040	2050	2060	2070	2080
STTGNQPEQG	LSACTTSSHY	AVIESEHPYK	PACVMHYKVT	FPECVRWMTI	EFDPQCGTAQ	SEDVLRLLIP	VRTVQNSGYG
2090	2100	2110	2120	2130	2140	2150	2160
PKLTSVHENL	NSWIELKKFS	GSSGWPTMVL	VLPGNEALFS	LETASDYVKD	DKASFYGFKC	FAIGYEFSPG	PDEGVIQLEK
2170	2180	2190	2200	2210	2220	2230	2240

Detailed Protein Report

Cmpd.	No. of Cmpds.	m/z meas.	Δ m/z [ppm]	z	Rt [min]	Score	P	Range	Sequence	Modification
1222	1	927.2551	104.81	3	44.9	11.3	1	1184-1209	R.ALTTRSHAALHILGCLDTLAAMQDLK.M	Oxidation: 22
1800	1	658.7973	-153.52	2	52.5	13.4	1	1909-1921	R.ALSVVSTVVRASK.D	
1461	1	799.6263	-94.96	3	48.0	13.1	1	4259-4279	K.AMVEFREHTGKPTTSSSEACR.F	Carbamidomethyl: 20; Oxidation: 2

Detailed Protein Report

Protein 66: low-density lipoprotein receptor-related protein 2 precursor [Homo sapiens]

**Accession:**	gi|126012573	**Score:**	61.9
**Database:**	refseq_human(refseq_human_20140103.fasta)	**MW [kDa]:**	521.6
**Database Date:**	2015-11-30	**pI:**	4.8
**Modification(s):**	Carbamidomethyl	**Sequence Coverage [%]:**	1.5
		**No. of unique Peptides:**	4

BRUKER

Detailed Protein Report

10	20	30	40	50	60	70	80
MDRGPAAVAC	TLLLALVACL	APASGQECDS	AHFRCGSGHC	IPADWRCDGT	KDCSDDADEI	GCAVVTCQQG	YFKCQSEGQC
90	100	110	120	130	140	150	160
IPNSWVCDQD	QDCDDGSDER	QDCSQSTCSS	HQITCSNGQC	IPSEYRCDHV	RDCPDGADEN	DCQYPTCEQL	TCDNGACYNT
170	180	190	200	210	220	230	240
SQKCDWKVDC	RDSSDEINCT	EICLHNEFSC	GNGECIPRAY	VCDHDNDCQD	GSDEHACNYP	TCGGYQFTCP	SGRCIYQNWV
250	260	270	280	290	300	310	320
CDGEDDCKDN	GDEDGCESGP	HDVHKCSPRE	WSCPESGRCI	SIYKVCDGIL	DCPGREDENN	TSTGKYCSMT	LCSALNCQYQ
330	340	350	360	370	380	390	400
CHETPYGGAC	FCPPGYIINH	NDSRTCVEFD	DCQIWGICDQ	KCESRPGRHL	CHCEEGYILE	RGQYCKANDS	FGEASIIFSN
410	420	430	440	450	460	470	480
GRDLLIGDIH	GRSFRILVES	QNRGVAVGVA	FHYHLQRVFW	TDTVQNKVFS	VDINGLNIQE	VLNVSVETPE	NLAVDWVNNK
490	500	510	520	530	540	550	560
IYLVETKVNR	IDMVNLDGSY	RVTLITENLG	HPRGIAVDPT	VGYLFFSDWE	SLSGEPKLER	AFMDGSNRKD	LVKTKLGWPA
570	580	590	600	610	620	630	640
GVTLDMISKR	VYWVDSRFDY	IETVTYDGIQ	RKTVVHGGSL	IPHPFGVSLF	EGQVFFTDWT	KMAVLKANKF	TETNPQVYYQ
650	660	670	680	690	700	710	720
ASLRPYGVTV	YHSLRQPYAT	NPCKDNNGGC	EQVCVLSHRT	DNDGLGFRCK	CTFGFQLDTD	ERHCIAVQNF	LIFSSQVAIR
730	740	750	760	770	780	790	800
GIPFTLSTQE	DVMVPVSGNP	SFFVGIDFDA	QDSTIFFSDM	SKHMIFKQKI	DGTGREILAA	NRVENVESLA	FDWISKNLYW
810	820	830	840	850	860	870	880
TDSHYKSISV	MRLADKTRRT	VVQYLNNPRS	VVVHPFAGYL	FFTDWFRPAK	IMRAWSDGSH	LLPVINTTLG	WPNGLAIDWA
890	900	910	920	930	940	950	960
ASRLYWVDAY	FDKIEHSTFD	GLDRRRLGHI	EQMTHPFGLA	IFGEHLFFTD	WRLGAIIRVR	KADGGEMTVI	RSGIAYILHL
970	980	990	1000	1010	1020	1030	1040
KSYDVNIQTG	SNACNQPTHP	NGDCSHFCFP	VPNFQRVCGC	PYGMRLASNH	LTCEGDPTNE	PPTEQCGLFS	FPCKNGRCVP
1050	1060	1070	1080	1090	1100	1110	1120
NYYLCDGVDD	CHDNSDEQLC	GTLNNTCSSS	AFTCGHGECI	PAHWRCDKRN	DCVDGSDEHN	CPTHAPASCL	DTQYTCDNHQ
1130	1140	1150	1160	1170	1180	1190	1200
CISKNWVCDT	DNDCGDGSDE	KNCNSTETCQ	PSQFNCPNHR	CIDLSFVCDG	DKDCVDGSDE	VGCVLNCTAS	QFKCASGDKC
1210	1220	1230	1240	1250	1260	1270	1280
IGVTNRCDGV	FDCSDNSDEA	GCPTRPPGMC	HSDEFQCQED	GICIPNFWEC	DGHPDCLYGS	DEHNACVPKT	CPSSYFHCDN
1290	1300	1310	1320	1330	1340	1350	1360
GNCIHRAWLC	DRDNDCGDMS	DEKDCPTQPF	RCPSWQWQCL	GHNICVNLSV	VCDGIFDCPN	GTDESPLCNG	NSCSDFNGGC
1370	1380	1390	1400	1410	1420	1430	1440
THECVQEPFG	AKCLCPLGFL	LANDSKTCED	IDECDILGSC	SQHCYNMRGS	FRCSCDTGYM	LESDGRTCKV	TASESLLLLV
1450	1460	1470	1480	1490	1500	1510	1520
ASQNKIIADS	VTSQVHNIYS	LVENGSYIVA	VDFDSISGRI	FWSDATQGKT	WSAFQNGTDR	RVVFDSSIIL	TETIAIDWVG
1530	1540	1550	1560	1570	1580	1590	1600
RNLYWTDYAL	ETIEVSKIDG	SHRTVLISKN	LTNPRGLALD	PRMNEHLLFW	SDWGHHPRIE	RASMDGSMRT	VIVQDKIFWP
1610	1620	1630	1640	1650	1660	1670	1680
CGLTIDYPNR	LLYFMDSYLD	YMDFCDYNGH	HRRQVIASDL	IIRHPYALTL	FEDSVYWTDR	ATRRVMRANK	WHGGNQSVVM
1690	1700	1710	1720	1730	1740	1750	1760
YNIQWPLGIV	AVHPSKQPNS	VNPCAFSRCS	HLCLLSSQGP	HFYSCVCPSG	WSLSPDLLNC	LRDDQPFLIT	VRQHIIFGIS
1770	1780	1790	1800	1810	1820	1830	1840
LNPEVKSNDA	MVPIAGIQNG	LDVEFDDAEQ	YIYWVENPGE	IHRVKTDGTN	RTVFASISMV	GPSMNLALDW	ISRNLYSTNP
1850	1860	1870	1880	1890	1900	1910	1920
RTQSIEVLTL	HGDIRYRKTL	IANDGTALGV	GFPIGITVDP	ARGKLYWSDQ	GTDSGVPAKI	ASANMDGTSV	KTLFTGNLEH
1930	1940	1950	1960	1970	1980	1990	2000
LECVTLDIEE	QKLYWAVTGR	GVIERGNVDG	TDRMILVHQL	SHPWGIAVHD	SFLYYTDEQY	EVIERVDKAT	GANKIVLRDN
2010	2020	2030	2040	2050	2060	2070	2080
VPNLRGLQVY	HRRNAAESSN	GCSNNMNACQ	QICLPVPGGL	FSCACATGFK	LNPDNRSCSP	YNSFIVVSML	SAIRGFSLEL
2090	2100	2110	2120	2130	2140	2150	2160
SDHSETMVPV	AGQGRNALHV	DVDVSSGFIY	WCDFSSSVAS	DNAIRRIKPD	GSSLMNIVTH	GIGENGVRGI	AVDWVAGNLY
2170	2180	2190	2200	2210	2220	2230	2240

Detailed Protein Report

Cmpd.	No. of Cmpds.	m/z meas.	Δ m/z [ppm]	z	Rt [min]	Score	P	Range	Sequence	Modification
573	1	656.8903	-140.87	3	35.8	15.5	1	279-295	R.CISIYKVCDGILDCPGR.E	Carbamidomethyl: 1, 8
1173	1	786.4695	28.78	1	44.3	12.4	0	776-782	R.EILAANR.V	
1012	1	974.7041	-35.28	3	41.2	13.1	1	1173-1199	K.DCVDGSDEVGCVLNCTASQFKCASGDK.C	Carbamidomethyl: 11, 15, 22
23	1	788.3643	33.00	3	29.6	20.9	0	3797-3816	R.TCHPEYFQCTSGHCVHSELK.C	Carbamidomethyl: 2

Detailed Protein Report

Protein 67: **PREDICTED: RANBP2-like and GRIP domain-containing protein 8 isoform X4 [Homo sapiens]**

Accession: gi|530369360
Database: refseq_human(refseq_human_20140103.fasta)
Database Date: 2015-11-30

Score: 61.3
MW [kDa]: 166.0
pI: 6.2
Sequence Coverage [%]: 4.7
No. of unique Peptides: 3

10	20	30	40	50	60	70	80
MKGHFYMYAG	SLLLKMGQHG	NNVQWRALSE	LAALCYLIAF	QVPRPKIKLR	EGKAGQNLLE	MMACDRLSQS	GHMLLSLSRG
**90**	**100**	**110**	**120**	**130**	**140**	**150**	**160**
KQDFLKEVVE	TFANKIGQSA	LYDALFSSQS	PKDTSFLGSD	DIGKIDVQEP	ELEDLARYDV	GAIRAHNGSL	QHLTWLGLQW
**170**	**180**	**190**	**200**	**210**	**220**	**230**	**240**
NSLPALPGIR	KWLKQLFHRL	PHETSRLETN	APESICILDL	EVFLLGVVYT	SHLQLKEKCN	SHHSSYQPLC	LPFPVCKQLC
**250**	**260**	**270**	**280**	**290**	**300**	**310**	**320**
TERQKSWWDA	VCTLIHRKAV	PGNLAKLRLL	VQHEINTLRA	QEKHGLQPAL	LVHWAKYLQK	TGSGLNSFYG	QLEYIGRSVH
**330**	**340**	**350**	**360**	**370**	**380**	**390**	**400**
YWKKVLPLLK	IIKKNSIPEP	IDPLFKHFHS	VDIQASEIVE	YEEDAHITFA	ILDAVNGNIE	DAVTAFESIK	SVVSYWNLAL
**410**	**420**	**430**	**440**	**450**	**460**	**470**	**480**
IFHRKAEDIE	NDALSPEEQE	ECRNYLTKTR	DYLIKIIDDG	DSNLSVVKKL	PVPLESVKQM	LNSVMQELED	YSEGGPLYKN
**490**	**500**	**510**	**520**	**530**	**540**	**550**	**560**
GSLRNADSEI	KHSTPSPTKY	SLSPSKSYKY	SPETPPRWTE	DRNSLLNMIC	QQVEAIKKEM	QELKLNSSKS	ASRHRWPTEN
**570**	**580**	**590**	**600**	**610**	**620**	**630**	**640**
YGPDSVPDGY	QGSQTFHGAP	LTVATTGPSV	YYSQSPAYNS	QYLLRPAANV	TPTKGSSNTE	FKSTKEGFSI	PVSADGFKFG
**650**	**660**	**670**	**680**	**690**	**700**	**710**	**720**
ISEPGNQEKK	REKPLENDTG	LQAQDIRGRK	KGRGVIFGQT	SSTFTFADVA	KSTSGEGFQF	GKKDLNFKGF	SGAGEKLFSS
**730**	**740**	**750**	**760**	**770**	**780**	**790**	**800**
RYGKMANKAN	TSGDFEKDDD	AYKTEDSDDI	HFEPVVQMPE	KVELVTGEEG	EKVLYSQGVK	LFRFDAEVRQ	WKERGLGNLK
**810**	**820**	**830**	**840**	**850**	**860**	**870**	**880**
ILKNEVNGKL	RMLMRREQVL	KVCANHWITT	TMNLKPLSGS	DRAWMWSASD	FSDGDAKLER	LAAKFKTPEL	AEEFKQKFEE
**890**	**900**	**910**	**920**	**930**	**940**	**950**	**960**
CQRLLLDIPL	QTPHKLVDTG	RAAKLIQRAE	EMKSGLKDFK	TFLTNDQTKV	TEEENKGSGT	GVAGASDTTI	KPNAENTGPT
**970**	**980**	**990**	**1000**	**1010**	**1020**	**1030**	**1040**
LEWDNYDLRE	DALDDSVSSS	SVHASPLASS	PVRKNLFRFD	ESTTGSNFSF	KSALSLSKSP	AKLNQSGTSV	GTDEESVVTQ
**1050**	**1060**	**1070**	**1080**	**1090**	**1100**	**1110**	**1120**
EEERDGQYFE	PVVPLPDLVE	VSSGEENEQV	VFSHRAEIYR	YDKDVGQWKE	RGIGDIKILQ	NYDNKQVRIV	MRRDQVLKLC
**1130**	**1140**	**1150**	**1160**	**1170**	**1180**	**1190**	**1200**
ANHRITPDMS	LQNMKGTERV	WVWTACDFAD	GERKVEHLAV	RFKLQDVADS	FKKIFDEAKT	AQEKDSLITP	HVSRSSTPRE
**1210**	**1220**	**1230**	**1240**	**1250**	**1260**	**1270**	**1280**
SPCGKIAVAV	LEEITRERTD	VIQGDDVADA	ASEVEVSSTS	ETTTKAVVSP	PKFVFVSESV	KRIFSSEKSK	PFAFGNSSAT
**1290**	**1300**	**1310**	**1320**	**1330**	**1340**	**1350**	**1360**
GSLFGFSFNA	PLKSNNSETS	SVAQSGSESK	VEPKKCELSK	NSDIEQSSDS	KVKNLSASFP	TEESSINYTF	KTPEKEPPLW
**1370**	**1380**	**1390**	**1400**	**1410**	**1420**	**1430**	**1440**
HAEFTKEELV	QKLRSTTKSA	DHLNGLLREI	EATNAVLMEQ	IKLLKSEIRR	LERNQEREKS	AANLEYLKNV	LLQFIFLKPG
**1450**	**1460**	**1470**	**1480**	**1490**			
SERERLLPVI	NTMLQLSPEE	KGKLAAVAQD	EEENPSRSSG				

Cmpd.	No. of Cmpds.	m/z meas.	Δ m/z [ppm]	z	Rt [min]	Score	P	Range	Sequence	Modification
2775	1	557.2986	22.82	3	63.1	14.9	1	623-638	K.STKEGFSIPVSADGFK.F	
1997	1	648.7895	-86.66	2	53.5	11.3	1	1114-1124	R.DQVLKLCANHR.I	
1535	1	1023.6824	101.59	2	49.0	12.2	1	1429-1445	K.NVLLQFIFLKPGSERER.L	

Detailed Protein Report

Protein 68: **PREDICTED: E3 ubiquitin-protein ligase HERC2 isoform X5 [Homo sapiens]**

**Accession:**	gi|578827509	**Score:**	59.6
**Database:**	refseq_human(refseq_human_20140103.fasta)	**MW [kDa]:**	517.6
**Database Date:**	2015-11-30	**pI:**	5.9
		**Sequence Coverage [%]:**	1.0
		**No. of unique Peptides:**	3

Detailed Protein Report

10	20	30	40	50	60	70	80
MPSESFCLAA	QARLDSKWLK	TDIQLAFTRD	GLCGLWNEMV	KDGEIVYTGT	ESTQNGELPP	RKDDSVEPSG	TKKEDLNDKE
90	100	110	120	130	140	150	160
KKDEEETPAP	IYRAKSILDS	WVWGKQPDVN	ELKECLSVLV	KEQQALAVQS	ATTTLSALRL	KQRLVILERY	FIALNRTVFQ
170	180	190	200	210	220	230	240
ENVKVKWKSS	GISLPPVDKK	RDVHGTPATK	GPGSIPLQDQ	HLALAILLEL	AVQRGTLSQM	LSAILLLLQL	WDSGAQETDN
250	260	270	280	290	300	310	320
ERSAQGTSAP	LLPLLQRFQS	IICRKDAPHS	EGDMHLLSGP	LSPNESFLRY	LTLPQDNELA	IDLRQTAVVV	MAHLDRLATP
330	340	350	360	370	380	390	400
CMPPLCSSPT	SHKGSLQEVI	GWGLIGWKYY	ANVIGPIQCE	GLANLGVTQI	ACAEKRFLIL	SRNGRVYTQA	YNSDTLAPQL
410	420	430	440	450	460	470	480
VQGLASRNIV	KIAAHSDGHH	YLALAATGEV	YSWGCGDGGR	LGHGDTVPLE	EPKVISAFSG	KQAGKHVVHI	ACGSTYSAAI
490	500	510	520	530	540	550	560
TAEGELYTWG	RGNYGRLGHG	SSEDEAIPML	VAGLKGLKVI	DVACGSGDAQ	TLAVTENGQV	WSWGDGDYGK	LGRGGSDGCK
570	580	590	600	610	620	630	640
TPKLIEKLQD	LDVVKVRCGS	QFSIALTKDG	QVYSWGKGDN	QRLGHGTEEH	VRYPKLLEGL	QGKKVIDVAA	GSTHCLALTE
650	660	670	680	690	700	710	720
DSEVHSWGSN	DQCQHFDTLR	VTKPEPAALP	GLDTKHIVGI	ACGPAQSFAW	SSCSEWSIGL	RVPFVVDICS	MTFEQLDLLL
730	740	750	760	770	780	790	800
RQVSEGMDGS	ADWPPPQEKE	CVAVATLNLL	RLQLHAAISH	QVDPEFLGLG	LGSILLNSLK	QTVVTLASSA	GVLSTVQSAA
810	820	830	840	850	860	870	880
QAVLQSGWSV	LLPTAEERAR	ALSALLPCAV	SGNEVNISPG	RRFMIDLLVG	SLMADGGLES	ALHAAITAEI	QDIEAKKEAQ
890	900	910	920	930	940	950	960
KEKEIDEQEA	NASTFHRSRT	PLDKDLINTG	ICESSGKQCL	PLVQLIQQLL	RNIASQTVAR	LKDVARRISS	CLDFEQHSRE
970	980	990	1000	1010	1020	1030	1040
RSASLDLLLR	FQRLLISKLY	PGESIGQTSD	ISSPELMGVG	SLLKKYTALL	CTHIGDILPV	AASIASTSWR	HFAEVAYIVE
1050	1060	1070	1080	1090	1100	1110	1120
GDFTGVLLPE	LVVSIVLLLS	KNAGLMQEAG	AVPLLGGLLE	HLDRFNHLAP	GKERDDHEEL	AWPGIMESFF	TGQNCRNNEE
1130	1140	1150	1160	1170	1180	1190	1200
VTLIRKADLE	NHNKDGGFWT	VIDGKVYDIK	DFQTQSLTGN	SILAQFAGED	PVVALEAALQ	FEDTRESMHA	FCVGQYLEPD
1210	1220	1230	1240	1250	1260	1270	1280
QEIVTIPDLG	SLSSPLIDTE	RNLGLLLGLH	ASYLAMSTPL	SPVEIECAKW	LQSSIFSGGL	QTSQIHYSYN	EEKDEDHCSS
1290	1300	1310	1320	1330	1340	1350	1360
PGGTPASKSR	LCSHRRALGD	HSQAFLQAIA	DNNIQDHNVK	DFLCQIERYC	RQCHLTTPIM	FPPEHPVEEV	GRLLLCCLLK
1370	1380	1390	1400	1410	1420	1430	1440
HEDLGHVALS	LVHAGALGIE	QVKHRTLPKS	VVDVCRVVYQ	AKCSLIKTHQ	EQGRSYKEVC	APVIERLRFL	FNELRPAVCN
1450	1460	1470	1480	1490	1500	1510	1520
DLSIMSKFKL	LSSLPRWRRI	AQKIIERRK	KRVPKKPEST	DDEEKIGNEE	SDLEEACILP	HSPINVDKRP	IAIKSPKDKW
1530	1540	1550	1560	1570	1580	1590	1600
QPLLSTVTGV	HKYKWLKQNV	QGLYPQSPLL	STIAEFALKE	EPVDVEKMRK	CLLKQLERAE	VRLEGIDTIL	KLASKNFLLP
1610	1620	1630	1640	1650	1660	1670	1680
SVQYAMFCGW	QRLIPEGIDI	GEPLTDCLKD	VDLIPPFNRM	LLEVTFGKLY	AWAVQNIRNV	LMDASAKFKE	LGIQPVPLQT
1690	1700	1710	1720	1730	1740	1750	1760
ITNENPSGPS	LGTIPQARFL	LVMLSMLTLQ	HGANNLDLLL	NSGMLALTQT	ALRLIGPSCD	NVEEDMNASA	QGASATVLEE
1770	1780	1790	1800	1810	1820	1830	1840
TRKETAPVQL	PVSGPELAAM	MKIGTRVMRG	VDWKWGDQDG	PPPGLGRVIG	ELGEDGWIRV	QWDTGSTNSY	RMGKEGKYDL
1850	1860	1870	1880	1890	1900	1910	1920
KLAELPAAAQ	PSAEDSDTED	DSEAEQTERN	IHPTAMMFTS	TINLLQTLCL	SAGVHAEIMQ	SEATKTLCGL	LRMLVESGTT
1930	1940	1950	1960	1970	1980	1990	2000
DKTSSPNRLV	YREQHRSWCT	LGFVRSIALT	PQVCGALSSP	QWITLLMKVV	EGHAPFTATS	LQRQILAVHL	LQAVLPSWDK
2010	2020	2030	2040	2050	2060	2070	2080
TERARDMKCL	VEKLFDFLGS	LLTTCSSDVP	LLRESTLRRR	RVRPQASLTA	THSSTLAEEV	VALLRTLHSL	TQWNGLINKY
2090	2100	2110	2120	2130	2140	2150	2160
INSQLRSITH	SFVGRPSEGA	QLEDYFPDSE	NPEVGGLMAV	LAVIGGIDGR	LRLGGQVMHD	EFGEGTVTRI	TPKGKITVQF
2170	2180	2190	2200	2210	2220	2230	2240

Detailed Protein Report

Cmpd.	No. of Cmpds.	m/z meas.	Δ m/z [ppm]	z	Rt [min]	Score	P	Range	Sequence	Modification
445	2	592.8266	-45.39	2	34.9	13.6	1	568-577	K.LQDLDVVKVR.C	
2175	1	1031.4134	-71.31	2	55.8	19.5	1	2684-2701	R.CHSSQPGMLLDSWSRMVK.S	
360	1	543.1311	-259.95	2	33.8	14.2	0	3920-3930	K.LYATGYGAGGR.L	

Detailed Protein Report

Protein 69: complement C3 precursor [Homo sapiens]

**Accession:**	gi|115298678	**Score:**	59.5
**Database:**	refseq_human(refseq_human_20140103.fasta)	**MW [kDa]:**	187.0
**Database Date:**	2015-11-30	**pI:**	6.0
		**Sequence Coverage [%]:**	2.8
		**No. of unique Peptides:**	3

10	20	30	40	50	60	70	80
MGPTSGPSLL	LLLLTHLPLA	LGSPMYSIIT	PNILRLESEE	TMVLEAHDAQ	GDVPVTVTVH	DFPGKKLVLS	SEKTVLTPAT
90	100	110	120	130	140	150	160
NHMGNVTFTI	PANREFKSEK	GRNKFVTVQA	TFGTQVVEKV	VLVSLQSGYL	FIQTDKTIYT	PGSTVLYRIF	TVNHKLLPVG
170	180	190	200	210	220	230	240
RTVMVNIENP	EGIPVKQDSL	SSQNQLGVLP	LSWDIPELVN	MGQWKIRAYY	ENSPQQVFST	EFEVKEYVLP	SFEVIVEPTE
250	260	270	280	290	300	310	320
KFYYIYNEKG	LEVTITARFL	YGKKVEGTAF	VIFGIQDGEQ	RISLPESLKR	IPIEDGSGEV	VLSRKVLLDG	VQNPRAEDLV
330	340	350	360	370	380	390	400
GKSLYVSATV	ILHSGSDMVQ	AERSGIPIVT	SPYQIHFTKT	PKYFKPGMPF	DLMVFVTNPD	GSPAYRVPVA	VQGEDTVQSL
410	420	430	440	450	460	470	480
TQGDGVAKLS	INTHPSQKPL	SITVRTKKQE	LSEAEQATRT	MQALPYSTVG	NSNNYLHLSV	LRTELRPGET	LNVNFLLRMD
490	500	510	520	530	540	550	560
RAHEAKIRYY	TYLIMNKGRL	LKAGRQVREP	GQDLVVLPLS	ITTDFIPSFR	LVAYYTLIGA	SGQREVVADS	VWVDVKDSCV
570	580	590	600	610	620	630	640
GSLVVKSGQS	EDRQPVPGQQ	MTLKIEGDHG	ARVVLVAVDK	GVFVLNKKNK	LTQSKIWDVV	EKADIGCTPG	SGKDYAGVFS
650	660	670	680	690	700	710	720
DAGLTFTSSS	GQQTAQRAEL	QCPQPAARRR	RSVQLTEKRM	DKVGKYPKEL	RKCCEDGMRE	NPMRFSCQRR	TRFISLGEAC
730	740	750	760	770	780	790	800
KKVFLDCCNY	ITELRRQHAR	ASHLGLARSN	LDEDIIAEEN	IVSRSEFPES	WLWNVEDLKE	PPKNGISTKL	MNIFLKDSIT
810	820	830	840	850	860	870	880
TWEILAVSMS	DKKGICVADP	FEVTVMQDFF	IDLRLPYSVV	RNEQVEIRAV	LYNYRQNQEL	KVRVELLHNP	AFCSLATTKR
890	900	910	920	930	940	950	960
RHQQTVTIPP	KSSLSVPYVI	VPLKTGLQEV	EVKAAVYHHF	ISDGVRKSLK	VVPEGIRMNK	TVAVRTLDPE	RLGREGVQKE
970	980	990	1000	1010	1020	1030	1040
DIPPADLSDQ	VPDTESETRI	LLQGTPVAQM	TEDAVDAERL	KHLIVTPSGC	GEQNMIGMTP	TVIAVHYLDE	TEQWEKFGLE
1050	1060	1070	1080	1090	1100	1110	1120
KRQGALELIK	KGYTQQLAFR	QPSSAFAAFV	KRAPSTWLTA	YVVKVFSLAV	NLIAIDSQVL	CGAVKWLILE	KQKPDGVFQE
1130	1140	1150	1160	1170	1180	1190	1200
DAPVIHQEMI	GGLRNNNEKD	MALTAFVLIS	LQEAKDICEE	QVNSLPGSIT	KAGDFLEANY	MNLQRSYTVA	IAGYALAQMG
1210	1220	1230	1240	1250	1260	1270	1280
RLKGPLLNKF	LTTAKDKNRW	EDPGKQLYNV	EATSYALLAL	LQLKDFDFVP	PVVRWLNEQR	YYGGGYGSTQ	ATFMVFQALA
1290	1300	1310	1320	1330	1340	1350	1360
QYQKDAPDHQ	ELNLDVSLQL	PSRSSKITHR	IHWESASLLR	SEETKENEGF	TVTAEGKGQG	TLSVVTMYHA	KAKDQLTCNK
1370	1380	1390	1400	1410	1420	1430	1440
FDLKVTIKPA	PETEKRPQDA	KNTMILEICT	RYRGDQDATM	SILDISMMTG	FAPDTDDLKQ	LANGVDRYIS	KYELDKAFSD
1450	1460	1470	1480	1490	1500	1510	1520
RNTLIIYLDK	VSHSEDDCLA	FKVHQYFNVE	LIQPGAVKVY	AYYNLEESCT	RFYHPEKEDG	KLNKLCRDEL	CRCAEENCFI
1530	1540	1550	1560	1570	1580	1590	1600
QKSDDKVTLE	ERLDKACEPG	VDYVYKTRLV	KVQLSNDFDE	YIMAIEQTIK	SGSDEVQVGQ	QRTFISPIKC	REALKLEEKK
1610	1620	1630	1640	1650	1660	1670	
HYLMWGLSSD	FWGEKPNLSY	IIGKDTWVEH	WPEEDECQDE	ENQKQCQDLG	AFTESMVVFG	CPN	

Cmpd.	No. of Cmpds.	m/z meas.	Δ m/z [ppm]	z	Rt [min]	Score	P	Range	Sequence	Modification
2933	1	940.0026	13.02	2	65.4	22.8	0	226-241	K.EYVLPSFEVIVEPTEK.F	
2727	4	701.3852	-51.38	2	62.5	16.6	0	892-904	K.SSLSVPYVIVPLK.T	
1782	3	1086.9427	-40.31	2	50.9	20.1	2	1505-1522	K.LCRDELCRCAEENCFIQK.S	

Detailed Protein Report

Protein 70: fibrinogen beta chain isoform 2 preproprotein [Homo sapiens]

**Accession:**	gi|296080754	**Score:**	59.1
**Database:**	refseq_human(refseq_human_20140103.fasta)	**MW [kDa]:**	49.9
**Database Date:**	2015-11-30	**pI:**	9.0
**Modification(s):**	Carbamidomethyl	**Sequence Coverage [%]:**	5.8
		**No. of unique Peptides:**	2

10	20	30	40	50	60	70	80
MKRMVSWSFH	KLKTMKHLLL	LLLCVFLVKS	QGVNDNEEGF	FSARGHRPLD	KKREEALLQQ	ERPIRNSVDE	LNNNVEAVSQ
**90**	**100**	**110**	**120**	**130**	**140**	**150**	**160**
TSSSSFQYMY	LLKDLWQKRQ	KQVKDNENVV	NEYSSELEKH	QLYIDETVNS	NIPTNLRVLR	SILENLRSKI	QKLESDVSAQ
**170**	**180**	**190**	**200**	**210**	**220**	**230**	**240**
MEYCRTPCTV	SCNIPVVSGK	ECEEIIRKGG	ETSEMYLIQP	DSSVKPYRVY	CDMNTENGGW	TVIQNRQDGS	VDFGRKWDPY
**250**	**260**	**270**	**280**	**290**	**300**	**310**	**320**
KQGFGNVATN	TDGKNYCGLP	GEYWLGNDKI	SQLTRMGPTE	LLIEMEDWKG	DKVKAHYGGF	TVQNEANKYQ	ISVNKYRGTA
**330**	**340**	**350**	**360**	**370**	**380**	**390**	**400**
GNALMDGASQ	LMGENRTMTI	HNGMFFSTYD	RDNDGWLTSD	PRKQCSKEDG	GGWWYNRCHA	ANPNGRYYWG	GQYTWDMAKH
**410**	**420**	**430**	**440**				
GTDDGVVWMN	WKGSWYSMRK	MSMKIRPFFP	QQ				

Cmpd.	No. of Cmpds.	m/z meas.	Δ m/z [ppm]	z	Rt [min]	Score	P	Range	Sequence	Modification
1961	3	893.2648	-149.91	2	53.0	20.5	0	255-269	K.NYCGLPGEYWLGNDK.I	Carbamidomethyl: 3
656	1	620.1188	-231.67	2	36.8	38.6	0	368-377	K.EDGGGWWYNR.C	

Detailed Protein Report

Protein 71: PDZ domain-containing protein 4 [Homo sapiens]

**Accession:**	gi|46559761	**Score:**	59.0
**Database:**	refseq_human(refseq_human_20140103.fasta)	**MW [kDa]:**	86.1
**Database Date:**	2015-11-30	**pI:**	5.7
**Modification(s):**	Carbamidomethyl, Oxidation	**Sequence Coverage [%]:**	10.5
		**No. of unique Peptides:**	2

10	20	30	40	50	60	70	80
MGCNMCVVQK	PEEQYKVMLQ	VNGKELSKLS	QEQTLQALRS	SKEPLVIQVL	RRSPRLRGDS	SCHDLQLVDS	GTQTDITFEH
**90**	**100**	**110**	**120**	**130**	**140**	**150**	**160**
IMALGKLRPP	TPPMVILEPP	PISHEYYDPA	EFMEGGPQEA	DRLDELEYEE	VELYKSSHRD	KLGLMVCYRT	DDEEDLGIYV
**170**	**180**	**190**	**200**	**210**	**220**	**230**	**240**
GEVNPNSIAA	KDGRIREGDR	IIQINGVDVQ	NREEAVAILS	QEENTNISLL	VARPESQLAK	RWKDSDRDDF	LDDFGSENEG
**250**	**260**	**270**	**280**	**290**	**300**	**310**	**320**
ELRARKLKSP	PAQQPGNEEE	KGAPDAGPGL	SNSQELDSGV	GRTDESTRNE	ESSEHDLLGD	EPPSSTNTPG	SLRKFGLQGD
**330**	**340**	**350**	**360**	**370**	**380**	**390**	**400**
ALQSRDFHFS	MDSLLAEGAG	LGGGDVPGLT	DEEYERYREL	LEIKCHLENG	NQLGLLFPRA	SGGNSALDVN	RNESLGHEMA
**410**	**420**	**430**	**440**	**450**	**460**	**470**	**480**
MLEEELRHLE	FKCRNILRAQ	KMQQLRERCM	KAWLLEEESL	YDLAASEPKK	HELSDISELP	EKSDKDSTSA	YNTGESCRST
**490**	**500**	**510**	**520**	**530**	**540**	**550**	**560**
PLLVEPLPES	PLRRAMAGNS	NLNRTPPGPA	VATPAKAAPP	PGSPAKFRSL	SRDPEAGRRQ	HAEERGRRNP	KTGLTLERVG
**570**	**580**	**590**	**600**	**610**	**620**	**630**	**640**
PESSPYLSRR	HRGQGQEGEH	YHSCVQLAPT	RGLEELGHGP	LSLAGGPRVG	GVAAAATEAP	RMEWKVKVRS	DGTRYVAKRP
**650**	**660**	**670**	**680**	**690**	**700**	**710**	**720**
VRDRLLKARA	LKIREERSGM	TTDDDAVSEM	KMGRYWSKEE	RKQHLIRARE	QRKRREFMMQ	SRLECLREQQ	NGDSKPELNI
**730**	**740**	**750**	**760**	**770**			
IALSHRKTMK	KRNKKILDNW	ITIQEMLAHG	ARSADGKRVY	NPLLSVTTV			

Cmpd.	No. of Cmpds.	m/z meas.	Δ m/z [ppm]	z	Rt [min]	Score	P	Range	Sequence	Modification
2759	1	905.4805	55.19	3	65.8	12.2	1	2-24	M.GCNMCVVQKPEEQYKVMLQVNGK.E	Carbamidomethyl: 2; Oxidation: 4, 17
450	1	1100.7316	-85.85	3	34.6	14.6	0	326-356	R.DFHFSMDSLLAEGAGLGGGDVPGLTDEEYER.Y	Oxidation: 6

Detailed Protein Report

Protein 72: tenascin-X isoform 1 precursor [Homo sapiens]

**Accession:**	gi|188528648	**Score:**	58.0
**Database:**	refseq_human(refseq_human_20140103.fasta)	**MW [kDa]:**	457.9
**Database Date:**	2015-11-30	**pI:**	4.9
**Modification(s):**	Carbamidomethyl	**Sequence Coverage [%]:**	1.6
		**No. of unique Peptides:**	4

Detailed Protein Report

10	20	30	40	50	60	70	80
MMPAQYALTS	SLVLLVLLST	ARAGPFSSRS	NVTLPAPRPP	PQPGGHTVGA	GVGSPSSQLY	EHTVEGGEKQ	VVFTHRINLP
90	100	110	120	130	140	150	160
PSTGCGCPPG	TEPPVLASEV	QALRVRLEIL	EELVKGLKEQ	CTGGCCPASA	QAGTGQTDVR	TLCSLHGVFD	LSRCTCSCEP
170	180	190	200	210	220	230	240
GWGGPTCSDP	TDAEIPPSSP	PSASGSCPDD	CNDQGRCVRG	RCVCFPGYTG	PSCGWPSCPG	DCQGRGRCVQ	GVCVCRAGFS
250	260	270	280	290	300	310	320
GPDCSQRSCP	RGCSQRGRCE	GGRCVCDPGY	TGDDCGMRSC	PRGCSQRGRC	ENGRCVCNPG	YTGEDCGVRS	CPRGCSQRGR
330	340	350	360	370	380	390	400
CKDGRCVCDP	GYTGEDCGTR	SCPWDCGEGG	RCVDGRCVCW	PGYTGEDCST	RTCPRDCRGR	GRCEDGECIC	DTGYSGDDCG
410	420	430	440	450	460	470	480
VRSCPGDCNQ	RGRCEDGRCV	CWPGYTGTDC	GSRACPRDCR	GRGRCENGVC	VCNAGYSGED	CGVRSCPGDC	RGRGRCESGR
490	500	510	520	530	540	550	560
CMCWPGYTGR	DCGTRACPGD	CRGRGRCVDG	RCVCNPGFTG	EDCGSRRCPG	DCRGHGLCED	GVCVCDAGYS	GEDCSTRSCP
570	580	590	600	610	620	630	640
GGCRGRGQCL	DGRCVCEDGY	SGEDCGVRQC	PNDCSQHGVC	QDGVCICWEG	YVSEDCSIRT	CPSNCHGRGR	CEEGRCLCDP
650	660	670	680	690	700	710	720
GYTGPTCATR	MCPADCRGRG	RCVQGVCLCH	VGYGGEDCGQ	EEPPASACPG	GCGPRELCRA	GQCVCVEGFR	GPDCAIQTCP
730	740	750	760	770	780	790	800
GDCRGRGECH	DGSCVCKDGY	AGEDCGEEVP	TIEGMRMHLL	EETTVRTEWT	PAPGPVDAYE	IQFIPTTEGA	SPPFTARVPS
810	820	830	840	850	860	870	880
SASAYDQRGL	APGQEYQVTV	RALRGTSWGL	PASKTITTMI	DGPQDLRVVA	VTPTTLELGW	LRPQAEVDRF	VVSYVSAGNQ
890	900	910	920	930	940	950	960
RVRLEVPPEA	DGTLLTDLMP	GVEYVVTVTA	ERGRAVSYPA	SVRANTGSSP	LGLLGTTDEP	PPSGPSTTQG	AQAPLLQQRP
970	980	990	1000	1010	1020	1030	1040
QELGELRVLG	RDETGRLRVV	WTAQPDTFAY	FQLRMRVPEG	PGAHEEVLPG	DVRQALVPPP	PPGTPYELSL	HGVPPGGKPS
1050	1060	1070	1080	1090	1100	1110	1120
DPIIYQGIMD	KDEEKPGKSS	GPPRLGELTV	TDRTSDSLLL	RWTVPEGEFD	SFVIQYKDRD	GQPQVVPVEG	PQRSAVITSL
1130	1140	1150	1160	1170	1180	1190	1200
DPGRKYKFVL	YGFVGKKRHG	PLVAEAKILP	QSDPSPGTPP	HLGNLWVTDP	TPDSLHLSWT	VPEGQFDTFM	VQYRDRDGRP
1210	1220	1230	1240	1250	1260	1270	1280
QVVPVEGPER	SFVVSSLDPD	HKYRFTLFGI	ANKKRYGPLT	ADGTTAPERK	EEPPRPEFLE	QPLLGELTVT	GVTPDSLRLS
1290	1300	1310	1320	1330	1340	1350	1360
WTVAQGPFDS	FMVQYKDAQG	QPQAVPVAGD	ENEVTVPGLD	PDRKYKMNLY	GLRGRQRVGP	ESVVAKTAPQ	EDVDETPSPT
1370	1380	1390	1400	1410	1420	1430	1440
ELGTEAPESP	EEPLLGELTV	TGSSPDSLSL	FWTVPQGSFD	SFTVQYKDRD	GRPRAVRVGG	KESEVTVGGL	EPGHKYKMHL
1450	1460	1470	1480	1490	1500	1510	1520
YGLHEGQRVG	PVSAVGVTAP	QQEETPPATE	SPLEPRLGEL	TVTDVTPNSV	GLSWTVPEGQ	FDSFIVQYKD	KDGQPQVVPV
1530	1540	1550	1560	1570	1580	1590	1600
AADQREVTVY	NLEPERKYKM	NMYGLHDGQR	MGPLSVVIVT	APLPPAPATE	ASKPPLEPRL	GELTVTDITP	DSVGLSWTVP
1610	1620	1630	1640	1650	1660	1670	1680
EGEFDSFVVQ	YKDRDGQPQV	VPVAADQREV	TIPDLEPSRK	YKFLLFGIQD	GKRRSPVSVE	AKTVARGDAS	PGAPPRLGEL
1690	1700	1710	1720	1730	1740	1750	1760
WVTDPTPDSL	RLSWTVPEGQ	FDSFVVQFKD	KDGPQVVPVE	GHERSVTVTP	LDAGRKYRFL	LYGLLGKKRH	GPLTADGTTE
1770	1780	1790	1800	1810	1820	1830	1840
ARSAMDDTGT	KRPPKPRLGE	ELQVTTVTQN	SVGLSWTVPE	GQFDSFVVQY	KDRDGQPQVV	PVEGSLREVS	VPGLDPAHRY
1850	1860	1870	1880	1890	1900	1910	1920
KLLLYGLHHG	KRVGPISAVA	ITAGREETET	ETTAPTPPAP	EPHLGELTVE	EATSHTLHLS	WMVTEGEFDS	FEIQYTDRDG
1930	1940	1950	1960	1970	1980	1990	2000
QLQMVRIGGD	RNDITLSGLE	SDHRYLVTLY	GFSDGKHVGP	VHVEALTVPE	EEKPSEPPTA	TPEPPIKPRL	GELTVTDATP
2010	2020	2030	2040	2050	2060	2070	2080
DSLSLSWTVP	EGQFDHFLVQ	YRNGDGQPKA	VRVPGHEEGV	TISGLEPDHK	YKMNLYGFHG	GQRMGPVSVV	GVTAAEEETP
2090	2100	2110	2120	2130	2140	2150	2160
SPTEPSMEAP	EPAEEPLLGE	LTVTGSSPDS	LSLSWTVPQG	RFDSFTVQYK	DRDGRPQVVR	VGGEESEVTV	GGLEPGRKYK
2170	2180	2190	2200	2210	2220	2230	2240

Detailed Protein Report

Cmpd.	No. of Cmpds.	m/z meas.	Δ m/z [ppm]	z	Rt [min]	Score	P	Range	Sequence	Modification
2799	1	732.8911	134.58	2	64.1	13.0	1	725-737	R.GRGECHDGSCVCK.D	Carbamidomethyl: 10, 12
1929	1	890.3591	-119.35	2	52.8	13.5	1	1418-1435	R.VGGKESEVTVGGLEPGHK.Y	
2906	1	557.9675	41.54	3	65.1	15.9	0	2141-2157	R.VGGEESEVTVGGLEPGR.K	
2882	1	981.9684	-29.71	2	65.2	15.6	1	2349-2366	R.VPGHEDRVTISGLEPDNK.Y	

Detailed Protein Report

Protein 73: PREDICTED: caspase-10 isoform X1 [Homo sapiens]

Accession: gi|530371069
Database: refseq_human(refseq_human_20140103.fasta)
Database Date: 2015-11-30
Modification(s): Carbamidomethyl

Score: 57.8
MW [kDa]: 58.8
pI: 6.2
Sequence Coverage [%]: 9.8
No. of unique Peptides: 3

10	20	30	40	50	60	70	80
MKSQGQHWYS	SSDKNCKVSF	REKLLIIDSN	LGVQDVENLK	FLCIGLVPNK	KLEKSSSASD	VFEHLLAEDL	LSEEDPFFLA
**90**	**100**	**110**	**120**	**130**	**140**	**150**	**160**
ELLYIIRQKK	LLQHLNCTKE	EVERLLPTRQ	RVSLFRNLLY	ELSEGIDSEN	LKDMIFLLKD	SLPKTEMTSL	SFLAFLEKQG
**170**	**180**	**190**	**200**	**210**	**220**	**230**	**240**
KIDEDNLTCL	EDLCKTVVPK	LLRNIEKYKR	EKAIQIVTPP	VDKEAESYQG	EEELVSQTDV	KTFLEALPES	WQNKHAGSNG
**250**	**260**	**270**	**280**	**290**	**300**	**310**	**320**
NRATNGAPSL	VSRGMQGASA	NTLNSETSTK	RAAVYRMNRN	HRGLCVIVNN	HSFTSLKDRQ	GTHKDAEILS	HVFQWLGFTV
**330**	**340**	**350**	**360**	**370**	**380**	**390**	**400**
HIHNNVTKVE	MEMVLQKQKC	NPAHADGDCF	VFCILTHGRF	GAVYSSDEAL	IPIREIMSHF	TALQCPRLAE	KPKLFFIQAC
**410**	**420**	**430**	**440**	**450**	**460**	**470**	**480**
QGEEIQPSVS	IEADALNPEQ	APTSLQDSIP	AEADFLLGLA	TVPGYVSFRH	VEEGSWYIQS	LCNHLKKLVP	RHEDILSILT
**490**	**500**	**510**	**520**	**530**			
AVNDDVSRRV	DKQGTKKQMP	QPAFTLRKKL	VFPVPLDALS	L			

Cmpd.	No. of Cmpds.	m/z meas.	Δ m/z [ppm]	z	Rt [min]	Score	P	Range	Sequence	Modification
2857	12	710.3665	-46.32	2	64.9	19.3	1	133-144	K.DMIFLLKDSLPK.T	
1677	1	933.3261	-154.07	2	50.9	26.9	1	235-253	K.HAGSNGNRATNGAPSLVSR.G	
2646	1	1145.5422	35.87	2	61.8	11.6	0	340-359	K.CNPAHADGDCFVFCILTHGR.F	Carbamidomethyl: 10, 14

Detailed Protein Report

Protein 74: PREDICTED: NEDD8 ultimate buster 1 isoform X1 [Homo sapiens]

**Accession:**	gi|530387030	**Score:**	57.3
**Database:**	refseq_human(refseq_human_20140103.fasta)	**MW [kDa]:**	73.2
**Database Date:**	2015-11-30	**pI:**	6.2
**Modification(s):**	Carbamidomethyl, Oxidation	**Sequence Coverage [%]:**	4.9
		**No. of unique Peptides:**	2

10	20	30	40	50	60	70	80
MKTNGGRCRI	RALCWSRREW	RGAGMAQKKY	LQAKLTQFLR	EDRIQLWKPP	YTDENKKVGL	ALKDLAKQYS	DRLECCENEV
**90**	**100**	**110**	**120**	**130**	**140**	**150**	**160**
EKVIEEIRCK	AIERGTGNDN	YRTTGIATIE	VFLPPRLKKD	RKNLLETRLH	ITGRELRSKI	AETFGLQENY	IKIVINKKQL
**170**	**180**	**190**	**200**	**210**	**220**	**230**	**240**
QLGKTLEEQG	VAHNVKAMVL	ELKQSEEDAR	KNFQLEEEEQ	NEAKLKEKQI	QRTKRGLEIL	AKRAETVVDP	EMTPYLDIAN
**250**	**260**	**270**	**280**	**290**	**300**	**310**	**320**
QTGRSIRIPP	SERKALMLAM	GYHEKGRAFL	KRKEYGIALP	CLLDADKYFC	ECCRELLDTV	DNYAVLQLDI	VWCYFRLEQL
**330**	**340**	**350**	**360**	**370**	**380**	**390**	**400**
ECLDDAEKKL	NLAQKCFKNC	YGENHQRLVH	IKGNCGKEKV	LFLRLYLLQG	IRNYHSGNDV	EAYEYLNKAR	QLFKELYIDP
**410**	**420**	**430**	**440**	**450**	**460**	**470**	**480**
SKVDNLLQLG	FTAQEARLGL	RACDGNVDHA	ATHITNRREE	LAQIRKEEKE	KKRRRLENIR	FLKGMGYSTH	AAQQVLHAAS
**490**	**500**	**510**	**520**	**530**	**540**	**550**	**560**
GNLDEALKIL	LSNPQMWWLN	DSNPETDNRQ	ESPSQENIDR	LVYMGFDALV	AEAALRVFRG	NVQLAAQTLA	HNGGSLPPEL
**570**	**580**	**590**	**600**	**610**	**620**	**630**	**640**
PLSPEDSLSP	PATSPSDSAG	TSSASTDEDM	ETEAVNEILE	DIPEHEEDYL	DSTLEDEEII	IAEYLSYVEN	RKSATKKN

Cmpd.	No. of Cmpds.	m/z meas.	Δ m/z [ppm]	z	Rt [min]	Score	P	Range	Sequence	Modification
2521	1	648.3547	69.36	2	62.0	10.7	0	255-265	K.ALMLAMGYHEK.G	Oxidation: 3, 6
2062	1	625.2581	-159.60	2	54.4	16.1	2	329-338	K.KLNLAQKCFK.N	Carbamidomethyl: 8

BRUKER

Detailed Protein Report

Protein 75: centrosomal protein of 290 kDa [Homo sapiens]

**Accession:**	gi|109255234	**Score:**	57.2
**Database:**	refseq_human(refseq_human_20140103.fasta)	**MW [kDa]:**	290.2
**Database Date:**	2015-11-30	**pI:**	5.7
**Modification(s):**	Oxidation	**Sequence Coverage [%]:**	2.0
		**No. of unique Peptides:**	5

Quantitation

mdown:qdown	**Median:** 0.41	**CV:** 41.53 %	**No. of Peptides:** 2

Detailed Protein Report

10	20	30	40	50	60	70	80
MPPNINWKEI	MKVDPDDLPR	QEELADNLLI	SLSKVEVNEL	KSEKQENVIH	LFRITQSLMK	MKAQEVELAL	EEVEKAGEEQ
90	100	110	120	130	140	150	160
AKFENQLKTK	VMKLENELEM	AQQSAGGRDT	RFLRNEICQL	EKQLEQKDRE	LEDMEKELEK	EKKVNEQLAL	RNEEAENENS
170	180	190	200	210	220	230	240
KLRRENKRLK	KKNEQLCQDI	IDYQKQIDSQ	KETLLSRRGE	DSDYRSQLSK	KNYELIQYLD	EIQTLTEANE	KIEVQNQEMR
250	260	270	280	290	300	310	320
KNLEESVQEM	EKMTDEYNRM	KAIVHQTDNV	IDQLKKENDH	YQLQVQELTD	LLKSKNEEDD	PIMVAVNAKV	EEWKLILSSK
330	340	350	360	370	380	390	400
DDEIIEYQQM	LHNLREKLKN	AQLDADKSNV	MALQQGIQER	DSQIKMLTEQ	VEQYTKEMEK	NTCIIEDLKN	ELQRNKGAST
410	420	430	440	450	460	470	480
LSQQTHMKIQ	STLDILKEKT	KEAERTAELA	EADAREKDKE	LVEALKRLKD	YESGVYGLED	AVVEIKNCKN	QIKIRDREIE
490	500	510	520	530	540	550	560
ILTKEINKLE	LKISDFLDEN	EALRERVGLE	PKTMIDLTEF	RNSKHLKQQQ	YRAENQILLK	EIESLEEERL	DLKKKIRQMA
570	580	590	600	610	620	630	640
QERGKRSATS	GLTTEDLNLT	ENISQGDRIS	ERKLDLLSLK	NMSEAQSKNE	FLSRELIEKE	RDLERSRTVI	AKFQNKLKEL
650	660	670	680	690	700	710	720
VEENKQLEEG	MKEILQAIKE	MQKDPDVKGG	ETSLIIPSLE	RLVNAIESKN	AEGIFDASLH	LKAQVDQLTG	RNEELRQELR
730	740	750	760	770	780	790	800
ESRKEAINYS	QQLAKANLKI	DHLEKETSLL	RQSEGSNVVF	KGIDLPDGIA	PSSASIINSQ	NEYLIHLLQE	LENKEKKLKN
810	820	830	840	850	860	870	880
LEDSLEDYNR	KFAVIRHQQS	LLYKEYLSEK	ETWKTESKTI	KEEKRKLEDQ	VQQDAIKVKE	YNNLLNALQM	DSDEMKKILA
890	900	910	920	930	940	950	960
ENSRKITVLQ	VNEKSLIRQY	TTLVELERQL	RKENEKQKNE	LLSMEAEVCE	KIGCLQRFKE	MAIFKIAALQ	KVVDNSVSLS
970	980	990	1000	1010	1020	1030	1040
ELELANKQYN	ELTAKYRDIL	QKDNMLVQRT	SNLEHLECEN	ISLKEQVESI	NKELEITKEK	LHTIEQAWEQ	ETKLGNESSM
1050	1060	1070	1080	1090	1100	1110	1120
DKAKKSITNS	DIVSISKKIT	MLEMKELNER	QRAEHCQKMY	EHLRTSLKQM	EERNFELETK	FAELTKINLD	AQKVEQMLRD
1130	1140	1150	1160	1170	1180	1190	1200
ELADSVSKAV	SDADRQRILE	LEKNEMELKV	EVSKLREISD	IARRQVEILN	AQQQSRDKEV	ESLRMQLLDY	QAQSDEKSLI
1210	1220	1230	1240	1250	1260	1270	1280
AKLHQHNVSL	QLSEATALGK	LESITSKLQK	MEAYNLRLEQ	KLDEKEQALY	YARLEGRNRA	KHLRQTIQSL	RRQFSGALPL
1290	1300	1310	1320	1330	1340	1350	1360
AQQEKFSKTM	IQLQNDKLKI	MQEMKNSQQE	HRNMENKTLE	MELKLKGLEE	LISTLKDTKG	AQKVINWHMK	IEELRLQELK
1370	1380	1390	1400	1410	1420	1430	1440
LNRELVKDKE	EIKYLNNIIS	EYERTISSLE	EEIVQQNKFH	EERQMAWDQR	EVDLERQLDI	FDRQQNEILN	AAQKFEEATG
1450	1460	1470	1480	1490	1500	1510	1520
SIPDPSLPLP	NQLEIALRKI	KENIRIILET	RATCKSLEEK	LKEKESALRL	AEQNILSRDK	VINELRLRLP	ATAEREKLIA
1530	1540	1550	1560	1570	1580	1590	1600
ELGRKEMEPK	SHHTLKIAHQ	TIANMQARLN	QKEEVLKKYQ	RLLEKAREEQ	REIVKKHEED	LHILHHRLEL	QADSSLNKFK
1610	1620	1630	1640	1650	1660	1670	1680
QTAWDLMKQS	PTPVPTNKHF	IRLAEMEQTV	AEQDDSLSSL	LVKLKKVSQD	LERQREITEL	KVKEFENIKL	QLQENHEDEV
1690	1700	1710	1720	1730	1740	1750	1760
KKVKAEVEDL	KYLLDQSQKE	SQCLKSELQA	QKEANSRAPT	TTMRNLVERL	KSQLALKEKQ	QKALSRALLE	LRAEMTAAAE
1770	1780	1790	1800	1810	1820	1830	1840
ERIISATSQK	EAHLNVQQIV	DRHTRELKTQ	VEDLNENLLK	LKEALKTSKN	RENSLTDNLN	DLNNELQKKQ	KAYNKILREK
1850	1860	1870	1880	1890	1900	1910	1920
EEIDQENDEL	KRQIKRLTSG	LQGKPLTDNK	QSLIEELQRK	VKKLENQLEG	KVEEVDLKPM	KEKNAKEELI	RWEEGKKWQA
1930	1940	1950	1960	1970	1980	1990	2000
KIEGIRNKLK	EKEGEVFTLT	KQLNTLKDLF	AKADKEKLTL	QRKLKTTGMT	VDQVLGIRAL	ESEKELEELK	KRNLDLENDI
2010	2020	2030	2040	2050	2060	2070	2080
LYMRAHQALP	RDSVVEDLHL	QNRYLQEKLH	ALEKQFSKDT	YSKPSISGIE	SDDHCQREQE	LQKENLKLSS	ENIELKFQLE
2090	2100	2110	2120	2130	2140	2150	2160
QANKDLPRLK	NQVRDLKEMC	EFLKKEKAEV	QRKLGHVRGS	GRSGKTIPEL	EKTIGLMKKV	VEKVQRENEQ	LKKASGILTS
2170	2180	2190	2200	2210	2220	2230	2240

Detailed Protein Report

Cmpd.	No. of Cmpds.	m/z meas.	Δ m/z [ppm]	z	Rt [min]	Score	P	Range	Sequence	Modification	Ratios
2146	2	937.4291	-49.48	2	55.4	15.4	2	115-129	R.NEICQLEKQLEQKDR.E		
1969	1	703.7594	-155.73	2	54.7	10.7	0	1966-1978	K.TTGMTVDQVLGIR.A	Oxidation: 4	
1615	1	494.1461	-276.33	2	50.1	10.7	2	2106-2113	K.EKAEVQRK.L		mdown:qdown 0.61
1421	1	846.6046	174.69	1	47.5	10.1	0	2387-2393	K.DLETQLK.M		mdown:qdown 0.28
1648	1	858.4049	-102.70	1	49.1	10.3	0	2438-2444	K.NILLEEK.V		

Detailed Protein Report

Protein 76: PREDICTED: ankyrin repeat domain-containing protein 11 isoform X3 [Homo sapiens]

**Accession:**	gi|578828957	**Score:**	56.1
**Database:**	refseq_human(refseq_human_20140103.fasta)	**MW [kDa]:**	293.2
**Database Date:**	2015-11-30	**pI:**	6.6
**Modification(s):**	Oxidation	**Sequence Coverage [%]:**	2.3
		**No. of unique Peptides:**	4

Quantitation

mdown:qdown	**Median:** 3.05	**CV:** 0.00 %	**No. of Peptides:** 1

Detailed Protein Report

10	20	30	40	50	60	70	80
MPPAQSLLWC	CKAGTLLEAP	WHEMEVPRSK	KKEKQGPERK	RIKKEPVTRK	AGLLFGMGLS	GIRAGYPLSE	RQQVALLMQM
90	100	110	120	130	140	150	160
TAEESANSPV	DTTPKHPSQS	TVCQKGTPNS	ASKTKDKVNK	RNERGETRLH	RAAIRGDARR	IKELISEGAD	VNVKDFAGWT
170	180	190	200	210	220	230	240
ALHEACNRGY	YDVAKQLLAA	GAEVNTKGLD	DDTPLHDAAN	NGHYKVVKLL	LRYGGNPQQS	NRKGETPLKV	ANSPTMVNLL
250	260	270	280	290	300	310	320
LGKGTYTSSE	ESSTESSEEE	DAPSFAPSSS	VDGNNTDSEF	EKGLKHKAKN	PEPQKATAPV	KDEYEFDEDD	EQDRVPPVDD
330	340	350	360	370	380	390	400
KHLLKKDYRK	ETKSNSFISI	PKMEVKSYTK	NNTIAPKKAS	HRILSDTSDE	EDASVTVGTG	EKLRLSAHTI	LPGSKTREPS
410	420	430	440	450	460	470	480
NAKQQKEKNK	VKKKRKKETK	GREVRFGKRS	DKFCSSESES	ESSESGEDDR	DSLGSSGCLK	GSPLVLKDPS	LFSSLSASST
490	500	510	520	530	540	550	560
SSHGSSAAQK	QNPSHTDQHT	KHWRTDNWKT	ISSPAWSEVS	SLSDSTRTRL	TSESDYSSEG	SSVESLKPVR	KRQEHRKRAS
570	580	590	600	610	620	630	640
LSEKKSPFLS	SAEGAVPKLD	KEGKVVKKHK	TKHKHKNKEK	GQCSISQELK	LKSFTYEYED	SKQKSDKAIL	LENDLSTENK
650	660	670	680	690	700	710	720
LKVLKHDRDH	FKKEEKLSKM	KLEEKEWLFK	DEKSLKRIKD	TNKDISRSFR	EEKDRSNKAE	KERSLKEKSP	KEEKLRLYKE
730	740	750	760	770	780	790	800
ERKKKSKDRP	SKLEKKNDLK	EDKISKEKEK	IFKEDKEKLK	KEKVYREDSA	FDEYCNKNQF	LENEDTKFSL	SDDQRDRWFS
810	820	830	840	850	860	870	880
DLSDSSFDFK	GEDSWDSPVT	DYRDMKSDSV	AKLILETVKE	DSKERRRDSR	AREKRDYREP	FFRKKDRDYL	DKNSEKRKEQ
890	900	910	920	930	940	950	960
TEKHKSVPGY	LSEKDKKRRE	SAEAGRDRKD	ALESCKERRD	GRAKPEEAHR	EELKECGCES	GFKDKSDGDF	GKGLEPWERH
970	980	990	1000	1010	1020	1030	1040
HPAREKEKKD	GPDKERKEKT	KPERYKEKSS	DKDKSEKSIL	EKCQKDKEFD	KCFKEKKDTK	EKHKDTHGKD	KERKASLDQG
1050	1060	1070	1080	1090	1100	1110	1120
KEKKEKAFPG	IISEDFSEKK	DDKKGKEKSW	YIADIFTDES	EDDRDSCMGS	GFKMGEASDL	PRTDGLQEKE	EGREAYASDR
1130	1140	1150	1160	1170	1180	1190	1200
HRKSSDKQHP	ERQKDKEPRD	RRKDRGAADA	GRDKKEKVFE	KHKEKKDKES	TEKYKDRKDR	ASVDSTQDKK	NKQKLPEKAE
1210	1220	1230	1240	1250	1260	1270	1280
KKHAAEDKAK	SKHKEKSDKE	HSKERKSSRS	ADAEKSLLEK	LEEEALHEYR	EDSNDKISEV	SSDSFTDRGQ	EPGLTAFLEV
1290	1300	1310	1320	1330	1340	1350	1360
SFTEPPGDDK	PRESACLPEK	LKEKERHRHS	SSSSKKSHDR	ERAKKEKAEK	KEKGEDYKEG	GSRKDSGQYE	KDFLEADAYG
1370	1380	1390	1400	1410	1420	1430	1440
VSYNMKADIE	DELDKTIELF	STEKKDKNDS	EREPSKKIEK	ELKPYGSSAI	NILKEKKKRE	KHREKWRDEK	ERHRDRHADG
1450	1460	1470	1480	1490	1500	1510	1520
LLRHHRDELL	RHHRDEQKPA	TRDKDSPPRV	LKDKSRDEGP	RLGDAKLKEK	FKDGAEKEKG	DPVKMSNGND	KVAPSKDPGK
1530	1540	1550	1560	1570	1580	1590	1600
KDARPREKLL	GDGDLMMTSF	ERMLSQKDLE	IEERHKRHKE	RMKQMEKLRH	RSGDPKLKEK	AKPADDGRKK	GLDIPAKKPP
1610	1620	1630	1640	1650	1660	1670	1680
GLDPPFKDKK	LKESTPIPPA	AENKLHPASG	ADSKDWLAGP	HMKEVLPASP	RPDQSRPTGV	PTPTSVLSCP	SYEEVMHTPR
1690	1700	1710	1720	1730	1740	1750	1760
TPSCSADDYA	DLVFDCADSQ	HSTPVPTAPT	SACSPSFFDR	FSVASSGLSE	NASQAPARPL	STNLYRSVSV	DIRRTPEEEF
1770	1780	1790	1800	1810	1820	1830	1840
SVGDKLFRQQ	SVPAASSYDS	PMPPSMEDRA	PLPPVPAEKF	ACLSPGYYSP	DYGLPSPKVD	ALHCPPAAVV	TVTPSPEGVF
1850	1860	1870	1880	1890	1900	1910	1920
SSLQAKPSPS	PRAELLVPSL	EGALPPDLDT	SEDQQATAAI	IPPEPSYLEP	LDEGPFSAVI	TEEPVEWAHP	SEQALASSLI
1930	1940	1950	1960	1970	1980	1990	2000
GGTSENPVSW	PVGSDLLLKS	PQRFPESPKR	FCPADPLHSA	APGPFSASEA	PYPAPPASPA	PYALPVAEPG	LEDVKDGVDA
2010	2020	2030	2040	2050	2060	2070	2080
VPAAISTSEA	APYAPPSGLE	SFFSNCKSLP	EAPLDVAPEP	ACVAAVAQVE	ALGPLENSFL	DGSRGLSHLG	QVEPVPWADA
2090	2100	2110	2120	2130	2140	2150	2160
FAGPEDDLDL	GPFSLPELPL	QTKDAADGEA	EPVEESLAPP	EEMPPGAPGV	INGGDVSTVV	AEEPPALPPD	QASTRLPAEL
2170	2180	2190	2200	2210	2220	2230	2240

Detailed Protein Report

Cmpd.	No. of Cmpds.	m/z meas.	Δ m/z [ppm]	z	Rt [min]	Score	P	Range	Sequence	Modification	Ratios
2918	1	1155.0468	-67.97	2	65.2	13.4	2	50-71	R. KAGLLFGMGLSGIRAGYPLSER. Q	Oxidation: 8	
1383	2	637.4051	150.05	3	45.8	14.2	0	510-527	K.TISSPAWSEVSSLSDSTR.T		mdown:qdown 3.05
313	2	501.1733	-259.03	2	33.0	13.5	2	1482-1490	R.LGDAKLKEK.F		
2584	1	658.2319	-130.00	2	61.0	15.1	0	2381-2391	K.LDAIEPYHSDR.A		

Detailed Protein Report

Protein 77: hemicentin-1 precursor [Homo sapiens]

**Accession:**	gi|118572606	**Score:**	56.1
**Database:**	refseq_human(refseq_human_20140103.fasta)	**MW [kDa]:**	613.0
**Database Date:**	2015-11-30	**pI:**	6.1
**Modification(s):**	Carbamidomethyl	**Sequence Coverage [%]:**	1.3
		**No. of unique Peptides:**	3

Quantitation

Wdown:Qdown	**Median:**	6.26	**CV:**	0.00 %	**No. of Peptides:**	1

Detailed Protein Report

10	20	30	40	50	60	70	80
MISWEVVHTV	FLFALLYSSL	AQDASPQSEI	RAEEIPEGAS	TLAFVFDVTG	SMYDDLVQVI	EGASKILETS	LKRPKRPLFN
90	100	110	120	130	140	150	160
FALVPFHDPE	IGPVTITTDP	KKFQYELREL	YVQGGGDCPE	MSIGAIKIAL	EISLPGSFIY	VFTDARSKDY	RLTHEVLQLI
170	180	190	200	210	220	230	240
QQKQSQVVFV	LTGDCDDRTH	IGYKVYEEIA	STSSGQVFHL	DKKQVNEVLK	WVEEAVQASK	VHLLSTDHLE	QAVNTWRIPF
250	260	270	280	290	300	310	320
DPSLKEVTVS	LSGPSPMIEI	RNPLGKLIKK	GFGLHELLNI	HNSAKVVNVK	EPEAGMWTVK	TSSSGRHSVR	ITGLSTIDFR
330	340	350	360	370	380	390	400
AGFSRKPTLD	FKKTVSRPVQ	GIPTYVLLNT	SGISTPARID	LLELLSISGS	SLKTIPVKYY	PHRKPYGIWN	ISDFVPPNEA
410	420	430	440	450	460	470	480
FFLKVTGYDK	DDYLFQRVSS	VSFSSIVPDA	PKVTMPEKTP	GYYLQPGQIP	CSVDSLLPFT	LSFVRNGVTL	GVDQYLKESA
490	500	510	520	530	540	550	560
SVNLDIAKVT	LSDEGFYECI	AVSSAGTGRA	QTFFDVSEPP	PVIQVPNNVT	VTPGERAVLT	CLIISAVDYN	LTWQRNDRDV
570	580	590	600	610	620	630	640
RLAEPARIRT	LANLSLELKS	VKFNDAGEYH	CMVSSEGGSS	AASVFLTVQE	PPKVTVMPKN	QSFTGGSEVS	IMCSATGYPK
650	660	670	680	690	700	710	720
PKIAWTVNDM	FIVGSHRYRM	TSDGTLFIKN	AAPKDAGIYG	CLASNSAGTD	KQNSTLRYIE	APKLMVVQSE	LLVALGDITV
730	740	750	760	770	780	790	800
MECKTSGIPP	PQVKWFKGDL	ELRPSTFLII	DPLLGLLKIQ	ETQDLDAGDY	TCVAINEAGR	ATGKITLDVG	SPPVFIQEPA
810	820	830	840	850	860	870	880
DVSMEIGSNV	TLPCYVQGYP	EPTIKWRRLD	NMPIFSRPFS	VSSISQLRTG	ALFILNLWAS	DKGTYICEAE	NQFGKIQSET
890	900	910	920	930	940	950	960
TVTVTGLVAP	LIGISPSVAN	VIEGQQLTLP	CTLLAGNPIP	ERRWIKNSAM	LLQNPYITVR	SDGSLHIERV	QLQDGGEYTC
970	980	990	1000	1010	1020	1030	1040
VASNVAGTNN	KTTSVVVHVL	PTIQHGQQIL	STIEGIPVTL	PCKASGNPKP	SVIWSKKGEL	ISTSSAKFSA	GADGSLYVVS
1050	1060	1070	1080	1090	1100	1110	1120
PGGEESGEYV	CTATNTAGYA	KRKVQLTVYV	RPRVFGDQRG	LSQDKPVEIS	VLAGEEVTLP	CEVKSLPPPI	ITWAKETQLI
1130	1140	1150	1160	1170	1180	1190	1200
SPFSPRHTFL	PSGSMKITET	RTSDSGMYLC	VATNIAGNVT	QAVKLNVHVP	PKIQRGPKHL	KVQVGQRVDI	PCNAQGTPLP
1210	1220	1230	1240	1250	1260	1270	1280
VITWSKGGST	MLVDGEHHVS	NPDGTLSIDQ	ATPSDAGIYT	CVATNIAGTD	ETEITLHVQE	PPTVEDLEPP	YNTTFQERVA
1290	1300	1310	1320	1330	1340	1350	1360
NQRIEFPCPA	KGTPKPTIKW	LHNGRELTGR	EPGISILEDG	TLLVIASVTP	YDNGEYICVA	VNEAGTTERK	YNLKVHVPPV
1370	1380	1390	1400	1410	1420	1430	1440
IKDKEQVTNV	SVLLNQLTNL	FCEVEGTPSP	IIMWYKDNVQ	VTESSTIQTV	NNGKILKLFR	ATPEDAGRYS	CKAINIAGTS
1450	1460	1470	1480	1490	1500	1510	1520
QKYFNIDVLV	PPTIIGTNFP	NEVSVVLNRD	VALECQVKGT	PFPDIHWFKD	GKPLFLGDPN	VELLDRGQVL	HLKNARRNDK
1530	1540	1550	1560	1570	1580	1590	1600
GRYQCTVSNA	AGKQAKDIKL	TIYIPPSIKG	GNVTTDISVL	INSLIKLECE	TRGLPMPAIT	WYKDGQPIMS	SSQALYIDKG
1610	1620	1630	1640	1650	1660	1670	1680
QYLHIPRAQV	SDSATYTCHV	ANVAGTAEKS	FHVDVYVPPM	IEGNLATPLN	KQVVIAHSLT	LECKAAGNPS	PILTWLKDGV
1690	1700	1710	1720	1730	1740	1750	1760
PVKANDNIRI	EAGGKKLEIM	SAQEIDRGQY	ICVATSVAGE	KEIKYEVDVL	VPPAIEGGDE	TSYFIVMVNN	LLELDCHVTG
1770	1780	1790	1800	1810	1820	1830	1840
SPPPTIMWLK	DGQLIDERDG	FKILLNGRKL	VIAQAQVSNT	GLYRCMAANT	AGDHKKEFEV	TVHVPPTIKS	SGLSERVVVK
1850	1860	1870	1880	1890	1900	1910	1920
YKPVALQCIA	NGIPNPSITW	LKDDQPVNTA	QGNLKIQSSG	RVLQIAKTLL	EDAGRYTCVA	TNAAGETQQH	IQLHVHEPPS
1930	1940	1950	1960	1970	1980	1990	2000
LEDAGKMLNE	TVLVSNPVQL	ECKAAGNPVP	VITWYKDNRL	LSGSTSMTFL	NRGQIIDIES	AQISDAGIYK	CVAINSAGAT
2010	2020	2030	2040	2050	2060	2070	2080
ELFYSLQVHV	APSISGSNNM	VAVVVNNPVR	LECEARGIPA	PSLTWLKDGS	PVSSFSNGLQ	VLSGGRILAL	TSAQISDTGR
2090	2100	2110	2120	2130	2140	2150	2160
YTCVAVNAAG	EKQRDIDLRV	YVPPNIMGEE	QNVSVLISQA	VELLCQSDAI	PPPTLTWLKD	GHPLLKKPGL	SISENRSVLK
2170	2180	2190	2200	2210	2220	2230	2240

Detailed Protein Report

Cmpd.	No. of Cmpds.	m/z meas.	Δ m/z [ppm]	z	Rt [min]	Score	P	Range	Sequence	Modification	Ratios
2962	1	1019.9724	-61.55	2	66.2	12.4	0	1188-1206	R.VDIPCNAQGTPLPVITWSK.G		
1984	1	883.9757	-72.13	3	53.5	15.0	1	5183-5205	R.RTSDGLSCQDINECQESSPCHQRC	Carbamidomethyl: 8	
1025	1	676.6792	37.90	3	41.3	16.6	2	5247-5264	K.NTRGGYKCIDLCPNGMTK.A	Carbamidomethyl: 8	Wdown:Qdown 6.26

Detailed Protein Report

Protein 78: coiled-coil domain-containing protein 168 [Homo sapiens]

**Accession:**	gi|226246554	**Score:**	56.0
**Database:**	refseq_human(refseq_human_20140103.fasta)	**MW [kDa]:**	801.4
**Database Date:**	2015-11-30	**pI:**	9.6
		**Sequence Coverage [%]:**	0.9
		**No. of unique Peptides:**	2

Detailed Protein Report

10	20	30	40	50	60	70	80
MSKQYYSFKK	GVGSGLEDNT	FMTLWDFLES	WIIQNDWVAI	FFIILLGIIF	EIILMKACAS	FWKKPTLPEK	GSSDVQETED
90	100	110	120	130	140	150	160
SCPKSRKLAP	ENWSVINSSS	GERVGTFLEK	RITSSLTSEE	KECNFEDRIL	FSREILWSGT	SESEDQVSPS	SESHVPSSNG
170	180	190	200	210	220	230	240
ISSSLPLFYS	EVEETCLSHT	EHPDREYETI	QFSSKKLFSM	MKTNKNKNSG	FSSDLSFSAS	RFTVENEDLD	VAPCPLAHLF
250	260	270	280	290	300	310	320
LSRDQVRLLE	ENVRNQIPSK	PKTKLGSRTT	YQCSRSQESL	NQNQPSVGMV	ISVQAQDSFP	GQNAFQNQGL	YEVQFTSQAQ
330	340	350	360	370	380	390	400
YINHNQESIK	SQPESKASNF	AQPEDVMKKP	FSSSTQDSFQ	SQDLDRNQHF	VEVPSIVEAK	YSVKGLESDE	HLGEDQHCVW
410	420	430	440	450	460	470	480
FIDSNKVKYS	IKGQDTIFKN	AEFLVLTLNP	NLVTEDMPQL	RSVKAQGQQQ	IVSSELNQDS	VYSSVPLLST	IKGQKNRRKT
490	500	510	520	530	540	550	560
PDSKSKLSLN	VPSLKAKKTP	TSQVFQITVC	HTLKNRNELG	CKNNTEKKEL	HERKDISDIA	LHLISVSKLI	LPYVKNYSRK
570	580	590	600	610	620	630	640
QLVKVMPGLI	KCGHFLQKQN	KSPDTEKINY	AGPLEETGIS	DITKKEKEYD	KENKRLKNIS	PKMLPQLEQS	FMVNTVQLKA
650	660	670	680	690	700	710	720
PCLLVETNGK	SKESLKDSIT	QAKGIGITEF	HVLNSKKPFD	LHIPKHKTSL	EEAISKPMQK	LVSSPEMESN	NRMKIQEDLQ
730	740	750	760	770	780	790	800
SSENSHLQLS	NGEELPTSTP	KTQRCFPREN	TQKQKDFLEL	VLELSNVGLL	ISPGSKMHKS	SEELEAIKIQ	VNTESVNLKE
810	820	830	840	850	860	870	880
SKPLILNVTE	DSDLRESEEL	ECNTGSNITN	MHQDKETSDA	FHSATYTTIS	QLPDTETHSI	SKAKADTLRI	IRLSHSASKQ
890	900	910	920	930	940	950	960
EKLPDEKETQ	NAEYIDKSCT	FKKPQQCDRK	EQEKEANSEL	TQGFRFSIHL	KQKPKYVKFQ	MEQISSGSSK	APNKEQEVQP
970	980	990	1000	1010	1020	1030	1040
QTLSTQTILE	NSPCPMMDPF	QVEKVKQSTD	RPTDRESAGD	PKNPLTMPEN	LPVGELLIET	TEYSVPFGGN	LQKTTDSHIA
1050	1060	1070	1080	1090	1100	1110	1120
EEKEDVKRYL	PAVALGSFNN	HLLTLPYFKR	QEIKKKLSET	KSVLSVKYVI	MKVKKPAISL	MPYINICGTS	NHRKKMGGNF
1130	1140	1150	1160	1170	1180	1190	1200
EIIIKQILQD	KIAAGMLLNV	IYPPMSILPN	TRMYSRLNAE	NHSHIKLVQE	ESQIEREEKY	PYFINEGNES	QNTLDAKLQD
1210	1220	1230	1240	1250	1260	1270	1280
EVKGVKETLP	KAVLHDSCNL	GLDAHLEKEI	KTEKEMHQPI	PFTETIIESV	VSPIMELSHA	ENVKSTQKTQ	TDCKCTADSE
1290	1300	1310	1320	1330	1340	1350	1360
TPSPISGKSL	IGDPLNQTRE	SYIPSNGSDT	REMGYCFAEE	KTEIPKDLPA	TSPETFNYCT	PVLSCSKVMK	KRVTFALTTS
1370	1380	1390	1400	1410	1420	1430	1440
TAKPKCVNTK	AVKPSISETV	SVTSHRKKSE	LDFKTKFKKI	NQTKGLVPEC	LNTLCSPMHS	RLQREFCLPA	SQLKQGETAD
1450	1460	1470	1480	1490	1500	1510	1520
KTYTDVFAKN	SISHDREEKL	QDGKEEEHKV	LLEAAPQLSQ	HLGSEAGQMK	EIHLESDPVL	NCLTLELHIN	GQRLQHQTGF
1530	1540	1550	1560	1570	1580	1590	1600
EQTTLETSLQ	MGPLEAEELQ	KANETENDIK	VLGGPKIPPP	KALQALENSD	GLILNAYQKD	NELVKSDEEL	NQPGSTNIQV
1610	1620	1630	1640	1650	1660	1670	1680
QPQTHFTQTI	LKSTSCPTLD	QFPFEKVESH	VRFSPLKSGE	AKVDEIIFYA	REGGISSDSS	HQKEQAGGTE	KKETAIFGSC
1690	1700	1710	1720	1730	1740	1750	1760
MPALSTPKTT	RNLKQFSDMK	TLVNPKCGII	KAKKPSISYM	LNIRAGAGPK	RRKELSCNLT	TKMKELHQGK	KGVDETYAFL
1770	1780	1790	1800	1810	1820	1830	1840
TMTPDINKYS	KVETEKDTLR	EKRLSSTQVK	QDTSPHEDSI	TSRDIKETLL	QDEEQEERKQ	EALLKVIPQH	LQHFMFRSGQ
1850	1860	1870	1880	1890	1900	1910	1920
GKDLDFHKLE	NQGSRKILFV	TKQDVPQQLQ	PAEPIQREET	KKCLQTQNGT	ICTVNSKLLP	LKSEDSVNGE	VLTGAIKRGV
1930	1940	1950	1960	1970	1980	1990	2000
PTDRKCMGEQ	HNSGKGEKAE	FNKDLQATVL	ELQKSPHGGE	AQKANLTDME	SGSSNAMNMN	VQHEREDKNI	QKMLTESVPC
2010	2020	2030	2040	2050	2060	2070	2080
YSQHLRFSTH	QMKDPDPCKS	GSEPKSPEGR	SWNLSHIVQK	TKQETHFRET	VLEPISGYMM	KQSPHMQEGI	KCMEGLKTSF
2090	2100	2110	2120	2130	2140	2150	2160
PKTGKSKIGS	IPRDTPWDEN	PRRKWDSSIS	EKTAWNQKNL	QTVLKPLDFS	SLMSSEYESR	SYTLEFIGKK	SMSPKCVTLK
2170	2180	2190	2200	2210	2220	2230	2240

Detailed Protein Report

Cmpd.	No. of Cmpds.	m/z meas.	Δ m/z [ppm]	z	Rt [min]	Score	P	Range	Sequence	Modification
2780	1	701.4146	-7.24	2	63.2	11.0	2	2385-2396	R.KIILNPKCLTMK.E	
2491	1	974.1939	35.72	3	59.5	10.7	2	5658-5682	R.DEDIYFTGFGTIRSGKRPEWLFTGK.K	

Detailed Protein Report

Protein 79: PREDICTED: centrosomal protein of 290 kDa isoform X4 [Homo sapiens]

**Accession:**	gi|530400856	**Score:**	55.9
**Database:**	refseq_human(refseq_human_20140103.fasta)	**MW [kDa]:**	234.7
**Database Date:**	2015-11-30	**pI:**	6.1
		**Sequence Coverage [%]:**	2.1
		**No. of unique Peptides:**	1

Detailed Protein Report

10	20	30	40	50	60	70	80
MQKLSLRQKS	AIFCQQIHEN	RADMDKSQVA	TLEEEQVHSQ	VKYADINLKE	DIIKSEVPLQ	TEILKNKLKV	NLPDPVSITA
90	100	110	120	130	140	150	160
QSKLSQINSL	ENLIEQLRRE	LVFLRSQNEI	IAQEFLIKEA	ECRNADIELE	HHRSQAEQNE	FLSRELIEKE	RDLERSRTVI
170	180	190	200	210	220	230	240
AKFQNKLKEL	VEENKQLEEG	MKEILQAIKE	MQKDPDVKGG	ETSLIIPSLE	RLVNAIESKN	AEGIFDASLH	LKAQVDQLTG
250	260	270	280	290	300	310	320
RNEELRQELR	ESRKEAINYS	QQLAKANLKI	DHLEKETSLL	RQSEGSNVVF	KGIDLPDGIA	PSSASIINSQ	NEYLIHLLQE
330	340	350	360	370	380	390	400
LENKEKKLKN	LEDSLEDYNR	KFAVIRHQQS	LLYKEYLSEK	ETWKTESKTI	KEEKRKLEDQ	VQQDAIKVKE	YNNLLNALQM
410	420	430	440	450	460	470	480
DSDEMKKILA	ENSRKITVLQ	VNEKSLIRQY	TTLVELERQL	RKENEKQKNE	LLSMEAEVCE	KIGCLQRFKE	MAIFKIAALQ
490	500	510	520	530	540	550	560
KVVDNSVSLS	ELELANKQYN	ELTAKYRDIL	QKDNMLVQRT	SNLEHLECEN	ISLKEQVESI	NKELEITKEK	LHTIEQAWEQ
570	580	590	600	610	620	630	640
ETKLGNESSM	DKAKKSITNS	DIVSISKKIT	MLEMKELNER	QRAEHCQKMY	EHLRTSLKQM	EERNFELETK	FAELTKINLD
650	660	670	680	690	700	710	720
AQKVEQMLRD	ELADSVSKAV	SDADRQRILE	LEKNEMELKV	EVSKLREISD	IARRQVEILN	AQQQSRDKEV	ESLRMQLLDY
730	740	750	760	770	780	790	800
QAQSDEKSLI	AKLHQHNVSL	QLSEATALGK	LESITSKLQK	MEAYNLRLEQ	KLDEKEQALY	YARLEGRNRA	KHLRQTIQSL
810	820	830	840	850	860	870	880
RRQFSGALPL	AQQEKFSKTM	IQLQNDKLKI	MQEMKNSQQE	HRNMENKTLE	MELKLKGLEE	LISTLKDTKG	AQKVINWHMK
890	900	910	920	930	940	950	960
IEELRLQELK	LNRELVKDKE	EIKYLNNIIS	EYERTISSLE	EEIVQQNKFH	EERQMAWDQR	EVDLERQLDI	FDRQQNEILN
970	980	990	1000	1010	1020	1030	1040
AAQKFEEATG	SIPDPSLPLP	NQLEIALRKI	KENIRIILET	RATCKSLEEK	LKEKESALRL	AEQNILSRDK	VINELRLRLP
1050	1060	1070	1080	1090	1100	1110	1120
ATAEREKLIA	ELGRKEMEPK	SHHTLKIAHQ	TIANMQARLN	QKEEVLKKYQ	RLLEKAREEQ	REIVKKHEED	LHILHHRLEL
1130	1140	1150	1160	1170	1180	1190	1200
QADSSLNKFK	QTAWDLMKQS	PTPVPTNKHF	IRLAEMEQTV	AEQDDSLSSL	LVKLKKVSQD	LERQREITEL	KVKEFENIKL
1210	1220	1230	1240	1250	1260	1270	1280
QLQENHEDEV	KKVKAEVEDL	KYLLDQSQKE	SQCLKSELQA	QKEANSRAPT	TTMRNLVERL	KSQLALKEKQ	QKALSRALLE
1290	1300	1310	1320	1330	1340	1350	1360
LRAEMTAAAE	ERIISATSQK	EAHLNVQQIV	DRHTRELKTQ	VEDLNENLLK	LKEALKTSKN	RENSLTDNLN	DLNNELQKKQ
1370	1380	1390	1400	1410	1420	1430	1440
KAYNKILREK	EEIDQENDEL	KRQIKRLTSG	LQGKPLTDNK	QSLIEELQRK	VKKLENQLEG	KVEEVDLKPM	KEKNAKEELI
1450	1460	1470	1480	1490	1500	1510	1520
RWEEGKKWQA	KIEGIRNKLK	EKEGEVFTLT	KQLNTLKDLF	AKADKEKLTL	QRKLKTTGMT	VDQVLGIRAL	ESEKELEELK
1530	1540	1550	1560	1570	1580	1590	1600
KRNLDLENDI	LYMRAHQALP	RDSVVEDLHL	QNRYLQEKLH	ALEKQFSKDT	YSKPSISGIE	SDDHCQREQE	LQKENLKLSS
1610	1620	1630	1640	1650	1660	1670	1680
ENIELKFQLE	QANKDLPRLK	NQVRDLKEMC	EFLKKEKAEV	QRKLGHVRGS	GRSGKTIPEL	EKTIGLMKKV	VEKVQRENEQ
1690	1700	1710	1720	1730	1740	1750	1760
LKKASGILTS	EKMANIEQEN	EKLKAELEKL	KAHLGHQLSM	HYESKTKGTE	KIIAENERLR	KELKKETDAA	EKLRIAKNNL
1770	1780	1790	1800	1810	1820	1830	1840
EILNEKMTVQ	LEETGKRLQF	AESRGPQLEG	ADSKSWKSIV	VTRMYETKLK	ELETDIAKKN	QSITDLKQLV	KEATEREQKV
1850	1860	1870	1880	1890	1900	1910	1920
NKYNEDLEQQ	IKILKHVPEG	AETEQGLKRE	LQVLRLANHQ	LDKEKAELIH	QIEANKDQSG	AESTIPDADQ	LKEKIKDLET
1930	1940	1950	1960	1970	1980	1990	2000
QLKMSDLEKQ	HLKEEIKKLK	KELENFDPSF	FEEIEDLKYN	YKEEVKKNIL	LEEKVKKLSE	QLGVELTSPV	AASEEFEDEE
2010							
ESPVNFPIY							

Cmpd.	No. of Cmpds.	m/z meas.	Δ m/z [ppm]	z	Rt [min]	Score	P	Range	Sequence	Modification

Detailed Protein Report

Cmpd.	No. of Cmpds.	m/z meas.	Δ m/z [ppm]	z	Rt [min]	Score	P	Range	Sequence	Modification
1190	3	500.7991	-35.31	2	43.5	14.1	2	2-9	M.QKLSLRQK.S	

Detailed Protein Report

**Protein 80:**	**PREDICTED: striated muscle preferentially expressed protein kinase isoform X14 [Homo sapiens]**		
**Accession:**	gi|578803490	**Score:**	55.8
**Database:**	refseq_human(refseq_human_20140103.fasta)	**MW [kDa]:**	283.5
**Database Date:**	2015-11-30	**pI:**	9.4
		**Sequence Coverage [%]:**	1.8
		**No. of unique Peptides:**	1

Quantitation

mdown:qdown	**Median:**	0.31	**CV:**	0.00 %	**No. of Peptides:**	1

Detailed Protein Report

10	20	30	40	50	60	70	80
MQKARGTRGE	DAGTRAPPSP	GVPPKRAKVG	AGGGAPVAVA	GAPVFLRPLK	NAAVCAGSDV	RLRVVVSGTP	QPSLRWFRDG
90	100	110	120	130	140	150	160
QLLPAPAPEP	SCLWLRRCGA	QDAGVYSCMA	QNERGRASCE	AVLTVLEVGD	SETAEDDISD	VQGTQRLELR	DDGAFSTPTG
170	180	190	200	210	220	230	240
GSDTLVGTSL	DTPPTSVTGT	SEEQVSWWGS	GQTVLEQEAG	SGGGTRRLPG	SPRQAQATGA	GPRHLGVEPL	VRASRANLVG
250	260	270	280	290	300	310	320
ASWGSEDSLS	VASDLYGSAF	SLYRGRALSI	HVSVPQSGLR	REEPDLQPQL	ASEAPRRPAQ	PPPSKSALLP	PPSPRVGKRS
330	340	350	360	370	380	390	400
PPGPPAQPAA	TPTSPHRRTQ	EPVLPEDTTT	EEKRGKKSKS	SGPSLAGTAE	SRPQTPLSEA	SGRLSALGRS	PRLVRAGSRI
410	420	430	440	450	460	470	480
LDKLQFFEER	RRSLERSDSP	PAPLRPWVPL	RKARSLEQPK	SERGAPWGTP	GASQEELRAP	GSVAERRRLF	QQKAASLDER
490	500	510	520	530	540	550	560
TRQRSPASDL	ELRFAQELGR	IRRSTSREEL	VRSHESLRAT	LQRAPSPREP	GEPPLFSRPS	TPKTSRAVSP	AAAQPPSPSS
570	580	590	600	610	620	630	640
AEKPGDEPGR	PRSRGPAGRT	EPGEGPQQEV	RRRDQFPLTR	SRAIQECRSP	VPPPAADPPE	ARTKAPPGRK	REPPAQAVRF
650	660	670	680	690	700	710	720
LPWATPGLEG	AAVPQTLEKN	RAGPEAEKRL	RRGPEEDGPW	GPWDRRGARS	QGKGRRARPT	SPELESSDDS	YVSAGEEPLE
730	740	750	760	770	780	790	800
APVFEIPLQN	VVVAPGADVL	LKCIITANPP	PQVSWHKDGS	ALRSEGRLLL	RAEGERHTLL	LREARAADAG	SYMATATNEL
810	820	830	840	850	860	870	880
GQATCAASLT	VRPGGSTSPF	SSPITSDEEY	LSPPEEFPEP	GETWPRTPTM	KPSPSQNRRS	SDTGSKAPPT	FKVSLMDQSV
890	900	910	920	930	940	950	960
REGQDVIMSI	RVQGEPKPVV	SWLRNRQPVR	PDQRRFAEEA	EGGLCRLRIL	AAERGDAGFY	TCKAVNEYGA	RQCEARLEVR
970	980	990	1000	1010	1020	1030	1040
AHPESRSLAV	LAPLQDVDVG	AGEMALFECL	VAGPTDVEVD	WLCRGRLLQP	ALLKCKMHFD	GRKCKLLLTS	VHEDDSGVYT
1050	1060	1070	1080	1090	1100	1110	1120
CKLSTAKDEL	TCSARLTVRP	SLAPLFTRLL	EDVEVLEGRA	ARFDCKISGT	PPPVVTWTHF	GCPMEESENL	RLRQDGGLHS
1130	1140	1150	1160	1170	1180	1190	1200
LHIAHVGSED	EGLYAVSAVN	THGQAHCSAQ	LYVEEPRTAA	SGPSSKLEKM	PSIPEEPEQG	ELERLSIPDF	LRPLQDLEVG
1210	1220	1230	1240	1250	1260	1270	1280
LAKEAMLECQ	VTGLPYPTIS	WFHNGHRIQS	SDDRRMTQYR	DVHRLVFPAV	GPQHAGVYKS	VIANKLGKAA	CYAHLYVTDV
1290	1300	1310	1320	1330	1340	1350	1360
VPGPPDGAPQ	VVAVTGRMVT	LTWNPPRSLD	MAIDPDSLTY	TVQHQVLGSD	QWTALVTGLR	EPGWAATGLR	KGVQHIFRVL
1370	1380	1390	1400	1410	1420	1430	1440
STTVKSSSKP	SPPSEPVQLL	EHGPTLEEAP	AMLDKPDIVY	VVEGQPASVT	VTFNHVEAQV	VWRSCRGALL	EARAGVYELS
1450	1460	1470	1480	1490	1500	1510	1520
QPDDDQYCLR	ICRVSRRDMG	ALTCTARNRH	GTQTCSVTLE	LAEAPRFESI	MEDVEVGAGE	TARFAVVVEG	KPLPDIMWYK
1530	1540	1550	1560	1570	1580	1590	1600
DEVLLTESSH	VSFVYEENEC	SLVVLSTGAQ	DGGVYTCTAQ	NLAGEVSCKA	ELAVHSAQTA	MEVEGVGEDE	DHRGRRLSDF
1610	1620	1630	1640	1650	1660	1670	1680
YDIHQEIGRG	AFSYLRRIVE	RSSGLEFAAK	FIPSQAKPKA	SARREARLLA	RLQHDCVLYF	HEAFERRRGL	VIVTELCTEE
1690	1700	1710	1720	1730	1740	1750	1760
LLERIARKPT	VCESEIRAYM	RQVLEGIHYL	HQSHVLHLDV	KPENLLVWDG	AAGEQQVRIC	DFGNAQELTP	GEPQYCQYGT
1770	1780	1790	1800	1810	1820	1830	1840
PEFVAPEIVN	QSPVSGVTDI	WPVGVVAFLC	LTGISPFVGE	NDRTTLMNIR	NYNVAFEETT	FLSLSREARG	FLIKVLVQDR
1850	1860	1870	1880	1890	1900	1910	1920
LRPTAEETLE	HPWFKTQAKG	AEVSTDHLKL	FLSRRRWQRS	QISYKCHLVL	RPIPELLRAP	PERVWVTMPR	RPPPSGGLSS
1930	1940	1950	1960	1970	1980	1990	2000
SSDSEEEELE	ELPSVPRPLQ	PEFSGSRVSL	TDIPTEDEAL	GTPETGAATP	MDWQEQGRAP	SQDQEAPSPE	ALPSPGQEPA
2010	2020	2030	2040	2050	2060	2070	2080
AGASPRRGEL	RRGSSAESAL	PRAGPRELGR	GLHKAASVEL	PQRRSPSPGA	TRLARGGLGE	GEYAQRLQAL	RQRLLRGGPE
2090	2100	2110	2120	2130	2140	2150	2160
DGKVSGLRGP	LLESLGGRAR	DPRMARAASS	EAAPHHQPPL	ENRGLQKSSS	FSQGEAEPRG	RHRRAGAPLE	IPVARLGARR
2170	2180	2190	2200	2210	2220	2230	2240

Detailed Protein Report

Cmpd.	No. of Cmpds.	m/z meas.	Δ m/z [ppm]	z	Rt [min]	Score	P	Range	Sequence	Modification	Ratios
92	1	531.9231	-79.62	3	30.5	25.4	2	2089-2103	R.GPLLESLGGRARDPR.M		mdown:qdown 0.31

Detailed Protein Report

Protein 81: **PREDICTED: LOW QUALITY PROTEIN: ubiquitin carboxyl-terminal hydrolase 17-like protein 20 [Homo sapiens]**

Accession: gi|578843530
Database: refseq_human(refseq_human_20140103.fasta)
Database Date: 2015-11-30
Modification(s): Carbamidomethyl

Score: 55.8
MW [kDa]: 38.1
pI: 9.8
Sequence Coverage [%]: 6.0
No. of unique Peptides: 3

10	20	30	40	50	60	70	80
MEDDSLYLGA	SAKVLILVLK	RFSDVTGNKI	AKNVQYPECL	DMQPYMSQPN	TGPLVYVLYA	VLVHAGWSCH	NGHYFSYVKA
90	100	110	120	130	140	150	160
QEGQWYKMDD	AEVTASSITS	VLSQQAYVLF	YIQKSEWERH	SESVSRGREP	RALGAEDTDR	RATQGELKRD	HPCLQAPELD
170	180	190	200	210	220	230	240
EHLVERATQE	STLDHWKFLQ	EQNKTKPEFN	VRKVEGTLPP	DVLVIHQSXY	KCGMKNHHPE	QQSSLLNLSS	TTPTHQESMN
250	260	270	280	290	300	310	320
TGTLASLRGR	ARRSKGKNKH	SKRALLVCQX	SQWKYRPTRT	VIKVDQPQRK	AAQGTTQGSV	EPQNLGRNPA	QAPKCAYEQG
330	340						
LRVTTLYIRH	RKPSDG						

Cmpd.	No. of Cmpds.	m/z meas.	Δ m/z [ppm]	z	Rt [min]	Score	P	Range	Sequence	Modification
1896	1	599.9585	-51.71	3	52.4	13.5	2	260-274	K.HSKRALLVCQNSQWK.Y	
2567	1	709.8660	-0.12	2	62.5	14.7	0	264-274	R.ALLVCQWSQWK.Y	Carbamidomethyl: 5
1491	1	981.6005	60.92	2	47.4	15.8	1	264-279	R.ALLVCQISQWKYRPTR.T	

Detailed Protein Report

Protein 82: intersectin-1 isoform ITSN-I [Homo sapiens]

**Accession:**	gi|47717123	**Score:**	55.5
**Database:**	refseq_human(refseq_human_20140103.fasta)	**MW [kDa]:**	195.3
**Database Date:**	2015-11-30	**pI:**	8.5
**Modification(s):**	Oxidation	**Sequence Coverage [%]:**	2.7
		**No. of unique Peptides:**	2

Quantitation

mdown:qdown **Median:** 0.83 **CV:** 0.00 % **No. of Peptides:** 1

10	20	30	40	50	60	70	80
MAQFPTPFGG	SLDIWAITVE	ERAKHDQQFH	SLKPISGFIT	GDQARNFFFQ	SGLPQPVLAQ	IWALADMNND	GRMDQVEFSI
90	100	110	120	130	140	150	160
AMKLIKLKLQ	GYQLPSALPP	VMKQQPVAIS	SAPAFGMGGI	ASMPPLTAVA	PVPMGSIPVV	GMSPTLVSSV	PTAAVPPLAN
170	180	190	200	210	220	230	240
GAPPVIQPLP	AFAHPAATLP	KSSSFSRSGP	GSQLNTKLQK	AQSFDVASVP	PVAEWAVPQS	SRLKYRQLFN	SHDKTMSGHL
250	260	270	280	290	300	310	320
TGPQARTILM	QSSLPQAQLA	SIWNLSDIDQ	DGKLTAEEFI	LAMHLIDVAM	SGQPLPPVLP	PEYIPPSFRR	VRSGSGISVI
330	340	350	360	370	380	390	400
SSTSVDQRLP	EEPVLEDEQQ	QLEKKLPVTF	EDKKRENFER	GNLELEKRRQ	ALLEQQRKEQ	ERLAQLERAE	QERKERERQE
410	420	430	440	450	460	470	480
QERKRQLELE	KQLEKQRELE	RQREEERRKE	IERREAAKRE	LERQRQLEWE	RNRRQELLNQ	RNKEQEDIVV	LKAKKKTLEF
490	500	510	520	530	540	550	560
ELEALNDKKH	QLEGKLQDIR	CRLTTQRQEI	ESTNKSRELR	IAEITHLQQQ	LQESQQMLGR	LIPEKQILND	QLKQVQQNSL
570	580	590	600	610	620	630	640
HRDSLVTLKR	ALEAKELARQ	HLRDQLDEVE	KETRSKLQEI	DIFNNQLKEL	REIHNKQQLQ	KQKSMEAERL	KQKEQERKII
650	660	670	680	690	700	710	720
ELEKQKEEAQ	RRAQERDKQW	LEHVQQEDEH	QRPRKLHEEE	KLKREESVKK	KDGEEKGKQE	AQDKLGRLFH	QHQEPAKPAV
730	740	750	760	770	780	790	800
QAPWSTAEKG	PLTISAQENV	KVVYYRALYP	FESRSHDEIT	IQPGDIVMVK	GEWVDESQTG	EPGWLGGELK	GKTGWFPANY
810	820	830	840	850	860	870	880
AEKIPENEVP	APVKPVTDST	SAPAPKLALR	ETPAPLAVTS	SEPSTTPNNW	ADFSSTWPTS	TNEKPETDNW	DAWAAQPSLT
890	900	910	920	930	940	950	960
VPSAGQLRQR	SAFTPATATG	SSPSPVLGQG	EKVEGLQAQA	LYPWRAKKDN	HLNFNKNDVI	TVLEQQDMWW	FGEVQGQKGW
970	980	990	1000	1010	1020	1030	1040
FPKSYVKLIS	GPIRKSTSMD	SGSSESPASL	KRVASPAAKP	VVSGEEFIAM	YTYESSEQGD	LTFQQGDVIL	VTKKDGDWWT
1050	1060	1070	1080	1090	1100	1110	1120
GTVGDKAGVF	PSNYVRLKDS	EGSGTAGKTG	SLGKKPEIAQ	VIASYTATGP	EQLTLAPGQL	ILIRKKNPGG	WWEGELQARG
1130	1140	1150	1160	1170	1180	1190	1200
KKRQIGWFPA	NYVKLLSPGT	SKITPTEPPK	STALAAVCQV	IGMYDYTAQN	DDELAFNKGQ	IINVLNKEDP	DWWKGEVNGQ
1210	1220	1230	1240	1250	1260	1270	1280
VGLFPSNYVK	LTTDMDPSQQ	WCSDLHLLDM	LTPTERKRQG	YIHELIVTEE	NYVNDLQLVT	EIFQKPLMES	ELLTEKEVAM
1290	1300	1310	1320	1330	1340	1350	1360
IFVNWKELIM	CNIKLLKALR	VRKKMSGEKM	PVKMIGDILS	AQLPHMQPYI	RFCSRQLNGA	ALIQQKTDEA	PDFKEFVKRL
1370	1380	1390	1400	1410	1420	1430	1440
AMDPRCKGMP	LSSFILKPMQ	RVTRYPLIIK	NILENTPENH	PDHSHLKHAL	EKAEELCSQV	NEGVREKENS	DRLEWIQAHV
1450	1460	1470	1480	1490	1500	1510	1520
QCEGLSEQLV	FNSVTNCLGP	RKFLHSGKLY	KAKSNKELYG	FLFNDFLLLT	QITKPLGSSG	TDKVFSPKSN	LQYKMYKTPI
1530	1540	1550	1560	1570	1580	1590	1600
FLNEVLVKLP	TDPSGDEPIF	HISHIDRVYT	LRAESINERT	AWVQKIKAAS	ELYIETEKKK	REKAYLVRSQ	RATGIGRLMV
1610	1620	1630	1640	1650	1660	1670	1680
NVVEGIELKP	CRSHGKSNPY	CEVTMGSQCH	ITKTIQDTLN	PKWNSNCQFF	IRDLEQEVLC	ITVFERDQFS	PDDFLGRTEI
1690	1700	1710	1720	1730			
RVADIKKDQG	SKGPVTKCLL	LHEVPTGEIV	VRLDLQLFDE	P			

Detailed Protein Report

Cmpd.	No. of Cmpds.	m/z meas.	Δ m/z [ppm]	z	Rt [min]	Score	P	Range	Sequence	Modification	Ratios
1569	1	788.7801	-144.71	2	49.5	15.7	2	584-596	R.DQLDEVEKETRSK.L		
2785	1	874.5334	88.10	1	63.9	14.2	1	1359-1365	K.RLAMDPR.C	Oxidation: 4	mdown:qdown 0.83

Detailed Protein Report

Protein 83: histone-lysine N-methyltransferase, H3 lysine-36 and H4 lysine-20 specific isoform b [Homo sapiens]

**Accession:**	gi|19923586	**Score:**	55.5
**Database:**	refseq_human(refseq_human_20140103.fasta)	**MW [kDa]:**	296.5
**Database Date:**	2015-11-30	**pI:**	9.5
**Modification(s):**	Carbamidomethyl	**Sequence Coverage [%]:**	1.1
		**No. of unique Peptides:**	2

Quantitation

mdown:qdown	**Median:**	0.50	**CV:**	0.00 %	**No. of Peptides:**	1
Wdown:Qdown	**Median:**	0.47	**CV:**	0.00 %	**No. of Peptides:**	1

Alias proteins:

Accession	Name	Description
gi|578811198	refseq_human (refseq_human_20140103.fasta)	PREDICTED: histone-lysine N-methyltransferase, H3 lysine-36 and H4 lysine-20 specific isoform X5 [Homo sapiens]
gi|530381144	refseq_human (refseq_human_20140103.fasta)	PREDICTED: histone-lysine N-methyltransferase, H3 lysine-36 and H4 lysine-20 specific isoform X1 [Homo sapiens]

Detailed Protein Report

10	20	30	40	50	60	70	80
MDQTCELPRR	NCLLPFSNPV	NLDAPEDKDS	PFGNGQSNFS	EPLNGCTMQL	STVSGTSQNA	YGQDSPSCYI	PLRRLQDLAS
90	100	110	120	130	140	150	160
MINVEYLNGS	ADGSESFQDP	EKSDSRAQTP	IVCTSLSPGG	PTALAMKQEP	SCNNSPELQV	KVTKTIKNGF	LHFENFTCVD
170	180	190	200	210	220	230	240
DADVDSEMDP	EQPVTEDESI	EEIFEETQTN	ATCNYETKSE	NGVKVAMGSE	QDSTPESRHG	AVKSPFLPLA	PQTETQKNKQ
250	260	270	280	290	300	310	320
RNEVDGSNEK	AALLPAPFSL	GDTNITIEEQ	LNSINLSFQD	DPDSSTSTLG	NMLELPGTSS	SSTSQELPFC	QPKKKSTPLK
330	340	350	360	370	380	390	400
YEVGDLIWAK	FKRRPWWPCR	ICSDPLINTH	SKMKVSNRRP	YRQYYVEAFG	DPSERAWVAG	KAIVMFEGRH	QFEELPVLRR
410	420	430	440	450	460	470	480
RGKQKEKGYR	HKVPQKILSK	WEASVGLAEQ	YDVPKGSKNR	KCIPGSIKLD	SEEDMPFEDC	TNDPESEHDL	LLNGCLKSLA
490	500	510	520	530	540	550	560
FDSEHSADEK	EKPCAKSRAR	KSSDNPKRTS	VKKGHIQFEA	HKDERRGKIP	ENLGLNFISG	DISDTQASNE	LSRIANSLTG
570	580	590	600	610	620	630	640
SNTAPGSFLF	SSCGKNTAKK	EFETSNGDSL	LGLPEGALIS	KCSREKNKPQ	RSLVCGSKVK	LCYIGAGDEE	KRSDSISICT
650	660	670	680	690	700	710	720
TSDDGSSDLD	PIEHSSESDN	SVLEIPDAFD	RTENMLSMQK	NEKIKYSRFA	ATNTRVKAKQ	KPLISNSHTD	HLMGCTKSAE
730	740	750	760	770	780	790	800
PGTETSQVNL	SDLKASTLVH	KPQSDFTNDA	LSPKFNLSSS	ISSENSLIKG	GAANQALLHS	KSKQPKFRSI	KCKHKENPVM
810	820	830	840	850	860	870	880
AEPPVINEEC	SLKCCSSDTK	GSPLASISKS	GKVDGLKLLN	NMHEKTRDSS	DIETAVVKHV	LSELKELSYR	SLGEDVSDSG
890	900	910	920	930	940	950	960
TSKPSKPLLF	SSASSQNHIP	IEPDYKFSTL	LMMLKDMHDS	KTKEQRLMTA	QNLVSYRSPG	RGDCSTNSPV	GVSKVLVSGG
970	980	990	1000	1010	1020	1030	1040
STHNSEKKGD	GTQNSANPSP	SGGDSALSGE	LSASLPGLLS	DKRDLPASGK	SRSDCVTRRN	CGRSKPSSKL	RDAFSAQMVK
1050	1060	1070	1080	1090	1100	1110	1120
NTVNRKALKT	ERKRKLNQLP	SVTLDAVLQG	DRERGGSLRG	GAEDPSKEDP	LQIMGHLTSE	DGDHFSDVHF	DSKVKQSDPG
1130	1140	1150	1160	1170	1180	1190	1200
KISEKGLSFE	NGKGPELDSV	MNSENDELNG	VNQVVPKKRW	QRLNQRRTKP	RKRMNRFKEK	ENSECAFRVL	LPSDPVQEGR
1210	1220	1230	1240	1250	1260	1270	1280
DEFPEHRTPS	ASILEEPLTE	QNHADCLDSA	GPRLNVCDKS	SASIGDMEKE	PGIPSLTPQA	ELPEPAVRSE	KKRLRKPSKW
1290	1300	1310	1320	1330	1340	1350	1360
LLEYTEEYDQ	IFAPKKKQKK	VQEQVHKVSS	RCEEESLLAR	GRSSAQNKQV	DENSLISTKE	EPPVLEREAP	FLEGPLAQSE
1370	1380	1390	1400	1410	1420	1430	1440
LGGGHAELPQ	LTLSVPVAPE	VSPRPALESE	ELLVKTPGNY	ESKRQRKPTK	KLLESNDLDP	GFMPKKGDLG	LSKKCYEAGH
1450	1460	1470	1480	1490	1500	1510	1520
LENGITESCA	TSYSKDFGGG	TTKIFDKPRK	RKRQRHAAAK	MQCKKVKNDD	SSKEIPGSEG	ELMPHRTATS	PKETVEEGVE
1530	1540	1550	1560	1570	1580	1590	1600
HDPGMPASKK	MQGERGGGAA	LKENVCQNCE	KLGELLLCEA	QCCGAFHLEC	LGLTEMPRGK	FICNECRTGI	HTCFVCKQSG
1610	1620	1630	1640	1650	1660	1670	1680
EDVKRCLLPL	CGKFYHEECV	QKYPPTVMQN	KGFRCSLHIC	ITCHAANPAN	VSASKGRLMR	CVRCPVAYHA	NDFCLAAGSK
1690	1700	1710	1720	1730	1740	1750	1760
ILASNSIICP	NHFTPRRGCR	NHEHVNVSWC	FVCSEGGSLL	CCDSCPAAFH	RECLNIDIPE	GNWYCNDCKA	GKKPHYREIV
1770	1780	1790	1800	1810	1820	1830	1840
WVKVGRYRWW	PAEICHPRAV	PSNIDKMRHD	VGEFPVLFFG	SNDYLWTHQA	RVFPYMEGDV	SSKDKMGKGV	DGTYKKALQE
1850	1860	1870	1880	1890	1900	1910	1920
AAARFEELKA	QKELRQLQED	RKNDKKPPPY	KHIKVNRPIG	RVQIFTADLS	EIPRCNCKAT	DENPCGIDSE	CINRMLLYEC
1930	1940	1950	1960	1970	1980	1990	2000
HPTVCPAGGR	CQNQCFSKRQ	YPEVEIFRTL	QRGWGLRTKT	DIKKGEFVNE	YVGELIDEEE	CRARIRYAQE	HDITNFYMLT
2010	2020	2030	2040	2050	2060	2070	2080
LDKDRIIDAG	PKGNYARFMN	HCCQPNCETQ	KWSVNGDTRV	GLFALSDIKA	GTELTFNYNL	ECLGNGKTVC	KCGAPNCSGF
2090	2100	2110	2120	2130	2140	2150	2160
LGVRPKNQPI	ATEEKSKKFK	KKQQGKRRTQ	GEITKEREDE	CFSCGDAGQL	VSCKKPGCPK	VYHADCLNLT	KRPAGKWECP
2170	2180	2190	2200	2210	2220	2230	2240

Detailed Protein Report

Cmpd.	No. of Cmpds.	m/z meas.	Δ m/z [ppm]	z	Rt [min]	Score	P	Range	Sequence	Modification	Ratios
1166	2	509.6492	-167.89	2	43.2	11.3	0	2-9	M.DQTCELPR.R	Carbamidomethyl: 4	
617	1	459.6161	-280.24	2	37.0	16.5	1	491-498	K.EKPCAKSR.A		mdown:qdown 0.50<br>Wdown:Qdown 0.47

Detailed Protein Report

Protein 84: PREDICTED: dynein heavy chain 12, axonemal isoform X1 [Homo sapiens]

**Accession:**	gi|578805765	**Score:**	55.1
**Database:**	refseq_human(refseq_human_20140103.fasta)	**MW [kDa]:**	454.9
**Database Date:**	2015-11-30	**pI:**	5.5
**Modification(s):**	Oxidation	**Sequence Coverage [%]:**	1.6
		**No. of unique Peptides:**	3

Quantitation

mdown:qdown	**Median:**	0.23	**CV:**	37.24 %	**No. of Peptides:**	2
Wdown:Qdown	**Median:**	0.34	**CV:**	56.08 %	**No. of Peptides:**	2

Detailed Protein Report

10	20	30	40	50	60	70	80
MSDANKAAIA	AEKEALNLKL	PPIVHLPENI	GVDTPTQSKL	LKYRRSKEQQ	QKINQLVIDG	AKRNLDRTLG	KRTPLLPPPD
90	100	110	120	130	140	150	160
YPQTMTSEMK	KKGFNYIYMK	QCVESSPLVP	IQQEWLDHML	RLIPESLKEG	KEREELLESL	INEVSSDFEN	SMKRYLVQSV
170	180	190	200	210	220	230	240
LVKPPVKSLE	DEGGPLPESP	VGLDYSNPWH	SSYVQARNQI	FSNLHIIHPT	MKMLLDLGYT	TFADTVLLDF	TGIRAKGPID
250	260	270	280	290	300	310	320
CESLKTDLSI	QTRNAEEKIM	NTWYPKVINL	FTKKEALEGV	KPEKLDAFYS	CVSTLMSNQL	KDLLRRTVEG	FVKLFDPKDQ
330	340	350	360	370	380	390	400
QRLPIFKIEL	TFDDDKMEFY	PTFQDLEDNV	LSLVERIAEA	LQNVQTIPSW	LSGTSTPVNL	DTELPEHVLH	WAVDTLKAAV
410	420	430	440	450	460	470	480
HRNLEGARKH	YETYVEKYNW	LLDGTAVENI	ETFQTEDHTF	DEYTEFIEKF	LSLASEIMLL	PQWIHYTMVR	LDCEDLKTGL
490	500	510	520	530	540	550	560
TNKAKAFANI	LLNDIASKYR	KENECICSEF	EAIKEHALKV	PETTEEMMDL	ISYVEKARTV	GIEELILRIQ	ESKRQMSYFL
570	580	590	600	610	620	630	640
DVFLFPQEDL	ALNATVLMWP	RKINPIFDEN	DELIENAKHK	KENELMAKRE	KLILEIEKES	RRMEEFTEFA	ELERMQQYVT
650	660	670	680	690	700	710	720
DVRQLQKRIQ	ESEEAVQFIN	KEEELFKWEL	TKYPELDKLK	VNIEPYQKFF	NFVLKWQRSE	KRWMDGGFLD	LNGESMEADV
730	740	750	760	770	780	790	800
EEFSREIFKT	LKFFQTKLKK	ELQEKRKAAR	KRSLEEEKIE	EEPKDNATIT	MCSTVMEQIK	AFKEYIPTVS	ILCNPGMRAR
810	820	830	840	850	860	870	880
HWKQISEIVG	YDLTPDSGTT	LRKVLKLNLT	PYLEQFEVIS	AGASKEFSLE	KAMNTMIGTW	EDIAFHISLY	RDTGVCILSS
890	900	910	920	930	940	950	960
VDEIQAILDD	QIIKTQTMRG	SPFIKPFEHE	IKAWEDRLIR	IQETIDEWLK	VQAQWLYLEP	IFCSEDIMQQ	MPEEGRQFQT
970	980	990	1000	1010	1020	1030	1040
VDRHWRDIMK	FCAKDPKVLA	ATSLTGLLEK	LQNCNELLEK	IMKGLNAYLE	KKRLFFPRFF	FLSNDEMLEI	LSETKDPLRV
1050	1060	1070	1080	1090	1100	1110	1120
QPHLKKCFEG	IAKLEFLPNL	DIKAMYSSEG	ERVELIALIS	TSAARGAVEK	WLIQVEDLML	RSVHDVIAAA	RLAYPESARR
1130	1140	1150	1160	1170	1180	1190	1200
DWVREWPGQV	VLCISQMFWT	SETQEVISGG	TEGLKKYYKE	LQNQLNEIVE	LVRGKLSKQT	RTTLGALVTI	DVHARDVVMD
1210	1220	1230	1240	1250	1260	1270	1280
MIKMGVSHDT	DFLWLAQLRY	YWENENARVR	IINCNVKYAY	EYLGNSPRLV	ITPLTDRCYR	TLIGAFYLNL	GGAPEGPAGT
1290	1300	1310	1320	1330	1340	1350	1360
GKTETTKDLA	KALAVQCVVF	NCSDGLDYLA	MGKFFKGLAS	SGAWACFDEF	NRIELEVLSV	VAQQILCIQR	AIQQKLVVFV
1370	1380	1390	1400	1410	1420	1430	1440
FEGTELKLNP	NCFVAITMNP	GYAGRSELPD	NLKVLFRTVA	MMVPNYALIA	EISLYSYGFL	NARPLSVKIV	MTYRLCSEQL
1450	1460	1470	1480	1490	1500	1510	1520
SSQFHYDYGM	RAVKAVLVAA	GNLKLKYPNE	NEDILLLRSI	KDVNEPKFLS	HDIPLFNGIT	SDLFPGIKLP	EADYHEFLEC
1530	1540	1550	1560	1570	1580	1590	1600
AHEACNVHNL	QPVKFFLEKI	IQTYEMMIVR	HGFMLVGEPF	AAKTKVLHVL	ADTLTLMNEH	GYGEEEKVIY	RTVNPKSITM
1610	1620	1630	1640	1650	1660	1670	1680
GQLFGQFDPV	SHEWTDGIVA	NTFREFALSE	TPDRKWVVFD	GPIDTLWIES	MNTVLDDNKK	LCLMSGEIIQ	MSPQMSLIFE
1690	1700	1710	1720	1730	1740	1750	1760
TMDLSQASPA	TVSRCGMIYL	EPSQLGWEPL	VSSWLNSLKG	PLCEPEYQAL	LRGLFAWLIP	PSLNQRVELF	QLNYLYTTIV
1770	1780	1790	1800	1810	1820	1830	1840
SKILKILITF	RISNYFKYVP	LKTQCTFIKF	FLHQQACFIF	SLIWSIGGSC	DTDGRRVFDT	FIRLIILGKD	DENPVPDSVG
1850	1860	1870	1880	1890	1900	1910	1920
KWECPFDEKG	LVYDYMYELK	NKGRWVHWNE	LIKNTNLGDK	QIKIQDIIVP	TMDTIRYTFL	MDLSITYAKP	LLFVGPTGTG
1930	1940	1950	1960	1970	1980	1990	2000
KSVYVKDKLM	NHLEKDQYFP	FYINLSARTS	ANQVQNIIMA	RLDKRRKGVF	GPPMGKKCII	FIDDMNMPAL	EKYGAQPPIE
2010	2020	2030	2040	2050	2060	2070	2080
LLRQFFDCGH	WYDLKDTSKI	TLVDIELIAA	MGPPGGGRNP	VTPRCIRHFN	ICSINSFSDE	TMVRIFSSIV	AFYLRTHEFP
2090	2100	2110	2120	2130	2140	2150	2160
PEYFVIGNQI	VNGTMEIYKQ	SVENLLPTPT	KSHYTFNLRD	FSRVIRGCLL	IERDAVANKH	TMIRLFVHEV	LRVFYDRLIN
2170	2180	2190	2200	2210	2220	2230	2240

Detailed Protein Report

Cmpd.	No. of Cmpds.	m/z meas.	Δ m/z [ppm]	z	Rt [min]	Score	P	Range	Sequence	Modification	Ratios
47	1	1103.4984	-39.62	2	29.9	12.9	1	73-91	R.TPLLPPPDYPQTMTSEMKK.K	Oxidation: 13, 17	Wdown:Qdown 0.57<br>mdown:qdown 0.33
1482	1	681.7771	64.45	3	47.3	18.6	2	267-284	K.VINLFTKKEALEGVKPEK.L		
1201	1	1070.0173	-20.70	2	43.7	12.3	1	583-600	K.INPIFDENDELIENAKHK.K		Wdown:Qdown 0.20<br>mdown:qdown 0.16

Detailed Protein Report

Protein 85: **fibrinogen gamma chain isoform gamma-A precursor [Homo sapiens]**

Accession: gi|70906437
Database: refseq_human(refseq_human_20140103.fasta)
Database Date: 2015-11-30

Score: 54.8
MW [kDa]: 49.5
pI: 5.7
Sequence Coverage [%]: 4.8
No. of unique Peptides: 2

Quantitation

mdown:qdown	**Median:**	0.52	**CV:**	98.68 %	**No. of Peptides:**	2
Wdown:Qdown	**Median:**	0.89	**CV:**	37.40 %	**No. of Peptides:**	2

10	20	30	40	50	60	70	80
MSWSLHPRNL	ILYFYALLFL	SSTCVAYVAT	RDNCCILDER	FGSYCPTTCG	IADFLSTYQT	KVDKDLQSLE	DILHQVENKT
**90**	**100**	**110**	**120**	**130**	**140**	**150**	**160**
SEVKQLIKAI	QLTYNPDESS	KPNMIDAATL	KSRKMLEEIM	KYEASILTHD	SSIRYLQEIY	NSNNQKIVNL	KEKVAQLEAQ
**170**	**180**	**190**	**200**	**210**	**220**	**230**	**240**
CQEPCKDTVQ	IHDITGKDCQ	DIANKGAKQS	GLYFIKPLKA	NQQFLVYCEI	DGSGNGWTVF	QKRLDGSVDF	KKNWIQYKEG
**250**	**260**	**270**	**280**	**290**	**300**	**310**	**320**
FGHLSPTGTT	EFWLGNEKIH	LISTQSAIPY	ALRVELEDWN	GRTSTADYAM	FKVGPEADKY	RLTYAYFAGG	DAGDAFDGFD
**330**	**340**	**350**	**360**	**370**	**380**	**390**	**400**
FGDDPSDKFF	TSHNGMQFST	WDNDNDKFEG	NCAEQDGSGW	WMNKCHAGHL	NGVYYQGGTY	SKASTPNGYD	NGIIWATWKT
**410**	**420**	**430**	**440**				
RWYSMKKTTM	KIIPFNRLTI	GEGQQHHLGG	AKQAGDV				

Cmpd.	No. of Cmpds.	m/z meas.	Δ m/z [ppm]	z	Rt [min]	Score	P	Range	Sequence	Modification	Ratios
176	1	757.3345	-43.89	2	31.4	25.9	0	135-146	R.YLQEIYNSNNQK.I		Wdown:Qdown 0.62<br>mdown:qdown 1.19
329	1	559.1487	-212.08	2	33.2	28.9	0	274-282	R.VELEDWNGR.T		Wdown:Qdown 1.28<br>mdown:qdown 0.23

Detailed Protein Report

Protein 86: stAR-related lipid transfer protein 9 [Homo sapiens]

**Accession:**	gi|270265793	**Score:**	54.8
**Database:**	refseq_human(refseq_human_20140103.fasta)	**MW [kDa]:**	516.0
**Database Date:**	2015-11-30	**pI:**	5.9
		**Sequence Coverage [%]:**	1.0
		**No. of unique Peptides:**	2

Detailed Protein Report

10	20	30	40	50	60	70	80
MANVQVAVRV	RPLSKRETKE	GGRIIVEVDG	KVAKIRNLKV	DNRPDGFGDS	REKVMAFGFD	YCYWSVNPED	PQYASQDVVF
90	100	110	120	130	140	150	160
QDLGMEVLSG	VAKGYNICLF	AYGQTGSGKT	YTMLGTPASV	GLTPRICEGL	FVREKDCASL	PSSCRIKVSF	LEIYNERVRD
170	180	190	200	210	220	230	240
LLKQSGQKKS	YTLRVREHPE	MGPYVQGLSQ	HVVTNYKQVI	QLLEEGIANR	ITAATHVHEA	SSRSHAIFTI	HYTQAILENN
250	260	270	280	290	300	310	320
LPSEMASKIN	LVDLAGSERA	DPSYCKDRIA	EGANINKSLV	TLGIVISTLA	QNSQVFSSCQ	SLNSSVSNGG	DSGILSSPSG
330	340	350	360	370	380	390	400
TSSGGAPSRR	QSYIPYRDSV	LTWLLKDSLG	GNSKTIMVAT	VSPAHTSYSE	TMSTLRYASS	AKNIINKPRV	NEDANLKLIR
410	420	430	440	450	460	470	480
ELREEIERLK	ALLLSFELRN	FSSLSDENLK	ELVLQNELKI	DQLTKDWTQK	WNDWQALMEH	YSVDINRRRA	GVVIDSSLPH
490	500	510	520	530	540	550	560
LMALEDDVLS	TGVVLYHLKE	GTTKIGRIDS	DQEQDIVLQG	QWIERDHCTI	TSACGVVVLR	PARGARCTVN	GREVTASCRL
570	580	590	600	610	620	630	640
TQGAVITLGK	AQKFRFNHPA	EAAVLRQRRQ	VGEAAAGRGS	LEWLDLDGDL	AASRLGLSPL	LWKERRALEE	QCDEDHQTPR
650	660	670	680	690	700	710	720
DGETSHRAQI	QQQQSYVEDL	RHQILAEEIR	AAKELEFDQA	WISQQIKENQ	QCLLREETWL	ASLQQQQQED	QVAEKELEAS
730	740	750	760	770	780	790	800
VALDAWLQTD	PEIQPSPFVQ	SQKRVVHLQL	LRRHTLRAAE	RNVRRKKVSF	QLERIIKKQR	LLEAQKRLEK	LTTLCWLQDD
810	820	830	840	850	860	870	880
STQEPPYQVL	SPDATVPRPP	CRSKLTSCSS	LSPQRLCSKH	MPQLHSIFLS	WDPSTTLPPR	PDPTHQTSEK	TSSEEHLPQA
890	900	910	920	930	940	950	960
ASYPARTGCL	RKNGLHSSGH	GQPCTARAAL	ARKGASAPDA	CLTMSPNSVG	IQEMEMGVKQ	PHQMVSQGLA	SLRKSANKLK
970	980	990	1000	1010	1020	1030	1040
PRHEPKIFTS	TTQTRGAKGL	ADPSHTQAGW	RKEGNLGTHK	AAKGASCNSL	YPHGPRQTAG	HGKAVKTFWT	EYKPPSPSRA
1050	1060	1070	1080	1090	1100	1110	1120
SKRHQRVLAT	RVRNITKKSS	HLPLGSPLKR	QQNTRDPDTM	VPLTDFSPVM	DHSREKDNDL	SDTDSNYSLD	SLSCVYAKAL
1130	1140	1150	1160	1170	1180	1190	1200
IEPLKPEERK	WDFPEPENSE	SDDSQLSEDS	LAEKRYQSPK	NRLGGNRPTN	NRGQPRTRTR	ASVRGFTAAS	DSDLLAQTHR
1210	1220	1230	1240	1250	1260	1270	1280
SFSLDSLIDA	EEELGEDQQE	EPFPGSADEI	PTETFWHLED	SSLPVMDQEA	ICRLGPINYR	TAARLDAVLP	MSSSFYLDPQ
1290	1300	1310	1320	1330	1340	1350	1360
FQPHCELQPH	CELQPHCELQ	PHCEQAESQV	EPSYSEQADS	LQGMQLSRES	PLMSMDSWFS	CDSKINPSSP	PGIVGSLCPS
1370	1380	1390	1400	1410	1420	1430	1440
PDMQEFHSCK	GERPGYWPNT	EELKPSDAET	VLPYSSKLHQ	GSTELLCSAR	DEHTASAADT	SRLSLWGIQR	LIQPGADGTF
1450	1460	1470	1480	1490	1500	1510	1520
QGRCIPDMTQ	QGSSEASHNS	SVSNVLAASA	TTLTHVGSTH	ERDWSALQQK	YLLELSCPVL	EAIGAPKPAY	PYLEEDSGSL
1530	1540	1550	1560	1570	1580	1590	1600
AQASSKGGDT	LLPVGPRVSS	NLNLNNFPVH	LSRIRRLRAE	KEQDSLNAKL	EGVSDFFSTS	EKEASYDETY	SADLESLSAS
1610	1620	1630	1640	1650	1660	1670	1680
RSTNAQVFAT	ENAIPDSMTE	ACEVKQNNLE	ECLQSCRKPG	LMTSSDEDFF	QKNACHSNVT	TATKADHWSQ	GWAPLRKNSA
1690	1700	1710	1720	1730	1740	1750	1760
VQPGQLSPDS	HYPLEEEKTD	CQESSKEAVR	RHINVSFALP	SGPELYLHSA	PWNPLSSSLQ	PPLLETFYVT	KSRDALTETA
1770	1780	1790	1800	1810	1820	1830	1840
LEIPACREVR	VPSPPPREAW	GFGHNHQALQ	GAYLKNNLPV	LLQNQNSKIA	SSQQVTAEIP	VDLNTREVIR	ESGKCPGNIT
1850	1860	1870	1880	1890	1900	1910	1920
EESHDSVYSS	VTQNRHFLPS	TSTKVCEFEN	QVVILNKKHS	FPALEGGEVT	AQSCCGASSD	STESGKSLLF	RESEAREEEE
1930	1940	1950	1960	1970	1980	1990	2000
LDQNTVLRQT	INVSLEKDMP	GESAVSLKSR	SVDRRVSSPV	MVAQGGGPTP	KWEGKNETGL	LEKGLRPKDS	SEEFKLPGTK
2010	2020	2030	2040	2050	2060	2070	2080
PAYERFQLVA	CPQERNPSEC	KSQEMLNPNR	EPSGKKQNKR	VNNTDEMARL	IRSVMQLENG	ILEIESKQNK	QVHASHTPGT
2090	2100	2110	2120	2130	2140	2150	2160
DKELVFQDQK	EQEKTDHAFR	PDSSGNPLPS	KDQPSSPRQT	DDTVFRDSEA	GAMEVNSIGN	HPQVQKITPN	PFRSREGVRE
2170	2180	2190	2200	2210	2220	2230	2240

Detailed Protein Report

Cmpd.	No. of Cmpds.	m/z meas.	Δ m/z [ppm]	z	Rt [min]	Score	P	Range	Sequence	Modification
2847	1	665.3691	57.41	2	64.1	17.6	0	2192-2202	R.EFTNTSLHPQR.M	
1886	1	863.9273	-37.56	2	52.2	12.3	1	2739-2754	K.KVVAALPSQAPYDDPR.V	

Detailed Protein Report

Protein 87: phosphatidylinositol 3,4,5-trisphosphate 5-phosphatase 1 isoform b [Homo sapiens]

Accession: gi|40254823
Database: refseq_human(refseq_human_20140103.fasta)
Database Date: 2015-11-30

Score: 54.3
MW [kDa]: 133.1
pI: 7.9
Sequence Coverage [%]: 4.0
No. of unique Peptides: 2

Quantitation

mdown:qdown	**Median:**	1.45	**CV:**	0.00 %	**No. of Peptides:**	1
Wdown:Qdown	**Median:**	1.00	**CV:**	0.00 %	**No. of Peptides:**	1

```
        10         20         30         40         50         60         70         80
MVPCWNHGNI TRSKAEELLS RTGKDGSFLV RASESISRAY ALCVLYRNCV YTYRILPNED DKFTVQASEG VSMRFFTKLD
        90        100        110        120        130        140        150        160
QLIEFYKKEN MGLVTHLQYP VPLEEEDTGD DPEEDTESVV SPPELPPRNI PLTASSCEAK EVPFSNENPR ATETSRPSLS
       170        180        190        200        210        220        230        240
ETLFQRLQSM DTSGLPEEHL KAIQDYLSTQ LAQDSEFVKT GSSSLPHLKK LTTLLCKELY GEVIRTLPSL ESLQRLFDQQ
       250        260        270        280        290        300        310        320
LSPGLRPRPQ VPGEANPINM VSKLSQLTSL LSSIEDKVKA LLHEGPESPH RPSLIPPVTF EVKAESLGIP QKMQLKVDVE
       330        340        350        360        370        380        390        400
SGKLIIKKSK DGSEDKFYSH KKILQLIKSQ KFLNKLVILV ETEKEKILRK EYVFADSKKR EGFCQLLQQM KNKHSEQPEP
       410        420        430        440        450        460        470        480
DMITIFIGTW NMGNAPPPKK ITSWFLSKGQ GKTRDDSADY IPHDIYVIGT QEDPLSEKEW LEILKHSLQE ITSVTFKTVA
       490        500        510        520        530        540        550        560
IHTLWNIRIV VLAKPEHENR ISHICTDNVK TGIANTLGNK GAVGVSFMFN GTSLGFVNSH LTSGSEKKLR RNQNYMNILR
       570        580        590        600        610        620        630        640
FLALGDKKLS PFNITHRFTH LFWFGDLNYR VDLPTWEAET IIQKIKQQQY ADLLSHDQLL TERREQKVFL HFEEEEITFA
       650        660        670        680        690        700        710        720
PTYRFERLTR DKYAYTKQKA TGMKYNLPSW CDRVLWKSYP LVHVVCQSYG STSDIMTSDH SPVFATFEAG VTSQFVSKNG
       730        740        750        760        770        780        790        800
PGTVDSQGQI EFLRCYATLK TKSQTKFYLE FHSSCLESFV KSQEGENEEG SEGELVVKFG ETLPKLKPII SDPEYLLDQH
       810        820        830        840        850        860        870        880
ILISIKSSDS DESYGEGCIA LRLEATETQL PIYTPLTHHG ELTGHFQGEI KLQTSQGKTR EKLYDFVKTE RDESSGPKTL
       890        900        910        920        930        940        950        960
KSLTSHDPMK QWEVTSRAPP CSGSSITEII NPNYMGVGPF GPPMPLHVKQ TLSPDQQPTA WSYDQPPKDS PLGPCRGESP
       970        980        990       1000       1010       1020       1030       1040
PTPPGQPPIS PKKFLPSTAN RGLPPRTQES RPSDLGKNAG DTLPQEDLPL TKPEMFENPL YGSLSSFPKP APRKDQESPK
      1050       1060       1070       1080       1090       1100       1110       1120
MPRKEPPPCP EPGILSPSIV LTKAQEADRG EGPGKQVPAP RLRSFTCSSS AEGRAAGGDK SQGKPKTPVS SQAPVPAKRP
      1130       1140       1150       1160       1170       1180       1190
IKPSRSEINQ QTPPTPTPRP PLPVKSPAVL HLQHSKGRDY RDNTELPHHG KHRPEEGPPG PLGRTAMQ
```

Cmpd.	No. of Cmpds.	m/z meas.	Δ m/z [ppm]	z	Rt [min]	Score	P	Range	Sequence	Modification	Ratios
499	3	584.8345	31.59	2	35.6	27.2	0	79-87	K.LDQLIEFYK.K		Wdown:Qdown 1.00<br>mdown:qdown 1.45
1543	1	1008.6543	64.47	2	49.1	16.6	1	1107-1125	K.TPVSSQAPVPAKRPIKPSR.S		

Detailed Protein Report

Protein 88: PREDICTED: integrin beta-7 isoform X3 [Homo sapiens]

**Accession:**	gi|578823673	**Score:**	54.2
**Database:**	refseq_human(refseq_human_20140103.fasta)	**MW [kDa]:**	85.0
**Database Date:**	2015-11-30	**pI:**	5.7
**Modification(s):**	Carbamidomethyl	**Sequence Coverage [%]:**	6.6
		**No. of unique Peptides:**	4

10	20	30	40	50	60	70	80
MVALPMVLVL	LLVLSRGESE	LDAKIPSTGD	ATEWRNPHLS	MLGSCQPAPS	CQKCILSHPS	CAWCKQLNFT	ASGEAEARRC
**90**	**100**	**110**	**120**	**130**	**140**	**150**	**160**
ARREELLARG	CPLEELEEPR	GQQEVLQDQP	LSQGARGEGA	TQLAPQRVRV	TLRPGEPQQL	QVRFLRAEGY	PVDLYYLMDL
**170**	**180**	**190**	**200**	**210**	**220**	**230**	**240**
SYSMKDDLER	VRQLGHALLV	RLQEVTHSVR	IGFGSFVDKT	VLPFVSTVPS	KLRHPCPTRL	ERCQSPFSFH	HVLSLTGDAQ
**250**	**260**	**270**	**280**	**290**	**300**	**310**	**320**
AFEREVGRQS	VSGNLDSPEG	GFDAILQAAL	CQEQIGWRNV	SRLLVFTSDD	TFHTAGDGKL	GGIFMPSDGH	CHLDSNGLYS
**330**	**340**	**350**	**360**	**370**	**380**	**390**	**400**
RSTEFDYPSV	GQVAQALSAA	NIQPIFAVTS	AALPVYQELS	KLIPKSAVGE	LSEDSSNVVQ	LIMDAYNSLS	STVTLEHSSL
**410**	**420**	**430**	**440**	**450**	**460**	**470**	**480**
PPGVHISYES	QCEGPEKREG	KAEDRGQCNH	VRINQTVTFW	VSLQATHCLP	EPHLLRLRAL	GFSEELIVEL	HTLCDCNCSD
**490**	**500**	**510**	**520**	**530**	**540**	**550**	**560**
TQPQAPHCSD	GQGHLQCGVC	SCAPGRLGRL	CECSVAELSS	PDLESGCRAP	NGTGPLCSGK	GHCQCGRCSC	SGQSSGHLCE
**570**	**580**	**590**	**600**	**610**	**620**	**630**	**640**
CDDASCERHE	GILCGGFGRC	QCGVCHCHAN	RTGRACECSG	DMDSCISPEG	GLCSGHGRCK	CNRCQCLDGY	YGALCDQCPG
**650**	**660**	**670**	**680**	**690**	**700**	**710**	**720**
CKTPCERHRD	CAECGAFRTG	PLATNCSTAC	AHTNVTLALA	PILDDGWCKE	RTLDNQLFFF	LVEDDARGTV	VLRVRPQEKG
**730**	**740**	**750**	**760**	**770**	**780**	**790**	
ADHTQAIVLG	CVGGIVAVGL	GLVLAYRLSV	EIYDRREYSR	FEKEQQQLNW	KQLLGFGSRL	AS	

Cmpd.	No. of Cmpds.	m/z meas.	Δ m/z [ppm]	z	Rt [min]	Score	P	Range	Sequence	Modification
2663	2	731.3721	63.85	2	64.5	13.4	0	54-65	K.CILSHPSCAWCK.Q	Carbamidomethyl: 1, 11
2734	1	772.4219	-54.41	2	65.4	16.0	1	200-213	K.TVLPFVSTVPSKLR.H	
2949	1	1019.4212	-21.13	3	66.0	11.9	2	569-594	R.HEGILCGGFGRCQCGVCHCHANRTGR.A	Carbamidomethyl: 6, 12, 14, 17, 19
143	1	722.7732	-4.06	2	30.5	13.0	0	580-591	R.CQCGVCHCHANR.T	Carbamidomethyl: 1, 3

Detailed Protein Report

Protein 89: PREDICTED: keratin, type I cytoskeletal 26 isoform X2 [Homo sapiens]

Accession: gi|578830847
Database: refseq_human(refseq_human_20140103.fasta)
Database Date: 2015-11-30
Modification(s): Oxidation

Score: 53.9
MW [kDa]: 51.6
pI: 4.7
Sequence Coverage [%]: 10.5
No. of unique Peptides: 4

Quantitation

mdown:qdown **Median:** 0.46 **CV:** 0.00 % **No. of Peptides:** 1

10	20	30	40	50	60	70	80
MSFRLSGGSR	RICSRTGSGR	LSGGGTGFVA	GNVCVGSGAR	SSFSCTLEGI	SSGGSFCNSG	GGLGSGACAG	FLGNEHSLLS
**90**	**100**	**110**	**120**	**130**	**140**	**150**	**160**
GNEKVTMQNL	NDRLASYLDH	VHALEEANAD	LEQKIKGWYE	KCEPGSSREH	DHDYSRYFSV	IEDLKRQIIS	ATICNASIVL
**170**	**180**	**190**	**200**	**210**	**220**	**230**	**240**
QNDNARLTAD	DFRLKYENEL	ALHHSVEADT	SGLRRVLDEL	TLCTTDLEIQ	CETLSEELTY	LKKSHEEEME	VLQYTAGGNV
**250**	**260**	**270**	**280**	**290**	**300**	**310**	**320**
NVEMNATPGV	DLTVLLNNMR	AEYEDLAEQN	RKDAEAWFNE	RSATLQQQIS	DHEGAATAAR	NELTELKRNL	QTLEIELQSL
**330**	**340**	**350**	**360**	**370**	**380**	**390**	**400**
MAVKHSYECS	LAETEGNYCN	QLQQIQDQIG	VMEEQLQQIR	TETEGQKLEY	EQLLDVKIFL	EKEIDIYCNL	LDGEERKSKS
**410**	**420**	**430**	**440**	**450**	**460**	**470**	
TCYKSKGYRP	VNSGNQAKEE	TIVKTVVEEL	DQIGNLLSLR	VHSVEEKSSK	ISNITVEQRV	PSKAP	

Cmpd.	No. of Cmpds.	m/z meas.	Δ m/z [ppm]	z	Rt [min]	Score	P	Range	Sequence	Modification	Ratios
108	1	557.2530	-43.29	2	30.6	12.5	1	1-10	-.MSFRLSGGSR.R	Oxidation: 1	
559	1	625.3768	63.05	2	35.6	11.4	0	368-377	K.LEYEQLLDVK.I		mdown:qdown 0.46
1697	1	905.3389	-109.42	2	49.8	10.8	1	383-397	K.EIDIYCNLLDGEERK.S		
2812	1	753.3845	-13.35	2	64.3	19.2	1	405-418	K.SKGYRPVNSGNQAK.E		

Detailed Protein Report

Protein 90: PREDICTED: zinc finger protein 729 isoform X1 [Homo sapiens]

**Accession:**	gi|578832877	**Score:**	53.6
**Database:**	refseq_human(refseq_human_20140103.fasta)	**MW [kDa]:**	144.0
**Database Date:**	2015-11-30	**pI:**	10.5
		**Sequence Coverage [%]:**	3.3
		**No. of unique Peptides:**	2

10	20	30	40	50	60	70	80
MAASLRDCAG	TTGGASGENP	DSGCGFMNGK	SFGPGPLTFR	DVTIEFSLEE	WQCLDTVQQN	LYRDVMLENY	RNLVFLGMAV
90	100	110	120	130	140	150	160
FKPDLITCLK	QGKEPWNMKR	HEMVTKPPVM	RSHFTQDLWP	DQSTKDSFQE	VILRTYARCG	HKNLRLRKDC	KSANEGKMHK
170	180	190	200	210	220	230	240
EGYNKLNQCR	TATQRKIFQC	NKHMKVFHKY	SNRNKVRHTK	KKTFKCIKCS	KSFFMLSCLI	RHKRIHIRQN	IYKCEERGKA
250	260	270	280	290	300	310	320
FKSFSTLTKH	KIIHTEDKPY	KYKKCGNAFK	FSSTFTKHKR	IHTGETPFRC	EECGKAFNQS	SNLTDHKRIH	TGEKTYKCEE
330	340	350	360	370	380	390	400
CGKAFKGSSN	FNAHKVIHTA	EKPYKCEDCG	KTFNHFSALR	KHKIIHTGKK	PYKREECGKA	FSQSSTLRKH	EIIHTGEKPY
410	420	430	440	450	460	470	480
KCEECGKAFK	WSSKLTVHKV	VHTGEKPYKC	EECGKAFSQF	STLKKHKIIH	TGKKPYKCEE	CGKAFNSSST	LMKHKIIHTG
490	500	510	520	530	540	550	560
EKPYKCEECG	KAFRQSSHLT	RHKAIHTGEK	PYKCEECGKA	FNHFSDLRRH	KIIHTGKKPY	KCEECGKAFS	QSSTLRNHQI
570	580	590	600	610	620	630	640
IHTGEKPYKC	EECGKAFKWS	SKLTVHKVIH	TGEKPCKCEE	CGKAFKHFSA	LRKHKVIHTR	EKLYKCEECG	KAFNNSSILA
650	660	670	680	690	700	710	720
KHKIIHTGKK	PYKCEECGKA	FRQSSHLTRH	KAIHTGEKPY	KCEECGKAFS	HFSALRRHKI	IHTGKKPYKC	EECGKAFSHF
730	740	750	760	770	780	790	800
SALRRHKIIH	TGEKPYKCEE	CGKAFKWSSK	LTVHKVIHTR	EKLYKCEECV	KAFNSFSALM	KHKVIHTGEK	PYKCEECGKA
810	820	830	840	850	860	870	880
FKWSSKLTVH	KVIHTGEKPC	KCEECGKAFK	HFSALRKHKV	IHTGKKPYKC	EECGKAFSQS	SSLRKHEIIH	SGEKPYKCEE
890	900	910	920	930	940	950	960
CGKAFKWLSK	LTVHKVIHTA	EKPCKCEECG	KAFKHFSALR	KHKIIHTGKK	PYKCEECGKA	FNDSSTLMKH	KIIHTGKKPY
970	980	990	1000	1010	1020	1030	1040
KCAECGKAFK	QSSHLTRHKA	IHTGEKPYKC	EECGKDFNNS	STLKKHKLIH	TREKLYKCEE	CVKAFNNFSA	LMKHKIIHTG
1050	1060	1070	1080	1090	1100	1110	1120
EKPYKCEECG	KAFKWSSKLT	EHKVIHTGEK	PCKCEECDKA	FKHFSALRKH	KVIHTGKKPY	QCDECGKAFN	NSSTLTKHKI
1130	1140	1150	1160	1170	1180	1190	1200
IHTGEKPYKC	EECGKAFSQS	SILTKHKIIH	SVEKPYKCEE	CGKAFNQSSH	LTRHKTIHTG	EKPYKCEECG	KAFIQCSYLI
1210	1220	1230	1240	1250			
RHKTIHTREK	PTNVKKVPKL	LSNPHTLLDK	TIHTGEKPYK	CEECAKAF			

Cmpd.	No. of Cmpds.	m/z meas.	Δ m/z [ppm]	z	Rt [min]	Score	P	Range	Sequence	Modification
1895	1	704.7670	-172.25	2	52.2	13.8	1	502-513	R.HKAIHTGEKPYK.C	
2677	1	665.3406	42.21	2	61.8	13.9	2	621-631	R.EKLYKCEECGK.A	

Detailed Protein Report

Protein 91: GRIP and coiled-coil domain-containing protein 1 [Homo sapiens]

Accession: gi|22208850
Database: refseq_human(refseq_human_20140103.fasta)
Database Date: 2015-11-30

Score: 53.4
MW [kDa]: 87.8
pI: 5.2
Sequence Coverage [%]: 6.5
No. of unique Peptides: 3

Quantitation

mdown:qdown	**Median:**	1.36	**CV:**	0.00 %	**No. of Peptides:**	1
Wdown:Qdown	**Median:**	0.90	**CV:**	41.33 %	**No. of Peptides:**	2

10	20	30	40	50	60	70	80
MEKFGMNFGG	GPSKKDLLET	IETQKKQLLQ	YQARLKDVVR	AYKSLLKEKE	ALEASIKVLS	VSHEADVGLA	GVQLPGLTFP
**90**	**100**	**110**	**120**	**130**	**140**	**150**	**160**
DSVDDRCSTH	SEDSTGTATS	LDTAASLTST	KGEFGVEDDR	PARGPPPPKS	EEASWSESGV	SSSSGDGPFA	GGEVDKRLHQ
**170**	**180**	**190**	**200**	**210**	**220**	**230**	**240**
LKTQLATLTS	SLATVTQEKS	RMEASYLADK	KKMKQDLEDA	SNKAEEERAR	LEGELKGLQE	QIAETKARLI	TQQHDRAQEQ
**250**	**260**	**270**	**280**	**290**	**300**	**310**	**320**
SDHALMLREL	QKLLQEERTQ	RQDLELRLEE	TREALAGRAY	AAEQMEGFEL	QTKQLTREVE	ELKSELQAIR	DEKNQPDPRL
**330**	**340**	**350**	**360**	**370**	**380**	**390**	**400**
QELQEEAARL	KSHFQAQLQQ	EMRKTALAED	QLRQQSQVEE	QRVAALENQI	SEVSELLGTY	EKAKQKDQLA	IQKLKERILQ
**410**	**420**	**430**	**440**	**450**	**460**	**470**	**480**
LDLENKTLAL	AASSRSPLDS	HGEESSLDVN	VLKDKMEKLK	RLLQVAARKS	QVTLDVEKLC	DLEIMPSSEA	ADGEKATALY
**490**	**500**	**510**	**520**	**530**	**540**	**550**	**560**
YQQELKQLKE	EFERYKMRAQ	VVLKSKNTKD	GNLGKELEAA	QEQLAELKEK	YISLRLSCEE	LEHQHQEAD	DWKQELARLQ
**570**	**580**	**590**	**600**	**610**	**620**	**630**	**640**
QLHRQELERC	QLDFRDRTLK	LEEELHKQRD	RALAVLTEKD	LELEQLRSVA	LASGLPGRRS	PVGGGGPGDP	ADTSSSDSLT
**650**	**660**	**670**	**680**	**690**	**700**	**710**	**720**
QALQLAAANE	PTFFLYAEQL	ARKEVEITSL	RKQKHRLEVE	VHQLQDRLLE	EGERHREEVA	ALQSHIEKNI	RDQSREGANL
**730**	**740**	**750**	**760**	**770**	**780**		
EYLKNIIYRF	LTLPDSLGRQ	QTLTAILTIL	HFSPEEKQVI	MRLPTSASWW	PSGKR		

Cmpd.	No. of Cmpds.	m/z meas.	Δ m/z [ppm]	z	Rt [min]	Score	P	Range	Sequence	Modification	Ratios
165	1	635.8027	-9.05	2	31.3	11.3	1	180-190	K.SRMEASYLADK.K		Wdown:Qdown 1.34<br>mdown:qdown 1.36
1918	2	572.7243	-166.46	2	54.0	17.8	1	344-353	R.KTALAEDQLR.Q		
55	1	1096.9659	-93.00	2	30.1	11.7	0	363-382	R.VAALENQISEVSELLGTYEK.A		Wdown:Qdown 0.61

Detailed Protein Report

Protein 92: PREDICTED: kinesin-like protein KIF13B isoform X2 [Homo sapiens]

**Accession:**	gi|530387685	**Score:**	53.3
**Database:**	refseq_human(refseq_human_20140103.fasta)	**MW [kDa]:**	195.4
**Database Date:**	2015-11-30	**pI:**	5.4
**Modification(s):**	Oxidation	**Sequence Coverage [%]:**	1.5
		**No. of unique Peptides:**	2

Alias proteins:

Accession	Name	Description
gi|578815259	refseq_human (refseq_human_20140103.fasta)	PREDICTED: kinesin-like protein KIF13B isoform X4 [Homo sapiens]
gi|578815257	refseq_human (refseq_human_20140103.fasta)	PREDICTED: kinesin-like protein KIF13B isoform X3 [Homo sapiens]

Detailed Protein Report

10	20	30	40	50	60	70	80
MDESVKEKYA	GQDIVFKCLG	ENILQNAFDG	YNACIFAYGQ	TGSGKSYTMM	GTADQPGLIP	RLCSGLFERT	QKEENEEQSF
90	100	110	120	130	140	150	160
KVEVSYMEIY	NEKVRDLLDP	KGSRQTLKVR	EHSVLGPYVD	GLSKLAVTSY	KDIESLMSEG	NKSRTVAATN	MNEESSRSHA
170	180	190	200	210	220	230	240
VFKITLTHTL	YDVKSGTSGE	KVGKLSLVDL	AGSERATKTG	AAGDRLKEGS	NINKSLTTLG	LVISALADQS	AGKNKNKFVP
250	260	270	280	290	300	310	320
YRDSVLTWLL	KDSLGGNSKT	AMVATVSPAA	DNYDETLSTL	RYADRAKHIV	NHAVVNEDPN	ARIIRDLREE	VEKLREQLTK
330	340	350	360	370	380	390	400
AEAMKSPELK	DRLEESEKLI	QEMTVTWEEK	LRKTEEIAQE	RQKQLESLGI	SLQSSGIKVG	DDKCFLVNLN	ADPALNELLV
410	420	430	440	450	460	470	480
YYLKEHTLIG	SANSQDIQLC	GMGILPEHCI	IDITSEGQVM	LTPQKNTRTF	VNGSSVSSPI	QLHHGDRILW	GNNHFFRLNL
490	500	510	520	530	540	550	560
PKKKKKAERE	DEDQDPSMKN	ENSSEQLDVD	GDSSSEVSSE	VNFNYEYAQM	EVTMKALGSN	DPMQSILNSL	EQQHEEEKRS
570	580	590	600	610	620	630	640
ALERQRLMYE	HELEQLRRRL	SPEKQNCRSM	DRFSFHSPSA	QQRLRQWAEE	REATLNNSLM	RLREQIVKAN	LLVREANYIA
650	660	670	680	690	700	710	720
EELDKRTEYK	VTLQIPASSL	DANRKRGSLL	SEPAIQVRRK	GKGKQIWSLE	KLDNRLLDMR	DLYQEWKECE	EDNPVIRSYF
730	740	750	760	770	780	790	800
KRADPFYDEQ	ENHSLIGVAN	VFLESLFYDV	KLQYAVPIIN	QKGEVAGRLH	VEVMRLSGDV	GERIAGGDEV	AEVSFEKETQ
810	820	830	840	850	860	870	880
ENKLVCMVKI	LQATGLPQHL	SHFVFCKYSF	WDQQEPVIVA	PEVDTSSSSV	SKEPHCMVVF	DHCNEFSVNI	TEDFIEHLSE
890	900	910	920	930	940	950	960
GALAIEVYGH	KINDPRKNPA	LWDLGIIQAK	TRSLRDRWSE	VTRKLEFWVQ	ILEQNENGEY	CPVEVISAKD	VPTGGIFQLR
970	980	990	1000	1010	1020	1030	1040
QGQSRRVQVE	VKSVQESGTL	PLMEECILSV	GIGCVKVRPL	RAPRTHETFH	EEEEDMDSYQ	DRDLERLRRK	WLNALTKRQE
1050	1060	1070	1080	1090	1100	1110	1120
YLDQQLQKLV	SKRDKTEDDA	DREAQLLEMR	LTLTEERNAV	MVPSAGSGIP	GAPAEWTPVP	GMETHIPVIF	LDLNADDFSS
1130	1140	1150	1160	1170	1180	1190	1200
QDNLDDPEAG	GWDATLTGEE	EEEFFELQIV	KQHDGEVKAE	ASWDSAVHGC	PQLSRGTPVD	ERLFLIVRVT	VQLSHPADMQ
1210	1220	1230	1240	1250	1260	1270	1280
LVLRKRICVN	VHGRQGFAQS	LLKKMSHRSS	IPGCGVTFEI	VSNIPEDAQG	VEEREALARM	AANVENPASA	DSEAYIEKYL
1290	1300	1310	1320	1330	1340	1350	1360
RSVLAVENLL	TLDRLRQEVA	VKEQLTGKGK	LSRRSISSPN	VNRLSGSRQD	LIPSYSLGSN	KGRWESQQDV	SQTTVSRGIA
1370	1380	1390	1400	1410	1420	1430	1440
PAPALSVSPQ	NNHSPDPGLS	NLAASYLNPV	KSFVPQMPKL	LKSLFPVRDE	KRGKRPSPLA	HQPVPRIMVQ	SASPDIRVTR
1450	1460	1470	1480	1490	1500	1510	1520
MEEAQPEMGP	DVLVQTMGAP	ALKICDKPAK	VPSPPPVIAV	TAVTPAPEAQ	DGPPSPLSEA	SSGYFSHSVS	TATLSDALGP
1530	1540	1550	1560	1570	1580	1590	1600
GLDAAAPPGS	MPTAPEAEPE	APISHPPPPT	AVPAEEPPGP	QQLVSPGRER	PDLEAPAPGS	PFRVRRVRAS	ELRSFSRMLA
1610	1620	1630	1640	1650	1660	1670	1680
GDPGCSPGAE	GNAPAPGAGG	QALASDSEEA	DEVPEWLREG	EFVTVGAHKT	GVVRYVGPAD	FQEGTWVGVE	LDLPSGKNDG
1690	1700	1710	1720	1730	1740	1750	1760
SIGGKQYFRC	NPGYGLLVRP	SRVRRATGPV	RRRSTGLRLG	APEARRSATL	SGSATNLASL	TAALAKADRS	HKNPENRKSW
1770							
AS							

Cmpd.	No. of Cmpds.	m/z meas.	Δ m/z [ppm]	z	Rt [min]	Score	P	Range	Sequence	Modification
978	1	955.6278	74.00	1	41.8	12.8	1	94-101	K.VRDLLDPK.G	
1541	1	513.1056	-215.54	2	48.0	12.4	1	585-592	K.QNCRSMDR.F	Oxidation: 6

Detailed Protein Report

Protein 93: PREDICTED: MAX gene-associated protein isoform X16 [Homo sapiens]

**Accession:**	gi|578826753	**Score:**	53.1
**Database:**	refseq_human(refseq_human_20140103.fasta)	**MW [kDa]:**	290.2
**Database Date:**	2015-11-30	**pI:**	8.7
**Modification(s):**	Oxidation	**Sequence Coverage [%]:**	2.6
		**No. of unique Peptides:**	2

Quantitation

Wdown:Qdown	**Median:**	1.85	**CV:**	0.00 %	**No. of Peptides:**	1

Detailed Protein Report

10	20	30	40	50	60	70	80
MFKLDTGKMP	VVYLEPCAVT	RSTVKISELP	DNMLSTSRKD	KSSMLAELEY	LPTYIENSNE	TAFCLGKESE	NGLRKHSPDL
90	100	110	120	130	140	150	160
RVVQKYPLLK	EPQWKYPDIS	DSISTERILD	DSKDSVGDSL	SGKEDLGRKR	TTMLKIATAA	KVVNANQNAS	PNVPGKRGRP
170	180	190	200	210	220	230	240
RKLKLCKAGR	PPKNTGKSLI	STKNTPVSPG	STFPDVKPDL	EDVDGVLFVS	FESKEALDIH	AVDGTTEESS	SLQASTTNDS
250	260	270	280	290	300	310	320
GYRARISQLE	KELIEDLKTL	RHKQVIHPGL	QEVGLKLNSV	DPTMSIDLKY	LGVQLPLAPA	TSFPFWNLTG	TNPASPDAGF
330	340	350	360	370	380	390	400
PFVSRTGKTN	DFTKIKGWRG	KFHSASASRN	EGGNSESSLK	NRSAFCSDKL	DEYLENEGKL	METSMGFSSN	APTSPVVYQL
410	420	430	440	450	460	470	480
PTKSTSYVRT	LDSVLKKQST	ISPSTSYSLK	PHSVPPVSRK	AKSQNRQATF	SGRTKSSYKS	ILPYPVSPKQ	KYSHVILGDK
490	500	510	520	530	540	550	560
VTKNSSGIIS	ENQANNFVVP	TLDENIFPKQ	ISLRQAQQQQ	QQQQGSRPPG	LSKSQVKLMD	LEDCALWEGK	PRTYITEERA
570	580	590	600	610	620	630	640
DVSLTTLLTA	QASLKTKPIH	TIIRKRAPPC	NNDFCRLGCV	CSSLALEKRQ	PAHCRRPDCM	FGCTCLKRKV	VLVKGGSKTK
650	660	670	680	690	700	710	720
HFQRKAAHRD	PVFYDTLGEE	AREEEEGIRE	EEEQLKEKKK	RKKLEYTICE	TEPEQPVRHY	PLWVKVEGEV	DPEPVYIPTP
730	740	750	760	770	780	790	800
SVIEPMKPLL	LPQPEVLSPT	VKGKLLTGIK	SPRSYTPKPN	PVIREEDKDP	VYLYFESMMT	CARVRVYERK	KEDQRQPSSS
810	820	830	840	850	860	870	880
SSPSPSFQQQ	TSCHSSPENH	NNAKEPDSEQ	QPLKQLTCDL	EDDSDKLQEK	SWKSSCNEGE	SSSTSYMHQR	SPGGPTKLIE
890	900	910	920	930	940	950	960
IISDCNWEED	RNKILSILSQ	HINSNMPQSL	KVGSFIIELA	SQRKSRGEKN	PPVYSSRVKI	SMPSCQDQDD	MAEKSGSETP
970	980	990	1000	1010	1020	1030	1040
DGPLSPGKME	DISPVQTDAL	DSVRERLHGG	KGLPFYAGLS	PAGKLVAYKR	KPSSSTSGLI	QVNGKSYPQA	KLLLGQMGAL
1050	1060	1070	1080	1090	1100	1110	1120
HPANRLAAYI	TGRLRPSVLD	LSTLSTVISK	VASNAKVAAS	RKPRTLLPST	SNSKMASSSG	TATNRPGKNL	KAFVPAKRPI
1130	1140	1150	1160	1170	1180	1190	1200
AARPSPGGVF	TQFVMSKVGA	LQQKIPGVST	PQTLAGTQKF	SIRPSPVMVV	TPVVSSEPVQ	VCSPVTAAVT	TTTPQVFLEN
1210	1220	1230	1240	1250	1260	1270	1280
TTAVTPMTAI	SDVETKETTY	SSGATTTGVV	EVSETNTSTS	VTSTQSTATV	NLTKTTGITT	PVASVAFPKS	LVASPSTITL
1290	1300	1310	1320	1330	1340	1350	1360
PVASTASTSL	VVVTAAASSS	MVTTPTSSLG	SVPIILSGIN	GSPPVSQRPE	NAAQIPVATP	QVSPNTVKRA	GPRLLLIPVQ
1370	1380	1390	1400	1410	1420	1430	1440
QGSPTLRPVS	NTQLQGHRMV	LQPVRSPSGM	NLFRHPNGQI	VQLLPLHQLR	GSNTQPNLQP	VMFRNPGSVM	GIRLPAPSKP
1450	1460	1470	1480	1490	1500	1510	1520
SETPPSSTSS	SAFSVMNPVI	QAVGSSSAVN	VITQAPSLLS	SGASFVSQAG	TLTLRISPPE	PQSFASKTGS	ETKITYSSGG
1530	1540	1550	1560	1570	1580	1590	1600
QPVGTASLIP	LQSGSFALLQ	LPGQKPVPSS	ILQHVASLQM	KRESQNPDQK	DETNSIKREQ	ETKKVLQSEG	EAVDPEANVI
1610	1620	1630	1640	1650	1660	1670	1680
KQNSGAATSE	ETLNDSLEDR	GDHLDEECLP	EEGCATVKPS	EHSCITGSHT	DQDYKDVNEE	YGARNRKSSK	EKVAVLEVRT
1690	1700	1710	1720	1730	1740	1750	1760
ISEKASNKTV	QNLSKVQHQK	LGDVKVEQQK	GFDNPEENSS	EFPVTFKEES	KFELSGSKVM	EQQSNLQPEA	KEKECGDSLE
1770	1780	1790	1800	1810	1820	1830	1840
KDRERWRKHL	KGPLTRKCVG	ASQECKKEAD	EQLIKETKTC	QENSDVFQQE	QGISDLLGKS	GITEDARVLK	TECDSWSRIS
1850	1860	1870	1880	1890	1900	1910	1920
NPSAFSIVPR	RAAKSSRGNG	HFQGHLLLPG	EQIQPKQEKK	GGRSSADFTV	LDLEEDDEDD	NEKTDDSIDE	IVDVVSDYQS
1930	1940	1950	1960	1970	1980	1990	2000
EEVDDVEKVN	NCVEYIEDDE	EHVDIETVEE	LSEEINVAHL	KTTAAHTQSF	KQPSCTHISA	DEKAAERSRK	APPIPLKLKP
2010	2020	2030	2040	2050	2060	2070	2080
DYWSDKLQKE	AEAFAYYRRT	HTANERRRRG	EMRDLFEKLK	ITLGLLHSSK	VSKSLILTRA	FSEIQGLTDQ	ADKLIGQKNL
2090	2100	2110	2120	2130	2140	2150	2160
LTRKRNILIR	KVSSLSGKTE	EVVLKKLEYI	YAKQQALEAQ	KRKKKMGSDE	FDISPRISKQ	QEGSSASSVD	LGQMFINNRR
2170	2180	2190	2200	2210	2220	2230	2240

Detailed Protein Report

Cmpd.	No. of Cmpds.	m/z meas.	Δ m/z [ppm]	z	Rt [min]	Score	P	Range	Sequence	Modification	Ratios
1840	1	899.4842	21.23	2	51.7	12.0	0	1585-1601	K.VLQSEGEAVDPEANVIK.Q		
238	1	897.4097	-91.61	2	31.7	17.0	1	2603-2620	K.VMPCLAPIAAKVGSVGHK.M	Oxidation: 2	Wdown:Qdown 1.85

Detailed Protein Report

Protein 94: E3 ubiquitin-protein ligase UBR5 isoform 2 [Homo sapiens]

**Accession:**	gi|544583486	**Score:**	53.1
**Database:**	refseq_human(refseq_human_20140103.fasta)	**MW [kDa]:**	309.0
**Database Date:**	2015-11-30	**pI:**	5.5
**Modification(s):**	Oxidation	**Sequence Coverage [%]:**	2.4
		**No. of unique Peptides:**	3

Quantitation

mdown:qdown	**Median:**	0.46	**CV:**	0.00 %	**No. of Peptides:**	1
Wdown:Qdown	**Median:**	7.90	**CV:**	0.00 %	**No. of Peptides:**	1

BRUKER

Detailed Protein Report

10	20	30	40	50	60	70	80
MTSIHFVVHP	LPGTEDQLND	RLREVSEKLN	KYNLNSHPPL	NVLEQATIKQ	CVVGPNHAAF	LLEDGRVCRI	GFSVQPDRLE
90	100	110	120	130	140	150	160
LGKPDNNDGS	KLNSNSGAGR	TSRPGRTSDS	PWFLSGSETL	GRLAGNTLGS	RWSSGVGGSG	GGSSGRSSAG	ARDSRRQTRV
170	180	190	200	210	220	230	240
IRTGRDRGSG	LLGSQPQPVI	PASVIPEELI	SQAQVVLQGK	SRSVIIRELQ	RTNLDVNLAV	NNLLSRDDED	GDDGDDTASE
250	260	270	280	290	300	310	320
SYLPGEDLMS	LLDADIHSAH	PSVIIDADAM	FSEDISYFGY	PSFRRSSLSR	LGSSRVLLLP	LERDSELLRE	RESVLRLRER
330	340	350	360	370	380	390	400
RWLDGASFDN	ERGSTSKEGE	PNLDKKNTPV	QSPVSLGEDL	QWWPDKDGTK	FICIGALYSE	LLAVSSKGEL	YQWKWSESEP
410	420	430	440	450	460	470	480
YRNAQNPSLH	HPRATFLGLT	NEKIVLLSAN	SIRATVATEN	NKVATWVDET	LSSVASKLEH	TAQTYSELQG	ERIVSLHCCA
490	500	510	520	530	540	550	560
LYTCAQLENS	LYWWGVVPFS	QRKKMLEKAR	AKNKKPKSSA	GISSMPNITV	GTQVCLRNNP	LYHAGAVAFS	ISAGIPKVGV
570	580	590	600	610	620	630	640
LMESVWNMND	SCRFQLRSPE	SLKNMEKASK	TTEAKPESKQ	EPVKTEMGPP	PSPASTCSDA	SSIASSASMP	YKRRRSTPAP
650	660	670	680	690	700	710	720
KEEEKVNEEQ	WSLREVVFVE	DVKNVPVGKV	LKVDGAYVAV	KFPGTSSNTN	CQNSSGPDAD	PSSLLQDCRL	LRIDELQVVK
730	740	750	760	770	780	790	800
TGGTPKVPDC	FQRTPKKLCI	PEKTEILAVN	VDSKGVHAVL	KTGNWVRYCI	FDLATGKAEQ	ENNFPTSSIA	FLGQNERNVA
810	820	830	840	850	860	870	880
IFTAGQESPI	ILRDGNGTIY	PMAKDCMGGI	RDPDWLDLPP	ISSLGMGVHS	LINLPANSTI	KKKAAVIIMA	VEKQTLMQHI
890	900	910	920	930	940	950	960
LRCDYEACRQ	YLMNLEQAVV	LEQNLQMLQT	FISHRCDGNR	NILHACVSVC	FPTSNKETKE	EEEAERSERN	TFAERLSAVE
970	980	990	1000	1010	1020	1030	1040
AIANAISVVS	SNGPGNRAGS	SSSRSLRLRE	MMRRSLRAAG	LGRHEAGASS	SDHQDPVSPP	IAPPSWVPDP	PAMDPDGDID
1050	1060	1070	1080	1090	1100	1110	1120
FILAPAVGSL	TTAATGTGQG	PSTSTIPGPS	TEPSVVESKD	RKANAHFILK	LLCDSVVLQP	YLRELLSAKD	ARGMTPFMSA
1130	1140	1150	1160	1170	1180	1190	1200
VSGRAYPAAI	TILETAQKIA	KAEISSSEKE	EDVFMGMVCP	SGTNPDDSPL	YVLCCNDTCS	FTWTGAEHIN	QDIFECRTCG
1210	1220	1230	1240	1250	1260	1270	1280
LLESLCCCTE	CARVCHKGHD	CKLKRTSPTA	YCDCWEKCKC	KTLIAGQKSA	RLDLLYRLLT	ATNLVTLPNS	RGEHLLLFLV
1290	1300	1310	1320	1330	1340	1350	1360
QTVARQTVEH	CQYRPPRIRE	DRNRKTASPE	DSDMPDHDLE	PPRFAQLALE	RVLQDWNALK	SMIMFGSQEN	KDPLSASSRI
1370	1380	1390	1400	1410	1420	1430	1440
GHLLPEEQVY	LNQQSGTIRL	DCFTHCLIVK	CTADILLLDT	LLGTLVKELQ	NKYTPGRREE	AIAVTMRFLR	SVARVFVILS
1450	1460	1470	1480	1490	1500	1510	1520
VEMASSKKKN	NFIPQPIGKC	KRVFQALLPY	AVEELCNVAE	SLIVPVRMGI	ARPTAPFTLA	STSIDAMQGS	EELFSVEPLP
1530	1540	1550	1560	1570	1580	1590	1600
PRPSSDQSSS	SSQSQSSYII	RNPQQRRISQ	SQPVRGRDEE	QDDIVSADVE	EVEVVEGVAG	EEDHHDEQEE	HGEENAEAEG
1610	1620	1630	1640	1650	1660	1670	1680
QHDEHDEDGS	DMELDLLAAA	ETESDSESNH	SNQDNASGRR	SVVTAATAGS	EAGASSVPAF	FSEDDSQSND	SSDSDSSSSQ
1690	1700	1710	1720	1730	1740	1750	1760
SDDIEQETFM	LDEPLERTTN	SSHANGAAQA	PRSMQWAVRN	TQHQRAASTA	PSSTSTPAAS	SAGLIYIDPS	NLRRSGTIST
1770	1780	1790	1800	1810	1820	1830	1840
SAAAAAAALE	ASNASSYLTS	ASSLARAYSI	VIRQISDLMG	LIPKYNHLVY	SQIPAAVKLT	YQDAVNLQNY	VEEKLIPTWN
1850	1860	1870	1880	1890	1900	1910	1920
WMVSIMDSTE	AQLRYGSALA	SAGDPGHPNH	PLHASQNSAR	RERMTAREEA	SLRTLEGRRR	ATLLSARQGM	MSARGDFLNY
1930	1940	1950	1960	1970	1980	1990	2000
ALSLMRSHND	EHSDVLPVLD	VCSLKHVAYV	FQALIYWIKA	MNQQTTLDTP	QLERKRTREL	LELGIDNEDS	EHENDDDTNQ
2010	2020	2030	2040	2050	2060	2070	2080
SATLNDKDDD	SLPAETGQNH	PFFRRSDSMT	FLGCIPPNPF	EVPLAEAIPL	ADQPHLLQPN	ARKEDLFGRP	SQGLYSSSAS
2090	2100	2110	2120	2130	2140	2150	2160
SGKCLMEVTV	DRNCLEVLPT	KMSYAANLKN	VMNMQNRQKK	EGEEQPVLPE	ETESSKPGPS	AHDLAAQLKS	SLLAEIGLTE
2170	2180	2190	2200	2210	2220	2230	2240

Detailed Protein Report

Cmpd.	No. of Cmpds.	m/z meas.	Δ m/z [ppm]	z	Rt [min]	Score	P	Range	Sequence	Modification	Ratios
1093	1	647.6752	-180.54	2	42.2	14.8	0	132-146	R.WSSGVGGSGGGSSGR.S		Wdown:Qdown 7.90<br>mdown:qdown 0.46
31	1	712.9884	0.22	3	29.6	11.4	1	1305-1323	R.KTASPEDSDMPDHDLEPPR.F		
2778	1	958.9830	42.28	2	66.1	16.7	1	2102-2117	K.MSYAANLKNVMNMQNR.Q	Oxidation: 11, 13	

Detailed Protein Report

Protein 95: neuralized-like protein 4 isoform 2 [Homo sapiens]

**Accession:**	gi|53829368	**Score:**	52.7
**Database:**	refseq_human(refseq_human_20140103.fasta)	**MW [kDa]:**	166.6
**Database Date:**	2015-11-30	**pI:**	5.5
		**Sequence Coverage [%]:**	2.4
		**No. of unique Peptides:**	4

Quantitation

mdown:qdown **Median:** 0.11 **CV:** 0.00 % **No. of Peptides:** 1

10	20	30	40	50	60	70	80
MAAGSGGSGG	SGGGPGPGPG	GGGGPSGSGS	GPGSNGGLGS	GGELHPRTGR	LVSLSACGRT	ARRQQPGQEF	NHGLVLSREP
90	100	110	120	130	140	150	160
LRDGRVFTVR	IDRKVNSWSG	SIEIGVTALD	PSVLDFPSSA	TGLKGGSWVV	SGCSVLRDGR	SVLEEYGQDL	DQLGEGDRVG
170	180	190	200	210	220	230	240
VERTVAGELR	LWVNGRDCGV	AATGLPPRVW	AVVDLYGKCT	QITVLPPEPG	FSPPTPIPTP	PLEPLAPTED	SALAEQGTSA
250	260	270	280	290	300	310	320
DEAFMVSPAQ	ARPETFPNSL	ESHNDFANME	LSEVVSNTIL	SAYNGGLLNV	NLSSPPAGEG	LGSSGAATSP	ILTSNDALLF
330	340	350	360	370	380	390	400
HEKCGTLIKL	SNNNKTAERR	RPLDEFNNGV	VMTNRPLRDN	EMFEIRIDKL	VDKWSGSIEI	GVTTHNPNSL	EYPATMTNLQ
410	420	430	440	450	460	470	480
SGTIMMSGCG	ILTNGKGTRR	EYCEFSLDEL	QEGDHIGLTR	KSNSALHFFI	NGIDQGVATP	LTPPVVYGVV	DLYGMAVKVT
490	500	510	520	530	540	550	560
IVHNNNHSDR	LRRNNAILRA	LSPEGALRRA	APAAQAEPER	LLFHPNCGQK	AAITHEGRTA	LRPHATDDFN	HGVVLSSRAL
570	580	590	600	610	620	630	640
RDGEVFQVRI	DKMVDKWAGS	IEIGVTTHNP	AYLQLPSTMT	NLRSGTWMMT	GNGVMHNGTT	ILDEYGHNLD	RLKAGDTVGV
650	660	670	680	690	700	710	720
VRREDGTLHF	FVNGMTQGPA	AWNVPPGVYA	VVDLYGQAAQ	ATIVDDVEVA	PVPEPLPEGN	NQVSPSSPSS	GAGGSDLRFH
730	740	750	760	770	780	790	800
QLHGSNAVIT	NGGRTALRHN	CRSEFNDAIV	ISNRALRDGE	LFEIVIQKMV	DRWSGSIEAG	VTAIRPEDLE	FPNTMTDIDY
810	820	830	840	850	860	870	880
DTWMLSGTAI	MQDGNTMRNN	YGCDLDALGT	GARIGMMRTA	KGDLHYFING	QDQGAACSGL	PPEVYAVVDL	YGQCVQVSIT
890	900	910	920	930	940	950	960
NATGPMDNSL	ATSNTATEKS	FPLHSPVAGV	AHRFHSTCGK	NVTLEEDGTR	AVRAAGYAHG	LVFSTKELRA	EEVFEVKVEE
970	980	990	1000	1010	1020	1030	1040
LDEKWAGSLR	LGLTTLAPGE	MGPGAGGGGP	GLPPSLPELR	TKTTWMVSSC	EVRRDGQLQR	MNYGRNLERL	GVGSRVGVRR
1050	1060	1070	1080	1090	1100	1110	1120
GADDTMHILV	DGEDMGPAAT	GIAKNVWAVL	DLYGPVRGVS	IVSSTRLEES	EGTQPPSPSS	DTGSEGEEDD	EGEEHGLGGQ
1130	1140	1150	1160	1170	1180	1190	1200
NEVGIIPTTL	EFLENHGKNI	LLSNGNRTAT	RVASYNQGIV	VINQPLVPQL	LVQVRIDFLN	RQWTSSLVLG	VITCAPERLN
1210	1220	1230	1240	1250	1260	1270	1280
FPASACALKR	AAWLLRGRGV	FHNGLKICEK	FGPNLDTCPE	GTILGLRLDS	SGGLHLHVNG	VDQGVAVPDV	PQPCHALVDL
1290	1300	1310	1320	1330	1340	1350	1360
YGQCEQVTIV	NPEPGAASGK	SAGTQGDMEK	ADMVDGIKES	VCWGPPPAAS	PLKSCEYHAL	CSRFQELLLL	PEDYFMPPPK
1370	1380	1390	1400	1410	1420	1430	1440
RSLCYCESCR	KLRGDEAHRR	RGEPPREYAL	PFGWCRFNLR	VNPRLEAGTL	TKKWHMAYHG	SNVAAVRRVL	DRGELGAGTA
1450	1460	1470	1480	1490	1500	1510	1520
SILSCRPLKG	EPGVGFEEPG	ENCAPPREEQ	PPPVLLSPSL	QYAGAETLAS	KVQFRDPKSQ	RTHQAQVAFQ	VCVRPGSYTP
1530	1540	1550	1560	1570			
GPPSAALGEP	PDPHFSPAEL	EWVTKEKGAT	LLCALLVRVE				

Cmpd.	No. of Cmpds.	m/z meas.	Δ m/z [ppm]	z	Rt [min]	Score	P	Range	Sequence	Modification	Ratios
760	1	856.2336	-324.05	1	38.9	11.7	1	493-499	R.RNNAILR.A		
512	1	457.1269	-288.36	2	35.0	10.5	0	500-508	R.ALSPEGALR.R		mdown:qdown 0.11
2675	1	715.3668	-42.44	2	61.8	10.6	1	1139-1151	K.NILLSNGNRTATR.V		

Detailed Protein Report

Cmpd.	No. of Cmpds.	m/z meas.	Δ m/z [ppm]	z	Rt [min]	Score	P	Range	Sequence	Modification	Ratios
1492	1	555.2127	-156.80	2	47.2	20.0	2	1372-1380	K.LRGDEAHRR.R		

Detailed Protein Report

Protein 96: **zinc finger protein 281 isoform 2 [Homo sapiens]**

**Accession:**	gi|526253066	**Score:**	52.7
**Database:**	refseq_human(refseq_human_20140103.fasta)	**MW [kDa]:**	93.2
**Database Date:**	2015-11-30	**pI:**	9.3
		**Sequence Coverage [%]:**	2.6
		**No. of unique Peptides:**	2

10	20	30	40	50	60	70	80
MKIGSGFLSG	GGGTGSSGGS	GSGGGGSGGG	GGGGSSGRRA	EMEPTFPQAP	AAEPPPPPAP	DMTFKKEPAA	SAAAFPSQRT
90	100	110	120	130	140	150	160
SWGFLQSLVS	IKQEKPADPE	EQQSHHHHHH	HHYGGLFAGA	EERSPGLGGG	EGGSHGVIQD	LSILHQHVQQ	QPAQHHRDVL
170	180	190	200	210	220	230	240
LSSSSRTDDH	HGTEEPKQDT	NVKKAKRPKP	ESQGIKAKRK	PSASSKPSLV	GDGEGAILSP	SQKPHICDHC	SAAFRSSYHL
250	260	270	280	290	300	310	320
RRHVLIHTGE	RPFQCSQCSM	GFIQKYLLQR	HEKIHSREKP	FGCDQCSMKF	IQKYHMERHK	RTHSGEKPYK	CDTCQQYFSR
330	340	350	360	370	380	390	400
TDRLLKHRRT	CGEVIVKGAT	SAEPGSSNHT	NMGNLAVLSQ	GNTSSSRRKT	KSKSIAIENK	EQKTGKTNES	QISNNINMQS
410	420	430	440	450	460	470	480
YSVEMPTVSS	SGGIIGTGID	ELQKRVPKLI	FKKGSRKNTD	KNYLNFVSPL	PDIVGQKSLS	GKPSGSLGIV	SNNSVETIGL
490	500	510	520	530	540	550	560
LQSTSGKQGQ	ISSNYDDAMQ	FSKKRRYLPT	ASSNSAFSIN	VGHMVSQQSV	IQSAGVSVLD	NEAPLSLIDS	SALNAEIKSC
570	580	590	600	610	620	630	640
HDKSGIPDEV	LQSILDQYSN	KSESQKEDPF	NIAEPRVDLH	TSGEHSELVQ	EENLSPGTQT	PSNDKASMLQ	EYSKYLQQAF
650	660	670	680	690	700	710	720
EKSTNASFTL	GHGFQFVSLS	SPLHNHTLFP	EKQIYTTSPL	ECGFGQSVTS	VLPSSLPKPP	FGMLFGSQPG	LYLSALDATH
730	740	750	760	770	780	790	800
QQLTPSQELD	DLIDSQKNLE	TSSAFQSSSQ	KLTSQKEQKN	LESSTGFQIP	SQELASQIDP	QKDIEPRTTY	QIENFAQAFG
810	820	830	840	850	860		
SQFKSGSRVP	MTFITNSNGE	VDHRVRTSVS	DFSGYTNMMS	DVSEPCSTRV	KTPTSQSYR		

Cmpd.	No. of Cmpds.	m/z meas.	Δ m/z [ppm]	z	Rt [min]	Score	P	Range	Sequence	Modification
2653	1	811.8976	73.50	2	64.2	24.6	1	311-323	K.CDTCQQYFSRTDR.L	
768	2	495.2668	-36.55	2	38.4	28.1	1	372-380	K.SKSIAIENK.E	

Detailed Protein Report

Protein 97: prolactin-inducible protein precursor [Homo sapiens]

**Accession:**	gi|4505821	**Score:**	52.5
**Database:**	refseq_human(refseq_human_20140103.fasta)	**MW [kDa]:**	16.6
**Database Date:**	2015-11-30	**pI:**	9.3
**Modification(s):**	Carbamidomethyl	**Sequence Coverage [%]:**	18.5
		**No. of unique Peptides:**	2

Quantitation

mdown:qdown	**Median:**	0.55	**CV:**	0.00 %	**No. of Peptides:**	1
Wdown:Qdown	**Median:**	1.61	**CV:**	0.00 %	**No. of Peptides:**	1

```
        10         20         30         40         50         60         70         80
MRLLQLLFRA SPATLLLVLC LQLGANKAQD NTRKIIIKNF DIPKSVRPND EVTAVLAVQT ELKECMVVKT YLISSIPLQG
        90        100        110        120        130        140        150
AFNYKYTACL CDDNPKTFYW DFYTNRTVQI AAVVDVIREL GICPDDAAVI PIKNNRFYTI EILKVE
```

Cmpd.	No. of Cmpds.	m/z meas.	Δ m/z [ppm]	z	Rt [min]	Score	P	Range	Sequence	Modification	Ratios
2176	2	642.3028	-132.15	2	57.4	28.1	0	107-118	R.TVQIAAVVDVIR.E		Wdown:Qdown 1.61<br>mdown:qdown 0.55
2317	1	805.9036	-28.46	2	57.6	24.3	0	119-133	R.ELGICPDDAAVIPIK.N	Carbamidomethyl: 5	

Detailed Protein Report

Protein 98: short transient receptor potential channel 4 isoform zeta [Homo sapiens]

Accession: gi|209863032
Database: refseq_human(refseq_human_20140103.fasta)
Database Date: 2015-11-30
Modification(s): Oxidation

Score: 52.2
MW [kDa]: 92.2
pI: 9.3
Sequence Coverage [%]: 4.9
No. of unique Peptides: 1

Quantitation

mdown:qdown	**Median:**	1.09	**CV:**	0.00 %	**No. of Peptides:**	1
Wdown:Qdown	**Median:**	1.24	**CV:**	0.00 %	**No. of Peptides:**	1

10	20	30	40	50	60	70	80
MAQFYYKRNV	NAPYRDRIPL	RIVRAESELS	PSEKAYLNAV	EKGDYASVKK	SLEEAEIYFK	ININCIDPLG	RTALLIAIEN
**90**	**100**	**110**	**120**	**130**	**140**	**150**	**160**
ENLELIELLL	SFNVYVGDAL	LHAIRKEVVG	AVELLLNHKK	PSGEKQFVAQ	PNCQQLLASR	WYDEFPGWRR	RHWAVKMVTC
**170**	**180**	**190**	**200**	**210**	**220**	**230**	**240**
FIIGLLFPVF	SVCYLIAPKS	PLGLFIRKPF	IKFICHTASY	LTFLFLLLLA	SQHIDRSDLN	RQGPPPTIVE	WMILPWVLGF
**250**	**260**	**270**	**280**	**290**	**300**	**310**	**320**
IWGEIKQMWD	GGLQDYIHDW	WNLMDFVMNS	LYLATISLKI	VAFVKYSALN	PRESWDMWHP	TLVAEALFAI	ANIFSSLRLI
**330**	**340**	**350**	**360**	**370**	**380**	**390**	**400**
SLFTANSHLG	PLQISLGRML	LDILKFLFIY	CLVLLAFANG	LNQLYFYYEE	TKGLTCKGIR	CEKQNNAFST	LFETLQSLFW
**410**	**420**	**430**	**440**	**450**	**460**	**470**	**480**
SIFGLINLYV	TNVKAQHEFT	EFVGATMFGT	YNVISLVVLL	NMLIAMMNNS	YQLIADHADI	EWKFARTKLW	MSYFEEGGTL
**490**	**500**	**510**	**520**	**530**	**540**	**550**	**560**
PTPFNVIPSP	KSLWYLIKWI	WTHLCKKKMR	RKPESFGTIG	RRAADNLRRH	HQYQEVMRNL	VKRYVAAMIR	DAKTEEGLTE
**570**	**580**	**590**	**600**	**610**	**620**	**630**	**640**
ENFKELKQDI	SSFRFEVLGL	LRGSKLSTIQ	SANASKESSN	SADSDEKSDS	EGNSKDKKKN	FSLFDLTTLI	HPRSAAIASE
**650**	**660**	**670**	**680**	**690**	**700**	**710**	**720**
RHNISNGSAL	VVQEPPREKQ	RKVNFVTDIK	NFGLFHRRSK	QNAAEQNANQ	IFSVSEEVAR	QQAAGPLERN	IQLESRGLAS
**730**	**740**	**750**	**760**	**770**	**780**	**790**	**800**
RGDLSIPGLS	EQCVLVDHRE	RNTDTLGLQV	GKRVCPFKSE	KVVVEDTVPI	IPKEKHAKEE	DSSIDYDLNL	PDTVTHEDYV
**810**							
TTRL							

Cmpd.	No. of Cmpds.	m/z meas.	Δ m/z [ppm]	z	Rt [min]	Score	P	Range	Sequence	Modification	Ratios
117	1	849.3965	-40.26	2	30.9	18.1	1	530-542	R.HHQYQEVMRNLVK.R	Oxidation: 8	Wdown:Qdown 1.24<br>mdown:qdown 1.09

Detailed Protein Report

Protein 99: desmoplakin isoform I [Homo sapiens]

**Accession:**	gi|58530840	**Score:**	52.0
**Database:**	refseq_human(refseq_human_20140103.fasta)	**MW [kDa]:**	331.6
**Database Date:**	2015-11-30	**pI:**	6.4
		**Sequence Coverage [%]:**	1.7
		**No. of unique Peptides:**	2

Quantitation

mdown:qdown	**Median:** 1.44	**CV:** 0.00 %	**No. of Peptides:** 1

Detailed Protein Report

10	20	30	40	50	60	70	80
MSCNGGSHPR	INTLGRMIRA	ESGPDLRYEV	TSGGGGTSRM	YYSRRGVITD	QNSDGYCQTG	TMSRHQNQNT	IQELLQNCSD
90	100	110	120	130	140	150	160
CLMRAELIVQ	PELKYGDGIQ	LTRSRELDEC	FAQANDQMEI	LDSLIREMRQ	MGQPCDAYQK	RLLQLQEQMR	ALYKAISVPR
170	180	190	200	210	220	230	240
VRRASSKGGG	GYTCQSGSGW	DEFTKHVTSE	CLGWMRQQRA	EMDMVAWGVD	LASVEQHINS	HRGIHNSIGD	YRWQLDKIKA
250	260	270	280	290	300	310	320
DLREKSAIYQ	LEEEYENLLK	ASFERMDHLR	QLQNIIQATS	REIMWINDCE	EEELLYDWSD	KNTNIAQKQE	AFSIRMSQLE
330	340	350	360	370	380	390	400
VKEKELNKLK	QESDQLVLNQ	HPASDKIEAY	MDTLQTQWSW	ILQITKCIDV	HLKENAAYFQ	FFEEAQSTEA	YLKGLQDSIR
410	420	430	440	450	460	470	480
KKYPCDKNMP	LQHLLEQIKE	LEKEREKILE	YKRQVQNLVN	KSKKIVQLKP	RNPDYRSNKP	IILRALCDYK	QDQKIVHKGD
490	500	510	520	530	540	550	560
ECILKDNNER	SKWYVTGPGG	VDMLVPSVGL	IIPPPNPLAV	DLSCKIEQYY	EAILALWNQL	YINMKSLVSW	HYCMIDIEKI
570	580	590	600	610	620	630	640
RAMTIAKLKT	MRQEDYMKTI	ADLELHYQEF	IRNSQGSEMF	GDDDKRKIQS	QFTDAQKHYQ	TLVIQLPGYP	QHQTVTTTEI
650	660	670	680	690	700	710	720
THHGTCQDVN	HNKVIETNRE	NDKQETWMLM	ELQKIRRQIE	HCEGRMTLKN	LPLADQGSSH	HITVKINELK	SVQNDSQAIA
730	740	750	760	770	780	790	800
EVLNQLKDML	ANFRGSEKYC	YLQNEVFGLF	QKLENINGVT	DGYLNSLCTV	RALLQAILQT	EDMLKVYEAR	LTEEETVCLD
810	820	830	840	850	860	870	880
LDKVEAYRCG	LKKIKNDLNL	KKSLLATMKT	ELQKAQQIHS	QTSQQYPLYD	LDLGKFGEKV	TQLTDRWQRI	DKQIDFRLWD
890	900	910	920	930	940	950	960
LEKQIKQLRN	YRDNYQAFCK	WLYDAKRRQD	SLESMKFGDS	NTVMRFLNEQ	KNLHSEISGK	RDKSEEVQKI	AELCANSIKD
970	980	990	1000	1010	1020	1030	1040
YELQLASYTS	GLETLLNIPI	KRTMIQSPSG	VILQEAADVH	ARYIELLTRS	GDYYRFLSEM	LKSLEDLKLK	NTKIEVLEEE
1050	1060	1070	1080	1090	1100	1110	1120
LRLARDANSE	NCNKNKFLDQ	NLQKYQAECS	QFKAKLASLE	ELKRQAELDG	KSAKQNLDKC	YGQIKELNEK	ITRLTYEIED
1130	1140	1150	1160	1170	1180	1190	1200
EKRRRKSVED	RFDQQKNDYD	QLQKARQCEK	ENLGWQKLES	EKAIKEKEYE	IERLRVLLQE	EGTRKREYEN	ELAKVRNHYN
1210	1220	1230	1240	1250	1260	1270	1280
EEMSNLRNKY	ETEINITKTT	IKEISMQKED	DSKNLRNQLD	RLSRENRDLK	DEIVRLNDSI	LQATEQRRRA	EENALQQKAC
1290	1300	1310	1320	1330	1340	1350	1360
GSEIMQKKQH	LEIELKQVMQ	QRSEDNARHK	QSLEEAAKTI	QDKNKEIERL	KAEFQEEAKR	RWEYENELSK	VRNNYDEEII
1370	1380	1390	1400	1410	1420	1430	1440
SLKNQFETEI	NITKTTIHQL	TMQKEEDTSG	YRAQIDNLTR	ENRSLSEEIK	RLKNTLTQTT	ENLRRVEEDI	QQQKATGSEV
1450	1460	1470	1480	1490	1500	1510	1520
SQRKQQLEVE	LRQVTQMRTE	ESVRYKQSLD	DAAKTIQDKN	KEIERLKQLI	DKETNDRKCL	EDENARLQRV	QYDLQKANSS
1530	1540	1550	1560	1570	1580	1590	1600
ATETINKLKV	QEQELTRLRI	DYERVSQERT	VKDQDITRFQ	NSLKELQLQK	QKVEEELNRL	KRTASEDSCK	RKKLEEELEG
1610	1620	1630	1640	1650	1660	1670	1680
MRRSLKEQAI	KITNLTQQLE	QASIVKKRSE	DDLRQQRDVL	DGHLREKQRT	QEELRRLSSE	VEALRRQLLQ	EQESVKQAHL
1690	1700	1710	1720	1730	1740	1750	1760
RNEHFQKAIE	DKSRSLNESK	IEIERLQSLT	ENLTKEHLML	EEELRNLRLE	YDDLRRGRSE	ADSDKNATIL	ELRSQLQISN
1770	1780	1790	1800	1810	1820	1830	1840
NRTLELQGLI	NDLQRERENL	RQEIEKFQKQ	ALEASNRIQE	SKNQCTQVVQ	ERESLLVKIK	VLEQDKARLQ	RLEDELNRAK
1850	1860	1870	1880	1890	1900	1910	1920
STLEAETRVK	QRLECEKQQI	QNDLNQWKTQ	YSRKEEAIRK	IESEREKSER	EKNSLRSEIE	RLQAEIKRIE	ERCRRKLEDS
1930	1940	1950	1960	1970	1980	1990	2000
TRETQSQLET	ERSRYQREID	KLRQRPYGSH	RETQTECEWT	VDTSKLVFDG	LRKKVTAMQL	YECQLIDKTT	LDKLLKGKKS
2010	2020	2030	2040	2050	2060	2070	2080
VEEVASEIQP	FLRGAGSIAG	ASASPKEKYS	LVEAKRKKLI	SPESTVMLLE	AQAATGGIID	PHRNEKLTVD	SAIARDLIDF
2090	2100	2110	2120	2130	2140	2150	2160
DDRQQIYAAE	KAITGFDDPF	SGKTVSVSEA	IKKNLIDRET	GMRLLEAQIA	SGGVVDPVNS	VFLPKDVALA	RGLIDRDLYR
2170	2180	2190	2200	2210	2220	2230	2240

Detailed Protein Report

Cmpd.	No. of Cmpds.	m/z meas.	Δ m/z [ppm]	z	Rt [min]	Score	P	Range	Sequence	Modification	Ratios
2423	1	837.8362	-126.41	2	60.6	16.4	2	1545-1558	R.VSQERTVKDQDITR.F		
1485	1	600.4356	127.57	2	47.3	10.5	2	1819-1828	K.IKVLEQDKAR.L		mdown:qdown 1.44

Detailed Protein Report

Protein 100: **guanine nucleotide-binding protein-like 3-like protein [Homo sapiens]**

**Accession:**	gi|9506611	**Score:**	51.9
**Database:**	refseq_human(refseq_human_20140103.fasta)	**MW [kDa]:**	65.5
**Database Date:**	2015-11-30	**pI:**	9.5
**Modification(s):**	Carbamidomethyl, Oxidation	**Sequence Coverage [%]:**	10.1
		**No. of unique Peptides:**	2

Alias proteins:

Accession	Name	Description
gi|296317324	refseq_human (refseq_human_20140103.fasta)	guanine nucleotide-binding protein-like 3-like protein [Homo sapiens]

10	20	30	40	50	60	70	80
MMKLRHKNKK	PGEGSKGHKK	ISWPYPQPAK	QNGKKATSKV	PSAPHFVHPN	DHANREAELK	KKWVEEMREK	QQAAREQERQ
**90**	**100**	**110**	**120**	**130**	**140**	**150**	**160**
KRRTIESYCQ	DVLRRQEEFE	HKEEVLQELN	MFPQLDDEAT	RKAYYKEFRK	VVEYSDVILE	VLDARDPLGC	RCFQMEEAVL
**170**	**180**	**190**	**200**	**210**	**220**	**230**	**240**
RAQGNKKLVL	VLNKIDLVPK	EVVEKWLDYL	RNELPTVAFK	ASTQHQVKNL	NRCSVPVDQA	SESLLKSKAC	FGAENLMRVL
**250**	**260**	**270**	**280**	**290**	**300**	**310**	**320**
GNYCRLGEVR	THIRVGVVGL	PNVGKSSLIN	SLKRSRACSV	GAVPGITKFM	QEVYLDKFIR	LLDAPGIVPG	PNSEVGTILR
**330**	**340**	**350**	**360**	**370**	**380**	**390**	**400**
NCVHVQKLAD	PVTPVETILQ	RCNLEEISNY	YGVSGFQTTE	HFLTAVAHRL	GKKKKGGLYS	QEQAAKAVLA	DWVSGKISFY
**410**	**420**	**430**	**440**	**450**	**460**	**470**	**480**
IPPPATHTLP	THLSAEIVKE	MTEVFDIEDT	EQANEDTMEC	LATGESDELL	GDTDPLEMEI	KLLHSPMTKI	ADAIENKTTV
**490**	**500**	**510**	**520**	**530**	**540**	**550**	**560**
YKIGDLTGYC	TNPNRHQMGW	AKRNVDHRPK	SNSMVDVCSV	DRRSVLQRIM	ETDPLQQGQA	LASALKNKKK	MQKRADKIAS
**570**	**580**	**590**					
KLSDSMMSAL	DLSGNADDGV	GD					

Cmpd.	No. of Cmpds.	m/z meas.	Δ m/z [ppm]	z	Rt [min]	Score	P	Range	Sequence	Modification
2071	1	1023.8928	-74.38	2	54.5	11.7	1	229-245	K.ACFGAENLMRVLGNYCR.L	Carbamidomethyl: 2, 16; Oxidation: 9
2097	1	701.2282	-87.20	3	56.4	10.9	0	562-582	K.LSDSMMSALDLSGNADDGVGD.-	Oxidation: 5, 6

Detailed Protein Report

Protein 101: PREDICTED: tetratricopeptide repeat protein 13 isoform X7 [Homo sapiens]

**Accession:**	gi|578802328	**Score:**	51.9
**Database:**	refseq_human(refseq_human_20140103.fasta)	**MW [kDa]:**	57.4
**Database Date:**	2015-11-30	**pI:**	9.3
		**Sequence Coverage [%]:**	10.8
		**No. of unique Peptides:**	3

Quantitation

mdown:qdown	**Median:**	2.05	**CV:**	0.00 %	**No. of Peptides:**	1
Wdown:Qdown	**Median:**	5.78	**CV:**	0.00 %	**No. of Peptides:**	1

```
        10         20         30         40         50         60         70         80
MMLYHHGSLQ EALKNFKRCL QLEPYNEVCQ YMKGLSHVAM GQFYEGIKAQ TKVMLNDPLP GQKASPEYLK VKYLREYSRY
        90        100        110        120        130        140        150        160
LHAHLDTPLT EYNIDVDLPG SFKDHWAKNL PFLIEDYEEQ PGLQPHIKDV LHQNFESYKP EVQELICVAD RLGSLMQYET
       170        180        190        200        210        220        230        240
PGFLPNKRIH RAMGLAALEV MQAVQRTWTN SKVRMNGKTR LMQWRDMFDI AVKWRRIADP DQPVLWLDQM PARSLSRGFN
       250        260        270        280        290        300        310        320
NHINLIRGQV INMRYLEYFE KILHFIKDRI LVYHGANNPK GLLEVREALE KVHKVEDLLP IMKQFNTKTK DGFTVNTKVP
       330        340        350        360        370        380        390        400
SLKDQGKEYD GFTITITGDK VGNILFSVET QTTEERTQLY HAEIDALYKD LTAKGKVLIL SSEFGEADAV CNLILSLVYY
       410        420        430        440        450        460        470        480
FYNLMPLSRG SSVIAYSVIV GALMASGKEV AGKIPKGKLV DFEAMTAPGS EAFSKVAKSW MNLKSISPSY KTLPSVSETF
       490        500        510
PTLRSMIEVL NTDSSPRCLK KL
```

Cmpd.	No. of Cmpds.	m/z meas.	Δ m/z [ppm]	z	Rt [min]	Score	P	Range	Sequence	Modification	Ratios
1705	1	820.8385	-139.02	2	49.9	10.6	1	234-247	R.SLSRGFNNHINLIR.G		
549	2	730.3654	19.41	2	36.2	15.9	0	328-340	K.EYDGFTITITGDK.V		Wdown:Qdown 5.78<br>mdown:qdown 2.05
2198	1	905.3670	-138.71	2	56.0	12.3	0	410-428	R.GSSVIAYSVIVGALMASGK.E		

Detailed Protein Report

Protein 102: PREDICTED: ubiquitin-protein ligase E3C isoform X3 [Homo sapiens]

**Accession:**	gi|530387311	**Score:**	51.9
**Database:**	refseq_human(refseq_human_20140103.fasta)	**MW [kDa]:**	116.7
**Database Date:**	2015-11-30	**pI:**	6.2
**Modification(s):**	Carbamidomethyl	**Sequence Coverage [%]:**	4.3
		**No. of unique Peptides:**	4

Quantitation

mdown:qdown **Median:** 1.08 **CV:** 0.00 % **No. of Peptides:** 1

10	20	30	40	50	60	70	80
MFSFEGDFKT	RPKVSLGGAS	RKEEKASLLH	RTQEERRKRE	EERRRLKNAI	IIQSFIRGYR	DRKQQYSIQR	SAFDRCATLS
**90**	**100**	**110**	**120**	**130**	**140**	**150**	**160**
QSGGAFPIAN	GPNLTLLVRQ	LLFFYKQNED	SKRLIWLYQN	LIKHSSLFVK	QLDGSERLTC	LFQIKRLMSL	CCRLLQNCND
**170**	**180**	**190**	**200**	**210**	**220**	**230**	**240**
DSLNVALPMR	MLEVFSSENT	YLPVLQDASY	VVSVIEQILH	YMIHNGYYRS	LYLLINSKLP	SSIEYSDLSR	VPIAKILLEN
**250**	**260**	**270**	**280**	**290**	**300**	**310**	**320**
VLKPLHFTYN	SCPEGARQQV	FTAFTEEFLA	APFTDQIFHF	IIPALADAQT	VFPYEPFLNA	LLLIESRCSR	KSGGAPWLFY
**330**	**340**	**350**	**360**	**370**	**380**	**390**	**400**
FVLTVGENYL	GALSEEGLLV	YLRVLQTFLS	QLPVSPASAS	CHDSASDSEE	ESEEADKPSS	PEDGRLSVSY	ITEECLKKLD
**410**	**420**	**430**	**440**	**450**	**460**	**470**	**480**
TKQQTNTLLN	LVWRDSASEE	VFTTMASVCH	TLMVQHRMMV	PKVRLLYSLA	FNARFLRHLW	FLISSMSTRM	ITGSMVPLLQ
**490**	**500**	**510**	**520**	**530**	**540**	**550**	**560**
VISRGSPMSF	EDSSRIIPLF	YLFSSLFSHS	LISIHDNEFF	GDPIEVVGQR	QSSMMPFTLE	ELIMLSRCLR	DACLGIIKLA
**570**	**580**	**590**	**600**	**610**	**620**	**630**	**640**
YPETKPEVRE	EYITAFQSIG	VTTSSEMQQC	IQMEQKRWIQ	LFKVITNLVK	MLKSRDTRRN	FCPPNHWLSE	QEDIKADKIF
**650**	**660**	**670**	**680**	**690**	**700**	**710**	**720**
QRLIYADKQE	VQGDGPFLDG	INVTIRRNYI	YEDAYDKLSP	ENEPDLKKRI	RVHLLNAHGL	DEAGIDGGGI	FREFLNELLK
**730**	**740**	**750**	**760**	**770**	**780**	**790**	**800**
SGFNPNQGFF	KTTNEGLLYP	NPAAQMLVGD	SFARHYYFLG	RMLGKALYEN	MLVELPFAGF	FLSKLLGTSA	DVDIHHLASL
**810**	**820**	**830**	**840**	**850**	**860**	**870**	**880**
DPEVYKNLLF	LKSYEDDVEE	LGLNFTVVNN	DLGEAQVVEL	KFGGKDIPVT	SANRIAYIHL	VADYRLNRQI	RQHCLAFRQG
**890**	**900**	**910**	**920**	**930**	**940**	**950**	**960**
LANVVSLEWL	RMFDQQEIQV	LISGAQVPIS	LEDLKSFTNY	SGGYSADHPV	IKVFWRVVEG	FTDEEKRKLL	KFVTSCSRPP
**970**	**980**	**990**	**1000**	**1010**	**1020**	**1030**	
LLGFKELYPA	FCIHNGGSDL	ERLPTASTCM	NLLKLPEFYD	ETLLRSKLLY	AIECAAGFEL	S	

Cmpd.	No. of Cmpds.	m/z meas.	Δ m/z [ppm]	z	Rt [min]	Score	P	Range	Sequence	Modification	Ratios
2476	4	817.2871	-207.41	1	61.3	12.6	0	124-130	K.HSSLFVK.Q		mdown:qdown 1.08
259	1	862.8548	57.47	3	32.6	16.7	2	689-712	K.RIRVHLLNAHGLDEAGIDGGGIFR.E		
2651	1	856.4088	-69.02	2	64.2	10.9	2	866-878	R.LNRQIRQHCLAFR.Q	Carbamidomethyl: 9	
2795	1	874.5325	111.23	1	64.0	11.6	0	872-878	R.QHCLAFR.Q		

Detailed Protein Report

Protein 103: PREDICTED: neuron navigator 2 isoform X5 [Homo sapiens]

**Accession:**	gi|578820908	**Score:**	51.7
**Database:**	refseq_human(refseq_human_20140103.fasta)	**MW [kDa]:**	264.1
**Database Date:**	2015-11-30	**pI:**	9.8
**Modification(s):**	Carbamidomethyl, Oxidation	**Sequence Coverage [%]:**	2.0
		**No. of unique Peptides:**	2

Detailed Protein Report

10	20	30	40	50	60	70	80
MPAILVASKM	KSGLPKPVHS	AAPILHVPPA	RAGPQPCYLK	LGSKVEVSKT	TYPSQIPLKS	QVLQGLQEPA	GEGLPLRKSG
90	100	110	120	130	140	150	160
SVENGFDTQI	YTDWANHYLA	KSGHKRLIRD	LQQDVTDGVL	LAQIIQVVAN	EKIEDINGCP	KNRSQMIENI	DACLNFLAAK
170	180	190	200	210	220	230	240
GINIQGLSAE	EIRNGNLKAI	LGLFFSLSRY	KQQQQQPQKQ	HLSSPLPPAV	SQVAGAPSQC	QAGTPQQQVP	VTPQAPCQPH
250	260	270	280	290	300	310	320
QPAPHQQSKA	QAEMQSSASS	KDSSQSKIIR	FTLGQKKISR	LPGPTARVSA	AGSEAKTRGG	STTANNRRSQ	SFNNYDKSKP
330	340	350	360	370	380	390	400
VTSPPPPPSS	HEKEPLASSA	SSHPGMSDNA	PASLESGSSS	TPTNCSTSSA	IPQPGAATKP	WRSKSLSVKH	SATVSMLSVK
410	420	430	440	450	460	470	480
PPGPEAPRPT	PEAMKPAPNN	QKSMLEKLKL	FNSKGGSKAG	EGPGSRDTSC	ERLETLPSFE	ESEELEAASR	MLTTVGPASS
490	500	510	520	530	540	550	560
SPKIALKGIA	QRTFSRALTN	KKSSLKGNEK	EKEKQQREKD	KEKSKDLAKR	ASVTERLDLK	EEPKEDPSGA	AVPEMPKKSS
570	580	590	600	610	620	630	640
KIASFIPKGG	KLNSAKKEPM	APSHSGIPKP	GMKSMPGKSP	SAPAPSKEGE	RSRSGKLSSG	LPQQKPQLDG	RHSSSSSSLA
650	660	670	680	690	700	710	720
SSEGKGPGGT	TLNHSISSQT	VSGSVGTTQT	TGSNTVSVQL	PQPQQQYNHP	NTATVAPFLY	RSQTDTEGNV	TAESSSTGVS
730	740	750	760	770	780	790	800
VEPSHFTKTG	QPALEELTGE	DPEARRLRTV	KNIADLRQNL	EETMSSLRGT	QVTHSTLETT	FDTNVTTEMS	GRSILSLTGR
810	820	830	840	850	860	870	880
PTPLSWRLGQ	SSPRLQAGDA	PSMGNGYPPR	ANASRFINTE	SGRYVYSAPL	RRQLASRGSS	VCHVDVSDKA	GDEMDLEGIS
890	900	910	920	930	940	950	960
MDAPGYMSDG	DVLSKNIRTD	DITSGYMTDG	GLGLYTRRLN	RLPDGMAVVR	ETLQRNTSLG	LGDADSWDDS	SSVSSGISDT
970	980	990	1000	1010	1020	1030	1040
IDNLSTDDIN	TSSSISSYAN	TPASSRKNLD	VQTDAEKHSQ	VERNSLWSGD	DVKKSDGGSD	SGIKMEPGSK	WRRNPSDVSD
1050	1060	1070	1080	1090	1100	1110	1120
ESDKSTSGKK	NPVISQTGSW	RRGMTAQVGI	TMPRTKPSAP	AGALKTPGTG	KTDDAKVSEK	GRLSPKASQV	KRSPSDAGRS
1130	1140	1150	1160	1170	1180	1190	1200
SGDESKKPLP	SSSRTPTANA	NSFGFKKQSG	SAAGLAMITA	SGVTVTSRSA	TLGKIPKSSA	LVSRSAGRKS	SMDGAQNQDD
1210	1220	1230	1240	1250	1260	1270	1280
GYLALSSRTN	LQYRSLPRPS	KSNSRNGAGN	RSSTSSIDSN	ISSKSAGLPV	PKLREPSKTA	LGSSLPGLVN	QTDKEKGISS
1290	1300	1310	1320	1330	1340	1350	1360
DNESVASCNS	VKVNPAAQPV	SSPAQTSLQP	GAKYPDVASP	TLRRLFGGKP	TKQVPIATAE	NMKNSVVISN	PHATMTQQGN
1370	1380	1390	1400	1410	1420	1430	1440
LDSPSGSGVL	SSGSSSPLYS	KNVDLNQSPL	ASSPSSAHSA	PSNSLTWGTN	ASSSSAVSKD	GLGFQSVSSL	HTSCESIDIS
1450	1460	1470	1480	1490	1500	1510	1520
LSSGGVPSHN	SSTGLIASSK	DDSLTPFVRT	NSVKTTLSES	PLSSPAASPK	FCRSTLPRKQ	DSDPHLDRNT	LPKKGLRYTP
1530	1540	1550	1560	1570	1580	1590	1600
TSQLRTQEDA	KEWLRSHSAG	GLQDTAANSP	FSSGSSVTSP	SGTRFNFSQL	ASPTTVTQMS	LSNPTMLRTH	SLSNADGQYD
1610	1620	1630	1640	1650	1660	1670	1680
PYTDSRFRNS	SMSLDEKSRT	MSRSGSFRDG	FEEVHGSSLS	LVSSTSSVYS	TPEEKCQSEI	RKLRRELDAS	QEKVSALTTQ
1690	1700	1710	1720	1730	1740	1750	1760
LTANAHLVAA	FEQSLGNMTI	RLQSLTMTAE	QKDSELNELR	KTIELLKKQN	AAAQAAINGV	INTPELNCKG	NGTAQSADLR
1770	1780	1790	1800	1810	1820	1830	1840
IRRQHSSDSV	SSINSATSHS	SVGSNIESDS	KKKKRKNWVN	ELRSSFKQAF	GKKKSPKSAS	SHSDIEEMTD	SSLPSSPKLP
1850	1860	1870	1880	1890	1900	1910	1920
HNGSTGSTPL	LRNSHSNSLI	SECMDSEAET	VMQLRNELRD	KEMKLTDIRL	EALSSAHQLD	QLREAMNRMQ	SEIEKLKAEN
1930	1940	1950	1960	1970	1980	1990	2000
DRLKSESQGS	GCSRAPSQVS	ISASPRQSMG	LSQHSLNLTE	STSLDMLLDD	TGECSARKEG	GRHVKIVVSF	QEEMKWKEDS
2010	2020	2030	2040	2050	2060	2070	2080
RPHLFLIGCI	GVSGKTKWDV	LDGVVRRLFK	EYIIHVDPVS	QLGLNSDSVL	GYSIGEIKRS	NTSETPELLP	CGYLVGENTT
2090	2100	2110	2120	2130	2140	2150	2160
ISVTVKGLAE	NSLDSLVFES	LIPKPILQRY	VSLLIEHRRI	ILSGPSGTGK	TYLANRLSEY	IVLREGRELT	DGVIATFNVD
2170	2180	2190	2200	2210	2220	2230	2240

Detailed Protein Report

Cmpd.	No. of Cmpds.	m/z meas.	Δ m/z [ppm]	z	Rt [min]	Score	P	Range	Sequence	Modification
183	1	904.3719	-34.40	2	31.7	10.6	2	435-452	K.GGSKAGEGPGSRDTSCER.L	Carbamidomethyl: 16
2463	1	647.1849	-205.84	2	61.2	24.0	0	1063-1074	R.GMTAQVGITMPR.T	Oxidation: 2, 10

Detailed Protein Report

Protein 104: Alstrom syndrome protein 1 [Homo sapiens]

**Accession:**	gi|110349786	**Score:**	51.6
**Database:**	refseq_human(refseq_human_20140103.fasta)	**MW [kDa]:**	460.9
**Database Date:**	2015-11-30	**pI:**	5.8
**Modification(s):**	Carbamidomethyl	**Sequence Coverage [%]:**	1.3
		**No. of unique Peptides:**	2

Detailed Protein Report

10	20	30	40	50	60	70	80
MEPEDLPWPG	ELEEEEEEEE	EEEEEEEEEA	AAAAAAANVDD	VVVVEEVEEE	AGRELDSDSH	YGPQHLESID	DEEDEEAKAW
90	100	110	120	130	140	150	160
LQAHPGRILP	PLSPPQHRYS	EGERTSLEKI	VPLTCHVWQQ	IVYQGNSRTQ	ISDTNVVCLE	TTAQRGSGDD	QKTESWHCLP
170	180	190	200	210	220	230	240
QEMDSSQTLD	TSQTRFNVRT	EDTEVTDFPS	LEEGILTQSE	NQVKEPNRDL	FCSPLLVIQD	SFASPDLPLL	TCLTQDQEFA
250	260	270	280	290	300	310	320
PDSLFHQSEL	SFAPLRGIPD	KSEDTEWSSR	PSEVSEALFQ	ATAEVASDLA	SSRFSVSQHP	LIGSTAVGSQ	CPFLPSEQGN
330	340	350	360	370	380	390	400
NEETISSVDE	LKIPKDCDRY	DDLCSYMSWK	TRKDTQWPEN	NLADKDQVSV	ATSFDITDEN	IATKRSDHFD	AARSYGQYWT
410	420	430	440	450	460	470	480
QEDSSKQAET	YLTKGLQGKV	ESDVITLDGL	NENAVVCSER	VAELQRKPTR	ESEYHSSDLR	MLRMSPDTVP	KAPKHLKAGD
490	500	510	520	530	540	550	560
TSKGGIAKVT	QSNLKSGITT	TPVDSDIGSH	LSLSLEDLSQ	LAVSSPLETT	TGQHTDTLNQ	KTLADTHLTE	ETLKVTAIPE
570	580	590	600	610	620	630	640
PADQKTATPT	VLSSSHSHRG	KPSIFYQQGL	PDSHLTEEAL	KVSAAPGLAD	QTTGMSTLTS	TSYSHREKPG	TFYQQELPES
650	660	670	680	690	700	710	720
NLTEEPLEVS	AAPGPVEQKT	GIPTVSSTSH	SHVEDLLFFY	RQTLPDGHLT	DQALKVSAVS	GPADQKTGTA	TVLSTPHSHR
730	740	750	760	770	780	790	800
EKPGIFYQQE	FADSHQTEET	LTKVSATPGP	ADQKTEIPAV	QSSSYSQREK	PSILYPQDLA	DSHLPEEGLK	VSAVAGPADQ
810	820	830	840	850	860	870	880
KTGLPTVPSS	AYSHREKLLV	FYQQALLDSH	LPEEALKVSA	VSGPADGKTG	TPAVTSTSSA	SSSLGEKPSA	FYQQTLPNSH
890	900	910	920	930	940	950	960
LTEEALKVSI	VPGPGDQKTG	IPSAPSSFYS	HREKPIIFSQ	QTLPDFLFPE	EALKVSAVSV	LAAQKTGTPT	VSSNSHSHSE
970	980	990	1000	1010	1020	1030	1040
KSSVFYQQEL	PDSDLPRESL	KMSAIPGLTD	QKTVPTPTVP	SGSFSHREKP	SIFYQQEWPD	SYATEKALKV	STGPGPADQK
1050	1060	1070	1080	1090	1100	1110	1120
TEIPAVQSSS	YPQREKPSVL	YPQVLSDSHL	PEESLKVSAF	PGPADQMTDT	PAVPSTFYSQ	REKPGIFYQQ	TLPESHLPKE
1130	1140	1150	1160	1170	1180	1190	1200
ALKISVAPGL	ADQKTGTPTV	TSTSYSQHRE	KPSIFHQQAL	PGTHIPEEAQ	KVSAVTGPGN	QKTWIPRVLS	TFYSQREKPG
1210	1220	1230	1240	1250	1260	1270	1280
IFYQQTLPGS	HIPEEAQKVS	PVLGPADQKT	GTPTPTSASY	SHTEKPGIFY	QQVLPDNHPT	EEALKISVAS	EPVDQTTGTP
1290	1300	1310	1320	1330	1340	1350	1360
AVTSTSYSQY	REKPSIFYQQ	SLPSSHLTEE	AKNVSAVPGP	ADQKTVIPIL	PSTFYSHTEK	PGVFYQQVLP	HSHPTEEALK
1370	1380	1390	1400	1410	1420	1430	1440
ISVASEPVDQ	TTGTPTVTST	SYSQHTEKPS	IFYQQSLPGS	HLTEEAKNVS	AVPGPGDRKT	GIPTLPSTFY	SHTEKPGSFY
1450	1460	1470	1480	1490	1500	1510	1520
QQVLPHSHLP	EEALEVSVAP	GPVDQTIGTP	TVTSPSSSFG	EKPIVIYKQA	FPEGHLPEES	LKVSVAPGPV	GQTTGAPTIT
1530	1540	1550	1560	1570	1580	1590	1600
SPSYSQHRAK	SGSFYQLALL	GSQIPEEALR	VSSAPGPADQ	TTGIPTITST	SYSFGEKPIV	NYKQAFPDGH	LPEEALKVSI
1610	1620	1630	1640	1650	1660	1670	1680
VSGPTEKKTD	IPAGPLGSSA	LGEKPITFYR	QALLDSPLNK	EVVKVSAAPG	PADQKTETLP	VHSTSYSNRG	KPVIFYQQTL
1690	1700	1710	1720	1730	1740	1750	1760
SDSHLPEEAL	KVPPVPGPDA	QKTETPSVSS	SLYSYREKPI	VFYQQALPDS	ELTQEALKVS	AVPQPADQKT	GLSTVTSSFY
1770	1780	1790	1800	1810	1820	1830	1840
SHTEKPNISY	QQELPDSHLT	EEALKVSNVP	GPADQKTGVS	TVTSTSYSHR	EKPIVSYQRE	LPHFTEAGLK	ILRVPGPADQ
1850	1860	1870	1880	1890	1900	1910	1920
KTGINILPSN	SYPQREHSVI	SYEQELPDLT	EVTLKAIGVP	GPADQKTGIQ	IASSSSYSNR	EKASIFHQQE	LPDVTEEALN
1930	1940	1950	1960	1970	1980	1990	2000
VFVVPGQGDR	KTEIPTVPLS	YYSRREKPSV	ISQQELPDSH	LTEEALKVSP	VSIPAEQKTG	IPIGLSSSYS	HSHKEKLKIS
2010	2020	2030	2040	2050	2060	2070	2080
TVHIPDDQKT	EFPAATLSSY	SQIEKPKIST	VIGPNDQKTP	SQTAFHSSYS	QTVKPNILFQ	QQLPDRDQSK	GILKISAVPE
2090	2100	2110	2120	2130	2140	2150	2160
LTDVNTGKPV	SLSSSYFHRE	KSNIFSPQEL	PGSHVTEDVL	KVSTIPGPAG	QKTVLPTALP	SSFSHREKPD	IFYQKDLPDR
2170	2180	2190	2200	2210	2220	2230	2240

Detailed Protein Report

Cmpd.	No. of Cmpds.	m/z meas.	Δ m/z [ppm]	z	Rt [min]	Score	P	Range	Sequence	Modification
312	1	862.8548	-22.20	2	33.0	10.7	1	340-352	R.YDDLCSYMSWKTR.K	Carbamidomethyl: 5
1836	4	863.4125	-60.24	2	51.6	18.6	1	3791-3805	R.LIQAFGHERVCLSPR.R	

Detailed Protein Report

Protein 105: E3 ubiquitin-protein ligase BRE1A [Homo sapiens]

Accession: gi|34878777
Database: refseq_human(refseq_human_20140103.fasta)
Database Date: 2015-11-30
Modification(s): Carbamidomethyl, Oxidation

Score: 51.4
MW [kDa]: 113.6
pI: 5.6
Sequence Coverage [%]: 6.4
No. of unique Peptides: 4

Quantitation

mdown:qdown	**Median:** 1.17	**CV:** 0.00 %	**No. of Peptides:**	1
Wdown:Qdown	**Median:** 3.32	**CV:** 0.00 %	**No. of Peptides:**	1

10	20	30	40	50	60	70	80
MSGIGNKRAA	GEPGTSMPPE	KKAAVEDSGT	TVETIKLGGV	SSTEELDIRT	LQTKNRKLAE	MLDQRQAIED	ELREHIEKLE
90	100	110	120	130	140	150	160
RRQATDDASL	LIVNRYWSQF	DENIRIILKR	YDLEQGLGDL	LTERKALVVP	EPEPDSDSNQ	ERKDDRERGE	GQEPAFSFLA
170	180	190	200	210	220	230	240
TLASSSSEEM	ESQLQERVES	SRRAVSQIVT	VYDKLQEKVE	LLSRKLNSGD	NLIVEEAVQE	LNSFLAQENM	RLQELTDLLQ
250	260	270	280	290	300	310	320
EKHRTMSQEF	SKLQSKVETA	ESRVSVLESM	IDDLQWDIDK	IRKREQRLNR	HLAEVLERVN	SKGYKVYGAG	SSLYGGTITI
330	340	350	360	370	380	390	400
NARKFEEMNA	ELEENKELAQ	NRLCELEKLR	QDFEEVTTQN	EKLKVELRSA	VEQVVKETPE	YRCMQSQFSV	LYNESLQLKA
410	420	430	440	450	460	470	480
HLDEARTLLH	GTRGTHQHQV	ELIERDEVSL	HKKLRTEVIQ	LEDTLAQVRK	EYEMLRIEFE	QTLAANEQAG	PINREMRHLI
490	500	510	520	530	540	550	560
SSLQNHNHQL	KGEVLRYKRK	LREAQSDLNK	TRLRSGSALL	QSQSSTEDPK	DEPAELKPDS	EDLSSQSSAS	KASQEDANEI
570	580	590	600	610	620	630	640
KSKRDEEERE	RERREKERER	EREREKEKER	EREKQKLKES	EKERDSAKDK	EKGKHDDGRK	KEAEIIKQLK	IELKKAQESQ
650	660	670	680	690	700	710	720
KEMKLLLDMY	RSAPKEQRDK	VQLMAAEKKS	KAELEDLRQR	LKDLEDKEKK	ENKKMADEDA	LRKIRAVEEQ	IEYLQKKLAM
730	740	750	760	770	780	790	800
AKQEEEALLS	EMDVTGQAFE	DMQEQNIRLM	QQLREKDDAN	FKLMSERIKS	NQIHKLLKEE	KEELADQVLT	LKTQVDAQLQ
810	820	830	840	850	860	870	880
VVRKLEEKEH	LLQSNIGTGE	KELGLRTQAL	EMNKRKAMEA	AQLADDLKAQ	LELAQKKLHD	FQDEIVENSV	TKEKDMFNFK
890	900	910	920	930	940	950	960
RAQEDISRLR	RKLETTKKPD	NVPKCDEILM	EEIKDYKARL	TCPCCNMRKK	DAVLTKCFHV	FCFECVKTRY	DTRQRKCPKC
970	980						
NAAFGANDFH	RIYIG						

Cmpd.	No. of Cmpds.	m/z meas.	Δ m/z [ppm]	z	Rt [min]	Score	P	Range	Sequence	Modification	Ratios
1337	1	926.7938	6.01	3	45.5	12.7	1	257-280	K.VETAESRVSVLESMIDDLQWDIDK.I		
463	1	475.6020	-277.84	2	35.1	11.6	0	827-834	R.TQALEMNK.R	Oxidation: 6	Wdown:Qdown 3.32<br>mdown:qdown 1.17
2961	1	788.3914	-25.63	2	65.8	15.7	2	835-848	K.RKAMEAAQLADDLK.A	Oxidation: 4	
1898	1	918.3471	-84.86	2	52.4	11.4	2	956-971	R.KCPKCNAAFGANDFHR.I	Carbamidomethyl: 5	

Detailed Protein Report

Protein 106: protein FAM115C isoform B [Homo sapiens]

**Accession:**	gi|293651621	**Score:**	51.4
**Database:**	refseq_human(refseq_human_20140103.fasta)	**MW [kDa]:**	90.6
**Database Date:**	2015-11-30	**pI:**	8.9
**Modification(s):**	Oxidation	**Sequence Coverage [%]:**	3.8
		**No. of unique Peptides:**	4

Quantitation

mdown:qdown	**Median:**	1.85	**CV:**	0.00 %	**No. of Peptides:**	1
Wdown:Qdown	**Median:**	0.60	**CV:**	0.00 %	**No. of Peptides:**	2

```
        10         20         30         40         50         60         70         80
MKIFQKEFKF ILKKCTPIQK YREENGNSSS SPHHQLQSHA SGPRYGEDVR QDQQQLLEGI SELDIRTGGV PSQLLVHGAL
        90        100        110        120        130        140        150        160
AFPLGLDASL NCFLAAAHYG RGRVVLAAHE CLLCAPKMGP FLLNAVRWLA RGQTGKVGVN TNLKDLCPLL SEHGLQCSLE
       170        180        190        200        210        220        230        240
PHLNSDLCVY CCKAYSDKEA KQLQEFVAEG GGLLIGGQAW WWASQNPGHC PLAGFPGNII LNCFGLSILP QTLKAGCFPV
       250        260        270        280        290        300        310        320
PTPEMRSYHF RKALSQFQAI LNHENGNLEK SCLAKLRVDG AAFLQIPAEG VPAYISLHRL LRKMLRGSGL PAVSRENPVA
       330        340        350        360        370        380        390        400
SDSYEAAVLS LATGLAHSGT DCSQLAQGLG TWTCSSSLYP SKHPITVEIN GINPESHSVI QVGMQWRDLS SCNLHLLGLS
       410        420        430        440        450        460        470        480
NSSLSASCVA GTTGTRHHAW LIFVFLVERE FHRKGNNDCW VSTGLYLLEG QNAEVSLSEA AASAGLRVQI GCHTDDLTKA
       490        500        510        520        530        540        550        560
RKLSRAPVVT HQCWMDRTER SVSCLWGGLL YVIVPKGSQL GPVPVTIRGA VPAPYYKLGK TSLEEWKRQM QENLAPWGEL
       570        580        590        600        610        620        630        640
ATDNIILTVP TTNLQALKDP EPVLRLWDEM MQAVARLAAE PFPFRRPERI VADVQISAGW MHSGYPIMCH LESVKEIINE
       650        660        670        680        690        700        710        720
MDMRSRGVWG PIHELGHNQQ RHGWEFPPHT TEATCNLWSV YVHETVLGIP RAQAHEALSP PERERRIKAH LGKGAPLCDW
       730        740        750        760        770        780        790        800
NVWTALETYL QLQEAFGWEP FTQLFAEYQT LSHLPKDNTG RMNLWVKKFS EKVKKNLVPF FEAWGWPIQK EVADSLASLP
       810        820
EWQENPMQVY LRARK
```

Cmpd.	No. of Cmpds.	m/z meas.	Δ m/z [ppm]	z	Rt [min]	Score	P	Range	Sequence	Modification	Ratios
7	1	689.3232	-60.71	1	29.3	10.0	0	15-20	K.CTPIQK.Y		
126	1	822.3680	-116.44	2	30.8	17.1	1	118-131	K.MGPFLLNAVRWLAR.G		
495	2	705.1921	-194.39	2	35.1	10.8	1	636-646	K.EIINEMDMRSR.G	Oxidation: 6	Wdown:Qdown 0.60
738	2	705.1981	-185.90	2	37.8	13.4	1	636-646	K.EIINEMDMRSR.G	Oxidation: 8	Wdown:Qdown 0.60

Detailed Protein Report

Protein 107: **PREDICTED: unhealthy ribosome biogenesis protein 2 homolog isoform X1 [Homo sapiens]**

**Accession:**	gi|530366678	**Score:**	51.3
**Database:**	refseq_human(refseq_human_20140103.fasta)	**MW [kDa]:**	166.5
**Database Date:**	2015-11-30	**pI:**	6.9
		**Sequence Coverage [%]:**	2.6
		**No. of unique Peptides:**	1

Quantitation

mdown:qdown **Median:** 1.55 **CV:** 0.00 % **No. of Peptides:** 1

10	20	30	40	50	60	70	80
MAAVYSGISL	KLKSKTTSWE	DKLKLAHFAW	ISHQCFLPNK	EQVLLDWARQ	SLVAFYKKKL	ELKEDIVERL	WIYIDNILHS
**90**	**100**	**110**	**120**	**130**	**140**	**150**	**160**
RKLQNLLKNG	KTINLQISLV	KIINERVAEF	SLSGSQRNIC	AVLRCCQGIL	STPALAVIYT	AKQELMVALL	SQLCWSACRQ
**170**	**180**	**190**	**200**	**210**	**220**	**230**	**240**
PEGAVVAQLF	EVIHLALGHY	LLILQQQVNP	RRAFGDVTAH	LLQPCLVLRH	LLSGGTWTQA	GQGQLRQVLS	RDIRSQIEAM
**250**	**260**	**270**	**280**	**290**	**300**	**310**	**320**
FRGGIFQPEL	LSSYKEGLLD	QQQGDVKTGA	MKNLLAPMDT	VLNRLVDAGY	CAASLHTSVV	ANSVALLYKL	FLDSYFKEGN
**330**	**340**	**350**	**360**	**370**	**380**	**390**	**400**
QLLCFQVLPR	LFGCLKISHL	QEEQSKALST	SDWTTELLVV	EQLLNSVANN	NIYNIAADRI	RHEEAQFRFY	RHVAELLINH
**410**	**420**	**430**	**440**	**450**	**460**	**470**	**480**
AQAPIPAWFR	CLKTLISLNH	LILEPDLDDL	LASAWIDAEV	TEFRTKKAQE	ALIRTVFQTY	AKLRQVPRLF	EEVLGVICRP
**490**	**500**	**510**	**520**	**530**	**540**	**550**	**560**
AAEALRQPVL	ASGPSTVLSA	CLLELPPSQI	LDTWSLVLEK	FQSLVLPYLQ	SDADMALKSL	SLSLLLHCIM	FNMRSLDSST
**570**	**580**	**590**	**600**	**610**	**620**	**630**	**640**
PLPIVRRTQC	MMERMMRELV	QPLLALLPDT	PGPEPELWLQ	KVSDSVLLLS	YTWAQVDAMF	SLNCSQYHSM	SGPLIGVALE
**650**	**660**	**670**	**680**	**690**	**700**	**710**	**720**
ISNLPSLLPG	VKTQHWKKIE	KFTAQFSSLG	TYCLEQLYLQ	KMKRTLMQTS	FRSEGAIQSL	RCDAAFIIGS	GRKSLNQRTT
**730**	**740**	**750**	**760**	**770**	**780**	**790**	**800**
ASWDGQVGMV	SGLTYPVAHW	HLIVSNLTIL	ISYLCPDDVG	YLASVLLRTL	PMGKAQEVSI	DEEAYITLEK	ISKAFLHSPL
**810**	**820**	**830**	**840**	**850**	**860**	**870**	**880**
FPEMQSLHSA	FLTCVTTSCS	SILCSGAQRD	SGLVSQQLPW	LFEKDHMVVG	HWENRFAKAG	PEGIEPRGEI	AQNLLSLVKS
**890**	**900**	**910**	**920**	**930**	**940**	**950**	**960**
DFPIQLEGEQ	LESILGLLEV	ISALQLDSLL	PPYHVHYFLV	LLSMAVTKLG	CSCSSSLALK	FLTTCYQLLG	YLQKGKSARS
**970**	**980**	**990**	**1000**	**1010**	**1020**	**1030**	**1040**
VFKIMYGSDI	FEVVLTSLFR	ASSRFLIEMD	DPAWLEFLQV	IGTFLEELMQ	MLIQMKLSLV	LNFRKITAFL	SSSKPYTEAA
**1050**	**1060**	**1070**	**1080**	**1090**	**1100**	**1110**	**1120**
SSKQLENQNP	QGRQLLLVSL	TRLCHVLGPF	LKEQKLGQEA	PAALSELLQQ	VVLQTGAVLQ	LCSVPGARGW	RLPSVLISSV
**1130**	**1140**	**1150**	**1160**	**1170**	**1180**	**1190**	**1200**
STLLEADLGQ	HCRDGGADIS	QGSDRTLLSH	VALYQGVYSQ	ILLELPALAG	HDQSFQAALQ	FLTLFFLAPE	LHPKKDSVFT
**1210**	**1220**	**1230**	**1240**	**1250**	**1260**	**1270**	**1280**
SMFHSVRRVL	ADPEIPVQVT	QDIEPHLGAL	FTQMLEVGTT	EDLRLVMQCI	LQGLDVSNMW	KADVQLLNRE	ASQEQPVSLT
**1290**	**1300**	**1310**	**1320**	**1330**	**1340**	**1350**	**1360**
VVGPVLDVLA	ALLRQGEEAI	GNPHHVSLAF	SILLTVPLDH	LKPLEYGSVF	PRLHNVLFSI	LQCHPKVMLK	AIPSFLNSFN
**1370**	**1380**	**1390**	**1400**	**1410**	**1420**	**1430**	**1440**
RLVFSVMREG	RQKDKGSIDD	LPTVLKCARL	VERMYSHIAA	RAEEFAVFSP	FMVAQYVLEV	QKVTLYPAVK	SLLQEGIYLI
**1450**	**1460**	**1470**	**1480**	**1490**			
LDLCIEPDVQ	FLRASLQPGM	RDIFKELYND	YLKYHKAKHE	GEKRYTA			

Cmpd.	No. of Cmpds.	m/z meas.	Δ m/z [ppm]	z	Rt [min]	Score	P	Range	Sequence	Modification	Ratios
598	1	661.8686	26.39	2	36.8	13.9	2	12-22	K.LKSKTTSWEDK.L		mdown:qdown 1.55

Detailed Protein Report

Protein 108: **PREDICTED: serine/threonine-protein kinase 31 isoform X3 [Homo sapiens]**

**Accession:**	gi|578813577	**Score:**	51.2
**Database:**	refseq_human(refseq_human_20140103.fasta)	**MW [kDa]:**	95.7
**Database Date:**	2015-11-30	**pI:**	4.8
**Modification(s):**	Carbamidomethyl, Oxidation	**Sequence Coverage [%]:**	4.2
		**No. of unique Peptides:**	3

10	20	30	40	50	60	70	80
MWVQGHSSRA	SATESVSFSG	IVQMDEDTHY	DKVEDVVGSH	IEDAVTFWAQ	SINRNKDIMK	IGCSLSEVCP	QASSVLGNLD
**90**	**100**	**110**	**120**	**130**	**140**	**150**	**160**
PNKIYGGLFS	EDQCWYRCKV	LKIISVEKCL	VRYIDYGNTE	ILNRSDIVEI	PLELQFSSVA	KKYKLWGLHI	PSDQEVTQFD
**170**	**180**	**190**	**200**	**210**	**220**	**230**	**240**
QQEKAAAVDL	TNHLEYTLKT	YIDTRMKNLA	AKMEILKEMR	HVDISVRFGK	DLSDAIQVLD	EGCFTTPASL	NGLEIIWAEY
**250**	**260**	**270**	**280**	**290**	**300**	**310**	**320**
SLAQENIKTC	EYVSEGNILI	AQRNEMQQKL	YMSVEDFILE	VDESSLNKRL	KTLQDLSVSL	EAVYGQAKEG	ANSDEILKKF
**330**	**340**	**350**	**360**	**370**	**380**	**390**	**400**
YDWKCDKREE	FTSVRSETDA	SLHRLVAWFQ	RTLKVFDLSV	EGSLISEDAM	DNIDEILEKT	ESSVCKELEI	ALVDQGDADK
**410**	**420**	**430**	**440**	**450**	**460**	**470**	**480**
EIISNTYSQV	LQKIHSEERL	IATVQAKYKD	SIEFKKQLIE	YLNKSPSVDH	LLSIKKTLKS	LKALLRWKLV	EKSNLEESDD
**490**	**500**	**510**	**520**	**530**	**540**	**550**	**560**
PDGSQIEKIK	EEITQLRNNV	FQEIYHEREE	YEMLTSLAQK	WFPELPLLHP	EIGLLKYMNS	GGLLTMSLER	DLLDAEPMKE
**570**	**580**	**590**	**600**	**610**	**620**	**630**	**640**
LSSKRPLVRS	EVNGQIILLK	GYSVDVDTEA	KVIERAATYH	RAWREAEGDS	GLLPLIFLFL	CKSDPMAYLM	VPYYPRANLN
**650**	**660**	**670**	**680**	**690**	**700**	**710**	**720**
AVQANMPLNS	EETLKVMKGV	AQGLHTLHKA	DIIHGSLHQN	NVFALNREQG	IVGDFDFTKS	VSQRASVNMM	VGDLSLMSPE
**730**	**740**	**750**	**760**	**770**	**780**	**790**	**800**
LKMGKPASPG	SDLYAYGCLL	LWLSVQNQEF	EINKDGIPKV	DQFHLDDKVK	SLLCSLICYR	SSMTAEQVLN	AECFLMPKEQ
**810**	**820**	**830**	**840**	**850**			
SVPNPEKDTE	YTLYKKEEEI	KTENLDKCME	KTRNGEANFD	C			

Cmpd.	No. of Cmpds.	m/z meas.	Δ m/z [ppm]	z	Rt [min]	Score	P	Range	Sequence	Modification
452	1	524.1225	-252.65	2	34.6	17.8	0	551-559	R.DLLDAEPMK.E	Oxidation: 8
249	3	592.1348	-240.88	2	32.3	18.3	0	581-591	K.GYSVDVDTEAK.V	
2669	1	948.3641	-79.89	2	64.6	15.1	2	817-831	K.EEEIKTENLDKCMEK.T	Carbamidomethyl: 12

Detailed Protein Report

Protein 109: Fanconi anemia group I protein isoform 2 [Homo sapiens]

**Accession:**	gi|82830440	**Score:**	51.0
**Database:**	refseq_human(refseq_human_20140103.fasta)	**MW [kDa]:**	142.5
**Database Date:**	2015-11-30	**pI:**	6.3
		**Sequence Coverage [%]:**	3.3
		**No. of unique Peptides:**	3

Quantitation

mdown:qdown	**Median:** 1.85	**CV:** 0.00 %	**No. of Peptides:**	1
Wdown:Qdown	**Median:** 1.67	**CV:** 0.00 %	**No. of Peptides:**	1

```
        10         20         30         40         50         60         70         80
MDQKILSLAA EKTADKLQEF LQTLREGDLT NLLQNQAVKG KVAGALLRAI FKGSPCSEEA GTLRRRKIYT CCIQLVESGD
        90        100        110        120        130        140        150        160
LQKEIASEII GLLMLEAHHF PGPLLVELAN EFISAVREGS LVNGKSLELL PIILTALATK KENLAYGKGV LSGEECKKQL
       170        180        190        200        210        220        230        240
INTLCSGRWD QQYVIQLTSM FKDVPLTAEE VEFVVEKALS MFSKMNLQEI PPLVYQLLVL SSKGSRKSVL EGIIAFFSAL
       250        260        270        280        290        300        310        320
DKQHNEEQSG DELLDVVTVP SGELRHVEGT IILHIVFAIK LDYELGRELV KHLKVGQQGD SNNNLSPFSI ALLLSVTRIQ
       330        340        350        360        370        380        390        400
RFQDQVLDLL KTSVVKSFKD LQLLQGSKFL QNLVPHRSYV STMILEVVKN SVHSWDHVTQ GLVELGFILM DSYGPKKVLD
       410        420        430        440        450        460        470        480
GKTIETSPSL SRMPNQHACK LGANILLETF KIHEMIRQEI LEQVLNRVVT RASSPISHFL DLLSNIVMYA PLVLQSCSSK
       490        500        510        520        530        540        550        560
VTEAFDYLSF LPLQTVQRLL KAVQPLLKVS MSMRDCLILV LRKAMFANQL DARKSAVAGF LLLLKNFKVL GSLSSSQCSQ
       570        580        590        600        610        620        630        640
SLSVSQVHVD VHSHYNSVAN ETFCLEIMDS LRRCLSQQAD VRLMLYEGFY DVLRRNSQLA NSVMQTLLSQ LKQFYEPKPD
       650        660        670        680        690        700        710        720
LLPPLKLEAC ILTQGDKISL QEPLDYLLCC IQHCLAWYKN TVIPLQQGEE EEEEEAFYE DLDDILESIT NRMIKSELED
       730        740        750        760        770        780        790        800
FELDKSADFS QSTSIGIKNN ICAFLVMGVC EVLIEYNFSI SSFSKNRFED ILSLFMCYKK LSDILNEKAG KAKTKMANKT
       810        820        830        840        850        860        870        880
SDSLLSMKFV SSLLTALFRV LLWRYTSIPT SVEESGKKEK GKSISLLCLE GLQKIFSAVQ QFYQPKIQQF LRALDVTDKE
       890        900        910        920        930        940        950        960
GEEREDADVS VTQRTAFQIR QFQRSLLNLL SSQEEDFNSK EALLLVTVLT SLSKLLEPSS PQFVQMLSWT SKICKENSRE
       970        980        990       1000       1010       1020       1030       1040
DALFCKSLMN LLFSLHVSYK SPVILLRDLS QDIHGHLGDI DQDVEVEKTN HFAIVNLRTA APTVCLLVLS QAEKVLEEVD
      1050       1060       1070       1080       1090       1100       1110       1120
WLITKLKGQV SQETLSEEAS SQATLPNQPV EKAIIMQLGT LLTFFHELVQ TALPSGSCVD TLLKDLCKMY TTLTALVRYY
      1130       1140       1150       1160       1170       1180       1190       1200
LQVCQSSGGI PKNMEKLVKL SGSHLTPLCY SFISYVQNKS KSLNYTGEKK EKPAAVATAM ARVLRETKPI PNLIFAIEQY
      1210       1220       1230       1240       1250       1260       1270
EKFLIHLSKK SKVNLMQHMK LSTSRDFKIK GNILDMVLRE DGEDENEEGT ASEHGGQNKE PAKKKRKK
```

Cmpd.	No. of Cmpds.	m/z meas.	Δ m/z [ppm]	z	Rt [min]	Score	P	Range	Sequence	Modification	Ratios
426	3	658.3901	11.29	2	34.4	22.4	2	780-791	K.KLSDILNEKAGK.A		
1909	2	918.3239	-154.66	2	52.5	13.3	1	885-900	R.EDADVSVTQRTAFQIR.Q		
13	1	814.3623	-84.24	2	29.4	15.2	1	1105-1118	K.DLCKMYTTLTALVR.Y		Wdown:Qdown 1.67<br>mdown:qdown 1.85

Detailed Protein Report

Protein 110: PREDICTED: histone-lysine N-methyltransferase 2A isoform X2 [Homo sapiens]

**Accession:**	gi|578822197	**Score:**	51.0
**Database:**	refseq_human(refseq_human_20140103.fasta)	**MW [kDa]:**	292.4
**Database Date:**	2015-11-30	**pI:**	6.6
		**Sequence Coverage [%]:**	1.3
		**No. of unique Peptides:**	2

Detailed Protein Report

10	20	30	40	50	60	70	80
MSRPLGLNPN	RPPLQLPGSQ	ASRSPSQHWS	SRLSHLLQDR	QEKKFPKPLL	VSPRKSSLHH	QNQEKPPPVN	KQENAGTLNI
90	100	110	120	130	140	150	160
LSTLSNGNSS	KQKIPADGVH	RIRVDFKEDC	EAENVWEMGG	LGILTSVPIT	PRVVCFLCAS	SGHVEFVYCQ	VCCEPFHKFC
170	180	190	200	210	220	230	240
LEENERPLED	QLENWCCRRC	KFCHVCGRQH	QATKQLLECN	KCRNSYHPEC	LGPNYPTKPT	KKKKVWICTK	CVRCKSCGST
250	260	270	280	290	300	310	320
TPGKGWDAQW	SHDFSLCHDC	AKLFAKGNFC	PLCDKCYDDD	DYESKMMQCG	KCDRWVHSKC	ENLSGTEDEM	YEILSNLPES
330	340	350	360	370	380	390	400
VAYTCVNCTE	RHPAEWRLAL	EKELQISLKQ	VLTALLNSRT	TSHLLRYRQA	AKPPDLNPET	EESIPSRSSP	EGPDPPVLTE
410	420	430	440	450	460	470	480
VSKQDDQQPL	DLEGVKRKMD	QGNYTSVLEF	SDDIVKIIQA	AINSDGGQPE	IKKANSMVKS	FFIRQMERVF	PWFSVKKSRF
490	500	510	520	530	540	550	560
WEPNKVSSNS	GMLPNAVLPP	SLDHNYAQWQ	EREENSHTEQ	PPLMKKIIPA	PKPKGPGEPD	SPTPLHPPTP	PILSTDRSRE
570	580	590	600	610	620	630	640
DSPELNPPPG	IEDNRQCALC	LTYGDDSAND	AGRLLYIGQN	EWTHVNCALW	SAEVFEDDDG	SLKNVHMAVI	RGKQLRCEFC
650	660	670	680	690	700	710	720
QKPGATVGCC	LTSCTSNYHF	MCSRAKNCVF	LDDKKVYCQR	HRDLIKGEVV	PENGFEVFRR	VFVDFEGISL	RRKFLNGLEP
730	740	750	760	770	780	790	800
ENIHMMIGSM	TIDCLGILND	LSDCEDKLFP	IGYQCSRVYW	STTDARKRCV	YTCKIVECRP	PVVEPDINST	VEHDENRTIA
810	820	830	840	850	860	870	880
HSPTSFTESS	SKESQNTAEI	ISPPSPDRPP	HSQTSGSCYY	HVISKVPRIR	TPSYSPTQRS	PGCRPLPSAG	SPTPTTHEIV
890	900	910	920	930	940	950	960
TVGDPLLSSG	LRSIGSRRHS	TSSLSPQRSK	LRIMSPMRTG	NTYSRNNVSS	VSTTGTATDL	ESSAKVVDHV	LGPLNSSTSL
970	980	990	1000	1010	1020	1030	1040
GQNTSTSSNL	QRTVVTVGNK	NSHLDGSSSS	EMKQSSASDL	VSKSSSLKGE	KTKVLSSKSS	EGSAHNVAYP	GIPKLAPQVH
1050	1060	1070	1080	1090	1100	1110	1120
NTTSRELNVS	KIGSFAEPSS	VSFSSKEALS	FPHLHLRGQR	NDRDQHTDST	QSANSSPDED	TEVKTLKLSG	MSNRSSIINE
1130	1140	1150	1160	1170	1180	1190	1200
HMGSSSRDRR	QKGKKSCKET	FKEKHSSKSF	LEPGQVTTGE	EGNLKPEFMD	EVLTPEYMGQ	RPCNNVSSDK	IGDKGLSMPG
1210	1220	1230	1240	1250	1260	1270	1280
VPKAPPMQVE	GSAKELQAPR	KRTVKVTLTP	LKMENESQSK	NALKESSPAS	PLQIESTSPT	EPISASENPG	DGPVAQPSPN
1290	1300	1310	1320	1330	1340	1350	1360
NTSCQDSQSN	NYQNLPVQDR	NLMLPDGPKP	QEDGSFKRRY	PRRSARARSN	MFFGLTPLYG	VRSYGEEDIP	FYSSSTGKKR
1370	1380	1390	1400	1410	1420	1430	1440
GKRSAEGQVD	GADDLSTSDE	DDLYYYNFTR	TVISSGGEER	LASHNLFREE	EQCDLPKISQ	LDGVDDGTES	DTSVTATTRK
1450	1460	1470	1480	1490	1500	1510	1520
SSQIPKRNGK	ENGTENLKID	RPEDAGEKEH	VTKSSVGHKN	EPKMDNCHSV	SRVKTQGQDS	LEAQLSSLES	SRRVHTSTPS
1530	1540	1550	1560	1570	1580	1590	1600
DKNLLDTYNT	ELLKSDSDNN	NSDDCGNILP	SDIMDFVLKN	TPSMQALGES	PESSSSELLN	LGEGLGLDSN	REKDMGLFEV
1610	1620	1630	1640	1650	1660	1670	1680
FSQQLPTTEP	VDSSVSSSIS	AEEQFELPLE	LPSDLSVLTT	RSPTVPSQNP	SRLAVISDSG	EKRVTITEKS	VASSESDPAL
1690	1700	1710	1720	1730	1740	1750	1760
LSPGVDPTPE	GHMTPDHFIQ	GHMDADHISS	PPCGSVEQGH	GNNQDLTRNS	STPGLQVPVS	PTVPIQNQKY	VPNSTDSPGP
1770	1780	1790	1800	1810	1820	1830	1840
SQISNAAVQT	TPPHLKPATE	KLIVVNQNMQ	PLYVLQTLPN	GVTQKIQLTS	SVSSTPSVME	TNTSVLGPMG	GGLTLTTGLN
1850	1860	1870	1880	1890	1900	1910	1920
PSLPTSQSLF	PSASKGLLPM	SHHQHLHSFP	AATQSSFPPN	ISNPPSGLLI	GVQPPPDPQL	LVSESSQRTD	LSTTVATPSS
1930	1940	1950	1960	1970	1980	1990	2000
GLKKRPISRL	QTRKNKKLAP	SSTPSNIAPS	DVVSNMTLIN	FTPSQLPNHP	SLLDLGSLNT	SSHRTVPNII	KRSKSSIMYF
2010	2020	2030	2040	2050	2060	2070	2080
EPAPLLPQSV	GGTAATAAGT	STISQDTSHL	TSGSVSGLAS	SSSVLNVVSM	QTTTTPTSSA	SVPGHVTLTN	PRLLGTPDIG
2090	2100	2110	2120	2130	2140	2150	2160
SISNLLIKAS	QQSLGIQDQP	VALPPSSGMF	PQLGTSQTPS	TAAITAASSI	CVLPSTQTTG	ITAASPSGEA	DEHYQLQHVN
2170	2180	2190	2200	2210	2220	2230	2240

Detailed Protein Report

Cmpd.	No. of Cmpds.	m/z meas.	Δ m/z [ppm]	z	Rt [min]	Score	P	Range	Sequence	Modification
11	1	820.3475	-49.73	1	29.3	12.9	1	2443-2448	K.HCRNYK.F	
2967	2	753.3836	9.44	2	66.3	16.1	1	2476-2488	R.KSAFDMFNFLASK.H	

Detailed Protein Report

Protein 111: mucin-19 precursor [Homo sapiens]

**Accession:**	gi|533112494	**Score:**	50.9
**Database:**	refseq_human(refseq_human_20140103.fasta)	**MW [kDa]:**	804.8
**Database Date:**	2015-11-30	**pI:**	4.8
**Modification(s):**	Carbamidomethyl	**Sequence Coverage [%]:**	0.9
		**No. of unique Peptides:**	3

Detailed Protein Report

10	20	30	40	50	60	70	80
MKLILWYLVV	ALWCFFKDVE	ALLYRQKSDG	KIAASRSGGF	SYGSSSSGDL	DRKKPLFSLE	FGSPGETEDK	SRQRQDAGSP
90	100	110	120	130	140	150	160
KSEDTPAGGF	FNSSSSSGDS	DRTKPFFSLG	LGAPGKAEDK	SGDSQDAGGS	KSEDTPPGGF	FYGSSSSGDS	DKKKPLFSFE
170	180	190	200	210	220	230	240
FGATGEDEDK	SRERWDAGNS	RSEDSPADST	NTRYGAGFSS	SGASLDVGFG	WGISDEKGLE	VSKADGRETR	GSGSAGGETI
250	260	270	280	290	300	310	320
VFGPDAGSSV	GTGSSGLKLG	AGKGDAAFGF	EVSDSNSFGD	TGISSKTVEG	NQTSSSGGSV	SIDLGDTSFR	SENQFVGGGS
330	340	350	360	370	380	390	400
LNSISNLWDS	GQEGFGINEI	GGNGMSGSVS	AEAGFKGFGS	DSSSSGDSSA	RNGFENSSGI	SEDSGVILGS	SDQHEVELSR
410	420	430	440	450	460	470	480
TGGNRKRSSD	PDEAGNLSPG	SDVSDSGGNT	WSSDSGSGGG	GVTSSSEYST	SGPLNTPEKG	SHIPEATPKY	SETNAIIGEI
490	500	510	520	530	540	550	560
STWSKGAYKS	FNGRIFFFES	SCPYTFCRHC	IESGGDFNIE	IKRNNDSEIE	KITVLIDNND	VSIFGDTILV	NGESVQIPYN
570	580	590	600	610	620	630	640
NKLIHIKKYG	EHNVLNSRRG	ILTLMWDKNN	KLSLTLHKQY	PTCGLCGNFN	STPGQDINEH	IANSKIPGDC	PNAVGKSYEV
650	660	670	680	690	700	710	720
CEDGIQHCNK	IIGTYFEKCG	KVAALSNDYK	MICIDEYCQT	RDKTSTCDTY	SELSRLCASD	GPGTFESWRS	DSDVVCGTQR
730	740	750	760	770	780	790	800
CPEQHIYKEC	GPSNPATCSN	VAPFQDSECV	SGCTCPEGYL	LDDIGEKGKC	VLKAECPCES	SGTVYQPGEV	REGPCGSQCT
810	820	830	840	850	860	870	880
CQDAKWSCTE	ALCPGRCKVE	GSSLTTFDGV	KYNFPGNCHF	LAVHNEDWSI	SVELRPCPSG	QTGTCLNSVT	LLLNSSVPVD
890	900	910	920	930	940	950	960
KYVFNSDGTV	TNDKIRNQGY	YYSDKIQIFN	ASSSYLQVET	YFHVKLQIQI	VPVMQLYVSM	PPNQFTDTVG	LCGSYNNKAE
970	980	990	1000	1010	1020	1030	1040
DDFMSSQNIL	EKTSQAFANS	WEMMSCPKGN	PSSCISIEKE	KFAERHCGIL	LDSSGPLASC	HPIVNPKPYH	EECKKYTCTC
1050	1060	1070	1080	1090	1100	1110	1120
ENSQDCLCTI	LGNYVKACAE	KETYIVGWRT	GLCEHSCPSG	LVFKYNVKAC	NSSCRSLSER	DRSCDVEDVP	VDGCTCPDAM
1130	1140	1150	1160	1170	1180	1190	1200
YQNNEGNCVL	KSQCDCYIND	EVMQPGKLIH	IDDNKCVCRD	GILLCQIPID	LTLQNCSGGA	EYVDCSDPKA	QRRTNRTCST
1210	1220	1230	1240	1250	1260	1270	1280
RNIPVFDENL	PCKRGCFCPE	GMVRNSKGIC	VFPNDCPCSF	GGREYDEGSV	TSVGCNECTC	IKGSWSCTQN	ECQTICHIYG
1290	1300	1310	1320	1330	1340	1350	1360
EGHVRTFDGK	SYSFDGLCQY	SFLEDYCGHE	NGTFRILTES	VPCCEDGLTC	SRKIIVAFQD	QNIVLQDGKV	TAVKSTESKK
1370	1380	1390	1400	1410	1420	1430	1440
CELNANAYSI	HTVGLYLILK	FQNGIIVIWD	KNTRLSVILD	PNWNGKVCGL	CGNNNGDLKD	DFTTRYSSVA	SGALEFGNSW
1450	1460	1470	1480	1490	1500	1510	1520
KTSQECSDTV	AQTFPCDSNP	YCKAWAVRKC	EILRDSTFRD	CHNKVDPSAY	HDACIEEACA	CDMEGKYLGF	CTAVAMYAEA
1530	1540	1550	1560	1570	1580	1590	1600
CSAVGVCVSW	RKPNLCPVYC	DYYNAPGECR	WHYEPCGTVT	AKTCKDQLVG	QKFSSLLEGC	YAKCPDSAPY	LDENTMKCVS
1610	1620	1630	1640	1650	1660	1670	1680
LSECSCFYND	VIPAGGVIED	NCGRTCYCIA	GQLECSETAP	TNSTFAVSTT	TATTILSTGA	AITLVTGGPS	TAASIPAITT
1690	1700	1710	1720	1730	1740	1750	1760
SSSETTGTTL	GPLTEPFTTG	ITETSVPIIS	TSGNAGMTGV	VSPTVTGASG	MAGTTGGVDA	ATTGAASENT	SERAGTPRVS
1770	1780	1790	1800	1810	1820	1830	1840
GETPAVGGGS	TPGEAGPGAT	VSGSTGVSAG	SITASPGASA	TSSESSKSGT	TGPSVGGKTG	ATSSEATSSE	GMSGVTGQSL
1850	1860	1870	1880	1890	1900	1910	1920
GSTAGSDSEI	TAKTSFTGSS	PPGKLTRPSP	GSPGHFSGGT	TEWGNVATTG	AAGENTSGAL	GSTEGSVEAT	TSAGSGNTAG
1930	1940	1950	1960	1970	1980	1990	2000
TSGTGDTGPG	NTAVSGTPVV	SPGATPGAPG	SSTPGEADIG	NTSFGKSGTP	TVSAASTTSS	PVSKHTDAAS	ATAVTISGSK
2010	2020	2030	2040	2050	2060	2070	2080
PGTPGTPGGA	TSGGKITSGW	SSSGTSTGAS	NTPGATGSST	GQTDTSGPSA	KVTGNYGQSS	EIPGTIKSSS	DVSGTMGQSD
2090	2100	2110	2120	2130	2140	2150	2160
TTSGPSVAVT	RTSEQSSGVT	VASEPSVGVS	GTTGPLAEIS	GTTRPLVSGL	RTTGSSAEGS	GTTGPSSRES	VTTRPLAEGS
2170	2180	2190	2200	2210	2220	2230	2240

Detailed Protein Report

Cmpd.	No. of Cmpds.	m/z meas.	Δ m/z [ppm]	z	Rt [min]	Score	P	Range	Sequence	Modification
2638	1	937.4152	-21.95	2	61.7	11.6	2	1085-1100	K.YNVKACNSSCRSLSER.D	Carbamidomethyl: 10
1442	1	1061.4940	-55.50	2	47.8	12.0	1	6074-6097	R.SSAGVTGTTGLSAKSGTSIPSAGK.T	
2090	1	1131.3695	-118.05	2	56.3	11.2	1	8209-8227	K.LVKFQSNDTCCEIGYCEPR.T	Carbamidomethyl: 10

Detailed Protein Report

Protein 112: sorting nexin-8 [Homo sapiens]

Accession: gi|23943858
Database: refseq_human(refseq_human_20140103.fasta)
Database Date: 2015-11-30
Modification(s): Oxidation

Score: 50.8
MW [kDa]: 52.5
pI: 7.2
Sequence Coverage [%]: 9.2
No. of unique Peptides: 1

10	20	30	40	50	60	70	80
MTGRAMDPLP	AAAVGAAAEA	EADEEADPPA	SDLPTPQAIE	PQAIVQQVPA	PSRMQMPQGN	PLLLSHTLQE	LLARDTVQVE
**90**	**100**	**110**	**120**	**130**	**140**	**150**	**160**
LIPEKKGLFL	KHVEYEVSSQ	RFKSSVYRRY	NDFVVFQEML	LHKFPYRMVP	ALPPKRMLGA	DREFIEARRR	ALKRFVNLVA
**170**	**180**	**190**	**200**	**210**	**220**	**230**	**240**
RHPLFSEDVV	LKLFLSFSGS	DVQNKLKESA	QCVGDEFLNC	KLATRAKDFL	PADIQAQFAI	SRELIRNIYN	SFHKLRDRAE
**250**	**260**	**270**	**280**	**290**	**300**	**310**	**320**
RIASRAIDNA	ADLLIFGKEL	SAIGSDTTPL	PSWAALNSST	WGSLKQALKG	LSVEFALLAD	KAAQQGKQEE	NDVVEKLNLF
**330**	**340**	**350**	**360**	**370**	**380**	**390**	**400**
LDLLQSYKDL	CERHEKGVLH	KHQRALHKYS	LMKRQMMSAT	AQNREPESVE	QLESRIVEQE	NAIQTMELRN	YFSLYCLHQE
**410**	**420**	**430**	**440**	**450**	**460**	**470**	
TQLIHVYLPL	TSHILRAFVN	SQIQGHKEMS	KVWNDLRPKL	SCLFAGPHST	LTPPCSPPED	GLCPH	

Cmpd.	No. of Cmpds.	m/z meas.	Δ m/z [ppm]	z	Rt [min]	Score	P	Range	Sequence	Modification
1502	1	978.1127	119.49	2	48.6	18.2	1	109-123	R.RYNDFVVFQEMLLHK.F	Oxidation: 11

Detailed Protein Report

Protein 113: PREDICTED: nesprin-2 isoform X6 [Homo sapiens]

**Accession:**	gi|530403362	**Score:**	50.7
**Database:**	refseq_human(refseq_human_20140103.fasta)	**MW [kDa]:**	795.8
**Database Date:**	2015-11-30	**pI:**	5.1
		**Sequence Coverage [%]:**	0.7
		**No. of unique Peptides:**	3

Detailed Protein Report

10	20	30	40	50	60	70	80
MASSPELPTE	DEQGSWGIDD	LHISLQAEQE	DTQKKAFTCW	INSQLARHTS	PSVISDLFTD	IKKGHVLLDL	LEVLSGQQLP
90	100	110	120	130	140	150	160
RDKGSNTFQC	RINIEHALTF	LRNRSIKLIN	IHVTDIIDGN	PSIILGLIWT	IILHFHIEKL	AQTLSCNYNQ	PSLDDVSVVD
170	180	190	200	210	220	230	240
SSPASSPPAK	KCSKVQARWQ	MSARKALLLW	AQEQCATYES	VNVTDFKSSW	RNGMAFLAII	HALRPDLIDM	KSVKHRSNKD
250	260	270	280	290	300	310	320
NLREAFRIAE	QELKIPRLLE	PEDVDVVDPD	EKSIMTYVAQ	FLQYSKDAPG	TGEEAQGKVK	DAMGWLTLQK	EKLQKLLKDS
330	340	350	360	370	380	390	400
ENDTYFKKYN	SLLSFMESFN	EEKKSFLDVL	SIKRDLDELD	KDHLQLREAW	DGLDHQINAW	KIKLNYALPP	PLHQTEAWLQ
410	420	430	440	450	460	470	480
EVEELMDEDL	SASQDHSQAV	TLIQEKMTLF	KSLMDRFEHH	SNILLTFENK	DENHLPLVPP	NKLEEMKRRI	NNILEKKFIL
490	500	510	520	530	540	550	560
LLEFHYYKCL	VLGLVDEVKS	KLDIWNIKYG	SRESVELLLE	DWHKFIEEKE	FLARLDTSFQ	KCGEIYKNLA	GECQNINKQY
570	580	590	600	610	620	630	640
MMVKSDVCMY	RKNIYNVKST	LQKVLACWAT	YVENLRLLRA	CFEETKKEEI	KEVPFETLAQ	WNLEHATLNE	AGNFLVEVSN
650	660	670	680	690	700	710	720
DVVGSSISKE	LRRLNKRWRK	LVSKTQLEMN	LPLMIKKQDQ	PTFDNSGNIL	SKEEKATVEF	STDMSVELPE	NYNQNIKAGE
730	740	750	760	770	780	790	800
KHEKENEEFT	GQLKVAKDVE	KLIGQVEIWE	AEAKSVLDQD	DVDTSMEESL	KHLIAKGSMF	DELMARSEDM	LQMDIQNISS
810	820	830	840	850	860	870	880
QESFQHVLTT	GLQAKIQEAK	EKVQINVVKL	IAALKNLTDV	SPDLDIRLKM	EESQKELESY	MMRAQQLLGQ	RESPGELISK
890	900	910	920	930	940	950	960
HKEALIISNT	KSLAKYLKAV	EELKNNVTED	IKMSLEEKSR	DVCAKWESLH	HELSLYVQQL	KIDIEKGKLS	DNILKLEKQI
970	980	990	1000	1010	1020	1030	1040
NKEKKLIRRG	RTKGLIKEHE	ACFSEEGCLY	QLNHHMEVLR	ELCEELPSQK	SQQEVKRLLK	DYEQKIERLL	KCASEIHMTL
1050	1060	1070	1080	1090	1100	1110	1120
QPTAGGTSKN	EGTITTSENR	GGDPHSEAPF	AKSDNQPSTE	KAMEPTMKFS	LASVLRPLQE	ESIMEKDYSA	SINSLLERYD
1130	1140	1150	1160	1170	1180	1190	1200
TYRDILEHHL	QNNKFRITSD	FSSEEDRSSS	CLQAKLTDLQ	VIKNETDARW	KEFEIISLKL	ENHVNDIKKP	FVIKERDTLK
1210	1220	1230	1240	1250	1260	1270	1280
ERERELQMTL	NTRMESLETA	LRLVLPVEKA	SLLLCGSDLP	LHKMAIQGFH	LIDADRIYQH	LRNIQDSIAK	QIEICNRLEE
1290	1300	1310	1320	1330	1340	1350	1360
PGNFVLKELH	PFDLHAMQNI	ILKYKTQFEG	MNHRVQRSED	TLKALEDFLA	SLRTAKLSAE	PVTDLSASDT	QVAQENTLTV
1370	1380	1390	1400	1410	1420	1430	1440
KNKEGEIHLM	KDKAKHLDKC	LKMLDMSFKD	AERGDDTSCE	NLLDAFSIKL	SETHGYGVQE	EFTEENKLLE	ACIFKNNELL
1450	1460	1470	1480	1490	1500	1510	1520
KNIQDVQSQI	SKIGLKDPTV	PAVKHRKKSL	IRLDKVLDEY	EEEKRHLQEM	ANSLPHFKDG	REKTVNQQCQ	NTVVLWENTK
1530	1540	1550	1560	1570	1580	1590	1600
ALVTECLEQC	GRVLELLKQY	QNFKSILTTL	IQKEESVISL	QASYMGKENL	KKRIAEIEIV	KEEFNEHLEV	VDKINQVCKN
1610	1620	1630	1640	1650	1660	1670	1680
LQFYLNKMKT	FEEPPFEKEA	NIIVDRWLDI	NEKTEDYYEN	LGRALALWDK	LFNLKNVIDE	WTEKALQKME	LHQLTEEDRE
1690	1700	1710	1720	1730	1740	1750	1760
RLKEELQVHE	QKTSEFSRRV	AEIQFLLQSS	EIPLELQVME	SSILNKMEHV	QKCLTGESNC	HALSGSTAEL	REDLDQAKTQ
1770	1780	1790	1800	1810	1820	1830	1840
IGMTESLLKA	LSPSDSLEIF	TKLEEIQQQI	LQQKHSMILL	ENQIGCLTPE	LSELKKQYES	VSDLFNTKKS	VLQDHFSKLL
1850	1860	1870	1880	1890	1900	1910	1920
NDQCKNFNDW	FSNIKVNLKE	CFESSETKKS	VEQKLQKLSD	FLTLEGRNSK	IKQVDSVLKH	VKKHLPKAHV	KELISWLVGQ
1930	1940	1950	1960	1970	1980	1990	2000
EFELEKMESI	CQARAKELED	SLQQLLRLQD	DHRNLRKWLT	NQEEKWKGME	EPGEKTELFC	QALARKREQF	ESVAQLNNSL
2010	2020	2030	2040	2050	2060	2070	2080
KEYGFTEEEE	IIMEATCLMD	RYQTLLRQLS	EIEEEDKLLP	TEDQSFNDLA	HDVIHWIKEI	KESLMVLNSS	EGKMPLEERI
2090	2100	2110	2120	2130	2140	2150	2160
QKIKEIILLK	PEGDARIETI	MKQAESSEAP	LVQKTLTDIS	NQWDNTLHLA	STYLSHQEKL	LLEGEKYLQS	KEDLRLMLIE
2170	2180	2190	2200	2210	2220	2230	2240

Detailed Protein Report

Cmpd.	No. of Cmpds.	m/z meas.	Δ m/z [ppm]	z	Rt [min]	Score	P	Range	Sequence	Modification
289	1	715.8764	45.86	2	32.3	10.0	0	1817-1828	K.QYESVSDLFNTK.K	
2908	1	922.9773	-1.53	2	65.5	11.3	1	3576-3590	K.ELVQTEIQERHSFTK.E	
1518	1	882.6077	144.13	2	48.8	11.3	2	3618-3632	K.SEQFEELQSILKKGK.L	

Detailed Protein Report

Protein 114: dynein heavy chain 6, axonemal [Homo sapiens]

**Accession:**	gi|194353966	**Score:**	50.7
**Database:**	refseq_human(refseq_human_20140103.fasta)	**MW [kDa]:**	475.7
**Database Date:**	2015-11-30	**pI:**	5.6
		**Sequence Coverage [%]:**	1.3
		**No. of unique Peptides:**	3

Alias proteins:

Accession	Name	Description
gi|578802753	refseq_human (refseq_human_20140103.fasta)	PREDICTED: dynein heavy chain 6, axonemal isoform X1 [Homo sapiens]

Detailed Protein Report

10	20	30	40	50	60	70	80
MTFRATDSEF	DLTNIEEYAE	NSALSRLNNI	KAKQRVSYVT	STENESDTQI	LTFRHITKAQ	EKTRKRQQPI	KLEPLPVLKV
90	100	110	120	130	140	150	160
YQDHKQPEYI	HEQNRFQLMT	AGIIKRPVSI	AKKSFATSST	QFLEHQDAVK	KMQIHRPYVE	VFSPSPPKLP	HTGIGKRGLF
170	180	190	200	210	220	230	240
GTRSSAYPKY	TFHDREEVVK	ANIRDPLQII	KIIRENEHLG	FLYMIPAVPR	SSIEYDTYNL	KVVSYENINK	NDYYTISQRA
250	260	270	280	290	300	310	320
VTHIYNEDIE	FIEIDRWEQE	YLYHRELTKI	PIFSLFRKWK	AFSVWRKNVR	SKKITGCQKS	LQKNLFIVNP	HLRPALLKIN
330	340	350	360	370	380	390	400
ELCYHLSFMG	LCYIEKCHTY	TLQEFKAAQV	IRLAEVTERL	GEFRNEAKYV	VRRACRFALR	AAGFVPDDCA	FGPFEDYHKV
410	420	430	440	450	460	470	480
QSSGSFINTP	HELPTYGDSE	KMTYTEQASK	RHYCMRLTCF	IRLNDYLIEN	TMHILTVNAV	NSLLNHLTDK	LKRTPSADVI
490	500	510	520	530	540	550	560
QKWITEEKPE	VPDKKGTLMV	EKQEEDESLI	PMFLTELMLT	VQSLLFEPSL	EDFLDGILGA	VNHCQNTVLS	VPNLVPDSYF
570	580	590	600	610	620	630	640
DAFTSPYINN	KLEGKTCGTG	PSLAAVFEDD	KNFHTIISQI	KETIQAAFES	ARIYAATFEK	FQIFFKENES	LDLQALKLQE
650	660	670	680	690	700	710	720
PDINFFSEQL	EKYHKQHKDA	VALRPTRNVG	LLLIDTRLLR	EKLIPSPLRC	LEVLNFMLPR	QSKKKVDAII	FEAQDAEYKL
730	740	750	760	770	780	790	800
EFVPTTTTEY	VHSLLFLDEI	QERIESLEDE	GNIVTQMYKL	MEQYQVPTPP	EDFAVFATMK	PSIVAVRNAI	DKSVGDRESS
810	820	830	840	850	860	870	880
IKQFCVHLGS	DLEELNNEVN	EVKLQAQDPQ	ILDISADQDK	IRLILNNLQS	VLADLQKRAF	QYKSYQKNFK	VEVSKFEALE
890	900	910	920	930	940	950	960
EVSAELKLKQ	LLWDSFSEWD	KLQQEWLKSK	FDCLDPEVLN	GQVSKYAKFV	TQLEKGLPPN	SVVPQLKYKV	EKMKEKLPVI
970	980	990	1000	1010	1020	1030	1040
IDLRNPTLKA	RHWAAIEQTV	DATLVDAEIP	LTLERLSQLH	VFDFGQEIQD	ISGQASGEAA	LEAILKKVED	SWKTTEFVIL
1050	1060	1070	1080	1090	1100	1110	1120
PHRDSKDVFI	LGGTDDIQVL	LDDSTINVAT	LASSRYLGPL	KTRVDEWQKQ	LALFNQTLEE	WLTCQRNWLY	LESIFNAPDI
1130	1140	1150	1160	1170	1180	1190	1200
QRQLPAEAKM	FLQVDKSWKE	IMRKVNRLPN	ALRAATQPGL	LETFQNNNAL	LDQIQKCLEA	YLESKRVIFP	RFYFLSNDEL
1210	1220	1230	1240	1250	1260	1270	1280
LEILAQTRNP	QAVQPHLRKC	FDSISKLEFA	LMPPAEGKIP	GIDGEPEKVY	TNDILAMLSP	EGERVSLGKG	LKARGNVEEW
1290	1300	1310	1320	1330	1340	1350	1360
LGKVEEAMFT	SLRRLCKAAI	ADYQGKLRTD	WVVAGHPSQV	ILTVSQIMWC	RDLTECLETE	HSNHIQALKN	FEKVNFERLN
1370	1380	1390	1400	1410	1420	1430	1440
ALAAIVQGSL	PKLHRNILTA	LITIDVHARD	IVTELVQSKV	ETVESFDWQR	QLRYYWDIDL	DNCVARMALS	QYTYGYEYLG
1450	1460	1470	1480	1490	1500	1510	1520
ACPRLVITPL	TDRCYLCLMG	ALQLDLGGAP	AGPAGTGKTE	TTKDLAKALA	IQCVVFNCSD	GLDYKMMGRF	FSGLAQSGAW
1530	1540	1550	1560	1570	1580	1590	1600
CCFDEFNRID	IEVLSVIAQQ	LITIRNAKAA	KLSRFMFEGR	EIKLVMTCAA	FITMNPGYAG	RTELPDNLKA	LFRPFAMMVP
1610	1620	1630	1640	1650	1660	1670	1680
NYALIAEVIL	YSEGFESSKI	LARKMTQMYK	LCSEQLSQQD	HYDFGMRAVK	SVLVMAGSLK	RENPDLNEDV	VLIRALQDSN
1690	1700	1710	1720	1730	1740	1750	1760
LPKFLTDDAL	LFSGIISDLF	PGVQIPEHDY	GILQSTIVDV	MNRQNLQPEM	CMVRKVIQFY	ETMLVRHGVM	LVGPTGGGKT
1770	1780	1790	1800	1810	1820	1830	1840
TVYRILAETL	GNLQKLGIEN	SFYQAVKTYV	LNPKSITMGE	LYGEVNNLTL	EWKDGLMALS	VRAAVNDTSE	DHKWIISDGP
1850	1860	1870	1880	1890	1900	1910	1920
VDALWIENMN	TVLDDNKMLC	LANSERIKLT	PQIHMLFEVQ	DLRVASPATV	SRCGMVFVDP	EELKWMPYVK	TWMKGISKKL
1930	1940	1950	1960	1970	1980	1990	2000
TEETQEYILN	LFQRYVDEGL	HFINKKCSQA	IPQVDISKVT	TLCCLLESLI	LGKDGVNLAM	EQTKLNTILC	QTFVFCYLWS
2010	2020	2030	2040	2050	2060	2070	2080
LGGNLTENYY	DSFDTFIRTQ	FDDNPDARLP	NSGDLWSIHM	DFDTKRLDPW	ERIIPTFKYN	RDVPFFEMLV	PTTDTVRYGY
2090	2100	2110	2120	2130	2140	2150	2160
LMEKLLAVKH	SVLFTGITGV	GKSVIAKGLL	NKIQESAGYV	PVYLNFSAQT	SSARTQEIIE	SKLERKRKNI	LGAPGNKRIV
2170	2180	2190	2200	2210	2220	2230	2240

Detailed Protein Report

Cmpd.	No. of Cmpds.	m/z meas.	Δ m/z [ppm]	z	Rt [min]	Score	P	Range	Sequence	Modification
2662	1	698.0710	213.61	2	62.1	10.4	0	1359-1372	R.LNALAAIVQGSLPK.L	
2264	1	1011.7667	170.21	1	56.8	10.2	1	2148-2157	R.KNILGAPGNK.R	
2924	1	1015.5128	-43.32	2	65.3	18.0	2	2905-2922	K.LRAAQAELDITMATLREK.Q	

Detailed Protein Report

Protein 115: **PREDICTED: obscurin isoform X31 [Homo sapiens]**

**Accession:**	gi|578802370	**Score:**	50.7
**Database:**	refseq_human(refseq_human_20140103.fasta)	**MW [kDa]:**	877.0
**Database Date:**	2015-11-30	**pI:**	5.6
**Modification(s):**	Carbamidomethyl, Oxidation	**Sequence Coverage [%]:**	0.7
		**No. of unique Peptides:**	4

Detailed Protein Report

10	20	30	40	50	60	70	80
MDQPQFSGAP	RFLTRPKAFV	VSVGKDATLS	CQIVGNPTPQ	VSWEKDQQPV	AAGARFRLAQ	DGDLYRLTIL	DLALGDSGQY
90	100	110	120	130	140	150	160
VCRARNAIGE	AFAAVGLQVD	AEAACAEQAP	HFLLRPTSIR	VREGSEATFR	CRVGGSPRPA	VSWSKDGRRL	GEPDGPRVRV
170	180	190	200	210	220	230	240
EELGEASALR	IRAARPRDGG	TYEVRAENPL	GAASAAAALV	VDSDAADTAS	RPGTSTAALL	AHLQRRREAM	RAEGAPASPP
250	260	270	280	290	300	310	320
STGTRTCTVT	EGKHARLSCY	VTGEPKPETV	WKKDGQLVTE	GRRHVVYEDA	QENFVLKILF	CKQSDRGLYT	CTASNLVGQT
330	340	350	360	370	380	390	400
YSSVLVVVRE	PAVPFKKRLQ	DLEVREKESA	TFLCEVPQPS	TEAAWFKEET	RLWASAKYGI	EEEGTERRLT	VRNVSADDDA
410	420	430	440	450	460	470	480
VYICETPEGS	RTVAELAVQG	NLLRKLPRKT	AVRVGDTAMF	CVELAVPVGP	VHWLRNQEEV	VAGGRVAISA	EGTRHTLTIS
490	500	510	520	530	540	550	560
QCCLEDVGQV	AFMAGDCQTS	TQFCVSAPRK	PPLQPPVDPV	VKARMESSVI	LSWSPPPHGE	RPVTIDGYLV	EKKKLGTYTW
570	580	590	600	610	620	630	640
IRCHEAEWVA	TPELTVADVA	EEGNFQFRVS	ALNSFGQSPY	LEFPGTVHLA	PKLAVRTPLK	AVQAVEGGEV	TFSVDLTVAS
650	660	670	680	690	700	710	720
AGEWFLDGQA	LKASSVYEIH	CDRTRHTLTI	REVPASLHGA	QLKFVANGIE	SSIRMEVRAA	PGLTANKPPA	AAAREVLARL
730	740	750	760	770	780	790	800
HEEAQLLAEL	SDQAAAVTWL	KDGRTLSPGP	KYEVQASAGR	RVLLVRDVAR	DDAGLYECVS	RGGRIAYQLS	VQGLARFLHK
810	820	830	840	850	860	870	880
DMAGSCVDAV	AGGPAQFECE	TSEAHVHVHW	YKDGMELGHS	GERFLQEDVG	TRHRLVAATV	TRQDEGTYSC	RVGEDSVDFR
890	900	910	920	930	940	950	960
LRVSEPKAVF	AKEQPACREV	QAEVGASATL	SCEVAQDQME	VTWYKDGKKL	SSSSKVHVEA	VGCMRRLVVQ	QVGQADSGEY
970	980	990	1000	1010	1020	1030	1040
SCEARGQRVS	FRLDVAEPKV	VFAKEQLARR	KLQAEAGASA	TLSCEVAQAQ	TEVTWYKDGK	KLSSSSKVCM	EATGCTRRLV
1050	1060	1070	1080	1090	1100	1110	1120
VQQAGQADAG	EYSCEAGGQR	LSFHLDVKEP	KVVFAKDQVA	HSEVQAEAGA	SATLSCEVAQ	AQTEVMWYKD	GKKLSSSLKV
1130	1140	1150	1160	1170	1180	1190	1200
HVEAKGCRRR	LVVQQAGKTD	AGDYSCEARG	QRVSFRLHIT	EPKMMFAKEQ	SVHNEVQAEA	GASAMLSCEV	AQAQTEVTWY
1210	1220	1230	1240	1250	1260	1270	1280
KDGKKLSSSS	KVGMEVKGCT	RRLVLPQAGK	ADAGEYSCEA	GGQRVSFHLH	ITEPKGVFAK	EQSVHNEVQA	EAGTTAMLSC
1290	1300	1310	1320	1330	1340	1350	1360
EVAQPQTEVT	WYKDGKKLSS	SSKVRMEVKG	CTRRLVVQQV	GKADAGEYSC	EAGGQRVSFQ	LHITEPKAVF	AKEQLVHNEV
1370	1380	1390	1400	1410	1420	1430	1440
RTEAGASATL	SCEVAQAQTE	VTWYKDGKKL	SSSSKVRIEA	AGCMRQLVVQ	QAGQADAGEY	TCEAGGQRLS	FHLDVSEPKA
1450	1460	1470	1480	1490	1500	1510	1520
VFAKEQLAHR	KVQAEAGAIA	TLSCEVAQAQ	TEVTWYKDGK	KLSSSSKVRM	EAVGCTRRLV	VQQACQADTG	EYSCEAGGQR
1530	1540	1550	1560	1570	1580	1590	1600
LSFSLDVAEP	KVVFAKEQPV	HREVQAQAGA	STTLSCEVAQ	AQTEVMWYKD	GKKLSFSSKV	RMEAVGCTRR	LVVQQAGQAV
1610	1620	1630	1640	1650	1660	1670	1680
AGEYSCEAGS	QRLSFHLHVA	EPKAVFAKEQ	PASREVQAEA	GTSATLSCEV	AQAQTEVTWY	KDGKKLSSSS	KVRMEAVGCT
1690	1700	1710	1720	1730	1740	1750	1760
RRLVVQEAGQ	ADAGEYSCKA	GDQRLSFHLH	VAEPKVVFAK	EQPAHREVQA	EAGASATLSC	EVAQAQTEVT	WYKDGKKLSS
1770	1780	1790	1800	1810	1820	1830	1840
SSKVRVEAVG	CTRRLVVQQA	GQAEAGEYSC	EAGGQQLSFR	LQVAEPKAVF	AKEQVVFAKD	QPVHREVQAE	AGTSTMLSCE
1850	1860	1870	1880	1890	1900	1910	1920
VAQAQTEVMW	YKDGKKLSSS	SKMRVEAVGC	TRRLVVQEAG	QADAGEYSCE	AGGQRLSFHL	HVAEPKVVFA	KEQPACREVQ
1930	1940	1950	1960	1970	1980	1990	2000
AEAGASATLS	CEVAQGQMEV	TWYKDGKKLS	SSSKVHMEAS	GYTRRLVVQQ	AGQADAGEYS	CEAGGQRLSF	RLHVAELEPQ
2010	2020	2030	2040	2050	2060	2070	2080
ISERPCRREP	LVVKEHEDII	LTATLATPSA	ATVTWLKDGV	EIRRSKRHET	ASQGDTHTLT	VHGAQVLDSA	IYSCRVGAEG
2090	2100	2110	2120	2130	2140	2150	2160
QDFPVQVEEV	AAKFCRLLEP	VCGELGGTVT	LACELSPACA	EVVWRCGNTQ	LRVGKRFQMV	AEGPVRSLTV	LGLRAEDAGE
2170	2180	2190	2200	2210	2220	2230	2240

Detailed Protein Report

Cmpd.	No. of Cmpds.	m/z meas.	Δ m/z [ppm]	z	Rt [min]	Score	P	Range	Sequence	Modification
1941	1	643.2925	-112.28	2	54.4	13.1	2	338-347	K.RLQDLEVREK.E	
2890	1	922.9785	45.19	2	65.3	10.6	2	1021-1037	K.KLSSSSKVCMEATGCTR.R	Carbamidomethyl: 15
2923	1	951.4595	12.61	2	65.7	13.9	2	1021-1037	K.KLSSSSKVCMEATGCTR.R	Carbamidomethyl: 9, 15
1563	1	927.4401	-8.97	3	48.2	13.0	1	5415-5440	R.EDSGQYAAYISNAMGAAYSSARLLVR.G	Oxidation: 14

Detailed Protein Report

Protein 116: sacsin isoform 2 [Homo sapiens]

**Accession:**	gi|491227401	**Score:**	50.5
**Database:**	refseq_human(refseq_human_20140103.fasta)	**MW [kDa]:**	504.2
**Database Date:**	2015-11-30	**pI:**	6.6
**Modification(s):**	Carbamidomethyl	**Sequence Coverage [%]:**	1.5
		**No. of unique Peptides:**	3

Detailed Protein Report

10	20	30	40	50	60	70	80
MAPYQGPALY	VYNNAVFTPE	DWHGIQEIAR	SRKKDDPLKV	GRFGIGFNSV	YHITDVPCIF	SGDQIGMLDP	HQTLFGPHES
90	100	110	120	130	140	150	160
GQCWNLKDDS	KEISELSDQF	APFVGIFGST	KETFINGNFP	GTFFRFPLRL	QPSQLSSNLY	NKQKVLELFE	SFRADADTVL
170	180	190	200	210	220	230	240
LFLKSVQDVS	LYVREADGTE	KLVFRVTSSE	SKALKHERPN	SIKILGTAIS	NYCKKTPSNN	ITCVTYHVNI	VLEEESTKDA
250	260	270	280	290	300	310	320
QKTSWLVCNS	VGGRGISSKL	DSLADELKFV	PIIGIAMPLS	SRDDEAKGAT	SDFSGKAFCF	LPLPPGEESS	TGLPVHISGF
330	340	350	360	370	380	390	400
FGLTDNRRSI	KWRELDQWRD	PAALWNEFLV	MNVVPKAYAT	LILDSIKRLE	MEKSSDFPLS	VDVIYKLWPE	ASKVKVHWQP
410	420	430	440	450	460	470	480
VLEPLFSELL	QNAVIYSISC	DWVRLEQVYF	SELDENLEYT	KTVLNYLQSS	GKQIAKVPGN	VDAAVQLTAA	SGTTPVRKVT
490	500	510	520	530	540	550	560
PAWVRQVLRK	CAHLGCAEEK	LHLLEFVLSD	QAYSELLGLE	LLPLQNGNFV	PFSSSVSDQD	VIYITSAEYP	RSLFPSLEGR
570	580	590	600	610	620	630	640
FILDNLKPHL	VAALKEAAQT	RGRPCTQLQL	LNPERFARLI	KEVMNTFWPG	RELIVQWYPF	DENRNHPSVS	WLKMVWKNLY
650	660	670	680	690	700	710	720
IHFSEDLTLF	DEMPLIPRTI	LEEGQTCVEL	IRLRIPSLVI	LDDESEAQLP	EFLADIVQKL	GGFVLKKLDA	SIQHPLIKKY
730	740	750	760	770	780	790	800
IHSPLPSAVL	QIMEKMPLQK	LCNQITSLLP	THKDALRKFL	ASLTDSSEKE	KRIIQELAIF	KRINHSSDQG	ISSYTKLKGC
810	820	830	840	850	860	870	880
KVLHHTAKLP	ADLRLSISVI	DSSDEATIRL	ANMLKIEQLK	TTSCLKLVLK	DIENAFYSHE	EVTQLMLWVL	ENLSSLKNEN
890	900	910	920	930	940	950	960
PNVLEWLTPL	KFIQISQEQM	VSAGELFDPD	IEVLKDLFCN	EEGTYFPPSV	FTSPDILHSL	RQIGLKNEAS	LKEKDVVQVA
970	980	990	1000	1010	1020	1030	1040
KKIEALQVGA	CPDQDVLLKK	AKTLLLVLNK	NHTLLQSSEG	KMTLKKIKWV	PACKERPPNY	PGSLVWKGDL	CNLCAPPDMC
1050	1060	1070	1080	1090	1100	1110	1120
DVGHAILIGS	SLPLVESIHV	NLEKALGIFT	KPSLSAVLKH	FKIVVDWYSS	KTFSDEDYYQ	FQHILLEIYG	FMHDHLNEGK
1130	1140	1150	1160	1170	1180	1190	1200
DSFRALKFPW	VWTGKKFCPL	AQAVIKPIHD	LDLQPYLHNV	PKTMAKFHQL	FKVCGSIEEL	TSDHISMVIQ	KIYLKSDQDL
1210	1220	1230	1240	1250	1260	1270	1280
SEQESKQNLH	LMLNIIRWLY	SNQIPASPNT	PVPIHHSKNP	SKLIMKPIHE	CCYCDIKVDD	LNDLLEDSVE	PIILVHEDIP
1290	1300	1310	1320	1330	1340	1350	1360
MKTAEWLKVP	CLSTRLINPE	NMGFEQSGQR	EPLTVRIKNI	LEEYPSVSDI	FKELLQNADD	ANATECSFLI	DMRRNMDIRE
1370	1380	1390	1400	1410	1420	1430	1440
NLLDPGMAAC	HGPALWSFNN	SQFSDSDFVN	ITRLGESLKR	GEVDKVGKFG	LGFNSVYHIT	DIPIIMSREF	MIMFDPNINH
1450	1460	1470	1480	1490	1500	1510	1520
ISKHIKDKSN	PGIKINWSKQ	QKRLRKFPNQ	FKPFIDVFGC	QLPLTVEAPY	SYNGTLFRLS	FRTQQEAKVS	EVSSTCYNTA
1530	1540	1550	1560	1570	1580	1590	1600
DIYSLVDEFS	LCGHRLIIFT	QSVKSMYLKY	LKIEETNPSL	AQDTVIIKKK	SCSSKALNTP	VLSVLKEAAK	LMKTCSSSNK
1610	1620	1630	1640	1650	1660	1670	1680
KLPSDEPKSS	CILQITVEEF	HHVFRRIADL	QSPLFRGPDD	DPAALFEMAK	SGQSKKPSDE	LSQKTVECTT	WLLCTCMDTG
1690	1700	1710	1720	1730	1740	1750	1760
EALKFSLSES	GRRLGLVPCG	AVGVQLSEIQ	DQKWTVKPHI	GEVFCYLPLR	IKTGLPVHIN	GCFAVTSNRK	EIWKTDTKGR
1770	1780	1790	1800	1810	1820	1830	1840
WNTTFMRHVI	VKAYLQVLSV	LRDLATSGEL	MDYTYYAVWP	DPDLVHDDFS	VICQGFYEDI	AHGKGKELTK	VFSDGSTWVS
1850	1860	1870	1880	1890	1900	1910	1920
MKNVRFLDDS	ILKRRDVGSA	AFKIFLKYLK	KTGSKNLCAV	ELPSSVKLGF	EEAGCKQILL	ENTFSEKQFF	SEVFFPNIQE
1930	1940	1950	1960	1970	1980	1990	2000
IEAELRDPLM	IFVLNEKVDE	FSGVLRVTPC	IPCSLEGHPL	VLPSRLIHPE	GRVAKLFDIK	DGRFPYGSTQ	DYLNPIILIK
2010	2020	2030	2040	2050	2060	2070	2080
LVQLGMAKDD	ILWDDMLERA	VSVAEINKSD	HVAACLRSSI	LLSLIDEKLK	IRDPRAKDFA	AKYQTIRFLP	FLTKPAGFSL
2090	2100	2110	2120	2130	2140	2150	2160
DWKGNSFKPE	TMFAATDLYT	AEHQDIVCLL	QPILNENSHS	FRGCGSVSLA	VKEFLGLLKK	PTVDLVINQL	KEVAKSVDDG
2170	2180	2190	2200	2210	2220	2230	2240

Detailed Protein Report

Cmpd.	No. of Cmpds.	m/z meas.	Δ m/z [ppm]	z	Rt [min]	Score	P	Range	Sequence	Modification
2078	1	1029.6855	128.38	2	56.1	10.5	0	1714-1730	K.WTVKPHIGEVFCYLPLR.I	
71	1	845.3572	-104.63	2	30.3	12.1	1	1872-1887	K.TGSKNLCAVELPSSVK.L	Carbamidomethyl: 7
2171	1	1022.8841	-140.49	2	57.3	14.3	2	2331-2348	K.DTTVKYCHADIPREVAVK.L	

Detailed Protein Report

Protein 117: peroxisome biogenesis factor 1 isoform 3 [Homo sapiens]

**Accession:**	gi|544186104	**Score:**	50.5
**Database:**	refseq_human(refseq_human_20140103.fasta)	**MW [kDa]:**	119.5
**Database Date:**	2015-11-30	**pI:**	5.7
		**Sequence Coverage [%]:**	3.4
		**No. of unique Peptides:**	1

10	20	30	40	50	60	70	80
MMKELQTKQL	QSNTVGITES	NENESEIPVD	SSSVASLWTM	IGSIFSFQSE	KKQETSWGLT	EINAFKNMQS	KVVPLDNIFR
**90**	**100**	**110**	**120**	**130**	**140**	**150**	**160**
VCKSQPPSIY	NASATSVFHK	HCAIHVFPWD	QEYFDVEPSF	TVTYGKLVKL	LSPKQQQSKT	KQNVLSPEKE	KQMSEPLDQK
**170**	**180**	**190**	**200**	**210**	**220**	**230**	**240**
KIRSDHNEED	EKACVLQVVW	NGLEELNNAI	KYTKNVEVLH	LGKVWIPDDL	RKRLNIEMHA	VVRITPVEVT	PKIPRSLKLQ
**250**	**260**	**270**	**280**	**290**	**300**	**310**	**320**
PRENLPKDIS	EEDIKTVFYS	WLQQSTTTML	PLVISEEEFI	KLETKDGLKE	FSLSIVHSWE	KEKDKNIFLL	SPNLLQKTTI
**330**	**340**	**350**	**360**	**370**	**380**	**390**	**400**
QVLLDPMVKE	ENSEEIDFIL	PFLKLSSLGG	VNSLGVSSLE	HITHSLLGRP	LSRQLMSLVA	GLRNGALLLT	GGKGSGKSTL
**410**	**420**	**430**	**440**	**450**	**460**	**470**	**480**
AKAICKEAFD	KLDAHVERVD	CKALRGKRLE	NIQKTLEVAF	SEAVWMQPSV	VLLDDLDLIA	GLPAVPEHEH	SPDAVQSQRL
**490**	**500**	**510**	**520**	**530**	**540**	**550**	**560**
AHALNDMIKE	FISMGSLVAL	IATSQSQQSL	HPLLVSAQGV	HIFQCVQHIQ	PPNQEQRCEI	LCNVIKNKLD	CDINKFTDLD
**570**	**580**	**590**	**600**	**610**	**620**	**630**	**640**
LQHVAKETGG	FVARDFTVLV	DRAIHSRLSR	QSISTREKLV	LTTLDFQKAL	RGFLPASLRS	VNLHKPRDLG	WDKIGGLHEV
**650**	**660**	**670**	**680**	**690**	**700**	**710**	**720**
RQILMDTIQL	PAKYPELFAN	LPIRQRTGIL	LYGPPGTGKT	LLAGVIARES	RMNFISVKGP	ELLSKYIGAS	EQAVRDIFIR
**730**	**740**	**750**	**760**	**770**	**780**	**790**	**800**
AQAAKPCILF	FDEFESIAPR	RGHDNTGVTD	RVVNQLLTQL	DGVEGLQGVY	VLAATSRPDL	IDPALLRPGR	LDKCVYCPPP
**810**	**820**	**830**	**840**	**850**	**860**	**870**	**880**
DQVSRLEILN	VLSDSLPLAD	DVDLQHVASV	TDSFTGADLK	ALLYNAQLEA	LHGMLLSSGL	QDGSSSSDSD	LSLSSMVFLN
**890**	**900**	**910**	**920**	**930**	**940**	**950**	**960**
HSSGSDDSAG	DGECGLDQSL	VSLEMSEILP	DESKFNMYRL	YFGSSYESEL	GNGTSSDLSS	QCLSAPSSMT	QDLPGVPGKD
**970**	**980**	**990**	**1000**	**1010**	**1020**	**1030**	**1040**
QLFSQPPVLR	TASQEGCQEL	TQEQRDQLRA	DISIIKGRYR	SQSGEDESMN	QPGPIKTRLA	ISQSHLMTAL	GHTRPSISED
**1050**	**1060**	**1070**	**1080**				
DWKNFAELYE	SFQNPKRRKN	QSGTMFRPGQ	KVTLA				

Cmpd.	No. of Cmpds.	m/z meas.	Δ m/z [ppm]	z	Rt [min]	Score	P	Range	Sequence	Modification
1127	6	1207.8838	99.74	1	42.8	28.3	2	233-242	K.IPRSLKLQPR.E	

Detailed Protein Report

Protein 118: peroxiredoxin-2 [Homo sapiens]

**Accession:**	gi|32189392	**Score:**	50.1
**Database:**	refseq_human(refseq_human_20140103.fasta)	**MW [kDa]:**	21.9
**Database Date:**	2015-11-30	**pI:**	5.6
		**Sequence Coverage [%]:**	5.6
		**No. of unique Peptides:**	1

10	20	30	40	50	60	70	80
MASGNARIGK	PAPDFKATAV	VDGAFKEVKL	SDYKGKYVVL	FFYPLDFTFV	CPTEIIAFSN	RAEDFRKLGC	EVLGVSVDSQ
**90**	**100**	**110**	**120**	**130**	**140**	**150**	**160**
FTHLAWINTP	RKEGGLGPLN	IPLLADVTRR	LSEDYGVLKT	DEGIAYRGLF	IIDGKGVLRQ	ITVNDLPVGR	SVDEALRLVQ
**170**	**180**	**190**	**200**				
AFQYTDEHGE	VCPAGWKPGS	DTIKPNVDDS	KEYFSKHN				

Cmpd.	No. of Cmpds.	m/z meas.	Δ m/z [ppm]	z	Rt [min]	Score	P	Range	Sequence	Modification
766	1	606.3268	-23.04	2	38.4	50.1	0	140-150	R.QITVNDLPVGR.S	

Detailed Protein Report

Protein 119: myotubularin-related protein 5 [Homo sapiens]

**Accession:**	gi|239735519	**Score:**	49.4
**Database:**	refseq_human(refseq_human_20140103.fasta)	**MW [kDa]:**	210.9
**Database Date:**	2015-11-30	**pI:**	6.7
**Modification(s):**	Carbamidomethyl, Oxidation	**Sequence Coverage [%]:**	2.0
		**No. of unique Peptides:**	3

Quantitation

mdown:qdown	**Median:**	0.57	**CV:**	11.37 %	**No. of Peptides:**	2
Wdown:Qdown	**Median:**	0.76	**CV:**	104.20 %	**No. of Peptides:**	3

Detailed Protein Report

10	20	30	40	50	60	70	80
MARLADYFVL	VAFGPHPRGS	GEGQGQILQR	FPEKDWEDNP	FPQGIELFCQ	PSGWQLCPER	NPPTFFVAVL	TDINSERHYC
90	100	110	120	130	140	150	160
ACLTFWEPAE	PSQETTRVED	ATEREEEGDE	GGQTHLSPTA	PAPSAQLFAP	KTLVLVSRLD	HTEVFRNSLG	LIYAIHVEGL
170	180	190	200	210	220	230	240
NVCLENVIGN	LLTCTVPLAG	GSQRTISLGA	GDRQVIQTPL	ADSLPVSRCS	VALLFRQLGI	TNVLSLFCAA	LTEHKVLFLS
250	260	270	280	290	300	310	320
RSYQRLADAC	RGLLALLFPL	RYSFTYVPIL	PAQLLEVLST	PTPFIIGVNA	AFQAETQELL	DVIVADLDGG	TVTIPECVHI
330	340	350	360	370	380	390	400
PPLPEPLQSQ	THSVLSMVLD	PELELADLAF	PPPTTSTSSL	KMQDKELRAV	FLRLFAQLLQ	GYRWCLHVVR	IHPEPVIRFH
410	420	430	440	450	460	470	480
KAAFLGQRGL	VEDDFLMKVL	EGMAFAGFVS	ERGVPYRPTD	LFDELVAHEV	ARMRADENHP	QRVLRHVQEL	AEQLYKNENP
490	500	510	520	530	540	550	560
YPAVAMHKVQ	RPGESSHLRR	VPRPFPRLDE	GTVQWIVDQA	AAKMQGAPPA	VKAERRTTVP	SGPPMTAILE	RCSGLHVNSA
570	580	590	600	610	620	630	640
RRLEVVRNCI	SYVFEGKMLE	AKKLLPAVLR	ALKGRAARRC	LAQELHLHVQ	QNRAVLDHQQ	FDFVVRMMNC	CLQDCTSLDE
650	660	670	680	690	700	710	720
HGIAAALLPL	VTAFCRKLSP	GVTQFAYSCV	QEHVVWSTPQ	FWEAMFYGDV	QTHIRALYLE	PTEDLAPAQE	VGEAPSQEDE
730	740	750	760	770	780	790	800
RSALDVASEQ	RRLWPTLSRE	KQQELVQKEE	STVFSQAIHY	ANRMSYLLLP	LDSSKSRLLR	ERAGLGDLES	ASNSLVTNSM
810	820	830	840	850	860	870	880
AGSVAESYDT	ESGFEDAETC	DVAGAVVRFI	NRFVDKVCTE	SGVTSDHLKG	LHVMVPDIVQ	MHIETLEAVQ	RESRRLPPIQ
890	900	910	920	930	940	950	960
KPKLLRPRLL	PGEECVLDGL	RVYLLPDGRE	EGAGGSAGGP	ALLPAEGAVF	LTTYRVIFTG	MPTDPLVGEQ	VVVRSFPVAA
970	980	990	1000	1010	1020	1030	1040
LTKEKRISVQ	TPVDQLLQDG	LQLRSCTFQL	LKMAFDEEVG	SDSAELFRKQ	LHKLRYPPDI	RATFAFTLGS	AHTPGRPPRV
1050	1060	1070	1080	1090	1100	1110	1120
TKDKGPSLRT	LSRNLVKNAK	KTIGRQHVTR	KKYNPPSWEH	RGQPPPEDQE	DEISVSEELE	PSTLTPSSAL	KPSDRMTMSS
1130	1140	1150	1160	1170	1180	1190	1200
LVERACCRDY	QRLGLGTLSS	SLSRAKSEPF	RISPVNRMYA	ICRSYPGLLI	VPQSVQDNAL	QRVSRCYRQN	RFPVVCWRSG
1210	1220	1230	1240	1250	1260	1270	1280
RSKAVLLRSG	GLHGKGVVGL	FKAQNAPSPG	QSQADSSSLE	QEKYLQAVVS	SMPRYADASG	RNTLSGFSSA	HMGSHVPSPR
1290	1300	1310	1320	1330	1340	1350	1360
ARVTTLSNPM	AASASRRTAP	RGKWGSVRTS	GRSSGLGTDV	GSRLAGRDAL	APPQANGGPP	DPGFLRPQRA	ALYILGDKAQ
1370	1380	1390	1400	1410	1420	1430	1440
LKGVRSDPLQ	QWELVPIEVF	EARQVKASFK	KLLKACVPGC	PAAEPSPASF	LRSLEDSEWL	IQIHKLLQVS	VLVVELLDSG
1450	1460	1470	1480	1490	1500	1510	1520
SSVLVGLEDG	WDITTQVVSL	VQLLSDPFYR	TLEGFRLLVE	KEWLSFGHRF	SHRGAHTLAG	QSSGFTPVFL	QFLDCVHQVH
1530	1540	1550	1560	1570	1580	1590	1600
LQFPMEFEFS	QFYLKFLGYH	HVSRRFRTFL	LDSDYERIEL	GLLYEEKGER	RGQVPCRSVW	EYVDRLSKRT	PVFHNYMYAP
1610	1620	1630	1640	1650	1660	1670	1680
EDAEVLRPYS	NVSNLKVWDF	YTEETLAEGP	PYDWELAQGP	PEPPEEERSD	GGAPQSRRRV	VWPCYDSCPR	AQPDAISRLL
1690	1700	1710	1720	1730	1740	1750	1760
EELQRLETEL	GQPAERWKDT	WDRVKAAQRL	EGRPDGRGTP	SSLLVSTAPH	HRRSLGVYLQ	EGPVGSTLSL	SLDSDQSSGS
1770	1780	1790	1800	1810	1820	1830	1840
TTSGSRQAAR	RSTSTLYSQF	QTAESENRSY	EGTLYKKGAF	MKPWKARWFV	LDKTKHQLRY	YDHRVDTECK	GVIDLAEVEA
1850	1860	1870	1880	1890	1900		
VAPGTPTMGA	PKTVDEKAFF	DVKTTRRVYN	FCAQDVPSAQ	QWVDRIQSCL	SDA		

Cmpd.	No. of Cmpds.	m/z meas.	Δ m/z [ppm]	z	Rt [min]	Score	P	Range	Sequence	Modification	Ratios
280	1	591.8074	33.08	2	32.6	12.3	1	242-251	R.SYQRLADACR.G		Wdown:Qdown 0.25<br>mdown:qdown 0.64
1064	3	751.8788	61.27	2	42.0	11.0	1	1116-1128	R.MTMSSLVERACCR.D	Oxidation: 3	Wdown:Qdown 0.87
348	1	800.7371	-149.37	2	33.7	13.1	1	1116-	R.MTMSSLVERACCR.D	Carbamidomethyl: 11, 12	mdown:qdown 0.51

Detailed Protein Report

Cmpd.	No. of Cmpds.	m/z meas.	Δ m/z [ppm]	z	Rt [min]	Score	P	Range	Sequence	Modification	Ratios
								1128			Wdown:Qdown 2.02

Detailed Protein Report

Protein 120: spectrin beta chain, non-erythrocytic 1 isoform 2 [Homo sapiens]

**Accession:**	gi|112382252	**Score:**	49.3
**Database:**	refseq_human(refseq_human_20140103.fasta)	**MW [kDa]:**	251.2
**Database Date:**	2015-11-30	**pI:**	5.3
		**Sequence Coverage [%]:**	1.9
		**No. of unique Peptides:**	2

Quantitation

mdown:qdown	**Median:**	0.88	**CV:**	0.00 %	**No. of Peptides:**	1
Wdown:Qdown	**Median:**	3.94	**CV:**	0.00 %	**No. of Peptides:**	1

Detailed Protein Report

10	20	30	40	50	60	70	80
MELQRTSSIS	GPLSPAYTGQ	VPYNYNQLEG	RFKQLQDERE	AVQKKTFTKW	VNSHLARVSC	RITDLYTDLR	DGRMLIKLLE
90	100	110	120	130	140	150	160
VLSGERLPKP	TKGRMRIHCL	ENVDKALQFL	KEQRVHLENM	GSHDIVDGNH	RLTLGLIWTI	ILRFQIQDIS	VETEDNKEKK
170	180	190	200	210	220	230	240
SAKDALLLWC	QMKTAGYPNV	NIHNFTTSWR	DGMAFNALIH	KHRPDLIDFD	KLKKSNAHYN	LQNAFNLAEQ	HLGLTKLLDP
250	260	270	280	290	300	310	320
EDISVDHPDE	KSIITYVVTY	YHYFSKMKAL	AVEGKRIGKV	LDNAIETEKM	IEKYESLASD	LLEWIEQTII	ILNNRKFANS
330	340	350	360	370	380	390	400
LVGVQQQLQA	FNTYRTVEKP	PKFTEKGNLE	VLLFTIQSKM	RANNQKVYMP	REGKLISDIN	KAWERLEKAE	HERELALRNE
410	420	430	440	450	460	470	480
LIRQEKLEQL	ARRFDRKAAM	RETWLSENQR	LVSQDNFGFD	LPAVEAATKK	HEAIETDIAA	YEERVQAVVA	VARELEAENY
490	500	510	520	530	540	550	560
HDIKRITARK	DNVIRLWEYL	LELLRARRQR	LEMNLGLQKI	FQEMLYIMDW	MDEMKVLVLS	QDYGKHLLGV	EDLLQKHTLV
570	580	590	600	610	620	630	640
EADIGIQAER	VRGVNASAQK	FATDGEGYKP	CDPQVIRDRV	AHMEFCYQEL	CQLAAERRAR	LEESRRLWKF	FWEMAEEEGW
650	660	670	680	690	700	710	720
IREKEKILSS	DDYGKDLTSV	MRLLSKHRAF	EDEMSGRSGH	FEQAIKEGED	MIAEEHFGSE	KIRERIIYIR	EQWANLEQLS
730	740	750	760	770	780	790	800
AIRKKRLEEA	SLLHQFQADA	DDIDAWMLDI	LKIVSSSDVG	HDEYSTQSLV	KKHKDVAEEI	ANYRPTLDTL	HEQASALPQE
810	820	830	840	850	860	870	880
HAESPDVRGR	LSGIEERYKE	VAELTRLRKQ	ALQDTLALYK	MFSEADACEL	WIDEKEQWLN	NMQIPEKLED	LEVIQHRFES
890	900	910	920	930	940	950	960
LEPEMNNQAS	RVAVVNQIAR	QLMHSGHPSE	KEIKAQQDKL	NTRWSQFREL	VDRKKDALLS	ALSIQNYHLE	CNETKSWIRE
970	980	990	1000	1010	1020	1030	1040
KTKVIESTQD	LGNDLAGVMA	LQRKLTGMER	DLVAIEAKLS	DLQKEAEKLE	SEHPDQAQAI	LSRLAEISDV	WEEMKTTLKN
1050	1060	1070	1080	1090	1100	1110	1120
REASLGEASK	LQQFLRDLDD	FQSWLSRTQT	AIASEDMPNT	LTEAEKLLTQ	HENIKNEIDN	YEEDYQKMRD	MGEMVTQGQT
1130	1140	1150	1160	1170	1180	1190	1200
DAQYMFLRQR	LQALDTGWNE	LHKMWENRQN	LLSQSHAYQQ	FLRDTKQAEA	FLNNQEYVLA	HTEMPTTLEG	AEAAIKKQED
1210	1220	1230	1240	1250	1260	1270	1280
FMTTMDANEE	KINAVVETGR	RLVSDGNINS	DRIQEKVDSI	DDRHRKNRET	ASELLMRLKD	NRDLQKFLQD	CQELSLWINE
1290	1300	1310	1320	1330	1340	1350	1360
KMLTAQDMSY	DEARNLHSKW	LKHQAFMAEL	ASNKEWLDKI	EKEGMQLISE	KPETEAVVKE	KLTGLHKMWE	VLESTTQTKA
1370	1380	1390	1400	1410	1420	1430	1440
QRLFDANKAE	LFTQSCADLD	KWLHGLESQI	QSDDYGKDLT	SVNILLKKQQ	MLENQMEVRK	KEIEELQSQA	QALSQEGKST
1450	1460	1470	1480	1490	1500	1510	1520
DEVDSKRLTV	QTKFMELLEP	LNERKHNLLA	SKEIHQFNRD	VEDEILWVGE	RMPLATSTDH	GHNLQTVQLL	IKKNQTLQKE
1530	1540	1550	1560	1570	1580	1590	1600
IQGHQPRIDD	IFERSQNIVT	DSSSLSAEAI	RQRLADLKQL	WGLLIEETEK	RHRRLEEAHR	AQQYYFDAAE	AEAWMSEQEL
1610	1620	1630	1640	1650	1660	1670	1680
YMMSEEKAKD	EQSAVSMLKK	HQILEQAVED	YAETVHQLSK	TSRALVADSH	PESERISMRQ	SKVDKLYAGL	KDLAEERRGK
1690	1700	1710	1720	1730	1740	1750	1760
LDERHRLFQL	NREVDDLEQW	IAEREVVAGS	HELGQDYEHV	TMLQERFREF	ARDTGNIGQE	RVDTVNHLAD	ELINSGHSDA
1770	1780	1790	1800	1810	1820	1830	1840
ATIAEWKDGL	NEAWADLLEL	IDTRTQILAA	SYELHKFYHD	AKEIFGRIQD	KHKKLPEELG	RDQNTVETLQ	RMHTTFEHDI
1850	1860	1870	1880	1890	1900	1910	1920
QALGTQVRQL	QEDAARLQAA	YAGDKADDIQ	KRENEVLEAW	KSLLDACESR	RVRLVDTGDK	FRFFSMVRDL	MLWMEDVIRQ
1930	1940	1950	1960	1970	1980	1990	2000
IEAQEKPRDV	SSVELLMNNH	QGIKAEIDAR	NDSFTTCIEL	GKSLLARKHY	ASEEIKEKLL	QLTEKRKEMI	DKWEDRWEWL
2010	2020	2030	2040	2050	2060	2070	2080
RLILEVHQFS	RDASVAEAWL	LGQEPYLSSR	EIGQSVDEVE	KLIKRHEAFE	KSAATWDERF	SALERLTTLE	LLEVRRQQEE
2090	2100	2110	2120	2130	2140	2150	2160
EERKRRPPSP	EPSTKVSEEA	ESQQQWDTSK	GEQVSQNGLP	AEQGSPRVSY	RSQTYQNYKN	FNSRRTASDQ	PWSGL

Detailed Protein Report

Cmpd.	No. of Cmpds.	m/z meas.	Δ m/z [ppm]	z	Rt [min]	Score	P	Range	Sequence	Modification	Ratios
418	6	607.8310	-55.05	2	34.3	14.1	2	820-829	K.EVAELTRLRK.Q		
601	2	548.1743	-250.21	2	36.1	12.2	0	1087-1095	K.LLTQHENIK.N		mdown:qdown 0.88<br>Wdown:Qdown 3.94

Detailed Protein Report

Protein 121: aldehyde oxidase [Homo sapiens]

Accession: gi|71773480
Database: refseq_human(refseq_human_20140103.fasta)
Database Date: 2015-11-30
Modification(s): Carbamidomethyl

Score: 49.1
MW [kDa]: 147.8
pI: 6.9
Sequence Coverage [%]: 3.7
No. of unique Peptides: 1

10	20	30	40	50	60	70	80
MDRASELLFY	VNGRKVIEKN	VDPETMLLPY	LRKKLRLTGT	KYGCGGGGCG	ACTVMISRYN	PITKRIRHHP	ANACLIPICS
90	100	110	120	130	140	150	160
LYGAAVTTVE	GIGSTHTRIH	PVQERIAKCH	GTQCGFCTPG	MVMSIYTLLR	NHPEPTLDQL	TDALGGNLCR	CTGYRPIIDA
170	180	190	200	210	220	230	240
CKTFCKTSGC	CQSKENGVCC	LDQGINGLPE	FEEGSKTSPK	LFAEEEFLPL	DPTQELIFPP	ELMIMAEKQS	QRTRVFGSER
250	260	270	280	290	300	310	320
MMWFSPVTLK	ELLEFKFKYP	QAPVIMGNTS	VGPEVKFKGV	FHPVIISPDR	IEELSVVNHA	YNGLTLGAGL	SLAQVKDILA
330	340	350	360	370	380	390	400
DVVQKLPEEK	TQMYHALLKH	LGTLAGSQIR	NMASLGGHII	SRHPDSDLNP	ILAVGNCTLN	LLSKEGKRQI	PLNEQFLSKC
410	420	430	440	450	460	470	480
PNADLKPQEI	LVSVNIPYSR	KWEFVSAFRQ	AQRQENALAI	VNSGMRVFFG	EGDGIIRELC	ISYGGVGPAT	ICAKNSCQKL
490	500	510	520	530	540	550	560
IGRHWNEQML	DIACRLILNE	VSLLGSAPGG	KVEFKRTLII	SFLFKFYLEV	SQILKKMDPV	HYPSLADKYE	SALEDLHSKH
570	580	590	600	610	620	630	640
HCSTLKYQNI	GPKQHPEDPI	GHPIMHLSGV	KHATGEAIYC	DDMPLVDQEL	FLTFVTSSRA	HAKIVSIDLS	EALSMPGVVD
650	660	670	680	690	700	710	720
IMTAEHLSDV	NSFCFFTEAE	KFLATDKVFC	VGQLVCAVLA	DSEVQAKRAA	KRVKIVYQDL	EPLILTIEES	IQHNSSFKPE
730	740	750	760	770	780	790	800
RKLEYGNVDE	AFKVVDQILE	GEIHMGGQEH	FYMETQSMLV	VPKGEDQEMD	VYVSTQFPKY	IQDIVASTLK	LPANKVMCHV
810	820	830	840	850	860	870	880
RRVGGAFGGK	VLKTGIIAAV	TAFAANKHGR	AVRCVLERGE	DMLITGGRHP	YLGKYKAGFM	NDGRILALDM	EHYSNAGASL
890	900	910	920	930	940	950	960
DESLFVIEMG	LLKMDNAYKF	PNLRCRGWAC	RTNLPSNTAF	RGFGFPQAAL	ITESCITEVA	AKCGLSPEKV	RIINMYKEID
970	980	990	1000	1010	1020	1030	1040
QTPYKQEINA	KNLIQCWREC	MAMSSYSLRK	VAVEKFNAEN	YWKKKGLAMV	PLKFPVGLGS	RAAGQAAALV	HIYLDGSVLV
1050	1060	1070	1080	1090	1100	1110	1120
THGGIEMGQG	VHTKMIQVVS	RELRMPMSNV	HLRGTSTETV	PNANISGGSV	VADLNGLAVK	DACQTLLKRL	EPIISKNPKG
1130	1140	1150	1160	1170	1180	1190	1200
TWKDWAQTAF	DESINLSAVG	YFRGYESDMN	WEKGEGQPFE	YFVYGAACSE	VEIDCLTGDH	KNIRTDIVMD	VGCSINPAID
1210	1220	1230	1240	1250	1260	1270	1280
IGQIEGAFIQ	GMGLYTIEEL	NYSPQGILHT	RGPDQYKIPA	ICDMPTELHI	ALLPPSQNSN	TLYSSKGLGE	SGVFLGCSVF
1290	1300	1310	1320	1330	1340		
FAIHDAVSAA	RQERGLHGPL	TLNSPLTPEK	IRMACEDKFT	KMIPRDEPGS	YVPWNVPI		

Cmpd.	No. of Cmpds.	m/z meas.	Δ m/z [ppm]	z	Rt [min]	Score	P	Range	Sequence	Modification
2110	1	938.3880	-65.37	2	55.0	10.6	1	151-166	R.CTGYRPIIDACKTFCK.T	Carbamidomethyl: 11

Detailed Protein Report

Protein 122: zinc finger protein 540 isoform a [Homo sapiens]

**Accession:**	gi|22749241	**Score:**	48.8
**Database:**	refseq_human(refseq_human_20140103.fasta)	**MW [kDa]:**	77.0
**Database Date:**	2015-11-30	**pI:**	10.5
		**Sequence Coverage [%]:**	7.4
		**No. of unique Peptides:**	3

Alias proteins:

Accession	Name	Description
gi|288557351	refseq_human (refseq_human_20140103.fasta)	zinc finger protein 540 isoform a [Homo sapiens]

10	20	30	40	50	60	70	80
MAHALVTFRD	VAIDFSQKEW	ECLDTTQRKL	YRDVMLENYN	NLVSLGYSGS	KPDVITLLEQ	GKEPCVVARD	VTGRQCPGLL
**90**	**100**	**110**	**120**	**130**	**140**	**150**	**160**
SRHKTKKLSS	EKDIHEISLS	KESIIEKSKT	LRLKGSIFRN	EWQNKSEFEG	QQGLKERSIS	QKKIVSKKMS	TDRKRPSFTL
**170**	**180**	**190**	**200**	**210**	**220**	**230**	**240**
NQRIHNSEKS	CDSHLVQHGK	IDSDVKHDCK	ECGSTFNNVY	QLTLHQKIHT	GEKSCKCEKC	GKVFSHSYQL	TLHQRFHTGE
**250**	**260**	**270**	**280**	**290**	**300**	**310**	**320**
KPYECQECGK	TFTLYPQLNR	HQKIHTGKKP	YMCKKCDKGF	FSRLELTQHK	RIHTGKKSYE	CKECGKVFQL	IFYFKEHERI
**330**	**340**	**350**	**360**	**370**	**380**	**390**	**400**
HTGKKPYECK	ECGKAFSVCG	QLTRHQKIHT	GVKPYECKEC	GKTFRLSFYL	TEHRRTHAGK	KPYECKECGK	SFNVRGQLNR
**410**	**420**	**430**	**440**	**450**	**460**	**470**	**480**
HKTIHTGIKP	FACKVCEKAF	SYSGDLRVHS	RIHTGEKPYE	CKECGKAFML	RSVLTEHQRL	HTGVKPYECK	ECGKTFRVRS
**490**	**500**	**510**	**520**	**530**	**540**	**550**	**560**
QISLHKKIHT	DVKPYKCVRC	GKTFRFGFYL	TEHQRIHTGE	KPYKCKECGK	AFIRRGNLKE	HLKIHSGLKP	YDCKECGKSF
**570**	**580**	**590**	**600**	**610**	**620**	**630**	**640**
SRRGQFTEHQ	KIHTGVKPYK	CKECGKAFSR	SVDLRIHQRI	HTGEKPYECK	QCGKAFRLNS	HLTEHQRIHT	GEKPYECKVC
**650**	**660**	**670**					
RKAFRQYSHL	YQHQKTHNVI						

Cmpd.	No. of Cmpds.	m/z meas.	Δ m/z [ppm]	z	Rt [min]	Score	P	Range	Sequence	Modification
2641	1	701.3872	28.83	2	61.3	12.6	1	70-82	R.DVTGRQCPGLLSR.H	
765	1	541.3095	42.61	3	38.4	11.9	1	303-315	K.ECGKVFQLIFYFK.E	
848	2	946.4705	3.74	2	39.4	11.1	2	331-347	K.ECGKAFSVCGQLTRHQK.I	

Detailed Protein Report

Protein 123: PREDICTED: extracellular matrix protein FRAS1 isoform X2 [Homo sapiens]

**Accession:**	gi|578809381	**Score:**	48.6
**Database:**	refseq_human(refseq_human_20140103.fasta)	**MW [kDa]:**	438.5
**Database Date:**	2015-11-30	**pI:**	5.2
**Modification(s):**	Carbamidomethyl	**Sequence Coverage [%]:**	1.4
		**No. of unique Peptides:**	2

10	20	30	40	50	60	70	80
MGVLKVWLGL	ALALAEFAVL	PHHSEGACVY	QDSLLADATI	WKPDSCQSCR	CHGDIVICKP	AVCRNPQCAF	EKGEVLQIAA
90	100	110	120	130	140	150	160
NQCCPECVLR	TPGSCHHEKK	IHEHGTEWAS	SPCSVCSCNH	GEVRCTPQPC	PPLSCGHQEL	AFIPEGSCCP	VCVGLGKPCS
170	180	190	200	210	220	230	240
YEGHVFQDGE	DWRLSRCAKC	LCRNGVAQCF	TAQCQPLFCN	QDETVVRVPG	KCCPQCSARS	CSAAGQVYEH	GEQWSENACT
250	260	270	280	290	300	310	320
TCICDRGEVR	CHKQACLPLR	CGKGQSRARR	HGQCCEECVS	PAGSCSYDGV	VRYQDEMWKG	SACEFCMCDH	GQVTCQTGEC
330	340	350	360	370	380	390	400
AKVECARDEE	LIHLDGKCCP	ECISRNGYCV	YEETGEFMSS	NASEVKRIPE	GEKWEDGPCK	VCECRGAQVT	CYEPSCPPCP
410	420	430	440	450	460	470	480
VGTLALEVKG	QCCPDCTSVH	CHPDCLTCSQ	SPDHCDLCQD	PTKLLQNGWC	VHSCGLGFYQ	AGSLCLACQP	QCSTCTSGLE
490	500	510	520	530	540	550	560
CSSCQPPLLM	RHGQCVPTCG	DGFYQDRHSC	AVCHESCAGC	WGPTEKHCLA	CRDPLHVLRD	GGCESSCGKG	FYNRQGTCSA
570	580	590	600	610	620	630	640
CDQSCDSCGP	SSPRCLTCTE	KTVLHDGKCM	SECPGGYYAD	ATGRCKACHS	SCLACMGPAP	SHCTGCKKPE	EGLQVEQLSD
650	660	670	680	690	700	710	720
VGIPSGECLA	QCRAHFYLES	TGICEACHQS	CFRCAGKSPH	NCTDCGPSHV	LLDGQCLSQC	PDGYFHQEGS	CTECHPTCRQ
730	740	750	760	770	780	790	800
CHGPLESDCI	SCYPHISLTN	GNCRTSCREE	QFLNLVGYCA	DCHHLCQHCA	ADLHNTGSIC	LRCQNAHYLL	LGDHCVPDCP
810	820	830	840	850	860	870	880
SGYYAERGAC	KKCHSSCRTC	QGRGPFSCSS	CDTNLVLSHT	GTCSTTCFPG	HYLDDNHVCQ	PCNTHCGSCD	SQASCTSCRD
890	900	910	920	930	940	950	960
PNKVLLFGEC	QYESCAPQYY	LDFSTNTCKE	CDWSCSACSG	PLKTDCLQCM	DGYVLQDGAC	VEQCLSSFYQ	DSGLCKNCDS
970	980	990	1000	1010	1020	1030	1040
YCLQCQGPHE	CTRCKGPFLL	LEAQCVQECG	KGYFADHAKH	KCTACPQGCL	QCSHRDRCHL	CDHGFFLKSG	LCVYNCVPGF
1050	1060	1070	1080	1090	1100	1110	1120
SVHTSNETCS	GKIHTPSLHV	NGSLILPIGS	IKPLDFSLLN	VQDQEGRVED	LLFHVVSTPT	NGQLVLSRNG	KEVQLDKAGR
1130	1140	1150	1160	1170	1180	1190	1200
FSWKDVNEKK	VRFVHSKEKL	RKGYLFLKIS	DQQFFSEPQL	INIQAFSTQA	PYVLRNEVLH	ISRGERATIT	TQMLDIRDDD
1210	1220	1230	1240	1250	1260	1270	1280
NPQDVVIEII	DPPLHGQLLQ	TLQSPATPIY	QFQLDELSRG	LLHYAHDGSD	STSDVAVLQA	NDGHSFHNIL	FQVKTVPQND
1290	1300	1310	1320	1330	1340	1350	1360
RGLQLVANSM	VWVPEGGMLQ	ITNRILQAEA	PGASAEEIIY	KITQDYPQFG	EVVLLVNMPA	DSPADEGQHL	PDGRTATPTS
1370	1380	1390	1400	1410	1420	1430	1440
TFTQQDINEG	IVWYRHSGAP	AQSDSFRFEV	SSASNAQTRL	ESHMFNIAIL	PQTPEAPKVS	LEASLHMTAR	EDGLTVIQPH
1450	1460	1470	1480	1490	1500	1510	1520
SLSFINSEKP	SGKIVYNITL	PLHPNQGIIE	HRDHPHSPIR	YFTQEDINQG	KVMYRPPPAA	PHLQELMAFS	FAGLPESVKF
1530	1540	1550	1560	1570	1580	1590	1600
HFTVSDGEHT	SPEMVLTIHL	LPSDQQLPVF	QVTAPRLAVS	PGGSTSVGLQ	VVVRDAETAP	KELFFELRRP	PQHGVLLKHT
1610	1620	1630	1640	1650	1660	1670	1680
AEFRRPMATG	DTFTYEDVEK	NALQYIHDGS	STREDSMEIS	VTDGLTVTML	EVRVEVSLSE	DRGPRLAAGS	SLSITVASKS
1690	1700	1710	1720	1730	1740	1750	1760
TAIITRSHLA	YVDDSSPDPE	IWIQLNYLPS	YGTLLRISGS	EVEELSEVSN	FTMEDINNKK	IRYSAVFETD	GHLVTDSFYF
1770	1780	1790	1800	1810	1820	1830	1840
SVSDMDHNHL	DNQIFTIMIT	PAENPPPVIA	FADLITVDEG	GRAPLSFHHF	FATDDDDNLQ	RDAIIKLSAL	PKYGCIENTG
1850	1860	1870	1880	1890	1900	1910	1920
TGDRFGPETA	SDLEASFPIQ	DVLENYIYYF	QSVHESIEPT	HDIFSFYVSD	GTSRSEIHSI	NITIERKNDE	PPRMTLQPLR
1930	1940	1950	1960	1970	1980	1990	2000
VQLSSGVVIS	NSSLSLQDLD	TPDNELIFVL	TKKPDHGHVL	WRQTASEPLE	NGRVLVQGST	FTYQDILAGL	VGYVPSVPGM
2010	2020	2030	2040	2050	2060	2070	2080
VVDEFQFSLT	DGLHVDTGRM	KIYTELPASD	TPHLAINQGL	QLSAGSVARI	TEQHLKVTDI	DSDDHQVMYI	MKEDPGAGRL
2090	2100	2110	2120	2130	2140	2150	2160
QMMKHGNLEQ	ISIKGPIRSF	TQADISQGHV	EYSHGTGEPG	GSFAFKFDVV	DGEGNRLIDK	SFSISILEDK	SPPVITTNKG
2170	2180	2190	2200	2210	2220	2230	2240

Detailed Protein Report

Cmpd.	No. of Cmpds.	m/z meas.	Δ m/z [ppm]	z	Rt [min]	Score	P	Range	Sequence	Modification
2609	1	1176.3241	-122.97	2	60.9	16.9	1	605-627	R.CKACHSSCLACMGPAPSHCTGCK.K	Carbamidomethyl: 1
30	1	1096.9812	-111.34	2	29.6	16.6	2	2080-2098	R.LQMMKHGNLEQISIKGPIR.S	

Detailed Protein Report

Protein 124: PREDICTED: collagen alpha-1(XIV) chain isoform X1 [Homo sapiens]

**Accession:**	gi|530389313	**Score:**	48.5
**Database:**	refseq_human(refseq_human_20140103.fasta)	**MW [kDa]:**	191.8
**Database Date:**	2015-11-30	**pI:**	5.1
		**Sequence Coverage [%]:**	2.7
		**No. of unique Peptides:**	3

Quantitation

mdown:qdown	**Median:**	0.60	**CV:**	0.00 %	**No. of Peptides:**	1
Wdown:Qdown	**Median:**	0.46	**CV:**	0.00 %	**No. of Peptides:**	1

Detailed Protein Report

10	20	30	40	50	60	70	80
MKIFQRKMRY	WLLPPFLAIV	YFCTIVQGQV	APPTRLRYNV	ISHDSIQISW	KAPRGKFGGY	KLLVTPTSGG	KTNQLNLQNT
90	100	110	120	130	140	150	160
ATKAIIQGLM	PDQNYTVQII	AYNKDKESKP	AQGQFRIKDL	EKRKDPKPRV	KVVDRGNGSR	PSSPEEVKFV	CQTPAIADIV
170	180	190	200	210	220	230	240
ILVDGSWSIG	RFNFRLVRHF	LENLVTAFDV	GSEKTRIGLA	QYSGDPRIEW	HLNAFSTKDE	VIEAVRNLPY	KGGNTLTGLA
250	260	270	280	290	300	310	320
LNYIFENSFK	PEAGSRTGVS	KIGILITDGK	SQDDIIPPSR	NLRESGVELF	AIGVKNADVN	ELQEIASEPD	STHVYNVAEF
330	340	350	360	370	380	390	400
DLMHTVVESL	TRTLCSRVEE	QDREIKASAH	AITGPPTELI	TSEVTARSFM	VNWTHAPGNV	EKYRVVYYPT	RGGKPDEVVV
410	420	430	440	450	460	470	480
DGTVSSTVLK	NLMSLTEYQI	AVFAIYAHTA	SEGLRGTETT	LALPMASDLL	LYDVTENSMR	VKWDAVPGAS	GYLILYAPLT
490	500	510	520	530	540	550	560
EGLAGDEKEM	KIGETHTDIE	LSGLLPNTEY	TVTVYAMFGE	EASDPVTGQE	TTLALSPPRN	LRISNVGSNS	ARLTWDPTSR
570	580	590	600	610	620	630	640
QINGYRIVYN	NADGTEINEV	EVDPITTFPL	KGLTPLTEYT	IAIFSIYDEG	QSEPLTGVFT	TEEVPAQQYL	EIDEVTTDSF
650	660	670	680	690	700	710	720
RVTWHPLSAD	EGLHKLMWIP	VYGGKTEEVV	LKEEQDSHVI	EGLEPGTEYE	VSLLAVLDDG	SESEVVTAVG	TTLDSFWTEP
730	740	750	760	770	780	790	800
ATTIVPTTSV	TSVFQTGIRN	LVVGDETTSS	LRVKWDISDS	DVQQFRVTYM	TAQGDPEEEV	IGTVMVPGSQ	NNLLLKPLLP
810	820	830	840	850	860	870	880
DTEYKVTVTP	IYTDGEGVSV	SAPGKTLPSS	GPQNLRVSEE	WYNRLRITWD	PPSSPVKGYR	IVYKPVSVPG	PTLETFVGAD
890	900	910	920	930	940	950	960
INTILITNLL	SGMDYNVKIF	ASQASGFSDA	LTGMVKTLFL	GVTNLQAKHV	EMTSLCAHWQ	VHRHATAYRV	VIESLQDRQK
970	980	990	1000	1010	1020	1030	1040
QESTVGGGTT	RHCFYGLQPD	SEYKISVYTK	LQEIEGPSVS	IMEKTQSLPT	RPPTFPPTIP	PAKEVCKAAK	ADLVFMVDGS
1050	1060	1070	1080	1090	1100	1110	1120
WSIGDENFNK	IISFLYSTVG	ALNKIGTDGT	QVAMVQFTDD	PRTEFKLNAY	KTKETLLDAI	KHISYKGGNT	KTGKAIKYVR
1130	1140	1150	1160	1170	1180	1190	1200
DTLFTAESGT	RRGIPKVIVV	ITDGRSQDDV	NKISREMQLD	GYSIFAIGVA	DADYSELVSI	GSKPSARHVF	FVDDFDAFKK
1210	1220	1230	1240	1250	1260	1270	1280
IEDELITFVC	ETASATCPVV	HKDGIDLAGF	KMMEMFGLVE	KDFSSVEGVS	MEPGTFNVFP	CYQLHKDALV	SQPTRYLHPE
1290	1300	1310	1320	1330	1340	1350	1360
GLPSDYTISF	LFRILPDTPQ	EPFALWEILN	KNSDPLVGVI	LDNGGKTLTY	FNYDQSGDFQ	TVTFEGPEIR	KIFYGSFHKL
1370	1380	1390	1400	1410	1420	1430	1440
HIVVSETLVK	VVIDCKQVGE	KAMNASANIT	SDGVEVLGKM	VRSRGPGGNS	APFQLQMFDI	VCSTSWANTD	KCCELPGLRD
1450	1460	1470	1480	1490	1500	1510	1520
DESCPDLPHS	CSCSETNEVA	LGPAGPPGGP	GLRGPKGQQG	EPGPKGPDGP	RGEIGLPGPQ	GPPGPQGPSG	LSIQGMPGMP
1530	1540	1550	1560	1570	1580	1590	1600
GEKGEKGDTG	LPGPQGIPGG	VGSPGRDGSP	GQRGLPGKDG	SSGPPGPPGP	IGIPGTPGVP	GITGSMGPQG	ALGPPGVPGA
1610	1620	1630	1640	1650	1660	1670	1680
KGERGERGDL	QSQAMVRSVA	RQVCEQLIQS	HMARYTAILN	QIPSHSSSIR	TVQGPPGEPG	RPGSPGAPGE	QGPPGTPGFP
1690	1700	1710	1720	1730	1740	1750	1760
GNAGVPGTPG	ERGLTGIKGE	KGNPGVGTQG	PRGPPGPAGP	SGESRPGSPG	PPGSPGPRGP	PGHLGVPGPQ	GPSGQPGYCD
1770	1780	1790					
PSSCSAYGVR	DLIPYNDYQH						

Cmpd.	No. of Cmpds.	m/z meas.	Δ m/z [ppm]	z	Rt [min]	Score	P	Range	Sequence	Modification	Ratios
1886	10	673.2683	-119.28	2	53.6	15.8	0	208-218	R.IEWHLNAFSTK.D		
64	1	809.3817	-76.89	2	30.0	10.1	1	740-754	R.NLVVGDETTSSLRVK.W		mdown:qdown 0.60<br>Wdown:Qdown 0.46
2197	1	585.1748	-243.43	2	57.7	10.6	0	826-836	K.TLPSSGPQNLR.V		

Detailed Protein Report

Protein 125: PREDICTED: A-kinase anchor protein 9 isoform X3 [Homo sapiens]

**Accession:**	gi|578813854	**Score:**	48.3
**Database:**	refseq_human(refseq_human_20140103.fasta)	**MW [kDa]:**	447.3
**Database Date:**	2015-11-30	**pI:**	4.8
**Modification(s):**	Oxidation	**Sequence Coverage [%]:**	0.8
		**No. of unique Peptides:**	2

Detailed Protein Report

10	20	30	40	50	60	70	80
MEDEERQKKL	EAGKAKLAQF	RQRKAQSDGQ	SPSKKQKKKR	KTSSSKHDVS	AHHDLNIDQS	QCNEMYINSS	QRVESTVIPE
90	100	110	120	130	140	150	160
STIMRTLHSG	EITSHEQGFS	VELESEISTT	ADDCSSEVNG	CSFVMRTGKP	TNLLREEEFG	VDDSYSEQGA	QDSPTHLEMM
170	180	190	200	210	220	230	240
ESELAGKQHE	IEELNRELEE	MRVTYGTEGL	QQLQEFEAAI	KQRDGIITQL	TANLQQARRE	KDETMREFLE	LTEQSQKLQI
250	260	270	280	290	300	310	320
QFQQLQASET	LRNSTHSSTA	ADLLQAKQQI	LTHQQQLEEQ	DHLLEDYQKK	KEDFTMQISF	LQEKIKVYEM	EQDKKVENSN
330	340	350	360	370	380	390	400
KEEIQEKETI	IEELNTKIIE	EEKKTLELKD	KLTTADKLLG	ELQEQIVQKN	QEIKNMKLEL	TNSKQKERQS	SEEIKQLMGT
410	420	430	440	450	460	470	480
VEELQKRNHK	DSQFETDIVQ	RMEQETQRKL	EQLRAELDEM	YGQQIVQMKQ	ELIRQHMAQM	EEMKTRHKGE	MENALRSYSN
490	500	510	520	530	540	550	560
ITVNEDQIKL	MNVAINELNI	KLQDTNSQKE	KLKEELGLIL	EEKCALQRQL	EDLVEELSFS	REQIQRARQT	IAEQESKLNE
570	580	590	600	610	620	630	640
AHKSLSTVED	LKAEIVSASE	SRKELELKHE	AEVTNYKIKL	EMLEKEKNAV	LDRMAESQEA	ELERLRTQLL	FSHEEELSKL
650	660	670	680	690	700	710	720
KEDLEIEHRI	NIEKLKDNLG	IHYKQQIDGL	QNEMSQKIET	MQFEKDNLIT	KQNQLILEIS	KLKDLQQSLV	NSKSEEMTLQ
730	740	750	760	770	780	790	800
INELQKEIEI	LRQEEKEKGT	LEQEVQELQL	KTELLEKQMK	EKENDLQEKF	AQLEAENSIL	KDEKKTLEDM	LKIHTPVSQE
810	820	830	840	850	860	870	880
ERLIFLDSIK	SKSKDSVWEK	EIEILIEENE	DLKQQCIQLN	EEIEKQRNTF	SFAEKNFEVN	YQELQEEYAC	LLKVKDDLED
890	900	910	920	930	940	950	960
SKNKQELEYK	SKLKALNEEL	HLQRINPTTV	KMKSSVFDED	KTFVAETLEM	GEVVEKDTTE	LMEKLEVTKR	EKLELSQRLS
970	980	990	1000	1010	1020	1030	1040
DLSEQLKQKH	GEISFLNEEV	KSLKQEKEQV	SLRCRELEII	INHNRAENVQ	SCDTQVSSLL	DGVVTMTSRG	AEGSVSKVNK
1050	1060	1070	1080	1090	1100	1110	1120
SFGEESKIMV	EDKVSFENMT	VGEESKQEQL	ILDHLPSVTK	ESSLRATQPS	ENDKLQKELN	VLKSEQNDLR	LQMEAQRICL
1130	1140	1150	1160	1170	1180	1190	1200
SLVYSTHVDQ	VREYMENEKD	KALCSLKEEL	IFAQEEKIKE	LQKIHQLELQ	TMKTQETGDE	GKPLHLLIGK	LQKAVSEECS
1210	1220	1230	1240	1250	1260	1270	1280
YFLQTLCSVL	GEYYTPALKC	EVNAEDKENS	GDYISENEDP	ELQDYRYEVQ	DFQENMHTLL	NKVTEEYNKL	LVLQTRLSKI
1290	1300	1310	1320	1330	1340	1350	1360
WGQQTDGMKL	EFGEENLPKE	ETEFLSIHSQ	MTNLEDIDVN	HKSKLSSLQD	LEKTKLEEQV	QELESLISSL	QQQLKETEQN
1370	1380	1390	1400	1410	1420	1430	1440
YEAEIHCLQK	RLQAVSESTV	PPSLPVDSVV	ITESDAQRTM	YPGSCVKKNI	DGTIEFSGEF	GVKEETNIVK	LLEKQYQEQL
1450	1460	1470	1480	1490	1500	1510	1520
EEEVAKVIVS	MSIAFAQQTE	LSRISGGKEN	TASSKQAHAV	CQQEQHYFNE	MKLSQDQIGF	QTFETVDVKF	KEEFKPLSKE
1530	1540	1550	1560	1570	1580	1590	1600
LGEHGKEILL	SNSDPHDIPE	SKDCVLTISE	EMFSKDKTFI	VRQSIHDEIS	VSSMDASRQL	MLNEEQLEDM	RQELVRQYQE
1610	1620	1630	1640	1650	1660	1670	1680
HQQATELLRQ	AHMRQMERQR	EDQEQLQEEI	KRLNRQLAQN	GNENQGEVEE	QTFKEKELDR	KPEDVPPEIL	SNERYALQKA
1690	1700	1710	1720	1730	1740	1750	1760
NNRLLKILLE	VVKTTAAVEE	TIGRHVLGIL	DRSSKSQSSA	SLIWRSEAEA	SVKSCVHEEH	TRVTDESIPS	YSGSDMPRND
1770	1780	1790	1800	1810	1820	1830	1840
INMWSKVTEE	GTELSQRLVR	SGFAGTEIDP	ENEELMLNIS	SRLQAAVEKL	LEAISETSSQ	LEHAKVTQTE	LMRESFRQKQ
1850	1860	1870	1880	1890	1900	1910	1920
EATESLKCQE	ELRERLHEES	RAREQLAVEL	SKAEGVIDGY	ADEKTLFERQ	IQEKTDIIDR	LEQELLCASN	RLQELEAEQQ
1930	1940	1950	1960	1970	1980	1990	2000
QIQEERELLS	RQKEAMKAEA	GPVEQQLLQE	TEKLMKEKLE	VQCQAEKVRD	DLQKQVKALE	IDVEEQVSRF	IELEQEKNTE
2010	2020	2030	2040	2050	2060	2070	2080
LMDLRQQNQA	LEKQLEKMRK	FLDEQAIDRE	HERDVFQQEI	QKLEQQLKVV	PRFQPISEHQ	TREVEQLANH	LKEKTDKCSE
2090	2100	2110	2120	2130	2140	2150	2160
LLLSKEQLQR	DIQERNEEIE	KLEFRVRELE	QALLVSADTF	QKVEDRKHFG	AVEAKPELSL	EVQLQAERDA	IDRKEKEITN
2170	2180	2190	2200	2210	2220	2230	2240

Detailed Protein Report

Cmpd.	No. of Cmpds.	m/z meas.	Δ m/z [ppm]	z	Rt [min]	Score	P	Range	Sequence	Modification
27	4	768.2815	-117.99	2	29.6	16.0	2	758-769	K.QMKEKENDLQEK.F	Oxidation: 2
49	1	819.8727	-87.95	2	29.3	12.5	1	3738-3751	R.WHRVTGSVSININR.D	

Detailed Protein Report

Protein 126: phospholipase B1, membrane-associated isoform 2 precursor [Homo sapiens]

**Accession:**	gi|283436114	**Score:**	48.1
**Database:**	refseq_human(refseq_human_20140103.fasta)	**MW [kDa]:**	161.6
**Database Date:**	2015-11-30	**pI:**	5.5
**Modification(s):**	Carbamidomethyl	**Sequence Coverage [%]:**	3.0
		**No. of unique Peptides:**	3

10	20	30	40	50	60	70	80
MGLRPGIFLL	ELLLLLGQGT	PQIHTSPRKS	TLEGQLWPET	LKNSPFPCNP	NKLGVNMPSK	SVHSLKPSDI	KFVAAIGNLE
90	100	110	120	130	140	150	160
IPPDPGTGDL	EKQDWTERPQ	QVCMGVMTVL	SDIIRYFSPS	VPMPVCHTGK	RVIPHDGAED	LWIQAQELVR	NMKENLQLDF
170	180	190	200	210	220	230	240
QFDWKLINVF	FSNASQCYLC	PSAQQAPSLS	TVLLSQNGLA	AGGVDELMGV	LDYLQQEVPR	AFVNLVDLSE	VAEVSRQYHG
250	260	270	280	290	300	310	320
TWLSPAPEPC	NCSEETTRLA	KVVMQWSYQE	AWNSLLASSR	YSEQESFTVV	FQPFFYETTP	SLHSEDPRLQ	DSTTLAWHLW
330	340	350	360	370	380	390	400
NRMMEPAGEK	DEPLSVKHGR	PMKCPSQESP	YLFSYRNSNY	LTRLQKPQDK	LEVREGAEIR	CPDKDPSDTV	PTSVHRLKPA
410	420	430	440	450	460	470	480
DINVIGALGD	SLTAGNGAGS	TPGNVLDVLT	QYRGLSWSVG	GDENIGTVTT	LANILREFNP	SLKGFSVGTG	KETSPNAFLN
490	500	510	520	530	540	550	560
QAVAGGRAED	LPVQARRLVD	LMKNDTRIHF	QEDWKIITLF	IGGNDLCDFC	NDLVPRAFVN	LVTVLEIVNL	RELYQEKKVY
570	580	590	600	610	620	630	640
CPRMILRSLC	PCVLKFDDNS	TELATLIEFN	KKFQEKTHQL	IESGRYDTRE	DFTVVVQPFF	ENVDMPKTSE	GLPDNSFFAP
650	660	670	680	690	700	710	720
DCFHFSSKSH	SRAASALWNN	MLEPVGQKTT	RHKFENKINI	TCPNQVQPFL	RTYKNSMQGH	GTWLPCRDRA	PSALHPTSVH
730	740	750	760	770	780	790	800
ALRPADIQVV	AALGDSLTAG	NGIGSKPDDL	PDVTTQYRGL	SYSAGGDGSL	ENVTTLPNIL	REFNRNLTGY	AVGTGDANDT
810	820	830	840	850	860	870	880
NAFLNQAVPG	AKAEDLMSQV	QTLMQKMKDD	HRVNFHEDWK	VITVLIGGSD	LCDYCTDSNL	YSAANFVHHL	RNALDVLHRE
890	900	910	920	930	940	950	960
VPRVLVNLVD	FLNPTIMRQV	FLGNPDKCPV	QQASVLCNCV	LTLRENSQEL	ARLEAFSRAY	RSSMRELVGS	GRYDTQEDFS
970	980	990	1000	1010	1020	1030	1040
VVLQPFFQNI	QLPVLADGLP	DTSFFAPDCI	HPNQKFHSQL	ARALWTNMLE	PLGSKTETLD	LRAEMPITCP	TQNEPFLRTP
1050	1060	1070	1080	1090	1100	1110	1120
RNSNYTYPIK	PAIENWGSDF	LCTEWKASNS	VPTSVHQLRP	ADIKVVAALG	DSLTTAVGAR	PNNSSDLPTS	WRGLSWSIGG
1130	1140	1150	1160	1170	1180	1190	1200
DGNLETHTTL	PNILKKFNPY	LLGFSTSTWE	GTAGLNVAAE	GARARDMPAQ	AWDLVERMKN	SPDINLEKDW	KLVTLFIGVN
1210	1220	1230	1240	1250	1260	1270	1280
DLCHYCENPE	AHLATEYVQH	IQQALDILSE	ELPRAFVNVV	EVMELASLYQ	GQGGKCAMLA	AQNNCTCLRH	SQSSLEKQEL
1290	1300	1310	1320	1330	1340	1350	1360
KKVNWNLQHG	ISSFSYWHQY	TQREDFAVVV	QPFFQNTLTP	LNERGDTDLT	FFSEDCFHFS	DRGHAEMAIA	LWNNMLEPVG
1370	1380	1390	1400	1410	1420	1430	1440
RKTTSNNFTH	SRAKLKCPSP	ESPYLYTLRN	SRLLPDQAEE	APEVLYWAVP	VAAGVGLVVG	IIGTVVWRCR	RGGRREDPPM
1450							
SLRTVAL							

Cmpd.	No. of Cmpds.	m/z meas.	Δ m/z [ppm]	z	Rt [min]	Score	P	Range	Sequence	Modification
428	3	587.8118	72.68	2	34.7	11.5	0	43-52	K.NSPFPCNPNK.L	Carbamidomethyl: 6
413	1	587.7939	24.16	2	34.5	11.6	1	375-384	R.EGAEIRCPDK.D	Carbamidomethyl: 7
2292	1	816.2731	-168.26	2	58.9	11.1	0	472-487	K.ETSPNAFLNQAVAGGR.A	

Detailed Protein Report

Protein 127: **eukaryotic translation initiation factor 4 gamma 3 isoform 1 [Homo sapiens]**

**Accession:**	gi|311771714	**Score:**	48.0
**Database:**	refseq_human(refseq_human_20140103.fasta)	**MW [kDa]:**	180.4
**Database Date:**	2015-11-30	**pI:**	5.2
**Modification(s):**	Carbamidomethyl, Oxidation	**Sequence Coverage [%]:**	2.8
		**No. of unique Peptides:**	3

10	20	30	40	50	60	70	80
MNSQPQTRSP	FFQRPQIQPP	RATIPNSSPS	IRPGAQTPTA	VYQANQHIMM	VNHLPMPYPV	PQGPQYCIPQ	YRHSGPPYVG
90	100	110	120	130	140	150	160
PPQQYPVQPP	GPGPFYPGPG	PGDFPNAYGT	PFYPSQPVYQ	SAPIIVPTQQ	QPPPAKREKK	TIRIRDPNQG	GKDITEEIMS
170	180	190	200	210	220	230	240
GGGSRNPTPP	IGRPTSTPTP	PQLPSQVPEH	SPVVYGTVES	AHLAASTPVT	AASDQKQEEK	PKPDPVLKSP	SPVLRLVLSG
250	260	270	280	290	300	310	320
EKKEQEGQTS	ETTAIVSIAE	LPLPPSPTTV	SSVARSTIAA	PTSSALSSQP	IFTTAIDDRC	ELSSPREDTI	PIPSLTSCTE
330	340	350	360	370	380	390	400
TSDPLPTNEN	DDDICKKPCS	VAPNDIPLVS	STNLINEING	VSEKLSATES	IVEIVKQEVL	PLTLELEILE	NPPEEMKLEC
410	420	430	440	450	460	470	480
IPAPITPSTV	PSFPPTPPTP	PASPPHTPVI	VPAAATTVSS	PSAAITVQRV	LEEDESIRTC	LSEDAKEIQN	KIEVEADGQT
490	500	510	520	530	540	550	560
EEILDSQNLN	SRRSPVPAQI	AITVPKTWKK	PKDRTRTTEE	MLEAELELKA	EEELSIDKVL	ESEQDKMSQG	FHPERDPSDL
570	580	590	600	610	620	630	640
KKVKAVEENG	EEAEPVRNGA	ESVSEGEGID	ANSGSTDSSG	DGVTFPFKPE	SWKPTDTEGK	KQYDREFLLD	FQFMPACIQK
650	660	670	680	690	700	710	720
PEGLPPISDV	VLDKINQPKL	PMRTLDPRIL	PRGPDFTPAF	ADFGRQTPGG	RGVPICKVQS	RHGLPILEQS	KAPTCPPLVM
730	740	750	760	770	780	790	800
SHPPMKSLPL	GLLNVGSRRS	QPGQRREPRK	IITVSVKEDV	HLKKAENAWK	PSQKRDSQAD	DPENIKTQEL	FRKVRSILNK
810	820	830	840	850	860	870	880
LTPQMFNQLM	KQVSGLTVDT	EERLKGVIDL	VFEKAIDEPS	FSVAYANMCR	CLVTLKVPMA	DKPGNTVNFR	KLLLNRCQKE
890	900	910	920	930	940	950	960
FEKDKADDDV	FEKKQKELEA	ASAPEERTRL	HDELEEAKDK	ARRRSIGNIK	FIGELFKLKM	LTEAIMHDCV	VKLLKNHDEE
970	980	990	1000	1010	1020	1030	1040
SLECLCRLLT	TIGKDLDFEK	AKPRMDQYFN	QMEKIVKERK	TSSRIRFMLQ	DVIDLRLCNW	VSRRADQGPK	TIEQIHKEAK
1050	1060	1070	1080	1090	1100	1110	1120
IEEQEEQRKV	QQLMTKEKRR	PGVQRVDEGG	WNTVQGAKNS	RVLDPSKFLK	ITKPTIDEKI	QLVPKAQLGS	WGKGSSGGAK
1130	1140	1150	1160	1170	1180	1190	1200
ASETDALRSS	ASSLNRFSAL	QPPAPSGSTP	STPVEFDSRR	TLTSRGSMGR	EKNDKPLPSA	TARPNTFMRG	GSSKDLLDNQ
1210	1220	1230	1240	1250	1260	1270	1280
SQEEQRREML	ETVKQLTGGV	DVERNSTEAE	RNKTRESAKP	EISAMSAHDK	AALSEEELER	KSKSIIDEFL	HINDFKEAMQ
1290	1300	1310	1320	1330	1340	1350	1360
CVEELNAQGL	LHVFVRVGVE	STLERSQITR	DHMGQLLYQL	VQSEKLSKQD	FFKGFSETLE	LADDMAIDIP	HIWLYLAELV
1370	1380	1390	1400	1410	1420	1430	1440
TPMLKEGGIS	MRELTIEFSK	PLLPVGRAGV	LLSEILHLLC	KQMSHKKVGA	LWREADLSWK	DFLPEGEDVH	NFLLEQKLDF
1450	1460	1470	1480	1490	1500	1510	1520
IESDSPCSSE	ALSKKELSAE	ELYKRLEKLI	IEDKANDEQI	FDWVEANLDE	IQMSSPTFLR	ALMTAVCKAA	IIADSSTFRV
1530	1540	1550	1560	1570	1580	1590	1600
DTAVIKQRVP	ILLKYLDSDT	EKELQALYAL	QASIVKLDQP	ANLLRMFFDC	LYDEEVISED	AFYKWESSKD	PAEQNGKGVA
1610	1620	1630					
LKSVTAFFTW	LREAEEESED	N					

Cmpd.	No. of Cmpds.	m/z meas.	Δ m/z [ppm]	z	Rt [min]	Score	P	Range	Sequence	Modification
2704	1	847.8687	-44.85	2	65.0	19.4	0	712-726	K.APTCPPLVMSHPPMK.S	Carbamidomethyl: 4; Oxidation: 9, 14
1921	1	781.2702	-157.47	2	52.7	13.1	0	857-870	K.VPMADKPGNTVNFR.K	Oxidation: 3
2882	1	938.5029	13.03	2	64.8	15.4	1	940-955	K.MLTEAIMHDCVVKLLK.N	Oxidation: 1, 7

Detailed Protein Report

Protein 128: transcription factor HIVEP3 isoform b [Homo sapiens]

**Accession:**	gi|189181750	**Score:**	47.9
**Database:**	refseq_human(refseq_human_20140103.fasta)	**MW [kDa]:**	259.2
**Database Date:**	2015-11-30	**pI:**	8.6
**Modification(s):**	Carbamidomethyl	**Sequence Coverage [%]:**	1.8
		**No. of unique Peptides:**	2

Quantitation

mdown:qdown	**Median:** 0.97	**CV:** 0.00 %	**No. of Peptides:** 1

10	20	30	40	50	60	70	80
MDPEQSVKGT	KKAEGSPRKR	LTKGEAIQTS	VSSSVPYPGS	GTAATQESPA	QELLAPQPFP	GPSSVLREGS	QEKTGQQQKP
90	100	110	120	130	140	150	160
PKRPPIEASV	HISQLPQHPL	TPAFMSPGKP	EHLLEGSTWQ	LVDPMRPGPS	GSFVAPGLHP	QSQLLPSHAS	IIPPEDLPGV
170	180	190	200	210	220	230	240
PKVFVPRPSQ	VSLKPTEEAH	KKERKPQKPG	KYICQYCSRP	CAKPSVLQKH	IRSHTGERPY	PCGPCGFSFK	TKSNLYKHRK
250	260	270	280	290	300	310	320
SHAHRIKAGL	ASGMGGEMYP	HGLEMERIPG	EEFEEPTEGE	STDSEEETSA	TSGHPAELSP	RPKQPLLSSG	LYSSGSHSSS
330	340	350	360	370	380	390	400
HERCSLSQSS	TAQSLEDPPP	FVEPSSEHPL	SHKPEDTHTI	KQKLALRLSE	RKKVIDEQAF	LSPGSKGSTE	SGYFSRSESA
410	420	430	440	450	460	470	480
EQQVSPPNTN	AKSYAEIIFG	KCGRIGQRTA	MLTATSTQPL	LPLSTEDKPS	LVPLSVPRTQ	VIEHITKLIT	INEAVVDTSE
490	500	510	520	530	540	550	560
IDSVKPRRSS	LSRRSSMESP	KSSLYREPLS	SHSEKTKPEQ	SLLSLQHPPS	TAPPVPLLRS	HSMPSAACTI	STPHHPFRGS
570	580	590	600	610	620	630	640
YSFDDHITDS	EALSHSSHVF	TSHPRMLKRQ	PAIELPLGGE	YSSEEPGPSS	KDTASKPSDE	VEPKESELTK	KTKKGLKTKG
650	660	670	680	690	700	710	720
VIYECNICGA	RYKKRDNYEA	HKKYYCSELQ	IAKPISAGTH	TSPEAEKSQI	EHEPWSQMMH	YKLGTTLELT	PLRKRRKEKS
730	740	750	760	770	780	790	800
LGDEEEPPAF	ESTKSQFGSP	GPSDAARNLP	LESTKSPAEP	SKSVPSLEGP	TGFQPRTPKP	GSGSESGKER	RTTSKEISVI
810	820	830	840	850	860	870	880
QHTSSFEKSD	SLEQPSGLEG	EDKPLAQFPS	PPPAPHGRSA	HSLQPKLVRQ	PNIQVPEILV	TEEPDRPDTE	PEPPPKEPEK
890	900	910	920	930	940	950	960
TEEFQWPQRS	QTLAQLPAEK	LPPKKKRLRL	AEMAQSSGES	SFESSVPLSR	SPSQESNVSL	SGSSRSASFE	RDDHGKAEAP
970	980	990	1000	1010	1020	1030	1040
SPSSDMRPKP	LGTHMLTVPS	HHPHAREMRR	SASEQSPNVS	HSAHMTETRS	KSFDYGSLSL	TGPSAPAPVA	PPARVAPPER
1050	1060	1070	1080	1090	1100	1110	1120
RKCFLVRQAS	LSRPPESELE	VAPKGRQESE	EPQPSSSKPS	AKSSLSQISS	AATSHGGPPG	GKGPGQDRPP	LGPTVPYTEA
1130	1140	1150	1160	1170	1180	1190	1200
LQVFHHPVAQ	TPLHEKPYLP	PPVSLFSFQH	LVQHEPGQSP	EFFSTQAMSS	LLSSPYSMPP	LPPSLFQAPP	LPLQPTVLHP
1210	1220	1230	1240	1250	1260	1270	1280
GQLHLPQLMP	HPANIPFRQP	PSFLPMPYPT	SSALSSGFFL	PLQSQFALQL	PGDVESHLPQ	IKTSLAPLAT	GSAGLSPSTE
1290	1300	1310	1320	1330	1340	1350	1360
YSSDIRLPPV	APPASSSAPT	SAPPLALPAC	PDTMVSLVVP	VRVQTNMPSY	GSAMYTTLSQ	ILVTQSQGSS	ATVALPKFEE
1370	1380	1390	1400	1410	1420	1430	1440
PPSKGTTVCG	ADVHEVGPGP	SGLSEEQSRA	FPTPYLRVPV	TLPERKGTSL	SSESILSLEG	SSSTAGGSKR	VLSPAGSLEL
1450	1460	1470	1480	1490	1500	1510	1520
TMETQQQKRV	KEEEASKADE	KLELVKPCSV	VLTSTEDGKR	PEKSHLGNQG	QGRRELEMLS	SLSSDPSDTK	EIPPLPHPAL
1530	1540	1550	1560	1570	1580	1590	1600
SHGTAPGSEA	LKEYPQPSGK	PHRRGLTPLS	VKKEDSKEQP	DLPSLAPPSS	LPLSETSSRP	AKSQEGTDSK	KVLQFPSLHT
1610	1620	1630	1640	1650	1660	1670	1680
TTNVSWCYLN	YIKPNHIQHA	DRRSSVYAGW	CISLYNPNLP	GVSTKAALSL	LRSKQKVSKE	TYTMATAPHP	EAGRLVPSSS
1690	1700	1710	1720	1730	1740	1750	1760
RKPRMTEVHL	PSLVSPEGQK	DLARVEKEEE	RRGEPEEDAP	ASQRGEPARI	KIFEGGYKSN	EEYVYVRGRG	RGKYVCEECG
1770	1780	1790	1800	1810	1820	1830	1840
IRCKKPSMLK	KHIRTHTDVR	PYVCKHCHFA	FKTKGNLTKH	MKSKAHSKKC	QETGVLEELE	AEEGTSDDLF	QDSEGREGSE
1850	1860	1870	1880	1890	1900	1910	1920
AVEEHQFSDL	EDSDSDSDLD	EDEDEDEEES	QDELSRPSSE	APPPGPPHAL	RADSSPILGP	QPPDAPASGT	EATRGSSVSE
1930	1940	1950	1960	1970	1980	1990	2000
AERLTASSCS	MSSQSMPGLP	WLGPAPLGSV	EKDTGSALSY	KPVSPRRPWS	PSKEAGSRPP	LARKHSLTKN	DSSPQRCSPA
2010	2020	2030	2040	2050	2060	2070	2080
REPQASAPSP	PGLHVDPGRG	MGALPCGSPR	LQLSPLTLCP	LGRELAPRAH	VLSKLEGTTD	PGLPRYSPTR	RWSPGQAESP
2090	2100	2110	2120	2130	2140	2150	2160
PRSAPPGKWA	LAGPGSPSAG	EHGPGLGLDP	RVLFPPAPLP	HKLLSRSPET	CASPWKAESR	SPSCSPGPAH	PLSSRPFSAL
2170	2180	2190	2200	2210	2220	2230	2240

Detailed Protein Report

Cmpd.	No. of Cmpds.	m/z meas.	Δ m/z [ppm]	z	Rt [min]	Score	P	Range	Sequence	Modification	Ratios
2516	1	941.4560	-33.22	2	61.9	13.2	2	635-651	K.GLKTKGVIYECNICGAR.Y	Carbamidomethyl: 11	
22	1	657.3901	133.33	2	28.9	15.8	1	1752-1762	R.GKYVCEECGIR.C	Carbamidomethyl: 5	mdown:qdown 0.97

Detailed Protein Report

Protein 129: PREDICTED: uncharacterized protein LOC101929845 [Homo sapiens]

**Accession:**	gi|578845877	**Score:**	47.5
**Database:**	refseq_human(refseq_human_20140103.fasta)	**MW [kDa]:**	90.2
**Database Date:**	2015-11-30	**pI:**	12.2
**Modification(s):**	Carbamidomethyl	**Sequence Coverage [%]:**	9.3
		**No. of unique Peptides:**	2

10	20	30	40	50	60	70	80
MEESAEQAPR	CDETRVGRRR	HSWRNTCGGV	GRAGTAVRRD	AASQSCCPTC	TGVATRRHSW	RRRQSRQTKE	FIFSELLSNL
**90**	**100**	**110**	**120**	**130**	**140**	**150**	**160**
YSRGNEQTLV	EALAEQADQG	VHLLRAAVQP	ALAWEREDTR	GGVGRAGRPR	SSSSQSCCPN	CTRVGTRRHS	WRSRQSRHRG
**170**	**180**	**190**	**200**	**210**	**220**	**230**	**240**
VTRLAWEEED	TPGGVGRAGR	PRSSSSRSCC	PTCTRVGTCR	HSWRRRQSRQ	TKEFIFSELL	SNLYSRGNEK	TLVEASAEQA
**250**	**260**	**270**	**280**	**290**	**300**	**310**	**320**
DQGVHLLRAA	VQPVLAWERE	DTRGGVGRAG	RPRSSSSQSC	CPTCTRVGTR	RHSWRSRPSR	HSGVTRLAWE	EEDTPGGVGR
**330**	**340**	**350**	**360**	**370**	**380**	**390**	**400**
AGRPRSSSSR	SCCPTCTRVG	TCRHLWRSRQ	SRHSGATRCC	FSELLSNLHW	RGNEKTLVEA	LAEQADQGVH	LLRAAVQPVL
**410**	**420**	**430**	**440**	**450**	**460**	**470**	**480**
AWERADTRGG	VGRAGRPRSS	SSQSCCPTCT	RVGTRRHSWR	RRQSRQTKEF	IFSELLSKLH	SRGNEKTLME	ESAEQAQRCD
**490**	**500**	**510**	**520**	**530**	**540**	**550**	**560**
ETRVGRRRHS	WRSRQSRQTK	KFIFSELLSN	LYSRRNLQTL	VEASAEQADQ	GVHLLRAAVQ	PVLAWEREDT	RGGVGRAGRP
**570**	**580**	**590**	**600**	**610**	**620**	**630**	**640**
RSSSSQSCCP	TCTRVGTRRH	SWRRRQSRQT	KEFIFSELLS	NLHSRGNEKT	LMEESAEQAQ	RRDETRVGRR	RHSWRSRQSR
**650**	**660**	**670**	**680**	**690**	**700**	**710**	**720**
QTKEFIFSEL	LSNLYSRRNL	QTLVEESAEQ	AQRCDKMLLL	RAAVQPALAW	EREDTRGGVG	RAGRPRSSSS	QSCCPTCPRV
**730**	**740**	**750**	**760**	**770**	**780**	**790**	**800**
GTRRHSWRRR	QSRQTKEFIF	SELLSNLHSR	GNEKTLVEAS	AEQADQGVHL	LRAAVQTALA	WEREDTHGGV	GRAGTEV

Cmpd.	No. of Cmpds.	m/z meas.	Δ m/z [ppm]	z	Rt [min]	Score	P	Range	Sequence	Modification
2221	1	1032.3950	-68.06	2	56.4	11.4	2	1-18	-.MEESAEQAPRCDETRVGR.R	
48	1	1220.0219	-37.76	2	30.0	11.4	2	33-56	R.AGTAVRRDAASQSCCPTCTGVATR.R	Carbamidomethyl: 14

Detailed Protein Report

Protein 130: mis18-binding protein 1 [Homo sapiens]

Accession: gi|42415492
Database: refseq_human(refseq_human_20140103.fasta)
Database Date: 2015-11-30
Modification(s): Oxidation

Score: 47.5
MW [kDa]: 129.0
pI: 9.9
Sequence Coverage [%]: 5.6
No. of unique Peptides: 3

Alias proteins:

Accession	Name	Description
gi|530404134	refseq_human (refseq_human_20140103.fasta)	PREDICTED: mis18-binding protein 1 isoform X1 [Homo sapiens]

```
        10         20         30         40         50         60         70         80
MIATPLKHSR IYLPPEASSQ RRNLPMDAIF FDSIPSGTLT PVKDLVKYQN SSLKLNDHKK NQFLKMTTFN NKNIFQSTML
        90        100        110        120        130        140        150        160
TEATTSNSSL DISAIKPNKD GLKNKANYES PGKIFLRMKE KVLRDKQEQP SRNSSLLEPQ KSGNNETFTP NRVEKKKLQH
       170        180        190        200        210        220        230        240
TYLCEEKENN KSFQSDDSSL RASVQGVPLE SSNNDIFLPV KQKIQCQQEK KAPLHNLTYE LPTLNQEQEN FLAVEARNKT
       250        260        270        280        290        300        310        320
LTRAQLAKQI FHSKESIVAT TKSKKDTFVL ESVDSADEQF QNTNAETLST NCIPIKNGSL LMVSDSERTT EGTSQQKVKE
       330        340        350        360        370        380        390        400
GNGKTVPGET GLPGSMKDTC KIVLATPRLH ITIPRRSKRN ISKLSPPRIF QTVTNGLKKN QVVQLQEWMI KSINNNTAIC
       410        420        430        440        450        460        470        480
VEGKLIDVTN IYWHSNVIIE RIEHNKLRTI SGNVYILKGM IDQISMKEAG YPNYLIRKFM FGFPENWKEH IDNFLEQLRA
       490        500        510        520        530        540        550        560
GEKNREKTKQ KQKTGRSVRD IRKSMKNDAR ENQTDTAQRA TTTYDFDCDN LELKSNKHSE SPGATELNMC HSNCQNKPTL
       570        580        590        600        610        620        630        640
RFPDDQVNNT IQNGGGDDLS NQELIGKKEY KMSSKKLKIG ERTNERIIKS QKQETTEELD VSIDILTSRE QFFSDEERKY
       650        660        670        680        690        700        710        720
MAINQKKAYI LVTPLKSRKV IEQRCMRYNL SAGTIKAVTD FVIPECQKKS PISKSMGTLE NTFEGHKSKN KEDCDERDLL
       730        740        750        760        770        780        790        800
TVNRKIKISN LEKEQMLTSD FKKNTRLLPK LKKIENQVAM SFYKHQSSPD LSSEESETEK EIKRKAEVKK TKAGNTKEAV
       810        820        830        840        850        860        870        880
VHLRKSTRNT SNIPVILEPE TEESENEFYI KQKKARPSVK ETLQKSGVRK EFPITEAVGS DKTNRHPLEC LPGLIQDKEW
       890        900        910        920        930        940        950        960
NEKELQKLHC AFASLPKHKP GFWSEVAAAV GSRSPEECQR KYMENPRGKG SQKHVTKKKP ANSKGQNGKR GDADQKQTIK
       970        980        990       1000       1010       1020       1030       1040
ITAKVGTLKR KQQMREFLEQ LPKDDHDDFF STTPLQHQRI LLPSFQDSED DDDILPNMDK NPTTPSSVIF PLVKTPQCQH
      1050       1060       1070       1080       1090       1100       1110       1120
VSPGMLGSIN RNDCDKYVFR MQKYHKSNGG IVWGNIKKKL VETDFSTPTP RRKTPFNTDL GENSGIGKLF TNAVESLDEE
      1130       1140
EKDYYFSNSD SA
```

Cmpd.	No. of Cmpds.	m/z meas.	Δ m/z [ppm]	z	Rt [min]	Score	P	Range	Sequence	Modification
1865	1	1057.5367	-22.09	2	52.0	12.7	0	182-201	R.ASVQGVPLESSNNDIFLPVK.Q	
2837	1	868.9237	11.97	2	64.0	11.5	1	325-341	K.TVPGETGLPGSMKDTCK.I	Oxidation: 12
2772	1	733.8764	59.69	2	63.7	13.0	0	695-707	K.SMGTLENTFEGHK.S	Oxidation: 2

Detailed Protein Report

Protein 131: OTU domain-containing protein 7B [Homo sapiens]

**Accession:**	gi|118026942	**Score:**	47.5
**Database:**	refseq_human(refseq_human_20140103.fasta)	**MW [kDa]:**	92.5
**Database Date:**	2015-11-30	**pI:**	6.3
**Modification(s):**	Oxidation	**Sequence Coverage [%]:**	6.2
		**No. of unique Peptides:**	3

Alias proteins:

Accession	Name	Description
gi|578801304	refseq_human (refseq_human_20140103.fasta)	PREDICTED: OTU domain-containing protein 7B isoform X5 [Homo sapiens]
gi|578801302	refseq_human (refseq_human_20140103.fasta)	PREDICTED: OTU domain-containing protein 7B isoform X4 [Homo sapiens]
gi|578801300	refseq_human (refseq_human_20140103.fasta)	PREDICTED: OTU domain-containing protein 7B isoform X3 [Homo sapiens]

10	20	30	40	50	60	70	80
MTLDMDAVLS	DFVRSTGAEP	GLARDLLEGK	NWDVNAALSD	FEQLRQVHAG	NLPPSFSEGS	GGSRTPEKGF	SDREPTRPPR
90	100	110	120	130	140	150	160
PILQRQDDIV	QEKRLSRGIS	HASSSIVSLA	RSHVSSNGGG	GGSNEHPLEM	PICAFQLPDL	TVYNEDFRSF	IERDLIEQSM
170	180	190	200	210	220	230	240
LVALEQAGRL	NWWVSVDPTS	QRLLPLATTG	DGNCLLHAAS	LGMWGFHDRD	LMLRKALYAL	MEKGVEKEAL	KRRWRWQQTQ
250	260	270	280	290	300	310	320
QNKESGLVYT	EDEWQKEWNE	LIKLASSEPR	MHLGTNGANC	GGVESSEEPV	YESLEEFHVF	VLAHVLRRPI	VVVADTMLRD
330	340	350	360	370	380	390	400
SGGEAFAPIP	FGGIYLPLEV	PASQCHRSPL	VLAYDQAHFS	ALVSMEQKEN	TKEQAVIPLT	DSEYKLLPLH	FAVDPGKGWE
410	420	430	440	450	460	470	480
WGKDDSDNVR	LASVILSLEV	KLHLLHSYMN	VKWIPLSSDA	QAPLAQPESP	TASAGDEPRS	TPESGDSDKE	SVGSSSTSNE
490	500	510	520	530	540	550	560
GGRRKEKSKR	DREKDKKRAD	SVANKLGSFG	KTLGSKLKKN	MGGLMHSKGS	KPGGVGTGLG	GSSGTETLEK	KKKNSLKSWK
570	580	590	600	610	620	630	640
GGKEEAAGDG	PVSEKPPAES	VGNGGSKYSQ	EVMQSLSILR	TAMQGEGKFI	FVGTLKMGHR	HQYQEEMIQR	YLSDAEERFL
650	660	670	680	690	700	710	720
AEQKQKEAER	KIMNGGIGGG	PPPAKKPEPD	AREEQPTGPP	AESRAMAFST	GYPGDFTIPR	PSGGGVHCQE	PRRQLAGGPC
730	740	750	760	770	780	790	800
VGGLPPYATF	PRQCPPGRPY	PHQDSIPSLE	PGSHSKDGLH	RGALLPPPYR	VADSYSNGYR	EPPEPDGWAG	GLRGLPPTQT
810	820	830	840	850			
KCKQPNCSFY	GHPETNNFCS	CCYREELRRR	EREPDGELLV	HRF			

Cmpd.	No. of Cmpds.	m/z meas.	Δ m/z [ppm]	z	Rt [min]	Score	P	Range	Sequence	Modification
2718	1	1175.5515	-33.60	2	62.4	11.6	0	348-368	R.SPLVLAYDQAHFSALVSMEQK.E	Oxidation: 18
2130	1	1052.4295	-112.31	2	56.8	17.0	1	529-551	K.GSKPGGVGTGLGGSSGTETLEKK.K	
917	1	491.6624	-132.38	2	40.3	18.8	0	631-638	R.YLSDAEER.F	

Detailed Protein Report

Protein 132: MAP/microtubule affinity-regulating kinase 4 isoform 2 [Homo sapiens]

**Accession:**	gi|33636756	**Score:**	47.4
**Database:**	refseq_human(refseq_human_20140103.fasta)	**MW [kDa]:**	75.3
**Database Date:**	2015-11-30	**pI:**	10.5
		**Sequence Coverage [%]:**	3.8
		**No. of unique Peptides:**	1

Quantitation

mdown:qdown **Median:** 0.40 **CV:** 0.00 % **No. of Peptides:** 1

```
        10         20         30         40         50         60         70         80
MSSRTVLAPG NDRNSDTHGT LGSGRSSDKG PSWSSRSLGA RCRNSIASCP EEQPHVGNYR LLRTIGKGNF AKVKLARHIL
        90        100        110        120        130        140        150        160
TGREVAIKII DKTQLNPSSL QKLFREVRIM KGLNHPNIVK LFEVIETEKT LYLVMEYASA GEVFDYLVSH GRMKEKEARA
       170        180        190        200        210        220        230        240
KFRQIVSAVH YCHQKNIVHR DLKAENLLLD AEANIKIADF GFSNEFTLGS KLDTFCGSPP YAAPELFQGK KYDGPEVDIW
       250        260        270        280        290        300        310        320
SLGVILYTLV SGSLPFDGHN LKELRERVLR GKYRVPFYMS TDCESILRRF LVLNPAKRCT LEQIMKDKWI NIGYEGEELK
       330        340        350        360        370        380        390        400
PYTEPEEDFG DTKRIEVMVG MGYTREEIKE SLTSQKYNEV TATYLLLGRK TEEGGDRGAP GLALARVRAP SDTTNGTSSS
       410        420        430        440        450        460        470        480
KGTSHSKGQR SSSSTYHRQR RHSDFCGPSP APLHPKRSPT STGEAELKEE RLPGRKASCS TAGSGSRGLP PSSPMVSSAH
       490        500        510        520        530        540        550        560
NPNKAEIPER RKDSTSTPNN LPPSMMTRRN TYVCTERPGA ERPSLLPNGK ENSSGTPRVP PASPSSHSLA PPSGERSRLA
       570        580        590        600        610        620        630        640
RGSTIRSTFH GGQVRDRRAG GGGGGGVQNG PPASPTLAHE AAPLPAGRPR PTTNLFTKLT SKLTRRVTLD PSKRQNSNRC
       650        660        670        680        690
VSGASLPQGS KIRSQTNLRE SGDLRSQVAI YLGIKRKPPP GCSDSPGV
```

Cmpd.	No. of Cmpds.	m/z meas.	Δ m/z [ppm]	z	Rt [min]	Score	P	Range	Sequence	Modification	Ratios
1367	1	388.0811	-379.40	2	45.6	16.7	1	106-111	R.EVRIMK.G		mdown:qdown 0.40

Detailed Protein Report

Protein 133: PREDICTED: dmX-like protein 2 isoform X3 [Homo sapiens]

**Accession:**	gi|530405585	**Score:**	47.4
**Database:**	refseq_human(refseq_human_20140103.fasta)	**MW [kDa]:**	291.5
**Database Date:**	2015-11-30	**pI:**	5.9
**Modification(s):**	Carbamidomethyl, Oxidation	**Sequence Coverage [%]:**	1.7
		**No. of unique Peptides:**	2

Detailed Protein Report

10	20	30	40	50	60	70	80
MHLHQVLTGA	VNPGDNCYSV	GSVGDVPFTA	YGSGCDIVIL	ANDFECVQII	PGAKHGNIQV	SCVECSNQQG	RIAASYGNAV
90	100	110	120	130	140	150	160
CIFEPLGINS	HKRNCQLKCQ	WLKTGQFFLS	SVTYNLAWDP	QDNRLLTATD	SIQLWAPPGD	DILEEEEEID	NTVPPVLNDW
170	180	190	200	210	220	230	240
KCVWQCKTSV	SVHLMEWSPD	GEYFATAGKD	DCLLKVWYPM	TGWKSSIIPQ	DHHEVKRRQS	STQFSFVYLA	HPRAVTGFSW
250	260	270	280	290	300	310	320
RKTSKYMPRG	SVCNVLLTSC	HDGVCRLWAE	TLLPEDCLLG	EQICETTTSS	IASSLSHAGR	HKDRIQHALE	TIHHLKNLRK
330	340	350	360	370	380	390	400
GQRRSSVLVT	HAELMPDQTA	MHEVQRHISH	HANALCHFHI	AASINPATDI	PNVLVGTAFN	VDDGNGGFVV	HWLNNKEFHF
410	420	430	440	450	460	470	480
TSSTEVFMHQ	LRKLSDKQVD	HENDDADRED	EEHSQEDRER	GLHMKLDHDL	SLDRESEAGT	GSSEHEDGER	EGSPRTYSRL
490	500	510	520	530	540	550	560
SVPMPLPTVL	LDRKIETLLT	EWNKNPDMLF	TIHPVDGTFL	VWHVKYLDEY	NPGIFRQVQV	SFSSRIPVAF	PSGDASSLSK
570	580	590	600	610	620	630	640
NIMMYACINA	TKDSHHTLLH	QEGMSVGSPH	GSQPHSRSHS	THMNILAPTV	MMISKHIDGS	LNQWAVTFAD	KSAFTTVLTV
650	660	670	680	690	700	710	720
SHKFRYCGHR	FHLNDLACHS	VLPLLLTSSH	HNALLTPELD	CQWDSDNKLS	RLMDPVKHIK	GSSKQPLRNA	ATRTFHDPNA
730	740	750	760	770	780	790	800
IYSELILWRV	DPIGPLSYTG	GVSELARINS	LHTSAFSNVA	WLPTLIPSYC	LGTYCNSASA	CFVASDGKNL	RLYQAVVDAR
810	820	830	840	850	860	870	880
KLLDELSDPE	SSKLIGEVFN	IVSQQSTARP	GCIIELDAIT	NQCGSNTQLL	HVFQEDFIIG	YKPHKEDMEK	KETEIFFQPS
890	900	910	920	930	940	950	960
QGYRPPPFSE	KFFLVVIEKD	SNNNSILHMW	HLHLKSVQAC	LAKASEGASS	ESLLSVPGQK	NVDSSPETSP	SVSPMPHSSS
970	980	990	1000	1010	1020	1030	1040
IANLQTASKL	ILSSRLVYSQ	PLDLPESVEV	IRATPSAGHL	SSSSIYPVCL	APYLVVTTCS	DNKVRFWKCC	MEANPECNKS
1050	1060	1070	1080	1090	1100	1110	1120
DEKEIYHWKR	WPLMNDEGED	NSSTVSIVGR	PVAVSCSYTG	RLAVAYKQPI	HHNGFVSKEF	SMHVCIFECE	STGGSEWVLE
1130	1140	1150	1160	1170	1180	1190	1200
QTIHLDDLVK	VGSVLDSRVS	VDSNLFVYSK	SDALLSKDRY	LIPNIKHLVH	LDWVSKEDGS	HILTVGVGAN	IFMYGRLSGI
1210	1220	1230	1240	1250	1260	1270	1280
VTEQTNSKDG	VAVITLPLGG	SIKQGVKSRW	VLLRSIDLVS	SVDGTPSLPV	SLSWVRDGIL	VVGMDCEMHV	YAQWKHAVKF
1290	1300	1310	1320	1330	1340	1350	1360
GDTEADSSNA	EEAAMQDHST	FKSNMLARKS	VVEGTAISDD	VFCSPTVIQD	GGLFEAAHVL	SPTLPQYHPT	QLLELMDLGK
1370	1380	1390	1400	1410	1420	1430	1440
VRRAKAILSH	LVKCIAGEVA	IVRDPDAGEG	TKRHLSRTIS	VSGSTAKETV	TVGKDGTRDY	TEIDSIPPLP	LYALLAADQD
1450	1460	1470	1480	1490	1500	1510	1520
TSYRISEEST	KIPQSYEDQT	VSQPEDQYSE	LFQIQDIPTD	DIDLEPEKRE	NKSKVINLSQ	YGPAYFGQEH	ARVLSSHLMH
1530	1540	1550	1560	1570	1580	1590	1600
SSLPGLTRLE	QMFLVALADT	VATTSTELDE	SRDKSCSGRD	TLDECGLRYL	LAMRLHTCLL	TSLPPLYRVQ	LLHQGVSTCH
1610	1620	1630	1640	1650	1660	1670	1680
FAWAFHSEAE	EELINMIPAI	QRGDPQWSEL	RAMGIGWWVR	NINTLRRCIE	KVAKASFQRN	NDALDAALFY	LSMKKKAVVW
1690	1700	1710	1720	1730	1740	1750	1760
GLFRSQHDEK	MTTFFSHNFN	EDRWRKAALK	NAFSLLGKQR	FEQSAAFFLL	AGSLKDAIEV	CLEKMEDIQL	AMVIARLYES
1770	1780	1790	1800	1810	1820	1830	1840
EFETSSTYIS	ILNQKILGCQ	KDGSGFSCKR	LHPDPFLRSL	AYWVMKDYTR	ALDTLLEQTP	KEDDEHQVII	KSCNPVAFSF
1850	1860	1870	1880	1890	1900	1910	1920
YNYLRTHPLL	IRRNLASPEG	TLATLGLKTE	KNFVDKINLI	ERKLFFTTAN	AHFKVGCPVL	ALEVLSKIPK	VTKTSALSAK
1930	1940	1950	1960	1970	1980	1990	2000
KDQPDFISHR	MDDVPSHSKA	LSDGNGSSGI	EWSNVTSSQY	DWSQPIVKVD	EEPLNLDWGE	DHDSALDEEE	DDAVGLVMKS
2010	2020	2030	2040	2050	2060	2070	2080
TDAREKDKQS	DQKASDPNML	LTPQEEDDPE	GDTEVDVIAE	QLKFRACLKI	LMTELRTLAT	GYEVDGGKLR	FQLYNWLEKE
2090	2100	2110	2120	2130	2140	2150	2160
IAALHEICNH	ESVIKEYSSK	TYSKVESDLL	DQEEMVDKPD	IGSYERHQIE	RRRLQAKREH	AERRKSWLQK	NQDLLRVFLS
2170	2180	2190	2200	2210	2220	2230	2240

Detailed Protein Report

Cmpd.	No. of Cmpds.	m/z meas.	Δ m/z [ppm]	z	Rt [min]	Score	P	Range	Sequence	Modification
951	1	677.3400	9.12	3	40.4	17.0	0	598-615	R.SHSTHMNILAPTVMMISK.H	Oxidation: 14, 15
1839	1	780.2904	-91.58	2	51.5	14.1	1	2574-2588	R.VSCSSHRGVPGGMGR.K	Carbamidomethyl: 3; Oxidation: 13

Detailed Protein Report

Protein 134: collagen alpha-2(XI) chain isoform 2 preproprotein [Homo sapiens]

**Accession:**	gi|111118972	**Score:**	47.3
**Database:**	refseq_human(refseq_human_20140103.fasta)	**MW [kDa]:**	162.1
**Database Date:**	2015-11-30	**pI:**	8.8
		**Sequence Coverage [%]:**	2.8
		**No. of unique Peptides:**	2

10	20	30	40	50	60	70	80
MERCSRCHRL	LLLLPLVLGL	SAAPGWAGAP	PVDVLRALRF	PSLPDGVRRA	KGICPADVAY	RVARPAQLSA	PTRQLFPGGF
90	100	110	120	130	140	150	160
PKDFSLLTVV	RTRPGLQAPL	LTLYSAQGVR	QLGLELGRPV	RFLYEDQTGR	PQPPSQPVFR	GLSLADGKWH	RVAVAVKGQS
170	180	190	200	210	220	230	240
VTLIVDCKKR	VTRPLPRSAR	PVLDTHGVII	FGARILDEEV	FEGDVQELAI	VPGVQAAYES	CEQKELECEG	GQRERPQNQQ
250	260	270	280	290	300	310	320
PHRAQRSPQQ	QPSRLHRPQN	QEPQSQDPTP	GEEEEILESS	LLPPLEEAAH	GPRGLKGEKG	EPAVLEPGML	VEGPPGPEGP
330	340	350	360	370	380	390	400
AGLIGPPGIQ	GNPGPVGDPG	ERGPPGRAGL	PGSDGAPGPP	GTSLMLPFRF	GSGGGDKGPV	VAAQEAQAQA	ILQQARLALR
410	420	430	440	450	460	470	480
GPPGPMGYTG	RPGPLGQPGS	PGLKGESGDL	GPQGPRGPQG	LTGPPGKAGR	RGRAGADGAR	GMPGDPGVKG	DRGFDGLPGL
490	500	510	520	530	540	550	560
PGEKGHRGDT	GAQGLPGPPG	EDGERGDDGE	IGPRGLPGES	GPRGLLGPKG	PPGIPGPPGV	RGMDGPQGPK	GSLGPQGEPG
570	580	590	600	610	620	630	640
PPGQQGTPGT	QGLPGPQGAI	GPHGEKGPQG	KPGLPGMPGS	DGPPGHPGKE	GPPGTKGNQG	PSGPQGPLGY	PGPRGVKGVD
650	660	670	680	690	700	710	720
GIRGLKGHKG	EKGEDGFPGF	KGDIGVKGDR	GEVGVPGSRG	EDGPEGPKGR	TGPTGDPGPP	GLMGEKGKLG	VPGLPGYPGR
730	740	750	760	770	780	790	800
QGPKGSLGFP	GFPGASGEKG	ARGLSGKSGP	RGERGPTGPR	GQRGPRGATG	KSGAKGTSGG	DGPHGPPGER	GLPGPQGPNG
810	820	830	840	850	860	870	880
FPGPKGPPGP	PGKDGLPGHP	GQRGEVGFQG	KTGPPGPPGV	VGPQGAAGET	GPMGERGHPG	PPGPPGEQGL	PGTAGKEGTK
890	900	910	920	930	940	950	960
GDPGPPGAPG	KDGPAGLRGF	PGERGLPGTA	GGPGLKGNEG	PSGPPGPAGS	PGERGAAGSG	GPIGPPGRPG	PQGPPGAAGE
970	980	990	1000	1010	1020	1030	1040
KGVPGEKGPI	GPTGRDGVQG	PVGLPGPAGP	PGVAGEDGDK	GEVGDPGQKG	TKGNKGEHGP	PGPPGPIGPV	GQPGAAGADG
1050	1060	1070	1080	1090	1100	1110	1120
EPGARGPQGH	FGAKGDEGTR	GFNGPPGPIG	LQGLPGPSGE	KGETGDVGPM	GPPGPPGPRG	PAGPNGADGP	QGPPGGVGNL
1130	1140	1150	1160	1170	1180	1190	1200
GPPGEKGEPG	ESGSPGIQGE	PGVKGPRGER	GEKGESGQPG	EPGPPGPKGP	TGDDGPKGNP	GPVGFPGDPG	PPGEGGPRGQ
1210	1220	1230	1240	1250	1260	1270	1280
DGAKGDRGED	GEPGQPGSPG	PTGENGPPGP	LGKRGPAGSP	GSEGRQGGKG	AKGDPGAIGA	PGKTGPVGPA	GPAGKPGPDG
1290	1300	1310	1320	1330	1340	1350	1360
LRGLPGSVGQ	QGRPGATGQA	GPPGPVGPPG	LPGLRGDAGA	KGEKGHPGLI	GLIGPPGEQG	EKGDRGLPGP	QGSPGQKGEM
1370	1380	1390	1400	1410	1420	1430	1440
GIPGASGPIG	PGGPPGLPGP	AGPKGAKGAT	GPGGPKGEKG	VQGPPGHPGP	PGEVIQPLPI	QMPKKTRRSV	DGSRLMQEDE
1450	1460	1470	1480	1490	1500	1510	1520
AIPTGGAPGS	PGGLEEIFGS	LDSLREEIEQ	MRRPTGTQDS	PARTCQDLKL	CHPELPDGEY	WVDPNQGCAR	DAFRVFCNFT
1530	1540	1550	1560	1570	1580	1590	1600
AGGETCVTPR	DDVTQFSYVD	SEGSPVGVVQ	LTFLRLLSVS	AHQDVSYPCS	GAARDGPLRL	RGANEDELSP	ETSPYVKEFR
1610	1620	1630	1640	1650	1660		
DGCQTQQGRT	VLEVRTPVLE	QLPVLDASFS	DLGAPPRRGG	VLLGPVCFMG			

Cmpd.	No. of Cmpds.	m/z meas.	Δ m/z [ppm]	z	Rt [min]	Score	P	Range	Sequence	Modification
2871	3	645.8309	-41.94	2	64.6	23.7	1	158-169	K.GQSVTLIVDCKK.R	
2503	1	862.3584	-77.51	2	61.7	12.3	1	689-706	K.GRTGPTGDPGPPGLMGEK.G	

Detailed Protein Report

Protein 135: PREDICTED: nesprin-1 isoform X17 [Homo sapiens]

**Accession:**	gi|578812600	**Score:**	47.2
**Database:**	refseq_human(refseq_human_20140103.fasta)	**MW [kDa]:**	1006.9
**Database Date:**	2015-11-30	**pI:**	5.3
**Modification(s):**	Oxidation	**Sequence Coverage [%]:**	0.6
		**No. of unique Peptides:**	4

Quantitation

mdown:qdown	**Median:** 1.08	**CV:** 0.00 %	**No. of Peptides:** 1

Detailed Protein Report

10	20	30	40	50	60	70	80
MATSRGASRC	PRDIANVMQR	LQDEQEIVQK	RTFTKWINSH	LAKRKPPMVV	DDLFEDMKDG	VKLLALLEVL	SGQKLPCEQG
90	100	110	120	130	140	150	160
RRMKRIHAVA	NIGTALKFLE	GRKSMHRGSP	IKLVNINSTD	IADGRPSIVL	GLMWTIILYF	QIEELTSNLP	QLQSLSSSAS
170	180	190	200	210	220	230	240
SVDSIVSSET	PSPPSKRKVT	TKIQGNAKKA	LLKWVQYTAG	KQTGIEVKDF	GKSWRSGVAF	HSVIHAIRPE	LVDLETVKGR
250	260	270	280	290	300	310	320
SNRENLEDAF	TIAETELGIP	RLLDPEDVDV	DKPDEKSIMT	YVAQFLKHYP	DIHNASTDGQ	EDDEILPGFP	SFANSVQNFK
330	340	350	360	370	380	390	400
REDRVIFKEM	KVWIEQFERD	LTRAQMVESN	LQDKYQSFKH	FRVQYEMKRK	QIEHLIQPLH	RDGKLSLDQA	LVKQSWDRVT
410	420	430	440	450	460	470	480
SRLFDWHIQL	DKSLPAPLGT	IGAWLYRAEV	ALREEITVQQ	VHEETANTIQ	RKLEQHKDLL	QNTDAHKRAF	HEIYRTRSVN
490	500	510	520	530	540	550	560
GIPVPPDQLE	DMAERFHFVS	STSELHLMKM	EFLELKYRLL	SLLVLAESKL	KSWIIKYGRR	ESVEQLLQNY	VSFIENSKFF
570	580	590	600	610	620	630	640
EQYEVTYQIL	KQTAEMYVKA	DGSVEEAENV	MKFMNETTAQ	WRNLSVEVRS	VRSMLEEVIS	NWDRYGNTVA	SLQAWLEDAE
650	660	670	680	690	700	710	720
KMLNQSENAK	KDFFRNLPHW	IQQHTAMNDA	GNFLIETCDE	MVSRDLKQQL	LLLNGRWREL	FMEVKQYAQA	DEMDRMKKEY
730	740	750	760	770	780	790	800
TDCVVTLSAF	ATEAHKKLSE	PLEVSFMNVK	LLIQDLEDIE	QRVPVMDAQY	KIITKTAHLI	TKESPQEEGK	EMFATMSKLK
810	820	830	840	850	860	870	880
EQLTKVKECY	SPLLYESQQL	LIPLEELEKQ	MTSFYDSLGK	INEIITVLER	EAQSSALFKQ	KHQELLACQE	NCKKTLTLIE
890	900	910	920	930	940	950	960
KGSQSVQKFV	TLSNVLKHFD	QTRLQRQIAD	IHVAFQSMVK	KTGDWKKHVE	TNSRLMKKFE	ESRAELEKVL	RIAQEGLEEK
970	980	990	1000	1010	1020	1030	1040
GDPEELLRRH	TEFFSQLDQR	VLNAFLKACD	ELTDILPEQE	QQGLQEAVRK	LHKQWKDLQG	EAPYHLLHLK	IDVEKNRFLA
1050	1060	1070	1080	1090	1100	1110	1120
SVEECRTELD	RETKLMPQEG	SEKIIKEHRV	FFSDKGPHHL	CEKRLQLIEE	LCVKLPVRDP	VRDTPGTCHV	TLKELRAAID
1130	1140	1150	1160	1170	1180	1190	1200
STYRKLMEDP	DKWKDYTSRF	SEFSSWISTN	ETQLKGIKGE	AIDTANHGEV	KRAVEEIRNG	VTKRGETLSW	LKSRLKVLTE
1210	1220	1230	1240	1250	1260	1270	1280
VSSENEAQKQ	GDELAKLSSS	FKALVTLLSE	VEKMLSNFGD	CVQYKEIVKN	SLEELISGSK	EVQEQAEKIL	DTENLFEAQQ
1290	1300	1310	1320	1330	1340	1350	1360
LLLHHQQKTK	RISAKKRDVQ	QQIAQAQQGE	GGLPDRGHEE	LRKLESTLDG	LERSRERQER	RIQVTLRKWE	RFETNKETVV
1370	1380	1390	1400	1410	1420	1430	1440
RYLFQTGSSH	ERFLSFSSLE	SLSSELEQTK	EFSKRTESIA	VQAENLVKEA	SEIPLGPQNK	QLLQQQAKSI	KEQVKKLEDT
1450	1460	1470	1480	1490	1500	1510	1520
LEEDIKTMEM	VKTKWDHFGS	NFETLSVWIT	EKEKELNALE	TSSSAMDMQI	SQIKVTIQEI	ESKLSSIVGL	EEEAQSFAQF
1530	1540	1550	1560	1570	1580	1590	1600
VTTGESARIK	AKLTQIRRYG	EELREHAQCL	EGTILGHLSQ	QQKFEENLRK	IQQSVSEFED	KLAVPIKICS	SATETYKVLQ
1610	1620	1630	1640	1650	1660	1670	1680
EHMDLCQALE	SLSSAITAFS	ASARKVVNRD	SCVQEAAALQ	QQYEDILRRA	KERQTALENL	LAHWQRLEKE	LSSFLTWLER
1690	1700	1710	1720	1730	1740	1750	1760
GEAKASSPEM	DISADRVKVE	GELQLIQALQ	NEVVSQASFY	SKLLQLKESL	FSVASKDDVK	MMKLHLEQLD	ERWRDLPQII
1770	1780	1790	1800	1810	1820	1830	1840
NKRINFLQSV	VAEHQQFDEL	LLSFSVWIKL	FLSELQTTSE	ISIMDHQVAL	TRHKDHAAEV	ESKKGELQSL	QGHLAKLGSL
1850	1860	1870	1880	1890	1900	1910	1920
GRAEDLHLLQ	GKAEDCFQLF	EEASQVVERR	QLALSHLAEF	LQSHASLSGI	LRQLRQTVEA	TNSMNKNESD	LIEKDLNDAL
1930	1940	1950	1960	1970	1980	1990	2000
QNAKALESAA	VSLDGILSKA	QYHLKIGSSE	QRTSCRATAD	QLCGEVERIQ	NLLGTKQSEA	DALAVLKKAF	QDQKEELLKS
2010	2020	2030	2040	2050	2060	2070	2080
IEDIEERTDK	ERLKEPTRQA	LQQRLRVFNQ	LEDELNSHEH	ELCWLKDKAK	QIAQKDVAFA	PEVDREINRL	EVTWDDTKRL
2090	2100	2110	2120	2130	2140	2150	2160
IHENQGQCCG	LIDLMREYQN	LKSAVSKVLE	NASSVIVTRT	TIKDQEDLKW	AFSKHETAKN	KMNYKQKDLD	NFTSKGKHLL
2170	2180	2190	2200	2210	2220	2230	2240

Detailed Protein Report

Cmpd.	No. of Cmpds.	m/z meas.	Δ m/z [ppm]	z	Rt [min]	Score	P	Range	Sequence	Modification	Ratios
1807	1	945.8341	-138.33	2	51.1	10.6	2	1681-1698	R.GEAKASSPEMDISADRVK.V		
742	1	524.3579	134.01	2	37.8	10.5	1	2443-2452	R.TGDSKVLEAK.L		
543	1	567.1646	-233.64	2	36.1	11.3	0	5483-5492	K.LMELDAAVQK.F	Oxidation: 2	mdown:qdown 1.08
2878	1	913.4666	-41.53	2	65.2	14.8	1	7089-7104	K.EVEKIEQNGLALIQNK.K		

Detailed Protein Report

Protein 136: PREDICTED: LOW QUALITY PROTEIN: hemicentin-2 [Homo sapiens]

**Accession:**	gi|578818143	**Score:**	47.1
**Database:**	refseq_human(refseq_human_20140103.fasta)	**MW [kDa]:**	546.0
**Database Date:**	2015-11-30	**pI:**	5.5
**Modification(s):**	Carbamidomethyl	**Sequence Coverage [%]:**	1.1
		**No. of unique Peptides:**	2

BRUKER

Detailed Protein Report

10	20	30	40	50	60	70	80
MMPGAPLLRL	LTAVSAAVAV	AVAGAPGTVM	PPTTGDATLA	FVFDVTGSMW	DELMQVIDGA	SRILERSLSR	RSQAIANYAL
90	100	110	120	130	140	150	160
VPFHDPDIGP	VTLTADPTVF	QRELRELYVQ	GGGDCPEMSV	GAIKAAVEVA	NPGSFIYVFS	DARAKDYHKK	EELLRLLQLK
170	180	190	200	210	220	230	240
QSQVVFVLTG	DCGDRTHPGY	LAYEEIAATS	SGQVFHLDKQ	QVTEVLKWVE	SAIQASKVHL	LSTDHEEEGE	HTWRLPFDPS
250	260	270	280	290	300	310	320
LKEVTISLSG	PGPEIEVQDP	LGRILQEDEG	LNVLLNIPDS	AKVVAFKPEH	PGLWSIKVYS	SGRHSVRITG	VSNIDFRAGF
330	340	350	360	370	380	390	400
STQPLLDLNH	TLEWPLQGVP	ISLVINSTGL	KAPGRLDSVE	LAQSSGKPLL	TLPTKPLSNG	STHQLWGGPP	FHTPKERFYL
410	420	430	440	450	460	470	480
KVKGKDHEGN	PLLRVSGVSY	SGVAPGAPLV	SMAPRIHGYL	HQPLLVSCSV	HSALPFRLQL	RRGEARLGEE	RHFQESGNSS
490	500	510	520	530	540	550	560
WEILRASKAE	EGTYECTAVS	RAGTGRAKAQ	IVVTDPPPQL	VPAPNVTVSP	GETAVLSCRV	LGEAPYNLTW	VRDWRVLPAS
570	580	590	600	610	620	630	640
TGRVAQLADL	SLEISGIIPT	DGGRYQCVAS	NANGVTRASV	WLLVREAPQV	SIHTSSQHFS	QGVEVKVSCS	ASGYPTPHIS
650	660	670	680	690	700	710	720
WSRESQALQE	DSRIHVDAQG	TLIIQGVAPE	DAGNYSCQAT	NEVGTDQETV	TLYYTDPPSV	SAVNAVVLVA	VGEEAVLVCE
730	740	750	760	770	780	790	800
ASGVPPPRVI	WYRGGLEMIL	APEGSSSGKL	RIPAAQERDA	GTYTCRAVNE	LGDASAEIQL	AVGHAPQLTE	LPRDVTVELG
810	820	830	840	850	860	870	880
RSALLACRAT	GRPPPTVTWR	RGDGQPLGLR	LGAGRGSRSR	QPDSGVLFFE	SVAPEDQAPY	VCEARNVFGK	VQAEARLIVT
890	900	910	920	930	940	950	960
GHAPPQIASS	APTVRVLEGQ	PVSLPCIVLA	GRPLPERHWL	KDGRPLPPGS	RHSIRADGSL	HLDRALQEHA	GRYSCVATNT
970	980	990	1000	1010	1020	1030	1040
AGSQHRDVEL	VVQVPPRIHP	TATHHITNEG	VAASLPCVAS	GVPAPTITWT	KETNALTSRG	PHYNVSKEGT	LLIAQPSAQD
1050	1060	1070	1080	1090	1100	1110	1120
AGAYVCTATN	TVGFSSQEMR	LSVNTKPRIH	MNGSRNADVP	LQVTAKAGEE	VTLDCEAKGS	PPPLVTWTKD	SRPVPPITNR
1130	1140	1150	1160	1170	1180	1190	1200
YGLLPSGSLR	LAQVQVGDSG	HYECTASNPA	GSASHRYVLG	VQVPPQVQPG	PRVLKVLVGE	ALDLNCVAEG	NPEPQLSWSK
1210	1220	1230	1240	1250	1260	1270	1280
DGVVLQGRGP	QGSVHFAAIR	TSDAGRYRCE	ASNSAGVDAW	EVELRVLEPP	HWGADETSGL	LERVAGENAS	LPCPARGTPK
1290	1300	1310	1320	1330	1340	1350	1360
PQVTWRKGPS	SEPLHGQPGV	AVLEEGSLFL	ASVSPADSGD	YECQATNEVG	STSRRAKLVV	YVPPSIREDG	RKANVSGMAG
1370	1380	1390	1400	1410	1420	1430	1440
QSLTLECDAN	GFPVPEIVWL	KDAQLIPKVG	GHRLLDEGQS	LHFPRIQEGD	SGLYSCRAEN	QAGTAQRDFH	LLVLTPPSVL
1450	1460	1470	1480	1490	1500	1510	1520
GAGAAQEVLG	LAGADVELQC	WTSGVPTPQV	EWTKDRQPVL	PGGPHLQVQE	DGQVLRITGS	HVGDEGRYQC	VAFSPAGQQA
1530	1540	1550	1560	1570	1580	1590	1600
RDFQLRVHAP	PTIWGSNETG	EVAVMEDHLV	QLLCEARGVP	TPNITWFKDG	ALLPTSTKVV	YTRGGRQLQL	GRAQSSDAGV
1610	1620	1630	1640	1650	1660	1670	1680
YTCKASNAVG	AAEKATRLDV	YVPPTIEGAG	GRPYVVKAVA	GRPVALECVA	RGHPSPTLSW	HHEGLPVAES	NESRLETDGS
1690	1700	1710	1720	1730	1740	1750	1760
VLRLESPGEA	SSGLYSCVAS	SPAGEAVLQY	SVEVQVPPQL	LVAEGLGQVT	TIVGQPLELP	CQASGSPVPT	IQWLQNGRPA
1770	1780	1790	1800	1810	1820	1830	1840
EELAGVQVAS	QGTTLHIDHV	ELDHSGLFAC	QATNEAGTAG	AEVEVSVHGE	WAPGVLEVPI	GQYHWGXEHH	SSFPAACDPP
1850	1860	1870	1880	1890	1900	1910	1920
VHRGWGAHPK	PPLVEGWCSP	GSLWGEPTGI	PVAYSLPPPL	QIEKVDLRDE	GIYTCAATNL	AGESKREVAL	KVLVPPNIEP
1930	1940	1950	1960	1970	1980	1990	2000
GPVNKAVLEN	ASVTLECLAS	GVPPPDVSWF	KGHQPVSSWM	GVTVSVDGRV	LRIEQAQLSD	AGSYRCVASN	VAGSTELRYG
2010	2020	2030	2040	2050	2060	2070	2080
LRVNVPPRIT	LPPSLPGPVL	VNTPVRLTCN	ATGAPSPTLM	WLKDGNPVSP	AGTPGLQVFP	GGRVLTLASA	RASDSGRYSC
2090	2100	2110	2120	2130	2140	2150	2160
VAVSAVGEDR	QDVVLQVHMP	PSILGEELNV	SVVANESVAL	ECQSHAMPPP	VLSWWKDGRP	LEPRPGVHLS	ADKALQVDR
2170	2180	2190	2200	2210	2220	2230	2240

Detailed Protein Report

Cmpd.	No. of Cmpds.	m/z meas.	Δ m/z [ppm]	z	Rt [min]	Score	P	Range	Sequence	Modification
2115	1	840.8591	-53.51	2	56.6	14.6	0	161-175	K.QSQVVFVLTGDCGDR.T	Carbamidomethyl: 12
61	1	623.8088	95.46	5	29.5	12.6	2	866-895	R.NVFGKVQAEARLIVTGHAPPQIASSAPTVR.V	

Detailed Protein Report

Protein 137: keratin, type II cuticular Hb4 [Homo sapiens]

Accession: gi|15431316
Database: refseq_human(refseq_human_20140103.fasta)
Database Date: 2015-11-30
Modification(s): Carbamidomethyl, Oxidation

Score: 47.1
MW [kDa]: 64.8
pI: 9.0
Sequence Coverage [%]: 5.8
No. of unique Peptides: 2

Quantitation

mdown:qdown **Median:** 1.41 **CV:** 0.00 % **No. of Peptides:** 1

10	20	30	40	50	60	70	80
MSCRSYRVSS	GHRVGNFSSC	SAMTPQNLNR	FRANSVSCWS	GPGFRGLGSF	GSRSVITFGS	YSPRIAAVGS	RPIHCGVRFG
**90**	**100**	**110**	**120**	**130**	**140**	**150**	**160**
AGCGMGFGDG	RGVGLGPRAD	SCVGLGFGAG	SGIGYGFGGP	GFGYRVGGVG	VPAAPSITAV	TVNKSLLTPL	NLEIDPNAQR
**170**	**180**	**190**	**200**	**210**	**220**	**230**	**240**
VKKDEKEQIK	TLNNKFASFI	DKVRFLEQQN	KLLETKWSFL	QEQKCIRSNL	EPLFESYITN	LRRQLEVLVS	DQARLQAERN
**250**	**260**	**270**	**280**	**290**	**300**	**310**	**320**
HLQDVLEGFK	KKYEEEVVCR	ANAENEFVAL	KKDVDAAFMN	KSDLEANVDT	LTQEIDFLKT	LYMEEIQLLQ	SHISETSVIV
**330**	**340**	**350**	**360**	**370**	**380**	**390**	**400**
KMDNSRDLNL	DGIIAEVKAQ	YEEVARRSRA	DAEAWYQTKY	EEMQVTAGQH	CDNLRNIRNE	INELTRLIQR	LKAEIEHAKA
**410**	**420**	**430**	**440**	**450**	**460**	**470**	**480**
QRAKLEAAVA	EAEQQGEATL	SDAKCKLADL	ECALQQAKQD	MARQLCEYQE	LMNAKLGLDI	EIATYRRLLE	GEESRLCEGV
**490**	**500**	**510**	**520**	**530**	**540**	**550**	**560**
GPVNISVSSS	RGGLVCGPEP	LVAGSTLSRG	GVTFSGSSSV	CATSGVLASC	GPSLGGARVA	PATGDLLSTG	TRSGSMLISE
**570**	**580**	**590**	**600**	**610**			
ACVPSVPCPL	PTQGGFSSCS	GGRSSSVRFV	STTTSCRTKY				

Cmpd.	No. of Cmpds.	m/z meas.	Δ m/z [ppm]	z	Rt [min]	Score	P	Range	Sequence	Modification	Ratios
553	1	607.3109	-5.53	2	35.5	13.1	0	54-64	R.SVITFGSYSPR.I		
603	2	652.6252	-207.98	2	36.9	13.1	0	79-91	R.FGAGCGMGFGDGR.G	Carbamidomethyl: 5; Oxidation: 7	mdown:qdown 1.41

Detailed Protein Report

Protein 138: **PREDICTED: PR domain zinc finger protein 16 isoform X3 [Homo sapiens]**

**Accession:**	gi|530360370	**Score:**	47.0
**Database:**	refseq_human(refseq_human_20140103.fasta)	**MW [kDa]:**	129.7
**Database Date:**	2015-11-30	**pI:**	5.9
**Modification(s):**	Carbamidomethyl	**Sequence Coverage [%]:**	3.5
		**No. of unique Peptides:**	2

10	20	30	40	50	60	70	80
MRSKARARKL	AKSDGDVVNN	MYEPNRDLLA	SHSAEDEAED	SAMSPIPVGP	PSPFPTSEDF	TPKEGSPYEA	PVYIPEDIPI
**90**	**100**	**110**	**120**	**130**	**140**	**150**	**160**
PADFELRESS	IPGAGLGVWA	KRKMEAGERL	GPCVVVPRAA	AKETDFGWEQ	ILTDVEVSPQ	EGCITKQISE	DLGSEKFCVD
**170**	**180**	**190**	**200**	**210**	**220**	**230**	**240**
ANQAGAGSWL	KYIRVACSCD	DQNLTMCQIS	EQIYYKVIKD	IEPGEELLVH	VKEGVYPLGT	VPPGLDEEPT	FRCDECDELF
**250**	**260**	**270**	**280**	**290**	**300**	**310**	**320**
QSKLDLRRHK	KYTCGSVGAA	LYEGLAEELK	PEGLGGGSGQ	AHECKDCERM	FPNKYSLEQH	MVIHTEEREY	KCDQCPKAFN
**330**	**340**	**350**	**360**	**370**	**380**	**390**	**400**
WKSNLIRHQM	SHDSGKRFEC	ENCVKVFTDP	SNLQRHIRSQ	HVGARAHACP	DCGKTFATSS	GLKQHKHIHS	TVKPFICEVC
**410**	**420**	**430**	**440**	**450**	**460**	**470**	**480**
HKSYTQFSNL	CRHKRMHADC	RTQIKCKDCG	QMFSTTSSLN	KHRRFCEGKN	HYTPGGIFAP	GLPLTPSPMM	DKAKPSPSLN
**490**	**500**	**510**	**520**	**530**	**540**	**550**	**560**
HASLGFNEYF	PSRPHPGSLP	FSTAPPTFPA	LTPGFPGIFP	PSLYPRPPLL	PPTSLLKSPL	NHTQDAKLPS	PLGNPALPLV
**570**	**580**	**590**	**600**	**610**	**620**	**630**	**640**
SAVSNSSQGT	TAAAGPEEKF	ESRLEDSCVE	KLKTRSSDMS	DGSDFEDVNT	TTGTDLDTTT	GTGSDLDSDV	DSDPDKDKGK
**650**	**660**	**670**	**680**	**690**	**700**	**710**	**720**
GKSAEGQPKF	GGGLAPPGAP	NSVAEVPVFY	SQHSFFPPPD	EQLLTATGAA	GDSIKAIASI	AEKYFGPGFM	GMQEKKLGSL
**730**	**740**	**750**	**760**	**770**	**780**	**790**	**800**
PYHSAFPFQF	LPNFPHSLYP	FTDRALAHNL	LVKAEPKSPR	DALKVGGPSA	ECPFDLTTKP	KDVKPILPMP	KGPSAPASGE
**810**	**820**	**830**	**840**	**850**	**860**	**870**	**880**
EQPLDLSIGS	RARASQNGGG	REPRKNHVYG	ERKLGAGEGL	PQVCPARMPQ	QPPLHYAKPS	PFFMDPIYSR	VEKRKVTDPV
**890**	**900**	**910**	**920**	**930**	**940**	**950**	**960**
GALKEKYLRP	SPLLFHPQMS	AIETMTEKLE	SFAAMKADSG	SSLQPLPHHP	FNFRSPPPTL	SDPILRKGKE	RYTCRYCGKI
**970**	**980**	**990**	**1000**	**1010**	**1020**	**1030**	**1040**
FPRSANLTRH	LRTHTGEQPY	RCKYCDRSFS	ISSNLQRHVR	NIHNKEKPFK	CHLCNRCFGQ	QTNLDRHLKK	HEHENAPVSQ
**1050**	**1060**	**1070**	**1080**	**1090**	**1100**	**1110**	**1120**
HPGVLTNHLG	TSASSPTSES	DNHALLDEKE	DSYFSEIRNF	IANSEMNQAS	TRTEKRADMQ	IVDGSAQCPG	LASEKQEDVE
**1130**	**1140**	**1150**	**1160**	**1170**	**1180**		
EEDDDDLEED	DEDSLAGKSQ	DDTVSPAPEP	QAAYEDEEDE	EPAASLAVGF	DHTRRHMQ		

Cmpd.	No. of Cmpds.	m/z meas.	Δ m/z [ppm]	z	Rt [min]	Score	P	Range	Sequence	Modification
1511	1	1005.9710	-61.06	2	47.6	21.5	2	104-122	K.MEAGERLGPCVVVPRAAAK.E	Carbamidomethyl: 10
2972	1	646.8226	-64.07	2	66.4	11.8	0	935-946	R.SPPPTLSDPILR.K	

Detailed Protein Report

Protein 139: myosin-9 [Homo sapiens]

**Accession:**	gi|12667788	**Score:**	47.0
**Database:**	refseq_human(refseq_human_20140103.fasta)	**MW [kDa]:**	226.4
**Database Date:**	2015-11-30	**pI:**	5.4
**Modification(s):**	Oxidation	**Sequence Coverage [%]:**	1.6
		**No. of unique Peptides:**	3

Quantitation

Wdown:Qdown	**Median:** 0.74	**CV:** 125.52 %	**No. of Peptides:** 2

Detailed Protein Report

10	20	30	40	50	60	70	80
MAQQAADKYL	YVDKNFINNP	LAQADWAAKK	LVWVPSDKSG	FEPASLKEEV	GEEAIVELVE	NGKKVKVNKD	DIQKMNPPKF
90	100	110	120	130	140	150	160
SKVEDMAELT	CLNEASVLHN	LKERYYSGLI	YTYSGLFCVV	INPYKNLPIY	SEEIVEMYKG	KKRHEMPPHI	YAITDTAYRS
170	180	190	200	210	220	230	240
MMQDREDQSI	LCTGESGAGK	TENTKKVIQY	LAYVASSHKS	KKDQGELERQ	LLQANPILEA	FGNAKTVKND	NSSRFGKFIR
250	260	270	280	290	300	310	320
INFDVNGYIV	GANIETYLLE	KSRAIRQAKE	ERTFHIFYYL	LSGAGEHLKT	DLLLEPYNKY	RFLSNGHVTI	PGQQDKDMFQ
330	340	350	360	370	380	390	400
ETMEAMRIMG	IPEEEQMGLL	RVISGVLQLG	NIVFKKERNT	DQASMPDNTA	AQKVSHLLGI	NVTDFTRGIL	TPRIKVGRDY
410	420	430	440	450	460	470	480
VQKAQTKEQA	DFAIEALAKA	TYERMFRWLV	LRINKALDKT	KRQGASFIGI	LDIAGFEIFD	LNSFEQLCIN	YTNEKLQQLF
490	500	510	520	530	540	550	560
NHTMFILEQE	EYQREGIEWN	FIDFGLDLQP	CIDLIEKPAG	PPGILALLDE	ECWFPKATDK	SFVEKVMQEQ	GTHPKFQKPK
570	580	590	600	610	620	630	640
QLKDKADFCI	IHYAGKVDYK	ADEWLMKNMD	PLNDNIATLL	HQSSDKFVSE	LWKDVDRIIG	LDQVAGMSET	ALPGAFKTRK
650	660	670	680	690	700	710	720
GMFRTVGQLY	KEQLAKLMAT	LRNTNPNFVR	CIIPNHEKKA	GKLDPHLVLD	QLRCNGVLEG	IRICRQGFPN	RVVFQEFRQR
730	740	750	760	770	780	790	800
YEILTPNSIP	KGFMDGKQAC	VLMIKALELD	SNLYRIGQSK	VFFRAGVLAH	LEEERDLKIT	DVIIGFQACC	RGYLARKAFA
810	820	830	840	850	860	870	880
KRQQQLTAMK	VLQRNCAAYL	KLRNWQWWRL	FTKVKPLLQV	SRQEEEMMAK	EEELVKVREK	QLAAENRLTE	METLQSQLMA
890	900	910	920	930	940	950	960
EKLQLQEQLQ	AETELCAEAE	ELRARLTAKK	QELEEICHDL	EARVEEEEER	CQHLQAEKKK	MQQNIQELEE	QLEEEESARQ
970	980	990	1000	1010	1020	1030	1040
KLQLEKVTTE	AKLKKLEEEQ	IILEDQNCKL	AKEKKLLEDR	IAEFTTNLTE	EEEKSKSLAK	LKNKHEAMIT	DLEERLRREE
1050	1060	1070	1080	1090	1100	1110	1120
KQRQELEKTR	RKLEGDSTDL	SDQIAELQAQ	IAELKMQLAK	KEEELQAALA	RVEEEAAQKN	MALKKIRELE	SQISELQEDL
1130	1140	1150	1160	1170	1180	1190	1200
ESERASRNKA	EKQKRDLGEE	LEALKTELED	TLDSTAAQQE	LRSKREQEVN	ILKKTLEEEA	KTHEAQIQEM	RQKHSQAVEE
1210	1220	1230	1240	1250	1260	1270	1280
LAEQLEQTKR	VKANLEKAKQ	TLENERGELA	NEVKVLLQGK	GDSEHKRKKV	EAQLQELQVK	FNEGERVRTE	LADKVTKLQV
1290	1300	1310	1320	1330	1340	1350	1360
ELDNVTGLLS	QSDSKSSKLT	KDFSALESQL	QDTQELLQEE	NRQKLSLSTK	LKQVEDEKNS	FREQLEEEEE	AKHNLEKQIA
1370	1380	1390	1400	1410	1420	1430	1440
TLHAQVADMK	KKMEDSVGCL	ETAEEVKRKL	QKDLEGLSQR	HEEKVAAYDK	LEKTKTRLQQ	ELDDLLVDLD	HQRQSACNLE
1450	1460	1470	1480	1490	1500	1510	1520
KKQKKFDQLL	AEEKTISAKY	AEERDRAEAE	AREKETKALS	LARALEEAME	QKAELERLNK	QFRTEMEDLM	SSKDDVGKSV
1530	1540	1550	1560	1570	1580	1590	1600
HELEKSKRAL	EQQVEEMKTQ	LEELEDELQA	TEDAKLRLEV	NLQAMKAQFE	RDLQGRDEQS	EEKKKQLVRQ	VREMEAELED
1610	1620	1630	1640	1650	1660	1670	1680
ERKQRSMAVA	ARKKLEMDLK	DLEAHIDSAN	KNRDEAIKQL	RKLQAQMKDC	MRELDDTRAS	REEILAQAKE	NEKKLKSMEA
1690	1700	1710	1720	1730	1740	1750	1760
EMIQLQEELA	AAERAKRQAQ	QERDELADEI	ANSSGKGALA	LEEKRRLEAR	IAQLEEELEE	EQGNTELIND	RLKKANLQID
1770	1780	1790	1800	1810	1820	1830	1840
QINTDLNLER	SHAQKNENAR	QQLERQNKEL	KVKLQEMEGT	VKSKYKASIT	ALEAKIAQLE	EQLDNETKER	QAACKQVRRT
1850	1860	1870	1880	1890	1900	1910	1920
EKKLKDVLLQ	VDDERRNAEQ	YKDQADKAST	RLKQLKRQLE	EAEEEAQRAN	ASRRKLQREL	EDATETADAM	NREVSSLKNK
1930	1940	1950	1960	1970			
LRRGDLPFVV	PRRMARKGAG	DGSDEEVDGK	ADGAEAKPAE				

Cmpd.	No. of Cmpds.	m/z meas.	Δ m/z [ppm]	z	Rt [min]	Score	P	Range	Sequence	Modification	Ratios
573	1	472.1766	-189.39	2	36.0	12.4	0	31-38	K.LVWVPSDK.S		Wdown:Qdown 0.28
1950	1	856.8003	-136.41	2	54.5	12.5	1	910-923	K.KQELEEICHDLEAR.V		
502	2	567.1670	-259.21	2	35.6	22.1	2	1604-	K.QRSMAVAARK.K	Oxidation: 4	Wdown:Qdown 1.96

Detailed Protein Report

Cmpd.	No. of Cmpds.	m/z meas.	Δ m/z [ppm]	z	Rt [min]	Score	P	Range	Sequence	Modification	Ratios
								1613			

Detailed Protein Report

Protein 140: PREDICTED: chromodomain-helicase-DNA-binding protein 7 isoform X4 [Homo sapiens]

**Accession:**	gi|578815680	**Score:**	47.0
**Database:**	refseq_human(refseq_human_20140103.fasta)	**MW [kDa]:**	214.1
**Database Date:**	2015-11-30	**pI:**	5.2
**Modification(s):**	Carbamidomethyl	**Sequence Coverage [%]:**	2.6
		**No. of unique Peptides:**	3

Detailed Protein Report

10	20	30	40	50	60	70	80
MILTDCPELR	NIPWRCVVID	EAHRLKNRNC	KLLEGLKMMD	LEHKVLLTGT	PLQNTVEELF	SLLHFLEPSR	FPSETTFMQE
90	100	110	120	130	140	150	160
FGDLKTEEQV	QKLQAILKPM	MLRRLKEDVE	KNLAPKEETI	IEVELTNIQK	KYYRAILEKN	FTFLSKGGGQ	ANVPNLLNTM
170	180	190	200	210	220	230	240
MELRKCCNHP	YLINGAEEKI	LEEFKETHNA	ESPDFQLQAM	IQAAGKLVLI	DKLLPKLKAG	GHRVLIFSQM	VRCLDILEDY
250	260	270	280	290	300	310	320
LIQRRYPYER	IDGRVRGNLR	QAAIDRFSKP	DSDRFVFLLC	TRAGGLGINL	TAADTCIIFD	SDWNPQNDLQ	AQARCHRIGQ
330	340	350	360	370	380	390	400
SKSVKIYRLI	TRNSYEREMF	DKASLKLGLD	KAVLQSMSGR	ENATNGVQQL	SKKEIEDLLR	KGAYGALMDE	EDEGSKFCEE
410	420	430	440	450	460	470	480
DIDQILLRRT	HTITIESEGK	GSTFAKASFV	ASGNRTDISL	DDPNFWQKWA	KKAELDIDAL	NGRNNLVIDT	PRVRKQTRLY
490	500	510	520	530	540	550	560
SAVKEDELME	FSDLESDSEE	KPCAKPRRPQ	DKSQGYARSE	CFRVEKNLLV	YGWGRWTDIL	SHGRYKRQLT	EQDVETICRT
570	580	590	600	610	620	630	640
ILVYCLNHYK	GDENIKSFIW	DLITPTADGQ	TRALVNHSGL	SAPVPRGRKG	KKVKAQSTQP	VVQDADWLAS	CNPDALFQED
650	660	670	680	690	700	710	720
SYKKHLKHHC	NKVLLRVRML	YYLRQEVIGD	QADKILEGAD	SSEADVWIPE	PFHAEVPADW	WDKEADKSLL	IGVFKHGYEK
730	740	750	760	770	780	790	800
YNSMRADPAL	CFLERVGMPD	AKAIAAEQRG	TDMLADGGDG	GEFDREDEDP	EYKPTRTPFK	DEIDEFANSP	SEDKEESMEI
810	820	830	840	850	860	870	880
HATGKHSESN	AELGQLYWPN	TSTLTTRLRR	LITAYQRSYK	RQQMRQEALM	KTDRRRRRPR	EEVRALEAER	EAIISEKRQK
890	900	910	920	930	940	950	960
WTRREEADFY	RVVSTFGVIF	DPVKQQFDWN	QFRAFARLDK	KSDESLEKYF	SCFVAMCRRV	CRMPVKPDDE	PPDLSSIIEP
970	980	990	1000	1010	1020	1030	1040
ITEERASRTL	YRIELLRKIR	EQVLHHPQLG	ERLKLCQPSL	DLPEWWECGR	HDRDLLVGAA	KHGVSRTDYH	ILNDPELSFL
1050	1060	1070	1080	1090	1100	1110	1120
DAHKNFAQNR	GAGNTSSLNP	LAVGFVQTPP	VISSAHIQDE	RVLEQAEGKV	EEPENPAAKE	KCEGKEEEEE	TDGSGKESKQ
1130	1140	1150	1160	1170	1180	1190	1200
ECEAEASSVK	NELKGVEVGA	DTGSKSISEK	GSEEDEEEKL	EDDDKSEESS	QPEAGAVSRG	KNFDEESNAS	MSTARDETRD
1210	1220	1230	1240	1250	1260	1270	1280
GFYMEDGDPS	VAQLLHERTF	AFSFWPKDRV	MINRLDNICE	AVLKGKWPVN	RRQMFDFQGL	IPGYTPTTVD	SPLQKRSFAE
1290	1300	1310	1320	1330	1340	1350	1360
LSMVGQASIS	GSEDITTSPQ	LSKEDALNLS	VPRQRRRRRR	KIEIEAERAA	KRRNLMEMVA	QLRESQVVSE	NGQEKVVDLS
1370	1380	1390	1400	1410	1420	1430	1440
KASREATSST	SNFSSLSSKF	ILPNVSTPVS	DAFKTQMELL	QAGLSRTPTR	HLLNGSLVDG	EPPMKRRRGR	RKNVEGLDLL
1450	1460	1470	1480	1490	1500	1510	1520
FMSHKRTSLS	AEDAEVTKAF	EEDIETPPTR	NIPSPGQLDP	DTRIPVINLE	DGTRLVGEDA	PKNKDLVEWL	KLHPTYTVDM
1530	1540	1550	1560	1570	1580	1590	1600
PSYVPKNADV	LFSSFQKPKQ	KRHRCRNPNK	LDINTLTGEE	RVPVVNKRNG	KKMGGAMAPP	MKDLPRWLEE	NPEFAVAPDW
1610	1620	1630	1640	1650	1660	1670	1680
TDIVKQSGFV	PESMFDRLLT	GPVVRGEGAS	RRGRRPKSEI	ARAAAAAAAV	ASTSGINPLL	VNSLFAGMDL	TSLQNLQNLQ
1690	1700	1710	1720	1730	1740	1750	1760
SLQLAGLMGF	PPGLATAATA	GGDAKNPAAV	LPLMLPGMAG	LPNVFGLGGL	LNNPLSAATG	NTTTASSQGE	PEDSTSKGEE
1770	1780	1790	1800	1810	1820	1830	1840
KGNENEDENK	DSEKSTDAVS	AADSANGSVG	AATAPAGLPS	NPLAFNPFLL	STMAPGLFYP	SMFLPPGLGG	LTLPGFPALA
1850	1860	1870	1880	1890	1900	1910	1920
GLQNAVGSSE	EKAADKAEGG	PFKDGETLEG	SDAEESLDKT	AESSLLEDEI	AQGEELDSLD	GGDEIENNEN	DE

Cmpd.	No. of Cmpds.	m/z meas.	Δ m/z [ppm]	z	Rt [min]	Score	P	Range	Sequence	Modification
2753	2	937.4586	-76.18	2	63.4	14.1	2	343-360	K.ASLKLGLDKAVLQSMSGR.E	
1713	1	806.6994	-162.85	2	51.4	15.5	0	750-765	R.GTDMLADGGDGGEFDR.E	
2811	1	995.0043	51.30	2	63.6	17.4	0	995-1010	K.LCQPSLDLPEWWECGR.H	Carbamidomethyl: 14

Detailed Protein Report

Protein 141: PREDICTED: rho guanine nucleotide exchange factor 5-like, partial [Homo sapiens]

**Accession:**	gi|578844357	**Score:**	46.6
**Database:**	refseq_human(refseq_human_20140103.fasta)	**MW [kDa]:**	165.0
**Database Date:**	2015-11-30	**pI:**	5.4
		**Sequence Coverage [%]:**	2.9
		**No. of unique Peptides:**	2

10	20	30	40	50	60	70	80
FSIIPEAPMR	SSQVSALGLE	AQEDEDPSYK	WREEHRLSAT	QQSELRDVCD	YAIETMPSFP	KEGSADVEPN	QESLVAEACD
90	100	110	120	130	140	150	160
TPEHWEAVPQ	SLAGRQARTL	APPELWACPI	QSEHLDMAPF	SSDLGSEEEE	VEFWPGLTSL	TLGSGQAEEE	EETSSDNSGQ
170	180	190	200	210	220	230	240
TRYYSPCEEH	PAETNQNEGS	ESGTIRQGEE	LPPEELQESQ	GLLHPQEVQV	LEEQGQQEAG	FRGEGTLRED	VCADGLLGEE
250	260	270	280	290	300	310	320
QMIEQVNDEK	GEQKQKQEQV	QDVMLGRQGE	RMGLTGEPEG	LNDGEWEQED	MERKAQGQGG	PEQGEERKRE	LQVPEENRAD
330	340	350	360	370	380	390	400
SQDEKSQIFL	GKSEEVTGKQ	EDHGIKEKGV	PVSGQEAKEP	ESWDGGRLGA	VGRARSREEE	NEHHGPSMPA	LIAPEDSPHC
410	420	430	440	450	460	470	480
DLFPGASYLM	TQIPGTQTES	RAEELSPAAL	SPSLEPIRCS	HQPISLLGSF	LTEESPDKEI	DQNSQQEGSR	LRKGTVSSQG
490	500	510	520	530	540	550	560
TEVVFASASV	TPPRTPDSAP	PSPAEAYPIT	PASVSARPPV	AFPRRETSCA	ARAPETASAP	LSMDDPSPCG	TSEMCPAALY
570	580	590	600	610	620	630	640
GFPSTGTSPP	RPPANSTGTV	QHLRSDSFPG	SHRTEQTPDL	VGMLLSYSHS	ELPQRPPKPA	IYSSVTPRRD	RRSGRDYSTV
650	660	670	680	690	700	710	720
SASPTALSTL	KQDSQESISN	LERPSSPPSI	QPWVSPHNPA	FATESPAYGS	SPSFVSMEDV	RIHEPLPPPP	PQRRDTHPSV
730	740	750	760	770	780	790	800
VETDGHARVV	VPTLKQHSHP	PPLALGSGLH	APHKGPLPQA	SDPAVARQHR	PLPSTPDSSH	HAQATPRWRY	NKPLPPTPDL
810	820	830	840	850	860	870	880
PQPHLPPISA	PGSSRIYRPL	PPLPIIDPPT	EPPPLPPKSR	GRSRSTRGGH	MNSGGHAKTR	PACQDWTVPL	PASAGRTSWP
890	900	910	920	930	940	950	960
PATARSTESF	TSTSRSKSEV	SPGMAFSNMT	NFLCPSSPTT	PWTPELQGPT	SKDEAGVSEH	PEAPAREPLR	RTTPQQGASG
970	980	990	1000	1010	1020	1030	1040
PGRSPVGQAR	QPEKPSHLHL	EKASSWPHRR	DSGRPPGDSS	GQAVAPSEGA	NKHKGWSRQG	LRRPSILPEG	SSDSRGPAVE
1050	1060	1070	1080	1090	1100	1110	1120
KHPGPSDTVV	FREKKPKEVM	GGFSRRCSKL	INSSQLLYQE	YSDVVLNKEI	QSQQRLESLS	ETPGPSSPRQ	PRKALVSSES
1130	1140	1150	1160	1170	1180	1190	1200
YLQRLSMASS	GSLWQEIPVV	RNSTVLLSMT	HEDQKLQEVK	FELIVSEASY	LRSLNIAVDH	FQLSTSLRAT	LSNQEHQWLF
1210	1220	1230	1240	1250	1260	1270	1280
SRLQDVRDVS	ATFLSDLEEN	FENNIFSFQV	CDVVLNHAPD	FRRVYLPYVT	NQTYQERTFQ	SLMNSNSNFR	EVLEKLESDP
1290	1300	1310	1320	1330	1340	1350	1360
VCQRLSLKSF	LILPFQRITR	LKLLLQNILK	RTQPGSSEEA	EATKAHHALE	QLIRDCNNNV	QSMRRTEELI	YLSQKIEFEC
1370	1380	1390	1400	1410	1420	1430	1440
KIFPLISQSR	WLVKSGELTA	LEFSASPGLR	RKLNTRPVHL	HLFNDCLLLS	RPREGSRFLV	FDHAPFSSIR	GEKCEMKLHG
1450	1460	1470	1480	1490	1500		
PHKNLFRLFL	RQNTQGAQAE	FLFRTETQSE	KLRWISALAM	PREELDLLEC	YTM		

Cmpd.	No. of Cmpds.	m/z meas.	Δ m/z [ppm]	z	Rt [min]	Score	P	Range	Sequence	Modification
2416	1	447.0974	-266.42	2	60.5	12.0	1	525-532	R.RETSCAAR.A	
1626	1	1058.4994	-29.00	2	49.0	12.0	2	933-951	K.DEAGVSEHPEAPAREPLRR.T	

Detailed Protein Report

Protein 142: numb-like protein [Homo sapiens]

**Accession:**	gi|10863899	**Score:**	46.6
**Database:**	refseq_human(refseq_human_20140103.fasta)	**MW [kDa]:**	64.9
**Database Date:**	2015-11-30	**pI:**	10.1
		**Sequence Coverage [%]:**	9.9
		**No. of unique Peptides:**	3

Quantitation

mdown:qdown **Median:** 1.04 **CV:** 0.00 % **No. of Peptides:** 1

10	20	30	40	50	60	70	80
MSRSAAASGG	PRRPERHLPP	APCGAPGPPE	TCRTEPDGAG	TMNKLRQSLR	RRKPAYVPEA	SRPHQWQADE	DAVRKGTCSF
90	100	110	120	130	140	150	160
PVRYLGHVEV	EESRGMHVCE	DAVKKLKAMG	RKSVKSVLWV	SADGLRVVDD	KTKDLLVDQT	IEKVSFCAPD	RNLDKAFSYI
170	180	190	200	210	220	230	240
CRDGTTRRWI	CHCFLALKDS	GERLSHAVGC	AFAACLERKQ	RREKECGVTA	AFDASRTSFA	REGSFRLSGG	GRPAEREAPD
250	260	270	280	290	300	310	320
KKKAEAAAAP	TVAPGPAQPG	HVSPTPATTS	PGEKGEAGTP	VAAGTTAAAI	PRRHAPLEQL	VRQGSFRGFP	ALSQKNSPFK
330	340	350	360	370	380	390	400
RQLSLRLNEL	PSTLQRRTDF	QVKGTVPEME	PPGAGDSDSI	NALCTQISSS	FASAGAPAPG	PPPATTGTSA	WGEPSVPPAA
410	420	430	440	450	460	470	480
AFQPGHKRTP	SEAERWLEEV	SQVAKAQQQQ	QQQQQQQQQQ	QQQQQQAASV	APVPTMPPAL	QPFPAPVGPF	DAAPAQVAVF
490	500	510	520	530	540	550	560
LPPPHMQPPF	VPAYPGLGYP	PMPRVPVVGI	TPSQMVANAF	CSAAQLQPQP	ATLLGKAGAF	PPPAIPSAPG	SQARPRPNGA
570	580	590	600	610			
PWPPEPAPAP	APELDPFEAQ	WAALEGKATV	EKPSNPFSGD	LQKTFEIEL			

Cmpd.	No. of Cmpds.	m/z meas.	Δ m/z [ppm]	z	Rt [min]	Score	P	Range	Sequence	Modification	Ratios
2693	1	1023.5273	37.29	2	64.9	10.3	2	203-221	R.EKECGVTAAFDASRTSFAR.E		
2525	1	834.4332	-13.93	2	62.0	11.3	2	227-242	R.LSGGGRPAEREAPDKK.K		
2024	2	788.7867	-185.87	2	53.8	13.7	2	308-321	R.GFPALSQKNSPFKR.Q		mdown:qdown 1.04

Detailed Protein Report

Protein 143: PREDICTED: zinc finger protein 708 isoform X1 [Homo sapiens]

Accession: gi|578833563
Database: refseq_human(refseq_human_20140103.fasta)
Database Date: 2015-11-30
Modification(s): Carbamidomethyl

Score: 46.4
MW [kDa]: 57.3
pI: 10.4
Sequence Coverage [%]: 6.0
No. of unique Peptides: 2

Quantitation

mdown:qdown	**Median:** 2.04	**CV:** 0.00 %	**No. of Peptides:**	1
Wdown:Qdown	**Median:** 1.15	**CV:** 0.00 %	**No. of Peptides:**	1

```
        10         20         30         40         50         60         70         80
MKRHEMAAKP PAMCSHFAKD LRPEQYIKNS FQQVILRRYG KCGYQKGCKS VDEHKLHKGG HKGLNRCVTT TQSKIVQCDK
        90        100        110        120        130        140        150        160
YVKVFHKYSN AKRHKIRHTG KNPFKCKECG KSFCMLSQLT QHEIIHTGEK PYKCEECGKA FKKSSNLTNH KIIHTGEKPY
       170        180        190        200        210        220        230        240
KCEECGKAFN QSSTLTRHKI IHTGEKLYKC EECGKAFNRS SNLTKHKIVH TGEKPYKCEE CGKAFKQSSN LTNHKKIHTG
       250        260        270        280        290        300        310        320
EKPYKCGECG KAFTLSSHLT THKRIHTGEK PYKCEECGKA FSVFSTLTKH KIIHTEEKPY KCEECGKAFN RSSHLTNHKV
       330        340        350        360        370        380        390        400
IHTGEKPYKC EECGKAFTKS STLTYHKVIH TGKKPYKCEE CGKAFSIFSI LTKHKVIHTE DKPYKCEECG KTFNYSSNFT
       410        420        430        440        450        460        470        480
NHKKIHTGEK PYKCEECGKS FILSSHLTTH KIIHTGEKPY KCKECGKAFN QSSTLMKHKI IHTGEKPYKC EECGKAFNQS
       490        500
PNLTKHKRIH TKEKPYKCK
```

Cmpd.	No. of Cmpds.	m/z meas.	Δ m/z [ppm]	z	Rt [min]	Score	P	Range	Sequence	Modification	Ratios
328	4	605.6684	-199.06	2	32.8	13.8	2	102-111	K.NPFKCKECGK.S	Carbamidomethyl: 8	Wdown:Qdown 1.15<br>mdown:qdown 2.04
1130	6	586.6095	-252.07	2	43.8	19.3	1	330-339	K.CEECGKAFTK.S	Carbamidomethyl: 4	

Detailed Protein Report

Protein 144: rap guanine nucleotide exchange factor 3 isoform b [Homo sapiens]

**Accession:**	gi|20070215	**Score:**	46.3
**Database:**	refseq_human(refseq_human_20140103.fasta)	**MW [kDa]:**	99.3
**Database Date:**	2015-11-30	**pI:**	8.6
**Modification(s):**	Carbamidomethyl, Oxidation	**Sequence Coverage [%]:**	2.5
		**No. of unique Peptides:**	2

Quantitation

mdown:qdown	**Median:** 0.50		**CV:** 6.46 %		**No. of Peptides:**	2

Alias proteins:

Accession	Name	Description
gi|148747862	refseq_human (refseq_human_20140103.fasta)	rap guanine nucleotide exchange factor 3 isoform b [Homo sapiens]

10	20	30	40	50	60	70	80
MVLRRMHRPR	SCSYQLLLEH	QRPSCIQGLR	WTPLTNSEES	LDFSESLEQA	STERVLRAGR	QLHRHLLATC	PNLIRDRKYH
**90**	**100**	**110**	**120**	**130**	**140**	**150**	**160**
LRLYRQCCSG	RELVDGILAL	GLGVHSRSQV	VGICQVLLDE	GALCHVKHDW	AFQDRDAQFY	RFPGPEPEPV	GTHEMEEELA
**170**	**180**	**190**	**200**	**210**	**220**	**230**	**240**
EAVALLSQRG	PDALLTVALR	KPPGQRTDEE	LDLIFEELLH	IKAVAHLSNS	VKRELAAVLL	FEPHSKAGTV	LFSQGDKGTS
**250**	**260**	**270**	**280**	**290**	**300**	**310**	**320**
WYIIWKGSVN	VVTHGKGLVT	TLHEGDDFGQ	LALVNDAPRA	ATIILREDNC	HFLRVDKQDF	NRIIKDVEAK	TMRLEEHGKV
**330**	**340**	**350**	**360**	**370**	**380**	**390**	**400**
VLVLERASQG	AGPSRPPTPG	RNRYTVMSGT	PEKILELLLE	AMGPDSSAHD	PTETFLSDFL	LTHRVFMPSA	QLCAALLHHF
**410**	**420**	**430**	**440**	**450**	**460**	**470**	**480**
HVEPAGGSEQ	ERSTYVCNKR	QQILRLVSQW	VALYGSMLHT	DPVATSFLQK	LSDLVGRDTR	LSNLLREQWP	ERRRCHRLEN
**490**	**500**	**510**	**520**	**530**	**540**	**550**	**560**
GCGNASPQMK	ARNLPVWLPN	QDEPLPGSSC	AIQVGDKVPY	DICRPDHSVL	TLQLPVTASV	REVMAALAQE	DGWTKGQVLV
**570**	**580**	**590**	**600**	**610**	**620**	**630**	**640**
KVNSAGDAIG	LQPDARGVAT	SLGLNERLFV	VNPQEVHELI	PHPDQLGPTV	GSAEGLDLVS	AKDLAGQLTD	HDWSLFNSIH
**650**	**660**	**670**	**680**	**690**	**700**	**710**	**720**
QVELIHYVLG	PQHLRDVTTA	NLERFMRRFN	ELQYWVATEL	CLCPVPGPRA	QLLRKFIKLA	AHLKEQKNLN	SFFAVMFGLS
**730**	**740**	**750**	**760**	**770**	**780**	**790**	**800**
NSAISRLAHT	WERLPHKVRK	LYSALERLLD	PSWNHRVYRL	ALAKLSPPVI	PFMPLLLKDM	TFIHEGNHTL	VENLINFEKM
**810**	**820**	**830**	**840**	**850**	**860**	**870**	**880**
RMMARAARML	HHCRSHNPVP	LSPLRSRVSH	LHEDSQVARI	STCSEQSLST	RSPASTWAYV	QQLKVIDNQR	ELSRLSRELE
**890**							
P							

Cmpd.	No. of Cmpds.	m/z meas.	Δ m/z [ppm]	z	Rt [min]	Score	P	Range	Sequence	Modification	Ratios
870	1	711.3337	31.93	2	39.7	13.9	0	478-490	R.LENGCGNASPQMK.A	Carbamidomethyl: 5; Oxidation: 12	mdown:qdown 0.47
997	3	555.1971	-145.78	2	41.0	15.0	2	800-808	K.MRMMARAAR.M	Oxidation: 1	mdown:qdown 0.54

Detailed Protein Report

Protein 145: transformation/transcription domain-associated protein isoform 2 [Homo sapiens]

**Accession:**	gi|4507691	**Score:**	46.3
**Database:**	refseq_human(refseq_human_20140103.fasta)	**MW [kDa]:**	434.1
**Database Date:**	2015-11-30	**pI:**	9.3
**Modification(s):**	Oxidation	**Sequence Coverage [%]:**	1.5
		**No. of unique Peptides:**	2

Detailed Protein Report

10	20	30	40	50	60	70	80
MAFVATQGAT	VVDQTTLMKK	YLQFVAALTD	VNTPDETKLK	MMQEVSENFE	NVTSSPQYST	FLEHIIPRFL	TFLQDGEVQF
90	100	110	120	130	140	150	160
LQEKPAQQLR	KLVLEIIHRI	PTNEHLRPHT	KNVLSVMFRF	LETENEENVL	ICLRIIIELH	KQFRPPITQE	IHHFLDFVKQ
170	180	190	200	210	220	230	240
IYKELPKVVN	RYFENPQVIP	ENTVPPPEMV	GMITTIAVKV	NPEREDSETR	THSIIPRGSL	SLKVLAELPI	IVVLMYQLYK
250	260	270	280	290	300	310	320
LNIHNVVAEF	VPLIMNTIAI	QVSAQARQHK	LYNKELYADF	IAAQIKTLSF	LAYIIRIYQE	LVTKYSQQMV	KGMLQLLSNC
330	340	350	360	370	380	390	400
PAETAHLRKE	LLIAAKHILT	TELRNQFIPC	MDKLFDESIL	IGSGYTARET	LRPLAYSTLA	DLVHHVRQHL	PLSDLSLAVQ
410	420	430	440	450	460	470	480
LFAKNIDDES	LPSSIQTMSC	KLLLNLVDCI	RSKSEQESGN	GRDVLMRMLE	VFVLKFHTIA	RYQLSAIFKK	CKPQSELGAV
490	500	510	520	530	540	550	560
EAALPGVPTA	PAAPGPAPSP	APVPAPPPPP	PPPPPATPVT	PAPVPPFEKQ	GEKDKEDKQT	FQVTDCRSLV	KTLVCGVKTI
570	580	590	600	610	620	630	640
TWGITSCKAP	GEAQFIPNKQ	LQPKETQIYI	KLVKYAMQAL	DIYQVQIAGN	GQTYIRVANC	QTVRMKEEKE	VLEHFAGVFT
650	660	670	680	690	700	710	720
MMNPLTFKEI	FQTTVPYMVE	RISKNYALQI	VANSFLANPT	TSALFATILV	EYLLDRLPEM	GSNVELSNLY	LKLFKLVFGS
730	740	750	760	770	780	790	800
VSLFAAENEQ	MLKPHLHKIV	NSSMELAQTA	KEPYNYFLLL	RALFRSIGGG	SHDLLYQEFL	PLLPNLLQGL	NMLQSGLHKQ
810	820	830	840	850	860	870	880
HMKDLFVELC	LTVPVRLSSL	LPYLPMLMDP	LVSALNGSQT	LVSQGLRTLE	LCVDNLQPDF	LYDHIQPVRA	ELMQALWRTL
890	900	910	920	930	940	950	960
RNPADSISHV	AYRVLGKFGG	SNRKMLKESQ	KLHYVVTEVQ	GPSITVEFSD	CKASLQLPME	KAIETALDCL	KSANTEPYYR
970	980	990	1000	1010	1020	1030	1040
RQAWEVIKCF	LVAMMSLEDN	KHALYQLLAH	PNFTEKTIPN	VIISHRYKAQ	DTPARKTFEQ	ALTGAFMSAV	IKDLRPSALP
1050	1060	1070	1080	1090	1100	1110	1120
FVASLIRHYT	MVAVAQQCGP	FLLPCYQVGS	QPSTAMFHSE	ENGSKGMDPL	VLIDAIAICM	AYEEKELCKI	GEVALAVIFD
1130	1140	1150	1160	1170	1180	1190	1200
VASIILGSKE	RACQLPLFSY	IVERLCACCY	EQAWYAKLGG	VVSIKFLMER	LPLTWVLQNQ	QTFLKALLFV	MMDLTGEVSN
1210	1220	1230	1240	1250	1260	1270	1280
GAVAMAKTTL	EQLLMRCATP	LKDEERAEEI	VAAQEKSFHH	VTHDLVREVT	SPNSTVRKQA	MHSLQVLAQV	TGKSVTVIME
1290	1300	1310	1320	1330	1340	1350	1360
PHKEVLQDMV	PPKKHLLRHQ	PANAQIGLME	GNTFCTTLQP	RLFTMDLNVV	EHKVFYTELL	NLCEAEDSAL	TKLPCYKSLP
1370	1380	1390	1400	1410	1420	1430	1440
SLVPLRIAAL	NALAACNYLP	QSREKIIAAL	FKALNSTNSE	LQEAGEACMR	KFLEGATIEV	DQIHTHMRPL	LMMLGDYRSL
1450	1460	1470	1480	1490	1500	1510	1520
TLNVVNRLTS	VTRLFPNSFN	DKFCDQMMQH	LRKWMEVVVI	THKGGQRSDG	NEMKICSAII	NLFHLIPAAP	QTLVKPLLEV
1530	1540	1550	1560	1570	1580	1590	1600
VMKTERAMLI	EAGSPFREPL	IKFLTRHPSQ	TVELFMMEAT	LNDPQWSRMF	MSFLKHKDAR	PLRDVLAANP	NRFITLLLPG
1610	1620	1630	1640	1650	1660	1670	1680
GAQTAVRPGS	PSTSTMRLDL	QFQAIKIISI	IVKNDDSWLA	SQHSLVSQLR	RVWVSENFQE	RHRKENMAAT	NWKEPKLLAY
1690	1700	1710	1720	1730	1740	1750	1760
CLLNYCKRNY	GDIELLFQLL	RAFTGRFLCN	MTFLKEYMEE	EIPKNYSIAQ	KRALFFRFVD	FNDPNFGDEL	KAKVLQHILN
1770	1780	1790	1800	1810	1820	1830	1840
PAFLYSFEKG	EGEQLLGPPN	PEGDNPESIT	SVFITKVLDP	EKQADMLDSL	RIYLLQYATL	LVEHAPHHIH	DNNKNRNSKL
1850	1860	1870	1880	1890	1900	1910	1920
RRLMTFAWPC	LLSKACVDPA	CKYSGHLLLA	HIIAKFAIHK	KIVLQVFHSL	LKAHAMEARA	IVRQAMAILT	PAVPARMEDG
1930	1940	1950	1960	1970	1980	1990	2000
HQMLTHWTRK	IIVEEGHTVP	QLVHILHLIV	QHFKVYYPVR	HHLVQHMVSA	MQRLGFTPSV	TIEQRRLAVD	LSEVVIKWEL
2010	2020	2030	2040	2050	2060	2070	2080
QRIKDQQPDS	DMDPNSSGEG	VNSVSSSIKR	GLSVDSAQEV	KRFRTATGAI	SAVFGRSQSL	PGADSLLAKP	IDKQHTDTVV
2090	2100	2110	2120	2130	2140	2150	2160
NFLIRVACQV	NDNTNTAGSP	GEVLSRRCVN	LLKTALRPDM	WPKSELKLQW	FDKLLMTVEQ	PNQVNYGNIC	TGLEVLSFLL
2170	2180	2190	2200	2210	2220	2230	2240
TVLQSPAILS	SFKPLQRGIA	ACMTCGNTKV	LRAVHSLLSR	LMSIFPTEPS	TSSVASKYEE	LECLYAAVGK	VIYEGLTNYE
2250	2260	2270	2280	2290	2300	2310	2320
KATNANPSQL	FGTLMILKSA	CSNNPSYIDR	LISVFMRSLQ	KMVREHLNPQ	AASGSTEATS	GTSELVMLSL	ELVKTRLAVM
2330	2340	2350	2360	2370	2380	2390	2400

Detailed Protein Report

Cmpd.	No. of Cmpds.	m/z meas.	Δ m/z [ppm]	z	Rt [min]	Score	P	Range	Sequence	Modification
873	1	946.4119	-18.65	3	40.4	15.0	1	2003-2029	R.IKDQQPDSDMDPNSSGEGVNSVSSSIK.R	Oxidation: 10
2507	1	913.4337	-85.65	2	61.8	14.5	2	2373-2387	R.EKSILLVKMMTYIEK.R	

Detailed Protein Report

Protein 146: alpha-1-acid glycoprotein 2 precursor [Homo sapiens]

Accession: gi|4505529
Database: refseq_human(refseq_human_20140103.fasta)
Database Date: 2015-11-30

Score: 46.2
MW [kDa]: 23.6
pI: 4.9
Sequence Coverage [%]: 5.0
No. of unique Peptides: 1

10	20	30	40	50	60	70	80
MALSWVLTVL	SLLPLLEAQI	PLCANLVPVP	ITNATLDRIT	GKWFYIASAF	RNEEYNKSVQ	EIQATFFYFT	PNKTEDTIFL
**90**	**100**	**110**	**120**	**130**	**140**	**150**	**160**
REYQTRQNQC	FYNSSYLNVQ	RENGTVSRYE	GGREHVAHLL	FLRDTKTLMF	GSYLDDEKNW	GLSFYADKPE	TTKEQLGEFY
**170**	**180**	**190**	**200**	**210**			
EALDCLCIPR	SDVMYTDWKK	DKCEPLEKQH	EKERKQEEGE	S			

Cmpd.	No. of Cmpds.	m/z meas.	Δ m/z [ppm]	z	Rt [min]	Score	P	Range	Sequence	Modification
1023	3	617.8379	-30.01	2	41.5	46.2	0	114-123	R.EHVAHLLFLR.D	

Detailed Protein Report

Protein 147: uncharacterized protein C4orf21 [Homo sapiens]

**Accession:**	gi|254826809	**Score:**	46.1
**Database:**	refseq_human(refseq_human_20140103.fasta)	**MW [kDa]:**	236.5
**Database Date:**	2015-11-30	**pI:**	5.8
		**Sequence Coverage [%]:**	2.1
		**No. of unique Peptides:**	2

Quantitation

mdown:qdown	**Median:** 1.15	**CV:** 0.00 %	**No. of Peptides:** 1

Alias proteins:

Accession	Name	Description
gi|530377937	refseq_human (refseq_human_20140103.fasta)	PREDICTED: uncharacterized protein C4orf21 isoform X1 [Homo sapiens]

Detailed Protein Report

10	20	30	40	50	60	70	80
MESQEFIVLY	THQKMKKSKV	WQDGILKITH	LGNKAILYDD	KGACLESLFL	KCLEVKPGDD	LESDRYLITV	EEVKVAGAIG
90	100	110	120	130	140	150	160
IVKQNVNKEA	PELNSRTFIS	SGRSLGCQPS	GLKRKFTGFQ	GPRQVPKKMV	IMESGESAAS	HEAKKTGPTI	FSPFCSMPPL
170	180	190	200	210	220	230	240
FPTVGKKDVN	NILADPENIV	TYKNRERNAM	DFSSVFSPSF	QINPEVLCEE	NYFCSPVNSG	NKLSDSLLTN	EPVKRDSLAS
250	260	270	280	290	300	310	320
HYSGVSQNIR	SKAQILALLK	SESSSSCEEL	NSEMTEHFPQ	KQPQGSLKIA	TKPKYLIQQE	ECAEMKSTEN	LYYQHQSENT
330	340	350	360	370	380	390	400
MRNKSRWAMY	LSSQSSPIHS	STVDGNDTER	KPKAQEDDVN	SNLKDLSLQK	IIQFVETYAE	ERKKYNVDQS	VGNNDPSWNQ
410	420	430	440	450	460	470	480
EVKLEIPSFN	ESSSLQVTCS	SAENDGILSE	SDIQEDNKIP	FNQNDKGCIK	GSVLIKENAQ	EVNTCGTLEK	EYEQSESSLP
490	500	510	520	530	540	550	560
ELKHLQIESS	NNSRISDDIT	DMISESKMDN	ESLNSIHESL	SNVTQPFLEV	TFNLNNFETS	DTEEESQESN	KISQDSESWV
570	580	590	600	610	620	630	640
KDILVNDGNS	CFQKRSENTN	CEEIEGEHLP	FLTSVSDKPT	VTFPVKETLP	SQFCDKTYVG	FDMGICKTEN	TGKEIEEYSD
650	660	670	680	690	700	710	720
TLSNFESFKW	TDAVYGDNKE	DANKPIQEVR	INYDFALPPN	KSKGINMNLH	IPHIQNQIAE	NSNLFSEDAQ	PQPFILGSDL
730	740	750	760	770	780	790	800
DKNDEHVLPS	TSSSDNSVQL	LNTNQNHYEC	IALDKSNTHI	SNSLFYPLGK	KHLISKDTEA	HISEPEDLGK	IRSPPPDHVE
810	820	830	840	850	860	870	880
VETAREGKQY	WNPRNSSELS	GLVNTISILK	SLCEHSTALD	SLEILKKKNT	VFQQGTQQTY	EPDSPPEVRK	PFITVVSPKS
890	900	910	920	930	940	950	960
PHLHKDSQQI	LKEDEVELSE	PLQSVQFSSS	GSKEETAFQA	VIPKQIERKT	CDPKPVEFQG	HQVKGSATSG	VMVRGHSSQL
970	980	990	1000	1010	1020	1030	1040
GCSQFPDSTE	YENFMTETPE	LPSTCMQIDF	LQVTSPEENI	STLSPVSTFS	LNSRDEDFMV	EFSETSLKAR	TLPDDLHFLN
1050	1060	1070	1080	1090	1100	1110	1120
LEGMKKSRSL	ENENLQRLSL	LSRTQVPLIT	LPRTDGPPDL	DSHSYMINSN	TYESSGSPML	NLCEKSAVLS	FSIEPEDQNE
1130	1140	1150	1160	1170	1180	1190	1200
TFFSEESREV	NPGDVSLNNI	STQSKWLKYQ	NTSQCNVATP	NRVDKRITDG	FFAEAVSGMH	FRDTSERQSD	AVNESSLDSV
1210	1220	1230	1240	1250	1260	1270	1280
HLQMIKGMLY	QQRQDFSSQD	SVSRKKVLSL	NLKQTSKTEE	IKNVLGGSTC	YNYSVKDLQE	ISGSELCFPS	GQKIKSAYLP
1290	1300	1310	1320	1330	1340	1350	1360
QRQIHIPAVF	QSPAHYKQTF	TSCLIEHLNI	LLFGLAQNLQ	KALSKVDISF	YTSLKGEKLK	NAENNVPSCH	HSQPAKLVMV
1370	1380	1390	1400	1410	1420	1430	1440
KKEGPNKGRL	FYTCDGPKAD	RCKFFKWLED	VTPGYSTQEG	ARPGMVLSDI	KSIGLYLRSQ	KIPLYEECQL	LVRKGFDFQR
1450	1460	1470	1480	1490	1500	1510	1520
KQYGKLKKFT	TVNPEFYNEP	KTKLYLKLSR	KERSSAYSKN	DLWVVSKTLD	FELDTFIACS	AFFGPSSINE	IEILPLKGYF
1530	1540	1550	1560	1570	1580	1590	1600
PSNWPTNMVV	HALLVCNAST	ELTTLKNIQD	YFNPATLPLT	QYLLTTSSPT	IVSNKRVSKR	KFIPPAFTNV	STKFELLSLG
1610	1620	1630	1640	1650	1660	1670	1680
ATLKLASELI	QVHKLNKDQA	TALIQIAQMM	ASHESIEEVK	ELQTHTFPIT	IIHGVFGAGK	SYLLAVVILF	FVQLFEKSEA
1690	1700	1710	1720	1730	1740	1750	1760
PTIGNARPWK	LLISSSTNVA	VDRVLLGLLS	LGFENFIRVG	SVRKIAKPIL	PYSLHAGSEN	ESEQLKELHA	LMKEDLTPTE
1770	1780	1790	1800	1810	1820	1830	1840
RVYVRKSIEQ	HKLGTNRTLL	KQVRVVGVTC	AACPFPCMND	LKFPVVVLDE	CSQITEPASL	LPIARFECEK	LILVGDPKQL
1850	1860	1870	1880	1890	1900	1910	1920
PPTIQGSDAA	HENGLEQTLF	DRLCLMGHKP	ILLRTQYRCH	PAISAIANDL	FYKGALMNGV	TEIERSPLLE	WLPTLCFYNV
1930	1940	1950	1960	1970	1980	1990	2000
KGLEQIERDN	SFHNVAEATF	TLKLIQSLIA	SGIAGSMIGV	ITLYKSQMYK	LCHLLSAVDF	HHPDIKTVQV	STVDAFQGAE
2010	2020	2030	2040	2050	2060	2070	2080
KEIIILSCVR	TRQVGFIDSE	KRMNVALTRG	KRHLLIVGNL	ACLRKNQLWG	RVIQHCEGRE	DGLQHANQYE	PQLNHLLKDY
2090	2100	2110					
FEKQVEEKQK	KKSEKEKSKD	KSHS					

Detailed Protein Report

Cmpd.	No. of Cmpds.	m/z meas.	Δ m/z [ppm]	z	Rt [min]	Score	P	Range	Sequence	Modification	Ratios
2940	2	973.5071	-8.25	2	65.5	10.8	1	167-183	K.KDVNNILADPENIVTYK.N		mdown:qdown 1.15
2930	7	698.3835	-51.56	2	65.8	21.0	1	282-294	K.QPQGSLKIATKPK.Y		

Detailed Protein Report

Protein 148: **serine/threonine-protein kinase Nek1 isoform 5 [Homo sapiens]**

**Accession:**	gi|313661434	**Score:**	45.9
**Database:**	refseq_human(refseq_human_20140103.fasta)	**MW [kDa]:**	138.0
**Database Date:**	2015-11-30	**pI:**	5.4
		**Sequence Coverage [%]:**	5.2
		**No. of unique Peptides:**	1

10	20	30	40	50	60	70	80
MEKYVRLQKI	GEGSFGKAIL	VKSTEDGRQY	VIKEINISRM	SSKEREESRR	EVAVLANMKH	PNIVQYRESF	EENGSLYIVM
**90**	**100**	**110**	**120**	**130**	**140**	**150**	**160**
DYCEGGDLFK	RINAQKGVLF	QEDQILDWFV	QICLALKHVH	DRKILHRDIK	SQNIFLTKDG	TVQLGDFGIA	RVLNSTVELA
**170**	**180**	**190**	**200**	**210**	**220**	**230**	**240**
RTCIGTPYYL	SPEICENKPY	NNKSDIWALG	CVLYELCTLK	HAFEAGSMKN	LVLKIISGSF	PPVSLHYSYD	LRSLVSQLFK
**250**	**260**	**270**	**280**	**290**	**300**	**310**	**320**
RNPRDRPSVN	SILEKGFIAK	RIEKFLSPQL	IAEEFCLKTF	SKFGSQPIPA	KRPASGQNSI	SVMPAQKITK	PAAKYGIPLA
**330**	**340**	**350**	**360**	**370**	**380**	**390**	**400**
YKKYGDKKLH	EKKPLQKHKQ	AHQTPEKRVN	TGEERRKISE	EAARKRRLEF	IEKEKKQKDQ	IISLMKAEQM	KRQEKERLER
**410**	**420**	**430**	**440**	**450**	**460**	**470**	**480**
INRAREQGWR	NVLSAGGSGE	VKAPFLGSGG	TIAPSSFSSR	GQYEHYHAIF	DQMQQQRAED	NEAKWKREIY	GRGLPERQKG
**490**	**500**	**510**	**520**	**530**	**540**	**550**	**560**
QLAVERAKQV	EEFLQRKREA	MQNKARAEGH	MVYLARLRQI	RLQNFNERQQ	IKAKLRGEKK	EANHSEGQEG	SEEADMRRKK
**570**	**580**	**590**	**600**	**610**	**620**	**630**	**640**
IESLKAHANA	RAAVLKEQLE	RKRKEAYERE	KKVWEEHLVA	KGVKSSDVSP	PLGQHETGGS	PSKQQMRSVI	SVTSALKEVG
**650**	**660**	**670**	**680**	**690**	**700**	**710**	**720**
VDSSLTDTRE	TSEEMQKTNN	AISSKREILR	RLNENLKAQE	DEKGKQNLSD	TFEINVHEDA	KEHEKEKSVS	SDRKKWEAGG
**730**	**740**	**750**	**760**	**770**	**780**	**790**	**800**
QLVIPLDELT	LDTSFSTTER	HTVGEVIKLG	PNGSPRRAWG	KSPTDSVLKI	LGEAELQLQT	ELLENTTIRS	EISPEGEKYK
**810**	**820**	**830**	**840**	**850**	**860**	**870**	**880**
PLITGEKKVQ	CISHEINPSA	IVDSPVETKS	PEFSEASPQM	SLKLEGNLEE	PDDLETEILQ	EPSGTNKDES	LPCTITDVWI
**890**	**900**	**910**	**920**	**930**	**940**	**950**	**960**
SEEKETKETQ	SADRITIQEN	EVSEDGVSST	VDQLSDIHIE	PGTNDSQHSK	CDVDKSVQPE	PFFHKVVHSE	HLNLVPQVQS
**970**	**980**	**990**	**1000**	**1010**	**1020**	**1030**	**1040**
VQCSPEESFA	FRSHSHLPPK	NKNKNSLLIG	LSTGLFDANN	PKMLRTCSLP	DLSKLFRTLM	DVPTVGDVRQ	DNLEIDEIED
**1050**	**1060**	**1070**	**1080**	**1090**	**1100**	**1110**	**1120**
ENIKEGPSDS	EDIVFEETDT	DLQELQASME	QLLREQPGEE	YSEEEESVLK	NSDVEPTANG	TDVADEDDNP	SSESALNEEW
**1130**	**1140**	**1150**	**1160**	**1170**	**1180**	**1190**	**1200**
HSDNSDGEIA	SECECDSVFN	HLEELRLHLE	QEMGFEKFFE	VYEKIKAIHE	DEDENIEICS	KIVQNILGNE	HQHLYAKILH
**1210**	**1220**						
LVMADGAYQE	DNDE						

Cmpd.	No. of Cmpds.	m/z meas.	Δ m/z [ppm]	z	Rt [min]	Score	P	Range	Sequence	Modification
894	1	946.4617	-25.43	3	40.0	16.1	1	411-440	R.NVLSAGGSGEVKAPFLGSGGTIAPSSFSSR.G	

Detailed Protein Report

Protein 149: neutrophil defensin 1 preproprotein [Homo sapiens]

Accession: gi|4758146
Database: refseq_human(refseq_human_20140103.fasta)
Database Date: 2015-11-30
Modification(s): Carbamidomethyl

Score: 45.9
MW [kDa]: 10.2
pI: 7.6
Sequence Coverage [%]: 9.6
No. of unique Peptides: 1

Quantitation

mdown:qdown **Median:** 1.15 **CV:** 0.00 % **No. of Peptides:** 1

Alias proteins:

Accession	Name	Description
gi|124248516	refseq_human (refseq_human_20140103.fasta)	neutrophil defensin 1 precursor [Homo sapiens]

```
        10         20         30         40         50         60         70         80
MRTLAILAAI LLVALQAQAE PLQARADEVA AAPEQIAADI PEVVVSLAWD ESLAPKHPGS RKNMACYCRI PACIAGERRY
        90        100
GTCIYQGRLW AFCC
```

Cmpd.	No. of Cmpds.	m/z meas.	Δ m/z [ppm]	z	Rt [min]	Score	P	Range	Sequence	Modification	Ratios
108	2	493.6493	-220.14	2	30.7	45.9	0	70-78	R.IPACIAGER.R	Carbamidomethyl: 4	mdown:qdown 1.15

Detailed Protein Report

Protein 150: PREDICTED: chromodomain-helicase-DNA-binding protein 4 isoform X6 [Homo sapiens]

**Accession:**	gi|578822562	**Score:**	45.3
**Database:**	refseq_human(refseq_human_20140103.fasta)	**MW [kDa]:**	216.7
**Database Date:**	2015-11-30	**pI:**	5.7
**Modification(s):**	Carbamidomethyl	**Sequence Coverage [%]:**	1.5
		**No. of unique Peptides:**	2

Detailed Protein Report

10	20	30	40	50	60	70	80
MASGLGSPSP	CSAGSEEEDM	DALLNNSLPP	PHPENEEDPE	EDLSETETPK	LKKKKKPKKP	RDPKIPKSKR	QKKERMLLCR
90	100	110	120	130	140	150	160
QLGDSSGEGP	EFVEEEEEVA	LRSDSEGSDY	TPGKKKKKKL	GPKKEKKSKS	KRKEEEEEED	DDDDSKEPKS	SAQLLEDWGM
170	180	190	200	210	220	230	240
EDIDHVFSEE	DYRTLTNYKA	FSQFVRPLIA	AKNPKIAVSK	MMMVLGAKWR	EFSTNNPFKG	SSGASVAAAA	AAAVAVVESM
250	260	270	280	290	300	310	320
VTATEVAPPP	PPVEVPIRKA	KTKEGKGPNA	RRKPKGSPRV	PDAKKPKPKK	VAPLKIKLGG	FGSKRKRSSS	EDDDLDVESD
330	340	350	360	370	380	390	400
FDDASINSYS	VSDGSTSRSS	RSRKKLRTTK	KKKKDHQDYC	EVCQQGGEII	LCDTCPRAYH	MVCLDPDMEK	APEGKWSCPH
410	420	430	440	450	460	470	480
CEKEGIQWEA	KEDNSEGEEI	LEEVGGDLEE	EDDHHMEFCR	VCKDGGELLC	CDTCPSSYHI	HCLNPPLPEI	PNGEWLCPRC
490	500	510	520	530	540	550	560
TCPALKGKVQ	KILIWKWGQP	PSPTPVPRPP	DADPNTPSPK	PLEGRPERQF	FVKWQGMSYW	HCSWVSELQL	ELHCQVMFRN
570	580	590	600	610	620	630	640
YQRKNDMDEP	PSGDFGGDEE	KSRKRKNKDP	KFAEMEERFY	RYGIKPEWMM	IHRILNHSVD	KKGHVHYLIK	WRDLPYDQAS
650	660	670	680	690	700	710	720
WESEDVEIQD	YDLFKQSYWN	HRELMRGEEG	RPGKKLKKVK	LRKLERPPET	PTVDPTVKYE	RQPEYLDATG	GTLHPYQMEG
730	740	750	760	770	780	790	800
LNWLRFSWAQ	GTDTILADEM	GLGKTVQTAV	FLYSLYKEGH	SKGPFLVSAP	LSTIINWERE	FEMWAPDMYV	VTYVGDKDSR
810	820	830	840	850	860	870	880
AIIRENEFSF	EDNAIRGGKK	ASRMKKEASV	KFHVLLTSYE	LITIDMAILG	SIDWACLIVD	EAHRLKNNQS	KFFRVLNGYS
890	900	910	920	930	940	950	960
LQHKLLLTGT	PLQNNLEELF	HLLNFLTPER	FHNLEGFLEE	FADIAKEDQI	KKLHDMLGPH	MLRRLKADVF	KNMPSKTELI
970	980	990	1000	1010	1020	1030	1040
VRVELSPMQK	KYYKYILTRN	FEALNARGGG	NQVSLLNVVM	DLKKCCNHPY	LFPVAAMEAP	KMPNGMYDGS	ALIRASGKLL
1050	1060	1070	1080	1090	1100	1110	1120
LLQKMLKNLK	EGGHRVLIFS	QMTKMLDLLE	DFLEHEGYKY	ERIDGGITGN	MRQEAIDRFN	APGAQQFCFL	LSTRAGGLGI
1130	1140	1150	1160	1170	1180	1190	1200
NLATADTVII	YDSDWNPHND	IQAFSRAHRI	GQNKKVMIYR	FVTRASVEER	ITQVAKKKMM	LTHLVVRPGL	GSKTGSMSKQ
1210	1220	1230	1240	1250	1260	1270	1280
ELDDILKFGT	EELFKDEATD	GGGDNKEGED	SSVIHYDDKA	IERLLDRNQD	ETEDTELQGM	NEYLSSFKVA	QYVVREEEMG
1290	1300	1310	1320	1330	1340	1350	1360
EEEEVEREII	KQEESVDPDY	WEKLLRHHYE	QQQEDLARNL	GKGKRIRKQV	NYNDGSQEDR	DWQDDQSDNQ	SDYSVASEEG
1370	1380	1390	1400	1410	1420	1430	1440
DEDFDERSEA	PRRPSRKGLR	NDKDKPLPPL	LARVGGNIEV	LGFNARQRKA	FLNAIMRYGM	PPQDAFTTQW	LVRDLRGKSE
1450	1460	1470	1480	1490	1500	1510	1520
KEFKAYVSLF	MRHLCEPGAD	GAETFADGVP	REGLSRQHVL	TRIGVMSLIR	KKVQEFEHVN	GRWSMPELAE	VEENKKMSQP
1530	1540	1550	1560	1570	1580	1590	1600
GSPSPKTPTP	STPGDTQPNT	PAPVPPAEDG	IKIEENSLKE	EESIEGEKEV	KSTAPETAIE	CTQAPAPASE	DEKVVVEPPE
1610	1620	1630	1640	1650	1660	1670	1680
GEEKVEKAEV	KERTEEPMET	EPKGKGAADV	EKVEEKSAID	LTPIVVEDKE	EKKEEEEKKE	VMLQNGETPK	DLNDEKQKKN
1690	1700	1710	1720	1730	1740	1750	1760
IKQRFMFNIA	DGGFTELHSL	WQNEERAATV	TKKTYEIWHR	RHDYWLLAGI	INHGYARWQD	IQNDPRYAIL	NEPFKGEMNR
1770	1780	1790	1800	1810	1820	1830	1840
GNFLEIKNKF	LARRFKLLEQ	ALVIEEQLRR	AAYLNMSEDP	SHPSMALNTR	FAEVECLAES	HQHLSKESMA	GNKPANAVLH
1850	1860	1870	1880	1890	1900	1910	
KGILKQLEEL	LSDMKADVTR	LPATIARIPP	VAVRLQMSER	NILSRLANRA	PEPTPQQVAQ	QQ	

Cmpd.	No. of Cmpds.	m/z meas.	Δ m/z [ppm]	z	Rt [min]	Score	P	Range	Sequence	Modification
2537	2	649.8872	-125.27	3	60.0	13.8	0	1005-1021	K.CCNHPYLFPVAAMEAPK.M	Carbamidomethyl: 2
2522	2	649.8743	-145.17	3	59.8	13.9	0	1005-1021	K.CCNHPYLFPVAAMEAPK.M	Carbamidomethyl: 1

Detailed Protein Report

Protein 151: PREDICTED: transmembrane channel-like protein 8 isoform X4 [Homo sapiens]

**Accession:**	gi|578830481	**Score:**	45.2
**Database:**	refseq_human(refseq_human_20140103.fasta)	**MW [kDa]:**	45.7
**Database Date:**	2015-11-30	**pI:**	10.7
		**Sequence Coverage [%]:**	4.2
		**No. of unique Peptides:**	1

10	20	30	40	50	60	70	80
MLLPRSVSSE	RAPGVPEPEE	LWEAEMERLR	GSGTPVRGLP	YAMMDKRLIW	QLREPAGVQT	LRWQRWQRRR	QTVERRLREA
**90**	**100**	**110**	**120**	**130**	**140**	**150**	**160**
AQRLARGLGL	WEGALYEIGG	LFGTGIRSYF	TFLRFLLLLN	LLSLLLTASF	VLLPLVWLRP	PDPGPTLNLT	LQCPGSRQSP
**170**	**180**	**190**	**200**	**210**	**220**	**230**	**240**
PGVLRFHNQL	WHVLTGRAFT	NTYLFYGAYR	VGPESSSVYS	IRLAYLLSPL	ACLLLCFCGT	LRRMVKGLPQ	KTLLGQGYQA
**250**	**260**	**270**	**280**	**290**	**300**	**310**	**320**
PLSAKVFSSW	DFCIRVQEAA	TIKKHEISNE	FKVELEEGRR	FQLMQQQTRA	QTACRLLSYL	RVNVLNGLLV	VGAISAIFWA
**330**	**340**	**350**	**360**	**370**	**380**	**390**	**400**
TKYSQDNKEV	SGVPVSAAPV	PAPWGHRPGQ	LPGSPAVHIS	GPAGELPSQH	GGQPHSDLVR	GAEAGQLGDV	LRLPGSDHTV
**410**							
HWQRQEQL							

Cmpd.	No. of Cmpds.	m/z meas.	Δ m/z [ppm]	z	Rt [min]	Score	P	Range	Sequence	Modification
704	1	457.7465	-33.68	2	37.6	18.2	1	79-86	R.EAAQRLAR.G	

Detailed Protein Report

Protein 152: dynein heavy chain 10, axonemal [Homo sapiens]

**Accession:**	gi|198442844	**Score:**	45.1
**Database:**	refseq_human(refseq_human_20140103.fasta)	**MW [kDa]:**	514.5
**Database Date:**	2015-11-30	**pI:**	5.6
		**Sequence Coverage [%]:**	1.0
		**No. of unique Peptides:**	2

Quantitation

mdown:qdown	**Median:**	0.91	**CV:**	0.00 %	**No. of Peptides:**	1
Wdown:Qdown	**Median:**	1.20	**CV:**	0.00 %	**No. of Peptides:**	1

BRUKER

Detailed Protein Report

10	20	30	40	50	60	70	80
MVPEEVEVEI	DEIPVLSEEG	EEEEETYSQK	VESVDKVRAK	RVSLRTESLG	QPLNREDEEM	DKEISEKLPS	KRTAKHIMEK
90	100	110	120	130	140	150	160
MHLHMLCTPL	PEEFLDQNVV	FFLRNTKEAI	SEATDMKEAM	EIMPETLEYG	IINANVLHFL	KNIICQVFLP	ALSFNQHRTS
170	180	190	200	210	220	230	240
TTVGVTSGEV	SNSSEHESDL	PPMPGEAVEY	HSIQLIRDEF	LMNVQKFASN	IQRTMQQLEG	EIKLEMPIIS	VEGEVSDLAA
250	260	270	280	290	300	310	320
DPETVDILEQ	CVINWLNQIS	TAVEAQLKKT	PQGKGPLAEI	EFWRERNATL	SALHEQTKLP	IVRKVLDVIK	ESDSMLVANL
330	340	350	360	370	380	390	400
QPVFTELFKF	HTEASDNVRF	LSTVERYFKN	ITHGSGFHVV	LDTIPAMMSA	LRMVWIISRH	YNKDERMIPL	MERIAWEIAE
410	420	430	440	450	460	470	480
RVCRVVNLRT	LFKENRASAQ	SKTLEARNTL	RLWKKAYFDT	RAKIEASGRE	DRWEFDRKRL	FERTDYMATI	CQDLSDVLQI
490	500	510	520	530	540	550	560
LEEFYNIFGP	ELKAVTGDPK	RIDDVLCRVD	GLVTPMENLT	FDPFSIKSSQ	FWKYVMDEFK	IEVLIDIINK	IFVQNLENPP
570	580	590	600	610	620	630	640
LYKNHPPVAG	AIYWERSLFF	RIKHTILRFQ	EVQEILDSDR	GQEVKQKYLE	VGRTMKEYED	RKYEQWMEVT	EQVLPALMKK
650	660	670	680	690	700	710	720
SLLTKSSIAT	EEPSTLERGA	VFAINFSPAL	REIINETKYL	EQLGFTVPEL	ARNVALQEDK	FLRYTAGIQR	MLDHYHMLIG
730	740	750	760	770	780	790	800
TLNDAESVLL	KDHSQELLRV	FRSGYKRLNW	NSLGIGDYIT	GCKQAIGKFE	SLVHQIHKNA	DDISSRLTLI	EAINLFKYPA
810	820	830	840	850	860	870	880
AKSEEELPGV	KEFFEHIERE	RASDVDHMVR	WYLAIGPLLT	KVEGLVVHTN	TGKAPKLASY	YKYWEKKIYE	VLTKLILKNL
890	900	910	920	930	940	950	960
QSFNSLILGN	VPLFHTETIL	TAPEIILHPN	TNEIDKMCFH	CVRNCVEITK	HFVRWMNGSC	IECPPQKGEE	EEVVIINFYN
970	980	990	1000	1010	1020	1030	1040
DISLNPQIIE	QAVMIPQNVH	RILINLMKYL	QKWKRYRPLW	KLDKAIVMEK	FAAKKPPCVA	YDEKLQFYSK	IAYEVMRHPL
1050	1060	1070	1080	1090	1100	1110	1120
IKDEHCIRLQ	LRHLANTVQE	NAKSWVISLG	KLLNESAKEE	LYNLHEEMEH	LAKNLRKIPN	TLEDLKFVLA	TIAEIRSKSL
1130	1140	1150	1160	1170	1180	1190	1200
VMELRYRDVQ	ERYRTMAMYN	LFPPDAEKEL	VDKIESIWSN	LFNDSVNVEH	ALGDIKRTFT	ELTRGEIMNY	RVQIEEFAKR
1210	1220	1230	1240	1250	1260	1270	1280
FYSEGPGSVG	DDLDKGVELL	GVYERELARH	EKSRQELANA	EKLFDLPITM	YPELLKVQKE	MSGLRMIYEL	YEGLKVAKEE
1290	1300	1310	1320	1330	1340	1350	1360
WSQTLWINLN	VQILQEGIEG	FLRALRKLPR	PVRGLSVTYY	LEAKMKAFKD	SIPLLLDLKN	EALRDRHWKE	LMEKTSVFFE
1370	1380	1390	1400	1410	1420	1430	1440
MTETFTLENM	FAMELHKHTD	VLNEIVTAAI	KEVAIEKAVK	EILDTWENMK	FTVVKYCKGT	QERGYILGSV	DEIIQSLDDN
1450	1460	1470	1480	1490	1500	1510	1520
TFNLQSISGS	RFVGPFLQTV	HKWEKTLSLI	GEVIEIWMLV	QRKWMYLESI	FIGGDIRSQL	PEEAKKFDNI	DKVFKRIMGE
1530	1540	1550	1560	1570	1580	1590	1600
TLKDPVIKRC	CEAPNRLSDL	QNVSEGLEKC	QKSLNDYLDS	KRNAFPRFFF	ISDDELLSIL	GSSDPLCVQE	HMIKMYDNIA
1610	1620	1630	1640	1650	1660	1670	1680
SLRFNDGDSG	EKLVSAMISA	EGEVMEFRKI	LRAEGRVEDW	MTAVLNEMRR	TNRLITKEAI	FRYCEDRSRV	DWMLLYQGMV
1690	1700	1710	1720	1730	1740	1750	1760
VLAASQVWWT	WEVEDVFHKA	QKGEKQAMKN	YGRKMHRQID	ELVTRITMPL	SKNDRKKYNT	VLIIDVHARD	IVDSFIRGSI
1770	1780	1790	1800	1810	1820	1830	1840
LEAREFDWES	QLRFYWDREP	DELNIRQCTG	TFGYGYEYMG	LNGRLVITPL	TDRIYLTLTQ	ALSMYLGGAP	AGPAGTGKTE
1850	1860	1870	1880	1890	1900	1910	1920
TTKDLAKALG	LLCVVTNCGE	GMDYRAVGKI	FSGLAQCGAW	GCFDEFNRID	ASVLSVISSQ	IQTIRNALIH	QLTTFQFEGQ
1930	1940	1950	1960	1970	1980	1990	2000
EISLDSRMGI	FITMNPGYAG	RTELPESVKA	LFRPVVVIVP	DLQQICEIML	FSEGFLEAKT	LAKKMTVLYK	LAREQLSKQY
2010	2020	2030	2040	2050	2060	2070	2080
HYDFGLRALK	SVLVMAGELK	RGSSDLREDV	VLMRALRDMN	LPKFVFEDVP	LFLGLISDLF	PGLDCPRVRY	PDFNDAVEQV
2090	2100	2110	2120	2130	2140	2150	2160
LEENGYAVLP	IQVDKVVQMF	ETMLTRHTTM	VVGPTRGGKS	VVINTLCQAQ	TKLGLTTKLY	ILNPKAVSVI	ELYGILDPTT
2170	2180	2190	2200	2210	2220	2230	2240

Detailed Protein Report

Cmpd.	No. of Cmpds.	m/z meas.	Δ m/z [ppm]	z	Rt [min]	Score	P	Range	Sequence	Modification	Ratios
1629	1	711.6687	-76.59	3	49.1	10.5	1	779-797	K.NADDISSRLTLIEAINLFK.Y		
115	1	679.8485	-38.99	2	30.8	24.0	0	2662-2673	R.VFNGLVLTNPER.F		Wdown:Qdown 1.20<br>mdown:qdown 0.91

Detailed Protein Report

Protein 153: **PREDICTED: pyridine nucleotide-disulfide oxidoreductase domain-containing protein 2 isoform X1 [Homo sapiens]**

**Accession:**	gi|578819959	**Score:**	45.1
**Database:**	refseq_human(refseq_human_20140103.fasta)	**MW [kDa]:**	61.0
**Database Date:**	2015-11-30	**pI:**	6.3
		**Sequence Coverage [%]:**	8.6
		**No. of unique Peptides:**	2

10	20	30	40	50	60	70	80
MAASGRGLCK	AVAASPFPAW	RRDNTEARGG	LKPEYDAVVI	GAGHNGLVAA	AYLQRLGVNT	AVFERRHVIG	GAAVTEEIIP
**90**	**100**	**110**	**120**	**130**	**140**	**150**	**160**
GFKFSRASYL	LSLLRPQIYT	DLELKKHGLR	LHLRNPYSFT	PMLEEGAGSK	VPRCLLLGTD	MAENQKQIAQ	FSQKDAQVFP
**170**	**180**	**190**	**200**	**210**	**220**	**230**	**240**
KYEEFMHRLA	LAIDPLLDAA	PVDMAAFQHG	SLLQRMRSLS	TLKPLLKAGR	ILGAQLPRYY	EVLTAPITKV	LDQWFESEPL
**250**	**260**	**270**	**280**	**290**	**300**	**310**	**320**
KATLATDAVI	GAMTSPHTPG	SGYVLLHHVM	GGLEGMQGAW	GYVQGGMGAL	SDAIASSATT	HGASIFTEKT	VAKVQVNSEG
**330**	**340**	**350**	**360**	**370**	**380**	**390**	**400**
CVQGVVLEDG	TEVRSKMVLS	NTSPQITFLK	LTPQEWLPEE	FLERISQLDT	RSPVTKINVA	VDRLPSFLAA	PNAPRGQPLP
**410**	**420**	**430**	**440**	**450**	**460**	**470**	**480**
HHQCSIHLNC	EDTLLLHQAF	EDAMDGLPSH	RPVIELCIPS	SLDPTLAPPG	CHVVSLFTQY	MPYTLAGGKA	WDEQERDAYA
**490**	**500**	**510**	**520**	**530**	**540**	**550**	**560**
DRVFDCIEVY	APGFKDSVVG	RDILTPPDLE	RIFGLPGGNI	FHCAMSLDQL	YFARPVPLHS	GYRCPLQGLY	LCGSGAHPGF
**570**							

Cmpd.	No. of Cmpds.	m/z meas.	Δ m/z [ppm]	z	Rt [min]	Score	P	Range	Sequence	Modification
2627	1	869.6010	138.74	2	61.1	14.0	0	67-83	R.HVIGGAAVTEEIIPGFK.F	
2482	1	836.1552	175.38	2	59.3	13.8	2	196-210	R.MRSLSTLKPLLKAGR.I	

Detailed Protein Report

Protein 154: astrotactin-1 isoform 2 precursor [Homo sapiens]

Accession: gi|46488921
Database: refseq_human(refseq_human_20140103.fasta)
Database Date: 2015-11-30

Score: 45.1
MW [kDa]: 135.0
pI: 4.9
Sequence Coverage [%]: 5.1
No. of unique Peptides: 1

10	20	30	40	50	60	70	80
MALAGLCALL	ACCWGPAAVL	ATAAGDVDPS	KELECKLKSI	TVSALPFLRE	NDLSIMHSPS	ASEPKLLFSV	RNDFPGEMVV
**90**	**100**	**110**	**120**	**130**	**140**	**150**	**160**
VDDLENTELP	YFVLEISGNT	EDIPLVRWRQ	QWLENGTLLF	HIHHQDGAPS	LPGQDPTEEP	QHESAEEELR	ILHISVMGGM
**170**	**180**	**190**	**200**	**210**	**220**	**230**	**240**
IALLLSILCL	VMILYTRRRW	CKRRRVPQPQ	KSASAEAANE	IHYIPSVLIG	GHGRESLRNA	RVQGHNSSGT	LSIRETPILD
**250**	**260**	**270**	**280**	**290**	**300**	**310**	**320**
GYEYDITDLR	HHLQRECMNG	GEDFASQVTR	TLDSLQGCNE	KSGMDLTPGS	DNAKLSLMNK	YKDNIIATSP	VDSNHQQATL
**330**	**340**	**350**	**360**	**370**	**380**	**390**	**400**
LSHTSSSQRK	RINNKARAGS	AFLNPEGDSG	TEAENDPQLT	FYTDPSRSRR	RSRVGSPRSP	VNKTTLTLIS	ITSCVIGLVC
**410**	**420**	**430**	**440**	**450**	**460**	**470**	**480**
SSHVNCPLVV	KITLHVPEHL	IADGSRFILL	EGSQLDASDW	LNPAQVVLFS	QQNSSGPWAM	DLCARRLLDP	CEHQCDPETG
**490**	**500**	**510**	**520**	**530**	**540**	**550**	**560**
ECLCYEGYMK	DPVHKHLCIR	NEWGTNQGPW	PYTIFQRGFD	LVLGEQPSDK	IFRFTYTLGE	GMWLPLSKSF	VIPPAELAIN
**570**	**580**	**590**	**600**	**610**	**620**	**630**	**640**
PSAKCKTDMT	VMEDAVEVRE	ELMTSSSFDS	LEVLLDSFGP	VRDCSKDNGG	CSKNFRCISD	RKLDSTGCVC	PSGLSPMKDS
**650**	**660**	**670**	**680**	**690**	**700**	**710**	**720**
SGCYDRHIGV	DCSDGFNGGC	EQLCLQQMAP	FPDDPTLYNI	LMFCGCIEDY	KLGVDGRSCQ	LITETCPEGS	DCGESRELPM
**730**	**740**	**750**	**760**	**770**	**780**	**790**	**800**
NQTLFGEMFF	GYNNHSKEVA	AGQVLKGTFR	QNNFARGLDQ	QLPDGLVVAT	VPLENQCLEE	ISEPTPDPDF	LTGMVNFSEV
**810**	**820**	**830**	**840**	**850**	**860**	**870**	**880**
SGYPVLQHWK	VRSVMYHIKL	NQVAISQALS	NALHSLDGAT	SRADFVALLD	QFGNHYIQEA	IYGFEESCSI	WYPNKQVQRR
**890**	**900**	**910**	**920**	**930**	**940**	**950**	**960**
LWLEYEDISK	GNSPSDESEE	RERDPKVLTF	PEYITSLSDS	GTKHMAAGVR	MECHSKGRCP	SSCPLCHVTS	SPDTPAEPVL
**970**	**980**	**990**	**1000**	**1010**	**1020**	**1030**	**1040**
LEVTKAAPIY	ELVTNNQTQR	LLQEATMSSL	WCSGTGDVIE	DWCRCDSTAF	GADGLPTCAP	LPQPVLRLST	VHEPSSTLVV
**1050**	**1060**	**1070**	**1080**	**1090**	**1100**	**1110**	**1120**
LEWEHSEPPI	GVQIVDYLLR	QEKVTDRMDH	SKVETETVLS	FVDDIISGAK	SPCAMPSQVP	DKQLTTISLI	IRCLEPDTIY
**1130**	**1140**	**1150**	**1160**	**1170**	**1180**	**1190**	**1200**
MFTLWGVDNT	GRRSRPSDVI	VKTPCPVVDD	VKAQEIADKI	YNLFNGYTSG	KEQQTAYNTL	LDLGSPTLHR	VLYHYNQHYE
**1210**	**1220**						
SFGEFTWRCE	DELGPR						

Cmpd.	No. of Cmpds.	m/z meas.	Δ m/z [ppm]	z	Rt [min]	Score	P	Range	Sequence	Modification
2260	1	1061.4977	-63.82	3	58.5	11.2	2	185-214	R.RVPQPQKSASAEAANEIHYIPSVLIGGHGR.E	

Detailed Protein Report

Protein 155: **PREDICTED: armadillo repeat-containing protein 3 isoform X3 [Homo sapiens]**

**Accession:**	gi|530392014	**Score:**	45.0
**Database:**	refseq_human(refseq_human_20140103.fasta)	**MW [kDa]:**	86.5
**Database Date:**	2015-11-30	**pI:**	5.8
		**Sequence Coverage [%]:**	2.6
		**No. of unique Peptides:**	1

10	20	30	40	50	60	70	80
MIFGILASNN	DVKKLLRELD	VMNSVIAQLA	PEEEVVIHEF	ASLCLANMSA	EYTSKVQIFE	HGGLEPLIRL	LSSPDPDVKK
**90**	**100**	**110**	**120**	**130**	**140**	**150**	**160**
NSMECIYNLV	QDFQCRAKLQ	ELNAIPPILD	LLKSEYPVIQ	LLALKTLGVI	ANDKESRTML	RDNQGLDHLI	KILETKELND
**170**	**180**	**190**	**200**	**210**	**220**	**230**	**240**
LHIEALAVIA	NCLEDMDTMV	QIQQTGGLKK	LLSFAENSTI	PDIQKNAAKA	ITKAAYDPEN	RKLFHEQEVE	KCLVALLGSE
**250**	**260**	**270**	**280**	**290**	**300**	**310**	**320**
NDGTKIAASQ	AISAMCENSG	SKDFFNNQGI	PQLIQLLKSD	NEEVREAAAL	ALANLTTCNP	ANANAAAEAD	GIDPLINLLS
**330**	**340**	**350**	**360**	**370**	**380**	**390**	**400**
SKRDGAIANA	ATVLTNMAMQ	EPLRLNIQNH	DIMHAIISPL	RSANTVVQSK	AALAVTATAC	DVEARTELRN	SGGLEPLVEL
**410**	**420**	**430**	**440**	**450**	**460**	**470**	**480**
LRSKNDEVRK	HASWAVMVCA	GDELTANELC	RLGALDILEE	VNVSGTRKNK	FSEAAYNKLL	NNNLSLKYSQ	TGYLSSSNII
**490**	**500**	**510**	**520**	**530**	**540**	**550**	**560**
NDGFYDYGRI	NPGTKLLPLK	ELCLQEPSDL	RAVLLINSKS	YVSPPSSMED	KSDVGYGRSI	SSSSSLRRSS	KEKNKKNSYH
**570**	**580**	**590**	**600**	**610**	**620**	**630**	**640**
FSAGFGSPIE	DKSEPASGRN	TVLSKSATKE	KGWRKSKGKK	EEEKVKEEEE	VMVVPKFVGE	GSSDKEWCPP	SDPDFSMYVY
**650**	**660**	**670**	**680**	**690**	**700**	**710**	**720**
EVTKSILPIT	NIKEQIEDLA	KYVAEKMGGK	IPKEKLPDFS	WELHISELKF	QLKSNVIPIG	HVKKGIFYHR	ALLFKALADR
**730**	**740**	**750**	**760**	**770**	**780**	**790**	
IGIGCSLVRG	EYGRAWNEVM	LQNDSRKGVI	GGLPAPEMYV	IDLMFHPGGL	MKLRSREADL	YRFI	

Cmpd.	No. of Cmpds.	m/z meas.	Δ m/z [ppm]	z	Rt [min]	Score	P	Range	Sequence	Modification
2698	1	709.8343	-97.82	2	64.9	17.6	1	2-14	M.IFGILASNNDVKK.L	

Detailed Protein Report

Protein 156: zinc finger protein 646 [Homo sapiens]

**Accession:**	gi|215820619	**Score:**	45.0
**Database:**	refseq_human(refseq_human_20140103.fasta)	**MW [kDa]:**	200.7
**Database Date:**	2015-11-30	**pI:**	6.8
**Modification(s):**	Carbamidomethyl, Oxidation	**Sequence Coverage [%]:**	2.2
		**No. of unique Peptides:**	3

Quantitation

mdown:qdown	**Median:**	1.04	**CV:**	0.00 %	**No. of Peptides:**	1
Wdown:Qdown	**Median:**	0.80	**CV:**	0.00 %	**No. of Peptides:**	1

Alias proteins:

Accession	Name	Description
gi|530409437	refseq_human (refseq_human_20140103.fasta)	PREDICTED: zinc finger protein 646 isoform X2 [Homo sapiens]
gi|530409435	refseq_human (refseq_human_20140103.fasta)	PREDICTED: zinc finger protein 646 isoform X1 [Homo sapiens]

Detailed Protein Report

10	20	30	40	50	60	70	80
MEDTPPSLSC	SDCQRHFPSL	PELSRHRELL	HPSPNQDSEE	ADSIPRPYRC	QQCGRGYRHP	GSLVNHRRTH	ETGLFPCTTC
90	100	110	120	130	140	150	160
GKDFSNPMAL	KSHMRTHAPE	GRRRHRPPRP	KEATPHLQGE	TVSTDSWGQR	LGSSEGWENQ	TKHTEETPDC	ESVPDPRAAS
170	180	190	200	210	220	230	240
GTWEDLPTRQ	REGLASHPGP	EDGADGWGPS	TNSARAPPLP	IPASSLLSNL	EQYLAESVVN	FTGGQEPTQS	PPAEEERRYK
250	260	270	280	290	300	310	320
CSQCGKTYKH	AGSLTNHRQS	HTLGIYPCAI	CFKEFSNLMA	LKNHSRLHAQ	YRPYHCPHCP	RVFRLPRELL	EHQQSHEGER
330	340	350	360	370	380	390	400
QEPRWEEKGM	PTTNGHTDES	SQDQLPSAQM	LNGSAELSTS	GELEDSGLEE	YRPFRCGDCG	RTYRHAGSLI	NHRKSHQTGV
410	420	430	440	450	460	470	480
YPCSLCSKQL	FNAAALKNHV	RAHHRPRQGV	GENGQPSVPP	APLLLAETTH	KEEEDPTTTL	DHRPYKCSEC	GRAYRHRGSL
490	500	510	520	530	540	550	560
VNHRHSHRTG	EYQCSLCPRK	YPNLMALRNH	VRVHCKAARR	SADIGAEGAP	SHLKVELPPD	PVEAEAAPHT	DQDHVCKHEE
570	580	590	600	610	620	630	640
EATDITPAAD	KTAAHICSIC	GLLFEDAESL	ERHGLTHGAG	EKENSRTETT	MSPPRAFACR	DCGKSYRHSG	SLINHRQTHQ
650	660	670	680	690	700	710	720
TGDFSCGACA	KHFHTMAAMK	NHLRRHSRRR	SRRHRKRAGG	ASGGREAKLL	AAESWTRELE	DNEGLESPQD	PSGESPHGAE
730	740	750	760	770	780	790	800
GNLESDGDCL	QAESEGDKCG	LERDETHFQG	DKESGGTGEG	LERKDASLLD	NLDIPGEEGG	GTHFCDSLTG	VDEDQKPATG
810	820	830	840	850	860	870	880
QPNSSSHSAN	AVTGWQAGAA	HTCSDCGHSF	PHATGLLSHR	PCHPPGIYQC	SLCPKEFDSL	PALRSHFQNH	RPGEATSAQP
890	900	910	920	930	940	950	960
FLCCLCGMIF	PGRAGYRLHR	RQAHSSSGMT	EGSEEEGEEE	GVAEAAPARS	PPLQLSEAEL	LNQLQREVEA	LDSAGYGHIC
970	980	990	1000	1010	1020	1030	1040
GCCGQTYDDL	GSLERHHQSQ	SSGTTADKAP	SPLGVAGDAM	EMVVDSVLED	IVNSVSGEGG	DAKSQEGAGT	PLGDSLCIQG
1050	1060	1070	1080	1090	1100	1110	1120
GESLLEAQPR	PFRCNQCGKT	YRHGGSLVNH	RKIHQTGDFL	CPVCSRCYPN	LAAYRNHLRN	HPRCKGSEPQ	VGPIPEAAGS
1130	1140	1150	1160	1170	1180	1190	1200
SELQVGPIPE	GGSNKPQHMA	EEGPGQAEVE	KLQEELKVEP	LEEVARVKEE	VWEETTVKGE	EIEPRLETAE	KGCQTEASSE
1210	1220	1230	1240	1250	1260	1270	1280
RPFSCEVCGR	SYKHAGSLIN	HRQSHQTGHF	GCQACSKGFS	NLMSLKNHRR	IHADPRRFRC	SECGKAFRLR	KQLASHQRVH
1290	1300	1310	1320	1330	1340	1350	1360
MERRGGGGTR	KATREDRPFR	CGQCGRTYRH	AGSLLNHRRS	HETGQYSCPT	CPKTYSNRMA	LKDHQRLHSE	NRRRRAGRSR
1370	1380	1390	1400	1410	1420	1430	1440
RTAVRCALCG	RSFPGRGSLE	RHLREHEETE	REPANGQGGL	DGTAASEANL	TGSQGLETQL	GGAEPVPHLE	DGVPRPGERS
1450	1460	1470	1480	1490	1500	1510	1520
QSPIRAASSE	APEPLSWGAG	KAGGWPVGGG	LGNHSGGWVP	QFLTRSEEPE	DSVHRSPCHA	GDCQLNGPTL	SHMDSWDNRD
1530	1540	1550	1560	1570	1580	1590	1600
NSSQLQPGSH	SSCSQCGKTY	CQSGSLLNHN	TNKTDRHYCL	LCSKEFLNPV	ATKSHSHNHI	DAQTFACPDC	GKAFESHQEL
1610	1620	1630	1640	1650	1660	1670	1680
ASHLQAHARG	HSQVPAQMEE	ARDPKAGTGE	DQVVLPGQGK	AQEAPSETPR	GPGESVERAR	GGQAVTSMAA	EDKERPFRCT
1690	1700	1710	1720	1730	1740	1750	1760
QCGRSYRHAG	SLLNHQKAHT	TGLYPCSLCP	KLLPNLLSLK	NHSRTHTDPK	RHCCSICGKA	FRTAARLEGH	GRVHAPREGP
1770	1780	1790	1800	1810	1820	1830	1840
FTCPHCPRHF	RRRISFVQHQ	QQHQEEWTVA	GSGAPVAPVT	GRGDLPLPPP	PTPTTPLLDP	SPQWPADLSF	SL

Cmpd.	No. of Cmpds.	m/z meas.	Δ m/z [ppm]	z	Rt [min]	Score	P	Range	Sequence	Modification	Ratios
2876	1	899.9560	111.44	2	64.7	16.6	0	1-15	-.MEDTPPSLSCSDCQR.H	Carbamidomethyl: 10, 13; Oxidation: 1	
2169	1	701.2467	-130.95	3	57.3	10.5	1	308-324	R.ELLEHQQSHEGERQEPR.W		
1307	1	1023.7335	252.51	1	46.0	17.9	0	1557-1564	R.HYCLLCSK.E	Carbamidomethyl: 3	mdown:qdown 1.04<br>Wdown:Qdown 0.80

Detailed Protein Report

Protein 157: DNA-dependent protein kinase catalytic subunit isoform 2 [Homo sapiens]

**Accession:**	gi|126032350	**Score:**	45.0
**Database:**	refseq_human(refseq_human_20140103.fasta)	**MW [kDa]:**	465.2
**Database Date:**	2015-11-30	**pI:**	6.9
**Modification(s):**	Carbamidomethyl, Oxidation	**Sequence Coverage [%]:**	0.8
		**No. of unique Peptides:**	3

Quantitation

mdown:qdown	**Median:**	1.23	**CV:**	0.00 %	**No. of Peptides:**	1

Detailed Protein Report

10	20	30	40	50	60	70	80
MAGSGAGVRC	SLLRLQETLS	AADRCGAALA	GHQLIRGLGQ	ECVLSSSPAV	LALQTSLVFS	RDFGLLVFVR	KSLNSIEFRE
90	100	110	120	130	140	150	160
CREEILKFLC	IFLEKMGQKI	APYSVEIKNT	CTSVYTKDRA	AKCKIPALDL	LIKLLQTFRS	SRLMDEFKIG	ELFSKFYGEL
170	180	190	200	210	220	230	240
ALKKKIPDTV	LEKVYELLGL	LGEVHPSEMI	NNAENLFRAF	LGELKTQMTS	AVREPKLPVL	AGCLKGLSSL	LCNFTKSMEE
250	260	270	280	290	300	310	320
DPQTSREIFN	FVLKAIRPQI	DLKRYAVPSA	GLRLFALHAS	QFSTCLLDNY	VSLFEVLLKW	CAHTNVELKK	AALSALESFL
330	340	350	360	370	380	390	400
KQVSNMVAKN	AEMHKNKLQY	FMEQFYGIIR	NVDSNNKELS	IAIRGYGLFA	GPCKVINAKD	VDFMYVELIQ	RCKQMFLTQT
410	420	430	440	450	460	470	480
DTGDDRVYQM	PSFLQSVASV	LLYLDTVPEV	YTPVLEHLVV	MQIDSFPQYS	PKMQLVCCRA	IVKVFLALAA	KGPVLRNCIS
490	500	510	520	530	540	550	560
TVVHQGLIRI	CSKPVVLPKG	PESESEDHRA	SGEVRTGKWK	VPTYKDYVDL	FRHLLSSDQM	MDSILADEAF	FSVNSSESL
570	580	590	600	610	620	630	640
NHLLYDEFVK	SVLKIVEKLD	LTLEIQTVGE	QENGDEAPGV	WMIPTSDPAA	NLHPAKPKDF	SAFINLVEFC	REILPEKQAE
650	660	670	680	690	700	710	720
FFEPWVYSFS	YELILQSTRL	PLISGFYKLL	SITVRNAKKI	KYFEGVSPKS	LKHSPEDPEK	YSCFALFVKF	GKEVAVKMKQ
730	740	750	760	770	780	790	800
YKDELLASCL	TFLLSLPHNI	IELDVRAYVP	ALQMAFKLGL	SYTPLAEVGL	NALEEWSIYI	DRHVMQPYYK	DILPCLDGYL
810	820	830	840	850	860	870	880
KTSALSDETK	NNWEVSALSR	AAQKGFNKVV	LKHLKKTKNL	SSNEAISLEE	IRIRVVQMLG	SLGGQINKNL	LTVTSSDEMM
890	900	910	920	930	940	950	960
KSYVAWDREK	RLSFAVPFRE	MKPVIFLDVF	LPRVTELALT	ASDRQTKVAA	CELLHSMVMF	MLGKATQMPE	GGQGAPPMYQ
970	980	990	1000	1010	1020	1030	1040
LYKRTFPVLL	RLACDVDQVT	RQLYEPLVMQ	LIHWFTNNKK	FESQDTVALL	EAILDGIVDP	VDSTLRDFCG	RCIREFLKWS
1050	1060	1070	1080	1090	1100	1110	1120
IKQITPQQQE	KSPVNTKSLF	KRLYSLALHP	NAFKRLGASL	AFNNIYREFR	EEESLVEQFV	FEALVIYMES	LALAHADEKS
1130	1140	1150	1160	1170	1180	1190	1200
LGTIQQCCDA	IDHLCRIIEK	KHVSLNKAKK	RRLPRGFPPS	ASLCLLDLVK	WLLAHCGRPQ	TECRHKSIEL	FYKFVPLLPG
1210	1220	1230	1240	1250	1260	1270	1280
NRSPNLWLKD	VLKEEGVSFL	INTFEGGGCG	QPSGILAQPT	LLYLRGPFSL	QATLCWLDLL	LAALECYNTF	IGERTVGALQ
1290	1300	1310	1320	1330	1340	1350	1360
VLGTEAQSSL	LKAVAFFLES	IAMHDIIAAE	KCFGTGAAGN	RTSPQEGERY	NYSKCTVVVR	IMEFTTTLLN	TSPEGWKLLK
1370	1380	1390	1400	1410	1420	1430	1440
KDLCNTHLMR	VLVQTLCEPA	SIGFNIGDVQ	VMAHLPDVCV	NLMKALKMSP	YKDILETHLR	EKITAQSIEE	LCAVNLYGPD
1450	1460	1470	1480	1490	1500	1510	1520
AQVDRSRLAA	VVSACKQLHR	AGLLHNILPS	QSTDLHHSVG	TELLSLVYKG	IAPGDERQCL	PSLDLSCKQL	ASGLLELAFA
1530	1540	1550	1560	1570	1580	1590	1600
FGGLCERLVS	LLLNPAVLST	ASLGSSQGSV	IHFSHGEYFY	SLFSETINTE	LLKNLDLAVL	ELMQSSVDNT	KMVSAVLNGM
1610	1620	1630	1640	1650	1660	1670	1680
LDQSFRERAN	QKHQGLKLAT	TILQHWKKCD	SWWAKDSPLE	TKMAVLALLA	KILQIDSSVS	FNTSHGSFPE	VFTTYISLLA
1690	1700	1710	1720	1730	1740	1750	1760
DTKLDLHLKG	QAVTLLPFFT	SLTGGSLEEL	RRVLEQLIVA	HFPMQSREFP	PGTPRFNNYV	DCMKKFLDAL	ELSQSPMLLE
1770	1780	1790	1800	1810	1820	1830	1840
LMTEVLCREQ	QHVMEELFQS	SFRRIARRGS	CVTQVGLLES	VYEMFRKDDP	RLSFTRQSFV	DRSLLTLLWH	CSLDALREFF
1850	1860	1870	1880	1890	1900	1910	1920
STIVVDAIDV	LKSRFTKLNE	STFDTQITKK	MGYYKILDVM	YSRLPKDDVH	AKESKINQVF	HGSCITEGNE	LTKTLIKLCY
1930	1940	1950	1960	1970	1980	1990	2000
DAFTENMAGE	NQLLERRRLY	HCAAYNCAIS	VICCVFNELK	FYQGFLFSEK	PEKNLLIFEN	LIDLKRRYNF	PVEVEVPMER
2010	2020	2030	2040	2050	2060	2070	2080
KKKYIEIRKE	AREAANGDSD	GPSYMSSLSY	LADSTLSEEM	SQFDFSTGVQ	SYSYSSQDPR	PATGRFRRRE	QRDPTVHDDV
2090	2100	2110	2120	2130	2140	2150	2160
LELEMDELNR	HECMAPLTAL	VKHMHRSLGP	PQGEEDSVPR	DLPSWMKFLH	GKLGNPIVPL	NIRLFLAKLV	INTEEVFRPY
2170	2180	2190	2200	2210	2220	2230	2240

Detailed Protein Report

Cmpd.	No. of Cmpds.	m/z meas.	Δ m/z [ppm]	z	Rt [min]	Score	P	Range	Sequence	Modification	Ratios
1341	1	473.1526	-298.22	2	45.3	12.1	0	1643-1651	K.MAVLALLAK.I	Oxidation: 1	
698	1	512.2692	-22.73	2	37.6	10.8	0	2434-2441	K.VCLDIIYK.M	Carbamidomethyl: 2	mdown:qdown 1.23
2232	1	937.4206	-14.54	2	56.5	22.1	1	3682-3696	K.ECSPWMSDFKVEFLR.N		

Detailed Protein Report

Protein 158: PREDICTED: cyclin-dependent kinase 7 isoform X1 [Homo sapiens]

**Accession:**	gi|530379082	**Score:**	44.9
**Database:**	refseq_human(refseq_human_20140103.fasta)	**MW [kDa]:**	32.7
**Database Date:**	2015-11-30	**pI:**	9.8
		**Sequence Coverage [%]:**	11.4
		**No. of unique Peptides:**	1

Alias proteins:

Accession	Name	Description
gi|530430719	refseq_human (refseq_human_20140103.fasta)	PREDICTED: cyclin-dependent kinase 7 isoform X1 [Homo sapiens]

10	20	30	40	50	60	70	80
MRSWTSLGRD	SLPPFTRPEI	RTPTKLSPLR	KSNLDIDQKL	KMVIIKDNSL	VLTPSHIKAY	MLMTLQGLEY	LHQHWILHRD
**90**	**100**	**110**	**120**	**130**	**140**	**150**	**160**
LKPNNLLLDE	NGVLKLADFG	LAKSFGSPNR	AYTHQVVTRW	YRAPELLFGA	RMYGVGVDMW	AVGCILAELL	LRVPFLPGDS
**170**	**180**	**190**	**200**	**210**	**220**	**230**	**240**
DLDQLTRIFE	TLGTPTEEQW	PDMCSLPDYV	TFKSFPGIPL	HHIFSAAGDD	LLDLIQGLFL	FNPCARITAT	QALKMKYFSN
**250**	**260**	**270**	**280**	**290**			
RPGPTPGCQL	PRPNCPVETL	KEQSNPALAI	KRKRTEALEQ	GGLPKKLIF			

Cmpd.	No. of Cmpds.	m/z meas.	Δ m/z [ppm]	z	Rt [min]	Score	P	Range	Sequence	Modification
50	1	662.2443	-185.28	2	29.3	10.8	0	47-58	K.DNSLVLTPSHIK.A	

Detailed Protein Report

Protein 159: PREDICTED: ankyrin repeat domain-containing protein 26 isoform X7 [Homo sapiens]

Accession: gi|578818368
Database: refseq_human(refseq_human_20140103.fasta)
Database Date: 2015-11-30

Score: 44.8
MW [kDa]: 187.0
pI: 5.3
Sequence Coverage [%]: 1.7
No. of unique Peptides: 1

10	20	30	40	50	60	70	80
MKKIFSKKGE	SPLGSFARRQ	RSSAGGGGEP	GEGAYSQPGY	HVRDRDLGKI	HKAASAGNVA	KVQQILLLRK	NGLNDRDKMN
90	100	110	120	130	140	150	160
RTALHLACAN	GHPEVVTLLV	DRKCQLNVCD	NENRTALMKA	VQCQEEKCAT	ILLEHGADPN	LADVHGNTAL	HYAVYNEDIS
170	180	190	200	210	220	230	240
VATKLLLYDA	NIEAKNKDDL	TPLLLAVSGK	KQQMVEFLIK	KKANVNAVDK	LESSHQLISE	YKEERIPKHS	SQNSNSVDES
250	260	270	280	290	300	310	320
SEDSLSRLSG	KPGVDDSWPT	SDDEDLNFDT	KNVPKPSLAK	LMTASQQSRK	NLEATYGTVR	TGNRTLFEDR	DSDSQDEVVV
330	340	350	360	370	380	390	400
ESLPTTSIKV	QCFSHPTYQS	PDLLPKPSHK	SLANPGLMKE	EPTKPGIAKK	ENGIDIIESA	PLEQTNNDNL	TYVDEVHKNN
410	420	430	440	450	460	470	480
RSDMMSALGL	GQEEDIESPW	DSESISENFP	QKYVDPLAGA	ADGKEKNIGN	EQAEDVFYIP	SCMSGSRNFK	MAKLEDTRNV
490	500	510	520	530	540	550	560
GMPVAHMESP	ERYLHLKPTI	EMKDSVPNKA	GGMKDVQTSK	AAEHDLEVAS	EEEQEREGSE	NNQPQDKVIL	KTCTLTEKTS
570	580	590	600	610	620	630	640
EKQNKQINRP	LSCLQKMSQE	PELNKECDRE	DVSVYSGLPC	VQNDEEMWTK	QGKLEWKNNL	KLITNELKQS	CGETCEKYKI
650	660	670	680	690	700	710	720
TASPGEESLH	DNSKGGTNLK	EIPSSLTNNI	LDCDEKDSPV	SVLFQALPEQ	KVLSLENGFS	FPSYSGSPEY	ACQSSSKPYL
730	740	750	760	770	780	790	800
NENKLGHENV	NKPDTEHVFN	TDENFYNDTE	NKKVRNPEVV	TGVMKEEFDK	TKNMNRNTTN	WKLDIRRVPQ	YSDPKRPFDL
810	820	830	840	850	860	870	880
ICSKEMNHMF	HIKRHSISAG	TDAYKKTKPI	QNLFQKPLYD	HCSANNYKSM	EPELENVRSS	PPRGDRTSKV	SLKEELQQDM
890	900	910	920	930	940	950	960
QRFKNEIGML	KVEFQALEKE	KVQLQKEVEE	ERKKHRNNEM	EVSANIHDGA	TDDAEDDDDD	DGLIQKRKSG	ETDHQQFPRK
970	980	990	1000	1010	1020	1030	1040
ENKEYASSGP	ALQMKEVKST	EKEKRTSKES	VNSPVFGKAS	LLTGGLLQVD	DDSSLSEIDE	DEGRPTKKTS	NEKNKVKNQI
1050	1060	1070	1080	1090	1100	1110	1120
QSMDDVDDLT	QSSETASEDC	ELPHSSYKNF	MLLIEQLGME	CKDSVSLLKI	QDAALSCERL	LELKKNHCEL	LTVKIKKMED
1130	1140	1150	1160	1170	1180	1190	1200
KVNVLQRELS	ETKEIKSQLE	HQKVEWEREL	CSLRFSLNQE	EEKRRNADTL	YEKIREQLRR	KEEQYRKEVE	VKQQLELSLQ
1210	1220	1230	1240	1250	1260	1270	1280
TLEMELRTVK	SNLNQVVQER	NDAQRQLSRE	QNARMLQDGI	LTNHLSKQKE	IEMAQKKMNS	ENSHSHEEEK	DLSHKNSMLQ
1290	1300	1310	1320	1330	1340	1350	1360
EEIAMLRLEI	DTIKNQNQEK	EKKCFEDLKI	VKEKNEDLQK	TIKQNEETLT	QTISQYNGRL	SVLTAENAML	NSKLENEKQS
1370	1380	1390	1400	1410	1420	1430	1440
KERLEAEVES	YHSRLAAAIH	DRDQSETSKR	ELELAFQRAR	DECSRLQDKM	NFDVSNLKDN	NEILSQQLFK	TESKLNSLEI
1450	1460	1470	1480	1490	1500	1510	1520
EFHHTRDALR	EKTLGLERVQ	KDLSQTQCQM	KEMEQKYQNE	QVKVNKYIGK	QESVEERLSQ	LQSENMLLRQ	QLDDAHNKAD
1530	1540	1550	1560	1570	1580	1590	1600
NKEKTVINIQ	DQFHAIVQKL	QAESEKQSLL	LEERNKELIS	ECNHLKERQY	QYENEKAERE	VVVRQLQQEL	ADTLKKQSMS
1610	1620	1630	1640				
EASLEVTSRY	RINLEDETQD	LKKKLGQIRN	QSWDP				

Cmpd.	No. of Cmpds.	m/z meas.	Δ m/z [ppm]	z	Rt [min]	Score	P	Range	Sequence	Modification
1740	1	555.2376	-81.69	2	51.7	17.4	1	1165-1173	R.RNADTLYEK.I	

Detailed Protein Report

Protein 160: PREDICTED: dolichyl-phosphate beta-glucosyltransferase isoform X1 [Homo sapiens]

**Accession:**	gi|530402309	**Score:**	44.8
**Database:**	refseq_human(refseq_human_20140103.fasta)	**MW [kDa]:**	32.8
**Database Date:**	2015-11-30	**pI:**	10.1
**Modification(s):**	Carbamidomethyl, Oxidation	**Sequence Coverage [%]:**	16.8
		**No. of unique Peptides:**	2

```
        10         20         30         40         50         60         70         80
MAPLLLQLAV LGAALAAAAL VLISIVAFTT ATKMPALHRH EEEKFFLNAK GQKETLPSIW DSPTKQLSVV VPSYNEEKRL
        90        100        110        120        130        140        150        160
PVMMDEALSY LEKRQKRDPA FTYEVIVVDD GSKDQTSKVA FKYCQKYGSD KVRVITLVKN RGKGGAIRMN QMAIACGSRA
       170        180        190        200        210        220        230        240
HLEKESIAQR SYFRTLLMYG FHFLVWFLCV KGIRDTQCGF KLFTREAASR TFSSLHVERW AFDVELLYIA QFFKIPIAEI
       250        260        270        280        290
AVNWTEIEGS KLVPFWSWLQ MGKDLLFIRL RYLTGAWRLE QTRKMN
```

Cmpd.	No. of Cmpds.	m/z meas.	Δ m/z [ppm]	z	Rt [min]	Score	P	Range	Sequence	Modification
2024	1	687.3070	-64.03	2	55.4	10.8	0	54-65	K.ETLPSIWDSPTK.Q	
153	1	635.8187	75.84	2	31.1	11.0	0	149-159	R.MNQMAIACGSR.A	Carbamidomethyl: 8; Oxidation: 1, 4

Detailed Protein Report

Protein 161: **PREDICTED: arf-GAP with Rho-GAP domain, ANK repeat and PH domain-containing protein 3 isoform X7 [Homo sapiens]**

**Accession:**	gi|578810904	**Score:**	44.6
**Database:**	refseq_human(refseq_human_20140103.fasta)	**MW [kDa]:**	149.1
**Database Date:**	2015-11-30	**pI:**	6.5
**Modification(s):**	Oxidation	**Sequence Coverage [%]:**	1.7
		**No. of unique Peptides:**	1

10	20	30	40	50	60	70	80
MDPGCLYYGV	QPVGTPGAPD	RRESRGVCQG	RAEHRLSRQD	LEAREDAGYA	SLELPGDSTL	LSPTLETEET	SDDLISPYAS
**90**	**100**	**110**	**120**	**130**	**140**	**150**	**160**
FSFTADRLTP	LLSGWLDKLS	PQGNYVFQRR	FVQFNGRSLM	YFGSDKDPFP	KGVIPLTAIE	MTRSSKDNKF	QVITGQRVFV
**170**	**180**	**190**	**200**	**210**	**220**	**230**	**240**
FRTESEAQRD	MWCSTLQSCL	KEQRLLGHPR	PPQPPRPLRT	GMLELRGHKA	KVFAALSPGE	LALYKSEQAF	SLGIGICFIE
**250**	**260**	**270**	**280**	**290**	**300**	**310**	**320**
LQGCSVRETK	SRSFDLLTPH	RCFSFTAESG	GARQSWAAAL	QEAVTETLSD	YEVAEKIWSN	RANRQCADCG	SSRPDWAAVN
**330**	**340**	**350**	**360**	**370**	**380**	**390**	**400**
LGVVICKQCA	GQHRALGSGI	SKVQSLKLDT	SVWSNEIVQL	FIVLGNDRAN	RFWAGTLPPG	EGLHPDATPG	PRGEFISRKY
**410**	**420**	**430**	**440**	**450**	**460**	**470**	**480**
RLGLFRKPHP	QYPDHSQLLQ	ALCAAVARPN	LLKNMTQLLC	VEAFEGEEPW	FPPAPDGSCP	GLLPSDPSPG	VYNEVVVRAT
**490**	**500**	**510**	**520**	**530**	**540**	**550**	**560**
YSGFLYCSPV	SNKAGPSPPR	RGRDAPPRLW	CVLGAALEMF	ASENSPEPLS	LIQPQDIVCL	GVSPPPTDPG	DRFPFSFELI
**570**	**580**	**590**	**600**	**610**	**620**	**630**	**640**
LAGGRIQHFG	TDGADSLEAW	TSAVGKWFSP	LSCHQLLGPG	LLRLGRLWLR	SPSHTAPAPG	LWLSGFGLLR	GDHLFLCSAP
**650**	**660**	**670**	**680**	**690**	**700**	**710**	**720**
GPGPPAPEDM	VHLRRLQEIS	VVSAADTPDK	KEHLVLVETG	RTLYLQGEGR	LDFTAWNAAI	GGAAGGGGTG	LQEQQMSRGD
**730**	**740**	**750**	**760**	**770**	**780**	**790**	**800**
IPIIVDACIS	FVTQHGLRLE	GVYRKGGARA	RSLRLLAEFR	RDARSVKLRP	GEHFVEDVTD	TLKRFFRELD	DPVTSARLLP
**810**	**820**	**830**	**840**	**850**	**860**	**870**	**880**
RWREAAELPQ	KNQRLEKYKD	VIGCLPRVNR	RTLATLIGHL	YRVQKCAALN	QMCTRNLALL	FAPSVFQTDG	RGEHEVRVLQ
**890**	**900**	**910**	**920**	**930**	**940**	**950**	**960**
ELIDGYISVF	DIDSDQVAQI	DLEVSLITTW	KDVQLSQAGD	LIMEVYIEQQ	LPDNCVTLKV	SPTLTAEELT	NQVLEMRGTA
**970**	**980**	**990**	**1000**	**1010**	**1020**	**1030**	**1040**
AGMDLWVTFE	IREHGELERP	LHPKEKVLEQ	ALQWCQLPEP	CSASLLLKKV	PLAQAGCLFT	GIRRESPRVG	LLRCREEPPR
**1050**	**1060**	**1070**	**1080**	**1090**	**1100**	**1110**	**1120**
LLGSRFQERF	FLLRGRCLLL	LKEKKSSKPE	REWPLEGAKV	YLGIRKKLKP	PTPWGFTLIL	EKMHLYLSCT	DEDEMWDWTT
**1130**	**1140**	**1150**	**1160**	**1170**	**1180**	**1190**	**1200**
SILKAQHDDQ	QPVVLRRHSS	SDLARQKFGT	MPLLPIRGDD	SGATLLSANQ	TLRRLHNRRT	LSMFFPMKSS	QGSVEEQEEL
**1210**	**1220**	**1230**	**1240**	**1250**	**1260**	**1270**	**1280**
EEPVYEEPVY	EEVGAFPELI	QDTSTSFSTT	REWTVKPENP	LTSQKSLDQP	FLSKSSTLGQ	EERPPEPPPG	PPSKSSPQAR
**1290**	**1300**	**1310**	**1320**	**1330**	**1340**	**1350**	
GSLEEQLLQE	LSSLILRKGE	TTAGLGSPSQ	PSSPQSPSPT	GLPTQTPGFP	TQPPCTSSPP	SSQPLT	

Cmpd.	No. of Cmpds.	m/z meas.	Δ m/z [ppm]	z	Rt [min]	Score	P	Range	Sequence	Modification
164	1	715.8153	8.42	2	31.4	24.0	0	170-181	R.DMWCSTLQSCLK.E	Oxidation: 2

Detailed Protein Report

Protein 162: protein FAM194B [Homo sapiens]

**Accession:**	gi|210147567	**Score:**	44.3
**Database:**	refseq_human(refseq_human_20140103.fasta)	**MW [kDa]:**	81.6
**Database Date:**	2015-11-30	**pI:**	4.6
		**Sequence Coverage [%]:**	4.9
		**No. of unique Peptides:**	1

10	20	30	40	50	60	70	80
MSAENNQLSG	ASPPHPPTTP	QYSTQNLPSE	KEDTEVELDE	ESLQDESPFS	PEGESLEDKE	YLEEEEDLEE	EEYLGKEEYL
**90**	**100**	**110**	**120**	**130**	**140**	**150**	**160**
KEEEYLGKEE	HLEEEEYLEK	AGYLEEEEYI	EEEEYLGKEG	YLEEEEYLGK	EEHLEEEEYL	GKEGYLEKED	YIEEVDYLGK
**170**	**180**	**190**	**200**	**210**	**220**	**230**	**240**
KAYLEEEEYL	GKKSYLEEEK	ALEKEENLEE	EEALEKEENL	DGKENLYKKY	LKEPKASYSS	QTMLLRDARS	PDAGPSQVTT
**250**	**260**	**270**	**280**	**290**	**300**	**310**	**320**
FLTVPLTFAT	PSPVSESATE	SSELLLTLYR	RSQASQTDWC	YDRTAVKSLK	SKSETEQETT	TKLAPEEHVN	TKVQQKKEEN
**330**	**340**	**350**	**360**	**370**	**380**	**390**	**400**
VLEFASKENF	WDGITDESID	KLEVEDLDEN	FLNSSYQTVF	KTIIKEMAAH	NELEEDFDIP	LTKLLESENR	WKLVIMLKKN
**410**	**420**	**430**	**440**	**450**	**460**	**470**	**480**
YEKFKETILR	IKRRREAQKL	TEMTSFTFHL	MSKPTPEKPE	TEEIQKPQRV	VHHRKKLERD	KEWIQKKTVV	HQGDGKLILY
**490**	**500**	**510**	**520**	**530**	**540**	**550**	**560**
PNKNVYQILF	PDGTGQIHYP	SGNLAMLILY	AKMKKFTYII	LEDSLEGRIR	ALINNSGNAT	FYDENSDIWL	NLSSNLGYYF
**570**	**580**	**590**	**600**	**610**	**620**	**630**	**640**
PKDKRQKAWN	WWNLNIHVHA	PPVQPISLKI	NEYIQVQIRS	QDKIIFCFTY	EQKQICLNLG	TRYKFVIPEV	LSEMKKKTIL
**650**	**660**	**670**	**680**	**690**	**700**		
EAEPGPTAQK	IRVLLGKMNR	LLNYATTPDL	ENFIEAVSIS	LMDNKYLKKM	LSKLWF		

Cmpd.	No. of Cmpds.	m/z meas.	Δ m/z [ppm]	z	Rt [min]	Score	P	Range	Sequence	Modification
2001	1	876.8626	-126.17	2	53.7	10.2	2	462-476	K.EWIQKKTVVHQGDGK.L	

Detailed Protein Report

Protein 163: ATP-binding cassette sub-family A member 13 [Homo sapiens]

**Accession:**	gi|31657092	**Score:**	44.3
**Database:**	refseq_human(refseq_human_20140103.fasta)	**MW [kDa]:**	575.8
**Database Date:**	2015-11-30	**pI:**	6.0
		**Sequence Coverage [%]:**	0.8
		**No. of unique Peptides:**	1

Detailed Protein Report

10	20	30	40	50	60	70	80
MGHAGCQFKA	LLWKNWLCRL	RNPVLFLAEF	FWPCILFVIL	TVLRFQEPPR	YRDICYLQPR	DLPSCGVIPF	VQSLLCNTGS
90	100	110	120	130	140	150	160
RCRNFSYEGS	MEHHFRLSRF	QTAADPKKVN	NLAFLKEIQD	LAEEIHGMMD	KAKNLKRLWV	ERSNTPDSSY	GSSFFTMDLN
170	180	190	200	210	220	230	240
KTEEVILKLE	SLHQQPHIWD	FLLLLPRLHT	SHDHVEDGMD	VAVNLLQTIL	NSLISLEDLD	WLPLNQTFSQ	VSELVLNVTI
250	260	270	280	290	300	310	320
STLTFLQQHG	VAVTEPVYHL	SMQNIVWDPQ	KVQYDLKSQF	GFDDLHTEQI	LNSSAELKEI	PTDTSLEKMV	CSVLSSTSED
330	340	350	360	370	380	390	400
EAEKWGHVGG	CHPKWSEAKN	YLVHAVSWLR	VYQQVFVQWQ	QGSLLQKTLT	GMGHSLEALR	NQFEEESKPW	KVVEALHTAL
410	420	430	440	450	460	470	480
LLLNDSLSAD	GPKDNHTFPK	ILQHLWKLQS	LLQNLPQWPA	LKRFLQLDGA	LRNAIAQNLH	FVQEVLICLE	TSANDFKWFE
490	500	510	520	530	540	550	560
LNQLKLEKDV	FFWELKQMLA	KNAVCPNGRF	SEKEVFLPPG	NSSIWGGLQG	LLCYCNSSET	SVLNKLLGSV	EDADRILQEV
570	580	590	600	610	620	630	640
ITWHKNMSVL	IPEEYLDWQE	LEMQLSEASL	SCTRLFLLLG	ADPSPENDVF	SSDCKHQLVS	TVIFHTLEKT	QFFLEQAYYW
650	660	670	680	690	700	710	720
KAFKKFIRKT	CEVAQYVNMQ	ESFQNRLLAF	PEESPCFEEN	MDWKMISDNY	FQFLNNLLKS	PTASISRALN	FTKHLLMMEK
730	740	750	760	770	780	790	800
KLHTLEDEQM	NFLLSFVEFF	EKLLLPNLFD	SSIVPSFHSL	PSLTEDILNI	SSLWTNHLKS	LKRDPSATDA	QKLLEFGNEV
810	820	830	840	850	860	870	880
IWKMQTLGSH	WIRKEPKNLL	RFIELILFEI	NPKLLELWAY	GISKGKRAKL	ENFFTLLNFS	VPENEILSTS	FNFSQLFHSD
890	900	910	920	930	940	950	960
WPKSPAMNID	FVRLSEAIIT	SLHEFGFLEQ	EQISEALNTV	YAIRNASDLF	SALSEPQKQE	VDKILTHIHL	NVFQDKDSAL
970	980	990	1000	1010	1020	1030	1040
LLQIYSSFYR	YIYELLNIQS	RGSSLTFLTQ	ISKHILDIIK	QFNFQNISKA	FAFLFKTAEV	LGGISNVSYC	QQLLSIFNFL
1050	1060	1070	1080	1090	1100	1110	1120
ELQAQSFMST	EGQELEVIHT	TLTGLKQLLI	IDEDFRISLF	QYMSQFFNSS	VEDLLDNKCL	ISDNKHISSV	NYSTSEESSF
1130	1140	1150	1160	1170	1180	1190	1200
VFPLAQIFSN	LSANVSVFNK	FMSIHCTVSW	LQMWTEIWET	ISQLFKFDMN	VFTSLHHGFT	QLLDELEDDV	KVSKSCQGIL
1210	1220	1230	1240	1250	1260	1270	1280
PTHNVARLIL	NLFKNVTQAN	DFHNWEDFLD	LRDFLVALGN	ALVSVKKLNL	EQVEKSLFTM	EAALHQLKTF	PFNESTSREF
1290	1300	1310	1320	1330	1340	1350	1360
LNSLLEVFIE	FSSTSEYIVR	NLDSINDFLS	NNLTNYGEKF	ENIITELREA	IVFLRNVSHD	RDLFSCADIF	QNVTECILED
1370	1380	1390	1400	1410	1420	1430	1440
GFLYVNTSQR	MLRILDTLNS	TFSSENTISS	LKGCIVWLDV	INHLYLLSNS	SFSQGHLQNI	LGNFRDIENK	MNSILKIVTW
1450	1460	1470	1480	1490	1500	1510	1520
VLNIKKPLCS	SNGSHINCVN	IYLKDVTDFL	NIVLTTVFEK	EKKPKFEILL	ALLNDSTKQV	RMSINNLTTD	FDFASQSNWR
1530	1540	1550	1560	1570	1580	1590	1600
YFTELILRPI	EMSDEIPNQF	QNIWLHLITL	GKEFQKLVKG	IYFNILENNS	SSKTENLLNI	FATSPKEKDV	NSVGNSIYHL
1610	1620	1630	1640	1650	1660	1670	1680
ASYLAFSLSH	DLQNSPKIII	SPEIMKATGL	GIQLIRDVFN	SLMPVVHHTS	PQNAGYMQAL	KKVTSVMRTL	KKADIDLLVD
1690	1700	1710	1720	1730	1740	1750	1760
QLEQVSVNLM	DFFKNISSVG	TGNLVVNLLV	GLMEKFADSS	HSWNVNHLLQ	LSRLFPKDVV	DAVIDVYYVL	PHAVRLLQGV
1770	1780	1790	1800	1810	1820	1830	1840
PGKNITEGLK	DVYSFTLLHG	ITISNITKED	FAIVIKILLD	TIELVSDKPD	IISEALACFP	VVWCWNHTNS	GFRQNSKIDP
1850	1860	1870	1880	1890	1900	1910	1920
CNVHGLMSSS	FYGKVASILD	HFHLSPQGED	SPCSNESSRM	EITRKVVCII	HELVDWNSIL	LELSEVFHVN	ISLVKTVQKF
1930	1940	1950	1960	1970	1980	1990	2000
WHKILPFVPP	SINQTRDSIS	ELCPSGSIKQ	VALQIIEKLK	NVNFTKVTSG	ENILDKLSSL	NKILNINEDT	ETSVQNIISS
2010	2020	2030	2040	2050	2060	2070	2080
NLERTVQLIS	EDWSLEKSTH	NLLSLFMMLQ	NANVTGSSLE	ALSSFIEKSE	TPYNFEELWP	KFQQIMKDLT	QDFRIRHLLS
2090	2100	2110	2120	2130	2140	2150	2160
EMNKGIKSIN	SMALQKITLQ	FAHFLEILDS	PSLKTLEIIE	DFLLVTKNWL	QEYANEDYSR	MIETLFIPVT	NESSTEDIAL
2170	2180	2190	2200	2210	2220	2230	2240
LAKAIATFWG	SLKNISRAGN	FDVAFLTHLL	NQEQLTNFSV	VQLLFENILI	NLINNLAGNS	QEAAWNLNDT	DLQIMNFINL
2250	2260	2270	2280	2290	2300	2310	2320
ILNHMQSETS	RKTVLSLRSI	VDFTEQFLKT	FFSLFLKEDS	ENKISLLLKY	FHKDVIAEMS	FVPKDKILEI	LKLDQFLTLM
2330	2340	2350	2360	2370	2380	2390	2400

Detailed Protein Report

Cmpd.	No. of Cmpds.	m/z meas.	Δ m/z [ppm]	z	Rt [min]	Score	P	Range	Sequence	Modification
2092	1	1030.3822	-211.70	1	56.3	15.0	1	3972-3981	K.QTRALSGGLK.R	

Detailed Protein Report

Protein 164: GDNF family receptor alpha-like precursor [Homo sapiens]

Accession: gi|148231468
Database: refseq_human(refseq_human_20140103.fasta)
Database Date: 2015-11-30
Modification(s): Carbamidomethyl, Oxidation

Score: 44.2
MW [kDa]: 44.5
pI: 9.6
Sequence Coverage [%]: 7.1
No. of unique Peptides: 3

10	20	30	40	50	60	70	80
MIVFIFLAMG	LSLENEYTSQ	TNNCTYLREQ	CLRDANGCKH	AWRVMEDACN	DSDPGDPCKM	RNSSYCNLSI	QYLVESNFQF
90	100	110	120	130	140	150	160
KECLCTDDFY	CTVNKLLGKK	CINKSDNVKE	DKFKWNLTTR	SHHGFKGMWS	CLEVAEACVG	DVVCNAQLAS	YLKACSANGN
170	180	190	200	210	220	230	240
PCDLKQCQAA	IRFFYQNIPF	NIAQMLAFCD	CAQSDIPCQQ	SKEALHSKTC	AVNMVPPPTC	LSVIRSCQND	ELCRRHYRTF
250	260	270	280	290	300	310	320
QSKCWQRVTR	KCHEDENCIS	TLSKQDLTCS	GSDDCKAAYI	DILGTVLQVQ	CTCRTITQSE	ESLCKIFQHM	LHRKSCFNYP
330	340	350	360	370	380	390	400
TLSNVKGMAL	YTRKHANKIT	LTGFHSPFNG	EVIYAAMCMT	VTCGILLLVM	VKLRTSRISS	KARDPSSIQI	PGEL

Cmpd.	No. of Cmpds.	m/z meas.	Δ m/z [ppm]	z	Rt [min]	Score	P	Range	Sequence	Modification
2904	2	937.4573	-13.81	2	65.5	12.0	0	209-225	K.TCAVNMVPPPTCLSVIR.S	Carbamidomethyl: 2; Oxidation: 6
2943	1	937.4648	-5.86	2	66.0	16.3	0	209-225	K.TCAVNMVPPPTCLSVIR.S	Carbamidomethyl: 12; Oxidation: 6
2830	1	648.3562	69.68	2	63.9	15.9	0	295-305	R.TITQSEESLCK.I	Carbamidomethyl: 10

BRUKER Detailed Protein Report

Protein 165: PREDICTED: autoimmune regulator isoform X1 [Homo sapiens]

**Accession:**	gi|530419216	**Score:**	44.0
**Database:**	refseq_human(refseq_human_20140103.fasta)	**MW [kDa]:**	36.5
**Database Date:**	2015-11-30	**pI:**	6.4
**Modification(s):**	Carbamidomethyl	**Sequence Coverage [%]:**	14.9
		**No. of unique Peptides:**	3

10	20	30	40	50	60	70	80
MWLVYSSGAP	GTQQPARNRV	FFPIGMAPGG	VCWRPDGWGT	GGQGRISGPG	SMGAGQRLGS	SGTQRCCWGS	CFGKEVALRR
**90**	**100**	**110**	**120**	**130**	**140**	**150**	**160**
VLHPSPVCMG	VSCLCQKNED	ECAVCRDGGE	LICCDGCPRA	FHLACLSPPL	REIPSGTWRC	SSCLQATVQE	VQPRAEEPRP
**170**	**180**	**190**	**200**	**210**	**220**	**230**	**240**
QEPPVETPLP	PGLRSAGEEV	RGPPGEPLAG	MDTTLVYKHL	PAPPSAAPLP	GLDSSALHPL	LCVGPEGQQN	LAPGARCGVC
**250**	**260**	**270**	**280**	**290**	**300**	**310**	**320**
GDGTDVLRCT	HCAAAFHWRC	HFPAGTSRPG	TGLRCRSCSG	DVTPAPVEGV	LAPSPARLAP	GPAKDDTASH	EPALHRDDLE
**330**	**340**	**350**					
SLLSEHTFDG	ILQWAIQSMA	RPAAPFPS					

Cmpd.	No. of Cmpds.	m/z meas.	Δ m/z [ppm]	z	Rt [min]	Score	P	Range	Sequence	Modification
1699	1	1011.4214	-66.62	3	51.2	12.9	1	18-45	R.NRVFFPIGMAPGGVCWRPDGWGTGGQGR.I	Carbamidomethyl: 15
1351	1	669.2815	20.48	2	45.7	14.6	0	107-119	R.DGGELICCDGCPR.A	
2864	1	651.8581	112.84	2	64.5	16.6	0	249-259	R.CTHCAAAFHWR.C	

Detailed Protein Report

Protein 166: PREDICTED: androglobin isoform X1 [Homo sapiens]

**Accession:**	gi|578812976	**Score:**	44.0
**Database:**	refseq_human(refseq_human_20140103.fasta)	**MW [kDa]:**	189.6
**Database Date:**	2015-11-30	**pI:**	9.1
		**Sequence Coverage [%]:**	2.3
		**No. of unique Peptides:**	2

10	20	30	40	50	60	70	80
MASKQTKKKE	VHRINSAHGS	DKSKDFYPFG	SNVQSGSTEQ	KKGKFPLWPE	WSEADINSEK	WDAGKGAKEK	DKTGKSPVFH
**90**	**100**	**110**	**120**	**130**	**140**	**150**	**160**
FFEDPEGKIE	LPPSLKIYSW	KRPQDILFSQ	TPVVVKNEIT	FDLFSANEHL	LCSELMRWII	SEIYAVWKIF	NGGILSNYFK
**170**	**180**	**190**	**200**	**210**	**220**	**230**	**240**
GTSGEPPLLP	WKPWEHIYSL	CKAVKGHMPL	FNSYGKYVVK	LYWMGCWRKI	TIDDFLPFDE	DNNLLLPATT	YEFELWPMLL
**250**	**260**	**270**	**280**	**290**	**300**	**310**	**320**
SKAIIKLANI	DIHVADRREL	GEFTVIHALT	GWLPEVISLH	PGYMDKVWEL	LKEILPEFKL	SDEASSESKI	AVLDSKLKEP
**330**	**340**	**350**	**360**	**370**	**380**	**390**	**400**
GKEGKEGKEI	KDGKEVKDVK	EFKPESSLTT	LKAPEKSDKV	PKEKADARDI	GKKRSKDGEK	EKFKFSLHGS	RPSSEVQYSV
**410**	**420**	**430**	**440**	**450**	**460**	**470**	**480**
QSLSDCSSAI	QTSHMVVYAT	FTPLYLFENK	IFSLEKMADS	AEKLREYGLS	HICSHPVLVT	RSRSCPLVAP	PKPPPLPPWK
**490**	**500**	**510**	**520**	**530**	**540**	**550**	**560**
LIRQKKETVI	TDEAQELIVK	KPERFLEISS	PFLNYRMTPF	TIPTEMHFVR	SLIKKGIPPG	SDLPSVSETD	ETATHSQTDL
**570**	**580**	**590**	**600**	**610**	**620**	**630**	**640**
SQITKATSQG	NTASQVILGK	GTDEQTDFGL	GDAHQSDGLN	LEREIVSQTT	ATQEKSQEEL	PTTNNSVSKE	IWLDFEDFCV
**650**	**660**	**670**	**680**	**690**	**700**	**710**	**720**
CFQNIYIFHK	PSSYCLNFQK	SEFKFSEERV	SYYLFVDSLK	PIELLVCFSA	LVRWGEYGAL	TKDSPPIEPG	LLTAETFSWK
**730**	**740**	**750**	**760**	**770**	**780**	**790**	**800**
SLKPGSLVLK	IHTYATKATV	VRLPVGRHML	LFNAYSPVGH	SIHICSMVSF	VIGDEHVVLP	NFEPESCRFT	EQSLLIMKAI
**810**	**820**	**830**	**840**	**850**	**860**	**870**	**880**
GNVIANFKDK	GKLSAALKDL	QTAHYPVPFH	DKELTAQHFR	VFHLSLWRLM	KKVQITKPPP	NFKFAFRAMV	LDLELLNSSL
**890**	**900**	**910**	**920**	**930**	**940**	**950**	**960**
EEVSLVEWLD	VKYCMPTSDK	EYSAEEVAAA	IKIQAMWRGT	YVRLLMKARI	PDTKENISVA	DTLQKVWAVL	EMNLEQYAVS
**970**	**980**	**990**	**1000**	**1010**	**1020**	**1030**	**1040**
LLRLMFKSKC	KSLESYPCYQ	DEETKIAFAD	YTVTYQEQPP	NSWFIVFRET	FLVHQDMILV	PKVYTTLPIC	ILHIVNNDTM
**1050**	**1060**	**1070**	**1080**	**1090**	**1100**	**1110**	**1120**
EQVPKVFQKV	VPYLYTKNKK	GYTFVAEAFT	GDTYVAASRW	KLRLIGSSAP	LPCLSRDSPC	NSFAIKEIRD	YYIPNDKKIL
**1130**	**1140**	**1150**	**1160**	**1170**	**1180**	**1190**	**1200**
FRYSVKVLTP	QPATIQVRTS	KPDAFIKLQV	LENEETMVSS	TGKGQAIIPA	FHFLKSEKGL	SSQSSKHILS	FHSASKKEQE
**1210**	**1220**	**1230**	**1240**	**1250**	**1260**	**1270**	**1280**
VYVKKKAAQG	IQKSPKGRAV	SAIQDIGLPL	VEEETTSTPT	REDSSSTPLQ	NYKYIIQCSV	LYNSWPLTES	QLTFVQALKD
**1290**	**1300**	**1310**	**1320**	**1330**	**1340**	**1350**	**1360**
LKKSNTKAYG	ERHEELINLG	SPDSHTISEG	QKSSVTSKTT	RKGKEKSSEK	EKTAKEKQAP	RFEPQISTVH	PQQEDPNKPY
**1370**	**1380**	**1390**	**1400**	**1410**	**1420**	**1430**	**1440**
WILRLVTEHN	ESELFEVKKD	TERADEIRAM	KQAWETTEPG	RAIKASQARL	HYLSGFIKKT	SDAESPPISE	SQTKPKEEVE
**1450**	**1460**	**1470**	**1480**	**1490**	**1500**	**1510**	**1520**
TAARGVKEPN	SKNSAGSESK	EMTQTGSGSA	VWKKWQLTKG	LRDVAKSTSS	ESGGVSSPGK	EEREQSTRKE	NIQTGPRTRS
**1530**	**1540**	**1550**	**1560**	**1570**	**1580**	**1590**	**1600**
PTILETSPRL	IRKALEFMDL	SQYVRKTDTD	PLLQTDELNQ	QQAMQKAEEI	HQFRQHRTRV	LSIRNIDQEE	RLKLKDEVLD
**1610**	**1620**	**1630**	**1640**	**1650**	**1660**	**1670**	
MYKEMQDSLD	EARQKIFDIR	EEYRNKLLEA	EHLKLEALSA	QEAAMKLETE	KMTPAPDTQK	KKKGKKK	

Cmpd.	No. of Cmpds.	m/z meas.	Δ m/z [ppm]	z	Rt [min]	Score	P	Range	Sequence	Modification
2723	1	921.1582	-5.50	3	65.3	17.8	1	138-160	R.WIISEIYAVWKIFNGGILSNYFK.G	
2707	2	933.4993	-17.55	2	65.1	26.2	2	913-927	K.IQAMWRGTYVRLLMK.A	

Detailed Protein Report

Protein 167: **putative ribosomal RNA methyltransferase NOP2 isoform 4 [Homo sapiens]**

**Accession:**	gi|385198063	**Score:**	43.8
**Database:**	refseq_human(refseq_human_20140103.fasta)	**MW [kDa]:**	69.6
**Database Date:**	2015-11-30	**pI:**	6.2
		**Sequence Coverage [%]:**	6.2
		**No. of unique Peptides:**	2

```
        10         20         30         40         50         60         70         80
MGRKLDPTKE KRGPGRKARK QKGAETELVR FLPAVSDENS KRLSSRARKR AAKRRLGSVE APKTNKSPEA KPLPGKLPKG
        90        100        110        120        130        140        150        160
AVQTAGKKGP QSLFNAPRGK KRPAPGSDEE EEEEDSEEDG MVNHGDLWGS EDDADTVDDY GADSNSEDEE EGEALLPIER
       170        180        190        200        210        220        230        240
AARKQKAREA AAGIQWSEEE TEDEEEEKEV TPESGPPKVE EADGGLQINV DEEPFVLPPA GEMEQDAQAP DLQRVHKRIQ
       250        260        270        280        290        300        310        320
DIVGILRDFG AQREEGRSRS EYLNRLKKDL AIYYSYGDFL LGKLMDLFPL SELVEFLEAN EVPRPVTLRT NTLKTRRRDL
       330        340        350        360        370        380        390        400
AQALINRGVN LDPLGKWSKT GLVVYDSSVP IGATPEYLAG HYMLQGASSM LPVMALAPQE HERILDMCCA PGGKTSYMAQ
       410        420        430        440        450        460        470        480
LMKNTGVILA NDANAERLKS VVGNLHRLGV TNTIISHYDG RQFPKVVGGF DRVLLDAPCS GTGVISKDPA VKTNKDEKDI
       490        500        510        520        530        540        550        560
LRCAHLQKEL LLSAIDSVNA TSKTGGYLVY CTCSITVEEN EWVVDYALKK RNVRLVPTGL DFGQEGFTRF RERRFHPSLR
       570        580        590        600        610        620        630
STRRFYPHTH NMDGFFIAKF KKFSNSIPQS QTDGVLLCRS GWTAVVQSQL IATSTFQVQA ILVPQTPK
```

Cmpd.	No. of Cmpds.	m/z meas.	Δ m/z [ppm]	z	Rt [min]	Score	P	Range	Sequence	Modification
2658	1	625.2870	-81.65	2	61.5	11.2	1	442-452	R.QFPKVVGGFDR.V	
2674	1	933.1060	151.43	2	64.6	13.8	0	583-599	K.FSNSIPQSQTDGVLLCR.S	

Detailed Protein Report

Protein 168: **FERM, RhoGEF and pleckstrin domain-containing protein 2 isoform b [Homo sapiens]**

**Accession:**	gi|545478372	**Score:**	43.7
**Database:**	refseq_human(refseq_human_20140103.fasta)	**MW [kDa]:**	73.2
**Database Date:**	2015-11-30	**pI:**	9.6
		**Sequence Coverage [%]:**	7.7
		**No. of unique Peptides:**	1

10	20	30	40	50	60	70	80
MGEIEGTYRV	LQTAGMRLGA	QTPVGVSTLE	PGQTLLPRMQ	EKHLHLRVKL	LDNTMEIFDI	EPKCDGQVLL	TQVWKRLNLV
90	100	110	120	130	140	150	160
ECDYFGMEFQ	NTQSYWIWLE	PMKPIIRQIR	RPKNVVLRLA	VKFFPPDPGQ	LQEEYTRYLF	ALQLKRDLLE	ERLTCADTTA
170	180	190	200	210	220	230	240
ALLTSHLLQS	EIGDYDETLD	REHLKVNEYL	PGQQHCLEKI	LEFHQKHVGQ	TPAESDFQVL	EIARKLEMYG	IRFHMASDRE
250	260	270	280	290	300	310	320
GTKIQLAVSH	MGVLVFQGTT	KINTFNWSKV	RKLSFKRKRF	LIKLHPEVHG	PYQDTLEFLL	GSRDECKNFW	KICVEYHTFF
330	340	350	360	370	380	390	400
RLLDQPKPKA	KAVFFSRGSS	FRYSGRTQKQ	LVDYFKDSGM	KRIPYERRHS	KTHTSVRALT	ADLPKQSISF	PEGLRTPASP
410	420	430	440	450	460	470	480
SSANAFYSLS	PSTLVPSGLP	EFKDSSSSLT	DPQVSYVKSP	AAERRSGAVA	GGPDTPSAQP	LGPPALQPGP	GLSTKSPQPS
490	500	510	520	530	540	550	560
PSSRKSPLSL	SPAFQVPLGP	AEQGSSPLLS	PVLSDAGGAG	MDCEEPRHKR	VPADEAYFIV	KEILATERTY	LKDLEVITVW
570	580	590	600	610	620	630	640
FRSAVVKEDA	MPATLMTLLF	SNIDPIYEFH	RGFLREVEQR	LALWEGPSKA	HTKGSHQRIG	DILLRNMRQL	KVFQLHEGHV
650							
AGVTKME							

Cmpd.	No. of Cmpds.	m/z meas.	Δ m/z [ppm]	z	Rt [min]	Score	P	Range	Sequence	Modification
22	1	1001.9616	-70.42	2	29.6	16.6	2	601-618	R.LALWEGPSKAHTKGSHQR.I	

Detailed Protein Report

Protein 169: **PREDICTED: intraflagellar transport protein 88 homolog isoform X7 [Homo sapiens]**

**Accession:**	gi|530402705	**Score:**	43.7
**Database:**	refseq_human(refseq_human_20140103.fasta)	**MW [kDa]:**	95.8
**Database Date:**	2015-11-30	**pI:**	9.3
		**Sequence Coverage [%]:**	4.4
		**No. of unique Peptides:**	1

10	20	30	40	50	60	70	80
MKVESGMKIK	VHNNAESETA	VGFLEVDQGC	PIFGFPGPHR	KKNCLGPHMK	FTNTKVQMMQ	NVHLAPETDE	DDLYSGYNDY
90	100	110	120	130	140	150	160
NPIYDIEELE	NDAAFQQAVR	TSHGRRPPIT	AKISSTAVTR	PIATGYGSKT	SLASSIGRPM	TGAIQDGVTR	PMTAVRAAGF
170	180	190	200	210	220	230	240
TKAALRGSAF	DPLSQSRGPA	SPLEAKKKDS	PEEKIKQLEK	EVNELVEESC	IANSCGDLKL	ALEKAKDAGR	KERVLVRQRE
250	260	270	280	290	300	310	320
QVTTPENINL	DLTYSVLFNL	ASQYSVNEMY	AEALNTYQVI	VKNKMFSNAG	ILKMNMGNIY	LKQRNYSKAI	KFYRMALDQV
330	340	350	360	370	380	390	400
PSVNKQMRIK	IMQNIGVTFI	QAGQYSDAIN	SYEHIMSMAP	NLKAGYNLTI	CYFAIGDREK	MKKAFQKLIT	VPLEIDEDKY
410	420	430	440	450	460	470	480
ISPSDDPHTN	LVTEAIKNDH	LRQMERERKA	MAEKYIMTSA	KLIAPVIETS	FAAGYDWCVE	VVKASQYVEL	ANDLEINKAV
490	500	510	520	530	540	550	560
TYLRQKDYNQ	AVEILKVLEK	KDSRVKSAAA	TNLSALYYMG	KDFAQASSYA	DIAVNSDRYN	PAALTNKGNT	VFANGDYEKA
570	580	590	600	610	620	630	640
AEFYKEALRN	DSSCTEALYN	IGLTYEKLNR	LDEALDCFLK	LHAILRNSAE	VLYQIANIYE	LMENPSQAIE	WLMQVVSVIP
650	660	670	680	690	700	710	720
TDPQVLSKLG	ELYDREGDKS	QAFQYYYESY	RYFPCNIEVI	EWLGAYYIDT	QFWEKAIQYF	ERASLIQPTQ	VKWQLMVASC
730	740	750	760	770	780	790	800
FRRSGNYQKA	LDTYKDTHRK	FPENVECSGS	VRTGHMERDP	LNLLPRKSVL	PGPEIAKCNY	FSGEKYYLSA	FLSSSLHRSW
810	820	830	840	850			
IKRCSRICQK	TEEVGKNERN	KGTAHKVRQR	WQWGLPWQKR	RKC			

Cmpd.	No. of Cmpds.	m/z meas.	Δ m/z [ppm]	z	Rt [min]	Score	P	Range	Sequence	Modification
1492	1	450.0889	-279.87	2	48.4	13.5	0	43-50	K.NCLGPHMK.F	

Detailed Protein Report

Protein 170: PREDICTED: coiled-coil domain-containing protein 91 isoform X6 [Homo sapiens]

**Accession:**	gi|578822930	**Score:**	43.6
**Database:**	refseq_human(refseq_human_20140103.fasta)	**MW [kDa]:**	51.6
**Database Date:**	2015-11-30	**pI:**	5.4
**Modification(s):**	Oxidation	**Sequence Coverage [%]:**	5.8
		**No. of unique Peptides:**	2

10	20	30	40	50	60	70	80
MWVQQHRPEN	TFGDQRETCN	FHCELPSRVY	GRTSLKLLMP	MWNKVSGVHL	SPSSPEIVLD	RDHSSSIGCL	SSDAIISSPE
90	100	110	120	130	140	150	160
NTHAANSIVS	QTIPKAQIQQ	STHTHLDISL	FPLGLTDEKS	NGTIALVDDS	EDPGANVSNI	QLQQKISSLE	IKLKVSEEEK
170	180	190	200	210	220	230	240
QRIKQDVESL	MEKHNVLEKG	FLKEKEQEAI	SFQDRYKELQ	EKHKQELEDM	RKAGHEALSI	IVDEYKALLQ	SSVKQQVEAI
250	260	270	280	290	300	310	320
EKQYISAIEK	QAHKCEELLN	AQHQRLLEML	DTEKELLKEK	IKEALIQQSQ	EQKEILEKCL	EEERQRNKEA	LVSAAKLEKE
330	340	350	360	370	380	390	400
AVKDAVLKVV	EEERKNLEKA	HAEERELWKT	EHAKDQEKVS	QEIQKAIQEQ	RKISQETVKA	AIIEEQKRSE	KAVEEAVKRT
410	420	430	440	450			
RDELIEYIKE	QKRLDQVIRQ	RSLSSLELFL	SCAQKQLSAL	IATEPVDIE			

Cmpd.	No. of Cmpds.	m/z meas.	Δ m/z [ppm]	z	Rt [min]	Score	P	Range	Sequence	Modification
1931	4	1022.8987	-73.55	2	54.2	16.6	0	1-16	-.MWVQQHRPENTFGDQR.E	Oxidation: 1
1996	1	665.3129	-29.71	2	55.1	11.2	2	203-212	K.HKQELEDMRK.A	Oxidation: 8

Detailed Protein Report

Protein 171: complement C4-A isoform 2 preproprotein [Homo sapiens]

**Accession:**	gi|356582273	**Score:**	43.6
**Database:**	refseq_human(refseq_human_20140103.fasta)	**MW [kDa]:**	187.6
**Database Date:**	2015-11-30	**pI:**	6.7
**Modification(s):**	Carbamidomethyl	**Sequence Coverage [%]:**	2.7
		**No. of unique Peptides:**	2

10	20	30	40	50	60	70	80
MRLLWGLIWA	SSFFTLSLQK	PRLLLFSPSV	VHLGVPLSVG	VQLQDVPRGQ	VVKGSVFLRN	PSRNNVPCSP	KVDFTLSSER
90	100	110	120	130	140	150	160
DFALLSLQVP	LKDAKSCGLH	QLLRGPEVQL	VAHSPWLKDS	LSRTTNIQGI	NLLFSSRRGH	LFLQTDQPIY	NPGQRVRYRV
170	180	190	200	210	220	230	240
FALDQKMRPS	TDTITVMVEN	SHGLRVRKKE	VYMPSSIFQD	DFVIPDISEP	GTWKISARFS	DGLESNSSTQ	FEVKKYVLPN
250	260	270	280	290	300	310	320
FEVKITPGKP	YILTVPGHLD	EMQLDIQARY	IYGKPVQGVA	YVRFGLLDED	GKKTFFRGLE	SQTKLVNGQS	HISLSKAEFQ
330	340	350	360	370	380	390	400
DALEKLNMGI	TDLQGLRLYV	AAAIIESPGG	EMEEAELTSW	YFVSSPFSLD	LSKTKRHLVP	GAPFLLQALV	REMSGSPASG
410	420	430	440	450	460	470	480
IPVKVSATVS	SPGSVPEVQD	IQQNTDGSGQ	VSIPIIIPQT	ISELQLSVSA	GSPHPAIARL	TVAAPPSGGP	GFLSIERPDS
490	500	510	520	530	540	550	560
RPPRVGDTLN	LNLRAVGSGA	TFSHYYYMIL	SRGQIVFMNR	EPKRTLTSVS	VFVDHHLAPS	FYFVAFYYHG	DHPVANSLRV
570	580	590	600	610	620	630	640
DVQAGACEGK	LELSVDGAKQ	YRNGESVKLH	LETDSLALVA	LGALDTALYA	AGSKSHKPLN	MGKVFEAMNS	YDLGCGPGGG
650	660	670	680	690	700	710	720
DSALQVFQAA	GLAFSDGDQW	TLSRKRLSCP	KEKTTRKKRN	VNFQKAINEK	LGQYASPTAK	RCCQDGVTRL	PMMRSCEQRA
730	740	750	760	770	780	790	800
ARVQQPDCRE	PFLSCCQFAE	SLRKKSRDKG	QAGLQRALEI	LQEEDLIDED	DIPVRSFFPE	NWLWRVETVD	RFQILTLWLP
810	820	830	840	850	860	870	880
DSLTTWEIHG	LSLSKTKGLC	VATPVQLRVF	REFHLHLRLP	MSVRRFEQLE	LRPVLYNYLD	KNLTVSVHVS	PVEGLCLAGG
890	900	910	920	930	940	950	960
GGLAQQVLVP	AGSARPVAFS	VVPTAAAAVS	LKVVARGSFE	FPVGDAVSKV	LQIEKEGAIH	REELVYELNP	LDHRGRTLEI
970	980	990	1000	1010	1020	1030	1040
PGNSDPNMIP	DGDFNSYVRV	TASDPLDTLG	SEGALSPGGV	ASLLRLPRGC	GEQTMIYLAP	TLAASRYLDK	TEQWSTLPPE
1050	1060	1070	1080	1090	1100	1110	1120
TKDHAVDLIQ	KGYMRIQQFR	KADGSYAAWL	SRDSSTWLTA	FVLKVLSLAQ	EQVGGSPEKL	QETSNWLLSQ	QQADGSFQDP
1130	1140	1150	1160	1170	1180	1190	1200
CPVLDRSMQG	GLVGNDETVA	LTAFVTIALH	HGLAVFQDEG	AEPLKQRVEA	SISKANSFLG	EKASAGLLGA	HAAAITAYAL
1210	1220	1230	1240	1250	1260	1270	1280
TLTKAPVDLL	GVAHNNLMAM	AQETGDNLYW	GSVTGSQSNA	VSPTPAPRNP	SDPMPQAPAL	WIETTAYALL	HLLLHEGKAE
1290	1300	1310	1320	1330	1340	1350	1360
MADQASAWLT	RQGSFQGGFR	STQDTVIALD	ALSAYWIASH	TTEERGLNVT	LSSTGRNGFK	SHALQLNNRQ	IRGLEEELQF
1370	1380	1390	1400	1410	1420	1430	1440
SLGSKINVKV	GGNSKGTLKV	LRTYNVLDMK	NTTCQDLQIE	VTVKGHVEYT	MEANEDYEDY	EYDELPAKDD	PDAPLQPVTP
1450	1460	1470	1480	1490	1500	1510	1520
LQLFEGRRNR	RRREAPKLTS	LSDRYVSHFE	TEGPHVLLYF	DSVPTSRECV	GFEAVQEVPV	GLVQPASATL	YDYYNPERRC
1530	1540	1550	1560	1570	1580	1590	1600
SVFYGAPSKS	RLLATLCSAE	VCQCAEGKCP	RQRRALERGL	QDEDGYRMKF	ACYYPRVEYG	FQVKVLREDS	RAAFRLFETK
1610	1620	1630	1640	1650	1660	1670	1680
ITQVLHFTKD	VKAAANQMRN	FLVRASCRLR	LEPGKEYLIM	GLDGATYDLE	GHPQYLLDSN	SWIEEMPSER	LCRSTRQRAA
1690	1700						
CAQLNDFLQE	YGTQGCQV						

Cmpd.	No. of Cmpds.	m/z meas.	Δ m/z [ppm]	z	Rt [min]	Score	P	Range	Sequence	Modification
1978	2	656.8089	-24.39	2	54.8	11.3	1	60-71	R.NPSRNNVPCSPK.V	
946	1	872.2583	-156.84	2	40.6	12.5	0	730-743	R.EPFLSCCQFAESLR.K	Carbamidomethyl: 6, 7

Detailed Protein Report

Protein 172: PREDICTED: protein PRR14L isoform X4 [Homo sapiens]

**Accession:**	gi|578837142	**Score:**	43.5
**Database:**	refseq_human(refseq_human_20140103.fasta)	**MW [kDa]:**	219.9
**Database Date:**	2015-11-30	**pI:**	6.2
**Modification(s):**	Carbamidomethyl	**Sequence Coverage [%]:**	2.4
		**No. of unique Peptides:**	3

Quantitation

mdown:qdown	**Median:**	0.17	**CV:**	0.00 %	**No. of Peptides:**	1
Wdown:Qdown	**Median:**	0.07	**CV:**	0.00 %	**No. of Peptides:**	1

Detailed Protein Report

10	20	30	40	50	60	70	80
MQSSKELSHV	DLPEDFLRSK	EGNVQITAET	LLKSAEVQGM	KVNGTKTDNN	EGHKNGNVSK	DLSAGCGEFQ	EVDKIMTSDE
90	100	110	120	130	140	150	160
VSETSTLVTP	EPLTFVDPVL	TEATPKEKEC	EELKSCPWLS	LPGNSAISNV	DNGKEELCKP	NLVCEADDNH	QQLHGHHNEQ
170	180	190	200	210	220	230	240
PSSTHDSPTA	TSPLKENSEV	SCFTSDLSGP	ESRTISLENC	GFEGGGLLKR	SAEKTDSSYF	YRGDDQGKNL	ASREENEERL
250	260	270	280	290	300	310	320
LIPRSERGGP	FLFNAREPEK	EISGRCSGEK	EPVVSPKENI	HNNCIQDSLH	TGNSSSLMPN	SFTEATEVML	NKNDLKITVH
330	340	350	360	370	380	390	400
VQGNLTNPED	HKETFTNMSH	PGGHSEESSF	SSLMQIEEAG	QTTPVEPNIL	SKSFYTKDCN	SLVSIQRNLE	GNTQLNEASC
410	420	430	440	450	460	470	480
NDFLFERKSI	VSLMPEDQIS	PVSEVLKPKQ	GTALLLPSPE	FDYRPESEKV	IQTSHDDIPL	LDEQSIACEM	NELSCTNELV
490	500	510	520	530	540	550	560
VNKVESECVL	NQQVSLNSQE	HANLPTDSLL	HLNKEMPLAT	GRDAHQSHHP	PLEGRADVIA	DIQTIPIQTK	IKDISPPGNQ
570	580	590	600	610	620	630	640
TCGASSNCPT	LNIKPVSLER	KKEMADSGTK	ALHSRLRSNK	REAAGFPQVV	SVIECHSVQS	QDISSCHRVR	KNVSQENMCS
650	660	670	680	690	700	710	720
ASAAFKSSKI	SLQVDNSLIT	KYENAFQHRD	HCCQGTGHSV	EKSSCKVSYT	SQERELDGKE	TNGSLPGDKI	RNKMVAGLLN
730	740	750	760	770	780	790	800
SGISNKTIHT	SSSIKLSEEG	LEGKEQDVSK	ETVFCKYNIS	DHAIQELNQT	VNIPGPEKVL	DQSPTVMFSS	FKNVKSVETL
810	820	830	840	850	860	870	880
DQKADEVLDC	QSNQNRPDEC	KSEGQSAKEM	LSSDQRETVT	EPHGEVNHNQ	KDLLVSSGSN	NSLPCGSPKK	CNLKGAFVKM
890	900	910	920	930	940	950	960
SGCDESTEGM	VDIVYTDCSN	KLAEGVLDVK	ASNLLDCGAR	QEKLAFQEDS	RSTLSRRELD	AAHTGTTGQD	SDFPVTAAST
970	980	990	1000	1010	1020	1030	1040
VDFLKIKKSC	EENVCRSLKD	CEMEKCPDSC	AHEMESVADH	EPNKRILGRV	NLSLNDSHYG	QQDKGTSLRE	TQEMTEGSRL
1050	1060	1070	1080	1090	1100	1110	1120
EPNSEFGKES	TFGISSKESM	SCHDESSVSL	RSLKSIEIMP	SQENSETNVN	SEETDLKNLC	KPKDGEMLCE	NVKDCTVLPE
1130	1140	1150	1160	1170	1180	1190	1200
MKEIVSRDWS	NSSDRDSVCT	CVEKNACKAC	HPHENSSDRH	LPLTVKTDIK	VKGEETEEHQ	RGRLGYLTVG	EQSEELVTRE
1210	1220	1230	1240	1250	1260	1270	1280
TGDGDPVSNI	SQTHFKCRGI	LNHAEKQQSP	EVLDYMLQKE	EKYIRQQKAH	TISQQCISSS	LLLDDAQNQN	QPKADKDEST
1290	1300	1310	1320	1330	1340	1350	1360
MINEITLAKL	AKDSIVAQTQ	KLEDQKEERL	HHPLRKDTES	CTSPCLLGAP	RKAQDPSSAG	CDQIHGAFAK	KGVLPLKKQP
1370	1380	1390	1400	1410	1420	1430	1440
HRTCKKVSYQ	EQIIVGRKIG	KIRSSAFLKS	SSNPIPTKAH	RLLSLCTLSA	PTRLEPETAP	TKSLVSHIPK	QMSTPCHPLR
1450	1460	1470	1480	1490	1500	1510	1520
SLNFRKTTKE	SALLNKLSIL	ASKLAPAMKT	QKLRYRRCSS	ELLPMAKSYK	RLRYKRLLDG	FSSSTEQLNP	YLAASGWDKR
1530	1540	1550	1560	1570	1580	1590	1600
PNSKPMALYS	LESIKMTFID	LSNKMPSLLF	GSEIFPVSFH	VKSSSSDCTT	ESSRTFPEHC	APARLALGEA	LQCPSQPPKW
1610	1620	1630	1640	1650	1660	1670	1680
TFSFFLSHGC	PGMATFREDT	GVHSQTHTQA	PPQPPAPLQD	YGGTAIVQTR	ADCSVLGLHT	LLALCSPGCY	RIWTKKRSFS
1690	1700	1710	1720	1730	1740	1750	1760
SHMPTMQRLF	MTQFTQGLKG	LRSPASIADK	VFCSLPYSVG	RVLSIWSQHG	PSVCSFEISS	LHSPHCKRQP	SLGTTSSHTM
1770	1780	1790	1800	1810	1820	1830	1840
LPYVPLPGME	ATYNTSGSQT	RLEPPFPALV	PKSCLVAESA	VSKLLLSASE	FQVRGLDELD	GVKAACPCPQ	SSPPEQKEAE
1850	1860	1870	1880	1890	1900	1910	1920
PEKRPKKVSQ	IRIRKTIPRP	DPNLTPMGLP	RPKRLKKKEF	SLEEIYTNKN	YKSPPANRCL	ETIFEEPKER	NGTLISISQQ
1930	1940	1950	1960	1970	1980	1990	
KRKRVLEFQD	FTVPRKRRAR	GKVKVAGSFT	RAQKAAVQSR	ELDALLIQKL	MELETFFAKE	EEQEQSSGC	

Cmpd.	No. of Cmpds.	m/z meas.	Δ m/z [ppm]	z	Rt [min]	Score	P	Range	Sequence	Modification	Ratios
1682	1	917.4150	-55.34	2	49.6	13.4	2	245-260	R.SERGGPFLFNAREPEK.E		Wdown:Qdown 0.07<br>mdown:qdown 0.17
140	1	793.8794	36.37	2	31.0	13.1	0	632-646	K.NVSQENMCSASAAFK.S		

Detailed Protein Report

Cmpd.	No. of Cmpds.	m/z meas.	Δ m/z [ppm]	z	Rt [min]	Score	P	Range	Sequence	Modification	Ratios
1702	2	867.8891	-25.32	2	49.8	17.0	1	1316-1331	R.KDTESCTSPCLLGAPR.K	Carbamidomethyl: 10	

Detailed Protein Report

Protein 173: RB1-inducible coiled-coil protein 1 isoform 2 [Homo sapiens]

**Accession:**	gi|134304846	**Score:**	43.2
**Database:**	refseq_human(refseq_human_20140103.fasta)	**MW [kDa]:**	182.7
**Database Date:**	2015-11-30	**pI:**	5.2
		**Sequence Coverage [%]:**	1.9
		**No. of unique Peptides:**	3

Alias proteins:

Accession	Name	Description
gi|578815756	refseq_human (refseq_human_20140103.fasta)	PREDICTED: RB1-inducible coiled-coil protein 1 isoform X1 [Homo sapiens]

10	20	30	40	50	60	70	80
MKLYVFLVNT	GTTLTFDTEL	TVQTVADLKH	AIQSKYKIAI	QHQVLVVNGG	ECMAADRRVC	TYSAGTDTNP	IFLFNKEMIL
90	100	110	120	130	140	150	160
CDRPPAIPKT	TFSTENDMEI	KVEESLMMPA	VFHTVASRTQ	LALEMYEVAK	KLCSFCEGLV	HDEHLQHQGW	AAIMANLEDC
170	180	190	200	210	220	230	240
SNSYQKLLFK	FESIYSNYLQ	SIEDIKLKLT	HLGTAVSVMA	KIPLLECLTR	HSYRECLGRL	DSLPEHEDSE	KAEMKRSTEL
250	260	270	280	290	300	310	320
VLSPDMPRTT	NESLLTSFPK	SVEHVSPDTA	DAESGKEIRE	SCQSTVHQQD	ETTIDTKDGD	LPFFNVSLLD	WINVQDRPND
330	340	350	360	370	380	390	400
VESLVRKCFD	SMSRLDPRII	RPFIAECRQT	IAKLDNQNMK	AIKGLEDRLY	ALDQMIASCG	RLVNEQKELA	QGFLANQKRA
410	420	430	440	450	460	470	480
ENLKDASVLP	DLCLSHANQL	MIMLQNHRKL	LDIKQKCTTA	KQELANNLHV	RLKWCCFVML	HADQDGEKLQ	ALLRLVIELL
490	500	510	520	530	540	550	560
ERVKIVEALS	TVPQMYCLAV	VEVVRRKMFI	KHYREWAGAL	VKDGKRLYEA	EKSKRESFGK	LFRKSFLRNR	LFRGLDSWPP
570	580	590	600	610	620	630	640
SFCTQKPRKF	DCELPDISLK	DLQFLQSFCP	SEVQPFLRVP	LLCDFEPLHQ	HVLALHNLVK	AAQSLDEMSQ	TITDLLSEQK
650	660	670	680	690	700	710	720
ASVSQTSPQS	ASSPRMESTA	GITTTTSPRT	PPPLTVQDPL	CPAVCPLEEL	SPDSIDAHTF	DFETIPHPNI	EQTIHQVSLD
730	740	750	760	770	780	790	800
LDSLAESPES	DFMSAVNEFV	IEENLSSPNP	ISDPQSPEMM	VESLYSSVIN	AIDSRRMQDT	NVCGKEDFGD	HTSLNVQLER
810	820	830	840	850	860	870	880
CRVVAQDSHF	SIQTIKEDLC	HFRTFVQKEQ	CDFSNSLKCT	AVEIRNIIEK	VKCSLEITLK	EKHQKELLSL	KNEYEGKLDG
890	900	910	920	930	940	950	960
LIKETEENEN	KIKKLKGELV	CLEEVLQNKD	NEFALVKHEK	EAVICLQNEK	DQKLLEMENI	MHSQNCEIKE	LKQSREIVLE
970	980	990	1000	1010	1020	1030	1040
DLKKLHVEND	EKLQLLRAEL	QSLEQSHLKE	LEDTLQVRHI	QEFEKVMTDH	RVSLEELKKE	NQQIINQIQE	SHAEIIQEKE
1050	1060	1070	1080	1090	1100	1110	1120
KQLQELKLKV	SDLSDTRCKL	EVELALKEAE	TDEIKILLEE	SRAQQKETLK	SLLEQETENL	RTEISKLNQK	IQDNNENYQV
1130	1140	1150	1160	1170	1180	1190	1200
GLAELRTLMT	IEKDQCISEL	ISRHEEESNI	LKAELNKVTS	LHNQAFEIEK	NLKEQIIELQ	SKLDSELSAL	ERQKDEKITQ
1210	1220	1230	1240	1250	1260	1270	1280
QEEKYEAIIQ	NLEKDRQKLV	SSQEQDREQL	IQKLNCEKDE	AIQTALKEFK	LEREVVEKEL	LEKVKHLENQ	IAKSPAIDST
1290	1300	1310	1320	1330	1340	1350	1360
RGDSSSLVAE	LQEKLQEEKA	KFLEQLEEQE	KRKNEEMQNV	RTSLIAEQQT	NFNTVLTREK	MRKENIINDL	SDKLKSTMQQ
1370	1380	1390	1400	1410	1420	1430	1440
QERDKDLIES	LSEDRARLLE	EKKKLEEEVS	KLRSSSFVPS	PYVATAPELY	GACAPELPGE	SDRSAVETAD	EGRVDSAMET
1450	1460	1470	1480	1490	1500	1510	1520
SMMSVQENIH	MLSEEKQRIM	LLERTLQLKE	EENKRLNQRL	MSQSMSSVSS	RHSEKIAIRD	FQVGDLVLII	LDERHDNYVL
1530	1540	1550	1560	1570	1580	1590	1600
FTVSPTLYFL	HSESLPALDL	KPASGASRRP	WVLGKVMEKE	YCQAKKAQNR	FKVPLGTKFY	RVKAVSWNKK	V

Cmpd.	No. of Cmpds.	m/z meas.	Δ m/z [ppm]	z	Rt [min]	Score	P	Range	Sequence	Modification
2032	1	687.8227	-80.45	2	55.5	10.2	1	388-399	K.ELAQGFLANQKR.A	
1335	1	497.7323	-72.04	2	46.4	14.6	1	508-514	K.MFIKHYR.E	

Detailed Protein Report

Cmpd.	No. of Cmpds.	m/z meas.	Δ m/z [ppm]	z	Rt [min]	Score	P	Range	Sequence	Modification
1272	1	673.3365	-36.20	2	45.6	18.3	1	1228-1238	R.EQLIQKLNCEK.D	

Detailed Protein Report

Protein 174: PREDICTED: leucine-rich repeat-containing protein 16C isoform X1 [Homo sapiens]

**Accession:**	gi|530423607	**Score:**	43.1
**Database:**	refseq_human(refseq_human_20140103.fasta)	**MW [kDa]:**	148.1
**Database Date:**	2015-11-30	**pI:**	6.1
**Modification(s):**	Oxidation	**Sequence Coverage [%]:**	2.8
		**No. of unique Peptides:**	2

Quantitation

mdown:qdown **Median:** 1.82 **CV:** 0.00 % **No. of Peptides:** 1

10	20	30	40	50	60	70	80
MAQTPDGISC	ELRGEITRFL	WPKEVELLLK	TWLPGEGAVQ	NHVLALLRWR	AYLLHTTCLP	LRVDCTFSYL	EVQAMALQET
90	100	110	120	130	140	150	160
PPQVTFELES	LRELVLEFPG	VAALEQLAQH	VAAAIKKVFP	RSTLGKLFRR	PTPASMLARL	ERSSPSESTD	PCSPCGGFLE
170	180	190	200	210	220	230	240
TYEALCDYNG	FPFREEIQWD	VDTIYHRQGC	RHFSLGDFSH	LGSRDLALSV	AALSYNLWFR	CLSCVDMKLS	LEVSEQILHM
250	260	270	280	290	300	310	320
MSQSSHLEEL	VLETCSLRGD	FVRRLAQALA	GHSSSGLREL	SLAGNLLDDR	GMTALSRHLE	RCPGALRRLS	LAQTGLTPRG
330	340	350	360	370	380	390	400
MRALGRALAT	NAAFDSTLTH	LDLSGNPGAL	GASEDSGGLY	SFLSRPNVLS	FLNLAGTDTA	LDTLFAAVSR	GCCTSLTHLD
410	420	430	440	450	460	470	480
ASRNVFSRTK	SRAAPAALQL	FLSRARTLRH	LGLAGCKLPP	DALRALLDGL	ALNTHLRDLH	LDLSACELRS	AGAQVIQDLV
490	500	510	520	530	540	550	560
CDAGAVSSLD	LADNGFGSDM	VTLVLAIGRS	RSLRHVALGR	NFNVRCKETL	DDVLHRIVQL	MQDDDCPLQS	LSVAESRLKL
570	580	590	600	610	620	630	640
GASVLLRALA	TNPNLTALDI	SGNAMGDAGA	KLLAKALRVN	SRLRSVVWDR	NHTSALGLLD	VAQALEQNHS	LKAMPLPLND
650	660	670	680	690	700	710	720
VAQAQRSRPE	LTARAVHQIQ	ACLLRNNRAD	PASSDHTTRL	QPLGLVSDPS	EQEVNELCQS	VQEHVELLGC	GAGPQGEAAV
730	740	750	760	770	780	790	800
RQAEDAIQNA	NFSLSILPIL	YEAGSSPSHH	WQLGQKLEGL	LRQVGEVCRQ	DIQDFTQATL	DTARSLCPQM	LQGSSWREQL
810	820	830	840	850	860	870	880
EGVLAGSRGL	PELLPEQLLQ	DAFTRLRDMR	LSITGTLAES	IVAQALAGLS	AARDQLVESL	AQQATVTMPP	ALPAPDGGEP
890	900	910	920	930	940	950	960
SLLEPGELEG	LFFPEEKEEE	KEKDDSPPQK	WPELSHGLHL	VPFIHSAAEE	AEPEPELAAP	GEDAEPQAGP	SARGSPSPAA
970	980	990	1000	1010	1020	1030	1040
PGPPAGPLPR	MDLPLAGQPL	RHPTRARPRP	RRQHHHRPPP	GGPQVPPALP	QEGNGLSARV	DEGVEEFFSK	RLIQQDRLWA
1050	1060	1070	1080	1090	1100	1110	1120
PEEDPATEGG	ATPVPRTLRK	KLGTLFAFKK	PRSTRGPRTD	LETSPGAAPR	TRKTTFGDLL	RPPTRPSRGE	ELGGAEGDTS
1130	1140	1150	1160	1170	1180	1190	1200
SPDPAGRSRP	RYTRDSKAYS	MILLPAEEEA	TLGARPDKRR	PLERGETELA	PSFEQRVQVM	LQRIGVSRGS	GGAEGKRKQS
1210	1220	1230	1240	1250	1260	1270	1280
KDGEIKKAGS	DGDIMDSSTE	APPISIKSRT	HSVSADPSCR	PGPGSQGPES	ATWKTLGQQL	NAELRSRGWG	QQDGPGPPSP
1290	1300	1310	1320	1330	1340	1350	1360
GQSPSPCRTS	PSPDSLGLPE	DPCLGPRNEE	RPLRLQRSPV	LKRRPKLEAP	PSPSLGSGLG	TEPLPPQPTE	PSSPERSPPS
1370	1380						
PATDQRGGGP	NP						

Cmpd.	No. of Cmpds.	m/z meas.	Δ m/z [ppm]	z	Rt [min]	Score	P	Range	Sequence	Modification	Ratios
2608	1	698.3486	-78.93	2	61.3	15.4	2	425-437	R.ARTLRHLGLAGCK.L		mdown:qdown 1.82
2909	1	682.3620	-10.00	3	65.1	17.7	2	1177-1196	R.VQVMLQRIGVSRGSGGAEGK.R	Oxidation: 4	

Detailed Protein Report

Protein 175: PREDICTED: amphiphysin isoform X1 [Homo sapiens]

Accession: gi|578813410
Database: refseq_human(refseq_human_20140103.fasta)
Database Date: 2015-11-30
Modification(s): Carbamidomethyl, Oxidation

Score: 43.1
MW [kDa]: 134.8
pI: 4.3
Sequence Coverage [%]: 3.7
No. of unique Peptides: 2

10	20	30	40	50	60	70	80
MADIKTGIFA	KNVQKRLNRA	QEKVLQKLGK	ADETKDEQFE	EYVQNFKRQE	AEGTRLQREL	RGYLAAIKGM	QEASMKLTES
**90**	**100**	**110**	**120**	**130**	**140**	**150**	**160**
LHEVYEPDWY	GREDVKMVGE	KCDVLWEDFH	QKLVDGSLLT	LDTYLGQFPD	IKNRIAKRSR	KLVDYDSARH	HLEALQSSKR
**170**	**180**	**190**	**200**	**210**	**220**	**230**	**240**
KDESRISKAE	EEFQKAQKVF	EEFNVDLQEE	LPSLWSRRVG	FYVNTFKNVS	SLEAKFHKEI	AVLCHKLYEV	MTKLGDQHAD
**250**	**260**	**270**	**280**	**290**	**300**	**310**	**320**
KAFTIQGAPS	DSGPLRIAKT	PSPPEEPSPL	PSPTASPNHT	LAPASPAPAR	PRSPSQTRKG	PPVPPLPKVT	PTKELQQENI
**330**	**340**	**350**	**360**	**370**	**380**	**390**	**400**
ISFFEDNFVP	EISVTTPSQN	EVPEVKKEET	LLDLDFDPFK	PEVTPAGSAG	VTHSPMSQTL	PWDLWTTDSS	ESLSLCNLIM
**410**	**420**	**430**	**440**	**450**	**460**	**470**	**480**
EETPDSGLAE	EIQRSQNDIG	AFTWGPDAST	DRVSQEVTSG	GFGEDSACPS	ETEQDIRLST	SLLSSADWPT	VAEESEHAPG
**490**	**500**	**510**	**520**	**530**	**540**	**550**	**560**
PAFPGGNEQL	PPKPAPEAGV	AIAACVEMEQ	LYDPLDSDMP	AMDTAGLFKE	SHEDMKKSDE	EEEKQKMEDS	LWAGVEACQK
**570**	**580**	**590**	**600**	**610**	**620**	**630**	**640**
VDTGAIDIKT	LEGTEEFEEK	ACERTGNRVV	ELLNTIGKED	HKTLKINEVE	TRKDLRAGYK	ESTNANENYF	EKTDLLEKDM
**650**	**660**	**670**	**680**	**690**	**700**	**710**	**720**
EGGDMAKELS	DRDQGLHDGI	QKVINNCGMI	EGFGAKPANH	PDILNPSLQT	YSVSNIEDGS	PEGWDLTRKD	IKKNAIDTPN
**730**	**740**	**750**	**760**	**770**	**780**	**790**	**800**
HDMIFYPAQD	STDAEEIQCE	TVLNALVGDR	DKNQAVLSIK	LEVEAQSRTD	LNYSLGRDAN	RRDLCSCKAE	SSLIEGSERT
**810**	**820**	**830**	**840**	**850**	**860**	**870**	**880**
VTENRSHTVN	LPRIAEIDSW	GTDPGQIWQP	SLDSAMNNLD	ITGFSGIPDG	QASGFASCLD	SINHWPIRDT	EGLDNSWTHS
**890**	**900**	**910**	**920**	**930**	**940**	**950**	**960**
DVVEHKEELK	VDLLAGDCRE	QAIASCWEQS	QEIARPLILG	TSWNASTESS	ASSRDQDINN	SDLSEDEIAN	QRYGLLYQEI
**970**	**980**	**990**	**1000**	**1010**	**1020**	**1030**	**1040**
EADKDEASGG	SFNGFTQPQD	TSLFTMQTDQ	SMICNLIIPG	ADADAAVGTL	VSAAEGAPGE	EAEAEKATVP	AGEGVSLEEA
**1050**	**1060**	**1070**	**1080**	**1090**	**1100**	**1110**	**1120**
KIGTETTEGA	ESAQPEAEEL	EATVPQEKVI	PSVVIEPASN	HEEEGENEIT	IGAEPKETTE	DAAPPGPTSE	TPELATEQKP
**1130**	**1140**	**1150**	**1160**	**1170**	**1180**	**1190**	**1200**
IQDPQPTPSA	PAMGAADQLA	SAREASQELP	PGFLYKVETL	HDFEAANSDE	LTLQRGDVVL	VVPSDSEADQ	DAGWLVGVKE
**1210**	**1220**	**1230**					
SDWLQYRDLA	TYKGLFPENF	TRRLD					

Cmpd.	No. of Cmpds.	m/z meas.	Δ m/z [ppm]	z	Rt [min]	Score	P	Range	Sequence	Modification
2902	1	836.7384	18.81	3	65.4	13.6	2	93-112	R.EDVKMVGEKCDVLWEDFHQK.L	Carbamidomethyl: 10; Oxidation: 5
1311	1	1115.7434	90.99	1	46.1	17.0	1	751-760	R.DKNQAVLSIK.L	

Detailed Protein Report

Protein 176: **PREDICTED: protein unc-13 homolog B isoform X1 [Homo sapiens]**

**Accession:**	gi|578816361	**Score:**	43.0
**Database:**	refseq_human(refseq_human_20140103.fasta)	**MW [kDa]:**	268.9
**Database Date:**	2015-11-30	**pI:**	5.4
**Modification(s):**	Carbamidomethyl, Oxidation	**Sequence Coverage [%]:**	1.7
		**No. of unique Peptides:**	3

Detailed Protein Report

10	20	30	40	50	60	70	80
MSLLCVRVKR	AKFQGSPDKF	NTYVTLKVQN	VKSTTVAVRG	DQPSWEQDFM	FEISRLDLGL	SVEVWNKGLI	WDTMVGTVWI
90	100	110	120	130	140	150	160
ALKTIRQSDE	EGPGEWSTLE	AETLMKDDEI	CGTRNPTPHK	ILLDTRFELP	FDIPEEEARY	WTYKWEQINA	LGADNEYSSQ
170	180	190	200	210	220	230	240
EESQRKPLPT	AAAQCCHWTY	LGWGEQQTFE	DPDSAVDDRD	SDYRSETSNS	FPPPYHTASQ	PNASVHQFPV	PVRSPQQLLL
250	260	270	280	290	300	310	320
QGSSRDSCND	SMQSYDLDYP	ERRAISPTSS	SRYGSSCNVS	QGSSQLSELD	QYHEQDDDHR	ETDSIHSCHS	SHSLSRDGQA
330	340	350	360	370	380	390	400
GFGEQEKPLE	VTGQAEKEAA	CEPKEMKEDA	TTHPPPDLVL	QKDHFLGPQE	SFPEENASSP	FTQARAHWIR	AVTKVRLQLQ
410	420	430	440	450	460	470	480
EVGNLFYILT	RNKRKKQRTP	QTLREKMKRL	LQESEEEIMV	TLGPSSRLSP	DKAKAETMCG	TKSKSSGPAG	SLPEDNSLPP
490	500	510	520	530	540	550	560
CCGSAALAIG	GDRDGDLAQL	CSFGQQNNSQ	LPLGSTAYVS	GSGSQDPSSV	SITTSCQEPS	ERNQTKTLLS	RGHGQGCSRE
570	580	590	600	610	620	630	640
QREPLGDVVE	YIIRELQGIS	RLQTEIAELQ	QHLSQVQGSV	DEVSSCVDSV	LSEIEGLQVS	TCSLAKVCEG	EKAQEPHVDR
650	660	670	680	690	700	710	720
PSEEAILYLY	GLPEQDGENT	MELVESFLAK	HLCVNGMQCN	RYIKKAYRAG	TSPSPRPTVV	KLAHLEHRDF	ILQKSILLQN
730	740	750	760	770	780	790	800
VGVRIATREE	PSCPQGNKNP	QKESISFFQQ	QHQDYSQTSL	NQDEPVLQME	TGDRGPITGT	YQMKAQDQQR	EHQAPEQQGP
810	820	830	840	850	860	870	880
CFLLKNNLSK	ESDVPKLGDE	IKGASRTSQV	ISGSCDELSE	KKASLSTPHQ	FEEPALVLIS	KEEASGKSQF	FKQYSQKHEA
890	900	910	920	930	940	950	960
CKVGKPENDC	HDKSEASSCL	SLSGLLKTER	INAEDKLLGC	EAGLDILSSK	ELEDLLADKS	RRLAALSCDS	MMEEIIIGPE
970	980	990	1000	1010	1020	1030	1040
TFSDMVHIDL	NEEEECAAHV	LKDVFDKSSC	VLGGSQEDED	VEIKFYTSKL	GRAIHHFRSA	LQGVFQKLEN	SGSISPEDLE
1050	1060	1070	1080	1090	1100	1110	1120
SNESGSQSEN	SDRLLGTVSS	GGAQDCSLES	PGSQGSESLL	SVVSGGVGVS	TQGEQTPQDP	STFSLASNNS	LPSVALAPCL
1130	1140	1150	1160	1170	1180	1190	1200
GSETCSRPGS	PKQGRLSLEQ	VCTETVYLNK	CINNFKNVLR	EKRLRQKKLL	HELVQKANRL	SVEDIHSEEK	RGALQIPDDG
1210	1220	1230	1240	1250	1260	1270	1280
DPSLPQWLPE	GPAGGLYGID	SMPDLRRKKP	LPLVSDLAMS	LVQSRKAGIT	SAMATRTSLK	DEELKSHVYK	KTLQALIYPI
1290	1300	1310	1320	1330	1340	1350	1360
SCTTPHNFEV	WTATTPTYCY	ECEGLLWGIA	RQGMRCSECG	VKCHEKCQDL	LNADCLQRAA	EKSCKHGAED	RTQNIIMAMK
1370	1380	1390	1400	1410	1420	1430	1440
DRMKIRERNK	PEIFEVIRDV	FTVNKAAHVQ	QMKTVKQSVL	DGTSKWSAKI	TITVVCAQGL	QAKDKTGSSD	PYVTVQVSKT
1450	1460	1470	1480	1490	1500	1510	1520
KKRTKTIFGN	LNPVWEEKFH	FECHNSSDRI	KVRVWDEDDD	IKSRVKQRLK	RESDDFLGQT	IIEVRTLSGE	MDVWYNLEKR
1530	1540	1550	1560	1570	1580	1590	1600
TDKSAVSGAI	RLQISVEIKG	EEKVAPYHVQ	YTCLHENLFH	YLTDIQGSGG	VRIPEARGDD	AWKVYFDETA	QEIVDEFAMR
1610	1620	1630	1640	1650	1660	1670	1680
YGIESIYQAM	THFACLSSKY	MCPGVPAVMS	TLLANINAYY	AHTTASTNVS	ASDRFAASNF	GKERFVKLLD	QLHNSLRIDL
1690	1700	1710	1720	1730	1740	1750	1760
STYRNNFPAG	SPERLQDLKS	TVDLLTSITF	FRMKVQELQS	PPRASQVVKD	CVKACLNSTY	EYIFNNCHDL	YSRQYQLKQE
1770	1780	1790	1800	1810	1820	1830	1840
LPPEEQGPSI	RNLDFWPKLI	TLIVSIIEED	KNSYTPVLNQ	FPQELNVGKV	SAEVMWHLFA	QDMKYALEEH	EKDHLCKSAD
1850	1860	1870	1880	1890	1900	1910	1920
YMNLHFKVKW	LHNEYVRDLP	VLQGQVPEYP	AWFEQFVLQW	LDENEDVSLE	FLRGALERDK	KDGFQQTSEH	ALFSCSVVDV
1930	1940	1950	1960	1970	1980	1990	2000
FTQLNQSFEI	IRKLECPDPS	ILAHYMRRFA	KTIGKVLMQY	ADILSKDFPA	YCTKEKLPCI	LMNNVQQLRV	QLEKMFEAMG
2010	2020	2030	2040	2050	2060	2070	2080
GKELDLEAAD	SLKELQVKLN	TVLDELSMVF	GNSFQVRIDE	CVRQMADILG	QVRGTGNASP	DARASAAQDA	DSVLRPLMDF
2090	2100	2110	2120	2130	2140	2150	2160
LDGNLTLFAT	VCEKTVLKRV	LKELWRVVMN	TMERMIVLPP	LTDQTGTQLI	FTAAKELSHL	SKLKDHMVRE	ETRNLTPKQC
2170	2180	2190	2200	2210	2220	2230	2240

Detailed Protein Report

Cmpd.	No. of Cmpds.	m/z meas.	Δ m/z [ppm]	z	Rt [min]	Score	P	Range	Sequence	Modification
2611	1	709.8596	-31.02	2	63.3	11.5	0	917-930	K.LLGCEAGLDILSSK.E	
2064	1	753.2991	-61.50	2	55.9	14.2	0	1327-1338	K.CQDLLNADCLQR.A	Carbamidomethyl: 1, 9
2797	1	937.4532	-3.05	2	64.1	17.3	1	1934-1948	K.LECPDPSILAHYMRR.F	Carbamidomethyl: 3; Oxidation: 13

Detailed Protein Report

Protein 177: PREDICTED: AP-3 complex subunit beta-2 isoform X2 [Homo sapiens]

Accession: gi|578827454
Database: refseq_human(refseq_human_20140103.fasta)
Database Date: 2015-11-30
Modification(s): Oxidation

Score: 43.0
MW [kDa]: 114.4
pI: 5.4
Sequence Coverage [%]: 2.9
No. of unique Peptides: 2

Alias proteins:

Accession	Name	Description
gi|578827456	refseq_human (refseq_human_20140103.fasta)	PREDICTED: AP-3 complex subunit beta-2 isoform X3 [Homo sapiens]

10	20	30	40	50	60	70	80
MLDTNKDSLK	LEAMKRIVAM	IARGKNASDL	FPAVVKNVAC	KNIEVKKLVY	VYLVRYAEEQ	QDLALLSIST	FQRGLKDPNQ
**90**	**100**	**110**	**120**	**130**	**140**	**150**	**160**
LIRASALRVL	SSIRVPIIVP	IMMLAIKEAA	SDMSPYVRKT	AAHAIPKLYS	LDSDQKDQLI	EVIEKLLADK	TTLVAGSVVM
**170**	**180**	**190**	**200**	**210**	**220**	**230**	**240**
AFEEVCPERI	DLIHKNYRKL	CNLLIDVEEW	GQVVIISMLT	RYARTQFLSP	TQNESLLEEN	AEKAFYGSEE	DEAKGAGSEE
**250**	**260**	**270**	**280**	**290**	**300**	**310**	**320**
TAAAAAPSRK	PYVMDPDHRL	LLRNTKPLLQ	SRSAAVVMAV	AQLYFHLAPK	AEVGVIAKAL	VRLLRSHSEV	QYVVLQNVAT
**330**	**340**	**350**	**360**	**370**	**380**	**390**	**400**
MSIKRRGMFE	PYLKSFYIRS	TDPTQIKILK	LEVLTNLANE	TNIPTVLREF	QTYIRSMDKD	FVAATIQAIG	RCATNIGRVR
**410**	**420**	**430**	**440**	**450**	**460**	**470**	**480**
DTCLNGLVQL	LSNRDELVVA	ESVVVIKKLL	QMQPAQHGEI	IKHLAKLTDN	IQVPMARASI	LWLIGEYCEH	VPRIAPDVLR
**490**	**500**	**510**	**520**	**530**	**540**	**550**	**560**
KMAKSFTAEE	DIVKLQVINL	AAKLYLTNSK	QTKLLTQYVL	SLAKYDQNYD	IRDRARFTRQ	LIVPSEQGGA	LSRHAKKLFL
**570**	**580**	**590**	**600**	**610**	**620**	**630**	**640**
APKPAPVLES	SFKDRDHFQL	GSLSHLLNAK	ATGYQELPDW	PEEAPDPSVR	NVEVPEWTKC	SNREKRKEKE	KPFYSDSEGE
**650**	**660**	**670**	**680**	**690**	**700**	**710**	**720**
SGPTESADSD	PESESESDSK	SSSESGSGES	SSESDNEDQD	EDEEKGRGSE	SEQSEEDGKR	KTKKKVPERK	GEASSSDEGS
**730**	**740**	**750**	**760**	**770**	**780**	**790**	**800**
DSSSSSSESE	MTSESEEEQL	EPASWSRKTP	PSSKSAPATK	EISLLDLEDF	TPPSVQPVSP	PAIVSTSLAA	DLEGLTLTDS
**810**	**820**	**830**	**840**	**850**	**860**	**870**	**880**
TLVPSLLSPV	SGVGRQELLH	RVAGEGLAVD	YTFSRQPFSG	DPHMVSVHIH	FSNSSDTPIK	GLHVGTPKLP	AGISIQEFPE
**890**	**900**	**910**	**920**	**930**	**940**	**950**	**960**
IESLAPGESA	TAVMGINFCD	STQAANFQLC	TQTRQFYVSI	QPPVGELMAP	VFMSENEFKK	EQGKLMGMNE	ITEKLMLPDT
**970**	**980**	**990**	**1000**	**1010**	**1020**	**1030**	**1040**
CRSDHIVVQK	VTATANLGRV	PCGTSDEYRF	AGRTLTGGSL	VLLTLDARPA	GAAQLTVNSE	KMVIGTMLVK	DVIQALTQ

Cmpd.	No. of Cmpds.	m/z meas.	Δ m/z [ppm]	z	Rt [min]	Score	P	Range	Sequence	Modification
2503	2	974.2875	-301.77	1	59.6	11.1	1	17-25	R.IVAMIARGK.N	Oxidation: 4
619	1	677.3962	18.75	2	36.6	11.7	1	77-88	K.DPNQLIRASALR.V	

Detailed Protein Report

Protein 178: PREDICTED: muskelin isoform X4 [Homo sapiens]

**Accession:**	gi|578814266	**Score:**	43.0
**Database:**	refseq_human(refseq_human_20140103.fasta)	**MW [kDa]:**	80.3
**Database Date:**	2015-11-30	**pI:**	6.1
		**Sequence Coverage [%]:**	6.3
		**No. of unique Peptides:**	2

10	20	30	40	50	60	70	80
MAAGGAVAAA	PECRLLPYAL	HKWSSFSSTY	LPENILVDKP	NDQSSRWSSE	SNYPPQYLIL	KLERPAIVQN	ITFGKYEKTH
90	100	110	120	130	140	150	160
VCNLKKFKVF	GGMNEENMTE	LLSSGLKNDY	NKETFTLKHK	IDEQMFPCRF	IKIVPLLSWG	PSFNFSIWYV	ELSGIDDPDI
170	180	190	200	210	220	230	240
VQPCLNWYSK	YREQEAIRLC	LKHFRQHNYT	EAFESLQKKT	KIALEHPMLT	DIHDKLVLKG	DFDACEELIE	KAVNDGLFNQ
250	260	270	280	290	300	310	320
YISQQEYKPR	WSQIIPKSTK	GDGEDNRPGM	RGGHQMVIDV	QTETVYLFGG	WDGTQDLADF	WAYSVKENQW	TCISRDTEKE
330	340	350	360	370	380	390	400
NGPSARSCHK	MCIDIQRRQI	YTLGRYLDSS	VRNSKSLKSD	FYRYDIDTNT	WMLLSEDTAA	DGGPKLVFDH	QMCMDSEKHM
410	420	430	440	450	460	470	480
IYTFGGRILT	CNGSVDDSRA	SEPQFSGLFA	FNCQCQTWKL	LREDSCNAGP	EDIQSRIGHC	MLFHSKNRCL	YVFGGQRSKT
490	500	510	520	530	540	550	560
YLNDFFSYDV	DSDHVDIISD	GTKKDSGMVP	MTGFTQRATI	DPELNEIHVL	SGLSKDKEKR	EENVRNSFWI	YDIVRNSWSC
570	580	590	600	610	620	630	640
VYKNDQAAKD	NPTKSLQEEE	PCPRFAHQLV	YDELHKVHYL	FGGNPGKSCS	PKMRLDDFWS	LKLCRPSKDY	LLRHCKYLIR
650	660	670	680	690	700		
KHRFEEKAQV	DPLSALKYLQ	NDLYITVDHS	DPEETKEFQL	LASALFKSGS	DFTALA		

Cmpd.	No. of Cmpds.	m/z meas.	Δ m/z [ppm]	z	Rt [min]	Score	P	Range	Sequence	Modification
1158	1	477.6545	-288.11	2	44.1	17.4	0	15-22	R.LLPYALHK.W	
2818	1	989.4652	-27.99	2	64.4	13.5	2	597-614	K.VHYLFGGNPGKSCSPKMR.L	

Detailed Protein Report

Protein 179: zinc finger protein 605 isoform 1 [Homo sapiens]

**Accession:**	gi|34222391	**Score:**	43.0
**Database:**	refseq_human(refseq_human_20140103.fasta)	**MW [kDa]:**	74.3
**Database Date:**	2015-11-30	**pI:**	10.4
**Modification(s):**	Carbamidomethyl	**Sequence Coverage [%]:**	9.0
		**No. of unique Peptides:**	4

Quantitation

mdown:qdown	**Median:** 0.80	**CV:** 0.00 %	**No. of Peptides:**	1
Wdown:Qdown	**Median:** 1.64	**CV:** 0.00 %	**No. of Peptides:**	1

```
        10         20         30         40         50         60         70         80
MIQSQISFED VAVDFTLEEW QLLNPTQKNL YRDVMLENYS NLVFLEVWLD NPKMWLRDNQ DNLKSMERGH KYDVFGKIFN
        90        100        110        120        130        140        150        160
SSINIVHVGL RSHKCGTGEK SLKCPFDLLI PKNNCERKKI DELNKKLLFC IKPGRTHGGI KYCDCSTCRK SSNEEPWLTA
       170        180        190        200        210        220        230        240
NHITHTGVYL CMECGRFFNK KSQLVIHQRT HTGEKPYQCS ECGKAFSQKS LLTVHQRTHS GEKPHGCSEC QKAFSRKSLL
       250        260        270        280        290        300        310        320
ILHQRIHTGE KPYGCSECGK AFSRKSQLKR HQITHTIEKP YSCSECGKAF SQKLKLITHQ RAHTGEKPYP CSHCGKAFFW
       330        340        350        360        370        380        390        400
KSQLITHQRT HTGKKPYGCG ECQKAFSRNS LLIRHQRIHT GEKPYECNEC GEAFIRKPQL IKHQITHTGE KNYRCSDCEE
       410        420        430        440        450        460        470        480
AFFKKSELIR HQKIHLGEKP YGCIQCGKTF FGKSQLLTHH RTHTGEKPYE CSECGKAFTQ KSSLISHQRT HTGEKPYECS
       490        500        510        520        530        540        550        560
ECRKTFSEKS SLIHHQRTHT GEKPFECSEC RKAFAWKPQL LRHQRIHTGE KPYECSECGK AFVQKVQLIK HQRNHTGEKT
       570        580        590        600        610        620        630        640
YGCSDCAKAF FEKAQLIIHQ RIHTGERPYK CGECGKSFTR KSHLMRHQRI HTGDKYYGCN ECGTTFNRKS QLMIHQRNHI
       650
I
```

Cmpd.	No. of Cmpds.	m/z meas.	Δ m/z [ppm]	z	Rt [min]	Score	P	Range	Sequence	Modification	Ratios
2421	1	863.4315	78.18	2	58.6	10.2	0	442-456	R.THTGEKPYECSECGK.A	Carbamidomethyl: 13	
1971	1	1079.8531	-128.17	2	53.3	10.5	1	523-540	R.HQRIHTGEKPYECSECGK.A	Carbamidomethyl: 16	
1972	1	665.2897	-23.77	2	54.8	11.9	2	591-601	K.CGECGKSFTRK.S	Carbamidomethyl: 1, 4	Wdown:Qdown 1.64<br>mdown:qdown 0.80
1767	1	856.7921	-80.88	2	52.1	10.3	1	616-629	K.YYGCNECGTTFNRK.S	Carbamidomethyl: 4	

Detailed Protein Report

Protein 180: PREDICTED: testis-specific gene 10 protein isoform X9 [Homo sapiens]

**Accession:**	gi|578804973	**Score:**	42.9
**Database:**	refseq_human(refseq_human_20140103.fasta)	**MW [kDa]:**	103.2
**Database Date:**	2015-11-30	**pI:**	5.3
		**Sequence Coverage [%]:**	3.9
		**No. of unique Peptides:**	1

Quantitation

mdown:qdown	**Median:** 1.21	**CV:** 0.00 %	**No. of Peptides:**	1
Wdown:Qdown	**Median:** 1.07	**CV:** 0.00 %	**No. of Peptides:**	1

10	20	30	40	50	60	70	80
MSSGQDGGPR	SPGRDPELQV	EAAEVTTKEY	DFGLGAPVSE	AENYQNTLQL	EQEVRNQDRF	ISTLKLQIED	LKQTNHGLEE
**90**	**100**	**110**	**120**	**130**	**140**	**150**	**160**
YVRKLLDSKE	VVSSQVDDLT	SHNEHLCKEL	IKIDQLAEQL	EKEKNFVVDS	ANKELEEAKI	DLICQQNNII	VLEDTIKRLK
**170**	**180**	**190**	**200**	**210**	**220**	**230**	**240**
SIILDTEKAQ	NKSPSRLDSF	VKTLEADKDH	YKSEAQHLRK	MMRSRSKSPR	RPSPTARGAN	CDVELLKTTT	RDREELKCML
**250**	**260**	**270**	**280**	**290**	**300**	**310**	**320**
EKYERHLAEI	QGNVKVLKSE	RDKIFLLYEQ	AQEEITRLRR	EMMKSCKSPK	STTAHAILRR	VETERDVAFT	DLRRMTTERD
**330**	**340**	**350**	**360**	**370**	**380**	**390**	**400**
SLRERLKIAQ	ETAFNEKAHL	EQRIEELECT	VHNLDDERME	QMSNMTLMKE	TISTVEKEMK	SLARKAMDTE	SELGRQKAEN
**410**	**420**	**430**	**440**	**450**	**460**	**470**	**480**
NSLRLLYENT	EKDLSDTQRH	LAKKKYELQL	TQEKIMCLDE	KIDNFTRQNI	AQREEISILG	GTLNDLAKEK	ECLQACLDKK
**490**	**500**	**510**	**520**	**530**	**540**	**550**	**560**
SENIASLGES	LAMKEKTISG	MKNIIAEMEQ	ASRQCTEALI	VCEQDVSRMR	RQLDETNDEL	AQIARERDIL	AHDNDNLQEQ
**570**	**580**	**590**	**600**	**610**	**620**	**630**	**640**
FAKAKQENQA	LSKKLNDTHN	ELNDIKQKVQ	DTNLEVNKLK	NILKSEESEN	RQMMEQLRKA	NEDAENWENK	ARQSEADNNT
**650**	**660**	**670**	**680**	**690**	**700**	**710**	**720**
LKLELITAEA	EGNRLKEKVD	SLNREVEQHL	NAERSYKSQI	STLHKSVVKM	EEELQKVQFE	KVSALADLSS	TRELCIKLDS
**730**	**740**	**750**	**760**	**770**	**780**	**790**	**800**
SKELLNRQLV	AKDQEIEMRE	NELDSAHSEI	ELLRSQMANE	RISMQNLEAL	LVANRDKEYQ	SQIALQEKES	EIQLLKEHLC
**810**	**820**	**830**	**840**	**850**	**860**	**870**	**880**
LAENKMAIQS	RDVAQFRNVV	TQLEADLDIT	KRQLGTERFE	RERAVQELRR	QNYSSNAYHM	SSTMKPNTKC	HSPERAHHRS
**890**	**900**						
PDRGLDRSLE	E						

Cmpd.	No. of Cmpds.	m/z meas.	Δ m/z [ppm]	z	Rt [min]	Score	P	Range	Sequence	Modification	Ratios
1178	1	760.8598	-40.38	2	43.4	12.7	1	218-231	R.GANCDVELLKTTTR.D		Wdown:Qdown 1.07<br>mdown:qdown 1.21

Detailed Protein Report

Protein 181: **PREDICTED: N-alpha-acetyltransferase 15, NatA auxiliary subunit isoform X1 [Homo sapiens]**

Accession: gi|530378196
Database: refseq_human(refseq_human_20140103.fasta)
Database Date: 2015-11-30
Modification(s): Oxidation

Score: 42.8
MW [kDa]: 101.1
pI: 7.8
Sequence Coverage [%]: 5.1
No. of unique Peptides: 1

10	20	30	40	50	60	70	80
MPAVSLPPKE	NALFKRILRC	YEHKQYRNGL	KFCKQILSNP	KFAEHGETLA	MKGLTLNCLG	KKEEAYELVR	RGLRNDLKSH
**90**	**100**	**110**	**120**	**130**	**140**	**150**	**160**
VCWHVYGLLQ	RSDKKYDEAI	KCYRNALKWD	KDNLQILRDL	SLLQIQMRDL	EGYRETRYQL	LQLRPAQRAS	WIGYAIAYHL
**170**	**180**	**190**	**200**	**210**	**220**	**230**	**240**
LEDYEMAAKI	LEEFRKTQQT	SPDKVDYEYS	ELLLYQNQVL	REAGLYREAL	EHLCTYEKQI	CDKLAVEETK	GELLLQLCRL
**250**	**260**	**270**	**280**	**290**	**300**	**310**	**320**
EDAADVYRGL	QERNPENWAY	YKGLEKALKP	ANMLERLKIY	EEAWTKYPRG	LVPRRLPLNF	LSGEKFKECL	DKFLRMNFSK
**330**	**340**	**350**	**360**	**370**	**380**	**390**	**400**
GCPPVFNTLR	SLYKDKEKVA	IIEELVVGYE	TSLKSCRLFN	PNDDGKEEPP	TTLLWVQYYL	AQHYDKIGQP	SIALEYINTA
**410**	**420**	**430**	**440**	**450**	**460**	**470**	**480**
IESTPTLIEL	FLVKAKIYKH	AGNIKEAARW	MDEAQALDTA	DRFINSKCAK	YMLKANLIKE	AEEMCSKFTR	EGTSAVENLN
**490**	**500**	**510**	**520**	**530**	**540**	**550**	**560**
EMQCMWFQTE	CAQAYKAMNK	FGEALKKCHE	IERHFIEITD	DQFDFHTYCM	RKITLRSYVD	LLKLEDVLRQ	HPFYFKAARI
**570**	**580**	**590**	**600**	**610**	**620**	**630**	**640**
AIEIYLKLHD	NPLTDENKEH	EADTANMSDK	ELKKLRNKQR	RAQKKAQIEE	EKKNAEKEKQ	QRNQKKKKDD	DDEEIGGPKE
**650**	**660**	**670**	**680**	**690**	**700**	**710**	**720**
ELIPEKLAKV	ETPLEEAIKF	LTPLKNLVKN	KIETHLFAFE	IYFRKEKFLL	MLQSVKRAFA	IDSSHPWLHE	CMIRLFNTVC
**730**	**740**	**750**	**760**	**770**	**780**	**790**	**800**
ESKDLSDTVR	TVLKQEMNRL	FGATNPKNFN	ETFLKRNSDS	LPHRLSAAKM	VYYLDPSSQK	RAIELATTLD	ESLTNRNLQT
**810**	**820**	**830**	**840**	**850**	**860**	**870**	
CMEVLEALYD	GSLGDCKEAA	EIYRANCHKL	FPYALAFMPP	GYEEDMKITV	NGDSSAEAEE	LANEI	

Cmpd.	No. of Cmpds.	m/z meas.	Δ m/z [ppm]	z	Rt [min]	Score	P	Range	Sequence	Modification
2297	1	863.8705	-61.74	2	57.2	20.5	1	316-330	R.MNFSKGCPPVFNTLR.S	Oxidation: 1

Detailed Protein Report

Protein 182: probable ATP-dependent RNA helicase DDX58 [Homo sapiens]

Accession: gi|27881482
Database: refseq_human(refseq_human_20140103.fasta)
Database Date: 2015-11-30
Modification(s): Oxidation

Score: 42.7
MW [kDa]: 106.5
pI: 6.0
Sequence Coverage [%]: 3.8
No. of unique Peptides: 1

10	20	30	40	50	60	70	80
MTTEQRRSLQ	AFQDYIRKTL	DPTYILSYMA	PWFREEEVQY	IQAEKNNKGP	MEAATLFLKF	LLELQEEGWF	RGFLDALDHA
90	100	110	120	130	140	150	160
GYSGLYEAIE	SWDFKKIEKL	EEYRLLLKRL	QPEFKTRIIP	TDIISDLSEC	LINQECEEIL	QICSTKGMMA	GAEKLVECLL
170	180	190	200	210	220	230	240
RSDKENWPKT	LKLALEKERN	KFSELWIVEK	GIKDVETEDL	EDKMETSDIQ	IFYQEDPECQ	NLSENSCPPS	EVSDTNLYSP
250	260	270	280	290	300	310	320
FKPRNYQLEL	ALPAMKGKNT	IICAPTGCGK	TFVSLLICEH	HLKKFPQGQK	GKVVFFANQI	PVYEQQKSVF	SKYFERHGYR
330	340	350	360	370	380	390	400
VTGISGATAE	NVPVEQIVEN	NDIIILTPQI	LVNNLKKGTI	PSLSIFTLMI	FDECHNTSKQ	HPYNMIMFNY	LDQKLGGSSG
410	420	430	440	450	460	470	480
PLPQVIGLTA	SVGVGDAKNT	DEALDYICKL	CASLDASVIA	TVKHNLEELE	QVVYKPQKFF	RKVESRISDK	FKYIIAQLMR
490	500	510	520	530	540	550	560
DTESLAKRIC	KDLENLSQIQ	NREFGTQKYE	QWIVTVQKAC	MVFQMPDKDE	ESRICKALFL	YTSHLRKYND	ALIISEHARM
570	580	590	600	610	620	630	640
KDALDYLKDF	FSNVRAAGFD	EIEQDLTQRF	EEKLQELESV	SRDPSNENPK	LEDLCFILQE	EYHLNPETIT	ILFVKTRALV
650	660	670	680	690	700	710	720
DALKNWIEGN	PKLSFLKPGI	LTGRGKTNQN	TGMTLPAQKC	ILDAFKASGD	HNILIATSVA	DEGIDIAQCN	LVILYEYVGN
730	740	750	760	770	780	790	800
VIKMIQTRGR	GRARGSKCFL	LTSNAGVIEK	EQINMYKEKM	MNDSILRLQT	WDEAVFREKI	LHIQTHEKFI	RDSQEKPKPV
810	820	830	840	850	860	870	880
PDKENKKLLC	RKCKALACYT	ADVRVIEECH	YTVLGDAFKE	CFVSRPHPKP	KQFSSFEKRA	KIFCARQNCS	HDWGIHVKYK
890	900	910	920	930			
TFEIPVIKIE	SFVVEDIATG	VQTLYSKWKD	FHFEKIPFDP	AEMSK			

Cmpd.	No. of Cmpds.	m/z meas.	Δ m/z [ppm]	z	Rt [min]	Score	P	Range	Sequence	Modification
1833	1	599.7809	-18.91	2	52.9	13.8	1	751-759	K.EQINMYKEK.M	Oxidation: 5

Detailed Protein Report

Protein 183: PREDICTED: uncharacterized protein LOC102724905 [Homo sapiens]

**Accession:**	gi|578835678	**Score:**	42.6
**Database:**	refseq_human(refseq_human_20140103.fasta)	**MW [kDa]:**	22.0
**Database Date:**	2015-11-30	**pI:**	10.3
**Modification(s):**	Carbamidomethyl	**Sequence Coverage [%]:**	16.7
		**No. of unique Peptides:**	2

```
        10         20         30         40         50         60         70         80
MKCSTSNCII SIKDYTSIRI NVAEVDKVTS RFNGQFKTYA FGGSPGTGVT GGKGREGGRS FCAGEGNERN GKGTAGCEAR
        90        100        110        120        130        140        150        160
DVPGSAAGPA RGRAAPLHPA PGPPLRGAIL QAAVAGTGGG GCSGHCGAEA RRQDGNHASC SQLGLDSTNG DFSYGFELGP
       170        180        190        200        210        220
WCISKCNSAN CNGKHMDRST PHHLGAKRCR LQASSLLENR GLQALEEPAD PGLLA
```

Cmpd.	No. of Cmpds.	m/z meas.	Δ m/z [ppm]	z	Rt [min]	Score	P	Range	Sequence	Modification
2384	1	1045.1618	131.45	2	60.1	16.4	2	2-19	M.KCSTSNCIISIKDYTSIR.I	Carbamidomethyl: 7
1810	1	641.7727	-58.27	2	52.6	13.4	0	3-13	K.CSTSNCIISIK.D	Carbamidomethyl: 1, 6

Detailed Protein Report

Protein 184: glutathione S-transferase P [Homo sapiens]

Accession: gi|4504183
Database: refseq_human(refseq_human_20140103.fasta)
Database Date: 2015-11-30

Score: 42.5
MW [kDa]: 23.3
pI: 5.3
Sequence Coverage [%]: 7.6
No. of unique Peptides: 1

10	20	30	40	50	60	70	80
MPPYTVVYFP	VRGRCAALRM	LLADQGQSWK	EEVVTVETWQ	EGSLKASCLY	GQLPKFQDGD	LTLYQSNTIL	RHLGRTLGLY
**90**	**100**	**110**	**120**	**130**	**140**	**150**	**160**
GKDQQEAALV	DMVNDGVEDL	RCKYISLIYT	NYEAGKDDYV	KALPGQLKPF	ETLLSQNQGG	KTFIVGDQIS	FADYNLLDLL
**170**	**180**	**190**	**200**	**210**	**220**		
LIHEVLAPGC	LDAFPLLSAY	VGRLSARPKL	KAFLASPEYV	NLPINGNGKQ			

Cmpd.	No. of Cmpds.	m/z meas.	Δ m/z [ppm]	z	Rt [min]	Score	P	Range	Sequence	Modification
1632	1	942.4899	12.06	2	49.1	42.5	0	56-71	K.FQDGDLTLYQSNTILR.H	

Detailed Protein Report

Protein 185: mitochondrial intermediate peptidase [Homo sapiens]

**Accession:**	gi|156105687	**Score:**	42.5
**Database:**	refseq_human(refseq_human_20140103.fasta)	**MW [kDa]:**	80.6
**Database Date:**	2015-11-30	**pI:**	6.6
**Modification(s):**	Oxidation	**Sequence Coverage [%]:**	5.2
		**No. of unique Peptides:**	2

10	20	30	40	50	60	70	80
MLCVGRLGGL	GARAAALPPR	RAGRGSLEAG	IRARRVSTSW	SPVGAAFNVK	PQGSRLDLFG	ERRGLFGVPE	LSAPEGFHIA
**90**	**100**	**110**	**120**	**130**	**140**	**150**	**160**
QEKALRKTEL	LVDRACSTPP	GPQTVLIFDE	LSDSLCRVAD	LADFVKIAHP	EPAFREAAEE	ACRSIGTMVE	KLNTNVDLYQ
**170**	**180**	**190**	**200**	**210**	**220**	**230**	**240**
SLQKLLADKK	LVDSLDPETR	RVAELFMFDF	EISGIHLDKE	KRKRAVDLNV	KILDLSSTFL	MGTNFPNKIE	KHLLPEHIRR
**250**	**260**	**270**	**280**	**290**	**300**	**310**	**320**
NFTSAGDHII	IDGLHAESPD	DLVREAAYKI	FLYPNAGQLK	CLEELLSSRD	LLAKLVGYST	FSHRALQGTI	AKNPETVMQF
**330**	**340**	**350**	**360**	**370**	**380**	**390**	**400**
LEKLSDKLSE	RTLKDFEMIR	GMKMKLNPQN	SEVMPWDPPY	YSGVIRAERY	NIEPSLYCPF	FSLGACMEGL	NILLNRLLGI
**410**	**420**	**430**	**440**	**450**	**460**	**470**	**480**
SLYAEQPAKG	EVWSEDVRKL	AVVHESEGLL	GYIYCDFFQR	ADKPHQDCHF	TIRGGRLKED	GDYQLPVVVL	MLNLPRSSRS
**490**	**500**	**510**	**520**	**530**	**540**	**550**	**560**
SPTLLTPGMM	ENLFHEMGHA	MHSMLGRTRY	QHVTGTRCPT	DFAEVPSILM	EYFANDYRVV	NQFARHYQTG	QPLPKNMVSR
**570**	**580**	**590**	**600**	**610**	**620**	**630**	**640**
LCESKKVCAA	ADMQLQVFYA	TLDQIYHGKH	PLRNSTTDIL	KETQEKFYGL	PYVPNTAWQL	RFSHLVGYGA	RYYSYLMSRA
**650**	**660**	**670**	**680**	**690**	**700**	**710**	**720**
VASMVWKECF	LQDPFNRAAG	ERYRREMLAH	GGGREPMLMV	EGMLQKCPSV	DDFVSALVSD	LDLDFETFLM	DSE

Cmpd.	No. of Cmpds.	m/z meas.	Δ m/z [ppm]	z	Rt [min]	Score	P	Range	Sequence	Modification
2189	1	993.8848	-92.03	2	56.0	11.7	1	632-647	R.YYSYLMSRAVASMVWK.E	Oxidation: 6, 13
3000	4	703.3599	16.84	2	66.4	13.5	0	675-686	R.EPMLMVEGMLQK.C	

Detailed Protein Report

Protein 186: PREDICTED: microtubule-actin cross-linking factor 1 isoform X21 [Homo sapiens]

**Accession:**	gi|578798822	**Score:**	42.0
**Database:**	refseq_human(refseq_human_20140103.fasta)	**MW [kDa]:**	853.1
**Database Date:**	2015-11-30	**pI:**	5.2
		**Sequence Coverage [%]:**	0.5
		**No. of unique Peptides:**	2

Quantitation

Wdown:Qdown	**Median:**	1.17	**CV:**	0.00 %	**No. of Peptides:**	1

Detailed Protein Report

10	20	30	40	50	60	70	80
MPLLDSSYLP	PTIFILTHVL	GVAGVLYWKR	HARGRADERD	RVQKKTFTKW	VNKHLMKVRK	HINDLYEDLR	DGHNLISLLE
90	100	110	120	130	140	150	160
VLSGIKLPRE	KGRMRFHRLQ	NVQIALDFLK	QRQVKLVNIR	NDDITDGNPK	LTLGLIWTII	LHFQISDIYI	SGESGDMSAK
170	180	190	200	210	220	230	240
EKLLLWTQKV	TAGYTGIKCT	NFSSCWSDGK	MFNALIHRYR	PDLVDMERVQ	IQSNRENLEQ	AFEVAERLGV	TRLLDAEDVD
250	260	270	280	290	300	310	320
VPSPDEKSVI	TYVSSIYDAF	PKVPEGGEGI	SATEVDSRWQ	EYQSRVDSLI	PWIKQHTILM	SDKTFPQNPV	ELKALYNQYI
330	340	350	360	370	380	390	400
HFKETEILAK	EREKGRIEEL	YKLLEVWIEF	GRIKLPQGYH	PNDVEEEWGK	LIIEMLEREK	SLRPAVERLE	LLLQIANKIQ
410	420	430	440	450	460	470	480
NGALNCEEKL	TLAKNTLQAD	AAHLESGQPV	QCESDVIMYI	QECEGLIRQL	QVDLQILRDE	NYYQLEELAF	RVMRLQDELV
490	500	510	520	530	540	550	560
TLRLECTNLY	RKGHFTSLEL	VPPSTLTTTH	LKAEPLTKAT	HSSSTSWFRK	PMTRAELVAI	SSSEDEGNLR	FVYELLSWVE
570	580	590	600	610	620	630	640
EMQMKLERAE	WGNDLPSVEL	QLETQQHIHT	SVEELGSSVK	EARLYEGKMS	QNFHTSYAET	LGKLETQYCK	LKETSSFRMR
650	660	670	680	690	700	710	720
HLQSLHKFVS	RATAELIWLN	EKEEEELAYD	WSDNNSNISA	KRNYFSELTM	ELEEKQDVFR	SLQDTAELLS	LENHPAKQTV
730	740	750	760	770	780	790	800
EAYSAAVQSQ	LQWMKQLCLC	VEQHVKENTA	YFQFFSDARE	LESFLRNLQD	SIKRKYSCDH	NTSLSRLEDL	LQDSMDEKEQ
810	820	830	840	850	860	870	880
LIQSKSSVAS	LVGRSKTIVQ	LKPRSPDHVL	KNTISVKAVC	DYRQIEITIC	KNDECVLEDN	SQRTKWKVIS	PTGNEAMVPS
890	900	910	920	930	940	950	960
VCFLIPPPNK	DAIEMASRVE	QSYQKVMALW	HQLHVNTKSL	ISWNYLRKDL	DLVQTWNLEK	LRSSAPGECH	QIMKNLQAHY
970	980	990	1000	1010	1020	1030	1040
EDFLQDSRDS	VLFSVADRLR	LEEEVEACKA	RFQHLMKSME	NEDKEETVAK	MYISELKNIR	LRLEEYEQRV	VKRIQSLASS
1050	1060	1070	1080	1090	1100	1110	1120
RTDRDAWQDN	ALRIAEQEHT	QEDLQQLRSD	LDAVSMKCDS	FLHQSPSSSS	VPTLRSELNL	LVEKMDHVYG	LSTVYLNKLK
1130	1140	1150	1160	1170	1180	1190	1200
TVDVIVRSIQ	DAELLVKGYE	IKLSQEEVVL	ADLSALEAHW	STLRHWLSDV	KDKNSVFSVL	DEEIAKAKVV	AEQMSRLTPE
1210	1220	1230	1240	1250	1260	1270	1280
RNLDLERYQE	KGSQLQERWH	RVIAQLEIRQ	SELESIQEVL	GDYRACHGTL	IKWIEETTAQ	QEMMKPGQAE	DSRVLSEQLS
1290	1300	1310	1320	1330	1340	1350	1360
QQTALFAEIE	RNQTKLDQCQ	KFSQQYSTIV	KDYELQLMTY	KAFVESQQKS	PGKRRRMLSS	SDAITQEFMD	LRTRYTALVT
1370	1380	1390	1400	1410	1420	1430	1440
LTTQHVKYIS	DALRRLEEEE	KVVEEEKQEH	VEKVKELLGW	VSTLARNTQG	KATSSETKES	TDIEKAILEQ	QVLSEELTTK
1450	1460	1470	1480	1490	1500	1510	1520
KEQVSEAIKT	SQIFLAKHGH	KLSEKEKKQI	SEQLNALNKA	YHDLCDGSAN	QLQQLQSQLA	HQTEQKECRA	VAGVIDLGTV
1530	1540	1550	1560	1570	1580	1590	1600
EIFPIFKAMQ	KGLLDQDTGL	VLLESQVIMS	GLIAPETGEN	LSLEEGIARN	LINPQMYQQL	RELQDALALI	SRLTESRGPL
1610	1620	1630	1640	1650	1660	1670	1680
SVVEAIEKRI	ISETVGLKIL	EAHLATGGFS	LSPSENCINL	EEAFHQGLIS	AWLHSVLESY	LRTSKNLIDP	NTAEKIGLLD
1690	1700	1710	1720	1730	1740	1750	1760
LMQRCIVHQE	SGFKLLPVKQ	LAGGMVSLKS	GRKVSIFRAV	QEGLIDRQVT	VRLLEAQLFA	GGIVDPRTGH	RLTVEEAVRH
1770	1780	1790	1800	1810	1820	1830	1840
NLIDQDMACA	ILIRQLQTGG	IIDTVTGQRL	TIDEAVSNDL	VAAKIALVIL	ESLWSFMGLL	WPESGEILPI	TDALEQGIVS
1850	1860	1870	1880	1890	1900	1910	1920
TELAHKILSN	RQHIKALFLP	ATTEILSWKK	AIESGILDRD	LANNLKSICI	PDVMPHMQLA	DSAEQNINPG	AAVLPCSKSH
1930	1940	1950	1960	1970	1980	1990	2000
PKATASQSEN	LLFQLMTHSY	INVQNGQRLL	LLDKELMETL	TSRDEYQTSP	PKVVEIGHQR	QKTPEGLQES	ANVKISGTFS
2010	2020	2030	2040	2050	2060	2070	2080
SGWTVRLPEF	QFSSQNKEYP	DREDCTTEKG	KKTTVETEDS	SVENPEQDLF	VEQKERNPNI	DALKVINKVK	LEVQRQLIGT
2090	2100	2110	2120	2130	2140	2150	2160
QREDQTAVSV	RENASRGHLL	TIPPAEAEGV	PLVVDKDVFS	VETPKKEHQP	LRNTSFTCQN	EQAHTLETEY	IHDETGGSHI
2170	2180	2190	2200	2210	2220	2230	2240

Detailed Protein Report

Cmpd.	No. of Cmpds.	m/z meas.	Δ m/z [ppm]	z	Rt [min]	Score	P	Range	Sequence	Modification	Ratios
324	1	755.2309	-145.08	2	33.4	13.7	1	998-1010	K.SMENEDKEETVAK.M		Wdown:Qdown 1.17
1103	1	1355.1276	184.19	1	42.5	16.9	2	2167-2178	K.LQVQVKKTLGIK.L		

Detailed Protein Report

Protein 187: **PREDICTED: zinc finger protein 554 isoform X2 [Homo sapiens]**

**Accession:**	gi|578832959	**Score:**	42.0
**Database:**	refseq_human(refseq_human_20140103.fasta)	**MW [kDa]:**	43.7
**Database Date:**	2015-11-30	**pI:**	9.8
**Modification(s):**	Carbamidomethyl	**Sequence Coverage [%]:**	10.8
		**No. of unique Peptides:**	2

10	20	30	40	50	60	70	80
MTVLRNQDST	YKKVALQEEP	ASGINMIKLI	REDGGWKQLE	DSHEDPQGLL	SQKASLHVVA	VPQEKATAWH	GFGENGNLSP
**90**	**100**	**110**	**120**	**130**	**140**	**150**	**160**
ALVLSQGSSK	GNHLCGSELD	ITSLASDSVL	NHHQLGYADR	RPCESNECGN	AIRQNSHFIQ	HGGKMFVYLE	NGQSLNHGMA
**170**	**180**	**190**	**200**	**210**	**220**	**230**	**240**
LTIHNKINTA	EKPFECHQCG	KVFNRRHSLS	EHQRIHTGEK	PYECQECGRA	FTHSSTLTRH	LRTHTGEKPY	GCGECGKAFN
**250**	**260**	**270**	**280**	**290**	**300**	**310**	**320**
RISSLTQHQR	IHTGEKPYKC	EDCGKSFCQS	SYLILHKRTH	TGEKPYECSE	CGKAFSDRSS	LNQHERTHTG	ENPYECKQCG
**330**	**340**	**350**	**360**	**370**	**380**	**390**	
RAFSQRSSLV	RHERTHTGEK	PYRCQECGKA	FSQSSSLVTH	QKTHSSQKTY	KIIDCGKAFY	QNRHLIGY	

Cmpd.	No. of Cmpds.	m/z meas.	Δ m/z [ppm]	z	Rt [min]	Score	P	Range	Sequence	Modification
2623	1	812.4211	89.86	2	63.6	11.3	0	223-237	R.THTGEKPYGCGECGK.A	Carbamidomethyl: 13
2493	4	741.8586	-20.96	2	61.6	20.5	0	266-277	K.SFCQSSYLILHK.R	Carbamidomethyl: 3

Detailed Protein Report

Protein 188: smoothelin isoform e [Homo sapiens]

Accession: gi|333360860
Database: refseq_human(refseq_human_20140103.fasta)
Database Date: 2015-11-30
Modification(s): Oxidation

Score: 42.0
MW [kDa]: 104.8
pI: 9.9
Sequence Coverage [%]: 3.9
No. of unique Peptides: 2

10	20	30	40	50	60	70	80
MGGMGTPGDL	HAACPSTTRR	DALCLGPEFE	FDLRHSAGCP	GDLGFFPLDP	DTEAGGSGCL	PTWLPLRRLA	RGRLEVTADL
90	100	110	120	130	140	150	160
AERRRIRSAI	RELQRQELER	EEEALASKRF	RAERQDNKEN	WLHSQQREAE	QRAALARLAG	QLESMNDVEE	LTALLRSAGE
170	180	190	200	210	220	230	240
YEERKLIRAA	IRRVRAQEIE	AATLAGRLYS	GRPNSGSRED	SKGLAAHRLE	QCEVPEREEQ	EQQAEVSKPT	PTPEGTSQDV
250	260	270	280	290	300	310	320
TTVTLLLRAP	PGSTSSSPAS	PSSSPTPASP	EPPLEPAEAQ	CLTAEVPGSP	EPPPSPPKTT	SPEPQESPTL	PSTEGQVVNK
330	340	350	360	370	380	390	400
LLSGPKETPA	AQSPTRGPSD	TKRADVAGPR	PCQRSLSVLS	PRQPAQNRES	TPLASGPSSF	QRAGSVRDRV	HKFTSDSPMA
410	420	430	440	450	460	470	480
ARLQDGTPQA	ALSPLTPARL	LGPSLTSTTP	ASSSSGSSSR	GPSDTSSRFS	KEQRGVAQPL	AQLRSCPQEE	GPRGRGLAAR
490	500	510	520	530	540	550	560
PLENRAGGPV	ARSEEPGAPL	PVAVGTAEPG	GSMKTTFTIE	IKDGRGQAST	GRVLLPTGNQ	RAELTLGLRA	PPTLLSTSSG
570	580	590	600	610	620	630	640
GKSTITRVNS	PGTLARLGSV	THVTSFSHAP	PSSRGGCSIK	MEAEPAEPLA	AAVEAANGAE	QTRVNKAPEG	RSPLSAEELM
650	660	670	680	690	700	710	720
TIEDEGVLDK	MLDQSTDFEE	RKLIRAALRE	LRQRKRDQRD	KERERRLQEA	RGRPGEGRGN	TATETTTRHS	QRAADGSAVS
730	740	750	760	770	780	790	800
TVTKTERLVH	SNDGTRTART	TTVESSFVRR	SENGSGSTMM	QTKTFSSSSS	SKKMGSIFDR	EDQASPRAGS	LAALEKRQAE
810	820	830	840	850	860	870	880
KKKELMKAQS	LPKTSASQAR	KAMIEKLEKE	GAAGSPGGPR	AAVQRSTSFG	VPNANSIKQM	LLDWCRAKTR	GYEHVDIQNF
890	900	910	920	930	940	950	960
SSSWSDGMAF	CALVHNFFPE	AFDYGQLSPQ	NRRQNFEVAF	SSAETHADCP	QLLDTEDMVR	LREPDWKCVY	TYIQEFYRCL
970	980						
VQKGLVKTKK	S						

Cmpd.	No. of Cmpds.	m/z meas.	Δ m/z [ppm]	z	Rt [min]	Score	P	Range	Sequence	Modification
2098	2	938.2855	-133.64	2	54.8	13.7	0	1-19	-.MGGMGTPGDLHAACPSTTR.R	Oxidation: 1
1621	1	502.6927	-108.16	2	50.1	15.3	1	523-532	K.DGRGQASTGR.V	

Detailed Protein Report

Protein 189: **TRIO and F-actin-binding protein isoform 1 [Homo sapiens]**

**Accession:**	gi|88501740	**Score:**	42.0
**Database:**	refseq_human(refseq_human_20140103.fasta)	**MW [kDa]:**	74.0
**Database Date:**	2015-11-30	**pI:**	6.5
		**Sequence Coverage [%]:**	8.9
		**No. of unique Peptides:**	2

10	20	30	40	50	60	70	80
MGGWKGPGQR	RGKEGPEARR	RAAERGGGGG	GGGVPAPRSP	AREPRPRSCL	LLPPPWGAAM	TPDLLNFKKG	WMSILDEPGE
**90**	**100**	**110**	**120**	**130**	**140**	**150**	**160**
PPSPSLTTTS	TSQWKKHWFV	LTDSSLKYYR	DSTAEEADEL	DGEIDLRSCT	DVTEYAVQRN	YGFQIHTKDA	VYTLSAMTSG
**170**	**180**	**190**	**200**	**210**	**220**	**230**	**240**
IRRNWIEALR	KTVRPTSAPD	VTKLSDSNKE	NALHSYSTQK	GPLKAGEQRA	GSEVISRGGP	RKADGQRQAL	DYVELSPLTQ
**250**	**260**	**270**	**280**	**290**	**300**	**310**	**320**
ASPQRARTPA	RTPDRLAKQE	ELERDLAQRS	EERRKWFEAT	DSRTPEVPAG	EGPRRGLGAP	LTEDQQNRLS	EEIEKKWQEL
**330**	**340**	**350**	**360**	**370**	**380**	**390**	**400**
EKLPLRENKR	VPLTALLNQS	RGERRGPPSD	GHEALEKEVQ	ALRAQLEAWR	LQGEAPQSAL	RSQEDGHIPP	GYISQEACER
**410**	**420**	**430**	**440**	**450**	**460**	**470**	**480**
SLAEMESSHQ	QVMEELQRHH	ERELQRLQQE	KEWLLAEETA	ATASAIEAMK	KAYQEELSRE	LSKTRSLQQG	PDGLRKQHQS
**490**	**500**	**510**	**520**	**530**	**540**	**550**	**560**
DVEALKRELQ	VLSEQYSQKC	LEIGALMRQA	EEREHTLRRC	QQEGQELLRH	NQELHGRLSE	EIDQLRGFIA	SQGMGNGCGR
**570**	**580**	**590**	**600**	**610**	**620**	**630**	**640**
SNERSSCELE	VLLRVKENEL	QYLKKEVQCL	RDELQMMQKD	KRFTSGKYQD	VYVELSHIKT	RSEREIEQLK	EHLRLAMAAL
**650**	**660**						
QEKESMRNSL	AE						

Cmpd.	No. of Cmpds.	m/z meas.	Δ m/z [ppm]	z	Rt [min]	Score	P	Range	Sequence	Modification
2143	1	1051.3799	-114.76	2	55.3	12.5	1	276-294	K.WFEATDSRTPEVPAGEGPR.R	
1759	1	920.8290	-90.65	2	52.0	12.2	1	547-564	R.GFIASQGMGNGCGRSNER.S	

Detailed Protein Report

Protein 190: ranBP2-like and GRIP domain-containing protein 4 [Homo sapiens]

Accession: gi|211059431
Database: refseq_human(refseq_human_20140103.fasta)
Database Date: 2015-11-30

Score: 41.9
MW [kDa]: 197.2
pI: 5.9
Sequence Coverage [%]: 2.7
No. of unique Peptides: 1

10	20	30	40	50	60	70	80
MSCSKAYGER	YVASVQGSAP	SPRKKSTRGF	YFAKLYYEAK	EYDLAKKYIC	TYINVREMDP	RAHRFLGLLY	ELEENTEKAV
**90**	**100**	**110**	**120**	**130**	**140**	**150**	**160**
ECYRRSVELN	PTQKDLVLKI	AELLCKNDVT	DGRAKYWVER	AAKLFPGSPA	IYKLKEQLLD	CEGEDGWNKL	FDLIQSELYV
**170**	**180**	**190**	**200**	**210**	**220**	**230**	**240**
RPDDVHVNIR	LVELYRSTKR	LKDAVARCHE	AERNIALRSS	LEWNSCVVQT	LKEYLESLQC	LESDKSDWRA	TNTDLLLAYA
**250**	**260**	**270**	**280**	**290**	**300**	**310**	**320**
NLMLLTLSTR	DVQESRELLE	SFDSALQSAK	SSLGGNDELS	ATFLEMKGHF	YMHAGSLLLK	MGQHGNNVQW	RALSELAALC
**330**	**340**	**350**	**360**	**370**	**380**	**390**	**400**
YLIAFQVPRP	KIKLIKGEAG	QNLLEMMACD	RLSQSGHMLL	NLSRGKQDFL	KVVVETFANK	SGQSALYDAL	FSSQSPKDTS
**410**	**420**	**430**	**440**	**450**	**460**	**470**	**480**
FLGSDDIGNI	DVQEPELEDL	ARYDVGAIRA	HNGSLQHLTW	LGLQWNSLPA	LPGIRKWLKQ	LFHHLPQETS	RLETNAPESI
**490**	**500**	**510**	**520**	**530**	**540**	**550**	**560**
CILDLEVFLL	GVVYTSHLQL	KEKCNSHHSS	YQPLCLPLPV	CKQLCTERQK	SWWDAVCTLI	HRKAVPGNSA	KLRLLVQHEI
**570**	**580**	**590**	**600**	**610**	**620**	**630**	**640**
NTLRAQEKHG	LQPALLVHWA	KCLQKMGSGL	NSFYDQREYI	GRSVHYWKKV	LPLLKIIKKK	NSIPEPIDPL	FKHFHSVDIQ
**650**	**660**	**670**	**680**	**690**	**700**	**710**	**720**
ASEIVEYEED	AHVTFAILDA	VNGNIEDAMT	AFESIKSVVS	YWNLALIFHR	KAEDIANDAL	SPEEQEECKN	YLRKTRGYLI
**730**	**740**	**750**	**760**	**770**	**780**	**790**	**800**
KILDDSDSNL	SVVKKLPVPL	ESVKEMLKSV	MQELENYSEG	DPLYKNGSLR	NADSEIKHST	PSPTKYSLSP	SKSYKYSPKT
**810**	**820**	**830**	**840**	**850**	**860**	**870**	**880**
PPRWAEDQNS	LLKMIRQEVK	AIKEEMQELK	LNSSKSASHH	RWPTENYGPD	SVPDGYQGSQ	TFHGAPLTVA	TTGPSVYYSQ
**890**	**900**	**910**	**920**	**930**	**940**	**950**	**960**
SPAYNSQYLL	RPAANVTPTK	GSSNTEFKST	KEGFSIPVSA	DGFKFGISEP	GNQEKESEKP	LENDTGFQAQ	DISGQKNGRG
**970**	**980**	**990**	**1000**	**1010**	**1020**	**1030**	**1040**
VIFGQTSSTF	TFADVAKSTS	GEGFQFGKKD	PNFKGFSGAG	EKLFSSQCGK	MANKANTSGD	FEKDDDAYKT	EDSDDIHFEP
**1050**	**1060**	**1070**	**1080**	**1090**	**1100**	**1110**	**1120**
VVQMPEKVEL	VIGEEGEKVL	YSQGVKLFRF	DAEVRQWKER	GLGNLKILKN	EVNGKPRMLM	RREQVLKVCA	NHWITTTMNL
**1130**	**1140**	**1150**	**1160**	**1170**	**1180**	**1190**	**1200**
KPLSGSDRAW	MWSASDFSDG	DAKLERLAAK	FKTPELAEEF	KQKFEECQQL	LLDIPLQTPH	KLVDTGRAAK	LIQRAEEMKS
**1210**	**1220**	**1230**	**1240**	**1250**	**1260**	**1270**	**1280**
GLKDFKTFLT	NDQTKVTEEE	NKGSGTGAAG	ASDTTIKPNP	ENTGPTLEWD	NCDLREDALD	DSVSSSSVHA	SPLASSPVRK
**1290**	**1300**	**1310**	**1320**	**1330**	**1340**	**1350**	**1360**
NLFHFGESTT	GSNFSFKSAL	SPSKSPAKLN	QSGTSVGTDE	ESDVTQEEER	DGQYFEPVVP	LPDLVEVSSG	EENEKVVFSH
**1370**	**1380**	**1390**	**1400**	**1410**	**1420**	**1430**	**1440**
RAELYRYDKD	VGQWKERGIG	DIKILQNYDN	KQVRIVMRRD	QVLKLCANHT	ITPDMSLQNM	KGTERVWVWT	ACDFADGERK
**1450**	**1460**	**1470**	**1480**	**1490**	**1500**	**1510**	**1520**
VEHLAVRFKL	QDVADSFKKI	FDEAKTAQEK	DSLITPHVSR	SSTPRESPCG	KIAVAVLEET	TRERTDVIQG	DDVADAASEV
**1530**	**1540**	**1550**	**1560**	**1570**	**1580**	**1590**	**1600**
EVSSTSETTT	KAVVSPPKFV	FGSESVKRIF	SSEKSKPFAF	GNSSATGSLF	GFSFNASLKS	NNSETSSVAQ	SGSESKVEPK
**1610**	**1620**	**1630**	**1640**	**1650**	**1660**	**1670**	**1680**
KCELSKNSDI	EQSSDSKVKN	LSASFPMEES	SINYTFKTPE	KEPPLWHAEF	TKEELVQKLS	STTKSADHLN	GLLREAEATS
**1690**	**1700**	**1710**	**1720**	**1730**	**1740**	**1750**	**1760**
AVLMEQIKLL	KSEIRRLERN	QEQEESAANV	EHLKNVLLQF	IFLKPGSERE	RLLPVINTML	QLSPEEKGKL	AAVAQGEE

Cmpd.	No. of Cmpds.	m/z meas.	Δ m/z [ppm]	z	Rt [min]	Score	P	Range	Sequence	Modification
2527	1	863.4144	10.20	2	62.0	14.8	0	1700-1714	R.NQEQEESAANVEHLK.N	

Detailed Protein Report

Protein 191: testis-specific serine/threonine-protein kinase 2 [Homo sapiens]

**Accession:**	gi|194294513	**Score:**	41.8
**Database:**	refseq_human(refseq_human_20140103.fasta)	**MW [kDa]:**	40.9
**Database Date:**	2015-11-30	**pI:**	9.8
**Modification(s):**	Carbamidomethyl	**Sequence Coverage [%]:**	2.8
		**No. of unique Peptides:**	1

Quantitation

mdown:qdown **Median:** 2.21 **CV:** 0.00 % **No. of Peptides:** 1

10	20	30	40	50	60	70	80
MDDATVLRKK	GYIVGINLGK	GSYAKVKSAY	SERLKFNVAV	KIIDRKKTPT	DFVERFLPRE	MDILATVNHG	SIIKTYEIFE
**90**	**100**	**110**	**120**	**130**	**140**	**150**	**160**
TSDGRIYIIM	ELGVQGDLLE	FIKCQGALHE	DVARKMFRQL	SSAVKYCHDL	DIVHRDLKCE	NLLLDKDFNI	KLSDFGFSKR
**170**	**180**	**190**	**200**	**210**	**220**	**230**	**240**
CLRDSNGRII	LSKTFCGSAA	YAAPEVLQSI	PYQPKVYDIW	SLGVILYIMV	CGSMPYDDSD	IRKMLRIQKE	HRVDFPRSKN
**250**	**260**	**270**	**280**	**290**	**300**	**310**	**320**
LTCECKDLIY	RMLQPDVSQR	LHIDEILSHS	WLQPPKPKAT	SSASFKREGE	GKYRAECKLD	TKTGLRPDHR	PDHKLGAKTQ
**330**	**340**	**350**	**360**				
HRLLVVPENE	NRMEDRLAET	SRAKDHHISG	AEVGKAST				

Cmpd.	No. of Cmpds.	m/z meas.	Δ m/z [ppm]	z	Rt [min]	Score	P	Range	Sequence	Modification	Ratios
174	2	642.2924	-49.65	2	31.4	20.1	2	293-302	K.YRAECKLDTK.T	Carbamidomethyl: 5	mdown:qdown 2.21

Detailed Protein Report

Protein 192: **PREDICTED: latrophilin-1 isoform X5 [Homo sapiens]**

**Accession:**	gi|578833089	**Score:**	41.8
**Database:**	refseq_human(refseq_human_20140103.fasta)	**MW [kDa]:**	96.9
**Database Date:**	2015-11-30	**pI:**	6.5
**Modification(s):**	Carbamidomethyl	**Sequence Coverage [%]:**	6.1
		**No. of unique Peptides:**	1

10	20	30	40	50	60	70	80
MARLAAVLWN	LCVTAVLVTS	ATQGLSRAGL	PFGLMRRELA	CEGYPIELRC	PGSDVIMVEN	ANYGRTDDKI	CDADPFQMEN
**90**	**100**	**110**	**120**	**130**	**140**	**150**	**160**
VQCYLPDAFK	IMSQRCNNRT	QCVVVAGSDA	FPDPCPGTYK	YLEVQYDCVP	YKVEQKVFVC	PGTLQKVLEP	TSTHESEHQS
**170**	**180**	**190**	**200**	**210**	**220**	**230**	**240**
GAWCKDPLQA	GDRIYVMPWI	PYRTDTLTEY	ASWEDYVAAR	HTTTYRLPNR	VDGTGFVVYD	GAVFYNKERT	RNIVKYDLRT
**250**	**260**	**270**	**280**	**290**	**300**	**310**	**320**
RIKSGETVIN	TANYHDTSPY	RWGGKTDIDL	AVDENGLWVI	YATEGNNGRL	VVSQLNPYTL	RFEGTWETGY	DKRSASNAFM
**330**	**340**	**350**	**360**	**370**	**380**	**390**	**400**
VCGVLYVLRS	VYVDDDSEAA	GNRVDYAFNT	NANREEPVSL	TFPNPYQFIS	SVDYNPRDNQ	LYVWNNYFVV	RYSLEFGPPD
**410**	**420**	**430**	**440**	**450**	**460**	**470**	**480**
PSAGPATSPP	LSTTTTARPT	PLTSTASPAA	TTPLRRAPLT	THPVGAINQL	GPDLPPATAP	VPSTRRPPAP	NLHVSPELFC
**490**	**500**	**510**	**520**	**530**	**540**	**550**	**560**
EPREVRRVQW	PATQQGMLVE	RPCPKGTRGI	ASFQCLPALG	LWNPRGPDLS	NCTSPWVNQV	AQKIKSGENA	ANIASELARH
**570**	**580**	**590**	**600**	**610**	**620**	**630**	**640**
TRGSIYAGDV	SSSVKLMEQL	LDILDAQLQA	LRPIERESAG	KNYNKMHKRE	RTCKDYIKAV	VETVDNLLRP	EALESWKDMN
**650**	**660**	**670**	**680**	**690**	**700**	**710**	**720**
ATEQVHTATM	LLDVLEEGAF	LLADNVREPA	RFLAAKENVV	LEVTVLNTEG	QVQELVFPQE	EYPRKNSIQL	SAKTIKQNSR
**730**	**740**	**750**	**760**	**770**	**780**	**790**	**800**
NGVVKVVFIL	YNNLGLFLST	ENATVKLAGE	AGPGGPGGAS	LVVNSQVIAA	SINKESSRVF	LMDPVIFTVA	HLEDKNHFNA
**810**	**820**	**830**	**840**	**850**	**860**	**870**	**880**
NCSFWNYSER	SMLGYWSTQG	CRLVESNKTH	TTCACSHLTN	FAVLMAHREI	ALWRSKLVLR	WSSLHKPSRA	

Cmpd.	No. of Cmpds.	m/z meas.	Δ m/z [ppm]	z	Rt [min]	Score	P	Range	Sequence	Modification
2050	1	900.3480	-63.50	3	55.8	14.3	1	96-120	R.CNNRTQCVVVAGSDAFPDPCPGTYK.Y	Carbamidomethyl: 7

Detailed Protein Report

Protein 193: xylosyltransferase 1 precursor [Homo sapiens]

**Accession:**	gi|28269693	**Score:**	41.8
**Database:**	refseq_human(refseq_human_20140103.fasta)	**MW [kDa]:**	107.5
**Database Date:**	2015-11-30	**pI:**	10.0
**Modification(s):**	Oxidation	**Sequence Coverage [%]:**	5.3
		**No. of unique Peptides:**	3

Quantitation

mdown:qdown	**Median:**	0.48	**CV:**	0.00 %	**No. of Peptides:**	1
Wdown:Qdown	**Median:**	1.47	**CV:**	0.00 %	**No. of Peptides:**	1

10	20	30	40	50	60	70	80
MVAAPCARRL	ARRSHSALLA	ALTVLLLQTL	VVWNFSSLDS	GAGERRGGAA	VGGGEQPPPA	PAPRRERRDL	PAEPAAARGG
**90**	**100**	**110**	**120**	**130**	**140**	**150**	**160**
GGGGGGGGGG	RGPQARARGG	GPGEPRGQQP	ASRGALPARA	LDPHPSPLIT	LETQDGYFSH	RPKEKVRTDS	NNENSVPKDF
**170**	**180**	**190**	**200**	**210**	**220**	**230**	**240**
ENVDNSNFAP	RTQKQKHQPE	LAKKPPSRQK	ELLKRKLEQQ	EKGKGHTFPG	KGPGEVLPPG	DRAAANSSHG	KDVSRPPHAR
**250**	**260**	**270**	**280**	**290**	**300**	**310**	**320**
KTGGSSPETK	YDQPPKCDIS	GKEAISALSR	AKSKHCRQEI	GETYCRHKLG	LLMPEKVTRF	CPLEGKANKN	VQWDEDSVEY
**330**	**340**	**350**	**360**	**370**	**380**	**390**	**400**
MPANPVRIAF	VLVVHGRASR	QLQRMFKAIY	HKDHFYYIHV	DKRSNYLHRQ	VLQVSRQYSN	VRVTPWRMAT	IWGGASLLST
**410**	**420**	**430**	**440**	**450**	**460**	**470**	**480**
YLQSMRDLLE	MTDWPWDFFI	NLSAADYPIR	TNDQLVAFLS	RYRDMNFLKS	HGRDNARFIR	KQGLDRLFLE	CDAHMWRLGD
**490**	**500**	**510**	**520**	**530**	**540**	**550**	**560**
RRIPEGIAVD	GGSDWFLLNR	RFVEYVTFST	DDLVTKMKQF	YSYTLLPAES	FFHTVLENSP	HCDTMVDNNL	RITNWNRKLG
**570**	**580**	**590**	**600**	**610**	**620**	**630**	**640**
CKCQYKHIVD	WCGCSPNDFK	PQDFHRFQQT	ARPTFFARKF	EAVVNQEIIG	QLDYYLYGNY	PAGTPGLRSY	WENVYDEPDG
**650**	**660**	**670**	**680**	**690**	**700**	**710**	**720**
IHSLSDVTLT	LYHSFARLGL	RRAETSLHTD	GENSCRYYPM	GHPASVHLYF	LADRFQGFLI	KHHATNLAVS	KLETLETWVM
**730**	**740**	**750**	**760**	**770**	**780**	**790**	**800**
PKKVFKIASP	PSDFGRLQFS	EVGTDWDAKE	RLFRNFGGLL	GPMDEPVGMQ	KWGKGPNVTV	TVIWVDPVNV	IAATYDILIE
**810**	**820**	**830**	**840**	**850**	**860**	**870**	**880**
STAEFTHYKP	PLNLPLRPGV	WTVKILHHWV	PVAETKFLVA	PLTFSNRQPI	KPEEALKLHN	GPLRNAYMEQ	SFQSLNPVLS
**890**	**900**	**910**	**920**	**930**	**940**	**950**	**960**
LPINPAQVEQ	ARRNAASTGT	ALEGWLDSLV	GGMWTAMDIC	ATGPTACPVM	QTCSQTAWSS	FSPDPKSELG	AVKPDGRLR

Cmpd.	No. of Cmpds.	m/z meas.	Δ m/z [ppm]	z	Rt [min]	Score	P	Range	Sequence	Modification	Ratios
87	1	669.7442	115.37	3	29.8	12.8	2	68-91	R. RDLPAEPAAARGGGGGGGGGGG G		
1789	1	916.3008	-236.11	1	52.4	13.7	0	289-296	K.LGLLMPEK.V	Oxidation: 5	Wdown:Qdown 1.47<br>mdown:qdown 0.48
2109	1	701.2880	-90.93	3	56.5	15.4	0	388-406	R.MATIWGGASLLSTYLQSMR.D	Oxidation: 18	

Detailed Protein Report

Protein 194: **PREDICTED: cleavage and polyadenylation specificity factor subunit 1 isoform X1 [Homo sapiens]**

**Accession:**	gi|578815943	**Score:**	41.8
**Database:**	refseq_human(refseq_human_20140103.fasta)	**MW [kDa]:**	160.4
**Database Date:**	2015-11-30	**pI:**	5.9
**Modification(s):**	Oxidation	**Sequence Coverage [%]:**	2.4
		**No. of unique Peptides:**	1

10	20	30	40	50	60	70	80
MYAVYKQAHP	PTGLEFSMYC	NFFNNSERNL	VVAGTSQLYV	YRLNRDAEAL	TKNDRSTEGK	AHREKLELAA	SFSFFGNVMS
**90**	**100**	**110**	**120**	**130**	**140**	**150**	**160**
MASVQLAGAK	RDALLLSFKD	AKLSVVEYDP	GTHDLKTLSL	HYFEEPELRD	GFVQNVHTPR	VRVDPDGRCA	AMLVYGTRLV
**170**	**180**	**190**	**200**	**210**	**220**	**230**	**240**
VLPFRRESLA	EEHEGLVGEG	QRSSFLPSYI	IDVRALDEKL	LNIIDLQFLH	GYYEPTLLIL	FEPNQTWPGR	VAVRQDTCSI
**250**	**260**	**270**	**280**	**290**	**300**	**310**	**320**
VAISLNITQK	VHPVIWSLTS	LPFDCTQALA	VPKPIGGVVV	FAVNSLLYLN	QSVPPYGVAL	NSLTTGTTAF	PLRTQEGVRI
**330**	**340**	**350**	**360**	**370**	**380**	**390**	**400**
TLDCAQATFI	SYDKMVISLK	GGEIYVLTLI	TDGMRSVRAF	HFDKAAASVL	TTSMVTMEPG	YLFLGSRLGN	SLLLKYTEKL
**410**	**420**	**430**	**440**	**450**	**460**	**470**	**480**
QEPPASAVRE	AADKEEPPSK	KKRVDATAGW	SAAGKSVPQD	EVDEIEVYGS	EAQSGTQLAT	YSFEVCDSIL	NIGPCANAAV
**490**	**500**	**510**	**520**	**530**	**540**	**550**	**560**
GEPAFLSEEF	QNSPEPDLEI	VVCSGHGKNG	ALSVLQKSIR	PQVVTTFELP	GCYDMWTVIA	PVRKEEEDNP	KGEGTEQEPS
**570**	**580**	**590**	**600**	**610**	**620**	**630**	**640**
TTPEADDDGR	RHGFLILSRE	DSTMILQTGQ	EIMELDTSGF	ATQGPTVFAG	NIGDNRYIVQ	VSPLGIRLLE	GVNQLHFIPV
**650**	**660**	**670**	**680**	**690**	**700**	**710**	**720**
DLGAPIVQCA	VADPYVVIMS	AEGHVTMFLL	KSDSYGGRHH	RLALHKPPLH	HQSKVITLCL	YRDLSGMFTT	ESRLGGARDE
**730**	**740**	**750**	**760**	**770**	**780**	**790**	**800**
LGGRSGPEAE	GLGSETSPTV	DDEEEMLYGD	SGSLFSPSKE	EARRSSQPPA	DRDPAPFRAE	PTHWCLLVRE	NGTMEIYQLP
**810**	**820**	**830**	**840**	**850**	**860**	**870**	**880**
DWRLVFLVKN	FPVGQRVLVD	SSFGQPTTQG	EARREEATRQ	GELPLVKEVL	LVALGSRQSR	PYLLVHVDQE	LLIYEAFPHD
**890**	**900**	**910**	**920**	**930**	**940**	**950**	**960**
SQLGQGNLKV	RFKKVPHNIN	FREKKPKPSK	KKAEGGGAEE	GAGARGRVAR	FRYFEDIYGY	SGVFICGPSP	HWLLVTGRGA
**970**	**980**	**990**	**1000**	**1010**	**1020**	**1030**	**1040**
LRLHPMAIDG	PVDSFAPFHN	VNCPRGFLYF	NRQGELRISV	LPAYLSYDAP	WPVRKIPLRC	TAHYVAYHVE	SKVYAVATST
**1050**	**1060**	**1070**	**1080**	**1090**	**1100**	**1110**	**1120**
NTPCARIPRM	TGEEKEFETI	ERDERYIHPQ	QEAFSIQLIS	PVSWEAIPNA	RIELQEWEHV	TCMKTVSLRS	EETVSGLKGY
**1130**	**1140**	**1150**	**1160**	**1170**	**1180**	**1190**	**1200**
VAAGTCLMQG	EEVTCRGRIL	IMDVIEVVPE	PGQPLTKNKF	KVLYEKEQKG	PVTALCHCNG	HLVSAIGQKI	FLWSLRASEL
**1210**	**1220**	**1230**	**1240**	**1250**	**1260**	**1270**	**1280**
TGMAFIDTQL	YIHQMISVKN	FILAADVMKS	ISLLRYQEES	KTLSLDAKPL	EVYSVDFMVD	NAQLGFLVSD	RDRNLMVYMY
**1290**	**1300**	**1310**	**1320**	**1330**	**1340**	**1350**	**1360**
LPEAKESFGG	MRLLRRADFH	VGAHVNTFWR	TPCRGATEGL	SKKSVVWENK	HITWFATLDG	GIGLLLPMQE	KTYRRLLMLQ
**1370**	**1380**	**1390**	**1400**	**1410**	**1420**	**1430**	**1440**
NALTTMLPHH	AGLNPRAFRM	LHVDRRTLQN	AVRNVLDGEL	LNRYLYLSTM	ERSELAKKIG	TTPDIILDDL	LETDRVTAHF
**1450**							

Cmpd.	No. of Cmpds.	m/z meas.	Δ m/z [ppm]	z	Rt [min]	Score	P	Range	Sequence	Modification
2457	1	596.2158	-123.37	2	61.1	10.4	0	1404-1412	R.YLYLSTMER.S	Oxidation: 7

Detailed Protein Report

Protein 195: talin-1 [Homo sapiens]

**Accession:**	gi|223029410	**Score:**	41.7
**Database:**	refseq_human(refseq_human_20140103.fasta)	**MW [kDa]:**	269.6
**Database Date:**	2015-11-30	**pI:**	5.7
**Modification(s):**	Oxidation	**Sequence Coverage [%]:**	1.4
		**No. of unique Peptides:**	2

Quantitation

mdown:qdown	**Median:**	0.35	**CV:**	0.00 %	**No. of Peptides:**	1
Wdown:Qdown	**Median:**	0.07	**CV:**	0.00 %	**No. of Peptides:**	1

Detailed Protein Report

10	20	30	40	50	60	70	80
MVALSLKISI	GNVVKTMQFE	PSTMVYDACR	IIRERIPEAP	AGPPSDFGLF	LSDDDPKKGI	WLEAGKALDY	YMLRNGDTME
90	100	110	120	130	140	150	160
YRKKQRPLKI	RMLDGTVKTI	MVDDSKTVTD	MLMTICARIG	ITNHDEYSLV	RELMEEKKEE	ITGTLRKDKT	LLRDEKKMEK
170	180	190	200	210	220	230	240
LKQKLHTDDE	LNWLDHGRTL	REQGVEEHET	LLLRRKFFYS	DQNVDSRDPV	QLNLLYVQAR	DDILNGSHPV	SFDKACEFAG
250	260	270	280	290	300	310	320
FQCQIQFGPH	NEQKHKAGFL	DLKDFLPKEY	VKQKGERKIF	QAHKNCGQMS	EIEAKVRYVK	LARSLKTYGV	SFFLVKEKMK
330	340	350	360	370	380	390	400
GKNKLVPRLL	GITKECVMRV	DEKTKEVIQE	WNLTNIKRWA	ASPKSFTLDF	GDYQDGYYSV	QTTEGEQIAQ	LIAGYIDIIL
410	420	430	440	450	460	470	480
KKKKSKDHFG	LEGDEESTML	EDSVSPKKST	VLQQQYNRVG	KVEHGSVALP	AIMRSGASGP	ENFQVGSMPP	AQQQITSGQM
490	500	510	520	530	540	550	560
HRGHMPPLTS	AQQALTGTIN	SSMQAVQAAQ	ATLDDFDTLP	PLGQDAASKA	WRKNKMDESK	HEIHSQVDAI	TAGTASVVNL
570	580	590	600	610	620	630	640
TAGDPAETDY	TAVGCAVTTI	SSNLTEMSRG	VKLLAALLED	EGGSGRPLLQ	AAKGLAGAVS	ELLRSAQPAS	AEPRQNLLQA
650	660	670	680	690	700	710	720
AGNVGQASGE	LLQQIGESDT	DPHFQDALMQ	LAKAVASAAA	ALVLKAKSVA	QRTEDSGLQT	QVIAAATQCA	LSTSQLVACT
730	740	750	760	770	780	790	800
KVVAPTISSP	VCQEQLVEAG	RLVAKAVEGC	VSASQAATED	GQLLRGVGAA	ATAVTQALNE	LLQHVKAHAT	GAGPAGRYDQ
810	820	830	840	850	860	870	880
ATDTILTVTE	NIFSSMGDAG	EMVRQARILA	QATSDLVNAI	KADAEGESDL	ENSRKLLSAA	KILADATAKM	VEAAKGAAAH
890	900	910	920	930	940	950	960
PDSEEQQQRL	REAAEGLRMA	TNAAAQNAIK	KKLVQRLEHA	AKQAAASATQ	TIAAAQHAAS	TPKASAGPQP	LLVQSCKAVA
970	980	990	1000	1010	1020	1030	1040
EQIPLLVQGV	RGSQAQPDSP	SAQLALIAAS	QSFLQPGGKM	VAAAKASVPT	IQDQASAMQL	SQCAKNLGTA	LAELRTAAQK
1050	1060	1070	1080	1090	1100	1110	1120
AQEACGPLEM	DSALSVVQNL	EKDLQEVKAA	ARDGKLKPLP	GETMEKCTQD	LGNSTKAVSS	AIAQLLGEVA	QGNENYAGIA
1130	1140	1150	1160	1170	1180	1190	1200
ARDVAGGLRS	LAQAARGVAA	LTSDPAVQAI	VLDTASDVLD	KASSLIEEAK	KAAGHPGDPE	SQQRLAQVAK	AVTQALNRCV
1210	1220	1230	1240	1250	1260	1270	1280
SCLPGQRDVD	NALRAVGDAS	KRLLSDSLPP	STGTFQEAQS	RLNEAAAGLN	QAATELVQAS	RGTPQDLARA	SGRFGQDFST
1290	1300	1310	1320	1330	1340	1350	1360
FLEAGVEMAG	QAPSQEDRAQ	VVSNLKGISM	SSSKLLLAAK	ALSTDPAAPN	LKSQLAAAAR	AVTDSINQLI	TMCTQQAPGQ
1370	1380	1390	1400	1410	1420	1430	1440
KECDNALREL	ETVRELLENP	VQPINDMSYF	GCLDSVMENS	KVLGEAMTGI	SQNAKNGNLP	EFGDAISTAS	KALCGFTEAA
1450	1460	1470	1480	1490	1500	1510	1520
AQAAYLVGVS	DPNSQAGQQG	LVEPTQFARA	NQAIQMACQS	LGEPGCTQAQ	VLSAATIVAK	HTSALCNSCR	LASARTTNPT
1530	1540	1550	1560	1570	1580	1590	1600
AKRQFVQSAK	EVANSTANLV	KTIKALDGAF	TEENRAQCRA	ATAPLLEAVD	NLSAFASNPE	FSSIPAQISP	EGRAAMEPIV
1610	1620	1630	1640	1650	1660	1670	1680
ISAKTMLESA	GGLIQTARAL	AVNPRDPPSW	SVLAGHSRTV	SDSIKKLITS	MRDKAPGQLE	CETAIAALNS	CLRDLDQASL
1690	1700	1710	1720	1730	1740	1750	1760
AAVSQQLAPR	EGISQEALHT	QMLTAVQEIS	HLIEPLANAA	RAEASQLGHK	VSQMAQYFEP	LTLAAVGAAS	KTLSHPQQMA
1770	1780	1790	1800	1810	1820	1830	1840
LLDQTKTLAE	SALQLLYTAK	EAGGNPKQAA	HTQEALEEAV	QMMTEAVEDL	TTTLNEAASA	AGVVGGMVDS	ITQAINQLDE
1850	1860	1870	1880	1890	1900	1910	1920
GPMGEPEGSF	VDYQTTMVRT	AKAIAVTVQE	MVTKSNTSPE	ELGPLANQLT	SDYGRLASEA	KPAAVAAENE	EIGSHIKHRV
1930	1940	1950	1960	1970	1980	1990	2000
QELGHGCAAL	VTKAGALQCS	PSDAYTKKEL	IECARRVSEK	VSHVLAALQA	GNRGTQACIT	AASAVSGIIA	DLDTTIMFAT
2010	2020	2030	2040	2050	2060	2070	2080
AGTLNREGTE	TFADHREGIL	KTAKVLVEDT	KVLVQNAAGS	QEKLAQAAQS	SVATITRLAD	VVKLGAASLG	AEDPETQVVL
2090	2100	2110	2120	2130	2140	2150	2160
INAVKDVAKA	LGDLISATKA	AAGKVGDDPA	VWQLKNSAKV	MVTNVTSLLK	TVKAVEDEAT	KGTRALEATT	EHIRQELAVF
2170	2180	2190	2200	2210	2220	2230	2240

Detailed Protein Report

Cmpd.	No. of Cmpds.	m/z meas.	Δ m/z [ppm]	z	Rt [min]	Score	P	Range	Sequence	Modification	Ratios
509	2	501.1523	-279.76	2	35.2	14.2	1	58-66	K.KGIWLEAGK.A		mdown:qdown 0.35<br>Wdown:Qdown 0.07
2760	1	698.3753	1.48	2	63.5	13.0	0	442-454	K.VEHGSVALPAIMR.S	Oxidation: 12	

Detailed Protein Report

Protein 196: sarcoplasmic/endoplasmic reticulum calcium ATPase 2 isoform a [Homo sapiens]

Accession: gi|4502285
Database: refseq_human(refseq_human_20140103.fasta)
Database Date: 2015-11-30
Modification(s): Carbamidomethyl, Oxidation

Score: 41.7
MW [kDa]: 109.6
pI: 5.1
Sequence Coverage [%]: 3.4
No. of unique Peptides: 2

10	20	30	40	50	60	70	80
MENAHTKTVE	EVLGHFGVNE	STGLSLEQVK	KLKERWGSNE	LPAEEGKTLL	ELVIEQFEDL	LVRILLLAAC	ISFVLAWFEE
**90**	**100**	**110**	**120**	**130**	**140**	**150**	**160**
GEETITAFVE	PFVILLILVA	NAIVGVWQER	NAENAIEALK	EYEPEMGKVY	RQDRKSVQRI	KAKDIVPGDI	VEIAVGDKVP
**170**	**180**	**190**	**200**	**210**	**220**	**230**	**240**
ADIRLTSIKS	TTLRVDQSIL	TGESVSVIKH	TDPVPDPRAV	NQDKKNMLFS	GTNIAAGKAM	GVVVATGVNT	EIGKIRDEMV
**250**	**260**	**270**	**280**	**290**	**300**	**310**	**320**
ATEQERTPLQ	QKLDEFGEQL	SKVISLICIA	VWIINIGHFN	DPVHGGSWIR	GAIYYFKIAV	ALAVAAIPEG	LPAVITTCLA
**330**	**340**	**350**	**360**	**370**	**380**	**390**	**400**
LGTRRMAKKN	AIVRSLPSVE	TLGCTSVICS	DKTGTLTTNQ	MSVCRMFILD	RVEGDTCSLN	EFTITGSTYA	PIGEVHKDDK
**410**	**420**	**430**	**440**	**450**	**460**	**470**	**480**
PVNCHQYDGL	VELATICALC	NDSALDYNEA	KGVYEKVGEA	TETALTCLVE	KMNVFDTELK	GLSKIERANA	CNSVIKQLMK
**490**	**500**	**510**	**520**	**530**	**540**	**550**	**560**
KEFTLEFSRD	RKSMSVYCTP	NKPSRTSMSK	MFVKGAPEGV	IDRCTHIRVG	STKVPMTSGV	KQKIMSVIRE	WGSGSDTLRC
**570**	**580**	**590**	**600**	**610**	**620**	**630**	**640**
LALATHDNPL	RREEMHLEDS	ANFIKYETNL	TFVGCVGMLD	PPRIEVASSV	KLCRQAGIRV	IMITGDNKGT	AVAICRRIGI
**650**	**660**	**670**	**680**	**690**	**700**	**710**	**720**
FGQDEDVTSK	AFTGREFDEL	NPSAQRDACL	NARCFARVEP	SHKSKIVEFL	QSFDEITAMT	GDGVNDAPAL	KKAEIGIAMG
**730**	**740**	**750**	**760**	**770**	**780**	**790**	**800**
SGTAVAKTAS	EMVLADDNFS	TIVAAVEEGR	AIYNNMKQFI	RYLISSNVGE	VVCIFLTAAL	GFPEALIPVQ	LLWVNLVTDG
**810**	**820**	**830**	**840**	**850**	**860**	**870**	**880**
LPATALGFNP	PDLDIMNKPP	RNPKEPLISG	WLFFRYLAIG	CYVGAATVGA	AAWWFIAADG	GPRVSFYQLS	HFLQCKEDNP
**890**	**900**	**910**	**920**	**930**	**940**	**950**	**960**
DFEGVDCAIF	ESPYPMTMAL	SVLVTIEMCN	ALNSLSENQS	LLRMPPWENI	WLVGSICLSM	SLHFLILYVE	PLPLIFQITP
**970**	**980**	**990**	**1000**				
LNVTQWLMVL	KISLPVILMD	ETLKFVARNY	LEPAILE				

Cmpd.	No. of Cmpds.	m/z meas.	Δ m/z [ppm]	z	Rt [min]	Score	P	Range	Sequence	Modification
1931	1	709.7683	-152.59	2	52.8	13.6	1	511-523	K.MFVKGAPEGVIDR.C	
2121	1	701.2646	-134.99	3	56.7	14.0	2	511-528	K.MFVKGAPEGVIDRCTHIR.V	Carbamidomethyl: 14; Oxidation: 1

Detailed Protein Report

Protein 197: ATPase family AAA domain-containing protein 2 [Homo sapiens]

Accession: gi|24497618
Database: refseq_human(refseq_human_20140103.fasta)
Database Date: 2015-11-30

Score: 41.6
MW [kDa]: 158.5
pI: 5.9
Sequence Coverage [%]: 2.8
No. of unique Peptides: 2

Quantitation

Wdown:Qdown **Median:** 0.88 **CV:** 0.00 % **No. of Peptides:** 1

10	20	30	40	50	60	70	80
MVVLRSSLEL	HNHSAASATG	SLDLSSDFLS	LEHIGRRRLR	SAGAAQKKPA	ATTAKAGDGS	SVKEVETYHR	TRALRSLRKD
90	100	110	120	130	140	150	160
AQNSSDSSFE	KNVEITEQLA	NGRHFTRQLA	RQQADKKKEE	HREDKVIPVT	RSLRARNIVQ	STEHLHEDNG	DVEVRRSCRI
170	180	190	200	210	220	230	240
RSRYSGVNQS	MLFDKLITNT	AEAVLQKMDD	MKKMRRQRMR	ELEDLGVFNE	TEESNLNMYT	RGKQKDIQRT	DEETTDNQEG
250	260	270	280	290	300	310	320
SVESSEEGED	QEHEDDGEDE	DDEDDDDDDD	DDDDDDDEDD	EDEEDGEEEN	QKRYYLRQRK	ATVYYQAPLE	KPRHQRKPNI
330	340	350	360	370	380	390	400
FYSGPASPAR	PRYRLSSAGP	RSPYCKRMNR	RRHAIHSSDS	TSSSSSEDEQ	HFERRRKRSR	NRAINRCLPL	NFRKDELKGI
410	420	430	440	450	460	470	480
YKDRMKIGAS	LADVDPMQLD	SSVRFDSVGG	LSNHIAALKE	MVVFPLLYPE	VFEKFKIQPP	RGCLFYGPPG	TGKTLVARAL
490	500	510	520	530	540	550	560
ANECSQGDKR	VAFFMRKGAD	CLSKWVGESE	RQLRLLFDQA	YQMRPSIIFF	DEIDGLAPVR	SSRQDQIHSS	IVSTLLALMD
570	580	590	600	610	620	630	640
GLDSRGEIVV	IGATNRLDSI	DPALRRPGRF	DREFLFSLPD	KEARKEILKI	HTRDWNPKPL	DTFLEELAEN	CVGYCGADIK
650	660	670	680	690	700	710	720
SICAEAALCA	LRRRYPQIYT	TSEKLQLDLS	SINISAKDFE	VAMQKMIPAS	QRAVTSPGQA	LSTVVKPLLQ	NTVDKILEAL
730	740	750	760	770	780	790	800
QRVFPHAEFR	TNKTLDSDIS	CPLLESDLAY	SDDDVPSVYE	NGLSQKSSHK	AKDNFNFLHL	NRNACYQPMS	FRPRILIVGE
810	820	830	840	850	860	870	880
PGFGQGSHLA	PAVIHALEKF	TVYTLDIPVL	FGVSTTSPEE	TCAQVIREAK	RTAPSIVYVP	HIHVWWEIVG	PTLKATFTTL
890	900	910	920	930	940	950	960
LQNIPSFAPV	LLLATSDKPH	SALPEEVQEL	FIRDYGEIFN	VQLPDKEERT	KFFEDLILKQ	AAKPPISKKK	AVLQALEVLP
970	980	990	1000	1010	1020	1030	1040
VAPPPEPRSL	TAEEVKRLEE	QEEDTFRELR	IFLRNVTHRL	AIDKRFRVFT	KPVDPDEVPD	YVTVIKQPMD	LSSVISKIDL
1050	1060	1070	1080	1090	1100	1110	1120
HKYLTVKDYL	RDIDLICSNA	LEYNPDRDPG	DRLIRHRACA	LRDTAYAIIK	EELDEDFEQL	CEEIQESRKK	RGCSSSKYAP
1130	1140	1150	1160	1170	1180	1190	1200
SYYHVMPKQN	STLVGDKRSD	PEQNEKLKTP	STPVACSTPA	QLKRKIRKKS	NWYLGTIKKR	RKISQAKDDS	QNAIDHKIES
1210	1220	1230	1240	1250	1260	1270	1280
DTEETQDTSV	DHNETGNTGE	SSVEENEKQQ	NASESKLELR	NNSNTCNIEN	ELEDSRKTTA	CTELRDKIAC	NGDASSSQII
1290	1300	1310	1320	1330	1340	1350	1360
HISDENEGKE	MCVLRMTRAR	RSQVEQQQLI	TVEKALAILS	QPTPSLVVDH	ERLKNLLKTV	VKKSQNYNIF	QLENLYAVIS
1370	1380	1390	1400				
QCIYRHRKDH	DKTSLIQKME	QEVENFSCSR					

Cmpd.	No. of Cmpds.	m/z meas.	Δ m/z [ppm]	z	Rt [min]	Score	P	Range	Sequence	Modification	Ratios
2101	1	1057.6924	66.12	1	54.8	10.3	2	38-47	R.RLRSAGAAQK.K		
2087	6	937.3467	-131.35	2	54.7	19.5	0	407-424	K.IGASLADVDPMQLDSSVR.F		Wdown:Qdown 0.88

Detailed Protein Report

Protein 198: endothelin-2 preproprotein [Homo sapiens]

Accession: gi|4503463
Database: refseq_human(refseq_human_20140103.fasta)
Database Date: 2015-11-30
Modification(s): Carbamidomethyl

Score: 41.5
MW [kDa]: 19.9
pI: 12.1
Sequence Coverage [%]: 10.1
No. of unique Peptides: 1

10	20	30	40	50	60	70	80
MVSVPTTWCS	VALALLVALH	EGKGQAAATL	EQPASSSHAQ	GTHLRLRRCS	CSSWLDKECV	YFCHLDIIWV	NTPEQTAPYG
**90**	**100**	**110**	**120**	**130**	**140**	**150**	**160**
LGNPPRRRRR	SLPRRCQCSS	ARDPACATFC	LRRPWTEAGA	VPSRKSPADV	FQTGKTGATT	GELLQRLRDI	STVKSLFAKR
**170**	**180**						
QQEAMREPRS	THSRWRKR						

Cmpd.	No. of Cmpds.	m/z meas.	Δ m/z [ppm]	z	Rt [min]	Score	P	Range	Sequence	Modification
2155	1	1051.4608	-0.17	2	55.5	16.2	2	95-112	R.RCQCSSARDPACATFCLR.R	Carbamidomethyl: 12, 16

Detailed Protein Report

Protein 199: Fanconi anemia group M protein [Homo sapiens]

**Accession:**	gi|74959747	**Score:**	41.4
**Database:**	refseq_human(refseq_human_20140103.fasta)	**MW [kDa]:**	232.0
**Database Date:**	2015-11-30	**pI:**	5.7
		**Sequence Coverage [%]:**	2.7
		**No. of unique Peptides:**	1

BRUKER

Detailed Protein Report

10	20	30	40	50	60	70	80
MSGRQRTLFQ	TWGSSISRSS	GTPGCSSGTE	RPQSPGSSKA	PLPAAAEAQL	ESDDDVLLVA	AYEAERQLCL	ENGGFCTSAG
90	100	110	120	130	140	150	160
ALWIYPTNCP	VRDYQLHISR	AALFCNTLVC	LPTGLGKTFI	AAVVMYNFYR	WFPSGKVVFM	APTKPLVTQQ	IEACYQVMGI
170	180	190	200	210	220	230	240
PQSHMAEMTG	STQASTRKEI	WCSKRVLFLT	PQVMVNDLSR	GACPAAEIKC	LVIDEAHKAL	GNYAYCQVVR	ELVKYTNHFR
250	260	270	280	290	300	310	320
ILALSATPGS	DIKAVQQVIT	NLLIGQIELR	SEDSPDILTY	SHERKVEKLI	VPLGEELAAI	QKTYIQILES	FARSLIQRNV
330	340	350	360	370	380	390	400
LMRRDIPNLT	KYQIILARDQ	FRKNPSPNIV	GIQQGIIEGE	FAICISLYHG	YELLQQMGMR	SLYFFLCGIM	DGTKGMTRSK
410	420	430	440	450	460	470	480
NELGRNEDFM	KLYNHLECMF	ARTRSTSANG	ISAIQQGDKN	KKFVYSHPKL	KKLEEVVIEH	FKSWNAENTT	EKKRDETRVM
490	500	510	520	530	540	550	560
IFSSFRDSVQ	EIAEMLSQHQ	PIIRVMTFVG	HASGKSTKGF	TQKEQLEVVK	QFRDGGYNTL	VSTCVGEEGL	DIGEVDLIIC
570	580	590	600	610	620	630	640
FDSQKSPIRL	VQRMGRTGRK	RQGRIVIILS	EGREERIYNQ	SQSNKRSIYK	AISSNRQVLH	FYQRSPRMVP	DGINPKLHKM
650	660	670	680	690	700	710	720
FITHGVYEPE	KPSRNLQRKS	SIFSYRDGMR	QSSLKKDWFL	SEEEFKLWNR	LYRLRDSDEI	KEITLPQVQF	SSLQNEENKP
730	740	750	760	770	780	790	800
AQESTTGIHQ	LSLSEWRLWQ	DHPLPTHQVD	HSDRCRHFIG	LMQMIEGMRH	EEGECSYELE	VESYLQMEDV	TSTFIAPRNE
810	820	830	840	850	860	870	880
SNNLASDTFI	THKKSSFIKN	INQGSSSSVI	ESDEECAEIV	KQTHIKPTKI	VSLKKKVSKE	IKKDQLKKEN	NHGIIDSVDN
890	900	910	920	930	940	950	960
DRNSTVENIF	QEDLPNDKRT	SDTDEIAATC	TINENVIKEP	CVLLTECQFT	NKSTSSLAGN	VLDSGYNSFN	DEKSVSSNLF
970	980	990	1000	1010	1020	1030	1040
LPFEEELYIV	RTDDQFYNCH	SLTKEVLANV	ERFLSYSPPP	LSGLSDLEYE	IAKGTALENL	LFLPCAEHLR	SDKCTCLLSH
1050	1060	1070	1080	1090	1100	1110	1120
SAVNSQQNLE	LNSLKCINYP	SEKSCLYDIP	NDNISDEPSL	CDCDVHKHNQ	NENLVPNNRV	QIHRSPAQNL	VGENNHDVDN
1130	1140	1150	1160	1170	1180	1190	1200
SDLPVLSTDQ	DESLLLFEDV	NTEFDDVSLS	PLNSKSESLP	VSDKTAISET	PLVSQFLISD	ELLLDNNSEL	QDQITRDANS
1210	1220	1230	1240	1250	1260	1270	1280
FKSRDQRGVQ	EEKVKNHEDI	FDCSRDLFSV	TFDLGFCSPD	SDDEILEHTS	DSNRPLDDLY	GRYLEIKEIS	DANYVSNQAL
1290	1300	1310	1320	1330	1340	1350	1360
IPRDHSKNFT	SGTVIIPSNE	DMQNPNYVHL	PLSAAKNEEL	LSPGYSQFSL	PVQKKVMSTP	LSKSNTLNSF	SKIRKEILKT
1370	1380	1390	1400	1410	1420	1430	1440
PDSSKEKVNL	QRFKEALNST	FDYSEFSLEK	SKSSGPMYLH	KSCHSVEDGQ	LLTSNESEDD	EIFRRKVKRA	KGNVLNSPED
1450	1460	1470	1480	1490	1500	1510	1520
QKNSEVDSPL	HAVKKRRFPI	NRSELSSSDE	SENFPKPCSQ	LEDFKVCNGN	ARRGIKVPKR	QSHLKHVARK	FLDDEAELSE
1530	1540	1550	1560	1570	1580	1590	1600
EDAEYVSSDE	NDESENEQDS	SLLDFLNDET	QLSQAINDSE	MRAIYMKSLR	SPMMNNKYKM	IHKTHKNINI	FSQIPEQDET
1610	1620	1630	1640	1650	1660	1670	1680
YLEDSFCVDE	EESCKGQSSE	EEVCVDFNLI	TDDCFANSKK	YKTRRAVMLK	EMMEQNCAHS	KKKLSRIILP	DDSSEEENNV
1690	1700	1710	1720	1730	1740	1750	1760
NDKRESNIAV	NPSTVKKNKQ	QDHCLNSVPS	GSSAQSKVRS	TPRVNPLAKQ	SKQTSLNLKD	TISEVSDFKP	QNHNEVQSTT
1770	1780	1790	1800	1810	1820	1830	1840
PPFTTVDSQK	DCRKFPVPQK	DGSALEDSST	SGASCSKSRP	HLAGTHTSLR	LPQEGKGTCI	LVGGHEITSG	LEVISSLRAI
1850	1860	1870	1880	1890	1900	1910	1920
HGLQVEVCPL	NGCDYIVSNR	MVVERRSQSE	MLNSVNKNKF	IEQIQHLQSM	FERICVIVEK	DREKTGDTSR	MFRRTKSYDS
1930	1940	1950	1960	1970	1980	1990	2000
LLTTLIGAGI	RILFSSCQEE	TADLLKELSL	VEQRKNVGIH	VPTVVNSNKS	EALQFYLSIP	NISYITALNM	CHQFSSVKRM
2010	2020	2030	2040	2050			
ANSSLQEISM	YAQVTHQKAE	EIYRYIHYVF	DIQMLPNDLN	QDRLKSDI			

Cmpd.	No. of Cmpds.	m/z meas.	Δ m/z [ppm]	z	Rt [min]	Score	P	Range	Sequence	Modification

Detailed Protein Report

Cmpd.	No. of Cmpds.	m/z meas.	Δ m/z [ppm]	z	Rt [min]	Score	P	Range	Sequence	Modification
2013	1	687.7835	-136.48	2	55.3	11.0	2	1356-1367	K.EILKTPDSSKEK.V	

Detailed Protein Report

Protein 200: cystatin-S precursor [Homo sapiens]

**Accession:**	gi|4503109	**Score:**	41.3
**Database:**	refseq_human(refseq_human_20140103.fasta)	**MW [kDa]:**	16.2
**Database Date:**	2015-11-30	**pI:**	4.8
		**Sequence Coverage [%]:**	7.8
		**No. of unique Peptides:**	1

10	20	30	40	50	60	70	80
MARPLCTLLL	LMATLAGALA	SSSKEENRII	PGGIYDADLN	DEWVQRALHF	AISEYNKATE	DEYYRRPLQV	LRAREQTFGG
**90**	**100**	**110**	**120**	**130**	**140**	**150**	
VNYFFDVEVG	RTICTKSQPN	LDTCAFHEQP	ELQKKQLCSF	EIYEVPWEDR	MSLVNSRCQE	A	

Cmpd.	No. of Cmpds.	m/z meas.	Δ m/z [ppm]	z	Rt [min]	Score	P	Range	Sequence	Modification
887	1	646.8226	-19.68	2	39.6	41.3	0	47-57	R.ALHFAISEYNK.A	

Detailed Protein Report

Protein 201: **PREDICTED: ATPase family AAA domain-containing protein 2B isoform X1 [Homo sapiens]**

**Accession:**	gi|578802946	**Score:**	41.2
**Database:**	refseq_human(refseq_human_20140103.fasta)	**MW [kDa]:**	166.4
**Database Date:**	2015-11-30	**pI:**	6.5
**Modification(s):**	Carbamidomethyl	**Sequence Coverage [%]:**	2.4
		**No. of unique Peptides:**	1

Quantitation

mdown:qdown	**Median:**	0.92	**CV:**	0.00 %	**No. of Peptides:**	1
Wdown:Qdown	**Median:**	4.19	**CV:**	0.00 %	**No. of Peptides:**	1

10	20	30	40	50	60	70	80
MVNTRKSSLR	LLGSKSPGPG	PGPGAGAEPG	ATGGSSHFIS	SRTRSSKTRA	ASCPAAKAGG	SGGAGVTLDE	ARKVEVDGSL
90	100	110	120	130	140	150	160
SDSHVSPPAK	RTLKQPDSVC	KDKSKSRSTG	QREEWNLSTG	QARLTSQPGA	TLPNGHSGLS	LRSHPLRGEK	KGDGDLSCIN
170	180	190	200	210	220	230	240
GDMEVRKSCR	SRKNRFESVN	QSLLFDQLVN	STAEAVLQEM	DNINIRRNRR	SGEVERLRMW	TDTEFENMDM	YSRVKRRRKS
250	260	270	280	290	300	310	320
LRRNSYGIQN	HHEVSTEGEE	EARTSKLLPL	EKISIESQEE	DGDIEVEEAE	GEENDRPYNL	RQRKTVDRYQ	APPIVPAHQK
330	340	350	360	370	380	390	400
KRENTLFDIH	RSPARRSHIR	RKKHAIHSSD	TTSSDEERFE	RRKSKSMARA	RNRCLPMNFR	AEDLASGILR	ERVKVGASLA
410	420	430	440	450	460	470	480
DVDPMNIDKS	VRFDSIGGLS	HHIHALKEMV	VFPLLYPEIF	EKFKIQPPRG	CLFYGPPGTG	KTLVARALAN	ECSQGDKKVA
490	500	510	520	530	540	550	560
FFMRKGADCL	SKWVGESERQ	LRLLFDQAYL	MRPSIIFFDE	IDGLAPVRSS	RQDQIHSSIV	STLLALMDGL	DNRGEIVVIG
570	580	590	600	610	620	630	640
ATNRLDSIDP	ALRRPGRFDR	EFLFNLPDQK	ARKHILQIHT	RDWNPKLSDA	FLGELAEKCV	GYCGADIKAL	CTEAALIALR
650	660	670	680	690	700	710	720
RRYPQIYASS	HKLQLDVSSI	VLSAQDFYHA	MQNIVPASQR	AVMSSGHALS	PIIRPLLERS	FNNILAVLQK	VFPHAEISQS
730	740	750	760	770	780	790	800
DKKEDIETLI	LEDSEDENAL	SIFETNCHSG	SPKKQSSSAA	IHKPYLHFTM	SPYHQPTSYR	PRLLLSGERG	SGQTSHLAPA
810	820	830	840	850	860	870	880
LLHTLERFSV	HRLDLPALYS	VSAKTPEESC	AQIFREARRT	VPSIVYMPHI	GDWWEAVSET	VRATFLTLLQ	DIPSFSPIFL
890	900	910	920	930	940	950	960
LSTSETMYSE	LPEEVKCIFR	IQYEEVLYIQ	RPIEEDRRKF	FQELILNQAS	MAPPRRKHAA	LCAMEVLPLA	LPSPPRQLSE
970	980	990	1000	1010	1020	1030	1040
SEKSRMEDQE	ENTLRELRLF	LRDVTKRLAT	DKRFNIFSKP	VDIEEVSDYL	EVIKEPMDLS	TVITKIDKHN	YLTAKDFLKD
1050	1060	1070	1080	1090	1100	1110	1120
IDLICSNALE	YNPDKDPGDK	IIRHRACTLK	DTAHAIIAAE	LDPEFNKLCE	EIKEARIKRG	LSVTSEQINP	HSTGARKTET
1130	1140	1150	1160	1170	1180	1190	1200
RVEEAFRHKQ	RNPMDVWHNS	ANKCAFRVRR	KSRRRSQWGK	GIIKKRKVNN	LKKDEEDTKF	ADYENHTEDR	KLLENGEFEV
1210	1220	1230	1240	1250	1260	1270	1280
STDCHEENGE	ETGDLSMTND	ESSCDIMDLD	QGQRLNNGAG	TKENFASTEE	ESSNESLLVN	SSSSLNPEQT	SRKETFLKGN
1290	1300	1310	1320	1330	1340	1350	1360
CLNGEASTDS	FEGIPVLECQ	NGKLEVVSFC	DSGDKCSSEQ	KILLEDQSKE	KPETSTENHG	DDLEKLEALE	CSNNEKLEPG
1370	1380	1390	1400	1410	1420	1430	1440
SDVEVKDAEL	DKEGASKVKK	YRKLILEQAK	TTSLELVPEE	PSEPVPPLIV	DRERLKKLLD	LLVDKSNNLA	VDQLERLYSL
1450	1460	1470	1480				
LSQCIYRHRK	DYDKSQLVEE	MERTVHMFET	FL				

Cmpd.	No. of Cmpds.	m/z meas.	Δ m/z [ppm]	z	Rt [min]	Score	P	Range	Sequence	Modification	Ratios
987	1	574.1653	-221.32	2	40.8	18.3	1	1088-1096	K.LCEEIKEAR.I	Carbamidomethyl: 2	mdown:qdown 0.92<br>Wdown:Qdown 4.19

Detailed Protein Report

Protein 202: PREDICTED: thyroglobulin isoform X10 [Homo sapiens]

**Accession:**	gi|578816124	**Score:**	41.2
**Database:**	refseq_human(refseq_human_20140103.fasta)	**MW [kDa]:**	302.4
**Database Date:**	2015-11-30	**pI:**	5.3
**Modification(s):**	Carbamidomethyl	**Sequence Coverage [%]:**	1.5
		**No. of unique Peptides:**	2

Quantitation

mdown:qdown	**Median:**	0.75	**CV:**	0.00 %	**No. of Peptides:**	1
Wdown:Qdown	**Median:**	0.79	**CV:**	118.24 %	**No. of Peptides:**	2

Detailed Protein Report

10	20	30	40	50	60	70	80
MALVLEIFTL	LASICWVSAN	IFEYQVDAQP	LRPCELQRET	AFLKQADYVP	QCAEDGSFQT	VQCQNDGRSC	WCVGANGSEV
90	100	110	120	130	140	150	160
LGSRQPGRPV	ACLSFCQLQK	QQILLSGYIN	STDTSYLPQC	QDSGDYAPVQ	CDVQQVQCWC	VDAEGMEVYG	TRQLGRPKRC
170	180	190	200	210	220	230	240
PRSCEIRNRR	LLHGVGDKSP	PQCSAEGEFM	PVQCKFVNTT	DMMIFDLVHS	YNRFPDAFVT	FSSFQRRFPE	VSGYCHCADS
250	260	270	280	290	300	310	320
QGRELAETGL	ELLLDEIYDT	IFAGLDLPST	FTETTLYRIL	QRRFLAVQSV	ISGRFRCPTK	CEVERFTATS	FGHPYVPSCR
330	340	350	360	370	380	390	400
RNGDYQAVQC	QTEGPCWCVD	AQGKEMHGTR	QQGEPPSCAE	GQSCASERQQ	ALSRLYFGTS	GYFSQHDLFS	SPEKRWASPR
410	420	430	440	450	460	470	480
VARFATSCPP	TIKELFVDSG	LLRPMVEGQS	QQFSVSENLL	KEAIRAIFPS	RGLARLALQF	TTNPKRLQQN	LFGGKFLVNV
490	500	510	520	530	540	550	560
GQFNLSGALG	TRGTFNFSQF	FQQLGLASFL	NGGRQEDLAK	PLSVGLDSNS	STGTPEAAKK	DGTMNKPTVG	SFGFEINLQE
570	580	590	600	610	620	630	640
NQNALKFLAS	LLELPEFLLF	LQHAISVPED	VARDLGDVME	TVLSSQTCEQ	TPERLFVPSC	TTEGSYEDVQ	CFSGECWCVN
650	660	670	680	690	700	710	720
SWGKELPGSR	VRGGQPRCPT	DCEKQRARMQ	SLMGSQPAGS	TLFVPACTSE	GHFLPVQCFN	SECYCVDAEG	QAIPGTRSAI
730	740	750	760	770	780	790	800
GKPKKCPTPC	QLQSEQAFLR	TVQALLSNSS	MLPTLSDTYI	PQCSTDGQWR	QVQCNGPPEQ	VFELYQRWEA	QNKGQDLTPA
810	820	830	840	850	860	870	880
KLLVKIMSYR	EAASGNFSLF	IQSLYEAGQQ	DVFPVLSQYP	SLQDVPLAAL	EGKRPQPREN	ILLEPYLFWQ	ILNGQLSQYP
890	900	910	920	930	940	950	960
GSYSDFSTPL	AHFDLRNCWC	VDEAGQELEG	MRSEPSKLPT	CPGSCEEAKL	RVLQFIRETE	EIVSASNSSR	FPLGESFLVA
970	980	990	1000	1010	1020	1030	1040
KGIRLRNEDL	GLPPLFPPRE	AFAEQFLRGS	DYAIRLAAQS	TLSFYQRRRF	SPDDSAGASA	LLRSGPYMPQ	CDAFGSWEPV
1050	1060	1070	1080	1090	1100	1110	1120
QCHAGTGHCW	CVDEKGGFIP	GSLTARSLQI	PQCPTTCEKS	RTSGLLSSWK	QARSQENPSP	KDLFVPACLE	TGEYARLQAS
1130	1140	1150	1160	1170	1180	1190	1200
GAGTWCVDPA	SGEELRPGSS	SSAQCPSLCN	VLKSGVLSRR	VSPGYVPACR	AEDGGFSPVQ	CDQAQGSCWC	VMDSGEEVPG
1210	1220	1230	1240	1250	1260	1270	1280
TRVTGGQPAC	ESPRCPLPFN	ASEVVGGTIL	CETISGPTGS	AMQQCQLLCR	QGSWSVFPPG	PLICSLESGR	WESQLPQPRA
1290	1300	1310	1320	1330	1340	1350	1360
CQRPQLWQTI	QTQGHFQLQL	PPGKMCSADY	ADLLQTFQVF	ILDELTARGF	CQIQVKTFGT	LVSIPVCNNS	SVQVGCLTRE
1370	1380	1390	1400	1410	1420	1430	1440
RLGVNVTWKS	RLEDIPVASL	PDLHDIERAL	VGKDLLGRFT	DLIQSGSFQL	HLDSKTFPAE	TIRFLQGDHF	GTSPRTWFGC
1450	1460	1470	1480	1490	1500	1510	1520
SEGFYQVLTS	EASQDGLGCV	KCPEGSYSQD	EECIPCPVGF	YQEQAGSLAC	VPCPVGRTTI	SAGAFSQTHC	VTDCQRNEAG
1530	1540	1550	1560	1570	1580	1590	1600
LQCDQNGQYR	ASQKDRGSGK	AFCVDGEGRR	LPWWETEAPL	EDSQCLMMQK	FEKVPESKVI	FDANAPVAVR	SKVPDSEFPV
1610	1620	1630	1640	1650	1660	1670	1680
MQCLTDCTED	EACSFFTVST	TEPEISCDFY	AWTSDNVACM	TSDQKRDALG	NSKATSFGSL	RCQVKVRSHG	QDSPAVYLKK
1690	1700	1710	1720	1730	1740	1750	1760
GQGSTTTLQK	RFEPTGFQNM	LSGLYNPIVF	SASGANLTDA	HLFCLLACDR	DLCCDGFVLT	QVQGGAIICG	LLSSPSVLLC
1770	1780	1790	1800	1810	1820	1830	1840
NVKDWMDPSE	AWANATCPGV	TYDQESHQVI	LRLGDQEFIK	SLTPLEGTQD	TFTNFQQVYL	WKDSDMGSRP	ESMGCRKDTV
1850	1860	1870	1880	1890	1900	1910	1920
PRPASPTEAG	LTTELFSPVD	LNQVIVNGNQ	SLSSQKHWLF	KHLFSAQQAN	LWCLSRCVQE	HSFCQLAEIT	ESASLYFTCT
1930	1940	1950	1960	1970	1980	1990	2000
LYPEAQVCDD	IMESNAQGCR	LILPQMPKAL	FRKKVILEDK	VKNFYTRLPF	QKLMGISIRN	KVPMSEKSIS	NGFFECERRC
2010	2020	2030	2040	2050	2060	2070	2080
DADPCCTGFG	FLNVSQLKGG	EVTCLTLNSL	GIQMCSEENG	GAWRILDCGS	PDIEVHTYPF	GWYQKPMSLD	SWQSLALSSV
2090	2100	2110	2120	2130	2140	2150	2160
VVDPSIRHFD	VAHVSTAATS	NFSAVRDLCL	SECSQHEACL	ITTLQTQPGA	VRCMFYADTQ	SCTHSLQGQN	CRLLLREEAT
2170	2180	2190	2200	2210	2220	2230	2240

Detailed Protein Report

Cmpd.	No. of Cmpds.	m/z meas.	Δ m/z [ppm]	z	Rt [min]	Score	P	Range	Sequence	Modification	Ratios
865	1	623.9049	-34.22	3	40.3	10.8	0	228-243	R.FPEVSGYCHCADSQGR.E	Carbamidomethyl: 8, 10	Wdown:Qdown 2.01<br>mdown:qdown 0.75
1861	1	719.7564	-125.05	2	51.8	13.2	2	1537-1550	R.GSGKAFCVDGEGRR.L		Wdown:Qdown 0.31

Detailed Protein Report

Protein 203: **structural maintenance of chromosomes protein 4 isoform 2 [Homo sapiens]**

**Accession:**	gi|570700827	**Score:**	41.0
**Database:**	refseq_human(refseq_human_20140103.fasta)	**MW [kDa]:**	144.4
**Database Date:**	2015-11-30	**pI:**	6.7
		**Sequence Coverage [%]:**	2.3
		**No. of unique Peptides:**	1

10	20	30	40	50	60	70	80
MPRKGTQPST	ARRREEGPPP	PSPDGASSDA	EPEPPSGRTE	SPATAAAMTN	EAGAPRLMIT	HIVNQNFKSY	AGEKILGPFH
90	100	110	120	130	140	150	160
KRFSCIIGPN	GSGKSNVIDS	MLFVFGYRAQ	KIRSKKLSVL	IHNSDEHKDI	QSCTVEVHFQ	KIIDKEGDDY	EVIPNSNFYV
170	180	190	200	210	220	230	240
SRTACRDNTS	VYHISGKKKT	FKDVGNLLRS	HGIDLDHNRF	LILQGEVEQI	AMMKPKGQTE	HDEGMLEYLE	DIIGCGRLNE
250	260	270	280	290	300	310	320
PIKVLCRRVE	ILNEHRGEKL	NRVKMVEKEK	DALEGEKNIA	IEFLTLENEI	FRKKNHVCQY	YIYELQKRIA	EMETQKEKIH
330	340	350	360	370	380	390	400
EDTKEINEKS	NILSNEMKAK	NKDVKDTEKK	LNKITKFIEE	NKEKFTQLDL	EDVQVREKLK	HATSKAKKLE	KQLQKDKEKV
410	420	430	440	450	460	470	480
EEFKSIPAKS	NNIINETTTR	NNALEKEKEK	EEKKLKEVMD	SLKQETQGLQ	KEKESREKEL	MGFSKSVNEA	RSKMDVAQSE
490	500	510	520	530	540	550	560
LDIYLSRHNT	AVSQLTKAKE	ALIAASETLK	ERKAAIRDIE	GKLPQTEQEL	KEKEKELQKL	TQEETNFKSL	VHDLFQKVEE
570	580	590	600	610	620	630	640
AKSSLAMNRS	RGKVLDAIIQ	EKKSGRIPGI	YGRLGDLGAI	DEKYDVAISS	CCHALDYIVV	DSIDIAQECV	NFLKRQNIGV
650	660	670	680	690	700	710	720
ATFIGLDKMA	VWAKKMTEIQ	TPENTPRLFD	LVKVKDEKIR	QAFYFALRDT	LVADNLDQAT	RVAYQKDRRW	RVVTLQGQII
730	740	750	760	770	780	790	800
EQSGTMTGGG	SKVMKGRMGS	SLVIEISEEE	VNKMESQLQN	DSKKAMQIQE	QKVQLEERVV	KLRHSEREMR	NTLEKFTASI
810	820	830	840	850	860	870	880
QRLIEQEEYL	NVQVKELEAN	VLATAPDKKK	QKLLEENVSA	FKTEYDAVAE	KAGKVEAEVK	RLHNTIVEIN	NHKLKAQQDK
890	900	910	920	930	940	950	960
LDKINKQLDE	CASAITKAQV	AIKTADRNLQ	KAQDSVLRTE	KEIKDTEKEV	DDLTAELKSL	EDKAAEVVKN	TNAAEESLPE
970	980	990	1000	1010	1020	1030	1040
IQKEHRNLLQ	ELKVIQENEH	ALQKDALSIK	LKLEQIDGHI	AEHNSKIKYW	HKEISKISLH	PIEDNPIEEI	SVLSPEDLEA
1050	1060	1070	1080	1090	1100	1110	1120
IKNPDSITNQ	IALLEARCHE	MKPNLGAIAE	YKKKEELYLQ	RVAELDKITY	ERDSFRQAYE	DLRKQRLNEF	MAGFYIITNK
1130	1140	1150	1160	1170	1180	1190	1200
LKENYQMLTL	GGDAELELVD	SLDPFSEGIM	FSVRPPKKSW	KKIFNLSGGE	KTLSSLALVF	ALHHYKPTPL	YFMDEIDAAL
1210	1220	1230	1240	1250	1260	1270	
DFKNVSIVAF	YIYEQTKNAQ	FIIISLRNNM	FEISDRLIGI	YKTYNITKSV	AVNPKEIASK	GLC	

Cmpd.	No. of Cmpds.	m/z meas.	Δ m/z [ppm]	z	Rt [min]	Score	P	Range	Sequence	Modification
452	5	862.8266	-126.10	2	34.3	30.1	2	457-471	R.EKELMGFSKSVNEAR.S	

Detailed Protein Report

Protein 204: small proline-rich protein 2E [Homo sapiens]

Accession: gi|83582817
Database: refseq_human(refseq_human_20140103.fasta)
Database Date: 2015-11-30
Modification(s): Carbamidomethyl

Score: 41.0
MW [kDa]: 7.8
pI: 10.6
Sequence Coverage [%]: 18.1
No. of unique Peptides: 1

Quantitation

mdown:qdown	**Median:**	1.02	**CV:**	0.00 %	**No. of Peptides:**	1
Wdown:Qdown	**Median:**	1.76	**CV:**	0.00 %	**No. of Peptides:**	1

```
        10         20         30         40         50         60         70         80
MSYQQQQCKQ PCQPPPVCPT PKCPEPCPPP KCPEPCPPPK CPQPCPPQQC QQKCPPVTPS PPCQPKCPPK SK
```

Cmpd.	No. of Cmpds.	m/z meas.	Δ m/z [ppm]	z	Rt [min]	Score	P	Range	Sequence	Modification	Ratios
85	3	753.3519	-18.26	2	30.5	41.0	0	10-22	K.QPCQPPPVCPTPK.C	Carbamidomethyl: 3, 9	Wdown:Qdown 1.76<br>mdown:qdown 1.02

Detailed Protein Report

Protein 205: **prosaposin isoform a preproprotein [Homo sapiens]**

**Accession:**	gi|11386147	**Score:**	40.9
**Database:**	refseq_human(refseq_human_20140103.fasta)	**MW [kDa]:**	58.1
**Database Date:**	2015-11-30	**pI:**	4.9
**Modification(s):**	Carbamidomethyl	**Sequence Coverage [%]:**	2.9
		**No. of unique Peptides:**	2

10	20	30	40	50	60	70	80
MYALFLLASL	LGAALAGPVL	GLKECTRGSA	VWCQNVKTAS	DCGAVKHCLQ	TVWNKPTVKS	LPCDICKDVV	TAAGDMLKDN
**90**	**100**	**110**	**120**	**130**	**140**	**150**	**160**
ATEEEILVYL	EKTCDWLPKP	NMSASCKEIV	DSYLPVILDI	IKGEMSRPGE	VCSALNLCES	LQKHLAELNH	QKQLESNKIP
**170**	**180**	**190**	**200**	**210**	**220**	**230**	**240**
ELDMTEVVAP	FMANIPLLLY	PQDGPRSKPQ	PKDNGDVCQD	CIQMVTDIQT	AVRTNSTFVQ	ALVEHVKEEC	DRLGPGMADI
**250**	**260**	**270**	**280**	**290**	**300**	**310**	**320**
CKNYISQYSE	IAIQMMMHMQ	PKEICALVGF	CDEVKEMPMQ	TLVPAKVASK	NVIPALELVE	PIKKHEVPAK	SDVYCEVCEF
**330**	**340**	**350**	**360**	**370**	**380**	**390**	**400**
LVKEVTKLID	NNKTEKEILD	AFDKMCSKLP	KSLSEECQEV	VDTYGSSILS	ILLEEVSPEL	VCSMLHLCSG	TRLPALTVHV
**410**	**420**	**430**	**440**	**450**	**460**	**470**	**480**
TQPKDGGFCE	VCKKLVGYLD	RNLEKNSTKQ	EILAALEKGC	SFLPDPYQKQ	CDQFVAEYEP	VLIEILVEVM	DPSFVCLKIG
**490**	**500**	**510**	**520**	**530**			
ACPSAHKPLL	GTEKCIWGPS	YWCQNTETAA	QCNAVEHCKR	HVWN			

Cmpd.	No. of Cmpds.	m/z meas.	Δ m/z [ppm]	z	Rt [min]	Score	P	Range	Sequence	Modification
2867	11	869.4159	28.09	2	64.6	19.3	0	93-107	K.TCDWLPKPNMSASCK.E	Carbamidomethyl: 2
1784	4	840.7706	-131.02	2	52.3	21.6	0	93-107	K.TCDWLPKPNMSASCK.E	

Detailed Protein Report

Protein 206: PREDICTED: hormone-sensitive lipase isoform X1 [Homo sapiens]

**Accession:**	gi|530416587	**Score:**	40.8
**Database:**	refseq_human(refseq_human_20140103.fasta)	**MW [kDa]:**	108.8
**Database Date:**	2015-11-30	**pI:**	6.2
		**Sequence Coverage [%]:**	3.6
		**No. of unique Peptides:**	2

10	20	30	40	50	60	70	80
MEPGSKSVSR	SDWQPEPHQR	PITPLEPGPE	KTPIAQPESK	TLQGSNTQQK	PASNQRPLTQ	QETPAQHDAE	SQKEPRAQQK
90	100	110	120	130	140	150	160
SASQEEFLAP	QKPAPQQSPY	IQRVLLTQQE	AASQQGPGLG	KESITQQEPA	LRQRHVAQPG	PGPGEPPPAQ	QEAESTPAAQ
170	180	190	200	210	220	230	240
AKPGAKREPS	APTESTSQET	PEQSDKQTTP	VQGAKSKQGS	LTELGFLTKL	QELSIQRSAL	EWKALSEWVT	DSESESDVGS
250	260	270	280	290	300	310	320
SSDTDSPATM	GGMVAQGVKL	GFKGKSGYKV	MSGYSGTSPH	EKTSARNHRH	YQDTASRLIH	NMDLRTMTQS	LVTLAEDNIA
330	340	350	360	370	380	390	400
FFSSQGPGET	AQRLSGVFAG	VREQALGLEP	ALGRLLGVAH	LFDLDPETPA	NGYRSLVHTA	RCCLAHLLHK	SRYVASNRRS
410	420	430	440	450	460	470	480
IFFRTSHNLA	ELEAYLAALT	QLRALVYYAQ	RLLVTNRPGV	LFFEGDEGLT	ADFLREYVTL	HKGCFYGRCL	GFQFTPAIRP
490	500	510	520	530	540	550	560
FLQTISIGLV	SFGEHYKRNE	TGLSVAASSL	FTSGRFAIDP	ELRGAEFERI	TQNLDVHFWK	AFWNITEMEV	LSSLANMASA
570	580	590	600	610	620	630	640
TVRVSRLLSL	PPEAFEMPLT	ADPTLTVTIS	PPLAHTGPGP	VLVRLISYDL	REGQDSEELS	SLIKSNGQRS	LELWPRPQQA
650	660	670	680	690	700	710	720
PRSRSLIVHF	HGGGFVAQTS	RSHEPYLKSW	AQELGAPIIS	IDYSLAPEAP	FPRALEECFF	AYCWAIKHCA	LLGAKTEDHS
730	740	750	760	770	780	790	800
NSDQKALGMM	GLVRRDTALL	LRDFRLGASS	WLNSFLELSG	RKSQKMSEPI	AEPMRRSVSE	AALAQPQGPL	GTDSLKNLTL
810	820	830	840	850	860	870	880
RDLSLRGNSE	TSSDTPEMSL	SAETLSPSTP	SDVNFLLPPE	DAGEEAEAKN	ELSPMDRGLG	VRAAFPEGFH	PRRSSQGATQ
890	900	910	920	930	940	950	960
MPLYSSPIVK	NPFMSPLLAP	DSMLKSLPPV	HIVACALDPM	LDDSVMLARR	LRNLGQPVTL	RVVEDLPHGF	LTLAALCRET
970	980	990	1000	1010			
RQAAELCVER	IRLVLTPPAG	AGPSGETGAA	GVDGGCGGRH				

Cmpd.	No. of Cmpds.	m/z meas.	Δ m/z [ppm]	z	Rt [min]	Score	P	Range	Sequence	Modification
2789	2	1023.0413	-12.46	2	63.3	21.9	2	375-392	R.SLVHTARCCLAHLLHKSR.Y	
1528	1	977.3724	-87.41	2	48.9	18.9	1	708-725	K.HCALLGAKTEDHSNSDQK.A	

Detailed Protein Report

Protein 207: **1-phosphatidylinositol 4,5-bisphosphate phosphodiesterase epsilon-1 isoform 2 [Homo sapiens]**

**Accession:**	gi|260166694	**Score:**	40.8
**Database:**	refseq_human(refseq_human_20140103.fasta)	**MW [kDa]:**	223.7
**Database Date:**	2015-11-30	**pI:**	6.2
**Modification(s):**	Carbamidomethyl, Oxidation	**Sequence Coverage [%]:**	1.7
		**No. of unique Peptides:**	2

Quantitation

mdown:qdown	**Median:**	0.51	**CV:**	0.00 %	**No. of Peptides:**	1

Detailed Protein Report

10	20	30	40	50	60	70	80
MVSEGSAAGR	DFAGMEEVRQ	LHVRFCKGIK	IWHQAWFLCS	LLGREPQERE	AGCQLWLCTL	SAVLKVGWLF	PLSEVPNFTL
90	100	110	120	130	140	150	160
LKDGCGCWRL	KEDQIYNAVR	REETENTVGS	LLHFLTKLPA	SETAHGRISV	GPCLKQCVRD	TVCEYRATLQ	RTSISQYITG
170	180	190	200	210	220	230	240
SLLEATTSLG	ARSGLLSTFG	GSTGRMMLKE	RQPGPSVANS	NALPSSSAGI	SKELIDLQPL	IQFPEEVASI	LMEQEQTIYR
250	260	270	280	290	300	310	320
RVLPVDYLCF	LTRDLGTPEC	QSSLPCLKAS	ISASILTTQN	GEHNALEDLV	MRFNEVSSWV	TWLILTAGSM	EEKREVFSYL
330	340	350	360	370	380	390	400
VHVAKCCWNM	GNYNAVMEFL	AGLRSRKVLK	MWQFMDQSDI	ETMRSLKDAM	AQHESSCEYR	KVVTRALHIP	GCKVVPFCGV
410	420	430	440	450	460	470	480
FLKELCEVLD	GASGLMKLCP	RYNSQEETLE	FVADYSGQDN	FLQRVGQNGL	KNSEKESTVN	SIFQVIRSCN	RSLETDEEDS
490	500	510	520	530	540	550	560
PSEGNSSRKS	SLKDKSRWQF	IIGDLLDSDN	DIFEQSKEYD	SHGSEDSQKA	FDHGTELIPW	YVLSIQADVH	QFLLQGATVI
570	580	590	600	610	620	630	640
HYDQDTHLSA	RCFLQLQPDN	STLTWVKPTT	ASPASSKAKL	GVLNNTAEPG	KFPLLGNAGL	SSLTEGVLDL	FAVKAVYMGH
650	660	670	680	690	700	710	720
PGIDIHTVCV	QNKLGSMFLS	ETGVTLLYGL	QTTDNRLLHF	VAPKHTAKML	FSGLLELTRA	VRKMRKFPDQ	RQQWLRKQYV
730	740	750	760	770	780	790	800
SLYQEDGRYE	GPTLAHAVEL	FGGRRWSARN	PSPGTSAKNA	EKPNMQRNNT	LGISTTKKKK	KILMRGESGE	VTDDEMATRK
810	820	830	840	850	860	870	880
AKMHKECRSR	SGSDPQDINE	QEESEVNAIA	NPPNPLPSRR	AHSLTTAGSP	NLAAGTSSPI	RPVSSPVLSS	SNKSPSSAWS
890	900	910	920	930	940	950	960
SSSWHGRIKG	GMKGFQSFMV	SDSNMSFVEF	VELFKSFSVR	SRKDLKDLFD	VYAVPCNRSG	SESAPLYTNL	TIDENTSDLQ
970	980	990	1000	1010	1020	1030	1040
PDLDLLTRNV	SDLGLFIKSK	QQLSDNQRQI	SDAIAAASIV	TNGTGIESTS	LGIFGVGILQ	LNDFLVNCQG	EHCTYDEILS
1050	1060	1070	1080	1090	1100	1110	1120
IIQKFEPSIS	MCHQGLMSFE	GFARFLMDKE	NFASKNDESQ	ENIKELQLPL	SYYYIESSHN	TYLTGHQLKG	ESSVELYSQV
1130	1140	1150	1160	1170	1180	1190	1200
LLQGCRSVEL	DCWDGDDGMP	IIYHGHTLTT	KIPFKEVVEA	IDRSAFINSD	LPIIISIENH	CSLPQQRKMA	EIFKTVFGEK
1210	1220	1230	1240	1250	1260	1270	1280
LVTKFLFETD	FSDDPMLPSP	DQLRKKVLLK	NKKLKAHQTP	VDILKQKAHQ	LASMQVQAYN	GGNANPRPAN	NEEEEDEEDE
1290	1300	1310	1320	1330	1340	1350	1360
YDYDYESLSD	DNILEDRPEN	KSCNDKLQFE	YNEEIPKRIK	KADNSACNKG	KVYDMELGEE	FYLDQNKKES	RQIAPELSDL
1370	1380	1390	1400	1410	1420	1430	1440
VIYCQAVKFP	GLSTLNASGS	SRGKERKSRK	SIFGNNPGRM	SPGETASFNK	TSGKSSCEGI	RQTWEESSSP	LNPTTSLSAI
1450	1460	1470	1480	1490	1500	1510	1520
IRTPKCYHIS	SLNENAAKRL	CRRYSQKLTQ	HTACQLLRTY	PAATRIDSSN	PNPLMFWLHG	IQLVALNYQT	DDLPLHLNAA
1530	1540	1550	1560	1570	1580	1590	1600
MFEANGGCGY	VLKPPVLWDK	NCPMYQKFSP	LERDLDSMDP	AVYSLTIVSG	QNVCPSNSMG	SPCIEVDVLG	MPLDSCHFRT
1610	1620	1630	1640	1650	1660	1670	1680
KPIHRNTLNP	MWNEQFLFHV	HFEDLVFLRF	AVVENNSSAV	TAQRIIPLKA	LKRGYRHLQL	RNLHNEVLEI	SSLFINSRRM
1690	1700	1710	1720	1730	1740	1750	1760
EENSSGNTMS	ASSMFNTEER	KCLQTHRVTV	HGVPGPEPFT	VFTINGGTKA	KQLLQQILTN	EQDIKPVTTD	YFLMEEKYFI
1770	1780	1790	1800	1810	1820	1830	1840
SKEKNECRKQ	PFQRAIGPEE	EIMQILSSWF	PEEGYMGRIV	LKTQQENLEE	KNIVQDDKEV	ILSSEEESFF	VQVHDVSPEQ
1850	1860	1870	1880	1890	1900	1910	1920
PRTVIKAPRV	STAQDVIQQT	LCKAKYSYSI	LSNPNPSDYV	LLEEVVKDTT	NKKTTTPKSS	QRVLLDQECV	FQAQSKWKGA
1930	1940	1950	1960	1970	1980	1990	2000
GKFILKLKEQ	VQASREDKKK	GISFASELKK	LTKSTKQPRG	LTSPSQLLTS	ESIQTKEEKP	VGGLSSSDTM	DYRQ

Cmpd.	No. of Cmpds.	m/z meas.	Δ m/z [ppm]	z	Rt [min]	Score	P	Range	Sequence	Modification	Ratios
2773	1	648.3579	4.41	2	63.1	11.0	0	689-699	K.MLFSGLLELTR.A	Oxidation: 1	
547	1	624.6856	-218.06	2	35.5	11.3	2	1319-1329	R.IKKADNSACNK.G	Carbamidomethyl: 9	mdown:qdown 0.51

Detailed Protein Report

Protein 208: cornifin-A [Homo sapiens]

**Accession:**	gi|45827734	**Score:**	40.7
**Database:**	refseq_human(refseq_human_20140103.fasta)	**MW [kDa]:**	9.9
**Database Date:**	2015-11-30	**pI:**	10.3
**Modification(s):**	Carbamidomethyl	**Sequence Coverage [%]:**	15.7
		**No. of unique Peptides:**	1

Quantitation

mdown:qdown	**Median:**	0.90	**CV:**	0.00 %	**No. of Peptides:**	1
Wdown:Qdown	**Median:**	2.67	**CV:**	0.00 %	**No. of Peptides:**	1

Alias proteins:

Accession	Name	Description
gi|315360635	refseq_human (refseq_human_20140103.fasta)	cornifin-A [Homo sapiens]

```
        10         20         30         40         50         60         70         80
MNSQQQKQPC TPPPQPQQQQ VKQPCQPPPQ EPCIPKTKEP CHPKVPEPCH PKVPEPCQPK VPEPCQPKVP EPCPSTVTPA
        90
PAQQKTKQK
```

Cmpd.	No. of Cmpds.	m/z meas.	Δ m/z [ppm]	z	Rt [min]	Score	P	Range	Sequence	Modification	Ratios
243	2	838.3874	-15.29	2	31.8	40.7	0	23-36	K.QPCQPPPQEPCIPK.T	Carbamidomethyl: 3, 11	Wdown:Qdown 2.67<br>mdown:qdown 0.90

Detailed Protein Report

Protein 209: PREDICTED: cytosolic carboxypeptidase 2 isoform X4 [Homo sapiens]

**Accession:**	gi|530395811	**Score:**	40.6
**Database:**	refseq_human(refseq_human_20140103.fasta)	**MW [kDa]:**	90.6
**Database Date:**	2015-11-30	**pI:**	9.4
**Modification(s):**	Oxidation	**Sequence Coverage [%]:**	5.6
		**No. of unique Peptides:**	2

10	20	30	40	50	60	70	80
MFPALETHLK	QTIPDPYEDF	MYRHLQYYGY	FKAQRGSLPN	SATHQHVRKN	NPQCLLNGSL	GEKDDLIPDT	LQKEKLLWPI
**90**	**100**	**110**	**120**	**130**	**140**	**150**	**160**
SLSSAVHRQI	EAINRDSHML	SLPHLRSRQL	LYDELDEVNP	RLREPQELFS	ILSTKRPLQA	PRWPIECEVI	KENIHHIEWA
**170**	**180**	**190**	**200**	**210**	**220**	**230**	**240**
PPQPEYFYQP	KGNEKVPEIV	GEKKGTVVYQ	LDSVPIEGSY	FTSSRVGGKR	GIVKELAVTL	QGPEDNTLLF	ESRFESGNLQ
**250**	**260**	**270**	**280**	**290**	**300**	**310**	**320**
KAVRVDTYEY	ELTLRTDLYT	NKHTQWFYFR	VQNTRKDATY	RFTIVNLLKP	KSLYTVGMKP	LLYSQLDANT	RNIGWRREGN
**330**	**340**	**350**	**360**	**370**	**380**	**390**	**400**
EIKYYKNNTD	DGQQPFYCLT	WTIQFPYDQD	TCFFAHFYPY	TYTDLQCYLL	SVANNPIQSQ	FCKLQTLCRS	LAGNTVYLLT
**410**	**420**	**430**	**440**	**450**	**460**	**470**	**480**
ITNPSQTPQE	AAAKKAVVLS	ARVHPGESNG	SWVMKGFLDF	ILSNSPDAQL	LRDIFVFKVL	PMLNPDGVIV	GNYRCSLAGR
**490**	**500**	**510**	**520**	**530**	**540**	**550**	**560**
DLNRHYKTIL	KESFPCIWYT	RNMIKRLLEE	REVLLYCDFH	GHSRKNNIFL	YGCNNNNRKY	WLHERVFPLM	LCKNAPDKFS
**570**	**580**	**590**	**600**	**610**	**620**	**630**	**640**
FHSCNFKVQK	CKEGTGRVVM	WRMGILNSYT	MESTFGGSTL	GNKRDTHFTI	EDLKSLGYHV	CDTLLDFCDP	DQMKFTQCLA
**650**	**660**	**670**	**680**	**690**	**700**	**710**	**720**
ELKELLRQEI	HKKFHELGQD	VDLEGSWSDI	SLSDIESSTS	GSDSSLSDGL	PVHLANIADE	TNLNRRDKDT	PLDPSMATLI
**730**	**740**	**750**	**760**	**770**	**780**	**790**	
LPKNKGRMQN	KKPGFTVSCS	PKRTINSSQE	PAPGMKPNWP	RSRYPATKRG	CAAMAAYPSL	HIYTYP	

Cmpd.	No. of Cmpds.	m/z meas.	Δ m/z [ppm]	z	Rt [min]	Score	P	Range	Sequence	Modification
179	1	879.8295	-147.14	2	31.6	13.6	1	33-48	K.AQRGSLPNSATHQHVR.K	
1977	1	746.3572	-41.97	2	54.8	11.3	1	546-558	R.VFPLMLCKNAPDK.F	Oxidation: 5

Detailed Protein Report

Protein 210: elongation factor 1-alpha 2 [Homo sapiens]

**Accession:**	gi|4503475	**Score:**	40.5
**Database:**	refseq_human(refseq_human_20140103.fasta)	**MW [kDa]:**	50.4
**Database Date:**	2015-11-30	**pI:**	9.7
		**Sequence Coverage [%]:**	4.3
		**No. of unique Peptides:**	1

Quantitation

Wdown:Qdown **Median:** 1.32 **CV:** 0.00 % **No. of Peptides:** 1

10	20	30	40	50	60	70	80
MGKEKTHINI	VVIGHVDSGK	STTTGHLIYK	CGGIDKRTIE	KFEKEAAEMG	KGSFKYAWVL	DKLKAERERG	ITIDISLWKF
90	100	110	120	130	140	150	160
ETTKYYITII	DAPGHRDFIK	NMITGTSQAD	CAVLIVAAGV	GEFEAGISKN	GQTREHALLA	YTLGVKQLIV	GVNKMDSTEP
170	180	190	200	210	220	230	240
AYSEKRYDEI	VKEVSAYIKK	IGYNPATVPF	VPISGWHGDN	MLEPSPNMPW	FKGWKVERKE	GNASGVSLLE	ALDTILPPTR
250	260	270	280	290	300	310	320
PTDKPLRLPL	QDVYKIGGIG	TVPVGRVETG	ILRPGMVVTF	APVNITTEVK	SVEMHHEALS	EALPGDNVGF	NVKNVSVKDI
330	340	350	360	370	380	390	400
RRGNVCGDSK	SDPPQEAAQF	TSQVIILNHP	GQISAGYSPV	IDCHTAHIAC	KFAELKEKID	RRSGKKLEDN	PKSLKSGDAA
410	420	430	440	450	460	470	
IVEMVPGKPM	CVESFSQYPP	LGRFAVRDMR	QTVAVGVIKN	VEKKSGGAGK	VTKSAQKAQK	AGK	

Cmpd.	No. of Cmpds.	m/z meas.	Δ m/z [ppm]	z	Rt [min]	Score	P	Range	Sequence	Modification	Ratios
668	1	488.2507	-58.30	2	36.9	28.2	0	248-255	R.LPLQDVYK.I		Wdown:Qdown 1.32

Detailed Protein Report

Protein 211: PREDICTED: serine/threonine-protein kinase mTOR isoform X2 [Homo sapiens]

**Accession:**	gi|530360520	**Score:**	40.5
**Database:**	refseq_human(refseq_human_20140103.fasta)	**MW [kDa]:**	263.7
**Database Date:**	2015-11-30	**pI:**	6.8
		**Sequence Coverage [%]:**	1.2
		**No. of unique Peptides:**	3

Quantitation

mdown:qdown	**Median:**	1.03	**CV:**	0.00 %	**No. of Peptides:**	1
Wdown:Qdown	**Median:**	14.16	**CV:**	0.00 %	**No. of Peptides:**	1

Detailed Protein Report

10	20	30	40	50	60	70	80
MQKPQWYRHT	FEEAEKGFDE	TLAKEKGMNR	DDRIHGALLI	LNELVRISSM	EGERLREEME	EITQQQLVHD	KYCKDLMGFG
90	100	110	120	130	140	150	160
TKPRHITPFT	SFQAVQPQQS	NALVGLLGYS	SHQGLMGFGT	SPSPAKSTLV	ESRCCRDLME	EKFDQVCQWV	LKCRNSKNSL
170	180	190	200	210	220	230	240
IQMTILNLLP	RLAAFRPSAF	TDTQYLQDTM	NHVLSCVKKE	KERTAAFQAL	GLLSVAVRSE	FKVYLPRVLD	IIRAALPPKD
250	260	270	280	290	300	310	320
FAHKRQKAMQ	VDATVFTCIS	MLARAMGPGI	QQDIKELLEP	MLAVGLSPAL	TAVLYDLSRQ	IPQLKKDIQD	GLLKMLSLVL
330	340	350	360	370	380	390	400
MHKPLRHPGM	PKGLAHQLAS	PGLTTLPEAS	DVGSITLALR	TLGSFEFEGH	SLTQFVRHCA	DHFLNSEHKE	IRMEAARTCS
410	420	430	440	450	460	470	480
RLLTPSIHLI	SGHAHVVSQT	AVQVVADVLS	KLLVVGITDP	DPDIRYCVLA	SLDERFDAHL	AQAENLQALF	VALNDQVFEI
490	500	510	520	530	540	550	560
RELAICTVGR	LSSMNPAFVM	PFLRKMLIQI	LTELEHSGIG	RIKEQSARML	GHLVSNAPRL	IRPYMEPILK	ALILKLKDPD
570	580	590	600	610	620	630	640
PDPNPGVINN	VLATIGELAQ	VSGLEMRKWV	DELFIIIMDM	LQDSSLLAKR	QVALWTLGQL	VASTGYVVEP	YRKYPTLLEV
650	660	670	680	690	700	710	720
LLNFLKTEQN	QGTRREAIRV	LGLLGALDPY	KHKVNIGMID	QSRDASAVSL	SESKSSQDSS	DYSTSEMLVN	MGNLPLDEFY
730	740	750	760	770	780	790	800
PAVSMVALMR	IFRDQSLSHH	HTMVVQAITF	IFKSLGLKCV	QFLPQVMPTF	LNVIRVCDGA	IREFLFQQLG	MLVSFVKSHI
810	820	830	840	850	860	870	880
RPYMDEIVTL	MREFWVMNTS	IQSTIILLIE	QIVVALGGEF	KLYLPQLIPH	MLRVFMHDNS	PGRIVSIKLL	AAIQLFGANL
890	900	910	920	930	940	950	960
DDYLHLLLPP	IVKLFDAPEA	PLPSRKAALE	TVDRLTESLD	FTDYASRIIH	PIVRTLDQSP	ELRSTAMDTL	SSLVFQLGKK
970	980	990	1000	1010	1020	1030	1040
YQIFIPMVNK	VLVRHRINHQ	RYDVLICRIV	KGYTLADEEE	DPLIYQHRML	RSGQGDALAS	GPVETGPMKK	LHVSTINLQK
1050	1060	1070	1080	1090	1100	1110	1120
AWGAARRVSK	DDWLEWLRRL	SLELLKDSSS	PSLRSCWALA	QAYNPMARDL	FNAAFVSCWS	ELNEDQQDEL	IRSIELALTS
1130	1140	1150	1160	1170	1180	1190	1200
QDIAEVTQTL	LNLAEFMEHS	DKGPLPLRDD	NGIVLLGERA	AKCRAYAKAL	HYKELEFQKG	PTPAILESLI	SINNKLQQPE
1210	1220	1230	1240	1250	1260	1270	1280
AAAGVLEYAM	KHFGELEIQA	TWYEKLHEWE	DALVAYDKKM	DTNKDDPELM	LGRMRCLEAL	GEWGQLHQQC	CEKWTLVNDE
1290	1300	1310	1320	1330	1340	1350	1360
TQAKMARMAA	AAAWGLGQWD	SMEEYTCMIP	RDTHDGAFYR	AVLALHQDLF	SLAQQCIDKA	RDLLDAELTA	MAGESYSRAY
1370	1380	1390	1400	1410	1420	1430	1440
GAMVSCHMLS	ELEEVIQYKL	VPERREIIRQ	IWWERLQGCQ	RIVEDWQKIL	MVRSLVVSPH	EDMRTWLKYA	SLCGKSGRLA
1450	1460	1470	1480	1490	1500	1510	1520
LAHKTLVLLL	GVDPSRQLDH	PLPTVHPQVT	YAYMKNMWKS	ARKIDAFQHM	QHFVQTMQQQ	AQHAIATEDQ	QHKQELHKLM
1530	1540	1550	1560	1570	1580	1590	1600
ARCFLKLGEW	QLNLQGINES	TIPKVLQYYS	AATEHDRSWY	KAWHAWAVMN	FEAVLHYKHQ	NQARDEKKKL	RHASGANITN
1610	1620	1630	1640	1650	1660	1670	1680
ATTAATTAAT	ATTTASTEGS	NSESEAESTE	NSPTPSPLQK	KVTEDLSKTL	LMYTVPAVQG	FFRSISLSRG	NNLQDTLRVL
1690	1700	1710	1720	1730	1740	1750	1760
TLWFDYGHWP	DVNEALVEGV	KAIQIDTWLQ	VIPQLIARID	TPRPLVGRLI	HQLLTDIGRY	HPQALIYPLT	VASKSTTTAR
1770	1780	1790	1800	1810	1820	1830	1840
HNAANKILKN	MCEHSNTLVQ	QAMMVSEELI	RVAILWHEMW	HEGLEEASRL	YFGERNVKGM	FEVLEPLHAM	MERGPQTLKE
1850	1860	1870	1880	1890	1900	1910	1920
TSFNQAYGRD	LMEAQEWCRK	YMKSGNVKDL	TQAWDLYYHV	FRRISKQLPQ	LTSLELQYVS	PKLLMCRDLE	LAVPGTYDPN
1930	1940	1950	1960	1970	1980	1990	2000
QPIIRIQSIA	PSLQVITSKQ	RPRKLTLMGS	NGHEFVFLLK	GHEDLRQDER	VMQLFGLVNT	LLANDPTSLR	KNLSIQRYAV
2010	2020	2030	2040	2050	2060	2070	2080
IPLSTNSGLI	GWVPHCDTLH	ALIRDYREKK	KILLNIEHRI	MLRMAPDYDH	LTLMQKVEVF	EHAVNNTAGD	DLAKLLWLKS
2090	2100	2110	2120	2130	2140	2150	2160
PSSEVWFDRR	TNYTRSLAVM	SMVGYILGLG	DRHPSNLMLD	RLSGKILHID	FGDCFEVAMT	REKFPEKIPF	RLTRMLTNAM
2170	2180	2190	2200	2210	2220	2230	2240

Detailed Protein Report

Cmpd.	No. of Cmpds.	m/z meas.	Δ m/z [ppm]	z	Rt [min]	Score	P	Range	Sequence	Modification	Ratios
1142	1	586.6322	-235.21	2	43.9	12.9	0	1840-1849	K.ETSFNQAYGR.D		mdown:qdown 1.03
1911	1	704.7631	-82.23	2	52.4	11.2	1	1850-1860	R.DLMEAQEWCRK.Y		
559	1	472.6946	-192.04	2	35.8	16.4	2	2277-2284	R.VRDKLTGR.D		

Detailed Protein Report

Protein 212: PREDICTED: bromodomain-containing protein 1 isoform X12 [Homo sapiens]

**Accession:**	gi|578837129	**Score:**	40.5
**Database:**	refseq_human(refseq_human_20140103.fasta)	**MW [kDa]:**	110.4
**Database Date:**	2015-11-30	**pI:**	9.9
**Modification(s):**	Carbamidomethyl	**Sequence Coverage [%]:**	5.3
		**No. of unique Peptides:**	2

10	20	30	40	50	60	70	80
MKSFTAPWER	VGFQGLLVVR	ATQDFAVEGK	MTGNHYQMRR	KGRCHRGSAA	RHPSSPCSVK	HSPTRETLTY	AQAQRMVEIE
**90**	**100**	**110**	**120**	**130**	**140**	**150**	**160**
IEGRLHRISI	FDPLEIILED	DLTAQEMSEC	NSNKENSERP	PVCLRTKRHK	NNRVKKKNEA	LPSAHGTPAS	ASALPEPKVR
**170**	**180**	**190**	**200**	**210**	**220**	**230**	**240**
IVEYSPPSAP	RRPPVYYKFI	EKSAEELDNE	VEYDMDEEDY	AWLEIVNEKR	KGDCVPAVSQ	SMFEFLMDRF	EKESHCENQK
**250**	**260**	**270**	**280**	**290**	**300**	**310**	**320**
QGEQQSLIDE	DAVCCICMDG	ECQNSNVILF	CDMCNLAVHQ	ECYGVPYIPE	GQWLCRHCLQ	SRARPADCVL	CPNKGGAFKK
**330**	**340**	**350**	**360**	**370**	**380**	**390**	**400**
TDDDRWGHVV	CALWIPEVGF	ANTVFIEPID	GVRNIPPARW	KLTCYLCKQK	GVGACIQCHK	ANCYTAFHVT	CAQKAGLYMK
**410**	**420**	**430**	**440**	**450**	**460**	**470**	**480**
MEPVKELTGG	GTTFSVRKTA	YCDVHTPPGC	TRRPLNIYGD	VEMKNGVCRK	ESSVKTVRST	SKVRKKAKKA	KKALAEPCAV
**490**	**500**	**510**	**520**	**530**	**540**	**550**	**560**
LPTVCAPYIP	PQRLNRIANQ	VAIQRKKQFV	ERAHSYWLLK	RLSRNGAPLL	RRLQSSLQSQ	RSSQQRENDE	EMKAAKEKLK
**570**	**580**	**590**	**600**	**610**	**620**	**630**	**640**
YWQRLRHDLE	RARLLIELLR	KREKLKREQV	KVEQVAMELR	LTPLTVLLRS	VLDQLQDKDP	ARIFAQPVSL	KEVPDYLDHI
**650**	**660**	**670**	**680**	**690**	**700**	**710**	**720**
KHPMDFATMR	KRLEAQGYKN	LHEFEEDFDL	IIDNCMKYNA	RDTVFYRAAV	RLRDQGGVVL	RQARREVDSI	GLEEASGMHL
**730**	**740**	**750**	**760**	**770**	**780**	**790**	**800**
PERPAAAPRR	PFSWEDVDRL	LDPANRAHLG	LEEQLRELLD	MLDLTCAMKS	SGSRSKRAKL	LKKEIALLRN	KLSQQHSQPL
**810**	**820**	**830**	**840**	**850**	**860**	**870**	**880**
PTGPGLEGFE	EDGAALGPEA	GEEGDKSPPK	LEPSDALPLP	SNSETNSEPP	TLKPVELNPE	QSKLFKRVTF	DNESHSACTQ
**890**	**900**	**910**	**920**	**930**	**940**	**950**	**960**
SALVSGRPPE	PTRASSGDVP	AAAASAVAEP	ASDVNRRTSV	LFCKSKSSFR	GWRLFCSQGK	GRGAHAETPR	WRRSPQESAW
**970**	**980**						
TQVLTLTTNV	LGSSLFGP						

Cmpd.	No. of Cmpds.	m/z meas.	Δ m/z [ppm]	z	Rt [min]	Score	P	Range	Sequence	Modification
832	1	902.3468	-117.98	2	39.2	13.7	1	303-319	R.ARPADCVLCPNKGGAFK.K	Carbamidomethyl: 6
1578	2	873.9352	-97.55	2	48.2	14.4	2	567-580	R.HDLERARLLIELLR.K	

Detailed Protein Report

Protein 213: PREDICTED: matrix extracellular phosphoglycoprotein isoform X1 [Homo sapiens]

**Accession:**	gi|578809281	**Score:**	39.9
**Database:**	refseq_human(refseq_human_20140103.fasta)	**MW [kDa]:**	62.1
**Database Date:**	2015-11-30	**pI:**	9.4
**Modification(s):**	Oxidation	**Sequence Coverage [%]:**	4.9
		**No. of unique Peptides:**	1

10	20	30	40	50	60	70	80
MRVFCVGLLL	FSVTWAAPTF	QPQTEKTKQS	CVEEQRITYK	GHYEKHGHYV	FKCVYMSPEK	KNQTDVKQEE	KNKDNIGFHH
**90**	**100**	**110**	**120**	**130**	**140**	**150**	**160**
LGKRINQELS	SKENIVQERK	KDLSLSEASE	NKGSSKSQNY	FTNRQRLNKE	YSISNKENTH	NGLRMSIYPK	STGNKGFEDG
**170**	**180**	**190**	**200**	**210**	**220**	**230**	**240**
DDAISKLHDQ	EEYGAALIRN	NMQHIMGPVT	AIKLLGEENK	ENTPRNVLNI	IPASMNYAKA	HSKDKKKPQR	DSQAQKSPVK
**250**	**260**	**270**	**280**	**290**	**300**	**310**	**320**
SKSTHRIQHN	IDYLKHLSKV	KKIPSDFEGS	GYTDLQERGD	NDISPFSGDG	QPFKDIPGKG	EATGPDLEGK	DIQTGFAGPS
**330**	**340**	**350**	**360**	**370**	**380**	**390**	**400**
EAESTHLDTK	KPGYNEIPER	EENGGNTIGT	RDETAKEADA	VDVSLVEGSN	DIMGSTNFKE	LPGREGNRVD	AGSQNAHQGK
**410**	**420**	**430**	**440**	**450**	**460**	**470**	**480**
VEFHYPPAPS	KEKRKEGSSD	AAESTNYNEI	PKNGKGSTRK	GVDHSNRNQA	TLNEKQRFPS	KGKSQGLPIP	SRGLDNEIKN
**490**	**500**	**510**	**520**	**530**	**540**	**550**	**560**
EMDSFNGPSH	ENIITHGRKY	HYVPHRQNNS	TRNKGMPQGK	GSWGRQPHSN	RRFSSRRRDD	SSESSDSGSS	SESDGD

Cmpd.	No. of Cmpds.	m/z meas.	Δ m/z [ppm]	z	Rt [min]	Score	P	Range	Sequence	Modification
2687	1	920.7982	-142.16	2	64.8	16.9	1	46-60	K.HGHYVFKCVYMSPEK.K	Oxidation: 11

Detailed Protein Report

Protein 214: hemopexin precursor [Homo sapiens]

Accession: gi|11321561
Database: refseq_human(refseq_human_20140103.fasta)
Database Date: 2015-11-30

Score: 39.8
MW [kDa]: 51.6
pI: 6.6
Sequence Coverage [%]: 9.5
No. of unique Peptides: 2

Alias proteins:

Accession	Name	Description
gi|530395269	r e f s e q _ h u m a n (refseq_human_20140103.fasta)	PREDICTED: hemopexin isoform X1 [Homo sapiens]

10	20	30	40	50	60	70	80
MARVLGAPVA	LGLWSLCWSL	AIATPLPPTS	AHGNVAEGET	KPDPDVTERC	SDGWSFDATT	LDDNGTMLFF	KGEFVWKSHK
**90**	**100**	**110**	**120**	**130**	**140**	**150**	**160**
WDRELISERW	KNFPSPVDAA	FRQGHNSVFL	IKGDKVWVYP	PEKKEKGYPK	LLQDEFPGIP	SPLDAAVECH	RGECQAEGVL
**170**	**180**	**190**	**200**	**210**	**220**	**230**	**240**
FFQGDREWFW	DLATGTMKER	SWPAVGNCSS	ALRWLGRYYC	FQGNQFLRFD	PVRGEVPPRY	PRDVRDYFMP	CPGRGHGHRN
**250**	**260**	**270**	**280**	**290**	**300**	**310**	**320**
GTGHGNSTHH	GPEYMRCSPH	LVLSALTSDN	HGATYAFSGT	HYWRLDTSRD	GWHSWPIAHQ	WPQGPSAVDA	AFSWEEKLYL
**330**	**340**	**350**	**360**	**370**	**380**	**390**	**400**
VQGTQVYVFL	TKGGYTLVSG	YPKRLEKEVG	TPHGIILDSV	DAAFICPGSS	RLHIMAGRRL	WWLDLKSGAQ	ATWTELPWPH
**410**	**420**	**430**	**440**	**450**	**460**	**470**	
EKVDGALCME	KSLGPNSCSA	NGPGLYLIHG	PNLYCYSDVE	KLNAAKALPQ	PQNVTSLLGC	TH	

Cmpd.	No. of Cmpds.	m/z meas.	Δ m/z [ppm]	z	Rt [min]	Score	P	Range	Sequence	Modification
2360	1	1073.7380	-84.18	3	59.8	17.0	0	290-317	R.DGWHSWPIAHQWPQGPSAVDAAFSWEEK.L	
1592	2	919.4423	-5.15	2	48.6	22.8	0	387-402	K.SGAQATWTELPWPHEK.V	

Detailed Protein Report

Protein 215: centrosomal protein of 55 kDa [Homo sapiens]

**Accession:**	gi|187608537	**Score:**	39.8
**Database:**	refseq_human(refseq_human_20140103.fasta)	**MW [kDa]:**	54.1
**Database Date:**	2015-11-30	**pI:**	6.6
		**Sequence Coverage [%]:**	5.0
		**No. of unique Peptides:**	2

Quantitation

mdown:qdown	**Median:**	0.36	**CV:**	26.65 %	**No. of Peptides:**	2
Wdown:Qdown	**Median:**	0.51	**CV:**	0.00 %	**No. of Peptides:**	1

Alias proteins:

Accession	Name	Description
gi|187608545	refseq_human (refseq_human_20140103.fasta)	centrosomal protein of 55 kDa [Homo sapiens]

```
        10         20         30         40         50         60         70         80
MSSRSTKDLI KSKWGSKPSN SKSETTLEKL KGEIAHLKTS VDEITSGKGK LTDKERHRLL EKIRVLEAEK EKNAYQLTEK
        90        100        110        120        130        140        150        160
DKEIQRLRDQ LKARYSTTAL LEQLEETTRE GERREQVLKA LSEEKDVLKQ QLSAATSRIA ELESKTNTLR LSQTVAPNCF
       170        180        190        200        210        220        230        240
NSSINNIHEM EIQLKDALEK NQQWLVYDQQ REVYVKGLLA KIFELEKKTE TAAHSLPQQT KKPESEGYLQ EEKQKCYNDL
       250        260        270        280        290        300        310        320
LASAKKDLEV ERQTITQLSF ELSEFRRKYE ETQKEVHNLN QLLYSQRRAD VQHLEDDRHK TEKIQKLREE NDIARGKLEE
       330        340        350        360        370        380        390        400
EKKRSEELLS QVQFLYTSLL KQQEEQTRVA LLEQQMQACT LDFENEKLDR QHVQHQLHVI LKELRKARNQ ITQLESLKQL
       410        420        430        440        450        460        470
HEFAITEPLV TFQGETENRE KVAASPKSPT AALNESLVEC PKCNIQYPAT EHRDLLVHVE YCSK
```

Cmpd.	No. of Cmpds.	m/z meas.	Δ m/z [ppm]	z	Rt [min]	Score	P	Range	Sequence	Modification	Ratios
1005	1	423.6388	-147.47	2	41.1	17.5	0	309-315	R.EENDIAR.G		mdown:qdown 0.28<br>Wdown:Qdown 0.51
398	1	587.3194	-13.57	2	34.3	10.3	0	389-398	R.NQITQLESLK.Q		mdown:qdown 0.46

Detailed Protein Report

Protein 216: PREDICTED: histone-lysine N-methyltransferase setd3 isoform X3 [Homo sapiens]

**Accession:**	gi|530404751	**Score:**	39.7
**Database:**	refseq_human(refseq_human_20140103.fasta)	**MW [kDa]:**	56.3
**Database Date:**	2015-11-30	**pI:**	5.7
		**Sequence Coverage [%]:**	3.4
		**No. of unique Peptides:**	1

Quantitation

mdown:qdown **Median:** 0.54 **CV:** 0.00 % **No. of Peptides:** 1

10	20	30	40	50	60	70	80
MCRSGLWLRK	YGKSKKAEEL	FLWVPRKLLM	TVESAKNSVL	GPLYSQDRIL	QAMGNIALAF	HLLCERASPN	SFWQPYIQTL
90	100	110	120	130	140	150	160
PSEYDTPLYF	EEDEVRYLQS	TQAIHDVFSQ	YKNTARQYAY	FYKVIQTHPH	ANKLPLKDSF	TYEDYRWAVS	SVMTRQNQIP
170	180	190	200	210	220	230	240
TEDGSRVTLA	LIPLWDMCNH	TNGLITTGYN	LEDDRCECVA	LQDFRAGEQI	YIFYGTRSNA	EFVIHSGFFF	DNNSHDRVKI
250	260	270	280	290	300	310	320
KLGVSKSDRL	YAMKAEVLAR	AGIPTSSVFA	LHFTEPPISA	QLLAFLRVFC	MTEEELKEHL	LGDSAIDRIF	TLGNSEFPVS
330	340	350	360	370	380	390	400
WDNEVKLWTF	LEDRASLLLK	TYKTTIEEDK	SVLKNHDLSV	RAKMAIKLRL	GEKEILEKAV	KSAAVNREYY	RQQMEEKAPL
410	420	430	440	450	460	470	480
PKYEESNLGL	LESSVGDSRL	PLVLRNLEEE	AGVQDALNIR	EAISKAKATE	NGLVNGENSI	PNGTRSENES	LNQESKRAVE
490	500						
DAKGSSSDST	AGVKE						

Cmpd.	No. of Cmpds.	m/z meas.	Δ m/z [ppm]	z	Rt [min]	Score	P	Range	Sequence	Modification	Ratios
832	1	491.6524	-172.14	2	39.8	26.5	0	117-123	R.QYAYFYK.V		mdown:qdown 0.54

Detailed Protein Report

Protein 217: protein piccolo isoform 2 [Homo sapiens]

**Accession:**	gi|150170670	**Score:**	39.7
**Database:**	refseq_human(refseq_human_20140103.fasta)	**MW [kDa]:**	537.6
**Database Date:**	2015-11-30	**pI:**	5.9
		**Sequence Coverage [%]:**	1.0
		**No. of unique Peptides:**	1

Detailed Protein Report

10	20	30	40	50	60	70	80
MGNEASLEGE	GLPEGLAAAA	AAGGGASGAG	SPSHTAIPAG	MEADLSQLSE	EERRQIAAVM	SRAQGLPKGS	VPPAAAESPS
90	100	110	120	130	140	150	160
MHRKQELDSS	HPPKQSGRPP	DPGRPAQPGL	SKSRTTDTFR	SEQKLPGRSP	STISLKESKS	RTDLKEEHKS	SMMPGFLSEV
170	180	190	200	210	220	230	240
NALSAVSSVV	NKFNPFDLIS	DSEASQEETT	KKQKVVQKEQ	GKPEGIIKPP	LQQQPPKPIP	KQQGPGRDPL	QQDGTPKSIS
250	260	270	280	290	300	310	320
SQQPEKIKSQ	PPGTGKPIQG	PTQTPQTDHA	KLPLQRDASR	PQTKQADIVR	GESVKPSLPS	PSKPPIQQPT	PGKPPAQQPG
330	340	350	360	370	380	390	400
HEKSQPGPAK	PPAQPSGLTK	PLAQQPGTVK	PPVQPPGTTK	PPAQPLGPAK	PPAQQTGSEK	PSSEQPGPKA	LAQPPGVGKT
410	420	430	440	450	460	470	480
PAQQPGPAKP	PTQQVGTPKP	LAQQPGLQSP	AKAPGPTKTP	VQQPGPGKIP	AQQAGPGKTS	AQQTGPTKPP	SQLPGPAKPP
490	500	510	520	530	540	550	560
PQQPGPAKPP	PQQPGSAKPP	SQQPGSTKPP	PQQPGPAKPS	PQQPGSTKPP	SQQPGSAKPS	AQQPSPAKPS	AQQSTKPVSQ
570	580	590	600	610	620	630	640
TGSGKPLQPP	TVSPSAKQPP	SQGLPKTICP	LCNTTELLLH	VPEKANFNTC	TECQTTVCSL	CGFNPNPHLT	EVKEWLCLNC
650	660	670	680	690	700	710	720
QMKRALGGDL	APVPSSPQPK	LKTAPVTTTS	AVSKSSPQPQ	QTSPKKDAAP	KQDLSKAPEP	KKPPPLVKQP	TLHGSPSAKA
730	740	750	760	770	780	790	800
KQPPEADSLS	KPAPPKEPSV	PSEQDKAPVA	DDKPKQPKMV	KPTTDLVSSS	SATTKPDIPS	SKVQSQAEEK	TTPPLKTDSA
810	820	830	840	850	860	870	880
KPSQSFPPTG	EKVSPFDSKA	IPRPASDSKI	ISHPGPSSES	KGQKQVDPVQ	KKEEPKKAQT	KMSPKPDAKP	MPKGSPTPPG
890	900	910	920	930	940	950	960
PRPTAGQTVP	TPQQSPKPQE	QSRRFSLNLG	SITDAPKSQP	TTPQETVTGK	LFGFGASIFS	QASNLISTAG	QPGPHSQSGP
970	980	990	1000	1010	1020	1030	1040
GAPMKQAPAP	SQPPTSQGPP	KSTGQAPPAP	AKSIPVKKET	KAPAAEKLEP	KAEQAPTVKR	TETEKKPPPI	KDSKSLTAEP
1050	1060	1070	1080	1090	1100	1110	1120
QKAVLPTKLE	KSPKPESTCP	LCKTELNIGS	KDPPNFNTCT	ECKNQVCNLC	GFNPTPHLTE	IQEWLCLNCQ	TQRAISGQLG
1130	1140	1150	1160	1170	1180	1190	1200
DIRKMPPAPS	GPKASPMPVP	TESSSQKTAV	PPQVKLVKKQ	EQEVKTEAEK	VILEKVKETL	SMEKIPPMVT	TDQKQEESKL
1210	1220	1230	1240	1250	1260	1270	1280
EKDKASALQE	KKPLPEEKKL	IPEEEKIRSE	EKKPLLEEKK	PTPEDKKLLP	EAKTSAPEEQ	KHDLLKSQVQ	IAEEKLEGRV
1290	1300	1310	1320	1330	1340	1350	1360
APKTVQEGKQ	PQTKMEGLPS	GTPQSLPKED	DKTTKTIKEQ	PQPPCTAKPD	QVEPGKEKTE	KEDDKSDTSS	SQQPKSPQGL
1370	1380	1390	1400	1410	1420	1430	1440
SDTGYSSDGI	SSSLGEIPSL	IPTDEKDILK	GLKKDSFSQE	SSPSSPSDLA	KLESTVLSIL	EAQASTLADE	KSEKKTQPHE
1450	1460	1470	1480	1490	1500	1510	1520
VSPEQPKDQE	KTQSLSETLE	ITISEEEIKE	SQEERKDTFK	KDSQQDIPSS	KDHKEKSEFV	DDITTRREPY	DSVEESSESE
1530	1540	1550	1560	1570	1580	1590	1600
NSPVPQRKRR	TSVGSSSSDE	YKQEDSQGSG	EEEDFIRKQI	IEMSADEDAS	GSEDDEFIRN	QLKEISSSTE	SQKKEETKGK
1610	1620	1630	1640	1650	1660	1670	1680
GKITAGKHRR	LTRKSSTSID	EDAGRRHSWH	DEDDEAFDES	PELKYRETKS	QESEELVVTG	GGGLRRFKTI	ELNSTIADKY
1690	1700	1710	1720	1730	1740	1750	1760
SAESSQKKTS	LYFDEEPELE	MESLTDSPED	RSRGEGSSSL	HASSFTPGTS	PTSVSSLDED	SDSSPSHKKG	ESKQQRKARH
1770	1780	1790	1800	1810	1820	1830	1840
RPHGPLLPTI	EDSSEEEELR	EEEELLKEQE	KQREIEQQQR	KSSSKKSKKD	KDELRAQRRR	ERPKTPPSNL	SPIEDASPTE
1850	1860	1870	1880	1890	1900	1910	1920
ELRQAAEMEE	LHRSSCSEYS	PSIESDPEGF	EISPEKIIEV	QKVYKLPTAV	SLYSPTDEQS	IMQKEGSQKA	LKSAEEMYEE
1930	1940	1950	1960	1970	1980	1990	2000
MMHKTHKYKA	FPAANERDEV	FEKEPLYGGM	LIEDYIYESL	VEDTYNGSVD	GSLLTRQEEE	NGFMQQKGRE	QKIRLSEQIY
2010	2020	2030	2040	2050	2060	2070	2080
EDPMQKITDL	QKEFYELESL	HSVVPQEDIV	SSSFIIPESH	EIVDLGTMVT	STEEERKLLD	ADAAYEELMK	RQQMQLTPGS
2090	2100	2110	2120	2130	2140	2150	2160
SPTQAPIGED	MTESTMDFDR	MPDASLTSSV	LSGASLTDST	SSATLSIPDV	KITQHFSTEE	IEDEYVTDYT	REIQEIIAHE
2170	2180	2190	2200	2210	2220	2230	2240
SLILTYSEPS	ESATSVPPSD	TPSLTSSVSS	VCTTDSSSPI	TTLDSITTVY	TEPVDMITKF	EDSEEISSST	YFPGSIIDYP
2250	2260	2270	2280	2290	2300	2310	2320
EEISVSLDRT	APPDGRASAD	HIVISLSDMA	SSIIESVVPK	PEGPVADTVS	TDLLISEKDP	VKKAKKETGN	GIILEVLEAY
2330	2340	2350	2360	2370	2380	2390	2400

Detailed Protein Report

Cmpd.	No. of Cmpds.	m/z meas.	Δ m/z [ppm]	z	Rt [min]	Score	P	Range	Sequence	Modification
1917	1	902.8454	-85.60	2	54.0	12.9	0	3678-3694	R.SMSDPKPLSPTADESSR.A	

Detailed Protein Report

Protein 218: **sodium-driven chloride bicarbonate exchanger isoform 2 [Homo sapiens]**

**Accession:**	gi|155722998	**Score:**	39.6
**Database:**	refseq_human(refseq_human_20140103.fasta)	**MW [kDa]:**	122.6
**Database Date:**	2015-11-30	**pI:**	5.9
**Modification(s):**	Oxidation	**Sequence Coverage [%]:**	2.8
		**No. of unique Peptides:**	1

Quantitation

Wdown:Qdown **Median:** 0.41 **CV:** 0.00 % **No. of Peptides:** 1

10	20	30	40	50	60	70	80
MEIKDQGAQM	EPLLPTRNDE	EAVVDRGGTR	SILKTHFEKE	DLEGHRTLFI	GVHVPLGGRK	SHRRHRHRGH	KHRKRDRERD
90	100	110	120	130	140	150	160
SGLEDGRESP	SFDTPSQRVQ	FILGTEDDDE	EHIPHDLFTE	LDEICWREGE	DAEWRETARW	LKFEEDVEDG	GERWSKPYVA
170	180	190	200	210	220	230	240
TLSLHSLFEL	RSCILNGTVL	LDMHANTLEE	IADMVLDQQV	SSGQLNEDVR	HRVHEALMKQ	HHHQNQKKLT	NRIPIVRSFA
250	260	270	280	290	300	310	320
DIGKKQSEPN	SMDKNAGQVV	SPQSAPACVE	NKNDVSRENS	TVDFSKVDLH	FMKKIPPGAE	ASNILVGELE	FLDRTVVAFV
330	340	350	360	370	380	390	400
RLSPAVLLQG	LAEVPIPTRF	LFILLGPLGK	GQQYHEIGRS	IATLMTDEVF	HDVAYKAKDR	NDLVSGIDEF	LDQVTVLPPG
410	420	430	440	450	460	470	480
EWDPSIRIEP	PKNVPSQEKR	KIPAVPNGTA	AHGEAEPHGG	HSGPELQRTG	RIFGGLILDI	KRKAPYFWSD	FRDAFSLQCL
490	500	510	520	530	540	550	560
ASFLFLYCAC	MSPVITFGGL	LGEATEGRIS	AIESLFGASM	TGIAYSLFGG	QPLTILGSTG	PVLVFEKILF	KFCKEYGLSY
570	580	590	600	610	620	630	640
LSLRASIGLW	TATLCIILVA	TDASSLVCYI	TRFTEEAFAS	LICIIFIYEA	LEKLFELSEA	YPINMHNDLE	LLTQYSCNCV
650	660	670	680	690	700	710	720
EPHNPSNGTL	KEWRESNISA	SDIIWENLTV	SECKSLHGEY	VGRACGHDHP	YVPDVLFWSV	ILFFSTVTLS	ATLKQFKTSR
730	740	750	760	770	780	790	800
YFPTKVRSIV	SDFAVFLTIL	CMVLIDYAIG	IPSPKLQVPS	VFKPTRDDRG	WFVTPLGPNP	WWTVIAAIIP	ALLCTILIFM
810	820	830	840	850	860	870	880
DQQITAVIIN	RKEHKLKKGC	GYHLDLLMVA	VMLGVCSIMG	LPWFVAATVL	SITHVNSLKL	ESECSAPGEQ	PKFLGIREQR
890	900	910	920	930	940	950	960
VTGLMIFILM	GSSVFMTSIL	KFIPMPVLYG	VFLYMGASSL	KGIQFFDRIK	LFWMPAKHQP	DFIYLRHVPL	RKVHLFTIIQ
970	980	990	1000	1010	1020	1030	1040
MSCLGLLWII	KVSRAAIVFP	MMVLALVFVR	KLMDLLFTKR	ELSWLDDLMP	ESKKKKLEDA	EKEEEQSMLA	MEDEGTVQLP
1050	1060	1070	1080	1090			
LEGHYRDDPS	VINISDEMSK	TALWRNLLIT	ADNSKDKESS	FPSKSSPS			

Cmpd.	No. of Cmpds.	m/z meas.	Δ m/z [ppm]	z	Rt [min]	Score	P	Range	Sequence	Modification	Ratios
1399	1	783.2967	-72.60	2	47.2	11.8	0	1047-1060	R.DDPSVINISDEMSK.T	Oxidation: 12	Wdown:Qdown 0.41

Detailed Protein Report

Protein 219: **PREDICTED: polyamine-modulated factor 1-binding protein 1 isoform X2 [Homo sapiens]**

**Accession:**	gi|530424400	**Score:**	39.6
**Database:**	refseq_human(refseq_human_20140103.fasta)	**MW [kDa]:**	94.2
**Database Date:**	2015-11-30	**pI:**	5.6
**Modification(s):**	Carbamidomethyl, Oxidation	**Sequence Coverage [%]:**	5.1
		**No. of unique Peptides:**	2

Quantitation

mdown:qdown	**Median:** 1.46	**CV:** 0.00 %	**No. of Peptides:**	1
Wdown:Qdown	**Median:** 2.39	**CV:** 0.00 %	**No. of Peptides:**	1

10	20	30	40	50	60	70	80
MKDEAGERDR	EVSSLNSKLL	SLQLDIKNLH	DVCKRQRKTL	QDNQLCMEEA	MNSSHDKKQA	QALAFEESEV	EFGSSKQCHL
**90**	**100**	**110**	**120**	**130**	**140**	**150**	**160**
RQLQQLKKKL	LVLQQELEFH	TEELQTSYYS	LRQYQSILEK	QTSDLVLLHH	HCKLKEDEVI	LYEEEMGNHN	ENTGEKLHLA
**170**	**180**	**190**	**200**	**210**	**220**	**230**	**240**
QEQLALAGDK	IASLERSLNL	YRDKYQSSLS	NIELLECQVK	MLQGELGGIM	GQEPENKGDH	SKVRIYTSPC	MIQEHQETQK
**250**	**260**	**270**	**280**	**290**	**300**	**310**	**320**
RLSEVWQKVS	QQDDLIQELR	NKLACSNALV	LEREKALIKL	QADFASCTAT	HRYPPSSSEE	CEDIKKILKH	LQEQKDSQCL
**330**	**340**	**350**	**360**	**370**	**380**	**390**	**400**
HVEEYQNLVK	DLRVELEAVS	EQKRNIMKDM	MKLELDLHGL	REETSAHIER	KDKDITILQC	RLQELQLEFT	ETQKLTLKKD
**410**	**420**	**430**	**440**	**450**	**460**	**470**	**480**
KFLQEKDEML	QELEKKLTQV	QNSLLKKEKE	LEKQQCMATE	LEMTVKEAKQ	DKSKEAECKA	LQAEVQKLKN	SLEEAKQQER
**490**	**500**	**510**	**520**	**530**	**540**	**550**	**560**
LAAQQAAQCK	EEAALAGCHL	EDTQRKLQKG	LLLDKQKADT	IQELQRELQM	LQKESSMAEK	EQTSNRKRVE	ELSLELSEAL
**570**	**580**	**590**	**600**	**610**	**620**	**630**	**640**
RKLENSDKEK	RQLQKTVAEQ	DMKMNDMLDR	IKHQHREQGS	IKCKLEEDLQ	EATKLLEDKR	EQLKKSKEHE	KLMEGELEAL
**650**	**660**	**670**	**680**	**690**	**700**	**710**	**720**
RQEFKKKDKT	LKENSRKLEE	ENENLRAELQ	CCSTQLESSL	NKYNTSQQVI	QDLNKEIALQ	KESLMSLQAQ	LDKALQKEKH
**730**	**740**	**750**	**760**	**770**	**780**	**790**	**800**
YLQTTITKEA	YDALSRKSAA	CQDDLTQALE	KLNHVTSETK	SLQQSLTQTQ	EKKAQLEEEI	IAYEERMKKL	NTELRKLRGF
**810**							
HQESELE							

Cmpd.	No. of Cmpds.	m/z meas.	Δ m/z [ppm]	z	Rt [min]	Score	P	Range	Sequence	Modification	Ratios
293	1	477.5768	-43.77	3	32.4	14.6	1	261-273	R.NKLACSNALVLER.E		mdown:qdown 1.46<br>Wdown:Qdown 2.39
1797	1	638.2204	-122.59	3	51.0	12.7	1	434-449	K.QQCMATELEMTVKEAK.Q	Carbamidomethyl: 3; Oxidation: 4	

Detailed Protein Report

Protein 220: PREDICTED: serine/arginine repetitive matrix protein 1 isoform X3 [Homo sapiens]

Accession: gi|530360787
Database: refseq_human(refseq_human_20140103.fasta)
Database Date: 2015-11-30
Modification(s): Oxidation

Score: 39.5
MW [kDa]: 103.7
pI: 12.4
Sequence Coverage [%]: 3.4
No. of unique Peptides: 2

10	20	30	40	50	60	70	80
MDAGFFRGTS	AEQDNRFSNK	QKKLLKQLKF	AECLEKKVDM	SKVNLEVIKP	WITKRVTEIL	GFEDDVVIEF	IFNQLEVKNP
**90**	**100**	**110**	**120**	**130**	**140**	**150**	**160**
DSKMMQINLT	GFLNGKNARE	FMGELWPLLL	SAQENIAGIP	SAFLELKKEE	IKQRQIEQEK	LASMKKQDED	KDKRDKEEKE
**170**	**180**	**190**	**200**	**210**	**220**	**230**	**240**
SSREKRERSR	SPRRRKSRSP	SPRRRSSPVR	RERKRSHSRS	PRHRTKSRSP	SPAPEKKEKT	PELPEPSVKV	KEPSVQEATS
**250**	**260**	**270**	**280**	**290**	**300**	**310**	**320**
TSDILKVPKP	EPIPEPKEPS	PEKNSKKEKE	KEKTRPRSRS	RSKSRSRTRS	RSPSHTRPRR	RHRSRSRSYS	PRRRPSPRRR
**330**	**340**	**350**	**360**	**370**	**380**	**390**	**400**
PSPRRRTPPR	RMPPPPRHRR	SRSPVRRRRR	SSASLSGSSS	SSSSSRSRSP	PKKPPKRTSS	PPRKTRRLSP	SASPPRRRHR
**410**	**420**	**430**	**440**	**450**	**460**	**470**	**480**
PSPPATPPPK	TRHSPTPQQS	NRTRKSRVSV	SPGRTSVTKH	KGTEKRESPS	PAPKPRKVEL	SESEEDKGGK	MAAADSVQQR
**490**	**500**	**510**	**520**	**530**	**540**	**550**	**560**
RQYRRQNQQS	SSDSGSSSSS	EDERPKRSHV	KNGEVGRRRR	HSPSRSASPS	PRKRQKETSP	RMQMGKRWQS	PVTKSGRRRR
**570**	**580**	**590**	**600**	**610**	**620**	**630**	**640**
SPSPPPTRRR	RSPSPAPPPR	RRRTPTPPPR	RRTPSPPPRR	RSPSPRRYSP	PIQRRYSPSP	PPKRRTASPP	PPPKRRASPS
**650**	**660**	**670**	**680**	**690**	**700**	**710**	**720**
PPPKRRVSHS	PPPKQRSSPV	TKRRSPSLSS	KHRKGSSPSR	STREARSPQP	NKRHSPSRP	RAPQTSSSPP	PVRRGASSSP
**730**	**740**	**750**	**760**	**770**	**780**	**790**	**800**
QRRQSPSPST	RPIRRVSRTP	EPKKIKKAAS	PSPQSVRRVS	SSRSVSGSPE	PAAKKPPAPP	SPVQSQSPST	NWSPAVPVKK
**810**	**820**	**830**	**840**	**850**	**860**	**870**	**880**
AKSPTPSPSP	PRNSDQEGGG	KKKKKKKDKK	HKKDKKHKKH	KKHKKEKAVA	AAAAAAVTPA	AIAAATTTLA	QEEPVAAPEP
**890**	**900**	**910**	**920**				
KKETESEAED	NLDDLEKHLR	EKALRSMRKA	QVSPQS				

Cmpd.	No. of Cmpds.	m/z meas.	Δ m/z [ppm]	z	Rt [min]	Score	P	Range	Sequence	Modification
2594	1	741.8530	-30.71	2	63.0	14.5	0	84-96	K.MMQINLTGFLNGK.N	Oxidation: 1
1844	1	590.7772	1.91	2	53.1	11.4	1	537-546	K.ETSPRMQMGK.R	Oxidation: 6

Detailed Protein Report

Protein 221: A disintegrin and metalloproteinase with thrombospondin motifs 20 preproprotein [Homo sapiens]

**Accession:**	gi|124430557	**Score:**	39.5
**Database:**	refseq_human(refseq_human_20140103.fasta)	**MW [kDa]:**	214.6
**Database Date:**	2015-11-30	**pI:**	7.2
**Modification(s):**	Carbamidomethyl	**Sequence Coverage [%]:**	1.7
		**No. of unique Peptides:**	1

Quantitation

mdown:qdown	**Median:**	0.57	**CV:**	0.00 %	**No. of Peptides:**	1
Wdown:Qdown	**Median:**	0.97	**CV:**	0.00 %	**No. of Peptides:**	1

Detailed Protein Report

10	20	30	40	50	60	70	80
MWVAKWLTGL	LYHLSLFITR	SWEVDFHPRQ	EALVRTLTSY	EVVIPERVNE	FGEVFPQSHH	FSRQKRSSEA	LEPMPFRTHY
90	100	110	120	130	140	150	160
RFTAYGQLFQ	LNLTADASFL	AAGYTEVHLG	TPERGAWESD	AGPSDLRHCF	YRGQVNSQED	YKAVVSLCGG	LTGTFKGQNG
170	180	190	200	210	220	230	240
EYFLEPIMKA	DGNEYEDGHN	KPHLIYRQDL	NNSFLQTLKY	CSVSESQIKE	TSLPFHTYSN	MNEDLNVMKE	RVLGHTSKNV
250	260	270	280	290	300	310	320
PLKDERRHSR	KKRLISYPRY	IEIMVTADAK	VVSAHGSNLQ	NYILTLMSIV	ATIYKDPSIG	NLIHIVVVKL	VMIHREEEGP
330	340	350	360	370	380	390	400
VINFDGATTL	KNFCSWQQTQ	NDLDDVHPSH	HDTAVLITRE	DICSSKEKCN	MLGLSYLGTI	CDPLQSCFIN	EEKGLISAFT
410	420	430	440	450	460	470	480
IAHELGHTLG	VQHDDNPRCK	EMKVTKYHVM	APALSFHMSP	WSWSNCSRKY	VTEFLDTGYG	ECLLDKPDEE	IYNLPSELPG
490	500	510	520	530	540	550	560
SRYDGNKQCE	LAFGPGSQMC	PHINICMHLW	CTSTEKLHKG	CFTQHVPPAD	GTDCGPGMHC	RHGLCVNKET	ETRPVNGEWG
570	580	590	600	610	620	630	640
PWEPYSSCSR	TCGGGIESAT	RRCNRPEPRN	GGNYCVGRRM	KFRSCNTDSC	PKGTQDFREK	QCSDFNGKHL	DISGIPSNVR
650	660	670	680	690	700	710	720
WLPRYSGIGT	KDRCKLYCQV	AGTNYFYLLK	DMVEDGTPCG	TETHDICVQG	QCMAAGCDHV	LNSSAKIDKC	GVCGGDNSSC
730	740	750	760	770	780	790	800
KTITGVFNSS	HYGYNVVVKI	PAGATNVDIR	QYSYSGQPDD	SYLALSDAEG	NFLFNGNFLL	STSKKEINVQ	GTRTVIEYSG
810	820	830	840	850	860	870	880
SNNAVERINS	TNRQEKELIL	QVLCVGNLYN	PDVHYSFNIP	LEERSDMFTW	DPYGPWEGCT	KMCQGLQRRN	ITCIHKSDHS
890	900	910	920	930	940	950	960
VVSDKECDHL	PLPSFVTQSC	NTDCELRWHV	IGKSECSSQC	GQGYRTLDIH	CMKYSIHEGQ	TVQVDDHYCG	DQLKPPTQEL
970	980	990	1000	1010	1020	1030	1040
CHGNCVFTRW	HYSEWSQCSR	SCGGGERSRE	SYCMNNFGHR	LADNECQELS	RVTRENCNEF	SCPSWAASEW	SECLVTCGKG
1050	1060	1070	1080	1090	1100	1110	1120
TKQRQVWCQL	NVDHLSDGFC	NSSTKPESLS	PCELHTCASW	QVGPWGPCTT	TCGHGYQMRD	VKCVNELASA	VLEDTECHEA
1130	1140	1150	1160	1170	1180	1190	1200
SRPSDRQSCV	LTPCSFISKL	ETALLPTVLI	KKMAQWRHGS	WTPCSVSCGR	GTQARYVSCR	DALDRIADES	YCAHLPRPAE
1210	1220	1230	1240	1250	1260	1270	1280
IWDCFTPCGE	WQAGDWSPCS	ASCGHGKTTR	QVLCMNYHQP	IDENYCDPEV	RPLMEQECSL	AACPPAHSHF	PSSPVQPSYY
1290	1300	1310	1320	1330	1340	1350	1360
LSTNLPLTQK	LEDNENQVVH	PSVRGNQWRT	GPWGSCSSSC	SGGLQHRAVV	CQDENGQSAS	YCDAASKPPE	LQQCGPGPCP
1370	1380	1390	1400	1410	1420	1430	1440
QWNYGNWGEC	SQTCGGGIKS	RLVICQFPNG	QILEDHNCEI	VNKPPSVIQC	HMHACPADVS	WHQEPWTSCS	ASCGKGRKYR
1450	1460	1470	1480	1490	1500	1510	1520
EVFCIDQFQR	KLEDTNCSQV	QKPPTHKACR	SVRCPSWKAN	SWNECSVTCG	SGVQQRDVYC	RLKGVGQVVE	EMCDQSTRPC
1530	1540	1550	1560	1570	1580	1590	1600
SQRRCWSQDC	VQHKGMERGR	LNCSTSCERK	DSHQRMECTD	NQIRQVNEIV	YNSSTISLTS	KNCRNPPCNY	IVVTADSSQC
1610	1620	1630	1640	1650	1660	1670	1680
ANNCGFSYRQ	RITYCTEIPS	TKKHKLHRLR	PIVYQECPVV	PSSQVYQCIN	SCLHLATWKV	GKWSKCSVTC	GIGIMKRQVK
1690	1700	1710	1720	1730	1740	1750	1760
CITKHGLSSD	LCLNHLKPGA	QKKCYANDCK	SFTTCKEIQV	KNHIRKDGDY	YLNIKGRIIK	IYCADMYLEN	PKEYLTLVQG
1770	1780	1790	1800	1810	1820	1830	1840
EENFSEVYGF	RLKNPYQCPF	NGSRREDCEC	DNGHLAAGYT	VFSKIRIDLT	SMQIKTTDLL	FSKTIFGNAV	PFATAGDCYS
1850	1860	1870	1880	1890	1900	1910	1920
AFRCPQGQFS	INLSGTGMKI	SSTAKWLTQG	SYTSVSIRRS	EDGTRFFGKC	GGYCGKCLPH	MTTGLPIQVI	

Cmpd.	No. of Cmpds.	m/z meas.	Δ m/z [ppm]	z	Rt [min]	Score	P	Range	Sequence	Modification	Ratios
1189	1	599.1520	-198.95	2	44.5	12.7	1	1541-1550	R.LNCSTSCERK.D	Carbamidomethyl: 7	Wdown:Qdown 0.97<br>mdown:qdown 0.57

Detailed Protein Report

Protein 222: PREDICTED: zinc finger protein DZIP1 isoform X5 [Homo sapiens]

**Accession:**	gi|578825194	**Score:**	39.5
**Database:**	refseq_human(refseq_human_20140103.fasta)	**MW [kDa]:**	101.9
**Database Date:**	2015-11-30	**pI:**	6.7
**Modification(s):**	Carbamidomethyl	**Sequence Coverage [%]:**	3.4
		**No. of unique Peptides:**	2

Quantitation

mdown:qdown **Median:** 1.04 **CV:** 0.00 % **No. of Peptides:** 1

10	20	30	40	50	60	70	80
MQAEAADWFS	SMVRGGRPGR	PRRGCRAGEN	RGFPGPPAPQ	PARPPSPPPA	ALSLCPPQPF	QKHVYYPLAS	GPEGPDVAVA
**90**	**100**	**110**	**120**	**130**	**140**	**150**	**160**
AAAAGAASMA	CAPPSAASGP	LPFFQFRPRL	ESVDWRRLSA	IDVDKVAGAV	DVLTLQENIM	NITFCKLEDE	KCPHCQSGVD
**170**	**180**	**190**	**200**	**210**	**220**	**230**	**240**
PVLLKLIRLA	QFTIEYLLHS	QEFLTSQLHT	LEERLRLSHC	DGEQSKKLLT	KQAGEIKTLK	EECKRRKKMI	STQQLMIEAK
**250**	**260**	**270**	**280**	**290**	**300**	**310**	**320**
ANYYQCHFCD	KAFMNQAFLQ	SHIQRRHTEE	NSHFEYQKNA	QIEKLRSEIV	VLKEELQLTR	SELEAAHHAS	AVRFSKEYEM
**330**	**340**	**350**	**360**	**370**	**380**	**390**	**400**
QKTKEEDFLK	LFDRWKEEEK	EKLVDEMEKV	KEMFMKEFKE	LTSKNSALEY	QLSEIQKSNM	QIKSNIGTLK	DAHEFKEDRS
**410**	**420**	**430**	**440**	**450**	**460**	**470**	**480**
PYPQDFHNVM	QLLDSQESKW	TARVQAIHQE	HKKEKGRLLS	HIEKLRTSMI	DDLNASNVFY	KKRIEELGQR	LQEQNELIIT
**490**	**500**	**510**	**520**	**530**	**540**	**550**	**560**
QRQQIKDFTC	NPLNSISEPK	EQAFSSHILE	PIEELSEEEK	GRENEQKLNN	NKMHLRKALK	SNSSLTKGLR	TMVEQNLMEK
**570**	**580**	**590**	**600**	**610**	**620**	**630**	**640**
LETLGINADI	RGISSDQLHR	VLKSVESERH	KQEREIPNFH	QIREFLEHQV	SCKIEEKALL	SSDQCSVSQM	DTLSTGEVPK
**650**	**660**	**670**	**680**	**690**	**700**	**710**	**720**
MIQLPSKNRQ	LIRQKAVSTD	RTSVPKIKKN	VMEDPFPRKS	STITTPPFSS	EEEQEDDDLI	RAYASPGPLP	VPPPQNKGSF
**730**	**740**	**750**	**760**	**770**	**780**	**790**	**800**
GKNTVKSDAD	GTEGSEIEDT	DDSPKPAGVA	VKTPTEKVEK	MFPHRKNVNK	PVGGTNVPEM	FIKKEELQEL	KCADVEDEDW
**810**	**820**	**830**	**840**	**850**	**860**	**870**	**880**
DISSLEEEIS	LGKKSGKEQK	EPPPAKNEPH	FAHVLNAWGA	FNPKGPKGEG	LLLSPLIQAR	SIFLLVVFHL	YKYLINLCWY
**890**	**900**						
IIRQKSCQCF	LYVI						

Cmpd.	No. of Cmpds.	m/z meas.	Δ m/z [ppm]	z	Rt [min]	Score	P	Range	Sequence	Modification	Ratios
1639	1	1023.6248	76.10	1	49.2	11.8	1	818-826	K.EQKEPPPAK.N		mdown:qdown 1.04
2531	1	595.3252	88.36	2	62.1	10.6	0	886-894	K.SCQCFLYVI.-	Carbamidomethyl: 2, 4	

Detailed Protein Report

Protein 223: RNA polymerase II transcription factor SIII subunit A2 [Homo sapiens]

Accession: gi|45439357
Database: refseq_human(refseq_human_20140103.fasta)
Database Date: 2015-11-30
Modification(s): Carbamidomethyl

Score: 39.4
MW [kDa]: 83.9
pI: 10.5
Sequence Coverage [%]: 4.9
No. of unique Peptides: 2

10	20	30	40	50	60	70	80
MAAGSTTLHA	VEKLQVRLAT	KTEPKKLEKY	LQKLSALPMT	ADILAETGIR	KTVKRLRKHQ	HVGDFARDLA	ARWKKLVLVD
**90**	**100**	**110**	**120**	**130**	**140**	**150**	**160**
RNTRPGPQDP	EESASRQRFG	EALQDQEKAW	GFPENATAPR	SPSHSPEHRR	TARRTPPGQQ	RPHPRSHSRE	PRAERKCPRI
**170**	**180**	**190**	**200**	**210**	**220**	**230**	**240**
APADSGRYRA	SPTRTAPLRM	PEGPEPAAPG	KQPGRGHTHA	AQGGPLLCPG	CQGQPQGKAV	VSHSKGHKSS	RQEKRPLCAQ
**250**	**260**	**270**	**280**	**290**	**300**	**310**	**320**
GDWHSPTLIR	EKSCGACLRE	ETPRMPSWAS	ARDRQPSDFK	TDKEGGQAGS	GQRVPALEEA	PDSHQKRPQH	SHSNKKRPSL
**330**	**340**	**350**	**360**	**370**	**380**	**390**	**400**
DGRDPGNGTH	GLSPEEKEQL	SNDRETQEGK	PPTAHLDRTS	VSSLSEVEEV	DMAEEFEQPT	LSCEKYLTYD	QLRKQKKKTG
**410**	**420**	**430**	**440**	**450**	**460**	**470**	**480**
KSATTALGDK	QRKANESKGT	RESWDSAKKL	PPVQESQSER	LQAAGADSAG	PKTVPSHVFS	ELWDLSEAWM	QANYDPLSDS
**490**	**500**	**510**	**520**	**530**	**540**	**550**	**560**
DSMTSQAKPE	ALSSPKFREE	AAFPGRRVNA	KMPVYSGSRP	ACQLQVPTLR	QQCAQVLRNN	PDALSDVGEV	PYWVLEPVLE
**570**	**580**	**590**	**600**	**610**	**620**	**630**	**640**
GWRPDQLYRR	KKDNHALVRE	TDELRRNHCF	QDFKEEKPQE	NKTWREQYLR	LPDAPEQRLR	VMTTNIRSAR	GNNPNGREAK
**650**	**660**	**670**	**680**	**690**	**700**	**710**	**720**
MICFKSVAKT	PYDTSRRQEK	SAGDADPENG	EIKPASKPAG	SSHTPSSQSS	SGGGRDSSSS	ILRWLPEKRA	NPCLSSSNEH
**730**	**740**	**750**	**760**				
AAPAAKTRKQ	AAKKVAPLMA	KAIRDYKRRF	SRR				

Cmpd.	No. of Cmpds.	m/z meas.	Δ m/z [ppm]	z	Rt [min]	Score	P	Range	Sequence	Modification
687	1	512.1344	-204.56	2	37.4	13.3	1	251-259	R.EKSCGACLR.E	Carbamidomethyl: 4
436	1	734.3141	-70.58	3	34.5	10.6	2	422-440	R.ESWDSAKKLPPVQESQSER.L	

Detailed Protein Report

Protein 224: protein inscuteable homolog isoform d [Homo sapiens]

Accession: gi|507417931
Database: refseq_human(refseq_human_20140103.fasta)
Database Date: 2015-11-30

Score: 39.4
MW [kDa]: 57.2
pI: 5.4
Sequence Coverage [%]: 9.6
No. of unique Peptides: 1

10	20	30	40	50	60	70	80
MMALPGGRHL	DSVTLPGQRL	HLMQVDSVQR	WMEDLKLMTE	CECMCVLQAK	PISLEEDAQG	DLILAGGPGP	GDPLQLLLKR
**90**	**100**	**110**	**120**	**130**	**140**	**150**	**160**
GWVISTELRR	IGQKLAQDRW	ARVHSMSVRL	TCHARSMVSE	YSAVSRNSLK	EMGEIEKLLM	EKCSELSAVT	ERCLQVENEH
**170**	**180**	**190**	**200**	**210**	**220**	**230**	**240**
VLKSMKACVS	ETLSMLGQHF	GQLLELALTR	EVQALVRKID	ASDNIYTTES	TTGNLFSLTQ	EGAPLCRIIA	KEGGVVALFK
**250**	**260**	**270**	**280**	**290**	**300**	**310**	**320**
VCRQDSFRCL	YPQALRTLAS	ICCVEEGVHQ	LEKVDGVLCL	ADILTDNSHS	EATRAEAAAV	VAQVTSPHLP	VTQHLSSFLE
**330**	**340**	**350**	**360**	**370**	**380**	**390**	**400**
SMEEIVTALV	KLCQEASSGE	VFLLASAALA	NITFFDTMAC	EMLLQLNAIR	VLLEACSDKQ	RVDTPYTRDQ	IVTILANMSV
**410**	**420**	**430**	**440**	**450**	**460**	**470**	**480**
LEQCASDIIQ	ENGVQLIMGM	LSEKPRSGTP	AEVAACERVQ	QKAAVTLARL	SRDPDVAREA	VRLSCMSRLI	ELCRSPSERN
**490**	**500**	**510**	**520**	**530**			
SSDAVLVACL	AMGIQLQVKK	TNISALLLWF	LFWHIQGCSA				

Cmpd.	No. of Cmpds.	m/z meas.	Δ m/z [ppm]	z	Rt [min]	Score	P	Range	Sequence	Modification
2332	1	952.9342	-79.33	2	57.6	13.0	1	2-19	M.MALPGGRHLDSVTLPGQR.L	

Detailed Protein Report

Protein 225: **PREDICTED: splicing regulatory glutamine/lysine-rich protein 1 isoform X2 [Homo sapiens]**

**Accession:**	gi|530379166	**Score:**	39.4
**Database:**	refseq_human(refseq_human_20140103.fasta)	**MW [kDa]:**	43.4
**Database Date:**	2015-11-30	**pI:**	11.1
		**Sequence Coverage [%]:**	6.4
		**No. of unique Peptides:**	1

10	20	30	40	50	60	70	80
MTPQAAAKEL	EEVMKRVREA	QSFISAAIEP	ESGKSNERKG	GRSRSHTRSK	SRSSSKSHSR	RKRSQSKHRS	RSHNRSRSRQ
90	100	110	120	130	140	150	160
KDRRRSKSPH	KKRSKSRERR	KSRSRSHSRD	KRKDTREKIK	EKERVKEKDR	EKEREREKER	EKEKERGKNK	DRDKEREKDR
170	180	190	200	210	220	230	240
EKDKEKDRER	EREKEHEKDR	DKEKEKEQDK	EKEREKDRSK	EIDEKRKKDK	KSRTPPRSYN	ASRRSRSSSR	ERRRRRSRSS
250	260	270	280	290	300	310	320
SRSPRTSKTI	KRKSSRSPSP	RSRNKKDKKR	EKERDHISER	RERERSTSMR	KSSNDRDGKE	KLEKNSTSLK	EKEHNKEPDS
330	340	350	360				
SVSKEVDDKD	APRTEENKIQ	HNGNCQLNEE	NLSTKTEAV				

Cmpd.	No. of Cmpds.	m/z meas.	Δ m/z [ppm]	z	Rt [min]	Score	P	Range	Sequence	Modification
2747	1	807.3929	1.22	2	65.6	17.1	2	311-324	K.EKEHNKEPDSSVSK.E	

BRUKER

Detailed Protein Report

Protein 226: latent-transforming growth factor beta-binding protein 4 isoform b [Homo sapiens]

**Accession:**	gi|110347412	**Score:**	39.3
**Database:**	refseq_human(refseq_human_20140103.fasta)	**MW [kDa]:**	169.3
**Database Date:**	2015-11-30	**pI:**	5.0
		**Sequence Coverage [%]:**	2.5
		**No. of unique Peptides:**	3

Quantitation

mdown:qdown **Median:** 0.11 **CV:** 0.00 % **No. of Peptides:** 1

10	20	30	40	50	60	70	80
MGDVKALLFV	AAARARRLGG	AAASESLAVS	EAFCRVRSCQ	PKKCAGPQRC	LNPVPAVPSP	SPSVRKRQVS	LNWQPLTLQE
90	100	110	120	130	140	150	160
ARALLKRRRP	RGPGGRGLLR	RRPPQRAPAG	KAPVLCPLIC	HNGGVCVKPD	RCLCPPDFAG	KFCQLHSSGA	RPPAPAVPGL
170	180	190	200	210	220	230	240
TRSVYTMPLA	NHRDDEHGVA	SMVSVHVEHP	QEASVVVHQV	ERVSGPWEEA	DAEAVARAEA	AARAEAAAPY	TVLAQSAPRE
250	260	270	280	290	300	310	320
DGYSDASGFG	YCFRELRGGE	CASPLPGLRT	QEVCCRGAGL	AWGVHDCQLC	SERLGNSERV	SAPDGPCPTG	FERVNGSCED
330	340	350	360	370	380	390	400
VDECATGGRC	QHGECANTRG	GYTCVCPDGF	LLDSSRSSCI	SQHVISEAKG	PCFRVLRDGG	CSLPILRNIT	KQICCCSRVG
410	420	430	440	450	460	470	480
KAWGRGCQLC	PPFGSEGFRE	ICPAGPGYHY	SASDLRYNTR	PLGQEPPRVS	LSQPRTLPAT	SRPSAGFLPT	HRLEPRPEPR
490	500	510	520	530	540	550	560
PDPRPGPELP	LPSIPAWTGP	EIPESGPSSG	MCQRNPQVCG	PGRCISRPSG	YTCACDSGFR	LSPQGTRCID	VDECRRVPPP
570	580	590	600	610	620	630	640
CAPGRCENSP	GSFRCVCGPG	FRAGPRAAEC	LDVDECHRVP	PPCDLGRCEN	TPGSFLCVCP	AGYQAAPHGA	SCQDVDECTQ
650	660	670	680	690	700	710	720
SPGLCGRGAC	KNLPGSFRCV	CPAGFRGSAC	EEDVDECAQE	PPPCGPGRCD	NTAGSFHCAC	PAGFRSRGPG	APCQDVDECA
730	740	750	760	770	780	790	800
RSPPPCTYGR	CENTEGSFQC	VCPMGFQPNT	AGSECEDVDE	CENHLACPGQ	ECVNSPGSFQ	CRTCPSGHHL	HRGRCTDVDE
810	820	830	840	850	860	870	880
CSSGAPPCGP	HGHCTNTEGS	FRCSCAPGYR	APSGRPGPCA	DVNECLEGDF	CFPHGECLNT	DGSFACTCAP	GYRPGPRGAS
890	900	910	920	930	940	950	960
CLDVDECSEE	DLCQSGICTN	TDGSFECICP	PGHRAGPDLA	SCLDVDECRE	RGPALCGSQR	CENSPGSYRC	VRDCDPGYHA
970	980	990	1000	1010	1020	1030	1040
GPEGTCDDVD	ECQEYGPEIC	GAQRCENTPG	SYRCTPACDP	GYQPTPGGGC	QDVDECRNRS	FCGAHAVCQN	LPGSFQCLCD
1050	1060	1070	1080	1090	1100	1110	1120
QGYEGARDGR	HCVDVNECET	LQGVCGAALC	ENVEGSFLCV	CPNSPEEFDP	MTGRCVPPRT	SAGTFPGSQP	QAPASPVLPA
1130	1140	1150	1160	1170	1180	1190	1200
RPPPPPLPRR	PSTPRQGPVG	SGRRECYFDT	AAPDACDNIL	ARNVTWQECC	CTVGEGWGSG	CRIQQCPGTE	TAEYQSLCPH
1210	1220	1230	1240	1250	1260	1270	1280
GRGYLAPSGD	LSLRRDVDEC	QLFRDQVCKS	GVCVNTAPGY	SCYCSNGYYY	HTQRLECIDN	DECADEEPAC	EGGRCVNTVG
1290	1300	1310	1320	1330	1340	1350	1360
SYHCTCEPPL	VLDGSQRRCV	SNESQSLDDN	LGVCWQEVGA	DLVCSHPRLD	RQATYTECCC	LYGEAWGMDC	ALCPAQDSDD
1370	1380	1390	1400	1410	1420	1430	1440
FEALCNVLRP	PAYSPPRPGG	FGLPYEYGPD	LGPPYQGLPY	GPELYPPPAL	PYDPYPPPPG	PFARREAPYG	APRFDMPDFE
1450	1460	1470	1480	1490	1500	1510	1520
DDGGPYGESE	APAPPGPGTR	WPYRSRDTRR	SFPEPEEPPE	GGSYAGSLAE	PYEELEAEEC	GILDGCTNGR	CVRVPEGFTC
1530	1540	1550	1560	1570	1580	1590	
RCFDGYRLDM	TRMACVDINE	CDEAEAASPL	CVNARCLNTD	GSFRCICRPG	FAPTHQPHHC	APARPRA	

Cmpd.	No. of Cmpds.	m/z meas.	Δ m/z [ppm]	z	Rt [min]	Score	P	Range	Sequence	Modification	Ratios
398	1	646.4124	27.70	2	34.0	10.5	2	89-100	R.RPRGPGGRGLLR.R		
1055	1	941.2810	-147.80	2	42.8	15.8	1	932-949	R.GPALCGSQRCENSPGSYR.C		
373	1	457.1205	-287.06	2	34.0	13.0	1	1136-1144	R.QGPVGSGRR.E		mdown:qdown 0.11

Detailed Protein Report

Protein 227: PREDICTED: neuroblastoma breakpoint family member 3 isoform X2 [Homo sapiens]

Accession: gi|578799908
Database: refseq_human(refseq_human_20140103.fasta)
Database Date: 2015-11-30
Modification(s): Oxidation

Score: 39.3
MW [kDa]: 67.0
pI: 4.2
Sequence Coverage [%]: 5.9
No. of unique Peptides: 1

Alias proteins:

Accession	Name	Description
gi|578799910	refseq_human (refseq_human_20140103.fasta)	PREDICTED: neuroblastoma breakpoint family member 3 isoform X3 [Homo sapiens]

```
        10         20         30         40         50         60         70         80
MVVSAGPWSG EKAEMNILEI NKKSRPQLAE NKQQFRNLKQ KCLVTQVAYF LANRQNNYDY EDCKDLIKSM LRDERLLTEE
        90        100        110        120        130        140        150        160
KLAEELGQAE ELRQYKVLVH SQERELTQLR EKLQEGRDAS RSLNQHLQAL LTPDEPDNSQ GRDLREQLAE GCRLAQHLVQ
       170        180        190        200        210        220        230        240
KLSPENDDDE DEDVKVEEAE KVQELYAPRE VQKAEEKEVP EDSLEECAIT CSNSHHPCES NQPYGNTRIT FEEDQVDSTL
       250        260        270        280        290        300        310        320
IDSSSHDEWL DAVCIIPENE SDHEQEEEKG PVSPRNLQES EEEEAPQESW DEGDWTLSIP PDMSASYQSD RSTFHSVEEQ
       330        340        350        360        370        380        390        400
QVGLALDIGR HWCDQVKKED QEATSPRLSR ELLDEKEPEV LQDSLDRFYS TPFEYLELPD LCQPYRSDFY SLQEQHLGLA
       410        420        430        440        450        460        470        480
LDLDRMKKDQ EEEEDQGPPC PRLSRELPEV VEPEDLQDSL DRWYSTPFSY PELPDSCQPY GSCFYSLEEE HVGFSLDVDE
       490        500        510        520        530        540        550        560
IEKYQEGEED QKPPCPRLNE VLMEAEEPEV LQDSLDRCYS TTSTYFQLHA SFQQYRSAFY SFEEQDVSLA LDVDNRFFTL
       570        580
TVIRHHLAFQ MGVIFPH
```

Cmpd.	No. of Cmpds.	m/z meas.	Δ m/z [ppm]	z	Rt [min]	Score	P	Range	Sequence	Modification
1974	2	696.2762	-133.49	2	54.8	13.9	2	65-75	K.DLIKSMLRDER.L	Oxidation: 6

Detailed Protein Report

Protein 228: PREDICTED: focal adhesion kinase 1 isoform X12 [Homo sapiens]

**Accession:**	gi|530389222	**Score:**	39.3
**Database:**	refseq_human(refseq_human_20140103.fasta)	**MW [kDa]:**	108.1
**Database Date:**	2015-11-30	**pI:**	5.7
**Modification(s):**	Oxidation	**Sequence Coverage [%]:**	4.2
		**No. of unique Peptides:**	2

Quantitation

mdown:qdown **Median:** 1.25 **CV:** 0.00 % **No. of Peptides:** 1

10	20	30	40	50	60	70	80
MEMLLMSGYE	LRIRYLPKGF	LNQFTEDKPT	LNFFYQQVKS	DYMLEIADQV	DQEIALKLGC	LEIRRSYWEM	RGNALEKKSN
**90**	**100**	**110**	**120**	**130**	**140**	**150**	**160**
YEVLEKDVGL	KRFFPKSLLD	SVKAKTLRKL	IQQTFRQFAN	LNREESILKF	FEILSPVYRF	DKECFKCALG	SSWIISVELA
**170**	**180**	**190**	**200**	**210**	**220**	**230**	**240**
IGPEEGISYL	TDKGCNPTHL	ADFTQVQTIQ	YSNSEDKDRK	GMLQLKIAGA	PEPLTVTAPS	LTIAENMADL	IDGYCRLVNG
**250**	**260**	**270**	**280**	**290**	**300**	**310**	**320**
TSQSFIIRPQ	KEGERALPSI	PKLANSEKQG	MRTHAVSVSD	EISGDETDDY	AEIIDEEDTY	TMPSKSYGID	EARDYEIQRE
**330**	**340**	**350**	**360**	**370**	**380**	**390**	**400**
RIELGRCIGE	GQFGDVHQGI	YMSPENPALA	VAIKTCKNCT	SDSVREKFLQ	EALTMRQFDH	PHIVKLIGVI	TENPVWIIME
**410**	**420**	**430**	**440**	**450**	**460**	**470**	**480**
LCTLGELRSF	LQVRKYSLDL	ASLILYAYQL	STALAYLESK	RFVHRDIAAR	NVLVSSNDCV	KLGDFGLSRY	MEDSTYYKAS
**490**	**500**	**510**	**520**	**530**	**540**	**550**	**560**
KGKLPIKWMA	PESINFRRFT	SASDVWMFGV	CMWEILMHGV	KPFQGVKNND	VIGRIENGER	LPMPPNCPPT	LYSLMTKCWA
**570**	**580**	**590**	**600**	**610**	**620**	**630**	**640**
YDPSRRPRFT	ELKAQLSTIL	EEEKAQQEER	MRMESRRQAT	VSWDSGGSDE	APPKPSRPGY	PSPRSSEGFY	PSPQHMVQTN
**650**	**660**	**670**	**680**	**690**	**700**	**710**	**720**
HYQVSGYPGS	HGITAMAGSI	YPGQASLLDQ	TDSWNHRPQE	IAMWQPNVED	STVLDLRGIG	QVLPTHLMEE	RLIRQQQEME
**730**	**740**	**750**	**760**	**770**	**780**	**790**	**800**
EDQRWLEKEE	RFLKPDVRLS	RGSIDREDGS	LQGPIGNQHI	YQPVGKPDPA	APPKKPPRPG	APGHLGSLAS	LSSPADSYNE
**810**	**820**	**830**	**840**	**850**	**860**	**870**	**880**
GVKPWRLQPQ	EISPPPTANL	DRSNDKVYEN	VTGLVKAVIE	MSSKIQPAPP	EEYVPMVKEV	GLALRTLLAT	VDETIPLLPA
**890**	**900**	**910**	**920**	**930**	**940**	**950**	**960**
STHREIEMAQ	KLLNSDLGEL	INKMKLAQQY	VMTSLQQEYK	KQMLTAAHAL	AVDAKNLLDV	IDQARLKMLG	QTRPH

Cmpd.	No. of Cmpds.	m/z meas.	Δ m/z [ppm]	z	Rt [min]	Score	P	Range	Sequence	Modification	Ratios
2249	1	937.4207	-94.13	2	56.6	13.7	2	484-498	K.LPIKWMAPESINFRR.F	Oxidation: 6	
2062	1	720.2626	-173.22	2	54.3	10.7	0	922-935	K.QMLTAAHALAVDAK.N		mdown:qdown 1.25

Detailed Protein Report

Protein 229: **PREDICTED: p21-activated protein kinase-interacting protein 1 isoform X2 [Homo sapiens]**

**Accession:**	gi|578811766	**Score:**	39.2
**Database:**	refseq_human(refseq_human_20140103.fasta)	**MW [kDa]:**	41.4
**Database Date:**	2015-11-30	**pI:**	10.1
**Modification(s):**	Carbamidomethyl	**Sequence Coverage [%]:**	7.6
		**No. of unique Peptides:**	1

10	20	30	40	50	60	70	80
MLEQQWTLVA	DFTHHAHTAS	LSAVAVNSRF	VVTGSKDETI	HIYDMKKKIE	HGALVHHSGT	ITCLKFYGNR	HLISGAEDGL
**90**	**100**	**110**	**120**	**130**	**140**	**150**	**160**
ICIWDAKKWE	CLKSIKAHKG	QVTFLSIHPS	GKLALSVGTD	KTLRTWNLVE	GRSAFIKNIK	QNAHIVEWSP	RGEQYVVIIQ
**170**	**180**	**190**	**200**	**210**	**220**	**230**	**240**
NKIDIYQLDT	ASISGTITNE	KRISSVKFLS	ESVLAVAGDE	EVIRFFDCDS	LVCLCEFKAH	ENRVKDMFSF	EIPEHHVIVS
**250**	**260**	**270**	**280**	**290**	**300**	**310**	**320**
ASSDGFIKMW	KLKQDKKVPP	SLLCEINTNA	RLTCLGVWLD	KVADMKESLP	PAAEPSPVSK	EQSKIGKKEP	GDTVHKEEKR
**330**	**340**	**350**	**360**	**370**			
SKPNTKKRGL	TGDSKKATKE	SGLISTKKRK	MVEMLEKKRK	KKKIKTMQ			

Cmpd.	No. of Cmpds.	m/z meas.	Δ m/z [ppm]	z	Rt [min]	Score	P	Range	Sequence	Modification
2184	1	778.2730	-90.09	3	55.8	13.6	1	205-223	R.FFDCDSLVCLCEFKAHENR.V	Carbamidomethyl: 11

Detailed Protein Report

Protein 230: protein unc-13 homolog A [Homo sapiens]

**Accession:**	gi|283837842	**Score:**	39.1
**Database:**	refseq_human(refseq_human_20140103.fasta)	**MW [kDa]:**	192.9
**Database Date:**	2015-11-30	**pI:**	5.1
**Modification(s):**	Oxidation	**Sequence Coverage [%]:**	2.5
		**No. of unique Peptides:**	2

10	20	30	40	50	60	70	80
MSLLCVGVKK	AKFDGAQEKF	NTYVTLKVQN	VKSTTIAVRG	SQPSWEQDFM	FEINRLDLGL	TVEVWNKGLI	WDTMVGTVWI
90	100	110	120	130	140	150	160
PLRTIRQSNE	EGPGEWLTLD	SQVIMADSEI	CGTKDPTFHR	ILLDTRFELP	LDIPEEEARY	WAKKLEQLNA	MRDQDEYSFQ
170	180	190	200	210	220	230	240
DEQDKPLPVP	SNQCCNWNYF	GWGEQHNDDP	DSAVDDRDSD	YRSETSNSIP	PPYYTTSQPN	ASVHQYSVRP	PPLGSRESYS
250	260	270	280	290	300	310	320
DSMHSYEEFS	EPQALSPTGS	SRYASSGELS	QGSSQLSEDF	DPDEHSLQGS	DMEDERDRDS	YHSCHSSVSY	HKDSPRWDQD
330	340	350	360	370	380	390	400
EEELEEDLED	FLEEEELPED	EEELEEEEEE	VPDDLGSYAQ	REDVAVAEPK	DFKRISLPPA	APGKEDKAPV	APTEAPDMAK
410	420	430	440	450	460	470	480
VAPKPATPDK	VPAAEQIPEA	EPPKDEESFR	PREDEEGQEG	QDSMSRAKAN	WLRAFNKVRM	QLQEARGEGE	MSKSLWFKGG
490	500	510	520	530	540	550	560
PGGGLIIIDS	MPDIRKRKPI	PLVSDLAMSL	VQSRKAGITS	ALASSTLNNE	ELKNHVYKKT	LQALIYPISC	TTPHNFEVWT
570	580	590	600	610	620	630	640
ATTPTYCYEC	EGLLWGIARQ	GMRCTECGVK	CHEKCQDLLN	ADCLQRAAEK	SSKHGAEDRT	QNIIMVLKDR	MKIRERNKPE
650	660	670	680	690	700	710	720
IFELIQEIFA	VTKTAHTQQM	KAVKQSVLDG	TSKWSAKISI	TVVCAQGLQA	KDKTGSSDPY	VTVQVGKTKK	RTKTIYGNLN
730	740	750	760	770	780	790	800
PVWEENFHFE	CHNSSDRIKV	RVWDEDDDIK	SRVKQRFKRE	SDDFLGQTII	EVRTLSGEMD	VWYNLDKRTD	KSAVSGAIRL
810	820	830	840	850	860	870	880
HISVEIKGEE	KVAPYHVQYT	CLHENLFHFV	TDVQNNGVVK	IPDAKGDDAW	KVYYDETAQE	IVDEFAMRYG	VESIYQAMTH
890	900	910	920	930	940	950	960
FACLSSKYMC	PGVPAVMSTL	LANINAYYAH	TTASTNVSAS	DRFAASNFGK	ERFVKLLDQL	HNSLRIDLSM	YRNNFPASSP
970	980	990	1000	1010	1020	1030	1040
ERLQDLKSTV	DLLTSITFFR	MKVQELQSPP	RASQVVKDCV	KACLNSTYEY	IFNNCHELYS	REYQTDPAKK	GEVLPEEQGP
1050	1060	1070	1080	1090	1100	1110	1120
SIKNLDFWSK	LITLIVSIIE	EDKNSYTPCL	NQFPQELNVG	KISAEVMWNL	FAQDMKYAME	EHDKHRLCKS	ADYMNLHFKV
1130	1140	1150	1160	1170	1180	1190	1200
KWLYNEYVTE	LPAFKDRVPE	YPAWFEPFVI	QWLDENEEVS	RDFLHGALER	DKKDGFQQTS	EHALFSCSVV	DVFSQLNQSF
1210	1220	1230	1240	1250	1260	1270	1280
EIIKKLECPD	PQIVGHYMRR	FAKTISNVLL	QYADIISKDF	ASYCSKEKEK	VPCILMNNTQ	QLRVQLEKMF	EAMGGKELDA
1290	1300	1310	1320	1330	1340	1350	1360
EASDILKELQ	VKLNNVLDEL	SRVFATSFQP	HIEECVKQMG	DILSQVKGTG	NVPASACSSV	AQDADNVLQP	IMDLLDSNLT
1370	1380	1390	1400	1410	1420	1430	1440
LFAKICEKTV	LKRVLKELWK	LVMNTMEKTI	VLPPLTDQTM	IGNLLRKHGK	GLEKGRVKLP	SHSDGTQMIF	NAAKELGQLS
1450	1460	1470	1480	1490	1500	1510	1520
KLKDHMVREE	AKSLTPKQCA	VVELALDTIK	QYFHAGGVGL	KKTFLEKSPD	LQSLRYALSL	YTQATDLLIK	TFVQTQSAQG
1530	1540	1550	1560	1570	1580	1590	1600
LGVEDPVGEV	SVHVELFTHP	GTGEHKVTVK	VVAANDLKWQ	TSGIFRPFIE	VNIIGPQLSD	KKRKFATKSK	NNSWAPKYNE
1610	1620	1630	1640	1650	1660	1670	1680
SFQFTLSADA	GPECYELQVC	VKDYCFARED	RTVGLAVLQL	RELAQRGSAA	CWLPLGRRIH	MDDTGLTVLR	ILSQRSNDEV
1690	1700	1710					
AKEFVKLKSD	TRSAEEGGAA	PAP					

Cmpd.	No. of Cmpds.	m/z meas.	Δ m/z [ppm]	z	Rt [min]	Score	P	Range	Sequence	Modification
2705	1	805.4372	84.57	2	65.1	14.8	1	460-473	R.MQLQEARGEGEMSK.S	Oxidation: 1
2215	1	682.1038	136.60	3	57.9	10.1	1	1122-1137	K.WLYNEYVTELPAFKDR.V	

Detailed Protein Report

Protein 231: **latent-transforming growth factor beta-binding protein 4 isoform c precursor [Homo sapiens]**

**Accession:**	gi|110347437	**Score:**	39.1
**Database:**	refseq_human(refseq_human_20140103.fasta)	**MW [kDa]:**	165.6
**Database Date:**	2015-11-30	**pI:**	4.8
**Modification(s):**	Carbamidomethyl	**Sequence Coverage [%]:**	2.8
		**No. of unique Peptides:**	1

10	20	30	40	50	60	70	80
MAGGVRLLWV	SLLVLLAQLG	PQPGLGRLGE	RLRVRFTPVV	CGLRCVHGPT	GSRCTPTCAP	RNATSVDSGA	PGGAAPGGPG
90	100	110	120	130	140	150	160
FRAFLCPLIC	HNGGVCVKPD	RCLCPPDFAG	KFCQLHSSGA	RPPAPAVPGL	TRSVYTMPLA	NHRDDEHGVA	SMVSVHVEHP
170	180	190	200	210	220	230	240
QEASVVVHQV	ERVSGPWEEA	DAEAVARAEA	AARAEAAAPY	TVLAQSAPRE	DGYSDASGFG	YCFRELRGGE	CASPLPGLRT
250	260	270	280	290	300	310	320
QEVCCRGAGL	AWGVHDCQLC	SERLGNSERV	SAPDGPCPTG	FERVNGSCED	VDECATGGRC	QHGECANTRG	GYTCVCPDGF
330	340	350	360	370	380	390	400
LLDSSRSSCI	SQHVISEAKG	PCFRVLRDGG	CSLPILRNIT	KQICCCSRVG	KAWGRGCQLC	PPFGSEGFRE	ICPAGPGYHY
410	420	430	440	450	460	470	480
SASDLRYNTR	PLGQEPPRVS	LSQPRTLPAT	SRPSAGFLPT	HRLEPRPEPR	PDPRPGPELP	LPSIPAWTGP	EIPESGPSSG
490	500	510	520	530	540	550	560
MCQRNPQVCG	PGRCISRPSG	YTCACDSGFR	LSPQGTRCID	VDECRRVPPP	CAPGRCENSP	GSFRCVCGPG	FRAGPRAAEC
570	580	590	600	610	620	630	640
LDVDECHRVP	PPCDLGRCEN	TPGSFLCVCP	AGYQAAPHGA	SCQDVDECTQ	SPGLCGRGAC	KNLPGSFRCV	CPAGFRGSAC
650	660	670	680	690	700	710	720
EEDVDECAQE	PPPCGPGRCD	NTAGSFHCAC	PAGFRSRGPG	APCQDVDECA	RSPPPCTYGR	CENTEGSFQC	VCPMGFQPNT
730	740	750	760	770	780	790	800
AGSECEDVDE	CENHLACPGQ	ECVNSPGSFQ	CRTCPSGHHL	HRGRCTDVDE	CSSGAPPCGP	HGHCTNTEGS	FRCSCAPGYR
810	820	830	840	850	860	870	880
APSGRPGPCA	DVNECLEGDF	CFPHGECLNT	DGSFACTCAP	GYRPGPRGAS	CLDVDECSEE	DLCQSGICTN	TDGSFECICP
890	900	910	920	930	940	950	960
PGHRAGPDLA	SCLDVDECRE	RGPALCGSQR	CENSPGSYRC	VRDCDPGYHA	GPEGTCDDVD	ECQEYGPEIC	GAQRCENTPG
970	980	990	1000	1010	1020	1030	1040
SYRCTPACDP	GYQPTPGGGC	QDVDECRNRS	FCGAHAVCQN	LPGSFQCLCD	QGYEGARDGR	HCVDVNECET	LQGVCGAALC
1050	1060	1070	1080	1090	1100	1110	1120
ENVEGSFLCV	CPNSPEEFDP	MTGRCVPPRT	SAGTFPGSQP	QAPASPVLPA	RPPPPPLPRR	PSTPRQGPVG	SGRRECYFDT
1130	1140	1150	1160	1170	1180	1190	1200
AAPDACDNIL	ARNVTWQECC	CTVGEGWGSG	CRIQQCPGTE	TAEYQSLCPH	GRGYLAPSGD	LSLRRDVDEC	QLFRDQVCKS
1210	1220	1230	1240	1250	1260	1270	1280
GVCVNTAPGY	SCYCSNGYYY	HTQRLECIDN	DECADEEPAC	EGGRCVNTVG	SYHCTCEPPL	VLDGSQRRCV	SNESQSLDDN
1290	1300	1310	1320	1330	1340	1350	1360
LGVCWQEVGA	DLVCSHPRLD	RQATYTECCC	LYGEAWGMDC	ALCPAQDSDD	FEALCNVLRP	PAYSPPRPGG	FGLPYEYGPD
1370	1380	1390	1400	1410	1420	1430	1440
LGPPYQGLPY	GPELYPPPAL	PYDPYPPPPG	PFARREAPYG	APRFDMPDFE	DDGGPYGESE	APAPPGPGTR	WPYRSRDTRR
1450	1460	1470	1480	1490	1500	1510	1520
SFPEPEEPPE	GGSYAGSLAE	PYEELEAEEC	GILDGCTNGR	CVRVPEGFTC	RCFDGYRLDM	TRMACVDINE	CDEAEAASPL
1530	1540	1550	1560				
CVNARCLNTD	GSFRCICRPG	FAPTHQPHHC	APARPRA				

Cmpd.	No. of Cmpds.	m/z meas.	Δ m/z [ppm]	z	Rt [min]	Score	P	Range	Sequence	Modification
1901	1	900.3202	-98.12	2	52.4	10.3	1	45-61	R.CVHGPTGSRCTPTCAPR.N	Carbamidomethyl: 10

Detailed Protein Report

Protein 232: SURP and G-patch domain-containing protein 2 [Homo sapiens]

**Accession:**	gi|224282117	**Score:**	39.0
**Database:**	refseq_human(refseq_human_20140103.fasta)	**MW [kDa]:**	120.1
**Database Date:**	2015-11-30	**pI:**	7.0
**Modification(s):**	Carbamidomethyl	**Sequence Coverage [%]:**	4.4
		**No. of unique Peptides:**	2

Alias proteins:

Accession	Name	Description
gi|224282119	refseq_human (refseq_human_20140103.fasta)	SURP and G-patch domain-containing protein 2 [Homo sapiens]

```
        10         20         30         40         50         60         70         80
MAARRITQET FDAVLQEKAK RYHMDASGEA VSETLQFKAQ DLLRAVPRSR AEMYDDVHSD GRYSLSGSVA HSRDAGREGL
        90        100        110        120        130        140        150        160
RSDVFPGPSF RSSNPSISDD SYFRKECGRD LEFSHSDSRD QVIGHRKLGH FRSQDWKFAL RGSWEQDFGH PVSQESSWSQ
       170        180        190        200        210        220        230        240
EYSFGPSAVL GDFGSSRLIE KECLEKESRD YDVDHPGEAD SVLRGGSQVQ ARGRALNIVD QEGSLLGKGE TQGLLTAKGG
       250        260        270        280        290        300        310        320
VGKLVTLRNV STKKIPTVNR ITPKTQGTNQ IQKNTPSPDV TLGTNPGTED IQFPIQKIPL GLDLKNLRLP RRKMSFDIID
       330        340        350        360        370        380        390        400
KSDVFSRFGI EIIKWAGFHT IKDDIKFSQL FQTLFELETE TCAKMLASFK CSLKPEHRDF CFFTIKFLKH SALKTPRVDN
       410        420        430        440        450        460        470        480
EFLNMLLDKG AVKTKNCFFE IIKPFDKYIM RLQDRLLKSV TPLLMACNAY ELSVKMKTLS NPLDLALALE TTNSLCRKSL
       490        500        510        520        530        540        550        560
ALLGQTFSLA SSFRQEKILE AVGLQDIAPS PAAFPNFEDS TLFGREYIDH LKAWLVSSGC PLQVKKAEPE PMREEEKMIP
       570        580        590        600        610        620        630        640
PTKPEIQAKA PSSLSDAVPQ RADHRVVGTI DQLVKRVIEG SLSPKERTLL KEDPAYWFLS DENSLEYKYY KLKLAEMQRM
       650        660        670        680        690        700        710        720
SENLRGADQK PTSADCAVRA MLYSRAVRNL KKKLLPWQRR GLLRAQGLRG WKARRATTGT QTLLSSGTRL KHHGRQAPGL
       730        740        750        760        770        780        790        800
SQAKPSLPDR NDAAKDCPPD PVGPSPQDPS LEASGPSPKP AGVDISEAPQ TSSPCPSADI DMKTMETAEK LARFVAQVGP
       810        820        830        840        850        860        870        880
EIEQFSIENS TDNPDLWFLH DQNSSAFKFY RKKVFELCPS ICFTSSPHNL HTGGGDTTGS QESPVDLMEG EAEFEDEPPP
       890        900        910        920        930        940        950        960
REAELESPEV MPEEEDEDDE DGGEEAPAPG GAGKSEGSTP ADGLPGEAAE DDLAGAPALS QASSGTCFPR KRISSKSLKV
       970        980        990       1000       1010       1020       1030       1040
GMIPAPKRVC LIQEPKVHEP VRIAYDRPRG RPMSKKKKPK DLDFAQQKLT DKNLGFQMLQ KMGWKEGHGL GSLGKGIREP
      1050       1060       1070       1080       1090
VSVGTPSEGE GLGADGQEHK EDTFDVFRQR MMQMYRHKRA NK
```

Cmpd.	No. of Cmpds.	m/z meas.	Δ m/z [ppm]	z	Rt [min]	Score	P	Range	Sequence	Modification
2696	1	835.4525	-15.26	2	62.1	11.4	1	213-228	R.GRALNIVDQEGSLLGK.G	
1971	6	948.3560	-134.88	2	53.1	17.6	0	439-455	K.SVTPLLMACNAYELSVK.M	Carbamidomethyl: 9

Detailed Protein Report

Protein 233: PREDICTED: protein FAM186A isoform X1 [Homo sapiens]

**Accession:**	gi|578823289	**Score:**	38.9
**Database:**	refseq_human(refseq_human_20140103.fasta)	**MW [kDa]:**	262.1
**Database Date:**	2015-11-30	**pI:**	9.1
		**Sequence Coverage [%]:**	1.4
		**No. of unique Peptides:**	1

Quantitation

Wdown:Qdown	**Median:** 0.34	**CV:** 0.00 %	**No. of Peptides:** 1

Detailed Protein Report

10	20	30	40	50	60	70	80
MFFKMKNEID	NDPESEKCIK	DSTIMRREPQ	NILSPLMLPN	LEIPFSVKDI	ISRIERAQLH	RAREDIDMQL	SEIMNNVHRI
90	100	110	120	130	140	150	160
MTRYTLVFNS	SSERNVSLTE	HKKKQRTNFL	EKMATYAKTI	EIREKTLANI	LAWLEEWNDV	LSEMTLMDVD	EHHHWIAQME
170	180	190	200	210	220	230	240
LLPDTLKAIE	NNVKILSRFS	TSFLDEKKKQ	KKKILSRGTL	WKSWKERVIK	RPSTARALRP	DQMISDQLAT	NTKVSEIQGM
250	260	270	280	290	300	310	320
LQELIGTTMF	STLENNAIKY	ISSTIVNLST	ALSMLNDELK	CVNFQSSTVY	AHETSEAEKE	LSLKIIRDLS	NENEMLQQKL
330	340	350	360	370	380	390	400
QDAEEKCEQL	IRSKIVIEQL	YAKLSTSSTL	KVLPGPSPQS	SRAIIKVGDT	EDNMDNILDK	ELENIVDEVQ	RKETKDSGIK
410	420	430	440	450	460	470	480
WDSTISYTAQ	AERTPDLTEL	RQQPVASEDI	SEDSTKDNVS	LKKGDFYQED	ETDEYQSWKR	SHKKATYVYE	TSGPNLSDNK
490	500	510	520	530	540	550	560
SGQKVSEAKP	SQYYELQVLK	KKRKEMKSFS	EDKSKSPTEA	KRKHLSLTET	KSQGGKSGTS	MMMLEQFRKV	KRESPFDKRP
570	580	590	600	610	620	630	640
TAAEIKVEPT	TESLDKEGKG	EIRSLVEPLS	MIQFDDTAEP	QKGKIKGKKH	HISSGTITSK	EEKTEEKEEL	TKQVKSHQLV
650	660	670	680	690	700	710	720
KSLSRVAKET	SESTRVLESP	DGKSEQSNLE	EFQEAIMAFL	KQKIDNIGKA	FDKKTVPKEE	ELLKRAEAEK	LGIIKAKMEE
730	740	750	760	770	780	790	800
YFQKVAETVT	KILRKYKDTK	KEEQVGEKPI	KQKKVVSFMP	GLHFQKSPIS	AKSESSTLLS	YESTDPVINN	LIQMILAEIE
810	820	830	840	850	860	870	880
SERDIPTVST	VQKDHKEKEK	QRQEQYLQEG	QEQMSGMSLK	QQLLGERNLL	KEHYEKISEN	WEEKKAWLQM	KEGKQEQQSQ
890	900	910	920	930	940	950	960
KQWQEEEMWK	EEQKQATPKQ	AEQEEKQKQR	GQEEEELPKS	SLQRLEEGTQ	KMKTQGLLLE	KENGQMRQIQ	KEAKHLGPHR
970	980	990	1000	1010	1020	1030	1040
RREKGKEKQK	PERGLEDLER	QIKTKDQMQM	KETQPKELEK	MVIQTPMTLS	PRWKSVLKDV	QRSYEGKEFQ	RNLKTLENLP
1050	1060	1070	1080	1090	1100	1110	1120
DEKEPISITP	PPSLQYSLPG	ALPISGQPLT	KCIHLTPQQA	QEVGITLTPQ	QAQAQGITLT	LQQAQELGIP	LTPQQAQALE
1130	1140	1150	1160	1170	1180	1190	1200
ILFTPQQAQA	LGIPLTPQQT	QVQGITLTPQ	QDQAPGISLT	TQQAQKLGIP	LTPQQAQALG	IPLTPQQAQE	LGIPLTPQQA
1210	1220	1230	1240	1250	1260	1270	1280
QALRVSLTPQ	QAQELGIPLT	PQQAQALGIT	LTLQQAQQLG	IPLTPQQAQA	LGITLTPKQV	QELGIPLTPQ	QAQALGITLT
1290	1300	1310	1320	1330	1340	1350	1360
PKQAQELGIP	LNPQQAQTLG	IPLTPKQAQA	LGIPFTPQQA	QALGIPLTPQ	QAQTQEITLT	PQQAQALGMP	LTTQQAQELG
1370	1380	1390	1400	1410	1420	1430	1440
IPLTPQHAQA	LGMPLTTQQA	QELGIPLTPQ	QAQALGMPLT	TQQAQELGIP	LTPQQAQELG	IPFTPQQAQA	QEITLTPQQA
1450	1460	1470	1480	1490	1500	1510	1520
QALGMPLTAQ	QAQELGITLT	PQQAQELGIP	LTPQQAQALG	IPLIPPQAQE	LGIPLTPQQA	QALGILLIPP	QAQELGIPLT
1530	1540	1550	1560	1570	1580	1590	1600
PQQAQALGIP	LIPPQAQELG	IPLTPQQVQA	LGIPLIPPQA	QELEIPLTPQ	QAQALGIPLT	PQQAQELGIP	LTPQQAQELG
1610	1620	1630	1640	1650	1660	1670	1680
IPLTPQQAQA	QGIPLTPQQA	QALGISLTPQ	QAQAQGITLT	PQQAQALGVP	ITPVNAWVSA	VTLTSEQTHA	LESPMNLEQA
1690	1700	1710	1720	1730	1740	1750	1760
QEQLLKLGVP	LTLDKAHTLG	SPLTLKQVQW	SHRPFQKSKA	SLPTGQSIIS	RLSPSLRLSL	ASSAPTAEKS	SIFGVSSTPL
1770	1780	1790	1800	1810	1820	1830	1840
QISRVPLNQG	PFAPGKPLEM	GILSEPGKLG	APQTLRSSGQ	TLVYGGQSTS	AQFPAPQAPP	SPGQLPISRA	PPTPGQPFIA
1850	1860	1870	1880	1890	1900	1910	1920
GVPPTSGQIP	SLWAPLSPGQ	PLVPEASSIP	GDLLESGPLT	FSEQLQEFQP	PATAEQSPYL	QAPSTPGQHL	ATWTLPGRAS
1930	1940	1950	1960	1970	1980	1990	2000
SLWIPPTSRH	PPTLWPSPAP	GKPQKSWSPS	VAKKRLAIIS	SLKSKSVLIH	PSAPDFKVAQ	VPFTTKKFQM	SEVSDTSEET
2010	2020	2030	2040	2050	2060	2070	2080
QILRDTFAIE	SFRTFQSHFT	KYRTPVYQTP	YTDERALLTL	MKPTTSPSSL	TTLLRTSQIS	PLEWYQKSRF	PPIDKPWILS
2090	2100	2110	2120	2130	2140	2150	2160
SVSDTKKPKV	MVPPSSPQEL	EEKRYFVDVE	AQKKNLILLN	QAIKTCGLPS	QLHTMARTLI	IEILHMDTVQ	LGYLFRKYIA
2170	2180	2190	2200	2210	2220	2230	2240

Detailed Protein Report

Cmpd.	No. of Cmpds.	m/z meas.	Δ m/z [ppm]	z	Rt [min]	Score	P	Range	Sequence	Modification	Ratios
82	1	724.8160	-30.23	2	30.3	10.0	0	308-319	R.DLSNENEMLQQK.L		Wdown:Qdown 0.34

Detailed Protein Report

Protein 234: replication factor C subunit 1 isoform 1 [Homo sapiens]

**Accession:**	gi|32528306	**Score:**	38.9
**Database:**	refseq_human(refseq_human_20140103.fasta)	**MW [kDa]:**	128.1
**Database Date:**	2015-11-30	**pI:**	9.9
		**Sequence Coverage [%]:**	3.5
		**No. of unique Peptides:**	2

10	20	30	40	50	60	70	80
MDIRKFFGVI	PSGKKLVSET	VKKNEKTKSD	EETLKAKKGI	KEIKVNSSRK	EDDFKQKQPS	KKKRIIYDSD	SESEETLQVK
90	100	110	120	130	140	150	160
NAKKPPEKLP	VSSKPGKISR	QDPVTYISET	DEEDDFMCKK	AASKSKENGR	STNSHLGTSN	MKKNEENTKT	KNKPLSPIKL
170	180	190	200	210	220	230	240
TPTSVLDYFG	TGSVQRSNKK	MVASKRKELS	QNTDESGLND	EAIAKQLQLD	EDAELERQLH	EDEEFARTLA	MLDEEPKTKK
250	260	270	280	290	300	310	320
ARKDTEAGET	FSSVQANLSK	AEKHKYPHKV	KTAQVSDERK	SYSPRKQSKY	ESSKESQQHS	KSSADKIGEV	SSPKASSKLA
330	340	350	360	370	380	390	400
IMKRKEESSY	KEIEPVASKR	KENAIKLKGE	TKTPKKTKSS	PAKKESVSPE	DSEKKRTNYQ	AYRSYLNREG	PKALGSKEIP
410	420	430	440	450	460	470	480
KGAENCLEGL	IFVITGVLES	IERDEAKSLI	ERYGGKVTGN	VSKKTNYLVM	GRDSGQSKSD	KAAALGTKII	DEDGLLNLIR
490	500	510	520	530	540	550	560
TMPGKKSKYE	IAVETEMKKE	SKLERTPQKN	VQGKRKISPS	KKESESKKSR	PTSKRDSLAK	TIKKETDVFW	KSLDFKEQVA
570	580	590	600	610	620	630	640
EETSGDSKAR	NLADDSSENK	VENLLWVDKY	KPTSLKTIIG	QQGDQSCANK	LLRWLRNWQK	SSSEDKKHAK	FGKFSGKDDG
650	660	670	680	690	700	710	720
SSFKAALLSG	PPGVGKTTTA	SLVCQELGYS	YVELNASDTR	SKSSLKAIVA	ESLNNTSIKG	FYSNGAASSV	STKHALIMDE
730	740	750	760	770	780	790	800
VDGMAGNEDR	GGIQELIGLI	KHTKIPIICM	CNDRNHPKIR	SLVHYCFDLR	FQRPRVEQIK	GAMMSIAFKE	GLKIPPPAMN
810	820	830	840	850	860	870	880
EIILGANQDI	RQVLHNLSMW	CARSKALTYD	QAKADSHRAK	KDIKMGPFDV	ARKVFAAGEE	TAHMSLVDKS	DLFFHDYSIA
890	900	910	920	930	940	950	960
PLFVQENYIH	VKPVAAGGDM	KKHLMLLSRA	ADSICDGDLV	DSQIRSKQNW	SLLPAQAIYA	SVLPGELMRG	YMTQFPTFPS
970	980	990	1000	1010	1020	1030	1040
WLGKHSSTGK	HDRIVQDLAL	HMSLRTYSSK	RTVNMDYLSL	LRDALVQPLT	SQGVDGVQDV	VALMDTYYLM	KEDFENIMEI
1050	1060	1070	1080	1090	1100	1110	1120
SSWGGKPSPF	SKLDPKVKAA	FTRAYNKEAH	LTPYSLQAIK	ASRHSTSPSL	DSEYNEELNE	DDSQSDEKDQ	DAIETDAMIK
1130	1140	1150					
KKTKSSKPSK	PEKDKEPRKG	KGKSSKK					

Cmpd.	No. of Cmpds.	m/z meas.	Δ m/z [ppm]	z	Rt [min]	Score	P	Range	Sequence	Modification
991	1	530.7704	-72.90	2	41.2	10.7	2	42-50	K.EIKVNSSRK.E	
6	1	776.7890	-162.05	2	28.8	17.4	1	776-789	R.VEQIKGAMMSIAFK.E	

Detailed Protein Report

Protein 235: zinc finger protein 275 [Homo sapiens]

Accession: gi|298231195
Database: refseq_human(refseq_human_20140103.fasta)
Database Date: 2015-11-30
Modification(s): Carbamidomethyl

Score: 38.9
MW [kDa]: 37.0
pI: 10.0
Sequence Coverage [%]: 9.7
No. of unique Peptides: 2

10	20	30	40	50	60	70	80
MMSHPCVSLL	GVPVLNPALV	PHLAQGQVLL	VSDPSPNTDP	AKYSESTSAT	RHQMKGEDAQ	PQEMASTSFP	RASGPSPEFR
90	100	110	120	130	140	150	160
QHGDSDGKRG	SPQNLPIEHH	FACKECGDTF	RLKVLLVQHQ	RVHSEEKGWE	CGDCGKVFRG	VAEFNEHRKS	HVAAEPQPGP
170	180	190	200	210	220	230	240
SRALENAAEK	REQMEREAKP	FECEECGKRF	KKNAGLSQHL	RVHSREKPFD	CEECGRSFKV	NTHLFRHQKL	HTSEKPFACK
250	260	270	280	290	300	310	320
ACSRDFLDRQ	ELLKHQRMHT	GHLPFDCDDC	GKSFRGVNGL	AEHQRIHSGA	KPYGCPHCGK	LFRRSSELTK	HRRIHTGEKP
330							
YACGLPWAL							

Cmpd.	No. of Cmpds.	m/z meas.	Δ m/z [ppm]	z	Rt [min]	Score	P	Range	Sequence	Modification
2741	1	806.4367	70.79	2	65.5	13.9	0	286-300	R.IHSGAKPYGCPHCGK.L	Carbamidomethyl: 13
803	1	820.2164	-287.56	1	39.4	12.4	1	304-310	R.RSSELTK.H	

Detailed Protein Report

Protein 236: zinc finger protein 841 [Homo sapiens]

Accession: gi|211057416
Database: refseq_human(refseq_human_20140103.fasta)
Database Date: 2015-11-30
Modification(s): Oxidation
Score: 38.7
MW [kDa]: 106.0
pI: 10.4
Sequence Coverage [%]: 6.1
No. of unique Peptides: 2

10	20	30	40	50	60	70	80
MALPQGSLTF	RDVAVEFSQE	EWKCLDPVQK	ALYRDVMLEN	YRNLGFLGLC	LPDLNIISML	EQGKEPWTVV	SQVKIARNPN
**90**	**100**	**110**	**120**	**130**	**140**	**150**	**160**
CGECMKGVIT	GISPKCVIKE	LPPIQNSNTG	EKFQAVMLEG	HESYDTENFY	FREIRKNLQE	VDFQWKDGEI	NYKEGPMTHK
**170**	**180**	**190**	**200**	**210**	**220**	**230**	**240**
NNLTGQRVRH	SQGDVENKHM	ENQLILRFQS	GLGELQKFQT	AEKIYGCNQI	ERTVNNCFLA	SPLQRIFPGV	QTNISRKYGN
**250**	**260**	**270**	**280**	**290**	**300**	**310**	**320**
DFLQLSLPTQ	DEKTHIREKP	YIGNECGKAF	RVSSSLINHQ	MIHTTEKPYR	CNESGKAFHR	GSLLTVHQIV	HTRGKPYQCD
**330**	**340**	**350**	**360**	**370**	**380**	**390**	**400**
VCGRIFRQNS	DLVNHRRSHT	GDKPYICNEC	GKSFSKSSHL	AVHQRIHTGE	KPYKCNRCGK	CFSQSSSLAT	HQTVHTGDKP
**410**	**420**	**430**	**440**	**450**	**460**	**470**	**480**
YKCNECGKTF	KRNSSLTAHH	IIHAGKKPYT	CDVCGKVFYQ	NSQLVRHQII	HTGETPYKCN	ECGKVFFQRS	RLAGHRRIHT
**490**	**500**	**510**	**520**	**530**	**540**	**550**	**560**
GEKPYKCNEC	GKVFSQHSHL	AVHQRVHTGE	KPYKCNECGK	AFNWGSLLTV	HQRIHTGEKP	YKCNVCGKVF	NYGGYLSVHM
**570**	**580**	**590**	**600**	**610**	**620**	**630**	**640**
RCHTGEKPLH	CNKCGMVFTY	YSCLARHQRM	HTGEKPYKCN	VCGKVFIDSG	NLSIHRRSHT	GEKPFQCNEC	GKVFSYYSCL
**650**	**660**	**670**	**680**	**690**	**700**	**710**	**720**
ARHRKIHTGE	KPYKCNDCGK	AYTQRSSLTK	HLVIHTGENP	YHCNEFGEAF	IQSSKLARYH	RNPTGEKPHK	CSECGRTFSH
**730**	**740**	**750**	**760**	**770**	**780**	**790**	**800**
KTSLVYHQRR	HTGEMPYKCI	ECGKVFNSTT	TLARHRRIHT	GEKPYKCNEC	GKVFRYRSGL	ARHWSIHTGE	KPYKCNECGK
**810**	**820**	**830**	**840**	**850**	**860**	**870**	**880**
AFRVRSILLN	HQMMHTGEKP	YKCNECGKAF	IERSNLVYHQ	RNHTGEKPYK	CMECGKAFGR	RSCLTKHQRI	HSSEKPYKCN
**890**	**900**	**910**	**920**	**930**			
ECGKSYISRS	GLTKHQIKHA	GENLTTKLNV	ERPLDVVLTS	GIPK			

Cmpd.	No. of Cmpds.	m/z meas.	Δ m/z [ppm]	z	Rt [min]	Score	P	Range	Sequence	Modification
2885	1	1129.0588	-29.01	2	64.8	11.2	1	179-197	K.HMENQLILRFQSGLGELQK.F	Oxidation: 2
1460	1	1011.7530	-79.39	3	48.0	16.8	2	590-616	R.MHTGEKPYKCNVCGKVFIDSGNLSIHR.R	

Detailed Protein Report

Protein 237: G2/mitotic-specific cyclin-B3 isoform 3 [Homo sapiens]

**Accession:**	gi|90669307	**Score:**	38.7
**Database:**	refseq_human(refseq_human_20140103.fasta)	**MW [kDa]:**	157.8
**Database Date:**	2015-11-30	**pI:**	6.3
		**Sequence Coverage [%]:**	3.4
		**No. of unique Peptides:**	3

Quantitation

mdown:qdown **Median:** 0.38 **CV:** 0.00 % **No. of Peptides:** 1

10	20	30	40	50	60	70	80
MLLPLPPQSS	KPVPKKSQSS	KIVPSHHDPS	EKTGENCQTK	ISPSSLQESP	SSLQGALKKR	SAFEDLTNAS	QCQPVQPKKE
**90**	**100**	**110**	**120**	**130**	**140**	**150**	**160**
ANKEFVKVVS	KKINRNTHAL	GLAKKNKRNL	KWHKLEVTPV	VASTTVVPNI	MEKPLILDIS	TTSKTPNTEE	ASLFRKPLVL
**170**	**180**	**190**	**200**	**210**	**220**	**230**	**240**
KEEPTIEDET	LINKSLSLKK	CSNHEEVSLL	EKLQPLQEES	DSDDAFVIEP	MTFKKTHKTE	EAAITKKTLS	LKKKMCASQR
**250**	**260**	**270**	**280**	**290**	**300**	**310**	**320**
KQSCQEESLA	VQDVNMEEDS	FFMESMSFKK	KPKTEESIPT	HKLSSLKKKC	TIYGKICHFR	KPPVLQTTIC	GAMSSIKKPT
**330**	**340**	**350**	**360**	**370**	**380**	**390**	**400**
TEKETLFQEL	SVLQEKHTTE	HEMSILKKSL	ALQKTNFKED	SLVKESLAFK	KKPSTEEAIM	MPVILKEQCM	TEGKRSRLKP
**410**	**420**	**430**	**440**	**450**	**460**	**470**	**480**
LVLQEITSGE	KSLIMKPLSI	KEKPSTEKES	FSQEPSALQK	KHTTQEEVSI	LKEPSSLLKS	PTEESPFDEA	LAFTKKCTIE
**490**	**500**	**510**	**520**	**530**	**540**	**550**	**560**
EAPPTKKPLI	LKRKHATQGT	MSHLKKPLIL	QTTSGEKSLI	KEPLPFKEEK	VSLKKKCTTQ	EMMSICPELL	DFQDMIGEDK
**570**	**580**	**590**	**600**	**610**	**620**	**630**	**640**
NSFFMEPMSF	RKNPTTEETV	LTKTSLSLQE	KKITQGKMSH	LKKPLVLQKI	TSEEESFYKK	LLPFKMKSTT	EEKFLSQEPS
**650**	**660**	**670**	**680**	**690**	**700**	**710**	**720**
ALKEKHTTLQ	EVSLSKESLA	IQEKATTEEE	FSQELFSLHV	KHTNKSGSLF	QEALVLQEKT	DAEEDSLKNL	LALQEKSTME
**730**	**740**	**750**	**760**	**770**	**780**	**790**	**800**
EESLINKLLA	LKEELSAEAA	TNIQTQLSLK	KKSTSHGKVF	FLKKQLALNE	TINEEEFLNK	QPLALEGYPS	IAEGETLFKK
**810**	**820**	**830**	**840**	**850**	**860**	**870**	**880**
LLAMQEEPSI	EKEAVLKEPT	IDTEAHFKEP	LALQEEPSTE	KEAVLKEPSV	DTEAHFKETL	ALQEKPSIEQ	EALFKRHSAL
**890**	**900**	**910**	**920**	**930**	**940**	**950**	**960**
WEKPSTEKET	IFKESLDLQE	KPSIKKETLL	KKPLALKMST	INEAVLFEDM	IALNEKPTTG	KELSFKEPLA	LQESPTYKED
**970**	**980**	**990**	**1000**	**1010**	**1020**	**1030**	**1040**
TFLKTLLVPQ	VGTSPNVSST	APESITSKSS	IATMTSVGKS	GTINEAFLFE	DMITLNEKPT	TGKELSFKEP	LALQESPTCK
**1050**	**1060**	**1070**	**1080**	**1090**	**1100**	**1110**	**1120**
EDTFLETFLI	PQIGTSPYVF	STTPESITEK	SSIATMTSVG	KSRTTTESSA	CESASDKPVS	PQAKGTPKEI	TPREDIDEDS
**1130**	**1140**	**1150**	**1160**	**1170**	**1180**	**1190**	**1200**
SDPSFNPMYA	KEIFSYMKER	EEQFILTDYM	NRQIEITSDM	RAILVDWLVE	VQVSFEMTHE	TLYLAVKLVD	LYLMKAVCKK
**1210**	**1220**	**1230**	**1240**	**1250**	**1260**	**1270**	**1280**
DKLQLLGATA	FMIAAKFEEH	NSPRVDDFVY	ICDDNYQRSE	VLSMEINILN	VLKCDINIPI	AYHFLRRYAR	CIHTNMKTLT
**1290**	**1300**	**1310**	**1320**	**1330**	**1340**	**1350**	**1360**
LSRYICEMTL	QEYHYVQEKA	SKLAAASLLL	ALYMKKLGYW	VPFLEHYSGY	SISELHPLVR	QLNKLLTFSS	YDSLKAVYYK
**1370**	**1380**	**1390**	**1400**				
YSHPVFFEVA	KIPALDMLKL	EEILNCDCEA	QGLVL				

Cmpd.	No. of Cmpds.	m/z meas.	Δ m/z [ppm]	z	Rt [min]	Score	P	Range	Sequence	Modification	Ratios
1030	1	670.7981	-53.16	2	41.6	10.6	1	290-300	K.CTIYGKICHFR.K		mdown:qdown 0.38
2723	1	639.3585	-37.42	2	62.4	12.0	2	348-358	K.KSLALQKTNFK.E		
1809	2	1011.7628	-33.19	3	51.3	16.1	2	535-560	K. KKCTTQEMMSICPELLDFQDMIGE N		

Detailed Protein Report

Protein 238: ankyrin-1 isoform 2 [Homo sapiens]

**Accession:**	gi|70780355	**Score:**	38.6
**Database:**	refseq_human(refseq_human_20140103.fasta)	**MW [kDa]:**	188.9
**Database Date:**	2015-11-30	**pI:**	6.2
		**Sequence Coverage [%]:**	1.4
		**No. of unique Peptides:**	2

Quantitation

mdown:qdown	**Median:**	1.76	**CV:**	0.00 %	**No. of Peptides:**	1
Wdown:Qdown	**Median:**	3.16	**CV:**	0.00 %	**No. of Peptides:**	1

10	20	30	40	50	60	70	80
MPYSVGFREA	DAATSFLRAA	RSGNLDKALD	HLRNGVDINT	CNQNGLNGLH	LASKEGHVKM	VVELLHKEII	LETTTKKGNT
90	100	110	120	130	140	150	160
ALHIAALAGQ	DEVVRELVNY	GANVNAQSQK	GFTPLYMAAQ	ENHLEVVKFL	LENGANQNVA	TEDGFTPLAV	ALQQGHENVV
170	180	190	200	210	220	230	240
AHLINYGTKG	KVRLPALHIA	ARNDDTRTAA	VLLQNDPNPD	VLSKTGFTPL	HIAAHYENLN	VAQLLLNRGA	SVNFTPQNGI
250	260	270	280	290	300	310	320
TPLHIASRRG	NVIMVRLLLD	RGAQIETKTK	DELTPLHCAA	RNGHVRISEI	LLDHGAPIQA	KTKNGLSPIH	MAAQGDHLDC
330	340	350	360	370	380	390	400
VRLLLQYDAE	IDDITLDHLT	PLHVAAHCGH	HRVAKVLLDK	GAKPNSRALN	GFTPLHIACK	KNHVRVMELL	LKTGASIDAV
410	420	430	440	450	460	470	480
TESGLTPLHV	ASFMGHLPIV	KNLLQRGASP	NVSNVKVETP	LHMAARAGHT	EVAKYLLQNK	AKVNAKAKDD	QTPLHCAARI
490	500	510	520	530	540	550	560
GHTNMVKLLL	ENNANPNLAT	TAGHTPLHIA	AREGHVETVL	ALLEKEASQA	CMTKKGFTPL	HVAAKYGKVR	VAELLLERDA
570	580	590	600	610	620	630	640
HPNAAGKNGL	TPLHVAVHHN	NLDIVKLLLP	RGGSPHSPAW	NGYTPLHIAA	KQNQVEVARS	LLQYGGSANA	ESVQGVTPLH
650	660	670	680	690	700	710	720
LAAQEGHAEM	VALLLSKQAN	GNLGNKSGLT	PLHLVAQEGH	VPVADVLIKH	GVMVDATTRM	GYTPLHVASH	YGNIKLVKFL
730	740	750	760	770	780	790	800
LQHQADVNAK	TKLGYSPLHQ	AAQQGHTDIV	TLLLKNGASP	NEVSSDGTTP	LAIAKRLGYI	SVTDVLKVVT	DETSFVLVSD
810	820	830	840	850	860	870	880
KHRMSFPETV	DEILDVSEDE	GEELISFKAE	RRDSRDVDEE	KELLDFVPKL	DQVVESPAIP	RIPCAMPETV	VIRSEEQEQA
890	900	910	920	930	940	950	960
SKEYDEDSLI	PSSPATETSD	NISPVASPVH	TGFLVSFMVD	ARGGSMRGSR	HNGLRVVIPP	RTCAAPTRIT	CRLVKPQKLS
970	980	990	1000	1010	1020	1030	1040
TPPPLAEEEG	LASRIIALGP	TGAQFLSPVI	VEIPHFASHG	RGDRELVVLR	SENGSVWKEH	RSRYGESYLD	QILNGMDEEL
1050	1060	1070	1080	1090	1100	1110	1120
GSLEELEKKR	VCRIITTDFP	LYFVIMSRLC	QDYDTIGPEG	GSLKSKLVPL	VQATFPENAV	TKRVKLALQA	QPVPDELVTK
1130	1140	1150	1160	1170	1180	1190	1200
LLGNQATFSP	IVTVEPRRRK	FHRPIGLRIP	LPPSWTDNPR	DSGEGDTTSL	RLLCSVIGGT	DQAQWEDITG	TTKLVYANEC
1210	1220	1230	1240	1250	1260	1270	1280
ANFTTNVSAR	FWLSDCPRTA	EAVNFATLLY	KELTAVPYMA	KFVIFAKMND	PREGRLRCYC	MTDDKVDKTL	EQHENFVEVA
1290	1300	1310	1320	1330	1340	1350	1360
RSRDIEVLEG	MSLFAELSGN	LVPVKKAAQQ	RSFHFQSFRE	NRLAMPVKVR	DSSREPGGSL	SFLRKAMKYE	DTQHILCHLN
1370	1380	1390	1400	1410	1420	1430	1440
ITMPPCAKGS	GAEDRRRTPT	PLALRYSILS	ESTPGSLSGT	EQAEMKMAVI	SEHLGLSWAE	LARELQFSVE	DINRIRVENP
1450	1460	1470	1480	1490	1500	1510	1520
NSLLEQSVAL	LNLWVIREGQ	NANMENLYTA	LQSIDRGEIV	NMLEGSGRQS	RNLKPDRRHT	DRDYSLSPSQ	MNGHQRGQAR
1530	1540	1550	1560	1570	1580	1590	1600
ITHSPTVSQV	TERSQDRLQD	WDADGSIVSY	LQDAAQGSWQ	EEVTQGPHSF	QGTSTMTEGL	EPGGSQEYEK	VLVSVSEHTW
1610	1620	1630	1640	1650	1660	1670	1680
TEQPEAESSQ	ADRDRRQQGQ	EEQVQEAKNT	FTQVVQGNEF	QNIPGEQVTE	EQFTDEQGNI	VTKKIIRKVV	RQIDLSSADA
1690	1700	1710	1720				
AQEHEEVTVE	GPLEDPSELE	VDIDYFMKHS	KDHTSTPNP				

Detailed Protein Report

Cmpd.	No. of Cmpds.	m/z meas.	Δ m/z [ppm]	z	Rt [min]	Score	P	Range	Sequence	Modification	Ratios
1714	2	917.4108	-124.71	2	50.0	23.4	1	287-303	R.ISEILLDHGAPIQAKTK.N		
2592	3	449.6181	-325.55	2	61.1	15.2	1	1492-1498	R.NLKPDRR.H		Wdown:Qdown 3.16<br>mdown:qdown 1.76

Detailed Protein Report

Protein 239: PREDICTED: testis-expressed sequence 2 protein isoform X4 [Homo sapiens]

**Accession:**	gi|530412523	**Score:**	38.6
**Database:**	refseq_human(refseq_human_20140103.fasta)	**MW [kDa]:**	125.0
**Database Date:**	2015-11-30	**pI:**	5.6
**Modification(s):**	Carbamidomethyl, Oxidation	**Sequence Coverage [%]:**	3.2
		**No. of unique Peptides:**	2

Quantitation

mdown:qdown	**Median:** 0.67	**CV:** 0.00 %	**No. of Peptides:**	1
Wdown:Qdown	**Median:** 0.16	**CV:** 0.00 %	**No. of Peptides:**	1

```
        10         20         30         40         50         60         70         80
MTSLYGRHAE KTTDMPKPSA PKVHVQRSVS RDTIAIHFSA SGEEEEEEEE EFREYFEEGL DDQSIVTGLE AKEDLYLEPQ
        90        100        110        120        130        140        150        160
VGHDPAGPAA SPVLADGLSV SQAPAILPVS KNTVKLLESP VPAAQVLSTV PLAVSPGSSS SGPLASSPSV SSLSEQKTSS
       170        180        190        200        210        220        230        240
SSPLSSPSKS PILSSSASTS TLSSAKPFMS LVKSLSTEVE PKESPHPARH RHLMKTLVKS LSTDTSRQES DTVSYKPPDS
       250        260        270        280        290        300        310        320
KLNLHLFKQF TQPRNTGGDS KTAPSSPLTS PSDTRSFFKV PEMEAKIEDT KRRLSEVIYE PFQLLSKIIG EESGSHRPKA
       330        340        350        360        370        380        390        400
LSSSASELSN LSSLNGHLES NNNYSIKEEE CDSEGDGYGS DSNIPRSDHP KSTGEPTREI ELKSSQGSSL KDLGLKTSSL
       410        420        430        440        450        460        470        480
VLEKCSLSAL VSKEDEEFCE LYTEDFDLET EGESKVDKLS DIPLKPEVLA EDGVVLDSED EVDSAVQHPE LPVKTLGFFI
       490        500        510        520        530        540        550        560
MCVYVYLILP LPHYVSGLFL GIGLGFMTAV CVIWFFTPPS AHKYHKLHKN LRHWNTRSLD IKEPEILKGW MNEIYNYDPE
       570        580        590        600        610        620        630        640
TYHATLTHSV FVRLEGGTLR LSKPNKNISR RASYNEPKPE VTYISQKIYD LSDSKIYLVP KTLARKRIWN KKYPICIELG
       650        660        670        680        690        700        710        720
QQDDFMSKAQ TDKETSEEKP PAEGSEDPKK PPRPQEGTRS SQRDQILYLF GRTGREKEEW FRRFILASKL KSEIKKSSGV
       730        740        750        760        770        780        790        800
SGGKPGLLPA HSRHNSPSGH LTHSRSSSKG SVEEIMSQPK QKELAGSVRQ KMLLDYSVYM GRCVPQESRS PQRSPLQSAE
       810        820        830        840        850        860        870        880
SSPTAGKKLP EVPPSEEEEQ EAWVNALLGR IFWDFLGEKY WSDLVSKKIQ MKLSKIKLPY FMNELTLTEL DMGVAVPKIL
       890        900        910        920        930        940        950        960
QAFKPYVDHQ GLWIDLEMSY NGSFLMTLET KMNLTKLGKE PLVEALKVGE IGKEGPRAFC LADSDEESSS AGSSEEDDAP
       970        980        990       1000       1010       1020       1030       1040
EPSGGDKQLL PGAEGYVGGH RTSKIMRFVD KITKSKYFQK ATETEFIKKK IEEVSNTPLL LTVEVQECRG TLAVNIPPPP
      1050       1060       1070       1080       1090       1100       1110       1120
TDRVWYGFRK PPHVELKARP KLGEREVTLV HVTDWIEKKL EQEFQKVFVM PNMDDVYITI MHSAMDPRST SCLLKDPPVE
      1130
AADQP
```

Cmpd.	No. of Cmpds.	m/z meas.	Δ m/z [ppm]	z	Rt [min]	Score	P	Range	Sequence	Modification	Ratios
1076	1	581.1694	-231.84	2	41.9	12.9	1	2-11	M.TSLYGRHAEK.T		mdown:qdown 0.67
2499	1	1044.5086	15.43	2	61.7	15.0	1	632-648	K.KYPICIELGQQDDFMSK.A	Carbamidomethyl: 5; Oxidation: 15	Wdown:Qdown 0.16

Detailed Protein Report

Protein 240: bone morphogenetic protein 1 isoform 3 precursor [Homo sapiens]

**Accession:**	gi|5453579	**Score:**	38.5
**Database:**	refseq_human(refseq_human_20140103.fasta)	**MW [kDa]:**	111.2
**Database Date:**	2015-11-30	**pI:**	6.5
		**Sequence Coverage [%]:**	3.4
		**No. of unique Peptides:**	3

Quantitation

mdown:qdown **Median:** 0.63 **CV:** 0.00 % **No. of Peptides:** 1

10	20	30	40	50	60	70	80
MPGVARLPLL	LGLLLLPRPG	RPLDLADYTY	DLAEEDDSEP	LNYKDPCKAA	AFLGDIALDE	EDLRAFQVQQ	AVDLRRHTAR
90	100	110	120	130	140	150	160
KSSIKAAVPG	NTSTPSCQST	NGQPQRGACG	RWRGRSRSRR	AATSRPERVW	PDGVIPFVIG	GNFTGSQRAV	FRQAMRHWEK
170	180	190	200	210	220	230	240
HTCVTFLERT	DEDSYIVFTY	RPCGCCSYVG	RRGGGPQAIS	IGKNCDKFGI	VVHELGHVVG	FWHEHTRPDR	DRHVSIVREN
250	260	270	280	290	300	310	320
IQPGQEYNFL	KMEPQEVESL	GETYDFDSIM	HYARNTFSRG	IFLDTIVPKY	EVNGVKPPIG	QRTRLSKGDI	AQARKLYKCP
330	340	350	360	370	380	390	400
ACGETLQDST	GNFSSPEYPN	GYSAHMHCVW	RISVTPGEKI	ILNFTSLDLY	RSRLCWYDYV	EVRDGFWRKA	PLRGRFCGSK
410	420	430	440	450	460	470	480
LPEPIVSTDS	RLWVEFRSSS	NWVGKGFFAV	YEAICGGDVK	KDYGHIQSPN	YPDDYRPSKV	CIWRIQVSEG	FHVGLTFQSF
490	500	510	520	530	540	550	560
EIERHDSCAY	DYLEVRDGHS	ESSTLIGRYC	GYEKPDDIKS	TSSRLWLKFV	SDGSINKAGF	AVNFFKEVDE	CSRPNRGGCE
570	580	590	600	610	620	630	640
QRCLNTLGSY	KCSCDPGYEL	APDKRRCEAA	CGGFLTKLNG	SITSPGWPKE	YPPNKNCIWQ	LVAPTQYRIS	LQFDFFETEG
650	660	670	680	690	700	710	720
NDVCKYDFVE	VRSGLTADSK	LHGKFCGSEK	PEVITSQYNN	MRVEFKSDNT	VSKKGFKAHF	FSDKDECSKD	NGGCQQDCVN
730	740	750	760	770	780	790	800
TFGSYECQCR	SGFVLHDNKH	DCKEAGCDHK	VTSTSGTITS	PNWPDKYPSK	KECTWAISST	PGHRVKLTFM	EMDIESQPEC
810	820	830	840	850	860	870	880
AYDHLEVFDG	RDAKAPVLGR	FCGSKKPEPV	LATGSRMFLR	FYSDNSVQRK	GFQASHATEC	GGQVRADVKT	KDLYSHAQFG
890	900	910	920	930	940	950	960
DNNYPGGVDC	EWVIVAEEGY	GVELVFQTFE	VEEETDCGYD	YMELFDGYDS	TAPRLGRYCG	SGPPEEVYSA	GDSVLVKFHS
970	980	990					
DDTITKKGFH	LRYTSTKFQD	TLHSRK					

Cmpd.	No. of Cmpds.	m/z meas.	Δ m/z [ppm]	z	Rt [min]	Score	P	Range	Sequence	Modification	Ratios
556	1	658.3907	22.79	2	35.8	11.5	2	303-314	R.TRLSKGDIAQAR.K		mdown:qdown 0.63
2634	3	753.8426	-101.90	2	61.7	16.4	1	525-537	R.LWLKFVSDGSINK.A		
2126	1	1131.3250	-234.65	1	56.8	10.6	1	978-986	K.FQDTLHSRK.-		

Detailed Protein Report

Protein 241: F-box only protein 41 [Homo sapiens]

**Accession:**	gi|289803020	**Score:**	38.4
**Database:**	refseq_human(refseq_human_20140103.fasta)	**MW [kDa]:**	94.4
**Database Date:**	2015-11-30	**pI:**	9.8
**Modification(s):**	Carbamidomethyl	**Sequence Coverage [%]:**	3.7
		**No. of unique Peptides:**	1

Quantitation

mdown:qdown **Median:** 1.17 **CV:** 0.00 % **No. of Peptides:** 1

Alias proteins:

Accession	Name	Description
gi|578802734	refseq_human (refseq_human_20140103.fasta)	PREDICTED: F-box only protein 41 isoform X2 [Homo sapiens]

10	20	30	40	50	60	70	80
MASLDLPYRC	PRCGEHKRFR	SLSSLRAHLE	YSHTYETLYI	LSKTNSICDG	AAAAAAAAAA	ASGFPLAPEP	AALLAVPGAR
90	100	110	120	130	140	150	160
REVFESTSFQ	GKEQAAGPSP	AAPHLLHHHH	HHAPLAHFPG	DLVPASLPCE	ELAEPGLVPA	AAARYALREI	EIPLGELFAR
170	180	190	200	210	220	230	240
KSVASSACST	PPPGPGPGPC	PGPASASPAS	PSPADVAYEE	GLARLKIRAL	EKLEVDRRLE	RLSEEVEQKI	AGQVGRLQAE
250	260	270	280	290	300	310	320
LERKAAELET	ARQESARLGR	EKEELEERAS	ELSRQVDVSV	ELLASLKQDL	VHKEQELSRK	QQEVVQIDQF	LKETAAREAS
330	340	350	360	370	380	390	400
AKLRLQQFIE	ELLERADRAE	RQLQVISSSC	GSTPSASLGR	GGGGGGAGPN	ARGPGRMREH	HVGPAVPNTY	AVSRHGSSPS
410	420	430	440	450	460	470	480
TGASSRVPAA	SQSSGCYDSD	SLELPRPEEG	APEDSGPGGL	GTRAQAANGG	SERSQPPRSS	GLRRQAIQNW	QRRPRRHSTE
490	500	510	520	530	540	550	560
GEEGDVSDVG	SRTTESEAEG	PLDAPRPGPA	MAGPLSSCRL	SARPEGGSGR	GRRAERVSPS	RSNEVISPEI	LKMRAALFCI
570	580	590	600	610	620	630	640
FTYLDTRTLL	HAAEVCRDWR	FVARHPAVWT	RVLLENARVC	SKFLAMLAQW	CTQAHSLTLQ	NLKPRQRGKK	ESKEEYARST
650	660	670	680	690	700	710	720
RGCLEAGLES	LLKAAGGNLL	ILRISHCPNI	LTDRSLWLAS	CYCRALQAVT	YRSATDPVGH	EVIWALGAGC	REIVSLQVAP
730	740	750	760	770	780	790	800
LHPCQQPTRF	SNRCLQMIGR	CWPHLRALGV	GGAGCGVQGL	ASLARNCMRL	QVLELDHVSE	ITQEVAAEVC	REGLKGLEML
810	820	830	840	850	860	870	880
VLTATPVTPK	ALLHFNSICR	NLKSIVVQIG	IADYFKEPSS	PEAQKLFEDM	VTKLQALRRR	PGFSKILHIK	VEGGC

Cmpd.	No. of Cmpds.	m/z meas.	Δ m/z [ppm]	z	Rt [min]	Score	P	Range	Sequence	Modification	Ratios
2879	2	813.9356	28.83	2	65.2	12.4	1	568-580	R.TLLHAAEVCRDWR.F	Carbamidomethyl: 9	mdown:qdown 1.17

Detailed Protein Report

Protein 242: chromosome-associated kinesin KIF4A [Homo sapiens]

**Accession:**	gi|116686122	**Score:**	38.4
**Database:**	refseq_human(refseq_human_20140103.fasta)	**MW [kDa]:**	139.8
**Database Date:**	2015-11-30	**pI:**	5.9
**Modification(s):**	Carbamidomethyl	**Sequence Coverage [%]:**	1.5
		**No. of unique Peptides:**	2

10	20	30	40	50	60	70	80
MKEEVKGIPV	RVALRCRPLV	PKEISEGCQM	CLSFVPGEPQ	VVVGTDKSFT	YDFVFDPSTE	QEEVFNTAVA	PLIKGVFKGY
90	100	110	120	130	140	150	160
NATVLAYGQT	GSGKTYSMGG	AYTAEQENEP	TVGVIPRVIQ	LLFKEIDKKS	DFEFTLKVSY	LEIYNEEILD	LLCPSREKAQ
170	180	190	200	210	220	230	240
INIREDPKEG	IKIVGLTEKT	VLVALDTVSC	LEQGNNSRTV	ASTAMNSQSS	RSHAIFTISL	EQRKKSDKNS	SFRSKLHLVD
250	260	270	280	290	300	310	320
LAGSERQKKT	KAEGDRLKEG	ININRGLLCL	GNVISALGDD	KKGGFVPYRD	SKLTRLLQDS	LGGNSHTLMI	ACVSPADSNL
330	340	350	360	370	380	390	400
EETLNTLRYA	DRARKIKNKP	IVNIDPQTAE	LNHLKQQVQQ	LQVLLLQAHG	GTLPGSITVE	PSENLQSLME	KNQSLVEENE
410	420	430	440	450	460	470	480
KLSRGLSEAA	GQTAQMLERI	ILTEQANEKM	NAKLEELRQH	AACKLDLQKL	VETLEDQELK	ENVEIICNLQ	QLITQLSDET
490	500	510	520	530	540	550	560
VACMAAAIDT	AVEQEAQVET	SPETSRSSDA	FTTQHALRQA	QMSKELVELN	KALALKEALA	RKMTQNDSQL	QPIQYQYQDN
570	580	590	600	610	620	630	640
IKELELEVIN	LQKEKEELVL	ELQTAKKDAN	QAKLSERRRK	RLQELEGQIA	DLKKKLNEQS	KLLKLKESTE	RTVSKLNQEI
650	660	670	680	690	700	710	720
RMMKNQRVQL	MRQMKEDAEK	FRQWKQKKDK	EVIQLKERDR	KRQYELLKLE	RNFQKQSNVL	RRKTEEAAAA	NKRLKDALQK
730	740	750	760	770	780	790	800
QREVADKRKE	TQSRGMEGTA	ARVKNWLGNE	IEVMVSTEEA	KRHLNDLLED	RKILAQDVAQ	LKEKKESGEN	PPPKLRRRTF
810	820	830	840	850	860	870	880
SLTEVRGQVS	ESEDSITKQI	ESLETEMEFR	SAQIADLQQK	LLDAESEDRP	KQRWENIATI	LEAKCALKYL	IGELVSSKIQ
890	900	910	920	930	940	950	960
VSKLESSLKQ	SKTSCADMQK	MLFEERNHFA	EIETELQAEL	VRMEQQHQEK	VLYLLSQLQQ	SQMAEKQLEE	SVSEKEQQLL
970	980	990	1000	1010	1020	1030	1040
STLKCQDEEL	EKMREVCEQN	QQLLRENEII	KQKLTLLQVA	SRQKHLPKDT	LLSPDSSFEY	VPPKPKPSRV	KEKFLEQSMD
1050	1060	1070	1080	1090	1100	1110	1120
IEDLKYCSEH	SVNEHEDGDG	DDDEGDDEEW	KPTKLVKVSR	KNIQGCSCKG	WCGNKQCGCR	KQKSDCGVDC	CCDPTKCRNR
1130	1140	1150	1160	1170	1180	1190	1200
QQGKDSLGTV	ERTQDSEGSF	KLEDPTEVTP	GLSFFNPVCA	TPNSKILKEM	CDVEQVLSKK	TPPAPSPFDL	PELKHVATEY
1210	1220	1230	1240				
QENKAPGKKK	KRALASNTSF	FSGCSPIEEE	AH				

Cmpd.	No. of Cmpds.	m/z meas.	Δ m/z [ppm]	z	Rt [min]	Score	P	Range	Sequence	Modification
2387	1	1051.5184	80.34	2	58.2	16.1	2	1082-1100	K.NIQGCSCKGWCGNKQCGCR.K	Carbamidomethyl: 11
2074	1	701.2275	-91.47	3	56.1	10.6	2	1082-1100	K.NIQGCSCKGWCGNKQCGCR.K	Carbamidomethyl: 18

Detailed Protein Report

Protein 243: ubiquilin-like protein [Homo sapiens]

**Accession:**	gi|145580580	**Score:**	38.3
**Database:**	refseq_human(refseq_human_20140103.fasta)	**MW [kDa]:**	52.9
**Database Date:**	2015-11-30	**pI:**	5.6
**Modification(s):**	Oxidation	**Sequence Coverage [%]:**	8.8
		**No. of unique Peptides:**	2

Quantitation

mdown:qdown	**Median:**	1.36	**CV:**	31.66 %	**No. of Peptides:**	2
Wdown:Qdown	**Median:**	0.78	**CV:**	0.00 %	**No. of Peptides:**	1

10	20	30	40	50	60	70	80
MWHAISRTSR	MSQSGCPSGL	LADKNISSSA	TRVIVKTAGN	QKDFMVADDI	SVRQFKEMLL	AHFQCQMDQL	VLVFMGCLLK
90	100	110	120	130	140	150	160
DHDTLSQRGI	MDGHTIYLVI	KSKQGSRSLA	HSFRDLPTND	PCHRDRNTKG	NSSRVHQPTG	MNQAPVELAH	FVGSDAPKVH
170	180	190	200	210	220	230	240
TQNLEVSHPE	CKAQMLENPS	IQRLLSNMEF	MWQFISEHLD	TQQLMQQNPE	VSRLLLDNSE	ILLQTLELAR	NLAMIQEIMQ
250	260	270	280	290	300	310	320
IQQPSQNLEY	PLNPQPYLGL	ETMPGGNNAL	GQNYADINDQ	MLNSMQDPFG	GNPFTALLAG	QVLEQVQSSP	PPPPPSQEQQ
330	340	350	360	370	380	390	400
DQLTQHPATR	VIYNSSGGFS	SNTSANDTLN	KVNHTSKANT	AMISTKGQSH	ICATRQPAWI	PALPSIELTQ	QLQEEYKDAT
410	420	430	440	450	460	470	480
VSLSSSRQTL	KGDLQLSDEQ	SSSQITGGMM	QLLMNNPYLA	AQIMLFTSMP	QLSEQWRQQL	PTFLQQTQIS	DLLSA

Cmpd.	No. of Cmpds.	m/z meas.	Δ m/z [ppm]	z	Rt [min]	Score	P	Range	Sequence	Modification	Ratios
177	1	642.3095	14.02	2	31.6	11.3	0	43-53	K.DFMVADDISVR.Q	Oxidation: 3	mdown:qdown 1.00<br>Wdown:Qdown 0.78
2144	1	953.3984	-72.07	2	57.0	10.6	1	358-375	K.ANTAMISTKGQSHICATR.Q	Oxidation: 5	mdown:qdown 1.85

Detailed Protein Report

Protein 244: PREDICTED: zinc finger protein 675 isoform X3 [Homo sapiens]

**Accession:**	gi|530414622	**Score:**	38.3
**Database:**	refseq_human(refseq_human_20140103.fasta)	**MW [kDa]:**	58.2
**Database Date:**	2015-11-30	**pI:**	10.2
**Modification(s):**	Carbamidomethyl	**Sequence Coverage [%]:**	5.6
		**No. of unique Peptides:**	1

10	20	30	40	50	60	70	80
MVNEPPVMCS	HFAQEFWPEQ	NIKDSFEKVT	LRRYEKCGND	NFQLKGCKSV	DECKLHKGGY	NGLNQCLPTM	QSKMFQCDKY
90	100	110	120	130	140	150	160
VKVFNKFSHS	DRHKIKHMEN	KPFKCKECGR	SFCMLSHLTR	HERNYTKVNF	CKCEECEKAV	NQSSKLTKHK	RIYTCEKLYK
170	180	190	200	210	220	230	240
CQECDRTFNQ	FSNLTEYKKD	YAREKPYKCE	ECGKAFNQSS	HLTTHKIIHT	GEKPYKCEEC	GKAFNQFSNL	TTHKKIHTGE
250	260	270	280	290	300	310	320
QPYICEECGK	AFTQSSTLTT	HKRIHTGEKP	YKCEECGKAF	NRSSKLTEHK	NIHTGEQPYK	CEECGKAFNR	SSNLTEHRKI
330	340	350	360	370	380	390	400
HTEEKPYKCK	ECGKAFKHSS	ALTTHKRIHT	GEKPYKCEEC	GKAFNRSSKL	TEHKKLHTGK	KPYKCEECGK	AFIQSSKLTE
410	420	430	440	450	460	470	480
HKKIHSGEIP	YKCEECGKAF	KHSSSLTTHK	RIHTGEKPYK	CEECGKAFSR	SSKLTEHKII	HTGEKPYKCE	RCDKAFNQSA
490	500						
NLTKHKKIHT	GEKLQNWNV						

Cmpd.	No. of Cmpds.	m/z meas.	Δ m/z [ppm]	z	Rt [min]	Score	P	Range	Sequence	Modification
1793	1	682.2364	-94.72	3	52.4	10.6	2	37-54	K.CGNDNFQLKGCKSVDECK.L	Carbamidomethyl: 17

Detailed Protein Report

Protein 245: death-inducer obliterator 1 isoform c [Homo sapiens]

**Accession:**	gi|301129165	**Score:**	38.3
**Database:**	refseq_human(refseq_human_20140103.fasta)	**MW [kDa]:**	243.7
**Database Date:**	2015-11-30	**pI:**	8.9
		**Sequence Coverage [%]:**	1.2
		**No. of unique Peptides:**	1

Alias proteins:

Accession	Name	Description
gi|301129170	refseq_human (refseq_human_20140103.fasta)	death-inducer obliterator 1 isoform c [Homo sapiens]

Detailed Protein Report

10	20	30	40	50	60	70	80
MDDKGDPSNE	EAPKAIKPTS	KEFRKTWGFR	RTTIAKREGA	GDAEADPLEP	PPPQQQLGLS	LRRSGRQPKR	TERVEQFLTI
90	100	110	120	130	140	150	160
ARRRGRRSMP	VSLEDSGEPT	SCPATDAETA	SEGSVESASE	TRSGPQSAST	AVKERPASSE	KVKGGDDHDD	TSDSDSDGLT
170	180	190	200	210	220	230	240
LKELQNRLRR	KREQEPTERP	LKGIQSRLRK	KRREEGPAET	VGSEASDTVE	GVLPSKQEPE	NDQGVVSQAG	KDDRESKLEG
250	260	270	280	290	300	310	320
KAAQDIKDEE	PGDLGRPKPE	CEGYDPNALY	CICRQPHNNR	FMICCDRCEE	WFHGDCVGIS	EARGRLLERN	GEDYICPNCT
330	340	350	360	370	380	390	400
ILQVQDETHS	ETADQQEAKW	RPGDADGTDC	TSIGTIEQKS	SEDQGIKGRI	EKAANPSGKK	KLKIFQPVIE	APGASKCIGP
410	420	430	440	450	460	470	480
GCCHVAQPDS	VYCSNDCILK	HAAATMKFLS	SGKEQKPKPK	EKMKMKPEKP	SLPKCGAQAG	IKISSVHKRP	APEKKETTVK
490	500	510	520	530	540	550	560
KAVVVPARSE	ALGKEAACES	STPSWASDHN	YNAVKPEKTA	APSPSLLYKS	TKEDRRSEEK	AAAMAASKKT	APPGSAVGKQ
570	580	590	600	610	620	630	640
PAPRNLVPKK	SSFANVAAAT	PAIKKPPSGF	KGTIPKRPWL	SATPSSGASA	ARQAGPAPAA	ATAASKKFPG	SAALVGAVRK
650	660	670	680	690	700	710	720
PVVPSVPMAS	PAPGRLGAMS	AAPSQPNSQI	RQNIRRSLKE	ILWKRVNDSD	DLIMTENEVG	KIALHIEKEM	FNLFQVTDNR
730	740	750	760	770	780	790	800
YKSKYRSIMF	NLKDPKNQGL	FHRVLREEIS	LAKLVRLKPE	ELVSKELSTW	KERPARSVME	SRTKLHNESK	KTAPRQEAIP
810	820	830	840	850	860	870	880
DLEDSPPVSD	SEEQQESARA	VPEKSTAPLL	DVFSSMLKDT	TSQHRAHLFD	LNCKICTGQV	PSAEDEPAPK	KQKLSASVKK
890	900	910	920	930	940	950	960
EDLKSKHDSS	APDPAPDSAD	EVMPEAVPEV	ASEPGLESAS	HPNVDRTYFP	GPPGDGHPEP	SPLEDLSPCP	ASCGSGVVTT
970	980	990	1000	1010	1020	1030	1040
VTVSGRDPRT	APSSSCTAVA	SAASRPDSTH	MVEARQDVPK	PVLTSVMVPK	SILAKPSSSP	DPRYLSVPPS	PNISTSESRS
1050	1060	1070	1080	1090	1100	1110	1120
PPEGDTTLFL	SRLSTIWKGF	INMQSVAKFV	TKAYPVSGCF	DYLSEDLPDT	IHIGGRIAPK	TVWDYVGKLK	SSVSKELCLI
1130	1140	1150	1160	1170	1180	1190	1200
RFHPATEEEE	VAYISLYSYF	SSRGRFGVVA	NNNRHVKDLY	LIPLSAQDPV	PSKLLPFEGP	GLESPRPNII	LGLVICQKIK
1210	1220	1230	1240	1250	1260	1270	1280
RPANSGELDK	MDEKRTRLQP	EEADVPAYPK	VATVPQSEKK	PSKYPLCSAD	AAVSTTPPGS	PPPPPPLPEP	PVLKVLSSLK
1290	1300	1310	1320	1330	1340	1350	1360
PAAPSPATAA	TTAAAASTAA	SSTASSASKT	ASPLEHILQT	LFGKKKSFDP	SAREPPGSTA	GLPQEPKTTA	EDGVPAPPLL
1370	1380	1390	1400	1410	1420	1430	1440
DPIVQQFGQF	SKDKALEEEE	DDRPYDPEEE	YDPERAFDTQ	LVERGRRHEV	ERAPEAAAAE	REEVAYDPED	ETILEEAKVT
1450	1460	1470	1480	1490	1500	1510	1520
VDDLPNRMCA	DVRRNSVERP	AEPVAGAATP	SLVEQQKMLE	ELNKQIEEQK	RQLEEQEEAL	RQQRAAVGVS	MAHFSVSDAL
1530	1540	1550	1560	1570	1580	1590	1600
MSPPPKSSLP	KAELFQQEQQ	SADKPASLPP	ASQASNHRDP	RQARRLATET	GEGEGEPLSR	LSARGAQGAL	PERDASRGGL
1610	1620	1630	1640	1650	1660	1670	1680
VGQAPMPVPE	EKEPASSPWA	SGEKPPAGSE	QDGWKAEPGE	GTRPATVGDS	SARPARRVLL	PTPPCGALQP	GFPLQHDGER
1690	1700	1710	1720	1730	1740	1750	1760
DPFTCPGFAS	QDKALGSAQY	EDPRNLHSAG	RSSSPAGETE	GDREPQARPG	EGTAPLPPPG	QKVGGSQPPF	QGQREPGPHA
1770	1780	1790	1800	1810	1820	1830	1840
LGMSGLHGPN	FPGPRGPAPP	FPEENIASND	GPRGPPPARF	GAQKGPIPSL	FSGQHGPPPY	GDSRGPSPSY	LGGPRGVAPS
1850	1860	1870	1880	1890	1900	1910	1920
QFEERKDPHG	EKREFQDAPY	NEVTGAPAQF	EGTEQAPFLG	SRGGAPFQFG	GQRRPLLSQL	KGPRGGPPPS	QFGGQRGPPP
1930	1940	1950	1960	1970	1980	1990	2000
GHFVGPRGPH	PSQFETARGP	HPNQFEGPRG	QAPNFMPGPR	GIQPQQFEDQ	RVHSPPRFTN	QRAPAPLQFG	GLRGSAPFSE
2010	2020	2030	2040	2050	2060	2070	2080
KNEQTPSRFH	FQGQAPQVMK	PGPRPLLELP	SHPPQHRKDR	WEEAGPPSAL	SSSAPGQGPE	ADGQWASADF	REGKGHEYRN
2090	2100	2110	2120	2130	2140	2150	2160
QTFEGRQRER	FDVGPKEKPL	EEPDAQGRAS	EDRRRERERG	RNWSRERDWD	RPREWDRHRD	KDSSRDWDRN	RERSANRDRE
2170	2180	2190	2200	2210	2220	2230	2240

Detailed Protein Report

Cmpd.	No. of Cmpds.	m/z meas.	Δ m/z [ppm]	z	Rt [min]	Score	P	Range	Sequence	Modification
62	1	675.3854	58.70	2	29.5	15.9	2	536-548	R.RSEEKAAAMAASK.K	

Detailed Protein Report

Protein 246: ubiquitin carboxyl-terminal hydrolase 43 isoform 2 [Homo sapiens]

Accession: gi|389616163
Database: refseq_human(refseq_human_20140103.fasta)
Database Date: 2015-11-30

Score: 38.2
MW [kDa]: 122.2
pI: 10.2
Sequence Coverage [%]: 2.5
No. of unique Peptides: 1

10	20	30	40	50	60	70	80
MDLGPGDAAG	GGPLAPRPRR	RRSLRRLFSR	FLLALGSRSR	PGDSPPRPQP	GHCDGDGEGG	FACAPGPVPA	APGSPGEERP
**90**	**100**	**110**	**120**	**130**	**140**	**150**	**160**
PGPQPQLQLP	AGDGARPPGA	QGLKNHGNTC	FMNAVVQCLS	NTDLLAEFLA	LGRYRAAPGR	AEVTEQLAAL	VRALWTREYT
**170**	**180**	**190**	**200**	**210**	**220**	**230**	**240**
PQLSAEFKNA	VSKYGSQFQG	NSQHDALEFL	LWLLDRVHED	LEGSSRGPVS	EKLPPEATKT	SENCLSPSAQ	LPLGQSFVQS
**250**	**260**	**270**	**280**	**290**	**300**	**310**	**320**
HFQAQYRSSL	TCPHCLKQSN	TFDPFLCVSL	PIPLRQTRFL	SVTLVFPSKS	QRFLRVGLAV	PILSTVAALR	KMVAEEGGVP
**330**	**340**	**350**	**360**	**370**	**380**	**390**	**400**
ADEVILVELY	PSGFQRSFFD	EEDLNTIAEG	DNVYAFQVPP	SPSQGTLSAH	PLGLSASPRL	AAREGQRFSL	SLHSESKVLI
**410**	**420**	**430**	**440**	**450**	**460**	**470**	**480**
LFCNLVGSGQ	QASRFGPPFL	IREDRAVSWA	QLQQSILSKV	RHLMKSEAPV	QNLGSLFSIR	VVGLSVACSY	LSPKDSRPLC
**490**	**500**	**510**	**520**	**530**	**540**	**550**	**560**
HWAVDRVLHL	RRPGGPPHVK	LAVEWDSSVK	ERLFGSLQEE	RAQDADSVWQ	QQQAHQQHSC	TLDECFQFYT	KEEQLAQDDA
**570**	**580**	**590**	**600**	**610**	**620**	**630**	**640**
WKCPHCQVLQ	QGMVKLSLWT	LPDILIIHLK	RFCQVGERRN	KLSTLVKFPL	SGLNMAPHVA	QRSTSPEAGL	GPWPSWKQPD
**650**	**660**	**670**	**680**	**690**	**700**	**710**	**720**
CLPTSYPLDF	LYDLYAVCNH	HGNLQAYCRN	SLDGQWYSYD	DSTVEPLRED	EVNTRGAYIL	FYQKRNSIPP	WSASSSMRGS
**730**	**740**	**750**	**760**	**770**	**780**	**790**	**800**
TSSSLSDHWL	LRLGSHAGST	RGSLLSWSSA	PCPSLPQVPD	SPIFTNSLCN	QEKGGLEPRR	LVRGVKGRSI	SMKAPTTSRA
**810**	**820**	**830**	**840**	**850**	**860**	**870**	**880**
KQGPFKTMPL	RWSFGSKEKP	PGASVELVEY	LESRRRPRST	SQSIVSLLTG	TAGEDEKSAS	PRSNVALPAN	SEDGGRAIER
**890**	**900**	**910**	**920**	**930**	**940**	**950**	**960**
GPAGVPCPSA	QPNHCLAPGN	SDGPNTARKL	KENAGQDIKL	PRKFDLPLTV	MPSVEHEKPA	RPEGQKAMNW	KESFQMGSKS
**970**	**980**	**990**	**1000**	**1010**	**1020**	**1030**	**1040**
SPPSPYMGFS	GNSKDSRRGT	SELDRPLQGT	LTLLRSVFRK	KENRRNERAE	VSPQVPPVSL	VSGGLSPAMD	GQAPGSPPAL
**1050**	**1060**	**1070**	**1080**	**1090**	**1100**	**1110**	**1120**
RIPEGLARGL	GSRLERDVWS	APSSLRLPRK	ASRAPRGSAL	GMSQRTVPGE	QASYGTFQRV	KYHTLSLGRK	KTLPESSF

Cmpd.	No. of Cmpds.	m/z meas.	Δ m/z [ppm]	z	Rt [min]	Score	P	Range	Sequence	Modification
2554	5	812.4077	-74.32	2	62.4	18.3	2	1042-1056	R.IPEGLARGLGSRLER.D	

Detailed Protein Report

Protein 247: PREDICTED: sperm flagellar protein 2 isoform X5 [Homo sapiens]

**Accession:**	gi|578810001	**Score:**	38.1
**Database:**	refseq_human(refseq_human_20140103.fasta)	**MW [kDa]:**	176.7
**Database Date:**	2015-11-30	**pI:**	5.7
		**Sequence Coverage [%]:**	3.4
		**No. of unique Peptides:**	3

Quantitation

mdown:qdown **Median:** 0.35 **CV:** 0.00 % **No. of Peptides:** 1

10	20	30	40	50	60	70	80
MSEILCQWLN	KELKVSRTVS	PKSFAKAFSS	GYLLGEVLHK	FELQDDFSEF	LDSRVSSAKL	NNFSRLEPTL	NLLGVQFDQN
90	100	110	120	130	140	150	160
VAHGIITEKP	GVATKLLYQL	YIALQKKKKS	GLTGVEMQTM	QRLTNLRLQN	MKSDTFQERL	RHMIPRQTDF	NLMRITYRFQ
170	180	190	200	210	220	230	240
EKYKHVKEDL	AHLHFEKLER	FQKLKEEQRC	FDIEKQYLNR	RRQNEIMAKI	QAAIIQIPKP	ASNRTLKALE	AQKMMKKKKE
250	260	270	280	290	300	310	320
AEDVADEIKK	FEALIKKDLQ	AKESASKTSL	DTAGQTTTDL	LNTYSDDEYI	KKIQKRLEED	AFAREQREKR	RRKLLMDQLI
330	340	350	360	370	380	390	400
AHEAQEEAYR	EEQLINRLMR	QSQQERRIAV	QLMHVRHEKE	VLWQNRIFRE	KQHEERRLKD	FQDALDREAA	LAKQAKIDFE
410	420	430	440	450	460	470	480
EQFLKEKRFH	DQIAVERAQA	RYEKHYSVCA	EILDQIVDLS	TKVADYRMLT	NNLIPYKLMH	DWKELFFNAK	PIYEQASVKT
490	500	510	520	530	540	550	560
LPANPSREQL	TELEKRDLLD	TNDYEEYKNM	VGEWALPEEM	VDNLPPSNNC	ILGHILHRLA	EKSLPPRAES	TTPELPSFAV
570	580	590	600	610	620	630	640
KGCLLGKTLS	GKTTILRSLQ	KDFPIQILSI	DTLVQEAIQA	FHDNEKVSEV	LPIQKNDEED	ALPVLQEEIK	ESQDPQHVFS
650	660	670	680	690	700	710	720
AGPVSDEVLP	ETEGETMLSA	NADKTPKAEE	VKSSDSFLKL	TTRAQLGAKS	EQLLKKGKSI	PDVLLVDIIV	NAINEIPVNQ
730	740	750	760	770	780	790	800
DCILDGFPMT	LNQAQLLEEA	LTGCNRNLTE	VERKKAQKST	LAIDPATSKE	IPLPSPAFDF	VILLDVSDTS	SMSRMNDIIA
810	820	830	840	850	860	870	880
EELSYKTAHE	DISQRVAAEN	QDKDGDQNLR	DQIQHRIIGF	LDNWPLLEQW	FSEPENILIK	INAEIDKESL	CEKVKEILTT
890	900	910	920	930	940	950	960
EIAKKKNKVE	KKLEEKEAEK	KAAASLAELP	LPTPPPAPPP	EPEKEKEIHQ	SHVASKTPTA	KGKPQSEAPH	GKQESLQEGK
970	980	990	1000	1010	1020	1030	1040
GKKGETALKR	KGSPKGKSSG	GKVPVKKSPA	DSTDTSPVAI	VPQPPKPGSE	EWVYVNEPVP	EEMPLFLVPY	WELIENSYIN
1050	1060	1070	1080	1090	1100	1110	1120
TIKTVLRHLR	EDQHTVLAYL	YEIRTSFQEF	LKRPDHKQDF	VAQWQADFNS	LPDDLWDDEE	TKAELHQRVN	DLRDRLWDIC
1130	1140	1150	1160	1170	1180	1190	1200
DARKEEAEQE	RLDIINESWL	QDTLGMTMNH	FFSLMQAELN	RFQDTKRLLQ	DYYWGMESKI	PVEDNKRFTR	IPLVQLDSKD
1210	1220	1230	1240	1250	1260	1270	1280
NSESQLRIPL	VPRISISLET	VTPKPKTKSV	LKGKMDNSLE	NVESNFEADE	KLVMDTWQQA	SLAVSHMVAA	EIHQRLMEEE
1290	1300	1310	1320	1330	1340	1350	1360
KENQPADPKE	KSPQMGANKK	VKKEPPKKKQ	EDKKPKGKSP	PMAEATPVIV	TTEEIAEIKR	KNELRVKIKE	EHLAALQFEE
1370	1380	1390	1400	1410	1420	1430	1440
IATQFRLELI	KTKALALLED	LVTKVVDVYK	LMEKWLGERY	LNEMASTEKL	TDVARYHIET	STKIQNELYL	SQEDFFINGN
1450	1460	1470	1480	1490	1500	1510	1520
IKVFPDPPPS	IRPPPVEKEE	DGTLTIEQLD	SLRDQFLDMA	PKECSENCFQ	RPSPSTCDAF	SNRRQEVASW	ITGSPPELSQ
1530	1540						
GISCNCSATA	LSRQRQK						

Cmpd.	No. of Cmpds.	m/z meas.	Δ m/z [ppm]	z	Rt [min]	Score	P	Range	Sequence	Modification	Ratios
2952	1	973.5048	-25.95	2	66.1	10.6	2	2-17	M.SEILCQWLNKELKVSR.T		
1496	1	1006.5889	17.49	3	47.4	12.9	2	1200-1226	K. DNSESQLRIPLVPRISISLETVTPK[illegible] T		

Detailed Protein Report

Cmpd.	No. of Cmpds.	m/z meas.	Δ m/z [ppm]	z	Rt [min]	Score	P	Range	Sequence	Modification	Ratios
1569	1	480.6396	-230.14	2	48.1	14.6	1	1292-1300	K.SPQMGANKK.V		mdown:qdown 0.35

Detailed Protein Report

Protein 248: PREDICTED: protein unc-45 homolog A isoform X3 [Homo sapiens]

**Accession:**	gi|530407244	**Score:**	38.1
**Database:**	refseq_human(refseq_human_20140103.fasta)	**MW [kDa]:**	87.2
**Database Date:**	2015-11-30	**pI:**	5.6
**Modification(s):**	Carbamidomethyl, Oxidation	**Sequence Coverage [%]:**	3.4
		**No. of unique Peptides:**	3

10	20	30	40	50	60	70	80
MSSTDAKVEQ	MFQILLDPEE	KGTEKKQKAS	QNLVVLARED	AGAEKIFRSN	GVQLLQRLLD	MGETDLMLAA	LRTLVGICSE
**90**	**100**	**110**	**120**	**130**	**140**	**150**	**160**
HQSRTVATLS	ILGTRRVVSI	LGVESQAVSL	AACHLLQVMF	DALKEGVKKG	FRGKEGAIIV	DPARELKVLI	SNLLDLLTEV
**170**	**180**	**190**	**200**	**210**	**220**	**230**	**240**
GVSGQGRDNA	LTLLIKAVPR	KSLKDPNNSL	TLWVIDQGLK	KILEVGGSLQ	DPPGELAVTA	NSRMSASILL	SKLFDDLKCD
**250**	**260**	**270**	**280**	**290**	**300**	**310**	**320**
AERENFHRLC	ENYIKSWFEG	QGLAGKLRAI	QTVSCLLQGP	CDAGNRALEL	SGVMESVIAL	CASEQEEEQL	VAVEALIHAA
**330**	**340**	**350**	**360**	**370**	**380**	**390**	**400**
GKAKRASFIT	ANGVSLLKDL	YKCSEKDSIR	IRALVGLCKL	GSAGGTDFSM	KQFAEGSTLK	LAKQCRKWLC	NDQIDAGTRR
**410**	**420**	**430**	**440**	**450**	**460**	**470**	**480**
WAVEGLAYLT	FDADVKEEFV	EDAAALKALF	QLSRLEERSV	LFAVASALVN	CTNSYDYEEP	DPKMVELAKY	AKQHVPEQHP
**490**	**500**	**510**	**520**	**530**	**540**	**550**	**560**
KDKPSFVRAR	VKKLLAAGVV	SAMVCMVKTE	SPVLTSSCRE	LLSRVFLALV	EEVEDRGTVV	AQGGGRALIP	LALEGTDVGQ
**570**	**580**	**590**	**600**	**610**	**620**	**630**	**640**
TKAAQALAKL	TITSNPEMTF	PGERIYEVVR	PLVSLLHLNC	SGLQNFEALM	ALTNLAGISE	RLRQKILKEK	AVPMIEGYMF
**650**	**660**	**670**	**680**	**690**	**700**	**710**	**720**
EEHEMIRRAA	TECMCNLAMS	KEVQDLFEAQ	GNDRLKLLVL	YSGEDDELLQ	RAAAGGLAML	TSMRPTLCSR	IPQVTTHWLE
**730**	**740**	**750**	**760**	**770**	**780**	**790**	**800**
ILQALLLSSN	QELQHRGAVV	VLNMVEASRE	IASTLMESEM	MEILSVLAKG	DHSPVTRAAA	ACLDKAVEYG	LIQPNQDGE

Cmpd.	No. of Cmpds.	m/z meas.	Δ m/z [ppm]	z	Rt [min]	Score	P	Range	Sequence	Modification
1687	1	793.3789	34.81	2	51.0	14.1	1	648-661	R.RAATECMCNLAMSK.E	Carbamidomethyl: 6
2677	1	731.3736	106.62	2	64.7	11.8	0	649-661	R.AATECMCNLAMSK.E	Carbamidomethyl: 5; Oxidation: 6, 11
150	1	977.7995	37.53	3	31.3	12.2	1	649-674	R.AATECMCNLAMSKEVQDLFEAQGNDR.L	Carbamidomethyl: 5

Detailed Protein Report

Protein 249: PREDICTED: UPF0505 protein C16orf62 isoform X2 [Homo sapiens]

Accession: gi|530408446
Database: refseq_human(refseq_human_20140103.fasta)
Database Date: 2015-11-30
Modification(s): Carbamidomethyl, Oxidation

Score: 38.1
MW [kDa]: 107.9
pI: 8.9
Sequence Coverage [%]: 5.7
No. of unique Peptides: 2

10	20	30	40	50	60	70	80
MAPAGVVTRA	VRSGELPLTS	MASAENEACA	VRSVACPSQA	WRLQKVLCGR	CGAASCPSQT	WRPRGAGSGG	VRMGSRADGP
90	100	110	120	130	140	150	160
RTSGHVTGKM	AVFPWHSRNR	NYKAEFASCR	LEAVPLEFGD	YHPLKPITVT	ESKTKKVNRK	GSTSSTSSSS	SSSVVDPLSS
170	180	190	200	210	220	230	240
VLDGTDPLSM	FAATADPAAL	AAAMDSSRRK	RDRDDNSVVG	SDFEPWTNKR	GEILARYTTT	EKLSINLFMG	SEKGKAGTAT
250	260	270	280	290	300	310	320
LAMSEKVRTR	LEELDDFEEG	SQKELLNLTQ	QDYVNRIEEL	NQSLKDAWAS	DQKVKALKIV	IQCSKLLSDT	SVIQFYPSKF
330	340	350	360	370	380	390	400
VLITDILDTF	GKLVYERIFS	MCVDSRSVLP	DHFSPENAND	TAKETCLNWF	FKIASIRELI	PRFYVEASIL	KCNKFLSKTG
410	420	430	440	450	460	470	480
ISECLPRLTC	MIRGIGDPLV	SVYARAYLCR	ALLTEMMERC	KKLGNNALLL	NSVMSAFRAE	FIATRSMDFI	GMIKECDESG
490	500	510	520	530	540	550	560
FPKHLLFRSL	GLNLALADPP	ESDRLQILNE	AWKVITKLKN	PQDYINCAEV	WVEYTCKHFT	KREVNTVLAD	VIKHMTPDRA
570	580	590	600	610	620	630	640
FEDSYPQLQL	IIKKVIAHFH	DFSVLFSVEK	FLPFLDMFQK	ESVRVEVCKC	IMDAFIKHQQ	EPTKDPVILN	ALLHVCKTMH
650	660	670	680	690	700	710	720
DSVNALTLED	EKRMLSYLIN	GFIKMVSFGR	DFEQQLSFYV	ESRSMFCNLE	PVLVQLIHAC	VAYCFITIPS	LAGIFTRLNL
730	740	750	760	770	780	790	800
YLHSGQVALA	NQCLSQADAF	FKAAISLVPE	VPKMINIDGK	MRPSESFLLE	FLCNFFSTLL	IVPDHPEHGV	LFLVRELLNV
810	820	830	840	850	860	870	880
IQDYTWEDNS	DEKIRIYTCV	LHLLSAMSQE	TYLYHIDKVD	SNDSLYGGDS	KFLAENNKLC	ETVMAQILEH	LKTLAKDEAL
890	900	910	920	930	940	950	960
KRQSSLGLSF	FNSILAHGDL	RNNKLNQLSV	NLWHLAQRHG	CADTRTMVKT	LEYIKKQSKQ	PDMTHLTELA	LRLPLQTRT

Cmpd.	No. of Cmpds.	m/z meas.	Δ m/z [ppm]	z	Rt [min]	Score	P	Range	Sequence	Modification
1659	1	1273.7346	60.72	2	50.7	10.5	2	554-574	K.HMTPDRAFEDSYPQLQLIIKK.V	Oxidation: 2
1481	2	949.1566	5.84	3	48.3	12.1	2	902-925	R.NNKLNQLSVNLWHLAQRHGCADTR.T	Carbamidomethyl: 20

Detailed Protein Report

Protein 250: biorientation of chromosomes in cell division protein 1-like 1 [Homo sapiens]

**Accession:**	gi|87299628	**Score:**	38.0
**Database:**	refseq_human(refseq_human_20140103.fasta)	**MW [kDa]:**	330.3
**Database Date:**	2015-11-30	**pI:**	4.9
		**Sequence Coverage [%]:**	0.9
		**No. of unique Peptides:**	1

Detailed Protein Report

10	20	30	40	50	60	70	80
MATNPQPQPP	PPAPPPPPPQ	PQPQPPPPPP	GPGAGPGAGG	AGGAGAGAGD	PQLVAMIVNH	LKSQGLFDQF	RRDCLADVDT
90	100	110	120	130	140	150	160
KPAYQNLRQR	VDNFVANHLA	THTWSPHLNK	NQLRNNIRQQ	VLKSGMLESG	IDRIISQVVD	PKINHTFRPQ	VEKAVHEFLA
170	180	190	200	210	220	230	240
TLNHKEEGSG	NTAPDDEKPD	TSLITQGVPT	PGPSANVAND	AMSILETITS	LNQEASAARA	STETSNAKTS	ERASKKLPSQ
250	260	270	280	290	300	310	320
PTTDTSTDKE	RTSEDMADKE	KSTADSGGEG	LETAPKSEEF	SDLPCPVEEI	KNYTKEHNNL	ILLNKDVQQE	SSEQKNKSTD
330	340	350	360	370	380	390	400
KGEKKPDSNE	KGERKKEKKE	KTEKKFDHSK	KSEDTQKVKD	EKQAKEKEVE	SLKLPSEKNS	NKAKTVEGTK	EDFSLIDSDV
410	420	430	440	450	460	470	480
DGLTDITVSS	VHTSDLSSFE	EDTEEEVVTS	DSMEEGEITS	DDEEKNKQNK	TKTQTSDSSE	GKTKSVRHAY	VHKPYLYSKY
490	500	510	520	530	540	550	560
YSDSDDELTV	EQRRQSIAKE	KEERLLRRQI	NREKLEEKRK	QKAEKTKSSK	TKGQGRSSVD	LEESSTKSLE	PKAARIKEVL
570	580	590	600	610	620	630	640
KERKVLEKKV	ALSKKRKKDS	RNVEENSKKK	QQYEEDSKET	LKTSEHCEKE	KISSSKELKH	VHAKSEPSKP	ARRLSESLHV
650	660	670	680	690	700	710	720
VDENKNESKL	EREHKRRTST	PVIMEGVQEE	TDTRDVKRQV	ERSEICTEEP	QKQKSTLKNE	KHLKKDDSET	PHLKSLLKKE
730	740	750	760	770	780	790	800
VKSSKEKPER	EKTPSEDKLS	VKHKYKGDCM	HKTGDETELH	SSEKGLKVEE	NIQKQSQQTK	LSSDDKTERK	SKHRNERKLS
810	820	830	840	850	860	870	880
VLGKDGKPVS	EYIIKTDENV	RKENNKKERR	LSAEKTKAEH	KSRRSSDSKI	QKDSLGSKQH	GITLQRRSES	YSEDKCDMDS
890	900	910	920	930	940	950	960
TNMDSNLKPE	EVVHKEKRRT	KSLLEEKLVL	KSKSKTQGKQ	VKVVETELQE	GATKQATTPK	PDKEKNTEEN	DSEKQRKSKV
970	980	990	1000	1010	1020	1030	1040
EDKPFEETGV	EPVLETASSS	AHSTQKDSSH	RAKLPLAKEK	YKSDKDSTST	RLERKLSDGH	KSRSLKHSSK	DIKKKDENKS
1050	1060	1070	1080	1090	1100	1110	1120
DDKDGKEVDS	SHEKARGNSS	LMEKKLSRRL	CENRRGSLSQ	EMAKGEEKLA	ANTLSTPSGS	SLQRPKKSGD	MTLIPEQEPM
1130	1140	1150	1160	1170	1180	1190	1200
EIDSEPGVEN	VFEVSKTQDN	RNNNSQQDID	SENMKQKTSA	TVQKDELRTC	TADSKATAPA	YKPGRGTGVN	SNSEKHADHR
1210	1220	1230	1240	1250	1260	1270	1280
STLTKKMHIQ	SAVSKMNPGE	KEPIHRGTTE	VNIDSETVHR	MLLSAPSEND	RVQKNLKNTA	AEEHVAQGDA	TLEHSTNLDS
1290	1300	1310	1320	1330	1340	1350	1360
SPSLSSVTVV	PLRESYDPDV	IPLFDKRTVL	EGSTASTSPA	DHSALPNQSL	TVRESEVLKT	SDSKEGGEGF	TVDTPAKASI
1370	1380	1390	1400	1410	1420	1430	1440
TSKRHIPEAH	QATLLDGKQG	KVIMPLGSKL	TGVIVENENI	TKEGGLVDMA	KKENDLNAEP	NLKQTIKATV	ENGKKDGIAV
1450	1460	1470	1480	1490	1500	1510	1520
DHVVGLNTEK	YAETVKLKHK	RSPGKVKDIS	IDVERRNENS	EVDTSAGSGS	APSVLHQRNG	QTEDVATGPR	RAEKTSVATS
1530	1540	1550	1560	1570	1580	1590	1600
TEGKDKDVTL	SPVKAGPATT	TSSETRQSEV	ALPCTSIEAD	EGLIIGTHSR	NNPLHVGAEA	SECTVFAAAE	EGGAVVTEGF
1610	1620	1630	1640	1650	1660	1670	1680
AESETFLTST	KEGESGECAV	AESEDRAADL	LAVHAVKIEA	NVNSVVTEEK	DDAVTSAGSE	EKCDGSLSRD	SEIVEGTITF
1690	1700	1710	1720	1730	1740	1750	1760
ISEVESDGAV	TSAGTEIRAG	SISSEEVDGS	QGNMMRMGPK	KETEGTVTCT	GAEGRSDNFV	ICSVTGAGPR	EERMVTGAGV
1770	1780	1790	1800	1810	1820	1830	1840
VLGDNDAPPG	TSASQEGDGS	VNDGTEGESA	VTSTGITEDG	EGPASCTGSE	DSSEGFAISS	ESEENGESAM	DSTVAKEGTN
1850	1860	1870	1880	1890	1900	1910	1920
VPLVAAGPCD	DEGIVTSTGA	KEEDEEGEDV	VTSTGRGNEI	GHASTCTGLG	EESEGVLICE	SAEGDSQIGT	VVEHVEAEAG
1930	1940	1950	1960	1970	1980	1990	2000
AAIMNANENN	VDSMSGTEKG	SKDTDICSSA	KGIVESSVTS	AVSGKDEVTP	VPGGCEGPMT	SAASDQSDSQ	LEKVEDTTIS
2010	2020	2030	2040	2050	2060	2070	2080
TGLVGGSYDV	LVSGEVPECE	VAHTSPSEKE	DEDIITSVEN	EECDGLMATT	ASGDITNQNS	LAGGKNQGKV	LIISTSTTND
2090	2100	2110	2120	2130	2140	2150	2160
YTPQVSAITD	VEGGLSDALR	TEENMEGTRV	TTEEFEAPMP	SAVSGDDSQL	TASRSEEKDE	CAMISTSIGE	EFELPISSAT
2170	2180	2190	2200	2210	2220	2230	2240

Detailed Protein Report

Cmpd.	No. of Cmpds.	m/z meas.	Δ m/z [ppm]	z	Rt [min]	Score	P	Range	Sequence	Modification
582	1	848.5905	146.58	2	36.6	10.8	2	853-867	K.DSLGSKQHGITLQRR.S	

Detailed Protein Report

Protein 251: **PREDICTED: disks large homolog 5 isoform X3 [Homo sapiens]**

**Accession:**	gi|578819987	**Score:**	38.0
**Database:**	refseq_human(refseq_human_20140103.fasta)	**MW [kDa]:**	176.9
**Database Date:**	2015-11-30	**pI:**	6.5
		**Sequence Coverage [%]:**	1.6
		**No. of unique Peptides:**	2

Quantitation

Wdown:Qdown	**Median:** 4.10	**CV:** 0.00 %	**No. of Peptides:** 1

Alias proteins:

Accession	Name	Description
gi|578839810	refseq_human (refseq_human_20140103.fasta)	PREDICTED: disks large homolog 5 isoform X3 [Homo sapiens]

10	20	30	40	50	60	70	80
MEPQRRELLA	QCQQSLAQAM	TEVEAVLGLL	EAAGALSPGE	RRQLDEEAGG	AKAELLLKLL	LAKERDHFQD	LRAALEKTQP
90	100	110	120	130	140	150	160
HLLPILYLNG	VVGPPQPAEG	AGSTYSVLST	MPSDSESSSS	LSSVGTTGKA	PSPPPLLTDQ	QVNEKVENLS	IQLRLMTRER
170	180	190	200	210	220	230	240
NELRKRLAFA	THGTAFDKRP	YHRLNPDYER	LKIQCVRAMS	DLQSLQNQHT	NALKRCEEVA	KETDFYHTLH	SRLLSDQTRL
250	260	270	280	290	300	310	320
KDDVDMLRRE	NGQLLRERNL	LQQSWEDMKR	LHEEDQKEIG	DLRAQQQQVL	KHNGSSEILN	KLYDTAMDKL	EVVKKDYDAL
330	340	350	360	370	380	390	400
RKRYSEKVAI	HNADLSRLEQ	LGEENQRLLK	QTEMLTQQRD	TAIQLQHQCA	LSLRRFEAIH	HELNKATAQN	KDLQWEMELL
410	420	430	440	450	460	470	480
QSELTELRTT	QVKTAKESEK	YREERDAVYS	EYKLIMSERD	QVISELDKLQ	TEVELAESKL	KSSTSEKKAA	NEEMEALRQI
490	500	510	520	530	540	550	560
KDTVTMDAGR	ANKEVEILRK	QCKALCQELK	EALQEADVAK	CRRDWAFQER	DKIVAERDSI	RTLCDNLRRE	RDRAVSELAE
570	580	590	600	610	620	630	640
ALRSLDDTRK	QKNDVSRELK	ELKEQMESQL	EKEARFRQLM	AHSSHDSAID	TDSMEWETEV	VEFERETEDI	DLKALGFDMA
650	660	670	680	690	700	710	720
EGVNEPCFPG	DCGIFVTKVD	KGSIADGRLR	VNDWLLRIND	VDLINKDKKQ	AIKALLNGEG	AINMVVRRRK	SLGGKVVTPL
730	740	750	760	770	780	790	800
HINLSGQKDS	GISLENGVYA	AAVLPGSPAA	KEGSLAVGDR	IVAINGIALD	NKSLNECESL	LRSCQDSLTL	SLLKEQKCVP
810	820	830	840	850	860	870	880
ASGELSPELQ	EWAPYSPGHS	SRHSNPPLYP	SRPSVGTVPR	SLTPSTTVSS	ILRNPIYTVR	SHRVGPCSSP	PAARDAGPQG
890	900	910	920	930	940	950	960
LHPSVQHQGR	LSLDLSHRTC	SDYSEMRATH	GSNSLPSSAR	LGSSSNLQFK	AERIKIPSTP	RYPRSVVGSE	RGSVSHSECS
970	980	990	1000	1010	1020	1030	1040
TPPQSPLNID	TLSSCSQSQT	SASTLPRIAV	NPASLGERRK	DRPYVEEPRH	VKVQKGSEPL	GISIVSGEKG	GIYVSKVTVG
1050	1060	1070	1080	1090	1100	1110	1120
SIAHQAGLEY	GDQLLEFNGI	NLRSATEQQA	RLIIGQQCDT	ITILAQYNPH	VHQLSSHSRS	SSHLDPAGTH	STLQGSGTTT
1130	1140	1150	1160	1170	1180	1190	1200
PEHPSVIDPL	MEQDEGPSTP	PAKQSSSRIA	GDANKKTLEP	RVVFIKKSQL	ELGVHLCGGN	LHGVFVAEVE	DDSPAKGPDG
1210	1220	1230	1240	1250	1260	1270	1280
LVPGDLILEY	GSLDVRNKTV	EEVYVEMLKP	RDGVRLKVQY	RPEEFTKAKG	LPGDSFYIRA	LYDRLADVEQ	ELSFKKDDIL
1290	1300	1310	1320	1330	1340	1350	1360
YVDDTLPQGT	FGSWMAWQLD	ENAQKIQRGQ	IPSKYVMDQE	FSRRLSMSEV	KDDNSATKTL	SAAARRSFFR	RKHKHKRSGS
1370	1380	1390	1400	1410	1420	1430	1440
KDGKDLLALD	AFSSDSIPLF	EDSVSLAYQR	VQKVDCTALR	PVLILGPLLD	VVKEMLVNEA	PGKFCRCPLE	VMKASQQAIE
1450	1460	1470	1480	1490	1500	1510	1520
RGVKDCLFVD	YKRRSGHFDV	TTVASIKEIT	EKNRHCLLDI	APHAIERLHH	MHIYPIVIFI	HYKSAKHIKE	QRDPIYLRDK
1530	1540	1550	1560	1570	1580		
VTQRHSKEQF	EAAQKLEQEY	SRYFTGVIQG	GALSSICTQI	LAMVNQEQNK	VLWIPACPL		

Detailed Protein Report

Cmpd.	No. of Cmpds.	m/z meas.	Δ m/z [ppm]	z	Rt [min]	Score	P	Range	Sequence	Modification	Ratios
2115	1	916.8144	-155.80	2	55.0	10.3	2	257-270	R.ERNLLQQSWEDMKR.L		Wdown:Qdown 4.10
1673	1	662.2991	-86.48	2	49.4	14.9	1	1248-1259	K.AKGLPGDSFYIR.A		

Detailed Protein Report

Protein 252: PREDICTED: cyclin-dependent kinase-like 1 isoform X1 [Homo sapiens]

Accession: gi|530404810
Database: refseq_human(refseq_human_20140103.fasta)
Database Date: 2015-11-30

Score: 37.9
MW [kDa]: 108.9
pI: 9.8
Sequence Coverage [%]: 3.9
No. of unique Peptides: 1

Quantitation

mdown:qdown	**Median:**	0.56	**CV:**	0.00 %	**No. of Peptides:**	1
Wdown:Qdown	**Median:**	0.15	**CV:**	0.00 %	**No. of Peptides:**	1

10	20	30	40	50	60	70	80
MELSTDTGWN	QKSNFPPCDS	RVSKEEAQLP	FPTPSSYGAD	SSNQTYAHKS	QSEREQHKEV	RAVLNHCSGL	DGGEESGLCG
**90**	**100**	**110**	**120**	**130**	**140**	**150**	**160**
TSTRIDAGTG	NGTDDKVTDQ	HRHRRCLQGT	KGNNRGSEVW	GLLLQGNVDR	SGGAPSAGVL	LRRRGYSCAL	HGLRKFANLA
**170**	**180**	**190**	**200**	**210**	**220**	**230**	**240**
GLLSRQQDSA	RGVSHHSRLK	IHFKKIYSSM	MEKYEKIGKI	GEGSYGVVFK	CRNRDTGQIV	AIKKFLESED	DPVIKKIALR
**250**	**260**	**270**	**280**	**290**	**300**	**310**	**320**
EIRMLKAPSP	YAAEPSLCGM	KMVRRGKKEF	LPAVAEKVDA	PSGVGGQGQD	SVTVGSLGRR	STYGRKQEKQ	VRQREGIIYC
**330**	**340**	**350**	**360**	**370**	**380**	**390**	**400**
YVAVLLRIYY	FDQGCVAREE	EQFQELVFGP	FCHIGSYFTG	HRTNVRPYIL	LLSRPSPFKT	AAGTYEAGLV	ILECSYFLAE
**410**	**420**	**430**	**440**	**450**	**460**	**470**	**480**
QEPYCPTQAL	QQPHPIIGPW	ALEGGGVESK	EDRHPPPKEA	PASCEGFLRS	AVPKQAYTPF	KTSPDKRLSD	CVATPPWAPP
**490**	**500**	**510**	**520**	**530**	**540**	**550**	**560**
TPLIISSGVL	VAICSMIDPV	PEFHSEGLLA	KATSGSAGIL	VWIFLCNDAF	IYGKYILRSG	VLWVRGLPGF	KSEAAALCHK
**570**	**580**	**590**	**600**	**610**	**620**	**630**	**640**
CYSSADPKRE	QQQDLLQRVK	EQSFHSVNGA	QARHKGSPSP	HQTQEPSWPH	PVDPTPGHRW	SCLPVPCRAP	ALLSPWVVDG
**650**	**660**	**670**	**680**	**690**	**700**	**710**	**720**
TGCCGAGGGS	DRGGSAAQEP	TQLKHPNLVN	LLEVFRRKRR	LHLVFEYCDH	TVLHELDRYQ	RGICNIFVCT	GRRLGEHTEA
**730**	**740**	**750**	**760**	**770**	**780**	**790**	**800**
LSKKKKKGGG	GPFLKLRAAS	CRITLFKNVG	CGLETTGDLS	LNSGGGAASR	GVAAALRALV	CGTELTSSDS	PQRCIHRDVK
**810**	**820**	**830**	**840**	**850**	**860**	**870**	**880**
PENILITKHS	VIKLCDFGFA	RLLTGPSDYY	TDYVATRWYR	SPELLVGDTQ	YGPPVDVWAI	GCVFAELLSG	VPLWPGKSDV
**890**	**900**	**910**	**920**	**930**	**940**	**950**	**960**
DQLYLIRKTL	GDLIPRHQQV	FSTNQYFSGV	KIPDPEDMSL	CLSVTLTEGG	LLASGAVKRS	QMGSSVSQAT	SWPHPDIVAE
**970**	**980**	**990**	**1000**				
TAELDDIAMA	RQTPVMLRFN	RQKEQEKYLS	YGA				

Cmpd.	No. of Cmpds.	m/z meas.	Δ m/z [ppm]	z	Rt [min]	Score	P	Range	Sequence	Modification	Ratios
317	1	442.6411	-276.80	2	33.0	10.9	0	889-896	K.TLGDLIPR.H		Wdown:Qdown 0.15<br>mdown:qdown 0.56

Detailed Protein Report

Protein 253: neuropeptides B/W receptor type 1 [Homo sapiens]

**Accession:**	gi|53828924	**Score:**	37.9
**Database:**	refseq_human(refseq_human_20140103.fasta)	**MW [kDa]:**	36.1
**Database Date:**	2015-11-30	**pI:**	10.1
		**Sequence Coverage [%]:**	7.0
		**No. of unique Peptides:**	1

10	20	30	40	50	60	70	80
MDNASFSEPW	PANASGPDPA	LSCSNASTLA	PLPAPLAVAV	PVVYAVICAV	GLAGNSAVLY	VLLRAPRMKT	VTNLFILNLA
**90**	**100**	**110**	**120**	**130**	**140**	**150**	**160**
IADELFTLVL	PINIADFLLR	QWPFGELMCK	LIVAIDQYNT	FSSLYFLTVM	SADRYLVVLA	TAESRRVAGR	TYSAARAVSL
**170**	**180**	**190**	**200**	**210**	**220**	**230**	**240**
AVWGIVTLVV	LPFAVFARLD	DEQGRRQCVL	VFPQPEAFWW	RASRLYTLVL	GFAIPVSTIC	VLYTTLLCRL	HAMRLDSHAK
**250**	**260**	**270**	**280**	**290**	**300**	**310**	**320**
ALERAKKRVT	FLVVAILAVC	LLCWTPYHLS	TVVALTTDLP	QTPLVIAISY	FITSLSYANS	CLNPFLYAFL	DASFRRNLRQ
**330**							
LITCRAAA							

Cmpd.	No. of Cmpds.	m/z meas.	Δ m/z [ppm]	z	Rt [min]	Score	P	Range	Sequence	Modification
408	1	604.3472	17.95	2	34.1	23.5	2	146-156	R.RVAGRTYSAAR.A	

Detailed Protein Report

Protein 254: histone H4 [Homo sapiens]

**Accession:**	gi|4504301	**Score:**	37.9
**Database:**	refseq_human(refseq_human_20140103.fasta)	**MW [kDa]:**	11.4
**Database Date:**	2015-11-30	**pI:**	11.8
		**Sequence Coverage [%]:**	7.8
		**No. of unique Peptides:**	1

Alias proteins:

Accession	Name	Description
gi|77539758	refseq_human (refseq_human_20140103.fasta)	histone H4 [Homo sapiens]
gi|28173560	refseq_human (refseq_human_20140103.fasta)	histone H4 [Homo sapiens]
gi|11415030	refseq_human (refseq_human_20140103.fasta)	histone H4 [Homo sapiens]
gi|4504323	refseq_human (refseq_human_20140103.fasta)	histone H4 [Homo sapiens]
gi|4504321	refseq_human (refseq_human_20140103.fasta)	histone H4 [Homo sapiens]
gi|4504317	refseq_human (refseq_human_20140103.fasta)	histone H4 [Homo sapiens]
gi|4504315	refseq_human (refseq_human_20140103.fasta)	histone H4 [Homo sapiens]
gi|4504313	refseq_human (refseq_human_20140103.fasta)	histone H4 [Homo sapiens]
gi|4504311	refseq_human (refseq_human_20140103.fasta)	histone H4 [Homo sapiens]
gi|4504309	refseq_human (refseq_human_20140103.fasta)	histone H4 [Homo sapiens]
gi|4504307	refseq_human (refseq_human_20140103.fasta)	histone H4 [Homo sapiens]
gi|4504305	refseq_human (refseq_human_20140103.fasta)	histone H4 [Homo sapiens]
gi|4504303	refseq_human (refseq_human_20140103.fasta)	histone H4 [Homo sapiens]

```
        10         20         30         40         50         60         70         80
MSGRGKGGKG LGKGGAKRHR KVLRDNIQGI TKPAIRRLAR RGGVKRISGL IYEETRGVLK VFLENVIRDA VTYTEHAKRK
        90        100        110
TVTAMDVVYA LKRQGRTLYG FGG
```

Cmpd.	No. of Cmpds.	m/z meas.	Δ m/z [ppm]	z	Rt [min]	Score	P	Range	Sequence	Modification
1347	10	495.2734	-38.66	2	45.6	37.9	0	61-68	K.VFLENVIR.D	

Detailed Protein Report

Protein 255: PREDICTED: centlein isoform X1 [Homo sapiens]

**Accession:**	gi|530389965	**Score:**	37.9
**Database:**	refseq_human(refseq_human_20140103.fasta)	**MW [kDa]:**	162.0
**Database Date:**	2015-11-30	**pI:**	8.8
		**Sequence Coverage [%]:**	2.6
		**No. of unique Peptides:**	3

Quantitation

mdown:qdown	**Median:** 1.15	**CV:** 55.25 %	**No. of Peptides:**	2
Wdown:Qdown	**Median:** 2.15	**CV:** 86.23 %	**No. of Peptides:**	2

```
        10         20         30         40         50         60         70         80
MAARSPPSPH PSPPARQLGP RSPRVGRGAE VHAMRSEASG FAGAAREVVA DESDKIWVGE EGSGGRRGPG GAAPAHAPLL
        90        100        110        120        130        140        150        160
SAPMGSRRLE GISVEEAMVT RTQLLEEELS SLKEELALCQ ADKEFVWSLW KRLQVTNPDL TQVVSLVVER EKQKSEAKDR
       170        180        190        200        210        220        230        240
KVLEILQVKD AKIQEFEQRE SVLKQEINDL VKRKIAVDEE NAFLRKEFSD LEKKFKDKSQ EIKDTKECVQ NKEEQNRLVI
       250        260        270        280        290        300        310        320
KNLEEENKKL STRCTDLLND LEKLRKQEAH LRKEKYSTDA KIKTFEDNLI EARKEVEVSQ SKYNALSLQL SNKQTELIQK
       330        340        350        360        370        380        390        400
DMDITLVRKE LQELQNLYKQ NSTHTAQQAE LIQQLQVLNM DTQKVLRNQE DVHTAESISY QKLYNELHIC FETTKSNEAM
       410        420        430        440        450        460        470        480
LRQSVTNLQD QLLQKEQENA KLKEKLQESQ GAPLPLPQES DPDYSAQVPH RPSLSSLETL MVSQKSEIEY LQEKLKIANE
       490        500        510        520        530        540        550        560
KLSENISANK GFSRKSIMTS AEGKHKEPPV KRSRSLSPKS SFTDSEELQK LRKAERKIEN LEKALQLKSQ ENDELRDAHE
       570        580        590        600        610        620        630        640
KRKERLQMLQ TNYRAVKEQL KQWEEGSGMT EIRKIKRADP QQLRQEDSDA VWNELAYFKR ENQELMIQKM NLEEELDELK
       650        660        670        680        690        700        710        720
VHISIDKAAI QELNRCVAER REEQLFRSGE DDEVKRSTPE KNGKEMLEQT LQKVTELENR LKSFEKRSRK LKEGNKKLMK
       730        740        750        760        770        780        790        800
ENDFLKSLLK QQQEDTETRE KELEQIIKGS KDVEKENTEL QVKISELETE VTSLRRQVAE ANALRNENEE LINPMEKSHQ
       810        820        830        840        850        860        870        880
SADRAKSEMA TMKVRSGRYD CKTTMTKVKF KAAKKNCSVG RHHTVLNHSI KVMSNVFENL SKDGWEDVSE SSDSEAQTSQ
       890        900        910        920        930        940        950        960
TLGTIIVETS QKISPTEDGK DQKESDPTED SQTQGKEIVQ TYLNIDGKTP KDYFHDKNAK KPTFQKKNCK MQKSSHTAVP
       970        980        990       1000       1010       1020       1030       1040
TRVNREKYKN ITAQKSSSNI ILLRERIISL QQQNSVLQNA KKTAELSVKE YKEVNEKLLH QQQVSDQRFQ TSRQTIKKLN
      1050       1060       1070       1080       1090       1100       1110       1120
LDLAGLRKEK EDLLKKLESS SEITSLAEEN SQVTFPRIQV TSLSPSRSMD LEMKQLQYKL KNATNELTKQ SSNVKTLKFE
      1130       1140       1150       1160       1170       1180       1190       1200
LLAKEEHIKE MHEKISRMER DITMKRHLIE DLKFRQKVNL ESNKSFSEML QNLDKKVKTL TEECSNKKVS IDSLKQRLNV
      1210       1220       1230       1240       1250       1260       1270       1280
AVKEKSQYEQ MYQKSKEELE KKDLKLTLLV SRISETESAM AEIETAASKQ LQELALQSEQ VLEGAQKTLL LANEKVEEFT
      1290       1300       1310       1320       1330       1340       1350       1360
TFVKALAKEL QNDVHVVRRQ IRELKKMKKN RDACKTSTHK AQTLAASILN ISRSDLEEIL DTEDQVEIEK TKIDAENDKE
      1370       1380       1390       1400       1410
WMLYIQKLLE GQLPFASYLL EAVLEKINEK KKLVEGYFTI MKDIR
```

Cmpd.	No. of Cmpds.	m/z meas.	Δ m/z [ppm]	z	Rt [min]	Score	P	Range	Sequence	Modification	Ratios
255	1	795.3881	-39.33	2	32.4	10.8	1	88-101	R.RLEGISVEEAMVTR.T		Wdown:Qdown 1.02<br>mdown:qdown 0.69
1953	1	643.3047	-169.45	1	54.5	11.9	0	1198-1203	R.LNVAVK.E		mdown:qdown 1.93<br>Wdown:Qdown 4.53
2736	1	683.3656	57.65	3	65.5	15.2	2	1206-1221	K.SQYEQMYQKSKEELEK.K		

Detailed Protein Report

Protein 256: PREDICTED: dedicator of cytokinesis protein 6 isoform X3 [Homo sapiens]

**Accession:**	gi|578833402	**Score:**	37.8
**Database:**	refseq_human(refseq_human_20140103.fasta)	**MW [kDa]:**	232.6
**Database Date:**	2015-11-30	**pI:**	6.3
		**Sequence Coverage [%]:**	1.4
		**No. of unique Peptides:**	2

Detailed Protein Report

10	20	30	40	50	60	70	80
MAASERRAFA	HKINRTVAAE	VRKQVSRERS	GSPHSSRRCS	SSLGVPLTEV	VEPLDFEDVL	LSRPPDAEPG	PLRDLVEFPA
90	100	110	120	130	140	150	160
DDLELLLQPR	ECRTTEPGIP	KDEKLDAQVR	AAVEMYIEDW	VIVHRRYQYL	SAAYSPVTTD	TQRERQKGLP	RQVFEQDASG
170	180	190	200	210	220	230	240
DERSGPEDSN	DSRRGSGSPE	DTPRSSGASS	IFDLRNLAAD	SLLPSLLERA	APEDVDRRNE	TLRRQHRPPA	LLTLYPAPDE
250	260	270	280	290	300	310	320
DEAVERCSRP	EPPREHFGQR	ILVKCLSLKF	EIEIEPIFGI	LALYDVREKK	KISENFYFDL	NSDSMKGLLR	AHGTHPAIST
330	340	350	360	370	380	390	400
LARSAIFSVT	YPSPDIFLVI	KLEKVLQQGD	ISECCEPYMV	LKEVDTAKNK	EKLEKLRLAA	EQFCTRLGRY	RMPFAWTAVH
410	420	430	440	450	460	470	480
LANIVSSAGQ	LDRDSDSEGE	RRPAWTDRRR	RGPQDRASSG	DDACSFSGFR	PATLTVTNFF	KQEAERLSDE	DLFKFLADMR
490	500	510	520	530	540	550	560
RPSSLLRRLR	PVTAQLKIDI	SPAPENPHFC	LSPELLHIKP	YPDPRGRPTK	EILEFPAREV	YAPHTSYRNL	LYVYPHSLNF
570	580	590	600	610	620	630	640
SSRQGSVRNL	AVRVQYMTGE	DPSQALPVIF	GKSSCSEFTR	EAFTPVVYHN	KSPEFYEEFK	LHLPACVTEN	HHLLFTFYHV
650	660	670	680	690	700	710	720
SCQPRPGTAL	ETPVGFTWIP	LLQHGRLRTG	PFCLPVSVDQ	PPPSYSVLTP	DVALPGMRWV	DGHKGVFSVE	LTAVSSVHPQ
730	740	750	760	770	780	790	800
DPYLDKFFTL	VHVLEEGAFP	FRLKDTVLSE	GNVEQELRAS	LAALRLASPE	PLVAFSHHVL	DKLVRLVIRP	PIISGQIVNL
810	820	830	840	850	860	870	880
GRGAFEAMAH	VVSLVHRSLE	AAQDARGHCP	QLAAYVHYAF	RLPGTEPSLP	DGAPPVTVQA	ATLARGSGRP	ASLYLARSKS
890	900	910	920	930	940	950	960
ISSSNPDLAV	APGSVDDEVS	RILASKAIDC	NSSRASSYLE	GSSSAPPATQ	LRPTVQKLLH	EELALQWVVS	SSAVREAILQ
970	980	990	1000	1010	1020	1030	1040
HAWFFFQLMV	KSMALHLLLG	QRLDTPRKLR	FPGRFLDDIT	ALVGSVGLEV	ITRVHKDVEL	AEHLNASLAF	FLSDLLSLVD
1050	1060	1070	1080	1090	1100	1110	1120
RGFVFSLVRA	HYKQVATRLQ	SSPNPAALLT	LRMEFTRILC	SHEHYVTLNL	PCCPLSPPAS	PSPSVSSTTS	QSSTFSSQAP
1130	1140	1150	1160	1170	1180	1190	1200
DPKVTSMFEL	SGPFRQQHFL	AGLLLTELAL	ALEPEAEGAF	LLHKKAISAV	HSLLCGHDTD	PRYAEATVKA	RVAELYLPLL
1210	1220	1230	1240	1250	1260	1270	1280
SIARDTLPRL	HDFAEGPGQR	SRLASMLDSD	TEGEGDIAGT	INPSVAMAIA	GGPLAPGSRA	SISQGPPTAS	RAGCALSAES
1290	1300	1310	1320	1330	1340	1350	1360
SRTLLACVLW	VLKNTEPALL	QRWATDLTLP	QLGRLLDLLY	LCLAAFEYKG	KKAFERINSL	TFKKSLDMKA	RLEEAILGTI
1370	1380	1390	1400	1410	1420	1430	1440
GARQEMVRRS	RERSPFGNPE	NVRWRKSVTH	WKQTSDRVDK	TKDEMEHEAL	VEGNLATEAS	LVVLDTLEII	VQTVMLSEAR
1450	1460	1470	1480	1490	1500	1510	1520
ESVLGAVLKV	VLYSLGSAQS	ALFLQHGLAT	QRALVSKFPE	LLFEEDTELC	ADLCLRLLRH	CGSRISTIRT	HASASLYLLM
1530	1540	1550	1560	1570	1580	1590	1600
RQNFEIGHNF	ARVKMQVTMS	LSSLVGTTQN	FSEEHLRRSL	KTILTYAEED	MGLRDSTFAE	QVQDLMFNLH	MILTDTVKMK
1610	1620	1630	1640	1650	1660	1670	1680
EHQEDPEMLI	DLMYRIARGY	QGSPDLRLTW	LQNMAGKHAE	LGNHAEAAQC	MVHAAALVAE	YLALLEDHRH	LPVGCVSFQN
1690	1700	1710	1720	1730	1740	1750	1760
ISSNVLEESA	ISDDILSPDE	EGFCSGKHFT	ELGLVGLLEQ	AAGYFTMGGL	YEAVNEVYKN	LIPILEAHRD	YKKLAAVHGK
1770	1780	1790	1800	1810	1820	1830	1840
LQEAFTKIMH	QSSGWERVFG	TYFRVGFYGA	HFGDLDEQEF	VYKEPSITKL	AEISHRLEEF	YTERFGDDVV	EIIKDSNPVD
1850	1860	1870	1880	1890	1900	1910	1920
KSKLDSQKAY	IQITYVEPYF	DTYELKDRVT	YFDRNYGLRT	FLFCTPFTPD	GRAHGELPEQ	HKRKTLLSTD	HAFPYIKTRI
1930	1940	1950	1960	1970	1980	1990	2000
RVCHREETVL	TPVEVAIEDM	QKKTRELAFA	TEQDPPDAKM	LQMVLQGSVG	PTVNQGPLEV	AQVFLAEIPE	DPKLFRHHNK
2010	2020	2030	2040	2050	2060	2070	2080
LRLCFKDFCK	KCEDALRKNK	ALIGPDQKEY	HRELERNYCR	LREALQPLLT	QRLPQLMAPT	PPGLRNSLNR	ASFRKADL

Cmpd.	No. of Cmpds.	m/z meas.	Δ m/z [ppm]	z	Rt [min]	Score	P	Range	Sequence	Modification

Detailed Protein Report

Cmpd.	No. of Cmpds.	m/z meas.	Δ m/z [ppm]	z	Rt [min]	Score	P	Range	Sequence	Modification
1750	1	628.2499	-105.50	2	51.8	11.8	2	28-38	R.ERSGSPHSSRR.C	
2956	1	753.3925	-14.65	2	66.1	10.4	0	1905-1917	K.TLLSTDHAFPYIK.T	

Detailed Protein Report

Protein 257: PREDICTED: utrophin isoform X8 [Homo sapiens]

**Accession:**	gi|578812959	**Score:**	37.8
**Database:**	refseq_human(refseq_human_20140103.fasta)	**MW [kDa]:**	287.1
**Database Date:**	2015-11-30	**pI:**	5.2
**Modification(s):**	Oxidation	**Sequence Coverage [%]:**	0.8
		**No. of unique Peptides:**	1

Detailed Protein Report

10	20	30	40	50	60	70	80
MSLMILPRWR	RPCKKKKTLD	EILENQKPAL	HKLAEETKAL	EKNVHPDVEK	LYKQEFDDVQ	GKWNKLKVLV	SKDLHLLEEI
90	100	110	120	130	140	150	160
ALTLRAFEAD	STVIEKWMDG	VKDFLMKQQA	AQGDDAGLQR	QLDQCSAFVN	EIETIESSLK	NMKEIETNLR	SGPVAGIKTW
170	180	190	200	210	220	230	240
VQTRLGDYQT	QLEKLSKEIA	TQKSRLSESQ	EKAANLKKDL	AEMQEWMTQA	EEEYLERDFE	YKSPEELESA	VEEMKRAKED
250	260	270	280	290	300	310	320
VLQKEVRVKI	LKDNIKLLAA	KVPSGGQELT	SELNVVLENY	QLLCNRIRGK	CHTLEEVWSC	WIELLHYLDL	ETTWLNTLEE
330	340	350	360	370	380	390	400
RMKSTEVLPE	KTDAVNEALE	SLESVLRHPA	DNRTQIRELG	QTLIDGGILD	DIISEKLEAF	NSRYEDLSHL	AESKQISLEK
410	420	430	440	450	460	470	480
QLQVLRETDQ	MLQVLQESLG	ELDKQLTTYL	TDRIDAFQVP	QEAQKIQAEI	SAHELTLEEL	RRNMRSQPLT	SPESRTARGG
490	500	510	520	530	540	550	560
SQMDVLQRKL	REVSTKFQLF	QKPANFEQRM	LDCKRVLDGV	KAELHVLDVK	DVDPDVIQTH	LDKCMKLYKT	LSEVKLEVET
570	580	590	600	610	620	630	640
VIKTGRHIVQ	KQQTDNPKGM	DEQLTSLKVL	YNDLGAQVTE	GKQDLERASQ	LARKMKKEAA	SLSEWLSATE	TELVQKSTSE
650	660	670	680	690	700	710	720
GLLGDLDTEI	SWAKNVLKDL	EKRKADLNTI	TESSAALQNL	IEGSEPILEE	RLCVLNAGWS	RVRTWTEDWC	NTLMNHQNQL
730	740	750	760	770	780	790	800
EIFDGNVAHI	STWLYQAEAL	LDEIEKKPTS	KQEEIVKRLV	SELDDANLQV	ENVRDQALIL	MNARGSSSRE	LVEPKLAELN
810	820	830	840	850	860	870	880
RNFEKVSQHI	KSAKLLIAQE	PLYQCLVTTE	TFETGVPFSD	LEKLENDIEN	MLKFVEKHLE	SSDEDEKMDE	ESAQIEEVLQ
890	900	910	920	930	940	950	960
RGEEMLHQPM	EDNKKEKIRL	QLLLLHTRYN	KIKAIPIQQR	KMGQLASGIR	SSLLPTDYLV	EINKILLCMD	DVELSLNVPE
970	980	990	1000	1010	1020	1030	1040
LNTAIYEDFS	FQEDSLKNIK	DQLDKLGEQI	AVIHEKQPDV	ILEASGPEAI	QIRDTLTQLN	AKWDRINRMY	SDRKGCFDRA
1050	1060	1070	1080	1090	1100	1110	1120
MEEWRQFHCD	LNDLTQWITE	AEELLVDTCA	PGGSLDLEKA	RIHQQELEVG	ISSHQPSFAA	LNRTGDGIVQ	KLSQADGSFL
1130	1140	1150	1160	1170	1180	1190	1200
KEKLAGLNQR	WDAIVAEVKD	RQPRLKGESK	QVMKYRHQLD	EIICWLTKAE	HAMQKRSTTE	LGENLQELRD	LTQEMEVHAE
1210	1220	1230	1240	1250	1260	1270	1280
KLKWLNRTEL	EMLSDKSLSL	PERDKISESL	RTVNMTWNKI	CREVPTTLKE	CIQEPSSVSQ	TRIAAHPNVQ	KVVLVSSASD
1290	1300	1310	1320	1330	1340	1350	1360
IPVQSHRTSE	ISIPADLDKT	ITELADWLVL	IDQMLKSNIV	TVGDVEEINK	TVSRMKITKA	DLEQRHPQLD	YVFTLAQNLK
1370	1380	1390	1400	1410	1420	1430	1440
NKASSSDMRT	AITEKLERVK	NQWDGTQHGV	ELRQQQLEDM	IIDSLQWDDH	REETEELMRK	YEARLYILQQ	ARRDPLTKQI
1450	1460	1470	1480	1490	1500	1510	1520
SDNQILLQEL	GPGDGIVMAF	DNVLQKLLEE	YGSDDTRNVK	ETTEYLKTSW	INLKQSIADR	QNALEAEWRT	VQASRRDLEN
1530	1540	1550	1560	1570	1580	1590	1600
FLKWIQEAET	TVNVLVDASH	RENALQDSIL	ARELKQQMQD	IQAEIDAHND	IFKSIDGNRQ	KMVKALGNSE	EATMLQHRLD
1610	1620	1630	1640	1650	1660	1670	1680
DMNQRWNDLK	AKSASIRAHL	EASAEKWNRL	LMSLEELIKW	LNMKDEELKK	QMPIGGDVPA	LQLQYDHCKA	LRRELKEKEY
1690	1700	1710	1720	1730	1740	1750	1760
SVLNAVDQAR	VFLADQPIEA	PEEPRRNLQS	KTELTPEERA	QKIAKAMRKQ	SSEVKEKWES	LNAVTSNWQK	QVDKALEKLR
1770	1780	1790	1800	1810	1820	1830	1840
DLQGAMDDLD	ADMKEAESVR	NGWKPVGDLL	IDSLQDHIEK	IMAFREEIAP	INFKVKTVND	LSSQLSPLDL	HPSLKMSRQL
1850	1860	1870	1880	1890	1900	1910	1920
DDLNMRWKLL	QVSVDDRLKQ	LQEAHRDFGP	SSQHFLSTSV	QLPWQRSISH	NKVPYYINHQ	TQTTCWDHPK	MTELFQSLAD
1930	1940	1950	1960	1970	1980	1990	2000
LNNVRFSAYR	TAIKIRRLQK	ALCLDLLELS	TTNEIFKQHK	LNQNDQLLSV	PDVINCLTTT	YDGLEQMHKD	LVNVPLCVDM
2010	2020	2030	2040	2050	2060	2070	2080
CLNWLLNVYD	TGRTGKIRVQ	SLKIGLMSLS	KGLLEEKYRY	LFKEVAGPTE	MCDQRQLGLL	LHDAIQIPRQ	LGEVAAFGGS
2090	2100	2110	2120	2130	2140	2150	2160
NIEPSVRSCF	QQNNNKPEIS	VKEFIDWMHL	EPQSMVWLPV	LHRVAAAETA	KHQAKCNICK	ECPIVGFRYR	SLKHFNYDVC
2170	2180	2190	2200	2210	2220	2230	2240
QSCFFSGRTA	KGHKLHYPMV	EYCIPTTSGE	DVRDFTKVLK	NKFRSKKYFA	KHPRLGYLPV	QTVLEGDNLE	TPITLISMWP
2250	2260	2270	2280	2290	2300	2310	2320
EHYDPSQSPQ	LFHDDTHSRI	EQYATRLAQM	ERTNGSFLTD	SSSTTGSVED	EHALIQQYCQ	TLGGESPVSQ	PQSPAQILKS
2330	2340	2350	2360	2370	2380	2390	2400

Detailed Protein Report

Cmpd.	No. of Cmpds.	m/z meas.	Δ m/z [ppm]	z	Rt [min]	Score	P	Range	Sequence	Modification
1249	1	764.3778	23.73	2	45.3	11.3	2	1023-1033	K.WDRINRMYSDR.K	Oxidation: 7

Detailed Protein Report

Protein 258: zinc finger CCCH domain-containing protein 13 [Homo sapiens]

Accession: gi|116008442
Database: refseq_human(refseq_human_20140103.fasta)
Database Date: 2015-11-30
Modification(s): Oxidation

Score: 37.8
MW [kDa]: 184.8
pI: 10.0
Sequence Coverage [%]: 1.9
No. of unique Peptides: 2

Alias proteins:

Accession	Name	Description
gi|530402208	refseq_human (refseq_human_20140103.fasta)	PREDICTED: zinc finger CCCH domain-containing protein 13 isoform X12 [Homo sapiens]

10	20	30	40	50	60	70	80
MSKIRRKVTV	ENTKTISDST	SRRPSVFERL	GPSTGSTAET	QCRNWLKTGN	CLYGNTCRFV	HGPSPRGKGY	SSNYRRSPER
90	100	110	120	130	140	150	160
PTGDLRERMK	NKRQDVDTEP	QKRNTEESSS	PVRKESSRGR	HREKEDIKIT	KERTPESEEE	NVEWETNRDD	SDNGDINYDY
170	180	190	200	210	220	230	240
VHELSLEMKR	QKIQRELMKL	EQENMEKREE	IIIKKEVSPE	VVRSKLSPSP	SLRKSSKSPK	RKSSPKSSSA	SKKDRKTSAV
250	260	270	280	290	300	310	320
SSPLLDQQRN	SKTNQSKKKG	PRTPSPPPPI	PEDIALGKKY	KEKYKVKDRI	EEKTRDGKDR	GRDFERQREK	RDKPRSTSPA
330	340	350	360	370	380	390	400
GQHHSPISSR	HHSSSSQSGS	SIQRHSPSPR	RKRTPSPSYQ	RTLTPPLRRS	ASPYPSHSLS	SPQRKQSPPR	HRSPMREKGR
410	420	430	440	450	460	470	480
HDHERTSQSH	DRRHERREDT	RGKRDREKDS	REEREYEQDQ	SSSRDHRDDR	EPRDGRDRRD	ARDTRDRREL	RDSRDMRDSR
490	500	510	520	530	540	550	560
EMRDYSRDTK	ESRDPRDSRS	TRDAHDYRDR	EGRDTHRKED	TYPEESRSYG	RNHLREESSR	TEIRNESRNE	SRSEIRNDRM
570	580	590	600	610	620	630	640
GRSRGRVPEL	PEKGSRGSRG	SQIDSHSSNS	NYHDSWETRS	SYPERDRYPE	RDNRDQARDS	SFERRHGERD	RRDNRERDQR
650	660	670	680	690	700	710	720
PSSPIRHQGR	NDELERDERR	EERRVDRVDD	RRDERARERD	RERERDRERE	RERERERDRE	REKERELERE	RARERERERE
730	740	750	760	770	780	790	800
KERDRERDRD	RDHDRERERE	RERDREKERE	REREERERER	ERERERERER	ERERERARER	DKERERQRDW	EDKDKGRDDR
810	820	830	840	850	860	870	880
REKREEIRED	RNPRDGHDER	KSKKRYRNEG	SPSPRQSPKR	RREHSPDSDA	YNSGDDKNEK	HRLLSQVVRP	QESRSLSPSH
890	900	910	920	930	940	950	960
LTEDRQGRWK	EEDRKPERKE	SSRRYEEQEL	KEKVSSVDKQ	REQTEILESS	RMRAQDIIGH	HQSEDRETSD	RAHDENKKKA
970	980	990	1000	1010	1020	1030	1040
KIQKKPIKKK	KEDDVGIERG	NIETTSEDGQ	VFSPKKGQKK	KSIEKKRKKS	KGDSDISDEE	AAQQSKKKRG	PRTPPITTKE
1050	1060	1070	1080	1090	1100	1110	1120
ELVEMCNGKN	GILEDSQKKE	DTAFSDWSDE	DVPDRTEVTE	AEHTATATTP	GSTPSPLSSL	LPPPPPVATA	TATTVPATLA
1130	1140	1150	1160	1170	1180	1190	1200
ATTAAAATSF	STSAITISTS	ATPTNTTNNT	FANEDSHRKC	HRTRVEKVET	PHVTIEDAQH	RKPMDQKRSS	SLGSNRSNRS
1210	1220	1230	1240	1250	1260	1270	1280
HTSGRLRSPS	NDSAHRSGDD	QSGRKRVLHS	GSRDREKTKS	LEITGERKSR	IDQLKRGEPS	RSTSSDRQDS	RSHSSRRSSP
1290	1300	1310	1320	1330	1340	1350	1360
ESDRQVHSRS	GSFDSRDRLQ	ERDRYEHDRE	RERERRDTRQ	REWDRDADKD	WPRNRDRDRL	REREREREREРD	KRRDLDRERE
1370	1380	1390	1400	1410	1420	1430	1440
RLISDSVERD	RDRDRDRTFE	SSQIESVKRC	EAKLEGEHER	DLESTSRDSL	ALDKERMDKD	LGSVQGFEET	NKSERTESLE
1450	1460	1470	1480	1490	1500	1510	1520
AGDDESKLDD	AHSLGSGAGE	GYEPISDDEL	DEILAGDAEK	REDQQDEEKM	PDPLDVIDVD	WSGLMPKHPK	EPREPGAALL
1530	1540	1550	1560	1570			
KFTPGAVMLR	VGISKKLAGS	ELFAKVKETC	QRLLEKPKGS	FILL			

Cmpd.	No. of Cmpds.	m/z meas.	Δ m/z [ppm]	z	Rt [min]	Score	P	Range	Sequence	Modification
1864	1	606.2622	-6.93	2	53.3	12.3	2	475-483	R.DMRDSREMR.D	Oxidation: 8
1287	1	795.9839	117.71	2	45.8	13.8	2	528-540	R.SYGRNHLREESSR.T	

Detailed Protein Report

Protein 259: **PREDICTED: 1-acyl-sn-glycerol-3-phosphate acyltransferase gamma isoform X1 [Homo sapiens]**

**Accession:**	gi|530419317	**Score:**	37.7
**Database:**	refseq_human(refseq_human_20140103.fasta)	**MW [kDa]:**	52.2
**Database Date:**	2015-11-30	**pI:**	9.9
**Modification(s):**	Carbamidomethyl, Oxidation	**Sequence Coverage [%]:**	6.0
		**No. of unique Peptides:**	2

```
        10         20         30         40         50         60         70         80
MCITALSVAP KGWVPAACRQ GAYHRGTSMP WGSLSSHGCA LGAVPGPSQK HRAGPAGPDA MGFCVCEPLE KDGCPQRGAV
        90        100        110        120        130        140        150        160
HPLLSSAMGL LAFLKTQFVL HLLVGFVFVV SGLVINFVQL CTLALWPVSK QLYRRLNCRL AYSLWSQLVM LLEWWSCTEC
       170        180        190        200        210        220        230        240
TLFTDQATVE RFGKEHAVII LNHNFEIDFL CGWTMCERFG VLGSSKVLAK KELLYVPLIG WTWYFLEIVF CKRKWEEDRD
       250        260        270        280        290        300        310        320
TVVEGLRRLS DYPEYMWFLL YCEGTRFTET KHRVSMEVAA AKGLPVLKYH LLPRTKGFTT AVKCLRGTVA AVYDVTLNFR
       330        340        350        360        370        380        390        400
GNKNPSLLGI LYGKKYEADM CVRRFPLEDI PLDEKEAAQW LHKLYQEKDA LQEIYNQKGM FPGEQFKPAR RPWTLLNFLS
       410        420        430        440        450        460        470
WATILLSPLF SFVLGVFASG SPLLILTFLG FVGAASFGVR RLIGVTEIEK GSSYGNQEFK KKE
```

Cmpd.	No. of Cmpds.	m/z meas.	Δ m/z [ppm]	z	Rt [min]	Score	P	Range	Sequence	Modification
2765	2	702.0547	47.21	3	65.9	20.2	1	1-19	-.MCITALSVAPKGWVPAACR.Q	Carbamidomethyl: 2, 18; Oxidation: 1
2735	1	905.4845	9.33	1	65.5	17.5	0	274-282	R.VSMEVAAAK.G	

Detailed Protein Report

Protein 260: **PREDICTED: WD repeat-containing protein 88 isoform X1 [Homo sapiens]**

Accession: gi|578833944
Database: refseq_human(refseq_human_20140103.fasta)
Database Date: 2015-11-30

Score: 37.7
MW [kDa]: 31.0
pI: 6.8
Sequence Coverage [%]: 9.4
No. of unique Peptides: 1

10	20	30	40	50	60	70	80
MASPPRCSPT	AHDRECKLPP	PSAPASEYCP	GKLSWGTMAR	ALGRFKLSIP	HTHLLATLDP	LALDREPPPH	LLPEKHQVPE
90	100	110	120	130	140	150	160
KLIWGDQDPL	SKIPFKILSG	HEHAVSTCHF	CVDDTKLLSG	SYDCTVKLWD	PVDGSVVRDF	EHRPKAPVVE	CSITGDSSRV
170	180	190	200	210	220	230	240
IAASYDKTVR	AWDLETGKLL	WKVRYDTFIV	SCKFSPDGKY	VVSGFDVDHG	ICIMDAENIT	TVSVIKDHHT	RSITSCCFDP
250	260	270	280				
DSQRVASVSL	DRCIKIWDVT	SQATLLTITK	LFSHFWRV				

Cmpd.	No. of Cmpds.	m/z meas.	Δ m/z [ppm]	z	Rt [min]	Score	P	Range	Sequence	Modification
2091	1	757.2566	-151.54	2	54.7	16.0	0	18-32	K.LPPPSAPASEYCPGK.L	

Detailed Protein Report

Protein 261: TPR and ankyrin repeat-containing protein 1 [Homo sapiens]

**Accession:**	gi|257467636	**Score:**	37.4
**Database:**	refseq_human(refseq_human_20140103.fasta)	**MW [kDa]:**	336.0
**Database Date:**	2015-11-30	**pI:**	6.3
**Modification(s):**	Carbamidomethyl	**Sequence Coverage [%]:**	1.6
		**No. of unique Peptides:**	1

Detailed Protein Report

10	20	30	40	50	60	70	80
MWDPRAARVP	PRDLAVLLCN	KSNAFFSLGK	WNEAFVAAKE	CLQWDPTYVK	GYYRAGYSLL	RLHQPYEAAR	MFFEGLRLVQ
90	100	110	120	130	140	150	160
RSQDQAPVAD	FLVGVFTTMS	SDSIVLQSFL	PCFDHIFTTG	FPTEVWQSVI	EKLAKKGLWH	SFLLLSAKKD	RLPRNIHVPE
170	180	190	200	210	220	230	240
LSLKSLFEKY	VFIGLYEKME	QVPKLVQWLI	SIGASVETIG	PYPLHALMRL	CIQARENHLF	RWLMDHKPEW	KGRINQKDGD
250	260	270	280	290	300	310	320
GCTVLHVVAA	HSPGYLVKRQ	TEDVQMLLRF	GADPTLLDRQ	SRSVVDVLKR	NKNFKAIEKI	NSHLEKLATC	SKDLSGFSNG
330	340	350	360	370	380	390	400
DGPTSENDIF	RKVLEQLVKY	MNSGNRLLHK	NFLKQEVVQR	FLRLLSTLQE	IPPDLVCDIN	QDCATTVFKF	LLEKQRWPEV
410	420	430	440	450	460	470	480
LLLLTRKVSG	EPPLGDCLIK	DCNFSDLDIC	TIIPHLSTWD	QRKKQLLGCL	IDSGALPDGL	QESQERPVVT	CLKHEDFELA
490	500	510	520	530	540	550	560
FLLLTKGADP	RAISLTEGDT	PLHAALHIFL	EIKADIGFSF	LSHLLDLFWS	NPTEFDYLNP	NVQDSNGNTL	MHILFQKGML
570	580	590	600	610	620	630	640
KRVKKLLDLL	VKFDINFNLK	NKEGKDARHR	IKKNDSLLLA	WNKALMENRR	RSRQDSAAHL	GKLSKSTAPG	HTSQLKSQGS
650	660	670	680	690	700	710	720
FKSVPCGATA	RTLPEGSAVP	DSWETLPGTQ	VTRKEPGALR	PCSLRDCLMQ	DITVLIQQVE	VDPSFPEDCL	QSSEPLEAGA
730	740	750	760	770	780	790	800
GKEGKKDDKP	TLGAGAPDCS	EVGEGHAQVG	LGALQLVPDD	NRGKEGNDDQ	DDWSTQEIEA	CLQDFDNMTW	EIECTSEMLK
810	820	830	840	850	860	870	880
KLSSKVMTKV	IKKKIILAIQ	QLGNGEWTQG	LQKRLKHLKG	SIQLFEAKLD	KGARMLWELA	IDFSPRCSEN	PEKIIATEQN
890	900	910	920	930	940	950	960
TCAMEKSGRI	YTEIIRIWDI	VLDHCKLADS	IKAICNAYNR	GLSCVLRKKL	KGINKGQVSA	NMKIQKRIPR	CYVEDTEAEK
970	980	990	1000	1010	1020	1030	1040
GREHVNPEYF	PPASAVETEY	NIMKFHSFST	NMAFNILNDT	TATVEYPFRV	GELEYAVIDL	NPRPLEPIIL	IGRSGTGKTT
1050	1060	1070	1080	1090	1100	1110	1120
CCLYRLWKKF	HVYWEKAEQA	GSPLLAKQVW	LKRRLEVEPG	KESPGGEEEE	EEEDEEEEDS	IEVETVESID	EQEYEACAGG
1130	1140	1150	1160	1170	1180	1190	1200
AGVEPAGDGQ	AAEVCAPEHP	HQLEHLHQIF	VTKNHVLCQE	VQRNFIELSK	STKATSHYKP	LDPNIHKLQD	LRDENFPLFV
1210	1220	1230	1240	1250	1260	1270	1280
TSKQLLLLLD	ASLPKPFFLR	NEDGSLKRTI	IGWSAQEEST	IPSWQEDEEE	AEVDGDYSEE	DKAVEMRTGD	SDPRVYVTFE
1290	1300	1310	1320	1330	1340	1350	1360
VFKNEIWPKM	TKGRTAYNPA	LIWKEIKSFL	KGSFEALSCP	HGRLTEEVYK	KLGRKRCPNF	KEDRSEIYSL	FSLYQQIRSQ
1370	1380	1390	1400	1410	1420	1430	1440
KGYFDEEDVL	YNISRRLSKL	RVLPWSIHEL	YGDEIQDFTQ	AELALLMKCI	NDPNSMFLTG	DTAQSIMKGV	AFRFSDLRSL
1450	1460	1470	1480	1490	1500	1510	1520
FHYASRNTID	KQCAVRKPKK	IHQLYQNYRS	HSGILNLASG	VVDLLQFYFP	ESFDRLPRDS	GLFDGPKPTV	LESCSVSDLA
1530	1540	1550	1560	1570	1580	1590	1600
ILLRGNKRKT	QPIEFGAHQV	ILVANETAKE	KIPEELGLAL	VLTIYEAKGL	EFDDVLLYNF	FTDSEAYKEW	KIISSFTPTS
1610	1620	1630	1640	1650	1660	1670	1680
TDSREENRPL	VEVPLDKPGS	SQGRSLMVNP	EMYKLLNGEL	KQLYTAITRA	RVNLWIFDEN	REKRAPAFKY	FIRRDFVQVV
1690	1700	1710	1720	1730	1740	1750	1760
KTDENKDFDD	SMFVKTSTPA	EWIAQGDYYA	KHQCWKVAAK	CYQKGGAFEK	EKLALAHDTA	LSMKSKKVSP	KEKQLEYLEL
1770	1780	1790	1800	1810	1820	1830	1840
AKTYLECKEP	TLSLKCLSYA	KEFQLSAQLC	ERLGKIRDAA	YFYKRSQCYK	DAFRCFEQIQ	EFDLALKMYC	QEELFEEAAI
1850	1860	1870	1880	1890	1900	1910	1920
AVEKYEEMLK	TKTLPISKLS	YSASQFYLEA	AAKYLSANKM	KEMMAVLSKL	DIEDQLVFLK	SRKRLAEAAD	LLNREGRREE
1930	1940	1950	1960	1970	1980	1990	2000
AALLMKQHGC	LLEAARLTAD	KDFQASCLLG	AARLNVARDS	DIEHTKDILR	EALDICYQTG	QLSGIAEAHF	LQGVILRDFQ
2010	2020	2030	2040	2050	2060	2070	2080
KLRDAFFKFD	TLNHSAGVVE	ALYEAASQCE	AEPEKILGLA	PGGLEILLSL	VRALKRVTNN	AEKEMVKSCF	EFFGISQVDA
2090	2100	2110	2120	2130	2140	2150	2160
KYCQIAQNDP	GPILRIIFDL	DLNLREKKTK	DHFLIMTDQV	KLALNKHLLG	RLCQITRSLL	GKTYRGVCMR	FIVGLKCEDE
2170	2180	2190	2200	2210	2220	2230	2240

Detailed Protein Report

Cmpd.	No. of Cmpds.	m/z meas.	Δ m/z [ppm]	z	Rt [min]	Score	P	Range	Sequence	Modification
770	1	573.6008	-82.93	3	39.0	12.7	2	2682-2695	R.ACLVVSLCISWRRR.V	Carbamidomethyl: 8

Detailed Protein Report

Protein 262: rapamycin-insensitive companion of mTOR isoform 3 [Homo sapiens]

**Accession:**	gi|550544215	**Score:**	37.4
**Database:**	refseq_human(refseq_human_20140103.fasta)	**MW [kDa]:**	155.8
**Database Date:**	2015-11-30	**pI:**	6.8
		**Sequence Coverage [%]:**	1.8
		**No. of unique Peptides:**	1

10	20	30	40	50	60	70	80
MGIIATFRSW	AGIINLCKPG	NSGIQSLIGV	LCIPNMEIRR	GLLEVLYDIF	RLPLPVVTEE	FIEALLSVDP	GRFQDSWRLS
**90**	**100**	**110**	**120**	**130**	**140**	**150**	**160**
DGFVAAEAKT	ILPHRARSRP	DLMDNYLALI	LSAFIRNGLL	EGLVEVITNS	DDHISVRATI	LLGELLHMAN	TILPHSHSHH
**170**	**180**	**190**	**200**	**210**	**220**	**230**	**240**
LHCLPTLMNM	AASFDIPKEK	RLRASAALNC	LKRFHEMKKR	GPKPYSLHLD	HIIQKAIATH	QKRDQYLRVQ	KDIFILKDTE
**250**	**260**	**270**	**280**	**290**	**300**	**310**	**320**
EALLINLRDS	QVLQHKENLE	WNWNLIGTIL	KWPNVNLRNY	KDEQLHRFVR	RLLYFYKPSS	KLYANLDLDF	AKAKQLTVVG
**330**	**340**	**350**	**360**	**370**	**380**	**390**	**400**
CQFTEFLLES	EEDGQGYLED	LVKDIVQWLN	ASSGMKPERS	LQNNGLLTTL	SQHYFLFIGT	LSCHPHGVKM	LEKCSVFQCL
**410**	**420**	**430**	**440**	**450**	**460**	**470**	**480**
LNLCSLKNQD	HLLKLTVSSL	DYSRDGLARV	ILSKILTAAT	DACRLYATKH	LRVLLRANVE	FFNNWGIELL	VTQLHDKNKT
**490**	**500**	**510**	**520**	**530**	**540**	**550**	**560**
ISSEALDILD	EACEDKANLH	ALIQMKPALS	HLGDKGLLLL	LRFLSIPKGF	SYLNERGYVA	KQLEKWHREY	NSKYVDLIEE
**570**	**580**	**590**	**600**	**610**	**620**	**630**	**640**
QLNEALTTYR	KPVDGDNYVR	RSNQRLQRPH	VYLPIHLYGQ	LVHHKTGCHL	LEVQNIITEL	CRNVRTPDLD	KWEEIKKLKA
**650**	**660**	**670**	**680**	**690**	**700**	**710**	**720**
SLWALGNIGS	SNWGLNLLQE	ENVIPDILKL	AKQCEVLSIR	GTCVYVLGLI	AKTKQGCDIL	KCHNWDAVRH	SRKHLWPVVP
**730**	**740**	**750**	**760**	**770**	**780**	**790**	**800**
DDVEQLCNEL	SSIPSTLSLN	SESTSSRHNS	ESESVPSSMF	ILEDDRFGSS	STSTFFLDIN	EDTEPTFYDR	SGPIKDKNSF
**810**	**820**	**830**	**840**	**850**	**860**	**870**	**880**
PFFASSKLVK	NRILNSLTLP	NKKHRSSSDP	KGGKLSSESK	TSNRRIRTLT	EPSVDFNHSD	DFTPISTVQK	TLQLETSFMG
**890**	**900**	**910**	**920**	**930**	**940**	**950**	**960**
NKHIEDTGST	PSIGENDLKF	TKNFGTENHR	ENTSRERLVV	ESSTSSHMKI	RSQSFNTDTT	TSGISSMSSS	PSRETVGVDA
**970**	**980**	**990**	**1000**	**1010**	**1020**	**1030**	**1040**
TTMDTDCGSM	STVVSTKTIK	TSHYLTPQSN	HLSLSKSNSV	SLVPPGSSHT	LPRRAQSLKA	PSIATIKSLA	DCNFSYTSSR
**1050**	**1060**	**1070**	**1080**	**1090**	**1100**	**1110**	**1120**
DAFGYATLKR	LQQQRMHPSL	SHSEALASPA	KDVLFTDTIT	MKANSFESRL	TPSRFMKALS	YASLDKEDLL	SPINQNTLQR
**1130**	**1140**	**1150**	**1160**	**1170**	**1180**	**1190**	**1200**
SSSVRSMVSS	ATYGGSDDYI	GLALPVDIND	IFQVKDIPYF	QTKNIPPHDD	RGARAFAHDA	GESTEDTGLQ	EHTDDNCLYC
**1210**	**1220**	**1230**	**1240**	**1250**	**1260**	**1270**	**1280**
VCIEILGFQP	SNQLSAICSH	SDFQDIPYSD	WCEQTIHNPL	EVVPSKFSGI	SGCSDGVSQE	GSASSTKSTE	LLLGVKTIPD
**1290**	**1300**	**1310**	**1320**	**1330**	**1340**	**1350**	**1360**
DTPMCRILLR	KEVLRLVINL	SSSVSTKCHE	TGLLTIKEKY	PQTFDDICLY	SEVSHLLSHC	TFRLPCRRFI	QELFQDVQFL
**1370**	**1380**	**1390**					
QMHEEAEAVL	ATPPKQPIVD	TSAES					

Cmpd.	No. of Cmpds.	m/z meas.	Δ m/z [ppm]	z	Rt [min]	Score	P	Range	Sequence	Modification
2622	1	658.3808	-6.32	2	61.5	16.9	2	182-193	R.LRASAALNCLKR.F	

Detailed Protein Report

Protein 263: **PREDICTED: receptor-type tyrosine-protein phosphatase delta isoform X16 [Homo sapiens]**

**Accession:**	gi|578816637	**Score:**	37.4
**Database:**	refseq_human(refseq_human_20140103.fasta)	**MW [kDa]:**	211.2
**Database Date:**	2015-11-30	**pI:**	6.2
**Modification(s):**	Oxidation	**Sequence Coverage [%]:**	2.0
		**No. of unique Peptides:**	2

BRUKER

Detailed Protein Report

10	20	30	40	50	60	70	80
MVHVARLLLL	LLTFFLRTDA	ETPPRFTRTP	VDQTGVSGGV	ASFICQATGD	PRPKIVWNKK	GKKVSNQRFE	VIEFDDGSGS
90	100	110	120	130	140	150	160
VLRIQPLRTP	RDEAIYECVA	SNNVGEISVS	TRLTVLREDQ	IPRGFPTIDM	GPQLKVVERT	RTATMLCAAS	GNPDPEITWF
170	180	190	200	210	220	230	240
KDFLPVDTSN	NNGRIKQLRS	VRRVPPRFSI	PPTNHEIMPG	GSVNITCVAV	GSPMPYVKWM	LGAEDLTPED	DMPIGRNVLE
250	260	270	280	290	300	310	320
LNDVRQSANY	TCVAMSTLGV	IEAIAQITVK	ALPKPPGTPV	VTESTATSIT	LTWDSGNPEP	VSYYIIQHKP	KNSEELYKEI
330	340	350	360	370	380	390	400
DGVATTRYSV	AGLSPYSDYE	FRVVAVNNIG	RGPPSEPVLT	QTSEQAPSSA	PRDVQARMLS	STTILVQWKE	PEEPNGQIQG
410	420	430	440	450	460	470	480
YRVYYTMDPT	QHVNNWMKHN	VADSQITTIG	NLVPQKTYSV	KVLAFTSIGD	GPLSSDIQVI	TQTGVPGQPL	NFKAEPESET
490	500	510	520	530	540	550	560
SILLSWTPPR	SDTIANYELV	YKDGEHGEEQ	RITIEPGTSY	RLQGLKPNSL	YYFRLAARSP	QGLGASTAEI	SARTMQSKPS
570	580	590	600	610	620	630	640
APPQDISCTS	PSSTSILVSW	QPPPVEKQNG	IITEYSIKYT	AVDGEDDKPH	EILGIPSDTT	KYLLEQLEKW	TEYRITVTAH
650	660	670	680	690	700	710	720
TDVGPGPESL	SVLIRTNEDV	PSGPPRKVEV	EAVNSTSVKV	SWRSPVPNKQ	HGQIRGYQVH	YVRMENGEPK	GQPMLKDVML
730	740	750	760	770	780	790	800
ADAQWEFDDT	TEHDMIISGL	QPETSYSLTV	TAYTTKGDGA	RSKPKLVSTT	GAVPGKPRLV	INHTQMNTAL	IQWHPPVDTF
810	820	830	840	850	860	870	880
GPLQGYRLKF	GRKDMEPLTT	LEFSEKEDHF	TATDIHKGAS	YVFRLSARNK	VGFGEEMVKE	ISIPEEVPTG	FPQNLHSEGT
890	900	910	920	930	940	950	960
TSTSVQLSWQ	PPVLAERNGI	ITKYTLLYRD	INIPLLPMEQ	LIVPADTTMT	LTGLKPDTTY	DVKVRAHTSK	GPGPYSPSVQ
970	980	990	1000	1010	1020	1030	1040
FRTLPVDQAV	FAKNFHVKAV	MKTSVLLSWE	IPENYNSAMP	FKILYDDGKM	VEEVDGRATQ	KLIVNLKPEK	SYSFVLTNRG
1050	1060	1070	1080	1090	1100	1110	1120
NSAGGLQHRV	TAKTAPDVLR	TKPAFIGKTN	LDGMITVQLP	EVPANENIKG	YYIIIVPLKK	SRGKFIKPWE	SPDEMELDEL
1130	1140	1150	1160	1170	1180	1190	1200
LKEISRKRRS	IRYGREVELK	PYIAAHFDVL	PTEFTLGDDK	HYGGFTNKQL	QSGQEYVFFV	LAVMEHAESK	MYATSPYSDP
1210	1220	1230	1240	1250	1260	1270	1280
VVSMDLDPQP	ITDEEEGLIW	VVGPVLAVVF	IICIVIAILL	YKSSKPDRKR	AESDSRKSSI	PNNKEIPSHH	PTDPVELRRL
1290	1300	1310	1320	1330	1340	1350	1360
NFQTPGSDDS	GYPGNLHSSS	MASHPPIPIL	ELADHIERLK	ANDNLKFSQE	YESIDPGQQF	TWEHSNLEVN	KPKNRYANVI
1370	1380	1390	1400	1410	1420	1430	1440
AYDHSRVLLS	AIEGIPGSDY	VNANYIDGYR	KQNAYIATQG	SLPETFGDFW	RMIWEQRSAT	VVMMTKLEER	SRVKCDQYWP
1450	1460	1470	1480	1490	1500	1510	1520
SRGTETHGLV	QVTLLDTVEL	ATYCVRTFAL	YKNGSSEKRE	VRQFQFTAWP	DHGVPEHPTP	FLAFLRRVKT	CNPPDAGPMV
1530	1540	1550	1560	1570	1580	1590	1600
VHCSAGVGRT	GCFIVIDAML	ERIKHEKTVD	IYGHVTLMRA	QRNYMVQTED	QYIFIHDALL	EAVTCGNTEV	PARNLYAYIQ
1610	1620	1630	1640	1650	1660	1670	1680
KLTQIETGEN	VTGMELEFKR	LASSKAHTSR	FISANLPCNK	FKNRLVNIMP	YESTRVCLQP	IRGVEGSDYI	NASFIDGYRQ
1690	1700	1710	1720	1730	1740	1750	1760
QKAYIATQGP	LAETTEDFWR	MLWEHNSTIV	VMLTKLREMG	REKCHQYWPA	ERSARYQYFV	VDPMAEYNMP	QYILREFKVT
1770	1780	1790	1800	1810	1820	1830	1840
DARDGQSRTV	RQFQFTDWPE	QGVPKSGEGF	IDFIGQVHKT	KEQFGQDGPI	SVHCSAGVGR	TGVFITLSIV	LERMRYEGVV
1850	1860	1870	1880	1890			
DIFQTVKMLR	TQRPAMVQTE	DQYQFSYRAA	LEYLGSFDHY	AT			

Cmpd.	No. of Cmpds.	m/z meas.	Δ m/z [ppm]	z	Rt [min]	Score	P	Range	Sequence	Modification
1512	1	682.1037	142.22	3	48.7	23.6	2	810-826	K.FGRKDMEPLTTLEFSEK.E	Oxidation: 6
1854	2	1074.5548	-28.36	2	51.8	13.8	2	1040-1060	R.GNSAGGLQHRVTAKTAPDVLR.T	

Detailed Protein Report

Protein 264: 14-3-3 protein zeta/delta [Homo sapiens]

**Accession:**	gi|4507953	**Score:**	37.3
**Database:**	refseq_human(refseq_human_20140103.fasta)	**MW [kDa]:**	27.7
**Database Date:**	2015-11-30	**pI:**	4.6
**Modification(s):**	Carbamidomethyl	**Sequence Coverage [%]:**	9.0
		**No. of unique Peptides:**	1

Alias proteins:

Accession	Name	Description
gi|530389321	refseq_human (refseq_human_20140103.fasta)	PREDICTED: 14-3-3 protein zeta/delta isoform X4 [Homo sapiens]
gi|530389319	refseq_human (refseq_human_20140103.fasta)	PREDICTED: 14-3-3 protein zeta/delta isoform X3 [Homo sapiens]
gi|530389317	refseq_human (refseq_human_20140103.fasta)	PREDICTED: 14-3-3 protein zeta/delta isoform X2 [Homo sapiens]
gi|208973244	refseq_human (refseq_human_20140103.fasta)	14-3-3 protein zeta/delta [Homo sapiens]
gi|208973242	refseq_human (refseq_human_20140103.fasta)	14-3-3 protein zeta/delta [Homo sapiens]
gi|208973240	refseq_human (refseq_human_20140103.fasta)	14-3-3 protein zeta/delta [Homo sapiens]
gi|208973238	refseq_human (refseq_human_20140103.fasta)	14-3-3 protein zeta/delta [Homo sapiens]
gi|21735625	refseq_human (refseq_human_20140103.fasta)	14-3-3 protein zeta/delta [Homo sapiens]

10	20	30	40	50	60	70	80
MDKNELVQKA	KLAEQAERYD	DMAACMKSVT	EQGAELSNEE	RNLLSVAYKN	VVGARRSSWR	VVSSIEQKTE	GAEKKQQMAR
**90**	**100**	**110**	**120**	**130**	**140**	**150**	**160**
EYREKIETEL	RDICNDVLSL	LEKFLIPNAS	QAESKVFYLK	MKGDYYRYLA	EVAAGDDKKG	IVDQSQQAYQ	EAFEISKKEM
**170**	**180**	**190**	**200**	**210**	**220**	**230**	**240**
QPTHPIRLGL	ALNFSVFYYE	ILNSPEKACS	LAKTAFDEAI	AELDTLSEES	YKDSTLIMQL	LRDNLTLWTS	DTQGDEAEAG
**250**							
EGGEN							

Cmpd.	No. of Cmpds.	m/z meas.	Δ m/z [ppm]	z	Rt [min]	Score	P	Range	Sequence	Modification
2523	1	709.8534	-14.08	2	62.0	10.3	0	92-103	R.DICNDVLSLLEK.F	Carbamidomethyl: 3

Detailed Protein Report

Protein 265: **PREDICTED: serine/threonine-protein phosphatase 4 regulatory subunit 4 isoform X1 [Homo sapiens]**

**Accession:**	gi|530404339	**Score:**	37.2
**Database:**	refseq_human(refseq_human_20140103.fasta)	**MW [kDa]:**	87.3
**Database Date:**	2015-11-30	**pI:**	9.4
		**Sequence Coverage [%]:**	7.0
		**No. of unique Peptides:**	2

Alias proteins:

Accession	Name	Description
gi|578839938	refseq_human (refseq_human_20140103.fasta)	PREDICTED: serine/threonine-protein phosphatase 4 regulatory subunit 4 isoform X3 [Homo sapiens]
gi|578839936	refseq_human (refseq_human_20140103.fasta)	PREDICTED: serine/threonine-protein phosphatase 4 regulatory subunit 4 isoform X2 [Homo sapiens]
gi|578839934	refseq_human (refseq_human_20140103.fasta)	PREDICTED: serine/threonine-protein phosphatase 4 regulatory subunit 4 isoform X1 [Homo sapiens]
gi|578826014	refseq_human (refseq_human_20140103.fasta)	PREDICTED: serine/threonine-protein phosphatase 4 regulatory subunit 4 isoform X3 [Homo sapiens]
gi|530404341	refseq_human (refseq_human_20140103.fasta)	PREDICTED: serine/threonine-protein phosphatase 4 regulatory subunit 4 isoform X2 [Homo sapiens]

10	20	30	40	50	60	70	80
MQLTAAMSFL	TILQDESVSI	HAYTHSFLQV	ILLHLEHRDT	GVSNAWLETL	LSVIEVLPKE	TLRHEILNPL	VSKAQLSQTV
**90**	**100**	**110**	**120**	**130**	**140**	**150**	**160**
QSRLVSCKIL	GKLTNKFDAH	TIKREILPLV	KSLCQDVEYE	VRSCMCRQLE	NIAQGIGTEL	TKSVVLPELI	ELSRDEGSSV
**170**	**180**	**190**	**200**	**210**	**220**	**230**	**240**
RLAAFETLVN	LLDIFDTDDR	SQTILPLVKS	FCEKSFKADE	SILISLSFHL	GKLCHGLYGI	FTPDQHLRFL	EFYKKLCTLG
**250**	**260**	**270**	**280**	**290**	**300**	**310**	**320**
LQQENGHNEN	QIPPQILEQE	KKYISVRKNC	AYNFPAMIVF	VDPKNFHMEL	YSTFFCLCHD	PEVPVRYTIA	ICFYEVSKLL
**330**	**340**	**350**	**360**	**370**	**380**	**390**	**400**
NSGVYLIHKE	LITLLQDESL	EVLDALIDHL	PEILELMSTG	GESSVQENKL	SSLPDLIPAL	TAAEQRAAAS	LKWRTHEKLL
**410**	**420**	**430**	**440**	**450**	**460**	**470**	**480**
QKYACLPHVI	SSDQIYYRFL	QRMFTIMMTN	NVLPVQKAAS	RTLCIFLRYN	RKQEQRHEVI	QKLIEQLGQG	KSYWNRLRFL
**490**	**500**	**510**	**520**	**530**	**540**	**550**	**560**
DTCEFIIEIF	SKSFFCKYFF	LPAIELTHDP	VANVRMKLCY	LLPKVKSTLK	IPADKHLLQQ	LEMCVRKLLC	QEKDKDVLAI
**570**	**580**	**590**	**600**	**610**	**620**	**630**	**640**
VKRTVLELDR	MEMSMDAFQK	KFYEKDLLDQ	EKEREELLLL	EMEQLEKEKQ	QNDGRPMSDK	MFEKKRRDTK	TPTQSLPKNI
**650**	**660**	**670**	**680**	**690**	**700**	**710**	**720**
PISVPGPSSV	TPSTSKEIKK	SKLIRSQSFN	NQAFHAKYGN	LEKCASKSST	TGYTTSVSGL	GKTSVLSLAD	DSFRTRNASS
**730**	**740**	**750**	**760**	**770**			
VPSSFSPNTP	LPSTSRGTGN	SVDPKSSGSK	DTQPRKATLK	SRKSNP			

Cmpd.	No. of Cmpds.	m/z meas.	Δ m/z [ppm]	z	Rt [min]	Score	P	Range	Sequence	Modification
119	1	566.9137	-154.93	3	30.9	13.4	2	89-103	K.ILGKLTNKFDAHTIK.R	
2420	1	701.3590	-21.14	3	58.6	13.0	0	498-515	K.YFFLPAIELTHDPVANVR.M	

Detailed Protein Report

Protein 266: E3 ubiquitin-protein ligase RNF213 isoform 3 [Homo sapiens]

**Accession:**	gi|366039979	**Score:**	37.2
**Database:**	refseq_human(refseq_human_20140103.fasta)	**MW [kDa]:**	591.0
**Database Date:**	2015-11-30	**pI:**	6.0
**Modification(s):**	Oxidation	**Sequence Coverage [%]:**	0.7
		**No. of unique Peptides:**	1

Detailed Protein Report

10	20	30	40	50	60	70	80
MECPSCQHVS	KEETPKFCSQ	CGERLPPAAP	IADSENNNST	MASASEGEME	CGQELKEEGG	PCLFPGSDSW	QENPEEPCSK
90	100	110	120	130	140	150	160
ASWTVQESKK	KKRKKKKKGN	KSASSELASL	PLSPASPCHL	TLLSNPWPQD	TALPHSQAQQ	SGPTGQPSQP	PGTATTPLEG
170	180	190	200	210	220	230	240
DGLSAPTEVG	DSPLQAQALG	EAGVATGSEA	QSSPQFQDHT	EGEDQDASIP	SGGRGLSQEG	TGPPTSAGEG	HSRTEDAAQE
250	260	270	280	290	300	310	320
LLLPESKGGS	SEPGTELQTT	EQQAGASASM	AVDAVAEPAN	AVKGAGKEMK	EKTQRMKQPP	ATTPPFKTHC	QEAETKTKDE
330	340	350	360	370	380	390	400
MAAAEEKVGK	NEQGEPEDLK	KPEGKNRSAA	AVKNEKEQKN	QEADVQEVKA	STLSPGGGVT	VFFHAIISLH	FPFNPDLHKV
410	420	430	440	450	460	470	480
FIRGGEEFGE	SKWDSNICEL	HYTRDLGHDR	VLVEGIVCIS	KKHLDKYIPY	KYVIYNGESF	EYEFIYKHQQ	KKGEYVNRCL
490	500	510	520	530	540	550	560
FIKSSLLGSG	DWHQYYDIVY	MKPHGRLQKV	MNHITDGPRK	DLVKGKQIAA	ALMLDSTFSI	LQTWDTINLN	SFFTQFEQFC
570	580	590	600	610	620	630	640
FVLQQPMIYE	GQAQLWTDLQ	YREKEVKRYL	WQHLKKHVVP	LPDGKSTDFL	PVDCPVRSKL	KTGLIVLFVV	EKIELLLEGS
650	660	670	680	690	700	710	720
LDWLCHLLTS	DASSPDEFHR	DLSHILGIPQ	SWRLYLVNLC	QRCMDTRTYT	WLGALPVLHC	CMELAPRHKD	AWRQPEDTWA
730	740	750	760	770	780	790	800
ALEGLSFSPF	REQMLDTSSL	LQFMREKQHL	LSIDEPLFRS	WFSLLPLSHL	VMYMENFIEH	LGRFPAHILD	CLSGIYYRLP
810	820	830	840	850	860	870	880
GLEQVLNTQD	VQDVQNVQNI	LEMLLRLLDT	YRDKIPEEAL	SPSYLTVCLK	LHEAICSSTK	LLKFYELPAL	SAEIVCRMIR
890	900	910	920	930	940	950	960
LLSLVDSAGQ	RDETGNNSVQ	TVFQGTLAAT	KRWLREVFTK	NMLTSSGASF	TYVKEIEVWR	RLVEIQFPAE	HGWKESLLGD
970	980	990	1000	1010	1020	1030	1040
MEWRLTKEEP	LSQITAYCNS	CWDTKGLEDS	VAKTFEKCII	EAVSSACQSQ	TSILQGFSYS	DLRKFGIVLS	AVITKSWPRT
1050	1060	1070	1080	1090	1100	1110	1120
ADNFNDILKH	LLTLADVKHV	FRLCGTDEKI	LANVTEDAKR	LIAVADSVLT	KVVGDLLSGT	ILVGQLELII	KHKNQFLDIW
1130	1140	1150	1160	1170	1180	1190	1200
QLREKSLSPQ	DEQCAVEEAL	DWRREELLLL	KKEKRCVDSL	LKMCGNVKHL	IQVDFGVLAV	RHSQDLSSKR	LNDTVTVRLS
1210	1220	1230	1240	1250	1260	1270	1280
TSSNSQRATH	YHLSSQVQEM	AGKIDLLRDS	HIFQLFWREA	AEPLSEPKED	QEAAELLSEP	EEESERHILE	LEEVYDYLYQ
1290	1300	1310	1320	1330	1340	1350	1360
PSYRKFIKLH	QDLKSGEVTL	AEIDVIFKDF	VNKYTDLDSE	LKIMCTVDHQ	DQRDWIKDRV	EQIKEYHHLH	QAVHAAKVIL
1370	1380	1390	1400	1410	1420	1430	1440
QVKESLGLNG	DFSVLNTLLN	FTDNFDDFRR	ETLDQINQEL	IQAKKLLQDI	SEARCKGLQA	LSLRKEFICW	VREALGGINE
1450	1460	1470	1480	1490	1500	1510	1520
LKVFVDLASI	SAGENDIDVD	RVACFHDAVQ	GYASLLFKLD	PSVDFSAFMK	HLKKLWKALD	KDQYLPRKLC	DSARNLEWLK
1530	1540	1550	1560	1570	1580	1590	1600
TVNESHGSVE	RSSLTLATAI	NQRGIYVIQA	PKGGQKISPD	TVLHLILPES	PGSHEESREY	SLEEVKELLN	KLMLMSGKKD
1610	1620	1630	1640	1650	1660	1670	1680
RNNTEVERFS	EVFCSVQRLS	QAFIDLHSAG	NMLFRTWIAM	AYCSPKQGVS	LQMDFGLDLV	TELKEGGDVT	ELLAALCRQM
1690	1700	1710	1720	1730	1740	1750	1760
EHFLDSWKRF	VTQKRMEHFY	LNFYTAEQLV	YLSTELRKQP	PSDAALTMLS	FIKSNCTLRD	VLRASVGCGS	EAARYRMRRV
1770	1780	1790	1800	1810	1820	1830	1840
MEELPLMLLS	EFSLVDKLRI	IMEQSMRCLP	AFLPDCLDLE	TLGHCLAHLA	GMGGSPVERC	LPRGLQVGQP	NLVVCGHSEV
1850	1860	1870	1880	1890	1900	1910	1920
LPAALAVYMQ	TPSQPLPTYD	EVLLCTPATT	FEEVALLLRR	CLTLGSLGHK	VYSLLFADQL	SYEVARQAEE	LFHNLCTQQH
1930	1940	1950	1960	1970	1980	1990	2000
REDYQLVMVC	DGDWEHCYLP	SAFSQHKVFV	TPQAPLEAIQ	AYLAGHYRVP	KQTLSAAAVF	NDRLCVGIVA	SERAGVGKSL
2010	2020	2030	2040	2050	2060	2070	2080
YVKRLHDKMK	MQLNVKNVPL	KTIRLIDPQV	DESRVLGALL	PFLDAQYQKV	PVLFHLDVTS	SVQTGIWVFL	FKLLILQYLM
2090	2100	2110	2120	2130	2140	2150	2160
DINGKMWLRN	PCHLYIVEIL	ERRTSVPSRS	SSALRTRVPQ	FSFLDIFPKV	TCRPPKEVID	MELSALRSDT	EPGMDLWEFC
2170	2180	2190	2200	2210	2220	2230	2240

Detailed Protein Report

Cmpd.	No. of Cmpds.	m/z meas.	Δ m/z [ppm]	z	Rt [min]	Score	P	Range	Sequence	Modification
2481	1	856.9085	-24.89	2	61.4	13.6	1	4661-4674	R.LLNFDTELSTKEMR.N	Oxidation: 13

Detailed Protein Report

Protein 267: condensin complex subunit 1 [Homo sapiens]

Accession: gi|178056552
Database: refseq_human(refseq_human_20140103.fasta)
Database Date: 2015-11-30
Modification(s): Carbamidomethyl

Score: 37.1
MW [kDa]: 157.1
pI: 6.2
Sequence Coverage [%]: 3.7
No. of unique Peptides: 2

10	20	30	40	50	60	70	80
MAPQMYEFHL	PLSPEELLKS	GGVNQYVVQE	VLSIKHLPPQ	LRAFQAAFRA	QGPLAMLQHF	DTIYSILHHF	RSIDPGLKED
**90**	**100**	**110**	**120**	**130**	**140**	**150**	**160**
TLQFLIKVVS	RHSQELPAIL	DDTTLSGSDR	NAHLNALKMN	CYALIRLLES	FETMASQTNL	VDLDLGGKGK	KARTKAAHGF
**170**	**180**	**190**	**200**	**210**	**220**	**230**	**240**
DWEEERQPIL	QLLTQLLQLD	IRHLWNHSII	EEEFVSLVTG	CCYRLLENPT	INHQKNRPTR	EAITHLLGVA	LTRYNHMLSA
**250**	**260**	**270**	**280**	**290**	**300**	**310**	**320**
TVKIIQMLQH	FEHLAPVLVA	AVSLWATDYG	MKSIVGEIVR	EIGQKCPQEL	SRDPSGTKGF	AAFLTELAER	VPAILMSSMC
**330**	**340**	**350**	**360**	**370**	**380**	**390**	**400**
ILLDHLDGEN	YMMRNAVLAA	MAEMVLQVLS	GDQLEAAARD	TRDQFLDTLQ	AHGHDVNSFV	RSRVLQLFTR	IVQQKALPLT
**410**	**420**	**430**	**440**	**450**	**460**	**470**	**480**
RFQAVVALAV	GRLADKSVLV	CKNAIQLLAS	FLANNPFSCK	LSDADLAGPL	QKETQKLQEM	RAQRRTAAAS	AVLDPEEEWE
**490**	**500**	**510**	**520**	**530**	**540**	**550**	**560**
AMLPELKSTL	QQLLQLPQGE	EEIPEQIANT	ETTEDVKGRI	YQLLAKASYK	KAIILTREAT	GHFQESEPFS	HIDPEESEET
**570**	**580**	**590**	**600**	**610**	**620**	**630**	**640**
RLLNILGLIF	KGPAASTQEK	NPRESTGNMV	TGQTVCKNKP	NMSDPEESRG	NDELVKQEML	VQYLQDAYSF	SRKITEAIGI
**650**	**660**	**670**	**680**	**690**	**700**	**710**	**720**
ISKMMYENTT	TVVQEVIEFF	VMVFQFGVPQ	ALFGVRRMLP	LIWSKEPGVR	EAVLNAYRQL	YLNPKGDSAR	AKAQALIQNL
**730**	**740**	**750**	**760**	**770**	**780**	**790**	**800**
SLLLVDASVG	TIQCLEEILC	EFVQKDELKP	AVTQLLWERA	TEKVACCPLE	RCSSVMLLGM	MARGKPEIVG	SNLDTLVSIG
**810**	**820**	**830**	**840**	**850**	**860**	**870**	**880**
LDEKFPQDYR	LAQQVCHAIA	NISDRRKPSL	GKRHPPFRLP	QEHRLFERLR	ETVTKGFVHP	DPLWIPFKEV	AVTLIYQLAE
**890**	**900**	**910**	**920**	**930**	**940**	**950**	**960**
GPEVICAQIL	QGCAKQALEK	LEEKRTSQED	PKESPAMLPT	FLLMNLLSLA	GDVALQQLVH	LEQAVSGELC	RRRVLREEQE
**970**	**980**	**990**	**1000**	**1010**	**1020**	**1030**	**1040**
HKTKDPKEKN	TSSETTMEEE	LGLVGATADD	TEAELIRGIC	EMELLDGKQT	LAAFVPLLLK	VCNNPGLYSN	PDLSAAASLA
**1050**	**1060**	**1070**	**1080**	**1090**	**1100**	**1110**	**1120**
LGKFCMISAT	FCDSQLRLLF	TMLEKSPLPI	VRSNLMVATG	DLAIRFPNLV	DPWTPHLYAR	LRDPAQQVRK	TAGLVMTHLI
**1130**	**1140**	**1150**	**1160**	**1170**	**1180**	**1190**	**1200**
LKDMVKVKGQ	VSEMAVLLID	PEPQIAALAK	NFFNELSHKG	NAIYNLLPDI	ISRLSDPELG	VEEEPFHTIM	KQLLSYITKD
**1210**	**1220**	**1230**	**1240**	**1250**	**1260**	**1270**	**1280**
KQTESLVEKL	CQRFRTSRTE	RQQRDLAYCV	SQLPLTERGL	RKMLDNFDCF	GDKLSDESIF	SAFLSVVGKL	RRGAKPEGKA
**1290**	**1300**	**1310**	**1320**	**1330**	**1340**	**1350**	**1360**
IIDEFEQKLR	ACHTRGLDGI	KELEIGQAGS	QRAPSAKKPS	TGSRYQPLAS	TASDNDFVTP	EPRRTTRRHP	NTQQRASKKK
**1370**	**1380**	**1390**	**1400**	**1410**			
PKVVFSSDES	SEEDLSAEMT	EDETPKKTTP	ILRASARRHR	S			

Cmpd.	No. of Cmpds.	m/z meas.	Δ m/z [ppm]	z	Rt [min]	Score	P	Range	Sequence	Modification
1765	1	788.2766	-109.58	2	52.0	10.1	1	154-166	R.TKAAHGFDWEEER.Q	
1660	1	927.4336	-29.14	3	49.5	11.3	2	286-310	K.CPQELSRDPSGTKGFAAFLTELAER.V	Carbamidomethyl: 1

Detailed Protein Report

Protein 268: zinc finger protein castor homolog 1 isoform a [Homo sapiens]

**Accession:**	gi|119709834	**Score:**	37.1
**Database:**	refseq_human(refseq_human_20140103.fasta)	**MW [kDa]:**	189.9
**Database Date:**	2015-11-30	**pI:**	6.6
**Modification(s):**	Carbamidomethyl	**Sequence Coverage [%]:**	2.4
		**No. of unique Peptides:**	1

Alias proteins:

Accession	Name	Description
gi|578799307	refseq_human (refseq_human_20140103.fasta)	PREDICTED: zinc finger protein castor homolog 1 isoform X2 [Homo sapiens]

Detailed Protein Report

10	20	30	40	50	60	70	80
MDLGTAEGTR	CTDPPAGKPA	MAPKRKGGLK	LNAICAKLSR	QVVVEKRADA	GSHTEGSPSQ	PRDQERSGPE	SGAARAPRSE
90	100	110	120	130	140	150	160
EDKRRAVIEK	WVNGEYSEEP	APTPVLGRIA	REGLELPPEG	VYMVQPQGCS	DEEDHAEEPS	KDGGALEEKD	SDGAASKEDS
170	180	190	200	210	220	230	240
GPSTRQASGE	ASSLRDYAAS	TMTEFLGMFG	YDDQNTRDEL	ARKISFEKLH	AGSTPEAATS	SMLPTSEDTL	SKRARFSKYE
250	260	270	280	290	300	310	320
EYIRKLKAGE	QLSWPAPSTK	TEERVGKEVV	GTLPGLRLPS	STAHLETKAT	ILPLPSHSSV	QMQNLVARAS	KYDFFIQKLK
330	340	350	360	370	380	390	400
TGENLRPQNG	STYKKPSKYD	LENVKYLHLF	KPGEGSPDMG	GAIAFKTGKV	GRPSKYDVRG	IQKPGPAKVP	PTPSLAPAPL
410	420	430	440	450	460	470	480
ASVPSAPSAP	GPGPEPPASL	SFNTPEYLKS	TFSKTDSITT	GTVSTVKNGL	PTDKPAVTED	VNIYQKYIAR	FSGSQHCGHI
490	500	510	520	530	540	550	560
HCAYQYREHY	HCLDPECNYQ	RFTSKQDVIR	HYNMHKKRDN	SLQHGFMRFS	PLDDCSVYYH	GCHLNGKSTH	YHCMQVGCNK
570	580	590	600	610	620	630	640
VYTSTSDVMT	HENFHKKNTQ	LINDGFQRFR	ATEDCGTADC	QFYGQKTTHF	HCRRPGCTFT	FKNKCDIEKH	KSYHIKDDAY
650	660	670	680	690	700	710	720
AKDGFKKFYK	YEECKYEGCV	YSKATNHFHC	IRAGCGFTFT	STSQMTSHKR	KHERRHIRSS	GALGLPPSLL	GAKDTEHEES
730	740	750	760	770	780	790	800
SNDDLVDFSA	LSSKNSSLSA	SPTSQQSSAS	LAAATAATEA	GPSATKPPNS	KISGLLPQGL	PGSIPLALAL	SNSGLPTPTP
810	820	830	840	850	860	870	880
YFPILAGRGS	TSLPVGTPSL	LGAVSSGSAA	SATPDTPTLV	ASGAGDSAPV	AAASVPAPPA	SIMERISASK	GLISPMMARL
890	900	910	920	930	940	950	960
AAAALKPSAT	FDPGSGQQVT	PARFPPAQVK	PEPGESTGAP	GPHEASQDRS	LDLTVKEPSN	ESNGHAVPAN	SSLLSSLMNK
970	980	990	1000	1010	1020	1030	1040
MSQGNPGLGS	LLNIKAEAEG	SPAAEPSPFL	GKAVKALVQE	KLAEPWKVYL	RRFGTKDFCD	GQCDFLHKAH	FHCVVEECGA
1050	1060	1070	1080	1090	1100	1110	1120
LFSTLDGAIK	HANFHFRTEG	GAAKGNTEAA	FPASAAETKP	PMAPSSPPVP	PVTTATVSSL	EGPAPSPASV	PSTPTLLAWK
1130	1140	1150	1160	1170	1180	1190	1200
QLASTIPQMP	QIPASVPHLP	ASPLATTSLE	NAKPQVKPGF	LQFQENDPCL	ATDCKYANKF	HFHCLFGNCK	YVCKTSGKAE
1210	1220	1230	1240	1250	1260	1270	1280
SHCLDHINPN	NNLVNVRDQF	AYYSLQCLCP	NQHCEFRMRG	HYHCLRTGCY	FVTNITTKLP	WHIKKHEKAE	RRAANGFKYF
1290	1300	1310	1320	1330	1340	1350	1360
TKREECGRLG	CKYNQVNSHF	HCIREGCQFS	FLLKHQMTSH	ARKHMRRMLG	KNFDRVPPSQ	GPPGLMDAET	DECMDYTGCS
1370	1380	1390	1400	1410	1420	1430	1440
PGAMSSESST	MDRSCSSTPV	GNESTAAGNT	ISMPTASGAK	KRFWIIEDMS	PFGKRRKTAS	SRKMLDEGMM	LEGFRRFDLY
1450	1460	1470	1480	1490	1500	1510	1520
EDCKDAACQF	SLKVTHYHCT	RENCGYKFCG	RTHMYKHAQH	HDRVDNLVLD	DFKRFKASLS	CHFADCPFSG	TSTHFHCLRC
1530	1540	1550	1560	1570	1580	1590	1600
RFRCTDSTKV	TAHRKHHGKQ	DVISAAGFCQ	FSSSADCAVP	DCKYKLKCSH	FHCTFPGCRH	TVVGMSQMDS	HKRKHEKQER
1610	1620	1630	1640	1650	1660	1670	1680
GEPAAEGPAP	GPPISLDGSL	SLGAEPGSLL	FLQSAAAGLG	LALGDADPG	PPDAAAPGPR	EGAAAAAAAA	GESSQEDEEE
1690	1700	1710	1720	1730	1740	1750	1760
ELELPEEEAE	DDEDEDDDED	DDDEDDDEDD	DDEDLRTDSE	ESLPEAAAEA	AGAGARTPAL	AALAALGAPG	PAPTAASSP

Cmpd.	No. of Cmpds.	m/z meas.	Δ m/z [ppm]	z	Rt [min]	Score	P	Range	Sequence	Modification
2568	2	673.3695	74.26	2	62.6	11.8	2	1279-1288	K.YFTKREECGR.L	Carbamidomethyl: 8

Detailed Protein Report

Protein 269: protein kinase C delta type [Homo sapiens]

Accession: gi|31377782
Database: refseq_human(refseq_human_20140103.fasta)
Database Date: 2015-11-30
Modification(s): Oxidation

Score: 36.8
MW [kDa]: 77.5
pI: 8.9
Sequence Coverage [%]: 6.4
No. of unique Peptides: 2

Alias proteins:

Accession	Name	Description
gi|578806373	refseq_human (refseq_human_20140103.fasta)	PREDICTED: protein kinase C delta type isoform X3 [Homo sapiens]
gi|578806371	refseq_human (refseq_human_20140103.fasta)	PREDICTED: protein kinase C delta type isoform X2 [Homo sapiens]
gi|47157325	refseq_human (refseq_human_20140103.fasta)	protein kinase C delta type [Homo sapiens]

10	20	30	40	50	60	70	80
MAPFLRIAFN	SYELGSLQAE	DEANQPFCAV	KMKEALSTER	GKTLVQKKPT	MYPEWKSTFD	AHIYEGRVIQ	IVLMRAAEEP
**90**	**100**	**110**	**120**	**130**	**140**	**150**	**160**
VSEVTVGVSV	LAERCKKNNG	KAEFWLDLQP	QAKVLMSVQY	FLEDVDCKQS	MRSEDEAKFP	TMNRRGAIKQ	AKIHYIKNHE
**170**	**180**	**190**	**200**	**210**	**220**	**230**	**240**
FIATFFGQPT	FCSVCKDFVW	GLNKQGYKCR	QCNAAIHKKC	IDKIIGRCTG	TAANSRDTIF	QKERFNIDMP	HRFKVHNYMS
**250**	**260**	**270**	**280**	**290**	**300**	**310**	**320**
PTFCDHCGSL	LWGLVKQGLK	CEDCGMNVHH	KCREKVANLC	GINQKLLAEA	LNQVTQRASR	RSDSASSEPV	GIYQGFEKKT
**330**	**340**	**350**	**360**	**370**	**380**	**390**	**400**
GVAGEDMQDN	SGTYGKIWEG	SSKCNINNFI	FHKVLGKGSF	GKVLLGELKG	RGEYFAIKAL	KKDVVLIDDD	VECTMVEKRV
**410**	**420**	**430**	**440**	**450**	**460**	**470**	**480**
LTLAAENPFL	THLICTFQTK	DHLFFVMEFL	NGGDLMYHIQ	DKGRFELYRA	TFYAAEIMCG	LQFLHSKGII	YRDLKLDNVL
**490**	**500**	**510**	**520**	**530**	**540**	**550**	**560**
LDRDGHIKIA	DFGMCKENIF	GESRASTFCG	TPDYIAPEIL	QGLKYTFSVD	WWSFGVLLYE	MLIGQSPFHG	DDEDELFESI
**570**	**580**	**590**	**600**	**610**	**620**	**630**	**640**
RVDTPHYPRW	ITKESKDILE	KLFEREPTKR	LGVTGNIKIH	PFFKTINWTL	LEKRRLEPPF	RPKVKSPRDY	SNFDQEFLNE
**650**	**660**	**670**	**680**				
KARLSYSDKN	LIDSMDQSAF	AGFSFVNPKF	EHLLED				

Cmpd.	No. of Cmpds.	m/z meas.	Δ m/z [ppm]	z	Rt [min]	Score	P	Range	Sequence	Modification
2648	1	937.4596	47.34	2	61.9	14.1	1	319-336	K.KTGVAGEDMQDNSGTYGK.I	Oxidation: 9
2810	1	648.3524	-55.50	2	63.6	10.4	2	614-623	K.RRLEPPFRPK.V	

Detailed Protein Report

Protein 270: ryanodine receptor 3 isoform 2 [Homo sapiens]

**Accession:**	gi|345842488	**Score:**	36.8
**Database:**	refseq_human(refseq_human_20140103.fasta)	**MW [kDa]:**	551.1
**Database Date:**	2015-11-30	**pI:**	5.4
**Modification(s):**	Carbamidomethyl, Oxidation	**Sequence Coverage [%]:**	1.0
		**No. of unique Peptides:**	3

Detailed Protein Report

10	20	30	40	50	60	70	80
MAEGGEGGED	EIQFLRTEDE	VVLQCIATIH	KEQRKFCLAA	EGLGNRLCFL	EPTSEAKYIP	PDLCVCNFVL	EQSLSVRALQ
90	100	110	120	130	140	150	160
EMLANTGENG	GEGAAQGGGH	RTLLYGHAVL	LRHSFSGMYL	TCLTTSRSQT	DKLAFDVGLR	EHATGEACWW	TIHPASKQRS
170	180	190	200	210	220	230	240
EGEKVRIGDD	LILVSVSSER	YLHLSVSNGN	IQVDASFMQT	LWNVHPTCSG	SSIEEGYLLG	GHVVRLFHGH	DECLTIPSTD
250	260	270	280	290	300	310	320
QNDSQHRRIF	YEAGGAGTRA	RSLWRVEPLR	ISWSGSNIRW	GQAFRLRHLT	TGHYLALTED	QGLILQDRAK	SDTKSTAFSF
330	340	350	360	370	380	390	400
RASKELKEKL	DSSHKRDIEG	MGVPEIKYGD	SVCFVQHIAS	GLWVTYKAQD	AKTSRLGPLK	RKVILHQEGH	MDDGLTLQRC
410	420	430	440	450	460	470	480
QREESQAARI	IRNTTALFSQ	FVSGNNRTAA	PITLPIEEVL	QTLQDLIAYF	QPPEEEMRHE	DKQNKLRSLK	NRQNLFKEEG
490	500	510	520	530	540	550	560
MLALVLNCID	RLNVYNSVAH	FAGIAREESG	MAWKEILNLL	YKLLAALIRG	NRNNCAQFSN	NLDWLISKLD	RLESSSGILE
570	580	590	600	610	620	630	640
VLHCILTESP	EALNLIAEGH	IKSIISLLDK	HGRNHKVLDI	LCSLCLCNGV	AVRANQNLIC	DNLLPRRNLL	LQTRLINDVT
650	660	670	680	690	700	710	720
SIRPNIFLGV	AEGSAQYKKW	YFELIIDQVD	PFLTAEPTHL	RVGWASSSGY	APYPGGGEGW	GGNGVGDDLY	SYGFDGLHLW
730	740	750	760	770	780	790	800
SGRIPRAVAS	INQHLLRSDD	VVSCCLDLGV	PSISFRINGQ	PVQGMFENFN	TDGLFFPVMS	FSAGVKVRFL	MGGRHGEFKF
810	820	830	840	850	860	870	880
LPPSGYAPCY	EALLPKEKMR	LEPVKEYKRD	ADGIRDLLGT	TQFLSQASFI	PCPVDTSQVI	LPPHLEKIRD	RLAENIHELW
890	900	910	920	930	940	950	960
GMNKIELGWT	FGKIRDDNKR	QHPCLVEFSK	LPETEKNYNL	QMSTETLKTL	LALGCHIAHV	NPAAEEDLKK	VKLPKNYMMS
970	980	990	1000	1010	1020	1030	1040
NGYKPAPLDL	SDVKLLPPQE	ILVDKLAENA	HNVWAKDRIK	QGWTYGIQQD	LKNKRNPRLV	PYALLDERTK	KSNRDSLREA
1050	1060	1070	1080	1090	1100	1110	1120
VRTFVGYGYN	IEPSDQELAD	SAVEKVSIDK	IRFFRVERSY	AVRSGKWYFE	FEVVTGGDMR	VGWARPGCRP	DVELGADDQA
1130	1140	1150	1160	1170	1180	1190	1200
FVFEGNRGQR	WHQGSGYFGR	TWQPGDVVGC	MINLDDASMI	FTLNGELLIT	NKGSELAFAD	YEIENGFVPI	CCLGLSQIGR
1210	1220	1230	1240	1250	1260	1270	1280
MNLGTDASTF	KFYTMCGLQE	GFEPFAVNMN	RDVAMWFSKR	LPTFVNVPKD	HPHIEVMRID	GTMDSPPCLK	VTHKTFGTQN
1290	1300	1310	1320	1330	1340	1350	1360
SNADMIYCRL	SMPVECHSSF	SHSPCLDSEA	FQKRKQMQEI	LSHTTTQCYY	AIRIFAGQDP	SCVWVGWVTP	DYHLYSEKFD
1370	1380	1390	1400	1410	1420	1430	1440
LNKNCTVTVT	LGDERGRVHE	SVKRSNCYMV	WGGDIVASSQ	RSNRSNVDLE	IGCLVDLAMG	MLSFSANGKE	LGTCYQVEPN
1450	1460	1470	1480	1490	1500	1510	1520
TKVFPAVFLQ	PTSTSLFQFE	LGKLKNAMPL	SAAIFRSEEK	NPVPQCPPRL	DVQTIQPVLW	SRMPNSFLKV	ETERVSERHG
1530	1540	1550	1560	1570	1580	1590	1600
WVVQCLEPLQ	MMALHIPEEN	RCVDILELCE	QEDLMRFHYH	TLRLYSAVCA	LGNSRVAYAL	CSHVDLSQLF	YAIDNKYLPG
1610	1620	1630	1640	1650	1660	1670	1680
LLRSGFYDLL	ISIHLASAKE	RKLMMKNEYI	IPITSTTRNI	RLFPDESKRH	GLPGVGLRTC	LKPGFRFSTP	CFVVTGEDHQ
1690	1700	1710	1720	1730	1740	1750	1760
KQSPEIPLES	LRTKALSMLT	EAVQCSGAHI	RDPVGGSVEF	QFVPVLKLIG	TLLVMGVFDD	DDVRQILLLI	DPSVFGEHSA
1770	1780	1790	1800	1810	1820	1830	1840
GTEEGAEKEE	VTQVEEKAVE	AGEKAGKEAP	VKGLLQTRLP	ESVKLQMCEL	LSYLCDCELQ	HRVEAIVAFG	DIYVSKLQAN
1850	1860	1870	1880	1890	1900	1910	1920
QKFRYNELMQ	ALNMSAALTA	RKTKEFRSPP	QEQINMLLNF	QLGENCPCPE	EIREELYDFH	EDLLLHCGVP	LEEEEEEEED
1930	1940	1950	1960	1970	1980	1990	2000
TSWTGKLCAL	VYKIKGPPKP	EKEQPTEEEE	RCPTTLKELI	SQTMICWAQE	DQIQDSELVR	MMFNLLRRQY	DSIGELLQAL
2010	2020	2030	2040	2050	2060	2070	2080
RKTYTISHTS	VSDTINLLAA	LGQIRSLLSV	RMGKEEELLM	INGLGDIMNN	KVFYQHPNLM	RVLGMHETVM	EVMVNVLGTE
2090	2100	2110	2120	2130	2140	2150	2160
KSQIAFPKMV	ASCCRFLCYF	CRISRQNQKA	MFEHLSYLLE	NSSVGLASPS	MRGSTPLDVA	ASSVMDNNEL	ALSLEEPDLE
2170	2180	2190	2200	2210	2220	2230	2240

Detailed Protein Report

Cmpd.	No. of Cmpds.	m/z meas.	Δ m/z [ppm]	z	Rt [min]	Score	P	Range	Sequence	Modification
58	1	845.3504	-92.64	3	29.9	13.1	2	382-402	R.KVILHQEGHMDDGLTLQRCQR.E	Carbamidomethyl: 19
833	1	575.1451	-229.53	2	39.8	12.5	0	1250-1258	K.DHPHIEVMR.I	Oxidation: 8
2682	1	1051.5508	66.61	2	64.7	11.2	1	1384-1401	K.RSNCYMVWGGDIVASSQR.S	Carbamidomethyl: 4; Oxidation: 6

Detailed Protein Report

Protein 271: toll-like receptor 9 precursor [Homo sapiens]

**Accession:**	gi|8394456	**Score:**	36.8
**Database:**	refseq_human(refseq_human_20140103.fasta)	**MW [kDa]:**	115.8
**Database Date:**	2015-11-30	**pI:**	9.7
		**Sequence Coverage [%]:**	3.2
		**No. of unique Peptides:**	3

Quantitation

mdown:qdown	**Median:** 0.74	**CV:** 0.00 %	**No. of Peptides:**	1
Wdown:Qdown	**Median:** 0.60	**CV:** 0.00 %	**No. of Peptides:**	1

```
        10         20         30         40         50         60         70         80
MGFCRSALHP LSLLVQAIML AMTLALGTLP AFLPCELQPH GLVNCNWLFL KSVPHFSMAA PRGNVTSLSL SSNRIHHLHD
        90        100        110        120        130        140        150        160
SDFAHLPSLR HLNLKWNCPP VGLSPMHFPC HMTIEPSTFL AVPTLEELNL SYNNIMTVPA LPKSLISLSL SHTNILMLDS
       170        180        190        200        210        220        230        240
ASLAGLHALR FLFMDGNCYY KNPCRQALEV APGALLGLGN LTHLSLKYNN LTVVPRNLPS SLEYLLLSYN RIVKLAPEDL
       250        260        270        280        290        300        310        320
ANLTALRVLD VGGNCRRCDH APNPCMECPR HFPQLHPDTF SHLSRLEGLV LKDSSLSWLN ASWFRGLGNL RVLDLSENFL
       330        340        350        360        370        380        390        400
YKCITKTKAF QGLTQLRKLN LSFNYQKRVS FAHLSLAPSF GSLVALKELD MHGIFFRSLD ETTLRPLARL PMLQTLRLQM
       410        420        430        440        450        460        470        480
NFINQAQLGI FRAFPGLRYV DLSDNRISGA SELTATMGEA DGGEKVWLQP GDLAPAPVDT PSSEDFRPNC STLNFTLDLS
       490        500        510        520        530        540        550        560
RNNLVTVQPE MFAQLSHLQC LRLSHNCISQ AVNGSQFLPL TGLQVLDLSH NKLDLYHEHS FTELPRLEAL DLSYNSQPFG
       570        580        590        600        610        620        630        640
MQGVGHNFSF VAHLRTLRHL SLAHNNIHSQ VSQQLCSTSL RALDFSGNAL GHMWAEGDLY LHFFQGLSGL IWLDLSQNRL
       650        660        670        680        690        700        710        720
HTLLPQTLRN LPKSLQVLRL RDNYLAFFKW WSLHFLPKLE VLDLAGNQLK ALTNGSLPAG TRLRRLDVSC NSISFVAPGF
       730        740        750        760        770        780        790        800
FSKAKELREL NLSANALKTV DHSWFGPLAS ALQILDVSAN PLHCACGAAF MDFLLEVQAA VPGLPSRVKC GSPGQLQGLS
       810        820        830        840        850        860        870        880
IFAQDLRLCL DEALSWDCFA LSLLAVALGL GVPMLHHLCG WDLWYCFHLC LAWLPWRGRQ SGRDEDALPY DAFVVFDKTQ
       890        900        910        920        930        940        950        960
SAVADWVYNE LRGQLEECRG RWALRLCLEE RDWLPGKTLF ENLWASVYGS RKTLFVLAHT DRVSGLLRAS FLLAQQRLLE
       970        980        990       1000       1010       1020       1030       1040
DRKDVVVLVI LSPDGRRSRY VRLRQRLCRQ SVLLWPHQPS GQRSFWAQLG MALTRDNHHF YNRNFCQGPT AE
```

Cmpd.	No. of Cmpds.	m/z meas.	Δ m/z [ppm]	z	Rt [min]	Score	P	Range	Sequence	Modification	Ratios
1901	1	698.8088	-121.07	2	52.3	10.8	0	235-247	K.LAPEDLANLTALR.V		
1454	1	1059.0764	13.42	2	48.0	15.1	1	788-807	R.VKCGSPGQLQGLSIFAQDLR.L		
399	1	945.4882	8.09	2	34.3	10.8	0	790-807	K.CGSPGQLQGLSIFAQDLR.L		mdown:qdown 0.74<br>Wdown:Qdown 0.60

Detailed Protein Report

Protein 272: tRNA wybutosine-synthesizing protein 1 homolog B isoform 1 [Homo sapiens]

**Accession:**	gi|224282123	**Score:**	36.7
**Database:**	refseq_human(refseq_human_20140103.fasta)	**MW [kDa]:**	76.9
**Database Date:**	2015-11-30	**pI:**	5.8
**Modification(s):**	Carbamidomethyl	**Sequence Coverage [%]:**	5.2
		**No. of unique Peptides:**	2

Quantitation

Wdown:Qdown **Median:** 1.27 **CV:** 0.00 % **No. of Peptides:** 1

10	20	30	40	50	60	70	80
MDPSADTWDL	SSPLISLWIN	RFYIYLGFAV	SISLWICVQI	VIEMQGFATV	LAEAVTSLDL	PVAIINLKEY	DPDDHLIEEV
**90**	**100**	**110**	**120**	**130**	**140**	**150**	**160**
TSKNVCVFLV	ATYTDGLPTE	SAEWFCKWLE	EASIDFRFGK	TYLKGMRDAV	FGLGNSAYAS	HFNKVGKNVD	KWLWMLGVHR
**170**	**180**	**190**	**200**	**210**	**220**	**230**	**240**
VMSRGEGDCD	VVKSKHGSIE	ANFRAWKTKF	ISQLQALQKG	ERKKSCGGHC	KKGKCESHQH	GSEEREEGSQ	EQDELHHRDT
**250**	**260**	**270**	**280**	**290**	**300**	**310**	**320**
KEEEPFESSS	EEEFGGEDHQ	SLNSIVDVED	LGKIMDHVKK	EKREKEQQEE	KSGLFRNMGR	NEDGERRAMI	TPALREALTK
**330**	**340**	**350**	**360**	**370**	**380**	**390**	**400**
QVDAPRERSL	LQTHILWNES	HRCMETTPSL	ACANKCVFCW	WHHNNPVGTE	WLWKMDQPEM	ILKEAIENHQ	NMIKQFKGVP
**410**	**420**	**430**	**440**	**450**	**460**	**470**	**480**
GVKAERFEEG	MTVKHCALSL	VGEPIMYPEI	NRFLKLLHQC	KISSFLVTNA	QFPAEIRNLE	PVTQLYVSVD	ASTKDSLKKI
**490**	**500**	**510**	**520**	**530**	**540**	**550**	**560**
DRPLFKDFWQ	QFLDSLKALA	VKQQRTVYRL	TLVKAWNVDE	LQAYAQLVSL	GNPDFIEVKG	VTYCRESSAS	SLTMAHVPWH
**570**	**580**	**590**	**600**	**610**	**620**	**630**	**640**
EEVVQFVREL	VDLIPEYEIA	CEHEHSNCLL	IAHRKFKIGG	EWWTWIDYNR	FQELIQEYED	SGGSKTFSAK	DYMARTPHWA
**650**	**660**	**670**					
LFGANERSFD	PKDTRHQRKN	KSKAISGC					

Cmpd.	No. of Cmpds.	m/z meas.	Δ m/z [ppm]	z	Rt [min]	Score	P	Range	Sequence	Modification	Ratios
912	1	697.8084	-22.08	2	39.9	10.4	1	161-173	R.VMSRGEGDCDVVK.S		Wdown:Qdown 1.27
2915	3	713.3417	41.37	2	65.2	16.0	0	343-355	R.CMETTPSLACANK.C	Carbamidomethyl: 10	

Detailed Protein Report

Protein 273: retinal dehydrogenase 1 [Homo sapiens]

Accession: gi|21361176
Database: refseq_human(refseq_human_20140103.fasta)
Database Date: 2015-11-30

Score: 36.7
MW [kDa]: 54.8
pI: 6.3
Sequence Coverage [%]: 6.0
No. of unique Peptides: 1

10	20	30	40	50	60	70	80
MSSSGTPDLP	VLLTDLKIQY	TKIFINNEWH	DSVSGKKFPV	FNPATEEELC	QVEEGDKEDV	DKAVKAARQA	FQIGSPWRTM
90	100	110	120	130	140	150	160
DASERGRLLY	KLADLIERDR	LLLATMESMN	GGKLYSNAYL	NDLAGCIKTL	RYCAGWADKI	QGRTIPIDGN	FFTYTRHEPI
170	180	190	200	210	220	230	240
GVCGQIIPWN	FPLVMLIWKI	GPALSCGNTV	VVKPAEQTPL	TALHVASLIK	EAGFPPGVVN	IVPGYGPTAG	AAISSHMDID
250	260	270	280	290	300	310	320
KVAFTGSTEV	GKLIKEAAGK	SNLKRVTLEL	GGKSPCIVLA	DADLDNAVEF	AHHGVFYHQG	QCCIAASRIF	VEESIYDEFV
330	340	350	360	370	380	390	400
RRSVERAKKY	ILGNPLTPGV	TQGPQIDKEQ	YDKILDLIES	GKKEGAKLEC	GGGPWGNKGY	FVQPTVFSNV	TDEMRIAKEE
410	420	430	440	450	460	470	480
IFGPVQQIMK	FKSLDDVIKR	ANNTFYGLSA	GVFTKDIDKA	ITISSALQAG	TVWVNCYGVV	SAQCPFGGFK	MSGNGRELGE
490	500	510					
YGFHEYTEVK	TVTVKISQKN	S					

Cmpd.	No. of Cmpds.	m/z meas.	Δ m/z [ppm]	z	Rt [min]	Score	P	Range	Sequence	Modification
2975	1	953.4552	-84.55	2	66.4	23.6	2	242-260	K.VAFTGSTEVGKLIKEAAGK.S	

Detailed Protein Report

Protein 274: PREDICTED: LIM/homeobox protein Lhx9 isoform X2 [Homo sapiens]

Accession: gi|530365098
Database: refseq_human(refseq_human_20140103.fasta)
Database Date: 2015-11-30
Modification(s): Carbamidomethyl, Oxidation

Score: 36.6
MW [kDa]: 37.1
pI: 10.2
Sequence Coverage [%]: 13.6
No. of unique Peptides: 2

Quantitation

mdown:qdown **Median:** 0.95 **CV:** 0.00 % **No. of Peptides:** 1

```
        10         20         30         40         50         60         70         80
MEIVGCRAED NSCPFRPPAM LFHGISGGHI QGIMEEMERR SKTEARLAKG AQLNGRDAGM PPLSPEKPAL CAGCGGKISD
        90        100        110        120        130        140        150        160
RYYLLAVDKQ WHLRCLKCCE CKLALESELT CFAKDGSIYC KEDYYRRFSV QRCARCHLGI SASEMVMRAR DSVYHLSCFT
       170        180        190        200        210        220        230        240
CSTCNKTLTT GDHFGMKDSL VYCRAHFETL LQGEYPPQLS YTELAAKSGG LALPYFNGTG TVQKGRPRKR KSPALGVDIV
       250        260        270        280        290        300        310        320
NYNSGCNENE ADHLDRDQQP YPPSQKTKRM RTSFKHHQLR TMKSYFAINH NPDAKDLKQL AQKTGLTKRV LQGEQILGHY
       330        340
SQTSRRLKIP
```

Cmpd.	No. of Cmpds.	m/z meas.	Δ m/z [ppm]	z	Rt [min]	Score	P	Range	Sequence	Modification	Ratios
2658	1	932.9537	71.73	2	64.4	15.8	0	151-166	R.DSVYHLSCFTCSTCNK.T	Carbamidomethyl: 11	
929	1	612.4347	236.48	2	41.1	10.6	0	167-177	K.TLTTGDHFGMK.D	Oxidation: 10	mdown:qdown 0.95

Detailed Protein Report

Protein 275: PREDICTED: NMDA receptor-regulated protein 2 isoform X4 [Homo sapiens]

**Accession:**	gi|530406441	**Score:**	36.5
**Database:**	refseq_human(refseq_human_20140103.fasta)	**MW [kDa]:**	86.5
**Database Date:**	2015-11-30	**pI:**	6.2
**Modification(s):**	Carbamidomethyl	**Sequence Coverage [%]:**	4.1
		**No. of unique Peptides:**	3

10	20	30	40	50	60	70	80
MSSKMVISEP	GLNWDISPKN	GLKTFFSREN	YKDHSMAPSL	KELRVLSNRR	IGENLNASAS	SVENEPAVSS	ATQAKEKVKT
**90**	**100**	**110**	**120**	**130**	**140**	**150**	**160**
TIGMVLLPKP	RVPYPRFSRF	SQREQRSYVD	LLVKYAKIPA	NSKAVGINKN	DYLQYLDMKK	HVNEEVTEFL	KFLQNSAKKC
**170**	**180**	**190**	**200**	**210**	**220**	**230**	**240**
AQDYNMLSDD	ARLFTEKILR	ACIEQVKKYS	EFYTLHEVTS	LMGFFPFRVE	MGLKLEKTLL	ALGSVKYVKT	VFPSMPIKLQ
**250**	**260**	**270**	**280**	**290**	**300**	**310**	**320**
LSKDDIATIE	TSEQTAEAMH	YDISKDPNAE	KLVSRYHPQI	ALTSQSLFTL	LNNHGPTYKE	QWEIPVCIQV	IPVAGSKPVK
**330**	**340**	**350**	**360**	**370**	**380**	**390**	**400**
VIYINSPLPQ	KKMTMRERNQ	IFHEVPLKFM	MSKNTSVPVS	AVFMDKPEEF	ISEMDMSCEV	NECRKIESLE	NLYLDFDDDV
**410**	**420**	**430**	**440**	**450**	**460**	**470**	**480**
TELETFGVTT	TKVSKSPSPA	STSTVPNMTD	APTAPKAGTT	TVAPSAPDIS	ANSRSLSQIL	MEQLQKEKQL	VTGMDGGPEE
**490**	**500**	**510**	**520**	**530**	**540**	**550**	**560**
CKNKDDQGFE	SCEKVSNSDK	PLIQDSDLKT	SDALQLENSQ	EIETSNKNDM	TIDILHADGE	RPNVLENLDN	SKEKTVGSEA
**570**	**580**	**590**	**600**	**610**	**620**	**630**	**640**
AKTEDTVLCS	SDTDEECLII	DTECKNNSDG	KTAVVGSNLS	SRPASPNSSS	GQASVGNQTN	TACSPEESCV	LKKPIKRVYK
**650**	**660**	**670**	**680**	**690**	**700**	**710**	**720**
KFDPVGEILK	MQDELLKPIS	RKVPELPLMN	LENSKQPSVS	EQLSGPSDSS	SWPKSGWPSA	FQKPKGRLPY	ELQDYVEDTS
**730**	**740**	**750**	**760**	**770**	**780**		
EYLAPQEGNF	VYKLFSLQDL	LLLVRCSVQR	IETRPRSKKR	KKIRRLTALR	NC		

Cmpd.	No. of Cmpds.	m/z meas.	Δ m/z [ppm]	z	Rt [min]	Score	P	Range	Sequence	Modification
1819	1	649.7840	-134.37	2	52.7	13.7	1	107-117	R.SYVDLLVKYAK.I	
9	1	650.7714	-185.46	2	29.3	12.0	2	178-188	K.ILRACIEQVKK.Y	
1355	1	607.6081	-208.99	2	45.5	10.8	0	485-494	K.DDQGFESCEK.V	Carbamidomethyl: 8

Detailed Protein Report

Protein 276: myeloid zinc finger 1 isoform 1 [Homo sapiens]

**Accession:**	gi|37622341	**Score:**	36.5
**Database:**	refseq_human(refseq_human_20140103.fasta)	**MW [kDa]:**	82.0
**Database Date:**	2015-11-30	**pI:**	10.3
**Modification(s):**	Carbamidomethyl	**Sequence Coverage [%]:**	7.2
		**No. of unique Peptides:**	1

Alias proteins:

Accession	Name	Description
gi|578834840	refseq_human (refseq_human_20140103.fasta)	PREDICTED: myeloid zinc finger 1 isoform X5 [Homo sapiens]
gi|578834838	refseq_human (refseq_human_20140103.fasta)	PREDICTED: myeloid zinc finger 1 isoform X4 [Homo sapiens]
gi|37622345	refseq_human (refseq_human_20140103.fasta)	myeloid zinc finger 1 isoform 1 [Homo sapiens]

```
        10         20         30         40         50         60         70         80
MRPAVLGSPD RAPPEDEGPV MVKLEDSEEE GEAALWDPGP EAARLRFRCF RYEEATGPQE ALAQLRELCR QWLRPEVRSK
        90        100        110        120        130        140        150        160
EQMLELLVLE QFLGALPPEI QARVQGQRPG SPEEAAALVD GLRREPGGPR RWVTVQVQGQ EVLSEKMEPS SFQPLPETEP
       170        180        190        200        210        220        230        240
PTPEPGPKTP PRTMQESPLG LQVKEESEVT EDSDFLESGP LAATQESVPT LLPEEAQRCG TVLDQIFPHS KTGPEGPSWR
       250        260        270        280        290        300        310        320
EHPRALWHEE AGGIFSPGFA LQLGSISAGP GSVSPHLHVP WDLGMAGLSG QIQSPSREGG FAHALLLPSD LRSEQDPTDE
       330        340        350        360        370        380        390        400
DPCRGVGPAL ITTRWRSPRG RSRGRPSTGG GVVRGGRCDV CGKVFSQRSN LLRHQKIHTG ERPFVCSECG RSFSRSSHLL
       410        420        430        440        450        460        470        480
RHQLTHTEER PFVCGDCGQG FVRSARLEEH RRVHTGEQPF RCAECGQSFR QRSNLLQHQR IHGDPPGPGA KPPAPPGAPE
       490        500        510        520        530        540        550        560
PPGPFPCSEC RESFARRAVL LEHQAVHTGD KSFGCVECGE RFGRRSVLLQ HRRVHSGERP FACAECGQSF RQRSNLTQHR
       570        580        590        600        610        620        630        640
RIHTGERPFA CAECGKAFRQ RPTLTQHLRV HTGEKPFACP ECGQRFSQRL KLTRHQRTHT GEKPYHCGEC GLGFTQVSRL
       650        660        670        680        690        700        710        720
TEHQRIHTGE RPFACPECGQ SFRQHANLTQ HRRIHTGERP YACPECGKAF RQRPTLTQHL RTHRREKPFA CQDCGRRFHQ
       730        740
STKLIQHQRV HSAE
```

Cmpd.	No. of Cmpds.	m/z meas.	Δ m/z [ppm]	z	Rt [min]	Score	P	Range	Sequence	Modification
2819	1	937.4520	9.17	2	64.4	12.8	1	673-688	R.RIHTGERPYACPECGK.A	Carbamidomethyl: 11

Detailed Protein Report

Protein 277: PREDICTED: probable E3 ubiquitin-protein ligase HERC1 isoform X6 [Homo sapiens]

**Accession:**	gi|530406610	**Score:**	36.5
**Database:**	refseq_human(refseq_human_20140103.fasta)	**MW [kDa]:**	524.9
**Database Date:**	2015-11-30	**pI:**	5.7
		**Sequence Coverage [%]:**	1.4
		**No. of unique Peptides:**	1

Detailed Protein Report

10	20	30	40	50	60	70	80
MATMIPPVKL	KWLEHLNSSW	ITEDSESIAT	REGVAVLYSK	LVSNKEVVPL	PQQVLCLKGP	QLPDFERESL	SSDEQDHYLD
90	100	110	120	130	140	150	160
ALLSSQLALA	KMVCSDSPFA	GALRKRLLVL	QRVFYALSNK	YHDKGKVKQQ	QHSPESSSGS	ADVHSVSERP	RSSTDALIEM
170	180	190	200	210	220	230	240
GVRTGLSLLF	ALLRQSWMMP	VSGPGLSLCN	DVIHTAIEVV	SSLPPLSLAN	ESKIPPMGLD	CLSQVTTFLK	GVTIPNSGAD
250	260	270	280	290	300	310	320
TLGRRLASEL	LLGLAAQRGS	LRYLLEWIEM	ALGASAVVHT	MEKGKLLSSQ	EGMISFDCFM	TILMQMRRSL	GSSADRSQWR
330	340	350	360	370	380	390	400
EPTRTSDGLC	SLYEAALCLF	EEIEAGQYCT	FVISTDGSVR	ACGKGSYGRL	GLGDSNNQST	LKKLTFEPHR	SIKKVSSSKG
410	420	430	440	450	460	470	480
SDGHTLAFTT	EGEVFSWGDG	DYGKLGHGNS	STQKYPKLIQ	GPLQGKVVVC	VSAGYRHSAA	VTEDGELYTW	GEGDFGRLGH
490	500	510	520	530	540	550	560
GDSNSRNIPT	LVKDISNVGE	VSCGSSHTIA	LSKDGRTVWS	FGGGDNGKLG	HGDTNRVYKP	KVIEALQGMF	IRKVCAGSQS
570	580	590	600	610	620	630	640
SLALTSTGQV	YAWGCGACLG	CGSSEATALR	PKLIEELAAT	RIVDVSIGDS	HCLALSHDNE	VYAWGNNSMG	QCGQGNSTGP
650	660	670	680	690	700	710	720
ITKPKKVSGL	DGIAIQQISA	GTSHSLAWTA	LPRDRQVVAW	HRPYCVDLEE	STFSHLRSFL	ERYCDKINSE	IPPLPFPSSR
730	740	750	760	770	780	790	800
EHHSFLKLCL	KLLSNHLALA	LAGGVATSIL	GRQAGPLRNL	LFRLMDSTVP	DEIQEVVIET	LSVGATMLLP	PLRERMELLH
810	820	830	840	850	860	870	880
SLLPQGPDRW	ESLSKGQRMQ	LDIILTSLQD	HTHVASLLGY	SSPSDAADLS	SVCTGYGNLS	DQPYGTQSCH	PDTHLAEILM
890	900	910	920	930	940	950	960
KTLLRNLGFY	TDQAFGELEK	NSDKFLLGTS	SSENSQPAHL	HELLCSLQKQ	LLAFCHINNI	SENSSSVALL	HKHLQLLLPH
970	980	990	1000	1010	1020	1030	1040
ATDIYSRSAN	LLKESPWNGS	VGEKLRDVIY	VSAAGSMLCQ	IVNSLLLLPV	SVARPLLSYL	LDLLPPLDCL	NRLLPAADLL
1050	1060	1070	1080	1090	1100	1110	1120
EDQELQWPLH	GGPELIDPAG	LPLPQPAQSW	VWLVDLERTI	ALLIGRCLGG	MLQGSPVSPE	EQDTAYWMKT	PLFSDGVEMD
1130	1140	1150	1160	1170	1180	1190	1200
TPQLDKCMSC	LLEVALSGNE	EQKPFDYKLR	PEIAVYVDLA	LGCSKEPARS	LWISMQDYAV	SKDWDSATLS	NESLLDTVSR
1210	1220	1230	1240	1250	1260	1270	1280
FVLAALLKHT	NLLSQACGES	RYQPGKHLSE	VYRCVYKVRS	RLLACKNLEL	IQTRSSSRDR	WISENQDSAD	VDPQEHSFTR
1290	1300	1310	1320	1330	1340	1350	1360
TIDEEAEMEE	QAERDREEGH	PEPEDEEEER	EHEVMTAGKI	FQCFLSAREV	ARSRDRDRMN	SGAGSGARAD	DPPPQSQQER
1370	1380	1390	1400	1410	1420	1430	1440
RVSTDLPEGQ	DVYTAACNSV	IHRCALLILG	VSPVIDELQK	RREEGQLQQP	STSASEGGGL	MTRSESLTAE	SRLVHTSPNY
1450	1460	1470	1480	1490	1500	1510	1520
RLIKSRSESD	LSQPESDEEG	YALSGRRNVD	LDLAASHRKR	GPMHSQLESL	SDSWARLKHS	RDWLCNSSYS	FESDFDLTKS
1530	1540	1550	1560	1570	1580	1590	1600
LGVHTLIENV	VSFVSGDVGN	APGFKEPEES	MSTSPQASII	AMEQQQLRAE	LRLEALHQIL	VLLSGMEEKG	SISLAGSRLS
1610	1620	1630	1640	1650	1660	1670	1680
SGFQSSTLLT	SVRLQFLAGC	FGLGTVGHTG	GKGESGRLHH	YQDGIRAAKR	NIQIEIQVAV	HKIYQQLSAT	LERALQANKH
1690	1700	1710	1720	1730	1740	1750	1760
HIEAQQRLLL	VTVFALSVHY	QPVDVSLAIS	TGLLNVLSQL	CGTDTMLGQP	LQLLPKTGVS	QLSTALKVAS	TRLLQILAIT
1770	1780	1790	1800	1810	1820	1830	1840
TGTYADKLSP	KVVQSLLDLL	CSQLKNLLSQ	TGVLHMASFG	EGEQEDGEEE	EKKVDSSGET	EKKDFRAALR	KQHAAELHLG
1850	1860	1870	1880	1890	1900	1910	1920
DFLVFLRRVV	SSKAIQSKMA	SPKWTEVLLN	IASQKCSSGI	PLVGNLRTRL	LALHVLEAVL	PACESGVEDD	QMAQIVERLF
1930	1940	1950	1960	1970	1980	1990	2000
SLLSDCMWET	PIAQAKHAIQ	IKEKEQEIKL	QKQGELEEED	ENLPIQEVSF	DPEKAQCCLV	ENGQILTHGS	GGKGYGLAST
2010	2020	2030	2040	2050	2060	2070	2080
GVTSGCYQWK	FYIVKENRGN	EGTCVGVSRW	PVHDFNHRTT	SDMWLYRAYS	GNLYHNGEQT	LTLSSFTQGD	FITCVLDMEA
2090	2100	2110	2120	2130	2140	2150	2160
RTISFGKNGE	EPKLAFEDVD	AAELYPCVMF	YSSNPGEKVK	ICDMQMRGTP	RDLLPGDPIC	SPVAAVLAEA	TIQLIRILHR
2170	2180	2190	2200	2210	2220	2230	2240

Detailed Protein Report

Cmpd.	No. of Cmpds.	m/z meas.	Δ m/z [ppm]	z	Rt [min]	Score	P	Range	Sequence	Modification
2832	1	923.8157	-4.48	3	64.6	12.0	1	487-513	R.NIPTLVKDISNVGEVSCGSSHTIALSK.D	

Detailed Protein Report

Protein 278: PREDICTED: peroxisomal acyl-coenzyme A oxidase 2 isoform X2 [Homo sapiens]

**Accession:**	gi|530373234	**Score:**	36.5
**Database:**	refseq_human(refseq_human_20140103.fasta)	**MW [kDa]:**	68.7
**Database Date:**	2015-11-30	**pI:**	8.8
**Modification(s):**	Oxidation	**Sequence Coverage [%]:**	6.5
		**No. of unique Peptides:**	2

10	20	30	40	50	60	70	80
MTQNERYKAA	MRRAFHIRLI	ARRLGWLEDG	RELGYAYRAL	SGDVALNIHR	VFVRALRSLG	SEEQIAKWDP	LCKNIQIIAT
**90**	**100**	**110**	**120**	**130**	**140**	**150**	**160**
YAQTELGHGT	YLQGLETEAT	YDAATQEFVI	HSPTLTATKW	WPGDLGRSAT	HALVQAQLIC	SGARRGMHAF	IVPIRSLQDH
**170**	**180**	**190**	**200**	**210**	**220**	**230**	**240**
TPLPGIIIGD	IGPKMDFDQT	DNGFLQLNHV	RVPRENMLSR	FAQVLPDGTY	VKLGTAQSNY	LPMVVVRVEL	LSGEILPILQ
**250**	**260**	**270**	**280**	**290**	**300**	**310**	**320**
KACVIAMRYS	VIRRQSRLRP	SDPEAKVLDY	QTQQQKLFPQ	LAISYAFHFL	AVSLLEFFQH	SYTAILNQDF	SFLPELHALS
**330**	**340**	**350**	**360**	**370**	**380**	**390**	**400**
TGMKAMMSEF	CTQGAEMCRR	ACGGHGYSKL	SGLPSLVTKL	SASCTYEGEN	TVLYLQVARF	LVKSYLQTQM	SPGSTPQRSL
**410**	**420**	**430**	**440**	**450**	**460**	**470**	**480**
SPSVAYLTAP	DLARCPAQRA	ADFLCPELYT	TAWAHVAVRL	IKDSVQHLQT	LTQSGADQHE	AWNQTTVIHL	QAAKVHCYYV
**490**	**500**	**510**	**520**	**530**	**540**	**550**	**560**
TVKGFTEALE	KLENEPAIQQ	VLKRLCDLHA	IHGILTNSGD	FLHDAFLSGA	QVDMARTAYL	DLLRLIRKDA	ILLTDAFDFT
**570**	**580**	**590**	**600**	**610**	**620**		
DQCLNSALGC	YDGNVYERLF	QWAQKSPTNT	QENPAYEEYI	RPLLQSWRSK	L		

Cmpd.	No. of Cmpds.	m/z meas.	Δ m/z [ppm]	z	Rt [min]	Score	P	Range	Sequence	Modification
1349	1	945.2230	-270.57	1	45.4	11.1	0	24-31	R.LGWLEDGR.E	
2448	1	863.7544	-77.93	2	58.9	15.3	0	325-339	K.AMMSEFCTQGAEMCR.R	Oxidation: 2, 13

BRUKER

Detailed Protein Report

Protein 279: PREDICTED: centrosome-associated protein CEP250 isoform X4 [Homo sapiens]

**Accession:**	gi|530417804	**Score:**	36.4
**Database:**	refseq_human(refseq_human_20140103.fasta)	**MW [kDa]:**	208.3
**Database Date:**	2015-11-30	**pI:**	4.9
**Modification(s):**	Oxidation	**Sequence Coverage [%]:**	2.7
		**No. of unique Peptides:**	2

10	20	30	40	50	60	70	80
MEAAEQARNA	LQVDLAEAEK	RREALWEKNT	HLEAQLQKAE	EAGAELQADL	RDIQEEKEEI	QKKLSESRHQ	QEAATTQLEQ
**90**	**100**	**110**	**120**	**130**	**140**	**150**	**160**
LHQEAKRQEE	VLARAVQEKE	ALVREKAALE	VRLQAVERDR	QDLAEQLQGL	SSAKELLESS	LFEAQQQNSV	IEVTKGQLEV
**170**	**180**	**190**	**200**	**210**	**220**	**230**	**240**
QIQTVTQAKE	VIQGEVRCLK	LELDTERSQA	EQERDAAARQ	LAQAEQEGKT	ALEQQKAAHE	KEVNQLREKW	EKERSWHQQE
**250**	**260**	**270**	**280**	**290**	**300**	**310**	**320**
LAKALESLER	EKMELEMRLK	EQQTEMEAIQ	AQREEERTQA	ESALCQMQLE	TEKERVSLLE	TLLQTQKELA	DASQQLERLR
**330**	**340**	**350**	**360**	**370**	**380**	**390**	**400**
QDMKVQKLKE	QETTGILQTQ	LQEAQRELKE	AARQHRDDLA	ALQEESSSLL	QDKMDLQKQV	EDLKSQLVAQ	DDSQRLVEQE
**410**	**420**	**430**	**440**	**450**	**460**	**470**	**480**
VQEKLRETQE	YNRIQKELER	EKASLTLSLM	EKEQRLLVLQ	EADSIRQQEL	SALRQDMQEA	QGEQKELSAQ	MELLRQEVKE
**490**	**500**	**510**	**520**	**530**	**540**	**550**	**560**
KEADFLAQEA	QLLEELEASH	ITEQQLRASL	WAQEAKAAQL	QLRLRSTESQ	LEALAAEQQP	GNQAQAQAQL	ASLYSALQQA
**570**	**580**	**590**	**600**	**610**	**620**	**630**	**640**
LGSVCESRPE	LSGGGDSAPS	VWGLEPDQNG	ARSLFKRGPL	LTALSAEAVA	SALHKLHQDL	WKTQQTRDVL	RDQVQKLEER
**650**	**660**	**670**	**680**	**690**	**700**	**710**	**720**
LTDTEAEKSQ	VHTELQDLQR	QLSQNQEEKS	KWEGKQNSLE	SELMELHETM	ASLQSRLRRA	ELQRMEAQGE	RELLQAAKEN
**730**	**740**	**750**	**760**	**770**	**780**	**790**	**800**
LTAQVEHLQA	AVVEARAQAS	AAGILEEDLR	TARSALKLKN	EEVESERERA	QALQEQGELK	VAQGKALQEN	LALLTQTLAE
**810**	**820**	**830**	**840**	**850**	**860**	**870**	**880**
REEEVETLRG	QIQELEKQRE	MQKAALELLS	LDLKKRNQEV	DLQQEQIQEL	EKCRSVLEHL	PMAVQEREQK	LTVQREQIRE
**890**	**900**	**910**	**920**	**930**	**940**	**950**	**960**
LEKDRETQRN	VLEHQLLELE	KKDQMIESQR	GQVQDLKKQL	VTLECLALEL	EENHHKMECQ	QKLIKELEGQ	RETQRVALTH
**970**	**980**	**990**	**1000**	**1010**	**1020**	**1030**	**1040**
LTLDLEERSQ	ELQAQSSQIH	DLESHSTVLA	RELQERDQEV	KSQREQIEEL	QRQKEHLTQD	LERRDQELML	QKERIQVLED
**1050**	**1060**	**1070**	**1080**	**1090**	**1100**	**1110**	**1120**
QRTRQTKILE	EDLEQIKLSL	RERGRELTTQ	RQLMQERAEE	GKGPSKAQRG	SLEHMKLILR	DKEKEVECQQ	EHIHELQELK
**1130**	**1140**	**1150**	**1160**	**1170**	**1180**	**1190**	**1200**
DQLEQQLQGL	HRKVGETSLL	LSQREQEIVV	LQQQLQEARE	QGELKEQSLQ	SQLDEAQRAL	AQRDQELEAL	QQEQQQAQGQ
**1210**	**1220**	**1230**	**1240**	**1250**	**1260**	**1270**	**1280**
EERVKEKADA	LQGALEQAHM	TLKERHGELQ	DHKEQARRLE	EELAVEGRRV	QALEEVLGDL	RAESREQEKA	LLALQQQCAE
**1290**	**1300**	**1310**	**1320**	**1330**	**1340**	**1350**	**1360**
QAQEHEVETR	ALQDSWLQAQ	AVLKERDQEL	EALRAESQSS	RHQEEAARAR	AEALQEALGK	AHAALQGKEQ	HLLEQAELSR
**1370**	**1380**	**1390**	**1400**	**1410**	**1420**	**1430**	**1440**
SLEASTATLQ	ASLDACQAHS	RQLEEALRIQ	EGEIQDQDLR	YQEDVQQLQQ	ALAQRDEELR	HQQEREQLLE	KSLAQRVQEN
**1450**	**1460**	**1470**	**1480**	**1490**	**1500**	**1510**	**1520**
MIQEKQNLGQ	EREEEEIRGL	HQSVRELQLT	LAQKEQEILE	LRETQQRNNL	EALPHSHKTS	PMEEQSLKLD	SLEPRLQREL
**1530**	**1540**	**1550**	**1560**	**1570**	**1580**	**1590**	**1600**
ERLQAALRQT	EAREIEWREK	AQDLALSLAQ	TKASVSSLQE	VAMFLQASVL	ERDSEQQRLQ	DELELTRRAL	EKERLHSPGA
**1610**	**1620**	**1630**	**1640**	**1650**	**1660**	**1670**	**1680**
TSTAELGSRG	EQGVQLGEVS	GVEAEPSPDG	MEKQSWRQRL	EHLQQAVARL	EIDRSRLQRH	NVQLRSTLEQ	VERERRKLKR
**1690**	**1700**	**1710**	**1720**	**1730**	**1740**	**1750**	**1760**
EAMRAAQAGS	LEISKATASS	PTQQDGRGQK	NSDAKCVAEL	QKEVVLLQAQ	LTLERKQKQD	YITRSAQTSR	ELAGLHHSLS
**1770**	**1780**	**1790**	**1800**	**1810**	**1820**		
HSLLAVAQAP	EATVLEAETR	RLDESLTQSL	TSPGPVLLHP	SPSTTQAASR			

Cmpd.	No. of	m/z meas.	Δ m/z	z	Rt	Score	P	Range	Sequence	Modification

Detailed Protein Report

	Cmpds.		[ppm]		[min]					
813	1	683.3344	-49.61	2	39.0	12.1	1	421-432	R.EKASLTLSLMEK.E	Oxidation: 10
1397	1	926.5056	55.39	3	46.2	12.2	2	1681-1707	R.EAMRAAQAGSLEISKATASSPTQQDGR.G	Oxidation: 3

Detailed Protein Report

Protein 280: T-complex protein 11 homolog isoform 3 [Homo sapiens]

**Accession:**	gi|387849264	**Score:**	36.3
**Database:**	refseq_human(refseq_human_20140103.fasta)	**MW [kDa]:**	56.8
**Database Date:**	2015-11-30	**pI:**	5.0
**Modification(s):**	Oxidation	**Sequence Coverage [%]:**	5.7
		**No. of unique Peptides:**	1

10	20	30	40	50	60	70	80
MTRGGGGGDT	ISKMPDVKES	VPPKYPGDSE	GRSCKPETSG	PPQEDKSGSE	DPPPCLTETV	NEVSKLSNKI	GMNCDYYMEE
90	100	110	120	130	140	150	160
KVLPPSSLEG	KVKETVHNAF	WDHLKEQLSA	TPPDFSCALE	LLKEIKEILL	SLLLPRQNRL	RIEIEEALDM	DLLKQEAEHG
170	180	190	200	210	220	230	240
ALKVLYLSKY	VLNMMALLCA	PVRDEAVQKL	ENITDPVWLL	RGIFQVLGRM	KMDMVNYTIQ	SLQPHLQEHS	IQYERAKFQE
250	260	270	280	290	300	310	320
LLNKQPSLLN	HTTKWLTQAA	GDLTMSPPTC	PDTSDSSSVA	GPSPNEAANN	PEPLSPTMVL	CQGFLNLLLW	DLENEEFPET
330	340	350	360	370	380	390	400
LLMDRTRLQE	LKSQLHQLTV	MASVLLVASS	FSGSVLFGSP	QFVDKLKRIT	KSLLEDFHSR	PEEAILTVSE	QVSQEIHQSL
410	420	430	440	450	460	470	480
KNMGLVALSS	DNTASLMGQL	QNIAKKENCV	CSVIDQRIHL	FLKCCLVLGV	QRSLLDLPGG	LTLIEAELAE	LGQKFVNLTH
490	500	510	520				
HNQQVFGPYY	TEILKTLISP	AQALETKVES	V				

Cmpd.	No. of Cmpds.	m/z meas.	Δ m/z [ppm]	z	Rt [min]	Score	P	Range	Sequence	Modification
2154	1	1052.4435	-57.89	2	57.1	16.6	2	14-32	K.MPDVKESVPPKYPGDSEGR.S	Oxidation: 1

Detailed Protein Report

Protein 281: kinesin-like protein KIF26B [Homo sapiens]

**Accession:**	gi|124430752	**Score:**	36.2
**Database:**	refseq_human(refseq_human_20140103.fasta)	**MW [kDa]:**	223.7
**Database Date:**	2015-11-30	**pI:**	9.7
**Modification(s):**	Carbamidomethyl	**Sequence Coverage [%]:**	2.5
		**No. of unique Peptides:**	1

Detailed Protein Report

10	20	30	40	50	60	70	80
MNSVAGNKER	LAVSTRGKKY	GVNEVCSPTK	PAAPFSPESW	YRKAYEESRA	GSRPTPEGAG	SALGSSGTPS	PGSGTSSPSS
90	100	110	120	130	140	150	160
FTGSPGPASP	GIGTSSPGSL	GGSPGFGTGS	PGSGSGGGSS	PGSDRGVWCE	NCNARLVELK	RQALRLLLPG	PFPGKDPAFS
170	180	190	200	210	220	230	240
AVIHDKLQVP	NTIRKAWNDR	DNRCDICATH	LNQLKQEAIQ	MVLTLEQAAG	SEHYDASPCS	PPPLSNIPTL	VGSRHVGGLQ
250	260	270	280	290	300	310	320
QPRDWAFVPA	PCATSNYTGF	ANKHGSKPSS	LGVSNGAEKK	SGSPTHQAKV	SLQMATSPSN	GNILNSVAIQ	AHQYLDGTWS
330	340	350	360	370	380	390	400
LSRTNGVTLY	PYQISQLMTE	SSREGLTEAV	LNRYNADKPS	ACSVPASQGS	CVASETSTGT	SVAASFFARA	AQKLNLSSKK
410	420	430	440	450	460	470	480
KKHRPSTSSA	AEPPLFATSF	SGILQTSPPP	APPCLLRAVN	KVKDTPGLGK	VKVMLRICST	LARDTSESSS	FLKVDPRKKQ
490	500	510	520	530	540	550	560
ITLYDPLTCG	GQNAFQKRGN	QVPPKMFAFD	AVFPQDASQA	EVCAGTVAEV	IQSVVNGADG	CVFCFGHAKL	GKSYTMIGKD
570	580	590	600	610	620	630	640
DSMQNLGIIP	CAISWLFKLI	NERKEKTGAR	FSVRVSAVEV	WGKEENLRDL	LSEVATGSLQ	DGQSPGVYLC	EDPICGTQLQ
650	660	670	680	690	700	710	720
NQSELRAPTA	EKAAFFLDAA	IASRRSHQQD	CDEDDHRNSH	VFFTLHIYQY	RMEKSGKGGM	SGGRSRLHLI	DLGSCVKALS
730	740	750	760	770	780	790	800
KNREGGSGLC	LSLSALGNVI	LALVNGSKHI	PYKESKLAML	LRESLGNMNC	RTTMIAHISA	AVGSYAETLS	TIQIASRVLR
810	820	830	840	850	860	870	880
MKKKKTKYTS	SSSGGESSCE	EGRMRRPTQL	RPFHTRATVD	PDFPIAHLSS	DPDYSSSSEQ	SCDTVIYIGP	NGTALSDKEL
890	900	910	920	930	940	950	960
TDNEGPPDFV	PIVPALQKTR	GDSRPAEAGE	AAAGKSERDC	LKCNTFAELQ	ERLDCIDGSE	EPSSFPFEEL	PAQFGPEQAS
970	980	990	1000	1010	1020	1030	1040
RGPRLSQAAG	ASPLSESDKE	DNGSEGQLTN	REGPELPASK	MQRSHSPVPA	AAPAHSPSPA	SPRSVPGSSS	QHSASPLVQS
1050	1060	1070	1080	1090	1100	1110	1120
PSLQSSRESL	NSCGFVEGKP	RPMGSPRLGI	ASLSKTSEYK	PPSSPSQRCK	VYTQKGVLPS	PAPLPPSSKD	SGVASRESLL
1130	1140	1150	1160	1170	1180	1190	1200
QPEVRTPPVG	MSPQVLKKSM	SAGSEGFPET	PVDDEQQAAT	PSESKKEILS	TTMVTVQQPL	ELNGEDELVF	TLVEELTISG
1210	1220	1230	1240	1250	1260	1270	1280
VLDSGRPTSI	ISFNSDCSAR	ALASGSRPVS	IISSISEDLE	CYSSTAPVSE	VSITQFLPLP	KMSLDEKAQD	AGSRRSSISS
1290	1300	1310	1320	1330	1340	1350	1360
WLSEMSAGSE	GEQSCHSFIA	QTCFGHGEAM	AEPVASEFVS	SLQNTAVVCR	EKPKASPDNL	LILSEMGDDS	FNKAAPIKGC
1370	1380	1390	1400	1410	1420	1430	1440
KISTVSKAMV	TISNTANLSS	CEGYIPMKTN	ITVYPCIAMS	PRNIQEPEAP	TATPKAGPTL	AQSRESKENS	AKKEMKFEDP
1450	1460	1470	1480	1490	1500	1510	1520
WLKREEEVKK	ETAHPNEEGM	MRCETATGPS	NAETRAEQEQ	DGKPSPGDRL	SSSSGEVSAS	PVTDNFRRVV	DGCEMALPGL
1530	1540	1550	1560	1570	1580	1590	1600
ATQSPVHPNK	SVKSSSLPRA	FQKASRQEEP	DSLSYYCAAE	TNGVGAASGT	PPSKATLEGK	VASPKHCVLA	RPKGTPPLPP
1610	1620	1630	1640	1650	1660	1670	1680
VRKSSLDQKN	RASPQHSASG	SGTSSPLNQP	AAFPAGLPDE	PSGKTKDASS	SSKLFSAKLE	QLASRSNSLG	RATVSHYECL
1690	1700	1710	1720	1730	1740	1750	1760
SLERAESLSS	VSSRLHAGKD	GTMPRAGRSL	GRSAGTSPPS	SGASPKAGQS	KISAVSRLLL	ASPRARGPSA	STTKTLSFST
1770	1780	1790	1800	1810	1820	1830	1840
KSLPQAVGQG	SSSPPGGKHT	PWSTQSLSRN	RSSGLASKLP	LRAVSGRISE	LLQGGAGARG	LQLRAGPEAE	ARGGALAEDE
1850	1860	1870	1880	1890	1900	1910	1920
PAAAHLLPSP	YSKITPPRRP	HRCSSGHGSD	NSSVLSGELP	PAMGKTALFY	HSGGSSGYES	VMRDSEATGS	ASSAQDSTSE
1930	1940	1950	1960	1970	1980	1990	2000
NSSSVGGRCR	SLKTPKKRSN	PGSQRRRLIP	ALSLDTSSPV	RKPPNSTGVR	WVDGPLRSSP	RGLGEPFEIK	VYEIDDVERL
2010	2020	2030	2040	2050	2060	2070	2080
QRRRGGASKE	AMCFNAKLKI	LEHRQQRIAE	VRAKYEWLMK	ELEATKQYLM	LDPNKWLSEF	DLEQVWELDS	LEYLEALECV
2090	2100	2110					
TERLESRVNF	CKAHLMMITC	FDITSRRR					

Detailed Protein Report

Cmpd.	No. of Cmpds.	m/z meas.	Δ m/z [ppm]	z	Rt [min]	Score	P	Range	Sequence	Modification
2229	1	597.6218	-9.28	3	56.3	11.5	1	126-140	R.GVWCENCNARLVELK.R	Carbamidomethyl: 7

Detailed Protein Report

Protein 282: PREDICTED: protein unc-13 homolog C isoform X2 [Homo sapiens]

**Accession:**	gi|530405878	**Score:**	36.2
**Database:**	refseq_human(refseq_human_20140103.fasta)	**MW [kDa]:**	247.7
**Database Date:**	2015-11-30	**pI:**	5.6
		**Sequence Coverage [%]:**	1.4
		**No. of unique Peptides:**	1

Quantitation

mdown:qdown	**Median:**	0.38	**CV:**	0.00 %	**No. of Peptides:**	1
Wdown:Qdown	**Median:**	1.31	**CV:**	0.00 %	**No. of Peptides:**	1

Detailed Protein Report

10	20	30	40	50	60	70	80
MVANFFKSLI	LPYIHKLCKG	MFTKKLGNTN	KNKEYRQQKK	DQDFPTAGQT	KSPKFSYTFK	STVKKIAKCS	STHNLSTEED
90	100	110	120	130	140	150	160
EASKEFSLSP	TFSYRVAIAN	GLQKNAKVTN	SDNEDLLQEL	SSIESSYSES	LNELRSSTEN	QAQSTHTMPV	RRNRKSSSSL
170	180	190	200	210	220	230	240
APSEGSSDGE	RTLHGLKLGA	LRKLRKWKKS	QECVSSDSEL	STMKKSWGIR	SKSLDRTVRN	PKTNALEPGF	SSSGCISQTH
250	260	270	280	290	300	310	320
DVMEMIFKEL	QGISQIETEL	SELRGHVNAL	KHSIDEISSS	VEVVQSEIEQ	LRTGFVQSRR	ETRDIHDYIK	HLGHMGSKAS
330	340	350	360	370	380	390	400
LRFLNVTEER	FEYVESVVYQ	ILIDKMGFSD	APNAIKIEFA	QRIGHQRDCP	NAKPRPILVY	FETPQQRDSV	LKKSYKLKGT
410	420	430	440	450	460	470	480
GIGISTDILT	HDIRERKEKG	IPSSQTYESM	AIKLSTPEPK	IKKNNWQSPD	DSDEDLESDL	NRNSYAVLSK	SELLTKGSTS
490	500	510	520	530	540	550	560
KPSSKSHSAR	SKNKTANSSR	ISNKSDYDKI	SSQLPESDIL	EKQTTTHYAD	ATPLWHSQSD	FFTAKLSRSE	SDFSKLCQSY
570	580	590	600	610	620	630	640
SEDFSENQFF	TRTNGSSLLS	SSDRELWQRK	QEGTATLYDS	PKDQHLNGGV	QGIQGQTETE	NTETVDSGMS	NGMVCASGDR
650	660	670	680	690	700	710	720
SHYSDSQLSL	HEDLSPWKEW	NQGADLGLDS	STQEGFDYET	NSLFDQQLDV	YNKDLEYLGK	CHSDLQDDSE	SYDLTQDDNS
730	740	750	760	770	780	790	800
SPCPGLDNEP	QGQWVGQYDS	YQGANSNELY	QNQNQLSMMY	RSQSELQSDD	SEDAPPKSWH	SRLSIDLSDK	TFSFPKFGST
810	820	830	840	850	860	870	880
LQRAKSALEV	VWNKSTQSLS	GYEDSGSSLM	GRFRTLSQST	ANESSTTLDS	DVYTEPYYYK	AEDEEDYTEP	VADNETDYVE
890	900	910	920	930	940	950	960
VMEQVLAKLE	NRTSITETDE	QMQAYDHLSY	ETPYETPQDE	GYDGPADDMV	SEEGLEPLNE	TSAEMEIRED	ENQNIPEQPV
970	980	990	1000	1010	1020	1030	1040
EITKPKRIRP	SFKEAALRAY	KKQMAELEEK	ILAGDSSSVD	EKARIVSGND	LDASKFSALQ	VCGGAGGGLY	GIDSMPDLRR
1050	1060	1070	1080	1090	1100	1110	1120
KKTLPIVRDV	AMTLAARKSG	LSLAMVIRTS	LNNEELKMHV	FKKTLQALIY	PMSSTIPHNF	EVWTATTPTY	CYECEGLLWG
1130	1140	1150	1160	1170	1180	1190	1200
IARQGMKCLE	CGVKCHEKCQ	DLLNADCLQR	AAEKSSKHGA	EDKTQTIITA	MKERMKIREK	NRPEVFEVIQ	EMFQISKEDF
1210	1220	1230	1240	1250	1260	1270	1280
VQFTKAAKQS	VLDGTSKWSA	KITITVVSAQ	GLQAKDKTGS	SDPYVTVQVG	KNKRRTKTIF	GNLNPVWDEK	FYFECHNSTD
1290	1300	1310	1320	1330	1340	1350	1360
RIKVRVWDED	DDIKSRVKQH	FKKESDDFLG	QTIVEVRTLS	GEMDVWYNLE	KRTDKSAVSG	AIRLKINVEI	KGEEKVAPYH
1370	1380	1390	1400	1410	1420	1430	1440
IQYTCLHENL	FHYLTEVKSN	GGVKIPEVKG	DEAWKVFFDD	ASQEIVDEFA	MRYGIESIYQ	AMTHFSCLSS	KYMCPGVPAV
1450	1460	1470	1480	1490	1500	1510	1520
MSTLLANINA	FYAHTTVSTN	IQVSASDRFA	ATNFGREKFI	KLLDQLHNSL	RIDLSKYREN	FPASNTERLQ	DLKSTVDLLT
1530	1540	1550	1560	1570	1580	1590	1600
SITFFRMKVL	ELQSPPKASM	VVKDCVRACL	DSTYKYIFDN	CHELYSQLTD	PSKKQDIPRE	DQGPTTKNLD	FWPQLITLMV
1610	1620	1630	1640	1650	1660	1670	1680
TIIDEDKTAY	TPVLNQFPQE	LNMGKISAEI	MWTLFALDMK	YALEEHENQR	LCKSTDYMNL	HFKVKWFYNE	YVRELPAFKD
1690	1700	1710	1720	1730	1740	1750	1760
AVPEYSLWFE	PFVMQWLDEN	EDVSMEFLHG	ALGRDKKDGF	QQTSEHALFS	CSVVDVFAQL	NQSFEIIKKL	ECPNPEALSH
1770	1780	1790	1800	1810	1820	1830	1840
LMRRFAKTIN	KVLLQYAAIV	SSDFSSHCDK	ENVLDSEAST	ILKELQVKLS	GVLDELSVTY	GESFQVIIEE	CIKQMSFELN
1850	1860	1870	1880	1890	1900	1910	1920
QMRANGNTTS	NKNSAAMDAE	IVLRSLMDFL	DKTLSLSAKI	CEKTVLKRVL	KELWKLVLNK	IEKQIVLPPL	TDQTGPQMIF
1930	1940	1950	1960	1970	1980	1990	2000
IAAKDLGQLS	KLKEHMIRED	ARGLTPRQCA	IMEVVLATIK	QYFHAGGNGL	KKNFLEKSPD	LQSLRYALSL	YTQTTDALIK
2010	2020	2030	2040	2050	2060	2070	2080
KFIDTQTSQS	RSSKDAVGQI	SVHVDITATP	GTGDHKVTVK	VIAINDLNWQ	TTAMFRPFVE	VCILGPNLGD	KKRKQGTKTK
2090	2100	2110	2120	2130	2140	2150	2160
SNTWSPKYNE	TFQFILGKEN	RPGAYELHLS	VKDYCFARED	RIIGMTVIQL	QNIAEKGSYG	AWYPLLKNIS	MDETGLTILR
2170	2180	2190					
ILSQRTSDDV	AKEFVRLKSE	TRSTEESA					

Cmpd.	No. of	m/z meas.	Δ m/z	z	Rt	Score	P	Range	Sequence	Modification	Ratios

Detailed Protein Report

	Cmpds.		[ppm]		[min]						
1046	1	742.4006	68.40	2	41.8	12.0	1	1497-<br>1508	K.YRENFPASNTER.L		Wdown:Qdown 1.31<br>mdown:qdown 0.38

Detailed Protein Report

Protein 283: PDZ domain-containing RING finger protein 4 isoform 2 [Homo sapiens]

Accession: gi|142976783
Database: refseq_human(refseq_human_20140103.fasta)
Database Date: 2015-11-30

Score: 36.1
MW [kDa]: 89.0
pI: 5.0
Sequence Coverage [%]: 3.2
No. of unique Peptides: 2

Quantitation

mdown:qdown	**Median:**	1.40	**CV:**	0.00 %	**No. of Peptides:**	1
Wdown:Qdown	**Median:**	1.42	**CV:**	0.00 %	**No. of Peptides:**	1

10	20	30	40	50	60	70	80
MGCNLCTFQK	REEHYKLLYE	VSQVNGKDLS	KATHEEAVEA	FRNAKEPIVV	QVLRRTPLSR	PAYGMASEVQ	LMNASTQTDI
**90**	**100**	**110**	**120**	**130**	**140**	**150**	**160**
TFEHIMALAK	LRPPTPPVPD	ICPFLLSDSC	HSLHPMEHEF	YEDNEYISSL	PADADRTEDF	EYEEVELCRV	SSQEKLGLTV
**170**	**180**	**190**	**200**	**210**	**220**	**230**	**240**
CYRTDDEEDT	GIYVSEVDPN	SIAAKDGRIR	EGDRILQING	EDVQNREEAV	ALLSNDECKR	IVLLVARPEI	QLDEGWLEDE
**250**	**260**	**270**	**280**	**290**	**300**	**310**	**320**
RNEFLEELNL	EMLEEEHNEA	MQPTANEVEQ	PKKQEEEEGT	TDTATSSSNN	HEKDSGVGRT	DESLRNDESS	EQENAAEDPN
**330**	**340**	**350**	**360**	**370**	**380**	**390**	**400**
STSLKSKRDL	GQSQDTLGSV	ELQYNESLVS	GEYIDSDCIG	NPDEDCERFR	QLLELKCKIR	NHGEYDLYYS	SSTIECNQGE
**410**	**420**	**430**	**440**	**450**	**460**	**470**	**480**
QEGVEHELQL	LNEELRNIEL	ECQNIMQAHR	LQKVTDQYGD	IWTLHDGGFR	NYNTSIDMQR	GKLDDIMEHP	EKSDKDSSSA
**490**	**500**	**510**	**520**	**530**	**540**	**550**	**560**
YNTAESCRST	PLTVDRSPDS	SLPRVINLTN	KKNLRSTMAA	TQSSSGQSSK	ESTSTKAKTT	EQGCSAESKE	KVLEGSKLPD
**570**	**580**	**590**	**600**	**610**	**620**	**630**	**640**
QEKAVSEHIP	YLSPYHSSSY	RYANIPAHAR	HYQSYMQLIQ	QKSAVEYAQS	QLSLVSMCKE	SQKCSEPKME	WKVKIRSDGT
**650**	**660**	**670**	**680**	**690**	**700**	**710**	**720**
RYITKRPVRD	RILKERALKI	KEERSGMTTD	DDTMSEMKMG	RYWSKEERKQ	HLVRAKEQRR	RREFMMRSRL	ECLKESPQSG
**730**	**740**	**750**	**760**	**770**	**780**		
SEGKKEINII	ELSHKKMMKK	RNKKILDNWM	TIQELMTHGA	KSPDGTRVHN	AFLSVTTV		

Cmpd.	No. of Cmpds.	m/z meas.	Δ m/z [ppm]	z	Rt [min]	Score	P	Range	Sequence	Modification	Ratios
652	2	639.3334	-62.76	2	37.0	25.9	2	369-378	R.FRQLLELKCK.I		Wdown:Qdown 1.42<br>mdown:qdown 1.40
2536	1	821.0789	154.65	2	60.0	10.2	1	497-511	R.SPDSSLPRVINLTNK.K		

Detailed Protein Report

Protein 284: nuclear receptor coactivator 3 isoform d [Homo sapiens]

Accession: gi|291490685
Database: refseq_human(refseq_human_20140103.fasta)
Database Date: 2015-11-30
Modification(s): Carbamidomethyl, Oxidation

Score: 36.1
MW [kDa]: 154.3
pI: 7.0
Sequence Coverage [%]: 4.2
No. of unique Peptides: 3

Quantitation

mdown:qdown	**Median:**	0.74	**CV:**	0.00 %	**No. of Peptides:**	1
Wdown:Qdown	**Median:**	0.60	**CV:**	0.00 %	**No. of Peptides:**	1

```
        10         20         30         40         50         60         70         80
MSGLGENLDP LASDSRKRKL PCDTPGQGLT CSGEKRRREQ ESKYIEELAE LISANLSDID NFNVKPDKCA ILKETVRQIR
        90        100        110        120        130        140        150        160
QIKEQGKTIS NDDDVQKADV SSTGQGVIDK DSLGPLLLQA LDGFLFVVNR DGNIVFVSEN VTQYLQYKQE DLVNTSVYNI
       170        180        190        200        210        220        230        240
LHEEDRKDFL KNLPKSTVNG VSWTNETQRQ KSHTFNCRML MKTPHDILED INASPEMRQR YETMQCFALS QPRAMMEEGE
       250        260        270        280        290        300        310        320
DLQSCMICVA RRITTGERTF PSNPESFITR HDLSGKVVNI DTNSLRSSMR PGFEDIIRRC IQRFFSLNDG QSWSQKRHYQ
       330        340        350        360        370        380        390        400
EVTSDGIFSP TAYLNGHAET PVYRFSLADG TIVTAQTKSK LFRNPVTNDR HGFVSTHFLQ REQNGYRPNP NPVGQGIRPP
       410        420        430        440        450        460        470        480
MAGCNSSVGG MSMSPNQGLQ MPSSRAYGLA DPSTTGQMSG ARYGGSSNIA SLTPGPGMQS PSSYQNNNYG LNMSSPPHGS
       490        500        510        520        530        540        550        560
PGLAPNQQNI MISPRNRGSP KIASHQFSPV AGVHSPMASS GNTGNHSFSS SSLSALQAIS EGVGTSLLST LSSPGPKLDN
       570        580        590        600        610        620        630        640
SPNMNITQPS KVSNQDSKSP LGFYCDQNPV ESSMCQSNSR DHLSDKESKE SSVEGAENQR GPLESKGHKK LLQLLTCSSD
       650        660        670        680        690        700        710        720
DRGHSSLTNS PLDSSCKESS VSVTSPSGVS SSTSGGVSST SNMHGSLLQE KHRILHKLLQ NGNSPAEVAK ITAEATGKDT
       730        740        750        760        770        780        790        800
SSITSCGDGN VVKQEQLSPK KKENNALLRY LLDRDDPSDA LSKELQPQVE GVDNKMSQCT SSTIPSSSQE KDPKIKTETS
       810        820        830        840        850        860        870        880
EEGSGDLDNL DAILGDLTSS DFYNNSISSN GSHLGTKQQV FQGTNSLGLK SSQSVQSIRP PYNRAVSLDS PVSVGSSPPV
       890        900        910        920        930        940        950        960
KNISAFPMLP KQPMLGGNPR MMDSQENYGS SMGDWGLPNS KAGRMEPMNS NSMGRPGGDY NTSLPRPALG GSIPTLPLRS
       970        980        990       1000       1010       1020       1030       1040
NSIPGARPVL QQQQQMLQMR PGEIPMGMGA NPYGQAAASN QLGSWPDGML SMEQVSHGTQ NRPLLRNSLD DLVGPPSNLE
      1050       1060       1070       1080       1090       1100       1110       1120
GQSDERALLD QLHTLLSNTD ATGLEEIDRA LGIPELVNQG QALEPKQDAF QGQEAAVMMD QKAGLYGQTY PAQGPPMQGG
      1130       1140       1150       1160       1170       1180       1190       1200
FHLQGQSPSF NSMMNQMNQQ GNFPLQGMHP RANIMRPRTN TPKQLRMQLQ QRLQGQQFLN QSRQALELKM ENPTAGGAAV
      1210       1220       1230       1240       1250       1260       1270       1280
MRPMMQPQQG FLNAQMVAQR SRELLSHHFR QQRVAMMMQQ QQQQQQQQQQ QQQQQQQQQQ QQQQQQQTQA FSPPPNVTAS
      1290       1300       1310       1320       1330       1340       1350       1360
PSMDGLLAGP TMPQAPPQQF PYQPNYGMGQ QPDPAFGRVS SPPNAMMSSR MGPSQNPMMQ HPQAASIYQS SEMKGWPSGN
      1370       1380       1390       1400       1410       1420
LARNSSFSQQ QFAHQGNPAV YSMVHMNGSS GHMGQMNMNP MPMSGMPMGP DQKYC
```

Cmpd.	No. of Cmpds.	m/z meas.	Δ m/z [ppm]	z	Rt [min]	Score	P	Range	Sequence	Modification	Ratios
544	1	630.6481	4.75	3	35.4	14.2	2	18-35	K.RKLPCDTPGQGLTCSGEK.R		mdown:qdown 0.74<br>Wdown:Qdown 0.60
286	1	881.1736	82.04	3	32.7	11.0	2	199-220	R.<br>MLMKTPHDILEDINASPEMRQR.<br>Y	Oxidation: 19	
787	1	1115.0348	46.39	2	38.7	10.9	1	234-252	R.AMMEEGEDLQSCMICVARR.I	Carbamidomethyl: 15	

Detailed Protein Report

Protein 285: PREDICTED: uncharacterized protein LOC102723569 isoform X1 [Homo sapiens]

**Accession:**	gi|578835246	**Score:**	36.1
**Database:**	refseq_human(refseq_human_20140103.fasta)	**MW [kDa]:**	28.7
**Database Date:**	2015-11-30	**pI:**	10.2
**Modification(s):**	Carbamidomethyl	**Sequence Coverage [%]:**	15.3
		**No. of unique Peptides:**	1

Quantitation

mdown:qdown	**Median:**	0.37	**CV:**	0.00 %	**No. of Peptides:**	1
Wdown:Qdown	**Median:**	1.53	**CV:**	0.00 %	**No. of Peptides:**	1

10	20	30	40	50	60	70	80
MCMASNAVCS	APETLAGYRI	IKMLPGSLKR	EDTRKQVKII	LLLFCIQQAS	YYGTAVMGQA	YLPELGTQWG	PPWLSLTGVT
**90**	**100**	**110**	**120**	**130**	**140**	**150**	**160**
TGCRQTPVLS	ILLARPAESS	ESRVGYWMPR	REEGAREVSP	KVLQGPQQAW	TEEVWTAWPC	PEPLCIITAV	PNLHPTLSLP
**170**	**180**	**190**	**200**	**210**	**220**	**230**	**240**
IPGPGALLLC	SRDSWSSLRQ	HTVLLWRRPS	PTHAGMLQPV	PEQVVAGGCW	QRRDNGPAPG	GKCWLQGTES	SPCHSPCYPL
**250**	**260**	**270**					
GNLKGISKNS	GSATLLSFLW	NM					

Cmpd.	No. of Cmpds.	m/z meas.	Δ m/z [ppm]	z	Rt [min]	Score	P	Range	Sequence	Modification	Ratios
169	1	826.3632	-11.95	3	30.9	10.2	0	223-244	K. CWLQGTESSPCHSPCYPLGNLK. G	Carbamidomethyl: 11	mdown:qdown 0.37<br>Wdown:Qdown 1.53

Detailed Protein Report

Protein 286: PREDICTED: collagen alpha-4(IV) chain isoform X9 [Homo sapiens]

**Accession:**	gi|578803629	**Score:**	36.0
**Database:**	refseq_human(refseq_human_20140103.fasta)	**MW [kDa]:**	135.0
**Database Date:**	2015-11-30	**pI:**	10.1
**Modification(s):**	Oxidation	**Sequence Coverage [%]:**	3.8
		**No. of unique Peptides:**	2

Quantitation

mdown:qdown	**Median:**	7.48	**CV:**	0.00 %	**No. of Peptides:**	1
Wdown:Qdown	**Median:**	1.74	**CV:**	0.00 %	**No. of Peptides:**	1

10	20	30	40	50	60	70	80
MWSLHIVLMR	CSFRLTKSLA	TGPWSLILIL	FSVQYVYGSG	KKYIGPCGGR	DCSVCHCVPE	KGSRGPPGPP	GPQGPIGPLG
90	100	110	120	130	140	150	160
APGPIGLSGE	KGMRGDRGPP	GAAGDKGDKG	PTGVPGFPGL	DGIPGHPGPP	GPRGKPGMSG	HNGSRGDPGF	PGGRGALGPG
170	180	190	200	210	220	230	240
GPLGHPGEKG	EKGNSVFILG	AVKGIQGDRG	DPGLPGLPGS	WGAGGPAGPT	GYPGEPGLVG	PPGQPGRPGL	KGNPGVGVKG
250	260	270	280	290	300	310	320
QMGDPGEVGQ	QGSPGPTLLV	EPPDFCLYKG	EKGIKGIPGM	VGLPGPPGRK	GESGIGAKGE	KGIPGFPGPR	GDPGSYGSPG
330	340	350	360	370	380	390	400
FPGLKGELGL	VGDPGLFGLI	GPKGDPGNRG	HPGPPGVLVT	PPLPLKGPPG	DPGFPGRYGE	TGDVGPPGPP	GLLGRPGEAC
410	420	430	440	450	460	470	480
AGMIGPPGPQ	GFPGLPGLPG	EAGIPGRPDS	APGKPGKPGS	PGLPGAPGLQ	GLPGSSVIYC	SVGNPGPQGI	KGKVGPPGGR
490	500	510	520	530	540	550	560
GPKGEKGNEG	LCACEPGPMG	PPGPPGLPGR	QGSKGDLGLP	GWLGTKGDPG	PPGAEGPPGL	PGKHGASGPP	GNKGAKGDMV
570	580	590	600	610	620	630	640
VSRVKGHKGE	RGPDGPPGFP	GQPGSHGRDG	HAGEKGDPGP	PGDHEDATPG	GKGFPGPLGP	PGKAGPVGPP	GLGFPGPPGE
650	660	670	680	690	700	710	720
RGHPGVPGHP	GVRGPDGLKG	QKGDTISCNV	TYPGRHGPPG	FDGPPGPKGF	PGPQGAPGLS	GSDGHKGRPG	TPGTAEIPGP
730	740	750	760	770	780	790	800
PGFRGDMGDP	GFGGEKGSSP	VGPPGPPGSP	GVNGQKGIPG	DPAFGHLGPP	GKRGLSGVPG	IKGPRGDPGC	PGAEGPAGIP
810	820	830	840	850	860	870	880
GFLGLKGPKG	REGHAGFPGV	PGPPGHSCER	GAPGIPGQPG	LPGYPGSPGA	PGGKGQPGDV	GPPGPAGMKG	LPGLPGRPGA
890	900	910	920	930	940	950	960
HGPPGLPGIP	GPFGDDGLPG	PPGPKGPRGL	PGFPGFPGER	GKPGAEGCPG	AKGEPGEKGM	SGLPGDRGLR	GAKGAIGPPG
970	980	990	1000	1010	1020	1030	1040
DEGEMAIISQ	KGTPGEPGPP	GDDGFPGERG	DKGTPGMQGR	RGEPGRYGPP	GFHRGEPGEK	GQPGPPGPPG	PPGSTGLRGF
1050	1060	1070	1080	1090	1100	1110	1120
IGFPGLPGDQ	GEPGSPGPPG	FSGIDGARGP	KGNKGDPASH	FGPPGPKGEP	GSPGCPGHFG	ASGEQGLPGI	QGPRGSPGRP
1130	1140	1150	1160	1170	1180	1190	1200
GPPGSSGPPG	CPGDHGMPGL	RGQPGEMGDP	GPRGLQGDPG	IPGPPGIKGP	SGSPGLNGLH	GLKGQKGTKG	ASGLHDVGPP
1210	1220	1230	1240	1250	1260	1270	1280
GPVGIPGLKG	ERGDPGSPGI	SPPGPRGKKG	PPGPPGSSGP	PGPAGATGRA	PKDIPDPGPP	GDQGPPGPDG	PRGAPGPPGL
1290	1300	1310	1320	1330	1340	1350	1360
PGSVDLLRGE	PGDCGLPGPP	GPPGPPGPPG	YKGFPGCDGK	DGQKGPVGFP	GPQGPHGFPG	PPGEKGLPGP	PGRKGPTGLP
1370	1380	1390	1400	1410	1420	1430	
GPRGEPGPPA	DVDDCPRIPG	LPGAPGMRGP	EGAMGLPGMR	GPSGPGGTRG	RQTALRKTCN		

Cmpd.	No. of Cmpds.	m/z meas.	Δ m/z [ppm]	z	Rt [min]	Score	P	Range	Sequence	Modification	Ratios
464	3	607.1279	-228.90	2	34.7	14.4	0	1142-1153	R.GQPGEMGDPGPR.G	Oxidation: 6	mdown:qdown 7.48<br>Wdown:Qdown 1.74
950	1	984.1390	132.77	2	41.4	10.5	1	1230-1252	K. GPPGPPGSSGPPGPAGATGRAP[illegible] D		

Detailed Protein Report

Protein 287: stromal interaction molecule 2 isoform 2 precursor [Homo sapiens]

**Accession:**	gi|281182822	**Score:**	36.0
**Database:**	refseq_human(refseq_human_20140103.fasta)	**MW [kDa]:**	83.9
**Database Date:**	2015-11-30	**pI:**	6.3
		**Sequence Coverage [%]:**	6.0
		**No. of unique Peptides:**	1

Quantitation

Wdown:Qdown **Median:** 1.24 **CV:** 0.00 % **No. of Peptides:** 1

10	20	30	40	50	60	70	80
MLVLGLLVAG	AADGCELVPR	HLRGRRATGS	AATAASSPAA	AAGDSPALMT	DPCMSLSPPC	FTEEDRFSLE	ALQTIHKQMD
**90**	**100**	**110**	**120**	**130**	**140**	**150**	**160**
DDKDGGIEVE	ESDEFIREDM	KYKDATNKHS	HLHREDKHIT	IEDLWKRWKT	SEVHNWTLED	TLQWLIEFVE	LPQYEKNFRD
**170**	**180**	**190**	**200**	**210**	**220**	**230**	**240**
NNVKGTTLPR	IAVHEPSFMI	SQLKISDRSH	RQKLQLKALD	VVLFGPLTRP	PHNWMKDFIL	TVSIVIGVGG	CWFAYTQNKT
**250**	**260**	**270**	**280**	**290**	**300**	**310**	**320**
SKEHVAKMMK	DLESLQTAEQ	SLMDLQERLE	KAQEENRNVA	VEKQNLERKM	MDEINYAKEE	ACRLRELREG	AECELSRRQY
**330**	**340**	**350**	**360**	**370**	**380**	**390**	**400**
AEQELEQVRM	ALKKAEKEFE	LRSSWSVPDA	LQKWLQLTHE	VEVQYYNIKR	QNAEMQLAIA	KDEAEKIKKK	RSTVFGTLHV
**410**	**420**	**430**	**440**	**450**	**460**	**470**	**480**
AHSSSLDEVD	HKILEAKKAL	SELTTCLRER	LFRWQQIEKI	CGFQIAHNSG	LPSLTSSLYS	DHSWVVMPRV	SIPPYPIAGG
**490**	**500**	**510**	**520**	**530**	**540**	**550**	**560**
VDDLDEDTPP	IVSQFPGTMA	KPPGSLARSS	SLCRSRRSIV	PSSPQPQRAQ	LAPHAPHPSH	PRHPHHPQHT	PHSLPSPDPD
**570**	**580**	**590**	**600**	**610**	**620**	**630**	**640**
ILSVSSCPAL	YRNEEEEEAI	YFSAEKQWEV	PDTASECDSL	NSSIGRKQSP	PLSLEIYQTL	SPRKISRDEV	SLEDSSRGDS
**650**	**660**	**670**	**680**	**690**	**700**	**710**	**720**
PVTVDVSWGS	PDCVGLTETK	SMIFSPASKV	YNGILEKSCS	MNQLSSGIPV	PKPRHTSCSS	AGNDSKPVQE	APSVARISSI
**730**	**740**	**750**					
PHDLCHNGEK	SKKPSKIKSL	FKKKSK					

Cmpd.	No. of Cmpds.	m/z meas.	Δ m/z [ppm]	z	Rt [min]	Score	P	Range	Sequence	Modification	Ratios
1585	1	942.4058	-100.34	2	49.7	13.0	1	661-677	K.SMIFSPASKVYNGILEK.S		Wdown:Qdown 1.24

Detailed Protein Report

Protein 288: exportin-5 [Homo sapiens]

**Accession:**	gi|22748937	**Score:**	36.0
**Database:**	refseq_human(refseq_human_20140103.fasta)	**MW [kDa]:**	136.2
**Database Date:**	2015-11-30	**pI:**	5.5
**Modification(s):**	Oxidation	**Sequence Coverage [%]:**	2.7
		**No. of unique Peptides:**	2

10	20	30	40	50	60	70	80
MAMDQVNALC	EQLVKAVTVM	MDPNSTQRYR	LEALKFCEEF	KEKCPICVPC	GLRLAEKTQV	AIVRHFGLQI	LEHVVKFRWN
90	100	110	120	130	140	150	160
GMSRLEKVYL	KNSVMELIAN	GTLNILEEEN	HIKDALSRIV	VEMIKREWPQ	HWPDMLIELD	TLSKQGETQT	ELVMFILLRL
170	180	190	200	210	220	230	240
AEDVVTFQTL	PPQRRRDIQQ	TLTQNMERIF	SFLLNTLQEN	VNKYQQVKTD	TSQESKAQAN	CRVGVAALNT	LAGYIDWVSM
250	260	270	280	290	300	310	320
SHITAENCKL	LEILCLLLNE	QELQLGAAEC	LLIAVSRKGK	LEDRKPLMVL	FGDVAMHYIL	SAAQTADGGG	LVEKHYVFLK
330	340	350	360	370	380	390	400
RLCQVLCALG	NQLCALLGAD	SDVETPSNFG	KYLESFLAFT	THPSQFLRSS	TQMTWGALFR	HEILSRDPLL	LAIIPKYLRA
410	420	430	440	450	460	470	480
SMTNLVKMGF	PSKTDSPSCE	YSRFDFDSDE	DFNAFFNSSR	AQQGEVMRLA	CRLDPKTSFQ	MAGEWLKYQL	STFLDAGSVN
490	500	510	520	530	540	550	560
SCSAVGTGEG	SLCSVFSPSF	VQWEAMTLFL	ESVITQMFRT	LNREEIPVND	GIELLQMVLN	FDTKDPLILS	CVLTNVSALF
570	580	590	600	610	620	630	640
PFVTYRPEFL	PQVFSKLFSS	VTFETVEESK	APRTRAVRNV	RRHACSSIIK	MCRDYPQLVL	PNFDMLYNHV	KQLLSNELLL
650	660	670	680	690	700	710	720
TQMEKCALME	ALVLISNQFK	NYERQKVFLE	ELMAPVASIW	LSQDMHRVLS	DVDAFIAYVG	TDQKSCDPGL	EDPCGLNRAR
730	740	750	760	770	780	790	800
MSFCVYSILG	VVKRTCWPTD	LEEAKAGGFV	VGYTSSGNPI	FRNPCTEQIL	KLLDNLLALI	RTHNTLYAPE	MLAKMAEPFT
810	820	830	840	850	860	870	880
KALDMLDAEK	SAILGLPQPL	LELNDSPVFK	TVLERMQRFF	STLYENCFHI	LGKAGPSMQQ	DFYTVEDLAT	QLLSSAFVNL
890	900	910	920	930	940	950	960
NNIPDYRLRP	MLRVFVKPLV	LFCPPEHYEA	LVSPILGPLF	TYLHMRLSQK	WQVINQRSLL	CGEDEAADEN	PESQEMLEEQ
970	980	990	1000	1010	1020	1030	1040
LVRMLTREVM	DLITVCCVSK	KGADHSSAPP	ADGDDEEMMA	TEVTPSAMAE	LTDLGKCLMK	HEDVCTALLI	TAFNSLAWKD
1050	1060	1070	1080	1090	1100	1110	1120
TLSCQRTTSQ	LCWPLLKQVL	SGTLLADAVT	WLFTSVLKGL	QMHGQHDGCM	ASLVHLAFQI	YEALRPRYLE	IRAVMEQIPE
1130	1140	1150	1160	1170	1180	1190	1200
IQKDSLDQFD	CKLLNPSLQK	VADKRRKDQF	KRLIAGCIGK	PLGEQFRKEV	HIKNLPSLFK	KTKPMLETEV	LDNDGGGLAT
1210							
IFEP							

Cmpd.	No. of Cmpds.	m/z meas.	Δ m/z [ppm]	z	Rt [min]	Score	P	Range	Sequence	Modification
629	1	459.6075	-259.30	2	37.2	12.3	0	441-448	R.AQQGEVMR.L	
1960	1	731.2880	-122.56	2	54.6	12.4	0	721-733	R.MSFCVYSILGVVK.R	Oxidation: 1

Detailed Protein Report

Protein 289: **PREDICTED: Fanconi anemia-associated protein of 100 kDa isoform X2 [Homo sapiens]**

**Accession:**	gi|578831470	**Score:**	35.9
**Database:**	refseq_human(refseq_human_20140103.fasta)	**MW [kDa]:**	77.0
**Database Date:**	2015-11-30	**pI:**	5.0
		**Sequence Coverage [%]:**	3.3
		**No. of unique Peptides:**	1

10	20	30	40	50	60	70	80
MQLFEQPCPG	EDPRPGGQIG	EVELSSYTPP	AGVPGKPAAP	HFLPVLCSVS	PSGSRVPHDL	LGGSGGFTLE	DALFGLLFGA
90	100	110	120	130	140	150	160
DATLLQSPVV	LCGLPDGQLC	CVILKALVTS	RSAPGDPNAL	VKILHHLEEP	VIFIGALKTE	PQAAEAAENF	LPDEDVHCDC
170	180	190	200	210	220	230	240
LVAFGHHGRM	LAIKASWDES	GKLVPELREY	CLPGPVLCAA	CGGGGRVYHS	TPSDLCVVDL	SRGSTPLGPE	QPEEGPGGLP
250	260	270	280	290	300	310	320
PMLCPASLNI	CSVVSLSASP	RTHEGGTKLL	ALSAKGRLMT	CSLDLDSEMP	GPARMTTESA	GQKIKELLSG	IGNISERVSF
330	340	350	360	370	380	390	400
LKKAVDQRNK	ALTSLNEAMN	VSCALLSSGT	GPRPISCTTS	TTWSRLQTQD	VLMATCVLEN	SSSFSLDQGW	TLCIQVLTSS
410	420	430	440	450	460	470	480
CALDLDSACS	AITYTIPVDQ	LGPGARREVT	LPLGPGENGG	LDLPVTVSCT	LFYSLREVVG	GALAPSDSED	PFLDECPSDV
490	500	510	520	530	540	550	560
LPEQEGVCLP	LSRHTVDMLQ	CLRFPGLAPP	HTRAPSPLGP	TRDPVATFLE	TCREPGSQPA	GPASLRAEYL	PPSVASIKVS
570	580	590	600	610	620	630	640
AELLRAALKD	GHSGVPLCCA	TLQWLLAENA	AVDVVRARAL	SSIQGVAPDG	ANVHLIVREV	AMTDLCPAGP	IQAVEIQVES
650	660	670	680	690	700	710	720
SSLADICRAH	HAVVGRMQTM	VTEQATQGSS	APDLRVQYLR	QIHANHETLL	REVQTLRDRL	CTEDEASSCA	TAQRLLQVYR
730	740						
QLRHPSLILL							

Cmpd.	No. of Cmpds.	m/z meas.	Δ m/z [ppm]	z	Rt [min]	Score	P	Range	Sequence	Modification
1236	1	765.0123	96.72	2	45.1	17.1	1	304-317	K.IKELLSGIGNISER.V	

Detailed Protein Report

Protein 290: **protein-tyrosine kinase 2-beta isoform b [Homo sapiens]**

**Accession:**	gi|27886588	**Score:**	35.9
**Database:**	refseq_human(refseq_human_20140103.fasta)	**MW [kDa]:**	111.1
**Database Date:**	2015-11-30	**pI:**	5.6
**Modification(s):**	Oxidation	**Sequence Coverage [%]:**	3.1
		**No. of unique Peptides:**	1

10	20	30	40	50	60	70	80
MSGVSEPLSR	VKLGTLRRPE	GPAEPMVVVP	VDVEKEDVRI	LKVCFYSNSF	NPGKNFKLVK	CTVQTEIREI	ITSILLSGRI
90	100	110	120	130	140	150	160
GPNIRLAECY	GLRLKHMKSD	EIHWLHPQMT	VGEVQDKYEC	LHVEAEWRYD	LQIRYLPEDF	MESLKEDRTT	LLYFYQQLRN
170	180	190	200	210	220	230	240
DYMQRYASKV	SEGMALQLGC	LELRRFFKDM	PHNALDKKSN	FELLEKEVGL	DLFFPKQMQE	NLKPKQFRKM	IQQTFQQYAS
250	260	270	280	290	300	310	320
LREEECVMKF	FNTLAGFANI	DQETYRCELI	QGWNITVDLV	IGPKGIRQLT	SQDAKPTCLA	EFKQIRSIRC	LPLEEGQAVL
330	340	350	360	370	380	390	400
QLGIEGAPQA	LSIKTSSLAE	AENMADLIDG	YCRLQGEHQG	SLIIHPRKDG	EKRNSLPQIP	MLNLEARRSH	LSESCSIESD
410	420	430	440	450	460	470	480
IYAEIPDETL	RRPGGPQYGI	AREDVVLNRI	LGEGFFGEVY	EGVYTNHKGE	KINVAVKTCK	KDCTLDNKEK	FMSEAVIMKN
490	500	510	520	530	540	550	560
LDHPHIVKLI	GIIEEEPTWI	IMELYPYGEL	GHYLERNKNS	LKVLTLVLYS	LQICKAMAYL	ESINCVHRDI	AVRNILVASP
570	580	590	600	610	620	630	640
ECVKLGDFGL	SRYIEDEDYY	KASVTRLPIK	WMSPESINFR	RFTTASDVWM	FAVCMWEILS	FGKQPFFWLE	NKDVIGVLEK
650	660	670	680	690	700	710	720
GDRLPKPDLC	PPVLYTLMTR	CWDYDPSDRP	RFTELVCSLS	DVYQMEKDIA	MEQERNARYR	TPKILEPTAF	QEPPPKPSRP
730	740	750	760	770	780	790	800
KYRPPPQTNL	LAPKLQFQEE	DFIQPSSREE	AQQLWEAEKV	KMRQILDKQQ	KQMVEDYQWL	RQEEKSLDPM	VYMNDKSPLT
810	820	830	840	850	860	870	880
PEKEVGYLEF	TGPPQKPPRL	GAQSIQPTAN	LDRTDDLVYL	NVMELVRAVL	ELKNELCQLP	PEGYVVVVKN	VGLTLRKLIG
890	900	910	920	930	940	950	960
SVDDLLPSLP	SSSRTEIEGT	QKLLNKDLAE	LINKMRLAQQ	NAVTSLSEEC	KRQMLTASHT	LAVDAKNLLD	AVDQAKVLAN
970							
LAHPPAE							

Cmpd.	No. of Cmpds.	m/z meas.	Δ m/z [ppm]	z	Rt [min]	Score	P	Range	Sequence	Modification
458	1	544.1530	-199.80	2	34.7	10.3	0	471-479	K.FMSEAVIMK.N	Oxidation: 2, 8

Detailed Protein Report

Protein 291: prolow-density lipoprotein receptor-related protein 1 precursor [Homo sapiens]

**Accession:**	gi|126012562	**Score:**	35.9
**Database:**	refseq_human(refseq_human_20140103.fasta)	**MW [kDa]:**	504.3
**Database Date:**	2015-11-30	**pI:**	5.0
**Modification(s):**	Carbamidomethyl, Oxidation	**Sequence Coverage [%]:**	1.1
		**No. of unique Peptides:**	1

Detailed Protein Report

10	20	30	40	50	60	70	80
MLTPPLLLLL	PLLSALVAAA	IDAPKTCSPK	QFACRDQITC	ISKGWRCDGE	RDCPDGSDEA	PEICPQSKAQ	RCQPNEHNCL
90	100	110	120	130	140	150	160
GTELCVPMSR	LCNGVQDCMD	GSDEGPHCRE	LQGNCSRLGC	QHHCVPTLDG	PTCYCNSSFQ	LQADGKTCKD	FDECSVYGTC
170	180	190	200	210	220	230	240
SQLCTNTDGS	FICGCVEGYL	LQPDNRSCKA	KNEPVDRPPV	LLIANSQNIL	ATYLSGAQVS	TITPTSTRQT	TAMDFSYANE
250	260	270	280	290	300	310	320
TVCWVHVGDS	AAQTQLKCAR	MPGLKGFVDE	HTINISLSLH	HVEQMAIDWL	TGNFYFVDDI	DDRIFVCNRN	GDTCVTLLDL
330	340	350	360	370	380	390	400
ELYNPKGIAL	DPAMGKVFFT	DYGQIPKVER	CDMDGQNRTK	LVDSKIVFPH	GITLDLVSRL	VYWADAYLDY	IEVVDYEGKG
410	420	430	440	450	460	470	480
RQTIIQGILI	EHLYGLTVFE	NYLYATNSDN	ANAQQKTSVI	RVNRFNSTEY	QVVTRVDKGG	ALHIYHQRRQ	PRVRSHACEN
490	500	510	520	530	540	550	560
DQYGKPGGCS	DICLLANSHK	ARTCRCRSGF	SLGSDGKSCK	KPEHELFLVY	GKGRPGIIRG	MDMGAKVPDE	HMIPIENLMN
570	580	590	600	610	620	630	640
PRALDFHAET	GFIYFADTTS	YLIGRQKIDG	TERETILKDG	IHNVEGVAVD	WMGDNLYWTD	DGPKKTISVA	RLEKAAQTRK
650	660	670	680	690	700	710	720
TLIEGKMTHP	RAIVVDPLNG	WMYWTDWEED	PKDSRRGRLE	RAWMDGSHRD	IFVTSKTVLW	PNGLSLDIPA	GRLYWVDAFY
730	740	750	760	770	780	790	800
DRIETILLNG	TDRKIVYEGP	ELNHAFGLCH	HGNYLFWTEY	RSGSVYRLER	GVGGAPPTVT	LLRSERPPIF	EIRMYDAQQQ
810	820	830	840	850	860	870	880
QVGTNKCRVN	NGGCSSLCLA	TPGSRQCACA	EDQVLDADGV	TCLANPSYVP	PPQCQPGEFA	CANSRCIQER	WKCDGDNDCL
890	900	910	920	930	940	950	960
DNSDEAPALC	HQHTCPSDRF	KCENNRCIPN	RWLCDGDNDC	GNSEDESNAT	CSARTCPPNQ	FSCASGRCIP	ISWTCDLDDD
970	980	990	1000	1010	1020	1030	1040
CGDRSDESAS	CAYPTCFPLT	QFTCNNGRCI	NINWRCDNDN	DCGDNSDEAG	CSHSCSSTQF	KCNSGRCIPE	HWTCDGDNDC
1050	1060	1070	1080	1090	1100	1110	1120
GDYSDETHAN	CTNQATRPPG	GCHTDEFQCR	LDGLCIPLRW	RCDGDTDCMD	SSDEKSCEGV	THVCDPSVKF	GCKDSARCIS
1130	1140	1150	1160	1170	1180	1190	1200
KAWVCDGDND	CEDNSDEENC	ESLACRPPSH	PCANNTSVCL	PPDKLCDGND	DCGDGSDEGE	LCDQCSLNNG	GCSHNCSVAP
1210	1220	1230	1240	1250	1260	1270	1280
GEGIVCSCPL	GMELGPDNHT	CQIQSYCAKH	LKCSQKCDQN	KFSVKCSCYE	GWVLEPDGES	CRSLDPFKPF	IIFSNRHEIR
1290	1300	1310	1320	1330	1340	1350	1360
RIDLHKGDYS	VLVPGLRNTI	ALDFHLSQSA	LYWTDVVEDK	IYRGKLLDNG	ALTSFEVVIQ	YGLATPEGLA	VDWIAGNIYW
1370	1380	1390	1400	1410	1420	1430	1440
VESNLDQIEV	AKLDGTLRTT	LLAGDIEHPR	AIALDPRDGI	LFWTDWDASL	PRIEAASMSG	AGRRTVHRET	GSGGWPNGLT
1450	1460	1470	1480	1490	1500	1510	1520
VDYLEKRILW	IDARSDAIYS	ARYDGSGHME	VLRGHEFLSH	PFAVTLYGGE	VYWTDWRTNT	LAKANKWTGH	NVTVVQRTNT
1530	1540	1550	1560	1570	1580	1590	1600
QPFDLQVYHP	SRQPMAPNPC	EANGGQGPCS	HLCLINYNRT	VSCACPHLMK	LHKDNTTCYE	FKKFLLYARQ	MEIRGVDLDA
1610	1620	1630	1640	1650	1660	1670	1680
PYYNYIISFT	VPDIDNVTVL	DYDAREQRVY	WSDVRTQAIK	RAFINGTGVE	TVVSADLPNA	HGLAVDWVSR	NLFWTSYDTN
1690	1700	1710	1720	1730	1740	1750	1760
KKQINVARLD	GSFKNAVVQG	LEQPHGLVVH	PLRGKLYWTD	GDNISMANMD	GSNRTLLFSG	QKGPVGLAID	FPESKLYWIS
1770	1780	1790	1800	1810	1820	1830	1840
SGNHTINRCN	LDGSGLEVID	AMRSQLGKAT	ALAIMGDKLW	WADQVSEKMG	TCSKADGSGS	VVLRNSTTLV	MHMKVYDESI
1850	1860	1870	1880	1890	1900	1910	1920
QLDHKGTNPC	SVNNGDCSQL	CLPTSETTRS	CMCTAGYSLR	SGQQACEGVG	SFLLYSVHEG	IRGIPLDPND	KSDALVPVSG
1930	1940	1950	1960	1970	1980	1990	2000
TSLAVGIDFH	AENDTIYWVD	MGLSTISRAK	RDQTWREDVV	TNGIGRVEGI	AVDWIAGNIY	WTDQGFDVIE	VARLNGSFRY
2010	2020	2030	2040	2050	2060	2070	2080
VVISQGLDKP	RAITVHPEKG	YLFWTEWGQY	PRIERSRLDG	TERVVLVNVS	ISWPNGISVD	YQDGKLYWCD	ARTDKIERID
2090	2100	2110	2120	2130	2140	2150	2160
LETGENREVV	LSSNNMDMFS	VSVFEDFIYW	SDRTHANGSI	KRGSKDNATD	SVPLRTGIGV	QLKDIKVFNR	DRQKGTNVCA
2170	2180	2190	2200	2210	2220	2230	2240

Detailed Protein Report

Cmpd.	No. of Cmpds.	m/z meas.	Δ m/z [ppm]	z	Rt [min]	Score	P	Range	Sequence	Modification
127	1	849.3809	-43.54	2	30.9	15.1	1	1560-1573	R.TVSCACPHLMKLHK.D	Carbamidomethyl: 4, 6; Oxidation: 10

Detailed Protein Report

Protein 292: PREDICTED: cortactin-binding protein 2 isoform X1 [Homo sapiens]

**Accession:**	gi|530386508	**Score:**	35.8
**Database:**	refseq_human(refseq_human_20140103.fasta)	**MW [kDa]:**	179.5
**Database Date:**	2015-11-30	**pI:**	9.5
**Modification(s):**	Carbamidomethyl	**Sequence Coverage [%]:**	2.1
		**No. of unique Peptides:**	2

Quantitation

mdown:qdown **Median:** 2.68 **CV:** 102.48 % **No. of Peptides:** 2

10	20	30	40	50	60	70	80
MIWCLHPQKK	KEFDVDTLSK	SELRMLLSVM	EGELEARDLV	IEALRARRKE	VFIQERYGRF	NLNDPFLALQ	RDYEAGAGDK
90	100	110	120	130	140	150	160
EKKPVCTNPL	SILEAVMAHC	KKMQERMSAQ	LAAAESRQKK	LEMEKLQLQA	LEQEHKKLAA	RLEEERGKNK	QVVLMLVKEC
170	180	190	200	210	220	230	240
KQLSGKVIEE	AQKLEDVMAK	LEEEKKKTNE	LEEELSAEKR	RSTEMEAQME	KQLSEFDTER	EQLRAKLNRE	EAHTTDLKEE
250	260	270	280	290	300	310	320
IDKMRKMIEQ	LKRGSDSKPS	LSLPRKTKDR	RLVSISVGTE	GTVTRSVACQ	TDLVTENADH	MKKLPLTMPV	KPSTGSPLVS
330	340	350	360	370	380	390	400
ANAKGSVCTS	ATMARPGIDR	QASYGDLIGA	SVPAFPPPSA	NKIEENGPST	GSTPDPTSST	PPLPSNAAPP	TAQTPGIAPQ
410	420	430	440	450	460	470	480
NSQAPPMHSL	HSPCANTSLH	PGLNPRIQAA	RFRFQGNAND	PDQNGNTTQS	PPSRDVSPTS	RDNLVAKQLA	RNTVTQALSR
490	500	510	520	530	540	550	560
FTSPQAGAPS	RPGVPPTGDV	GTHPPVGRTS	LKTHGVARVD	RGNPPPIPPK	KPGLSQTPSP	PHPQLKVIID	SSRASNTGAK
570	580	590	600	610	620	630	640
VDNKTVASTP	SSLPQGNRVI	NEENLPKSSS	PQLPPKPSID	LTVAPAGCAV	SALATSQVGA	WPAATPGLNQ	PACSDSSLVI
650	660	670	680	690	700	710	720
PTTIAFCSSI	NPVSASSCRP	GASDSLLVTA	SGWSPSLTPL	LMSGGPAPLA	GRPTLLQQAA	AQGNVTLLSM	LLNEEGLDIN
730	740	750	760	770	780	790	800
YSCEDGHSAL	YSAAKNGHTD	CVRLLLSAEA	QVNAADKNGF	TPLCAAAAQG	HFECVELLIS	YDANINHAAD	GGQTPLYLAC
810	820	830	840	850	860	870	880
KNGNKECIKL	LLEAGTNRSV	KTTDGWTPVH	AAVDTGNVDS	LKLLMYHRIP	AHGNSFNEEE	SESSVFDLDG	GEESPEGISK
890	900	910	920	930	940	950	960
PVVPADLINH	ANREGWTAAH	IAASKGFKNC	LEILCRHGGL	EPERRDKCNR	TVHDVATDDC	KHLLENLNAL	KIPLRISVGE
970	980	990	1000	1010	1020	1030	1040
IEPSNYGSDD	LECENTICAL	NIRKQTSWDD	FSKAVSQALT	NHFQAISSDG	WWSLEDVTCN	NTTDSNIGLS	ARSIRSITLG
1050	1060	1070	1080	1090	1100	1110	1120
NVPWSVGQSF	AQSPWDFMRK	NKAEHITVLL	SGPQEGCLSS	VTYASMIPLQ	MMQNYLRLVE	QYHNVIFHGP	EGSLQDYIVH
1130	1140	1150	1160	1170	1180	1190	1200
QLALCLKHRQ	MAAGFSCEIV	RAEVDAGFSK	EQLLDLFISS	ACLIPVKQSP	SKKKIIIILE	NLEKSSLSEL	LRDFLAPLEN
1210	1220	1230	1240	1250	1260	1270	1280
RSTESPCTFQ	KGNGLSECYY	FHENCFLMGT	IAKACLQGSD	LLVQQHFRWV	QLRWDGEPMQ	GLLQRFLRRK	VVNKFKGQAP
1290	1300	1310	1320	1330	1340	1350	1360
SPCDPVCKIV	DWALSVWRQL	NSCLARLGTP	EALLGPKYFL	SCPVVPGHAQ	VTVKWMSKLW	NGVIAPRVQE	AILSRASVKR
1370	1380	1390	1400	1410	1420	1430	1440
QPGFGQTTAK	RHPSQGQQAV	VKAALSILLN	KAVLHGCPLP	RAELDQHTAD	FKGGSFPLSI	VSSYNTCNKK	KGESGAWRKV
1450	1460	1470	1480	1490	1500	1510	1520
NTSPRRKSGR	FSLPTWNKPD	LSTEGMKNKT	ISQLNCNRNA	SLSKQKSLEN	DLSLTLNLDQ	RLSLGSDDEA	DLVKELQSMC
1530	1540	1550	1560	1570	1580	1590	1600
SSKSESDISK	IADSRDDLRM	FDSSGNNPVL	SATINNLRMP	VSQKEVSPLS	SHQTTECSNS	KSKTELGVSR	VKSFLPVPRS
1610	1620	1630	1640	1650			
KVTQCSQNTK	RSSSSSNTRQ	IEINNNSKEV	NWNLHKNEHL	EKPNK			

Cmpd.	No. of Cmpds.	m/z meas.	Δ m/z [ppm]	z	Rt [min]	Score	P	Range	Sequence	Modification	Ratios
493	1	464.6529	-266.41	2	34.8	10.9	0	38-45	R.DLVIEALR.A		mdown:qdown 6.24

Detailed Protein Report

Cmpd.	No. of Cmpds.	m/z meas.	Δ m/z [ppm]	z	Rt [min]	Score	P	Range	Sequence	Modification	Ratios
2703	3	973.4604	-78.66	2	65.0	13.9	0	1151-1167	K.EQLLDLFISSACLIPVK.Q	Carbamidomethyl: 12	mdown:qdown 1.15

Detailed Protein Report

Protein 293: sodium channel protein type 7 subunit alpha [Homo sapiens]

**Accession:**	gi|111378393	**Score:**	35.8
**Database:**	refseq_human(refseq_human_20140103.fasta)	**MW [kDa]:**	193.4
**Database Date:**	2015-11-30	**pI:**	9.1
		**Sequence Coverage [%]:**	1.4
		**No. of unique Peptides:**	1

Quantitation

Wdown:Qdown	**Median:** 4.16	**CV:** 0.00 %	**No. of Peptides:** 1

Alias proteins:

Accession	Name	Description
gi|578804726	refseq_human (refseq_human_20140103.fasta)	PREDICTED: sodium channel protein type 7 subunit alpha isoform X3 [Homo sapiens]
gi|578804724	refseq_human (refseq_human_20140103.fasta)	PREDICTED: sodium channel protein type 7 subunit alpha isoform X2 [Homo sapiens]
gi|578804722	refseq_human (refseq_human_20140103.fasta)	PREDICTED: sodium channel protein type 7 subunit alpha isoform X1 [Homo sapiens]

Detailed Protein Report

10	20	30	40	50	60	70	80
MLASPEPKGL	VPFTKESFEL	IKQHIAKTHN	EDHEEEDLKP	TPDLEVGKKL	PFIYGNLSQG	MVSEPLEDVD	PYYYKKKNTF
90	100	110	120	130	140	150	160
IVLNKNRTIF	RFNAASILCT	LSPFNCIRRT	TIKVLVHPFF	QLFILISVLI	DCVFMSLTNL	PKWRPVLENT	LLGIYTFEIL
170	180	190	200	210	220	230	240
VKLFARGVWA	GSFSFLGDPW	NWLDFSVTVF	EVIIRYSPLD	FIPTLQTART	LRILKIIPLN	QGLKSLVGVL	IHCLKQLIGV
250	260	270	280	290	300	310	320
IILTLFFLSI	FSLIGMGLFM	GNLKHKCFRW	PQENENETLH	NRTGNPYYIR	ETENFYYLEG	ERYALLCGNR	TDAGQCPEGY
330	340	350	360	370	380	390	400
VCVKAGINPD	QGFTNFDSFG	WALFALFRLM	AQDYPEVLYH	QILYASGKVY	MIFFVVVSFL	FSFYMASLFL	GILAMAYEEE
410	420	430	440	450	460	470	480
KQRVGEISKK	IEPKFQQTGK	ELQEGNETDE	AKTIQIEMKK	RSPISTDTSL	DVLEDATLRH	KEELEKSKKI	CPLYWYKFAK
490	500	510	520	530	540	550	560
TFLIWNCSPC	WLKLKEFVHR	IIMAPFTDLF	LIICIILNVC	FLTLEHYPMS	KQTNTLLNIG	NLVFIGIFTA	EMIFKIIAMH
570	580	590	600	610	620	630	640
PYGYFQVGWN	IFDSMIVFHG	LIELCLANVA	GMALLRLFRM	LRIFKLGKYW	PTFQILMWSL	SNSWVALKDL	VLLLFTFIFF
650	660	670	680	690	700	710	720
SAAFGMKLFG	KNYEEFVCHI	DKDCQLPRWH	MHDFFHSFLN	VFRILCGEWV	ETLWDCMEVA	GQSWCIPFYL	MVILIGNLLV
730	740	750	760	770	780	790	800
LYLFLALVSS	FSSCKDVTAE	ENNEAKNLQL	AVARIKKGIN	YVLLKILCKT	QNVPKDTMDH	VNEVYVKEDI	SDHTLSELSN
810	820	830	840	850	860	870	880
TQDFLKDKEK	SSGTEKNATE	NESQSLIPSP	SVSETVPIAS	GESDIENLDN	KEIQSKSGDG	GSKEKIKQSS	SSECSTVDIA
890	900	910	920	930	940	950	960
ISEEEEMFYG	GERSKHLKNG	CRRGSSLGQI	SGASKKGKIW	QNIRKTCCKI	VENNWFKCFI	GLVTLLSTGT	LAFEDIYMDQ
970	980	990	1000	1010	1020	1030	1040
RKTIKILLEY	ADMIFTYIFI	LEMLLKWMAY	GFKAYFSNGW	YRLDFVVVIV	FCLSLIGKTR	EELKPLISMK	FLRPLRVLSQ
1050	1060	1070	1080	1090	1100	1110	1120
FERMKVVVRA	LIKTTLPTLN	VFLVCLMIWL	IFSIMGVDLF	AGRFYECIDP	TSGERFPSSE	VMNKSRCESL	LFNESMLWEN
1130	1140	1150	1160	1170	1180	1190	1200
AKMNFDNVGN	GFLSLLQVAT	FNGWITIMNS	AIDSVAVNIQ	PHFEVNIYMY	CYFINFIIFG	VFLPLSMLIT	VIIDNFNKHK
1210	1220	1230	1240	1250	1260	1270	1280
IKLGGSNIFI	TVKQRKQYRR	LKKLMYEDSQ	RPVPRPLNKL	QGFIFDVVTS	QAFNVIVMVL	ICFQAIAMMI	DTDVQSLQMS
1290	1300	1310	1320	1330	1340	1350	1360
IALYWINSIF	VMLYTMECIL	KLIAFRCFYF	TIAWNIFDFM	VVIFSITGLC	LPMTVGSYLV	PPSLVQLILL	SRIIHMLRLG
1370	1380	1390	1400	1410	1420	1430	1440
KGPKVFHNLM	LPLMLSLPAL	LNIILLIFLV	MFIYAVFGMY	NFAYVKKEAG	INDVSNFETF	GNSMLCLFQV	AIFAGWDGML
1450	1460	1470	1480	1490	1500	1510	1520
DAIFNSKWSD	CDPDKINPGT	QVRGDCGNPS	VGIFYFVSYI	LISWLIIVNM	YIVVVMEFLN	IASKKKNKTL	SEDDFRKFFQ
1530	1540	1550	1560	1570	1580	1590	1600
VWKRFDPDRT	QYIDSSKLSD	FAAALDPPLF	MAKPNKGQLI	ALDLPMAVGD	RIHCLDILLA	FTKRVMGQDV	RMEKVVSEIE
1610	1620	1630	1640	1650	1660	1670	1680
SGFLLANPFK	ITCEPITTTL	KRKQEAVSAT	IIQRAYKNYR	LRRNDKNTSD	IHMIDGDRDV	HATKEGAYFD	KAKEKSPIQS
1690							
QI							

Cmpd.	No. of Cmpds.	m/z meas.	Δ m/z [ppm]	z	Rt [min]	Score	P	Range	Sequence	Modification	Ratios
235	1	627.3257	-128.41	2	32.1	15.7	2	747-757	K.NLQLAVARIKK.G		Wdown:Qdown 4.16

Detailed Protein Report

Protein 294: PREDICTED: dedicator of cytokinesis protein 11 isoform X2 [Homo sapiens]

**Accession:**	gi|530422336	**Score:**	35.8
**Database:**	refseq_human(refseq_human_20140103.fasta)	**MW [kDa]:**	235.7
**Database Date:**	2015-11-30	**pI:**	8.1
**Modification(s):**	Oxidation	**Sequence Coverage [%]:**	0.5
		**No. of unique Peptides:**	2

Detailed Protein Report

10	20	30	40	50	60	70	80
MFKEKAKVVE	PLDYENVIAQ	RKTQIYSDPL	RDLLMFPMED	ISISVIGRQR	RTVQSTVPED	AEKRAQSLFV	KECIKTYSTD
90	100	110	120	130	140	150	160
WHVVNYKYED	FSGDFRMLPC	KSLRPEKIPN	HVFEIDEDCE	KDEDSSSLCS	QKGGVIKQGW	LHKANVNSTI	TVTMKVFKRR
170	180	190	200	210	220	230	240
YFYLTQLPDG	SYILNSYKDE	KNSKESKGCI	YLDACIDVVQ	CPKMRRHAFE	LKMLDKYSHY	LAAETEQEME	EWLITLKKII
250	260	270	280	290	300	310	320
QINTDSLVQE	KKETVETAQD	DETSSQGKAE	NIMASLERSM	HPELMKYGRE	TEQLNKLSRG	DGRQNLFSFD	SEVQRLDFSG
330	340	350	360	370	380	390	400
IEPDIKPFEE	KCNKRFLVNC	HDLTFNILGQ	IGDNAKGPPT	NVEPFFINLA	LFDVKNNCKI	SADFHVDLNP	PSVREMLWGS
410	420	430	440	450	460	470	480
STQLASDGSP	KGSSPESYIH	GIAESQLRYI	QQGIFSVTNP	HPEIFLVARI	EKVLQGNITH	CAEPYIKNSD	PVKTAQKVHR
490	500	510	520	530	540	550	560
TAKQVCSRLG	QYRMPFAWAA	RPIFKDTQGS	LDLDGRFSPL	YKQDSSKLSS	EDILKLLSEY	KKPEKTKLQI	IPGQLNITVE
570	580	590	600	610	620	630	640
CVPVDLSNCI	TSSYVPLKPF	EKNCQNITVE	VEEFVPEMTK	YCYPFTIYKN	HLYVYPLQLK	YDSQKTFAKA	RNIAVCVEFR
650	660	670	680	690	700	710	720
DSDESDASAL	KCIYGKPAGS	VFTTNAYAVV	SHHNQNPEFY	DEIKIELPIH	LHQKHHLLFT	FYHVSCEINT	KGTTKKQDTV
730	740	750	760	770	780	790	800
ETPVGFAWVP	LLKDGRIITF	EQQLPVSANL	PPGYLNLNDA	ESRRQCNVDI	KWVDGAKPLL	KIKSHLESTI	YTQDLHVHKF
810	820	830	840	850	860	870	880
FHHCQLIQSG	SKEVPGELIK	YLKCLHAMEI	QVMIQFLPVI	LMQLFRVLTN	MTHEDDVPIN	CTMVLLHIVS	KCHEEGLDSY
890	900	910	920	930	940	950	960
LRSFIKYSFR	PEKPSAPQAQ	LIHETLATTM	IAILKQSADF	LSINKLLKYS	WFFFEIIAKS	MATYLLEENK	IKLPRGQRFP
970	980	990	1000	1010	1020	1030	1040
ETYHHVLHSL	LLAIIPHVTI	RYAEIPDESR	NVNYSLASFL	KRCLTLMDRG	FIFNLINDYI	SGFSPKDPKV	LAEYKFEFLQ
1050	1060	1070	1080	1090	1100	1110	1120
TICNHEHYIP	LNLPMAFAKP	KLQRVQDFFS	FAVDRLTSVD	SNLEYSLSDE	YCKHHFLVGL	LLRETSIALQ	DNYEIRYTAI
1130	1140	1150	1160	1170	1180	1190	1200
SVIKNLLIKH	AFDTRYQHKN	QQAKIAQLYL	PFVGLLLENI	QRLAGRDTLY	SCAAMPNSAS	RDEFPCGFTS	PANRGSLSTD
1210	1220	1230	1240	1250	1260	1270	1280
KDTAYGSFQN	GHGIKREDSR	GSLIPEGATG	FPDQGNTGEN	TRQSSTRSSV	SQYNRLDQYE	IRSLLMCYLY	IVKMISEDTL
1290	1300	1310	1320	1330	1340	1350	1360
LTYWNKVSPQ	ELINILILLE	VCLFHFRYMG	KRNIARVHDA	WLSKHFGIDR	KSQTMPALRN	RSGVMQARLQ	HLSSLESSFT
1370	1380	1390	1400	1410	1420	1430	1440
LNHSSTTTEA	DIFHQALLEG	NTATEVSLTV	LDTISFFTQC	FKTQLLNNDG	HNPLMKKVFD	IHLAFLKNGQ	SEVSLKHVFA
1450	1460	1470	1480	1490	1500	1510	1520
SLRAFISKFP	SAFFKGRVNM	CAAFCYEVLK	CCTSKISSTR	NEASALLYLL	MRNNFEYTKR	KTFLRTHLQI	IIAVSQLIAD
1530	1540	1550	1560	1570	1580	1590	1600
VALSGGSRFQ	ESLFIINNFA	NSDRPMKATA	FPAEVKDLTK	RIRTVLMATA	QMKEHEKDPE	MLIDLQYSLA	KSYASTPELR
1610	1620	1630	1640	1650	1660	1670	1680
KTWLDSMAKI	HVKNGDFSEA	AMCYVHVAAL	VAEFLHRKKL	FPNGCSAFKK	ITPNIDEEGA	MKEDAGMMDV	HYSEEVLLEL
1690	1700	1710	1720	1730	1740	1750	1760
LEQCVDGLWK	AERYEIISEI	SKLIVPIYEK	RREFEKLTQV	YRTLHGAYTK	ILEVMHTKKR	LLGTFFRVAF	YGQSFFEEED
1770	1780	1790	1800	1810	1820	1830	1840
GKEYIYKEPK	LTGLSEISLR	LVKLYGEKFG	TENVKIIQDS	DKVNAKELDP	KYAHIQVTYV	KPYFDDKELT	ERKTEFERNH
1850	1860	1870	1880	1890	1900	1910	1920
NISRFVFEAP	YTLSGKKQGC	IEEQCKRRTI	LTTSNSFPYV	KKRIPINCEQ	QINLKPIDVA	TDEIKDKTAE	LQKLCSSTDV
1930	1940	1950	1960	1970	1980	1990	2000
DMIQLQLKLQ	GCVSVQVNAG	PLAYARAFLN	DSQASKYPPK	KVSELKDMFR	KFIQACSIAL	ELNERLIKED	QVEYHEGLKS
2010	2020	2030	2040	2050	2060		
NFRDMVKELS	DIIHEQILQE	DTMHSPWMSN	TLHVFCAISG	TSSDRGYGSP	RYAEV		

Cmpd.	No. of	m/z meas.	Δ m/z	z	Rt	Score	P	Range	Sequence	Modification

Detailed Protein Report

	Cmpds.		[ppm]		[min]					
2375	3	682.7793	-68.50	2	58.1	17.0	1	279-289	R.SMHPELMKYGR.E	Oxidation: 7
1995	1	682.7325	-137.16	2	53.4	18.8	1	279-289	R.SMHPELMKYGR.E	Oxidation: 2

Detailed Protein Report

Protein 295: **PREDICTED: GRAM domain-containing protein 1C isoform X2 [Homo sapiens]**

**Accession:**	gi|530374810	**Score:**	35.8
**Database:**	refseq_human(refseq_human_20140103.fasta)	**MW [kDa]:**	69.6
**Database Date:**	2015-11-30	**pI:**	6.9
		**Sequence Coverage [%]:**	4.8
		**No. of unique Peptides:**	2

10	20	30	40	50	60	70	80
MEGAPTVRQV	MNEGDSSLAT	DLQEDVEENP	SPTVEENNVV	VKKQGPNLHN	WSGDWSFWIS	SSTYKDRNEE	YRRQFTHLPD
**90**	**100**	**110**	**120**	**130**	**140**	**150**	**160**
TERLIADYAC	ALQRDILLQG	RLYLSENWLC	FYSNIFRWET	TISIALKNIT	FMTKEKTARL	IPNAIQIVTE	SEKFFFTSFG
**170**	**180**	**190**	**200**	**210**	**220**	**230**	**240**
ARDRSYLSIF	RLWQNVLLDK	SLTRQEFWQL	LQQNYGTELG	LNAEEMENLS	LSIEDVQPRS	PGRSSLDDSG	ERDEKLSKSI
**250**	**260**	**270**	**280**	**290**	**300**	**310**	**320**
SFTSESISRV	SETESFDGNS	SKGGLGKEES	QNEKQTKKSL	LPTLEKKLTR	VPSKSLDLNK	NEYLSLDKSS	TSDSVDEENV
**330**	**340**	**350**	**360**	**370**	**380**	**390**	**400**
PEKDLHGRLF	INRIFHISAD	RMFELLFTSS	RFMQKFASSR	NIIDVVSTPW	TAELGGDQLR	TMTYTIVLNS	PLTGKCTAAT
**410**	**420**	**430**	**440**	**450**	**460**	**470**	**480**
EKQTLYKESR	EARFYLVDSE	VLTHDVPYHD	YFYTVNRYCI	IRSSKQKCRL	RVSTDLKYRK	QPWGLVKSLI	EKNSWSSLED
**490**	**500**	**510**	**520**	**530**	**540**	**550**	**560**
YFKQLGKKKE	MENYNVTLIV	VMSIFVLLLV	LLNVTLFLKL	SKIEHAAQSF	YRLRLQEEKS	LNLASDMVSR	AETIQKNKDQ
**570**	**580**	**590**	**600**	**610**			
AHRLKGVLRD	SIVMLEQLKS	SLIMLQKTFD	LLNKNKTGMA	VES			

Cmpd.	No. of Cmpds.	m/z meas.	Δ m/z [ppm]	z	Rt [min]	Score	P	Range	Sequence	Modification
1154	1	981.6192	184.16	1	44.1	11.5	1	66-72	K.DRNEEYR.R	
2537	1	884.5766	142.07	2	62.2	14.2	2	588-603	K.TFDLLNKNKTGMAVES.-	

Detailed Protein Report

Protein 296: PREDICTED: coiled-coil domain-containing protein 146 isoform X3 [Homo sapiens]

**Accession:**	gi|530386260	**Score:**	35.8
**Database:**	refseq_human(refseq_human_20140103.fasta)	**MW [kDa]:**	97.2
**Database Date:**	2015-11-30	**pI:**	9.8
**Modification(s):**	Oxidation	**Sequence Coverage [%]:**	3.2
		**No. of unique Peptides:**	3

Quantitation

mdown:qdown	**Median:** 0.50	**CV:** 14.84 %	**No. of Peptides:**	2
Wdown:Qdown	**Median:** 0.88	**CV:** 0.00 %	**No. of Peptides:**	1

```
        10         20         30         40         50         60         70         80
MQTSSPTTRL FLRIKEKSLK EEKIIIVKEF EKITKPGEME KKMKILREST EELRKEIMQK KLEIKNLRED LASKQKQLLK
        90        100        110        120        130        140        150        160
EQKELEELLG HQVVLKDEVA HHQTIPVQIG KEIEKITRKK VEMEKKKIVL EQEVKTLNDS LKKVENKVSA IVDEKENVIK
       170        180        190        200        210        220        230        240
EVEGKRALLE IKEREHNQLV KLLELARENE ATSLTERGIL DLNLRNSLID KQNYHDELSR KQREKERDFR NLRKMELLLK
       250        260        270        280        290        300        310        320
VSWDALRQTQ ALHQRLLLEM EAIPKDDSTL SERRRELHKE VEVAKRNLAQ QKIISEMESK LVEQQLAEEN KLLKEQENMK
       330        340        350        360        370        380        390        400
ELVVNLLRMT QIKIDEKEQK SKDFLKAQQK YTNIVKEMKA KDLEIRIHKK KKCEIYRRLR EFAKLYDTIR NERNKFVNLL
       410        420        430        440        450        460        470        480
HKAHQKVNEI KERHKMSLNE LEILRNSAVS QERKLQNSML KHANNVTIRE SMQNDVRKIV SKLQEMKEKK EAQLNNIDRL
       490        500        510        520        530        540        550        560
ANTITMIEEE MVQLRKRYEK AVQHRNESGV QLIEREEEIC IFYEKINIQE KMKLNGEIEI HLLEEKIQFL KMKIAEKQRQ
       570        580        590        600        610        620        630        640
ICVTQKLLPA KRSLDADLAV LQIQFSQCTD RIKDLEKQFV KPDGENRARF LPGKDLTEKE MIQKLDKLEL QLAKKEEKLL
       650        660        670        680        690        700        710        720
EKDFIYEQVS RLTDRLCSKT QGCKQDTLLL AKKMNGYQRR IKNATEKMMA LVAELSMKQA LTIELQKEVR EKEDFIFTCN
       730        740        750        760        770        780        790        800
SRIEKGLPLN KEIEKEWLKV LRDEEMHALA IAEKSQEFLE ADNRQLPNGV YTTAEQRPNA YIPEADATLP LPKPYGALAP
       810        820        830
FKPSEPGANM RHIRKPVIKP VEI
```

Cmpd.	No. of Cmpds.	m/z meas.	Δ m/z [ppm]	z	Rt [min]	Score	P	Range	Sequence	Modification	Ratios
1670	1	524.8066	68.22	2	50.8	14.1	0	33-41	K.ITKPGEMEK.K	Oxidation: 7	mdown:qdown 0.58
1783	1	870.2900	-263.69	1	52.3	10.4	0	396-402	K.FVNLLHK.A		
595	1	572.7739	-48.16	2	36.8	11.3	0	506-515	R.NESGVQLIER.E		Wdown:Qdown 0.88<br>mdown:qdown 0.43

Detailed Protein Report

Protein 297: replication factor C subunit 4 [Homo sapiens]

**Accession:**	gi|4506491	**Score:**	35.6
**Database:**	refseq_human(refseq_human_20140103.fasta)	**MW [kDa]:**	39.7
**Database Date:**	2015-11-30	**pI:**	9.3
		**Sequence Coverage [%]:**	7.4
		**No. of unique Peptides:**	2

Alias proteins:

Accession	Name	Description
gi|31881687	refseq_human (refseq_human_20140103.fasta)	replication factor C subunit 4 [Homo sapiens]

10	20	30	40	50	60	70	80
MQAFLKGTSI	STKPPLTKDR	GVAASAGSSG	ENKKAKPVPW	VEKYRPKCVD	EVAFQEEVVA	VLKKSLEGAD	LPNLLFYGPP
**90**	**100**	**110**	**120**	**130**	**140**	**150**	**160**
GTGKTSTILA	AARELFGPEL	FRLRVLELNA	SDERGIQVVR	EKVKNFAQLT	VSGSRSDGKP	CPPFKIVILD	EADSMTSAAQ
**170**	**180**	**190**	**200**	**210**	**220**	**230**	**240**
AALRRTMEKE	SKTTRFCLIC	NYVSRIIEPL	TSRCSKFRFK	PLSDKIQQQR	LLDIAKKENV	KISDEGIAYL	VKVSEGDLRK
**250**	**260**	**270**	**280**	**290**	**300**	**310**	**320**
AITFLQSATR	LTGGKEITEK	VITDIAGVIP	AEKIDGVFAA	CQSGSFDKLE	AVVKDLIDEG	HAATQLVNQL	HDVVVENNLS
**330**	**340**	**350**	**360**	**370**			
DKQKSIITEK	LAEVDKCLAD	GADEHLQLIS	LCATVMQQLS	QNC			

Cmpd.	No. of Cmpds.	m/z meas.	Δ m/z [ppm]	z	Rt [min]	Score	P	Range	Sequence	Modification
2686	2	703.3641	38.67	2	64.8	15.8	1	19-33	K.DRGVAASAGSSGENK.K	
1817	2	727.2971	-204.84	2	51.2	19.9	2	206-217	K.IQQQRLLDIAKK.E	

Detailed Protein Report

Protein 298: CCR4-NOT transcription complex subunit 1 isoform a [Homo sapiens]

**Accession:**	gi|42716275	**Score:**	35.5
**Database:**	refseq_human(refseq_human_20140103.fasta)	**MW [kDa]:**	266.8
**Database Date:**	2015-11-30	**pI:**	6.7
		**Sequence Coverage [%]:**	1.3
		**No. of unique Peptides:**	1

Detailed Protein Report

10	20	30	40	50	60	70	80
MNLDSLSLAL	SQISYLVDNL	TKKNYRASQQ	EIQHIVNRHG	PEADRHLLRC	LFSHVDFSGD	GKSSGKDFHQ	TQFLIQECAL
90	100	110	120	130	140	150	160
LITKPNFIST	LSYAIDNPLH	YQKSLKPAPH	LFAQLSKVLK	LSKVQEVIFG	LALLNSSSSD	LRGFAAQFIK	QKLPDLLRSY
170	180	190	200	210	220	230	240
IDADVSGNQE	GGFQDIAIEV	LHLLLSHLLF	GQKGAFGVGQ	EQIDAFLKTL	RRDFPQERCP	VVLAPLLYPE	KRDILMDRIL
250	260	270	280	290	300	310	320
PDSGGVAKTM	MESSLADFMQ	EVGYGFCASI	EECRNIIVQF	GVREVTAAQV	ARVLGMMART	HSGLTDGIPL	QSISAPGSGI
330	340	350	360	370	380	390	400
WSDGKDKSDG	AQAHTWNVEV	LIDVLKELNP	SLNFKEVTYE	LDHPGFQIRD	SKGLHNVVYG	IQRGLGMEVF	PVDLIYRPWK
410	420	430	440	450	460	470	480
HAEGQLSFIQ	HSLINPEIFC	FADYPCHTVA	TDILKAPPED	DNREIATWKS	LDLIESLLRL	AEVGQYEQVK	QLFSFPIKHC
490	500	510	520	530	540	550	560
PDMLVLALLQ	INTSWHTLRH	ELISTLMPIF	LGNHPNSAII	LHYAWHGQGQ	SPSIRQLIMH	AMAEWYMRGE	QYDQAKLSRI
570	580	590	600	610	620	630	640
LDVAQDLKAL	SMLLNGTPFA	FVIDLAALAS	RREYLKLDKW	LTDKIREHGE	PFIQACMTFL	KRRCPSILGG	LAPEKDQPKS
650	660	670	680	690	700	710	720
AQLPPETLAT	MLACLQACAG	SVSQELSETI	LTMVANCSNV	MNKARQPPPG	VMPKGRPPSA	SSLDAISPVQ	IDPLAGMTSL
730	740	750	760	770	780	790	800
SIGGSAAPHT	QSMQGFPPNL	GSAFSTPQSP	AKAFPPLSTP	NQTTAFSGIG	GLSSQLPVGG	LGTGSLTGIG	TGALGLPAVN
810	820	830	840	850	860	870	880
NDPFVQRKLG	TSGLNQPTFQ	QSKMKPSDLS	QVWPEANQHF	SKEIDDEANS	YFQRIYNHPP	HPTMSVDEVL	EMLQRFKDST
890	900	910	920	930	940	950	960
IKREREVFNC	MLRNLFEEYR	FFPQYPDKEL	HITACLFGGI	IEKGLVTYMA	LGLALRYVLE	ALRKPFGSKM	YYFGIAALDR
970	980	990	1000	1010	1020	1030	1040
FKNRLKDYPQ	YCQHLASISH	FMQFPHHLQE	YIEYGQQSRD	PPVKMQGSIT	TPGSIALAQA	QAQAQVPAKA	PLAGQVSTMV
1050	1060	1070	1080	1090	1100	1110	1120
TTSTTTTVAK	TVTVTRPTGV	SFKKDVPPSI	NTTNIDTLLV	ATDQTERIVE	PPENIQEKIA	FIFNNLSQSN	MTQKVEELKE
1130	1140	1150	1160	1170	1180	1190	1200
TVKEEFMPWV	SQYLVMKRVS	IEPNFHSLYS	NFLDTLKNPE	FNKMVLNETY	RNIKVLLTSD	KAAANFSDRS	LLKNLGHWLG
1210	1220	1230	1240	1250	1260	1270	1280
MITLAKNKPI	LHTDLDVKSL	LLEAYVKGQQ	ELLYVVPFVA	KVLESSIRSV	VFRPPNPWTM	AIMNVLAELH	QEHDLKLNLK
1290	1300	1310	1320	1330	1340	1350	1360
FEIEVLCKNL	ALDINELKPG	NLLKDKDRLK	NLDEQLSAPK	KDVKQPEELP	PITTTTTSTT	PATNTTCTAT	VPPQPQYSYH
1370	1380	1390	1400	1410	1420	1430	1440
DINVYSLAGL	APHITLNPTI	PLFQAHPQLK	QCVRQAIERA	VQELVHPVVD	RSIKIAMTTC	EQIVRKDFAL	DSEESRMRIA
1450	1460	1470	1480	1490	1500	1510	1520
AHHMMRNLTA	GMAMITCREP	LLMSISTNLK	NSFASALRTA	SPQQREMMDQ	AAAQLAQDNC	ELACCFIQKT	AVEKAGPEMD
1530	1540	1550	1560	1570	1580	1590	1600
KRLATEFELR	KHARQEGRRY	CDPVVLTYQA	ERMPEQIRLK	VGGVDPKQLA	VYEEFARNVP	GFLPTNDLSQ	PTGFLAQPMK
1610	1620	1630	1640	1650	1660	1670	1680
QAWATDDVAQ	IYDKCITELE	QHLHAIPPTL	AMNPQAQALR	SLLEVVVLSR	NSRDAIAALG	LLQKAVEGLL	DATSGADADL
1690	1700	1710	1720	1730	1740	1750	1760
LLRYRECHLL	VLKALQDGRA	YGSPWCNKQI	TRCLIECRDE	YKYNVEAVEL	LIRNHLVNMQ	QYDLHLAQSM	ENGLNYMAVA
1770	1780	1790	1800	1810	1820	1830	1840
FAMQLVKILL	VDERSVAHVT	EADLFHTIET	LMRINAHSRG	NAPEGLPQLM	EVVRSNYEAM	IDRAHGGPNF	MMHSGISQAS
1850	1860	1870	1880	1890	1900	1910	1920
EYDDPPGLRE	KAEYLLREWV	NLYHSAAAGR	DSTKAFSAFV	GQMHQQGILK	TDDLITRFFR	LCTEMCVEIS	YRAQAEQQHN
1930	1940	1950	1960	1970	1980	1990	2000
PAANPTMIRA	KCYHNLDAFV	RLIALLVKHS	GEATNTVTKI	NLLNKVLGIV	VGVLLQDHDV	RQSEFQQLPY	HRIFIMLLLE
2010	2020	2030	2040	2050	2060	2070	2080
LNAPEHVLET	INFQTLTAFC	NTFHILRPTK	APGFVYAWLE	LISHRIFIAR	MLAHTPQQKG	WPMYAQLLID	LFKYLAPFLR
2090	2100	2110	2120	2130	2140	2150	2160
NVELTKPMQI	LYKGTLRVLL	VLLHDFPEFL	CDYHYGFCDV	IPPNCIQLRN	LILSAFPRNM	RLPDPFTPNL	KVDMLSEINI
2170	2180	2190	2200	2210	2220	2230	2240

Detailed Protein Report

Cmpd.	No. of Cmpds.	m/z meas.	Δ m/z [ppm]	z	Rt [min]	Score	P	Range	Sequence	Modification
585	1	603.8101	58.84	2	36.2	10.4	0	2365-2376	K.QAQQVMEGTGAS.-	

Detailed Protein Report

Protein 299: **PREDICTED: LOW QUALITY PROTEIN: double homeobox protein 4-like protein 4 [Homo sapiens]**

Accession: gi|578798033
Database: refseq_human(refseq_human_20140103.fasta)
Database Date: 2015-11-30
Modification(s): Carbamidomethyl

Score: 35.5
MW [kDa]: 45.9
pI: 10.8
Sequence Coverage [%]: 5.0
No. of unique Peptides: 2

10	20	30	40	50	60	70	80
MTLPTPSEST	LPAEARGWGQ	RRRLVWTPRQ	REALXACFYR	NPYMGIATRV	QLAQSIGIPE	TRVXIYFQNE	RSRQLRQHRR
**90**	**100**	**110**	**120**	**130**	**140**	**150**	**160**
ESRPWPVRRG	PQEGRTAVIG	SQTALLLRAF	EKDRLPGIEA	REELARERGL	LESRIQIWFQ	IRRARHQGQA	GRAPAQAGGR
**170**	**180**	**190**	**200**	**210**	**220**	**230**	**240**
SNAAPAXCHP	ARSWVAFAHT	GRWGRVFPHP	XVPCAPGALP	QGAFVNQGAR	AFLVFQHSQA	APVEGISQPA	LGRGDFSYTA
**250**	**260**	**270**	**280**	**290**	**300**	**310**	**320**
PAPLEGALSH	PQSPPXPPHT	GKSRENRDPQ	RDGLLGTCVV	GQPGPAQAGP	QGQGVLAPPA	SQGIPWWGWA	WGPQASGAAW
**330**	**340**	**350**	**360**	**370**	**380**	**390**	**400**
EPQARAASPR	QPMPPEDSAR	QGQMQGMPAP	SQALQELGRS	SALPSGLLLD	KFLASLVFLQ	QVQSFLETET	PGELEALEEA
**410**	**420**						
VSLEEPLSEQ	EYRALLEEI						

Cmpd.	No. of Cmpds.	m/z meas.	Δ m/z [ppm]	z	Rt [min]	Score	P	Range	Sequence	Modification
2748	1	826.4475	66.34	3	65.6	12.9	2	0-0	.QREALCACFYRNPYMGIATR.	Carbamidomethyl: 6, 8
2114	1	1131.4444	-110.45	1	56.6	10.9	0	173-182	R.SWVAFAHTGR.W	

Detailed Protein Report

Protein 300: pogo transposable element with ZNF domain isoform 3 [Homo sapiens]

Accession: gi|302699211
Database: refseq_human(refseq_human_20140103.fasta)
Database Date: 2015-11-30
Modification(s): Carbamidomethyl, Oxidation

Score: 35.5
MW [kDa]: 145.2
pI: 6.8
Sequence Coverage [%]: 2.1
No. of unique Peptides: 3

Quantitation

mdown:qdown **Median:** 0.84 **CV:** 0.00 % **No. of Peptides:** 1

10	20	30	40	50	60	70	80
MADTDLFMEC	EEEELEPWQK	ISDVIEDSVV	EDYNSVDKTT	TVSVSQQPVS	APVPIAAHAS	VAGHLSTSTT	VSSSGAQNSD
90	100	110	120	130	140	150	160
STKKTLVTLI	ANNNAPGTQF	VKPTVGVPQV	FSQMTPVRPG	STMPVRPTTN	TFTTVIPATL	TIRSTVPQSQ	SQQTKSTPST
170	180	190	200	210	220	230	240
STTPTATQPT	SLGQLAVQSP	GQSNQTTNPK	LAPSFPSPPA	VSIASFVTVK	RPGVTGENSN	EVAKLVNTLN	TIPSLGQSPG
250	260	270	280	290	300	310	320
PVVVSNNSSA	HGSQRTSGPE	SSMKVTSSIP	VFDLQDGGRK	ICPRCNAQFR	VTEALRGHMC	YCCPEMVEYQ	KKGKSLDSEP
330	340	350	360	370	380	390	400
SVPSAAKPPS	PEKTAPVAST	PSSTPIPALS	PPTKVPEPNE	NVGDAVQTKL	IMLVDDFYYG	RDGGKVAQLT	NFPKVATSFR
410	420	430	440	450	460	470	480
CPHCTKRLKN	NIRFMNHMKH	HVELDQQNGE	VDGHTICQHC	YRQFSTPFQL	QCHLENVHSP	YESTTKCKIC	EWAFESEPLF
490	500	510	520	530	540	550	560
LQHMKDTHKP	GEMPYVCQVC	QYRSSLYSEV	DVHFRMIHED	TRHLLCPYCL	KVFKNGNAFQ	QHYMRHQKRN	VYHCNKCRLQ
570	580	590	600	610	620	630	640
FLFAKDKIEH	KLQHHKTFRK	PKQLEGLKPG	TKVTIRASRG	QPRTVPVSSN	DTPPSALQEA	APLTSSMDPL	PVFLYPPVQR
650	660	670	680	690	700	710	720
SIQKRAVRKM	SVMGRQTCLE	CSFEIPDFPN	HFPTYVHCSL	CRYSTCCSRA	YANHMINNHV	PRKSPKYLAL	FKNSVSGIKL
730	740	750	760	770	780	790	800
ACTSCTFVTS	VGDAMAKHLV	FNPSHRSSSI	LPRGLTWIAH	SRHGQTRDRV	HDRNVKNMYP	PPSFPTNKAA	TVKSAGATPA
810	820	830	840	850	860	870	880
EPEELLTPLA	PALPSPASTA	TPPPTPTHPQ	ALALPPLATE	GAECLNVDDQ	DEGSPVTQEP	ELASGGGGSG	GVGKKEQLSV
890	900	910	920	930	940	950	960
KKLRVVLFAL	CCNTEQAAEH	FRNPQRRIRR	WLRRFQASQG	ENLEGKYLSF	EAEEKLAEWV	LTQREQQLPV	NEETLFQKAT
970	980	990	1000	1010	1020	1030	1040
KIGRSLEGGF	KISYEWAVRF	MLRHHLTPHA	RRAVAHTLPK	DVAENAGLFI	DFVQRQIHNQ	DLPLSMIVAI	DEISLFLDTE
1050	1060	1070	1080	1090	1100	1110	1120
VLSSDDRKEN	ALQTVGTGEP	WCDVVLAILA	DGTVLPTLVF	YRGQMDQPAN	MPDSILLEAK	ESGYSDDEIM	ELWSTRVWQK
1130	1140	1150	1160	1170	1180	1190	1200
HTACQRSKGM	LVMDCHRTHL	SEEVLAMLSA	SSTLPAVVPA	GCSSKIQPLD	VCIKRTVKNF	LHKKWKEQAR	EMADTACDSD
1210	1220	1230	1240	1250	1260	1270	1280
VLLQLVLVWL	GEVLGVIGDC	PELVQRSFLV	ASVLPGPDGN	INSPTRNADM	QEELIASLEE	QLKLSGEHSE	SSTPRPRSSP
1290	1300	1310	1320				
EETIEPESLH	QLFEGESETE	SFYGFEEADL	DLMEI				

Cmpd.	No. of Cmpds.	m/z meas.	Δ m/z [ppm]	z	Rt [min]	Score	P	Range	Sequence	Modification	Ratios
240	1	732.8262	-63.03	2	31.7	14.2	1	285-296	R.CNAQFRVTEALR.G	Carbamidomethyl: 1	mdown:qdown 0.84
2117	1	990.8950	2.39	2	55.0	10.3	1	297-312	R.GHMCYCCPEMVEYQKK.G	Oxidation: 3, 10	
2186	1	1031.8206	-95.59	2	55.9	11.0	1	297-312	R.GHMCYCCPEMVEYQKK.G	Carbamidomethyl: 4, 6	

Detailed Protein Report

Protein 301: testis- and ovary-specific PAZ domain-containing protein 1 [Homo sapiens]

**Accession:**	gi|222537754	**Score:**	35.5
**Database:**	refseq_human(refseq_human_20140103.fasta)	**MW [kDa]:**	190.8
**Database Date:**	2015-11-30	**pI:**	9.1
**Modification(s):**	Carbamidomethyl	**Sequence Coverage [%]:**	3.2
		**No. of unique Peptides:**	3

Quantitation

mdown:qdown	**Median:**	1.11	**CV:**	0.00 %	**No. of Peptides:**	1
Wdown:Qdown	**Median:**	1.14	**CV:**	0.00 %	**No. of Peptides:**	1

10	20	30	40	50	60	70	80
MRRPPPLGPT	TASGPEGNVR	NLQKRQAPGP	GAAGGCGPEA	GGCRENKQKR	RMVARATPGR	GEVESDKSVA	ASGAGKAARR
90	100	110	120	130	140	150	160
QVEGRRGPVS	PSDSSDPRGL	EAAKEAELPL	QTERHTKEKR	KVTEASSDDP	QPGLDLVRKE	SLTSSESFQT	VECLQSLGKE
170	180	190	200	210	220	230	240
SIIEGIKRRI	RNKKLKSLEN	PPLKITENEA	TQNIKVEFQD	ELYKNTPKYS	CNILSPEVEN	NSVLKLRDCN	CFPHSKGCND
250	260	270	280	290	300	310	320
ENNLPYKPDG	GCMHVAENFS	KKENLRSLAE	KSDTNSIPQL	LQTEENVMGV	NKLLPEESDL	YQSKTNGLLS	CLQHEKNKYS
330	340	350	360	370	380	390	400
IEESSVGRKP	RKRMKLSEKA	DETVTEMNFS	NEYNKSELML	QENQMIADGK	EAETKSPLNV	LRKVSHNTVS	LMDHLLSVPE
410	420	430	440	450	460	470	480
TVEKETSSEH	HVNAVFQKTI	EPLLKEETEN	ASEPLGYESM	ASKEDFKSMK	SFIGKSPNEY	HIERRSSRED	LRSASEELKL
490	500	510	520	530	540	550	560
SCQRTIPMTG	KRTWPYYSCA	RISAWCWKKA	SLPESSYFLR	GSQESCRQVD	VPKHQTNQTH	LTDSKLLLQS	SLTETNTESS
570	580	590	600	610	620	630	640
SKEKLDSNSN	CLSSVSAVEP	TLMVIKEPII	KDDKKIKSEE	LSRRGSEVIS	NTTEDTQLTS	ETQSLTGNKK	KARGNLTKLN
650	660	670	680	690	700	710	720
LTATSKDGQE	ANNSAGKTIH	RKACIAQQTF	IVPDLVKILN	TGRLTNFKIP	LLKNKSEKRK	EVNAKSSERE	AYSPLELLDN
730	740	750	760	770	780	790	800
LSGADVRQNR	SKENVSMMML	GPQTLSIRNS	VTPVQASSDS	FYNKKSYSIS	PSFTKQGNNS	KPSNHVSEPG	NIVSNKEVAS
810	820	830	840	850	860	870	880
LTVENNAFSC	DPGYVEKSPS	FCCNEQETFR	PVSSEVRGRK	ITKNFSEVGF	PDILKAYEDD	VLLIDVIQDD	PDLFGVSNEG
890	900	910	920	930	940	950	960
ELSFTSEVPK	ISQEPNVAGE	HQSTDSKYME	TPVKKEPSDD	LRELPVLDCG	WIKPDICASN	SAESEIKRDP	KDVNTSLGEV
970	980	990	1000	1010	1020	1030	1040
ANETSENETL	GDFSEQIKGS	DLDEKHRFTD	KVITKEEKEN	IYEVCKSKDS	RNADFMVGEC	QFAVPVPKPL	CLLVPPLNLS
1050	1060	1070	1080	1090	1100	1110	1120
GRQEDTILNT	WMNDFRFLGK	HSVLKLQNPE	TCEIFKREKN	VGVFQKSLGL	MIPYKYCKFH	FNTLRGCERP	LCKFAHVPEQ
1130	1140	1150	1160	1170	1180	1190	1200
GDEKVCMDVF	KKYININELC	LLQRAVNIFM	EYYRKFPPGV	YFDLQVLNDL	LNSLLKHCLL	KEVFQIVNLS	IMVKMLPSLK
1210	1220	1230	1240	1250	1260	1270	1280
ILLNIFEYVA	TMKLRNAVPA	LIDIFCKLVE	AGMVLDPEHF	NYIVKLLYQV	QASKQEITAV	LEMKSRLQMR	RFKKNWKCDL
1290	1300	1310	1320	1330	1340	1350	1360
DSALNKLEHC	KEKGDWTKLG	KLYINVKMGC	EKFADFQTFC	ACIAETLTKN	YEDERPDIPF	CEFAETVSKD	PQNSKVDKGV
1370	1380	1390	1400	1410	1420	1430	1440
LGRIGISAMY	FYHKLLQWSK	GRKVLEKLYE	LKIHFTSLKG	LIGPEKLASR	CQIVNVAAEI	FLKSGSLDGA	IWVMRESEWI
1450	1460	1470	1480	1490	1500	1510	1520
INTPLWPCDR	LDVLNRHNLL	CTIAHEILAK	SLYRQTFEVL	QNLPGFQNSQ	ETVEVSQYSL	LFNKLLGSCI	ESSSLGMSSS
1530	1540	1550	1560	1570	1580	1590	1600
VAEFMISKSI	PIDFSFLRRL	ITSLGRSRLW	LKARAHYKSA	LSLGCYPPLE	GNLYRKLLLI	PSYLSEIEML	LAIEIFMVSN
1610	1620	1630	1640	1650	1660	1670	1680
ASSIQSPGTS	TQILQIVLKR	CEDNQSRSND	DYQAAVERLI	MAARISDPKL	FVKHMTVNVN	KEQVYSLEHC	SALKWLKENM
1690	1700						
KWAGKVWLFS	NH						

Detailed Protein Report

Cmpd.	No. of Cmpds.	m/z meas.	Δ m/z [ppm]	z	Rt [min]	Score	P	Range	Sequence	Modification	Ratios
2728	1	913.4776	71.07	2	62.5	11.3	1	25-44	K. RQAPGPGAAGGCGPEAGGCR. E	Carbamidomethyl: 19	
934	1	872.4494	41.89	2	40.5	10.6	0	749-764	R.NSVTPVQASSDSFYNK.K		
64	1	1068.4736	-22.86	2	30.2	13.6	1	1114-1131	K.FAHVPEQGDEKVCMDVFK.K	Carbamidomethyl: 13	Wdown:Qdown 1.14<br>mdown:qdown 1.11

Detailed Protein Report

Protein 302: PREDICTED: protein FAM45A isoform X1 [Homo sapiens]

Accession: gi|530393695
Database: refseq_human(refseq_human_20140103.fasta)
Database Date: 2015-11-30

Score: 35.4
MW [kDa]: 39.8
pI: 6.3
Sequence Coverage [%]: 10.6
No. of unique Peptides: 2

10	20	30	40	50	60	70	80
MLSSDHLSTV	EKDTNGEVLW	VWCYPSTTAT	LRNLLLRKCC	LTDENKLLHP	FVFGQYRRTW	FYITTIEVPD	SSILKKVTHF
**90**	**100**	**110**	**120**	**130**	**140**	**150**	**160**
SIVLTAKDFN	PEKYAAFTRI	LCRMYLKHGS	PVKMMESYIA	VLTKGICQSE	ENGSFLSKDF	DARKAYLAGS	IKDIVSQFGM
**170**	**180**	**190**	**200**	**210**	**220**	**230**	**240**
ETVILHTALM	LKKRIVVYHP	KIEAVQEFTR	TLPALVWHRQ	DWTILHSYVH	LNADELEALQ	MCTGYVAGFV	DLEVSNRPDL
**250**	**260**	**270**	**280**	**290**	**300**	**310**	**320**
YDVFVNLAES	EITIAPLAKE	AMAMGKLHKE	MGQLIVQSAE	DPEKSESHVI	QDIALKTREI	FTNLAPFSEV	SADGEKRVLN
**330**	**340**	**350**					
LEALKQKRFP	PATENFLYHL	AAAEQMLKI					

Cmpd.	No. of Cmpds.	m/z meas.	Δ m/z [ppm]	z	Rt [min]	Score	P	Range	Sequence	Modification
2766	1	804.4142	11.70	3	63.0	10.7	2	38-57	R.KCCLTDENKLLHPFVFGQYR.R	
2881	2	973.5257	3.04	2	64.8	12.2	1	77-93	K.VTHFSIVLTAKDFNPEK.Y	

Detailed Protein Report

Protein 303: cytoplasmic dynein 1 heavy chain 1 [Homo sapiens]

**Accession:**	gi|33350932	**Score:**	35.1
**Database:**	refseq_human(refseq_human_20140103.fasta)	**MW [kDa]:**	532.1
**Database Date:**	2015-11-30	**pI:**	6.0
**Modification(s):**	Oxidation	**Sequence Coverage [%]:**	0.6
		**No. of unique Peptides:**	2

Quantitation

Wdown:Qdown	**Median:** 2.42	**CV:** 0.00 %	**No. of Peptides:** 1

Detailed Protein Report

10	20	30	40	50	60	70	80
MSEPGGGGGE	DGSAGLEVSA	VQNVADVSVL	QKHLRKLVPL	LLEDGGEAPA	ALEAALEEKS	ALEQMRKFLS	DPQVHTVLVE
90	100	110	120	130	140	150	160
RSTLKEDVGD	EGEEEKEFIS	YNINIDIHYG	VKSNSLAFIK	RTPVIDADKP	VSSQLRVLTL	SEDSPYETLH	SFISNAVAPF
170	180	190	200	210	220	230	240
FKSYIRESGK	ADRDGDKMAP	SVEKKIAELE	MGLLHLQQNI	EIPEISLPIH	PMITNVAKQC	YERGEKPKVT	DFGDKVEDPT
250	260	270	280	290	300	310	320
FLNQLQSGVN	RWIREIQKVT	KLDRDPASGT	ALQEISFWLN	LERALYRIQE	KRESPEVLLT	LDILKHGKRF	HATVSFDTDT
330	340	350	360	370	380	390	400
GLKQALETVN	DYNPLMKDFP	LNDLLSATEL	DKIRQALVAI	FTHLRKIRNT	KYPIQRALRL	VEAISRDLSS	QLLKVLGTRK
410	420	430	440	450	460	470	480
LMHVAYEEFE	KVMVACFEVF	QTWDDEYEKL	QVLLRDIVKR	KREENLKMVW	RINPAHRKLQ	ARLDQMRKFR	RQHEQLRAVI
490	500	510	520	530	540	550	560
VRVLRPQVTA	VAQQNQGEVP	EPQDMKVAEV	LFDAADANAI	EEVNLAYENV	KEVDGLDVSK	EGTEAWEAAM	KRYDERIDRV
570	580	590	600	610	620	630	640
ETRITARLRD	QLGTAKNANE	MFRIFSRFNA	LFVRPHIRGA	IREYQTQLIQ	RVKDDIESLH	DKFKVQYPQS	QACKMSHVRD
650	660	670	680	690	700	710	720
LPPVSGSIIW	AKQIDRQLTA	YMKRVEDVLG	KGWENHVEGQ	KLKQDGDSFR	MKLNTQEIFD	DWARKVQQRN	LGVSGRIFTI
730	740	750	760	770	780	790	800
ESTRVRGRTG	NVLKLKVNFL	PEIITLSKEV	RNLKWLGFRV	PLAIVNKAHQ	ANQLYPFAIS	LIESVRTYER	TCEKVEERNT
810	820	830	840	850	860	870	880
ISLLVAGLKK	EVQALIAEGI	ALVWESYKLD	PYVQRLAETV	FNFQEKVDDL	LIIEEKIDLE	VRSLETCMYD	HKTFSEILNR
890	900	910	920	930	940	950	960
VQKAVDDLNL	HSYSNLPIWV	NKLDMEIERI	LGVRLQAGLR	AWTQVLLGQA	EDKAEVDMDT	DAPQVSHKPG	GEPKIKNVVH
970	980	990	1000	1010	1020	1030	1040
ELRITNQVIY	LNPPIEECRY	KLYQEMFAWK	MVVLSLPRIQ	SQRYQVGVHY	ELTEEEKFYR	NALTRMPDGP	VALEESYSAV
1050	1060	1070	1080	1090	1100	1110	1120
MGIVSEVEQY	VKVWLQYQCL	WDMQAENIYN	RLGEDLNKWQ	ALLVQIRKAR	GTFDNAETKK	EFGPVVIDYG	KVQSKVNLKY
1130	1140	1150	1160	1170	1180	1190	1200
DSWHKEVLSK	FGQMLGSNMT	EFHSQISKSR	QELEQHSVDT	ASTSDAVTFI	TYVQSLKRKI	KQFEKQVELY	RNGQRLLEKQ
1210	1220	1230	1240	1250	1260	1270	1280
RFQFPPSWLY	IDNIEGEWGA	FNDIMRRKDS	AIQQQVANLQ	MKIVQEDRAV	ESRTTDLLTD	WEKTKPVTGN	LRPEEALQAL
1290	1300	1310	1320	1330	1340	1350	1360
TIYEGKFGRL	KDDREKCAKA	KEALELTDTG	LLSGSEERVQ	VALEELQDLK	GVWSELSKVW	EQIDQMKEQP	WVSVQPRKLR
1370	1380	1390	1400	1410	1420	1430	1440
QNLDALLNQL	KSFPARLRQY	ASYEFVQRLL	KGYMKINMLV	IELKSEALKD	RHWKQLMKRL	HVNWVVSELT	LGQIWDVDLQ
1450	1460	1470	1480	1490	1500	1510	1520
KNEAIVKDVL	LVAQGEMALE	EFLKQIREVW	NTYELDLVNY	QNKCRLIRGW	DDLFNKVKEH	INSVSAMKLS	PYYKVFEEDA
1530	1540	1550	1560	1570	1580	1590	1600
LSWEDKLNRI	MALFDVWIDV	QRRWVYLEGI	FTGSADIKHL	LPVETQRFQS	ISTEFLALMK	KVSKSPLVMD	VLNIQGVQRS
1610	1620	1630	1640	1650	1660	1670	1680
LERLADLLGK	IQKALGEYLE	RERSSFPRFY	FVGDEDLLEI	IGNSKNVAKL	QKHFKKMFAG	VSSIILNEDN	SVVLGISSRE
1690	1700	1710	1720	1730	1740	1750	1760
GEEVMFKTPV	SITEHPKINE	WLTLVEKEMR	VTLAKLLAES	VTEVEIFGKA	TSIDPNTYIT	WIDKYQAQLV	VLSAQIAWSE
1770	1780	1790	1800	1810	1820	1830	1840
NVETALSSMG	GGGDAAPLHS	VLSNVEVTLN	VLADSVLMEQ	PPLRRRKLEH	LITELVHQRD	VTRSLIKSKI	DNAKSFEWLS
1850	1860	1870	1880	1890	1900	1910	1920
QMRFYFDPKQ	TDVLQQLSIQ	MANAKFNYGF	EYLGVQDKLV	QTPLTDRCYL	TMTQALEARL	GGSPFGPAGT	GKTESVKALG
1930	1940	1950	1960	1970	1980	1990	2000
HQLGRFVLVF	NCDETFDFQA	MGRIFVGLCQ	VGAWGCFDEF	NRLEERMLSA	VSQQVQCIQE	ALREHSNPNY	DKTSAPITCE
2010	2020	2030	2040	2050	2060	2070	2080
LLNKQVKVSP	DMAIFITMNP	GYAGRSNLPD	NLKKLFRSLA	MTKPDRQLIA	QVMLYSQGFR	TAEVLANKIV	PFFKLCDEQL
2090	2100	2110	2120	2130	2140	2150	2160
SSQSHYDFGL	RALKSVLVSA	GNVKRERIQK	IKREKEERGE	AVDEGEIAEN	LPEQEILIQS	VCETMVPKLV	AEDIPLLFSL
2170	2180	2190	2200	2210	2220	2230	2240

Detailed Protein Report

Cmpd.	No. of Cmpds.	m/z meas.	Δ m/z [ppm]	z	Rt [min]	Score	P	Range	Sequence	Modification	Ratios
1836	1	805.7966	-107.51	2	53.0	18.5	1	570-583	R.DQLGTAKNANEMFR.I	Oxidation: 12	Wdown:Qdown 2.42
1465	1	671.4099	30.99	2	47.1	16.6	2	705-716	R.KVQQRNLGVSGR.I		

Detailed Protein Report

Protein 304: PREDICTED: protein THEMIS isoform X2 [Homo sapiens]

**Accession:**	gi|578812733	**Score:**	35.1
**Database:**	refseq_human(refseq_human_20140103.fasta)	**MW [kDa]:**	62.5
**Database Date:**	2015-11-30	**pI:**	5.8
		**Sequence Coverage [%]:**	4.0
		**No. of unique Peptides:**	2

Quantitation

mdown:qdown **Median:** 1.29 **CV:** 0.00 % **No. of Peptides:** 1

Alias proteins:

Accession	Name	Description
gi|578839551	refseq_human (refseq_human_20140103.fasta)	PREDICTED: protein THEMIS isoform X2 [Homo sapiens]

10	20	30	40	50	60	70	80
MEEITRTIHI	GPSRLGHPCF	YHQKDIKLEN	LIIKQGEQIM	LNSVEEIDGE	IMVSCAVARN	HQTHSFNLPL	SQEGEFYECE
**90**	**100**	**110**	**120**	**130**	**140**	**150**	**160**
DERIYTLKEI	VEWKIPKNRT	RTVNLTDFSN	KWDSTNPFPK	DFYGTLILKP	VYEIQGVMKF	RKDIIRILPS	LDVEVKDITD
**170**	**180**	**190**	**200**	**210**	**220**	**230**	**240**
SYDANWFLQL	LSTEDLFEMT	SKEFPIVTEV	IEAPEGNHLP	QSILQPGKTI	VIHKKYQASR	ILASEIRSNF	PKRHFLIPTS
**250**	**260**	**270**	**280**	**290**	**300**	**310**	**320**
YKGKFKRRPR	EFPTAYDLEI	AKSEKEPLHV	VATKAFHSPH	DKLSSVSVGD	QFLVHQSETT	EVLCEGIKKV	VNVLACEKIL
**330**	**340**	**350**	**360**	**370**	**380**	**390**	**400**
KKSYEAALLP	LYMEGGFVEV	IHDKKQYPIS	ELCKQFRLPF	NVKVSVRDLS	IEEDVLAATP	GLQLEEDITD	SYLLISDFAN
**410**	**420**	**430**	**440**	**450**	**460**	**470**	**480**
PTECWEIPVG	RLNMTVQLVS	NFSRDAEPFL	VRTLVEEITE	EQYYMMRRYE	SSASHPPPRP	PKHPSVEETK	LTLLTLAEER
**490**	**500**	**510**	**520**	**530**	**540**	**550**	
TVDLPKSPKR	HHVDITKKLH	PNQAGLDSKV	LIGSQNDLVD	EEKERSNRGA	TAIAETFKNE	KHQK	

Cmpd.	No. of Cmpds.	m/z meas.	Δ m/z [ppm]	z	Rt [min]	Score	P	Range	Sequence	Modification	Ratios
500	1	599.8540	-33.23	2	35.6	16.2	1	25-34	K.DIKLENLIIK.Q		mdown:qdown 1.29
2974	2	698.3723	10.43	2	66.4	18.9	1	100-111	R.TRTVNLTDFSNK.W		

Detailed Protein Report

Protein 305: A-kinase anchor protein 2 isoform 2 [Homo sapiens]

Accession: gi|211971074
Database: refseq_human(refseq_human_20140103.fasta)
Database Date: 2015-11-30

Score: 35.1
MW [kDa]: 94.6
pI: 4.9
Sequence Coverage [%]: 3.0
No. of unique Peptides: 1

10	20	30	40	50	60	70	80
MEIEVSVAEC	KSVPGITSTP	HPMDHPSAFY	SPPHNGLLTD	HHESLDNDVA	REIRYLDEVL	EANCCDSAVD	GTYNGTSSPE
90	100	110	120	130	140	150	160
PGAVVLVGGL	SPPVHEATQP	EPTERTASRQ	APPHIELSNS	SPDPMAEAER	TNGHSPSQPR	DALGDSLQVP	VSPSSTTSSR
170	180	190	200	210	220	230	240
CSSRDGEFTL	TTLKKEAKFE	LRAFHEDKKP	SKLFEDDEHE	KEQYCIRKVR	PSEEMLELEK	ERRELIRSQA	VKKNPGIAAK
250	260	270	280	290	300	310	320
WWNPPQEKTI	EEQLDEEHLE	SHKKYKERKE	RRAQQEQLLL	QKQLQQQQQQ	PPSQLCTAPA	SSHERASMID	KAKEDIVTEQ
330	340	350	360	370	380	390	400
IDFSAARKQF	QLMENSRQAV	AKGQSTPRLF	SIKPFYRPLG	SVNSDKPLTN	PRPPSVGGPP	EDSGASAAKG	QKSPGALETP
410	420	430	440	450	460	470	480
SAAGSQGNTA	SQGKEGPYSE	PSKRGPLSKL	WAEDGEFTSA	RAVLTVVKDD	DHGILDQFSR	SVNVSLTQEE	LDSGLDELSV
490	500	510	520	530	540	550	560
RSQDTTVLET	LSNDFSMDNI	SDSGASNETT	NALQENSLAD	FSLPQTPQTD	NPSEGRGEGV	SKSFSDHGFY	SPSSTLGDSP
570	580	590	600	610	620	630	640
LVDDPLEYQA	GLLVQNAIQQ	AIAEQVDKAV	SKTSRDGAEQ	QGPEATVEEA	EAAAFGSEKP	QSMFEPPQVS	SPVQEKRDVL
650	660	670	680	690	700	710	720
PKILPAEDRA	LRERGPPQPL	PAVQPSGPIN	MEETRPEGSY	FSKYSEAAEL	RSTASLLATQ	ESDVMVGPFK	LRSRKQRTLS
730	740	750	760	770	780	790	800
MIEEEIRAAQ	EREEELKRQR	QVLQSTQSPR	TKNAPSLPSR	TCYKTAPGKI	EKVKPPPSPT	TEGPSLQPDL	APEEAAGTQR
810	820	830	840	850	860		
PKNLMQTLME	DYETHKSKRR	ERMDDSSVLE	ATRVNRRKSA	LALRWEAGIY	ANQEEEDNE		

Cmpd.	No. of Cmpds.	m/z meas.	Δ m/z [ppm]	z	Rt [min]	Score	P	Range	Sequence	Modification
2188	1	796.7740	-143.67	2	57.6	14.4	1	823-836	R.MDDSSVLEATRVNR.R	

Detailed Protein Report

Protein 306: **guanylate cyclase soluble subunit alpha-3 isoform D [Homo sapiens]**

Accession: gi|194595482
Database: refseq_human(refseq_human_20140103.fasta)
Database Date: 2015-11-30

Score: 35.1
MW [kDa]: 70.1
pI: 7.8
Sequence Coverage [%]: 5.6
No. of unique Peptides: 2

Quantitation

mdown:qdown	**Median:**	0.88	**CV:**	0.00 %	**No. of Peptides:**	1
Wdown:Qdown	**Median:**	3.94	**CV:**	0.00 %	**No. of Peptides:**	1

```
        10         20         30         40         50         60         70         80
MFCTKLKDLK ITGECPFSLL APGQVPNESS EEAAGSSESC KATVPICQDI PEKNIQESLP QRKTSRSRVY LHTLAESICK
        90        100        110        120        130        140        150        160
LIFPEFERLN VALQRTLAKH KIKESRKSLE REDFEKTIAE QAVAAGVPVE VIKESLGEEV FKICYEEDEN ILGVVGGTLK
       170        180        190        200        210        220        230        240
DFLNSFSTLL KQSSHCQEAG KRGRLEDASI LCLDKEDDFL HVYYFFPKRT TSLILPGIIK AAAHVLYETE VEVSLMPPCF
       250        260        270        280        290        300        310        320
HNDCSEFVNQ PYLLYSVHMK STKPSLSPSK PQSSLVIPTS LFCKTFPFHF MFDKDMTILQ FGNGIRRLMN RRDFQGKPNF
       330        340        350        360        370        380        390        400
EEYFEILTPK INQTFSGIMT MLNMQFVVRV RRWDNSVKKS SRVMDLKGQM IYIVESSAIL FLGSPCVDRL EDFTGRGLYL
       410        420        430        440        450        460        470        480
SDIPIHNALR DVVLIGEQAR AQDGLKKRLG KLKATLEQAH QALEEEKKKT VDLLCSIFPC EVAQQLWQGQ VVQAKKFSNV
       490        500        510        520        530        540        550        560
TMLFSDIVGF TAICSQCSPL QVITMLNALY TRFDQQCGEL DVYKVETIGD AYCVAGGLHK ESDTHAVQIA LMALKMMELS
       570        580        590        600        610        620        630
DEVMSPHGEP IKMRIGLHSG SVFAGVVGVK MPRYCLFGNN VTLANKFESC SVPRKINVSP TTYR
```

Cmpd.	No. of Cmpds.	m/z meas.	Δ m/z [ppm]	z	Rt [min]	Score	P	Range	Sequence	Modification	Ratios
289	1	548.1845	-238.23	2	32.7	11.4	2	2-10	M.FCTKLKDLK.I		mdown:qdown 0.88<br>Wdown:Qdown 3.94
2692	1	848.1258	162.49	2	64.9	11.8	0	117-133	K.TIAEQAVAAGVPVEVIK.E		

Detailed Protein Report

Protein 307: glutamate receptor ionotropic, kainate 3 precursor [Homo sapiens]

**Accession:**	gi|28605145	**Score:**	35.0
**Database:**	refseq_human(refseq_human_20140103.fasta)	**MW [kDa]:**	104.0
**Database Date:**	2015-11-30	**pI:**	7.8
**Modification(s):**	Oxidation	**Sequence Coverage [%]:**	2.7
		**No. of unique Peptides:**	1

10	20	30	40	50	60	70	80
MTAPWRRLRS	LVWEYWAGLL	VCAFWIPDSR	GMPHVIRIGG	IFEYADGPNA	QVMNAEEHAF	RFSANIINRN	RTLLPNTTLT
90	100	110	120	130	140	150	160
YDIQRIHFHD	SFEATKKACD	QLALGVVAIF	GPSQGSCTNA	VQSICNALEV	PHIQLRWKHH	PLDNKDTFYV	NLYPDYASLS
170	180	190	200	210	220	230	240
HAILDLVQYL	KWRSATVVYD	DSTGLIRLQE	LIMAPSRYNI	RLKIRQLPID	SDDSRPLLKE	MKRGREFRII	FDCSHTMAAQ
250	260	270	280	290	300	310	320
ILKQAMAMGM	MTEYYHFIFT	TLDLYALDLE	PYRYSGVNLT	GFRILNVDNP	HVSAIVEKWS	MERLQAAPRS	ESGLLDGVMM
330	340	350	360	370	380	390	400
TDAALLYDAV	HIVSVCYQRA	PQMTVNSLQC	HRHKAWRFGG	RFMNFIKEAQ	WEGLTGRIVF	NKTSGLRTDF	DLDIISLKED
410	420	430	440	450	460	470	480
GLEKVGVWSP	ADGLNITEVA	KGRGPNVTDS	LTNRSLIVTT	VLEEPFVMFR	KSDRTLYGND	RFEGYCIDLL	KELAHILGFS
490	500	510	520	530	540	550	560
YEIRLVEDGK	YGAQDDKGQW	NGMVKELIDH	KADLAVAPLT	ITHVREKAID	FSKPFMTLGV	SILYRKPNGT	NPSVFSFLNP
570	580	590	600	610	620	630	640
LSPDIWMYVL	LAYLGVSCVL	FVIARFSPYE	WYDAHPCNPG	SEVVENNFTL	LNSFWFGMGS	LMQQGSELMP	KALSTRIIGG
650	660	670	680	690	700	710	720
IWWFFTLIII	SSYTANLAAF	LTVERMESPI	DSADDLAKQT	KIEYGAVKDG	ATMTFFKKSK	ISTFEKMWAF	MSSKPSALVK
730	740	750	760	770	780	790	800
NNEEGIQRAL	TADYALLMES	TTIEYVTQRN	CNLTQIGGLI	DSKGYGIGTP	MGSPYRDKIT	IAILQLQEED	KLHIMKEKWW
810	820	830	840	850	860	870	880
RGSGCPEEEN	KEASALGIQK	IGGIFIVLAA	GLVLSVLVAV	GEFVYKLRKT	AEREQRSFCS	TVADEIRFSL	TCQRRVKHKP
890	900	910	920				
QPPMMVKTDA	VINMHTFNDR	RLPGKDSMAC	STSLAPVFP				

Cmpd.	No. of Cmpds.	m/z meas.	Δ m/z [ppm]	z	Rt [min]	Score	P	Range	Sequence	Modification
2002	1	704.2753	-62.09	2	55.1	13.0	0	666-678	R.MESPIDSADDLAK.Q	Oxidation: 1

Detailed Protein Report

Protein 308: aldo-keto reductase family 1 member C2 isoform 2 [Homo sapiens]

Accession: gi|207028673
Database: refseq_human(refseq_human_20140103.fasta)
Database Date: 2015-11-30

Score: 35.0
MW [kDa]: 15.7
pI: 8.9
Sequence Coverage [%]: 23.7
No. of unique Peptides: 1

10	20	30	40	50	60	70	80
MDSKYQCVKL	NDGHFMPVLG	FGTYAPAEVP	KSKALEAVKL	AIEAGFHHID	SAHVYNNEEQ	VGLAIRSKIA	DGSVKREDIF
**90**	**100**	**110**	**120**	**130**	**140**		
YTSKLWSNSH	RPELVRPALE	RSLKNLQLDY	VDLYLIHFPV	SVKEDIGILT	WKKSPKHNS		

Cmpd.	No. of Cmpds.	m/z meas.	Δ m/z [ppm]	z	Rt [min]	Score	P	Range	Sequence	Modification
2935	1	859.1202	17.35	3	65.9	17.7	1	10-33	K.LNDGHFMPVLGFGTYAPAEVPKSK.A	

Detailed Protein Report

Protein 309: dnaJ homolog subfamily C member 14 [Homo sapiens]

Accession: gi|119943096
Database: refseq_human(refseq_human_20140103.fasta)
Database Date: 2015-11-30
Modification(s): Carbamidomethyl, Oxidation

Score: 34.9
MW [kDa]: 78.5
pI: 9.4
Sequence Coverage [%]: 5.7
No. of unique Peptides: 3

Quantitation

mdown:qdown	**Median:** 1.37	**CV:** 10.31 %	**No. of Peptides:**	2
Wdown:Qdown	**Median:** 1.17	**CV:** 81.46 %	**No. of Peptides:**	3

```
        10         20         30         40         50         60         70         80
MAQKHPGERG LYGAHHSGGA SLRTLGPSVD PEIPSFSGLR DSAGTAPNGT RCLTEHSGPK HTQHPNPAHW LDPSHGPPGG
        90        100        110        120        130        140        150        160
PGPPRDAEDP DQSETSSEEE SGVDQELSKE NETGNQKDGN SFLSIPSACN CQGTPGIPEG PYSEGGNGSS SNFCHHCTSP
       170        180        190        200        210        220        230        240
ALGEDELEEE YDDEESLKFP SDFSRVSSGK KPPSRRQRHR FPTKEDTREG GRRDPRSPGR HRLGRKRSQA DKRKGLGLWG
       250        260        270        280        290        300        310        320
AEELCQLGQA GFWWLIELLV LVGEYVETCG HLIYACRQLK SSDLDLFRVW MGVWTGRLGG WAQVMFQFLS QGFYCGVGLF
       330        340        350        360        370        380        390        400
TRFLKLLGAL LLLALALFLG FLQLGWRFLV GLGDRLGWRD KATWLFSWLD SPALQRCLTL LRDSRPWQRL VRIVQWGWLE
       410        420        430        440        450        460        470        480
LPWVKQNINR QGNAPVASGR YCQPEEEVAR LLTMAGVPED ELNPFHVLGV EATASDVELK KAYRQLAVMV HPDKNHHPRA
       490        500        510        520        530        540        550        560
EEAFKVLRAA WDIVSNAEKR KEYEMKRMAE NELSRSVNEF LSKLQDDLKE AMNTMMCSRC QGKHRRFEMD REPKSARYCA
       570        580        590        600        610        620        630        640
ECNRLHPAEE GDFWAESSML GLKITYFALM DGKVYDITEW AGCQRVGISP DTHRVPYHIS FGSRIPGTRG RQRATPDAPP
       650        660        670        680        690        700        710
ADLQDFLSRI FQVPPGQMPN GNFFAAPQPA PGAAAASKPN STVPKGEAKP KRRKKVRRPF QR
```

Cmpd.	No. of Cmpds.	m/z meas.	Δ m/z [ppm]	z	Rt [min]	Score	P	Range	Sequence	Modification	Ratios
2138	1	661.2888	-106.46	2	55.3	10.6	1	278-288	R.QLKSSDLDLFR.V		Wdown:Qdown 2.63
2317	1	951.3323	-86.76	2	57.4	10.0	1	524-539	K.LQDDLKEAMNTMMCSR.C	Oxidation: 12	Wdown:Qdown 0.46<br>mdown:qdown 1.52
1717	3	820.8378	-35.57	2	50.0	14.3	2	552-564	R.EPKSARYCAECNR.L	Carbamidomethyl: 8, 11	Wdown:Qdown 1.30<br>mdown:qdown 1.23

Detailed Protein Report

Protein 310: PREDICTED: inositol 1,4,5-trisphosphate receptor type 2 isoform X1 [Homo sapiens]

**Accession:**	gi|578822836	**Score:**	34.9
**Database:**	refseq_human(refseq_human_20140103.fasta)	**MW [kDa]:**	212.9
**Database Date:**	2015-11-30	**pI:**	6.3
**Modification(s):**	Oxidation	**Sequence Coverage [%]:**	2.2
		**No. of unique Peptides:**	2

10	20	30	40	50	60	70	80
MTEKMSSFLY	IGDIVSLYAE	GSVNGFISTL	GLVDDRCVVH	PEAGDLANPP	KKFRDCLFKV	CPMNRYSAQK	QYWKAKQAKQ
90	100	110	120	130	140	150	160
GNHTEAALLK	KLQHAAELEQ	KQNESENKKL	LGEIVKYSNV	IQLLHIKSNK	YLTVNKRLPA	LLEKNAMRVS	LDAAGNEGSW
170	180	190	200	210	220	230	240
FYIHPFWKLR	SEGDNIVVGD	KVVLMPVNAG	QPLHASNIEL	LDNPGCKEVN	AVNCNTSWKI	TLFMKYSSYR	EDVLKGGDVV
250	260	270	280	290	300	310	320
RLFHAEQEKF	LTCDEYEKKQ	HIFLRTTLRQ	SATSATSSKA	LWEIEVVHHD	PCRGGAGQWN	SLFRFKHLAT	GNYLAAELNP
330	340	350	360	370	380	390	400
DYRDAQNEGK	NVRDGVPPTS	KKKRQAGEKI	MYTLVSVPHG	NDIASLFELD	ATTLQRADCL	VPRNSYVRLR	HLCTNTWVTS
410	420	430	440	450	460	470	480
TSIPIDTDEE	RPVMLKIGTC	QTKEDKEAFA	IVSVPLSEVR	DLDFANDANK	VLATTVKKLE	NGTITQNERR	FVTKLLEDLI
490	500	510	520	530	540	550	560
FFVADVPNNG	QEVLDVVITK	PNRERQKLMR	EQNILAQVFG	ILKAPFKEKA	GEGSMLRLED	LGDQRYAPYK	YMLRLCYRVL
570	580	590	600	610	620	630	640
RHSQQDYRKN	QEYIAKNFCV	MQSQIGYDIL	AEDTITALLH	NNRKLLEKHI	TAKEIETFVS	LLRRNREPRF	LDYLSDLCVS
650	660	670	680	690	700	710	720
NTTAIPVTQE	LICKFMLSPG	NADILIQTKV	VSMQADNPME	SSILSDDIDD	EEVWLYWIDS	NKEPHGKAIR	HLAQEAKEGT
730	740	750	760	770	780	790	800
KADLEVLTYY	RYQLNLFARM	CLDRQYLAIN	QISTQLSVDL	ILRCVSDESL	PFDLRASFCR	LMLHMHVDRD	PQESVVPVRY
810	820	830	840	850	860	870	880
ARLWTEIPTK	ITIHEYDSIT	DSSRNDMKRK	FALTMEFVEE	YLKEVVNQPF	PFGDKEKNKL	TFEVVHLARN	LIYFGFYSFS
890	900	910	920	930	940	950	960
ELLRLTRTLL	AILDIVQAPM	SSYFERLSKF	QDGGNNVMRT	IHGVGEMMTQ	MVLSRGSIFP	MSVPDVPPSI	HPSKQGSPTE
970	980	990	1000	1010	1020	1030	1040
HEDVTVMDTK	LKIIEILQFI	LSVRLDYRIS	YMLSIYKKEF	GEDNDNAETS	ASGSPDTLLP	SAIVPDIDEI	AAQAETMFAG
1050	1060	1070	1080	1090	1100	1110	1120
RKEKNPVQLD	DEGGRTFLRV	LIHLIMHDYP	PLLSGALQLL	FKHFSQRAEV	LQAFKQVQLL	VSNQDVDNYK	QIKADLDQLR
1130	1140	1150	1160	1170	1180	1190	1200
LTVEKSELWV	EKSSNYENGE	IGESQVKGGE	EPIEESNILS	PVQDGTKKPQ	IDSNKSNNYR	IVKEILIRLS	KLCVQNKKCR
1210	1220	1230	1240	1250	1260	1270	1280
NQHQRLLKNM	GAHSVVLDLL	QIPYEKNDEK	MNEVMNLAHT	FLQNFCRGNP	QNQVLLHKHL	NLFLTPGLLE	AETMRHIFMN
1290	1300	1310	1320	1330	1340	1350	1360
NYHLCNEISE	RVVQHFVHCI	ETHGRHVEYL	RFLQTIVKAD	GKYVKKCQDM	VMTELINGGE	DVLIFYNDRA	SFPILLHMMC
1370	1380	1390	1400	1410	1420	1430	1440
SERDRGDESG	PLAYHITLVE	LLAACTEGKN	VYTEIKCNSL	LPLDDIVRVV	THDDCIPEVK	IAYVNFVNHC	YVDTEVEMKE
1450	1460	1470	1480	1490	1500	1510	1520
IYTSNHIWKL	FENFLVDMAR	VCNTTTDRKH	ADIFLEKCVT	ESIMNIVSGF	FNSPFSDNST	SLQTHQPVFI	QLLQSAFRIY
1530	1540	1550	1560	1570	1580	1590	1600
NCTWPNPAQK	ASVESCIRTL	AEVAKNRGIA	IPVDLDSQVN	TLFMKSHSNM	VQRAAMGWRL	SARSGPRFKE	ALGGPAWDYR
1610	1620	1630	1640	1650	1660	1670	1680
NIIEKLQDVV	ASLEHQFSPM	MQAEFSVLVD	VLYSPELLFP	EGSDARIRCG	AFMSKLINHT	KKLMEKEEKL	CIKILQTLRE
1690	1700	1710	1720	1730	1740	1750	1760
MLEKKDSFVE	EGNTLRKILL	NRYFKGDYSI	GVNGHLSGAY	SKTAQVGGSF	SGQDSDKMGI	SMSDIQCLLD	KEGASELVID
1770	1780	1790	1800	1810	1820	1830	1840
VIVNTKNDRI	FSEGIFLGIA	LLEGGNTQTQ	YSFYQQLHEQ	KKSEKFFKVL	YDRMKAAQKE	IRSTVTVNTI	DLGNKKRDDD
1850	1860	1870					
NELMTSGPRM	REFWSWGDPK	TALQD					

Detailed Protein Report

Cmpd.	No. of Cmpds.	m/z meas.	Δ m/z [ppm]	z	Rt [min]	Score	P	Range	Sequence	Modification
2130	3	648.2779	-125.55	2	55.2	13.9	2	1199-1208	K.CRNQHQRLLK.N	
1156	1	867.9132	-54.78	2	43.1	10.9	2	1647-1661	R.IRCGAFMSKLINHTK.K	Oxidation: 7

Detailed Protein Report

Protein 311: dynein heavy chain 2, axonemal [Homo sapiens]

**Accession:**	gi|75677365	**Score:**	34.9
**Database:**	refseq_human(refseq_human_20140103.fasta)	**MW [kDa]:**	507.4
**Database Date:**	2015-11-30	**pI:**	5.9
		**Sequence Coverage [%]:**	0.9
		**No. of unique Peptides:**	2

Alias proteins:

Accession	Name	Description
gi|530409951	refseq_human (refseq_human_20140103.fasta)	PREDICTED: dynein heavy chain 2, axonemal isoform X1 [Homo sapiens]

BRUKER

Detailed Protein Report

10	20	30	40	50	60	70	80
MSSKAEKKQR	LSGRGSSQAS	WSGRATRAAV	ATQEQGNAPA	VSEPELQAEL	PKEEPEPRLE	GPQAQSEESV	EPEADVKPLF
90	100	110	120	130	140	150	160
LSRAALTGLA	DAVWTQEHDA	ILEHFAQDPT	ESILTIFIDP	CFGLKLELGM	PVQTQNQLVY	FIRQAPVPIT	WENFEATVQF
170	180	190	200	210	220	230	240
GTVRGPYIPA	LLRLLGGVFA	PQIFANTGWP	ESIRNHFASH	LHKFLACLTD	TRYKLEGHTV	LYIPAEAMNM	KPEMVIKDKE
250	260	270	280	290	300	310	320
LVQRLETSMI	HWTRQIKEML	SAQETVETGE	NLGPLEEIEF	WRNRCMDLSG	ISKQLVKKGV	KHVESILHLA	KSSYLAPFMK
330	340	350	360	370	380	390	400
LAQQIQDGSR	QAQSNLTFLS	ILKEPYQELA	FMKPKDISSK	LPKLISLIRI	IWVNSPHYNT	RERLTSLFRK	VCDCQYHFAR
410	420	430	440	450	460	470	480
WEDGKQGPLP	CFFGAQGPQI	TRNLLEIEDI	FHKNLHTLRA	VRGGILDVKN	TCWHEDYNKF	RAGIKDLEVM	TQNLITSAFE
490	500	510	520	530	540	550	560
LVRDVPHGVL	LLDTFHRLAS	REAIKRTYDK	KAVDLYMLFN	SELALVNRER	NKKWPDLEPY	VAQYSGKARW	VHILRRRIDR
570	580	590	600	610	620	630	640
VMTCLAGAHF	LPRIGTGKES	VHTYQQMVQA	IDELVRKTFQ	EWTSSLDKDC	IRRLDTPLLR	ISQEKAGMLD	VNFDKSLLIL
650	660	670	680	690	700	710	720
FAEIDYWERL	LFETPHYVVN	VAERAEDLRI	LRENLLLVAR	DYNRIIAMLS	PDEQALFKER	IRLLDKKIHP	GLKKLHWALK
730	740	750	760	770	780	790	800
GASAFFITEC	RIHASKVQMI	VNEFKASTLT	IGWRAQEMSE	KLLVRISGKR	VYRDLEFEED	QREHRAAVQQ	KLMNLHQDVV
810	820	830	840	850	860	870	880
TIMTNSYEVF	KNDGPEIQQQ	WMLYMIRLDR	MMEDALRLNV	KWSLLELSKA	INGDGKTSPN	PLFQVLVILK	NDLQGSVAQV
890	900	910	920	930	940	950	960
EFSPTLQTLA	GVVNDIGNHL	FSTISVFCHL	PDILTKRKLH	REPIQTVVEQ	DEDIKKIQTQ	ISSGMTNNAS	LLQNYLKTWD
970	980	990	1000	1010	1020	1030	1040
MYREIWEINK	DSFIHRYQRL	NPPVSSFVAD	IARYTEVANN	VQKEETVTNI	QFVLLDCSHL	KFSLVQHCNE	WQNKFATLLR
1050	1060	1070	1080	1090	1100	1110	1120
EMAAGRLLEL	HTYLKENAEK	ISRPPQTLEE	LGVSLQLVDA	LKHDLANVET	QIPPIHEQFA	ILEKYEVPVE	DSVLEMLDSL
1130	1140	1150	1160	1170	1180	1190	1200
NGEWVVFQQT	LLDSKQMLKK	HKEKFKTGLI	HSADDFKKKA	HTLLEDFEFK	GHFTSNVGYM	SALDQITQVR	AMLMAMREEE
1210	1220	1230	1240	1250	1260	1270	1280
NSLRANLGIF	KIEQPPSKDL	QNLEKELDAL	QQIWEIARDW	EENWNEWKTG	RFLILQTETM	ETTAHGLFRR	LTKLAKEYKD
1290	1300	1310	1320	1330	1340	1350	1360
RNWEIIETTR	SKIEQFKRTM	PLISDLRNPA	LRERHWDQVR	DEIQREFDQE	SESFTLEQIV	ELGMDQHVEK	IGEISASATK
1370	1380	1390	1400	1410	1420	1430	1440
ELAIEVALQN	IAKTWDVTQL	DIVPYKDKGH	HRLRGTEEVF	QALEDNQVAL	STMKASRFVK	AFEKDVDHWE	RCLSLILEVI
1450	1460	1470	1480	1490	1500	1510	1520
EMILTVQRQW	MYLENIFLGE	DIRKQLPNES	TLFDQVNSNW	KAIMDRMNKD	NNALRSTHHP	GLLDTLIEMN	TILEDIQKSL
1530	1540	1550	1560	1570	1580	1590	1600
DMYLETKRHI	FPRFYFLSND	DLLEILGQSR	NPEAVQPHLK	KCFDNIKLLR	IQKVGGPSSK	WEAVGMFSGD	GEYIDFLHSV
1610	1620	1630	1640	1650	1660	1670	1680
FLEGPVESWL	GDVEQTMRVT	LRDLLRNCHL	ALRKFLNKRD	KWVKEWAGQV	VITASQIQWT	ADVTKCLLTA	KERADKKILK
1690	1700	1710	1720	1730	1740	1750	1760
VMKKNQVSIL	NKYSEAIRGN	LTKIMRLKIV	ALVTIEIHAR	DVLEKLYKSG	LMDVNSFDWL	SQLRFYWEKD	LDDCVIRQTN
1770	1780	1790	1800	1810	1820	1830	1840
TQFQYNYEYL	GNSGRLVITP	LTDRCYMTLT	TALHLHRGGS	PKGPAGTGKT	ETVKDLGKAL	GIYVIVVNCS	EGLDYKSMGR
1850	1860	1870	1880	1890	1900	1910	1920
MYSGLAQTGA	WGCFDEFNRI	NIEVLSVVAH	QILCILSALA	AGLTHFHFDG	FEINLVWSCG	IFITMNPGYA	GRTELPENLK
1930	1940	1950	1960	1970	1980	1990	2000
SMFRPIAMVV	PDSTLIAEII	LFGEGFGNCK	ILAKKVYTLY	SLAVQQLSRQ	DHYDFGLRAL	TSLLRYAGKK	RRLQPDLTDE
2010	2020	2030	2040	2050	2060	2070	2080
EVLLLSMRDM	NIAKLTSVDA	PLFNAIVQDL	FPNIELPVID	YGKLRETVEQ	EIRDMGLQST	PFTLTKVFQL	YETKNSRHST
2090	2100	2110	2120	2130	2140	2150	2160
MIVGCTGSGK	TASWRILQAS	LSSLCRAGDP	NFNIVREFPL	NPKALSLGEL	YGEYDLSTNE	WTDGILSSVM	RTACADEKPD
2170	2180	2190	2200	2210	2220	2230	2240

Detailed Protein Report

Cmpd.	No. of Cmpds.	m/z meas.	Δ m/z [ppm]	z	Rt [min]	Score	P	Range	Sequence	Modification
58	1	827.0195	-82.92	3	30.1	13.2	1	1393-1414	R.LRGTEEVFQALEDNQVALSTMK.A	
312	1	712.8533	-32.33	2	33.2	10.2	2	3248-3258	K.LEMLKKQYDEK.L	

Detailed Protein Report

Protein 312: **PREDICTED: hephaestin isoform X3 [Homo sapiens]**

**Accession:**	gi|578838557	**Score:**	34.8
**Database:**	refseq_human(refseq_human_20140103.fasta)	**MW [kDa]:**	97.3
**Database Date:**	2015-11-30	**pI:**	5.8
**Modification(s):**	Oxidation	**Sequence Coverage [%]:**	4.1
		**No. of unique Peptides:**	2

10	20	30	40	50	60	70	80
MWAMESGHLL	WALLFMQSLW	PQLTDGATRV	YYLGIRDVQW	NYAPKGRNVI	TNQPLDSDIV	ASSFLKSDKN	RIGGTYKKTI
**90**	**100**	**110**	**120**	**130**	**140**	**150**	**160**
YKEYKDDSYT	DEVAQPAWLG	FLGPVLQAEV	GDVILIHLKN	FATRPYTIHP	HGVFYEKDSE	GSLYPDGSSG	PLKADDSVPP
**170**	**180**	**190**	**200**	**210**	**220**	**230**	**240**
GGSHIYNWTI	PEGHAPTDAD	PACLTWIYHS	HVDAPRDIAT	GLIGPLITCK	RGALDGNSPP	QRQDVDHDFF	LLFSVVDENL
**250**	**260**	**270**	**280**	**290**	**300**	**310**	**320**
SWHLNENIAT	YCSDPASVDK	EDETFQESNR	MHAINGFVFG	NLPELNMCAQ	KRVAWHLFGM	GNEIDVHTAF	FHGQMLTTRG
**330**	**340**	**350**	**360**	**370**	**380**	**390**	**400**
HHTDVANIFP	ATFVTAEMVP	WEPGTWLISC	QVNSHFRDGM	QALYKVKSCS	MAPPVDLLTG	KVRQYFIEAH	EIQWDYGPMG
**410**	**420**	**430**	**440**	**450**	**460**	**470**	**480**
HDGSTGKNLR	EPGSISDKFF	QKSSSRIGGT	YWKVRYEAFQ	DETFQEKMHL	EEDRHLGILG	PVIRAEVGDT	IQVVFYNRAS
**490**	**500**	**510**	**520**	**530**	**540**	**550**	**560**
QPFSMQPHGV	FYEKDYEGTV	YNDGTFEIYC	QAGSHREAGM	RAIYNVSQCP	GHQATPRQRY	QAARIYYIMA	EEVEWDYCPD
**570**	**580**	**590**	**600**	**610**	**620**	**630**	**640**
RSWEREWHNQ	SEKDSYGYIF	LSNKDGLLGS	RYKKAVFREY	TDGTFRIPRP	RTGPEEHLGI	LGPLIKGEVG	DILTVVFKNN
**650**	**660**	**670**	**680**	**690**	**700**	**710**	**720**
ASRPYSVHAH	GVLESTTVWP	LAAEPAINGK	LYANLRGLTM	YQGERVAWYM	LAMGQDVDLH	TIHFHAESFL	YRNGENYRAD
**730**	**740**	**750**	**760**	**770**	**780**	**790**	**800**
VVDLFPGTFE	VVEMVASNPG	TWLMHCHVTD	HVHAGMETLF	TVFSRTEHLS	PLTVITKETE	KAVPPRDIEE	GNVKMLGMQI
**810**	**820**	**830**	**840**	**850**	**860**		
PIKNVEMLAS	VLVAISVTLL	LVVLALGGVV	WYQHRQRKLR	RNRRSILDDS	FKLLSFKQ		

Cmpd.	No. of Cmpds.	m/z meas.	Δ m/z [ppm]	z	Rt [min]	Score	P	Range	Sequence	Modification
2643	1	784.0056	-47.59	3	61.3	13.5	0	271-291	R.MHAINGFVFGNLPELNMCAQK.R	Oxidation: 17
1824	2	805.8049	-133.19	2	52.8	21.4	0	465-478	R.AEVGDTIQVVFYNR.A	

Detailed Protein Report

Protein 313: acyl-protein thioesterase 2 [Homo sapiens]

**Accession:**	gi|9966764	**Score:**	34.8
**Database:**	refseq_human(refseq_human_20140103.fasta)	**MW [kDa]:**	24.7
**Database Date:**	2015-11-30	**pI:**	6.9
**Modification(s):**	Oxidation	**Sequence Coverage [%]:**	12.6
		**No. of unique Peptides:**	2

10	20	30	40	50	60	70	80
MCGNTMSVPL	LTDAATVSGA	ERETAAVIFL	HGLGDTGHSW	ADALSTIRLP	HVKYICPHAP	RIPVTLNMKM	VMPSWFDLMG
**90**	**100**	**110**	**120**	**130**	**140**	**150**	**160**
LSPDAPEDEA	GIKKAAENIK	ALIEHEMKNG	IPANRIVLGG	FSQGGALSLY	TALTCPHPLA	GIVALSCWLP	LHRAFPQAAN
**170**	**180**	**190**	**200**	**210**	**220**	**230**	**240**
GSAKDLAILQ	CHGELDPMVP	VRFGALTAEK	LRSVVTPARV	QFKTYPGVMH	SSCPQEMAAV	KEFLEKLLPP	V

Cmpd.	No. of Cmpds.	m/z meas.	Δ m/z [ppm]	z	Rt [min]	Score	P	Range	Sequence	Modification
1333	1	1061.5792	39.36	1	46.4	21.3	0	154-164	R.AFPQAANGSAK.D	
1865	2	984.3384	-99.79	2	51.8	13.6	0	204-221	K.TYPGVMHSSCPQEMAAVK.E	Oxidation: 6, 14

Detailed Protein Report

Protein 314: PREDICTED: integrin beta-8 isoform X2 [Homo sapiens]

**Accession:**	gi|530384769	**Score:**	34.8
**Database:**	refseq_human(refseq_human_20140103.fasta)	**MW [kDa]:**	71.2
**Database Date:**	2015-11-30	**pI:**	9.2
**Modification(s):**	Carbamidomethyl	**Sequence Coverage [%]:**	4.7
		**No. of unique Peptides:**	1

Alias proteins:

Accession	Name	Description
gi|530384771	refseq_human (refseq_human_20140103.fasta)	PREDICTED: integrin beta-8 isoform X3 [Homo sapiens]

10	20	30	40	50	60	70	80
MLKVHPLKKY	PVDLYYLVDV	SASMHNNIEK	LNSVGNDLSR	KMAFFSRDFR	LGFGSYVDKT	VSPYISIHPE	RIHNQCSDYN
**90**	**100**	**110**	**120**	**130**	**140**	**150**	**160**
LDCMPPHGYI	HVLSLTENIT	EFEKAVHRQK	ISGNIDTPEG	GFDAMLQAAV	CESHIGWRKE	AKRLLLVMTD	QTSHLALDSK
**170**	**180**	**190**	**200**	**210**	**220**	**230**	**240**
LAGIVVPNDG	NCHLKNNVYV	KSTTMEHPSL	GQLSEKLIDN	NINVIFAVQG	KQFHWYKDLL	PLLPGTIAGE	IESKAANLNN
**250**	**260**	**270**	**280**	**290**	**300**	**310**	**320**
LVVEAYQKLI	SEVKVQVENQ	VQGIYFNITA	ICPDGSRKPG	MEGCRNVTSN	DEVLFNVTVT	MKKCDVTGGK	NYAIIKPIGF
**330**	**340**	**350**	**360**	**370**	**380**	**390**	**400**
NETAKIHIHR	NCSCQCEDNR	GPKGKCVDET	FLDSKCFQCD	ENKCHFDEDQ	FSSESCKSHK	DQPVCSGRGV	CVCGKCSCHK
**410**	**420**	**430**	**440**	**450**	**460**	**470**	**480**
IKLGKVYGKY	CEKDDFSCPY	HHGNLCAGHG	ECEAGRCQCF	SGWEGDRCQC	PSAAAQHCVN	SKGQVCSGRG	TCVCGRCECT
**490**	**500**	**510**	**520**	**530**	**540**	**550**	**560**
DPRSIGRFCE	HCPTCYTACK	ENWNCMQCLH	PHNLSQAILD	QCKTSCALME	QQHYVDQTSE	CFSSPSYLRI	FFIIFIVTFL
**570**	**580**	**590**	**600**	**610**	**620**	**630**	**640**
IGLLKVLIIR	QVILQWNSNK	IKSSSDYRVS	ASKKDKLILQ	SVCTRAVTYR	REKPEEIKMD	ISKLNAHETF	RCNF

Cmpd.	No. of Cmpds.	m/z meas.	Δ m/z [ppm]	z	Rt [min]	Score	P	Range	Sequence	Modification
2775	1	746.3843	84.07	3	66.0	16.7	2	381-400	K.DQPVCSGRGVCVCGKCSCHK.I	Carbamidomethyl: 5, 11, 18

Detailed Protein Report

Protein 315: scm-like with four MBT domains protein 1 [Homo sapiens]

**Accession:**	gi|53692193	**Score:**	34.8
**Database:**	refseq_human(refseq_human_20140103.fasta)	**MW [kDa]:**	98.1
**Database Date:**	2015-11-30	**pI:**	5.8
**Modification(s):**	Oxidation	**Sequence Coverage [%]:**	4.5
		**No. of unique Peptides:**	1

Quantitation

Wdown:Qdown	**Median:**	1.64	**CV:**	0.00 %	**No. of Peptides:**	1	

Alias proteins:

Accession	Name	Description
gi|578806223	refseq_human (refseq_human_20140103.fasta)	PREDICTED: scm-like with four MBT domains protein 1 isoform X4 [Homo sapiens]
gi|578806221	refseq_human (refseq_human_20140103.fasta)	PREDICTED: scm-like with four MBT domains protein 1 isoform X3 [Homo sapiens]
gi|530372640	refseq_human (refseq_human_20140103.fasta)	PREDICTED: scm-like with four MBT domains protein 1 isoform X2 [Homo sapiens]

10	20	30	40	50	60	70	80
MNGEQQLDAD	AGSGMEEVEL	SWEDYLEETG	STAVPYGSFK	HVDTRLQNGF	APGMKLEVAV	RTDPETYWVA	TVITTCEQLL
90	100	110	120	130	140	150	160
LLRYDGYGED	RRADFWCDIR	KADLYPIGWC	EQNKKTLEAP	EGIRDKVSDW	DEFLRQTLIG	ACSPPVPLLE	GLRNGRNPLD
170	180	190	200	210	220	230	240
LIAPGSRLEC	QAFQDSLSTW	IVTVVENIGG	RLKLRYEGLE	SSDNYEHWLY	YLDPFLHHVG	WAAQQGYELQ	PPSAIRHLKN
250	260	270	280	290	300	310	320
EAEWQEILAK	VKEEEEEPLP	SYLFKDKQVI	GIHTFSVNMK	LEAVDPWSPF	GISPATVVKV	FDEKYFLVEM	DDLRPENHAR
330	340	350	360	370	380	390	400
RSFVCHADSP	GIFPVQWSLK	NGLHISPPPG	YPSQDFDWAD	YLKQCGAEAA	PQRCFPPLIS	EHEFKENMKL	EAVNPILPEE
410	420	430	440	450	460	470	480
VCVATITAVR	GSYLWLQLEG	SKKPIPECIV	SVESMDIFPL	GWCETNGHPL	STPRRARVYK	QRKIAVVQPE	KQVPSSRTVH
490	500	510	520	530	540	550	560
EGLRNQELNS	TESVMINGKY	CCPKIYFNHR	CFSGPYLNKG	RIAELPQCVG	PGNCVLVLRE	VLTLLINAAY	KPSRVLRELQ
570	580	590	600	610	620	630	640
LDKDSVWHGC	GEVLKAKYKG	KSYRATVEIV	KTADRVTEFC	RQTCIKLECC	PNLFGPRMVL	DKCSENCSVL	TKTKYTHYYG
650	660	670	680	690	700	710	720
KKKNKRIGRP	PGGHSNLACA	LKKASKRRKR	RKNVFVHKKK	RSSASVDNTP	AGSPQGSGGE	DEDDPDEGDD	DSLSEGSTSE
730	740	750	760	770	780	790	800
QQDELQEESE	MSEKKSCSSS	PTQSEISTSL	PPDRQRRKRE	LRTFSFSDDE	NKPPSPKEIR	IEVAERLHLD	SNPLKWSVAD
810	820	830	840	850	860	870	
VVRFIRSTDC	APLARIFLDQ	EIDGQALLLL	TLPTVQECMD	LKLGPAIKLC	HHIERIKFAF	YEQFAN	

Cmpd.	No. of Cmpds.	m/z meas.	Δ m/z [ppm]	z	Rt [min]	Score	P	Range	Sequence	Modification	Ratios
2349	1	873.4186	-61.16	2	57.8	11.0	1	46-61	R.LQNGFAPGMKLEVAVR.T	Oxidation: 9	Wdown:Qdown 1.64

Detailed Protein Report

Protein 316: PREDICTED: dedicator of cytokinesis protein 5 isoform X2 [Homo sapiens]

**Accession:**	gi|530388077	**Score:**	34.8
**Database:**	refseq_human(refseq_human_20140103.fasta)	**MW [kDa]:**	119.2
**Database Date:**	2015-11-30	**pI:**	7.9
**Modification(s):**	Carbamidomethyl, Oxidation	**Sequence Coverage [%]:**	3.9
		**No. of unique Peptides:**	3

Quantitation

mdown:qdown **Median:** 1.06 **CV:** 0.00 % **No. of Peptides:** 1

10	20	30	40	50	60	70	80
MDLIRPRVSL	VCQIVRVGHM	ELKEGKKHTC	GLRRPFGVAV	MDITDIIHGK	VDDEEKQHFI	PFQQIAMETY	IRQRQLIMSP
**90**	**100**	**110**	**120**	**130**	**140**	**150**	**160**
LITSHVIGEN	EPLTSVLNKV	IAAKEVNHKG	QGLWVSLKLL	PGDLTQVQKN	FSHLVDRSTA	IARKMGFPEI	ILPGDVRNDI
**170**	**180**	**190**	**200**	**210**	**220**	**230**	**240**
YVTLIHGEFD	KGKKKTPKNV	EVTMSVHDEE	GKLLEKAIHP	GAGYEGISEY	KSVVYYQVKQ	PCWYETVKVS	IAIEEVTRCH
**250**	**260**	**270**	**280**	**290**	**300**	**310**	**320**
IRFTFRHRSS	QETRDKSERA	FGVAFVKLMN	PDGTTLQDGR	HDLVVYKGDN	KKMEDAKFYL	TLPGTKMEME	EKELQASKNL
**330**	**340**	**350**	**360**	**370**	**380**	**390**	**400**
VTFTPSKDST	KDSFQIATLI	CSTKLTQNVD	LLGLLNWRSN	SQNIKHNLKK	LMEVDGGEIV	KFLQDTLDAL	FNIMMEMSDS
**410**	**420**	**430**	**440**	**450**	**460**	**470**	**480**
ETYDFLVFDA	LVFIISLIGD	IKFQHFNPVL	ETYIYKHFSA	TLAYVKLSKV	LNFYVANADD	SSKTELLFAA	LKALKYLFRF
**490**	**500**	**510**	**520**	**530**	**540**	**550**	**560**
IIQSRVLYLR	FYGQSKDGDE	FNNSIRQLFL	AFNMLMDRPL	EEAVKIKGAA	LKYLPSIIND	VKLVFDPVEL	SVLFCKFIQS
**570**	**580**	**590**	**600**	**610**	**620**	**630**	**640**
IPDNQLVRQK	LNCMTKIVES	TLFRQSECRE	VLLPLLTDQL	SGQLDDNSNK	PDHEASSQLL	SNILEVLDRK	DVGATAVHIQ
**650**	**660**	**670**	**680**	**690**	**700**	**710**	**720**
LIMERLLRRI	NRTVIGMNRQ	SPHIGSFVAC	MIALLQQMDD	SHYSHYISTF	KTRQDIIDFL	METFIMFKDL	IGKNVYAKDW
**730**	**740**	**750**	**760**	**770**	**780**	**790**	**800**
MVMNMTQNRV	FLRAINQFAE	VLTRFFMDQA	SFELQLWNNY	FHLAVAFLTH	ESLQLETFSQ	AKRNKIVKKY	GDMRKEIGFR
**810**	**820**	**830**	**840**	**850**	**860**	**870**	**880**
IRDMWYNLGP	HKIKFIPSMV	GPILEVTLTP	EVELRKATIP	IFFDMMQCEF	NFSGNGNFHM	FENELITKLD	QEVEGGRGDE
**890**	**900**	**910**	**920**	**930**	**940**	**950**	**960**
QYKVLLEKLL	LEHCRKHKYL	SSSGEVFALL	VSSLLENLLD	YRTIIMQDES	KENRMSCTVN	VLNFYKEKKR	EDIYIRYLYK
**970**	**980**	**990**	**1000**	**1010**	**1020**	**1030**	**1040**
LRDLHRDCEN	YTEAAYTLLL	HAELLQVNGS	ESLFQQGGVQ	CPFRLNSIHF	MILMGKNFIP	GISFYTSFFS	SF

Cmpd.	No. of Cmpds.	m/z meas.	Δ m/z [ppm]	z	Rt [min]	Score	P	Range	Sequence	Modification	Ratios
1026	1	583.6927	-110.67	2	41.3	11.4	0	497-506	K.DGDEFNNSIR.Q		
1143	2	630.9795	-7.44	3	43.0	11.9	0	694-708	R.QDIIDFLMETFIMFK.D		
862	1	945.8909	-57.70	2	40.2	11.6	1	932-946	K.ENRMSCTVNVLNFYK.E	Carbamidomethyl: 6; Oxidation: 4	mdown:qdown 1.06

Detailed Protein Report

Protein 317: trinucleotide repeat-containing gene 6B protein isoform 3 [Homo sapiens]

**Accession:**	gi|67782330	**Score:**	34.8
**Database:**	refseq_human(refseq_human_20140103.fasta)	**MW [kDa]:**	109.3
**Database Date:**	2015-11-30	**pI:**	7.0
**Modification(s):**	Oxidation	**Sequence Coverage [%]:**	2.3
		**No. of unique Peptides:**	2

Quantitation

mdown:qdown	**Median:**	1.02	**CV:**	0.00 %	**No. of Peptides:**	1
Wdown:Qdown	**Median:**	0.13	**CV:**	0.00 %	**No. of Peptides:**	1

10	20	30	40	50	60	70	80
MQTNEGEVSE	ESSSKVEQED	FVMEGHGKTP	PPGEESKQEK	EQEREEQLME	DKKRKKEDKK	KKEATQKVTE	QKTKVPEVTK
90	100	110	120	130	140	150	160
PSLSQPTAAS	PIGSSPSPPV	NGGNNAKRVA	VPNGQPPSAA	RYMPREVPPR	FRCQQDHKVL	LKRGQPPPPS	CMLLGGGAGP
170	180	190	200	210	220	230	240
PPCTAPGANP	NNAQVTGALL	QSESGTAPVW	SKSTPPAPDN	GTSAWGEPNE	SSPGWGEMDD	TGASTTGWGN	TPANAPNAMK
250	260	270	280	290	300	310	320
PNSKSMQDGW	GESDGPVTGA	RHPSWEEEED	GGVWNTTGSQ	GSASSHNSAS	WGQGGKKQMK	CSLKGGNNDS	WMNPLAKQFS
330	340	350	360	370	380	390	400
NMGLLSQTED	NPSSKMDLSV	GSLSDKKFDV	DKRAMNLGDF	NDIMRKDRSG	FRPPNSKDMG	TTDSGPYFEK	GGSHGLFGNS
410	420	430	440	450	460	470	480
TAQSRGLHTP	VQPLNSSPSL	RAQVPPQFIS	PQVSASMLKQ	FPNSGLSPGL	FNVGPQLSPQ	QIAMLSQLPQ	IPQFQLACQL
490	500	510	520	530	540	550	560
LLQQQQQQQL	LQNQRKISQA	VRQQQEQQLA	RMVSALQQQQ	QQQQRQPGMK	HSPSHPVGPK	PHLDNMVPNA	LNVGLPDLQT
570	580	590	600	610	620	630	640
KGPIPGYGSG	FSSGGMDYGM	VGGKEAGTES	RFKQWTSMME	GLPSVATQEA	NMHKNGAIVA	PGKTRGGSPY	NQFDIIPGDT
650	660	670	680	690	700	710	720
LGGHTGPAGD	SWLPAKSPPT	NKIGSKSSNA	SWPPEFQPGV	PWKGIQNIDP	ESDPYVTPGS	VLGGTATSPI	VDTDHQLLRD
730	740	750	760	770	780	790	800
NTTGSNSSLN	TSLPSPGAWP	YSASDNSFTN	VHSTSAKFPD	YKSTWSPDPI	GHNPTHLSNK	MWKNHISSRN	TTPLPRPPPG
810	820	830	840	850	860	870	880
LTNPKPSSPW	SSTAPRSVRG	WGTQDSRLAS	ASTWSDGGSV	RPSYWLVLHN	LTPQIDGSTL	RTICMQHGPL	LTFHLNLTQG
890	900	910	920	930	940	950	960
TALIRYSTKQ	EAAKAQTALH	MCVLGNTTIL	AEFATDDEVS	RFLAQAQPPT	PAATPSAPAA	GWQSLETGQN	QSDPVGPALN
970	980	990	1000	1010	1020	1030	
LFGGSTGLGQ	WSSSAGGSSG	ADLAGASLWG	PPNYSSSLWG	VPTVEDPHRM	GSPAPLLPGD	LLGGGSDSI	

Cmpd.	No. of Cmpds.	m/z meas.	Δ m/z [ppm]	z	Rt [min]	Score	P	Range	Sequence	Modification	Ratios
1277	1	829.2282	-142.65	2	45.6	10.2	0	1-15	-.MQTNEGEVSEESSSK.V	Oxidation: 1	
433	6	564.6888	-181.74	2	34.7	24.6	0	503-511	R.QQQEQQLAR.M		Wdown:Qdown 0.13<br>mdown:qdown 1.02

Detailed Protein Report

Protein 318: uncharacterized aarF domain-containing protein kinase 2 [Homo sapiens]

Accession: gi|32261307
Database: refseq_human(refseq_human_20140103.fasta)
Database Date: 2015-11-30
Modification(s): Oxidation

Score: 34.8
MW [kDa]: 68.9
pI: 10.0
Sequence Coverage [%]: 7.7
No. of unique Peptides: 3

Quantitation

mdown:qdown **Median:** 0.21 **CV:** 0.00 % **No. of Peptides:** 1

10	20	30	40	50	60	70	80
MVAPWRVSVR	VCLSHLRCFE	LRQGLSLLRP	SECPRDARLC	WLLLGTLPKV	VSLCGDVGEG	APDVLSRRRV	RCSGAAGAGP
**90**	**100**	**110**	**120**	**130**	**140**	**150**	**160**
AESLPRAGPL	GGVFLHLRLW	LRAGALLVKF	FPLLLLYPLT	YLAPSVSTLW	LHLLLKATET	SGPTYIKLGQ	WASTRRDLFS
**170**	**180**	**190**	**200**	**210**	**220**	**230**	**240**
EAFCAQFSKL	HVRVTPHPWT	HTERFLRQAF	GDDWGSILSF	ENREPVGSGC	VAQVYKAYAN	TAFLETDSVQ	RLGRASCLPP
**250**	**260**	**270**	**280**	**290**	**300**	**310**	**320**
FSHTGAVGGL	RELFGYLGNG	RKPPENLADQ	SFLERLLLPK	ADLVGSNAGV	SRAQVPGHQP	EATNLISVAV	KVLHPGLLAQ
**330**	**340**	**350**	**360**	**370**	**380**	**390**	**400**
VHMDLLLMKI	GSRVLGVLPG	IKWLSLPEIV	EEFEKLMVQQ	IDLRYEAQNL	EHFQVNFRNV	KAVKFPTPLR	PFVTREVLVE
**410**	**420**	**430**	**440**	**450**	**460**	**470**	**480**
TYEESVPVSS	YQQAGIPVDL	KRKIARLGIN	MLLKMIFVDN	FVHADLHPGN	ILVQGANGLS	SSQEAQLQQA	DICDTLVVAV
**490**	**500**	**510**	**520**	**530**	**540**	**550**	**560**
PSSLCPLRLV	LLDAGIVAEL	QAPDLRNFRA	VFMAVVMGQG	QRVAELILHH	ARASECRDVE	GFKTEMAMLV	TQARKNTITL
**570**	**580**	**590**	**600**	**610**	**620**	**630**	
EKLHVSSLLS	SVFKLLMTHK	VKLESNFASI	VFAIMVLEGL	GRSLDPKLDI	LEAARPFLLT	GPVCPP	

Cmpd.	No. of Cmpds.	m/z meas.	Δ m/z [ppm]	z	Rt [min]	Score	P	Range	Sequence	Modification	Ratios
2782	2	835.4333	6.80	2	63.2	13.6	0	235-251	R.ASCLPPFSHTGAVGGLR.E		
2955	1	953.4514	-107.61	2	66.1	10.5	0	489-506	R.LVLLDAGIVAELQAPDLR.N		
2880	1	713.3447	-10.93	2	64.7	10.8	0	510-522	R.AVFMAVVMGQGQR.V	Oxidation: 4, 8	mdown:qdown 0.21

Detailed Protein Report

Protein 319: **microtubule-associated protein 6 isoform 1 [Homo sapiens]**

Accession: gi|48375173
Database: refseq_human(refseq_human_20140103.fasta)
Database Date: 2015-11-30
Modification(s): Oxidation

Score: 34.8
MW [kDa]: 86.5
pI: 9.8
Sequence Coverage [%]: 3.8
No. of unique Peptides: 2

10	20	30	40	50	60	70	80
MAWPCITRAC	CIARFWNQLD	KADIAVPLVF	TKYSEATEHP	GAPPQPPPPQ	QQAQPALAPP	SARAVAIETQ	PAQGELDAVA
90	100	110	120	130	140	150	160
RATGPAPGPT	GEREPAAGPG	RSGPGPGLGS	GSTSGPADSV	MRQDYRAWKV	QRPEPSCRPR	SEYQPSDAPF	ERETQYQKDF
170	180	190	200	210	220	230	240
RAWPLPRRGD	HPWIPKPVQI	SAASQASAPI	LGAPKRRPQS	QERWPVQAAA	EAREQEAAPG	GAGGLAAGKA	SGADERDTRR
250	260	270	280	290	300	310	320
KAGPAWIVRR	AEGLGHEQTP	LPAAQAQVQA	TGPEAGRGRA	AADALNRQIR	EEVASAVSSS	YRNEFRAWTD	IKPVKPIKAK
330	340	350	360	370	380	390	400
PQYKPPDDKM	VHETSYSAQF	KGEASKPTTA	DNKVIDRRRI	RSLYSEPFKE	PPKVEKPSVQ	SSKPKKTSAS	HKPTRKAKDK
410	420	430	440	450	460	470	480
QAVSGQAAKK	KSAEGPSTTK	PDDKEQSKEM	NNKLAEAKES	LAQPVSDSSK	TQGPVATEPD	KDQGSVVPGL	LKGQGPMVQE
490	500	510	520	530	540	550	560
PLKKQGSVVP	GPPKDLGPMI	PLPVKDQDHT	VPEPLKNESP	VISAPVKDQG	PSVPVPPKNQ	SPMVPAKVKD	QGSVVPESLK
570	580	590	600	610	620	630	640
DQGPRIPEPV	KNQAPMVPAP	VKDEGPMVSA	SVKDQGPMVS	APVKDQGPIV	PAPVKGEGPI	VPAPVKDEGP	MVSAPIKDQD
650	660	670	680	690	700	710	720
PMVPEHPKDE	SAMATAPIKN	QGSMVSEPVK	NQGLVVSGPV	KDQDVVVPEH	AKVHDSAVVA	PVKNQGPVVP	ESVKNQDPIL
730	740	750	760	770	780	790	800
PVLVKDQGPT	VLQPPKNQGR	IVPEPLKNQV	PIVPVPLKDQ	DPLVPVPAKD	QGPAVPEPLK	TQGPRDPQLP	TVSPLPRVMI
810	820						
PTAPHTEYIE	SSP						

Cmpd.	No. of Cmpds.	m/z meas.	Δ m/z [ppm]	z	Rt [min]	Score	P	Range	Sequence	Modification
1658	1	1045.0497	8.34	2	49.4	18.6	1	528-547	K.DQGPSVPVPPKNQSPMVPAK.V	Oxidation: 16
1905	5	572.7262	-106.04	2	53.9	16.3	0	594-604	K.DQGPMVSAPVK.D	Oxidation: 5

Detailed Protein Report

Protein 320: uncharacterized protein C9orf172 [Homo sapiens]

Accession: gi|148762978
Database: refseq_human(refseq_human_20140103.fasta)
Database Date: 2015-11-30

Score: 34.8
MW [kDa]: 106.6
pI: 10.2
Sequence Coverage [%]: 3.1
No. of unique Peptides: 2

10	20	30	40	50	60	70	80
MTRTDPPDLL	VSTVYQDIKV	ATPGPASKCS	PCERSVARPA	EPAPFNKRHC	RSFDFLEALD	GPAMETLPEP	PPPESAVPRA
90	100	110	120	130	140	150	160
RTREAEPRRR	ARSKSAPRAP	PGLTPAPASP	PVLPRRGREA	QRAARAEASP	RREPAYPALR	ALANELHPIK	LQPQRGGPGR
170	180	190	200	210	220	230	240
VAPLCAAAGR	CAPPEPPAGP	APHVRCRLDI	KPDDAVLQHA	TRGSRSCGPT	EAAHWARPAP	QFHGLTVPGP	RHMALSRTPT
250	260	270	280	290	300	310	320
PSDSYCADPR	AFYCDGPLPG	PRDYAERRSL	PFTTPPGPTQ	FFYTEEPQGF	RGSFAASPGP	TFDAYYPRPY	PSEELSGPSP
330	340	350	360	370	380	390	400
RRMGGYYAGE	VRTFPIQEPP	SRSYYGEAPR	AYGLPYGPRY	VPEEPRAHST	ARPFYTEDFG	RYRERDVLAR	TYPHPRSSPA
410	420	430	440	450	460	470	480
WADWGPRPYR	TLQVVPPSDP	DPLLASWHGG	TGTSPPRLAT	DSRHYSRSWD	NILAPGPRRE	DPLGRGRSYE	NLLGREVREP
490	500	510	520	530	540	550	560
RGVSPEGRRP	PVVVNLSTSP	RRYAALSLSE	TSLTEKGRAG	EGLGRNWYVT	PEITITDNDL	RATERPSARA	WELPGGRTRP
570	580	590	600	610	620	630	640
PPHAAPDGPT	SGRQRSLEQL	DELITDLVID	SRPTAGQASE	PAADCLGPQL	RRLLDSRPAG	SGAPALAPPR	SPPASAGSAE
650	660	670	680	690	700	710	720
EPAAPGEAAD	ASPEPSADED	DLMTCSNARC	RRTETMFNAC	LYFKSCHSCY	TYYCSRLCRR	EDWDAHKARC	VYGRVGSVCR
730	740	750	760	770	780	790	800
HVLQFCRDSG	PVHRAFSRIA	RVGFLSRGRG	VLFLGFPSPG	SADNFLRFGL	EGLLLSPTYL	SLRELATHAA	PLGSYARELA
810	820	830	840	850	860	870	880
AAGRLYEPAE	CFLLSVSVAV	GPGTAPPGTP	ALPAPAPRSH	GPTVRKFAKV	ALAAGSPARP	PPARSREPDM	ETLILTPPPG
890	900	910	920	930	940	950	960
TAGLDQDGEA	GRRAREVAFI	HIQRELRLRG	VFLRHEFPRV	YEQLCEFVEA	NRRFTPTTIY	PTDRRTGRPF	MCMIMAASEP
970	980						
RALDWVASAN	LLDDIM						

Cmpd.	No. of Cmpds.	m/z meas.	Δ m/z [ppm]	z	Rt [min]	Score	P	Range	Sequence	Modification
2104	1	977.4132	-55.55	2	54.9	10.8	1	945-961	R.RTGRPFMCMIMAASEPR.A	
1808	3	599.9802	55.20	3	51.3	13.7	0	946-961	R.TGRPFMCMIMAASEPR.A	

Detailed Protein Report

Protein 321: integrin beta-4 isoform 3 precursor [Homo sapiens]

**Accession:**	gi|54607033	**Score:**	34.8
**Database:**	refseq_human(refseq_human_20140103.fasta)	**MW [kDa]:**	194.9
**Database Date:**	2015-11-30	**pI:**	5.7
**Modification(s):**	Carbamidomethyl	**Sequence Coverage [%]:**	1.2
		**No. of unique Peptides:**	1

Alias proteins:

Accession	Name	Description
gi|530412114	refseq_human (refseq_human_20140103.fasta)	PREDICTED: integrin beta-4 isoform X4 [Homo sapiens]

Detailed Protein Report

10	20	30	40	50	60	70	80
MAGPRPSPWA	RLLLAALISV	SLSGTLANRC	KKAPVKSCTE	CVRVDKDCAY	CTDEMFRDRR	CNTQAELLAA	GCQRESIVVM
90	100	110	120	130	140	150	160
ESSFQITEET	QIDTTLRRSQ	MSPQGLRVRL	RPGEERHFEL	EVFEPLESPV	DLYILMDFSN	SMSDDLDNLK	KMGQNLARVL
170	180	190	200	210	220	230	240
SQLTSDYTIG	FGKFVDKVSV	PQTDMRPEKL	KEPWPNSDPP	FSFKNVISLT	EDVDEFRNKL	QGERISGNLD	APEGGFDAIL
250	260	270	280	290	300	310	320
QTAVCTRDIG	WRPDSTHLLV	FSTESAFHYE	ADGANVLAGI	MSRNDERCHL	DTTGTYTQYR	TQDYPSVPTL	VRLLAKHNII
330	340	350	360	370	380	390	400
PIFAVTNYSY	SYYEKLHTYF	PVSSLGVLQE	DSSNIVELLE	EAFNRIRSNL	DIRALDSPRG	LRTEVTSKMF	QKTRTGSFHI
410	420	430	440	450	460	470	480
RRGEVGIYQV	QLRALEHVDG	THVCQLPEDQ	KGNIHLKPSF	SDGLKMDAGI	ICDVCTCELQ	KEVRSARCSF	NGDFVCGQCV
490	500	510	520	530	540	550	560
CSEGWSGQTC	NCSTGSLSDI	QPCLREGEDK	PCSGRGECQC	GHCVCYGEGR	YEGQFCEYDN	FQCPRTSGFL	CNDRGRCSMG
570	580	590	600	610	620	630	640
QCVCEPGWTG	PSCDCPLSNA	TCIDSNGGIC	NGRGHCECGR	CHCHQQSLYT	DTICEINYSA	IHPGLCEDLR	SCVQCQAWGT
650	660	670	680	690	700	710	720
GEKKGRTCEE	CNFKVKMVDE	LKRAEEVVVR	CSFRDEDDDC	TYSYTMEGDG	APGPNSTVLV	HKKKDCPPGS	FWWLIPLLLL
730	740	750	760	770	780	790	800
LLPLLALLLL	LCWKYCACCK	ACLALLPCCN	RGHMVGFKED	HYMLRENLMA	SDHLDTPMLR	SGNLKGRDVV	RWKVTNNMQR
810	820	830	840	850	860	870	880
PGFATHAASI	NPTELVPYGL	SLRLARLCTE	NLLKPDTREC	AQLRQEVEEN	LNEVYRQISG	VHKLQQTKFR	QQPNAGKKQD
890	900	910	920	930	940	950	960
HTIVDTVLMA	PRSAKPALLK	LTEKQVEQRA	FHDLKVAPGY	YTLTADQDAR	GMVEFQEGVE	LVDVRVPLFI	RPEDDDEKQL
970	980	990	1000	1010	1020	1030	1040
LVEAIDVPAG	TATLGRRLVN	ITIIKEQARD	VVSFEQPEFS	VSRGDQVARI	PVIRRVLDGG	KSQVSYRTQD	GTAQGNRDYI
1050	1060	1070	1080	1090	1100	1110	1120
PVEGELLFQP	GEAWKELQVK	LLELQEVDSL	LRGRQVRRFH	VQLSNPKFGA	HLGQPHSTTI	IIRDPDELDR	SFTSQMLSSQ
1130	1140	1150	1160	1170	1180	1190	1200
PPPHGDLGAP	QNPNAKAAGS	RKIHFNWLPP	SGKPMGYRVK	YWIQGDSESE	AHLLDSKVPS	VELTNLYPYC	DYEMKVCAYG
1210	1220	1230	1240	1250	1260	1270	1280
AQGEGPYSSL	VSCRTHQEVP	SEPGRLAFNV	VSSTVTQLSW	AEPAETNGEI	TAYEVCYGLV	NDDNRPIGPM	KKVLVDNPKN
1290	1300	1310	1320	1330	1340	1350	1360
RMLLIENLRE	SQPYRYTVKA	RNGAGWGPER	EAIINLATQP	KRPMSIPIIP	DIPIVDAQSG	EDYDSFLMYS	DDVLRSPSGS
1370	1380	1390	1400	1410	1420	1430	1440
QRPSVSDDTE	HLVNGRMDFA	FPGSTNSLHR	MTTTSAAAYG	THLSPHVPHR	VLSTSSTLTR	DYNSLTRSEH	SHSTTLPRDY
1450	1460	1470	1480	1490	1500	1510	1520
STLTSVSSHD	SRLTAGVPDT	PTRLVFSALG	PTSLRVSWQE	PRCERPLQGY	SVEYQLLNGG	ELHRLNIPNP	AQTSVVVEDL
1530	1540	1550	1560	1570	1580	1590	1600
LPNHSYVFRV	RAQSQEGWGR	EREGVITIES	QVHPQSPLCP	LPGSAFTLST	PSAPGPLVFT	ALSPDSLQLS	WERPRRPNGD
1610	1620	1630	1640	1650	1660	1670	1680
IVGYLVTCEM	AQGGGPATAF	RVDGDSPESR	LTVPGLSENV	PYKFKVQART	TEGFGPEREG	IITIESQDGG	PFPQLGSRAG
1690	1700	1710	1720	1730	1740	1750	1760
LFQHPLQSEY	SSITTTHTSA	TEPFLVDGLT	LGAQHLEAGG	SLTRHVTQEF	VSRTLTTSGT	LSTHMDQQFF	QT

Cmpd.	No. of Cmpds.	m/z meas.	Δ m/z [ppm]	z	Rt [min]	Score	P	Range	Sequence	Modification
2157	1	841.3003	-27.93	2	57.2	16.5	1	47-59	K.DCAYCTDEMFRDR.R	Carbamidomethyl: 2

Detailed Protein Report

Protein 322: tumor protein D54 isoform g [Homo sapiens]

**Accession:**	gi|345197262	**Score:**	34.7
**Database:**	refseq_human(refseq_human_20140103.fasta)	**MW [kDa]:**	19.5
**Database Date:**	2015-11-30	**pI:**	6.2
		**Sequence Coverage [%]:**	9.9
		**No. of unique Peptides:**	1

10	20	30	40	50	60	70	80
MDSAGQDINL	NSPNKGLLSD	SMTDVPVDTG	VEEEIVTLRQ	VLAAKERHCG	ELKRRLGLST	LGELKQNLSR	SWHDVQVSSA
**90**	**100**	**110**	**120**	**130**	**140**	**150**	**160**
YVKTSEKLGE	WNEKVTQSDL	YKKTQETLSQ	AGQKTSAALS	TVGSAISRKL	GDMRNSATFK	SFEDRVGTIK	SKVVGDRENG
**170**	**180**	**190**					
SDNLPSSAGS	GDKPLSDPAP	F					

Cmpd.	No. of Cmpds.	m/z meas.	Δ m/z [ppm]	z	Rt [min]	Score	P	Range	Sequence	Modification
2523	1	1052.4103	-115.77	2	59.8	34.7	1	66-83	K.QNLSRSWHDVQVSSAYVK.T	

Detailed Protein Report

Protein 323: PREDICTED: latent-transforming growth factor beta-binding protein 1 isoform X5 [Homo sapiens]

Accession: gi|530367595
Database: refseq_human(refseq_human_20140103.fasta)
Database Date: 2015-11-30
Modification(s): Carbamidomethyl, Oxidation

Score: 34.7
MW [kDa]: 134.0
pI: 9.1
Sequence Coverage [%]: 4.3
No. of unique Peptides: 1

10	20	30	40	50	60	70	80
MAGAWLRWGL	LLWAGLLASS	AHGRLRRITY	VVHPGPGLAA	GALPLSGPPR	SRTFNVALNA	RYSRSSAAAG	APSRASPGVP
90	100	110	120	130	140	150	160
SERTRRTSKP	GGAALQGLRP	PPPPPPEPAR	PAVPGGQLHP	NPGGHPAAAP	FTKQGRQVVR	SKVPQETQSG	GGSRLQVHQK
170	180	190	200	210	220	230	240
QQLQGVNVCG	GRCCHGWSKA	PGSQRCTKPS	CVPPCQNGGM	CLRPQLCVCK	PGTKGKACET	IAAQDTSSPV	FGGQSPGAAS
250	260	270	280	290	300	310	320
SWGPPEQAAK	HTSSKKADTL	PRVSPVAQMT	LTLKPKPSVG	LPQQIHSQVT	PLSSQSVVIH	HGQTQEYVLK	PKYFPAQKGI
330	340	350	360	370	380	390	400
SGEQSTEGSF	PLRYVQDQVA	APFQLSNHTG	RIKVVFTPSI	CKVTCTKGSC	QNSCEKGNTT	TLISENGHAA	DTLTATNFRV
410	420	430	440	450	460	470	480
VICHLPCMNG	GQCSSRDKCQ	CPPNFTGKLC	QIPVHGASVP	KLYQHSQQPG	KALGTHVIHS	THTLPLTVTS	QQGVKVKFPP
490	500	510	520	530	540	550	560
NIVNIHVKHP	PEASVQIHQV	SRIDGPTGQK	TKEAQPGQSQ	VSYQGLPVQK	TQTIHSTYSH	QQVIPHVYPV	AAKTQLGRCF
570	580	590	600	610	620	630	640
QETIGSQCGK	ALPGLSKQED	CCGTVGTSWG	FNKCQKCPKK	PSYHGYNQMM	ECLPGYKRVN	NTFCQDINEC	QLQGVCPNGE
650	660	670	680	690	700	710	720
CLNTMGSYRC	TCKIGFGPDP	TFSSCVPDPP	VISEEKGPCY	RLVSSGRQCM	HPLSVHLTKQ	LCCCSVGKAW	GPHCEKCPLP
730	740	750	760	770	780	790	800
GTAAFKEICP	GGMGYTVSGV	HRRRPIHHHV	GKGPVFVKPK	NTQPVAKSTH	PPPLPAKEEP	VEALTFSREH	GPGVAEPEVA
810	820	830	840	850	860	870	880
TAPPEKEIPS	LDQEKTKLEP	GQPQLSPGIS	TIHLHPQFPV	VIEKTSPPVP	VEVAPEASTS	SASQVIAPTQ	VTEINECTVN
890	900	910	920	930	940	950	960
PDICGAGHCI	NLPVRYTCIC	YEGYRFSEQQ	RKCVDIDECT	QVQHLCSQGR	CENTEGSFLC	ICPAGFMASE	EGTNCIDVDE
970	980	990	1000	1010	1020	1030	1040
CLRPDVCGEG	HCVNTVGAFR	CEYCDSGYRM	TQRGRCEDID	ECLNPSTCPD	EQCVNSPGSY	QCVPCTEGFR	GWNGQCLDVD
1050	1060	1070	1080	1090	1100	1110	1120
ECLEPNVCAN	GDCSNLEGSY	MCSCHKGYTR	TPDHKHCRDI	DECQQGNLCV	NGQCKNTEGS	FRCTCGQGYQ	LSAAKDQCED
1130	1140	1150	1160	1170	1180	1190	1200
IDECQHRHLC	AHGQCRNTEG	SFQCVCDQGY	RASGLGDHCE	DINECLEDKS	VCQRGDCINT	AGSYDCTCPD	GFQLDDNKTC
1210	1220	1230	1240	1250			
QDINECEHPG	LCGPQGECLN	TEGSFHCVCQ	QGFSISADGR	TCEDVNE			

Cmpd.	No. of Cmpds.	m/z meas.	Δ m/z [ppm]	z	Rt [min]	Score	P	Range	Sequence	Modification
65	1	1240.0095	73.75	3	30.2	22.4	1	180-214	K.APGSQRCTKPSCVPPCQNGGMCLRPQLCVCKPGTK.G	Carbamidomethyl: 7; Oxidation: 21

Detailed Protein Report

Protein 324: transcription regulator protein BACH1 [Homo sapiens]

**Accession:**	gi|4502353	**Score:**	34.6
**Database:**	refseq_human(refseq_human_20140103.fasta)	**MW [kDa]:**	81.9
**Database Date:**	2015-11-30	**pI:**	4.8
**Modification(s):**	Carbamidomethyl	**Sequence Coverage [%]:**	3.7
		**No. of unique Peptides:**	2

Quantitation

mdown:qdown	**Median:**	0.64	**CV:**	0.00 %	**No. of Peptides:**	1
Wdown:Qdown	**Median:**	2.40	**CV:**	0.00 %	**No. of Peptides:**	1

Alias proteins:

Accession	Name	Description
gi|530418993	refseq_human (refseq_human_20140103.fasta)	PREDICTED: transcription regulator protein BACH1 isoform X3 [Homo sapiens]
gi|530418991	refseq_human (refseq_human_20140103.fasta)	PREDICTED: transcription regulator protein BACH1 isoform X2 [Homo sapiens]
gi|530418989	refseq_human (refseq_human_20140103.fasta)	PREDICTED: transcription regulator protein BACH1 isoform X1 [Homo sapiens]
gi|45827690	refseq_human (refseq_human_20140103.fasta)	transcription regulator protein BACH1 [Homo sapiens]

10	20	30	40	50	60	70	80
MSLSENSVFA	YESSVHSTNV	LLSLNDQRKK	DVLCDVTIFV	EGQRFRAHRS	VLAACSSYFH	SRIVGQADGE	LNITLPEEVT
**90**	**100**	**110**	**120**	**130**	**140**	**150**	**160**
VKGFEPLIQF	AYTAKLILSK	ENVDEVCKCV	EFLSVHNIEE	SCFQFLKFKF	LDSTADQQEC	PRKKCFSSHC	QKTDLKLSLL
**170**	**180**	**190**	**200**	**210**	**220**	**230**	**240**
DQRDLETDEV	EEFLENKNVQ	TPQCKLRRYQ	GNAKASPPLQ	DSASQTYESM	CLEKDAALAL	PSLCPKYRKF	QKAFGTDRVR
**250**	**260**	**270**	**280**	**290**	**300**	**310**	**320**
TGESSVKDIH	ASVQPNERSE	NECLGGVPEC	RDLQVMLKCD	ESKLAMEPEE	TKKDPASQCP	TEKSEVTPFP	HNSSIDPHGL
**330**	**340**	**350**	**360**	**370**	**380**	**390**	**400**
YSLSLLHTYD	QYGDLNFAGM	QNTTVLTEKP	LSGTDVQEKT	FGESQDLPLK	SDLGTREDSS	VASSDRSSVE	REVAEHLAKG
**410**	**420**	**430**	**440**	**450**	**460**	**470**	**480**
FWSDICSTDT	PCQMQLSPAV	AKDGSEQISQ	KRSECPWLGI	RISESPEPGQ	RTFTTLSSVN	CPFISTLSTE	GCSSNLEIGN
**490**	**500**	**510**	**520**	**530**	**540**	**550**	**560**
DDYVSEPQQE	PCPYACVISL	GDDSETDTEG	DSESCSAREQ	ECEVKLPFNA	QRIISLSRND	FQSLLKMHKL	TPEQLDCIHD
**570**	**580**	**590**	**600**	**610**	**620**	**630**	**640**
IRRRSKNRIA	AQRCRKRKLD	CIQNLESEIE	KLQSEKESLL	KERDHILSTL	GETKQNLTGL	CQKVCKEAAL	SQEQIQILAK
**650**	**660**	**670**	**680**	**690**	**700**	**710**	**720**
YSAADCPLSF	LISEKDKSTP	DGELALPSIF	SLSDRPPAVL	PPCARGNSEP	GYARGQESQQ	MSTATSEQAG	PAEQCRQSGG
**730**	**740**						
ISDFCQQMTD	KCTTDE						

Cmpd.	No. of Cmpds.	m/z meas.	Δ m/z [ppm]	z	Rt [min]	Score	P	Range	Sequence	Modification	Ratios
504	1	667.7055	-215.81	2	35.6	22.1	1	615-626	K.QNLTGLCQKVCK.E		mdown:qdown 0.64<br>Wdown:Qdown 2.40
1770	1	851.2192	-170.05	2	52.1	12.5	0	717-731	R.QSGGISDFCQQMTDK.C	Carbamidomethyl: 9	

Detailed Protein Report

Protein 325: **PREDICTED: DNA polymerase kappa isoform X3 [Homo sapiens]**

Accession: gi|530379363
Database: refseq_human(refseq_human_20140103.fasta)
Database Date: 2015-11-30
Modification(s): Oxidation

Score: 34.5
MW [kDa]: 57.6
pI: 9.2
Sequence Coverage [%]: 10.1
No. of unique Peptides: 2

10	20	30	40	50	60	70	80
MDSTKEKCDS	YKDDLLLRMG	LNDNKAGMEG	LDKEKINKII	MEATKGSRFY	GNELKKEKQV	NQRIENMMQQ	KAQITSQQLR
**90**	**100**	**110**	**120**	**130**	**140**	**150**	**160**
KAQLQVDRFA	MELEQSRNLS	NTIVHIDMDA	FYAAVEMRDN	PELKDKPIAV	GSMSMLSTSN	YHARRFGVRA	AMPGFIAKRL
**170**	**180**	**190**	**200**	**210**	**220**	**230**	**240**
CPQLIIVPPN	FDKYRAVSKE	VKEILADYDP	NFMAMSLDEA	YLNITKHLEE	RQNWPEDKRR	YFIKMGSSVE	NDLQNLYIQR
**250**	**260**	**270**	**280**	**290**	**300**	**310**	**320**
SITFTDNPGK	EVNKLSEHER	SISPLLFEES	PSDVQPPGDP	FQVNFEEQNN	PQILQNSVVF	GTSAQEVVKE	IRFRIEQKTT
**330**	**340**	**350**	**360**	**370**	**380**	**390**	**400**
LTASAGIAPN	TMLAKVCSDK	NKPNGQYQIL	PNRQAVMDFI	KDLPIRKVSG	IGKVTEKMLK	ALGIITCTEL	YQQRALLSLL
**410**	**420**	**430**	**440**	**450**	**460**	**470**	**480**
FSETSWHYFL	HISLGLGSTH	LTRDGERKSM	SVERTFSEIN	KAEEQYSLCQ	ELCSELAQDL	QKERLKVFGY	LVFPMKRTGN
**490**	**500**	**510**					
TNKGALLAFY	RLETKPCQPL	SVH					

Cmpd.	No. of Cmpds.	m/z meas.	Δ m/z [ppm]	z	Rt [min]	Score	P	Range	Sequence	Modification
2396	1	1045.2249	47.66	3	60.3	12.4	1	180-206	K.EVKEILADYDPNFMAMSLDEAYLNITK.H	
1772	1	807.4927	33.69	2	52.1	10.9	2	465-477	R.LKVFGYLVFPMKR.T	Oxidation: 11

Detailed Protein Report

Protein 326: retinoic acid-induced protein 1 [Homo sapiens]

**Accession:**	gi|40807477	**Score:**	34.5
**Database:**	refseq_human(refseq_human_20140103.fasta)	**MW [kDa]:**	203.2
**Database Date:**	2015-11-30	**pI:**	9.9
		**Sequence Coverage [%]:**	0.8
		**No. of unique Peptides:**	1

Quantitation

mdown:qdown	**Median:** 0.69	**CV:** 0.00 %	**No. of Peptides:** 1

BRUKER

Detailed Protein Report

10	20	30	40	50	60	70	80
MQSFRERCGF	HGKQQNYQQT	SQETSRLENY	RQPSQAGLSC	DRQRLLAKDY	YNPQPYPSYE	GGAGTPSGTA	AAVAADKYHR
90	100	110	120	130	140	150	160
GSKALPTQQG	LQGRPAFPGY	GVQDSSPYPG	RYAGEESLQA	WGAPQPPPPQ	PQPLPAGVAK	YDENLMKKTA	VPPSRQYAEQ
170	180	190	200	210	220	230	240
GAQVPFRTHS	LHVQQPPPPQ	QPLAYPKLQR	QKLQNDIASP	LPFPQGTHFP	QHSQSFPTSS	TYSSSVQGGG	QGAHSYKSCT
250	260	270	280	290	300	310	320
APTAQPHDRP	LTASSSLAPG	QRVQNLHAYQ	SGRLSYDQQQ	QQQQQQQQQQ	QALQSRHHAQ	ETLHYQNLAK	YQHYGQQGQG
330	340	350	360	370	380	390	400
YCQPDAAVRT	PEQYYQTFSP	SSSHSPARSV	GRSPSYSSTP	SPLMPNLENF	PYSQQPLSTG	AFPAGITDHS	HFMPLLNPSP
410	420	430	440	450	460	470	480
TDATSSVDTQ	AGNCKPLQKD	KLPENLLSDL	SLQSLTALTS	QVENISNTVQ	QLLLSKAAVP	QKKGVKNLVS	RTPEQHKSQH
490	500	510	520	530	540	550	560
CSPEGSGYSA	EPAGTPLSEP	PSSTPQSTHA	EPQEADYLSG	SEDPLERSFL	YCNQARGSPA	RVNSNSKAKP	ESVSTCSVTS
570	580	590	600	610	620	630	640
PDDMSTKSDD	SFQSLHGSLP	LDSFSKFVAG	ERDCPRLLLS	ALAQEDLASE	ILGLQEAIGE	KADKAWAEAP	SLVKDSSKPP
650	660	670	680	690	700	710	720
FSLENHSACL	DSVAKSAWPR	PGEPEALPDS	LQLDKGGNAK	DFSPGLFEDP	SVAFATPDPK	KTTGPLSFGT	KPTLGVPAPD
730	740	750	760	770	780	790	800
PTTAAFDCFP	DTTAASSADS	ANPFAWPEEN	LGDACPRWGL	HPGELTKGLE	QGGKASDGIS	KGDTHEASAC	LGFQEEDPPG
810	820	830	840	850	860	870	880
EKVASLPGDF	KQEEVGGVKE	EAGGLLQCPE	VAKADRWLED	SRHCCSTADF	GDLPLLPPTS	RKEDLEAEEE	YSSLCELLGS
890	900	910	920	930	940	950	960
PEQRPGMQDP	LSPKAPLICT	KEEVEEVLDS	KAGWGSPCHL	SGESVILLGP	TVGTESKVQS	WFESSLSHMK	PGEEGPDGER
970	980	990	1000	1010	1020	1030	1040
APGDSTTSDA	SLAQKPNKPA	VPEAPIAKKE	PVPRGKSLRS	RRVHRGLPEA	EDSPCRAPVL	PKDLLLPESC	TGPPQGQMEG
1050	1060	1070	1080	1090	1100	1110	1120
AGAPGRGASE	GLPRMCTRSL	TALSEPRTPG	PPGLTTTPAP	PDKLGGKQRA	AFKSGKRVGK	PSPKAASSPS	NPAALPVASD
1130	1140	1150	1160	1170	1180	1190	1200
SSPMGSKTKE	TDSPSTPGKD	QRSMILRSRT	KTQEIFHSKR	RRPSEGRLPN	CRATKKLLDN	SHLPATFKVS	SSPQKEGRVS
1210	1220	1230	1240	1250	1260	1270	1280
QRARVPKPGA	GSKLSDRPLH	ALKRKSAFMA	PVPTKKRNLV	LRSRSSSSSN	ASGNGGDGKE	ERPEGSPTLF	KRMSSPKKAK
1290	1300	1310	1320	1330	1340	1350	1360
PTKGNGEPAT	KLPPPETPDA	CLKLASRAAF	QGAMKTKVLP	PRKGRGLKLE	AIVQKITSPS	LKKFACKAPG	ASPGNPLSPS
1370	1380	1390	1400	1410	1420	1430	1440
LSDKDRGLKG	AGGSPVGVEE	GLVNVGTGQK	LPTSGADPLC	RNPTNRSLKG	KLMNSKKLSS	TDCFKTEAFT	SPEALQPGGT
1450	1460	1470	1480	1490	1500	1510	1520
ALAPKKRSRK	GRAGAHGLSK	GPLEKRPYLG	PALLLTPRDR	ASGTQGASED	NSGGGGKKPK	MEELGLASQP	PEGRPCQPQT
1530	1540	1550	1560	1570	1580	1590	1600
RAQKQPGHTN	YSSYSKRKRL	TRGRAKNTTS	SPCKGRAKRR	RQQQVLPLDP	AEPEIRLKYI	SSCKRLRSDS	RTPAFSPFVR
1610	1620	1630	1640	1650	1660	1670	1680
VEKRDAFTTI	CTVVNSPGDA	PKPHRKPSSS	ASSSSSSSF	SLDAAGASLA	TLPGGSILQP	RPSLPLSSTM	HLGPVVSKAL
1690	1700	1710	1720	1730	1740	1750	1760
STSCLVCCLC	QNPANFKDLG	DLCGPYYPEH	CLPKKKPKLK	EKVRPEGTCE	EASLPLERTL	KGPECAAAAT	AGKPPRPDGP
1770	1780	1790	1800	1810	1820	1830	1840
ADPAKQGPLR	TSARGLSRRL	QSCYCCDGRE	DGGEEAAPAD	KGRKHECSKE	APAEPGGEAQ	EHWVHEACAV	WTGGVYLVAG
1850	1860	1870	1880	1890	1900	1910	
KLFGLQEAMK	VAVDMMCSSC	QEAGATIGCC	HKGCLHTYHY	PCASDAGCIF	IEENFSLKCP	KHKRLP	

Cmpd.	No. of Cmpds.	m/z meas.	Δ m/z [ppm]	z	Rt [min]	Score	P	Range	Sequence	Modification	Ratios
95	1	510.4790	-193.63	3	30.4	18.7	2	1790-1804	R.EDGGEEAAPADKGRK.H		mdown:qdown 0.69

Detailed Protein Report

Protein 327: MAP7 domain-containing protein 2 isoform 4 [Homo sapiens]

**Accession:**	gi|270483747	**Score:**	34.4
**Database:**	refseq_human(refseq_human_20140103.fasta)	**MW [kDa]:**	76.6
**Database Date:**	2015-11-30	**pI:**	9.4
		**Sequence Coverage [%]:**	3.5
		**No. of unique Peptides:**	1

10	20	30	40	50	60	70	80
MEGFLKSDER	QRLAKERREE	REKCLAAREQ	QILEKQKRAR	LQYEKQMEER	WRKLEEQRQR	EDQKRAAVEE	KRKQKLREEE
**90**	**100**	**110**	**120**	**130**	**140**	**150**	**160**
ERLEAMMRRS	LERTQQLELK	KKYSWGAPLA	IGPGGHDACD	KLSTSTMSLP	KPTEPPMNKR	LSSSTVAISY	SPDRAPLGPL
**170**	**180**	**190**	**200**	**210**	**220**	**230**	**240**
NPSYKSSPTR	NIEKKKATST	STSGAGDVGK	EALSGGEASL	VEKVKRGQRT	ATSLPVVNFG	SPLRRCEFSG	GIPKRPSSPV
**250**	**260**	**270**	**280**	**290**	**300**	**310**	**320**
ISKTATKAYP	QSPKTTKPPY	PGSPVKYRLP	ALSGQDMPKR	KAEKEKSNKE	REGTLAQQAA	GPQGEEALEK	HVVDKHASEK
**330**	**340**	**350**	**360**	**370**	**380**	**390**	**400**
HAAAAGGKAE	NSAALGKPTA	GTTDAGEAAK	ILAEKRRQAR	LQKEQEEQER	LEKEEQDRLE	REELKRKAEE	ERLRLEEEAR
**410**	**420**	**430**	**440**	**450**	**460**	**470**	**480**
KQEEERKRQE	EEKKKQEGEE	KRKAGEEAKR	KAEEELLLKE	KQEQEKQEKA	MIEKQKEAAE	TKAREVAEQM	RLEREQIMLQ
**490**	**500**	**510**	**520**	**530**	**540**	**550**	**560**
IEQERLERKK	RIDEIMKRTR	KSDVSPQVKK	EDPKVGVQPA	VCVEKKTKLV	VPNKMEINGL	NTCQEVNGVD	HAAPETYPQD
**570**	**580**	**590**	**600**	**610**	**620**	**630**	**640**
IFSNGLKPAG	GLIHLDALDG	KSNSLDDSTE	EVQSMDVSPV	SKEELISIPE	FSPVSEMIPG	VSLDQNGTGN	ARALQDLLDF
**650**	**660**	**670**	**680**	**690**			
TGPPTFPKRS	SENLSLDDCN	KNLIEGFNSP	GQETPLNTFC				

Cmpd.	No. of Cmpds.	m/z meas.	Δ m/z [ppm]	z	Rt [min]	Score	P	Range	Sequence	Modification
1648	1	575.1737	-226.87	2	50.5	13.4	1	311-320	K.HVVDKHASEK.H	

Detailed Protein Report

Protein 328: mucin-2 precursor [Homo sapiens]

**Accession:**	gi|557129045	**Score:**	34.4
**Database:**	refseq_human(refseq_human_20140103.fasta)	**MW [kDa]:**	539.8
**Database Date:**	2015-11-30	**pI:**	5.4
**Modification(s):**	Carbamidomethyl	**Sequence Coverage [%]:**	0.8
		**No. of unique Peptides:**	1

Detailed Protein Report

10	20	30	40	50	60	70	80
MGLPLARLAA	VCLALSLAGG	SELQTEGRTR	NHGHNVCSTW	GNFHYKTFDG	DVFRFPGPCD	YNFASDCRGS	YKEFAVHLKR
90	100	110	120	130	140	150	160
GPGQAEAPAG	VESILLTIKD	DTIYLTRHLA	VLNGAVVSTP	HYSPGLLIEK	SDAYTKVYSR	AGLTLMWNRE	DALMLELDTK
170	180	190	200	210	220	230	240
FRNHTCGLCG	DYNGLQSYSE	FLSDGVLFSP	LEFGNMQKIN	QPDVVCEDPE	EEVAPASCSE	HRAECERLLT	AEAFADCQDL
250	260	270	280	290	300	310	320
VPLEPYLRAC	QQDRCRCPGG	DTCVCSTVAE	FSRQCSHAGG	RPGNWRTATL	CPKTCPGNLV	YLESGSPCMD	TCSHLEVSSL
330	340	350	360	370	380	390	400
CEEHRMDGCF	CPEGTVYDDI	GDSGCVPVSQ	CHCRLHGHLY	TPGQEITNDC	EQCVCNAGRW	VCKDLPCPGT	CALEGGSHIT
410	420	430	440	450	460	470	480
TFDGKTYTFH	GDCYYVLAKG	DHNDSYALLG	ELAPCGSTDK	QTCLKTVVLL	ADKKKNVVVF	KSDGSVLLNE	LQVNLPHVTA
490	500	510	520	530	540	550	560
SFSVFRPSSY	HIMVSMAIGV	RLQVQLAPVM	QLFVTLDQAS	QGQVQGLCGN	FNGLEGDDFK	TASGLVEATG	AGFANTWKAQ
570	580	590	600	610	620	630	640
STCHDKLDWL	DDPCSLNIES	ANYAEHWCSL	LKKTETPFGR	CHSAVDPAEY	YKRCKYDTCN	CQNNEDCLCA	ALSSYARACT
650	660	670	680	690	700	710	720
AKGVMLWGWR	EHVCNKDVGS	CPNSQVFLYN	LTTCQQTCRS	LSEADSHCLE	GFAPVDGCGC	PDHTFLDEKG	RCVPLAKCSC
730	740	750	760	770	780	790	800
YHRGLYLEAG	DVVVRQEERC	VCRDGRLHCR	QIRLIGQSCT	APKIHMDCSN	LTALATSKPR	ALSCQTLAAG	YYHTECVSGC
810	820	830	840	850	860	870	880
VCPDGLMDDG	RGGCVVEKEC	PCVHNNDLYS	SGAKIKVDCN	TCTCKRGRWV	CTQAVCHGTC	SIYGSGHYIT	FDGKYYDFDG
890	900	910	920	930	940	950	960
HCSYVAVQDY	CGQNSSLGSF	SIITENVPCG	TTGVTCSKAI	KIFMGRTELK	LEDKHRVVIQ	RDEGHHVAYT	TREVGQYLVV
970	980	990	1000	1010	1020	1030	1040
ESSTGIIVIW	DKRTTVFIKL	APSYKGTVCG	LCGNFDHRSN	NDFTTRDHMV	VSSELDFGNS	WKEAPTCPDV	STNPEPCSLN
1050	1060	1070	1080	1090	1100	1110	1120
PHRRSWAEKQ	CSILKSSVFS	ICHSKVDPKP	FYEACVHDSC	SCDTGGDCEC	FCSAVASYAQ	ECTKEGACVF	WRTPDLCPIF
1130	1140	1150	1160	1170	1180	1190	1200
CDYYNPPHEC	EWHYEPCGNR	SFETCRTING	IHSNISVSYL	EGCYPRCPKD	RPIYEEDLKK	CVTADKCGCY	VEDTHYPPGA
1210	1220	1230	1240	1250	1260	1270	1280
SVPTEETCKS	CVCTNSSQVV	CRPEEGKILN	QTQDGAFCYW	EICGPNGTVE	KHFNICSITT	RPSTLTTFTT	ITLPTTPTTF
1290	1300	1310	1320	1330	1340	1350	1360
TTTTTTTTPT	SSTVLSTTPK	LCCLWSDWIN	EDHPSSGSDD	GDRETFDGVC	GAPEDIECRS	VKDPHLSLEQ	LGQKVQCDVS
1370	1380	1390	1400	1410	1420	1430	1440
VGFICKNEDQ	FGNGPFGLCY	DYKIRVNCCW	PMDKCITTPS	PPTTTPSPPP	TSTTTLPPTT	TPSPPTTTTT	TPPPTTTPSP
1450	1460	1470	1480	1490	1500	1510	1520
PITTTTTPPP	TTTPSPPIST	TTTPPPTTTP	SPPTTTPSPP	TTTPSPPTTT	TTTPPPTTTP	SPPTTTPITP	PASTTTLPPT
1530	1540	1550	1560	1570	1580	1590	1600
TTPSPPTTTT	TTPPPTTTPS	PPTTTPITPP	TSTTTLPPTT	TPSPPPTTTT	TPPPTTTPSP	PTTTTPSPPT	ITTTTPPPTT
1610	1620	1630	1640	1650	1660	1670	1680
TPSPPTTTTT	TPPPTTTPSP	PTTTPITPPT	STTTLPPTTT	PSPPPTTTTT	PPPTTTPSPP	TTTTPSPPIT	TTTTPPPTTT
1690	1700	1710	1720	1730	1740	1750	1760
PSSPITTTPS	PPTTTMTTPS	PTTTPSSPIT	TTTTPSSTTT	PSPPPTTMTT	PSPTTTPSPP	TTTMTTLPPT	TTSSPLTTTP
1770	1780	1790	1800	1810	1820	1830	1840
LPPSITPPTF	SPFSTTTPTT	PCVPLCNWTG	WLDSGKPNFH	KPGGDTELIG	DVCGPGWAAN	ISCRATMYPD	VPIGQLGQTV
1850	1860	1870	1880	1890	1900	1910	1920
VCDVSVGLIC	KNEDQKPGGV	IPMAFCLNYE	INVQCCECVT	QPTTMTTTTT	ENPTPPTTTP	ITTTTTVTPT	PTPTGTQTPT
1930	1940	1950	1960	1970	1980	1990	2000
TTPITTTTTV	TPTPTPTGTQ	TPTTTPITTT	TTVTPTPTPT	GTQTPTTTPI	TTTTTVTPTP	TPTGTQTPTT	TPITTTTTVT
2010	2020	2030	2040	2050	2060	2070	2080
PTPTPTGTQT	PTTTPITTTT	TVTPTPTPTG	TQTPTTTPIT	TTTTVTPTPT	PTGTQTPTTT	PITTTTTVTP	TPTPTGTQTP
2090	2100	2110	2120	2130	2140	2150	2160
TTTPITTTTT	VTPTPTPTGT	QTPTTTPITT	TTTVTPTPTP	TGTQTPTTTP	ITTTTTVTPT	PTPTGTQTPT	TTPITTTTTV
2170	2180	2190	2200	2210	2220	2230	2240
TPTPTPTGTQ	TPTTTPITTT	TTVTPTPTPT	GTQTPTTTPI	TTTTTVTPTP	TPTGTQTPTT	TPITTTTTVT	PTPTPTGTQT
2250	2260	2270	2280	2290	2300	2310	2320
PTTTPITTTT	TVTPTPTPTG	TQTPTTTPIT	TTTTVTPTPT	PTGTQTPTTT	PITTTTTVTP	TPTPTGTQTP	TTTPITTTTT
2330	2340	2350	2360	2370	2380	2390	2400

Detailed Protein Report

Cmpd.	No. of Cmpds.	m/z meas.	Δ m/z [ppm]	z	Rt [min]	Score	P	Range	Sequence	Modification
2678	1	857.6944	26.58	3	64.7	10.2	0	616-637	K.YDTCNCQNNEDCLCAALSSYAR.A	Carbamidomethyl: 4, 12

Detailed Protein Report

Protein 329: DNA-directed RNA polymerase II subunit RPB1 [Homo sapiens]

**Accession:**	gi|4505939	**Score:**	34.4
**Database:**	refseq_human(refseq_human_20140103.fasta)	**MW [kDa]:**	217.1
**Database Date:**	2015-11-30	**pI:**	7.1
		**Sequence Coverage [%]:**	2.2
		**No. of unique Peptides:**	1

Detailed Protein Report

10	20	30	40	50	60	70	80
MHGGGPPSGD	SACPLRTIKR	VQFGVLSPDE	LKRMSVTEGG	IKYPETTEGG	RPKLGGLMDP	RQGVIERTGR	CQTCAGNMTE
90	100	110	120	130	140	150	160
CPGHFGHIEL	AKPVFHVGFL	VKTMKVLRCV	CFFCSKLLVD	SNNPKIKDIL	AKSKGQPKKR	LTHVYDLCKG	KNICEGGEEM
170	180	190	200	210	220	230	240
DNKFGVEQPE	GDEDLTKEKG	HGGCGRYQPR	IRRSGLELYA	EWKHVNEDSQ	EKKILLSPER	VHEIFKRISD	EECFVLGMEP
250	260	270	280	290	300	310	320
RYARPEWMIV	TVLPVPPLSV	RPAVVMQGSA	RNQDDLTHKL	ADIVKINNQL	RRNEQNGAAA	HVIAEDVKLL	QFHVATMVDN
330	340	350	360	370	380	390	400
ELPGLPRAMQ	KSGRPLKSLK	QRLKGKEGRV	RGNLMGKRVD	FSARTVITPD	PNLSIDQVGV	PRSIAANMTF	AEIVTPFNID
410	420	430	440	450	460	470	480
RLQELVRRGN	SQYPGAKYII	RDNGDRIDLR	FHPKPSDLHL	QTGYKVERHM	CDGDIVIFNR	QPTLHKMSMM	GHRVRILPWS
490	500	510	520	530	540	550	560
TFRLNLSVTT	PYNADFDGDE	MNLHLPQSLE	TRAEIQELAM	VPRMIVTPQS	NRPVMGIVQD	TLTAVRKFTK	RDVFLERGEV
570	580	590	600	610	620	630	640
MNLLMFLSTW	DGKVPQPAIL	KPRPLWTGKQ	IFSLIIPGHI	NCIRTHSTHP	DDEDSGPYKH	ISPGDTKVVV	ENGELIMGIL
650	660	670	680	690	700	710	720
CKKSLGTSAG	SLVHISYLEM	GHDITRLFYS	NIQTVINNWL	LIEGHTIGIG	DSIADSKTYQ	DIQNTIKKAK	QDVIEVIEKA
730	740	750	760	770	780	790	800
HNNELEPTPG	NTLRQTFENQ	VNRILNDARD	KTGSSAQKSL	SEYNNFKSMV	VSGAKGSKIN	ISQVIAVVGQ	QNVEGKRIPF
810	820	830	840	850	860	870	880
GFKHRTLPHF	IKDDYGPESR	GFVENSYLAG	LTPTEFFFHA	MGGREGLIDT	AVKTAETGYI	QRRLIKSMES	VMVKYDATVR
890	900	910	920	930	940	950	960
NSINQVVQLR	YGEDGLAGES	VEFQNLATLK	PSNKAFEKKF	RFDYTNERAL	RRTLQEDLVK	DVLSNAHIQN	ELEREFERMR
970	980	990	1000	1010	1020	1030	1040
EDREVLRVIF	PTGDSKVVLP	CNLLRMIWNA	QKIFHINPRL	PSDLHPIKVV	EGVKELSKKL	VIVNGDDPLS	RQAQENATLL
1050	1060	1070	1080	1090	1100	1110	1120
FNIHLRSTLC	SRRMAEEFRL	SGEAFDWLLG	EIESKFNQAI	AHPGEMVGAL	AAQSLGEPAT	QMTLNTFHYA	GVSAKNVTLG
1130	1140	1150	1160	1170	1180	1190	1200
VPRLKELINI	SKKPKTPSLT	VFLLGQSARD	AERAKDILCR	LEHTTLRKVT	ANTAIYYDPN	PQSTVVAEDQ	EWVNVYYEMP
1210	1220	1230	1240	1250	1260	1270	1280
DFDVARISPW	LLRVELDRKH	MTDRKLTMEQ	IAEKINAGFG	DDLNCIFNDD	NAEKLVLRIR	IMNSDENKMQ	EEEEVVDKMD
1290	1300	1310	1320	1330	1340	1350	1360
DDVFLRCIES	NMLTDMTLQG	IEQISKVYMH	LPQTDNKKKI	IITEDGEFKA	LQEWILETDG	VSLMRVLSEK	DVDPVRTTSN
1370	1380	1390	1400	1410	1420	1430	1440
DIVEIFTVLG	IEAVRKALER	ELYHVISFDG	SYVNYRHLAL	LCDTMTCRGH	LMAITRHGVN	RQDTGPLMKC	SFEETVDVLM
1450	1460	1470	1480	1490	1500	1510	1520
EAAAHGESDP	MKGVSENIML	GQLAPAGTGC	FDLLLDAEKC	KYGMEIPTNI	PGLGAAGPTG	MFFGSAPSPM	GGISPAMTPW
1530	1540	1550	1560	1570	1580	1590	1600
NQGATPAYGA	WSPSVGSGMT	PGAAGFSPSA	ASDASGFSPG	YSPAWSPTPG	SPGSPGPSSP	YIPSPGGAMS	PSYSPTSPAY
1610	1620	1630	1640	1650	1660	1670	1680
EPRSPGGYTP	QSPSYSPTSP	SYSPTSPSYS	PTSPNYSPTS	PSYSPTSPSY	SPTSPSYSPT	SPSYSPTSPS	YSPTSPSYSP
1690	1700	1710	1720	1730	1740	1750	1760
TSPSYSPTSP	SYSPTSPSYS	PTSPSYSPTS	PSYSPTSPSY	SPTSPSYSPT	SPSYSPTSPS	YSPTSPNYSP	TSPNYTPTSP
1770	1780	1790	1800	1810	1820	1830	1840
SYSPTSPSYS	PTSPNYTPTS	PNYSPTSPSY	SPTSPSYSPT	SPSYSPSSPR	YTPQSPTYTP	SSPSYSPSSP	SYSPTSPKYT
1850	1860	1870	1880	1890	1900	1910	1920
PTSPSYSPSS	PEYTPTSPKY	SPTSPKYSPT	SPKYSPTSPT	YSPTTPKYSP	TSPTYSPTSP	VYTPTSPKYS	PTSPTYSPTS
1930	1940	1950	1960	1970	1980		
PKYSPTSPTY	SPTSPKGSTY	SPTSPGYSPT	SPTYSLTSPA	ISPDDSDEEN			

Cmpd.	No. of Cmpds.	m/z meas.	Δ m/z [ppm]	z	Rt [min]	Score	P	Range	Sequence	Modification
652	1	918.1693	7.00	3	37.5	17.7	2	358-382	K.RVDFSARTVITPDPNLSIDQVGVPR.S	

Detailed Protein Report

Protein 330: PREDICTED: CAD protein isoform X4 [Homo sapiens]

**Accession:**	gi|530368098	**Score:**	34.2
**Database:**	refseq_human(refseq_human_20140103.fasta)	**MW [kDa]:**	235.9
**Database Date:**	2015-11-30	**pI:**	6.1
**Modification(s):**	Carbamidomethyl, Oxidation	**Sequence Coverage [%]:**	2.7
		**No. of unique Peptides:**	2

Detailed Protein Report

10	20	30	40	50	60	70	80
MAALVLEDGS	VLRGQPFGAA	VSTAGEVVFQ	TGMVGYPEAL	TDPSYKAQIL	VLTYPLIGNY	GIPPDEMDEF	GLCKWFESSG
90	100	110	120	130	140	150	160
IHVAALVVGE	CCPTPSHWSA	TRTLHEWLQQ	HGIPGLQGVD	TRELTKKLRE	QGSLLGKLVQ	NGTEPSSLPF	LDPNARPLVP
170	180	190	200	210	220	230	240
EVSIKTPRVF	NTGGAPRILA	LDCGLKYNQI	RCLCQRGAEV	TVVPWDHALD	SQEYEGLFLS	NGPGDPASYP	SVVSTLSRVL
250	260	270	280	290	300	310	320
SEPNPRPVFG	ICLGHQLLAL	AIGAKTYKMR	YGNRGHNQPC	LLVGSGRCFL	TSQNHGFAVE	TDSLPADWAP	LFTNANDGSN
330	340	350	360	370	380	390	400
EGIVHNSLPF	FSVQFHPEHQ	AGPSDMELLF	DIFLETVKEA	TAGNPGGQTV	RERLTERLCP	PGIPTPGSGL	PPPRKVLILG
410	420	430	440	450	460	470	480
SGGLSIGQAG	EFDYSGSQAI	KALKEENIQT	LLINPNIATV	QTSQGLADKV	YFLPITPHYV	TQVIRNERPD	GVLLTFGGQT
490	500	510	520	530	540	550	560
ALNCGVELTK	AGVLARYGVR	VLGTPVETIE	LTEDRRAFAA	RMAEIGEHVA	PSEAANSLEQ	AQAAAERLGY	PVLVRAAFAL
570	580	590	600	610	620	630	640
GGLGSGFASN	REELSALVAP	AFAHTSQVLV	DKSLKGWKEI	EYEVVRDAYG	NCVTYYIIEV	NARLSRSSAL	ASKATGYPLA
650	660	670	680	690	700	710	720
YVAAKLALGI	PLPELRNSVT	GGTAAFEPSV	DYCVVKIPRW	DLSKFLRVST	KIGSCMKSVG	EVMGIGRSFE	EAFQKALRMV
730	740	750	760	770	780	790	800
DENCVGFDHT	VKPVSDMELE	TPTDKRIFVV	AAALWAGYSV	DRLYELTRID	RWFLHRMKRI	IAHAQLLEQH	RGQPLPPDLL
810	820	830	840	850	860	870	880
QQAKCLGFSD	KQIALAVLST	ELAVRKLRQE	LGICPAVKQI	DTVAAEWPAQ	TNYLYLTYWG	TTHDLTFRTP	HVLVLGSGVY
890	900	910	920	930	940	950	960
RIGSSVEFDW	CAVGCIQQLR	KMGYKTIMVN	YNPETVSTDY	DMCDRLYFDE	ISFEVVMDIY	ELENPEGVIL	SMGGQLPNNM
970	980	990	1000	1010	1020	1030	1040
AMALHRQQCR	VLGTSPEAID	SAENRFKFSR	LLDTIGISQP	QWRELSDLES	ARQFCQTVGY	PCVVRPSYVL	SGAAMNVAYT
1050	1060	1070	1080	1090	1100	1110	1120
DGDLERFLSS	AAAVSKEHPV	VISKFIQEAK	EIDVDAVASD	GVVAAIAISE	HVENAGVHSG	DATLVTPPQD	ITAKTLERIK
1130	1140	1150	1160	1170	1180	1190	1200
AIVHAVGQEL	QVTGPFNLQL	IAKDDQLKVI	ECNVRVSRSF	PFVSKTLGVD	LVALATRVIM	GEEVEPVGLM	TGSGVVGVKV
1210	1220	1230	1240	1250	1260	1270	1280
PQFSFSRLAG	ADVVLGVEMT	STGEVAGFGE	SRCEAYLKAM	LSTGFKIPKK	NILLTIGSYK	NKSELLPTVR	LLESLGYSLY
1290	1300	1310	1320	1330	1340	1350	1360
ASLGTADFYT	EHGVKVTAVD	WHFEEAVDGE	CPPQRSILEQ	LAEKNFELVI	NLSMRGAGGR	RLSSFVTKGY	RTRRLAADFS
1370	1380	1390	1400	1410	1420	1430	1440
VPLIIDIKCT	KLFVEALGQI	GPAPPLKVHV	DCMTSQKLVR	LPGLIDVHVH	LREPGGTHKE	DFASGTAAAL	AGGITMVCAM
1450	1460	1470	1480	1490	1500	1510	1520
PNTRPPIIDA	PALALAQKLA	EAGARCDFAL	FLGASSENAG	TLGTVAGSAA	GLKLYLNETF	SELRLDSVVQ	WMEHFETWPS
1530	1540	1550	1560	1570	1580	1590	1600
HLPIVAHAEQ	QTVAAVLMVA	QLTQRSVHIC	HVARKEEILL	IKAAKARGLP	VTCEVAPHHL	FLSHDDLERL	GPGKGEVRPE
1610	1620	1630	1640	1650	1660	1670	1680
LGSRQDVEAL	WENMAVIDCF	ASDHAPHTLE	EKCGSRPPPG	FPGLETMLPL	LLTAVSEGRL	SLDDLLQRLH	HNPRRIFHLP
1690	1700	1710	1720	1730	1740	1750	1760
PQEDTYVEVD	LEHEWTIPSH	MPFSKAHWTP	FEGQKVKGTV	RRVVLRGEVA	YIDGQVLVPP	GYGQDVRKWP	QGAVPQLPPS
1770	1780	1790	1800	1810	1820	1830	1840
APATSEMTTT	PERPRRGIPG	LPDGRFHLPP	RIHRASDPGL	PAEEPKEKSS	RKVAEPELMG	TPDGTCYPPP	PVPRQASPQN
1850	1860	1870	1880	1890	1900	1910	1920
LGTPGLLHPQ	TSPLLHSLVG	QHILSVQQFT	KDQMSHLFNV	AHTLRMMVQK	ERSLDILKGK	VMASMFYEVS	TRTSSSFAAA
1930	1940	1950	1960	1970	1980	1990	2000
MARLGGAVLS	FSEATSSVQK	GESLADSVQT	MSCYADVVVL	RHPQPGAVEL	AAKHCRRPVI	NAGDGVGEHP	TQALLDIFTI
2010	2020	2030	2040	2050	2060	2070	2080
REELGTVNGM	TITMVGDLKH	GRTVHSLACL	LTQYRVSLRY	VAPPSLRMPP	TVRAFVASRG	TKQEEFESIE	EALPDTDVLY
2090	2100	2110	2120	2130	2140	2150	2160
MTRIQKERFG	STQEYEACFG	QFILTPHIMT	RAKKKMVVMH	PMPRVNEISV	EVDSDPRAAY	FRQAENGMYI	RMALLATVLG
2170							

Detailed Protein Report

Cmpd.	No. of Cmpds.	m/z meas.	Δ m/z [ppm]	z	Rt [min]	Score	P	Range	Sequence	Modification
2542	1	836.1207	149.60	2	60.1	10.3	1	1354-1368	R.RLAADFSVPLIIDIK.C	
1509	1	1061.8026	-76.29	3	48.7	10.6	2	1369-1397	K.CTKLFVEALGQIGPAPPLKVHVDCMTSQK.L	Carbamidomethyl: 1; Oxidation: 25

Detailed Protein Report

Protein 331: PREDICTED: centriolin isoform X3 [Homo sapiens]

**Accession:**	gi|578817016	**Score:**	34.2
**Database:**	refseq_human(refseq_human_20140103.fasta)	**MW [kDa]:**	258.5
**Database Date:**	2015-11-30	**pI:**	5.3
**Modification(s):**	Oxidation	**Sequence Coverage [%]:**	2.3
		**No. of unique Peptides:**	1

Detailed Protein Report

10	20	30	40	50	60	70	80
MKKGSQQKIF	SKAKIPSSSH	SPIPSSMSNM	RSRSLSPLIG	SETLPFHSGG	QWCEQVEIAD	ENNMLLDYQD	HKGADSHAGV
90	100	110	120	130	140	150	160
RYITEALIKK	LTKQDNLALI	KSLNLSLSKD	GGKKFKYIEN	LEKCVKLEVL	NLSYNLIGKI	EKLDKLLKLR	ELNLSYNKIS
170	180	190	200	210	220	230	240
KIEGIENMCN	LQKLNLAGNE	IEHIPVWLGK	KLKSLRVLNL	KGNKISSLQD	ISKLKPLQDL	ISLILVENPV	VTLPHYLQFT
250	260	270	280	290	300	310	320
IFHLRSLESL	EGQPVTTQDR	QEAFERFSLE	EVERLERDLE	KKMIETEELK	SKQTRFLEEI	KNQDKLNKSL	KEEAMLQKQS
330	340	350	360	370	380	390	400
CEELKSDLNT	KNELLKQKTI	ELTRACQKQY	ELEQELAFYK	IDAKFEPLNY	YPSEYAEIDK	APDESPYIGK	SRYKRNMFAT
410	420	430	440	450	460	470	480
ESYIIDSAQA	VQIKKMEPDE	QLRNDHMNLR	GHTPLDTQLE	DKEKKISAAQ	TRLSELHDEI	EKAEQQILRA	TEEFKQLEEA
490	500	510	520	530	540	550	560
IQLKKISEAG	KDLLYKQLSG	RLQLVNKLRQ	EALDLELQME	KQKQEIAGKQ	KEIKDLQIAI	DSLDSKDPKH	SHMKAQKSGK
570	580	590	600	610	620	630	640
EQQLDIMNKQ	YQQLESRLDE	ILSRIAKETE	EIKDLEEQLT	EGQIAANEAL	KKDLEGVISG	LQEYLGTIKG	QATQAQNECR
650	660	670	680	690	700	710	720
KLRDEKETLL	QRLTEVEQER	DQLEIVAMDA	ENMRKELAEL	ESALQEQHEV	NASLQQTQGD	LSAYEAELEA	RLNLRDAEAN
730	740	750	760	770	780	790	800
QLKEELEKVT	RLTQLEQSAL	QAELEKERQA	LKNALGKAQF	SEEKEQENSE	LHAKLKHLQD	DNNLLKQQLK	DFQNHLNHVV
810	820	830	840	850	860	870	880
DGLVRPEEVA	ARVDELRRKL	KLGTGEMNIH	SPSDVLGKSL	ADLQKQFSEI	LARSKWERDE	AQVRERKLQE	EMALQQEKLA
890	900	910	920	930	940	950	960
TGQEEFRQAC	ERALEARMNF	DKRQHEARIQ	QMENEIHYLQ	ENLKSMEEIQ	GLTDLQLQEA	DEEKERILAQ	LRELEKKKKL
970	980	990	1000	1010	1020	1030	1040
EDAKSQEQVF	GLDKELKKLK	KAVATSDKLA	TAELTIAKDQ	LKSLHGTVMK	INQERAEELQ	EAERFSRKAA	QAARDLTRAE
1050	1060	1070	1080	1090	1100	1110	1120
AEIELLQNLL	RQKGEQFRLE	MEKTGVGTGA	NSQVLEIEKL	NETMERQRTE	IARLQNVLDL	TGSDNKGGFE	NVLEEIAELR
1130	1140	1150	1160	1170	1180	1190	1200
REVSYQNDYI	SSMADPFKRR	GYWYFMPPPP	SSKVSSHSSQ	ATKDSGVGLK	YSASTPVRKP	RPGQQDGKEG	SQPPPASGYW
1210	1220	1230	1240	1250	1260	1270	1280
VYSPIRSGLH	KLFPSRDADS	GGDSQEESEL	DDQEEPPFVP	PPGYMMYTVL	PDGSPVPQGM	ALYAPPPPLP	NNSRPLTPGT
1290	1300	1310	1320	1330	1340	1350	1360
VVYGPPPAGA	PMVYGPPPPN	FSIPFIPMGV	LHCNVPEHHN	LENEVSRLED	IMQHLKSKKR	EERWMRASKR	QSEKEMEELH
1370	1380	1390	1400	1410	1420	1430	1440
HNIDDLLQEK	KSLECEVEEL	HRTVQKRQQQ	KDFIDGNVES	LMTELEIEKS	LKHHEDIVDE	IECIEKTLLK	RRSELREADR
1450	1460	1470	1480	1490	1500	1510	1520
LLAEAESELS	CTKEKTKNAV	EKFTDAKRSL	LQTESDAEEL	ERRAQETAVN	LVKADQQLRS	LQADAKDLEQ	HKIKQEEILK
1530	1540	1550	1560	1570	1580	1590	1600
EINKIVAAKD	SDFQCLSKKK	EKLTEELQKL	QKDIEMAERN	EDHHLQVLKE	SEVLLQAKRA	ELEKLKSQVT	SQQQEMAVLD
1610	1620	1630	1640	1650	1660	1670	1680
RQLGHKKEEL	HLLQGSMVQA	KADLQEALRL	GETEVTEKCN	HIREVKSLLE	ELSFQKGELN	VQISERKTQL	TLIKQEIEKE
1690	1700	1710	1720	1730	1740	1750	1760
EENLQVVLRQ	MSKHKTELKN	ILDMLQLENH	ELQGLKLQHD	QRVSELEKTQ	VAVLEEKLEL	ENLQQISQQQ	KGEIEWQKQL
1770	1780	1790	1800	1810	1820	1830	1840
LERDKREIER	MTAESRALQS	CVECLSKEKE	DLQEKCDIWE	KKLAQTKRVL	AAAEENSKME	QSNLEKLELN	VRKLQQELDQ
1850	1860	1870	1880	1890	1900	1910	1920
LNRDKLSLHN	DISAMQQQLQ	EKREAVNSLQ	EELANVQDHL	NLAKQDLLHT	TKHQDVLLSE	QTRLQKDISE	WANRFEDCQK
1930	1940	1950	1960	1970	1980	1990	2000
EEETKQQQLQ	VLQNEIEENK	LKLVQQEMMF	QRLQKERESE	ESKLETSKVT	LKEQQHQLEK	ELTDQKSKLD	QVLSKVLAAE
2010	2020	2030	2040	2050	2060	2070	2080
ERVRTLQEEE	RWCESLEKTL	SQTKRQLSER	EQQLVEKSGE	LLALQKEADS	MRADFSLLRN	QFLTERKKAE	KQVASLKEAL
2090	2100	2110	2120	2130	2140	2150	2160
KIQRSQLEKN	LLFLPELPAD	LEAILERNEN	LEGELESLKE	NLPFTMNEGP	FEEKLNFSQV	HIMDEHWRGE	ALREKLRHRE
2170	2180	2190	2200	2210	2220	2230	2240
DRLKAQLRHC	MSKQAEVLIK	GKRQTEGTLH	SLRRQVDALG	ELVTSTSADS	ASSPSLSQLE	SSLTEDSQLG	QNQEKNASAR
2250							

Detailed Protein Report

Cmpd.	No. of Cmpds.	m/z meas.	Δ m/z [ppm]	z	Rt [min]	Score	P	Range	Sequence	Modification
2056	1	1029.5727	91.82	2	55.8	13.5	2	415-430	K.KMEPDEQLRNDHMNLR.G	Oxidation: 2, 13

Detailed Protein Report

Protein 332: PREDICTED: rootletin-like [Homo sapiens]

**Accession:**	gi|578843302	**Score:**	34.1
**Database:**	refseq_human(refseq_human_20140103.fasta)	**MW [kDa]:**	147.5
**Database Date:**	2015-11-30	**pI:**	5.5
		**Sequence Coverage [%]:**	3.4
		**No. of unique Peptides:**	1

10	20	30	40	50	60	70	80
MRAAIERRWR	REQELCLQLK	SSQALVASLQ	EQLSESRREL	WAAQKLQQER	AREQAREREA	LRGQLEAQRL	EVQQCRASCK
**90**	**100**	**110**	**120**	**130**	**140**	**150**	**160**
LLGREKAALE	MVVEELKGKA	DAADAEKQGL	EAEAAELQRS	LLLQAERREE	LALRRERSCR	ALETSQGRLQ	QLEEKVSGLR
**170**	**180**	**190**	**200**	**210**	**220**	**230**	**240**
EELASVREAL	STAQLQRDVV	ESEREGLRSA	LARAECSNAD	LELLVRRLKS	EGVEQRDSLA	AMAALMEGLA	QDKSALNHLA
**250**	**260**	**270**	**280**	**290**	**300**	**310**	**320**
LQLEQERDQL	REQRKTLEQE	RARAGEQLAQ	AEQQLALERA	ERRGLQQACG	RLEQRQEQLE	GQAALLGREK	AQLQEQVGQV
**330**	**340**	**350**	**360**	**370**	**380**	**390**	**400**
TCQKQALEEQ	LAQSLQDQEA	QMGTLQQALQ	GKDALSEERA	QLLAKQEALE	RQGRLAAEEA	ADLRVERDSL	ESSLLEAQQL
**410**	**420**	**430**	**440**	**450**	**460**	**470**	**480**
ATKLQEQLEE	EARSAGLARQ	ALQVEMEQLQ	SDWEVQEMKL	RQDTVRLQRQ	VAQQEREAQR	ALESQALAHR	EALAQLQREK
**490**	**500**	**510**	**520**	**530**	**540**	**550**	**560**
ETLSLTLAEE	KEVARCQLEQ	EKELVTKSAA	EREALKGEIQ	SLKQERDESL	LQLEHKMQQA	LSLKETERSL	LSEELSRARR
**570**	**580**	**590**	**600**	**610**	**620**	**630**	**640**
TLERVQQEAQ	SQQEQAQATI	SATTEELKAL	QAQFEDAITA	HQRETTALRE	SLQDLAAERG	DVEREVRGRA	GGLLGMPEDR
**650**	**660**	**670**	**680**	**690**	**700**	**710**	**720**
RKQGRRKVSF	QQISKSGPVS	DHTGKQNEIA	KLGAAGGGGG	GGVERAERLR	AQLTVAQEGL	AALRQELQGV	EESREGLHRE
**730**	**740**	**750**	**760**	**770**	**780**	**790**	**800**
AQEARRALSD	EAREKDVLLL	FNSELRATIC	RAEQEKASFK	RSKEEKEQKL	LILEEAQAAL	QQEASALRAH	LWELEQAGGD
**810**	**820**	**830**	**840**	**850**	**860**	**870**	**880**
ARQELRELHR	QVLGLQRKLA	EVEAAGEAHG	QRLQEHLRES	RGAEQTLRAE	LHSVTRKLQE	ASGVADALQA	RLDQACHRIH
**890**	**900**	**910**	**920**	**930**	**940**	**950**	**960**
SLEQELAQAE	GARQDAEAQL	GRLCSTLRRG	LGLQRQSPWA	SPEQPGSPTK	GSDSSQALPG	QQGTSPPARP	HSPLRWPSPT
**970**	**980**	**990**	**1000**	**1010**	**1020**	**1030**	**1040**
PGGRSSELMD	VATVQDILRD	FVQKLREAQR	ERDDSRIQMA	TLSSRLSEAE	CRCARAQSRV	GQLQKALAEA	EEGQRRVEGA
**1050**	**1060**	**1070**	**1080**	**1090**	**1100**	**1110**	**1120**
LSSARAARAL	QKEALRRLEL	EHLASVRAAG	QEKRRLQEQL	ETLRQALEES	RRHSQGLAKQ	GKLLEEQLTN	LEHRCQKAEV
**1130**	**1140**	**1150**	**1160**	**1170**	**1180**	**1190**	**1200**
SLEPLRQVLC	RPQRLSGGQE	AAEAQAERRV	LQEQTAALRT	ERARLQGELA	ALRAQLAQME	QETLKREEDV	ARLGAEKEQL
**1210**	**1220**	**1230**	**1240**	**1250**	**1260**	**1270**	**1280**
DQSLNSLHQE	VDGALRQNQQ	LQAQMTEMEQ	AHTQRLQDLT	AQHQRDLATE	AERLHGARPQ	ATQALESQEW	THQQRVKVLE
**1290**	**1300**	**1310**	**1320**				
EQVASLKEQL	DQEVQWRQQA	HLGQAFQTGQ					

Cmpd.	No. of Cmpds.	m/z meas.	Δ m/z [ppm]	z	Rt [min]	Score	P	Range	Sequence	Modification
2904	2	973.5046	-21.62	2	65.1	13.6	2	479-495	R.EKETLSLTLAEEKEVAR.C	

Detailed Protein Report

Protein 333: **PREDICTED: transforming acidic coiled-coil-containing protein 3 isoform X2 [Homo sapiens]**

**Accession:**	gi|530375987	**Score:**	34.1
**Database:**	refseq_human(refseq_human_20140103.fasta)	**MW [kDa]:**	53.9
**Database Date:**	2015-11-30	**pI:**	5.2
**Modification(s):**	Carbamidomethyl	**Sequence Coverage [%]:**	5.2
		**No. of unique Peptides:**	1

Quantitation

mdown:qdown **Median:** 1.72 **CV:** 0.00 % **No. of Peptides:** 1

```
        10         20         30         40         50         60         70         80
MSLQVLNDKN VSNEKNTENC DFLFSPPEVT GRSSVLRVSQ KENVPPKNLA KAMKVTFQTP LRDPQTHRIL SPSMASKLEA
        90        100        110        120        130        140        150        160
PFTQDDTLGL ENSHPVWTQK EKQLHSASAE DTPVVQLAAE TPTAESKERA LNSASTSLPT SCPGSEPVPT HQQGQPALEL
       170        180        190        200        210        220        230        240
KEESFRDPAE VLGTGAEVDY LEQFGTSSFK ESALRKQSLY LKFDPLLRDS PGRPVPVATE TSSMHGANET PSGRPREAKL
       250        260        270        280        290        300        310        320
VEFDFLGALD IPVPGPPPGV PAPGGPPLST GPIVDLLQYS QKDLDAVVRT QVKATQEENR ELRSRCEELH GKNLELGKIM
       330        340        350        360        370        380        390        400
DRFEEVVYQA MEEVQKQKEL SKAEIQKVLK EKDQLTTDLN SMEKSFSDLF KRFEKQKEVI EGYRKNEESL KKCVEDYLAR
       410        420        430        440        450        460        470        480
ITQEGQRYQA LKAHAEEKLQ LANEEIAQVR SKAQAEALAL QASLRKEQMR IQSLEKTVEQ KTKENEELTR ICDDLISKME
       490
KI
```

Cmpd.	No. of Cmpds.	m/z meas.	Δ m/z [ppm]	z	Rt [min]	Score	P	Range	Sequence	Modification	Ratios
1875	1	917.8351	-120.99	2	53.5	12.8	1	464-478	K.ENEELTRICDDLISK.M	Carbamidomethyl: 9	mdown:qdown 1.72

Detailed Protein Report

Protein 334: PREDICTED: F-box/WD repeat-containing protein 8 isoform X2 [Homo sapiens]

**Accession:**	gi|530401261	**Score:**	34.0
**Database:**	refseq_human(refseq_human_20140103.fasta)	**MW [kDa]:**	46.4
**Database Date:**	2015-11-30	**pI:**	5.5
**Modification(s):**	Carbamidomethyl, Oxidation	**Sequence Coverage [%]:**	7.7
		**No. of unique Peptides:**	2

Alias proteins:

Accession	Name	Description
gi|530401263	refseq_human (refseq_human_20140103.fasta)	PREDICTED: F-box/WD repeat-containing protein 8 isoform X3 [Homo sapiens]

```
        10         20         30         40         50         60         70         80
MRCCGTGCAS RKGTFRIAAS LTILAGSSSS KSAEPRNTCY EPTGRYTSGD VRVWDTRTWD YVAPFLESED EEDEPGMQPN
        90        100        110        120        130        140        150        160
VSFVRINSSL AVAAYEDGFL NIWDLRTGKY PVHRFEHDAR IQALALSQDD ATVATASAFD VVMLSPNEEG YWQIAAEFEV
       170        180        190        200        210        220        230        240
PKLVQYLEIV PETRRYPVAV AAAGDLMYLL KAEDSARTLL YAHGPPVTCL DVSANQVAFG VQGLGWVYEG SKILVYSLEA
       250        260        270        280        290        300        310        320
GRRLLKLGNV LRDFTCVNLS DSPPNLMVSG NMDGRVRIHD LRSGNIALSL SAHQLRVSAV QMDDWKIVSG GEEGLVSVWD
       330        340        350        360        370        380        390        400
YRMNQKLWEV YSGHPVQHIS FSSHSLITAN VPYQTVMRNA DLDSFTTHRR HRGLIRAYEF AVDQLAFQSP LPVCRSSCDA
       410        420
MATHYYDLAL AFPYNHV
```

Cmpd.	No. of Cmpds.	m/z meas.	Δ m/z [ppm]	z	Rt [min]	Score	P	Range	Sequence	Modification
2655	2	673.3551	106.75	2	64.3	12.0	2	1-12	-.MRCCGTGCASRK.G	Carbamidomethyl: 3; Oxidation: 1
2701	1	973.4995	-32.63	2	62.2	10.8	1	17-36	R.IAASLTILAGSSSSKSAEPR.N	

Detailed Protein Report

Protein 335: **insulin receptor-related protein precursor [Homo sapiens]**

Accession: gi|31657140
Database: refseq_human(refseq_human_20140103.fasta)
Database Date: 2015-11-30
Modification(s): Carbamidomethyl

Score: 34.0
MW [kDa]: 143.6
pI: 6.0
Sequence Coverage [%]: 2.1
No. of unique Peptides: 2

10	20	30	40	50	60	70	80
MAVPSLWPWG	ACLPVIFLSL	GFGLDTVEVC	PSLDIRSEVA	ELRQLENCSV	VEGHLQILLM	FTATGEDFRG	LSFPRLTQVT
**90**	**100**	**110**	**120**	**130**	**140**	**150**	**160**
DYLLLFRVYG	LESLRDLFPN	LAVIRGTRLF	LGYALVIFEM	PHLRDVALPA	LGAVLRGAVR	VEKNQELCHL	STIDWGLLQP
**170**	**180**	**190**	**200**	**210**	**220**	**230**	**240**
APGANHIVGN	KLGEECADVC	PGVLGAAGEP	CAKTTFSGHT	DYRCWTSSHC	QRVCPCPHGM	ACTARGECCH	TECLGGCSQP
**250**	**260**	**270**	**280**	**290**	**300**	**310**	**320**
EDPRACVACR	HLYFQGACLW	ACPPGTYQYE	SWRCVTAERC	ASLHSVPGRA	STFGIHQGSC	LAQCPSGFTR	NSSSIFCHKC
**330**	**340**	**350**	**360**	**370**	**380**	**390**	**400**
EGLCPKECKV	GTKTIDSIQA	AQDLVGCTHV	EGSLILNLRQ	GYNLEPQLQH	SLGLVETITG	FLKIKHSFAL	VSLGFFKNLK
**410**	**420**	**430**	**440**	**450**	**460**	**470**	**480**
LIRGDAMVDG	NYTLYVLDNQ	NLQQLGSWVA	AGLTIPVGKI	YFAFNPRLCL	EHIYRLEEVT	GTRGRQNKAE	INPRTNGDRA
**490**	**500**	**510**	**520**	**530**	**540**	**550**	**560**
ACQTRTLRFV	SNVTEADRIL	LRWERYEPLE	ARDLLSFIVY	YKESPFQNAT	EHVGPDACGT	QSWNLLDVEL	PLSRTQEPGV
**570**	**580**	**590**	**600**	**610**	**620**	**630**	**640**
TLASLKPWTQ	YAVFVRAITL	TTEEDSPHQG	AQSPIVYLRT	LPAAPTVPQD	VISTSNSSSH	LLVRWKPPTQ	RNGNLTYYLV
**650**	**660**	**670**	**680**	**690**	**700**	**710**	**720**
LWQRLAEDGD	LYLNDYCHRG	LRLPTSNNDP	RFDGEDGDPE	AEMESDCCPC	QHPPPGQVLP	PLEAQEASFQ	KKFENFLHNA
**730**	**740**	**750**	**760**	**770**	**780**	**790**	**800**
ITIPISPWKV	TSINKSPQRD	SGRHRRAAGP	LRLGGNSSDF	EIQEDKVPRE	RAVLSGLRHF	TEYRIDIHAC	NHAAHTVGCS
**810**	**820**	**830**	**840**	**850**	**860**	**870**	**880**
AATFVFARTM	PHREADGIPG	KVAWEASSKN	SVLLRWLEPP	DPNGLILKYE	IKYRRLGEEA	TVLCVSRLRY	AKFGGVHLAL
**890**	**900**	**910**	**920**	**930**	**940**	**950**	**960**
LPPGNYSARV	RATSLAGNGS	WTDSVAFYIL	GPEEEDAGGL	HVLLTATPVG	LTLLIVLAAL	GFFYGKKRNR	TLYASVNPEY
**970**	**980**	**990**	**1000**	**1010**	**1020**	**1030**	**1040**
FSASDMYVPD	EWEVPREQIS	IIRELGQGSF	GMVYEGLARG	LEAGEESTPV	ALKTVNELAS	PRECIEFLKE	ASVMKAFKCH
**1050**	**1060**	**1070**	**1080**	**1090**	**1100**	**1110**	**1120**
HVVRLLGVVS	QGQPTLVIME	LMTRGDLKSH	LRSLRPEAEN	NPGLPQPALG	EMIQMAGEIA	DGMAYLAANK	FVHRDLAARN
**1130**	**1140**	**1150**	**1160**	**1170**	**1180**	**1190**	**1200**
CMVSQDFTVK	IGDFGMTRDV	YETDYYRKGG	KGLLPVRWMA	PESLKDGIFT	THSDVWSFGV	VLWEIVTLAE	QPYQGLSNEQ
**1210**	**1220**	**1230**	**1240**	**1250**	**1260**	**1270**	**1280**
VLKFVMDGGV	LEELEGCPLQ	LQELMSRCWQ	PNPRLRPSFT	HILDSIQEEL	RPSFRLLSFY	YSPECRGARG	SLPTTDAEPD
**1290**	**1300**						
SSPTPRDCSP	QNGGPGH						

Cmpd.	No. of Cmpds.	m/z meas.	Δ m/z [ppm]	z	Rt [min]	Score	P	Range	Sequence	Modification
561	2	730.2206	-117.87	2	36.3	16.7	0	213-225	R.VCPCPHGMACTAR.G	Carbamidomethyl: 2, 4
2751	1	922.4677	-48.57	2	65.7	17.3	2	499-512	R.ILLRWERYEPLEAR.D	

Detailed Protein Report

Protein 336: storkhead-box protein 1 isoform a [Homo sapiens]

**Accession:**	gi|63025200	**Score:**	33.9
**Database:**	refseq_human(refseq_human_20140103.fasta)	**MW [kDa]:**	110.9
**Database Date:**	2015-11-30	**pI:**	8.7
**Modification(s):**	Carbamidomethyl	**Sequence Coverage [%]:**	3.2
		**No. of unique Peptides:**	2

Alias proteins:

Accession	Name	Description
gi|194328691	refseq_huma (refseq_human_20140103.fasta)	storkhead-box protein 1 isoform a [Homo sapiens]

10	20	30	40	50	60	70	80
MARPVQLAPG	SLALVLCRLE	AQKAAGAAEE	PGGRAVFRAF	RRANARCFWN	ARLARAASRL	AFQGWLRRGV	LLVRAPPACL
**90**	**100**	**110**	**120**	**130**	**140**	**150**	**160**
QVLRDAWRRR	ALRPPRGFRI	RAVGDVFPVQ	MNPITQSQFV	PLGEVLCCAI	SDMNTAQIVV	TQESLLERLM	KHYPGIAIPS
**170**	**180**	**190**	**200**	**210**	**220**	**230**	**240**
EDILYTTLGT	LIKERKIYHT	GEGYFIVTPQ	TYFITNTTTQ	ENKRMLPSDE	SRLMPASMTY	LVSMESCAES	AQENAAPISH
**250**	**260**	**270**	**280**	**290**	**300**	**310**	**320**
CQSCQCFRDM	HTQDVQEAPV	AAEVTRKSHR	GLGESVSWVQ	NGAVSVSAEH	HICESTKPLP	YTRDKEKGKK	FGFSLLWRSL
**330**	**340**	**350**	**360**	**370**	**380**	**390**	**400**
SRKEKPKTEH	SSFSAQFPPE	EWPVRDEDDL	DNIPRDVEHE	IIKRINPILT	VDNLIKHTVL	MQKYEEQKKY	NSQGTSTDML
**410**	**420**	**430**	**440**	**450**	**460**	**470**	**480**
TIGHKYPSKE	GVKKRQGLSA	KPQGQGHSRR	DRHKARNQGS	EFQPGSIRLE	KHPKLPATQP	IPRIKSPNEM	VGQKPLGEIT
**490**	**500**	**510**	**520**	**530**	**540**	**550**	**560**
TVLGSHLIYK	KRISNPFQGL	SHRGSTISKG	HKIQKTSDLK	PSQTGPKEKP	FQKPRSLDSS	RIFDGKAKEP	YAEQPNDKME
**570**	**580**	**590**	**600**	**610**	**620**	**630**	**640**
AESIYINDPT	VKPINDDFRG	HLFSHPQQSM	LQNDGKCCPF	MESMLRYEVY	GGENEVIPEV	LRKSHSHFDK	LGETKQTPHS
**650**	**660**	**670**	**680**	**690**	**700**	**710**	**720**
LPSRGASFSD	RTPSACRLVD	NTIHQFQNLG	LLDYPVGVNP	LRQAARQDKD	SEELLRKGFV	QDAETTSLEN	EQLSNDDQAL
**730**	**740**	**750**	**760**	**770**	**780**	**790**	**800**
YQNEVEDDDG	ACSSLYLEED	DISENDDLRQ	MLPGHSQYSF	TGGSQGNHLG	KQKVIERSLT	EYNSTMERVE	SQVLKRNECY
**810**	**820**	**830**	**840**	**850**	**860**	**870**	**880**
KPTGLHATPG	ESQEPNLSAE	SCGLNSGAQF	GFNYEEEPSV	AKCVQASAPA	DERIFDYYSA	RKASFEAEVI	QDTIGDTGKK
**890**	**900**	**910**	**920**	**930**	**940**	**950**	**960**
PASWSQSPQN	QEMRKHFPQK	FQLFNTSHMP	VLAQDVQYEH	SHLEGTENHS	MAGDSGIDSP	RTQSLGSNNS	VILDGLKRRQ
**970**	**980**	**990**					
NFLQNVEGTK	SSQPLTSNSL	LPLTPVINV					

Cmpd.	No. of Cmpds.	m/z meas.	Δ m/z [ppm]	z	Rt [min]	Score	P	Range	Sequence	Modification
1506	1	881.6342	118.58	2	48.6	18.4	1	69-84	R.GVLLVRAPPACLQVLR.D	Carbamidomethyl: 11
2951	1	823.4152	-31.89	2	66.1	15.5	0	942-957	R.TQSLGSNNSVILDGLK.R	

Detailed Protein Report

Protein 337: ubiquitin carboxyl-terminal hydrolase 6 [Homo sapiens]

**Accession:**	gi|109638751	**Score:**	33.9
**Database:**	refseq_human(refseq_human_20140103.fasta)	**MW [kDa]:**	158.6
**Database Date:**	2015-11-30	**pI:**	8.8
**Modification(s):**	Oxidation	**Sequence Coverage [%]:**	4.1
		**No. of unique Peptides:**	3

Alias proteins:

Accession	Name	Description
gi|530410741	refseq_human (refseq_human_20140103.fasta)	PREDICTED: ubiquitin carboxyl-terminal hydrolase 6 isoform X1 [Homo sapiens]

```
        10         20         30         40         50         60         70         80
MDMVENADSL QAQERKDILM KYDKGHRAGL PEDKGPEPVG INSSIDRFGI LHETELPPVT AREAKKIRRE MTRTSKWMEM
        90        100        110        120        130        140        150        160
LGEWETYKHS SKLIDRVYKG IPMNIRGPVW SVLLNIQEIK LKNPGRYQIM KERGKRSSEH IHHIDLDVRT TLRNHVFFRD
       170        180        190        200        210        220        230        240
RYGAKQRELF YILLAYSEYN PEVGYCRDLS HITALFLLYL PEEDAFWALV QLLASERHSL PGFHSPNGGT VQGLQDQQEH
       250        260        270        280        290        300        310        320
VVPKSQPKTM WHQDKEGLCG QCASLGCLLR NLIDGISLGL TLRLWDVYLV EGEQVLMPIT SIALKVQQKR LMKTSRCGLW
       330        340        350        360        370        380        390        400
ARLRNQFFDT WAMNDDTVLK HLRASTKKLT RKQGDLPPPA KREQGSLAPR PVPASRGGKT LCKGYRQAPP GPPAQFQRPI
       410        420        430        440        450        460        470        480
CSASPPWASR FSTPCPGGAV REDTYPVGTQ GVPSLALAQG GPQGSWRFLE WKSMPRLPTD LDIGGPWFPH YDFEWSCWVR
       490        500        510        520        530        540        550        560
AISQEDQLAT CWQAEHCGEV HNKDMSWPEE MSFTANSSKI DRQKVPTEKG ATGLSNLGNT CFMNSSIQCV SNTQPLTQYF
       570        580        590        600        610        620        630        640
ISGRHLYELN RTNPIGMKGH MAKCYGDLVQ ELWSGTQKSV APLKLRRTIA KYAPKFDGFQ QQDSQELLAF LLDGLHEDLN
       650        660        670        680        690        700        710        720
RVHEKPYVEL KDSDGRPDWE VAAEAWDNHL RRNRSIIVDL FHGQLRSQVK CKTCGHISVR FDPFNFLSLP LPMDSYMDLE
       730        740        750        760        770        780        790        800
ITVIKLDGTT PVRYGLRLNM DEKYTGLKKQ LRDLCGLNSE QILLAEVHDS NIKNFPQDNQ KVQLSVSGFL CAFEIPVPSS
       810        820        830        840        850        860        870        880
PISASSPTQI DFSSSPSTNG MFTLTTNGDL PKPIFIPNGM PNTVVPCGTE KNFTNGMVNG HMPSLPDSPF TGYIIAVHRK
       890        900        910        920        930        940        950        960
MMRTELYFLS PQENRPSLFG MPLIVPCTVH TRKKDLYDAV WIQVSWLARP LPPQEASIHA QDRDNCMGYQ YPFTLRVVQK
       970        980        990       1000       1010       1020       1030       1040
DGNSCAWCPQ YRFCRGCKID CGEDRAFIGN AYIAVDWHPT ALHLRYQTSQ ERVVDKHESV EQSRRAQAEP INLDSCLRAF
      1050       1060       1070       1080       1090       1100       1110       1120
TSEEELGESE MYYCSKCKTH CLATKKLDLW RLPPFLIIHL KRFQFVNDQW IKSQKIVRFL RESFDPSAFL VPRDPALCQH
      1130       1140       1150       1160       1170       1180       1190       1200
KPLTPQGDEL SKPRILAREV KKVDAQSSAG KEDMLLSKSP SSLSANISSS PKGSPSSSRK SGTSCPSSKN SSPNSSPRTL
      1210       1220       1230       1240       1250       1260       1270       1280
GRSKGRLRLP QIGSKNKPSS SKKNLDASKE NGAGQICELA DALSRGHMRG GSQPELVTPQ DHEVALANGF LYEHEACGNG
      1290       1300       1310       1320       1330       1340       1350       1360
CGDGYSNGQL GNHSEEDSTD DQREDTHIKP IYNLYAISCH SGILSGGHYI TYAKNPNCKW YCYNDSSCEE LHPDEIDTDS
      1370       1380       1390       1400       1410
AYILFYEQQG IDYAQFLPKI DGKKMADTSS TDEDSESDYE KYSMLQ
```

Cmpd.	No. of Cmpds.	m/z meas.	Δ m/z [ppm]	z	Rt [min]	Score	P	Range	Sequence	Modification
1774	8	673.2348	-219.99	2	52.2	11.9	2	89-99	K.HSSKLIDRVYK.G	
2932	1	965.1226	-27.88	3	65.8	10.3	0	218-244	R.HSLPGFHSPNGGTVQGLQDQQEHVVPK.S	
2697	1	1175.5508	58.82	2	62.1	11.6	1	1039-1058	R.AFTSEEELGESEMYYCSKCK.T	Oxidation: 13

Detailed Protein Report

Protein 338: prostate tumor-overexpressed gene 1 protein [Homo sapiens]

**Accession:**	gi|33695090	**Score:**	33.7
**Database:**	refseq_human(refseq_human_20140103.fasta)	**MW [kDa]:**	46.8
**Database Date:**	2015-11-30	**pI:**	11.6
**Modification(s):**	Carbamidomethyl, Oxidation	**Sequence Coverage [%]:**	9.6
		**No. of unique Peptides:**	1

Alias proteins:

Accession	Name	Description
gi|530416718	refseq_human (refseq_human_20140103.fasta)	PREDICTED: prostate tumor-overexpressed gene 1 protein isoform X3 [Homo sapiens]
gi|530416716	refseq_human (refseq_human_20140103.fasta)	PREDICTED: prostate tumor-overexpressed gene 1 protein isoform X2 [Homo sapiens]

```
        10         20         30         40         50         60         70         80
MVRPRRAPYR SGAGGPLGGR GRPPRPLVVR AVRSRSWPAS PRGPQPPRIR ARSAPPMEGA RVFGALGPIG PSSPGLTLGG
        90        100        110        120        130        140        150        160
LAVSEHRLSN KLLAWSGVLE WQEKRRPYSD STAKLKRTLP CQAYVNQGEN LETDQWPQKL IMQLIPQQLL TTLGPLFRNS
       170        180        190        200        210        220        230        240
QLAQFHFTNR DCDSLKGLCR IMGNGFAGCM LFPHISPCEV RVLMLLYSSK KKIFMGLIPY DQSGFVSAIR QVITTRKQAV
       250        260        270        280        290        300        310        320
GPGGVNSGPV QIVNNKFLAW SGVMEWQEPR PEPNSRSKRW LPSHVYVNQG EILRTEQWPR KLYMQLIPQQ LLTTLVPLFR
       330        340        350        360        370        380        390        400
NSRLVQFHFT KDLETLKSLC RIMDNGFAGC VHFSYKASCE IRVLMLLYSS EKKIFIGLIP HDQGNFVNGI RRVIANQQQV
       410        420
LQRNLEQEQQ QRGMGG
```

Cmpd.	No. of Cmpds.	m/z meas.	Δ m/z [ppm]	z	Rt [min]	Score	P	Range	Sequence	Modification
2316	1	974.1925	62.96	3	59.2	21.5	2	332-356	K.DLETLKSLCRIMDNGFAGCVHFSYK.A	Carbamidomethyl: 19; Oxidation: 12

Detailed Protein Report

Protein 339: glycerol-3-phosphate dehydrogenase, mitochondrial precursor [Homo sapiens]

**Accession:**	gi|285002231	**Score:**	33.7
**Database:**	refseq_human(refseq_human_20140103.fasta)	**MW [kDa]:**	80.8
**Database Date:**	2015-11-30	**pI:**	8.4
**Modification(s):**	Carbamidomethyl	**Sequence Coverage [%]:**	5.9
		**No. of unique Peptides:**	3

Alias proteins:

Accession	Name	Description
gi|530370176	refseq_human (refseq_human_20140103.fasta)	PREDICTED: glycerol-3-phosphate dehydrogenase, mitochondrial isoform X1 [Homo sapiens]
gi|285002233	refseq_human (refseq_human_20140103.fasta)	glycerol-3-phosphate dehydrogenase, mitochondrial precursor [Homo sapiens]

10	20	30	40	50	60	70	80
MAFQKAVKGT	ILVGGGALAT	VLGLSQFAHY	RRKQMNLAYV	KAADCISEPV	NREPPSREAQ	LLTLQNTSEF	DILVIGGGAT
**90**	**100**	**110**	**120**	**130**	**140**	**150**	**160**
GSGCALDAVT	RGLKTALVER	DDFSSGTSSR	STKLIHGGVR	YLQKAIMKLD	IEQYRMVKEA	LHERANLLEI	APHLSAPLPI
**170**	**180**	**190**	**200**	**210**	**220**	**230**	**240**
MLPVYKWWQL	PYYWVGIKLY	DLVAGSNCLK	SSYVLSKSRA	LEHFPMLQKD	KLVGAIVYYD	GQHNDARMNL	AIALTAARYG
**250**	**260**	**270**	**280**	**290**	**300**	**310**	**320**
AATANYMEVV	SLLKKTDPQT	GKVRVSGARC	KDVLTGQEFD	VRAKCVINAT	GPFTDSVRKM	DDKDAAAICQ	PSAGVHIVMP
**330**	**340**	**350**	**360**	**370**	**380**	**390**	**400**
GYYSPESMGL	LDPATSDGRV	IFFLPWQKMT	IAGTTDTPTD	VTHHPIPSEE	DINFILNEVR	NYLSCDVEVR	RGDVLAAWSG
**410**	**420**	**430**	**440**	**450**	**460**	**470**	**480**
IRPLVTDPKS	ADTQSISRNH	VVDISESGLI	TIAGGKWTTY	RSMAEDTINA	AVKTHNLKAG	PSRTVGLFLQ	GGKDWSPTLY
**490**	**500**	**510**	**520**	**530**	**540**	**550**	**560**
IRLVQDYGLE	SEVAQHLAAT	YGDKAFEVAK	MASVTGKRWP	IVGVRLVSEF	PYIEAEVKYG	IKEYACTAVD	MISRRTRLAF
**570**	**580**	**590**	**600**	**610**	**620**	**630**	**640**
LNVQAAEEAL	PRIVELMGRE	LNWDDYKKQE	QLETARKFLY	YEMGYKSRSE	QLTDRSEISL	LPSDIDRYKK	RFHKFDADQK
**650**	**660**	**670**	**680**	**690**	**700**	**710**	**720**
GFITIVDVQR	VLESINVQMD	ENTLHEILNE	VDLNKNGQVE	LNEFLQLMSA	IQKGRVSGSR	LAILMKTAEE	NLDRRVPIPV
**730**							
DRSCGGL							

Cmpd.	No. of Cmpds.	m/z meas.	Δ m/z [ppm]	z	Rt [min]	Score	P	Range	Sequence	Modification
1028	1	863.9268	-97.49	2	41.6	10.0	2	114-128	K.LIHGGVRYLQKAIMK.L	
29	1	706.3122	-81.53	3	29.6	11.9	1	179-197	K.LYDLVAGSNCLKSSYVLSK.S	Carbamidomethyl: 10
2822	1	607.3358	86.08	2	64.4	11.7	0	598-606	K.FLYYEMGYK.S	

Detailed Protein Report

Protein 340: leukocyte receptor cluster member 1 [Homo sapiens]

Accession: gi|24308289
Database: refseq_human(refseq_human_20140103.fasta)
Database Date: 2015-11-30

Score: 33.6
MW [kDa]: 30.5
pI: 10.1
Sequence Coverage [%]: 7.6
No. of unique Peptides: 1

10	20	30	40	50	60	70	80
MNILPKKSWH	VRNKDNVARV	RRDEAQAREE	EKERERRVLL	AQQEARTEFL	RKKARHQNSL	PELEAAEAGA	PGSGPVDLFR
**90**	**100**	**110**	**120**	**130**	**140**	**150**	**160**
ELLEEGKGVI	RGNKEYEEEK	RQEKERQEKA	LGILTYLGQS	AAEAQTQPPW	YQLPPGRGGP	PPGPAPDEKI	KSRLDPLREM
**170**	**180**	**190**	**200**	**210**	**220**	**230**	**240**
QKHLGKKRQH	GGDEGSRSRK	EKEGSEKQRP	KEPPSLDQLR	AERLRREAAE	RSRAEALLAR	VQGRALQEGQ	PEEDETDDRR
**250**	**260**	**270**					
RRYNSQFNPQ	LARRPRQQDP	HLTH					

Cmpd.	No. of Cmpds.	m/z meas.	Δ m/z [ppm]	z	Rt [min]	Score	P	Range	Sequence	Modification
575	1	565.3015	-6.83	2	36.5	11.1	1	154-162	R.LDPLREMQK.H	

Detailed Protein Report

Protein 341: PREDICTED: regulator of G-protein signaling 3 isoform X5 [Homo sapiens]

**Accession:**	gi|578817739	**Score:**	33.6
**Database:**	refseq_human(refseq_human_20140103.fasta)	**MW [kDa]:**	68.1
**Database Date:**	2015-11-30	**pI:**	9.9
**Modification(s):**	Carbamidomethyl, Oxidation	**Sequence Coverage [%]:**	6.9
		**No. of unique Peptides:**	3

10	20	30	40	50	60	70	80
MEWLSPDIAL	PRRDEWTQTS	PARKRITHAK	VQGAGQLRLS	IDAQDRVLLL	HIIEGKGLIS	KQPGTCDPYV	KISLIPEDSR
90	100	110	120	130	140	150	160
LRHQKTQTVP	DCRDPAFHEH	FFFPVQEEDD	QKRLLVTVWN	RASQSRQSGL	IGCMSFGVKS	LLTPDKEISG	WYYLLGEHLG
170	180	190	200	210	220	230	240
RTKHLKVARR	RLRPLRDPLL	RMPGGGDTEN	GKKLKITIPR	GKDGFGFTIC	CDSPVRVQAV	DSGGPAERAG	LQQLDTVLQL
250	260	270	280	290	300	310	320
NERPVEHWKC	VELAHEIRSC	PSEIILLVWR	MVPQVKPGPD	GGVLRRASCK	STHDLQSPPN	KREKNCTHGV	QARPEQRHSC
330	340	350	360	370	380	390	400
HLVCDSSDGL	LLGGWERYTE	VAKRGGQHTL	PALSRATAPT	DPNYIILAPL	NPGSQLLRPV	YQEDTIPEES	GSPSKGKSYT
410	420	430	440	450	460	470	480
GLGKKSRLMK	TVQTMKGHGN	YQNCPVVRPH	ATHSSYGTYV	TLAPKVLVFP	VFVQPLDLCN	PARTLLLSEE	LLLYEGRNKA
490	500	510	520	530	540	550	560
AEVTLFAYSD	LLLFTKEDEP	GRCDVLRNPL	YLQSVKLQEG	SSEDLKFCVL	YLAEKAECLF	TLEAHSQEQK	KRVCWCLSEN
570	580	590	600	610	620		
IAKQQQLAAS	PPDSKKLHPF	GSLQQEMGPV	NSTNATQDRS	FTSPGQTLIG			

Cmpd.	No. of Cmpds.	m/z meas.	Δ m/z [ppm]	z	Rt [min]	Score	P	Range	Sequence	Modification
2171	1	576.2313	-138.85	3	55.7	10.6	1	31-46	K.VQGAGQLRLSIDAQDR.V	
27	4	819.8677	-73.87	2	29.0	12.2	2	81-93	R.LRHQKTQTVPDCR.D	Carbamidomethyl: 12
1945	1	700.2356	-147.65	2	54.4	10.8	0	127-139	R.QSGLIGCMSFGVK.S	Carbamidomethyl: 7; Oxidation: 8

Detailed Protein Report

Protein 342: KRR1 small subunit processome component homolog [Homo sapiens]

**Accession:**	gi|117676403	**Score:**	33.5
**Database:**	refseq_human(refseq_human_20140103.fasta)	**MW [kDa]:**	43.6
**Database Date:**	2015-11-30	**pI:**	10.3
		**Sequence Coverage [%]:**	6.3
		**No. of unique Peptides:**	1

Quantitation

mdown:qdown	**Median:**	1.15	**CV:**	0.00 %	**No. of Peptides:**	1
Wdown:Qdown	**Median:**	1.28	**CV:**	0.00 %	**No. of Peptides:**	1

10	20	30	40	50	60	70	80
MASPSLERPE	KGAGKSEFRN	QKPKPENQDE	SELLTVPDGW	KEPAFSKEDN	PRGLLEESSF	ATLFPKYREA	YLKECWPLVQ
**90**	**100**	**110**	**120**	**130**	**140**	**150**	**160**
KALNEHHVNA	TLDLIEGSMT	VCTTKKTFDP	YIIIRARDLI	KLLARSVSFE	QAVRILQDDV	ACDIIKIGSL	VRNKERFVKR
**170**	**180**	**190**	**200**	**210**	**220**	**230**	**240**
RQRLIGPKGS	TLKALELLTN	CYIMVQGNTV	SAIGPFSGLK	EVRKVVLDTM	KNIHPIYNIK	SLMIKRELAK	DSELRSQSWE
**250**	**260**	**270**	**280**	**290**	**300**	**310**	**320**
RFLPQFKHKN	VNKRKEPKKK	TVKKEYTPFP	PPQPESQIDK	ELASGEYFLK	ANQKKRQKME	AIKAKQAEAI	SKRQEERNKA
**330**	**340**	**350**	**360**	**370**	**380**	**390**	
FIPPKEKPIV	KPKEASTETK	IDVASIKEKV	KKAKNKKLGA	LTAEEIALKM	EADEKKKKKK	K	

Cmpd.	No. of Cmpds.	m/z meas.	Δ m/z [ppm]	z	Rt [min]	Score	P	Range	Sequence	Modification	Ratios
2913	2	973.5096	1.50	2	65.6	13.8	2	2-19	M.ASPSLERPEKGAGKSEFR.N		Wdown:Qdown 1.28<br>mdown:qdown 1.15

Detailed Protein Report

Protein 343: PREDICTED: centrosomal protein KIAA1731 isoform X7 [Homo sapiens]

**Accession:**	gi|578822447	**Score:**	33.5
**Database:**	refseq_human(refseq_human_20140103.fasta)	**MW [kDa]:**	268.9
**Database Date:**	2015-11-30	**pI:**	5.4
		**Sequence Coverage [%]:**	1.0
		**No. of unique Peptides:**	2

Detailed Protein Report

10	20	30	40	50	60	70	80
MERFEKAHVR	GFQAMKKIHL	AQNQEKLMKE	LKQLQQEDLA	RRRQTVAQMP	PQLVELPYKR	SEMKEDWQRE	LEFAFEDMYN
90	100	110	120	130	140	150	160
ADRKVKGNLI	LHLEPEPLPT	VTNQIQDEEL	DLSMEQENLG	AAEDLPVTEA	EICSSETDVP	LVMKTQQIPS	KVLFKKLLNK
170	180	190	200	210	220	230	240
IRSQKSLWTI	KSMSEDESEM	ITTVSEIESK	APTVESGTIA	SKERTLSSGQ	EQVVESDTLT	IESGPLASED	KPLSCGTNSG
250	260	270	280	290	300	310	320
KEQEINETLP	ITTVAQSSVL	LHPQEAAARI	RMSARQKQIM	EIEEQKQKQL	ELLEQIEQQK	LRLETDCFRA	QLEEEKRKKT
330	340	350	360	370	380	390	400
QPTGVGCRKS	HHYINHLVGI	APASCPVISD	EDSHRQMIRN	YQHQLLQQNR	LHRQSVETAR	KQLLEYQTML	KGRCPSVSAP
410	420	430	440	450	460	470	480
SLITDSVISV	PSWKSERPTA	ISEHWDQGQR	LKLSPNKYQP	IQPIQTSKLE	QDHFQVARQN	HFPQRQVETT	ETLRASDILT
490	500	510	520	530	540	550	560
NQALESQEHL	RQFSQTETQQ	RDYKLVPKDS	ETLSRALSHD	RQLISQDARK	ISETFGATTF	QSLESQQLFS	ENSENISYHL
570	580	590	600	610	620	630	640
TEPSSFVPLV	PQHSFSSLPV	KVESGKIQEP	FSAMSKSTVS	TSHSIISQMH	DRPLLPSENI	TAQQGNMKAL	QEQLDLQKKV
650	660	670	680	690	700	710	720
LQATQEAQEQ	LLLCKQKEVE	QQTGLSVFLP	LVTPDSSALL	PSAKADLGRI	QESSPTKNNI	AVSSDHHVIS	QLQDKRLSLS
730	740	750	760	770	780	790	800
QPILSQQNNF	KFLQEQLNIQ	KDSLQARREA	QEVLYVHKQS	ELDRRVCSEQ	AEPSFPFQVA	QHTFTSLPSA	DTKSGKIQEQ
810	820	830	840	850	860	870	880
HSSKSEKGLV	SCQSDIPISQ	DGSLSFLQQF	LPLHDSLKLL	QEQLTKQRDT	LQARHEAQVE	LLLHRQRDLG	DSKSGLVSSS
890	900	910	920	930	940	950	960
SSPVVVQHSV	ASQASAKAEP	RRIQELYLSE	KENVGPSCHL	IIPTFQDKSL	SFPQHSLAQQ	ENLTILQEQS	QIQRVILGAK
970	980	990	1000	1010	1020	1030	1040
EGTQEFVHTE	SELEKRISSE	QTGTSSSLSQ	VDESERFQEC	ISIKSDSTIP	LSHPKIPRCQ	ERLLRVSQHM	LPLQDNLEEH
1050	1060	1070	1080	1090	1100	1110	1120
QAWLDTEKEA	FHFSQKTQEN	TSSEQTGSSS	FIPQLVQLSF	TSLASAESGT	ILEPLFTESE	SKIFSSHLQI	PQLQDRLLRI
1130	1140	1150	1160	1170	1180	1190	1200
SQLIQPQQDN	LKALQEQLAT	QREAIILARQ	EAREELLLHQ	SEWEGRISPE	QVDTSSLPLV	PQHSFASLPL	NESERNQEPC
1210	1220	1230	1240	1250	1260	1270	1280
SINSDNIVSS	GHSEIPTLPD	GLLGLSHLVL	PQQDNLIALE	EHLHAQTDFL	PSIEKTQKEL	VLSKPCKFEE	KVSSEHFIQS
1290	1300	1310	1320	1330	1340	1350	1360
HHGDLQALQQ	QLDTQKKAIR	SIQEVQEELL	LQRLSELEKR	VSSEQVCSSS	FVSQVPVADS	ERTQKSFPTK	SNDTLPSSHR
1370	1380	1390	1400	1410	1420	1430	1440
EIPRLQDRLL	SLSKPILPQQ	DNMTAQLDAQ	REVMYSYEKP	QEELSLNKQR	KLNKSESAEH	TIPSLFLPKE	TEHSFIPLPF
1450	1460	1470	1480	1490	1500	1510	1520
AEAKPKSTCE	LYSSQNEHAA	PPSNPVIPGF	QDRLLSFSQS	VLTQQDNLGL	QKQLDLQREV	LHYSQKAQEK	LLVQRQTALQ
1530	1540	1550	1560	1570	1580	1590	1600
QQIQKHEETL	KDFFKDSQIS	KPTVENDLKT	QKMGQLRDWF	PNTQDLAGND	QENIRHADRN	NSDDNHLASE	DTSAKQSGEH
1610	1620	1630	1640	1650	1660	1670	1680
LEKDLGRRSS	KPPVAKVKCG	LDLNQHELSA	IQEVESPAIG	RTSILGKPGI	YEDRDPLRVS	ISREQSFFGS	PLAHDPFSCL
1690	1700	1710	1720	1730	1740	1750	1760
QLVGQENVCG	DDYDEAVKLK	ESVVENHAVL	SYAVEEEHAY	LGPTVKPDDK	AKTLSYEPLS	SATVSTGSLL	SYENTDLSLT
1770	1780	1790	1800	1810	1820	1830	1840
DPESFSEHMD	DSKQESTTSK	EEETNIISSI	VPSTQDIYQR	QNSSDVHKSL	LPAVDETTCG	HTHFQQMIDK	YINEANLIPE
1850	1860	1870	1880	1890	1900	1910	1920
KTDLQELEHI	FPNLHHQLFK	PLEPHPDFDL	SSSSSGISPD	NRDFYQRSDS	SSESHCATGL	SKSTVYFTAL	RRTSMHSSLN
1930	1940	1950	1960	1970	1980	1990	2000
TSPNQQPDTN	LAHVGAHSFA	TENIIGGSEQ	CFEQLQPEYS	SQEESQHADL	PSIFSIEARD	SSQGMKNQNY	PSEEHTEILQ
2010	2020	2030	2040	2050	2060	2070	2080
NKKKIVHFQL	SIGNLSSVYS	SSDEANVFDQ	LNVQHSTPCG	SNSSECSTKH	QLESRKESMG	FEELSKRGVV	TMLQSQGLIE
2090	2100	2110	2120	2130	2140	2150	2160
DNKNETCRVL	DINPQVEETD	SRLCVRTVEM	GTSIQAPYSL	TTQNEKYFEN	SAETDIPKIT	KKLSQLGESE	LFASSGSFSL
2170	2180	2190	2200	2210	2220	2230	2240

Detailed Protein Report

Cmpd.	No. of Cmpds.	m/z meas.	Δ m/z [ppm]	z	Rt [min]	Score	P	Range	Sequence	Modification
131	1	608.2888	-54.72	2	30.9	17.7	0	749-758	R.EAQEVLYVHK.Q	
2043	1	709.3053	-139.25	2	55.7	15.7	1	2299-2311	K.EKPSISSSVSRLK.G	

Detailed Protein Report

Protein 344: keratin, type II cytoskeletal 8 isoform 2 [Homo sapiens]

Accession: gi|4504919
Database: refseq_human(refseq_human_20140103.fasta)
Database Date: 2015-11-30

Score: 33.4
MW [kDa]: 53.7
pI: 5.4
Sequence Coverage [%]: 4.6
No. of unique Peptides: 1

Quantitation

mdown:qdown **Median:** 1.08 **CV:** 0.00 % **No. of Peptides:** 1

Alias proteins:

Accession	Name	Description
gi|372466577	refseq_human (refseq_human_20140103.fasta)	keratin, type II cytoskeletal 8 isoform 2 [Homo sapiens]

```
        10         20         30         40         50         60         70         80
MSIRVTQKSY KVSTSGPRAF SSRSYTSGPG SRISSSSFSR VGSSNFRGGL GGGYGGASGM GGITAVTVNQ SLLSPLVLEV
        90        100        110        120        130        140        150        160
DPNIQAVRTQ EKEQIKTLNN KFASFIDKVR FLEQQNKMLE TKWSLLQQQK TARSNMDNMF ESYINNLRRQ LETLGQEKLK
       170        180        190        200        210        220        230        240
LEAELGNMQG LVEDFKNKYE DEINKRTEME NEFVLIKKDV DEAYMNKVEL ESRLEGLTDE INFLRQLYEE EIRELQSQIS
       250        260        270        280        290        300        310        320
DTSVVLSMDN SRSLDMDSII AEVKAQYEDI ANRSRAEAES MYQIKYEELQ SLAGKHGDDL RRTKTEISEM NRNISRLQAE
       330        340        350        360        370        380        390        400
IEGLKGQRAS LEAAIADAEQ RGELAIKDAN AKLSELEAAL QRAKQDMARQ LREYQELMNV KLALDIEIAT YRKLLEGEES
       410        420        430        440        450        460        470        480
RLESGMQNMS IHTKTTSGYA GGLSSAYGGL TSPGLSYSLG SSFGSGAGSS SFSRTSSSRA VVVKKIETRD GKLVSESSDV
       490
LPK
```

Cmpd.	No. of Cmpds.	m/z meas.	Δ m/z [ppm]	z	Rt [min]	Score	P	Range	Sequence	Modification	Ratios
447	1	738.2345	-160.13	2	34.2	11.1	0	402-414	R.LESGMQNMSIHTK.T		mdown:qdown 1.08

Detailed Protein Report

Protein 345: **PREDICTED: EGF-containing fibulin-like extracellular matrix protein 1 isoform X2 [Homo sapiens]**

**Accession:**	gi|530367362	**Score:**	33.4
**Database:**	refseq_human(refseq_human_20140103.fasta)	**MW [kDa]:**	59.3
**Database Date:**	2015-11-30	**pI:**	9.5
		**Sequence Coverage [%]:**	9.8
		**No. of unique Peptides:**	2

Quantitation

Wdown:Qdown **Median:** 1.24 **CV:** 0.00 % **No. of Peptides:** 1

```
        10         20         30         40         50         60         70         80
MSTCPLWRGV PASLGNEELA REGKRGRQRG PLEQGARTSL RCALYRRARN AGLSARASLS SLLRPPSPSP FPSLSPPPPA
        90        100        110        120        130        140        150        160
AAAAAGLRQI RPTGLPSPAH SPRCPGPERS AAAQVFLLCC ARNSASSRFT MLKALFLTML TLALVKSQDT EETITYTQCT
       170        180        190        200        210        220        230        240
DGYEWDPVRQ QCKDIDECDI VPDACKGGMK CVNHYGGYLC LPKTAQIIVN NEQPQQETQP AEGTSGATTG VVAASSMATS
       250        260        270        280        290        300        310        320
GVLPGGGFVA SAAAVAGPEM QTGRNNFVIR RNPADPQRIP SNPSHRIQCA AGYEQSEHNV CQDIDECTAG THNCRADQVC
       330        340        350        360        370        380        390        400
INLRGSFACQ CPPGYQKRGE QCVDIDECRT SSYLCQYQCV NEPGKFSCMC PQGYQVVRSR TCQDINECET TNECREDEMC
       410        420        430        440        450        460        470        480
WNYHGGFRCY PRNPCQDPYI LTPENRCVCP VSNAMCRELP QSIVYKYMSI RSDRSVPSDI FQIQATTIYA NTINTFRIKS
       490        500        510        520        530        540        550
GNENGEFYLR QTSPVSAMLV LVKSLSGPRE HIVDLEMLTV SSIGTFRTSS VLRLTIIVGP FSF
```

Cmpd.	No. of Cmpds.	m/z meas.	Δ m/z [ppm]	z	Rt [min]	Score	P	Range	Sequence	Modification	Ratios
952	1	824.0174	-86.17	3	40.4	12.7	2	2-24	M. STCPLWRGVPASLGNEELAREGK R		
555	1	676.8735	43.07	2	36.3	10.5	0	110-122	R.SAAAQVFLLCCAR.N		Wdown:Qdown 1.24

Detailed Protein Report

Protein 346: **PREDICTED: keratin, type II cuticular Hb3 isoform X1 [Homo sapiens]**

**Accession:**	gi|530400232	**Score:**	33.4
**Database:**	refseq_human(refseq_human_20140103.fasta)	**MW [kDa]:**	49.6
**Database Date:**	2015-11-30	**pI:**	5.3
**Modification(s):**	Carbamidomethyl	**Sequence Coverage [%]:**	7.4
		**No. of unique Peptides:**	1

10	20	30	40	50	60	70	80
MTCGFNSIGC	GFRPGNFSCV	SACGPRPSRC	CITAAPYRGI	SCYRGLTGGF	GSHSVCGGFR	AGSCGRSFGY	RSGGVCGPSP
**90**	**100**	**110**	**120**	**130**	**140**	**150**	**160**
PCITTVSVNE	SLLTPLNLEI	DPNAQCVKQE	EKEQIKSLNS	RFAAFIDKVR	FLEQQNKLLE	TKLQFYQNRE	CCQSNLEPLF
**170**	**180**	**190**	**200**	**210**	**220**	**230**	**240**
AGYIETLRRE	AECVEADSGR	LASELNHVQE	VLEGYKKKYE	EEVALRATAE	NEFVALKKDV	DCAYLRKSDL	EANVEALIQE
**250**	**260**	**270**	**280**	**290**	**300**	**310**	**320**
IDFLRRLYEE	EIRILQSHIS	DTSVVVKLDN	SRDLNMDCIV	AEIKAQYDDI	ATRSRAEAES	WYRSKCEEMK	ATVIRHGETL
**330**	**340**	**350**	**360**	**370**	**380**	**390**	**400**
RRTKEEINEL	NRMIQRLTAE	VENAKCQNSK	LEAAVAQSEQ	QGEAALSDAR	CKLAELEGAL	QKAKQDMACL	IREYQEVMNS
**410**	**420**	**430**	**440**	**450**			
KLGLDIEIAT	YRRLLEGEEQ	RLCEGVEAAA	PGVGLCAGIS	ACRAPGR			

Cmpd.	No. of Cmpds.	m/z meas.	Δ m/z [ppm]	z	Rt [min]	Score	P	Range	Sequence	Modification
2096	1	1052.4199	-78.76	2	54.8	12.5	0	422-443	R.LCEGVEAAAPGVGLCAGISACR.A	Carbamidomethyl: 2

Detailed Protein Report

Protein 347: PREDICTED: filamin-B isoform X6 [Homo sapiens]

**Accession:**	gi|530372129	**Score:**	33.3
**Database:**	refseq_human(refseq_human_20140103.fasta)	**MW [kDa]:**	233.6
**Database Date:**	2015-11-30	**pI:**	5.2
		**Sequence Coverage [%]:**	1.1
		**No. of unique Peptides:**	1

Detailed Protein Report

10	20	30	40	50	60	70	80
MPVTEKDLAE	DAPWKKIQQN	TFTRWCNEHL	KCVNKRIGNL	QTDLSDGLRL	IALLEVLSQK	RMYRKYHQRP	TFRQMQLENV
90	100	110	120	130	140	150	160
SVALEFLDRE	SIKLVSIDSK	AIVDGNLKLI	LGLVWTLILH	YSISMPVWED	EGDDDAKKQT	PKQRLLGWIQ	NKIPYLPITN
170	180	190	200	210	220	230	240
FNQNWQDGKA	LGALVDSCAP	GLCPDWESWD	PQKPVDNARE	AMQQADDWLG	VPQVITPEEI	IHPDVDEHSV	MTYLSQFPKA
250	260	270	280	290	300	310	320
KLKPGAPLKP	KLNPKKARAY	GRGIEPTGNM	VKQPAKFTVD	TISAGQGDVM	VFVEDPEGNK	EEAQVTPDSD	KNKTYSVEYL
330	340	350	360	370	380	390	400
PKVTGLHKVT	VLFAGQHISK	SPFEVSVDKA	QGDASKVTAK	GPGLEAVGNI	ANKPTYFDIY	TAGAGVGDIG	VEVEDPQGKN
410	420	430	440	450	460	470	480
TVELLVEDKG	NQVYRCVYKP	MQPGPHVVKI	FFAGDTIPKS	PFVVQVGEAC	NPNACRASGR	GLQPKGVRIR	ETTDFKVDTK
490	500	510	520	530	540	550	560
AAGSGELGVT	MKGPKGLEEL	VKQKDFLDGV	YAFEYYPSTP	GRYSIAITWG	GHHIPKSPFE	VQVGPEAGMQ	KVRAWGPGLH
570	580	590	600	610	620	630	640
GGIVGRSADF	VVESIGSEVG	SLGFAIEGPS	QAKIEYNDQN	DGSCDVKYWP	KEPGEYAVHI	MCDDEDIKDS	PYMAFIHPAT
650	660	670	680	690	700	710	720
GGYNPDLVRA	YGPGLEKSGC	IVNNLAEFTV	DPKDAGKAPL	KIFAQDGEGQ	RIDIQMKNRM	DGTYACSYTP	VKAIKHTIAV
730	740	750	760	770	780	790	800
VWGGVNIPHS	PYRVNIGQGS	HPQKVKVFGP	GVERSGLKAN	EPTHFTVDCT	EAGEGDVSVG	IKCDARVLSE	DEEDVDFDII
810	820	830	840	850	860	870	880
HNANDTFTVK	YVPPAAGRYT	IKVLFASQEI	PASPFRVKVD	PSHDASKVKA	EGPGLSKAGV	ENGKPTHFTV	YTKGAGKAPL
890	900	910	920	930	940	950	960
NVQFNSPLPG	DAVKDLDIID	NYDYSHTVKY	TPTQQGNMQV	LVTYGGDPIP	KSPFTVGVAA	PLDLSKIKLN	GLENRVEVGK
970	980	990	1000	1010	1020	1030	1040
DQEFTVDTRG	AGGQGKLDVT	ILSPSRKVVP	CLVTPVTGRE	NSTAKFIPRE	EGLYAVDVTY	DGHPVPGSPY	TVEASLPPDP
1050	1060	1070	1080	1090	1100	1110	1120
SKVKAHGPGL	EGGLVGKPAE	FTIDTKGAGT	GGLGLTVEGP	CEAKIECSDN	GDGTCSVSYL	PTKPGEYFVN	ILFEEVHIPG
1130	1140	1150	1160	1170	1180	1190	1200
SPFKADIEMP	FDPSKVVASG	PGLEHGKVGE	AGLLSVDCSE	AGPGALGLEA	VSDSGTKAEV	SIQNNKDGTY	AVTYVPLTAG
1210	1220	1230	1240	1250	1260	1270	1280
MYTLTMKYGG	ELVPHFPARV	KVEPAVDTSR	IKVFGPGIEG	KDVFREATTD	FTVDSRPLTQ	VGGDHIKAHI	ANPSGASTEC
1290	1300	1310	1320	1330	1340	1350	1360
FVTDNADGTY	QVEYTPFEKG	LHVVEVTYDD	VPIPNSPFKV	AVTEGCQPSR	VQAQGPGLKE	AFTNKPNVFT	VVTRGAGIGG
1370	1380	1390	1400	1410	1420	1430	1440
LGITVEGPSE	SKINCRDNKD	GSCSAEYIPF	APGDYDVNIT	YGGAHIPGSP	FRVPVKDVVD	PSKVKIAGPG	LGSGVRARVL
1450	1460	1470	1480	1490	1500	1510	1520
QSFTVDSSKA	GLAPLEVRVL	GPRADDTDSQ	SWRSPLKALS	EFFKGDPKGD	FNKTGLVEPV	NVVDNGDGTH	TVTYTPSQEG
1530	1540	1550	1560	1570	1580	1590	1600
PYMVSVKYAD	EEIPRSPFKV	KVLPTYDASK	VTASGPGLSS	YGVPASLPVD	FAIDARDAGE	GLLAVQITDQ	EGKPKRAIVH
1610	1620	1630	1640	1650	1660	1670	1680
DNKDGTYAVT	YIPDKTGRYM	IGVTYGGDDI	PLSPYRIRAT	QTGDASKCLA	TGPGIASTVK	TGEEVGFVVD	AKTAGKGKVT
1690	1700	1710	1720	1730	1740	1750	1760
CTVLTPDGTE	AEADVIENED	GTYDIFYTAA	KPGTYVIYVR	FGGVDIPNSP	FTVMATDGEV	TAVEEAPVNA	CPPGFRPWVT
1770	1780	1790	1800	1810	1820	1830	1840
EEAYVPVSDM	NGLGFKPFDL	VIPFAVRKGE	ITGEVHMPSG	KTATPEIVDN	KDGTVTVRYA	PTEVGLHEMH	IKYMGSHIPE
1850	1860	1870	1880	1890	1900	1910	1920
SPLQFYVNYP	NSGSVSAYGP	GLVYGVANKT	ATFTIVTEDA	GEGGLDLAIE	GPSKAEISCI	DNKDGTCTVT	YLPTLPGDYS
1930	1940	1950	1960	1970	1980	1990	2000
ILVKYNDKHI	PGSPFTAKIT	DDSRRCSQVK	LGSAADFLLD	ISETDLSSLT	ASIKAPSGRD	EPCLLKRLPN	NHIGISFIPR
2010	2020	2030	2040	2050	2060	2070	2080
EVGEHLVSIK	KNGNHVANSP	VSIMVVQSEI	GDARRAKVYG	RGLSEGRTFE	MSDFIVDTRD	AGYGGISLAV	EGPSKVDIQT
2090	2100	2110	2120	2130	2140	2150	2160
EDLEDGTCKV	SYFPTVPGVY	IVSTKFADEH	VPGSPFTVKI	SGEGRVKESI	TRTSRAPSVA	TVGSICDLNL	KIPGVRVMNC
2170	2180						

Detailed Protein Report

Cmpd.	No. of Cmpds.	m/z meas.	Δ m/z [ppm]	z	Rt [min]	Score	P	Range	Sequence	Modification
171	1	614.7837	-134.87	2	30.9	13.4	1	977-987	K.LDVTILSPSRK.V	

Detailed Protein Report

Protein 348: PREDICTED: diacylglycerol kinase iota isoform X2 [Homo sapiens]

**Accession:**	gi|578814735	**Score:**	33.2
**Database:**	refseq_human(refseq_human_20140103.fasta)	**MW [kDa]:**	113.9
**Database Date:**	2015-11-30	**pI:**	9.1
**Modification(s):**	Carbamidomethyl	**Sequence Coverage [%]:**	6.0
		**No. of unique Peptides:**	3

Quantitation

mdown:qdown	**Median:** 1.70	**CV:** 0.00 %	**No. of Peptides:**	1
Wdown:Qdown	**Median:** 1.24	**CV:** 0.00 %	**No. of Peptides:**	1

10	20	30	40	50	60	70	80
MNPSSSAGEE	KGATGGSSSS	GSGAGSCCLG	AEGGADPRGA	GSAAAAGAAA	LDEPAAAGQK	EKDEALEEKL	RNLTFRKQVS
90	100	110	120	130	140	150	160
YRKAISRAGL	QHLAPAHPLS	LPVANGPAKE	PRATLDWSEN	AVNGEHLWLE	TNVSGDLCYL	GEENCQVRFA	KSALRRKCAV
170	180	190	200	210	220	230	240
CKIVVHTACI	EQLEKINFRC	KPTFREGGSR	SPRENFVRHH	WVHRRRQEGK	CKQCGKGFQQ	KFSFHSKEIV	AISCSWCKQA
250	260	270	280	290	300	310	320
FHNKVTCFML	HHIEEPCSLG	AHAAVIVPPT	WIIKVKKPQN	SLKASNRKKK	RTSFKRKASK	RGMEQENKGR	PFVIKPISSP
330	340	350	360	370	380	390	400
LMKPLLVFVN	PKSGGNQGTK	VLQMFMWYLN	PRQVFDLSQE	GPKDALELYR	KVPNLRILAC	GGDGTVGWIL	SILDELQLSP
410	420	430	440	450	460	470	480
QPPVGVLPLG	TGNDLARTLN	WGGGYTDEPV	SKILCQVEDG	TVVQLDRWNL	HVERNPDLPP	EELEDGVCKK	LDRKQLKYKE
490	500	510	520	530	540	550	560
EYHMDILPLN	VFNNYFSLGF	DAHVTLEFHE	SREANPEKFN	SRFRNKMFYA	GAAFSDFLQR	SSRDLSKHVK	VVCDGTDLTP
570	580	590	600	610	620	630	640
KIQELKFQCI	VFLNIPRYCA	GTMPWGNPGD	HHDFEPQRHD	DGYIEVIGFT	MASLAALQVG	GHGERLHQCR	EVMLLTYKSI
650	660	670	680	690	700	710	720
PMQVDGEPCR	LAPAMIRISL	RNQANMVQKS	KRRTSMPLLN	DPQSVPDRLR	IRVNKISLQD	YEGFHYDKEK	LREASIPLGI
730	740	750	760	770	780	790	800
LVVRGDCDLE	TCRMYIDRLQ	EDLQSVSSGS	QRVHYQDHET	SFPRALSAQR	LSPRWCFLDA	TSADRFYRID	RSQEHLHFVM
810	820	830	840	850	860	870	880
EISQDEIFIL	DPDMVVSQPA	GTPPGMPDLV	VEQASGISDW	WNPALRKRML	SDSGLGMIAP	YYEDSDLKDL	SHSRVLQSPV
890	900	910	920	930	940	950	960
SSEDHAILQA	VIAGDLMKLI	ESYKNGGSLL	IQGPDHCSLL	HYAAKTGNGE	IVKYILDHGP	SELLDMADSE	TGETALHKAA
970	980	990	1000	1010	1020	1030	
CQRNRAVCQL	LVDAGASLRK	TDSKGKTPQE	RAQQAGDPDL	AAYLESRQNY	KVIGHEDLET	AV	

Cmpd.	No. of Cmpds.	m/z meas.	Δ m/z [ppm]	z	Rt [min]	Score	P	Range	Sequence	Modification	Ratios
1635	1	1100.4411	-20.69	3	50.3	12.6	1	2-38	M. NPSSSAGEEKGATGGSSSSGSGA G	Carbamidomethyl: 26	
800	2	451.4498	-220.69	3	38.5	10.1	0	228-238	K.EIVAISCSWCK.Q	Carbamidomethyl: 7, 10	Wdown:Qdown 1.24<br>mdown:qdown 1.70
2495	1	735.4148	-22.06	2	61.6	10.5	2	275-287	K.VKKPQNSLKASNR.K		

Detailed Protein Report

Protein 349: **serine/threonine-protein kinase 3 isoform 2 [Homo sapiens]**

**Accession:**	gi|372622373	**Score:**	33.2
**Database:**	refseq_human(refseq_human_20140103.fasta)	**MW [kDa]:**	59.4
**Database Date:**	2015-11-30	**pI:**	5.0
		**Sequence Coverage [%]:**	5.6
		**No. of unique Peptides:**	1

10	20	30	40	50	60	70	80
MLQLMDSGIT	ICLRNGAASV	FKKKEWSTQG	EENKQDSKLK	KLSEDSLTKQ	PEEVFDVLEK	LGEGSYGSVF	KAIHKESGQV
**90**	**100**	**110**	**120**	**130**	**140**	**150**	**160**
VAIKQVPVES	DLQEIIKEIS	IMQQCDSPYV	VKYYGSYFKN	TDLWIVMEYC	GAGSVSDIIR	LRNKTLIEDE	IATILKSTLK
**170**	**180**	**190**	**200**	**210**	**220**	**230**	**240**
GLEYLHFMRK	IHRDIKAGNI	LLNTEGHAKL	ADFGVAGQLT	DTMAKRNTVI	GTPFWMAPEV	IQEIGYNCVA	DIWSLGITSI
**250**	**260**	**270**	**280**	**290**	**300**	**310**	**320**
EMAEGKPPYA	DIHPMRAIFM	IPTNPPPTFR	KPELWSDDFT	DFVKKCLVKN	PEQRATATQL	LQHPFIKNAK	PVSILRDLIT
**330**	**340**	**350**	**360**	**370**	**380**	**390**	**400**
EAMEIKAKRH	EEQQRELEEE	EENSDEDELD	SHTMVKTSVE	SVGTMRATST	MSEGAQTMIE	HNSTMLESDL	GTMVINSEDE
**410**	**420**	**430**	**440**	**450**	**460**	**470**	**480**
EEEDGTMKRN	ATSPQVQRPS	FMDYFDKQDF	KNKSHENCNQ	NMHEPFPMSK	NVFPDNWKVP	QDGDFDFLKN	LSLEELQMRL
**490**	**500**	**510**	**520**				
KALDPMMERE	IEELRQRYTA	KRQPILDAMD	AKKRRQQNF				

Cmpd.	No. of Cmpds.	m/z meas.	Δ m/z [ppm]	z	Rt [min]	Score	P	Range	Sequence	Modification
2001	2	731.8177	-100.79	2	55.1	18.2	0	2-14	M.LQLMDSGITICLR.N	

Detailed Protein Report

Protein 350: uncharacterized protein C2orf78 [Homo sapiens]

**Accession:**	gi|122937341	**Score:**	33.1
**Database:**	refseq_human(refseq_human_20140103.fasta)	**MW [kDa]:**	100.1
**Database Date:**	2015-11-30	**pI:**	9.7
		**Sequence Coverage [%]:**	2.7
		**No. of unique Peptides:**	1

Quantitation

mdown:qdown	**Median:**	0.43	**CV:**	0.00 %	**No. of Peptides:**	1
Wdown:Qdown	**Median:**	0.88	**CV:**	0.00 %	**No. of Peptides:**	1

Alias proteins:

Accession	Name	Description
gi|530367561	refseq_human (refseq_human_20140103.fasta)	PREDICTED: uncharacterized protein C2orf78 isoform X1 [Homo sapiens]

10	20	30	40	50	60	70	80
MHWLASATQT	SASIVSSSLL	SAVDVSSSLT	MSEYFQNTSL	PGTANSRQFS	LPVVSNAAFL	TGSISNFSRA	SAPAISSAWL
**90**	**100**	**110**	**120**	**130**	**140**	**150**	**160**
QPSASGTSFQ	PLMGSAYLYQ	HSSTTMLSGV	TGQSHICTSA	ASYPGVFEWD	STASTVKKSS	SLRDFTVTVI	DQNTAVSSMS
**170**	**180**	**190**	**200**	**210**	**220**	**230**	**240**
MTAQYYKTSD	TNTMVPLYPS	LSASLVQGTL	TQIPNQQGHN	LSLPCQIGSQ	VYYYNQGTLG	PQLSCLQSYG	SVSYTGYRAS
**250**	**260**	**270**	**280**	**290**	**300**	**310**	**320**
AHQPEMVMVL	KEVQPTNVLP	PVSTSGMYYS	VSSQPITETS	VQVMETSLGM	DTSLGLQSPS	QTFCLPQTPE	FSKSFSSRNT
**330**	**340**	**350**	**360**	**370**	**380**	**390**	**400**
QTLESNPSPE	LGDISITPVQ	SPTNLLTLSP	APSQEKNENE	NLDEIKTNLS	KPLDVHQILI	GNQDPPLLPV	EIPDIHPLLA
**410**	**420**	**430**	**440**	**450**	**460**	**470**	**480**
CIDPLGQEEQ	PGSENANLRN	KSLSLEDQGI	FENGIESSSD	LADITTWVED	TYLPPIFSSL	QDLDQPESPS	AKKAKDTSAI
**490**	**500**	**510**	**520**	**530**	**540**	**550**	**560**
KVNQVQEKSC	VIKGHSDQVR	KNKHKASEPI	QGAPKAKIQP	KNPECLLERE	VVVGSATVSN	SASVNKAKHS	SNKPHKAASS
**570**	**580**	**590**	**600**	**610**	**620**	**630**	**640**
RISKTKSHGQ	EKTKGNRKNS	SKKSEESKQS	GKKVKVEEKQ	TIPNMKRKKN	QPELSQKTLK	KPRSSLGMHM	LESVQVFHAL
**650**	**660**	**670**	**680**	**690**	**700**	**710**	**720**
GKKIDMKTGF	SSSRTLGSSS	NTQNRQPFPA	LKPWLDIQHE	GKGPEKIQVK	AQKLDGSAEK	ECTSPSHSEL	PPPGKVKLIP
**730**	**740**	**750**	**760**	**770**	**780**	**790**	**800**
LPFLTLDQPQ	ARHVSRRPNP	LASRRPAVAY	PARPDSTNSA	QSNAVNPSRP	APTNTSLTGP	ATPAQPISAK	ATQPSSANPT
**810**	**820**	**830**	**840**	**850**	**860**	**870**	**880**
QPTVPQSAAS	RPSAYKTSSC	SSLQREPVST	AVTSLRSLPK	PQNQFLIQDF	SLQPRPWRKP	TVPEPVMSTP	ITEEQRPERE
**890**	**900**	**910**	**920**	**930**			
AMKRKAQQER	ENAAKYTSLG	KVQFFIERER	DMEIAEYYGY	TI			

Cmpd.	No. of Cmpds.	m/z meas.	Δ m/z [ppm]	z	Rt [min]	Score	P	Range	Sequence	Modification	Ratios
273	1	572.6538	-235.90	2	32.1	17.4	1	886-895	K.AQQERENAAK.Y		Wdown:Qdown 0.88<br>mdown:qdown 0.43

Detailed Protein Report

Protein 351: E3 SUMO-protein ligase CBX4 [Homo sapiens]

Accession: gi|55770830
Database: refseq_human(refseq_human_20140103.fasta)
Database Date: 2015-11-30
Modification(s): Oxidation

Score: 33.1
MW [kDa]: 61.3
pI: 10.0
Sequence Coverage [%]: 6.1
No. of unique Peptides: 2

10	20	30	40	50	60	70	80
MELPAVGEHV	FAVESIEKKR	IRKGRVEYLV	KWRGWSPKYN	TWEPEENILD	PRLLIAFQNR	ERQEQLMGYR	KRGPKPKPLV
**90**	**100**	**110**	**120**	**130**	**140**	**150**	**160**
VQVPTFARRS	NVLTGLQDSS	TDNRAKLDLG	AQGKGQGHQY	ELNSKKHHQY	QPHSKERAGK	PPPPGKSGKY	YYQLNSKKHH
**170**	**180**	**190**	**200**	**210**	**220**	**230**	**240**
PYQPDPKMYD	LQYQGGHKEA	PSPTCPDLGA	KSHPPDKWAQ	GAGAKGYLGA	VKPLAGAAGA	PGKGSEKGPP	NGMMPAPKEA
**250**	**260**	**270**	**280**	**290**	**300**	**310**	**320**
VTGNGIGGKM	KIVKNKNKNG	RIVIVMSKYM	ENGMQAVKIK	SGEVAEGEAR	SPSHKKRAAD	ERHPPADRTF	KKAAGAEEKK
**330**	**340**	**350**	**360**	**370**	**380**	**390**	**400**
VEAPPKRREE	EVSGVSDPQP	QDAGSRKLSP	TKEAFGEQPL	QLTTKPDLLA	WDPARNTHPP	SHHPHPHPHH	HHHHHHHHHH
**410**	**420**	**430**	**440**	**450**	**460**	**470**	**480**
AVGLNLSHVR	KRCLSETHGE	REPCKKRLTA	RSISTPTCLG	GSPAAERPAD	LPPAAALPQP	EVILLDSDLD	EPIDLRCVKT
**490**	**500**	**510**	**520**	**530**	**540**	**550**	**560**
RSEAGEPPSS	LQVKPETPAS	AAVAVAAAAA	PTTTAEKPPA	EAQDEPAESL	SEFKPFFGNI	IITDVTANCL	TVTFKEYVTV
**570**							

Cmpd.	No. of Cmpds.	m/z meas.	Δ m/z [ppm]	z	Rt [min]	Score	P	Range	Sequence	Modification
1064	1	500.6481	-289.29	2	42.9	11.2	1	105-114	R.AKLDLGAQGK.G	
443	1	699.2695	-110.76	3	34.5	10.6	1	228-249	K.GPPNGMMPAPKEAVTGNGIGGK.M	Oxidation: 6

Detailed Protein Report

Protein 352: membrane-bound transcription factor site-1 protease preproprotein [Homo sapiens]

**Accession:**	gi|4506775	**Score:**	33.1
**Database:**	refseq_human(refseq_human_20140103.fasta)	**MW [kDa]:**	117.7
**Database Date:**	2015-11-30	**pI:**	9.5
**Modification(s):**	Oxidation	**Sequence Coverage [%]:**	3.4
		**No. of unique Peptides:**	2

Quantitation

mdown:qdown	**Median:**	1.41	**CV:**	0.00 %	**No. of Peptides:**	1
Wdown:Qdown	**Median:**	1.20	**CV:**	0.00 %	**No. of Peptides:**	1

```
        10         20         30         40         50         60         70         80
MKLVNIWLLL LVVLLCGKKH LGDRLEKKSF EKAPCPGCSH LTLKVEFSST VVEYEYIVAF NGYFTAKARN SFISSALKSS
        90        100        110        120        130        140        150        160
EVDNWRIIPR NNPSSDYPSD FEVIQIKEKQ KAGLLTLEDH PNIKRVTPQR KVFRSLKYAE SDPTVPCNET RWSQKWQSSR
       170        180        190        200        210        220        230        240
PLRRASLSLG SGFWHATGRH SSRRLLRAIP RQVAQTLQAD VLWQMGYTGA NVRVAVFDTG LSEKHPHFKN VKERTNWTNE
       250        260        270        280        290        300        310        320
RTLDDGLGHG TFVAGVIASM RECQGFAPDA ELHIFRVFTN NQVSYTSWFL DAFNYAILKK IDVLNLSIGG PDFMDHPFVD
       330        340        350        360        370        380        390        400
KVWELTANNV IMVSAIGNDG PLYGTLNNPA DQMDVIGVGG IDFEDNIARF SSRGMTTWEL PGGYGRMKPD IVTYGAGVRG
       410        420        430        440        450        460        470        480
SGVKGGCRAL SGTSVASPVV AGAVTLLVST VQKRELVNPA SMKQALIASA RRLPGVNMFE QGHGKLDLLR AYQILNSYKP
       490        500        510        520        530        540        550        560
QASLSPSYID LTECPYMWPY CSQPIYYGGM PTVVNVTILN GMGVTGRIVD KPDWQPYLPQ NGDNIEVAFS YSSVLWPWSG
       570        580        590        600        610        620        630        640
YLAISISVTK KAASWEGIAQ GHVMITVASP AETESKNGAE QTSTVKLPIK VKIIPTPPRS KRVLWDQYHN LRYPPGYFPR
       650        660        670        680        690        700        710        720
DNLRMKNDPL DWNGDHIHTN FRDMYQHLRS MGYFVEVLGA PFTCFDASQY GTLLMVDSEE EYFPEEIAKL RRDVDNGLSL
       730        740        750        760        770        780        790        800
VIFSDWYNTS VMRKVKFYDE NTRQWWMPDT GGANIPALNE LLSVWNMGFS DGLYEGEFTL ANHDMYYASG CSIAKFPEDG
       810        820        830        840        850        860        870        880
VVITQTFKDQ GLEVLKQETA VVENVPILGL YQIPAEGGGR IVLYGDSNCL DDSHRQKDCF WLLDALLQYT SYGVTPPSLS
       890        900        910        920        930        940        950        960
HSGNRQRPPS GAGSVTPERM EGNHLHRYSK VLEAHLGDPK PRPLPACPRL SWAKPQPLNE TAPSNLWKHQ KLLSIDLDKV
       970        980        990       1000       1010       1020       1030       1040
VLPNFRSNRP QVRPLSPGES GAWDIPGGIM PGRYNQEVGQ TIPVFAFLGA MVVLAFFVVQ INKAKSRPKR RKPRVKRPQL
      1050       1060
MQQVHPPKTP SV
```

Cmpd.	No. of Cmpds.	m/z meas.	Δ m/z [ppm]	z	Rt [min]	Score	P	Range	Sequence	Modification	Ratios
1660	1	612.4256	151.20	3	49.3	12.0	1	387-404	R.MKPDIVTYGAGVRGSGVK.G		Wdown:Qdown 1.20<br>mdown:qdown 1.41
1464	1	742.6304	-61.81	3	48.1	21.1	1	645-662	R.MKNDPLDWNGDHIHTNFR.D	Oxidation: 1	

BRUKER

Detailed Protein Report

Protein 353: zinc finger protein 674 isoform 1 [Homo sapiens]

**Accession:**	gi|89994742	**Score:**	33.1
**Database:**	refseq_human(refseq_human_20140103.fasta)	**MW [kDa]:**	67.2
**Database Date:**	2015-11-30	**pI:**	10.2
**Modification(s):**	Carbamidomethyl	**Sequence Coverage [%]:**	8.6
		**No. of unique Peptides:**	2

Quantitation

Wdown:Qdown **Median:** 0.33 **CV:** 0.00 % **No. of Peptides:** 1

```
        10         20         30         40         50         60         70         80
MAMSQESLTF KDVFVDFTLE EWQQLDSAQK NLYRDVMLEN YSHLVSVGHL VGKPDVIFRL GPGDESWMAD GGTPVRTCAG
        90        100        110        120        130        140        150        160
EDRPEVWEVD EQIDHYKESQ DKFLWQAAFI GKETLKDESG QECKICRKII YLNTDFVSVK QRLPKYYSWE RCSKHHLNFL
       170        180        190        200        210        220        230        240
GQNRSYVRKK DDGCKAYWKV CLHYNLHKAQ PAERFFDPNQ RGKALHQKQA LRKSQRSQTG EKLYKCTECG KVFIQKANLV
       250        260        270        280        290        300        310        320
VHQRTHTGEK PYECCECAKA FSQKSTLIAH QRTHTGEKPY ECSECGKTFI QKSTLIKHQR THTGEKPFVC DKCPKAFKSS
       330        340        350        360        370        380        390        400
YHLIRHEKTH IRQAFYKGIK CTTSSLIYQR IHTSEKPQCS EHGKASDEKP SPTKHWRTHT KENIYECSKC GKSFRGKSHL
       410        420        430        440        450        460        470        480
SVHQRIHTGE KPYECSICGK TFSGKSHLSV HHRTHTGEKP YECRRCGKAF GEKSTLIVHQ RMHTGEKPYK CNECGKAFSE
       490        500        510        520        530        540        550        560
KSPLIKHQRI HTGERPYECT DCKKAFSRKS TLIKHQRIHT GEKPYKCSEC GKAFSVKSTL IVHHRTHTGE KPYECRDCGK
       570        580        590
AFSGKSTLIK HQRSHTGDKN L
```

Cmpd.	No. of Cmpds.	m/z meas.	Δ m/z [ppm]	z	Rt [min]	Score	P	Range	Sequence	Modification	Ratios
2101	1	599.9147	-53.58	3	56.4	11.0	2	378-392	R.THTKENIYECSKCGK.S	Carbamidomethyl: 10	Wdown:Qdown 0.33
1639	1	1136.1099	81.18	2	50.4	11.8	2	546-565	R.THTGEKPYECRDCGKAFSGK.S	Carbamidomethyl: 13	

Detailed Protein Report

Protein 354: PREDICTED: serine/arginine repetitive matrix protein 2 isoform X3 [Homo sapiens]

**Accession:**	gi|530407861	**Score:**	32.8
**Database:**	refseq_human(refseq_human_20140103.fasta)	**MW [kDa]:**	281.3
**Database Date:**	2015-11-30	**pI:**	12.6
		**Sequence Coverage [%]:**	0.9
		**No. of unique Peptides:**	2

Quantitation

mdown:qdown	**Median:**	0.54	**CV:**	0.00 %	**No. of Peptides:**	1
Wdown:Qdown	**Median:**	0.36	**CV:**	0.00 %	**No. of Peptides:**	1

Detailed Protein Report

10	20	30	40	50	60	70	80
MYNGIGLPTP	RGSGTNGYVQ	RNLSLVRGRR	GERPDYKGEE	ELRRLEAALV	KRPNPDILDH	ERKRRVELRC	LELEEMMEEQ
90	100	110	120	130	140	150	160
GYEEQQIQEK	VATFRLMLLE	KDVNPGGKEE	TPGQRPAVTE	THQLAELNEK	KNERLRAAFG	ISDSYVDGSS	FDPQRRAREA
170	180	190	200	210	220	230	240
KQPAPEPPKP	YSLVRESSSS	RSPTPKQKKK	KKKKDRGRRS	ESSSPRRERK	KSSKKKKHRS	ESESKKRKHR	SPTPKSKRKS
250	260	270	280	290	300	310	320
KDKKRKRSRS	TTPAPKSRRA	HRSTSADSAS	SSDTSRSRSR	SAAAKTHTTA	LAGRSPSPAS	GRRGEGDAPF	SEPGTTSTQR
330	340	350	360	370	380	390	400
PSSPETATKQ	PSSPYEDKDK	DKKEKSATRP	SPSPERSSTG	PEPPAPTPLL	AERHGGSPQP	LATTPLSQEP	VNPPSEASPT
410	420	430	440	450	460	470	480
RDRSPPKSPE	KLPQSSSSES	SPPSPQPTKV	SRHASSSPES	PKPAPAPGSH	REISSSPTSK	NRSHGRAKRD	KSHSHTPSRR
490	500	510	520	530	540	550	560
MGRSRSPATA	KRGRSRSRTP	TKRGHSRSRS	PQWRRSRSAQ	RWGRSRSPQR	RGRSRSPQRP	GWSRSRNTQR	RGRSRSARRG
570	580	590	600	610	620	630	640
RSHSRSPATR	GRSRSRTPAR	RGRSRSRTPA	RRRSRSRTPT	RRRSRSRTPA	RRGRSRSRTP	ARRRSRTRSP	VRRRSRSRSP
650	660	670	680	690	700	710	720
ARRSGRSRSR	TPARRGRSRS	RTPARRGRSR	SRTPARRSGR	SRSRTPARRG	RSRSRTPRRG	RSRSRSLVRR	GRSHSRTPQR
730	740	750	760	770	780	790	800
RGRSGSSSER	KNKSRTSQRR	SRSNSSPEMK	KSRISSRRSR	SLSSPRSKAK	SRLSLRRSLS	GSSPCPKQKS	QTPPRRSRSG
810	820	830	840	850	860	870	880
SSQPKAKSRT	PPRRSRSSSS	PPPKQKSKTP	SRQSHSSSSP	HPKVKSGTPP	RQGSITSPQA	NEQSVTPQRR	SCFESSPDPE
890	900	910	920	930	940	950	960
LKSRTPSRHS	CSGSSPPRVK	SSTPPRQSPS	RSSSPQPKVK	AIISPRQRSH	SGSSSPSPSR	VTSRTTPRRS	RSVSPCSNVE
970	980	990	1000	1010	1020	1030	1040
SRLLPRYSHS	GSSSPDTKVK	PETPPRQSHS	GSISPYPKVK	AQTPPGPSLS	GSKSPCPQEK	SKDSLVQSCP	GSLSLCAGVK
1050	1060	1070	1080	1090	1100	1110	1120
SSTPPGESYF	GVSSLQLKGQ	SQTSPDHRSD	TSSPEVRQSH	SESPSLQSKS	QTSPKGGRSR	SSSPVTELAS	RSPIRQDRGE
1130	1140	1150	1160	1170	1180	1190	1200
FSASPMLKSG	MSPEQSRFQS	DSSSYPTVDS	NSLLGQSRLE	TAESKEKMAL	PPQEDATASP	PRQKDKFSPF	PVQDRPESSL
1210	1220	1230	1240	1250	1260	1270	1280
VFKDTLRTPP	RERSGAGSSP	ETKEQNSALP	TSSQDEELME	VVEKSEEPAG	QILSHLSSEL	KEMSTSNFES	SPEVEERPAV
1290	1300	1310	1320	1330	1340	1350	1360
SLTLDQSQSQ	ASLEAVEVPS	MASSWGGPHF	SPEHKELSNS	PLRENSFGSP	LEFRNSGPLG	TEMNTGFSSE	VKEDLNGPFL
1370	1380	1390	1400	1410	1420	1430	1440
NQLETDPSLD	MKEQSTRSSG	HSSSELSPDA	VEKAGMSSNQ	SISSPVLDAV	PRTPSRERSS	SASSPEMKDG	LPRTPSRRSR
1450	1460	1470	1480	1490	1500	1510	1520
SGSSPGLRDG	SGTPSRHSLS	GSSPGMKDIP	RTPSRGRSEC	DSSPEPKALP	QTPRPRSRSP	SSPELNNKCL	TPQRERSGSE
1530	1540	1550	1560	1570	1580	1590	1600
SSVDQKTVAR	TPLGQRSRSG	SSQELDVKPS	ASPQERSESD	SSPDSKAKTR	TPLRQRSRSG	SSPEVDSKSR	LSPRRSRSGS
1610	1620	1630	1640	1650	1660	1670	1680
SPEVKDKPRA	APRAQSGSDS	SPEPKAPAPR	ALPRRSRSGS	SSKGRGPSPE	GSSSTESSPE	HPPKSRTARR	GSRSSPEPKT
1690	1700	1710	1720	1730	1740	1750	1760
KSRTPPRRRS	SRSSPELTRK	ARLSRRSRSA	SSSPETRSRT	PPRHRRSPSV	SSPEPAEKSR	SSRRRRSASS	PRTKTTSRRG
1770	1780	1790	1800	1810	1820	1830	1840
RSPSPKPRGL	QRSRSRSRRE	KTRTTRRRDR	SGSSQSTSRR	RQRSRSRSRV	TRRRRGGSGY	HSRSPARQES	SRTSSRRRRG
1850	1860	1870	1880	1890	1900	1910	1920
RSRTPPTSRK	RSRSRTSPAP	WKRSRSRASP	ATHRRSRSRT	PLISRRRSRS	RTSPVSRRRS	RSRTSVTRRR	SRSRASPVSR
1930	1940	1950	1960	1970	1980	1990	2000
RRSRSRTPPV	TRRRSRSRTP	TTRRRSRSRT	PPVTRRRSRS	RTPPVTRRRS	RSRTSPITRR	RSRSRTSPVT	RRRSRSRTSP
2010	2020	2030	2040	2050	2060	2070	2080
VTRRRSRSRT	SPVTRRRSRS	RTPPAIRRRS	RSRTPLLPRK	RSRSRSPLAI	RRRSRSRTPR	TARGKRSLTR	SPPAIRRRSA
2090	2100	2110	2120	2130	2140	2150	2160
SGSSSDRSRS	ATPPATRNHS	GSRTPPVALN	SSRMSCFSRP	SMSPTPLDRC	RSPGMLEPLG	SSRTPMSVLQ	QAGGSMMDGP
2170	2180	2190	2200	2210	2220	2230	2240

Detailed Protein Report

Cmpd.	No. of Cmpds.	m/z meas.	Δ m/z [ppm]	z	Rt [min]	Score	P	Range	Sequence	Modification	Ratios
2951	7	703.3519	-15.42	2	65.6	16.8	1	833-845	R.QSHSSSSPHPKVK.S		
873	1	491.7878	51.39	2	39.7	16.0	1	1866-1874	R.SRASPATHR.R		Wdown:Qdown 0.36<br>mdown:qdown 0.54

Detailed Protein Report

Protein 355: PREDICTED: mucin-6 isoform X1 [Homo sapiens]

**Accession:**	gi|578840955	**Score:**	32.8
**Database:**	refseq_human(refseq_human_20140103.fasta)	**MW [kDa]:**	141.7
**Database Date:**	2015-11-30	**pI:**	5.7
**Modification(s):**	Carbamidomethyl	**Sequence Coverage [%]:**	1.1
		**No. of unique Peptides:**	2

10	20	30	40	50	60	70	80
MVQRWLLLSC	CGALLSAGLA	NTSYTSPGLQ	RLKDSPQTAP	DKGQCSTWGA	GHFSTFDHHV	YDFSGTCNYI	FAATCKDAFP
**90**	**100**	**110**	**120**	**130**	**140**	**150**	**160**
TFSVQLRRGP	DGSISRIIVE	LGASVVTVSE	AIISVKDIGV	ISLPYTSNGL	QITPFGQSVR	LVAKQLELEL	EVVWGPDSHL
**170**	**180**	**190**	**200**	**210**	**220**	**230**	**240**
MVLVERKYMG	QMCGLCGNFD	GKVTNEFVSE	EGKFLEPHKF	AALQKLDDPG	EICTFQDIPS	THVRQAQHAR	ICTQLLTLVA
**250**	**260**	**270**	**280**	**290**	**300**	**310**	**320**
PECSVSKEPF	VLSCQADVAA	APQPGPQNSS	CATLSEYSRQ	CSMVGQPVRR	WRSPGLCSVG	QCPANQVYQE	CGSACVKTCS
**330**	**340**	**350**	**360**	**370**	**380**	**390**	**400**
NPQHSCSSSC	TFGCFCPEGT	VLNDLSNNHT	CVPVTQCPCV	LHGAMYAPGE	VTIAACQTCR	CTLGRWVCTE	RPCPGHCSLE
**410**	**420**	**430**	**440**	**450**	**460**	**470**	**480**
GGSFVTTFDA	RPYRFHGTCT	YILLQSPQLP	EDGALMAVYD	KSGVSHSETS	LVAVVYLSRQ	DKIVISQDEV	VTNNGEAKWL
**490**	**500**	**510**	**520**	**530**	**540**	**550**	**560**
PYKTRNITVF	RQTSTHLQMA	TSFGLELVVQ	LRPIFQAYVT	VGPQFRGQTR	GLCGNFNGDT	TDDFTTSMGI	AEGTASLFVD
**570**	**580**	**590**	**600**	**610**	**620**	**630**	**640**
SWRAGNCPAA	LERETDPCSM	SQLNKVCAET	HCSMLLRTGT	VFERCHATVN	PAPFYKRCMY	QACNYEETFP	HICAALGDYV
**650**	**660**	**670**	**680**	**690**	**700**	**710**	**720**
HACSLRGVLL	WGWRSSVDNC	TIPCTGNTTF	SYNSQACERT	CLSLSDRATE	CHHSAVPVDG	CNCPDGTYLN	QKGECVRKAQ
**730**	**740**	**750**	**760**	**770**	**780**	**790**	**800**
CPCILEGYKF	ILAEQSTVIN	GITCHCINGR	LSCPQRPQMF	LASCQAPKTF	KSCSQSSENK	FGAACAPTCQ	MLATGVACVP
**810**	**820**	**830**	**840**	**850**	**860**	**870**	**880**
TKCEPGCVCA	EGLYENADGQ	CVPPEECPCE	FSGVSYPGGA	ELHTDCRTCS	CSRGRWACQQ	GTHCPSTCTL	YGEGHVITFD
**890**	**900**	**910**	**920**	**930**	**940**	**950**	**960**
GQRFVFDGNC	EYILATDVCG	VNDSQPTFKI	LTENVICGNS	GVTCSRAIKI	FLGGLSVVLA	DRNYTVTGEE	PHVQLGVTPG
**970**	**980**	**990**	**1000**	**1010**	**1020**	**1030**	**1040**
ALSLVVDISI	PGRYNLTLIW	NRHMTILIRI	ARASQDPLCG	LCGNFNGNMK	DDFETRSRYV	ASSELELVNS	WKESPLCGDV
**1050**	**1060**	**1070**	**1080**	**1090**	**1100**	**1110**	**1120**
SFVTDPCSLN	AFRRSWAERK	CSVINSQTFA	TCHSKVYHLP	YYEACVRDAC	GCDSGGDCEC	LCDAVAAYAQ	ACLDKGVCVD
**1130**	**1140**	**1150**	**1160**	**1170**	**1180**	**1190**	**1200**
WRTPAFCPIY	CGFYNTHTQD	GHGEYQYTQE	ANCTWHYQPC	LCPSQPQSVP	GSNIEGCYNC	SQDEYFDHEE	GVCVPCMPPT
**1210**	**1220**	**1230**	**1240**	**1250**	**1260**	**1270**	**1280**
TPQPPTTPQL	PTTGSRPTQV	WPMTGTSTTI	GLLSSTGPSP	SSNHTPASPT	QTPLLPATLT	SSKPTASSGG	KEPPAEPMER
**1290**	**1300**	**1310**	**1320**				
AAAGGPRYTS	GVGLGMALLP	GSHLPLSRFP	A				

Cmpd.	No. of Cmpds.	m/z meas.	Δ m/z [ppm]	z	Rt [min]	Score	P	Range	Sequence	Modification
2899	3	840.9173	97.31	2	65.0	13.7	0	168-182	K.YMGQMCGLCGNFDGK.V	Carbamidomethyl: 6
1896	3	840.7895	-54.74	2	53.8	19.2	0	168-182	K.YMGQMCGLCGNFDGK.V	Carbamidomethyl: 9

Detailed Protein Report

Protein 356: PREDICTED: pleckstrin homology domain-containing family A member 4 isoform X3 [Homo sapiens]

**Accession:**	gi|530416942	**Score:**	32.8
**Database:**	refseq_human(refseq_human_20140103.fasta)	**MW [kDa]:**	65.5
**Database Date:**	2015-11-30	**pI:**	10.7
		**Sequence Coverage [%]:**	7.4
		**No. of unique Peptides:**	3

Quantitation

mdown:qdown	**Median:**	0.66	**CV:**	30.90 %	**No. of Peptides:**	3
Wdown:Qdown	**Median:**	4.13	**CV:**	0.00 %	**No. of Peptides:**	1

10	20	30	40	50	60	70	80
MEGSRPRSSL	SLASSASTIS	SLSSLSPKKP	TRAVNKIHAF	GKRGNALRRD	PNLPVHIRGW	LHKQDSSGLR	LWKRRWFVLS
**90**	**100**	**110**	**120**	**130**	**140**	**150**	**160**
GHCLFYYKDS	REESVLGSVL	LPSYNIRPDG	PGAPRGRRFT	FTAEHPGMRT	YVLAADTLED	LRGWLRALGR	ASRAEGDDYG
**170**	**180**	**190**	**200**	**210**	**220**	**230**	**240**
QPRSPARPQP	GEGPGGPGGP	PEVSRGEEGR	ISESPEVTRL	SRGRGRPRLL	TPSPTTDLHS	GLQMRRARSP	DLFTPLSRPP
**250**	**260**	**270**	**280**	**290**	**300**	**310**	**320**
SPLSLPRPRS	APARRPPAPS	GDTAPPARPH	TPLSRIDVRP	PLDWGPQRQT	LSRPPTPRRG	PPSEAGGGKP	PRSPQHWSQE
**330**	**340**	**350**	**360**	**370**	**380**	**390**	**400**
PRTQAHSGSP	TYLQLPPRPP	GTRASMVLLP	GPPLESTFHQ	SLETDTLLTK	LCGQDRLLRR	LQEEIDQKQE	EKEQLEAALE
**410**	**420**	**430**	**440**	**450**	**460**	**470**	**480**
LTRQQLGQAT	REAGAPGRAW	GRQRLLQDRL	VSVRATLCHL	TQERERVWDT	YSGLEQELGT	LRETLEYLLH	LGSPQDRVSA
**490**	**500**	**510**	**520**	**530**	**540**	**550**	**560**
QQQLWMVEDT	LAGLGGPQKP	PPHTEPDSPS	PVLQGEESSE	RESLPESLEL	SSPRSPETDW	GRPPGGDKDL	ASPHLGLGSP
**570**	**580**	**590**	**600**				
RVSRASSPEG	RHLPSPQLGT	KKYGSITDWP	ALRSM				

Cmpd.	No. of Cmpds.	m/z meas.	Δ m/z [ppm]	z	Rt [min]	Score	P	Range	Sequence	Modification	Ratios
361	2	711.1755	-208.54	2	33.6	11.6	1	151-163	R.ASRAEGDDYGQPR.S		Wdown:Qdown 4.13<br>mdown:qdown 0.43
375	1	603.6767	-228.84	2	33.8	10.1	0	300-312	R.GPPSEAGGGKPPR.S		mdown:qdown 0.88
1037	1	945.4829	-32.03	2	41.7	11.1	2	565-582	R.ASSPEGRHLPSPQLGTKK.Y		mdown:qdown 0.74

Detailed Protein Report

Protein 357: H/ACA ribonucleoprotein complex subunit 4 isoform 3 [Homo sapiens]

**Accession:**	gi|570700851	**Score:**	32.8
**Database:**	refseq_human(refseq_human_20140103.fasta)	**MW [kDa]:**	47.6
**Database Date:**	2015-11-30	**pI:**	10.0
**Modification(s):**	Oxidation	**Sequence Coverage [%]:**	6.7
		**No. of unique Peptides:**	1

10	20	30	40	50	60	70	80
MADAEVIILP	KKHKKKKERK	SLPEEDVAEI	QHAEEFLIKP	ESKVAKLDTS	QWPLLLKNFD	KLNVRTTHYT	PLACGSNPLK
**90**	**100**	**110**	**120**	**130**	**140**	**150**	**160**
REIGDYIRTG	FINLDKPSNP	SSHEVVAWIR	RILRVEKTGH	SGTLDPKVTG	CLIVCIERAT	RLVKSQQSAG	KEYVGIVRLH
**170**	**180**	**190**	**200**	**210**	**220**	**230**	**240**
NAIEGGTQLS	RALETLTGAL	FQRPPLIAAV	KRQLRVRTIY	ESKMIEYDPE	RRLGIFWVSC	EAGTYIRTLC	VHLGLLLGVG
**250**	**260**	**270**	**280**	**290**	**300**	**310**	**320**
GQMQELRRVR	SGVMSEKDHM	VTMHDVLDAQ	WLYDNHKDES	YLRRVVYPLE	KLLTSHKRLV	MKDSAVNAIC	YGAKIMLPGV
**330**	**340**	**350**	**360**	**370**	**380**	**390**	**400**
LRYEDGIEVN	QEIVVITTKG	EAICMAIALM	TTAVISTCDH	GIVAKIKRVI	MERDTYPRKW	GLGPKASQKK	LMIKQGLLDK
**410**	**420**	**430**					
HGKPTDSTPA	TWKQEYVDYR						

Cmpd.	No. of Cmpds.	m/z meas.	Δ m/z [ppm]	z	Rt [min]	Score	P	Range	Sequence	Modification
1944	1	618.9252	-105.31	3	53.0	18.6	2	298-314	K.RLVMKDSAVNAICYGAK.I	Oxidation: 4

Detailed Protein Report

Protein 358: PREDICTED: kinesin-like protein KIF23 isoform X8 [Homo sapiens]

**Accession:**	gi|578827640	**Score:**	32.8
**Database:**	refseq_human(refseq_human_20140103.fasta)	**MW [kDa]:**	56.1
**Database Date:**	2015-11-30	**pI:**	9.9
**Modification(s):**	Carbamidomethyl	**Sequence Coverage [%]:**	10.7
		**No. of unique Peptides:**	1

10	20	30	40	50	60	70	80
MSFPPLPSCE	ILDINDEQTL	PRLIEALEKR	HNLRQMMIDE	FNKQSNAFKA	LLQEFDNAVL	SKENHMQGKL	NEKEKMISGQ
**90**	**100**	**110**	**120**	**130**	**140**	**150**	**160**
KLEIERLEKK	NKTLEYKIEI	LEKTTTIYEE	DKRNLQQELE	TQNQKLQRQF	SDKRRLEARL	QGMVTETTMK	WEKECERRVA
**170**	**180**	**190**	**200**	**210**	**220**	**230**	**240**
AKQLEMQNKL	WVKDEKLKQL	KAIVTEPKTE	KPERPSRERD	REKVTQRSVS	PSPVPLSSNY	IAQISNGQQL	MSQPQLHRRS
**250**	**260**	**270**	**280**	**290**	**300**	**310**	**320**
NSCSSISVAS	CISEWEQKIP	TYNTPLKVTS	IARRRQQEPG	QSKTCIVSDR	RRGMYWTEGR	EVVPTFRNEI	EIEEDHCGRL
**330**	**340**	**350**	**360**	**370**	**380**	**390**	**400**
LFQPDQNAPP	IRLRHRRSRS	AGDRWVDHKP	ASNMQTETVM	QPHVPHAITV	SVANEKALAK	CEKYMLTHQE	LASDGEIETK
**410**	**420**	**430**	**440**	**450**	**460**	**470**	**480**
LIKGDIYKTR	GGGQSVQFTD	IETLKQESPN	GSRKRRSSTV	APAQPDGAES	EWTDVETRCS	VAVEMRAGSQ	LGPGYQHHAQ
**490**							
PKRKKP							

Cmpd.	No. of Cmpds.	m/z meas.	Δ m/z [ppm]	z	Rt [min]	Score	P	Range	Sequence	Modification
2419	1	1051.5349	69.41	2	58.6	10.1	0	240-258	R.SNSCSSISVASCISEWEQK.I	Carbamidomethyl: 12

Detailed Protein Report

Protein 359: fanconi-associated nuclease 1 isoform b [Homo sapiens]

Accession: gi|226246525
Database: refseq_human(refseq_human_20140103.fasta)
Database Date: 2015-11-30
Modification(s): Carbamidomethyl

Score: 32.7
MW [kDa]: 59.5
pI: 6.3
Sequence Coverage [%]: 7.3
No. of unique Peptides: 1

Alias proteins:

Accession	Name	Description
gi|226246529	refseq_human (refseq_human_20140103.fasta)	fanconi-associated nuclease 1 isoform b [Homo sapiens]
gi|226246527	refseq_human (refseq_human_20140103.fasta)	fanconi-associated nuclease 1 isoform b [Homo sapiens]

10	20	30	40	50	60	70	80
MMSEGKPPDK	KRPRRSLSIS	KNKKKASNSI	ISCFNNAPPA	KLACPVCSKM	VPRYDLNRHL	DEMCANNDFV	QVDPGQVGLI
**90**	**100**	**110**	**120**	**130**	**140**	**150**	**160**
NSNVSMVDLT	SVTLEDVTPK	KSPPPKTNLT	PGQSDSAKRE	VKQKISPYFK	SNDVVCKNQD	ELRNRSVKVI	CLGSLASKLS
**170**	**180**	**190**	**200**	**210**	**220**	**230**	**240**
RKYVKAKKSI	DKDEEFAGSS	PQSSKSTVVK	SLIDNSSEIE	DEDQILENSS	QKENVFKCDS	LKEECIPEHM	VRGSKIMEAE
**250**	**260**	**270**	**280**	**290**	**300**	**310**	**320**
SQKATRECEK	SALTPGFSDN	AIMLFSPDFT	LRNTLKSTSE	DSLVKQECIK	EVVEKREACH	CEEVKMTVAS	EAKIQLSDSE
**330**	**340**	**350**	**360**	**370**	**380**	**390**	**400**
AKSHSSADDA	SAWSNIQEAP	LQDDSCLNND	IPHSIPLEQG	SSCNGPGQTT	GHPYYLRSFL	VVLKTVLENE	DDMLLFDEQE
**410**	**420**	**430**	**440**	**450**	**460**	**470**	**480**
KGIVTKFYQL	SATGQKLYVR	LFQRKLSWIK	MTKLEYEEIA	LDLTPVIEEL	TNAGFLQTES	ELQELSEVLE	LLSAPELKSL
**490**	**500**	**510**	**520**	**530**	**540**		
AKTFHLVNPN	GQKQQLVDAF	LKLAKQRSVC	TWGKNKPGIG	AVILKRFCWL	LLQ		

Cmpd.	No. of Cmpds.	m/z meas.	Δ m/z [ppm]	z	Rt [min]	Score	P	Range	Sequence	Modification
443	1	874.1657	67.87	3	34.2	13.4	2	25-49	K.KASNSIISCFNNAPPAKLACPVCSK.M	Carbamidomethyl: 20

Detailed Protein Report

Protein 360: cystatin-B [Homo sapiens]

Accession: gi|4503117
Database: refseq_human(refseq_human_20140103.fasta)
Database Date: 2015-11-30

Score: 32.7
MW [kDa]: 11.1
pI: 7.9
Sequence Coverage [%]: 12.2
No. of unique Peptides: 1

Quantitation

mdown:qdown	**Median:**	0.39	**CV:**	0.00 %	**No. of Peptides:**	1
Wdown:Qdown	**Median:**	2.00	**CV:**	0.00 %	**No. of Peptides:**	1

```
         10         20         30         40         50         60         70         80
MMCGAPSATQ PATAETQHIA DQVRSQLEEK ENKKFPVFKA VSFKSQVVAG TNYFIKVHVG DEDFVHLRVF QSLPHENKPL
         90        100
TLSNYQTNKA KHDELTYF
```

Cmpd.	No. of Cmpds.	m/z meas.	Δ m/z [ppm]	z	Rt [min]	Score	P	Range	Sequence	Modification	Ratios
953	2	663.8439	-18.59	2	40.4	32.7	0	45-56	K.SQVVAGTNYFIK.V		Wdown:Qdown 2.00<br>mdown:qdown 0.39

Detailed Protein Report

Protein 361: keratin, type I cuticular Ha2 [Homo sapiens]

Accession: gi|116488398
Database: refseq_human(refseq_human_20140103.fasta)
Database Date: 2015-11-30
Modification(s): Carbamidomethyl

Score: 32.5
MW [kDa]: 50.3
pI: 4.6
Sequence Coverage [%]: 4.5
No. of unique Peptides: 2

10	20	30	40	50	60	70	80
MTSSCCVTNN	LQASLKSCPR	PASVCSSGVN	CRPELCLGYV	CQPMACLPSV	CLPTTFRPAS	CLSKTYLSSS	CQAASGISGS
**90**	**100**	**110**	**120**	**130**	**140**	**150**	**160**
MGPGSWYSEG	AFNGNEKETM	QFLNDRLASY	LTRVRQLEQE	NAELESRIQE	ASHSQVLTMT	PDYQSHFRTI	EELQQKILCT
**170**	**180**	**190**	**200**	**210**	**220**	**230**	**240**
KAENARMVVN	IDNAKLAADD	FRAKYEAELA	MRQLVEADIN	GLRRILDDLT	LCKADLEAQV	ESLKEELMCL	KKNHEEEVGS
**250**	**260**	**270**	**280**	**290**	**300**	**310**	**320**
LRCQLGDRLN	IEVDAAPPVD	LTRVLEEMRC	QYEAMVEANR	RDVEEWFNMQ	MEELNQQVAT	SSEQLQNYQS	DIIDLRRTVN
**330**	**340**	**350**	**360**	**370**	**380**	**390**	**400**
TLEIELQAQH	SLRDSLENTL	TESEARYSSQ	LAQMQCMITN	VEAQLAEIRA	DLERQNQEYQ	VLLDVRARLE	GEINTYRSLL
**410**	**420**	**430**	**440**	**450**			
ENEDCKLPCN	PCSTPSCTTC	VPSPCVPRTV	CVPRTVGMPC	SPCPQGRY			

Cmpd.	No. of Cmpds.	m/z meas.	Δ m/z [ppm]	z	Rt [min]	Score	P	Range	Sequence	Modification
2706	1	1051.5703	74.71	2	65.1	11.7	1	429-447	R.TVCVPRTVGMPCSPCPQGR.Y	Carbamidomethyl: 12, 15
63	1	518.2825	104.44	3	30.0	10.0	1	435-448	R.TVGMPCSPCPQGRY.-	Carbamidomethyl: 6

Detailed Protein Report

Protein 362: elongator complex protein 1 [Homo sapiens]

**Accession:**	gi|38569394	**Score:**	32.5
**Database:**	refseq_human(refseq_human_20140103.fasta)	**MW [kDa]:**	150.2
**Database Date:**	2015-11-30	**pI:**	5.6
**Modification(s):**	Carbamidomethyl	**Sequence Coverage [%]:**	2.3
		**No. of unique Peptides:**	2

10	20	30	40	50	60	70	80
MRNLKLFRTL	EFRDIQGPGN	PQCFSLRTEQ	GTVLIGSEHG	LIEVDPVSRE	VKNEVSLVAE	GFLPEDGSGR	IVGVQDLLDQ
90	100	110	120	130	140	150	160
ESVCVATASG	DVILCSLSTQ	QLECVGSVAS	GISVMSWSPD	QELVLLATGQ	QTLIMMTKDF	EPILEQQIHQ	DDFGESKFIT
170	180	190	200	210	220	230	240
VGWGRKETQF	HGSEGRQAAF	QMQMHESALP	WDDHRPQVTW	RGDGQFFAVS	VVCPETGARK	VRVWNREFAL	QSTSEPVAGL
250	260	270	280	290	300	310	320
GPALAWKPSG	SLIASTQDKP	NQQDIVFFEK	NGLLHGHFTL	PFLKDEVKVN	DLLWNADSSV	LAVWLEDLQR	EESSIPKTCV
330	340	350	360	370	380	390	400
QLWTVGNYHW	YLKQSLSFST	CGKSKIVSLM	WDPVTPYRLH	VLCQGWHYLA	YDWHWTTDRS	VGDNSSDLSN	VAVIDGNRVL
410	420	430	440	450	460	470	480
VTVFRQTVVP	PPMCTYQLLF	PHPVNQVTFL	AHPQKSNDLA	VLDASNQISV	YKCGDCPSAD	PTVKLGAVGG	SGFKVCLRTP
490	500	510	520	530	540	550	560
HLEKRYKIQF	ENNEDQDVNP	LKLGLLTWIE	EDVFLAVSHS	EFSPRSVIHH	LTAASSEMDE	EHGQLNVSSS	AAVDGVIISL
570	580	590	600	610	620	630	640
CCNSKTKSVV	LQLADGQIFK	YLWESPSLAI	KPWKNSGGFP	VRFPYPCTQT	ELAMIGEEEC	VLGLTDRCRF	FINDIEVASN
650	660	670	680	690	700	710	720
ITSFAVYDEF	LLLTTHSHTC	QCFCLRDASF	KTLQAGLSSN	HVSHGEVLRK	VERGSRIVTV	VPQDTKLVLQ	MPRGNLEVVH
730	740	750	760	770	780	790	800
HRALVLAQIR	KWLDKLMFKE	AFECMRKLRI	NLNLIYDHNP	KVFLGNVETF	IKQIDSVNHI	NLFFTELKEE	DVTKTMYPAP
810	820	830	840	850	860	870	880
VTSSVYLSRD	PDGNKIDLVC	DAMRAVMESI	NPHKYCLSIL	TSHVKKTTPE	LEIVLQKVHE	LQGNAPSDPD	AVSAEEALKY
890	900	910	920	930	940	950	960
LLHLVDVNEL	YDHSLGTYDF	DLVLMVAEKS	QKDPKEYLPF	LNTLKKMETN	YQRFTIDKYL	KRYEKAIGHL	SKCGPEYFPE
970	980	990	1000	1010	1020	1030	1040
CLNLIKDKNL	YNEALKLYSP	SSQQYQDISI	AYGEHLMQEH	MYEPAGLMFA	RCGAHEKALS	AFLTCGNWKQ	ALCVAAQLNF
1050	1060	1070	1080	1090	1100	1110	1120
TKDQLVGLGR	TLAGKLVEQR	KHIDAAMVLE	ECAQDYEEAV	LLLLEGAAWE	EALRLVYKYN	RLDIIETNVK	PSILEAQKNY
1130	1140	1150	1160	1170	1180	1190	1200
MAFLDSQTAT	FSRHKKRLLV	VRELKEQAQQ	AGLDDEVPHG	QESDLFSETS	SVVSGSEMSG	KYSHSNSRIS	ARSSKNRRKA
1210	1220	1230	1240	1250	1260	1270	1280
ERKKHSLKEG	SPLEDLALLE	ALSEVVQNTE	NLKDEVYHIL	KVLFLFEFDE	QGRELQKAFE	DTLQLMERSL	PEIWTLTYQQ
1290	1300	1310	1320	1330	1340		
NSATPVLGPN	STANSIMASY	QQQKTSVPVL	DAELFIPPKI	NRRTQWKLSL	LD		

Cmpd.	No. of Cmpds.	m/z meas.	Δ m/z [ppm]	z	Rt [min]	Score	P	Range	Sequence	Modification
2081	1	938.3438	-121.46	2	56.2	11.3	0	53-70	K.NEVSLVAEGFLPEDGSGR.I	
2670	2	625.3045	66.40	2	61.7	21.2	0	453-464	K.CGDCPSADPTVK.L	Carbamidomethyl: 4

Detailed Protein Report

Protein 363: **PREDICTED: multiple epidermal growth factor-like domains protein 6 isoform X4 [Homo sapiens]**

**Accession:**	gi|578798522	**Score:**	32.4
**Database:**	refseq_human(refseq_human_20140103.fasta)	**MW [kDa]:**	135.9
**Database Date:**	2015-11-30	**pI:**	5.9
**Modification(s):**	Carbamidomethyl	**Sequence Coverage [%]:**	4.4
		**No. of unique Peptides:**	2

```
        10         20         30         40         50         60         70         80
MGASRDRGLA ALWCLGLLGG LARVAGTHYR YLWRGCYPCH LGQAGYPVSA GDQRPDVDEC RTHNGGCQHR CVNTPGSYLC
        90        100        110        120        130        140        150        160
ECKPGFRLHT DSRTCLAINS CALGNGGCQH HCVQLTITRH RCQCRPGFQL QEDGRHCVRR SPCANRNGSC MHRCQVVRGL
       170        180        190        200        210        220        230        240
ARCECHVGYQ LAADGKACED VDECAAGLAQ CAHGCLNTQG SFKCVCHAGY ELGADGRQCY RIEMEIVNSC EANNGGCSHG
       250        260        270        280        290        300        310        320
CSHTSAGPLC TCPRGYELDT DQRTCIDVDD CADSPCCQQV CTNNPGGYEC GCYAGYRLSA DGCGCEDVDE CASSRGGCEH
       330        340        350        360        370        380        390        400
HCTNLAGSFQ CSCEAGYRLH EDRRGCSPLE EPMVDLDGEL PFVRPLPHIA VLQDELPQLF QDDDVGADEE EAELRGEHTL
       410        420        430        440        450        460        470        480
TEKFVCLDDS FGHDCSLTCD DCRNGGTCLL GLDGCDCPEG WTGLICNETC PPDTFGKNCS FSCSCQNGGT CDSVTGACRC
       490        500        510        520        530        540        550        560
PPGVSGTNCE DGCPKGYYGK HCRKKCNCAN RGRCHRLYGA CLCDPGLYGR FCHLTCPPWA FGPGCSEECQ CVQPHTQSCD
       570        580        590        600        610        620        630        640
KRDGSCSCKA GFRGERCQAE CELGYFGPGC WQACTCPVGV ACDSVSGECG KRCPAGFQGE DCGQECPVGT FGVNCSSSCS
       650        660        670        680        690        700        710        720
CGGAPCHGVT GQCRCPPGRT GEDCEAECPQ GHFGPGCEQR CQCQHGAACD HVSGACTCPA GWRGTFCEHA CPAGFFGLDC
       730        740        750        760        770        780        790        800
RSACNCTAGA ACDAVNGSCL CPAGRRGPRC AETCPAHTYG HNCSQACACF NGASCDPVHG QCHCAPGWMG PSCLQACPAG
       810        820        830        840        850        860        870        880
LYGDNCRHSC LCQNGGTCDP VSGHCACPEG WAGLACEKEC LPRDVRAGCR HSGGCLNGGL CDPHTGRCLC PAGWTGDKCQ
       890        900        910        920        930        940        950        960
SPCLRGWFGE ACAQRCSCPP GAACHHVTGA CRCPPGFTGS GCEQACPPGS FGEDCAQMCQ CPGENPACHP ATGTCSCAAG
       970        980        990       1000       1010       1020       1030       1040
YHGPSCQQRC PPGRYGPGCE QLCGCLNGGS CDAATGACRC PTGFLGTDCN LTCPQGRFGP NCTHVCGCGQ GAACDPVTGT
      1050       1060       1070       1080       1090       1100       1110       1120
CLCPPGRAGV RCERGCPQNR FGVGCEHTCS CRNGGLCHAS NGSCSCGLGW TGRHCELACP PGRYGAACHL ECSCHNNSTC
      1130       1140       1150       1160       1170       1180       1190       1200
EPATGTCRCG PGFYGQACEH PCPPGFHGAG CQGLCWCQHG APCDPISGRC LCPAGFHGHF CERGCEPGSF GEGCHQRCDC
      1210       1220       1230       1240       1250       1260       1270       1280
DGGAPCDPVT GLCLCPPGRS GATCNLDCRR GQFGPSCTLH CDCGGGADCD PVSGQCHCVD GYMGPTCREG GPLRLPENPS
      1290       1300       1310
LAQGSAGTLP ASSRPTSRSG GPARH
```

Cmpd.	No. of Cmpds.	m/z meas.	Δ m/z [ppm]	z	Rt [min]	Score	P	Range	Sequence	Modification
1769	1	682.2402	-68.51	3	52.1	11.4	0	704-721	R.GTFCEHACPAGFFGLDCR.S	Carbamidomethyl: 4, 17
12	1	1103.5102	57.86	2	29.4	10.6	0	1198-1219	R.CDCDGGAPCDPVTGLCLCPPGR.S	Carbamidomethyl: 16

Detailed Protein Report

Protein 364: C3 and PZP-like alpha-2-macroglobulin domain-containing protein 8 [Homo sapiens]

**Accession:**	gi|118600977	**Score:**	32.4
**Database:**	refseq_human(refseq_human_20140103.fasta)	**MW [kDa]:**	211.2
**Database Date:**	2015-11-30	**pI:**	6.2
		**Sequence Coverage [%]:**	1.8
		**No. of unique Peptides:**	1

Quantitation

mdown:qdown	**Median:**	0.69	**CV:**	0.00 %	**No. of Peptides:**	1
Wdown:Qdown	**Median:**	1.61	**CV:**	0.00 %	**No. of Peptides:**	1

Detailed Protein Report

10	20	30	40	50	60	70	80
MAPGALRGGC	EQRAPGGGAR	TAALGPWPWP	SPGPAPSPGA	APPRSRKMSG	ALLWPLLPLL	LLLLSARDGV	RAAQPQAPGY
90	100	110	120	130	140	150	160
LIAAPSVFRA	GVEEVISVTI	FNSPREVTVQ	AQLVAQGEPV	VQSQGAILDK	GTIKLKVPTG	LRGQALLKVW	GRGWQAEEGP
170	180	190	200	210	220	230	240
LFHNQTSVTV	DGRGASVFIQ	TDKPVYRPQH	RVLISIFTVS	PNLRPVNEKL	EAYILDPRGS	RMIEWRHLKP	FCCGITNMSF
250	260	270	280	290	300	310	320
PLSDQPVLGE	WFIFVEMQGH	AYNKSFEVQK	YVLPKFELLI	DPPRYIQDLD	ACETGTVRAR	YTFGKPVAGA	LMINMTVNGV
330	340	350	360	370	380	390	400
GYYSHEVGRP	VLRTTKILGS	RDFDICVRDM	IPADVPEHFR	GRVSIWAMVT	SVDGSQQVAF	DDSTPVQRQL	VDIRYSKDTR
410	420	430	440	450	460	470	480
KQFKPGLAYV	GKVELSYPDG	SPAEGVTVQI	KAELTPKDNI	YTSEVVSQRG	LVGFEIPSIP	TSAQHVWLET	KVMALNGKPV
490	500	510	520	530	540	550	560
GAQYLPSYLS	LGSWYSPSQC	YLQLQPPSHP	LQVGEEAYFS	VKSTCPCNFT	LYYEVAARGN	IVLSGQQPAH	TTQQRSKRAA
570	580	590	600	610	620	630	640
PALEKPIRLT	HLSETEPPPA	PEAEVDVCVT	SLHLAVTPSM	VPLGRLLVFY	VRENGEGVAD	SLQFAVETFF	ENQVSVTYSA
650	660	670	680	690	700	710	720
NETQPGEVVD	LRIRAARGSC	VCVAAVDKSV	YLLRSGFRLT	PAQVFQELED	YDVSDSFGVS	REDGPFWWAG	LTAQRRRRSS
730	740	750	760	770	780	790	800
VFPWPWGITK	DSGFAFTETG	LVVMTDRVSL	NHRQDGGLYT	DEAVPAFQPH	TGSLVAVAPS	RHPPRTEKRK	RTFFPETWIW
810	820	830	840	850	860	870	880
HCLNISDPSG	EGTLSVKVPD	SITSWVGEAV	ALSTSQGLGI	AEPSLLKTFK	PFFVDFMLPA	LIIRGEQVKI	PLSVYNYMGT
890	900	910	920	930	940	950	960
CAEVYMKLSV	PKGIQFVGHP	GKRHVTKKMC	VAPGEAEPIW	VVLSFSDLGL	NNITAKALAY	GDTNCCRDGR	SSKHPEENHA
970	980	990	1000	1010	1020	1030	1040
DRRVPIGVDH	VRRSVMVEAE	GVPRAYTYSA	FFCPSERVHI	STPNKYEFQY	VQRPLRLTRF	DVAVRAHNDA	RVALSSGPQD
1050	1060	1070	1080	1090	1100	1110	1120
TAGMIEIVLG	GHQNTRSWIS	TSKMGEPVAS	AHTAKILSWD	EFRTFWISWR	GGLIQVGHGP	EPSNESVIVA	WTLPRPPEVQ
1130	1140	1150	1160	1170	1180	1190	1200
FIGFSTGWGS	MGEFRIWRKM	EVDESYSEAF	TLGVPHGAIP	GSERATASII	GDVMGPTLNH	LNNLLRLPFG	CGEQNMIHFA
1210	1220	1230	1240	1250	1260	1270	1280
PNVFVLKYLQ	KTQQLSPEVE	RETTDYLVQG	YQRQLTYKRQ	DGSYSAFGER	DASGSMWLTA	FVLKSFAQAR	SFIFVDPREL
1290	1300	1310	1320	1330	1340	1350	1360
AAAKSWIIQQ	QQADGSFLAV	GRVLNKDIQG	GIHGTVPLTA	YVVVALLETG	TASEEERGST	DKARHFLESA	APLAMDPYSC
1370	1380	1390	1400	1410	1420	1430	1440
ALTTYALTLL	RSPAAPEALR	KLRSLAIMRD	GVTHWSLSNS	WDVDKGTFLS	FSDRVSQSVV	SAEVEMTAYA	LLTYTLLGDV
1450	1460	1470	1480	1490	1500	1510	1520
AAALPVVKWL	SQQRNALGGF	SSTQDTCVAL	QALAEYAILS	YAGGINLTVS	LASTNLDYQE	TFELHRTNQK	VLQTAAIPSL
1530	1540	1550	1560	1570	1580	1590	1600
PTGLFVSAKG	DGCCLMQIDV	TYNVPDPVAK	PAFQLLVSLQ	EPEAQGRPPP	MPASAAEGSR	GDWPPADDDD	PAADQHHQEY
1610	1620	1630	1640	1650	1660	1670	1680
KVMLEVCTRW	LHAGSSNMAV	LEVPLLSGFR	ADIESLEQLL	LDKHMGMKRY	EVAGRRVLFY	FDEIPSRCLT	CVRFRALREC
1690	1700	1710	1720	1730	1740	1750	1760
VVGRTSALPV	SVYDYYEPAF	EATRFYNVST	HSPLARELCA	GPACNEVERA	PARGPGWFPG	ESGPAVAPEE	GAAIARCGCD
1770	1780	1790	1800	1810	1820	1830	1840
HDCGAQGNPV	CGSDGVVYAS	ACRLREAACR	QAAPLEPAPP	SCCALEQRLP	ASSSSTYGDD	LASVAPGPLQ	QDVKLNGAGL
1850	1860	1870	1880	1890	1900	1910	1920
EVEDSDPEPE	GEAEDRVTAG	PRPPVSSGNL	ESSTQSASPF	HRWGQTPAPQ	RHSGRVVGAH	RPGLLSPVFV	YSPAFQSGGE
1930	1940						
EGLWMSNTCT	LR						

Cmpd.	No. of Cmpds.	m/z meas.	Δ m/z [ppm]	z	Rt [min]	Score	P	Range	Sequence	Modification	Ratios
968	1	820.0912	-24.82	3	40.6	16.4	1	1285-1306	K.SWIIQQQQADGSFLAVGRVLNK.D		Wdown:Qdown 1.61<br>mdown:qdown 0.69

Detailed Protein Report

Protein 365: PREDICTED: serine/threonine-protein kinase B-raf isoform X2 [Homo sapiens]

Accession: gi|530387108
Database: refseq_human(refseq_human_20140103.fasta)
Database Date: 2015-11-30
Modification(s): Oxidation

Score: 32.4
MW [kDa]: 83.9
pI: 7.9
Sequence Coverage [%]: 2.1
No. of unique Peptides: 1

Quantitation

mdown:qdown	**Median:**	0.64	**CV:**	0.00 %	**No. of Peptides:**	1
Wdown:Qdown	**Median:**	0.48	**CV:**	0.00 %	**No. of Peptides:**	1

10	20	30	40	50	60	70	80
MAALSGGGGG	GAEPGQALFN	GDMEPEAGAG	AGAAASSAAD	PAIPEEVWNI	KQMIKLTQEH	IEALLDKFGG	EHNPPSIYLE
90	100	110	120	130	140	150	160
AYEEYTSKLD	ALQQREQQLL	ESLGNGTDFS	VSSSASMDTV	TSSSSSSLSV	LPSSLSVFQN	PTDVARSNPK	SPQKPIVRVF
170	180	190	200	210	220	230	240
LPNKQRTVVP	ARCGVTVRDS	LKKALMMRGL	IPECCAVYRI	QDGEKKPIGW	DTDISWLTGE	ELHVEVLENV	PLTTHNFVRK
250	260	270	280	290	300	310	320
TFFTLAFCDF	CRKLLFQGFR	CQTCGYKFHQ	RCSTEVPLMC	VNYDQLDLLF	VSKFFEHHPI	PQEEASLAET	ALTSGSSPSA
330	340	350	360	370	380	390	400
PASDSIGPQI	LTSPSPSKSI	PIPQPFRPAD	EDHRNQFGQR	DRSSSAPNVH	INTIEPVNID	DLIRDQGFRG	DGGSTTGLSA
410	420	430	440	450	460	470	480
TPPASLPGSL	TNVKALQKSP	GPQRERKSSS	SSEDRNRMKT	LGRRDSSDDW	EIPDGQITVG	QRIGSGSFGT	VYKGKWHGDV
490	500	510	520	530	540	550	560
AVKMLNVTAP	TPQQLQAFKN	EVGVLRKTRH	VNILLFMGYS	TKPQLAIVTQ	WCEGSSLYHH	LHIIETKFEM	IKLIDIARQT
570	580	590	600	610	620	630	640
AQGMDYLHAK	SIIHRDLKSN	NIFLHEDLTV	KIGDFGLATV	KSRWSGSHQF	EQLSGSILWM	APEVIRMQDK	NPYSFQSDVY
650	660	670	680	690	700	710	720
AFGIVLYELM	TGQLPYSNIN	NRDQIIFMVG	RGYLSPDLSK	VRSNCPKAMK	RLMAECLKKK	RDERPLFPQE	NLQPSSSHHH
730	740	750	760				
GSICSYFLSL	VFVQFVNIKT	QFCSSNLFLK	IQNFQCIS				

Cmpd.	No. of Cmpds.	m/z meas.	Δ m/z [ppm]	z	Rt [min]	Score	P	Range	Sequence	Modification	Ratios
116	1	929.4622	17.68	2	30.9	16.0	1	184-199	K.ALMMRGLIPECCAVYR.I	Oxidation: 3, 4	Wdown:Qdown 0.48<br>mdown:qdown 0.64

Detailed Protein Report

Protein 366: protein FAM104A isoform 3 [Homo sapiens]

Accession: gi|574956994
Database: refseq_human(refseq_human_20140103.fasta)
Database Date: 2015-11-30

Score: 32.4
MW [kDa]: 9.8
pI: 12.7
Sequence Coverage [%]: 40.8
No. of unique Peptides: 1

```
        10         20         30         40         50         60         70         80
MGGRGADAGS SGGTGPTEGY SPPAASTRAA ARAKARGGGR GGRRNTTPSV PSLRGAAPRS FHPPAAMSER LRPSLQAVTV
        90        100        110
VGAAAAAAAA SIARTGPAGR KAA
```

Cmpd.	No. of Cmpds.	m/z meas.	Δ m/z [ppm]	z	Rt [min]	Score	P	Range	Sequence	Modification
2594	1	974.3959	-62.39	3	60.7	16.5	2	1-32	-.MGGRGADAGSSGGTGPTEGYSPPAASTRAAAR.A	

Detailed Protein Report

Protein 367: PREDICTED: HMG box transcription factor BBX isoform X10 [Homo sapiens]

**Accession:**	gi|578807613	**Score:**	32.4
**Database:**	refseq_human(refseq_human_20140103.fasta)	**MW [kDa]:**	98.5
**Database Date:**	2015-11-30	**pI:**	9.6
**Modification(s):**	Oxidation	**Sequence Coverage [%]:**	3.2
		**No. of unique Peptides:**	1

10	20	30	40	50	60	70	80
MKGSNRNKDH	SAEGEGVGKR	PKRKCLQWHP	LLAKKLLDFS	EEEEEEDEEE	DIDKVQLLGA	DGLEQDVGET	EDDESPEQRA
**90**	**100**	**110**	**120**	**130**	**140**	**150**	**160**
RRPMNAFLLF	CKRHRSLVRQ	EHPRLDNRGA	TKILADWWAV	LDPKEKQKYT	DMAKEYKDAF	MKANPGYKWC	PTTNKPVKSP
**170**	**180**	**190**	**200**	**210**	**220**	**230**	**240**
TPTVNPRKKL	WAFPSDSSRD	LPSPKKAKTE	EMPQLNFGMA	DPTQMGGLSM	LLLAGEHALG	TPEVSSGTCR	PDVSESPELR
**250**	**260**	**270**	**280**	**290**	**300**	**310**	**320**
QKSPLFQFAE	ISSSTSHSDA	STKQCQTSAL	FQFAEISSNT	SQLGGAEPVK	RCGKSALFQL	AEMCLASEGM	KMEESKLIKA
**330**	**340**	**350**	**360**	**370**	**380**	**390**	**400**
KESDGGRIKE	LEKGKEEKEI	KMEKTDETRL	QKEAEFEKSA	KENLRDSKEL	RNFEALQIDD	IMAIKMEDPK	EIRKEELEED
**410**	**420**	**430**	**440**	**450**	**460**	**470**	**480**
HKCSHFPDFS	YSASSKIIIS	DVPSRKDHMC	HPHGIMIIED	PAALNKPEKL	KKKKKKSKMD	RHGNDKSTPK	KTCKKRQSSE
**490**	**500**	**510**	**520**	**530**	**540**	**550**	**560**
SDIESVIYTI	EAVAKGDWGI	EKLGDTPRKK	VRTSSSGKGS	ILDAKPPKKK	VKSREKKMSK	EKSSDTTKES	RPPDFISISA
**570**	**580**	**590**	**600**	**610**	**620**	**630**	**640**
SKNISGETPE	GIKAEPLTPM	EDALPPSLSG	QAKPEDSDCH	RKIETCGSRK	SERSCKGALY	KTLVSEGMLT	SLRANVDRGK
**650**	**660**	**670**	**680**	**690**	**700**	**710**	**720**
RSSGKGNSSD	HEGCWNEESW	TFSQSGTSGS	KKFKKTKPKE	DCLLGSAKLD	EEFEKKFNSL	PQYSPVTFDR	KCVPVPRKKK
**730**	**740**	**750**	**760**	**770**	**780**	**790**	**800**
KTGNVSSEPT	KTSKGSGDKW	SNKQLFLDAI	HPTEEPTTTQ	EPLVGSQKRK	ARKTKITHLV	RTADGRVSPA	GGTLDDKPKE
**810**	**820**	**830**	**840**	**850**	**860**	**870**	**880**
QLQRSLPKAT	ETDCNDKCSH	NTEVGETRSS	TPEMPAVSAF	FSLAALAEVA	AMENVHRGQR	STPLTHDGQP	KEMPQAPVLI
**890**							
SCADQ							

Cmpd.	No. of Cmpds.	m/z meas.	Δ m/z [ppm]	z	Rt [min]	Score	P	Range	Sequence	Modification
2908	1	939.4942	3.35	2	65.1	16.7	1	622-638	K.TLVSEGMLTSLRANVDR.G	Oxidation: 7

Detailed Protein Report

Protein 368: PREDICTED: cytoplasmic tyrosine-protein kinase BMX isoform X1 [Homo sapiens]

**Accession:**	gi|578837945	**Score:**	32.3
**Database:**	refseq_human(refseq_human_20140103.fasta)	**MW [kDa]:**	77.9
**Database Date:**	2015-11-30	**pI:**	9.3
**Modification(s):**	Oxidation	**Sequence Coverage [%]:**	5.3
		**No. of unique Peptides:**	2

10	20	30	40	50	60	70	80
MDTKSILEEL	LLKRSQQKKK	MSPNNYKERL	FVLTKTNLSY	YEYDKMKRGS	RKGSIEIKKI	RCVEKVNLEE	QTPVERQYPF
**90**	**100**	**110**	**120**	**130**	**140**	**150**	**160**
QIVYKDGLLY	VYASNEESRS	QWLKALQKEI	RGNPHLLVKY	HSGFFVDGKF	LCCQQSCKAA	PGCTLWEAYA	NLHTAVNEEK
**170**	**180**	**190**	**200**	**210**	**220**	**230**	**240**
HRVPTFPDRV	LKIPRAVPVL	KMDAPSSSTT	LAQYDNESKK	NYGSQPPSSS	TSLAQYDSNS	KKIYGSQPNF	NMQYIPREDF
**250**	**260**	**270**	**280**	**290**	**300**	**310**	**320**
PDWWQVRKLK	SSSSEDVASS	NQKERNVNHT	TSKISWEFPE	SSSSEEEENL	DDYDWFAGNI	SRSQSEQLLR	QKGKEGAFMV
**330**	**340**	**350**	**360**	**370**	**380**	**390**	**400**
RNSSQVGMYT	VSLFSKAVND	KKGTVKHYHV	HTNAENKLYL	AENYCFDSIP	KLIHYHQHNS	AGMITRLRHP	VSTKANKVPD
**410**	**420**	**430**	**440**	**450**	**460**	**470**	**480**
SVSLGNGIWE	LKREEITLLK	ELGSGQFGVV	QLGKWKGQYD	VAVKMIKEGS	MSEDEFFQEA	QTMMKLSHPK	LVKFYGVCSK
**490**	**500**	**510**	**520**	**530**	**540**	**550**	**560**
EYPIYIVTEY	ISNGCLLNYL	RSHGKGLEPS	QLLEMCYDVC	EGMAFLESHQ	FIHRDLAARN	CLVDRDLCVK	VSDFGMTRYV
**570**	**580**	**590**	**600**	**610**	**620**	**630**	**640**
LDDQYVSSVG	TKFPVKWSAP	EVFHYFKYSS	KSDVWAFGIL	MWEVFSLGKQ	PYDLYDNSQV	VLKVSQGHRL	YRPHLASDTI
**650**	**660**	**670**	**680**				
YQIMYSCWHE	LPEKRPTFQQ	LLSSIEPLRE	KDKH				

Cmpd.	No. of Cmpds.	m/z meas.	Δ m/z [ppm]	z	Rt [min]	Score	P	Range	Sequence	Modification
1584	1	1022.9716	-60.15	2	49.7	16.6	1	559-576	R.YVLDDQYVSSVGTKFPVK.W	
2432	1	701.1001	113.81	3	58.7	15.7	0	592-609	K.SDVWAFGILMWEVFSLGK.Q	Oxidation: 10

Detailed Protein Report

Protein 369: **PREDICTED: conserved oligomeric Golgi complex subunit 7 isoform X2 [Homo sapiens]**

**Accession:**	gi|530409377	**Score:**	32.3
**Database:**	refseq_human(refseq_human_20140103.fasta)	**MW [kDa]:**	79.1
**Database Date:**	2015-11-30	**pI:**	5.2
		**Sequence Coverage [%]:**	5.7
		**No. of unique Peptides:**	1

10	20	30	40	50	60	70	80
MPKVLRDVEA	LKQEASFLKE	QMILVKEDIK	KFEQDTSQSM	QVLVEIDQVK	SRMQLAAESL	QEADKWSTLS	ADIEETFKTQ
90	100	110	120	130	140	150	160
DIAVISAKLT	GMQNSLMMLV	DTPDYSEKCV	HLEALKNRLE	ALASPQIVAA	FTSQAVDQSK	VFVKVFTEID	RMPQLLAYYY
170	180	190	200	210	220	230	240
KCHKVQLLAA	WQELCQSDLS	LDRQLTGLYD	ALLGAWHTQI	QWATQVFQKP	HEVVMVLLIQ	TLGALMPSLP	SCLSNGVERA
250	260	270	280	290	300	310	320
GPEQELTRLL	EFYDATAHFA	KGLEMALLPH	LHEHNLVKVT	ELVDAVYDPY	KPYQLKYGDM	EESNLLIQMS	AVPLEHGEVI
330	340	350	360	370	380	390	400
DCVQELSHSV	NKLFGLASAA	VDRCVRFTNG	LGTCGLLSAL	KSLFAKYVSD	FTSTLQSIRK	KCKLDHIPPN	SLFQEDWTAF
410	420	430	440	450	460	470	480
QNSIRIIATC	GELLRHCGDF	EQQLANRILS	TAGKYLSDSC	SPRSLAGFQE	SILTDKKNSA	KNPWQEYNYL	QKDNPAEYAS
490	500	510	520	530	540	550	560
LMEILYTLKE	KGSSNHNLLA	APRAALTRLN	QQAHQLAFDS	VFLRIKQQLL	LISKMDSWNT	AGIGETLTDE	LPAFSLTPLE
570	580	590	600	610	620	630	640
YISNIGQYIM	SLPLNLEPFV	TQEDSALELA	LHAGKLPFPP	EQGDELPELD	NMADNWLGSI	ARATMQTYCD	AILQIPELSP
650	660	670	680	690	700	710	
HSAKQLATDI	DYLINVMDAL	GLQPSRTLQH	IVTLLKTRPE	DYRQVSKGLP	RRLATTVATM	RSVNY	

Cmpd.	No. of Cmpds.	m/z meas.	Δ m/z [ppm]	z	Rt [min]	Score	P	Range	Sequence	Modification
1449	1	946.3990	-84.94	3	46.6	13.1	2	27-50	K.EDIKKFEQDTSQSMQVLVEIDQVK.S	

Detailed Protein Report

Protein 370: PREDICTED: insulin-like growth factor 1 receptor isoform X2 [Homo sapiens]

Accession: gi|530407102
Database: refseq_human(refseq_human_20140103.fasta)
Database Date: 2015-11-30
Modification(s): Carbamidomethyl, Oxidation

Score: 32.3
MW [kDa]: 141.1
pI: 5.4
Sequence Coverage [%]: 3.7
No. of unique Peptides: 3

10	20	30	40	50	60	70	80
MTNLKDIGLY	NLRNITRGAI	RIEKNADLCY	LSTVDWSLIL	DAVSNNYIVG	NKPPKECGDL	CPGTMEEKPM	CEKTTINNEY
**90**	**100**	**110**	**120**	**130**	**140**	**150**	**160**
NYRCWTTNRC	QKMCPSTCGK	RACTENNECC	HPECLGSCSA	PDNDTACVAC	RHYYYAGVCV	PACPPNTYRF	EGWRCVDRDF
**170**	**180**	**190**	**200**	**210**	**220**	**230**	**240**
CANILSAESS	DSEGFVIHDG	ECMQECPSGF	IRNGSQSMYC	IPCEGPCPKV	CEEEKKTKTI	DSVTSAQMLQ	GCTIFKGNLL
**250**	**260**	**270**	**280**	**290**	**300**	**310**	**320**
INIRRGNNIA	SELENFMGLI	EVVTGYVKIR	HSHALVSLSF	LKNLRLILGE	EQLEGNYSFY	VLDNQNLQQL	WDWDHRNLTI
**330**	**340**	**350**	**360**	**370**	**380**	**390**	**400**
KAGKMYFAFN	PKLCVSEIYR	MEEVTGTKGR	QSKGDINTRN	NGERASCESD	VLHFTSTTTS	KNRIIITWHR	YRPPDYRDLI
**410**	**420**	**430**	**440**	**450**	**460**	**470**	**480**
SFTVYYKEAP	FKNVTEYDGQ	DACGSNSWNM	VDVDLPPNKD	VEPGILLHGL	KPWTQYAVYV	KAVTLTMVEN	DHIRGAKSEI
**490**	**500**	**510**	**520**	**530**	**540**	**550**	**560**
LYIRTNASVP	SIPLDVLSAS	NSSSQLIVKW	NPPSLPNGNL	SYYIVRWQRQ	PQDGYLYRHN	YCSKDKIPIR	KYADGTIDIE
**570**	**580**	**590**	**600**	**610**	**620**	**630**	**640**
EVTENPKTEV	CGGEKGPCCA	CPKTEAEKQA	EKEEAEYRKV	FENFLHNSIF	VPRPERKRRD	VMQVANTTMS	SRSRNTTAAD
**650**	**660**	**670**	**680**	**690**	**700**	**710**	**720**
TYNITDPEEL	ETEYPFFESR	VDNKERTVIS	NLRPFTLYRI	DIHSCNHEAE	KLGCSASNFV	FARTMPAEGA	DDIPGPVTWE
**730**	**740**	**750**	**760**	**770**	**780**	**790**	**800**
PRPENSIFLK	WPEPENPNGL	ILMYEIKYGS	QVEDQRECVS	RQEYRKYGGA	KLNRLNPGNY	TARIQATSLS	GNGSWTDPVF
**810**	**820**	**830**	**840**	**850**	**860**	**870**	**880**
FYVQAKTGYE	NFIHLIIALP	VAVLLIVGGL	VIMLYVFHRK	RNNSRLGNGV	LYASVNPEYF	SAADVYVPDE	WEVAREKITM
**890**	**900**	**910**	**920**	**930**	**940**	**950**	**960**
SRELGQGSFG	MVYEGVAKGV	VKDEPETRVA	IKTVNEAASM	RERIEFLNEA	SVMKEFNCHH	VVRLLGVVSQ	GQPTLVIMEL
**970**	**980**	**990**	**1000**	**1010**	**1020**	**1030**	**1040**
MTRGDLKSYL	RSLRPEMENN	PVLAPPSLSK	MIQMAGEIAD	GMAYLNANKF	VHRDLAARNC	MVAEDFTVKI	GDFGMTRDIY
**1050**	**1060**	**1070**	**1080**	**1090**	**1100**	**1110**	**1120**
ETDYYRKGGK	GLLPVRWMSP	ESLKDGVFTT	YSDVWSFGVV	LWEIATLAEQ	PYQGLSNEQV	LRFVMEGGLL	DKPDNCPDML
**1130**	**1140**	**1150**	**1160**	**1170**	**1180**	**1190**	**1200**
FELMRMCWQY	NPKMRPSFLE	IISSIKEEME	PGFREVSFYY	SEENKLPEPE	ELDLEPENME	SVPLDPSASS	SSLPLPDRHS
**1210**	**1220**	**1230**	**1240**	**1250**			
GHKAENGPGP	GVLVLRASFD	ERQPYAHMNG	GRKNERALPL	PQSSTC			

Cmpd.	No. of Cmpds.	m/z meas.	Δ m/z [ppm]	z	Rt [min]	Score	P	Range	Sequence	Modification
1818	1	697.0290	127.81	3	51.4	10.3	0	56-73	K.ECGDLCPGTMEEKPMCEK.T	Carbamidomethyl: 16; Oxidation: 10, 15
1420	1	669.8545	-38.10	2	46.5	11.2	0	271-282	R.HSHALVSLSFLK.N	
2019	1	660.9088	-130.71	3	53.8	10.8	1	325-340	K.MYFAFNPKLCVSEIYR.M	

Detailed Protein Report

Protein 371: PHD and RING finger domain-containing protein 1 isoform 4 [Homo sapiens]

**Accession:**	gi|557440810	**Score:**	32.2
**Database:**	refseq_human(refseq_human_20140103.fasta)	**MW [kDa]:**	178.1
**Database Date:**	2015-11-30	**pI:**	10.1
**Modification(s):**	Oxidation	**Sequence Coverage [%]:**	1.3
		**No. of unique Peptides:**	1

10	20	30	40	50	60	70	80
MDDDSLDELV	ARSPGPDGHP	QVGPADPAES	SVGSSGDSGD	DSDSEHGDGT	DGEDEGASEE	EDLEDRSGSE	DSEDDGETLL
**90**	**100**	**110**	**120**	**130**	**140**	**150**	**160**
EVAGTQGKLE	AAGSFNSDDD	AESCPICLNA	FRDQAVGTPE	NCAHYFCLDC	IVEWSKNANS	CPVDRTLFKC	ICIRAQFGGK
**170**	**180**	**190**	**200**	**210**	**220**	**230**	**240**
ILRKIPVENT	KASEEEEDPT	FCEVCGRSDR	EDRLLLCDGC	DAGYHMECLD	PPLQEVPVDE	WFCPECAAPG	VVLAADAGPV
**250**	**260**	**270**	**280**	**290**	**300**	**310**	**320**
SEEEVSLLLA	DVVPTTSRLR	PRAGRTRAIA	RTRQSERVRA	TVNRNRISTA	RRVQHTPGRL	GSSLLDEAIE	AVATGLSTAV
**330**	**340**	**350**	**360**	**370**	**380**	**390**	**400**
YQRPLTPRTP	ARRKRKTRRR	KKVPGRKKTP	SGPSAKSKSS	ATRSKKRQHR	VKKRRGKKVK	SEATTRSRIA	RTLGLRRPVH
**410**	**420**	**430**	**440**	**450**	**460**	**470**	**480**
SSCIPSVLKP	VEPSLGLLRA	DIGAASLSLF	GDPYELDPFD	SSEELSANPL	SPLSAKRRAL	SRSALQSHQP	VARPVSVGLS
**490**	**500**	**510**	**520**	**530**	**540**	**550**	**560**
RRRLPAAVPE	PDLEEEPVPD	LLGSILSGQS	LLMLGSSDVI	IHRDGSLSAK	RAAPVSFQRN	SGSLSRGEEG	FKGCLQPRAL
**570**	**580**	**590**	**600**	**610**	**620**	**630**	**640**
PSGSPAQGPS	GNRPQSTGLS	CQGRSRTPAR	TAGAPVRLDL	PAAPGAVQAR	NLSNGSVPGF	RQSHSPWFNG	TNKHTLPLAS
**650**	**660**	**670**	**680**	**690**	**700**	**710**	**720**
AASKISSRDS	KPPCRSVVPG	PPLKPAPRRT	DISELPRIPK	IRRDDGGGRR	DAAPAHGQSI	EIPSACISRL	TGREGTGQPG
**730**	**740**	**750**	**760**	**770**	**780**	**790**	**800**
RGTRAESEAS	SRVPREPGVH	TGSSRPPAPS	SHGSLAPLGP	SRGKGVGSTF	ESFRINIPGN	MAHSSQLSSP	GFCNTFRPVD
**810**	**820**	**830**	**840**	**850**	**860**	**870**	**880**
DKEQRKENPS	PLFSIKKTKQ	LRSEVYDPSD	PTGSDSSAPG	SSPERSGPGL	LPSEITRTIS	INSPKAQTVQ	AVRCVTSYTV
**890**	**900**	**910**	**920**	**930**	**940**	**950**	**960**
ESIFGTEPEP	PLGPSSAMSK	LRGAVAAEGA	SDTEREEPTE	SQGLAARLRR	PSPPEPWDEE	DGASCSTFFG	SEERTVTCVT
**970**	**980**	**990**	**1000**	**1010**	**1020**	**1030**	**1040**
VVEPEAPPSP	DVLQAATHRV	VELRPPSRSR	STSSSRSRKK	AKRKRVSREH	GRTRSGTRSE	SRDRSSRSAS	PSVGEERPRR
**1050**	**1060**	**1070**	**1080**	**1090**	**1100**	**1110**	**1120**
QRSKAKSRRS	SSDRSSSRER	AKRKKAKDKS	REHRRGPWGH	SRRTSRSRSG	SPGSSSYEHY	ESRKKKKRRS	ASRPRGRECS
**1130**	**1140**	**1150**	**1160**	**1170**	**1180**	**1190**	**1200**
PTSSLERLCR	HKHQRERSHE	RPDRKESVAW	PRDRRKRRSR	SPSSEHRARE	HRRPRSREKW	PQTRSHSPER	KGAVREASPA
**1210**	**1220**	**1230**	**1240**	**1250**	**1260**	**1270**	**1280**
PLAQGEPGRE	DLPTRLPALG	EAHVSPEVAT	ADKAPLQAPP	VLEVAAECEP	DDLDLDYGDS	VEAGHVFDDF	SSDAVFIQLD
**1290**	**1300**	**1310**	**1320**	**1330**	**1340**	**1350**	**1360**
DMSSPPSPES	TDSSPERDFP	LKPALPPASL	AVAAIQREVS	LMHDEDPSQP	PPLPEGTQEP	HLLRPDAAEK	AEAPSSPDVA
**1370**	**1380**	**1390**	**1400**	**1410**	**1420**	**1430**	**1440**
PAGKEDSPSA	SGRVQEAARP	EEVVSQTPLL	RSRALVKRVT	WNLQESESSA	PAEDRAPRAP	LHRPQKPREG	AWDMEDVAPT
**1450**	**1460**	**1470**	**1480**	**1490**	**1500**	**1510**	**1520**
GVRQVFSELP	FPSHVLPEPG	FPDTDPSQVY	SPGLPPAPAQ	PSSIPPCALV	SQPTVQFILQ	GSLPLVGCGA	AQTLAPVPAA
**1530**	**1540**	**1550**	**1560**	**1570**	**1580**	**1590**	**1600**
LTPASEPASQ	ATAASNSEEK	TPAPRLAAEK	TKKEEYMKKL	HMQERAVEEV	KLAIKPFYQK	REVTKEEYKD	ILRKAVQKIC
**1610**	**1620**	**1630**	**1640**	**1650**			
HSKSGEINPV	KVANLVKAYV	DKYRHMRRHK	KPEAGEEPPT	QGAEG			

Cmpd.	No. of Cmpds.	m/z meas.	Δ m/z [ppm]	z	Rt [min]	Score	P	Range	Sequence	Modification
1686	1	697.6744	-192.66	2	51.0	20.4	0	1-12	-.MDDDSLDELVAR.S	Oxidation: 1

Detailed Protein Report

Protein 372: NACHT, LRR and PYD domains-containing protein 2 isoform 3 [Homo sapiens]

**Accession:**	gi|291463280	**Score:**	32.1
**Database:**	refseq_human(refseq_human_20140103.fasta)	**MW [kDa]:**	117.8
**Database Date:**	2015-11-30	**pI:**	5.8
**Modification(s):**	Carbamidomethyl	**Sequence Coverage [%]:**	2.4
		**No. of unique Peptides:**	3

Quantitation

Wdown:Qdown **Median:** 0.59 **CV:** 0.00 % **No. of Peptides:** 1

```
        10         20         30         40         50         60         70         80
MVSSAQMGFN LQALLEQLSQ DELSKFKYLI TTFSLAHELQ KIPHKEMASL QVFEKMHRMD LSERAKDEVR EAALKSFNKR
        90        100        110        120        130        140        150        160
KPLSLGITRK ERPPLDVDEM LERFKTEAQA FTETKGNVIC LGKEVFKGKK PDKDNRCRYI LKTKFREMWK SWPGDSKEVQ
       170        180        190        200        210        220        230        240
VMAERYKMLI PFSNPRVLPG PFSYTVVLYG PAGLGKTTLA QKLMLDWAED NLIHKFKYAF YLSCRELSRL GPCSFAELVF
       250        260        270        280        290        300        310        320
RDWPELQDDI PHILAQARKI LFVIDGFDEL GAAPGALIED ICGDWEKKKP VPVLLGSLLN RVMLPKAALL VTTRPRALRD
       330        340        350        360        370        380        390        400
LRILAEEPIY IRVEGFLEED RRAYFLRHFG DEDQAMRAFE LMRSNAALFQ LGSAPAVCWI VCTTLKLQME KGEDPVPTCL
       410        420        430        440        450        460        470        480
TRTGLFLRFL CSRFPQGAQL RGALRTLSLL AAQGLWAQTS VLHREDLERL GVQESDLRLF LDGDILRQDR VSKGCYSFIH
       490        500        510        520        530        540        550        560
LSFQQFLTAL FYTLEKEEEE DRDGHTWDIG DVQKLLSGVE RLRNPDLIQA GYYSFGLANE KRAKELEATF GCRMSPDIKQ
       570        580        590        600        610        620        630        640
ELLRCDISCK GGHSTVTDLQ ELLGCLYESQ EEELVKEVMA QFKEISLHLN AVDVVPSSFC VKHCRNLQKM SLQVIKENLP
       650        660        670        680        690        700        710        720
ENVTASESDA EVERSQDDQH MLPFWTDLCS IFGSNKDLMG LAINDSFLSA SLVRILCEQI ASDTCHLQRV VFKNISPADA
       730        740        750        760        770        780        790        800
HRNLCLALRG HKTVTYLTLQ GNDQDDMFPA LCEVLRHPEC NLRYLGLVSC SATTQQWADL SLALEVNQSL TCVNLSDNEL
       810        820        830        840        850        860        870        880
LDEGAKLLYT TLRHPKCFLQ RLSLENCHLT EANCKDLAAV LVVSRELTHL CLAKNPIGNT GVKFLCEGLR YPECKLQTLV
       890        900        910        920        930        940        950        960
LWNCDITSDG CCDLTKLLQE KSSLLCLDLG LNHIGVKGMK FLCEALRKPL CNLRCLWLWG CSIPPFSCED LCSALSCNQS
       970        980        990       1000       1010       1020       1030       1040
LVTLDLGQNP LGSSGVKMLF ETLTCSSGTL RTLRLKIDDF NDELNKLLEE IEEKNPQLII DTEKHHPWAE RPSSHDFMI
```

Cmpd.	No. of Cmpds.	m/z meas.	Δ m/z [ppm]	z	Rt [min]	Score	P	Range	Sequence	Modification	Ratios
1358	1	727.3750	-33.15	2	45.7	10.3	1	710-722	R.VVFKNISPADAHR.N		Wdown:Qdown 0.59
1270	1	419.0769	-336.69	2	44.6	11.0	0	864-870	K.FLCEGLR.Y		
1197	1	786.4909	152.63	2	44.6	10.8	1	864-875	K.FLCEGLRYPECK.L	Carbamidomethyl: 3, 11	

Detailed Protein Report

Protein 373: integrin beta-1-binding protein 2 [Homo sapiens]

Accession: gi|6912438
Database: refseq_human(refseq_human_20140103.fasta)
Database Date: 2015-11-30

Score: 32.0
MW [kDa]: 38.4
pI: 4.9
Sequence Coverage [%]: 8.6
No. of unique Peptides: 1

10	20	30	40	50	60	70	80
MSLLCRNKGC	GQHFDPNTNL	PDSCCHHPGV	PIFHDALKGW	SCCRKRTVDF	SEFLNIKGCT	MGPHCAEKLP	EAPQPEGPAT
**90**	**100**	**110**	**120**	**130**	**140**	**150**	**160**
SSSLQEQKPL	NVIPKSAETL	RRERPKSELP	LKLLPLNISQ	ALEMALEQKE	LDQEPGAGLD	SLIRTGSSCQ	NPGCDAVYQG
**170**	**180**	**190**	**200**	**210**	**220**	**230**	**240**
PESDATPCTY	HPGAPRFHEG	MKSWSCCGIQ	TLDFGAFLAQ	PGCRVGRHDW	GKQLPASCRH	DWHQTDSLVV	VTVYGQIPLP
**250**	**260**	**270**	**280**	**290**	**300**	**310**	**320**
AFNWVKASQT	ELHVHIVFDG	NRVFQAQMKL	WGVINVEQSS	VFLMPSRVEI	SLVKADPGSW	AQLEHPDALA	KKARAGVVLE
**330**	**340**	**350**					
MDEEESDDSD	DDLSWTEEEE	EEEAMGE					

Cmpd.	No. of Cmpds.	m/z meas.	Δ m/z [ppm]	z	Rt [min]	Score	P	Range	Sequence	Modification
297	1	861.8427	115.33	3	32.8	21.3	2	46-68	K.RTVDFSEFLNIKGCTMGPHCAEK.L	

Detailed Protein Report

Protein 374: **deoxyribonuclease-1-like 2 precursor [Homo sapiens]**

**Accession:**	gi|4503347	**Score:**	32.0
**Database:**	refseq_human(refseq_human_20140103.fasta)	**MW [kDa]:**	32.8
**Database Date:**	2015-11-30	**pI:**	4.6
**Modification(s):**	Oxidation	**Sequence Coverage [%]:**	8.0
		**No. of unique Peptides:**	2

Alias proteins:

Accession	Name	Description
gi|578828006	refseq_human (refseq_human_20140103.fasta)	PREDICTED: deoxyribonuclease-1-like 2 isoform X3 [Homo sapiens]
gi|530407699	refseq_human (refseq_human_20140103.fasta)	PREDICTED: deoxyribonuclease-1-like 2 isoform X2 [Homo sapiens]

10	20	30	40	50	60	70	80
MGGPRALLAA	LWALEAAGTA	ALRIGAFNIQ	SFGDSKVSDP	ACGSIIAKIL	AGYDLALVQE	VRDPDLSAVS	ALMEQINSVS
**90**	**100**	**110**	**120**	**130**	**140**	**150**	**160**
EHEYSFVSSQ	PLGRDQYKEM	YLFVYRKDAV	SVVDTYLYPD	PEDVFSREPF	VVKFSAPGTG	ERAPPLPSRR	ALTPPPLPAA
**170**	**180**	**190**	**200**	**210**	**220**	**230**	**240**
AQNLVLIPLH	AAPHQAVAEI	DALYDVYLDV	IDKWGTDDML	FLGDFNADCS	YVRAQDWAAI	RLRSSEVFKW	LIPDSADTTV
**250**	**260**	**270**	**280**	**290**	**300**		
GNSDCAYDRI	VACGARLRRS	LKPQSATVHD	FQEEFGLDQT	QALAISDHFP	VEVTLKFHR		

Cmpd.	No. of Cmpds.	m/z meas.	Δ m/z [ppm]	z	Rt [min]	Score	P	Range	Sequence	Modification
1794	1	568.7414	-60.72	2	52.4	15.0	0	99-106	K.EMYLFVYR.K	Oxidation: 2
1326	1	939.1023	103.59	2	45.3	17.0	2	214-229	R.AQDWAAIRLRSSEVFK.W	

Detailed Protein Report

Protein 375: Golgi apparatus protein 1 isoform 3 precursor [Homo sapiens]

Accession: gi|224586817
Database: refseq_human(refseq_human_20140103.fasta)
Database Date: 2015-11-30
Modification(s): Carbamidomethyl, Oxidation

Score: 32.0
MW [kDa]: 134.5
pI: 6.5
Sequence Coverage [%]: 1.4
No. of unique Peptides: 1

10	20	30	40	50	60	70	80
MAACGRVRRM	FRLSAALHLL	LLFAAGAEKL	PGQGVHSQGQ	GPGANFVSFV	GQAGGGGPAG	QQLPQLPQSS	QLQQQQQQQQ
90	100	110	120	130	140	150	160
QQQQPQPPQP	PFPAGGPPAR	RGGAGAGGGW	KLAEEESCRE	DVTRVCPKHT	WSNNLAVLEC	LQDVREPENE	ISSDCNHLLW
170	180	190	200	210	220	230	240
NYKLNLTTDP	KFESVAREVC	KSTITEIKEC	ADEPVGKGYM	VSCLVDHRGN	ITEYQCHQYI	TKMTAIIFSD	YRLICGFMDD
250	260	270	280	290	300	310	320
CKNDINILKC	GSIRLGEKDA	HSQGEVVSCL	EKGLVKEAEE	REPKIQVSEL	CKKAILRVAE	LSSDDFHLDR	HLYFACRDDR
330	340	350	360	370	380	390	400
ERFCENTQAG	EGRVYKCLFN	HKFEESMSEK	CREALTTRQK	LIAQDYKVSY	SLAKSCKSDL	KKYRCNVENL	PRSREARLSY
410	420	430	440	450	460	470	480
LLMCLESAVH	RGRQVSSECQ	GEMLDYRRML	MEDFSLSPEI	ILSCRGEIEH	HCSGLHRKGR	TLHCLMKVVR	GEKGNLGMNC
490	500	510	520	530	540	550	560
QQALQTLIQE	TDPGADYRID	RALNEACESV	IQTACKHIRS	GDPMILSCLM	EHLYTEKMVE	DCEHRLLELQ	YFISRDWKLD
570	580	590	600	610	620	630	640
PVLYRKCQGD	ASRLCHTHGW	NETSEFMPQG	AVFSCLYRHA	YRTEEQGRRL	SRECRAEVQR	ILHQRAMDVK	LDPALQDKCL
650	660	670	680	690	700	710	720
IDLGKWCSEK	TETGQELECL	QDHLDDLVVE	CRDIVGNLTE	LESEDIQIEA	LLMRACEPII	QNFCHDVADN	QIDSGDLMEC
730	740	750	760	770	780	790	800
LIQNKHQKDM	NEKCAIGVTH	FQLVQMKDFR	FSYKFKMACK	EDVLKLCPNI	KKKVDVVICL	STTVRNDTLQ	EAKEHRVSLK
810	820	830	840	850	860	870	880
CRRQLRVEEL	EMTEDIRLEP	DLYEACKSDI	KNFCSAVQYG	NAQIIECLKE	NKKQLSTRCH	QKVFKLQETE	MMDPELDYTL
890	900	910	920	930	940	950	960
MRVCKQMIKR	FCPEADSKTM	LQCLKQNKNS	ELMDPKCKQM	ITKRQITQNT	DYRLNPMLRK	ACKADIPKFC	HGILTKAKDD
970	980	990	1000	1010	1020	1030	1040
SELEGQVISC	LKLRYADQRL	SSDCEDQIRI	IIQESALDYR	LDPQLQLHCS	DEISSLCAEE	AAAQEQTGQV	EECLKVNLLK
1050	1060	1070	1080	1090	1100	1110	1120
IKTELCKKEV	LNMLKESKAD	IFVDPVLHTA	CALDIKHHCA	AITPGRGRQM	SCLMEALEDK	RVRLQPECKK	RLNDRIEMWS
1130	1140	1150	1160	1170	1180		
YAAKVAPADG	FSDLAMQVMT	SPSKNYILSV	ISGSICILFL	IGLMCGRITK	RVTRELKDR		

Cmpd.	No. of Cmpds.	m/z meas.	Δ m/z [ppm]	z	Rt [min]	Score	P	Range	Sequence	Modification
484	1	546.2461	55.39	2	34.7	15.7	0	538-545	K.MVEDCEHR.L	Carbamidomethyl: 5; Oxidation: 1

Detailed Protein Report

Protein 376: DNA endonuclease RBBP8 isoform b [Homo sapiens]

**Accession:**	gi|42718017	**Score:**	31.9
**Database:**	refseq_human(refseq_human_20140103.fasta)	**MW [kDa]:**	98.4
**Database Date:**	2015-11-30	**pI:**	6.2
**Modification(s):**	Carbamidomethyl	**Sequence Coverage [%]:**	2.5
		**No. of unique Peptides:**	1

10	20	30	40	50	60	70	80
MNISGSSCGS	PNSADTSSDF	KDLWTKLKEC	HDREVQGLQV	KVTKLKQERI	LDAQRLEEFF	TKNQQLREQQ	KVLHETIKVL
90	100	110	120	130	140	150	160
EDRLRAGLCD	RCAVTEEHMR	KKQQEFENIR	QQNLKLITEL	MNERNTLQEE	NKKLSEQLQQ	KIENDQQHQA	AELECEEDVI
170	180	190	200	210	220	230	240
PDSPITAFSF	SGVNRLRRKE	NPHVRYIEQT	HTKLEHSVCA	NEMRKVSKSS	THPQHNPNEN	EILVADTYDQ	SQSPMAKAHG
250	260	270	280	290	300	310	320
TSSYTPDKSS	FNLATVVAET	LGLGVQEESE	TQGPMSPLGD	ELYHCLEGNH	KKQPFEESTR	NTEDSLRFSD	STSKTPPQEE
330	340	350	360	370	380	390	400
LPTRVSSPVF	GATSSIKSGL	DLNTSLSPSL	LQPGKKKHLK	TLPFSNTCIS	RLEKTRSKSE	DSALFTHHSL	GSEVNKIIIQ
410	420	430	440	450	460	470	480
SSNKQILINK	NISESLGEQN	RTEYGKDSNT	DKHLEPLKSL	GGRTSKRKKT	EEESEHEVSC	PQASFDKENA	FPFPMDNQFS
490	500	510	520	530	540	550	560
MNGDCVMDKP	LDLSDRFSAI	QRQEKSQGSE	TSKNKFRQVT	LYEALKTIPK	GFSSSRKASD	GNCTLPKDSP	GEPCSQECII
570	580	590	600	610	620	630	640
LQPLNKCSPD	NKPSLQIKEE	NAVFKIPLRP	RESLETENVL	DDIKSAGSHE	PIKIQTRSDH	GGCELASVLQ	LNPCRTGKIK
650	660	670	680	690	700	710	720
SLQNNQDVSF	ENIQWSIDPG	ADLSQYKMDV	TVIDTKDGSQ	SKLGGETVDM	DCTLVSETVL	LKMKKQEQKG	EKSSNEERKM
730	740	750	760	770	780	790	800
NDSLEDMFDR	TTHEEYESCL	ADSFSQAADE	EEELSTATKK	LHTHGDKQDK	VKQKAFVEPY	FKGDESIMQI	CQQKKEKRNW
810	820	830	840	850	860	870	
LPAQDTDSAT	FHPTHQRIFG	KLVFLPLRLV	WKEVILRKIL	ILVLVQKDVS	LTTQYFLQKA	RSRRHRR	

Cmpd.	No. of Cmpds.	m/z meas.	Δ m/z [ppm]	z	Rt [min]	Score	P	Range	Sequence	Modification
2621	1	673.3610	86.09	2	63.5	12.5	0	194-204	K.LEHSVCANEMR.K	Carbamidomethyl: 6

Detailed Protein Report

Protein 377: **PREDICTED: mediator of RNA polymerase II transcription subunit 12 isoform X3 [Homo sapiens]**

**Accession:**	gi|530421861	**Score:**	31.8
**Database:**	refseq_human(refseq_human_20140103.fasta)	**MW [kDa]:**	240.6
**Database Date:**	2015-11-30	**pI:**	6.7
		**Sequence Coverage [%]:**	1.5
		**No. of unique Peptides:**	1

Detailed Protein Report

10	20	30	40	50	60	70	80
MAAFGILSYE	HRPLKRPRLG	PPDVYPQDPK	QKEDELTALN	VKQGFNNQPA	VSGDEHGSAK	NVSFNPAKIS	SNFSSIIAEK
90	100	110	120	130	140	150	160
LRCNTLPDTG	RRKPQVNQKD	NFWLVTARSQ	SAINTWFTDL	AGTKPLTQLA	KKVPIFSKKE	EVFGYLAKYT	VPVMRAAWLI
170	180	190	200	210	220	230	240
KMTCAYYAAI	SETKVKKRHV	DPFMEWTQII	TKYLWEQLQK	MAEYYRPGPA	GSGGCGSTIG	PLPHDVEVAI	RQWDYTEKLA
250	260	270	280	290	300	310	320
MFMFQDGMLD	RHEFLTWVLE	CFEKIRPGED	ELLKLLLPLL	LRYSGEFVQS	AYLSRRLAYF	CTRRLALQLD	GVSSHSSHVI
330	340	350	360	370	380	390	400
SAQSTSTLPT	TPAPQPPTSS	TPSTPFSDLL	MCPQHRPLVF	GLSCILQTIL	LCCPSALVWH	YSLTDSRIKT	GSPLDHLPIA
410	420	430	440	450	460	470	480
PSNLPMPEGN	SAFTQQVRAK	LREIEQQIKE	RGQAVEVRWS	FDKCQEATAG	FTIGRVLHTL	EVLDSHSFER	SDFSNSLDSL
490	500	510	520	530	540	550	560
CNRIFGLGPS	KDGHEISSDD	DAVVSLLCEW	AVSCKRSGRH	RAMVVAKLLE	KRQAEIEAER	CGESEAADEK	GSIASGSLSA
570	580	590	600	610	620	630	640
PSAPIFQDVL	LQFLDTQAPM	LTDPRSESER	VEFFNLVLLF	CELIRHDVFS	HNMYTCTLIS	RGDLAFGAPG	PRPPSPFDDP
650	660	670	680	690	700	710	720
ADDPEHKEAE	GSSSSKLEDP	GLSESMDIDP	SSSVLFEDME	KPDFSLFSPT	MPCEGKGSPS	PEKPDVEKEV	KPPPKEKIEG
730	740	750	760	770	780	790	800
TLGVLYDQPR	HVQYATHFPI	PQEESCSHEC	NQRLVVLFGV	GKQRDDARHA	IKKITKDILK	VLNRKGTAET	DQLAPIVPLN
810	820	830	840	850	860	870	880
PGDLTFLGGE	DGQKRRRNRP	EAFPTAEDIF	AKFQHLSHYD	QHQVTAQVSR	NVLEQITSFA	LGMSYHLPLV	QHVQFIFDLM
890	900	910	920	930	940	950	960
EYSLSISGLI	DFAIQLLNEL	SVVEAELLLK	SSDLVGSYTT	SLCLCIVAVL	RHYHACLILN	QDQMAQVFEG	LCGVVKHGMN
970	980	990	1000	1010	1020	1030	1040
RSDGSSAERC	ILAYLYDLYT	SCSHLKNKFG	ELFSDFCSKV	KNTIYCNVEP	SESNMRWAPE	FMIDTLENPA	AHTFTYTGLG
1050	1060	1070	1080	1090	1100	1110	1120
KSLSENPANR	YSFVCNALMH	VCVGHHDPDR	VNDIAILCAE	LTGYCKSLSA	EWLGVLKALC	CSSNNGTCGF	NDLLCNVDVS
1130	1140	1150	1160	1170	1180	1190	1200
DLSFHDSLAT	FVAILIARQC	LLLEDLIRCA	AIPSLLNAAC	SEQDSEPGAR	LTCRILLHLF	KTPQLNPCQS	DGNKPTVGIR
1210	1220	1230	1240	1250	1260	1270	1280
SSCDRHLLAA	SQNRIVDGAV	FAVLKAVFVL	GDAELKGSGF	TVTGGTEELP	EEEGGGGSGG	RRQGGRNISV	ETASLDVYAK
1290	1300	1310	1320	1330	1340	1350	1360
YVLRSICQQE	WVGERCLKSL	CEDSNDLQDP	VLSSAQAQRL	MQLICYPHRL	LDNEDGENPQ	RQRIKRILQN	LDQWTMRQSS
1370	1380	1390	1400	1410	1420	1430	1440
LELQLMIKQT	PNNEMNSLLE	NIAKATIEVF	QQSAETGSSS	GSTASNMPSS	SKTKPVLSSL	ERSGVWLVAP	LIAKLPTSVQ
1450	1460	1470	1480	1490	1500	1510	1520
GHVLKAAGEE	LEKGQHLGSS	SRKERDRQKQ	KSMSLLSQQP	FLSLVLTCLK	GQDEQREGLL	TSLYSQVHQI	VNNWRDDQYL
1530	1540	1550	1560	1570	1580	1590	1600
DDCKPKQLMH	EALKLRLNLV	GGMFDTVQRS	TQQTTEWAML	LLEIIISGTV	DMQSNNELFT	TVLDMLSVLI	NGTLAADMSS
1610	1620	1630	1640	1650	1660	1670	1680
ISQGSMEENK	RAYMNLAKKL	QKELGERQSD	SLEKVRQLLP	LPKQTRDVIT	CEPQGSLIDT	KGNKIAGFDS	IFKKEGLQVS
1690	1700	1710	1720	1730	1740	1750	1760
TKQKISPWDL	FEGLKPSAPL	SWGWFGTVRV	DRRVARGEEQ	QRLLLYHTHL	RPRPRAYYLE	PLPLPPEDEE	PPAPTLLEPE
1770	1780	1790	1800	1810	1820	1830	1840
KKAPEPPKTD	KPGAAPPSTE	ERKKKSTKGK	KRSQPATKTE	DYGMGPGRSG	PYGVTVPPDL	LHHPNPGSIT	HLNYRQGSIG
1850	1860	1870	1880	1890	1900	1910	1920
LYTQNQPLPA	GGPRVDPYRP	VRLPMQKLPT	RPTYPGVLPT	TMTGVMGLEP	SSYKTSVYRQ	QQPAVPQGQR	LRQQLQAKIQ
1930	1940	1950	1960	1970	1980	1990	2000
SQGMLGQSSV	HQMTPSSSYG	LQTSQGYTPY	VSHVGLQQHT	GPADPTRHLQ	QRPSGYVHQQ	APTYGHGLTS	TQRFSHQTLQ
2010	2020	2030	2040	2050	2060	2070	2080
QTPMISTMTP	MSAQGVQAGV	RSTAILPEQQ	QQQQQQQQQQ	QQQQQQQQQQ	QQQQYHIRQQ	QQQQILRQQQ	QQQQQQQQQQ
2090	2100	2110	2120	2130	2140	2150	2160
QQQQQQQQQQ	QQQHQQQQQQ	QAAPPQPQPQ	SQPQFQRQGL	QQTQQQQQTA	ALVRQLQQQL	SNTQPQPSTN	IFGRY

Detailed Protein Report

Cmpd.	No. of Cmpds.	m/z meas.	Δ m/z [ppm]	z	Rt [min]	Score	P	Range	Sequence	Modification
1964	5	753.7894	-104.99	2	54.7	14.5	1	989-1001	K.FGELFSDFCSKVK.N	

Detailed Protein Report

Protein 378: **PREDICTED: uncharacterized protein LOC102725205, partial [Homo sapiens]**

**Accession:**	gi|578845222	**Score:**	31.8
**Database:**	refseq_human(refseq_human_20140103.fasta)	**MW [kDa]:**	95.5
**Database Date:**	2015-11-30	**pI:**	6.0
**Modification(s):**	Oxidation	**Sequence Coverage [%]:**	3.3
		**No. of unique Peptides:**	3

```
        10         20         30         40         50         60         70         80
TLSHTLHPPT HTPNLTHSTH THTTPPTHTP HPTHHSTPLT HSTPHTSHTH STPLTHSTPH SWAAKSLERL LWVVASSPPR
        90        100        110        120        130        140        150        160
RTAVVGRIPG AGMPAGAGGP ACRCPCLSRV GWRDLDAEPS SQEFRQLSLD SHQVAAFQTE MECWARGSGM DATEPLCCDC
       170        180        190        200        210        220        230        240
QTSDLVCWSQ QGHQGPERGA LGSLEEPGLL SAREAWPVSC SSIFLEVSGR RLESRMPPGG AAINISPGDK RTNQSPAGYR
       250        260        270        280        290        300        310        320
GEDKSGKRLM SELWLQVAAA RDGLQDFPGL LVAGRGSSSG DWSERAYGWE LEELMEKRGR VGWSSREALW SGEGEEEPNK
       330        340        350        360        370        380        390        400
EKQQSTWNIS IPPSKAVLEE QPQSPIVPKS GKSVKTFIRN RYEPAKRCEE LICAELIRMN KVTTDSAMGI ADKDLSEELQ
       410        420        430        440        450        460        470        480
HRLQHALASL QVAQGEDLTA DIMYSPSASE PLPFMQCDGE QETGTASPIW LWNLWAGESH PHGVHALATL GWEGPMVNAG
       490        500        510        520        530        540        550        560
EKVQEEEGAP ATGPSCGQHR EGCCGGEDVG VEGCGGGHLL ALGCPSKTLT ARGILPVGGA ASTTSLQEAM FVFRTTLIRA
       570        580        590        600        610        620        630        640
VGCKEQRPLK GEGGAVKDVE SRRCAGLWFR EPCGRQVKME LHKSAGETPG KLKVEYADGG MPEARLWEGT HIFETKVKLS
       650        660        670        680        690        700        710        720
TVIHEAAVQW PSGDIEKTHL SSGLFSELIR GLCSFDMFLP DTVVCWPLPW SAFHPVLLQT MSAPGLSPAL DPLCPQLLAD
       730        740        750        760        770        780        790        800
LGSEKQSCPS REFGWGLAEV PSVSNLCKGG LALQAADGGS GGGVRTPSYY LNECMGITAA LYEHLKTTLS NPSFSKEKES
       810        820        830        840        850        860        870        880
VNFPFSMHEV TGVCHSGGPV FKKNREETVR LSAGGAARQK ELMLYPVVSF PKGLHQLPSF LALIMTISVV TSLLSKESIF
       890
SSCQE
```

Cmpd.	No. of Cmpds.	m/z meas.	Δ m/z [ppm]	z	Rt [min]	Score	P	Range	Sequence	Modification
2662	1	933.9882	34.09	2	64.5	10.3	0	194-210	R.EAWPVSCSSIFLEVSGR.R	
992	2	647.6724	-181.96	2	40.9	10.3	0	614-625	K.VEYADGGMPEAR.L	
2897	1	655.6487	-212.02	2	65.0	11.2	0	614-625	K.VEYADGGMPEAR.L	Oxidation: 8

Detailed Protein Report

Protein 379: transient receptor potential cation channel subfamily M member 8 [Homo sapiens]

Accession: gi|109689695
Database: refseq_human(refseq_human_20140103.fasta)
Database Date: 2015-11-30
Modification(s): Carbamidomethyl

Score: 31.7
MW [kDa]: 127.6
pI: 7.0
Sequence Coverage [%]: 1.5
No. of unique Peptides: 1

10	20	30	40	50	60	70	80
MSFRAARLSM	RNRRNDTLDS	TRTLYSSASR	STDLSYSESD	LVNFIQANFK	KRECVFFTKD	SKATENVCKC	GYAQSQHMEG
90	100	110	120	130	140	150	160
TQINQSEKWN	YKKHTKEFPT	DAFGDIQFET	LGKKGKYIRL	SCDTDAEILY	ELLTQHWHLK	TPNLVISVTG	GAKNFALKPR
170	180	190	200	210	220	230	240
MRKIFSRLIY	IAQSKGAWIL	TGGTHYGLMK	YIGEVVRDNT	ISRSSEENIV	AIGIAAWGMV	SNRDTLIRNC	DAEGYFLAQY
250	260	270	280	290	300	310	320
LMDDFTRDPL	YILDNNHTHL	LLVDNGCHGH	PTVEAKLRNQ	LEKYISERTI	QDSNYGGKIP	IVCFAQGGGK	ETLKAINTSI
330	340	350	360	370	380	390	400
KNKIPCVVVE	GSGQIADVIA	SLVEVEDALT	SSAVKEKLVR	FLPRTVSRLP	EEETESWIKW	LKEILECSHL	LTVIKMEEAG
410	420	430	440	450	460	470	480
DEIVSNAISY	ALYKAFSTSE	QDKDNWNGQL	KLLLEWNQLD	LANDEIFTND	RRWESADLQE	VMFTALIKDR	PKFVRLFLEN
490	500	510	520	530	540	550	560
GLNLRKFLTH	DVLTELFSNH	FSTLVYRNLQ	IAKNSYNDAL	LTFVWKLVAN	FRRGFRKEDR	NGRDEMDIEL	HDVSPITRHP
570	580	590	600	610	620	630	640
LQALFIWAIL	QNKKELSKVI	WEQTRGCTLA	ALGASKLLKT	LAKVKNDINA	AGESEELANE	YETRAVELFT	ECYSSDEDLA
650	660	670	680	690	700	710	720
EQLLVYSCEA	WGGSNCLELA	VEATDQHFIA	QPGVQNFLSK	QWYGEISRDT	KNWKIILCLF	IIPLVGCGFV	SFRKKPVDKH
730	740	750	760	770	780	790	800
KKLLWYYVAF	FTSPFVVFSW	NVVFYIAFLL	LFAYVLLMDF	HSVPHPPELV	LYSLVFVLFC	DEVRQWYVNG	VNYFTDLWNV
810	820	830	840	850	860	870	880
MDTLGLFYFI	AGIVFRLHSS	NKSSLYSGRV	IFCLDYIIFT	LRLIHIFTVS	RNLGPKIIML	QRMLIDVFFF	LFLFAVWMVA
890	900	910	920	930	940	950	960
FGVARQGILR	QNEQRWRWIF	RSVIYEPYLA	MFGQVPSDVD	GTTYDFAHCT	FTGNESKPLC	VELDEHNLPR	FPEWITIPLV
970	980	990	1000	1010	1020	1030	1040
CIYMLSTNIL	LVNLLVAMFG	YTVGTVQENN	DQVWKFQRYF	LVQEYCSRLN	IPFPFIVFAY	FYMVVKKCFK	CCCKEKNMES
1050	1060	1070	1080	1090	1100	1110	
SVCCFKNEDN	ETLAWEGVMK	ENYLVKINTK	ANDTSEEMRH	RFRQLDTKLN	DLKGLLKEIA	NKIK	

Cmpd.	No. of Cmpds.	m/z meas.	Δ m/z [ppm]	z	Rt [min]	Score	P	Range	Sequence	Modification
2072	1	731.2846	-37.73	2	56.1	12.0	1	1035-1046	K.EKNMESSVCCFK.N	Carbamidomethyl: 9

Detailed Protein Report

Protein 380: PREDICTED: transcription factor TFIIIB component B" homolog isoform X2 [Homo sapiens]

**Accession:**	gi|578840763	**Score:**	31.7
**Database:**	refseq_human(refseq_human_20140103.fasta)	**MW [kDa]:**	289.3
**Database Date:**	2015-11-30	**pI:**	4.9
**Modification(s):**	Carbamidomethyl	**Sequence Coverage [%]:**	1.5
		**No. of unique Peptides:**	2

Quantitation

mdown:qdown	**Median:**	1.23	**CV:**	0.00 %	**No. of Peptides:**	1
Wdown:Qdown	**Median:**	1.02	**CV:**	0.00 %	**No. of Peptides:**	1

Detailed Protein Report

10	20	30	40	50	60	70	80
MFRRARLSVK	PNVRPGVGAR	GSTASNPQRG	RESPRPPEPA	TDSASKPAEP	TDVPTVDFGG	AEPQEKAPRS	STEKTGGDND
90	100	110	120	130	140	150	160
VEESSRSSST	VSQRRKRISS	TSSLVKSSVS	VPSESHPLST	INQEAPQPTA	TSTKEKQPCS	DRYRIYKAQK	LREMLKEELR
170	180	190	200	210	220	230	240
KEKKQWKNKY	AINESQRPPD	RSKMTMRDFI	YYLPDNNPMT	SSLEQEKKTE	KPSTPVQTRE	QEGKSTPNAE	DNEMEEETDD
250	260	270	280	290	300	310	320
GPLLVPRVKV	AEDGSIILDE	ESLTVEVLRT	KGPCVVEEND	PIFERGSTTT	YSSFRKNYYS	KPWSNKETDM	FFLAISMVGT
330	340	350	360	370	380	390	400
DFSMIGQLFP	HRARIEIKNK	FKREEKTNGW	RIDKAFQEKR	PFDFDFFAHL	LQKVLAEEEK	RKQKSVKNHS	LKEKKSTKPR
410	420	430	440	450	460	470	480
KNVKVKKVAC	EGVNNDPDES	MSSRISDTER	SQKDAQTVEE	ESLTLSREDA	EQVALEVDLN	QKKRRRKKQD	GANELGVNNL
490	500	510	520	530	540	550	560
LENATVQAGP	SKGEKHKNKC	QAIRPELKEG	ECSKEQMLSC	TQNIDGIVGF	ASTEKVEKRT	DPILSLSNQQ	DATSVATESS
570	580	590	600	610	620	630	640
ESSTSDLPSF	EVGIRALCEV	NNAEGSCIEE	RNVDLKNNSL	EIDQTENVKP	MLRGRFQRPK	PNLSRAGKKS	VLSQGKTESE
650	660	670	680	690	700	710	720
SKNSHSKTSV	EKNHVEKDKM	NTLDILRMET	TERENPEAET	VSVLGEKNCL	QEGSQLKALR	PVQVRGRLQK	PKPNAGKAAE
730	740	750	760	770	780	790	800
RKEILISQEE	IGANVEKNEN	ESCADRDTPQ	HMEDQSRKDF	EEEDVILQPE	KNDSFQNVQP	DEPKVLNECL	SVQENNKANK
810	820	830	840	850	860	870	880
LNQVPILRTR	FQKPKPNIGR	GTGRREISSK	EEVLEKILVS	GEMAAALRET	VRLDTSPKEM	VPAEINTKEM	QSDLKETGRR
890	900	910	920	930	940	950	960
AISPREKILD	VIDDTIEMET	GLKAMGREIC	LREKTPEVID	ATEEIDKDLE	EAGRREISPQ	KNGPEEVKPL	GEVETDLKAT
970	980	990	1000	1010	1020	1030	1040
GNESSPREKT	PEVTDATEEI	DKNLEETGRR	KISPRENGPE	EVKPVDEMET	DLNATGRESS	PREKTPEVID	ATEEIDLEET
1050	1060	1070	1080	1090	1100	1110	1120
EREVSPQENG	LEEVKPLGEM	ETDLKATGRD	SFPRGKTPEV	IDAIEEIEID	LEETEREISP	QENGLEEVKP	LGEMQTDLKA
1130	1140	1150	1160	1170	1180	1190	1200
TGREISPREK	TPEVIDATEE	IDKDLEETGR	REISPEENGP	EEVKPVDEME	TDLKTTGREG	SSREKTREVI	DAAEVIETDL
1210	1220	1230	1240	1250	1260	1270	1280
EETEREISPQ	ENGPEEVKPV	GKMETDLKEI	REEISQREKV	LAEFSAIREK	EIDLKETGKR	DIPIMEKVSG	KMAVVEEMEA
1290	1300	1310	1320	1330	1340	1350	1360
DLKETGKENF	RERGSEEICV	TEEKVAELKQ	TGKTDISPRE	NELEETSTSR	QTDTHLMQSG	SNDFSAVPSL	DIQNISSEVL
1370	1380	1390	1400	1410	1420	1430	1440
SMMHTPVEEK	RNSEKEVSSH	FSHFKISSQT	HESDKTEVQG	IQSPDVPEQF	SDINLSKSLP	QEQKPLEIKP	APFVRSRFKR
1450	1460	1470	1480	1490	1500	1510	1520
PKPNLARAAL	KRETTESEKY	IYEKKSETKK	METIVMQENN	EQTDTLPSQH	DEASLMISRE	KDTLGHRNEE	AVILPCTQTE
1530	1540	1550	1560	1570	1580	1590	1600
RNLSPSNSCE	PKEESQSAPV	QKNDSVVSVG	TNNVNTFQQE	MKESVIQTAR	QVRGRLQRPR	PNIRKTGQRQ	IVDKGEAKGI
1610	1620	1630	1640	1650	1660	1670	1680
IKEGRTILPK	DETEKKVLTV	SNSQIETEIE	VPSSAVPEHR	MYENQSQVVL	VENLHVNKTN	ETIRHENKPY	VPSSAQMTRR
1690	1700	1710	1720	1730	1740	1750	1760
KFQKAKPNLG	RAHSKKEEPV	LEKVTTDQSK	EGKPEDHLLQ	KGASNTQLLL	KEKAELLTSL	EVSARKDCVG	SKESALAKID
1770	1780	1790	1800	1810	1820	1830	1840
AELEEVGPSR	RVGEETVGDN	SPSSVVEEQY	LNKLTSCPQP	LNETSYSKIA	LDGKTTISST	SEYERNRGER	RSHKKFKPNV
1850	1860	1870	1880	1890	1900	1910	1920
TRGRGSKRVR	GKTSKKEPRA	SKAMLVTLRA	SQEEDDDADD	FESDYEEESY	HLAPEEVNKA	PVFVPVGLRS	PEPVSAQIEE
1930	1940	1950	1960	1970	1980	1990	2000
TMEELEITVN	VPDVGCIAVV	EHELPNTDVT	TEEMKQEENL	SVPFEMTTSE	HIQDEPGTND	GSTEAAITLL	TMGDLVLQSE
2010	2020	2030	2040	2050	2060	2070	2080
ISSEQGDVGV	CIIPHVHSKD	KSHIPSSLDN	VNHKIVHECQ	ELSSPVITTS	PASFEENKIV	LEEQSSREEI	SLMEKVKENA
2090	2100	2110	2120	2130	2140	2150	2160
TPTRNTISKV	TSNLRIRSRL	AKPKPNLEKT	LGTNRLDDYQ	EVSSLCVTKG	AEMETQRETE	KNASKATELE	NKNLGPVTTA
2170	2180	2190	2200	2210	2220	2230	2240
ENKDQSKLAC	VHGIKGTSIS	SEVNLTERNE	NQEESSQEVH	MLSVAPVASS	ETGPCTLGLD	RGLGENSVEE	PQIKDSKGDS
2250	2260	2270	2280	2290	2300	2310	2320
VLTLPVPEYT	PTSIPEVQQE	NIINPQDLTV	NLVANVPQDG	EDEQAFILTL	VEIPANAVEE	FTDATAQFMP	NPLLPAPILV
2330	2340	2350	2360	2370	2380	2390	2400

Detailed Protein Report

Cmpd.	No. of Cmpds.	m/z meas.	Δ m/z [ppm]	z	Rt [min]	Score	P	Range	Sequence	Modification	Ratios
138	1	613.7562	-197.90	2	31.0	10.7	1	1438-1447	R.FKRPKPNLAR.A		Wdown:Qdown 1.02<br>mdown:qdown 1.23
2728	1	806.1966	-169.47	2	65.4	11.0	0	2392-2404	R.DDCQEYTTEVHSK.E	Carbamidomethyl: 3	

Detailed Protein Report

Protein 381: **PREDICTED: arf-GAP with GTPase, ANK repeat and PH domain-containing protein 3 isoform X2 [Homo sapiens]**

**Accession:**	gi|530386889	**Score:**	31.7
**Database:**	refseq_human(refseq_human_20140103.fasta)	**MW [kDa]:**	61.7
**Database Date:**	2015-11-30	**pI:**	11.8
		**Sequence Coverage [%]:**	5.2
		**No. of unique Peptides:**	1

10	20	30	40	50	60	70	80
MERGWPQGDS	CPGERPAACR	RAHSVCDSLD	LHGASAGRAA	AALQAALCAA	SEQPARPRSV	CSGGPEPPPT	GARGLLLGLL
**90**	**100**	**110**	**120**	**130**	**140**	**150**	**160**
RPRLGRRGLA	PSGPPVSPAP	SPASSPAPTR	RSRTRGEPTP	RPRPASMTFL	EVNRLELAAA	EAPGAGLGRA	GSAGFLRGAA
**170**	**180**	**190**	**200**	**210**	**220**	**230**	**240**
LWSSQRWPVL	RGGRGPEGPR	RGLAALRKSF	SFRLRRGQEV	RRSESGLLAR	PPRARTRSDG	DAGSLGAFPS	RRDLLGSDAP
**250**	**260**	**270**	**280**	**290**	**300**	**310**	**320**
RAAPEPGRPR	TAAGLWRLLT	SRFRRREPAP	AAPLWGRRAA	AAPELLRAPS	DSFVNSQEWT	LSRSVPELKV	GIVGNLSSGK
**330**	**340**	**350**	**360**	**370**	**380**	**390**	**400**
SALVHRYLTG	TYVQEESPEG	GRFKKEIVVD	GQSYLLLIRD	EGGPPELQFA	AWVDAVVFVF	SLEDEISFQT	VYNYFLRLCS
**410**	**420**	**430**	**440**	**450**	**460**	**470**	**480**
FRNASEVPMV	LVGTQDAISA	ANPRVIDDSR	ARKLSTDLKR	CTYYETCATY	GLNVERVFQD	VAQKVVALRK	KQQLAIGPCK
**490**	**500**	**510**	**520**	**530**	**540**	**550**	**560**
SLPNSPSHSA	VSAASIPAVH	INQATNGGGS	AFSDYSSSVP	STPSISQREL	RIETIAASST	PTPIRKQSKR	RSNIFTICAT
**570**	**580**						
VSNFSSTKRP	FQLLPN						

Cmpd.	No. of Cmpds.	m/z meas.	Δ m/z [ppm]	z	Rt [min]	Score	P	Range	Sequence	Modification
1530	1	873.6409	147.98	2	47.7	10.1	1	345-359	K.KEIVVDGQSYLLLIR.D	

Detailed Protein Report

Protein 382: ras and Rab interactor 2 isoform 2 [Homo sapiens]

**Accession:**	gi|18466802	**Score:**	31.7
**Database:**	refseq_human(refseq_human_20140103.fasta)	**MW [kDa]:**	100.1
**Database Date:**	2015-11-30	**pI:**	6.1
		**Sequence Coverage [%]:**	2.0
		**No. of unique Peptides:**	1

Alias proteins:

Accession	Name	Description
gi|578835435	refseq_human (refseq_human_20140103.fasta)	PREDICTED: ras and Rab interactor 2 isoform X10 [Homo sapiens]
gi|578835433	refseq_human (refseq_human_20140103.fasta)	PREDICTED: ras and Rab interactor 2 isoform X9 [Homo sapiens]
gi|578835431	refseq_human (refseq_human_20140103.fasta)	PREDICTED: ras and Rab interactor 2 isoform X8 [Homo sapiens]
gi|578835429	refseq_human (refseq_human_20140103.fasta)	PREDICTED: ras and Rab interactor 2 isoform X7 [Homo sapiens]
gi|530425753	refseq_human (refseq_human_20140103.fasta)	PREDICTED: ras and Rab interactor 2 isoform X3 [Homo sapiens]

```
        10         20         30         40         50         60         70         80
MTAWTMGARG LDKRGSFFKL IDTIASEIGE LKQEMVRTDV NLENGLEPAE THSMVRHKDG GYSEEEDVKT CARDSGYDSL
        90        100        110        120        130        140        150        160
SNRLSILDRL LHTHPIWLQL SLSEEEAAEV LQAQPPGIFL VHKSTKMQKK VLSLRLPCEF GAPLKEFAIK ESTYTFSLEG
       170        180        190        200        210        220        230        240
SGISFADLFR LIAFYCISRD VLPFTLKLPY AISTAKSEAQ LEELAQMGLN FWSSPADSKP PNLPPPHRPL SSDGVCPASL
       250        260        270        280        290        300        310        320
RQLCLINGVH SIKTRTPSEL ECSQTNGALC FINPLFLKVH SQDLSGGLKR PSTRTPNANG TERTRSPPPR PPPPAINSLH
       330        340        350        360        370        380        390        400
TSPRLARTET QTSMPETVNH NKHGNVALPG TKPTPIPPPR LKKQASFLEA EGGAKTLSGG RPGAGPELEL GTAGSPGGAP
       410        420        430        440        450        460        470        480
PEAAPGDCTR APPPSSESRP PCHGGRQRLS DMSISTSSSD SLEFDRSMPL FGYEADTNSS LEDYEGESDQ ETMAPPIKSK
       490        500        510        520        530        540        550        560
KKRSSSFVLP KLVKSQLQKV SGVFSSFMTP EKRMVRRIAE LSRDKCTYFG CLVQDYVSFL QENKECHVSS TDMLQTIRQF
       570        580        590        600        610        620        630        640
MTQVKNYLSQ SSELDPPIES LIPEDQIDVV LEKAMHKCIL KPLKGHVEAM LKDFHMADGS WKQLKENLQL VRQRNPQELG
       650        660        670        680        690        700        710        720
VFAPTPDFVD VEKIKVKFMT MQKMYSPEKK VMLLLRVCKL IYTVMENNSG RMYGADDFLP VLTYVIAQCD MLELDTEIEY
       730        740        750        760        770        780        790        800
MMELLDPSLL HGEGGYYLTS AYGALSLIKN FQEEQAARLL SSETRDTLRQ WHKRRTTNRT IPSVDDFQNY LRVAFQEVNS
       810        820        830        840        850        860        870        880
GCTGKTLLVR PYITTEDVCQ ICAEKFKVGD PEEYSLFLFV DETWQQLAED TYPQKIKAEL HSRPQPHIFH FVYKRIKNDP
       890        900
YGIIFQNGEE DLTTS
```

Cmpd.	No. of Cmpds.	m/z meas.	Δ m/z [ppm]	z	Rt [min]	Score	P	Range	Sequence	Modification
2319	1	719.3506	-2.64	2	57.7	16.1	1	1-13	-.MTAWTMGARGLDK.R	

Detailed Protein Report

Protein 383: mothers against decapentaplegic homolog 3 isoform 1 [Homo sapiens]

**Accession:**	gi|5174513	**Score:**	31.7
**Database:**	refseq_human(refseq_human_20140103.fasta)	**MW [kDa]:**	48.0
**Database Date:**	2015-11-30	**pI:**	6.8
**Modification(s):**	Carbamidomethyl, Oxidation	**Sequence Coverage [%]:**	7.1
		**No. of unique Peptides:**	2

Quantitation

mdown:qdown **Median:** 0.97 **CV:** 0.00 % **No. of Peptides:** 1

10	20	30	40	50	60	70	80
MSSILPFTPP	IVKRLLGWKK	GEQNGQEEKW	CEKAVKSLVK	KLKKTGQLDE	LEKAITTQNV	NTKCITIPRS	LDGRLQVSHR
**90**	**100**	**110**	**120**	**130**	**140**	**150**	**160**
KGLPHVIYCR	LWRWPDLHSH	HELRAMELCE	FAFNMKKDEV	CVNPYHYQRV	ETPVLPPVLV	PRHTEIPAEF	PPLDDYSHSI
**170**	**180**	**190**	**200**	**210**	**220**	**230**	**240**
PENTNFPAGI	EPQSNIPETP	PPGYLSEDGE	TSDHQMNHSM	DAGSPNLSPN	PMSPAHNNLD	LQPVTYCEPA	FWCSISYYEL
**250**	**260**	**270**	**280**	**290**	**300**	**310**	**320**
NQRVGETFHA	SQPSMTVDGF	TDPSNSERFC	LGLLSNVNRN	AAVELTRRHI	GRGVRLYYIG	GEVFAECLSD	SAIFVQSPNC
**330**	**340**	**350**	**360**	**370**	**380**	**390**	**400**
NQRYGWHPAT	VCKIPPGCNL	KIFNNQEFAA	LLAQSVNQGF	EAVYQLTRMC	TIRMSFVKGW	GAEYRRQTVT	STPCWIELHL
**410**	**420**	**430**					
NGPLQWLDKV	LTQMGSPSIR	CSSVS					

Cmpd.	No. of Cmpds.	m/z meas.	Δ m/z [ppm]	z	Rt [min]	Score	P	Range	Sequence	Modification	Ratios
1610	1	1100.1365	-2.93	2	50.0	20.3	2	1-19	-.MSSILPFTPPIVKRLLGWK.K	Oxidation: 1	
1152	2	644.3310	-22.52	2	43.1	11.4	1	64-74	K.CITIPRSLDGR.L	Carbamidomethyl: 1	mdown:qdown 0.97

Detailed Protein Report

Protein 384: serine/threonine-protein kinase WNK3 isoform 2 [Homo sapiens]

**Accession:**	gi|50845416	**Score:**	31.6
**Database:**	refseq_human(refseq_human_20140103.fasta)	**MW [kDa]:**	191.7
**Database Date:**	2015-11-30	**pI:**	5.4
		**Sequence Coverage [%]:**	2.8
		**No. of unique Peptides:**	2

Alias proteins:

Accession	Name	Description
gi|578838231	refseq_human (refseq_human_20140103.fasta)	PREDICTED: serine/threonine-protein kinase WNK3 isoform X2 [Homo sapiens]
gi|578838229	refseq_human (refseq_human_20140103.fasta)	PREDICTED: serine/threonine-protein kinase WNK3 isoform X1 [Homo sapiens]

Detailed Protein Report

10	20	30	40	50	60	70	80
MATDSGDPAS	TEDSEKPDGI	SFENRVPQVA	ATLTVEARLK	EKNSTFSASG	ETVERKRFFR	KSVEMTEDDK	VAESSPKDER
90	100	110	120	130	140	150	160
IKAAMNIPRV	DKLPSNVLRG	GQEVKYEQCS	KSTSEISKDC	FKEKNEKEME	EEAEMKAVAT	SPSGRFLKFD	IELGRGAFKT
170	180	190	200	210	220	230	240
VYKGLDTETW	VEVAWCELQD	RKLTKAEQQR	FKEEAEMLKG	LQHPNIVRFY	DSWESILKGK	KCIVLVTELM	TSGTLKTYLK
250	260	270	280	290	300	310	320
RFKVMKPKVL	RSWCRQILKG	LQFLHTRTPP	IIHRDLKCDN	IFITGPTGSV	KIGDLGLATL	MRTSFAKSVI	GTPEFMAPEM
330	340	350	360	370	380	390	400
YEEHYDESVD	VYAFGMCMLE	MATSEYPYSE	CQNAAQIYRK	VTSGIKPASF	NKVTDPEVKE	IIEGCIRQNK	SERLSIRDLL
410	420	430	440	450	460	470	480
NHAFFAEDTG	LRVELAEEDD	CSNSSLALRL	WVEDPKKLKG	KHKDNEAIEF	SFNLETDTPE	EVAYEMVKSG	FFHESDSKAV
490	500	510	520	530	540	550	560
AKSIRDRVTP	IKKTREKKPA	GCLEERRDSQ	CKSMGNVFPQ	PQNTTLPLAP	AQQTGAECEE	TEVDQHVRQQ	LLQRKPQQHC
570	580	590	600	610	620	630	640
SSVTGDNLSE	AGAASVIHSD	TSSQPSVAYS	SNQTMGSQMV	SNIPQAEVNV	PGQIYSSQQL	VGHYQQVSGL	QKHSKLTQPQ
650	660	670	680	690	700	710	720
ILPLVQGQST	VLPVHVLGPT	VVSQPQVSPL	TVQKVPQIKP	VSQPVGAEQQ	AALLKPDLVR	SLNQDVATTK	ENVSSPDNPS
730	740	750	760	770	780	790	800
GNGKQDRIKQ	RRASCPRPEK	GTKFQLTVLQ	VSTSGDNMVE	CQLETHNNKM	VTFKFDVDGD	APEDIADYMV	EDNFVLESEK
810	820	830	840	850	860	870	880
EKFVEELRAI	VGQAQEILHV	HFATERATGV	DSITVDSNSS	QTGSSEQVQI	NSTSTQTSNE	SAPQSSPVGR	WRFCINQTIR
890	900	910	920	930	940	950	960
NRETQSPPSL	QHSMSAVPGR	HPLPSPKNTS	NKEISRDTLL	TIENNPCHRA	LFTSKSEHKD	VVDGKISECA	SVETKQPAIL
970	980	990	1000	1010	1020	1030	1040
YQVEDNRQIM	APVTNSSSYS	TTSVRAVPAE	CEGLTKQASI	FIPVYPCHQT	ASQADALMSH	PGESTQTSGN	SLTTLAFDQK
1050	1060	1070	1080	1090	1100	1110	1120
PQTLSVQQPA	MDAEFISQEG	ETTVNTEASS	PKTVIPTQTP	GLEPTTLQPT	TVLESDGERP	PKLEFADNRI	KTLDEKLRNL
1130	1140	1150	1160	1170	1180	1190	1200
LYQEHSISSI	YPESQKDTQS	IDSPFSSSAE	DTLSCPVTEV	IAISHCGIKD	SPVQSPNFQQ	TGSKLLSNVA	ASQPANISVF
1210	1220	1230	1240	1250	1260	1270	1280
KRDLNVITSV	PSELCLHEMS	SDASLPGDPE	AYPAAVSSGG	AIHLQTGVET	EEMRSAIAPD	PIPLTRESTA	DTRALNRCKA
1290	1300	1310	1320	1330	1340	1350	1360
MSGSFQRGRF	QVITIPQQQS	AKMTSFGIEH	ISVFSETNHS	SEEAFIKTAK	SQLVEIEPAT	QNPKTSFSYE	KLQALQETCK
1370	1380	1390	1400	1410	1420	1430	1440
ENKGVPKQGD	NFLSFSAACE	TDVSSVTPEK	EFEETSATGS	SMQSGSELLL	KEREILTAGK	QPSSDSEFSA	SLAGSGKSVA
1450	1460	1470	1480	1490	1500	1510	1520
KTGPESNQCL	PHHEEQAYAQ	TQSSLFYSPS	SPMSSDDESE	IEDEDLKVEL	QRLREKHIQE	VVNLQTQQNK	ELQELYERLR
1530	1540	1550	1560	1570	1580	1590	1600
SIKDSKTQST	EIPLPPASPR	RPRSFKSKLR	SRPQSLTHVD	NGIVATDPLC	VESNAASCQQ	SPASKKGMFT	DDLHKLVDDW
1610	1620	1630	1640	1650	1660	1670	1680
TKEAVGNSLI	KPSLNQLKQS	QHKLETENWN	KVSENTPSTM	GYTSTWISSL	SQIRGAVPTS	LPQGLSLPSF	PGPLSSYGMP
1690	1700	1710	1720	1730	1740	1750	
HVCQYNAVAG	AGYPVQWVGI	SGTTQQSVVI	PAQSGGPFQP	GMNMQAFPTS	SVQNPATIPP	GPK	

Cmpd.	No. of Cmpds.	m/z meas.	Δ m/z [ppm]	z	Rt [min]	Score	P	Range	Sequence	Modification
1147	1	956.4746	-119.29	1	43.0	10.6	2	486-493	R.DRVTPIKK.T	
1472	1	918.1282	-10.85	3	46.9	10.8	2	1414-1441	R.EILTAGKQPSSDSEFSASLAGSGKSVAK.T	

Detailed Protein Report

Protein 385: PREDICTED: putative tripartite motif-containing protein 61 isoform X1 [Homo sapiens]

**Accession:**	gi|578809113	**Score:**	31.6
**Database:**	refseq_human(refseq_human_20140103.fasta)	**MW [kDa]:**	55.4
**Database Date:**	2015-11-30	**pI:**	9.5
		**Sequence Coverage [%]:**	6.4
		**No. of unique Peptides:**	1

Alias proteins:

Accession	Name	Description
gi|578809117	refseq_human (refseq_human_20140103.fasta)	PREDICTED: putative tripartite motif-containing protein 61 isoform X3 [Homo sapiens]
gi|578809115	refseq_human (refseq_human_20140103.fasta)	PREDICTED: putative tripartite motif-containing protein 61 isoform X2 [Homo sapiens]

10	20	30	40	50	60	70	80
MEFVTALADL	RAEASCPICL	DYLKDPVTIS	CGHNFCLSCI	IMSWKDLHDS	FPCPFCHFCC	PERKFISNPQ	LGSLTEIAKQ
**90**	**100**	**110**	**120**	**130**	**140**	**150**	**160**
LQIRSKKRKR	QEEKHVCKKH	NQVLTFFCQK	DLELLCPRCS	LSTDHQHHCV	WPIKKAASYH	RKKLEEYNAP	WKERVELIEK
**170**	**180**	**190**	**200**	**210**	**220**	**230**	**240**
VITMQTRKSL	ELKKKVKHRA	EEVKSEFEQL	RLFLQNEQET	VLRQLQDEEM	DILAQLNESL	TKFSDYTSSL	KYLLKEIESI
**250**	**260**	**270**	**280**	**290**	**300**	**310**	**320**
YVKSELELLA	NVKDIYHRYE	NLKFPELFLF	KLKKYGYHLP	PQYSGLDKII	KRFQVDVILD	PETAHRKLIV	SEDRKTVRYG
**330**	**340**	**350**	**360**	**370**	**380**	**390**	**400**
NTTQNVPHNP	RRFYLLPAVL	GSKGYSCGRQ	YWEVEVKDKP	EWILGVCNDC	LPRRRKSQPI	LVQDGLWGIW	RSSQNNYIVL
**410**	**420**	**430**	**440**	**450**	**460**	**470**	**480**
GHREIILLPQ	VIPSKIGIFL	DYEMNEVSFY	NLNDRSLLYT	FNDNFTGALW	PYFYTGTDSK	PLKISTVTDS	E

Cmpd.	No. of Cmpds.	m/z meas.	Δ m/z [ppm]	z	Rt [min]	Score	P	Range	Sequence	Modification
1483	1	620.0670	154.80	3	47.3	17.2	0	358-373	K.DKPEWILGVCNDCLPR.R	

Detailed Protein Report

Protein 386: tetratricopeptide repeat protein 37 [Homo sapiens]

Accession: gi|7662078
Database: refseq_human(refseq_human_20140103.fasta)
Database Date: 2015-11-30
Modification(s): Carbamidomethyl

Score: 31.5
MW [kDa]: 175.4
pI: 8.1
Sequence Coverage [%]: 1.5
No. of unique Peptides: 1

Quantitation

mdown:qdown **Median:** 1.29 **CV:** 0.00 % **No. of Peptides:** 1

10	20	30	40	50	60	70	80
MSSKEVKTAL	KSARDAIRNK	EYKEALKHCK	TVLKQEKNNY	NAWVFIGVAA	AELEQPDQAQ	SAYKKAAELE	PDQLLAWQGL
90	100	110	120	130	140	150	160
ANLYEKYNHI	NAKDDLPGVY	QKLLDLYESV	DKQKWCDVCK	KLVDLYYQEK	KHLEVARTWH	KLIKTRQEQG	AENEELHQLW
170	180	190	200	210	220	230	240
RKLTQFLAES	TEDQNNETQQ	LLFTAFENAL	GLSDKIPSED	HQVLYRHFIQ	SLSKFPHESA	RLKKACEGMI	NIYPTVQYPL
250	260	270	280	290	300	310	320
EVLCLHLIES	GNLTDEGQQY	CCRLVEMDSK	SGPGLIGLGI	KALQDKKYED	AVRNLTEGLK	ESPVCTSGWY	HLAEAQVKMH
330	340	350	360	370	380	390	400
RPKEAVLSCS	QALKIVDNLG	ASGNSLYQRN	LCLHLKAEAL	IKLSDYDSSE	EAIRTLDQIS	DADNIPGLLV	LKSLAYRNKG
410	420	430	440	450	460	470	480
SFDEAAKIME	DLLSSYPDLA	EVHALEALIH	FTKKDYLQAE	KCFQRALEKD	TEVAEYHYQL	GLTYWFMGEE	TRKDKTKALT
490	500	510	520	530	540	550	560
HFLKAARLDT	YMGKVFCYLG	HYYRDVVGDK	NRARGCYRKA	FELDDTDAES	GAAAVDLSVE	LEDMEMALAI	LTTVTQKASA
570	580	590	600	610	620	630	640
GTAKWAWLRR	GLYYLKAGQH	SQAVADLQAA	LRADPKDFNC	WESLGEAYLS	RGGYTTALKS	FTKASELNPE	SIYSVFKVAA
650	660	670	680	690	700	710	720
IQQILGKYKE	AVAQYQMIIK	KKEDYVPALK	GLGECHLMMA	KAALVDYLDG	KAVDYIEKAL	EYFTCALQHR	ADVSCLWKLA
730	740	750	760	770	780	790	800
GDACTCLYAV	APSKVNVHVL	GVLLGQKEGK	QVLKKNELLH	LGGRCYGRAL	KLMSTSNTWC	DLGINYYRQA	QHLAETGSNM
810	820	830	840	850	860	870	880
NDLKELLEKS	LHCLKKAVRL	DSNNHLYWNA	LGVVACYSGI	GNYALAQHCF	IKSIQSEQIN	AVAWTNLGVL	YLTNENIEQA
890	900	910	920	930	940	950	960
HEAFKMAQSL	DPSYLMCWIG	QALIAEAVGS	YDTMDLFRHT	TELNMHTEGA	LGYAYWVCTT	LQDKSNRETE	LYQYNILQMN
970	980	990	1000	1010	1020	1030	1040
AIPAAQVILN	KYVERIQNYA	PAFTMLGYLN	EHLQLKKEAA	NAYQRAILLL	QTAEDQDTYN	VAIRNYGRLL	CSTGEYDKAI
1050	1060	1070	1080	1090	1100	1110	1120
QAFKSTPLEV	LEDIIGFALA	LFMKGLYKES	SKAYERALSI	VESEQDKAHI	LTALAITEYK	QGKTDVAKTL	LFKCSILKEP
1130	1140	1150	1160	1170	1180	1190	1200
TTESLQALCA	LGLAMQDATL	SKAALNELLK	HIKHKDSNYQ	RCLLTSAIYA	LQGRSVAVQK	QISKAVHSNP	GDPALWSLLS
1210	1220	1230	1240	1250	1260	1270	1280
RVVAQYAQRN	AKGGVVAGNV	AHILDSNHGK	KALLYTAVNQ	LAMGSSSAED	EKNTALKTIQ	KAALLSPGDP	AIWAGLMAAC
1290	1300	1310	1320	1330	1340	1350	1360
HADDKLALVN	NTQPKRIDLY	LALLSAVSAS	IKDEKFFENY	NQSLEKWSLS	QAVTGLIDTG	RISEAETLCT	KNLKSNPDQP
1370	1380	1390	1400	1410	1420	1430	1440
AVILLLRQVQ	CKPLLESQKP	LPDAVLEELQ	KTVMSNSTSV	PAWQWLAHVY	QSQGMMRAAE	MCYRKSLQLA	SQRGSWSGKL
1450	1460	1470	1480	1490	1500	1510	1520
SSLLRLALLA	LKVCMANISN	DHWPSLVQEA	TTEALKLCFC	PLAVLLQALL	QFKRKMGARE	TRRLLERVVY	QPGYPKSIAS
1530	1540	1550	1560	1570			
TARWYLLRHL	YAKDDYELID	VLVNNAKTHG	DTRALELNQR	LSSQ			

Cmpd.	No. of Cmpds.	m/z meas.	Δ m/z [ppm]	z	Rt [min]	Score	P	Range	Sequence	Modification	Ratios
1224	1	533.6546	-167.61	2	44.0	14.5	1	113-120	K.QKWCDVCK.K	Carbamidomethyl: 7	mdown:qdown 1.29

Detailed Protein Report

Protein 387: keratin, type II cuticular Hb2 [Homo sapiens]

**Accession:**	gi|27477127	**Score:**	31.4
**Database:**	refseq_human(refseq_human_20140103.fasta)	**MW [kDa]:**	56.6
**Database Date:**	2015-11-30	**pI:**	6.4
**Modification(s):**	Carbamidomethyl	**Sequence Coverage [%]:**	5.7
		**No. of unique Peptides:**	1

10	20	30	40	50	60	70	80
MSYHSFQPGS	RCGSQSFSSY	SAVMPRMVTH	YAVSKGPCRP	GGGRGLRALG	CLGSRSLCNV	GFGRPRVASR	CGGTLPGFGY
**90**	**100**	**110**	**120**	**130**	**140**	**150**	**160**
RLGATCGPSA	CITPVTINES	LLVPLALEID	PTVQRVKRDE	KEQIKCLNNR	FASFINKVRF	LEQKNKLLET	KWNFMQQQRC
**170**	**180**	**190**	**200**	**210**	**220**	**230**	**240**
CQTNIEPIFE	GYISALRRQL	DCVSGDRVRL	ESELCSLQAA	LEGYKKKYEE	ELSLRPCVEN	EFVALKKDVD	TAFLMKADLE
**250**	**260**	**270**	**280**	**290**	**300**	**310**	**320**
TNAEALVQEI	DFLKSLYEEE	ICLLQSQISE	TSVIVKMDNS	RELDVDGIIA	EIKAQYDDIA	SRSKAEAEAW	YQCRYEELRV
**330**	**340**	**350**	**360**	**370**	**380**	**390**	**400**
TAGNHCDNLR	NRKNEILEMN	KLIQRLQQET	ENVKAQRCKL	EGAIAEAEQQ	GEAALNDAKC	KLAGLEEALQ	KAKQDMACLL
**410**	**420**	**430**	**440**	**450**	**460**	**470**	**480**
KEYQEVMNSK	LGLDIEIATY	RRLLEGEEHR	LCEGIGPVNI	SVSSSKGAFL	YEPCGVSTPV	LSTGVLRSNG	GCSIVGTGEL
**490**	**500**	**510**	**520**				
YVPCEPQGLL	SCGSGRKSSM	TLGAGGSSPS	HKH				

Cmpd.	No. of Cmpds.	m/z meas.	Δ m/z [ppm]	z	Rt [min]	Score	P	Range	Sequence	Modification
1841	1	1057.4972	-8.19	2	51.7	10.5	0	160-177	R.CCQTNIEPIFEGYISALR.R	Carbamidomethyl: 2

Detailed Protein Report

Protein 388: centromere-associated protein E isoform 2 [Homo sapiens]

**Accession:**	gi|557878612	**Score:**	31.3
**Database:**	refseq_human(refseq_human_20140103.fasta)	**MW [kDa]:**	301.6
**Database Date:**	2015-11-30	**pI:**	5.3
**Modification(s):**	Carbamidomethyl	**Sequence Coverage [%]:**	1.0
		**No. of unique Peptides:**	1

10	20	30	40	50	60	70	80
MAEEGAVAVC	VRVRPLNSRE	ESLGETAQVY	WKTDNNVIYQ	VDGSKSFNFD	RVFHGNETTK	NVYEEIAAPI	IDSAIQGYNG
90	100	110	120	130	140	150	160
TIFAYGQTAS	GKTYTMMGSE	DHLGVIPRAI	HDIFQKIKKF	PDREFLLRVS	YMEIYNETIT	DLLCGTQKMK	PLIIREDVNR
170	180	190	200	210	220	230	240
NVYVADLTEE	VVYTSEMALK	WITKGEKSRH	YGETKMNQRS	SRSHTIFRMI	LESREKGEPS	NCEGSVKVSH	LNLVDLAGSE
250	260	270	280	290	300	310	320
RAAQTGAAGV	RLKEGCNINR	SLFILGQVIK	KLSDGQVGGF	INYRDSKLTR	ILQNSLGGNA	KTRIICTITP	VSFDETLTAL
330	340	350	360	370	380	390	400
QFASTAKYMK	NTPYVNEVST	DEALLKRYRK	EIMDLKKQLE	EVSLETRAQA	MEKDQLAQLL	EEKDLLQKVQ	NEKIENLTRM
410	420	430	440	450	460	470	480
LVTSSSLTLQ	QELKAKRKRR	VTWCLGKINK	MKNSNYADQF	NIPTNITTKT	HKLSINLLRE	IDESVCSESD	VFSNTLDTLS
490	500	510	520	530	540	550	560
EIEWNPATKL	LNQENIESEL	NSLRADYDNL	VLDYEQLRTE	KEEMELKLKE	KNDLDEFEAL	ERKTKKDQEN	ELSSKVELLR
570	580	590	600	610	620	630	640
EKEDQIKKLQ	EYIDSQKLEN	IKMDLSYSLE	SIEDPKQMKQ	TLFDAETVAL	DAKRESAFLR	SENLELKEKM	KELATTYKQM
650	660	670	680	690	700	710	720
ENDIQLYQSQ	LEAKKKMQVD	LEKELQSAFN	EITKLTSLID	GKVPKDLLCN	LELEGKITDL	QKELNKEVEE	NEALREEVIL
730	740	750	760	770	780	790	800
LSELKSLPSE	VERLRKEIQD	KSEELHIITS	EKDKLFSEVV	HKESRVQGLL	EEIGKTKDDL	ATTQSNYKST	DQEFQNFKTL
810	820	830	840	850	860	870	880
HMDFEQKYKM	VLEENERMNQ	EIVNLSKEAQ	KFDSSLGALK	TELSYKTQEL	QEKTREVQER	LNEMEQLKEQ	LENRDSTLQT
890	900	910	920	930	940	950	960
VEREKTLITE	KLQQTLEEVK	TLTQEKDDLK	QLQESLQIER	DQLKSDIHDT	VNMNIDTQEQ	LRNALESLKQ	HQETINTLKS
970	980	990	1000	1010	1020	1030	1040
KISEEVSRNL	HMEENTGETK	DEFQQKMVGI	DKKQDLEAKN	TQTLTADVKD	NEIIEQQRKI	FSLIQEKNEL	QQMLESVIAE
1050	1060	1070	1080	1090	1100	1110	1120
KEQLKTDLKE	NIEMTIENQE	ELRLLGDELK	KQQEIVAQEK	NHAIKKEGEL	SRTCDRLAEV	EEKLKEKSQQ	LQEKQQQLLN
1130	1140	1150	1160	1170	1180	1190	1200
VQEEMSEMQK	KINEIENLKN	ELKNKELTLE	HMETERLELA	QKLNENYEEV	KSITKERKVL	KELQKSFETE	RDHLRGYIRE
1210	1220	1230	1240	1250	1260	1270	1280
IEATGLQTKE	ELKIAHIHLK	EHQETIDELR	RSVSEKTAQI	INTQDLEKSH	TKLQEEIPVL	HEEQELLPNV	KEVSETQETM
1290	1300	1310	1320	1330	1340	1350	1360
NELELLTEQS	TTKDSTTLAR	IEMERLRLNE	KFQESQEEIK	SLTKERDNLK	TIKEALEVKH	DQLKEHIRET	LAKIQESQSK
1370	1380	1390	1400	1410	1420	1430	1440
QEQSLNMKEK	DNETTKIVSE	MEQFKPKDSA	LLRIEIEMLG	LSKRLQESHD	EMKSVAKEKD	DLQRLQEVLQ	SESDQLKENI
1450	1460	1470	1480	1490	1500	1510	1520
KEIVAKHLET	EEELKVAHCC	LKEQEETINE	LRVNLSEKET	EISTIQKQLE	AINDKLQNKI	QEIYEKEEQF	NIKQISEVQE
1530	1540	1550	1560	1570	1580	1590	1600
KVNELKQFKE	HRKAKDSALQ	SIESKMLELT	NRLQESQEEI	QIMIKEKEEM	KRVQEALQIE	RDQLKENTKE	IVAKMKESQE
1610	1620	1630	1640	1650	1660	1670	1680
KEYQFLKMTA	VNETQEKMCE	IEHLKEQFET	QKLNLENIET	ENIRLTQILH	ENLEEMRSVT	KERDDLRSVE	ETLKVERDQL
1690	1700	1710	1720	1730	1740	1750	1760
KENLRETITR	DLEKQEELKI	VHMHLKEHQE	TIDKLRGIVS	EKTNEISNMQ	KDLEHSNDAL	KAQDLKIQEE	LRIAHMHLKE
1770	1780	1790	1800	1810	1820	1830	1840
QQETIDKLRG	IVSEKTDKLS	NMQKDLENSN	AKLQEKIQEL	KANEHQLITL	KKDVNETQKK	VSEMEQLKKQ	IKDQSLTLSK
1850	1860	1870	1880	1890	1900	1910	1920
LEIENLNLAQ	KLHENLEEMK	SVMKERDNLR	RVEETLKLER	DQLKESLQET	KARDLEIQQE	LKTARMLSKE	HKETVDKLRE
1930	1940	1950	1960	1970	1980	1990	2000
KISEKTIQIS	DIQKDLDKSK	DELQKKQDRQ	NHQVKPEKRL	LSDGQQHLTE	SLREKCSRIK	ELLKRYSEMD	DHYECLNRLS
2010	2020	2030	2040	2050	2060	2070	2080
LDLEKEIEFQ	KELSMRVKAN	LSLPYLQTKH	IEKLFTANQR	CSMEFHRIMK	KLKYVLSYVT	KIKEEQHESI	NKFEMDFIDE
2090	2100	2110	2120	2130	2140	2150	2160
VEKQKELLIK	IQHLQQDCDV	PSRELRDLKL	NQNMDLHIEE	ILKDFSESEF	PSIKTEFQQV	LSNRKEMTQF	LEEWLNTRFD
2170	2180	2190	2200	2210	2220	2230	2240
IEKLKNGIQK	ENDRICQVNN	FFNNRIIAIM	NESTEFEERS	ATISKEWEQD	LKSLKEKNEK	LFKNYQTLKT	SLASGAQVNP
2250	2260	2270	2280	2290	2300	2310	2320
TTQDNKNPHV	TSRATQLTTE	KIRELENSLH	EAKESAMHKE	SKIIKMQKEL	EVTNDIIAKL	QAKVHESNKC	LEKTKETIQV
2330	2340	2350	2360	2370	2380	2390	2400

Detailed Protein Report

Cmpd.	No. of Cmpds.	m/z meas.	Δ m/z [ppm]	z	Rt [min]	Score	P	Range	Sequence	Modification
1821	1	920.3533	-110.00	2	52.8	20.0	2	2474-2489	R.EAHKQVTCENSPKSPK.V	Carbamidomethyl: 8

Detailed Protein Report

Protein 389: beta-crystallin B1 [Homo sapiens]

Accession: gi|4503061
Database: refseq_human(refseq_human_20140103.fasta)
Database Date: 2015-11-30

Score: 31.3
MW [kDa]: 28.0
pI: 9.2
Sequence Coverage [%]: 13.5
No. of unique Peptides: 2

10	20	30	40	50	60	70	80
MSQAAKASAS	ATVAVNPGPD	TKGKGAPPAG	TSPSPGTTLA	PTTVPITSAK	AAELPPGNYR	LVVFELENFQ	GRRAEFSGEC
**90**	**100**	**110**	**120**	**130**	**140**	**150**	**160**
SNLADRGFDR	VRSIIVSAGP	WVAFEQSNFR	GEMFILEKGE	YPRWNTWSSS	YRSDRLMSFR	PIKMDAQEHK	ISLFEGANFK
**170**	**180**	**190**	**200**	**210**	**220**	**230**	**240**
GNTIEIQGDD	APSLWVYGFS	DRVGSVKVSS	GTWVGYQYPG	YRGYQYLLEP	GDFRHWNEWG	AFQPQMQSLR	RLRDKQWHLE
**250**	**260**						
GSFPVLATEP	PK						

Cmpd.	No. of Cmpds.	m/z meas.	Δ m/z [ppm]	z	Rt [min]	Score	P	Range	Sequence	Modification
2177	1	1051.4295	-96.54	2	55.7	20.8	1	1-22	-.MSQAAKASASATVAVNPGPDTK.G	
2974	1	729.3714	20.28	2	65.9	10.5	0	203-214	R.GYQYLLEPGDFR.H	

Detailed Protein Report

Protein 390: **wee1-like protein kinase 2 [Homo sapiens]**

**Accession:**	gi|157738687	**Score:**	31.3
**Database:**	refseq_human(refseq_human_20140103.fasta)	**MW [kDa]:**	62.9
**Database Date:**	2015-11-30	**pI:**	6.1
		**Sequence Coverage [%]:**	5.5
		**No. of unique Peptides:**	2

10	20	30	40	50	60	70	80
MDDKDIDKEL	RQKLNFSYCE	ETEIEGQKKV	EESREASSQT	PEKGEVQDSE	AKGTPPWTPL	SNVHELDTSS	EKDKESPDQI
**90**	**100**	**110**	**120**	**130**	**140**	**150**	**160**
LRTPVSHPLK	CPETPAQPDS	RSKLLPSDSP	STPKTMLSRL	VISPTGKLPS	RGPKHLKLTP	APLKDEMTSL	ALVNINPFTP
**170**	**180**	**190**	**200**	**210**	**220**	**230**	**240**
ESYKKLFLQS	GGKRKIRGDL	EEAGPEEGKG	GLPAKRCVLR	ETNMASRYEK	EFLEVEKIGV	GEFGTVYKCI	KRLDGCVYAI
**250**	**260**	**270**	**280**	**290**	**300**	**310**	**320**
KRSMKTFTEL	SNENSALHEV	YAHAVLGHHP	HVVRYYSSWA	EDDHMIIQNE	YCNGGSLQAA	ISENTKSGNH	FEEPKLKDIL
**330**	**340**	**350**	**360**	**370**	**380**	**390**	**400**
LQISLGLNYI	HNSSMVHLDI	KPSNIFICHK	MQSESSGVIE	EVENEADWFL	SANVMYKIGD	LGHATSINKP	KVEEGDSRFL
**410**	**420**	**430**	**440**	**450**	**460**	**470**	**480**
ANEILQEDYR	HLPKADIFAL	GLTIAVAAGA	ESLPTNGAAW	HHIRKGNFPD	VPQELSESFS	SLLKNMIQPD	AEQRPSAAAL
**490**	**500**	**510**	**520**	**530**	**540**	**550**	**560**
ARNTVLRPSL	GKTEELQQQL	NLEKFKTATL	ERELREAQQA	QSPQGYTHHG	DTGVSGTHTG	SRSTKRLVGG	KSARSSSFTS
**570**							
GEREPLH							

Cmpd.	No. of Cmpds.	m/z meas.	Δ m/z [ppm]	z	Rt [min]	Score	P	Range	Sequence	Modification
1295	2	1023.3898	-86.03	2	45.9	14.1	1	12-28	R.QKLNFSYCEETEIEGQK.K	
135	1	725.9700	90.61	2	30.9	17.2	0	378-391	K.IGDLGHATSINKPK.V	

Detailed Protein Report

Protein 391: cytoplasmic phosphatidylinositol transfer protein 1 isoform b [Homo sapiens]

**Accession:**	gi|32307142	**Score:**	31.3
**Database:**	refseq_human(refseq_human_20140103.fasta)	**MW [kDa]:**	31.7
**Database Date:**	2015-11-30	**pI:**	5.3
		**Sequence Coverage [%]:**	11.9
		**No. of unique Peptides:**	1

10	20	30	40	50	60	70	80
MLLKEYRICM	PLTVDEYKIG	QLYMISKHSH	EQSDRGEGVE	VVQNEPFEDP	HHGNGQFTEK	RVYLNSKLPS	WARAVVPKIF
**90**	**100**	**110**	**120**	**130**	**140**	**150**	**160**
YVTEKAWNYY	PYTITEYTCS	FLPKFSIHIE	TKYEDNKGSN	DTIFDNEAKD	VEREVCFIDI	ACDEIPERYY	KESEDPKHFK
**170**	**180**	**190**	**200**	**210**	**220**	**230**	**240**
SEKTGRGQLR	EGWRDSHQPI	MCSYKLVTVK	FEVWGLQTRV	EQFVHKVVRD	ILLIGHRQAF	AWVDEWYDMT	MDDVREYEKN
**250**	**260**	**270**					
MHEQTNIKVC	NQHSSPVDDI	ESHAQTST					

Cmpd.	No. of Cmpds.	m/z meas.	Δ m/z [ppm]	z	Rt [min]	Score	P	Range	Sequence	Modification
38	1	918.9134	2.10	2	29.2	16.1	1	171-185	R.EGWRDSHQPIMCSYK.L	

Detailed Protein Report

Protein 392: zinc finger protein 486 [Homo sapiens]

Accession: gi|150170665
Database: refseq_human(refseq_human_20140103.fasta)
Database Date: 2015-11-30
Modification(s): Carbamidomethyl

Score: 31.2
MW [kDa]: 53.6
pI: 10.1
Sequence Coverage [%]: 9.5
No. of unique Peptides: 3

10	20	30	40	50	60	70	80
MPGPLRSLEM	ESLQFRDVAV	EFSLEEWHCL	DTAQQNLYRD	VMLENYRHLV	FLGIIVSKPD	LITCLEQGIK	PLTMKRHEMI
90	100	110	120	130	140	150	160
AKPPVVCSHF	AQDLWPEQSI	KDSYQKVILR	KFEKCGHGNL	HFKKGCESVD	ECKLHKRGYN	GLNQCLTTTQ	SKIFQCGKYV
170	180	190	200	210	220	230	240
KVFHQFSNSK	RHKRRHTEKK	PLKYIEGDKA	FNQSSTHTTH	KKIDTGEKPY	KCEECGKAFN	RSSHLTTHKI	THTREKPYKC
250	260	270	280	290	300	310	320
EECGKVFKYF	SSFTTHKKIH	SGEKPYICEE	CGKAFMYPYT	LTTHKIIHTG	EQPYKCKECD	KAFNHPATLS	SHKKIHTGEK
330	340	350	360	370	380	390	400
PYTCDKCGKA	FISSSILSKH	EKIHTGEKPY	KCEECGKAFT	RSSHLTMHKI	IHTGEKPYKC	EECGKAFTWS	AGLHKHRRTH
410	420	430	440	450	460	470	
TGEKPYKCEE	CGKAYTTSSN	LTEHKTTHTG	EKPYKCKECG	KAFNWSSDLN	KHKRIHIGQK	PRT	

Cmpd.	No. of Cmpds.	m/z meas.	Δ m/z [ppm]	z	Rt [min]	Score	P	Range	Sequence	Modification
2109	1	1065.5116	14.23	2	54.9	10.5	2	296-313	K.CKECDKAFNHPATLSSHK.K	Carbamidomethyl: 1, 4
2242	1	961.4221	-46.22	2	56.7	10.2	2	314-329	K.KIHTGEKPYTCDKCGK.A	Carbamidomethyl: 11, 14
1341	1	699.4714	135.76	2	46.5	10.5	1	327-339	K.CGKAFISSSILSK.H	Carbamidomethyl: 1

Detailed Protein Report

Protein 393: pyrroline-5-carboxylate reductase 2 isoform 2 [Homo sapiens]

**Accession:**	gi|410991931	**Score:**	31.1
**Database:**	refseq_human(refseq_human_20140103.fasta)	**MW [kDa]:**	25.9
**Database Date:**	2015-11-30	**pI:**	10.2
		**Sequence Coverage [%]:**	14.2
		**No. of unique Peptides:**	1

10	20	30	40	50	60	70	80
MSVGFIGAGQ	LAYALARGFT	AAGILSAHKI	IASSPEMNLP	TVSALRKMGV	NLTRSNKETV	KHSDVLFLAV	KPHIIPFILD
**90**	**100**	**110**	**120**	**130**	**140**	**150**	**160**
EIGADVQARH	IVVSCAAGVT	ISSVEKAFMA	LDALADGGVK	MGLPRRLAIQ	LGAQALLGAA	KMLLDSEQHP	CQLKDNVCSP
**170**	**180**	**190**	**200**	**210**	**220**	**230**	**240**
GGATIHALHF	LESGGFRSLL	INAVEASCIR	TRELQSMADQ	EKISPAALKK	TLLDRVKLES	PTVSTLTPSS	PGKLLTRSLA
**250**							
LGGKKD							

Cmpd.	No. of Cmpds.	m/z meas.	Δ m/z [ppm]	z	Rt [min]	Score	P	Range	Sequence	Modification
290	1	1074.7498	98.90	2	33.0	17.1	2	121-141	K.MGLPRRLAIQLGAQALLGAAK.M	

Detailed Protein Report

Protein 394: **PREDICTED: zinc finger protein 649 isoform X1 [Homo sapiens]**

**Accession:**	gi|530417068	**Score:**	31.1
**Database:**	refseq_human(refseq_human_20140103.fasta)	**MW [kDa]:**	40.8
**Database Date:**	2015-11-30	**pI:**	10.5
		**Sequence Coverage [%]:**	10.8
		**No. of unique Peptides:**	2

10	20	30	40	50	60	70	80
MPTEIEFPES	RKPISTKSQF	LKHQQTHNIE	KAHECTDCGK	AFLKKSQLTE	HKRIHTGKKP	HVCSLCGKAF	YKKYRLTEHE
**90**	**100**	**110**	**120**	**130**	**140**	**150**	**160**
RAHRGEKPHG	CSLCGKAFYK	RYRLTEHERA	HKGEKPYGCS	ECGKAFPRKS	ELTEHQRIHT	GIKPHQCSEC	GRAFSRKSLL
**170**	**180**	**190**	**200**	**210**	**220**	**230**	**240**
VVHQRTHTGE	KPHTCSECGK	GFIQKGNLNI	HQRTHTGEKP	YGCIDCGKAF	SQKSCLVAHQ	RYHTGKTPFV	CPECGQPCSQ
**250**	**260**	**270**	**280**	**290**	**300**	**310**	**320**
KSGLIRHQKI	HSGEKPYKCS	DCGKAFLTKT	MLIVHHRTHT	GERPYGCDEC	EKAYFYMSCL	VKHKRIHSRE	KRGDSVKVEN
**330**	**340**	**350**	**360**	**370**			
PSTASHSLSP	SEHVQGKSPV	NMVTVAMVAG	QCEFAHILHS				

Cmpd.	No. of Cmpds.	m/z meas.	Δ m/z [ppm]	z	Rt [min]	Score	P	Range	Sequence	Modification
2142	1	882.4021	-87.85	2	55.4	12.7	2	53-68	K.RIHTGKKPHVCSLCGK.A	
1729	1	848.5007	100.59	3	51.6	18.4	2	227-249	K.TPFVCPECGQPCSQKSGLIRHQK.I	

Detailed Protein Report

Protein 395: PREDICTED: zinc finger protein 468 isoform X2 [Homo sapiens]

Accession: gi|578835091
Database: refseq_human(refseq_human_20140103.fasta)
Database Date: 2015-11-30

Score: 31.0
MW [kDa]: 54.3
pI: 10.3
Sequence Coverage [%]: 4.5
No. of unique Peptides: 2

Quantitation

Wdown:Qdown **Median:** 0.19 **CV:** 0.00 % **No. of Peptides:** 1

10	20	30	40	50	60	70	80
MLKTLSSTGQ	GNTEVIHTGT	LHRQASHHIG	EFCFHEIEKD	IHGFEFQWKE	DETNGHAAPM	TEIKELAGST	GQHDQRHAGN
**90**	**100**	**110**	**120**	**130**	**140**	**150**	**160**
KRIKDQLGSS	FHLHLPEPHI	FQSEGKIGNQ	VEKSINNASS	VSTSQRICCR	PKTHISNKYG	NNSLHSSLLT	QKWEVHMREK
**170**	**180**	**190**	**200**	**210**	**220**	**230**	**240**
SFECIQSFKS	FNCSSLLKKH	QIIHLEEKQC	KCDVCGKVFN	QKRYLACHRR	CHTGEKPYKC	NECGKTFGHN	SSLFIHKALH
**250**	**260**	**270**	**280**	**290**	**300**	**310**	**320**
TGEKPYECEE	CDKVFSRKSH	LERHKRIHTG	EKPYKCKVCD	EAFAYNSYLA	KHTILHTGEK	PYTCNECGKV	FNRLSTLARH
**330**	**340**	**350**	**360**	**370**	**380**	**390**	**400**
HRLHTGEKPY	KCEECDKVFS	RKSHLERHRR	IHSGEKPYKC	EECCKVFSRK	SNLERHRRIH	TGEKPYKCKV	CDKAFQRDSH
**410**	**420**	**430**	**440**	**450**	**460**	**470**	
LAQHQRVHTG	EKPYKCNECG	KTFGQTSSLI	IHRRLHTGEK	PYKCNECGKT	FSQMSSLVYH	HRLHSGEKP	

Cmpd.	No. of Cmpds.	m/z meas.	Δ m/z [ppm]	z	Rt [min]	Score	P	Range	Sequence	Modification	Ratios
1828	4	673.2559	-104.81	2	52.9	19.8	1	159-169	R.EKSFECIQSFK.S		
232	1	563.7487	-90.74	2	32.3	11.2	1	170-179	K.SFNCSSLLKK.H		Wdown:Qdown 0.19

Detailed Protein Report

Protein 396: **PREDICTED: zinc finger CCCH domain-containing protein 7A isoform X4 [Homo sapiens]**

**Accession:**	gi|530407964	**Score:**	30.9
**Database:**	refseq_human(refseq_human_20140103.fasta)	**MW [kDa]:**	67.6
**Database Date:**	2015-11-30	**pI:**	9.6
**Modification(s):**	Carbamidomethyl	**Sequence Coverage [%]:**	3.6
		**No. of unique Peptides:**	2

10	20	30	40	50	60	70	80
MNGPGSLFAS	ENFLGISSQP	RNDFGNFFGS	AVTKPSSSVT	PRHPLEGTHE	LRQACQICFV	KSGPKLMDFT	YHANIDHKCK
**90**	**100**	**110**	**120**	**130**	**140**	**150**	**160**
KDILIGRIKN	VEDKSWKKIR	PRPTKTNYEG	PYYICKDVAA	EEECRYSGHC	TFAYCQEEID	VWTLERKGAF	SREAFFGGNG
**170**	**180**	**190**	**200**	**210**	**220**	**230**	**240**
KINLTVFKLL	QEHLGEFIFL	CEKCFDHKPR	MISKRNKDNS	TACSHPVTKH	EFEDNKCLVH	ILRETTVKYS	KIRSFHGQCQ
**250**	**260**	**270**	**280**	**290**	**300**	**310**	**320**
LDLCRHEVRY	GCLREDECFY	AHSLVELKVW	IMQNETGISH	DAIAQESKRY	WQNLEANVPG	AQVLGNQIMP	GFLNMKIKFV
**330**	**340**	**350**	**360**	**370**	**380**	**390**	**400**
CAQCLRNGQV	IEPDKNRKYC	SAKARHSWTK	DRRAMRVMSI	ERKKWMNIRP	LPTKKQMPLQ	FDLCNHIASG	KKCQYVGNCS
**410**	**420**	**430**	**440**	**450**	**460**	**470**	**480**
FAHSPEEREV	WTYMKENGIQ	DMEQFYELWL	KSQKNEKSED	IASQSNKENG	KQIHMPTDYA	EVTVDFHCWM	CGKNCNSEKQ
**490**	**500**	**510**	**520**	**530**	**540**	**550**	**560**
WQGHISSEKH	KEKVFHTEDD	QYCWQHRFPT	GYFSICDRYM	NGTCPEGNSC	KFAHGNAELH	EWEERRDALK	MKLNKARKDH
**570**	**580**	**590**					
LIGPNDNDFG	KYSFLFKDLN						

Cmpd.	No. of Cmpds.	m/z meas.	Δ m/z [ppm]	z	Rt [min]	Score	P	Range	Sequence	Modification
2596	1	658.6717	-192.67	2	61.1	11.2	0	198-209	K.DNSTACSHPVTK.H	Carbamidomethyl: 6
202	1	999.6888	178.38	1	31.7	19.7	0	327-335	R.NGQVIEPDK.N	

Detailed Protein Report

Protein 397: **LIM/homeobox protein Lhx1 [Homo sapiens]**

**Accession:**	gi|30410785	**Score:**	30.9
**Database:**	refseq_human(refseq_human_20140103.fasta)	**MW [kDa]:**	44.8
**Database Date:**	2015-11-30	**pI:**	9.1
**Modification(s):**	Carbamidomethyl	**Sequence Coverage [%]:**	7.6
		**No. of unique Peptides:**	1

10	20	30	40	50	60	70	80
MVHCAGCKRP	ILDRFLLNVL	DRAWHVKCVQ	CCECKCNLTE	KCFSREGKLY	CKNDFFRCFG	TKCAGCAQGI	SPSDLVRRAR
**90**	**100**	**110**	**120**	**130**	**140**	**150**	**160**
SKVFHLNCFT	CMMCNKQLST	GEELYIIDEN	KFVCKEDYLS	NSSVAKENSL	HSATTGSDPS	LSPDSQDPSQ	DDAKDSESAN
**170**	**180**	**190**	**200**	**210**	**220**	**230**	**240**
VSDKEAGSNE	NDDQNLGAKR	RGPRTTIKAK	QLETLKAAFA	ATPKPTRHIR	EQLAQETGLN	MRVIQVWFQN	RRSKERRMKQ
**250**	**260**	**270**	**280**	**290**	**300**	**310**	**320**
LSALGARRHA	FFRSPRRMRP	LVDRLEPGEL	IPNGPFSFYG	DYQSEYYGPG	GNYDFFPQGP	PSSQAQTPVD	LPFVPSSGPS
**330**	**340**	**350**	**360**	**370**	**380**	**390**	**400**
GTPLGGLEHP	LPGHHPSSEA	QRFTDILAHP	PGDSPSPEPS	LPGPLHSMSA	EVFGPSPPFS	SLSVNGGASY	GNHLSHPPEM
**410**							
NEAAVW							

Cmpd.	No. of Cmpds.	m/z meas.	Δ m/z [ppm]	z	Rt [min]	Score	P	Range	Sequence	Modification
2236	1	916.4377	77.30	2	56.6	12.2	1	28-41	K.CVQCCECKCNLTEK.C	Carbamidomethyl: 4, 5, 7, 9

Detailed Protein Report

Protein 398: **PREDICTED: short transient receptor potential channel 7 isoform X1 [Homo sapiens]**

**Accession:**	gi|530380051	**Score:**	30.8
**Database:**	refseq_human(refseq_human_20140103.fasta)	**MW [kDa]:**	55.5
**Database Date:**	2015-11-30	**pI:**	9.0
		**Sequence Coverage [%]:**	4.8
		**No. of unique Peptides:**	1

10	20	30	40	50	60	70	80
MKFVAHAVSF	TIFLGLLVVN	ASDRFEGVKT	LPNETFTDYP	KQIFRVKTTQ	FSWTEMLIMK	WVLGMIWSEC	KEIWEEGPRE
90	100	110	120	130	140	150	160
YVLHLWNLLD	FGMLSIFVAS	FTARFMAFLK	ATEAQLYVDQ	HVQDDTLHNV	SLPPEVAYFT	YARDKWWPSD	PQIISEGLYA
170	180	190	200	210	220	230	240
IAVVLSFSRI	AYILPANESF	GPLQISLGRT	VKDIFKFMVI	FIMVFVAFMI	GMFNLYSYYR	GAKYNPAFTT	VEESFKTLFW
250	260	270	280	290	300	310	320
SIFGLSEVIS	VVLKYDHKFI	ENIGYVLYGV	YNVTMVVVLL	NMLIAMINNS	YQEIEEDADV	EWKFARAKLW	LSYFDEGRTL
330	340	350	360	370	380	390	400
PAPFNLVPSP	KSFYYLIMRI	KMCLIKLCKS	KAKSCENDLE	MGMLNSKFKK	TRYQAGMRNS	ENLTANNTLS	KPTRYQKIMK
410	420	430	440	450	460	470	480
RLIKRYVLKA	QVDRENDEVN	EGELKEIKQD	ISSLRYELLE	EKSQATGELA	DLIQQLSEKF	GKNLNKDHLR	VNKGKDI

Cmpd.	No. of Cmpds.	m/z meas.	Δ m/z [ppm]	z	Rt [min]	Score	P	Range	Sequence	Modification
965	1	607.1283	-333.12	1	41.6	14.2	0	342-346	K.MCLIK.L	

Detailed Protein Report

Protein 399: pre-mRNA 3' end processing protein WDR33 isoform 1 [Homo sapiens]

**Accession:**	gi|56243590	**Score:**	30.8
**Database:**	refseq_human(refseq_human_20140103.fasta)	**MW [kDa]:**	145.8
**Database Date:**	2015-11-30	**pI:**	9.9
		**Sequence Coverage [%]:**	2.5
		**No. of unique Peptides:**	1

10	20	30	40	50	60	70	80
MATEIGSPPR	FFHMPRFQHQ	APRQLFYKRP	DFAQQQAMQQ	LTFDGKRMRK	AVNRKTIDYN	PSVIKYLENR	IWQRDQRDMR
**90**	**100**	**110**	**120**	**130**	**140**	**150**	**160**
AIQPDAGYYN	DLVPPIGMLN	NPMNAVTTKF	VRTSTNKVKC	PVFVVRWTPE	GRRLVTGASS	GEFTLWNGLT	FNFETILQAH
**170**	**180**	**190**	**200**	**210**	**220**	**230**	**240**
DSPVRAMTWS	HNDMWMLTAD	HGGYVKYWQS	NMNNVKMFQA	HKEAIREASF	SPTDNKFATC	SDDGTVRIWD	FLRCHEERIL
**250**	**260**	**270**	**280**	**290**	**300**	**310**	**320**
RGHGADVKCV	DWHPTKGLVV	SGSKDSQQPI	KFWDPKTGQS	LATLHAHKNT	VMEVKLNLNG	NWLLTASRDH	LCKLFDIRNL
**330**	**340**	**350**	**360**	**370**	**380**	**390**	**400**
KEELQVFRGH	KKEATAVAWH	PVHEGLFASG	GSDGSLLFWH	VGVEKEVGGM	EMAHEGMIWS	LAWHPLGHIL	CSGSNDHTSK
**410**	**420**	**430**	**440**	**450**	**460**	**470**	**480**
FWTRNRPGDK	MRDRYNLNLL	PGMSEDGVEY	DDLEPNSLAV	IPGMGIPEQL	KLAMEQEQMG	KDESNEIEMT	IPGLDWGMEE
**490**	**500**	**510**	**520**	**530**	**540**	**550**	**560**
VMQKDQKKVP	QKKVPYAKPI	PAQFQQAWMQ	NKVPIPAPNE	VLNDRKEDIK	LEEKKKTQAE	IEQEMATLQY	TNPQLLEQLK
**570**	**580**	**590**	**600**	**610**	**620**	**630**	**640**
IERLAQKQVE	QIQPPPSSGT	PLLGPQPFPG	QGPMSQIPQG	FQQPHPSQQM	PMNMAQMGPP	GPQGQFRPPG	PQGQMGPQGP
**650**	**660**	**670**	**680**	**690**	**700**	**710**	**720**
PLHQGGGGPQ	GFMGPQGPQG	PPQGLPRPQD	MHGPQGMQRH	PGPHGPLGPQ	GPPGPQGSSG	PQGHMGPQGP	PGPQGHIGPQ
**730**	**740**	**750**	**760**	**770**	**780**	**790**	**800**
GPPGPQGHLG	PQGPPGTQGM	QGPPGPRGMQ	GPPHPHGIQG	GPGSQGIQGP	VSQGPLMGLN	PRGMQGPPGP	RENQGPAPQG
**810**	**820**	**830**	**840**	**850**	**860**	**870**	**880**
MIMGHPPQEM	RGPHPPGGLL	GHGPQEMRGP	QEIRGMQGPP	PQGSMLGPPQ	ELRGPPGSQS	QQGPPQGSLG	PPPQGGMQGP
**890**	**900**	**910**	**920**	**930**	**940**	**950**	**960**
PGPQGQQNPA	RGPHPSQGPI	PFQQQKTPLL	GDGPRAPFNQ	EGQSTGPPPL	IPGLGQQGAQ	GRIPPLNPGQ	GPGPNKGDSR
**970**	**980**	**990**	**1000**	**1010**	**1020**	**1030**	**1040**
GPPNHHMGPM	SERRHEQSGG	PEHGPERGPF	RGGQDCRGPP	DRRGPHPDFP	DDFSRPDDFH	PDKRFGHRLR	EFEGRGGPLP
**1050**	**1060**	**1070**	**1080**	**1090**	**1100**	**1110**	**1120**
QEEKWRRGGP	GPPFPPDHRE	FSEGDGRGAA	RGPPGAWEGR	RPGDERFPRD	PEDPRFRGRR	EESFRRGAPP	RHEGRAPPRG
**1130**	**1140**	**1150**	**1160**	**1170**	**1180**	**1190**	**1200**
RDGFPGPEDF	GPEENFDASE	EAARGRDLRG	RGRGTPRGGR	KGLLPTPDEF	PRFEGGRKPD	SWDGNREPGP	GHEHFRDTPR
**1210**	**1220**	**1230**	**1240**	**1250**	**1260**	**1270**	**1280**
PDHPPHDGHS	PASRERSSSL	QGMDMASLPP	RKRPWHDGPG	TSEHREMEAP	GGPSEDRGGK	GRGGPGPAQR	VPKSGRSSSL
**1290**	**1300**	**1310**	**1320**	**1330**	**1340**		
DGEHHDGYHR	DEPFGGPPGS	GTPSRGGRSG	SNWGRGSNMN	SGPPRRGASR	GGGRGR		

Cmpd.	No. of Cmpds.	m/z meas.	Δ m/z [ppm]	z	Rt [min]	Score	P	Range	Sequence	Modification
2698	1	937.4703	7.09	2	62.7	13.8	1	1-16	-.MATEIGSPPRFFHMPR.F	

Detailed Protein Report

Protein 400: thrombospondin type-1 domain-containing protein 7B [Homo sapiens]

**Accession:**	gi|122937257	**Score:**	30.8
**Database:**	refseq_human(refseq_human_20140103.fasta)	**MW [kDa]:**	175.5
**Database Date:**	2015-11-30	**pI:**	9.1
**Modification(s):**	Carbamidomethyl, Oxidation	**Sequence Coverage [%]:**	2.5
		**No. of unique Peptides:**	2

Quantitation

mdown:qdown	**Median:**	2.43	**CV:**	0.00 %	**No. of Peptides:**	1
Wdown:Qdown	**Median:**	0.71	**CV:**	0.00 %	**No. of Peptides:**	1

10	20	30	40	50	60	70	80
MHNTADEVVL	YHKLAGPWGR	CTGDCGPGGV	QSRAVWCFHV	DGWTSHLSNC	GESNRPPKER	SCFRVCDWHS	DLFQWEVSDW
90	100	110	120	130	140	150	160
HHCVLVPYAR	GEVKPRTAEC	VTAQHGLQHR	MVRCIQKLNR	TVVANEICEH	FALQPPTEQA	CLIPCPRDCV	VSEFLPWSNC
170	180	190	200	210	220	230	240
SKGCGKKLQH	RTRAVIAPPL	FGGLQCPNLT	ESRACDAPIS	CPLGEEEYTF	SLKVGPWSKC	RLPHLKEINP	SGRTVLDFNS
250	260	270	280	290	300	310	320
DSNERVTFKH	QSYKAHHHSK	SWAIEIGYQT	RQVSCTRSDG	QNAMLSLCLQ	DSFPLTVQSC	IMPKDCETSQ	WSSWSPCSKT
330	340	350	360	370	380	390	400
CRSGSLLPGF	RSRSRNVKHM	AIGGGKECPE	LLEKEACIVE	GELLQQCPRY	SWRTSEWKEC	QVSLLLEQQD	PHWHVTGPVC
410	420	430	440	450	460	470	480
GGGIQTREVY	CAQSVPAAAA	LRAKEVSRPV	EKALCVGPAP	LPSQLCNIPC	STDCIVSSWS	AWGLCIHENC	HDPQGKKGFR
490	500	510	520	530	540	550	560
TRQRHVLMES	TGPAGHCPHL	VESVPCEDPM	CYRWLASEGI	CFPDHGKCGL	GHRILKAVCQ	NDRGEDVSGS	LCPVPPPPER
570	580	590	600	610	620	630	640
KSCEIPCRMD	CVLSEWTEWS	SCSQSCSNKN	SDGKQTRSRT	ILALAGEGGK	PCPPSQALQE	HRLCNDHSCM	QLHWETSPWG
650	660	670	680	690	700	710	720
PCSEDTLVTA	LNATIGWNGE	ATCGVGIQTR	RVFCVKSHVG	QVMTKRCPDS	TRPETVRPCF	LPCKKDCIVT	AFSEWTPCPR
730	740	750	760	770	780	790	800
MCQAGNATVK	QSRYRIIIQE	AANGGQECPD	TLYEERECED	VSLCPVYRWK	PQKWSPCILV	PESVWQGITG	SSEACGKGLQ
810	820	830	840	850	860	870	880
TRAVSCISDD	NRSAEMMECL	KQTNGMPLLV	QECTVPCRED	CTFTAWSKFT	PCSTNCEATK	SRRRQLTGKS	RKKEKCQDSD
890	900	910	920	930	940	950	960
LYPLVETELC	PCDEFISQPY	GNWSDCILPE	GRREPHRGLR	VQADSKECGE	GLRFRAVACS	DKNGRPVDPS	FCSSSGYIQE
970	980	990	1000	1010	1020	1030	1040
KCVIPCPFDC	KLSDWSSWGS	CSSSCGIGVR	IRSKWLKEKP	YNGGRPCPKL	DLKNQAQVHE	AVPCYSECNQ	YSWVVEHWSS
1050	1060	1070	1080	1090	1100	1110	1120
CKINNELRSL	RCGGGTQSRK	IRCVNTADGE	GGAVDSNLCN	QDEIPPETQS	CSLMCPNECV	MSEWGLWSKC	PQSCDPHTMQ
1130	1140	1150	1160	1170	1180	1190	1200
RRTRHLLRPS	LNSRTCAEDS	QVQPCLLNEN	CFQFQYNLTE	WSTCQLSENA	PCGQGVRTRL	LSCVCSDGKP	VSMDQCEQHN
1210	1220	1230	1240	1250	1260	1270	1280
LEKPQRMSIP	CLVECVVNCQ	LSGWTAWTEC	SQTCGHGGRM	SRTRFIIMPT	QGEGRPCPTE	LTQEKTCPVT	PCYSWVLGNW
1290	1300	1310	1320	1330	1340	1350	1360
SACKLEGGDC	GEGVQIRSLS	CMVHSGSISH	AAGRVEDALC	GEMPFQDSIL	KQLCSVPCPG	DCHLTEWSEW	STCELTCIDG
1370	1380	1390	1400	1410	1420	1430	1440
RSFETVGRQS	RSRTFIIQSF	ENQDSCPQQV	LETRPCTGGK	CYHYTWKASL	WNNNERTVWC	QRSDGVNVTG	GCSPQARPAA
1450	1460	1470	1480	1490	1500	1510	1520
IRQCIPACRK	PFSYCTQGGV	CGCEKGYTEI	MKSNGFLDYC	MKVPGSEDKK	ADVKNLSGKN	RPVNSKIHDI	FKGWSLQPLD
1530	1540	1550	1560	1570	1580		
PDGRVKIWVY	GVSGGAFLIM	IFLIFTSYLV	CKKPKPHQST	PPQQKPLTLA	YDGDLDM		

Cmpd.	No. of Cmpds.	m/z meas.	Δ m/z [ppm]	z	Rt [min]	Score	P	Range	Sequence	Modification	Ratios
122	1	981.4639	-1.83	2	30.8	11.7	0	822-838	K.QTNGMPLLVQECTVPCR.E	Carbamidomethyl: 16; Oxidation: 5	Wdown:Qdown 0.71<br>mdown:qdown 2.43
1180	1	883.7188	-3.03	3	44.4	19.1	1	1443-	R.	Carbamidomethyl: 2, 6, 13	

Detailed Protein Report

Cmpd.	No. of Cmpds.	m/z meas.	Δ m/z [ppm]	z	Rt [min]	Score	P	Range	Sequence	Modification	Ratios
								1465	QCIPACRKPFSYCTQGGVCGCEKG		

Detailed Protein Report

Protein 401: centrosome-associated protein 350 [Homo sapiens]

**Accession:**	gi|171184451	**Score:**	30.8
**Database:**	refseq_human(refseq_human_20140103.fasta)	**MW [kDa]:**	350.7
**Database Date:**	2015-11-30	**pI:**	5.9
		**Sequence Coverage [%]:**	1.2
		**No. of unique Peptides:**	2

Quantitation

mdown:qdown	**Median:**	0.70	**CV:**	0.00 %	**No. of Peptides:**	1
Wdown:Qdown	**Median:**	0.75	**CV:**	0.00 %	**No. of Peptides:**	1

Detailed Protein Report

10	20	30	40	50	60	70	80
MRSSKSKEVP	LPNPRNSQSK	DTVQADITTS	WDALSQTKAA	LRHIENKLEV	APTSTAVCDS	VMDTKKSSTS	ATRKISRKDG
90	100	110	120	130	140	150	160
RYLDDSWVNA	PISKSTKSRK	EKSRSPLRAT	TLESNVKKNN	RVEFREPLVS	YREIHGAPSN	FSSSHLESKH	VYCVDVNEEK
170	180	190	200	210	220	230	240
TESGNWMIGS	REERNIRSCD	FESSQSSVIN	DTVVRFLNDR	PAIDALQNSE	CLIRMGASMR	TEEEMPNRTK	GSENNLKLSV
250	260	270	280	290	300	310	320
NNMAHDTDPK	ALRLTDSSPS	STSTSNSQRL	DILKRRQHDV	KLEKLKERIR	KQWEHSEETN	GRGQKLGHID	HPVMVVNVDN
330	340	350	360	370	380	390	400
SVTAKVRKVA	TAPPAPAYKG	FNPSETKIRT	PDGKVWQEAE	FQNMSRELYR	DLALHFADDI	SIKEKPAEKS	KEKKVVKPVR
410	420	430	440	450	460	470	480
KVQKVAQLSS	TECRTGSSHL	ISTSSWRDGQ	KLVKKILGPA	PRMEPKEQRT	ASSDRGGRER	TAKSGGHIGR	AESDPRLDVL
490	500	510	520	530	540	550	560
HRHLQRNSER	SRSKSRSENN	IKKLASSLPD	NKQEENTALN	KDFLPIEIRG	ILDDLQLDST	AHTAKQDTVE	LQNQKSSAPV
570	580	590	600	610	620	630	640
HAPRSHSPVK	RKPDKITANE	DPPVISKRRH	YDTDEVRQYI	VRQQEERKRK	QNEEKKAQKE	ATEQKNKRLQ	ELYRKQKEAF
650	660	670	680	690	700	710	720
TKVKNVPPSE	PSATRRLQET	YSKLLLEKTL	LEEPSHQHVT	QETQAKPGYQ	PSGESDKENK	VQERPPSASS	SSDMSLSEPP
730	740	750	760	770	780	790	800
QPLARKDLME	STWMQPERLS	PQVHHSQPQP	FAGTAGSLLS	HLLSLEHVGI	LHKDFESILP	TRKNHNMASR	PLTFTPQPYV
810	820	830	840	850	860	870	880
TSPAAYTDAL	LKPSASQYKS	KLDRIEALKA	TAASLSSRIE	SEAKKLAGAS	INYGSAWNTE	YDVQQAPQED	GPWTKAVTPP
890	900	910	920	930	940	950	960
VKDDNEDVFS	ARIQKMLGSC	VSHATFDDDL	PGVGNLSEFK	KLPEMIRPQS	AISSFRVRSP	GPKPEGLLAQ	LCKRQTDSSS
970	980	990	1000	1010	1020	1030	1040
SDMQACSQDK	AKISLGSSID	SVSEGPLLSE	GSLSEEEGDQ	DGQPLLKVAE	ILKEKEFCPG	ERNSYEPIKE	FQKEAEKFLP
1050	1060	1070	1080	1090	1100	1110	1120
LFGHIGGTQS	KGPWEELAKG	SPHSVINIFT	KSYQLYGKGF	EDKLDRGTST	SRPLNATATP	LSGVSYEDDF	VSSPGTGTST
1130	1140	1150	1160	1170	1180	1190	1200
EKKSTLEPHS	TLSPQEDHSN	RKSAYDPSSV	DVTSQHSSGA	QSAASSRSST	SSKGKKGKKE	KTEWLDSFTG	NVQNSLLDEE
1210	1220	1230	1240	1250	1260	1270	1280
KAERGSHQGK	KSGTSSKLSV	KDFEQTLDTD	STLEDLSGHS	VSVSSDKGRS	QKTPTSPLSP	SSQKSLQFDV	AGTSSERSKS
1290	1300	1310	1320	1330	1340	1350	1360
SVMPPTITGF	KPNAPLTDLN	PAASRTTTEN	MAPIPGSKRF	SPAGLHHRMA	AELSYLNAIE	ESVRQLSDVE	RVRGISLAQQ
1370	1380	1390	1400	1410	1420	1430	1440
ESVSLAQIIK	AQQQRHERDL	ALLKLKAEQE	ALESQRQLEE	TRNKAAQVHA	ESLQQVVQSQ	REVTEVLQEA	TCKIAAQQSE
1450	1460	1470	1480	1490	1500	1510	1520
TARLTTDAAR	QICEMAELTR	THISDAVVAS	GAPLAILYDH	QRQHLPDFVK	QLRTRTETDR	KSPSVSLSQS	KEGTLDSKHQ
1530	1540	1550	1560	1570	1580	1590	1600
KYSASYDSYS	ESSGYKNHDR	RSSSGSSRQE	SPSVPSCKEN	EKKLNGEKIE	SSIDEQVQTA	ADDSLRSDSV	PSLPDEKDST
1610	1620	1630	1640	1650	1660	1670	1680
SIATEYSLKF	DESMTEDEIE	EQSFRSLLPS	ESHRRFNMEK	RRGHHDDSDE	EASPEKTTLS	TAKELNMPFS	GGQDSFSKFT
1690	1700	1710	1720	1730	1740	1750	1760
MEMVRQYMKE	EEMRAAHQSS	LLRLREKALK	EKTKAELAWL	EHQKKHLRDK	GEDDKMPPLR	KKQRGLLLRL	QQEKAEIKRL
1770	1780	1790	1800	1810	1820	1830	1840
QEANKAARKE	RQLILKQQEE	IEKIRQTTIK	LQEKLKSAGE	SKLDSHSDDD	TKDNKATSPG	PTDLETRSPS	PISISSSETS
1850	1860	1870	1880	1890	1900	1910	1920
SIMQKLKKMR	SRMDEKFLTK	REQKLMQRRQ	HAEELLEWKR	RLDAEEAEIR	QMEKQALAAW	DKELIKPKTP	KKELEDQRTE
1930	1940	1950	1960	1970	1980	1990	2000
QKEIASEEES	PVPLYSHLNS	ESSIPEELGS	PAVEYVPSES	IGQEQPGSPD	HSILTEEMIC	SQELESSTSP	SKHSLPKSCT
2010	2020	2030	2040	2050	2060	2070	2080
SVSKQESSKG	SHRTGGQCHL	PIKSHQHCYS	WSDESLSMTQ	SETTSDQSDI	EGRIRALKDE	LRKRKSVVNQ	LKKEQKKRQK
2090	2100	2110	2120	2130	2140	2150	2160
ERLKAQEASL	IKQLESYDEF	IKKTEAELSQ	DLETSPTAKP	QIKTLSSASE	KPKIKPLTPL	HRSETAKNWK	SLTESERSRG
2170	2180	2190	2200	2210	2220	2230	2240

Detailed Protein Report

Cmpd.	No. of Cmpds.	m/z meas.	Δ m/z [ppm]	z	Rt [min]	Score	P	Range	Sequence	Modification	Ratios
2836	5	973.5061	1.61	2	64.6	19.7	1	329-347	K.VATAPPAPAYKGFNPSETK.I		
316	1	966.9340	-27.85	2	33.3	11.1	2	1795-1812	K.LKSAGESKLDSHSDDDTK.D		Wdown:Qdown 0.75<br>mdown:qdown 0.70

Detailed Protein Report

Protein 402: **myosin-IIIb isoform 1 [Homo sapiens]**

**Accession:**	gi|284172510	**Score:**	30.7
**Database:**	refseq_human(refseq_human_20140103.fasta)	**MW [kDa]:**	148.5
**Database Date:**	2015-11-30	**pI:**	8.7
		**Sequence Coverage [%]:**	3.0
		**No. of unique Peptides:**	1

10	20	30	40	50	60	70	80
MKHLYGLFHY	NPMMLGLESL	PDPTDTWEII	ETIGKGTYGK	VYKVTNKRDG	SLAAVKILDP	VSDMDEEIEA	EYNILQFLPN
**90**	**100**	**110**	**120**	**130**	**140**	**150**	**160**
HPNVVKFYGM	FYKADHCVGG	QLWLVLELCN	GGSVTELVKG	LLRCGQRLDE	AMISYILYGA	LLGLQHLHNN	RIIHRDVKGN
**170**	**180**	**190**	**200**	**210**	**220**	**230**	**240**
NILLTTEGGV	KLVDFGVSAQ	LTSTRLRRNT	SVGTPFWMAP	EVIACEQQYD	SSYDARCDVW	SLGITAIELG	DGDPPLFDMH
**250**	**260**	**270**	**280**	**290**	**300**	**310**	**320**
PVKTLFKIPR	NPPPTLLHPE	KWCEEFNHFI	SQCLIKDFER	RPSVTHLLDH	PFIKGVHGKV	LFLQKQLAKV	LQDQKHQNPV
**330**	**340**	**350**	**360**	**370**	**380**	**390**	**400**
AKTRHERMHT	RRPYHVEDAE	KYCLEDDLVN	LEVLDEDTII	HQLQKRYADL	LIYTYVGDIL	IALNPFQNLS	IYSPQFSRLY
**410**	**420**	**430**	**440**	**450**	**460**	**470**	**480**
HGVKRASNPP	HIFASADAAY	QCMVTLSKDQ	CIVISGESGS	GKTESAHLIV	QHLTFLGKAN	NQTLREKILQ	VNSLVEAFGN
**490**	**500**	**510**	**520**	**530**	**540**	**550**	**560**
SCTAINDNSS	RFGKYLEMMF	TPTGVVMGAR	ISEYLLEKSR	VIKQAAREKN	FHIFYYIYAG	LHHQKKLSDF	RLPEEKPPRY
**570**	**580**	**590**	**600**	**610**	**620**	**630**	**640**
IADETGRVMH	DITSKESYRR	QFEAIQHCFR	IIGFTDKEVH	SVYRILAGIL	NIGNIEFAAI	SSQHQTDKSE	VPNAEALQNA
**650**	**660**	**670**	**680**	**690**	**700**	**710**	**720**
ASVLCISPEE	LQEALTSHCV	VTRGETIIRA	NTVDRAADVR	DAMSKALYGR	LFSWIVNRIN	TLLQPDENIC	SAGGGMNVGI
**730**	**740**	**750**	**760**	**770**	**780**	**790**	**800**
LDIFGFENFQ	RNSFEQLCIN	IANEQIQYYF	NQHVFALEQM	EYQNEGIDAV	PVEYEDNRPL	LDMFLQKPLG	LLALLDEESR
**810**	**820**	**830**	**840**	**850**	**860**	**870**	**880**
FPQATDQTLV	DKFEDNLRCK	YFWRPKGVEL	CFGIQHYAGK	VLYDASGVLE	KNRDTLPADV	VVVLRTSENK	LLQQLFSIPL
**890**	**900**	**910**	**920**	**930**	**940**	**950**	**960**
TKTGNLAQTR	ARITVASSSL	PPHFSAGKAK	VDTLEVIRHP	EETTNMKRQT	VASYFRYSLM	DLLSKMVVGQ	PHFVRCIKPN
**970**	**980**	**990**	**1000**	**1010**	**1020**	**1030**	**1040**
DDREALQFSR	ERVLAQLRST	GILETVSIRR	QGYSHRILFE	EFVKRYYYLA	FTAHQTPLAS	KESCVAILEK	SRLDHWVLGK
**1050**	**1060**	**1070**	**1080**	**1090**	**1100**	**1110**	**1120**
TKVFLKYYHV	EQLNLLLREV	IGRVVVLQAY	TKGWLGARRY	KRVREKREKG	AIAIQSGDTS	NQSSGPHSPV	AAGTRGSAEV
**1130**	**1140**	**1150**	**1160**	**1170**	**1180**	**1190**	**1200**
QDCSEPGDHK	VLRGSVHRRS	HSQAESNNGR	TQTSSNSPAV	TEKNGHSQAQ	SSPKGCDIFA	GHANKHSVSG	TDLLSSRICH
**1210**	**1220**	**1230**	**1240**	**1250**	**1260**	**1270**	**1280**
PAPDQQGLSL	WGAPQKPGSE	NGLAQKHRTP	RRRCQQPKML	SSPEDTMYYN	QLNGTLEYQG	SKRKPRKLGQ	IKVLDGEDEY
**1290**	**1300**	**1310**	**1320**				
YKSLSPVDCI	PEENNSAHPS	FFSSSSKGDS	FAQH				

Cmpd.	No. of Cmpds.	m/z meas.	Δ m/z [ppm]	z	Rt [min]	Score	P	Range	Sequence	Modification
1349	2	926.7733	-30.95	3	45.6	14.9	1	495-518	K.YLEMMFTPTGVVMGARISEYLLEK.S	

Detailed Protein Report

**Protein 403:**	**PREDICTED: probable JmjC domain-containing histone demethylation protein 2C isoform X6 [Homo sapiens]**		
**Accession:**	gi|530393271	**Score:**	30.6
**Database:**	refseq_human(refseq_human_20140103.fasta)	**MW [kDa]:**	250.9
**Database Date:**	2015-11-30	**pI:**	9.0
		**Sequence Coverage [%]:**	0.9
		**No. of unique Peptides:**	1

Detailed Protein Report

10	20	30	40	50	60	70	80
MNSQAAVPKQ	NTHQQQQQRS	IRPNKRKGSD	SSIPDEEKMK	EEKYDYISRG	ENPKGKNKHL	MNKRRKPEED	EKKLNMKRLR
90	100	110	120	130	140	150	160
TDNVSDFSES	SDSENSNKRI	IDNSSEQKPE	NELKNKNTSK	INGEEGKPHN	NEKAGEETLK	NSQPPWDQIQ	EDKKHEEAEK
170	180	190	200	210	220	230	240
RKSVDTQLQE	DMIIHSSEQS	TVSDHNSNDL	LPQECNMDKT	HTMELLPKEK	FVSRPPTPKC	VIDITNDTNL	EKVAQENSST
250	260	270	280	290	300	310	320
FGLQTLQKMD	PNVSDSKHSI	ANAKFLETAK	KDSDQSWVSD	VVKVDLTQSS	VTNASSGNDH	LNMEKEKYVS	YISPLSAVSV
330	340	350	360	370	380	390	400
MEDKLHKRSP	PPETIKSKLN	TSVDTHKIKS	SPSPEVVKPK	ITHSPDSVKS	KATYVNSQAT	GERRLANKIE	HELSRCSFHP
410	420	430	440	450	460	470	480
IPTRSSTLET	TKSPLIIDKN	EHFTVYRDPA	LIGSETGANH	ISPFLSQHPF	PLHSSSHRTC	LNPGTHHPAL	TPAPHLLAGS
490	500	510	520	530	540	550	560
SSQTPLPTIN	THPLTSGPHH	AVHHPHLLPT	VLPGVPTASL	LGGHPRLESA	HASSLSHLAL	AHQQQQQLLQ	HQSPHLLGQA
570	580	590	600	610	620	630	640
HPSASYNQLG	LYPIIWQYPN	GTHAYSGLGL	PSSKWVHPEN	AVNAEASLRR	NSPSPWLHQP	TPVTSADGIG	LLSHIPVRPS
650	660	670	680	690	700	710	720
SAEPHRPLKI	TAHSSPPLTK	TLVDHHKEEL	ERKAFMEPLR	SVASTSAKND	LDLNRSQTGK	DCHLHRHFVD	PVLNQLQRPP
730	740	750	760	770	780	790	800
QETGERLNKY	KEEHRRILQE	SIDVAPFTTK	IKGLEGEREN	YSRVASSSSS	PKSHIIKQDM	DVERSVSDLY	KMKHSVPQSL
810	820	830	840	850	860	870	880
PQSNYFTTLS	NSVVNEPPRS	YPSKEVSNIY	GDKQSNALAA	AAANPQTLTS	FITSLSKPPP	LIKHQPESEG	LVGKIPEHLP
890	900	910	920	930	940	950	960
HQIASHSVTT	FRNDCRSPTH	LTVSSTNTLR	SMPALHRAPV	FHPPIHHSLE	RKEGSYSSLS	PPTLTPVMPV	NAGGKVQESQ
970	980	990	1000	1010	1020	1030	1040
KPPTLIPEPK	DSQANFKSSS	EQSLTEMWRP	NNNLSKEKTE	WHVEKSSGKL	QAAMASVIVR	PSSSTKTDSM	PAMQLASKDR
1050	1060	1070	1080	1090	1100	1110	1120
VSERSSAGAH	KTDCLKLAEA	GETGRIILPN	VNSDSVHTKS	EKNFQAVSQG	SVPSSVMSAV	NTMCNTKTDV	ITSAADTTSV
1130	1140	1150	1160	1170	1180	1190	1200
SSWGGSEVIS	SLSNTILAST	SSECVSSKSV	SQPVAQKQEC	KVSTTAPVTL	ASSKTGSVVQ	PSSGFSGTTD	FIHLKKHKAA
1210	1220	1230	1240	1250	1260	1270	1280
LAAAQYKSSN	ASETEPNAIK	NQTLSASLPL	DSTVICSTIN	KANSVGNGQA	SQTSQPNYHT	KLKKAWLTRH	SEEDKNTNKM
1290	1300	1310	1320	1330	1340	1350	1360
ENSGNSVSEI	IKPCSVNLIA	STSSDIQNSV	DSKIIVDKYV	KDDKVNRRKA	KRTYESGSES	GDSDESESKS	EQRTKRQPKP
1370	1380	1390	1400	1410	1420	1430	1440
TYKKKQNDLQ	KRKGEIEEDL	KPNGVLSRSA	KERSKLKLQS	NSNTGIPRSV	LKDWRKVKKL	KQTGESFLQD	DSCCEIGPNL
1450	1460	1470	1480	1490	1500	1510	1520
QKCRECRLIR	SKKGEEPAHS	PVFCRFYYFR	RLSFSKNGVV	RIDGFSSPDQ	YDDEAMSLWT	HENFEDDELD	IETSKYILDI
1530	1540	1550	1560	1570	1580	1590	1600
IGDKFCQLVT	SEKTALSWVK	KDAKIAWKRA	VRGVREMCDA	CEATLFNIHW	VCQKCGFVVC	LDCYKAKERK	SSRDKELYAW
1610	1620	1630	1640	1650	1660	1670	1680
MKCVKGQPHD	HKHLMPTQII	PGSVLTDLLD	AMHTLREKYG	IKSHCHCTNK	QNLQVGNFPT	MNGVSQVLQN	VLNHSNKISL
1690	1700	1710	1720	1730	1740	1750	1760
CMPESQQQNT	PPKSEKNGGS	SPESDVGTDN	KLTPPESQSP	LHWLADLAEQ	KAREEKKENK	ELTLENQIKE	EREQDNSESP
1770	1780	1790	1800	1810	1820	1830	1840
NGRTSPLVSQ	NNEQGSTLRD	LLTTTAGKLR	VGSTDAGIAF	APVYSMGAPS	SKSGRTMPNI	LDDIIASVVE	NKIPPSKTSK
1850	1860	1870	1880	1890	1900	1910	1920
INVKPELKEE	PEESIISAVD	ENNKLYSDIP	HSWICEKHIL	WLKDYKNSSN	WKLFKECWKQ	GQPAVVSGVH	KKMNISLWKA
1930	1940	1950	1960	1970	1980	1990	2000
ESISLDFGDH	QADLLNCKDS	IISNANVKEF	WDGFEEVSKR	QKNKSGETVV	LKLKDWPSGE	DFKTMMPARY	EDLLKSLPLP
2010	2020	2030	2040	2050	2060	2070	2080
EYCNPEGKFN	LASHLPGFFV	RPDLGPRLCS	AYGVVAAKDH	DIGTTNLHIE	VSDVVNILVY	VGIAKGNGIL	SKAGILKKFE
2090	2100	2110	2120	2130	2140	2150	2160
EEDLDDILRK	RLKDSSEIPG	ALWHIYAGKD	VDKIREFLQK	ISKEQGLEVL	PEHDPIRDQS	WYVNKKLRQR	LLEEYGVRTC
2170	2180	2190	2200	2210	2220	2230	2240

Detailed Protein Report

Cmpd.	No. of Cmpds.	m/z meas.	Δ m/z [ppm]	z	Rt [min]	Score	P	Range	Sequence	Modification
1263	1	1019.5782	7.40	1	45.5	17.4	1	1912-1919	K.KMNISLWK.A	

Detailed Protein Report

Protein 404: **PREDICTED: probable tubulin polyglutamylase TTLL2 isoform X1 [Homo sapiens]**

**Accession:**	gi|578812991	**Score:**	30.6
**Database:**	refseq_human(refseq_human_20140103.fasta)	**MW [kDa]:**	59.5
**Database Date:**	2015-11-30	**pI:**	9.4
		**Sequence Coverage [%]:**	5.2
		**No. of unique Peptides:**	2

Quantitation

mdown:qdown **Median:** 2.82 **CV:** 0.00 % **No. of Peptides:** 1

```
        10         20         30         40         50         60         70         80
MAEDEPSGAL LKPLVFRVDE TTPAVVQSVL LERGWNKFDK QEQNAEDWNL YWRTSSFRMT EHNSVKPWQQ LNHHPGTTKL
        90        100        110        120        130        140        150        160
TRKDCLAKHL KHMRRMYGTS LYQFIPLTFV MPNDYTKFVA EYFQERQMLG TKHSYWICKP AELSRGRGIL IFSDFKDFIF
       170        180        190        200        210        220        230        240
DDMYIVQKYI SNPLLIGRYK CDLRIYVCVT GFKPLTIYVY QEGLVRFATE KFDLSNLQNN YAHLTNSSIN KSGASYEKIK
       250        260        270        280        290        300        310        320
EVIGHGCKWT LSRFFSYLRS WDVDDLLLWK KIHRMVILTI LAIAPSVPFA ANCFELFGFD ILIDDNLKPW LLEVNYSPAL
       330        340        350        360        370        380        390        400
TLDCSTDVLV KRKLVHDIID LIYLNGLRNE GREASNATHG NSNIDAAKSD RGGLDAPDCL PYDSLSFTSR MYNEDDSVVE
       410        420        430        440        450        460        470        480
KAVSVRPEAA PASQLEGEMS GQDFHLSTRE MPQSKPKLRS RHTPHKTLMP YASLFQSHSC KTKTSPCVLS DRGKAPDPQA
       490        500        510        520
GNFVLVFPFN EATLGASRNG LNVKRIIQEL QKLMNKQHS
```

Cmpd.	No. of Cmpds.	m/z meas.	Δ m/z [ppm]	z	Rt [min]	Score	P	Range	Sequence	Modification	Ratios
531	1	542.2992	-6.22	2	35.3	12.3	1	239-248	K.IKEVIGHGCK.W		mdown:qdown 2.82
89	1	647.9192	-109.54	3	30.5	18.2	1	447-463	K.TLMPYASLFQSHSCKTK.T		

Detailed Protein Report

Protein 405: zinc-binding alcohol dehydrogenase domain-containing protein 2 [Homo sapiens]

**Accession:**	gi|28557745	**Score:**	30.4
**Database:**	refseq_human(refseq_human_20140103.fasta)	**MW [kDa]:**	40.1
**Database Date:**	2015-11-30	**pI:**	9.2
**Modification(s):**	Oxidation	**Sequence Coverage [%]:**	9.0
		**No. of unique Peptides:**	1

10	20	30	40	50	60	70	80
MLRLVPTGAR	AIVDMSYARH	FLDFQGSAIP	QAMQKLVVTR	LSPNFREAVT	LSRDCPVPLP	GDGDLLVRNR	FVGVNASDIN
**90**	**100**	**110**	**120**	**130**	**140**	**150**	**160**
YSAGRYDPSV	KPPFDIGFEG	IGEVVALGLS	ASARYTVGQA	VAYMAPGSFA	EYTVVPASIA	TPVPSVKPEY	LTLLVSGTTA
**170**	**180**	**190**	**200**	**210**	**220**	**230**	**240**
YISLKELGGL	SEGKKVLVTA	AAGGTGQFAM	QLSKKAKCHV	IGTCSSDEKS	AFLKSLGCDR	PINYKTEPVG	TVLKQEYPEG
**250**	**260**	**270**	**280**	**290**	**300**	**310**	**320**
VDVVYESVGG	AMFDLAVDAL	ATKGRLIVIG	FISGYQTPTG	LSPVKAGTLP	AKLLKKSASV	QGFFLNHYLS	KYQAAMSHLL
**330**	**340**	**350**	**360**	**370**	**380**		
EMCVSGDLVC	EVDLGDLSPE	GRFTGLESIF	RAVNYMYMGK	NTGKIVVELP	HSVNSKL		

Cmpd.	No. of Cmpds.	m/z meas.	Δ m/z [ppm]	z	Rt [min]	Score	P	Range	Sequence	Modification
26	6	1068.4724	-95.51	2	29.6	14.6	2	1-19	-.MLRLVPTGARAIVDMSYAR.H	Oxidation: 1

Detailed Protein Report

Protein 406: DNA-directed RNA polymerase I subunit RPA49 isoform 2 [Homo sapiens]

**Accession:**	gi|544346178	**Score:**	30.4
**Database:**	refseq_human(refseq_human_20140103.fasta)	**MW [kDa]:**	39.8
**Database Date:**	2015-11-30	**pI:**	9.6
**Modification(s):**	Carbamidomethyl, Oxidation	**Sequence Coverage [%]:**	6.3
		**No. of unique Peptides:**	1

Quantitation

mdown:qdown	**Median:** 4.15	**CV:** 0.00 %	**No. of Peptides:**	1
Wdown:Qdown	**Median:** 1.18	**CV:** 0.00 %	**No. of Peptides:**	1

```
        10         20         30         40         50         60         70         80
MMLNCSTCSH YFQVFHQESK IIFWKEPRTC FLILSLPLPG ELTWDVSVES ELALESQTKT YREKMDSCIE AFGTTKQKRA
        90        100        110        120        130        140        150        160
LNTRRMNRVG NESLNRAVAK AAETIIDTKG VTALVSDAIH NDLQDDSLYL PPCYDDAAKP EDVYKFEDLL SPAEYEALQS
       170        180        190        200        210        220        230        240
PSEAFRNVTS EEILKMIEEN SHCTFVIEAL KSLPSDVESR DRQARCIWFL DTLIKFRAHR VVKRKSALGP GVPHIINTKL
       250        260        270        280        290        300        310        320
LKHFTCLTYN NGRLRNLISD SMKAKITAYV IILALHIHDF QIDLTVLQRD LKLSEKRMME IAKAMRLKIS KRRVSVAAGS
       330        340        350
EEDHKLGTLS LPLPPAQTSD RLAKRRKIT
```

Cmpd.	No. of Cmpds.	m/z meas.	Δ m/z [ppm]	z	Rt [min]	Score	P	Range	Sequence	Modification	Ratios
182	1	688.3002	4.63	2	31.0	12.9	0	65-76	K.MDSCIEAFGTTK.Q	Carbamidomethyl: 4; Oxidation: 1	Wdown:Qdown 1.18<br>mdown:qdown 4.15

Detailed Protein Report

Protein 407: calcium-binding mitochondrial carrier protein SCaMC-1 isoform 2 [Homo sapiens]

**Accession:**	gi|47458041	**Score:**	30.3
**Database:**	refseq_human(refseq_human_20140103.fasta)	**MW [kDa]:**	51.3
**Database Date:**	2015-11-30	**pI:**	5.6
**Modification(s):**	Oxidation	**Sequence Coverage [%]:**	7.2
		**No. of unique Peptides:**	1

Quantitation

mdown:qdown	**Median:**	0.57	**CV:**	0.00 %	**No. of Peptides:**	1
Wdown:Qdown	**Median:**	1.08	**CV:**	0.00 %	**No. of Peptides:**	1

10	20	30	40	50	60	70	80
MDSLYGDLFW	YLDYNKDGTL	DIFELQEGLE	DVGAIQSLEE	AKKIFTTGDV	NKDGKLDFEE	FMKYLKDHEK	KMKLAFKSLD
**90**	**100**	**110**	**120**	**130**	**140**	**150**	**160**
KNNDGKIEAS	EIVQSLQTLG	LTISEQQAEL	ILQSIDVDGT	MTVDWNEWRD	YFLFNPVTDI	EEIIRFWKHS	TGIDIGDSLT
**170**	**180**	**190**	**200**	**210**	**220**	**230**	**240**
IPDEFTEDEK	KSGQWWRQLL	AGGIAGAVSR	TSTAPLDRLK	IMMQVHGSKS	DKMNIFGGFR	QMVKEGGIRS	LWRGNGTNVI
**250**	**260**	**270**	**280**	**290**	**300**	**310**	**320**
KIAPETAVKF	WAYEQYKKLL	TEEGQKIGTF	ERFISGSMAG	ATAQTFIYPM	EVMKTRLAVG	KTGQYSGIYD	CAKKILKHEG
**330**	**340**	**350**	**360**	**370**	**380**	**390**	**400**
LGAFYKGYVP	NLLGIIPYAG	IDLAVYELLK	SYWLDNFAKD	SVNPGVMVLL	GCGALSSTCG	QLASYPLALV	RTRMQAQAML
**410**	**420**	**430**	**440**	**450**	**460**		
EGSPQLNMVG	LFRRIISKEG	IPGLYRGITP	NFMKVLPAVG	ISYVVYENMK	QTLGVTQK		

Cmpd.	No. of Cmpds.	m/z meas.	Δ m/z [ppm]	z	Rt [min]	Score	P	Range	Sequence	Modification	Ratios
9	1	803.7023	-37.89	3	29.3	14.8	1	394-414	R. MQAQAMLEGSPQLNMVGLFRR. I	Oxidation: 1, 15	mdown:qdown 0.57<br>Wdown:Qdown 1.08

Detailed Protein Report

Protein 408: armadillo repeat-containing X-linked protein 2 [Homo sapiens]

**Accession:**	gi|7662162	**Score:**	30.3
**Database:**	refseq_human(refseq_human_20140103.fasta)	**MW [kDa]:**	65.6
**Database Date:**	2015-11-30	**pI:**	9.3
		**Sequence Coverage [%]:**	5.7
		**No. of unique Peptides:**	1

Alias proteins:

Accession	Name	Description
gi|532691767	refseq_human (refseq_human_20140103.fasta)	armadillo repeat-containing X-linked protein 2 [Homo sapiens]
gi|530433311	refseq_human (refseq_human_20140103.fasta)	PREDICTED: armadillo repeat-containing X-linked protein 2 isoform X9 [Homo sapiens]
gi|530433309	refseq_human (refseq_human_20140103.fasta)	PREDICTED: armadillo repeat-containing X-linked protein 2 isoform X8 [Homo sapiens]
gi|530433307	refseq_human (refseq_human_20140103.fasta)	PREDICTED: armadillo repeat-containing X-linked protein 2 isoform X7 [Homo sapiens]
gi|530433305	refseq_human (refseq_human_20140103.fasta)	PREDICTED: armadillo repeat-containing X-linked protein 2 isoform X6 [Homo sapiens]
gi|530433303	refseq_human (refseq_human_20140103.fasta)	PREDICTED: armadillo repeat-containing X-linked protein 2 isoform X5 [Homo sapiens]
gi|530433299	refseq_human (refseq_human_20140103.fasta)	PREDICTED: armadillo repeat-containing X-linked protein 2 isoform X3 [Homo sapiens]
gi|530433297	refseq_human (refseq_human_20140103.fasta)	PREDICTED: armadillo repeat-containing X-linked protein 2 isoform X2 [Homo sapiens]
gi|530433295	refseq_human (refseq_human_20140103.fasta)	PREDICTED: armadillo repeat-containing X-linked protein 2 isoform X1 [Homo sapiens]
gi|29540562	refseq_human (refseq_human_20140103.fasta)	armadillo repeat-containing X-linked protein 2 [Homo sapiens]

10	20	30	40	50	60	70	80
MSRVRDAGCV	AAGIVIGAGA	WYCVYKYTRG	RDQTKKRMAK	PKNRAVAGTG	ARARAGLRAG	FTIDLGSGFS	PPTPVRAEAE
**90**	**100**	**110**	**120**	**130**	**140**	**150**	**160**
DRAQDEASAL	DTVGAEAVAP	AASSAEAQSG	AGSQAQEADG	AGVGPKAESV	VGAAMASAIA	PPPGVTEALG	AAEAPAMAGA
**170**	**180**	**190**	**200**	**210**	**220**	**230**	**240**
PKVAEAPREA	ETSRAAVPPG	TVVPTEAAAP	TEVTEGPGVA	APTKVAEAPG	VASPTEAAEA	PVPATPTGAA	APTGAAESPG
**250**	**260**	**270**	**280**	**290**	**300**	**310**	**320**
TSGSPRTAVV	PGTSAAKKAT	PGAHTGAIPK	ATSATGAVPK	GGGKGVTRSR	NGGKGKGKKS	KVEVDELGMG	FRPGDGAAAA
**330**	**340**	**350**	**360**	**370**	**380**	**390**	**400**
AAASANGGQA	FLAEVPDSEE	GESGWTDTES	DSDSEPETQR	RGRGRRPVAM	QKRPFPYEID	EILGVRDLRK	VLALLQKSDD
**410**	**420**	**430**	**440**	**450**	**460**	**470**	**480**
PFIQQVALLT	LSNNANYSCN	QETIRKLGGL	PIIANMINKT	DPHIKEKALM	AMNNLSENYE	NQGRLQVYMN	KVMDDIMASN
**490**	**500**	**510**	**520**	**530**	**540**	**550**	**560**
LNSAVQVVGL	KFLTNMTITN	DYQHLLVNSI	ANFFRLLSQG	GGKIKVEILK	ILSNFAENPD	MLKKLLSTQV	PASFSSLYNS
**570**	**580**	**590**	**600**	**610**	**620**	**630**	**640**
YVESEILINA	LTLFEIIYDN	LRAEVFNYRE	FNKGSLFYLC	TTSGVCVKKI	RALANHHDLL	VKVKVIKLVN	KF

Cmpd.	No. of Cmpds.	m/z meas.	Δ m/z [ppm]	z	Rt [min]	Score	P	Range	Sequence	Modification
2790	1	853.4116	-28.91	2	63.3	15.1	1	594-609	K.GSLFYLCTTSGVCVKK.I	

Detailed Protein Report

Protein 409: intraflagellar transport protein 122 homolog isoform 7 [Homo sapiens]

**Accession:**	gi|526253060	**Score:**	30.3
**Database:**	refseq_human(refseq_human_20140103.fasta)	**MW [kDa]:**	118.4
**Database Date:**	2015-11-30	**pI:**	6.8
		**Sequence Coverage [%]:**	2.3
		**No. of unique Peptides:**	2

10	20	30	40	50	60	70	80
MFNGIISIRN	KNGEEKVKIE	RPGGSLSPIW	SICWNPSREE	RNDILAVADW	GQKVSFYQLS	GKQIGKDRAL	NFDPCCISYF
90	100	110	120	130	140	150	160
TKGEYILLGG	SDKQVSLFTK	DGVRLGTVGE	QNSWVWTCQA	KPDSNYVVVG	CQDGTISFYQ	LIFSTVHGLY	KDRYAYRDSM
170	180	190	200	210	220	230	240
TDVIVQHLIT	EQKVRIKCKE	LVKKIAIYRN	RLAIQLPEKI	LIYELYSEDL	SDMHYRVKEK	IIKKFECNLL	VVCANHIILC
250	260	270	280	290	300	310	320
QEKRLQCLSF	SGVKEREWQM	ESLIRYIKVI	GGPPGREGLL	VGLKNGQILK	IFVDNLFAIV	LLKQATAVRC	LDMSASRKKL
330	340	350	360	370	380	390	400
AVVDENDTCL	VYDIDTKELL	FQEPNANSVA	WNTQCEDMLC	FSGGGYLNIK	ASTFPVHRQK	LQGFVVGYNG	SKIFCLHVFS
410	420	430	440	450	460	470	480
ISAVEVPQSA	PMYQYLDRKL	FKEAYQIACL	GVTDTDWREL	AMEALEGLDF	ETAKKAFIRV	QDLRYLELIS	SIEERKKRGE
490	500	510	520	530	540	550	560
TNNDLFLADV	FSYQGKFHEA	AKLYKRSGHE	NLALEMYTDL	CMFEYAKDFL	GSGDPKETKM	LITKQADWAR	NIKEPKAAVE
570	580	590	600	610	620	630	640
MYISAGEHVK	AIEICGDHGW	VDMLIDIARK	LDKAEREPLL	LCATYLKKLD	SPGYAAETYL	KMGDLKSLVQ	LHVETQRWDE
650	660	670	680	690	700	710	720
AFALGEKHPE	FKDDIYMPYA	QWLAENDRFE	EAQKAFHKAG	RQREAVQVLE	QLTNNAVAES	RFNDAAYYYW	MLSMQCLDIA
730	740	750	760	770	780	790	800
QDPAQKDTML	GKFYHFQRLA	ELYHGYHAIH	RHTEDPFSVH	RPETLFNISR	FLLHSLPKDT	PSGISKVKIL	FTLAKQSKAL
810	820	830	840	850	860	870	880
GAYRLARHAY	DKLRGLYIPA	RFQKSIELGT	LTIRAKPFHD	SEELVPLCYR	CSTNNPLLNN	LGNVCINCRQ	PFIFSASSYD
890	900	910	920	930	940	950	960
VLHLVEFYLE	EGITDEEAIS	LIDLEVLRPK	RDDRQLEIAN	NSSQILRLVE	TKDSIGDEDP	FTAKLSFEQG	GSEFVPVVVS
970	980	990	1000	1010	1020	1030	1040
RLVLRSMSRR	DVLIKRWPPP	LRWQYFRSLL	PDASITMCPS	CFQMFHSEDY	ELLVLQHGCC	PYCRRCKDDP	GP

Cmpd.	No. of Cmpds.	m/z meas.	Δ m/z [ppm]	z	Rt [min]	Score	P	Range	Sequence	Modification
2627	3	665.3628	29.07	2	61.5	19.4	0	42-53	R.NDILAVADWGQK.V	
2085	1	731.3255	-153.98	2	56.2	10.9	2	813-824	K.LRGLYIPARFQK.S	

Detailed Protein Report

Protein 410: protein limb expression 1 homolog [Homo sapiens]

**Accession:**	gi|167466286	**Score:**	30.3
**Database:**	refseq_human(refseq_human_20140103.fasta)	**MW [kDa]:**	31.9
**Database Date:**	2015-11-30	**pI:**	5.3
		**Sequence Coverage [%]:**	8.5
		**No. of unique Peptides:**	1

10	20	30	40	50	60	70	80
MDRTLESLRH	IIAQVLPHRD	PALVFKDLNV	VSMLQEFWES	KQQQKAAFPS	EGVVVYESLP	APGPPFVSYV	TLPGGSCFGN
**90**	**100**	**110**	**120**	**130**	**140**	**150**	**160**
FQCCLSRAEA	RRDAAKVALI	NSLFNELPSR	RITKEFIMES	VQEAVASTSG	TLDDADDPST	SVGAYHYMLE	SNMGKTMLEF
**170**	**180**	**190**	**200**	**210**	**220**	**230**	**240**
QELMTIFQLL	HWNGSLKALR	ETKCSRQEVI	SYYSQYSLDE	KMRSHMALDW	IMKERDSPGI	VSQELRMALR	QLEEARKAGQ
**250**	**260**	**270**	**280**	**290**			
ELRFYKEKKE	ILSLALTQIC	SDPDTSSPSD	DQLSLTALCG	YH			

Cmpd.	No. of Cmpds.	m/z meas.	Δ m/z [ppm]	z	Rt [min]	Score	P	Range	Sequence	Modification
2110	1	902.3468	-108.11	2	56.5	14.0	2	202-215	K.MRSHMALDWIMKER.D	

Detailed Protein Report

Protein 411: **CMP-N-acetylneuraminate-beta-1,4-galactoside alpha-2,3-sialyltransferase isoform n [Homo sapiens]**

**Accession:**	gi|394953753	**Score:**	30.3
**Database:**	refseq_human(refseq_human_20140103.fasta)	**MW [kDa]:**	26.7
**Database Date:**	2015-11-30	**pI:**	10.2
**Modification(s):**	Carbamidomethyl	**Sequence Coverage [%]:**	10.4
		**No. of unique Peptides:**	2

10	20	30	40	50	60	70	80
MGLLVFVRNL	LLALCLFLVL	GFLYYSAWKL	HLLQWEEDSK	YDRLGFLLNL	DSKLFSKPAP	MFLDDSFRKW	ARIREFVPPF
**90**	**100**	**110**	**120**	**130**	**140**	**150**	**160**
GIKGQDNLIK	AILSVTKEYR	LTPALDSLRC	RRCIIVGNGG	VLANKSLGSR	IDDYDIVVRL	NSAPVKGFEK	DVGSKTTLRI
**170**	**180**	**190**	**200**	**210**	**220**	**230**	**240**
TYPEGAMQRP	EQYERDSLFV	LAGFKWQDFK	WLKYIVYKER	VSWTHNIQRE	KEFLRKLVKA	RVITDLSSGI	

Cmpd.	No. of Cmpds.	m/z meas.	Δ m/z [ppm]	z	Rt [min]	Score	P	Range	Sequence	Modification
146	2	657.8165	-71.56	2	31.1	10.8	0	113-125	R.CIIVGNGGVLANK.S	Carbamidomethyl: 1
2295	3	1131.4541	-161.50	1	57.1	19.5	1	220-230	K.ARVITDLSSGI.-	

Detailed Protein Report

Protein 412: PREDICTED: PR domain zinc finger protein 1 isoform X1 [Homo sapiens]

**Accession:**	gi|530383954	**Score:**	30.2
**Database:**	refseq_human(refseq_human_20140103.fasta)	**MW [kDa]:**	73.8
**Database Date:**	2015-11-30	**pI:**	9.9
**Modification(s):**	Oxidation	**Sequence Coverage [%]:**	3.6
		**No. of unique Peptides:**	1

10	20	30	40	50	60	70	80
MRYVNPAHSP	REQNLAACQN	GMNIYFYTIK	PIPANQELLV	WYCRDFAERL	HYPYPGELTM	MNLTQTQSSL	KQPSTEKNEL
**90**	**100**	**110**	**120**	**130**	**140**	**150**	**160**
CPKNVPKREY	SVKEILKLDS	NPSKGKDLYR	SNISPLTSEK	DLDDFRRRGS	PEMPFYPRVV	YPIRAPLPED	FLKASLAYGI
**170**	**180**	**190**	**200**	**210**	**220**	**230**	**240**
ERPTYITRSP	IPSSTTPSPS	ARSSPDQSLK	SSSPHSSPGN	TVSPVGPGSQ	EHRDSYAYLN	ASYGTEGLGS	YPGYAPLPHL
**250**	**260**	**270**	**280**	**290**	**300**	**310**	**320**
PPAFIPSYNA	HYPKFLLPPY	GMNCNGLSAV	SSMNGINNFG	LFPRLCPVYS	NLLGGGSLPH	PMLNPTSLPS	SLPSDGARRL
**330**	**340**	**350**	**360**	**370**	**380**	**390**	**400**
LQPEHPREVL	VPAPHSAFSF	TGAAASMKDK	ACSPTSGSPT	AGTAATAEHV	VQPKATSAAM	AAPSSDEAMN	LIKNKRNMTG
**410**	**420**	**430**	**440**	**450**	**460**	**470**	**480**
YKTLPYPLKK	QNGKIKYECN	VCAKTFGQLS	NLKVHLRVHS	GERPFKCQTC	NKGFTQLAHL	QKHYLVHTGE	KPHECQVCHK
**490**	**500**	**510**	**520**	**530**	**540**	**550**	**560**
RFSSTSNLKT	HLRLHSGEKP	YQCKVCPAKF	TQFVHLKLHK	RLHTRERPHK	CSQCHKNYIH	LCSLKVHLKG	NCAAAPAPGL
**570**	**580**	**590**	**600**	**610**	**620**	**630**	**640**
PLEDLTRINE	EIEKFDISDN	ADRLEDVEDD	ISVISVVEKE	ILAVVRKEKE	ETGLKVSLQR	NMGNGLLSSG	CSLYESSDLP
**650**	**660**	**670**					
LMKLPPSNPL	PLVPVKVKQE	TVEPMDP					

Cmpd.	No. of Cmpds.	m/z meas.	Δ m/z [ppm]	z	Rt [min]	Score	P	Range	Sequence	Modification
1540	3	598.8105	61.20	2	47.8	11.5	0	129-138	R.GSPEMPFYPR.V	Oxidation: 5

Detailed Protein Report

Protein 413: **PREDICTED: nuclear receptor subfamily 1 group D member 2 isoform X1 [Homo sapiens]**

**Accession:**	gi|530373510	**Score:**	30.2
**Database:**	refseq_human(refseq_human_20140103.fasta)	**MW [kDa]:**	45.6
**Database Date:**	2015-11-30	**pI:**	9.9
**Modification(s):**	Oxidation	**Sequence Coverage [%]:**	9.8
		**No. of unique Peptides:**	1

10	20	30	40	50	60	70	80
MEVNAGGVIA	YISSSSSASS	PASCHSEGSE	NSFQSSSSSV	PSSPNSSNSD	TNGNPKNGDL	ANIEGILKND	RIDCSMKTSK
**90**	**100**	**110**	**120**	**130**	**140**	**150**	**160**
SSAPGMTKSH	SGVTKFSGMV	LLCKVCGDVA	SGFHYGVHAC	EGCKGFFRRS	IQQNIQYKKC	LKNENCSIMR	MNRNRCQQCR
**170**	**180**	**190**	**200**	**210**	**220**	**230**	**240**
FKKCLSVGMS	RDAVRFGRIP	KREKQRMLIE	MQSAMKTMMN	SQFSGHLQND	TLVEHHEQTA	LPAQEQLRPK	PQLEQENIKS
**250**	**260**	**270**	**280**	**290**	**300**	**310**	**320**
SSPPSSDFAK	EEVIGMVTRA	HKDTFMYNQE	QQENSAESMQ	PQRGERIPKN	MEQYNLNHDH	CGNGLSSHFP	CSESQQHLNG
**330**	**340**	**350**	**360**	**370**	**380**	**390**	**400**
QFKGRNIMHY	PNGHAICIAN	GHCMNFSNAY	TQRVCDRVPI	DGFSQNENKN	SYLCNTGGRM	HLYSVHIQPK	FHQKQQYHTI
**410**							
SSLNHEVL							

Cmpd.	No. of Cmpds.	m/z meas.	Δ m/z [ppm]	z	Rt [min]	Score	P	Range	Sequence	Modification
1576	1	1011.7169	-72.08	3	49.6	10.7	1	96-124	K.FSGMVLLCKVCGDVASGFHYGVHACEGCK.G	Oxidation: 4

Detailed Protein Report

Protein 414: telomerase protein component 1 [Homo sapiens]

**Accession:**	gi|21536371	**Score:**	30.1
**Database:**	refseq_human(refseq_human_20140103.fasta)	**MW [kDa]:**	290.3
**Database Date:**	2015-11-30	**pI:**	9.2
		**Sequence Coverage [%]:**	0.9
		**No. of unique Peptides:**	1

Alias proteins:

Accession	Name	Description
gi|530404542	refseq_human (refseq_human_20140103.fasta)	PREDICTED: telomerase protein component 1 isoform X1 [Homo sapiens]

Detailed Protein Report

10	20	30	40	50	60	70	80
MEKLHGHVSA	HPDILSLENR	CLAMLPDLQP	LEKLHQHVST	HSDILSLKNQ	CLATLPDLKT	MEKPHGYVSA	HPDILSLENQ
90	100	110	120	130	140	150	160
CLATLSDLKT	MEKPHGHVSA	HPDILSLENR	CLATLSSLKS	TVSASPLFQS	LQISHMTQAD	LYRVNNSNCL	LSEPPSWRAQ
170	180	190	200	210	220	230	240
HFSKGLDLST	CPIALKSISA	TETAQEATLG	RWFDSEEKKG	AETQMPSYSL	SLGEEEEVED	LAVKLTSGDS	ESHPEPTDHV
250	260	270	280	290	300	310	320
LQEKKMALLS	LLCSTLVSEV	NMNNTSDPTL	AAIFEICREL	ALLEPEFILK	ASLYARQQLN	VRNVANNILA	IAAFLPACRP
330	340	350	360	370	380	390	400
HLRRYFCAIV	QLPSDWIQVA	ELYQSLAEGD	KNKLVPLPAC	LRTAMTDKFA	QFDEYQLAKY	NPRKHRAKRH	PRRPPRSPGM
410	420	430	440	450	460	470	480
EPPFSHRCFP	RYIGFLREEQ	RKFEKAGDTV	SEKKNPPRFT	LKKLVQRLHI	HKPAQHVQAL	LGYRYPSNLQ	LFSRSRLPGP
490	500	510	520	530	540	550	560
WDSSRAGKRM	KLSRPETWER	ELSLRGNKAS	VWEELIENGK	LPFMAMLRNL	CNLLRVGISS	RHHELILQRL	QHAKSVIHSR
570	580	590	600	610	620	630	640
QFPFRFLNAH	DAIDALEAQL	RNQALPFPSN	ITLMRRILTR	NEKNRPRRRF	LCHLSRQQLR	MAMRIPVLYE	QLKREKLRVH
650	660	670	680	690	700	710	720
KARQWKYDGE	MLNRYRQALE	TAVNLSVKHS	LPLLPGRTVL	VYLTDANADR	LCPKSNPQGP	PLNYALLLIG	MMITRAEQVD
730	740	750	760	770	780	790	800
VVLCGGDTLK	TAVLKAEEGI	LKTAIKLQAQ	VQEFDENDGW	SLNTFGKYLL	SLAGQRVPVD	RVILLGQSMD	DGMINVAKQL
810	820	830	840	850	860	870	880
YWQRVNSKCL	FVGILLRRVQ	YLSTDLNPND	VTLSGCTDAI	LKFIAEHGAS	HLLEHVGQMD	KIFKIPPPPG	KTGVQSLRPL
890	900	910	920	930	940	950	960
EEDTPSPLAP	VSQQGWRSIR	LFISSTFRDM	HGERDLLLRS	VLPALQARAA	PHRISLHGID	LRWGVTEEET	RRNRQLEVCL
970	980	990	1000	1010	1020	1030	1040
GEVENAQLFV	GILGSRYGYI	PPSYNLPDHP	HFHWAQQYPS	GRSVTEMEVM	QFLNRNQRLQ	PSAQALIYFR	DSSFLSSVPD
1050	1060	1070	1080	1090	1100	1110	1120
AWKSDFVSES	EEAARRISEL	KSYLSRQKGI	TCRRYPCEWG	GVAAGRPYVG	GLEEFGQLVL	QDVWNMIQKL	YLQPGALLEQ
1130	1140	1150	1160	1170	1180	1190	1200
PVSIPDDDLV	QATFQQLQKP	PSPARPRLLQ	DTVQRLMLPH	GRLSLVTGQS	GQGKTAFLAS	LVSALQAPDG	AKVASLVFFH
1210	1220	1230	1240	1250	1260	1270	1280
FSGARPDQGL	ALTLLRRLCT	YLRGQLKEPG	ALPSTYRSLV	WELQQRLLPK	SAESLHPGQT	QVLIIDGADR	LVDQNGQLIS
1290	1300	1310	1320	1330	1340	1350	1360
DWIPKKLPRC	VHLVLSVSSD	AGLGETLEQS	QGAHVLALGP	LEASARARLV	REELALYGKR	LEESPFNNQM	RLLLVKRESG
1370	1380	1390	1400	1410	1420	1430	1440
RPLYLRLVTD	HLRLFTLYEQ	VSERLRTLPA	TVPLLLQHIL	STLEKEHGPD	VLPQALTALE	VTRSGLTVDQ	LHGVLSVWRT
1450	1460	1470	1480	1490	1500	1510	1520
LPKGTKSWEE	AVAAGNSGDP	YPMGPFACLV	QSLRSLLGEG	PLERPGARLC	LPDGPLRTAA	KRCYGKRPGL	EDTAHILIAA
1530	1540	1550	1560	1570	1580	1590	1600
QLWKTCDADA	SGTFRSCPPE	ALGDLPYHLL	QSGNRGLLSK	FLTNLHVVAA	HLELGLVSRL	LEAHALYASS	VPKEEQKLPE
1610	1620	1630	1640	1650	1660	1670	1680
ADVAVFRTFL	RQQASILSQY	PRLLPQQAAN	QPLDSPLCHQ	ASLLSRRWHL	QHTLRWLNKP	RTMKNQQSSS	LSLAVSSSPT
1690	1700	1710	1720	1730	1740	1750	1760
AVAFSTNGQR	AAVGTANGTV	YLLDLRTWQE	EKSVVSGCDG	ISACLFLSDD	TLFLTAFDGL	LELWDLQHGC	RVLQTKAHQY
1770	1780	1790	1800	1810	1820	1830	1840
QITGCCLSPD	CRLLATVCLG	GCLKLWDTVR	GQLAFQHTYP	KSLNCVAFHP	EGQVIATGSW	AGSISFFQVD	GLKVTKDLGA
1850	1860	1870	1880	1890	1900	1910	1920
PGASIRTLAF	NVPGGVVAVG	RLDSMVELWA	WREGARLAAF	PAHHGFVAAA	LFLHAGCQLL	TAGEDGKVQV	WSGSLGRPRG
1930	1940	1950	1960	1970	1980	1990	2000
HLGSLSLSPA	LSVALSPDGD	RVAVGYRADG	IRIYKISSGS	QGAQGQALDV	AVSALAWLSP	KVLVSGAEDG	SLQGWALKEC
2010	2020	2030	2040	2050	2060	2070	2080
SLQSLWLLSR	FQKPVLGLAT	SQELLASASE	DFTVQLWPRQ	LLTRPHKAED	FPCGTELRGH	EGPVSCCSFS	TDGGSLATGG
2090	2100	2110	2120	2130	2140	2150	2160
RDRSLLCWDV	RTPKTPVLIH	SFPACHRDWV	TGCAWTKDNL	LISCSSDGSV	GLWDPESGQR	LGQFLGHQSA	VSAVAAVEEH
2170	2180	2190	2200	2210	2220	2230	2240

Detailed Protein Report

Cmpd.	No. of Cmpds.	m/z meas.	Δ m/z [ppm]	z	Rt [min]	Score	P	Range	Sequence	Modification
1409	1	630.7535	142.03	3	46.2	11.3	2	610-624	R.FLCHLSRQQLRMAMR.I	

Detailed Protein Report

Protein 415: uncharacterized protein C5orf55 precursor [Homo sapiens]

Accession: gi|156523054
Database: refseq_human(refseq_human_20140103.fasta)
Database Date: 2015-11-30
Modification(s): Carbamidomethyl

Score: 30.0
MW [kDa]: 12.7
pI: 12.0
Sequence Coverage [%]: 27.7
No. of unique Peptides: 1

10	20	30	40	50	60	70	80
MPAVFMLASS	SALQCGRGVP	RFPRTEVGAG	HSVNEETKAE	KVGNQTSVIP	ATSRQAALGT	SWTQRRTQPL	QERSHWHPRG
**90**	**100**	**110**	**120**				
NNASGMGGHR	MFPGPLRGPA	AQVLENECGS	LGRAAEGRS				

Cmpd.	No. of Cmpds.	m/z meas.	Δ m/z [ppm]	z	Rt [min]	Score	P	Range	Sequence	Modification
1854	1	829.2700	-158.89	2	51.7	10.9	0	98-113	R.GPAAQVLENECGSLGR.A	Carbamidomethyl: 11

Detailed Protein Report

Protein 416: PREDICTED: pericentriolar material 1 protein isoform X28 [Homo sapiens]

**Accession:**	gi|578815347	**Score:**	30.0
**Database:**	refseq_human(refseq_human_20140103.fasta)	**MW [kDa]:**	180.4
**Database Date:**	2015-11-30	**pI:**	5.3
		**Sequence Coverage [%]:**	2.1
		**No. of unique Peptides:**	2

10	20	30	40	50	60	70	80
MATGGGPFED	GMNDQDLPNW	SNENVDDRLN	NMDWGAQQKK	ANRSSEKNKK	KFGVESDKRV	TNDISPESSP	GVGRRRTKTP
90	100	110	120	130	140	150	160
HTFPHSRYMS	QMSVPEQAEL	EKLKQRINFS	DLDQRSIGSD	SQGRATAANN	KRQLSENRKP	FNFLPMQINT	NKSKDASTNP
170	180	190	200	210	220	230	240
PNRETIGSAQ	CKELFASALS	NDLLQNCQVS	EEDGRGEPAM	ESSQIVSRLV	QIRDYITKAS	SMREDLVEKN	ERSANVERLT
250	260	270	280	290	300	310	320
HLIDHLKEQE	KSYMKFLKKI	LARENEEEDV	RTIDSAVGSG	SVAESTSLNI	DVQSEASDTT	EEASFSLRIR	PCIEDKLGNS
330	340	350	360	370	380	390	400
ASQEQVSDID	VTTSPKGKGD	RPQNDRELRP	NRKYSQKRGF	PSKARDPQQE	PMEEIENLKK	QHDLLKRMLQ	QQEQLRALQG
410	420	430	440	450	460	470	480
RQAALLALQH	KAEQAIAVMD	DSVVAETAGS	LSGVSITSEL	NEELNDLIQR	FHNQLRDSQP	PAVPDNRRQA	ESLSLTREVS
490	500	510	520	530	540	550	560
QSRKPSASER	LPDEKVELFS	KMRVLQEKKQ	KMDKLLGELH	TLRDQHLNNS	SSSPQRSVDQ	RSTSAPSASV	GLAPVVNGES
570	580	590	600	610	620	630	640
NSLTSSVPYP	TASLVSQNES	ENEGHLNPSE	KLQKLNEVRK	RLNELRELVH	YYEQTSDMMT	DAVNENRKDE	ETEESEYDSE
650	660	670	680	690	700	710	720
HENSEPVTNI	RNPQVASTWN	EVNSHSNAQC	VSNNRDGRTV	NSNCEINNRS	AANIRALNMP	PSLADCRYNR	EGEQEIHVAQ
730	740	750	760	770	780	790	800
GEDDEEEEEE	AEEEGVSGAS	LSSHRSSLVD	EHPEDAEFEQ	KINRLMAAKQ	KLRQLQDLVA	MVQDDDAAQG	VISASASNLD
810	820	830	840	850	860	870	880
DFYPAEEDTK	QNSNNTRGNA	NKTQKDTGVN	EKAREKFYEA	KLQQQQRELK	QLQEERKKLI	DIQEKIQALQ	TACPDLQLSA
890	900	910	920	930	940	950	960
ASVGNCPTKK	YMPAVTSTPT	VNQHETSTSK	SVFEPEDSSI	VDNELWSEMR	RHEMLREELR	QRRKQLEALM	AEHQRRQGLA
970	980	990	1000	1010	1020	1030	1040
ETASPVAVSL	RSDGSENLCT	PQQSRTEKTM	ATWGGSTQCA	LDEEGDEDGY	LSEGIVRTDE	EEEEEQDASS	NDNFSVCPSN
1050	1060	1070	1080	1090	1100	1110	1120
SVNHNSYNGK	ETKNRWKNNC	PFSADENYRP	LAKTRQQNIS	MQRQENLRWV	SELSYVEEKE	QWQEQINQLK	KQLDFSVSIC
1130	1140	1150	1160	1170	1180	1190	1200
QTLMQDQQTL	SCLLQTLLTG	PYSVMPSNVA	SPQVHFIMHQ	LNQCYTQLTW	QQNNVQRLKQ	MLNELMRQQN	QHPEKPGGKE
1210	1220	1230	1240	1250	1260	1270	1280
RGSSASHPPS	PSLFCPFSFP	TQPVNLFNIP	GFTNFSSFAP	GMNFSPLFPS	NFGDFSQNIS	TPSEQQQPLA	QNSSGKTEYM
1290	1300	1310	1320	1330	1340	1350	1360
AFPKPFESSS	SIGAEKPRNK	KLPEEEVESS	RTPWLYEQEG	EVEKPFIKTG	FSVSVEKSTS	SNRKNQLDTN	GRRRQFDEES
1370	1380	1390	1400	1410	1420	1430	1440
LESFSSMPDP	VDPTTVTKTF	KTRKASAQAS	LASKDKTPKS	KSKKRNSTQL	KSRVKNIRYE	SASMSSTCEP	CKSRNRHSAQ
1450	1460	1470	1480	1490	1500	1510	1520
TEEPVQAKVF	SRKNHEQLEK	IIKCNRSTEI	SSAHARRILQ	QSNRNACNEA	PETGSDFSMF	EALRDTIYSE	VATLISQNES
1530	1540	1550	1560	1570	1580	1590	1600
RPHFLIELFH	ELQLLNTDYL	RQRALYALQD	IVSRHISESH	EKGENVKSVN	SGTWIASNSE	LTPSESLATT	DDVFIQEK

Cmpd.	No. of Cmpds.	m/z meas.	Δ m/z [ppm]	z	Rt [min]	Score	P	Range	Sequence	Modification
54	2	809.3848	-118.90	2	29.9	12.2	1	397-411	R.ALQGRQAALLALQHK.A	
1791	1	732.8049	117.99	3	52.4	17.8	1	1544-1562	R.ALYALQDIVSRHISESHEK.G	

Detailed Protein Report

Protein 417: protein FAM83G [Homo sapiens]

**Accession:**	gi|115392150	**Score:**	30.0
**Database:**	refseq_human(refseq_human_20140103.fasta)	**MW [kDa]:**	90.8
**Database Date:**	2015-11-30	**pI:**	5.9
		**Sequence Coverage [%]:**	2.3
		**No. of unique Peptides:**	2

Quantitation

mdown:qdown	**Median:** 1.11	**CV:** 0.00 %	**No. of Peptides:** 1
Wdown:Qdown	**Median:** 0.80	**CV:** 0.00 %	**No. of Peptides:** 1

```
        10         20         30         40         50         60         70         80
MAFSQVQCLD DNHVNWRSSE SKPEFFYSEE QRLALEALVA RGRDAFYEVL KRENIRDFLS ELELKRILET IEVYDPGSED
        90        100        110        120        130        140        150        160
PRGTGPSQGP EDNGVGDGEE ASGADGVPIE AEPLPSLEYW PQKSDRSIPQ LDLGWPDTIA YRGVTRASVY MQPPIDGQAH
       170        180        190        200        210        220        230        240
IKEVVRKMIS QAQKVIAVVM DMFTDVDIFK DLLDAGFKRK VAVYIIVDES NVKYFLHMCE RACMHLGHLK NLRVRSSGGT
       250        260        270        280        290        300        310        320
EFFTRSATKF KGALAQKFMF VDGDRAVCGS YSFTWSAART DRNVISVLSG QVVEMFDRQF QELYLMSHSV SLKGIPMEKE
       330        340        350        360        370        380        390        400
PEPEPIVLPS VVPLVPAGTV AKKLVNPKYA LVKAKSVDEI AKISSEKQEA KKPLGLKGPA LAEHPGELPE LLPPIHPGLL
       410        420        430        440        450        460        470        480
HLERANMFEY LPTWVEPDPE PGSDILGYIN IIDPNIWNPQ PSQMNRIKIR DTSQASAQHQ LWKQSQDSRP RPEPCPPPEP
       490        500        510        520        530        540        550        560
SAPQDGVPAE NGLPQGDPEP LPPVPKPRTV PVADVLARDS SDIGWVLELP KEEAPQNGTD HRLPRMAGPG HAPLQRQLSV
       570        580        590        600        610        620        630        640
TQDDPESLGV GLPNGLDGVE EEDDDDYVTL SDQDSHSGSS GRGPGPRRPS VASSVSEEYF EVREHSVPLR RRHSEQVANG
       650        660        670        680        690        700        710        720
PTPPPRRQLS APHITRGTFV GPQGGSPWAQ SRGREEADAL KRMQAQRSTD KEAQGQQFHH HRVPASGTRD KDGFPGPPRY
       730        740        750        760        770        780        790        800
RSAADSVQSS TRNAGPAMAG PHHWQAKGGQ VPRLLPDPGS PRLAQNARPM TDGRATEEHP SPFGIPYSKL SQSKHLKART
       810        820        830
GGSQWASSDS KRRAQAPRDR KDP
```

Cmpd.	No. of Cmpds.	m/z meas.	Δ m/z [ppm]	z	Rt [min]	Score	P	Range	Sequence	Modification	Ratios
1617	1	712.0414	-43.20	3	48.9	13.8	2	33-51	R.LALEALVARGRDAFYEVLK.R		
568	5	492.7372	-37.03	2	35.9	16.2	0	44-51	R.DAFYEVLK.R		Wdown:Qdown 0.80<br>mdown:qdown 1.11

Detailed Protein Report

Protein 418: **nuclear receptor coactivator 4 isoform 3 [Homo sapiens]**

**Accession:**	gi|14149617	**Score:**	30.0
**Database:**	refseq_human(refseq_human_20140103.fasta)	**MW [kDa]:**	69.7
**Database Date:**	2015-11-30	**pI:**	5.7
**Modification(s):**	Carbamidomethyl	**Sequence Coverage [%]:**	5.2
		**No. of unique Peptides:**	1

Alias proteins:

**Accession**	**Name**	**Description**
gi|223890287	refseq_human (refseq_human_20140103.fasta)	nuclear receptor coactivator 4 isoform 3 [Homo sapiens]
gi|223890284	refseq_human (refseq_human_20140103.fasta)	nuclear receptor coactivator 4 isoform 3 [Homo sapiens]

10	20	30	40	50	60	70	80
MNTFQDQSGS	SSNREPLLRC	SDARRDLELA	IGGVLRAEQQ	IKDNLREVKA	QIHSCISRHL	ECLRSREVWL	YEQVDLIYQL
90	100	110	120	130	140	150	160
KEETLQQQAQ	QLYSLLGQFN	CLTHQLECTQ	NKDLANQVSV	CLERLGSLTL	KPEDSTVLLF	EADTITLRQT	ITTFGSLKTI
170	180	190	200	210	220	230	240
QIPEHLMAHA	SSANIGPFLE	KRGCISMPEQ	KSASGIVAVP	FSEWLLGSKP	ASGYQAPYIP	STDPQDWLTQ	KQTLENSQTS
250	260	270	280	290	300	310	320
SRACNFFNNV	GGNLKGLENW	LLKSEKSSYQ	KCNSHSTTSS	FSIEMEKVGD	QELPDQDEMD	LSDWLVTPQE	SHKLRKPENG
330	340	350	360	370	380	390	400
SRETSEKFKL	LFQSYNVNDW	LVKTDSCTNC	QGNQPKGVEI	ENLGNLKCLN	DHLEAKKPLS	TPSMVTEDWL	VQNHQDPCKV
410	420	430	440	450	460	470	480
EEVCRANEPC	TSFAECVCDE	NCEKEALYKW	LLKKEGKDKN	GMPVEPKPEP	EKHKDSLNMW	LCPRKEVIEQ	TKAPKAMTPS
490	500	510	520	530	540	550	560
RIADSFQVIK	NSPLSEWLIR	PPYKEGSPKE	VPGTEDRAGK	QKFKSPMNTS	WCSFNTADWV	LPGKKMGNLS	QLSSGEDKWL
570	580	590	600	610	620		
LRKKAQEVLL	NSPLQEEHNF	PPDHYGLPAV	CDLFACMQLK	VDKEKWLYRT	PLQM		

Cmpd.	No. of Cmpds.	m/z meas.	Δ m/z [ppm]	z	Rt [min]	Score	P	Range	Sequence	Modification
88	1	1103.5131	90.47	2	29.8	12.6	0	406-424	R.ANEPCTSFAECVCDENCEK.E	Carbamidomethyl: 5, 17

Detailed Protein Report

Protein 419: MLX-interacting protein [Homo sapiens]

**Accession:**	gi|111955326	**Score:**	30.0
**Database:**	refseq_human(refseq_human_20140103.fasta)	**MW [kDa]:**	101.1
**Database Date:**	2015-11-30	**pI:**	9.5
**Modification(s):**	Oxidation	**Sequence Coverage [%]:**	3.2
		**No. of unique Peptides:**	1

10	20	30	40	50	60	70	80
MAADVFMCSP	RRPRSRGRQV	LLKPQVSEDD	DDSDTDEPSP	PPASGAATPA	RAHASAAPPP	PRAGPGREEP	PRRQQIIHSG
**90**	**100**	**110**	**120**	**130**	**140**	**150**	**160**
HFMVSSPHRE	HPPKKGYDFD	TVNKQTCQTY	SFGKTSSCHL	SIDASLTKLF	ECMTLAYSGK	LVSPKWKNFK	GLKLQWRDKI
**170**	**180**	**190**	**200**	**210**	**220**	**230**	**240**
RLNNAIWRAW	YMQYLEKRKN	PVCHFVTPLD	GSVDVDEHRR	PEAITTEGKY	WKSRIEIVIR	EYHKWRTYFK	KRLQQHKDED
**250**	**260**	**270**	**280**	**290**	**300**	**310**	**320**
LSSLVQDDDM	LYWHKHGDGW	KTPVPMEEDP	LLDTDMLMSE	FSDTLFSTLS	SHQPVAWPNP	REIAHLGNAD	MIQPGLIPLQ
**330**	**340**	**350**	**360**	**370**	**380**	**390**	**400**
PNLDFMDTFE	PFQDLFSSSR	SIFGSMLPAS	ASAPVPDPNN	PPAQESILPT	TALPTVSLPD	SLIAPPTAPS	LAHMDEQGCE
**410**	**420**	**430**	**440**	**450**	**460**	**470**	**480**
HTSRTEDPFI	QPTDFGPSEP	PLSVPQPFLP	VFTMPLLSPS	PAPPPISPVL	PLVPPPATAL	NPPAPPTFHQ	PQKFAGVNKA
**490**	**500**	**510**	**520**	**530**	**540**	**550**	**560**
PSVITHTASA	TLTHDAPATT	FSQSQGLVIT	THHPAPSAAP	CGLALSPVTR	PPQPRLTFVH	PKPVSLTGGR	PKQPHKIVPA
**570**	**580**	**590**	**600**	**610**	**620**	**630**	**640**
PKPEPVSLVL	KNARIAPAAF	SGQPQAVIMT	SGPLKREGML	ASTVSQSNVV	IAPAAIARAP	GVPEFHSSIL	VTDLGHGTSS
**650**	**660**	**670**	**680**	**690**	**700**	**710**	**720**
PPAPVSRLFP	STAQDPLGKG	EQVPLHGGSP	QVTVTGPSRD	CPNSGQASPC	ASEQSPSPQS	PQNNCSGKSD	PKNVAALKNR
**730**	**740**	**750**	**760**	**770**	**780**	**790**	**800**
QMKHISAEQK	RRFNIKMCFD	MLNSLISNNS	KLTSHAITLQ	KTVEYITKLQ	QERGQMQEEA	RRLREEIEEL	NATIISCQQL
**810**	**820**	**830**	**840**	**850**	**860**	**870**	**880**
LPATGVPVTR	RQFDHMKDMF	DEYVKTRTLQ	NWKFWIFSII	IKPLFESFKG	MVSTSSLEEL	HRTALSWLDQ	HCSLPILRPM
**890**	**900**	**910**	**920**				
VLSTLRQLST	STSILTDPAQ	LPEQASKAVT	RIGKRLGES				

Cmpd.	No. of Cmpds.	m/z meas.	Δ m/z [ppm]	z	Rt [min]	Score	P	Range	Sequence	Modification
717	1	866.9167	32.26	2	37.8	13.5	0	737-751	K.MCFDMLNSLISNNSK.L	Oxidation: 1

Detailed Protein Report

Protein 420: PREDICTED: neurexin-2-beta isoform X15 [Homo sapiens]

Accession: gi|578821888
Database: refseq_human(refseq_human_20140103.fasta)
Database Date: 2015-11-30
Modification(s): Carbamidomethyl

Score: 29.9
MW [kDa]: 133.0
pI: 5.5
Sequence Coverage [%]: 2.0
No. of unique Peptides: 1

10	20	30	40	50	60	70	80
MEGPAHLTLN	SEVGSLLFSE	GGAGRGGAGD	VHQPTKGKEE	FVATFKGNEF	FCYDLSHNPI	QSSTDEITLA	FRTLQRNGLM
90	100	110	120	130	140	150	160
LHTGKSADYV	NLSLKSGAVW	LVINLGSGAF	EALVEPVNGK	FNDNAWHDVR	VTRNLRQVTI	SVDGILTTTG	YTQEDYTMLG
170	180	190	200	210	220	230	240
SDDFFYIGGS	PNTADLPGSP	VSNNFMGCLK	DVVYKNNDFK	LELSRLAKEG	DPKMKLQGDL	SFRCEDVAAL	DPVTFESPEA
250	260	270	280	290	300	310	320
FVALPRWSAK	RTGSISLDFR	TTEPNGLLLF	SQGRRAGGGA	GSHSSAQRAD	YFAMELLDGH	LYLLLDMGSG	GIKLRASSRK
330	340	350	360	370	380	390	400
VNDGEWCHVD	FQRDGRKGSI	SVNSRSTPFL	ATGDSEILDL	ESELYLGGLP	EGGRVDLPLP	PEVWTAALRA	GYVGCVRDLF
410	420	430	440	450	460	470	480
IDGRSRDLRG	LAEAQGAVGV	APFCSRETLK	QCASAPCRNG	GVCREGWNRF	ICDCIGTGFL	GRVCEREATV	LSYDGSMYMK
490	500	510	520	530	540	550	560
IMLPNAMHTE	AEDVSLRFMS	QRAYGLMMAT	TSRESADTLR	LELDGGQMKL	TVNLDCLRVG	CAPSKGPETL	FAGHKLNDNE
570	580	590	600	610	620	630	640
WHTVRVVRRG	KSLQLSVDNV	TVEGQMAGAH	MRLEFHNIET	GIMTERRFIS	VVPSNFIGHL	SGLVFNGQPY	MDQCKDGDIT
650	660	670	680	690	700	710	720
YCELNARFGL	RAIVADPVTF	KSRSSYLALA	TLQAYASMHL	FFQFKTTAPD	GLLLFNSGNG	NDFIVIELVK	GYIHYVFDLG
730	740	750	760	770	780	790	800
NGPSLMKGNS	DKPVNDNQWH	NVVVSRDPGN	VHTLKIDSRT	VTQHSNGARN	LDLKGELYIG	GLSKNMFSNL	PKLVASRDGF
810	820	830	840	850	860	870	880
QGCLASVDLN	GRLPDLIADA	LHRIGQVERG	CDGPSTTCTE	ESCANQGVCL	QQWDGFTCDC	TMTSYGGPVC	NDPGTTYIFG
890	900	910	920	930	940	950	960
KGGALITYTW	PPNDRPSTRM	DRLAVGFSTH	QRSAVLVRVD	SASGLGDYLQ	LHIDQGTVGV	IFNVGTDDIT	IDEPNAIVSD
970	980	990	1000	1010	1020	1030	1040
GKYHVVRFTR	SGGNATLQVD	SWPVNERYPA	GRQLTIFNSQ	AAIKIGGRDQ	GRPFQGQVSG	LYYNGLKVLA	LAAESDPNVR
1050	1060	1070	1080	1090	1100	1110	1120
TEGHLRLVGE	GPSVLLSAET	TATTLLADMA	TTIMETTTTM	ATTTTRRGRS	PTLRDSTTQN	TDDLLVASAE	CPSDDEDLEE
1130	1140	1150	1160	1170	1180	1190	1200
CEPSTANPTG	PGERGPPGAV	EVIRESSSTT	GMVVGIVAAA	ALCILILLYA	MYKYRNRDEG	SYQVDQSRNY	ISNSAQSNGA
1210	1220	1230					
VVKEKAPAAP	KTPSKAKKNK	DKEYYV					

Cmpd.	No. of Cmpds.	m/z meas.	Δ m/z [ppm]	z	Rt [min]	Score	P	Range	Sequence	Modification
2853	1	650.6072	-52.95	3	64.3	10.2	2	427-444	R.ETLKQCASAPCRNGGVCR.E	Carbamidomethyl: 6

Detailed Protein Report

Protein 421: FCH domain only protein 1 isoform a [Homo sapiens]

**Accession:**	gi|239049440	**Score:**	29.9
**Database:**	refseq_human(refseq_human_20140103.fasta)	**MW [kDa]:**	97.0
**Database Date:**	2015-11-30	**pI:**	6.5
**Modification(s):**	Carbamidomethyl	**Sequence Coverage [%]:**	2.7
		**No. of unique Peptides:**	2

10	20	30	40	50	60	70	80
MSYFGEHFWG	EKNHGFEVLY	HSVKQGPIST	KELADFIRER	ATIEETYSKA	MAKLSKLASN	GTPMGTFAPL	WEVFRVSSDK
**90**	**100**	**110**	**120**	**130**	**140**	**150**	**160**
LALCHLELTR	KLQDLIKDVL	RYGEEQLKTH	KKCKEEVVST	LDAVQVLSGV	SQLLPKSREN	YLNRCMDQER	LRRESTSQKE
**170**	**180**	**190**	**200**	**210**	**220**	**230**	**240**
MDKAETKTKK	AAESLRRSVE	KYNSARADFE	QKMLDSALRF	QAMEETHLRH	MKALLGSYAH	SVEDTHVQIG	QVHEEFKQNI
**250**	**260**	**270**	**280**	**290**	**300**	**310**	**320**
ENVSVEMLLR	KFAESKGTGR	EKPGPLDFEA	YSAAALQEAM	KRLRGAKAFR	LPGLSRRERE	PEPPAAVDFL	EPDSGTCPEV
**330**	**340**	**350**	**360**	**370**	**380**	**390**	**400**
DEEGFTVRPD	VTQNSTAEPS	RFSSSDSDFD	DEEPRKFYVH	IKPAPARAPA	CSPEAAAAQL	RATAGSLILP	PGPGGTMKRH
**410**	**420**	**430**	**440**	**450**	**460**	**470**	**480**
SSRDAAGKPQ	RPRSAPRTSS	CAERLQSEEQ	VSKNLFGPPL	ESAFDHEDFT	GSSSLGFTSS	PSPFSSSSPE	NVEDSGLDSP
**490**	**500**	**510**	**520**	**530**	**540**	**550**	**560**
SHAAPGPSPD	SWVPRPGTPQ	SPPSCRAPPP	EARGIRAPPL	PDSPQPLASS	PGPWGLEALA	GGDLMPAPAD	PTAREGLAAP
**570**	**580**	**590**	**600**	**610**	**620**	**630**	**640**
PRRLRSRKVS	CPLTRSNGDL	SRSLSPSPLG	SSAASTALER	PSFLSQTGHG	VSRGPSPVVL	GSQDALPIAT	AFTEYVHAYF
**650**	**660**	**670**	**680**	**690**	**700**	**710**	**720**
RGHSPSCLAR	VTGELTMTFP	AGIVRVFSGT	PPPPVLSFRL	VHTTAIEHFQ	PNADLLFSDP	SQSDPETKDF	WLNMAALTEA
**730**	**740**	**750**	**760**	**770**	**780**	**790**	**800**
LQRQAEQNPT	ASYYNVVLLR	YQFSRPGPQS	VPLQLSAHWQ	CGATLTQVSV	EYGYRPGATA	VPTPLTNVQI	LLPVGEPVTN
**810**	**820**	**830**	**840**	**850**	**860**	**870**	**880**
VRLQPAATWN	LEEKRLTWRL	PDVSEAGGSG	RLSASWEPLS	GPSTPSPVAA	QFTSEGTTLS	GVDLELVGSG	YRMSLVKRRF
**890**	**900**						
ATAAPPQGCT	W						

Cmpd.	No. of Cmpds.	m/z meas.	Δ m/z [ppm]	z	Rt [min]	Score	P	Range	Sequence	Modification
1900	1	693.7929	-23.70	2	53.8	12.5	0	2-12	M.SYFGEHFWGEK.N	
2590	1	731.8881	54.25	2	62.9	17.4	1	879-891	R.RFATAAPPQGCTW.-	Carbamidomethyl: 11

Detailed Protein Report

Protein 422: PREDICTED: zinc finger protein 280D isoform X2 [Homo sapiens]

**Accession:**	gi|530406054	**Score:**	29.9
**Database:**	refseq_human(refseq_human_20140103.fasta)	**MW [kDa]:**	110.5
**Database Date:**	2015-11-30	**pI:**	10.7
		**Sequence Coverage [%]:**	1.7
		**No. of unique Peptides:**	1

10	20	30	40	50	60	70	80
MTFSLSKPTD	WGSLHTRRAR	NARLLACKFR	AVRILGLPPG	HTSQQLAPTW	RQGPGPRPGP	ARGGARGSSR	ARDAAHMPVP
**90**	**100**	**110**	**120**	**130**	**140**	**150**	**160**
ASRVGEKTGS	SDTGRLTRSL	PMRQRAGCTR	APPFPRLGCQ	LRNGSRRRRR	SVVFRAPPFP	PPLSPPPQGS	SQADPLRSRK
**170**	**180**	**190**	**200**	**210**	**220**	**230**	**240**
ENRNNLLACL	ESHGNSKMAE	LFMECEEEEL	EPWQKKVKEV	EDDDDDEPIF	VGEISSSKPA	ISNILNRVNP	SSYSRGLKNG
**250**	**260**	**270**	**280**	**290**	**300**	**310**	**320**
ALSRGITAAF	KPTSQHYTNP	TSNPVPASPI	NFHPESRSSD	SSVIVQPFSK	PGYITNSSRV	VSNKSSELLF	DLTQDTGLSH
**330**	**340**	**350**	**360**	**370**	**380**	**390**	**400**
YQGGPTLSMA	GMSESSFLSK	RPSTSEVNNV	NPKKPKPSES	VSGANSSAVL	PSVKSPSVTS	SQAMLAKGTN	TSSNQSKNGT
**410**	**420**	**430**	**440**	**450**	**460**	**470**	**480**
PFPRACPKCN	IHFNLLDPLK	NHMKYCCPDM	INNFLGLAKT	EFSSTVNKNT	TIDSEKGKLI	MLVNDFYYGK	HEGDVQEEQK
**490**	**500**	**510**	**520**	**530**	**540**	**550**	**560**
THTTFKCFSC	LKILKNNIRF	MNHMKHHLEL	EKQSSESWEN	HTTCQHCYRQ	FPTPFQLQCH	IESTHTPHEF	STICKICELS
**570**	**580**	**590**	**600**	**610**	**620**	**630**	**640**
FETEHVLLQH	MKDNHKPGEM	PYVCQVCNYR	SSSFSDVETH	FRTSHENTKN	LLCPFCLKVI	KIATPYMHHY	MKHQKKGIHR
**650**	**660**	**670**	**680**	**690**	**700**	**710**	**720**
CTKCRLQFLT	CKEKMDHKTQ	HHRTFIKPKQ	LEGLPPGTKV	TIRASVGPLQ	SGASPTPSIS	ASASTLQLSP	PRTKNITAKN
**730**	**740**	**750**	**760**	**770**	**780**	**790**	**800**
PAKSNTSKPN	TVKSNASKPN	TSKPNGSKSK	YKPKISNMQK	KQSTLASSNK	KSKVNTALRN	LRYRRGIHKC	IECCSEIKDF
**810**	**820**	**830**	**840**	**850**	**860**	**870**	**880**
ANHFPTYVHC	SFCRYNTSCS	KAYVNHMMSF	HSNRPSKRFC	IFKKHSENLR	GITLVCLNCD	FLSDVSGLDN	MATHLSQHKT
**890**	**900**	**910**	**920**	**930**	**940**	**950**	**960**
HTCQVVMQKV	SVCIPTSEHL	SECRSSPEIS	KNIQGIPGQC	PPSMDTTDAC	SLFPRNVNNL	TVGPALRLWP	QEIRSWGDSL
**970**	**980**	**990**	**1000**				
STWLASHKAD	VVWPEMALRP	TKLSILPTGG	FGL				

Cmpd.	No. of Cmpds.	m/z meas.	Δ m/z [ppm]	z	Rt [min]	Score	P	Range	Sequence	Modification
44	1	634.4553	151.04	2	29.2	11.9	0	936-947	R.NVNNLTVGPALR.L	

Detailed Protein Report

Protein 423: PREDICTED: RING finger protein 17 isoform X9 [Homo sapiens]

**Accession:**	gi|578824965	**Score:**	29.9
**Database:**	refseq_human(refseq_human_20140103.fasta)	**MW [kDa]:**	174.6
**Database Date:**	2015-11-30	**pI:**	5.1
**Modification(s):**	Carbamidomethyl	**Sequence Coverage [%]:**	2.6
		**No. of unique Peptides:**	2

10	20	30	40	50	60	70	80
MLDTNTAEEI	DEALNTAHHS	FEQLSIAGKA	LEHMQKQTIE	ERERVIEVVE	KQFDQLLAFF	DSRKKNLCEE	FARTTDDYLS
**90**	**100**	**110**	**120**	**130**	**140**	**150**	**160**
NLIKAKSYIE	EKKNNLNAAM	NIARALQLSP	SLRTYCDLNQ	IIRTLQLTSD	SELAQVSSPQ	LRNPPRLSVN	CSEIICMFNN
**170**	**180**	**190**	**200**	**210**	**220**	**230**	**240**
MGKIEFRDST	KCYPQENEIR	QNVQKKYNNK	KELSCYDTYP	PLEKKKVDMS	VLTSEAPPPP	LQPETNDVHL	EAKNFQPQKD
**250**	**260**	**270**	**280**	**290**	**300**	**310**	**320**
VATASPKTIA	VLPQMGSSPD	VIIEEIIEDN	VESSAELVFV	SHVIDPCHFY	IRKYSQIKDA	KVLEKKVNEF	CNRSSHLDPS
**330**	**340**	**350**	**360**	**370**	**380**	**390**	**400**
DILELGARIF	VSSIKNGMWC	RGTITELIPI	EGRNTRKPCS	PTRLFVHEVA	LIQIFMVDFG	NSEVLIVTGV	VDTHVRPEHS
**410**	**420**	**430**	**440**	**450**	**460**	**470**	**480**
AKQHIALNDL	CLVLRKSEPY	TEGLLKDIQP	LAQPCSLKDI	VPQNSNEGWE	EEAKVEFLKM	VNNKAVSMKV	FREEDGVLIV
**490**	**500**	**510**	**520**	**530**	**540**	**550**	**560**
DLQKPPPNKI	SSDMPVSLRD	ALVFMELAKF	KSQSLRSHFE	KNTTLHYHPP	ILPKEMTDVS	VTVCHINSPG	DFYLQLIEGL
**570**	**580**	**590**	**600**	**610**	**620**	**630**	**640**
DILFLLKTIE	EFYKSEDGEN	LEILCPVQDQ	ACVAKFEDGI	WYRAKVIELN	HWESCWKSCI	DRTKQQKLII	QKPVNSSLML
**650**	**660**	**670**	**680**	**690**	**700**	**710**	**720**
TVCLSHAILI	VKVQIKGLPG	HQEVEVKYVD	FGNTAKITIK	DVRKIKDEFL	NAPEKAIKCK	LAYIEPYKRT	MQWSKEAKEK
**730**	**740**	**750**	**760**	**770**	**780**	**790**	**800**
FEEKAQDKFM	TCSVIKILED	NVLLVELFDS	LGAPEMTTTS	INDQLVKEGL	ASYEIGYILK	DNSQKHIEVW	DPSPEEIISN
**810**	**820**	**830**	**840**	**850**	**860**	**870**	**880**
EVHNLNPVSA	KSLPNENFQS	LYNKELPVHI	CNVISPEKIY	VQWLLTENLL	NSLEEKMIAA	YENSKWEPVK	WENDMHCAVK
**890**	**900**	**910**	**920**	**930**	**940**	**950**	**960**
IQDKNQWRRG	QIIRMVTDTL	VEVLLYDVGV	ELVVNVDCLR	KLEENLKTMG	RLSLECSLVD	IRPAGGSDKW	TATACDCLSL
**970**	**980**	**990**	**1000**	**1010**	**1020**	**1030**	**1040**
YLTGAVATII	LQVDSEENNT	TWPLPVKIFC	RDEKGERVDV	SKYLIKKGLA	LRERRINNLD	NSHSLSEKSL	EVPLEQEDSV
**1050**	**1060**	**1070**	**1080**	**1090**	**1100**	**1110**	**1120**
VTNCIKTNFD	PDKKTADIIS	EQKVSEFQEK	ILEPRTTRGY	KPPAIPNMNV	FEATVSCVGD	DGTIFVVPKL	SEFELIKMTN
**1130**	**1140**	**1150**	**1160**	**1170**	**1180**	**1190**	**1200**
EIQSNLKCLG	LLEPYFWKKG	EACAVRGSDT	LWYRGKVMEV	VGGAVRVQYL	DHGFTEKIPQ	CHLYPILLYP	DIPQFCIPCQ
**1210**	**1220**	**1230**	**1240**	**1250**	**1260**	**1270**	**1280**
LHNTTPVGNV	WQPDAIEVLQ	QLLSKRQVDI	HIMELPKNPW	EKLSIHLYFD	GMSLSYFMAY	YKYCTSEHTE	EMLKEKPRSD
**1290**	**1300**	**1310**	**1320**	**1330**	**1340**	**1350**	**1360**
HDKKYEEEQW	EIRFEELLSA	ETDTPLLPPY	LSSSLPSPGE	LYAVQVKHVV	SPNEVYICLD	SIETSNQSNQ	HSDTDDSGVS
**1370**	**1380**	**1390**	**1400**	**1410**	**1420**	**1430**	**1440**
GESESESLDE	ALQRVNKKVE	ALPPLTDFRT	EMPCLAEYDD	GLWYRAKIVA	IKEFNPLSIL	VQFVDYGSTA	KLTLNRLCQI
**1450**	**1460**	**1470**	**1480**	**1490**	**1500**	**1510**	**1520**
PSHLMRYPAR	AIKVLLAGFK	PPLRDLGETR	IPYCPKWSME	ALWAMIDCLQ	GKQLYAVSMA	PAPEQIVTLY	DDEQHPVHMP
**1530**	**1540**						
LVEMGLADKD	E						

Cmpd.	No. of Cmpds.	m/z meas.	Δ m/z [ppm]	z	Rt [min]	Score	P	Range	Sequence	Modification
2739	1	698.3923	-31.29	4	63.2	12.7	0	628-652	K.LIIQKPVNSSLMLTVCLSHAILIVK.V	Carbamidomethyl: 16
2279	4	863.8727	-142.91	2	57.0	17.3	2	1375-1389	R.VNKKVEALPPLTDFR.T	

Detailed Protein Report

Protein 424: homeobox protein cut-like 2 [Homo sapiens]

**Accession:**	gi|116517292	**Score:**	29.9
**Database:**	refseq_human(refseq_human_20140103.fasta)	**MW [kDa]:**	161.6
**Database Date:**	2015-11-30	**pI:**	5.3
**Modification(s):**	Carbamidomethyl	**Sequence Coverage [%]:**	1.5
		**No. of unique Peptides:**	1

Quantitation

mdown:qdown	**Median:** 0.45	**CV:** 0.00 %	**No. of Peptides:**	1
Wdown:Qdown	**Median:** 1.05	**CV:** 0.00 %	**No. of Peptides:**	1

10	20	30	40	50	60	70	80
MAANVGSMFQ	YWKRFDLRRL	QKELNSVASE	LSARQEESEH	SHKHLIELRR	EFKKNVPEEI	REMVAPVLKS	FQAEVVALSK
90	100	110	120	130	140	150	160
RSQEAEAAFL	SVYKQLIEAP	DPVPVFEAAR	SLDDRLQPPS	FDPSGQPRRD	LHTSWKRNPE	LLSPKEQREG	TSPAGPTLTE
170	180	190	200	210	220	230	240
GSRLPGIPGK	ALLTETLLQR	NEAEKQKGLQ	EVQITLAARL	GEAEEKIKVL	HSALKATQAE	LLELRRKYDE	EAASKADEVG
250	260	270	280	290	300	310	320
LIMTNLEKAN	QRAEAAQREV	ESLREQLASV	NSSIRLACCS	PQGPSGDKVN	FTLCSGPRLE	AALASKDREI	LRLLKDVQHL
330	340	350	360	370	380	390	400
QSSLQELEEA	SANQIADLER	QLTAKSEAIE	KLEEKLQAQS	DYEEIKTELS	ILKAMKLASS	TCSLPQGMAK	PEDSLLIAKE
410	420	430	440	450	460	470	480
AFFPTQKFLL	EKPSLLASPE	EDPSEDDSIK	DSLGTEQSYP	SPQQLPPPPG	PEDPLSPSPG	QPLLGPSLGP	DGTRTFSLSP
490	500	510	520	530	540	550	560
FPSLASGERL	MMPPAAFKGE	AGGLLVFPPA	FYGAKPPTAP	ATPAPGPEPL	GGPEPADGGG	GGAAGPGAEE	EQLDTAEIAF
570	580	590	600	610	620	630	640
QVKEQLLKHN	IGQRVFGHYV	LGLSQGSVSE	ILARPKPWRK	LTVKGKEPFI	KMKQFLSDEQ	NVLALRTIQV	RQRGSITPRI
650	660	670	680	690	700	710	720
RTPETGSDDA	IKSILEQAKK	EIESQKGGEP	KTSVAPLSIA	NGTTPASTSE	DAIKSILEQA	RREMQAQQQA	LLEMEVAPRG
730	740	750	760	770	780	790	800
RSVPPSPPER	PSLATASQNG	APALVKQEEG	SGGPAQAPLP	VLSPAAFVQS	IIRKVKSEIG	DAGYFDHHWA	SDRGLLSRPY
810	820	830	840	850	860	870	880
ASVSPSLSSS	SSSGYSGQPN	GRAWPRGDEA	PVPPEDEAAA	GAEDEPPRTG	ELKAEGATAE	AGARLPYYPA	YVPRTLKPTV
890	900	910	920	930	940	950	960
PPLTPEQYEL	YMYREVDTLE	LTRQVKEKLA	KNGICQRIFG	EKVLGLSQGS	VSDMLSRPKP	WSKLTQKGRE	PFIRMQLWLS
970	980	990	1000	1010	1020	1030	1040
DQLGQAVGQQ	PGASQASPTE	PRSSPSPPPS	PTEPEKSSQE	PLSLSLESSK	ENQQPEGRSS	SSLSGKMYSG	SQAPGGIQEI
1050	1060	1070	1080	1090	1100	1110	1120
VAMSPELDTY	SITKRVKEVL	TDNNLGQRLF	GESILGLTQG	SVSDLLSRPK	PWHKLSLKGR	EPFVRMQLWL	NDPHNVEKLR
1130	1140	1150	1160	1170	1180	1190	1200
DMKKLEKKAY	LKRRYGLIST	GSDSESPATR	SECPSPCLQP	QDLSLLQIKK	PRVVLAPEEK	EALRKAYQLE	PYPSQQTIEL
1210	1220	1230	1240	1250	1260	1270	1280
LSFQLNLKTN	TVINWFHNYR	SRMRREMLVE	GTQDEPDLDP	SGGPGILPPG	HSHPDPTPQS	PDSETEDQKP	TVKELELQEG
1290	1300	1310	1320	1330	1340	1350	1360
PEENSTPLTT	QDKAQVRIKQ	EQMEEDAEEE	AGSQPQDSGE	LDKGQGPPKE	EHPDPPGNDG	LPKVAPGPLL	PGGSTPDCPS
1370	1380	1390	1400	1410	1420	1430	1440
LHPQQESEAG	ERLHPDPLSF	KSASESSRCS	LEVSLNSPSA	ASSPGLMMSV	SPVPSSSAPI	SPSPPGAPPA	KVPSASPTAD
1450	1460	1470	1480	1490			
MAGALHPSAK	VNPNLQRRHE	KMANLNNIIY	RVERAANREE	ALEWEF			

Cmpd.	No. of Cmpds.	m/z meas.	Δ m/z [ppm]	z	Rt [min]	Score	P	Range	Sequence	Modification	Ratios
561	2	661.2196	-178.61	2	35.9	14.0	1	912-922	K.NGICQRIFGEK.V	Carbamidomethyl: 4	Wdown:Qdown 1.05<br>mdown:qdown 0.45

Detailed Protein Report

Protein 425: treslin [Homo sapiens]

Accession: gi|118421085
Database: refseq_human(refseq_human_20140103.fasta)
Database Date: 2015-11-30

Score: 29.9
MW [kDa]: 210.7
pI: 9.8
Sequence Coverage [%]: 1.0
No. of unique Peptides: 1

10	20	30	40	50	60	70	80
MACCHKVMLL	LDTAGGAARH	SRVRRAALRL	LTYLSCRFGL	ARVHWAFKFF	DSQGARSRPS	RVSDFRELGS	RSWEDFEEEL
90	100	110	120	130	140	150	160
EARLEDRAHL	PGPAPRATHT	HGALMETLLD	YQWDRPEITS	PTKPILRSSG	RRLLDVESEA	KEAEAALGGL	VNAVFLLAPC
170	180	190	200	210	220	230	240
PHSQRELLQF	VSGCEAQAQR	LPPTPKQVME	KLLPKRVREV	MVARKITFYW	VDTTEWSKLW	ESPDHLGYWT	VCELLHHGGG
250	260	270	280	290	300	310	320
TVLPSESFSW	DFAQAGEMLL	RSGIKLSSEP	HLSPWISMLP	TDATLNRLLY	NSPEYEASFP	RMEGMLFLPV	EAGKEIQETW
330	340	350	360	370	380	390	400
TVTLEPLAMH	QRHFQKPVRI	FLKGSVAQWS	LPTSSTLGTD	SWMLGSPEES	TATQRLLFQQ	LVSRLTAEEL	HLVADVDPGE
410	420	430	440	450	460	470	480
GRPPITGVIS	PLSASAMILT	VCRTKEAEFQ	RHVLQTAVAD	SPRDTASLFS	DVVDSILNQT	HDSLADTASA	ASPVPEWAQQ
490	500	510	520	530	540	550	560
ELGHTTPWSP	AVVEKWFPFC	NISGASSDLM	ESFGLLQAAS	ANKEESSKTE	GELIHCLAEL	YQRKSREEST	IAHQEDSKKK
570	580	590	600	610	620	630	640
RGVPRTPVRQ	KMNTMCRSLK	MLNVARLNVK	AQKLHPDGSP	DVAGEKGIQK	IPSGRTVDKL	EDRGRTLRSS	KPKDFKTEEE
650	660	670	680	690	700	710	720
LLSYIRENYQ	KTVATGEIML	YACARNMIST	VKMFLKSKGT	KELEVNCLNQ	VKSSLLKTSK	SLRQNLGKKL	DKEDKVRECQ
730	740	750	760	770	780	790	800
LQVFLRLEMC	LQCPSINEST	DDMEQVVEEV	TDLLRMVCLT	EDSAYLAEFL	EEILRLYIDS	IPKTLGNLYN	SLGFVIPQKL
810	820	830	840	850	860	870	880
AGVLPTDFFS	DDSMTQENKS	PLLSVPFLSS	ARRSVSGSPE	SDELQELRTR	SAKKRRKNAL	IRHKSIAEVS	QNLRQIEIPK
890	900	910	920	930	940	950	960
VSKRATKKEN	SHPAPQQPSQ	PVKDTVQEVT	KVRRNLFNQE	LLSPSKRSLK	RGLPRSHSVS	AVDGLEDKLD	NFKKNKGYHK
970	980	990	1000	1010	1020	1030	1040
LLTKSVAETP	VHKQISKRLL	HRQIKGRSSD	PGPDIGVVEE	SPEKGDEISL	RRSPRIKQLS	FSRTHSASFY	SVSQPKSRSV
1050	1060	1070	1080	1090	1100	1110	1120
QRVHSFQQDK	SDQRENSPVQ	SIRSPKSLLF	GAMSEMISPS	EKGSARMKKR	SRNTLDSEVP	AAYQTPKKSH	QKSLSFSKTT
1130	1140	1150	1160	1170	1180	1190	1200
PRRISHTPQT	PLYTPERLQK	SPAKMTPTKQ	AAFKESLKDS	SSPGHDSPLD	SKITPQKRHT	QAGEGTSLET	KTPRTPKRQG
1210	1220	1230	1240	1250	1260	1270	1280
TQPPGFLPNC	TWPHSVNSSP	ESPSCPAPPT	SSTAQPRREC	LTPIRDPLRT	PPRAAAFMGT	PQNQTHQQPH	VLRAARAEEP
1290	1300	1310	1320	1330	1340	1350	1360
AQKLKDKAIK	TPKRPGNSTV	TSSPPVTPKK	LFTSPLCDVS	KKSPFRKSKI	ECPSPGELDQ	KEPQMSPSVA	ASLSCPVPST
1370	1380	1390	1400	1410	1420	1430	1440
PPELSQRATL	DTVPPPPPSK	VGKRCRKTSD	PRRSIVECQP	DASATPGVGT	ADSPAAPTDS	RDDQKGLSLS	PQSPPERRGY
1450	1460	1470	1480	1490	1500	1510	1520
PGPGLRSDWH	ASSPLLITSD	TEHVTLLSEA	EHHGIGDLKS	NVLSVEEGEG	LRTADAEKSS	LSHPGIPPSP	PSCGPGSPLM
1530	1540	1550	1560	1570	1580	1590	1600
PSRDVHCTTD	GRQCQASAQL	DNLPASAWHS	TDSASPQTYE	VELEMQASGL	PKLRIKKIDP	SSSLEAEPLS	KEESSLGEES
1610	1620	1630	1640	1650	1660	1670	1680
FLPALSMPRA	SRSLSKPEPT	YVSPPCPRLS	HSTPGKSRGQ	TYICQACTPT	HGPSSTPSPF	QTDGVPWTPS	PKHSGKTTPD
1690	1700	1710	1720	1730	1740	1750	1760
IIKDWPRRKR	AVGCGAGSSS	GRGEVGADLP	GSLSLLESEG	KDHGLELSIH	RTPILEDFEL	EGVCQLPDQS	PPRNSMPKAE
1770	1780	1790	1800	1810	1820	1830	1840
EASSWGQFGL	SSRKRVLLAK	EEADRGAKRI	CDLREDSEVS	KSKEGSPSWS	AWQLPSTGDE	EVFVSGSTPP	PSCAVRSCLS
1850	1860	1870	1880	1890	1900	1910	1920
ASALQALTQS	PLLFQGKTPS	SQSKDPRDED	VDVLPSTVED	SPFSRAFSRR	RPISRTYTRK	KLMGTWLEDL	

Detailed Protein Report

Cmpd.	No. of Cmpds.	m/z meas.	Δ m/z [ppm]	z	Rt [min]	Score	P	Range	Sequence	Modification
949	1	473.1120	-311.20	2	41.4	19.1	1	1005-1012	K.GDEISLRR.S	

Detailed Protein Report

Protein 426: caspase recruitment domain-containing protein 9 isoform 2 [Homo sapiens]

**Accession:**	gi|16554562	**Score:**	29.8
**Database:**	refseq_human(refseq_human_20140103.fasta)	**MW [kDa]:**	56.6
**Database Date:**	2015-11-30	**pI:**	5.5
		**Sequence Coverage [%]:**	3.3
		**No. of unique Peptides:**	1

10	20	30	40	50	60	70	80
MSDYENDDEC	WSVLEGFRVT	LTSVIDPSRI	TPYLRQCKVL	NPDDEEQVLS	DPNLVIRKRK	VGVLLDILQR	TGHKGYVAFL
**90**	**100**	**110**	**120**	**130**	**140**	**150**	**160**
ESLELYYPQL	YKKVTGKEPA	RVFSMIIDAS	GESGLTQLLM	TEVMKLQKKV	QDLTALLSSK	DDFIKELRVK	DSLLRKHQER
**170**	**180**	**190**	**200**	**210**	**220**	**230**	**240**
VQRLKEECEA	GSRELKRCKE	ENYDLAMRLA	HQSEEKGAAL	MRNRDLQLEI	DQLKHSLMKA	EDDCKVERKH	TLKLRHAMEQ
**250**	**260**	**270**	**280**	**290**	**300**	**310**	**320**
RPSQELLWEL	QQEKALLQAR	VQELEASVQE	GKLDRSSPYI	QVLEEDWRQA	LRDHQEQANT	IFSLRKDLRQ	GEARRLRCME
**330**	**340**	**350**	**360**	**370**	**380**	**390**	**400**
EKEMFELQCL	ALRKDSKMYK	DRIEAILLQM	EEVAIERDQA	IATREELHAQ	HARGLQEKDA	LRKQVRELGE	KADELQLQVF
**410**	**420**	**430**	**440**	**450**	**460**	**470**	**480**
QCEAQLLAVE	GRLRRQQLET	LVLSSDLEDG	SPRRSQELSL	PQDLEDTQLS	DKGCLAGGGS	PKQPFAALHQ	EQVLRNPHDA
**490**	**500**						
GPAGLPGIGA	VC						

Cmpd.	No. of Cmpds.	m/z meas.	Δ m/z [ppm]	z	Rt [min]	Score	P	Range	Sequence	Modification
1767	1	627.2880	-179.52	2	50.6	12.0	1	60-70	R.KVGVLLDILQR.T	

Detailed Protein Report

Protein 427: zinc finger protein ZIC 5 [Homo sapiens]

**Accession:**	gi|118766337	**Score:**	29.7
**Database:**	refseq_human(refseq_human_20140103.fasta)	**MW [kDa]:**	68.4
**Database Date:**	2015-11-30	**pI:**	9.9
**Modification(s):**	Oxidation	**Sequence Coverage [%]:**	4.5
		**No. of unique Peptides:**	2

10	20	30	40	50	60	70	80
MFLKAGRGNK	VPPVRVYGPD	CVVLMEPPLS	KRNPPALRLA	DLATAQVQPL	QNMTGFPALA	GPPAHSQLRA	AVAHLRLRDL
**90**	**100**	**110**	**120**	**130**	**140**	**150**	**160**
GADPGVATTP	LGPEHMAQAS	TLGLSPPSQA	FPAHPEAPAA	AARAAALVAH	PGAGSYPCGG	GSSGAQPSAP	PPPAPPLPPT
**170**	**180**	**190**	**200**	**210**	**220**	**230**	**240**
PSPPPPPPPP	PPPALSGYTT	TNSGGGGSSG	KGHSRDFVLR	RDLSATAPAA	AMHGAPLGGE	QRSGTGSPQH	PAPPPHSAGM
**250**	**260**	**270**	**280**	**290**	**300**	**310**	**320**
FISASGTYAG	PDGSGGPALF	PALHDTPGAP	GGHPHPLNGQ	MRLGLAAAAA	AAAAELYGRA	EPPFAPRSGD	AHYGAVAAAA
**330**	**340**	**350**	**360**	**370**	**380**	**390**	**400**
AAALHGYGAV	NLNLNLAAAA	AAAAAGPGPH	LQHHAPPPAP	PPPPAPAQHP	HQHHPHLPGA	AGAFLRYMRQ	PIKQELICKW
**410**	**420**	**430**	**440**	**450**	**460**	**470**	**480**
IDPDELAGLP	PPPPPPPPPP	PPPPAGGAKP	CSKTFGTMHE	LVNHVTVEHV	GGPEQSSHVC	FWEDCPREGK	PFKAKYKLIN
**490**	**500**	**510**	**520**	**530**	**540**	**550**	**560**
HIRVHTGEKP	FPCPFPGCGK	VFARSENLKI	HKRTHTGEKP	FKCEFDGCDR	KFANSSDRKK	HSHVHTSDKP	YYCKIRGCDK
**570**	**580**	**590**	**600**	**610**	**620**	**630**	**640**
SYTHPSSLRK	HMKIHCKSPP	PSPGPLGYSS	VGTPVGAPLS	PVLDPARSHS	STLSPQVTNL	NEWYVCQASG	APSHLHTPSS
**650**	**660**	**670**					
NGTTSETEDE	EIYGNPEVVR	TIH					

Cmpd.	No. of Cmpds.	m/z meas.	Δ m/z [ppm]	z	Rt [min]	Score	P	Range	Sequence	Modification
2925	1	1018.9798	-14.49	2	65.7	18.1	0	202-222	R.DLSATAPAAAMHGAPLGGEQR.S	Oxidation: 11
2142	1	1052.3280	-209.35	1	57.0	11.7	2	531-539	R.KFANSSDRK.K	

Detailed Protein Report

Protein 428: rho GTPase-activating protein 11A isoform 3 [Homo sapiens]

**Accession:**	gi|557128992	**Score:**	29.7
**Database:**	refseq_human(refseq_human_20140103.fasta)	**MW [kDa]:**	92.6
**Database Date:**	2015-11-30	**pI:**	9.9
		**Sequence Coverage [%]:**	5.4
		**No. of unique Peptides:**	2

Alias proteins:

Accession	Name	Description
gi|578827655	refseq_human (refseq_human_20140103.fasta)	PREDICTED: rho GTPase-activating protein 11A isoform X3 [Homo sapiens]
gi|557129007	refseq_human (refseq_human_20140103.fasta)	rho GTPase-activating protein 11A isoform 3 [Homo sapiens]

```
        10         20         30         40         50         60         70         80
MDSSNLAVIF APNLLQTSEG HEKMSSNTEK KLRLQAAVVQ TLIDYASDIG RVPDFILEKI PAMLGIDGLC ATPSLEGFEE
        90        100        110        120        130        140        150        160
GEYETPGEYK RKRRQSVGDF VSGALNKFKP NRTPSITPQE ERIAQLSESP VILTPNAKRT LPVDSSHGFS SKKRKSIKHN
       170        180        190        200        210        220        230        240
FNFELLPSNL FNSSSTPVSV HIDTSSEGSS QSSLSPVLIG GNHLITAGVP RRSKRIAGKK VCRVESGKAG CFSPKISHKE
       250        260        270        280        290        300        310        320
KVRRSLRLKF NLGKNGREVN GCSGVNRYES VGWRLANQQS LKNRIESVKT GLLFSPDVDE KLPKKGSEKI SKSEETLLTP
       330        340        350        360        370        380        390        400
ERLVGTNYRM SWTGPNNSSF QEVDANEASS MVENLEVENS LEPDIMVEKS PATSCELTPS NLNNKHNSNI TSSPLSGDEN
       410        420        430        440        450        460        470        480
NMTKETLVKV QKAFSESGSN LHALMNQRQS SVTNVGKVKL TEPSYLEDSP EENLFETNDL TIVESKEKYE HHTGKGEKCF
       490        500        510        520        530        540        550        560
SERDFSPLQT QTFNRETTIK CYSTQMKMEH EKDIHSNMPK DYLSKQEFSS DEEIKKQQSP KDKLNNKLKE NENMMEGNLP
       570        580        590        600        610        620        630        640
KCAAHSKDEA RSSFSQQSTC VVTNLSKPRP MRIAKQQSLE TCEKTVSESS QMTEHRKVSD HIQWFNKLSL NEPNRIKVKS
       650        660        670        680        690        700        710        720
PLKFQRTPVR QSVRRINSLL EYSRQPTGHK LASLGDTASP LVKSVSCDGA LSSCIESASK DSSVSCIKSG PKEQKSMSCE
       730        740        750        760        770        780        790        800
ESNIGAISKS SMELPSKSFL KMRKHPDSVN ASLRSTTVYK QKILSDGQVK VPLDDLTNHD IVKPVVNNNM GISSGINNRV
       810        820        830        840
LRRPSERGRA WYKGSPKHPI GKTQLLPTSK PVDL
```

Cmpd.	No. of Cmpds.	m/z meas.	Δ m/z [ppm]	z	Rt [min]	Score	P	Range	Sequence	Modification
2089	3	682.6083	-53.17	3	54.7	11.6	0	386-404	K.HNSNITSSPLSGDENNMTK.E	
2804	1	698.3677	40.53	4	64.2	18.2	2	716-741	K.SMSCEESNIGAISKSSMELPSKSFLK.M	

Detailed Protein Report

Protein 429: pyruvate carboxylase, mitochondrial precursor [Homo sapiens]

**Accession:**	gi|106049292	**Score:**	29.6
**Database:**	refseq_human(refseq_human_20140103.fasta)	**MW [kDa]:**	129.6
**Database Date:**	2015-11-30	**pI:**	6.4
		**Sequence Coverage [%]:**	2.1
		**No. of unique Peptides:**	1

Alias proteins:

Accession	Name	Description
gi|578821479	refseq_human (refseq_human_20140103.fasta)	PREDICTED: pyruvate carboxylase, mitochondrial isoform X5 [Homo sapiens]
gi|578821475	refseq_human (refseq_human_20140103.fasta)	PREDICTED: pyruvate carboxylase, mitochondrial isoform X4 [Homo sapiens]
gi|530396856	refseq_human (refseq_human_20140103.fasta)	PREDICTED: pyruvate carboxylase, mitochondrial isoform X3 [Homo sapiens]
gi|530396854	refseq_human (refseq_human_20140103.fasta)	PREDICTED: pyruvate carboxylase, mitochondrial isoform X2 [Homo sapiens]
gi|106049528	refseq_human (refseq_human_20140103.fasta)	pyruvate carboxylase, mitochondrial precursor [Homo sapiens]
gi|106049295	refseq_human (refseq_human_20140103.fasta)	pyruvate carboxylase, mitochondrial precursor [Homo sapiens]

```
        10         20         30         40         50         60         70         80
MLKFRTVHGG LRLLGIRRTS TAPAASPNVR RLEYKPIKKV MVANRGEIAI RVFRACTELG IRTVAIYSEQ DTGQMHRQKA
        90        100        110        120        130        140        150        160
DEAYLIGRGL APVQAYLHIP DIIKVAKENN VDAVHPGYGF LSERADFAQA CQDAGVRFIG PSPEVVRKMG DKVEARAIAI
       170        180        190        200        210        220        230        240
AAGVPVVPGT DAPITSLHEA HEFSNTYGFP IIFKAAYGGG GRGMRVVHSY EELEENYTRA YSEALAAFGN GALFVEKFIE
       250        260        270        280        290        300        310        320
KPRHIEVQIL GDQYGNILHL YERDCSIQRR HQKVVEIAPA AHLDPQLRTR LTSDSVKLAK QVGYENAGTV EFLVDRHGKH
       330        340        350        360        370        380        390        400
YFIEVNSRLQ VEHTVTEEIT DVDLVHAQIH VAEGRSLPDL GLRQENIRIN GCAIQCRVTT EDPARSFQPD TGRIEVFRSG
       410        420        430        440        450        460        470        480
EGMGIRLDNA SAFQGAVISP HYDSLLVKVI AHGKDHPTAA TKMSRALAEF RVRGVKTNIA FLQNVLNNQQ FLAGTVDTQF
       490        500        510        520        530        540        550        560
IDENPELFQL RPAQNRAQKL LHYLGHVMVN GPTTPIPVKA SPSPTDPVVP AVPIGPPPAG FRDILLREGP EGFARAVRNH
       570        580        590        600        610        620        630        640
PGLLLMDTTF RDAHQSLLAT RVRTHDLKKI APYVAHNFSK LFSMENWGGA TFDVAMRFLY ECPWRRLQEL RELIPNIPFQ
       650        660        670        680        690        700        710        720
MLLRGANAVG YTNYPDNVVF KFCEVAKENG MDVFRVFDSL NYLPNMLLGM EAAGSAGGVV EAAISYTGDV ADPSRTKYSL
       730        740        750        760        770        780        790        800
QYYMGLAEEL VRAGTHILCI KDMAGLLKPT ACTMLVSSLR DRFPDLPLHI HTHDTSGAGV AAMLACAQAG ADVVDVAADS
       810        820        830        840        850        860        870        880
MSGMTSQPSM GALVACTRGT PLDTEVPMER VFDYSEYWEG ARGLYAAFDC TATMKSGNSD VYENEIPGGQ YTNLHFQAHS
       890        900        910        920        930        940        950        960
MGLGSKFKEV KKAYVEANQM LGDLIKVTPS SKIVGDLAQF MVQNGLSRAE AEAQAEELSF PRSVVEFLQG YIGVPHGGFP
       970        980        990       1000       1010       1020       1030       1040
EPFRSKVLKD LPRVEGRPGA SLPPLDLQAL EKELVDRHGE EVTPEDVLSA AMYPDVFAHF KDFTATFGPL DSLNTRLFLQ
      1050       1060       1070       1080       1090       1100       1110       1120
GPKIAEEFEV ELERGKTLHI KALAVSDLNR AGQRQVFFEL NGQLRSILVK DTQAMKEMHF HPKALKDVKG QIGAPMPGKV
      1130       1140       1150       1160       1170       1180
IDIKVVAGAK VAKGQPLCVL SAMKMETVVT SPMEGTVRKV HVTKDMTLEG DDLILEIE
```

Cmpd.	No. of Cmpds.	m/z meas.	Δ m/z [ppm]	z	Rt [min]	Score	P	Range	Sequence	Modification
511	1	706.0223	94.27	2	35.3	14.9	2	1120-1133	K.VIDIKVVAGAKVAK.G	

Detailed Protein Report

Protein 430: **basement membrane-specific heparan sulfate proteoglycan core protein precursor [Homo sapiens]**

**Accession:**	gi|126012571	**Score:**	29.6
**Database:**	refseq_human(refseq_human_20140103.fasta)	**MW [kDa]:**	468.5
**Database Date:**	2015-11-30	**pI:**	6.1
		**Sequence Coverage [%]:**	0.4
		**No. of unique Peptides:**	1

Detailed Protein Report

10	20	30	40	50	60	70	80
MGWRAAGALL	LALLLHGRLL	AVTHGLRAYD	GLSLPEDIET	VTASQMRWTH	SYLSDDEDML	ADSISGDDLG	SGDLGSGDFQ
90	100	110	120	130	140	150	160
MVYFRALVNF	TRSIEYSPQL	EDAGSREFRE	VSEAVVDTLE	SEYLKIPGDQ	VVSVVFIKEL	DGWVFVELDV	GSEGNADGAQ
170	180	190	200	210	220	230	240
IQEMLLRVIS	SGSVASYVTS	PQGFQFRRLG	TVPQFPRACT	EAEFACHSYN	ECVALEYRCD	RRPDCRDMSD	ELNCEEPVLG
250	260	270	280	290	300	310	320
ISPTFSLLVE	TTSLPPRPET	TIMRQPPVTH	APQPLLPGSV	RPLPCGPQEA	ACRNGHCIPR	DYLCDGQEDC	EDGSDELDCG
330	340	350	360	370	380	390	400
PPPPCEPNEF	PCGNGHCALK	LWRCDGDFDC	EDRTDEANCP	TKRPEEVCGP	TQFRCVSTNM	CIPASFHCDE	ESDCPDRSDE
410	420	430	440	450	460	470	480
FGCMPPQVVT	PPRESIQASR	GQTVTFTCVA	IGVPTPIINW	RLNWGHIPSH	PRVTVTSEGG	RGTLIIRDVK	ESDQGAYTCE
490	500	510	520	530	540	550	560
AMNARGMVFG	IPDGVLELVP	QRGPCPDGHF	YLEHSAACLP	CFCFGITSVC	QSTRRFRDQI	RLRFDQPDDF	KGVNVTMPAQ
570	580	590	600	610	620	630	640
PGTPPLSSTQ	LQIDPSLHEF	QLVDLSRRFL	VHDSFWALPE	QFLGNKVDSY	GGSLRYNVRY	ELARGMLEPV	QRPDVVLMGA
650	660	670	680	690	700	710	720
GYRLLSRGHT	PTQPGALNQR	QVQFSEEHWV	HESGRPVQRA	ELLQVLQSLE	AVLIQTVYNT	KMASVGLSDI	AMDTTVTHAT
730	740	750	760	770	780	790	800
SHGRAHSVEE	CRCPIGYSGL	SCESCDAHFT	RVPGGPYLGT	CSGCNCNGHA	SSCDPVYGHC	LNCQHNTEGP	QCNKCKAGFF
810	820	830	840	850	860	870	880
GDAMKATATS	CRPCPCPYID	ASRRFSDTCF	LDTDGQATCD	ACAPGYTGRR	CESCAPGYEG	NPIQPGGKCR	PVNQEIVRCD
890	900	910	920	930	940	950	960
ERGSMGTSGE	ACRCKNNVVG	RLCNECADGS	FHLSTRNPDG	CLKCFCMGVS	RHCTSSSWSR	AQLHGASEEP	GHFSLTNAAS
970	980	990	1000	1010	1020	1030	1040
THTTNEGIFS	PTPGELGFSS	FHRLLSGPYF	WSLPSRFLGD	KVTSYGGELR	FTVTQRSQPG	STPLHGQPLV	VLQGNNIILE
1050	1060	1070	1080	1090	1100	1110	1120
HHVAQEPSPG	QPSTFIVPFR	EQAWQRPDGQ	PATREHLLMA	LAGIDTLLIR	ASYAQQPAES	RVSGISMDVA	VPEETGQDPA
1130	1140	1150	1160	1170	1180	1190	1200
LEVEQCSCPP	GYRGPSCQDC	DTGYTRTPSG	LYLGTCERCS	CHGHSEACEP	ETGACQGCQH	HTEGPRCEQC	QPGYYGDAQR
1210	1220	1230	1240	1250	1260	1270	1280
GTPQDCQLCP	CYGDPAAGQA	AHTCFLDTDG	HPTCDACSPG	HSGRHCERCA	PGYYGNPSQG	QPCQRDSQVP	GPIGCNCDPQ
1290	1300	1310	1320	1330	1340	1350	1360
GSVSSQCDAA	GQCQCKAQVE	GLTCSHCRPH	HFHLSASNPD	GCLPCFCMGI	TQQCASSAYT	RHLISTHFAP	GDFQGFALVN
1370	1380	1390	1400	1410	1420	1430	1440
PQRNSRLTGE	FTVEPVPEGA	QLSFGNFAQL	GHESFYWQLP	ETYQGDKVAA	YGGKLRYTLS	YTAGPQGSPL	SDPDVQITGN
1450	1460	1470	1480	1490	1500	1510	1520
NIMLVASQPA	LQGPERRSYE	IMFREEFWRR	PDGQPATREH	LLMALADLDE	LLIRATFSSV	PLAASISAVS	LEVAQPGPSN
1530	1540	1550	1560	1570	1580	1590	1600
RPRALEVEEC	RCPPGYIGLS	CQDCAPGYTR	TGSGLYLGHC	ELCECNGHSD	LCHPETGACS	QCQHNAAGEF	CELCAPGYYG
1610	1620	1630	1640	1650	1660	1670	1680
DATAGTPEDC	QPCACPLTNP	ENMFSRTCES	LGAGGYRCTA	CEPGYTGQYC	EQCGPGYVGN	PSVQGGQCLP	ETNQAPLVVE
1690	1700	1710	1720	1730	1740	1750	1760
VHPARSIVPQ	GGSHSLRCQV	SGSPPHYFYW	SREDGRPVPS	GTQQRHQGSE	LHFPSVQPSD	AGVYICTCRN	LHQSNTSRAE
1770	1780	1790	1800	1810	1820	1830	1840
LLVTEAPSKP	ITVTVEEQRS	QSVRPGADVT	FICTAKSKSP	AYTLVWTRLH	NGKLPTRAMD	FNGILTIRNV	QLSDAGTYVC
1850	1860	1870	1880	1890	1900	1910	1920
TGSNMFAMDQ	GTATLHVQAS	GTLSAPVVSI	HPPQLTVQPG	QLAEFRCSAT	GSPTPTLEWT	GGPGGQLPAK	AQIHGGILRL
1930	1940	1950	1960	1970	1980	1990	2000
PAVEPTDQAQ	YLCRAHSSAG	QQVARAVLHV	HGGGGPRVQV	SPERTQVHAG	RTVRLYCRAA	GVPSATITWR	KEGGSLPPQA
2010	2020	2030	2040	2050	2060	2070	2080
RSERTDIATL	LIPAITTADA	GFYLCVATSP	AGTAQARIQV	VVLSASDASP	PPVKIESSSP	SVTEGQTLDL	NCVVAGSAHA
2090	2100	2110	2120	2130	2140	2150	2160
QVTWYRRGGS	LPPHTQVHGS	RLRLPQVSPA	DSGEYVCRVE	NGSGPKEASI	TVSVLHGTHS	GPSYTPVPGS	TRPIRIEPSS
2170	2180	2190	2200	2210	2220	2230	2240

Detailed Protein Report

Cmpd.	No. of Cmpds.	m/z meas.	Δ m/z [ppm]	z	Rt [min]	Score	P	Range	Sequence	Modification
362	1	457.1238	-295.03	2	33.9	17.6	0	4004-4012	R.SAEPLALGR.W	

Detailed Protein Report

Protein 431: **PREDICTED: thrombospondin type-1 domain-containing protein 7A isoform X4 [Homo sapiens]**

Accession: gi|578813343
Database: refseq_human(refseq_human_20140103.fasta)
Database Date: 2015-11-30
Modification(s): Carbamidomethyl

Score: 29.6
MW [kDa]: 169.7
pI: 9.0
Sequence Coverage [%]: 2.4
No. of unique Peptides: 2

10	20	30	40	50	60	70	80
MGLQARRWAS	GSRGAAGPRR	GVLQLLPLPL	PLPLLLLLLL	RPGAGRAAAQ	GEAEAPTLYL	WKTGPWGRCM	GDECGPGGIQ
90	100	110	120	130	140	150	160
TRAVWCAHVE	GWTTLHTNCK	QAERPNNQQN	CFKVCDWHKE	LYDWRLGPWN	QCQPVISKSL	EKPLECIKGE	EGIQVREIAC
170	180	190	200	210	220	230	240
IQKDKDIPAE	DIICEYFEPK	PLLEQACLIP	CQQDCIVSEF	SAWSECSKTC	GSGLQHRTRH	VVAPPQFGGS	GCPNLTEFQV
250	260	270	280	290	300	310	320
CQSSPCEAEE	LRYSLHVGPW	STCSMPHSRQ	VRQARRRGKN	KEREKDRSKG	VKDPEARELI	KKKRNRNRQN	RQENKYWDIQ
330	340	350	360	370	380	390	400
IGYQTREVMC	INKTGKAADL	SFCQQEKLPM	TFQSCVITKE	CQVSEWSEWS	PCSKTCHDMV	SPAGTRVRTR	TIRQFPIGSE
410	420	430	440	450	460	470	480
KECPEFEEKE	PCLSQGDGVV	PCATYGWRTT	EWTECRVDPL	LSQQDKRRGN	QTALCGGGIQ	TREVYCVQAN	ENLLSQLSTH
490	500	510	520	530	540	550	560
KNKEASKPMD	LKLCTGPIPN	TTQLCHIPCP	TECEVSPWSA	WGPCTYENCN	DQQGKKGFKL	RKRRITNEPT	GGSGVTGNCP
570	580	590	600	610	620	630	640
HLLEAIPCEE	PACYDWKAVR	LGNCEPDNGK	ECGPGTQVQE	VVCINSDGEE	VDRQLCRDAI	FPIPVACDAP	CPKDCVLSTW
650	660	670	680	690	700	710	720
STWSSCSHTC	SGKTTEGKQI	RARSILAYAG	EEGGIRCPNS	SALQEVRSCN	EHPCTVYHWQ	TGPWGQCIED	TSVSSFNTTT
730	740	750	760	770	780	790	800
TWNGEASCSV	GMQTRKVICV	RVNVGQVGPK	KCPESLRPET	VRPCLLPCKK	DCIVTPYSDW	TSCPSSCKEG	DSSIRKQSRH
810	820	830	840	850	860	870	880
RVIIQLPANG	GRDCTDPLYE	EKACEAPQAC	QSYRWKTHKW	RRCQLVPWSV	QQDSPGAQEG	CGPGRQARAI	TCRKQDGGQA
890	900	910	920	930	940	950	960
GIHECLQYAG	PVPALTQACQ	IPCQDDCQLT	SWSKFSSCNG	DCGAVRTRKR	TLVGKSKKKE	KCKNSHLYPL	IETQYCPCDK
970	980	990	1000	1010	1020	1030	1040
YNAQPVGNWS	DCILPEGKVE	VLLGMKVQGD	IKECGQGYRY	QAMACYDQNG	RLVETSRCNS	HGYIEEACII	PCPSDCKLSE
1050	1060	1070	1080	1090	1100	1110	1120
WSNWSRCSKS	CGSGVKVRSK	WLREKPYNGG	RPCPKLDHVN	QAQVYEVVPC	HSDCNQYLWV	TEPWSICKVT	FVNMRENCGE
1130	1140	1150	1160	1170	1180	1190	1200
GVQTRKVRCM	QNTADGPSEH	VEDYLCDPEE	MPLGSRVCKL	PCPEDCVISE	WGPWTQCVLP	CNQSSFRQRS	ADPIRQPADE
1210	1220	1230	1240	1250	1260	1270	1280
GRSCPNAVEK	EPCNLNKNCY	HYDYNVTDWS	TCQLSEKAVC	GNGIKTRMLD	CVRSDGKSVD	LKYCEALGLE	KNWQMNTSCM
1290	1300	1310	1320	1330	1340	1350	1360
VECPVNCQLS	DWSPWSECSQ	TCGLTGKMIR	RRTVTQPFQG	DGRPCPSLMD	QSKPCPVKPC	YRWQYGQWSP	CQVQEAQCGE
1370	1380	1390	1400	1410	1420	1430	1440
GTRTRNISCV	VSDGSADDFS	KVVDEEFCAD	IELIIDGNKN	MVLEESCSQP	CPGDCYLKDW	SSWSLCQLTC	VNGEDLGFGG
1450	1460	1470	1480	1490	1500	1510	1520
IQVRSRPVII	QELENQHLCP	EQMLETKSCY	DGQCYEYKWM	ASAWKGSSRT	VWCQRSDGIN	VTAKSQRNPK	EGKTTD

Cmpd.	No. of Cmpds.	m/z meas.	Δ m/z [ppm]	z	Rt [min]	Score	P	Range	Sequence	Modification
2861	1	795.7251	11.18	3	64.4	16.9	1	253-272	R.YSLHVGPWSTCSMPHSRQVR.Q	Carbamidomethyl: 11
1536	2	977.3535	-97.31	2	47.7	12.7	1	394-409	R.QFPIGSEKECPEFEEK.E	Carbamidomethyl: 10

Detailed Protein Report

Protein 432: PREDICTED: zinc finger protein 33B isoform X3 [Homo sapiens]

**Accession:**	gi|578819760	**Score:**	29.5
**Database:**	refseq_human(refseq_human_20140103.fasta)	**MW [kDa]:**	92.3
**Database Date:**	2015-11-30	**pI:**	9.9
		**Sequence Coverage [%]:**	4.4
		**No. of unique Peptides:**	1

10	20	30	40	50	60	70	80
MPHPLATSAF	LRFRLSFCLS	HVLRGRRSRD	FRGLPALLWR	MQPEERVSCI	FRVVSVFPRT	EQNEQGYCAH	KPEVIFRLEQ
**90**	**100**	**110**	**120**	**130**	**140**	**150**	**160**
GEEPWRLEEE	FPSQSFPVWT	ADHLKERSQE	NQSKHLWEVV	FINNEMLTKE	QGNVIGIPFN	MDVSSFPSRK	MFCQYDSRGM
**170**	**180**	**190**	**200**	**210**	**220**	**230**	**240**
SFNTVSELVI	SKINYLGKKS	DEFNACGKLL	LNIKHDETHT	REKNEVLKNR	NTLSHRENTL	QHEKIQTLDH	NFEYSICQET
**250**	**260**	**270**	**280**	**290**	**300**	**310**	**320**
LLEKAVFNTR	KRENAEENNC	DYNEFGRTFC	DSSSLLFHQI	PPSKDSHYEF	SDCEKFLCVK	STLSKHDGVP	VKHYDCGESG
**330**	**340**	**350**	**360**	**370**	**380**	**390**	**400**
NNFRRKLCLS	QLQKGDKGEK	HFECNECGKA	FWEKSHLTRH	QRVHTGEKHF	QCNQCGKTFW	EKSNLTKHQR	SHTGEKPFEC
**410**	**420**	**430**	**440**	**450**	**460**	**470**	**480**
NECGKAFSHK	SALTLHQRTH	TGEKPYQCNA	CGKTFYQKSD	LTKHQRTHTG	QKPYECYECG	KSFCMNSHLT	VHQRTHTGEK
**490**	**500**	**510**	**520**	**530**	**540**	**550**	**560**
PFECLECGKS	FCQKSHLTQH	QRTHIGDKPY	ECNACGKTFY	HKSVLTRHQI	IHTGLKPYEC	YECGKTFCLK	SDLTIHQRTH
**570**	**580**	**590**	**600**	**610**	**620**	**630**	**640**
TGEKPFACPE	CGKFFSHKST	LSQHYRTHTG	EKPYECHECG	KIFYNKSYLT	KHNRTHTGEK	PYECNECGKT	FCQKSQLTQH
**650**	**660**	**670**	**680**	**690**	**700**	**710**	**720**
QRIHIGEKPY	ECNECGKAFC	HKSALIVHQR	THTQEKPYKC	NECGKSFCVK	SGLILHERKH	TGEKPYECNE	CGKSFSHKSS
**730**	**740**	**750**	**760**	**770**	**780**	**790**	**800**
LTVHHRAHTG	EKSCQCNECG	KIFYRKSDLA	KHQRSHTGEK	PYECNCTCRKT	FSQKSNLIVH	QRTHIGEKPY	E

Cmpd.	No. of Cmpds.	m/z meas.	Δ m/z [ppm]	z	Rt [min]	Score	P	Range	Sequence	Modification
2070	1	682.5545	-123.95	3	56.0	12.2	1	752-768	K.HQRSHTGEKPYECNTCR.K	

Detailed Protein Report

Protein 433: PREDICTED: integrin alpha-11 isoform X2 [Homo sapiens]

**Accession:**	gi|530405525	**Score:**	29.5
**Database:**	refseq_human(refseq_human_20140103.fasta)	**MW [kDa]:**	122.0
**Database Date:**	2015-11-30	**pI:**	6.0
		**Sequence Coverage [%]:**	2.5
		**No. of unique Peptides:**	1

10	20	30	40	50	60	70	80
MRLGLSLATN	PKDNSFLACS	PLWSHECGSS	YYTTGMCSRV	NSNFRFSKTV	APALQRCQTY	MDIVIVLDGS	NSIYPWVEVQ
**90**	**100**	**110**	**120**	**130**	**140**	**150**	**160**
HFLINILKKF	YIGPGQIQVG	VVQYGEDVVH	EFHLNDYRSV	KDVVEAASHI	EQRGGTETRT	AFGIEFARSE	AFQKGGRKGA
**170**	**180**	**190**	**200**	**210**	**220**	**230**	**240**
KKVMIVITDG	ESHDSPDLEK	VIQQSERDNV	TRYAVAVLGY	YNRRGINPET	FLNEIKYIAS	DPDDKHFFNV	TDEAALKDIV
**250**	**260**	**270**	**280**	**290**	**300**	**310**	**320**
DALGDRIFSL	EGTNKNETSF	GLEMSQTGFS	SHVVEDGVLL	GAVGAYDWNG	AVLKETSAGK	VIPLRESYLK	EFPEELKNHG
**330**	**340**	**350**	**360**	**370**	**380**	**390**	**400**
AYLGYTVTSV	VSSRQGRVYV	AGAPRFNHTG	KVILFTMHNN	RSLTIHQAMR	GQQIGSYFGS	EITSVDIDGD	GVTDVLLVGA
**410**	**420**	**430**	**440**	**450**	**460**	**470**	**480**
PMYFNEGRER	GKVYVYELRQ	NLFVYNGTLK	DSHSYQNARF	GSSIASVRDL	NQDSYNDVVV	GAPLEDNHAG	AIYIFHGFRG
**490**	**500**	**510**	**520**	**530**	**540**	**550**	**560**
SILKTPKQRI	TASELATGLQ	YFGCSIHGQL	DLNEDGLIDL	AVGALGNAVI	LWSRPVVQIN	ASLHFEPSKI	NIFHRDCKRS
**570**	**580**	**590**	**600**	**610**	**620**	**630**	**640**
GRDATCLAAF	LCFTPIFLAP	HFQTTTVGIR	YNATMDERRY	TPRAHLDEGG	DRFTNRAVLL	SSGQELCERI	NFHVLDTADY
**650**	**660**	**670**	**680**	**690**	**700**	**710**	**720**
VKPVTFSVEY	SLEDPDHGPM	LDDGWPTTLR	VSVPFWNGCN	EDEHCVPDLV	LDARSDLPTA	MEYCQRVLRK	PAQDCSAYTL
**730**	**740**	**750**	**760**	**770**	**780**	**790**	**800**
SFDTTVFIIE	STRQRVAVEA	TLENRGENAY	STVLNISQSA	NLQFASLIQK	EDSDGSIECV	NEERRLQKQV	CNVSYPFFRA
**810**	**820**	**830**	**840**	**850**	**860**	**870**	**880**
KAKVAFRLDF	EFSKSIFLHH	LEIELAAGSD	SNERDSTKED	NVAPLRFHLK	YEADVLFTRS	SSLSHYEVKP	NSSLERYDGI
**890**	**900**	**910**	**920**	**930**	**940**	**950**	**960**
GPPFSCIFRI	QNLGLFPIHG	MMMKITIPIA	TRSGNRLLKL	RDFLTDEANT	SCNIWGNSTE	YRPTPVEEDL	RRAPQLNHSN
**970**	**980**	**990**	**1000**	**1010**	**1020**	**1030**	**1040**
SDVVSINCNI	RLVPNQEINF	HLLGNLWLRS	LKALKYKSMK	IMVNAALQRQ	FHSPFIFREE	DPSRQIVFEI	SKQEDWQVPI
**1050**	**1060**	**1070**	**1080**	**1090**			
WIIVGSTLGG	LLLLALLVLA	LWKLGFFRSA	RRRREPGLDP	TPKVLE			

Cmpd.	No. of Cmpds.	m/z meas.	Δ m/z [ppm]	z	Rt [min]	Score	P	Range	Sequence	Modification
294	1	680.1976	-193.61	2	33.0	14.9	0	789-799	K.QVCNVSYPFFR.A	

Detailed Protein Report

Protein 434: kinesin light chain 4 isoform c [Homo sapiens]

Accession: gi|19923891
Database: refseq_human(refseq_human_20140103.fasta)
Database Date: 2015-11-30

Score: 29.4
MW [kDa]: 35.1
pI: 5.3
Sequence Coverage [%]: 12.7
No. of unique Peptides: 1

10	20	30	40	50	60	70	80
MSGLVLGQRD	EPAGHRLSQE	EILGSTRLVS	QGLEALRSEH	QAVLQSLSQT	IECLQQGGHE	EGLVHEKARQ	LRRSMENIEL
**90**	**100**	**110**	**120**	**130**	**140**	**150**	**160**
GLSEAQVMLA	LASHLSTVES	EKQKLRAQVR	RLCQENQWLR	DELAGTQQRL	QRSEQAVAQL	EEEKKHLEFL	GQLRQYDEDG
**170**	**180**	**190**	**200**	**210**	**220**	**230**	**240**
HTSEEKEGDA	TKDSLDDLFP	NEEEEDPSNG	LSRGQGATAA	QQGGYEIPAR	LRTLHNLVIQ	YAAQGRYEVA	VPLCKQALED
**250**	**260**	**270**	**280**	**290**	**300**	**310**	**320**
LERTSGRGHP	DVATMLNILA	LVYRDQNKYK	EAAHLLNDAL	SIRESTLGPD	HPAVSIPCPP	HPTPRTPHHC	CFGLS

Cmpd.	No. of Cmpds.	m/z meas.	Δ m/z [ppm]	z	Rt [min]	Score	P	Range	Sequence	Modification
1691	1	1293.5862	-85.42	2	49.8	17.1	1	213-235	R.TLHNLVIQYAAQGRYEVAVPLCK.Q	

Detailed Protein Report

Protein 435: matrix metalloproteinase-27 precursor [Homo sapiens]

**Accession:**	gi|73808268	**Score:**	29.4
**Database:**	refseq_human(refseq_human_20140103.fasta)	**MW [kDa]:**	59.0
**Database Date:**	2015-11-30	**pI:**	9.4
		**Sequence Coverage [%]:**	5.7
		**No. of unique Peptides:**	1

10	20	30	40	50	60	70	80
MKRLLLLFLF	FITFSSAFPL	VRMTENEENM	QLAQAYLNQF	YSLEIEGNHL	VQSKNRSLID	DKIREMQAFF	GLTVTGKLDS
90	100	110	120	130	140	150	160
NTLEIMKTPR	CGVPDVGQYG	YTLPGWRKYN	LTYRIINYTP	DMARAAVDEA	IQEGLEVWSK	VTPLKFTKIS	KGIADIMIAF
170	180	190	200	210	220	230	240
RTRVHGRCPR	YFDGPLGVLG	HAFPPGPGLG	GDTHFDEDEN	WTKDGAGFNL	FLVAAHEFGH	ALGLSHSNDQ	TALMFPNYVS
250	260	270	280	290	300	310	320
LDPRKYPLSQ	DDINGIQSIY	GGLPKEPAKP	KEPTIPHACD	PDLTFDAITT	FRREVMFFKG	RHLWRIYYDI	TDVEFELIAS
330	340	350	360	370	380	390	400
FWPSLPADLQ	AAYENPRDKI	LVFKDENFWM	IRGYAVLPDY	PKSIHTLGFP	GRVKKIDAAV	CDKTTRKTYF	FVGIWCWRFD
410	420	430	440	450	460	470	480
EMTQTMDKGF	PQRVVKHFPG	ISIRVDAAFQ	YKGFFFFSRG	SKQFEYDIKT	KNITRIMRTN	TWFQCKEPKN	SSFGFDINKE
490	500	510	520				
KAHSGGIKIL	YHKSLSLFIF	GIVHLLKNTS	IYQ				

Cmpd.	No. of Cmpds.	m/z meas.	Δ m/z [ppm]	z	Rt [min]	Score	P	Range	Sequence	Modification
2773	1	1051.5696	39.22	2	66.0	16.6	2	425-442	R.VDAAFQYKGFFFFSRGSK.Q	

Detailed Protein Report

Protein 436: PREDICTED: sp110 nuclear body protein isoform X3 [Homo sapiens]

Accession: gi|530370295
Database: refseq_human(refseq_human_20140103.fasta)
Database Date: 2015-11-30

Score: 29.4
MW [kDa]: 58.5
pI: 10.2
Sequence Coverage [%]: 4.5
No. of unique Peptides: 1

Quantitation

mdown:qdown **Median:** 1.30 **CV:** 0.00 % **No. of Peptides:** 1

10	20	30	40	50	60	70	80
MNAEEDSEEM	PSLLTSTVQV	ASDNLIPQIR	DKEDPQEMPH	SPLGSMPEIR	DNSPEPNDPE	EPQEVSSTPS	DKKGKKRKRC
90	100	110	120	130	140	150	160
IWSTPKRRHK	KKSLPGGTAS	SRHGIQKKLK	RVDQVPQKKD	DSTCNSTVET	RAQKARTECA	RKSRSEEIID	GTSEMNEGKR
170	180	190	200	210	220	230	240
SQKTPSTPRR	VTQGAASPGH	GIQEKLQVVD	KVTQRKDDST	WNSEVMMRVQ	KARTKCARKS	RLKEKKKEKD	ICSSSKRRFQ
250	260	270	280	290	300	310	320
KNIHRRGKPK	SDTVDFHCSK	LPVTCGEAKG	ILYKKKMKHG	SSVKCIRNED	GTWLTPNEFE	VEGKGRNAKN	WKRNIRCEGM
330	340	350	360	370	380	390	400
TLGELLKRKN	SDECEVCCQG	GQLLCCGTCP	RVFHEDCHIP	PVEAKRMLWS	CTFCRMKRSS	GSQQCHHVSK	TLERQMQPQD
410	420	430	440	450	460	470	480
QLKCEFLLLK	AYCHPQSSFF	TGIPFNIRDY	GEPFQEAMWL	DLVKERLITE	MYTVAWFVRD	MRLMFRNHKT	FYKASDFGQV
490	500	510	520				
GLDLEAEFEK	DLKDVLGFHE	ANDGGFWTLP					

Cmpd.	No. of Cmpds.	m/z meas.	Δ m/z [ppm]	z	Rt [min]	Score	P	Range	Sequence	Modification	Ratios
2672	1	484.7588	30.75	2	61.7	13.8	1	460-466	R.DMRLMFR.N		mdown:qdown 1.30

Detailed Protein Report

Protein 437: **PREDICTED: janus kinase and microtubule-interacting protein 3 isoform X5 [Homo sapiens]**

**Accession:**	gi|530394820	**Score:**	29.4
**Database:**	refseq_human(refseq_human_20140103.fasta)	**MW [kDa]:**	105.7
**Database Date:**	2015-11-30	**pI:**	6.1
**Modification(s):**	Carbamidomethyl, Oxidation	**Sequence Coverage [%]:**	3.9
		**No. of unique Peptides:**	1

10	20	30	40	50	60	70	80
MSKRGMSSRA	KGDKAEALAA	LQAANEDLRA	KLTDIQIELQ	QEKSKVSKVE	REKNQELRQV	REHEQHKTAV	LLTELKTKLH
90	100	110	120	130	140	150	160
EEKMKELQAV	RETLLRQHEA	ELLRVIKIKD	NENQRLQALL	SALRDGGPEK	VKTVLLSEAK	EEAKKGFEVE	KVKMQQEISE
170	180	190	200	210	220	230	240
LKGAKRQVEE	ALTLVIQADK	IKAAEIRSVY	HLHQEEITRI	KKECEREIRR	LQLDEKDARR	FQLKIAELSA	IIRKLEDRNA
250	260	270	280	290	300	310	320
LLSEERNELL	KRVREAESQY	KPLLDKNKRL	SRKNEDLSHA	LRRMENKLKF	VTQENIEMRQ	RAGIIRRPSS	LNDLDQSQDE
330	340	350	360	370	380	390	400
REVDFLKLQI	VEQQNLIDEL	SKTLETAGYV	KSVLERDKLL	RFRKQRKKMA	KLPKPVVVET	FFGYDEEASL	ESDGSSVSYQ
410	420	430	440	450	460	470	480
TDRTDQTPCT	PDDDLEEGMA	KEETELRFRQ	LTMEYQALQR	AYALLQEQVG	GTLDAEREVK	TREQLQAEVQ	RAQARIEDLE
490	500	510	520	530	540	550	560
KALAEQGQDM	KWIEEKQALY	RRNQELVEKI	KQMETEEARL	RHEVQDARDQ	NELLEFRILE	LEERERKSPA	ISFHHTPFVD
570	580	590	600	610	620	630	640
GKSPLQVYCE	AEGVTDIVVA	ELMKKLDILG	DNAVSNLTNE	EQVVVIQART	VLTLAEKWLQ	QIEETEAALQ	RKMVDLESEK
650	660	670	680	690	700	710	720
ELFSKQKGYL	DEELDYRKQA	LDQANKHILE	LEAMLYDALQ	QEAGAKVAEL	LSEEEREKLK	VAVEQWKRQV	MSELRERDAQ
730	740	750	760	770	780	790	800
ILRERMELLQ	LAQQRIKELE	ERIEAQKRQI	KELEEKLSFS	GHSPSWHPDV	PHIESDPFPP	VGPESRDKMG	RRVSILKTQG
810	820	830	840	850	860	870	880
DLVSARPSRG	GRVRRGSVWG	VELPSTWDVP	ELELALPTSR	GLLRDGPGLG	CWTLGPFSRT	AAPIPFPGMC	SIPCQAGVRT
890	900	910	920				
ASVQKHQPRV	PSSLLVLTVA	ATGVWRKIFR	NNRGLA				

Cmpd.	No. of Cmpds.	m/z meas.	Δ m/z [ppm]	z	Rt [min]	Score	P	Range	Sequence	Modification
1669	1	1045.0250	19.09	2	50.8	18.3	0	860-879	R.TAAPIPFPGMCSIPCQAGVR.T	Carbamidomethyl: 15; Oxidation: 10

Detailed Protein Report

Protein 438: interferon-induced guanylate-binding protein 2 [Homo sapiens]

**Accession:**	gi|38327558	**Score:**	29.3
**Database:**	refseq_human(refseq_human_20140103.fasta)	**MW [kDa]:**	67.2
**Database Date:**	2015-11-30	**pI:**	5.4
**Modification(s):**	Oxidation	**Sequence Coverage [%]:**	5.2
		**No. of unique Peptides:**	1

10	20	30	40	50	60	70	80
MAPEINLPGP	MSLIDNTKGQ	LVVNPEALKI	LSAITQPVVV	VAIVGLYRTG	KSYLMNKLAG	KKNGFSLGST	VKSHTKGIWM
**90**	**100**	**110**	**120**	**130**	**140**	**150**	**160**
WCVPHPKKPE	HTLVLLDTEG	LGDIEKGDNE	NDSWIFALAI	LLSSTFVYNS	MGTINQQAMD	QLHYVTELTD	RIKANSSPGN
**170**	**180**	**190**	**200**	**210**	**220**	**230**	**240**
NSVDDSADFV	SFFPAFVWTL	RDFTLELEVD	GEPITADDYL	ELSLKLRKGT	DKKSKSFNDP	RLCIRKFFPK	RKCFVFDWPA
**250**	**260**	**270**	**280**	**290**	**300**	**310**	**320**
PKKYLAHLEQ	LKEEELNPDF	IEQVAEFCSY	ILSHSNVKTL	SGGIPVNGPR	LESLVLTYVN	AISSGDLPCM	ENAVLALAQI
**330**	**340**	**350**	**360**	**370**	**380**	**390**	**400**
ENSAAVEKAI	AHYEQQMGQK	VQLPTETLQE	LLDLHRDSER	EAIEVFMKNS	FKDVDQMFQR	KLGAQLEARR	DDFCKQNSKA
**410**	**420**	**430**	**440**	**450**	**460**	**470**	**480**
SSDCCMALLQ	DIFGPLEEDV	KQGTFSKPGG	YRLFTQKLQE	LKNKYYQVPR	KGIQAKEVLK	KYLESKEDVA	DALLQTDQSL
**490**	**500**	**510**	**520**	**530**	**540**	**550**	**560**
SEKEKAIEVE	RIKAESAEAA	KKMLEEIQKK	NEEMMEQKEK	SYQEHVKQLT	EKMERDRAQL	MAEQEKTLAL	KLQEQERLLK
**570**	**580**	**590**	**600**				
EGFENESKRL	QKDIWDIQMR	SKSLEPICNI	L				

Cmpd.	No. of Cmpds.	m/z meas.	Δ m/z [ppm]	z	Rt [min]	Score	P	Range	Sequence	Modification
2870	1	1027.5482	62.66	2	65.0	11.1	2	503-518	K.MLEEIQKKNEEMMEQK.E	Oxidation: 12

Detailed Protein Report

Protein 439: zinc finger and SCAN domain-containing protein 29 [Homo sapiens]

**Accession:**	gi|109715825	**Score:**	29.3
**Database:**	refseq_human(refseq_human_20140103.fasta)	**MW [kDa]:**	96.7
**Database Date:**	2015-11-30	**pI:**	6.6
		**Sequence Coverage [%]:**	4.0
		**No. of unique Peptides:**	1

Alias proteins:

Accession	Name	Description
gi|578826641	refseq_human (refseq_human_20140103.fasta)	PREDICTED: zinc finger and SCAN domain-containing protein 29 isoform X1 [Homo sapiens]

10	20	30	40	50	60	70	80
MMAKSALREN	GTNSETFRQR	FRRFHYQEVA	GPREAFSQLW	ELCCRWLRPE	VRTKEQIVEL	LVLEQFLTVL	PGEIQNWVQE
**90**	**100**	**110**	**120**	**130**	**140**	**150**	**160**
QCPENGEEAV	TLVEDLEREP	GRPRSSVTVS	VKGQEVRLEK	MTPPKSSQEL	LSVRQESVEP	QPRGVPKKER	ARSPDLGPQE
**170**	**180**	**190**	**200**	**210**	**220**	**230**	**240**
QMNPKEKLKP	FQRSGLPFPK	SGVVSRLEQG	EPWIPDLLGS	KEKELPSGSH	IGDRRVHADL	LPSKKDRRSW	VEQDHWSFED
**250**	**260**	**270**	**280**	**290**	**300**	**310**	**320**
EKVAGVHWGY	EETRTLLAIL	SQTEFYEALR	NCHRNSQVYG	AVAERLREYG	FLRTLEQCRT	KFKGLQKSYR	KVKSGHPPET
**330**	**340**	**350**	**360**	**370**	**380**	**390**	**400**
CPFFEEMEAL	MSAQVIALPS	NGLEAAASHS	GLVGSDAETE	EPGQRGWQHE	EGAEEAVAQE	SDSDDMDLEA	TPQDPNSAAP
**410**	**420**	**430**	**440**	**450**	**460**	**470**	**480**
VVFRSPGGVH	WGYEETKTYL	AILSETQFYE	ALRNCHRNSQ	LYGAVAERLW	EYGFLRTPEQ	CRTKFKSLQT	SYRKVKNGQA
**490**	**500**	**510**	**520**	**530**	**540**	**550**	**560**
PETCPFFEEM	DALVSVRVAA	PPNDGQEETA	SCPVQGTSEA	EAQKQAEEAD	EATEEDSDDD	EEDTEIPPGA	VITRAPVLFQ
**570**	**580**	**590**	**600**	**610**	**620**	**630**	**640**
SPRGFEAGFE	NEDNSKRDIS	EEVQLHRTLL	ARSERKIPRY	LHQGKGNESD	CRSGRQWAKT	SGEKRGKLTL	PEKSLSEVLS
**650**	**660**	**670**	**680**	**690**	**700**	**710**	**720**
QQRPCLGERP	YKYLKYSKSF	GPNSLLMHQV	SHQVENPYKC	ADCGKSFSRS	ARLIRHRRIH	TGEKPYKCLD	CGKSFRDSSN
**730**	**740**	**750**	**760**	**770**	**780**	**790**	**800**
FITHRRIHTG	EKPYQCGECG	KCFNQSSSLI	IHQRTHTGEK	PYQCEECGKS	FNNSSHFSAH	RRIHTGERPH	VCPDCGKSFS
**810**	**820**	**830**	**840**	**850**	**860**		
KSSDLRAHHR	THTGEKPYGC	HDCGKCFSKS	SALNKHGEIH	AREKLLTQSA	PK		

Cmpd.	No. of Cmpds.	m/z meas.	Δ m/z [ppm]	z	Rt [min]	Score	P	Range	Sequence	Modification
1688	1	1136.5634	-13.85	2	49.6	11.2	1	438-456	R.NSQLYGAVAERLWEYGFLR.T	

Detailed Protein Report

Protein 440: CDK5 regulatory subunit-associated protein 2 isoform c [Homo sapiens]

**Accession:**	gi|440309853	**Score:**	29.2
**Database:**	refseq_human(refseq_human_20140103.fasta)	**MW [kDa]:**	189.5
**Database Date:**	2015-11-30	**pI:**	5.4
		**Sequence Coverage [%]:**	1.1
		**No. of unique Peptides:**	1

10	20	30	40	50	60	70	80
MMDLVLEEDV	TVPGTLSGCS	GLVPSVPDDL	DGINPNAGLG	NGLLPNVSEE	TVSPTRARNM	KDFENQITEL	KKENFNLKLR
90	100	110	120	130	140	150	160
IYFLEERMQQ	EFHGPTEHIY	KTNIELKVEV	ESLKRELQER	EQLLIKASKA	VESLAEAGGS	EIQRVKEDAR	KKVQQVEDLL
170	180	190	200	210	220	230	240
TKRILLLEKD	VTAAQAELEK	AFAGTETEKA	LRLRLESKLS	EMKKMHEGDL	AMALVLDEKD	RLIEELKLSL	KSKEALIQCL
250	260	270	280	290	300	310	320
KEEKSQMACP	DENVSSGELR	GLCAAPREEK	ERETEAAQME	HQKERNSFEE	RIQALEEDLR	EKEREIATEK	KNSLKRDKAI
330	340	350	360	370	380	390	400
QGLTMALKSK	EKKVEELNSE	IEKLSAAFAK	AREALQKAQT	QEFQGSEDYE	TALSGKEALS	AALRSQNLTK	STENHRLRRS
410	420	430	440	450	460	470	480
IKKITQELSD	LQQERERLEK	DLEEAHREKS	KGDCTIRDLR	NEVEKLRNEV	NEREKAMENR	YKSLLSESNK	KLHNQEQVIK
490	500	510	520	530	540	550	560
HLTESTNQKD	VLLQKFNEKD	LEVIQQNCYL	MAAEDLELRS	EGLITEKCSS	QQPPGSKTIF	SKEKKQSSDY	EELIQVLKKE
570	580	590	600	610	620	630	640
QDIYTHLVKS	LQESDSINNL	QAELNKIFAL	RKQLEQDVLS	YQNLRKTLEE	QISEIRRREE	SFSLYSDQTS	YLSICLEENN
650	660	670	680	690	700	710	720
RFQVEHFSQE	ELKKKVSDLI	QLVKELYTDN	QHLKKTIFDL	SCMGFQGNGF	PDRLASTEQT	EEAKKSRLPI	LIKPSRSLGN
730	740	750	760	770	780	790	800
MYRLPATQEV	VTQLQSQILE	LQGELKEFKT	CNKQLHQKLI	LAEAVMEGRP	TPDKTLLNAQ	PPVGAAYQDS	PGEQKGIKTT
810	820	830	840	850	860	870	880
SSVWRDKEMD	SDQQRSYEID	SEICPPDDLA	SLPSCKENPE	DVLSPTSVAT	YLSSKSQPSA	KVSVMGTDQS	ESINTSNETE
890	900	910	920	930	940	950	960
YLKQKIHDLE	TELEGYQNFI	FQLQKHSQCS	EAIITVLCGT	EGAQDGLSKP	KNGSDGEEMT	FSSLHQVRYV	KHVKILGPLA
970	980	990	1000	1010	1020	1030	1040
PEMIDSRVLE	NLKQQLEEQE	YKLQKEQNLN	MQLFSEIHNL	QNKFRDLSPP	RYDSLVQSQA	RELSLQRQQI	KDGHGICVIS
1050	1060	1070	1080	1090	1100	1110	1120
RQHMNTMIKA	FEELLQASDV	DYCVAEGFQE	QLNQCAELLE	KLEKLFLNGK	SVGVEMNTQN	ELMERIEEDN	LTYQHLLPES
1130	1140	1150	1160	1170	1180	1190	1200
PEPSASHALS	DYETSEKSFF	SRDQKQDNET	EKTSVMVNSF	SQDLLMEHIQ	EIRTLRKRLE	ESIKTNEKLR	KQLERQGSEF
1210	1220	1230	1240	1250	1260	1270	1280
VQGSTSIFAS	GSELHSSLTS	EIHFLRKQNQ	ALNAMLIKGS	RDKQKENDKL	RESLSRKTVS	LEHLQREYAS	VKEENERLQK
1290	1300	1310	1320	1330	1340	1350	1360
EGSEKERHNQ	QLIQEVRCSG	QELSRVQEEV	KLRQQLLSQN	DKLLQSLRVE	LKAYEKLDEE	HRRLREASGE	GWKGQDPFRD
1370	1380	1390	1400	1410	1420	1430	1440
LHSLLMEIQA	LRLQLERSIE	TSSTLQSRLK	EQLARGAEKA	QEGALTLAVQ	AVSIPEVPLQ	PDKHDGDKYP	MESDNSFDLF
1450	1460	1470	1480	1490	1500	1510	1520
DSSQAVTPKS	VSETPPLSGN	DTDSLSCDSG	SSATSTPCVS	RLVTGHHLWA	SKNGRHVLGL	IEDYEALLKQ	ISQGQRLLAE
1530	1540	1550	1560	1570	1580	1590	1600
MDIQTQEAPS	STSQELGTKG	PHPAPLSKFV	SSVSTAKLTL	EEAYRRLKLL	WRVSLPEDGQ	CPLHCEQIGE	MKAEVTKLHK
1610	1620	1630	1640	1650	1660	1670	
KLFEQEKKLQ	NTMKLLQLSK	RQEKVIFDQL	VVTHKILRKA	RGNLELRPGG	AHPGTCSPSR	PGS	

Cmpd.	No. of Cmpds.	m/z meas.	Δ m/z [ppm]	z	Rt [min]	Score	P	Range	Sequence	Modification
578	1	575.8071	-12.69	2	36.1	15.6	1	1558-1566	K.LTLEEAYRR.L	

Detailed Protein Report

Protein 441: adenylate cyclase type 9 [Homo sapiens]

**Accession:**	gi|50959205	**Score:**	29.2
**Database:**	refseq_human(refseq_human_20140103.fasta)	**MW [kDa]:**	150.6
**Database Date:**	2015-11-30	**pI:**	7.3
		**Sequence Coverage [%]:**	2.0
		**No. of unique Peptides:**	1

10	20	30	40	50	60	70	80
MASPPHQQLL	HHHSTEVSCD	SSGDSNSVRV	KINPKQLSSN	SHPKHCKYSI	SSSCSSSGDS	GGVPRRVGGG	GRLRRQKKLP
**90**	**100**	**110**	**120**	**130**	**140**	**150**	**160**
QLFERASSRW	WDPKFDSVNL	EEACLERCFP	QTQRRFRYAL	FYIGFACLLW	SIYFAVHMRS	RLIVMVAPAL	CFLLVCVGFF
**170**	**180**	**190**	**200**	**210**	**220**	**230**	**240**
LFTFTKLYAR	HYAWTSLALT	LLVFALTLAA	QFQVLTPVSG	RGDSSNLTAT	ARPTDTCLSQ	VGSFSMCIEV	LFLLYTVMHL
**250**	**260**	**270**	**280**	**290**	**300**	**310**	**320**
PLYLSLCLGV	AYSVLFETFG	YHFRDEACFP	SPGAGALHWE	LLSRGLLHGC	IHAIGVHLFV	MSQVRSRSTF	LKVGQSIMHG
**330**	**340**	**350**	**360**	**370**	**380**	**390**	**400**
KDLEVEKALK	ERMIHSVMPR	IIADDLMKQG	DEESENSVKR	HATSSPKNRK	KKSSIQKAPI	AFRPFKMQQI	EEVSILFADI
**410**	**420**	**430**	**440**	**450**	**460**	**470**	**480**
VGFTKMSANK	SAHALVGLLN	DLFGRFDRLC	EETKCEKIST	LGDCYYCVAG	CPEPRADHAY	CCIEMGLGMI	KAIEQFCQEK
**490**	**500**	**510**	**520**	**530**	**540**	**550**	**560**
KEMVNMRVGV	HTGTVLCGIL	GMRRFKFDVW	SNDVNLANLM	EQLGVAGKVH	ISEATAKYLD	DRYEMEDGKV	IERLGQSVVA
**570**	**580**	**590**	**600**	**610**	**620**	**630**	**640**
DQLKGLKTYL	ISGQRAKESR	CSCAEALLSG	FEVIDGSQVS	SGPRGQGTAS	SGNVSDLAQT	VKTFDNLKTC	PSCGITFAPK
**650**	**660**	**670**	**680**	**690**	**700**	**710**	**720**
SEAGAEGGAP	QNGCQDEHKN	STKASGGPNP	KTQNGLLSPP	QEEKLTNSQT	SLCEILQEKG	RWAGVSLDQS	ALLPLRFKNI
**730**	**740**	**750**	**760**	**770**	**780**	**790**	**800**
REKTDAHFVD	VIKEDSLMKD	YFFKPPINQF	SLNFLDQELE	RSYRTSYQEE	VIKNSPVKTF	ASPTFSSLLD	VFLSTTVFLT
**810**	**820**	**830**	**840**	**850**	**860**	**870**	**880**
LSTTCFLKYE	AATVPPPPAA	LAVFSAALLL	EVLSLAVSIR	MVFFLEDVMA	CTKRLLEWIA	GWLPRHCIGA	ILVSLPALAV
**890**	**900**	**910**	**920**	**930**	**940**	**950**	**960**
YSHVTSEYET	NIHFPVFTGS	AALIAVVHYC	NFCQLSSWMR	SSLATVVGAG	PLLLLYVSLC	PDSSVLTSPL	DAVQNFSSER
**970**	**980**	**990**	**1000**	**1010**	**1020**	**1030**	**1040**
NPCNSSVPRD	LRRPASLIGQ	EVVLVFFLLL	LLVWFLNREF	EVSYRLHYHG	DVEADLHRTK	IQSMRDQADW	LLRNIIPYHV
**1050**	**1060**	**1070**	**1080**	**1090**	**1100**	**1110**	**1120**
AEQLKVSQTY	SKNHDSGGVI	FASIVNFSEF	YEENYEGGKE	CYRVLNELIG	DFDELLSKPD	YSSIEKIKTI	GATYMAASGL
**1130**	**1140**	**1150**	**1160**	**1170**	**1180**	**1190**	**1200**
NTAQAQDGSH	PQEHLQILFE	FAKEMMRVVD	DFNNNMLWFN	FKLRVGFNHG	PLTAGVIGTT	KLLYDIWGDT	VNIASRMDTT
**1210**	**1220**	**1230**	**1240**	**1250**	**1260**	**1270**	**1280**
GVECRIQVSE	ESYRVLSKMG	YDFDYRGTVN	VKGKGQMKTY	LYPKCTDHRV	IPQHQLSISP	DIRVQVDGSI	GRSPTDEIAN
**1290**	**1300**	**1310**	**1320**	**1330**	**1340**	**1350**	**1360**
LVPSVQYVDK	TSLGSDSSTQ	AKDAHLSPKR	PWKEPVKAEE	RGRFGKAIEK	DDCDETGIEE	ANELTKLNVS	KSV

Cmpd.	No. of Cmpds.	m/z meas.	Δ m/z [ppm]	z	Rt [min]	Score	P	Range	Sequence	Modification
1840	1	920.8515	-101.83	2	53.0	12.7	2	1235-1249	K.GQMKTYLYPKCTDHR.V	

Detailed Protein Report

Protein 442: zinc finger protein 740 [Homo sapiens]

Accession: gi|51972182
Database: refseq_human(refseq_human_20140103.fasta)
Database Date: 2015-11-30
Modification(s): Carbamidomethyl

Score: 29.2
MW [kDa]: 21.8
pI: 10.5
Sequence Coverage [%]: 5.2
No. of unique Peptides: 2

10	20	30	40	50	60	70	80
MAQASLLACE	GLAGVSLVPT	AASKKMMLSQ	IASKQAENGE	RAGSPDVLRC	SSQGHRKDSD	KSRSRKDDDS	LSEASHSKKT
**90**	**100**	**110**	**120**	**130**	**140**	**150**	**160**
VKKVVVVEQN	GSFQVKIPKN	FVCEHCFGAF	RSSYHLKRHI	LIHTGEKPFE	CDICDMRFIQ	KYHLERHKRV	HSGEKPYQCE
**170**	**180**	**190**	**200**				
RCHQCFSRTD	RLLRHKRMCQ	GCQSKTSDGQ	FSL				

Cmpd.	No. of Cmpds.	m/z meas.	Δ m/z [ppm]	z	Rt [min]	Score	P	Range	Sequence	Modification
1915	2	655.2467	-50.69	2	52.6	13.1	1	162-171	R.CHQCFSRTDR.L	Carbamidomethyl: 4
1903	2	655.2746	-8.02	2	52.5	16.0	1	162-171	R.CHQCFSRTDR.L	Carbamidomethyl: 1

Detailed Protein Report

Protein 443: **PREDICTED: phosphatidylinositol-glycan-specific phospholipase D isoform X3 [Homo sapiens]**

**Accession:**	gi|530381810	**Score:**	29.2
**Database:**	refseq_human(refseq_human_20140103.fasta)	**MW [kDa]:**	73.7
**Database Date:**	2015-11-30	**pI:**	5.7
		**Sequence Coverage [%]:**	2.5
		**No. of unique Peptides:**	1

Quantitation

mdown:qdown	**Median:**	0.80	**CV:**	0.00 %	**No. of Peptides:**	1
Wdown:Qdown	**Median:**	0.71	**CV:**	0.00 %	**No. of Peptides:**	1

10	20	30	40	50	60	70	80
MSAGIVWALN	KDSLGPWELY	VPVKDLLGIY	EKLYGRKVIT	ENVIVDCSHI	QFLEMYGEML	AVSKLYPTYS	TKSPFLVEQF
**90**	**100**	**110**	**120**	**130**	**140**	**150**	**160**
QEYFLGGLDD	MAFWSTNIYH	LTSFMLENGT	SDCNLPENPL	FIACGGQQNH	TQGSKMQKND	FHRNLTTSLT	ESVDRNINYT
**170**	**180**	**190**	**200**	**210**	**220**	**230**	**240**
ERGVFFSVNS	WTPDSMSFIY	KALERNIRTM	FIGGSQLSQK	HVSSPLASYF	LSFPYARLGW	AMTSADLNQD	GHGDLVVGAP
**250**	**260**	**270**	**280**	**290**	**300**	**310**	**320**
GYSRPGHIHI	GRVYLIYGND	LGLPPVDLDL	DKEAHRILEG	FQPSGRFGSA	LAVLDFNVDG	VPDLAVGAPS	VGSEQLTYKG
**330**	**340**	**350**	**360**	**370**	**380**	**390**	**400**
AVYVYFGSKQ	GGMSSSPNIT	ISCQDIYCNL	GWTLLAADVN	GDSEPDLVIG	SPFAPGGGKQ	KGIVAAFYSG	PSLSDKEKLN
**410**	**420**	**430**	**440**	**450**	**460**	**470**	**480**
VEAANWTVRG	EEDFSWFGYS	LHGVTVDNRT	LLLVGSPTWK	NASRLGHLLH	IRDEKKSLGR	VYGYFPPNGQ	SWFTISGDKA
**490**	**500**	**510**	**520**	**530**	**540**	**550**	**560**
MGKLGTSLSS	GHVLMNGTLK	QVLLVGAPTY	DDVSKVAFLT	VTLHQGGATR	MYALTSDAQP	LLLSTFSGDR	RFSRFGGVLH
**570**	**580**	**590**	**600**	**610**	**620**	**630**	**640**
LSDLDDDGLD	EIIMAAPLRI	ADVTSGLIGG	EDGRVYVYNG	KETTLGDMTG	KCKSWITPCP	EEKAQYVLIS	PEASSRFGSS
**650**	**660**	**670**	**680**				
LITVRSKAKN	QVVIAAGRSS	LGARLSGALH	VYSLGSD				

Cmpd.	No. of Cmpds.	m/z meas.	Δ m/z [ppm]	z	Rt [min]	Score	P	Range	Sequence	Modification	Ratios
257	1	625.9672	-15.19	3	32.4	29.2	1	595-611	R.VYVYNGKETTLGDMTGK.C		Wdown:Qdown 0.71<br>mdown:qdown 0.80

Detailed Protein Report

Protein 444: ADP/ATP translocase 1 [Homo sapiens]

**Accession:**	gi|55749577	**Score:**	29.1
**Database:**	refseq_human(refseq_human_20140103.fasta)	**MW [kDa]:**	33.0
**Database Date:**	2015-11-30	**pI:**	10.3
**Modification(s):**	Carbamidomethyl, Oxidation	**Sequence Coverage [%]:**	11.7
		**No. of unique Peptides:**	1

10	20	30	40	50	60	70	80
MGDHAWSFLK	DFLAGGVAAA	VSKTAVAPIE	RVKLLLQVQH	ASKQISAEKQ	YKGIIDCVVR	IPKEQGFLSF	WRGNLANVIR
90	100	110	120	130	140	150	160
YFPTQALNFA	FKDKYKQLFL	GGVDRHKQFW	RYFAGNLASG	GAAGATSLCF	VYPLDFARTR	LAADVGKGAA	QREFHGLGDC
170	180	190	200	210	220	230	240
IIKIFKSDGL	RGLYQGFNVS	VQGIIIYRAA	YFGVYDTAKG	MLPDPKNVHI	FVSWMIAQSV	TAVAGLVSYP	FDTVRRRMMM
250	260	270	280	290	300		
QSGRKGADIM	YTGTVDCWRK	IAKDEGAKAF	FKGAWSNVLR	GMGGAFVLVL	YDEIKKYV		

Cmpd.	No. of Cmpds.	m/z meas.	Δ m/z [ppm]	z	Rt [min]	Score	P	Range	Sequence	Modification
2202	1	1051.1584	137.54	2	57.7	12.3	2	246-263	K.GADIMYTGTVDCWRKIAK.D	Carbamidomethyl: 12; Oxidation: 5

Detailed Protein Report

Protein 445: 5'-nucleotidase isoform 1 preproprotein [Homo sapiens]

**Accession:**	gi|4505467	**Score:**	29.0
**Database:**	refseq_human(refseq_human_20140103.fasta)	**MW [kDa]:**	63.3
**Database Date:**	2015-11-30	**pI:**	6.6
**Modification(s):**	Carbamidomethyl, Oxidation	**Sequence Coverage [%]:**	3.7
		**No. of unique Peptides:**	1

10	20	30	40	50	60	70	80
MCPRAARAPA	TLLLALGAVL	WPAAGAWELT	ILHTNDVHSR	LEQTSEDSSK	CVNASRCMGG	VARLFTKVQQ	IRRAEPNVLL
**90**	**100**	**110**	**120**	**130**	**140**	**150**	**160**
LDAGDQYQGT	IWFTVYKGAE	VAHFMNALRY	DAMALGNHEF	DNGVEGLIEP	LLKEAKFPIL	SANIKAKGPL	ASQISGLYLP
**170**	**180**	**190**	**200**	**210**	**220**	**230**	**240**
YKVLPVGDEV	VGIVGYTSKE	TPFLSNPGTN	LVFEDEITAL	QPEVDKLKTL	NVNKIIALGH	SGFEMDKLIA	QKVRGVDVVV
**250**	**260**	**270**	**280**	**290**	**300**	**310**	**320**
GGHSNTFLYT	GNPPSKEVPA	GKYPFIVTSD	DGRKVPVVQA	YAFGKYLGYL	KIEFDERGNV	ISSHGNPILL	NSSIPEDPSI
**330**	**340**	**350**	**360**	**370**	**380**	**390**	**400**
KADINKWRIK	LDNYSTQELG	KTIVYLDGSS	QSCRFRECNM	GNLICDAMIN	NNLRHTDEMF	WNHVSMCILN	GGGIRSPIDE
**410**	**420**	**430**	**440**	**450**	**460**	**470**	**480**
RNNGTITWEN	LAAVLPFGGT	FDLVQLKGST	LKKAFEHSVH	RYGQSTGEFL	QVGGIHVVYD	LSRKPGDRVV	KLDVLCTKCR
**490**	**500**	**510**	**520**	**530**	**540**	**550**	**560**
VPSYDPLKMD	EVYKVILPNF	LANGGDGFQM	IKDELLRHDS	GDQDINVVST	YISKMKVIYP	AVEGRIKFST	GSHCHGSFSL
**570**	**580**						
IFLSLWAVIF	VLYQ						

Cmpd.	No. of Cmpds.	m/z meas.	Δ m/z [ppm]	z	Rt [min]	Score	P	Range	Sequence	Modification
1850	1	727.3153	-15.31	2	51.6	13.1	1	51-63	K.CVNASRCMGGVAR.L	Carbamidomethyl: 1, 7; Oxidation: 8

Detailed Protein Report

Protein 446: putative Polycomb group protein ASXL3 [Homo sapiens]

**Accession:**	gi|149944526	**Score:**	29.0
**Database:**	refseq_human(refseq_human_20140103.fasta)	**MW [kDa]:**	241.8
**Database Date:**	2015-11-30	**pI:**	5.8
**Modification(s):**	Oxidation	**Sequence Coverage [%]:**	1.7
		**No. of unique Peptides:**	1

Detailed Protein Report

10	20	30	40	50	60	70	80
MKDKRKKKDR	TWAEAARLAL	EKHPNSPMTA	KQILEVIQKE	GLKETSGTSP	LACLNAMLHT	NTRIGDGTFF	KIPGKSGLYA
90	100	110	120	130	140	150	160
LKKEESSCPA	DGTLDLVCES	ELDGTDMAEA	NAHGEENGVC	SKQVTDEASS	TRDSSLTNTA	VQSKLVSSFQ	QHTKKALKQA
170	180	190	200	210	220	230	240
LRQQQKRRNG	VSMMVNKTVP	RVVLTPLKVS	DEQSDSPSGS	ESKNGEADSS	DKEMKHGQKS	PTGKQTSQHL	KRLKKSGLGH
250	260	270	280	290	300	310	320
LKWTKAEDID	IETPGSILVN	TNLRALINKH	TFASLPQHFQ	QYLLLLLPEV	DRQMGSDGIL	RLSTSALNNE	FFAYAAQGWK
330	340	350	360	370	380	390	400
QRLAEGEFTP	EMQLRIRQEI	EKEKKTEPWK	EKFFERFYGE	KLGMSREESV	KLTTGPNNAG	AQSSSSCGTS	GLPVSAQTAL
410	420	430	440	450	460	470	480
AEQQPKSMKS	PASPEPGFCA	TLCPMVEIPP	KDIMAELESE	DILIPEESVI	QEEIAEEVET	SICECQDENH	KTIPEFSEEA
490	500	510	520	530	540	550	560
ESLTNSHEEP	QIAPPEDNLE	SCVMMNDVLE	TLPHIEVKIE	GKSESPQEEM	TVVIDQLEVC	DSLIPSTSSM	THVSDTEHKE
570	580	590	600	610	620	630	640
SETAVETSTP	KIKTGSSSLE	GQFPNEGIAI	DMELQSDPEE	QLSENACISE	TSFSSESPEG	ACTSLPSPGG	ETQSTSEESC
650	660	670	680	690	700	710	720
TPASLETTFC	SEVSSTENTD	KYNQRNSTDE	NFHASLMSEI	SPISTSPEIS	EASLMSNLPL	TSEASPVSNL	PLTSETSPMS
730	740	750	760	770	780	790	800
DLPLTSETSS	VSSMLLTSET	TFVSSLPLPS	ETSPISNSSI	NERMAHQQRK	SPSVSEEPLS	PQKDESSATA	KPLGENLTSQ
810	820	830	840	850	860	870	880
QKNLSNTPEP	IIMSSSSIAP	EAFPSEDLHN	KTLSQQTCKS	HVDTEKPYPA	SIPELASTEM	IKVKNHSVLQ	RTEKKVLPSP
890	900	910	920	930	940	950	960
LELSVFSEGT	DNKGNELPSA	KLQDKQYISS	VDKAPFSEGS	RNKTHKQGST	QSRLETSHTS	KSSEPSKSPD	GIRNESRDSE
970	980	990	1000	1010	1020	1030	1040
ISKRKTAEQH	SFGICKEKRA	RIEDDQSTRN	ISSSSPPEKE	QPPREEPRVP	PLKIQLSKIG	PPFIIKSQPV	SKPESRASTS
1050	1060	1070	1080	1090	1100	1110	1120
TSVSGGRNTG	ARTLADIKAR	AQQARAQREA	AAAAAVAAAA	SIVSGAMGSP	GEGGKTRTLA	HIKEQTKAKL	FAKHQARAHL
1130	1140	1150	1160	1170	1180	1190	1200
FQTSKETRLP	PPLSSKEGPP	NLEVSSTPET	KMEGSTGVII	VNPNCRSPSN	KSAHLRETTT	VLQQSLNPSK	LPETATDLSV
1210	1220	1230	1240	1250	1260	1270	1280
HSSDENIPVS	HLSEKIVSST	SSENSSVPML	FNKNSVPVSV	CSTAISGAIK	EHPFVSSVDK	SSVLMSVDSA	NTTISACNIS
1290	1300	1310	1320	1330	1340	1350	1360
MLKTIQGTDT	PCIAIIPKCI	ESTPISATTE	GSSISSSMDD	KQLLISSSSA	SNLVSTQYTS	VPTPSIGNNL	PNLSTSSVLI
1370	1380	1390	1400	1410	1420	1430	1440
PPMGINNRFP	SEKIAIPGSE	EQATVSMGTT	VRAALSCSDS	VAVTDSLVAH	PTVAMFTGNM	LTINSYDSPP	KLSAESLDKN
1450	1460	1470	1480	1490	1500	1510	1520
SGPRNRADNS	GKPQQPPGGF	APAAINRSIP	CKVIVDHSTT	LTSSLSLTVS	VESSEASLDL	QGRPVRTEAS	VQPVACPQVS
1530	1540	1550	1560	1570	1580	1590	1600
VISRPEPVAN	EGIDHSSTFI	AASAAKQDSK	TLPATCTSLR	ELPLVPDKLN	EPTAPSHNFA	EQARGPAPFK	SEADTTCSNQ
1610	1620	1630	1640	1650	1660	1670	1680
YNPSNRICWN	DDGMRSTGQP	LVTHSGSSKQ	KEYLEQSCPK	AIKTEHANYL	NVSELHPRNL	VTNVALPVKS	ELHEADKGFR
1690	1700	1710	1720	1730	1740	1750	1760
MDTEDFPGPE	LPPPAAEGAS	SVQQTQNMKA	STSSPMEEAI	SLATDALKRV	PGAGSSGCRL	SSVEANNPLV	TQLLQGNLPL
1770	1780	1790	1800	1810	1820	1830	1840
EKVLPQPRLG	AKLEINRLPL	PLQTTSVGKT	APERNVEIPP	SSPNPDGKGY	LAGTLAPLQM	RKRENHPKKR	VARTVGEHTQ
1850	1860	1870	1880	1890	1900	1910	1920
VKCEPGKLLV	EPDVKGVPCV	ISSGISQLGH	SQPFKQEWLN	KHSMQNRIVH	SPEVKQQKRL	LPSCSFQQNL	FHVDKNGGFH
1930	1940	1950	1960	1970	1980	1990	2000
TDAGTSHRQQ	FYQMPVAARG	PIPTAALLQA	SSKTPVGCNA	FAFNRHLEQK	GLGEVSLSSA	PHQLRLANML	SPNMPMKEGD
2010	2020	2030	2040	2050	2060	2070	2080
EVGGTAHTMP	NKALVHPPPP	PPPPPPPPLA	LPPPPPPPPP	LPPPLPNAEV	PSDQKQPPVT	METTKRLSWP	QSTGICSNIK
2090	2100	2110	2120	2130	2140	2150	2160
SEPLSFEEGL	SSSCELGMKQ	VSYDQNEMKE	QLKAFALKSA	DFSSYLLSEP	QKPFTQLAAQ	KMQVQQQQQL	CGNYPTIHFG
2170	2180	2190	2200	2210	2220	2230	2240
STSFKRAASA	IEKSIGILGS	GSNPATGLSG	QNAQMPVQNF	ADSSNADELE	LKCSCRLKAM	IVCKGCGAFC	HDDCIGPSKL
2250							
CVACLVVR							

Detailed Protein Report

Cmpd.	No. of Cmpds.	m/z meas.	Δ m/z [ppm]	z	Rt [min]	Score	P	Range	Sequence	Modification
1390	1	1030.1245	-9.16	3	47.1	12.9	0	1681-1709	R.MDTEDFPGPELPPPAAEGASSVQQTQNMK.A	Oxidation: 1

Detailed Protein Report

Protein 447: PREDICTED: histone-lysine N-methyltransferase SETD2 isoform X3 [Homo sapiens]

**Accession:**	gi|578806017	**Score:**	29.0
**Database:**	refseq_human(refseq_human_20140103.fasta)	**MW [kDa]:**	253.1
**Database Date:**	2015-11-30	**pI:**	6.6
		**Sequence Coverage [%]:**	1.1
		**No. of unique Peptides:**	1

Detailed Protein Report

10	20	30	40	50	60	70	80
MKQLQPQPPP	KMGDFYDPEH	PTPEEEENEA	KIENVQKTGF	IKGPMFKGVA	SSRFLPKGTK	TKVNLEEQGR	QKVSFSFSLT
90	100	110	120	130	140	150	160
KKTLQNRFLT	ALGNEKQSDT	PNPPAVPLQV	DSTPKMKMEI	GDTLSTAEES	SPPKSRVELG	KIHFKKHLLH	VTSRPLLATT
170	180	190	200	210	220	230	240
TAVASPPTHA	APLPAVIAES	TTVDSPPSSP	PPPPPPAQAT	TLSSPAPVTE	PVALPHTPIT	VLMAAPVPLP	VDVAVRSLKE
250	260	270	280	290	300	310	320
PPIIIVPESL	EADTKQDTIS	NSLEEHVTQI	LNEQADISSK	KEDSHIGKDE	EIPDSSKISL	SCKKTGSKKK	SSQSEGIFLG
330	340	350	360	370	380	390	400
SESDEDSVRT	SSSQRSHDLK	FSASIEKERD	FKKSSAPLKS	EDLGKPSRSK	TDRDDKYFSY	SKLERDTRYV	SSRCRSERER
410	420	430	440	450	460	470	480
RRSRSHSRSE	RGSRTNLSYS	RSERSHYYDS	DRRYHRSSPY	RERTRYSRPY	TDNRARESSD	SEEEYKKTYS	RRTSSHSSSY
490	500	510	520	530	540	550	560
RDLRTSSYSK	SDRDCKTETS	YLEMERRGKY	SSKLERESKR	TSENEAIKRC	CSPPNELGFR	RGSSYSKHDS	SASRYKSTLS
570	580	590	600	610	620	630	640
KPIPKSDKFK	NSFCCTELNE	EIKQSHSFSL	QTPCSKGSEL	RMINKNPERE	KAGSPAPSNR	LNDSPTLKKL	DELPIFKSEF
650	660	670	680	690	700	710	720
ITHDSHDSIK	ELDSLSKVKN	DQLRSFCPIE	LNINGSPGAE	SDLATFCTSK	TDAVLMTSDD	SVTGSELSPL	VKACMLSSNG
730	740	750	760	770	780	790	800
FQNISRCKEK	DLDDTCMLHK	KSESPFRETE	PLVSPHQDKL	MSMPVMTVDY	SKTVVKEPVD	TRVSCCKTKD	SDIYCTLNDS
810	820	830	840	850	860	870	880
NPSLCNSEAE	NIEPSVMKIS	SNSFMNVHLE	SKPVICDSRN	LTDHSKFACE	EYKQSIGSTS	SASVNHFDDL	YQPIGSSGIA
890	900	910	920	930	940	950	960
SSLQSLPPGI	KVDSLTLLKC	GENTSPVLDA	VLKSKKSSEF	LKHAGKETIV	EVGSDLPDSG	KGFASRENRR	NNGLSGKCLQ
970	980	990	1000	1010	1020	1030	1040
EAQEEGNSIL	PERRGRPEIS	LDERGEGGHV	HTSDDSEVVF	SSCDLNLTME	DSDGVTYALK	CDSSGHAPEI	VSTVHEDYSG
1050	1060	1070	1080	1090	1100	1110	1120
SSESSNDESD	SEDTDSDDSS	IPRNRLQSVV	VVPKNSTLPM	EETSPCSSRS	SQSYRHYSDH	WEDERLESRR	HLYEEKFESI
1130	1140	1150	1160	1170	1180	1190	1200
ASKACPQTDK	FFLHKGTEKN	PEISFTQSSR	KQIDNRLPEL	SHPQSDGVDS	TSHTDVKSDP	LGHPNSEETV	KAKIPSRQQE
1210	1220	1230	1240	1250	1260	1270	1280
ELPIYSSDFE	DVPNKSWQQT	TFQNRPDSRL	GKTELSFSSS	CEIPHVDGLH	SSEELRNLGW	DFSQEKPSTT	YQQPDSSYGA
1290	1300	1310	1320	1330	1340	1350	1360
CGGHKYQQNA	EQYGGTRDYW	QGNGYWDPRS	GRPPGTGVVY	DRTQGQVPDS	LTDDREEEEN	WDQQDGSHFS	DQSDKFLLSL
1370	1380	1390	1400	1410	1420	1430	1440
QKDKGSVQAP	EISSNSIKDT	LAVNEKKDFS	KNLEKNDIKD	RGPLKKRRQE	IESDSESDGE	LQDRKKVRVE	VEQGETSVPP
1450	1460	1470	1480	1490	1500	1510	1520
GSALVGPSCV	MDDFRDPQRW	KECAKQGKMP	CYFDLIEENV	YLTERKKNKS	HRDIKRMQCE	CTPLSKDERA	QGEIACGEDC
1530	1540	1550	1560	1570	1580	1590	1600
LNRLLMIECS	SRCPNGDYCS	NRRFQRKQHA	DVEVILTEKK	GWGLRAAKDL	PSNTFVLEYC	GEVLDHKEFK	ARVKEYARNK
1610	1620	1630	1640	1650	1660	1670	1680
NIHYYFMALK	NDEIIDATQK	GNCSRFMNHS	CEPNCETQKW	TVNGQLRVGF	FTTKLVPSGS	ELTFDYQFQR	YGKEAQKCFC
1690	1700	1710	1720	1730	1740	1750	1760
GSANCRGYLG	GENRVSIRAA	GGKMKKERSR	KKDSEVYRIP	KKSQTEKENT	TTERGRDAVG	FRDQTPAPKT	PNRSRERDPD
1770	1780	1790	1800	1810	1820	1830	1840
KQTQNKEKRK	RRSSLSPPSS	AYERGTKRPD	DRYDTPTSKK	KVRIKDRNKL	STEERRKLFE	QEVAQREAQK	QQQQMQNLGM
1850	1860	1870	1880	1890	1900	1910	1920
TSPLPYDSLG	YNAPHHPFAG	YPPGYPMQAY	VDPSNPNAGK	VLLPTPSMDP	VCSPAPYDHA	QPLVGHSTEP	LSAPPPVPVV
1930	1940	1950	1960	1970	1980	1990	2000
PHVAAPVEVS	SSQYVAQSDG	VVHQDSSVAV	LPVPAPGPVQ	GQNYSVWDSN	QQSVSVQQQY	SPAQSQATIY	YQGQTCPTVY
2010	2020	2030	2040	2050	2060	2070	2080
GVTSPYSQTT	PPIVQSYAQP	SLQYIQGQQI	FTAHPQGVVV	QPAAAVTTIV	APGQPQPLQP	SEMVVTNNLL	DLPPPSPPKP
2090	2100	2110	2120	2130	2140	2150	2160
KTIVLPPNWK	TARDPEGKIY	YYHVITRQTQ	WDPPTWESPG	DDASLEHEAE	MDLGTPTYDE	NPMKASKKPK	TAEADTSSEL
2170	2180	2190	2200	2210	2220	2230	2240

Detailed Protein Report

Cmpd.	No. of Cmpds.	m/z meas.	Δ m/z [ppm]	z	Rt [min]	Score	P	Range	Sequence	Modification
2100	1	707.7227	-113.09	2	54.8	14.1	1	422-432	R.SERSHYYDSDR.R	

Detailed Protein Report

Protein 448: PREDICTED: putative Polycomb group protein ASXL1 isoform X6 [Homo sapiens]

**Accession:**	gi|578835836	**Score:**	28.9
**Database:**	refseq_human(refseq_human_20140103.fasta)	**MW [kDa]:**	159.1
**Database Date:**	2015-11-30	**pI:**	5.6
**Modification(s):**	Carbamidomethyl, Oxidation	**Sequence Coverage [%]:**	2.4
		**No. of unique Peptides:**	2

10	20	30	40	50	60	70	80
MLSFPNLNQV	LENYSDAPMT	PKQILQVIEA	EGLKEMRSGT	SPLACLNAML	HSNSRGGEGL	FYKLPGRISL	FTLKKDALQW
90	100	110	120	130	140	150	160
SRHPATVEGE	EPEDTADVES	CGSNEASTVS	GENDVSLDET	SSNASCSTES	QSRPLSNPRD	SYRASSQANK	QKKKTGVMLP
170	180	190	200	210	220	230	240
RVVLTPLKVN	GAHVESASGQ	MKRNRGEEID	FETPGSILVN	TNLRALINSR	TFHALPSHFQ	QQLLFLLPEV	DRQVGTDGLL
250	260	270	280	290	300	310	320
RLSSSALNNE	FFTHAAQSWR	ERLADGEFTH	EMQVRIRQEM	EKEKKVEQWK	EKFFEDYYGQ	KLGLTKEESL	QQNVGQEEAE
330	340	350	360	370	380	390	400
IKSGLCVPGE	SVRIQRGPAT	RQRDGHFKKR	SRPDLRTRAR	RNLYKKQESE	QAGVAKDAKS	VASDVPLYKD	GEAKTDPAGL
410	420	430	440	450	460	470	480
SSPHLPGTSS	AAPDLEGPEF	PVESVASRIQ	AEPDNLARAS	ASPDRIPSLP	QETVDQEPKD	QKRKSFEQAA	SASFPEKKPR
490	500	510	520	530	540	550	560
LEDRQSFRNT	IESVHTEKPQ	PTKEEPKVPP	IRIQLSRIKP	PWVVKGQPTY	QICPRIIPTT	ESSCRGWTGA	RTLADIKARA
570	580	590	600	610	620	630	640
LQVRGARGHH	CHREAATTAI	GGGGGPGGGG	GGATDEGGGR	GSSSGDGGEA	CGHPEPRGGP	STPGKCTSDL	QRTQLLPPYP
650	660	670	680	690	700	710	720
LNGEHTQAGT	AMSRARREDL	PSLRKEESCL	LQRATVGLTD	GLGDASQLPV	APTGDQPCQA	LPLLSSQTSV	AERLVEQPQL
730	740	750	760	770	780	790	800
HPDVRTECES	GTTSWESDDE	EQGPTVPADN	GPIPSLVGDD	TLEKGTGQAL	DSHPTMKDPV	NVTPSSTPES	SPTDCLQNRA
810	820	830	840	850	860	870	880
FDDELGLGGS	CPPMRESDTR	QENLKTKALV	SNSSLHWIPI	PSNDEVVKQP	KPESREHIPS	VEPQVGEEWE	KAAPTPPALP
890	900	910	920	930	940	950	960
GDLTAEEGLD	PLDSLTSLWT	VPSRGGSDSN	GSYCQQVDIE	KLKINGDSEA	LSPHGESTDT	ASDFEGHLTE	DSSEADTREA
970	980	990	1000	1010	1020	1030	1040
AVTKGSSVDK	DEKPNWNQSA	PLSKVNGDMR	LVTRTDGMVA	PQSWVSRVCA	VRQKIPDSLL	LASTEYQPRA	VCLSMPGSSV
1050	1060	1070	1080	1090	1100	1110	1120
EATNPLVMQL	LQGSLPLEKV	LPPAHDDSMS	ESPQVPLTKD	QSHGSLRMGS	LHGLGKNSGM	VDGSSPSSLR	ALKEPLLPDS
1130	1140	1150	1160	1170	1180	1190	1200
CETGTGLARI	EATQAPGAPQ	KNCKAVPSFD	SLHPVTNPIT	SSRKLEEMDS	KEQFSSFSCE	DQKEVRAMSQ	DSNSNAAPGK
1210	1220	1230	1240	1250	1260	1270	1280
SPGDLTTSRT	PRFSSPNVIS	FGPEQTGRAL	GDQSNVTGQG	KKLFGSGNVA	ATLQRPRPAD	PMPLPAEIPP	VFPSGKLGPS
1290	1300	1310	1320	1330	1340	1350	1360
TNSMSGGVQT	PREDWAPKPH	AFVGSVKNEK	TFVGGPLKAN	AENRKATGHS	PLELVGHLEG	MPFVMDLPFW	KLPREPGKGL
1370	1380	1390	1400	1410	1420	1430	1440
SEPLEPSSLP	SQLSIKQAFY	GKLSKLQLSS	TSFNYSSSSP	TFPKGLAGSV	VQLSHKANFG	ASHSASLSLQ	MFTDSSTVES
1450	1460	1470	1480	1490			
ISLQCACSLK	AMIMCQGCGA	FCHDDCIGPS	KLCVLCLVVR				

Cmpd.	No. of Cmpds.	m/z meas.	Δ m/z [ppm]	z	Rt [min]	Score	P	Range	Sequence	Modification
2381	1	937.9548	9.34	2	58.5	11.6	0	38-55	R.SGTSPLACLNAMLHSNSR.G	Oxidation: 12
2764	2	922.4660	80.47	2	65.9	17.3	0	905-921	R.GGSDSNGSYCQQVDIEK.L	Carbamidomethyl: 10

Detailed Protein Report

Protein 449: **PREDICTED: notchless protein homolog 1 isoform X5 [Homo sapiens]**

**Accession:**	gi|578831085	**Score:**	28.9
**Database:**	refseq_human(refseq_human_20140103.fasta)	**MW [kDa]:**	42.7
**Database Date:**	2015-11-30	**pI:**	10.1
**Modification(s):**	Carbamidomethyl	**Sequence Coverage [%]:**	4.1
		**No. of unique Peptides:**	1

10	20	30	40	50	60	70	80
MRAGSCWVPR	STCPWTSPRT	GCSSCATRYW	PRYLASGSGD	TTVRFWDLST	ETPHFTCKGH	RHWVLSISWS	PDGRKLASGC
**90**	**100**	**110**	**120**	**130**	**140**	**150**	**160**
KNGQILLWDP	STGKQVGRTL	AGHSKWITGL	SWEPLHANPE	CRYVASSSKD	GSVRIWDTTA	GRCERILTGH	TQSVTCLRWG
**170**	**180**	**190**	**200**	**210**	**220**	**230**	**240**
GDGLLYSASQ	DRTIKVWRAH	DGVLCRTLQG	HGHWVNTMAL	STDYALRTGA	FEPAEASVNP	QDLQGSLQEL	KERALSRYNL
**250**	**260**	**270**	**280**	**290**	**300**	**310**	**320**
VRGQGPERLV	SGSDDFTLFL	WSPAEDKKPL	TRMTGHQALI	NQVLFSPDSR	IVASASFDKS	IKLWDGRTGK	YLASLRGHVA
**330**	**340**	**350**	**360**	**370**	**380**	**390**	
AVYQIAWSAD	SRLLVSGSSD	STLKVWDVKA	QKLAMDLPGH	ADEVYAVDWS	PDGQRVASGG	KDKCLRM	

Cmpd.	No. of Cmpds.	m/z meas.	Δ m/z [ppm]	z	Rt [min]	Score	P	Range	Sequence	Modification
2764	1	937.4600	-13.04	2	63.6	12.4	1	143-158	R.CERILTGHTQSVTCLR.W	Carbamidomethyl: 1

Detailed Protein Report

Protein 450: PREDICTED: ryanodine receptor 1 isoform X3 [Homo sapiens]

**Accession:**	gi|578834746	**Score:**	28.9
**Database:**	refseq_human(refseq_human_20140103.fasta)	**MW [kDa]:**	563.6
**Database Date:**	2015-11-30	**pI:**	5.1
**Modification(s):**	Carbamidomethyl	**Sequence Coverage [%]:**	0.6
		**No. of unique Peptides:**	1

Detailed Protein Report

10	20	30	40	50	60	70	80
MGDAEGEDEV	QFLRTDDEVV	LQCSATVLKE	QLKLCLAAEG	FGNRLCFLEP	TSNAQNVPPD	LAICCFVLEQ	SLSVRALQEM
90	100	110	120	130	140	150	160
LANTVEAGVE	SSQGGGHRTL	LYGHAILLRH	AHSRMYLSCL	TTSRSMTDKL	AFDVGLQEDA	TGEACWWTMH	PASKQRSEGE
170	180	190	200	210	220	230	240
KVRVGDDIIL	VSVSSERYLH	LSTASGELQV	DASFMQTLWN	MNPICSRCEE	GFVTGGHVLR	LFHGHMDECL	TISPADSDDQ
250	260	270	280	290	300	310	320
RRLVYYEGGA	VCTHARSLWR	LEPLRISWSG	SHLRWGQPLR	VRHVTTGQYL	ALTEDQGLVV	VDASKAHTKA	TSFCFRISKE
330	340	350	360	370	380	390	400
KLDVAPKRDV	EGMGPPEIKY	GESLCFVQHV	ASGLWLTYAA	PDPKALRLGV	LKKKAMLHQE	GHMDDALSLT	RCQQEESQAA
410	420	430	440	450	460	470	480
RMIHSTNGLY	NQFIKSLDSF	SGKPRGSGPP	AGTALPIEGV	ILSLQDLIIY	FEPPSEDLQH	EEKQSKLRSL	RNRQSLFQEE
490	500	510	520	530	540	550	560
GMLSMVLNCI	DRLNVYTTAA	HFAEFAGEEA	AESWKEIVNL	LYELLASLIR	GNRSNCALFS	TNLDWLVSKL	DRLEASSGIL
570	580	590	600	610	620	630	640
EVLYCVLIES	PEVLNIIQEN	HIKSIISLLD	KHGRNHKVLD	VLCSLCVCNG	VAVRSNQDLI	TENLLPGREL	LLQTNLINYV
650	660	670	680	690	700	710	720
TSIRPNIFVG	RAEGTTQYSK	WYFEVMVDEV	TPFLTAQATH	LRVGWALTEG	YTPYPGAGEG	WGGNGVGDDL	YSYGFDGLHL
730	740	750	760	770	780	790	800
WTGHVARPVT	SPGQHLLAPE	DVISCCLDLS	VPSISFRING	CPVQGVFESF	NLDGLFFPVV	SFSAGVKVRF	LLGGRHGEFK
810	820	830	840	850	860	870	880
FLPPPGYAPC	HEAVLPRERL	HLEPIKEYRR	EGPRGPHLVG	PSRCLSHTDF	VPCPVDTVQI	VLPPHLERIR	EKLAENIHEL
890	900	910	920	930	940	950	960
WALTRIEQGW	TYGPVRDDNK	RLHPCLVDFH	SLPEPERNYN	LQMSGETLKT	LLALGCHVGM	ADEKAEDNLK	KTKLPKTYMM
970	980	990	1000	1010	1020	1030	1040
SNGYKPAPLD	LSHVRLTPAQ	TTLVDRLAEN	GHNVWARDRV	GQGWSYSAVQ	DIPARRNPRL	VPYRLLDEAT	KRSNRDSLCQ
1050	1060	1070	1080	1090	1100	1110	1120
AVRTLLGYGY	NIEPPDQEPS	QVENQSRCDR	VRIFRAEKSY	TVQSGRWYFE	FEAVTTGEMR	VGWARPELRP	DVELGADELA
1130	1140	1150	1160	1170	1180	1190	1200
YVFNGHRGQR	WHLGSEPFGR	PWQPGDVVGC	MIDLTENTII	FTLNGEVLMS	DSGSETAFRE	IEIGDGFLPV	CSLGPGQVGH
1210	1220	1230	1240	1250	1260	1270	1280
LNLGQDVSSL	RFFAICGLQE	GFEPFAINMQ	RPVTTWFSKG	LPQFEPVPLE	HPHYEVSRVD	GTVDTPPCLR	LTHRTWGSQN
1290	1300	1310	1320	1330	1340	1350	1360
SLVEMLFLRL	SLPVQFHQHF	RCTAGATPLA	PPGLQPPAED	EARAAEPDPD	YENLRRSAGG	WSEAENGKEG	TAKEGAPGGT
1370	1380	1390	1400	1410	1420	1430	1440
PQAGGEAQPA	RAENEKDATT	EKNKKRGFLF	KAKKVAMMTQ	PPATPTLPRL	PHDVVPADNR	DDPEIILNTT	TYYYSVRVFA
1450	1460	1470	1480	1490	1500	1510	1520
GQEPSCVWAG	WVTPDYHQHD	MSFDLSKVRV	VTVTMGDEQG	NVHSSLKCSN	CYMVWGGDFV	SPGQQGRISH	TDLVIGCLVD
1530	1540	1550	1560	1570	1580	1590	1600
LATGLMTFTA	NGKESNTFFQ	VEPNTKLFPA	VFVLPTHQNV	IQFELGKQKN	IMPLSAAMFQ	SERKNPAPQC	PPRLEMQMLM
1610	1620	1630	1640	1650	1660	1670	1680
PVSWSRMPNH	FLQVETRRAG	ERLGWAVQCQ	EPLTMMALHI	PEENRCMDIL	ELSERLDLQR	FHSHTLRLYR	AVCALGNNRV
1690	1700	1710	1720	1730	1740	1750	1760
AHALCSHVDQ	AQLLHALEDA	HLPGPLRAGY	YDLLISIHLE	SACRSRRSML	SEYIVPLTPE	TRAITLFPPG	RSTENGHPRH
1770	1780	1790	1800	1810	1820	1830	1840
GLPGVGVTTS	LRPPHHFSPP	CFVAALPAAG	AAEAPARLSP	AIPLEALRDK	ALRMLGEAVR	DGGQHARDPV	GGSVEFQFVP
1850	1860	1870	1880	1890	1900	1910	1920
VLKLVSTLLV	MGIFGDEDVK	QILKMIEPEV	FTEEEEEEDE	EEEGEEEDEE	EKEEDEEETA	QEKEDEEKEE	EEAAEGEKEE
1930	1940	1950	1960	1970	1980	1990	2000
GLEEGLLQMK	LPESVKLQMC	HLLEYFCDQE	LQHRVESLAA	FAERYVDKLQ	ANQRSRYGLL	IKAFSMTAAE	TARRTREFRS
2010	2020	2030	2040	2050	2060	2070	2080
PPQEQINMLL	QFKDGTDEED	CPLPEEIRQD	LLDFHQDLLA	HCGIQLDGEE	EEPEEETTLG	SRLMSLLEKV	RLVKKKEEKP
2090	2100	2110	2120	2130	2140	2150	2160
EEERSAEESK	PRSLQELVSH	MVVRWAQEDF	VQSPELVRAM	FSLLHRQYDG	LGELLRALPR	AYTISPSSVE	DTMSLLECLG
2170	2180	2190	2200	2210	2220	2230	2240

Detailed Protein Report

Cmpd.	No. of Cmpds.	m/z meas.	Δ m/z [ppm]	z	Rt [min]	Score	P	Range	Sequence	Modification
2128	1	1051.4436	-85.59	2	55.2	12.7	1	901-917	K.RLHPCLVDFHSLPEPER.N	Carbamidomethyl: 5

Detailed Protein Report

Protein 451: **PREDICTED: protein TBATA isoform X13 [Homo sapiens]**

**Accession:**	gi|578819092	**Score:**	28.9
**Database:**	refseq_human(refseq_human_20140103.fasta)	**MW [kDa]:**	35.1
**Database Date:**	2015-11-30	**pI:**	6.4
		**Sequence Coverage [%]:**	9.2
		**No. of unique Peptides:**	1

10	20	30	40	50	60	70	80
MGIPTISVPI	GDPQSNRNPQ	LSSAWKKELK	ELASRVAFLT	KEDELKKKED	TTSLNWNCKS	RRPQAPGLSG	TDLGAAELGI
**90**	**100**	**110**	**120**	**130**	**140**	**150**	**160**
KKEQKEEPLR	EQGAKYSAET	GRLIPASTRA	VGRRSHQGQ	QSQSSSRHEG	VQAFLLQDQE	LLVLELLCRI	LETDLLSAIQ
**170**	**180**	**190**	**200**	**210**	**220**	**230**	**240**
FWLLYAPPKE	KDLALGLLQT	AVAQLLPQPL	VSIPTEKLLS	QLPEVHEPPQ	EKQEPPCRWA	PHPALPFCSL	LSSQSIPEEN
**250**	**260**	**270**	**280**	**290**	**300**	**310**	**320**
EDITFYKKRK	TRVHWRSSSP	PDAFKPEHRE	EDIEAEGREL	RGPNTCTCLA	QEQPQGFRGV	SVSTTFTAVP	DSLP

Cmpd.	No. of Cmpds.	m/z meas.	Δ m/z [ppm]	z	Rt [min]	Score	P	Range	Sequence	Modification
2340	2	953.1627	151.14	2	57.7	17.7	2	96-113	K.YSAETGRLIPASTRAVGR.R	

BRUKER

Detailed Protein Report

Protein 452: PREDICTED: eukaryotic translation initiation factor 5B isoform X1 [Homo sapiens]

**Accession:**	gi|530368915	**Score:**	28.9
**Database:**	refseq_human(refseq_human_20140103.fasta)	**MW [kDa]:**	138.6
**Database Date:**	2015-11-30	**pI:**	5.3
		**Sequence Coverage [%]:**	1.9
		**No. of unique Peptides:**	1

10	20	30	40	50	60	70	80
MGKKQKNKSE	DSTKDDIDLD	ALAAEIEGAG	AAKEQEPQKS	KGKKKKEKKK	QDFDEDDILK	ELEELSLEAQ	GIKADRETVA
90	100	110	120	130	140	150	160
VKPTENNEEE	FTSKDKKKKG	QKGKKQSFDD	NDSEELEDKD	SKSKKTAKPK	VEMYSGSDDD	DDFNKLPKKA	KGKAQKSNKK
170	180	190	200	210	220	230	240
WDGSEEDEDN	SKKIKERSRI	NSSGESGDES	DEFLQSRKGQ	KKNQKNKPGP	NIESGNEDDD	ASFKIKTVAQ	KKAEKKERER
250	260	270	280	290	300	310	320
KKRDEEKAKL	RKLKEKEELE	TGKKDQSKQK	ESQRKFEEET	VKSKVTVDTG	VIPASEEKAE	TPTAAEDDNE	GDKKKKDKKK
330	340	350	360	370	380	390	400
KKGEKEEKEK	EKKKGPSKAT	VKAMQEALAK	LKEEEERQKR	EEEERIKRLE	ELEAKRKEEE	RLEQEKRERK	KQKEKERKER
410	420	430	440	450	460	470	480
LKKEGKLLTK	SQREARARAE	ATLKLLQAQG	VEVPSKDSLP	KKRPIYEDKK	RKKIPQQLES	KEVSESMELC	AAVEVMEQGV
490	500	510	520	530	540	550	560
PEKEETPPPV	EPEEEDTEDA	GLDDWEAMAS	DEETEKVEGN	KVHIEVKENP	EEEEEEEEEE	EEDEESEEEE	EEEGESEGSE
570	580	590	600	610	620	630	640
GDEEDEKVSD	EKDSGKTLDK	KPSKEMSSDS	EYDSDDDRTK	EERAYDKAKR	RIEKRRLEHS	KNVNTEKLRA	PIICVLGHVD
650	660	670	680	690	700	710	720
TGKTKILDKL	RHTHVQDGEA	GGITQQIGAT	NVPLEAINEQ	TKMIKNFDRE	NVRIPGMLII	DTPGHESFSN	LRNRGSSLCD
730	740	750	760	770	780	790	800
IAILVVDIMH	GLEPQTIESI	NLLKSKKCPF	IVALNKIDRL	YDWKKSPDSD	VAATLKKQKK	NTKDEFEERA	KAIIVEFAQQ
810	820	830	840	850	860	870	880
GLNAALFYEN	KDPRTFVSLV	PTSAHTGDGM	GSLIYLLVEL	TQTMLSKRLA	HCEELRAQVM	EVKALPGMGT	TIDVILINGR
890	900	910	920	930	940	950	960
LKEGDTIIVP	GVEGPIVTQI	RGLLLPPPMK	ELRVKNQYEK	HKEVEAAQGV	KILGKDLEKT	LAGLPLLVAY	KEDEIPVLKD
970	980	990	1000	1010	1020	1030	1040
ELIHELKQTL	NAIKLEEKGV	YVQASTLGSL	EALLEFLKTS	EVPYAGINIG	PVHKKDVMKA	SVMLEHDPQY	AVILAFDVRI
1050	1060	1070	1080	1090	1100	1110	1120
ERDAQEMADS	LGVRIFSAEI	IYHLFDAFTK	YRQDYKKQKQ	EEFKHIAVFP	CKIKILPQYI	FNSRDPIVMG	VTVEAGQVKQ
1130	1140	1150	1160	1170	1180	1190	1200
GTPMCVPSKN	FVDIGIVTSI	EINHKQVDVA	KKGQEVCVKI	EPIPGESPKM	FGRHFEATDI	LVSKISRQSI	DALKDWFRDE
1210	1220						
MQKSDWQLIV	ELKKVFEII						

Cmpd.	No. of Cmpds.	m/z meas.	Δ m/z [ppm]	z	Rt [min]	Score	P	Range	Sequence	Modification
129	1	680.3267	-74.77	2	31.0	13.0	2	765-777	K.KSPDSDVAATLKK.Q	

Detailed Protein Report

Protein 453: zinc finger protein 581 [Homo sapiens]

**Accession:**	gi|7705495	**Score:**	28.8
**Database:**	refseq_human(refseq_human_20140103.fasta)	**MW [kDa]:**	22.0
**Database Date:**	2015-11-30	**pI:**	10.6
**Modification(s):**	Carbamidomethyl	**Sequence Coverage [%]:**	13.7
		**No. of unique Peptides:**	2

Alias proteins:

Accession	Name	Description
gi|578834534	refseq_human (refseq_human_20140103.fasta)	PREDICTED: zinc finger protein 581 isoform X1 [Homo sapiens]

```
        10         20         30         40         50         60         70         80
MLVLPSPCPQ PLAFSSVETM EGPPRRTCRS PEPGPSSSIG SPQASSPPRP NHYLLIDTQG VPYTVLVDEE SQREPGASGA
        90        100        110        120        130        140        150        160
PGQKKCYSCP VCSRVFEYMS YLQRHSITHS EVKPFECDIC GKAFKRASHL ARHHSIHLAG GGRPHGCPLC PRRFRDAGEL
       170        180        190        200
AQHSRVHSGE RPFQCPHCPR RFMEQNTLQK HTRWKHP
```

Cmpd.	No. of Cmpds.	m/z meas.	Δ m/z [ppm]	z	Rt [min]	Score	P	Range	Sequence	Modification
1072	2	984.1823	6.75	3	42.1	11.2	2	127-153	R.ASHLARHHSIHLAGGGRPHGCPLCPRR.F	Carbamidomethyl: 24
1056	2	984.2136	38.53	3	41.9	17.6	2	127-153	R.ASHLARHHSIHLAGGGRPHGCPLCPRR.F	Carbamidomethyl: 21

Detailed Protein Report

Protein 454: follistatin-related protein 1 precursor [Homo sapiens]

Accession: gi|5901956
Database: refseq_human(refseq_human_20140103.fasta)
Database Date: 2015-11-30
Modification(s): Carbamidomethyl

Score: 28.8
MW [kDa]: 35.0
pI: 5.3
Sequence Coverage [%]: 8.4
No. of unique Peptides: 1

10	20	30	40	50	60	70	80
MWKRWLALAL	ALVAVAWVRA	EEELRSKSKI	CANVFCGAGR	ECAVTEKGEP	TCLCIEQCKP	HKRPVCGSNG	KTYLNHCELH
**90**	**100**	**110**	**120**	**130**	**140**	**150**	**160**
RDACLTGSKI	QVDYDGHCKE	KKSVSPSASP	VVCYQSNRDE	LRRRIIQWLE	AEIIPDGWFS	KGSNYSEILD	KYFKNFDNGD
**170**	**180**	**190**	**200**	**210**	**220**	**230**	**240**
SRLDSSEFLK	FVEQNETAIN	ITTYPDQENN	KLLRGLCVDA	LIELSDENAD	WKLSFQEFLK	CLNPSFNPPE	KKCALEDETY
**250**	**260**	**270**	**280**	**290**	**300**	**310**	
ADGAETEVDC	NRCVCACGNW	VCTAMTCDGK	NQKGAQTQTE	EEMTRYVQEL	QKHQETAEKT	KRVSTKEI	

Cmpd.	No. of Cmpds.	m/z meas.	Δ m/z [ppm]	z	Rt [min]	Score	P	Range	Sequence	Modification
2625	1	671.8235	5.72	2	61.5	11.9	0	72-81	K.TYLNHCELHR.D	Carbamidomethyl: 6

Detailed Protein Report

Protein 455: tyrosine-protein kinase TXK [Homo sapiens]

**Accession:**	gi|148596974	**Score:**	28.8
**Database:**	refseq_human(refseq_human_20140103.fasta)	**MW [kDa]:**	61.2
**Database Date:**	2015-11-30	**pI:**	9.1
**Modification(s):**	Carbamidomethyl, Oxidation	**Sequence Coverage [%]:**	6.5
		**No. of unique Peptides:**	2

Quantitation

mdown:qdown	**Median:**	0.34	**CV:**	0.00 %	**No. of Peptides:**	1
Wdown:Qdown	**Median:**	1.88	**CV:**	171.26 %	**No. of Peptides:**	2

```
        10         20         30         40         50         60         70         80
MILSSYNTIQ SVFCCCCCCS VQKRQMRTQI SLSTDEELPE KYTQRRRPWL SQLSNKKQSN TGRVQPSKRK PLPPLPPSEV
        90        100        110        120        130        140        150        160
AEEKIQVKAL YDFLPREPCN LALRRAEEYL ILEKYNPHWW KARDRLGNEG LIPSNYVTEN KITNLEIYEW YHRNITRNQA
       170        180        190        200        210        220        230        240
EHLLRQESKE GAFIVRDSRH LGSYTISVFM GARRSTEAAI KHYQIKKNDS GQWYVAERHA FQSIPELIWY HQHNAAGLMT
       250        260        270        280        290        300        310        320
RLRYPVGLMG SCLPATAGFS YEKWEIDPSE LAFIKEIGSG QFGVVHLGEW RSHIQVAIKA INEGSMSEED FIEEAKVMMK
       330        340        350        360        370        380        390        400
LSHSKLVQLY GVCIQRKPLY IVTEFMENGC LLNYLRENKG KLRKEMLLSV CQDICEGMEY LERNGYIHRD LAARNCLVSS
       410        420        430        440        450        460        470        480
TCIVKISDFG MTRYVLDDEY VSSFGAKFPI KWSPPEVFLF NKYSSKSDVW SFGVLMWEVF TEGKMPFENK SNLQVVEAIS
       490        500        510        520        530
EGFRLYRPHL APMSIYEVMY SCWHEKPEGR PTFAELLRAV TEIAETW
```

Cmpd.	No. of Cmpds.	m/z meas.	Δ m/z [ppm]	z	Rt [min]	Score	P	Range	Sequence	Modification	Ratios
169	1	898.4349	69.51	3	31.3	11.6	0	1-23	-.MILSSYNTIQSVFCCCCCCSVQK.R	Carbamidomethyl: 14, 15; Oxidation: 1	Wdown:Qdown 0.58
866	2	583.6973	-174.23	2	40.3	17.2	0	395-405	R.NCLVSSTCIVK.I		Wdown:Qdown 6.04<br>mdown:qdown 0.34

Detailed Protein Report

Protein 456: ATP-sensitive inward rectifier potassium channel 1 isoform a [Homo sapiens]

**Accession:**	gi|4504837	**Score:**	28.6
**Database:**	refseq_human(refseq_human_20140103.fasta)	**MW [kDa]:**	44.8
**Database Date:**	2015-11-30	**pI:**	9.9
**Modification(s):**	Oxidation	**Sequence Coverage [%]:**	9.0
		**No. of unique Peptides:**	1

10	20	30	40	50	60	70	80
MNASSRNVFD	TLIRVLTESM	FKHLRKWVVT	RFFGHSRQRA	RLVSKDGRCN	IEFGNVEAQS	RFIFFVDIWT	TVLDLKWRYK
**90**	**100**	**110**	**120**	**130**	**140**	**150**	**160**
MTIFITAFLG	SWFFFGLLWY	AVAYIHKDLP	EFHPSANHTP	CVENINGLTS	AFLFSLETQV	TIGYGFRCVT	EQCATAIFLL
**170**	**180**	**190**	**200**	**210**	**220**	**230**	**240**
IFQSILGVII	NSFMCGAILA	KISRPKKRAK	TITFSKNAVI	SKRGGKLCLL	IRVANLRKSL	LIGSHIYGKL	LKTTVTPEGE
**250**	**260**	**270**	**280**	**290**	**300**	**310**	**320**
TIILDQININ	FVVDAGNENL	FFISPLTIYH	VIDHNSPFFH	MAAETLLQQD	FELVVFLDGT	VESTSATCQV	RTSYVPEEVL
**330**	**340**	**350**	**360**	**370**	**380**	**390**	**400**
WGYRFAPIVS	KTKEGKYRVD	FHNFSKTVEV	ETPHCAMCLY	NEKDVRARMK	RGYDNPNFIL	SEVNETDDTK	M

Cmpd.	No. of Cmpds.	m/z meas.	Δ m/z [ppm]	z	Rt [min]	Score	P	Range	Sequence	Modification
2895	1	859.1037	-1.61	3	65.4	17.8	2	1-22	-.MNASSRNVFDTLIRVLTESMFK.H	Oxidation: 20

Detailed Protein Report

Protein 457: protein MROH8 isoform 1 [Homo sapiens]

**Accession:**	gi|194595501	**Score:**	28.6
**Database:**	refseq_human(refseq_human_20140103.fasta)	**MW [kDa]:**	119.2
**Database Date:**	2015-11-30	**pI:**	9.5
		**Sequence Coverage [%]:**	2.7
		**No. of unique Peptides:**	1

Quantitation

mdown:qdown **Median:** 0.60 **CV:** 0.00 % **No. of Peptides:** 1

10	20	30	40	50	60	70	80
MPLSRRGRGS	RLAGSRAATS	NLPRATALSP	KSAGRGALSI	SHAQLLTRRR	HSSEQVPPES	EPRADFRSGK	WLQEPATGDA
**90**	**100**	**110**	**120**	**130**	**140**	**150**	**160**
RDSRQALRAR	MSSKHRICSQ	EEVVIPCAYD	SDSESVDLEL	SNLEIIKKGS	SSIELTDLDI	PDIPGLHCEP	LSHSPRHLTQ
**170**	**180**	**190**	**200**	**210**	**220**	**230**	**240**
QDPLSEAIVE	KLIQSIQKVF	NGELKGELEK	LKFLGDLSSL	SQALPYDETA	KSFIHSHIAD	IVHTLNVLVQ	EERPHSLSSS
**250**	**260**	**270**	**280**	**290**	**300**	**310**	**320**
MRQEVFVTIA	DLSYQDVHLL	LGSEDRAELF	SLTIKSIITL	PSVRTLTQIQ	EIMPNGTCNT	ECLYRQTFQA	FSEMLQSLVV
**330**	**340**	**350**	**360**	**370**	**380**	**390**	**400**
KDPHLENLDT	IIKHLVPWLQ	SVKDHERERA	TASMAQVLKC	LSKHLNLKLP	LRFQRLGHLV	ALMALLCGDP	QEKVAEEAAE
**410**	**420**	**430**	**440**	**450**	**460**	**470**	**480**
GIHSLLHITL	RLKYITHDKK	DQQNLKRALT	KCREFLELHS	SAAKCFYNCP	FRIAQVFEGF	LDSNELCQFI	MTTFDTLKTL
**490**	**500**	**510**	**520**	**530**	**540**	**550**	**560**
KHPCIQRSAG	ELLLTLAKNT	ESQFEKVPEI	MGVICAQLSI	ISQPRVRQQI	INTVSLFISR	PKYTDIVLSF	LLCHPVPYNR
**570**	**580**	**590**	**600**	**610**	**620**	**630**	**640**
HLAEVWRMLS	VELPSTTWIL	WRLLRKLQKC	HNEPAQEKMA	YVAVAATDAL	YEVFLGNRLR	AATFRLFPQL	LMTLLIQIHH
**650**	**660**	**670**	**680**	**690**	**700**	**710**	**720**
SIGLTMSDVD	IPSGLYTEQE	VPSEVTPLCF	AMQATKTLLL	RTCCLQEFNI	MEKNKGWALL	GGKDGHLQGL	FLLANALLER
**730**	**740**	**750**	**760**	**770**	**780**	**790**	**800**
NQLLAQKVMY	LLVPLLNRGN	DKHKLTSAGF	FVELLRSPVA	KRLPSIYSVA	RFKDWLQDGN	HLFRILGLRG	LYNLVGHQEM
**810**	**820**	**830**	**840**	**850**	**860**	**870**	**880**
REDIKSLLPY	IVDSLRETDE	KIVLSAIQIL	LQLVRTMDFT	TLAAMMRTLF	SLFGDVRSDV	HRFSVTLFGA	AIKSVKNPDK
**890**	**900**	**910**	**920**	**930**	**940**	**950**	**960**
KSIENQVLDS	LVPLLLYSQD	ENDAVAEESR	QVLTICAQFL	KWKLPQEVYS	KDPWHIKPTE	AGTICRFFEK	KCKGKINILE
**970**	**980**	**990**	**1000**	**1010**	**1020**	**1030**	**1040**
QTLMYSKNPK	LPIRRSAVLF	VGLLSKYMDH	NELRRMGTDW	IEDDLRDLLC	DPEPSLCIIA	SQTLLLVQMA	RAEPKPKQRV
**1050**	**1060**						
NWLQKLMGRS	SA						

Cmpd.	No. of Cmpds.	m/z meas.	Δ m/z [ppm]	z	Rt [min]	Score	P	Range	Sequence	Modification	Ratios
1490	1	977.1102	85.91	2	48.4	10.9	1	774-789	K.DWLQDGNHLFRILGLR.G		mdown:qdown 0.60

Detailed Protein Report

Protein 458: integrin alpha-E precursor [Homo sapiens]

**Accession:**	gi|148728188	**Score:**	28.6
**Database:**	refseq_human(refseq_human_20140103.fasta)	**MW [kDa]:**	130.1
**Database Date:**	2015-11-30	**pI:**	5.4
**Modification(s):**	Oxidation	**Sequence Coverage [%]:**	3.3
		**No. of unique Peptides:**	2

10	20	30	40	50	60	70	80
MWLFHTLLCI	ASLALLAAFN	VDVARPWLTP	KGGAPFVLSS	LLHQDPSTNQ	TWLLVTSPRT	KRTPGPLHRC	SLVQDEILCH
90	100	110	120	130	140	150	160
PVEHVPIPKG	RHRGVTVVRS	HHGVLICIQV	LVRRPHSLSS	ELTGTCSLLG	PDLRPQAQAN	FFDLENLLDP	DARVDTGDCY
170	180	190	200	210	220	230	240
SNKEGGGEDD	VNTARQRRAL	EKEEEEDKEE	EEDEEEEEAG	TEIAIILDGS	GSIDPPDFQR	AKDFISNMMR	NFYEKCFECN
250	260	270	280	290	300	310	320
FALVQYGGVI	QTEFDLRDSQ	DVMASLARVQ	NITQVGSVTK	TASAMQHVLD	SIFTSSHGSR	RKASKVMVVL	TDGGIFEDPL
330	340	350	360	370	380	390	400
NLTTVINSPK	MQGVERFAIG	VGEEFKSART	ARELNLIASD	PDETHAFKVT	NYMALDGLLS	KLRYNIISME	GTVGDALHYQ
410	420	430	440	450	460	470	480
LAQIGFSAQI	LDERQVLLGA	VGAFDWSGGA	LLYDTRSRRG	RFLNQTAAAA	ADAEAAQYSY	LGYAVAVLHK	TCSLSYIAGA
490	500	510	520	530	540	550	560
PRYKHHGAVF	ELQKEGREAS	FLPVLEGEQM	GSYFGSELCP	VDIDMDGSTD	FLLVAAPFYH	VHGEEGRVYV	YRLSEQDGSF
570	580	590	600	610	620	630	640
SLARILSGHP	GFTNARFGFA	MAAMGDLSQD	KLTDVAIGAP	LEGFGADDGA	SFGSVYIYNG	HWDGLSASPS	QRIRASTVAP
650	660	670	680	690	700	710	720
GLQYFGMSMA	GGFDISGDGL	ADITVGTLGQ	AVVFRSRPVV	RLKVSMAFTP	SALPIGFNGV	VNVRLCFEIS	SVTTASESGL
730	740	750	760	770	780	790	800
REALLNFTLD	VDVGKQRRRL	QCSDVRSCLG	CLREWSSGSQ	LCEDLLLMPT	EGELCEEDCF	SNASVKVSYQ	LQTPEGQTDH
810	820	830	840	850	860	870	880
PQPILDRYTE	PFAIFQLPYE	KACKNKLFCV	AELQLATTVS	QQELVVGLTK	ELTLNINLTN	SGEDSYMTSM	ALNYPRNLQL
890	900	910	920	930	940	950	960
KRMQKPPSPN	IQCDDPQPVA	SVLIMNCRIG	HPVLKRSSAH	VSVVWQLEEN	AFPNRTADIT	VTVTNSNERR	SLANETHTLQ
970	980	990	1000	1010	1020	1030	1040
FRHGFVAVLS	KPSIMYVNTG	QGLSHHKEFL	FHVHGENLFG	AEYQLQICVP	TKLRGLQVVA	VKKLTRTQAS	TVCTWSQERA
1050	1060	1070	1080	1090	1100	1110	1120
CAYSSVQHVE	EWHSVSCVIA	SDKENVTVAA	EISWDHSEEL	LKDVTELQIL	GEISFNKSLY	EGLNAENHRT	KITVVFLKDE
1130	1140	1150	1160	1170	1180		
KYHSLPIIIK	GSVGGLLVLI	VILVILFKCG	FFKRKYQQLN	LESIRKAQLK	SENLLEEEN		

Cmpd.	No. of Cmpds.	m/z meas.	Δ m/z [ppm]	z	Rt [min]	Score	P	Range	Sequence	Modification
1655	1	896.8887	87.41	3	49.2	13.3	0	306-330	K.VMVVLTDGGIFEDPLNLTTVINSPK.M	Oxidation: 2
2277	1	816.2926	-144.34	2	58.7	15.3	1	1098-1111	K.SLYEGLNAENHRTK.I	

Detailed Protein Report

Protein 459: WD repeat-containing protein 89 [Homo sapiens]

**Accession:**	gi|18087841	**Score:**	28.6
**Database:**	refseq_human(refseq_human_20140103.fasta)	**MW [kDa]:**	43.2
**Database Date:**	2015-11-30	**pI:**	5.7
		**Sequence Coverage [%]:**	7.5
		**No. of unique Peptides:**	2

Alias proteins:

Accession	Name	Description
gi|384871679	refseq_human (refseq_human_20140103.fasta)	WD repeat-containing protein 89 [Homo sapiens]
gi|57165359	refseq_human (refseq_human_20140103.fasta)	WD repeat-containing protein 89 [Homo sapiens]

```
        10         20         30         40         50         60         70         80
MEKIEEQFAN LHIVKCSLGT KEPTYLLGID TSKTVQAGKE NLVAVLCSNG SIRIYDKERL NVLREFSGYP GLLNGVRFAN
        90        100        110        120        130        140        150        160
SCDSVYSACT DGTVKCWDAR VAREKPVQLF KGYPSNIFIS FDINCNDHII CAGTEKVDDD ALLVFWDARM NSQNLSTTKD
       170        180        190        200        210        220        230        240
SLGAYSETHS DDVTQVRFHP SNPNMVVSGS SDGLVNVFDI NIDNEEDALV TTCNSISSVS CIGWSGKGYK QIYCMTHDEG
       250        260        270        280        290        300        310        320
FYWWDLNHLD TDEPVTRLNI QDVREVVNMK EDALDYLIGG LYHEKTDTLH VIGGTNKGRI HLMNCSMSGL THVTSLQGGH
       330        340        350        360        370        380        390
AATVRSFCWN VQDDSLLTGG EDAQLLLWKP GAIEKTFTKK ESMKIASSVH QRVRVHSNDS YKRRKKQ
```

Cmpd.	No. of Cmpds.	m/z meas.	Δ m/z [ppm]	z	Rt [min]	Score	P	Range	Sequence	Modification
2497	1	709.8401	-97.59	2	61.6	14.1	2	54-64	R.IYDKERLNVLR.E	
633	1	1042.1848	135.88	2	37.2	14.5	2	365-382	K.IASSVHQRVRVHSNDSYK.R	

Detailed Protein Report

Protein 460: LIM domain only protein 3 isoform 1 [Homo sapiens]

Accession: gi|21361802
Database: refseq_human(refseq_human_20140103.fasta)
Database Date: 2015-11-30
Modification(s): Carbamidomethyl

Score: 28.6
MW [kDa]: 16.6
pI: 9.9
Sequence Coverage [%]: 26.2
No. of unique Peptides: 2

Quantitation

Wdown:Qdown **Median:** 1.61 **CV:** 0.00 % **No. of Peptides:** 1

Alias proteins:

Accession	Name	Description
gi|578822949	refseq_human (refseq_human_20140103.fasta)	PREDICTED: LIM domain only protein 3 isoform X2 [Homo sapiens]
gi|578822947	refseq_human (refseq_human_20140103.fasta)	PREDICTED: LIM domain only protein 3 isoform X1 [Homo sapiens]
gi|343790979	refseq_human (refseq_human_20140103.fasta)	LIM domain only protein 3 isoform 1 [Homo sapiens]
gi|343790975	refseq_human (refseq_human_20140103.fasta)	LIM domain only protein 3 isoform 1 [Homo sapiens]
gi|47778935	refseq_human (refseq_human_20140103.fasta)	LIM domain only protein 3 isoform 1 [Homo sapiens]

10	20	30	40	50	60	70	80
MLSVQPDTKP	KGCAGCNRKI	KDRYLLKALD	KYWHEDCLKC	ACCDCRLGEV	GSTLYTKANL	ILCRRDYLRL	FGVTGNCAAC
90	100	110	120	130	140	150	
SKLIPAFEMV	MRAKDNVYHL	DCFACQLCNQ	RFCVGDKFFL	KNNMILCQTD	YEEGLMKEGY	APQVR	

Cmpd.	No. of Cmpds.	m/z meas.	Δ m/z [ppm]	z	Rt [min]	Score	P	Range	Sequence	Modification	Ratios
2251	1	952.9306	80.50	2	56.8	15.2	1	32-46	K.YWHEDCLKCACCDCR.L	Carbamidomethyl: 14	
459	1	820.1336	71.06	3	35.1	13.3	1	70-92	R. LFGVTGNCAACSKLIPAFEMVMR. A		Wdown:Qdown 1.61

Detailed Protein Report

Protein 461: PREDICTED: protein ELYS isoform X2 [Homo sapiens]

**Accession:**	gi|578802192	**Score:**	28.5
**Database:**	refseq_human(refseq_human_20140103.fasta)	**MW [kDa]:**	252.3
**Database Date:**	2015-11-30	**pI:**	6.2
		**Sequence Coverage [%]:**	1.4
		**No. of unique Peptides:**	1

Detailed Protein Report

10	20	30	40	50	60	70	80
MRDLRAQVTS	GLLPFPEVTL	QALGEDEITL	ESVLRGKFAA	GKNGLACLAC	GPQLEVVNSI	TGERLSAYRF	SGVNEQPPVV
90	100	110	120	130	140	150	160
LAVKEFSWQK	RTGLLIGLEE	TEGSVLCLYD	LGISKVVKAV	VLPGRVTAIE	PIINHGGASA	STQHLHPSLR	WLFGVAAVVT
170	180	190	200	210	220	230	240
DVGQILLVDL	CLDDLSCNQN	EVEASDLEVL	TGIPAEVPHI	RESVMRQGRH	LCFQLVSPTG	TAVSTLSYIS	RTNQLAVGFS
250	260	270	280	290	300	310	320
DGYLALWNMK	SMKREYYIQL	ESGQVPVYAV	TFQEPENDPR	NCCYLWAVQS	TQDSEGDVLS	LHLLQLAFGN	RKCLASGQIL
330	340	350	360	370	380	390	400
YEGLEYCEER	YTLDLTGGMF	PLRGQTSNTK	LLGCQSIEKF	RSHGDREEGV	NEALSPDTSV	SVFTWQVNIY	GQGKPSVYLG
410	420	430	440	450	460	470	480
LFDINRWYHA	QMPDSLRSGE	YLHNCSYFAL	WSLESVVSRT	SPHGILDILV	HERSLNRGVP	PSYPPPEQFF	NPSTYNFDAT
490	500	510	520	530	540	550	560
CLLNSGVVHL	TCTGFQKETL	TFLKKSGPSL	NELIPDGYNR	CLVAGLLSPR	FVDVQPSSLS	QEEQLEAILS	AAIQTSSLGL
570	580	590	600	610	620	630	640
LTGYIRRWIT	EEQPNSATNL	RFVLEWTWNK	VVLTKEEFDR	LCVPLFDGSC	HFMDPQTIQS	IQQCYLLLSN	LNIVLSCFAS
650	660	670	680	690	700	710	720
EAREITERGL	IDLSNKFVVS	HLICQYAQVV	LWFSHSGLLP	EGIDDSVQLS	RLCYNYPVIQ	NYYTSRRQKF	ERLSRGKWNP
730	740	750	760	770	780	790	800
DCLMIDGLVS	QLGERIEKLW	KRDEGGTGKY	PPASLHAVLD	MYLLDGVTEA	AKHSITIYLL	LDIMYSFPNK	TDTPIESFPT
810	820	830	840	850	860	870	880
VFAISWGQVK	LIQGFWLIDH	NDYESGLDLL	FHPATAKPLS	WQHSKIIQAF	MSQGEHRQAL	RYIQTMKPTV	SSGNDVILHL
890	900	910	920	930	940	950	960
TVLLFNRCMV	EAWNFLRQHC	NRLNIEELLK	HMYEVCQEMG	LMEDLLKLPF	TDTEQECLVK	FLQSSASVQN	HEFLLVHHLQ
970	980	990	1000	1010	1020	1030	1040
RANYVPALKL	NQTLKINVMN	DRDPRLRERS	LARNSILDQY	GKILPRVHRK	LAIERAKPYH	LSTSSVFRLV	SRPKPLSAVP
1050	1060	1070	1080	1090	1100	1110	1120
KQVVTGTVLT	RSVFINNVLS	KIGEVWASKE	PINSTTPFNS	SKIEEPSPIV	YSLPAPELPE	AFFGTPISKA	SQKISRLLDL
1130	1140	1150	1160	1170	1180	1190	1200
VVQPVPRPSQ	CSEFIQQSSM	KSPLYLVSRS	LPSSSQLKGS	PQAISRASEL	HLLETPLVVK	KAKSLAMSVT	TSGFSEFTPQ
1210	1220	1230	1240	1250	1260	1270	1280
SILRSTLRST	PLASPSPSPG	RSPQRLKETR	ISFVEEDVHP	KWIPGAADDS	KLEVFTTPKK	CAVPVETEWL	KSKDRTTSFF
1290	1300	1310	1320	1330	1340	1350	1360
LNSPEKEHQE	MDEGSQSLEK	LDVSKGNSSV	SITSDETTLE	YQDAPSPEDL	EETVFTASKP	KSSSTALTTN	VTEQTEKDGD
1370	1380	1390	1400	1410	1420	1430	1440
KDVFASEVTP	SDLQKQMGNL	EDAETKDLLV	AAEAFSELNH	LSPVQGTEAS	LCAPSVYEGK	IFTQKSKVPV	LDEGLTSVET
1450	1460	1470	1480	1490	1500	1510	1520
YTPAIRANDN	KSMADVLGDG	GNSSLTISEG	PIVSERRLNQ	EVALNLKEDH	EVEVGVLKES	VDLPEEKLPI	SDSPPDTQEI
1530	1540	1550	1560	1570	1580	1590	1600
HVIEQEKLEA	QDSGEEARNL	SFNELYPSGT	LKLQYNFDTI	DQQFCDLADN	KDTAECDIAE	VDGELFVAQS	NFTLILEGEE
1610	1620	1630	1640	1650	1660	1670	1680
GEVEPGDFAS	SDVLPKAANT	ATEEKLVCSG	ENDNHGQIAN	LPSAVTSDQK	SQKVDTLPYV	PEPIKVAIAE	NLLDVIKDTR
1690	1700	1710	1720	1730	1740	1750	1760
SKEITSDTME	QSIHETIPLV	SQNIMCPTKL	VKSAFKTAQE	TSTMTMNVSQ	VDDVVSSKTR	TRGQRIQNVN	VKSAQQEASA
1770	1780	1790	1800	1810	1820	1830	1840
DVATPKMPGQ	SVRKKTRKAK	EISEASENIY	SDVRGLSQNQ	QIPQNSVTPR	RGRRKKEVNQ	DILENTSSVE	QELQITTGRE
1850	1860	1870	1880	1890	1900	1910	1920
SKRLKSSQLL	EPAVEETTKK	EVKVSSVTKR	TPRRIKRSVE	NQESVEIIND	LKVSTVTSPS	RMIRKLRSTN	LDASENTGNK
1930	1940	1950	1960	1970	1980	1990	2000
QDDKSSDKQL	RIKHVRRVRG	REVSPSDVRE	DSNLESSQLT	VQAEFDMSAI	PRKRGRPRKI	NPSEDVGSKA	VKEERSPKKK
2010	2020	2030	2040	2050	2060	2070	2080
EAPSIRRRST	RNTPAKSENV	DVGKPALGKS	ILVPNEELSM	VMSSKKKLTK	KTESQSQKRS	LHSVSEERTD	EMTHKETNEQ
2090	2100	2110	2120	2130	2140	2150	2160
EERLLATASF	TKSSRSSRTR	SSKAILLPDL	SEPNNEPLFS	PASEVPRKAK	AKKIEVPAQL	KELVSDLSSQ	FVISPPALRS
2170	2180	2190	2200	2210	2220	2230	2240
RQKNTSNKNK	LEDELKDDAQ	SVETLGKPKA	KRIRTSKTKQ	ASKNTEKESA	WSPPPIEIRL	ISPLASPADG	VKSKPRKTTE
2250	2260	2270					
VTGTGLGRNR	KKLSSYPKQI	LRRKML					

Detailed Protein Report

Cmpd.	No. of Cmpds.	m/z meas.	Δ m/z [ppm]	z	Rt [min]	Score	P	Range	Sequence	Modification
2789	1	735.0657	-23.60	3	66.4	12.6	2	1010-1028	R.KLAIERAKPYHLSTSSVFR.L	

Detailed Protein Report

Protein 462: ephrin type-A receptor 6 isoform a [Homo sapiens]

**Accession:**	gi|145312251	**Score:**	28.4
**Database:**	refseq_human(refseq_human_20140103.fasta)	**MW [kDa]:**	126.5
**Database Date:**	2015-11-30	**pI:**	6.5
		**Sequence Coverage [%]:**	2.7
		**No. of unique Peptides:**	1

10	20	30	40	50	60	70	80
MQFPSPPAAR	SSPAPQAASS	SEAAAPATGQ	PGPSCPVPGT	SRRGRPGTPP	AGRVEEEEEE	EEEDVDKDPH	PTQNTCLRCR
90	100	110	120	130	140	150	160
HFSLRERKRE	PRRTMGGCEV	REFLLQFGFF	LPLLTAWPGD	CSHVSNNQVV	LLDTTTVLGE	LGWKTYPLNG	WDAITEMDEH
170	180	190	200	210	220	230	240
NRPIHTYQVC	NVMEPNQNNW	LRTNWISRDA	AQKIYVEMKF	TLRDCNSIPW	VLGTCKETFN	LFYMESDESH	GIKFKPNQYT
250	260	270	280	290	300	310	320
KIDTIAADES	FTQMDLGDRI	LKLNTEIREV	GPIERKGFYL	AFQDIGACIA	LVSVRVFYKK	CPFTVRNLAM	FPDTIPRVDS
330	340	350	360	370	380	390	400
SSLVEVRGSC	VKSAEERDTP	KLYCGADGDW	LVPLGRCICS	TGYEEIEGSC	HACRPGFYKA	FAGNTKCSKC	PPHSLTYMEA
410	420	430	440	450	460	470	480
TSVCQCEKGY	FRAEKDPPSM	ACTRPPSAPR	NVVFNINETA	LILEWSPPSD	TGGRKDLTYS	VICKKCGLDT	SQCEDCGGGL
490	500	510	520	530	540	550	560
RFIPRHTGLI	NNSVIVLDFV	SHVNYTFEIE	AMNGVSELSF	SPKPFTAITV	TTDQDAPSLI	GVVRKDWASQ	NSIALSWQAP
570	580	590	600	610	620	630	640
AFSNGAILDY	EIKYYEKEHE	QLTYSSTRSK	APSVIITGLK	PATKYVFHIR	VRTATGYSGY	SQKFEFETGD	ETSDMAAEQG
650	660	670	680	690	700	710	720
QILVIATAAV	GGFTLLVILT	LFFLITGRCQ	WYIKAKMKSE	EKRRNHLQNG	HLRFPGIKTY	IDPDTYEDPS	LAVHEFAKEI
730	740	750	760	770	780	790	800
DPSRIRIERV	IGAGEFGEVC	SGRLKTPGKR	EIPVAIKTLK	GGHMDRQRRD	FLREASIMGQ	FDHPNIIRLE	GVVTKRSFPA
810	820	830	840	850	860	870	880
IGVEAFCPSF	LRAGFLNSIQ	APHPVPGGGS	LPPRIPAGRP	VMIVVEYMEN	GSLDSFLRKH	DGHFTVIQLV	GMLRGIASGM
890	900	910	920	930	940	950	960
KYLSDMGYVH	RDLAARNILV	NSNLVCKVSD	FGLSRVLEDD	PEAAYTTTGG	KIPIRWTAPE	AIAYRKFSSA	SDAWSYGIVM
970	980	990	1000	1010	1020	1030	1040
WEVMSYGERP	YWEMSNQDVI	LSIEEGYRLP	APMGCPASLH	QLMLHCWQKE	RNHRPKFTDI	VSFLDKLIRN	PSALHTLVED
1050	1060	1070	1080	1090	1100	1110	1120
ILVMPESPGE	VPEYPLFVTV	GDWLDSIKMG	QYKNNFVAAG	FTTFDLISRM	SIDDIRRIGV	ILIGHQRRIV	SSIQTLRLHM
1130	1140						
MHIQEKGFHV							

Cmpd.	No. of Cmpds.	m/z meas.	Δ m/z [ppm]	z	Rt [min]	Score	P	Range	Sequence	Modification
2727	2	698.3840	-68.94	2	63.0	12.0	0	591-604	K.APSVIITGLKPATK.Y	

Detailed Protein Report

Protein 463: **PREDICTED: ankyrin repeat domain-containing protein 24 isoform X6 [Homo sapiens]**

**Accession:**	gi|578833058	**Score:**	28.4
**Database:**	refseq_human(refseq_human_20140103.fasta)	**MW [kDa]:**	121.1
**Database Date:**	2015-11-30	**pI:**	4.8
		**Sequence Coverage [%]:**	2.6
		**No. of unique Peptides:**	1

10	20	30	40	50	60	70	80
MKTLRARFKK	TESQDWGKSD	ERLLQAVENN	DAPRVAALIA	RKGLVPTKLD	PEGKSAFHLA	AMRGAASCLE	VMIAHGSNVM
90	100	110	120	130	140	150	160
SADGAGYNAL	HLAAKYGHPQ	CLKQLLQASC	VVDVVDSSGW	TALHHAAAGG	CLSCSEVLCS	FKAHLNPQDR	SGATPLIIAA
170	180	190	200	210	220	230	240
QMCHTDLCRL	LLQQGAAAND	QDLQGRTALM	LACEGASPET	VEVLLQGGAQ	PGITDALGQD	AAHYGALAGD	KLILHLLQEA
250	260	270	280	290	300	310	320
AQRPSPPSAL	TEDDSGEASS	QNSMSSHGKQ	GAPKKRKAPP	PPASIPMPDD	RDAYEEIVRL	RQERGRLLQK	IRGLEQHKER
330	340	350	360	370	380	390	400
RQQESPEASS	LHILERQVQE	LQQLLVERQE	EKESLGREVE	SLQSRLSLLE	NERENTSYDV	TTLQDEEGEL	PDLPGAEVLL
410	420	430	440	450	460	470	480
SRQLSPSAQE	HLASLQEQVA	VLTRQNQELM	EKVQILENFE	KDETQMEVEA	LAEVIPLALY	DSLRAEFDQL	RRQHAEALQA
490	500	510	520	530	540	550	560
LRQQETREVP	REEGAACGES	EVAGATATKN	GPTHMELNGS	VAPETKVNGA	ETIDEEAAGD	ETMEARTMEA	EATGAEATGA
570	580	590	600	610	620	630	640
EATGAKVTET	KPTGAEVREM	ETTEEEANME	TKPTGAQATD	TETTGVEAMG	VEATKTKAEE	AEMQAYGVGA	GQAEPPVTGT
650	660	670	680	690	700	710	720
TNMEATGSRA	TGMESTGVSA	TGVENPGVEA	TVPGISAGPI	LHPGAAEASE	KLQVELETRI	RGLEEALRQR	EREAAAELEA
730	740	750	760	770	780	790	800
ALGKCEAAEA	EAGRLRERVR	EAEGSGASGG	GGGDTTQLRA	ALEQAREDLR	DRDSRLRELE	AASACLDEAR	ASRLLAEEEA
810	820	830	840	850	860	870	880
RGLRAELAQR	EEARLEQSRE	LEVLREQLAT	ARATGEQQRT	AAAELGRARD	AAEARVAELP	AACEEARQGL	AELREASEAL
890	900	910	920	930	940	950	960
RQSVVPASEH	RRLQEEALEL	RGRAASLEQE	VVATGKEAAR	LRAELERERV	CSVALSEHER	IVGTLQANVA	QLEGQLEELG
970	980	990	1000	1010	1020	1030	1040
RRHEKTSAEV	FQVQREALFM	KSERHAAEAQ	LATAEQQLRG	LRTEAERARQ	AQSRAQEALD	KAKEKDKKIT	ELSKEVFNLK
1050	1060	1070	1080	1090	1100	1110	1120
EALKEQPAAL	ATPEVEALRD	QVKDLQQQLQ	EAARDHSSVV	ALYRSHLLYA	IQGQMDEDVQ	RILSQILQMQ	RLQAQGR

Cmpd.	No. of Cmpds.	m/z meas.	Δ m/z [ppm]	z	Rt [min]	Score	P	Range	Sequence	Modification
1938	1	780.7925	-113.31	2	52.9	10.6	2	725-738	K.CEAAEAEAGRLRER.V	

Detailed Protein Report

Protein 464: PREDICTED: contactin-associated protein-like 3B isoform X6 [Homo sapiens]

**Accession:**	gi|578816699	**Score:**	28.4
**Database:**	refseq_human(refseq_human_20140103.fasta)	**MW [kDa]:**	127.3
**Database Date:**	2015-11-30	**pI:**	9.3
		**Sequence Coverage [%]:**	3.0
		**No. of unique Peptides:**	2

10	20	30	40	50	60	70	80
MASVAWAVLK	VLLLLPTQTW	SPVGAGNPPD	CDSPLASALP	RSSFSSSSEL	SSSHGPGFSR	LNRRDGAGGW	TPLVSNKYQW
**90**	**100**	**110**	**120**	**130**	**140**	**150**	**160**
LQIDLGERME	VTAVATQGGY	GSSDWVTSYL	LMFSDGGRNW	KQYRREESIW	GFPGNTNADS	VVHYRLQPPF	EARFLRFLPL
**170**	**180**	**190**	**200**	**210**	**220**	**230**	**240**
AWNPRGRIGM	RIEVYGCAYK	SEVVYFDGQS	ALLYTLDKKP	LKPIRDVISL	KFKAMQSNGI	LLHREGQHGN	HITLELIKGK
**250**	**260**	**270**	**280**	**290**	**300**	**310**	**320**
LVFFLNSGNA	KLPSTIAPVT	LTLGSLLDDQ	HWHSVLIELL	DTQVNFTVDK	HTHHFQAKGD	SSNLDLNFEI	SFGGILSPGR
**330**	**340**	**350**	**360**	**370**	**380**	**390**	**400**
SRAFTRKSFH	GCLENLYYNG	VDVTELAKKH	KPQILMMGNV	SFSCPQPQTV	PVTFLSSRSY	LALPGNSGED	KVSVTFQFRT
**410**	**420**	**430**	**440**	**450**	**460**	**470**	**480**
WNRAGHLLFG	ELQRGSGSFV	LFLKDGKLKL	SLFQAGQSPR	NVTAGAGLND	GQWHSVSFSA	KWSHMNVVVD	DDTAVQPLVA
**490**	**500**	**510**	**520**	**530**	**540**	**550**	**560**
VLIDSGDTYY	FGDSAWTVVR	HGGPDAVTLR	GAPSGHPLSA	VSFAYAAGAG	QLRAAVNLAE	RCEQRLALRC	GTARRPDSRD
**570**	**580**	**590**	**600**	**610**	**620**	**630**	**640**
GTPLSWWVGR	TNETHTSWGG	SLPDAQKCTC	GLEGNCIDSQ	YYCNCDAGQN	EWTSDTIVLS	QKEHLPVTQI	VMTDTGQPHS
**650**	**660**	**670**	**680**	**690**	**700**	**710**	**720**
EADYTLGPLL	CRGDKSFWNS	ASFNTETSYL	HFPAFHGELT	ADVCFFFKTT	VSSGVFMENL	GITDFIRIEL	RGRLATLDKS
**730**	**740**	**750**	**760**	**770**	**780**	**790**	**800**
QAPTEVTFSF	DVGNGPCEVT	VQSPTPFNDN	QWHHVRAERN	VKGASLQVDQ	LPQKMQPAPA	DGHVRLQLNS	QLFIGGTATR
**810**	**820**	**830**	**840**	**850**	**860**	**870**	**880**
QRGFLGCIRS	LQLNGVALDL	EERATVTPGV	EPGCAGHCST	YGHLCRNGGR	CREKRRGVTC	DCAFSAYDGP	FCSNEISAYF
**890**	**900**	**910**	**920**	**930**	**940**	**950**	**960**
ATGSSMTYHF	QEHYTLSENS	SSLVSSLHRD	VTLTREMITL	SFRTTRTPSL	LLYVSSFYEE	YLSVILANNG	SLQIRYKLDR
**970**	**980**	**990**	**1000**	**1010**	**1020**	**1030**	**1040**
HQNPDAFTFD	FKNMADGQLH	QVKINREEAV	VMVEVNQSAK	KQVILSSGTE	FNAVKSLILG	KVLEAAGADP	DTRRAATSGF
**1050**	**1060**	**1070**	**1080**	**1090**	**1100**	**1110**	**1120**
TGCLSAVRFG	CAAPLKAALR	PSGPSRVTVR	GHVAPMARCA	AGAASGSPAR	ELAPRLAGGA	GRSGPVDEGE	PLVNADRRDS
**1130**	**1140**	**1150**	**1160**	**1170**			
AVIGGVIAVE	IFILLCITAI	AIRIYQQRKL	RKENESKVSK	KEEC			

Cmpd.	No. of Cmpds.	m/z meas.	Δ m/z [ppm]	z	Rt [min]	Score	P	Range	Sequence	Modification
1888	1	899.4768	-2.42	2	52.3	14.7	1	400-414	R.TWNRAGHLLFGELQR.G	
1567	1	919.5953	159.08	2	49.4	13.7	1	1071-1090	R.GHVAPMARCAAGAASGSPAR.E	

Detailed Protein Report

Protein 465: metallothionein-1G [Homo sapiens]

**Accession:**	gi|10835230	**Score:**	28.4
**Database:**	refseq_human(refseq_human_20140103.fasta)	**MW [kDa]:**	6.1
**Database Date:**	2015-11-30	**pI:**	10.5
**Modification(s):**	Carbamidomethyl	**Sequence Coverage [%]:**	62.3
		**No. of unique Peptides:**	2

```
        10         20         30         40         50         60         70
MDPNCSCAAG VSCTCASSCK CKECKCTSCK KSCCSCCPVG CAKCAQGCIC KGASEKCSCC A
```

Cmpd.	No. of Cmpds.	m/z meas.	Δ m/z [ppm]	z	Rt [min]	Score	P	Range	Sequence	Modification
834	1	703.6768	119.69	3	39.8	13.8	0	1-20	-.MDPNCSCAAGVSCTCASSCK.C	Carbamidomethyl: 5, 15, 19
2136	1	1019.7667	-122.67	2	56.9	14.5	2	26-43	K.CTSCKKSCCSCCPVGCAK.C	Carbamidomethyl: 1, 11, 12, 16

Detailed Protein Report

Protein 466: mediator of RNA polymerase II transcription subunit 24 isoform 2 [Homo sapiens]

**Accession:**	gi|119220575	**Score:**	28.3
**Database:**	refseq_human(refseq_human_20140103.fasta)	**MW [kDa]:**	108.9
**Database Date:**	2015-11-30	**pI:**	6.5
**Modification(s):**	Oxidation	**Sequence Coverage [%]:**	2.3
		**No. of unique Peptides:**	1

Alias proteins:

Accession	Name	Description
gi|392583926	refseq_human (refseq_human_20140103.fasta)	mediator of RNA polymerase II transcription subunit 24 isoform 2 [Homo sapiens]

10	20	30	40	50	60	70	80
MKVVNLKQAI	LQAWKERWSD	YQWAINMKKF	FPKGATWDIL	NLADALLEQA	MIGPSPNPLI	LSYLKYAISS	QFDDFSRDLC
**90**	**100**	**110**	**120**	**130**	**140**	**150**	**160**
VQALLDIMDM	FCDRLSCHGK	AEECIGLCRA	LLSALHWLLR	CTAASAERLR	EGLEAGTPAA	GEKQLAMCLQ	RLEKTLSSTK
**170**	**180**	**190**	**200**	**210**	**220**	**230**	**240**
NRALLHIAKL	EEASSWTAIE	HSLLKLGEIL	ANLSNPQLRS	QAEQCGTLIR	SIPTMLSVHA	EQMHKTGFPT	VHAVILLEGT
**250**	**260**	**270**	**280**	**290**	**300**	**310**	**320**
MNLTGETQSL	VEQLTMVKRM	QHIPTPLFVL	EIWKACFVGL	IESPEGTEEL	KWTAFTFLKI	PQVLVKLKKY	SHGDKDFTED
**330**	**340**	**350**	**360**	**370**	**380**	**390**	**400**
VNCAFEFLLK	LTPLLDKADQ	RCNCDCTNFL	LQECGKQGLL	SEASVNNLMA	KRKADREHAP	QQKSGENANI	QPNIQLILRA
**410**	**420**	**430**	**440**	**450**	**460**	**470**	**480**
EPTVTNILKT	MDADHSKSPE	GLLGVLGHML	SGKSLDLLLA	AAAATGKLKS	FARKFINLNE	FTTYGSEEST	KPASVRALLF
**490**	**500**	**510**	**520**	**530**	**540**	**550**	**560**
DISFLMLCHV	AQTYGSEVIL	SESRTGAEVP	FFETWMQTCM	PEEGKILNPD	HPCFRPDSTK	VESLVALLNN	SSEMKLVQMK
**570**	**580**	**590**	**600**	**610**	**620**	**630**	**640**
WHEACLSISA	AILEILNAWE	NGVLAFESIQ	KITDNIKGKV	CSLAVCAVAW	LVAHVRMLGL	DEREKSLQMI	RQLAGPLFSE
**650**	**660**	**670**	**680**	**690**	**700**	**710**	**720**
NTLQFYNERV	VIMNSILERM	CADVLQQTAT	QIKFPSTGVD	TMPYWNLLPP	KRPIKEVLTD	IFAKVLEKGW	VDSRSIHIFD
**730**	**740**	**750**	**760**	**770**	**780**	**790**	**800**
TLLHMGGVYW	FCNNLIKELL	KETRKEHTLR	AVELLYSIFC	LDMQQVTLVL	LGHILPGLLT	DSSKWHSLMD	PPGTALAKLA
**810**	**820**	**830**	**840**	**850**	**860**	**870**	**880**
VWCALSSYSS	HKGQASTRQK	KRHREDIEDY	ISLFPLDDVQ	PSKLMRLLSS	NEDDANILSS	PTDRSMSSSL	SASQLHTVNM
**890**	**900**	**910**	**920**	**930**	**940**	**950**	**960**
RDPLNRVLAN	LFLLISSILG	SRTAGPHTQF	VQWFMEECVD	CLEQGGRGSV	LQFMPFTTVS	ELVKVSAMSS	PKVVLAITDL
**970**	**980**						
SLPLGRQVAA	KAIAAL						

Cmpd.	No. of Cmpds.	m/z meas.	Δ m/z [ppm]	z	Rt [min]	Score	P	Range	Sequence	Modification
1772	1	793.3983	26.85	2	50.8	14.1	1	18-29	R.WSDYQWAINMKK.F	Oxidation: 10

Detailed Protein Report

Protein 467: lysine-specific demethylase 3A [Homo sapiens]

**Accession:**	gi|20357522	**Score:**	28.3
**Database:**	refseq_human(refseq_human_20140103.fasta)	**MW [kDa]:**	147.2
**Database Date:**	2015-11-30	**pI:**	9.4
**Modification(s):**	Carbamidomethyl	**Sequence Coverage [%]:**	1.8
		**No. of unique Peptides:**	1

Alias proteins:

Accession	Name	Description
gi|226442779	refseq_human (refseq_human_20140103.fasta)	lysine-specific demethylase 3A [Homo sapiens]

10	20	30	40	50	60	70	80
MVLTLGESWP	VLVGRRFLSL	SAADGSDGSH	DSWDVERVAE	WPWLSGTIRA	VSHTDVTKKD	LKVCVEFDGE	SWRKRRWIEV
**90**	**100**	**110**	**120**	**130**	**140**	**150**	**160**
YSLLRRAFLV	EHNLVLAERK	SPEISERIVQ	WPAITYKPLL	DKAGLGSITS	VRFLGDQQRV	FLSKDLLKPI	QDVNSLRLSL
**170**	**180**	**190**	**200**	**210**	**220**	**230**	**240**
TDNQIVSKEF	QALIVKHLDE	SHLLKGDKNL	VGSEVKIYSL	DPSTQWFSAT	VINGNPASKT	LQVNCEEIPA	LKIVDPSLIH
**250**	**260**	**270**	**280**	**290**	**300**	**310**	**320**
VEVVHDNLVT	CGNSARIGAV	KRKSSENNGT	LVSKQAKSCS	EASPSMCPVQ	SVPTTVFKEI	LLGCTAATPP	SKDPRQQSTP
**330**	**340**	**350**	**360**	**370**	**380**	**390**	**400**
QAANSPPNLG	AKIPQGCHKQ	SLPEEISSCL	NTKSEALRTK	PDVCKAGLLS	KSSQIGTGDL	KILTEPKGSC	TQPKTNTDQE
**410**	**420**	**430**	**440**	**450**	**460**	**470**	**480**
NRLESVPQAL	TGLPKECLPT	KASSKAELEI	ANPPELQKHL	EHAPSPSDVS	NAPEVKAGVN	SDSPNNCSGK	KVEPSALACR
**490**	**500**	**510**	**520**	**530**	**540**	**550**	**560**
SQNLKESSVK	VDNESCCSRS	NNKIQNAPSR	KSVLTDPAKL	KKLQQSGEAF	VQDDSCVNIV	AQLPKCRECR	LDSLRKDKEQ
**570**	**580**	**590**	**600**	**610**	**620**	**630**	**640**
QKDSPVFCRF	FHFRRLQFNK	HGVLRVEGFL	TPNKYDNEAI	GLWLPLTKNV	VGIDLDTAKY	ILANIGDHFC	QMVISEKEAM
**650**	**660**	**670**	**680**	**690**	**700**	**710**	**720**
STIEPHRQVA	WKRAVKGVRE	MCDVCDTTIF	NLHWVCPRCG	FGVCVDCYRM	KRKNCQQGAA	YKTFSWLKCV	KSQIHEPENL
**730**	**740**	**750**	**760**	**770**	**780**	**790**	**800**
MPTQIIPGKA	LYDVGDIVHS	VRAKWGIKAN	CPCSNRQFKL	FSKPASKEDL	KQTSLAGEKP	TLGAVLQQNP	SVLEPAAVGG
**810**	**820**	**830**	**840**	**850**	**860**	**870**	**880**
EAASKPAGSM	KPACPASTSP	LNWLADLTSG	NVNKENKEKQ	PTMPILKNEI	KCLPPLPPLS	KSSTVLHTFN	STILTPVSNN
**890**	**900**	**910**	**920**	**930**	**940**	**950**	**960**
NSGFLRNLLN	SSTGKTENGL	KNTPKILDDI	FASLVQNKTT	SDLSKRPQGL	TIKPSILGFD	TPHYWLCDNR	LLCLQDPNNK
**970**	**980**	**990**	**1000**	**1010**	**1020**	**1030**	**1040**
SNWNVFRECW	KQGQPVMVSG	VHHKLNSELW	KPESFRKEFG	EQEVDLVNCR	TNEIITGATV	GDFWDGFEDV	PNRLKNEKEP
**1050**	**1060**	**1070**	**1080**	**1090**	**1100**	**1110**	**1120**
MVLKLKDWPP	GEDFRDMMPS	RFDDLMANIP	LPEYTRRDGK	LNLASRLPNY	FVRPDLGPKM	YNAYGLITPE	DRKYGTTNLH
**1130**	**1140**	**1150**	**1160**	**1170**	**1180**	**1190**	**1200**
LDVSDAANVM	VYVGIPKGQC	EQEEEVLKTI	QDGDSDELTI	KRFIEGKEKP	GALWHIYAAK	DTEKIREFLK	KVSEEQGQEN
**1210**	**1220**	**1230**	**1240**	**1250**	**1260**	**1270**	**1280**
PADHDPIHDQ	SWYLDRSLRK	RLHQEYGVQG	WAIVQFLGDV	VFIPAGAPHQ	VHNLYSCIKV	AEDFVSPEHV	KHCFWLTQEF
**1290**	**1300**	**1310**	**1320**	**1330**			
RYLSQTHTNH	EDKLQVKNVI	YHAVKDAVAM	LKASESSFGK	P			

Cmpd.	No. of Cmpds.	m/z meas.	Δ m/z [ppm]	z	Rt [min]	Score	P	Range	Sequence	Modification
81	1	821.0339	-27.87	3	30.4	17.2	2	457-480	K.AGVNSDSPNNCSGKKVEPSALACR.S	Carbamidomethyl: 23

Detailed Protein Report

Protein 468: zinc finger protein 441 [Homo sapiens]

**Accession:**	gi|193083160	**Score:**	28.3
**Database:**	refseq_human(refseq_human_20140103.fasta)	**MW [kDa]:**	80.1
**Database Date:**	2015-11-30	**pI:**	9.9
**Modification(s):**	Carbamidomethyl	**Sequence Coverage [%]:**	4.6
		**No. of unique Peptides:**	2

Quantitation

mdown:qdown **Median:** 1.65 **CV:** 0.00 % **No. of Peptides:** 1

10	20	30	40	50	60	70	80
MDSVAFEDVA	INFTCEEWAL	LGPSQKSLYR	DVMQETIRNL	DCIGMIWQNH	DIEEDQYKDL	RRNLRCHMVE	RACEIKDNSQ
**90**	**100**	**110**	**120**	**130**	**140**	**150**	**160**
CGGPFTQTQD	SIVNEKIPGV	DPWESSECTD	VLMGRSSLNC	YVRVDSEHKP	CEYQEYGEKP	YTHTQCGTAF	SYQPCFQIHE
**170**	**180**	**190**	**200**	**210**	**220**	**230**	**240**
RPQHGKKLYD	CKECASFSSL	ENLQRHMAAH	HGDGPRICKL	CGNAFIWPSL	FHMLRRTHTE	EKPYEYEQCS	TAFPAYSSTL
**250**	**260**	**270**	**280**	**290**	**300**	**310**	**320**
RHERTHSGEK	PYQCKQCGKA	FSCSCYTQLY	ERTHTGEQSY	ECKQCGKAFY	HLGSFQRHMI	VHTGDGPHKC	KICGKGFLSP
**330**	**340**	**350**	**360**	**370**	**380**	**390**	**400**
SSVRRHKRTH	TGEKPYECKY	CGKAFSDCTG	FRRHMITHTG	DGPHKCKVCG	KAFDSPSLCR	RHETTHTGEK	PYKCECGKAF
**410**	**420**	**430**	**440**	**450**	**460**	**470**	**480**
SDFYYFRNHE	TTHTGEKPYK	CKQCGKAFIC	CTYLQIHERI	HTGERPYKCK	QCGKAFRSSN	YIRVHEKTHT	GEKPYECKQC
**490**	**500**	**510**	**520**	**530**	**540**	**550**	**560**
GKALSHLKSF	QRHMIMHTGD	GPHKCKICGK	SFDSPSSFRR	HERIHTGERP	YKCKLCGKGF	RSSSYIQLHE	RTHTGEKPYG
**570**	**580**	**590**	**600**	**610**	**620**	**630**	**640**
CQQCGKALSD	LSSFRRHMIT	HTGNGPHKCK	ICGKGFDYPS	SVQRHERTHT	GEKPYECKEC	GKAFSHSSYL	RIHERVHTGE
**650**	**660**	**670**	**680**	**690**	**700**		
KPYKCKECGK	PFHCPSAFHK	HERTHSMEKP	YKCKECGEAF	HCISSFHKHE	MTH		

Cmpd.	No. of Cmpds.	m/z meas.	Δ m/z [ppm]	z	Rt [min]	Score	P	Range	Sequence	Modification	Ratios
2763	1	1022.5215	80.04	2	63.0	13.2	1	256-272	K.QCGKAFSCSCYTQLYER.T	Carbamidomethyl: 2	
864	1	893.3321	-112.97	2	40.2	15.1	1	464-478	R.VHEKTHTGEKPYECK.Q		mdown:qdown 1.65

Detailed Protein Report

Protein 469: PREDICTED: ubiquitin carboxyl-terminal hydrolase 24 isoform X1 [Homo sapiens]

**Accession:**	gi|530362258	**Score:**	28.2
**Database:**	refseq_human(refseq_human_20140103.fasta)	**MW [kDa]:**	275.5
**Database Date:**	2015-11-30	**pI:**	6.1
**Modification(s):**	Carbamidomethyl, Oxidation	**Sequence Coverage [%]:**	1.0
		**No. of unique Peptides:**	1

Quantitation

mdown:qdown	**Median:**	1.09	**CV:**	0.00 %	**No. of Peptides:**	1
Wdown:Qdown	**Median:**	1.24	**CV:**	0.00 %	**No. of Peptides:**	1

Detailed Protein Report

10	20	30	40	50	60	70	80
MDRCMPEAFK	KLLTSSAVHK	WGTEIHEGIY	NMLMLLIELV	AERIKQDPIP	TGLLGVLTMA	FNPDNEYHFK	NRMKVSQRNW
90	100	110	120	130	140	150	160
AEVFGEGNMF	AVSPVSTFQK	EPHGWVVDLV	NKFGELGGFA	AIQAKLHSED	IELGAVSALI	QPLGVCAEYL	NSSVVQPMLD
170	180	190	200	210	220	230	240
PVILTTIQDV	RSVEEKDLKD	KRLVSIPELL	SAVKLLCMRF	QPDLVTIVDD	LRLDILLRML	KSPHFSAKMN	SLKEVTKLIE
250	260	270	280	290	300	310	320
DSTLSKSVKN	AIDTDRLLDW	LVENSVLSIA	LEGNIDQAQY	CDRIKGIIEL	LGSKLSLDEL	TKIWKIQSGQ	SSTVIENIHT
330	340	350	360	370	380	390	400
IIAAAAVKFN	SDQLNHLFVL	IQKSWETESD	RVRQKLLSLI	GRIGREARFE	TTSGKVLDVL	WELAHLPTLP	SSLIQQALEE
410	420	430	440	450	460	470	480
HLTILSDAYA	VKEAIKRSYI	IKCIEDIKRP	GEWSGLEKNK	KDGFKSSQLN	NPQFVWVVPA	LRQLHEITRS	FIKQTYQKQD
490	500	510	520	530	540	550	560
KSIIQDLKKN	FEIVKLVTGS	LIACHRLAAA	VAGPGGLSGS	TLVDGRYTYR	EYLEAHLKFL	AFFLQEATLY	LGWNRAKEIW
570	580	590	600	610	620	630	640
ECLVTGQDVC	ELDREMCFEW	FTKGQHDLES	DVQQQLFKEK	ILKLESYEIT	MNGFNLFKTF	FENVNLCDHR	LKRQGAQLYV
650	660	670	680	690	700	710	720
EKLELIGMDF	IWKIAMESPD	EEIANEAIQL	IINYSYINLN	PRLKKDSVSL	HKKFIADCYT	RLEAASSALG	GPTLTHAVTR
730	740	750	760	770	780	790	800
ATKMLTATAM	PTVATSVQSP	YRSTKLVIIE	RLLLLAERYV	ITIEDFYSVP	RTILPHGASF	HGHLLTLNVT	YESTKDTFTV
810	820	830	840	850	860	870	880
EAHSNETIGS	VRWKIAKQLC	SPVDNIQIFT	NDSLLTVNKD	QKLLHQLGFS	DEQILTVKTS	GSGTPSGSSA	DSSTSSSSSS
890	900	910	920	930	940	950	960
SGVFSSSYAM	EQEKSLPGVV	MALVCNVFDM	LYQLANLEEP	RITLRVRKLL	LLIPTDPAIQ	EALDQLDSLG	RKKTLLSESS
970	980	990	1000	1010	1020	1030	1040
SQSSKSPSLS	SKQQHQPSAS	SILESLFRSF	APGMSTFRVL	YNLEVLSSKL	MPTADDDMAR	SCAKSFCENF	LKAGGLSLVV
1050	1060	1070	1080	1090	1100	1110	1120
NVMQRDSIPS	EVDYETRQGV	YSICLQLARF	LLVGQTMPTL	LDEDLTKDGI	EALSSRPFRN	VSRQTSRQMS	LCGTPEKSSY
1130	1140	1150	1160	1170	1180	1190	1200
RQLSVSDRSS	IRVEEIIPAA	RVAIQTMEVS	DFTSTVACFM	RLSWAAAAGR	LDLVGSSQPI	KESNSLCPAG	IRNRLSSSGS
1210	1220	1230	1240	1250	1260	1270	1280
NCSSGSEGEP	VALHAGICVR	QQSVSTKDSL	IAGEALSLLV	TCLQLRSQQL	ASFYNLPCVA	DFIIDILLGS	PSAEIRRVAC
1290	1300	1310	1320	1330	1340	1350	1360
DQLYTLSQTD	TSAHPDVQKP	NQFLLGVILT	AQLPLWSPTS	IMRGVNQRLL	SQCMEYFDLR	CQLLDDLTTS	EMEQLRISPA
1370	1380	1390	1400	1410	1420	1430	1440
TMLEDEITWL	DNFEPNRTAE	CETSEADNIL	LAGHLRLIKT	LLSLCGAEKE	MLGSSLIKPL	LDDFLFRASR	IILNSHSPAG
1450	1460	1470	1480	1490	1500	1510	1520
SAAISQQDFH	PKCSTANSRL	AAYEVLVMLA	DSSPSNLQII	IKELLSMHHQ	PDPALTKEFD	YLPPVDSRSS	SGFVGLRNGG
1530	1540	1550	1560	1570	1580	1590	1600
ATCYMNAVFQ	QLYMQPGLPE	SLLSVDDDTD	NPDDSVFYQV	QSLFGHLMES	KLQYYVPENF	WKIFKMWNKE	LYVREQQDAY
1610	1620	1630	1640	1650	1660	1670	1680
EFFTSLIDQM	DEYLKKMGRD	QIFKNTFQGI	YSDQKICKDC	PHRYEREEAF	MALNLGVTSC	QSLEISLDQF	VRGEVLEGSN
1690	1700	1710	1720	1730	1740	1750	1760
AYYCEKCKEK	RITVKRTCIK	SLPSVLVIHL	MRFGFDWESG	RSIKYDEQIR	FPWMLNMEPY	TVSGMARQDS	SSEVGENGRS
1770	1780	1790	1800	1810	1820	1830	1840
VDQGGGGSPR	KKVALTENYE	LVGVIVHSGQ	AHAGHYYSFI	KDRRGCGKGK	WYKFNDTVIE	EFDLNDETLE	YECFGGEYRP
1850	1860	1870	1880	1890	1900	1910	1920
KVYDQTNPYT	DVRRRYWNAY	MLFYQRVSDQ	NSPVLPKKSR	VSVVRQEAED	LSLSAPSSPE	ISPQSSPRPH	RPNNDRLSIL
1930	1940	1950	1960	1970	1980	1990	2000
TKLVKKGEKK	GLFVEKMPAR	IYQMVRDENL	KFMKNRDVYS	SDYFSFVLSL	ASLNATKLKH	PYYPCMAKVS	LQLAIQFLFQ
2010	2020	2030	2040	2050	2060	2070	2080
TYLRTKKKLR	VDTEEWIATI	EALLSKSFDA	CQWLVEYFIS	SEGRELIKIF	LLECNVREVR	VAVATILEKT	LDSALFYQDK
2090	2100	2110	2120	2130	2140	2150	2160
LKSLHQLLEV	LLALLDKDVP	ENCKNCAQYF	FLFNTFVQKQ	GIRAGDLLLR	HSALRHMISF	LLGASRQNNQ	IRRWSSAQAR
2170	2180	2190	2200	2210	2220	2230	2240
EFGNLHNTVA	LLVLHSDVSS	QRNVAPGIFK	QRPPISIAPS	SPLLPLHEEV	EALLFMSEGK	PYLLEVMFAL	RELTGSLLAL
2250	2260	2270	2280	2290	2300	2310	2320
IEMVVYCCFC	NEHFSFTMLH	FIKNQLETAP	PHELKNTFQL	LHEILVIEDP	IQVERVKFVF	ETENGLLALM	HHSNHVDSSR
2330	2340	2350	2360	2370	2380	2390	2400

Detailed Protein Report

Cmpd.	No. of Cmpds.	m/z meas.	Δ m/z [ppm]	z	Rt [min]	Score	P	Range	Sequence	Modification	Ratios
143	2	849.3662	-2.35	2	31.0	11.7	1	1010-1024	K.LMPTADDDMARSCAK.S	Carbamidomethyl: 13; Oxidation: 2	Wdown:Qdown 1.24<br>mdown:qdown 1.09

Detailed Protein Report

Protein 470: PREDICTED: RING finger protein 112 isoform X3 [Homo sapiens]

**Accession:**	gi|578830030	**Score:**	28.2
**Database:**	refseq_human(refseq_human_20140103.fasta)	**MW [kDa]:**	66.2
**Database Date:**	2015-11-30	**pI:**	9.8
**Modification(s):**	Carbamidomethyl	**Sequence Coverage [%]:**	3.4
		**No. of unique Peptides:**	1

10	20	30	40	50	60	70	80
MGNSGNSWSH	TPFPKLELGL	GPQPMAPREL	PTCSICLERL	RDPISLDCGH	DFCIRCFSTH	RLPGCEPPCC	PECRKICKQK
**90**	**100**	**110**	**120**	**130**	**140**	**150**	**160**
RGLRSLGEKM	KLLPQRPLPP	ALQETCPVRA	EPLLLVRINA	SGGLILRMGA	INRCLKHPLA	RDTPVCLLAV	LGEQHSGKSF
**170**	**180**	**190**	**200**	**210**	**220**	**230**	**240**
LLNHLLQGLP	GLESGEGGRP	RGGEASLQGC	RWGANGLARG	IWMWSHPFLL	GKEGKKVAVF	LVDTGDAMSP	ELSRETRIKL
**250**	**260**	**270**	**280**	**290**	**300**	**310**	**320**
CALTTMLSSY	QILSTSQELK	DTDLDYLEMF	VHVAEVMGKH	YGMVPIQHLD	LLVRDSSHPN	KAGQGHVGNI	FQSTPLAASQ
**330**	**340**	**350**	**360**	**370**	**380**	**390**	**400**
RLSGRYPKVQ	ELLQGKRARC	CLLPAPGRRR	MNQGHASPGD	TDDDFRHLLG	AYVSDVLSAA	PQHAKSRCQG	YWNEGRAVAR
**410**	**420**	**430**	**440**	**450**	**460**	**470**	**480**
GDRRLLTGQQ	LAQEIKNLSG	WMGRTGPGFT	SPDEMAAQLH	DLRKVEAAKR	EFEEYVRQQD	VATKRIFSAL	RVLPDTMRNL
**490**	**500**	**510**	**520**	**530**	**540**	**550**	**560**
LSTQKDAILA	RHGVALLCKG	RDQTLEALEA	ELQATAKAFM	DSYTMRFCGH	LAAVGGAVGA	GLMGLAGGVV	GAGMAAAALA
**570**	**580**	**590**	**600**	**610**	**620**		
AEAGMVAAGA	AVGATGAAVV	GGGVGAGLAA	TVGCMEKEED	ERLLEGDREP	LLQEE		

Cmpd.	No. of Cmpds.	m/z meas.	Δ m/z [ppm]	z	Rt [min]	Score	P	Range	Sequence	Modification
2148	1	1062.3966	-148.73	1	57.0	17.9	1	128-136	R.MGAINRCLK.H	Carbamidomethyl: 7

Detailed Protein Report

Protein 471: AP-3 complex subunit delta-1 isoform 2 [Homo sapiens]

**Accession:**	gi|117553580	**Score:**	28.2
**Database:**	refseq_human(refseq_human_20140103.fasta)	**MW [kDa]:**	130.1
**Database Date:**	2015-11-30	**pI:**	9.4
		**Sequence Coverage [%]:**	2.3
		**No. of unique Peptides:**	2

Quantitation

mdown:qdown **Median:** 0.50 **CV:** 0.00 % **No. of Peptides:** 1

10	20	30	40	50	60	70	80
MALKMVKGSI	DRMFDKNLQD	LVRGIRNHKE	DEAKYISQCI	DEIKQELKQD	NIAVKANAVC	KLTYLQMLGY	DISWAAFNII
90	100	110	120	130	140	150	160
EVMSASKFTF	KRIGYLAASQ	SFHEGTDVIM	LTTNQIRKDL	SSPSQYDTGV	ALTGLSCFVT	PDLARDLAND	IMTLMSHTKP
170	180	190	200	210	220	230	240
YIRKKAVLIM	YKVFLKYPES	LRPAFPRLKE	KLEDPDPGVQ	SAAVNVICEL	ARRNPKNYLS	LAPLFFKLMT	SSTNNWVLIK
250	260	270	280	290	300	310	320
IIKLFGALTP	LEPRLGKKLI	EPLTNLIHST	SAMSLLYECV	NTVIAVLISL	SSGMPNHSAS	IQLCVQKLRI	LIEDSDQNLK
330	340	350	360	370	380	390	400
YLGLLAMSKI	LKTHPKSVQS	HKDLILQCLD	DKDESIRLRA	LDLLYGMVSK	KNLMEIVKKL	MTHVDKAEGT	TYRDELLTKI
410	420	430	440	450	460	470	480
IDICSQSNYQ	YITNFEWYIS	ILVELTRLEG	TRHGHLIAAQ	MLDVAIRVKA	IRKFAVSQMS	ALLDSAHLLA	SSTQRNGICE
490	500	510	520	530	540	550	560
VLYAAAWICG	EFSEHLQEPH	HTLEAMLRPR	VTTLPGHIQA	VYVQNVVKLY	ASILQQKEQA	GEAEGAQAVT	QLMVDRLPQF
570	580	590	600	610	620	630	640
VQSADLEVQE	RASCILQLVK	HIQKLQAKDV	PVAEEVSALF	AGELNPVAPK	AQKKVPVPEG	LDLDAWINEP	LSDSESEDER
650	660	670	680	690	700	710	720
PRAVFHEEEQ	RRPKHRPSEA	DEEELARRRE	ARKQEQANNP	FYIKSSPSPQ	KRYQDTPGVE	HIPVVQIDLS	VPLKVPGLPM
730	740	750	760	770	780	790	800
SDQYVKLEEE	RRHRQKLEKD	KRRKKRKEKE	KKGKRRHSSL	PTESDEDIAP	AQQVDIVTEE	MPENALPSDE	DDKDPNDPYR
810	820	830	840	850	860	870	880
ALDIDLDKPL	ADSEKLPIQK	HRNTETSKSP	EKDVPMVEKK	SKKPKKKEKK	HKEKERDKEK	KKEKEKKKSP	KPKKKKHRKE
890	900	910	920	930	940	950	960
KEERTKGKKK	SKKQPPGSEE	AAGEPVQNGA	PEEEQLPPES	SYSLLAENSY	VKMTCDIRGS	LQEDSQVTVA	IVLENRSSSI
970	980	990	1000	1010	1020	1030	1040
LKGMELSVLD	SLNARMARPQ	GSSVHDGVPV	PFQLPPGVSN	EAQYVFTIQS	IVMAQKLKGT	LSFIAKNDEG	ATHEKLDFRL
1050	1060	1070	1080	1090	1100	1110	1120
HFSCSSYLIT	TPCYSDAFAK	LLESGDLSMS	SIKVDGIRMS	FQNLLAKICF	HHHFSVVERV	DSCASMYSRS	IQGHHVCLLV
1130	1140	1150	1160				
KKGENSVSVD	GKCSDSTLLS	NLLEEMKATL	AKC				

Cmpd.	No. of Cmpds.	m/z meas.	Δ m/z [ppm]	z	Rt [min]	Score	P	Range	Sequence	Modification	Ratios
545	1	592.3161	-55.37	2	35.4	15.2	1	17-26	K.NLQDLVRGIR.N		mdown:qdown 0.50
1829	1	682.2682	-136.57	3	52.9	13.0	2	343-359	K.DLILQCLDDKDESIRLR.A		

Detailed Protein Report

Protein 472: von Willebrand factor D and EGF domain-containing protein precursor [Homo sapiens]

**Accession:**	gi|209571555	**Score:**	28.1
**Database:**	refseq_human(refseq_human_20140103.fasta)	**MW [kDa]:**	176.7
**Database Date:**	2015-11-30	**pI:**	5.3
**Modification(s):**	Carbamidomethyl	**Sequence Coverage [%]:**	2.4
		**No. of unique Peptides:**	2

10	20	30	40	50	60	70	80
MPGGACVLVI	ALMFLAWGEA	QECSPGGHQF	LRSPYRSVRF	DSWHLQQSAV	QDLICDHSLS	PGWYRFLILD	RPAEMPTKCV
**90**	**100**	**110**	**120**	**130**	**140**	**150**	**160**
EMNHCGTQAP	IWLSLRDSET	LPSPGEIKQL	TACATWQFLF	STTKDCCLFQ	IPVSVRNCGN	FSVYLLQPTQ	GCMGYCAEAI
**170**	**180**	**190**	**200**	**210**	**220**	**230**	**240**
SDARLHPCGS	DETETGGDCV	RQLAASLPPP	PAGRPEVLVE	LIESRLFCRC	SFDVPATKNS	VGFHIAWSRL	SSQEVKEELT
**250**	**260**	**270**	**280**	**290**	**300**	**310**	**320**
QETTVQAFSL	LELDGINLRL	GDRIFCSASV	FFLENPHVQS	VAIESQEFFA	GFKLQPELST	ISEDGKEYYL	RIESTVPIIC
**330**	**340**	**350**	**360**	**370**	**380**	**390**	**400**
SEFSELDQEC	KISLKLKTIG	QGREHLGLNL	ALSSCHVDLL	QTSSCANGTC	SHTFVYYTAV	TDFSRDGDRV	SNIVVQPIVN
**410**	**420**	**430**	**440**	**450**	**460**	**470**	**480**
EDFLWNNYIP	DSIQIKVKDV	PTAYCYTFTD	PHIITFDGRV	YDNFKTGTFV	LYKSMSRDFE	VHVRQWDCRS	LHYPVSCNCG
**490**	**500**	**510**	**520**	**530**	**540**	**550**	**560**
FVAQEGGDIV	TFDMCNGQLR	ESQPYLFIKS	QDVTRNIKIS	ESYLGRKVTI	WFSSGAFIRA	DLGEWGMSLT	IRAPSVDYRN
**570**	**580**	**590**	**600**	**610**	**620**	**630**	**640**
TLGLCGTFDE	NPENDFHDKN	GMQIDQNFNN	YVAFINEWRI	LPGKSMSDTL	PVSMTSPGKP	SYCSCSLDTA	AYPSSEDLDS
**650**	**660**	**670**	**680**	**690**	**700**	**710**	**720**
VSRSEIALGC	KDLNHVSLSS	LIPELDVTSE	YINSDTLVRE	INKHTSPEEY	NLNLFLQEKK	HINLTKLGLN	VQKHPGNEKE
**730**	**740**	**750**	**760**	**770**	**780**	**790**	**800**
DSLQYLANKK	YTQGRGSHSQ	EMRYNRQNRW	KRQNFHEFPP	LFAFPSLSQT	DLEELTYFFP	EDHAEDVQQE	FFPSWPTPSG
**810**	**820**	**830**	**840**	**850**	**860**	**870**	**880**
LTEYSTLTLC	QETLANSSIG	RLCLAFLGKR	LDSVIEMCVK	DVLLKDDLSW	AEAGVALLEN	ECEKRIVEEG	KYNTEEYGTS
**890**	**900**	**910**	**920**	**930**	**940**	**950**	**960**
IEDILSVLKC	PNLCSGNGQC	MEWGCACSPS	FSSYDCSDSY	DKAPEITELG	NAGFCDVQKY	NCMMVRVFGK	GFKELPSIKC
**970**	**980**	**990**	**1000**	**1010**	**1020**	**1030**	**1040**
EVTKLQYNSS	EWMPGEPIYT	QTVFHNSRAV	DCQLPTDVQQ	FDTMDLVGGK	PTGKWQLKVS	NDGYKFSNPK	ITVIYDGACQ
**1050**	**1060**	**1070**	**1080**	**1090**	**1100**	**1110**	**1120**
VCGLYKNDSC	TIKENVCIID	GLCYVEGDKN	PTSPCLICRP	KISRFTWSFL	ENNQPPVIQA	LQDKLQTFYG	ENFEYQFVAF
**1130**	**1140**	**1150**	**1160**	**1170**	**1180**	**1190**	**1200**
DPEGSDIHFT	LDSGPEGASV	SSAGLFMWKT	DLLTTQQITV	RLNDDCDAET	RVTIEVTVKS	CDCLNGGSCV	SDRNFSPGSG
**1210**	**1220**	**1230**	**1240**	**1250**	**1260**	**1270**	**1280**
VYLCVCLPGF	HGSLCEVDIS	GCQSNPCGLG	SYISGFHSYS	CDCPPELKVE	TQFVNQFTTQ	TVVLTRSDKS	VNKEEDDKNA
**1290**	**1300**	**1310**	**1320**	**1330**	**1340**	**1350**	**1360**
QGRKRHVKPT	SGNAFTICKY	PCGKSRECVA	PNICKCKPGY	IGSNCQTALC	DPDCKNHGKC	IKPNICQCLP	GHGGATCDEE
**1370**	**1380**	**1390**	**1400**	**1410**	**1420**	**1430**	**1440**
HCNPPCQHGG	TCLAGNLCTC	PYGFVGPRCE	TMVCNRHCEN	GGQCLTPDIC	QCKPGWYGPT	CSTALCDPVC	LNGGSCNKPN
**1450**	**1460**	**1470**	**1480**	**1490**	**1500**	**1510**	**1520**
TCLCPNGFFG	EHCQNAFCHP	PCKNGGHCMR	NNVCVCREGY	TGRRFQKSIC	DPTCMNGGKC	VGPSTCSCPS	GWSGKRCNTP
**1530**	**1540**	**1550**	**1560**	**1570**	**1580**	**1590**	**1600**
ICLQKCKNGG	ECIAPSICHC	PSSWEGVRCQ	IPICNPKCLY	GGRCIFPNVC	SCRTEYSGVK	CEKKIQIRRH	

Cmpd.	No. of Cmpds.	m/z meas.	Δ m/z [ppm]	z	Rt [min]	Score	P	Range	Sequence	Modification
2213	1	916.8887	8.94	2	56.3	16.8	0	165-181	R.LHPCGSDETETGGDCVR.Q	Carbamidomethyl: 15
2186	1	1159.9541	-107.87	2	55.8	11.4	1	1026-1046	K.FSNPKITVIYDGACQVCGLYK.N	

Detailed Protein Report

Protein 473: eyes absent homolog 1 isoform 5 [Homo sapiens]

Accession: gi|568384804
Database: refseq_human(refseq_human_20140103.fasta)
Database Date: 2015-11-30

Score: 28.1
MW [kDa]: 51.8
pI: 5.7
Sequence Coverage [%]: 5.5
No. of unique Peptides: 1

10	20	30	40	50	60	70	80
MQQATAYATY	PQPGQPYGIS	SYGIKTEGGL	SQSQSPGQTG	FLSYGTSFST	PQPGQAPYSY	QMQGSSFTTS	SGIYTGNNSL
**90**	**100**	**110**	**120**	**130**	**140**	**150**	**160**
TNSSGFNSSQ	QDYPSYPSFG	QGQYAQYYNS	SPYPAHYMTS	SNTSPTTPST	NATYQLQEPP	SGITSQAVTD	PTAEYSTIHS
**170**	**180**	**190**	**200**	**210**	**220**	**230**	**240**
PSTPIKDSDS	DRLRRGSDGK	SRGRGRRNNN	PSPPPDSDLE	RVFIWDLDET	IIVFHSLLTG	SYANRYGRDP	PTSVSLGLRM
**250**	**260**	**270**	**280**	**290**	**300**	**310**	**320**
EEMIFNLADT	HLFFNDLEEC	DQVHIDDVSS	DDNGQDLSTY	NFGTDGFPAA	ATSANLCLAT	GVRGGVDWMR	KLAFRYRRVK
**330**	**340**	**350**	**360**	**370**	**380**	**390**	**400**
EIYNTYKNNV	GGLLGPAKRE	AWLQLRAEIE	ALTDSWLTLA	LKALSLIHSR	TNCVNILVTT	TQLIPALAKV	LLYGLGIVFP
**410**	**420**	**430**	**440**	**450**	**460**	**470**	**480**
IENIYSATKI	GKESCFERII	QRFGRKVVYV	VIGDGVEEEQ	GAKKHAMPFW	RISSHSDLMA	LHHALELEYL	

Cmpd.	No. of Cmpds.	m/z meas.	Δ m/z [ppm]	z	Rt [min]	Score	P	Range	Sequence	Modification
2746	1	683.0052	-51.66	3	65.6	13.5	2	426-444	R.KVVYVVIGDGVEEEQGAKK.H	

Detailed Protein Report

Protein 474: SCO-spondin precursor [Homo sapiens]

**Accession:**	gi|134031945	**Score:**	28.1
**Database:**	refseq_human(refseq_human_20140103.fasta)	**MW [kDa]:**	547.1
**Database Date:**	2015-11-30	**pI:**	5.6
**Modification(s):**	Carbamidomethyl	**Sequence Coverage [%]:**	0.7
		**No. of unique Peptides:**	2

Detailed Protein Report

10	20	30	40	50	60	70	80
MLLPALLFGM	AWALADGRWC	EWTETIRVEE	EVAPRQEDLV	PCASLDHYSR	LGWRLDLPWS	GRSGLTRSPA	PGLCPIYKPP
90	100	110	120	130	140	150	160
ETRPAKWNRT	VRTCCPGWGG	AHCTEALAKA	SPEGHCFAMW	QCQLQAGSAN	ASAGSLEECC	ARPWGQSWWD	GSSQACRSCS
170	180	190	200	210	220	230	240
SRHLPGSASS	PALLQPLAGA	VGQLWSQHQR	PSATCASWSG	FHYRTFDGRH	YHFLGRCTYL	LAGAADSTWA	VHLTPGDRCP
250	260	270	280	290	300	310	320
QPGHCQRVTM	GPEEVLIQAG	NVSVKGQLVP	EGQSWLLHGL	SLQWLGDWLV	LSGGLGVVVR	LDRTGSISIS	VDHELWGQTQ
330	340	350	360	370	380	390	400
GLCGLYNGWP	EDDFMEPGGG	LAMLAATFGN	SWRLPGSESG	CLDAVEVAQG	CDPLGLIDAD	VEPGHLRAEA	QDVCHQLLEG
410	420	430	440	450	460	470	480
PFGQCHAQVS	PAEYHEACLF	AYCAGAMAGS	GQEGRQQAVC	ATFASYVQAC	ARRHIHIRWR	KPGFCERLCP	GGQLYSDCVS
490	500	510	520	530	540	550	560
LCPPSCEAVG	QGEEESCREE	CVSGCECPRG	LFWNGTLCVP	AAHCPCYYCR	QRYVPGDTVR	QLCNPCVCRD	GRWHCAQALC
570	580	590	600	610	620	630	640
PAECAVGGDG	HYLTFDGRSY	SFWGGQGCRY	SLVQDYVKGQ	LLILLEHGAC	DAGSCLHAIS	VSLEDTHIQL	RDSGAVLVNG
650	660	670	680	690	700	710	720
QDVGLPWIGA	EGLSVRRASS	AFLLLRWPGA	QVLWGLSDPV	AYITLDPRHA	HQVQGLCGTF	TQNQQDDFLT	PAGDVETSIA
730	740	750	760	770	780	790	800
AFASKFQVAG	KGRCPSEDSA	LLSPCTTHSQ	RHAFAEAACA	ILHSSVFQEC	HRLVDKEPFY	LRCLAAVCGC	DPGSDCLCPV
810	820	830	840	850	860	870	880
LSAYARRCAQ	EGASPPWRNQ	TLCPVMCPGG	QEYRECAPAC	GQHCGKPEDC	GELGSCVAGC	NCPLGLLWDP	EGQCVPPSLC
890	900	910	920	930	940	950	960
PCQLGARRYA	PGSATMKECN	RCICQERGLW	NCTARHCPSQ	AFCPRELVYA	PGACLLTCDS	PSANHSCPAG	STDGCVCPPG
970	980	990	1000	1010	1020	1030	1040
TVLLDERCVP	PDLCPCRHSG	QWYLPNATIQ	EDCNVCVCRG	RQWHCTGQRR	SGRCQASGAP	HYVTFDGLAF	TYPGACEYLL
1050	1060	1070	1080	1090	1100	1110	1120
VREASGLFTV	SAQNLPCGAS	GLTCTKALAV	RLEGTVVHML	RGRAVTVNGV	SVTPPKVYTG	PGLSLRRAGL	FLLLSTHLGL
1130	1140	1150	1160	1170	1180	1190	1200
TLLWDGGTRV	LVQLSPQFRG	RVAGLCGDFD	GDASNDLRSR	QGVLEPTAEL	AAHSWRLSPL	CPEPGDLPHP	CTMNTHRAGW
1210	1220	1230	1240	1250	1260	1270	1280
ARARCGALLQ	PLFTLCHAEV	PPQQHYEWCL	YDACGCDSGG	DCECLCSAIA	TYADECARHG	HHVRWRSQEL	CSLQCEGGQV
1290	1300	1310	1320	1330	1340	1350	1360
YEACGPTCPP	TCHEQHPEPG	WHCQVVACVE	GCFCPEGTLL	HGGACLEPAS	CPCEWGRNSF	PPGSVLQKDC	GNCTCQEGQW
1370	1380	1390	1400	1410	1420	1430	1440
HCGGDGGHCE	ELVPACAEGE	ALCQENGHCV	PHGWLCDNQD	DCGDGSDEEG	CAAPGCGEGQ	MTCSSGHCLP	LALLCDRQDD
1450	1460	1470	1480	1490	1500	1510	1520
CGDGTDEPSY	PCPQGLLACA	DGRCLPPALL	CDGHPDCLDA	ADEESCLGQV	TCVPGEVSCV	DGTCLGAIQL	CDGVWDCPDG
1530	1540	1550	1560	1570	1580	1590	1600
ADEGPGHCPL	PSLPTPPAST	LPGPSPGSLD	TASSPLASAS	PAPPCGPFEF	RCGSGECTPR	GWRCDQEEDC	ADGSDERGCG
1610	1620	1630	1640	1650	1660	1670	1680
GPCAPHHAPC	ARGPHCVSPE	QLCDGVRQCP	DGSDEGPDAC	GGLPALGGPN	RTGLPCPEYT	CPNGTCIGFQ	LVCDGQPDCG
1690	1700	1710	1720	1730	1740	1750	1760
RPGQVGPSPE	EQGCGAWGPW	SPWGPCSRTC	GPWGQGRSRR	CSPLGLLVLQ	NCPGPEHQSQ	ACFTAACPVD	GEWSTWSPWS
1770	1780	1790	1800	1810	1820	1830	1840
VCSEPCRGTM	TRQRQCHSPQ	NGGRTCAALP	GGLHSTRQTK	PCPQDGCPNA	TCSGELMFQP	CAPCPLTCDD	ISGQVTCPPD
1850	1860	1870	1880	1890	1900	1910	1920
WPCGSPGCWC	PEGQVLGSEG	WCVWPRQCPC	LVDGARYWPG	QRIKADCQLC	ICQDGRPRRC	RLNPDCAVDC	GWSSWSPWAK
1930	1940	1950	1960	1970	1980	1990	2000
CLGPCGSQSI	QWSFRSSNNP	RPSGRGRQCR	GIHRKARRCQ	TEPCEGCEHQ	GQVHRVGERW	HGGPCRVCQC	LHNLTAHCSP
2010	2020	2030	2040	2050	2060	2070	2080
YCPLGSCPQG	WVLVEGTGES	CCHCALPGEN	QTVQPMATPA	AAPAPSPQIR	FPLATYILPP	SGDPCYSPLG	LAGLAEGSLH
2090	2100	2110	2120	2130	2140	2150	2160
ASSQQLEHPT	QAALLGAPTQ	GPSPQGWHAG	GDAYAKWHTR	PHYLQLDLLQ	PRNLTGILVP	ETGSSNAYAS	SFSLQFSSNG
2170	2180	2190	2200	2210	2220	2230	2240

Detailed Protein Report

Cmpd.	No. of Cmpds.	m/z meas.	Δ m/z [ppm]	z	Rt [min]	Score	P	Range	Sequence	Modification
1675	2	1136.5319	16.36	2	49.5	14.8	1	2854-2875	R.GRSCSSLAPGDTTCPGPHSQTR.D	Carbamidomethyl: 4
1364	1	669.3350	-3.76	2	45.8	13.3	1	3671-3682	R.RCARPPCPGPAR.Q	Carbamidomethyl: 2

Detailed Protein Report

Protein 475: growth arrest-specific protein 7 isoform a [Homo sapiens]

**Accession:**	gi|41406076	**Score:**	28.1
**Database:**	refseq_human(refseq_human_20140103.fasta)	**MW [kDa]:**	39.2
**Database Date:**	2015-11-30	**pI:**	6.3
		**Sequence Coverage [%]:**	8.3
		**No. of unique Peptides:**	1

10	20	30	40	50	60	70	80
MSNMENSFDD	VSCLSPQNLG	SSSPSKKQSK	ENTITINCVT	FPHPDTMPEQ	QLLKPTEWSY	CDYFWADKKD	PQGNGTVAGF
**90**	**100**	**110**	**120**	**130**	**140**	**150**	**160**
ELLLQKQLKG	KQMQKEMSEF	IRERIKIEED	YAKNLAKLSQ	NSLASQEEGS	LGEAWAQVKK	SLADEAEVHL	KFSAKLHSEV
**170**	**180**	**190**	**200**	**210**	**220**	**230**	**240**
EKPLMNFREN	FKKDMKKCDH	HIADLRKQLA	SRYASVEKAR	KALTERQRDL	EMKTQQLEIK	LSNKTEEDIK	KARRKSTQAG
**250**	**260**	**270**	**280**	**290**	**300**	**310**	**320**
DDLMRCVDLY	NQAQSKWFEE	MVTTTLELER	LEVERVEMIR	QHLCQYTQLR	HETDMFNQST	VEPVDQLLRK	VDPAKDRELW
**330**	**340**						
VREHKTGNIR	PVDMEI						

Cmpd.	No. of Cmpds.	m/z meas.	Δ m/z [ppm]	z	Rt [min]	Score	P	Range	Sequence	Modification
2697	1	596.2791	-61.86	3	62.6	14.1	0	70-86	K.DPQGNGTVAGFELLLQK.Q	

Detailed Protein Report

Protein 476: transmembrane protein 151A [Homo sapiens]

Accession: gi|23397566
Database: refseq_human(refseq_human_20140103.fasta)
Database Date: 2015-11-30
Modification(s): Carbamidomethyl, Oxidation

Score: 28.0
MW [kDa]: 51.2
pI: 9.3
Sequence Coverage [%]: 9.4
No. of unique Peptides: 2

10	20	30	40	50	60	70	80
MPEDGAGDGG	EVPALIPDGE	PLREEQRPLK	QSLGSSLCRE	SHWKCLLLTL	LIHACGAVVA	WCRLATVPRL	VLGPEAALAR
90	100	110	120	130	140	150	160
GAGGPPPTYP	ASPCSDGYLY	IPLAFVSLLY	LLYLAECWHC	HVRSCQAPRT	DAHTVLALIR	RLQQAPPCVW	WKATSYHYVR
170	180	190	200	210	220	230	240
RTRQITRYRN	GDAYTTTQVY	HERADSRTAR	GEFDYSAHGV	RDVSKELVGL	AEHAATRLRF	TKCFSFGSAE	AEASYLTQRA
250	260	270	280	290	300	310	320
RFFSANEGLD	DYLEAREGMH	LKDVDFRESL	MVFADPRSPP	WYARAWVFWL	VSAATLSWPL	RVVAAYGTAH	VHYQVEKLFG
330	340	350	360	370	380	390	400
ASSPPPGAVP	SGPPLSRVAT	VDFTELEWHI	CSNRQLVPSY	SEAVVMGAGS	GAYLRGCQRC	RRSVSSNSLP	PARPSGPRLP
410	420	430	440	450	460	470	
FSRSRLSLGA	GGRATPGVFR	SLSGGPLGRR	GEDTEPLESP	PCYEDALYFP	VLIVHGDSGC	QGDGQGAL	

Cmpd.	No. of Cmpds.	m/z meas.	Δ m/z [ppm]	z	Rt [min]	Score	P	Range	Sequence	Modification
136	1	977.7569	-49.80	3	31.1	10.2	2	355-381	R.QLVPSYSEAVVMGAGSGAYLRGCQRCR.R	Carbamidomethyl: 23; Oxidation: 12
1863	1	864.4736	-15.26	2	52.0	17.8	2	414-430	R.ATPGVFRSLSGGPLGRR.G	

Detailed Protein Report

Protein 477: **SWI/SNF complex subunit SMARCC2 isoform b [Homo sapiens]**

**Accession:**	gi|21237808	**Score:**	28.0
**Database:**	refseq_human(refseq_human_20140103.fasta)	**MW [kDa]:**	124.8
**Database Date:**	2015-11-30	**pI:**	5.3
**Modification(s):**	Carbamidomethyl, Oxidation	**Sequence Coverage [%]:**	2.0
		**No. of unique Peptides:**	1

10	20	30	40	50	60	70	80
MAVRKKDGGP	NVKYYEAADT	VTQFDNVRLW	LGKNYKKYIQ	AEPPTNKSLS	SLVVQLLQFQ	EEVFGKHVSN	APLTKLPIKC
90	100	110	120	130	140	150	160
FLDFKAGGSL	CHILAAAYKF	KSDQGWRRYD	FQNPSRMDRN	VEMFMTIEKS	LVQNNCLSRP	NIFLCPEIEP	KLLGKLKDII
170	180	190	200	210	220	230	240
KRHQGTVTED	KNNASHVVYP	VPGNLEEEEW	VRPVMKRDKQ	VLLHWGYYPD	SYDTWIPASE	IEASVEDAPT	PEKPRKVHAK
250	260	270	280	290	300	310	320
WILDTDTFNE	WMNEEDYEVN	DDKNPVSRRK	KISAKTLTDE	VNSPDSDRRD	KKGGNYKKRK	RSPSPSPTPE	AKKKNAKKGP
330	340	350	360	370	380	390	400
STPYTKSKRG	HREEEQEDLT	KDMDEPSPVP	NVEEVTLPKT	VNTKKDSESA	PVKGGTMTDL	DEQEDESMET	TGKDEDENST
410	420	430	440	450	460	470	480
GNKGEQTKNP	DLHEDNVTEQ	THHIIIPSYA	AWFDYNSVHA	IERRALPEFF	NGKNKSKTPE	IYLAYRNFMI	DTYRLNPQEY
490	500	510	520	530	540	550	560
LTSTACRRNL	AGDVCAIMRV	HAFLEQWGLI	NYQVDAESRP	TPMGPPPTSH	FHVLADTPSG	LVPLQPKTPQ	GRQVDADTKA
570	580	590	600	610	620	630	640
GRKGKELDDL	VPETAKGKPE	LQTSASQQML	NFPDKGKEKP	TDMQNFGLRT	DMYTKKNVPS	KSKAAASATR	EWTEQETLLL
650	660	670	680	690	700	710	720
LEALEMYKDD	WNKVSEHVGS	RTQDECILHF	LRLPIEDPYL	EDSEASLGPL	AYQPIPFSQS	GNPVMSTVAF	LASVVDPRVA
730	740	750	760	770	780	790	800
SAAAKSALEE	FSKMKEEVPT	ALVEAHVRKV	EEAAKVTGKA	DPAFGLESSG	IAGTTSDEPE	RIEESGNDEA	RVEGQATDEK
810	820	830	840	850	860	870	880
KEPKEPREGG	GAIEEEAKEK	TSEAPKKDEE	KGKEGDSEKE	SEKSDGDPIV	DPEKEKEPKE	GQEEVLKEVV	ESEGERKTKV
890	900	910	920	930	940	950	960
ERDIGEGNLS	TAAAAALAAA	AVKAKHLAAV	EERKIKSLVA	LLVETQMKKL	EIKLRHFEEL	ETIMDREREA	LEYQRQQLLA
970	980	990	1000	1010	1020	1030	1040
DRQAFHMEQL	KYAEMRARQQ	HFQQMHQQQQ	QPPPALPPGS	QPIPPTGAAG	PPAVHGLAVA	PASVVPAPAG	SGAPPGSLGP
1050	1060	1070	1080	1090	1100	1110	1120
SEQIGQAGST	AGPQQQQPAG	APQPGAVPPG	VPPPGPHGPS	PFPNQQTPPS	MMPGAVPGSG	HPGVADPGTP	LPPDPTAPSP
1130	1140						
GTVTPVPPPQ							

Cmpd.	No. of Cmpds.	m/z meas.	Δ m/z [ppm]	z	Rt [min]	Score	P	Range	Sequence	Modification
1963	2	696.2530	-136.00	2	54.6	12.3	1	488-499	R.RNLAGDVCAIMR.V	Carbamidomethyl: 8; Oxidation: 11

Detailed Protein Report

Protein 478: **PREDICTED: probable ATP-dependent RNA helicase DDX11 isoform X15 [Homo sapiens]**

**Accession:**	gi|578822806	**Score:**	28.0
**Database:**	refseq_human(refseq_human_20140103.fasta)	**MW [kDa]:**	94.9
**Database Date:**	2015-11-30	**pI:**	6.8
**Modification(s):**	Oxidation	**Sequence Coverage [%]:**	4.1
		**No. of unique Peptides:**	2

10	20	30	40	50	60	70	80
MPLDGVRDSG	MASRGPAAKD	GENMVSASQK	KGEIWSMANE	TQKVGAIHFP	FPFTPYSIQE	DFMAELYRVL	EAGKIGIFES
90	100	110	120	130	140	150	160
PTGTGKSLSL	ICGALSWLRD	FEQKKREEEA	RLLETGTGPL	HDEKDESLCL	SSSCEGAAGT	PRPAGEPAWV	TQFVQKKEER
170	180	190	200	210	220	230	240
DLVDRLKAEQ	ARRKQREERL	QQLQHRVQLK	YAAKRLRQEE	EERENLLRLS	REMLETGPEA	ERLEQLESGE	EELVLAEYES
250	260	270	280	290	300	310	320
DEEKKVASRV	DEDEDDLEEE	HITKIYYCSR	THSQLAQFVH	EVKKSPFGKD	VRLVSLGSRQ	NLCVNEDVKS	LGSVQLINDR
330	340	350	360	370	380	390	400
CVDMQRSRHE	KKKGAEEEKP	KRRRQEKQAA	CPFYNHEQMG	LLRDEALAEV	KDMEQLLALG	KEARACPYYG	SRLAIPAAQL
410	420	430	440	450	460	470	480
VVLPYQMLLH	AATRQAAGIR	LQDQVVIIDE	AHNLIDTITG	MHSVEVSGSQ	LCQAHSQLLQ	YVERYGKRLK	AKNLMYLKQI
490	500	510	520	530	540	550	560
LYLLEKFVAV	LGGNIKQNPN	TQSLSQTGTE	LKTINDFLFQ	SQIDNINLFK	VQRYCEKSMI	SRKLFGFTER	YGAVFSSREQ
570	580	590	600	610	620	630	640
PKLAGFQQFL	QSLQPRTTEA	LAAPADESQA	STLRPASPLM	HIQGFLAALT	TANQDGRVIL	SRQGSLSQST	LKFLLLNPAV
650	660	670	680	690	700	710	720
HFAQVVKECR	AVVIAGGTMQ	PVSDFRQQLL	ACAGVEAERV	VEFSCGHVIP	PDNILPLVIC	SGISNQPLEF	TFQKRELPQM
730	740	750	760	770	780	790	800
MDEVGRILCN	LCGVVPGGVV	CFFPSYEYLR	QVHAHWEKGG	LLGRLAARKK	IFQEPKSAHQ	VEQVLLAYSR	CIQACGQERG
810	820	830	840	850			
QVTGALLLSV	VGGKMSEGIN	FSDNLGRPEP	PARHPQGRLW	WRTCA			

Cmpd.	No. of Cmpds.	m/z meas.	Δ m/z [ppm]	z	Rt [min]	Score	P	Range	Sequence	Modification
2190	1	966.3871	-90.93	2	57.6	10.3	2	1-19	-.MPLDGVRDSGMASRGPAAK.D	Oxidation: 11
1689	1	932.9135	-80.83	2	51.0	17.7	2	543-558	R.KLFGFTERYGAVFSSR.E	

Detailed Protein Report

Protein 479: **PREDICTED: putative dehydrogenase/reductase SDR family member 4-like 2 isoform X1 [Homo sapiens]**

**Accession:**	gi|578826096	**Score:**	28.0
**Database:**	refseq_human(refseq_human_20140103.fasta)	**MW [kDa]:**	26.3
**Database Date:**	2015-11-30	**pI:**	10.7
		**Sequence Coverage [%]:**	10.2
		**No. of unique Peptides:**	1

Quantitation

Wdown:Qdown **Median:** 3.09 **CV:** 0.00 % **No. of Peptides:** 1

10	20	30	40	50	60	70	80
MHKARLRGHC	ARAGKSVRLA	SSGMTRRDPL	TNKVALVTAS	TDWIGFAVAQ	RLAQDGAHVV	VSRRKQQNVD	QAVATLQGEG
90	100	110	120	130	140	150	160
LSMTGTVCHV	GKMKDWERLV	ATNLDINGKA	LALMIKAVVP	EMEKRGGGSV	GFLASVAAFR	PLPGFSPYNV	SKTALLGLNK
170	180	190	200	210	220	230	240
TLAIELAPRN	IRVNCLAPGL	IKTSFSRMLW	MDKEKEESMK	ETLRIRRLGE	PEDSLGIVSF	LCSEDASYLT	GETVMVGGGT
250							
PSRL							

Cmpd.	No. of Cmpds.	m/z meas.	Δ m/z [ppm]	z	Rt [min]	Score	P	Range	Sequence	Modification	Ratios
36	1	660.7253	-206.67	2	29.1	13.6	2	16-27	K.SVRLASSGMTRR.D		Wdown:Qdown 3.09

Detailed Protein Report

Protein 480: PREDICTED: egl nine homolog 1 isoform X1 [Homo sapiens]

**Accession:**	gi|530366273	**Score:**	28.0
**Database:**	refseq_human(refseq_human_20140103.fasta)	**MW [kDa]:**	41.1
**Database Date:**	2015-11-30	**pI:**	9.8
		**Sequence Coverage [%]:**	11.7
		**No. of unique Peptides:**	1

10	20	30	40	50	60	70	80
MANDSGGPGG	PSPSERDRQY	CELCGKMENL	LRCSRCRSSF	YCCKEHQRQD	WKKHKLVCQG	SEGALGHGVG	PHQHSGPAPP
**90**	**100**	**110**	**120**	**130**	**140**	**150**	**160**
AAVPPPRAGA	REPRKAAARR	DNASGDAAKG	KVKAKPPADP	AAAASPCRAA	AGGQGSAVAA	EAEPGKEEPP	ARSSLFQEKA
**170**	**180**	**190**	**200**	**210**	**220**	**230**	**240**
NLYPPSNTPG	DALSPGGGLR	PNGQTKPLPA	LKLALEYIVP	CMNKHGICVV	DDFLGKETGQ	QIGDEVRALH	DTGKFTDGQL
**250**	**260**	**270**	**280**	**290**	**300**	**310**	**320**
VSQKSDSSKD	IRGDKITWIE	GKEPGCETIG	LLMSSMDDLI	RHCNGKLGSY	KINGRTKAMV	ACYPGNGTGY	VRHVDNPNGD
**330**	**340**	**350**	**360**	**370**	**380**	**390**	
GRCVTCIYYL	NKDWDAKVSG	GILRIFPEGK	AQFADIEPKF	DRLLFFWSDR	RNPHEVQPAY	ATR	

Cmpd.	No. of Cmpds.	m/z meas.	Δ m/z [ppm]	z	Rt [min]	Score	P	Range	Sequence	Modification
1622	1	1100.2202	-32.79	3	50.2	17.8	0	160-192	K.ANLYPPSNTPGDALSPGGGLRPNGQTKPLPALK.L	

Detailed Protein Report

Protein 481: **PREDICTED: RANBP2-like and GRIP domain-containing protein 2 isoform X2 [Homo sapiens]**

**Accession:**	gi|530368052	**Score:**	27.9
**Database:**	refseq_human(refseq_human_20140103.fasta)	**MW [kDa]:**	185.5
**Database Date:**	2015-11-30	**pI:**	5.8
		**Sequence Coverage [%]:**	1.7
		**No. of unique Peptides:**	1

10	20	30	40	50	60	70	80
MRRSKAYGER	YLASVQGSAP	SPGKKLRGFY	FAKLYYEAKE	YDLAKKYVCT	YLSVQERDPR	AHRFLGLLYE	LEENTEKAVE
90	100	110	120	130	140	150	160
CYRRSLELNP	PQKDLVLKIA	ELLCKNDVTD	GRAKYWVERA	AKLFPGSPAI	YKLKEHLLDC	EGEDGWNKLF	DWIQSELYVR
170	180	190	200	210	220	230	240
PDDVHMNIRL	VELYRSNKRL	KDAVARCHEA	ERNIALRSSL	EWNSCVVQTL	KEYLESLQCL	ESDKSDWRAT	NTDLLLAYAN
250	260	270	280	290	300	310	320
LMLLTLSTRD	VQESRELLES	FDSALQSAKS	SLGGNDELSA	TFLEMKGHFY	MHAGSLLLKM	GQHGNNVQWQ	ALSELAALCY
330	340	350	360	370	380	390	400
VIAFQVPRPK	IKLIKGEAGQ	NLLEMMACDR	LSQSGHMLLN	LSRGKQDFLK	EVVETFANKS	GQSVLYNALF	SSQSSKDTSF
410	420	430	440	450	460	470	480
LGSDDIGNID	VQEPELEDLA	RYDVGAIQAH	NGSLQHLTWL	GLQWNSLPAL	PGIRKWLKQL	FHHLPQETSR	LETNAPESIC
490	500	510	520	530	540	550	560
ILDLEVFLLG	VVYTSHLQLK	EKCNSHHSSY	QPLCLPLPVC	KRLCTERQKS	WWDAVCTLIH	RKAVPGNSAE	LRLVVQHEIN
570	580	590	600	610	620	630	640
TLRAQEKHGL	QPALLVHWAK	CLQKMGRGLN	SSYDQQEYIG	RSVHYWKKVL	PLLKIIKKNS	IPEPIDPLFK	HFHSVDIQAS
650	660	670	680	690	700	710	720
EIVEYEEDAH	ITFAILDAVH	GNIEDAVTAF	ESIKSVVSYW	NLALIFHRKA	EDIENDAVFP	EEQEECKNYL	RKTRDYLIKI
730	740	750	760	770	780	790	800
IDDSDSNLSV	VKKLPVPLES	VKEMLKSVMQ	ELEDYSEGGP	LYKNGSLRNA	DSEIKHSTPS	PTKYSLSPSK	SYKYSPKTPP
810	820	830	840	850	860	870	880
RWAEDQNSLR	KMICQEVKAI	TKLNSSKSAS	RHRWPTENYG	PDSVPDGYQG	SQTFHGAPLT	VATTGPSVYY	SQSPAYNSQY
890	900	910	920	930	940	950	960
LLRPAANVTP	TKGSSNTEFK	STKEGFSIAV	SADGFKFGIS	EPGNQEKKSE	KPLENDTGFQ	AQDISGQKNG	RGVIFGQTSS
970	980	990	1000	1010	1020	1030	1040
TFTFADVAKS	TSGEGFQFGK	KDPNFKGFSG	AGEKLFSSQC	GKMANKANTS	GDFEKDDDAC	KTEDSDDIHF	EPVVQMPEKV
1050	1060	1070	1080	1090	1100	1110	1120
ELVTGEEGEK	VLYSQGVKLF	RFDAEISQWK	ERGLGNLKIL	KNEVNGKPRM	LMRRDQVLKV	CANHWITTTM	NLKPLSGSDR
1130	1140	1150	1160	1170	1180	1190	1200
AWMWLASDFS	DGDAKLERLA	AQFKTPELAE	EFKQKFEECQ	RLLLDIPLQT	PHKLVDTGRA	AKLIQRAEEM	KSGLKDFKTF
1210	1220	1230	1240	1250	1260	1270	1280
LTNDQTKVTE	EENKGSGTGA	AGASDTTIKP	NPENTGPTLE	WDNYDLREDA	LDDNVSSSSV	HDSPLASSPV	RKNIFRFDES
1290	1300	1310	1320	1330	1340	1350	1360
TTGFNFSFKS	ALSLSKSPAK	LNQSGTSVGT	DEESDVTQEE	ERDGQYFEPV	VPLPDLVEVS	SGEENEQVVF	SHMAELYRYD
1370	1380	1390	1400	1410	1420	1430	1440
KDVGQWKERG	IGDIKILQNY	DNKQVRIVMR	RDQVLKLCAN	HRITPDMSLQ	NMKGTERVWV	WTACDFADGE	RKVEHLAVRF
1450	1460	1470	1480	1490	1500	1510	1520
KLQDVADSFK	KIFDEAKTAQ	EKDSLITPHV	SRSSTPRESP	CGKIAVAVLE	ETTRERTDVI	QGDDVADAAS	EVEVSSTSET
1530	1540	1550	1560	1570	1580	1590	1600
TTKAVVSPPK	FVFGSESVKR	IFSSEKSNPF	AFGNSSATGS	LFGFSFNAPL	KSNDSETSSV	AQSGSESKVE	PKKCELSKNS
1610	1620	1630	1640	1650	1660		
DIEQSSDSKV	KNLSASFPME	ESSINYTFKT	PEKAKEKKKP	EDSPSDSLGL	LTS		

Cmpd.	No. of Cmpds.	m/z meas.	Δ m/z [ppm]	z	Rt [min]	Score	P	Range	Sequence	Modification
2905	1	1023.0342	20.17	2	65.1	16.6	2	794-810	K.YSPKTPPRWAEDQNSLR.K	

Detailed Protein Report

Protein 482: **PREDICTED: membrane-associated guanylate kinase, WW and PDZ domain-containing protein 2 isoform X1 [Homo sapiens]**

**Accession:**	gi|530386693	**Score:**	27.9
**Database:**	refseq_human(refseq_human_20140103.fasta)	**MW [kDa]:**	157.1
**Database Date:**	2015-11-30	**pI:**	5.9
		**Sequence Coverage [%]:**	1.8
		**No. of unique Peptides:**	1

10	20	30	40	50	60	70	80
MSKSLKKKSH	WTSKVHESVI	GRNPEGQLGF	ELKGGAENGQ	FPYLGEVKPG	KVAYESGSKL	VSEELLLEVN	ETPVAGLTIR
90	100	110	120	130	140	150	160
DVLAVIKHCK	DPLRLKCVKQ	GGIVDKDLRH	YLNLRFQKGS	VDHELQQIIR	DNLYLRTVPC	TTRPHKEGEV	PGVDYIFITV
170	180	190	200	210	220	230	240
EDFMELEKSG	ALLESGTYED	NYYGTPKPPA	EPAPLLLNVT	DQILPGATPS	AEGKRKRNKS	VSNMEKASIE	PPEEEEEERP
250	260	270	280	290	300	310	320
VVNGNGVVVT	PESSEHEDKS	AGASGEMPSQ	PYPAPVYSQP	EELKEQMDDT	KPTKPEDNEE	PDPLPDNWEM	AYTEKGEVYF
330	340	350	360	370	380	390	400
IDHNTKTTSW	LDPRLAKKAK	PPEECKENEL	PYGWEKIDDP	IYGTYYVDHI	NRRTQFENPV	LEAKRKLQQH	NMPHTELGTK
410	420	430	440	450	460	470	480
PLQAPGFREK	PLFTRDASQL	KGTFLSTTLK	KSNMGFGFTI	IGGDEPDEFL	QVKSVIPDGP	AAQDGKMETG	DVIVYINEVC
490	500	510	520	530	540	550	560
VLGHTHADVV	KLFQSVPIGQ	SVNLVLCRGY	PLPFDPEDPA	NSMVPPLAIM	ERPPPVMVNG	RHNYETYLEY	ISRTSQSVPD
570	580	590	600	610	620	630	640
ITDRPPHSLH	SMPTDGQLDG	TYPPPVHDDN	VSMASSGATQ	AELMTLTIVK	GAQGFGFTIA	DSPTGQRVKQ	ILDIQGCPGL
650	660	670	680	690	700	710	720
CEGDLIVEIN	QQNVQNLSHT	EVVDILKDCP	IGSETSLIIH	RGGFFSPWKT	PKPIMDRWEN	QGSPQTSLSA	PAIPQNLPFP
730	740	750	760	770	780	790	800
PALHRSSFPD	STEAFDPRKP	DPYELYEKSR	AIYESRRPDY	KELDVHLRRM	ESGFGFRILG	GDEPGQPILI	GAVIAMGSAD
810	820	830	840	850	860	870	880
RDGRLHPGDE	LVYVDGIPVA	GKTHRYVIDL	MHHAARNGQV	NLTVRRKVLC	GGEPCPENGR	SPGSVSTHHS	SPRSDYATYT
890	900	910	920	930	940	950	960
NSNHAAPSSN	ASPPEGFASH	SLQTSDVVIH	RKENEGFGFV	IISSLNRPES	GSTITVPHKI	GRIIDGSPAD	RCAKLKVGDR
970	980	990	1000	1010	1020	1030	1040
ILAVNGQSII	NMPHADIVKL	IKDAGLSVTL	RIIPQEELNS	PTSAPSSEKQ	SPMAQQSPLA	QQSPLAQPSP	ATPNSPIAQP
1050	1060	1070	1080	1090	1100	1110	1120
APPQPLQLQG	HENSYRSEVK	ARQDVKPDIR	QPPFTDYRQP	PLDYRQPPGG	DYQQPPPLDY	RQPPLLDYRQ	HSPDTRQYPL
1130	1140	1150	1160	1170	1180	1190	1200
SDYRQPQDFD	YFTVDMEKGA	KGFGFSIRGG	REYKMDLYVL	RLAEDGPAIR	NGRMRVGDQI	IEINGESTRD	MTHARAIELI
1210	1220	1230	1240	1250	1260	1270	1280
KSGGRRVRLL	LKRGTGQVPE	YDEPAPWSSP	AAAAPGLPEV	GVSLDDGLAP	FSPSHPAPPS	DPSHQISPGP	TWDIKREHDV
1290	1300	1310	1320	1330	1340	1350	1360
RKPKELSACG	QKKQRLGEQR	ERSASPQRAA	RPRLEEAPGG	QGRPEAGRPA	SEARAPGLAA	ADAADAARAG	GKEAPRAAAG
1370	1380	1390	1400	1410	1420	1430	1440
SELCRREGPG	AAPAFAGPGG	GGSGALEAEG	RAGARAGPRP	GPRPPGGAPA	RKAAVAPGPW	KVPGSDKLPS	VLKPGASAAS
1450							
R							

Cmpd.	No. of Cmpds.	m/z meas.	Δ m/z [ppm]	z	Rt [min]	Score	P	Range	Sequence	Modification
1930	1	643.2948	-156.54	2	54.2	17.3	1	980-991	K.LIKDAGLSVTLR.I	

Detailed Protein Report

Protein 483: uncharacterized protein KIAA0408 [Homo sapiens]

**Accession:**	gi|59806363	**Score:**	27.9
**Database:**	refseq_human(refseq_human_20140103.fasta)	**MW [kDa]:**	79.1
**Database Date:**	2015-11-30	**pI:**	9.6
**Modification(s):**	Oxidation	**Sequence Coverage [%]:**	3.7
		**No. of unique Peptides:**	1

Quantitation

Wdown:Qdown **Median:** 2.32 **CV:** 0.00 % **No. of Peptides:** 1

10	20	30	40	50	60	70	80
MDLHKQWENT	ETNWHKEKME	LLDQFDNERK	EWESQWKIMQ	KKIEELCREV	KLWRKININE	SAKIIDLYHE	KTIPEKVIES
90	100	110	120	130	140	150	160
SPNYPDLGQS	EFIRTNHKDG	LRKENKREQS	LVSGGNQMCK	EQKATKKSKV	GFLDPLATDN	QKECEAWPDL	RTSEEDSKSC
170	180	190	200	210	220	230	240
SGALSTALEE	LAKVSEELCS	FQEEIRKRSN	HRRMKSDSFL	QEMPNVTNIP	HGDPMINNDQ	CILPISLEKE	KQKNRKNLSC
250	260	270	280	290	300	310	320
TNVLQSNSTK	KCGIDTIDLK	RNETPPVPPP	RSTSRNFPSS	DSEQAYERWK	ERLDHNSWVP	HEGRSKRNYN	PHFPLRQQEM
330	340	350	360	370	380	390	400
SMLYPNEGKT	SKDGIIFSSL	VPEVKIDSKP	PSNEDVGLSM	WSCDIGIGAK	RSPSTSWFQK	TCSTPSNPKY	EMVIPDHPAK
410	420	430	440	450	460	470	480
SHPDLHVSND	CSSSVAESSS	PLRNFSCGFE	RTTRNEKLAA	KTDEFNRTVF	RTDRNCQAIQ	QNHSCSKSSE	DLKPCDTSST
490	500	510	520	530	540	550	560
HTGSISQSND	VSGIWKTNAH	MPVPMENVPD	NPTKKSTTGL	VRQMQGHLSP	RSYRNMLHEH	DWRPSNLSGR	PRSADPRSNY
570	580	590	600	610	620	630	640
GVVEKLLKTY	ETATESALQN	SKCFQDNWTK	CNSDVSGGAT	LSQHLEMLQM	EQQFQQKTAV	WGGQEVKQGI	DPKKITEESM
650	660	670	680	690	700		
SVNASHGKGF	SRPARPANRR	LPSRWASRSP	SAPPALRRTT	HNYTISLRSE	ALMV		

Cmpd.	No. of Cmpds.	m/z meas.	Δ m/z [ppm]	z	Rt [min]	Score	P	Range	Sequence	Modification	Ratios
2682	2	841.8664	-25.81	2	61.9	15.5	1	17-29	K.EKMELLDQFDNER.K	Oxidation: 3	Wdown:Qdown 2.32

Detailed Protein Report

Protein 484: transmembrane protein 2 isoform b [Homo sapiens]

**Accession:**	gi|209447096	**Score:**	27.9
**Database:**	refseq_human(refseq_human_20140103.fasta)	**MW [kDa]:**	147.3
**Database Date:**	2015-11-30	**pI:**	9.4
**Modification(s):**	Carbamidomethyl	**Sequence Coverage [%]:**	1.4
		**No. of unique Peptides:**	1

10	20	30	40	50	60	70	80
MYATDSRGHS	PAFLQPQNGN	SRHPSGYVPG	KVVPLRPPPP	PKSQASAKFT	SIRREDRATF	AFSPEEQQAQ	RESQKQKRHK
90	100	110	120	130	140	150	160
NTFICFAITS	FSFFIALAII	LGISSKYAPD	ENCPDQNPRL	RNWDPGQDSA	KQVVIKEGDM	LRLTSDATVH	SIVIQDGGLL
170	180	190	200	210	220	230	240
VFGDNKDGSR	NITLRTHYIL	IQDGGALHIG	AEKCRYKSKA	TITLYGKSDE	GESMPTFGKK	FIGVEAGGTL	ELHGARKASW
250	260	270	280	290	300	310	320
TLLARTLNSS	GLPFGSYTFE	KDFSRGLNVR	VIDQDTAKIL	ESERFDTHEY	RNESRRLQEF	LRFQDPGRIV	AIAVGDSAAK
330	340	350	360	370	380	390	400
SLLQGTIQMI	QERLGSELIQ	GLGYRQAWAL	VGVIDGGSTS	CNESVRNYEN	HSSGGKALAQ	REFYTVDGQK	FSVTAYSEWI
410	420	430	440	450	460	470	480
EETPQFLHMG	EIIDGVDMRA	EVGILTRNIV	IQGEVEDSCY	AENQCQFFDY	DTFGGHIMIM	KNFTSVHLSY	VELKHMGQQQ
490	500	510	520	530	540	550	560
MGRYPVHFHL	CGDVDYKGGY	RHATFVDGLS	IHHSFSRCIT	VHGTNGLLIK	DTIGFDTLGH	CFFLEDGIEQ	RNTLFHNLGL
570	580	590	600	610	620	630	640
LTKPGTLLPT	DRNNSMCTTM	RDKVFGNYIP	VPATDCMAVS	TFWIAHPNNN	LINNAAAGSQ	DAGIWYLFHK	EPTGESSGLQ
650	660	670	680	690	700	710	720
LLAKPELTPL	GIFYNNRVHS	NFKAGLFIDK	GVKTTNSSAA	DPREYLCLDN	SARFRPHQDA	NPEKPRVAAL	IDRLIAFKNN
730	740	750	760	770	780	790	800
DNGAWVRGGD	IIVQNSAFAD	NGIGLTFASD	GSFPSDEGSS	QEVSESLFVG	ESRNYGFQGG	QNKYVGTGGI	DQKPRTLPRN
810	820	830	840	850	860	870	880
RTFPIRGFQI	YDGPIHLTRS	TFKKYVPTPD	RYSSAIGFLM	KNSWQITPRN	NISLVKFGPH	VSLNVFFGKP	GPWFEDCEMD
890	900	910	920	930	940	950	960
GDKNSIFHDI	DGSVTGYKDA	YVGRMDNYLI	RHPSCVNVSK	WNAVICSGTY	AQVYVQTWST	QNLSMTITRD	EYPSNPMVLR
970	980	990	1000	1010	1020	1030	1040
GINQKAAFPQ	YQPVVMLEKG	YTIHWNGPAP	RTTFLYLVNF	NKNDWIRVGL	CYPSNTSFQV	TFGYLQRQNG	SLSKIEEYEP
1050	1060	1070	1080	1090	1100	1110	1120
VHSLEELQRK	QSERKFYFDS	STGLLFLYLK	AKSHRHGHSY	CSSQGCERVK	IQAATDSKDI	SNCMAKAYPQ	YYRKPSVVKR
1130	1140	1150	1160	1170	1180	1190	1200
MPAMLTGLCQ	GCGTRQVVFT	SDPHKSYLPV	QFQSPDKAET	QRGDPSVISV	NGTDFTFRSA	GVLLLVVDPC	SVPFRLTEKT
1210	1220	1230	1240	1250	1260	1270	1280
VFPLADVSRI	EEYLKTGIPP	RSIVLLSTRG	EIKQLNISHL	LVPLGLAKPA	HLYDKGSTIF	LGFSGNFKPS	WTKLFTSPAG
1290	1300	1310	1320	1330			
QGLGVLEQFI	PLQLDEYGCP	RATTVRRRDL	ELLKQASKAH				

Cmpd.	No. of Cmpds.	m/z meas.	Δ m/z [ppm]	z	Rt [min]	Score	P	Range	Sequence	Modification
2384	1	841.6330	235.44	1	58.2	16.4	2	194-199	K.CRYKSK.A	Carbamidomethyl: 1

Detailed Protein Report

Protein 485: zinc finger protein 182 isoform 2 [Homo sapiens]

**Accession:**	gi|55769561	**Score:**	27.9
**Database:**	refseq_human(refseq_human_20140103.fasta)	**MW [kDa]:**	71.5
**Database Date:**	2015-11-30	**pI:**	10.0
		**Sequence Coverage [%]:**	3.7
		**No. of unique Peptides:**	1

10	20	30	40	50	60	70	80
MAKPQGLVTF	EDVAVDFTQE	EWQYLNPPQR	TLYRDVMLET	YSNLVFVGQQ	VTKPNLILKL	EVEECPAEGK	IPFWNFPEVC
90	100	110	120	130	140	150	160
QVDEQIERQH	QDDQDKCLLM	QVGFSDKKTI	ITKSARDCHE	FGNILHLSTN	LVASIQRPDK	HESFGNNMVD	NLDLFSRSSA
170	180	190	200	210	220	230	240
ENKYDNGCAK	LFFHTEYEKT	NPGMKPYGYK	ECGKGLRRKK	GLSLHQRIKN	GEKPFECTAC	RKTFSKKSHL	IVHWRTHTGE
250	260	270	280	290	300	310	320
KPFGCTECGK	AFSQKSQLII	HLRTHTGERP	FECPECGKAF	REKSTVIIHY	RTHTGEKPYE	CNECGKAFTQ	KSNLIVHQKT
330	340	350	360	370	380	390	400
HTGEKTYECT	KCGESFIQKL	DLIIHHSTHT	GKKPHECNEC	KKTFSDKSTL	IIHQRTHTGE	KPHKCTECGK	SFNEKSTLIV
410	420	430	440	450	460	470	480
HQRTHTGEKP	YECDVCGKTF	TQKSNLGVHQ	RTHSGEKPFE	CNECEKAFSQ	KSYLMLHQRG	HTGEKPYECN	ECEKAFSQKS
490	500	510	520	530	540	550	560
YLIIHQRTHT	EEKPYKCNEC	GKAFREKSKL	IIHQRIHTGE	KPYECPVCWK	AFSQKSQLII	HQRTHTGEKP	YACTECGKAF
570	580	590	600	610	620	630	
REKSTFTVHQ	RTHTGEKPYK	CTECGKAFTQ	KSNLIVHQRT	HAGKKAHGRG	HTRKSKFMAH		

Cmpd.	No. of Cmpds.	m/z meas.	Δ m/z [ppm]	z	Rt [min]	Score	P	Range	Sequence	Modification
2247	2	820.1114	178.53	2	58.3	14.6	2	223-235	K.TFSKKSHLIVHWR.T	

Detailed Protein Report

Protein 486: **PREDICTED: inactive ribonuclease-like protein 10 isoform X1 [Homo sapiens]**

**Accession:**	gi|530403621	**Score:**	27.9
**Database:**	refseq_human(refseq_human_20140103.fasta)	**MW [kDa]:**	27.0
**Database Date:**	2015-11-30	**pI:**	5.0
		**Sequence Coverage [%]:**	3.3
		**No. of unique Peptides:**	1

10	20	30	40	50	60	70	80
MWGAPLPRRP	VWDVRSASAG	PQPCLGGKMK	LNLVQIFFML	LMLLLGLGMG	LGLGLHMATA	VLEESDQPLN	EFWSSDSQDK
90	100	110	120	130	140	150	160
AEATEEGDGT	QTTETLVLSN	KEVVQPGWPE	DPILGEDEVG	GNKMLRASAL	FQSNKDYLRL	DQTDRECNDM	MAHKMKEPSQ
170	180	190	200	210	220	230	240
SCIAQYAFIH	EDLNTVKAVC	NSPVIACELK	GGKCHKSSRP	FDLTLCELSQ	PDQVTPNCNY	LTSVIKKHII	ITCNDMKRQL
250							
PTGQ							

Cmpd.	No. of Cmpds.	m/z meas.	Δ m/z [ppm]	z	Rt [min]	Score	P	Range	Sequence	Modification
1593	1	927.7563	290.39	1	49.8	27.9	0	1-8	-.MWGAPLPR.R	

Detailed Protein Report

Protein 487: kinesin-like protein KIF1C [Homo sapiens]

**Accession:**	gi|40254834	**Score:**	27.9
**Database:**	refseq_human(refseq_human_20140103.fasta)	**MW [kDa]:**	122.9
**Database Date:**	2015-11-30	**pI:**	6.5
		**Sequence Coverage [%]:**	2.8
		**No. of unique Peptides:**	1

Alias proteins:

Accession	Name	Description
gi|530409731	refseq_human (refseq_human_20140103.fasta)	PREDICTED: kinesin-like protein KIF1C isoform X1 [Homo sapiens]

10	20	30	40	50	60	70	80
MAGASVKVAV	RVRPFNARET	SQDAKCVVSM	QGNTTSIINP	KQSKDAPKSF	TFDYSYWSHT	STEDPQFASQ	QQVYRDIGEE
90	100	110	120	130	140	150	160
MLLHAFEGYN	VCIFAYGQTG	AGKSYTMMGR	QEPGQQGIVP	QLCEDLFSRV	SENQSAQLSY	SVEVSYMEIY	CERVRDLLNP
170	180	190	200	210	220	230	240
KSRGSLRVRE	HPILGPYVQD	LSKLAVTSYA	DIADLMDCGN	KARTVAATNM	NETSSRSHAV	FTIVFTQRCH	DQLTGLDSEK
250	260	270	280	290	300	310	320
VSKISLVDLA	GSERADSSGA	RGMRLKEGAN	INKSLTTLGK	VISALADMQS	KKRKSDFIPY	RDSVLTWLLK	ENLGGNSRTA
330	340	350	360	370	380	390	400
MIAALSPADI	NYEETLSTLR	YADRTKQIRC	NAIINEDPNA	RLIRELQEEV	ARLRELLMAQ	GLSASALEGL	KTEEGSVRGA
410	420	430	440	450	460	470	480
LPAVSSPPAP	VSPSSPTTHN	GELEPSFSPN	TESQIGPEEA	MERLQETEKI	IAELNETWEE	KLRKTEALRM	EREALLAEMG
490	500	510	520	530	540	550	560
VAVREDGGTV	GVFSPKKTPH	LVNLNEDPLM	SECLLYHIKD	GVTRVGQVDM	DIKLTGQFIR	EQHCLFRSIP	QPDGEVVVTL
570	580	590	600	610	620	630	640
EPCEGAETYV	NGKLVTEPLV	LKSGNRIVMG	KNHVFRFNHP	EQARLERERG	VPPPPGPPSE	PVDWNFAQKE	LLEQQGIDIK
650	660	670	680	690	700	710	720
LEMEKRLQDL	ENQYRKEKEE	ADLLLEQQRL	YADSDSGDDS	DKRSCEESWR	LISSLREQLP	PTTVQTIVKR	CGLPSSGKRR
730	740	750	760	770	780	790	800
APRRVYQIPQ	RRRLQGKDPR	WATMADLKMQ	AVKEICYEVA	LADFRHGRAE	IEALAALKMR	ELCRTYGKPD	GPGDAWRAVA
810	820	830	840	850	860	870	880
RDVWDTVGEE	EGGGAGSGGG	SEEGARGAEV	EDLRAHIDKL	TGILQEVKLQ	NSSKDRELQA	LRDRMLRMER	VIPLAQDHED
890	900	910	920	930	940	950	960
ENEEGGEVPW	APPEGSEAAE	EAAPSDRMPS	ARPPSPPLSS	WERVSRLMEE	DPAFRRGRLR	WLKQEQLRLQ	GLQGSGGRGG
970	980	990	1000	1010	1020	1030	1040
GLRRPPARFV	PPHDCKLRFP	FKSNPQHRES	WPGMGSGEAP	TPLQPPEEVT	PHPATPARRP	PSPRRSHHPR	RNSLDGGGRS
1050	1060	1070	1080	1090	1100	1110	
RGAGSAQPEP	QHFQPKKHNS	YPQPPQPYPA	QRPPGPRYPP	YTTPPRMRRQ	RSAPDLKESG	AAV	

Cmpd.	No. of Cmpds.	m/z meas.	Δ m/z [ppm]	z	Rt [min]	Score	P	Range	Sequence	Modification
1891	1	902.3321	-145.45	2	53.7	11.9	1	244-261	K.ISLVDLAGSERADSSGAR.G	

Detailed Protein Report

Protein 488: trafficking protein particle complex subunit 9 isoform a [Homo sapiens]

**Accession:**	gi|238624122	**Score:**	27.9
**Database:**	refseq_human(refseq_human_20140103.fasta)	**MW [kDa]:**	139.3
**Database Date:**	2015-11-30	**pI:**	6.9
		**Sequence Coverage [%]:**	2.7
		**No. of unique Peptides:**	1

Quantitation

mdown:qdown	**Median:**	2.97	**CV:**	0.00 %	**No. of Peptides:**	1
Wdown:Qdown	**Median:**	1.74	**CV:**	0.00 %	**No. of Peptides:**	1

10	20	30	40	50	60	70	80
MVPAGDQDRA	PHRGKPAQAG	ARTSRASRAL	RSWRRSQAAR	ATVTHPRGGH	DRGSHGGYRE	GHRGCRRDPQ	WASAGPPPLS
90	100	110	120	130	140	150	160
FTEEVKFELR	ALKDWDFKMS	VPDYMQCAED	HQTLLVVVQP	VGIVSEENFF	RIYKRICSVS	QISVRDSQRV	LYIRYRHHYP
170	180	190	200	210	220	230	240
PENNEWGDFQ	THRKVVGLIT	ITDCFSAKDW	PQTFEKFHVQ	KEIYGSTLYD	SRLFVFGLQG	EIVEQPRTDV	AFYPNYEDCQ
250	260	270	280	290	300	310	320
TVEKRIEDFI	ESLFIVLESK	RLDRATDKSG	DKIPLLCVPF	EKKDFVGLDT	DSRHYKKRCQ	GRMRKHVGDL	CLQAGMLQDS
330	340	350	360	370	380	390	400
LVHYHMSVEL	LRSVNDFLWL	GAALEGLCSA	SVIYHYPGGT	GGKSGARRFQ	GSTLPAEAAN	RHRPGAQEVL	IDPGALTTNG
410	420	430	440	450	460	470	480
INPDTSTEIG	RAKNCLSPED	IIDKYKEAIS	YYSKYKNAGV	IELEACIKAV	RVLAIQKRSM	EASEFLQNAV	YINLRQLSEE
490	500	510	520	530	540	550	560
EKIQRYSILS	ELYELIGFHR	KSAFFKRVAA	MQCVAPSIAE	PGWRACYKLL	LETLPGYSLS	LDPKDFSRGT	HRGWAAVQMR
570	580	590	600	610	620	630	640
LLHELVYASR	RMGNPALSVR	HLSFLLQTML	DFLSDQEKKD	VAQSLENYTS	KCPGTMEPIA	LPGGLTLPPV	PFTKLPIVRH
650	660	670	680	690	700	710	720
VKLLNLPASL	RPHKMKSLLG	QNVSTKSPFI	YSPIIAHNRG	EERNKKIDFQ	WVQGDVCEVQ	LMVYNPMPFE	LRVENMGLLT
730	740	750	760	770	780	790	800
SGVEFESLPA	ALSLPAESGL	YPVTLVGVPQ	TTGTITVNGY	HTTVFGVFSD	CLLDNLPGIK	TSGSTVEVIP	ALPRLQISTS
810	820	830	840	850	860	870	880
LPRSAHSLQP	SSGDEISTNV	SVQLYNGESQ	QLIIKLENIG	MEPLEKLEVT	SKVLTTKEKL	YGDFLSWKLE	ETLAQFPLQP
890	900	910	920	930	940	950	960
GKVATFTINI	KVKLDFSCQE	NLLQDLSDDG	ISVSGFPLSS	PFRQVVRPRV	EGKPVNPPES	NKAGDYSHVK	TLEAVLNFKY
970	980	990	1000	1010	1020	1030	1040
SGGPGHTEGY	YRNLSLGLHV	EVEPSVFFTR	VSTLPATSTR	QCHLLLDVFN	STEHELTVST	RSSEALILHA	GECQRMAIQV
1050	1060	1070	1080	1090	1100	1110	1120
DKFNFESFPE	SPGEKGQFAN	PKQLEEERRE	ARGLEIHSKL	GICWRIPSLK	RSGEASVEGL	LNQLVLEHLQ	LAPLQWDVLV
1130	1140	1150	1160	1170	1180	1190	1200
DGQPCDREAV	AACQVGDPVR	LEVRLTNRSP	RSVGPFALTV	VPFQDHQNGV	HNYDLHDTVS	FVGSSTFYLD	AVQPSGQSAC
1210	1220	1230	1240	1250			
LGALLFLYTG	DFFLHIRFHE	DSTSKELPPS	WFCLPSVHVC	ALEAQA			

Cmpd.	No. of Cmpds.	m/z meas.	Δ m/z [ppm]	z	Rt [min]	Score	P	Range	Sequence	Modification	Ratios
69	1	820.3460	-71.39	3	30.1	12.0	1	951-972	K.TLEAVLNFKYSGGPGHTEGYYR.N		Wdown:Qdown 1.74<br>mdown:qdown 2.97

Detailed Protein Report

Protein 489: PREDICTED: regulator of G-protein signaling 22 isoform X8 [Homo sapiens]

**Accession:**	gi|578815920	**Score:**	27.8
**Database:**	refseq_human(refseq_human_20140103.fasta)	**MW [kDa]:**	121.2
**Database Date:**	2015-11-30	**pI:**	6.5
**Modification(s):**	Carbamidomethyl	**Sequence Coverage [%]:**	2.1
		**No. of unique Peptides:**	1

10	20	30	40	50	60	70	80
MPEKRLTAEP	PTITEEEFED	SLATDDFLVD	YFNEFLSLPT	FSEAIRFNAD	YGVFEVANDA	PQFLEKQLKK	ILQNQQPRNP
**90**	**100**	**110**	**120**	**130**	**140**	**150**	**160**
IYDVVRKGKN	EVKPVQMNAP	DEDETINVNY	NIMCLSREEG	IKWIKKERLP	AFLESDCYFE	YRLAKLVSQV	RWSKSGMNFT
**170**	**180**	**190**	**200**	**210**	**220**	**230**	**240**
VGSNFSPWIV	KKPPSLPPPA	TEEDNLVIMK	KFYVSLGEAS	YTQTKDWFAL	AKQSQQTVST	FSLPCCVPYN	KLKSPAISSV
**250**	**260**	**270**	**280**	**290**	**300**	**310**	**320**
SENFIFDDGV	HPRTKKDPSK	TNKLISEFEE	EEGEEEEVSV	SLQDTPSQAL	LRVYLEKKQD	VDESLTMHFS	TCEEFLSSYI
**330**	**340**	**350**	**360**	**370**	**380**	**390**	**400**
YFILRGAIQQ	IVGKPVGETP	DYINFNNITK	VSFDDCFESI	HGKNFLSELV	QTTKERSEEI	EQTSLSSKNE	SAGPESRADW
**410**	**420**	**430**	**440**	**450**	**460**	**470**	**480**
CISHRTYDIG	NRKEFERFKK	FIKGTLGERY	WWLWMDIERL	KVLKDPGRHQ	RHLEKMKKCY	LVSNGDYYLS	AEILSKFKLL
**490**	**500**	**510**	**520**	**530**	**540**	**550**	**560**
DGSQWNEEHL	RNIQSEVLKP	LLLYWAPRFC	VTHSASTKYA	SAELKFWHLR	QAKPRKDIDP	FPQMATLLPL	RPKSCIPQIP
**570**	**580**	**590**	**600**	**610**	**620**	**630**	**640**
EIQKEEFSLS	QPPKSPNKSP	EVKTATQKPW	KRELLYPGSS	KDDVIEKGSK	YMSESSKVIH	LTSFTDISEC	LKPQLDRRYA
**650**	**660**	**670**	**680**	**690**	**700**	**710**	**720**
YTEEPRVKTV	SDVGALGGSD	MENLLQSLYV	ENRAGFFFTK	FCEHSGNKLW	KNSVYFWFDL	QAYHQLFYQE	TLQPFKVCKQ
**730**	**740**	**750**	**760**	**770**	**780**	**790**	**800**
AQYLFATYVA	PSATLDIGLQ	QEKKKEIYMK	IQPPFEDLFD	TAEEYILLLL	LEPWTKMVKS	DQIAYKKVEL	VEETRQLDST
**810**	**820**	**830**	**840**	**850**	**860**	**870**	**880**
YFRKLQALHK	ETFSKKAEVQ	MKDIAEELLL	QKAEKKIGVW	KDLCHSHCDE	SVIQKKITTI	INCFINSSIP	PALQIDIPVE
**890**	**900**	**910**	**920**	**930**	**940**	**950**	**960**
QAQKIIEHRK	ELGPYVFREA	QMTIFGVLFK	FWPQFCEFRK	NLTDENIMSV	LERRQEYNKQ	KKKLAVLEDE	KSGKDGIKQY
**970**	**980**	**990**	**1000**	**1010**	**1020**	**1030**	**1040**
ANTSVPAIKT	ALLSDSFLGL	QPYGRQPTWC	YSKYIEALEQ	ERILLKIQEE	LEKKLFAGLQ	PLTNFKASSS	TMSLKKNMSA
**1050**							
HSSQK							

Cmpd.	No. of Cmpds.	m/z meas.	Δ m/z [ppm]	z	Rt [min]	Score	P	Range	Sequence	Modification
2278	1	557.5521	-50.47	3	58.7	12.0	0	842-855	K.DLCHSHCDESVIQK.K	Carbamidomethyl: 7

Detailed Protein Report

Protein 490: **putative pre-mRNA-splicing factor ATP-dependent RNA helicase DHX16 isoform 1 [Homo sapiens]**

Accession: gi|255982614
Database: refseq_human(refseq_human_20140103.fasta)
Database Date: 2015-11-30
Modification(s): Carbamidomethyl, Oxidation

Score: 27.8
MW [kDa]: 119.2
pI: 6.4
Sequence Coverage [%]: 3.0
No. of unique Peptides: 1

10	20	30	40	50	60	70	80
MATPAGLERW	VQDELHSVLG	LSERHVAQFL	IGTAQRCTSA	EEFVQRLRDT	DTLDLSGPAR	DFALRLWNKV	PRKAVVEKPA
**90**	**100**	**110**	**120**	**130**	**140**	**150**	**160**
RAAEREARAL	LEKNRSYRLL	EDSEESSEET	VSRAGSSLQK	KRKKRKHLRK	KREEEEEEEA	SEKGKKKTGG	SKQQTEKPES
**170**	**180**	**190**	**200**	**210**	**220**	**230**	**240**
EDEWERTERE	RLQDLEERDA	FAERVRQRDK	DRTRNVLERS	DKKAYEEAQK	RLKMAEEDRK	AMVPELRKKS	RREYLAKRER
**250**	**260**	**270**	**280**	**290**	**300**	**310**	**320**
EKLEDLEAEL	ADEEFLFGDV	ELSRHERQEL	KYKRRVRDLA	REYRAAGEQE	KLEATNRYHM	PKETRGQPAR	AVDLVEEESG
**330**	**340**	**350**	**360**	**370**	**380**	**390**	**400**
APGEEQRRWE	EARLGAASLK	FGARDAASQE	PKYQLVLEEE	ETIEFVRATQ	LQGDEEPSAP	PTSTQAQQKE	SIQAVRRSLP
**410**	**420**	**430**	**440**	**450**	**460**	**470**	**480**
VFPFREELLA	AIANHQVLII	EGETGSGKTT	QIPQYLFEEG	YTNKGMKIAC	TQPRRVAAMS	VAARVAREMG	VKLGNEVGYS
**490**	**500**	**510**	**520**	**530**	**540**	**550**	**560**
IRFEDCTSER	TVLRYMTDGM	LLREFLSEPD	LASYSVVMVD	EAHERTLHTD	ILFGLIKDVA	RFRPELKVLV	ASATMDTARF
**570**	**580**	**590**	**600**	**610**	**620**	**630**	**640**
STFFDDAPVF	RIPGRRFPVD	IFYTKAPEAD	YLEACVVSVL	QIHVTQPPGD	ILVFLTGQEE	IEAACEMLQD	RCRRLGSKIR
**650**	**660**	**670**	**680**	**690**	**700**	**710**	**720**
ELLVLPIYAN	LPSDMQARIF	QPTPPGARKV	VVATNIAETS	LTIEGIIYVL	DPGFCKQKSY	NPRTGMESLT	VTPCSKASAN
**730**	**740**	**750**	**760**	**770**	**780**	**790**	**800**
QRAGRAGRVA	AGKCFRLYTA	WAYQHELEET	TVPEIQRTSL	GNVVLLLKSL	GIHDLMHFDF	LDPPPYETLL	LALEQLYALG
**810**	**820**	**830**	**840**	**850**	**860**	**870**	**880**
ALNHLGELTT	SGRKMAELPV	DPMLSKMILA	SEKYSCSEEI	LTVAAMLSVN	NSIFYRPKDK	VVHADNARVN	FFLPGGDHLV
**890**	**900**	**910**	**920**	**930**	**940**	**950**	**960**
LLNVYTQWAE	SGYSSQWCYE	NFVQFRSMRR	ARDVREQLEG	LLERVEVGLS	SCQGDYIRVR	KAITAGYFYH	TARLTRSGYR
**970**	**980**	**990**	**1000**	**1010**	**1020**	**1030**	**1040**
TVKQQQTVFI	HPNSSLFEQQ	PRWLLYHELV	LTTKEFMRQV	LEIESSWLLE	VAPHYYKAKE	LEDPHAKKMP	KKIGKTREEL
**1050**							
G							

Cmpd.	No. of Cmpds.	m/z meas.	Δ m/z [ppm]	z	Rt [min]	Score	P	Range	Sequence	Modification
2134	1	937.3539	-151.57	2	55.3	12.3	2	448-464	K.IACTQPRRVAAMSVAAR.V	Carbamidomethyl: 3; Oxidation: 12

Detailed Protein Report

Protein 491: **PREDICTED: zinc finger protein 778 isoform X4 [Homo sapiens]**

**Accession:**	gi|578828903	**Score:**	27.8
**Database:**	refseq_human(refseq_human_20140103.fasta)	**MW [kDa]:**	66.5
**Database Date:**	2015-11-30	**pI:**	9.6
		**Sequence Coverage [%]:**	4.1
		**No. of unique Peptides:**	1

Quantitation

mdown:qdown **Median:** 0.60 **CV:** 0.00 % **No. of Peptides:** 1

10	20	30	40	50	60	70	80
MAAPDLAHGG	HVSRDSVCLH	EEQTQAAGMV	AGWLINCYQD	AVTFDDVAVD	FTQEEWTLLD	PSQRDLYRDV	MLENYENLAS
**90**	**100**	**110**	**120**	**130**	**140**	**150**	**160**
VGHHLFQPSV	IYWLEQEEEL	RAGRRAVLQE	WRLKTKGPAL	RQDRSWFRAS	NETQTARSHN	GGQLCDRTQC	GEAFSEHSGL
**170**	**180**	**190**	**200**	**210**	**220**	**230**	**240**
STHVRTQNTG	DSCVSNHYER	DFFIPCQKTL	FKIGEQFSVL	GQCGKAFSST	PNVVSQQACT	RDRSLDYSSC	GEVFLNQSYL
**250**	**260**	**270**	**280**	**290**	**300**	**310**	**320**
QARAGSHNGE	ETWKWKPCGK	ALTHSMGCAT	PVEMHAVRNP	HVCRECGKAF	RYTAYLTGRV	QVHPGEKPCE	LEECGKASPV
**330**	**340**	**350**	**360**	**370**	**380**	**390**	**400**
SSSLTQHVRI	HAAEKPCECK	ECGKAFTGLS	GLSKHVQTDP	GQKPYECKDC	GKAYNRVYLL	NEHVKTHTEE	KPFICTVCRK
**410**	**420**	**430**	**440**	**450**	**460**	**470**	**480**
SFRNSSCLNK	HIQIHTGIKP	YECKDCGKTF	TVSSSLTEHI	RTHTGEKPYE	CKVCGKAFTT	SSHLIVHIRT	HTGEKPYICK
**490**	**500**	**510**	**520**	**530**	**540**	**550**	**560**
ECGKAFASSS	HLIEHRRTHT	GEKPYICNEC	GKAFRASSHL	HKHGRIHTGQ	KPYKCKECGK	AYNRFYLLKE	HLKTYTEEQV
**570**	**580**	**590**					
FVCKDCGKSF	KNSSCLNHHT	QIHTDEKPF					

Cmpd.	No. of Cmpds.	m/z meas.	Δ m/z [ppm]	z	Rt [min]	Score	P	Range	Sequence	Modification	Ratios
1559	1	977.1043	137.72	2	48.2	11.4	2	122-137	R.QDRSWFRASNETQTAR.S		mdown:qdown 0.60

Detailed Protein Report

Protein 492: PREDICTED: cullin-9 isoform X3 [Homo sapiens]

**Accession:**	gi|578811520	**Score:**	27.8
**Database:**	refseq_human(refseq_human_20140103.fasta)	**MW [kDa]:**	268.2
**Database Date:**	2015-11-30	**pI:**	5.3
**Modification(s):**	Oxidation	**Sequence Coverage [%]:**	1.3
		**No. of unique Peptides:**	2

Detailed Protein Report

10	20	30	40	50	60	70	80
MVGERHAGDL	MVPLGPRLQA	YPEELIRQRP	GHDGHPEYLI	RWSVLKCGEV	GKVGVEEGKA	EHILMWLSAP	EVYANCPGLL
90	100	110	120	130	140	150	160
GERALSKGLQ	HEPAGVSGSF	PRDPGGLDEV	AMGEMEADVQ	ALVRRAARQL	AESGTPSLTA	AVLHTIHVLS	AYASIGPLTG
170	180	190	200	210	220	230	240
VFRETGALDL	LMHMLCNPEP	QIRRSAGKML	QALAAHDAGS	RAHVLLSLSQ	QDGIEQHMDF	DSRYTLLELF	AETTSSEEHC
250	260	270	280	290	300	310	320
MAFEGIHLPQ	IPGKLLFSLV	KRYLCVTSLL	DQLNSSPELG	AGDQSSPCAT	REKSRGQREL	EFSMAVGNLI	SELVRSMGWA
330	340	350	360	370	380	390	400
RNLSEQGMSP	PRPTRSIFQP	YISGPSLLLP	TIVTTPRRQG	WVFRQRSEFS	SRSGYGEYVQ	QTLQPGMRVR	MLDDYEEISA
410	420	430	440	450	460	470	480
GDEGEFRQSN	NGIPPVQTLG	EKALGEISVS	VEMAESLLQV	LSSRFEGSTL	NDLLNSQIYT	KYGLLSNEPS	SSSTSRNHSC
490	500	510	520	530	540	550	560
TPDPEEESKS	EASFSEEETE	SLKAKAEAPK	TEAEPTKTRT	ETPMAQSDSQ	LFNQLLVTEG	MTLPTEMKEA	ASEMARALRG
570	580	590	600	610	620	630	640
PGPRSSLDQH	VAAVVATVQI	SSLDTNLQLS	GLSALSQAVE	EVTERDHPLV	RPDRSLREKL	VKMLVELLTN	QVGEKMVVVQ
650	660	670	680	690	700	710	720
ALRLLYLLMT	KHEWRPLFAR	EGGIYAVLVC	MQEYKTSVLV	QQAGLAALKM	LAVASSSEIP	TFVTGRDSIH	SLFDAQMTRE
730	740	750	760	770	780	790	800
IFASIDSATR	PGSESLLLTV	PAAVILMLNT	EGCSSAARNG	LLLLNLLLCN	HHTLGDQIIT	QELRDTLFRH	SGIAPRTEPM
810	820	830	840	850	860	870	880
PTTRTILMML	LNRYSEPPGS	PERAALETPI	IQGQDGSPEL	LIRSLVGGPS	AELLLDLERV	LCREGSPGGA	VRPLLKRLQQ
890	900	910	920	930	940	950	960
ETQPFLLLLR	TLDAPGPNKT	LLLSVLRVIT	RLLDFPEAMV	LPWHEVLEPC	LNCLSGPSSD	SEIVQELTCF	LHRLASMHKD
970	980	990	1000	1010	1020	1030	1040
YAVVLCCLGA	KEILSKVLDK	HSAQLLLGCE	LRDLVTECEK	YAQLYSNLTS	SILAGCIQMV	LGQIEDHRRT	HQPINIPFFD
1050	1060	1070	1080	1090	1100	1110	1120
VFLRHLCQGS	SVEVKEDKCW	EKVEVSSNPH	RASKLTDHNP	KTYWESNGST	GSHYITLHMH	RGVLVRQLTL	LVASEDSSYM
1130	1140	1150	1160	1170	1180	1190	1200
PARVVVFGGD	STSCIGTELN	TVNVMPSASR	VILLENLNRF	WPIIQIRIKR	CQQGGIDTRV	RGVEVLGPKP	TFWPLFREQL
1210	1220	1230	1240	1250	1260	1270	1280
CRRTCLFYTI	RAQAWSRDIA	EDHRRLLQLC	PRLNRVLRHE	QNFADRFLPD	DEAAQALGKT	CWEALVSPLV	QNITSPDAEG
1290	1300	1310	1320	1330	1340	1350	1360
VSALGWLLDQ	YLEQRETSRN	PLSRAASFAS	RVRRLCHLLV	HVEPPPGPSP	EPSTRPFSKN	SKGRDRSPAP	SPVLPSSSLR
1370	1380	1390	1400	1410	1420	1430	1440
NITQCWLSVV	QEQVSRFLAA	AWRAPDFVPR	YCKLYEHLQR	AGSELFGPRA	AFMLALRSGF	SGALLQQSFL	TAAHMSEQFA
1450	1460	1470	1480	1490	1500	1510	1520
RYIDQQIQGG	LIGGAPGVEM	LGQLQRHLEP	IMVLSGLELA	TTFEHFYQHY	MADRLLSFGS	SWLEGAVLEQ	IGLCFPNRLP
1530	1540	1550	1560	1570	1580	1590	1600
QLMLQSLSTS	EELQRQFHLF	QLQRLDKLFL	EQEDEEEKRL	EEEEEEEEEE	EAEKELFIED	PSPAISILVL	SPRCWPVSPL
1610	1620	1630	1640	1650	1660	1670	1680
CYLYHPRKCL	PTEFCDALDR	FSSFYSQSQN	HPVLDMGPHR	RLQWTWLGRA	ELQFGKQILH	VSTVQMWLLL	KFNQTEEVSV
1690	1700	1710	1720	1730	1740	1750	1760
ETLLKDSDLS	PELLLQALVP	LTSGNGPLTL	HEGQDFPHGG	VLRLHEPGPQ	RSGEALWLIP	PQAYLNVEKD	EGRTLEQKRN
1770	1780	1790	1800	1810	1820	1830	1840
LLSCLLVRIL	KAHGEKGLHI	DQLVCLVLEA	WQKGPNPPGT	LGHTVAGGVA	CTSTDVLSCI	LHLLGQGYVK	RRDDRPQILM
1850	1860	1870	1880	1890	1900	1910	1920
YAAPEPMGPC	RGQADVPFCG	SQSETSKPSP	EAVATLASLQ	LPAGRTMSPQ	EVEGLMKQTV	RQVQETLNLE	PDVAQHLLAH
1930	1940	1950	1960	1970	1980	1990	2000
SHWGAEQLLQ	SYSEDPEPLL	LAAGLCVHQA	QAVPVRPDHC	PVCVSPLGCD	DDLPSLCCMH	YCCKSCWNEY	LTTRIEQNLV
2010	2020	2030	2040	2050	2060	2070	2080
LNCTCPIADC	PAQPTGAFIR	AIVSSPEVIS	KYEKALLRGY	VESCSNLTWC	TNPQGCDRIL	CRQGLGCGTT	CSKCGWASCF
2090	2100	2110	2120	2130	2140	2150	2160
NCSFPEAHYP	ASCGHMSQWV	DDGGYYDGMS	VEAQSKHLAK	LISKRCPSCQ	APIEKNEGCL	HMTCAKCNHG	FCWRCLKSWK
2170	2180	2190	2200	2210	2220	2230	2240

Detailed Protein Report

Cmpd.	No. of Cmpds.	m/z meas.	Δ m/z [ppm]	z	Rt [min]	Score	P	Range	Sequence	Modification
2528	1	938.1784	46.28	3	60.3	13.1	2	164-188	R.ETGALDLLMHMLCNPEPQIRRSAGK.M	Oxidation: 9, 11
949	1	737.3420	-85.80	1	40.4	14.7	0	790-796	R.HSGIAPR.T	

Detailed Protein Report

Protein 493: probable guanine nucleotide exchange factor MCF2L2 [Homo sapiens]

**Accession:**	gi|540344580	**Score:**	27.8
**Database:**	refseq_human(refseq_human_20140103.fasta)	**MW [kDa]:**	126.9
**Database Date:**	2015-11-30	**pI:**	6.0
		**Sequence Coverage [%]:**	3.1
		**No. of unique Peptides:**	1

10	20	30	40	50	60	70	80
MLSCLKEEMP	PQELTRRLAT	VITHVDEIMQ	QEVRPLMAVE	IIEQLHRQFA	ILSGGRGEDG	APIITFPEFS	GFKHIPDEDF
90	100	110	120	130	140	150	160
LNVMTYLTSI	PSVEAASIGF	IVVIDRRRDK	WSSVKASLTR	IAVAFPGNLQ	LIFILRPSRF	IQRTFTDIGI	KYYRNEFKTK
170	180	190	200	210	220	230	240
VPIIMVNSVS	DLHGYIDKSQ	LTRELGGTLE	YRHGQWVNHR	TAIENFALTL	KTTAQMLQTF	GSCLATAELP	RSMLSTEDLL
250	260	270	280	290	300	310	320
MSHTRQRDKL	QDELKLLGKQ	GTTLLSCIQE	PATKCPNSKL	NLNQLENVTT	MERLLVQLDE	TEKAFSHFWS	EHHLKLNQCL
330	340	350	360	370	380	390	400
QLQHFEHDFC	KAKLALDNLL	EEQAEFTGIG	DSVMHVEQIL	KEHKKLEEKS	QEPLEKAQLL	ALVGDQLIQS	HHYAADAIRP
410	420	430	440	450	460	470	480
RCVELRHLCD	DFINGNKKKW	DILGKSLEFH	RQLDKVSQWC	EAGIYLLASQ	AVDKCQSREG	VDIALNDIAT	FLGTVKEYPL
490	500	510	520	530	540	550	560
LSPKEFYNEF	ELLLTLDAKA	KAQKVLQRLD	DVQEIFHKRQ	VSLMKLAAKQ	TRPVQPVAPH	PESSPKWVSS	KTSQPSTSVP
570	580	590	600	610	620	630	640
LARPLRTSEE	PYTETELNSR	GKEDDETKFE	VKSEEIFESH	HERGNPELEQ	QARLGDLSPR	RRIIRDLLET	EEIYIKEIKS
650	660	670	680	690	700	710	720
IIDGYITPMD	FIWLKHLIPD	VLQNNKDFLF	GNIRELYEFH	NRTFLKELEK	CAENPELLAH	CFLKRKEDLQ	IYFKYHKNLP
730	740	750	760	770	780	790	800
RARAIWQECQ	DCAYFGVCQR	QLDHNLPLFK	YLKGPSQRLI	KYQMLLKGLL	DFESPEDMEI	DPGELGGSAK	DGPKRTKDSA
810	820	830	840	850	860	870	880
FSTELQQALA	VIEDLIKSCE	LAVDLAAVTE	CPDDIGKLGK	LLLHGPFSVW	TIHKDRYKMK	DLIRFKPSQR	QIYLFERGIV
890	900	910	920	930	940	950	960
FCKIRMEPGD	QGLSPHYSFK	KAMKLMTLSI	RQLGRGSHRK	FEIASRNGLE	KYILQAASKE	IRDCWFSEIS	KLLMEQQNNI
970	980	990	1000	1010	1020	1030	1040
KDQGNPQFEM	STSKGSGAGS	GPWIKNMERA	TTSKEDPASS	TGGIKGCSSR	EFSSMDTFED	CEGAEDMEKE	SSALSLAGLF
1050	1060	1070	1080	1090	1100	1110	1120
QSDDSHETCS	SKSAFLERGE	SSQGEKEERD	EEETATRSTE	EERAGASTGR	LAPAGATAGF	QARALRPRTS	AQES

Cmpd.	No. of Cmpds.	m/z meas.	Δ m/z [ppm]	z	Rt [min]	Score	P	Range	Sequence	Modification
2845	1	938.5013	-5.79	2	64.1	16.3	0	459-476	R.EGVDIALNDIATFLGTVK.E	

Detailed Protein Report

Protein 494: PREDICTED: uncharacterized protein LOC101928647 [Homo sapiens]

Accession: gi|530368444
Database: refseq_human(refseq_human_20140103.fasta)
Database Date: 2015-11-30
Modification(s): Carbamidomethyl, Oxidation

Score: 27.8
MW [kDa]: 25.5
pI: 12.1
Sequence Coverage [%]: 13.3
No. of unique Peptides: 2

10	20	30	40	50	60	70	80
MEISLAGISA	CLRGLALLRG	EVTVLTLKSR	FQEAGDEAAP	FPNPRFCHLP	LLPFSKVQIT	GYPSGPATPS	FLIPDPSKYE
**90**	**100**	**110**	**120**	**130**	**140**	**150**	**160**
APRIWVSSCF	PCLVTRPVCR	GQRRELLPPH	GGAREPPKDR	AATAPHLLPI	PAPPARAQKE	PRPQPSRAGQ	PGAHLEGPGS
**170**	**180**	**190**	**200**	**210**	**220**	**230**	**240**
GRVAAASSPC	GSCCGLRVAP	APGSSSSRSP	TLHPFSTPLC	KSKAFRSSLG	AAAGAAKAAG	GAQQPSGSRG	GVARAAGEAP
**250**							
RCPARPPS							

Cmpd.	No. of Cmpds.	m/z meas.	Δ m/z [ppm]	z	Rt [min]	Score	P	Range	Sequence	Modification
593	1	690.2064	-214.88	2	36.3	15.6	0	1-13	-.MEISLAGISACLR.G	Oxidation: 1
27	1	1182.5709	-22.85	2	29.6	12.2	1	84-103	R.IWVSSCFPCLVTRPVCRGQR.R	Carbamidomethyl: 9

Detailed Protein Report

Protein 495: PREDICTED: nephronectin isoform X3 [Homo sapiens]

**Accession:**	gi|530377475	**Score:**	27.8
**Database:**	refseq_human(refseq_human_20140103.fasta)	**MW [kDa]:**	50.2
**Database Date:**	2015-11-30	**pI:**	9.8
		**Sequence Coverage [%]:**	4.3
		**No. of unique Peptides:**	1

Alias proteins:

Accession	Name	Description
gi|578808996	refseq_human (refseq_human_20140103.fasta)	PREDICTED: nephronectin isoform X4 [Homo sapiens]

10	20	30	40	50	60	70	80
MNTYGSYKCY	CLNGYMLMPD	GSCSSALTCS	MANCQYGCDV	VKGQIRCQCP	SPGLQLAPDG	RTCVDVDECA	TGRASCPRFR
**90**	**100**	**110**	**120**	**130**	**140**	**150**	**160**
QCVNTFGSYI	CKCHKGFDLM	YIGGKYQCHD	IDECSLGQYQ	CSSFARCYNI	RGSYKCKCKE	GYQGDGLTCV	YIPKVMIEPS
**170**	**180**	**190**	**200**	**210**	**220**	**230**	**240**
GPIHVPKGNG	TILKGDTGNN	NWIPDVGSTW	WPPKTPYIPP	IITNRPTSKP	TTRPTPKPTP	IPTPPPPPPL	PTELRTPLPP
**250**	**260**	**270**	**280**	**290**	**300**	**310**	**320**
TTPERPTTGL	TTIAPAASTP	PGGITVDNRV	QTDPQKPRGD	VFIPRQPSND	LFEIFEIERG	VSADDEAKDD	PGVLVHSCNF
**330**	**340**	**350**	**360**	**370**	**380**	**390**	**400**
DHGLCGWIRE	KDNDLHWEPI	RDPAGGQYLT	VSAAKAPGGK	AARLVLPLGR	LMHSGDLCLS	FRHKVTGLHS	GTLQVFVRKH
**410**	**420**	**430**	**440**	**450**	**460**	**470**	
GAHGAALWGR	NGGHGWRQTQ	ITLRGADIKS	VVFKGEKRRG	HTGEIGLDDV	SLKKGHCSEE	R	

Cmpd.	No. of Cmpds.	m/z meas.	Δ m/z [ppm]	z	Rt [min]	Score	P	Range	Sequence	Modification
2444	1	1044.1842	92.38	2	60.9	14.4	1	155-174	K.VMIEPSGPIHVPKGNGTILK.G	

Detailed Protein Report

Protein 496: mitochondrial dynamic protein MID49 isoform 3 [Homo sapiens]

**Accession:**	gi|222080060	**Score:**	27.8
**Database:**	refseq_human(refseq_human_20140103.fasta)	**MW [kDa]:**	20.5
**Database Date:**	2015-11-30	**pI:**	10.7
		**Sequence Coverage [%]:**	17.6
		**No. of unique Peptides:**	1

Quantitation

mdown:qdown	**Median:** 1.43	**CV:** 0.00 %	**No. of Peptides:** 1
Wdown:Qdown	**Median:** 0.30	**CV:** 0.00 %	**No. of Peptides:** 1

```
        10         20         30         40         50         60         70         80
MAEFSQKRGK RRSDEGLGSM VDFLLANARL VLGVGGAAVL GIATLAVKRF IDRATSPRDE DDTKADSWKE LSLLKATPHL
        90        100        110        120        130        140        150        160
QPRPPPAALS QPVLPLAPSS SAPGEAAGLR AGPCDHPSSP GGFGQTAGWR HRPGAAGLLS EQVPGTALWG IRAWGAALRR
       170        180        190        200        210
AAGGGCGPCA SPGATGAGAG PVEPGAGRGH CGEGPSLLGR AQDAA
```

Cmpd.	No. of Cmpds.	m/z meas.	Δ m/z [ppm]	z	Rt [min]	Score	P	Range	Sequence	Modification	Ratios
773	1	450.7679	13.60	2	38.5	10.8	1	153-160	R.AWGAALRR.A		mdown:qdown 1.43<br>Wdown:Qdown 0.30

Detailed Protein Report

Protein 497: **PREDICTED: T-lymphoma invasion and metastasis-inducing protein 1 isoform X4 [Homo sapiens]**

**Accession:**	gi|530419050	**Score:**	27.7
**Database:**	refseq_human(refseq_human_20140103.fasta)	**MW [kDa]:**	161.7
**Database Date:**	2015-11-30	**pI:**	6.4
**Modification(s):**	Carbamidomethyl	**Sequence Coverage [%]:**	2.4
		**No. of unique Peptides:**	1

Quantitation

Wdown:Qdown **Median:** 1.24 **CV:** 0.00 % **No. of Peptides:** 1

10	20	30	40	50	60	70	80
MGNAESQHVE	HEFYGEKHAS	LGRKHTSRSL	RLSHKTRRTR	HASSGKVIHR	NSEVSTRSSS	TPSIPQSLAE	NGLEPFSQDG
90	100	110	120	130	140	150	160
TLEDFGSPIW	VDRVDMGLRP	VSYTDSSVTP	SVDSSIVLTA	ASVQSMPDTE	ESRLYGDDAT	YLAEGGRRQH	SYTSNGPTFM
170	180	190	200	210	220	230	240
ETASFKKKRS	KSADIWREDS	LEFSLSDLSQ	EHLTSNEEIL	GSAEEKDCEE	ARGMETRASP	RQLSTCQRAN	SLGDLYAQKN
250	260	270	280	290	300	310	320
SGVTANGGPG	SKFAGYCRNL	VSDIPNLANH	KMPPAAAEET	PPYSNYNTLP	CRKSHCLSEG	ATNPQISHSN	SMQGRRAKTT
330	340	350	360	370	380	390	400
QDVNAGEGSE	FADSGIEGAT	TDTDLLSRRS	NATNSSYSPT	TGRAFVGSDS	GSSSTGDAAR	QGVYENFRRE	LEMSTTNSES
410	420	430	440	450	460	470	480
LEEAGSAHSD	EQSSGTLSSP	GQSDILLTAA	QGTVRKAGAL	AVKNFLVHKK	NKKVESATRR	KWKHYWVSLK	GCTLFFYESD
490	500	510	520	530	540	550	560
GRSGIDHNSI	PKHAVWVENS	IVQAVPEHPK	KDFVFCLSNS	LGDAFLFQTT	SQTELENWIT	AIHSACATAV	ARHHHKEDTL
570	580	590	600	610	620	630	640
RLLKSEIKKL	EQKIDMDEKM	KKMGEMQLSS	VTDSKKKKTI	LDQIFVWEQN	LEQFQMDLFR	FRCYLASLQG	GELPNPKRLL
650	660	670	680	690	700	710	720
AFASRPTKVA	MGRLGIFSVS	SFHALVAART	GETGVRRRTQ	AMSRSASKRR	SRFSSLWGLD	TTSKKKQGRP	SINQVFGEGT
730	740	750	760	770	780	790	800
EAVKKSLEGI	FDDIVPDGKR	EKEVVLPNVH	QHNPDCDIWV	HEYFTPSWFC	LPNNQPALTV	VRPGDTARDT	LELICKTHQL
810	820	830	840	850	860	870	880
DHSAHYLRLK	FLIENKMQLY	VPQPEEDIYE	LLYKEIEICP	KVTQSIHIEK	SDTAADTYGF	SLSSVEEDGI	RRLYVNSVKE
890	900	910	920	930	940	950	960
TGLASKKGLK	AGDEILEINN	RAADALNSSM	LKDFLSQPSL	GLLVRTYPEL	EEGVELLESP	PHRVDGPADL	GESPLAFLTS
970	980	990	1000	1010	1020	1030	1040
NPGHSLCSEQ	GSSAETAPEE	TEGPDLESSD	ETDHSSKSTE	QVAAFCRSLH	EMNPSDQSPS	PQDSTGPQLA	TMRQLSDADK
1050	1060	1070	1080	1090	1100	1110	1120
LRKVICELLE	TERTYVKDLN	CLMERYLKPL	QKETFLTQDE	LDVLFGNLTE	MVEFQVEFLK	TLEDGVRLVP	DLEKLEKVDQ
1130	1140	1150	1160	1170	1180	1190	1200
FKKVLFSLGG	SFLYYADRFK	LYSAFCASHT	KVPKVLVKAK	TDTAFKAFLD	AQNPKQQHSS	TLESYLIKPI	QRILKYPLLL
1210	1220	1230	1240	1250	1260	1270	1280
RELFALTDAE	SEEHYHLDVA	IKTMNKVASH	INEMQKIHEE	FGAVFDQLIA	EQTGEKKEVA	DLSMGDLLLH	TTVIWLNPPA
1290	1300	1310	1320	1330	1340	1350	1360
SLGKWKKEPE	LAAFVFKTAV	VLVYKDGSKQ	KKKLVGSHRL	SIYEDWDPFR	FRHMIPTEAL	QVRALASADA	EANAVCEIVH
1370	1380	1390	1400	1410	1420	1430	1440
VKSESEGRPE	RVFHLCCSSP	ESRKDFLKAV	HSILRDKHRR	QLLKTESLPS	SQQYVPFGGK	RLCALKGARP	AMSRAGTVGI
1450	1460						
RTFKIPVTPT	PQ						

Cmpd.	No. of Cmpds.	m/z meas.	Δ m/z [ppm]	z	Rt [min]	Score	P	Range	Sequence	Modification	Ratios
1363	1	942.5111	16.59	2	45.8	11.1	2	1139-1154	R.FKLYSAFCASHTKVPK.V	Carbamidomethyl: 8	Wdown:Qdown 1.24

Detailed Protein Report

Protein 498: **PREDICTED: serine/threonine-protein kinase Nek4 isoform X1 [Homo sapiens]**

**Accession:**	gi|578806508	**Score:**	27.7
**Database:**	refseq_human(refseq_human_20140103.fasta)	**MW [kDa]:**	89.9
**Database Date:**	2015-11-30	**pI:**	8.7
		**Sequence Coverage [%]:**	4.0
		**No. of unique Peptides:**	1

10	20	30	40	50	60	70	80
MPLAAYCYLR	VVGKGSYGEV	TLVKHRRDGK	QYVIKKLNLR	NASSRERRAA	EQEAQLLSQL	KHPNIVTYKE	SWEGGDGLLY
90	100	110	120	130	140	150	160
IVMGFCEGGD	LYRKLKEQKG	QLLPENQVVE	WFVQIAMALQ	YLHEKHILHR	DLKTQNVFLT	RTNIIKVGDL	GIARVLENHC
170	180	190	200	210	220	230	240
DMASTLIGTP	YYMSPELFSN	KPYNYKSDVW	ALGCCVYEMA	TLKHAFNAKD	MNSLVYRIIE	GKLPPMPRDY	SPELAELIRT
250	260	270	280	290	300	310	320
MLSKRPEERP	SVRSILRQPY	IKRQISFFLE	ATKIKTSKNN	IKNGDSQSKP	FATVVSGEAE	SNHEVIHPQP	LSSEGSQTYI
330	340	350	360	370	380	390	400
MGEGKCLSQE	KPRASGLLKS	PASLKAHTCK	QDLSNTTELA	TISSVNIDIL	PAKGRDSVSD	GFVQENQPRY	LDASNELGGI
410	420	430	440	450	460	470	480
CSISQVEEEM	LQDNTKSSAQ	PENLIPMWSS	DIVTGEKNEP	VKPLQPLIKE	QKPKDQDQVA	GECIIEKQGR	IHPDLQPHNS
490	500	510	520	530	540	550	560
GSEPSLSRQR	RQKRREQTEH	RGEKRQVRRD	LFAFQESPPR	FLPSHPIVGK	VDVTSTQKEA	ENQRRVVTGS	VSSSRSSEMS
570	580	590	600	610	620	630	640
SSKDRPLSAR	ERRRLKQSQE	EMSSSGPSVR	KASLSVAGPG	KPQEEDQPLP	ARRLSSDCSV	TQERKQIHCL	SEDELSSSTS
650	660	670	680	690	700	710	720
STDKSDGDYG	EGKGQTNEIN	ALVQLMTQTL	KLDSKESCED	VPVANPVSEF	KLHRKYRDTL	ILHGKVAEEA	EEIHFKELPS
730	740	750	760	770	780	790	800
AIMPGSEKIR	RLVEVLRTDV	IRGLGVQLLE	QVYDLLEEED	EFDREVRLRE	HMGEKYTTYS	VKARQLKFFE	ENMNF

Cmpd.	No. of Cmpds.	m/z meas.	Δ m/z [ppm]	z	Rt [min]	Score	P	Range	Sequence	Modification
1866	1	716.4002	35.77	3	52.0	13.8	0	592-612	K.ASLSVAGPGKPQEEDQPLPAR.R	

Detailed Protein Report

Protein 499: PREDICTED: histone acetyltransferase KAT2A isoform X2 [Homo sapiens]

**Accession:**	gi|578830713	**Score:**	27.7
**Database:**	refseq_human(refseq_human_20140103.fasta)	**MW [kDa]:**	54.1
**Database Date:**	2015-11-30	**pI:**	9.6
		**Sequence Coverage [%]:**	4.2
		**No. of unique Peptides:**	1

10	20	30	40	50	60	70	80
MLEEEIYGAN	SPIWESGFTM	PPSEGTQLVP	RPASVSAAVV	PSTPIFSPSM	GGGSNSSLSL	DSAGAEPMPG	EKRTLPENLT
90	100	110	120	130	140	150	160
LEDAKRLRVM	GDIPMELVNE	VMLTITDPAA	MLGPETSLLS	ANAARDETAR	LEERRGIIEF	HVIGNSLTPK	ANRRVLLWLV
170	180	190	200	210	220	230	240
GLQNVFSHQL	PRMPKEYIAR	LVFDPKHKTL	ALIKDGRVIG	GICFRMFPTQ	GFTEIVFCAV	TSNEQVKGYG	THLMNHLKEY
250	260	270	280	290	300	310	320
HIKHNILYFL	TYADEYAIGY	FKKQGFSKDI	KVPKSRYLGY	IKDYEGATLM	ECELNPRIPY	TELSHIIKKQ	KEVIIKKLIE
330	340	350	360	370	380	390	400
RKQAQIRKVY	PGLSCFKEGV	RQIPVESVPG	IRETGWKPLG	KEKGKELKDP	DQLYTTLKNL	LAQIKSHPSA	WPFMEPVKKS
410	420	430	440	450	460	470	480
EAPDYYEVIR	FPIDLKTMTE	RLRSRYYVTR	KLFVADLQRV	IANCREYNPP	DSEYCRCASA	LEKFFYFKLK	EGGLIDK

Cmpd.	No. of Cmpds.	m/z meas.	Δ m/z [ppm]	z	Rt [min]	Score	P	Range	Sequence	Modification
1555	1	699.2025	-339.50	1	49.3	15.6	1	269-274	K.DIKVPK.S	

Detailed Protein Report

Protein 500: engulfment and cell motility protein 1 isoform 2 [Homo sapiens]

**Accession:**	gi|18765702	**Score:**	27.7
**Database:**	refseq_human(refseq_human_20140103.fasta)	**MW [kDa]:**	28.7
**Database Date:**	2015-11-30	**pI:**	6.0
		**Sequence Coverage [%]:**	10.1
		**No. of unique Peptides:**	1

Alias proteins:

Accession	Name	Description
gi|86788140	refseq_human (refseq_human_20140103.fasta)	engulfment and cell motility protein 1 isoform 2 [Homo sapiens]

```
        10         20         30         40         50         60         70         80
MQVVKEQVMR ALTTKPSSLD QFKSKLQNLS YTEILKIRQS ERMNQEDFQS RPILELKEKI QPEILELIKQ QRLNRLVEGT
        90        100        110        120        130        140        150        160
CFRKLNARRR QDKFWYCRLS PNHKVLHYGD LEESPQGEVP HDSLQDKLPV ADIKAVVTGK DCPHMKEKGA LKQNKEVLEL
       170        180        190        200        210        220        230        240
AFSILYDSNC QLNFIAPDKH EYCIWTDGLN ALLGKDMMSD LTRNDLDTLL SMEIKLRLLD LENIQIPDAP PPIPKEPSNY
       250
DFVYDCN
```

Cmpd.	No. of Cmpds.	m/z meas.	Δ m/z [ppm]	z	Rt [min]	Score	P	Range	Sequence	Modification
1550	1	1011.5446	-2.78	2	49.2	15.4	2	128-146	K.LPVADIKAVVTGKDCPHMK.E	

Detailed Protein Report

Protein 501: dynein heavy chain 9, axonemal isoform 2 [Homo sapiens]

**Accession:**	gi|114155133	**Score:**	27.7
**Database:**	refseq_human(refseq_human_20140103.fasta)	**MW [kDa]:**	511.6
**Database Date:**	2015-11-30	**pI:**	5.6
**Modification(s):**	Oxidation	**Sequence Coverage [%]:**	0.7
		**No. of unique Peptides:**	1

Detailed Protein Report

10	20	30	40	50	60	70	80
MRLAEERAAL	AAENADGEPG	ADRRLRLLGT	YVAMSLRPAA	GAWERCAGSA	EAEQLLQAFL	GRDAAEGPRP	LLVVRPGPRG
90	100	110	120	130	140	150	160
LAIRPGLEVG	PESGLAGAKA	LFFLRTGPEP	PGPDSFRGAV	VCGDLPAAPL	EHLAALFSEV	VLPVLANEKN	RLNWPHMICE
170	180	190	200	210	220	230	240
DVRRHAHSLQ	CDLSVILEQV	KGKTLLPLPA	GSEKMEFADS	KSETVLDSID	KSVIYAIESA	VIKWSYQVQV	VLKRESSQPL
250	260	270	280	290	300	310	320
LQGENPTPKV	ELEFWKSRYE	DLKYIYNQLR	TITVRGMAKL	LDKLQSSYFP	AFKAMYRDVV	AALAEAQDIH	VHLIPLQRHL
330	340	350	360	370	380	390	400
EALENAEFPE	VKPQLRPLLH	VVCLIWATCK	SYRSPGRLTV	LLQEICNLLI	QQASNYLSPE	DLLRSEVEES	QRKLQVVSDT
410	420	430	440	450	460	470	480
LSFFKQEFQD	RRENLHTYFK	ENQEVKEWDF	QSSLVFVRLD	GFLGQLHVVE	GLLKTALDFH	KLGKVEFSGV	RGNALSQQVQ
490	500	510	520	530	540	550	560
QMHEEFQEMY	RLLSGSSSDC	LYLQSTDFEN	DVSEFNQKVE	DLDRRLGTIF	IQAFDDAPGL	EHAFKLLDIA	GNLLERPLVA
570	580	590	600	610	620	630	640
RDTSDKYLVL	IQMFNKDLDA	VRMIYSQHVQ	EEAELGFSPV	HKNMPTVAGG	LRWAQELRQR	IQGPFSNFGR	ITHPCMESAE
650	660	670	680	690	700	710	720
GKRMQQKYED	MLSLLEKYET	RLYEDWCRTV	SEKSQYNLSQ	PLLKRDPETK	EITINFNPQL	ISVLKEMSYL	EPREMKHMPE
730	740	750	760	770	780	790	800
TAAAMFSSRD	FYRQLVANLE	LMANWYNKVM	KTLLEVEFPL	VEEELQNIDL	RLRAAEETLN	WKTEGICDYV	TEITSSIHDL
810	820	830	840	850	860	870	880
EQRIQKTKDN	VEEIQNIMKT	WVTPIFKTKD	GKRESLLSLD	DRHDRMEKYY	NLIKESGLKI	HALVQENLGL	FSADPTSNIW
890	900	910	920	930	940	950	960
KTYVNSIDNL	LLNGFFLAIE	CSLKYLLENT	ECKAGLTPIF	EAQLSLAIPE	LVFYPSLESG	VKGGFCDIVE	GLITSIFRIP
970	980	990	1000	1010	1020	1030	1040
SLVPRLSPQN	GSPHYQVDLD	GIPDLANMRR	TLMERVQRMM	GLCCGYQSTF	SQYSYLYVED	RKEVLGQFLL	YGHILTPEEI
1050	1060	1070	1080	1090	1100	1110	1120
EDHVEDGIPE	NPPLLSQFKV	QIDSYETLYE	EVCRLEPIKV	FDGWMKIDIR	PFKASLLNII	KRWSLLFKQH	LVDHVTHSLA
1130	1140	1150	1160	1170	1180	1190	1200
NLDAFIKKSE	SGLLKKVEKG	DFQGLVEIMG	HLMAVKERQS	NTDEMFEPLK	QTIELLKTYE	QELPETVFKQ	LEELPEKWNN
1210	1220	1230	1240	1250	1260	1270	1280
IKKVAITVKQ	QVAPLQANEV	TLLRQRCTAF	DAEQQQFWEQ	FHKEAPFRFD	SIHPHQMLDA	RHIEIQQMES	TMASISESAS
1290	1300	1310	1320	1330	1340	1350	1360
LFEVNVPDYK	QLRQCRKEVC	QLKELWDTIG	MVTSSIHAWE	TTPWRNINVE	AMELECKQFA	RHIRNLDKEV	RAWDAFTGLE
1370	1380	1390	1400	1410	1420	1430	1440
STVWNTLSSL	RAVAELQNPA	IRERHWRQLM	QATGVSFTMD	QDTTLAHLLQ	LQLHHYEDEV	RGIVDKAAKE	MGMEKTLKEL
1450	1460	1470	1480	1490	1500	1510	1520
QTTWAGMEFQ	YEPHPRTNVP	LLCSDEDLIE	VLEDNQVQLQ	NLVMSKYVAF	FLEEVSGWQK	KLSTVDAVIS	IWFEVQRTWT
1530	1540	1550	1560	1570	1580	1590	1600
HLESIFTGSE	DIRAQLPQDS	KRFEGIDIDF	KELAYDAQKI	PNVVQTTNKP	GLYEKLEDIQ	GRLCLCEKAL	AEYLDTKRLA
1610	1620	1630	1640	1650	1660	1670	1680
FPRFYFLSSS	DLLDILSNGT	APQQVQRHLS	KLFDNMAKMR	FQLDASGEPT	KTSLGMYSKE	EEYVAFSEPC	DCSGQVEIWL
1690	1700	1710	1720	1730	1740	1750	1760
NHVLGHMKAT	VRHEMTEGVT	AYEEKPREQW	LFDHPAQVAL	TCTQIWWTTE	VGMAFARLEE	GYESAMKDYY	KKQVAQLKTL
1770	1780	1790	1800	1810	1820	1830	1840
ITMLIGQLSK	GDRQKIMTIC	TIDVHARDVV	AKMIAQKVDN	AQAFLWLSQL	RHRWDDEVKH	CFANICDAQF	LYSYEYLGNT
1850	1860	1870	1880	1890	1900	1910	1920
PRLVITPLTD	RCYITLTQSL	HLTMSGAPAG	PAGTGKTETT	KDLGRALGIL	VYVFNCSEQM	DYKSCGNIYK	GLAQTGAWGC
1930	1940	1950	1960	1970	1980	1990	2000
FDEFNRISVE	VLSVVAVQVK	SIQDAIRDKK	QWFSFLGEEI	SLNPSVGIFI	TMNPGYAGRT	ELPENLKSLF	RPCAMVVPDF
2010	2020	2030	2040	2050	2060	2070	2080
ELICEIMLVA	EGFIEAQSLA	RKFITLYQLC	KELLSKQDHY	DWGLRAIKSV	LVVAGSLKRG	DPDRPEDQVL	MRSLRDFNIP
2090	2100	2110	2120	2130	2140	2150	2160
KIVTDDMPIF	MGLIGDLFPA	LDVPRRRDPN	FEALVRKAIV	DLKLQAEDNF	VLKVVQLEEL	LAVRHSVFVV	GGAGTGKSQV
2170	2180	2190	2200	2210	2220	2230	2240

Detailed Protein Report

Cmpd.	No. of Cmpds.	m/z meas.	Δ m/z [ppm]	z	Rt [min]	Score	P	Range	Sequence	Modification
106	1	1068.1367	75.32	2	30.1	13.8	2	182-201	K.GKTLLPLPAGSEKMEFADSK.S	Oxidation: 14

Detailed Protein Report

Protein 502: PERQ amino acid-rich with GYF domain-containing protein 2 isoform c [Homo sapiens]

Accession: gi|156766047
Database: refseq_human(refseq_human_20140103.fasta)
Database Date: 2015-11-30

Score: 27.6
MW [kDa]: 149.4
pI: 5.3
Sequence Coverage [%]: 1.6
No. of unique Peptides: 2

Quantitation

mdown:qdown **Median:** 2.00 **CV:** 94.41 % **No. of Peptides:** 2

10	20	30	40	50	60	70	80
MAAETQTLNF	GPEWLRALSS	GGSITSPPLS	PALPKYKLAD	YRYGREEMLA	LFLKDNKIPS	DLLDKEFLPI	LQEEPLPPLA
90	100	110	120	130	140	150	160
LVPFTEEEQR	NFSMSVNSAA	VLRLTGRGGG	GTVVGAPRGR	SSSRGRGRGR	GECGFYQRSF	DEVEGVFGRG	GGREMHRSQS
170	180	190	200	210	220	230	240
WEERGDRRFE	KPGRKDVGRP	NFEEGGPTSV	GRKHEFIRSE	SENWRIFREE	QNGEDEDGGW	RLAGSRRDGE	RWRPHSPGWR
250	260	270	280	290	300	310	320
EHMERRRRFE	FDFRDRDDER	GYRRVRSGSG	SIDDDRDSLP	EWCLEDAEEE	MGTFDSSGAF	LSLKKVQKEP	IPEEQEMDFR
330	340	350	360	370	380	390	400
PVDEGEECSD	SEGSHNEEAK	EPDKTNKKEG	EKTDRVGVEA	SEETPQTSSS	SARPGTPSDH	QSQEASQFER	KDEPKTEQTE
410	420	430	440	450	460	470	480
KAEEETRMEN	SLPAKVPSRG	DEMVADVQQP	LSQIPSDTAS	PLLILPPPVP	NPSPTLRPVE	TPVVGAPGMG	SVSTEPDDEE
490	500	510	520	530	540	550	560
GLKHLEQQAE	KMVAYLQDSA	LDDERLASKL	QEHRAKGVSI	PLMHEAMQKW	YYKDPQGEIQ	GPFNNQEMAE	WFQAGYFTMS
570	580	590	600	610	620	630	640
LLVKRACDES	FQPLGDIMKM	WGRVPFSPGP	APPPHMGELD	QERLTRQQEL	TALYQMQHLQ	YQQFLIQQQY	AQVLAQQQKA
650	660	670	680	690	700	710	720
ALSSQQQQQL	ALLLQQFQTL	KMRISDQNII	PSVTRSVSVP	DTGSIWELQP	TASQPTVWEG	GSVWDLPLDT	TTPGPALEQL
730	740	750	760	770	780	790	800
QQLEKAKAAK	LEQERREAEM	RAKREEEERK	RQEELRRQQE	EILRRQQEEE	RKRREEEELA	RRKQEEALRR	QREQEIALRR
810	820	830	840	850	860	870	880
QREEEERQQQ	EEALRRLEER	RREEEERRKQ	EELLRKQEEE	AAKWAREEEE	AQRRLEENRL	RMEEEAARLR	HEEEERKRKE
890	900	910	920	930	940	950	960
LEVQRQKELM	RQRQQQQEAL	RRLQQQQQQQ	QLAQMKLPSS	STWGQQSNTT	ACQSQATLSL	AEIQKLEEER	ERQLREEQRR
970	980	990	1000	1010	1020	1030	1040
QQRELMKALQ	QQQQQQQQKL	SGWGNVSKPS	GTTKSLLEIQ	QEEARQMQKQ	QQQQQQHQQP	NRARNNTHSN	LHTSIGNSVW
1050	1060	1070	1080	1090	1100	1110	1120
GSINTGPPNQ	WASDLVSSIW	SNADTKNSNM	GFWDDAVKEV	GPRNSTNKNK	NNASLSKSVG	VSNRQNKKVE	EEEKLLKLFQ
1130	1140	1150	1160	1170	1180	1190	1200
GVNKAQDGFT	QWCEQMLHAL	NTANNLDVPT	FVSFLKEVES	PYEVHDYIRA	YLGDTSEAKE	FAKQFLERRA	KQKANQQRQQ
1210	1220	1230	1240	1250	1260	1270	1280
QQLPQQQQQQ	PPQQPPQQPQ	QQDSVWGMNH	STLHSVFQTN	QSNNQQSNFE	AVQSGKKKKK	QKMVRADPSL	LGFSVNASSE
1290	1300						
RLNMGEIETL	DDY						

Cmpd.	No. of Cmpds.	m/z meas.	Δ m/z [ppm]	z	Rt [min]	Score	P	Range	Sequence	Modification	Ratios
1628	1	1071.6266	33.70	1	50.2	10.0	1	758-765	R.QQEEILRR.Q		mdown:qdown 4.44
175	2	838.3909	-15.70	2	31.6	17.6	0	1010-1022	K.QQQQQQQHQQPNR.A		mdown:qdown 0.90

Detailed Protein Report

Protein 503: dnaJ homolog subfamily A member 3, mitochondrial isoform 3 [Homo sapiens]

**Accession:**	gi|557357763	**Score:**	27.6
**Database:**	refseq_human(refseq_human_20140103.fasta)	**MW [kDa]:**	33.1
**Database Date:**	2015-11-30	**pI:**	10.1
**Modification(s):**	Oxidation	**Sequence Coverage [%]:**	9.3
		**No. of unique Peptides:**	1

10	20	30	40	50	60	70	80
MAEPQAERPR	LCVFPDLLRP	PSAADIETWC	QPYRKIFGEF	SSSSFGDFQT	VFDQPQEYFM	ELTFNQAAKG	VNKEFTVNIM
**90**	**100**	**110**	**120**	**130**	**140**	**150**	**160**
DTCERCNGKG	NEPGTKVQHC	HYCGGSGMET	INTGPFVMRS	TCRRCGGRGS	IIISPCVVCR	GAGQAKQKKR	VMIPVPAGVE
**170**	**180**	**190**	**200**	**210**	**220**	**230**	**240**
DGQTVRMPVG	KREIFITFRV	QKSPVFRRDG	ADIHSDLFIS	IAQALLGGTA	RAQGLYETIN	VTIPPGTQTD	QKIRMGGKGI
**250**	**260**	**270**	**280**	**290**	**300**	**310**	
PRINSYGYGD	HYIHIKIRVP	KRLTSRQQSL	ILSYAEDETD	VEGTVNGVTL	TSSGKRSTGN		

Cmpd.	No. of Cmpds.	m/z meas.	Δ m/z [ppm]	z	Rt [min]	Score	P	Range	Sequence	Modification
2705	1	842.5419	120.53	2	62.2	10.1	0	151-166	R.VMIPVPAGVEDGQTVR.M	Oxidation: 2

Detailed Protein Report

Protein 504: laminin subunit gamma-1 precursor [Homo sapiens]

Accession: gi|145309326
Database: refseq_human(refseq_human_20140103.fasta)
Database Date: 2015-11-30
Modification(s): Carbamidomethyl

Score: 27.6
MW [kDa]: 177.5
pI: 4.9
Sequence Coverage [%]: 1.6
No. of unique Peptides: 2

Quantitation

mdown:qdown	**Median:** 1.02	**CV:** 0.00 %	**No. of Peptides:**	1
Wdown:Qdown	**Median:** 1.63	**CV:** 0.00 %	**No. of Peptides:**	1

10	20	30	40	50	60	70	80
MRGSHRAAPA	LRPRGRLWPV	LAVLAAAAAA	GCAQAAMDEC	TDEGGRPQRC	MPEFVNAAFN	VTVVATNTCG	TPPEEYCVQT
90	100	110	120	130	140	150	160
GVTGVTKSCH	LCDAGQPHLQ	HGAAFLTDYN	NQADTTWWQS	QTMLAGVQYP	SSINLTLHLG	KAFDITYVRL	KFHTSRPESF
170	180	190	200	210	220	230	240
AIYKRTREDG	PWIPYQYYSG	SCENTYSKAN	RGFIRTGGDE	QQALCTDEFS	DISPLTGGNV	AFSTLEGRPS	AYNFDNSPVL
250	260	270	280	290	300	310	320
QEWVTATDIR	VTLNRLNTFG	DEVFNDPKVL	KSYYYAISDF	AVGGRCKCNG	HASECMKNEF	DKLVCNCKHN	TYGVDCEKCL
330	340	350	360	370	380	390	400
PFFNDRPWRR	ATAESASECL	PCDCNGRSQE	CYFDPELYRS	TGHGGHCTNC	QDNTDGAHCE	RCRENFFRLG	NNEACSSCHC
410	420	430	440	450	460	470	480
SPVGSLSTQC	DSYGRCSCKP	GVMGDKCDRC	QPGFHSLTEA	GCRPCSCDPS	GSIDECNIET	GRCVCKDNVE	GFNCERCKPG
490	500	510	520	530	540	550	560
FFNLESSNPR	GCTPCFCFGH	SSVCTNAVGY	SVYSISSTFQ	IDEDGWRAEQ	RDGSEASLEW	SSERQDIAVI	SDSYFPRYFI
570	580	590	600	610	620	630	640
APAKFLGKQV	LSYGQNLSFS	FRVDRRDTRL	SAEDLVLEGA	GLRVSVPLIA	QGNSYPSETT	VKYVFRLHEA	TDYPWRPALT
650	660	670	680	690	700	710	720
PFEFQKLLNN	LTSIKIRGTY	SERSAGYLDD	VTLASARPGP	GVPATWVESC	TCPVGYGGQF	CEMCLSGYRR	ETPNLGPYSP
730	740	750	760	770	780	790	800
CVLCACNGHS	ETCDPETGVC	NCRDNTAGPH	CEKCSDGYYG	DSTAGTSSDC	QPCPCPGGSS	CAVVPKTKEV	VCTNCPTGTT
810	820	830	840	850	860	870	880
GKRCELCDDG	YFGDPLGRNG	PVRLCRLCQC	SDNIDPNAVG	NCNRLTGECL	KCIYNTAGFY	CDRCKDGFFG	NPLAPNPADK
890	900	910	920	930	940	950	960
CKACNCNLYG	TMKQQSSCNP	VTGQCECLPH	VTGQDCGACD	PGFYNLQSGQ	GCERCDCHAL	GSTNGQCDIR	TGQCECQPGI
970	980	990	1000	1010	1020	1030	1040
TGQHCERCEV	NHFGFGPEGC	KPCDCHPEGS	LSLQCKDDGR	CECREGFVGN	RCDQCEENYF	YNRSWPGCQE	CPACYRLVKD
1050	1060	1070	1080	1090	1100	1110	1120
KVADHRVKLQ	ELESLIANLG	TGDEMVTDQA	FEDRLKEAER	EVMDLLREAQ	DVKDVDQNLM	DRLQRVNNTL	SSQISRLQNI
1130	1140	1150	1160	1170	1180	1190	1200
RNTIEETGNL	AEQARAHVEN	TERLIEIASR	ELEKAKVAAA	NVSVTQPEST	GDPNNMTLLA	EEARKLAERH	KQEADDIVRV
1210	1220	1230	1240	1250	1260	1270	1280
AKTANDTSTE	AYNLLLRTLA	GENQTAFEIE	ELNRKYEQAK	NISQDLEKQA	ARVHEEAKRA	GDKAVEIYAS	VAQLSPLDSE
1290	1300	1310	1320	1330	1340	1350	1360
TLENEANNIK	MEAENLEQLI	DQKLKDYEDL	REDMRGKELE	VKNLLEKGKT	EQQTADQLLA	RADAAKALAE	EAAKKGRDTL
1370	1380	1390	1400	1410	1420	1430	1440
QEANDILNNL	KDFDRRVNDN	KTAAEEALRK	IPAINQTITE	ANEKTREAQQ	ALGSAAADAT	EAKNKAHEAE	RIASAVQKNA
1450	1460	1470	1480	1490	1500	1510	1520
TSTKAEAERT	FAEVTDLDNE	VNNMLKQLQE	AEKELKRKQD	DADQDMMMAG	MASQAAQEAE	INARKAKNSV	TSLLSIINDL
1530	1540	1550	1560	1570	1580	1590	1600
LEQLGQLDTV	DLNKLNEIEG	TLNKAKDEMK	VSDLDRKVSD	LENEAKKQEA	AIMDYNRDIE	EIMKDIRNLE	DIRKTLPSGC
1610							
FNTPSIEKP							

Cmpd.	No. of Cmpds.	m/z meas.	Δ m/z [ppm]	z	Rt [min]	Score	P	Range	Sequence	Modification	Ratios
2687	2	941.9781	66.10	2	62.0	14.3	1	303-318	K.LVCNCKHNTYGVDCEK.C	Carbamidomethyl: 14	
1858	1	590.7710	-122.60	2	53.3	13.3	2	1037-1046	R.LVKDKVADHR.V		Wdown:Qdown 1.63<br>mdown:qdown 1.02

Detailed Protein Report

Protein 505: PREDICTED: calpastatin isoform X10 [Homo sapiens]

Accession: gi|578810591
Database: refseq_human(refseq_human_20140103.fasta)
Database Date: 2015-11-30

Score: 27.6
MW [kDa]: 80.1
pI: 4.9
Sequence Coverage [%]: 4.3
No. of unique Peptides: 1

10	20	30	40	50	60	70	80
MSQPGQKPAA	SPRPRRAAAA	RRTHEHVSEK	TSESPSKPGE	KKGSDEKKAA	SLGSSQSSRT	YAGGTASATK	VSASSGATSK
**90**	**100**	**110**	**120**	**130**	**140**	**150**	**160**
SSSMNPTETK	AVKTEPEKKS	QSTKPKSLPK	QASDTGSNDA	HNKKAVSRSA	EQQPSEKSTE	PKTKPQDMIS	AGGESVAGIT
**170**	**180**	**190**	**200**	**210**	**220**	**230**	**240**
AISGKPGDKK	KEKKSLTPAV	PVESKPDKPS	GKSGMDAALD	DLIDTLGGPE	ETEEENTTYT	GPEVSDPMSS	TYIEELGKRE
**250**	**260**	**270**	**280**	**290**	**300**	**310**	**320**
VTIPPKYREL	LAKKEGITGP	PADSSKPIGP	DDAIDALSSD	FTCGSPTAAG	KKTEKEESTE	VLKAQSAGTV	RSAAPPQEKK
**330**	**340**	**350**	**360**	**370**	**380**	**390**	**400**
RKVEKDTMSD	QALEALSASL	GTRQAEPELD	LRSIKEVDEA	KAKEEKLEKC	GEDDETIPSE	YRLKPATDKD	GKPLLPEPEE
**410**	**420**	**430**	**440**	**450**	**460**	**470**	**480**
KPKPRSESEL	IDELSEDFDR	SECKEKPSKP	TEKTEESKAA	APAPVSEAVC	RTSMCSIQSA	PPEPATLKGT	VPDDAVEALA
**490**	**500**	**510**	**520**	**530**	**540**	**550**	**560**
DSLGKKEADP	EDGKPVMDKV	KEKAKEEDRE	KLGEKEETIP	PDYRLEEVKD	KDGKPLLPKE	SKEQLPPMSE	DFLLDALSED
**570**	**580**	**590**	**600**	**610**	**620**	**630**	**640**
FSGPQNASSL	KFEDAKLAAA	ISEVVSQTPA	STTQAGAPPR	DTSSDKDLDD	ALDKLSDSLG	QRQPDPDENK	PMEDKVKEKA
**650**	**660**	**670**	**680**	**690**	**700**	**710**	**720**
KAEHRDKLGE	RDDTIPPEYR	HLLDDNGQDK	PVKPPTKKSE	DSKKPADDQD	PIDALSGDLD	SCPSTTETSQ	NTAKDKCKKA
**730**	**740**	**750**					
ASSSKAPKNG	GKAKDSAKTT	EETSKPKDD					

Cmpd.	No. of Cmpds.	m/z meas.	Δ m/z [ppm]	z	Rt [min]	Score	P	Range	Sequence	Modification
2971	1	816.9085	-57.77	2	65.9	16.1	1	2-16	M.SQPGQKPAASPRPRR.A	

Detailed Protein Report

Protein 506: TRAF-type zinc finger domain-containing protein 1 [Homo sapiens]

**Accession:**	gi|5729828	**Score:**	27.5
**Database:**	refseq_human(refseq_human_20140103.fasta)	**MW [kDa]:**	64.8
**Database Date:**	2015-11-30	**pI:**	5.1
**Modification(s):**	Carbamidomethyl, Oxidation	**Sequence Coverage [%]:**	2.9
		**No. of unique Peptides:**	1

Alias proteins:

Accession	Name	Description
gi|219879806	refseq_human (refseq_human_20140103.fasta)	TRAF-type zinc finger domain-containing protein 1 [Homo sapiens]

```
        10         20         30         40         50         60         70         80
MAEFLDDQET RLCDNCKKEI PVFNFTIHEI HCQRNIGMCP TCKEPFPKSD METHMAAEHC QVTCKCNKKL EKRLLKKHEE
        90        100        110        120        130        140        150        160
TECPLRLAVC QHCDLELSIL KLKEHEDYCG ARTELCGNCG RNVLVKDLKT HPEVCGREGE EKRNEVAIPP NAYDESWGQD
       170        180        190        200        210        220        230        240
GIWIASQLLR QIEALDPPMR LPRRPLRAFE SDVFHNRTTN QRNITAQVSI QNNLFEEQER QERNRGQQPP KEGGEESANL
       250        260        270        280        290        300        310        320
DFMLALSLQN EGQASSVAEQ DFWRAVCEAD QSHGGPRSLS DIKGAADEIM LPCEFCEELY PEELLIDHQT SCNPSRALPS
       330        340        350        360        370        380        390        400
LNTGSSSPRG VEEPDVIFQN FLQQAASNQL DSLMGLSNSH PVEESIIIPC EFCGVQLEEE VLFHHQDQCD QRPATATNHV
       410        420        430        440        450        460        470        480
TEGIPRLDSQ PQETSPELPR RRVRHQGDLS SGYLDDTKQE TANGPTSCLP PSRPINNMTA TYNQLSRSTS GPRPGCQPSS
       490        500        510        520        530        540        550        560
PCVPKLSNSD SQDIQGRNRD SQNGAIAPGH VSVIRPPQNL YPENIVPSFS PGPSGRYGAS GRSEGGRNSR VTPAAANYRS
       570        580        590
RTAKAKPSKQ QGAGDAEEEE EE
```

Cmpd.	No. of Cmpds.	m/z meas.	Δ m/z [ppm]	z	Rt [min]	Score	P	Range	Sequence	Modification
2118	1	1052.3339	-103.36	2	56.6	14.2	1	1-17	-.MAEFLDDQETRLCDNCK.K	Carbamidomethyl: 13; Oxidation: 1

Detailed Protein Report

Protein 507: beta-defensin 119 isoform a precursor [Homo sapiens]

**Accession:**	gi|37704382	**Score:**	27.5
**Database:**	refseq_human(refseq_human_20140103.fasta)	**MW [kDa]:**	9.8
**Database Date:**	2015-11-30	**pI:**	10.1
**Modification(s):**	Carbamidomethyl	**Sequence Coverage [%]:**	20.2
		**No. of unique Peptides:**	1

10	20	30	40	50	60	70	80
MKLLYLFLAI	LLAIEEPVIS	GKRHILRCMG	NSGICRASCK	KNEQPYLYCR	NCQSCCLQSY	MRISISGKEE	NTDWSYEKQW
90							
PRLP							

Cmpd.	No. of Cmpds.	m/z meas.	Δ m/z [ppm]	z	Rt [min]	Score	P	Range	Sequence	Modification
1509	1	981.9713	5.72	2	47.6	13.7	2	24-40	R.HILRCMGNSGICRASCK.K	Carbamidomethyl: 12, 16

Detailed Protein Report

Protein 508: retinitis pigmentosa 9 protein [Homo sapiens]

Accession: gi|42718020
Database: refseq_human(refseq_human_20140103.fasta)
Database Date: 2015-11-30

Score: 27.5
MW [kDa]: 26.1
pI: 10.4
Sequence Coverage [%]: 9.5
No. of unique Peptides: 2

Quantitation

Wdown:Qdown **Median:** 0.61 **CV:** 0.00 % **No. of Peptides:** 1

10	20	30	40	50	60	70	80
MSSRPGREDV	GAAGARRPRE	PPEQELQRRR	EQKRRRHDAQ	QLQQLKHLES	FYEKPPPGLI	KEDETKPEDC	IPDVPGNEHA
90	100	110	120	130	140	150	160
REFLAHAPTK	GLWMPLGKEV	KVMQCWRCKR	YGHRTGDKEC	PFFIKGNQKL	EQFRVAHEDP	MYDIIRDNKR	HEKDVRIQQL
170	180	190	200	210	220	230	
KQLLEDSTSD	EDRSSSSSSE	GKEKHKKKKK	KEKHKKRKKE	KKKKKKRKHK	SSKSNEGSDS	E	

Cmpd.	No. of Cmpds.	m/z meas.	Δ m/z [ppm]	z	Rt [min]	Score	P	Range	Sequence	Modification	Ratios
1338	1	845.6519	284.75	1	46.4	14.4	0	8-16	R.EDVGAAGAR.R		Wdown:Qdown 0.61
2386	1	704.1871	-182.11	2	58.5	13.1	0	162-173	K.QLLEDSTSDEDR.S		

Detailed Protein Report

Protein 509: **dehydrogenase/reductase SDR family member on chromosome X precursor [Homo sapiens]**

**Accession:**	gi|193804850	**Score:**	27.5
**Database:**	refseq_human(refseq_human_20140103.fasta)	**MW [kDa]:**	36.4
**Database Date:**	2015-11-30	**pI:**	9.7
		**Sequence Coverage [%]:**	11.2
		**No. of unique Peptides:**	1

10	20	30	40	50	60	70	80
MSPLSAARAA	LRVYAVGAAV	ILAQLLRRCR	GGFLEPVFPP	RPDRVAIVTG	GTDGIGYSTA	KHLARLGMHV	IIAGNNDSKA
**90**	**100**	**110**	**120**	**130**	**140**	**150**	**160**
KQVVSKIKEE	TLNDKVEFLY	CDLASMTSIR	QFVQKFKMKK	IPLHVLINNA	GVMMVPQRKT	RDGFEEHFGL	NYLGHFLLTN
**170**	**180**	**190**	**200**	**210**	**220**	**230**	**240**
LLLDTLKESG	SPGHSARVVT	VSSATHYVAE	LNMDDLQSSA	CYSPHAAYAQ	SKLALVLFTY	HLQRLLAAEG	SHVTANVVDP
**250**	**260**	**270**	**280**	**290**	**300**	**310**	**320**
GVVNTDVYKH	VFWATRLAKK	LLGWLLFKTP	DEGAWTSIYA	AVTPELEGVG	GHYLYNEKET	KSLHVTYNQK	LQQQLWSKSC
**330**	**340**						
EMTGVLDVTL							

Cmpd.	No. of Cmpds.	m/z meas.	Δ m/z [ppm]	z	Rt [min]	Score	P	Range	Sequence	Modification
1586	1	1073.1340	41.86	2	48.5	14.7	2	62-81	K.HLARLGMHVIIAGNNDSKAK.Q	

Detailed Protein Report

Protein 510: **PREDICTED: coiled-coil domain-containing protein 125 isoform X5 [Homo sapiens]**

**Accession:**	gi|530379210	**Score:**	27.5
**Database:**	refseq_human(refseq_human_20140103.fasta)	**MW [kDa]:**	48.2
**Database Date:**	2015-11-30	**pI:**	9.5
		**Sequence Coverage [%]:**	5.5
		**No. of unique Peptides:**	1

Alias proteins:

Accession	Name	Description
gi|530430737	refseq_human (refseq_human_20140103.fasta)	PREDICTED: coiled-coil domain-containing protein 125 isoform X5 [Homo sapiens]

10	20	30	40	50	60	70	80
MKRVFSIPSI	RASKIHSLKC	PEFPITDDKV	ALEVEMLKTE	LEASQRQLRG	KEEALKILQS	MAILGKATSH	TQAVLQKTME
**90**	**100**	**110**	**120**	**130**	**140**	**150**	**160**
QNRSLEKEIN	ALQWEIEFDH	NRFKNIEESW	IQKYDRLNCE	NAVLKENLKV	KTEEIKMLKS	DNAVLNQRYL	EALAMLDIKQ
**170**	**180**	**190**	**200**	**210**	**220**	**230**	**240**
QKMAQENMCC	DKSGFAEASG	LELAVLGACL	CHGPGGNPCS	CARMAASTRK	LLLQLKQELE	ILQKSKEEAY	VMADAFRIAF
**250**	**260**	**270**	**280**	**290**	**300**	**310**	**320**
EQQLMRKNDQ	ALQLTQMDKM	HKKATKWMNW	KHLKEDGFPS	PRSKKTFGQR	LLGMLPSENS	SKRMEDQDSP	QEVLKMLIDL
**330**	**340**	**350**	**360**	**370**	**380**	**390**	**400**
LNDKEEALAH	QRKVSYMLAR	ALEDKDTASN	ENKEKNPIKE	NFPFNNPWRK	TSEFSVLGDP	IHSSVCILNS	VGCICSIQHS
**410**	**420**	**430**					
QIDPNYRTLK	RSHSLPSSII	F					

Cmpd.	No. of Cmpds.	m/z meas.	Δ m/z [ppm]	z	Rt [min]	Score	P	Range	Sequence	Modification
2803	1	973.5138	-3.08	2	64.1	15.5	1	30-46	K.VALEVEMLKTELEASQR.Q	

Detailed Protein Report

Protein 511: PREDICTED: Hermansky-Pudlak syndrome 3 protein isoform X3 [Homo sapiens]

Accession: gi|578807815
Database: refseq_human(refseq_human_20140103.fasta)
Database Date: 2015-11-30

Score: 27.5
MW [kDa]: 106.3
pI: 5.8
Sequence Coverage [%]: 3.5
No. of unique Peptides: 1

Quantitation

mdown:qdown **Median:** 1.76 **CV:** 0.00 % **No. of Peptides:** 1

10	20	30	40	50	60	70	80
MVQLYNLHPF	GSQQVVPCKL	EPDRFCGGGR	DALFVAAGCK	VEAFAVAGQE	LCQPRCAFST	LGRVLRLAYS	EAGDYLVAIE
90	100	110	120	130	140	150	160
EKNKATFLRA	YVNWRNKRTE	NSRVCIRMIG	HNVEGPFSKA	FRDQMYIIEM	PLSEAPLCIS	CCPVKGDLLV	GCTNKLVLFS
170	180	190	200	210	220	230	240
LKYQIINEEF	SLLDFERSLI	IHIDNITPVE	VSFCVGYVAV	MSDLEVLIVK	LESGPKNGER	VHHHPHKTNN	RIRRTEEGIS
250	260	270	280	290	300	310	320
NEISQLESDD	FVICQKPLEL	LGEKSEQSGL	SVTLESTGLA	DEKRKYSHFQ	HLLYRRFAPD	ISSYVLSDDI	KLHSLQLLPI
330	340	350	360	370	380	390	400
YQTGSLTSDG	KNLSQEKELL	SLFCFFSLPH	VGYLYMVVKS	VELMSVYQYP	EKSQQAVLTP	QFLHVITSNN	LQCFTVRCSA
410	420	430	440	450	460	470	480
AAAREEDPYM	DTTLKACPPV	SMDVCALRIQ	LFIGLKAICH	FKNHIILLTK	AEPEAIPERR	QSPKRLLSRK	DTSVKIKIPP
490	500	510	520	530	540	550	560
VAEAGWNLYI	VNTISPVQLY	KEMVDYSNTY	KTVKTQSCIH	LLSEAHLLVR	AALMDASQLE	PGEKAELLEA	FKESCGHLGD
570	580	590	600	610	620	630	640
CYSRLDSQHS	HLTLPYYKMS	GLSMAEVLAR	TDWTVEDGLQ	KYERGLIFYI	NHSLYENLDE	ELNEELAAKV	VQMFYVAEPK
650	660	670	680	690	700	710	720
QVPHILCSPS	MKNINPLTAM	SYLRKLDTSG	FSSILVTLTK	AAVALKMGDL	DMHRNEMKSH	SEMKLVCGFI	LEPRLLIQQR
730	740	750	760	770	780	790	800
KGQIVPTELA	LHLKETQPGL	LVASVLGLQK	NNKIGIEEAD	SFFKVLCAKD	EDTIPQLLVD	FWEAQLVACL	PDVVLQELFF
810	820	830	840	850	860	870	880
KLTSQYIWRL	SKRQPPDTTP	LRTSEDLINA	CSHYGLIYPW	VHVVISSDSL	ADKNYTEDLS	KLQSLICGPS	FDIASIIPFL
890	900	910	920	930	940	950	
EPLSEDTIAG	LSVHVLCRTR	LKEYEQCIDI	LLERCPEAVI	PYANHELKEE	NRIDSVVEKT	VA	

Cmpd.	No. of Cmpds.	m/z meas.	Δ m/z [ppm]	z	Rt [min]	Score	P	Range	Sequence	Modification	Ratios
2610	1	449.6838	-247.84	2	60.9	14.6	1	715-721	R.LLIQQRK.G		mdown:qdown 1.76

Detailed Protein Report

Protein 512: calcium/calmodulin-dependent protein kinase kinase 2 isoform 6 [Homo sapiens]

Accession: gi|27437023
Database: refseq_human(refseq_human_20140103.fasta)
Database Date: 2015-11-30

Score: 27.4
MW [kDa]: 54.1
pI: 7.0
Sequence Coverage [%]: 8.4
No. of unique Peptides: 2

10	20	30	40	50	60	70	80
MSSCVSSQPS	SNRAAPQDEL	GGRGSSSSES	QKPCEALRGL	SSLSIHLGME	SFIVVTECEP	GCAVDLGLAR	DRPLEADGQE
**90**	**100**	**110**	**120**	**130**	**140**	**150**	**160**
VPLDTSGSQA	RPHLSGRKLS	LQERSQGGLA	AGGSLDMNGR	CICPSLPYSP	VSSPQSSPRL	PRRPTVESHH	VSITGMQDCV
**170**	**180**	**190**	**200**	**210**	**220**	**230**	**240**
QLNQYTLKDE	IGKGSYGVVK	LAYNENDNTY	YAMKVLSKKK	LIRQAGFPRR	PPPRGTRPAP	GGCIQPRGPI	EQVYQEIAIL
**250**	**260**	**270**	**280**	**290**	**300**	**310**	**320**
KKLDHPNVVK	LVEVLDDPNE	DHLYMVFELV	NQGPVMEVPT	LKPLSEDQAR	FYFQDLIKGI	EYLHYQKIIH	RDIKPSNLLV
**330**	**340**	**350**	**360**	**370**	**380**	**390**	**400**
GEDGHIKIAD	FGVSNEFKGS	DALLSNTVGT	PAFMAPESLS	ETRKIFSGKA	LDVWAMGVTL	YCFVFGQCPF	MDERIMCLHS
**410**	**420**	**430**	**440**	**450**	**460**	**470**	**480**
KIKSQALEFP	DQPDIAEDLK	DLITRMLDKN	PESRIVVPEI	KILVKTMIRK	RSFGNPFEGS	RREERSLSAP	GNLLTKKPTR
**490**	**500**						
ECESLSELKT							

Cmpd.	No. of Cmpds.	m/z meas.	Δ m/z [ppm]	z	Rt [min]	Score	P	Range	Sequence	Modification
2098	1	966.7709	-4.81	3	56.4	10.8	0	370-394	K.ALDVWAMGVTLYCFVFGQCPFMDER.I	
1288	2	917.4243	-149.73	2	44.9	16.6	2	430-445	K.NPESRIVVPEIKILVK.T	

Detailed Protein Report

Protein 513: kinesin heavy chain isoform 5A [Homo sapiens]

**Accession:**	gi|45446749	**Score:**	27.4
**Database:**	refseq_human(refseq_human_20140103.fasta)	**MW [kDa]:**	117.3
**Database Date:**	2015-11-30	**pI:**	5.6
**Modification(s):**	Carbamidomethyl	**Sequence Coverage [%]:**	2.6
		**No. of unique Peptides:**	2

Quantitation

mdown:qdown **Median:** 0.21 **CV:** 0.00 % **No. of Peptides:** 1

10	20	30	40	50	60	70	80
MAETNNECSI	KVLCRFRPLN	QAEILRGDKF	IPIFQGDDSV	VIGGKPYVFD	RVFPPNTTQE	QVYHACAMQI	VKDVLAGYNG
90	100	110	120	130	140	150	160
TIFAYGQTSS	GKTHTMEGKL	HDPQLMGIIP	RIARDIFNHI	YSMDENLEFH	IKVSYFEIYL	DKIRDLLDVT	KTNLSVHEDK
170	180	190	200	210	220	230	240
NRVPFVKGCT	ERFVSSPEEI	LDVIDEGKSN	RHVAVTNMNE	HSSRSHSIFL	INIKQENMET	EQKLSGKLYL	VDLAGSEKVS
250	260	270	280	290	300	310	320
KTGAEGAVLD	EAKNINKSLS	ALGNVISALA	EGTKSYVPYR	DSKMTRILQD	SLGGNCRTTM	FICCSPSSYN	DAETKSTLMF
330	340	350	360	370	380	390	400
GQRAKTIKNT	ASVNLELTAE	QWKKKYEKEK	EKTKAQKETI	AKLEAELSRW	RNGENVPETE	RLAGEEAALG	AELCEETPVN
410	420	430	440	450	460	470	480
DNSSIVVRIA	PEERQKYEEE	IRRLYKQLDD	KDDEINQQSQ	LIEKLKQQML	DQEELLVSTR	GDNEKVQREL	SHLQSENDAA
490	500	510	520	530	540	550	560
KDEVKEVLQA	LEELAVNYDQ	KSQEVEEKSQ	QNQLLVDELS	QKVATMLSLE	SELQRLQEVS	GHQRKRIAEV	LNGLMKDLSE
570	580	590	600	610	620	630	640
FSVIVGNGEI	KLPVEISGAI	EEEFTVARLY	ISKIKSEVKS	VVKRCRQLEN	LQVECHRKME	VTGRELSSCQ	LLISQHEAKI
650	660	670	680	690	700	710	720
RSLTEYMQSV	ELKKRHLEES	YDSLSDELAK	LQAQETVHEV	ALKDKEPDTQ	DADEVKKALE	LQMESHREAH	HRQLARLRDE
730	740	750	760	770	780	790	800
INEKQKTIDE	LKDLNQKLQL	ELEKLQADYE	KLKSEEHEKS	TKLQELTFLY	ERHEQSKQDL	KGLEETVARE	LQTLHNLRKL
810	820	830	840	850	860	870	880
FVQDVTTRVK	KSAEMEPEDS	GGIHSQKQKI	SFLENNLEQL	TKVHKQLVRD	NADLRCELPK	LEKRLRATAE	RVKALEGALK
890	900	910	920	930	940	950	960
EAKEGAMKDK	RRYQQEVDRI	KEAVRYKSSG	KRGHSAQIAK	PVRPGHYPAS	SPTNPYGTRS	PECISYTNSL	FQNYQNLYLQ
970	980	990	1000	1010	1020	1030	1040
ATPSSTSDMY	FANSCTSSGA	TSSGGPLASY	QKANMDNGNA	TDINDNRSDL	PCGYEAEDQA	KLFPLHQETA	AS

Cmpd.	No. of Cmpds.	m/z meas.	Δ m/z [ppm]	z	Rt [min]	Score	P	Range	Sequence	Modification	Ratios
2155	1	713.2696	-111.00	2	57.1	15.2	0	607-617	R.QLENLQVECHR.K	Carbamidomethyl: 9	mdown:qdown 0.21
1747	1	851.2332	-164.00	2	51.8	12.2	0	812-827	K.SAEMEPEDSGGIHSQK.Q		

Detailed Protein Report

Protein 514: aspartyl aminopeptidase [Homo sapiens]

**Accession:**	gi|156416028	**Score:**	27.4
**Database:**	refseq_human(refseq_human_20140103.fasta)	**MW [kDa]:**	53.4
**Database Date:**	2015-11-30	**pI:**	8.0
		**Sequence Coverage [%]:**	7.6
		**No. of unique Peptides:**	1

10	20	30	40	50	60	70	80
MSGHSPTRGA	MQVAMNGKAR	KEAVQTAAKE	LLKFVNRSPS	PFHAVAECRN	RLLQAGFSEL	KETEKWNIKP	ESKYFMTRNS
**90**	**100**	**110**	**120**	**130**	**140**	**150**	**160**
STIIAFAVGG	QYVPGNGFSL	IGAHTDSPCL	RVKRRSRRSQ	VGFQQVGVET	YGGGIWSTWF	DRDLTLAGRV	IVKCPTSGRL
**170**	**180**	**190**	**200**	**210**	**220**	**230**	**240**
EQQLVHVERP	ILRIPHLAIH	LQRNINENFG	PNTEMHLVPI	LATAIQEELE	KGTPEPGPLN	AVDERHHSVL	MSLLCAHLGL
**250**	**260**	**270**	**280**	**290**	**300**	**310**	**320**
SPKDIVEMEL	CLADTQPAVL	GGAYDEFIFA	PRLDNLHSCF	CALQALIDSC	AGPGSLATEP	HVRMVTLYDN	EEVGSESAQG
**330**	**340**	**350**	**360**	**370**	**380**	**390**	**400**
AQSLLTELVL	RRISASCQHP	TAFEEAIPKS	FMISADMAHA	VHPNYLDKHE	ENHRPLFHKG	PVIKVNSKQR	YASNAVSEAL
**410**	**420**	**430**	**440**	**450**	**460**	**470**	**480**
IREVANKVKV	PLQDLMVRND	TPCGTTIGPI	LASRLGLRVL	DLGSPQLAMH	SIREMACTTG	VLQTLTLFKG	FFELFPSLSH
**490**							
NLLVD							

Cmpd.	No. of Cmpds.	m/z meas.	Δ m/z [ppm]	z	Rt [min]	Score	P	Range	Sequence	Modification
2198	1	1031.5929	23.90	2	56.1	12.4	2	391-409	R.YASNAVSEALIREVANKVK.V	

Detailed Protein Report

Protein 515: **PREDICTED: suppressor of tumorigenicity 7 protein-like isoform X10 [Homo sapiens]**

**Accession:**	gi|530362849	**Score:**	27.4
**Database:**	refseq_human(refseq_human_20140103.fasta)	**MW [kDa]:**	35.9
**Database Date:**	2015-11-30	**pI:**	9.9
**Modification(s):**	Oxidation	**Sequence Coverage [%]:**	5.6
		**No. of unique Peptides:**	2

10	20	30	40	50	60	70	80
MADRGGVGEA	AAVGASPASV	PGLNPTLGWR	ERLRAGLAGT	GASLWFVAGL	GLLYALRIPL	RLCENLAAVT	VFLNSLTPKF
**90**	**100**	**110**	**120**	**130**	**140**	**150**	**160**
YVALTGTSSL	ISGLIFIFEW	WYFHKHGTSF	IEQVSVSHLQ	PLMGGTESSI	SEPGSPSRNR	ENETSRQNLS	ECKVWRNPLN
**170**	**180**	**190**	**200**	**210**	**220**	**230**	**240**
LFRGAEYRRY	TWVTGKEPLT	YYDMNLSAQD	HQTFFTCDTD	FLRPSDTVMQ	KAWRERNPPA	RIKAAYQALE	LNNDCATAYV
**250**	**260**	**270**	**280**	**290**	**300**	**310**	**320**
LLAEEEATTI	VDAERLFKQA	LKAGETIYRQ	SQQCQHQSPQ	HEAQLRRDTN	VLVYIKRRLA	MCARKLGRIR	EAVKIMRDI

Cmpd.	No. of Cmpds.	m/z meas.	Δ m/z [ppm]	z	Rt [min]	Score	P	Range	Sequence	Modification
2047	1	625.2681	-128.78	2	54.2	13.8	1	259-269	K.QALKAGETIYR.Q	
2581	2	836.1729	-298.97	1	60.5	13.6	1	298-304	R.RLAMCAR.K	Oxidation: 4

Detailed Protein Report

Protein 516: voltage-dependent R-type calcium channel subunit alpha-1E isoform 2 [Homo sapiens]

**Accession:**	gi|329663531	**Score:**	27.3
**Database:**	refseq_human(refseq_human_20140103.fasta)	**MW [kDa]:**	254.4
**Database Date:**	2015-11-30	**pI:**	9.3
**Modification(s):**	Oxidation	**Sequence Coverage [%]:**	1.3
		**No. of unique Peptides:**	2

Quantitation

Wdown:Qdown	**Median:** 1.28	**CV:** 0.00 %	**No. of Peptides:** 1

Detailed Protein Report

10	20	30	40	50	60	70	80
MARFGEAVVA	RPGSGDGDSD	QSRNRQGTPV	PASGQAAAYK	QTKAQRARTM	ALYNPIPVRQ	NCFTVNRSLF	IFGEDNIVRK
90	100	110	120	130	140	150	160
YAKKLIDWPP	FEYMILATII	ANCIVLALEQ	HLPEDDKTPM	SRRLEKTEPY	FIGIFCFEAG	IKIVALGFIF	HKGSYLRNGW
170	180	190	200	210	220	230	240
NVMDFIVVLS	GILATAGTHF	NTHVDLRTLR	AVRVLRPLKL	VSGIPSLQIV	LKSIMKAMVP	LLQIGLLLFF	AILMFAIIGL
250	260	270	280	290	300	310	320
EFYSGKLHRA	CFMNNSGILE	GFDPPHPCGV	QGCPAGYECK	DWIGPNDGIT	QFDNILFAVL	TVFQCITMEG	WTTVLYNTND
330	340	350	360	370	380	390	400
ALGATWNWLY	FIPLIIIGSF	FVLNLVLGVL	SGEFAKERER	VENRRAFMKL	RRQQQIEREL	NGYRAWIDKA	EEVMLAEENK
410	420	430	440	450	460	470	480
NAGTSALEVL	RRATIKRSRT	EAMTRDSSDE	HCVDISSVGT	PLARASIKSA	KVDGVSYFRH	KERLLRISIR	HMVKSQVFYW
490	500	510	520	530	540	550	560
IVLSLVALNT	ACVAIVHHNQ	PQWLTHLLYY	AEFLFLGLFL	LEMSLKMYGM	GPRLYFHSSF	NCFDFGVTVG	SIFEVVWAIF
570	580	590	600	610	620	630	640
RPGTSFGISV	LRALRLLRIF	KITKYWASLR	NLVVSLMSSM	KSIISLLFLL	FLFIVVFALL	GMQLFGGRFN	FNDGTPSANF
650	660	670	680	690	700	710	720
DTFPAAIMTV	FQILTGEDWN	EVMYNGIRSQ	GGVSSGMWSA	IYFIVLTLFG	NYTLLNVFLA	IAVDNLANAQ	ELTKDEQEEE
730	740	750	760	770	780	790	800
EAFNQKHALQ	KAKEVSPMSA	PNMPSIERER	RRRHHMSVWE	QRTSQLRKHM	QMSSQEALNR	EEAPTMNPLN	PLNPLSSLNP
810	820	830	840	850	860	870	880
LNAHPSLYRR	PRAIEGLALG	LALEKFEEER	ISRGGSLKGD	GGDRSSALDN	QRTPLSLGQR	EPPWLARPCH	GNCDPTQQEA
890	900	910	920	930	940	950	960
GGGEAVVTFE	DRARHRQSQR	RSRHRRVRTE	GKESSSASRS	RSASQERSLD	EAMPTEGEKD	HELRGNHGAK	EPTIQEERAQ
970	980	990	1000	1010	1020	1030	1040
DLRRTNSLMV	SRGSGLAGGL	DEADTPLVLP	HPELEVGKHV	VLTEQEPEGS	SEQALLGNVQ	LDMGRVISQS	EPDLSCITAN
1050	1060	1070	1080	1090	1100	1110	1120
TDKATTESTS	VTVAIPDVDP	LVDSTVVHIS	NKTDGEASPL	KEAEIREDEE	EVEKKKQKKE	KRETGKAMVP	HSSMFIFSTT
1130	1140	1150	1160	1170	1180	1190	1200
NPIRRACHYI	VNLRYFEMCI	LLVIAASSIA	LAAEDPVLTN	SERNKVLRYF	DYVFTGVFTF	EMVIKMIDQG	LILQDGSYFR
1210	1220	1230	1240	1250	1260	1270	1280
DLWNILDFVV	VVGALVAFAL	ANALGTNKGR	DIKTIKSLRV	LRVLRPLKTI	KRLPKLKAVF	DCVVTSLKNV	FNILIVYKLF
1290	1300	1310	1320	1330	1340	1350	1360
MFIFAVIAVQ	LFKGKFFYCT	DSSKDTEKEC	IGNYVDHEKN	KMEVKGREWK	RHEFHYDNII	WALLTLFTVS	TGEGWPQVLQ
1370	1380	1390	1400	1410	1420	1430	1440
HSVDVTEEDR	GPSRSNRMEM	SIFYVVYFVV	FPFFFVNIFV	ALIIITFQEQ	GDKMMEECSL	EKNERACIDF	AISAKPLTRY
1450	1460	1470	1480	1490	1500	1510	1520
MPQNRHTFQY	RVWHFVVSPS	FEYTIMAMIA	LNTVVLMMKY	YSAPCTYELA	LKYLNIAFTM	VFSLECVLKV	IAFGFLNYFR
1530	1540	1550	1560	1570	1580	1590	1600
DTWNIFDFIT	VIGSITEIIL	TDSKLVNTSG	FNMSFLKLFR	AARLIKLLRQ	GYTIRILLWT	FVQSFKALPY	VCLLIAMLFF
1610	1620	1630	1640	1650	1660	1670	1680
IYAIIGMQVF	GNIKLDEESH	INRHNNFRSF	FGSLMLLFRS	ATGEAWQEIM	LSCLGEKGCE	PDTTAPSGQN	ENERCGTDLA
1690	1700	1710	1720	1730	1740	1750	1760
YVYFVSFIFF	CSFLMLNLFV	AVIMDNFEYL	TRDSSILGPH	HLDEFVRVWA	EYDRAACGRI	HYTEMYEMLT	LMSPPLGLGK
1770	1780	1790	1800	1810	1820	1830	1840
RCPSKVAYKR	LVLMNMPVAE	DMTVHFTSTL	MALIRTALDI	KIAKGGADRQ	QLDSELQKET	LAIWPHLSQK	MLDLLVPMPK
1850	1860	1870	1880	1890	1900	1910	1920
ASDLTVGKIY	AAMMIMDYYK	QSKVKKQRQQ	LEEQKNAPMF	QRMEPSSLPQ	EIIANAKALP	YLQQDPVSGL	SGRSGYPSMS
1930	1940	1950	1960	1970	1980	1990	2000
PLSPQDIFQL	ACMDPADDGQ	FQERQSLVVT	DPSSMRRSFS	TIRDKRSNSS	WLEEFSMERS	SENTYKSRRR	SYHSSLRLSA
2010	2020	2030	2040	2050	2060	2070	2080
HRLNSDSGHK	SDTHRSGGRE	RGRSKERKHL	LSPDVSRCNS	EERGTQADWE	SPERRQSRSP	SEGRSQTPNR	QGTGSLSESS
2090	2100	2110	2120	2130	2140	2150	2160
IPSVSDTSTP	RRSRRQLPPV	PPKPRPLLSY	SSLIRHAGSI	SPPADGSEEG	SPLTSQALES	NNACLTESSN	SPHPQQSQHA
2170	2180	2190	2200	2210	2220	2230	2240

Detailed Protein Report

Cmpd.	No. of Cmpds.	m/z meas.	Δ m/z [ppm]	z	Rt [min]	Score	P	Range	Sequence	Modification	Ratios
2194	1	973.4542	-4.91	2	57.6	16.3	1	734-750	K.EVSPMSAPNMPSIERER.R	Oxidation: 10	Wdown:Qdown 1.28
1976	1	698.7954	-42.34	2	53.2	11.0	1	1728-1739	R.VWAEYDRAACGR.I		

Detailed Protein Report

Protein 517: unconventional myosin-IXb isoform 2 [Homo sapiens]

**Accession:**	gi|194272142	**Score:**	27.3
**Database:**	refseq_human(refseq_human_20140103.fasta)	**MW [kDa]:**	229.0
**Database Date:**	2015-11-30	**pI:**	9.4
**Modification(s):**	Carbamidomethyl, Oxidation	**Sequence Coverage [%]:**	1.3
		**No. of unique Peptides:**	1

Detailed Protein Report

10	20	30	40	50	60	70	80
MSVKEAGSSG	RREQAAYHLH	IYPQLSTTES	QASCRVTATK	DSTTSDVIKD	AIASLRLDGT	KCYVLVEVKE	SGGEEWVLDA
90	100	110	120	130	140	150	160
NDSPVHRVLL	WPRRAQDEHP	QEDGYYFLLQ	ERNADGTIKY	VHMQLVAQAT	ATRRLVERGL	LPRQQADFDD	LCNLPELTEG
170	180	190	200	210	220	230	240
NLLKNLKHRF	LQQKIYTYAG	SILVAINPFK	FLPIYNPKYV	KMYENQQLGK	LEPHVFALAD	VAYYTMLRKR	VNQCIVISGE
250	260	270	280	290	300	310	320
SGSGKTQSTN	FLIHCLTALS	QKGYASGVER	TILGAGPVLE	AFGNAKTAHN	NNSSRFGKFI	QVSYLESGIV	RGAVVEKYLL
330	340	350	360	370	380	390	400
EKSRLVSQEK	DERNYHVFYY	LLLGVSEEER	QEFQLKQPED	YFYLNQHNLK	IEDGEDLKHD	FERLKQAMEM	VGFLPATKKQ
410	420	430	440	450	460	470	480
IFAVLSAILY	LGNVTYKKRA	TGREEGLEVG	PPEVLDTLSQ	LLKVKREILV	EVLTKRKTVT	VNDKLILPYS	LSEAITARDS
490	500	510	520	530	540	550	560
MAKSLYSALF	DWIVLRINHA	LLNKKDVEEA	VSCLSIGVLD	IFGFEDFERN	SFEQFCINYA	NEQLQYYFNQ	HIFKLEQEEY
570	580	590	600	610	620	630	640
QGEGITWHNI	GYTDNVGCIH	LISKKPTGLF	YLLDEESNFP	HATSQTLLAK	FKQQHEDNKY	FLGTPVMEPA	FIIQHFAGKV
650	660	670	680	690	700	710	720
KYQIKDFREK	NMDYMRPDIV	ALLRGSDSSY	VRELIGMDPV	AVFRWAVLRA	AIRAMAVLRE	AGRLRAERAE	KAAGMSSPGA
730	740	750	760	770	780	790	800
QSHPEELPRG	ASTPSEKLYR	DLHNQMIKSI	KGLPWQGEDP	RSLLQSLSRL	QKPRAFILKS	KGIKQKQIIP	KNLLDSKSLK
810	820	830	840	850	860	870	880
LIISMTLHDR	TTKSLLHLHK	KKKPPSISAQ	FQTSLNKLLE	ALGKAEPFFI	RCIRSNAEKK	ELCFDDELVL	QQLRYTGMLE
890	900	910	920	930	940	950	960
TVRIRRSGYS	AKYTFQDFTE	QFQVLLPKDA	QPCREVISTL	LEKMKIDKRN	YQIGKTKVFL	KETERQALQE	TLHREVVRKI
970	980	990	1000	1010	1020	1030	1040
LLLQSWFRMV	LERRHFLQMK	RAAVTIQACW	RSYRVRRALE	RTQAAVYLQA	SWRGYWQRKL	YRHQKQSIIR	LQSLCRGHLQ
1050	1060	1070	1080	1090	1100	1110	1120
RKSFSQMISE	KQKAEEKERE	ALEAARAGAE	EGGQGQAAGG	QQVAEQGPEP	AEDGGHLASE	PEVQPSDRSP	LEHSSPEKEA
1130	1140	1150	1160	1170	1180	1190	1200
PSPEKTLPPQ	KTVAAESHEK	VPSSREKRES	RRQRGLEHVK	FQNKHIQSCK	EESALREPSR	RVTQEQGVSL	LEDKKESRED
1210	1220	1230	1240	1250	1260	1270	1280
ETLLVVETEA	ENTSQKQPTE	QPQAMAVGKV	SEETEKTLPS	GSPRPGQLER	PTSLALDSRV	SPPAPGSAPE	TPEDKSKPCG
1290	1300	1310	1320	1330	1340	1350	1360
SPRVQEKPDS	PGGSTQIQRY	LDAERLASAV	ELWRGKKLVA	AASPSAMLSQ	SLDLSDRHRA	TGAALTPTEE	RRTSFSTSDV
1370	1380	1390	1400	1410	1420	1430	1440
SKLLPSLAKA	QPAAETTDGE	RSAKKPAVQK	KKPGDASSLP	DAGLSPGSQV	DSKSTFKRLF	LHKTKDKKYS	LEGAEELENA
1450	1460	1470	1480	1490	1500	1510	1520
VSGHVVLEAT	TMKKGLEAPS	GQQHRHAAGE	KRTKEPGGKG	KKNRNVKIGK	ITVSEKWRES	VFRQITNANE	LKYLDEFLLN
1530	1540	1550	1560	1570	1580	1590	1600
KINDLRSQKT	PIESLFIEAT	EKFRSNIKTM	YSVPNGKIHV	GYKDLMENYQ	IVVSNLATER	GQKDTNLVLN	LFQSLLDEFT
1610	1620	1630	1640	1650	1660	1670	1680
RGYTKNDFEP	VKQSKAQKKK	RKQERAVQEH	NGHVFASYQV	SIPQSCEQCL	SYIWLMDKAL	LCSVCKMTCH	KKCVHKIQSH
1690	1700	1710	1720	1730	1740	1750	1760
CSYTYGRKGE	PGVEPGHFGV	CVDSLTSDKA	SVPIVLEKLL	EHVEMHGLYT	EGLYRKSGAA	NRTRELRQAL	QTDPAAVKLE
1770	1780	1790	1800	1810	1820	1830	1840
NFPIHAITGV	LKQWLRELPE	PLMTFAQYGD	FLRAVELPEK	QEQLAAIYAV	LEHLPEANHN	SLERLIFHLV	KVALLEDVNR
1850	1860	1870	1880	1890	1900	1910	1920
MSPGALAIIF	APCLLRCPDN	SDPLTSMKDV	LKITTCVEML	IKEQMRKYKV	KMEEISQLEA	AESIAFRRLS	LLRQNAPWPL
1930	1940	1950	1960	1970	1980	1990	2000
KLGFSSPYEG	VLNKSPKTRD	IQEEELEVLL	EEEAAGGDED	REKEILIERI	QSIKEEKEDI	TYRLPELDPR	GSDEENLDSE
2010	2020	2030					
TSASTESLLE	ERAGRGASEG	QY					

Cmpd.	No. of Cmpds.	m/z meas.	Δ m/z [ppm]	z	Rt [min]	Score	P	Range	Sequence	Modification

Detailed Protein Report

Cmpd.	No. of Cmpds.	m/z meas.	Δ m/z [ppm]	z	Rt [min]	Score	P	Range	Sequence	Modification
1951	1	918.3471	-89.50	2	53.1	10.7	1	1857-1872	R.CPDNSDPLTSMKDVLK.I	Carbamidomethyl: 1; Oxidation: 11

Detailed Protein Report

Protein 518: **PREDICTED: iron-sulfur cluster assembly enzyme ISCU, mitochondrial isoform X1 [Homo sapiens]**

**Accession:**	gi|530400013	**Score:**	27.3
**Database:**	refseq_human(refseq_human_20140103.fasta)	**MW [kDa]:**	16.9
**Database Date:**	2015-11-30	**pI:**	10.5
**Modification(s):**	Oxidation	**Sequence Coverage [%]:**	16.0
		**No. of unique Peptides:**	2

10	20	30	40	50	60	70	80
MAAAGAFRLR	RAASALLLRS	PRLPARELSA	PARLYHKKVV	DHYENPRNVG	SLDKTSKNVG	TGLVGAPACG	DVMKLQIQVD
**90**	**100**	**110**	**120**	**130**	**140**	**150**	**160**
EKGKIVDARF	KTFGCGSAIA	SSSLATEWVK	GKTVEEALTI	KNTDIAKELC	LPPVKLHCSR	KEGMRNISLN	ASMEVY

Cmpd.	No. of Cmpds.	m/z meas.	Δ m/z [ppm]	z	Rt [min]	Score	P	Range	Sequence	Modification
2838	1	485.7928	-38.39	2	64.0	10.4	1	11-19	R.RAASALLLR.S	
2964	1	619.9693	5.46	3	66.2	16.9	2	141-156	R.KEGMRNISLNASMEVY.-	Oxidation: 4

Detailed Protein Report

Protein 519: PREDICTED: DNA (cytosine-5)-methyltransferase 3A isoform X4 [Homo sapiens]

**Accession:**	gi|578802756	**Score:**	27.3
**Database:**	refseq_human(refseq_human_20140103.fasta)	**MW [kDa]:**	89.3
**Database Date:**	2015-11-30	**pI:**	5.6
		**Sequence Coverage [%]:**	3.2
		**No. of unique Peptides:**	1

10	20	30	40	50	60	70	80
MPAMPSSGPG	DTSSSAAERE	EDRKDGEEQE	EPRGKEERQE	PSTTARKVGR	PGRKRKHPPV	ESGDTPKDPA	VISKSPSMAQ
**90**	**100**	**110**	**120**	**130**	**140**	**150**	**160**
DSGASELLPN	GDLEKRSEPQ	PEEGSPAGGQ	KGGAPAEGEG	AAETLPEASR	AVENGCCTPK	EGRGAPAEAG	KEQKETNIES
**170**	**180**	**190**	**200**	**210**	**220**	**230**	**240**
MKMEGSRGRL	RGGLGWESSL	RQRPMPRLTF	QAGDPYYISK	RKRDEWLARW	KREAEKKAKV	IAGMNAVEEN	QGPGESQKVE
**250**	**260**	**270**	**280**	**290**	**300**	**310**	**320**
EASPPAVQQP	TDPASPTVAT	TPEPVGSDAG	DKNATKAGDD	EPEYEDGRGF	GIGELVWGKL	RGFSWWPGRI	VSWWMTGRSR
**330**	**340**	**350**	**360**	**370**	**380**	**390**	**400**
AAEGTRWVMW	FGDGKFSVVC	VEKLMPLSSF	CSAFHQATYN	KQPMYRKAIY	EVLQVASSRA	GKLFPVCHDS	DESDTAKAVE
**410**	**420**	**430**	**440**	**450**	**460**	**470**	**480**
VQNKPMIEWA	LGGFQPSGPK	GLEPPEEEKN	PYKEVYTDMW	VEPEAAAYAP	PPPAKKPRKS	TAEKPKVKEI	IDERTRERLV
**490**	**500**	**510**	**520**	**530**	**540**	**550**	**560**
YEVRQKCRNI	EDICISCGSL	NVTLEHPLFV	GGMCQNCKNC	FLECAYQYDD	DGYQSYCTIC	CGGREVLMCG	NNNCCRCFCV
**570**	**580**	**590**	**600**	**610**	**620**	**630**	**640**
ECVDLLVGPG	AAQAAIKEDP	WNCYMCGHKG	TYGLLRRRED	WPSRLQMFFA	NNHDQEFDPP	KVYPPVPAEK	RKPIRVLSLF
**650**	**660**	**670**	**680**	**690**	**700**	**710**	**720**
DGIATGLLVL	KDLGIQVDRY	IASEVCEDSI	TVGMVRHQGK	IMYVGDVRSV	TQKHIQEWGP	FDLVIGGSPC	NDLSIVNPAR
**730**	**740**	**750**	**760**	**770**	**780**	**790**	**800**
KGLYEGTGRL	FFEFYRLLHD	ARPKEGDDRP	FFWLFENVVA	MGVSDKRDIS	RFLESNPVMI	DAKEVSAAHR	ARYFWGNLPG
**810**							
MNSSAK							

Cmpd.	No. of Cmpds.	m/z meas.	Δ m/z [ppm]	z	Rt [min]	Score	P	Range	Sequence	Modification
2336	1	873.3793	-3.43	2	57.6	10.1	2	20-33	R.EEDRKDGEEQEEPR.G	

Detailed Protein Report

Protein 520: G protein-regulated inducer of neurite outgrowth 1 [Homo sapiens]

**Accession:**	gi|112821681	**Score:**	27.2
**Database:**	refseq_human(refseq_human_20140103.fasta)	**MW [kDa]:**	102.3
**Database Date:**	2015-11-30	**pI:**	9.3
		**Sequence Coverage [%]:**	3.4
		**No. of unique Peptides:**	2

Alias proteins:

Accession	Name	Description
gi|530380833	refseq_human (refseq_human_20140103.fasta)	PREDICTED: G protein-regulated inducer of neurite outgrowth 1 isoform X1 [Homo sapiens]

10	20	30	40	50	60	70	80
MDTAEDPAWL	QLLQKDSSPP	GPRPTAFFCP	QDGSLGAGSS	AMRDYCPSQQ	KASPAPPRHT	PDQSPGMESR	HRSPSGAGEG
**90**	**100**	**110**	**120**	**130**	**140**	**150**	**160**
ASCSDGPRGS	LACPSPTCFS	PQESPSKETL	EAHGASISGT	PEATTSGKPE	PVSSVKTEPK	SSDDRNPMFL	EKMDFKSSKQ
**170**	**180**	**190**	**200**	**210**	**220**	**230**	**240**
ADSTSIGKED	PGSSRKADPM	FTGKAEPEIL	GKGDPVAPGR	MDPMTVRKED	LGSLGKVDPL	CSSKTYTVSP	RKEDPGSLRK
**250**	**260**	**270**	**280**	**290**	**300**	**310**	**320**
VDPVSSDKVD	PVFPRKEEPR	YSGKEHPVSS	EKVAPTSAEK	VDLVLSGKRD	PGPSGKADPM	PLESMDSAST	GKTEPGLLGK
**330**	**340**	**350**	**360**	**370**	**380**	**390**	**400**
LIPGSSGKNG	PVSSGTGAPG	SLGRLDPTCL	GMADPASVGN	VETVPATKED	SRFLGKMDPA	SSGEGRPVSG	HTDTTASAKT
**410**	**420**	**430**	**440**	**450**	**460**	**470**	**480**
DLTSLKNVDP	MSSGKVDPVS	LGKMDPMCSG	KPELLSPGQA	ERVSVGKAGT	VSPGKEDPVS	SRREDPISAG	SRKTSSEKVN
**490**	**500**	**510**	**520**	**530**	**540**	**550**	**560**
PESSGKTNPV	SSGPGDPRSL	GTAGPPSAVK	AEPATGGKGD	PLSSEKAGLV	ASGKAAPTAS	GKAEPLAVGK	EDPVSKGKAD
**570**	**580**	**590**	**600**	**610**	**620**	**630**	**640**
AGPSGQGDSV	SIGKVVSTPG	KTVPVPSGKV	DPVSLGKAEA	IPEGKVGSLP	LEKGSPVTTT	KADPRASGKA	QPQSGGKAET
**650**	**660**	**670**	**680**	**690**	**700**	**710**	**720**
KLPGQEGAAA	PGEAGAVCLK	KETPQASEKV	DPGSCRKAEP	LASGKGEPVS	LGKADSAPSR	KTESPSLGKV	VPLSLEKTKP
**730**	**740**	**750**	**760**	**770**	**780**	**790**	**800**
SSSSRQLDRK	ALGSARSPEG	ARGSEGRVEP	KAEPVSSTEA	SSLGQKDLEA	AGAERSPCPE	AAAPPPGPRT	RDNFTKAPSW
**810**	**820**	**830**	**840**	**850**	**860**	**870**	**880**
EASAPPPPRE	DAGTQAGAQA	CVSVAVSPMS	PQDGAGGSAF	SFQAAPRAPS	PPSRRDAGLQ	VSLGAAETRS	VATGPMTPQA
**890**	**900**	**910**	**920**	**930**	**940**	**950**	**960**
AAPPAFPEVR	VRPGSALAAA	VAPPEPAEPV	RDVSWDEKGM	TWEVYGAAME	VEVLGMAIQK	HLERQIEEHG	RQGAPAPPPA
**970**	**980**	**990**	**1000**	**1010**			
ARAGPGRSGS	VRTAPPDGAA	KRPPGLFRAL	LQSVRRPRCC	SRAGPTAE			

Cmpd.	No. of Cmpds.	m/z meas.	Δ m/z [ppm]	z	Rt [min]	Score	P	Range	Sequence	Modification
2305	1	853.3930	-63.26	2	57.5	15.6	2	225-239	K.TYTVSPRKEDPGSLR.K	
2330	1	682.5941	-86.77	3	57.6	11.6	0	424-442	K.MDPMCSGKPELLSPGQAER.V	

Detailed Protein Report

Protein 521: PREDICTED: sodium/hydrogen exchanger 10 isoform X2 [Homo sapiens]

**Accession:**	gi|578807311	**Score:**	27.2
**Database:**	refseq_human(refseq_human_20140103.fasta)	**MW [kDa]:**	126.9
**Database Date:**	2015-11-30	**pI:**	7.8
**Modification(s):**	Oxidation	**Sequence Coverage [%]:**	2.6
		**No. of unique Peptides:**	1

10	20	30	40	50	60	70	80
MSPDLFFRIF	TPVVFFTTAF	DMDTYMLQKL	FWQILLISIP	GFLVNYILVL	WHLASVNQLL	LKPTQWLLFS	AILVSSDPML
**90**	**100**	**110**	**120**	**130**	**140**	**150**	**160**
TAAAIRDLGL	SRSLISLING	ESLMTSVISL	ITFTSIMDFD	QRLQSKRNHT	LAEEIVGGIC	SYIIASFLFG	ILSSKLIQFW
**170**	**180**	**190**	**200**	**210**	**220**	**230**	**240**
MSTVFGDDVN	HISLIFSILY	LIFYICELVG	MSGIFTLAIV	GLLLNSTSFK	AAIEETLLLE	FWTFLSRIAF	LMVFTFFGLL
**250**	**260**	**270**	**280**	**290**	**300**	**310**	**320**
IPAHTYLYIE	FVDIYYSLNI	YLTLIVLRFL	TLLLISPVLS	RVGHEFSWRW	IFIMVCSEMK	GMPNINMALL	LAYSDLYFGS
**330**	**340**	**350**	**360**	**370**	**380**	**390**	**400**
DKEKSQILFH	GVLVCLITLV	VNRFILPVAV	TILGLRDATS	TKYKSVCCTF	QHFQELTKSA	ASALKFDKDL	ANADWNMIEK
**410**	**420**	**430**	**440**	**450**	**460**	**470**	**480**
AITLENPYML	NEEETTEHQK	VKCPHCNKEI	DEIFNTEAME	LANRRLLSAQ	IASYQRQYRN	EILSQSAVQV	LVGAAESFGE
**490**	**500**	**510**	**520**	**530**	**540**	**550**	**560**
KKGKCMSLDT	IKNYSESQKT	VTFARKLLLN	WVYNTRKEKE	GPSKYFFFRI	CHTIVFTEEF	EHVGYLVILM	NIFPFIISWI
**570**	**580**	**590**	**600**	**610**	**620**	**630**	**640**
SQLNVIYHSE	LKHTNYCFLT	LYILEALLKI	AAMRKDFFSH	AWNIFELAIT	LIGILHVILI	EIDTIKYIFN	ETEVIVFIKV
**650**	**660**	**670**	**680**	**690**	**700**	**710**	**720**
VQFFRILRIF	KLIAPKLLQI	IDKRMSHQKT	FWYGILKGYV	QGEADIMTII	DQITSSKQIK	QMLLKQVIRN	MEHAIKELGY
**730**	**740**	**750**	**760**	**770**	**780**	**790**	**800**
LEYDHPEIAV	TVKTKEEINV	MLNMATEILK	AFGLKGIISK	TEGAGINKLI	MAKKKEVLDS	QSIIRPLTVE	EVLYHIPWLD
**810**	**820**	**830**	**840**	**850**	**860**	**870**	**880**
KNKDYINFIQ	EKAKVVTFDC	GNDIFEEGDE	PKGIYIIISG	MVKLEKSKPG	LGIDQMVESK	EKDFPIIDTD	YMLSGEIIGE
**890**	**900**	**910**	**920**	**930**	**940**	**950**	**960**
INCLTNEPMK	YSATCKTVVE	TCFIPKTHLY	DAFEQCSPLI	KQKMWLKLGL	AITARKIREH	LSYEDWNYNM	QLKLSNIYVV
**970**	**980**	**990**	**1000**	**1010**	**1020**	**1030**	**1040**
DIPMSTKTDI	YDENLIYVIL	IHGAVEDCLL	RKTYRAPFLI	PITCHQIQSI	EDFTKVVIIQ	TPINMKTFRR	NIRKFVPKHK
**1050**	**1060**	**1070**	**1080**	**1090**	**1100**	**1110**	
SYLTPGLIGS	VGTLEEGIQE	ERNVKEDGAH	SAATARSPQP	CSLLGTKFNC	KESPRINLRK	VRKE	

Cmpd.	No. of Cmpds.	m/z meas.	Δ m/z [ppm]	z	Rt [min]	Score	P	Range	Sequence	Modification
1365	1	817.1004	44.43	3	46.8	12.7	0	301-322	K.GMPNINMALLLAYSDLYFGSDK.E	Oxidation: 2

Detailed Protein Report

Protein 522: coiled-coil domain-containing protein 38 [Homo sapiens]

Accession: gi|226491199
Database: refseq_human(refseq_human_20140103.fasta)
Database Date: 2015-11-30

Score: 27.2
MW [kDa]: 65.3
pI: 9.4
Sequence Coverage [%]: 5.9
No. of unique Peptides: 2

Quantitation

mdown:qdown **Median:** 1.82 **CV:** 0.00 % **No. of Peptides:** 1

10	20	30	40	50	60	70	80
MSSNLLPTLN	SGGKVKDGST	KEDRPYKIFF	RDLFLVKENE	MAAKETEKFM	NRNMKVYQKT	TFSSRMKSHS	YLSQLAFYPK
**90**	**100**	**110**	**120**	**130**	**140**	**150**	**160**
RSGRSFEKFG	PGPAPIPRLI	EGSDTKRTVH	EFINDQRDRF	LLEYALSTKR	NTIKKFEKDI	AMRERQLKKA	EKKLQDDALA
**170**	**180**	**190**	**200**	**210**	**220**	**230**	**240**
FEEFLRENDQ	RSVDALKMAA	QETINKLQMT	AELKKASMEV	QAVKSEIAKT	EFLLREYMKY	GFFLLQMSPK	HWQIQQALKR
**250**	**260**	**270**	**280**	**290**	**300**	**310**	**320**
AQASKSKANI	ILPKILAKLS	LHSSNKEGIL	EESGRTAVLS	EDASQGRDSQ	GKPSRSLTRT	PEKKKSNLAE	SFGSEDSLEF
**330**	**340**	**350**	**360**	**370**	**380**	**390**	**400**
LLDDEMDVDL	EPALYFKEPE	ELLQVLRELE	EQNLTLFQYS	QDVDENLEEV	NKREKVIQDK	TNSNIEFLLE	QEKMLKANCV
**410**	**420**	**430**	**440**	**450**	**460**	**470**	**480**
REEEKAAELQ	LKSKLFSFGE	FNSDAQEILI	DSLSKKITQV	YKVCIGDAED	DGLNPIQKLV	KVESRLVELC	DLIESIPKEN
**490**	**500**	**510**	**520**	**530**	**540**	**550**	**560**
VEAIERMKQK	EWRQKFRDEK	MKEKQRHQQE	RLKAALEKAV	AQPKKKLGRR	LVFHSKPPSG	NKQQLPLVNE	TKTKSQEEEY
**570**							
FFT							

Cmpd.	No. of Cmpds.	m/z meas.	Δ m/z [ppm]	z	Rt [min]	Score	P	Range	Sequence	Modification	Ratios
39	1	750.3346	-92.35	2	29.8	15.7	1	85-98	R.SFEKFGPGPAPIPR.L		
353	1	676.4443	133.31	3	33.1	11.6	1	443-461	K.VCIGDAEDDGLNPIQKLVK.V		mdown:qdown 1.82

Detailed Protein Report

Protein 523: PREDICTED: histone-lysine N-methyltransferase ASH1L isoform X3 [Homo sapiens]

**Accession:**	gi|530365072	**Score:**	27.2
**Database:**	refseq_human(refseq_human_20140103.fasta)	**MW [kDa]:**	191.3
**Database Date:**	2015-11-30	**pI:**	10.4
		**Sequence Coverage [%]:**	1.8
		**No. of unique Peptides:**	1

10	20	30	40	50	60	70	80
MDPRNTAMLG	LGSDSEGFSR	KSPSAISTGT	LVSKREVELE	KNTKEEEDLR	KRNRERNIEA	GKDDGLTDAQ	QQFSVKETNF
90	100	110	120	130	140	150	160
SEGNLKLKIG	LQAKRTKKPP	KNLENYVCRP	AIKTTIKHPR	KALKSGKMTD	EKNEHCPSKR	DPSKLYKKAD	DVAAIECQSE
170	180	190	200	210	220	230	240
EVIRLHSQGE	NNPLSKKLSP	VHSEMADYIN	ATPSTLLGSR	DPDLKDRALL	NGGTSVTEKL	AQLIATCPPS	KSSKTKPKKL
250	260	270	280	290	300	310	320
GTGTTAGLVS	KDLIRKAGVG	SVAGIIHKDL	IKKPTISTAV	GLVTKDPGKK	PVFNAAVGLV	NKDSVKKLGT	GTTAVFINKN
330	340	350	360	370	380	390	400
LGKKPGTITT	VGLLSKDSGK	KLGIGIVPGL	VHKESGKKLG	LGTVVGLVNK	DLGKKLGSTV	GLVAKDCAKK	IVASSAMGLV
410	420	430	440	450	460	470	480
NKDIGKKLMS	CPLAGLISKD	AINLKAEALL	PTQEPLKASC	STNINNQESQ	ELSESLKDSA	TSKTFEKNVV	RQNKESILEK
490	500	510	520	530	540	550	560
FSVRKEIINL	EKEMFNEGTC	IQQDSFSSSE	KGSYETSKHE	KQPPVYCTSP	DFKMGGASDV	STAKSPFSAV	GESNLPSPSP
570	580	590	600	610	620	630	640
TVSVNPLTRS	PPETSSQLAP	NPLLLSSTTE	LIEEISESVG	KNQFTSESTH	LNVGHRSVGH	SISIECKGID	KEVNDSKTTH
650	660	670	680	690	700	710	720
IDIPRISSSL	GKKPSLTSES	SIHTITPSVV	NFTSLFSNKP	FLKLGAVSAS	DKHCQVAESL	STSLQSKPLK	KRKGRKPRWT
730	740	750	760	770	780	790	800
KVVARSTCRS	PKGLELERSE	LFKNVSCSSL	SNSNSEPAKF	MKNIGPPSFV	DHDFLKRRLP	KLSKSTAPSL	ALLADSEKPS
810	820	830	840	850	860	870	880
HKSFATHKLS	SSMCVSSDLL	SDIYKPKRGR	PKSKEMPQLE	GPPKRTLKIP	ASKVFSLQSK	EEQEPPILQP	EIEIPSFKQG
890	900	910	920	930	940	950	960
LSVSPFPKKR	GRPKRQMRSP	VKMKPPVLSV	APFVATESPS	KLESESDNHR	SSSDFFESED	QLQDPDDLDD	SHRPSVCSMS
970	980	990	1000	1010	1020	1030	1040
DLEMEPDKKI	TKRNNGQLMK	TIIRKINKMK	TLKRKKLLNQ	ILSSSVESSN	KGKVQSKLHN	TVSSLAATFG	SKLGQQINVS
1050	1060	1070	1080	1090	1100	1110	1120
KKGTIYIGKR	RGRKPKTVLN	GILSGSPTSL	AVLEQTAQQA	AGSALGQILP	PLLPSSASSS	EILPSPICSQ	SSGTSGGQSP
1130	1140	1150	1160	1170	1180	1190	1200
VSSDAGFVEP	SSVPYLHLHS	RQGSMIQTLA	MKKASKGRRR	LSPPTLLPNS	PSHLSELTSL	KEATPSPISE	SHSDETIPSD
1210	1220	1230	1240	1250	1260	1270	1280
SGIGTDNNST	SDRAEKFCGQ	KKRRHSFEHV	SLIPPETSTV	LSSLKEKHKH	KCKRRNHDYL	SYDKMKRQKR	KRKKKYPQLR
1290	1300	1310	1320	1330	1340	1350	1360
NRQDPDFIAE	LEELISRLSE	IRITHRSHHF	IPRDLLPTIF	RINFNSFYTH	PSFPLDPLHY	IRKPDLKKKR	GRPPKMREAM
1370	1380	1390	1400	1410	1420	1430	1440
AEMPFMHSLS	FPLSSTGFYP	SYGMPYSPSP	LTAAPIGLGY	YGRYPPTLYP	PPPSPSFTTP	LPPPSYMHAG	HLLLNPAKYH
1450	1460	1470	1480	1490	1500	1510	1520
KKKHKLLRQE	AFLTTSRTPL	LSMSTYPSVP	PEMAYGWMVE	HKHRHRHKHR	EHRSSEQPQV	SMDTGSSRSV	LESLKRYRFG
1530	1540	1550	1560	1570	1580	1590	1600
KDAVGERYKH	KEKHRCHMSC	PHLSPSKSLI	NREEQWVHRE	PSESSPLALG	LQTPLQIDCS	ESSPSLSLGG	FTPNSEPASS
1610	1620	1630	1640	1650	1660	1670	1680
DEHTNLFTSA	IGSCRVSNPN	SSGRKKLTDS	PGLFSAQDTS	LNRLHRKESL	PSNERAVQTL	AGSQPTSDKP	SQRPSESTNC
1690	1700	1710	1720	1730	1740	1750	
SPTRKRSSSE	STSSTDKTED	WYEYLEVTYI	LMIIETMRIN	MFPVGNKRSS	LSKSKISCFW	G	

Cmpd.	No. of Cmpds.	m/z meas.	Δ m/z [ppm]	z	Rt [min]	Score	P	Range	Sequence	Modification
2429	1	856.6272	147.06	2	60.7	15.4	1	273-289	K.KPTISTAVGLVTKDPGK.K	

Detailed Protein Report

Protein 524: ral GTPase-activating protein subunit alpha-2 [Homo sapiens]

**Accession:**	gi|118600961	**Score:**	27.2
**Database:**	refseq_human(refseq_human_20140103.fasta)	**MW [kDa]:**	210.6
**Database Date:**	2015-11-30	**pI:**	5.7
**Modification(s):**	Oxidation	**Sequence Coverage [%]:**	2.1
		**No. of unique Peptides:**	2

10	20	30	40	50	60	70	80
MFSRRSHGDV	KKSTQKVLDP	KKDVLTRLKH	LRALLDNVDA	NDLKQFFETN	YSQIYFIFYE	NFIALENSLK	LKGNNKSQRE
90	100	110	120	130	140	150	160
ELDSILFLFE	KILQFLPERI	FFRWHYQSIG	STLKKLLHTG	NSIKIRCEGI	RLFLLWLQAL	QTNCAEEQVL	IFACLVPGFP
170	180	190	200	210	220	230	240
AVMSSRGPCT	LETLINPSPS	VADVKIYPEE	ITPLLPAISG	EKIAEDQTCF	FLQILLKYMV	IQAASLEWKN	KENQDTGFKF
250	260	270	280	290	300	310	320
LFTLFRKYYL	PHLFPSFTKL	TNIYKPVLDI	PHLRPKPVYI	TTTRDNENIY	STKIPYMAAR	VVFIKWIVTF	FLEKKYLTAT
330	340	350	360	370	380	390	400
QNTKNGVDVL	PKIIQTVGGG	AVQERAPELD	GGGPTEQDKS	HSNSSTLSDR	RLSNSSLCSI	EEEHRMVYEM	VQRILLSTRG
410	420	430	440	450	460	470	480
YVNFVNEVFH	QAFLLPSCEI	AVTRKVVQVY	RKWILQDKPV	FMEEPDRKDV	AQEDAEKLGF	SETDSKEASS	ESSGHKRSSS
490	500	510	520	530	540	550	560
WGRTYSFTSA	MSRGCVTEEE	NTNVKAGVQA	LLQVFLTNSA	NIFLLEPCAE	VPVLLKEQVD	ACKAVLIIFR	RMIMELTMNK
570	580	590	600	610	620	630	640
KTWEQMLQIL	LRITEAVMQK	PKDKQIKDLF	AQSLAGLLFR	TLMVAWIRAN	LCVYISRELW	DDFLGVLSSL	TEWEELINEW
650	660	670	680	690	700	710	720
ANIMDSLTAV	LARTVYGVEM	TNLPLDKLSE	QKEKKQRGKG	CVLDPQKGTT	VGRSFSLSWR	SHPDVTEPMR	FRSATTSGAP
730	740	750	760	770	780	790	800
GVEKARNIVR	QKATEVEECQ	QSENAPAAGS	GHLTVGQQQQ	VLRSSSTSDI	PEPLCSDSSQ	GQKAENTQNS	SSSEPQPIQE
810	820	830	840	850	860	870	880
NKGHVKREHE	GITILVRRSS	SPAELDLKDD	LQQTQGKCRE	RQKSESTNSD	TTLGCTNEAE	LSMGPWQTCE	EDPELNTPTD
890	900	910	920	930	940	950	960
VVADADARHW	LQLSPTDASN	LTDSSECLTD	DCSIIAGGSL	TGWHPDSAAV	LWRRVLGILG	DVNNIQSPKI	HARVFCYLYE
970	980	990	1000	1010	1020	1030	1040
LWYKLAKIRD	NLAISLDNQS	SPSPPVLIPP	LRMFASWLFK	AATLPNEYKE	GKLQAYRLIC	AMMTRRQDVL	PNSDFLVHFY
1050	1060	1070	1080	1090	1100	1110	1120
LVMHLGLTSE	DQDILNTIIR	HCPPRFFSLG	FPGFSMLVGD	FITAAARVLS	TDILTAPRSE	AVTVLGSLVC	FPNTYQEIPL
1130	1140	1150	1160	1170	1180	1190	1200
LQSVPEVNEA	ITGTEDVKHY	LINILLKNAT	EEPNEYARCI	AVCSLGVWIC	EELAQCTSHP	QVKEAINVIG	VTLKFPNKIV
1210	1220	1230	1240	1250	1260	1270	1280
AQVACDVLQL	LVSYWEKLQM	FETSLPRKMA	EILVATVAFL	LPSAEYSSVE	TDKKFIVSLL	LCLLDWCMAL	PVSVLLHPVS
1290	1300	1310	1320	1330	1340	1350	1360
TAVLEEQHSA	RAPLLDYIYR	VLHCCVCGSS	TYTQQSHYIL	TLADLSSTDY	DPFLPLANVK	SSEPVQYHSS	AELGNLLTVE
1370	1380	1390	1400	1410	1420	1430	1440
EEKKRRSLEL	IPLTARMVMA	HLVNHLGHYP	LSGGPAILHS	LVSENHDNAH	VEGSELSFEV	FRSPNLQLFV	FNDSTLISYL
1450	1460	1470	1480	1490	1500	1510	1520
QTPTEGPVGG	SPVGSLSDVR	VIVRDISGKY	SWDGKVLYGP	LEGCLAPNGR	NPSFLISSWH	RDTFGPQKDS	SQVEEGDDVL
1530	1540	1550	1560	1570	1580	1590	1600
DKLLENIGHT	SPECLLPSQL	NLNEPSLTPC	GMNYDQEKEI	IEVILRQNAQ	EDEYIQSHNF	DSAMKVTSQG	QPSPVEPRGP
1610	1620	1630	1640	1650	1660	1670	1680
FYFCRLLLDD	LGMNSWDRRK	NFHLLKKNSK	LLRELKNLDS	RQCRETHKIA	VFYIAEGQED	KCSILSNERG	SQAYEDFVAG
1690	1700	1710	1720	1730	1740	1750	1760
LGWEVDLSTH	CGFMGGLQRN	GSTGQTAPYY	ATSTVEVIFH	VSTRMPSDSD	DSLTKKLRHL	GNDEVHIVWS	EHSRDYRRGI
1770	1780	1790	1800	1810	1820	1830	1840
IPTAFGDVSI	IIYPMKNHMF	FIAITKKPEV	PFFGPLFDGA	IVSGKLLPSL	VCATCINASR	AVKCLIPLYQ	SFYEERALYL
1850	1860	1870	1880				
EAIIQNHREV	MTFEDFAAQV	FSPSPSYSLS	GTD				

Cmpd.	No. of Cmpds.	m/z meas.	Δ m/z [ppm]	z	Rt [min]	Score	P	Range	Sequence	Modification

Detailed Protein Report

Cmpd.	No. of Cmpds.	m/z meas.	Δ m/z [ppm]	z	Rt [min]	Score	P	Range	Sequence	Modification
2782	2	1024.0405	20.51	2	66.1	16.6	1	433-448	K.WILQDKPVFMEEPDRK.D	Oxidation: 10
1722	1	1273.4940	-96.18	2	51.5	10.6	0	1341-1363	K.SSEPVQYHSSAELGNLLTVEEEK.K	

Detailed Protein Report

Protein 525: **tumor necrosis factor receptor superfamily member 21 precursor [Homo sapiens]**

**Accession:**	gi|7657039	**Score:**	27.2
**Database:**	refseq_human(refseq_human_20140103.fasta)	**MW [kDa]:**	71.8
**Database Date:**	2015-11-30	**pI:**	9.3
		**Sequence Coverage [%]:**	4.6
		**No. of unique Peptides:**	1

Quantitation

Wdown:Qdown **Median:** 0.67 **CV:** 0.00 % **No. of Peptides:** 1

10	20	30	40	50	60	70	80
MGTSPSSSTA	LASCSRIARR	ATATMIAGSL	LLLGFLSTTT	AQPEQKASNL	IGTYRHVDRA	TGQVLTCDKC	PAGTYVSEHC
90	100	110	120	130	140	150	160
TNTSLRVCSS	CPVGTFTRHE	NGIEKCHDCS	QPCPWPMIEK	LPCAALTDRE	CTCPPGMFQS	NATCAPHTVC	PVGWGVRKKG
170	180	190	200	210	220	230	240
TETEDVRCKQ	CARGTFSDVP	SSVMKCKAYT	DCLSQNLVVI	KPGTKETDNV	CGTLPSFSSS	TSPSPGTAIF	PRPEHMETHE
250	260	270	280	290	300	310	320
VPSSTYVPKG	MNSTESNSSA	SVRPKVLSSI	QEGTVPDNTS	SARGKEDVNK	TLPNLQVVNH	QQGPHHRHIL	KLLPSMEATG
330	340	350	360	370	380	390	400
GEKSSTPIKG	PKRGHPRQNL	HKHFDINEHL	PWMIVLFLLL	VLVVIVVCSI	RKSSRTLKKG	PRQDPSAIVE	KAGLKKSMTP
410	420	430	440	450	460	470	480
TQNREKWIYY	CNGHGIDILK	LVAAQVGSQW	KDIYQFLCNA	SEREVAAFSN	GYTADHERAY	AALQHWTIRG	PEASLAQLIS
490	500	510	520	530	540	550	560
ALRQHRRNDV	VEKIRGLMED	TTQLETDKLA	LPMSPSPLSP	SPIPSPNAKL	ENSALLTVEP	SPQDKNKGFF	VDESEPLLRC
570	580	590	600	610	620	630	640
DSTSSGSSAL	SRNGSFITKE	KKDTVLRQVR	LDPCDLQPIF	DDMLHFLNPE	ELRVIEEIPQ	AEDKLDRLFE	IIGVKSQEAS
650	660						
QTLLDSVYSH	LPDLL						

Cmpd.	No. of Cmpds.	m/z meas.	Δ m/z [ppm]	z	Rt [min]	Score	P	Range	Sequence	Modification	Ratios
2722	1	941.9859	28.38	2	62.4	13.6	1	1-19	-.MGTSPSSSTALASCSRIAR.R		Wdown:Qdown 0.67

Detailed Protein Report

Protein 526: PREDICTED: neuroligin-2 isoform X1 [Homo sapiens]

**Accession:**	gi|530410532	**Score:**	27.1
**Database:**	refseq_human(refseq_human_20140103.fasta)	**MW [kDa]:**	88.9
**Database Date:**	2015-11-30	**pI:**	5.7
		**Sequence Coverage [%]:**	4.5
		**No. of unique Peptides:**	1

10	20	30	40	50	60	70	80
MWLLALCLVG	LAGAQRGGGG	PGGGAPGGPG	LGLGSLGEER	FPVVNTAYGR	VRGVRRELNN	EILGPVVQFL	GVPYATPPLG
90	100	110	120	130	140	150	160
ARRFQPPEAP	ASWPGVRNAT	TLPPACPQNL	HGALPAIMLP	VWFTDNLEAA	ATYVQNQSED	CLYLNLYVPT	EDDIRDPGKK
170	180	190	200	210	220	230	240
PVMLFLHGGS	YMEGTGNMFD	GSVLAAYGNV	IVATLNYRLG	VLGFLSTGDQ	AAKGNYGLLD	QIQALRWLSE	NIAHFGGDPE
250	260	270	280	290	300	310	320
RITIFGSGAG	ASCVNLLILS	HHSEGLFQKA	IAQSGTAISS	WSVNYQPLKY	TRLLAAKVGC	DREDSAEAVE	CLRRKPSREL
330	340	350	360	370	380	390	400
VDQDVQPARY	HIAFGPVVDG	DVVPDDPEIL	MQQGEFLNYD	MLIGVNQGEG	LKFVEDSAES	EDGVSASAFD	FTVSNFVDNL
410	420	430	440	450	460	470	480
YGYPEGKDVL	RETIKFMYTD	WADRDNGEMR	RKTLLALFTD	HQWVAPAVAT	AKLHADYQSP	VYFYTFYHHC	QAEGRPEWAD
490	500	510	520	530	540	550	560
AAHGDELPYV	FGVPMVGATD	LFPCNFSKND	VMLSAVVMTY	WTNFAKTGDP	NQPVPQDTKF	IHTKPNRFEE	VVWSKFNSKE
570	580	590	600	610	620	630	640
KQYLHIGLKP	RVRDNYRANK	VAFWLELVPH	LHNLHTELFT	TTTRLPPYAT	RWPPRPPAGA	PGTRRPPPPA	TLPPEPEPEP
650	660	670	680	690	700	710	720
GPRAYDRFPG	DSRDYSTELS	VTVAVGASLL	FLNILAFAAL	YYKRDRRQEL	RCRRLSPPGG	SGSGVPGGGP	LLPAAGRELP
730	740	750	760	770	780	790	800
PEEELVSLQL	KRGGGVGADP	AEALRPACPP	DYTLALRRAP	DDVPLLAPGA	LTLLPSGLGP	PPPPPPPSLH	PFGPFPPPPP
810	820						
TATSHNNTLP	HPHSTTRV						

Cmpd.	No. of Cmpds.	m/z meas.	Δ m/z [ppm]	z	Rt [min]	Score	P	Range	Sequence	Modification
1419	1	981.9479	-37.80	2	47.5	11.8	0	17-40	R.GGGGPGGGAPGGPGLGLGSLGEER.F	

Detailed Protein Report

Protein 527: **PREDICTED: U2 snRNP-associated SURP motif-containing protein isoform X3 [Homo sapiens]**

Accession: gi|530374190
Database: refseq_human(refseq_human_20140103.fasta)
Database Date: 2015-11-30
Modification(s): Oxidation

Score: 27.1
MW [kDa]: 111.5
pI: 6.5
Sequence Coverage [%]: 2.1
No. of unique Peptides: 2

10	20	30	40	50	60	70	80
MADKTPGGSQ	KASSKTRSSD	VHSSGSSDAH	MDASGPSDSD	MPSRTRPKSP	RKHNYRNESA	RESLCDSPHQ	NLSRPLLENK
90	100	110	120	130	140	150	160
LKAFSIGKMS	TAKRTLSKKE	QEELKKKEDE	KAAAEIYEEF	LAAFEGSDGN	KVKTFVRGGV	VNAAKEEHET	DEKRGKIYKP
170	180	190	200	210	220	230	240
SSRFADQKNP	PNQSSNERPP	SLLVIETKKP	PLKKGEKEKK	KSNLELFKEE	LKQIQEERDE	RHKTKGRLSR	FEPPQSDSDG
250	260	270	280	290	300	310	320
QRRSMDAPSR	RNRSSGVLDD	YAPGSHDVGD	PSTTNLYLGN	INPQMNEEML	CQEFGRFGPL	ASVKIMWPRT	DEERARERNC
330	340	350	360	370	380	390	400
GFVAFMNRRD	AERALKNLNG	KMIMSFEMKL	GWGKAVPIPP	HPIYIPPSMM	EHTLPPPPSG	LPFNAQPRER	LKNPNAPMLP
410	420	430	440	450	460	470	480
PPKNKEDFEK	TLSQAIVKVV	IPTERNLLAL	IHRMIEFVVR	EGPMFEAMIM	NREINNPMFR	FLFENQTPAH	VYYRWKLYSI
490	500	510	520	530	540	550	560
LQGDSPTKWR	TEDFRMFKNG	SFWRPPPLNP	YLHGMSEEQE	TEAFVEEPSK	KGALKEEQRD	KLEEILRGLT	PRKNDIGDAM
570	580	590	600	610	620	630	640
VFCLNNAEAA	EEIVDCITES	LSILKTPLPK	KIARLYLVSD	VLYNSSAKVA	NASYYRKFFE	TKLCQIFSDL	NATYRTIQGH
650	660	670	680	690	700	710	720
LQSENFKQRV	MTCFRAWEDW	AIYPEPFLIK	LQNIFLGLVN	IIEEKETEDV	PDDLDGAPIE	EELDGAPLED	VDGIPIDATP
730	740	750	760	770	780	790	800
IDDLDGVPIK	SLDDDLDGVP	LDATEDSKKN	EPIFKVAPSK	WEAVDESELE	AQAVTTSKWE	LFDQHEESEE	EENQNQEEES
810	820	830	840	850	860	870	880
EDEEDTQSSK	SEEHHLYSNP	IKEEMTESKF	SKYSEMSEEK	RAKLREIELK	VMKFQDELES	GKRPKKPGQS	FQEQVEHYRD
890	900	910	920	930	940	950	960
KLLQREKEKE	LERERERDKK	DKEKLESRSK	DKKEKDECTP	TRKERSPSGS	RTPKRSRRSR	SRSPKKSGKK	SRSQSRSPHR
970	980						
SHKKSKKNKH							

Cmpd.	No. of Cmpds.	m/z meas.	Δ m/z [ppm]	z	Rt [min]	Score	P	Range	Sequence	Modification
662	1	460.1225	-221.33	2	37.1	12.9	1	244-251	R.SMDAPSRR.N	
2921	1	729.3717	78.12	2	65.3	14.3	0	441-452	R.EGPMFEAMIMNR.E	Oxidation: 4, 10

Detailed Protein Report

Protein 528: PREDICTED: gametocyte-specific factor 1-like isoform X1 [Homo sapiens]

Accession: gi|530417873
Database: refseq_human(refseq_human_20140103.fasta)
Database Date: 2015-11-30

Score: 27.1
MW [kDa]: 11.9
pI: 9.1
Sequence Coverage [%]: 20.4
No. of unique Peptides: 1

Quantitation

mdown:qdown	**Median:** 0.79	**CV:** 0.00 %	**No. of Peptides:**	1
Wdown:Qdown	**Median:** 2.92	**CV:** 0.00 %	**No. of Peptides:**	1

```
        10         20         30         40         50         60         70         80
MEPEAFEICP YDPHHRIPLS RFQYHLASCR RKNPKKAKKM ATCKYNACHV VPIKNLEEHE AVCVNRSAVE EEDTENPLKV
        90        100        110
VCENDTKESA RETSPQKILR PGQ
```

Cmpd.	No. of Cmpds.	m/z meas.	Δ m/z [ppm]	z	Rt [min]	Score	P	Range	Sequence	Modification	Ratios
675	1	562.6665	-181.99	2	37.0	16.1	0	22-30	R.FQYHLASCR.R		mdown:qdown 0.79<br>Wdown:Qdown 2.92

Detailed Protein Report

Protein 529: **PREDICTED: gamma-aminobutyric acid type B receptor subunit 2 isoform X1 [Homo sapiens]**

**Accession:**	gi|530391788	**Score:**	27.1
**Database:**	refseq_human(refseq_human_20140103.fasta)	**MW [kDa]:**	77.5
**Database Date:**	2015-11-30	**pI:**	9.6
**Modification(s):**	Oxidation	**Sequence Coverage [%]:**	2.6
		**No. of unique Peptides:**	1

10	20	30	40	50	60	70	80
MAAKVFCCAY	EENMYGSKYQ	WIIPGWYEPS	WWEQVHTEAN	SSRCLRKNLL	AAMEGYIGVD	FEPLSSKQIK	TISGKTPQQY
90	100	110	120	130	140	150	160
EREYNNKRSG	VGPSKFHGYA	YDGIWVIAKT	LQRAMETLHA	SSRHQRIQDF	NYTDHTLGRI	ILNAMNETNF	FGVTGQVVFR
170	180	190	200	210	220	230	240
NGERMGTIKF	TQFQDSREVK	VGEYNAVADT	LEIINDTIRF	QGSEPPKDKT	IILEQLRKIS	LPLYSILSAL	TILGMIMASA
250	260	270	280	290	300	310	320
FLFFNIKNRN	QKLIKMSSPY	MNNLIILGGM	LSYASIFLFG	LDGSFVSEKT	FETLCTVRTW	ILTVGYTTAF	GAMFAKTWRV
330	340	350	360	370	380	390	400
HAIFKNVKMK	KKIIKDQKLL	VIVGGMLLID	LCILICWQAV	DPLRRTVEKY	SMEPDPAGRD	ISIRPLLEHC	ENTHMTIWLG
410	420	430	440	450	460	470	480
IVYAYKGLLM	LFGCFLAWET	RNVSIPALND	SKYIGMSVYN	VGIMCIIGAA	VSFLTRDQPN	VQFCIVALVI	IFCSTITLCL
490	500	510	520	530	540	550	560
VFVPKLITLR	TNPDAATQNR	RFQFTQNQKK	EDSKTSTSVT	SVNQASTSRL	EGLQSENHRL	RMKITELDKD	LEEVTMQLQD
570	580	590	600	610	620	630	640
TPEKTTYIKQ	NHYQELNDIL	NLGNFTESTD	GGKAILKNHL	DQNPQLQWNT	TEPSRTCKDP	IEDINSPEHI	QRRLSLQLPI
650	660	670	680	690			
LHHAYLPSIG	GVDASCVSPC	VSPTASPRHR	HVPPSFRVMV	SGL			

Cmpd.	No. of Cmpds.	m/z meas.	Δ m/z [ppm]	z	Rt [min]	Score	P	Range	Sequence	Modification
814	1	965.4561	44.99	2	39.0	10.5	1	2-18	M.AAKVFCCAYEENMYGSK.Y	Oxidation: 13

Detailed Protein Report

Protein 530: **PREDICTED: TBC domain-containing protein kinase-like protein isoform X1 [Homo sapiens]**

**Accession:**	gi|578809649	**Score:**	27.1
**Database:**	refseq_human(refseq_human_20140103.fasta)	**MW [kDa]:**	84.9
**Database Date:**	2015-11-30	**pI:**	5.8
**Modification(s):**	Carbamidomethyl	**Sequence Coverage [%]:**	3.3
		**No. of unique Peptides:**	1

10	20	30	40	50	60	70	80
MFPLKDAEMG	AFTFFASALP	HDVCGSNGLP	LTPNSIKILG	RFQILKTITH	PRLCQYVDIS	RGKHERLVVV	AEHCERSLED
**90**	**100**	**110**	**120**	**130**	**140**	**150**	**160**
LLRERKPVSC	STVLCIAFEV	LQGLQYMNKH	GIVHRALSPH	NILLDRKGHI	KLAKFGLYHM	TAHGDDVDFP	IGYPSYLAPE
**170**	**180**	**190**	**200**	**210**	**220**	**230**	**240**
VIAQGIFKTT	DHMPSKKPLP	SGPKSDVWSL	GIILFELCVG	RKLFQSLDIS	ERLKFLLTLD	CVDDTLIVLA	EEHGCLDIIK
**250**	**260**	**270**	**280**	**290**	**300**	**310**	**320**
ELPETVIDLL	NKCLTFHPSK	RPTPDQLMKD	KVFSEVSPLY	TPFTKPASLF	SSSLRCADLT	LPEDISQLCK	DINNDYLAER
**330**	**340**	**350**	**360**	**370**	**380**	**390**	**400**
SIEEVYYLWC	LAGGDLEKEL	VNKEIIRSKP	PICTLPNFLF	EDGESFGQGR	DRSSLLDDTT	VTLSLCQLRN	RLKDVGGEAF
**410**	**420**	**430**	**440**	**450**	**460**	**470**	**480**
YPLLEDDQSN	LPHSNSNNEL	SAAATLPLII	REKDTEYQLN	RIILFDRLLK	AYPYKKNQIW	KEARVDIPPL	MRGLTWAALL
**490**	**500**	**510**	**520**	**530**	**540**	**550**	**560**
GVEGAIHAKY	DAIDKDTPIP	TDRQIEVDIP	RCHQYDELLS	SPEGHAKFRR	VLKAWVVSHP	DLVYWQGLDS	LCAPFLYLNF
**570**	**580**	**590**	**600**	**610**	**620**	**630**	**640**
NNEALAYACM	SAFIPKYLYN	FFLKDNSHVI	QEYLTVFSQM	IAFHDPELSN	HLNEIGFIPD	LYAIPWFLTM	FTHVFPLHKI
**650**	**660**	**670**	**680**	**690**	**700**	**710**	**720**
FHLWDTLLLG	NSSFPFCIGV	AILQQLRDRL	LANGFNECIL	LFSDLPEIDI	ERCVRESINL	FCWTPKSATY	RQHAQPPKPS
**730**	**740**	**750**					
SDSSGGRSSA	PYFSAECPDP	PKTDLKIH					

Cmpd.	No. of Cmpds.	m/z meas.	Δ m/z [ppm]	z	Rt [min]	Score	P	Range	Sequence	Modification
1977	3	876.8617	-79.82	2	53.4	16.4	1	693-706	R.CVRESINLFCWTPK.S	Carbamidomethyl: 10

Detailed Protein Report

Protein 531: **PREDICTED: inhibitor of Bruton tyrosine kinase isoform X3 [Homo sapiens]**

**Accession:**	gi|578812678	**Score:**	27.1
**Database:**	refseq_human(refseq_human_20140103.fasta)	**MW [kDa]:**	148.9
**Database Date:**	2015-11-30	**pI:**	8.5
**Modification(s):**	Oxidation	**Sequence Coverage [%]:**	2.2
		**No. of unique Peptides:**	2

10	20	30	40	50	60	70	80
MSSPMPDCTS	KCRSLKHALD	VLSVVTKGSE	NQIKAFLSSH	CYNAATIKDV	FGRNALHLVS	SCGKKGVLDW	LIQKGVDLLV
**90**	**100**	**110**	**120**	**130**	**140**	**150**	**160**
KDKESGWTAL	HRSIFYGHID	CVWSLLKHGV	SLYIQDKEGL	SALDLVMKDR	PTHVVFKNTD	PTDVYTWGDN	TNFTLGHGSQ
**170**	**180**	**190**	**200**	**210**	**220**	**230**	**240**
NSKHHPELVD	LFSRSGIYIK	QVVLCKFHSV	FLSQKGQVYT	CGHGPGGRLG	HGDEQTCLVP	RLVEGLNGHN	CSQVAAAKDH
**250**	**260**	**270**	**280**	**290**	**300**	**310**	**320**
TVVLTEDGCV	YTFGLNIFHQ	LGIIPPPSSC	NVPRQIQAKY	LKGRTIIGVA	AGRFHTVLWT	REAVYTMGLN	GGQLGCLLDP
**330**	**340**	**350**	**360**	**370**	**380**	**390**	**400**
NGEKCVTAPR	QVSALHHKDI	ALSLVAASDG	ATVCVTTRGD	IYLLADYQCK	KMASKQLNLK	KVLVSGGHME	YKVDPEHLKE
**410**	**420**	**430**	**440**	**450**	**460**	**470**	**480**
NGGQKICILA	MDGAGRVFCW	RSVNSSLKQC	RWAYPRQVFI	SDIALNRNEI	LFVTQDGEGF	RGRWFEEKRK	SSEKKEILSN
**490**	**500**	**510**	**520**	**530**	**540**	**550**	**560**
LHNSSSDVSY	VSDINSVYER	IRLEKLTFAH	RAVSVSTDPS	GCNFAILQSD	PKTSLYEIPA	VSSSSFFEEF	GKLLREADEM
**570**	**580**	**590**	**600**	**610**	**620**	**630**	**640**
DSIHDVTFQV	GNRLFPAHKY	ILAVHSDFFQ	KLFLSDGNTS	EFTDIYQKDE	DSAGCHLFVV	EKVHPDMFEY	LLQFIYTDTC
**650**	**660**	**670**	**680**	**690**	**700**	**710**	**720**
DFLTHGFKPR	IHLNKNPEEY	QGTLNSHLNK	VNFHEDDNQK	SAFEVYKSNQ	AQTVSERQKS	KPKSCKKGKN	IREDDPVRML
**730**	**740**	**750**	**760**	**770**	**780**	**790**	**800**
QTVAKKFDFS	NLSSRLDGVR	FENEKINVIA	KNTGNKLKLS	QKKCSFLCDV	TMKSVDGKEF	PCHKCVLCAR	LEYFHSMLSS
**810**	**820**	**830**	**840**	**850**	**860**	**870**	**880**
SWIEASSCAA	LEMPIHSDIL	KVILDYLYTD	EAVVIKESQN	VDFICSVLVV	ADQLLITRLK	EICEVALTEK	LTLKNAAMLL
**890**	**900**	**910**	**920**	**930**	**940**	**950**	**960**
EFAAMYSAKQ	LKLSCLQFIG	LNMAALLEAR	SLDVLSDGVL	KDLSEFYRKM	IPAMDRRVIT	PYQDGPDISY	LEVEDGDIFL
**970**	**980**	**990**	**1000**	**1010**	**1020**	**1030**	**1040**
KEEINMEQNH	SETMFKKAKT	KAKKKPRKRS	DSSGGYNLSD	IIQSPSSTEL	LTSDSEGSYA	GVGSPRDLQS	PDFTTGFHSD
**1050**	**1060**	**1070**	**1080**	**1090**	**1100**	**1110**	**1120**
KIEAKVKPYV	NGTSPVYSRE	DLKPWEKSPI	LKISAPQPIP	SNRIDTTSSA	SWVAGSFSPV	SPPVVDLRTI	MEIEESRQKC
**1130**	**1140**	**1150**	**1160**	**1170**	**1180**	**1190**	**1200**
GATPKSHLGK	TVSHGVKLSQ	KQRKMIALTT	KENNSGMNSM	ETVLFTPSKA	PKPVNAWASS	LHSVSSKSFR	DFLLEEKKSV
**1210**	**1220**	**1230**	**1240**	**1250**	**1260**	**1270**	**1280**
TSHSSGDHVK	KVSFKGIENS	QAPKIVRCST	HGTPGPEGNH	ISDLPLLDSP	NPWLSSSVTA	PSMVAPVTFA	SIVEEELQQE
**1290**	**1300**	**1310**	**1320**	**1330**	**1340**		
AALIRSREKP	LALIQIEEHA	IQDLLVFYEA	FGNPEEFVIV	ERTPQGPLAV	PMWNKHGC		

Cmpd.	No. of Cmpds.	m/z meas.	Δ m/z [ppm]	z	Rt [min]	Score	P	Range	Sequence	Modification
1342	1	388.0955	-190.68	3	45.3	14.3	0	764-773	K.CSFLCDVTMK.S	Oxidation: 9
2082	1	701.2889	-119.01	3	54.6	12.7	1	871-889	K.LTLKNAAMLLEFAAMYSAK.Q	Oxidation: 15

Detailed Protein Report

Protein 532: spermatogenesis-associated protein 31E1 [Homo sapiens]

Accession: gi|155029550
Database: refseq_human(refseq_human_20140103.fasta)
Database Date: 2015-11-30

Score: 27.1
MW [kDa]: 157.0
pI: 10.3
Sequence Coverage [%]: 1.7
No. of unique Peptides: 2

10	20	30	40	50	60	70	80
MGNLVIPLGK	GRAGRVESGQ	RIPPPAPRPS	VECTGDDIAL	QMEKMLFPLK	SPSATWLSPS	STPWMMDFIL	TSVCGLVLLF
90	100	110	120	130	140	150	160
LLLLYVHSDP	PSPPPGRKRS	SREPQRERSG	RSRSRKISAL	KACRILLREL	EETRDLNYLL	ESHLRKLAGE	GSSHLPLGGD
170	180	190	200	210	220	230	240
PLGDVCKPVP	AKAHQPHGKC	MQDPSPASLS	PPAPPAPLAS	TLSPGPMTFS	EPFGPHSTLS	ASGPPEPLLP	LKCPATQPHV
250	260	270	280	290	300	310	320
VFPPSPQPHG	PLASSPPPPD	SSLAGLQCGS	TTCPVPQSSP	LHNQVLPPPT	RVISGLGCSS	DPIWDLYCWR	EAATTWGLST
330	340	350	360	370	380	390	400
YSHGKSQPRH	LPDHTSEASF	WGDPTPKHME	VGGCTFIHPD	VQKLLETLIA	KRALMKMWQE	KERKRADHPH	MTSLGKEWDI
410	420	430	440	450	460	470	480
TTLNPFWNVS	TQPQQLPRPQ	QVSDATTVGN	HLQQKRSQLF	WDLPSLNSES	LATTVWVSRN	PSSQNAHSVP	LDKASTSLPG
490	500	510	520	530	540	550	560
EPEVEASSQL	SQAPPQPHHM	AQPQHFTPAW	PQSQPPPLAE	IQTQAHLSPP	VPSLGCSSPP	QIRGCGASYP	TSQERTQSVI
570	580	590	600	610	620	630	640
PTGKEYLEWP	LKKRPKWKRV	LPSLLKKSQA	VLSQPTAHLP	QERPASWSPK	SAPILPGVVT	SPELPEHWWQ	GRNAIHQEQS
650	660	670	680	690	700	710	720
CGPPSRLQAS	GDLLQPDGEF	PGRPQSQAED	TQQALLPSQP	SDFAGKGRKD	VQKTGFRSSG	RFSDKGCLGS	KLGPDPSRDQ
730	740	750	760	770	780	790	800
GSGRTSVKAL	DEDKEAEGDL	RRSWKYQSVS	STPRDPDKEH	LENKLQIHLA	RKVGEIKEGW	IPMPVRRSWL	MAKCAVPKSD
810	820	830	840	850	860	870	880
THRKPGKLAS	WRGGKAHVNT	SQELSFLHPC	TQQILEVHLV	RFCVRHSWGT	DLQSLEPINV	WSGEAQAPPF	PQSTFTPWAS
890	900	910	920	930	940	950	960
WVSRVESVPK	VPIFLGKRPQ	NGPGDNRTTS	KSVPTVSGPL	AAPPPEQEGV	QRPPRGSQSA	DTHGRSEAFP	TGHKGRGCSQ
970	980	990	1000	1010	1020	1030	1040
PPTCSLVGRT	WQSRTVLESG	KPKPRLEGSM	GSEMAGNEAW	LESESMSPGD	PCSSRALQVL	SIGSQWARAE	DALQALKVGE
1050	1060	1070	1080	1090	1100	1110	1120
KPPTWEVTLG	ASVRASSGSV	QEDLRSTGAL	GTTGNPSASS	VCVAQDPEQL	HLKAQVVSEI	ALIVQVDSEE	QLPGRAPGIL
1130	1140	1150	1160	1170	1180	1190	1200
LQDGATGLCL	PGRHMDMLTA	ADRLPTQAPL	STSQSVSGKN	MTASQGPCAL	LWKGGDSPGQ	QEPGSPKAKA	PQKSQKTLGC
1210	1220	1230	1240	1250	1260	1270	1280
ADKGEAHRRP	RTGEQGHRSK	GPRTSEASGR	SHPAQAREIG	DKQERKYNQL	QLEKGQTPPE	SHFQRKISHH	PQGLHPRKGG
1290	1300	1310	1320	1330	1340	1350	1360
TRWEDVLQKG	KPGADAFQSW	GSGPPRQFMD	CMADKAWTIS	RVVGQILVDK	LGLQWGRGPS	EVNRHKGDFR	AQENVPSCCH
1370	1380	1390	1400	1410	1420	1430	1440
RGHCHQERSR	EMRALACSPK	ATPKGHHCPV	KNRGIRDRDS	SWAPPPREPV	SPAGPHHHRP	RMASTSGGPH	PQLQELMSAQ
1450							
RCLAS							

Cmpd.	No. of Cmpds.	m/z meas.	Δ m/z [ppm]	z	Rt [min]	Score	P	Range	Sequence	Modification
2526	1	574.6432	-239.26	2	60.3	12.8	0	1055-1065	R.ASSGSVQEDLR.S	
1848	1	805.3101	-128.16	2	53.1	14.3	2	1394-1407	R.GIRDRDSSWAPPPR.E	

Detailed Protein Report

Protein 533: SH3 and multiple ankyrin repeat domains protein 3 [Homo sapiens]

**Accession:**	gi|380748963	**Score:**	27.1
**Database:**	refseq_human(refseq_human_20140103.fasta)	**MW [kDa]:**	184.6
**Database Date:**	2015-11-30	**pI:**	9.6
**Modification(s):**	Oxidation	**Sequence Coverage [%]:**	1.4
		**No. of unique Peptides:**	2

10	20	30	40	50	60	70	80
MDGPGASAVV	VRVGIPDLQQ	TKCLRLDPAA	PVWAAKQRVL	CALNHSLQDA	LNYGLFQPPS	RGRAGKFLDE	ERLLQEYPPN
90	100	110	120	130	140	150	160
LDTPLPYLEF	RYKRRVYAQN	LIDDKQFAKL	HTKANLKKFM	DYVQLHSTDK	VARLLDKGLD	PNFHDPDSGE	CPLSLAAQLD
170	180	190	200	210	220	230	240
NATDLLKVLK	NGGAHLDFRT	RDGLTAVHCA	TRQRNAAALT	TLLDLGASPD	YKDSRGLTPL	YHSALGGGDA	LCCELLLHDH
250	260	270	280	290	300	310	320
AQLGITDENG	WQEIHQACRF	GHVQHLEHLL	FYGADMGAQN	ASGNTALHIC	ALYNQESCAR	VLLFRGANRD	VRNYNSQTAF
330	340	350	360	370	380	390	400
QVAIIAGNFE	LAEVIKTHKD	SDVVPFRETP	SYAKRRRLAG	PSGLASPRPL	QRSASDINLK	GEAQPAASPG	PSLRSLPHQL
410	420	430	440	450	460	470	480
LLQRLQEEKD	RDRDADQESN	ISGPLAGRAG	QSKISPSGPG	GPGPAPGPGP	APPAPPAPPP	RGPKRKLYSA	VPGRKFIAVK
490	500	510	520	530	540	550	560
AHSPQGEGEI	PLHRGEAVKV	LSIGEGGFWE	GTVKGRTGWF	PADCVEEVQM	RQHDTRPETR	EDRTKRLFRH	YTVGSYDSLT
570	580	590	600	610	620	630	640
SHSDYVIDDK	VAVLQKRDHE	GFGFVLRGAK	AETPIEEFTP	TPAFPALQYL	ESVDVEGVAW	RAGLRTGDFL	IEVNGVNVVK
650	660	670	680	690	700	710	720
VGHKQVVALI	RQGGNRLVMK	VVSVTRKPEE	DGARRRAPPP	PKRAPSTTLT	LRSKSMTAEL	EELASIRRRK	GEKLDEMLAA
730	740	750	760	770	780	790	800
AAEPTLRPDI	ADADSRAATV	KQRPTSRRIT	PAEISSLFER	QGLPGPEKLP	GSLRKGIPRT	KSVGEDEKLA	SLLEGRFPRS
810	820	830	840	850	860	870	880
TSMQDPVREG	RGIPPPPQTA	PPPPPAPYYF	DSGPPPAFSP	PPPPGRAYDT	VRSSFKPGLE	ARLGAGAAGL	YEPGAALGPL
890	900	910	920	930	940	950	960
PYPERQKRAR	SMIILQDSAP	ESGDAPRPPP	AATPPERPKR	RPRPPGPDSP	YANLGAFSAS	LFAPSKPQRR	KSPLVKQLQV
970	980	990	1000	1010	1020	1030	1040
EDAQERAALA	VGSPGPGGGS	FAREPSPTHR	GPRPGGLDYG	AGDGPGLAFG	GPGPAKDRRL	EERRRSTVFL	SVGAIEGSAP
1050	1060	1070	1080	1090	1100	1110	1120
GADLPSLQPS	RSIDERLLGT	GPTAGRDLLL	PSPVSALKPL	VSGPSLGPSG	STFIHPLTGK	PLDPSSPLAL	ALAARERALA
1130	1140	1150	1160	1170	1180	1190	1200
SQAPSRSPTP	VHSPDADRPG	PLFVDVQARD	PERGSLASPA	FSPRSPAWIP	VPARREAEKV	PREERKSPED	KKSMILSVLD
1210	1220	1230	1240	1250	1260	1270	1280
TSLQRPAGLI	VVHATSNGQE	PSRLGGAEEE	RPGTPELAPA	PMQSAAVAEP	LPSPRAQPPG	GTPADAGPGQ	GSSEEEPELV
1290	1300	1310	1320	1330	1340	1350	1360
FAVNLPPAQL	SSSDEETREE	LARIGLVPPP	EEFANGVLLA	TPLAGPGPSP	TTVPSPASGK	PSSEPPPAPE	SAADSGVEEA
1370	1380	1390	1400	1410	1420	1430	1440
DTRSSSDPHL	ETTSTISTVS	SMSTLSSESG	ELTDTHTSFA	DGHTFLLEKP	PVPPKPKLKS	PLGKGPVTFR	DPLLKQSSDS
1450	1460	1470	1480	1490	1500	1510	1520
ELMAQQHHAA	SAGLASAAGP	ARPRYLFQRR	SKLWGDPVES	RGLPGPEDDK	PTVISELSSR	LQQLNKDTRS	LGEEPVGGLG
1530	1540	1550	1560	1570	1580	1590	1600
SLLDPAKKSP	IAAARLFSSL	GELSSISAQR	SPGGPGGGAS	YSVRPSGRYP	VARRAPSPVK	PASLERVEGL	GAGAGGAGRP
1610	1620	1630	1640	1650	1660	1670	1680
FGLTPPTILK	SSSLSIPHEP	KEVRFVVRSV	SARSRSPSPS	PLPSPASGPG	PGAPGPRRPF	QQKPLQLWSK	FDVGDWLESI
1690	1700	1710	1720	1730	1740		
HLGEHRDRFE	DHEIEGAHLP	ALTKDDFVEL	GVTRVGHRMN	IERALRQLDG	S		

Cmpd.	No. of Cmpds.	m/z meas.	Δ m/z [ppm]	z	Rt [min]	Score	P	Range	Sequence	Modification
1868	1	840.7873	-166.49	2	53.4	13.8	1	693-707	R.SKSMTAELEELASIR.R	Oxidation: 4
498	1	546.2697	-47.83	2	34.8	13.2	0	853-862	R.SSFKPGLEAR.L	

Detailed Protein Report

Protein 534: spatacsin isoform 2 [Homo sapiens]

**Accession:**	gi|237681069	**Score:**	27.1
**Database:**	refseq_human(refseq_human_20140103.fasta)	**MW [kDa]:**	266.5
**Database Date:**	2015-11-30	**pI:**	5.6
**Modification(s):**	Carbamidomethyl	**Sequence Coverage [%]:**	1.2
		**No. of unique Peptides:**	1

Detailed Protein Report

10	20	30	40	50	60	70	80
MAAEEGVASA	ASAGGSWGTA	AMGRVLPMLL	VPVPAEAMGQ	LGSRAQLRTQ	PEALGSLTAA	GSLQVLSLTP	GSRGGGRCCL
90	100	110	120	130	140	150	160
EGPFWHFLWE	DSRNSSTPTE	KPKLLALGEN	YELLIYEFNL	KDGRCDATIL	YSCSREALQK	LIDDQDISIS	LLSLRILSFH
170	180	190	200	210	220	230	240
NNTSLLFINK	CVILHIIFPE	RDAAIRVLNC	FTLPLPAQAV	DMIIDTQLCR	GILFVLSSLG	WIYIFDVVDG	TYVAHVDLAL
250	260	270	280	290	300	310	320
HKEDMCNEQQ	QEPAKISSFT	SLKVSQDLDV	AVIVSSSNSA	VALNLNLYFR	QHPGHLLCER	ILEDLPIQGP	KGVDEDDPVN
330	340	350	360	370	380	390	400
SAYNMKLAKF	SFQIDRSWKA	QLSSLNETIK	NSKLEVSCCA	PWFQDILHLE	SPESGNHSTS	VQSWAFIPQD	IMHGQYNVLQ
410	420	430	440	450	460	470	480
KDHAKTSDPG	RSWKIMHISE	QEEPIELKCV	SVTGFTALFT	WEVERMGYTI	TLWDLETQGM	QCFSLGTKCI	PVDSSGDQQL
490	500	510	520	530	540	550	560
CFVLTENGLS	LILFGLTQEE	FLNRLMIHGS	ASTVDTLCHL	NGWGRCSIPI	HALEAGIENR	QLDTVNFFLK	SKENLFNPSS
570	580	590	600	610	620	630	640
KSSVSDQFDH	LSSHLYLRNV	EELIPALDLL	CSAIRESYSE	PQSKHFSEQL	LNLTLSFLNN	QIKELFIHTE	ELDEHLQKGV
650	660	670	680	690	700	710	720
NILTSYINEL	RTFMIKFPWK	LTDAIDEYDV	HENVPKVKES	NIWKKLSFEE	VIASAILNNK	IPEAQTFFRI	DSHSAQKLEE
730	740	750	760	770	780	790	800
LIGIGLNLVF	DNLKKNNIKE	ASELLKNMGF	DVKGQLLKIC	FYTTNKNIRD	FLVEILKEKN	YFSEKEKRTI	DFVHQVEKLY
810	820	830	840	850	860	870	880
LGHFQENMQI	QSFPRYWIKE	QDFFKHKSVL	DSFLKYDCKD	EFNKQDHRIV	LNWALWWDQL	TQESILLPRI	SPEEYKSYSP
890	900	910	920	930	940	950	960
EALWRYLTAR	HDWLNIILWI	GEFQTQHSYA	SLQQNKWPLL	TVDVINQNTS	CNNYMRNEIL	DKLARNGVFL	ASELEDFECF
970	980	990	1000	1010	1020	1030	1040
LLRLSRIGGV	IQDTLPVQNY	KTKEGWDFHS	QFILYCLEHS	LQHLLYVYLD	CYKLSPENCP	FLEKKELHEA	HPWFEFLVQC
1050	1060	1070	1080	1090	1100	1110	1120
RQVASNLTDP	KLIFQASLAN	AQILIPTNQA	SVSSMLLEGH	TLLALATTMY	SPGGVSQVVQ	NEENENCLKK	VDPQLLKMAL
1130	1140	1150	1160	1170	1180	1190	1200
TPYPKLKTAL	FPQCTPPSVL	PSDITIYHLI	QSLSPFDPSR	LFGWQSANTL	AIGDAWSHLP	HFSSPDLVNK	YAIVERLNFA
1210	1220	1230	1240	1250	1260	1270	1280
YYLHNGRPSF	AFGTFLVQEL	IKSKTPKQLI	QQVGNEAYVI	GLSSFHIPSI	GAACVCFLEL	LGLDSLKLRV	DMKVANIILS
1290	1300	1310	1320	1330	1340	1350	1360
YKCRNEDAQY	SFIRESVAEK	LSKLADGEKT	TTEELLVLLE	EGTWNSIQQQ	EIKRLSSESS	SQWALVVQFC	RLHNMKLSIS
1370	1380	1390	1400	1410	1420	1430	1440
YLRECAKAND	WLQFIIHSQL	HNYHPAEVKS	LIQYFSPVIQ	DHLRLAFENL	PSVPTSKMDS	DQVCNKCPQE	LQGSKQEMTD
1450	1460	1470	1480	1490	1500	1510	1520
LFEILLQCSE	EPDSWHWLLV	EAVKQQAPIL	SVLASCLQGA	SAISCLCVWI	ITSVEDNVAT	EAMGHIQDST	EDHTWNLEDL
1530	1540	1550	1560	1570	1580	1590	1600
SVIWRTLLTR	QKSKTLIRGF	QLFFKDSPLL	LVMEMYELCM	FFRNYKEAEA	KLLEFQKSLE	TLNTAATKVH	PVIPAMWLED
1610	1620	1630	1640	1650	1660	1670	1680
QVCFLLKLML	QQCKTQYELG	KLLQLFVERE	HLFSDGPDVK	KLCILCQILK	DTSIAINHTI	ITSYSIENLQ	HECRSILERL
1690	1700	1710	1720	1730	1740	1750	1760
QTDGQFALAR	RVAELAELPV	DNLVIKEITQ	EMQTLKHIEQ	WSLKQARIDF	WKKCHENFKK	NSISSKAASS	FFSTQAHVAC
1770	1780	1790	1800	1810	1820	1830	1840
EHPTGWSSME	ERHLLLTLAG	HWLAQEDVVP	LDKLEELEKQ	IWLCRITQHT	LGRNQEETEP	RFSRQISTSG	ELSFDSLASE
1850	1860	1870	1880	1890	1900	1910	1920
FSFSKLAALN	TSKYLELNSL	PSKETCENRL	DWKEQESLNF	LIGRLLDDGC	VHEASRVCRY	FHFYNPDVAL	VLHCRALASG
1930	1940	1950	1960	1970	1980	1990	2000
EASMEDLHPE	IHALLQSAEL	LEEEAPDIPL	RRVHSRHKQM	FNPTEESQTF	LQLTTLCQDR	TLVGMKLLDK	ISSVPHGELS
2010	2020	2030	2040	2050	2060	2070	2080
CTTELLILAH	HCFTLTCHME	GIIRVLQAAH	MLTDNHLAPS	EEYGLVVRLL	TGIGRYNEMT	YIFDLLHKKH	YFEVLMRKKL
2090	2100	2110	2120	2130	2140	2150	2160
DPSGTLKTAL	LDYIKRCRPG	DSEKHNMIAL	CFSMCREIGE	NHEAAARIQL	KLIESQPWED	SLKDGHQLKQ	LLLKALTLML
2170	2180	2190	2200	2210	2220	2230	2240

Detailed Protein Report

Cmpd.	No. of Cmpds.	m/z meas.	Δ m/z [ppm]	z	Rt [min]	Score	P	Range	Sequence	Modification
1898	1	686.2375	-119.80	2	52.2	12.6	0	1885-1896	R.LLDDGCVHEASR.V	Carbamidomethyl: 6

Detailed Protein Report

Protein 535: PREDICTED: ubiquitin thioesterase ZRANB1 isoform X3 [Homo sapiens]

**Accession:**	gi|530393888	**Score:**	27.0
**Database:**	refseq_human(refseq_human_20140103.fasta)	**MW [kDa]:**	44.2
**Database Date:**	2015-11-30	**pI:**	4.7
		**Sequence Coverage [%]:**	5.5
		**No. of unique Peptides:**	2

```
        10         20         30         40         50         60         70         80
MLAILLTEVS QQAAKCIPAM VCPELTEQIR REIAASLHQR KGDFACYFLT DLVTFTLPAD IEDLPPTVQE KLFDEVLDRD
        90        100        110        120        130        140        150        160
VQKELEEESP IINWSLELAT RLDSRLYALW NRTAGDCLLD SVLQATWGIY DKDSVLRKAL HDSLHDCSHW FYTRWKDWES
       170        180        190        200        210        220        230        240
WYSQSFGLHF SLREEQWQED WAFILSLASQ PGASLEQTHI FVLAHILRRP IIVYGVKYYK SFRGETLGYT RFQGVYLPLL
       250        260        270        280        290        300        310        320
WEQSFCWKSP IALGYTRGHF SALVAMENDG YGNRGAGANL NTDDDVTITF LPLVDSERKL LHVHFLSAQE LGNEEQQEKL
       330        340        350        360        370        380        390
LREWLDCCVT EGGVLVAMQK SSRRRNHPLV TQMVEKWLDR YRQIRPCTSL SDGEEDEDDE DE
```

Cmpd.	No. of Cmpds.	m/z meas.	Δ m/z [ppm]	z	Rt [min]	Score	P	Range	Sequence	Modification
1033	2	590.8060	-42.13	2	41.7	16.3	1	31-40	R.REIAASLHQR.K	
2725	1	648.3400	-3.80	2	62.5	10.7	0	346-356	R.NHPLVTQMVEK.W	

Detailed Protein Report

Protein 536: probable ATP-dependent RNA helicase DDX20 [Homo sapiens]

**Accession:**	gi|256223453	**Score:**	27.0
**Database:**	refseq_human(refseq_human_20140103.fasta)	**MW [kDa]:**	92.2
**Database Date:**	2015-11-30	**pI:**	6.5
		**Sequence Coverage [%]:**	1.6
		**No. of unique Peptides:**	1

10	20	30	40	50	60	70	80
MAAAFEASGA	LAAVATAMPA	EHVAVQVPAP	EPTPGPVRIL	RTAQDLSSPR	TRTGDVLLAE	PADFESLLLS	RPVLEGLRAA
**90**	**100**	**110**	**120**	**130**	**140**	**150**	**160**
GFERPSPVQL	KAIPLGRCGL	DLIVQAKSGT	GKTCVFSTIA	LDSLVLENLS	TQILILAPTR	EIAVQIHSVI	TAIGIKMEGL
**170**	**180**	**190**	**200**	**210**	**220**	**230**	**240**
ECHVFIGGTP	LSQDKTRLKK	CHIAVGSPGR	IKQLIELDYL	NPGSIRLFIL	DEADKLLEEG	SFQEQINWIY	SSLPASKQML
**250**	**260**	**270**	**280**	**290**	**300**	**310**	**320**
AVSATYPEFL	ANALTKYMRD	PTFVRLNSSD	PSLIGLKQYY	KVVNSYPLAH	KVFEEKTQHL	QELFSRIPFN	QALVFSNLHS
**330**	**340**	**350**	**360**	**370**	**380**	**390**	**400**
RAQHLADILS	SKGFPAECIS	GNMNQNQRLD	AMAKLKHFHC	RVLISTDLTS	RGIDAEKVNL	VVNLDVPLDW	ETYMHRIGRA
**410**	**420**	**430**	**440**	**450**	**460**	**470**	**480**
GRFGTLGLTV	TYCCRGEEEN	MMMRIAQKCN	INLLPLPDPI	PSGLMEECVD	WDVEVKAAVH	TYGIASVPNQ	PLKKQIQKIE
**490**	**500**	**510**	**520**	**530**	**540**	**550**	**560**
RTLQIQKAHG	DHMASSRNNS	VSGLSVKSKN	NTKQKLPVKS	HSECGIIEKA	TSPKELGCDR	QSEEQMKNSV	QTPVENSTNS
**570**	**580**	**590**	**600**	**610**	**620**	**630**	**640**
QHQVKEALPV	SLPQIPCLSS	FKIHQPYTLT	FAELVEDYEH	YIKEGLEKPV	EIIRHYTGPG	DQTVNPQNGF	VRNKVIEQRV
**650**	**660**	**670**	**680**	**690**	**700**	**710**	**720**
PVLASSSQSG	DSESDSDSYS	SRTSSQSKGN	KSYLEGSSDN	QLKDSESTPV	DDRISLEQPP	NGSDTPNPEK	YQESPGIQMK
**730**	**740**	**750**	**760**	**770**	**780**	**790**	**800**
TRLKEGASQR	AKQSRRNLPR	RSSFRLQTEA	QEDDWYDCHR	EIRLSFSDTY	QDYEEYWRAY	YRAWQEYYAA	ASHSYYWNAQ
**810**	**820**	**830**					
RHPSWMAAYH	MNTIYLQEMM	HSNQ					

Cmpd.	No. of Cmpds.	m/z meas.	Δ m/z [ppm]	z	Rt [min]	Score	P	Range	Sequence	Modification
827	1	683.3392	-78.48	2	39.2	27.0	2	178-190	R.LKKCHIAVGSPGR.I	

Detailed Protein Report

Protein 537: **ankyrin repeat and BTB/POZ domain-containing protein BTBD11 isoform a [Homo sapiens]**

**Accession:**	gi|65786661	**Score:**	27.0
**Database:**	refseq_human(refseq_human_20140103.fasta)	**MW [kDa]:**	120.8
**Database Date:**	2015-11-30	**pI:**	6.4
		**Sequence Coverage [%]:**	0.9
		**No. of unique Peptides:**	1

10	20	30	40	50	60	70	80
MARRGKKPVV	RTLEDLTLDS	GYGGAADSVR	SSNLSLCCSD	SHPASPYGGS	CWPPLADSMH	SRHNSFDTVN	TALVEDSEGL
90	100	110	120	130	140	150	160
DCAGQHCSRL	LPDLDEVPWT	LQELEALLLR	SRDPRAGPAV	PGGLPKDALA	KLSTLVSRAL	VRIAKEAQRL	SLRFAKCTKY
170	180	190	200	210	220	230	240
EIQSAMEIVL	SWGLAAHCTA	AALAALSLYN	MSSAGGDRLG	RGKSARCGLT	FSVGRVYRWM	VDSRVALRIH	EHAAIYLTAC
250	260	270	280	290	300	310	320
MESLFRDIYS	RVVASGVPRS	CSGPGSGSGS	GPGPSSGPGA	APAADKEREA	PGGGAASGGA	CSAASSASGG	SSCCAPPAAA
330	340	350	360	370	380	390	400
AAAVPPAAAA	NHHHHHHHAL	HEAPKFTVET	LEHTVNNDSE	IWGLLQPYQH	LICGKNASGV	LCLPDSLNLH	RDPQRSNKPG
410	420	430	440	450	460	470	480
ELPMFSQSEL	RTIEQSLLAT	RVGSIAELSD	LVSRAMHHLQ	PLNAKHHGNG	TPLHHKQGAL	YWEPEALYTL	CYFMHCPQME
490	500	510	520	530	540	550	560
WENPNVEPSK	VNLQVERPFL	VLPPLMEWIR	VAVAHAGHRR	SFSMDSDDVR	QAARLLLPGV	DCEPRQLRAD	DCFCASRKLD
570	580	590	600	610	620	630	640
AVAIEAKFKQ	DLGFRMLNCG	RTDLVKQAVS	LLGPDGINTM	SEQGMTPLMY	ACVRGDEAMV	QMLLDAGADL	NVEVVSTPHK
650	660	670	680	690	700	710	720
YPSVHPETRH	WTALTFAVLH	GHIPVVQLLL	DAGAKVEGSV	EHGEENYSET	PLQLAAAVGN	FELVSLLLER	GADPLIGTMY
730	740	750	760	770	780	790	800
RNGISTTPQG	DMNSFSQAAA	HGHRNVFRKL	LAQPEKEKSD	ILSLEEILAE	GTDLAETAPP	PLCASRNSKA	KLRALREAMY
810	820	830	840	850	860	870	880
HSAEHGYVDV	TIDIRSIGVP	WTLHTWLESL	RIAFQQHRRP	LIQCLLKEFK	TIQEEEYTEE	LVTQGLPLMF	EILKASKNEV
890	900	910	920	930	940	950	960
ISQQLCVIFT	HCYGPYPIPK	LTEIKRKQTS	RLDPHFLNNK	EMSDVTFLVE	GRPFYAHKVL	LFTASPRFKA	LLSSKPTNDG
970	980	990	1000	1010	1020	1030	1040
TCIEIGYVKY	SIFQLVMQYL	YYGGPESLLI	KNNEIMELLS	AAKFFQLEAL	QRHCEIICAK	SINTDNCVDI	YNHAKFLGVT
1050	1060	1070	1080	1090	1100	1110	
ELSAYCEGYF	LKNMMVLIEN	EAFKQLLYDK	NGEGTGQDVL	QDLQRTLAIR	IQSIHLSSSK	GSVV	

Cmpd.	No. of Cmpds.	m/z meas.	Δ m/z [ppm]	z	Rt [min]	Score	P	Range	Sequence	Modification
1645	1	583.7942	-77.28	2	50.5	27.0	2	150-159	R.LSLRFAKCTK.Y	

Detailed Protein Report

Protein 538: zinc finger protein 610 isoform b [Homo sapiens]

Accession: gi|239787106
Database: refseq_human(refseq_human_20140103.fasta)
Database Date: 2015-11-30

Score: 27.0
MW [kDa]: 48.6
pI: 10.2
Sequence Coverage [%]: 7.2
No. of unique Peptides: 1

10	20	30	40	50	60	70	80
MLCDEEAQKR	KAKESGMALP	QGRLTFMDVA	IEFSQEEWKS	LDPGQRALYR	DVMLENYRNL	VFLGRSCVLG	SNAENKPIKN
**90**	**100**	**110**	**120**	**130**	**140**	**150**	**160**
QLGLTLEAHL	SELQLFQAGR	KIYRSNQVEK	FTNHRSSVSP	LQKISSSFTT	HIFNKYRNDL	IDFPLLPQEE	KAYIRGKSYE
**170**	**180**	**190**	**200**	**210**	**220**	**230**	**240**
YECSEDGEVF	RVRASLTNHQ	VIHTAEKPYK	CTECGKVFSR	NSHLVEHWRI	HTGQKPYKCS	ECDKVFNRNS	NLARHQRIHT
**250**	**260**	**270**	**280**	**290**	**300**	**310**	**320**
GEKPHKCNEC	GKAFRECSGL	TTHLVIHTGE	KPYKCNECGK	NFRHKFSLTN	HQRSHTAEKP	YKCNECGKVF	SLLSYLARHQ
**330**	**340**	**350**	**360**	**370**	**380**	**390**	**400**
IIHSTEKPYK	CNECGRAFHK	RPGLMAHLLI	HTGEKPYKCN	ECDKVFGRKL	YLTNHQRIHT	GERPYKCNAC	GKVFNQNPHL
**410**	**420**						
SRHRKIHAGE	NSLRTLQME						

Cmpd.	No. of Cmpds.	m/z meas.	Δ m/z [ppm]	z	Rt [min]	Score	P	Range	Sequence	Modification
1493	1	949.3248	-78.23	2	48.5	12.6	1	156-171	R.GKSYEYECSEDGEVFR.V	

Detailed Protein Report

Protein 539: zinc finger protein 177 isoform b [Homo sapiens]

**Accession:**	gi|289547607	**Score:**	27.0
**Database:**	refseq_human(refseq_human_20140103.fasta)	**MW [kDa]:**	36.4
**Database Date:**	2015-11-30	**pI:**	9.8
		**Sequence Coverage [%]:**	5.3
		**No. of unique Peptides:**	2

Alias proteins:

Accession	Name	Description
gi|289547609	refseq_human (refseq_human_20140103.fasta)	zinc finger protein 177 isoform b [Homo sapiens]

10	20	30	40	50	60	70	80
MAAGWLTTWS	QNSVTFQEVA	VDFSQEEWAL	LDPAQKNLYK	DVMLENFRNL	ASVGYQLCRH	SLISKVDQEQ	LKTDERGILQ
**90**	**100**	**110**	**120**	**130**	**140**	**150**	**160**
GDCADWETQL	KPKDTIAMQN	IPGGKTSNGI	NTNCVRTHSG	EMPYECSDCG	KAFIFQSSLK	KHMRSHTGEK	PYECDHCGKS
**170**	**180**	**190**	**200**	**210**	**220**	**230**	**240**
FSQSSHLNVH	KRTHTGEKPY	DCKECGKAFT	VPSSLQKHVR	THTGEKPYEC	SDCGKAFIDQ	SSLKKHTRSH	TGEKPYECNQ
**250**	**260**	**270**	**280**	**290**	**300**	**310**	**320**
CGKSFSTGSY	LIVHKRTHTG	EKTYECKECG	KAFRNSSCLR	VHVRTHTGEK	PYKCIQCEKA	FSTSTNLIMH	KRIHNGQKLH
**330**							
E							

Cmpd.	No. of Cmpds.	m/z meas.	Δ m/z [ppm]	z	Rt [min]	Score	P	Range	Sequence	Modification
977	1	512.1767	-143.10	2	41.0	10.1	0	41-48	K.DVMLENFR.N	
1517	1	1008.6274	90.62	1	48.8	16.8	0	216-224	K.AFIDQSSLK.K	

Detailed Protein Report

Protein 540: poly [ADP-ribose] polymerase 10 [Homo sapiens]

Accession: gi|157738665
Database: refseq_human(refseq_human_20140103.fasta)
Database Date: 2015-11-30
Modification(s): Carbamidomethyl

Score: 26.9
MW [kDa]: 109.9
pI: 4.8
Sequence Coverage [%]: 3.8
No. of unique Peptides: 2

Quantitation

mdown:qdown	**Median:**	0.70	**CV:**	29.92 %	**No. of Peptides:**	2
Wdown:Qdown	**Median:**	0.74	**CV:**	0.00 %	**No. of Peptides:**	1

10	20	30	40	50	60	70	80
MVAMAEAEAG	VAVEVRGLPP	AVPDELLTLY	FENRRRSGGG	PVLSWQRLGC	GGVLTFREPA	DAERVLAQAD	HELHGAQLSL
**90**	**100**	**110**	**120**	**130**	**140**	**150**	**160**
RPAPPRAPAR	LLLQGLPPGT	TPQRLEQHVQ	ALLRASGLPV	QPCCALASPR	PDRALVQLPK	PLSEADVRVL	EEQAQNLGLE
**170**	**180**	**190**	**200**	**210**	**220**	**230**	**240**
GTLVSLARVP	QARAVRVVGD	GASVDLLLLE	LYLENERRSG	GGPLEDLQRL	PGPLGTVASF	QQWQVAERVL	QQEHRLQGSE
**250**	**260**	**270**	**280**	**290**	**300**	**310**	**320**
LSLVPHYDIL	EPEELAENTS	GGDHPSTQGP	RATKHALLRT	GGLVTALQGA	GTVTMGSGEE	PGQSGASLRT	GPMVQGRGIM
**330**	**340**	**350**	**360**	**370**	**380**	**390**	**400**
TTGSGQEPGQ	SGTSLRTGPM	GSLGQAEQVS	SMPMGSLEHE	GLVSLRPVGL	QEQEGPMSLG	PVGSAGPVET	SKGLLGQEGL
**410**	**420**	**430**	**440**	**450**	**460**	**470**	**480**
VEIAMDSPEQ	EGLVGPMEIT	MGSLEKAGPV	SPGCVKLAGQ	EGLVEMVLLM	EPGAMRFLQL	YHEDLLAGLG	DVALLPLEGP
**490**	**500**	**510**	**520**	**530**	**540**	**550**	**560**
DMTGFRLCGA	QASCQAAEEF	LRSLLGSISC	HVLCLEHPGS	ARFLLGPEGQ	HLLQGLEAQF	QCVFGTERLA	TATLDTGLEE
**570**	**580**	**590**	**600**	**610**	**620**	**630**	**640**
VDPTEALPVL	PGNAHTLWTP	DSTGGDQEDV	SLEEVRELLA	TLEGLDLDGE	DWLPRELEEE	GPQEQPEEEV	TPGHEEEEPV
**650**	**660**	**670**	**680**	**690**	**700**	**710**	**720**
APSTVAPRWL	EEEAALQLAL	HRSLEPQGQV	AEQEEAAALR	QALTLSLLEQ	PPLEAEEPPD	GGTDGKAQLV	VHSAFEQDVE
**730**	**740**	**750**	**760**	**770**	**780**	**790**	**800**
ELDRALRAAL	EVHVQEETVG	PWRRTLPAEL	RARLERCHGV	SVALRGDCTI	LRGFGAHPAR	AARHLVALLA	GPWDQSLAFP
**810**	**820**	**830**	**840**	**850**	**860**	**870**	**880**
LAASGPTLAG	QTLKGPWNNL	ERLAENTGEF	QEVVRAFYDT	LDAARSSIRV	VRVERVSHPL	LQQQYELYRE	RLLQRCERRP
**890**	**900**	**910**	**920**	**930**	**940**	**950**	**960**
VEQVLYHGTT	APAVPDICAH	GFNRSFCGRN	ATVYGKGVYF	ARRASLSVQD	RYSPPNADGH	KAVFVARVLT	GDYGQGRRGL
**970**	**980**	**990**	**1000**	**1010**	**1020**	**1030**	
RAPPLRGPGH	VLLRYDSAVD	CICQPSIFVI	FHDTQALPTH	LITCEHVPRA	SPDDPSGLPG	RSPDT	

Cmpd.	No. of Cmpds.	m/z meas.	Δ m/z [ppm]	z	Rt [min]	Score	P	Range	Sequence	Modification	Ratios
1670	1	932.4465	-0.39	2	49.6	13.6	0	318-336	R.GIMTTGSGQEPGQSGTSLR.T		mdown:qdown 0.94<br>Wdown:Qdown 0.74
45	1	1096.9782	-64.39	2	29.9	13.4	0	503-522	R.SLLGSISCHVLCLEHPGSAR.F	Carbamidomethyl: 8, 12	mdown:qdown 0.52

Detailed Protein Report

Protein 541: **PREDICTED: MAM domain-containing glycosylphosphatidylinositol anchor protein 1 isoform X2 [Homo sapiens]**

Accession: gi|578811587
Database: refseq_human(refseq_human_20140103.fasta)
Database Date: 2015-11-30
Modification(s): Carbamidomethyl

Score: 26.9
MW [kDa]: 59.5
pI: 9.6
Sequence Coverage [%]: 7.8
No. of unique Peptides: 1

10	20	30	40	50	60	70	80
MEVTCLLLLA	LIPFHCRGQG	VYAPAQAQIV	HAGQACVVKE	DNISERVYTI	REGDTLMLQC	LVTGHPRPQV	RWTKTAGSAS
**90**	**100**	**110**	**120**	**130**	**140**	**150**	**160**
DKFQETSVFN	ETLRIERIAR	TQGGRYYCKA	ENGVGVPAIK	SIRVDVQYLD	EPMLTVHQTV	SDVRGNFYQE	KTVFLRCTVN
**170**	**180**	**190**	**200**	**210**	**220**	**230**	**240**
SNPPARFIWK	RGSDTLSHSQ	DNGVDIYEPL	YTQGETKVLK	LKNLRPQDYA	SYTCQVSVRN	VCGIPDKAIT	FRLTNTTAPP
**250**	**260**	**270**	**280**	**290**	**300**	**310**	**320**
ALKLSVNETL	VVNPGENVTV	QCLLTGGDPL	PQLQWSHGPG	PLPLGALAQG	GTLSIPSVQA	RDSGYYNCTA	TNNVGNPAKK
**330**	**340**	**350**	**360**	**370**	**380**	**390**	**400**
TVNLLVRSMK	NATFQITPDV	IKESENIQLG	QDLKLSCHVD	AVPQEKVTYQ	WFKNGKPARM	SKRLLVTRND	PELPAVTSSL
**410**	**420**	**430**	**440**	**450**	**460**	**470**	**480**
ELIDLHFSDY	GTYLCMASFP	GAPVPDLSVE	VNISSETVPP	TISVPKGRAV	VTVREGSPAE	LQCEVRGKPR	PPVLWSRVDK
**490**	**500**	**510**	**520**	**530**	**540**		
EAALLPSGLP	LEETPDGKLR	LERVSRDMSG	TYRCQTARYN	GFNVRPREAQ	VQLNVQWTR		

Cmpd.	No. of Cmpds.	m/z meas.	Δ m/z [ppm]	z	Rt [min]	Score	P	Range	Sequence	Modification
1451	1	652.4568	150.96	3	46.7	16.5	0	2-17	M.EVTCLLLLALIPFHCR.G	Carbamidomethyl: 4, 15

Detailed Protein Report

Protein 542: **PREDICTED: beta/gamma crystallin domain-containing protein 3 isoform X1 [Homo sapiens]**

**Accession:**	gi|530373918	**Score:**	26.9
**Database:**	refseq_human(refseq_human_20140103.fasta)	**MW [kDa]:**	298.2
**Database Date:**	2015-11-30	**pI:**	5.0
		**Sequence Coverage [%]:**	0.7
		**No. of unique Peptides:**	1

Detailed Protein Report

10	20	30	40	50	60	70	80
MKGNLLEGPL	EDSDCSKTSF	NKENSLTNNP	ELQNIASSNN	LLNKNAWGSI	ERNRSSPSSV	TNSSYDGESD	SQHHLSCEPV
90	100	110	120	130	140	150	160
SQTNRNLVCS	ALLTGSNHRK	VPCSPDFQRV	TTTENTIKEN	STVMSNRTLV	QREELVEPQG	PAISDFSCSK	SDGSDTTEQE
170	180	190	200	210	220	230	240
STNLPSPNKS	IRHEHLQLPE	SECSDKQTID	SSSKQAATHT	NIIALQRHAV	TDTEFVNEGK	RLSAQDSQKN	VAVREIRRET
250	260	270	280	290	300	310	320
ESASAGESIA	SSHVKAPEDK	IESLPKDTDQ	YFETKAKKLD	FRSHDKIPHI	RMNKKDLASL	NYISESAVVA	SLGNENAPEL
330	340	350	360	370	380	390	400
KFELNRSHIS	ETPLDSESPQ	QAEVSPDAKT	SLSLDCKKLN	FSISPPTFVS	GVGMLSKLDI	PDLMNEGSPV	PIETGNVNIV
410	420	430	440	450	460	470	480
GISYQPRKCK	EENVKNHVEA	AGRKSPPPSF	CLEYTSAIFE	FKEVLSNSEK	CQVLPGSEAS	GPHLTGLELL	SFDSGNLSKD
490	500	510	520	530	540	550	560
CSSILSQDPN	RVELVSSNTK	ANMSIIEKSD	SLSLEAKTAN	IVSKAEIDGQ	NNVLVESHSG	RGKTISLSKV	SLSKVEPRNI
570	580	590	600	610	620	630	640
SQDKMSSFPL	KITHVPEKPI	LSELTFLEVE	QGKRFQSINH	NEIGEKCSDA	GLKENCQAEL	SPAASKYEDK	PEPEVDALGS
650	660	670	680	690	700	710	720
PPALLKSNIS	WILPPIHDEK	ISRQMAQNCE	AHTCVFHQSL	DICGTKKISG	HSEMAELSLT	NISPKFQETG	SMKVNSPFLD
730	740	750	760	770	780	790	800
SDSSLEKNSS	ASEDSSFLKV	PSVLKLEKKS	SSYRKKENIH	FLNGGIDSVS	SSSSYPEEVS	MIVNSHKPQN	NLDSIQVTKD
810	820	830	840	850	860	870	880
LTHEGTSVTN	LLYPTTSYLE	FETSVSIGTE	VTPFQEHFGI	YTGKISIDFP	TAAQFDNLVE	AETGAVAGPA	ASVNSSGQQC
890	900	910	920	930	940	950	960
SEASAEHIEA	RRRAHDQLLD	LKSSLLKKAD	TLIGEIFNSV	REELKFKHTV	STCQEHIAIE	GIMNLGTLKE	DISEKNPSEV
970	980	990	1000	1010	1020	1030	1040
TLTEIQQTEG	LEEQGMENMS	EVKEKPCVSP	TVGEKNLLVD	PNSMNVSCLL	EDKARELVNE	IIYVAQEKLR	NDTFEDTEDT
1050	1060	1070	1080	1090	1100	1110	1120
WDSELQANTS	KILNSDSVKP	HDVVREFLVS	EQPVNQSTQI	SENKVLNEFF	SLSNLASGTE	SIKGGEIVLY	QKSLFSGNGS
1130	1140	1150	1160	1170	1180	1190	1200
GLSDSINLQE	SDTVLLAEDM	SHKRLDDRVK	THLFRSEDCN	ETMEIENVDN	NKTETEDRRT	LVLNFKWPPL	VNDDIHAPGT
1210	1220	1230	1240	1250	1260	1270	1280
SKSSLSDSLV	CISEKNLPGH	SKNTPLAMSD	VGKVHKKDNE	INIGKIELIP	SMLETGKTNK	KDAELNILKY	EAVPPMIEMG
1290	1300	1310	1320	1330	1340	1350	1360
RIHKMDAELN	VTKTEPKANV	FKMGEVYQMD	AESCIEKTEG	SAVILGMEKA	YKMKDTEGDI	GKIEVIPMMP	EVKNIHQKDA
1370	1380	1390	1400	1410	1420	1430	1440
EGDIVKTEMT	PVTVDMENIY	QTHAEGDIGK	TGTIALSEVE	NIHQKGGEGI	SEKAEVIPVT	LAMENTYQKD	AEGDIGKAEV
1450	1460	1470	1480	1490	1500	1510	1520
MPVRLEMENT	YPKDTERDGG	KTEVMPLALE	VVNTYQKNAK	GFTGNTEGSV	LKMEATYRKT	AEEVIKNTEI	VPCVLKVKEA
1530	1540	1550	1560	1570	1580	1590	1600
HETAPAPLEM	EKACKRDVKE	TIGATVSTPS	VIEMEKISPE	DRGENIGKHK	VLPAVVDIEK	IHGTGLELTT	KQGEAMLPAF
1610	1620	1630	1640	1650	1660	1670	1680
ESKTPQEYAE	GSVEETKEEP	TEIKEGLIAH	ENRLPTYFRG	YESPTLSKDY	EGYPAPAMPD	FQPGDTTVRL	DKRMSLTAIY
1690	1700	1710	1720	1730	1740	1750	1760
DKRRETDYSD	KGYNLAFVSQ	DEQENSSFTI	LYEEPLQEED	KYASAEARQT	QSVLFHDTSA	DSMPVLACER	SESRTDLVHH
1770	1780	1790	1800	1810	1820	1830	1840
FEKGTKLGET	FDSDSSEMFL	SVEAKRYKIY	PLALSPIYED	DSSQEDILSS	EVSPGHHGPR	KSRDSENQSS	SVLSLLQSVS
1850	1860	1870	1880	1890	1900	1910	1920
ERLKMNFDED	DREAADEEEE	EEEAAVLHKG	DLRAGSGERV	TFQLPDPSIT	FYPDDQESVG	ISKNSYVMPN	EPTTSNLQVG
1930	1940	1950	1960	1970	1980	1990	2000
LWPEKTSFLQ	KSDLTSKLHS	SLKSAYHQYL	QTSQSHSSEK	GARFGGIFQE	PVSKYFRVQD	SPGRLSPFIE	NVDKQTLRCN
2010	2020	2030	2040	2050	2060	2070	2080
PRPGKMVIYD	LHESTYKQEV	YCNIPDATSW	SFPNGVLIKV	VRGCWILYEK	PHFRGQKCVL	EEGEKVLNRD	WILQNRRHPQ
2090	2100	2110	2120	2130	2140	2150	2160
RNFILGSLKR	VLKDCSIPEI	ELFPQSDPAC	CPVYIQRAVP	NLEELNISKS	VSFTVKSGVW	LAYPDINFKG	QATVLEEDHG
2170	2180	2190	2200	2210	2220	2230	2240

Detailed Protein Report

Cmpd.	No. of Cmpds.	m/z meas.	Δ m/z [ppm]	z	Rt [min]	Score	P	Range	Sequence	Modification
2896	1	1012.4872	-151.04	1	65.4	13.7	1	740-748	K.VPSVLKLEK.K	

Detailed Protein Report

Protein 543: **PREDICTED: tubulin polymerization-promoting protein family member 3 isoform X1 [Homo sapiens]**

**Accession:**	gi|530423965	**Score:**	26.9
**Database:**	refseq_human(refseq_human_20140103.fasta)	**MW [kDa]:**	28.3
**Database Date:**	2015-11-30	**pI:**	10.1
**Modification(s):**	Carbamidomethyl	**Sequence Coverage [%]:**	6.8
		**No. of unique Peptides:**	1

Quantitation

mdown:qdown **Median:** 0.59 **CV:** 0.00 % **No. of Peptides:** 1

10	20	30	40	50	60	70	80
METEKQGIIR	RSRLGCSPTR	EPAGSGAAEP	PGLPRPSVHS	CVVLSFRTHT	SGAAARSEHC	TQDPPSAWAK	HPVASKLPWT
90	100	110	120	130	140	150	160
LPLVNQGGMA	ASTDMAGLEE	SFRKFAIHGD	PKASGQEMNG	KNWAKLCKDC	KVADGKSVTG	TDVDIVFSKV	KGKSARVINY
170	180	190	200	210	220	230	240
EEFKKALEEL	ATKRFKGKSK	EEAFDAICQL	VAGKEPANVG	VTKAKTGGAV	DRLTDTSRYT	GSHKERFDES	GKGKGIAGRQ
250	260	270					
DILDDSGYVS	AYKNAGTYDA	KVKK					

Cmpd.	No. of Cmpds.	m/z meas.	Δ m/z [ppm]	z	Rt [min]	Score	P	Range	Sequence	Modification	Ratios
396	1	595.2630	-86.70	2	34.3	12.8	2	11-20	R.RSRLGCSPTR.E	Carbamidomethyl: 6	mdown:qdown 0.59

Detailed Protein Report

Protein 544: FERM domain-containing protein 6 isoform 1 [Homo sapiens]

**Accession:**	gi|22748721	**Score:**	26.9
**Database:**	refseq_human(refseq_human_20140103.fasta)	**MW [kDa]:**	70.9
**Database Date:**	2015-11-30	**pI:**	7.4
**Modification(s):**	Carbamidomethyl	**Sequence Coverage [%]:**	4.4
		**No. of unique Peptides:**	1

Alias proteins:

Accession	Name	Description
gi|578825482	refseq_human (refseq_human_20140103.fasta)	PREDICTED: FERM domain-containing protein 6 isoform X2 [Homo sapiens]
gi|109715866	refseq_human (refseq_human_20140103.fasta)	FERM domain-containing protein 6 isoform 1 [Homo sapiens]

10	20	30	40	50	60	70	80
MNKLNFHNNR	VMQDRRSVCI	FLPNDESLNI	IINVKILCHQ	LLVQVCDLLR	LKDCHLFGLS	VIQNNEHVYM	ELSQKLYKYC
**90**	**100**	**110**	**120**	**130**	**140**	**150**	**160**
PKEWKKEASK	GIDQFGPPMI	IHFRVQYYVE	NGRLISDRAA	RYYYYWHLRK	QVLHSQCVLR	EEAYFLLAAF	ALQADLGNFK
**170**	**180**	**190**	**200**	**210**	**220**	**230**	**240**
RNKHYGKYFE	PEAYFPSWVV	SKRGKDYILK	HIPNMHKDQF	ALTASEAHLK	YIKEAVRLDD	VAVHYYRLYK	DKREIEASLT
**250**	**260**	**270**	**280**	**290**	**300**	**310**	**320**
LGLTMRGIQI	FQNLDEEKQL	LYDFPWTNVG	KLVFVGKKFE	ILPDGLPSAR	KLIYYTGCPM	RSRHLLQLLS	NSHRLYMNLQ
**330**	**340**	**350**	**360**	**370**	**380**	**390**	**400**
PVLRHIRKLE	ENEEKKQYRE	SYISDNLDLD	MDQLEKRSRA	SGSSAGSMKH	KRLSRHSTAS	HSSSHTSGIE	ADTKPRDTGP
**410**	**420**	**430**	**440**	**450**	**460**	**470**	**480**
EDSYSSSAIH	RKLKTCSSMT	SHGSSHTSGV	ESGGKDRLEE	DLQDDEIEML	VDDPRDLEQM	NEESLEVSPD	MCIYITEDML
**490**	**500**	**510**	**520**	**530**	**540**	**550**	**560**
MSRKLNGHSG	LIVKEIGSST	SSSSETVVKL	RGQSTDSLPQ	TICRKPKTST	DRHSLSLDDI	RLYQKDFLRI	AGLCQDTAQS
**570**	**580**	**590**	**600**	**610**	**620**		
YTFGCGHELD	EEGLYCNSCL	AQQCINIQDA	FPVKRTSKYF	SLDLTHDEVP	EFVV		

Cmpd.	No. of Cmpds.	m/z meas.	Δ m/z [ppm]	z	Rt [min]	Score	P	Range	Sequence	Modification
1818	1	731.8030	-66.05	2	52.7	13.2	0	512-524	R.GQSTDSLPQTICR.K	Carbamidomethyl: 12

Detailed Protein Report

Protein 545: PREDICTED: SNW domain-containing protein 1 isoform X2 [Homo sapiens]

**Accession:**	gi|530403266	**Score:**	26.8
**Database:**	refseq_human(refseq_human_20140103.fasta)	**MW [kDa]:**	65.4
**Database Date:**	2015-11-30	**pI:**	10.2
**Modification(s):**	Oxidation	**Sequence Coverage [%]:**	5.6
		**No. of unique Peptides:**	1

10	20	30	40	50	60	70	80
MALTSFLPAP	TQLSQDQLEA	EEKARSQRSR	QTSLVSSRRE	PPPYGYRKGW	IPRLLEDFGD	GGAFPEIHVA	QYPLDMGRKK
90	100	110	120	130	140	150	160
KMSNALAIQV	DSEGKIKYDA	IARQGQSKDK	VIYSKYTDLV	PKEVMNADDP	DLQRPDEEAI	KEITEKTRVA	LEKSVSQKVA
170	180	190	200	210	220	230	240
AAMPVRAADK	LAPAQYIRYT	PSQQGVAFNS	GAKQRVIRMV	EMQKDPMEPP	RFKINKKIPR	GPPSPPAPVM	HSPSRKMTVK
250	260	270	280	290	300	310	320
EQQEWKIPPC	ISNWKNAKGY	TIPLDKRLAA	DGRGLQTVHI	NENFAKLAEA	LYIADRKARE	AVEMRAQVER	KMAQKEKEKH
330	340	350	360	370	380	390	400
EEKLREMAQK	ARERRAGIKT	HVEKEDGEAR	ERDEIRHDRR	KERQHDRNLS	RAAPDKRSKL	QRNENRDISE	VIALGVPNPR
410	420	430	440	450	460	470	480
TSNEVQYDQR	LFNQSKGMDS	GFAGGEDEIY	NVYDQAWRGG	KDMAQSIYRP	SKNLDKDMYG	DDLEARIKTN	RCQAIQLNFS
490	500	510	520	530	540	550	560
VYTGESKVVH	SFFSFSLDLF	PTRSFLVQTV	DREAEKDQCS	LRKILLVWTS	FWKKPNSMVA	LKDPQIAAAP	RNTSMKARRG
570	580						
GRNRHRSLQS	E						

Cmpd.	No. of Cmpds.	m/z meas.	Δ m/z [ppm]	z	Rt [min]	Score	P	Range	Sequence	Modification
1677	1	956.4573	-2.25	2	49.5	13.3	2	453-468	K.NLDKDMYGDDLEARIK.T	Oxidation: 6

Detailed Protein Report

Protein 546: PREDICTED: uncharacterized protein LOC100996701 [Homo sapiens]

Accession: gi|397139476
Database: refseq_human(refseq_human_20140103.fasta)
Database Date: 2015-11-30
Modification(s): Carbamidomethyl

Score: 26.8
MW [kDa]: 22.6
pI: 12.1
Sequence Coverage [%]: 15.0
No. of unique Peptides: 1

10	20	30	40	50	60	70	80
MAALCLRKGP	LPLCPRPSHG	LKAMGGEGSC	QDDTESGALI	FLPSDDAQPG	KALRPQRSGP	GGSERRGRGW	GRAGALEEQV
**90**	**100**	**110**	**120**	**130**	**140**	**150**	**160**
RQGPSAQRHP	RTQACSRPCS	PSPHCSCGKG	KHGALPQGQC	SAWLELTMVT	VPCCHHCSHC	PGGQPGPQLH	CAWTVWSWAV
**170**	**180**	**190**	**200**	**210**	**220**		
PSSASRACGD	GHRRSTCQAQ	GSCTGLPPLR	GCLSRLVPGC	PCPHLRQQDK	GKWN		

Cmpd.	No. of Cmpds.	m/z meas.	Δ m/z [ppm]	z	Rt [min]	Score	P	Range	Sequence	Modification
1676	1	916.3092	-152.23	2	50.9	15.0	1	174-190	R.RSTCQAQGSCTGLPPLR.G	Carbamidomethyl: 10

Detailed Protein Report

Protein 547: proteoglycan 4 isoform D precursor [Homo sapiens]

**Accession:**	gi|189181724	**Score:**	26.8
**Database:**	refseq_human(refseq_human_20140103.fasta)	**MW [kDa]:**	136.5
**Database Date:**	2015-11-30	**pI:**	10.1
		**Sequence Coverage [%]:**	1.4
		**No. of unique Peptides:**	2

Quantitation

mdown:qdown	**Median:** 1.04	**CV:** 0.00 %	**No. of Peptides:**	1
Wdown:Qdown	**Median:** 1.77	**CV:** 0.00 %	**No. of Peptides:**	1

10	20	30	40	50	60	70	80
MAWKTLPIYL	LLLLSVFVIQ	QVSSQELSCK	GRCFESFERG	RECDCDAQCK	KYDKCCPDYE	SFCAEVKDNK	KNRTKKKPTP
**90**	**100**	**110**	**120**	**130**	**140**	**150**	**160**
KPPVVDEAGS	GLDNGDFKVT	TPDTSTTQHN	KVSTSPKITT	AKPINPRPSL	PPNSDTSKET	SLTVNKETTV	ETKETTTTNK
**170**	**180**	**190**	**200**	**210**	**220**	**230**	**240**
QTSTDGKEKT	TSAKETQSIE	KTSAKDLAPT	SKVLAKPTPK	AETTTKGPAL	TTPKEPTPTT	PKEPASTTPK	EPTPTTIKSA
**250**	**260**	**270**	**280**	**290**	**300**	**310**	**320**
PTTPKEPAPT	TTKSAPTTPK	EPAPTTTKEP	APTTPKEPAP	TTTKEPAPTT	TKSAPTTPKE	PAPTTPKKPA	PTTPKEPAPT
**330**	**340**	**350**	**360**	**370**	**380**	**390**	**400**
TPKEPTPTTP	KEPAPTTKEP	APTTPKEPAP	TAPKKPAPTT	PKEPAPTTPK	EPAPTTTKEP	SPTTPKEPAP	TTTKSAPTTT
**410**	**420**	**430**	**440**	**450**	**460**	**470**	**480**
KEPAPTTTKS	APTTPKEPSP	TTTKEPAPTT	PKEPAPTTPK	KPAPTTPKEP	APTTPKEPAP	TTTKKPAPTT	PKEPAPTTPK
**490**	**500**	**510**	**520**	**530**	**540**	**550**	**560**
ETAPTTPKKL	TPTTPEKLAP	TTPEKPAPTT	PEELAPTTPE	EPTPTTPEEP	APTTPKAAAP	NTPKEPAPTT	PKEPAPTTPK
**570**	**580**	**590**	**600**	**610**	**620**	**630**	**640**
EPAPTTPKET	APTTPKGTAP	TTLKEPAPTT	PKKPAPKELA	PTTTKEPTST	TCDKPAPTTP	KGTAPTTPKE	PAPTTPKEPA
**650**	**660**	**670**	**680**	**690**	**700**	**710**	**720**
PTTPKGTAPT	TLKEPAPTTP	KKPAPKELAP	TTTKGPTSTT	SDKPAPTTPK	ETAPTTPKEP	APTTPKKPAP	TTPETPPPTT
**730**	**740**	**750**	**760**	**770**	**780**	**790**	**800**
SEVSTPTTTK	EPTTIHKSPD	ESTPELSAEP	TPKALENSPK	EPGVPTTKTP	AATKPEMTTT	AKDKTTERDL	RTTPETTTAA
**810**	**820**	**830**	**840**	**850**	**860**	**870**	**880**
PKMTKETATT	TEKTTESKIT	ATTTQVTSTT	TQDTTPFKIT	TLKTTTLAPK	VTTTKKTITT	TEIMNKPEET	AKPKDRATNS
**890**	**900**	**910**	**920**	**930**	**940**	**950**	**960**
KATTPKPQKP	TKAPKKPTST	KKPKTMPRVR	KPKTTPTPRK	MTSTMPELNP	TSRIAEAMLQ	TTTRPNQTPN	SKLVEVNPKS
**970**	**980**	**990**	**1000**	**1010**	**1020**	**1030**	**1040**
EDAGGAEGET	PHMLLRPHVF	MPEVTPDMDY	LPRVPNQGII	INPMLSDETN	ICNGKPVDGL	TTLRNGTLVA	FRGHYFWMLS
**1050**	**1060**	**1070**	**1080**	**1090**	**1100**	**1110**	**1120**
PFSPPSPARR	ITEVWGIPSP	IDTVFTRCNC	EGKTFFFKDS	QYWRFTNDIK	DAGYPKPIFK	GFGGLTGQIV	AALSTAKYKN
**1130**	**1140**	**1150**	**1160**	**1170**	**1180**	**1190**	**1200**
WPESVYFFKR	GGSIQQYIYK	QEPVQKCPGR	RPALNYPVYG	ETTQVRRRRF	ERAIGPSQTH	TIRIQYSPAR	LAYQDKGVLH
**1210**	**1220**	**1230**	**1240**	**1250**	**1260**	**1270**	**1280**
NEVKVSILWR	GLPNVVTSAI	SLPNIRKPDG	YDYYAFSKDQ	YYNIDVPSRT	ARAITTRSGQ	TLSKVWYNCP	

Cmpd.	No. of Cmpds.	m/z meas.	Δ m/z [ppm]	z	Rt [min]	Score	P	Range	Sequence	Modification	Ratios
2386	1	994.9816	-34.55	2	58.2	14.8	2	785-802	K.TTERDLRTTPETTTAAPK.M		Wdown:Qdown 1.77
285	1	559.1440	-261.71	2	32.7	12.0	0	792-802	R.TTPETTTAAPK.M		mdown:qdown 1.04

Detailed Protein Report

Protein 548: PREDICTED: autophagy-related protein 2 homolog B isoform X1 [Homo sapiens]

**Accession:**	gi|578825925	**Score:**	26.8
**Database:**	refseq_human(refseq_human_20140103.fasta)	**MW [kDa]:**	232.5
**Database Date:**	2015-11-30	**pI:**	5.4
		**Sequence Coverage [%]:**	1.2
		**No. of unique Peptides:**	1

Quantitation

mdown:qdown	**Median:**	0.87	**CV:**	0.00 %	**No. of Peptides:**	1
Wdown:Qdown	**Median:**	0.18	**CV:**	0.00 %	**No. of Peptides:**	1

Detailed Protein Report

10	20	30	40	50	60	70	80
MPWPFSESIK	KRACRYLLQR	YLGHFLQEKL	SLEQLSLDLY	QGTGSLAQVP	LDKWCLNEIL	ESADAPLEVT	EGFIQSISLS
90	100	110	120	130	140	150	160
VPWGSLLQDN	CALEVRGLEM	VFRPRPRPAT	GSEPMYWSSF	MTSSMQLAKE	CLSQKLTDEQ	GEGSQPFEGL	EKFAETIETV
170	180	190	200	210	220	230	240
LRRVKVTFID	TVLRIEHVPE	NSKTGTALEI	RIERTVYCDE	TADESSGINV	HQPTAFAHKL	LQLSGVSLFW	DEFSASAKSS
250	260	270	280	290	300	310	320
PVCSTAPVET	EPKLSPSWNP	KIIYEPHPQL	TRNLPEIAPS	DPVQIGRLIG	RLELSLTLKQ	NEVLPGAKLD	VDGQIDSIHL
330	340	350	360	370	380	390	400
LLSPRQVHLL	LDMLAAIAGP	ENSSKIGLAN	KDRKNRPMQQ	EDEYRIQMEL	NRYYLRKDSL	SVGVSSEQSF	YETETARTPS
410	420	430	440	450	460	470	480
SREEVFFSMA	DMDMSHSLSS	LPPLGDPPNM	DLELSLTSTY	TNTPAGSPLS	ATVLQPTWGE	FLDHHKEQPV	RGSTFPSNLV
490	500	510	520	530	540	550	560
HPTPLQKTSL	PSRSVSVDES	RPELIFRLAV	GTFSISVLHI	DPLSPPETSQ	NLNPLTPMAV	AFFTCIEKID	PARFSTEDFK
570	580	590	600	610	620	630	640
SFRAVFAEAC	SHDHLRFIGT	GIKVSYEQRQ	RSASRYFSTD	MSIGQMEFLE	CLFPTDFHSV	PPHYTELLTF	HSKEETGSHS
650	660	670	680	690	700	710	720
PVCLQLHYKH	SENRGPQGNQ	ARLSSVPHKA	ELQIKLNPVC	CELDISIVDR	LNSLLQPQKL	ATVEMMASHM	YTSYNKHISL
730	740	750	760	770	780	790	800
HKAFTEVFLD	DSHSPANCRI	SVQVATPALN	LSVRFPIPDL	RSDQERGPWF	KKSLQKEILY	LAFTDLEFKT	EFIGGSTPEQ
810	820	830	840	850	860	870	880
IKLELTFREL	IGSFQEEKGD	PSIKFFHVSS	GVDGDTTSSD	DFDWPRIVLK	INPPAMHSIL	ERIAAEEEEE	NDGHYQEEEE
890	900	910	920	930	940	950	960
GGAHSLKDVC	DLRRPAPSPF	SSRRVMFENE	QMVMPGDPVE	MTEFQDKAIS	NSHYVLELTL	PNIYVTLPNK	SFYEKLYNRI
970	980	990	1000	1010	1020	1030	1040
FNDLLLWEPT	APSPVETFEN	ISYGIGLSVA	SQLINTFNKD	SFSAFKSAVH	YDEESGSEEE	TLQYFSTVDP	NYRSRRKKKL
1050	1060	1070	1080	1090	1100	1110	1120
DSQNKNSQSF	LSVLLNINHG	LIAVFTDVKQ	DNGDLLENKH	GEFWLEFNSG	SLFCVTKYEG	FDDKHYICLH	SSSFSLYHKG
1130	1140	1150	1160	1170	1180	1190	1200
IVNGVILPTE	TRLPSSTRPH	WLEPTIYSSE	EDGLSKTSSD	GVGGDSLNML	SVAVKILSDK	SESNTKEFLI	AVGLKGATLQ
1210	1220	1230	1240	1250	1260	1270	1280
HRMLPSGLSW	HEQILYFLNI	ADEPVLGYNP	PTSFTTFHVH	LWSCALDYRP	LYLPIRSLLT	VETFSVSSSV	ALDKSSSTLR
1290	1300	1310	1320	1330	1340	1350	1360
IILDEAALHL	SDKCNTVTIN	LSRDYVRVMD	MGLLELTITA	VKSDSDGEQT	EPRFELHCSS	DVVHIRTCSD	SCAALMNLIQ
1370	1380	1390	1400	1410	1420	1430	1440
YIASYGDLQT	PNKADMKPGA	FQRRSKVDSS	GRSSSRGPVL	PEADQQMLRD	LMSDAMEEID	MQQGTSSVKP	QANGVLDEKS
1450	1460	1470	1480	1490	1500	1510	1520
QIQEPCCSDL	FLFPDESGNV	SQESGPTYAS	FSHHFISDAM	TGVPTENDDF	CILFAPKAAM	QEKEEEPVIK	IMVDDAIVIR
1530	1540	1550	1560	1570	1580	1590	1600
DNYFSLPVNK	TDTSKAPLHF	PIPVIRYVVK	EVSLVWHLYG	GKDFGIVPPT	SPAKSYISPH	SSPSHTPTRH	GRNTVCGGKG
1610	1620	1630	1640	1650	1660	1670	1680
RNHDFLMEIQ	LSKVKFQHEV	YPPCKPDCDS	SLSEHPVSRQ	VFIVQDLEIR	DRLATSQMNK	FLYLYCSKEM	PRKAHSNMLT
1690	1700	1710	1720	1730	1740	1750	1760
VKALHVCPES	GRSPQECCLR	VSLMPLRLNI	DQDALFFLKD	FFTSLSAEVE	LQMTPDPEVK	KSPGADVTCS	LPRHLSTSKE
1770	1780	1790	1800	1810	1820	1830	1840
PNLVISFSGP	KQPSQNDSAN	SVEVVNGMEE	KNFSAEEASF	RDQPVFFREF	RFTSEVPIRL	DYHGKHVSMD	QGTLAGILIG
1850	1860	1870	1880	1890	1900	1910	1920
LAQLNCSELK	LKRLSYRHGL	LGVDKLFSYA	ITEWLNDIKK	NQLPGILGGV	GPMHSLVQLV	QGLKDLVWLP	IEQYRKDGRI
1930	1940	1950	1960	1970	1980	1990	2000
VRGFQRGAAS	FGTSTAMAAL	ELTNRMVQTI	QAAAETAYDM	VSPGTLSIEP	KKTKRFPHHR	LAHQPVDLRE	GVAKAYSVVK
2010	2020	2030	2040	2050	2060	2070	2080
EGITDTAQTI	YETAAREHES	RGVTGAVGEV	LRQIPPAVVK	PLIVATEATS	NVLGGMRNQI	RPDVRQDESQ	KWRHGDD

Cmpd.	No. of Cmpds.	m/z meas.	Δ m/z [ppm]	z	Rt [min]	Score	P	Range	Sequence	Modification	Ratios

Detailed Protein Report

Cmpd.	No. of Cmpds.	m/z meas.	Δ m/z [ppm]	z	Rt [min]	Score	P	Range	Sequence	Modification	Ratios
2179	1	766.3372	-41.60	2	55.9	12.0	0	239-253	K.SSPVCSTAPVETEPK.L		Wdown:Qdown 0.18<br>mdown:qdown 0.87

Detailed Protein Report

Protein 549: **PREDICTED: peripheral plasma membrane protein CASK isoform X4 [Homo sapiens]**

**Accession:**	gi|578838134	**Score:**	26.8
**Database:**	refseq_human(refseq_human_20140103.fasta)	**MW [kDa]:**	100.7
**Database Date:**	2015-11-30	**pI:**	5.9
**Modification(s):**	Carbamidomethyl	**Sequence Coverage [%]:**	4.0
		**No. of unique Peptides:**	1

10	20	30	40	50	60	70	80
MADDDVLFED	VYELCEVIGK	GPFSVVRRCI	NRETGQQFAV	KIVDVAKFTS	SPGLSTEDLK	REASICHMLK	HPHIVELLET
90	100	110	120	130	140	150	160
YSSDGMLYMV	FEFMDGADLC	FEIVKRADAG	FVYSEAVASH	YMRQILEALR	YCHDNNIIHR	DVKPHCVLLA	SKENSAPVKL
170	180	190	200	210	220	230	240
GGFGVAIQLG	ESGLVAGGRV	GTPHFMAPEV	VKREPYGKPV	DVWGCGVILF	ILLSGCLPFY	GTKERLFEGI	IKGKYKMNPR
250	260	270	280	290	300	310	320
QWSHISESAK	DLVRRMLMLD	PAERITVYEA	LNHPWLKERD	RYAYKIHLPE	TVEQLRKFNA	RRKLKGAVLA	AVSSHKFNSF
330	340	350	360	370	380	390	400
YGDPPEELPD	FSEDPTSSGA	VSQVLDSLEE	IHALTDCSEK	DLDFLHSVFQ	DQHLHTLLDL	YDKINTKSSP	QIRNPPSDAV
410	420	430	440	450	460	470	480
QRAKEVLEEI	SCYPENNDAK	ELKRILTQPH	FMALLQTHDV	VAHEVYSDEA	LRVTPPPTSP	YLNGDSPESA	NGDMDMENVT
490	500	510	520	530	540	550	560
RVRLVQFQKN	TDEPMGITLK	MNELNHCIVA	RIMHGGMIHR	QGTLHVGDEI	REINGISVAN	QTVEQLQKML	REMRGSITFK
570	580	590	600	610	620	630	640
IVPSYRTQSS	SCEIYVRAQF	EYDPAKDDLI	PCKEAGIRFR	VGDIIQIISK	DDHNWWQGKL	ENSKNGTAGL	IPSPELQEWR
650	660	670	680	690	700	710	720
VACIAMEKTK	QEQQASCTWF	GKKKKQYKDK	YLAKHNAVFD	QLDLVTYEEV	VKLPAFKRKT	LVLLGAHGVG	RRHIKNTLIT
730	740	750	760	770	780	790	800
KHPDRFAYPI	PHTTRPPKKD	EENGKNYYFV	SHDQMMQDIS	NNEYLEYGSH	EDAMYGTKLE	TIRKIHEQGL	IAILDVEPQA
810	820	830	840	850	860	870	880
LKVLRTAEFA	PFVVFIAAPT	ITPGLNEDES	LQRLQKESDI	LQRTYAHYFD	LTIINNEIDE	TIRHLEEAVE	LVCTAPQWVP
890							
VSWVY							

Cmpd.	No. of Cmpds.	m/z meas.	Δ m/z [ppm]	z	Rt [min]	Score	P	Range	Sequence	Modification
2304	1	1022.8910	-115.95	2	59.1	14.3	1	561-577	K.IVPSYRTQSSSCEIYVR.A	Carbamidomethyl: 12

Detailed Protein Report

Protein 550: RNA polymerase-associated protein RTF1 homolog [Homo sapiens]

Accession: gi|195976782
Database: refseq_human(refseq_human_20140103.fasta)
Database Date: 2015-11-30

Score: 26.8
MW [kDa]: 80.3
pI: 8.9
Sequence Coverage [%]: 4.4
No. of unique Peptides: 2

10	20	30	40	50	60	70	80
MRGRLCVGRA	AAAAAAVAVP	LAGGQEGSPG	GGRRGSRGTT	MVKKRKGRVV	IDSDTEDSGS	DENLDQELLS	LAKRKRSDSE
**90**	**100**	**110**	**120**	**130**	**140**	**150**	**160**
EKEPPVSQPA	ASSDSETSDS	DDEWTFGSNK	NKKKGKARKI	EKKGTMKKQA	NKTASSGSSD	KDSSAESSAP	EEGEVSDSDS
**170**	**180**	**190**	**200**	**210**	**220**	**230**	**240**
NSSSSSSDSD	SSSEDEEFHD	GYGEDLMGDE	EDRARLEQMT	EKEREQELFN	RIEKREVLKR	RFEIKKKLKT	AKKKEKKEKK
**250**	**260**	**270**	**280**	**290**	**300**	**310**	**320**
KKQEEEQEKK	KLTQIQESQV	TSHNKERRSK	RDEKLDKKSQ	AMEELKAERE	KRKNRTAELL	AKKQPLKTSE	VYSDDEEEEE
**330**	**340**	**350**	**360**	**370**	**380**	**390**	**400**
DDKSSEKSDR	SSRTSSSDEE	EEKEEIPPKS	QPVSLPEELN	RVRLSRHKLE	RWCHMPFFAK	TVTGCFVRIG	IGNHNSKPVY
**410**	**420**	**430**	**440**	**450**	**460**	**470**	**480**
RVAEITGVVE	TAKVYQLGGT	RTNKGLQLRH	GNDQRVFRLE	FVSNQEFTES	EFMKWKEAMF	SAGMQLPTLD	EINKKELSIK
**490**	**500**	**510**	**520**	**530**	**540**	**550**	**560**
EALNYKFNDQ	DIEEIVKEKE	RFRKAPPNYA	MKKTQLLKEK	AMAEDLGDQD	KAKQIQDQLN	ELEERAEALD	RQRTKNISAI
**570**	**580**	**590**	**600**	**610**	**620**	**630**	**640**
SYINQRNREW	NIVESEKALV	AESHNMKNQQ	MDPFTRRQCK	PTIVSNSRDP	AVQAAILAQL	NAKYGSGVLP	DAPKEMSKGQ
**650**	**660**	**670**	**680**	**690**	**700**	**710**	**720**
GKDKDLNSKS	ASDLSEDLFK	VHDFDVKIDL	QVPSSESKAL	AITSKAPPAK	DGAPRRSLNL	EDYKKRRGLI	

Cmpd.	No. of Cmpds.	m/z meas.	Δ m/z [ppm]	z	Rt [min]	Score	P	Range	Sequence	Modification
255	1	530.8805	-123.29	3	32.6	11.9	2	203-214	K.EREQELFNRIEK.R	
90	1	650.3247	-10.31	3	29.8	14.9	2	624-642	K.YGSGVLPDAPKEMSKGQGK.D	

Detailed Protein Report

Protein 551: **PREDICTED: double zinc ribbon and ankyrin repeat-containing protein 1 isoform X3 [Homo sapiens]**

Accession: gi|530425777
Database: refseq_human(refseq_human_20140103.fasta)
Database Date: 2015-11-30
Modification(s): Carbamidomethyl

Score: 26.8
MW [kDa]: 63.6
pI: 9.4
Sequence Coverage [%]: 6.5
No. of unique Peptides: 1

10	20	30	40	50	60	70	80
MLTLESFQSP	GFAHVSGQKC	LTSTEIMRIQ	RETDFLKCAH	CLAPRPSDPF	ARFCQECGSP	VPPIFGCRLP	PPEGAQMGLC
**90**	**100**	**110**	**120**	**130**	**140**	**150**	**160**
AECRSLVPMN	TPICVVCEAP	LALQLQPQAS	LHLKEKVICR	ACGTGNPAHL	RYCVTCEGAL	PSSQESMCSG	DKAPPPPTQK
**170**	**180**	**190**	**200**	**210**	**220**	**230**	**240**
GGTISCYRCG	RWNLWEASFC	GWCGAMLGIP	AGCSVCPKCG	ASNHLSARFC	GSCGICVKSL	VKLSLDRSLA	LAAEEPRPFS
**250**	**260**	**270**	**280**	**290**	**300**	**310**	**320**
EPRCAWQSLN	IPLPRSDVGT	KRDIGTQTVG	LFYPSGKLLA	KKEQELASQK	QRQEKMSDHK	PLLTAISPGR	GYWRRQLDHI
**330**	**340**	**350**	**360**	**370**	**380**	**390**	**400**
SAHLRCYAQN	NPEFRALIAE	PRMGKLISAT	VHEDGCEVSI	RLNYSQVSNK	NLYLNKAVNF	SDHLLSSAAE	GDGGLCGSRS
**410**	**420**	**430**	**440**	**450**	**460**	**470**	**480**
SWVSDYSQST	SDTIEKIKRI	KNFKTKTFQE	KKEQLIPENR	LLLKEVGPTG	EGRVSVIEQL	LDEGADPNCC	DEDNRPVITV
**490**	**500**	**510**	**520**	**530**	**540**	**550**	**560**
AVMNKHHEAI	PVLVQRGADI	DQQWGPLRNT	ALHEATLLGL	AGRESTATLL	GCNASIQKKN	AGGQTAYDLA	LNTGDDLVTS
**570**	**580**	**590**					
LFAAKFGQGL	EDQLAQTRSL	SLDDC					

Cmpd.	No. of Cmpds.	m/z meas.	Δ m/z [ppm]	z	Rt [min]	Score	P	Range	Sequence	Modification
2106	1	1052.4447	-67.35	3	56.5	13.1	1	1-28	-.MLTLESFQSPGFAHVSGQKCLTSTEIMR.I	Carbamidomethyl: 20

Detailed Protein Report

Protein 552: PREDICTED: RAS guanyl-releasing protein 2 isoform X5 [Homo sapiens]

**Accession:**	gi|578821082	**Score:**	26.8
**Database:**	refseq_human(refseq_human_20140103.fasta)	**MW [kDa]:**	52.4
**Database Date:**	2015-11-30	**pI:**	7.0
		**Sequence Coverage [%]:**	8.8
		**No. of unique Peptides:**	2

Alias proteins:

Accession	Name	Description
gi|578821084	refseq_human (refseq_human_20140103.fasta)	PREDICTED: RAS guanyl-releasing protein 2 isoform X6 [Homo sapiens]

10	20	30	40	50	60	70	80
MSLLFDHLEP	MELAEHLTYL	EYRSFCKILF	QDYHSFVTHG	CTVDNPVLER	FISLFNSVSQ	WVQLMILSKP	TAPQRALVIT
**90**	**100**	**110**	**120**	**130**	**140**	**150**	**160**
HFVHVAEKLL	QLQNFNTLMA	VVGGLSHSSI	SRLKETHSHV	SPETIKLWEG	LTELVTATGN	YGNYRRRLAA	CVGFRFPILG
**170**	**180**	**190**	**200**	**210**	**220**	**230**	**240**
VHLKDLVALQ	LALPDWLDPA	RTRLNGAKMK	QLFSILEELA	MVTSLRPPVQ	ANPDLLSLLT	VSLDQYQTED	ELYQLSLQRE
**250**	**260**	**270**	**280**	**290**	**300**	**310**	**320**
PRSKSSPTSP	TSCTPPPRPP	VLEEWTSAAK	PKLDQALVVE	HIEKMVESVF	RNFDVDGDGH	ISQEEFQIIR	GNFPYLSAFG
**330**	**340**	**350**	**360**	**370**	**380**	**390**	**400**
DLDQNQDGCI	SREEMVSYFL	RSSSVLGGRM	GFVHNFQESN	SLRPVACRHC	KALILGIYKQ	GLKCRACGVN	CHKQCKDRLS
**410**	**420**	**430**	**440**	**450**	**460**	**470**	
VECRRRAQSV	SLEGSAPSPS	PMHSHHHRAF	SFSLPRPGRR	GSRPPEIREE	EVQTVEDGVF	DIHL	

Cmpd.	No. of Cmpds.	m/z meas.	Δ m/z [ppm]	z	Rt [min]	Score	P	Range	Sequence	Modification
1804	1	1056.6673	56.24	3	52.5	10.2	2	156-183	R.FPILGVHLKDLVALQLALPDWLDPARTR.L	
2600	1	731.3793	68.68	2	63.1	16.6	2	386-398	R.ACGVNCHKQCKDR.L	

Detailed Protein Report

Protein 553: **PREDICTED: double zinc ribbon and ankyrin repeat-containing protein 1 isoform X7 [Homo sapiens]**

**Accession:**	gi|578835454	**Score:**	26.7
**Database:**	refseq_human(refseq_human_20140103.fasta)	**MW [kDa]:**	76.5
**Database Date:**	2015-11-30	**pI:**	9.7
**Modification(s):**	Oxidation	**Sequence Coverage [%]:**	3.4
		**No. of unique Peptides:**	1

Quantitation

mdown:qdown	**Median:**	7.99	**CV:**	0.00 %	**No. of Peptides:**	1
Wdown:Qdown	**Median:**	0.15	**CV:**	0.00 %	**No. of Peptides:**	1

10	20	30	40	50	60	70	80
MTAGSVCVPQ	IIPLRVPQPG	KANHEIDNNT	LLEMKSDTPD	VNIYYTLDGS	KPEFLKRIGY	GENNTFKYIK	PITLPDGKIQ
**90**	**100**	**110**	**120**	**130**	**140**	**150**	**160**
VKAIAVSKDC	RQSGIVTKVF	HVDYEPPNIV	SPEDNVENVL	KDSSRQEFKN	GFVGSKLKKK	YKNSENQRSW	NVNLRKFPVP
**170**	**180**	**190**	**200**	**210**	**220**	**230**	**240**
RFCTRKRSEV	FDKHGDNENS	KGDRLSQMGL	CAECRSLVPM	NTPICVVCEA	PLALQLQPQA	SLHLKEKVIC	RACGTGNPAH
**250**	**260**	**270**	**280**	**290**	**300**	**310**	**320**
LRYCVTCEGA	LPSSQESMCS	GDKAPPPPTQ	KGGTISCYRC	GRWNLWEASF	CGWCGAMLGI	PAGCSVCPKC	GASNHLSARF
**330**	**340**	**350**	**360**	**370**	**380**	**390**	**400**
CGSCGICVKS	LVKLSLDRSL	ALAAEEPRPF	SEPRCAWQSL	NIPLPRSDVG	TKRDIGTQTV	GLFYPSGKLL	AKKEQELASQ
**410**	**420**	**430**	**440**	**450**	**460**	**470**	**480**
KQRQEKMSDH	KPLLTAISPG	RGYWRRQLDH	ISAHLRCYAQ	NNPEFRALIA	EPRMGKLISA	TVHEDGCEVS	IRLNYSQVSN
**490**	**500**	**510**	**520**	**530**	**540**	**550**	**560**
KNLYLNKAVN	FSDHLLSSAA	EGDGGLCGSR	SSWVSDYSQS	TSDTIEKIKR	IKNFKTKTFQ	EKKEQLIPEN	RLLLKEVGPT
**570**	**580**	**590**	**600**	**610**	**620**	**630**	**640**
GEGRVSVIEQ	LLDEGADPNC	CDEDNRPVIT	VAVMNKHHEA	IPVLVQRGAD	IDQQWGPLRN	TALHEATLLG	LAGRESTATL
**650**	**660**	**670**	**680**	**690**	**700**		
LGCNASIQKK	NAGGQTAYDL	ALNTGDDLVT	SLFAAKFGQG	LEDQLAQTRS	LSLDDC		

Cmpd.	No. of Cmpds.	m/z meas.	Δ m/z [ppm]	z	Rt [min]	Score	P	Range	Sequence	Modification	Ratios
1524	2	829.3909	-6.37	2	47.8	13.0	0	22-35	K.ANHEIDNNTLLEMK.S	Oxidation: 13	Wdown:Qdown 0.15

Detailed Protein Report

Protein 554: ubiquitin carboxyl-terminal hydrolase 32 [Homo sapiens]

Accession: gi|22550104
Database: refseq_human(refseq_human_20140103.fasta)
Database Date: 2015-11-30

Score: 26.7
MW [kDa]: 181.5
pI: 6.0
Sequence Coverage [%]: 1.9
No. of unique Peptides: 1

10	20	30	40	50	60	70	80
MGAKESRIGF	LSYEEALRRV	TDVELKRLKD	AFKRTCGLSY	YMGQHCFIRE	VLGDGVPPKV	AEVIYCSFGG	TSKGLHFNNL
90	100	110	120	130	140	150	160
IVGLVLLTRG	KDEEKAKYIF	SLFSSESGNY	VIREEMERML	HVVDGKVPDT	LRKCFSEGEK	VNYEKFRNWL	FLNKDAFTFS
170	180	190	200	210	220	230	240
RWLLSGGVYV	TLTDDSDTPT	FYQTLAGVTH	LEESDIIDLE	KRYWLLKAQS	RTGRFDLETF	GPLVSPPIRP	SLSEGLFNAF
250	260	270	280	290	300	310	320
DENRDNHIDF	KEISCGLSAC	CRGPLAERQK	FCFKVFDVDR	DGVLSRVELR	DMVVALLEVW	KDNRTDDIPE	LHMDLSDIVE
330	340	350	360	370	380	390	400
GILNAHDTTK	MGHLTLEDYQ	IWSVKNVLAN	EFLNLLFQVC	HIVLGLRPAT	PEEEGQIIRG	WLERESRYGL	QAGHNWFIIS
410	420	430	440	450	460	470	480
MQWWQQWKEY	VKYDANPVVI	EPSSVLNGGK	YSFGTAAHPM	EQVEDRIGSS	LSYVNTTEEK	FSDNISTASE	ASETAGSGFL
490	500	510	520	530	540	550	560
YSATPGADVC	FARQHNTSDN	NNQCLLGANG	NILLHLNPQK	PGAIDNQPLV	TQEPVKATSL	TLEGGRLKRT	PQLIHGRDYE
570	580	590	600	610	620	630	640
MVPEPVWRAL	YHWYGANLAL	PRPVIKNSKT	DIPELELFPR	YLLFLRQQPA	TRTQQSNIWV	NMGNVPSPNA	PLKRVLAYTG
650	660	670	680	690	700	710	720
CFSRMQTIKE	IHEYLSQRLR	IKEEDMRLWL	YNSENYLTLL	DDEDHKLEYL	KIQDEQHLVI	EVRNKDMSWP	EEMSFIANSS
730	740	750	760	770	780	790	800
KIDRHKVPTE	KGATGLSNLG	NTCFMNSSIQ	CVSNTQPLTQ	YFISGRHLYE	LNRTNPIGMK	GHMAKCYGDL	VQELWSGTQK
810	820	830	840	850	860	870	880
NVAPLKLRWT	IAKYAPRFNG	FQQQDSQELL	AFLLDGLHED	LNRVHEKPYV	ELKDSDGRPD	WEVAAEAWDN	HLRRNRSIVV
890	900	910	920	930	940	950	960
DLFHGQLRSQ	VKCKTCGHIS	VRFDPFNFLS	LPLPMDSYMH	LEITVIKLDG	TTPVRYGLRL	NMDEKYTGLK	KQLSDLCGLN
970	980	990	1000	1010	1020	1030	1040
SEQILLAEVH	GSNIKNFPQD	NQKVRLSVSG	FLCAFEIPVP	VSPISASSPT	QTDFSSSPST	NEMFTLTTNG	DLPRPIFIPN
1050	1060	1070	1080	1090	1100	1110	1120
GMPNTVVPCG	TEKNFTNGMV	NGHMPSLPDS	PFTGYIIAVH	RKMMRTELYF	LSSQKNRPSL	FGMPLIVPCT	VHTRKKDLYD
1130	1140	1150	1160	1170	1180	1190	1200
AVWIQVSRLA	SPLPPQEASN	HAQDCDDSMG	YQYPFTLRVV	QKDGNSCAWC	PWYRFCRGCK	IDCGEDRAFI	GNAYIAVDWD
1210	1220	1230	1240	1250	1260	1270	1280
PTALHLRYQT	SQERVVDEHE	SVEQSRRAQA	EPINLDSCLR	AFTSEEELGE	NEMYYCSKCK	THCLATKKLD	LWRLPPILII
1290	1300	1310	1320	1330	1340	1350	1360
HLKRFQFVNG	RWIKSQKIVK	FPRESFDPSA	FLVPRDPALC	QHKPLTPQGD	ELSEPRILAR	EVKKVDAQSS	AGEEDVLLSK
1370	1380	1390	1400	1410	1420	1430	1440
SPSSLSANII	SSPKGSPSSS	RKSGTSCPSS	KNSSPNSSPR	TLGRSKGRLR	LPQIGSKNKL	SSSKENLDAS	KENGAGQICE
1450	1460	1470	1480	1490	1500	1510	1520
LADALSRGHV	LGGSQPELVT	PQDHEVALAN	GFLYEHEACG	NGYSNGQLGN	HSEEDSTDDQ	REDTRIKPIY	NLYAISCHSG
1530	1540	1550	1560	1570	1580	1590	1600
ILGGGHYVTY	AKNPNCKWYC	YNDSSCKELH	PDEIDTDSAY	ILFYEQQGID	YAQFLPKTDG	KKMADTSSMD	EDFESDYKKY
1610							
CVLQ							

Cmpd.	No. of Cmpds.	m/z meas.	Δ m/z [ppm]	z	Rt [min]	Score	P	Range	Sequence	Modification
2778	1	1023.5416	14.56	2	63.2	11.2	1	1361-1381	K.SPSSLSANIISSPKGSPSSSR.K	

Detailed Protein Report

Protein 555: PREDICTED: zinc finger protein 124 isoform X3 [Homo sapiens]

Accession: gi|578802322
Database: refseq_human(refseq_human_20140103.fasta)
Database Date: 2015-11-30

Score: 26.7
MW [kDa]: 35.5
pI: 10.4
Sequence Coverage [%]: 10.0
No. of unique Peptides: 1

10	20	30	40	50	60	70	80
MQETFRNLAS	IGNKGEDQSI	EDQYKNSSRN	LRHIISHSGN	NPYGCEECGK	KPCTCKQCQK	TSLSVTRVHR	DTVMHTGNGH
**90**	**100**	**110**	**120**	**130**	**140**	**150**	**160**
YGCTICEKVF	NIPSSFQIHQ	RNHTGEKPYE	CMECGKALGF	SRSLNRHKRI	HTGEKRYECK	QCGKAFSRSS	HLRDHERTHT
**170**	**180**	**190**	**200**	**210**	**220**	**230**	**240**
GEKPYECKHC	GKAFRYSNCL	HYHERTHTGE	KPYVCMECGK	AFSCLSSLQG	HIKAHAGEEP	YPCKQCGKAF	RYASSLQKHE
**250**	**260**	**270**	**280**	**290**	**300**	**310**	**320**
KTHIAQKPYV	CNNCGKGFRC	SSSLRDHERT	HTGEKPYECQ	KCGKAFSRAS	TLWKHKKTHT	GEKPYKCKKM	

Cmpd.	No. of Cmpds.	m/z meas.	Δ m/z [ppm]	z	Rt [min]	Score	P	Range	Sequence	Modification
2539	1	787.0551	174.57	2	60.0	14.7	0	89-101	K.VFNIPSSFQIHQR.N	

Detailed Protein Report

Protein 556: **PREDICTED: probable global transcription activator SNF2L1 isoform X5 [Homo sapiens]**

**Accession:**	gi|578838748	**Score:**	26.7
**Database:**	refseq_human(refseq_human_20140103.fasta)	**MW [kDa]:**	121.1
**Database Date:**	2015-11-30	**pI:**	9.0
**Modification(s):**	Oxidation	**Sequence Coverage [%]:**	2.2
		**No. of unique Peptides:**	2

Quantitation

Wdown:Qdown **Median:** 8.01 **CV:** 0.00 % **No. of Peptides:** 1

10	20	30	40	50	60	70	80
MEQDTAAVAA	TVAAADATAT	IVVIEDEQPG	PSTSQEEGAA	AAATEATAAT	EKGEKKKEKN	VSSFQLKLAA	KAPKSEKEMD
**90**	**100**	**110**	**120**	**130**	**140**	**150**	**160**
PEYEEKMKAD	RAKRFEFLLK	QTELFAHFIQ	PSAQKSPTSP	LNMKLGRPRI	KKDEKQSLIS	AGDYRHRRTE	QEEDEELLSE
**170**	**180**	**190**	**200**	**210**	**220**	**230**	**240**
SRKTSNVCIR	FEVSPSYVKG	GPLRDYQIRG	LNWLISLYEN	GVNGILADEM	GLGKTLQTIA	LLGYLKHYRN	IPGPHMVLVP
**250**	**260**	**270**	**280**	**290**	**300**	**310**	**320**
KSTLHNWMNE	FKRWVPSLRV	ICFVGDKDAR	AAFIRDEMMP	GEWDVCVTSY	EMVIKEKSVF	KKFHWRYLVI	DEAHRIKNEK
**330**	**340**	**350**	**360**	**370**	**380**	**390**	**400**
SKLSEIVREF	KSTNRLLLTG	TPLQNNLHEL	WALLNFLLPD	VFNSADDFDS	WFDTKNCLGD	QKLVERLHAV	LKPFLLRRIK
**410**	**420**	**430**	**440**	**450**	**460**	**470**	**480**
TDVEKSLPPK	KEIKIYLGLS	KMQREWYTKI	LMKDIDVLNS	SGKMDKMRLL	NILMQLRKCC	NHPYLFDGAE	PGPPYTTDEH
**490**	**500**	**510**	**520**	**530**	**540**	**550**	**560**
IVSNSGKMVV	LDKLLAKLKE	QGSRVLIFSQ	MTRLLDILED	YCMWRGYEYC	RLDGQTPHEE	REEAIEAFNA	PNSSKFIFML
**570**	**580**	**590**	**600**	**610**	**620**	**630**	**640**
STRAGGLGIN	LASADVVILY	DSDWNPQVDL	QAMDRAHRIG	QKKPVRVFRL	ITDNTVEERI	VERAEIKLRL	DSIVIQQGRL
**650**	**660**	**670**	**680**	**690**	**700**	**710**	**720**
IDQQSNKLAK	EEMLQMIRHG	ATHVFASKES	ELTDEDITTI	LERGEKKTAE	MNERLQKMGE	SSLRNFRMDI	EQSLYKFEGE
**730**	**740**	**750**	**760**	**770**	**780**	**790**	**800**
DYREKQKLGM	VEWIEPPKRE	RKANYAVDAY	FREALRVSEP	KIPKAPRPPK	QPNVQDFQFF	PPRLFELLEK	EILYYRKTIG
**810**	**820**	**830**	**840**	**850**	**860**	**870**	**880**
YKVPRNPDIP	NPALAQREEQ	KKIDGAEPLT	PEETEEKEKL	LTQGFTNWTK	RDFNQFIKAN	EKYGRDDIDN	IAREVEGKSP
**890**	**900**	**910**	**920**	**930**	**940**	**950**	**960**
EEVMEYSAVF	WERCNELQDI	EKIMAQIERG	EARIQRRISI	KKALDAKIAR	YKAPFHQLRI	QYGTSKGKNY	TEEEDRFLIC
**970**	**980**	**990**	**1000**	**1010**	**1020**	**1030**	**1040**
MLHKMGFDRE	NVYEELRQCV	RNAPQFRFDW	FIKSRTAMEF	QRRCNTLISL	IEKENMEIEE	RERAEKKKRA	TKTPMVKFSA
**1050**							
FS							

Cmpd.	No. of Cmpds.	m/z meas.	Δ m/z [ppm]	z	Rt [min]	Score	P	Range	Sequence	Modification	Ratios
1545	1	765.2820	-48.68	2	49.2	16.1	1	75-86	K.SEKEMDPEYEEK.M	Oxidation: 5	
2348	1	646.6880	-199.21	2	58.0	10.6	2	684-694	R.GEKKTAEMNER.L		Wdown:Qdown 8.01

Detailed Protein Report

Protein 557: DNA polymerase zeta catalytic subunit isoform b [Homo sapiens]

**Accession:**	gi|556695361	**Score:**	26.7
**Database:**	refseq_human(refseq_human_20140103.fasta)	**MW [kDa]:**	343.9
**Database Date:**	2015-11-30	**pI:**	9.5
		**Sequence Coverage [%]:**	1.1
		**No. of unique Peptides:**	2

Alias proteins:

Accession	Name	Description
gi|578812913	refseq_human (refseq_human_20140103.fasta)	PREDICTED: DNA polymerase zeta catalytic subunit isoform X6 [Homo sapiens]
gi|556695404	refseq_human (refseq_human_20140103.fasta)	DNA polymerase zeta catalytic subunit isoform b [Homo sapiens]

Detailed Protein Report

10	20	30	40	50	60	70	80
MAFSIDRALN	VALGNPSSTA	QHVFKVSLVS	GMPFYGYHEK	ERHFMKIYLY	NPTMVKRICE	LLQSGAIMNK	FYQPHEAHIP
90	100	110	120	130	140	150	160
YLLQLFIDYN	LYGMNLINLA	AVKFRKARRK	SNTLHATGSC	KNHLSGNSLA	DTLFRWEQDE	IPSSLILEGV	EPQSTCELEV
170	180	190	200	210	220	230	240
DAVAADILNR	LDIEAQIGGN	PGLQAIWEDE	KQRRRNRNET	SQMSQPESQD	HRFVPATESE	KKFQKRLQEI	LKQNDFSVTL
250	260	270	280	290	300	310	320
SGSVDYSDGS	QEFSAELTLH	SEVLSPEMLQ	CTPANMVEVH	KDKESSKGHT	RHKVEEALIN	EEAILNLMEN	SQTFQPLTQR
330	340	350	360	370	380	390	400
LSESPVFMDS	SPDEALVHLL	AGLESDGYRG	ERNRMPSPCR	SFGNNKYPQN	SDDEENEPQI	EKEEMELSLV	MSQRWDSNIE
410	420	430	440	450	460	470	480
EHCAKKRSLC	RNTHRSSTED	DDSSSGEEME	WSDNSLLLAS	LSIPQLDGTA	DENSDNPLNN	ENSRTHSSVI	ATSKLSVKPS
490	500	510	520	530	540	550	560
IFHKDAATLE	PSSSAKITFQ	CKHTSALSSH	VLNKEDLIED	LSQTNKNTEK	GLDNSVTSFT	NESTYSMKYP	GSLSSTVHSE
570	580	590	600	610	620	630	640
NSHKENSKKE	ILPVSSCESS	IFDYEEDIPS	VTRQVPSRKY	TNIRKIEKDS	PFIHMHRHPN	ENTLGKNSFN	FSDLNHSKNK
650	660	670	680	690	700	710	720
VSSEGNEKGN	STALSSLFPS	SFTENCELLS	CSGENRTMVH	SLNSTADESG	LNKLKIRYEE	FQEHKTEKPS	LSQQAAHYMF
730	740	750	760	770	780	790	800
FPSVVLSNCL	TRPQKLSPVT	YKLQPGNKPS	RLKLNKRKLA	GHQETSTKSS	ETGSTKDNFI	QNNPCNSNPE	KDNALASDLT
810	820	830	840	850	860	870	880
KTTRGAFENK	TPTDGFIDCH	FGDGTLETEQ	SFGLYGNKYT	LRAKRKVNYE	TEDSESSFVT	HNSKISLPHP	MEIGESLDGT
890	900	910	920	930	940	950	960
LKSRKRRKMS	KKLPPVIIKY	IIINRFRGRK	NMLVKLGKID	SKEKQVILTE	EKMELYKKLA	PLKDFWPKVP	DSPATKYPIY
970	980	990	1000	1010	1020	1030	1040
PLTPKKSHRR	KSKHKSAKKK	TGKQQRTNNE	NIKRTLSFRK	KRSHAILSPP	SPSYNAETED	CDLNYSDVMS	KLGFLSERST
1050	1060	1070	1080	1090	1100	1110	1120
SPINSSPPRC	WSPTDPRAEE	IMAAAEKEAM	LFKGPNVYKK	TVNSRIGKTS	RARAQIKKSK	AKLANPSIVT	KKRNKRNQTN
1130	1140	1150	1160	1170	1180	1190	1200
KLVDDGKKKP	RAKQKTNEKG	TSRKHTTLKD	EKIKSQSGAE	VKFVLKHQNV	SEFASSSGGS	QLLFKQKDMP	LMGSAVDHPL
1210	1220	1230	1240	1250	1260	1270	1280
SASLPTGINA	QQKLSGCFSS	FLESKKSVDL	QTFPSSRDDL	HPSVVCNSIG	PGVSKINVQR	PHNQSAMFTL	KESTLIQKNI
1290	1300	1310	1320	1330	1340	1350	1360
FDLSNHLSQV	AQNTQISSGM	SSKIEDNANN	IQRNYLSSIG	KLSEYRNSLE	SKLDQAYTPN	FLHCKDSQQQ	IVCIAEQSKH
1370	1380	1390	1400	1410	1420	1430	1440
SETCSPGNTA	SEESQMPNNC	FVTSLRSPIK	QIAWEQKQRG	FILDMSNFKP	ERVKPRSLSE	AISQTKALSQ	CKNRNVSTPS
1450	1460	1470	1480	1490	1500	1510	1520
AFGEGQSGLA	VLKELLQKRQ	QKAQNANTTQ	DPLSNKHQPN	KNISGSLEHN	KANKRTRSVT	SPRKPRTPRS	TKQKEKIPKL
1530	1540	1550	1560	1570	1580	1590	1600
LKVDSLNLQN	SSQLDNSVSD	DSPIFFSDPG	FESCYSLEDS	LSPEHNYNFD	INTIGQTGFC	SFYSGSQFVP	ADQNLPQKFL
1610	1620	1630	1640	1650	1660	1670	1680
SDAVQDLFPG	QAIEKNEFLS	HDNQKCDEDK	HHTTDSASWI	RSGTLSPEIF	EKSTIDSNEN	RRHNQWKNSF	HPLTTRSNSI
1690	1700	1710	1720	1730	1740	1750	1760
MDSFCVQQAE	DCLSEKSRLN	RSSVSKEVFL	SLPQPNNSDW	IQGHTRKEMG	QSLDSANTSF	TAILSSPDGE	LVDVACEDLE
1770	1780	1790	1800	1810	1820	1830	1840
LYVSRNNDML	TPTPDSSPRS	TSSPSQSKNG	SFTPRTANIL	KPLMSPPSRE	EIMATLLDHD	LSETIYQEPF	CSNPSDVPEK
1850	1860	1870	1880	1890	1900	1910	1920
PREIGGRLLM	VETRLANDLA	EFEGDFSLEG	LRLWKTAFSA	MTQNPRPGSP	LRSGQGVVNK	GSSNSPKMVE	DKKIVIMPCK
1930	1940	1950	1960	1970	1980	1990	2000
CAPSRQLVQV	WLQAKEEYER	SKKLPKTKPT	GVVKSAENFS	SSVNPDDKPV	VPPKMDVSPC	ILPTTAHTKE	DVDNSQIALQ
2010	2020	2030	2040	2050	2060	2070	2080
APTTGCSQTA	SESQMLPPVA	SASDPEKDED	DDDNYYISYS	SPDSPVIPPW	QQPISPDSKA	LNGDDRPSSP	VEELPSLAFE
2090	2100	2110	2120	2130	2140	2150	2160
NFLKPIKDGI	QKSPCSEPQE	PLVISPINTR	ARTGKCESLC	FHSTPIIQRK	LLERLPEAPG	LSPLSTEPKT	QKLSNKKGSN
2170	2180	2190	2200	2210	2220	2230	2240

Detailed Protein Report

Cmpd.	No. of Cmpds.	m/z meas.	Δ m/z [ppm]	z	Rt [min]	Score	P	Range	Sequence	Modification
2757	1	710.3801	13.75	2	63.5	13.2	0	58-70	R.ICELLQSGAIMNK.F	
1743	1	786.4023	-29.21	3	50.5	13.6	1	2643-2664	R.QLGLKLIANVTFGYTSANFSGR.M	

Detailed Protein Report

Protein 558: pleckstrin homology domain-containing family N member 1 isoform b [Homo sapiens]

Accession: gi|237820624
Database: refseq_human(refseq_human_20140103.fasta)
Database Date: 2015-11-30

Score: 26.7
MW [kDa]: 62.6
pI: 9.4
Sequence Coverage [%]: 5.0
No. of unique Peptides: 1

Quantitation

mdown:qdown	**Median:** 0.67	**CV:** 0.00 %	**No. of Peptides:**	1
Wdown:Qdown	**Median:** 1.36	**CV:** 0.00 %	**No. of Peptides:**	1

```
        10         20         30         40         50         60         70         80
MGNSHCVPQA PRRLRASFSR KPSLKGNRED SARMSAGLPG PEAARSGDAA ANKLFHYIPG TDILDLENQR ENLEQPFLSV
        90        100        110        120        130        140        150        160
FKKGRRRVPV RNLGKVVHYA KVQLRFQHSQ DVSDCYLELF PAHLYFQAHG SEGLTFQGLL PLTELSVCPL EGSREHAFQI
       170        180        190        200        210        220        230        240
TGPLPAPLLV LCPSRAELDR WLYHLEKQTA LLGGPRRCHS APPQGSCGDE LPWTLQRRLT RLRTASGHEP GGSAVCASRV
       250        260        270        280        290        300        310        320
KLQHLPAQEQ WDRLLVLYPT SLAIFSEELD GLCFKGELPL RAVHINLEEK EKQIRSFLIE GPLINTIRVV CASYEDYGHW
       330        340        350        360        370        380        390        400
LLCLRAVTHR EGAPPLPGAE SFPGSQVMGS GRGSLSSGGQ TSWDSGCLAP PSTRTSHSLP ESSVPSTVGC SSQHTPLHRL
       410        420        430        440        450        460        470        480
SLESSPDAPD HTSETSHSPL YADPYTPPAT SHRRVTDVRG LEEFLSAMQS ARGPTPSSPL PSVPVSVPAS DPRSCSSGPA
       490        500        510        520        530        540        550        560
GPYLLSKKGA LQSRAAQRHR GSAKDGGPQP PDAPQLVSSA REGSPEPWLP LTDGRSPRRS RDPGYDHLWD ETLSSSHQKC
       570        580
PQLGGPEASG GLVQWI
```

Cmpd.	No. of Cmpds.	m/z meas.	Δ m/z [ppm]	z	Rt [min]	Score	P	Range	Sequence	Modification	Ratios
94	1	846.3548	-78.26	2	30.6	15.7	0	505-521	K.DGGPQPPDAPQLVSSAR.E		Wdown:Qdown 1.36<br>mdown:qdown 0.67

Detailed Protein Report

Protein 559: protein-glutamine gamma-glutamyltransferase 2 isoform a [Homo sapiens]

Accession: gi|39777597
Database: refseq_human(refseq_human_20140103.fasta)
Database Date: 2015-11-30

Score: 26.7
MW [kDa]: 77.3
pI: 5.0
Sequence Coverage [%]: 4.4
No. of unique Peptides: 1

10	20	30	40	50	60	70	80
MAEELVLERC	DLELETNGRD	HHTADLCREK	LVVRRGQPFW	LTLHFEGRNY	EASVDSLTFS	VVTGPAPSQE	AGTKARFPLR
90	100	110	120	130	140	150	160
DAVEEGDWTA	TVVDQQDCTL	SLQLTTPANA	PIGLYRLSLE	ASTGYQGSSF	VLGHFILLFN	AWCPADAVYL	DSEEERQEYV
170	180	190	200	210	220	230	240
LTQQGFIYQG	SAKFIKNIPW	NFGQFEDGIL	DICLILLDVN	PKFLKNAGRD	CSRRSSPVYV	GRVVSGMVNC	NDDQGVLLGR
250	260	270	280	290	300	310	320
WDNNYGDGVS	PMSWIGSVDI	LRRWKNHGCQ	RVKYGQCWVF	AAVACTVLRC	LGIPTRVVTN	YNSAHDQNSN	LLIEYFRNEF
330	340	350	360	370	380	390	400
GEIQGDKSEM	IWNFHCWVES	WMTRPDLQPG	YEGWQALDPT	PQEKSEGTYC	CGPVPVRAIK	EGDLSTKYDA	PFVFAEVNAD
410	420	430	440	450	460	470	480
VVDWIQQDDG	SVHKSINRSL	IVGLKISTKS	VGRDEREDIT	HTYKYPEGSS	EEREAFTRAN	HLNKLAEKEE	TGMAMRIRVG
490	500	510	520	530	540	550	560
QSMNMGSDFD	VFAHITNNTA	EEYVCRLLLC	ARTVSYNGIL	GPECGTKYLL	NLNLEPFSEK	SVPLCILYEK	YRDCLTESNL
570	580	590	600	610	620	630	640
IKVRALLVEP	VINSYLLAER	DLYLENPEIK	IRILGEPKQK	RKLVAEVSLQ	NPLPVALEGC	TFTVEGAGLT	EEQKTVEIPD
650	660	670	680	690			
PVEAGEEVKV	RMDLLPLHMG	LHKLVVNFES	DKLKAVKGFR	NVIIGPA			

Cmpd.	No. of Cmpds.	m/z meas.	Δ m/z [ppm]	z	Rt [min]	Score	P	Range	Sequence	Modification
2534	1	742.5453	192.31	2	62.1	11.8	1	541-552	K.SVPLCILYEKYR.D	

Detailed Protein Report

Protein 560: general transcription factor 3C polypeptide 1 isoform 2 [Homo sapiens]

**Accession:**	gi|555943794	**Score:**	26.6
**Database:**	refseq_human(refseq_human_20140103.fasta)	**MW [kDa]:**	236.1
**Database Date:**	2015-11-30	**pI:**	8.2
**Modification(s):**	Carbamidomethyl, Oxidation	**Sequence Coverage [%]:**	1.6
		**No. of unique Peptides:**	2

Detailed Protein Report

10	20	30	40	50	60	70	80
MDALESLLDE	VALEGLDGLC	LPALWSRLET	RVPPFPLPLE	PCTQEFLWRA	LATHPGISFY	EEPRERPDLQ	LQDRYEEIDL
90	100	110	120	130	140	150	160
ETGILESRRD	PVALEDVYPI	HMILENKDGI	QGSCRYFKER	KNITNDIRTK	SLQPRCTMVE	AFDRWGKKLI	IVASQAMRYR
170	180	190	200	210	220	230	240
ALIGQEGDPD	LKLPDFSYCI	LERLGRSRWQ	GELQRDLHTT	AFKVDAGKLH	YHRKILNKNG	LITMQSHVIR	LPTGAQQHSI
250	260	270	280	290	300	310	320
LLLLNRFHVD	RRSKYDILME	KLSVMLSTRT	NHIETLGKLR	EELGLCERTF	KRLYQYMLNA	GLAKVVSLRL	QEIHPECGPC
330	340	350	360	370	380	390	400
KTKKGTDVMV	RCLKLLKEFK	RNDHDDDEDE	EVISKTVPPV	DIVFERDMLT	QTYDLIERRG	TKGISQAEIR	VAMNVGKLEA
410	420	430	440	450	460	470	480
RMLCRLLQRF	KVVKGFMEDE	GRQRTTKYIS	CVFAEESDLS	RQYQREKARS	ELLTTVSLAS	MQEESLLPEG	EDTFLSESDS
490	500	510	520	530	540	550	560
EEERSSSKRR	GRGSQKDTRA	SANLRPKTQP	HHSTPTKGGW	KVVNLHPLKK	QPPSFPGAAE	ERACQSLASR	DSLLDTSSVS
570	580	590	600	610	620	630	640
EPNVSFVSHC	ADSNSGDIAV	IEEVRMENPK	ESSSSLKTGR	HSSGQDKPHE	TYRLLKRRNL	IIEAVTNLRL	IESLFTIQKM
650	660	670	680	690	700	710	720
IMDQEKQEGV	STKCCKKSIV	RLVRNLSEEG	LLRLYRTTVI	QDGIKKKVDL	VVHPSMDQND	PLVRSAIEQV	RFRISNSSTA
730	740	750	760	770	780	790	800
NRVKTSQPPV	PQGEAEEDSQ	GKEGPSGSGD	SQLSASSRSE	SGRMKKSDNK	MGITPLRNYH	PIVVPGLGRS	LGFLPKMPRL
810	820	830	840	850	860	870	880
RVVHMFLWYL	IYGHPASNTV	EKPSFISERR	TIKQESGRAG	VRPSSSGSAW	EACSEAPSKG	SQDGVTWEAE	VELATETVYV
890	900	910	920	930	940	950	960
DDASWMRYIP	PIPVHRDFGF	GWALVSDILL	CLPLSIFIQI	VQVSYKVDNL	EEFLNDPLKK	HTLIRFLPRP	IRQQLLYKRR
970	980	990	1000	1010	1020	1030	1040
YIFSVVENLQ	RLCYMGLLQF	GPTEKFQDKD	QVFIFLKKNA	VIVDTTICDP	HYNLARSSRP	FERRLYVLNS	MQDVENYWFD
1050	1060	1070	1080	1090	1100	1110	1120
LQCVCLNTPL	GVVRCPRVRK	NSSTDQGSDE	EGSLQKEQES	AMDKHNLERK	CAMLEYTTGS	REVVDEGLIP	GDGLGAAGLD
1130	1140	1150	1160	1170	1180	1190	1200
SSFYGHLKRN	WIWTSYIINQ	AKKENTAAEN	GLTVRLQTFL	SKRPMPLSAR	GNSRLNIWGE	ARVGSELCAG	WEEQFEVDRE
1210	1220	1230	1240	1250	1260	1270	1280
PSLDRNRRVR	GGKSQKRKRL	KKDPGKKIKR	KKKGEFPGEK	SKRLRYHDEA	DQSALQRMTR	LRVTWSMQED	GLLVLCRIAS
1290	1300	1310	1320	1330	1340	1350	1360
NVLNTKVKGP	FVTWQVVRDI	LHATFEESLD	KTSHSVGRRA	RYIVKNPQAY	LNYKVCLAEV	YQDKALVGDF	MNRRGDYDDP
1370	1380	1390	1400	1410	1420	1430	1440
KVCANEFKEF	VEKLKEKFSS	ALRNSNLEIP	DTLQELFARY	RVLAIGDEKD	QTRKEDELNS	VDDIHFLVLQ	NLIQSTLALS
1450	1460	1470	1480	1490	1500	1510	1520
DSQMKSYQSF	QTFRLYREYK	DHVLVKAFME	CQKRSLVNRR	RVNHTLGPKK	NRALPFVPMS	YQLSQTYYRI	FTWRFPSTIC
1530	1540	1550	1560	1570	1580	1590	1600
TESFQFLDRM	RAAGKLDQPD	RFSFKDQDNN	EPTNDMVAFS	LDGPGGNCVA	VLTLFSLGLI	SVDVRIPEQI	IVVDSSMVEN
1610	1620	1630	1640	1650	1660	1670	1680
EVIKSLGKDG	SLEDDEDEED	DLDEGVGGKR	RSMEVKPAQA	SHTNYLLMRG	YYSPGIVSTR	NLNPNDSIVV	NSCQMKFQLR
1690	1700	1710	1720	1730	1740	1750	1760
CTPVPARLRP	AAAPLEELTM	GTSCLPDTFT	KLINPQENTC	SLEEFVLQLE	LSGYSPEDLT	AALEILEAII	ATGCFGIDKE
1770	1780	1790	1800	1810	1820	1830	1840
ELRRRFSALE	KAGGGRTRTF	ADCIQALLEQ	HQVLEVGGNT	ARLVAMGSAW	PWLLHSVRLK	DREDADIQRE	DPQARPLEGS
1850	1860	1870	1880	1890	1900	1910	1920
SSEDSPPEGQ	APPSHSPRGT	KRRASWASEN	GETDAEGTQM	TPAKRPALQD	SNLAPSLGPG	AEDGAEAQAP	SPPPALEDTA
1930	1940	1950	1960	1970	1980	1990	2000
AAGAAQEDQE	GVGFTESFGA	ANISQAARER	DCESVCFIGR	PWRVVDGHLN	LPVCKGMMEA	MLYHIMTRPG	IPESSLLRHY
2010	2020	2030	2040	2050	2060	2070	2080
QGVLQPVAVL	ELLQGLESLG	CIRKRWLRKP	RPVSLFSTPV	VEEVEVPSSL	DESPMAFYEP	TLDCTLRLGR	VFPHEVNWNK
2090							
WIHL							

Detailed Protein Report

Cmpd.	No. of Cmpds.	m/z meas.	Δ m/z [ppm]	z	Rt [min]	Score	P	Range	Sequence	Modification
2192	1	1052.3938	-65.32	2	55.9	13.3	2	640-656	K.MIMDQEKQEGVSTKCCK.K	Carbamidomethyl: 15, 16; Oxidation: 1, 3
1476	1	977.4972	62.60	2	47.0	13.3	0	1183-1199	R.VGSELCAGWEEQFEVDR.E	

Detailed Protein Report

Protein 561: NF-X1-type zinc finger protein NFXL1 [Homo sapiens]

**Accession:**	gi|89363020	**Score:**	26.6
**Database:**	refseq_human(refseq_human_20140103.fasta)	**MW [kDa]:**	101.3
**Database Date:**	2015-11-30	**pI:**	10.5
		**Sequence Coverage [%]:**	3.2
		**No. of unique Peptides:**	1

Alias proteins:

Accession	Name	Description
gi|520261838	refseq_human (refseq_human_20140103.fasta)	NF-X1-type zinc finger protein NFXL1 [Homo sapiens]
gi|520261712	refseq_human (refseq_human_20140103.fasta)	NF-X1-type zinc finger protein NFXL1 [Homo sapiens]

10	20	30	40	50	60	70	80
MEASWRQVAG	GRGRSRGRAT	AAPSGNGVHL	RGAGGGREKG	SVGAVPSGTS	PGGVATTAAA	GSRHSPAGSQ	ALQTTAASEL
90	100	110	120	130	140	150	160
MSQKKFEEIK	KANQAAARKL	VEEQFSSSSE	EGDEDFEGKQ	GKILANTFIT	YTTQTDGDTR	ELERTKQYVN	EAFQAGAMTC
170	180	190	200	210	220	230	240
LICIASVKRN	QAVWSCSGCF	CIFHMPCIQK	WAKDSQFLVS	SVTDDDFGKK	DCPWPCPKCR	FEYKRSETPS	RYYCYCGKVE
250	260	270	280	290	300	310	320
DPPLDPWLVP	HSCGQVCERE	FKPPCGHKCL	LLCHPGPCPP	CPKMVTTTCY	CKKAKPIPRR	CSAKEWSCQL	PCGQKLLCGQ
330	340	350	360	370	380	390	400
HKCENPCHAG	SCQPCPRVSR	QKCVCGKKVA	ERSCASPLWH	CDQVCGKTLP	CGNHTCEQVC	HVGACGECPR	SGKRFCPCQK
410	420	430	440	450	460	470	480
SKFSLPCTED	VPTCGDSCDK	VLECGIHRCS	QRCHRGPCET	CRQEVEKHCR	CGKHTKRMPC	HKPYLCETKC	VKMRDCQKHQ
490	500	510	520	530	540	550	560
CRRKCCPGNC	PPCDQNCGRT	LGCRNHKCPS	VCHRGSCYPC	PETVDVKCNC	GNTKVTVPCG	RERTTRPPKC	KEQCSRPPTC
570	580	590	600	610	620	630	640
HHTSQEKHRC	HFGSCPPCHQ	PCQKVLEKCG	HLCPAPCHDQ	ALIKQTGRHQ	PTGPWEQPSE	PAFIQTALPC	PPCQVPIPME
650	660	670	680	690	700	710	720
CLGKHEVSPL	PCHAVGPYSC	KRVCGRILDC	QNHTCMKECH	KVTKTDGCTG	KNKAGPECLH	CEEGCSKSRP	LGCLHPCILR
730	740	750	760	770	780	790	800
CHPGECPPCV	QMLRIKCHCK	ITSLYVECRK	ITTADVNEKN	LLSCCKNQCP	KELPCGHRCK	EMCHPGECPF	NCNQKVKLRC
810	820	830	840	850	860	870	880
PCKRIKKELQ	CNKVRENQVS	IECDTTCKEM	KRKASEIKEA	EAKAALEEEK	RRQQAELEAF	ENRLKGRRKK	NRKRDEVAVE
890	900	910	920				
LSLWQKHKYY	LISVCGVVVV	VFAWYITHDV	N				

Cmpd.	No. of Cmpds.	m/z meas.	Δ m/z [ppm]	z	Rt [min]	Score	P	Range	Sequence	Modification
490	1	587.1540	-200.57	2	35.5	10.2	2	475-483	R.DCQKHQCRR.K	

Detailed Protein Report

Protein 562: protein phosphatase 1A isoform 3 [Homo sapiens]

**Accession:**	gi|193211600	**Score:**	26.5
**Database:**	refseq_human(refseq_human_20140103.fasta)	**MW [kDa]:**	51.3
**Database Date:**	2015-11-30	**pI:**	6.8
**Modification(s):**	Oxidation	**Sequence Coverage [%]:**	8.1
		**No. of unique Peptides:**	2

10	20	30	40	50	60	70	80
MFCSGRKWVA	EATICTKLMK	REKRRMGKRR	AKKAKREEKK	KGGERRRNEK	RGNQMKRMCE	RKKYETDLED	QDIMGAFLDK
**90**	**100**	**110**	**120**	**130**	**140**	**150**	**160**
PKMEKHNAQG	QGNGLRYGLS	SMQGWRVEME	DAHTAVIGLP	SGLESWSFFA	VYDGHAGSQV	AKYCCEHLLD	HITNNQDFKG
**170**	**180**	**190**	**200**	**210**	**220**	**230**	**240**
SAGAPSVENV	KNGIRTGFLE	IDEHMRVMSE	KKHGADRSGS	TAVGVLISPQ	HTYFINCGDS	RGLLCRNRKV	HFFTQDHKPS
**250**	**260**	**270**	**280**	**290**	**300**	**310**	**320**
NPLEKERIQN	AGGSVMIQRV	NGSLAVSRAL	GDFDYKCVHG	KGPTEQLVSP	EPEVHDIERS	EEDDQFIILA	CDGIWDVMGN
**330**	**340**	**350**	**360**	**370**	**380**	**390**	**400**
EELCDFVRSR	LEVTDDLEKV	CNEVVDTCLY	KGSRDNMSVI	LICFPNAPKV	SPEAVKKEAE	LDKYLECRVE	EIIKKQGEGV
**410**	**420**	**430**	**440**	**450**	**460**		
PDLVHVMRTL	ASENIPSLPP	GGELASKRNV	IEAVYNRLNP	YKNDDTDSTS	TDDMW		

Cmpd.	No. of Cmpds.	m/z meas.	Δ m/z [ppm]	z	Rt [min]	Score	P	Range	Sequence	Modification
2669	1	974.0887	123.98	2	62.2	10.6	2	1-17	-.MFCSGRKWVAEATICTK.L	Oxidation: 1
2937	1	1018.9845	-66.59	2	65.9	15.9	1	409-428	R.TLASENIPSLPPGGELASKR.N	

Detailed Protein Report

Protein 563: **PREDICTED: immunoglobulin-like domain-containing receptor 2 isoform X6 [Homo sapiens]**

**Accession:**	gi|578800910	**Score:**	26.5
**Database:**	refseq_human(refseq_human_20140103.fasta)	**MW [kDa]:**	65.5
**Database Date:**	2015-11-30	**pI:**	9.4
**Modification(s):**	Carbamidomethyl	**Sequence Coverage [%]:**	2.8
		**No. of unique Peptides:**	2

10	20	30	40	50	60	70	80
MDRVLLRWIS	LFWLTAMVEG	LQVTVPDKKK	VAMLFQPTVL	RCHFSTSSHQ	PAVVQWKFKS	YCQDRMGESL	GMSSTRAQSL
**90**	**100**	**110**	**120**	**130**	**140**	**150**	**160**
SKRNLEWDPY	LDCLDSRRTV	RVVASKQGST	VTLGDFYRGR	EITIVHDADL	QIGKLMWGDS	GLYYCIITTP	DDLEGKNEDS
**170**	**180**	**190**	**200**	**210**	**220**	**230**	**240**
VELLVLGRTG	LLADLLPSFA	VEIMPEWVFV	GLVLLGVFLF	FVLVGICWCQ	CCPHSCCCYV	RCPCCPDSCC	CPQAFRKGYR
**250**	**260**	**270**	**280**	**290**	**300**	**310**	**320**
IQADKERDSM	KVLYYVEKEL	AQFDPARRMR	GRYNNTISEL	SSLHEEDSNF	RQSFHQMRSK	QFPVSGDLES	NPDYWSGVMG
**330**	**340**	**350**	**360**	**370**	**380**	**390**	**400**
GSSGASRGPS	AMEYNKEDRE	SFRHSQPRSK	SEMLSRKNFA	TGVPAVSMDE	LAAFADSYGQ	RPRRADGNSH	EARGGSRFER
**410**	**420**	**430**	**440**	**450**	**460**	**470**	**480**
SESRAHSGFY	QDDSLEEYYG	QRSRSREPLT	DADRGWAFSP	ARRRPAEDAH	LPRLVSRTPG	TAPKYDHSYL	GSARERQARP
**490**	**500**	**510**	**520**	**530**	**540**	**550**	**560**
EGASRGGSLE	TPSKRSAQLG	PRSASYYAWS	PPGTYKAGSS	QDDQEDASDD	ALPPYSELEL	TRGPSYRGRD	LPYHSNSEKK
**570**	**580**	**590**					
RKKEPAKKTN	DFPTRMSLVV						

Cmpd.	No. of Cmpds.	m/z meas.	Δ m/z [ppm]	z	Rt [min]	Score	P	Range	Sequence	Modification
1141	2	902.1920	-134.75	2	42.8	15.4	0	222-236	R.CPCCPDSCCCPQAFR.K	Carbamidomethyl: 3, 4, 8
813	1	625.4938	-120.64	3	38.7	11.2	1	222-237	R.CPCCPDSCCCPQAFRK.G	Carbamidomethyl: 8, 10

Detailed Protein Report

Protein 564: spectrin beta chain, erythrocytic isoform b [Homo sapiens]

**Accession:**	gi|67782319	**Score:**	26.5
**Database:**	refseq_human(refseq_human_20140103.fasta)	**MW [kDa]:**	246.3
**Database Date:**	2015-11-30	**pI:**	5.0
**Modification(s):**	Oxidation	**Sequence Coverage [%]:**	1.0
		**No. of unique Peptides:**	1

Detailed Protein Report

10	20	30	40	50	60	70	80
MTSATEFENV	GNQPPYSRIN	ARWDAPDDEL	DNDNSSARLF	ERSRIKALAD	EREVVQKKTF	TKWVNSHLAR	VSCRITDLYK
90	100	110	120	130	140	150	160
DLRDGRMLIK	LLEVLSGEML	PKPTKGKMRI	HCLENVDKAL	QFLKEQRVHL	ENMGSHDIVD	GNHRLVLGLI	WTIILRFQIQ
170	180	190	200	210	220	230	240
DIVVQTQEGR	ETRSAKDALL	LWCQMKTAGY	PHVNVTNFTS	SWKDGLAFNA	LIHKHRPDLI	DFDKLKDSNA	RHNLEHAFNV
250	260	270	280	290	300	310	320
AERQLGIIPL	LDPEDVFTEN	PDEKSIITYV	VAFYHYFSKM	KVLAVEGKRV	GKVIDHAIET	EKMIEKYSGL	ASDLLTWIEQ
330	340	350	360	370	380	390	400
TITVLNSRKF	ANSLTGVQQQ	LQAFSTYRTV	EKPPKFQEKG	NLEVLLFTIQ	SRMRANNQKV	YTPHDGKLVS	DINRAWESLE
410	420	430	440	450	460	470	480
EAEYRRELAL	RNELIRQEKL	EQLARRFDRK	AAMRETWLSE	NQRLVAQDNF	GYDLAAVEAA	KKKHEAIETD	TAAYEERVRA
490	500	510	520	530	540	550	560
LEDLAQELEK	ENYHDQKRIT	ARKDNILRLW	SYLQELLQSR	RQRLETTLAL	QKLFQDMLHS	IDWMDEIKAH	LLSAEFGKHL
570	580	590	600	610	620	630	640
LEVEDLLQKH	KLMEADIAIQ	GDKVKAITAA	TLKFTEGKGY	QPCDPQVIQD	RISHLEQCFE	ELSNMAAGRK	AQLEQSKRLW
650	660	670	680	690	700	710	720
KFFWEMDEAE	SWIKEKEQIY	SSLDYGKDLT	SVLILQRKHK	AFEDELRGLD	AHLEQIFQEA	HGMVARKQFG	HPQIEARIKE
730	740	750	760	770	780	790	800
VSAQWDQLKD	LAAFCKKNLQ	DAENFFQFQG	DADDLKAWLQ	DAHRLLSGED	VGQDEGATRA	LGKKHKDFLE	ELEESRGVME
810	820	830	840	850	860	870	880
HLEQQAQGFP	EEFRDSPDVT	HRLQALRELY	QQVVAQADLR	QQRLQEALDL	YTVFGETDAC	ELWMGEKEKW	LAEMEMPDTL
890	900	910	920	930	940	950	960
EDLEVVQHRF	DILDQEMKTL	MTQIDGVNLA	ANSLVESGHP	RSREVKQYQD	HLNTRWQAFQ	TLVSERREAV	DSALRVHNYC
970	980	990	1000	1010	1020	1030	1040
VDCEETSKWI	TDKTKVVEST	KDLGRDLAGI	IAIQRKLSGL	ERDVAAIQAR	VDALERESQQ	LMDSHPEQKE	DIGQRQKHLE
1050	1060	1070	1080	1090	1100	1110	1120
ELWQGLQQSL	QGQEDLLGEV	SQLQAFLQDL	DDFQAWLSIT	QKAVASEDMP	ESLPEAEQLL	QQHAGIKDEI	DGHQDSYQRV
1130	1140	1150	1160	1170	1180	1190	1200
KESGEKVIQG	QTDPEYLLLG	QRLEGLDTGW	NALGRMWESR	SHTLAQCLGF	QEFQKDAKQA	EAILSNQEYT	LAHLEPPDSL
1210	1220	1230	1240	1250	1260	1270	1280
EAAEAGIRKF	EDFLGSMENN	RDKVLSPVDS	GNKLVAEGNL	YSDKIKEKVQ	LIEDRHRKNN	EKAQEASVLL	RDNLELQNFL
1290	1300	1310	1320	1330	1340	1350	1360
QNCQELTLWI	NDKLLTSQDV	SYDEARNLHN	KWLKHQAFVA	ELASHEGWLE	NIDAEGKQLM	DEKPQFTALV	SQKLEALHRL
1370	1380	1390	1400	1410	1420	1430	1440
WDELQATTKE	KTQHLSAARS	SDLRLQTHAD	LNKWISAMED	QLRSDDPGKD	LTSVNRMLAK	LKRVEDQVNV	RKEELGELFA
1450	1460	1470	1480	1490	1500	1510	1520
QVPSMGEEGG	DADLSIEKRF	LDLLEPLGRR	KKQLESSRAK	LQISRDLEDE	TLWVEERLPL	AQSADYGTNL	QTVQLFMKKN
1530	1540	1550	1560	1570	1580	1590	1600
QTLQNEILGH	TPRVEDVLQR	GQQLVEAAEI	DCQDLEERLG	HLQSSWDRLR	EAAAGRLQRL	RDANEAQQYY	LDADEAEAWI
1610	1620	1630	1640	1650	1660	1670	1680
GEQELYVISD	EIPKDEEGAI	VMLKRHLRQQ	RAVEDYGRNI	KQLASRAQGL	LSAGHPEGEQ	IIRLQGQVDK	HYAGLKDVAE
1690	1700	1710	1720	1730	1740	1750	1760
ERKRKLENMY	HLFQLKRETD	DLEQWISEKE	LVASSPEMGQ	DFDHVTLLRD	KFRDFARETG	AIGQERVDNV	NAFIERLIDA
1770	1780	1790	1800	1810	1820	1830	1840
GHSEAATIAE	WKDGLNEMWA	DLLELIDTRM	QLLAASYDLH	RYFYTGAEIL	GLIDEKHREL	PEDVGLDAST	AESFHRVHTA
1850	1860	1870	1880	1890	1900	1910	1920
FERELHLLGV	QVQQFQDVAT	RLQTAYAGEK	AEAIQNKEQE	VSAAWQALLD	ACAGRRTQLV	DTADKFRFFS	MARDLLSWME
1930	1940	1950	1960	1970	1980	1990	2000
SIIRQIETQE	RPRDVSSVEL	LMKYHQGINA	EIETRSKNFS	ACLELGESLL	QRQHQASEEI	REKLQQVMSR	RKEMNEKWEA
2010	2020	2030	2040	2050	2060	2070	2080
RWERLRMLLE	VCQFSRDASV	AEAWLIAQEP	YLASGDFGHT	VDSVEKLIKR	HEAFEKSTAS	WAERFAALEK	PTTLELKERQ
2090	2100	2110	2120	2130	2140		
IAERPAEETG	PQEEEGETAG	EAPVSHHAAT	ERTSPVSLWS	RLSSSWESLQ	PEPSHPY		

Cmpd.	No. of Cmpds.	m/z meas.	Δ m/z [ppm]	z	Rt [min]	Score	P	Range	Sequence	Modification
2770	1	905.4785	51.90	3	66.0	26.5	1	868-889	K.EKWLAEMEMPDTLEDLEVVQHR.F	Oxidation: 9

Detailed Protein Report

Protein 565: zinc finger protein 483 isoform a [Homo sapiens]

**Accession:**	gi|190014620	**Score:**	26.5
**Database:**	refseq_human(refseq_human_20140103.fasta)	**MW [kDa]:**	85.0
**Database Date:**	2015-11-30	**pI:**	9.9
**Modification(s):**	Carbamidomethyl	**Sequence Coverage [%]:**	4.6
		**No. of unique Peptides:**	1

10	20	30	40	50	60	70	80
MQAVVPLNKM	TAISPEPQTL	ASTEQNEVPR	VVTSGEQEAI	LRGNAADAES	FRQRFRWFCY	SEVAGPRKAL	SQLWELCNQW
**90**	**100**	**110**	**120**	**130**	**140**	**150**	**160**
LRPDIHTKEQ	ILELLVFEQF	LTILPGEIRI	WVKSQHPESS	EEVVTLIEDL	TQMLEEKDPV	SQDSTVSQEE	NSKEDKMVTV
**170**	**180**	**190**	**200**	**210**	**220**	**230**	**240**
CPNTESCESI	TLKDVAVNFS	RGEWKKLEPF	QKELYKEVLL	ENLRNLEFLD	FPVSKLELIS	QLKWVELPWL	LEEVSKSSRL
**250**	**260**	**270**	**280**	**290**	**300**	**310**	**320**
DESALDKIIE	RCLRDDDHGL	MEESQQYCGS	SEEDHGNQGN	SKGRVAQNKT	LGSGSRGKKF	DPDKSPFGHN	FKETSDLIKH
**330**	**340**	**350**	**360**	**370**	**380**	**390**	**400**
LRVYLRKKSR	RYNESKKPFS	FHSDLVLNRK	EKTAGEKSRK	SNDGGKVLSH	SSALTEHQKR	QKIHLGDRSQ	KCSKCGIIFI
**410**	**420**	**430**	**440**	**450**	**460**	**470**	**480**
RRSTLSRRKT	PMCEKCRKDS	CQEAALNKDE	GNESGEKTHK	CSKCGKAFGY	SASLTKHRRI	HTGEKPYMCN	ECGKAFSDSS
**490**	**500**	**510**	**520**	**530**	**540**	**550**	**560**
SLTPHHRTHS	GEKPFKCDDC	GKGFTLSAHL	IKHQRIHTGE	KPYKCKDCGR	PFSDSSSLIQ	HQRIHTGEKP	YTCSNCGKSF
**570**	**580**	**590**	**600**	**610**	**620**	**630**	**640**
SHSSSLSKHQ	RIHTGEKPYK	CGECGKAFRQ	NSCLTRHQRI	HTGEKPYLCN	DCGMTFSHFT	SVIYHQRLHS	GEKPYKCNQC
**650**	**660**	**670**	**680**	**690**	**700**	**710**	**720**
EKAFPTHSLL	SRHQRIHTGV	KPYKCKECGK	SFSQSSSLNE	HHRIHTGEKP	YECNYCGATF	SRSSILVEHL	KIHTGRREYE
**730**	**740**	**750**					
CNECEKTFKS	NSGLIRHRGF	HSAE					

Cmpd.	No. of Cmpds.	m/z meas.	Δ m/z [ppm]	z	Rt [min]	Score	P	Range	Sequence	Modification
173	1	1318.5770	-87.18	2	31.6	12.9	1	68-88	R.KALSQLWELCNQWLRPDIHTK.E	Carbamidomethyl: 10

Detailed Protein Report

Protein 566: protein sidekick-2 precursor [Homo sapiens]

**Accession:**	gi|222352127	**Score:**	26.5
**Database:**	refseq_human(refseq_human_20140103.fasta)	**MW [kDa]:**	239.2
**Database Date:**	2015-11-30	**pI:**	6.6
		**Sequence Coverage [%]:**	1.5
		**No. of unique Peptides:**	1

Quantitation

mdown:qdown	**Median:** 2.45	**CV:** 0.00 %	**No. of Peptides:** 1

Detailed Protein Report

10	20	30	40	50	60	70	80
MWGLLIWTLL	ALHQIRAARA	QDDVSPYFKT	EPVRTQVHLE	GNRLVLTCMA	EGSWPLEFKW	LHNNRELTKF	SLEYRYMITS
90	100	110	120	130	140	150	160
LDRTHAGFYR	CIVRNRMGAL	LQRQTEVQVA	YMGSFEEGEK	HQSVSHGEAA	VIRAPRIASF	PQPQVTWFRD	GRKIPPSSRI
170	180	190	200	210	220	230	240
AITLENTLVI	LSTVAPDAGR	YYVQAVNDKN	GDNKTSQPIT	LTVENVGGPA	DPIAPTIIIP	PKNTSVVAGT	SEVTLECVAN
250	260	270	280	290	300	310	320
ARPLIKLHII	WKKDGVLLSG	GISDHNRRLT	IPNPTGSDAG	YYECEAVLRS	SSVPSVVRGA	YLSVLEPPQF	VKEPERHITA
330	340	350	360	370	380	390	400
EMEKVVDIPC	QAKGVPPPSI	TWYKDAAVVE	VEKLTRFRQR	NDGGLQISGL	VPDDTGMFQC	FARNAAGEVQ	TSTYLAVTSI
410	420	430	440	450	460	470	480
APNITRGPLD	STVIDGMSVV	LACETSGAPR	PAITWQKGER	ILASGSVQLP	RFTPLESGSL	LISPTHISDA	GTYTCLATNS
490	500	510	520	530	540	550	560
RGVDEASADL	VVWARTRITK	PPQDQSVIKG	TQASMVCGVT	HDPRVTIRYI	WEKDGATLGT	ESHPRIRLDR	NGSLHISQTW
570	580	590	600	610	620	630	640
SGDIGTYTCR	VISAGGNDSR	SAHLRVRQLP	HAPEHPVATL	STVERRAINL	TWTKPFDGNS	PLIRYILEMS	ENNAPWTVLL
650	660	670	680	690	700	710	720
ASVDPKATSV	TVKGLVPARS	YQFRLCAVND	VGKGQFSKDT	ERVSLPEEPP	TAPPQNVIAS	GRTNQSIMIQ	WQPPPESHQN
730	740	750	760	770	780	790	800
GILKGYIIRY	CLAGLPVGYQ	FKNITDADVN	NLLLEDLIIW	TNYEIEVAAY	NSAGLGVYSS	KVTEWTLQGV	PTVPPGNVHA
810	820	830	840	850	860	870	880
EATNSTTIRF	TWNAPSPQFI	NGINQGYKLI	AWEPEQEEEV	TMVTARPNFQ	DSIHVGFVSG	LKKFTEYFTS	VLCFTTPGDG
890	900	910	920	930	940	950	960
PRSTPQLVRT	HEDVPGPVGH	LSFSEILDTS	LKVSWQEPGE	KNGILTGYRI	SWEEYNRTNT	RVTHYLPNVT	LEYRVTGLTA
970	980	990	1000	1010	1020	1030	1040
LTTYTIEVAA	MTSKGQGQVS	ASTISSGVPP	ELPGPPTNLG	ISNIGPRSVT	LQFRPGYDGK	TSISRWLVEA	QVGVVGEGEE
1050	1060	1070	1080	1090	1100	1110	1120
WLLIHQLSNE	PDARSMEVPD	LNPFTCYSFR	MRQVNIVGTS	PPSQPSRKIQ	TLQAPPDMAP	ANVSLRTASE	TSLWLRWMPL
1130	1140	1150	1160	1170	1180	1190	1200
PEMEYNGNPE	SVGYKIKYSR	SDGHGKTLSH	VVQDRVERDY	TIEDLEEWTE	YRVQVQAFNA	IGSGPWSQTV	VGRTRESVPS
1210	1220	1230	1240	1250	1260	1270	1280
SGPTNVSALA	TTSSSMLVRW	SEVPEADRNG	LVLGYKVMYK	EKDSDTQPRF	WLVEGNSSRS	AQLTGLGKYV	LYEVQVLAFT
1290	1300	1310	1320	1330	1340	1350	1360
RIGDGSPSHP	PILERTLDDV	PGPPMGILFP	EVRTTSVRLI	WQPPAAPNGI	ILAYQITHRL	NTTTANTATV	EVLAPSARQY
1370	1380	1390	1400	1410	1420	1430	1440
TATGLKPESV	YLFRITAQTR	KGWGEAAEAL	VVTTEKRDRP	QPPSRPMVQQ	EDVRARSVLL	SWEPGSDGLS	PVRYYTIQTR
1450	1460	1470	1480	1490	1500	1510	1520
ELPSGRWALH	SASVSHNASS	FIVDRLKPFT	SYKFRVKATN	DIGDSEFSEE	SESLTTLQAA	PDEAPTILSV	TPHTTTSVLI
1530	1540	1550	1560	1570	1580	1590	1600
RWQPPAEDKI	NGILLGFRIR	YRELLYEGLR	GFTLRGINNP	GATWAELTSM	YSMRNLSRPS	LTQYELDNLN	KHRRYEIRMS
1610	1620	1630	1640	1650	1660	1670	1680
VYNAVGEGPS	SPPQEVFVGE	AVPTAAPRNV	VVHGATATQL	DVTWEPPPLD	SQNGDIQGYK	IYFWEAQRGN	LTERVKTLFL
1690	1700	1710	1720	1730	1740	1750	1760
AENSVKLKNL	TGYTAYMVSV	AAFNAAGDGP	RSTPTQGQTQ	QAAPSAPSSV	KFSELTTTSV	NVSWEAPQFP	NGILEGYRLV
1770	1780	1790	1800	1810	1820	1830	1840
YEPCSPVDGV	SKIVTVDVKG	NSPLWLKVKD	LAEGVTYRFR	IRAKTFTYGP	EIEANVTTGP	GEGAPGPPGV	PIIVRYSSAI
1850	1860	1870	1880	1890	1900	1910	1920
AIHWSSGDPG	KGPITRYVIE	ARPSDEGLWD	ILIKDIPKEV	SSYTFSMDIL	KPGVSYDFRV	IAVNDYGFGT	PSSPSQSVPA
1930	1940	1950	1960	1970	1980	1990	2000
QKANPFYEEW	WFLVVIALVG	LIFILLLVFV	LIIRGQSKKY	AKKTDSGNSA	KSGALGHSEM	MSLDESSFPA	LELNNRRLSV
2010	2020	2030	2040	2050	2060	2070	2080
KNSFCRKNGL	YTRSPPRPSP	GSLHYSDEDV	TKYNDLIPAE	SSSLTEKPSE	ISDSQGSDSE	YEVDSNHQKA	HSFVNHYISD
2090	2100	2110	2120	2130	2140	2150	2160
PTYYNSWRRQ	QKGISRAQAY	SYTESDSGEP	DHTTVTNSTS	TQQGSLFRPK	ASRTPTPQNP	PNPPSQQSTL	YRPPSSLAPG
2170	2180						

Detailed Protein Report

Cmpd.	No. of Cmpds.	m/z meas.	Δ m/z [ppm]	z	Rt [min]	Score	P	Range	Sequence	Modification	Ratios
2022	1	1134.9008	-126.03	2	53.8	15.1	0	269-289	R.LTIPNPTGSDAGYYECEAVLR.S		mdown:qdown 2.45

Detailed Protein Report

Protein 567: origin recognition complex subunit 1 isoform 2 [Homo sapiens]

Accession: gi|299890795
Database: refseq_human(refseq_human_20140103.fasta)
Database Date: 2015-11-30

Score: 26.4
MW [kDa]: 96.7
pI: 10.1
Sequence Coverage [%]: 2.5
No. of unique Peptides: 1

10	20	30	40	50	60	70	80
MAHYPTRLKT	RKTYSWVGRP	LLDRKLHYQT	YREMCVKTEG	CSTEIHIQIG	QFVLIEGDDD	ENPYVAKLLE	LFEDDSDPPP
**90**	**100**	**110**	**120**	**130**	**140**	**150**	**160**
KKRARVQWFV	RFCEVPACKR	HLLGRKPGAQ	EIFWYDYPAC	DSNINAETII	GLVRVIPLAP	KDVVPTNLKN	EKTLFVKLSW
**170**	**180**	**190**	**200**	**210**	**220**	**230**	**240**
NEKKFRPLSS	ELFAELNKPQ	ESAAKCQKPV	RAKSKSAESP	SWTPAEHVAK	RIESRHSASK	SRQTPTHPLT	PRARKRLELG
**250**	**260**	**270**	**280**	**290**	**300**	**310**	**320**
NLGNPQMSQQ	TSCASLDSPG	RIKRKVAFSE	ITSPSKRSQP	DKLQTLSPAL	KAPEKTRETG	LSYTEDDKKA	SPEHRIILRT
**330**	**340**	**350**	**360**	**370**	**380**	**390**	**400**
RIAASKTIDI	REERTLTPIS	GGQRSSVVPS	VILKPENIKK	RDAKEAKAQN	EATSTPHRIR	RKSSVLTMNR	IRQQLRFLGN
**410**	**420**	**430**	**440**	**450**	**460**	**470**	**480**
SKSDQEEKEI	LPAAEISDSS	SDEEEASTPP	LPRRAPRTVS	RNLRSSLKSS	LHTLTKLKPR	TPRCAAPQIR	SRSLAAQEPA
**490**	**500**	**510**	**520**	**530**	**540**	**550**	**560**
SVLEEARLRL	HVSAVPESLP	CREQEFQDIY	NFVESKLLDH	TGGCMYISGV	PGTGKTATVH	EVIRCLQQAA	QANDVPPFQY
**570**	**580**	**590**	**600**	**610**	**620**	**630**	**640**
IEVNGMKLTE	PHQVYVQILQ	KLTGQKATAN	HAAELLAKQF	CTRGSPQETT	VLLVDELDLL	WTHKQDIMYN	LFDWPTHKEA
**650**	**660**	**670**	**680**	**690**	**700**	**710**	**720**
RLVVLAIANT	MDLPERIMMN	RVSSRLGLTR	MCFQPYTYSQ	LQQILRSRLK	HLKAFEDDAI	QLVARKVAAL	SGDARRCLDI
**730**	**740**	**750**	**760**	**770**	**780**	**790**	**800**
CRRATEICEF	SQQKPDSPGL	VTIAHSMEAV	DEMFSSSYIT	AIKNSSVLEQ	SFLRAILAEF	RRSGLEEATF	QQIYSQHVAL
**810**	**820**	**830**	**840**	**850**	**860**		
CRMEGLPYPT	MSETMAVCSH	LGSCRLLLVE	PSRNDLLLRV	RLNVSQDDVL	YALKDE		

Cmpd.	No. of Cmpds.	m/z meas.	Δ m/z [ppm]	z	Rt [min]	Score	P	Range	Sequence	Modification
1039	1	516.1512	-244.92	2	41.5	10.2	1	327-334	K.TIDIREER.T	

Detailed Protein Report

Protein 568: X-ray repair cross-complementing protein 6 isoform 3 [Homo sapiens]

**Accession:**	gi|573014819	**Score:**	26.4
**Database:**	refseq_human(refseq_human_20140103.fasta)	**MW [kDa]:**	64.2
**Database Date:**	2015-11-30	**pI:**	9.8
**Modification(s):**	Oxidation	**Sequence Coverage [%]:**	7.2
		**No. of unique Peptides:**	1

10	20	30	40	50	60	70	80
MKKQRKNKKR	TLKQVCIQSV	YISKIISSDR	DLLAVVFYGT	EKDKNSVNFK	NIYVLQELDN	PGAKRILELD	QFKGQQGQKR
**90**	**100**	**110**	**120**	**130**	**140**	**150**	**160**
FQDMMGHGSD	YSLSEVLWVC	ANLFSDVQFK	MSHKRIMLFT	NEDNPHGNDS	AKASRARTKA	GDLRDTGIFL	DLMHLKKPGG
**170**	**180**	**190**	**200**	**210**	**220**	**230**	**240**
FDISLFYRDI	ISIAEDEDLR	VHFEESSKLE	DLLRKVRAKE	TRKRALSRLK	LKLNKDIVIS	VGIYNLVQKA	LKPPPIKLYR
**250**	**260**	**270**	**280**	**290**	**300**	**310**	**320**
ETNEPVKTKT	RTFNTSTGGL	LLPSDTKRSQ	IYGSRQIILE	KEETEELKRF	DDPGLMLMGF	KPLVLLKKHH	YLRPSLFVYP
**330**	**340**	**350**	**360**	**370**	**380**	**390**	**400**
EESLVIGSST	LFSALLIKCL	EKEVAALCRY	TPRRNIPPYF	VALVPQEEEL	DDQKIQVTPP	GFQLVFLPFA	DDKRKMPFTE
**410**	**420**	**430**	**440**	**450**	**460**	**470**	**480**
KIMATPEQVG	KMKAIVEKLR	FTYRSDSFEN	PVLQQHFRNL	EALALDLMEP	EQAVDLTLPK	VEAMNKRLGS	LVDEFKELVY
**490**	**500**	**510**	**520**	**530**	**540**	**550**	**560**
PPDYNPEGKV	TKRKHDNEGS	GSKRPKVEYS	EEELKTHISK	GTLGKFTVPM	LKEACRAYGL	KSGLKKQELL	EALTKHFQD

Cmpd.	No. of Cmpds.	m/z meas.	Δ m/z [ppm]	z	Rt [min]	Score	P	Range	Sequence	Modification
50	1	813.6863	-91.93	3	30.0	12.2	0	439-460	R.NLEALALDLMEPEQAVDLTLPK.V	Oxidation: 10

Detailed Protein Report

Protein 569: **serine/threonine-protein kinase TAO3 [Homo sapiens]**

**Accession:**	gi|148746218	**Score:**	26.4
**Database:**	refseq_human(refseq_human_20140103.fasta)	**MW [kDa]:**	105.3
**Database Date:**	2015-11-30	**pI:**	6.9
		**Sequence Coverage [%]:**	2.8
		**No. of unique Peptides:**	1

10	20	30	40	50	60	70	80
MRKGVLKDPE	IADLFYKDDP	EELFIGLHEI	GHGSFGAVYF	ATNAHTSEVV	AIKKMSYSGK	QTHEKWQDIL	KEVKFLRQLK
**90**	**100**	**110**	**120**	**130**	**140**	**150**	**160**
HPNTIEYKGC	YLKEHTAWLV	MEYCLGSASD	LLEVHKKPLQ	EVEIAAITHG	ALHGLAYLHS	HALIHRDIKA	GNILLTEPGQ
**170**	**180**	**190**	**200**	**210**	**220**	**230**	**240**
VKLADFGSAS	MASPANSFVG	TPYWMAPEVI	LAMDEGQYDG	KVDIWSLGIT	CIELAERKPP	LFNMNAMSAL	YHIAQNDSPT
**250**	**260**	**270**	**280**	**290**	**300**	**310**	**320**
LQSNEWTDSF	RRFVDYCLQK	IPQERPTSAE	LLRHDFVRRD	RPLRVLIDLI	QRTKDAVREL	DNLQYRKMKK	ILFQETRNGP
**330**	**340**	**350**	**360**	**370**	**380**	**390**	**400**
LNESQEDEED	SEHGTSLNRE	MDSLGSNHSI	PSMSVSTGSQ	SSSVNSMQEV	MDESSSELVM	MHDDESTINS	SSSVVHKKDH
**410**	**420**	**430**	**440**	**450**	**460**	**470**	**480**
VFIRDEAGHG	DPRPEPRPTQ	SVQSQALHYR	NRERFATIKS	ASLVTRQIHE	HEQENELREQ	MSGYKRMRRQ	HQKQLIALEN
**490**	**500**	**510**	**520**	**530**	**540**	**550**	**560**
KLKAEMDEHR	LKLQKEVETH	ANNSSIELEK	LAKKQVAIIE	KEAKVAAADE	KKFQQQILAQ	QKKDLTTFLE	SQKKQYKICK
**570**	**580**	**590**	**600**	**610**	**620**	**630**	**640**
EKIKEEMNED	HSTPKKEKQE	RISKHKENLQ	HTQAEEEAHL	LTQQRLYYDK	NCRFFKRKIM	IKRHEVEQQN	IREELNKKRT
**650**	**660**	**670**	**680**	**690**	**700**	**710**	**720**
QKEMEHAMLI	RHDESTRELE	YRQLHTLQKL	RMDLIRLQHQ	TELENQLEYN	KRRERELHRK	HVMELRQQPK	NLKAMEMQIK
**730**	**740**	**750**	**760**	**770**	**780**	**790**	**800**
KQFQDTCKVQ	TKQYKALKNH	QLEVTPKNEH	KTILKTLKDE	QTRKLAILAE	QYEQSINEMM	ASQALRLDEA	QEAECQALRL
**810**	**820**	**830**	**840**	**850**	**860**	**870**	**880**
QLQQEMELLN	AYQSKIKMQT	EAQHERELQK	LEQRVSLRRA	HLEQKIEEEL	AALQKERSER	IKNLLERQER	EIETFDMESL
**890**	**900**						
RMGFGNLVTL	DFPKEDYR						

Cmpd.	No. of Cmpds.	m/z meas.	Δ m/z [ppm]	z	Rt [min]	Score	P	Range	Sequence	Modification
2240	1	927.9733	43.59	2	56.5	10.8	1	643-657	K.EMEHAMLIRHDESTR.E	

Detailed Protein Report

Protein 570: protein-glutamine gamma-glutamyltransferase 5 isoform 2 [Homo sapiens]

**Accession:**	gi|4759230	**Score:**	26.4
**Database:**	refseq_human(refseq_human_20140103.fasta)	**MW [kDa]:**	71.9
**Database Date:**	2015-11-30	**pI:**	5.9
**Modification(s):**	Carbamidomethyl	**Sequence Coverage [%]:**	6.7
		**No. of unique Peptides:**	2

10	20	30	40	50	60	70	80
MAQGLEVALT	DLQSSRNNVR	HHTEEITVDH	LLVRRGQAFN	LTLYFRNRSF	QPGLDNIIFV	VETEDAVYLD	SEPQRQEYVM
**90**	**100**	**110**	**120**	**130**	**140**	**150**	**160**
NDYGFIYQGS	KNWIRPCPWN	YGQFEDKIID	ICLKLLDKSL	HFQTDPATDC	ALRGSPVYVS	RVVCAMINSN	DDNGVLNGNW
**170**	**180**	**190**	**200**	**210**	**220**	**230**	**240**
SENYTDGANP	AEWTGSVAIL	KQWNATGCQP	VRYGQCWVFA	AVMCTVMRCL	GIPTRVITNF	DSGHDTDGNL	IIDEYYDNTG
**250**	**260**	**270**	**280**	**290**	**300**	**310**	**320**
RILGNKKKDT	IWNFHVWNEC	WMARKDLPPA	YGGWQVLDAT	PQEMSNGVYC	CGPASVRAIK	EGEVDLNYDT	PFVFSMVNAD
**330**	**340**	**350**	**360**	**370**	**380**	**390**	**400**
CMSWLVQGGK	EQKLHQDTSS	VGNFISTKSI	QSDERDDITE	NYKYEEGSLQ	ERQVFLKALQ	KLKARSFHGS	QRGAELQPSR
**410**	**420**	**430**	**440**	**450**	**460**	**470**	**480**
PTSLSQDSPR	SLHTPSLRPS	DVVQVSLKFK	LLDPPNMGQD	ICFVLLALNM	SSQFKDLKVN	LSAQSLLHDG	SPLSPFWQDT
**490**	**500**	**510**	**520**	**530**	**540**	**550**	**560**
AFITLSPKEA	KTYPCKISYS	QYSQYLSTDK	LIRISALGEE	KSSPEKILVN	KIITLSYPSI	TINVLGAAVV	NQPLSIQVIF
**570**	**580**	**590**	**600**	**610**	**620**	**630**	**640**
SNPLSEQVED	CVLTVEGSGL	FKKQQKVFLG	VLKPQHQASI	ILETVPFKSG	QRQIQANMRS	NKFKDIKGYR	NVYVDFAL

Cmpd.	No. of Cmpds.	m/z meas.	Δ m/z [ppm]	z	Rt [min]	Score	P	Range	Sequence	Modification
2196	1	961.3531	-82.76	2	56.1	16.4	0	193-208	R.YGQCWVFAAVMCTVMR.C	Carbamidomethyl: 4
1787	1	1023.8570	-1.46	3	52.3	10.0	1	429-455	K.FKLLDPPNMGQDICFVLLALNMSSQFK.D	

Detailed Protein Report

Protein 571: **PREDICTED: histone-lysine N-methyltransferase EHMT1 isoform X7 [Homo sapiens]**

**Accession:**	gi|530426806	**Score:**	26.3
**Database:**	refseq_human(refseq_human_20140103.fasta)	**MW [kDa]:**	99.7
**Database Date:**	2015-11-30	**pI:**	5.5
**Modification(s):**	Carbamidomethyl	**Sequence Coverage [%]:**	3.5
		**No. of unique Peptides:**	1

10	20	30	40	50	60	70	80
MAAADAEAVP	ARGEPQQDCC	VKTELLGEET	PMAADEGSAE	KQAGEAHMAA	DGETNGSCEN	SDASSHANAA	KHTQDSARVN
**90**	**100**	**110**	**120**	**130**	**140**	**150**	**160**
PQDGTNTLTR	IAENGVSERD	SEAAKQNHVT	ADDFVQTSVI	GSNGYILNKP	ALQAQPLRTT	STLASSLPGH	AAKTLPGGAG
**170**	**180**	**190**	**200**	**210**	**220**	**230**	**240**
KGRTPSAFPQ	TPAAPPATLG	EGSADTEDRK	LPAPGADVKV	HRARKTMPKS	VVGLHAASKD	PREVREARDH	KEPKEEINKN
**250**	**260**	**270**	**280**	**290**	**300**	**310**	**320**
ISDFGRQQLL	PPFPSLHQSL	PQNQCYMATT	KSQTACLPFV	LAAAVSRKKK	RRMGTYSLVP	KKKTKVLKQR	TVIEMFKSIT
**330**	**340**	**350**	**360**	**370**	**380**	**390**	**400**
HSTVGSKGEK	DLGASSLHVN	GESLEMDSDE	DDSEELEEDD	GHGAEQAAAF	PTEDSRTSKE	SMSEADRAQK	MDGESEEEQE
**410**	**420**	**430**	**440**	**450**	**460**	**470**	**480**
SVDTGEEEEG	GDESDLSSES	SIKKKFLKRK	GKTDSPWIKP	ARKRRRRSRK	KPSGALGSES	YKSSAGSAEQ	TAPGDSTGYM
**490**	**500**	**510**	**520**	**530**	**540**	**550**	**560**
EVSLDSLDLR	VKGILSSQAE	GLANGPDVLE	TDGLQEVPLC	SCRMETPKSR	EITTLANNQC	MATESVDHEL	GRCTNSVVKY
**570**	**580**	**590**	**600**	**610**	**620**	**630**	**640**
ELMRPSNKAP	LLVLCEDHRG	RMVKHQCCPG	CGYFCTAGNF	MECQPESSIS	HRFHKDCASR	VNNASYCPHC	GEESSKAKEV
**650**	**660**	**670**	**680**	**690**	**700**	**710**	**720**
TIAKADTTST	VTPVPGQEKG	SALEGRADTT	TGSAAGPPLS	EDDKLQGAAS	HVPEGFDPTG	PAGLGRPTPG	LSQGPGKETL
**730**	**740**	**750**	**760**	**770**	**780**	**790**	**800**
ESALIALDSE	KPKKLRFHPK	QLYFSARQGE	LQKVLLMLVD	GIDPNFKMEH	QNKRSPLHAA	AEAGHVDICH	MLVQAGANID
**810**	**820**	**830**	**840**	**850**	**860**	**870**	**880**
TCSEDQRTPL	MEAAENNHLE	AVKYLIKAGA	LVDPKDAEGS	TCLHLAAKKG	HYEVVQYLLS	NGQMDVNCQD	DGGWTPMIWA
**890**	**900**	**910**	**920**	**930**			
TEYKHVDLVK	LLLSKGSDIN	IRDNDDGTMK	RGALSRQKEE	KREAEEP			

Cmpd.	No. of Cmpds.	m/z meas.	Δ m/z [ppm]	z	Rt [min]	Score	P	Range	Sequence	Modification
1764	1	990.9536	23.24	2	50.6	10.5	1	621-638	R.VNNASYCPHCGEESSKAK.E	Carbamidomethyl: 7

Detailed Protein Report

Protein 572: mitochondrial inner membrane protein OXA1L [Homo sapiens]

Accession: gi|223718097
Database: refseq_human(refseq_human_20140103.fasta)
Database Date: 2015-11-30

Score: 26.3
MW [kDa]: 55.2
pI: 10.6
Sequence Coverage [%]: 3.2
No. of unique Peptides: 1

10	20	30	40	50	60	70	80
MVTWLYRFLP	TSNMAAKLRS	LLPPDLRLQF	WLHARLQKCF	LSRGCGSYCA	GAKASPLPGK	MAMGLMCGRR	ELLRLLQSGR
90	100	110	120	130	140	150	160
RVHSVAGPSQ	WLGKPLTTRL	LFPAAPCCCR	PHYLFLAASG	PRSLSTSAIS	FAEVQVQAPP	VVAATPSPTA	VPEVASGETA
170	180	190	200	210	220	230	240
DVVQTAAEQS	FAELGLGSYT	PVGLIQNLLE	FMHVDLGLPW	WGAIAACTVF	ARCLIFPLIV	TGQREAARIH	NHLPEIQKFS
250	260	270	280	290	300	310	320
SRIREAKLAG	DHIEYYKASS	EMALYQKKHG	IKLYKPLILP	VTQAPIFISF	FIALREMANL	PVPSLQTGGL	WWFQDLTVSD
330	340	350	360	370	380	390	400
PIYILPLAVT	ATMWAVLELG	AETGVQSSDL	QWMRNVIRMM	PLITLPITMH	FPTAVFMYWL	SSNLFSLVQV	SCLRIPAVRT
410	420	430	440	450	460	470	480
VLKIPQRVVH	DLDKLPPREG	FLESFKKGWK	NAEMTRQLRE	REQRMRNQLE	LAARGPLRQT	FTHNPLLQPG	KDNPPNIPSS
490	500						
SSKPKSKYPW	HDTLG						

Cmpd.	No. of Cmpds.	m/z meas.	Δ m/z [ppm]	z	Rt [min]	Score	P	Range	Sequence	Modification
1726	1	546.7057	-111.00	2	51.5	15.2	1	428-436	K.GWKNAEMTR.Q	

Detailed Protein Report

Protein 573: helicase SRCAP [Homo sapiens]

**Accession:**	gi|146219843	**Score:**	26.3
**Database:**	refseq_human(refseq_human_20140103.fasta)	**MW [kDa]:**	343.3
**Database Date:**	2015-11-30	**pI:**	5.6
		**Sequence Coverage [%]:**	1.1
		**No. of unique Peptides:**	1

Detailed Protein Report

10	20	30	40	50	60	70	80
MQSSPSPAHP	QLPVLQTQMV	SDGMTGSNPV	SPASSSSPAS	SGAGGISPQH	IAQDSSLDGP	PGPPDGATVP	LEGFSLSQAA
90	100	110	120	130	140	150	160
DLANKGPKWE	KSHAEIAEQA	KHEAEIETRI	AELRKEGFWS	LKRLPKVPEP	PRPKGHWDYL	CEEMQWLSAD	FAQERRWKRG
170	180	190	200	210	220	230	240
VARKVVRMVI	RHHEEQRQKE	ERARREEQAK	LRRIASTMAK	DVRQFWSNVE	KVVQFKQQSR	LEEKRKKALD	LHLDFIVGQT
250	260	270	280	290	300	310	320
EKYSDLLSQS	LNQPLTSSKA	GSSPCLGSSS	AASSPPPPAS	RLDDEDGDFQ	PQEDEEEDDE	ETIEVEEQQE	GNDAEAQRRE
330	340	350	360	370	380	390	400
IELLRREGEL	PLEELLRSLP	PQLLEGPSSP	SQTPSSHDSD	TRDGPEEGAE	EEPPQVLEIK	PPPSAVTQRN	KQPWHPDEDD
410	420	430	440	450	460	470	480
EEFTANEEEA	EDEEDTIAAE	EQLEGEVDHA	MELSELAREG	ELSMEELLQQ	YAGAYAPGSG	SSEDEDEDEV	DANSSDCEPE
490	500	510	520	530	540	550	560
GPVEAEEPPQ	EDSSSQSDSV	EDRSEDEEDE	HSEEEETSGS	SASEESESEE	SEDAQSQSQA	DEEEEDDDFG	VEYLLARDEE
570	580	590	600	610	620	630	640
QSEADAGSGP	PTPGPTTLGP	KKEITDIAAA	AESLQPKGYT	LATTQVKTPI	PLLLRGQLRE	YQHIGLDWLV	TMYEKKLNGI
650	660	670	680	690	700	710	720
LADEMGLGKT	IQTISLLAHL	ACEKGNWGPH	LIIVPTSVML	NWEMELKRWC	PSFKILTYYG	AQKERKLKRQ	GWTKPNAFHV
730	740	750	760	770	780	790	800
CITSYKLVLQ	DHQAFRRKNW	RYLILDEAQN	IKNFKSQRWQ	SLLNFNSQRR	LLLTGTPLQN	SLMELWSLMH	FLMPHVFQSH
810	820	830	840	850	860	870	880
REFKEWFSNP	LTGMIEGSQE	YNEGLVKRLH	KVLRPFLLRR	VKVDVEKQMP	KKYEHVIRCR	LSKRQRCLYD	DFMAQTTTKE
890	900	910	920	930	940	950	960
TLATGHFMSV	INILMQLRKV	CNHPNLFDPR	PVTSPFITPG	ICFSTASLVL	RATDVHPLQR	IDMGRFDLIG	LEGRVSRYEA
970	980	990	1000	1010	1020	1030	1040
DTFLPRHRLS	RRVLLEVATA	PDPPPRPKPV	KMKVNRMLQP	VPKQEGRTVV	VVNNPRAPLG	PVPVRPPPGP	ELSAQPTPGP
1050	1060	1070	1080	1090	1100	1110	1120
VPQVLPASLM	VSASPAGPPL	IPASRPPGPV	LLPPLQPNSG	SLPQVLPSPL	GVLSGTSRPP	TPTLSLKPTP	PAPVRLSPAP
1130	1140	1150	1160	1170	1180	1190	1200
PPGSSSLLKP	LTVPPGYTFP	PAAATTTSTT	TATATTTAVP	APTPAPQRLI	LSPDMQARLP	SGEVVSIGQL	ASLAQRPVAN
1210	1220	1230	1240	1250	1260	1270	1280
AGGSKPLTFQ	IQGNKLTLTG	AQVRQLAVGQ	PRPLQRNVVH	LVSAGGQHHL	ISQPAHVALI	QAVAPTPGPT	PVSVLPSSTP
1290	1300	1310	1320	1330	1340	1350	1360
STTPAPTGLS	LPLAANQVPP	TMVNNTGVVK	IVVRQAPRDG	LTPVPPLAPA	PRPPSSGLPA	VLNPRPTLTP	GRLPTPTLGT
1370	1380	1390	1400	1410	1420	1430	1440
ARAPMPTPTL	VRPLLKLVHS	PSPEVSASAP	GAAPLTISSP	LHVPSSLPGP	ASSPMPIPNS	SPLASPVSST	VSVPLSSSLP
1450	1460	1470	1480	1490	1500	1510	1520
ISVPTTLPAP	ASAPLTIPIS	APLTVSASGP	ALLTSVTPPL	APVVPAAPGP	PSLAPSGASP	SASALTLGLA	TAPSLSSSQT
1530	1540	1550	1560	1570	1580	1590	1600
PGHPLLLAPT	SSHVPGLNST	VAPACSPVLV	PASALASPFP	SAPNPAPAQA	SLLAPASSAS	QALATPLAPM	AAPQTAILAP
1610	1620	1630	1640	1650	1660	1670	1680
SPAPPLAPLP	VLAPSPGAAP	VLASSQTPVP	VMAPSSTPGT	SLASASPVPA	PTPVLAPSST	QTMLPAPVPS	PLPSPASTQT
1690	1700	1710	1720	1730	1740	1750	1760
LALAPALAPT	LGGSSPSQTL	SLGTGNPQGP	FPTQTLSLTP	ASSLVPTPAQ	TLSLAPGPPL	GPTQTLSLAP	APPLAPASPV
1770	1780	1790	1800	1810	1820	1830	1840
GPAPAHTLTL	APASSSASLL	APASVQTLTL	SPAPVPTLGP	AAAQTLALAP	ASTQSPASQA	SSLVVSASGA	APLPVTMVSR
1850	1860	1870	1880	1890	1900	1910	1920
LPVSKDEPDT	LTLRSGPPSP	PSTATSFGGP	RPRRQPPPPP	RSPFYLDSLE	EKRKRQRSER	LERIFQLSEA	HGALAPVYGT
1930	1940	1950	1960	1970	1980	1990	2000
EVLDFCTLPQ	PVASPIGPRS	PGPSHPTFWT	YTEAAHRAVL	FPQQRLDQLS	EIIERFIFVM	PPVEAPPPSL	HACHPPPWLA
2010	2020	2030	2040	2050	2060	2070	2080
PRQAAFQEQL	ASELWPRARP	LHRIVCNMRT	QFPDLRLIQY	DCGKLQTLAV	LLRQLKAEGH	RVLIFTQMTR	MLDVLEQFLT
2090	2100	2110	2120	2130	2140	2150	2160
YHGHLYLRLD	GSTRVEQRQA	LMERFNADKR	IFCFILSTRS	GGVGVNLTGA	DTVVFYDSDW	NPTMDAQAQD	RCHRIGQTRD
2170	2180	2190	2200	2210	2220	2230	2240

Detailed Protein Report

Cmpd.	No. of Cmpds.	m/z meas.	Δ m/z [ppm]	z	Rt [min]	Score	P	Range	Sequence	Modification
1437	1	1087.6372	55.64	2	46.7	13.2	0	880-898	K.ETLATGHFMSVINILMQLR.K	

Detailed Protein Report

Protein 574: nucleolar RNA helicase 2 isoform 2 [Homo sapiens]

Accession: gi|379317177
Database: refseq_human(refseq_human_20140103.fasta)
Database Date: 2015-11-30
Modification(s): Carbamidomethyl, Oxidation

Score: 26.3
MW [kDa]: 79.6
pI: 10.0
Sequence Coverage [%]: 2.9
No. of unique Peptides: 1

10	20	30	40	50	60	70	80
MNSPKSKKAK	KKEEPSQNDI	SPKTKSLRKK	KEPIEKKVVS	SKTKKVTKNE	EPSEEEIDAP	KPKKMKKEKE	MNGETREKSP
**90**	**100**	**110**	**120**	**130**	**140**	**150**	**160**
KLKNGFPHPE	PDCNPSEAAS	EESNSEIEQE	IPVEQKEGAF	SNFPISEETI	KLLKGRGVTF	LFPIQAKTFH	HVYSGKDLIA
**170**	**180**	**190**	**200**	**210**	**220**	**230**	**240**
QARTGTGKTF	SFAIPLIEKL	HGELQDRKRG	RAPQVLVLAP	TRELANQVSK	DFSDITKKLS	VACFYGGTPY	GGQFERMRNG
**250**	**260**	**270**	**280**	**290**	**300**	**310**	**320**
IDILVGTPGR	IKDHIQNGKL	DLTKLKHVVL	DEVDQMLDMG	FADQVEEILS	VAYKKDSEDN	PQTLLFSATC	PHWVFNVAKK
**330**	**340**	**350**	**360**	**370**	**380**	**390**	**400**
YMKSTYEQVD	LIGKKTQKTA	ITVEHLAIKC	HWTQRAAVIG	DVIRVYSGHQ	GRTIIFCETK	KEAQELSQNS	AIKQDAQSLH
**410**	**420**	**430**	**440**	**450**	**460**	**470**	**480**
GDIPQKQREI	TLKGFRNGSF	GVLVATNVAA	RGLDIPEVDL	VIQSSPPKDV	ESYIHRSGRT	GRAGRTGVCI	CFYQHKEEYQ
**490**	**500**	**510**	**520**	**530**	**540**	**550**	**560**
LVQVEQKAGI	KFKRIGVPSA	TEIIKASSKD	AIRLLDSVPP	TAISHFKQSA	EKLIEEKGAV	EALAAALAHI	SGATSVDQRS
**570**	**580**	**590**	**600**	**610**	**620**	**630**	**640**
LINSNVGFVT	MILQCSIEMP	NISYAWKELK	EQLGEEIDSK	VKGMVFLKGK	LGVCFDVPTA	SVTEIQEKWH	DSRRWQLSVA
**650**	**660**	**670**	**680**	**690**	**700**	**710**	**720**
TEQPELEGPR	EGYGGFRGQR	EGSRGFRGQR	DGNRRFRGQR	EGSRGPRGQR	SGGGNKSNRS	QNKGQKRSFS	KAFGQ

Cmpd.	No. of Cmpds.	m/z meas.	Δ m/z [ppm]	z	Rt [min]	Score	P	Range	Sequence	Modification
63	1	814.0267	-36.10	3	30.1	14.9	2	218-238	K.KLSVACFYGGTPYGGQFERMR.N	Carbamidomethyl: 6; Oxidation: 20

Detailed Protein Report

Protein 575: queuine tRNA-ribosyltransferase [Homo sapiens]

**Accession:**	gi|13654276	**Score:**	26.2
**Database:**	refseq_human(refseq_human_20140103.fasta)	**MW [kDa]:**	44.0
**Database Date:**	2015-11-30	**pI:**	7.0
**Modification(s):**	Oxidation	**Sequence Coverage [%]:**	6.5
		**No. of unique Peptides:**	1

Quantitation

mdown:qdown	**Median:**	0.33	**CV:**	0.00 %	**No. of Peptides:**	1
Wdown:Qdown	**Median:**	1.10	**CV:**	0.00 %	**No. of Peptides:**	1

10	20	30	40	50	60	70	80
MAGAATQASL	ESAPRIMRLV	AECSRSRARA	GELWLPHGTV	ATPVFMPVGT	QATMKGITTE	QLDALGCRIC	LGNTYHLGLR
**90**	**100**	**110**	**120**	**130**	**140**	**150**	**160**
PGPELIQKAN	GLHGFMNWPH	NLLTDSGGFQ	MVSLVSLSEV	TEEGVRFRSP	YDGNETLLSP	EKSVQIQNAL	GSDIIMQLDD
**170**	**180**	**190**	**200**	**210**	**220**	**230**	**240**
VVSSTVTGPR	VEEAMYRSIR	WLDRCIAAHQ	RPDKQNLFAI	IQGGLDADLR	ATCLEEMTKR	DVPGFAIGGL	SGGESKSQFW
**250**	**260**	**270**	**280**	**290**	**300**	**310**	**320**
RMVALSTSRL	PKDKPRYLMG	VGYATDLVVC	VALGCDMFDC	VFPTRTARFG	SALVPTGNLQ	LRKKVFEKDF	GPIDPECTCP
**330**	**340**	**350**	**360**	**370**	**380**	**390**	**400**
TCQKHSRAFL	HALLHSDNTA	ALHHLTVHNI	AYQLQLMSAV	RTSIVEKRFP	DFVRDFMGAM	YGDPTLCPTW	ATDALASVGI
**410**							
TLG							

Cmpd.	No. of Cmpds.	m/z meas.	Δ m/z [ppm]	z	Rt [min]	Score	P	Range	Sequence	Modification	Ratios
512	1	719.0204	199.94	2	35.3	10.4	2	16-27	R.IMRLVAECSRSR.A	Oxidation: 2	Wdown:Qdown 1.10<br>mdown:qdown 0.33

Detailed Protein Report

Protein 576: eukaryotic translation initiation factor 3 subunit B [Homo sapiens]

**Accession:**	gi|33239445	**Score:**	26.2
**Database:**	refseq_human(refseq_human_20140103.fasta)	**MW [kDa]:**	92.4
**Database Date:**	2015-11-30	**pI:**	4.7
		**Sequence Coverage [%]:**	2.8
		**No. of unique Peptides:**	1

Quantitation

mdown:qdown **Median:** 0.43 **CV:** 0.00 % **No. of Peptides:** 1

Alias proteins:

Accession	Name	Description
gi|83367072	refseq_human (refseq_human_20140103.fasta)	eukaryotic translation initiation factor 3 subunit B [Homo sapiens]

10	20	30	40	50	60	70	80
MQDAENVAVP	EAAEERAEPG	QQQPAAEPPP	AEGLLRPAGP	GAPEAAGTEA	SSEEVGIAEA	GPESEVRTEP	AAEAEAASGP
**90**	**100**	**110**	**120**	**130**	**140**	**150**	**160**
SESPSPPAAE	ELPGSHAEPP	VPAQGEAPGE	QARDERSDSR	AQAVSEDAGG	NEGRAAEAEP	RALENGDADE	PSFSDPEDFV
**170**	**180**	**190**	**200**	**210**	**220**	**230**	**240**
DDVSEEELLG	DVLKDRPQEA	DGIDSVIVVD	NVPQVGPDRL	EKLKNVIHKI	FSKFGKITND	FYPEEDGKTK	GYIFLEYASP
**250**	**260**	**270**	**280**	**290**	**300**	**310**	**320**
AHAVDAVKNA	DGYKLDKQHT	FRVNLFTDFD	KYMTISDEWD	IPEKQPFKDL	GNLRYWLEEA	ECRDQYSVIF	ESGDRTSIFW
**330**	**340**	**350**	**360**	**370**	**380**	**390**	**400**
NDVKDPVSIE	ERARWTETYV	RWSPKGTYLA	TFHQRGIALW	GGEKFKQIQR	FSHQGVQLID	FSPCERYLVT	FSPLMDTQDD
**410**	**420**	**430**	**440**	**450**	**460**	**470**	**480**
PQAIIIWDIL	TGHKKRGFHC	ESSAHWPIFK	WSHDGKFFAR	MTLDTLSIYE	TPSMGLLDKK	SLKISGIKDF	SWSPGGNIIA
**490**	**500**	**510**	**520**	**530**	**540**	**550**	**560**
FWVPEDKDIP	ARVTLMQLPT	RQEIRVRNLF	NVVDCKLHWQ	KNGDYLCVKV	DRTPKGTQGV	VTNFEIFRMR	EKQVPVDVVE
**570**	**580**	**590**	**600**	**610**	**620**	**630**	**640**
MKETIIAFAW	EPNGSKFAVL	HGEAPRISVS	FYHVKNNGKI	ELIKMFDKQQ	ANTIFWSPQG	QFVVLAGLRS	MNGALAFVDT
**650**	**660**	**670**	**680**	**690**	**700**	**710**	**720**
SDCTVMNIAE	HYMASDVEWD	PTGRYVVTSV	SWWSHKVDNA	YWLWTFQGRL	LQKNNKDRFC	QLLWRPRPPT	LLSQEQIKQI
**730**	**740**	**750**	**760**	**770**	**780**	**790**	**800**
KKDLKKYSKI	FEQKDRLSQS	KASKELVERR	RTMMEDFRKY	RKMAQELYME	QKNERLELRG	GVDTDELDSN	VDDWEEETIE
**810**	**820**						
FFVTEEIIPL	GNQE						

Cmpd.	No. of Cmpds.	m/z meas.	Δ m/z [ppm]	z	Rt [min]	Score	P	Range	Sequence	Modification	Ratios
450	1	680.6717	-200.25	2	34.9	12.9	0	121-134	R.AQAVSEDAGGNEGR.A		mdown:qdown 0.43

Detailed Protein Report

Protein 577: PREDICTED: death ligand signal enhancer isoform X6 [Homo sapiens]

Accession: gi|530380768
Database: refseq_human(refseq_human_20140103.fasta)
Database Date: 2015-11-30

Score: 26.2
MW [kDa]: 42.7
pI: 10.1
Sequence Coverage [%]: 6.2
No. of unique Peptides: 1

10	20	30	40	50	60	70	80
MWRLPGLLGR	ALPRTLGPSL	WRVTPKSTSP	DGPQTTSSTL	LVPVPNLDRS	GPHGPGTSGG	PRSHGWKDAF	QWMSSRVSPN
90	100	110	120	130	140	150	160
TLWDAISWGT	LAVLALQLAR	QIHFQASLPA	GPQRVEHCSW	HSPLDRFFSS	PLWHPCSSLR	QHILPSPDGP	APRHTGLREP
170	180	190	200	210	220	230	240
RLGQEEASAQ	PRNFSHNSLR	GARPQDPSEE	GPGDFGFLHA	SSSIESEAKP	AQPQPTGEKE	QDKSKTLSLE	EAVTSIQQLF
250	260	270	280	290	300	310	320
QLSVSIAFNF	LGTENMKSGD	HTAAFSYFQK	AAARGYSKAQ	YNAGLCHEHG	RGTPRDISKA	VLYYQLAASQ	GHSLAQYRYA
330	340	350	360	370	380	390	
RCLLRDPASS	WNPERQRAVS	LLKQAADSGL	REAQAFLGVL	FTKEPYLDEQ	RAVKYLWLAA	NNGPRGPAT	

Cmpd.	No. of Cmpds.	m/z meas.	Δ m/z [ppm]	z	Rt [min]	Score	P	Range	Sequence	Modification
2125	1	775.3350	-103.58	2	56.7	14.8	0	101-114	R.QIHFQASLPAGPQR.V	

Detailed Protein Report

Protein 578: PREDICTED: dehydrogenase/reductase SDR family member 11 isoform X3 [Homo sapiens]

Accession: gi|578831391
Database: refseq_human(refseq_human_20140103.fasta)
Database Date: 2015-11-30

Score: 26.2
MW [kDa]: 14.1
pI: 10.1
Sequence Coverage [%]: 32.3
No. of unique Peptides: 1

Alias proteins:

Accession	Name	Description
gi|578840409	refseq_human (refseq_human_20140103.fasta)	PREDICTED: dehydrogenase/reductase SDR family member 11 isoform X3 [Homo sapiens]

```
        10         20         30         40         50         60         70         80
MARPGMERWR DRLALVTGAS GGIGAAVARA LVQQGLKVVG CARTVGNIEE LAAECKSAGY PGTLIPYRCD LSNEEDILSM
        90        100        110        120        130        140
FSAIRSQHSG VDICINNAGL ARPDTLLSGS TSGWKDMFNG LSSLPHKPTL PRR
```

Cmpd.	No. of Cmpds.	m/z meas.	Δ m/z [ppm]	z	Rt [min]	Score	P	Range	Sequence	Modification
2671	1	1079.8373	-43.87	3	64.6	12.8	2	38-68	K.VVGCARTVGNIEELAAECKSAGYPGTLIPYR.C	

Detailed Protein Report

Protein 579: PREDICTED: protein CASP isoform X6 [Homo sapiens]

**Accession:**	gi|578813926	**Score:**	26.2
**Database:**	refseq_human(refseq_human_20140103.fasta)	**MW [kDa]:**	172.0
**Database Date:**	2015-11-30	**pI:**	5.9
**Modification(s):**	Carbamidomethyl, Oxidation	**Sequence Coverage [%]:**	1.5
		**No. of unique Peptides:**	1

10	20	30	40	50	60	70	80
MERAAGALGD	AFSGRVLESR	APGARWDRGM	PGGGPRAESP	APRGDRAGAP	RPAPESPPCP	RGECLPATPR	PEERRTLRVR
90	100	110	120	130	140	150	160
PARASSAPAA	PGQPRDSARW	MLCVAGARLK	RELDATATVL	ANRQDESEQS	RKRLIEQSRE	FKKNTPEDLR	KQVAPLLKSF
170	180	190	200	210	220	230	240
QGEIDALSKR	SKEAEAAFLN	VYKRLIDVPD	PVPALDLGQQ	LQLKVQRLHD	IETENQKLRE	TLEEYNKEFA	EVKNQEVTIK
250	260	270	280	290	300	310	320
ALKEKIREYE	QTLKNQAETI	ALEKEQKLQN	DFAEKERKLQ	ETQMSTTSKL	EEAEHKVQSL	QTALEKTRTE	LFDLKTKYDE
330	340	350	360	370	380	390	400
ETTAKADEIE	MIMTDLERAN	QRAEVAQREA	ETLREQLSSA	NHSLQLASQI	QKAPDVEQAI	EVLTRSSLEV	ELAAKEREIA
410	420	430	440	450	460	470	480
QLVEDVQRLQ	ASLTKLRENS	ASQISQLEQQ	LSAKNSTLKQ	LEEKLKGQAD	YEEVKKELNI	LKSMEFAPSE	GAGTQDAAKP
490	500	510	520	530	540	550	560
LEVLLLEKNR	SLQSENAALR	ISNSDLSGSA	RRKGKDQPES	RRPGSLPAPP	PSQLPRNPGE	QASNTNGTHQ	FSPAGLSQDF
570	580	590	600	610	620	630	640
FSSSLASPSL	PLASTGKFAL	NSLLQRQLMQ	SFYSKAMQEA	GSTSMIFSTG	PYSTNSISSQ	SPLQQSPDVN	GMAPSPSQSE
650	660	670	680	690	700	710	720
SAGSVSEGEE	MDTAEIARQV	KEQLIKHNIG	QRIFGHYVLG	LSQGSVSEIL	ARPKPWNKLT	VRGKEPFHKM	KQFLSDEQNI
730	740	750	760	770	780	790	800
LALRSIQGRQ	RGNITTRIRA	SETGSDEAIK	SILEQAKREL	QVQKTAEPAQ	PSSASGSGNS	DDAIRSILQQ	ARREMEAQQA
810	820	830	840	850	860	870	880
ALDPALKQAP	LSQSDITILT	PKLLSTSPMP	TVSSYPPLAI	SLKKPSAAPE	AGASALPNPP	ALKKEAQDAP	GLDPQGAADC
890	900	910	920	930	940	950	960
AQGVLRQVKN	EVGRSGAWKD	HWWSAVQPER	RNAASSEEAK	AEETGGGKEK	GSGGSGGGSQ	PRAERSQLQG	PSSSEYWKEW
970	980	990	1000	1010	1020	1030	1040
PSAESPYSQS	SELSLTGASR	SETPQNSPLP	SSPIVPMSKP	TKPSVPPLTP	EQYEVYMYQE	VDTIELTRQV	KEKLAKNGIC
1050	1060	1070	1080	1090	1100	1110	1120
QRIFGEKVLG	LSQGSVSDML	SRPKPWSKLT	QKGREPFIRM	QLWLNGELGQ	GVLPVQGQQQ	GPVLHSVTSL	QDPLQQGCVS
1130	1140	1150	1160	1170	1180	1190	1200
SESTPKTSAS	CSPAPESPMS	SSESVKSLTE	LVQQPCPPIE	ASKDSKPPEP	SDPPASDSQP	TTPLPLSGHS	ALSIQELVAM
1210	1220	1230	1240	1250	1260	1270	1280
SPELDTYGIT	KRVKEVLTDN	NLGQRLFGET	ILGLTQGSVS	DLLARPKPWH	KLSLKGREPF	VRMQLWLNDP	NNVEKLMDMK
1290	1300	1310	1320	1330	1340	1350	1360
RMEKKAYMKR	RHSSVSDSQP	CEPPSVGTEY	SQGASPQPQH	QLKKPRVVLA	PEEKEALKRA	YQQKPYPSPK	TIEDLATQLN
1370	1380	1390	1400	1410	1420	1430	1440
LKTSTVINWF	HNYRSRIRRE	LFIEEIQAGS	QGQAGASDSP	SARSGRAAPS	SEGDSCDGVE	ATEGPGSADT	EEPKSQGEAE
1450	1460	1470	1480	1490	1500	1510	1520
REEVPRPAEQ	TEPPPSGTPG	PDDARDDDHE	GGPVEGPGPL	PSPASATATA	APAAPEDAAT	SAAAAPGEGP	AAPSSAPPPS
1530	1540	1550	1560	1570	1580	1590	
NSSSSSAPRR	PSSLQSLFGL	PEAAGARDSR	DNPLRKKKAA	NLNSIIHRLE	KAASREEPIE	WEF	

Cmpd.	No. of Cmpds.	m/z meas.	Δ m/z [ppm]	z	Rt [min]	Score	P	Range	Sequence	Modification
1855	1	738.7765	-167.06	2	53.2	12.2	2	100-111	R.WMLCVAGARLKR.E	Carbamidomethyl: 4; Oxidation: 2

Detailed Protein Report

Protein 580: sperm-specific antigen 2 isoform 4 [Homo sapiens]

**Accession:**	gi|566559898	**Score:**	26.1
**Database:**	refseq_human(refseq_human_20140103.fasta)	**MW [kDa]:**	119.1
**Database Date:**	2015-11-30	**pI:**	5.1
**Modification(s):**	Oxidation	**Sequence Coverage [%]:**	2.7
		**No. of unique Peptides:**	1

10	20	30	40	50	60	70	80
MNSTGSGKSS	GTVSSVSELL	ELYEEDPEEI	LYNLGFGRDE	PDIASKIPSR	FFNSSSFAKG	IDIKVFLSAQ	MQRMEVENPN
90	100	110	120	130	140	150	160
YALTSRFRQI	EVLTTVANAF	SSLYSQVSGT	PLQRIGSMSS	VTSNKETDPP	PPLTRSNTAN	RLMKTLSKLN	LCVDKTEKGE
170	180	190	200	210	220	230	240
SSSPSPSAEK	GKILNVSVIE	ESGNKNDQKS	QKIMKKKESS	SMLATVKEEV	SGSSAAVTEN	ADSDRISDEA	NSNFNQGTEN
250	260	270	280	290	300	310	320
EQSKETQSHE	SKLGEESGIV	ESKLDSDFNI	SSHSELENSS	ELKSVHISTP	EKEPCAPLTI	PSIRNIMTQQ	KDSFEMEEVQ
330	340	350	360	370	380	390	400
STEGEAPHVP	ATYQLGLTKS	KRDHLLRTAS	QHSDSSGFAE	DSTDCLSLNH	LQVQESLQAM	GSSADSCDSE	TTVTSLGEDL
410	420	430	440	450	460	470	480
ATPTAQDQPY	FNESEEESLV	PLQKGLEKAA	AVADKRKSGS	QDFPQCNTIE	NTGTKQSTCS	PGDHIIEITE	VEEDLFPAET
490	500	510	520	530	540	550	560
VELLREASAE	SDVGKSSESE	FTQYTTHHIL	KSLASIEAKC	SDMSSENTTG	PPSSMDRVNT	ALQRAQMKVC	SLSNQRMGRS
570	580	590	600	610	620	630	640
LLKSKDLLKQ	RYLFAKAGYP	LRRSQSLPTT	LLSPVRVVSS	VNVRLSPGKE	TRCSPPSFTY	KYTPEEEQEL	EKRVMEHDGQ
650	660	670	680	690	700	710	720
SLVKSTIFIS	PSSVKKEEAP	QSEAPRVEEC	HHGRTPTCSR	LAPPPMSQST	CSLHSIHSEW	QERPLCEHTR	TLSTHSVPNI
730	740	750	760	770	780	790	800
SGATCSAFAS	PFGCPYSHRH	ATYPYRVCSV	NPPSAIEMQL	RRVLHDIRNS	LQNLSQYPMM	RGPDPAAAPY	STQKSSVLPL
810	820	830	840	850	860	870	880
YENTFQELQV	MRRSLNLFRT	QMMDLELAML	RQQTMVYHHM	TEEERFEVDQ	LQGLRNSVRM	ELQDLELQLE	ERLLGLEEQL
890	900	910	920	930	940	950	960
RAVRMPSPFR	SSALMVTELM	QEQSYLKSEL	GLGLGEMGFE	IPPGESSESV	FSQATSESSS	VCSGPSHANR	RTGVPSTASV
970	980	990	1000	1010	1020	1030	1040
GKSKTPLVAR	KKVFRASVAL	TPTAPSRTGS	VQTPPDLESS	EEVDAAEGAP	EVVGPKSEVE	EGHGKLPSMP	AAEEMHKNVE
1050	1060	1070	1080	1090			
QDELQQVIRE	IKESIVGEIR	REIVSGLLAA	VSSSKASNSK	QDYH			

Cmpd.	No. of Cmpds.	m/z meas.	Δ m/z [ppm]	z	Rt [min]	Score	P	Range	Sequence	Modification
1851	1	917.8276	-74.46	2	53.2	12.3	0	832-845	R.QQTMVYHHMTEEER.F	Oxidation: 4

Detailed Protein Report

Protein 581: 1-phosphatidylinositol 4,5-bisphosphate phosphodiesterase eta-1 isoform c [Homo sapiens]

**Accession:**	gi|195972873	**Score:**	26.1
**Database:**	refseq_human(refseq_human_20140103.fasta)	**MW [kDa]:**	114.4
**Database Date:**	2015-11-30	**pI:**	8.3
		**Sequence Coverage [%]:**	3.2
		**No. of unique Peptides:**	1

```
        10         20         30         40         50         60         70         80
MADLEVYKNL SPEKVERCMS VMQSGTQMIK LKRGTKGLVR LFYLDEHRTR LRWRPSRKSE KAKILIDSIY KVTEGRQSEI
        90        100        110        120        130        140        150        160
FHRQAEGNFD PSCCFTIYHG NHMESLDLIT SNPEEARTWI TGLKYLMAGI SDEDSLAKRQ RTHDQWVKQT FEEADKNGDG
       170        180        190        200        210        220        230        240
LLNIEEIHQL MHKLNVNLPR RKVRQMFQEA DTDENQGTLT FEEFCVFYKM MSLRRDLYLL LLSYSDKKDH LTVEELAQFL
       250        260        270        280        290        300        310        320
KVEQKMNNVT TDYCLDIIKK FEVSEENKVK NVLGIEGFTN FMRSPACDIF NPLHHEVYQD MDQPLCNYYI ASSHNTYLTG
       330        340        350        360        370        380        390        400
DQLLSQSKVD MYARVLQEGC RCVEVDCWDG PDGEPVVHHG YTLTSKILFR DVVETINKHA FVKNEFPVIL SIENHCSIQQ
       410        420        430        440        450        460        470        480
QRKIAQYLKG IFGDKLDLSS VDTGECKQLP SPQSLKGKIL VKGKKLPYHL GDDAEEGEVS DEDSADEIED ECKFKLHYSN
       490        500        510        520        530        540        550        560
GTTEHQVESF IRKKLESLLK ESQIRDKEDP DSFTVRALLK ATHEGLNAHL KQSPDVKESG KKSHGRSLMT NFGKHKKTTK
       570        580        590        600        610        620        630        640
SRSKSYSTDD EEDTQQSTGK EGGQLYRLGR RRKTMKLCRE LSDLVVYTNS VAAQDIVDDG TTGNVLSFSE TRAHQVVQQK
       650        660        670        680        690        700        710        720
SEQFMIYNQK QLTRIYPSAY RIDSSNFNPL PYWNAGCQLV ALNYQSEGRM MQLNRAKFKA NGNCGYVLKP QQMCKGTFNP
       730        740        750        760        770        780        790        800
FSGDPLPANP KKQLILKVIS GQQLPKPPDS MFGDRGEIID PFVEVEIIGL PVDCCKDQTR VVDDNGFNPV WEETLTFTVH
       810        820        830        840        850        860        870        880
MPEIALVRFL VWDHDPIGRD FVGQRTVTFS SLVPGYRHVY LEGLTEASIF VHITINEIYG KWSPLILNPS YTILHFLGAT
       890        900        910        920        930        940        950        960
KNRQLQGLKG LFNKNPRHSS SENNSHYVRK RSIGDRILRR TASAPAKGRK KSKMGFQEMV EIKDSVSEAT RDQDGVLRRT
       970        980        990       1000       1010
TRSLQARPVS MPVDRNLLGA LSLPVSETAK DIEGKENSLV QI
```

Cmpd.	No. of Cmpds.	m/z meas.	Δ m/z [ppm]	z	Rt [min]	Score	P	Range	Sequence	Modification
845	1	1419.3283	69.70	2	39.4	12.2	1	379-402	K.HAFVKNEFPVILSIENHCSIQQQR.K	

Detailed Protein Report

Protein 582: AP-1 complex subunit mu-1 isoform 2 [Homo sapiens]

**Accession:**	gi|14210504	**Score:**	26.1
**Database:**	refseq_human(refseq_human_20140103.fasta)	**MW [kDa]:**	48.6
**Database Date:**	2015-11-30	**pI:**	7.0
**Modification(s):**	Carbamidomethyl	**Sequence Coverage [%]:**	6.4
		**No. of unique Peptides:**	2

Quantitation

Wdown:Qdown **Median:** 1.64 **CV:** 0.00 % **No. of Peptides:** 1

10	20	30	40	50	60	70	80
MSASAVYVLD	LKGKVLICRN	YRGDVDMSEV	EHFMPILMEK	EEEGMLSPIL	AHGGVRFMWI	KHNNLYLVAT	SKKNACVSLV
**90**	**100**	**110**	**120**	**130**	**140**	**150**	**160**
FSFLYKVVQV	FSEYFKELEE	ESIRDNFVII	YELLDELMDF	GYPQTTDSKI	LQEYITQEGH	KLETGAPRPP	ATVTNAVSWR
**170**	**180**	**190**	**200**	**210**	**220**	**230**	**240**
SEGIKYRKNE	VFLDVIESVN	LLVSANGNVL	RSEIVGSIKM	RVFLSGMPEL	RLGLNDKVLF	DNTGRGKSKS	VELEDVKFHQ
**250**	**260**	**270**	**280**	**290**	**300**	**310**	**320**
CVRLSRFEND	RTISFIPPDG	EFELMSYRLN	THVKPLIWIE	SVIEKHSHSR	IEYMIKAKSQ	FKRRSTANNV	EIHIPVPNDA
**330**	**340**	**350**	**360**	**370**	**380**	**390**	**400**
DSPKFKTTVG	SVKWVPENSE	IVWSIKSFPG	GKEYLMRAHF	GLPSVEAEDK	EGKPPISVKF	EIPYFTTSGI	QVRYLKIIEK
**410**	**420**	**430**					
SGYQALPWVR	YITQNGDYQL	RTQ					

Cmpd.	No. of Cmpds.	m/z meas.	Δ m/z [ppm]	z	Rt [min]	Score	P	Range	Sequence	Modification	Ratios
1641	1	873.4079	-31.68	2	49.0	15.9	1	230-243	K.SVELEDVKFHQCVR.L	Carbamidomethyl: 12	Wdown:Qdown 1.64
1957	1	700.2365	-156.66	2	54.6	10.2	0	358-370	R.AHFGLPSVEAEDK.E		

Detailed Protein Report

Protein 583: nuclear receptor subfamily 1 group D member 1 [Homo sapiens]

Accession: gi|13430848
Database: refseq_human(refseq_human_20140103.fasta)
Database Date: 2015-11-30
Modification(s): Carbamidomethyl, Oxidation

Score: 26.1
MW [kDa]: 66.8
pI: 9.9
Sequence Coverage [%]: 2.9
No. of unique Peptides: 1

10	20	30	40	50	60	70	80
MTTLDSNNNT	GGVITYIGSS	GSSPSRTSPE	SLYSDNSNGS	FQSLTQGCPT	YFPPSPTGSL	TQDPARSFGS	IPPSLSDDGS
**90**	**100**	**110**	**120**	**130**	**140**	**150**	**160**
PSSSSSSSSS	SSSFYNGSPP	GSLQVAMEDS	SRVSPSKSTS	NITKLNGMVL	LCKVCGDVAS	GFHYGVHACE	GCKGFFRRSI
**170**	**180**	**190**	**200**	**210**	**220**	**230**	**240**
QQNIQYKRCL	KNENCSIVRI	NRNRCQQCRF	KKCLSVGMSR	DAVRFGRIPK	REKQRMLAEM	QSAMNLANNQ	LSSQCPLETS
**250**	**260**	**270**	**280**	**290**	**300**	**310**	**320**
PTQHPTPGPM	GPSPPPAPVP	SPLVGFSQFP	QQLTPPRSPS	PEPTVEDVIS	QVARAHREIF	TYAHDKLGSS	PGNFNANHAS
**330**	**340**	**350**	**360**	**370**	**380**	**390**	**400**
GSPPATTPHR	WENQGCPPAP	NDNNTLAAQR	HNEALNGLRQ	APSSYPPTWP	PGPAHHSCHQ	SNSNGHRLCP	THVYAAPEGK
**410**	**420**	**430**	**440**	**450**	**460**	**470**	**480**
APANSPRQGN	SKNVLLACPM	NMYPHGRSGR	TVQEIWEDFS	MSFTPAVREV	VEFAKHIPGF	RDLSQHDQVT	LLKAGTFEVL
**490**	**500**	**510**	**520**	**530**	**540**	**550**	**560**
MVRFASLFNV	KDQTVMFLSR	TTYSLQELGA	MGMGDLLSAM	FDFSEKLNSL	ALTEEELGLF	TAVVLVSADR	SGMENSASVE
**570**	**580**	**590**	**600**	**610**	**620**		
QLQETLLRAL	RALVLKNRPL	ETSRFTKLLL	KLPDLRTLNN	MHSEKLLSFR	VDAQ		

Cmpd.	No. of Cmpds.	m/z meas.	Δ m/z [ppm]	z	Rt [min]	Score	P	Range	Sequence	Modification
116	1	1044.8604	-131.76	2	30.7	26.1	1	413-430	K.NVLLACPMNMYPHGRSGR.T	Carbamidomethyl: 6; Oxidation: 10

Detailed Protein Report

Protein 584: nucleolar transcription factor 1 isoform b [Homo sapiens]

**Accession:**	gi|115529449	**Score:**	26.1
**Database:**	refseq_human(refseq_human_20140103.fasta)	**MW [kDa]:**	84.9
**Database Date:**	2015-11-30	**pI:**	5.3
		**Sequence Coverage [%]:**	3.9
		**No. of unique Peptides:**	1

Alias proteins:

Accession	Name	Description
gi|115529451	refseq_human (refseq_human_20140103.fasta)	nucleolar transcription factor 1 isoform b [Homo sapiens]

10	20	30	40	50	60	70	80
MNGEADCPTD	LEMAAPKGQD	RWSQEDMLTL	LECMKNNLPS	NDSSKFKTTE	SHMDWEKVAF	KDFSGDMCKL	KWVEISNEVR
**90**	**100**	**110**	**120**	**130**	**140**	**150**	**160**
KFRTLTELIL	DAQEHVKNPY	KGKKLKKHPD	FPKKPLTPYF	RFFMEKRAKY	AKLHPEMSNL	DLTKILSKKY	KELPEKKKMK
**170**	**180**	**190**	**200**	**210**	**220**	**230**	**240**
YIQDFQREKQ	EFERNLARFR	EDHPDLIQNA	KKSDIPEKPK	TPQQLWYTHE	KKVYLKVRPD	EIMRDYIQKH	PELNISEEGI
**250**	**260**	**270**	**280**	**290**	**300**	**310**	**320**
TKSTLTKAER	QLKDKFDGRP	TKPPPNSYSL	YCAELMANMK	DVPSTERMVL	CSQQWKLLSQ	KEKDAYHKKC	DQKKKDYEVE
**330**	**340**	**350**	**360**	**370**	**380**	**390**	**400**
LLRFLESLPE	EEQQRVLGEE	KMLNINKKQA	TSPASKKPAQ	EGGKGGSEKP	KRPVSAMFIF	SEEKRRQLQE	ERPELSESEL
**410**	**420**	**430**	**440**	**450**	**460**	**470**	**480**
TRLLARMWND	LSEKKKAKYK	AREAALKAQS	ERKPGGEREE	RGKLPESPKR	AEEIWQQSVI	GDYLARFKND	RVKALKAMEM
**490**	**500**	**510**	**520**	**530**	**540**	**550**	**560**
TWNNMEKKEK	LMWIKKAAED	QKRYERELSE	MRAPPAATNS	SKKMKFQGEP	KKPPMNGYQK	FSQELLSNGE	LNHLPLKERM
**570**	**580**	**590**	**600**	**610**	**620**	**630**	**640**
VEIGSRWQRI	SQSQKEHYKK	LAEEQQKQYK	VHLDLWVKSL	SPQDRAAYKE	YISNKRKSMT	KLRGPNPKSS	RTTLQSKSES
**650**	**660**	**670**	**680**	**690**	**700**	**710**	**720**
EEDDEEDEDD	EDEDEEEEDD	ENGDSSEDGG	DSSESSSEDE	SEDGDENEED	DEDEDDDEDD	DEDEDNESEG	SSSSSSSSGD
**730**							
SSDSDSN							

Cmpd.	No. of Cmpds.	m/z meas.	Δ m/z [ppm]	z	Rt [min]	Score	P	Range	Sequence	Modification
1966	1	871.8504	-139.97	2	54.7	11.1	1	108-121	K.HPDFPKKPLTPYFR.F	

Detailed Protein Report

Protein 585: PREDICTED: 6-phosphofructokinase, liver type isoform X4 [Homo sapiens]

**Accession:**	gi|530419271	**Score:**	26.1
**Database:**	refseq_human(refseq_human_20140103.fasta)	**MW [kDa]:**	82.2
**Database Date:**	2015-11-30	**pI:**	7.9
**Modification(s):**	Carbamidomethyl	**Sequence Coverage [%]:**	4.3
		**No. of unique Peptides:**	2

10	20	30	40	50	60	70	80
MNAAVRAVTR	MGIYVGAKVF	LIYEGYEGLV	EGGENIKQAN	WLSVSNIIQL	GGTIIGSARC	KAFTTREGRR	AAAYNLVQHG
**90**	**100**	**110**	**120**	**130**	**140**	**150**	**160**
ITNLCVIGGD	GSLTGANIFR	SEWGSLLEEL	VAEGKISETT	ARTYSHLNIA	GLVGSIDNDF	CGTDMTIGTD	SALHRIMEVI
**170**	**180**	**190**	**200**	**210**	**220**	**230**	**240**
DAITTTAQSH	QRTFVLEVMG	RHCGYLALVS	ALASGADWLF	IPEAPPEDGW	ENFMCERLGE	TRSRGSRLNI	IIIAEGAIDR
**250**	**260**	**270**	**280**	**290**	**300**	**310**	**320**
NGKPISSSYV	KDLVVQRLGF	DTRVTVLGHV	QRGGTPSAFD	RILSSKMGME	AVMALLEATP	DTPACVVTLS	GNQSVRLPLM
**330**	**340**	**350**	**360**	**370**	**380**	**390**	**400**
ECVQMTKEVQ	KAMDDKRFDE	ATQLRGGSFE	NNWNIYKLLA	HQKPPKEKSN	FSLAILNVGA	PAAGMNAAVR	SAVRTGISHG
**410**	**420**	**430**	**440**	**450**	**460**	**470**	**480**
HTVYVVHDGF	EGLAKGQVQE	VGWHDVAGWL	GRGGSMLGTK	RTLPKGQLES	IVENIRIYGI	HALLVVGGFE	AYEGVLQLVE
**490**	**500**	**510**	**520**	**530**	**540**	**550**	**560**
ARGRYEELCI	VMCVIPATIS	NNVPGTDFSL	GSDTAVNAAM	ESCDRIKQSA	SGTKRRVFIV	ETMGGYCGYL	ATVTGIAVGA
**570**	**580**	**590**	**600**	**610**	**620**	**630**	**640**
DAAYVFEDPF	NIHDLKVNVE	HMTEKMKTDI	QRGLVLRNEK	CHDYYTTEFL	YNLYSSEGKG	VFDCRTNVLG	HLQQGGAPTP
**650**	**660**	**670**	**680**	**690**	**700**	**710**	**720**
FDRNYGTKLG	VKAMLWLSEK	LREVYRKGRV	FANAPDSACV	IGLKKKAVAF	SPVTELKKDT	DFEHRMPREQ	WWLSLRLMLK
**730**	**740**	**750**	**760**				
MLAQYRISMA	AYVSGELEHV	TRRTLSMDKG	F				

Cmpd.	No. of Cmpds.	m/z meas.	Δ m/z [ppm]	z	Rt [min]	Score	P	Range	Sequence	Modification
1744	1	647.3021	-75.58	4	50.5	11.6	1	38-61	K.QANWLSVSNIIQLGGTIIGSARCK.A	Carbamidomethyl: 23
156	1	584.6910	-183.35	2	31.3	14.4	2	60-69	R.CKAFTTREGR.R	

Detailed Protein Report

Protein 586: long-chain-fatty-acid--CoA ligase 3 [Homo sapiens]

**Accession:**	gi|42794752	**Score:**	26.1
**Database:**	refseq_human(refseq_human_20140103.fasta)	**MW [kDa]:**	80.4
**Database Date:**	2015-11-30	**pI:**	9.4
		**Sequence Coverage [%]:**	4.9
		**No. of unique Peptides:**	2

Alias proteins:

Accession	Name	Description
gi|42794754	refseq_human (refseq_human_20140103.fasta)	long-chain-fatty-acid--CoA ligase 3 [Homo sapiens]

```
        10         20         30         40         50         60         70         80
MNNHVSSKPS TMKLKHTINP ILLYFIHFLI SLYTILTYIP FYFFSESRQE KSNRIKAKPV NSKPDSAYRS VNSLDGLASV
        90        100        110        120        130        140        150        160
LYPGCDTLDK VFTYAKNKFK NKRLLGTREV LNEEDEVQPN GKIFKKVILG QYNWLSYEDV FVRAFNFGNG LQMLGQKPKT
       170        180        190        200        210        220        230        240
NIAIFCETRA EWMIAAQACF MYNFQLVTLY ATLGGPAIVH ALNETEVTNI ITSKELLQTK LKDIVSLVPR LRHIITVDGK
       250        260        270        280        290        300        310        320
PPTWSEFPKG IIVHTMAAVE ALGAKASMEN QPHSKPLPSD IAVIMYTSGS TGLPKGVMIS HSNIIAGITG MAERIPELGE
       330        340        350        360        370        380        390        400
EDVYIGYLPL AHVLELSAEL VCLSHGCRIG YSSPQTLADQ SSKIKKGSKG DTSMLKPTLM AAVPEIMDRI YKNVMNKVSE
       410        420        430        440        450        460        470        480
MSSFQRNLFI LAYNYKMEQI SKGRNTPLCD SFVFRKVRSL LGGNIRLLLC GGAPLSATTQ RFMNICFCCP VGQGYGLTES
       490        500        510        520        530        540        550        560
AGAGTISEVW DYNTGRVGAP LVCCEIKLKN WEEGGYFNTD KPHPRGEILI GGQSVTMGYY KNEAKTKADF FEDENGQRWL
       570        580        590        600        610        620        630        640
CTGDIGEFEP DGCLKIIDRK KDLVKLQAGE YVSLGKVEAA LKNLPLVDNI CAYANSYHSY VIGFVVPNQK ELTELARKKG
       650        660        670        680        690        700        710        720
LKGTWEELCN SCEMENEVLK VLSEAAISAS LEKFEIPVKI RLSPEPWTPE TGLVTDAFKL KRKELKTHYQ ADIERMYGRK
       730
```

Cmpd.	No. of Cmpds.	m/z meas.	Δ m/z [ppm]	z	Rt [min]	Score	P	Range	Sequence	Modification
1801	1	785.8979	-30.88	2	51.2	15.7	1	2-15	M.NNHVSSKPSTMKLK.H	
2875	1	1193.0834	-51.05	2	64.7	10.4	2	682-702	R.LSPEPWTPETGLVTDAFKLKR.K	

Detailed Protein Report

Protein 587: vacuolar protein sorting-associated protein 13B isoform 1 [Homo sapiens]

**Accession:**	gi|35493701	**Score:**	26.0
**Database:**	refseq_human(refseq_human_20140103.fasta)	**MW [kDa]:**	445.7
**Database Date:**	2015-11-30	**pI:**	6.0
		**Sequence Coverage [%]:**	0.9
		**No. of unique Peptides:**	1

Detailed Protein Report

10	20	30	40	50	60	70	80
MLESYVTPIL	MSYVNRYIKN	LKPSDLQLSL	WGGDVVLSKL	ELKLDVLEQE	LKLPFTFLSG	HIHELRIHVP	WTKLGSEPVV
90	100	110	120	130	140	150	160
ITINTMECIL	KLKDGIQDDH	ESCGSNSTNR	STAESTKSSI	KPRRMQQAAP	TDPDLPPGYV	QSLIRRVVNN	VNIVINNLIL
170	180	190	200	210	220	230	240
KYVEDDIVLS	VNITSAECYT	VGELWDRAFM	DISATDLVLR	KVINFSDCTV	CLDKRNASGK	IEFYQDPLLY	KCSFRTRLHF
250	260	270	280	290	300	310	320
TYENLNSKMP	SVIKIHTLVE	SLKLSITDQQ	LPMFIRIMQL	GIALYYGEIG	NFKEGEIEDL	TCHNKDMLGN	ITGSEDETRI
330	340	350	360	370	380	390	400
DMQYPAQHKG	QELYSQQDEE	QPQGWVSWAW	SFVPAIVSYD	DGEEDFVGND	PASTMHQQKA	QTLKDPIVSI	GFYCTKATVT
410	420	430	440	450	460	470	480
FKLTEMQVES	SYYSPQKVKS	KEVLCWEQEG	TTVEALMMGE	PFFDCQIGFV	GCRAMCLKGI	MGVKDFEENM	NRSETEACFF
490	500	510	520	530	540	550	560
ICGDNLSTKG	FTYLTNSLFD	YRSPENNGTR	AEFILDSTHH	KETYTEIAGM	QRFGAFYMDY	LYTMENTSGK	GSTNQQDFSS
570	580	590	600	610	620	630	640
GKSEDLGTVQ	EKSTKSLVIG	PLDFRLDSSA	VHRILKMIVC	ALEHEYEPYS	RLKSDIKDEN	ETILNPEEVA	LLEEYIPTRH
650	660	670	680	690	700	710	720
TSVTLLKCTC	TISMAEFNLL	DHLLPVIMGE	KNSSNFMNTT	NFQSLRPLPS	IRILVDKINL	EHSVPMYAEQ	LVHVVSSLTQ
730	740	750	760	770	780	790	800
PSDNLLHYCY	VHCYLKIFGF	QAGLTSLDCS	GSYCLPVPVI	PSFSTALYGK	LLKLPTCWTK	RSQIAITEGI	FELPNLTIQA
810	820	830	840	850	860	870	880
TRAQTLLLQA	IYQSWSHLGN	VSSSAVIEAL	INEIFLSIGV	KSKNPLPTLE	GSIQNVELKY	CSTSLVKCAS	GTMGSIKICA
890	900	910	920	930	940	950	960
KAPVDSGKEK	LIPLLQGPSD	TKDLHSTKWL	NESRKPESLL	APDLMAFTIQ	VPQYIDYCHN	SGAVLLCSIQ	GLAVNIDPIL
970	980	990	1000	1010	1020	1030	1040
YTWLIYQPQK	RTSRHMQQQP	VVAVPLVMPV	CRRKEDEVSI	GSAPLAKQQS	YQASEYASSP	VKTKTVTESR	PLSVPVKAML
1050	1060	1070	1080	1090	1100	1110	1120
NISESCRSPE	ERMKEFIGIV	WNAVKHLTLQ	LEVQSCCVFI	PNDSLPSPST	IVSGDIPGTV	RSWYHGQTSM	PGTLVLCLPQ
1130	1140	1150	1160	1170	1180	1190	1200
IKIISAGHKY	MEPLQEIPFV	IPRPILEEGD	AFPWTISLHN	FSIYTLLGKQ	VTLCLVEPMG	CTSTLAVTSQ	KLLATGPDTR
1210	1220	1230	1240	1250	1260	1270	1280
HSFVVCLHVD	LESLEIKCSN	PQVQLFYELT	DIMNKVWNKI	QKRGNLNLSP	TSPETMAGPV	PTSPVRSSIG	TAPPDTSTCS
1290	1300	1310	1320	1330	1340	1350	1360
PSADIGTTTE	GDSIQAGEES	PFSDSVTLEQ	TTSNIGGTSG	RVSLWMQWVL	PKITIKLFAP	DPENKGTEVC	MVSELEDLSA
1370	1380	1390	1400	1410	1420	1430	1440
SIDVQDVYTK	VKCKIESFNI	DHYRSRPGEG	WQSGHFEGVF	LQCKEKSVTT	TKLLDGTHQQ	HGFLSLTYTK	AVTKNVRHKL
1450	1460	1470	1480	1490	1500	1510	1520
TSRNERRSFH	KLSEGLMDGS	PHFLHEILLS	AQAFDIVLYF	PLLNAIASIF	QAKLPKTQKE	KRKSPGQPMR	THTLTSRNLP
1530	1540	1550	1560	1570	1580	1590	1600
LIYVNTSVIR	IFIPKTEEMQ	PTVEANQAAK	EDTVVLKIGS	VAMAPQADNP	LGRSVLRKDI	YQRALNLGIL	RDPGSEIEDR
1610	1620	1630	1640	1650	1660	1670	1680
QYQIDLQSIN	IGTAQWHQLK	PEKESVSGGV	VTETERNSQN	PALEWNMASS	IRRHQERRAI	LTPVLTDFSV	RITGAPAVIF
1690	1700	1710	1720	1730	1740	1750	1760
TKVVSPENLH	TEEILVCGHS	LEVNITTNLD	FFLSVAQVQL	LHQLIVANMT	GLEPSNKAAE	ISKQEQKKVD	IFDGGMAETS
1770	1780	1790	1800	1810	1820	1830	1840
SRYSGAQDSG	IGSDSVKIRI	VQIEQHSGAS	QHRIARPSRQ	SSIVKNLNFI	PFDIFITASR	ISLMTYSCMA	LSKSKSQEQK
1850	1860	1870	1880	1890	1900	1910	1920
NNEKTDKSSL	NLPEVDSDVA	KPNQACISTV	TAEDLLRSSI	SFPSGKKIGV	LSLESLHAST	RSSARQALGI	TIVRQPGRRG
1930	1940	1950	1960	1970	1980	1990	2000
TGDLQLEPFL	YFIVSQPSLL	LSCHHRKQRV	EVSIFDAVLK	GVASDYKCID	PGKTLPEALD	YCTVWLQTVP	GEIDSKSGIP
2010	2020	2030	2040	2050	2060	2070	2080
PSFITLQIKD	FLNGPADVNL	DISKPLKANL	SFTKLDQINL	FLKKIKNAHS	LAHSEETSAM	SNTMVNKDDL	PVSKYYRGKL
2090	2100	2110	2120	2130	2140	2150	2160
SKPKIHGDGV	QKISAQENMW	RAVSCFQKIS	VQTTQIVISM	ETVPHTSKPC	LLASLSNLNG	SLSVKATQKV	PGIILGSSFL
2170	2180	2190	2200	2210	2220	2230	2240
LSINDFLLKT	SLKERSRILI	GPCCATANLE	AKWCKHSGNP	GPEQSIPKIS	IDLRGGLLQV	FWGQEHLNCL	VLLHELLNGY
2250	2260	2270	2280	2290	2300	2310	2320
LNEEGNFEVQ	VSEPVPQMSS	PVEKNQTFKS	EQSSDDLRTG	LFQYVQDAES	LKLPGVYEVL	FYNETEDCPG	MMLWRYPEPR
2330	2340	2350	2360	2370	2380	2390	2400

Detailed Protein Report

Cmpd.	No. of Cmpds.	m/z meas.	Δ m/z [ppm]	z	Rt [min]	Score	P	Range	Sequence	Modification
1721	1	682.3288	-51.59	3	51.5	13.2	1	2196-2214	K.HSGNPGPEQSIPKISIDLR.G	

Detailed Protein Report

Protein 588: 2-oxoglutarate dehydrogenase-like, mitochondrial isoform c [Homo sapiens]

**Accession:**	gi|221316669	**Score:**	26.0
**Database:**	refseq_human(refseq_human_20140103.fasta)	**MW [kDa]:**	91.2
**Database Date:**	2015-11-30	**pI:**	6.4
		**Sequence Coverage [%]:**	3.1
		**No. of unique Peptides:**	1

10	20	30	40	50	60	70	80
MFINDVEQCQ	WIRQKFETPG	VMQFSSEEKR	TLLARLVRSM	RFEDFLARKW	SSEKRFGLEG	CEVMIPALKT	IIDKSSEMGI
**90**	**100**	**110**	**120**	**130**	**140**	**150**	**160**
ENVILGMPHR	GRLNVLANVI	RKDLEQIFCQ	FDPKLEAADE	GSGDVKYHLG	MYHERINRVT	NRNITLSLVA	NPSHLEAVDP
**170**	**180**	**190**	**200**	**210**	**220**	**230**	**240**
VVQGKTKAEQ	FYRGDAQGKK	VMSILVHGDA	AFAGQGVVYE	TFHLSDLPSY	TTNGTVHVVV	NNQIGFTTDP	RMARSSPYPT
**250**	**260**	**270**	**280**	**290**	**300**	**310**	**320**
DVARVVNAPI	FHVNADDPEA	VIYVCSVAAE	WRNTFNKDVV	VDLVCYRRRG	HNEMDEPMFT	QPLMYKQIHR	QVPVLKKYAD
**330**	**340**	**350**	**360**	**370**	**380**	**390**	**400**
KLIAEGTVTL	QEFEEEIAKY	DRICEEAYGR	SKDKKILHIK	HWLDSPWPGF	FNVDGEPKSM	TCPATGIPED	MLTHIGSVAS
**410**	**420**	**430**	**440**	**450**	**460**	**470**	**480**
SVPLEDFKIH	TGLSRILRGR	ADMTKNRTVD	WALAEYMAFG	SLLKEGIHVR	LSGQDVERGT	FSHRHHVLHD	QEVDRRTCVP
**490**	**500**	**510**	**520**	**530**	**540**	**550**	**560**
MNHLWPDQAP	YTVCNSSLSE	YGVLGFELGY	AMASPNALVL	WEAQFGDFHN	TAQCIIDQFI	STGQAKWVRH	NGIVLLLPHG
**570**	**580**	**590**	**600**	**610**	**620**	**630**	**640**
MEGMGPEHSS	ARPERFLQMS	NDDSDAYPAF	TKDFEVSQLY	DCNWIVVNCS	TPANYFHVLR	RQILLPFRKP	LIIFTPKSLL
**650**	**660**	**670**	**680**	**690**	**700**	**710**	**720**
RHPEAKSSFD	QMVSGTSFQR	VIPEDGAAAR	APEQVQRLIF	CTGKVYYDLV	KERSSQDLEE	KVAITRLEQI	SPFPFDLIKQ
**730**	**740**	**750**	**760**	**770**	**780**	**790**	**800**
EAEKYPGAEL	AWCQEEHKNM	GYYDYISPRF	MTILRRARPI	WYVGRDPAAA	PATGNRNTHL	VSLKKFLDTA	FNLQAFEGKT
**810**							
F							

Cmpd.	No. of Cmpds.	m/z meas.	Δ m/z [ppm]	z	Rt [min]	Score	P	Range	Sequence	Modification
865	1	676.8710	-83.95	2	39.3	10.8	2	91-102	R.GRLNVLANVIRK.D	

Detailed Protein Report

Protein 589: speckle-type POZ protein [Homo sapiens]

Accession: gi|4507183
Database: refseq_human(refseq_human_20140103.fasta)
Database Date: 2015-11-30

Score: 26.0
MW [kDa]: 42.1
pI: 5.5
Sequence Coverage [%]: 7.8
No. of unique Peptides: 1

Quantitation

mdown:qdown	**Median:**	1.45	**CV:**	0.00 %	**No. of Peptides:**	1
Wdown:Qdown	**Median:**	1.52	**CV:**	0.00 %	**No. of Peptides:**	1

Alias proteins:

Accession	Name	Description
gi|530412967	refseq_human (refseq_human_20140103.fasta)	PREDICTED: speckle-type POZ protein isoform X3 [Homo sapiens]
gi|530412965	refseq_human (refseq_human_20140103.fasta)	PREDICTED: speckle-type POZ protein isoform X2 [Homo sapiens]
gi|56117836	refseq_human (refseq_human_20140103.fasta)	speckle-type POZ protein [Homo sapiens]
gi|56117834	refseq_human (refseq_human_20140103.fasta)	speckle-type POZ protein [Homo sapiens]
gi|56117832	refseq_human (refseq_human_20140103.fasta)	speckle-type POZ protein [Homo sapiens]
gi|56117830	refseq_human (refseq_human_20140103.fasta)	speckle-type POZ protein [Homo sapiens]
gi|56117828	refseq_human (refseq_human_20140103.fasta)	speckle-type POZ protein [Homo sapiens]

10	20	30	40	50	60	70	80
MSRVPSPPPP	AEMSSGPVAE	SWCYTQIKVV	KFSYMWTINN	FSFCREEMGE	VIKSSTFSSG	ANDKLKWCLR	VNPKGLDEES
**90**	**100**	**110**	**120**	**130**	**140**	**150**	**160**
KDYLSLYLLL	VSCPKSEVRA	KFKFSILNAK	GEETKAMESQ	RAYRFVQGKD	WGFKKFIRRD	FLLDEANGLL	PDDKLTLFCE
**170**	**180**	**190**	**200**	**210**	**220**	**230**	**240**
VSVVQDSVNI	SGQNTMNMVK	VPECRLADEL	GGLWENSRFT	DCCLCVAGQE	FQAHKAILAA	RSPVFSAMFE	HEMEESKKNR
**250**	**260**	**270**	**280**	**290**	**300**	**310**	**320**
VEINDVEPEV	FKEMMCFIYT	GKAPNLDKMA	DDLLAAADKY	ALERLKVMCE	DALCSNLSVE	NAAEILILAD	LHSADQLKTQ
**330**	**340**	**350**	**360**	**370**	**380**		
AVDFINYHAS	DVLETSGWKS	MVVSHPHLVA	EAYRSLASAQ	CPFLGPPRKR	LKQS		

Cmpd.	No. of Cmpds.	m/z meas.	Δ m/z [ppm]	z	Rt [min]	Score	P	Range	Sequence	Modification	Ratios
1325	1	508.1630	-228.12	2	45.1	14.8	1	67-74	K.WCLRVNPK.G		mdown:qdown 1.45<br>Wdown:Qdown 1.52

Detailed Protein Report

Protein 590: methylcytosine dioxygenase TET1 [Homo sapiens]

**Accession:**	gi|156139122	**Score:**	26.0
**Database:**	refseq_human(refseq_human_20140103.fasta)	**MW [kDa]:**	235.2
**Database Date:**	2015-11-30	**pI:**	9.4
**Modification(s):**	Oxidation	**Sequence Coverage [%]:**	1.5
		**No. of unique Peptides:**	1

Quantitation

mdown:qdown	**Median:** 1.06	**CV:** 0.00 %	**No. of Peptides:** 1

Detailed Protein Report

10	20	30	40	50	60	70	80
MSRSRHARPS	RLVRKEDVNK	KKKNSQLRKT	TKGANKNVAS	VKTLSPGKLK	QLIQERDVKK	KTEPKPPVPV	RSLLTRAGAA
90	100	110	120	130	140	150	160
RMNLDRTEVL	FQNPESLTCN	GFTMALRSTS	LSRRLSQPPL	VVAKSKKVPL	SKGLEKQHDC	DYKILPALGV	KHSENDSVPM
170	180	190	200	210	220	230	240
QDTQVLPDIE	TLIGVQNPSL	LKGKSQETTQ	FWSQRVEDSK	INIPTHSGPA	AEILPGPLEG	TRCGEGLFSE	ETLNDTSGSP
250	260	270	280	290	300	310	320
KMFAQDTVCA	PFPQRATPKV	TSQGNPSIQL	EELGSRVESL	KLSDSYLDPI	KSEHDCYPTS	SLNKVIPDLN	LRNCLALGGS
330	340	350	360	370	380	390	400
TSPTSVIKFL	LAGSKQATLG	AKPDHQEAFE	ATANQQEVSD	TTSFLGQAFG	AIPHQWELPG	ADPVHGEALG	ETPDLPEIPG
410	420	430	440	450	460	470	480
AIPVQGEVFG	TILDQQETLG	MSGSVVPDLP	VFLPVPPNPI	ATFNAPSKWP	EPQSTVSYGL	AVQGAIQILP	LGSGHTPQSS
490	500	510	520	530	540	550	560
SNSEKNSLPP	VMAISNVENE	KQVHISFLPA	NTQGFPLAPE	RGLFHASLGI	AQLSQAGPSK	SDRGSSQVSV	TSTVHVVNTT
570	580	590	600	610	620	630	640
VVTMPVPMVS	TSSSSYTTLL	PTLEKKKRKR	CGVCEPCQQK	TNCGECTYCK	NRKNSHQICK	KRKCEELKKK	PSVVVPLEVI
650	660	670	680	690	700	710	720
KENKRPQREK	KPKVLKADFD	NKPVNGPKSE	SMDYSRCGHG	EEQKLELNPH	TVENVTKNED	SMTGIEVEKW	TQNKKSQLTD
730	740	750	760	770	780	790	800
HVKGDFSANV	PEAEKSKNSE	VDKKRTKSPK	LFVQTVRNGI	KHVHCLPAET	NVSFKKFNIE	EFGKTLENNS	YKFLKDTANH
810	820	830	840	850	860	870	880
KNAMSSVATD	MSCDHLKGRS	NVLVFQQPGF	NCSSIPHSSH	SIINHHASIH	NEGDQPKTPE	NIPSKEPKDG	SPVQPSLLSL
890	900	910	920	930	940	950	960
MKDRRLTLEQ	VVAIEALTQL	SEAPSENSSP	SKSEKDEESE	QRTASLLNSC	KAILYTVRKD	LQDPNLQGEP	PKLNHCPSLE
970	980	990	1000	1010	1020	1030	1040
KQSSCNTVVF	NGQTTTLSNS	HINSATNQAS	TKSHEYSKVT	NSLSLFIPKS	NSSKIDTNKS	IAQGIITLDN	CSNDLHQLPP
1050	1060	1070	1080	1090	1100	1110	1120
RNNEVEYCNQ	LLDSSKKLDS	DDLSCQDATH	TQIEEDVATQ	LTQLASIIKI	NYIKPEDKKV	ESTPTSLVTC	NVQQKYNQEK
1130	1140	1150	1160	1170	1180	1190	1200
GTIQQKPPSS	VHNNHGSSLT	KQKNPTQKKT	KSTPSRDRRK	KKPTVVSYQE	NDRQKWEKLS	YMYGTICDIW	IASKFQNFGQ
1210	1220	1230	1240	1250	1260	1270	1280
FCPHDFPTVF	GKISSSTKIW	KPLAQTRSIM	QPKTVFPPLT	QIKLQRYPES	AEEKVKVEPL	DSLSLFHLKT	ESNGKAFTDK
1290	1300	1310	1320	1330	1340	1350	1360
AYNSQVQLTV	NANQKAHPLT	QPSSPPNQCA	NVMAGDDQIR	FQQVVKEQLM	HQRLPTLPGI	SHETPLPESA	LTLRNVNVVC
1370	1380	1390	1400	1410	1420	1430	1440
SGGITVVSTK	SEEEVCSSSF	GTSEFSTVDS	AQKNFNDYAM	NFFTNPTKNL	VSITKDSELP	TCSCLDRVIQ	KDKGPYYTHL
1450	1460	1470	1480	1490	1500	1510	1520
GAGPSVAAVR	EIMENRYGQK	GNAIRIEIVV	YTGKEGKSSH	GCPIAKWVLR	RSSDEEKVLC	LVRQRTGHHC	PTAVMVVLIM
1530	1540	1550	1560	1570	1580	1590	1600
VWDGIPLPMA	DRLYTELTEN	LKSYNGHPTD	RRCTLNENRT	CTCQGIDPET	CGASFSFGCS	WSMYFNGCKF	GRSPSPRRFR
1610	1620	1630	1640	1650	1660	1670	1680
IDPSSPLHEK	NLEDNLQSLA	TRLAPIYKQY	APVAYQNQVE	YENVARECRL	GSKEGRPFSG	VTACLDFCAH	PHRDIHNMNN
1690	1700	1710	1720	1730	1740	1750	1760
GSTVVCTLTR	EDNRSLGVIP	QDEQLHVLPL	YKLSDTDEFG	SKEGMEAKIK	SGAIEVLAPR	RKKRTCFTQP	VPRSGKKRAA
1770	1780	1790	1800	1810	1820	1830	1840
MMTEVLAHKI	RAVEKKPIPR	IKRKNNSTTT	NNSKPSSLPT	LGSNTETVQP	EVKSETEPHF	ILKSSDNTKT	YSLMPSAPHP
1850	1860	1870	1880	1890	1900	1910	1920
VKEASPGFSW	SPKTASATPA	PLKNDATASC	GFSERSSTPH	CTMPSGRLSG	ANAAAADGPG	ISQLGEVAPL	PTLSAPVMEP
1930	1940	1950	1960	1970	1980	1990	2000
LINSEPSTGV	TEPLTPHQPN	HQPSFLTSPQ	DLASSPMEED	EQHSEADEPP	SDEPLSDDPL	SPAEEKLPHI	DEYWSDSEHI
2010	2020	2030	2040	2050	2060	2070	2080
FLDANIGGVA	IAPAHGSVLI	ECARRELHAT	TPVEHPNRNH	PTRLSLVFYQ	HKNLNKPQHG	FELNKIKFEA	KEAKNKKMKA
2090	2100	2110	2120	2130	2140		
SEQKDQAANE	GPEQSSEVNE	LNQIPSHKAL	TLTHDNVVTV	SPYALTHVAG	PYNHWV		

Detailed Protein Report

Cmpd.	No. of Cmpds.	m/z meas.	Δ m/z [ppm]	z	Rt [min]	Score	P	Range	Sequence	Modification	Ratios
1153	1	945.9628	20.41	2	43.1	13.0	0	1674-1690	R.DIHNMNNGSTVVCTLTR.E	Oxidation: 5	mdown:qdown 1.06

Detailed Protein Report

Protein 591: PREDICTED: MORC family CW-type zinc finger protein 4 isoform X2 [Homo sapiens]

**Accession:**	gi|578838478	**Score:**	26.0
**Database:**	refseq_human(refseq_human_20140103.fasta)	**MW [kDa]:**	92.9
**Database Date:**	2015-11-30	**pI:**	6.4
		**Sequence Coverage [%]:**	3.6
		**No. of unique Peptides:**	1

10	20	30	40	50	60	70	80
MLLYRGAPAG	PGAPGCGLAR	PGGGPQAFGI	RLSTMSPRYL	QSNSSSHTRP	FSAIAELLDN	AVDPDVSART	VFIDVEEVKN
**90**	**100**	**110**	**120**	**130**	**140**	**150**	**160**
KSCLTFTDDG	CGMTPHKLHR	MLRNKNGKSE	LDFDTDQYDI	LVSDFDTEEK	MTGGVTSELP	ETEYSLRAFC	GILYMKPRMK
**170**	**180**	**190**	**200**	**210**	**220**	**230**	**240**
IFLRQKKVTT	QMIAKSLANV	EYDTYKPTFT	NKQVRITFGF	SCKNSNQFGI	MMYHNNRLIK	SFEKVGCQVK	PTRGEGVGVI
**250**	**260**	**270**	**280**	**290**	**300**	**310**	**320**
GVIECNFLKP	AYNKQDFEYT	KEYRLTINAL	AQKLNAYWKE	KTSQDNFETS	TVARPIPKVP	DQTWVQCDEC	LKWRKLPGKI
**330**	**340**	**350**	**360**	**370**	**380**	**390**	**400**
DPSMLPARWF	CYYNSHPKYR	RCSVPEEQEL	TDEDLCLSKA	KKQEQTVEEK	KKMPMENENH	QVFSNPPKIL	TVQEMAGLNN
**410**	**420**	**430**	**440**	**450**	**460**	**470**	**480**
KTIGYEGIHS	PSVLPSGGEE	SRSPSLQLKP	LDSSVLQFSS	KYKWILGEEP	VEKRRRLQNE	MTTPSLDYSM	PAPYRRVEAP
**490**	**500**	**510**	**520**	**530**	**540**	**550**	**560**
VAYPEGENSH	DKSSSERSTP	PYLFPEYPEA	SKNTGQNREV	SILYPGAKDQ	RQGSLLPEEL	EDQMPRLVAE	ESNRGSTTIN
**570**	**580**	**590**	**600**	**610**	**620**	**630**	**640**
KEEVNKGPFV	AVVGVAKGVR	DSGAPIQLIP	FNREELAERR	KAVESWNPVP	YSVASAAIPA	AAIGEKARGY	EESEGHNTPK
**650**	**660**	**670**	**680**	**690**	**700**	**710**	**720**
LKNQRELEEL	KRTTEKLERV	LAERNLFQQK	VEELEQERNH	WQSEFKKVQH	ELVIYSTQEA	EGLYWSKKHM	GYRQAEFQIL
**730**	**740**	**750**	**760**	**770**	**780**	**790**	**800**
KAELERTKEE	KQELKEKLKE	TETHLEMLQK	AQVSYRTPEG	DDLERALAKL	TRLRIHVSYL	LTSVLPHLEL	REIGYDSEQV
**810**	**820**						
DGILYTVLEA	NHILD						

Cmpd.	No. of Cmpds.	m/z meas.	Δ m/z [ppm]	z	Rt [min]	Score	P	Range	Sequence	Modification
2715	1	856.8870	34.41	2	65.2	15.1	0	82-97	K.SCLTFTDDGCGMTPHK.L	

Detailed Protein Report

Protein 592: **PREDICTED: phosphatidylinositol 4,5-bisphosphate 3-kinase catalytic subunit beta isoform isoform X3 [Homo sapiens]**

**Accession:**	gi|530374778	**Score:**	26.0
**Database:**	refseq_human(refseq_human_20140103.fasta)	**MW [kDa]:**	121.0
**Database Date:**	2015-11-30	**pI:**	7.1
**Modification(s):**	Oxidation	**Sequence Coverage [%]:**	2.1
		**No. of unique Peptides:**	1

```
        10         20         30         40         50         60         70         80
MCFSFIMPPA MADILDIWAV DSQIASDGSI PVDFLLPTGI YIQLEVPREA TISYIKQMLW KQVHNYPMFN LLMDIDSYMF
        90        100        110        120        130        140        150        160
ACVNQTAVYE ELEDETRRLC DVRPFLPVLK LVTRSCDPGE KLDSKIGVLI GKGLHEFDSL KDPEVNEFRR KMRKFSEEKI
       170        180        190        200        210        220        230        240
LSLVGLSWMD WLKQTYPPEH EPSIPENLED KLYGGKLIVA VHFENCQDVF SFQVSPNMNP IKVNELAIQK RLTIHGKEDE
       250        260        270        280        290        300        310        320
VSPYDYVLQV SGRVEYVFGD HPLIQFQYIR NCVMNRALPH FILVECCKIK KMYEQEMIAI EAAINRNSSN LPLPLPPKKT
       330        340        350        360        370        380        390        400
RIISHVWENN NPFQIVLVKG NKLNTEETVK VHVRAGLFHG TELLCKTIVS SEVSGKNDHI WNEPLEFDIN ICDLPRMARL
       410        420        430        440        450        460        470        480
CFAVYAVLDK VKTKKSTKTI NPSKYQTIRK AGKVHYPVAW VNTMVFDFKG QLRTGDIILH SWSSFPDELE EMLNPMGTVQ
       490        500        510        520        530        540        550        560
TNPYTENATA LHVKFPENKK QPYYYPPFDK SRGGKKFLPV LKEILDRDPL SQLCENEMDL IWTLRQDCRE IFPQSLPKLL
       570        580        590        600        610        620        630        640
LSIKWNKLED VAQLQALLQI WPKLPPREAL ELLDFNYPDQ YVREYAVGCL RQMSDEELSQ YLLQLVQVLK YEPFLDCALS
       650        660        670        680        690        700        710        720
RFLLERALGN RRIGQFLFWH LRSEVHIPAV SVQFGVILEA YCRGSVGHMK VLSKQVEALN KLKTLNSLIK LNAVKLNRAK
       730        740        750        760        770        780        790        800
GKEAMHTCLK QSAYREALSD LQSPLNPCVI LSELYVEKCK YMDSKMKPLW LVYNNKVFGE DSVGVIFKNG DDLRQDMLTL
       810        820        830        840        850        860        870        880
QMLRLMDLLW KEAGLDLRML PYGCLATGDR SGLIEVVSTS ETIADIQLNS SNVAAAAAFN KDALLNWLKE YNSGDDLDRA
       890        900        910        920        930        940        950        960
IEEFTLSCAG YCVASYVLGI GDRHSDNIMV KKTGQLFHID FGHILGNFKS KFGIKRERVP FILTYDFIHV IQQGKTGNTE
       970        980        990       1000       1010       1020       1030       1040
KFGRFRQCCE DAYLILRRHG NLFITLFALM LTAGLPELTS VKDIQYLKDS LALGKSEEEA LKQFKQKFDE ALRESWTTKV
      1050       1060
NWMAHTVRKD YRS
```

Cmpd.	No. of Cmpds.	m/z meas.	Δ m/z [ppm]	z	Rt [min]	Score	P	Range	Sequence	Modification
2763	1	959.4928	32.66	1	65.8	10.7	0	904-911	R.HSDNIMVK.K	Oxidation: 6

Detailed Protein Report

Protein 593: TNF receptor-associated factor 6 [Homo sapiens]

**Accession:**	gi|4759254	**Score:**	25.9
**Database:**	refseq_human(refseq_human_20140103.fasta)	**MW [kDa]:**	59.5
**Database Date:**	2015-11-30	**pI:**	6.0
**Modification(s):**	Carbamidomethyl	**Sequence Coverage [%]:**	3.8
		**No. of unique Peptides:**	2

Alias proteins:

Accession	Name	Description
gi|22027630	refseq_human (refseq_human_20140103.fasta)	TNF receptor-associated factor 6 [Homo sapiens]

```
        10         20         30         40         50         60         70         80
MSLLNCENSC GSSQSESDCC VAMASSCSAV TKDDSVGGTA STGNLSSSFM EEIQGYDVEF DPPLESKYEC PICLMALREA
        90        100        110        120        130        140        150        160
VQTPCGHRFC KACIIKSIRD AGHKCPVDNE ILLENQLFPD NFAKREILSL MVKCPNEGCL HKMELRHLED HQAHCEFALM
       170        180        190        200        210        220        230        240
DCPQCQRPFQ KFHINIHILK DCPRRQVSCD NCAASMAFED KEIHDQNCPL ANVICEYCNT ILIREQMPNH YDLDCPTAPI
       250        260        270        280        290        300        310        320
PCTFSTFGCH EKMQRNHLAR HLQENTQSHM RMLAQAVHSL SVIPDSGYIS EVRNFQETIH QLEGRLVRQD HQIRELTAKM
       330        340        350        360        370        380        390        400
ETQSMYVSEL KRTIRTLEDK VAEIEAQQCN GIYIWKIGNF GMHLKCQEEE KPVVIHSPGF YTGKPGYKLC MRLHLQLPTA
       410        420        430        440        450        460        470        480
QRCANYISLF VHTMQGEYDS HLPWPFQGTI RLTILDQSEA PVRQNHEEIM DAKPELLAFQ RPTIPRNPKG FGYVTFMHLE
       490        500        510        520        530
ALRQRTFIKD DTLLVRCEVS TRFDMGSLRR EGFQPRSTDA GV
```

Cmpd.	No. of Cmpds.	m/z meas.	Δ m/z [ppm]	z	Rt [min]	Score	P	Range	Sequence	Modification
2891	1	713.3518	19.27	2	64.9	11.3	0	68-78	K.YECPICLMALR.E	Carbamidomethyl: 3, 6
1778	1	1057.3158	-132.00	1	52.2	14.7	0	134-142	K.CPNEGCLHK.M	Carbamidomethyl: 1

Detailed Protein Report

Protein 594: **PREDICTED: zinc finger protein with KRAB and SCAN domains 4 isoform X3 [Homo sapiens]**

**Accession:**	gi|530381975	**Score:**	25.9
**Database:**	refseq_human(refseq_human_20140103.fasta)	**MW [kDa]:**	49.6
**Database Date:**	2015-11-30	**pI:**	9.3
**Modification(s):**	Carbamidomethyl	**Sequence Coverage [%]:**	7.5
		**No. of unique Peptides:**	2

Alias proteins:

Accession	Name	Description
gi|530381977	refseq_human (refseq_human_20140103.fasta)	PREDICTED: zinc finger protein with KRAB and SCAN domains 4 isoform X4 [Homo sapiens]

10	20	30	40	50	60	70	80
MAREPRKNAA	LDAQSAEDQT	GLLTVKVEKE	EASALTAEVP	VGDQGQELLC	CKMALLTQTQ	GSQSSQCQPM	KALFKHESLG
**90**	**100**	**110**	**120**	**130**	**140**	**150**	**160**
SQPLHDRVLQ	VPGLAQGGCC	REDAMVASRL	TPGSQGLLKM	EDVALTLTPG	WTQLDSSQVN	LYRDEKQENH	SSLVSLGGEI
**170**	**180**	**190**	**200**	**210**	**220**	**230**	**240**
QTKSRDLPPV	KKLPEKEHGK	ICHLREDIAQ	IPTHAEAGEQ	EGRLQRKQKN	AIGSRRHYCH	ECGKSFAQSS	GLTKHRRIHT
**250**	**260**	**270**	**280**	**290**	**300**	**310**	**320**
GEKPYECEDC	GKTFIGSSAL	VIHQRVHTGE	KPYECEECGK	VFSHSSNLIK	HQRTHTGEKP	YECDDCGKTF	SQSCSLLEHH
**330**	**340**	**350**	**360**	**370**	**380**	**390**	**400**
KIHTGEKPYQ	CNMCGKAFRR	NSHLLRHQRI	HGDKNVQNPE	HGESWESQGR	TESQWENTEA	PVSYKCNECE	RSFTRNRSLI
**410**	**420**	**430**	**440**	**450**			
EHQKIHTGEK	PYQCDTCGKG	FTRTSYLVQH	QRSHVGKKTL	SQ			

Cmpd.	No. of Cmpds.	m/z meas.	Δ m/z [ppm]	z	Rt [min]	Score	P	Range	Sequence	Modification
2714	1	973.1242	130.42	2	62.3	15.6	0	8-26	K.NAALDAQSAEDQTGLLTVK.V	
2444	1	729.3737	3.44	2	58.9	10.3	0	88-101	R.VLQVPGLAQGGCCR.E	Carbamidomethyl: 12

Detailed Protein Report

Protein 595: kinesin-like protein KIF3B [Homo sapiens]

**Accession:**	gi|4758646	**Score:**	25.9
**Database:**	refseq_human(refseq_human_20140103.fasta)	**MW [kDa]:**	85.1
**Database Date:**	2015-11-30	**pI:**	7.8
		**Sequence Coverage [%]:**	4.3
		**No. of unique Peptides:**	1

10	20	30	40	50	60	70	80
MSKLKSSESV	RVVVRCRPMN	GKEKAASYDK	VVDVDVKLGQ	VSVKNPKGTA	HEMPKTFTFD	AVYDWNAKQF	ELYDETFRPL
**90**	**100**	**110**	**120**	**130**	**140**	**150**	**160**
VDSVLQGFNG	TIFAYGQTGT	GKTYTMEGIR	GDPEKRGVIP	NSFDHIFTHI	SRSQNQQYLV	RASYLEIYQE	EIRDLLSKDQ
**170**	**180**	**190**	**200**	**210**	**220**	**230**	**240**
TKRLELKERP	DTGVYVKDLS	SFVTKSVKEI	EHVMNVGNQN	RSVGATNMNE	HSSRSHAIFV	ITIECSEVGL	DGENHIRVGK
**250**	**260**	**270**	**280**	**290**	**300**	**310**	**320**
LNLVDLAGSE	RQAKTGAQGE	RLKEATKINL	SLSALGNVIS	ALVDGKSTHI	PYRDSKLTRL	LQDSLGGNAK	TVMVANVGPA
**330**	**340**	**350**	**360**	**370**	**380**	**390**	**400**
SYNVEETLTT	LRYANRAKNI	KNKPRVNEDP	KDALLREFQE	EIARLKAQLE	KRSIGRRKRR	EKRREGGGSG	GGGEEEEEEG
**410**	**420**	**430**	**440**	**450**	**460**	**470**	**480**
EEGEEEGDDK	DDYWREQQEK	LEIEKRAIVE	DHSLVAEEKM	RLLKEKEKKM	EDLRREKDAA	EMLGAKIKAM	ESKLLVGGKN
**490**	**500**	**510**	**520**	**530**	**540**	**550**	**560**
IVDHTNEQQK	ILEQKRQEIA	EQKRREREIQ	QQMESRDEET	LELKETYSSL	QQEVDIKTKK	LKKLFSKLQA	VKAEIHDLQE
**570**	**580**	**590**	**600**	**610**	**620**	**630**	**640**
EHIKERQELE	QTQNELTREL	KLKHLIIENF	IPLEEKSKIM	NRAFFDEEED	HWKLHPITRL	ENQQMMKRPV	SAVGYKRPLS
**650**	**660**	**670**	**680**	**690**	**700**	**710**	**720**
QHARMSMMIR	PEARYRAENI	VLLELDMPSR	TTRDYEGPAI	APKVQAALDA	ALQDEDEIQV	DASSFESTAN	KKSKARPKSG
**730**	**740**	**750**					
RKSGSSSSSS	GTPASQLYPQ	SRGLVPK					

Cmpd.	No. of Cmpds.	m/z meas.	Δ m/z [ppm]	z	Rt [min]	Score	P	Range	Sequence	Modification
850	1	911.1296	-3.32	3	39.4	14.3	2	178-201	K.DLSSFVTKSVKEIEHVMNVGNQNR.S	

Detailed Protein Report

Protein 596: PREDICTED: ryanodine receptor 2 isoform X7 [Homo sapiens]

**Accession:**	gi|578802309	**Score:**	25.9
**Database:**	refseq_human(refseq_human_20140103.fasta)	**MW [kDa]:**	557.1
**Database Date:**	2015-11-30	**pI:**	5.6
		**Sequence Coverage [%]:**	0.5
		**No. of unique Peptides:**	1

Detailed Protein Report

10	20	30	40	50	60	70	80
MADGGEGEDE	IQFLRTDDEV	VLQCTATIHK	EQQKLCLAAE	GFGNRLCFLE	STSNSKNVPP	DLSICTFVLE	QSLSVRALQE
90	100	110	120	130	140	150	160
MLANTVEKSE	GQVDVEKWKF	MMKTAQGGGH	RTLLYGHAIL	LRHSYSGMYL	CCLSTSRSST	DKLAFDVGLQ	EDTTGEACWW
170	180	190	200	210	220	230	240
TIHPASKQRS	EGEKVRVGDD	LILVSVSSER	YLHLSYGNGS	LHVDAAFQQT	LWSVAPISSG	SEAAQGYLIG	GDVLRLLHGH
250	260	270	280	290	300	310	320
MDECLTVPSG	EHGEEQRRTV	HYEGGAVSVH	ARSLWRLETL	RVAWSGSHIR	WGQPFRLRHV	TTGKYLSLME	DKNLLLMDKE
330	340	350	360	370	380	390	400
KADVKSTAFT	FRSSKEKLDV	GVRKEVDGMG	TSEIKYGDSV	CYIQHVDTGL	WLTYQSVDVK	SVRMGSIQRK	AIMHHEGHMD
410	420	430	440	450	460	470	480
DGISLSRSQH	EESRTARVIR	STVFLFNRFI	RGLDALSKKA	KASTVDLPIE	SVSLSLQDLI	GYFHPPDEHL	EHEDKQNRLR
490	500	510	520	530	540	550	560
ALKNRQNLFQ	EEGMINLVLE	CIDRLHVYSS	AAHFADVAGR	EAGESWKSIL	NSLYELLAAL	IRGNRKNCAQ	FSGSLDWLIS
570	580	590	600	610	620	630	640
RLERLEASSG	ILEVLHCVLV	ESPEALNIIK	EGHIKSIISL	LDKHGRNHKV	LDVLCSLCVC	HGVAVRSNQH	LICDNLLPGR
650	660	670	680	690	700	710	720
DLLLQTRLVN	HVSSMRPNIF	LGVSEGSAQY	KKWYYELMVD	HTEPFVTAEA	THLRVGWAST	EGYSPYPGGG	EEWGGNGVGD
730	740	750	760	770	780	790	800
DLFSYGFDGL	HLWSGCIART	VSSPNQHLLR	TDDVISCCLD	LSAPSISFRI	NGQPVQGMFE	NFNIDGLFFP	VVSFSAGIKV
810	820	830	840	850	860	870	880
RFLLGGRHGE	FKFLPPPGYA	PCYEAVLPKE	KLKVEHSREY	KQERTYTRDL	LGPTVSLTQA	AFTPIPVDTS	QIVLPPHLER
890	900	910	920	930	940	950	960
IREKLAENIH	ELWVMNKIEL	GWQYGPVRDD	NKRQHPCLVE	FSKLPEQERN	YNLQMSLETL	KTLLALGCHV	GISDEHAEDK
970	980	990	1000	1010	1020	1030	1040
VKKMKLPKNY	QLTSGYKPAP	MDLSFIKLTP	SQEAMVDKLA	ENAHNVWARD	RIRQGWTYGI	QQDVKNRRNP	RLVPYTLLDD
1050	1060	1070	1080	1090	1100	1110	1120
RTKKSNKDSL	REAVRTLLGY	GYNLEAPDQD	HAARAEVCSG	TGERFRIFRA	EKTYAVKAGR	WYFEFETVTA	GDMRVGWSRP
1130	1140	1150	1160	1170	1180	1190	1200
GCQPDQELGS	DERAFAFDGF	KAQRWHQGNE	HYGRSWQAGD	VVGCMVDMNE	HTMMFTLNGE	ILLDDSGSEL	AFKDFDVGDG
1210	1220	1230	1240	1250	1260	1270	1280
FIPVCSLGVA	QVGRMNFGKD	VSTLKYFTIC	GLQEGYEPFA	VNTNRDITMW	LSKRLPQFLQ	VPSNHEHIEV	TRIDGTIDSS
1290	1300	1310	1320	1330	1340	1350	1360
PCLKVTQKSF	GSQNSNTDIM	FYRLSMPIEC	AEVFSKTVAG	GLPGAGLFGP	KNDLEDYDAD	SDFEVLMKTA	HGHLVPDRVD
1370	1380	1390	1400	1410	1420	1430	1440
KDKEATKPEF	NNHKDYAQEK	PSRLKQRFLL	RRTKPDYSTS	HSARLTEDVL	ADDRDDYDFL	MQTSTYYYSV	RIFPGQEPAN
1450	1460	1470	1480	1490	1500	1510	1520
VWVGWITSDF	HQYDTGFDLD	RVRTVTVTLG	DEKGKVHESF	AIDSLCGFGI	KRSNCYMVCA	GESMSPGQGR	NNNGLEIGCV
1530	1540	1550	1560	1570	1580	1590	1600
VDAASGLLTF	IANGKELSTY	YQVEPSTKLF	PAVFAQATSP	NVFQFELGRI	KNVMPLSAGL	FKSEHKNPVP	QCPPRLHVQF
1610	1620	1630	1640	1650	1660	1670	1680
LSHVLWSRMP	NQFLKVDVSR	ISERQGWLVQ	CLDPLQFMSL	HIPEENRSVD	ILELTEQEEL	LKFHYHTLRL	YSAVCALGNH
1690	1700	1710	1720	1730	1740	1750	1760
RVAHALCSHV	DEPQLLYAIE	NKYMPGLLRA	GYYDLLIDIH	LSSYATARLM	MNNEYIVPMT	EETKSITLFP	DENKKHGLPG
1770	1780	1790	1800	1810	1820	1830	1840
IGLSTSLRPR	MQFSSPSFVS	ISNECYQYSP	EFPLDILKSK	TIQMLTEAVK	EGSLHARDPV	GGTTEFLFVP	LIKLFYTLLI
1850	1860	1870	1880	1890	1900	1910	1920
MGIFHNEDLK	HILQLIEPSV	FKEAATPEEE	SDTLEKELSV	DDAKLQGAGE	EEAKGGKRPK	EGLLQMKLPE	PVKLQMCLLL
1930	1940	1950	1960	1970	1980	1990	2000
QYLCDCQVRH	RIEAIVAFSD	DFVAKLQDNQ	RFRYNEVMQA	LNMSAALTAR	KTKEFRSPPQ	EQINMLLNFK	DDKSECPCPE
2010	2020	2030	2040	2050	2060	2070	2080
EIRDQLLDFH	EDLMTHCGIE	LDEDGSLDGN	SDLTIRGRLL	SLVEKVTYLK	KKQAEKPVES	DSKKSSTLQQ	LISETMVRWA
2090	2100	2110	2120	2130	2140	2150	2160
QESVIEDPEL	VRAMFVLLHR	QYDGIGGLVR	ALPKTYTING	VSVEDTINLL	ASLGQIRSLL	SVRMGKEEEK	LMIRGLGDIM
2170	2180	2190	2200	2210	2220	2230	2240

Detailed Protein Report

Cmpd.	No. of Cmpds.	m/z meas.	Δ m/z [ppm]	z	Rt [min]	Score	P	Range	Sequence	Modification
1437	1	946.4677	46.36	2	46.5	13.9	2	4372-4388	K.EETKSEPEKAEGEDGEK.E	

Detailed Protein Report

Protein 597: protein AKNAD1 [Homo sapiens]

Accession: gi|91754185
Database: refseq_human(refseq_human_20140103.fasta)
Database Date: 2015-11-30
Modification(s): Carbamidomethyl, Oxidation

Score: 25.9
MW [kDa]: 92.8
pI: 6.4
Sequence Coverage [%]: 4.7
No. of unique Peptides: 2

10	20	30	40	50	60	70	80
MDEADFSEHT	TYKQEDLPYD	GDLSQIKIGN	DYSFTSKKDG	LEVLNQIIFI	ADDPQEKAMH	SETCGNTAVT	IPLGKITENA
**90**	**100**	**110**	**120**	**130**	**140**	**150**	**160**
ANKKDEKEKQ	CTAALHIPAN	EGDASKSSIS	DILLHHLSKE	PFLRGQGIDC	ETLPEISNAD	SFEEEAIIKS	IISCYNKNSW
**170**	**180**	**190**	**200**	**210**	**220**	**230**	**240**
PKEQTPELTD	QLNPKRDGEN	SNKPGSATTT	EENTSDLEGP	VAAGDSSHQE	NVNVLTKTKG	PGDKQKSYQG	QSPQKQQTEK
**250**	**260**	**270**	**280**	**290**	**300**	**310**	**320**
ANSGNTFKYG	QGQVHYQLPD	FSKIAPKVKI	PKNKIINKPL	AIAKQASFSS	KSRDKPTLVQ	DSLETTPESN	CVEKQHQEQK
**330**	**340**	**350**	**360**	**370**	**380**	**390**	**400**
GKITEPSQQI	QMEPIVHIHQ	ELLTGIESEA	SLSKLSPTSQ	KGTSSSSSYI	FQKISQGKQM	CQKLKEQTDQ	LKTKVQEFSK
**410**	**420**	**430**	**440**	**450**	**460**	**470**	**480**
RIKQDSPYHL	QDKKLVLEKL	QGHLELLEQN	FLATKDKHLT	LQQQVHKHES	TIVGDFDPER	KVEGEIFKLE	MLLEDVKEKM
**490**	**500**	**510**	**520**	**530**	**540**	**550**	**560**
DESKYTSAPS	LPVSSPVTLD	DLASTFSSLS	NEIPKEHPGH	PSGPRGSGGS	EVTGTPQGGP	QEAPNEELCE	LAPQTYLNGH
**570**	**580**	**590**	**600**	**610**	**620**	**630**	**640**
YGDAAAQNKP	DQVAMRLSSN	SGEDPNGTPR	RQDCAEMTAP	SPSCAFCRRL	LEWKQNVEKK	GHGRINCGRF	SIVLHEKAPH
**650**	**660**	**670**	**680**	**690**	**700**	**710**	**720**
SDSTPNSDTG	HSFCSDSGTE	MQSNKCQDCG	TKIPTSRRAC	RKEPTKEFHY	RYNTPGQNYS	NHSKRGAFVQ	PHSLDESKNS
**730**	**740**	**750**	**760**	**770**	**780**	**790**	**800**
SPSFLKPKRI	CSQRVNSKSF	KGEHEPTPGK	KKLQAFMTYS	SDPATPSPHF	YSCRISGSKS	LCDFDSTEEI	KSEILNSALD
**810**	**820**	**830**	**840**				
HALRTATILK	ETTDQMIKTI	AEDLAKAQRW	RNRLKY				

Cmpd.	No. of Cmpds.	m/z meas.	Δ m/z [ppm]	z	Rt [min]	Score	P	Range	Sequence	Modification
2577	1	1202.2445	83.07	2	60.9	12.0	2	249-269	K.YGQGQVHYQLPDFSKIAPKVK.I	
2161	1	1023.4064	-11.81	2	55.6	13.9	1	592-609	R.QDCAEMTAPSPSCAFCRR.L	Carbamidomethyl: 16; Oxidation: 6

Detailed Protein Report

Protein 598: tetratricopeptide repeat protein 40 [Homo sapiens]

**Accession:**	gi|359385708	**Score:**	25.9
**Database:**	refseq_human(refseq_human_20140103.fasta)	**MW [kDa]:**	303.3
**Database Date:**	2015-11-30	**pI:**	7.2
**Modification(s):**	Oxidation	**Sequence Coverage [%]:**	1.3
		**No. of unique Peptides:**	2

Quantitation

Wdown:Qdown	**Median:** 1.22	**CV:** 0.00 %	**No. of Peptides:** 1

Detailed Protein Report

10	20	30	40	50	60	70	80
MDLVITQELA	RAESQQDAAS	LKKAYELIKS	ANLGKSEFDP	SESFSPDLFV	LCAEQALKMR	QPEVSEDCIQ	MYFKVKAPIT
90	100	110	120	130	140	150	160
QFLGRAHLCR	AQMCAPKSAE	NLEEFENCVT	EYMKAINFAK	GEPRYYFLVY	NASVLYWQMV	RPFLKPGYRH	HLIPSLSQII
170	180	190	200	210	220	230	240
NVLSQTEEED	KEWRAELMLE	LLECYLQAGR	KEEAARFCST	AAPFIKSHVP	QKYRQIFSVM	VRHELMDELQ	LKEEKKNSIS
250	260	270	280	290	300	310	320
LSVTFYINML	KAKAEQNDLP	GDISVILRKA	YRHLGHYNHQ	RFPSISEEKM	LLLFELARFS	LTLKCMEISS	ACLSDLKKME
330	340	350	360	370	380	390	400
SKDPGKLIEM	ECLECESEAL	RLESKMKVYN	RAAVEAQLDI	IQRLDVALQR	AVRLGDPRVI	HVVCATQWNT	CLPLLQHNLR
410	420	430	440	450	460	470	480
HHLRKPLAGV	ADVLEKLDSL	MTLLRCQVHM	EMAQIEEDED	RLEPATEHLR	KAARLDSLGL	YRDRIQMAST	RLRLCTTLYQ
490	500	510	520	530	540	550	560
APERAEDKAI	MAVEQAKKAT	PKDSVRKKRA	LLVNAGLALA	PDAFQIVLDS	ENEAKVSTGK	NRGRFTYLCA	KAWHHTVSVD
570	580	590	600	610	620	630	640
KAAGHLRRLG	NENDKERIQI	WAELAKVARK	QGVWDVCRTA	SRFCLLYDNV	KVKKLRLRRG	KKKRGRDGSV	QDTWSQPEVV
650	660	670	680	690	700	710	720
LQRQVCPDLL	RKFAEVGFIH	AEATVHLLRS	EGVELNDRAI	PPEDLSQHPA	GYVPEPPEVN	AEWITYRTWI	ESLSRCAMNN
730	740	750	760	770	780	790	800
WLRSAEIGQE	IQEAWIVQNA	VVYVLNHNHH	LILAGRQKEL	VDALYHLLSI	VKATGHSGDP	VMLVTLCNTL	ARGLIISWIP
810	820	830	840	850	860	870	880
VQAAEKSRKF	MRPNAFHSPL	DAGATSEIKT	AVEVCEFALN	LTNGSAPEET	VPTGTRQQLI	ATWVKAKQLL	QQQIGPRLGT
890	900	910	920	930	940	950	960
EEQGTNEDVS	SVTRVLVALE	MYSCNGLGLM	DFTVPSLAQL	VKMASECNWS	DPLVELQTLT	RLTHFAHAAR	DHETTMACAH
970	980	990	1000	1010	1020	1030	1040
RALEMGIKYL	KKFGPEESRL	VAEMLCTATA	IQGRSIMENL	KGRKQLRLVA	AKAFTESARF	GGIAGSSALV	MLAARHYWNA
1050	1060	1070	1080	1090	1100	1110	1120
WLPLLSSAVY	RKKAKGALKR	LIGIINKTEA	RKQEKGKTLL	LHQWPTADFQ	GGGTTEGYFL	PGAEDDLALR	AALYGLLFHS
1130	1140	1150	1160	1170	1180	1190	1200
HADQDDWEGG	LKVLDEAVQV	LPRTAHRLLI	FKHMVIVKAK	LGQNFSMEIQ	KFKAESEDYL	ARMWHRLALN	SPSVSGELAC
1210	1220	1230	1240	1250	1260	1270	1280
YNNAIQALQK	PEMEWQKVEY	LMEFGQWLHH	RHFPLEDVVF	HLRWAVEILL	AMKPPGDVPE	PQPTPDGEYV	AVEMPPRSPV
1290	1300	1310	1320	1330	1340	1350	1360
SEAEEAVSLE	QLRSVRQLEA	LARVHILLAL	VLSPGAEGYE	DCCLAAYAFF	RHIWQVSLMT	AGKSVLENRP	LAATSSHLLL
1370	1380	1390	1400	1410	1420	1430	1440
PKKEKENERS	KEKEKERSKE	KENERSKEKD	KEKGKEEKVK	EPKQSQSPAP	IKQLEDLPMS	IEEWASYSCP	EEVLSVLKQD
1450	1460	1470	1480	1490	1500	1510	1520
RSDSTVNPSS	IQKPTYSLYF	LDHLVKALQK	MCLHELTVPV	LQLGVLISDS	VVGSKGLSDL	YHLRLAHACS	ELKLREAAAR
1530	1540	1550	1560	1570	1580	1590	1600
HEEAVGQVCV	SELEQASCRK	EIALKKEKNK	EPLLEESLPA	LNEQTLPVQP	GEIKPLDAKD	KILKMNGETG	RDLDGTSFPH
1610	1620	1630	1640	1650	1660	1670	1680
LWMLKAEVLL	EMNLYQPARL	LLSEAYLAFQ	ELDEPCAEAQ	CLLLLAQLAN	KEKNYGQAKK	MIAQAQHLGG	SEEFWYNSTL
1690	1700	1710	1720	1730	1740	1750	1760
TLAEALLSME	HSGREATVCH	IFQKLINAFK	ILKKERPNRL	PLLEFMITDL	EARCLSLRVR	VAQHSAVTEP	TECSLLLKEM
1770	1780	1790	1800	1810	1820	1830	1840
DDGLLEIERK	FIDCGCKENC	VDVKLERAKI	KRLRAQNEKD	EEQKTAYYLE	AYGLAQGAVA	EEEGRLHSIQ	GLYGLAQGAM
1850	1860	1870	1880	1890	1900	1910	1920
AEEEGRLHSV	QGLLSLQDLQ	NVNTPLMRKL	ARLKLGLVEM	ALDMLQFIWE	EAHGQQSEQG	SLEKLLADYL	QNTSDYTSVG
1930	1940	1950	1960	1970	1980	1990	2000
LQWFTLKRTL	AHGALAQLGS	LQPLSVGCVE	IRARLLGLAG	RALHLLAMQA	DPVHPTCYWE	AGPSVGAKLS	GLKSLELEVE
2010	2020	2030	2040	2050	2060	2070	2080
EEGATKSSRD	PPASRAAPEE	HCRRGEDLKR	RMVLAQQYLA	QASEVLLQCL	QVALGSGLLD	VAAAASLEMV	ECVGTLDPAT
2090	2100	2110	2120	2130	2140	2150	2160
TCQFLALSQS	CSASETMRDV	LLAATANTSS	SQLAALLQLQ	HQLRCQDRTT	TSLGARVEQR	LAAVSKAWQN	LCVTEQHFNL
2170	2180	2190	2200	2210	2220	2230	2240

Detailed Protein Report

Cmpd.	No. of Cmpds.	m/z meas.	Δ m/z [ppm]	z	Rt [min]	Score	P	Range	Sequence	Modification	Ratios
2308	1	841.6632	-116.58	3	57.3	10.2	2	972-994	K. KFGPEESRLVAEMLCTATAIQGR. S	Oxidation: 13	
114	1	793.3810	-31.26	2	30.8	15.7	1	1161-1173	K.LGQNFSMEIQKFK.A	Oxidation: 7	Wdown:Qdown 1.22

Detailed Protein Report

Protein 599: E3 ubiquitin-protein ligase RNF103 isoform 2 [Homo sapiens]

**Accession:**	gi|312147324	**Score:**	25.9
**Database:**	refseq_human(refseq_human_20140103.fasta)	**MW [kDa]:**	78.6
**Database Date:**	2015-11-30	**pI:**	5.5
**Modification(s):**	Oxidation	**Sequence Coverage [%]:**	4.6
		**No. of unique Peptides:**	1

10	20	30	40	50	60	70	80
MSRLRPRPPL	IHGCGGFFEA	IVWYETGIFA	TQLVDPVALS	FKKLKTILEC	RGLGYSGLPE	KKDVRELVEK	SGDLMEGELY
**90**	**100**	**110**	**120**	**130**	**140**	**150**	**160**
SALKEEEASE	SVSSTNFSGE	MHFYELVEDT	KDGIWLVQVI	ANDRSPLVGK	IHWEKMVKKV	SRFGIRTGTF	NCSSDPRYCR
**170**	**180**	**190**	**200**	**210**	**220**	**230**	**240**
RRGWVRSTLI	MSVPQTSTSK	GKVMLKEYSG	RKIEVEHIFK	WITAHAASRI	KTIYNAEHLK	EEWNKSDQYW	LKIYLFANLD
**250**	**260**	**270**	**280**	**290**	**300**	**310**	**320**
QPPAFFSALS	IKFTGRVEFI	FVNVENWDNK	SYMTDIGIYN	MPSYILRTPE	GIYRYGNHTG	EFISLQAMDS	FLRSLQPEVN
**330**	**340**	**350**	**360**	**370**	**380**	**390**	**400**
DLFVLSLVLV	NLMAWMDLFI	TQGATIKRFV	VLISTLGTYN	SLLIISWLPV	LGFLQLPYLD	SFYEYSLKLL	RYSNTTTLAS
**410**	**420**	**430**	**440**	**450**	**460**	**470**	**480**
WVRADWMFYS	SHPALFLSTY	LGHGLLIDYF	EKKRRRNNNN	DEVNANNLEW	LSSLWDWYTS	YLFHPIASFQ	NFPVESDWDE
**490**	**500**	**510**	**520**	**530**	**540**	**550**	**560**
DPDLFLERLA	FPDLWLHPLI	PTDYIKNLPM	WRFKCLGVQS	EEEMSEGSQD	TENDSESENT	DTLSSEKEVF	EDKQSVLHNS
**570**	**580**	**590**	**600**	**610**	**620**	**630**	**640**
PGTASHCDAE	ACSCANKYCQ	TSPCERKGRS	YGSYNTNEDM	EPDWLTWPAD	MLHCTECVVC	LENFENGCLL	MGLPCGHVFH
**650**	**660**	**670**	**680**	**690**			
QNCIVMWLAG	GRHCCPVCRW	PSYKKKQPYA	QHQPLSNDVP	S			

Cmpd.	No. of Cmpds.	m/z meas.	Δ m/z [ppm]	z	Rt [min]	Score	P	Range	Sequence	Modification
2911	1	840.9107	-41.30	2	65.2	10.8	1	167-182	R.STLIMSVPQTSTSKGK.V	Oxidation: 5

Detailed Protein Report

Protein 600: 1-phosphatidylinositol 3-phosphate 5-kinase isoform 2 [Homo sapiens]

**Accession:**	gi|121583483	**Score:**	25.9
**Database:**	refseq_human(refseq_human_20140103.fasta)	**MW [kDa]:**	237.0
**Database Date:**	2015-11-30	**pI:**	6.2
		**Sequence Coverage [%]:**	1.2
		**No. of unique Peptides:**	1

Quantitation

mdown:qdown	**Median:** 1.96	**CV:** 0.00 %	**No. of Peptides:** 1

Detailed Protein Report

10	20	30	40	50	60	70	80
MATDDKTSPT	LDSANDLPRS	PTSPSHLTHF	KPLTPDQDEP	PFKSAYSSFV	NLFRFNKERA	EGGQGEQQPL	SGSWTSPQLP
90	100	110	120	130	140	150	160
SRTQSVRSPT	PYKKQLNEEL	QRRSSALDTR	RKAEPTFGGH	DPRTAVQLRS	LSTVLKRLKE	IMEGKSQDSD	LKQYWMPDSQ
170	180	190	200	210	220	230	240
CKECYDCSEK	FTTFRRRHHC	RLCGQIFCSR	CCNQEIPGKF	MGYTGDLRAC	TYCRKIALSY	AHSTDSNSIG	EDLNALSDSA
250	260	270	280	290	300	310	320
CSVSVLDPSE	PRTPVGSRKA	SRNIFLEDDL	AWQSLIHPDS	SNTPLSTRLV	SVQEDAGKSP	ARNRSASITN	LSLDRSGSPM
330	340	350	360	370	380	390	400
VPSYETSVSP	QANRTYVRTE	TTEDERKILL	DSVQLKDLWK	KICHHSSGME	FQDHRYWLRT	HPNCIVGKEL	VNWLIRNGHI
410	420	430	440	450	460	470	480
ATRAQAIAIG	QAMVDGRWLD	CVSHHDQLFR	DEYALYRPLQ	STEFSETPSP	DSDSVNSVEG	HSEPSWFKDI	KFDDSDTEQI
490	500	510	520	530	540	550	560
AEEGDDNLAN	SASPSKRTSV	SSFQSTVDSD	SAASISLNVE	LDNVNFHIKK	PSKYPHVPPH	PADQKEYLIS	DTGGQQLSIS
570	580	590	600	610	620	630	640
DAFIKESLFN	RRVEEKSKEL	PFTPLGWHHN	NLELLREENG	EKQAMERLLS	ANHNHMMALL	QQLLHSDSLS	SSWRDIIVSL
650	660	670	680	690	700	710	720
VCQVVQTVRP	DVKNQDDDMD	IRQFVHIKKI	PGGKKFDSVV	VNGFVCTKNI	AHKKMSSCIK	NPKILLLKCS	IEYLYREETK
730	740	750	760	770	780	790	800
FTCIDPIVLQ	EREFLKNYVQ	RIVDVRPTLV	LVEKTVSRIA	QDMLLEHGIT	LVINVKSQVL	ERISRMTQGD	LVMSMDQLLT
810	820	830	840	850	860	870	880
KPHLGTCHKF	YMQIFQLPNE	QTKTLMFFEG	CPQHLGCTIK	LRGGSDYELA	RVKEILIFMI	CVAYHSQLEI	SFLMDEFAMP
890	900	910	920	930	940	950	960
PTLMQNPSFH	SLIEGRGHEG	AVQEQYGGGS	IPWDPDIPPE	SLPCDDSSLL	ELRIVFEKGE	QENKNLPQAV	ASVKHQEHST
970	980	990	1000	1010	1020	1030	1040
TACPAGLPCA	FFAPVPESLL	PLPVDDQQDA	LGSEQPETLQ	QTVVLQDPKS	QIRAFRDPLQ	DDTGLYVTEE	VTSSEDKRKT
1050	1060	1070	1080	1090	1100	1110	1120
YSLAFKQELK	DVILCISPVI	TFREPFLLTE	KGMRCSTRDY	FAEQVYWSPL	LNKEFKEMEN	RRKKQLLRDL	SGLQGMNGSI
1130	1140	1150	1160	1170	1180	1190	1200
QAKSIQVLPS	HELVSTRIAE	HLGDSQSLGR	MLADYRARGG	RIQPKNSDPF	AHSKDASSTS	SGQSGSKNEG	DEERGLILSD
1210	1220	1230	1240	1250	1260	1270	1280
AVWSTKVDCL	NPINHQRLCV	LFSSSSAQSS	NAPSACVSPW	IVTMEFYGKN	DLTLGIFLER	YCFRPSYQCP	SMFCDTPMVH
1290	1300	1310	1320	1330	1340	1350	1360
HIRRFVHGQG	CVQIILKELD	SPVPGYQHTI	LTYSWCRICK	QVTPVVALSN	ESWSMSFAKY	LELRFYGHQY	TRRANAEPCG
1370	1380	1390	1400	1410	1420	1430	1440
HSIHHDYHQY	FSYNQMVASF	SYSPIRLLEV	CVPLPKIFIK	RQAPLKVSLL	QDLKDFFQKV	SQVYVAIDER	LASLKTDTFS
1450	1460	1470	1480	1490	1500	1510	1520
KTREEKMEDI	FAQKEMEEGE	FKNWIEKMQA	RLMSSSVDTP	QQLQSVFESL	IAKKQSLCEV	LQAWNNRLQD	LFQQEKGRKR
1530	1540	1550	1560	1570	1580	1590	1600
PSVPPSPGRL	RQGEESKISA	MDASPRNISP	GLQNGEKEDR	FLTTLSSQSS	TSSTHLQLPT	PPEVMSEQSV	GGPPELDTAS
1610	1620	1630	1640	1650	1660	1670	1680
SSEDVFDGHL	LGSTDSQVKE	KSTMKAIFAN	LLPGNSYNPI	PFPFDPDKHY	LMYEHERVPI	AVCEKEPSSI	IAFALSCKEY
1690	1700	1710	1720	1730	1740	1750	1760
RNALEELSKA	TQWNSAEEGL	PTNSTSDSRP	KSSSPIRLPE	MSGGQTNRTT	ETEPQPTKKA	SGMLSFFRGT	AGKSPDLSSQ
1770	1780	1790	1800	1810	1820	1830	1840
KRETLRGADS	AYYQVGQTGK	EGTENQGVEP	QDEVDGGDTQ	KKQLINPHVE	LQFSDANAKF	YCRLYYAGEF	HKMREVILDS
1850	1860	1870	1880	1890	1900	1910	1920
SEEDFIRSLS	HSSPWQARGG	KSGAAFYATE	DDRFILKQMP	RLEVQSFLDF	APHYFNYITN	AVQQKRPTAL	AKILGVYRIG
1930	1940	1950	1960	1970	1980	1990	2000
YKNSQNNTEK	KLDLLVMENL	FYGRKMAQVF	DLKGSLRNRN	VKTDTGKESC	DVVLLDENLL	KMVRDNPLYI	RSHSKAVLRT
2010	2020	2030	2040	2050	2060	2070	2080
SIHSDSHFLS	SHLIIDYSLL	VGRDDTSNEL	VVGIIDYIRT	FTWDKKLEMV	VKSTGILGGQ	GKMPTVVSPE	LYRTRFCEAM
2090	2100						
DKYFLMVPDH	WTGLGLNC						

Detailed Protein Report

Cmpd.	No. of Cmpds.	m/z meas.	Δ m/z [ppm]	z	Rt [min]	Score	P	Range	Sequence	Modification	Ratios
596	1	656.3887	96.05	2	36.0	14.0	1	112-123	R.KAEPTFGGHDPR.T		mdown:qdown 1.96

Detailed Protein Report

Protein 601: zinc finger protein 268 isoform c [Homo sapiens]

**Accession:**	gi|259490323	**Score:**	25.9
**Database:**	refseq_human(refseq_human_20140103.fasta)	**MW [kDa]:**	98.7
**Database Date:**	2015-11-30	**pI:**	10.1
**Modification(s):**	Carbamidomethyl	**Sequence Coverage [%]:**	3.5
		**No. of unique Peptides:**	1

10	20	30	40	50	60	70	80
MDVFVDFTWE	EWQLLDPAQK	CLYRSVMLEN	YSNLVSLGYQ	HTKPDIIFKL	EQGEELCMVQ	AQVPNQTCPN	TVWKIDDLMD
90	100	110	120	130	140	150	160
WHQENKDKLG	STAKSFECTT	FGKLCLLSTK	YLSRQKPHKC	GTHGKSLKYI	DFTSDYARNN	PNGFQVHGKS	FFHSKHEQTV
170	180	190	200	210	220	230	240
IGIKYCESIE	SGKTVNKKSQ	LMCQQMYMGE	KPFGCSCCEK	AFSSKSYLLV	HQQTHAEEKP	YGCNECGKDF	SSKSYLIVHQ
250	260	270	280	290	300	310	320
RIHTGEKLHE	CSECRKTFSF	HSQLVIHQRI	HTGENPYECC	ECGKVFSRKD	QLVSHQKTHS	GQKPYVCNEC	GKAFGLKSQL
330	340	350	360	370	380	390	400
IIHERIHTGE	KPYECNECQK	AFNTKSNLMV	HQRTHTGEKP	YVCSDCGKAF	TFKSQLIVHQ	GIHTGVKPYG	CIQCGKGFSL
410	420	430	440	450	460	470	480
KSQLIVHQRS	HTGMKPYVCN	ECGKAFRSKS	YLIIHTRTHT	GEKLHECNNC	GKAFSFKSQL	IIHQRIHTGE	NPYECHECGK
490	500	510	520	530	540	550	560
AFSRKYQLIS	HQRTHAGEKP	YECTDCGKAF	GLKSQLIIHQ	RTHTGEKPFE	CSECQKAFNT	KSNLIVHQRT	HTGEKPYSCN
570	580	590	600	610	620	630	640
ECGKAFTFKS	QLIVHKGVHT	GVKPYGCSQC	AKTFSLKSQL	IVHQRSHTGV	KPYGCSECGK	AFRSKSYLII	HMRTHTGEKP
650	660	670	680	690	700	710	720
HECRECGKSF	SFNSQLIVHQ	RIHTGENPYE	CSECGKAFNR	KDQLISHQRT	HAGEKPYGCS	ECGKAFSSKS	YLIIHMRTHS
730	740	750	760	770	780	790	800
GEKPYECNEC	GKAFIWKSLL	IVHERTHAGV	NPYKCSQCEK	SFSGKLRLLV	HQRMHTREKP	YECSECGKAF	IRNSQLIVHQ
810	820	830	840	850	860	870	
RTHSGEKPYG	CNECGKTFSQ	KSILSAHQRT	HTGEKPCKCT	ECGKAFCWKS	QLIMHQRTHV	DDKH	

Cmpd.	No. of Cmpds.	m/z meas.	Δ m/z [ppm]	z	Rt [min]	Score	P	Range	Sequence	Modification
2187	1	618.9601	39.18	3	55.8	10.5	1	774-788	R.MHTREKPYECSECGK.A	Carbamidomethyl: 13

Detailed Protein Report

Protein 602: ubiquitin carboxyl-terminal hydrolase 35 [Homo sapiens]

**Accession:**	gi|148746183	**Score:**	25.9
**Database:**	refseq_human(refseq_human_20140103.fasta)	**MW [kDa]:**	113.3
**Database Date:**	2015-11-30	**pI:**	5.3
**Modification(s):**	Carbamidomethyl	**Sequence Coverage [%]:**	2.1
		**No. of unique Peptides:**	1

Quantitation

mdown:qdown	**Median:**	0.20	**CV:**	0.00 %	**No. of Peptides:**	1
Wdown:Qdown	**Median:**	0.22	**CV:**	0.00 %	**No. of Peptides:**	1

10	20	30	40	50	60	70	80
MDKILEAVVT	SSYPVSVKQG	LVRRVLEAAR	QPLEREQCLA	LLALGARLYV	GGAEELPRRV	GCQLLHVAGR	HHPDVFAEFF
**90**	**100**	**110**	**120**	**130**	**140**	**150**	**160**
SARRVLRLLQ	GGAGPPGPRA	LACVQLGLQL	LPEGPAADEV	FALLRREVLR	TVCERPGPAA	CAQVARLLAR	HPRCVPDGPH
**170**	**180**	**190**	**200**	**210**	**220**	**230**	**240**
RLLFCQQLVR	CLGRFRCPAE	GEEGAVEFLE	QAQQVSGLLA	QLWRAQPAAI	LPCLKELFAV	ISCAEEEPPS	SALASVVQHL
**250**	**260**	**270**	**280**	**290**	**300**	**310**	**320**
PLELMDGVVR	NLSNDDSVTD	SQMLTAISRM	IDWVSWPLGK	NIDKWIIALL	KGLAAVKKFS	ILIEVSLTKI	EKVFSKLLYP
**330**	**340**	**350**	**360**	**370**	**380**	**390**	**400**
IVRGAALSVL	KYMLLTFQHS	HEAFHLLLPH	IPPMVASLVK	EDSNSGTSCL	EQLAELVHCM	VFRFPGFPDL	YEPVMEAIKD
**410**	**420**	**430**	**440**	**450**	**460**	**470**	**480**
LHVPNEDRIK	QLLGQDAWTS	QKSELAGFYP	RLMAKSDTGK	IGLINLGNTC	YVNSILQALF	MASDFRHCVL	RLTENNSQPL
**490**	**500**	**510**	**520**	**530**	**540**	**550**	**560**
MTKLQWLFGF	LEHSQRPAIS	PENFLSASWT	PWFSPGTQQD	CSEYLKYLLD	RLHEEEKTGT	RICQKLKQSS	SPSPPEEPPA
**570**	**580**	**590**	**600**	**610**	**620**	**630**	**640**
PSSTSVEKMF	GGKIVTRICC	LCCLNVSSRE	EAFTDLSLAF	PPPERCRRRR	LGSVMRPTED	ITARELPPPT	SAQGPGRVGP
**650**	**660**	**670**	**680**	**690**	**700**	**710**	**720**
RRQRKHCITE	DTPPTSLYIE	GLDSKEAGGQ	SSQEERIERE	EEGKEERTEK	EEVGEEEEST	RGEGEREKEE	EVEEEEEKVE
**730**	**740**	**750**	**760**	**770**	**780**	**790**	**800**
KETEKEAEQE	KEEDSLGAGT	HPDAAIPSGE	RTCGSEGSRS	VLDLVNYFLS	PEKLTAENRY	YCESCASLQD	AEKVVELSQG
**810**	**820**	**830**	**840**	**850**	**860**	**870**	**880**
PCYLILTLLR	FSFDLRTMRR	RKILDDVSIP	LLLRLPLAGG	RGQAYDLCSV	VVHSGVSSES	GHYYCYAREG	AARPAASLGT
**890**	**900**	**910**	**920**	**930**	**940**	**950**	**960**
ADRPEPENQW	YLFNDTRVSF	SSFESVSNVT	SFFPKDTAYV	LFYRQRPREG	PEAELGSSRV	RTEPTLHKDL	MEAISKDNIL
**970**	**980**	**990**	**1000**	**1010**	**1020**		
YLQEQEKEAR	SRAAYISALP	TSPHWGRGFD	EDKDEDEGSP	GGCNPAGGNG	GDFHRLVF		

Cmpd.	No. of Cmpds.	m/z meas.	Δ m/z [ppm]	z	Rt [min]	Score	P	Range	Sequence	Modification	Ratios
574	1	469.1409	-166.57	2	36.0	10.2	0	154-161	R.CVPDGPHR.L	Carbamidomethyl: 1	mdown:qdown 0.20<br>Wdown:Qdown 0.22

Detailed Protein Report

Protein 603: **tumor necrosis factor receptor superfamily member 10B isoform 2 precursor [Homo sapiens]**

**Accession:**	gi|224494026	**Score:**	25.8
**Database:**	refseq_human(refseq_human_20140103.fasta)	**MW [kDa]:**	45.1
**Database Date:**	2015-11-30	**pI:**	5.4
**Modification(s):**	Carbamidomethyl	**Sequence Coverage [%]:**	4.6
		**No. of unique Peptides:**	2

10	20	30	40	50	60	70	80
MEQRGQNAPA	ASGARKRHGP	GPREARGARP	GPRVPKTLVL	VVAAVLLLVS	AESALITQQD	LAPQQRAAPQ	QKRSSPSEGL
90	100	110	120	130	140	150	160
CPPGHHISED	GRDCISCKYG	QDYSTHWNDL	LFCLRCTRCD	SGEVELSPCT	TTRNTVCQCE	EGTFREEDSP	EMCRKCRTGC
170	180	190	200	210	220	230	240
PRGMVKVGDC	TPWSDIECVH	KESGIIIGVT	VAAVVLIVAV	FVCKSLLWKK	VLPYLKGICS	GGGGDPERVD	RSSQRPGAED
250	260	270	280	290	300	310	320
NVLNEIVSIL	QPTQVPEQEM	EVQEPAEPTG	VNMLSPGESE	HLLEPAEAER	SQRRRLLVPA	NEGDPTETLR	QCFDDFADLV
330	340	350	360	370	380	390	400
PFDSWEPLMR	KLGLMDNEIK	VAKAEAAGHR	DTLYTMLIKW	VNKTGRDASV	HTLLDALETL	GERLAKQKIE	DHLLSSGKFM
410	420						
YLEGNADSAM	S						

Cmpd.	No. of Cmpds.	m/z meas.	Δ m/z [ppm]	z	Rt [min]	Score	P	Range	Sequence	Modification
2928	1	973.4951	-17.22	2	65.4	11.7	2	210-228	K.KVLPYLKGICSGGGGDPER.V	
2235	1	937.9180	-60.88	2	56.4	14.1	1	211-228	K.VLPYLKGICSGGGGDPER.V	Carbamidomethyl: 9

Detailed Protein Report

Protein 604: zinc finger protein 688 isoform b [Homo sapiens]

**Accession:**	gi|67010013	**Score:**	25.8
**Database:**	refseq_human(refseq_human_20140103.fasta)	**MW [kDa]:**	28.9
**Database Date:**	2015-11-30	**pI:**	11.6
		**Sequence Coverage [%]:**	6.9
		**No. of unique Peptides:**	1

10	20	30	40	50	60	70	80
MPRSPLRSSL	STEASGPRGP	FKCPASPRPI	TAQLLPGSSP	IIGRCVLRVQ	RGFPGPKPAL	ISWMEQESEA	WSPAAQDPEK
**90**	**100**	**110**	**120**	**130**	**140**	**150**	**160**
GERLGGARRG	DVPNRKEEEP	EEVPRAKGPR	KAPVKESPEV	LVERNPDPAI	SVAPARAQPP	KNAAWDPTTG	AQPPAPIPSM
**170**	**180**	**190**	**200**	**210**	**220**	**230**	**240**
DAQAGQRRHV	CTDCGRRFTY	PSLLVSHRRM	HSGERPFPCP	ECGMRFKRKF	AVEAHQWIHR	SCSGGRRGRR	PGIRAVPRAP
**250**	**260**	**270**					
VRGDRDPPVL	FRHYPDIFEE	CG					

Cmpd.	No. of Cmpds.	m/z meas.	Δ m/z [ppm]	z	Rt [min]	Score	P	Range	Sequence	Modification
1884	1	600.0016	18.70	3	52.2	14.1	2	2-18	M.PRSPLRSSLSTEASGPR.G	

Detailed Protein Report

Protein 605: lysine-specific demethylase 4C isoform 3 [Homo sapiens]

**Accession:**	gi|226442893	**Score:**	25.7
**Database:**	refseq_human(refseq_human_20140103.fasta)	**MW [kDa]:**	91.8
**Database Date:**	2015-11-30	**pI:**	5.3
**Modification(s):**	Carbamidomethyl	**Sequence Coverage [%]:**	3.2
		**No. of unique Peptides:**	1

10	20	30	40	50	60	70	80
MEVAEVESPL	NPSCKIMTFR	PSMEEFREFN	KYLAYMESKG	AHRAGLAKVI	PPKEWKPRQC	YDDIDNLLIP	APIQQMVTGQ
**90**	**100**	**110**	**120**	**130**	**140**	**150**	**160**
SGLFTQYNIQ	KKAMTVKEFR	QLANSGKYCT	PRYLDYEDLE	RKYWKNLTFV	APIYGADING	SIYDEGVDEW	NIARLNTVLD
**170**	**180**	**190**	**200**	**210**	**220**	**230**	**240**
VVEEECGISI	EGVNTPYLYF	GMWKTTFAWH	TEDMDLYSIN	YLHFGEPKSW	YAIPPEHGKR	LERLAQGFFP	SSSQGCDAFL
**250**	**260**	**270**	**280**	**290**	**300**	**310**	**320**
RHKMTLISPS	VLKKYGIPFD	KITQEAGEFM	ITFPYGYHAG	FNHGFNCAES	TNFATVRWID	YGKVAKLCTC	RKDMVKISMD
**330**	**340**	**350**	**360**	**370**	**380**	**390**	**400**
IFVRKFQPDR	YQLWKQGKDI	YTIDHTKPTP	ASTPEVKAWL	QRRRKVRKAS	RSFQCARSTS	KRPKADEEEE	VSDEVDGAEV
**410**	**420**	**430**	**440**	**450**	**460**	**470**	**480**
PNPDSVTDDL	KVSEKSEAAV	KLRNTEASSE	EESSASRMQV	EQNLSDHIKL	SGNSCLSTSV	TEDIKTEDDK	AYAYRSVPSI
**490**	**500**	**510**	**520**	**530**	**540**	**550**	**560**
SSEADDSIPL	SSGYEKPEKS	DPSELSWPKS	PESCSSVAES	NGVLTEGEES	DVESHGNGLE	PGEIPAVPSG	ERNSFKVPSI
**570**	**580**	**590**	**600**	**610**	**620**	**630**	**640**
AEGENKTSKS	WRHPLSRPPA	RSPMTLVKQQ	APSDEELPEV	LSIEEEVEET	ESWAKPLIHL	WQTKSPNFAA	EQEYNATVAR
**650**	**660**	**670**	**680**	**690**	**700**	**710**	**720**
MKPHCAICTL	LMPYHKPDSS	NEENDARWET	KLDEVVTSEG	KTKPLIPEMC	FIYSEENIEY	SPPNAFLEED	GTSLLISCAK
**730**	**740**	**750**	**760**	**770**	**780**	**790**	**800**
CCVRVHASCY	GIPSHEICDG	WLCARCKRNA	WTAECCLCNL	RGGALKQTKN	NKWAHVMCAV	AVPEVRFTNV	PERTQIDVGR
**810**	**820**						
IPLQRLKLGR	LGI						

Cmpd.	No. of Cmpds.	m/z meas.	Δ m/z [ppm]	z	Rt [min]	Score	P	Range	Sequence	Modification
149	1	855.2460	-164.97	2	31.1	10.1	1	748-761	K.RNAWTAECCLCNLR.G	Carbamidomethyl: 8

Detailed Protein Report

Protein 606: integrin beta-like protein 1 isoform 3 [Homo sapiens]

Accession: gi|418203907
Database: refseq_human(refseq_human_20140103.fasta)
Database Date: 2015-11-30
Modification(s): Carbamidomethyl

Score: 25.7
MW [kDa]: 43.9
pI: 5.0
Sequence Coverage [%]: 10.0
No. of unique Peptides: 1

10	20	30	40	50	60	70	80
MNLVAVMGAF	NKGHGKCDCG	KCKCDQGWYG	DACQYPTNCD	LTKKKSNQMC	KNSQDIICSN	AGTCHCGRCK	CDNSDGSGLV
90	100	110	120	130	140	150	160
YGKFCECDDR	ECIDDETEEI	CGGHGKCYCG	NCYCKAGWHG	DKCEFQCDIT	PWESKRRCTS	PDGKICSNRG	TCVCGECTCH
170	180	190	200	210	220	230	240
DVDPTGDWGD	IHGDTCECDE	RDCRAVYDRY	SDDFCSGHGQ	CNCGRCDCKA	GWYGKKCEHP	QSCTLSAEES	IRKCQGSSDL
250	260	270	280	290	300	310	320
PCSGRGKCEC	GKCTCYPPGD	RRVYGKTCEC	DDRRCEDLDG	VVCGGHGTCS	CGRCVCERGW	FGKLCQHPRK	CNMTEEQSKN
330	340	350	360	370	380	390	400
LCESADGILC	SGKGSCHCGK	CICSAEEWYI	SGEFCDCDDR	DCDKHDGLIC	TGNGICSCGN	CECWDGWNGN	ACEIWLGSEY
410							
P							

Cmpd.	No. of Cmpds.	m/z meas.	Δ m/z [ppm]	z	Rt [min]	Score	P	Range	Sequence	Modification
2134	1	1062.3814	-60.33	2	56.8	10.7	1	52-70	K.NSQDIICSNAGTCHCGRCK.C	Carbamidomethyl: 13, 15

Detailed Protein Report

Protein 607: PREDICTED: centromere protein T isoform X3 [Homo sapiens]

**Accession:**	gi|578829227	**Score:**	25.7
**Database:**	refseq_human(refseq_human_20140103.fasta)	**MW [kDa]:**	34.4
**Database Date:**	2015-11-30	**pI:**	5.0
**Modification(s):**	Carbamidomethyl, Oxidation	**Sequence Coverage [%]:**	4.7
		**No. of unique Peptides:**	1

10	20	30	40	50	60	70	80
MSWCCLWLCL	SSVGRTGSAG	PSLPFSELCS	LGLLRLRPVF	SPLHSGPGKP	AQFLAGEAEE	VNAFALGFLS	TSSGVSGEDE
**90**	**100**	**110**	**120**	**130**	**140**	**150**	**160**
VEPLHDGVEE	AEKKMEEEGV	SVSEMEATGA	QGPSRVEEAE	GHTEVTEAEG	SQGTAEADGP	GASSGDEDAS	GRAASPESAS
**170**	**180**	**190**	**200**	**210**	**220**	**230**	**240**
STPESLQARR	HHQFLEPAPA	PGAAVLSSEP	AEPLLVRHPP	RPRTTGPRPR	QDPHKAGLSH	YVKLFSFYAK	MPMERKALEM
**250**	**260**	**270**	**280**	**290**	**300**	**310**	**320**
VEKCLDKYFQ	HLCDDLEVFA	AHAGRKTVKP	EDLELLMRRQ	GLVTDQVSLH	VLVERHLPLE	YRQLLIPCAY	SGNSVFPAQ

Cmpd.	No. of Cmpds.	m/z meas.	Δ m/z [ppm]	z	Rt [min]	Score	P	Range	Sequence	Modification
2474	1	965.7758	-154.68	2	59.6	14.1	0	1-15	-.MSWCCLWLCLSSVGR.T	Carbamidomethyl: 4, 5, 9; Oxidation: 1

Detailed Protein Report

Protein 608: **sushi domain-containing protein 1 isoform 3 precursor [Homo sapiens]**

**Accession:**	gi|544063419	**Score:**	25.7
**Database:**	refseq_human(refseq_human_20140103.fasta)	**MW [kDa]:**	80.4
**Database Date:**	2015-11-30	**pI:**	6.0
**Modification(s):**	Carbamidomethyl	**Sequence Coverage [%]:**	4.0
		**No. of unique Peptides:**	1

10	20	30	40	50	60	70	80
MGRGPWDAGP	SRRLLPLLLL	LGLARGAAGA	PGPDGLDVCA	TCHEHATCQQ	REGKKICICN	YGFVGNGRTQ	CVDKNECQFG
**90**	**100**	**110**	**120**	**130**	**140**	**150**	**160**
ATLVCGNHTS	CHNTPGGFYC	ICLEGYRATN	NNKTFIPNDG	TFCTDIDECE	VSGLCRHGGR	CVNTHGSFEC	YCMDGYLPRN
**170**	**180**	**190**	**200**	**210**	**220**	**230**	**240**
GPEPFHPTTD	ATSCTEIDCG	TPPEVPDGYI	IGNYTSSLGS	QVRYACREGF	FSVPEDTVSS	CTGLGTWESP	KLHCQEINCG
**250**	**260**	**270**	**280**	**290**	**300**	**310**	**320**
NPPEMRHAIL	VGNHSSRLGG	VARYVCQEGF	ESPGGKITSV	CTEKGTWRES	TLTCTEILTK	INDVSLFNDT	CVRWQINSRR
**330**	**340**	**350**	**360**	**370**	**380**	**390**	**400**
INPKISYVIS	IKGQRLDPME	SVREETVNLT	TDSRTPEVCL	ALYPGTNYTV	NISTAPPRRS	MPAVIGFQTA	EVDLLEDDGS
**410**	**420**	**430**	**440**	**450**	**460**	**470**	**480**
FNISIFNETC	LKLNRRSRKV	GSEHMYQFTV	LGQRWYLANF	SHATSFNFTT	REQVPVVCLD	LYPTTDYTVN	VTLLRSPKRH
**490**	**500**	**510**	**520**	**530**	**540**	**550**	**560**
SVQITIATPP	AVKQTISNIS	GFNETCLRWR	SIKTADMEEM	YLFHIWGQRW	YQKEFAQEMT	FNISSSSRDP	EVCLDLRPGT
**570**	**580**	**590**	**600**	**610**	**620**	**630**	**640**
NYNVSLRALS	SELPVVISLT	TQITEPPLPE	VEFFTVHRGP	LPRLRLRKAK	EKNGPISSYQ	VLVLPLALQS	TFSCDSEGAS
**650**	**660**	**670**	**680**	**690**	**700**	**710**	**720**
SFFSNASDAD	GYVAAELLAK	DVPDDAMEIP	IGDRLYYGEY	YNAPLKRGSD	YCIILRITSE	WNKIRHSCCC	RWRVLDWVPW
**730**							
LL							

Cmpd.	No. of Cmpds.	m/z meas.	Δ m/z [ppm]	z	Rt [min]	Score	P	Range	Sequence	Modification
844	1	776.9364	79.23	2	39.3	10.6	0	301-313	K.INDVSLFNDTCVR.W	Carbamidomethyl: 11

Detailed Protein Report

Protein 609: PREDICTED: kinesin-like protein KIF21A isoform X9 [Homo sapiens]

**Accession:**	gi|530400550	**Score:**	25.7
**Database:**	refseq_human(refseq_human_20140103.fasta)	**MW [kDa]:**	179.8
**Database Date:**	2015-11-30	**pI:**	6.1
		**Sequence Coverage [%]:**	1.5
		**No. of unique Peptides:**	1

Quantitation

mdown:qdown	**Median:** 0.95	**CV:** 0.00 %	**No. of Peptides:**	1
Wdown:Qdown	**Median:** 1.95	**CV:** 0.00 %	**No. of Peptides:**	1

```
        10         20         30         40         50         60         70         80
MLGAPDESSV RVAVRIRPQL AKEKIEGCHI CTSVTPGEPQ VFLGKDKAFT FDYVFDIDSQ QEQIYIQCIE KLIEGCFEGY
        90        100        110        120        130        140        150        160
NATVFAYGQT GAGKTYTMGT GFDVNIVEEE LGIISRAVKH LFKSIEEKKH IAIKNGLPAP DFKVNAQFLE LYNEEVLDLF
       170        180        190        200        210        220        230        240
DTTRDIDAKS KKSNIRIHED STGGIYTVGV TTRTVNTESE MMQCLKLGAL SRTTASTQMN VQSSRSHAIF TIHVCQTRVC
       250        260        270        280        290        300        310        320
PQIDADNATD NKIISESAQM NEFETLTAKF HFVDLAGSER LKRTGATGER AKEGISINCG LLALGNVISA LGDKSKRATH
       330        340        350        360        370        380        390        400
VPYRDSKLTR LLQDSLGGNS QTIMIACVSP SDRDFMETLN TLKYANRARN IKNKVMVNQD RASQQINALR SEITRLQMEL
       410        420        430        440        450        460        470        480
MEYKTGKRII DEEGVESIND MFHENAMLQT ENNNLRVRIK AMQETVDALR SRITQLVSDQ ANHVLARAGE GNEEISNMIH
       490        500        510        520        530        540        550        560
SYIKEIEDLR AKLLESEAVN ENLRKNLTRA TARAPYFSGS STFSPTILSS DKETIEIIDL AKKDLEKLKR KEKRKKKRLQ
       570        580        590        600        610        620        630        640
KLEESNREER SVAGKEDNTD TDQEKKEEKG VSERENNELE VEESQEVSDH EDEEEEEEEE EDDIDGGESS DESDSESDEK
       650        660        670        680        690        700        710        720
ANYQADLANI TCEIAIKQKL IDELENSQKR LQTLKKQYEE KLMMLQHKIR DTQLERDQVL QNLGSVESYS EEKAKKVRSE
       730        740        750        760        770        780        790        800
YEKKLQAMNK ELQRLQAAQK EHARLLKNQS QYEKQLKKLQ QDVMEMKKTK VRLMKQMKEE QEKARLTESR RNREIAQLKK
       810        820        830        840        850        860        870        880
DQRKRDHQLR LLEAQKRNQE VVLRRKTEEV TALRRQVRPM SDKVAGKVTR KLSSSDAPAQ DTGSSAAAVE TDASRTGAQQ
       890        900        910        920        930        940        950        960
KMRIPVARVQ ALPTPATNGN RKKYQRKGLT GRVFISKTAR MKWQLLERRV TDIIMQKMTI SNMEADMNRL LKQREELTKR
       970        980        990       1000       1010       1020       1030       1040
REKLSKRREK IVKENGEGDK NVANINEEME SLTANIDYIN DSISDCQANI MQMEEAKEEG ETLDVTAVIN ACTLTEARYL
      1050       1060       1070       1080       1090       1100       1110       1120
LDHFLSMGIN KGLQAAQKEA QIKVLEGRLK QTEITSATQN QLLFHMLKEK AELNPELDAL LGHALQENVE DSTDEDAPLN
      1130       1140       1150       1160       1170       1180       1190       1200
SPGSEGSTLS SDLMKLCGEV KPKNKARRRT TTQMELLYAD SSELASDTST GDASLPGPLT PVAEGQEIGM NTETSGTSAR
      1210       1220       1230       1240       1250       1260       1270       1280
EKELSPPPGL PSKIGSISRQ SSLSEKKIPE PSPVTRRKAY EKAEKSKAKE QKQGIINPFP ASKGIRAFPL QCIHIAEGHT
      1290       1300       1310       1320       1330       1340       1350       1360
KAVLCVDSTD DLLFTGSKDR TCKVWNLVTG QEIMSLGGHP NNVVSVKYCN YTSLVFTVST SYIKVWDIRD SAKCIRTLTS
      1370       1380       1390       1400       1410       1420       1430       1440
SGQVTLGDAC SASTSRTVAI PSGENQINQI ALNPTGTFLY AASGNAVRMW DLKRFQSTGK LTGHLGPVMC LTVDQISSGQ
      1450       1460       1470       1480       1490       1500       1510       1520
DLIITGSKDH YIKMFDVTEG ALGTVSPTHN FEPPHYDGIE ALTIQGDNLF SGSRDNGIKK WDLTQKDLLQ QVPNAHKDWV
      1530       1540       1550       1560       1570       1580       1590       1600
CALGVVPDHP VLLSGCRGGI LKVWNMDTFM PVGEMKGHDS PINAICVNST HIFTAADDRT VRIWKARNLQ DGQISDTGDL
      1610
GEDIASN
```

Cmpd.	No. of Cmpds.	m/z meas.	Δ m/z [ppm]	z	Rt [min]	Score	P	Range	Sequence	Modification	Ratios

Detailed Protein Report

Cmpd.	No. of Cmpds.	m/z meas.	Δ m/z [ppm]	z	Rt [min]	Score	P	Range	Sequence	Modification	Ratios
770	1	839.4295	-37.29	2	38.4	14.8	2	731-744	K.ELQRLQAAQKEHAR.L		Wdown:Qdown 1.95<br>mdown:qdown 0.95

Detailed Protein Report

Protein 610: **methyl-CpG-binding domain protein 5 [Homo sapiens]**

**Accession:**	gi|38202209	**Score:**	25.6
**Database:**	refseq_human(refseq_human_20140103.fasta)	**MW [kDa]:**	159.8
**Database Date:**	2015-11-30	**pI:**	10.0
		**Sequence Coverage [%]:**	1.7
		**No. of unique Peptides:**	1

10	20	30	40	50	60	70	80
MNGGKECDGG	DKEGGLPAIQ	VPVGWQRRVD	QNGVLYVSPS	GSLLSCLEQV	KTYLLTDGTC	KCGLECPLIL	PKVFNFDPGA
90	100	110	120	130	140	150	160
AVKQRTAEDV	KADEDVTKLC	IHKRKIIAVA	TLHKSMEAPH	PSLVLTSPGG	GTNATPVVPS	RAATPRSVRN	KSHEGITNSV
170	180	190	200	210	220	230	240
MPECKNPFKL	MIGSSNAMGR	LYVQELPGSQ	QQELHPVYPR	QRLGSSEHGQ	KSPFRGSHGG	LPSPASSGSQ	IYGDGSISPR
250	260	270	280	290	300	310	320
TDPLGSPDVF	TRSNPGFHGA	PNSSPIHLNR	TPLSPPSVML	HGSPVQSSCA	MAGRTNIPLS	PTLTTKSPVM	KKPMCNFSTN
330	340	350	360	370	380	390	400
MEIPRAMFHH	KPPQGPPPPP	PPSCALQKKP	LTSEKDPLGI	LDPIPSKPVN	QNPVIINPTS	FHSNVHSQVP	MMNVSMPPAV
410	420	430	440	450	460	470	480
VPLPSNLPLP	TVKPGHMNHG	SHVQRVQHSA	STSLSPSPVT	SPVHMMGTGI	GRIEASPQRS	RSSSTSSDHG	NFMMPPVGPQ
490	500	510	520	530	540	550	560
ATSSGIKVPP	RSPRSTIGSP	RPSMPSSPST	KSDGHHQYKD	IPNPLIAGIS	NVLNTPSSAA	FPTASAGSSS	VKSQPGLLGM
570	580	590	600	610	620	630	640
PLNQILNQHN	AASFPASSLL	SAAAKAQLAN	QNKLAGNNSS	SSSNSGAVAG	SGNTEGHSTL	NTMFPPTANM	LLPTGEGQSG
650	660	670	680	690	700	710	720
RAALRDKLMS	QQKDALRKRK	QPPTTVLSLL	RQSQMDSSAV	PKPGPDLLRK	QGQGSFPISS	MSQLLQSMSC	QSSHLSSNST
730	740	750	760	770	780	790	800
PGCGASNTAL	PCSANQLHFT	DPSMNSSVLQ	NIPLRGEAVH	CHNANTNFVH	SNSPVPNHHL	AGLINQIQAS	GNCGMLSQSG
810	820	830	840	850	860	870	880
MALGNSLHPN	PPQSRISTSS	TPVIPNSIVS	SYNQTSSEAG	GSGPSSSIAI	AGTNHPAITK	TTSVLQDGVI	VTTAAGNPLQ
890	900	910	920	930	940	950	960
SQLPIGSDFP	FVGQEHALHF	PSNSTSNNHL	PHPLNPSLLS	SLPISLPVNQ	QHLLNQNLLN	ILQPSAGEGD	MSSINNTLSN
970	980	990	1000	1010	1020	1030	1040
HQLTHLQSLL	NNNQMFPPNQ	QQQQLLQGYQ	NLQAFQGQST	IPCPANNNPM	ACLFQNFQVR	MQEDAALLNK	RISTQPGLTA
1050	1060	1070	1080	1090	1100	1110	1120
LPENPNTTLP	PFQDTPCELQ	PRIDPSLGQQ	VKDGLVVGGP	GDASVDAIYK	AVVDAASKGM	QVVITTAVNS	TTQISPIPAL
1130	1140	1150	1160	1170	1180	1190	1200
SAMSAFTASI	GDPLNLSSAV	SAVIHGRNMG	GVDHDGRLRN	SRGARLPKNL	DHGKNVNEGD	GFEYFKSASC	HTSKKQWDGE
1210	1220	1230	1240	1250	1260	1270	1280
QSPRGERNRW	KYEEFLDHPG	HIHSSPCHER	PNNVSTLPFL	PGEQHPILLP	PRNCPGDKIL	EENFRYNNYK	RTMMSFKERL
1290	1300	1310	1320	1330	1340	1350	1360
ENTVERCAHI	NGNRPRQSRG	FGELLSTAKQ	DLVLEEQSPS	SSNSLENSLV	KDYIHYNGDF	NAKSVNGCVP	SPSDAKSISS
1370	1380	1390	1400	1410	1420	1430	1440
EDDLRNPDSP	SSNELIHYRP	RTFNVGDLVW	GQIKGLTSWP	GKLVREDDVH	NSCQQSPEEG	KVEPEKLKTL	TEGLEAYSRV
1450	1460	1470	1480	1490	1500		
RKRNRKSGKL	NNHLEAAIHE	AMSELDKMSG	TVHQIPQGDR	QMRPPKPKRR	KISR		

Cmpd.	No. of Cmpds.	m/z meas.	Δ m/z [ppm]	z	Rt [min]	Score	P	Range	Sequence	Modification
2584	1	834.4230	41.03	2	62.8	12.5	0	312-325	K.KPMCNFSTNMEIPR.A	

Detailed Protein Report

Protein 611: zinc finger and BTB domain-containing protein 5 [Homo sapiens]

**Accession:**	gi|7662074	**Score:**	25.6
**Database:**	refseq_human(refseq_human_20140103.fasta)	**MW [kDa]:**	74.2
**Database Date:**	2015-11-30	**pI:**	5.8
**Modification(s):**	Carbamidomethyl	**Sequence Coverage [%]:**	5.2
		**No. of unique Peptides:**	2

Alias proteins:

Accession	Name	Description
gi|530390257	refseq_human (refseq_human_20140103.fasta)	PREDICTED: zinc finger and BTB domain-containing protein 5 isoform X1 [Homo sapiens]

10	20	30	40	50	60	70	80
MDFPGHFEQI	FQQLNYQRLH	GQLCDCVIVV	GNRHFKAHRS	VLAACSTHFR	ALFSVAEGDQ	TMNMIQLDSE	VVTAEAFAAL
**90**	**100**	**110**	**120**	**130**	**140**	**150**	**160**
IDMMYTSTLM	LGESNVMDVL	LAASHLHLNS	VVKACKHYLT	TRTLPMSPPS	ERVQEQSARM	QRSFMLQQLG	LSIVSSALNS
**170**	**180**	**190**	**200**	**210**	**220**	**230**	**240**
SQNGEEQPAP	MSSSMRSNLD	QRTPFPMRRL	HKRKQSAEER	ARQRLRPSID	ESAISDVTPE	NGPSGVHSRE	EFFSPDSLKI
**250**	**260**	**270**	**280**	**290**	**300**	**310**	**320**
VDNPKADGMT	DNQEDSAIMF	DQSFGTQEDA	QVPSQSDNSA	GNMAQLSMAS	RATQVETSFD	QEAAPEKSSF	QCENPEVGLG
**330**	**340**	**350**	**360**	**370**	**380**	**390**	**400**
EKEHMRVVVK	SEPLSSPEPQ	DEVSDVTSQA	EGSESVEVEG	VVVSAEKIDL	SPESSDRSFS	DPQSSTDRVG	DIHILEVTNN
**410**	**420**	**430**	**440**	**450**	**460**	**470**	**480**
LEHKSTFSIS	NFLNKSRGNN	FTANQNNDDN	IPNTTSDCRL	ESEAPYLLSP	EAGPAGGPSS	APGSHVENPF	SEPADSHFVR
**490**	**500**	**510**	**520**	**530**	**540**	**550**	**560**
PMQEVMGLPC	VQTSGYQGGE	QFGMDFSRSG	LGLHSSFSRV	MIGSPRGGAS	NFPYYRRIAP	KMPVVTSVRS	SQIPENSTSS
**570**	**580**	**590**	**600**	**610**	**620**	**630**	**640**
QLMMNGATSS	FENGHPSQPG	PPQLTRASAD	VLSKCKKALS	EHNVLVVEGA	RKYACKICCK	TFLTLTDCKK	HIRVHTGEKP
**650**	**660**	**670**	**680**				
YACLKCGKRF	SQSSHLYKHS	KTTCLRWQSS	NLPSTLL				

Cmpd.	No. of Cmpds.	m/z meas.	Δ m/z [ppm]	z	Rt [min]	Score	P	Range	Sequence	Modification
2671	1	699.0932	93.54	3	62.2	11.2	1	19-36	R.LHGQLCDCVIVVGNRHFK.A	Carbamidomethyl: 6
1742	1	689.9841	-17.31	3	50.5	14.5	2	613-629	K.YACKICCKTFLTLTDCK.K	Carbamidomethyl: 6, 7

Detailed Protein Report

Protein 612: prosaposin receptor GPR37 precursor [Homo sapiens]

Accession: gi|4885323
Database: refseq_human(refseq_human_20140103.fasta)
Database Date: 2015-11-30
Modification(s): Oxidation

Score: 25.6
MW [kDa]: 67.1
pI: 9.9
Sequence Coverage [%]: 3.8
No. of unique Peptides: 1

Quantitation

mdown:qdown **Median:** 1.22 **CV:** 0.00 % **No. of Peptides:** 1
Wdown:Qdown **Median:** 5.84 **CV:** 0.00 % **No. of Peptides:** 1

10	20	30	40	50	60	70	80
MRAPGALLAR	MSRLLLLLLL	KVSASSALGV	APASRNETCL	GESCAPTVIQ	RRGRDAWGPG	NSARDVLRAR	APREEQGAAF
90	100	110	120	130	140	150	160
LAGPSWDLPA	APGRDPAAGR	GAEASAAGPP	GPPTRPPGPW	RWKGARGQEP	SETLGRGNPT	ALQLFLQISE	EEEKGPRGAG
170	180	190	200	210	220	230	240
ISGRSQEQSV	KTVPGASDLF	YWPRRAGKLQ	GSHHKPLSKT	ANGLAGHEGW	TIALPGRALA	QNGSLGEGIH	EPGGPRRGNS
250	260	270	280	290	300	310	320
TNRRVRLKNP	FYPLTQESYG	AYAVMCLSVV	IFGTGIIGNL	AVMCIVCHNY	YMRSISNSLL	ANLAFWDFLI	IFFCLPLVIF
330	340	350	360	370	380	390	400
HELTKKWLLE	DFSCKIVPYI	EVASLGVTTF	TLCALCIDRF	RAATNVQMYY	EMIENCSSTT	AKLAVIWVGA	LLLALPEVVL
410	420	430	440	450	460	470	480
RQLSKEDLGF	SGRAPAERCI	IKISPDLPDT	IYVLALTYDS	ARLWWYFGCY	FCLPTLFTIT	CSLVTARKIR	KAEKACTRGN
490	500	510	520	530	540	550	560
KRQIQLESQM	NCTVVALTIL	YGFCIIPENI	CNIVTAYMAT	GVSQQTMDLL	NIISQFLLFF	KSCVTPVLLF	CLCKPFSRAF
570	580	590	600	610	620		
MECCCCCCEE	CIQKSSTVTS	DDNDNEYTTE	LELSPFSTIR	REMSTFASVG	THC		

Cmpd.	No. of Cmpds.	m/z meas.	Δ m/z [ppm]	z	Rt [min]	Score	P	Range	Sequence	Modification	Ratios
2128	2	721.2347	-109.30	2	56.8	13.5	1	601-613	R.REMSTFASVGTHC.-	Oxidation: 3	Wdown:Qdown 5.84<br>mdown:qdown 1.22

Detailed Protein Report

Protein 613: abnormal spindle-like microcephaly-associated protein isoform 1 [Homo sapiens]

**Accession:**	gi|126116596	**Score:**	25.6
**Database:**	refseq_human(refseq_human_20140103.fasta)	**MW [kDa]:**	409.5
**Database Date:**	2015-11-30	**pI:**	11.2
		**Sequence Coverage [%]:**	0.7
		**No. of unique Peptides:**	1

Quantitation

mdown:qdown	**Median:** 0.47	**CV:** 0.00 %	**No. of Peptides:** 1

Detailed Protein Report

10	20	30	40	50	60	70	80
MANRRVGRGC	WEVSPTERRP	PAGLRGPAAE	EEASSPPVLS	LSHFCRSPFL	CFGDVLLGAS	RTLSLALDNP	NEEVAEVKIS
90	100	110	120	130	140	150	160
HFPAADLGFS	VSQRCFVLQP	KEKIVISVNW	TPLKEGRVRE	IMTFLVNDVL	KHQAILLGNA	EEQKKKKRSL	WDTIKKKKIS
170	180	190	200	210	220	230	240
ASTSHNRRVS	NIQNVNKTFS	VSQKVDRVRS	PLQACENLAM	NEGGPPTENN	SLILEENKIP	ISPISPAFNE	CHGATCLPLS
250	260	270	280	290	300	310	320
VRRSTTYSSL	HASENRELLN	VHSANVSKVS	FNEKAVTETS	FNSVNVNGQR	GENSKLSLTP	NCSSTLNITQ	SQIHFLSPDS
330	340	350	360	370	380	390	400
FVNNSHGANN	ELELVTCLSS	DMFMKDNSQP	VHLESTIAHE	IYQKILSPDS	FIKDNYGLNQ	DLESESVNPI	LSPNQFLKDN
410	420	430	440	450	460	470	480
MAYMCTSQQT	CKVPLSNENS	QVPQSPEDWR	KSEVSPRIPE	CQGSKSPKAI	FEELVEMKSN	YYSFIKQNNP	KFSAVQDISS
490	500	510	520	530	540	550	560
HSHNKQPKRR	PILSATVTKR	KATCTRENQT	EINKPKAKRC	LNSAVGEHEK	VINNQKEKED	FHSYLPIIDP	ILSKSKSYKN
570	580	590	600	610	620	630	640
EVTPSSTTAS	VARKRKSDGS	MEDANVRVAI	TEHTEVREIK	RIHFSPSEPK	TSAVKKTKNV	TTPISKRISN	REKLNLKKKT
650	660	670	680	690	700	710	720
DLSIFRTPIS	KTNKRTKPII	AVAQSSLTFI	KPLKTDIPRH	PMPFAAKNMF	YDERWKEKQE	QGFTWWLNFI	LTPDDFTVKT
730	740	750	760	770	780	790	800
NISEVNAATL	LLGIENQHKI	SVPRAPTKEE	MSLRAYTARC	RLNRLRRAAC	RLFTSEKMVK	AIKKLEIEIE	ARRLIVRKDR
810	820	830	840	850	860	870	880
HLWKDVGERQ	KVLNWLLSYN	PLWLRIGLET	TYGELISLED	NSDVTGLAMF	ILNRLLWNPD	IAAEYRHPTV	PHLYRDGHEE
890	900	910	920	930	940	950	960
ALSKFTLKKL	LLLVCFLDYA	KISRLIDHDP	CLFCKDAEFK	ASKEILLAFS	RDFLSGEGDL	SRHLGLLGLP	VNHVQTPFDE
970	980	990	1000	1010	1020	1030	1040
FDFAVTNLAV	DLQCGVRLVR	TMELLTQNWD	LSKKLRIPAI	SRLQKMHNVD	IVLQVLKSRG	IELSDEHGNT	ILSKDIVDRH
1050	1060	1070	1080	1090	1100	1110	1120
REKTLRLLWK	IAFAFQVDIS	LNLDQLKEEI	AFLKHTKSIK	KTISLLSCHS	DDLINKKKGK	RDSGSFEQYS	ENIKLLMDWV
1130	1140	1150	1160	1170	1180	1190	1200
NAVCAFYNKK	VENFTVSFSD	GRVLCYLIHH	YHPCYVPFDA	ICQRTTQTVE	CTQTGSVVLN	SSSESDDSSL	DMSLKAFDHE
1210	1220	1230	1240	1250	1260	1270	1280
NTSELYKELL	ENEKKNFHLV	RSAVRDLGGI	PAMINHSDMS	NTIPDEKVVI	TYLSFLCARL	LDLRKEIRAA	RLIQTTWRKY
1290	1300	1310	1320	1330	1340	1350	1360
KLKTDLKRHQ	EREKAARIIQ	LAVINFLAKQ	RLRKRVNAAL	VIQKYWRRVL	AQRKLLMLKK	EKLEKVQNKA	ASLIQGYWRR
1370	1380	1390	1400	1410	1420	1430	1440
YSTRQRFLKL	KYYSIILQSR	IRMIIAVTSY	KRYLWATVTI	QRHWRAYLRR	KQDQQRYEML	KSSTLIIQSM	FRKWKQRKMQ
1450	1460	1470	1480	1490	1500	1510	1520
SQVKATVILQ	RAFREWHLRK	QAKEENSAII	IQSWYRMHKE	LRKYIYIRSC	VVIIQKRFRC	FQAQKLYKRR	KESILTIQKY
1530	1540	1550	1560	1570	1580	1590	1600
YKAYLKGKIE	RTNYLQKRAA	AIQLQAAFRR	LKAHNLCRQI	RAACVIQSYW	RMRQDRVRFL	NLKKTIIKFQ	AHVRKHQQRQ
1610	1620	1630	1640	1650	1660	1670	1680
KYKKMKKAAV	IIQTHFRAYI	FAMKVLASYQ	KTRSAVIVLQ	SAYRGMQARK	MYIHILTSVI	KIQSYYRAYV	SKKEFLSLKN
1690	1700	1710	1720	1730	1740	1750	1760
ATIKLQSTVK	MKQTRKQYLH	LRAAALFIQQ	CYRSKKIAAQ	KREEYMQMRE	SCIKLQAFVR	GYLVRKQMRL	QRKAVISLQS
1770	1780	1790	1800	1810	1820	1830	1840
YFRMRKARQY	YLKMYKAIIV	IQNYYHAYKA	QVNQRKNFLQ	VKKAATCLQA	AYRGYKVRQL	IKQQSIAALK	IQSAFRGYNK
1850	1860	1870	1880	1890	1900	1910	1920
RVKYQSVLQS	IIKIQRWYRA	YKTLHDTRTH	FLKTKAAVIS	LQSAYRGWKV	RKQIRREHQA	ALKIQSAFRM	AKAQKQFRLF
1930	1940	1950	1960	1970	1980	1990	2000
KTAALVIQQN	FRAWTAGRKQ	CMEYIELRHA	VLVLQSMWKG	KTLRRQLQRQ	HKCAIIIQSY	YRMHVQQKKW	KIMKKAALLI
2010	2020	2030	2040	2050	2060	2070	2080
QKYYRAYSIG	REQNHLYLKT	KAAVVTLQSA	YRGMKVRKRI	KDCNKAAVTI	QSKYRAYKTK	KKYATYRASA	IIIQRWYRGI
2090	2100	2110	2120	2130	2140	2150	2160
KITNHQHKEY	LNLKKTAIKI	QSVYRGIRVR	RHIQHMHRAA	TFIKAMFKMH	QSRISYHTMR	KAAIVIQVRC	RAYYQGKMQR
2170	2180	2190	2200	2210	2220	2230	2240
EKYLTILKAV	KVLQASFRGV	RVRRTLRKMQ	TAATLIQSNY	RRYRQQTYFN	KLKKITKTVQ	QRYWAMKERN	IQFQRYNKLR
2250	2260	2270	2280	2290	2300	2310	2320
HSVIYIQAIF	RGKKARRHLK	MMHIAATLIQ	RRFRTLMMRR	RFLSLKKTAI	LIQRKYRAHL	CTKHHLQFLQ	VQNAVIKIQS
2330	2340	2350	2360	2370	2380	2390	2400

Detailed Protein Report

Cmpd.	No. of Cmpds.	m/z meas.	Δ m/z [ppm]	z	Rt [min]	Score	P	Range	Sequence	Modification	Ratios
402	1	711.3339	-88.13	2	34.3	14.6	2	178-189	K.TFSVSQKVDRVR.S		mdown:qdown 0.47

Detailed Protein Report

Protein 614: **PREDICTED: leucine-rich repeat and coiled-coil domain-containing protein 1 isoform X6 [Homo sapiens]**

**Accession:**	gi|530388606	**Score:**	25.6
**Database:**	refseq_human(refseq_human_20140103.fasta)	**MW [kDa]:**	77.0
**Database Date:**	2015-11-30	**pI:**	6.0
		**Sequence Coverage [%]:**	3.2
		**No. of unique Peptides:**	1

10	20	30	40	50	60	70	80
MKPPYLKELY	VSSSLANCPM	LQESEKPKTE	IIKVDQSHSE	DNTYQSLVEQ	LDQEREKRWR	AEQAENKLMD	YIDELHKHAN
**90**	**100**	**110**	**120**	**130**	**140**	**150**	**160**
EKEDIHSLAL	LTTDRLKEII	FRERNSKGQL	EVMVHKLQNE	IKKLTVELMK	AKDQQEDHLK	HLRTLEKTLE	KMERQKRQQQ
**170**	**180**	**190**	**200**	**210**	**220**	**230**	**240**
AAQIRLIQEV	ELKASAADRE	IYLLRTSLHR	EREQAQQLHQ	LLALKEQEHR	KELETREFFT	DADFQDALAK	EIAKEEKKHE
**250**	**260**	**270**	**280**	**290**	**300**	**310**	**320**
QMIKEYQEKI	DVLSQQYMDL	ENEFRIALTV	EARRFQDVKD	GFENVATELA	KSKHALIWAQ	RKENESSSLI	KDLTCMVKEQ
**330**	**340**	**350**	**360**	**370**	**380**	**390**	**400**
KTKLAEVSKL	KQETAANLQN	QINTLEILIE	DDKQKSIQIE	LLKHEKVQLI	SELAAKESLI	FGLRTERKVW	GHELAQQGSS
**410**	**420**	**430**	**440**	**450**	**460**	**470**	**480**
LAQNRGKLEA	QIESLSRENE	CLRKTNESDS	DALRIKCKII	DDQTETIRKL	KDCLQEKDEH	IKRLQEKITE	IEKCTQEQLD
**490**	**500**	**510**	**520**	**530**	**540**	**550**	**560**
EKSSQLDEVL	EKLERHNERK	EKLKQQLKGK	EVELEEIRKA	YSTLNRKWHD	KGELLCHLET	QVKEVKEKFE	NKEKKLKAER
**570**	**580**	**590**	**600**	**610**	**620**	**630**	**640**
DKSIELQKNA	MEKLHSMDDA	FKRQVDAIVE	AHQAEIAQLA	NEKQKCIDSA	NLKVHQIEKE	MRELLEETCK	NKKTMEAKIK
**650**	**660**						
QLAFALNEIQ	QDM						

Cmpd.	No. of Cmpds.	m/z meas.	Δ m/z [ppm]	z	Rt [min]	Score	P	Range	Sequence	Modification
2425	1	679.2638	-89.31	2	60.7	11.3	1	452-462	K.DCLQEKDEHIK.R	

Detailed Protein Report

Protein 615: PREDICTED: collagen alpha-1(XXII) chain isoform X2 [Homo sapiens]

Accession: gi|578815852
Database: refseq_human(refseq_human_20140103.fasta)
Database Date: 2015-11-30
Modification(s): Oxidation

Score: 25.6
MW [kDa]: 129.6
pI: 7.8
Sequence Coverage [%]: 3.7
No. of unique Peptides: 1

10	20	30	40	50	60	70	80
MAGLRGNAVA	GLLWMLLLWS	GGGGCQAQRA	GCKSVHYDLV	FLLDTSSSVG	KEDFEKVRQW	VANLVDTFEV	GPDRTRVGVV
**90**	**100**	**110**	**120**	**130**	**140**	**150**	**160**
RYSDRPTTAF	ELGLFGSQEE	VKAAARRLAY	HGGNTNTGDA	LRYITARSFS	PHAGGRPRDR	AYKQVAILLT	DGRSQDLVLD
**170**	**180**	**190**	**200**	**210**	**220**	**230**	**240**
AAAAAHRAGI	RIFAVGVGEA	LKEELEEIAS	EPKSAHVFHV	SDFNAIDKIR	GKLRRRLCEN	VLCPSVRVEG	DRFKHTNGGT
**250**	**260**	**270**	**280**	**290**	**300**	**310**	**320**
KEITGFDLMD	LFSVKEILGK	RENGAQSSYV	RMGSFPVVQS	TEDVFPQGLP	DEYAFVTTFR	FRKTSRKEDW	YIWQVIDQYS
**330**	**340**	**350**	**360**	**370**	**380**	**390**	**400**
IPQVSIRLDG	ENKAVEYNAV	AGPQGEKGDV	GPAGPPGVPG	SVVQQEGLKG	EQGAPGPRGH	QGAPGPPGAR	GPIGPEGRDG
**410**	**420**	**430**	**440**	**450**	**460**	**470**	**480**
PPGLQGLRGK	KGDMGPPGIP	GLLGLQGPPG	PPGVPGPPGP	GGSPGLPGEI	GFPGKPGPPG	PTGPPGKDGP	NGPPGPPGTK
**490**	**500**	**510**	**520**	**530**	**540**	**550**	**560**
GEPGERGEDG	LPGKPGLRGE	IGEQGLAGRP	GEKGEAGLPG	APGFPGVRGE	KGDQGEKGEL	GLPGLKGDRG	EKGEAGPAGP
**570**	**580**	**590**	**600**	**610**	**620**	**630**	**640**
PGLPGTTSLF	TPHPRMPGEQ	GPKGEKGDPG	LPGEPGLQGR	PGELGPQGPT	GPPGAKGQEG	AHGAPGAAGN	PGAPGHVGAP
**650**	**660**	**670**	**680**	**690**	**700**	**710**	**720**
GPSGPPGSVG	APGLRGTPGK	DGERGEKGAA	GEEGSPGPVG	PRGDPGAPGL	PGPPGKGKDG	EPGLRGSPGL	PGPLGTKGDR
**730**	**740**	**750**	**760**	**770**	**780**	**790**	**800**
GAPGIPGSPG	SRGDPGIGVA	GPPGPSGPPG	DKGSPGSRGL	PGFPGPQGPA	GRDGAPGNPG	ERGPPGKPGL	SSLLSPGDIN
**810**	**820**	**830**	**840**	**850**	**860**	**870**	**880**
LLAKDVCNDC	PPGPPGLPGL	PGFKGDKGVP	GKPGREGTEG	KKGEAGPPGL	PGPPGIAGPQ	GSQGERGADG	EVGQKGDQGH
**890**	**900**	**910**	**920**	**930**	**940**	**950**	**960**
PGVPGFMGPP	GNPGPPGADG	IAGAAGPPGI	QGSPGKEGPP	GPQGPSGLPG	IPGEEGKEGR	DGKPGPPGEP	GKAGEPGLPG
**970**	**980**	**990**	**1000**	**1010**	**1020**	**1030**	**1040**
PEGARGPPGF	KGHTGDSGAP	GPRGESGAMG	LPGQEGLPGK	DGDTGPTGPQ	GPQGPRGPPG	KNGSPGSPGE	PGPSGTPGQK
**1050**	**1060**	**1070**	**1080**	**1090**	**1100**	**1110**	**1120**
GSKGENGSPG	LPGFLGPRGP	PGEPGEKGVP	GKEGVPGKPG	EPGFKGERGD	PGIKGDKGPP	GGKGQPGDPG	IPGHKGHTGL
**1130**	**1140**	**1150**	**1160**	**1170**	**1180**	**1190**	**1200**
MGPQGLPGEN	GPVGPPGPPG	QPGFPGLRGE	SPSMETLRRL	IQEELGKQLE	TRLAYLLAQM	PPAYMKSSQG	RPGPPGPPGK
**1210**	**1220**	**1230**	**1240**	**1250**	**1260**	**1270**	**1280**
DGLPGRAGPM	GEPGRPGQGG	LEGPSGPIGP	KGERGAKGDP	GAPGVGLRGE	MGPPGIPGQP	GEPGYAKDGL	PGIPGPQGET
**1290**	**1300**	**1310**	**1320**				
GPAGHPGLPG	PPGPPGQCDP	SQCAYFASLA	ARPGNVKGP				

Cmpd.	No. of Cmpds.	m/z meas.	Δ m/z [ppm]	z	Rt [min]	Score	P	Range	Sequence	Modification
1945	1	927.8884	-53.65	2	53.0	13.2	0	1249-1267	R.GEMGPPGIPGQPGEPGYAK.D	Oxidation: 3

Detailed Protein Report

Protein 616: PREDICTED: alpha-tectorin isoform X1 [Homo sapiens]

**Accession:**	gi|530398338	**Score:**	25.6
**Database:**	refseq_human(refseq_human_20140103.fasta)	**MW [kDa]:**	238.8
**Database Date:**	2015-11-30	**pI:**	5.1
**Modification(s):**	Carbamidomethyl	**Sequence Coverage [%]:**	1.3
		**No. of unique Peptides:**	1

Quantitation

Wdown:Qdown	**Median:** 1.63	**CV:** 0.00 %	**No. of Peptides:** 1

Detailed Protein Report

10	20	30	40	50	60	70	80
MNYSSFLRIW	VSFIFALVQH	QAQPRELMYP	FWQNDTKTPK	VDDGSSSEIK	LAIPVFFFGV	PYRTVYVNNN	GVVSFNVLVS
90	100	110	120	130	140	150	160
QFTPESFPLT	DGRAFVAPFW	ADVHNGIRGE	IYYRETMEPA	ILKRATKDIR	KYFKDMATFS	ATWVFIVTWE	EVTFYGGSST
170	180	190	200	210	220	230	240
TPVNTFQAVL	VSDGSYTFTL	FNYYEINWTT	GTASGGDPLT	GLGGVMAQAG	FNGGNLTNFF	SLPGSRTPEI	VNIQETTNVN
250	260	270	280	290	300	310	320
VPGRWAFKVD	GKEIDPANGC	TSRGQFLRRG	EVFWDDLNCT	VKCRCLDFNN	EIYCQEASCS	PYEVCEPKGK	FFYCSAVETS
330	340	350	360	370	380	390	400
TCVVFGEPHY	HTFDGFLFHF	QGSCAYLLAR	QCLQTSSLPF	FSVEAKNEHR	RGSAVSWVKE	LSVEVNGYKI	LIPKGSYGRV
410	420	430	440	450	460	470	480
KVNDLVTSLP	VTLDLGTVKI	YQSGISTAVE	TDFGLLVTFD	GQHYASISVP	GSYINSTCGL	CGNYNKNPLD	DFLRPDGRPA
490	500	510	520	530	540	550	560
MSVLDLGESW	RVYHADWKCD	SGCVDNCTQC	DAATEALYFG	SDYCGFLNKT	DGPLWECGTV	VDPTAFVHSC	VYDLCSVRDN
570	580	590	600	610	620	630	640
GTLLCQAIQA	YALVCQALGI	PIGDWRTQTG	CVSTVQCPSF	SHYSVCTSSC	PDTCSDLTAS	RNCATPCTEG	CECNQGFVLS
650	660	670	680	690	700	710	720
TSQCVPLHKC	GCDFDGHYYT	MGEFFWATAN	CTVQCLCEEG	GDVYCFNKTC	GSGEVCAVED	GYQGCFPKRE	TVCLLSQNQV
730	740	750	760	770	780	790	800
LHTFDGASYA	FPSEFSYTLL	KTCPERPEYL	EIDINKKKPD	AGPAWLRGLR	ILVADQEVKI	GGIGASEVKL	NGQEVELPFF
810	820	830	840	850	860	870	880
HPSGKLEIYR	NKNSTTVESK	GVVTVQYSDI	GLLYIRLSTT	YFNCTGGLCG	FYNANASDEF	CLPNGKCTDN	LAVFLESWTT
890	900	910	920	930	940	950	960
FEEICNGECG	DLLKACNNDS	ELLKFYRSRS	RCGIINDPSN	SSFLECHGVV	NVTAYYRTCL	FRLCQSGGNE	SELCDSVARY
970	980	990	1000	1010	1020	1030	1040
ASACKNADVE	VGPWRTYDFC	PLECPENSHF	EECITCTETC	ETLTLGPICV	DSCSEGCQCD	EGYALLGSQC	VTRSECGCNF
1050	1060	1070	1080	1090	1100	1110	1120
EGHQLATNET	FWVDLDCQIF	CYCSGTDNRV	HCETIPCKDD	EYCMEEGGLY	YCQARTDASC	IVSGYGHYLT	FDGFPFDFQT
1130	1140	1150	1160	1170	1180	1190	1200
SCPLILCTTG	SRPSSDSFPK	FVVTAKNEDR	DPSLALWVKQ	VDVTVFGYSI	VIHRAYKHTV	LVNSERLYLP	LKLGQGKINI
1210	1220	1230	1240	1250	1260	1270	1280
FSFGFHVVVE	TDFGLKVVYD	WKTFLSITVP	RSMQNSTYGL	CGRYNGNPDD	DLEMPMGLLA	SSVNEFGQSW	VKRDTFCQVG
1290	1300	1310	1320	1330	1340	1350	1360
CGDRCPSCAK	VEGFSKVQQL	CSLIPNQNAA	FSKCHSKVNP	TFFYKNCLFD	SCIDGGAVQT	ACSWLQNYAS	TCQTQGITVT
1370	1380	1390	1400	1410	1420	1430	1440
GWRNYTSCTV	TCPPNSHYES	CVSVCQPRCA	AIRLKSDCSH	YCVEGCHCDA	GYVLNGKSCI	LPHSCGCYSD	GKYYEPKQLF
1450	1460	1470	1480	1490	1500	1510	1520
WNSDCTRRCR	CFRRNVIQCD	PRQCKSDEEC	ALRNGVRGCF	STKTSYCLAA	GGGVFRTFDG	AFLRFPANCA	FVLSTICQKL
1530	1540	1550	1560	1570	1580	1590	1600
PDISFQLIIN	FDKWSAPNLT	IISPVYFYIN	EEQILINDRN	TVKVNGTQVN	VPFITGLATK	IYSSEGFLVI	DTSPDIQIYY
1610	1620	1630	1640	1650	1660	1670	1680
NGFNVIKISI	SERLQNKVCG	LCGNFNGDLT	DDYVTLRGKP	VVSSVVLAQS	WKTNGMQKSC	NELQFSQYAA	MCDNVHIQKM
1690	1700	1710	1720	1730	1740	1750	1760
QGDGYCLKLT	DMKGFFQPCY	GLLDPLPFYE	SCYLDGCYSH	KKFQLCGSLA	AYGEACRSFG	ILSTEWIEKE	NCSGVVEDPC
1770	1780	1790	1800	1810	1820	1830	1840
VGADCPNRTC	ELGNGRELCG	CIEPPPYGNN	SHDIIDAEVT	CKAAQMEVSI	SKCKLFQLGF	EREGVRINDR	QCTGIEGEDF
1850	1860	1870	1880	1890	1900	1910	1920
ISFQINNTKG	NCGNIVQSNG	THIMYKNTLW	IESANNTGNI	ITRDRTINVE	FSCAYELDIK	ISLDSVVKPM	LSVINLTVPT
1930	1940	1950	1960	1970	1980	1990	2000
QEGSFITKMA	LYKNASYKHP	YRQGEVVLTT	RDVLYVGVFV	VGADATHLIL	TLNKCYATPT	RDSNDKLRYF	IIEGGCQNLK
2010	2020	2030	2040	2050	2060	2070	2080
DNTIGIEENA	VSLTCRFHVT	VFKFIGDYDE	VHLHCAVSLC	DSEKYSCKIT	CPHNSRIATD	YTKEPKEQII	SVGPIRRKRL
2090	2100	2110	2120	2130	2140	2150	2160
DWCEDNGGCE	QICTSRVDGP	LCSCVTGTLQ	EDGKSCRASN	SSMELQVWTL	LLIMIQISLW	HFVYKSGTTS	

Detailed Protein Report

Cmpd.	No. of Cmpds.	m/z meas.	Δ m/z [ppm]	z	Rt [min]	Score	P	Range	Sequence	Modification	Ratios
132	1	673.3206	73.69	3	30.4	10.7	1	1274-1290	R.DTFCQVGCGDRCPSCAK.V	Carbamidomethyl: 4, 8, 12, 15	Wdown:Qdown 1.63

Detailed Protein Report

Protein 617: PREDICTED: protein TANC2 isoform X9 [Homo sapiens]

**Accession:**	gi|578830697	**Score:**	25.6
**Database:**	refseq_human(refseq_human_20140103.fasta)	**MW [kDa]:**	154.7
**Database Date:**	2015-11-30	**pI:**	8.9
**Modification(s):**	Oxidation	**Sequence Coverage [%]:**	2.4
		**No. of unique Peptides:**	1

10	20	30	40	50	60	70	80
MFRNSLKMLL	TGGKSSRKNR	SSDGGSEEPP	DRRQSSVDSR	QSRSGQGGIS	TESDCAFEPD	YAVPPLPVSE	GMQHIRIMEG
**90**	**100**	**110**	**120**	**130**	**140**	**150**	**160**
MSRSLPSSPL	LTHQSISVRL	QPVKKLTAPL	RKAKFVESPR	IPESELGSPT	LTSAQKLDVD	AYCPGDAEQE	LGPPPSVDEA
**170**	**180**	**190**	**200**	**210**	**220**	**230**	**240**
ANTLMTRLGF	LLGEKVTEVQ	PGDQYSMEVQ	DENQTSAITQ	RISPCSTLTS	STASPPASSP	CSTLPPISTN	ATAKDCSYGA
**250**	**260**	**270**	**280**	**290**	**300**	**310**	**320**
VTSPTSTLES	RDSGIIATLT	SYSENVERTK	YAGESSKELG	SGGNIKPWQS	QKSSMDSCLY	RVDENMTAST	YSLNKIPERN
**330**	**340**	**350**	**360**	**370**	**380**	**390**	**400**
LETVLSQSVQ	SIPLYLMPRP	NSVAATSSAH	LEDLAYLDEQ	RHTPLRTSLR	MPRQSMGGAR	TQQDLRVRFA	PYRPPDISLK
**410**	**420**	**430**	**440**	**450**	**460**	**470**	**480**
PLLFEVPSIT	TESVFVGRDW	VFHEIDAQLQ	SSNASVNQGV	VIVGNIGFGK	TAIISRLVAL	SCHGTRMRQI	ASDSPHASPK
**490**	**500**	**510**	**520**	**530**	**540**	**550**	**560**
HVDANRELPL	TQPPSAHSSI	TSGSCPGTPE	MRRRQEEAMR	RLASQVVAYH	YCQADNAYTC	LVPEFVHNVA	ALLCRSPQLT
**570**	**580**	**590**	**600**	**610**	**620**	**630**	**640**
AYREQLLREP	HLQSMLSLRS	CVQDPMASFR	RGVLEPLENL	HKERKIPDED	FIILIDGLNE	AEFHKPDYGD	TIVSFLSKMI
**650**	**660**	**670**	**680**	**690**	**700**	**710**	**720**
GKFPSWLKLI	VTVRTSLQEI	TKLLPFHRIF	LDRLEENEAI	DQDLQAYILH	RIHSSSEIQN	NISLNGKMDN	TTFGKLSSHL
**730**	**740**	**750**	**760**	**770**	**780**	**790**	**800**
KTLSQGSYLY	LKLTFDLIEK	GYLVLKSSSY	KVVPVSLSEV	YLLQCNMKFP	TQSSFDRVMP	LLNVAVASLH	PLTDEHIFQA
**810**	**820**	**830**	**840**	**850**	**860**	**870**	**880**
INAGSIEGTL	EWEDFQQRME	NLSMFLIKRR	DMTRMFVHPS	FREWLIWREE	GEKTKFLCDP	RSGHTLLAFW	FSRQEGKLNR
**890**	**900**	**910**	**920**	**930**	**940**	**950**	**960**
QQTIELGHHI	LKAHIFKGLS	KKVGVSSSIL	QGLWISYSTE	GLSMALASLR	NLYTPNIKVS	RLLILGGANI	NYRTEVLNNA
**970**	**980**	**990**	**1000**	**1010**	**1020**	**1030**	**1040**
PILCVQSHLG	YTEMVALLLE	FGANVDASSE	SGLTPLGYAA	AAGYLSIVVL	LCKKRAKVDH	LDKNGQCALV	HAALRGHLEV
**1050**	**1060**	**1070**	**1080**	**1090**	**1100**	**1110**	**1120**
VKFLIQCDWT	MAGQQQGVFK	KSHAIQQALI	AAASMGYTEI	VSYLLDLPEK	DEEEVERAQI	NSFDSLWGET	ALTAAAGRGK
**1130**	**1140**	**1150**	**1160**	**1170**	**1180**	**1190**	**1200**
LEVCRLLLEQ	GAAVAQPNRR	GAVPLFSTVR	QGHWQIVDLL	LTHGADVNMA	DKQGRTPLMM	AASEGHLGTV	DFLLAQGASI
**1210**	**1220**	**1230**	**1240**	**1250**	**1260**	**1270**	**1280**
ALMDKEGLTA	LSWACLKGHL	SVVRSLVDNG	AATDHADKNG	RTPLDLAAFY	GDAEVVQFLV	DHGAMIEHVD	YSGMRPLDRA
**1290**	**1300**	**1310**	**1320**	**1330**	**1340**	**1350**	**1360**
VGCRNTSVVV	TLLKKGAKIG	PATWAMATSK	PDIMIILLSK	LMEEGDMFYK	KGKVKEAAQR	YQYALKKFPR	EGFGEDLKTF
**1370**	**1380**	**1390**	**1400**				
RELKVSLLLN	LSRCRRKMNT	VRSSLRGPER	GHQAVSQQP				

Cmpd.	No. of Cmpds.	m/z meas.	Δ m/z [ppm]	z	Rt [min]	Score	P	Range	Sequence	Modification
1044	1	983.4577	-74.07	2	41.8	11.9	1	564-579	R.EQLLREPHLQSMLSLR.S	Oxidation: 12

Detailed Protein Report

Protein 618: kinase D-interacting substrate of 220 kDa [Homo sapiens]

**Accession:**	gi|55741641	**Score:**	25.5
**Database:**	refseq_human(refseq_human_20140103.fasta)	**MW [kDa]:**	196.4
**Database Date:**	2015-11-30	**pI:**	6.2
**Modification(s):**	Oxidation	**Sequence Coverage [%]:**	1.9
		**No. of unique Peptides:**	1

10	20	30	40	50	60	70	80
MSVLISQSVI	NYVEEENIPA	LKALLEKCKD	VDERNECGQT	PLMIAAEQGN	LEIVKELIKN	GANCNLEDLD	NWTALISASK
90	100	110	120	130	140	150	160
EGHVHIVEEL	LKCGVNLEHR	DMGGWTALMW	ACYKGRTDVV	ELLLSHGANP	SVTGLYSVYP	IIWAAGRGHA	DIVHLLLQNG
170	180	190	200	210	220	230	240
AKVNCSDKYG	TTPLVWAARK	GHLECVKHLL	AMGADVDQEG	ANSMTALIVA	VKGGYTQSVK	EILKRNPNVN	LTDKDGNTAL
250	260	270	280	290	300	310	320
MIASKEGHTE	IVQDLLDAGT	YVNIPDRSGD	TVLIGAVRGG	HVEIVRALLQ	KYADIDIRGQ	DNKTALYWAV	EKGNATMVRD
330	340	350	360	370	380	390	400
ILQCNPDTEI	CTKDGETPLI	KATKMRNIEV	VELLLDKGAK	VSAVDKKGDT	PLHIAIRGRS	RKLAELLLRN	PKDGRLLYRP
410	420	430	440	450	460	470	480
NKAGETPYNI	DCSHQKSILT	QIFGARHLSP	TETDGDMLGY	DLYSSALADI	LSEPTMQPPI	CVGLYAQWGS	GKSFLLKKLE
490	500	510	520	530	540	550	560
DEMKTFAGQQ	IEPLFQFSWL	IVFLTLLLCG	GLGLLFAFTV	HPNLGIAVSL	SFLALLYIFF	IVIYFGGRRE	GESWNWAWVL
570	580	590	600	610	620	630	640
STRLARHIGY	LELLLKLMFV	NPPELPEQTT	KALPVRFLFT	DYNRLSSVGG	ETSLAEMIAT	LSDACEREFG	FLATRLFRVF
650	660	670	680	690	700	710	720
KTEDTQGKKK	WKKTCCLPSF	VIFLFIIGCI	ISGITLLAIF	RVDPKHLTVN	AVLISIASVV	GLAFVLNCRT	WWQVLDSLLN
730	740	750	760	770	780	790	800
SQRKRLHNAA	SKLHKLKSEG	FMKVLKCEVE	LMARMAKTID	SFTQNQTRLV	VIIDGLDACE	QDKVLQMLDT	VRVLFSKGPF
810	820	830	840	850	860	870	880
IAIFASDPHI	IIKAINQNLN	SVLRDSNING	HDYMRNIVHL	PVFLNSRGLS	NARKFLVTSA	TNGDVPCSDT	TGIQEDADRR
890	900	910	920	930	940	950	960
VSQNSLGEMT	KLGSKTALNR	RDTYRRRQMQ	RTITRQMSFD	LTKLLVTEDW	FSDISPQTMR	RLLNIVSVTG	RLLRANQISF
970	980	990	1000	1010	1020	1030	1040
NWDRLASWIN	LTEQWPYRTS	WLILYLEETE	GIPDQMTLKT	IYERISKNIP	TTKDVEPLLE	IDGDIRNFEV	FLSSRTPVLV
1050	1060	1070	1080	1090	1100	1110	1120
ARDVKVFLPC	TVNLDPKLRE	IIADVRAARE	QISIGGLAYP	PLPLHEGPPR	APSGYSQPPS	VCSSTSFNGP	FAGGVVSPQP
1130	1140	1150	1160	1170	1180	1190	1200
HSSYYSGMTG	PQHPFYNRPF	FAPYLYTPRY	YPGGSQHLIS	RPSVKTSLPR	DQNNGLEVIK	EDAAEGLSSP	TDSSRGSGPA
1210	1220	1230	1240	1250	1260	1270	1280
PGPVVLLNSL	NVDAVCEKLK	QIEGLDQSML	PQYCTTIKKA	NINGRVLAQC	NIDELKKEMN	MNFGDWHLFR	STVLEMRNAE
1290	1300	1310	1320	1330	1340	1350	1360
SHVVPEDPRF	LSESSSGPAP	HGEPARRASH	NELPHTELSS	QTPYTLNFSF	EELNTLGLDE	GAPRHSNLSW	QSQTRRTPSL
1370	1380	1390	1400	1410	1420	1430	1440
SSLNSQDSSI	EISKLTDKVQ	AEYRDAYREY	IAQMSQLEGG	PGSTTISGRS	SPHSTYYMGQ	SSSGGSIHSN	LEQEKGKDSE
1450	1460	1470	1480	1490	1500	1510	1520
PKPDDGRKSF	LMKRGDVIDY	SSSGVSTNDA	SPLDPITEED	EKSDQSGSKL	LPGKKSSERS	SLFQTDLKLK	GSGLRYQKLP
1530	1540	1550	1560	1570	1580	1590	1600
SDEDESGTEE	SDNTPLLKDD	KDRKAEGKVE	RVPKSPEHSA	EPIRTFIKAK	EYLSDALLDK	KDSSDSGVRS	SESSPNHSLH
1610	1620	1630	1640	1650	1660	1670	1680
NEVADDSQLE	KANLIELEDD	SHSGKRGIPH	SLSGLQDPII	ARMSICSEDK	KSPSECSLIA	SSPEENWPAC	QKAYNLNRTP
1690	1700	1710	1720	1730	1740	1750	1760
STVTLNNNSA	PANRANQNFD	EMEGIRETSQ	VILRPSSSPN	PTTIQNENLK	SMTHKRSQRS	SYTRLSKDPP	ELHAAASSES
1770	1780						
TGFGEERESI	L						

Cmpd.	No. of	m/z meas.	Δ m/z	z	Rt	Score	P	Range	Sequence	Modification

Detailed Protein Report

	Cmpds.		[ppm]		[min]					
1150	1	1137.7077	130.83	2	44.0	14.9	0	35-55	R.NECGQTPLMIAAEQGNLEIVK.E	Oxidation: 9

Detailed Protein Report

Protein 619: sterile alpha motif domain-containing protein 9 [Homo sapiens]

**Accession:**	gi|38201706	**Score:**	25.5
**Database:**	refseq_human(refseq_human_20140103.fasta)	**MW [kDa]:**	184.2
**Database Date:**	2015-11-30	**pI:**	8.7
**Modification(s):**	Carbamidomethyl, Oxidation	**Sequence Coverage [%]:**	2.1
		**No. of unique Peptides:**	2

Quantitation

mdown:qdown	**Median:**	1.09	**CV:**	0.00 %	**No. of Peptides:**	1
Wdown:Qdown	**Median:**	1.76	**CV:**	0.00 %	**No. of Peptides:**	1

Alias proteins:

Accession	Name	Description
gi|300863105	refseq_human (refseq_human_20140103.fasta)	sterile alpha motif domain-containing protein 9 [Homo sapiens]

10	20	30	40	50	60	70	80
MAKQLNLPEN	TDDWTKEDVN	QWLESHKIDQ	KHREILTEQD	VNGAVLKWLK	KEHLVDMGIT	HGPAIQIEEL	FKELRKTAIE
90	100	110	120	130	140	150	160
DSIQTSKMGK	PSKNAPKDQT	VSQKERRETS	KQKQKGKENP	DMANPSAMST	TAKGSKSLKV	ELIEDKIDYT	KERQPSIDLT
170	180	190	200	210	220	230	240
CVSYPFDEFS	NPYRYKLDFS	LQPETGPGNL	IDPIHEFKAF	TNTATATEED	VKMKFSNEVF	RFASACMNSR	TNGTIHFGVK
250	260	270	280	290	300	310	320
DKPHGKIVGI	KVTNDTKEAL	INHFNLMINK	YFEDHQVQQA	KKCIREPRFV	EVLLPNSTLS	DRFVIEVDII	PQFSECQYDY
330	340	350	360	370	380	390	400
FQIKMQNYNN	KIWEQSKKFS	LFVRDGTSSK	DITKNKVDFR	AFKADFKTLA	ESRKAAEEKF	RAKTNKKERE	GPKLVKLLTG
410	420	430	440	450	460	470	480
NQDLLDNSYY	EQYILVTNKC	HPDQTKHLDF	LKEIKWFAVL	EFDPESNING	VVKAYKESRV	ANLHFPSVYV	EQKTTPNETI
490	500	510	520	530	540	550	560
STLNLYHQPS	WIFCNGRLDL	DSEKYKPFDP	SSWQRERASD	VRKLISFLTH	EDIMPRGKFL	VVFLLLSSVD	DPRDPLIETF
570	580	590	600	610	620	630	640
CAFYQDLKGM	ENILCICVHP	HIFQGWKDLL	EARLIKHQDE	ISSQCISALS	LEEINGTILK	LKSVTQSSKR	LLPSIGLSTV
650	660	670	680	690	700	710	720
LLKKEEDIMT	ALEIICENEC	EGTLLEKDKN	KFLEFKASKE	EDFYRGGKVS	WWNFYFSSES	YSSPFVKRDK	YERLEAMIQN
730	740	750	760	770	780	790	800
CADSSKPTST	KIIHLYHHPG	CGGTTLAMHI	LWELRKKFRC	AVLKNKTVDF	SEIGEQVTSL	ITYGAMNRQE	YVPVLLLVDD
810	820	830	840	850	860	870	880
FEEQDNVYLL	QYSIQTAIAK	KYIRYEKPLV	IILNCMRSQN	PEKSARIPDS	IAVIQQLSPK	EQRAFELKLK	EIKEQHKNFE
890	900	910	920	930	940	950	960
DFYSFMIMKT	NFNKEYIENV	VRNILKGQNI	FTKEAKLFSF	LALLNSYVPD	TTISLSQCEK	FLGIGNKKAF	WGTEKFEDKM
970	980	990	1000	1010	1020	1030	1040
GTYSTILIKT	EVIECGNYCG	VRIIHSLIAE	FSLEELKKSY	HLNKSQIMLD	MLTENLFFDT	GMGKSKFLQD	MHTLLLTRHR
1050	1060	1070	1080	1090	1100	1110	1120
DEHEGETGNW	FSPFIEALHK	DEGNEAVEAV	LLESIHRFNP	NAFICQALAR	HFYIKKKDFG	NALNWAKQAK	IIEPDNSYIS
1130	1140	1150	1160	1170	1180	1190	1200
DTLGQVYKSK	IRWWIEENGG	NGNISVDDLI	ALLDLAEHAS	SAFKESQQQS	EDREYEVKER	LYPKSKRRYD	TYNIAGYQGE
1210	1220	1230	1240	1250	1260	1270	1280
IEVGLYTIQI	LQLIPFFDNK	NELSKRYMVN	FVSGSSDIPG	DPNNEYKLAL	KNYIPYLTKL	KFSLKKSFDF	FDEYFVLLKP
1290	1300	1310	1320	1330	1340	1350	1360
RNNIKQNEEA	KTRRKVAGYF	KKYVDIFCLL	EESQNNTGLG	SKFSEPLQVE	RCRRNLVALK	ADKFSGLLEY	LIKSQEDAIS
1370	1380	1390	1400	1410	1420	1430	1440
TMKCIVNEYT	FLLEQCTVKI	QSKEKLNFIL	ANIILSCIQP	TSRLVKPVEK	LKDQLREVLQ	PIGLTYQFSE	PYFLASLLFW
1450	1460	1470	1480	1490	1500	1510	1520
PENQQLDQHS	EQMKEYAQAL	KNSFKGQYKH	MHRTKQPIAY	FFLGKGKRLE	RLVHKGKIDQ	CFKKTPDINS	LWQSGDVWKE
1530	1540	1550	1560	1570	1580	1590	
EKVQELLLRL	QGRAENNCLY	IEYGINEKIT	IPITPAFLGQ	LRSGRSIEKV	SFYLGFSIGG	PLAYDIEIV	

Detailed Protein Report

Cmpd.	No. of Cmpds.	m/z meas.	Δ m/z [ppm]	z	Rt [min]	Score	P	Range	Sequence	Modification	Ratios
2028	2	840.7762	-104.20	2	55.5	13.9	0	118-133	K.ENPDMANPSAMSTTAK.G	Oxidation: 5	
1785	1	660.9025	-122.86	3	50.8	11.7	0	714-731	R.LEAMIQNCADSSKPTSTK.I	Carbamidomethyl: 8	Wdown:Qdown 1.76<br>mdown:qdown 1.09

Detailed Protein Report

Protein 620: regulator of G-protein signaling 12 isoform 2 [Homo sapiens]

**Accession:**	gi|4506509	**Score:**	25.5
**Database:**	refseq_human(refseq_human_20140103.fasta)	**MW [kDa]:**	149.5
**Database Date:**	2015-11-30	**pI:**	8.0
		**Sequence Coverage [%]:**	2.3
		**No. of unique Peptides:**	1

Quantitation

mdown:qdown	**Median:**	1.47	**CV:**	0.00 %	**No. of Peptides:**	1
Wdown:Qdown	**Median:**	1.74	**CV:**	0.00 %	**No. of Peptides:**	1

10	20	30	40	50	60	70	80
MFRAGEASKR	PLPGPSPPRV	RSVEVARGRA	GYGFTLSGQA	PCVLSCVMRG	SPADFVGLRA	GDQILAVNEI	NVKKASHEDV
90	100	110	120	130	140	150	160
VKLIGKCSGV	LHMVIAEGVG	RFESCSSDEE	GGLYEGKGWL	KPKLDSKALG	INRAERVVEE	MQSGGIFNMI	FENPSLCASN
170	180	190	200	210	220	230	240
SEPLKLKQRS	LSESAATRFD	VGHESINNPN	PNMLSKEEIS	KVIHDDSVFS	IGLESHDDFA	LDASILNVAM	IVGYLGSIEL
250	260	270	280	290	300	310	320
PSTSSNLESD	SLQAIRGCMR	RLRAEQKIHS	LVTMKIMHDC	VQLSTDKAGV	VAEYPAEKLA	FSAVCPDDRR	FFGLVTMQTN
330	340	350	360	370	380	390	400
DDGSLAQEEE	GALRTSCHVF	MVDPDLFNHK	IHQGIARRFG	FECTADPDTN	GCLEFPASSL	PVLQFISVLY	RDMGELIEGM
410	420	430	440	450	460	470	480
RARAFLDGDA	DAHQNNSTSS	NSDSGIGNFH	QEEKSNRVLV	VDLGGSSSRH	GPGGSAWDGV	GGRGAQPWGA	PWTGPFCPDP
490	500	510	520	530	540	550	560
EGSPPFEAAH	QTDRFWDLNK	HLGPASPVEV	PPASLRSSVP	PSKRGTVGAG	CGFNQRWLPV	HVLREWQCGH	TSDQDSYTDS
570	580	590	600	610	620	630	640
TDGWSSINCG	TLPPPMSKIP	ADRYRVEGSF	AQPPLNAPKR	EWSRKAFGMQ	SIFGPHRNVR	KTKEDKKGSK	FGRGTGLTQP
650	660	670	680	690	700	710	720
SQRTSARRSF	GRSKRFSITR	SLDDLESATV	SDGELTGADL	KDCVSNNSLS	SNASLPSVQS	CRRLRERRVA	SWAVSFERLL
730	740	750	760	770	780	790	800
QDPVGVRYFS	DFLRKEFSEE	NILFWQACEY	FNHVPAHDKK	ELSYRAREIF	SKFLCSKATT	PVNIDSQAQL	ADDVLRAPHP
810	820	830	840	850	860	870	880
DMFKEQQLQI	FNLMKFDSYT	RFLKSPLYQE	CILAEVEGRA	LPDSQQVPSS	PASKHSLGSD	HSSVSTPKKL	SGKSKSGRSL
890	900	910	920	930	940	950	960
NEELGDEDSE	KKRKGAFFSW	SRTRSTGRSQ	KKREHGDHAD	DALHANGGLC	RRESQGSVSS	AGSLDLSEAC	RTLAPEKDKA
970	980	990	1000	1010	1020	1030	1040
TKHCCIHLPD	GTSCVVAVKA	GFSIKDILSG	LCERHGINGA	AADLFLVGGD	KPLVLHQDSS	ILESRDLRLE	KRTLFRLDLV
1050	1060	1070	1080	1090	1100	1110	1120
PINRSVGLKA	KPTKPVTEVL	RPVVARYGLD	LSGLLVRLSG	EKEPLDLGAP	ISSLDGQRVV	LEEKDPSRGK	ASADKQKGVP
1130	1140	1150	1160	1170	1180	1190	1200
VKQNTAVNSS	SRNHSATGEE	RTLGKSNSIK	IKGENGKNAR	DPRLSKREES	IAKIGKKKYQ	KINLDEAEEF	FELISKAQSN
1210	1220	1230	1240	1250	1260	1270	1280
RADDQRGLLR	KEDLVLPEFL	RLPPGSTELT	LPTPAAVAKG	FSKRSATGNG	RESASQPGEQ	WEPVQESSDS	PSTSPGSASS
1290	1300	1310	1320	1330	1340	1350	1360
PPGPPGTTPP	GQKSPSGPFC	TPQSPVSLAQ	EGTAQIWKRQ	SQEVEAGGIQ	TVEDEHVAEL	TLMGEGDISS	PNSTLLPPPS
1370	1380						
TPQEVPGPSR	PGTSRF						

Cmpd.	No. of Cmpds.	m/z meas.	Δ m/z [ppm]	z	Rt [min]	Score	P	Range	Sequence	Modification	Ratios
592	1	607.1554	-246.73	2	36.0	14.3	2	1153-1163	K.GENGKNARDPR.L		mdown:qdown 1.47<br>Wdown:Qdown 1.74

Detailed Protein Report

Protein 621: PREDICTED: protein MAATS1 isoform X2 [Homo sapiens]

**Accession:**	gi|578807867	**Score:**	25.5
**Database:**	refseq_human(refseq_human_20140103.fasta)	**MW [kDa]:**	62.4
**Database Date:**	2015-11-30	**pI:**	9.2
		**Sequence Coverage [%]:**	3.0
		**No. of unique Peptides:**	1

10	20	30	40	50	60	70	80
MIERAREKRA	WEASLPALSD	TSQFEKRRKM	MNEMERKEWA	FREQEIEKLQ	EIRLEVLKEL	LRKREENQNE	VNMKHLNARW
**90**	**100**	**110**	**120**	**130**	**140**	**150**	**160**
SKLQEGKEAK	MAKIQRTHVS	SNAIRKLVGK	RKNIEGKLER	RNIIKDYSDY	ASQVYGPLSR	LGCFPDNNSE	DFVVKNYYLN
**170**	**180**	**190**	**200**	**210**	**220**	**230**	**240**
TYEGLVELES	CLPDFVTQPQ	IRAPKPKVIT	TKAGFLKRAA	RLDYELAEVH	KALLDKKNKV	LEVKKPPRFL	QRNPIPQPRL
**250**	**260**	**270**	**280**	**290**	**300**	**310**	**320**
PTPTLEMTSN	EEEEMEMAVI	YLQKLLRGRV	VQNMMFEGKE	KRLELIQELR	TCHALQEDEK	LVKKAEKQVT	LALQRQRNLH
**330**	**340**	**350**	**360**	**370**	**380**	**390**	**400**
EHKVSLVENH	LAGLEGRALA	DMFDFLSKEL	VRLQEERRIH	AFVMLAERQR	RVREAEESGR	RQVEKQRLRE	EDEIFKEVVK
**410**	**420**	**430**	**440**	**450**	**460**	**470**	**480**
VHHSTISSYL	EDIILNTEAN	TAEEQARAEI	EKMAEKINDI	AYEMESRRTY	LQSEEIVAEL	VYSFLIPEVQ	KYFVKEKVRN
**490**	**500**	**510**	**520**	**530**	**540**		
AQRKHILAAH	QIIHSYTESM	VQKKLTEGEQ	DEASNAAMLL	EKETQNENNS			

Cmpd.	No. of Cmpds.	m/z meas.	Δ m/z [ppm]	z	Rt [min]	Score	P	Range	Sequence	Modification
2124	1	652.6392	-14.46	3	55.1	11.1	2	433-448	K.MAEKINDIAYEMESRR.T	

Detailed Protein Report

Protein 622: catenin delta-2 isoform 4 [Homo sapiens]

**Accession:**	gi|570359551	**Score:**	25.5
**Database:**	refseq_human(refseq_human_20140103.fasta)	**MW [kDa]:**	87.2
**Database Date:**	2015-11-30	**pI:**	9.4
**Modification(s):**	Carbamidomethyl	**Sequence Coverage [%]:**	5.8
		**No. of unique Peptides:**	1

10	20	30	40	50	60	70	80
MRSLSQSQGD	PLPPAHTGTY	RTSTAPSSPG	VDSVPLQRTG	SQHGPQNAAA	ATFQRASYAA	GPASNYADPY	RQLQYCPSVE
**90**	**100**	**110**	**120**	**130**	**140**	**150**	**160**
SPYSKSGPAL	PPEGTLARSP	SIDSIQKDPR	EFGWRDPELP	EVIQMLQHQF	PSVQSNAAAY	LQHLCFGDNK	IKAEIRRQGG
**170**	**180**	**190**	**200**	**210**	**220**	**230**	**240**
IQLLVDLLDH	RMTEVHRSAC	GALRNLVYGK	ANDDNKIALK	NCGGIPALVR	LLRKTTDLEI	RELVTGVLWN	LSSCDALKMP
**250**	**260**	**270**	**280**	**290**	**300**	**310**	**320**
IIQDALAVLT	NAVIIPHSGW	ENSPLQDDRK	IQLHSSQVLR	NATGCLRNVS	SAGEEARRRM	RECDGLTDAL	LYVIQSALGS
**330**	**340**	**350**	**360**	**370**	**380**	**390**	**400**
SEIDSKTVEN	CVCILRNLSY	RLAAETSQGQ	HMGTDELDGL	LCGEANGKDA	ESSGCWGKKK	KKKKSQDQWD	GVGPLPDCAE
**410**	**420**	**430**	**440**	**450**	**460**	**470**	**480**
PPKGIQMLWH	PSIVKPYLTL	LSECSNPDTL	EGAAGALQNL	AAGSWKWSVY	IRAAVRKEKG	LPILVELLRI	DNDRVVCAVA
**490**	**500**	**510**	**520**	**530**	**540**	**550**	**560**
TALRNMALDV	RNKELIGKYA	MRDLVHRLPG	GNNSNNTASK	AMSDDTVTAV	CCTLHEVITK	NMENAKALRD	AGGIEKLVGI
**570**	**580**	**590**	**600**	**610**	**620**	**630**	**640**
SKSKGDKHSP	KVVKAASQVL	NSMWQYRDLR	SLYKKDGWSQ	YHFVASSSTI	ERDRQRPYSS	SRTPSISPVR	VSPNNRSASA
**650**	**660**	**670**	**680**	**690**	**700**	**710**	**720**
PASPREMISL	KERKTDYECT	GSNATYHGAK	GEHTSRKDAM	TAQNTGISTL	YRNSYGAPAE	DIKHNQVSAQ	PVPQEPSRKD
**730**	**740**	**750**	**760**	**770**	**780**	**790**	**800**
YETYQPFQNS	TRNYDESFFE	DQVHHRPPAS	EYTMHLGLKS	TGNYVDFYSA	ARPYSELNYE	TSHYPASPDS	WV

Cmpd.	No. of Cmpds.	m/z meas.	Δ m/z [ppm]	z	Rt [min]	Score	P	Range	Sequence	Modification
292	1	1293.8398	-46.50	3	32.8	12.9	1	342-378	R.LAAETSQGQHMGTDELDGLLCGEANGKDAESSGCWGK.K	Carbamidomethyl: 21, 34

Detailed Protein Report

Protein 623: vacuole membrane protein 1 [Homo sapiens]

**Accession:**	gi|20070349	**Score:**	25.4
**Database:**	refseq_human(refseq_human_20140103.fasta)	**MW [kDa]:**	46.2
**Database Date:**	2015-11-30	**pI:**	6.5
**Modification(s):**	Carbamidomethyl, Oxidation	**Sequence Coverage [%]:**	7.1
		**No. of unique Peptides:**	1

10	20	30	40	50	60	70	80
MAENGKNCDQ	RRVAMNKEHH	NGNFTDPSSV	NEKKRREREE	RQNIVLWRQP	LITLQYFSLE	ILVILKEWTS	KLWHRQSIVV
**90**	**100**	**110**	**120**	**130**	**140**	**150**	**160**
SFLLLLAVLI	ATYYVEGVHQ	QYVQRIEKQF	LLYAYWIGLG	ILSSVGLGTG	LHTFLLYLGP	HIASVTLAAY	ECNSVNFPEP
**170**	**180**	**190**	**200**	**210**	**220**	**230**	**240**
PYPDQIICPD	EEGTEGTISL	WSIISKVRIE	ACMWGIGTAI	GELPPYFMAR	AARLSGAEPD	DEEYQEFEEM	LEHAESAQDF
**250**	**260**	**270**	**280**	**290**	**300**	**310**	**320**
ASRAKLAVQK	LVQKVGFFGI	LACASIPNPL	FDLAGITCGH	FLVPFWTFFG	ATLIGKAIIK	MHIQKIFVII	TFSKHIVEQM
**330**	**340**	**350**	**360**	**370**	**380**	**390**	**400**
VAFIGAVPGI	GPSLQKPFQE	YLEAQRQKLH	HKSEMGTPQG	ENWLSWMFEK	LVVVMVCYFI	LSIINSMAQS	YAKRIQQRLN
**410**							
SEEKTK							

Cmpd.	No. of Cmpds.	m/z meas.	Δ m/z [ppm]	z	Rt [min]	Score	P	Range	Sequence	Modification
2411	1	669.6353	-215.87	2	58.5	12.4	1	1-11	-.MAENGKNCDQR.R	Carbamidomethyl: 8; Oxidation: 1

BRUKER

Detailed Protein Report

Protein 624: ubiquitin-like-conjugating enzyme ATG10 [Homo sapiens]

**Accession:**	gi|18594496	**Score:**	25.4
**Database:**	refseq_human(refseq_human_20140103.fasta)	**MW [kDa]:**	25.3
**Database Date:**	2015-11-30	**pI:**	5.3
**Modification(s):**	Carbamidomethyl, Oxidation	**Sequence Coverage [%]:**	6.8
		**No. of unique Peptides:**	2

Quantitation

Wdown:Qdown **Median:** 0.93 **CV:** 0.00 % **No. of Peptides:** 1

Alias proteins:

Accession	Name	Description
gi|530379522	refseq_human (refseq_human_20140103.fasta)	PREDICTED: ubiquitin-like-conjugating enzyme ATG10 isoform X2 [Homo sapiens]
gi|530379520	refseq_human (refseq_human_20140103.fasta)	PREDICTED: ubiquitin-like-conjugating enzyme ATG10 isoform X1 [Homo sapiens]
gi|196162717	refseq_human (refseq_human_20140103.fasta)	ubiquitin-like-conjugating enzyme ATG10 [Homo sapiens]

10	20	30	40	50	60	70	80
MEEDEFIGEK	TFQRYCAEFI	KHSQQIGDSW	EWRPSKDCSD	GYMCKIHFQI	KNGSVMSHLG	ASTHGQTCLP	MEEAFELPLD
**90**	**100**	**110**	**120**	**130**	**140**	**150**	**160**
DCEVIETAAA	SEVIKYEYHV	LYSCSYQVPV	LYFRASFLDG	RPLTLKDIWE	GVHECYKMRL	LQGPWDTITQ	QEHPILGQPF
**170**	**180**	**190**	**200**	**210**	**220**	**230**	
FVLHPCKTNE	FMTPVLKNSQ	KINKNVNYIT	SWLSIVGPVV	GLNLPLSYAK	ATSQDERNVP		

Cmpd.	No. of Cmpds.	m/z meas.	Δ m/z [ppm]	z	Rt [min]	Score	P	Range	Sequence	Modification	Ratios
1635	1	576.3013	186.62	2	49.0	10.1	0	37-45	K.DCSDGYMCK.I	Carbamidomethyl: 2, 8; Oxidation: 7	
2921	2	922.9870	83.40	2	65.7	15.3	1	37-51	K.DCSDGYMCKIHFQIK.N	Carbamidomethyl: 2	Wdown:Qdown 0.93

Detailed Protein Report

Protein 625: anaphase-promoting complex subunit 2 [Homo sapiens]

**Accession:**	gi|7019327	**Score:**	25.4
**Database:**	refseq_human(refseq_human_20140103.fasta)	**MW [kDa]:**	93.8
**Database Date:**	2015-11-30	**pI:**	5.0
		**Sequence Coverage [%]:**	3.2
		**No. of unique Peptides:**	1

10	20	30	40	50	60	70	80
MAAAVVVAEG	DSDSRPGQEL	LVAWNTVSTG	LVPPAALGLV	SSRTSGAVPP	KEEELRAAVE	VLRGHGLHSV	LEEWFVEVLQ
**90**	**100**	**110**	**120**	**130**	**140**	**150**	**160**
NDLQANISPE	FWNAISQCEN	SADEPQCLLL	LLDAFGLLES	RLDPYLRSLE	LLEKWTRLGL	LMGTGAQGLR	EEVHTMLRGV
**170**	**180**	**190**	**200**	**210**	**220**	**230**	**240**
LFFSTPRTFQ	EMIQRLYGCF	LRVYMQSKRK	GEGGTDPELE	GELDSRYARR	RYYRLLQSPL	CAGCSSDKQQ	CWCRQALEQF
**250**	**260**	**270**	**280**	**290**	**300**	**310**	**320**
HQLSQVLHRL	SLLERVSAEA	VTTTLHQVTR	ERMEDRCRGE	YERSFLREFH	KWIERVVGWL	GKVFLQDGPA	RPASPEAGNT
**330**	**340**	**350**	**360**	**370**	**380**	**390**	**400**
LRRWRCHVQR	FFYRIYASLR	IEELFSIVRD	FPDSRPAIED	LKYCLERTDQ	RQQLLVSLKA	ALETRLLHPG	VNTCDIITLY
**410**	**420**	**430**	**440**	**450**	**460**	**470**	**480**
ISAIKALRVL	DPSMVILEVA	CEPIRRYLRT	REDTVRQIVA	GLTGDSDGTG	DLAVELSKTD	PASLETGQDS	EDDSGEPEDW
**490**	**500**	**510**	**520**	**530**	**540**	**550**	**560**
VPDPVDADPG	KSSSKRRSSD	IISLLVSIYG	SKDLFINEYR	SLLADRLLHQ	FSFSPEREIR	NVELLKLRFG	EAPMHFCEVM
**570**	**580**	**590**	**600**	**610**	**620**	**630**	**640**
LKDMADSRRI	NANIREEDEK	RPAEEQPPFG	VYAVILSSEF	WPPFKDEKLE	VPEDIRAALE	AYCKKYEQLK	AMRTLSWKHT
**650**	**660**	**670**	**680**	**690**	**700**	**710**	**720**
LGLVTMDVEL	ADRTLSVAVT	PVQAVILLYF	QDQASWTLEE	LSKAVKMPVA	LLRRRMSVWL	QQGVLREEPP	GTFSVIEEER
**730**	**740**	**750**	**760**	**770**	**780**	**790**	**800**
PQDRDNMVLI	DSDDESDSGM	ASQADQKEEE	LLLFWTYIQA	MLTNLESLSL	DRIYNMLRMF	VVTGPALAEI	DLQELQGYLQ
**810**	**820**	**830**					
KKVRDQQLVY	SAGVYRLPKN	CS					

Cmpd.	No. of Cmpds.	m/z meas.	Δ m/z [ppm]	z	Rt [min]	Score	P	Range	Sequence	Modification
2369	1	665.7303	-155.03	2	59.9	12.8	1	570-580	R.INANIREEDEK.R	

Detailed Protein Report

Protein 626: **multiple epidermal growth factor-like domains protein 8 isoform 2 precursor [Homo sapiens]**

**Accession:**	gi|145701025	**Score:**	25.4
**Database:**	refseq_human(refseq_human_20140103.fasta)	**MW [kDa]:**	295.7
**Database Date:**	2015-11-30	**pI:**	6.5
**Modification(s):**	Carbamidomethyl, Oxidation	**Sequence Coverage [%]:**	1.6
[truncated: 350,909 more chars]
