# Supplementary material for: Exploration of key factors in Gingival Crevicular fluids from patients undergoing Periodontally Accelerated Osteogenic Orthodontics (PAOO) using proteome analysis
Source: BMC Oral Health. 2023 Nov 27;23:934. doi: 10.1186/s12903-023-03606-7 (PMC10683118; doi:10.1186/s12903-023-03606-7)
Supplement: Supplementary file 5 — Supplementary Material 5 [file 12903_2023_3606_MOESM5_ESM.pdf]

Detailed Protein Report

Project Info

Name: teeth-151208 **Date:** Dec 8, 2015

Note:

Sample Info & Protocols

Name: id-UP **Date:** Dec 10, 2015

Fraction:

Volume: **Concentration:**

Note:

Buffer: **Organism:**

Search Result Info

Search Result	Location	Search Engine	Database
ZT_human_PE_2015-12-10 16:24:42	/teeth-151208/id-UP/"Combined MS/MS spectra"	Mascot	refseq_human

Protein 1: serum albumin preproprotein [Homo sapiens]

Accession: gi|4502027

Database: refseq_human(refseq_human_20140103.fasta)

Database Date: 2015-11-30

Modification(s): Carbamidomethyl, Oxidation

Score: 1475.0

MW [kDa]: 69.3

pI: 5.9

Sequence Coverage [%]: 54.5

No. of unique Peptides: 31

Quantitation

QU:MU	**Median:**	1.11	**CV:**	102.80 %	**No. of Peptides:**	15
WUP:QUP	**Median:**	0.82	**CV:**	70.70 %	**No. of Peptides:**	17

```
        10         20         30         40         50         60         70         80
MKWVTFISLL FLFSSAYSRG VFRRDAHKSE VAHRFKDLGE ENFKALVLIA FAQYLQQCPF EDHVKLVNEV TEFAKTCVAD
        90        100        110        120        130        140        150        160
ESAENCDKSL HTLFGDKLCT VATLRETYGE MADCCAKQEP ERNECFLQHK DDNPNLPRLV RPEVDVMCTA FHDNEETFLK
       170        180        190        200        210        220        230        240
KYLYEIARRH PYFYAPELLF FAKRYKAAFT ECCQAADKAA CLLPKLDELR DEGKASSAKQ RLKCASLQKF GERAFKAWAV
       250        260        270        280        290        300        310        320
ARLSQRFPKA EFAEVSKLVT DLTKVHTECC HGDLLECADD RADLAKYICE NQDSISSKLK ECCEKPLLEK SHCIAEVEND
       330        340        350        360        370        380        390        400
EMPADLPSLA ADFVESKDVC KNYAEAKDVF LGMFLYEYAR RHPDYSVVLL LRLAKTYETT LEKCCAAADP HECYAKVFDE
       410        420        430        440        450        460        470        480
FKPLVEEPQN LIKQNCELFE QLGEYKFQNA LLVRYTKKVP QVSTPTLVEV SRNLGKVGSK CCKHPEAKRM PCAEDYLSVV
       490        500        510        520        530        540        550        560
LNQLCVLHEK TPVSDRVTKC CTESLVNRRP CFSALEVDET YVPKEFNAET FTFHADICTL SEKERQIKKQ TALVELVKHK
       570        580        590        600        610
PKATKEQLKA VMDDFAAFVE KCCKADDKET CFAEEGKKLV AASQAALGL
```

Cmpd.	No. of Cmpds.	m/z meas.	Δ m/z [ppm]	z	Rt [min]	Score	P	Range	Sequence	Modification	Ratios
250	12	613.8261	32.39	2	33.1	70.4	1	35-44	R.FKDLGEENFK.A		WUP:QUP 1.90<br>QU:MU 1.13
514	15	575.2390	-125.46	2	34.9	63.4	0	66-75	K.LVNEVTEFAK.T		
529	11	509.2667	-10.16	2	36.6	49.5	0	89-97	K.SLHTLFGDK.L		QU:MU 0.23
299	2	467.2609	-4.27	2	33.7	52.0	0	98-105	K.LCTVATLR.E	Carbamidomethyl: 2	
265	3	879.3978	-18.24	3	33.3	25.7	2	118-138	K.QEPERNECFLQHKDDNPNLPR.L	Carbamidomethyl: 8	WUP:QUP 1.09<br>QU:MU 1.47
279	2	666.3028	-17.84	3	33.5	40.0	1	123-138	R.NECFLQHKDDNPNLPR.L	Carbamidomethyl: 3	WUP:QUP 0.81<br>QU:MU 1.34
											WUP:QUP 2.55

Detailed Protein Report

Cmpd.	No. of Cmpds.	m/z meas.	Δ m/z [ppm]	z	Rt [min]	Score	P	Range	Sequence	Modification	Ratios
350	3	528.2959	-3.73	2	34.4	39.6	1	161-168	K.KYLYEIAR.R		QU:MU 0.82
326	11	464.1239	-272.44	2	32.8	30.1	0	162-168	K.YLYEIAR.R		WUP:QUP 0.87<br>QU:MU 1.39
18	2	686.2378	-71.67	2	29.2	64.2	0	187-198	K.AAFTECCQAADK.A	Carbamidomethyl: 6, 7	
1590	1	977.4188	-47.42	2	50.1	11.8	1	187-205	K.AAFTECCQAADKAACLLPK.L		
31	4	537.7767	3.24	2	30.1	45.4	1	206-214	K.LDELRDEGK.A		QU:MU 2.86<br>WUP:QUP 0.31
143	2	696.2767	-10.48	3	31.5	53.1	0	265-281	K.VHTECCHGDLLECADDR.A	Carbamidomethyl: 5, 6, 13	WUP:QUP 0.63<br>QU:MU 1.67
45	8	722.3225	-3.01	2	30.4	77.4	0	287-298	K.YICENQDSISSK.L	Carbamidomethyl: 3	WUP:QUP 0.50<br>QU:MU 1.82
104	1	773.8655	-47.15	2	31.0	20.6	1	299-310	K.LKECCEKPLLEK.S	Carbamidomethyl: 4, 5	WUP:QUP 0.64
2459	1	997.4538	2.48	3	59.0	27.7	0	311-337	K.<br>SHCIAEVENDEMPADLPSLAADFV<br>D	Carbamidomethyl: 3;<br>Oxidation: 12	
2841	5	812.4044	8.52	2	65.9	42.2	0	348-360	K.DVFLGMFLYEYAR.R		
2516	10	820.4094	17.73	2	59.7	54.5	0	348-360	K.DVFLGMFLYEYAR.R	Oxidation: 6	WUP:QUP 2.21
8	5	776.7890	-17.37	2	29.1	42.3	0	384-396	K.CCAAADPHECYAK.V	Carbamidomethyl: 1, 2, 10	
2964	40	682.3684	-2.26	3	65.8	51.0	0	397-413	K.VFDEFKPLVEEPQNLIK.Q		
1272	12	829.3522	-33.47	2	45.5	60.5	0	414-426	K.QNCELFEQLGEYK.F	Carbamidomethyl: 3	WUP:QUP 0.90<br>QU:MU 1.12
742	18	480.7783	-13.77	2	39.3	53.1	0	427-434	K.FQNALLVR.Y		QU:MU 1.46
694	1	547.3131	-7.95	3	37.1	59.0	1	438-452	K.KVPQVSTPTLVEVSR.N		WUP:QUP 0.50<br>QU:MU 1.51
930	1	756.4093	-20.82	2	40.0	32.0	0	439-452	K.VPQVSTPTLVEVSR.N		WUP:QUP 0.70
78	4	569.7455	-12.45	2	30.7	62.5	0	500-508	K.CCTESLVNR.R	Carbamidomethyl: 1, 2	WUP:QUP 0.31<br>QU:MU 3.50
1168	1	637.6405	-12.97	3	44.2	54.8	0	509-524	R.RPCFSALEVDETYVPK.E	Carbamidomethyl: 3	WUP:QUP 0.36
2419	11	753.9221	-119.82	3	58.5	64.2	0	525-543	K.EFNAETFTFHADICTLSEK.E	Carbamidomethyl: 14	WUP:QUP 1.24
628	1	564.7514	-179.84	2	37.9	25.5	1	549-558	K.KQTALVELVK.H		QU:MU 1.02<br>WUP:QUP 1.60
807	12	500.7995	-11.94	2	38.5	46.8	0	550-558	K.QTALVELVK.H		
2104	14	671.7654	-82.73	2	56.7	98.0	0	570-581	K.AVMDDFAAFVEK.C		QU:MU 0.11
27	1	543.2457	-2.64	3	30.0	35.7	2	585-598	K.ADDKETCFAEEGKK.L	Carbamidomethyl: 7	
895	1	571.3207	-52.32	2	39.6	22.1	1	598-609	K.KLVAASQAALGL.-		

Detailed Protein Report

Protein 2: hemoglobin subunit beta [Homo sapiens]

**Accession:**	gi|4504349	**Score:**	703.6
**Database:**	refseq_human(refseq_human_20140103.fasta)	**MW [kDa]:**	16.0
**Database Date:**	2015-11-30	**pI:**	6.9
**Modification(s):**	Carbamidomethyl	**Sequence Coverage [%]:**	84.4
		**No. of unique Peptides:**	12

Quantitation

QU:MU	**Median:**	0.65	**CV:**	69.99 %	**No. of Peptides:**	7
WUP:QUP	**Median:**	0.51	**CV:**	49.14 %	**No. of Peptides:**	10

```
        10         20         30         40         50         60         70         80
MVHLTPEEKS AVTALWGKVN VDEVGGEALG RLLVVYPWTQ RFFESFGDLS TPDAVMGNPK VKAHGKKVLG AFSDGLAHLD
        90        100        110        120        130        140        150
NLKGTFATLS ELHCDKLHVD PENFRLLGNV LVCVLAHHFG KEFTPPVQAA YQKVVAGVAN ALAHKYH
```

Cmpd.	No. of Cmpds.	m/z meas.	Δ m/z [ppm]	z	Rt [min]	Score	P	Range	Sequence	Modification	Ratios
63	3	476.7531	-11.35	2	30.5	38.7	0	2-9	M.VHLTPEEK.S		QU:MU 1.43<br>WUP:QUP 0.36
629	13	466.7591	-9.74	2	36.3	58.8	0	10-18	K.SAVTALWGK.V		WUP:QUP 1.66<br>QU:MU 0.23
496	9	657.8496	20.65	2	36.2	81.3	0	19-31	K.VNVDEVGGEALGR.L		WUP:QUP 0.57
2284	26	637.8143	-81.79	2	56.9	61.8	0	32-41	R.LLVVYPWTQR.F		WUP:QUP 0.46<br>QU:MU 0.52
2107	7	1029.8517	-122.16	2	56.7	64.9	0	42-60	R.FFESFGDLSTPDAVMGNPK.V		
2980	23	599.9999	-0.41	3	66.0	82.5	1	67-83	K.KVLGAFSDGLAHLDNLK.G		WUP:QUP 0.28
2515	57	835.4601	13.21	2	59.7	95.3	0	68-83	K.VLGAFSDGLAHLDNLK.G		WUP:QUP 0.75
527	3	739.8504	-0.54	2	35.0	36.5	0	84-96	K.GTFATLSELHCDK.L	Carbamidomethyl: 11	WUP:QUP 0.38<br>QU:MU 0.70
187	13	563.6579	-226.49	2	31.2	45.5	0	97-105	K.LHVDPENFR.L		QU:MU 1.24<br>WUP:QUP 0.42
532	2	689.8492	-6.46	2	36.6	44.0	0	122-133	K.EFTPPVQAAYQK.V		WUP:QUP 0.46<br>QU:MU 0.97
307	6	575.3418	2.06	2	33.8	76.7	0	134-145	K.VVAGVANALAHK.Y		QU:MU 0.32
430	1	725.3587	-59.21	2	35.4	17.7	1	134-147	K.VVAGVANALAHKYH.-		WUP:QUP 0.49

Detailed Protein Report

Protein 3: keratin, type I cytoskeletal 13 isoform a [Homo sapiens]

Accession: gi|131412225
Database: refseq_human(refseq_human_20140103.fasta)
Database Date: 2015-11-30
Modification(s): Carbamidomethyl

Score: 575.0
MW [kDa]: 49.5
pI: 4.8
Sequence Coverage [%]: 33.4
No. of unique Peptides: 12

Quantitation

QU:MU	**Median:**	1.04	**CV:**	25.84 %	**No. of Peptides:**	7
WUP:QUP	**Median:**	1.09	**CV:**	110.84 %	**No. of Peptides:**	8

```
        10         20         30         40         50         60         70         80
MSLRLQSSSA SYGGGFGGGS CQLGGGRGVS TCSTRFVSGG SAGGYGGGVS CGFGGGAGSG FGGGYGGGLG GGYGGGLGGG
        90        100        110        120        130        140        150        160
FGGGFAGGFV DFGACDGGLL TGNEKITMQN LNDRLASYLE KVRALEEANA DLEVKIRDWH LKQSPASPER DYSPYYKTIE
       170        180        190        200        210        220        230        240
ELRDKILTAT IENNRVILEI DNARLAADDF RLKYENELAL RQSVEADING LRRVLDELTL SKTDLEMQIE SLNEELAYMK
       250        260        270        280        290        300        310        320
KNHEEEMKEF SNQVVGQVNV EMDATPGIDL TRVLAEMREQ YEAMAERNRR DAEEWFHAKS AELNKEVSTN TAMIQTSKTE
       330        340        350        360        370        380        390        400
ITELRRTLQG LEIELQSQLS MKAGLENTVA ETECRYALQL QQIQGLISSI EAQLSELRSE MECQNQEYKM LLDIKTRLEQ
       410        420        430        440        450        460
EIATYRSLLE GQDAKMIGFP SSAGSVSPRS TSVTTTSSAS VTTTSNASGR RTSDVRRP
```

Cmpd.	No. of Cmpds.	m/z meas.	Δ m/z [ppm]	z	Rt [min]	Score	P	Range	Sequence	Modification	Ratios
337	2	1073.9554	-24.90	2	34.2	51.4	0	5-27	R.LQSSSASYGGGFGGGSCQLGGGG	Carbamidomethyl: 17	WUP:QUP 1.88<br>QU:MU 0.75
323	3	651.3296	-4.86	2	34.0	78.4	0	124-135	R.ALEEANADLEVK.I		
264	4	572.8011	-32.35	2	33.3	62.0	0	166-175	K.ILTATIENNR.V		WUP:QUP 0.13
547	1	521.6684	-248.82	2	36.4	53.1	0	176-184	R.VILEIDNAR.L		QU:MU 1.21<br>WUP:QUP 2.66
518	1	624.8405	-16.71	2	36.5	46.5	1	192-201	R.LKYENELALR.Q		WUP:QUP 1.23
322	1	679.3584	-6.47	2	34.0	14.0	1	202-213	R.QSVEADINGLRR.V		WUP:QUP 1.52<br>QU:MU 1.32
727	1	509.2783	-32.74	2	39.1	53.0	0	214-222	R.VLDELTLSK.T		
434	2	566.7484	-14.34	2	35.4	31.5	0	291-299	R.DAEEWFHAK.S		QU:MU 0.74
153	4	725.3478	16.83	2	31.6	68.8	0	343-355	K.AGLENTVAETECR.Y	Carbamidomethyl: 12	WUP:QUP 1.76<br>QU:MU 1.13
269	3	690.3609	-9.58	2	33.1	55.3	1	396-406	K.TRLEQEIATYR.S		WUP:QUP 1.45<br>QU:MU 1.45
942	3	696.8484	-3.18	2	41.8	28.0	0	416-429	K.MIGFPSSAGSVSPR.S		
38	3	1001.9629	-16.86	2	30.2	32.9	0	430-450	R.STSVTTTSSASVTTTSNASGR.R		WUP:QUP 0.63<br>QU:MU 0.88

Detailed Protein Report

Protein 4: apolipoprotein A-I preproprotein [Homo sapiens]

**Accession:**	gi|4557321	**Score:**	524.0
**Database:**	refseq_human(refseq_human_20140103.fasta)	**MW [kDa]:**	30.8
**Database Date:**	2015-11-30	**pI:**	5.5
		**Sequence Coverage [%]:**	43.8
		**No. of unique Peptides:**	12

Quantitation

QU:MU	**Median:** 1.32	**CV:** 17.55 %	**No. of Peptides:**	7
WUP:QUP	**Median:** 1.01	**CV:** 24.03 %	**No. of Peptides:**	6

Alias proteins:

Accession	Name	Description
gi|530398069	refseq_human (refseq_human_20140103.fasta)	PREDICTED: apolipoprotein A-I isoform X2 [Homo sapiens]
gi|530398067	refseq_human (refseq_human_20140103.fasta)	PREDICTED: apolipoprotein A-I isoform X1 [Homo sapiens]

```
        10         20         30         40         50         60         70         80
MKAAVLTLAV LFLTGSQARH FWQQDEPPQS PWDRVKDLAT VYVDVLKDSG RDYVSQFEGS ALGKQLNLKL LDNWDSVTST
        90        100        110        120        130        140        150        160
FSKLREQLGP VTQEFWDNLE KETEGLRQEM SKDLEEVKAK VQPYLDDFQK KWQEEMELYR QKVEPLRAEL QEGARQKLHE
       170        180        190        200        210        220        230        240
LQEKLSPLGE EMRDRARAHV DALRTHLAPY SDELRQRLAA RLEALKENGG ARLAEYHAKA TEHLSTLSEK AKPALEDLRQ
       250        260        270
GLLPVLESFK VSFLSALEEY TKKLNTQ
```

Cmpd.	No. of Cmpds.	m/z meas.	Δ m/z [ppm]	z	Rt [min]	Score	P	Range	Sequence	Modification	Ratios
2017	8	731.8316	-133.68	2	53.6	39.6	1	35-47	R.VKDLATVYVDVLK.D		
2150	1	618.2001	-238.81	2	55.2	52.2	0	37-47	K.DLATVYVDVLK.D		WUP:QUP 0.79<br>QU:MU 1.39
893	2	700.8159	-31.87	2	39.6	45.2	0	52-64	R.DYVSQFEGSALGK.Q		QU:MU 1.22
1864	18	806.8865	-12.14	2	51.7	89.9	0	70-83	K.LLDNWDSVTSTFSK.L		WUP:QUP 0.78<br>QU:MU 1.13
2426	2	966.9435	-27.92	2	58.6	21.1	0	86-101	R.EQLGPVTQEFWDNLEK.E		
497	2	626.6875	-201.93	2	34.7	29.3	0	121-130	K.VQPYLDDFQK.K		QU:MU 1.15<br>WUP:QUP 1.02
257	4	690.8471	-20.89	2	32.0	53.5	1	121-131	K.VQPYLDDFQKK.W		QU:MU 1.36<br>WUP:QUP 0.91
774	1	642.2953	8.43	2	39.7	17.4	0	132-140	K.WQEEMELYR.Q		QU:MU 1.94<br>WUP:QUP 1.54
1475	3	896.3833	-111.83	1	48.6	11.9	0	158-164	K.LHELQEK.L		
283	3	651.3276	-0.37	2	33.5	58.6	0	185-195	R.THLAPYSDELR.Q		WUP:QUP 1.18<br>QU:MU 1.19
2876	19	615.8555	-4.42	2	64.7	47.2	0	240-250	R.QGLLPVLESFK.V		
2827	22	693.8609	-0.42	2	65.8	57.9	0	251-262	K.VSFLSALEEYTK.K		

Detailed Protein Report

Protein 5: keratin, type II cytoskeletal 6C [Homo sapiens]

**Accession:**	gi|155969697	**Score:**	467.0
**Database:**	refseq_human(refseq_human_20140103.fasta)	**MW [kDa]:**	60.0
**Database Date:**	2015-11-30	**pI:**	8.9
**Modification(s):**	Carbamidomethyl	**Sequence Coverage [%]:**	20.7
		**No. of unique Peptides:**	10

Quantitation

QU:MU	**Median:**	1.28	**CV:**	85.94 %	**No. of Peptides:**	4
WUP:QUP	**Median:**	1.38	**CV:**	64.36 %	**No. of Peptides:**	5

10	20	30	40	50	60	70	80
MASTSTTIRS	HSSSRRGFSA	NSARLPGVSR	SGFSSISVSR	SRGSGGLGGA	CGGAGFGSRS	LYGLGGSKRI	SIGGGSCAIS
90	100	110	120	130	140	150	160
GGYGSRAGGS	YGFGGAGSGF	GFGGGAGIGF	GLGGGAGLAG	GFGGPGFPVC	PPGGIQEVTV	NQSLLTPLNL	QIDPAIQRVR
170	180	190	200	210	220	230	240
AEEREQIKTL	NNKFASFIDK	VRFLEQQNKV	LDTKWTLLQE	QGTKTVRQNL	EPLFEQYINN	LRRQLDSIVG	ERGRLDSELR
250	260	270	280	290	300	310	320
NMQDLVEDLK	NKYEDEINKR	TAAENEFVTL	KKDVDAAYMN	KVELQAKADT	LTDEINFLRA	LYDAELSQMQ	THISDTSVVL
330	340	350	360	370	380	390	400
SMDNNRNLDL	DSIIAEVKAQ	YEEIAQRSRA	EAESWYQTKY	EELQVTAGRH	GDDLRNTKQE	IAEINRMIQR	LRSEIDHVKK
410	420	430	440	450	460	470	480
QCASLQAAIA	DAEQRGEMAL	KDAKNKLEGL	EDALQKAKQD	LARLLKEYQE	LMNVKLALDV	EIATYRKLLE	GEECRLNGEG
490	500	510	520	530	540	550	560
VGQVNVSVVQ	STISSGYGGA	SGVGSGLGLG	GGSSYSYGSG	LGIGGGFSSS	SGRAIGGGLS	SVGGGSSTIK	YTTTSSSSRK
570							
SYKH							

Cmpd.	No. of Cmpds.	m/z meas.	Δ m/z [ppm]	z	Rt [min]	Score	P	Range	Sequence	Modification	Ratios
152	1	712.8348	20.14	2	31.9	14.7	0	43-59	R.GSGGLGGACGGAGFGSR.S	Carbamidomethyl: 9	
470	1	799.8881	6.12	2	35.9	18.2	0	70-86	R.ISIGGGSCAISGGYGSR.A	Carbamidomethyl: 8	WUP:QUP 2.01
808	1	602.3214	-1.12	2	40.1	44.0	0	195-204	K.WTLLQEQGTK.T		
2908	8	945.9990	10.55	2	65.1	35.8	0	208-222	R.QNLEPLFEQYINNLR.R		
2813	2	682.9825	-67.79	3	65.6	26.1	1	208-223	R.QNLEPLFEQYINNLRR.Q		
328	2	675.8743	11.03	2	34.1	67.2	1	261-272	R.TAAENEFVTLKK.D		WUP:QUP 1.73<br>QU:MU 0.56
1975	11	704.2826	-108.99	2	55.1	74.7	0	288-299	K.ADTLTDEINFLR.A		QU:MU 0.74<br>WUP:QUP 2.35
2923	15	665.3592	-11.18	2	65.3	86.6	0	327-338	R.NLDLDSIIAEVK.A		
167	4	583.1671	-220.95	2	31.8	56.5	0	360-369	K.YEELQVTAGR.H		WUP:QUP 1.38<br>QU:MU 1.71
1633	4	632.2990	-81.98	2	50.2	43.1	0	456-466	K.LALDVEIATYR.K		WUP:QUP 0.45<br>QU:MU 3.73

Detailed Protein Report

Protein 6: hemoglobin subunit delta [Homo sapiens]

Accession: gi|4504351
Database: refseq_human(refseq_human_20140103.fasta)
Database Date: 2015-11-30

Score: 461.0
MW [kDa]: 16.0
pI: 9.1
Sequence Coverage [%]: 48.3
No. of unique Peptides: 1

Quantitation

QU:MU	**Median:** 1.14	**CV:** 0.00 %	**No. of Peptides:** 1
WUP:QUP	**Median:** 0.16	**CV:** 0.00 %	**No. of Peptides:** 1

10	20	30	40	50	60	70	80
MVHLTPEEKT	AVNALWGKVN	VDAVGGEALG	RLLVVYPWTQ	RFFESFGDLS	SPDAVMGNPK	VKAHGKKVLG	AFSDGLAHLD
90	100	110	120	130	140	150	
NLKGTFSQLS	ELHCDKLHVD	PENFRLLGNV	LVCVLARNFG	KEFTPQMQAA	YQKVVAGVAN	ALAHKYH	

Cmpd.	No. of Cmpds.	m/z meas.	Δ m/z [ppm]	z	Rt [min]	Score	P	Range	Sequence	Modification	Ratios
467	1	628.8213	-19.13	2	35.8	42.8	0	19-31	K.VNVDAVGGEALGR.L		QU:MU 1.14<br>WUP:QUP 0.16

Detailed Protein Report

Protein 7: hemoglobin subunit alpha [Homo sapiens]

**Accession:**	gi|4504345	**Score:**	438.9
**Database:**	refseq_human(refseq_human_20140103.fasta)	**MW [kDa]:**	15.2
**Database Date:**	2015-11-30	**pI:**	9.4
**Modification(s):**	Oxidation	**Sequence Coverage [%]:**	50.7
		**No. of unique Peptides:**	8

Quantitation

QU:MU	**Median:**	0.55	**CV:**	45.17 %	**No. of Peptides:**	3
WUP:QUP	**Median:**	0.28	**CV:**	94.03 %	**No. of Peptides:**	5

Alias proteins:

Accession	Name	Description
gi|4504347	refseq_human (refseq_human_20140103.fasta)	hemoglobin subunit alpha [Homo sapiens]

```
        10         20         30         40         50         60         70         80
MVLSPADKTN VKAAWGKVGA HAGEYGAEAL ERMFLSFPTT KTYFPHFDLS HGSAQVKGHG KKVADALTNA VAHVDDMPNA
        90        100        110        120        130        140        150
LSALSDLHAH KLRVDPVNFK LLSHCLLVTL AAHLPAEFTP AVHASLDKFL ASVSTVLTSK YR
```

Cmpd.	No. of Cmpds.	m/z meas.	Δ m/z [ppm]	z	Rt [min]	Score	P	Range	Sequence	Modification	Ratios
4	1	729.4098	-5.99	1	29.0	28.6	0	2-8	M.VLSPADK.T		
31	2	586.3232	-24.65	2	29.3	50.8	1	2-12	M.VLSPADKTNVK.A		WUP:QUP 0.72
146	11	765.3477	-30.12	2	31.6	78.8	0	18-32	K.VGAHAGEYGAEALER.M		WUP:QUP 0.74<br>QU:MU 0.96
1126	1	544.1344	-264.30	2	42.3	39.7	0	33-41	R.MFLSFPTTK.T	Oxidation: 1	WUP:QUP 0.19
1845	38	536.1590	-227.09	2	51.4	57.9	0	33-41	R.MFLSFPTTK.T		
1189	4	917.4299	-21.44	2	45.0	51.1	0	42-57	K.TYFPHFDLSHGSAQVK.G		QU:MU 0.50
592	6	544.3095	-13.04	2	37.4	51.7	1	92-100	K.LRVDPVNFK.L		WUP:QUP 0.13<br>QU:MU 0.34
1648	39	626.8403	-33.03	2	48.9	80.4	0	129-140	K.FLASVSTVLTSK.Y		WUP:QUP 0.13

Detailed Protein Report

Protein 8: keratin, type I cytoskeletal 15 [Homo sapiens]

Accession: gi|24430190
Database: refseq_human(refseq_human_20140103.fasta)
Database Date: 2015-11-30

Score: 385.3
MW [kDa]: 49.2
pI: 4.6
Sequence Coverage [%]: 16.0
No. of unique Peptides: 2

Quantitation

QU:MU	**Median:**	0.36	**CV:**	29.32 %	**No. of Peptides:**	2
WUP:QUP	**Median:**	3.83	**CV:**	0.00 %	**No. of Peptides:**	1

```
        10         20         30         40         50         60         70         80
MTTTFLQTSS STFGGGSTRG GSLLAGGGGF GGGSLSGGGG SRSISASSAR FVSSGSGGGY GGGMRVCGFG GGAGSVFGGG
        90        100        110        120        130        140        150        160
FGGGVGGGFG GGFGGGDGGL LSGNEKITMQ NLNDRLASYL DKVRALEEAN ADLEVKIHDW YQKQTPTSPE CDYSQYFKTI
       170        180        190        200        210        220        230        240
EELRDKIMAT TIDNSRVILE IDNARLAADD FRLKYENELA LRQGVEADIN GLRRVLDELT LARTDLEMQI EGLNEELAYL
       250        260        270        280        290        300        310        320
KKNHEEEMKE FSSQLAGQVN VEMDAAPGVD LTRVLAEMRE QYEAMAEKNR RDVEAWFFSK TEELNKEVAS NTEMIQTSKT
       330        340        350        360        370        380        390        400
EITDLRRTMQ ELEIELQSQL SMKAGLENSL AETECRYATQ LQQIQGLIGG LEAQLSELRC EMEAQNQEYK MLLDIKTRLE
       410        420        430        440        450        460
QEIATYRSLL EGQDAKMAGI GIREASSGGG GSSSNFHINV EESVDGQVVS SHKREI
```

Cmpd.	No. of Cmpds.	m/z meas.	Δ m/z [ppm]	z	Rt [min]	Score	P	Range	Sequence	Modification	Ratios
296	1	532.8004	-15.38	2	33.7	58.7	1	116-124	R.LASYLDKVR.A		QU:MU 0.27
853	1	515.2848	-30.68	2	40.2	40.1	0	215-223	R.VLDELTLAR.T		QU:MU 0.48<br>WUP:QUP 3.83

Detailed Protein Report

Protein 9: serotransferrin precursor [Homo sapiens]

**Accession:**	gi|4557871	**Score:**	352.0
**Database:**	refseq_human(refseq_human_20140103.fasta)	**MW [kDa]:**	77.0
**Database Date:**	2015-11-30	**pI:**	7.0
**Modification(s):**	Carbamidomethyl	**Sequence Coverage [%]:**	18.2
		**No. of unique Peptides:**	10

Quantitation

QU:MU	**Median:** 1.30	**CV:** 33.87 %	**No. of Peptides:**	7
WUP:QUP	**Median:** 0.94	**CV:** 38.12 %	**No. of Peptides:**	6

10	20	30	40	50	60	70	80
MRLAVGALLV	CAVLGLCLAV	PDKTVRWCAV	SEHEATKCQS	FRDHMKSVIP	SDGPSVACVK	KASYLDCIRA	IAANEADAVT
90	100	110	120	130	140	150	160
LDAGLVYDAY	LAPNNLKPVV	AEFYGSKEDP	QTFYYAVAVV	KKDSGFQMNQ	LRGKKSCHTG	LGRSAGWNIP	IGLLYCDLPE
170	180	190	200	210	220	230	240
PRKPLEKAVA	NFFSGSCAPC	ADGTDFPQLC	QLCPGCGCST	LNQYFGYSGA	FKCLKDGAGD	VAFVKHSTIF	ENLANKADRD
250	260	270	280	290	300	310	320
QYELLCLDNT	RKPVDEYKDC	HLAQVPSHTV	VARSMGGKED	LIWELLNQAQ	EHFGKDKSKE	FQLFSSPHGK	DLLFKDSAHG
330	340	350	360	370	380	390	400
FLKVPPRMDA	KMYLGYEYVT	AIRNLREGTC	PEAPTDECKP	VKWCALSHHE	RLKCDEWSVN	SVGKIECVSA	ETTEDCIAKI
410	420	430	440	450	460	470	480
MNGEADAMSL	DGGFVYIAGK	CGLVPVLAEN	YNKSDNCEDT	PEAGYFAVAV	VKKSASDLTW	DNLKGKKSCH	TAVGRTAGWN
490	500	510	520	530	540	550	560
IPMGLLYNKI	NHCRFDEFFS	EGCAPGSKKD	SSLCKLCMGS	GLNLCEPNNK	EGYYGYTGAF	RCLVEKGDVA	FVKHQTVPQN
570	580	590	600	610	620	630	640
TGGKNPDPWA	KNLNEKDYEL	LCLDGTRKPV	EEYANCHLAR	APNHAVVTRK	DKEACVHKIL	RQQQHLFGSN	VTDCSGNFCL
650	660	670	680	690	700		
FRSETKDLLF	RDDTVCLAKL	HDRNTYEKYL	GEEYVKAVGN	LRKCSTSSLL	EACTFRRP		

Cmpd.	No. of Cmpds.	m/z meas.	Δ m/z [ppm]	z	Rt [min]	Score	P	Range	Sequence	Modification	Ratios
568	2	708.3474	-22.90	2	37.1	42.7	0	47-60	K.SVIPSDGPSVACVK.K	Carbamidomethyl: 12	WUP:QUP 1.25<br>QU:MU 1.10
431	2	637.3141	-25.64	2	35.0	51.8	0	226-236	K.HSTIFENLANK.A		WUP:QUP 0.82<br>QU:MU 1.39
302	1	850.4388	3.54	3	33.8	10.5	1	252-273	R.<br>KPVDEYKDCHLAQVPSHTVVAR.<br>S	Carbamidomethyl: 9	WUP:QUP 0.63
498	1	746.3968	18.25	2	36.2	42.7	1	298-310	K.SKEFQLFSSPHGK.D		QU:MU 0.82
678	2	638.8051	-22.74	2	38.0	31.2	0	300-310	K.EFQLFSSPHGK.D		QU:MU 2.12
172	3	761.3492	-29.85	2	31.0	78.7	1	372-384	R.LKCDEWSVNSVGK.I	Carbamidomethyl: 3	QU:MU 1.29<br>WUP:QUP 0.57
255	1	863.4074	23.48	2	33.2	14.5	0	385-399	K.IECVSAETTEDCIAK.I	Carbamidomethyl: 3, 12	WUP:QUP 1.20<br>QU:MU 0.93
840	1	853.8816	-5.85	2	40.5	22.5	0	516-530	K.LCMGSGLNLCEPNNK.E	Carbamidomethyl: 2, 10	
448	1	642.1562	-205.50	2	34.1	34.3	0	531-541	K.EGYYGYTGAFR.C		QU:MU 1.94<br>WUP:QUP 1.54
845	1	766.3399	-10.08	2	39.0	23.0	0	684-696	K.CSTSSLLEACTFR.R	Carbamidomethyl: 1, 10	

Detailed Protein Report

Protein 10: PREDICTED: alpha-2-macroglobulin isoform X1 [Homo sapiens]

**Accession:**	gi|578822814	**Score:**	332.9
**Database:**	refseq_human(refseq_human_20140103.fasta)	**MW [kDa]:**	167.4
**Database Date:**	2015-11-30	**pI:**	6.3
**Modification(s):**	Carbamidomethyl	**Sequence Coverage [%]:**	10.3
		**No. of unique Peptides:**	12

Quantitation

QU:MU	**Median:**	1.75	**CV:**	37.08 %	**No. of Peptides:**	5
WUP:QUP	**Median:**	0.63	**CV:**	31.85 %	**No. of Peptides:**	4

10	20	30	40	50	60	70	80
MGKNKLLHPS	LVLLLLVLLP	TDASVSGKPQ	YMVLVPSLLH	TETTEKGCVL	LSYLNETVTV	SASLESVRGN	RSLFTDLEAE
90	100	110	120	130	140	150	160
NDVLHCVAFA	VPKSSSNEEV	MFLTVQVKGP	TQEFKKRTTV	MVKNEDSLVF	VQTDKSIYKP	GQTVKFRVVS	MDENFHPLNE
170	180	190	200	210	220	230	240
LIPLVYIQDP	KGNRIAQWQS	FQLEGGLKQF	SFPLSSEPFQ	GSYKVVVQKK	SGGRTEHPFT	VEEFVLPKFE	VQVTVPKIIT
250	260	270	280	290	300	310	320
ILEEEMNVSV	CGLYTYGKPV	PGHVTVSICR	KYSDASDCHG	EDSQAFCEKF	SGQLNSHGCF	YQQVKTKVFQ	LKRKEYEMKL
330	340	350	360	370	380	390	400
HTEAQIQEEG	TVVELTGRQS	SEITRTITKL	SFVKVDSHFR	QGIPFFGQVR	LVDGKGVPIP	NKVIFIRGNE	ANYYSNATTD
410	420	430	440	450	460	470	480
EHGLVQFSIN	TTNVMGTSLT	VRVNYKDRSP	CYGYQWVSEE	HEEAHHTAYL	VFSPSKSFVH	LEPMSHELPC	GHTQTVQAHY
490	500	510	520	530	540	550	560
ILNGGTLLGL	KKLSFYYLIM	AKGGIVRTGT	HGLLVKQEDM	KGHFSISIPV	KSDIAPVARL	LIYAVLPTGD	VIGDSAKYDV
570	580	590	600	610	620	630	640
ENCLANKVDL	SFSPSQSLPA	SHAHLRVTAA	PQSVCALRAV	DQSVLLMKPD	AELSASSVYN	LLPEKDLTGF	PGPLNDQDNE
650	660	670	680	690	700	710	720
DCINRHNVYI	NGITYTPVSS	TNEKDMYSFL	EDMGLKAFTN	SKIRKPKMCP	QLQQYEMHGP	EGLRVGFYES	DVMGRGHARL
730	740	750	760	770	780	790	800
VHVEEPHTET	VRKYFPETWI	WDLVVVNSAG	VAEVGVTVPD	TITEWKAGAF	CLSEDAGLGI	SSTASLRAFQ	PFFVELTMPY
810	820	830	840	850	860	870	880
SVIRGEAFTL	KATVLNYLPK	CIRVSVQLEA	SPAFLAVPVE	KEQAPHCICA	NGRQTVSWAV	TPKSLGNVNF	TVSAEALESQ
890	900	910	920	930	940	950	960
ELCGTEVPSV	PEHGRKDTVI	KPLLVEPEGL	EKETTFNSLL	CPSGGEVSEE	LSLKLPPNVV	EESARASVSV	LGDILGSAMQ
970	980	990	1000	1010	1020	1030	1040
NTQNLLQMPY	GCGEQNMVLF	APNIYVLDYL	NETQQLTPEI	KSKAIGYLNT	GYQRQLNYKH	YDGSYSTFGE	RYGRNQGNTW
1050	1060	1070	1080	1090	1100	1110	1120
LTAFVLKTFA	QARAYIFIDE	AHITQALIWL	SQRQKDNGCF	RSSGSLLNNA	IKGGVEDEVT	LSAYITIALL	EIPLTVTHPV
1130	1140	1150	1160	1170	1180	1190	1200
VRNALFCLES	AWKTAQEGDH	GSHVYTKALL	AYAFALAGNQ	DKRKEVLKSL	NEEAVKKDNS	VHWERPQKPK	APVGHFYEPQ
1210	1220	1230	1240	1250	1260	1270	1280
APSAEVEMTS	YVLLAYLTAQ	PAPTSEDLTS	ATNIVKWITK	QQNAQGGFSS	TQDTVVALHA	LSKYGAATFT	RTGKAAQVTI
1290	1300	1310	1320	1330	1340	1350	1360
QSSGTFSSKF	QVDNNNRLLL	QQVSLPELPG	EYSMKVTGEG	CVYLQTSLKY	NILPEKEEFP	FALGVQTLPQ	TCDEPKAHTS
1370	1380	1390	1400	1410	1420	1430	1440
FQISLSVSYT	GSRSASNMAI	VDVKMVSGFI	PLKPTVKMLE	RSNHVSRTEV	SSNHVLIYLD	KVSNQTLSLF	FTVLQDVPVR
1450	1460	1470	1480	1490	1500	1510	1520
DLKPAIVKVY	DYYETDEFAI	AEYNAPCSKG	KPLTLLQKAV	RAPSACPPNL	LLHKTLGKYL	SKTSNYSHCT	LL

Cmpd.	No. of Cmpds.	m/z meas.	Δ m/z [ppm]	z	Rt [min]	Score	P	Range	Sequence	Modification	Ratios
743	1	697.8370	-9.33	2	39.3	19.9	0	124-135	K.NEDSLVFVQTDK.S		
1703	3	802.9034	-27.20	2	49.6	35.7	0	175-188	R.IAQWQSFQLEGGLK.Q		WUP:QUP 1.07<br>QU:MU 1.50
2299	2	924.9110	-35.46	2	57.0	20.1	0	189-204	K.QFSFPLSSEPFQGSYK.V		WUP:QUP 0.55<br>QU:MU 1.41
2052	5	574.7391	-130.69	2	54.0	26.2	0	361-370	R.QGIPFFGQVR.L		

Detailed Protein Report

Cmpd.	No. of Cmpds.	m/z meas.	Δ m/z [ppm]	z	Rt [min]	Score	P	Range	Sequence	Modification	Ratios
898	1	542.6752	-250.41	2	39.6	25.9	0	522-531	K.GHFSISIPVK.S		
276	2	636.8299	-16.03	2	33.4	30.1	0	587-598	R.VTAAPQSVCALR.A	Carbamidomethyl: 9	QU:MU 1.12
2461	2	942.5232	-4.52	2	59.1	21.9	0	824-841	R.VSVQLEASPAFLAVPVEK.E		
305	2	558.6600	-261.38	2	32.5	48.3	0	854-863	R.QTVSWAVTPK.S		WUP:QUP 0.48<br>QU:MU 2.34
189	1	605.6838	-232.96	2	31.2	17.9	0	935-945	K.LPPNVVEESAR.A		WUP:QUP 0.55<br>QU:MU 2.98
2462	1	669.8332	5.93	2	59.1	43.0	0	1123-1133	R.NALFCLESAWK.T	Carbamidomethyl: 5	
2729	1	783.4212	1.90	2	65.2	28.1	0	1148-1162	K.ALLAYAFALAGNQDK.R		
2649	1	973.5082	-44.93	2	64.0	15.9	1	1385-1401	K.MVSGFIPLKPTVKMLER.S		

Detailed Protein Report

Protein 11: keratin, type I cytoskeletal 14 [Homo sapiens]

**Accession:**	gi|15431310	**Score:**	315.6
**Database:**	refseq_human(refseq_human_20140103.fasta)	**MW [kDa]:**	51.6
**Database Date:**	2015-11-30	**pI:**	4.9
		**Sequence Coverage [%]:**	14.2
		**No. of unique Peptides:**	3

10	20	30	40	50	60	70	80
MTTCSRQFTS	SSSMKGSCGI	GGGIGGGSSR	ISSVLAGGSC	RAPSTYGGGL	SVSSSRFSSG	GAYGLGGGYG	GGFSSSSSSF
**90**	**100**	**110**	**120**	**130**	**140**	**150**	**160**
GSGFGGGYGG	GLGAGLGGGF	GGGFAGGDGL	LVGSEKVTMQ	NLNDRLASYL	DKVRALEEAN	ADLEVKIRDW	YQRQRPAEIK
**170**	**180**	**190**	**200**	**210**	**220**	**230**	**240**
DYSPYFKTIE	DLRNKILTAT	VDNANVLLQI	DNARLAADDF	RTKYETELNL	RMSVEADING	LRRVLDELTL	ARADLEMQIE
**250**	**260**	**270**	**280**	**290**	**300**	**310**	**320**
SLKEELAYLK	KNHEEEMNAL	RGQVGGDVNV	EMDAAPGVDL	SRILNEMRDQ	YEKMAEKNRK	DAEEWFFTKT	EELNREVATN
**330**	**340**	**350**	**360**	**370**	**380**	**390**	**400**
SELVQSGKSE	ISELRRTMQN	LEIELQSQLS	MKASLENSLE	ETKGRYCMQL	AQIQEMIGSV	EEQLAQLRCE	MEQQNQEYKI
**410**	**420**	**430**	**440**	**450**	**460**	**470**	**480**
LLDVKTRLEQ	EIATYRRLLE	GEDAHLSSSQ	FSSGSQSSRD	VTSSSRQIRT	KVMDVHDGKV	VSTHEQVLRT	KN

Cmpd.	No. of Cmpds.	m/z meas.	Δ m/z [ppm]	z	Rt [min]	Score	P	Range	Sequence	Modification
88	1	713.3252	-37.61	2	30.0	39.9	0	42-56	R.APSTYGGGLSVSSSR.F	
282	1	633.8578	31.13	2	33.5	20.5	1	202-211	R.TKYETELNLR.M	
246	2	512.5147	-194.60	3	33.1	22.6	2	406-417	K.TRLEQEIATYRR.L	

Detailed Protein Report

Protein 12: titin isoform N2-A [Homo sapiens]

**Accession:**	gi|291045225	**Score:**	283.3
**Database:**	refseq_human(refseq_human_20140103.fasta)	**MW [kDa]:**	3711.3
**Database Date:**	2015-11-30	**pI:**	6.1
**Modification(s):**	Carbamidomethyl, Oxidation	**Sequence Coverage [%]:**	0.8
		**No. of unique Peptides:**	18

Quantitation

QU:MU	**Median:**	1.93	**CV:**	119.44 %	**No. of Peptides:**	2
WUP:QUP	**Median:**	0.85	**CV:**	65.88 %	**No. of Peptides:**	5

Detailed Protein Report

10	20	30	40	50	60	70	80
MTTQAPTFTQ	PLQSVVVLEG	STATFEAHIS	GFPVPEVSWF	RDGQVISTST	LPGVQISFSD	GRAKLTIPAV	TKANSGRYSL
90	100	110	120	130	140	150	160
KATNGSGQAT	STAELLVKAE	TAPPNFVQRL	QSMTVRQGSQ	VRLQVRVTGI	PTPVVKFYRD	GAEIQSSLDF	QISQEGDLYS
170	180	190	200	210	220	230	240
LLIAEAYPED	SGTYSVNATN	SVGRATSTAE	LLVQGEEEVP	AKKTKTIVST	AQISESRQTR	IEKKIEAHFD	ARSIATVEMV
250	260	270	280	290	300	310	320
IDGAAGQQLP	HKTPPRIPPK	PKSRSPTPPS	IAAKAQLARQ	QSPSPIRHSP	SPVRHVRAPT	PSPVRSVSPA	ARISTSPIRS
330	340	350	360	370	380	390	400
VRSPLLMRKT	QASTVATGPE	VPPPWKQEGY	VASSSEAEMR	ETTLTTSTQI	RTEERWEGRY	GVQEQVTISG	AAGAAASVSA
410	420	430	440	450	460	470	480
SASYAAEAVA	TGAKEVKQDA	DKSAAVATVV	AAVDMARVRE	PVISAVEQTA	QRTTTTAVHI	QPAQEQVRKE	AEKTAVTKVV
490	500	510	520	530	540	550	560
VAADKAKEQE	LKSRTKEVIT	TKQEQMHVTH	EQIRKETEKT	FVPKVVISAA	KAKEQETRIS	EEITKKQKQV	TQEAIRQETE
570	580	590	600	610	620	630	640
ITAASMVVVA	TAKSTKLETV	PGAQEETTTQ	QDQMHLSYEK	IMKETRKTVV	PKVIVATPKV	KEQDLVSRGR	EGITTKREQV
650	660	670	680	690	700	710	720
QITQEKMRKE	AEKTALSTIA	VATAKAKEQE	TILRTRETMA	TRQEQIQVTH	GKVDVGKKAE	AVATVVAAVD	QARVREPREP
730	740	750	760	770	780	790	800
GHLEESYAQQ	TTLEYGYKER	ISAAKVAEPP	QRPASEPHVV	PKAVKPRVIQ	APSETHIKTT	DQKGMHISSQ	IKKTTDLTTE
810	820	830	840	850	860	870	880
RLVHVDKRPR	TASPHFTVSK	ISVPKTEHGY	EASIAGSAIA	TLQKELSATS	SAQKITKSVK	APTVKPSETR	VRAEPTPLPQ
890	900	910	920	930	940	950	960
FPFADTPDTY	KSEAGVEVKK	EVGVSITGTT	VREERFEVLH	GREAKVTETA	RVPAPVEIPV	TPPTLVSGLK	NVTVIEGESV
970	980	990	1000	1010	1020	1030	1040
TLECHISGYP	SPTVTWYRED	YQIESSIDFQ	ITFQSGIARL	MIREAFAEDS	GRFTCSAVNE	AGTVSTSCYL	AVQVSEEFEK
1050	1060	1070	1080	1090	1100	1110	1120
ETTAVTEKFT	TEEKRFVESR	DVVMTDTSLT	EEQAGPGEPA	APYFITKPVV	QKLVEGGSVV	FGCQVGGNPK	PHVYWKKSGV
1130	1140	1150	1160	1170	1180	1190	1200
PLTTGYRYKV	SYNKQTGECK	LVISMTFADD	AGEYTIVVRN	KHGETSASAS	LLEEADYELL	MKSQQEMLYQ	TQVTAFVQEP
1210	1220	1230	1240	1250	1260	1270	1280
KVGETAPGFV	YSEYEKEYEK	EQALIRKKMA	KDTVVVRTYV	EDQEFHISSF	EERLIKEIEY	RIIKTTLEEL	LEEDGEEKMA
1290	1300	1310	1320	1330	1340	1350	1360
VDISESEAVE	SGFDSRIKNY	RILEGMGVTF	HCKMSGYPLP	KIAWYKDGKR	IKHGERYQMD	FLQDGRASLR	IPVVLPEDEG
1370	1380	1390	1400	1410	1420	1430	1440
IYTAFASNIK	GNAICSGKLY	VEPAAPLGAP	TYIPTLEPVS	RIRSLSPRSV	SRSPIRMSPA	RMSPARMSPA	RMSPARMSPG
1450	1460	1470	1480	1490	1500	1510	1520
RRLEETDESQ	LERLYKPVFV	LKPVSFKCLE	GQTARFDLKV	VGRPMPETFW	FHDGQQIVND	YTHKVVIKED	GTQSLIIVPA
1530	1540	1550	1560	1570	1580	1590	1600
TPSDSGEWTV	VAQNRAGRSS	ISVILTVEAV	EHQVKPMFVE	KLKNVNIKEG	SRLEMKVRAT	GNPNPDIVWL	KNSDIIVPHK
1610	1620	1630	1640	1650	1660	1670	1680
YPKIRIEGTK	GEAALKIDST	VSQDSAWYTA	TAINKAGRDT	TRCKVNVEVE	FAEPEPERKL	IIPRGTYRAK	EIAAPELEPL
1690	1700	1710	1720	1730	1740	1750	1760
HLRYGQEQWE	EGDLYDKEKQ	QKPFFKKKLT	SLRLKRFGPA	HFECRLTPIG	DPTMVVEWLH	DGKPLEAANR	LRMINEFGYC
1770	1780	1790	1800	1810	1820	1830	1840
SLDYGVAYSR	DSGIITCRAT	NKYGTDHTSA	TLIVKDEKSL	VEESQLPEGR	KGLQRIEELE	RMAHEGALTG	VTTDQKEKQK
1850	1860	1870	1880	1890	1900	1910	1920
PDIVLYPEPV	RVLEGETARF	RCRVTGYPQP	KVNWYLNGQL	IRKSKRFRVR	YDGIHYLDIV	DCKSYDTGEV	KVTAENPEGV
1930	1940	1950	1960	1970	1980	1990	2000
IEHKVKLEIQ	QREDFRSVLR	RAPEPRPEFH	VHEPGKLQFE	VQKVDRPVDT	TETKEVVKLK	RAERITHEKV	PEESEELRSK
2010	2020	2030	2040	2050	2060	2070	2080
FKRRTEEGYY	EAITAVELKS	RKKDESYEEL	LRKTKDELLH	WTKELTEEEK	KALAEEGKIT	IPTFKPDKIE	LSPSMEAPKI
2090	2100	2110	2120	2130	2140	2150	2160
FERIQSQTVG	QGSDAHFRVR	VVGKPDPECE	WYKNGVKIER	SDRIYWYWPE	DNVCELVIRD	VTAEDSASIM	VKAINIAGET
2170	2180	2190	2200	2210	2220	2230	2240

Detailed Protein Report

Cmpd.	No. of Cmpds.	m/z meas.	Δ m/z [ppm]	z	Rt [min]	Score	P	Range	Sequence	Modification	Ratios
619	1	690.3860	77.00	2	37.3	10.5	0	4467-4479	R.VTNEVGSSICSAR.V	Carbamidomethyl: 10	
2868	1	805.9212	74.64	2	66.3	17.2	0	5591-5605	K.AQNEVGSDTCVCTVK.L	Carbamidomethyl: 10	
2735	1	1045.0652	51.04	2	64.6	11.1	1	6628-6646	K.NDAGMRECSAVLTVLEPAR.I	Carbamidomethyl: 8	
1761	1	785.8716	-131.54	2	51.8	13.2	2	9274-9287	K.IHVAISKRVEPPPK.V		
1331	1	640.2131	-156.95	2	45.0	10.2	1	9719-9728	K.EVTIMEEKER.A	Oxidation: 5	WUP:QUP 1.16
1495	1	620.4184	75.49	3	48.4	11.8	2	10442-10458	K.GPEISEKIIPPKKPPTK.V		
1106	7	555.2011	-153.95	2	43.9	16.9	0	11321-11331	K.GTAIFACDIAK.D		
1925	1	661.3041	-146.74	2	54.4	13.5	1	12630-12641	R.AAAHLTVIEKLR.I		WUP:QUP 0.93
958	10	481.1097	-309.72	2	41.5	15.0	0	13135-13142	K.TWVLATDR.A		
1842	1	740.3191	-71.84	2	53.4	15.5	2	13621-13633	K.GYIVERCPRGSDK.W		WUP:QUP 2.11<br>QU:MU 0.75
2512	3	813.3358	-60.53	2	61.3	13.8	0	17073-17085	R.VPDLLEGCQYEFR.V	Carbamidomethyl: 8	
2614	2	701.8585	19.90	2	62.7	22.1	1	27706-27717	K.MVCSSVARTTFK.V	Carbamidomethyl: 3;<br>Oxidation: 1	
69	1	731.7932	-168.95	2	30.8	10.2	1	28600-28611	K.QLTFTVERLVEK.T		
560	1	607.8375	45.47	2	37.0	10.1	1	29711-29721	R.AQNEKGVSEPR.E		WUP:QUP 0.40
658	1	677.6643	-16.40	3	38.2	11.8	2	31095-31112	K.DAGFYVVCAKNRFGIDQK.T		
2593	1	616.1731	-223.16	2	60.7	11.0	1	31970-31979	R.MPYDVPEPRK.Y		
2811	1	1052.5183	-55.98	2	65.6	16.6	0	32274-32291	R.SLSPTYIELMRPVSELIR.S		
27	1	731.6483	-99.39	3	29.3	11.9	2	32802-32820	K.TNLQFMGQAFKSIHEKVSK.I		WUP:QUP 0.49<br>QU:MU 4.95

Detailed Protein Report

Protein 13: keratin, type II cytoskeletal 5 [Homo sapiens]

Accession: gi|119395754
Database: refseq_human(refseq_human_20140103.fasta)
Database Date: 2015-11-30
Modification(s): Carbamidomethyl

Score: 273.1
MW [kDa]: 62.3
pI: 8.6
Sequence Coverage [%]: 13.7
No. of unique Peptides: 2

Quantitation

QU:MU	**Median:**	1.06	**CV:**	13.88 %	**No. of Peptides:**	2
WUP:QUP	**Median:**	1.54	**CV:**	18.38 %	**No. of Peptides:**	2

10	20	30	40	50	60	70	80
MSRQSSVSFR	SGGSRSFSTA	SAITPSVSRT	SFTSVSRSGG	GGGGGFGRVS	LAGACGVGGY	GSRSLYNLGG	SKRISISTSG
**90**	**100**	**110**	**120**	**130**	**140**	**150**	**160**
GSFRNRFGAG	AGGGYGFGGG	AGSGFGFGGG	AGGGFGLGGG	AGFGGGFGGP	GFPVCPPGGI	QEVTVNQSLL	TPLNLQIDPS
**170**	**180**	**190**	**200**	**210**	**220**	**230**	**240**
IQRVRTEERE	QIKTLNNKFA	SFIDKVRFLE	QQNKVLDTKW	TLLQEQGTKT	VRQNLEPLFE	QYINNLRRQL	DSIVGERGRL
**250**	**260**	**270**	**280**	**290**	**300**	**310**	**320**
DSELRNMQDL	VEDFKNKYED	EINKRTTAEN	EFVMLKKDVD	AAYMNKVELE	AKVDALMDEI	NFMKMFFDAE	LSQMQTHVSD
**330**	**340**	**350**	**360**	**370**	**380**	**390**	**400**
TSVVLSMDNN	RNLDLDSIIA	EVKAQYEEIA	NRSRTEAESW	YQTKYEELQQ	TAGRHGDDLR	NTKHEISEMN	RMIQRLRAEI
**410**	**420**	**430**	**440**	**450**	**460**	**470**	**480**
DNVKKQCANL	QNAIADAEQR	GELALKDARN	KLAELEEALQ	KAKQDMARLL	REYQELMNTK	LALDVEIATY	RKLLEGEECR
**490**	**500**	**510**	**520**	**530**	**540**	**550**	**560**
LSGEGVGPVN	ISVVTSSVSS	GYGSGSGYGG	GLGGGLGGGL	GGGLAGGSSG	SYYSSSSGGV	GLGGGLSVGG	SGFSASSGRG
**570**	**580**	**590**	**600**				
LGVGFGSGGG	SSSSVKFVST	TSSSRKSFKS					

Cmpd.	No. of Cmpds.	m/z meas.	Δ m/z [ppm]	z	Rt [min]	Score	P	Range	Sequence	Modification	Ratios
585	1	851.2523	-176.11	2	36.9	23.4	0	406-420	K.QCANLQNAIADAEQR.G	Carbamidomethyl: 2	WUP:QUP 1.85<br>QU:MU 0.92
455	1	720.3680	11.23	2	35.7	13.9	0	560-576	R.GLGVGFGSGGGSSSSVK.F		WUP:QUP 1.28<br>QU:MU 1.21

Detailed Protein Report

Protein 14: annexin A1 [Homo sapiens]

**Accession:**	gi|4502101	**Score:**	224.8
**Database:**	refseq_human(refseq_human_20140103.fasta)	**MW [kDa]:**	38.7
**Database Date:**	2015-11-30	**pI:**	6.7
**Modification(s):**	Oxidation	**Sequence Coverage [%]:**	24.0
		**No. of unique Peptides:**	5

Quantitation

QU:MU	**Median:**	0.79	**CV:**	25.20 %	**No. of Peptides:**	3
WUP:QUP	**Median:**	1.22	**CV:**	0.79 %	**No. of Peptides:**	2

```
        10         20         30         40         50         60         70         80
MAMVSEFLKQ AWFIENEEQE YVQTVKSSKG GPGSAVSPYP TFNPSSDVAA LHKAIMVKGV DEATIIDILT KRNNAQRQQI
        90        100        110        120        130        140        150        160
KAAYLQETGK PLDETLKKAL TGHLEEVVLA LLKTPAQFDA DELRAAMKGL GTDEDTLIEI LASRTNKEIR DINRVYREEL
       170        180        190        200        210        220        230        240
KRDLAKDITS DTSGDFRNAL LSLAKGDRSE DFGVNEDLAD SDARALYEAG ERRKGTDVNV FNTILTTRSY PQLRRVFQKY
       250        260        270        280        290        300        310        320
TKYSKHDMNK VLDLELKGDI EKCLTAIVKC ATSKPAFFAE KLHQAMKGVG TRHKALIRIM VSRSEIDMND IKAFYQKMYG
       330        340        350
ISLCQAILDE TKGDYEKILV ALCGGN
```

Cmpd.	No. of Cmpds.	m/z meas.	Δ m/z [ppm]	z	Rt [min]	Score	P	Range	Sequence	Modification	Ratios	
1254	2	786.0316	-32.49	3	45.3	18.2	0	30-53	K. GGPGSAVSPYPTFNPSSDVAALH A		QU:MU	0.59
2994	1	973.5095	-39.14	2	66.2	10.2	1	54-71	K.AIMVKGVDEATIIDILTK.R	Oxidation: 3		
422	1	631.7779	-41.67	2	35.3	82.8	0	114-124	K.TPAQFDADELR.A		QU:MU<br>WUP:QUP	0.78<br>1.21
2959	4	851.9284	-21.25	2	65.8	81.3	0	129-144	K.GLGTDEDTLIEILASR.T			
2421	1	775.8147	-125.84	2	58.5	32.3	0	215-228	K.GTDVNVFNTILTTR.S		QU:MU<br>WUP:QUP	1.08<br>1.23

BRUKER

Detailed Protein Report

Protein 15: actin, cytoplasmic 2 [Homo sapiens]

**Accession:**	gi|4501887	**Score:**	217.8
**Database:**	refseq_human(refseq_human_20140103.fasta)	**MW [kDa]:**	41.8
**Database Date:**	2015-11-30	**pI:**	5.2
		**Sequence Coverage [%]:**	18.4
		**No. of unique Peptides:**	5

Quantitation

QU:MU	**Median:**	0.66	**CV:**	11.30 %	**No. of Peptides:**	2
WUP:QUP	**Median:**	2.75	**CV:**	23.69 %	**No. of Peptides:**	4

Alias proteins:

Accession	Name	Description
gi|316659409	refseq_human (refseq_human_20140103.fasta)	actin, cytoplasmic 2 [Homo sapiens]

```
        10         20         30         40         50         60         70         80
MEEEIAALVI DNGSGMCKAG FAGDDAPRAV FPSIVGRPRH QGVMVGMGQK DSYVGDEAQS KRGILTLKYP IEHGIVTNWD
        90        100        110        120        130        140        150        160
DMEKIWHHTF YNELRVAPEE HPVLLTEAPL NPKANREKMT QIMFETFNTP AMYVAIQAVL SLYASGRTTG IVMDSGDGVT
       170        180        190        200        210        220        230        240
HTVPIYEGYA LPHAILRLDL AGRDLTDYLM KILTERGYSF TTTAEREIVR DIKEKLCYVA LDFEQEMATA ASSSSLEKSY
       250        260        270        280        290        300        310        320
ELPDGQVITI GNERFRCPEA LFQPSFLGME SCGIHETTFN SIMKCDVDIR KDLYANTVLS GGTTMYPGIA DRMQKEITAL
       330        340        350        360        370        380
APSTMKIKII APPERKYSVW IGGSILASLS TFQQMWISKQ EYDESGPSIV HRKCF
```

Cmpd.	No. of Cmpds.	m/z meas.	Δ m/z [ppm]	z	Rt [min]	Score	P	Range	Sequence	Modification	Ratios
962	2	599.7074	-248.44	2	42.1	46.8	0	29-39	R.AVFPSIVGRPR.H		WUP:QUP 3.30
1159	2	651.9980	-43.48	3	44.1	29.5	0	96-113	R.VAPEEHPVLLTEAPLNPK.A		
1450	4	895.8455	-116.24	2	48.3	44.3	0	239-254	K.SYELPDGQVITIGNER.F		WUP:QUP 3.60<br>QU:MU 0.59
563	1	581.2929	-34.36	2	37.0	51.0	0	316-326	K.EITALAPSTMK.I		WUP:QUP 2.03
102	3	758.7219	-175.32	2	31.0	46.3	0	360-372	K.QEYDESGPSIVHR.K		WUP:QUP 2.39<br>QU:MU 0.74

Detailed Protein Report

Protein 16: **keratin, type II cytoskeletal 2 epidermal [Homo sapiens]**

**Accession:**	gi|47132620	**Score:**	200.1
**Database:**	refseq_human(refseq_human_20140103.fasta)	**MW [kDa]:**	65.4
**Database Date:**	2015-11-30	**pI:**	8.9
		**Sequence Coverage [%]:**	5.5
		**No. of unique Peptides:**	1

10	20	30	40	50	60	70	80
MSCQISCKSR	GRGGGGGGFR	GFSSGSAVVS	GGSRRSTSSF	SCLSRHGGGG	GGFGGGGFGS	RSLVGLGGTK	SISISVAGGG
**90**	**100**	**110**	**120**	**130**	**140**	**150**	**160**
GGFGAAGGFG	GRGGGFGGGS	SFGGGSGFSG	GGFGGGGFGG	GRFGGFGGPG	GVGGLGGPGG	FGPGGYPGGI	HEVSVNQSLL
**170**	**180**	**190**	**200**	**210**	**220**	**230**	**240**
QPLNVKVDPE	IQNVKAQERE	QIKTLNNKFA	SFIDKVRFLE	QQNQVLQTKW	ELLQQMNVGT	RPINLEPIFQ	GYIDSLKRYL
**250**	**260**	**270**	**280**	**290**	**300**	**310**	**320**
DGLTAERTSQ	NSELNNMQDL	VEDYKKKYED	EINKRTAAEN	DFVTLKKDVD	NAYMIKVELQ	SKVDLLNQEI	EFLKVLYDAE
**330**	**340**	**350**	**360**	**370**	**380**	**390**	**400**
ISQIHQSVTD	TNVILSMDNS	RNLDLDSIIA	EVKAQYEEIA	QRSKEEAEAL	YHSKYEELQV	TVGRHGDSLK	EIKIEISELN
**410**	**420**	**430**	**440**	**450**	**460**	**470**	**480**
RVIQRLQGEI	AHVKKQCKNV	QDAIADAEQR	GEHALKDARN	KLNDLEEALQ	QAKEDLARLL	RDYQELMNVK	LALDVEIATY
**490**	**500**	**510**	**520**	**530**	**540**	**550**	**560**
RKLLEGEECR	MSGDLSSNVT	VSVTSSTISS	NVASKAAFGG	SGGRGSSSGG	GYSSGSSSYG	SGGRQSGSRG	GSGGGGSISG
**570**	**580**	**590**	**600**	**610**	**620**	**630**	**640**
GGYGSGGGSG	GRYGSGGGSK	GGSISGGGYG	SGGGKHSSGG	GSRGGSSSGG	GYGSGGGGSS	SVKGSSGEAF	GSSVTFSFR

Cmpd.	No. of Cmpds.	m/z meas.	Δ m/z [ppm]	z	Rt [min]	Score	P	Range	Sequence	Modification
412	1	738.3807	-21.14	2	35.1	70.4	0	198-209	R.FLEQQNQVLQTK.W	

Detailed Protein Report

Protein 17: **keratin, type II cytoskeletal 75 [Homo sapiens]**

**Accession:**	gi|153791158	**Score:**	197.6
**Database:**	refseq_human(refseq_human_20140103.fasta)	**MW [kDa]:**	59.5
**Database Date:**	2015-11-30	**pI:**	8.7
		**Sequence Coverage [%]:**	8.2
		**No. of unique Peptides:**	1

10	20	30	40	50	60	70	80
MSRQSSITFQ	SGSRRGFSTT	SAITPAAGRS	RFSSVSVARS	AAGSGGLGRI	SSAGASFGSR	SLYNLGGAKR	VSINGCGSSC
**90**	**100**	**110**	**120**	**130**	**140**	**150**	**160**
RSGFGGRASN	RFGVNSGFGY	GGGVGGGFSG	PSFPVCPPGG	IQEVTVNQSL	LTPLHLQIDP	TIQRVRAEER	EQIKTLNNKF
**170**	**180**	**190**	**200**	**210**	**220**	**230**	**240**
ASFIDKVRFL	EQQNKVLETK	WALLQEQGSR	TVRQNLEPLF	DSYTSELRRQ	LESITTERGR	LEAELRNMQD	VVEDFKVRYE
**250**	**260**	**270**	**280**	**290**	**300**	**310**	**320**
DEINKRTAAE	NEFVALKKDV	DAAYMNKVEL	EAKVKSLPEE	INFIHSVFDA	ELSQLQTQVG	DTSVVLSMDN	NRNLDLDSII
**330**	**340**	**350**	**360**	**370**	**380**	**390**	**400**
AEVKAQYEDI	ANRSRAEAES	WYQTKYEELQ	VTAGRHGDDL	RNTKQEISEM	NRMIQRLRAE	IDSVKKQCSS	LQTAIADAEQ
**410**	**420**	**430**	**440**	**450**	**460**	**470**	**480**
RGELALKDAR	AKLVDLEEAL	QKAKQDMARL	LREYQELMNI	KLALDVEIAT	YRKLLEGEEC	RLSGEGVSPV	NISVVTSTLS
**490**	**500**	**510**	**520**	**530**	**540**	**550**	**560**
SGYGSGSSIG	GGNLGLGGGS	GYSFTTSGGH	SLGAGLGGSG	FSATSNRGLG	GSGSSVKFVS	TTSSSQKSYT	H

Cmpd.	No. of Cmpds.	m/z meas.	Δ m/z [ppm]	z	Rt [min]	Score	P	Range	Sequence	Modification
300	1	678.9411	74.83	2	32.5	11.4	1	413-424	K.LVDLEEALQKAK.Q	

Detailed Protein Report

Protein 18: **haptoglobin isoform 2 preproprotein [Homo sapiens]**

**Accession:**	gi|186910296	**Score:**	190.9
**Database:**	refseq_human(refseq_human_20140103.fasta)	**MW [kDa]:**	38.4
**Database Date:**	2015-11-30	**pI:**	6.1
**Modification(s):**	Carbamidomethyl	**Sequence Coverage [%]:**	15.6
		**No. of unique Peptides:**	4

Quantitation

QU:MU	**Median:**	0.99	**CV:**	43.43 %	**No. of Peptides:**	4
WUP:QUP	**Median:**	0.74	**CV:**	66.89 %	**No. of Peptides:**	4

```
        10         20         30         40         50         60         70         80
MSALGAVIAL LLWGQLFAVD SGNDVTDIAD DGCPKPPEIA HGYVEHSVRY QCKNYYKLRT EGDGVYTLNN EKQWINKAVG
        90        100        110        120        130        140        150        160
DKLPECEAVC GKPKNPANPV QRILGGHLDA KGSFPWQAKM VSHHNLTTGA TLINEQWLLT TAKNLFLNHS ENATAKDIAP
       170        180        190        200        210        220        230        240
TLTLYVGKKQ LVEIEKVVLH PNYSQVDIGL IKLKQKVSVN ERVMPICLPS KDYAEVGRVG YVSGWGRNAN FKFTDHLKYV
       250        260        270        280        290        300        310        320
MLPVADQDQC IRHYEGSTVP EKKTPKSPVG VQPILNEHTF CAGMSKYQED TCYGDAGSAF AVHDLEEDTW YATGILSFDK
       330        340        350
SCAVAEYGVY VKVTSIQDWV QKTIAEN
```

Cmpd.	No. of Cmpds.	m/z meas.	Δ m/z [ppm]	z	Rt [min]	Score	P	Range	Sequence	Modification	Ratios
107	1	854.9264	-2.66	2	30.3	16.6	1	58-72	K.LRTEGDGVYTLNNEK.Q		WUP:QUP 0.74<br>QU:MU 1.81
73	2	619.9745	-5.86	3	29.9	61.3	1	78-94	K.AVGDKLPECEAVCGKPK.N	Carbamidomethyl: 9, 13	WUP:QUP 0.73<br>QU:MU 1.02
1754	7	645.8334	-54.84	2	50.3	65.3	0	157-168	K.DIAPTLTLYVGK.K		QU:MU 0.94<br>WUP:QUP 0.32
741	2	602.2951	-44.79	2	39.2	47.7	0	333-342	K.VTSIQDWVQK.T		QU:MU 0.56<br>WUP:QUP 1.76

Detailed Protein Report

Protein 19: actin, aortic smooth muscle [Homo sapiens]

**Accession:**	gi|4501883	**Score:**	174.6
**Database:**	refseq_human(refseq_human_20140103.fasta)	**MW [kDa]:**	42.0
**Database Date:**	2015-11-30	**pI:**	5.1
**Modification(s):**	Carbamidomethyl	**Sequence Coverage [%]:**	19.9
		**No. of unique Peptides:**	2

Alias proteins:

Accession	Name	Description
gi|213688375	refseq_human (refseq_human_20140103.fasta)	actin, aortic smooth muscle [Homo sapiens]

10	20	30	40	50	60	70	80
MCEEEDSTAL	VCDNGSGLCK	AGFAGDDAPR	AVFPSIVGRP	RHQGVMVGMG	QKDSYVGDEA	QSKRGILTLK	YPIEHGIITN
**90**	**100**	**110**	**120**	**130**	**140**	**150**	**160**
WDDMEKIWHH	SFYNELRVAP	EEHPTLLTEA	PLNPKANREK	MTQIMFETFN	VPAMYVAIQA	VLSLYASGRT	TGIVLDSGDG
**170**	**180**	**190**	**200**	**210**	**220**	**230**	**240**
VTHNVPIYEG	YALPHAIMRL	DLAGRDLTDY	LMKILTERGY	SFVTTAEREI	VRDIKEKLCY	VALDFENEMA	TAASSSSLEK
**250**	**260**	**270**	**280**	**290**	**300**	**310**	**320**
SYELPDGQVI	TIGNERFRCP	ETLFQPSFIG	MESAGIHETT	YNSIMKCDID	IRKDLYANNV	LSGGTTMYPG	IADRMQKEIT
**330**	**340**	**350**	**360**	**370**	**380**		
ALAPSTMKIK	IIAPPERKYS	VWIGGSILAS	LSTFQQMWIS	KQEYDEAGPS	IVHRKCF		

Cmpd.	No. of Cmpds.	m/z meas.	Δ m/z [ppm]	z	Rt [min]	Score	P	Range	Sequence	Modification
2365	1	1015.4376	26.15	2	60.0	17.3	0	2-20	M.CEEEDSTALVCDNGSGLCK.A	Carbamidomethyl: 11
1391	1	652.6877	2.54	3	47.5	15.3	0	98-115	R.VAPEEHPTLLTEAPLNPK.A	

Detailed Protein Report

Protein 20: lactotransferrin isoform 2 [Homo sapiens]

**Accession:**	gi|312433998	**Score:**	170.8
**Database:**	refseq_human(refseq_human_20140103.fasta)	**MW [kDa]:**	73.1
**Database Date:**	2015-11-30	**pI:**	9.3
**Modification(s):**	Carbamidomethyl	**Sequence Coverage [%]:**	11.6
		**No. of unique Peptides:**	6

Quantitation

QU:MU	**Median:**	0.73	**CV:**	42.10 %	**No. of Peptides:**	3
WUP:QUP	**Median:**	3.59	**CV:**	25.66 %	**No. of Peptides:**	2

```
        10         20         30         40         50         60         70         80
MRKVRGPPVS CIKRDSPIQC IQAIAENRAD AVTLDGGFIY EAGLAPYKLR PVAAEVYGTE RQPRTHYYAV AVVKKGGSFQ
        90        100        110        120        130        140        150        160
LNELQGLKSC HTGLRRTAGW NVPIGTLRPF LNWTGPPEPI EAAVARFFSA SCVPGADKGQ FPNLCRLCAG TGENKCAFSS
       170        180        190        200        210        220        230        240
QEPYFSYSGA FKCLRDGAGD VAFIRESTVF EDLSDEAERD EYELLCPDNT RKPVDKFKDC HLARVPSHAV VARSVNGKED
       250        260        270        280        290        300        310        320
AIWNLLRQAQ EKFGKDKSPK FQLFGSPSGQ KDLLFKDSAI GFSRVPPRID SGLYLGSGYF TAIQNLRKSE EEVAARRARV
       330        340        350        360        370        380        390        400
VWCAVGEQEL RKCNQWSGLS EGSVTCSSAS TTEDCIALVL KGEADAMSLD GGYVYTAGKC GLVPVLAENY KSQQSSDPDP
       410        420        430        440        450        460        470        480
NCVDRPVEGY LAVAVVRRSD TSLTWNSVKG KKSCHTAVDR TAGWNIPMGL LFNQTGSCKF DEYFSQSCAP GSDPRSNLCA
       490        500        510        520        530        540        550        560
LCIGDEQGEN KCVPNSNERY YGYTGAFRCL AENAGDVAFV KDVTVLQNTD GNNNEAWAKD LKLADFALLC LDGKRKPVTE
       570        580        590        600        610        620        630        640
ARSCHLAMAP NHAVVSRMDK VERLKQVLLH QQAKFGRNGS DCPDKFCLFQ SETKNLLFND NTECLARLHG KTTYEKYLGP
       650        660        670
QYVAGITNLK KCSTSPLLEA CEFLRK
```

Cmpd.	No. of Cmpds.	m/z meas.	Δ m/z [ppm]	z	Rt [min]	Score	P	Range	Sequence	Modification	Ratios
739	1	787.3768	-43.94	2	39.2	13.3	1	320-332	R.VVWCAVGEQELRK.C	Carbamidomethyl: 4	
1575	3	681.8469	-16.13	2	49.9	23.1	0	380-391	K.CGLVPVLAENYK.S	Carbamidomethyl: 1	
458	1	619.2938	-20.74	2	35.7	45.8	0	419-429	R.SDTSLTWNSVK.G		QU:MU 1.20
593	1	904.3991	-2.21	2	37.4	38.9	0	476-491	R.SNLCALCIGDEQGENK.C	Carbamidomethyl: 4, 7	
2333	1	668.3646	18.05	2	59.0	28.6	0	543-554	K.LADFALLCLDGK.R	Carbamidomethyl: 8	QU:MU 0.73<br>WUP:QUP 2.79
1229	1	790.3991	23.83	2	45.5	20.9	0	615-627	K.NLLFNDNTECLAR.L	Carbamidomethyl: 10	WUP:QUP 4.62<br>QU:MU 0.44

Detailed Protein Report

BRUKER

Protein 21: POTE ankyrin domain family member F [Homo sapiens]

**Accession:**	gi|153791352	**Score:**	148.1
**Database:**	refseq_human(refseq_human_20140103.fasta)	**MW [kDa]:**	121.4
**Database Date:**	2015-11-30	**pI:**	5.8
		**Sequence Coverage [%]:**	4.7
		**No. of unique Peptides:**	1

10	20	30	40	50	60	70	80
MVVEVDSMPA	ASSVKKPFGL	RSKMGKWCCR	CFPCCRESGK	SNVGTSGDHD	DSAMKTLRSK	MGKWCRHCFP	CCRGSGKSNV
90	100	110	120	130	140	150	160
GASGDHDDSA	MKTLRNKMGK	WCCHCFPCCR	GSSKSKVGAW	GDYDDSAFME	PRYHVRGEDL	DKLHRAAWWG	KVPRKDLIVM
170	180	190	200	210	220	230	240
LRDTDVNKQD	KQKRTALHLA	SANGNSEVVK	LLLDRRCQLN	VLDNKKRTAL	IKAVQCQEDE	CALMLLEHGT	DPNIPDEYGN
250	260	270	280	290	300	310	320
TTLHYAIYNE	DKLMAKALLL	YGADIESKNK	HGLTPLLLGV	HEQKQQVVKF	LIKKKANLNA	LDRYGRTALI	LAVCCGSASI
330	340	350	360	370	380	390	400
VSLLLEQNID	VSSQDLSGQT	AREYAVSSHH	HVICQLLSDY	KEKQMLKISS	ENSNPEQDLK	LTSEEESQRF	KGSENSQPEK
410	420	430	440	450	460	470	480
MSQEPEINKD	GDREVEEEMK	KHESNNVGLL	ENLTNGVTAG	NGDNGLIPQR	KSRTPENQQF	PDNESEEYHR	ICELLSDYKE
490	500	510	520	530	540	550	560
KQMPKYSSEN	SNPEQDLKLT	SEEESQRLKG	SENGQPEKRS	QEPEINKDGD	RELENFMAIE	EMKKHRSTHV	GFPENLTNGA
570	580	590	600	610	620	630	640
TAGNGDDGLI	PPRKSRTPES	QQFPDTENEE	YHSDEQNDTQ	KQFCEEQNTG	ILHDEILIHE	EKQIEVVEKM	NSELSLSCKK
650	660	670	680	690	700	710	720
EKDILHENST	LREEIAMLRL	ELDTMKHQSQ	LREKKYLEDI	ESVKKRNDNL	LKALQLNELT	MDDDTAVLVI	DNGSGMCKAG
730	740	750	760	770	780	790	800
FAGDDAPRAV	FPSIVGRPRQ	QGMMGGMHQK	ESYVGKEAQS	KRGILTLKYP	MEHGIITNWD	DMEKIWHHTF	YNELRVAPEE
810	820	830	840	850	860	870	880
HPVLLTEATL	NPKANREKMT	QIMFETFNTP	AMYVAIQAVL	SLYTSGRTTG	IVMDSGDGVT	HTVPIYEGNA	LPHATLRLDL
890	900	910	920	930	940	950	960
AGRELPDYLM	KILTEHGYRF	TTMAEREIVR	DIKEKLCYVA	LDFEQEMATV	ASSSSLEKSY	ELPDGQVITI	GNERFRCPEA
970	980	990	1000	1010	1020	1030	1040
LFQPCFLGME	SCGIHETTFN	SIMKSDVDIR	KDLYTNTVLS	GGTTMYPGMA	HRMQKEIAAL	APSMMKIRII	APPKRKYSVW
1050	1060	1070	1080				
VGGSILASLS	TFQQMWISKQ	EYDESGPSIV	HRKCL				

Cmpd.	No. of Cmpds.	m/z meas.	Δ m/z [ppm]	z	Rt [min]	Score	P	Range	Sequence	Modification
1070	1	670.5538	205.94	2	43.5	10.8	2	152-162	K.VPRKDLIVMLR.D	

Detailed Protein Report

Protein 22: alpha-1-antitrypsin precursor [Homo sapiens]

**Accession:**	gi|50363217	**Score:**	141.1
**Database:**	refseq_human(refseq_human_20140103.fasta)	**MW [kDa]:**	46.7
**Database Date:**	2015-11-30	**pI:**	5.3
		**Sequence Coverage [%]:**	15.8
		**No. of unique Peptides:**	3

Quantitation

QU:MU	**Median:**	2.56	**CV:**	0.00 %	**No. of Peptides:**	1
WUP:QUP	**Median:**	0.71	**CV:**	28.96 %	**No. of Peptides:**	2

Alias proteins:

Accession	Name	Description
gi|189163542	refseq_huma (refseq_human_20140103.fasta)	alpha-1-antitrypsin precursor [Homo sapiens]
gi|189163540	refseq_huma (refseq_human_20140103.fasta)	alpha-1-antitrypsin precursor [Homo sapiens]
gi|189163538	refseq_huma (refseq_human_20140103.fasta)	alpha-1-antitrypsin precursor [Homo sapiens]
gi|189163536	refseq_huma (refseq_human_20140103.fasta)	alpha-1-antitrypsin precursor [Homo sapiens]
gi|189163534	refseq_huma (refseq_human_20140103.fasta)	alpha-1-antitrypsin precursor [Homo sapiens]
gi|189163532	refseq_huma (refseq_human_20140103.fasta)	alpha-1-antitrypsin precursor [Homo sapiens]
gi|189163530	refseq_huma (refseq_human_20140103.fasta)	alpha-1-antitrypsin precursor [Homo sapiens]
gi|189163528	refseq_huma (refseq_human_20140103.fasta)	alpha-1-antitrypsin precursor [Homo sapiens]
gi|50363221	refseq_huma (refseq_human_20140103.fasta)	alpha-1-antitrypsin precursor [Homo sapiens]
gi|50363219	refseq_huma (refseq_human_20140103.fasta)	alpha-1-antitrypsin precursor [Homo sapiens]

10	20	30	40	50	60	70	80
MPSSVSWGIL	LLAGLCCLVP	VSLAEDPQGD	AAQKTDTSHH	DQDHPTFNKI	TPNLAEFAFS	LYRQLAHQSN	STNIFFSPVS
**90**	**100**	**110**	**120**	**130**	**140**	**150**	**160**
IATAFAMLSL	GTKADTHDEI	LEGLNFNLTE	IPEAQIHEGF	QELLRTLNQP	DSQLQLTTGN	GLFLSEGLKL	VDKFLEDVKK
**170**	**180**	**190**	**200**	**210**	**220**	**230**	**240**
LYHSEAFTVN	FGDTEEAKKQ	INDYVEKGTQ	GKIVDLVKEL	DRDTVFALVN	YIFFKGKWER	PFEVKDTEEE	DFHVDQVTTV
**250**	**260**	**270**	**280**	**290**	**300**	**310**	**320**
KVPMMKRLGM	FNIQHCKKLS	SWVLLMKYLG	NATAIFFLPD	EGKLQHLENE	LTHDIITKFL	ENEDRRSASL	HLPKLSITGT
**330**	**340**	**350**	**360**	**370**	**380**	**390**	**400**
YDLKSVLGQL	GITKVFSNGA	DLSGVTEEAP	LKLSKAVHKA	VLTIDEKGTE	AAGAMFLEAI	PMSIPPEVKF	NKPFVFLMIE
**410**	**420**						
QNTKSPLFMG	KVVNPTQK						

Cmpd.	No. of Cmpds.	m/z meas.	Δ m/z [ppm]	z	Rt [min]	Score	P	Range	Sequence	Modification	Ratios
2521	1	821.3964	-47.50	2	61.5	24.3	0	50-63	K.ITPNLAEFAFSLYR.Q		
2391	1	1287.6505	-18.34	2	58.2	20.0	0	126-149	R. TLNQPDSQLQLTTGNGLFLSEGLK L		WUP:QUP 0.94
677	3	555.6562	-269.06	2	36.9	69.2	0	315-324	K.LSITGTYDLK.S		QU:MU 2.56<br>WUP:QUP 0.53

Detailed Protein Report

Protein 23: keratin, type II cytoskeletal 79 [Homo sapiens]

**Accession:**	gi|32567786	**Score:**	140.4
**Database:**	refseq_human(refseq_human_20140103.fasta)	**MW [kDa]:**	57.8
**Database Date:**	2015-11-30	**pI:**	6.9
**Modification(s):**	Oxidation	**Sequence Coverage [%]:**	6.7
		**No. of unique Peptides:**	1

Quantitation

QU:MU **Median:** 0.66 **CV:** 0.00 % **No. of Peptides:** 1

10	20	30	40	50	60	70	80
MRSSVSRQTY	STKGGFSSNS	ASGGSGSQAR	TSFSSVTVSR	SSGSGGGAHC	GPGTGGFGSR	SLYNLGGHKS	ISVSVAGGAL
90	100	110	120	130	140	150	160
LGRALGGFGF	GSRAFMGQGA	GRQTFGPACP	PGGIQEVTVN	QSLLTPLHVE	IDPEIQRVRT	QEREQIKTLN	NKFASFIDKV
170	180	190	200	210	220	230	240
RFLEQQNKVL	ETKWALLQEQ	GQNLGVTRNN	LEPLFEAYLG	SMRSTLDRLQ	SERGRLDSEL	RNVQDLVEDF	KNKYEDEINK
250	260	270	280	290	300	310	320
HTAAENEFVV	LKKDVDAAYM	GRMDLHGKVG	TLTQEIDFLQ	QLYEMELSQV	QTHVSNTNVV	LSMDNNRNLD	LDSIIAEVKA
330	340	350	360	370	380	390	400
QYELIAQRSR	AEAEAWYQTK	YEELQVTAGK	HGDNLRDTKN	EIAELTRTIQ	RLQGEADAAK	KQCQQLQTAI	AEAEQRGELA
410	420	430	440	450	460	470	480
LKDAQKKLGD	LDVALHQAKE	DLTRLLRDYQ	ELMNVKLALD	VEIATYRKLL	ESEESRMSGE	CPSAVSISVT	GNSTTVCGGG
490	500	510	520	530	540		
AASFGGGISL	GGSGGATKGG	FSTNVGYSTV	KGGPVSAGTS	ILRKTTTVKT	SSQRY		

Cmpd.	No. of Cmpds.	m/z meas.	Δ m/z [ppm]	z	Rt [min]	Score	P	Range	Sequence	Modification	Ratios	
140	1	773.9092	30.36	2	31.7	10.7	2	1-13	-.MRSSVSRQTYSTK.G	Oxidation: 1	QU:MU	0.66

Detailed Protein Report

Protein 24: fibrinogen gamma chain isoform gamma-A precursor [Homo sapiens]

**Accession:**	gi|70906437	**Score:**	122.9
**Database:**	refseq_human(refseq_human_20140103.fasta)	**MW [kDa]:**	49.5
**Database Date:**	2015-11-30	**pI:**	5.7
		**Sequence Coverage [%]:**	10.8
		**No. of unique Peptides:**	4

Quantitation

QU:MU	**Median:** 1.76	**CV:** 0.00 %	**No. of Peptides:**	1
WUP:QUP	**Median:** 0.36	**CV:** 0.00 %	**No. of Peptides:**	1

```
        10         20         30         40         50         60         70         80
MSWSLHPRNL ILYFYALLFL SSTCVAYVAT RDNCCILDER FGSYCPTTCG IADFLSTYQT KVDKDLQSLE DILHQVENKT
        90        100        110        120        130        140        150        160
SEVKQLIKAI QLTYNPDESS KPNMIDAATL KSRKMLEEIM KYEASILTHD SSIRYLQEIY NSNNQKIVNL KEKVAQLEAQ
       170        180        190        200        210        220        230        240
CQEPCKDTVQ IHDITGKDCQ DIANKGAKQS GLYFIKPLKA NQQFLVYCEI DGSGNGWTVF QKRLDGSVDF KKNWIQYKEG
       250        260        270        280        290        300        310        320
FGHLSPTGTT EFWLGNEKIH LISTQSAIPY ALRVELEDWN GRTSTADYAM FKVGPEADKY RLTYAYFAGG DAGDAFDGFD
       330        340        350        360        370        380        390        400
FGDDPSDKFF TSHNGMQFST WDNDNDKFEG NCAEQDGSGW WMNKCHAGHL NGVYYQGGTY SKASTPNGYD NGIIWATWKT
       410        420        430        440
RWYSMKKTTM KIIPFNRLTI GEGQQHHLGG AKQAGDV
```

Cmpd.	No. of Cmpds.	m/z meas.	Δ m/z [ppm]	z	Rt [min]	Score	P	Range	Sequence	Modification	Ratios
127	1	757.2233	-190.62	2	30.5	61.4	0	135-146	R.YLQEIYNSNNQK.I		QU:MU 1.76<br>WUP:QUP 0.36
1191	1	647.3732	-13.52	2	43.1	26.2	0	189-199	K.QSGLYFIKPLK.A		
2104	2	841.8525	-155.05	2	54.7	23.4	0	259-273	K.IHLISTQSAIPYALR.V		
353	1	559.1708	-172.44	2	34.1	11.9	0	274-282	R.VELEDWNGR.T		

Detailed Protein Report

Protein 25: transthyretin precursor [Homo sapiens]

**Accession:**	gi|4507725	**Score:**	120.5
**Database:**	refseq_human(refseq_human_20140103.fasta)	**MW [kDa]:**	15.9
**Database Date:**	2015-11-30	**pI:**	5.4
		**Sequence Coverage [%]:**	18.4
		**No. of unique Peptides:**	3

Quantitation

QU:MU **Median:** 1.43 **CV:** 0.00 % **No. of Peptides:** 1

```
        10         20         30         40         50         60         70         80
MASHRLLLLC LAGLVFVSEA GPTGTGESKC PLMVKVLDAV RGSPAINVAV HVFRKAADDT WEPFASGKTS ESGELHGLTT
        90        100        110        120        130        140        150
EEEFVEGIYK VEIDTKSYWK ALGISPFHEH AEVVFTANDS GPRRYTIAAL LSPYSYSTTA VVTNPKE
```

Cmpd.	No. of Cmpds.	m/z meas.	Δ m/z [ppm]	z	Rt [min]	Score	P	Range	Sequence	Modification	Ratios
1401	2	683.8722	-15.95	2	45.9	76.1	0	42-54	R.GSPAINVAVHVFR.K		
594	2	761.8596	-3.46	2	37.4	15.4	1	55-68	R.KAADDTWEPFASGK.T		QU:MU 1.43
810	1	697.8221	10.50	2	40.1	29.0	0	56-68	K.AADDTWEPFASGK.T		

Detailed Protein Report

Protein 26: neutrophil defensin 1 preproprotein [Homo sapiens]

Accession: gi|4758146
Database: refseq_human(refseq_human_20140103.fasta)
Database Date: 2015-11-30
Modification(s): Carbamidomethyl

Score: 108.8
MW [kDa]: 10.2
pI: 7.6
Sequence Coverage [%]: 19.1
No. of unique Peptides: 2

Quantitation

QU:MU	**Median:** 0.71	**CV:** 0.00 %	**No. of Peptides:** 1
WUP:QUP	**Median:** 1.65	**CV:** 0.00 %	**No. of Peptides:** 1

Alias proteins:

Accession	Name	Description
gi|124248516	refseq_human (refseq_human_20140103.fasta)	neutrophil defensin 1 precursor [Homo sapiens]

```
        10         20         30         40         50         60         70         80
MRTLAILAAI LLVALQAQAE PLQARADEVA AAPEQIAADI PEVVVSLAWD ESLAPKHPGS RKNMACYCRI PACIAGERRY
        90        100
GTCIYQGRLW AFCC
```

Cmpd.	No. of Cmpds.	m/z meas.	Δ m/z [ppm]	z	Rt [min]	Score	P	Range	Sequence	Modification	Ratios
147	1	493.7604	4.77	2	31.8	52.9	0	70-78	R.IPACIAGER.R	Carbamidomethyl: 4	QU:MU 0.71
108	3	559.2630	8.21	2	31.3	55.9	0	80-88	R.YGTCIYQGR.L	Carbamidomethyl: 4	WUP:QUP 1.65

Detailed Protein Report

Protein 27: laminin subunit alpha-2 isoform a precursor [Homo sapiens]

**Accession:**	gi|28559088	**Score:**	104.3
**Database:**	refseq_human(refseq_human_20140103.fasta)	**MW [kDa]:**	343.7
**Database Date:**	2015-11-30	**pI:**	6.0
**Modification(s):**	Carbamidomethyl, Oxidation	**Sequence Coverage [%]:**	2.8
		**No. of unique Peptides:**	8

Quantitation

QU:MU	**Median:**	0.80	**CV:**	107.92 %	**No. of Peptides:**	3
WUP:QUP	**Median:**	1.64	**CV:**	49.37 %	**No. of Peptides:**	2

Detailed Protein Report

10	20	30	40	50	60	70	80
MPGAAGVLLL	LLLSGGLGGV	QAQRPQQQRQ	SQAHQQRGLF	PAVLNLASNA	LITTNATCGE	KGPEMYCKLV	EHVPGQPVRN
90	100	110	120	130	140	150	160
PQCRICNQNS	SNPNQRHPIT	NAIDGKNTWW	QSPSIKNGIE	YHYVTITLDL	QQVFQIAYVI	VKAANSPRPG	NWILERSLDD
170	180	190	200	210	220	230	240
VEYKPWQYHA	VTDTECLTLY	NIYPRTGPPS	YAKDDEVICT	SFYSKIHPLE	NGEIHISLIN	GRPSADDPSP	ELLEFTSARY
250	260	270	280	290	300	310	320
IRLRFQRIRT	LNADLMMFAH	KDPREIDPIV	TRRYYYSVKD	ISVGGMCICY	GHARACPLDP	ATNKSRCECE	HNTCGDSCDQ
330	340	350	360	370	380	390	400
CCPGFHQKPW	RAGTFLTKTE	CEACNCHGKA	EECYYDENVA	RRNLSLNIRG	KYIGGGVCIN	CTQNTAGINC	ETCTDGFFRP
410	420	430	440	450	460	470	480
KGVSPNYPRP	CQPCHCDPIG	SLNEVCVKDE	KHARRGLAPG	SCHCKTGFGG	VSCDRCARGY	TGYPDCKACN	CSGLGSKNED
490	500	510	520	530	540	550	560
PCFGPCICKE	NVEGGDCSRC	KSGFFNLQED	NWKGCDECFC	SGVSNRCQSS	YWTYGKIQDM	SGWYLTDLPG	RIRVAPQQDD
570	580	590	600	610	620	630	640
LDSPQQISIS	NAEARQALPH	SYYWSAPAPY	LGNKLPAVGG	QLTFTISYDL	EEEEEDTERV	LQLMIILEGN	DLSISTAQDE
650	660	670	680	690	700	710	720
VYLHPSEEHT	NVLLLKEESF	TIHGTHFPVR	RKEFMTVLAN	LKRVLLQITY	SFGMDAIFRL	SSVNLESAVS	YPTDGSIAAA
730	740	750	760	770	780	790	800
VEVCQCPPGY	TGSSCESCWP	RHRRVNGTIF	GGICEPCQCF	GHAESCDDVT	GECLNCKDHT	GGPYCDKCLP	GFYGEPTKGT
810	820	830	840	850	860	870	880
SEDCQPCACP	LNIPSNNFSP	TCHLDRSLGL	ICDGCPVGYT	GPRCERCAEG	YFGQPSVPGG	SCQPCQCNDN	LDFSIPGSCD
890	900	910	920	930	940	950	960
SLSGSCLICK	PGTTGRYCEL	CADGYFGDAV	DAKNCQPCRC	NAGGSFSEVC	HSQTGQCECR	ANVQGQRCDK	CKAGTFGLQS
970	980	990	1000	1010	1020	1030	1040
ARGCVPCNCN	SFGSKSFDCE	ESGQCWCQPG	VTGKKCDRCA	HGYFNFQEGG	CTACECSHLG	NNCDPKTGRC	ICPPNTIGEK
1050	1060	1070	1080	1090	1100	1110	1120
CSKCAPNTWG	HSITTGCKAC	NCSTVGSLDF	QCNVNTGQCN	CHPKFSGAKC	TECSRGHWNY	PRCNLCDCFL	PGTDATTCDS
1130	1140	1150	1160	1170	1180	1190	1200
ETKKCSCSDQ	TGQCTCKVNV	EGIHCDRCRP	GKFGLDAKNP	LGCSSCYCFG	TTTQCSEAKG	LIRTWVTLKA	EQTILPLVDE
1210	1220	1230	1240	1250	1260	1270	1280
ALQHTTTKGI	VFQHPEIVAH	MDLMREDLHL	EPFYWKLPEQ	FEGKKLMAYG	GKLKYAIYFE	AREETGFSTY	NPQVIIRGGT
1290	1300	1310	1320	1330	1340	1350	1360
PTHARIIVRH	MAAPLIGQLT	RHEIEMTEKE	WKYYGDDPRV	HRTVTREDFL	DILYDIHYIL	IKATYGNFMR	QSRISEISME
1370	1380	1390	1400	1410	1420	1430	1440
VAEQGRGTTM	TPPADLIEKC	DCPLGYSGLS	CEACLPGFYR	LRSQPGGRTP	GPTLGTCVPC	QCNGHSSLCD	PETSICQNCQ
1450	1460	1470	1480	1490	1500	1510	1520
HHTAGDFCER	CALGYYGIVK	GLPNDCQQCA	CPLISSSNNF	SPSCVAEGLD	DYRCTACPRG	YEGQYCERCA	PGYTGSPGNP
1530	1540	1550	1560	1570	1580	1590	1600
GGSCQECECD	PYGSLPVPCD	PVTGFCTCRP	GATGRKCDGC	KHWHAREGWE	CVFCGDECTG	LLLGDLARLE	QMVMSINLTG
1610	1620	1630	1640	1650	1660	1670	1680
PLPAPYKMLY	GLENMTQELK	HLLSPQRAPE	RLIQLAEGNL	NTLVTEMNEL	LTRATKVTAD	GEQTGQDAER	TNTRAKSLGE
1690	1700	1710	1720	1730	1740	1750	1760
FIKELARDAE	AVNEKAIKLN	ETLGTRDEAF	ERNLEGLQKE	IDQMIKELRR	KNLETQKEIA	EDELVAAEAL	LKKVKKLFGE
1770	1780	1790	1800	1810	1820	1830	1840
SRGENEEMEK	DLREKLADYK	NKVDDAWDLL	REATDKIREA	NRLFAVNQKN	MTALEKKKEA	VESGKRQIEN	TLKEGNDILD
1850	1860	1870	1880	1890	1900	1910	1920
EANRLADEIN	SIIDYVEDIQ	TKLPPMSEEL	NDKIDDLSQE	IKDRKLAEKV	SQAESHAAQL	NDSSAVLDGI	LDEAKNISFN
1930	1940	1950	1960	1970	1980	1990	2000
ATAAFKAYSN	IKDYIDEAEK	VAKEAKDLAH	EATKLATGPR	GLLKEDAKGC	LQKSFRILNE	AKKLANDVKE	NEDHLNGLKT
2010	2020	2030	2040	2050	2060	2070	2080
RIENADARNG	DLLRTLNDTL	GKLSAIPNDT	AAKLQAVKDK	ARQANDTAKD	VLAQITELHQ	NLDGLKKNYN	KLADSVAKTN
2090	2100	2110	2120	2130	2140	2150	2160
AVVKDPSKNK	IIADADATVK	NLEQEADRLI	DKLKPIKELE	DNLKKNISEI	KELINQARKQ	ANSIKVSVSS	GGDCIRTYKP
2170	2180	2190	2200	2210	2220	2230	2240

Detailed Protein Report

Cmpd.	No. of Cmpds.	m/z meas.	Δ m/z [ppm]	z	Rt [min]	Score	P	Range	Sequence	Modification	Ratios
2885	1	1023.0269	2.26	2	64.8	14.8	2	248-264	R.IRTLNADLMMFAHKDPR.E	Oxidation: 9	
2021	1	665.3031	-44.41	2	55.2	14.0	1	295-306	R.ACPLDPATNKSR.C	Carbamidomethyl: 2	QU:MU 0.31
892	1	739.3524	121.81	2	39.5	10.1	0	1125-1137	K.CSCSDQTGQCTCK.V	Carbamidomethyl: 1, 10	
1065	1	836.1966	-317.81	1	42.9	11.8	1	1756-1762	K.KLFGESR.G		
445	1	475.6547	-205.40	2	34.1	10.7	1	1810-1817	K.NMTALEKK.K	Oxidation: 2	WUP:QUP 1.03<br>QU:MU 2.57
1656	1	803.8745	-81.91	2	50.5	10.4	2	1969-1982	K.GCLQKSFRILNEAK.K		
1760	1	568.7537	-31.99	2	52.3	14.8	0	2146-2156	K.VSVSSGGDCIR.T	Carbamidomethyl: 9	
2430	1	531.0471	-454.49	1	60.8	17.8	0	2291-2295	K.ADAVR.V		WUP:QUP 2.62<br>QU:MU 0.65

Detailed Protein Report

Protein 28: heat shock protein beta-1 [Homo sapiens]

Accession: gi|4504517
Database: refseq_human(refseq_human_20140103.fasta)
Database Date: 2015-11-30

Score: 97.8
MW [kDa]: 22.8
pI: 6.0
Sequence Coverage [%]: 21.5
No. of unique Peptides: 3

Quantitation

QU:MU	**Median:**	0.88	**CV:**	0.00 %	**No. of Peptides:**	1
WUP:QUP	**Median:**	2.40	**CV:**	0.00 %	**No. of Peptides:**	1

```
        10         20         30         40         50         60         70         80
MTERRVPFSL LRGPSWDPFR DWYPHSRLFD QAFGLPRLPE EWSQWLGGSS WPGYVRPLPP AAIESPAVAA PAYSRALSRQ
        90        100        110        120        130        140        150        160
LSSGVSEIRH TADRWRVSLD VNHFAPDELT VKTKDGVVEI TGKHEERQDE HGYISRCFTR KYTLPPGVDP TQVSSSLSPE
       170        180        190        200        210
GTLTVEAPMP KLATQSNEIT IPVTFESRAQ LGGPEAAKSD ETAAK
```

Cmpd.	No. of Cmpds.	m/z meas.	Δ m/z [ppm]	z	Rt [min]	Score	P	Range	Sequence	Modification	Ratios
1772	7	582.3088	-9.00	2	50.5	62.7	0	28-37	R.LFDQAFGLPR.L		
1635	1	953.4046	-99.49	2	50.2	22.4	0	172-188	K.LATQSNEITIPVTFESR.A		WUP:QUP 2.40<br>QU:MU 0.88
2630	2	822.4504	42.56	2	63.7	12.6	1	189-205	R.AQLGGPEAAKSDETAAK.-		

Detailed Protein Report

Protein 29: **apolipoprotein A-II preproprotein [Homo sapiens]**

Accession: gi|4502149
Database: refseq_human(refseq_human_20140103.fasta)
Database Date: 2015-11-30

Score: 97.3
MW [kDa]: 11.2
pI: 7.1
Sequence Coverage [%]: 32.0
No. of unique Peptides: 2

10	20	30	40	50	60	70	80
MKLLAATVLL	LTICSLEGAL	VRRQAKEPCV	ESLVSQYFQT	VTDYGKDLME	KVKSPELQAE	AKSYFEKSKE	QLTPLIKKAG
**90**	**100**	**110**					
TELVNFLSYF	VELGTQPATQ						

Cmpd.	No. of Cmpds.	m/z meas.	Δ m/z [ppm]	z	Rt [min]	Score	P	Range	Sequence	Modification
457	3	578.8364	-24.29	2	35.7	60.5	1	68-77	K.SKEQLTPLIK.K	
2890	1	1193.0548	-39.81	2	64.9	36.7	0	79-100	K.AGTELVNFLSYFVELGTQPATQ.-	

Detailed Protein Report

Protein 30: keratin, type II cytoskeletal 1 [Homo sapiens]

Accession: gi|119395750
Database: refseq_human(refseq_human_20140103.fasta)
Database Date: 2015-11-30

Score: 94.9
MW [kDa]: 66.0
pI: 8.8
Sequence Coverage [%]: 3.7
No. of unique Peptides: 1

10	20	30	40	50	60	70	80
MSRQFSSRSG	YRSGGGFSSG	SAGIINYQRR	TTSSSTRRSG	GGGGRFSSCG	GGGGSFGAGG	GFGSRSLVNL	GGSKSISISV
**90**	**100**	**110**	**120**	**130**	**140**	**150**	**160**
ARGGGRGSGF	GGGYGGGGFG	GGGFGGGGFG	GGGIGGGGFG	GFGSGGGGFG	GGGFGGGGYG	GGYGPVCPPG	GIQEVTINQS
**170**	**180**	**190**	**200**	**210**	**220**	**230**	**240**
LLQPLNVEID	PEIQKVKSRE	REQIKSLNNQ	FASFIDKVRF	LEQQNQVLQT	KWELLQQVDT	STRTHNLEPY	FESFINNLRR
**250**	**260**	**270**	**280**	**290**	**300**	**310**	**320**
RVDQLKSDQS	RLDSELKNMQ	DMVEDYRNKY	EDEINKRTNA	ENEFVTIKKD	VDGAYMTKVD	LQAKLDNLQQ	EIDFLTALYQ
**330**	**340**	**350**	**360**	**370**	**380**	**390**	**400**
AELSQMQTQI	SETNVILSMD	NNRSLDLDSI	IAEVKAQYED	IAQKSKAEAE	SLYQSKYEEL	QITAGRHGDS	VRNSKIEISE
**410**	**420**	**430**	**440**	**450**	**460**	**470**	**480**
LNRVIQRLRS	EIDNVKKQIS	NLQQSISDAE	QRGENALKDA	KNKLNDLEDA	LQQAKEDLAR	LLRDYQELMN	TKLALDLEIA
**490**	**500**	**510**	**520**	**530**	**540**	**550**	**560**
TYRTLLEGEE	SRMSGECAPN	VSVSVSTSHT	TISGGGSRGG	GGGGYGSGGS	SYGSGGGSYG	SGGGGGGGRG	SYGSGGSSYG
**570**	**580**	**590**	**600**	**610**	**620**	**630**	**640**
SGGGSYGSGG	GGGGHGSYGS	GSSSGGYRGG	SGGGGGGSSG	GRGSGGGSSG	GSIGGRGSSS	GGVKSSGGSS	SVKFVSTTYS
**650**							
GVTR							

Cmpd.	No. of Cmpds.	m/z meas.	Δ m/z [ppm]	z	Rt [min]	Score	P	Range	Sequence	Modification
1554	1	692.3258	-33.18	2	49.6	24.5	0	186-197	K.SLNNQFASFIDK.V	

Detailed Protein Report

Protein 31: immunoglobulin lambda-like polypeptide 5 isoform 1 [Homo sapiens]

**Accession:**	gi|295986608	**Score:**	94.5
**Database:**	refseq_human(refseq_human_20140103.fasta)	**MW [kDa]:**	23.0
**Database Date:**	2015-11-30	**pI:**	10.0
**Modification(s):**	Carbamidomethyl	**Sequence Coverage [%]:**	23.8
		**No. of unique Peptides:**	3

Quantitation

WUP:QUP **Median:** 0.90 **CV:** 0.00 % **No. of Peptides:** 1

```
        10         20         30         40         50         60         70         80
MRPKTGQVGC ETPEELGPGP RQRWPLLLLG LAMVAHGLLR PMVAPQSGDP DPGASVGSSR SSLRSLWGRL LLQPSPQRAD
        90        100        110        120        130        140        150        160
PRCWPRGFWS EPQSLCYVFG TGTKVTVLGQ PKANPTVTLF PPSSEELQAN KATLVCLISD FYPGAVTVAW KADGSPVKAG
       170        180        190        200        210        220
VETTKPSKQS NNKYAASSYL SLTPEQWKSH RSYSCQVTHE GSTVEKTVAP TECS
```

Cmpd.	No. of Cmpds.	m/z meas.	Δ m/z [ppm]	z	Rt [min]	Score	P	Range	Sequence	Modification	Ratios
2042	1	1045.4503	-102.49	2	55.9	14.4	2	70-86	R.LLLQPSPQRADPRCWPR.G	Carbamidomethyl: 14	
1468	2	1022.0062	-16.69	2	48.0	16.1	0	113-131	K.ANPTVTLFPPSSEELQANK.A		
1789	12	872.4180	-17.25	2	50.7	64.0	0	174-188	K.YAASSYLSLTPEQWK.S		WUP:QUP 0.90

Detailed Protein Report

Protein 32: PREDICTED: nesprin-2 isoform X8 [Homo sapiens]

**Accession:**	gi|578825626	**Score:**	91.8
**Database:**	refseq_human(refseq_human_20140103.fasta)	**MW [kDa]:**	553.3
**Database Date:**	2015-11-30	**pI:**	5.1
**Modification(s):**	Oxidation	**Sequence Coverage [%]:**	2.1
		**No. of unique Peptides:**	7

Quantitation

QU:MU	**Median:**	0.75	**CV:**	0.00 %	**No. of Peptides:**	1
WUP:QUP	**Median:**	6.20	**CV:**	0.00 %	**No. of Peptides:**	1

Detailed Protein Report

10	20	30	40	50	60	70	80
MASSPELPTE	DEQGSWGIDD	LHISLQAEQE	DTQKKAFTCW	INSQLARHTS	PSVISDLFTD	IKKGHVLLDL	LEVLSGQQLP
**90**	**100**	**110**	**120**	**130**	**140**	**150**	**160**
RDKGSNTFQC	RINIEHALTF	LRNRSIKLIN	IHVTDIIDGN	PSIILGLIWT	IILHFHIEKL	AQTLSCNYNQ	PSLDDVSVVD
**170**	**180**	**190**	**200**	**210**	**220**	**230**	**240**
SSPASSPPAK	KCSKVQARWQ	MSARKALLLW	AQEQCATYES	VNVTDFKSSW	RNGMAFLAII	HALRPDLIDM	KSVKHRSNKD
**250**	**260**	**270**	**280**	**290**	**300**	**310**	**320**
NLREAFRIAE	QELKIPRLLE	PEDVDVVDPD	EKSIMTYVAQ	FLQYSKDAPG	TGEEAQGKVK	DAMGWLTLQK	EKLQKLLKDS
**330**	**340**	**350**	**360**	**370**	**380**	**390**	**400**
ENDTYFKKYN	SLLSFMESFN	EEKKSFLDVL	SIKRDLDELD	KDHLQLREAW	DGLDHQINAW	KIKLNYALPP	PLHQTEAWLQ
**410**	**420**	**430**	**440**	**450**	**460**	**470**	**480**
EVEELMDEDL	SASQDHSQAV	TLIQEKMTLF	KSLMDRFEHH	SNILLTFENK	DENHLPLVPP	NKLEEMKRRI	NNILEKKFIL
**490**	**500**	**510**	**520**	**530**	**540**	**550**	**560**
LLEFHYYKCL	VLGLVDEVKS	KLDIWNIKYG	SRESVELLLE	DWHKFIEEKE	FLARLDTSFQ	KCGEIYKNLA	GECQNINKQY
**570**	**580**	**590**	**600**	**610**	**620**	**630**	**640**
MMVKSDVCMY	RKNIYNVKST	LQKVLACWAT	YVENLRLLRA	CFEETKKEEI	KEVPFETLAQ	WNLEHATLNE	AGNFLVEVSN
**650**	**660**	**670**	**680**	**690**	**700**	**710**	**720**
DVVGSSISKE	LRRLNKRWRK	LVSKTQLEMN	LPLMIKKQDQ	PTFDNSGNIL	SKEEKATVEF	STDMSVELPE	NYNQNIKAGE
**730**	**740**	**750**	**760**	**770**	**780**	**790**	**800**
KHEKENEEFT	GQLKVAKDVE	KLIGQVEIWE	AEAKSVLDQD	DVDTSMEESL	KHLIAKGSMF	DELMARSEDM	LQMDIQNISS
**810**	**820**	**830**	**840**	**850**	**860**	**870**	**880**
QESFQHVLTT	GLQAKIQEAK	EKVQINVVKL	IAALKNLTDV	SPDLDIRLKM	EESQKELESY	MMRAQQLLGQ	RESPGELISK
**890**	**900**	**910**	**920**	**930**	**940**	**950**	**960**
HKEALIISNT	KSLAKYLKAV	EELKNNVTED	IKMSLEEKSR	DVCAKWESLH	HELSLYVQQL	KIDIEKGKLS	DNILKLEKQI
**970**	**980**	**990**	**1000**	**1010**	**1020**	**1030**	**1040**
NKEKKLIRRG	RTKGLIKEHE	ACFSEEGCLY	QLNHHMEVLR	ELCEELPSQK	SQQEVKRLLK	DYEQKIERLL	KCASEIHMTL
**1050**	**1060**	**1070**	**1080**	**1090**	**1100**	**1110**	**1120**
QPTAGGTSKN	EGTITTSENR	GGDPHSEAPF	AKSDNQPSTE	KAMEPTMKFS	LASVLRPLQE	ESIMEKDYSA	SINSLLERYD
**1130**	**1140**	**1150**	**1160**	**1170**	**1180**	**1190**	**1200**
TYRDILEHHL	QNNKFRITSD	FSSEEDRSSS	CLQAKLTDLQ	VIKNETDARW	KEFEIISLKL	ENHVNDIKKP	FVIKERDTLK
**1210**	**1220**	**1230**	**1240**	**1250**	**1260**	**1270**	**1280**
ERERELQMTL	NTRMESLETA	LRLVLPVEKA	SLLLCGSDLP	LHKMAIQGFH	LIDADRIYQH	LRNIQDSIAK	QIEICNRLEE
**1290**	**1300**	**1310**	**1320**	**1330**	**1340**	**1350**	**1360**
PGNFVLKELH	PFDLHAMQNI	ILKYKTQFEG	MNHRVQRSED	TLKALEDFLA	SLRTAKLSAE	PVTDLSASDT	QVAQENTLTV
**1370**	**1380**	**1390**	**1400**	**1410**	**1420**	**1430**	**1440**
KNKEGEIHLM	KDKAKHLDKC	LKMLDMSFKD	AERGDDTSCE	NLLDAFSIKL	SETHGYGVQE	EFTEENKLLE	ACIFKNNELL
**1450**	**1460**	**1470**	**1480**	**1490**	**1500**	**1510**	**1520**
KNIQDVQSQI	SKIGLKDPTV	PAVKHRKKSL	IRLDKVLDEY	EEEKRHLQEM	ANSLPHFKDG	REKTVNQQCQ	NTVVLWENTK
**1530**	**1540**	**1550**	**1560**	**1570**	**1580**	**1590**	**1600**
ALVTECLEQC	GRVLELLKQY	QNFKSILTTL	IQKEESVISL	QASYMGKENL	KKRIAEIEIV	KEEFNEHLEV	VDKINQVCKN
**1610**	**1620**	**1630**	**1640**	**1650**	**1660**	**1670**	**1680**
LQFYLNKMKT	FEEPPFEKEA	NIIVDRWLDI	NEKTEDYYEN	LGRALALWDK	LFNLKNVIDE	WTEKALQKME	LHQLTEEDRE
**1690**	**1700**	**1710**	**1720**	**1730**	**1740**	**1750**	**1760**
RLKEELQVHE	QKTSEFSRRV	AEIQFLLQSS	EIPLELQVME	SSILNKMEHV	QKCLTGESNC	HALSGSTAEL	REDLDQAKTQ
**1770**	**1780**	**1790**	**1800**	**1810**	**1820**	**1830**	**1840**
IGMTESLLKA	LSPSDSLEIF	TKLEEIQQQI	LQQKHSMILL	ENQIGCLTPE	LSELKKQYES	VSDLFNTKKS	VLQDHFSKLL
**1850**	**1860**	**1870**	**1880**	**1890**	**1900**	**1910**	**1920**
NDQCKNFNDW	FSNIKVNLKE	CFESSETKKS	VEQKLQKLSD	FLTLEGRNSK	IKQVDSVLKH	VKKHLPKAHV	KELISWLVGQ
**1930**	**1940**	**1950**	**1960**	**1970**	**1980**	**1990**	**2000**
EFELEKMESI	CQARAKELED	SLQQLLRLQD	DHRNLRKWLT	NQEEKWKGME	EPGEKTELFC	QALARKREQF	ESVAQLNNSL
**2010**	**2020**	**2030**	**2040**	**2050**	**2060**	**2070**	**2080**
KEYGFTEEEE	IIMEATCLMD	RYQTLLRQLS	EIEEEDKLLP	TEDQSFNDLA	HDVIHWIKEI	KESLMVLNSS	EGKMPLEERI
**2090**	**2100**	**2110**	**2120**	**2130**	**2140**	**2150**	**2160**
QKIKEIILLK	PEGDARIETI	MKQAESSEAP	LVQKTLTDIS	NQWDNTLHLA	STYLSHQEKL	LLEGEKYLQS	KEDLRLMLIE
**2170**	**2180**	**2190**	**2200**	**2210**	**2220**	**2230**	**2240**

Detailed Protein Report

Cmpd.	No. of Cmpds.	m/z meas.	Δ m/z [ppm]	z	Rt [min]	Score	P	Range	Sequence	Modification	Ratios
2639	1	686.8883	34.43	2	63.0	14.6	0	451-462	K.DENHLPLVPPNK.L		
2757	1	849.0563	-8.59	3	65.6	10.4	0	696-717	K.<br>ATVEFSTDMSVELPENYNQNIK.<br>A	Oxidation: 9	
2961	1	927.4882	-20.92	2	65.8	15.8	1	864-880	R.AQQLLGQRESPGELISK.H		
133	1	679.8336	-94.89	2	31.4	13.6	1	2192-2203	K.AQDLTSLLKELK.S		WUP:QUP 6.20<br>QU:MU 0.75
1892	1	628.2831	-31.74	2	54.0	13.1	0	2204-2214	K.SQGNYLLECTK.N		
1662	1	882.4096	-80.38	2	51.0	13.3	2	3618-3632	K.SEQFEELQSILKKGK.L		
1911	1	568.7711	-62.35	2	52.2	11.0	1	4627-4636	K.SLKAGLDYNR.S		

Detailed Protein Report

Protein 33: **alpha-enolase isoform 1 [Homo sapiens]**

**Accession:**	gi|4503571	**Score:**	85.2
**Database:**	refseq_human(refseq_human_20140103.fasta)	**MW [kDa]:**	47.1
**Database Date:**	2015-11-30	**pI:**	7.7
		**Sequence Coverage [%]:**	6.9
		**No. of unique Peptides:**	2

Quantitation

QU:MU	**Median:** 0.82	**CV:** 24.50 %	**No. of Peptides:**	2
WUP:QUP	**Median:** 1.59	**CV:** 16.64 %	**No. of Peptides:**	2

10	20	30	40	50	60	70	80
MSILKIHARE	IFDSRGNPTV	EVDLFTSKGL	FRAAVPSGAS	TGIYEALELR	DNDKTRYMGK	GVSKAVEHIN	KTIAPALVSK
**90**	**100**	**110**	**120**	**130**	**140**	**150**	**160**
KLNVTEQEKI	DKLMIEMDGT	ENKSKFGANA	ILGVSLAVCK	AGAVEKGVPL	YRHIADLAGN	SEVILPVPAF	NVINGGSHAG
**170**	**180**	**190**	**200**	**210**	**220**	**230**	**240**
NKLAMQEFMI	LPVGAANFRE	AMRIGAEVYH	NLKNVIKEKY	GKDATNVGDE	GGFAPNILEN	KEGLELLKTA	IGKAGYTDKV
**250**	**260**	**270**	**280**	**290**	**300**	**310**	**320**
VIGMDVAASE	FFRSGKYDLD	FKSPDDPSRY	ISPDQLADLY	KSFIKDYPVV	SIEDPFDQDD	WGAWQKFTAS	AGIQVVGDDL
**330**	**340**	**350**	**360**	**370**	**380**	**390**	**400**
TVTNPKRIAK	AVNEKSCNCL	LLKVNQIGSV	TESLQACKLA	QANGWGVMVS	HRSGETEDTF	IADLVVGLCT	GQIKTGAPCR
**410**	**420**	**430**	**440**				
SERLAKYNQL	LRIEEELGSK	AKFAGRNFRN	PLAK				

Cmpd.	No. of Cmpds.	m/z meas.	Δ m/z [ppm]	z	Rt [min]	Score	P	Range	Sequence	Modification	Ratios
1942	1	902.8469	-142.55	2	54.2	18.2	0	33-50	R.AAVPSGASTGIYEALELR.D		WUP:QUP 1.35<br>QU:MU 1.04
1618	5	713.3563	-14.47	2	50.5	67.0	0	270-281	R.YISPDQLADLYK.S		WUP:QUP 1.87<br>QU:MU 0.64

Detailed Protein Report

Protein 34: usherin isoform B [Homo sapiens]

**Accession:**	gi|219842266	**Score:**	84.9
**Database:**	refseq_human(refseq_human_20140103.fasta)	**MW [kDa]:**	575.2
**Database Date:**	2015-11-30	**pI:**	6.4
**Modification(s):**	Carbamidomethyl	**Sequence Coverage [%]:**	1.9
		**No. of unique Peptides:**	6

Quantitation

QU:MU	**Median:**	1.49	**CV:**	0.00 %	**No. of Peptides:**	1
WUP:QUP	**Median:**	1.15	**CV:**	0.00 %	**No. of Peptides:**	1

Detailed Protein Report

10	20	30	40	50	60	70	80
MNCPVLSLGS	GFLFQVIEML	IFAYFASISL	TESRGLFPRL	ENVGAFKKVS	IVPTQAVCGL	PDRSTFCHSS	AAAESIQFCT
90	100	110	120	130	140	150	160
QRFCIQDCPY	RSSHPTYTAL	FSAGLSSCIT	PDKNDLHPNA	HSNSASFIFG	NHKSCFSSPP	SPKLMASFTL	AVWLKPEQQG
170	180	190	200	210	220	230	240
VMCVIEKTVD	GQIVFKLTIS	EKETMFYYRT	VNGLQPPIKV	MTLGRILVKK	WIHLSVQVHQ	TKISFFINGV	EKDHTPFNAR
250	260	270	280	290	300	310	320
TLSGSITDFA	SGTVQIGQSL	NGLEQFVGRM	QDFRLYQVAL	TNREILEVFS	GDLLRLHAQS	HCRCPGSHPR	VHPLAQRYCI
330	340	350	360	370	380	390	400
PNDAGDTADN	RVSRLNPEAH	PLSFVNDNDV	GTSWVSNVFT	NITQLNQGVT	ISVDLENGQY	QVFYIIIQFF	SPQPTEIRIQ
410	420	430	440	450	460	470	480
RKKENSLDWE	DWQYFARNCG	AFGMKNNGDL	EKPDSVNCLQ	LSNFTPYSRG	NVTFSILTPG	PNYRPGYNNF	YNTPSLQEFV
490	500	510	520	530	540	550	560
KATQIRFHFH	GQYYTTETAV	NLRHRYYAVD	EITISGRCQC	HGHADNCDTT	SQPYRCLCSQ	ESFTEGLHCD	RCLPLYNDKP
570	580	590	600	610	620	630	640
FRQGDQVYAF	NCKPCQCNSH	SKSCHYNISV	DPFPFEHFRG	GGGVCDDCEH	NTTGRNCELC	KDYFFRQVGA	DPSAIDVCKP
650	660	670	680	690	700	710	720
CDCDTVGTRN	GSILCDQIGG	QCNCKRHVSG	RQCNQCQNGF	YNLQELDPDG	CSPCNCNTSG	TVDGDITCHQ	NSGQCKCKAN
730	740	750	760	770	780	790	800
VIGLRCDHCN	FGFKFLRSFN	DVGCEPCQCN	LHGSVNKFCN	PHSGQCECKK	EAKGLQCDTC	RENFYGLDVT	NCKACDCDTA
810	820	830	840	850	860	870	880
GSLPGTVCNA	KTGQCICKPN	VEGRQCNKCL	EGNFYLRQNN	SFLCLPCNCD	KTGTINGSLL	CNKSTGQCPC	KLGVTGLRCN
890	900	910	920	930	940	950	960
QCEPHRYNLT	IDNFQHCQMC	ECDSLGTLPG	TICDPISGQC	LCVPNRQGRR	CNQCQPGFYI	SPGNATGCLP	CSCHTTGAVN
970	980	990	1000	1010	1020	1030	1040
HICNSLTGQC	VCQDASIAGQ	RCDQCKDHYF	GFDPQTGRCQ	PCNCHLSGAL	NETCHLVTGQ	CFCKQFVTGS	KCDACVPSAS
1050	1060	1070	1080	1090	1100	1110	1120
HLDVNNLLGC	SKTPFQQPPP	RGQVQSSSAI	NLSWSPPDSP	NAHWLTYSLL	RDGFEIYTTE	DQYPYSIQYF	LDTDLLPYTK
1130	1140	1150	1160	1170	1180	1190	1200
YSYYIETTNV	HGSTRSVAVT	YKTKPGVPEG	NLTLSYIIPI	GSDSVTLTWT	TLSNQSGPIE	KYILSCAPLA	GGQPCVSYEG
1210	1220	1230	1240	1250	1260	1270	1280
HETSATIWNL	VPFAKYDFSV	QACTSGGCLH	SLPITVTTAQ	APPQRLSPPK	MQKISSTELH	VEWSPPAELN	GIIIRYELYM
1290	1300	1310	1320	1330	1340	1350	1360
RRLRSTKETT	SEESRVFQSS	GWLSPHSFVE	SANENALKPP	QTMTTITGLE	PYTKYEFRVL	AVNMAGSVSS	AWVSERTGES
1370	1380	1390	1400	1410	1420	1430	1440
APVFMIPPSV	FPLSSYSLNI	SWEKPADNVT	RGKVVGYDIN	MLSEQSPQQS	IPMAFSQLLH	TAKSQELSYT	VEGLKPYRIY
1450	1460	1470	1480	1490	1500	1510	1520
EFTITLCNSV	GCVTSASGAG	QTLAAAPAQL	RPPLVKGINS	TTIHLKWFPP	EELNGPSPIY	QLERRESSLP	ALMTTMMKGI
1530	1540	1550	1560	1570	1580	1590	1600
RFIGNGYCKF	PSSTHPVNTD	FTGIKASFRT	KVPEGLIVFA	ASPGNQEEYF	ALQLKKGRLY	FLFDPQGSPV	EVTTTNDHGK
1610	1620	1630	1640	1650	1660	1670	1680
QYSDGKWHEI	IAIRHQAFGQ	ITLDGIYTGS	SAILNGSTVI	GDNTGVFLGG	LPRSYTILRK	DPEIIQKGFV	GCLKDVHFMK
1690	1700	1710	1720	1730	1740	1750	1760
NYNPSAIWEP	LDWQSSEEQI	NVYNSWEGCP	ASLNEGAQFL	GAGFLELHPY	MFHGGMNFEI	SFKFRTDQLN	GLLLFVYNKD
1770	1780	1790	1800	1810	1820	1830	1840
GPDFLAMELK	SGILTFRLNT	SLAFTQVDLL	LGLSYCNGKW	NKVIIKKEGS	FISASVNGLM	KHASESGDQP	LVVNSPVYVG
1850	1860	1870	1880	1890	1900	1910	1920
GIPQELLNSY	QHLCLEQGFG	GCMKDVKFTR	GAVVNLASVS	SGAVRVNLDG	CLSTDSAVNC	RGNDSILVYQ	GKEQSVYEGG
1930	1940	1950	1960	1970	1980	1990	2000
LQPFTEYLYR	VIASHEGGSV	YSDWSRGRTT	GAAPQSVPTP	SRVRSLNGYS	IEVTWDEPVV	RGVIEKYILK	AYSEDSTRPP
2010	2020	2030	2040	2050	2060	2070	2080
RMPSASAEFV	NTSNLTGILT	GLLPFKNYAV	TLTACTLAGC	TESSHALNIS	TPQEAPQEVQ	PPVAKSLPSS	LLLSWNPPKK
2090	2100	2110	2120	2130	2140	2150	2160
ANGIITQYCL	YMDGRLIYSG	SEENYTVTDL	AVFTPHQFLL	SACTHVGCTN	SSWVLLYTAQ	LPPEHVDSPV	LTVLDSRTIH
2170	2180	2190	2200	2210	2220	2230	2240

Detailed Protein Report

Cmpd.	No. of Cmpds.	m/z meas.	Δ m/z [ppm]	z	Rt [min]	Score	P	Range	Sequence	Modification	Ratios
449	1	565.3223	-66.47	2	35.3	13.2	1	200-209	K.VMTLGRILVK.K		
2191	1	863.3484	-20.01	2	55.8	14.2	0	794-811	K.ACDCDTAGSLPGTVCNAK.T		
2688	2	885.4613	98.77	2	63.8	13.3	0	838-851	R.QNNSFLCLPCNCDK.T	Carbamidomethyl: 7, 10, 12	
1593	1	1172.1148	23.38	2	50.1	15.7	0	3098-3119	K.SNGTQITTVEDTPSDIPTPTIR.G		
864	1	471.1407	-194.52	2	39.2	18.2	0	3546-3554	R.GTSLSFSDK.E		
206	1	898.4111	-54.24	3	32.3	10.4	2	5085-5108	K.RMSPLNVYPPGENHMGLADTKIPI S		WUP:QUP 1.15<br>QU:MU 1.49

Detailed Protein Report

Protein 35: **PREDICTED: keratin, type I cytoskeletal 10 isoform X1 [Homo sapiens]**

**Accession:**	gi|530412176	**Score:**	84.1
**Database:**	refseq_human(refseq_human_20140103.fasta)	**MW [kDa]:**	63.3
**Database Date:**	2015-11-30	**pI:**	5.0
		**Sequence Coverage [%]:**	5.6
		**No. of unique Peptides:**	1

10	20	30	40	50	60	70	80
MSVRYSSSKH	YSSSRSGGGG	GGGGCGGGGG	VSSLRISSSK	GSLGGGFSSG	GFSGGSFSRG	SSGGGCFGGS	SGGYGGLGGF
90	100	110	120	130	140	150	160
GGGSFRGSYG	SSSFGGSYGG	IFGGGSFGGG	SFGGGSFGGG	GFGGGGFGGG	FGGGFGGDGG	LLSGNEKVTM	QNLNDRLASY
170	180	190	200	210	220	230	240
LDKVRALEES	NYELEGKIKE	WYEKHGNSHQ	GEPRDYSKYY	KTIDDLKNQI	LNLTTDNANI	LLQIDNARLA	ADDFRLKYEN
250	260	270	280	290	300	310	320
EVALRQSVEA	DINGLRRVLD	ELTLTKADLE	MQIESLTEEL	AYLKKNHEEE	MKDLRNVSTG	DVNVEMNAAP	GVDLTQLLNN
330	340	350	360	370	380	390	400
MRSQYEQLAE	QNRKDAEAWF	NEKSKELTTE	IDNNIEQISS	YKSEITELRR	NVQALEIELQ	SQLALKQSLE	ASLAETEGRY
410	420	430	440	450	460	470	480
CVQLSQIQAQ	ISALEEQLQQ	IRAETECQNT	EYQQLLDIKI	RLENEIQTYR	SLLEGEGSSG	GGGRGGGSFG	GGYGGGSSGG
490	500	510	520	530	540	550	560
GSSGGGHGGG	HGGSSGGGYG	GGSSGGGSSG	GGYGGGSSSG	GHGGSSSGGY	GGGSSGGGGG	GYGGGSSGGG	SSSGGGYGGG
570	580	590	600	610	620	630	
SSSGGHKSSS	SGSVGESSSK	GPRSAETSWD	TNKTRVIKTI	IEEVAPDGRV	LSSMVESETK	KHYY	

Cmpd.	No. of Cmpds.	m/z meas.	Δ m/z [ppm]	z	Rt [min]	Score	P	Range	Sequence	Modification
348	1	806.4041	46.58	2	34.3	11.3	1	185-198	K.HGNSHQGEPRDYSK.Y	

Detailed Protein Report

Protein 36: trinucleotide repeat-containing gene 18 protein [Homo sapiens]

**Accession:**	gi|169658378	**Score:**	83.3
**Database:**	refseq_human(refseq_human_20140103.fasta)	**MW [kDa]:**	314.3
**Database Date:**	2015-11-30	**pI:**	9.6
**Modification(s):**	Carbamidomethyl, Oxidation	**Sequence Coverage [%]:**	3.1
		**No. of unique Peptides:**	4

Quantitation

QU:MU	**Median:**	1.13	**CV:**	0.00 %	**No. of Peptides:**	1
WUP:QUP	**Median:**	1.90	**CV:**	0.00 %	**No. of Peptides:**	1

Alias proteins:

Accession	Name	Description
gi|578813684	refseq_human (refseq_human_20140103.fasta)	PREDICTED: trinucleotide repeat-containing gene 18 protein isoform X1 [Homo sapiens]

Detailed Protein Report

10	20	30	40	50	60	70	80
MDGRDFGPQR	SVHGPPPPLL	SGLAMDSHRV	GAATAGRLPA	SGLPGPLPPG	KYMAGLNLHP	HPGEAFLGSF	VASGMGPSAS
90	100	110	120	130	140	150	160
SHGSPVPLPS	DLSFRSPTPS	NLPMVQLWAA	HAHEGFSHLP	SGLYPSYLHL	NHLEPPSSGS	PLLSQLGQPS	IFDTQKGQGP
170	180	190	200	210	220	230	240
GGDGFYLPTA	GAPGSLHSHA	PSARTPGGGH	SSGAPAKGSS	SRDGPAKERA	GRGGEPPPLF	GKKDPRARGE	EASGPRGVVD
250	260	270	280	290	300	310	320
LTQEARAEGR	QDRGPPRLAE	RLSPFLAESK	TKNAALQPSV	LTMCNGGAGD	VGLPALVAEA	GRGGAKEAAR	QDEGARLLRR
330	340	350	360	370	380	390	400
TETLLPGPRP	CPSPLPPPPA	PPKGPPAPPA	ATPAGVYTVF	REQGREHRVV	APTFVPSVEA	FDERPGPIQI	ASQARDARAR
410	420	430	440	450	460	470	480
EREAGRPGVL	QAPPGSPRPL	DRPEGLREKN	SVIRSLKRPP	PADAPTVRAT	RASPDPRAYV	PAKELLKPEA	DPRPCERAPR
490	500	510	520	530	540	550	560
GPAGPAAQQA	AKLFGLEPGR	PPPTGPEHKW	KPFELGNFAA	TQMAVLAAQH	HHSRAEEEAA	VVAASSSKKA	YLDPGAVLPR
570	580	590	600	610	620	630	640
SAATCGRPVA	DMHSAAHGSG	EASAMQSLIK	YSGSFARDAV	AVRPGGCGKK	SPFGGLGTMK	PEPAPTSAGA	SRAQARLPHS
650	660	670	680	690	700	710	720
GGPAAGGGRQ	LKRDPERPES	AKAFGREGSG	AQGEAEVRHP	PVGIAVAVAR	QKDSGGSGRL	GPGLVDQERS	LSLSNVKGHG
730	740	750	760	770	780	790	800
RADEDCVDDR	ARHREERLLG	ARLDRDQEKL	LRESKELADL	ARLHPTSCAP	NGLNPNLMVT	GGPALAGSGR	WSADPAAHLA
810	820	830	840	850	860	870	880
THPWLPRSGN	ASMWLAGHPY	GLGPPSLHQG	MAPAFPPGLG	GSLPSAYQFV	RDPQSGQLVV	IPSDHLPHFA	ELMERATVPP
890	900	910	920	930	940	950	960
LWPALYPPGR	SPLHHAQQLQ	LFSQQHFLRQ	QEFLYLQQQA	AQALELQRSA	QLVQERLKAQ	EHRAEMEEKG	SKRGLEAAGK
970	980	990	1000	1010	1020	1030	1040
AGLATAGPGL	LPRKPPGLAA	GPAGTYGKAV	SPPPSPRASP	VAALKAKVIQ	KLEDVSKPPA	YAYPATPSSH	PTSPPPASPP
1050	1060	1070	1080	1090	1100	1110	1120
PTPGITRKEE	APENVVEKKD	LELEKEAPSP	FQALFSDIPP	RYPFQALPPH	YGRPYPFLLQ	PTAAADADGL	APDVPLPADG
1130	1140	1150	1160	1170	1180	1190	1200
PERLALSPED	KPIRLSPSKI	TEPLREGPEE	EPLAEREVKA	EVEDMDEGPT	ELPPLESPLP	LPAAEAMATP	SPAGGCGGGL
1210	1220	1230	1240	1250	1260	1270	1280
LEAQALSATG	QSCAEPSECP	DFVEGPEPRV	DSPGRTEPCT	AALDLGVQLT	PETLVEAKEE	PVEVPVAVPV	VEAVPEEGLA
1290	1300	1310	1320	1330	1340	1350	1360
QVAPSESQPT	LEMSDCDVPA	GEGQCPSLEP	QEAVPVLGST	CFLEEASSDQ	FLPSLEDPLA	GMNALAAAAE	LPQARPLPSP
1370	1380	1390	1400	1410	1420	1430	1440
GAAGAQALEK	LEAAESLVLE	QSFLHGITLL	SEIAELELER	RSQEMGGAER	ALVARPSLES	LLAAGSHMLR	EVLDGPVVDP
1450	1460	1470	1480	1490	1500	1510	1520
LKNLRLPREL	KPNKKYSWMR	KKEERMYAMK	SSLEDMDALE	LDFRMRLAEV	QRQYKEKQRE	LVKLQRRRDS	EDRREEPHRS
1530	1540	1550	1560	1570	1580	1590	1600
LARRGPGRPR	KRTHAPSALS	PPRKRGKSGH	SSGKLSSKSL	LTSDDYELGA	GIRKRHKGSE	EEHDALIGMG	KARGRNQTWD
1610	1620	1630	1640	1650	1660	1670	1680
EHEASSDFIS	QLKIKKKKMA	SDQEQLASKL	DKALSLTKQD	KLKSPFKFSD	SAGGKSKTSG	GCGRYLTPYD	SLLGKNRKAL
1690	1700	1710	1720	1730	1740	1750	1760
AKGLGLSLKS	SREGKHKRAA	KTRKMEVGFK	ARGQPKSAHS	PFASEVSSYS	YNTDSEEDEE	FLKDEWPAQG	PSSSKLTPSL
1770	1780	1790	1800	1810	1820	1830	1840
LCSMVAKNSK	AAGGPKLTKR	GLAAPRTLKP	KPATSRKQPF	CLLLREAEAR	SSFSDSSEES	FDQDESSEEE	DEEEELEEED
1850	1860	1870	1880	1890	1900	1910	1920
EASGGGYRLG	ARERALSPGL	EESGLGLLAR	FAASALPSPT	VGPSLSVVQL	EAKQKARKKE	ERQSLLGTEF	EYTDSESEVK
1930	1940	1950	1960	1970	1980	1990	2000
VRKRSPAGLL	RPKKGLGEPG	PSLAAPTPGA	RGPDPSSPDK	AKLAVEKGRK	ARKLRGPKEP	GFEAGPEASD	DDLWTRRRSE
2010	2020	2030	2040	2050	2060	2070	2080
RIFLHDASAA	APAPVSTAPA	TKTSRCAKGG	PLSPRKDAGR	AKDRKDPRKK	KKGKEAGPGA	GLPPPRAPAL	PSEARAPHAS
2090	2100	2110	2120	2130	2140	2150	2160
SLTAAKRSKA	KAKGKEVKKE	NRGKGGAVSK	LMESMAAEED	FEPNQDSSFS	EDEHLPRGGA	VERPLTPAPR	SCIIDKDELK
2170	2180	2190	2200	2210	2220	2230	2240

Detailed Protein Report

Cmpd.	No. of Cmpds.	m/z meas.	Δ m/z [ppm]	z	Rt [min]	Score	P	Range	Sequence	Modification	Ratios
1629	1	1052.2731	65.37	3	50.1	21.1	1	271-302	K. TKNAALQPSVLTMCNGGAGDVGL G	Carbamidomethyl: 14; Oxidation: 13	
2682	1	734.8888	-6.21	2	63.7	11.3	2	1642-1655	K.LKSPFKFSDSAGGK.S		
177	1	613.7776	-171.05	2	31.9	22.8	1	1787-1797	R.TLKPKPATSRK.Q		WUP:QUP 1.90<br>QU:MU 1.13
1643	1	713.3402	-67.63	2	50.8	15.8	2	2464-2476	K.LDHEGVTSPKSKK.A		

Detailed Protein Report

Protein 37: zinc finger protein 622 [Homo sapiens]

Accession: gi|15529978
Database: refseq_human(refseq_human_20140103.fasta)
Database Date: 2015-11-30
Modification(s): Carbamidomethyl, Oxidation

Score: 81.8
MW [kDa]: 54.2
pI: 5.8
Sequence Coverage [%]: 16.1
No. of unique Peptides: 6

Quantitation

QU:MU	**Median:** 0.93	**CV:** 0.00 %	**No. of Peptides:**	1
WUP:QUP	**Median:** 1.47	**CV:** 0.00 %	**No. of Peptides:**	1

```
        10         20         30         40         50         60         70         80
MATYTCITCR VAFRDADMQR AHYKTDWHRY NLRRKVASMA PVTAEGFQER VRAQRAVAEE ESKGSATYCT VCSKKFASFN
        90        100        110        120        130        140        150        160
AYENHLKSRR HVELEKKAVQ AVNRKVEMMN EKNLEKGLGV DSVDKDAMNA AIQQAIKAQP SMSPKKAPPA PAKEARNVVA
       170        180        190        200        210        220        230        240
VGTGGRGTHD RDPSEKPPRL QWFEQQAKKL AKQQEEDSEE EEDLDGDDW  EDIDSDEELE CEDTEAMDDV VEQDAEEEEA
       250        260        270        280        290        300        310        320
EEGPPLGAIP ITDCLFCSHH SSSLMKNVAH MTKDHSFFIP DIEYLSDIKG LIKYLGEKVG VGKICLWCNE KGKSFYSTEA
       330        340        350        360        370        380        390        400
VQAHMNDKSH CKLFTDGDAA LEFADFYDFR SSYPDHKEGE DPNKAEELPS EKNLEYDDET MELILPSGAR VGHRSLMRYY
       410        420        430        440        450        460        470        480
KQRFGLSRAV AVAKNRKAVG RVLQQYRALG WTGSTGAALM RERDMQYVQR MKSKWMLKTG MKNNATKQMH FRVQVRF
```

Cmpd.	No. of Cmpds.	m/z meas.	Δ m/z [ppm]	z	Rt [min]	Score	P	Range	Sequence	Modification	Ratios
2592	1	822.7237	16.97	3	62.4	10.6	2	1-20	-.MATYTCITCRVAFRDADMQR.A	Carbamidomethyl: 6, 9	
2687	2	973.5040	65.98	2	64.7	14.9	2	15-29	R.DADMQRAHYKTDWHR.Y	Oxidation: 4	WUP:QUP 1.47<br>QU:MU 0.93
1830	3	938.9541	-36.71	2	51.2	19.3	2	34-50	R.RKVASMAPVTAEGFQER.V		
1757	1	560.2452	1.92	2	52.3	12.6	0	64-74	K.GSATYCTVCSK.K		
2237	1	753.3462	-49.90	2	56.3	10.4	1	299-311	K.VGVGKICLWCNEK.G	Carbamidomethyl: 7	
1586	1	480.6461	-220.76	2	49.5	14.0	1	395-401	R.SLMRYYK.Q		

Detailed Protein Report

Protein 38: **PREDICTED: dynein heavy chain 5, axonemal isoform X1 [Homo sapiens]**

**Accession:**	gi|530378724	**Score:**	79.8
**Database:**	refseq_human(refseq_human_20140103.fasta)	**MW [kDa]:**	526.8
**Database Date:**	2015-11-30	**pI:**	5.7
**Modification(s):**	Carbamidomethyl, Oxidation	**Sequence Coverage [%]:**	2.1
		**No. of unique Peptides:**	4

Detailed Protein Report

10	20	30	40	50	60	70	80
MDKLQRLKGE	KEAKRALLDA	RHNYLFAIVA	SCLDLNKTEV	EDAILEGNQI	ERIDQLFAVG	GLRHLMFYYQ	DVEEAETGQL
90	100	110	120	130	140	150	160
GSLGGVNLVS	GKIKKPKVFV	TEGNDVALTG	VCVFFIRTDP	SKAITPDNIH	QEVSFNMLDA	ADGGLLNSVR	RLLSDIFIPA
170	180	190	200	210	220	230	240
LRATSHGWGE	LEGLQDAANI	RQEFLSSLEG	FVNVLSGAQE	SLKEKVNLRK	CDILELKTLK	EPTDYLTLAN	NPETLGKIED
250	260	270	280	290	300	310	320
CMKVWIKQTE	QVLAENNQLL	KEADDVGPRA	ELEHWKKRLS	KFNYLLEQLK	SPDVKAVLAV	LAAAKSKLLK	TWREMDIRIT
330	340	350	360	370	380	390	400
DATNEAKDNV	KYLYTLEKCC	DPLYSSDPLS	MMDAIPTLIN	AIKMIYSISH	YYNTSEKITS	LFVKVTNQII	SACKAYITNN
410	420	430	440	450	460	470	480
GTASIWNQPQ	DVVEEKILSA	IKLKQEYQLC	FHKTKQKLKQ	NPNAKQFDFS	EMYIFGKFET	FHRRLAKIID	IFTTLKTYSV
490	500	510	520	530	540	550	560
LQDSTIEGLE	DMATKYQGIV	ATIKKKEYNF	LDQRKMDFDQ	DYEEFCKQTN	DLHNELRKFM	DVTFAKIQNT	NQALRMLKKF
570	580	590	600	610	620	630	640
ERLNIPNLGI	DDKYQLILEN	YGADIDMISK	LYTKQKYDPP	LARNQPPIAG	KILWARQLFH	RIQQPMQLFQ	QHPAVLSTAE
650	660	670	680	690	700	710	720
AKPIIRSYNR	MAKVLLEFEV	LFHRAWLRQI	EEIHVGLEAS	LLVKAPGTGE	LFVNFDPQIL	ILFRETECMA	QMGLEVSPLA
730	740	750	760	770	780	790	800
TSLFQKRDRY	KRNFSNMKMM	LAEYQRVKSK	IPAAIEQLIV	PHLAKVDEAL	QPGLAALTWT	SLNIEAYLEN	TFAKIKDLEL
810	820	830	840	850	860	870	880
LLDRVNDLIE	FRIDAILEEM	SSTPLCQLPQ	EEPLTCEEFL	QMTKDLCVNG	AQILHFKSSL	VEEAVNELVN	MLLDVEVLSE
890	900	910	920	930	940	950	960
EESEKISNEN	SVNYKNESSA	KREEGNFDTL	TSSINARANA	LLLTTVTRKK	KETEMLGEEA	RELLSHFNHQ	NMDALLKVTR
970	980	990	1000	1010	1020	1030	1040
NTLEAIRKRI	HSSHTINFRD	SNSASNMKQN	SLPIFRASVT	LAIPNIVMAP	ALEDVQQTLN	KAVECIISVP	KGVRQWSSEL
1050	1060	1070	1080	1090	1100	1110	1120
LSKKKIQERK	MAALQSNEDS	DSDVEMGENE	LQDTLEIASV	NLPIPVQTKN	YYKNVSENKE	IVKLVSVLST	IINSTKKEVI
1130	1140	1150	1160	1170	1180	1190	1200
TSMDCFKRYN	HIWQKGKEEA	IKTFITQSPL	LSEFESQILY	FQNLEQEINA	EPEYVCVGSI	ALYTADLKFA	LTAETKAWMV
1210	1220	1230	1240	1250	1260	1270	1280
VIGRHCNKKY	RSEMENIFML	IEEFNKKLNR	PIKDLDDIRI	AMAALKEIRE	EQISIDFQVG	PIEESYALLN	RYGLLIAREE
1290	1300	1310	1320	1330	1340	1350	1360
IDKVDTLHYA	WEKLLARAGE	VQNKLVSLQP	SFKKELISAV	EVFLQDCHQF	YLDYDLNGPM	ASGLKPQEAS	DRLIMFQNQF
1370	1380	1390	1400	1410	1420	1430	1440
DNIYRKYITY	TGGEELFGLP	ATQYPQLLEI	KKQLNLLQKI	YTLYNSVIET	VNSYYDILWS	EVNIEKINNE	LLEFQNRCRK
1450	1460	1470	1480	1490	1500	1510	1520
LPRALKDWQA	FLDLKKIIDD	FSECCPLLEY	MASKAMMERH	WERITTLTGH	SLDVGNESFK	LRNIMEAPLL	KYKEEIEDIC
1530	1540	1550	1560	1570	1580	1590	1600
ISAVKERDIE	QKLKQVINEW	DNKTFTFGSF	KTRGELLLRG	DSTSEIIANM	EDSLMLLGSL	LSNRYNMPFK	AQIQKWVQYL
1610	1620	1630	1640	1650	1660	1670	1680
SNSTDIIESW	MTVQNLWIYL	EAVFVGGDIA	KQLPKEAKRF	SNIDKSWVKI	MTRAHEVPSV	VQCCVGDETL	GQLLPHLLDQ
1690	1700	1710	1720	1730	1740	1750	1760
LEICQKSLTG	YLEKKRLCFP	RFFFVSDPAL	LEILGQASDS	HTIQAHLLNV	FDNIKSVKFH	EKIYDRILSI	SSQEGETIEL
1770	1780	1790	1800	1810	1820	1830	1840
DKPVMAEGNV	EVWLNSLLEE	SQSSLHLVIR	QAAANIQETG	FQLTEFLSSF	PAQVGLLGIQ	MIWTRDSEEA	LRNAKFDKKI
1850	1860	1870	1880	1890	1900	1910	1920
MQKTNQAFLE	LLNTLIDVTT	RDLSSTERVK	YETLITIHVH	QRDIFDDLCH	MHIKSPMDFE	WLKQCRFYFN	EDSDKMMIHI
1930	1940	1950	1960	1970	1980	1990	2000
TDVAFIYQNE	FLGCTDRLVI	TPLTDRCYIT	LAQALGMSMG	GAPAGPAGTG	KTETTKDMGR	CLGKYVVVFN	CSDQMDFRGL
2010	2020	2030	2040	2050	2060	2070	2080
GRIFKGLAQS	GSWGCFDEFN	RIDLPVLSVA	AQQISIILTC	KKEHKKSFIF	TDGDNVTMNP	EFGLFLTMNP	GYAGRQELPE
2090	2100	2110	2120	2130	2140	2150	2160
NLKINFRSVA	MMVPDRQIII	RVKLASCGFI	DNVVLARKFF	TLYKLCEEQL	SKQVHYDFGL	RNILSVLRTL	GAAKRANPMD
2170	2180	2190	2200	2210	2220	2230	2240

Detailed Protein Report

Cmpd.	No. of Cmpds.	m/z meas.	Δ m/z [ppm]	z	Rt [min]	Score	P	Range	Sequence	Modification
1349	1	991.5669	90.75	3	47.0	28.2	1	1947-1976	R.CYITLAQALGMSMGGAPAGPAGTGKTETTK.D	Carbamidomethyl: 1; Oxidation: 11, 13
2802	1	849.0671	-21.12	3	66.2	12.2	2	1999-2021	R.GLGRIFKGLAQSGSWGCFDEFNR.I	
78	1	529.5845	-42.73	3	29.9	11.4	2	3729-3741	R.THLMEDVTANKRR.M	Oxidation: 4
2750	1	905.4797	-0.39	2	65.5	16.2	2	3993-4008	R.TIAQARKYIVDSMGEK.Y	

Detailed Protein Report

Protein 39: coiled-coil domain-containing protein 168 [Homo sapiens]

**Accession:**	gi|226246554	**Score:**	79.6
**Database:**	refseq_human(refseq_human_20140103.fasta)	**MW [kDa]:**	801.4
**Database Date:**	2015-11-30	**pI:**	9.6
**Modification(s):**	Carbamidomethyl, Oxidation	**Sequence Coverage [%]:**	1.5
		**No. of unique Peptides:**	6

Quantitation

QU:MU	**Median:**	3.74	**CV:**	0.00 %	**No. of Peptides:**	1
WUP:QUP	**Median:**	0.50	**CV:**	0.00 %	**No. of Peptides:**	1

Detailed Protein Report

10	20	30	40	50	60	70	80
MSKQYYSFKK	GVGSGLEDNT	FMTLWDFLES	WIIQNDWVAI	FFIILLGIIF	EIILMKACAS	FWKKPTLPEK	GSSDVQETED
90	100	110	120	130	140	150	160
SCPKSRKLAP	ENWSVINSSS	GERVGTFLEK	RITSSLTSEE	KECNFEDRIL	FSREILWSGT	SESEDQVSPS	SESHVPSSNG
170	180	190	200	210	220	230	240
ISSSLPLFYS	EVEETCLSHT	EHPDREYETI	QFSSKKLFSM	MKTNKNKNSG	FSSDLSFSAS	RFTVENEDLD	VAPCPLAHLF
250	260	270	280	290	300	310	320
LSRDQVRLLE	ENVRNQIPSK	PKTKLGSRTT	YQCSRSQESL	NQNQPSVGMV	ISVQAQDSFP	GQNAFQNQGL	YEVQFTSQAQ
330	340	350	360	370	380	390	400
YINHNQESIK	SQPESKASNF	AQPEDVMKKP	FSSSTQDSFQ	SQDLDRNQHF	VEVPSIVEAK	YSVKGLESDE	HLGEDQHCVW
410	420	430	440	450	460	470	480
FIDSNKVKYS	IKGQDTIFKN	AEFLVLTLNP	NLVTEDMPQL	RSVKAQGQQQ	IVSSELNQDS	VYSSVPLLST	IKGQKNRRKT
490	500	510	520	530	540	550	560
PDSKSKLSLN	VPSLKAKKTP	TSQVFQITVC	HTLKNRNELG	CKNNTEKKEL	HERKDISDIA	LHLISVSKLI	LPYVKNYSRK
570	580	590	600	610	620	630	640
QLVKVMPGLI	KCGHFLQKQN	KSPDTEKINY	AGPLEETGIS	DITKKEKEYD	KENKRLKNIS	PKMLPQLEQS	FMVNTVQLKA
650	660	670	680	690	700	710	720
PCLLVETNGK	SKESLKDSIT	QAKGIGITEF	HVLNSKKPFD	LHIPKHKTSL	EEAISKPMQK	LVSSPEMESN	NRMKIQEDLQ
730	740	750	760	770	780	790	800
SSENSHLQLS	NGEELPTSTP	KTQRCFPREN	TQKQKDFLEL	VLELSNVGLL	ISPGSKMHKS	SEELEAIKIQ	VNTESVNLKE
810	820	830	840	850	860	870	880
SKPLILNVTE	DSDLRESEEL	ECNTGSNITN	MHQDKETSDA	FHSATYTTIS	QLPDTETHSI	SKAKADTLRI	IRLSHSASKQ
890	900	910	920	930	940	950	960
EKLPDEKETQ	NAEYIDKSCT	FKKPQQCDRK	EQEKEANSEL	TQGFRFSIHL	KQKPKYVKFQ	MEQISSGSSK	APNKEQEVQP
970	980	990	1000	1010	1020	1030	1040
QTLSTQTILE	NSPCPMMDPF	QVEKVKQSTD	RPTDRESAGD	PKNPLTMPEN	LPVGELLIET	TEYSVPFGGN	LQKTTDSHIA
1050	1060	1070	1080	1090	1100	1110	1120
EEKEDVKRYL	PAVALGSFNN	HLLTLPYFKR	QEIKKKLSET	KSVLSVKYVI	MKVKKPAISL	MPYINICGTS	NHRKKMGGNF
1130	1140	1150	1160	1170	1180	1190	1200
EIIIKQILQD	KIAAGMLLNV	IYPPMSILPN	TRMYSRLNAE	NHSHIKLVQE	ESQIEREEKY	PYFINEGNES	QNTLDAKLQD
1210	1220	1230	1240	1250	1260	1270	1280
EVKGVKETLP	KAVLHDSCNL	GLDAHLEKEI	KTEKEMHQPI	PFTETIIESV	VSPIMELSHA	ENVKSTQKTQ	TDCKCTADSE
1290	1300	1310	1320	1330	1340	1350	1360
TPSPISGKSL	IGDPLNQTRE	SYIPSNGSDT	REMGYCFAEE	KTEIPKDLPA	TSPETFNYCT	PVLSCSKVMK	KRVTFALTTS
1370	1380	1390	1400	1410	1420	1430	1440
TAKPKCVNTK	AVKPSISETV	SVTSHRKKSE	LDFKTKFKKI	NQTKGLVPEC	LNTLCSPMHS	RLQREFCLPA	SQLKQGETAD
1450	1460	1470	1480	1490	1500	1510	1520
KTYTDVFAKN	SISHDREEKL	QDGKEEEHKV	LLEAAPQLSQ	HLGSEAGQMK	EIHLESDPVL	NCLTLELHIN	GQRLQHQTGF
1530	1540	1550	1560	1570	1580	1590	1600
EQTTLETSLQ	MGPLEAEELQ	KANETENDIK	VLGGPKIPPP	KALQALENSD	GLILNAYQKD	NELVKSDEEL	NQPGSTNIQV
1610	1620	1630	1640	1650	1660	1670	1680
QPQTHFTQTI	LKSTSCPTLD	QFPFEKVESH	VRFSPLKSGE	AKVDEIIFYA	REGGISSDSS	HQKEQAGGTE	KKETAIFGSC
1690	1700	1710	1720	1730	1740	1750	1760
MPALSTPKTT	RNLKQFSDMK	TLVNPKCGII	KAKKPSISYM	LNIRAGAGPK	RRKELSCNLT	TKMKELHQGK	KGVDETYAFL
1770	1780	1790	1800	1810	1820	1830	1840
TMTPDINKYS	KVETEKDTLR	EKRLSSTQVK	QDTSPHEDSI	TSRDIKETLL	QDEEQEERKQ	EALLKVIPQH	LQHFMFRSGQ
1850	1860	1870	1880	1890	1900	1910	1920
GKDLDFHKLE	NQGSRKILFV	TKQDVPQQLQ	PAEPIQREET	KKCLQTQNGT	ICTVNSKLLP	LKSEDSVNGE	VLTGAIKRGV
1930	1940	1950	1960	1970	1980	1990	2000
PTDRKCMGEQ	HNSGKGEKAE	FNKDLQATVL	ELQKSPHGGE	AQKANLTDME	SGSSNAMNMN	VQHEREDKNI	QKMLTESVPC
2010	2020	2030	2040	2050	2060	2070	2080
YSQHLRFSTH	QMKDPDPCKS	GSEPKSPEGR	SWNLSHIVQK	TKQETHFRET	VLEPISGYMM	KQSPHMQEGI	KCMEGLKTSF
2090	2100	2110	2120	2130	2140	2150	2160
PKTGKSKIGS	IPRDTPWDEN	PRRKWDSSIS	EKTAWNQKNL	QTVLKPLDFS	SLMSSEYESR	SYTLEFIGKK	SMSPKCVTLK
2170	2180	2190	2200	2210	2220	2230	2240

Detailed Protein Report

Cmpd.	No. of Cmpds.	m/z meas.	Δ m/z [ppm]	z	Rt [min]	Score	P	Range	Sequence	Modification	Ratios
2367	1	805.3600	-33.80	2	60.0	10.6	0	1883-1897	K.CLQTQNGTICTVNSK.L		
270	1	642.3049	-15.58	2	33.4	11.3	0	2241-2251	R.QNLDGHITEEK.E		QU:MU 3.74<br>WUP:QUP 0.50
2490	1	610.2540	-89.47	2	61.1	10.6	2	3706-3716	K.QEGKMQEGKGK.S		
1634	1	803.8641	-92.41	2	50.2	10.9	0	5427-5440	K.MCILFSKPLPSNLK.L	Oxidation: 1	
2371	8	974.2361	79.07	3	60.1	12.7	2	5658-5682	R.DEDIYFTGFGTIRSGKRPEWLFTGK		
1685	1	916.4063	-73.78	2	50.8	10.7	2	6846-6860	R.ECRKETLIITPPSCK.S	Carbamidomethyl: 2, 14	

Detailed Protein Report

Protein 40: hemoglobin subunit epsilon [Homo sapiens]

**Accession:**	gi|4885393	**Score:**	77.9
**Database:**	refseq_human(refseq_human_20140103.fasta)	**MW [kDa]:**	16.2
**Database Date:**	2015-11-30	**pI:**	9.4
		**Sequence Coverage [%]:**	15.0
		**No. of unique Peptides:**	1

10	20	30	40	50	60	70	80
MVHFTAEEKA	AVTSLWSKMN	VEEAGGEALG	RLLVVYPWTQ	RFFDSFGNLS	SPSAILGNPK	VKAHGKKVLT	SFGDAIKNMD
**90**	**100**	**110**	**120**	**130**	**140**	**150**	
NLKPAFAKLS	ELHCDKLHVD	PENFKLLGNV	MVIILATHFG	KEFTPEVQAA	WQKLVSAVAI	ALAHKYH	

Cmpd.	No. of Cmpds.	m/z meas.	Δ m/z [ppm]	z	Rt [min]	Score	P	Range	Sequence	Modification
2297	6	596.8075	-111.81	2	57.0	16.1	0	134-145	K.LVSAVAIALAHK.Y	

Detailed Protein Report

Protein 41: **PREDICTED: DNA-directed RNA polymerase, mitochondrial isoform X1 [Homo sapiens]**

**Accession:**	gi|530425287	**Score:**	77.9
**Database:**	refseq_human(refseq_human_20140103.fasta)	**MW [kDa]:**	145.0
**Database Date:**	2015-11-30	**pI:**	10.3
**Modification(s):**	Carbamidomethyl, Oxidation	**Sequence Coverage [%]:**	5.4
		**No. of unique Peptides:**	4

Quantitation

QU:MU	**Median:** 1.41	**CV:** 0.00 %	**No. of Peptides:**	1
WUP:QUP	**Median:** 1.07	**CV:** 0.00 %	**No. of Peptides:**	1

```
        10         20         30         40         50         60         70         80
MPSRLRCILG VYFRLGFPFA ASVDHETTTP GRRRGRMRRR GPVGWPGAAC VMSALCWGRG AAGLKRALRP CGRPGLPGKE
        90        100        110        120        130        140        150        160
GTAGGVCGPR RSSSASPQEQ DQDRRKDWGH VELLEVLQAR VRQLQAESVS EVVVNRVDVA RLPECGSGDG SLQPPRKVQM
       170        180        190        200        210        220        230        240
GAKDATPVPC GRWAKILEKD KRTQQMRMQR LKAKLQMPFQ SGEFKALTRR LQVEPRLLSK QMAGCLEDCT RQAPESPWEE
       250        260        270        280        290        300        310        320
QLARLLQEAP GKLSLDVEQA PSGQHSQAQL SGQQQRLLAF FKCCLLTDQL PLAHHLLVVH HGQRQKRKLL TLDMYNAVML
       330        340        350        360        370        380        390        400
GWARQGAFKE LVYVLFMVKD AGLTPDLLSY AAALQCMGRQ DQDAGTIERC LEQMSQEGLK LQALFTAVLL SEEDRATVLK
       410        420        430        440        450        460        470        480
AVHKVKPTFS LPPQLPPPVN TSKLLRDVYA KDGRVSYPKL HLPLKTLQCL FEKQLHMELA SRVCVVSVEK PTLPSKEVKH
       490        500        510        520        530        540        550        560
ARKTLKTLRD QWEKALCRAL RETKNRLERE VYEGRFSLYP FLCLLDEREV VRMLLQVLQA LPAQGESFTT LARELSARTF
       570        580        590        600        610        620        630        640
SRHVVQRQRV SGQVQALQNH YRKYLCLLAS DAEVPEPCLP RQYWEELGAP EALREQPWPL PVQMELGKLL AEMLVQATQM
       650        660        670        680        690        700        710        720
PCSLDKPHRS SRLVPVLYHV YSFRNVQQIG ILKPHPAYVQ LLEKAAEPTL TFEAVDVPML CPPLPWTSPH SGAFLLSPTK
       730        740        750        760        770        780        790        800
LMRTVEGATQ HQELLETCPP TALHGALDAL TQLGNCAWRV NGRVLDLVLQ LFQAKGCPQL GVPAPPSEAP QPPEAHLPHS
       810        820        830        840        850        860        870        880
AAPARKAELR RELAHCQKVA REMHSLRAEA LYRLSLAQHL RDRVFWLPHN MDFRGRTYPC PPHFNHLGSD VARALLEFAQ
       890        900        910        920        930        940        950        960
GRPLGPHGLD WLKIHLVNLT GLKKREPLRK RLAFAEEVMD DILDSADQPL TGRKWWMGAE EPWQTLACCM EVANAVRASD
       970        980        990       1000       1010       1020       1030       1040
PAAYVSHLPV HQDGSCNGLQ HYAALGRDSV GAASVNLEPS DVPQDVYSGV AAQVEVFRRQ DAQRGMRVAQ VLEGFITRKV
      1050       1060       1070       1080       1090       1100       1110       1120
VKQTVMTVVY GVTRYGGRLQ IEKRLRELSD FPQEFVWEAS HYLVRQVFKS LQEMFSGTRA IQHWLTESAR LISHMGSVVE
      1130       1140       1150       1160       1170       1180       1190       1200
WVTPLGVPVI QPYRLDSKVK QIGGGIQSIT YTHNGDISRK PNTRKQKNGF PPNFIHSLDS SHMMLTALHC YRSPPLCHRK
      1210       1220       1230       1240       1250       1260       1270       1280
GLTFVSVHDC YWTHAADVSV MNQVCREQFV RLHSEPILQD LSRFLVKRFC SEPQKILEAS QLKETLQAVP KPGAFDLEQV
      1290
KRSTYFFS
```

Cmpd.	No. of Cmpds.	m/z meas.	Δ m/z [ppm]	z	Rt [min]	Score	P	Range	Sequence	Modification	Ratios
3	1	1244.4602	-117.14	2	28.9	10.3	1	41-65	R. GPVGWPGAACVMSALCWGRGA/ R	Carbamidomethyl: 10; Oxidation: 12	
77	1	793.8731	-24.51	2	29.9	14.5	1	158-172	K.VQMGAKDATPVPCGR.W	Carbamidomethyl: 13	
2861	1	682.6750	3.46	3	66.2	16.6	0	584-601	K.YLCLLASDAEVPEPCLPR.Q	Carbamidomethyl: 3	
390	1	685.3636	4.04	2	34.8	10.5	0	1043-1054	K.QTVMTVVYGVTR.Y	Oxidation: 4	WUP:QUP 1.07<br>QU:MU 1.41

Detailed Protein Report

Protein 42: obscurin isoform b [Homo sapiens]

**Accession:**	gi|403501446	**Score:**	77.3
**Database:**	refseq_human(refseq_human_20140103.fasta)	**MW [kDa]:**	867.9
**Database Date:**	2015-11-30	**pI:**	5.6
		**Sequence Coverage [%]:**	0.7
		**No. of unique Peptides:**	5

Quantitation

QU:MU	**Median:**	0.57	**CV:**	58.49 %	**No. of Peptides:**	2
WUP:QUP	**Median:**	1.62	**CV:**	48.00 %	**No. of Peptides:**	2

Detailed Protein Report

10	20	30	40	50	60	70	80
MDQPQFSGAP	RFLTRPKAFV	VSVGKDATLS	CQIVGNPTPQ	VSWEKDQQPV	AAGARFRLAQ	DGDLYRLTIL	DLALGDSGQY
90	100	110	120	130	140	150	160
VCRARNAIGE	AFAAVGLQVD	AEAACAEQAP	HFLLRPTSIR	VREGSEATFR	CRVGGSPRPA	VSWSKDGRRL	GEPDGPRVRV
170	180	190	200	210	220	230	240
EELGEASALR	IRAARPRDGG	TYEVRAENPL	GAASAAAALV	VDSDAADTAS	RPGTSTAALL	AHLQRRREAM	RAEGAPASPP
250	260	270	280	290	300	310	320
STGTRTCTVT	EGKHARLSCY	VTGEPKPETV	WKKDGQLVTE	GRRHVVYEDA	QENFVLKILF	CKQSDRGLYT	CTASNLVGQT
330	340	350	360	370	380	390	400
YSSVLVVVRE	PAVPFKKRLQ	DLEVREKESA	TFLCEVPQPS	TEAAWFKEET	RLWASAKYGI	EEEGTERRLT	VRNVSADDDA
410	420	430	440	450	460	470	480
VYICETPEGS	RTVAELAVQG	NLLRKLPRKT	AVRVGDTAMF	CVELAVPVGP	VHWLRNQEEV	VAGGRVAISA	EGTRHTLTIS
490	500	510	520	530	540	550	560
QCCLEDVGQV	AFMAGDCQTS	TQFCVSAPRK	PPLQPPVDPV	VKARMESSVI	LSWSPPPHGE	RPVTIDGYLV	EKKKLGTYTW
570	580	590	600	610	620	630	640
IRCHEAEWVA	TPELTVADVA	EEGNFQFRVS	ALNSFGQSPY	LEFPGTVHLA	PKLAVRTPLK	AVQAVEGGEV	TFSVDLTVAS
650	660	670	680	690	700	710	720
AGEWFLDGQA	LKASSVYEIH	CDRTRHTLTI	REVPASLHGA	QLKFVANGIE	SSIRMEVRAA	PGLTANKPPA	AAAREVLARL
730	740	750	760	770	780	790	800
HEEAQLLAEL	SDQAAAVTWL	KDGRTLSPGP	KYEVQASAGR	RVLLVRDVAR	DDAGLYECVS	RGGRIAYQLS	VQGLARFLHK
810	820	830	840	850	860	870	880
DMAGSCVDAV	AGGPAQFECE	TSEAHVHVHW	YKDGMELGHS	GERFLQEDVG	TRHRLVAATV	TRQDEGTYSC	RVGEDSVDFR
890	900	910	920	930	940	950	960
LRVSEPKVVF	AKEQLARRKL	QAEAGASATL	SCEVAQAQTE	VTWYKDGKKL	SSSSKVCMEA	TGCTRRLVVQ	QAGQADAGEY
970	980	990	1000	1010	1020	1030	1040
SCEAGGQRLS	FHLDVKEPKV	VFAKDQVAHS	EVQAEAGASA	TLSCEVAQAQ	TEVMWYKDGK	KLSSSLKVHV	EAKGCRRRLV
1050	1060	1070	1080	1090	1100	1110	1120
VQQAGKTDAG	DYSCEARGQR	VSFRLHITEP	KMMFAKEQSV	HNEVQAEAGA	SAMLSCEVAQ	AQTEVTWYKD	GKKLSSSSKV
1130	1140	1150	1160	1170	1180	1190	1200
GMEVKGCTRR	LVLPQAGKAD	AGEYSCEAGG	QRVSFHLHIT	EPKGVFAKEQ	SVHNEVQAEA	GTTAMLSCEV	AQPQTEVTWY
1210	1220	1230	1240	1250	1260	1270	1280
KDGKKLSSSS	KVRMEVKGCT	RRLVVQQVGK	ADAGEYSCEA	GGQRVSFQLH	ITEPKAVFAK	EQLVHNEVRT	EAGASATLSC
1290	1300	1310	1320	1330	1340	1350	1360
EVAQAQTEVT	WYKDGKKLSS	SSKVRIEAAG	CMRQLVVQQA	GQADAGEYTC	EAGGQRLSFH	LDVSEPKAVF	AKEQLAHRKV
1370	1380	1390	1400	1410	1420	1430	1440
QAEAGAIATL	SCEVAQAQTE	VTWYKDGKKL	SSSSKVRMEA	VGCTRRLVVQ	QACQADTGEY	SCEAGGQRLS	FSLDVAEPKV
1450	1460	1470	1480	1490	1500	1510	1520
VFAKEQPVHR	EVQAQAGAST	TLSCEVAQAQ	TEVMWYKDGK	KLSFSSKVRM	EAVGCTRRLV	VQQAGQAVAG	EYSCEAGSQR
1530	1540	1550	1560	1570	1580	1590	1600
LSFHLHVAEP	KAVFAKEQPA	SREVQAEAGT	SATLSCEVAQ	AQTEVTWYKD	GKKLSSSSKV	RMEAVGCTRR	LVVQEAGQAD
1610	1620	1630	1640	1650	1660	1670	1680
AGEYSCKAGD	QRLSFHLHVA	EPKVVFAKEQ	PAHREVQAEA	GASATLSCEV	AQAQTEVTWY	KDGKKLSSSS	KVRVEAVGCT
1690	1700	1710	1720	1730	1740	1750	1760
RRLVVQQAGQ	AEAGEYSCEA	GGQQLSFRLQ	VAELEPQISE	RPCRREPLVV	KEHEDIILTA	TLATPSAATV	TWLKDGVEIR
1770	1780	1790	1800	1810	1820	1830	1840
RSKRHETASQ	GDTHTLTVHG	AQVLDSAIYS	CRVGAEGQDF	PVQVEEVAAK	FCRLLEPVCG	ELGGTVTLAC	ELSPACAEVV
1850	1860	1870	1880	1890	1900	1910	1920
WRCGNTQLRV	GKRFQMVAEG	PVRSLTVLGL	RAEDAGEYVC	ESRDDHTSAQ	LTVSVPRVVK	FMSGLSTVVA	EEGGEATFQC
1930	1940	1950	1960	1970	1980	1990	2000
VVSPSDVAVV	WFRDGALLQP	SEKFAISQSG	ASHSLTISDL	VLEDAGQITV	EAEGASSSAA	LRVREAPVLF	KKKLEPQTVE
2010	2020	2030	2040	2050	2060	2070	2080
ERSSVTLEVE	LTRPWPELRW	TRNATALAPG	KNVEIHAEGA	RHRLVLHNVG	FADRGFFGCE	TPDDKTQAKL	TVEMRQVRLV
2090	2100	2110	2120	2130	2140	2150	2160
RGLQAVEARE	QGTATMEVQL	SHADVDGSWT	RDGLRFQQGP	TCHLAVRGPM	HTLTLSGLRP	EDSGLMVFKA	EGVHTSARLV
2170	2180	2190	2200	2210	2220	2230	2240

Detailed Protein Report

Cmpd.	No. of Cmpds.	m/z meas.	Δ m/z [ppm]	z	Rt [min]	Score	P	Range	Sequence	Modification	Ratios
1991	1	643.3110	-83.65	2	54.8	19.9	2	338-347	K.RLQDLEVREK.E		WUP:QUP 2.55<br>QU:MU 0.33
961	1	512.1292	-217.77	2	42.1	10.5	0	872-880	R.VGEDSVDFR.L		WUP:QUP 1.03<br>QU:MU 0.98
2661	2	686.8980	-30.78	2	63.3	13.1	2	881-892	R.LRVSEPKVVFAK.E		
2091	1	709.3658	-116.08	2	56.0	20.8	2	5981-5992	R.KYLLQARTAIIK.S		
2727	1	846.9286	-83.14	2	65.2	13.0	2	7693-7708	K.ASGRALAAKIIPYHPK.D		

Detailed Protein Report

Protein 43: PREDICTED: ceruloplasmin isoform X3 [Homo sapiens]

**Accession:**	gi|578807065	**Score:**	75.8
**Database:**	refseq_human(refseq_human_20140103.fasta)	**MW [kDa]:**	122.1
**Database Date:**	2015-11-30	**pI:**	5.4
**Modification(s):**	Carbamidomethyl	**Sequence Coverage [%]:**	2.6
		**No. of unique Peptides:**	2

10	20	30	40	50	60	70	80
MKILILGIFL	FLCSTPAWAK	EKHYYIGIIE	TTWDYASDHG	EKKLISVDTE	HSNIYLQNGP	DRIGRLYKKA	LYLQYTDETF
90	100	110	120	130	140	150	160
RTTIEKPVWL	GFLGPIIKAE	TGDKVYVHLK	NLASRPYTFH	SHGITYYKEH	EGAIYPDNTT	DFQRADDKVY	PGEQYTYMLL
170	180	190	200	210	220	230	240
ATEEQSPGEG	DGNCVTRIYH	SHIDAPKDIA	SGLIGPLIIC	KKDSLDKEKE	KHIDREFVVM	FSVVDENFSW	YLEDNIKTYC
250	260	270	280	290	300	310	320
SEPEKVDKDN	EDFQESNRMY	SVNGYTFGSL	PGLSMCAEDR	VKWYLFGMGN	EVDVHAAFFH	GQALTNKNYR	IDTINLFPAT
330	340	350	360	370	380	390	400
LFDAYMVAQN	PGEWMLSCQN	LNHLKAGLQA	FFQVQECNKS	SSKDNIRGKH	VRHYYIAAEE	IIWNYAPSGI	DIFTKENLTA
410	420	430	440	450	460	470	480
PGSDSAVFFE	QGTTRIGGSY	KKLVYREYTD	ASFTNRKERG	PEEEHLGILG	PVIWAEVGDT	IRVTFHNKGA	YPLSIEPIGV
490	500	510	520	530	540	550	560
RFNKNNEGTY	YSPNYNPQSR	SVPPSASHVA	PTETFTYEWT	VPKEVGPTNA	DPVCLAKMYY	SAVEPTKDIF	TGLIGPMKIC
570	580	590	600	610	620	630	640
KKGSLHANGR	QKDVDKEFYL	FPTVFDENES	LLLEDNIRMF	TTAPDQVDKE	DEDFQESNKM	HSMNGFMYGN	QPGLTMCKGD
650	660	670	680	690	700	710	720
SVVWYLFSAG	NEADVHGIYF	SGNTYLWRGE	RRDTANLFPQ	TSLTLHMWPD	TEGTFNVECL	TTDHYTGGMK	QKYTVNQCRR
730	740	750	760	770	780	790	800
QSEDSTFYLG	ERTYYIAAVE	VEWDYSPQRE	WEKELHHLQE	QNVSNAFLDK	GEFYIGSKYK	KVVYRQYTDS	TFRVPVERKA
810	820	830	840	850	860	870	880
EEEHLGILGP	QLHADVGDKV	KIIFKNMATR	PYSIHAHGVQ	TESSTVTPTL	PGETLTYVWK	IPERSGAGTE	DSACIPWAYY
890	900	910	920	930	940	950	960
STVDQVKDLY	SGLIGPLIVC	RRPYLKVFNP	RRKLEFALLF	LVFDENESWY	LDDNIKTYSD	HPEKVNKDDE	EFIESNKMHA
970	980	990	1000	1010	1020	1030	1040
INGRMFGNLQ	GLTMHVGDEV	NWYLMGMGNE	IDLHTVHFHG	HSFQYKHRGV	YSSDVFDIFP	GTYQTLEMFP	RTPGIWLLHC
1050	1060	1070					
HVTDHIHAGM	ETTYTVLQNE	GGTSM					

Cmpd.	No. of Cmpds.	m/z meas.	Δ m/z [ppm]	z	Rt [min]	Score	P	Range	Sequence	Modification
2714	5	735.4243	1.19	2	65.0	45.6	0	188-201	K.DIASGLIGPLIICK.K	Carbamidomethyl: 13
2789	2	788.4137	-22.96	2	65.3	30.2	0	888-901	K.DLYSGLIGPLIVCR.R	Carbamidomethyl: 13

Detailed Protein Report

Protein 44: probable helicase senataxin [Homo sapiens]

**Accession:**	gi|113722133	**Score:**	74.7
**Database:**	refseq_human(refseq_human_20140103.fasta)	**MW [kDa]:**	302.7
**Database Date:**	2015-11-30	**pI:**	6.9
**Modification(s):**	Carbamidomethyl, Oxidation	**Sequence Coverage [%]:**	3.2
		**No. of unique Peptides:**	5

Quantitation

QU:MU	**Median:**	0.43	**CV:**	0.00 %	**No. of Peptides:**	1

Detailed Protein Report

10	20	30	40	50	60	70	80
MSTCCWCTPG	GASTIDFLKR	YASNTPSGEF	QTADEDLCYC	LECVAEYHKA	RDELPFLHEV	LWELETLRLI	NHFEKSMKAE
90	100	110	120	130	140	150	160
IGDDDELYIV	DNNGEMPLFD	ITGQDFENKL	RVPLLEILKY	PYLLLHERVN	ELCVEALCRM	EQANCSFQVF	DKHPGIYLFL
170	180	190	200	210	220	230	240
VHPNEMVRRW	AILTARNLGK	VDRDDYYDLQ	EVLLCLFKVI	ELGLLESPDI	YTSSVLEKGK	LILLPSHMYD	TTNYKSYWLG
250	260	270	280	290	300	310	320
ICMLLTILEE	QAMDSLLLGS	DKQNDFMQSI	LHTMEREADD	DSVDPFWPAL	HCFMVILDRL	GSKVWGQLMD	PIVAFQTIIN
330	340	350	360	370	380	390	400
NASYNREIRH	IRNSSVRTKL	EPESYLDDMV	TCSQIVYNYN	PEKTKKDSGW	RTAICPDYCP	NMYEEMETLA	SVLQSDIGQD
410	420	430	440	450	460	470	480
MRVHNSTFLW	FIPFVQSLMD	LKDLGVAYIA	QVVNHLYSEV	KEVLNQTDAV	CDKVTEFFLL	ILVSVIELHR	NKKCLHLLWV
490	500	510	520	530	540	550	560
SSQQWVEAVV	KCAKLPTTAF	TRSSEKSSGN	CSKGTAMISS	LSLHSMPSNS	VQLAYVQLIR	SLLKEGYQLG	QQSLCKRFWD
570	580	590	600	610	620	630	640
KLNLFLRGNL	SLGWQLTSQE	THELQSCLKQ	IIRNIKFKAP	PCNTFVDLTS	ACKISPASYN	KEESEQMGKT	SRKDMHCLEA
650	660	670	680	690	700	710	720
SSPTFSKEPM	KVQDSVLIKA	DNTIEGDNNE	QNYIKDVKLE	DHLLAGSCLK	QSSKNIFTER	AEDQIKISTR	KQKSVKEISS
730	740	750	760	770	780	790	800
YTPKDCTSRN	GPERGCDRGI	IVSTRLLTDS	STDALEKVST	SNEDFSLKDD	ALAKTSKRKT	KVQKDEICAK	LSHVIKKQHR
810	820	830	840	850	860	870	880
KSTLVDNTIN	LDENLTVSNI	ESFYSRKDTG	VQKGDGFIHN	LSLDPSGVLD	DKNGEQKSQN	NVLPKEKQLK	NEELVIFSFH
890	900	910	920	930	940	950	960
ENNCKIQEFH	VDGKELIPFT	EMTNASEKKS	SPFKDLMTVP	ESRDEEMSNS	TSVIYSNLTR	EQAPDISPKS	DTLTDSQIDR
970	980	990	1000	1010	1020	1030	1040
DLHKLSLLAQ	ASVITFPSDS	PQNSSQLQRK	VKEDKRCFTA	NQNNVGDTSR	GQVIIISDSD	DDDDERILSL	EKLTKQDKIC
1050	1060	1070	1080	1090	1100	1110	1120
LEREHPEQHV	STVNSKEEKN	PVKEEKTETL	FQFEESDSQC	FEFESSSEVF	SVWQDHPDDN	NSVQDGEKKC	LAPIANTTNG
1130	1140	1150	1160	1170	1180	1190	1200
QGCTDYVSEV	VKKGAEGIEE	HTRPRSISVE	EFCEIEVKKP	KRKRSEKPMA	EDPVRPSSSV	RNEGQSDTNK	RDLVGNDFKS
1210	1220	1230	1240	1250	1260	1270	1280
IDRRTSTPNS	RIQRATTVSQ	KKSSKLCTCT	EPIRKVPVSK	TPKKTHSDAK	KGQNRSSNYL	SCRTTPAIVP	PKKFRQCPEP
1290	1300	1310	1320	1330	1340	1350	1360
TSTAEKLGLK	KGPRKAYELS	QRSLDYVAQL	RDHGKTVGVV	DTRKKTKLIS	PQNLSVRNNK	KLLTSQELQM	QRQIRPKSQK
1370	1380	1390	1400	1410	1420	1430	1440
NRRRLSDCES	TDVKRAGSHT	AQNSDIFVPE	SDRSDYNCTG	GTEVLANSNR	KQLIKCMPSE	PETIKAKHGS	PATDDACPLN
1450	1460	1470	1480	1490	1500	1510	1520
QCDSVVLNGT	VPTNEVIVST	SEDPLGGGDP	TARHIEMAAL	KEGEPDSSSD	AEEDNLFLTQ	NDPEDMDLCS	QMENDNYKLI
1530	1540	1550	1560	1570	1580	1590	1600
ELIHGKDTVE	VEEDSVSRPQ	LESLSGTKCK	YKDCLETTKN	QGEYCPKHSE	VKAADEDVFR	KPGLPPPASK	PLRPTTKIFS
1610	1620	1630	1640	1650	1660	1670	1680
SKSTSRIAGL	SKSLETSSAL	SPSLKNKSKG	IQSILKVPQP	VPLIAQKPVG	EMKNSCNVLH	PQSPNNSNRQ	GCKVPFGESK
1690	1700	1710	1720	1730	1740	1750	1760
YFPSSSPVNI	LLSSQSVSDT	FVKEVLKWKY	EMFLNFGQCG	PPASLCQSIS	RPVPVRFHNY	GDYFNVFFPL	MVLNTFETVA
1770	1780	1790	1800	1810	1820	1830	1840
QEWLNSPNRE	NFYQLQVRKF	PADYIKYWEF	AVYLEECELA	KQLYPKENDL	VFLAPERINE	EKKDTERNDI	QDLHEYHSGY
1850	1860	1870	1880	1890	1900	1910	1920
VHKFRRTSVM	RNGKTECYLS	IQTQENFPAN	LNELVNCIVI	SSLVTTQRKL	KAMSLLGSRN	QLARAVLNPN	PMDFCTKDLL
1930	1940	1950	1960	1970	1980	1990	2000
TTTSERIIAY	LRDFNEDQKK	AIETAYAMVK	HSPSVAKICL	IHGPPGTGKS	KTIVGLLYRL	LTENQRKGHS	DENSNAKIKQ
2010	2020	2030	2040	2050	2060	2070	2080
NRVLVCAPSN	AAVDELMKKI	ILEFKEKCKD	KKNPLGNCGD	INLVRLGPEK	SINSEVLKFS	LDSQVNHRMK	KELPSHVQAM
2090	2100	2110	2120	2130	2140	2150	2160
HKRKEFLDYQ	LDELSRQRAL	CRGGREIQRQ	ELDENISKVS	KERQELASKI	KEVQGRPQKT	QSIIILESHI	ICCTLSTSGG
2170	2180	2190	2200	2210	2220	2230	2240

Detailed Protein Report

Cmpd.	No. of Cmpds.	m/z meas.	Δ m/z [ppm]	z	Rt [min]	Score	P	Range	Sequence	Modification	Ratios
896	1	576.9688	-1.76	3	41.3	12.0	1	679-694	K.LEDHLLAGSCLKQSSK.N		
2700	1	856.9282	-25.41	2	64.9	12.2	2	853-867	K.NGEQKSQNNVLPKEK.Q		QU:MU 0.43
160	1	896.7503	-91.72	3	31.7	17.5	1	1573-1597	K. AADEDVFRKPGLPPPASKPLRPTT I		
1617	1	781.3954	-38.20	2	48.5	10.2	1	1613-1627	K.SLETSSALSPSLKNK.S		
2158	1	682.2802	-2.49	3	57.4	22.7	1	2220-2235	K.AQEYGYDQSMMARFCR.L	Carbamidomethyl: 15; Oxidation: 10, 11	

Detailed Protein Report

Protein 45: glyceraldehyde-3-phosphate dehydrogenase isoform 2 [Homo sapiens]

**Accession:**	gi|378404908	**Score:**	73.6
**Database:**	refseq_human(refseq_human_20140103.fasta)	**MW [kDa]:**	31.5
**Database Date:**	2015-11-30	**pI:**	7.9
**Modification(s):**	Carbamidomethyl	**Sequence Coverage [%]:**	10.6
		**No. of unique Peptides:**	2

Quantitation

QU:MU	**Median:** 0.52	**CV:** 14.71 %	**No. of Peptides:**	2
WUP:QUP	**Median:** 2.83	**CV:** 0.00 %	**No. of Peptides:**	1

10	20	30	40	50	60	70	80
MVYMFQYDST	HGKFHGTVKA	ENGKLVINGN	PITIFQERDP	SKIKWGDAGA	EYVVESTGVF	TTMEKAGAHL	QGGAKRVIIS
**90**	**100**	**110**	**120**	**130**	**140**	**150**	**160**
APSADAPMFV	MGVNHEKYDN	SLKIISNASC	TTNCLAPLAK	VIHDNFGIVE	GLMTTVHAIT	ATQKTVDGPS	GKLWRDGRGA
**170**	**180**	**190**	**200**	**210**	**220**	**230**	**240**
LQNIIPASTG	AAKAVGKVIP	ELNGKLTGMA	FRVPTANVSV	VDLTCRLEKP	AKYDDIKKVV	KQASEGPLKG	ILGYTEHQVV
**250**	**260**	**270**	**280**	**290**	**300**		
SSDFNSDTHS	STFDAGAGIA	LNDHFVKLIS	WYDNEFGYSN	RVVDLMAHMA	SKE		

Cmpd.	No. of Cmpds.	m/z meas.	Δ m/z [ppm]	z	Rt [min]	Score	P	Range	Sequence	Modification	Ratios
676	3	917.4657	2.44	2	38.0	53.0	0	104-120	K.IISNASCTTNCLAPLAK.V	Carbamidomethyl: 7, 11	WUP:QUP 2.83<br>QU:MU 0.45
1648	4	882.4064	1.76	2	50.9	20.6	0	268-281	K.LISWYDNEFGYSNR.V		QU:MU 0.61

Detailed Protein Report

Protein 46: dynein heavy chain 9, axonemal isoform 2 [Homo sapiens]

**Accession:**	gi|114155133	**Score:**	73.3
**Database:**	refseq_human(refseq_human_20140103.fasta)	**MW [kDa]:**	511.6
**Database Date:**	2015-11-30	**pI:**	5.6
		**Sequence Coverage [%]:**	1.1
		**No. of unique Peptides:**	4

Quantitation

QU:MU	**Median:**	0.47	**CV:**	0.00 %	**No. of Peptides:**	1
WUP:QUP	**Median:**	2.21	**CV:**	0.00 %	**No. of Peptides:**	1

Detailed Protein Report

10	20	30	40	50	60	70	80
MRLAEERAAL	AAENADGEPG	ADRRLRLLGT	YVAMSLRPAA	GAWERCAGSA	EAEQLLQAFL	GRDAAEGPRP	LLVVRPGPRG
90	100	110	120	130	140	150	160
LAIRPGLEVG	PESGLAGAKA	LFFLRTGPEP	PGPDSFRGAV	VCGDLPAAPL	EHLAALFSEV	VLPVLANEKN	RLNWPHMICE
170	180	190	200	210	220	230	240
DVRRHAHSLQ	CDLSVILEQV	KGKTLLPLPA	GSEKMEFADS	KSETVLDSID	KSVIYAIESA	VIKWSYQVQV	VLKRESSQPL
250	260	270	280	290	300	310	320
LQGENPTPKV	ELEFWKSRYE	DLKYIYNQLR	TITVRGMAKL	LDKLQSSYFP	AFKAMYRDVV	AALAEAQDIH	VHLIPLQRHL
330	340	350	360	370	380	390	400
EALENAEFPE	VKPQLRPLLH	VVCLIWATCK	SYRSPGRLTV	LLQEICNLLI	QQASNYLSPE	DLLRSEVEES	QRKLQVVSDT
410	420	430	440	450	460	470	480
LSFFKQEFQD	RRENLHTYFK	ENQEVKEWDF	QSSLVFVRLD	GFLGQLHVVE	GLLKTALDFH	KLGKVEFSGV	RGNALSQQVQ
490	500	510	520	530	540	550	560
QMHEEFQEMY	RLLSGSSSDC	LYLQSTDFEN	DVSEFNQKVE	DLDRRLGTIF	IQAFDDAPGL	EHAFKLLDIA	GNLLERPLVA
570	580	590	600	610	620	630	640
RDTSDKYLVL	IQMFNKDLDA	VRMIYSQHVQ	EEAELGFSPV	HKNMPTVAGG	LRWAQELRQR	IQGPFSNFGR	ITHPCMESAE
650	660	670	680	690	700	710	720
GKRMQQKYED	MLSLLEKYET	RLYEDWCRTV	SEKSQYNLSQ	PLLKRDPETK	EITINFNPQL	ISVLKEMSYL	EPREMKHMPE
730	740	750	760	770	780	790	800
TAAAMFSSRD	FYRQLVANLE	LMANWYNKVM	KTLLEVEFPL	VEEELQNIDL	RLRAAEETLN	WKTEGICDYV	TEITSSIHDL
810	820	830	840	850	860	870	880
EQRIQKTKDN	VEEIQNIMKT	WVTPIFKTKD	GKRESLLSLD	DRHDRMEKYY	NLIKESGLKI	HALVQENLGL	FSADPTSNIW
890	900	910	920	930	940	950	960
KTYVNSIDNL	LLNGFFLAIE	CSLKYLLENT	ECKAGLTPIF	EAQLSLAIPE	LVFYPSLESG	VKGGFCDIVE	GLITSIFRIP
970	980	990	1000	1010	1020	1030	1040
SLVPRLSPQN	GSPHYQVDLD	GIPDLANMRR	TLMERVQRMM	GLCCGYQSTF	SQYSYLYVED	RKEVLGQFLL	YGHILTPEEI
1050	1060	1070	1080	1090	1100	1110	1120
EDHVEDGIPE	NPPLLSQFKV	QIDSYETLYE	EVCRLEPIKV	FDGWMKIDIR	PFKASLLNII	KRWSLLFKQH	LVDHVTHSLA
1130	1140	1150	1160	1170	1180	1190	1200
NLDAFIKKSE	SGLLKKVEKG	DFQGLVEIMG	HLMAVKERQS	NTDEMFEPLK	QTIELLKTYE	QELPETVFKQ	LEELPEKWNN
1210	1220	1230	1240	1250	1260	1270	1280
IKKVAITVKQ	QVAPLQANEV	TLLRQRCTAF	DAEQQQFWEQ	FHKEAPFRFD	SIHPHQMLDA	RHIEIQQMES	TMASISESAS
1290	1300	1310	1320	1330	1340	1350	1360
LFEVNVPDYK	QLRQCRKEVC	QLKELWDTIG	MVTSSIHAWE	TTPWRNINVE	AMELECKQFA	RHIRNLDKEV	RAWDAFTGLE
1370	1380	1390	1400	1410	1420	1430	1440
STVWNTLSSL	RAVAELQNPA	IRERHWRQLM	QATGVSFTMD	QDTTLAHLLQ	LQLHHYEDEV	RGIVDKAAKE	MGMEKTLKEL
1450	1460	1470	1480	1490	1500	1510	1520
QTTWAGMEFQ	YEPHPRTNVP	LLCSDEDLIE	VLEDNQVQLQ	NLVMSKYVAF	FLEEVSGWQK	KLSTVDAVIS	IWFEVQRTWT
1530	1540	1550	1560	1570	1580	1590	1600
HLESIFTGSE	DIRAQLPQDS	KRFEGIDIDF	KELAYDAQKI	PNVVQTTNKP	GLYEKLEDIQ	GRLCLCEKAL	AEYLDTKRLA
1610	1620	1630	1640	1650	1660	1670	1680
FPRFYFLSSS	DLLDILSNGT	APQQVQRHLS	KLFDNMAKMR	FQLDASGEPT	KTSLGMYSKE	EEYVAFSEPC	DCSGQVEIWL
1690	1700	1710	1720	1730	1740	1750	1760
NHVLGHMKAT	VRHEMTEGVT	AYEEKPREQW	LFDHPAQVAL	TCTQIWWTTE	VGMAFARLEE	GYESAMKDYY	KKQVAQLKTL
1770	1780	1790	1800	1810	1820	1830	1840
ITMLIGQLSK	GDRQKIMTIC	TIDVHARDVV	AKMIAQKVDN	AQAFLWLSQL	RHRWDDEVKH	CFANICDAQF	LYSYEYLGNT
1850	1860	1870	1880	1890	1900	1910	1920
PRLVITPLTD	RCYITLTQSL	HLTMSGAPAG	PAGTGKTETT	KDLGRALGIL	VYVFNCSEQM	DYKSCGNIYK	GLAQTGAWGC
1930	1940	1950	1960	1970	1980	1990	2000
FDEFNRISVE	VLSVVAVQVK	SIQDAIRDKK	QWFSFLGEEI	SLNPSVGIFI	TMNPGYAGRT	ELPENLKSLF	RPCAMVVPDF
2010	2020	2030	2040	2050	2060	2070	2080
ELICEIMLVA	EGFIEAQSLA	RKFITLYQLC	KELLSKQDHY	DWGLRAIKSV	LVVAGSLKRG	DPDRPEDQVL	MRSLRDFNIP
2090	2100	2110	2120	2130	2140	2150	2160
KIVTDDMPIF	MGLIGDLFPA	LDVPRRRDPN	FEALVRKAIV	DLKLQAEDNF	VLKVVQLEEL	LAVRHSVFVV	GGAGTGKSQV
2170	2180	2190	2200	2210	2220	2230	2240

Detailed Protein Report

Cmpd.	No. of Cmpds.	m/z meas.	Δ m/z [ppm]	z	Rt [min]	Score	P	Range	Sequence	Modification	Ratios
2873	2	731.8707	-28.75	2	66.4	25.7	1	1075-1086	R.LEPIKVFDGWMK.I		
1912	2	634.8166	-103.44	2	54.3	17.9	0	2134-2144	K.VVQLEELLAVR.H		WUP:QUP 2.21<br>QU:MU 0.47
54	1	796.7965	-120.13	2	30.4	11.9	0	3448-3461	R.MSVENATILINCER.W		
1679	4	648.2912	-180.05	2	51.3	17.9	0	4475-4486	K.WVLAGVALLLQI.-		

Detailed Protein Report

Protein 47: PREDICTED: histone-lysine N-methyltransferase 2A isoform X1 [Homo sapiens]

**Accession:**	gi|578822195	**Score:**	72.9
**Database:**	refseq_human(refseq_human_20140103.fasta)	**MW [kDa]:**	323.9
**Database Date:**	2015-11-30	**pI:**	9.2
**Modification(s):**	Carbamidomethyl, Oxidation	**Sequence Coverage [%]:**	3.0
		**No. of unique Peptides:**	5

Quantitation

QU:MU	**Median:**	2.24	**CV:**	0.00 %	**No. of Peptides:**	1
WUP:QUP	**Median:**	0.78	**CV:**	38.96 %	**No. of Peptides:**	2

Detailed Protein Report

10	20	30	40	50	60	70	80
MLAQADKLPM	TDKRVASLLK	KAKAQLCKIE	KSKSLKQTDQ	PKAQGQESDS	SETSVRGPRI	KHVCRRAAVA	LGRKRAVFPD
90	100	110	120	130	140	150	160
DMPTLSALPW	EEREKILSSM	GNDDKSSIAG	SEDAEPLAPP	IKPIKPVTRN	KAPQEPPVKK	GRRSRRCGQC	PGCQVPEDCG
170	180	190	200	210	220	230	240
VCTNCLDKPK	FGGRNIKKQC	CKMRKCQNLQ	WMPSKAYLQK	QAKAVKKKEK	KSKTSEKKDS	KESSVVKNVV	DSSQKPTPSA
250	260	270	280	290	300	310	320
REDPAPKKSS	SEPPPRKPVE	EKSEEGNVSA	PGPESKQATT	PASRKSSKQV	SQPALVIPPQ	PPTTGPPRKE	VPKTTPSEPK
330	340	350	360	370	380	390	400
KKQPPPPESG	PEQSKQKKVA	PRPSIPVKQK	PKEKEKPPPV	NKQENAGTLN	ILSTLSNGNS	SKQKIPADGV	HRIRVDFKED
410	420	430	440	450	460	470	480
CEAENVWEMG	GLGILTSVPI	TPRVVCFLCA	SSGHVEFVYC	QVCCEPFHKF	CLEENERPLE	DQLENWCCRR	CKFCHVCGRQ
490	500	510	520	530	540	550	560
HQATKQLLEC	NKCRNSYHPE	CLGPNYPTKP	TKKKKVWICT	KCVRCKSCGS	TTPGKGWDAQ	WSHDFSLCHD	CAKLFAKGNF
570	580	590	600	610	620	630	640
CPLCDKCYDD	DDYESKMMQC	GKCDRWVHSK	CENLSGTEDE	MYEILSNLPE	SVAYTCVNCT	ERHPAEWRLA	LEKELQISLK
650	660	670	680	690	700	710	720
QVLTALLNSR	TTSHLLRYRQ	AAKPPDLNPE	TEESIPSRSS	PEGPDPPVLT	EVSKQDDQQP	LDLEGVKRKM	DQGNYTSVLE
730	740	750	760	770	780	790	800
FSDDIVKIIQ	AAINSDGGQP	EIKKANSMVK	SFFIRQMERV	FPWFSVKKSR	FWEPNKVSSN	SGMLPNAVLP	PSLDHNYAQW
810	820	830	840	850	860	870	880
QEREENSHTE	QPPLMKKIIP	APKPKGPGEP	DSPTPLHPPT	PPILSTDRSR	EDSPELNPPP	GIEDNRQCAL	CLTYGDDSAN
890	900	910	920	930	940	950	960
DAGRLLYIGQ	NEWTHVNCAL	WSAEVFEDDD	GSLKNVHMAV	IRGKQLRCEF	CQKPGATVGC	CLTSCTSNYH	FMCSRAKNCV
970	980	990	1000	1010	1020	1030	1040
FLDDKKVYCQ	RHRDLIKGEV	VPENGFEVFR	RVFVDFEGIS	LRRKFLNGLE	PENIHMMIGS	MTIDCLGILN	DLSDCEDKLF
1050	1060	1070	1080	1090	1100	1110	1120
PIGYQCSRVY	WSTTDARKRC	VYTCKIVECR	PPVVEPDINS	TVEHDENRTI	AHSPTSFTES	SSKESQNTAE	IISPPSPDRP
1130	1140	1150	1160	1170	1180	1190	1200
PHSQTSGSCY	YHVISKVPRI	RTPSYSPTQR	SPGCRPLPSA	GSPTPTTHEI	VTVGDPLLSS	GLRSIGSRRH	STSSLSPQRS
1210	1220	1230	1240	1250	1260	1270	1280
KLRIMSPMRT	GNTYSRNNVS	SVSTTGTATD	LESSAKVVDH	VLGPLNSSTS	LGQNTSTSSN	LQRTVVTVGN	KNSHLDGSSS
1290	1300	1310	1320	1330	1340	1350	1360
SEMKQSSASD	LVSKSSSLKG	EKTKVLSSKS	SEGSAHNVAY	PGIPKLAPQV	HNTTSRELNV	SKIGSFAEPS	SVSFSSKEAL
1370	1380	1390	1400	1410	1420	1430	1440
SFPHLHLRGQ	RNDRDQHTDS	TQSANSSPDE	DTEVKTLKLS	GMSNRSSIIN	EHMGSSSRDR	RQKGKKSCKE	TFKEKHSSKS
1450	1460	1470	1480	1490	1500	1510	1520
FLEPGQVTTG	EEGNLKPEFM	DEVLTPEYMG	QRPCNNVSSD	KIGDKGLSMP	GVPKAPPMQV	EGSAKELQAP	RKRTVKVTLT
1530	1540	1550	1560	1570	1580	1590	1600
PLKMENESQS	KNALKESSPA	SPLQIESTSP	TEPISASENP	GDGPVAQPSP	NNTSCQDSQS	NNYQNLPVQD	RNLMLPDGPK
1610	1620	1630	1640	1650	1660	1670	1680
PQEDGSFKRR	YPRRSARARS	NMFFGLTPLY	GVRSYGEEDI	PFYSSSTGKK	RGKRSAEGQV	DGADDLSTSD	EDDLYYYNFT
1690	1700	1710	1720	1730	1740	1750	1760
RTVISSGGEE	RLASHNLFRE	EEQCDLPKIS	QLDGVDDGTE	SDTSVTATTR	KSSQIPKRNG	KENGTENLKI	DRPEDAGEKE
1770	1780	1790	1800	1810	1820	1830	1840
HVTKSSVGHK	NEPKMDNCHS	VSRVKTQGQD	SLEAQLSSLE	SSRRVHTSTP	SDKNLLDTYN	TELLKSDSDN	NNSDDCGNIL
1850	1860	1870	1880	1890	1900	1910	1920
PSDIMDFVLK	NTPSMQALGE	SPESSSSELL	NLGEGLGLDS	NREKDMGLFE	VFSQQLPTTE	PVDSSVSSSI	SAEEQFELPL
1930	1940	1950	1960	1970	1980	1990	2000
ELPSDLSVLT	TRSPTVPSQN	PSRLAVISDS	GEKRVTITEK	SVASSESDPA	LLSPGVDPTP	EGHMTPDHFI	QGHMDADHIS
2010	2020	2030	2040	2050	2060	2070	2080
SPPCGSVEQG	HGNNQDLTRN	SSTPGLQVPV	SPTVPIQNQK	YVPNSTDSPG	PSQISNAAVQ	TTPPHLKPAT	EKLIVVNQNM
2090	2100	2110	2120	2130	2140	2150	2160
QPLYVLQTLP	NGVTQKIQLT	SSVSSTPSVM	ETNTSVLGPM	GGGLTLTTGL	NPSLPTSQSL	FPSASKGLLP	MSHHQHLHSF
2170	2180	2190	2200	2210	2220	2230	2240

Detailed Protein Report

Cmpd.	No. of Cmpds.	m/z meas.	Δ m/z [ppm]	z	Rt [min]	Score	P	Range	Sequence	Modification	Ratios	
1751	1	1061.1573	33.03	3	51.7	15.9	2	144-170	R. SRRCGQCPGCQVPEDCGVCTNC F	Carbamidomethyl: 4, 10, 16, 19, 22		
908	1	541.3264	90.29	3	41.4	12.3	2	242-256	R.EDPAPKKSSSEPPPR.K		WUP:QUP<br>QU:MU	0.54<br>2.24
1836	13	682.2601	12.95	3	52.8	16.3	1	567-582	K.CYDDDDYESKMMQCGK.C	Carbamidomethyl: 1, 14		
53	1	1096.9888	-34.10	2	30.4	17.5	1	1264-1284	R. TVVTVGNKNSHLDGSSSSEMK. Q	Oxidation: 20		
395	1	625.3909	142.58	2	34.6	10.9	2	2950-2960	K.LPCNCGAKKCR.K	Carbamidomethyl: 5	WUP:QUP	1.14

Detailed Protein Report

Protein 48: PREDICTED: ankyrin repeat domain-containing protein 11 isoform X4 [Homo sapiens]

**Accession:**	gi|578828959	**Score:**	72.9
**Database:**	refseq_human(refseq_human_20140103.fasta)	**MW [kDa]:**	286.8
**Database Date:**	2015-11-30	**pI:**	6.4
**Modification(s):**	Oxidation	**Sequence Coverage [%]:**	2.6
		**No. of unique Peptides:**	6

Quantitation

QU:MU	**Median:** 1.14	**CV:** 0.00 %	**No. of Peptides:** 1

Detailed Protein Report

10	20	30	40	50	60	70	80
MGLSGIRAGY	PLSERQQVAL	LMQMTAEESA	NSPVDTTPKH	PSQSTVCQKG	TPNSASKTKD	KVNKRNERGE	TRLHRAAIRG
90	100	110	120	130	140	150	160
DARRIKELIS	EGADVNVKDF	AGWTALHEAC	NRGYYDVAKQ	LLAAGAEVNT	KGLDDDTPLH	DAANNGHYKV	VKLLLRYGGN
170	180	190	200	210	220	230	240
PQQSNRKGET	PLKVANSPTM	VNLLLGKGTY	TSSEESSTES	SEEEDAPSFA	PSSSVDGNNT	DSEFEKGLKH	KAKNPEPQKA
250	260	270	280	290	300	310	320
TAPVKDEYEF	DEDDEQDRVP	PVDDKHLLKK	DYRKETKSNS	FISIPKMEVK	SYTKNNTIAP	KKASHRILSD	TSDEEDASVT
330	340	350	360	370	380	390	400
VGTGEKLRLS	AHTILPGSKT	REPSNAKQQK	EKNKVKKKRK	KETKGREVRF	GKRSDKFCSS	ESESESSESG	EDDRDSLGSS
410	420	430	440	450	460	470	480
GCLKGSPLVL	KDPSLFSSLS	ASSTSSHGSS	AAQKQNPSHT	DQHTKHWRTD	NWKTISSPAW	SEVSSLSDST	RTRLTSESDY
490	500	510	520	530	540	550	560
SSEGSSVESL	KPVRKRQEHR	KRASLSEKKS	PFLSSAEGAV	PKLDKEGKVV	KKHKTKHKHK	NKEKGQCSIS	QELKLKSFTY
570	580	590	600	610	620	630	640
EYEDSKQKSD	KAILLENDLS	TENKLKVLKH	DRDHFKKEEK	LSKMKLEEKE	WLFKDEKSLK	RIKDTNKDIS	RSFREEKDRS
650	660	670	680	690	700	710	720
NKAEKERSLK	EKSPKEEKLR	LYKEERKKKS	KDRPSKLEKK	NDLKEDKISK	EKEKIFKEDK	EKLKKEKVYR	EDSAFDEYCN
730	740	750	760	770	780	790	800
KNQFLENEDT	KFSLSDDQRD	RWFSDLSDSS	FDFKGEDSWD	SPVTDYRDMK	SDSVAKLILE	TVKEDSKERR	RDSRAREKRD
810	820	830	840	850	860	870	880
YREPFFRKKD	RDYLDKNSEK	RKEQTEKHKS	VPGYLSEKDK	KRRESAEAGR	DRKDALESCK	ERRDGRAKPE	EAHREELKEC
890	900	910	920	930	940	950	960
GCESGFKDKS	DGDFGKGLEP	WERHHPAREK	EKKDGPDKER	KEKTKPERYK	EKSSDKDKSE	KSILEKCQKD	KEFDKCFKEK
970	980	990	1000	1010	1020	1030	1040
KDTKEKHKDT	HGKDKERKAS	LDQGKEKKEK	AFPGIISEDF	SEKKDDKKGK	EKSWYIADIF	TDESEDDRDS	CMGSGFKMGE
1050	1060	1070	1080	1090	1100	1110	1120
ASDLPRTDGL	QEKEEGREAY	ASDRHRKSSD	KQHPERQKDK	EPRDRRKDRG	AADAGRDKKE	KVFEKHKEKK	DKESTEKYKD
1130	1140	1150	1160	1170	1180	1190	1200
RKDRASVDST	QDKKNKQKLP	EKAEKKHAAE	DKAKSKHKEK	SDKEHSKERK	SSRSADAEKS	LLEKLEEEAL	HEYREDSNDK
1210	1220	1230	1240	1250	1260	1270	1280
ISEVSSDSFT	DRGQEPGLTA	FLEVSFTEPP	GDDKPRESAC	LPEKLKEKER	HRHSSSSSKK	SHDRERAKKE	KAEKKEKGED
1290	1300	1310	1320	1330	1340	1350	1360
YKEGGSRKDS	GQYEKDFLEA	DAYGVSYNMK	ADIEDELDKT	IELFSTEKKD	KNDSEREPSK	KIEKELKPYG	SSAINILKEK
1370	1380	1390	1400	1410	1420	1430	1440
KKREKHREKW	RDEKERHRDR	HADGLLRHHR	DELLRHHRDE	QKPATRDKDS	PPRVLKDKSR	DEGPRLGDAK	LKEKFKDGAE
1450	1460	1470	1480	1490	1500	1510	1520
KEKGDPVKMS	NGNDKVAPSK	DPGKKDARPR	EKLLGDGDLM	MTSFERMLSQ	KDLEIEERHK	RHKERMKQME	KLRHRSGDPK
1530	1540	1550	1560	1570	1580	1590	1600
LKEKAKPADD	GRKKGLDIPA	KKPPGLDPPF	KDKKLKESTP	IPPAAENKLH	PASGADSKDW	LAGPHMKEVL	PASPRPDQSR
1610	1620	1630	1640	1650	1660	1670	1680
PTGVPTPTSV	LSCPSYEEVM	HTPRTPSCSA	DDYADLVFDC	ADSQHSTPVP	TAPTSACSPS	FFDRFSVASS	GLSENASQAP
1690	1700	1710	1720	1730	1740	1750	1760
ARPLSTNLYR	SVSVDIRRTP	EEEFSVGDKL	FRQQSVPAAS	SYDSPMPPSM	EDRAPLPPVP	AEKFACLSPG	YYSPDYGLPS
1770	1780	1790	1800	1810	1820	1830	1840
PKVDALHCPP	AAVVTVTPSP	EGVFSSLQAK	PSPSPRAELL	VPSLEGALPP	DLDTSEDQQA	TAAIIPPEPS	YLEPLDEGPF
1850	1860	1870	1880	1890	1900	1910	1920
SAVITEEPVE	WAHPSEQALA	SSLIGGTSEN	PVSWPVGSDL	LLKSPQRFPE	SPKRFCPADP	LHSAAPGPFS	ASEAPYPAPP
1930	1940	1950	1960	1970	1980	1990	2000
ASPAPYALPV	AEPGLEDVKD	GVDAVPAAIS	TSEAAPYAPP	SGLESFFSNC	KSLPEAPLDV	APEPACVAAV	AQVEALGPLE
2010	2020	2030	2040	2050	2060	2070	2080
NSFLDGSRGL	SHLGQVEPVP	WADAFAGPED	DLDLGPFSLP	ELPLQTKDAA	DGEAEPVEES	LAPPEEMPPG	APGVINGGDV
2090	2100	2110	2120	2130	2140	2150	2160
STVVAEEPPA	LPPDQASTRL	PAELEPEPSG	EPKLDVALEA	AVEAETVPEE	RARGDPDSSV	EPAPVPPEQR	PLGSGDQGAE
2170	2180	2190	2200	2210	2220	2230	2240

Detailed Protein Report

Cmpd.	No. of Cmpds.	m/z meas.	Δ m/z [ppm]	z	Rt [min]	Score	P	Range	Sequence	Modification	Ratios
793	1	1105.5255	-96.76	2	39.9	11.9	2	167-187	R.KGETPLKVANSPTMVNLLLGK.G		
462	1	475.6124	-248.29	2	34.3	11.4	1	1171-1179	K.SSRSADAEK.S		QU:MU 1.14
2600	1	741.8765	-22.04	2	62.5	14.8	2	1379-1390	R.DRHADGLLRHHR.D		
255	1	501.2790	-48.16	2	31.9	12.8	2	1426-1434	R.LGDAKLKEK.F		
2267	1	937.3359	-108.02	2	56.7	10.6	1	1471-1486	R.EKLLGDGDLMMTSFER.M	Oxidation: 10, 11	
1507	1	619.9684	9.93	3	48.5	11.3	1	1471-1486	R.EKLLGDGDLMMTSFER.M	Oxidation: 11	

Detailed Protein Report

Protein 49: dynein heavy chain 1, axonemal [Homo sapiens]

**Accession:**	gi|197927452	**Score:**	71.3
**Database:**	refseq_human(refseq_human_20140103.fasta)	**MW [kDa]:**	487.2
**Database Date:**	2015-11-30	**pI:**	5.5
**Modification(s):**	Oxidation	**Sequence Coverage [%]:**	1.6
		**No. of unique Peptides:**	4

Quantitation

QU:MU	**Median:**	1.67	**CV:**	0.00 %	**No. of Peptides:**	1
WUP:QUP	**Median:**	1.04	**CV:**	0.00 %	**No. of Peptides:**	1

Alias proteins:

Accession	Name	Description
gi|578805960	refseq_human (refseq_human_20140103.fasta)	PREDICTED: dynein heavy chain 1, axonemal isoform X1 [Homo sapiens]

Detailed Protein Report

10	20	30	40	50	60	70	80
MEQPNSKGYS	LGRTPQGPEC	SSAPAVQVGT	HRGLEYNPGK	ILPGSDYGLG	NPPALDPKLP	HLPLPPAPPT	LSDLGQPRKS
90	100	110	120	130	140	150	160
PLTGTDKKYP	LMKQRGFYSD	ILSPGTLDQL	GEVCRGPRMS	QNLLRQADLD	KFTPRVGSFE	VPEDFQERME	QQCIGSTTRL
170	180	190	200	210	220	230	240
LAQTDFPLQA	YEPKMQVPFQ	VLPGQHPRKI	EIERRKQQYL	SLDIEQLLFS	QGIDSNKLMP	RHLDHQHPQT	IEQGHDPIFP
250	260	270	280	290	300	310	320
IYLPLKVFDN	EDFDCRTPRE	WINMGLEPGS	LDRKPVPGKA	LLPTDDFLGH	EDPKSQKLKY	KWCEVGVLDY	DEEKKLYLVH
330	340	350	360	370	380	390	400
KTDEKGLVRD	EMGRPILNAG	VTTEGRPPLQ	VCQYWVPRIQ	LLFCAEDPCM	FAQRVVQANA	LRKNTEALLL	YNLYVDCMPS
410	420	430	440	450	460	470	480
DGQHVISEQS	LSKIKQWALS	TPRMRKGPSV	LEHLSSLARE	VSLDYERSMN	KINFDHVVSS	KPETFSYVTL	PKKEEEQVPE
490	500	510	520	530	540	550	560
RGLVSVPKYH	FWEQKEDFTF	VSLLTRPEVI	TALSKVRAEC	NKVTAMSLFH	SSLSKYSHLE	EFEQIQSQTF	SQVQMFLKDS
570	580	590	600	610	620	630	640
WISSLKVAMR	SSLRDMSKGW	YNLYETNWEV	YLMSKLRKLM	ELVKYMLQDT	LRFLVQDSLA	SFSQFISDTC	CSVLNCTDDM
650	660	670	680	690	700	710	720
VWGDDLINSP	YRPRKNPLFI	MDLVLDSSGV	HYSTPLEQFE	ASLLNLFDKG	ILATHAVPQL	EKLVMEDIFI	SGDPLLESVG
730	740	750	760	770	780	790	800
LHEPLVEELR	ATIASAVSKA	MIPLQAYAKE	YRKYLELNNN	DIASFLKTYQ	TQGLLAQEVR	EVVLTHLREK	EILDSSLPSS
810	820	830	840	850	860	870	880
IIIGPFYINT	DNVKQSLSKK	RKALATSVLD	ILAKNLHKEV	DSICEEFRSI	SRKIYEKPNS	IEELAELREW	MKGIPERLVG
890	900	910	920	930	940	950	960
LEERIVKVMD	DYQVMDEFLY	NLSSDDFNDK	WIASNWPSKI	LGQIELVQQQ	HVEDEEKFRK	IQIMDQNNFQ	EKLEGLQLVV
970	980	990	1000	1010	1020	1030	1040
AGFSIHVEIS	RAHEIANEVR	RVKKQLKDCQ	QLAMLYNNRE	RIFSLPITNY	DKLSRMVKEF	QPYLDLWTTA	SDWLRWSESW
1050	1060	1070	1080	1090	1100	1110	1120
MNDPLSAIDA	EQLEKNVVEA	FKTMHKCVKQ	FKDMPACQEV	ALDIRARIEE	FKPYIPLIQG	LRNPGMRIRH	WETLSNQINI
1130	1140	1150	1160	1170	1180	1190	1200
NVRPKANLTF	ARCLEMNLQD	HIESISKVAE	VAGKEYAIEQ	ALDKMEKEWS	TILFNVLPYK	ATDTYILKSP	DEASQLLDDH
1210	1220	1230	1240	1250	1260	1270	1280
IVMTQNMSFS	PYKKPFEQRI	NSWENKLKLT	QEVLEEWLNC	QRSWLYLEPI	FSSEDINQQL	PVESKRYQTM	ERIWKKIMKN
1290	1300	1310	1320	1330	1340	1350	1360
AYENREVINV	CSDLRMLDSL	RDCNKILDLV	QKGLSEYLET	KRSAFPRFYF	LSDDELLEIL	SQTKDPTAVQ	PHLRKCFENI
1370	1380	1390	1400	1410	1420	1430	1440
ARLLFQEDLE	ITHMYSAEGE	EVQLCFSIYP	SSNVEDWLRE	VERSMKASVH	DIIEKAIRAY	PTMPRTQWVL	NWPGQVTIAG
1450	1460	1470	1480	1490	1500	1510	1520
CQTYWTMEVA	EALEAGNLRS	QLFPQLCQQL	SDLVALVRGK	LSRMQRAVLS	ALIVIEVHAK	DVVSKLIQEN	VVSVNDFQWI
1530	1540	1550	1560	1570	1580	1590	1600
SQLRYYWTNN	DLYIRAVNAE	FIYGYEYLGN	SGRLVITPLT	DRCYLTLTGA	LHLKFGGAPA	GPAGTGKTET	TKDLGKALAI
1610	1620	1630	1640	1650	1660	1670	1680
QTVVFNCSDQ	LDFMAMGKFF	KGLASAGAWA	CFDEFNRIDI	EVLSVVAQQI	TTIQKAQQQR	VERFMFEGVE	IPLVPSCAVF
1690	1700	1710	1720	1730	1740	1750	1760
ITMNPGYAGR	TELPDNLKAL	FRPVAMMVPD	YAMITEISLY	SFGFNEASVL	AKKITTTFKL	SSEQLSSQDH	YDFGMRAVKT
1770	1780	1790	1800	1810	1820	1830	1840
VISAAGNLKR	ENPSMNEELI	CLRAIRDVNV	PKFLQEDLKL	FSGIVSDLFP	TIKEEDTDYG	ILDEAIREAC	RNSNLKDVEG
1850	1860	1870	1880	1890	1900	1910	1920
FLTKCIQLYE	TTVVRHGLML	VGPTGSGKST	CYRVLAAAMT	SLKGQPSISG	GMYEAVNYYV	LNPKSITMGQ	LYGEFDLLTH
1930	1940	1950	1960	1970	1980	1990	2000
EWTDGIFSSF	IRAGAITSDT	NKKWYMFDGP	VDAIWIENMN	TVLDDNKKLC	LSSGEIIKLT	EAMTMMFEVQ	DLAVASPATV
2010	2020	2030	2040	2050	2060	2070	2080
SRCGMVYLEP	SILGLMPFIE	CWLRKLPPLL	KPYEEHFKAL	FVSFLEESIS	FVRSSVKEVI	ASTNCNLTMS	LLKLLDCFFK
2090	2100	2110	2120	2130	2140	2150	2160
PFLPREGLKK	IPSEKLSRIV	ELIEPWFIFS	LIWSVGATGD	SSGRTSFSHW	LRLKMENEQL	TLLFPEEGLV	FDYRLEDAGI
2170	2180	2190	2200	2210	2220	2230	2240

Detailed Protein Report

Cmpd.	No. of Cmpds.	m/z meas.	Δ m/z [ppm]	z	Rt [min]	Score	P	Range	Sequence	Modification	Ratios	
1891	2	918.3580	-147.46	2	53.5	15.4	2	559-574	K.DSWISSLKVAMRSSLR.D		WUP:QUP	1.04
178	1	715.8622	76.35	2	31.9	12.3	0	2496-2506	R.CMEQWEVTFNK.V	Oxidation: 2	QU:MU	1.67
2816	1	788.8905	-16.60	2	65.6	10.2	1	3081-3094	R.LREVEDGIATMQAK.Y	Oxidation: 11		
51	1	706.3163	-75.95	3	30.3	15.4	2	3699-3718	K.KLSAISLGQGQGPRAEAMMR.S	Oxidation: 18		

Detailed Protein Report

Protein 50: protocadherin Fat 2 precursor [Homo sapiens]

**Accession:**	gi|13787217	**Score:**	71.0
**Database:**	refseq_human(refseq_human_20140103.fasta)	**MW [kDa]:**	479.0
**Database Date:**	2015-11-30	**pI:**	4.9
		**Sequence Coverage [%]:**	1.7
		**No. of unique Peptides:**	3

Alias proteins:

Accession	Name	Description
gi|578810809	refseq_human (refseq_human_20140103.fasta)	PREDICTED: protocadherin Fat 2 isoform X1 [Homo sapiens]

Detailed Protein Report

10	20	30	40	50	60	70	80
MTIALLGFAI	FLLHCATCEK	PLEGILSSSA	WHFTHSHYNA	TIYENSSPKT	YVESFEKMGI	YLAEPQWAVR	YRIISGDVAN
90	100	110	120	130	140	150	160
VFKTEEYVVG	NFCFLRIRTK	SSNTALLNRE	VRDSYTLIIQ	ATEKTLELEA	LTRVVVHILD	QNDLKPLFSP	PSYRVTISED
170	180	190	200	210	220	230	240
MPLKSPICKV	TATDADLGQN	AEFYYAFNTR	SEMFAIHPTS	GVVTVAGKLN	VTWRGKHELQ	VLAVDRMRKI	SEGNGFGSLA
250	260	270	280	290	300	310	320
ALVVHVEPAL	RKPPAIASVV	VTPPDSNDGT	TYATVLVDAN	SSGAEVESVE	VVGGDPGKHF	KAIKSYARSN	EFSLVSVKDI
330	340	350	360	370	380	390	400
NWMEYLHGFN	LSLQARSGSG	PYFYSQIRGF	HLPPSKLSSL	KFEKAVYRVQ	LSEFSPPGSR	VVMVRVTPAF	PNLQYVLKPS
410	420	430	440	450	460	470	480
SENVGFKLNA	RTGLITTTKL	MDFHDRAHYQ	LHIRTSPGQA	STVVVIDIVD	CNNHAPLFNR	SSYDGTLDEN	IPPGTSVLAV
490	500	510	520	530	540	550	560
TATDRDHGEN	GYVTYSIAGP	KALPFSIDPY	LGIISTSKPM	DYELMKRIYT	FRVRASDWGS	PFRREKEVSI	FLQLRNLNDN
570	580	590	600	610	620	630	640
QPMFEEVNCT	GSIRQDWPVG	KSIMTMSAID	VDELQNLKYE	IVSGNELEYF	DLNHFSGVIS	LKRPFINLTA	GQPTSYSLKI
650	660	670	680	690	700	710	720
TASDGKNYAS	PTTLNITVVK	DPHFEVPVTC	DKTGVLTQFT	KTILHFIGLQ	NQESSDEEFT	SLSTYQINHY	TPQFEDHFPQ
730	740	750	760	770	780	790	800
SIDVLESVPI	NTPLARLAAT	DPDAGFNGKL	VYVIADGNEE	GCFDIELETG	LLTVAAPLDY	EATNFYILNV	TVYDLGTPQK
810	820	830	840	850	860	870	880
SSWKLLTVNV	KDWNDNAPRF	PPGGYQLTIS	EDTEVGTTIA	ELTTKDADSE	DNGRVRYTLL	SPTEKFSLHP	LTGELVVTGH
890	900	910	920	930	940	950	960
LDRESEPRYI	LKVEARDQPS	KGHQLFSVTD	LIITLEDVND	NSPQCITEHN	RLKVPEDLPP	GTVLTFLDAS	DPDLGPAGEV
970	980	990	1000	1010	1020	1030	1040
RYVLMDGAHG	TFRVDLMTGA	LILERELDFE	RRAGYNLSLW	ASDGGRPLAR	RTLCHVEVIV	LDVNENLHPP	HFASFVHQGQ
1050	1060	1070	1080	1090	1100	1110	1120
VQENSPSGTQ	VIVVAAQDDD	SGLDGELQYF	LRAGTGLAAF	SINQDTGMIQ	TLAPLDREFA	SYYWLTVLAV	DRGSVPLSSV
1130	1140	1150	1160	1170	1180	1190	1200
TEVYIEVTDA	NDNPPQMSQA	VFYPSIQEDA	PVGTSVLQLD	AWDPDSSSKG	KLTFNITSGN	YMGFFMIHPV	TGLLSTAQQL
1210	1220	1230	1240	1250	1260	1270	1280
DRENKDEHIL	EVTVLDNGEP	SLKSTSRVVV	GILDVNDNPP	IFSHKLFNVR	LPERLSPVSP	GPVYRLVASD	LDEGLNGRVT
1290	1300	1310	1320	1330	1340	1350	1360
YSIEDSDEEA	FSIDLVTGVV	SSSSTFTAGE	YNILTIKATD	SGQPPLSASV	RLHIEWIPWP	RPSSIPLAFD	ETYYSFTVME
1370	1380	1390	1400	1410	1420	1430	1440
TDPVNHMVGV	ISVEGRPGLF	WFNISGGDKD	MDFDIEKTTG	SIVIARPLDT	RRRSNYNLTV	EVTDGSRTIA	TQVHIFMIAN
1450	1460	1470	1480	1490	1500	1510	1520
INHHRPQFLE	TRYEVRVPQD	TVPGVELLRV	QAIDQDKGKS	LIYTIHGSQD	PGSASLFQLD	PSSGVLVTVG	KLDLGSGPSQ
1530	1540	1550	1560	1570	1580	1590	1600
HTLTVMVRDQ	EIPIKRNFVW	VTIHVEDGNL	HPPRFTQLHY	EASVPDTIAP	GTELLQVRAM	DADRGVNAEV	HYSLLKGNSE
1610	1620	1630	1640	1650	1660	1670	1680
GFFNINALLG	IITLAQKLDQ	ANHAPHTLTV	KAEDQGSPQW	HDLATVIIHV	YPSDRSAPIF	SKSEYFVEIP	ESIPVGSPIL
1690	1700	1710	1720	1730	1740	1750	1760
LVSAMSPSEV	TYELREGNKD	GVFSMNSYSG	LISTQKKLDH	EKISSYQLKI	RGSNMAGAFT	DVMVVVDIID	ENDNAPMFLK
1770	1780	1790	1800	1810	1820	1830	1840
STFVGQISEA	APLYSMIMDK	NNNPFVIHAS	DSDKEANSLL	VYKILEPEAL	KFFKIDPSMG	TLTIVSEMDY	ESMPSFQFCV
1850	1860	1870	1880	1890	1900	1910	1920
YVHDQGSPVL	FAPRPAQVII	HVRDVNDSPP	RFSEQIYEVA	IVGPIHPGME	LLMVRASDED	SEVNYSIKTG	NADEAVTIHP
1930	1940	1950	1960	1970	1980	1990	2000
VTGSISVLNP	AFLGLSRKLT	IRASDGLYQD	TALVKISLTQ	VLDKSLQFDQ	DVYWAAVKEN	LQDRKALVIL	GAQGNHLNDT
2010	2020	2030	2040	2050	2060	2070	2080
LSYFLLNGTD	MFHMVQSAGV	LQTRGVAFDR	EQQDTHELAV	EVRDNRTPQR	VAQGLVRVSI	EDVNDNPPKF	KHLPYYTIIQ
2090	2100	2110	2120	2130	2140	2150	2160
DGTEPGDVLF	QVSATDEDLG	TNGAVTYEFA	EDYTYFRIDP	YLGDISLKKP	FDYQALNKYH	LKVIARDGGT	PSLQSEEEVL
2170	2180	2190	2200	2210	2220	2230	2240
VTVRNKSNPL	FQSPYYKVRV	PENITLYTPI	LHTQARSPEG	LRLIYNIVEE	EPLMLFTTDF	KTGVLTVTGP	LDYESKTKHV
2250	2260	2270	2280	2290	2300	2310	2320
FTVRATDTAL	GSFSEATVEV	LVEDVNDNPP	TFSQLVYTTS	ISEGLPAQTP	VIQLLASDQD	SGRNRDVSYQ	IVEDGSDVSK
2330	2340	2350	2360	2370	2380	2390	2400

Detailed Protein Report

Cmpd.	No. of Cmpds.	m/z meas.	Δ m/z [ppm]	z	Rt [min]	Score	P	Range	Sequence	Modification
2633	1	741.3552	-72.85	2	63.0	15.7	1	71-83	R.YRIISGDVANVFK.T	
1721	1	1030.6326	87.08	2	51.3	16.6	1	993-1011	R.AGYNLSLWASDGGRPLARR.T	
65	2	1096.9820	-88.55	2	30.5	12.9	2	3376-3395	K.GELQVAKALDREQASSYSLK.L	

Detailed Protein Report

Protein 51: PREDICTED: protein AHNAK2 isoform X1 [Homo sapiens]

**Accession:**	gi|530403029	**Score:**	69.9
**Database:**	refseq_human(refseq_human_20140103.fasta)	**MW [kDa]:**	605.3
**Database Date:**	2015-11-30	**pI:**	5.1
**Modification(s):**	Oxidation	**Sequence Coverage [%]:**	1.5
		**No. of unique Peptides:**	5

Quantitation

QU:MU	**Median:**	1.02	**CV:**	0.00 %	**No. of Peptides:**	1	

Detailed Protein Report

10	20	30	40	50	60	70	80
MSRPEAVQEA	TEVTLKTEVE	AGASGYSVTG	GGDQGIFVKQ	VLKDSSAAKL	FNLREGDQLL	STTVFFENIK	YEDALKILQY
90	100	110	120	130	140	150	160
SEPYKVQFKI	RRQLPAPQDE	EWASSDAQHG	PQGKEKEDTD	VADGCRETPT	KTLEGDGDQE	RLISKPRVGR	GRQSQRERLS
170	180	190	200	210	220	230	240
WPKFQSIKSK	RGPGPQRSHS	SSEAYEPRDA	HDVSPTSTDT	EAQLTVERQE	QKAGPGSQRR	RKFLNLRFRT	GSGQGPSSTG
250	260	270	280	290	300	310	320
QPGRGFQSGV	GRAGVLEELG	PWGDSLEETG	AATGSRREER	AEQDREVMPA	QSMPLPTELG	DPRLCEGTPQ	EGGLRAARLH
330	340	350	360	370	380	390	400
GKTLEGQAQE	TAVAQRKPRA	QPTPGMSREG	EGEGLQSLEI	GIARLSLRDT	TEGGTQIGPP	EIRVRVHDLK	TPKFAFSTEK
410	420	430	440	450	460	470	480
EPERERRLST	PQRGKRQDAS	SKAGTGLKGE	EVEGAGWMPG	REPTTHAEAQ	GDEGDGEEGL	QRTRITEEQD	KGREDTEGQI
490	500	510	520	530	540	550	560
RMPKFKIPSL	GWSPSKHTKT	GREKATEDTE	QGREGEATAT	ADRREQRRTE	EGLKDKEDSD	SMTNTTKIQL	IHDEKRLKKE
570	580	590	600	610	620	630	640
QILTEKEVAT	KDSKFKMPKF	KMPLFGASAP	GKSMEASVDV	SAPKVEADVS	LLSMQGDLKT	TDLSVQTPSA	DLEVQDGQVD
650	660	670	680	690	700	710	720
VKLPEGPLPE	GASLKGHLPK	VQRPSLKMPK	VDLKGPKLDL	KGPKAEVTAP	DVKMSLSSME	VDVQAPRAKL	DGARLEGDLS
730	740	750	760	770	780	790	800
LADKEVTAKD	SKFKMPKFKM	PSFGVSAPGK	SMEDSVDVSA	PKVEADVSLS	SMQGDLKATD	LSIQPPSADL	EVQAGQVDVK
810	820	830	840	850	860	870	880
LPEGPVPEGA	GPKVHLPKVE	MPSFKMPKVD	LKGPQIDVKG	PKLDLKGPKA	EVTAPDGEVS	LPSMEVDVQA	QKAKLDGAWL
890	900	910	920	930	940	950	960
EGDLSLADKD	VTAKDSKFKM	PKFKMPSFGV	SAPGKSIKAL	VDVSAPKVEA	DLSLPSMQGD	LKTTDLSIQP	ASTDLKVQAD
970	980	990	1000	1010	1020	1030	1040
QVDVKLPEGH	LPEGAGLKGH	LPKVEMPSFK	MPKVALKGPQ	VDVKGPKLDL	KSPKAEVTAP	DVEVSLPSVE	VDVEAPGAKL
1050	1060	1070	1080	1090	1100	1110	1120
DSARLEGELS	LADKDVTAKD	SRFKMPKFKM	PSFGASAPGK	SIEASVDVSA	PKVEADVSLP	SMQGDLKTTD	LSIQPPSADL
1130	1140	1150	1160	1170	1180	1190	1200
EVHAGQVDVK	LLEGHVPEGA	GFKGHLPKVQ	MPSLKMPKVD	LKGPQVEVRG	PKLDLKGHKA	EVTAHEVAVS	LPSVEVDMQA
1210	1220	1230	1240	1250	1260	1270	1280
PGAKLDGAQL	DGDLSLADKD	VTAKDSKFKM	PKFKMPSFGV	SAPGKSIEAS	VDLSAPKVEA	DMSLPSMQGD	LKTTDLSIQP
1290	1300	1310	1320	1330	1340	1350	1360
PSTDLELQAG	QLDVKLPEGP	VPEGAGLKGH	LPKLQMPSFK	VPKVDLKGPE	IDIKGPKLDL	KDPKVEVTAP	DVEVSLPSVE
1370	1380	1390	1400	1410	1420	1430	1440
VDVEAPGAKL	DGGRLEEDMS	LADKDLTTKD	SKFKMPKFKM	PSFGVSAPGK	SIEASVDVSA	PKVEADVSLP	SMQGDLKATD
1450	1460	1470	1480	1490	1500	1510	1520
LSIQPPSADL	EVQAGQVDVK	LPEGPVSEGA	GLKGHLPKVQ	MPSFKMPKVD	LKGPQIDVKG	PKLDLKGPKV	EVTAPDVKMS
1530	1540	1550	1560	1570	1580	1590	1600
LSSMEVDVQA	PRAKLDGAQL	EGDLSLADKA	VTAKDSKFKM	PKFKMPSFGV	SAPGKSIEAS	VDVSEPKVEA	DVSLPSMQGD
1610	1620	1630	1640	1650	1660	1670	1680
LKTTDLSIQS	PSADLEVQAG	QVNVKLPEGP	LPEGAGFKGH	LPKVQMPSLK	MPKVALKGPQ	MDVKGPKLDL	KGPKAEVMAP
1690	1700	1710	1720	1730	1740	1750	1760
DVEVSLPSVE	VDVEAPGAKL	DSVRLEGDLS	LADKDVTAKD	SKFKMPKFKM	PSFGVSAPGK	SIEASVDVSA	PKVEAEVSLP
1770	1780	1790	1800	1810	1820	1830	1840
SMQGDLKTTD	LCIPLPSADL	VVQAGQVDMK	LPEGQVPEGA	GLKGHLPKVD	MPSFKMPKVD	LKGPQTDVKG	AKLDLKGPKA
1850	1860	1870	1880	1890	1900	1910	1920
EVTAPDVEVS	LPSMEVDVQA	QKAKLDGARL	EGDLSLADKD	MTAKDSKFKM	PKFKMPSFGV	SAPGRSIEAS	VDVPAPKVEA
1930	1940	1950	1960	1970	1980	1990	2000
DVSLPSMQGD	LKTTDLSIQP	PSADLKVQTG	QVDVKLPEGH	VPEGAGLKGH	LPKVEMPSLK	MPKVDLKGPQ	VDIKGPKLDL
2010	2020	2030	2040	2050	2060	2070	2080
KDPKVEMRVP	DVEVSLPSME	VDVQAPRAKL	DSAHLQGDLT	LANKDLTTKD	SKFKMPKFKM	PSFGVSAPGK	SIEASVDVSP
2090	2100	2110	2120	2130	2140	2150	2160
PKVEADMSLP	SMQGDLKTTD	LSIQPLSADV	KVQAGQVDVK	LLEGPVPEEV	GLKGHLPKLQ	MPSFKVPKVD	LKGPEIDIKG
2170	2180	2190	2200	2210	2220	2230	2240

Detailed Protein Report

Cmpd.	No. of Cmpds.	m/z meas.	Δ m/z [ppm]	z	Rt [min]	Score	P	Range	Sequence	Modification	Ratios
2046	1	596.7306	-219.53	2	56.0	16.0	2	384-393	R.VRVHDLKTPK.F		QU:MU 1.02
267	1	862.8828	-39.08	2	33.3	11.9	1	1375-1389	R.LEEDMSLADKDLTTK.D	Oxidation: 5	
738	1	769.3703	-105.01	2	39.2	11.2	2	3308-3321	K.SPQVDIKGPKLDLK.V		
1307	2	926.0984	-40.13	3	46.5	19.2	1	3391-3417	R. SIEASLDVSAPKVEADVSLSSMQG A		
1329	1	1023.0254	-12.68	2	46.8	11.6	1	5662-5679	R.VMVTSAARTELILPEQDR.K	Oxidation: 2	

Detailed Protein Report

Protein 52: 14-3-3 protein zeta/delta [Homo sapiens]

**Accession:**	gi|4507953	**Score:**	69.6
**Database:**	refseq_human(refseq_human_20140103.fasta)	**MW [kDa]:**	27.7
**Database Date:**	2015-11-30	**pI:**	4.6
**Modification(s):**	Carbamidomethyl	**Sequence Coverage [%]:**	9.0
		**No. of unique Peptides:**	2

Alias proteins:

Accession	Name	Description
gi|530389321	refseq_human (refseq_human_20140103.fasta)	PREDICTED: 14-3-3 protein zeta/delta isoform X4 [Homo sapiens]
gi|530389319	refseq_human (refseq_human_20140103.fasta)	PREDICTED: 14-3-3 protein zeta/delta isoform X3 [Homo sapiens]
gi|530389317	refseq_human (refseq_human_20140103.fasta)	PREDICTED: 14-3-3 protein zeta/delta isoform X2 [Homo sapiens]
gi|208973244	refseq_human (refseq_human_20140103.fasta)	14-3-3 protein zeta/delta [Homo sapiens]
gi|208973242	refseq_human (refseq_human_20140103.fasta)	14-3-3 protein zeta/delta [Homo sapiens]
gi|208973240	refseq_human (refseq_human_20140103.fasta)	14-3-3 protein zeta/delta [Homo sapiens]
gi|208973238	refseq_human (refseq_human_20140103.fasta)	14-3-3 protein zeta/delta [Homo sapiens]
gi|21735625	refseq_human (refseq_human_20140103.fasta)	14-3-3 protein zeta/delta [Homo sapiens]

```
        10         20         30         40         50         60         70         80
MDKNELVQKA KLAEQAERYD DMAACMKSVT EQGAELSNEE RNLLSVAYKN VVGARRSSWR VVSSIEQKTE GAEKKQQMAR
        90        100        110        120        130        140        150        160
EYREKIETEL RDICNDVLSL LEKFLIPNAS QAESKVFYLK MKGDYYRYLA EVAAGDDKKG IVDQSQQAYQ EAFEISKKEM
       170        180        190        200        210        220        230        240
QPTHPIRLGL ALNFSVFYYE ILNSPEKACS LAKTAFDEAI AELDTLSEES YKDSTLIMQL LRDNLTLWTS DTQGDEAEAG
       250
EGGEN
```

Cmpd.	No. of Cmpds.	m/z meas.	Δ m/z [ppm]	z	Rt [min]	Score	P	Range	Sequence	Modification
2739	1	709.8489	-20.41	2	64.6	22.3	0	92-103	R.DICNDVLSLLEK.F	Carbamidomethyl: 3
2725	2	595.3333	-1.39	2	65.2	47.3	0	213-222	K.DSTLIMQLLR.D	

Detailed Protein Report

Protein 53: **voltage-dependent P/Q-type calcium channel subunit alpha-1A isoform 3 [Homo sapiens]**

**Accession:**	gi|187828880	**Score:**	69.2
**Database:**	refseq_human(refseq_human_20140103.fasta)	**MW [kDa]:**	256.6
**Database Date:**	2015-11-30	**pI:**	9.3
**Modification(s):**	Oxidation	**Sequence Coverage [%]:**	3.1
		**No. of unique Peptides:**	5

Detailed Protein Report

10	20	30	40	50	60	70	80
MARFGDEMPA	RYGGGGSGAA	AGVVVGSGGG	RGAGGSRQGG	QPGAQRMYKQ	SMAQRARTMA	LYNPIPVRQN	CLTVNRSLFL
90	100	110	120	130	140	150	160
FSEDNVVRKY	AKKITEWPPF	EYMILATIIA	NCIVLALEQH	LPDDDKTPMS	ERLDDTEPYF	IGIFCFEAGI	KIIALGFAFH
170	180	190	200	210	220	230	240
KGSYLRNGWN	VMDFVVVLTG	ILATVGTEFD	LRTLRAVRVL	RPLKLVSGIP	SLQVVLKSIM	KAMIPLLQIG	LLLFFAILIF
250	260	270	280	290	300	310	320
AIIGLEFYMG	KFHTTCFEEG	TDDIQGESPA	PCGTEEPART	CPNGTKCQPY	WEGPNNGITQ	FDNILFAVLT	VFQCITMEGW
330	340	350	360	370	380	390	400
TDLLYNSNDA	SGNTWNWLYF	IPLIIIGSFF	MLNLVLGVLS	GEFAKERERV	ENRRAFLKLR	RQQQIERELN	GYMEWISKAE
410	420	430	440	450	460	470	480
EVILAEDETD	GEQRHPFDGA	LRRTTIKKSK	TDLLNPEEAE	DQLADIASVG	SPFARASIKS	AKLENSTFFH	KKERRMRFYI
490	500	510	520	530	540	550	560
RRMVKTQAFY	WTVLSLVALN	TLCVAIVHYN	QPEWLSDFLY	YAEFIFLGLF	MSEMFIKMYG	LGTRPYFHSS	FNCFDCGVII
570	580	590	600	610	620	630	640
GSIFEVIWAV	IKPGTSFGIS	VLRALRLLRI	FKVTKYWASL	RNLVVSLLNS	MKSIISLLFL	LFLFIVVFAL	LGMQLFGGQF
650	660	670	680	690	700	710	720
NFDEGTPPTN	FDTFPAAIMT	VFQILTGEDW	NEVMYDGIKS	QGGVQGGMVF	SIYFIVLTLF	GNYTLLNVFL	AIAVDNLANA
730	740	750	760	770	780	790	800
QELTKDEQEE	EEAANQKLAL	QKAKEVAEVS	PLSAANMSIA	VKEQQKNQKP	AKSVWEQRTS	EMRKQNLLAS	REALYNEMDP
810	820	830	840	850	860	870	880
DERWKAAYTR	HLRPDMKTHL	DRPLVVDPQE	NRNNNTNKSR	AAEPTVDQRL	GQQRAEDFLR	KQARYHDRAR	DPSGSAGLDA
890	900	910	920	930	940	950	960
RRPWAGSQEA	ELSREGPYGR	ESDHHAREGS	LEQPGFWEGE	AERGKAGDPH	RRHVHRQGGS	RESRSGSPRT	GADGEHRRHR
970	980	990	1000	1010	1020	1030	1040
AHRRPGEEGP	EDKAERRARH	REGSRPARGG	EGEGEGPDGG	ERRRRHRHGA	PATYEGDARR	EDKERRHRRR	KENQGSGVPV
1050	1060	1070	1080	1090	1100	1110	1120
SGPNLSTTRP	IQQDLGRQDP	PLAEDIDNMK	NNKLATAESA	APHGSLGHAG	LPQSPAKMGN	STDPGPMLAI	PAMATNPQNA
1130	1140	1150	1160	1170	1180	1190	1200
ASRRTPNNPG	NPSNPGPPKT	PENSLIVTNP	SGTQTNSAKT	ARKPDHTTVD	IPPACPPPLN	HTVVQVNKNA	NPDPLPKKEE
1210	1220	1230	1240	1250	1260	1270	1280
EKKEEEEDDR	GEDGPKPMPP	YSSMFILSTT	NPLRRLCHYI	LNLRYFEMCI	LMVIAMSSIA	LAAEDPVQPN	APRNNVLRYF
1290	1300	1310	1320	1330	1340	1350	1360
DYVFTGVFTF	EMVIKMIDLG	LVLHQGAYFR	DLWNILDFIV	VSGALVAFAF	TGNSKGKDIN	TIKSLRVLRV	LRPLKTIKRL
1370	1380	1390	1400	1410	1420	1430	1440
PKLKAVFDCV	VNSLKNVFNI	LIVYMLFMFI	FAVVAVQLFK	GKFFHCTDES	KEFEKDCRGK	YLLYEKNEVK	ARDREWKKYE
1450	1460	1470	1480	1490	1500	1510	1520
FHYDNVLWAL	LTLFTVSTGE	GWPQVLKHSV	DATFENQGPS	PGYRMEMSIF	YVVYFVVFPF	FFVNIFVALI	IITFQEQGDK
1530	1540	1550	1560	1570	1580	1590	1600
MMEEYSLEKN	ERACIDFAIS	AKPLTRHMPQ	NKQSFQYRMW	QFVVSPPFEY	TIMAMIALNT	IVLMMKFYGA	SVAYENALRV
1610	1620	1630	1640	1650	1660	1670	1680
FNIVFTSLFS	LECVLKVMAF	GILNYFRDAW	NIFDFVTVLG	SITDILVTEF	GNNFINLSFL	RLFRAARLIK	LLRQGYTIRI
1690	1700	1710	1720	1730	1740	1750	1760
LLWTFVQSFK	ALPYVCLLIA	MLFFIYAIIG	MQVFGNIGID	VEDEDSDEDE	FQITEHNNFR	TFFQALMLLF	RSATGEAWHN
1770	1780	1790	1800	1810	1820	1830	1840
IMLSCLSGKP	CDKNSGILTR	ECGNEFAYFY	FVSFIFLCSF	LMLNLFVAVI	MDNFEYLTRD	SSILGPHHLD	EYVRVWAEYD
1850	1860	1870	1880	1890	1900	1910	1920
PAACGRIHYK	DMYSLLRVIS	PPLGLGKKCP	HRVACKRLLR	MDLPVADDNT	VHFNSTLMAL	IRTALDIKIA	KGGADKQQMD
1930	1940	1950	1960	1970	1980	1990	2000
AELRKEMMAI	WPNLSQKTLD	LLVTPHKSTD	LTVGKIYAAM	MIMEYYRQSK	AKKLQAMREE	QDRTPLMFQR	MEPPSPTQEG
2010	2020	2030	2040	2050	2060	2070	2080
GPGQNALPST	QLDPGGALMA	HESGLKESPS	WVTQRAQEMF	QKTGTWSPEQ	GPPTDMPNSQ	PNSQSVEMRE	MGRDGYSDSE
2090	2100	2110	2120	2130	2140	2150	2160
HYLPMEGQGR	AASMPRLPAE	NQRRRGRPRG	NNLSTISDTS	PMKRSASVLG	PKARRLDDYS	LERVPPEENQ	RHHQRRRDRS
2170	2180	2190	2200	2210	2220	2230	2240

Detailed Protein Report

Cmpd.	No. of Cmpds.	m/z meas.	Δ m/z [ppm]	z	Rt [min]	Score	P	Range	Sequence	Modification
35	2	796.7935	-124.10	2	29.4	12.9	0	12-31	R.YGGGGSGAAAGVVVGSGGGR.G	
2300	2	822.4329	20.49	2	57.1	15.3	1	1124-1139	R.RTPNNPGNPSNPGPPK.T	
118	1	753.3697	-54.70	2	31.4	13.4	0	1533-1546	R.ACIDFAISAKPLTR.H	
2664	1	701.8434	-53.81	2	63.4	12.1	0	1741-1751	R.TFFQALMLLFR.S	Oxidation: 7
2837	1	913.4693	21.67	1	64.1	15.5	1	1869-1876	K.CPHRVACK.R	

Detailed Protein Report

Protein 54: PREDICTED: striated muscle preferentially expressed protein kinase isoform X4 [Homo sapiens]

**Accession:**	gi|530369685	**Score:**	68.3
**Database:**	refseq_human(refseq_human_20140103.fasta)	**MW [kDa]:**	339.0
**Database Date:**	2015-11-30	**pI:**	9.7
**Modification(s):**	Carbamidomethyl	**Sequence Coverage [%]:**	1.7
		**No. of unique Peptides:**	3

Quantitation

QU:MU	**Median:**	0.69	**CV:**	86.96 %	**No. of Peptides:**	2
WUP:QUP	**Median:**	1.26	**CV:**	205.34 %	**No. of Peptides:**	2

Detailed Protein Report

10	20	30	40	50	60	70	80
MKKLWVKKRF	QKTGHSRRAF	GRLTHVFRSC	RKQSYDSETA	EDDISDVQGT	QRLELRDDGA	FSTPTGGSDT	LVGTSLDTPP
90	100	110	120	130	140	150	160
TSVTGTSEEQ	VSWWGSGQTV	LEQEAGSGGG	TRRLPGSPSS	VPQSGLRREE	PDLQPQLASE	APRRPAQPPP	SKSALLPPPS
170	180	190	200	210	220	230	240
PRVGKRSPPG	PPAQPAATPT	SPHRRTQEPV	LPEDTTTEEK	RGKKSKSSGP	SLAGTAESRP	QTPLSEASGR	LSALGRSPRL
250	260	270	280	290	300	310	320
VRAGSRILDK	LQFFEERRRS	LERSDSPPAP	LRPWVPLRKA	RSLEQPKSER	GAPWGTPGAS	QEELRAPGSV	AERRRLFQQK
330	340	350	360	370	380	390	400
AASLDERTRQ	RSPASDLELR	FAQELGRIRR	STSREELVRS	HESLRATLQR	APSPREPGEP	PLFSRPSTPK	TSRAVSPAAA
410	420	430	440	450	460	470	480
QPPSPSSAEK	PGDEPGRPRS	RGPAGRTEPG	EGPQQEVRRR	DQFPLTRSRA	IQECRSPVPP	PAADPPEART	KAPPGRKREP
490	500	510	520	530	540	550	560
PAQAVRFLPW	ATPGLEGAAV	PQTLEKNRAG	PEAEKRLRRG	PEEDGPWGPW	DRRGARSQGK	GRRARPTSPE	LESSDDSYVS
570	580	590	600	610	620	630	640
AGEEPLEAPV	FEIPLQNVVV	APGADVLLKC	IITANPPPQV	SWHKDGSALR	SEGRLLLRAE	GERHTLLLRE	ARAADAGSYM
650	660	670	680	690	700	710	720
ATATNELGQA	TCAASLTVRP	GGSTSPFSSP	ITSDEEYLSP	PEEFPEPGET	WPRTPTMKPS	PSQNRRSSDT	GSKAPPTFKV
730	740	750	760	770	780	790	800
SLMDQSVREG	QDVIMSIRVQ	GEPKPVVSWL	RNRQPVRPDQ	RRFAEEAEGG	LCRLRILAAE	RGDAGFYTCK	AVNEYGARQC
810	820	830	840	850	860	870	880
EARLEVRAHP	ESRSLAVLAP	LQDVDVGAGE	MALFECLVAG	PTDVEVDWLC	RGRLLQPALL	KCKMHFDGRK	CKLLLTSVHE
890	900	910	920	930	940	950	960
DDSGVYTCKL	STAKDELTCS	ARLTVRPSLA	PLFTRLLEDV	EVLEGRAARF	DCKISGTPPP	VVTWTHFGCP	MEESENLRLR
970	980	990	1000	1010	1020	1030	1040
QDGGLHSLHI	AHVGSEDEGL	YAVSAVNTHG	QAHCSAQLYV	EEPRTAASGP	SSKLEKMPSI	PEEPEQGELE	RLSIPDFLRP
1050	1060	1070	1080	1090	1100	1110	1120
LQDLEVGLAK	EAMLECQVTG	LPYPTISWFH	NGHRIQSSDD	RRMTQYRDVH	RLVFPAVGPQ	HAGVYKSVIA	NKLGKAACYA
1130	1140	1150	1160	1170	1180	1190	1200
HLYVTDVVPG	PPDGAPQVVA	VTGRMVTLTW	NPPRSLDMAI	DPDSLTYTVQ	HQVLGSDQWT	ALVTGLREPG	WAATGLRKGV
1210	1220	1230	1240	1250	1260	1270	1280
QHIFRVLSTT	VKSSSKPSPP	SEPVQLLEHG	PTLEEAPAML	DKPDIVYVVE	GQPASVTVTF	NHVEAQVVWR	SCRGALLEAR
1290	1300	1310	1320	1330	1340	1350	1360
AGVYELSQPD	DDQYCLRICR	VSRRDMGALT	CTARNRHGTQ	TCSVTLELAE	APRFESIMED	VEVGAGETAR	FAVVVEGKPL
1370	1380	1390	1400	1410	1420	1430	1440
PDIMWYKDEV	LLTESSHVSF	VYEENECSLV	VLSTGAQDGG	VYTCTAQNLA	GEVSCKAELA	VHSAQTAMEV	EGVGEDEDHR
1450	1460	1470	1480	1490	1500	1510	1520
GRRLSDFYDI	HQEIGRGAFS	YLRRIVERSS	GLEFAAKFIP	SQAKPKASAR	REARLLARLQ	HDCVLYFHEA	FERRRGLVIV
1530	1540	1550	1560	1570	1580	1590	1600
TELCTEELLE	RIARKPTVCE	SEIRAYMRQV	LEGIHYLHQS	HVLHLDVKPE	NLLVWDGAAG	EQQVRICDFG	NAQELTPGEP
1610	1620	1630	1640	1650	1660	1670	1680
QYCQYGTPEF	VAPEIVNQSP	VSGVTDIWPV	GVVAFLCLTG	ISPFVGENDR	TTLMNIRNYN	VAFEETTFLS	LSREARGFLI
1690	1700	1710	1720	1730	1740	1750	1760
KVLVQDRLRP	TAEETLEHPW	FKTQAKGAEV	STDHLKLFLS	RRRWQRSQIS	YKCHLVLRPI	PELLRAPPER	VWVTMPRRPP
1770	1780	1790	1800	1810	1820	1830	1840
PSGGLSSSSD	SEEEELEELP	SVPRPLQPEF	SGSRVSLTDI	PTEDEALGTP	ETGAATPMDW	QEQGRAPSQD	QEAPSPEALP
1850	1860	1870	1880	1890	1900	1910	1920
SPGQEPAAGA	SPRRGELRRG	SSAESALPRA	GPRELGRGLH	KAASVELPQR	RSPSPGATRL	ARGGLGEGEY	AQRLQALRQR
1930	1940	1950	1960	1970	1980	1990	2000
LLRGGPEDGK	VSGLRGPLLE	SLGGRARDPR	MARAASSEAA	PHHQPPLENR	GLQKSSSFSQ	GEAEPRGRHR	RAGAPLEIPV
2010	2020	2030	2040	2050	2060	2070	2080
ARLGARRLQE	SPSLSALSEA	QPSSPARPSA	PKPSTPKSAE	PSATTPSDAP	QPPAPQPAQD	KAPEPRPEPV	RASKPAPPPQ
2090	2100	2110	2120	2130	2140	2150	2160
ALQTLALPLT	PYAQIIQSLQ	LSGHAQGPSQ	GPAAPPSEPK	PHAAVFARVA	SPPPGAPEKR	VPSAGGPPVL	AEKARVPTVP
2170	2180	2190	2200	2210	2220	2230	2240

Detailed Protein Report

Cmpd.	No. of Cmpds.	m/z meas.	Δ m/z [ppm]	z	Rt [min]	Score	P	Range	Sequence	Modification	Ratios	
661	2	480.6345	-248.82	2	37.8	10.5	1	10-17	R.FQKTGHSR.R		QU:MU<br>WUP:QUP	1.46<br>0.35
146	1	531.9457	-37.14	3	31.8	25.5	2	1936-1950	R.GPLLESLGGRARDPR.M		WUP:QUP<br>QU:MU	4.55<br>0.33
43	1	796.8047	-128.81	2	30.2	14.7	2	2824-2837	R.FGVVRACRENATGR.T	Carbamidomethyl: 7		

Detailed Protein Report

Protein 55: PREDICTED: collagen alpha-1(XIV) chain isoform X1 [Homo sapiens]

**Accession:**	gi|530389313	**Score:**	67.8
**Database:**	refseq_human(refseq_human_20140103.fasta)	**MW [kDa]:**	191.8
**Database Date:**	2015-11-30	**pI:**	5.1
**Modification(s):**	Carbamidomethyl	**Sequence Coverage [%]:**	3.4
		**No. of unique Peptides:**	4

Quantitation

QU:MU	**Median:**	2.11	**CV:**	0.00 %	**No. of Peptides:**	1
WUP:QUP	**Median:**	0.41	**CV:**	0.00 %	**No. of Peptides:**	1

Detailed Protein Report

10	20	30	40	50	60	70	80
MKIFQRKMRY	WLLPPFLAIV	YFCTIVQGQV	APPTRLRYNV	ISHDSIQISW	KAPRGKFGGY	KLLVTPTSGG	KTNQLNLQNT
90	100	110	120	130	140	150	160
ATKAIIQGLM	PDQNYTVQII	AYNKDKESKP	AQGQFRIKDL	EKRKDPKPRV	KVVDRGNGSR	PSSPEEVKFV	CQTPAIADIV
170	180	190	200	210	220	230	240
ILVDGSWSIG	RFNFRLVRHF	LENLVTAFDV	GSEKTRIGLA	QYSGDPRIEW	HLNAFSTKDE	VIEAVRNLPY	KGGNTLTGLA
250	260	270	280	290	300	310	320
LNYIFENSFK	PEAGSRTGVS	KIGILITDGK	SQDDIIPPSR	NLRESGVELF	AIGVKNADVN	ELQEIASEPD	STHVYNVAEF
330	340	350	360	370	380	390	400
DLMHTVVESL	TRTLCSRVEE	QDREIKASAH	AITGPPTELI	TSEVTARSFM	VNWTHAPGNV	EKYRVVYYPT	RGGKPDEVVV
410	420	430	440	450	460	470	480
DGTVSSTVLK	NLMSLTEYQI	AVFAIYAHTA	SEGLRGTETT	LALPMASDLL	LYDVTENSMR	VKWDAVPGAS	GYLILYAPLT
490	500	510	520	530	540	550	560
EGLAGDEKEM	KIGETHTDIE	LSGLLPNTEY	TVTVYAMFGE	EASDPVTGQE	TTLALSPPRN	LRISNVGSNS	ARLTWDPTSR
570	580	590	600	610	620	630	640
QINGYRIVYN	NADGTEINEV	EVDPITTFPL	KGLTPLTEYT	IAIFSIYDEG	QSEPLTGVFT	TEEVPAQQYL	EIDEVTTDSF
650	660	670	680	690	700	710	720
RVTWHPLSAD	EGLHKLMWIP	VYGGKTEEVV	LKEEQDSHVI	EGLEPGTEYE	VSLLAVLDDG	SESEVVTAVG	TTLDSFWTEP
730	740	750	760	770	780	790	800
ATTIVPTTSV	TSVFQTGIRN	LVVGDETTSS	LRVKWDISDS	DVQQFRVTYM	TAQGDPEEEV	IGTVMVPGSQ	NNLLLKPLLP
810	820	830	840	850	860	870	880
DTEYKVTVTP	IYTDGEGVSV	SAPGKTLPSS	GPQNLRVSEE	WYNRLRITWD	PPSSPVKGYR	IVYKPVSVPG	PTLETFVGAD
890	900	910	920	930	940	950	960
INTILITNLL	SGMDYNVKIF	ASQASGFSDA	LTGMVKTLFL	GVTNLQAKHV	EMTSLCAHWQ	VHRHATAYRV	VIESLQDRQK
970	980	990	1000	1010	1020	1030	1040
QESTVGGGTT	RHCFYGLQPD	SEYKISVYTK	LQEIEGPSVS	IMEKTQSLPT	RPPTFPPTIP	PAKEVCKAAK	ADLVFMVDGS
1050	1060	1070	1080	1090	1100	1110	1120
WSIGDENFNK	IISFLYSTVG	ALNKIGTDGT	QVAMVQFTDD	PRTEFKLNAY	KTKETLLDAI	KHISYKGGNT	KTGKAIKYVR
1130	1140	1150	1160	1170	1180	1190	1200
DTLFTAESGT	RRGIPKVIVV	ITDGRSQDDV	NKISREMQLD	GYSIFAIGVA	DADYSELVSI	GSKPSARHVF	FVDDFDAFKK
1210	1220	1230	1240	1250	1260	1270	1280
IEDELITFVC	ETASATCPVV	HKDGIDLAGF	KMMEMFGLVE	KDFSSVEGVS	MEPGTFNVFP	CYQLHKDALV	SQPTRYLHPE
1290	1300	1310	1320	1330	1340	1350	1360
GLPSDYTISF	LFRILPDTPQ	EPFALWEILN	KNSDPLVGVI	LDNGGKTLTY	FNYDQSGDFQ	TVTFEGPEIR	KIFYGSFHKL
1370	1380	1390	1400	1410	1420	1430	1440
HIVVSETLVK	VVIDCKQVGE	KAMNASANIT	SDGVEVLGKM	VRSRGPGGNS	APFQLQMFDI	VCSTSWANTD	KCCELPGLRD
1450	1460	1470	1480	1490	1500	1510	1520
DESCPDLPHS	CSCSETNEVA	LGPAGPPGGP	GLRGPKGQQG	EPGPKGPDGP	RGEIGLPGPQ	GPPGPQGPSG	LSIQGMPGMP
1530	1540	1550	1560	1570	1580	1590	1600
GEKGEKGDTG	LPGPQGIPGG	VGSPGRDGSP	GQRGLPGKDG	SSGPPGPPGP	IGIPGTPGVP	GITGSMGPQG	ALGPPGVPGA
1610	1620	1630	1640	1650	1660	1670	1680
KGERGERGDL	QSQAMVRSVA	RQVCEQLIQS	HMARYTAILN	QIPSHSSSIR	TVQGPPGEPG	RPGSPGAPGE	QGPPGTPGFP
1690	1700	1710	1720	1730	1740	1750	1760
GNAGVPGTPG	ERGLTGIKGE	KGNPGVGTQG	PRGPPGPAGP	SGESRPGSPG	PPGSPGPRGP	PGHLGVPGPQ	GPSGQPGYCD
1770	1780	1790					
PSSCSAYGVR	DLIPYNDYQH						

Cmpd.	No. of Cmpds.	m/z meas.	Δ m/z [ppm]	z	Rt [min]	Score	P	Range	Sequence	Modification	Ratios
2684	5	673.3700	31.83	2	63.7	19.1	0	208-218	R.IEWHLNAFSTK.D		
74	1	809.3972	-57.78	2	30.6	10.2	1	740-754	R.NLVVGDETTSSLRVK.W		WUP:QUP 0.41<br>QU:MU 2.11
834	1	683.9031	-31.20	2	40.0	13.1	1	1133-1145	R.GIPKVIVVITDGR.S		
2428	2	637.7139	-201.25	2	58.6	10.6	1	1371-1381	K.VVIDCKQVGEK.A	Carbamidomethyl: 5	

Detailed Protein Report

Protein 56: DNA-dependent protein kinase catalytic subunit isoform 2 [Homo sapiens]

**Accession:**	gi|126032350	**Score:**	66.7
**Database:**	refseq_human(refseq_human_20140103.fasta)	**MW [kDa]:**	465.2
**Database Date:**	2015-11-30	**pI:**	6.9
**Modification(s):**	Carbamidomethyl, Oxidation	**Sequence Coverage [%]:**	1.7
		**No. of unique Peptides:**	5

Quantitation

QU:MU	**Median:**	0.97	**CV:**	59.76 %	**No. of Peptides:**	2
WUP:QUP	**Median:**	2.35	**CV:**	0.00 %	**No. of Peptides:**	1

Detailed Protein Report

10	20	30	40	50	60	70	80
MAGSGAGVRC	SLLRLQETLS	AADRCGAALA	GHQLIRGLGQ	ECVLSSSPAV	LALQTSLVFS	RDFGLLVFVR	KSLNSIEFRE
90	100	110	120	130	140	150	160
CREEILKFLC	IFLEKMGQKI	APYSVEIKNT	CTSVYTKDRA	AKCKIPALDL	LIKLLQTFRS	SRLMDEFKIG	ELFSKFYGEL
170	180	190	200	210	220	230	240
ALKKKIPDTV	LEKVYELLGL	LGEVHPSEMI	NNAENLFRAF	LGELKTQMTS	AVREPKLPVL	AGCLKGLSSL	LCNFTKSMEE
250	260	270	280	290	300	310	320
DPQTSREIFN	FVLKAIRPQI	DLKRYAVPSA	GLRLFALHAS	QFSTCLLDNY	VSLFEVLLKW	CAHTNVELKK	AALSALESFL
330	340	350	360	370	380	390	400
KQVSNMVAKN	AEMHKNKLQY	FMEQFYGIIR	NVDSNNKELS	IAIRGYGLFA	GPCKVINAKD	VDFMYVELIQ	RCKQMFLTQT
410	420	430	440	450	460	470	480
DTGDDRVYQM	PSFLQSVASV	LLYLDTVPEV	YTPVLEHLVV	MQIDSFPQYS	PKMQLVCCRA	IVKVFLALAA	KGPVLRNCIS
490	500	510	520	530	540	550	560
TVVHQGLIRI	CSKPVVLPKG	PESESEDHRA	SGEVRTGKWK	VPTYKDYVDL	FRHLLSSDQM	MDSILADEAF	FSVNSSESL
570	580	590	600	610	620	630	640
NHLLYDEFVK	SVLKIVEKLD	LTLEIQTVGE	QENGDEAPGV	WMIPTSDPAA	NLHPAKPKDF	SAFINLVEFC	REILPEKQAE
650	660	670	680	690	700	710	720
FFEPWVYSFS	YELILQSTRL	PLISGFYKLL	SITVRNAKKI	KYFEGVSPKS	LKHSPEDPEK	YSCFALFVKF	GKEVAVKMKQ
730	740	750	760	770	780	790	800
YKDELLASCL	TFLLSLPHNI	IELDVRAYVP	ALQMAFKLGL	SYTPLAEVGL	NALEEWSIYI	DRHVMQPYYK	DILPCLDGYL
810	820	830	840	850	860	870	880
KTSALSDETK	NNWEVSALSR	AAQKGFNKVV	LKHLKKTKNL	SSNEAISLEE	IRIRVVQMLG	SLGGQINKNL	LTVTSSDEMM
890	900	910	920	930	940	950	960
KSYVAWDREK	RLSFAVPFRE	MKPVIFLDVF	LPRVTELALT	ASDRQTKVAA	CELLHSMVMF	MLGKATQMPE	GGQGAPPMYQ
970	980	990	1000	1010	1020	1030	1040
LYKRTFPVLL	RLACDVDQVT	RQLYEPLVMQ	LIHWFTNNKK	FESQDTVALL	EAILDGIVDP	VDSTLRDFCG	RCIREFLKWS
1050	1060	1070	1080	1090	1100	1110	1120
IKQITPQQQE	KSPVNTKSLF	KRLYSLALHP	NAFKRLGASL	AFNNIYREFR	EEESLVEQFV	FEALVIYMES	LALAHADEKS
1130	1140	1150	1160	1170	1180	1190	1200
LGTIQQCCDA	IDHLCRIIEK	KHVSLNKAKK	RRLPRGFPPS	ASLCLLDLVK	WLLAHCGRPQ	TECRHKSIEL	FYKFVPLLPG
1210	1220	1230	1240	1250	1260	1270	1280
NRSPNLWLKD	VLKEEGVSFL	INTFEGGGCG	QPSGILAQPT	LLYLRGPFSL	QATLCWLDLL	LAALECYNTF	IGERTVGALQ
1290	1300	1310	1320	1330	1340	1350	1360
VLGTEAQSSL	LKAVAFFLES	IAMHDIIAAE	KCFGTGAAGN	RTSPQEGERY	NYSKCTVVVR	IMEFTTTLLN	TSPEGWKLLK
1370	1380	1390	1400	1410	1420	1430	1440
KDLCNTHLMR	VLVQTLCEPA	SIGFNIGDVQ	VMAHLPDVCV	NLMKALKMSP	YKDILETHLR	EKITAQSIEE	LCAVNLYGPD
1450	1460	1470	1480	1490	1500	1510	1520
AQVDRSRLAA	VVSACKQLHR	AGLLHNILPS	QSTDLHHSVG	TELLSLVYKG	IAPGDERQCL	PSLDLSCKQL	ASGLLELAFA
1530	1540	1550	1560	1570	1580	1590	1600
FGGLCERLVS	LLLNPAVLST	ASLGSSQGSV	IHFSHGEYFY	SLFSETINTE	LLKNLDLAVL	ELMQSSVDNT	KMVSAVLNGM
1610	1620	1630	1640	1650	1660	1670	1680
LDQSFRERAN	QKHQGLKLAT	TILQHWKKCD	SWWAKDSPLE	TKMAVLALLA	KILQIDSSVS	FNTSHGSFPE	VFTTYISLLA
1690	1700	1710	1720	1730	1740	1750	1760
DTKLDLHLKG	QAVTLLPFFT	SLTGGSLEEL	RRVLEQLIVA	HFPMQSREFP	PGTPRFNNYV	DCMKKFLDAL	ELSQSPMLLE
1770	1780	1790	1800	1810	1820	1830	1840
LMTEVLCREQ	QHVMEELFQS	SFRRIARRGS	CVTQVGLLES	VYEMFRKDDP	RLSFTRQSFV	DRSLLTLLWH	CSLDALREFF
1850	1860	1870	1880	1890	1900	1910	1920
STIVVDAIDV	LKSRFTKLNE	STFDTQITKK	MGYYKILDVM	YSRLPKDDVH	AKESKINQVF	HGSCITEGNE	LTKTLIKLCY
1930	1940	1950	1960	1970	1980	1990	2000
DAFTENMAGE	NQLLERRRLY	HCAAYNCAIS	VICCVFNELK	FYQGFLFSEK	PEKNLLIFEN	LIDLKRRYNF	PVEVEVPMER
2010	2020	2030	2040	2050	2060	2070	2080
KKKYIEIRKE	AREAANGDSD	GPSYMSSLSY	LADSTLSEEM	SQFDFSTGVQ	SYSYSSQDPR	PATGRFRRRE	QRDPTVHDDV
2090	2100	2110	2120	2130	2140	2150	2160
LELEMDELNR	HECMAPLTAL	VKHMHRSLGP	PQGEEDSVPR	DLPSWMKFLH	GKLGNPIVPL	NIRLFLAKLV	INTEEVFRPY
2170	2180	2190	2200	2210	2220	2230	2240

Detailed Protein Report

Cmpd.	No. of Cmpds.	m/z meas.	Δ m/z [ppm]	z	Rt [min]	Score	P	Range	Sequence	Modification	Ratios
1135	1	473.1745	-251.80	2	44.3	15.1	0	1643-1651	K.MAVLALLAK.I	Oxidation: 1	QU:MU 0.56
9	1	798.7834	5.94	3	29.1	17.8	2	2314-2334	K.EVYAAAAEVLGLILRYVMERK.N		
935	1	512.1405	-273.82	2	41.8	10.3	0	2434-2441	K.VCLDIIYK.M	Carbamidomethyl: 2	WUP:QUP 2.35<br>QU:MU 1.68
272	1	898.1750	46.75	3	33.4	12.1	2	3030-3050	K.IWSEPFYQETYLPYMIRSKLK.L		
2214	1	722.7106	-205.39	2	56.0	11.5	2	3236-3247	K.FSMKMKMIDSAR.K		

Detailed Protein Report

Protein 57: semaphorin-5B isoform 3 precursor [Homo sapiens]

**Accession:**	gi|373432662	**Score:**	65.9
**Database:**	refseq_human(refseq_human_20140103.fasta)	**MW [kDa]:**	116.0
**Database Date:**	2015-11-30	**pI:**	9.0
**Modification(s):**	Carbamidomethyl	**Sequence Coverage [%]:**	6.1
		**No. of unique Peptides:**	2

10	20	30	40	50	60	70	80
MVLAGPLAVS	LLLPSLTLLV	SHLSSSQDVS	SEPSSEQQLC	ALSKHPTVAF	EDLQPWVSNF	TYPGARDFSQ	LALDPSGNQL
90	100	110	120	130	140	150	160
IVGARNYLFR	LSLANVSLLQ	ATEWASSEDT	RRSCQSKGKT	EEECQNYVRV	LIVAGRKVFM	CGTNAFSPMC	TSRQVGNLSR
170	180	190	200	210	220	230	240
TIEKINGVAR	CPYDPRHNST	AVISSQGELY	AATVIDFSGR	DPAIYRSLGS	GPPLRTAQYN	SKWLNEPNFV	AAYDIGLFAY
250	260	270	280	290	300	310	320
FFLRENAVEH	DCGRTVYSRV	ARVCKNDVGG	RFLLEDTWTT	FMKARLNCSR	PGEVPFYYNE	LQSAFHLPEQ	DLIYGVFTTN
330	340	350	360	370	380	390	400
VNSIAASAVC	AFNLSAISQA	FNGPFRYQEN	PRAAWLPIAN	PIPNFQCGTL	PETGPNENLT	ERSLQDAQRL	FLMSEAVQPV
410	420	430	440	450	460	470	480
TPEPCVTQDS	VRFSHLVVDL	VQAKDTLYHV	LYIGTESGTI	LKALSTASRS	LHGCYLEELH	VLPPGRREPL	RSLRILHSAR
490	500	510	520	530	540	550	560
ALFVGLRDGV	LRVPLERCAA	YRSQGACLGA	RDPYCGWDGK	QQRCSTLEDS	SNMSLWTQNI	TACPVRNVTR	DGGFGPWSPW
570	580	590	600	610	620	630	640
QPCEHLDGDN	SGSCLCRARS	CDSPRPRCGG	LDCLGPAIHI	ANCSRNGAWT	PWSSWALCST	SCGIGFQVRQ	RSCSNPAPRH
650	660	670	680	690	700	710	720
GGRICVGKSR	EERFCNENTP	CPVPIFWASW	GSWSKCSSNC	GGGMQSRRRA	CENGNSCLGC	GVEFKTCNPE	GCPEVRRNTP
730	740	750	760	770	780	790	800
WTPWLPVNVT	QGGARQEQRF	RFTCRAPLAD	PHGLQFGRRR	TETRTCPADG	SGSCDTDALV	EVLLRSGSTS	PHTVSGGWAA
810	820	830	840	850	860	870	880
WGPWSSCSRD	CELGFRVRKR	TCTNPEPRNG	GLPCVGDAAE	YQDCNPQACP	GEDICLGLHT	EEALCATQAC	PEGWSPWSEW
890	900	910	920	930	940	950	960
SKCTDDGAQS	RSRHCEELLP	GSSACAGNSS	QSRPCPYSEI	PVILPASSME	EATDCAGFNL	IHLVATGISC	FLGSGLLTLA
970	980	990	1000	1010	1020	1030	1040
VYLSCQHCQR	QSQESTLVHP	ATPNHLHYKG	GGTPKNEKYT	PMEFKTLNKN	NLIPDDRANF	YPLQQTNVYT	TTYYPSPLNK
1050	1060						
HSFRPEASPG	QRCFPNS						

Cmpd.	No. of Cmpds.	m/z meas.	Δ m/z [ppm]	z	Rt [min]	Score	P	Range	Sequence	Modification
2242	7	682.6902	75.49	3	58.4	22.5	2	113-129	R.SCQSKGKTEEECQNYVR.V	Carbamidomethyl: 2
1478	1	1061.4566	-34.44	2	48.1	20.8	0	765-785	R.TCPADGSGSCDTDALVEVLLR.S	

Detailed Protein Report

Protein 58: chromodomain-helicase-DNA-binding protein 2 isoform 1 [Homo sapiens]

**Accession:**	gi|118421089	**Score:**	65.7
**Database:**	refseq_human(refseq_human_20140103.fasta)	**MW [kDa]:**	211.2
**Database Date:**	2015-11-30	**pI:**	8.8
**Modification(s):**	Oxidation	**Sequence Coverage [%]:**	4.0
		**No. of unique Peptides:**	5

Quantitation

QU:MU	**Median:**	1.42	**CV:**	0.00 %	**No. of Peptides:**	1
WUP:QUP	**Median:**	1.05	**CV:**	0.00 %	**No. of Peptides:**	1

Detailed Protein Report

10	20	30	40	50	60	70	80
MMRNKDKSQE	EDSSLHSNAS	SHSASEEASG	SDSGSQSESE	QGSDPGSGHG	SESNSSSESS	ESQSESESES	AGSKSQPVLP
90	100	110	120	130	140	150	160
EAKEKPASKK	ERIADVKKMW	EEYPDVYGVR	RSNRSRQEPS	RFNIKEEASS	GSESGSPKRR	GQRQLKKQEK	WKQEPSEDEQ
170	180	190	200	210	220	230	240
EQGTSAESEP	EQKKVKARRP	VPRRTVPKPR	VKKQPKTQRG	KRKKQDSSDE	DDDDDEAPKR	QTRRRAAKNV	SYKEDDDFET
250	260	270	280	290	300	310	320
DSDDLIEMTG	EGVDEQQDNS	ETIEKVLDSR	LGKKGATGAS	TTVYAIEANG	DPSGDFDTEK	DEGEIQYLIK	WKGWSYIHST
330	340	350	360	370	380	390	400
WESEESLQQQ	KVKGLKKLEN	FKKKEDEIKQ	WLGKVSPEDV	EYFNCQQELA	SELNKQYQIV	ERVIAVKTSK	STLGQTDFPA
410	420	430	440	450	460	470	480
HSRKPAPSNE	PEYLCKWMGL	PYSECSWEDE	ALIGKKFQNC	IDSFHSRNNS	KTIPTRECKA	LKQRPRFVAL	KKQPAYLGGE
490	500	510	520	530	540	550	560
NLELRDYQLE	GLNWLAHSWC	KNNSVILADE	MGLGKTIQTI	SFLSYLFHQH	QLYGPFLIVV	PLSTLTSWQR	EFEIWAPEIN
570	580	590	600	610	620	630	640
VVVYIGDLMS	RNTIREYEWI	HSQTKRLKFN	ALITTYEILL	KDKTVLGSIN	WAFLGVDEAH	RLKNDDSLLY	KTLIDFKSNH
650	660	670	680	690	700	710	720
RLLITGTPLQ	NSLKELWSLL	HFIMPEKFEF	WEDFEEDHGK	GRENGYQSLH	KVLEPFLLRR	VKKDVEKSLP	AKVEQILRVE
730	740	750	760	770	780	790	800
MSALQKQYYK	WILTRNYKAL	AKGTRGSTSG	FLNIVMELKK	CCNHCYLIKP	PEENERENGQ	EILLSLIRSS	GKLILLDKLL
810	820	830	840	850	860	870	880
TRLRERGNRV	LIFSQMVRML	DILAEYLTIK	HYPFQRLDGS	IKGEIRKQAL	DHFNADGSED	FCFLLSTRAG	GLGINLASAD
890	900	910	920	930	940	950	960
TVVIFDSDWN	PQNDLQAQAR	AHRIGQKKQV	NIYRLVTKGT	VEEEIIERAK	KKMVLDHLVI	QRMDTTGRTI	LENNSGRSNS
970	980	990	1000	1010	1020	1030	1040
NPFNKEELTA	ILKFGAEDLF	KELEGEESEP	QEMDIDEILR	LAETRENEVS	TSATDELLSQ	FKVANFATME	DEEELEERPH
1050	1060	1070	1080	1090	1100	1110	1120
KDWDEIIPEE	QRKKVEEEER	QKELEEIYML	PRIRSSTKKA	QTNDSDSDTE	SKRQAQRSSA	SESETEDSDD	DKKPKRRGRP
1130	1140	1150	1160	1170	1180	1190	1200
RSVRKDLVEG	FTDAEIRRFI	KAYKKFGLPL	ERLECIARDA	ELVDKSVADL	KRLGELIHNS	CVSAMQEYEE	QLKENASEGK
1210	1220	1230	1240	1250	1260	1270	1280
GPGKRRGPTI	KISGVQVNVK	SIIQHEEEFE	MLHKSIPVDP	EEKKKYCLTC	RVKAAHFDVE	WGVEDDSRLL	LGIYEHGYGN
1290	1300	1310	1320	1330	1340	1350	1360
WELIKTDPEL	KLTDKILPVE	TDKKPQGKQL	QTRADYLLKL	LRKGLEKKGA	VTGGEEAKLK	KRKPRVKKEN	KVPRLKEEHG
1370	1380	1390	1400	1410	1420	1430	1440
IELSSPRHSD	NPSEEGEVKD	DGLEKSPMKK	KQKKKENKEN	KEKQMSSRKD	KEGDKERKKS	KDKKEKPKSG	DAKSSSKSKR
1450	1460	1470	1480	1490	1500	1510	1520
SQGPVHITAG	SEPVPIGEDE	DDDLDQETFS	ICKERMRPVK	KALKQLDKPD	KGLNVQEQLE	HTRNCLLKIG	DRIAECLKAY
1530	1540	1550	1560	1570	1580	1590	1600
SDQEHIKLWR	RNLWIFVSKF	TEFDARKLHK	LYKMAHKKRS	QEEEEQKKKD	DVTGGKKPFR	PEASGSSRDS	LISQSHTSHN
1610	1620	1630	1640	1650	1660	1670	1680
LHPQKPHLPA	SHGPQMHGHP	RDNYNHPNKR	HFSNADRGDW	QRERKFNYGG	GNNNPPWGSD	RHHQYEQHWY	KDHHYGDRRH
1690	1700	1710	1720	1730	1740	1750	1760
MDAHRSGSYR	PNNMSRKRPY	DQYSSDRDHR	GHRDYYDRHH	HDSKRRRSDE	FRPQNYHQQD	FRRMSDHRPA	MGYHGQGPSD
1770	1780	1790	1800	1810	1820	1830	
HYRSFHTDKL	GEYKQPLPPL	HPAVSDPRSP	PSQKSPHDSK	SPLDHRSPLE	RSLEQKNNPD	YNWNVRKT	

Cmpd.	No. of Cmpds.	m/z meas.	Δ m/z [ppm]	z	Rt [min]	Score	P	Range	Sequence	Modification	Ratios
2644	1	913.4624	-16.46	2	63.1	14.1	1	743-759	K.GTRGSTSGFLNIVMELK.K	Oxidation: 14	
1777	2	1022.8922	-139.78	2	52.6	14.7	2	932-948	K.KMVLDHLVIQRMDTTGR.T	Oxidation: 2, 12	
1792	1	898.7912	-12.20	3	52.2	11.6	1	1212-1234	K. ISGVQVNVKSIIQHEEEFEMLHK. S		
2192	1	885.2814	-167.85	2	57.8	12.6	0	1221-1234	K.SIIQHEEEFEMLHK.S		
1619	1	1015.0002	-35.61	2	50.0	12.7	2	1531-	R.RNLWIFVSKFTEFDAR.K		WUP:QUP 1.05

Detailed Protein Report

Cmpd.	No. of Cmpds.	m/z meas.	Δ m/z [ppm]	z	Rt [min]	Score	P	Range	Sequence	Modification	Ratios
								1546			QU:MU 1.42

Detailed Protein Report

Protein 59: **voltage-dependent P/Q-type calcium channel subunit alpha-1A isoform 4 [Homo sapiens]**

**Accession:**	gi|187828892	**Score:**	65.3
**Database:**	refseq_human(refseq_human_20140103.fasta)	**MW [kDa]:**	282.4
**Database Date:**	2015-11-30	**pI:**	9.6
		**Sequence Coverage [%]:**	3.0
		**No. of unique Peptides:**	1

Detailed Protein Report

10	20	30	40	50	60	70	80
MARFGDEMPA	RYGGGGSGAA	AGVVVGSGGG	RGAGGSRQGG	QPGAQRMYKQ	SMAQRARTMA	LYNPIPVRQN	CLTVNRSLFL
90	100	110	120	130	140	150	160
FSEDNVVRKY	AKKITEWPPF	EYMILATIIA	NCIVLALEQH	LPDDDKTPMS	ERLDDTEPYF	IGIFCFEAGI	KIIALGFAFH
170	180	190	200	210	220	230	240
KGSYLRNGWN	VMDFVVVLTG	ILATVGTEFD	LRTLRAVRVL	RPLKLVSGIP	SLQVVLKSIM	KAMIPLLQIG	LLLFFAILIF
250	260	270	280	290	300	310	320
AIIGLEFYMG	KFHTTCFEEG	TDDIQGESPA	PCGTEEPART	CPNGTKCQPY	WEGPNNGITQ	FDNILFAVLT	VFQCITMEGW
330	340	350	360	370	380	390	400
TDLLYNSNDA	SGNTWNWLYF	IPLIIIGSFF	MLNLVLGVLS	GEFAKERERV	ENRRAFLKLR	RQQQIERELN	GYMEWISKAE
410	420	430	440	450	460	470	480
EVILAEDETD	GEQRHPFDAL	RRTTIKKSKT	DLLNPEEAED	QLADIASVGS	PFARASIKSA	KLENSTFFHK	KERRMRFYIR
490	500	510	520	530	540	550	560
RMVKTQAFYW	TVLSLVALNT	LCVAIVHYNQ	PEWLSDFLYY	AEFIFLGLFM	SEMFIKMYGL	GTRPYFHSSF	NCFDCGVIIG
570	580	590	600	610	620	630	640
SIFEVIWAVI	KPGTSFGISV	LRALRLLRIF	KVTKYWASLR	NLVVSLLNSM	KSIISLLFLL	FLFIVVFALL	GMQLFGGQFN
650	660	670	680	690	700	710	720
FDEGTPPTNF	DTFPAAIMTV	FQILTGEDWN	EVMYDGIKSQ	GGVQGGMVFS	IYFIVLTLFG	NYTLLNVFLA	IAVDNLANAQ
730	740	750	760	770	780	790	800
ELTKDEQEEE	EAANQKLALQ	KAKEVAEVSP	LSAANMSIAV	KEQQKNQKPA	KSVWEQRTSE	MRKQNLLASR	EALYNEMDPD
810	820	830	840	850	860	870	880
ERWKAAYTRH	LRPDMKTHLD	RPLVVDPQEN	RNNNTNKSRA	AEPTVDQRLG	QQRAEDFLRK	QARYHDRARD	PSGSAGLDAR
890	900	910	920	930	940	950	960
RPWAGSQEAE	LSREGPYGRE	SDHHAREGSL	EQPGFWEGEA	ERGKAGDPHR	RHVHRQGGSR	ESRSGSPRTG	ADGEHRRHRA
970	980	990	1000	1010	1020	1030	1040
HRRPGEEGPE	DKAERRARHR	EGSRPARGGE	GEGEGPDGGE	RRRRHRHGAP	ATYEGDARRE	DKERRHRRRK	ENQGSGVPVS
1050	1060	1070	1080	1090	1100	1110	1120
GPNLSTTRPI	QQDLGRQDPP	LAEDIDNMKN	NKLATAESAA	PHGSLGHAGL	PQSPAKMGNS	TDPGPMLAIP	AMATNPQNAA
1130	1140	1150	1160	1170	1180	1190	1200
SRRTPNNPGN	PSNPGPPKTP	ENSLIVTNPS	GTQTNSAKTA	RKPDHTTVDI	PPACPPPLNH	TVVQVNKNAN	PDPLPKKEEE
1210	1220	1230	1240	1250	1260	1270	1280
KKEEEEDDRG	EDGPKPMPPY	SSMFILSTTN	PLRRLCHYIL	NLRYFEMCIL	MVIAMSSIAL	AAEDPVQPNA	PRNNVLRYFD
1290	1300	1310	1320	1330	1340	1350	1360
YVFTGVFTFE	MVIKMIDLGL	VLHQGAYFRD	LWNILDFIVV	SGALVAFAFT	GNSKGKDINT	IKSLRVLRVL	RPLKTIKRLP
1370	1380	1390	1400	1410	1420	1430	1440
KLKAVFDCVV	NSLKNVFNIL	IVYMLFMFIF	AVVAVQLFKG	KFFHCTDESK	EFEKDCRGKY	LLYEKNEVKA	RDREWKKYEF
1450	1460	1470	1480	1490	1500	1510	1520
HYDNVLWALL	TLFTVSTGEG	WPQVLKHSVD	ATFENQGPSP	GYRMEMSIFY	VVYFVVFPFF	FVNIFVALII	ITFQEQGDKM
1530	1540	1550	1560	1570	1580	1590	1600
MEEYSLEKNE	RACIDFAISA	KPLTRHMPQN	KQSFQYRMWQ	FVVSPPFEYT	IMAMIALNTI	VLMMKFYGAS	VAYENALRVF
1610	1620	1630	1640	1650	1660	1670	1680
NIVFTSLFSL	ECVLKVMAFG	ILNYFRDAWN	IFDFVTVLGS	ITDILVTEFG	NNFINLSFLR	LFRAARLIKL	LRQGYTIRIL
1690	1700	1710	1720	1730	1740	1750	1760
LWTFVQSFKA	LPYVCLLIAM	LFFIYAIIGM	QVFGNIGIDV	EDEDSDEDEF	QITEHNNFRT	FFQALMLLFR	SATGEAWHNI
1770	1780	1790	1800	1810	1820	1830	1840
MLSCLSGKPC	DKNSGILTRE	CGNEFAYFYF	VSFIFLCSFL	MLNLFVAVIM	DNFEYLTRDS	SILGPHHLDE	YVRVWAEYDP
1850	1860	1870	1880	1890	1900	1910	1920
AAWGRMPYLD	MYQMLRHMSP	PLGLGKKCPA	RVAYKRLLRM	DLPVADDNTV	HFNSTLMALI	RTALDIKIAK	GGADKQQMDA
1930	1940	1950	1960	1970	1980	1990	2000
ELRKEMMAIW	PNLSQKTLDL	LVTPHKSTDL	TVGKIYAAMM	IMEYYRQSKA	KKLQAMREEQ	DRTPLMFQRM	EPPSPTQEGG
2010	2020	2030	2040	2050	2060	2070	2080
PGQNALPSTQ	LDPGGALMAH	ESGLKESPSW	VTQRAQEMFQ	KTGTWSPEQG	PPTDMPNSQP	NSQSVEMREM	GRDGYSDSEH
2090	2100	2110	2120	2130	2140	2150	2160
YLPMEGQGRA	ASMPRLPAEN	QRRRGRPRGN	NLSTISDTSP	MKRSASVLGP	KARRLDDYSL	ERVPPEENQR	HHQRRRDRSH
2170	2180	2190	2200	2210	2220	2230	2240
RASERSLGRY	TDVDTGLGTD	LSMTTQSGDL	PSKERDQERG	RPKDRKHRQH	HHHHHHHHHP	PPPDKDRYAQ	ERPDHGRARA
2250	2260	2270	2280	2290	2300	2310	2320
RDQRWSRSPS	EGREHMAHRQ	GSSSVSGSPA	PSTSGTSTPR	RGRRQLPQTP	STPRPHVSYS	PVIRKAGGSG	PPQQQQQQQQ
2330	2340	2350	2360	2370	2380	2390	2400

Detailed Protein Report

Cmpd.	No. of Cmpds.	m/z meas.	Δ m/z [ppm]	z	Rt [min]	Score	P	Range	Sequence	Modification
2706	1	741.8621	-28.64	2	64.1	11.6	2	2371-2384	R.VPGPARSESPRACR.H	

Detailed Protein Report

Protein 60: **PREDICTED: CDK5 regulatory subunit-associated protein 2 isoform X4 [Homo sapiens]**

**Accession:**	gi|578817652	**Score:**	64.9
**Database:**	refseq_human(refseq_human_20140103.fasta)	**MW [kDa]:**	211.3
**Database Date:**	2015-11-30	**pI:**	5.4
**Modification(s):**	Oxidation	**Sequence Coverage [%]:**	3.3
		**No. of unique Peptides:**	4

Quantitation

QU:MU	**Median:**	1.34	**CV:**	15.04 %	**No. of Peptides:**	2
WUP:QUP	**Median:**	0.32	**CV:**	8.26 %	**No. of Peptides:**	2

Detailed Protein Report

10	20	30	40	50	60	70	80
MMDLVLEEDV	TVPGTLSGCS	GLVPSVPDDL	DGINPNAGLG	NGLLPNVSEE	TVSPTRARNM	KDFENQITEL	KKENFNLKLR
90	100	110	120	130	140	150	160
IYFLEERMQQ	EFHGPTEHIY	KTNIELKVEV	ESLKRELQER	EQLLIKASKA	VESLAEAGGS	EIQRVKEDAR	KKVQQVEDLL
170	180	190	200	210	220	230	240
TKRILLLEKD	VTAAQAELEK	AFAGTETEKA	LRLRLESKLS	EMKKMHEGDL	AMALVLDEKD	RLIEELKLSL	KSKEALIQCL
250	260	270	280	290	300	310	320
KEEKSQMACP	DENVSSGELR	GLCAAPREEK	ERETEAAQME	HQKERNSFEE	RIQALEEDLR	EKEREIATEK	KNSLKRDKAI
330	340	350	360	370	380	390	400
QGLTMALKSK	EKKVEELNSE	IEKLSAAFAK	AREALQKAQT	QEFQGSEDYE	TALSGKEALS	AALRSQNLTK	STENHRLRRS
410	420	430	440	450	460	470	480
IKKITQELSD	LQQERERLEK	DLEEAHREKS	KGDCTIRDLR	NEVEKLRNEV	NEREKAMENR	YKSLLSESNK	KLHNQEQVIK
490	500	510	520	530	540	550	560
HLTESTNQKD	VLLQKFNEKD	LEVIQQNCYL	MAAEDLELRS	EGLITEKCSS	QQPPGSKTIF	SKEKKQSSDY	EELIQVLKKE
570	580	590	600	610	620	630	640
QDIYTHLVKS	LQESDSINNL	QAELNKIFAL	RKQLEQDVLS	YQNLRKTLEE	QISEIRRREE	ESFSLYSDQT	SYLSICLEEN
650	660	670	680	690	700	710	720
NRFQVEHFSQ	EELKKKVSDL	IQLVKELYTD	NQHLKKTIFD	LSCMGFQGNG	FPDRLASTEQ	TEIMKDLSKG	GCKNGYLRHT
730	740	750	760	770	780	790	800
ESKISDCDGA	HAPGCLEEGA	FINLLAPLFN	EKATLLLESR	PDLLKVVREL	LLGQLFLTEQ	EVSGEHLDGK	TEKTPKQKGE
810	820	830	840	850	860	870	880
LVHFVQTNSF	SKPHDELKLS	CEAQLVKAGE	VPKVGLKDAS	VQTVATEGDL	LRFKHEATRE	AWEEKPINTA	LSAEHRPENL
890	900	910	920	930	940	950	960
HGVPGWQAAL	LSLPGITNRE	AKKSRLPILI	KPSRSLGNMY	RLPATQEVVT	QLQSQILELQ	GELKEFKTCN	KQLHQKLILA
970	980	990	1000	1010	1020	1030	1040
EAVMEGRPTP	DKTLLNAQPP	VGAAYQDSPG	EQKGIKTTSS	VWRDKEMDSD	QQRSYEIDSE	ICPPDDLASL	PSCKENPEDV
1050	1060	1070	1080	1090	1100	1110	1120
LSPTSVATYL	SSKSQPSAKV	SVMGTDQSES	INTSNETEYL	KQKIHDLETE	LEGYQNFIFQ	LQKHSQCSEA	IITVLCGTEG
1130	1140	1150	1160	1170	1180	1190	1200
AQDGLSKPKN	GSDGEEMTFS	SLHQVRYVKH	VKILGPLAPE	MIDSRVLENL	KQQLEEQEYK	LQKEQNLNMQ	LFSEIHNLQN
1210	1220	1230	1240	1250	1260	1270	1280
KFRDLSPPRY	DSLVQSQARE	LSLQRQQIKD	GHGICVISRQ	HMNTMIKAFE	ELLQASDVDY	CVAEGFQEQL	NQCAELLEKL
1290	1300	1310	1320	1330	1340	1350	1360
EKLFLNGKSV	GVEMNTQNEL	MERIEEDNLT	YQHLLPESPE	PSASHALSDY	ETSEKSFFSR	DQKQDNETEK	TSVMVNSFSQ
1370	1380	1390	1400	1410	1420	1430	1440
DLLMEHIQEI	RTLRKRLEES	IKTNEKLRKQ	LERQGSEFVQ	GSTSIFASGS	ELHSSLTSEI	HFLRKQNQAL	NAMLIKGSRD
1450	1460	1470	1480	1490	1500	1510	1520
KQKENDKLRE	SLSRKTVSLE	HLQREYASVK	EENERLQKEG	SEKERHNQQL	IQEVRCSGQE	LSRVQEEVKL	RQQLLSQNDK
1530	1540	1550	1560	1570	1580	1590	1600
LLQSLRVELK	AYEKLDEEHR	RLREASGEGW	KGQDPFRDLH	SLLMEIQALR	LQLERSIETS	STLQSRLKEQ	LARGAEKAQE
1610	1620	1630	1640	1650	1660	1670	1680
GALTLAVQAV	SIPEVPLQPD	KHDGDKYPME	SDNSFDLFDS	SQAVTPKSVS	ETPPLSGNDT	DSLSCDSGSS	ATSTPCVSRL
1690	1700	1710	1720	1730	1740	1750	1760
VTGHHLWASK	NGRHVLGLIE	DYEALLKQIS	QGQRLLAEMD	IQTQEAPSST	SQELGTKGPH	PAPLSKFVSS	VSTAKLTLEE
1770	1780	1790	1800	1810	1820	1830	1840
AYRRLKLLWR	VSLPEDGQCP	LHCEQIGEMK	AEVTKLHKKL	FEQEKKLQNT	MKLLQLSKRQ	EKVIFDQLVV	THKILRKARG
1850	1860	1870					
NLELRPGGAH	PGTCSPSRPG	S					

Cmpd.	No. of Cmpds.	m/z meas.	Δ m/z [ppm]	z	Rt [min]	Score	P	Range	Sequence	Modification	Ratios
93	1	529.5810	3.65	3	30.1	13.6	1	271-283	K.ERETEAAQMEHQK.E		WUP:QUP 0.30<br>QU:MU 1.55
2305	1	937.4652	-77.24	2	57.1	11.9	1	906-921	R.LPILIKPSRSLGNMYR.L	Oxidation: 14	
268	2	501.1673	-209.52	2	32.1	13.8	1	1589-1597	K.EQLARGAEK.A		WUP:QUP 0.35<br>QU:MU 1.15
667	1	575.7990	-26.64	2	38.3	15.2	1	1756-	K.LTLEEAYRR.L		

Detailed Protein Report

Cmpd.	No. of Cmpds.	m/z meas.	Δ m/z [ppm]	z	Rt [min]	Score	P	Range	Sequence	Modification	Ratios
								1764			

Detailed Protein Report

Protein 61: PREDICTED: E3 ubiquitin-protein ligase UBR4 isoform X11 [Homo sapiens]

**Accession:**	gi|578798772	**Score:**	64.8
**Database:**	refseq_human(refseq_human_20140103.fasta)	**MW [kDa]:**	574.7
**Database Date:**	2015-11-30	**pI:**	5.6
**Modification(s):**	Carbamidomethyl, Oxidation	**Sequence Coverage [%]:**	1.6
		**No. of unique Peptides:**	4

Detailed Protein Report

10	20	30	40	50	60	70	80
MATSGGEEAA	AAAPAPGTPA	TGADTTPGWE	VAVRPLLSAS	YSAFEMKELP	QLVASVIESE	SEILHHEKQY	EPFYSSFVAL
90	100	110	120	130	140	150	160
STHYITTVCS	LIPRNQLQSV	AAACKVLIEF	SLLRLENPDE	ACAVSQKHLI	LLIKGLCTGC	SRLDRTEIIT	FTAMMKSAKL
170	180	190	200	210	220	230	240
PQTVKTLSDV	EDQKELASPV	SPELRQKEVQ	MNFLNQLTSV	FNPRTVASQP	ISTQTLVEGE	NDEQSSTDQA	SAIKTKNVFI
250	260	270	280	290	300	310	320
AQNVASLQEL	GGSEKLLRVC	LNLPYFLRYI	NRFQDAVLAN	SFFIMPATVA	DATAVRNGFH	SLVIDVTMAL	DTLSLPVLEP
330	340	350	360	370	380	390	400
LNPSRLQDVT	VLSLSCLYAG	VSVATCMAIL	HVGSAQQVRT	GSTSSKEDDY	ESDAATIVQK	CLEIYDMIGQ	AISSSRRAGG
410	420	430	440	450	460	470	480
EHYQNFQLLG	AWCLLNSLFL	ILNLSPTALA	DKGKEKDPLA	ALRVRDILSR	TKEGVGSPKL	GPGKGHQGFG	VLSVILANHA
490	500	510	520	530	540	550	560
IKLLTSLFQD	LQVEALHKGW	ETDGPPAALS	IMAQSTSIQR	IQRLIDSVPL	MNLLLTLLST	SYRKACVLQR	QRKGSMSSDA
570	580	590	600	610	620	630	640
SASTDSNTYY	EDDFSSTEED	SSQDDDSEPI	LGQWFEETIS	PSKEKAAPPP	PPPPPPLESS	PRVKSPSKQA	PGEKGNILAS
650	660	670	680	690	700	710	720
RKDPELFLGL	ASNILNFITS	SMLNSRNNFI	RNYLSVSLSE	HHMATLASII	KEVDKDGLKG	SSDEEFAAAL	YHFNHSLVTS
730	740	750	760	770	780	790	800
DLQSPNLQNT	LLQQLGVAPF	SEGPWPLYIH	PQSLSVLSRL	LLIWQHKASA	QGDPDVPECL	KVWDRFLSTM	KQNALQGVVP
810	820	830	840	850	860	870	880
SETEDLNVEH	LQMLLLIFHN	FTETGRRAIL	SLFVQIIQEL	SVNMDAQMRF	VPLILARLLL	IFDYLLHQYS	KAPVYLFEQV
890	900	910	920	930	940	950	960
QHNLLSPPFG	WASGSQDSNS	RRATTPLYHG	FKEVEENWSK	HFSSDAVPHP	RFYCVLSPEA	SEDDLNRLDS	VACDVLFSKL
970	980	990	1000	1010	1020	1030	1040
VKYDELYAAL	TALLAAGSQL	DTVRRKENKN	VTALEACALQ	YYFLILWRIL	GILPPSKTYI	NQLSMNSPEM	SECDILHTLR
1050	1060	1070	1080	1090	1100	1110	1120
WSSRLRISSY	VNWIKDHLIK	QGMKAEHASS	LLELASTTKC	SSVKYDVEIV	EEYFARQISS	FCSIDCTTIL	QLHEIPSLQS
1130	1140	1150	1160	1170	1180	1190	1200
IYTLDAAISK	VQVSLDEHFS	KMAAETDPHK	SSEITKNLLP	ATLQLIDTYA	SFTRAYLLQN	FNEEGTTEKP	SKEKLQGFAA
1210	1220	1230	1240	1250	1260	1270	1280
VLAIGSSRCK	ANTLGPTLVQ	NLPSSVQTVC	ESWNNINTNE	FPNIGSWRNA	FANDTIPSES	YISAVQAAHL	GTLCSQSLPL
1290	1300	1310	1320	1330	1340	1350	1360
AASLKHTLLS	LVRLTGDLIV	WSDEMNPPQV	IRTLLPLLLE	SSTESVAEIS	SNSLERILGP	AESDEFLARV	YEKLITGCYN
1370	1380	1390	1400	1410	1420	1430	1440
ILANHADPNS	GLDESILEEC	LQYLEKQLES	SQARKAMEEF	FSDSGELVQI	MMATANENLS	AKFCNRVLKF	FTKLFQLTEK
1450	1460	1470	1480	1490	1500	1510	1520
SPNPSLLHLC	GSLAQLACVE	PVRLQAWLTR	MTTSPPKDSD	QLDVIQENRQ	LLQLLTTYIV	RENSQVGEGV	CAVLLGTLTP
1530	1540	1550	1560	1570	1580	1590	1600
MATEMLANGD	GTGFPELMVV	MATLASAGQG	AGHLQLHNAA	VDWLSRCKKY	LSQKNVVEKL	NANVMHGKHV	MILECTCHIM
1610	1620	1630	1640	1650	1660	1670	1680
SYLADVTNAL	SQSNGQGPSH	LSVDGEERAI	EVDSDWVEEL	AVEEEDSQAE	DSDEDSLCNK	LCTFTITQKE	FMNQHWYHCH
1690	1700	1710	1720	1730	1740	1750	1760
TCKMVDGVGV	CTVCAKVCHK	DHEISYAKYG	SFFCDCGAKE	DGSCLALVKR	TPSSGMSSTM	KESAFQSEPR	ISESLVRHAS
1770	1780	1790	1800	1810	1820	1830	1840
TSSPADKAKV	TISDGKVADE	EKPKKSSLCR	TVEGCREELQ	NQANFSFAPL	VLDMLNFLMD	AIQTNFQQAS	AVGSSSRAQQ
1850	1860	1870	1880	1890	1900	1910	1920
ALSELHTVEK	AVEMTDQLMV	PTLGSQEGAF	ENVRMNYSGD	QGQTIRQLIS	AHVLRRVAMC	VLSSPHGRRQ	HLAVSHEKGK
1930	1940	1950	1960	1970	1980	1990	2000
ITVLQLSALL	KQADSSKRKL	TLTRLASAPV	PFTVLSLTGN	PCKEDYLAVC	GLKDCHVLTF	SSSGSVSDHL	VLHPQLATGN
2010	2020	2030	2040	2050	2060	2070	2080
FIIKAVWLPG	SQTELAIVTA	DFVKIYDLCV	DALSPTFYFL	LPSSKIRDVT	FLFNEEGKNI	IVIMSSAGYI	YTQLMEEASS
2090	2100	2110	2120	2130	2140	2150	2160
AQQGPFYVTN	VLEINHEDLK	QDSNSQVAGG	GVSVYYSHVL	QMLFFSYCQG	KSFAATISRT	TLEVLQLFPI	NIKSSNGGSK
2170	2180	2190	2200	2210	2220	2230	2240

Detailed Protein Report

Cmpd.	No. of Cmpds.	m/z meas.	Δ m/z [ppm]	z	Rt [min]	Score	P	Range	Sequence	Modification
270	1	530.5999	-29.12	3	33.1	13.6	2	1777-1790	K.VADEEKPKKSSLCR.T	
1928	1	917.3279	-102.08	2	54.5	15.2	1	2608-2623	K.LPQMETGMDEGKEPQK.Q	Oxidation: 4
1934	1	745.7866	-161.90	2	54.6	10.5	1	3240-3252	R.DLHTLDSHVRGIK.K	
375	1	878.7841	3.73	3	34.7	12.0	2	4668-4690	K.VFLDCFCKIAAGIKNNSNGHQLK.D	Carbamidomethyl: 5, 7

Detailed Protein Report

Protein 62: centromere protein F [Homo sapiens]

**Accession:**	gi|55770834	**Score:**	64.2
**Database:**	refseq_human(refseq_human_20140103.fasta)	**MW [kDa]:**	357.3
**Database Date:**	2015-11-30	**pI:**	4.9
**Modification(s):**	Oxidation	**Sequence Coverage [%]:**	1.7
		**No. of unique Peptides:**	3

Detailed Protein Report

10	20	30	40	50	60	70	80
MSWALEEWKE	GLPTRALQKI	QELEGQLDKL	KKEKQQRQFQ	LDSLEAALQK	QKQKVENEKT	EGTNLKRENQ	RLMEICESLE
90	100	110	120	130	140	150	160
KTKQKISHEL	QVKESQVNFQ	EGQLNSGKKQ	IEKLEQELKR	CKSELERSQQ	AAQSADVSLN	PCNTPQKIFT	TPLTPSQYYS
170	180	190	200	210	220	230	240
GSKYEDLKEK	YNKEVEERKR	LEAEVKALQA	KKASQTLPQA	TMNHRDIARH	QASSSVFSWQ	QEKTPSHLSS	NSQRTPIRRD
250	260	270	280	290	300	310	320
FSASYFSGEQ	EVTPSRSTLQ	IGKRDANSSF	FDNSSSPHLL	DQLKAQNQEL	RNKINELELR	LQGHEKEMKG	QVNKFQELQL
330	340	350	360	370	380	390	400
QLEKAKVELI	EKEKVLNKCR	DELVRTTAQY	DQASTKYTAL	EQKLKKLTED	LSCQRQNAES	ARCSLEQKIK	EKEKEFQEEL
410	420	430	440	450	460	470	480
SRQQRSFQTL	DQECIQMKAR	LTQELQQAKN	MHNVLQAELD	KLTSVKQQLE	NNLEEFKQKL	CRAEQAFQAS	QIKENELRRS
490	500	510	520	530	540	550	560
MEEMKKENNL	LKSHSEQKAR	EVCHLEAELK	NIKQCLNQSQ	NFAEEMKAKN	TSQETMLRDL	QEKINQQENS	LTLEKLKLAV
570	580	590	600	610	620	630	640
ADLEKQRDCS	QDLLKKREHH	IEQLNDKLSK	TEKESKALLS	ALELKKKEYE	ELKEEKTLFS	CWKSENEKLL	TQMESEKENL
650	660	670	680	690	700	710	720
QSKINHLETC	LKTQQIKSHE	YNERVRTLEM	DRENLSVEIR	NLHNVLDSKS	VEVETQKLAY	MELQQKAEFS	DQKHQKEIEN
730	740	750	760	770	780	790	800
MCLKTSQLTG	QVEDLEHKLQ	LLSNEIMDKD	RCYQDLHAEY	ESLRDLLKSK	DASLVTNEDH	QRSLLAFDQQ	PAMHHSFANI
810	820	830	840	850	860	870	880
IGEQGSMPSE	RSECRLEADQ	SPKNSAILQN	RVDSLEFSLE	SQKQMNSDLQ	KQCEELVQIK	GEIEENLMKA	EQMHQSFVAE
890	900	910	920	930	940	950	960
TSQRISKLQE	DTSAHQNVVA	ETLSALENKE	KELQLLNDKV	ETEQAEIQEL	KKSNHLLEDS	LKELQLLSET	LSLEKKEMSS
970	980	990	1000	1010	1020	1030	1040
IISLNKREIE	ELTQENGTLK	EINASLNQEK	MNLIQKSESF	ANYIDEREKS	ISELSDQYKQ	EKLILLQRCE	ETGNAYEDLS
1050	1060	1070	1080	1090	1100	1110	1120
QKYKAAQEKN	SKLECLLNEC	TSLCENRKNE	LEQLKEAFAK	EHQEFLTKLA	FAEERNQNLM	LELETVQQAL	RSEMTDNQNN
1130	1140	1150	1160	1170	1180	1190	1200
SKSEAGGLKQ	EIMTLKEEQN	KMQKEVNDLL	QENEQLMKVM	KTKHECQNLE	SEPIRNSVKE	RESERNQCNF	KPQMDLEVKE
1210	1220	1230	1240	1250	1260	1270	1280
ISLDSYNAQL	VQLEAMLRNK	ELKLQESEKE	KECLQHELQT	IRGDLETSNL	QDMQSQEISG	LKDCEIDAEE	KYISGPHELS
1290	1300	1310	1320	1330	1340	1350	1360
TSQNDNAHLQ	CSLQTTMNKL	NELEKICEIL	QAEKYELVTE	LNDSRSECIT	ATRKMAEEVG	KLLNEVKILN	DDSGLLHGEL
1370	1380	1390	1400	1410	1420	1430	1440
VEDIPGGEFG	EQPNEQHPVS	LAPLDESNSY	EHLTLSDKEV	QMHFAELQEK	FLSLQSEHKI	LHDQHCQMSS	KMSELQTYVD
1450	1460	1470	1480	1490	1500	1510	1520
SLKAENLVLS	TNLRNFQGDL	VKEMQLGLEE	GLVPSLSSSC	VPDSSSLSSL	GDSSFYRALL	EQTGDMSLLS	NLEGAVSANQ
1530	1540	1550	1560	1570	1580	1590	1600
CSVDEVFCSS	LQEENLTRKE	TPSAPAKGVE	ELESLCEVYR	QSLEKLEEKM	ESQGIMKNKE	IQELEQLLSS	ERQELDCLRK
1610	1620	1630	1640	1650	1660	1670	1680
QYLSENEQWQ	QKLTSVTLEM	ESKLAAEKKQ	TEQLSLELEV	ARLQLQGLDL	SSRSLLGIDT	EDAIQGRNES	CDISKEHTSE
1690	1700	1710	1720	1730	1740	1750	1760
TTERTPKHDV	HQICDKDAQQ	DLNLDIEKIT	ETGAVKPTGE	CSGEQSPDTN	YEPPGEDKTQ	GSSECISELS	FSGPNALVPM
1770	1780	1790	1800	1810	1820	1830	1840
DFLGNQEDIH	NLQLRVKETS	NENLRLLHVI	EDRDRKVESL	LNEMKELDSK	LHLQEVQLMT	KIEACIELEK	IVGELKKENS
1850	1860	1870	1880	1890	1900	1910	1920
DLSEKLEYFS	CDHQELLQRV	ETSEGLNSDL	EMHADKSSRE	DIGDNVAKVN	DSWKERFLDV	ENELSRIRSE	KASIEHEALY
1930	1940	1950	1960	1970	1980	1990	2000
LEADLEVVQT	EKLCLEKDNE	NKQKVIVCLE	EELSVVTSER	NQLRGELDTM	SKKTTALDQL	SEKMKEKTQE	LESHQSECLH
2010	2020	2030	2040	2050	2060	2070	2080
CIQVAEAEVK	EKTELLQTLS	SDVSELLKDK	THLQEKLQSL	EKDSQALSLT	KCELENQIAQ	LNKEKELLVK	ESESLQARLS
2090	2100	2110	2120	2130	2140	2150	2160
ESDYEKLNVS	KALEAALVEK	GEFALRLSST	QEEVHQLRRG	IEKLRVRIEA	DEKKQLHIAE	KLKERERENP	SLKDKVENLE
2170	2180	2190	2200	2210	2220	2230	2240

Detailed Protein Report

Cmpd.	No. of Cmpds.	m/z meas.	Δ m/z [ppm]	z	Rt [min]	Score	P	Range	Sequence	Modification
2707	1	821.4735	62.12	2	64.1	11.9	2	301-314	R.LQGHEKEMKGQVNK.F	Oxidation: 8
1276	1	917.4679	30.80	2	46.1	11.7	0	2267-2283	K.ELNEAVAALCGDQEIMK.A	
552	1	600.8199	-67.28	2	35.3	13.6	1	2316-2325	K.KQLCVLQQLK.E	

Detailed Protein Report

Protein 63: Alstrom syndrome protein 1 [Homo sapiens]

**Accession:**	gi|110349786	**Score:**	64.1
**Database:**	refseq_human(refseq_human_20140103.fasta)	**MW [kDa]:**	460.9
**Database Date:**	2015-11-30	**pI:**	5.8
**Modification(s):**	Oxidation	**Sequence Coverage [%]:**	1.2
		**No. of unique Peptides:**	2

Quantitation

QU:MU	**Median:**	2.56	**CV:**	0.00 %	**No. of Peptides:**	1
WUP:QUP	**Median:**	0.53	**CV:**	0.00 %	**No. of Peptides:**	1

BRUKER

Detailed Protein Report

10	20	30	40	50	60	70	80
MEPEDLPWPG	ELEEEEEEEE	EEEEEEEEEA	AAAAAAANVDD	VVVVEEVEEE	AGRELDSDSH	YGPQHLESID	DEEDEEAKAW
90	100	110	120	130	140	150	160
LQAHPGRILP	PLSPPQHRYS	EGERTSLEKI	VPLTCHVWQQ	IVYQGNSRTQ	ISDTNVVCLE	TTAQRGSGDD	QKTESWHCLP
170	180	190	200	210	220	230	240
QEMDSSQTLD	TSQTRFNVRT	EDTEVTDFPS	LEEGILTQSE	NQVKEPNRDL	FCSPLLVIQD	SFASPDLPLL	TCLTQDQEFA
250	260	270	280	290	300	310	320
PDSLFHQSEL	SFAPLRGIPD	KSEDTEWSSR	PSEVSEALFQ	ATAEVASDLA	SSRFSVSQHP	LIGSTAVGSQ	CPFLPSEQGN
330	340	350	360	370	380	390	400
NEETISSVDE	LKIPKDCDRY	DDLCSYMSWK	TRKDTQWPEN	NLADKDQVSV	ATSFDITDEN	IATKRSDHFD	AARSYGQYWT
410	420	430	440	450	460	470	480
QEDSSKQAET	YLTKGLQGKV	ESDVITLDGL	NENAVVCSER	VAELQRKPTR	ESEYHSSDLR	MLRMSPDTVP	KAPKHLKAGD
490	500	510	520	530	540	550	560
TSKGGIAKVT	QSNLKSGITT	TPVDSDIGSH	LSLSLEDLSQ	LAVSSPLETT	TGQHTDTLNQ	KTLADTHLTE	ETLKVTAIPE
570	580	590	600	610	620	630	640
PADQKTATPT	VLSSSHSHRG	KPSIFYQQGL	PDSHLTEEAL	KVSAAPGLAD	QTTGMSTLTS	TSYSHREKPG	TFYQQELPES
650	660	670	680	690	700	710	720
NLTEEPLEVS	AAPGPVEQKT	GIPTVSSTSH	SHVEDLLFFY	RQTLPDGHLT	DQALKVSAVS	GPADQKTGTA	TVLSTPHSHR
730	740	750	760	770	780	790	800
EKPGIFYQQE	FADSHQTEET	LTKVSATPGP	ADQKTEIPAV	QSSSYSQREK	PSILYPQDLA	DSHLPEEGLK	VSAVAGPADQ
810	820	830	840	850	860	870	880
KTGLPTVPSS	AYSHREKLLV	FYQQALLDSH	LPEEALKVSA	VSGPADGKTG	TPAVTSTSSA	SSSLGEKPSA	FYQQTLPNSH
890	900	910	920	930	940	950	960
LTEEALKVSI	VPGPGDQKTG	IPSAPSSFYS	HREKPIIFSQ	QTLPDFLFPE	EALKVSAVSV	LAAQKTGTPT	VSSNSHSHSE
970	980	990	1000	1010	1020	1030	1040
KSSVFYQQEL	PDSDLPRESL	KMSAIPGLTD	QKTVPTPTVP	SGSFSHREKP	SIFYQQEWPD	SYATEKALKV	STGPGPADQK
1050	1060	1070	1080	1090	1100	1110	1120
TEIPAVQSSS	YPQREKPSVL	YPQVLSDSHL	PEESLKVSAF	PGPADQMTDT	PAVPSTFYSQ	REKPGIFYQQ	TLPESHLPKE
1130	1140	1150	1160	1170	1180	1190	1200
ALKISVAPGL	ADQKTGTPTV	TSTSYSQHRE	KPSIFHQQAL	PGTHIPEEAQ	KVSAVTGPGN	QKTWIPRVLS	TFYSQREKPG
1210	1220	1230	1240	1250	1260	1270	1280
IFYQQTLPGS	HIPEEAQKVS	PVLGPADQKT	GTPTPTSASY	SHTEKPGIFY	QQVLPDNHPT	EEALKISVAS	EPVDQTTGTP
1290	1300	1310	1320	1330	1340	1350	1360
AVTSTSYSQY	REKPSIFYQQ	SLPSSHLTEE	AKNVSAVPGP	ADQKTVIPIL	PSTFYSHTEK	PGVFYQQVLP	HSHPTEEALK
1370	1380	1390	1400	1410	1420	1430	1440
ISVASEPVDQ	TTGTPTVTST	SYSQHTEKPS	IFYQQSLPGS	HLTEEAKNVS	AVPGPGDRKT	GIPTLPSTFY	SHTEKPGSFY
1450	1460	1470	1480	1490	1500	1510	1520
QQVLPHSHLP	EEALEVSVAP	GPVDQTIGTP	TVTSPSSSFG	EKPIVIYKQA	FPEGHLPEES	LKVSVAPGPV	GQTTGAPTIT
1530	1540	1550	1560	1570	1580	1590	1600
SPSYSQHRAK	SGSFYQLALL	GSQIPEEALR	VSSAPGPADQ	TTGIPTITST	SYSFGEKPIV	NYKQAFPDGH	LPEEALKVSI
1610	1620	1630	1640	1650	1660	1670	1680
VSGPTEKKTD	IPAGPLGSSA	LGEKPITFYR	QALLDSPLNK	EVVKVSAAPG	PADQKTETLP	VHSTSYSNRG	KPVIFYQQTL
1690	1700	1710	1720	1730	1740	1750	1760
SDSHLPEEAL	KVPPVPGPDA	QKTETPSVSS	SLYSYREKPI	VFYQQALPDS	ELTQEALKVS	AVPQPADQKT	GLSTVTSSFY
1770	1780	1790	1800	1810	1820	1830	1840
SHTEKPNISY	QQELPDSHLT	EEALKVSNVP	GPADQKTGVS	TVTSTSYSHR	EKPIVSYQRE	LPHFTEAGLK	ILRVPGPADQ
1850	1860	1870	1880	1890	1900	1910	1920
KTGINILPSN	SYPQREHSVI	SYEQELPDLT	EVTLKAIGVP	GPADQKTGIQ	IASSSSYSNR	EKASIFHQQE	LPDVTEEALN
1930	1940	1950	1960	1970	1980	1990	2000
VFVVPGQGDR	KTEIPTVPLS	YYSRREKPSV	ISQQELPDSH	LTEEALKVSP	VSIPAEQKTG	IPIGLSSSYS	HSHKEKLKIS
2010	2020	2030	2040	2050	2060	2070	2080
TVHIPDDQKT	EFPAATLSSY	SQIEKPKIST	VIGPNDQKTP	SQTAFHSSYS	QTVKPNILFQ	QQLPDRDQSK	GILKISAVPE
2090	2100	2110	2120	2130	2140	2150	2160
LTDVNTGKPV	SLSSSYFHRE	KSNIFSPQEL	PGSHVTEDVL	KVSTIPGPAG	QKTVLPTALP	SSFSHREKPD	IFYQKDLPDR
2170	2180	2190	2200	2210	2220	2230	2240

Detailed Protein Report

Cmpd.	No. of Cmpds.	m/z meas.	Δ m/z [ppm]	z	Rt [min]	Score	P	Range	Sequence	Modification	Ratios
881	1	555.8212	17.82	2	40.6	26.2	0	1219-1229	K.VSPVLGPADQK.T		QU:MU 2.56<br>WUP:QUP 0.53
1890	1	1036.8846	-93.94	2	54.0	16.6	2	2503-2519	R.NAEEEESRVRAHAWNMK.F	Oxidation: 16	

Detailed Protein Report

Protein 64: PREDICTED: Golgin subfamily A member 4 isoform X7 [Homo sapiens]

**Accession:**	gi|530372330	**Score:**	64.0
**Database:**	refseq_human(refseq_human_20140103.fasta)	**MW [kDa]:**	259.0
**Database Date:**	2015-11-30	**pI:**	5.2
**Modification(s):**	Oxidation	**Sequence Coverage [%]:**	2.8
		**No. of unique Peptides:**	5

Detailed Protein Report

10	20	30	40	50	60	70	80
MFKKLKQKIS	EEQQQLQQAL	APAQASSNSS	TPTRMRSRTS	SFTEQLDEGT	PNRESGDTQS	FAQKLQLRVP	SVESLFRSPI
90	100	110	120	130	140	150	160
KESLFRSSSK	ESLVRTSSRE	SLNRLDLDSS	TASFDPPSDM	DSEAEDLVGN	SDSLNKEQLI	QRLRRMERSL	SSYRGKYSEG
170	180	190	200	210	220	230	240
ILSQSQDKSL	RRIAELREEL	QMDQQAKKHL	QEEFDASLEE	KDQYISVLQT	QVSLLKQRLR	NGPMNVDVLK	PLPQLEPQAE
250	260	270	280	290	300	310	320
VFTKEENPES	DGEPVVEDGT	SVKTLETLQQ	RVKRQENLLK	RCKETIQSHK	EQCTLLTSEK	EALQEQLDER	LQELEKIKDL
330	340	350	360	370	380	390	400
HMAEKTKLIT	QLRDAKNLIE	QLEQDKGMVI	AETKRQMHET	LEMKEEEIAQ	LRSRIKQMTT	QGEELREQKE	KSERAAFEEL
410	420	430	440	450	460	470	480
EKALSTAQKT	EEARRKLKAE	MDEQIKTIEK	TSEEERISLQ	QELSRVKQEV	VDVMKKSSEE	QIAKLQKLHE	KELARKEQEL
490	500	510	520	530	540	550	560
TKKLQTRERE	FQEQMKVALE	KSQSEYLKIS	QEKEQQESLA	LEELELQKKA	ILTESENKLR	DLQQEAETYR	TRILELESSL
570	580	590	600	610	620	630	640
EKSLQENKNQ	SKDLAVHLEA	EKNKHNKEIT	VMVEKHKTEL	ESLKHQQDAL	WTEKLQVLKQ	QYQTEMEKLR	EKCEQEKETL
650	660	670	680	690	700	710	720
LKDKEIIFQA	HIEEMNEKTL	EKLDVKQTEL	ESLSSELSEV	LKARHKLEEE	LSVLKDQTDK	MKQELEAKMD	EQKNHHQQQV
730	740	750	760	770	780	790	800
DSIIKEHEVS	IQRTEKALKD	QINQLELLLK	ERDKHLKEHQ	AHVENLEADI	KRSEGELQQA	SAKLDVFQSY	QSATHEQTKA
810	820	830	840	850	860	870	880
YEEQLAQLQQ	KLLDLETERI	LLTKQVAEVE	AQKKDVCTEL	DAHKIQVQDL	MQQLEKQNSE	MEQKVKSLTQ	VYESKLEDGN
890	900	910	920	930	940	950	960
KEQEQTKQIL	VEKENMILQM	REGQKKEIEI	LTQKLSAKED	SIHILNEEYE	TKFKNQEKKM	EKVKQKAKEM	QETLKKKLLD
970	980	990	1000	1010	1020	1030	1040
QEAKLKKELE	NTALELSQKE	KQFNAKMLEM	AQANSAGISD	AVSRLETNQK	EQIESLTEVH	RRELNDVISI	WEKKLNQQAE
1050	1060	1070	1080	1090	1100	1110	1120
ELQEIHEIQL	QEKEQEVAEL	KQKILLFGCE	KEEMNKEITW	LKEEGVKQDT	TLNELQEQLK	QKSAHVNSLA	QDETKLKAHL
1130	1140	1150	1160	1170	1180	1190	1200
EKLEVDLNKS	LKENTFLQEQ	LVELKMLAEE	DKRKVSELTS	KLKTTDEEFQ	SLKSSHEKSN	KSLEDKSLEF	KKLSEELAIQ
1210	1220	1230	1240	1250	1260	1270	1280
LDICCKKTEA	LLEAKTNELI	NISSSKTNAI	LSRISHCQHR	TTKVKEALLI	KTCTVSELEA	QLRQLTEEQN	TLNISFQQAT
1290	1300	1310	1320	1330	1340	1350	1360
HQLEEKENQI	KSMKADIESL	VTEKEALQKE	GGNQQQAASE	KESCITQLKK	ELSENINAVT	LMKEELKEKK	VEISSLSKQL
1370	1380	1390	1400	1410	1420	1430	1440
TDLNVQLQNS	ISLSEKEAAI	SSLRKQYDEE	KCELLDQVQD	LSFKVDTLSK	EKISALEQVD	DWSNKFSEWK	KKAQSRFTQH
1450	1460	1470	1480	1490	1500	1510	1520
QNTVKELQIQ	LELKSKEAYE	KDEQINLLKE	ELDQQNKRFD	CLKGEMEDDK	SKMEKKESNL	ETELKSQTAR	IMELEDHITQ
1530	1540	1550	1560	1570	1580	1590	1600
KTIEIESLNE	VLKNYNQQKD	IEHKELVQKL	QHFQELGEEK	DNRVKEAEEK	ILTLENQVYS	MKAELETKKK	ELEHVNLSVK
1610	1620	1630	1640	1650	1660	1670	1680
SKEEELKALE	DRLESESAAK	LAELKRKAEQ	KIAAIKKQLL	SQMEEKEEQY	KKGTESHLSE	LNTKLQERER	EVHILEEKLK
1690	1700	1710	1720	1730	1740	1750	1760
SVESSQSETL	IVPRSAKNVA	AYTEQEEADS	QGCVQKTYEE	KISVLQRNLT	EKEKLLQRVG	QEKEETVSSH	FEMRCQYQER
1770	1780	1790	1800	1810	1820	1830	1840
LIKLEHAEAK	QHEDQSMIGH	LQEELEEKNK	KYSLIVAQHV	EKEGGKNNIQ	AKQNLENVFD	DVQKTLQEKE	LTCQILEQKI
1850	1860	1870	1880	1890	1900	1910	1920
KELDSCLVRQ	KEVHRVEMEE	LTSKYEKLQA	LQQMDGRNKP	TELLEENTEE	KSKSHLVQPK	LLSNMEAQHN	DLEFKLAGAE
1930	1940	1950	1960	1970	1980	1990	2000
REKQKLGKEI	VRLQKDLRML	RKEHQQELEI	LKKEYDQERE	EKIKQEQEDL	ELKHNSTLKQ	LMREFNTQLA	QKEQELEMTI
2010	2020	2030	2040	2050	2060	2070	2080
KETINKAQEV	EAELLESHQE	ETNQLLKKIA	EKDDDLKRTA	KRYEEILDAR	EEEMTAKVRD	LQTQLEELQK	KYQQKLEQEE
2090	2100	2110	2120	2130	2140	2150	2160
NPGNDNVTIM	ELQTQLAQKT	TLISDSKLKE	QEFREQIHNL	EDRLKKYEKN	VYATTVGTPY	KGGNLYHTDV	SLFGEPTEFE
2170	2180	2190	2200	2210	2220		

Detailed Protein Report

Cmpd.	No. of Cmpds.	m/z meas.	Δ m/z [ppm]	z	Rt [min]	Score	P	Range	Sequence	Modification
362	2	539.2298	-100.57	2	33.2	13.3	1	947-955	K.AKEMQETLK.K	
2557	1	715.3634	85.34	2	61.9	10.2	1	1479-1490	R.FDCLKGEMEDDK.S	
2790	2	683.3645	24.15	3	65.3	12.8	1	1813-1829	K.QNLENVFDDVQKTLQEK.E	
190	1	716.5084	147.04	2	32.1	12.0	2	1840-1851	K.IKELDSCLVRQK.E	
1522	1	620.4115	154.27	3	48.7	15.7	2	1850-1864	R.QKEVHRVEMEELTSK.Y	Oxidation: 9

Detailed Protein Report

Protein 65: **smoothelin isoform e [Homo sapiens]**

**Accession:**	gi|333360860	**Score:**	63.9
**Database:**	refseq_human(refseq_human_20140103.fasta)	**MW [kDa]:**	104.8
**Database Date:**	2015-11-30	**pI:**	9.9
**Modification(s):**	Carbamidomethyl, Oxidation	**Sequence Coverage [%]:**	3.0
		**No. of unique Peptides:**	1

10	20	30	40	50	60	70	80
MGGMGTPGDL	HAACPSTTRR	DALCLGPEFE	FDLRHSAGCP	GDLGFFPLDP	DTEAGGSGCL	PTWLPLRRLA	RGRLEVTADL
**90**	**100**	**110**	**120**	**130**	**140**	**150**	**160**
AERRRIRSAI	RELQRQELER	EEEALASKRF	RAERQDNKEN	WLHSQQREAE	QRAALARLAG	QLESMNDVEE	LTALLRSAGE
**170**	**180**	**190**	**200**	**210**	**220**	**230**	**240**
YEERKLIRAA	IRRVRAQEIE	AATLAGRLYS	GRPNSGSRED	SKGLAAHRLE	QCEVPEREEQ	EQQAEVSKPT	PTPEGTSQDV
**250**	**260**	**270**	**280**	**290**	**300**	**310**	**320**
TTVTLLLRAP	PGSTSSSPAS	PSSSPTPASP	EPPLEPAEAQ	CLTAEVPGSP	EPPPSPPKTT	SPEPQESPTL	PSTEGQVVNK
**330**	**340**	**350**	**360**	**370**	**380**	**390**	**400**
LLSGPKETPA	AQSPTRGPSD	TKRADVAGPR	PCQRSLSVLS	PRQPAQNRES	TPLASGPSSF	QRAGSVRDRV	HKFTSDSPMA
**410**	**420**	**430**	**440**	**450**	**460**	**470**	**480**
ARLQDGTPQA	ALSPLTPARL	LGPSLTSTTP	ASSSSGSSSR	GPSDTSSRFS	KEQRGVAQPL	AQLRSCPQEE	GPRGRGLAAR
**490**	**500**	**510**	**520**	**530**	**540**	**550**	**560**
PLENRAGGPV	ARSEEPGAPL	PVAVGTAEPG	GSMKTTFTIE	IKDGRGQAST	GRVLLPTGNQ	RAELTLGLRA	PPTLLSTSSG
**570**	**580**	**590**	**600**	**610**	**620**	**630**	**640**
GKSTITRVNS	PGTLARLGSV	THVTSFSHAP	PSSRGGCSIK	MEAEPAEPLA	AAVEAANGAE	QTRVNKAPEG	RSPLSAEELM
**650**	**660**	**670**	**680**	**690**	**700**	**710**	**720**
TIEDEGVLDK	MLDQSTDFEE	RKLIRAALRE	LRQRKRDQRD	KERERRLQEA	RGRPGEGRGN	TATETTTRHS	QRAADGSAVS
**730**	**740**	**750**	**760**	**770**	**780**	**790**	**800**
TVTKTERLVH	SNDGTRTART	TTVESSFVRR	SENGSGSTMM	QTKTFSSSSS	SKKMGSIFDR	EDQASPRAGS	LAALEKRQAE
**810**	**820**	**830**	**840**	**850**	**860**	**870**	**880**
KKKELMKAQS	LPKTSASQAR	KAMIEKLEKE	GAAGSPGGPR	AAVQRSTSFG	VPNANSIKQM	LLDWCRAKTR	GYEHVDIQNF
**890**	**900**	**910**	**920**	**930**	**940**	**950**	**960**
SSSWSDGMAF	CALVHNFFPE	AFDYGQLSPQ	NRRQNFEVAF	SSAETHADCP	QLLDTEDMVR	LREPDWKCVY	TYIQEFYRCL
**970**	**980**						
VQKGLVKTKK	S						

Cmpd.	No. of Cmpds.	m/z meas.	Δ m/z [ppm]	z	Rt [min]	Score	P	Range	Sequence	Modification
1663	1	1044.8761	-92.00	2	50.6	17.6	1	1-20	-.MGGMGTPGDLHAACPSTTRR.D	Carbamidomethyl: 14; Oxidation: 1

Detailed Protein Report

Protein 66: PREDICTED: 14-3-3 protein epsilon isoform X1 [Homo sapiens]

**Accession:**	gi|530410617	**Score:**	63.4
**Database:**	refseq_human(refseq_human_20140103.fasta)	**MW [kDa]:**	27.4
**Database Date:**	2015-11-30	**pI:**	4.6
**Modification(s):**	Oxidation	**Sequence Coverage [%]:**	11.3
		**No. of unique Peptides:**	1

Alias proteins:

Accession	Name	Description
gi|578840292	refseq_human (refseq_human_20140103.fasta)	PREDICTED: 14-3-3 protein epsilon isoform X1 [Homo sapiens]

```
        10         20         30         40         50         60         70         80
MDDREDLVYQ AKLAEQAERY DEMVESMKKV AGMDVELTVE ERNLLSVAYK NVIGARRASW RIISSIEQKE ENKGGEDKLK
        90        100        110        120        130        140        150        160
MIREYRQMVE TELKLICCDI LDVLDKHLIP AANTGESKVF YYKMKGDYHR YLAEFATGND RKEAAENSLV AYKAASDIAM
       170        180        190        200        210        220        230        240
TELPPTHPIR LGLALNFSVF YYEILNSPDR ACRLAKAAFD DAIAELDTLS EESYKDSTLI MQLLRDNLTL WTSDMQGDDS
       250
```

Cmpd.	No. of Cmpds.	m/z meas.	Δ m/z [ppm]	z	Rt [min]	Score	P	Range	Sequence	Modification
2589	1	1045.0814	94.73	2	62.4	16.1	2	13-29	K.LAEQAERYDEMVESMKK.V	Oxidation: 11, 15

Detailed Protein Report

Protein 67: PREDICTED: dynein heavy chain 10, axonemal isoform X1 [Homo sapiens]

**Accession:**	gi|530401632	**Score:**	63.2
**Database:**	refseq_human(refseq_human_20140103.fasta)	**MW [kDa]:**	528.4
**Database Date:**	2015-11-30	**pI:**	5.5
**Modification(s):**	Oxidation	**Sequence Coverage [%]:**	1.3
		**No. of unique Peptides:**	4

Detailed Protein Report

10	20	30	40	50	60	70	80
MDDLRVLWMR	DRVYAAFGIT	DPQLFEDLLN	RDDGQGEDLI	LHFLNQASEE	EGPSALFIYR	TMVPEEVEVE	IDEIPVLSEE
90	100	110	120	130	140	150	160
GEEEEETYSQ	KVESVDKVRA	KRVSLRTESL	GQPLNREDEE	MDKEISEKLP	SKRTAKHIME	KMHLHMLCTP	LPEEFLDQNV
170	180	190	200	210	220	230	240
VFFLRNTKEA	ISEATDMKEA	MEIMPETLEY	GIINANVLHF	LKNIICQVFL	PALSFNQHRT	STTVGVTSGE	VSNSSEHESD
250	260	270	280	290	300	310	320
LPPMPGEAVE	YHSIQLIRDE	FLMNVQKFAS	NIQRTMQQLE	GEIKLEMPII	SVEGEVSDLA	ADPETVDILE	QCVINWLNQI
330	340	350	360	370	380	390	400
STAVEAQLKK	TPQGKGPLAE	IEFWRERNAT	LSALHEQTKL	PIVRKVLDVI	KESDSMLVAN	LQPVFTELFK	FHTEASDNVR
410	420	430	440	450	460	470	480
FLSTVERYFK	NITHGSGFHV	VLDTIPAMMS	ALRMVWIISR	HYNKDERMIP	LMERIAWEIA	ERVCRVVNLR	TLFKENRASA
490	500	510	520	530	540	550	560
QSKTLEARNT	LRLWKKAYFD	TRAKIEASGR	EDRWEFDRKR	LFERTDYMAT	ICQDLSDVLQ	ILEEFYNIFG	PELKAVTGDP
570	580	590	600	610	620	630	640
KRIDDVLCRV	DGLVTPMENL	TFDPFSIKSS	QFWKYVMDEF	KIEVLVIEKE	AKHFIDESFK	TLRSAEAAFD	MLLKFKHIRS
650	660	670	680	690	700	710	720
REAVNRQMMM	KFNDILAQYC	KEIDIINKIF	VQNLENPPLY	KNHPPVAGAI	YWERSLFFRI	KHTILRFQEV	QEILDSDRGQ
730	740	750	760	770	780	790	800
EVKQKYLEVG	RTMKEYEDRK	YEQWMEVTEQ	VLPALMKKSL	LTKSSIATEE	PSTLERGAVF	AINFSPALRE	IINETKYLEQ
810	820	830	840	850	860	870	880
LGFTVPELAR	NVALQEDKFL	RYTAGIQRML	DHYHMLIGTL	NDAESVLLKD	HSQELLRVFR	SGYKRLNWNS	LGIGDYITGC
890	900	910	920	930	940	950	960
KQAIGKFESL	VHQIHKNADD	ISSRLTLIEA	INLFKYPAAK	SEEELPGVKE	FFEHIERERA	SDVDHMVRWY	LAIGPLLTKV
970	980	990	1000	1010	1020	1030	1040
EGLVVHTNTG	KAPKLASYYK	YWEKKIYEVL	TKLILKNLQS	FNSLILGNVP	LFHTETILTA	PEIILHPNTN	EIDKMCFHCV
1050	1060	1070	1080	1090	1100	1110	1120
RNCVEITKHF	VRWMNGSCIE	CPPQKGEEEE	VVIINFYNDI	SLNPQIIEQA	VMIPQNVHRI	LINLMKYLQK	WKRYRPLWKL
1130	1140	1150	1160	1170	1180	1190	1200
DKAIVMEKFA	AKKPPCVAYD	EKLQFYSKIA	YEVMRHPLIK	DEHCIRLQLR	HLANTVQENA	KSWVISLGKL	LNESAKEELY
1210	1220	1230	1240	1250	1260	1270	1280
NLHEEMEHLA	KNLRKIPNTL	EDLKFVLATI	AEIRSKSLVM	ELRYRDVQER	YRTMAMYNLF	PPDAEKELVD	KIESIWSNLF
1290	1300	1310	1320	1330	1340	1350	1360
NDSVNVEHAL	GDIKRTFTEL	TRGEIMNYRV	QIEEFAKRFY	SEGPGSVGDD	LDKGVELLGV	YERELARHEK	SRQELANAEK
1370	1380	1390	1400	1410	1420	1430	1440
LFDLPITMYP	ELLKVQKEMS	GLRMIYELYE	GLKVAKEEWS	QTLWINLNVQ	ILQEGIEGFL	RALRKLPRPV	RGLSVTYYLE
1450	1460	1470	1480	1490	1500	1510	1520
AKMKAFKDSI	PLLLDLKNEA	LRDRHWKELM	EKTSVFFEMT	ETFTLENMFA	MELHKHTDVL	NEIVTAAIKE	VAIEKAVKEI
1530	1540	1550	1560	1570	1580	1590	1600
LDTWENMKFT	VVKYCKGTQE	RGYILGSVDE	IIQSLDDNTF	NLQSISGSRF	VGPFLQTVHK	WEKTLSLIGE	VIEIWMLVQR
1610	1620	1630	1640	1650	1660	1670	1680
KWMYLESIFI	GGDIRSQLPE	EAKKFDNIDK	VFKRIMGETL	KDPVIKRCCE	APNRLSDLQN	VSEGLEKCQK	SLNDYLDSKR
1690	1700	1710	1720	1730	1740	1750	1760
NAFPRFFFIS	DDELLSILGS	SDPLCVQEHM	IKMYDNIASL	RFNDGDSGEK	LVSAMISAEG	EVMEFRKILR	AEGRVEDWMT
1770	1780	1790	1800	1810	1820	1830	1840
AVLNEMRRTN	RLITKEAIFR	YCEDRSRVDW	MLLYQGMVVL	AASQVWWTWE	VEDVFHKAQK	GEKQAMKNYG	RKMHRQIDEL
1850	1860	1870	1880	1890	1900	1910	1920
VTRITMPLSK	NDRKKYNTVL	IIDVHARDIV	DSFIRGSILE	AREFDWESQL	RFYWDREPDE	LNIRQCTGTF	GYGYEYMGLN
1930	1940	1950	1960	1970	1980	1990	2000
GRLVITPLTD	RIYLTLTQAL	SMYLGGAPAG	PAGTGKTETT	KDLAKALGLL	CVVTNCGEGM	DYRAVGKIFS	GLAQCGAWGC
2010	2020	2030	2040	2050	2060	2070	2080
FDEFNRIDAS	VLSVISSQIQ	TIRNALIHQL	TTFQFEGQEI	SLDSRMGIFI	TMNPGYAGRT	ELPESVKALF	RPVVVIVPDL
2090	2100	2110	2120	2130	2140	2150	2160
QQICEIMLFS	EGFLEAKTLA	KKMTVLYKLA	REQLSKQYHY	DFGLRALKSV	LVMAGELKRG	SSDLREDVVL	MRALRDMNLP
2170	2180	2190	2200	2210	2220	2230	2240

Detailed Protein Report

Cmpd.	No. of Cmpds.	m/z meas.	Δ m/z [ppm]	z	Rt [min]	Score	P	Range	Sequence	Modification
1643	1	938.9588	-53.24	2	50.3	11.9	2	624-639	R.SAEAAFDMLLKFKHIR.S	
2459	1	1045.2266	-235.83	1	61.2	14.2	0	940-948	R.ASDVDHMVR.W	Oxidation: 7
2662	1	835.4217	30.04	2	61.6	11.7	1	2634-2646	K.RLLVFMDDMNMPR.V	Oxidation: 6, 9
1556	1	502.7809	-7.71	2	49.7	11.3	1	3742-3750	K.ATEVSEKLK.L	

Detailed Protein Report

Protein 68: 14-3-3 protein sigma [Homo sapiens]

Accession: gi|5454052
Database: refseq_human(refseq_human_20140103.fasta)
Database Date: 2015-11-30

Score: 62.7
MW [kDa]: 27.8
pI: 4.5
Sequence Coverage [%]: 11.3
No. of unique Peptides: 1

10	20	30	40	50	60	70	80
MERASLIQKA	KLAEQAERYE	DMAAFMKGAV	EKGEELSCEE	RNLLSVAYKN	VVGGQRAAWR	VLSSIEQKSN	EEGSEEKGPE
**90**	**100**	**110**	**120**	**130**	**140**	**150**	**160**
VREYREKVET	ELQGVCDTVL	GLLDSHLIKE	AGDAESRVFY	LKMKGDYYRY	LAEVATGDDK	KRIIDSARSA	YQEAMDISKK
**170**	**180**	**190**	**200**	**210**	**220**	**230**	**240**
EMPPTNPIRL	GLALNFSVFH	YEIANSPEEA	ISLAKTTFDE	AMADLHTLSE	DSYKDSTLIM	QLLRDNLTLW	TADNAGEEGG
**250**							
EAPQEPQS							

Cmpd.	No. of Cmpds.	m/z meas.	Δ m/z [ppm]	z	Rt [min]	Score	P	Range	Sequence	Modification
2035	3	1051.3758	-123.72	2	55.8	15.4	2	10-27	K.AKLAEQAERYEDMAAFMK.G	

Detailed Protein Report

Protein 69: dynein heavy chain 2, axonemal [Homo sapiens]

**Accession:**	gi|75677365	**Score:**	62.4
**Database:**	refseq_human(refseq_human_20140103.fasta)	**MW [kDa]:**	507.4
**Database Date:**	2015-11-30	**pI:**	5.9
**Modification(s):**	Carbamidomethyl, Oxidation	**Sequence Coverage [%]:**	1.4
		**No. of unique Peptides:**	5

Alias proteins:

Accession	Name	Description
gi|530409951	refseq_human (refseq_human_20140103.fasta)	PREDICTED: dynein heavy chain 2, axonemal isoform X1 [Homo sapiens]

Detailed Protein Report

10	20	30	40	50	60	70	80
MSSKAEKKQR	LSGRGSSQAS	WSGRATRAAV	ATQEQGNAPA	VSEPELQAEL	PKEEPEPRLE	GPQAQSEESV	EPEADVKPLF
90	100	110	120	130	140	150	160
LSRAALTGLA	DAVWTQEHDA	ILEHFAQDPT	ESILTIFIDP	CFGLKLELGM	PVQTQNQLVY	FIRQAPVPIT	WENFEATVQF
170	180	190	200	210	220	230	240
GTVRGPYIPA	LLRLLGGVFA	PQIFANTGWP	ESIRNHFASH	LHKFLACLTD	TRYKLEGHTV	LYIPAEAMNM	KPEMVIKDKE
250	260	270	280	290	300	310	320
LVQRLETSMI	HWTRQIKEML	SAQETVETGE	NLGPLEEIEF	WRNRCMDLSG	ISKQLVKKGV	KHVESILHLA	KSSYLAPFMK
330	340	350	360	370	380	390	400
LAQQIQDGSR	QAQSNLTFLS	ILKEPYQELA	FMKPKDISSK	LPKLISLIRI	IWVNSPHYNT	RERLTSLFRK	VCDCQYHFAR
410	420	430	440	450	460	470	480
WEDGKQGPLP	CFFGAQGPQI	TRNLLEIEDI	FHKNLHTLRA	VRGGILDVKN	TCWHEDYNKF	RAGIKDLEVM	TQNLITSAFE
490	500	510	520	530	540	550	560
LVRDVPHGVL	LLDTFHRLAS	REAIKRTYDK	KAVDLYMLFN	SELALVNRER	NKKWPDLEPY	VAQYSGKARW	VHILRRRIDR
570	580	590	600	610	620	630	640
VMTCLAGAHF	LPRIGTGKES	VHTYQQMVQA	IDELVRKTFQ	EWTSSLDKDC	IRRLDTPLLR	ISQEKAGMLD	VNFDKSLLIL
650	660	670	680	690	700	710	720
FAEIDYWERL	LFETPHYVVN	VAERAEDLRI	LRENLLLVAR	DYNRIIAMLS	PDEQALFKER	IRLLDKKIHP	GLKKLHWALK
730	740	750	760	770	780	790	800
GASAFFITEC	RIHASKVQMI	VNEFKASTLT	IGWRAQEMSE	KLLVRISGKR	VYRDLEFEED	QREHRAAVQQ	KLMNLHQDVV
810	820	830	840	850	860	870	880
TIMTNSYEVF	KNDGPEIQQQ	WMLYMIRLDR	MMEDALRLNV	KWSLLELSKA	INGDGKTSPN	PLFQVLVILK	NDLQGSVAQV
890	900	910	920	930	940	950	960
EFSPTLQTLA	GVVNDIGNHL	FSTISVFCHL	PDILTKRKLH	REPIQTVVEQ	DEDIKKIQTQ	ISSGMTNNAS	LLQNYLKTWD
970	980	990	1000	1010	1020	1030	1040
MYREIWEINK	DSFIHRYQRL	NPPVSSFVAD	IARYTEVANN	VQKEETVTNI	QFVLLDCSHL	KFSLVQHCNE	WQNKFATLLR
1050	1060	1070	1080	1090	1100	1110	1120
EMAAGRLLEL	HTYLKENAEK	ISRPPQTLEE	LGVSLQLVDA	LKHDLANVET	QIPPIHEQFA	ILEKYEVPVE	DSVLEMLDSL
1130	1140	1150	1160	1170	1180	1190	1200
NGEWVVFQQT	LLDSKQMLKK	HKEKFKTGLI	HSADDFKKKA	HTLLEDFEFK	GHFTSNVGYM	SALDQITQVR	AMLMAMREEE
1210	1220	1230	1240	1250	1260	1270	1280
NSLRANLGIF	KIEQPPSKDL	QNLEKELDAL	QQIWEIARDW	EENWNEWKTG	RFLILQTETM	ETTAHGLFRR	LTKLAKEYKD
1290	1300	1310	1320	1330	1340	1350	1360
RNWEIIETTR	SKIEQFKRTM	PLISDLRNPA	LRERHWDQVR	DEIQREFDQE	SESFTLEQIV	ELGMDQHVEK	IGEISASATK
1370	1380	1390	1400	1410	1420	1430	1440
ELAIEVALQN	IAKTWDVTQL	DIVPYKDKGH	HRLRGTEEVF	QALEDNQVAL	STMKASRFVK	AFEKDVDHWE	RCLSLILEVI
1450	1460	1470	1480	1490	1500	1510	1520
EMILTVQRQW	MYLENIFLGE	DIRKQLPNES	TLFDQVNSNW	KAIMDRMNKD	NNALRSTHHP	GLLDTLIEMN	TILEDIQKSL
1530	1540	1550	1560	1570	1580	1590	1600
DMYLETKRHI	FPRFYFLSND	DLLEILGQSR	NPEAVQPHLK	KCFDNIKLLR	IQKVGGPSSK	WEAVGMFSGD	GEYIDFLHSV
1610	1620	1630	1640	1650	1660	1670	1680
FLEGPVESWL	GDVEQTMRVT	LRDLLRNCHL	ALRKFLNKRD	KWVKEWAGQV	VITASQIQWT	ADVTKCLLTA	KERADKKILK
1690	1700	1710	1720	1730	1740	1750	1760
VMKKNQVSIL	NKYSEAIRGN	LTKIMRLKIV	ALVTIEIHAR	DVLEKLYKSG	LMDVNSFDWL	SQLRFYWEKD	LDDCVIRQTN
1770	1780	1790	1800	1810	1820	1830	1840
TQFQYNYEYL	GNSGRLVITP	LTDRCYMTLT	TALHLHRGGS	PKGPAGTGKT	ETVKDLGKAL	GIYVIVVNCS	EGLDYKSMGR
1850	1860	1870	1880	1890	1900	1910	1920
MYSGLAQTGA	WGCFDEFNRI	NIEVLSVVAH	QILCILSALA	AGLTHFHFDG	FEINLVWSCG	IFITMNPGYA	GRTELPENLK
1930	1940	1950	1960	1970	1980	1990	2000
SMFRPIAMVV	PDSTLIAEII	LFGEGFGNCK	ILAKKVYTLY	SLAVQQLSRQ	DHYDFGLRAL	TSLLRYAGKK	RRLQPDLTDE
2010	2020	2030	2040	2050	2060	2070	2080
EVLLLSMRDM	NIAKLTSVDA	PLFNAIVQDL	FPNIELPVID	YGKLRETVEQ	EIRDMGLQST	PFTLTKVFQL	YETKNSRHST
2090	2100	2110	2120	2130	2140	2150	2160
MIVGCTGSGK	TASWRILQAS	LSSLCRAGDP	NFNIVREFPL	NPKALSLGEL	YGEYDLSTNE	WTDGILSSVM	RTACADEKPD
2170	2180	2190	2200	2210	2220	2230	2240

Detailed Protein Report

Cmpd.	No. of Cmpds.	m/z meas.	Δ m/z [ppm]	z	Rt [min]	Score	P	Range	Sequence	Modification
271	1	701.3595	-80.46	2	33.1	11.1	2	1681-1692	K.VMKKNQVSILNK.Y	
2974	1	826.4375	-16.40	2	65.9	10.8	2	1693-1706	K.YSEAIRGNLTKIMR.L	
1377	1	976.4425	-17.17	2	46.8	10.3	1	2078-2095	R.HSTMIVGCTGSGKTASWR.I	Carbamidomethyl: 8; Oxidation: 4
2410	1	1023.8441	-100.67	2	60.0	14.9	2	4373-4389	K.KSAKGMYSCPCYYYPNR.A	Oxidation: 6
2695	1	1044.5484	84.19	2	63.9	15.3	2	4373-4389	K.KSAKGMYSCPCYYYPNR.A	Carbamidomethyl: 9

Detailed Protein Report

Protein 70: PREDICTED: nebulin isoform X22 [Homo sapiens]

**Accession:**	gi|530370465	**Score:**	62.1
**Database:**	refseq_human(refseq_human_20140103.fasta)	**MW [kDa]:**	961.5
**Database Date:**	2015-11-30	**pI:**	9.5
**Modification(s):**	Oxidation	**Sequence Coverage [%]:**	0.8
		**No. of unique Peptides:**	3

Detailed Protein Report

10	20	30	40	50	60	70	80
MADDEDYEEV	VEYYTEEVVY	EEVPGETITK	IYETTTTRTS	DYEQSETSKP	ALAQPALAQP	ASAKPVERRK	VIRKKVDPSK
90	100	110	120	130	140	150	160
FMTPYIAHSQ	KMQDLFSPNK	YKEKFEKTKG	QPYASTTDTP	ELRRIKKVQD	QLSEVKYRMD	GDVAKTICHV	DEKAKDIEHA
170	180	190	200	210	220	230	240
KKVSQQVSKV	LYKQNWEDTK	DKYLLPPDAP	ELVQAVKNTA	MFSKKLYTED	WEADKSLFYP	YNDSPELRRV	AQAQKALSDV
250	260	270	280	290	300	310	320
AYKKGLAEQQ	AQFTPLADPP	DIEFAKKVTN	QVSKQKYKED	YENKIKGKWS	ETPCFEVANA	RMNADNISTR	KYQEDFENMK
330	340	350	360	370	380	390	400
DQIYFMQTET	PEYKMNKKAG	VAASKVKYKE	DYEKNKGKAD	YNVLPASENP	QLRQLKAAGD	ALSDKLYKEN	YEKTKAKSIN
410	420	430	440	450	460	470	480
YCETPKFKLD	TVLQNFSSDK	KYKDSYLKDI	LGHYVGSFED	PYHSHCMKVT	AQNSDKNYKA	EYEEDRGKGF	FPQTITQEYE
490	500	510	520	530	540	550	560
AIKKLDQCKD	HTYKVHPDKT	KFTQVTDSPV	LLQAQVNSKQ	LSDLNYKAKH	ESEKFKCHIP	PDTPAFIQHK	VNAYNLSDNL
570	580	590	600	610	620	630	640
YKQDWEKSKA	KKFDIKVDAI	PLLAAKANTK	NTSDVMYKKD	YEKNKGKMIG	VLSINDDPKM	LHSLKVAKNQ	SDRLYKENYE
650	660	670	680	690	700	710	720
KTKAKSMNYC	ETPKYQLDTQ	LKNFSEARYK	DLYVKDVLGH	YVGSMEDPYH	THCMKVAAQN	SDKSYKAEYE	EDKGKCYFPQ
730	740	750	760	770	780	790	800
TITQEYEAIK	KLDQCKDHTY	KVHPDKTKFT	AVTDSPVLLQ	AQLNTKQLSD	LNYKAKHEGE	KFKCHIPADA	PQFIQHRVNA
810	820	830	840	850	860	870	880
YNLSDNVYKQ	DWEKSKAKKF	DIKVDAIPLL	AAKANTKNTS	DVMYKKDYEK	SKGKMIGALS	INDDPKMLHS	LKTAKNQSDR
890	900	910	920	930	940	950	960
EYRKDYEKSK	TIYTAPLDML	QVTQAKKSQA	IASDVDYKHI	LHSYSYPPDS	INVDLAKKAY	ALQSDVEYKA	DYNSWMKGCG
970	980	990	1000	1010	1020	1030	1040
WVPFGSLEME	KAKRASDILN	EKKYRQHPDT	LKFTSIEDAP	ITVQSKINQA	QRSDIAYKAK	GEEIIHKYNL	PPDLPQFIQA
1050	1060	1070	1080	1090	1100	1110	1120
KVNAYNISEN	MYKADLKDLS	KKGYDLRTDA	IPIRAAKAAR	QAASDVQYKK	DYEKAKGKMV	GFQSLQDDPK	LVHYMNVAKI
1130	1140	1150	1160	1170	1180	1190	1200
QSDREYKKDY	EKTKSKYNTP	HDMFNVVAAK	KAQDVVSNVN	YKHSLHHYTY	LPDAMDLELS	KNMMQIQSDN	VYKEDYNNWM
1210	1220	1230	1240	1250	1260	1270	1280
KGIGWIPIGS	LDVEKVKKAG	DALNEKKYRQ	HPDTLKFTSI	VDSPVMVQAK	QNTKQVSDIL	YKAKGEDVKH	KYTMSPDLPQ
1290	1300	1310	1320	1330	1340	1350	1360
FLQAKCNAYN	ISDVCYKRDW	YDLIAKGNNV	LGDAIPITAA	KASRNIASDY	KYKEAYEKSK	GKHVGFRSLQ	DDPKLVHYMN
1370	1380	1390	1400	1410	1420	1430	1440
VAKLQSDREY	KKNYENTKTS	YHTPGDMVSI	TAAKMAQDVA	TNVNYKQPLH	HYTYLPDAMS	LEHTRNVNQI	QSDNVYKDEY
1450	1460	1470	1480	1490	1500	1510	1520
NSFLKGIGWI	PIGSLEVEKV	KKAGDALNER	KYRQHPDTVK	FTSVPDSMGM	VLAQHNTKQL	SDLNYKVEGE	KLKHKYTIDP
1530	1540	1550	1560	1570	1580	1590	1600
ELPQFIQAKV	NALNMSDAHY	KADWKKTIAK	GYDLRPDAIP	IVAAKSSRNI	ASDCKYKEAY	EKAKGKQVGF	LSLQDDPKLV
1610	1620	1630	1640	1650	1660	1670	1680
HYMNVAKIQS	DREYKKGYEA	SKTKYHTPLD	MVSVTAAKKS	QEVATNANYR	QSYHHYTLLP	DALNVEHSRN	AMQIQSDNLY
1690	1700	1710	1720	1730	1740	1750	1760
KSDFTNWMKG	IGWVPIESLE	VEKAKKAGEI	LSEKKYRQHP	EKLKFTYAMD	TMEQALNKSN	KLNMDKRLYT	EKWNKDKTTI
1770	1780	1790	1800	1810	1820	1830	1840
HVMPDTPDIL	LSRVNQITMS	DKLYKAGWEE	EKKKGYDLRP	DAIAIKAARA	SRDIASDYKY	KKAYEQAKGK	HIGFRSLEDD
1850	1860	1870	1880	1890	1900	1910	1920
PKLVHFMQVA	KMQSDREYKK	GYEKSKTSFH	TPVDMLSVVA	AKKSQEVATN	ANYRNVIHTY	NMLPDAMSFE	LAKNMMQIQS
1930	1940	1950	1960	1970	1980	1990	2000
DNQYKADYAD	FMKGIGWLPL	GSLEAEKNKK	AMEIISEKKY	RQHPDTLKYS	TLMDSMNMVL	AQNNAKIMNE	HLYKQAWEAD
2010	2020	2030	2040	2050	2060	2070	2080
KTKVHIMPDI	PQIILAKANA	INMSDKLYKL	SLEESKKKGY	DLRPDAIPIK	AAKASRDIAS	DYKYKYNYEK	GKGKMVGFRS
2090	2100	2110	2120	2130	2140	2150	2160
LEDDPKLVHS	MQVAKMQSDR	EYKKNYENTK	TSYHTPADML	SVTAAKDAQA	NITNTNYKHL	IHKYILLPDA	MNIELTRNMN
2170	2180	2190	2200	2210	2220	2230	2240

Detailed Protein Report

Cmpd.	No. of Cmpds.	m/z meas.	Δ m/z [ppm]	z	Rt [min]	Score	P	Range	Sequence	Modification
457	2	544.7651	77.28	2	35.3	13.1	0	646-654	K.SMNYCETPK.Y	Oxidation: 2
2640	1	701.8709	47.97	2	63.1	10.8	0	6260-6270	R.CQYILSDLEYR.H	
2657	1	994.4910	10.30	2	63.3	10.6	1	8000-8015	K.LNQENFSSVLYKENMR.K	Oxidation: 15

Detailed Protein Report

Protein 71: small proline-rich protein 2A [Homo sapiens]

Accession: gi|5174693
Database: refseq_human(refseq_human_20140103.fasta)
Database Date: 2015-11-30
Modification(s): Carbamidomethyl

Score: 62.0
MW [kDa]: 8.0
pI: 10.3
Sequence Coverage [%]: 36.1
No. of unique Peptides: 3

Quantitation

QU:MU	**Median:**	1.33	**CV:**	0.00 %	**No. of Peptides:**	1
WUP:QUP	**Median:**	1.12	**CV:**	29.24 %	**No. of Peptides:**	2

```
        10         20         30         40         50         60         70         80
MSYQQQQCKQ PCQPPPVCPT PKCPEPCPPP KCPEPCPPPK CPQPCPPQQC QQKYPPVTPS PPCQSKYPPK SK
```

Cmpd.	No. of Cmpds.	m/z meas.	Δ m/z [ppm]	z	Rt [min]	Score	P	Range	Sequence	Modification	Ratios
2928	1	724.8814	36.56	2	65.4	11.5	0	10-22	K.QPCQPPPVCPTPK.C	Carbamidomethyl: 3	
88	3	753.3275	-50.62	2	30.8	36.7	0	10-22	K.QPCQPPPVCPTPK.C	Carbamidomethyl: 3, 9	WUP:QUP 1.49<br>QU:MU 1.33
171	1	729.5012	195.91	2	31.9	13.8	0	54-66	K.YPPVTPSPPCQSK.Y	Carbamidomethyl: 10	WUP:QUP 0.84

Detailed Protein Report

Protein 72: **PREDICTED: UDP-glucose:glycoprotein glucosyltransferase 1 isoform X1 [Homo sapiens]**

**Accession:**	gi|578804601	**Score:**	60.9
**Database:**	refseq_human(refseq_human_20140103.fasta)	**MW [kDa]:**	176.5
**Database Date:**	2015-11-30	**pI:**	5.3
**Modification(s):**	Carbamidomethyl, Oxidation	**Sequence Coverage [%]:**	5.9
		**No. of unique Peptides:**	5

10	20	30	40	50	60	70	80
MGCKGDASGA	CAAGVCYKMG	VLVVLTVLWL	FSSVKADSKA	ITTSLTTKWF	STPLLLEASE	FLAEDSQEKF	WNFVEASQNI
90	100	110	120	130	140	150	160
GSSDHDGTDY	SYYHAILEAA	FQFLSPLQQN	LFKFCLSLRS	YSATIQAFQQ	IAADEPPPEG	CNSFFSVHGK	KTCESDTLEA
170	180	190	200	210	220	230	240
LLLTASERPK	PLLFKGDHRY	PSSNPESPVV	IFYSEIGSEE	FSNFHRQLIS	KSNAGKINYV	FRHYIFNPRK	EPVYLSGYGV
250	260	270	280	290	300	310	320
ELAIKSTEYK	AKDDTQVKGT	EVNTTVIGEN	DPIDEVQGFL	FGKLRDLHPD	LEGQLKELRK	HLVESTNEMA	PLKVWQLQDL
330	340	350	360	370	380	390	400
SFQTAARILA	SPVELALVVM	KDLSQNFPTK	ARAITKTAVS	SELRTEVEEN	QKYFKGTLGL	QPGDSALFIN	GLHMDLDTQD
410	420	430	440	450	460	470	480
IFSLFDVLRN	EARVMEGLHR	LGIEGLSLHN	VLKLNIQPSE	ADYAVDIRSP	AISWVNNLEV	DSRYNSWPSS	LQELLRPTFP
490	500	510	520	530	540	550	560
GVIRQIRKNL	HNMVFIVDPA	HETTAELMNT	AEMFLSNHIP	LRIGFIFVVN	DSEDVDGMQD	AGVAVLRAYN	YVAQEVDDYH
570	580	590	600	610	620	630	640
AFQTLTHIYN	KVRTGEKVKV	EHVVSVLEKK	YPYVEVNSIL	GIDSAYDRNR	KEARGYYEQT	GVGPLPVVLF	NGMPFEREQL
650	660	670	680	690	700	710	720
DPDELETITM	HKILETTTFF	QRAVYLGELP	HDQDVVEYIM	NQPNVVPRIN	SRILTAERDY	LDLTASNNFF	VDDYARFTIL
730	740	750	760	770	780	790	800
DSQGKTAAVA	NSMNYLTKKG	MSSKEIYDDS	FIRPVTFWIV	GDFDSPSGRQ	LLYDAIKHQK	SSNNVRISMI	NNPAKEISYE
810	820	830	840	850	860	870	880
NTQISRAIWA	ALQTQTSNAA	KNFITKMAKE	GAAEALAAGA	DIAEFSVGGM	DFSLFKEVFE	SSKMDFILSH	AVYCRDVLKL
890	900	910	920	930	940	950	960
KKGQRAVISN	GRIIGPLEDS	ELFNQDDFHL	LENIILKTSG	QKIKSHIQQL	RVEEDVASDL	VMKVDALLSA	QPKGDPRIEY
970	980	990	1000	1010	1020	1030	1040
QFFEDRHSAI	KLRPKEGETY	FDVVAVVDPV	TREAQRLAPL	LLVLAQLINM	NLRVFMNCQS	KLSDMPLKSF	YRYVLEPEIS
1050	1060	1070	1080	1090	1100	1110	1120
FTSDNSFAKG	PIAKFLDMPQ	SPLFTLNLNT	PESWMVESVR	TPYDLDNIYL	EEVDSVVAAE	YELEYLLLEG	HCYDITTGQP
1130	1140	1150	1160	1170	1180	1190	1200
PRGLQFTLGT	SANPVIVDTI	VMANLGYFQL	KANPGAWILR	LRKGRSEDIY	RIYSHDGTDS	PPDADEVVIV	LNNFKSKIIK
1210	1220	1230	1240	1250	1260	1270	1280
VKVQKKADMV	NEDLLSDGTS	ENESGFWDSF	KWGFTGQKTE	EVKQDKDDII	NIFSVASGHL	YERFLRIMML	SVLKNTKTPV
1290	1300	1310	1320	1330	1340	1350	1360
KFWFLKNYLS	PTFKEFIPYM	ANEYNFQYEL	VQYKWPRWLH	QQTEKQRIIW	GYKILFLDVL	FPLVVDKFLF	VDADQIVRTD
1370	1380	1390	1400	1410	1420	1430	1440
LKELRDFNLD	GAPYGYTPFC	DSRREMDGYR	FWKSGYWASH	LAGRKYHISA	LYVVDLKKFR	KIAAGDRLRG	QYQGLSQDPN
1450	1460	1470	1480	1490	1500	1510	1520
SLSNLDQDLP	NNMIHQVPIK	SLPQEWLWCE	TWCDDASKKR	AKTIDLCNNP	MTKEPKLEAA	VRIVPEWQDY	DQEIKQLQIR
1530	1540	1550					
FQKEKETGAL	YKEKTKEPSR	EGPQKREEL					

Cmpd.	No. of Cmpds.	m/z meas.	Δ m/z [ppm]	z	Rt [min]	Score	P	Range	Sequence	Modification
1283	1	882.7587	-117.26	2	45.6	10.6	1	1-18	-.MGCKGDASGACAAGVCYK.M	Carbamidomethyl: 11; Oxidation: 1
1565	1	1018.8365	-18.21	3	49.3	12.6	2	611-637	R.KEARGYYEQTGVGPLPVVLFNGMPFER.E	
1888	1	600.2525	-61.03	3	53.5	13.2	0	638-652	R.EQLDPDELETITMHK.I	
2009	1	599.9210	-67.30	3	53.5	11.7	1	1014-1028	R.VFMNCQSKLSDMPLK.S	Carbamidomethyl: 5

Detailed Protein Report

Cmpd.	No. of Cmpds.	m/z meas.	Δ m/z [ppm]	z	Rt [min]	Score	P	Range	Sequence	Modification
2056	1	973.8963	-78.49	2	54.1	12.8	0	1033-1049	R.YVLEPEISFTSDNSFAK.G	

Detailed Protein Report

Protein 73: **PREDICTED: biorientation of chromosomes in cell division protein 1-like 1 isoform X1 [Homo sapiens]**

**Accession:**	gi|530376284	**Score:**	60.5
**Database:**	refseq_human(refseq_human_20140103.fasta)	**MW [kDa]:**	335.3
**Database Date:**	2015-11-30	**pI:**	4.9
**Modification(s):**	Oxidation	**Sequence Coverage [%]:**	1.4
		**No. of unique Peptides:**	2

Quantitation

QU:MU	**Median:**	0.83	**CV:**	0.00 %	**No. of Peptides:**	1
WUP:QUP	**Median:**	1.83	**CV:**	0.00 %	**No. of Peptides:**	1

10	20	30	40	50	60	70	80
MATNPQPQPP	PPAPPPPPPQ	PQPQPPPPPP	GPGAGPGAGG	AGGAGAGAGD	PQLVAMIVNH	LKSQGLFDQF	RRDCLADVDT
90	100	110	120	130	140	150	160
KPAYQNLRQR	VDNFVANHLA	THTWSPHLNK	NQLRNNIRQQ	VLKSGMLESG	IDRIISQVVD	PKINHTFRPQ	VEKAVHEFLA
170	180	190	200	210	220	230	240
TLNHKEEGSG	NTAPDDEKPD	TSLITQGVPT	PGPSANVAND	AMSILETITS	LNQEASAARA	STETSNAKTS	ERASKKLPSQ
250	260	270	280	290	300	310	320
PTTDTSTDKE	RTSEDMADKE	KSTADSGGEG	LETAPKSEEF	SDLPCPVEEI	KNYTKEHNNL	ILLNKDVQQE	SSEQKNKSTD
330	340	350	360	370	380	390	400
KGEKKPDSNE	KGERKKEKKE	KTEKKFDHSK	KSEDTQKVKD	EKQAKEKEVE	SLKLPSEKNS	NKAKTVEGTK	EDFSLIDSDV
410	420	430	440	450	460	470	480
DGLTDITVSS	VHTSDLSSFE	EDTEEEVVTS	DSMEEGEITS	DDEEKNKQNK	TKTQTSDSSE	GKTKSVRHAY	VHKPYLYSKY
490	500	510	520	530	540	550	560
YSDSDDELTV	EQRRQSIAKE	KEERLLRRQI	NREKLEEKRK	QKAEKTKSSK	TKGQGRSSVD	LEESSTKSLE	PKAARIKEVL
570	580	590	600	610	620	630	640
KERKVLEKKV	ALSKKRKKDS	RNVEENSKKK	QQYEEDSKET	LKTSEHCEKE	KISSSKELKH	VHAKSEPSKP	ARRLSESLHV
650	660	670	680	690	700	710	720
VDENKNESKL	EREHKRRTST	PVIMEGVQEE	TDTRDVKRQV	ERSEICTEEP	QKQKSTLKNE	KHLKKDDSET	PHLKSLLKKE
730	740	750	760	770	780	790	800
VKSSKEKPER	EKTPSEDKLS	VKHKYKGDCM	HKTGDETELH	SSEKGLKVEE	NIQKQSQQTK	LSSDDKTERK	SKHRNERKLS
810	820	830	840	850	860	870	880
VLGKDGKPVS	EYIIKTDENV	RKENNKKERR	LSAEKTKAEH	KSRRSSDSKI	QKDSLGSKQH	GITLQRRSES	YSEDKCDMDS
890	900	910	920	930	940	950	960
TNMDSNLKPE	EVVHKEKRRT	KSLLEEKLVL	KSKSKTQGKQ	VKVVETELQE	GATKQATTPK	PDKEKNTEEN	DSEKQRKSKV
970	980	990	1000	1010	1020	1030	1040
EDKPFEETGV	EPVLETASSS	AHSTQKDSSH	RAKLPLAKEK	YKSDKDSTST	RLERKLSDGH	KSRSLKHSSK	DIKKKDENKS
1050	1060	1070	1080	1090	1100	1110	1120
DDKDGKEVDS	SHEKARGNSS	LMEKKLSRRL	CENRRGSLSQ	EMAKGEEKLA	ANTLSTPSGS	SLQRPKKSGD	MTLIPEQEPM
1130	1140	1150	1160	1170	1180	1190	1200
EIDSEPGVEN	VFEVSKTQDN	RNNNSQQDID	SENMKQKTSA	TVQKDELRTC	TADSKATAPA	YKPGRGTGVN	SNSEKHADHR
1210	1220	1230	1240	1250	1260	1270	1280
STLTKKMHIQ	SAVSKMNPGE	KEPIHRGTTE	VNIDSETVHR	MLLSAPSEND	RVQKNLKNTA	AEEHVAQGDA	TLEHSTNLDS
1290	1300	1310	1320	1330	1340	1350	1360
SPSLSSVTVV	PLRESYDPDV	IPLFDKRTVL	EGSTASTSPA	DHSALPNQSL	TVRESEVLKT	SDSKEGGEGF	TVDTPAKASI
1370	1380	1390	1400	1410	1420	1430	1440
TSKRHIPEAH	QATLLDGKQG	KVIMPLGSKL	TGVIVENENI	TKEGGLVDMA	KKENDLNAEP	NLKQTIKATV	ENGKKDGIAV
1450	1460	1470	1480	1490	1500	1510	1520
DHVVGLNTEK	YAETVKLKHK	RSPGKVKDIS	IDVERRNENS	EVDTSAGSGS	APSVLHQRNG	QTEDVATGPR	RAEKTSVATS
1530	1540	1550	1560	1570	1580	1590	1600
TEGKDKDVTL	SPVKAGPATT	TSSETRQSEV	ALPCTSIEAD	EGLIIGTHSR	NNPLHVGAEA	SECTVFAAAE	EGGAVVTEGF
1610	1620	1630	1640	1650	1660	1670	1680
AESETFLTST	KEGESGECAV	AESEDRAADL	LAVHAVKIEA	NVNSVVTEEK	DDAVTSAGSE	EKCDGSLSRD	SEIVEGTITF
1690	1700	1710	1720	1730	1740	1750	1760
ISEVESDGAV	TSAGTEIRAG	SISSEEVDGS	QGNMMRMGPK	KETEGTVTCT	GAEGRSDNFV	ICSVTGAGPR	EERMVTGAGV
1770	1780	1790	1800	1810	1820	1830	1840
VLGDNDAPPG	TSASQEGDGS	VNDGTEGESA	VTSTGITEDG	EGPASCTGSE	DSSEGFAISS	ESEENGESAM	DSTVAKEGTN
1850	1860	1870	1880	1890	1900	1910	1920
VPLVAAGPCD	DEGIVTSTGA	KEEDEEGEDV	VTSTGRGNEI	GHASTCTGLG	EESEGVLICE	SAEGDSQIGT	VVEHVEAEAG
1930	1940	1950	1960	1970	1980	1990	2000
AAIMNANENN	VDSMSGTEKG	SKDTDICSSA	KGIVESSVTS	AVSGKDEVTP	VPGGCEGPMT	SAASDQSDSQ	LEKVEDTTIS
2010	2020	2030	2040	2050	2060	2070	2080
TGLVGGSYDV	LVSGEVPECE	VAHTSPSEKE	DEDIITSVEN	EECDGLMATT	ASGDITNQNS	LAGGKNQGKV	LIISTSTTND
2090	2100	2110	2120	2130	2140	2150	2160
YTPQVSAITD	VEGGLSDALR	TEENMEGTRV	TTEEFEAPMP	SAVSGDDSQL	TASRSEEKDE	CAMISTSIGE	EFELPISSAT
2170	2180	2190	2200	2210	2220	2230	2240

Detailed Protein Report

Cmpd.	No. of Cmpds.	m/z meas.	Δ m/z [ppm]	z	Rt [min]	Score	P	Range	Sequence	Modification	Ratios
1228	1	954.9820	37.30	2	45.5	23.8	0	658-674	R.TSTPVIMEGVQEETDTR.D	Oxidation: 7	QU:MU 0.83<br>WUP:QUP 1.83
2965	1	557.6376	40.39	3	65.8	10.0	2	2881-2894	K.YPVETTLKMKDDSK.T	Oxidation: 9	

Detailed Protein Report

Protein 74: **PREDICTED: striated muscle preferentially expressed protein kinase isoform X14 [Homo sapiens]**

**Accession:**	gi|578803490	**Score:**	59.3
**Database:**	refseq_human(refseq_human_20140103.fasta)	**MW [kDa]:**	283.5
**Database Date:**	2015-11-30	**pI:**	9.4
		**Sequence Coverage [%]:**	1.8
		**No. of unique Peptides:**	1

Quantitation

WUP:QUP	**Median:** 0.74	**CV:** 0.00 %	**No. of Peptides:** 1

Detailed Protein Report

10	20	30	40	50	60	70	80
MQKARGTRGE	DAGTRAPPSP	GVPPKRAKVG	AGGGAPVAVA	GAPVFLRPLK	NAAVCAGSDV	RLRVVVSGTP	QPSLRWFRDG
90	100	110	120	130	140	150	160
QLLPAPAPEP	SCLWLRRCGA	QDAGVYSCMA	QNERGRASCE	AVLTVLEVGD	SETAEDDISD	VQGTQRLELR	DDGAFSTPTG
170	180	190	200	210	220	230	240
GSDTLVGTSL	DTPPTSVTGT	SEEQVSWWGS	GQTVLEQEAG	SGGGTRRLPG	SPRQAQATGA	GPRHLGVEPL	VRASRANLVG
250	260	270	280	290	300	310	320
ASWGSEDSLS	VASDLYGSAF	SLYRGRALSI	HVSVPQSGLR	REEPDLQPQL	ASEAPRRPAQ	PPPSKSALLP	PPSPRVGKRS
330	340	350	360	370	380	390	400
PPGPPAQPAA	TPTSPHRRTQ	EPVLPEDTTT	EEKRGKKSKS	SGPSLAGTAE	SRPQTPLSEA	SGRLSALGRS	PRLVRAGSRI
410	420	430	440	450	460	470	480
LDKLQFFEER	RRSLERSDSP	PAPLRPWVPL	RKARSLEQPK	SERGAPWGTP	GASQEELRAP	GSVAERRRLF	QQKAASLDER
490	500	510	520	530	540	550	560
TRQRSPASDL	ELRFAQELGR	IRRSTSREEL	VRSHESLRAT	LQRAPSPREP	GEPPLFSRPS	TPKTSRAVSP	AAAQPPSPSS
570	580	590	600	610	620	630	640
AEKPGDEPGR	PRSRGPAGRT	EPGEGPQQEV	RRRDQFPLTR	SRAIQECRSP	VPPPAADPPE	ARTKAPPGRK	REPPAQAVRF
650	660	670	680	690	700	710	720
LPWATPGLEG	AAVPQTLEKN	RAGPEAEKRL	RRGPEEDGPW	GPWDRRGARS	QGKGRRARPT	SPELESSDDS	YVSAGEEPLE
730	740	750	760	770	780	790	800
APVFEIPLQN	VVVAPGADVL	LKCIITANPP	PQVSWHKDGS	ALRSEGRLLL	RAEGERHTLL	LREARAADAG	SYMATATNEL
810	820	830	840	850	860	870	880
GQATCAASLT	VRPGGSTSPF	SSPITSDEEY	LSPPEEFPEP	GETWPRTPTM	KPSPSQNRRS	SDTGSKAPPT	FKVSLMDQSV
890	900	910	920	930	940	950	960
REGQDVIMSI	RVQGEPKPVV	SWLRNRQPVR	PDQRRFAEEA	EGGLCRLRIL	AAERGDAGFY	TCKAVNEYGA	RQCEARLEVR
970	980	990	1000	1010	1020	1030	1040
AHPESRSLAV	LAPLQDVDVG	AGEMALFECL	VAGPTDVEVD	WLCRGRLLQP	ALLKCKMHFD	GRKCKLLLTS	VHEDDSGVYT
1050	1060	1070	1080	1090	1100	1110	1120
CKLSTAKDEL	TCSARLTVRP	SLAPLFTRLL	EDVEVLEGRA	ARFDCKISGT	PPPVVTWTHF	GCPMEESENL	RLRQDGGLHS
1130	1140	1150	1160	1170	1180	1190	1200
LHIAHVGSED	EGLYAVSAVN	THGQAHCSAQ	LYVEEPRTAA	SGPSSKLEKM	PSIPEEPEQG	ELERLSIPDF	LRPLQDLEVG
1210	1220	1230	1240	1250	1260	1270	1280
LAKEAMLECQ	VTGLPYPTIS	WFHNGHRIQS	SDDRRMTQYR	DVHRLVFPAV	GPQHAGVYKS	VIANKLGKAA	CYAHLYVTDV
1290	1300	1310	1320	1330	1340	1350	1360
VPGPPDGAPQ	VVAVTGRMVT	LTWNPPRSLD	MAIDPDSLTY	TVQHQVLGSD	QWTALVTGLR	EPGWAATGLR	KGVQHIFRVL
1370	1380	1390	1400	1410	1420	1430	1440
STTVKSSSKP	SPPSEPVQLL	EHGPTLEEAP	AMLDKPDIVY	VVEGQPASVT	VTFNHVEAQV	VWRSCRGALL	EARAGVYELS
1450	1460	1470	1480	1490	1500	1510	1520
QPDDDQYCLR	ICRVSRRDMG	ALTCTARNRH	GTQTCSVTLE	LAEAPRFESI	MEDVEVGAGE	TARFAVVVEG	KPLPDIMWYK
1530	1540	1550	1560	1570	1580	1590	1600
DEVLLTESSH	VSFVYEENEC	SLVVLSTGAQ	DGGVYTCTAQ	NLAGEVSCKA	ELAVHSAQTA	MEVEGVGEDE	DHRGRRLSDF
1610	1620	1630	1640	1650	1660	1670	1680
YDIHQEIGRG	AFSYLRRIVE	RSSGLEFAAK	FIPSQAKPKA	SARREARLLA	RLQHDCVLYF	HEAFERRRGL	VIVTELCTEE
1690	1700	1710	1720	1730	1740	1750	1760
LLERIARKPT	VCESEIRAYM	RQVLEGIHYL	HQSHVLHLDV	KPENLLVWDG	AAGEQQVRIC	DFGNAQELTP	GEPQYCQYGT
1770	1780	1790	1800	1810	1820	1830	1840
PEFVAPEIVN	QSPVSGVTDI	WPVGVVAFLC	LTGISPFVGE	NDRTTLMNIR	NYNVAFEETT	FLSLSREARG	FLIKVLVQDR
1850	1860	1870	1880	1890	1900	1910	1920
LRPTAEETLE	HPWFKTQAKG	AEVSTDHLKL	FLSRRRWQRS	QISYKCHLVL	RPIPELLRAP	PERVWVTMPR	RPPPSGGLSS
1930	1940	1950	1960	1970	1980	1990	2000
SSDSEEEELE	ELPSVPRPLQ	PEFSGSRVSL	TDIPTEDEAL	GTPETGAATP	MDWQEQGRAP	SQDQEAPSPE	ALPSPGQEPA
2010	2020	2030	2040	2050	2060	2070	2080
AGASPRRGEL	RRGSSAESAL	PRAGPRELGR	GLHKAASVEL	PQRRSPSPGA	TRLARGGLGE	GEYAQRLQAL	RQRLLRGGPE
2090	2100	2110	2120	2130	2140	2150	2160
DGKVSGLRGP	LLESLGGRAR	DPRMARAASS	EAAPHHQPPL	ENRGLQKSSS	FSQGEAEPRG	RHRRAGAPLE	IPVARLGARR
2170	2180	2190	2200	2210	2220	2230	2240

Detailed Protein Report

Cmpd.	No. of Cmpds.	m/z meas.	Δ m/z [ppm]	z	Rt [min]	Score	P	Range	Sequence	Modification	Ratios	
257	1	765.3459	-79.23	2	33.2	16.1	0	2578-2592	R.SESVPSLGLEAAIEK.L		WUP:QUP	0.74

Detailed Protein Report

Protein 75: myosin-9 [Homo sapiens]

**Accession:**	gi|12667788	**Score:**	59.2
**Database:**	refseq_human(refseq_human_20140103.fasta)	**MW [kDa]:**	226.4
**Database Date:**	2015-11-30	**pI:**	5.4
**Modification(s):**	Oxidation	**Sequence Coverage [%]:**	2.6
		**No. of unique Peptides:**	3

BRUKER

Detailed Protein Report

10	20	30	40	50	60	70	80
MAQQAADKYL	YVDKNFINNP	LAQADWAAKK	LVWVPSDKSG	FEPASLKEEV	GEEAIVELVE	NGKKVKVNKD	DIQKMNPPKF
90	100	110	120	130	140	150	160
SKVEDMAELT	CLNEASVLHN	LKERYYSGLI	YTYSGLFCVV	INPYKNLPIY	SEEIVEMYKG	KKRHEMPPHI	YAITDTAYRS
170	180	190	200	210	220	230	240
MMQDREDQSI	LCTGESGAGK	TENTKKVIQY	LAYVASSHKS	KKDQGELERQ	LLQANPILEA	FGNAKTVKND	NSSRFGKFIR
250	260	270	280	290	300	310	320
INFDVNGYIV	GANIETYLLE	KSRAIRQAKE	ERTFHIFYYL	LSGAGEHLKT	DLLLEPYNKY	RFLSNGHVTI	PGQQDKDMFQ
330	340	350	360	370	380	390	400
ETMEAMRIMG	IPEEEQMGLL	RVISGVLQLG	NIVFKKERNT	DQASMPDNTA	AQKVSHLLGI	NVTDFTRGIL	TPRIKVGRDY
410	420	430	440	450	460	470	480
VQKAQTKEQA	DFAIEALAKA	TYERMFRWLV	LRINKALDKT	KRQGASFIGI	LDIAGFEIFD	LNSFEQLCIN	YTNEKLQQLF
490	500	510	520	530	540	550	560
NHTMFILEQE	EYQREGIEWN	FIDFGLDLQP	CIDLIEKPAG	PPGILALLDE	ECWFPKATDK	SFVEKVMQEQ	GTHPKFQKPK
570	580	590	600	610	620	630	640
QLKDKADFCI	IHYAGKVDYK	ADEWLMKNMD	PLNDNIATLL	HQSSDKFVSE	LWKDVDRIIG	LDQVAGMSET	ALPGAFKTRK
650	660	670	680	690	700	710	720
GMFRTVGQLY	KEQLAKLMAT	LRNTNPNFVR	CIIPNHEKKA	GKLDPHLVLD	QLRCNGVLEG	IRICRQGFPN	RVVFQEFRQR
730	740	750	760	770	780	790	800
YEILTPNSIP	KGFMDGKQAC	VLMIKALELD	SNLYRIGQSK	VFFRAGVLAH	LEEERDLKIT	DVIIGFQACC	RGYLARKAFA
810	820	830	840	850	860	870	880
KRQQQLTAMK	VLQRNCAAYL	KLRNWQWWRL	FTKVKPLLQV	SRQEEEMMAK	EEELVKVREK	QLAAENRLTE	METLQSQLMA
890	900	910	920	930	940	950	960
EKLQLQEQLQ	AETELCAEAE	ELRARLTAKK	QELEEICHDL	EARVEEEEER	CQHLQAEKKK	MQQNIQELEE	QLEEEESARQ
970	980	990	1000	1010	1020	1030	1040
KLQLEKVTTE	AKLKKLEEEQ	IILEDQNCKL	AKEKKLLEDR	IAEFTTNLTE	EEEKSKSLAK	LKNKHEAMIT	DLEERLRREE
1050	1060	1070	1080	1090	1100	1110	1120
KQRQELEKTR	RKLEGDSTDL	SDQIAELQAQ	IAELKMQLAK	KEEELQAALA	RVEEEAAQKN	MALKKIRELE	SQISELQEDL
1130	1140	1150	1160	1170	1180	1190	1200
ESERASRNKA	EKQKRDLGEE	LEALKTELED	TLDSTAAQQE	LRSKREQEVN	ILKKTLEEEA	KTHEAQIQEM	RQKHSQAVEE
1210	1220	1230	1240	1250	1260	1270	1280
LAEQLEQTKR	VKANLEKAKQ	TLENERGELA	NEVKVLLQGK	GDSEHKRKKV	EAQLQELQVK	FNEGERVRTE	LADKVTKLQV
1290	1300	1310	1320	1330	1340	1350	1360
ELDNVTGLLS	QSDSKSSKLT	KDFSALESQL	QDTQELLQEE	NRQKLSLSTK	LKQVEDEKNS	FREQLEEEEE	AKHNLEKQIA
1370	1380	1390	1400	1410	1420	1430	1440
TLHAQVADMK	KKMEDSVGCL	ETAEEVKRKL	QKDLEGLSQR	HEEKVAAYDK	LEKTKTRLQQ	ELDDLLVDLD	HQRQSACNLE
1450	1460	1470	1480	1490	1500	1510	1520
KKQKKFDQLL	AEEKTISAKY	AEERDRAEAE	AREKETKALS	LARALEEAME	QKAELERLNK	QFRTEMEDLM	SSKDDVGKSV
1530	1540	1550	1560	1570	1580	1590	1600
HELEKSKRAL	EQQVEEMKTQ	LEELEDELQA	TEDAKLRLEV	NLQAMKAQFE	RDLQGRDEQS	EEKKKQLVRQ	VREMEAELED
1610	1620	1630	1640	1650	1660	1670	1680
ERKQRSMAVA	ARKKLEMDLK	DLEAHIDSAN	KNRDEAIKQL	RKLQAQMKDC	MRELDDTRAS	REEILAQAKE	NEKKLKSMEA
1690	1700	1710	1720	1730	1740	1750	1760
EMIQLQEELA	AAERAKRQAQ	QERDELADEI	ANSSGKGALA	LEEKRRLEAR	IAQLEEELEE	EQGNTELIND	RLKKANLQID
1770	1780	1790	1800	1810	1820	1830	1840
QINTDLNLER	SHAQKNENAR	QQLERQNKEL	KVKLQEMEGT	VKSKYKASIT	ALEAKIAQLE	EQLDNETKER	QAACKQVRRT
1850	1860	1870	1880	1890	1900	1910	1920
EKKLKDVLLQ	VDDERRNAEQ	YKDQADKAST	RLKQLKRQLE	EAEEEAQRAN	ASRRKLQREL	EDATETADAM	NREVSSLKNK
1930	1940	1950	1960	1970			
LRRGDLPFVV	PRRMARKGAG	DGSDEEVDGK	ADGAEAKPAE				

Cmpd.	No. of Cmpds.	m/z meas.	Δ m/z [ppm]	z	Rt [min]	Score	P	Range	Sequence	Modification
2655	1	822.4643	30.49	2	64.2	12.9	2	1213-1226	K.ANLEKAKQTLENER.G	
187	1	849.4195	-61.73	2	32.3	21.9	2	1358-	K.QIATLHAQVADMKKK.M	Oxidation: 12

Detailed Protein Report

Cmpd.	No. of Cmpds.	m/z meas.	Δ m/z [ppm]	z	Rt [min]	Score	P	Range	Sequence	Modification
								1372		
2426	1	625.6164	-235.71	2	60.8	13.5	0	1593-1602	R.EMEAELEDER.K	

Detailed Protein Report

Protein 76: 14-3-3 protein eta [Homo sapiens]

**Accession:**	gi|4507951	**Score:**	59.1
**Database:**	refseq_human(refseq_human_20140103.fasta)	**MW [kDa]:**	28.2
**Database Date:**	2015-11-30	**pI:**	4.6
**Modification(s):**	Oxidation	**Sequence Coverage [%]:**	11.4
		**No. of unique Peptides:**	1

10	20	30	40	50	60	70	80
MGDREQLLQR	ARLAEQAERY	DDMASAMKAV	TELNEPLSNE	DRNLLSVAYK	NVVGARRSSW	RVISSIEQKT	MADGNEKKLE
**90**	**100**	**110**	**120**	**130**	**140**	**150**	**160**
KVKAYREKIE	KELETVCNDV	LSLLDKFLIK	NCNDFQYESK	VFYLKMKGDY	YRYLAEVASG	EKKNSVVEAS	EAAYKEAFEI
**170**	**180**	**190**	**200**	**210**	**220**	**230**	**240**
SKEQMQPTHP	IRLGLALNFS	VFYYEIQNAP	EQACLLAKQA	FDDAIAELDT	LNEDSYKDST	LIMQLLRDNL	TLWTSDQQDE
**250**							
EAGEGN							

Cmpd.	No. of Cmpds.	m/z meas.	Δ m/z [ppm]	z	Rt [min]	Score	P	Range	Sequence	Modification
2722	1	1044.5326	52.39	2	65.2	11.7	2	11-28	R.ARLAEQAERYDDMASAMK.A	Oxidation: 13, 17

Detailed Protein Report

Protein 77: fibronectin isoform 6 preproprotein [Homo sapiens]

**Accession:**	gi|47132549	**Score:**	59.0
**Database:**	refseq_human(refseq_human_20140103.fasta)	**MW [kDa]:**	239.5
**Database Date:**	2015-11-30	**pI:**	5.5
**Modification(s):**	Carbamidomethyl	**Sequence Coverage [%]:**	2.6
		**No. of unique Peptides:**	3

Detailed Protein Report

10	20	30	40	50	60	70	80
MLRGPGPGLL	LLAVQCLGTA	VPSTGASKSK	RQAQQMVQPQ	SPVAVSQSKP	GCYDNGKHYQ	INQQWERTYL	GNALVCTCYG
90	100	110	120	130	140	150	160
GSRGFNCESK	PEAEETCFDK	YTGNTYRVGD	TYERPKDSMI	WDCTCIGAGR	GRISCTIANR	CHEGGQSYKI	GDTWRRPHET
170	180	190	200	210	220	230	240
GGYMLECVCL	GNGKGEWTCK	PIAEKCFDHA	AGTSYVVGET	WEKPYQGWMM	VDCTCLGEGS	GRITCTSRNR	CNDQDTRTSY
250	260	270	280	290	300	310	320
RIGDTWSKKD	NRGNLLQCIC	TGNGRGEWKC	ERHTSVQTTS	SGSGPFTDVR	AAVYQPQPHP	QPPPYGHCVT	DSGVVYSVGM
330	340	350	360	370	380	390	400
QWLKTQGNKQ	MLCTCLGNGV	SCQETAVTQT	YGGNSNGEPC	VLPFTYNGRT	FYSCTTEGRQ	DGHLWCSTTS	NYEQDQKYSF
410	420	430	440	450	460	470	480
CTDHTVLVQT	RGGNSNGALC	HFPFLYNNHN	YTDCTSEGRR	DNMKWCGTTQ	NYDADQKFGF	CPMAAHEEIC	TTNEGVMYRI
490	500	510	520	530	540	550	560
GDQWDKQHDM	GHMMRCTCVG	NGRGEWTCIA	YSQLRDQCIV	DDITYNVNDT	FHKRHEEGHM	LNCTCFGQGR	GRWKCDPVDQ
570	580	590	600	610	620	630	640
CQDSETGTFY	QIGDSWEKYV	HGVRYQCYCY	GRGIGEWHCQ	PLQTYPSSSG	PVEVFITETP	SQPNSHPIQW	NAPQPSHISK
650	660	670	680	690	700	710	720
YILRWRPKNS	VGRWKEATIP	GHLNSYTIKG	LKPGVVYEGQ	LISIQQYGHQ	EVTRFDFTTT	STSTPVTSNT	VTGETTPFSP
730	740	750	760	770	780	790	800
LVATSESVTE	ITASSFVVSW	VSASDTVSGF	RVEYELSEEG	DEPQYLDLPS	TATSVNIPDL	LPGRKYIVNV	YQISEDGEQS
810	820	830	840	850	860	870	880
LILSTSQTTA	PDAPPDPTVD	QVDDTSIVVR	WSRPQAPITG	YRIVYSPSVE	GSSTELNLPE	TANSVTLSDL	QPGVQYNITI
890	900	910	920	930	940	950	960
YAVEENQEST	PVVIQQETTG	TPRSDTVPSP	RDLQFVEVTD	VKVTIMWTPP	ESAVTGYRVD	VIPVNLPGEH	GQRLPISRNT
970	980	990	1000	1010	1020	1030	1040
FAEVTGLSPG	VTYYFKVFAV	SHGRESKPLT	AQQTTKLDAP	TNLQFVNETD	STVLVRWTPP	RAQITGYRLT	VGLTRRGQPR
1050	1060	1070	1080	1090	1100	1110	1120
QYNVGPSVSK	YPLRNLQPAS	EYTVSLVAIK	GNQESPKATG	VFTTLQPGSS	IPPYNTEVTE	TTIVITWTPA	PRIGFKLGVR
1130	1140	1150	1160	1170	1180	1190	1200
PSQGGEAPRE	VTSDSGSIVV	SGLTPGVEYV	YTIQVLRDGQ	ERDAPIVNKV	VTPLSPPTNL	HLEANPDTGV	LTVSWERSTT
1210	1220	1230	1240	1250	1260	1270	1280
PDITGYRITT	TPTNGQQGNS	LEEVVHADQS	SCTFDNLSPG	LEYNVSVYTV	KDDKESVPIS	DTIIPAVPPP	TDLRFTNIGP
1290	1300	1310	1320	1330	1340	1350	1360
DTMRVTWAPP	PSIDLTNFLV	RYSPVKNEED	VAELSISPSD	NAVVLTNLLP	GTEYVVSVSS	VYEQHESTPL	RGRQKTGLDS
1370	1380	1390	1400	1410	1420	1430	1440
PTGIDFSDIT	ANSFTVHWIA	PRATITGYRI	RHHPEHFSGR	PREDRVPHSR	NSITLTNLTP	GTEYVVSIVA	LNGREESPLL
1450	1460	1470	1480	1490	1500	1510	1520
IGQQSTVSDV	PRDLEVVAAT	PTSLLISWDA	PAVTVRYYRI	TYGETGGNSP	VQEFTVPGSK	STATISGLKP	GVDYTITVYA
1530	1540	1550	1560	1570	1580	1590	1600
VTGRGDSPAS	SKPISINYRT	EIDKPSQMQV	TDVQDNSISV	KWLPSSSPVT	GYRVTTTPKN	GPGPTKTKTA	GPDQTEMTIE
1610	1620	1630	1640	1650	1660	1670	1680
GLQPTVEYVV	SVYAQNPSGE	SQPLVQTAVT	TIPAPTDLKF	TQVTPTSLSA	QWTPPNVQLT	GYRVRVTPKE	KTGPMKEINL
1690	1700	1710	1720	1730	1740	1750	1760
APDSSSVVVS	GLMVATKYEV	SVYALKDTLT	SRPAQGVVTT	LENVSPPRRA	RVTDATETTI	TISWRTKTET	ITGFQVDAVP
1770	1780	1790	1800	1810	1820	1830	1840
ANGQTPIQRT	IKPDVRSYTI	TGLQPGTDYK	IYLYTLNDNA	RSSPVVIDAS	TAIDAPSNLR	FLATTPNSLL	VSWQPPRARI
1850	1860	1870	1880	1890	1900	1910	1920
TGYIIKYEKP	GSPPREVVPR	PRPGVTEATI	TGLEPGTEYT	IYVIALKNNQ	KSEPLIGRKK	TGQEALSQTT	ISWAPFQDTS
1930	1940	1950	1960	1970	1980	1990	2000
EYIISCHPVG	TDEEPLQFRV	PGTSTSATLT	GLTRGATYNI	IVEALKDQQR	HKVREEVVTV	GNSVNEGLNQ	PTDDSCFDPY
2010	2020	2030	2040	2050	2060	2070	2080
TVSHYAVGDE	WERMSESGFK	LLCQCLGFGS	GHFRCDSSRW	CHDNGVNYKI	GEKWDRQGEN	GQMMSCTCLG	NGKGEFKCDP
2090	2100	2110	2120	2130	2140	2150	2160
HEATCYDDGK	TYHVGEQWQK	EYLGAICSCT	CFGGQRGWRC	DNCRRPGGEP	SPEGTTGQSY	NQYSQRYHQR	TNTNVNCPIE
2170	2180						

Detailed Protein Report

Cmpd.	No. of Cmpds.	m/z meas.	Δ m/z [ppm]	z	Rt [min]	Score	P	Range	Sequence	Modification
1763	1	1044.9953	44.20	2	51.9	16.6	1	534-550	K.RHEEGHMLNCTCFGQGR.G	Carbamidomethyl: 10, 12
2765	1	904.4689	14.28	2	65.7	16.9	0	923-938	K.VTIMWTPPESAVTGYR.V	
1706	1	796.3662	-51.72	2	51.1	15.3	0	1525-1539	R.GDSPASSKPISINYR.T	

Detailed Protein Report

Protein 78: **polyribonucleotide nucleotidyltransferase 1, mitochondrial precursor [Homo sapiens]**

Accession: gi|188528628
Database: refseq_human(refseq_human_20140103.fasta)
Database Date: 2015-11-30

Score: 58.9
MW [kDa]: 85.9
pI: 8.8
Sequence Coverage [%]: 5.5
No. of unique Peptides: 3

Quantitation

WUP:QUP **Median:** 0.72 **CV:** 0.00 % **No. of Peptides:** 1

10	20	30	40	50	60	70	80
MAACRYCCSC	LRLRPLSDGP	FLLPRRDRAL	TQLQVRALWS	SAGSRAVAVD	LGNRKLEISS	GKLARFADGS	AVVQSGDTAV
**90**	**100**	**110**	**120**	**130**	**140**	**150**	**160**
MVTAVSKTKP	SPSQFMPLVV	DYRQKAAAAG	RIPTNYLRRE	IGTSDKEILT	SRIIDRSIRP	LFPAGYFYDT	QVLCNLLAVD
**170**	**180**	**190**	**200**	**210**	**220**	**230**	**240**
GVNEPDVLAI	NGASVALSLS	DIPWNGPVGA	VRIGIIDGEY	VVNPTRKEMS	SSTLNLVVAG	APKSQIVMLE	ASAENILQQD
**250**	**260**	**270**	**280**	**290**	**300**	**310**	**320**
FCHAIKVGVK	YTQQIIQGIQ	QLVKETGVTK	RTPQKLFTPS	PEIVKYTHKL	AMERLYAVFT	DYEHDKVSRD	EAVNKIRLDT
**330**	**340**	**350**	**360**	**370**	**380**	**390**	**400**
EEQLKEKFPE	ADPYEIIESF	NVVAKEVFRS	IVLNEYKRCD	GRDLTSLRNV	SCEVDMFKTL	HGSALFQRGQ	TQVLCTVTFD
**410**	**420**	**430**	**440**	**450**	**460**	**470**	**480**
SLESGIKSDQ	VITAINGIKD	KNFMLHYEFP	PYATNEIGKV	TGLNRRELGH	GALAEKALYP	VIPRDFPFTI	RVTSEVLESN
**490**	**500**	**510**	**520**	**530**	**540**	**550**	**560**
GSSSMASACG	GSLALMDSGV	PISSAVAGVA	IGLVTKTDPE	KGEIEDYRLL	TDILGIEDYN	GDMDFKIAGT	NKGITALQAD
**570**	**580**	**590**	**600**	**610**	**620**	**630**	**640**
IKLPGIPIKI	VMEAIQQASV	AKKEILQIMN	KTISKPRASR	KENGPVVETV	QVPLSKRAKF	VGPGGYNLKK	LQAETGVTIS
**650**	**660**	**670**	**680**	**690**	**700**	**710**	**720**
QVDEETFSVF	APTPSAMHEA	RDFITEICKD	DQEQQLEFGA	VYTATITEIR	DTGVMVKLYP	NMTAVLLHNT	QLDQRKIKHP
**730**	**740**	**750**	**760**	**770**	**780**	**790**	
TALGLEVGQE	IQVKYFGRDP	ADGRMRLSRK	VLQSPATTVV	RTLNDRSSIV	MGEPISQSSS	NSQ	

Cmpd.	No. of Cmpds.	m/z meas.	Δ m/z [ppm]	z	Rt [min]	Score	P	Range	Sequence	Modification	Ratios
2849	8	973.4915	-40.84	2	64.3	15.3	2	120-136	R.EIGTSDKEILTSRIIDR.S		
80	1	586.3280	116.43	2	30.9	28.4	0	369-378	R.NVSCEVDMFK.T		WUP:QUP 0.72
2737	1	885.4690	-31.70	2	64.6	15.2	1	751-766	K.VLQSPATTVVRTLNDR.S		

BRUKER

Detailed Protein Report

Protein 79: ATP-binding cassette sub-family A member 3 [Homo sapiens]

**Accession:**	gi|116734710	**Score:**	58.7
**Database:**	refseq_human(refseq_human_20140103.fasta)	**MW [kDa]:**	191.2
**Database Date:**	2015-11-30	**pI:**	8.2
**Modification(s):**	Carbamidomethyl	**Sequence Coverage [%]:**	2.3
		**No. of unique Peptides:**	3

Quantitation

QU:MU	**Median:**	0.87	**CV:**	0.00 %	**No. of Peptides:**		1
WUP:QUP	**Median:**	1.16	**CV:**	176.78 %	**No. of Peptides:**		2

10	20	30	40	50	60	70	80
MAVLRQLALL	LWKNYTLQKR	KVLVTVLELF	LPLLFSGILI	WLRLKIQSEN	VPNATIYPGQ	SIQELPLFFT	FPPPGDTWEL
90	100	110	120	130	140	150	160
AYIPSHSDAA	KTVTETVRRA	LVINMRVRGF	PSEKDFEDYI	RYDNCSSSVL	AAVVFEHPFN	HSKEPLPLAV	KYHLRFSYTR
170	180	190	200	210	220	230	240
RNYMWTQTGS	FFLKETEGWH	TTSLFPLFPN	PGPREPTSPD	GGEPGYIREG	FLAVQHAVDR	AIMEYHADAA	TRQLFQRLTV
250	260	270	280	290	300	310	320
TIKRFPYPPF	IADPFLVAIQ	YQLPLLLLLS	FTYTALTIAR	AVVQEKERRL	KEYMRMMGLS	SWLHWSAWFL	LFFLFLLIAA
330	340	350	360	370	380	390	400
SFMTLLFCVK	VKPNVAVLSR	SDPSLVLAFL	LCFAISTISF	SFMVSTFFSK	ANMAAAFGGF	LYFFTYIPYF	FVAPRYNWMT
410	420	430	440	450	460	470	480
LSQKLCSCLL	SNVAMAMGAQ	LIGKFEAKGM	GIQWRDLLSP	VNVDDDFCFG	QVLGMLLLDS	VLYGLVTWYM	EAVFPGQFGV
490	500	510	520	530	540	550	560
PQPWYFFIMP	SYWCGKPRAV	AGKEEEDSDP	EKALRNEYFE	AEPEDLVAGI	KIKHLSKVFR	VGNKDRAAVR	DLNLNLYEGQ
570	580	590	600	610	620	630	640
ITVLLGHNGA	GKTTTLSMLT	GLFPPTSGRA	YISGYEISQD	MVQIRKSLGL	CPQHDILFDN	LTVAEHLYFY	AQLKGLSRQK
650	660	670	680	690	700	710	720
CPEEVKQMLH	IIGLEDKWNS	RSRFLSGGMR	RKLSIGIALI	AGSKVLILDE	PTSGMDAISR	RAIWDLLQRQ	KSDRTIVLTT
730	740	750	760	770	780	790	800
HFMDEADLLG	DRIAIMAKGE	LQCCGSSLFL	KQKYGAGYHM	TLVKEPHCNP	EDISQLVHHH	VPNATLESSA	GAELSFILPR
810	820	830	840	850	860	870	880
ESTHRFEGLF	AKLEKKQKEL	GIASFGASIT	TMEEVFLRVG	KLVDSSMDIQ	AIQLPALQYQ	HERRASDWAV	DSNLCGAMDP
890	900	910	920	930	940	950	960
SDGIGALIEE	ERTAVKLNTG	LALHCQQFWA	MFLKKAAYSW	REWKMVAAQV	LVPLTCVTLA	LLAINYSSEL	FDDPMLRLTL
970	980	990	1000	1010	1020	1030	1040
GEYGRTVVPF	SVPGTSQLGQ	QLSEHLKDAL	QAEGQEPREV	LGDLEEFLIF	RASVEGGGFN	ERCLVAASFR	DVGERTVVNA
1050	1060	1070	1080	1090	1100	1110	1120
LFNNQAYHSP	ATALAVVDNL	LFKLLCGPHA	SIVVSNFPQP	RSALQAAKDQ	FNEGRKGFDI	ALNLLFAMAF	LASTFSILAV
1130	1140	1150	1160	1170	1180	1190	1200
SERAVQAKHV	QFVSGVHVAS	FWLSALLWDL	ISFLIPSLLL	LVVFKAFDVR	AFTRDGHMAD	TLLLLLLYGW	AIIPLMYLMN
1210	1220	1230	1240	1250	1260	1270	1280
FFFLGAATAY	TRLTIFNILS	GIATFLMVTI	MRIPAVKLEE	LSKTLDHVFL	VLPNHCLGMA	VSSFYENYET	RRYCTSSEVA
1290	1300	1310	1320	1330	1340	1350	1360
AHYCKKYNIQ	YQENFYAWSA	PGVGRFVASM	AASGCAYLIL	LFLIETNLLQ	RLRGILCALR	RRRTLTELYT	RMPVLPEDQD
1370	1380	1390	1400	1410	1420	1430	1440
VADERTRILA	PSPDSLLHTP	LIIKELSKVY	EQRVPLLAVD	RLSLAVQKGE	CFGLLGFNGA	GKTTTFKMLT	GEESLTSGDA
1450	1460	1470	1480	1490	1500	1510	1520
FVGGHRISSD	VGKVRQRIGY	CPQFDALLDH	MTGREMLVMY	ARLRGIPERH	IGACVENTLR	GLLLEPHANK	LVRTYSGGNK
1530	1540	1550	1560	1570	1580	1590	1600
RKLSTGIALI	GEPAVIFLDE	PSTGMDPVAR	RLLWDTVARA	RESGKAIIIT	SHSMEECEAL	CTRLAIMVQG	QFKCLGSPQH
1610	1620	1630	1640	1650	1660	1670	1680
LKSKFGSGYS	LRAKVQSEGQ	QEALEEFKAF	VDLTFPGSVL	EDEHQGMVHY	HLPGRDLSWA	KVFGILEKAK	EKYGVDDYSV
1690	1700	1710					
SQISLEQVFL	SFAHLQPPTA	EEGR					

Detailed Protein Report

Cmpd.	No. of Cmpds.	m/z meas.	Δ m/z [ppm]	z	Rt [min]	Score	P	Range	Sequence	Modification	Ratios	
198	1	424.2139	-143.01	3	32.2	15.7	1	672-684	R.KLSIGIALIAGSK.V			
32	1	877.8644	-76.86	2	29.3	10.4	1	739-753	K.GELQCCGSSLFLKQK.Y	Carbamidomethyl: 5, 6	WUP:QUP	0.35
535	3	607.3148	34.29	2	36.7	32.6	0	988-998	K.DALQAEGQEPR.E		QU:MU<br>WUP:QUP	0.87<br>3.82

Detailed Protein Report

Protein 80: sp110 nuclear body protein isoform a [Homo sapiens]

Accession: gi|190343010
Database: refseq_human(refseq_human_20140103.fasta)
Database Date: 2015-11-30

Score: 58.6
MW [kDa]: 78.3
pI: 10.1
Sequence Coverage [%]: 10.2
No. of unique Peptides: 4

10	20	30	40	50	60	70	80
MFTMTRAMEE	ALFQHFMHQK	LGIAYAIHKP	FPFFEGLLDN	SIITKRMYME	SLEACRNLIP	VSRVVHNILT	QLERTFNLSL
**90**	**100**	**110**	**120**	**130**	**140**	**150**	**160**
LVTLFSQINL	REYPNLVTIY	RSFKRVGASY	ERQSRDTPIL	LEAPTGLAEG	SSLHTPLALP	PPQPPQPSCS	PCAPRVSEPG
**170**	**180**	**190**	**200**	**210**	**220**	**230**	**240**
TSSQQSDEIL	SESPSPSDPV	LPLPALIQEG	RSTSVTNDKL	TSKMNAEEDS	EEMPSLLTST	VQVASDNLIP	QIRDKEDPQE
**250**	**260**	**270**	**280**	**290**	**300**	**310**	**320**
MPHSPLGSMP	EIRDNSPEPN	DPEEPQEVSS	TPSDKKGKKR	KRCIWSTPKR	RHKKKSLPGG	TASSRHGIQK	KLKRVDQVPQ
**330**	**340**	**350**	**360**	**370**	**380**	**390**	**400**
KKDDSTCNST	VETRAQKART	ECARKSRSEE	IIDGTSEMNE	GKRSQKTPST	PRRVTQGAAS	PGHGIQEKLQ	VVDKVTQRKD
**410**	**420**	**430**	**440**	**450**	**460**	**470**	**480**
DSTWNSEVMM	RVQKARTKCA	RKSRSKEKKK	EKDICSSSKR	RFQKNIHRRG	KPKSDTVDFH	CSKLPVTCGE	AKGILYKKKM
**490**	**500**	**510**	**520**	**530**	**540**	**550**	**560**
KHGSSVKCIR	NEDGTWLTPN	EFEVEGKGRN	AKNWKRNIRC	EGMTLGELLK	RKNSDECEVC	CQGGQLLCCG	TCPRVFHEDC
**570**	**580**	**590**	**600**	**610**	**620**	**630**	**640**
HIPPVEAKRM	LWSCTFCRMK	RSSGSQQCHH	VSKTLERQMQ	PQDQLIRDYG	EPFQEAMWLD	LVKERLITEM	YTVAWFVRDM
**650**	**660**	**670**	**680**	**690**			
RLMFRNHKTF	YKASDFGQVG	LDLEAEFEKD	LKDVLGFHEA	NDGGFWTLP			

Cmpd.	No. of Cmpds.	m/z meas.	Δ m/z [ppm]	z	Rt [min]	Score	P	Range	Sequence	Modification
1618	1	882.8999	95.52	3	48.5	11.3	2	374-398	R.VTQGAASPGHGIQEKLQVVDKVTQR.K	
220	1	978.1311	-3.65	3	32.7	10.8	2	482-507	K.HGSSVKCIRNEDGTWLTPNEFEVEGK.G	
1829	1	781.3008	-84.17	2	53.2	11.5	2	569-580	K.RMLWSCTFCRMK.R	
2660	1	484.7692	52.33	2	61.6	14.6	1	639-645	R.DMRLMFR.N	

Detailed Protein Report

Protein 81: filaggrin [Homo sapiens]

**Accession:**	gi|60097902	**Score:**	58.5
**Database:**	refseq_human(refseq_human_20140103.fasta)	**MW [kDa]:**	434.9
**Database Date:**	2015-11-30	**pI:**	9.6
		**Sequence Coverage [%]:**	1.5
		**No. of unique Peptides:**	3

BRUKER

Detailed Protein Report

10	20	30	40	50	60	70	80
MSTLLENIFA	IINLFKQYSK	KDKNTDTLSK	KELKELLEKE	FRQILKNPDD	PDMVDVFMDH	LDIDHNKKID	FTEFLLMVFK
90	100	110	120	130	140	150	160
LAQAYYESTR	KENLPISGHK	HRKHSHHDKH	EDNKQEENKE	NRKRPSSLER	RNNRKGNKGR	SKSPRETGGK	RHESSSEKKE
170	180	190	200	210	220	230	240
RKGYSPTHRE	EEYGKNHHNS	SKKEKNKTEN	TRLGDNRKRL	SERLEEKEDN	EEGVYDYENT	GRMTQKWIQS	GHIATYYTIQ
250	260	270	280	290	300	310	320
DEAYDTTDSL	LEENKIYERS	RSSDGKSSSQ	VNRSRHENTS	QVPLQESRTR	KRRGSRVSQD	RDSEGHSEDS	ERHSGSASRN
330	340	350	360	370	380	390	400
HHGSAWEQSR	DGSRHPRSHD	EDRASHGHSA	DSSRQSGTRH	AETSSRGQTA	SSHEQARSSP	GERHGSGHQQ	SADSSRHSAT
410	420	430	440	450	460	470	480
GRGQASSAVS	DRGHRGSSGS	QASDSEGHSE	NSDTQSVSGH	GKAGLRQQSH	QESTRGRSGE	RSGRSGSSLY	QVSTHEQPDS
490	500	510	520	530	540	550	560
AHGRTGTSTG	GRQGSHHEQA	RDSSRHSASQ	EGQDTIRGHP	GSSRGGRQGS	HHEQSVNRSG	HSGSHHSHTT	SQGRSDASHG
570	580	590	600	610	620	630	640
QSGSRSASRQ	TRNEEQSGDG	TRHSGSRHHE	ASSQADSSRH	SQVGQGQSSG	PRTSRNQGSS	VSQDSDSQGH	SEDSERWSGS
650	660	670	680	690	700	710	720
ASRNHHGSAQ	EQSRDGSRHP	RSHHEDRAGH	GHSADSSRKS	GTRHTQNSSS	GQAASSHEQA	RSSAGERHGS	RHQLQSADSS
730	740	750	760	770	780	790	800
RHSGTGHGQA	SSAVRDSGHR	GSSGSQATDS	EGHSEDSDTQ	SVSGHGQAGH	HQQSHQESAR	DRSGERSRRS	GSFLYQVSTH
810	820	830	840	850	860	870	880
KQSESSHGWT	GPSTGVRQGS	HHEQARDNSR	HSASQDGQDT	IRGHPGSSRR	GRQGSHHEQS	VDRSGHSGSH	HSHTTSQGRS
890	900	910	920	930	940	950	960
DASRGQSGSR	SASRTTRNEE	QSRDGSRHSG	SRHHEASSHA	DISRHSQAGQ	GQSEGSRTSR	RQGSSVSQDS	DSEGHSEDSE
970	980	990	1000	1010	1020	1030	1040
RWSGSASRNH	RGSAQEQSRH	GSRHPRSHHE	DRAGHGHSAD	SSRQSGTPHA	ETSSGGQAAS	SHEQARSSPG	ERHGSRHQQS
1050	1060	1070	1080	1090	1100	1110	1120
ADSSRHSGIP	RRQASSAVRD	SGHWGSSGSQ	ASDSEGHSEE	SDTQSVSGHG	QDGPHQQSHQ	ESARDWSGGR	SGRSGSFIYQ
1130	1140	1150	1160	1170	1180	1190	1200
VSTHEQSESA	HGRTRTSTGR	RQGSHHEQAR	DSSRHSASQE	GQDTIRAHPG	SRRGGRQGSH	HEQSVDRSGH	SGSHHSHTTS
1210	1220	1230	1240	1250	1260	1270	1280
QGRSDASHGQ	SGSRSASRQT	RKDKQSGDGS	RHSGSRHHEA	ASWADSSRHS	QVGQEQSSGS	RTSRHQGSSV	SQDSDSERHS
1290	1300	1310	1320	1330	1340	1350	1360
DDSERLSGSA	SRNHHGSSRE	QSRDGSRHPG	FHQEDRASHG	HSADSSRQSG	THHTESSSHG	QAVSSHEQAR	SSPGERHGSR
1370	1380	1390	1400	1410	1420	1430	1440
HQQSADSSRH	SGIGHRQASS	AVRDSGHRGS	SGSQVTNSEG	HSEDSDTQSV	SAHGQAGPHQ	QSHKESARGQ	SGESSGRSRS
1450	1460	1470	1480	1490	1500	1510	1520
FLYQVSSHEQ	SESTHGQTAP	STGGRQGSRH	EQARNSSRHS	ASQDGQDTIR	GHPGSSRGGR	QGSYHEQSVD	RSGHSGYHHS
1530	1540	1550	1560	1570	1580	1590	1600
HTTPQGRSDA	SHGQSGPRSA	SRQTRNEEQS	GDGSRHSGSR	HHEPSTRAGS	SRHSQVGQGE	SAGSKTSRRQ	GSSVSQDRDS
1610	1620	1630	1640	1650	1660	1670	1680
EGHSEDSERR	SESASRNHYG	SAREQSRHGS	RNPRSHQEDR	ASHGHSAESS	RQSGTRHAET	SSGGQAASSQ	EQARSSPGER
1690	1700	1710	1720	1730	1740	1750	1760
HGSRHQQSAD	SSTDSGTGRR	QDSSVVGDSG	NRGSSGSQAS	DSEGHSEESD	TQSVSAHGQA	GPHQQSHQES	TRGQSGERSG
1770	1780	1790	1800	1810	1820	1830	1840
RSGSFLYQVS	THEQSESAHG	RTGPSTGGRQ	RSRHEQARDS	SRHSASQEGQ	DTIRGHPGSS	RGGRQGSHYE	QSVDSSGHSG
1850	1860	1870	1880	1890	1900	1910	1920
SHHSHTTSQE	RSDVSRGQSG	SRSVSRQTRN	EKQSGDGSRH	SGSRHHEASS	RADSSRHSQV	GQGQSSGPRT	SRNQGSSVSQ
1930	1940	1950	1960	1970	1980	1990	2000
DSDSQGHSED	SERWSGSASR	NHLGSAWEQS	RDGSRHPGSH	HEDRAGHGHS	ADSSRQSGTR	HTESSSRGQA	ASSHEQARSS
2010	2020	2030	2040	2050	2060	2070	2080
AGERHGSHHQ	LQSADSSRHS	GIGHGQASSA	VRDSGHRGYS	GSQASDSEGH	SEDSDTQSVS	AQGKAGPHQQ	SHKESARGQS
2090	2100	2110	2120	2130	2140	2150	2160
GESSGRSGSF	LYQVSTHEQS	ESTHGQSAPS	TGGRQGSHYD	QAQDSSRHSA	SQEGQDTIRG	HPGPSRGGRQ	GSHQEQSVDR
2170	2180	2190	2200	2210	2220	2230	2240

Detailed Protein Report

Cmpd.	No. of Cmpds.	m/z meas.	Δ m/z [ppm]	z	Rt [min]	Score	P	Range	Sequence	Modification
2478	1	589.2111	-88.51	2	60.9	15.3	1	898-907	R.NEEQSRDGSR.H	
46	1	731.3139	-20.71	3	30.3	16.4	1	3577-3596	R.HPTSHHEDRAGHGHSAESSR.Q	
371	1	938.4376	1.41	2	34.6	15.1	2	3797-3814	R.SDASHGQSGSRSASRETR.N	

Detailed Protein Report

Protein 82: desmoplakin isoform I [Homo sapiens]

**Accession:**	gi|58530840	**Score:**	58.4
**Database:**	refseq_human(refseq_human_20140103.fasta)	**MW [kDa]:**	331.6
**Database Date:**	2015-11-30	**pI:**	6.4
**Modification(s):**	Carbamidomethyl, Oxidation	**Sequence Coverage [%]:**	2.5
		**No. of unique Peptides:**	4

Quantitation

QU:MU	**Median:** 2.24	**CV:** 0.00 %	**No. of Peptides:** 1

Detailed Protein Report

10	20	30	40	50	60	70	80
MSCNGGSHPR	INTLGRMIRA	ESGPDLRYEV	TSGGGGTSRM	YYSRRGVITD	QNSDGYCQTG	TMSRHQNQNT	IQELLQNCSD
90	100	110	120	130	140	150	160
CLMRAELIVQ	PELKYGDGIQ	LTRSRELDEC	FAQANDQMEI	LDSLIREMRQ	MGQPCDAYQK	RLLQLQEQMR	ALYKAISVPR
170	180	190	200	210	220	230	240
VRRASSKGGG	GYTCQSGSGW	DEFTKHVTSE	CLGWMRQQRA	EMDMVAWGVD	LASVEQHINS	HRGIHNSIGD	YRWQLDKIKA
250	260	270	280	290	300	310	320
DLREKSAIYQ	LEEEYENLLK	ASFERMDHLR	QLQNIIQATS	REIMWINDCE	EEELLYDWSD	KNTNIAQKQE	AFSIRMSQLE
330	340	350	360	370	380	390	400
VKEKELNKLK	QESDQLVLNQ	HPASDKIEAY	MDTLQTQWSW	ILQITKCIDV	HLKENAAYFQ	FFEEAQSTEA	YLKGLQDSIR
410	420	430	440	450	460	470	480
KKYPCDKNMP	LQHLLEQIKE	LEKEREKILE	YKRQVQNLVN	KSKKIVQLKP	RNPDYRSNKP	IILRALCDYK	QDQKIVHKGD
490	500	510	520	530	540	550	560
ECILKDNNER	SKWYVTGPGG	VDMLVPSVGL	IIPPPNPLAV	DLSCKIEQYY	EAILALWNQL	YINMKSLVSW	HYCMIDIEKI
570	580	590	600	610	620	630	640
RAMTIAKLKT	MRQEDYMKTI	ADLELHYQEF	IRNSQGSEMF	GDDDKRKIQS	QFTDAQKHYQ	TLVIQLPGYP	QHQTVTTTEI
650	660	670	680	690	700	710	720
THHGTCQDVN	HNKVIETNRE	NDKQETWMLM	ELQKIRRQIE	HCEGRMTLKN	LPLADQGSSH	HITVKINELK	SVQNDSQAIA
730	740	750	760	770	780	790	800
EVLNQLKDML	ANFRGSEKYC	YLQNEVFGLF	QKLENINGVT	DGYLNSLCTV	RALLQAILQT	EDMLKVYEAR	LTEEETVCLD
810	820	830	840	850	860	870	880
LDKVEAYRCG	LKKIKNDLNL	KKSLLATMKT	ELQKAQQIHS	QTSQQYPLYD	LDLGKFGEKV	TQLTDRWQRI	DKQIDFRLWD
890	900	910	920	930	940	950	960
LEKQIKQLRN	YRDNYQAFCK	WLYDAKRRQD	SLESMKFGDS	NTVMRFLNEQ	KNLHSEISGK	RDKSEEVQKI	AELCANSIKD
970	980	990	1000	1010	1020	1030	1040
YELQLASYTS	GLETLLNIPI	KRTMIQSPSG	VILQEAADVH	ARYIELLTRS	GDYYRFLSEM	LKSLEDLKLK	NTKIEVLEEE
1050	1060	1070	1080	1090	1100	1110	1120
LRLARDANSE	NCNKNKFLDQ	NLQKYQAECS	QFKAKLASLE	ELKRQAELDG	KSAKQNLDKC	YGQIKELNEK	ITRLTYEIED
1130	1140	1150	1160	1170	1180	1190	1200
EKRRRKSVED	RFDQQKNDYD	QLQKARQCEK	ENLGWQKLES	EKAIKEKEYE	IERLRVLLQE	EGTRKREYEN	ELAKVRNHYN
1210	1220	1230	1240	1250	1260	1270	1280
EEMSNLRNKY	ETEINITKTT	IKEISMQKED	DSKNLRNQLD	RLSRENRDLK	DEIVRLNDSI	LQATEQRRRA	EENALQQKAC
1290	1300	1310	1320	1330	1340	1350	1360
GSEIMQKKQH	LEIELKQVMQ	QRSEDNARHK	QSLEEAAKTI	QDKNKEIERL	KAEFQEEAKR	RWEYENELSK	VRNNYDEEII
1370	1380	1390	1400	1410	1420	1430	1440
SLKNQFETEI	NITKTTIHQL	TMQKEEDTSG	YRAQIDNLTR	ENRSLSEEIK	RLKNTLTQTT	ENLRRVEEDI	QQQKATGSEV
1450	1460	1470	1480	1490	1500	1510	1520
SQRKQQLEVE	LRQVTQMRTE	ESVRYKQSLD	DAAKTIQDKN	KEIERLKQLI	DKETNDRKCL	EDENARLQRV	QYDLQKANSS
1530	1540	1550	1560	1570	1580	1590	1600
ATETINKLKV	QEQELTRLRI	DYERVSQERT	VKDQDITRFQ	NSLKELQLQK	QKVEEELNRL	KRTASEDSCK	RKKLEEELEG
1610	1620	1630	1640	1650	1660	1670	1680
MRRSLKEQAI	KITNLTQQLE	QASIVKKRSE	DDLRQQRDVL	DGHLREKQRT	QEELRRLSSE	VEALRRQLLQ	EQESVKQAHL
1690	1700	1710	1720	1730	1740	1750	1760
RNEHFQKAIE	DKSRSLNESK	IEIERLQSLT	ENLTKEHLML	EEELRNLRLE	YDDLRRGRSE	ADSDKNATIL	ELRSQLQISN
1770	1780	1790	1800	1810	1820	1830	1840
NRTLELQGLI	NDLQRERENL	RQEIEKFQKQ	ALEASNRIQE	SKNQCTQVVQ	ERESLLVKIK	VLEQDKARLQ	RLEDELNRAK
1850	1860	1870	1880	1890	1900	1910	1920
STLEAETRVK	QRLECEKQQI	QNDLNQWKTQ	YSRKEEAIRK	IESEREKSER	EKNSLRSEIE	RLQAEIKRIE	ERCRRKLEDS
1930	1940	1950	1960	1970	1980	1990	2000
TRETQSQLET	ERSRYQREID	KLRQRPYGSH	RETQTECEWT	VDTSKLVFDG	LRKKVTAMQL	YECQLIDKTT	LDKLLKGKKS
2010	2020	2030	2040	2050	2060	2070	2080
VEEVASEIQP	FLRGAGSIAG	ASASPKEKYS	LVEAKRKKLI	SPESTVMLLE	AQAATGGIID	PHRNEKLTVD	SAIARDLIDF
2090	2100	2110	2120	2130	2140	2150	2160
DDRQQIYAAE	KAITGFDDPF	SGKTVSVSEA	IKKNLIDRET	GMRLLEAQIA	SGGVVDPVNS	VFLPKDVALA	RGLIDRDLYR
2170	2180	2190	2200	2210	2220	2230	2240

Detailed Protein Report

Cmpd.	No. of Cmpds.	m/z meas.	Δ m/z [ppm]	z	Rt [min]	Score	P	Range	Sequence	Modification	Ratios
2092	1	1045.4569	7.22	2	56.0	12.2	0	46-64	R.GVITDQNSDGYCQTGTMSR.H	Carbamidomethyl: 12	
2178	4	596.7430	-119.04	2	57.6	11.1	1	1330-1339	R.LKAEFQEEAK.R		QU:MU 2.24
1858	1	616.2551	-101.28	2	53.1	10.2	1	1488-1497	K.QLIDKETNDR.K		
2393	1	1176.1976	83.64	2	59.8	12.7	2	2465-2484	R.RVVIVDPETNKEMSVQEAYK.K	Oxidation: 13	

Detailed Protein Report

Protein 83: PREDICTED: glutamine-rich protein 2 isoform X2 [Homo sapiens]

Accession: gi|578831523
Database: refseq_human(refseq_human_20140103.fasta)
Database Date: 2015-11-30
Modification(s): Oxidation

Score: 57.7
MW [kDa]: 72.2
pI: 9.9
Sequence Coverage [%]: 8.1
No. of unique Peptides: 4

Quantitation

WUP:QUP **Median:** 1.84 **CV:** 0.00 % **No. of Peptides:** 1

```
        10         20         30         40         50         60         70         80
MRSRPAKPTW RRSSSCWHRW VERLQRILEG EGNQEAGKEL KAGELRLQLG VLRVTVADIE KELAELRESQ DRGKAAMENS
        90        100        110        120        130        140        150        160
VSEASLYLQD QLDKLRMIIE SMLTSSSTLL SMSMAPHKAH TLAPGQIDPE ATCPACSLDV SHQVSTLVRR YEQLQDMVNS
       170        180        190        200        210        220        230        240
LAVSRPSKKA KLQRQDEELL GRVQSAILQV QGDCEKLNIT TSNLIEDHRQ KQKDIAMLYQ GLEKLEKEKA NREHLEMEID
       250        260        270        280        290        300        310        320
VKADKSALAT KVSRVQFDAT TEQLNHMMQE LVAKMSGQEQ DWQKMLDRLL TEMDNKLDRL ELDPVKQLLE DRWKSLRQQL
       330        340        350        360        370        380        390        400
RERPPLYQAD EAAAMRRQLL AHFHCLSCDR PLETPVTGHA IPVTPAGPGL PGHHSIRPYT VFELEQVRQH SRNLKLGSAF
       410        420        430        440        450        460        470        480
PRGDLAQMEQ SVGRLRSMHS KMLMNIEKVQ IHFGGSTKAS SQIIRELLHA QCLGSPCYKR VTDMADYTYS TVPRRCGGSH
       490        500        510        520        530        540        550        560
TLTYPYHRSR PQHLPRGLYP TEEIQIAMKH DEVDILGLDG HIYKGRMDTR LPGILRKDSS GTSKRKSQQP RPHVHRPPSL
       570        580        590        600        610        620        630        640
SSNGQLPSRP QSAQISAGNT SVSSRQQKDR PSSEGRLSQP NTAHPPSSAA VANRGLERHV DMPPGEGLEE PTRGPRSSTA
       650
Q
```

Cmpd.	No. of Cmpds.	m/z meas.	Δ m/z [ppm]	z	Rt [min]	Score	P	Range	Sequence	Modification	Ratios
2513	1	410.0863	-280.49	2	61.4	19.3	1	68-74	R.ESQDRGK.A		
592	1	1105.2058	124.92	2	35.8	10.7	1	151-169	R.YEQLQDMVNSLAVSRPSKK.A	Oxidation: 7	
2139	1	937.3370	-142.40	2	56.6	10.9	1	322-337	R.ERPPLYQADEAAAMRR.Q		WUP:QUP 1.84
16	1	537.2110	-148.15	2	29.8	16.9	0	429-438	K.VQIHFGGSTK.A		

BRUKER

Detailed Protein Report

Protein 84: complement C3 precursor [Homo sapiens]

**Accession:**	gi|115298678	**Score:**	57.2
**Database:**	refseq_human(refseq_human_20140103.fasta)	**MW [kDa]:**	187.0
**Database Date:**	2015-11-30	**pI:**	6.0
**Modification(s):**	Carbamidomethyl	**Sequence Coverage [%]:**	2.5
		**No. of unique Peptides:**	3

10	20	30	40	50	60	70	80
MGPTSGPSLL	LLLLTHLPLA	LGSPMYSIIT	PNILRLESEE	TMVLEAHDAQ	GDVPVTVTVH	DFPGKKLVLS	SEKTVLTPAT
90	100	110	120	130	140	150	160
NHMGNVTFTI	PANREFKSEK	GRNKFVTVQA	TFGTQVVEKV	VLVSLQSGYL	FIQTDKTIYT	PGSTVLYRIF	TVNHKLLPVG
170	180	190	200	210	220	230	240
RTVMVNIENP	EGIPVKQDSL	SSQNQLGVLP	LSWDIPELVN	MGQWKIRAYY	ENSPQQVFST	EFEVKEYVLP	SFEVIVEPTE
250	260	270	280	290	300	310	320
KFYYIYNEKG	LEVTITARFL	YGKKVEGTAF	VIFGIQDGEQ	RISLPESLKR	IPIEDGSGEV	VLSRKVLLDG	VQNPRAEDLV
330	340	350	360	370	380	390	400
GKSLYVSATV	ILHSGSDMVQ	AERSGIPIVT	SPYQIHFTKT	PKYFKPGMPF	DLMVFVTNPD	GSPAYRVPVA	VQGEDTVQSL
410	420	430	440	450	460	470	480
TQGDGVAKLS	INTHPSQKPL	SITVRTKKQE	LSEAEQATRT	MQALPYSTVG	NSNNYLHLSV	LRTELRPGET	LNVNFLLRMD
490	500	510	520	530	540	550	560
RAHEAKIRYY	TYLIMNKGRL	LKAGRQVREP	GQDLVVLPLS	ITTDFIPSFR	LVAYYTLIGA	SGQREVVADS	VWVDVKDSCV
570	580	590	600	610	620	630	640
GSLVVKSGQS	EDRQPVPGQQ	MTLKIEGDHG	ARVVLVAVDK	GVFVLNKKNK	LTQSKIWDVV	EKADIGCTPG	SGKDYAGVFS
650	660	670	680	690	700	710	720
DAGLTFTSSS	GQQTAQRAEL	QCPQPAARRR	RSVQLTEKRM	DKVGKYPKEL	RKCCEDGMRE	NPMRFSCQRR	TRFISLGEAC
730	740	750	760	770	780	790	800
KKVFLDCCNY	ITELRRQHAR	ASHLGLARSN	LDEDIIAEEN	IVSRSEFPES	WLWNVEDLKE	PPKNGISTKL	MNIFLKDSIT
810	820	830	840	850	860	870	880
TWEILAVSMS	DKKGICVADP	FEVTVMQDFF	IDLRLPYSVV	RNEQVEIRAV	LYNYRQNQEL	KVRVELLHNP	AFCSLATTKR
890	900	910	920	930	940	950	960
RHQQTVTIPP	KSSLSVPYVI	VPLKTGLQEV	EVKAAVYHHF	ISDGVRKSLK	VVPEGIRMNK	TVAVRTLDPE	RLGREGVQKE
970	980	990	1000	1010	1020	1030	1040
DIPPADLSDQ	VPDTESETRI	LLQGTPVAQM	TEDAVDAERL	KHLIVTPSGC	GEQNMIGMTP	TVIAVHYLDE	TEQWEKFGLE
1050	1060	1070	1080	1090	1100	1110	1120
KRQGALELIK	KGYTQQLAFR	QPSSAFAAFV	KRAPSTWLTA	YVVKVFSLAV	NLIAIDSQVL	CGAVKWLILE	KQKPDGVFQE
1130	1140	1150	1160	1170	1180	1190	1200
DAPVIHQEMI	GGLRNNNEKD	MALTAFVLIS	LQEAKDICEE	QVNSLPGSIT	KAGDFLEANY	MNLQRSYTVA	IAGYALAQMG
1210	1220	1230	1240	1250	1260	1270	1280
RLKGPLLNKF	LTTAKDKNRW	EDPGKQLYNV	EATSYALLAL	LQLKDFDFVP	PVVRWLNEQR	YYGGGYGSTQ	ATFMVFQALA
1290	1300	1310	1320	1330	1340	1350	1360
QYQKDAPDHQ	ELNLDVSLQL	PSRSSKITHR	IHWESASLLR	SEETKENEGF	TVTAEGKGQG	TLSVVTMYHA	KAKDQLTCNK
1370	1380	1390	1400	1410	1420	1430	1440
FDLKVTIKPA	PETEKRPQDA	KNTMILEICT	RYRGDQDATM	SILDISMMTG	FAPDTDDLKQ	LANGVDRYIS	KYELDKAFSD
1450	1460	1470	1480	1490	1500	1510	1520
RNTLIIYLDK	VSHSEDDCLA	FKVHQYFNVE	LIQPGAVKVY	AYYNLEESCT	RFYHPEKEDG	KLNKLCRDEL	CRCAEENCFI
1530	1540	1550	1560	1570	1580	1590	1600
QKSDDKVTLE	ERLDKACEPG	VDYVYKTRLV	KVQLSNDFDE	YIMAIEQTIK	SGSDEVQVGQ	QRTFISPIKC	REALKLEEKK
1610	1620	1630	1640	1650	1660	1670	
HYLMWGLSSD	FWGEKPNLSY	IIGKDTWVEH	WPEEDECQDE	ENQKQCQDLG	AFTESMVVFG	CPN	

Cmpd.	No. of Cmpds.	m/z meas.	Δ m/z [ppm]	z	Rt [min]	Score	P	Range	Sequence	Modification
2396	4	701.3860	-50.24	2	59.8	21.6	0	892-904	K.SSLSVPYVIVPLK.T	
1824	2	1087.0109	22.47	2	51.1	15.7	2	1505-1522	K.LCRDELCRCAEENCFIQK.S	
192	1	650.8121	22.36	2	31.2	19.9	0	1536-1546	K.ACEPGVDYVYK.T	Carbamidomethyl: 2

Detailed Protein Report

Protein 85: fatty acid-binding protein, epidermal [Homo sapiens]

Accession: gi|4557581
Database: refseq_human(refseq_human_20140103.fasta)
Database Date: 2015-11-30
Modification(s): Carbamidomethyl

Score: 57.2
MW [kDa]: 15.2
pI: 7.5
Sequence Coverage [%]: 10.4
No. of unique Peptides: 1

```
        10         20         30         40         50         60         70         80
MATVQQLEGR WRLVDSKGFD EYMKELGVGI ALRKMGAMAK PDCIITCDGK NLTIKTESTL KTTQFSCTLG EKFEETTADG
        90        100        110        120        130        140
RKTQTVCNFT DGALVQHQEW DGKESTITRK LKDGKLVVEC VMNNVTCTRI YEKVE
```

Cmpd.	No. of Cmpds.	m/z meas.	Δ m/z [ppm]	z	Rt [min]	Score	P	Range	Sequence	Modification
1139	2	847.8676	-43.89	2	44.4	57.2	0	116-129	K.LVVECVMNNVTCTR.I	Carbamidomethyl: 5, 12

Detailed Protein Report

Protein 86: uncharacterized protein C4orf21 [Homo sapiens]

**Accession:**	gi|254826809	**Score:**	57.2
**Database:**	refseq_human(refseq_human_20140103.fasta)	**MW [kDa]:**	236.5
**Database Date:**	2015-11-30	**pI:**	5.8
		**Sequence Coverage [%]:**	3.1
		**No. of unique Peptides:**	2

Alias proteins:

Accession	Name	Description
gi|530377937	refseq_human (refseq_human_20140103.fasta)	PREDICTED: uncharacterized protein C4orf21 isoform X1 [Homo sapiens]

BRUKER

Detailed Protein Report

10	20	30	40	50	60	70	80
MESQEFIVLY	THQKMKKSKV	WQDGILKITH	LGNKAILYDD	KGACLESLFL	KCLEVKPGDD	LESDRYLITV	EEVKVAGAIG
90	100	110	120	130	140	150	160
IVKQNVNKEA	PELNSRTFIS	SGRSLGCQPS	GLKRKFTGFQ	GPRQVPKKMV	IMESGESAAS	HEAKKTGPTI	FSPFCSMPPL
170	180	190	200	210	220	230	240
FPTVGKKDVN	NILADPENIV	TYKNRERNAM	DFSSVFSPSF	QINPEVLCEE	NYFCSPVNSG	NKLSDSLLTN	EPVKRDSLAS
250	260	270	280	290	300	310	320
HYSGVSQNIR	SKAQILALLK	SESSSSCEEL	NSEMTEHFPQ	KQPQGSLKIA	TKPKYLIQQE	ECAEMKSTEN	LYYQHQSENT
330	340	350	360	370	380	390	400
MRNKSRWAMY	LSSQSSPIHS	STVDGNDTER	KPKAQEDDVN	SNLKDLSLQK	IIQFVETYAE	ERKKYNVDQS	VGNNDPSWNQ
410	420	430	440	450	460	470	480
EVKLEIPSFN	ESSSLQVTCS	SAENDGILSE	SDIQEDNKIP	FNQNDKGCIK	GSVLIKENAQ	EVNTCGTLEK	EYEQSESSLP
490	500	510	520	530	540	550	560
ELKHLQIESS	NNSRISDDIT	DMISESKMDN	ESLNSIHESL	SNVTQPFLEV	TFNLNNFETS	DTEEESQESN	KISQDSESWV
570	580	590	600	610	620	630	640
KDILVNDGNS	CFQKRSENTN	CEEIEGEHLP	FLTSVSDKPT	VTFPVKETLP	SQFCDKTYVG	FDMGICKTEN	TGKEIEEYSD
650	660	670	680	690	700	710	720
TLSNFESFKW	TDAVYGDNKE	DANKPIQEVR	INYDFALPPN	KSKGINMNLH	IPHIQNQIAE	NSNLFSEDAQ	PQPFILGSDL
730	740	750	760	770	780	790	800
DKNDEHVLPS	TSSSDNSVQL	LNTNQNHYEC	IALDKSNTHI	SNSLFYPLGK	KHLISKDTEA	HISEPEDLGK	IRSPPPDHVE
810	820	830	840	850	860	870	880
VETAREGKQY	WNPRNSSELS	GLVNTISILK	SLCEHSTALD	SLEILKKKNT	VFQQGTQQTY	EPDSPPEVRK	PFITVVSPKS
890	900	910	920	930	940	950	960
PHLHKDSQQI	LKEDEVELSE	PLQSVQFSSS	GSKEETAFQA	VIPKQIERKT	CDPKPVEFQG	HQVKGSATSG	VMVRGHSSQL
970	980	990	1000	1010	1020	1030	1040
GCSQFPDSTE	YENFMTETPE	LPSTCMQIDF	LQVTSPEENI	STLSPVSTFS	LNSRDEDFMV	EFSETSLKAR	TLPDDLHFLN
1050	1060	1070	1080	1090	1100	1110	1120
LEGMKKSRSL	ENENLQRLSL	LSRTQVPLIT	LPRTDGPPDL	DSHSYMINSN	TYESSGSPML	NLCEKSAVLS	FSIEPEDQNE
1130	1140	1150	1160	1170	1180	1190	1200
TFFSEESREV	NPGDVSLNNI	STQSKWLKYQ	NTSQCNVATP	NRVDKRITDG	FFAEAVSGMH	FRDTSERQSD	AVNESSLDSV
1210	1220	1230	1240	1250	1260	1270	1280
HLQMIKGMLY	QQRQDFSSQD	SVSRKKVLSL	NLKQTSKTEE	IKNVLGGSTC	YNYSVKDLQE	ISGSELCFPS	GQKIKSAYLP
1290	1300	1310	1320	1330	1340	1350	1360
QRQIHIPAVF	QSPAHYKQTF	TSCLIEHLNI	LLFGLAQNLQ	KALSKVDISF	YTSLKGEKLK	NAENNVPSCH	HSQPAKLVMV
1370	1380	1390	1400	1410	1420	1430	1440
KKEGPNKGRL	FYTCDGPKAD	RCKFFKWLED	VTPGYSTQEG	ARPGMVLSDI	KSIGLYLRSQ	KIPLYEECQL	LVRKGFDFQR
1450	1460	1470	1480	1490	1500	1510	1520
KQYGKLKKFT	TVNPEFYNEP	KTKLYLKLSR	KERSSAYSKN	DLWVVSKTLD	FELDTFIACS	AFFGPSSINE	IEILPLKGYF
1530	1540	1550	1560	1570	1580	1590	1600
PSNWPTNMVV	HALLVCNAST	ELTTLKNIQD	YFNPATLPLT	QYLLTTSSPT	IVSNKRVSKR	KFIPPAFTNV	STKFELLSLG
1610	1620	1630	1640	1650	1660	1670	1680
ATLKLASELI	QVHKLNKDQA	TALIQIAQMM	ASHESIEEVK	ELQTHTFPIT	IIHGVFGAGK	SYLLAVVILF	FVQLFEKSEA
1690	1700	1710	1720	1730	1740	1750	1760
PTIGNARPWK	LLISSSTNVA	VDRVLLGLLS	LGFENFIRVG	SVRKIAKPIL	PYSLHAGSEN	ESEQLKELHA	LMKEDLTPTE
1770	1780	1790	1800	1810	1820	1830	1840
RVYVRKSIEQ	HKLGTNRTLL	KQVRVVGVTC	AACPFPCMND	LKFPVVVLDE	CSQITEPASL	LPIARFECEK	LILVGDPKQL
1850	1860	1870	1880	1890	1900	1910	1920
PPTIQGSDAA	HENGLEQTLF	DRLCLMGHKP	ILLRTQYRCH	PAISAIANDL	FYKGALMNGV	TEIERSPLLE	WLPTLCFYNV
1930	1940	1950	1960	1970	1980	1990	2000
KGLEQIERDN	SFHNVAEATF	TLKLIQSLIA	SGIAGSMIGV	ITLYKSQMYK	LCHLLSAVDF	HHPDIKTVQV	STVDAFQGAE
2010	2020	2030	2040	2050	2060	2070	2080
KEIIILSCVR	TRQVGFIDSE	KRMNVALTRG	KRHLLIVGNL	ACLRKNQLWG	RVIQHCEGRE	DGLQHANQYE	PQLNHLLKDY
2090	2100	2110					
FEKQVEEKQK	KKSEKEKSKD	KSHS					

Detailed Protein Report

Cmpd.	No. of Cmpds.	m/z meas.	Δ m/z [ppm]	z	Rt [min]	Score	P	Range	Sequence	Modification
1998	1	902.8620	-77.52	2	55.4	13.3	1	128-144	K.KMVIMESGESAASHEAK.K	
2085	1	698.2938	-180.04	2	56.0	10.9	1	282-294	K.QPQGSLKIATKPK.Y	

Detailed Protein Report

Protein 87: **PREDICTED: beta/gamma crystallin domain-containing protein 3 isoform X1 [Homo sapiens]**

**Accession:**	gi|530373918	**Score:**	57.1
**Database:**	refseq_human(refseq_human_20140103.fasta)	**MW [kDa]:**	298.2
**Database Date:**	2015-11-30	**pI:**	5.0
**Modification(s):**	Oxidation	**Sequence Coverage [%]:**	2.5
		**No. of unique Peptides:**	3

Quantitation

WUP:QUP	**Median:** 1.04	**CV:** 0.00 %	**No. of Peptides:** 1

Detailed Protein Report

10	20	30	40	50	60	70	80
MKGNLLEGPL	EDSDCSKTSF	NKENSLTNNP	ELQNIASSNN	LLNKNAWGSI	ERNRSSPSSV	TNSSYDGESD	SQHHLSCEPV
90	100	110	120	130	140	150	160
SQTNRNLVCS	ALLTGSNHRK	VPCSPDFQRV	TTTENTIKEN	STVMSNRTLV	QREELVEPQG	PAISDFSCSK	SDGSDTTEQE
170	180	190	200	210	220	230	240
STNLPSPNKS	IRHEHLQLPE	SECSDKQTID	SSSKQAATHT	NIIALQRHAV	TDTEFVNEGK	RLSAQDSQKN	VAVREIRRET
250	260	270	280	290	300	310	320
ESASAGESIA	SSHVKAPEDK	IESLPKDTDQ	YFETKAKKLD	FRSHDKIPHI	RMNKKDLASL	NYISESAVVA	SLGNENAPEL
330	340	350	360	370	380	390	400
KFELNRSHIS	ETPLDSESPQ	QAEVSPDAKT	SLSLDCKKLN	FSISPPTFVS	GVGMLSKLDI	PDLMNEGSPV	PIETGNVNIV
410	420	430	440	450	460	470	480
GISYQPRKCK	EENVKNHVEA	AGRKSPPPSF	CLEYTSAIFE	FKEVLSNSEK	CQVLPGSEAS	GPHLTGLELL	SFDSGNLSKD
490	500	510	520	530	540	550	560
CSSILSQDPN	RVELVSSNTK	ANMSIIEKSD	SLSLEAKTAN	IVSKAEIDGQ	NNVLVESHSG	RGKTISLSKV	SLSKVEPRNI
570	580	590	600	610	620	630	640
SQDKMSSFPL	KITHVPEKPI	LSELTFLEVE	QGKRFQSINH	NEIGEKCSDA	GLKENCQAEL	SPAASKYEDK	PEPEVDALGS
650	660	670	680	690	700	710	720
PPALLKSNIS	WILPPIHDEK	ISRQMAQNCE	AHTCVFHQSL	DICGTKKISG	HSEMAELSLT	NISPKFQETG	SMKVNSPFLD
730	740	750	760	770	780	790	800
SDSSLEKNSS	ASEDSSFLKV	PSVLKLEKKS	SSYRKKENIH	FLNGGIDSVS	SSSSYPEEVS	MIVNSHKPQN	NLDSIQVTKD
810	820	830	840	850	860	870	880
LTHEGTSVTN	LLYPTTSYLE	FETSVSIGTE	VTPFQEHFGI	YTGKISIDFP	TAAQFDNLVE	AETGAVAGPA	ASVNSSGQQC
890	900	910	920	930	940	950	960
SEASAEHIEA	RRRAHDQLLD	LKSSLLKKAD	TLIGEIFNSV	REELKFKHTV	STCQEHIAIE	GIMNLGTLKE	DISEKNPSEV
970	980	990	1000	1010	1020	1030	1040
TLTEIQQTEG	LEEQGMENMS	EVKEKPCVSP	TVGEKNLLVD	PNSMNVSCLL	EDKARELVNE	IIYVAQEKLR	NDTFEDTEDT
1050	1060	1070	1080	1090	1100	1110	1120
WDSELQANTS	KILNSDSVKP	HDVVREFLVS	EQPVNQSTQI	SENKVLNEFF	SLSNLASGTE	SIKGGEIVLY	QKSLFSGNGS
1130	1140	1150	1160	1170	1180	1190	1200
GLSDSINLQE	SDTVLLAEDM	SHKRLDDRVK	THLFRSEDCN	ETMEIENVDN	NKTETEDRRT	LVLNFKWPPL	VNDDIHAPGT
1210	1220	1230	1240	1250	1260	1270	1280
SKSSLSDSLV	CISEKNLPGH	SKNTPLAMSD	VGKVHKKDNE	INIGKIELIP	SMLETGKTNK	KDAELNILKY	EAVPPMIEMG
1290	1300	1310	1320	1330	1340	1350	1360
RIHKMDAELN	VTKTEPKANV	FKMGEVYQMD	AESCIEKTEG	SAVILGMEKA	YKMKDTEGDI	GKIEVIPMMP	EVKNIHQKDA
1370	1380	1390	1400	1410	1420	1430	1440
EGDIVKTEMT	PVTVDMENIY	QTHAEGDIGK	TGTIALSEVE	NIHQKGGEGI	SEKAEVIPVT	LAMENTYQKD	AEGDIGKAEV
1450	1460	1470	1480	1490	1500	1510	1520
MPVRLEMENT	YPKDTERDGG	KTEVMPLALE	VVNTYQKNAK	GFTGNTEGSV	LKMEATYRKT	AEEVIKNTEI	VPCVLKVKEA
1530	1540	1550	1560	1570	1580	1590	1600
HETAPAPLEM	EKACKRDVKE	TIGATVSTPS	VIEMEKISPE	DRGENIGKHK	VLPAVVDIEK	IHGTGLELTT	KQGEAMLPAF
1610	1620	1630	1640	1650	1660	1670	1680
ESKTPQEYAE	GSVEETKEEP	TEIKEGLIAH	ENRLPTYFRG	YESPTLSKDY	EGYPAPAMPD	FQPGDTTVRL	DKRMSLTAIY
1690	1700	1710	1720	1730	1740	1750	1760
DKRRETDYSD	KGYNLAFVSQ	DEQENSSFTI	LYEEPLQEED	KYASAEARQT	QSVLFHDTSA	DSMPVLACER	SESRTDLVHH
1770	1780	1790	1800	1810	1820	1830	1840
FEKGTKLGET	FDSDSSEMFL	SVEAKRYKIY	PLALSPIYED	DSSQEDILSS	EVSPGHHGPR	KSRDSENQSS	SVLSLLQSVS
1850	1860	1870	1880	1890	1900	1910	1920
ERLKMNFDED	DREAADEEEE	EEEAAVLHKG	DLRAGSGERV	TFQLPDPSIT	FYPDDQESVG	ISKNSYVMPN	EPTTSNLQVG
1930	1940	1950	1960	1970	1980	1990	2000
LWPEKTSFLQ	KSDLTSKLHS	SLKSAYHQYL	QTSQSHSSEK	GARFGGIFQE	PVSKYFRVQD	SPGRLSPFIE	NVDKQTLRCN
2010	2020	2030	2040	2050	2060	2070	2080
PRPGKMVIYD	LHESTYKQEV	YCNIPDATSW	SFPNGVLIKV	VRGCWILYEK	PHFRGQKCVL	EEGEKVLNRD	WILQNRRHPQ
2090	2100	2110	2120	2130	2140	2150	2160
RNFILGSLKR	VLKDCSIPEI	ELFPQSDPAC	CPVYIQRAVP	NLEELNISKS	VSFTVKSGVW	LAYPDINFKG	QATVLEEDHG
2170	2180	2190	2200	2210	2220	2230	2240

Detailed Protein Report

Cmpd.	No. of Cmpds.	m/z meas.	Δ m/z [ppm]	z	Rt [min]	Score	P	Range	Sequence	Modification	Ratios
1963	1	918.3504	-135.25	2	54.9	12.1	1	501-517	K.ANMSIIEKSDSLSLEAK.T		WUP:QUP 1.04
137	1	793.8712	-30.92	2	31.4	10.2	1	1430-1444	K.DAEGDIGKAEVMPVR.L		
1726	1	900.4572	47.30	3	49.9	14.8	1	1649-1672	K.DYEGYPAPAMPDFQPGDTTVRLDR	Oxidation: 10	

Detailed Protein Report

Protein 88: **PREDICTED: protein KIAA1199 isoform X3 [Homo sapiens]**

**Accession:**	gi|578827243	**Score:**	56.3
**Database:**	refseq_human(refseq_human_20140103.fasta)	**MW [kDa]:**	90.7
**Database Date:**	2015-11-30	**pI:**	9.6
		**Sequence Coverage [%]:**	5.8
		**No. of unique Peptides:**	3

10	20	30	40	50	60	70	80
MYSHGNLEIP	WSLPVLITPC	TRQKSSRCFP	ADPAPPTRSK	WQIKDVVGYN	SLGHCFFTED	GPEERNTFDH	CLGLLVKSGT
**90**	**100**	**110**	**120**	**130**	**140**	**150**	**160**
LLPSDRDSKM	CKMITEDSYP	GYIPKPRQDC	NAVSTFWMAN	PNNNLINCAA	AGSEETGFWF	IFHHVPTGPS	VGMYSPGYSE
**170**	**180**	**190**	**200**	**210**	**220**	**230**	**240**
HIPLGKFYNN	RAHSNYRAGM	IIDNGVKTTE	ASAKDKRPFL	SIISARYSPH	QDADPLKPRE	PAIIRHFIAY	KNQDHGAWLR
**250**	**260**	**270**	**280**	**290**	**300**	**310**	**320**
GGDVWLDSCR	FADNGIGLTL	ASGGTFPYDD	GSKQEIKNSL	FVGESGNVGT	EMMDNRIWGP	GGLDHSGRTL	PIGQNFPIRG
**330**	**340**	**350**	**360**	**370**	**380**	**390**	**400**
IQLYDGPINI	QNCTFRKFVA	LEGRHTSALA	FRLNNAWQSC	PHNNVTGIAF	EDVPITSRVF	FGEPGPWFNQ	LDMDGDKTSV
**410**	**420**	**430**	**440**	**450**	**460**	**470**	**480**
FHDVDGSVSE	YPGSYLTKND	NWLVRHPDCI	NVPDWRGAIC	SGCYAQMYIQ	AYKTSNLRMK	IIKNDFPSHP	LYLEGALTRS
**490**	**500**	**510**	**520**	**530**	**540**	**550**	**560**
THYQQYQPVV	TLQKGYTIHW	DQTAPAELAI	WLINFNKGDW	IRVGLCYPRG	TTFSILSDVH	NRLLKQTSKT	GVFVRTLQMD
**570**	**580**	**590**	**600**	**610**	**620**	**630**	**640**
KVEQSYPGRS	HYYWDEDSGL	LFLKLKAQNE	REKFAFCSMK	GCERIKIKAL	IPKNAGVSDC	TATAYPKFTE	RAVVDVPMPK
**650**	**660**	**670**	**680**	**690**	**700**	**710**	**720**
KLFGSQLKTK	DHFLEVKMES	SKQHFFHLWN	DFAYIEVDGK	KYPSSEDGIQ	VVVIDGNQGR	VVSHTSFRNS	ILQGIPWQLF
**730**	**740**	**750**	**760**	**770**	**780**	**790**	**800**
NYVATIPDNS	IVLMASKGRY	VSRGPWTRVL	EKLGADRGLK	LKEQMAFVGF	KGSFRPIWVT	LDTEDHKAKI	FQVVPIPVVK
**810**							
KKKL							

Cmpd.	No. of Cmpds.	m/z meas.	Δ m/z [ppm]	z	Rt [min]	Score	P	Range	Sequence	Modification
1123	3	473.1123	-298.74	2	44.2	17.0	0	78-86	K.SGTLLPSDR.D	
737	1	936.9549	11.85	2	39.2	10.8	0	570-584	R.SHYYWDEDSGLLFLK.L	
143	1	796.0835	178.80	2	30.7	10.0	2	740-752	R.YVSRGPWTRVLEK.L	

Detailed Protein Report

Protein 89: PREDICTED: rapamycin-insensitive companion of mTOR isoform X2 [Homo sapiens]

**Accession:**	gi|530378756	**Score:**	56.3
**Database:**	refseq_human(refseq_human_20140103.fasta)	**MW [kDa]:**	159.9
**Database Date:**	2015-11-30	**pI:**	6.8
		**Sequence Coverage [%]:**	3.0
		**No. of unique Peptides:**	2

Quantitation

QU:MU	**Median:** 2.42	**CV:** 4.84 %	**No. of Peptides:**	2
WUP:QUP	**Median:** 0.71	**CV:** 68.39 %	**No. of Peptides:**	2

10	20	30	40	50	60	70	80
MGIIATFRSW	AGIINLCKPG	NSGIQSLIGV	LCIPNMEIRR	GLLEVLYDIF	RLPLPVVTEE	FIEALLSVDP	GRFQDSWRLS
**90**	**100**	**110**	**120**	**130**	**140**	**150**	**160**
DGFVAAEAKT	ILPHRARSRP	DLMDNYLALI	LSAFIRNGLL	EGLVEVITNS	DDHISVRATI	LLGELLHMAN	TILPHSHSHH
**170**	**180**	**190**	**200**	**210**	**220**	**230**	**240**
LHCLPTLMNM	AASFDIPKEK	RLRASAALNC	LKRFHEMKKR	GPKPYSLHLD	HIIQKAIATH	QKRDQYLRVQ	KDIFILKDTE
**250**	**260**	**270**	**280**	**290**	**300**	**310**	**320**
EALLINLRDS	QVLQHKENLE	WNWNLIGTIL	KWPNVNLRNY	KDEQLHRFVR	RLLYFYKPSS	KLYANLDLDF	AKAKQLTVVG
**330**	**340**	**350**	**360**	**370**	**380**	**390**	**400**
CQFTEFLLES	EEDGQGYLED	LVKDIVQWLN	ASSGMKPERS	LQNNGLLTTL	SQHYFLFIGT	LSCHPHGVKM	LEKCSVFQCL
**410**	**420**	**430**	**440**	**450**	**460**	**470**	**480**
LNLCSLKNQD	HLLKLTVSSL	DYSRDGLARV	ILSKILTAAT	DACRLYATKH	LRVLLRANVE	FFNNWGIELL	VTQLHDKNKT
**490**	**500**	**510**	**520**	**530**	**540**	**550**	**560**
ISSEALDILD	EACEDKANLH	ALIQMKPALS	HLGDKGLLLL	LRFLSIPKGF	SYLNERGYVA	KQLEKWHREY	NSKYVDLIEE
**570**	**580**	**590**	**600**	**610**	**620**	**630**	**640**
QLNEALTTYR	KPVDGDNYVR	RSNQRLQRPH	VYLPIHLYGQ	LVHHKTGCHL	LEVQNIITEL	CRNVRTPDLD	KWEEIKKLKA
**650**	**660**	**670**	**680**	**690**	**700**	**710**	**720**
SLWALGNIGS	SNWGLNLLQE	ENVIPDILKL	AKQCEVLSIR	GTCVYVLGLI	AKTKQGCDIL	KCHNWDAVRH	SRKHLWPVVP
**730**	**740**	**750**	**760**	**770**	**780**	**790**	**800**
DDVEQLCNEL	SSIPSTLSLN	SESTSSRHNS	ESESVPSSMF	ILEDDRFGSS	STSTFFLDIN	EDTEPTFYDR	SGPIKDKNSF
**810**	**820**	**830**	**840**	**850**	**860**	**870**	**880**
PFFASSKLVK	NRILNSLTLP	NKKHRSSSDP	KGGKLSSESK	TSNRRIRTLT	EPSVDFNHSD	DFTPISTVQK	TLQLETSFMG
**890**	**900**	**910**	**920**	**930**	**940**	**950**	**960**
NKHIEDTGST	PSIGENDLKF	TKNFGTENHR	ENTSRERLVV	ESSTSSHMKI	RSQSFNTDTT	TSGISSMSSS	PSRETVGVDA
**970**	**980**	**990**	**1000**	**1010**	**1020**	**1030**	**1040**
TTMDTDCGSM	STVVSTKTIK	TSHYLTPQSN	HLSLSKSNSV	SLVPPGSSHT	LPRRAQSLKA	PSIATIKSLA	DCNFSYTSSR
**1050**	**1060**	**1070**	**1080**	**1090**	**1100**	**1110**	**1120**
DAFGYATLKR	LQQQRMHPSL	SHSEALASPA	KDVLFTDTIT	MKANSFESRL	TPSRFMKALS	YASLDKEDLL	SPINQNTLQR
**1130**	**1140**	**1150**	**1160**	**1170**	**1180**	**1190**	**1200**
SSSVRSMVSS	ATYGGSDDYI	GLALPVDIND	IFQVKDIPYF	QTKNIPPHDD	RGARAFAHDA	GGLPSGTGGL	VKNSFHLLRQ
**1210**	**1220**	**1230**	**1240**	**1250**	**1260**	**1270**	**1280**
QMSLTEIMNS	IHSDASLFLE	STEDTGLQEH	TDDNCLYCVC	IEILGFQPSN	QLSAICSHSD	FQDIPYSDWC	EQTIHNPLEV
**1290**	**1300**	**1310**	**1320**	**1330**	**1340**	**1350**	**1360**
VPSKFSGISG	CSDGVSQEGS	ASSTKSTELL	LGVKTIPDDT	PMCRILLRKE	VLRLVINLSS	SVSTKCHETG	LLTIKEKYPQ
**1370**	**1380**	**1390**	**1400**	**1410**	**1420**	**1430**	
TFDDICLYSE	VSHLLSHCTF	RLPCRRFIQE	LFQDVQFLQM	HEEAEAVLAT	PPKQPIVDTS	AES	

Cmpd.	No. of Cmpds.	m/z meas.	Δ m/z [ppm]	z	Rt [min]	Score	P	Range	Sequence	Modification	Ratios
2751	1	805.9143	-22.12	2	65.5	23.4	1	435-449	K.ILTAATDACRLYATK.H		WUP:QUP 1.32<br>QU:MU 2.54
275	1	795.2750	-158.71	2	33.1	12.4	1	916-929	R.ERLVVESSTSSHMK.I		WUP:QUP 0.38<br>QU:MU 2.31

Detailed Protein Report

Protein 90: dynein heavy chain 11, axonemal [Homo sapiens]

**Accession:**	gi|459642365	**Score:**	56.1
**Database:**	refseq_human(refseq_human_20140103.fasta)	**MW [kDa]:**	520.0
**Database Date:**	2015-11-30	**pI:**	6.0
**Modification(s):**	Carbamidomethyl	**Sequence Coverage [%]:**	1.0
		**No. of unique Peptides:**	1

Detailed Protein Report

10	20	30	40	50	60	70	80
MAAQVAAREA	RDFREAPTLR	LTSGAGLEAV	GAVELEEEEE	NEEEAAARRA	RSFAQDARVR	FLGGRLAMML	GFTEEKWSQY
90	100	110	120	130	140	150	160
LESEDNRQVL	GEFLESTSPA	CLVFSFAASG	RLAASQEIPR	DANHKLVFIS	KKITESIGVN	DFSQVVLFGE	LPALSLGHVS
170	180	190	200	210	220	230	240
AFLDEILVPV	LSNKNNHKSW	SCFTSQDMEY	HIEVMKKKMY	IFRGKMSRRT	LLPIPTVAGK	MDLDQNCSEN	KPPSNERIIL
250	260	270	280	290	300	310	320
HAIESVVIEW	SHQIQEIIER	DSVQRLLNGL	HLSPQAELDF	WMMRRENLSC	IYDQLQAPVV	LKMVKILTTK	QSSYFPTLKD
330	340	350	360	370	380	390	400
IFLAVENALL	EAQDVELYLR	PLRRHIQCLQ	ETEFPQTRIL	IAPLFHTICL	IWSHSKFYNT	PARVIVLLQE	FCNLFINQAT
410	420	430	440	450	460	470	480
AYLSPEDLLR	GEIEESLEKV	QVAVNILKTF	KNSFFNYRKK	LASYFMGRKL	RPWDFQSHLV	FCRFDKFLDR	LIKIEDIFAT
490	500	510	520	530	540	550	560
TLEFEKLERL	EFGGTKGAIL	NGQVHEMSEE	LMELCKLFKQ	STYDPSDCTN	MEFESDYVAF	KSKTLEFDRR	LGTIICEAFF
570	580	590	600	610	620	630	640
NCNGLEAAFK	LLTIFGNFLE	KPVVMEIFSL	HYSTLVHMFN	TELDVCKQLY	NEHMKQIECG	HVVLNKNMPF	TSGNMKWAQQ
650	660	670	680	690	700	710	720
VLQRLQMFWS	NFASLRYLFL	GNPDHALVYQ	KYVEMTTLLD	QFESRIYNEW	KSNVDEICEF	NLNQPLVKFS	AINGLLCVNF
730	740	750	760	770	780	790	800
DPKLVAVLRE	VKYLLMLKKQ	DIPDSALAIF	KKRNTILKYI	GNLDLLVQGY	NKLKQTLLEV	EYPLIEDELR	AIDEQLTAAT
810	820	830	840	850	860	870	880
TWLTWQDDCW	GYIERVRAAT	SELEHRVERT	QKNVKVIQQT	MRGWARCVLP	PRREHRREAA	FTLEDKGDLF	TKKYKLIQGD
890	900	910	920	930	940	950	960
GCKIHNLVEE	NRKLFKANPS	LDTWKIYVEF	IDDIVVEGFF	QAIMHDLDFF	LKNTEKQLKP	APFFQAQMIL	LPPEIVFKPS
970	980	990	1000	1010	1020	1030	1040
LDREAGDGFY	DLVEEMLCNS	FRMSAQMNRI	ATHLEIKNYQ	NDMDNMLGLA	EVRQEIMNRV	VNVINKVLDF	RNTLETHTYL
1050	1060	1070	1080	1090	1100	1110	1120
WVDDRAEFMK	HFLLYGHAVS	SDEMDAHANE	EIPEQPPTLE	QFKEQIDIYE	ALYVQMSKFE	DFRVFDSWFK	VDMKPFKVSL
1130	1140	1150	1160	1170	1180	1190	1200
LTIIKKWSWM	FQEHLLRFVI	DSLNELQEFI	KETDSGLQRE	LNEGDHDGLV	DIMVHLLAVR	SRQRATDELF	EPLKETITLL
1210	1220	1230	1240	1250	1260	1270	1280
ESYGQKMPEQ	VYIQLEELPE	RWETTKKIAA	TVRHEVSPLH	NAEVTLIRKK	CILFDAKQAE	FRERFRHYAP	LGFNAENPYT
1290	1300	1310	1320	1330	1340	1350	1360
ALDKANEELE	ALEEEMLQMQ	ESTRLFEVAL	PEYKQMKQCR	KEIKLLKGLW	DVIIYVRRSI	DNWTKTQWRQ	IHVEQMDVEL
1370	1380	1390	1400	1410	1420	1430	1440
RRFAKEIWSL	NKEVRVWDAY	TGLEGTVKDM	TASLRAITEL	QSPALRDRHW	HQLMKAIGVK	FLINEATTLA	DLLALRLHRV
1450	1460	1470	1480	1490	1500	1510	1520
EDDVRRIVDK	AVKELGTEKV	ITEISQTWAT	MKFSYEVHYR	TGIPLLKSDE	QLFETLEHNQ	VQLQTLLQSK	YVEYFIEQVL
1530	1540	1550	1560	1570	1580	1590	1600
SWQNKLNIAD	LVIFTWMEVQ	RTWSHLESIF	VCSEDIRIQL	VKDARRFDGV	DAEFKELMFK	TAKVENVLEA	TCRPNLYEKL
1610	1620	1630	1640	1650	1660	1670	1680
KDLQSRLSLC	EKALAEYLET	KRIAFPRFYF	VSSADLLDIL	SKGAQPKQVT	CHLAKLFDSI	ADLQFEDNQD	VSAHRAVGMY
1690	1700	1710	1720	1730	1740	1750	1760
SKEKEYVPFQ	AECECVGHVE	TWLLQLEQTM	QETVRHSITE	AIVAYEEKPR	ELWIFDFPAQ	VALTSSQIWW	TTDVGIAFSR
1770	1780	1790	1800	1810	1820	1830	1840
LEEGYETALK	DFHKKQISQL	NTLITLLLGE	LPPGDRQKIM	TICTIDVHAR	DVVAKLISQK	VVSPQAFTWL	SQLRHRWEDT
1850	1860	1870	1880	1890	1900	1910	1920
QKHCFVNICD	AQFQYFYEYL	GNSPRLVITP	LTDRCYITLT	QSLHLTMSGA	PAGPAGTGKT	ETTKDLGRAL	GMMVYVFNCS
1930	1940	1950	1960	1970	1980	1990	2000
EQMDYKSIGN	IYKGLVQTGA	WGCFDEFNRI	SVEVLSVVAV	QVKMIHDAIR	NRKKRFVFLG	EAITLKPSVG	IFITMNPGYA
2010	2020	2030	2040	2050	2060	2070	2080
GRTELPENLK	ALFRPCAMVA	PDIELICEIL	LVAEGFVDAR	ALARKFITLY	TLCKELLSKQ	DHYDWGLRAI	KSVLVVAGSL
2090	2100	2110	2120	2130	2140	2150	2160
KRGDKNRPED	QVLMRALRDF	NMPKIVTDDI	PVFLGLVGDL	FPALDVPRRR	KLHFEQMVRQ	STLELRLQPE	ESFILKVVQL
2170	2180	2190	2200	2210	2220	2230	2240

Detailed Protein Report

Cmpd.	No. of Cmpds.	m/z meas.	Δ m/z [ppm]	z	Rt [min]	Score	P	Range	Sequence	Modification
2496	1	822.4302	-68.99	2	61.8	12.6	1	2977-2990	R.LQLKIILCFSPVGR.T	Carbamidomethyl: 8

Detailed Protein Report

Protein 91: rap guanine nucleotide exchange factor 3 isoform a [Homo sapiens]

Accession: gi|148747859
Database: refseq_human(refseq_human_20140103.fasta)
Database Date: 2015-11-30
Modification(s): Carbamidomethyl, Oxidation

Score: 55.9
MW [kDa]: 103.6
pI: 7.3
Sequence Coverage [%]: 4.1
No. of unique Peptides: 2

Quantitation

QU:MU	**Median:**	1.98	**CV:**	0.00 %	**No. of Peptides:**	1
WUP:QUP	**Median:**	1.01	**CV:**	0.00 %	**No. of Peptides:**	1

10	20	30	40	50	60	70	80
MKVGWPGESC	WQVGLAVEDS	PALGAPRVGA	LPDVVPEGTL	LNMVLRRMHR	PRSCSYQLLL	EHQRPSCIQG	LRWTPLTNSE
90	100	110	120	130	140	150	160
ESLDFSESLE	QASTERVLRA	GRQLHRHLLA	TCPNLIRDRK	YHLRLYRQCC	SGRELVDGIL	ALGLGVHSRS	QVVGICQVLL
170	180	190	200	210	220	230	240
DEGALCHVKH	DWAFQDRDAQ	FYRFPGPEPE	PVGTHEMEEE	LAEAVALLSQ	RGPDALLTVA	LRKPPGQRTD	EELDLIFEEL
250	260	270	280	290	300	310	320
LHIKAVAHLS	NSVKRELAAV	LLFEPHSKAG	TVLFSQGDKG	TSWYIIWKGS	VNVVTHGKGL	VTTLHEGDDF	GQLALVNDAP
330	340	350	360	370	380	390	400
RAATIILRED	NCHFLRVDKQ	DFNRIIKDVE	AKTMRLEEHG	KVVLVLERAS	QGAGPSRPPT	PGRNRYTVMS	GTPEKILELL
410	420	430	440	450	460	470	480
LEAMGPDSSA	HDPTETFLSD	FLLTHRVFMP	SAQLCAALLH	HFHVEPAGGS	EQERSTYVCN	KRQQILRLVS	QWVALYGSML
490	500	510	520	530	540	550	560
HTDPVATSFL	QKLSDLVGRD	TRLSNLLREQ	WPERRRCHRL	ENGCGNASPQ	MKARNLPVWL	PNQDEPLPGS	SCAIQVGDKV
570	580	590	600	610	620	630	640
PYDICRPDHS	VLTLQLPVTA	SVREVMAALA	QEDGWTKGQV	LVKVNSAGDA	IGLQPDARGV	ATSLGLNERL	FVVNPQEVHE
650	660	670	680	690	700	710	720
LIPHPDQLGP	TVGSAEGLDL	VSAKDLAGQL	TDHDWSLFNS	IHQVELIHYV	LGPQHLRDVT	TANLERFMRR	FNELQYWVAT
730	740	750	760	770	780	790	800
ELCLCPVPGP	RAQLLRKFIK	LAAHLKEQKN	LNSFFAVMFG	LSNSAISRLA	HTWERLPHKV	RKLYSALERL	LDPSWNHRVY
810	820	830	840	850	860	870	880
RLALAKLSPP	VIPFMPLLLK	DMTFIHEGNH	TLVENLINFE	KMRMMARAAR	MLHHCRSHNP	VPLSPLRSRV	SHLHEDSQVA
890	900	910	920	930			
RISTCSEQSL	STRSPASTWA	YVQQLKVIDN	QRELSRLSRE	LEP			

Cmpd.	No. of Cmpds.	m/z meas.	Δ m/z [ppm]	z	Rt [min]	Score	P	Range	Sequence	Modification	Ratios
1402	1	727.3976	-11.71	2	45.9	15.2	0	256-268	R.ELAAVLLFEPHSK.A		QU:MU 1.98<br>WUP:QUP 1.01
1805	1	937.7928	-121.06	2	52.4	13.0	1	517-532	R.CHRLENGCGNASPQMK.A	Carbamidomethyl: 1, 8;<br>Oxidation: 15	

Detailed Protein Report

Protein 92: fanconi-associated nuclease 1 isoform a [Homo sapiens]

**Accession:**	gi|226246523	**Score:**	55.4
**Database:**	refseq_human(refseq_human_20140103.fasta)	**MW [kDa]:**	114.2
**Database Date:**	2015-11-30	**pI:**	7.4
**Modification(s):**	Oxidation	**Sequence Coverage [%]:**	4.7
		**No. of unique Peptides:**	3

Quantitation

QU:MU	**Median:**	0.83	**CV:**	0.00 %	**No. of Peptides:**	1
WUP:QUP	**Median:**	1.53	**CV:**	0.00 %	**No. of Peptides:**	1

Alias proteins:

Accession	Name	Description
gi|578840975	refseq_human (refseq_human_20140103.fasta)	PREDICTED: fanconi-associated nuclease 1 isoform X4 [Homo sapiens]
gi|578840973	refseq_human (refseq_human_20140103.fasta)	PREDICTED: fanconi-associated nuclease 1 isoform X3 [Homo sapiens]
gi|578840971	refseq_human (refseq_human_20140103.fasta)	PREDICTED: fanconi-associated nuclease 1 isoform X2 [Homo sapiens]
gi|578840969	refseq_human (refseq_human_20140103.fasta)	PREDICTED: fanconi-associated nuclease 1 isoform X1 [Homo sapiens]
gi|530405539	refseq_human (refseq_human_20140103.fasta)	PREDICTED: fanconi-associated nuclease 1 isoform X4 [Homo sapiens]
gi|530405537	refseq_human (refseq_human_20140103.fasta)	PREDICTED: fanconi-associated nuclease 1 isoform X3 [Homo sapiens]
gi|530405535	refseq_human (refseq_human_20140103.fasta)	PREDICTED: fanconi-associated nuclease 1 isoform X2 [Homo sapiens]
gi|530405533	refseq_human (refseq_human_20140103.fasta)	PREDICTED: fanconi-associated nuclease 1 isoform X1 [Homo sapiens]

Detailed Protein Report

10	20	30	40	50	60	70	80
MMSEGKPPDK	KRPRRSLSIS	KNKKKASNSI	ISCFNNAPPA	KLACPVCSKM	VPRYDLNRHL	DEMCANNDFV	QVDPGQVGLI
90	100	110	120	130	140	150	160
NSNVSMVDLT	SVTLEDVTPK	KSPPPKTNLT	PGQSDSAKRE	VKQKISPYFK	SNDVVCKNQD	ELRNRSVKVI	CLGSLASKLS
170	180	190	200	210	220	230	240
RKYVKAKKSI	DKDEEFAGSS	PQSSKSTVVK	SLIDNSSEIE	DEDQILENSS	QKENVFKCDS	LKEECIPEHM	VRGSKIMEAE
250	260	270	280	290	300	310	320
SQKATRECEK	SALTPGFSDN	AIMLFSPDFT	LRNTLKSTSE	DSLVKQECIK	EVVEKREACH	CEEVKMTVAS	EAKIQLSDSE
330	340	350	360	370	380	390	400
AKSHSSADDA	SAWSNIQEAP	LQDDSCLNND	IPHSIPLEQG	SSCNGPGQTT	GHPYYLRSFL	VVLKTVLENE	DDMLLFDEQE
410	420	430	440	450	460	470	480
KGIVTKFYQL	SATGQKLYVR	LFQRKLSWIK	MTKLEYEEIA	LDLTPVIEEL	TNAGFLQTES	ELQELSEVLE	LLSAPELKSL
490	500	510	520	530	540	550	560
AKTFHLVNPN	GQKQQLVDAF	LKLAKQRSVC	TWGKNKPGIG	AVILKRAKAL	AGQSVRICKG	PRAVFSRILL	LFSLTDSMED
570	580	590	600	610	620	630	640
EDAACGGQGQ	LSTVLLVNLG	RMEFPSYTIN	RKTHIFQDRD	DLIRYAAATH	MLSDISSAMA	NGNWEEAKEL	AQCAKRDWNR
650	660	670	680	690	700	710	720
LKNHPSLRCH	EDLPLFLRCF	TVGWIYTRIL	SRFVEILQRL	HMYEEAVREL	ESLLSQRIYC	PDSRGRWWDR	LALNLHQHLK
730	740	750	760	770	780	790	800
RLEPTIKCIT	EGLADPEVRT	GHRLSLYQRA	VRLRESPSCK	KFKHLFQQLP	EMAVQDVKHV	TITGRLCPQR	GMCKSVFVME
810	820	830	840	850	860	870	880
AGEAADPTTV	LCSVEELALA	HYRRSGFDQG	IHGEGSTFST	LYGLLLWDII	FMDGIPDVFR	NACQAFPLDL	CTDSFFTSRR
890	900	910	920	930	940	950	960
PALEARLQLI	HDAPEESLRA	WVAATWHEQE	GRVASLVSWD	RFTSLQQAQD	LVSCLGGPVL	SGVCRHLAAD	FRHCRGGLPD
970	980	990	1000	1010	1020		
LVVWNSQSRH	FKLVEVKGPN	DRLSHKQMIW	LAELQKLGAE	VEVCHVVAVG	AKSQSLS		

Cmpd.	No. of Cmpds.	m/z meas.	Δ m/z [ppm]	z	Rt [min]	Score	P	Range	Sequence	Modification	Ratios
691	1	765.8997	57.54	2	38.6	18.5	1	223-235	K.EECIPEHMVRGSK.I	Oxidation: 8	
60	5	768.2851	-144.27	2	30.4	19.5	2	233-246	R.GSKIMEAESQKATR.E		QU:MU 0.83<br>WUP:QUP 1.53
97	2	826.6883	-79.67	3	31.1	17.4	0	922-945	R.<br>FTSLQQAQDLVSCLGGPVLSGVC<br>H		

Detailed Protein Report

Protein 93: replication factor C subunit 1 isoform 1 [Homo sapiens]

**Accession:**	gi|32528306	**Score:**	55.2
**Database:**	refseq_human(refseq_human_20140103.fasta)	**MW [kDa]:**	128.1
**Database Date:**	2015-11-30	**pI:**	9.9
**Modification(s):**	Carbamidomethyl	**Sequence Coverage [%]:**	7.5
		**No. of unique Peptides:**	5

10	20	30	40	50	60	70	80
MDIRKFFGVI	PSGKKLVSET	VKKNEKTKSD	EETLKAKKGI	KEIKVNSSRK	EDDFKQKQPS	KKKRIIYDSD	SESEETLQVK
**90**	**100**	**110**	**120**	**130**	**140**	**150**	**160**
NAKKPPEKLP	VSSKPGKISR	QDPVTYISET	DEEDDFMCKK	AASKSKENGR	STNSHLGTSN	MKKNEENTKT	KNKPLSPIKL
**170**	**180**	**190**	**200**	**210**	**220**	**230**	**240**
TPTSVLDYFG	TGSVQRSNKK	MVASKRKELS	QNTDESGLND	EAIAKQLQLD	EDAELERQLH	EDEEFARTLA	MLDEEPKTKK
**250**	**260**	**270**	**280**	**290**	**300**	**310**	**320**
ARKDTEAGET	FSSVQANLSK	AEKHKYPHKV	KTAQVSDERK	SYSPRKQSKY	ESSKESQQHS	KSSADKIGEV	SSPKASSKLA
**330**	**340**	**350**	**360**	**370**	**380**	**390**	**400**
IMKRKEESSY	KEIEPVASKR	KENAIKLKGE	TKTPKKTKSS	PAKKESVSPE	DSEKKRTNYQ	AYRSYLNREG	PKALGSKEIP
**410**	**420**	**430**	**440**	**450**	**460**	**470**	**480**
KGAENCLEGL	IFVITGVLES	IERDEAKSLI	ERYGGKVTGN	VSKKTNYLVM	GRDSGQSKSD	KAAALGTKII	DEDGLLNLIR
**490**	**500**	**510**	**520**	**530**	**540**	**550**	**560**
TMPGKKSKYE	IAVETEMKKE	SKLERTPQKN	VQGKRKISPS	KKESESKKSR	PTSKRDSLAK	TIKKETDVFW	KSLDFKEQVA
**570**	**580**	**590**	**600**	**610**	**620**	**630**	**640**
EETSGDSKAR	NLADDSSENK	VENLLWVDKY	KPTSLKTIIG	QQGDQSCANK	LLRWLRNWQK	SSSEDKKHAK	FGKFSGKDDG
**650**	**660**	**670**	**680**	**690**	**700**	**710**	**720**
SSFKAALLSG	PPGVGKTTTA	SLVCQELGYS	YVELNASDTR	SKSSLKAIVA	ESLNNTSIKG	FYSNGAASSV	STKHALIMDE
**730**	**740**	**750**	**760**	**770**	**780**	**790**	**800**
VDGMAGNEDR	GGIQELIGLI	KHTKIPIICM	CNDRNHPKIR	SLVHYCFDLR	FQRPRVEQIK	GAMMSIAFKE	GLKIPPPAMN
**810**	**820**	**830**	**840**	**850**	**860**	**870**	**880**
EIILGANQDI	RQVLHNLSMW	CARSKALTYD	QAKADSHRAK	KDIKMGPFDV	ARKVFAAGEE	TAHMSLVDKS	DLFFHDYSIA
**890**	**900**	**910**	**920**	**930**	**940**	**950**	**960**
PLFVQENYIH	VKPVAAGGDM	KKHLMLLSRA	ADSICDGDLV	DSQIRSKQNW	SLLPAQAIYA	SVLPGELMRG	YMTQFPTFPS
**970**	**980**	**990**	**1000**	**1010**	**1020**	**1030**	**1040**
WLGKHSSTGK	HDRIVQDLAL	HMSLRTYSSK	RTVNMDYLSL	LRDALVQPLT	SQGVDGVQDV	VALMDTYYLM	KEDFENIMEI
**1050**	**1060**	**1070**	**1080**	**1090**	**1100**	**1110**	**1120**
SSWGGKPSPF	SKLDPKVKAA	FTRAYNKEAH	LTPYSLQAIK	ASRHSTSPSL	DSEYNEELNE	DDSQSDEKDQ	DAIETDAMIK
**1130**	**1140**	**1150**					
KKTKSSKPSK	PEKDKEPRKG	KGKSSKK					

Cmpd.	No. of Cmpds.	m/z meas.	Δ m/z [ppm]	z	Rt [min]	Score	P	Range	Sequence	Modification
967	1	530.7788	-57.17	2	40.4	11.1	2	42-50	K.EIKVNSSRK.E	
1884	1	974.4729	4.34	2	51.9	11.6	2	125-142	K.SKENGRSTNSHLGTSNMK.K	
1563	1	949.5033	-22.26	2	49.7	10.2	1	469-485	K.IIDEDGLLNLIRTMPGK.K	
2929	1	965.1417	7.65	3	65.4	10.9	1	657-682	K.TTTASLVCQELGYSYVELNASDTRSK.S	Carbamidomethyl: 8
1265	1	990.4490	-59.66	2	45.4	11.3	2	745-760	K.IPIICMCNDRNHPKIR.S	Carbamidomethyl: 5

Detailed Protein Report

Protein 94: protein S100-A11 [Homo sapiens]

Accession: gi|5032057
Database: refseq_human(refseq_human_20140103.fasta)
Database Date: 2015-11-30
Modification(s): Carbamidomethyl

Score: 54.9
MW [kDa]: 11.7
pI: 7.5
Sequence Coverage [%]: 10.5
No. of unique Peptides: 1

```
         10         20         30         40         50         60         70         80
MAKISSPTET ERCIESLIAV FQKYAGKDGY NYTLSKTEFL SFMNTELAAF TKNQKDPGVL DRMMKKLDTN SDGQLDFSEF
         90        100        110
LNLIGGLAMA CHDSFLKAVP SQKRT
```

Cmpd.	No. of Cmpds.	m/z meas.	Δ m/z [ppm]	z	Rt [min]	Score	P	Range	Sequence	Modification
2752	3	654.3389	-24.71	2	65.5	54.9	0	13-23	R.CIESLIAVFQK.Y	Carbamidomethyl: 1

Detailed Protein Report

Protein 95: collagen alpha-1(XXVIII) chain precursor [Homo sapiens]

**Accession:**	gi|154759255	**Score:**	54.2
**Database:**	refseq_human(refseq_human_20140103.fasta)	**MW [kDa]:**	116.6
**Database Date:**	2015-11-30	**pI:**	6.1
**Modification(s):**	Oxidation	**Sequence Coverage [%]:**	6.6
		**No. of unique Peptides:**	2

Quantitation

WUP:QUP **Median:** 2.39 **CV:** 0.00 % **No. of Peptides:** 1

10	20	30	40	50	60	70	80
MWNRYFVFYL	LLLSAFTSQT	VSGQRKKGPK	SNLLARKSDV	QGSICFIDIV	FIVDSSESSK	IALFDKQKDF	VDSLSDKIFQ
**90**	**100**	**110**	**120**	**130**	**140**	**150**	**160**
LTPGRSLEYD	IKLAALQFSS	SVQIDPPFSS	WKDLQTFKQK	VKSMNLIGQG	TFSYYAISNA	TRLLKREGRK	DGVKVVLLMT
**170**	**180**	**190**	**200**	**210**	**220**	**230**	**240**
DGIDHPKNPD	VQSISEDARI	SGISFITIAL	STVVNEAKLR	LISGDSSSEP	TLLLSDPTLV	DKIQDRLDIL	FEKKCERKIC
**250**	**260**	**270**	**280**	**290**	**300**	**310**	**320**
ECEKGDPGDP	GPPGTHGNPG	IKGERGPKGN	PGNAQKGEAG	ERGPGGIPGY	KGDKGERGEC	GKPGIKGDKG	SPGPYGPKGP
**330**	**340**	**350**	**360**	**370**	**380**	**390**	**400**
RGIQGITGPP	GDPGPKGFQG	NKGEPGPPGP	YGSPGAPGIG	QQGIKGERGQ	EGRPGAPGPI	GVGEPGQPGP	RGPEGVPGER
**410**	**420**	**430**	**440**	**450**	**460**	**470**	**480**
GLPGEGFPGP	KGEKGSEGPT	GPQGLQGLSI	KGEKGDIGPV	GPQGPMGIPG	IGSQGEQGIQ	GPIGPPGPQG	PAGQGLPGSK
**490**	**500**	**510**	**520**	**530**	**540**	**550**	**560**
GEVGQMGPTG	PRGPVGIGVQ	GPKGEPGSIG	LPGQPGVPGE	DGAAGKKGEA	GLPGARGPEG	PPGKGQPGPK	GDEGKKGSKG
**570**	**580**	**590**	**600**	**610**	**620**	**630**	**640**
NQGQRGLPGP	EGPKGEPGIM	GPFGMPGTSI	PGPPGPKGDR	GGPGIPGFKG	EPGLSIRGPK	GVQGPRGPVG	APGLKGDGYP
**650**	**660**	**670**	**680**	**690**	**700**	**710**	**720**
GVPGPRGLPG	PPGPMGLRGV	GDTGAKGEPG	VRGPPGPSGP	RGVGTQGPKG	DTGQKGLPGP	PGPPGYGSQG	IKGEQGPQGF
**730**	**740**	**750**	**760**	**770**	**780**	**790**	**800**
PGPKGTMGHG	LPGQKGEHGE	RGDVGKKGDK	GEIGEPGSPG	KQGLQGPKGD	LGLTKEEIIK	LITEICGCGP	KCKETPLELV
**810**	**820**	**830**	**840**	**850**	**860**	**870**	**880**
FVIDSSESVG	PENFQIIKNF	VKTMADRVAL	DLATARIGII	NYSHKVEKVA	NLKQFSSKDD	FKLAVDNMQY	LGEGTYTATA
**890**	**900**	**910**	**920**	**930**	**940**	**950**	**960**
LQAANDMFED	ARPGVKKVAL	VITDGQTDSR	DKEKLTEVVK	NASDTNVEIF	VIGVVKKNDP	NFEIFHKEMN	LIATDPEHVY
**970**	**980**	**990**	**1000**	**1010**	**1020**	**1030**	**1040**
QFDDFFTLQD	TLKQKLFQKI	CEDFDSYLVQ	IFGSSSPQPG	FGMSGEELSE	STPEPQKEIS	ESLSVTRDQD	EDDKAPEPTW
**1050**	**1060**	**1070**	**1080**	**1090**	**1100**	**1110**	**1120**
ADDLPATTSS	EATTTPRPLL	STPVDGAEDP	RCLEALKPGN	CGEYVVRWYY	DKQVNSCARF	WFSGCNGSGN	RFNSEKECQE
**1130**							
TCIQG							

Cmpd.	No. of Cmpds.	m/z meas.	Δ m/z [ppm]	z	Rt [min]	Score	P	Range	Sequence	Modification	Ratios
1277	1	990.9708	-72.85	2	45.6	13.8	2	150-167	R.KDGVKVVLLMTDGIDHPK.N	Oxidation: 10	WUP:QUP 2.39
201	1	714.3696	-12.54	3	32.5	18.4	2	667-689	K. GEPGVRGPPGPSGPRGVGTQGP G		

Detailed Protein Report

Protein 96: ATP-binding cassette sub-family A member 12 isoform b [Homo sapiens]

**Accession:**	gi|27881501	**Score:**	54.2
**Database:**	refseq_human(refseq_human_20140103.fasta)	**MW [kDa]:**	256.8
**Database Date:**	2015-11-30	**pI:**	7.6
**Modification(s):**	Oxidation	**Sequence Coverage [%]:**	2.2
		**No. of unique Peptides:**	4

Quantitation

WUP:QUP	**Median:** 1.33	**CV:** 0.00 %	**No. of Peptides:** 1

Detailed Protein Report

10	20	30	40	50	60	70	80
MFTYIKIITS	GDSDNITHVW	NEDDGQTLSP	SSLAAQLLIL	ENFEDALLNI	SANSPYIPYL	ACVRNVTDSL	ARGSPENLRL
90	100	110	120	130	140	150	160
LQSTIRFKKS	FLRNGSYEDY	FPPVPEVLKS	KLSQLRNLTE	LLCESETFSL	IEKSCQLSDM	SFGSLCEESE	FDLQLLEAAE
170	180	190	200	210	220	230	240
LGTEIAASLL	YHDNVISKKV	RDLLTGDPSK	INLNMDQFLE	QALQMNYLEN	ITQLIPIIEA	MLHVNNSADA	SEKPGQLLEM
250	260	270	280	290	300	310	320
FKNVEELKED	LRRTTGMSNR	TIDKLLAIPI	PDNRAEIISQ	VFWLHSCDTN	ITTPKLEDAM	KEFCNLSLSE	RSRQSYLIGL
330	340	350	360	370	380	390	400
TLLHYLNIYN	FTYKVFFPRK	DQKPVEKMME	LFIRLKEILN	QMASGTHPLL	DKMRSLKQMH	LPRSVPLTQA	MYRSNRMNTP
410	420	430	440	450	460	470	480
QGSFSTISQA	LCSQGITTEY	LTAMLPSSQR	PKGNHTKDFL	TYKLTKEQIA	SKYGIPINST	PFCFSLYKDI	INMPAGPVIW
490	500	510	520	530	540	550	560
AFLKPMLLGR	ILYAPYNPVT	KAIMEKSNVT	LRQLAELREK	SQEWMDKSPL	FMNSFHLLNQ	AIPMLQNTLR	NPFVQVFVKF
570	580	590	600	610	620	630	640
SVGLDAVELL	KQIDELDILR	LKLENNIDII	DQLNTLSSLT	VNISSCVLYD	RIQAAKTIDE	MEREAKRLYK	SNELFGSVIF
650	660	670	680	690	700	710	720
KLPSNRSWHR	GYDSGNVFLP	PVIKYTIRMS	LKTAQTTRSL	RTKIWAPGPH	NSPSHNQIYG	RAFIYLQDSI	ERAIIELQTG
730	740	750	760	770	780	790	800
RNSQEIAVQV	QAIPYPCFMK	DNFLTSVSYS	LPIVLMVAWV	VFIAAFVKKL	VYEKDLRLHE	YMKMMGVNSC	SHFFAWLIES
810	820	830	840	850	860	870	880
VGFLLVTIVI	LIIILKFGNI	LPKTNGFILF	LYFSDYSFSV	IAMSYLISVF	FNNTNIAALI	GSLIYIIAFF	PFIVLVTVEN
890	900	910	920	930	940	950	960
ELSYVLKVFM	SLLSPTAFSY	ASQYIARYEE	QGIGLQWENM	YTSPVQDDTT	SFGWLCCLIL	ADSFIYFLIA	WYVRNVFPGT
970	980	990	1000	1010	1020	1030	1040
YGMAAPWYFP	ILPSYWKERF	GCAEVKPEKS	NGLMFTNIMM	QNTNPSASPE	YMFSSNIEPE	PKDLTVGVAL	HGVTKIYGSK
1050	1060	1070	1080	1090	1100	1110	1120
VAVDNLNLNF	YEGHITSLLG	PNGAGKTTTI	SMLTGLFGAS	AGTIFVYGKD	IKTDLHTVRK	NMGVCMQHDV	LFSYLTTKEH
1130	1140	1150	1160	1170	1180	1190	1200
LLLYGSIKVP	HWTKKQLHEE	VKRTLKDTGL	YSHRHKRVGT	LSGGMKRKLS	ISIALIGGSR	VVILDEPSTG	VDPCSRRSIW
1210	1220	1230	1240	1250	1260	1270	1280
DVISKNKTAR	TIILSTHHLD	EAEVLSDRIA	FLEQGGLRCC	GSPFYLKEAF	GDGYHLTLTK	KKSPNLNANA	VCDTMAVTAM
1290	1300	1310	1320	1330	1340	1350	1360
IQSHLPEAYL	KEDIGGELVY	VLPPFSTKVS	GAYLSLLRAL	DNGMGDLNIG	CYGISDTTVE	EVFLNLTKES	QKNSAMSLEH
1370	1380	1390	1400	1410	1420	1430	1440
LTQKKIGNSN	ANGISTPDDL	SVSSSNFTDR	DDKILTRGER	LDGFGLLLKK	IMAILIKRFH	HTRRNWKGLI	AQVILPIVFV
1450	1460	1470	1480	1490	1500	1510	1520
TTAMGLGTLR	NSSNSYPEIQ	ISPSLYGTSE	QTAFYANYHP	STEALVSAMW	DFPGIDNMCL	NTSDLQCLNK	DSLEKWNTSG
1530	1540	1550	1560	1570	1580	1590	1600
EPITNFGVCS	CSENVQECPK	FNYSPPHRRT	YSSQVIYNLT	GQRVENYLIS	TANEFVQKRY	GGWSFGLPLT	KDLRFDITGV
1610	1620	1630	1640	1650	1660	1670	1680
PANRTLAKVW	YDPEGYHSLP	AYLNSLNNFL	LRVNMSKYDA	ARHGIIMYSH	PYPGVQDQEQ	ATISSLIDIL	VALSILMGYS
1690	1700	1710	1720	1730	1740	1750	1760
VTTASFVTYV	VREHQTKAKQ	LQHISGIGVT	CYWVTNFIYD	MVFYLVPVAF	SIGIIAIFKL	PAFYSENNLG	AVSLLLLLFG
1770	1780	1790	1800	1810	1820	1830	1840
YATFSWMYLL	AGLFHETGMA	FITYVCVNLF	FGINSIVSLS	VVYFLSKEKP	NDPTLELISE	TLKRIFLIFP	QFCFGYGLIE
1850	1860	1870	1880	1890	1900	1910	1920
LSQQQSVLDF	LKAYGVEYPN	ETFEMNKLGA	MFVALVSQGT	MFFSLRLLIN	ESLIKKLRLF	FRKFNSSHVR	ETIDEDEDVR
1930	1940	1950	1960	1970	1980	1990	2000
AERLRVESGA	AEFDLVQLYC	LTKTYQLIHK	KIIAVNNISI	GIPAGECFGL	LGVNGAGKTT	IFKMLTGDII	PSSGNILIRN
2010	2020	2030	2040	2050	2060	2070	2080
KTGSLGHVDS	HSSLVGYCPQ	EDALDDLVTV	EEHLYFYARV	HGIPEKDIKE	TVHKLLRRLH	LMPFKDRATS	MCSYGTKRKL
2090	2100	2110	2120	2130	2140	2150	2160
STALALIGKP	SILLLDEPSS	GMDPKSKRHL	WKIISEEVQN	KCSVILTSHS	MEECEALCTR	LAIMVNGKFQ	CIGSLQHIKS
2170	2180	2190	2200	2210	2220	2230	2240

Detailed Protein Report

Cmpd.	No. of Cmpds.	m/z meas.	Δ m/z [ppm]	z	Rt [min]	Score	P	Range	Sequence	Modification	Ratios
1898	1	890.3676	-104.02	2	53.6	10.7	1	341-354	K.DQKPVEKMMELFIR.L	Oxidation: 8	WUP:QUP 1.33
1953	1	590.7822	22.74	2	52.8	13.4	1	519-527	R.EKSQEWMDK.S		
2766	1	702.8107	-71.83	2	65.0	19.2	1	612-623	R.IQAAKTIDEMER.E		
2087	1	753.3822	-28.39	2	56.5	10.9	0	651-664	R.GYDSGNVFLPPVIK.Y		

Detailed Protein Report

Protein 97: **PREDICTED: pericentrin isoform X6 [Homo sapiens]**

**Accession:**	gi|530419254	**Score:**	54.0
**Database:**	refseq_human(refseq_human_20140103.fasta)	**MW [kDa]:**	355.7
**Database Date:**	2015-11-30	**pI:**	5.2
		**Sequence Coverage [%]:**	1.2
		**No. of unique Peptides:**	2

Detailed Protein Report

10	20	30	40	50	60	70	80
MFTVSDHPPE	QHGMFTVGDH	PPEQRGMFTV	SDHPPEQHGM	FTVSDHPPEQ	RGMFTISDHQ	PEQRGMFTVS	DHTPEQRGIF
90	100	110	120	130	140	150	160
TISDHPAEQR	GMFTKECEQE	CELAITDLES	GREDEAGLHQ	SQAVHGLELE	ALRLSLSNMH	TAQLELTQAN	LQKEKETALT
170	180	190	200	210	220	230	240
ELREMLNSRR	AQELALLQSR	QQHELELLRE	QHAREKEEVV	LRCGQEAAEL	KEKLQSEMEK	NAQIVKTLKE	DWESEKDLCL
250	260	270	280	290	300	310	320
ENLRKELSAK	HQSEMEDLQN	QFQKELAEQR	AELEKIFQDK	NQAERALRNL	ESHHQAAIEK	LREDLQSEHG	RCLEDLEFKF
330	340	350	360	370	380	390	400
KESEKEKQLE	LENLQASYED	LKAQSQEEIR	RLWSQLDSAR	TSRQELSELH	EQLLARTSRV	EDLEQLKQRE	KTQHESELEQ
410	420	430	440	450	460	470	480
LRIYFEKKLR	DAEKTYQEDL	TLLQQRLQGA	REDALLDSVE	VGLSCVGLEE	KPEKGRKDHV	DELEPERHKE	SLPRFQAELE
490	500	510	520	530	540	550	560
ESHRHQLEAL	ESPLCIQHEG	HVSDRCCVET	SALGHEWRLE	PSEGHSQELP	WVHLQGVQDG	DLEADTERAA	RVLGLETEHK
570	580	590	600	610	620	630	640
VQLSLLQTEL	KEEIELLKIE	NRNLYGKLQH	ETRLKDDLEK	VKHNLIEDHQ	KELNNAKQKT	ELMKQEFQRK	ETDWKVMKEE
650	660	670	680	690	700	710	720
LQREAEEKLT	LMLLELREKA	ESEKQTIINK	FELREAEMRQ	LQDQQAAQIL	DLERSLTEQQ	GRLQQLEQDL	TSDDALHCSQ
730	740	750	760	770	780	790	800
CGREPPTAQD	GELAALHVKE	DCALQLMLAR	SRFLEERKEI	TEKFSAEQDA	FLQEAQEQHA	RELQLLQERH	QQQLLSVTAE
810	820	830	840	850	860	870	880
LEARHQAALG	ELTASLESKQ	GALLAARVAE	LQTKHAADLG	ALETRHLSSL	DSLESCYLSE	FQTIREEHRQ	ALELLRADFE
890	900	910	920	930	940	950	960
EQLWKKDSLH	QTILTQELEK	LKRKHEGELQ	SVRDHLRTEV	STELAGTVAH	ELQGVHQGEF	GSEKKTALHE	KEETLRLQSA
970	980	990	1000	1010	1020	1030	1040
QAQPFHQEEK	ESLSLQLQKK	NHQVQQLKDQ	VLSLSHEIEE	CRSELEVLQQ	RRERENREGA	NLLSMLKADV	NLSHSERGAL
1050	1060	1070	1080	1090	1100	1110	1120
QDALRRLLGL	FGETLRAAVT	LRSRIGERVG	LCLDDAGAGL	ALSTAPALEE	TWSDVALPEL	DRTLSECAEM	SSVAEISSHM
1130	1140	1150	1160	1170	1180	1190	1200
RESFLMSPES	VRECEQPIRR	VFQSLSLAVD	GLMEMALDSS	RQLEEARQIH	SRFEKEFSFK	NEETAQVVRK	HQELLECLKE
1210	1220	1230	1240	1250	1260	1270	1280
ESAAKAELAL	ELHKTQGTLE	GFKVETADLK	EVLAGKEDSE	HRLVLELESL	RRQLQQAAQE	QAALREECTR	LWSRGEATAT
1290	1300	1310	1320	1330	1340	1350	1360
DAEAREAALR	KEVEDLTKEQ	SETRKQAEKD	RSALLSQMKI	LESELEEQLS	QHRGCAKQAE	AVTALEQQVA	SLDKHLRNQR
1370	1380	1390	1400	1410	1420	1430	1440
QFMDEQAAER	EHEREEFQQE	IQRLEGQLRQ	AAKPQPWGPR	DSQQAPLDGE	VELLQQKLRE	KLDEFNELAI	QKESADRQVL
1450	1460	1470	1480	1490	1500	1510	1520
MQEEEIKRLE	EMNINIRKKV	AQLQEEVEKQ	KNIVKGLEQD	KEVLKKQQMS	SLLLASTLQS	TLDAGRCPEP	PSGSPPEGPE
1530	1540	1550	1560	1570	1580	1590	1600
IQLEVTQRAL	LRRESEVLDL	KEQLEKMKGD	LESKNEEILH	LNLKLDMQNS	QTAVSLRELE	EENTSLKVIY	TRSSEIEELK
1610	1620	1630	1640	1650	1660	1670	1680
ATIENLQENQ	KRLQKEKAEE	IEQLHEVIEK	LQHELSLMGP	VVHEVSDSQA	GSLQSELLCS	QAGGPRGQAL	QGELEAALEA
1690	1700	1710	1720	1730	1740	1750	1760
KEALSRLLAD	QERRHSQALE	ALQQRLQGAE	EAAELQLAEL	ERNVALREAE	VEDMASRIQE	FEAALKAKEA	TIAERNLEID
1770	1780	1790	1800	1810	1820	1830	1840
ALNQRKAAHS	AELEAVLLAL	ARIRRALEQQ	PLAAGAAPPE	LQWLRAQCAR	LSRQLQVLHQ	RFLRCQVELD	RRQARRATAH
1850	1860	1870	1880	1890	1900	1910	1920
TRVPGAHPQP	RMDGGAKAQV	TGDVEASHDA	ALEPVVPDPQ	GDLQPVLVTL	KDAPLCKQEG	VMSVLTVCQR	QLQSELLLVK
1930	1940	1950	1960	1970	1980	1990	2000
NEMRLSLEDG	GKGKEKVLED	CQLPKVDLVA	QVKQLQEKLN	RLLYSMTFQN	VDAADTKSLW	PMASAHLLES	SWSDDSCDGE
2010	2020	2030	2040	2050	2060	2070	2080
EPDISPHIDT	CDANTATGGV	TDVIKNQAID	ACDANTTPGG	VTDVIKNWDS	LIPDEMPDSP	IQEKSECQDM	SLSSPTSVLG
2090	2100	2110	2120	2130	2140	2150	2160
GSRHQSHTAE	AGPRKSPVGM	LDLSSWSSPE	VLRKDWTLEP	WPSLPVTPHS	GALSLCSADT	SLGDRADTSL	PQTQGPGLLC
2170	2180	2190	2200	2210	2220	2230	2240

Detailed Protein Report

Cmpd.	No. of Cmpds.	m/z meas.	Δ m/z [ppm]	z	Rt [min]	Score	P	Range	Sequence	Modification
2710	4	1044.5270	3.44	2	65.0	25.0	2	1173-1189	R.FEKEFSFKNEETAQVVR.K	
490	2	472.1760	-159.67	2	34.6	13.3	1	3049-3056	R.TRESPPTR.D	

Detailed Protein Report

Protein 98: glutathione S-transferase P [Homo sapiens]

Accession: gi|4504183
Database: refseq_human(refseq_human_20140103.fasta)
Database Date: 2015-11-30

Score: 53.5
MW [kDa]: 23.3
pI: 5.3
Sequence Coverage [%]: 7.6
No. of unique Peptides: 1

Quantitation

QU:MU **Median:** 0.64 **CV:** 0.00 % **No. of Peptides:** 1

10	20	30	40	50	60	70	80
MPPYTVVYFP	VRGRCAALRM	LLADQGQSWK	EEVVTVETWQ	EGSLKASCLY	GQLPKFQDGD	LTLYQSNTIL	RHLGRTLGLY
**90**	**100**	**110**	**120**	**130**	**140**	**150**	**160**
GKDQQEAALV	DMVNDGVEDL	RCKYISLIYT	NYEAGKDDYV	KALPGQLKPF	ETLLSQNQGG	KTFIVGDQIS	FADYNLLDLL
**170**	**180**	**190**	**200**	**210**	**220**		
LIHEVLAPGC	LDAFPLLSAY	VGRLSARPKL	KAFLASPEYV	NLPINGNGKQ			

Cmpd.	No. of Cmpds.	m/z meas.	Δ m/z [ppm]	z	Rt [min]	Score	P	Range	Sequence	Modification	Ratios
1649	4	942.4739	-4.87	2	50.9	53.5	0	56-71	K.FQDGDLTLYQSNTILR.H		QU:MU 0.64

Detailed Protein Report

Protein 99: PREDICTED: complement receptor type 1 isoform X4 [Homo sapiens]

**Accession:**	gi|578800553	**Score:**	53.4
**Database:**	refseq_human(refseq_human_20140103.fasta)	**MW [kDa]:**	224.1
**Database Date:**	2015-11-30	**pI:**	6.7
**Modification(s):**	Carbamidomethyl	**Sequence Coverage [%]:**	2.4
		**No. of unique Peptides:**	1

Quantitation

QU:MU	**Median:**	0.52	**CV:**	0.00 %	**No. of Peptides:**	1
WUP:QUP	**Median:**	4.24	**CV:**	0.00 %	**No. of Peptides:**	1

BRUKER

Detailed Protein Report

10	20	30	40	50	60	70	80
MCLGRMGASS	PRSPEPVGPP	APGLPFCCGG	SLLAVVVLLA	LPVAWGQCNA	PEWLPFARPT	NLTDEFEFPI	GTYLNYECRP
90	100	110	120	130	140	150	160
GYSGRPFSII	CLKNSVWTGA	KDRCRRKSCR	NPPDPVNGMV	HVIKGIQFGS	QIKYSCTKGY	RLIGSSSATC	IISGDTVIWD
170	180	190	200	210	220	230	240
NETPICDRIP	CGLPPTITNG	DFISTNRENF	HYGSVVTYRC	NPGSGGRKVF	ELVGEPSIYC	TSNDDQVGIW	SGPAPQCIIP
250	260	270	280	290	300	310	320
NKCTPPNVEN	GILVSDNRSL	FSLNEVVEFR	CQPGFVMKGP	RRVKCQALNK	WEPELPSCSR	VCQPPPDVLH	AERTQRDKDN
330	340	350	360	370	380	390	400
FSPGQEVFYS	CEPGYDLRGA	ASMRCTPQGD	WSPAAPTCEV	KSCDDFMGQL	LNGRVLFPVN	LQLGAKVDFV	CDEGFQLKGS
410	420	430	440	450	460	470	480
SASYCVLAGM	ESLWNSSVPV	CEQIFCPSPP	VIPNGRHTGK	PLEVFPFGKT	VNYTCDPHPD	RGTSFDLIGE	STIRCTSDPQ
490	500	510	520	530	540	550	560
GNGVWSSPAP	RCGILGHCQA	PDHFLFAKLK	TQTNASDFPI	GTSLKYECRP	EYYGRPFSIT	CLDNLVWSSP	KDVCKRKSCK
570	580	590	600	610	620	630	640
TPPDPVNGMV	HVITDIQVGS	RINYSCTTGH	RLIGHSSAEC	ILSGNAAHWS	TKPPICQRIP	CGLPPTIANG	DFISTNRENF
650	660	670	680	690	700	710	720
HYGSVVTYRC	NPGSGGRKVF	ELVGEPSIYC	TSNDDQVGIW	SGPAPQCIIP	NKCTPPNVEN	GILVSDNRSL	FSLNEVVEFR
730	740	750	760	770	780	790	800
CQPGFVMKGP	RRVKCQALNK	WEPELPSCSR	VCQPPPDVLH	AERTQRDKDN	FSPGQEVFYS	CEPGYDLRGA	ASMRCTPQGD
810	820	830	840	850	860	870	880
WSPAAPTCEV	KSCDDFMGQL	LNGRVLFPVN	LQLGAKVDFV	CDEGFQLKGS	SASYCVLAGM	ESLWNSSVPV	CEQIFCPSPP
890	900	910	920	930	940	950	960
VIPNGRHTGK	PLEVFPFGKT	VNYTCDPHPD	RGTSFDLIGE	STIRCTSDPQ	GNGVWSSPAP	RCGILGHCQA	PDHFLFAKLK
970	980	990	1000	1010	1020	1030	1040
TQTNASDFPI	GTSLKYECRP	EYYGRPFSIT	CLDNLVWSSP	KDVCKRKSCK	TPPDPVNGMV	HVITDIQVGS	RINYSCTTGH
1050	1060	1070	1080	1090	1100	1110	1120
RLIGHSSAEC	ILSGNAAHWS	TKPPICQRIP	CGLPPTIANG	DFISTNRENF	HYGSVVTYRC	NPGSGGRKVF	ELVGEPSIYC
1130	1140	1150	1160	1170	1180	1190	1200
TSNDDQVGIW	SGPAPQCIIP	NKCTPPNVEN	GILVSDNRSL	FSLNEVVEFR	CQPGFVMKGP	RRVKCQALNK	WEPELPSCSR
1210	1220	1230	1240	1250	1260	1270	1280
VCQPPPDVLH	AERTQRDKDN	FSPGQEVFYS	CEPGYDLRGA	ASLHCTPQGD	WSPEAPRCAV	KSCDDFLGQL	PHGRVLFPLN
1290	1300	1310	1320	1330	1340	1350	1360
LQLGAKVSFV	CDEGFRLKGS	SVSHCVLVGM	RSLWNNSVPV	CEHIFCPNPP	AILNGRHTGT	PSGDIPYGKE	ISYTCDPHPD
1370	1380	1390	1400	1410	1420	1430	1440
RGMTFNLIGE	STIRCTSDPH	GNGVWSSPAP	RCELSVRAGH	CKTPEQFPFA	SPTIPINDFE	FPVGTSLNYE	CRPGYFGKMF
1450	1460	1470	1480	1490	1500	1510	1520
SISCLENLVW	SSVEDNCRRK	SCGPPPEPFN	GMVHINTDTQ	FGSTVNYSCN	EGFRLIGSPS	TTCLVSGNNV	TWDKKAPICE
1530	1540	1550	1560	1570	1580	1590	1600
IISCEPPPTI	SNGDFYSNNR	TSFHNGTVVT	YQCHTGPDGE	QLFELVGERS	IYCTSKDDQV	GVWSSPPPRC	ISTNKCTAPE
1610	1620	1630	1640	1650	1660	1670	1680
VENAIRVPGN	RSFFTLTEII	RFRCQPGFVM	VGSHTVQCQT	NGRWGPKLPH	CSRVCQPPPE	ILHGEHTLSH	QDNFSPGQEV
1690	1700	1710	1720	1730	1740	1750	1760
FYSCEPSYDL	RGAASLHCTP	QGDWSPEAPR	CTVKSCDDFL	GQLPHGRVLL	PLNLQLGAKV	SFVCDEGFRL	KGRSASHCVL
1770	1780	1790	1800	1810	1820	1830	1840
AGMKALWNSS	VPVCEQIFCP	NPPAILNGRH	TGTPFGDIPY	GKEISYACDT	HPDRGMTFNL	IGESSIRCTS	DPQGNGVWSS
1850	1860	1870	1880	1890	1900	1910	1920
PAPRCELSVP	AACPHPPKIQ	NGHYIGGHVS	LYLPGMTISY	ICDPGYLLVG	KGFIFCTDQG	IWSQLDHYCK	EVNCSFPLFM
1930	1940	1950	1960	1970	1980	1990	2000
NGISKELEMK	KVYHYGDYVT	LKCEDGYTLE	GSPWSQCQAD	DRWDPPLAKC	TSRTHDALIV	GTLSGTIFFI	LLIIFLSWII
2010	2020	2030	2040	2050			
LKHRKGNNAH	ENPKEVAIHL	HSQGGSSVHP	RTLQTNEENS	RVLP			

Cmpd.	No. of Cmpds.	m/z meas.	Δ m/z [ppm]	z	Rt [min]	Score	P	Range	Sequence	Modification	Ratios
73	2	826.3528	-22.94	3	30.6	30.9	1	339-361	R.GAASMRCTPQGDWSPAAPTCEVS	Carbamidomethyl: 7, 20	WUP:QUP 4.24<br>QU:MU 0.52

Detailed Protein Report

Protein 100: PREDICTED: sterol regulatory element-binding protein 1 isoform X1 [Homo sapiens]

**Accession:**	gi|530410590	**Score:**	53.4
**Database:**	refseq_human(refseq_human_20140103.fasta)	**MW [kDa]:**	121.4
**Database Date:**	2015-11-30	**pI:**	9.4
**Modification(s):**	Oxidation	**Sequence Coverage [%]:**	4.3
		**No. of unique Peptides:**	2

Quantitation

QU:MU	**Median:**	1.57	**CV:**	0.00 %	**No. of Peptides:**	1
WUP:QUP	**Median:**	1.19	**CV:**	0.00 %	**No. of Peptides:**	1

10	20	30	40	50	60	70	80
MDEPPFSEAA	LEQALGEPCD	LDAALLTDIE	DMLQLINNQD	SDFPGLFDPP	YAGSGAGGTD	PASPDTSSPG	SLSPPPATLS
90	100	110	120	130	140	150	160
SSLEAFLSGP	QAAPSPLSPP	QPAPTPLKMY	PSMPAFSPGP	GIKEESVPLS	ILQTPTPQPL	PGALLPQSFP	APAPPQFSST
170	180	190	200	210	220	230	240
PVLGYPSPPG	GFSTGSPPGN	TQQPLPGLPL	ASPPGVPPVS	LHTQVQSVVP	QQLLTVTAAP	TAAPVTTTVT	SQIQQVLLQP
250	260	270	280	290	300	310	320
HFIKADSLLL	TAMKTDGATV	KAAGLSPLVS	GTTVQTGPLP	TLVSGGTILA	TVPLVVDAEK	LPINRLAAGS	KAPASAQSRG
330	340	350	360	370	380	390	400
EKRTAHNAIE	KRYRSSINDK	IIELKDLVVG	TEAKLNKSAV	LRKAIDYIRF	LQHSNQKLKQ	ENLSLRTAVH	KSKSLKDLVS
410	420	430	440	450	460	470	480
ACGSGGNTDV	LMEGVKTEVE	DTLTPPPSDA	GSPFQSSPLS	LGSRGSGSGG	SGSDSEPDSP	VFEDSKAKPE	QRPSLHSRGM
490	500	510	520	530	540	550	560
LDRSRLALCT	LVFLCLSCNP	LASLLGARGL	PSPSDTTSVY	HSPGRNVLGT	ESRDGPGWAQ	WLLPPVVWLL	NGLLVLVSLV
570	580	590	600	610	620	630	640
LLFVYGEPVT	RPHSGPAVYF	WRHRKQADLD	LARGDFAQAA	QQLWLALRAL	GRPLPTSHLD	LACSLLWNLI	RHLLQRLWVG
650	660	670	680	690	700	710	720
RWLAGRAGGL	QQDCALRVDA	SASARDAALV	YHKLHQLHTM	GKHTGGHLTA	TNLALSALNL	AECAGDAVSV	ATLAEIYVAA
730	740	750	760	770	780	790	800
ALRVKTSLPR	ALHFLTRFFL	SSARQACLAQ	SGSVPPAMQW	LCHPVGHRFF	VDGDWSVLST	PWESLYSLAG	NPVDPLAQVT
810	820	830	840	850	860	870	880
QLFREHLLER	ALNCVTQPNP	SPGSADGDKE	FSDALGYLQL	LNSCSDAAGA	PAYSFSISSS	MATTTGVDPV	AKWWASLTAV
890	900	910	920	930	940	950	960
VIHWLRRDEE	AAERLCPLVE	HLPRVLQESE	RPLPRAALHS	FKAARALLGC	AKAESGPASL	TICEKASGYL	QDSLATTPAS
970	980	990	1000	1010	1020	1030	1040
SSIDKAVQLF	LCDLLLVVRT	SLWRQQQPPA	PAPAAQGTSS	RPQASALELR	GFQRDLSSLR	RLAQSFRPAM	RRVFLHEATA
1050	1060	1070	1080	1090	1100	1110	1120
RLMAGASPTR	THQLLDRSLR	RRAGPGGKGG	AVAELEPRPT	RREHAEALLL	ASCYLPPGFL	SAPGQRVGML	AEAARTLEKL
1130	1140	1150					
GDRRLLHDCQ	QMLMRLGGGT	TVTSS					

Cmpd.	No. of Cmpds.	m/z meas.	Δ m/z [ppm]	z	Rt [min]	Score	P	Range	Sequence	Modification	Ratios
1578	1	798.3570	-33.20	2	49.4	11.7	0	109-123	K.MYPSMPAFSPGPGIK.E	Oxidation: 5	
783	1	676.8811	19.88	2	39.8	12.5	0	1069-1081	K.GGAVAELEPRPTR.R		WUP:QUP 1.19<br>QU:MU 1.57

Detailed Protein Report

Protein 101: **PREDICTED: guanine nucleotide exchange factor VAV3 isoform X4 [Homo sapiens]**

Accession: gi|530361574
Database: refseq_human(refseq_human_20140103.fasta)
Database Date: 2015-11-30
Modification(s): Carbamidomethyl, Oxidation

Score: 52.8
MW [kDa]: 72.1
pI: 6.9
Sequence Coverage [%]: 6.8
No. of unique Peptides: 3

10	20	30	40	50	60	70	80
MEPWKQCAQW	LIHCKVLPTN	HRVTWDSAQV	FDLAQTLRDG	VLLCQLLNNL	RAHSINLKEI	NLRPQMSQFL	CLKNIRTFLT
**90**	**100**	**110**	**120**	**130**	**140**	**150**	**160**
ACCETFGMRK	SELFEAFDLF	DVRDFGKVIE	TLSRLSRTPI	ALATGIRPFP	TEESINDEDI	YKGLPDLIDE	TLVEDEEDLY
**170**	**180**	**190**	**200**	**210**	**220**	**230**	**240**
DCVYGEDEGG	EVYEDLMKAE	EAHQPKCPEN	DIRSCCLAEI	KQTEEKYTET	LESIEKYFMA	PLKRFLTAAE	FDSVFINIPE
**250**	**260**	**270**	**280**	**290**	**300**	**310**	**320**
LVKLHRNLMQ	EIHDSIVNKN	DQNLYQVFIN	YKERLVIYGQ	YCSGVESAIS	SLDYISKTKE	DVKLKLEECS	KRANNGKFTL
**330**	**340**	**350**	**360**	**370**	**380**	**390**	**400**
RDLLVVPMQR	VLKYHLLLQE	LVKHTTDPTE	KANLKLALDA	MKDLAQYVNE	VKRDNETLRE	IKQFQLSIEN	LNQPVLLFGR
**410**	**420**	**430**	**440**	**450**	**460**	**470**	**480**
PQGDGEIRIT	TLDKHTKQER	HIFLFDLAVI	VCKRKGDNYE	MKEIIDLQQY	KIANNPTTDK	ENKKWSYGFY	LIHTQGQNGL
**490**	**500**	**510**	**520**	**530**	**540**	**550**	**560**
EFYCKTKDLK	KKWLEQFEMA	LSNIRPDYAD	SNFHDFKMHT	FTRVTSCKVC	QMLLRGTFYQ	GYLCFKCGAR	AHKECLGRVD
**570**	**580**	**590**	**600**	**610**	**620**	**630**	
NCGRVNSGEQ	GTLKLPEKRT	NGLRRTPKQV	DPDVPCLLHF	FISMAPATRS	IVKSQKKNKK	F	

Cmpd.	No. of Cmpds.	m/z meas.	Δ m/z [ppm]	z	Rt [min]	Score	P	Range	Sequence	Modification
154	1	968.4666	19.86	2	31.6	10.4	1	74-89	K.NIRTFLTACCETFGMR.K	Carbamidomethyl: 10; Oxidation: 15
877	1	705.9834	77.48	2	41.0	16.1	1	322-333	R.DLLVVPMQRVLK.Y	
103	1	807.7358	-182.29	2	31.0	26.3	2	551-564	R.AHKECLGRVDNCGR.V	Carbamidomethyl: 12

Detailed Protein Report

Protein 102: PREDICTED: extracellular sulfatase Sulf-1 isoform X1 [Homo sapiens]

**Accession:**	gi|578815628	**Score:**	52.7
**Database:**	refseq_human(refseq_human_20140103.fasta)	**MW [kDa]:**	129.6
**Database Date:**	2015-11-30	**pI:**	9.5
**Modification(s):**	Oxidation	**Sequence Coverage [%]:**	5.7
		**No. of unique Peptides:**	3

Alias proteins:

Accession	Name	Description
gi|578815632	refseq_human (refseq_human_20140103.fasta)	PREDICTED: extracellular sulfatase Sulf-1 isoform X3 [Homo sapiens]
gi|578815630	refseq_human (refseq_human_20140103.fasta)	PREDICTED: extracellular sulfatase Sulf-1 isoform X2 [Homo sapiens]

10	20	30	40	50	60	70	80
MKYSCCALVL	AVLGTELLGS	LCSTVRSPRF	RGRIQQERKN	IRPNIILVLT	DDQDVELGSL	QVMNKTRKIM	EHGGATFINA
**90**	**100**	**110**	**120**	**130**	**140**	**150**	**160**
FVTTPMCCPS	RSSMLTGKYV	HNHNVYTNNE	NCSSPSWQAM	HEPRTFAVYL	NNTGYRTAFF	GKYLNEYNGS	YIPPGWREWL
**170**	**180**	**190**	**200**	**210**	**220**	**230**	**240**
GLIKNSRFYN	YTVCRNGIKE	KHGFDYAKDY	FTDLITNESI	NYFKMSKRMY	PHRPVMMVIS	HAAPHGPEDS	APQFSKLYPN
**250**	**260**	**270**	**280**	**290**	**300**	**310**	**320**
ASQHITPSYN	YAPNMDKHWI	MQYTGPMLPI	HMEFTNILQR	KRLQTLMSVD	DSVERLYNML	VETGELENTY	IIYTADHGYH
**330**	**340**	**350**	**360**	**370**	**380**	**390**	**400**
IGQFGLVKGK	SMPYDFDIRV	PFFIRGPSVE	PGSIVPQIVL	NIDLAPTILD	IAGLDTPPDV	DGKSVLKLLD	PEKPGNRFRT
**410**	**420**	**430**	**440**	**450**	**460**	**470**	**480**
NKKAKIWRDT	FLVERGKFLR	KKEESSKNIQ	QSNHLPKYER	VKELCQQARY	QTACEQPGQK	WQCIEDTSGK	LRIHKCKGPS
**490**	**500**	**510**	**520**	**530**	**540**	**550**	**560**
DLLTVRQSTR	NLYARGFHDK	DKECSCRESG	YRASRSQRKS	QRQFLRNQGT	PKYKPRFVHT	RQTRSLSVEF	EGEIYDINLE
**570**	**580**	**590**	**600**	**610**	**620**	**630**	**640**
EEEELQVLQP	RNIAKRHDEG	HKGPRDLQAS	SGGNRGRMLA	DSSNAVGPPT	TVRVTHKCFI	LPNDSIHCER	ELYQSARAWK
**650**	**660**	**670**	**680**	**690**	**700**	**710**	**720**
DHKAYIDKEI	EALQDKIKNL	REVRGHLKRR	KPEECSCSKQ	SYYNKEKGVK	KQEKLKSHLH	PFKEAAQEVD	SKLQLFKENN
**730**	**740**	**750**	**760**	**770**	**780**	**790**	**800**
RRRKKERKEK	RRQRKGEECS	LPGLTCFTHD	NNHWQTAPFW	NLGSFCACTS	SNNNTYWCLR	TVNETHNFLF	CEFATGFLEY
**810**	**820**	**830**	**840**	**850**	**860**	**870**	**880**
FDMNTDPYQL	TNTVHTVERG	ILNQLHVQLM	ELRSCQGYKQ	CNPRPKNLDV	EDSYGMDGKV	NQPRLTADIN	WQGLEELHSV
**890**	**900**	**910**	**920**	**930**	**940**	**950**	**960**
NENIYEYRQN	YRLSLVDWTN	YLKDLDRVFA	LLKSHYEQNK	TNKTQTAQSD	GFLVVSAEHA	VSMEMASADS	DEDPRHKVGK
**970**	**980**	**990**	**1000**	**1010**	**1020**	**1030**	**1040**
TPHLTLPADL	QTLHLNRPTL	SPESKLEWNN	DIPEVNHLNS	EHWRKTEKWT	GHEETNHLET	DFSGDGMTEL	ELGPSPRLQP
**1050**	**1060**	**1070**	**1080**	**1090**	**1100**	**1110**	**1120**
IRRHPKELPQ	YGGPGKDIFE	DQLYLPVHSD	GISVHQMFTM	ATAEHRSNSS	IAGKMLTKVE	KNHEKEKSQH	LEGSASSSLS
**1130**							
SD							

Cmpd.	No. of Cmpds.	m/z meas.	Δ m/z [ppm]	z	Rt [min]	Score	P	Range	Sequence	Modification
2819	1	701.9509	-109.35	3	65.7	16.7	2	329-345	K.GKSMPYDFDIRVPFFIR.G	Oxidation: 4
2524	1	813.8725	-59.59	2	61.5	11.6	1	428-440	K.NIQQSNHLPKYER.V	
2488	1	673.3201	-4.49	3	61.7	13.8	2	577-595	R.HDEGHKGPRDLQASSGGNR.G	

Detailed Protein Report

Protein 103: transcription initiation factor TFIID subunit 1-like [Homo sapiens]

**Accession:**	gi|24429572	**Score:**	52.5
**Database:**	refseq_human(refseq_human_20140103.fasta)	**MW [kDa]:**	207.2
**Database Date:**	2015-11-30	**pI:**	5.1
**Modification(s):**	Oxidation	**Sequence Coverage [%]:**	3.3
		**No. of unique Peptides:**	4

Quantitation

WUP:QUP	**Median:** 0.54	**CV:** 0.00 %	**No. of Peptides:** 1

Detailed Protein Report

10	20	30	40	50	60	70	80
MRPGCDLLLR	AAATVTAAIM	SDSDSEEDSS	GGGPFTLAGI	LFGNISGAGQ	LEGESVLDDE	CKKHLAGLGA	LGLGSLITEL
90	100	110	120	130	140	150	160
TANEELTGTG	GALVNDEGWI	RSTEDAVDYS	DINEVAEDES	QRHQQTMGSL	QPLYHSDYDE	DDYDADCEDI	DCKLMPPPPP
170	180	190	200	210	220	230	240
PPGPMKKDKD	QDAITCVSES	GEDIILPSII	APSFLASEKV	DFSSYSDSES	EMGPQEATQA	ESEDGKLTLP	LAGIMQHDAT
250	260	270	280	290	300	310	320
KLLPSVTELF	PEFRPGKVLR	FLHLFGPGKN	VPSVWRSARR	KRKKHRELIQ	EEQIQEVECS	VESEVSQKSL	WNYDYAPPPP
330	340	350	360	370	380	390	400
PEQCLADDEI	TMMVPVESKF	SQSTGDVDKV	TDTKPRVAEW	RYGPARLWYD	MLGVSEDGSG	FDYGFKLRKT	QHEPVIKSRM
410	420	430	440	450	460	470	480
MEEFRKLEES	NGTDLLADEN	FLMVTQLHWE	DSIIWDGEDI	KHKGTKPQGA	SLAGWLPSIK	TRNVMAYNVQ	QGFAPTLDDD
490	500	510	520	530	540	550	560
KPWYSIFPID	NEDLVYGRWE	DNIIWDAQAM	PRLLEPPVLA	LDPNDENLIL	EIPDEKEEAT	SNSPSKESKK	ESSLKKSRIL
570	580	590	600	610	620	630	640
LGKTGVIREE	PQQNMSQPEV	KDPWNLSNDE	YYFPKQQGLR	GTFGGNIIQH	SIPAMELWQP	FFPTHMGPIK	IRQFHRPPLK
650	660	670	680	690	700	710	720
KYSFGALSQP	GPHSVQPLLK	HIKKKAKMRE	QERQASGGGE	LFFMRTPQDL	TGKDGDLILA	EYSEENGPLM	MQVGMATKIK
730	740	750	760	770	780	790	800
NYYKRKPGKD	PGAPDCKYGE	TVYCHTSPFL	GSLHPGQLLQ	ALENNLFRAP	VYLHKMPETD	FLIIRTRQGY	YIRELVDIFV
810	820	830	840	850	860	870	880
VGQQCPLFEV	PGPNSRRANM	HIRDFLQVFI	YRLFWKSKDR	PRRIRMEDIK	KAFPSHSESS	IRKRLKLCAD	FKRTGMDSNW
890	900	910	920	930	940	950	960
WVLKSDFRLP	TEEEIRAKVS	PEQCCAYYSM	IAAKQRLKDA	GYGEKSFFAP	EEENEEDFQM	KIDDEVHAAP	WNTTRAFIAA
970	980	990	1000	1010	1020	1030	1040
MKGKCLLEVT	GVADPTGCGE	GFSYVKIPNK	PTQQKDDKEP	QAVKKTVTGT	DADLRRLSLK	NAKQLLRKFG	VPEEEIKKLS
1050	1060	1070	1080	1090	1100	1110	1120
RWEVIDVVRT	MSTEQAHSGE	GPMSKFARGS	RFSVAEHQER	YKEECQRIFD	LQNKVLSSTE	VLSTDTDSIS	AEDSDFEEMG
1130	1140	1150	1160	1170	1180	1190	1200
KNIENMLQNK	KTSSQLSREW	EEQERKELRR	MLLVAGSAAS	GNNHRDDVTA	SMTSLKSSAT	GHCLKIYRTF	RDEEGKEYVR
1210	1220	1230	1240	1250	1260	1270	1280
CETVRKPAVI	DAYVRIRTTK	DEKFIQKFAL	FDEKHREEMR	KERRRIQEQL	RRLKRNQEKE	KLKGPPEKKP	KKMKERPDLK
1290	1300	1310	1320	1330	1340	1350	1360
LKCGACGAIG	HMRTNKFCPL	YYQTNVPPSK	PVAMTEEQEE	ELEKTVIHND	NEELIKVEGT	KIVFGKQLIE	NVHEVRRKSL
1370	1380	1390	1400	1410	1420	1430	1440
VLKFPKQQLP	PKKKRRVGTT	VHCDYLNIPH	KSIHRRRTDP	MVTLSSILES	IINDMRDLPN	THPFHTPVNA	KVVKDYYKII
1450	1460	1470	1480	1490	1500	1510	1520
TRPMDLQTLR	ENVRKCLYPS	REEFREHLEL	IVKNSATYNG	PKHSLTQISQ	SMLDLCDEKL	KEKEDKLARL	EKAINPLLDD
1530	1540	1550	1560	1570	1580	1590	1600
DDQVAFSFIL	DNIVTQKMMA	VPDSWPFHHP	VNKKFVPDYY	KMIVNPVDLE	TIRKNISKHK	YQSRESFLDD	VNLILANSVK
1610	1620	1630	1640	1650	1660	1670	1680
YNGPESQYTK	TAQEIVNICY	QTITEYDEHL	TQLEKDICTA	KEAALEEAEL	ESLDPMTPGP	YTSQPPDMYD	TNTSLSTSRD
1690	1700	1710	1720	1730	1740	1750	1760
ASVFQDESNL	SVLDISTATP	EKQMCQGQGR	LGEEDSDVDV	EGYDDEEEDG	KPKPPAPEGG	DGDLADEEEG	TVQQPEASVL
1770	1780	1790	1800	1810	1820	1830	
YEDLLISEGE	DDEEDAGSDE	EGDNPFSAIQ	LSESGSDSDV	GYGGIRPKQP	FMLQHASGEH	KDGHGK	

Cmpd.	No. of Cmpds.	m/z meas.	Δ m/z [ppm]	z	Rt [min]	Score	P	Range	Sequence	Modification	Ratios
2709	1	813.0615	149.49	2	62.3	13.8	0	227-241	K.LTLPLAGIMQHDATK.L	Oxidation: 9	
1183	1	839.3834	23.08	2	44.9	11.4	0	1050-1065	R.TMSTEQAHSGEGPMSK.F		
14	1	847.2854	-163.39	2	29.8	15.5	2	1069-1082	R.GSRFSVAEHQERYK.E		
2649	1	835.4339	3.24	2	61.4	11.7	1	1150-1165	R.RMLLVAGSAASGNNHR.D	Oxidation: 2	WUP:QUP 0.54

Detailed Protein Report

Protein 104: origin recognition complex subunit 6 [Homo sapiens]

**Accession:**	gi|7657427	**Score:**	52.2
**Database:**	refseq_human(refseq_human_20140103.fasta)	**MW [kDa]:**	28.1
**Database Date:**	2015-11-30	**pI:**	9.8
**Modification(s):**	Carbamidomethyl	**Sequence Coverage [%]:**	20.6
		**No. of unique Peptides:**	3

Quantitation

QU:MU **Median:** 0.94 **CV:** 0.00 % **No. of Peptides:** 1

10	20	30	40	50	60	70	80
MGSELIGRLA	PRLGLAEPDM	LRKAEEYLRL	SRVKCVGLSA	RTTETSSAVM	CLDLAASWMK	CPLDRAYLIK	LSGLNKETYQ
**90**	**100**	**110**	**120**	**130**	**140**	**150**	**160**
SCLKSFECLL	GLNSNIGIRD	LAVQFSCIEA	VNMASKILKS	YESSLPQTQQ	VDLDLSRPLF	TSAALLSACK	ILKLKVDKNK
**170**	**180**	**190**	**200**	**210**	**220**	**230**	**240**
MVATSGVKKA	IFDRLCKQLE	KIGQQVDREP	GDVATPPRKR	KKIVVEAPAK	EMEKVEEMPH	KPQKDEDLTQ	DYEEWKRKIL
**250**	**260**						
ENAASAQKAT	AE						

Cmpd.	No. of Cmpds.	m/z meas.	Δ m/z [ppm]	z	Rt [min]	Score	P	Range	Sequence	Modification	Ratios
2162	1	701.2632	-88.04	3	57.4	14.6	0	42-60	R.TTETSSAVMCLDLAASWMK.C	Carbamidomethyl: 10	QU:MU 0.94
55	1	930.9009	-129.85	2	30.4	14.2	2	61-76	K.CPLDRAYLIKLSGLNK.E	Carbamidomethyl: 1	
2540	1	941.9343	-22.57	2	61.7	12.6	0	100-116	R.DLAVQFSCIEAVNMASK.I	Carbamidomethyl: 8	

Detailed Protein Report

Protein 105: PREDICTED: zinc finger protein 510 isoform X3 [Homo sapiens]

Accession: gi|530390743
Database: refseq_human(refseq_human_20140103.fasta)
Database Date: 2015-11-30
Modification(s): Carbamidomethyl

Score: 51.9
MW [kDa]: 72.1
pI: 10.2
Sequence Coverage [%]: 8.5
No. of unique Peptides: 1

10	20	30	40	50	60	70	80
MAPVQKNLYR	DVMLENYSNL	VSVGYCCFKP	EVIFKLEQGE	EPWFSEEEFS	NQSHPKDYRG	DDLIKQNKKI	KDKHLEQAIC
**90**	**100**	**110**	**120**	**130**	**140**	**150**	**160**
INNKTLTTEE	EKVLGKPFTL	HVAAVASTKM	SCKCNSWEVN	LQSISEFIIN	NRNYSTKKIG	CGNVCENSPF	KINFEKTQTG
**170**	**180**	**190**	**200**	**210**	**220**	**230**	**240**
EKFYEHNKNM	KALNYNENLP	KHPKFQTLEQ	AFECNKIGKA	FNDKANCVKH	NSSHTGETSS	KDDEFRKNCD	KKTLFDHRRT
**250**	**260**	**270**	**280**	**290**	**300**	**310**	**320**
GTGKKHLHLN	QCGKSFEKST	VEEYNKLNMG	IKHYELNPSG	NNFNRKAHLT	DPQTAVIEEN	PLVSNDRTQT	WVKSSEYHEN
**330**	**340**	**350**	**360**	**370**	**380**	**390**	**400**
KKSYQTSVHR	VRRRSHSMMK	PYKCNECGKS	FCQKGHLIQH	QRTHTGEKPF	ECSECGKTFS	QKSHLSTHQR	IHTAEKPYKC
**410**	**420**	**430**	**440**	**450**	**460**	**470**	**480**
NECGKTFVQK	STLRGHQRIH	TGEKPYECSE	CGKTFVQKST	LRDHHRIHTG	EKSFQCNQCG	KTFGQKSNLR	IHQRTHTGEK
**490**	**500**	**510**	**520**	**530**	**540**	**550**	**560**
TYQCNECEKS	FWRKDHLIQH	QKTHTGEKPF	KCNECGKTFA	RTSTLRVHQR	IHTGEKPFKC	NECGKKFVRK	AILSDHQRIH
**570**	**580**	**590**	**600**	**610**	**620**	**630**	
TGEKPFQCNK	CGKTFGQKSN	LRIHQRTHSG	EKSYECNEYG	KLCKKSTLSL	YQKIQGEGNP	Y	

Cmpd.	No. of Cmpds.	m/z meas.	Δ m/z [ppm]	z	Rt [min]	Score	P	Range	Sequence	Modification
282	1	627.0085	98.28	3	33.2	10.2	2	481-494	K.TYQCNECEKSFWRK.D	Carbamidomethyl: 7

Detailed Protein Report

Protein 106: PREDICTED: protein odd-skipped-related 2 isoform X2 [Homo sapiens]

**Accession:**	gi|530388731	**Score:**	51.8
**Database:**	refseq_human(refseq_human_20140103.fasta)	**MW [kDa]:**	43.8
**Database Date:**	2015-11-30	**pI:**	10.9
**Modification(s):**	Carbamidomethyl	**Sequence Coverage [%]:**	6.0
		**No. of unique Peptides:**	1

Quantitation

WUP:QUP **Median:** 4.54 **CV:** 0.00 % **No. of Peptides:** 1

10	20	30	40	50	60	70	80
MGLRAEEGKR	KPPRSVRGEV	SPSQGPLAQE	DEARRLRSCT	PACHPGKRGK	GGHRARRQRS	AWDLTTHPLT	HRSQELRGAA
**90**	**100**	**110**	**120**	**130**	**140**	**150**	**160**
ATEGFLYVLL	SHWVFVGAPR	PPASDSWKKG	LVPSAPPASR	KMGSKALPAP	IPLHPSLQLT	NYSFLQAVNT	FPATVDHLQG
**170**	**180**	**190**	**200**	**210**	**220**	**230**	**240**
LYGLSAVQTM	HMNHWTLGYP	NVHEITRSTI	TEMAAAQGLV	DARFPFPALP	FTTHLFHPKQ	GAIAHVLPAL	HKDRPRFDFA
**250**	**260**	**270**	**280**	**290**	**300**	**310**	**320**
NLAVAATQED	PPKMGDLSKL	SPGLGSPISG	LSKLTPDRKP	SRGRLPSKTK	KEFICKFCGR	HFTKSYNLLI	HERTHTDERP
**330**	**340**	**350**	**360**	**370**	**380**	**390**	**400**
YTCDICHKAF	RRQDHLRDHR	YIHSKEKPFK	CQECGKGFCQ	SRTLAVHKTL	HMQTSSPTAA	SSAAKCSGET	VICGGTA

Cmpd.	No. of Cmpds.	m/z meas.	Δ m/z [ppm]	z	Rt [min]	Score	P	Range	Sequence	Modification	Ratios
857	2	663.6905	-200.92	2	40.8	13.1	1	36-47	R.LRSCTPACHPGK.R	Carbamidomethyl: 8	WUP:QUP 4.54

Detailed Protein Report

Protein 107: protein SOGA1 isoform 1 [Homo sapiens]

Accession: gi|257467639
Database: refseq_human(refseq_human_20140103.fasta)
Database Date: 2015-11-30
Modification(s): Carbamidomethyl

Score: 51.8
MW [kDa]: 183.7
pI: 6.1
Sequence Coverage [%]: 2.4
No. of unique Peptides: 3

10	20	30	40	50	60	70	80
MEAPAAEPPV	RGCGPQPAPA	PAPAPERKKS	HRAPSPARPK	DVAGWSLAKG	RRGPGPGSAV	ACSAAFSSRP	DKKGRAVAPG
**90**	**100**	**110**	**120**	**130**	**140**	**150**	**160**
ARGAGVRVAG	VRTGVRAKGR	PRSGAGPRPP	PPPPSLTDSS	SEVSDCASEE	ARLLGLELAL	SSDAESAAGG	PAGVRTGQPA
**170**	**180**	**190**	**200**	**210**	**220**	**230**	**240**
QPAPSAQQPP	RPPASPDEPS	VAASSVGSSR	LPLSASLAFS	DLTEEMLDCG	PSGLVRELEE	LRSENDYLKD	EIEELRAEML
**250**	**260**	**270**	**280**	**290**	**300**	**310**	**320**
EMRDVYMEED	VYQLQELRQQ	LDQASKTCRI	LQYRLRKAER	RSLRAAQTGQ	VDGELIRGLE	QDVKVSKDIS	MRLHKELEVV
**330**	**340**	**350**	**360**	**370**	**380**	**390**	**400**
EKKRARLEEE	NEELRQRLIE	TELAKQVLQT	ELERPREHSL	KKRGTRSLGK	ADKKTLVQED	SADLKCQLHF	AKEESALMCK
**410**	**420**	**430**	**440**	**450**	**460**	**470**	**480**
KLTKLAKEND	SMKEELLKYR	SLYGDLDSAL	SAEELADAPH	SRETELKVHL	KLVEEEANLL	SRRIVELEVE	NRGLRAEMDD
**490**	**500**	**510**	**520**	**530**	**540**	**550**	**560**
MKDHGGGCGG	PEARLAFSAL	GGGECGGESLA	ELRRHLQFVE	EEAELLRRSS	AELEDQNKLL	LNELAKFRSE	HELDVALSED
**570**	**580**	**590**	**600**	**610**	**620**	**630**	**640**
SCSVLSEPSQ	EELAAAKLQI	GELSGKVKKL	QYENRVLLSN	LQRCDLASCQ	STRPMLETDA	EAGDSAQCVP	APLGETHESH
**650**	**660**	**670**	**680**	**690**	**700**	**710**	**720**
AVRLCRAREA	EVLPGLREQA	ALVSKAIDVL	VADANGFTAG	LRLCLDNECA	DFRLHEAPDN	SEGPRDTKLI	HAILVRLSVL
**730**	**740**	**750**	**760**	**770**	**780**	**790**	**800**
QQELNAFTRK	ADAVLGCSVK	EQQESFSSLP	PLGSQGLSKE	ILLAKDLGSD	FQPPDFRDLP	EWEPRIREAF	RTGDLDSKPD
**810**	**820**	**830**	**840**	**850**	**860**	**870**	**880**
PSRSFRPYRA	EDNDSYASEI	KELQLVLAEA	HDSLRGLQEQ	LSQERQLRKE	EADNFNQKMV	QLKEDQQRAL	LRREFELQSL
**890**	**900**	**910**	**920**	**930**	**940**	**950**	**960**
SLQRRLEQKF	WSQEKNMLVQ	ESQQFKHNFL	LLFMKLRWFL	KRWRQGKVLP	SEGDDFLEVN	SMKELYLLME	EEEINAQHSD
**970**	**980**	**990**	**1000**	**1010**	**1020**	**1030**	**1040**
NKACTGDSWT	QNTPNEYIKT	LADMKVTLKE	LCWLLRDERR	GLTELQQQFA	KAKATWETER	AELKGHTSQM	ELKTGKGAGE
**1050**	**1060**	**1070**	**1080**	**1090**	**1100**	**1110**	**1120**
RAGPDWKAAL	QREREEQQHL	LAESYSAVME	LTRQLQISER	NWSQEKLQLV	ERLQGEKQQV	EQQVKELQNR	LSQLQKAADP
**1130**	**1140**	**1150**	**1160**	**1170**	**1180**	**1190**	**1200**
WVLKHSELEK	QDNSWKETRS	EKIHDKEAVS	EVELGGNGLK	RTKSVSSMSE	FESLLDCSPY	LAGGDARGKK	LPNNPAFGFV
**1210**	**1220**	**1230**	**1240**	**1250**	**1260**	**1270**	**1280**
SSEPGDPEKD	TKEKPGLSSR	DCNHLGALAC	QDPPGRQMQR	SYTAPDKTGI	RVYYSPPVAR	RLGVPVVHDK	EGKIIIEPGF
**1290**	**1300**	**1310**	**1320**	**1330**	**1340**	**1350**	**1360**
LFTTAKPKES	AEADGLAESS	YGRWLCNFSR	QRLDGGSAGS	PSAAGPGFPA	ALHDFEMSGN	MSDDMKEITN	CVRQAMRSGS
**1370**	**1380**	**1390**	**1400**	**1410**	**1420**	**1430**	**1440**
LERKVKSTSS	QTVGLASVGT	QTIRTVSVGL	QTDPPRSSLH	GKAWSPRSSS	LVSVRSKQIS	SSLDKVHSRI	ERPCCSPKYG
**1450**	**1460**	**1470**	**1480**	**1490**	**1500**	**1510**	**1520**
SPKLQRRSVS	KLDSSKDRSL	WNLHQGKQNG	SAWARSTTTR	DSPVLRNIND	GLSSLFSVVE	HSGSTESVWK	LGMSETRAKP
**1530**	**1540**	**1550**	**1560**	**1570**	**1580**	**1590**	**1600**
EPPKYGIVQE	FFRNVCGRAP	SPTSSAGEEG	TKKPEPLSPA	SYHQPEGVAR	ILNKKAAKLG	SSEEVRLTML	PQVGKDGVLR
**1610**	**1620**	**1630**	**1640**	**1650**	**1660**	**1670**	
DGDGAVVLPN	EDAVCDCSTQ	SLTSCFARSS	RSAIRHSPSK	CRLHPSESSW	GGEERALPPS	E	

Cmpd.	No. of Cmpds.	m/z meas.	Δ m/z [ppm]	z	Rt [min]	Score	P	Range	Sequence	Modification
63	1	1097.5505	-104.35	1	30.7	13.5	2	93-102	R.TGVRAKGRPR.S	
2190	1	701.1048	122.16	3	57.8	13.2	2	1426-1443	K.VHSRIERPCCSPKYGSPK.L	Carbamidomethyl: 9
2282	1	701.1125	133.17	3	58.4	14.5	2	1426-1443	K.VHSRIERPCCSPKYGSPK.L	Carbamidomethyl: 10

Detailed Protein Report

Protein 108: PAS domain-containing protein 1 [Homo sapiens]

Accession: gi|157785548
Database: refseq_human(refseq_human_20140103.fasta)
Database Date: 2015-11-30
Modification(s): Carbamidomethyl

Score: 51.6
MW [kDa]: 87.4
pI: 4.8
Sequence Coverage [%]: 4.3
No. of unique Peptides: 2

10	20	30	40	50	60	70	80
MKMRGEKRRD	KVNPKSSQRK	LNWIPSFPTY	DYFNQVTLQL	LDGFMITLST	DGVIICVAEN	ISSLLGHLPA	EIVGKKLLSL
90	100	110	120	130	140	150	160
LPDEEKDEVY	QKIILKFPLL	NSETHIEFCC	HLKRGNVEHG	DSSAYENVKF	IVNVRDICNE	FPVVFSGLFS	SHLCADFAAC
170	180	190	200	210	220	230	240
VPQEDRLYLV	GNVCILRTQL	LQQLYTSKAV	SDEAVLTQDS	DEEPFVGELS	SSQGQRGHTS	MKAVYVEPAA	AAAAAAISDD
250	260	270	280	290	300	310	320
QIDIAEVEQY	GPQENVHMFV	DSDSTYCSST	VFLDTMPESP	ALSLQDFRGE	PEVNPLYRAD	PVDLEFSVDQ	VDSVDQEGPM
330	340	350	360	370	380	390	400
DQQDPENPVA	PLDQAGLMDP	VDPEDSVDLG	AAGASAQPLQ	PSSPVAYDII	SQELELMKKL	KEQLEERTWL	LHDAIQNQQN
410	420	430	440	450	460	470	480
ALELMMDHLQ	KQPNTLRHVV	IPDLQSSEAV	PKKQQKQHAG	QVKRPLPHPK	DVKCFCGLSL	SNSLKNTGEL	QEPCVAFNQQ
490	500	510	520	530	540	550	560
QLVQQEQHLK	EQQRQLREQL	QQLREQRKVQ	KQKKMQEKKK	LQEQKMQEKK	KLQEQRRQKK	KKLQERKKWQ	GQMLQKEPEE
570	580	590	600	610	620	630	640
EQQKQQLQEQ	PLKHNVIVGN	ERVQICLQNP	RDVSVPLCNH	PVRFLQAQPI	VPVQRAAEQQ	PSGFYQDENC	GQQEDESQSF
650	660	670	680	690	700	710	720
YPEAYQGPPV	NQLPLIDTSN	SEAISSSSIP	QFPITSDSTI	STLETPQDYI	RLWQELSDSL	GPVVQVNTWS	CDEQGTLHGQ
730	740	750	760	770	780		
PTYHQVQVSE	VGVEGPPDPQ	AFQGPAAYQP	DQMRSAEQTR	LMPAEQRDSN	KPC		

Cmpd.	No. of Cmpds.	m/z meas.	Δ m/z [ppm]	z	Rt [min]	Score	P	Range	Sequence	Modification
2772	2	557.6353	40.20	3	65.8	22.5	1	451-465	K.DVKCFCGLSLSNSLK.N	Carbamidomethyl: 4
2612	1	1045.0458	-11.45	2	62.7	10.6	1	574-591	K.HNVIVGNERVQICLQNPR.D	

Detailed Protein Report

Protein 109: **zinc finger protein 92 isoform 1 [Homo sapiens]**

Accession: gi|37537684
Database: refseq_human(refseq_human_20140103.fasta)
Database Date: 2015-11-30
Modification(s): Carbamidomethyl, Oxidation

Score: 51.5
MW [kDa]: 60.2
pI: 10.3
Sequence Coverage [%]: 8.9
No. of unique Peptides: 3

10	20	30	40	50	60	70	80
MVDKTPVMCS	HFAQDVWPEH	SIKDSFQKVI	LRTYGKYGHE	NLQLRKDHKS	VDACKVYKGG	YNGLNQCLTT	TDSKIFQCDK
90	100	110	120	130	140	150	160
YVKVFHKFPN	VNRNKIRHTG	KKPFKCKNRG	KSFCMLSQLT	QHKKIHTREY	SYKCEECGKA	FNWSSTLTKH	KIIHTGEKPY
170	180	190	200	210	220	230	240
KCEECGKAFN	RSSNLTKHKI	IHTGEKPYKC	EECGKAFNRS	STLTKHKRIH	TEEKPYKCEE	CGKAFNQFSI	LNKHKRIHME
250	260	270	280	290	300	310	320
DKPYKCEECG	KAFRVFSILK	KHKIIHTGEK	PYKCEECGKA	FNQFSNLTKH	KIIHTGEKPY	KCDECGKAFN	QSSTLTKHKR
330	340	350	360	370	380	390	400
IHTGEKPYKC	EECGKAFKQS	STLTEHKIIH	TGEKPYKCEK	CGKAFSWSSA	FTKHKRNHME	DKPYKCEECG	KAFSVFSTLT
410	420	430	440	450	460	470	480
KHKIIHTREK	PYKCEECGKA	FNQSSIFTKH	KIIHTEGKSY	KCEKCGNAFN	QSSNLTARKI	IYTGEKPYKY	EECDKAFNKF
490	500	510	520				
STLITHQIIY	TGEKPCKHEC	GRAFNKSSNY	TKEKLQT				

Cmpd.	No. of Cmpds.	m/z meas.	Δ m/z [ppm]	z	Rt [min]	Score	P	Range	Sequence	Modification
2753	1	920.1206	24.93	3	65.6	10.2	1	1-23	-.MVDKTPVMCSHFAQDVWPEHSIK.D	Carbamidomethyl: 9; Oxidation: 8
1095	1	540.7909	-75.68	2	41.9	25.1	1	252-260	K.AFRVFSILK.K	
2728	6	741.8636	30.01	2	64.4	16.2	0	445-458	K.CGNAFNQSSNLTAR.K	

Detailed Protein Report

Protein 110: PREDICTED: supervillin isoform X13 [Homo sapiens]

**Accession:**	gi|578818553	**Score:**	51.5
**Database:**	refseq_human(refseq_human_20140103.fasta)	**MW [kDa]:**	217.6
**Database Date:**	2015-11-30	**pI:**	6.6
**Modification(s):**	Oxidation	**Sequence Coverage [%]:**	3.4
		**No. of unique Peptides:**	3

Quantitation

WUP:QUP	**Median:** 0.55	**CV:** 0.00 %	**No. of Peptides:** 1

Detailed Protein Report

10	20	30	40	50	60	70	80
MKPNVFGREK	SDFNKKNLSF	LGFPRQRKVS	SFQKEFSLED	KEQLANHERG	IDAQLLVALP	KVAELRQIFE	PKKKEFLEMK
90	100	110	120	130	140	150	160
RKERIARRLE	GIENDTQPIL	LQSCTGLVTH	RLLEEDTPRY	MRASDPASPH	IGRSNEEEET	SDSSLEKQTR	SKYCTETSGV
170	180	190	200	210	220	230	240
HGDSPYGSGT	MDTHSLESKA	ERIARYKAER	RRQLAEKYGL	TLDPEADSEY	LSRYTKSRKE	PDAVEKRGGK	SDKQEESSRD
250	260	270	280	290	300	310	320
ASSLYPGTET	MGLRTCAGES	KDYALHVGDG	SSDPEVLLNI	ENQRRGQELS	ATRQAHDLSP	AAESSSTFSF	SGRDSSFTEV
330	340	350	360	370	380	390	400
PRSPKHAHSS	SLQQAASRSP	SFGDPQLSPE	ARPRCTSHSE	TPTVDDEEKV	DERAKLSVAA	KRLLFREMEK	SFDEQNVPKR
410	420	430	440	450	460	470	480
RSRNTAVEQR	LRRLQDRSLT	QPITTEEVVI	AATEPIPASC	SGGTHPVMAR	LPSPTVARSA	VQPARLQASA	HQKALAKDQT
490	500	510	520	530	540	550	560
NEGKELAEQG	EPDSSTLSLA	EKLALFNKLS	QPVSKAISTR	NRIDTRQRRM	NARYQTQPVT	LGEVEQVQSG	KLIPFSPAVN
570	580	590	600	610	620	630	640
TSVSTVASTV	APMYAGDLRT	KPPLDHNASA	TDYKFSSSIE	NSDSPVRSIL	KSQAWQPLVE	GSENKGMLRE	YGETESKRAL
650	660	670	680	690	700	710	720
TGRDSGMEKY	GSFEEAEASY	PILNRAREGD	SHKESKYAVP	RRGSLERANP	PITHLGDEPK	EFSMAKMNAQ	GNLDLRDRLP
730	740	750	760	770	780	790	800
FEEKVEVENV	MKRKFSLRAA	EFGEPTSEQT	GTAAGKTIAQ	TTAPVSWKPQ	DSSEQPQEKL	CKNPCAMFAA	GEIKTPTGEG
810	820	830	840	850	860	870	880
LLDSPSKTMS	IKERLALLKK	SGEEDWRNRL	SRRQEGGKAP	ASSLHTQEAG	RSLIKKEEGG	VADDSAISNL	LWEPVYASTY
890	900	910	920	930	940	950	960
SPAIPAAHKY	LSFVSINQRV	TESRESQMTI	EERKQLITVR	EEAWKTRGRG	AANDSTQFTV	AGRMVKKGLA	SPTAITPVAS
970	980	990	1000	1010	1020	1030	1040
PICGKTRGTT	PVSKPLEDIE	ARPDMQLESD	LKLDRLETFL	RRLNNKVGGM	HETVLTVTGK	SVKEVMKPDD	DETFAKFYRS
1050	1060	1070	1080	1090	1100	1110	1120
VDYNMPRSPV	EMDEDFDVIF	DPYAPKLTSS	VAEHKRAVRP	KRRVQASKNP	LKMLAAREDL	LQEYTEQRLN	VAFMESKRMK
1130	1140	1150	1160	1170	1180	1190	1200
VEKMSSNSNF	SEVTLAGLAS	KENFSNVSLR	SVNLTEQNSN	NSAVPYKRLM	LLQIKGRRHV	QTRLVEPRAS	ALNSGDCFLL
1210	1220	1230	1240	1250	1260	1270	1280
LSPHCCFLWV	GEFANVIEKA	KASELATLIQ	TKRELGCRAT	YIQTIEEGIN	THTHAAKDFW	KLLGGQTSYQ	SAGDPKEDEL
1290	1300	1310	1320	1330	1340	1350	1360
YEAAIIETNC	IYRLMDDKLV	PDDDYWGKIP	KCSLLQPKEV	LVFDFGSEVY	VWHGKEVTLA	QRKIAFQLAK	HLWNGTFDYE
1370	1380	1390	1400	1410	1420	1430	1440
NCDINPLDPG	ECNPLIPRKG	QGRPDWAIFG	RLTEHNETIL	FKEKFLDWTE	LKRSNEKNPG	ELAQHKEDPR	TDVKAYDVTR
1450	1460	1470	1480	1490	1500	1510	1520
MVSMPQTTAG	TILDGVNVGR	GYGLVEGHDR	RQFEITSVSV	DVWHILEFDY	SRLPKQSIGQ	FHEGDAYVVK	WKFMVSTAVG
1530	1540	1550	1560	1570	1580	1590	1600
SRQKGEHSVR	AAGKEKCVYF	FWQGRHSTVS	EKGTSALMTV	ELDEERGAQV	QVLQGKEPPC	FLQCFQGGMV	VHSGRREEEE
1610	1620	1630	1640	1650	1660	1670	1680
ENVQSEWRLY	CVRGEVPVEG	NLLEVACHCS	SLRSRTSMVV	LNVNKALIYL	WHGCKAQAHT	KEVGRTAANK	IKEQCPLEAG
1690	1700	1710	1720	1730	1740	1750	1760
LHSSSKVTIH	ECDEGSEPLG	FWDALGRRDR	KAYDCMLQDP	GSFNFAPRLF	ILSSSSGDFA	ATEFVYPARA	PSVVSSMPFL
1770	1780	1790	1800	1810	1820	1830	1840
QEDLYSAPQP	ALFLVDNHHE	VYLWQGWWPI	ENKITGSARI	RWASDRKSAM	ETVLQYCKGK	NLKKPAPKSY	LIHAGLEPLT
1850	1860	1870	1880	1890	1900	1910	1920
FTNMFPSWEH	REDIAEITEM	DTEVSNQITL	VEDVLAKLCK	TIYPLADLLA	RPLPEGVDPL	KLEIYLTDED	FEFALDMTRD
1930	1940	1950					
EYNALPAWKQ	VNLKKAKGLF						

Cmpd.	No. of Cmpds.	m/z meas.	Δ m/z [ppm]	z	Rt [min]	Score	P	Range	Sequence	Modification	Ratios
874	1	903.4160	-143.87	2	40.5	11.0	1	50-66	R.GIDAQLLVALPKVAELR.Q		
2793	1	846.4331	0.98	2	66.1	19.3	1	323-338	R.SPKHAHSSSLQQAASR.S		WUP:QUP 0.55
2904	1	974.3155	-109.14	2	65.1	10.9	0	1712-	K.AYDCMLQDPGSFNFAPR.L	Oxidation: 5	

Detailed Protein Report

Cmpd.	No. of Cmpds.	m/z meas.	Δ m/z [ppm]	z	Rt [min]	Score	P	Range	Sequence	Modification	Ratios
								1728			

Detailed Protein Report

Protein 111: testis-specific serine/threonine-protein kinase 2 [Homo sapiens]

**Accession:**	gi|194294513	**Score:**	51.3
**Database:**	refseq_human(refseq_human_20140103.fasta)	**MW [kDa]:**	40.9
**Database Date:**	2015-11-30	**pI:**	9.8
**Modification(s):**	Carbamidomethyl	**Sequence Coverage [%]:**	5.3
		**No. of unique Peptides:**	2

Quantitation

QU:MU	**Median:**	3.74	**CV:**	0.00 %	**No. of Peptides:**	1
WUP:QUP	**Median:**	0.50	**CV:**	0.00 %	**No. of Peptides:**	1

10	20	30	40	50	60	70	80
MDDATVLRKK	GYIVGINLGK	GSYAKVKSAY	SERLKFNVAV	KIIDRKKTPT	DFVERFLPRE	MDILATVNHG	SIIKTYEIFE
**90**	**100**	**110**	**120**	**130**	**140**	**150**	**160**
TSDGRIYIIM	ELGVQGDLLE	FIKCQGALHE	DVARKMFRQL	SSAVKYCHDL	DIVHRDLKCE	NLLLDKDFNI	KLSDFGFSKR
**170**	**180**	**190**	**200**	**210**	**220**	**230**	**240**
CLRDSNGRII	LSKTFCGSAA	YAAPEVLQSI	PYQPKVYDIW	SLGVILYIMV	CGSMPYDDSD	IRKMLRIQKE	HRVDFPRSKN
**250**	**260**	**270**	**280**	**290**	**300**	**310**	**320**
LTCECKDLIY	RMLQPDVSQR	LHIDEILSHS	WLQPPKPKAT	SSASFKREGE	GKYRAECKLD	TKTGLRPDHR	PDHKLGAKTQ
**330**	**340**	**350**	**360**				
HRLLVVPENE	NRMEDRLAET	SRAKDHHISG	AEVGKAST				

Cmpd.	No. of Cmpds.	m/z meas.	Δ m/z [ppm]	z	Rt [min]	Score	P	Range	Sequence	Modification	Ratios
1388	1	570.1598	-180.58	2	45.7	10.7	1	238-246	R.SKNLTCECK.D	Carbamidomethyl: 6, 8	
136	2	642.2923	-49.74	2	30.6	19.6	2	293-302	K.YRAECKLDTK.T	Carbamidomethyl: 5	QU:MU 3.74<br>WUP:QUP 0.50

Detailed Protein Report

Protein 112: PREDICTED: ankyrin repeat domain-containing protein 12 isoform X6 [Homo sapiens]

**Accession:**	gi|578832112	**Score:**	51.3
**Database:**	refseq_human(refseq_human_20140103.fasta)	**MW [kDa]:**	202.9
**Database Date:**	2015-11-30	**pI:**	6.5
**Modification(s):**	Oxidation	**Sequence Coverage [%]:**	3.4
		**No. of unique Peptides:**	3

Quantitation

QU:MU	**Median:**	0.51	**CV:**	0.00 %	**No. of Peptides:**	1
WUP:QUP	**Median:**	4.73	**CV:**	0.00 %	**No. of Peptides:**	1

Detailed Protein Report

10	20	30	40	50	60	70	80
MVNVPELLLG	QSPSRNDTKI	INSEEAQSVN	PSSVDENIDS	ETEKDSLICE	SKQILPSKTP	LPSALDEYEF	KDDDDEEINK
90	100	110	120	130	140	150	160
MIDDRHILRK	EQRKENEPEA	EKTHLFAKQE	KAFYPKSFKS	KKQKPSRVLY	SSTESSDEEA	LQNKKISTSC	SVIPETSNSD
170	180	190	200	210	220	230	240
MQTKKEYVVS	GEHKQKGKVK	RKLKNQNKNK	ENQELKQEKE	GKENTRITNL	TVNTGLDCSE	KTREEGNFRK	SFSPKDDTSL
250	260	270	280	290	300	310	320
HLFHISTGKS	PKHSCGLSEK	QSTPLKQEHT	KTCLSPGSSE	MSLQPDLVRY	DNTESEFLPE	SSSVKSCKHK	EKSKHQKDFH
330	340	350	360	370	380	390	400
LEFGEKSNAK	IKDEDHSPTF	ENSDCTLKKM	DKEGKTLKKH	KLKHKEREKE	KHKKEIEGEK	EKYKTKDSAK	ELQRSVEFDR
410	420	430	440	450	460	470	480
EFWKENFFKS	DETEDLFLNM	EHESLTLEKK	SKLEKNIKDD	KSTKEKHVSK	ERNFKEERDK	IKKESEKSFR	EEKIKDLKEE
490	500	510	520	530	540	550	560
RENIPTDKDS	EFTSLGMSAI	EESIGLHLVE	KEIDIEKQEK	HIKESKEKPE	KRSQIKEKDI	EKMERKTFEK	EKKIKHEHKS
570	580	590	600	610	620	630	640
EKDKLDLSEC	VDKIKEKDKL	YSHHTEKCHK	EGEKSKNTAA	IKKTDDREKS	REKMDRKHDK	EKPEKERHLA	ESKEKHLMEK
650	660	670	680	690	700	710	720
KNKQSDNSEY	SKSEKGKNKE	KDRELDKKEK	SRDKESINIT	NSKHIQEEKK	SSIVDGNKAQ	HEKPLSLKEK	TKDEPLKTPD
730	740	750	760	770	780	790	800
GKEKDKKDKD	IDRYKERDKH	KDKIQINSLL	KLKSEADKPK	PKSSPASKDT	RPKEKRLVND	DLMQTSFERM	LSLKDLEIEQ
810	820	830	840	850	860	870	880
WHKKHKEKIK	QKEKERLRNR	NCLELKIKDK	EKTKHTPTES	KNKELTRSKS	SEVTDAYTKE	KQPKDAVSNR	SQSVDTKNVM
890	900	910	920	930	940	950	960
TLGKSSFVSD	NSLNRSPRSE	NEKPGLSSRS	VSMISVASSE	DSCHTTVTTP	RPPVEYDSDF	MLESSESQMS	FSQSPFLSIA
970	980	990	1000	1010	1020	1030	1040
KSPALHEREL	DSLADLPERI	KPPYANRLST	SHLRSSSVED	VKLIISEGRP	TIEVRRCSMP	SVICEHTKQF	QTISEESNQG
1050	1060	1070	1080	1090	1100	1110	1120
SLLTVPGDTS	PSPKPEVFSN	VPERDLSNVS	NIHSSFATSP	TGASNSKYVS	ADRNLIKNTA	PVNTVMDSPV	HLEPSSQVGV
1130	1140	1150	1160	1170	1180	1190	1200
IQNKSWEMPV	DRLETLSTRD	FICPNSNIPD	QESSLQSFCN	SENKVLKENA	DFLSLRQTEL	PGNSCAQDPA	SFMPPQQPCS
1210	1220	1230	1240	1250	1260	1270	1280
FPSQSLSDAE	SISKHMSLSY	VANQEPGILQ	QKNAVQIISS	ALDTDNESTK	DTENTFVLGD	VQKTDAFVPV	YSDSTIQEAS
1290	1300	1310	1320	1330	1340	1350	1360
PNFEKAYTLP	VLPSEKDFNG	SDASTQLNTH	YAFSKLTYKS	SSGHEVENST	TDTQVISHEK	ENKLESLVLT	HLSRCDSDLC
1370	1380	1390	1400	1410	1420	1430	1440
EMNAGMPKGN	LNEQDPKHCP	ESEKCLLSIE	DEESQQSILS	SLENHSQQST	QPEMHKYGQL	VKVELEENAE	DDKTENQIPQ
1450	1460	1470	1480	1490	1500	1510	1520
RMTRNKANTM	ANQSKQILAS	CTLLSEKDSE	SSSPRGRIRL	TEDDDPQIHH	PRKRKVSRVP	QPVQVSPSLL	QAKEKTQQSL
1530	1540	1550	1560	1570	1580	1590	1600
AAIVDSLKLD	EIQPYSSERA	NPYFEYLHIR	KKIEEKRKLL	CSVIPQAPQY	YDEYVTFNGS	YLLDGNPLSK	ICIPTITPPP
1610	1620	1630	1640	1650	1660	1670	1680
SLSDPLKELF	RQQEVVRMKL	RLQHSIEREK	LIVSNEQEVL	RVHYRAARTL	ANQTLPFSAC	TVLLDAEVYN	VPLDSQSDDS
1690	1700	1710	1720	1730	1740	1750	1760
KTSVRDRFNA	RQFMSWLQDV	DDKFDKLKTC	LLMRQQHEAA	ALNAVQRLEW	QLKLQELDPA	TYKSISIYEI	QEFYVPLVDV
1770							
NDDFELTPI							

Cmpd.	No. of Cmpds.	m/z meas.	Δ m/z [ppm]	z	Rt [min]	Score	P	Range	Sequence	Modification	Ratios	
468	1	572.6830	-230.04	2	34.3	15.5	2	447-455	K.HVSKERNFK.E		WUP:QUP<br>QU:MU	4.73<br>0.51
110	1	1083.4576	-80.32	2	31.3	13.6	2	865-884	K.DAVSNRSQSVDTKNVMTLGK.S	Oxidation: 16		
623	1	824.1088	-20.46	3	37.4	10.9	2	995-1016	R.SSSVEDVKLIISEGRPTIEVRR.C			

Detailed Protein Report

Protein 113: fibroblast growth factor receptor substrate 2 [Homo sapiens]

**Accession:**	gi|110347408	**Score:**	51.0
**Database:**	refseq_human(refseq_human_20140103.fasta)	**MW [kDa]:**	57.0
**Database Date:**	2015-11-30	**pI:**	5.6
**Modification(s):**	Carbamidomethyl	**Sequence Coverage [%]:**	9.6
		**No. of unique Peptides:**	4

Alias proteins:

Accession	Name	Description
gi|507834075	refseq_human (refseq_human_20140103.fasta)	fibroblast growth factor receptor substrate 2 [Homo sapiens]
gi|507834073	refseq_human (refseq_human_20140103.fasta)	fibroblast growth factor receptor substrate 2 [Homo sapiens]
gi|507834071	refseq_human (refseq_human_20140103.fasta)	fibroblast growth factor receptor substrate 2 [Homo sapiens]
gi|507834069	refseq_human (refseq_human_20140103.fasta)	fibroblast growth factor receptor substrate 2 [Homo sapiens]
gi|507834066	refseq_human (refseq_human_20140103.fasta)	fibroblast growth factor receptor substrate 2 [Homo sapiens]
gi|507834061	refseq_human (refseq_human_20140103.fasta)	fibroblast growth factor receptor substrate 2 [Homo sapiens]
gi|110347414	refseq_human (refseq_human_20140103.fasta)	fibroblast growth factor receptor substrate 2 [Homo sapiens]

```
        10         20         30         40         50         60         70         80
MGSCCSCPDK DTVPDNHRNK FKVINVDDDG NELGSGIMEL TDTELILYTR KRDSVKWHYL CLRRYGYDSN LFSFESGRRC
        90        100        110        120        130        140        150        160
QTGQGIFAFK CARAEELFNM LQEIMQNNSI NVVEEPVVER NNHQTELEVP RTPRTPTTPG FAAQNLPNGY PRYPSFGDAS
       170        180        190        200        210        220        230        240
SHPSSRHPSV GSARLPSVGE ESTHPLLVAE EQVHTYVNTT GVQEERKNRT SVHVPLEARV SNAESSTPKE EPSSIEDRDP
       250        260        270        280        290        300        310        320
QILLEPEGVK FVLGPTPVQK QLMEKEKLEQ LGRDQVSGSG ANNTEWDTGY DSDERRDAPS VNKLVYENIN GLSIPSASGV
       330        340        350        360        370        380        390        400
RRGRLTSTST SDTQNINNSA QRRTALLNYE NLPSLPPVWE ARKLSRDEDD NLGPKTPSLN GYHNNLDPMH NYVNTENVTV
       410        420        430        440        450        460        470        480
PASAHKIEYS RRRDCTPTVF NFDIRRPSLE HRQLNYIQVD LEGGSDSDNP QTPKTPTTPL PQTPTRRTEL YAVIDIERTA
       490        500        510
AMSNLQKALP RDDGTSRKTR HNSTDLPM
```

Cmpd.	No. of Cmpds.	m/z meas.	Δ m/z [ppm]	z	Rt [min]	Score	P	Range	Sequence	Modification
1454	1	450.0854	-157.84	2	48.4	13.2	0	2-10	M.GSCCSCPDK.D	
390	1	524.3566	177.97	2	33.5	14.0	0	57-63	K.WHYLCLR.R	Carbamidomethyl: 5
2420	1	822.3542	-46.73	2	60.7	12.7	1	80-93	R.CQTGQGIFAFKCAR.A	Carbamidomethyl: 1, 12
1513	1	1023.0018	-57.43	2	49.1	11.1	1	304-322	K.LVYENINGLSIPSASGVRR.G	

Detailed Protein Report

Protein 114: **PREDICTED: ral GTPase-activating protein subunit alpha-2 isoform X1 [Homo sapiens]**

**Accession:**	gi|530425829	**Score:**	50.8
**Database:**	refseq_human(refseq_human_20140103.fasta)	**MW [kDa]:**	216.1
**Database Date:**	2015-11-30	**pI:**	5.8
**Modification(s):**	Oxidation	**Sequence Coverage [%]:**	3.5
		**No. of unique Peptides:**	3

Detailed Protein Report

10	20	30	40	50	60	70	80
MFSRRSHGDV	KKSTQKVLDP	KKDVLTRLKH	LRALLDNVDA	NDLKQFFETN	YSQIYFIFYE	NFIALENSLK	LKGNNKSQRE
90	100	110	120	130	140	150	160
ELDSILFLFE	KILQFLPERI	FFRWHYQSIG	STLKKLLHTG	NSIKIRCEGI	RLFLLWLQAL	QTNCAEEQVL	IFACLVPGFP
170	180	190	200	210	220	230	240
AVMSSRGPCT	LETLINPSPS	VADVKIYPEE	ITPLLPAISG	EKIAEDQTCF	FLQILLKYMV	IQAASLEWKN	KENQDTGFKF
250	260	270	280	290	300	310	320
LFTLFRKYYL	PHLFPSFTKL	TNIYKPVLDI	PHLRPKPVYI	TTTRDNENIY	STKIPYMAAR	VVFIKWIVTF	FLEKKYLTAT
330	340	350	360	370	380	390	400
QNTKNGVDVL	PKIIQTVGGG	AVQERAPELD	GGGPTEQDKS	HSNSSTLSDR	RLSNSSLCSI	EEEHRMVYEM	VQRILLSTRG
410	420	430	440	450	460	470	480
YVNFVNEVFH	QAFLLPSCEI	AVTRKVVQVY	RKWILQDKPV	FMEEPDRKDV	AQEDAEKLGF	SETDSKEASS	ESSGHKRSSS
490	500	510	520	530	540	550	560
WGRTYSFTSA	MSRGCVTEEE	NTNVKAGVQA	LLQVFLTNSA	NIFLLEPCAE	VPVLLKEQVD	ACKAVLIIFR	RMIMELTMNK
570	580	590	600	610	620	630	640
KTWEQMLQIL	LRITEAVMQK	PKDKQIKDLF	AQSLAGLLFR	TLMVAWIRAN	LCVYISRELW	DDFLGVLSSL	TEWEELINEW
650	660	670	680	690	700	710	720
ANIMDSLTAV	LARTVYGVEM	TNLPLDKLSE	QKEKKQRGKG	CVLDPQKGTT	VGRSFSLSWR	SHPDVTEPMR	FRSATTSGAP
730	740	750	760	770	780	790	800
GVEKARNIVR	QKATEVEECQ	QSENAPAAGS	GHLTVGQQQQ	VLRSSSTSDI	PEPLCSDSSQ	GQKAENTQNS	SSSEPQPIQE
810	820	830	840	850	860	870	880
NKGHVKREHE	GITILVRRSS	SPAELDLKDD	LQQTQGKCRE	RQKSESTNSD	TTLGCTNEAE	LSMGPWQTCE	EDPELNTPTD
890	900	910	920	930	940	950	960
VVADADARHW	LQLSPTDASN	LTDSSECLTD	DCSIIAGGSL	TGWHPDSAAV	LWRRVLGILG	DVNNIQSPKI	HARVFCYLYE
970	980	990	1000	1010	1020	1030	1040
LWYKLAKIRD	NLAISLDNQS	SPSPPVLIPP	LRMFASWLFK	AATLPNEYKE	GKLQAYRLIC	AMMTRRQDVL	PNSDFLVHFY
1050	1060	1070	1080	1090	1100	1110	1120
LVMHLGLTSE	DQDILNTIIR	HCPPRFFSLG	FPGFSMLVGD	FITAAARVLS	TDILTAPRSE	AVTVLGSLVC	FPNTYQEIPL
1130	1140	1150	1160	1170	1180	1190	1200
LQSVPEVNEA	ITGTEDVKHY	LINILLKNAT	EEPNEYARCI	AVCSLGVWIC	EELAQCTSHP	QVKEAINVIG	VTLKFPNKIV
1210	1220	1230	1240	1250	1260	1270	1280
AQVACDVLQL	LVSYWEKLQM	FETSLPRKMA	EILVATVAFL	LPSAEYSSVE	TDKKFIVSLL	LCLLDWCMAL	PVSVLLHPVS
1290	1300	1310	1320	1330	1340	1350	1360
TAVLEEQHSA	RAPLLDYIYR	VLHCCVCGSS	TYTQQSHYIL	TLADLSSTDY	DPFLPLANVK	SSEPVQYHSS	AELGNLLTVE
1370	1380	1390	1400	1410	1420	1430	1440
EEKKRRSLEL	IPLTARMVMA	HLVNHLGHYP	LSGGPAILHS	LVSENHDNAH	VEGSELSFEV	FRSPNLQLFV	FNDSTLISYL
1450	1460	1470	1480	1490	1500	1510	1520
QTPTEGPVGG	SPVGSLSDVR	VIVRDISGKY	SWDGKVLYGP	LEGCLAPNGR	NPSFLISSWH	RDTFGPQKDS	SQVEEGDDVL
1530	1540	1550	1560	1570	1580	1590	1600
DKLLENIGHT	SPECLLPSQL	NLNEPSLTPC	GMNYDQEKEI	IEVILRQNAQ	EDEYIQSHNF	DSAMKVTSQG	QPSPVEPRGP
1610	1620	1630	1640	1650	1660	1670	1680
FYFCRLLLDD	LGMNSWDRRK	NFHLLKKNSK	LLRELKNLDS	RQCRETHKIA	VFYIAEGQED	KCSILSNERG	SQAYEDFVAG
1690	1700	1710	1720	1730	1740	1750	1760
LGWEVDLSTH	CGFMGGLQRN	GSTGQTAPYY	ATSTVEVIFH	VSTRMPSDSD	DSLTKKLRHL	GNDEVHIVWS	EHSRDYRRGI
1770	1780	1790	1800	1810	1820	1830	1840
IPTAFGDVSI	IIYPMKNHMF	FIAITKKPEV	PFFGPLFDGA	IVSGKLLPSL	VCATCINASR	AVKCLIPLYQ	SFYEERALYL
1850	1860	1870	1880	1890	1900	1910	1920
EAIIQNHREV	MTFEDFAAQV	FSPSPSYSLS	GTGALASSLS	ADLEEPLSEE	ETEHPLLPLP	RATKPRISRK	FRCCTSALSR
1930							
SSH							

Cmpd.	No. of Cmpds.	m/z meas.	Δ m/z [ppm]	z	Rt [min]	Score	P	Range	Sequence	Modification
1746	2	1023.9594	-58.70	2	51.6	16.7	1	433-448	K.WILQDKPVFMEEPDRK.D	Oxidation: 10
584	1	666.3548	8.18	2	37.3	10.6	1	713-726	R.SATTSGAPGVEKAR.N	
1736	1	1273.5367	-62.63	2	51.5	13.3	0	1341-	K.SSEPVQYHSSAELGNLLTVEEEK.K	

Detailed Protein Report

Cmpd.	No. of Cmpds.	m/z meas.	Δ m/z [ppm]	z	Rt [min]	Score	P	Range	Sequence	Modification
								1363		

BRUKER

Detailed Protein Report

Protein 115: zinc finger protein 281 isoform 1 [Homo sapiens]

**Accession:**	gi|6912752	**Score:**	50.8
**Database:**	refseq_human(refseq_human_20140103.fasta)	**MW [kDa]:**	96.9
**Database Date:**	2015-11-30	**pI:**	9.5
**Modification(s):**	Carbamidomethyl, Oxidation	**Sequence Coverage [%]:**	5.3
		**No. of unique Peptides:**	3

Quantitation

QU:MU	**Median:** 0.61	**CV:** 0.00 %	**No. of Peptides:**	1
WUP:QUP	**Median:** 0.43	**CV:** 0.00 %	**No. of Peptides:**	1

Alias proteins:

Accession	Name	Description
gi|526253064	refseq_huma (refseq_human_20140103.fasta)	zinc finger protein 281 isoform 1 [Homo sapiens]

10	20	30	40	50	60	70	80
MKIGSGFLSG	GGGTGSSGGS	GSGGGGSGGG	GGGGSSGRRA	EMEPTFPQGM	VMFNHRLPPV	TSFTRPAGSA	APPPQCVLSS
**90**	**100**	**110**	**120**	**130**	**140**	**150**	**160**
STSAAPAAEP	PPPPAPDMTF	KKEPAASAAA	FPSQRTSWGF	LQSLVSIKQE	KPADPEEQQS	HHHHHHHHYG	GLFAGAEERS
**170**	**180**	**190**	**200**	**210**	**220**	**230**	**240**
PGLGGGEGGS	HGVIQDLSIL	HQHVQQQPAQ	HHRDVLLSSS	SRTDDHHGTE	EPKQDTNVKK	AKRPKPESQG	IKAKRKPSAS
**250**	**260**	**270**	**280**	**290**	**300**	**310**	**320**
SKPSLVGDGE	GAILSPSQKP	HICDHCSAAF	RSSYHLRRHV	LIHTGERPFQ	CSQCSMGFIQ	KYLLQRHEKI	HSREKPFGCD
**330**	**340**	**350**	**360**	**370**	**380**	**390**	**400**
QCSMKFIQKY	HMERHKRTHS	GEKPYKCDTC	QQYFSRTDRL	LKHRRTCGEV	IVKGATSAEP	GSSNHTNMGN	LAVLSQGNTS
**410**	**420**	**430**	**440**	**450**	**460**	**470**	**480**
SSRRKTKSKS	IAIENKEQKT	GKTNESQISN	NINMQSYSVE	MPTVSSSGGI	IGTGIDELQK	RVPKLIFKKG	SRKNTDKNYL
**490**	**500**	**510**	**520**	**530**	**540**	**550**	**560**
NFVSPLPDIV	GQKSLSGKPS	GSLGIVSNNS	VETIGLLQST	SGKQGQISSN	YDDAMQFSKK	RRYLPTASSN	SAFSINVGHM
**570**	**580**	**590**	**600**	**610**	**620**	**630**	**640**
VSQQSVIQSA	GVSVLDNEAP	LSLIDSSALN	AEIKSCHDKS	GIPDEVLQSI	LDQYSNKSES	QKEDPFNIAE	PRVDLHTSGE
**650**	**660**	**670**	**680**	**690**	**700**	**710**	**720**
HSELVQEENL	SPGTQTPSND	KASMLQEYSK	YLQQAFEKST	NASFTLGHGF	QFVSLSSPLH	NHTLFPEKQI	YTTSPLECGF
**730**	**740**	**750**	**760**	**770**	**780**	**790**	**800**
GQSVTSVLPS	SLPKPPFGML	FGSQPGLYLS	ALDATHQQLT	PSQELDDLID	SQKNLETSSA	FQSSSQKLTS	QKEQKNLESS
**810**	**820**	**830**	**840**	**850**	**860**	**870**	**880**
TGFQIPSQEL	ASQIDPQKDI	EPRTTYQIEN	FAQAFGSQFK	SGSRVPMTFI	TNSNGEVDHR	VRTSVSDFSG	YTNMMSDVSE
**890**	**900**						
PCSTRVKTPT	SQSYR						

Cmpd.	No. of Cmpds.	m/z meas.	Δ m/z [ppm]	z	Rt [min]	Score	P	Range	Sequence	Modification	Ratios
2793	1	840.4111	74.23	2	65.3	14.1	1	347-359	K.CDTCQQYFSRTDR.L	Carbamidomethyl: 4	
698	2	495.2768	-16.50	2	37.2	25.1	1	408-416	K.SKSIAIENK.E		QU:MU 0.61<br>WUP:QUP 0.43
2862	1	693.8668	91.67	4	66.2	11.6	1	861-885	R.<br>VRTSVSDFSGYTNMMSDVSEPCS<br>V	Oxidation: 14	

Detailed Protein Report

Protein 116: PREDICTED: midasin isoform X3 [Homo sapiens]

**Accession:**	gi|578812559	**Score:**	50.8
**Database:**	refseq_human(refseq_human_20140103.fasta)	**MW [kDa]:**	627.0
**Database Date:**	2015-11-30	**pI:**	5.4
		**Sequence Coverage [%]:**	0.9
		**No. of unique Peptides:**	2

Detailed Protein Report

10	20	30	40	50	60	70	80
MEHFLLEVAA	APLRLIAAKN	EKSRSELGRF	LAKQVWTPQD	RQCVLSTLAQ	LLLDKDCTVL	VGRQLRPLLL	DLLERNAEAI
90	100	110	120	130	140	150	160
KAGGQINHDL	HERLCVSMSK	LIGNHPDVLP	FALRYFKDTS	PVFQRLFLES	SDANPVRYGR	RRMKLRDLME	AAFKFLQQEQ
170	180	190	200	210	220	230	240
SVFRELWDWS	VCVPLLRSHD	TLVRWYTANC	LALVTCMNEE	HKLSFLKKIF	NSDELIHFRL	RLLEEAQLQD	LEKALVLANP
250	260	270	280	290	300	310	320
EVSLWRKQKE	LQYLQGHLVS	SDLSPRVTAV	CGVVLPGQLP	APGELGGNRS	SSREQELALR	SYVLVESVCK	SLQTLAMAVA
330	340	350	360	370	380	390	400
SQNAVLLEGP	IGCGKTSLVE	YLAAVTGRTK	PPQLLKVQLG	DQTDSKMLLG	MYRCTDVPGE	FVWQPGTLTQ	AATMGHWILL
410	420	430	440	450	460	470	480
EDIDYAPLDV	VSVLIPLLEN	GELLIPGRGD	CLKVAPGFQF	FATRRLLSCG	GNWYRPLNSH	ATLLDKYWTK	IHLDNLDKRE
490	500	510	520	530	540	550	560
LNEVLQSRYP	SLLAVVDHLL	DIYIQLTGEK	HHSWSDSSVG	CEQAPEEVSE	ARRENKRPTL	EGRELSLRDL	LNWCNRIAHS
570	580	590	600	610	620	630	640
FDSSSLSASL	NIFQEALDCF	TAMLSEHTSK	LKMAEVIGSK	LNISRKKAEF	FCQLYKPEIV	INELDLQVGR	VRLLRKQSEA
650	660	670	680	690	700	710	720
VHLQREKFTF	AATRPSSVLI	EQLAVCVSKG	EPVLLVGETG	TGKTSTIQYL	AHITGHRLRV	VNMNQQSDTA	DLLGGYKPVD
730	740	750	760	770	780	790	800
HKLIWLPLRE	AFEELFAQTF	SKKQNFTFLG	HIQTCYRQKR	WHDLLRLMQH	VHKSAVNKDG	KDSETGLLIK	EKWEAFGLRL
810	820	830	840	850	860	870	880
NHAQQQMKMT	ENTLLFAFVE	GTLAQAVKKG	EWILLDEINL	AAPEILECLS	GLLEGSSGSL	VLLDRGDTEP	LVRHPDFRLF
890	900	910	920	930	940	950	960
ACMNPATDVG	KRNLPPGIRN	RFTELYVEEL	ESKEDLQVLI	VDYLKGLSVN	KNTVQGIINF	YTALRKESGT	KLVDGTGHRP
970	980	990	1000	1010	1020	1030	1040
HYSLRTLCRA	LRFAASNPCG	NIQRSLYEGF	CLGFLTQLDR	ASHPIVQKLI	CQHIVPGNVK	SLLKQPIPEP	KGGRLIQVEG
1050	1060	1070	1080	1090	1100	1110	1120
YWIAVGDKEP	TIDETYILTS	SVKLNLRDIV	RVVSAGTYPV	LIQGETSVGK	TSLIQWLAAA	TGNHCVRINN	HEHTDIQEYI
1130	1140	1150	1160	1170	1180	1190	1200
GCYTSDSSGK	LVFKEGVLID	AMRKGYWIIL	DELNLAPTDV	LEALNRLLDD	NRELLVTETQ	EVVKAHPRFM	LFATQNPPGL
1210	1220	1230	1240	1250	1260	1270	1280
YGGRKVLSRA	FRNRFVELHF	DELPSSELET	ILHKRCSLPP	SYCSKLVKVM	LDLQSYRRSS	SVFAGKQGFI	TLRDLFRWAE
1290	1300	1310	1320	1330	1340	1350	1360
RYRLAEPTEK	EYDWLQHLAN	DGYMLLAGRV	RKQEEIDVIQ	EVLEKHFKKK	LCPQSLFSKE	NVLKLLGKLS	TQISTLECNF
1370	1380	1390	1400	1410	1420	1430	1440
GHIVWTEGMR	RLAMLVGRAL	EFGEPVLLVG	DTGCGKTTIC	QVFAALANQK	LYSVSCHLHM	ETSDFLGGLR	PVRQKPNDKE
1450	1460	1470	1480	1490	1500	1510	1520
EIDTSRLFEW	HDGPLVQAMK	EDGFFLLDEI	SLADDSVLER	LNSVLEVEKS	LVLAEKGSPE	DKDSEIELLT	AGKKFRILAT
1530	1540	1550	1560	1570	1580	1590	1600
MNPGGDFGKK	ELSPALRNRF	TEIWCPQSTS	REDLIQIISH	NLRPGLCLGR	IDPKGSDIPE	VMLDFIDWLT	HQEFGRKCVV
1610	1620	1630	1640	1650	1660	1670	1680
SIRDILSWVN	FMNKMGEEAA	LKRPEIISTV	TSFVHAACLV	YIDGIGSGVT	SSGFGTALLA	RKECLKFLIK	RLAKIVRLTE
1690	1700	1710	1720	1730	1740	1750	1760
YQKNELKIYD	RMKAKEFTGI	DNLWGIHPFF	IPRGPVLHRN	NIADYALSAG	TTAMNAQRLL	RATKLKKPIL	LEGSPGVGKT
1770	1780	1790	1800	1810	1820	1830	1840
SLVGALAKAS	GNTLVRINLS	EQTDITDLFG	ADLPVEGGKG	GEFAWRDGPL	LAALKAGHWV	VLDELNLASQ	SVLEGLNACF
1850	1860	1870	1880	1890	1900	1910	1920
DHRGEIYVPE	LGMSFQVQHE	KTKIFGCQNP	FRQGGGRKGL	PRSFLNRFTQ	VFVDPLTVID	MEFIASTLFP	AIEKNIVKKM
1930	1940	1950	1960	1970	1980	1990	2000
VAFNNQIDHE	VTVEKKWGQK	GGPWEFNLRD	LFRWCQLMLV	DQSPGCYDPG	QHVFLVYGER	MRTEEDKKKV	IAVFKDVFGS
2010	2020	2030	2040	2050	2060	2070	2080
NSNPYMGTRL	FRITPYDVQL	GYSVLSRGSC	VPHPSRHPLL	LLHQSFQPLE	SIMKCVQMSW	MVILVGPASV	GKTSLVQLLA
2090	2100	2110	2120	2130	2140	2150	2160
HLTGHTLKIM	AMNSAMDTTE	LLGGFEQVDL	IRPWRRLLEK	VEGTVRALLR	DSLLISADDA	EVVLRAWSHF	LLTYKPKCLG
2170	2180	2190	2200	2210	2220	2230	2240

Detailed Protein Report

Cmpd.	No. of Cmpds.	m/z meas.	Δ m/z [ppm]	z	Rt [min]	Score	P	Range	Sequence	Modification
558	1	512.6910	-181.41	2	35.4	13.3	2	1688-1695	K.IYDRMKAK.E	
1915	1	918.3456	-31.31	2	53.8	12.2	1	4832-4848	K.EEKEEAEADDGGQGEDK.I	

Detailed Protein Report

Protein 117: PREDICTED: integrin beta-4 isoform X9 [Homo sapiens]

**Accession:**	gi|578830859	**Score:**	50.7
**Database:**	refseq_human(refseq_human_20140103.fasta)	**MW [kDa]:**	198.8
**Database Date:**	2015-11-30	**pI:**	5.8
**Modification(s):**	Oxidation	**Sequence Coverage [%]:**	1.6
		**No. of unique Peptides:**	2

10	20	30	40	50	60	70	80
MVVAVRNRAG	SEGLESPSWG	LREGQQEGQE	GRRKRMAGPR	PSPWARLLLA	ALISVSLSGT	LANRCKKAPV	KSCTECVRVD
90	100	110	120	130	140	150	160
KDCAYCTDEM	FRDRRCNTQA	ELLAAGCQRE	SIVVMESSFQ	ITEETQIDTT	LRRSQMSPQG	LRVRLRPGEE	RHFELEVFEP
170	180	190	200	210	220	230	240
LESPVDLYIL	MDFSNSMSDD	LDNLKKMGQN	LARVLSQLTS	DYTIGFGKFV	DKVSVPQTDM	RPEKLKEPWP	NSDPPFSFKN
250	260	270	280	290	300	310	320
VISLTEDVDE	FRNKLQGERI	SGNLDAPEGG	FDAILQTAVC	TRDIGWRPDS	THLLVFSTES	AFHYEADGAN	VLAGIMSRND
330	340	350	360	370	380	390	400
ERCHLDTTGT	YTQYRTQDYP	SVPTLVRLLA	KHNIIPIFAV	TNYSYSYYEK	LHTYFPVSSL	GVLQEDSSNI	VELLEEAFNR
410	420	430	440	450	460	470	480
IRSNLDIRAL	DSPRGLRTEV	TSKMFQKTRT	GSFHIRRGEV	GIYQVQLRAL	EHVDGTHVCQ	LPEDQKGNIH	LKPSFSDGLK
490	500	510	520	530	540	550	560
MDAGIICDVC	TCELQKEVRS	ARCSFNGDFV	CGQCVCSEGW	SGQTCNCSTG	SLSDIQPCLR	EGEDKPCSGR	GECQCGHCVC
570	580	590	600	610	620	630	640
YGEGRYEGQF	CEYDNFQCPR	TSGFLCNDRG	RCSMGQCVCE	PGWTGPSCDC	PLSNATCIDS	NGGICNGRGH	CECGRCHCHQ
650	660	670	680	690	700	710	720
QSLYTDTICE	INYSAIHPGL	CEDLRSCVQC	QAWGTGEKKG	RTCEECNFKV	KMVDELKRAE	EVVVRCSFRD	EDDDCTYSYT
730	740	750	760	770	780	790	800
MEGDGAPGPN	STVLVHKKKD	CPPGSFWWLI	PLLLLLLPLL	ALLLLLCWKY	CACCKACLAL	LPCCNRGHMV	GFKEDHYMLR
810	820	830	840	850	860	870	880
ENLMASDHLD	TPMLRSGNLK	GRDVVRWKVT	NNMQRPGFAT	HAASINPTEL	VPYGLSLRLA	RLCTENLLKP	DTRECAQLRQ
890	900	910	920	930	940	950	960
EVEENLNEVY	RQISGVHKLQ	QTKFRQQPNA	GKKQDHTIVD	TVLMAPRSAK	PALLKLTEKQ	VEQRAFHDLK	VAPGYYTLTA
970	980	990	1000	1010	1020	1030	1040
DQDARGMVEF	QEGVELVDVR	VPLFIRPEDD	DEKQLLVEAI	DVPAGTATLG	RRLVNITIIK	EQARDVVSFE	QPEFSVSRGD
1050	1060	1070	1080	1090	1100	1110	1120
QVARIPVIRR	VLDGGKSQVS	YRTQDGTAQG	NRDYIPVEGE	LLFQPGEAWK	ELQVKLLELQ	EVDSLLRGRQ	VRRFHVQLSN
1130	1140	1150	1160	1170	1180	1190	1200
PKFGAHLGQP	HSTTIIIRDP	DELDRSFTSQ	MLSSQPPPHG	DLGAPQNPNA	KAAGSRKIHF	NWLPPSGKPM	GYRVKYWIQG
1210	1220	1230	1240	1250	1260	1270	1280
DSESEAHLLD	SKVPSVELTN	LYPYCDYEMK	VCAYGAQGEG	PYSSLVSCRT	HQEVPSEPGR	LAFNVVSSTV	TQLSWAEPAE
1290	1300	1310	1320	1330	1340	1350	1360
TNGEITAYEV	CYGLVNDDNR	PIGPMKKVLV	DNPKNRMLLI	ENLRESQPYR	YTVKARNGAG	WGPEREAIIN	LATQPKRPMS
1370	1380	1390	1400	1410	1420	1430	1440
IPIIPDIPIV	DAQSGEDYDS	FLMYSDDVLR	SPSGSQRPSV	SDDTEHLVNG	RMDFAFPGST	NSLHRMTTTS	AAAYGTHLSP
1450	1460	1470	1480	1490	1500	1510	1520
HVPHRVLSTS	STLTRDYNSL	TRSEHSHSTT	LPRDYSTLTS	VSSHDSRLTA	GVPDTPTRLV	FSALGPTSLR	VSWQEPRCER
1530	1540	1550	1560	1570	1580	1590	1600
PLQGYSVEYQ	LLNGGELHRL	NIPNPAQTSV	VVEDLLPNHS	YVFRVRAQSQ	EGWGREREGV	ITIESQVHPQ	SPLCPLPGSA
1610	1620	1630	1640	1650	1660	1670	1680
FTLSTPSAPG	PLVFTALSPD	SLQLSWERPR	RPNGDIVGYL	VTCEMAQGGG	PATAFRVDGD	SPESRLTVPG	LSENVPYKFK
1690	1700	1710	1720	1730	1740	1750	1760
VQARTTEGFG	PEREGIITIE	SQDGGPFPQL	GSRAGLFQHP	LQSEYSSITT	THTSATEPFL	VDGLTLGAQH	LEAGGSLTRH
1770	1780	1790					
VTQEFVSRTL	TTSGTLSTHM	DQQFFQT					

Cmpd.	No. of	m/z meas.	Δ m/z	z	Rt	Score	P	Range	Sequence	Modification

Detailed Protein Report

	Cmpds.		[ppm]		[min]					
1596	1	480.7905	34.21	2	49.7	13.2	1	1-8	-.MVVAVRNR.A	Oxidation: 1
522	1	678.9701	154.68	2	36.5	13.0	1	403-414	R.SNLDIRALDSPR.G	

Detailed Protein Report

**Protein 118:**	**PREDICTED: basement membrane-specific heparan sulfate proteoglycan core protein isoform X10 [Homo sapiens]**		
**Accession:**	gi|578799003	**Score:**	50.6
**Database:**	refseq_human(refseq_human_20140103.fasta)	**MW [kDa]:**	433.5
**Database Date:**	2015-11-30	**pI:**	6.1
**Modification(s):**	Oxidation	**Sequence Coverage [%]:**	2.1
		**No. of unique Peptides:**	4

Detailed Protein Report

10	20	30	40	50	60	70	80
MGWRAAGALL	LALLLHGRLL	AVTHGLRAYD	GLSLPEDIET	VTASQMRWTH	SYLSDDEDML	ADSISGDDLG	SGDLGSGDFQ
90	100	110	120	130	140	150	160
MVYFRALVNF	TRSIEYSPQL	EDAGSREFRE	VSEAVVDTLE	SEYLKIPGDQ	VVSVVFIKEL	DGWVFVELDV	GSEGNADGAQ
170	180	190	200	210	220	230	240
IQEMLLRVIS	SGSVASYVTS	PQGFQFRRLG	TAQTPPPPPA	AVAVAVTPVP	QFPRACTEAE	FACHSYNECV	ALEYRCDRRP
250	260	270	280	290	300	310	320
DCRDMSDELN	CEEPVLGISP	TFSLLVETTS	LPPRPETTIM	RQPPVTHAPQ	PLLPGSVRPL	PCGPQEAACR	NGHCIPRDYL
330	340	350	360	370	380	390	400
CDGQEDCEDG	SDELDCGPPP	PCEPNEFPCG	NGHCALKLWR	CDGDFDCEDR	TDEANCPTKR	PEEVCGPTQF	RCVSTNMCIP
410	420	430	440	450	460	470	480
ASFHCDEESD	CPDRSDEFGC	MPPQVVTPPR	ESIQASRGQT	VTFTCVAIGV	PTPIINWRLN	WGHIPSHPRV	TVTSEGGRGT
490	500	510	520	530	540	550	560
LIIRDVKESD	QGAYTCEAMN	ARGMVFGIPD	GVLELVPQRA	GPCPDGHFYL	EHSAACLPCF	CFGITSVCQS	TRRFRDQIRL
570	580	590	600	610	620	630	640
RFDQPDDFKG	VNVTMPAQPG	TPPLSSTQLQ	IDPSLHEFQL	VDLSRRFLVH	DSFWALPEQF	LGNKVDSYGG	SLRYNVRYEL
650	660	670	680	690	700	710	720
ARGMLEPVQR	PDVVLMGAGY	RLLSRGHTPT	QPGALNQRQV	QFSEEHWVHE	SGRPVQRAEL	LQVLQSLEAV	LIQTVYNTKM
730	740	750	760	770	780	790	800
ASVGLSDIAM	DTTVTHATSH	GRAHSVEECR	CPIGYSGLSC	ESCDAHFTRV	PGGPYLGTCS	GCNCNGHASS	CDPVYGHCLN
810	820	830	840	850	860	870	880
CQHNTEGPQC	NKCKAGFFGD	AMKATATSCR	PCPCPYIDAS	RRFSDTCFLD	TDGQATCDAC	APGYTGRRCE	SCAPGYEGNP
890	900	910	920	930	940	950	960
IQPGGKCRPV	NQEIVRCDER	GSMGTSGEAC	RCKNNVVGRL	CNECADGSFH	LSTRNPDGCL	KCFCMGVSRH	CTSSSWSRAQ
970	980	990	1000	1010	1020	1030	1040
LHGASEEPGH	FSLTNAASTH	TTNEGIFSPT	PGELGFSSFH	RLLSGPYFWS	LPSRFLGDKV	TSYGGELRFT	VTQRSQPGST
1050	1060	1070	1080	1090	1100	1110	1120
PLHGQPLVVL	QGNNIILEHH	VAQEPSPGQP	STFIVPFREQ	AWQRPDGQPA	TREHLLMALA	GIDTLLIRAS	YAQQPAESRV
1130	1140	1150	1160	1170	1180	1190	1200
SGISMDVAVP	EETGQDPALE	VEQCSCPPGY	RGPSCQDCDT	GYTRTPSGLY	LGTCERCSCH	GHSEACEPET	GACQGCQHHT
1210	1220	1230	1240	1250	1260	1270	1280
EGPRCEQCQP	GYYGDAQRGT	PQDCQLCPCY	GDPAAGQAAH	TCFLDTDGHP	TCDACSPGHS	GRHCERCAPG	YYGNPSQGQP
1290	1300	1310	1320	1330	1340	1350	1360
CQRDSQVPGP	IGCNCDPQGS	VSSQCDAAGQ	CQCKAQVEGL	TCSHCRPHHF	HLSASNPDGC	LPCFCMGITQ	QCASSAYTRH
1370	1380	1390	1400	1410	1420	1430	1440
LISTHFAPGD	FQGFALVNPQ	RNSRLTGEFT	VEPVPEGAQL	SFGNFAQLGH	ESFYWQLPET	YQGDKGEAYF	ARMRRAHQRT
1450	1460	1470	1480	1490	1500	1510	1520
LPMALAPGLW	EVLARLLPPF	HQNRTSLSEE	QLRAAVTAGR	IPEPPEGRDW	AQRASQVDEA	QRRMDAEIWQ	LLSSFAAQPQ
1530	1540	1550	1560	1570	1580	1590	1600
PPPQGLSPHP	QPAAALRAAP	PPSSSSSSSS	SSASLSFSPG	SQFSLSYEGF	SLLPGSLYYW	QLPRAFLGDK	VAAYGGKLRY
1610	1620	1630	1640	1650	1660	1670	1680
TLSYTAGPQG	SPLSDPDVQI	TGNNIMLVAS	QPALQGPERR	SYEIMFREEF	WRRPDGQPAT	REHLLMALAD	LDELLIRATF
1690	1700	1710	1720	1730	1740	1750	1760
SSVPLAASIS	AVSLEVAQPG	PSNRPRALEV	EECRCPPGYI	GLSCQDCAPG	YTRTGSGLYL	GHCELCECNG	HSDLCHPETG
1770	1780	1790	1800	1810	1820	1830	1840
ACSQCQHNAA	GEFCELCAPG	YYGDATAGTP	EDCQPCACPL	TNPENMFSRT	CESLGAGGYR	CTACEPGYTG	QYCEQCGPGY
1850	1860	1870	1880	1890	1900	1910	1920
VGNPSVQGGQ	CLPETNQAPL	VVEVHPARSI	VPQGGSHSLR	CQVSGSPPHY	FYWSREDGRP	VPSGTQQRHQ	GSELHFPSVQ
1930	1940	1950	1960	1970	1980	1990	2000
PSDAGVYICT	CRNLHQSNTS	RAELLVTEAP	SKPITVTVEE	QRSQSVRPGA	DVTFICTAKS	KSPAYTLVWT	RLHNGKLPTR
2010	2020	2030	2040	2050	2060	2070	2080
AMDFNGILTI	RNVQLSDAGT	YVCTGSNMFA	MDQGTATLHV	QASGTLSAPV	VSIHPPQLTV	QPGQLAEFRC	SATGSPTPTL
2090	2100	2110	2120	2130	2140	2150	2160
EWTGGPGGQL	PAKAQIHGGI	LRLPAVEPTD	QAQYLCRAHS	SAGQQVARAV	LHVHGGGGPR	VQVSPERTQV	HAGRTVRLYC
2170	2180	2190	2200	2210	2220	2230	2240

Detailed Protein Report

Cmpd.	No. of Cmpds.	m/z meas.	Δ m/z [ppm]	z	Rt [min]	Score	P	Range	Sequence	Modification
2417	1	1175.2100	-20.32	2	60.1	10.2	1	5-27	R.AAGALLLALLLHGRLLAVTHGLR.A	
2083	1	1052.3950	-135.43	2	56.4	13.6	0	643-661	R.GMLEPVQRPDVVLMGAGYR.L	Oxidation: 2
85	1	827.0046	173.12	2	31.0	13.9	0	920-934	R.LCNECADGSFHLSTR.N	
1714	1	1011.6020	66.80	3	51.7	12.9	1	3926-3953	R.FDAGSGMATIRHPTPLALGHFHTVTLLR.S	Oxidation: 7

Detailed Protein Report

Protein 119: fibrillin-1 precursor [Homo sapiens]

**Accession:**	gi|281485550	**Score:**	50.6
**Database:**	refseq_human(refseq_human_20140103.fasta)	**MW [kDa]:**	312.1
**Database Date:**	2015-11-30	**pI:**	4.7
**Modification(s):**	Carbamidomethyl, Oxidation	**Sequence Coverage [%]:**	1.8
		**No. of unique Peptides:**	3

Detailed Protein Report

10	20	30	40	50	60	70	80
MRRGRLLEIA	LGFTVLLASY	TSHGADANLE	AGNVKETRAS	RAKRRGGGGH	DALKGPNVCG	SRYNAYCCPG	WKTLPGGNQC
90	100	110	120	130	140	150	160
IVPICRHSCG	DGFCSRPNMC	TCPSGQIAPS	CGSRSIQHCN	IRCMNGGSCS	DDHCLCQKGY	IGTHCGQPVC	ESGCLNGGRC
170	180	190	200	210	220	230	240
VAPNRCACTY	GFTGPQCERD	YRTGPCFTVI	SNQMCQGQLS	GIVCTKTLCC	ATVGRAWGHP	CEMCPAQPHP	CRRGFIPNIR
250	260	270	280	290	300	310	320
TGACQDVDEC	QAIPGLCQGG	NCINTVGSFE	CKCPAGHKLN	EVSQKCEDID	ECSTIPGICE	GGECTNTVSS	YFCKCPPGFY
330	340	350	360	370	380	390	400
TSPDGTRCID	VRPGYCYTAL	TNGRCSNQLP	QSITKMQCCC	DAGRCWSPGV	TVAPEMCPIR	ATEDFNKLCS	VPMVIPGRPE
410	420	430	440	450	460	470	480
YPPPPLGPIP	PVLPVPPGFP	PGPQIPVPRP	PVEYLYPSRE	PPRVLPVNVT	DYCQLVRYLC	QNGRCIPTPG	SYRCECNKGF
490	500	510	520	530	540	550	560
QLDLRGECID	VDECEKNPCA	GGECINNQGS	YTCQCRAGYQ	STLTRTECRD	IDECLQNGRI	CNNGRCINTD	GSFHCVCNAG
570	580	590	600	610	620	630	640
FHVTRDGKNC	EDMDECSIRN	MCLNGMCINE	DGSFKCICKP	GFQLASDGRY	CKDINECETP	GICMNGRCVN	TDGSYRCECF
650	660	670	680	690	700	710	720
PGLAVGLDGR	VCVDTHMRST	CYGGYKRGQC	IKPLFGAVTK	SECCCASTEY	AFGEPCQPCP	AQNSAEYQAL	CSSGPGMTSA
730	740	750	760	770	780	790	800
GSDINECALD	PDICPNGICE	NLRGTYKCIC	NSGYEVDSTG	KNCVDINECV	LNSLLCDNGQ	CRNTPGSFVC	TCPKGFIYKP
810	820	830	840	850	860	870	880
DLKTCEDIDE	CESSPCINGV	CKNSPGSFIC	ECSSESTLDP	TKTICIETIK	GTCWQTVIDG	RCEININGAT	LKSQCCSSLG
890	900	910	920	930	940	950	960
AAWGSPCTLC	QVDPICGKGY	SRIKGTQCED	IDECEVFPGV	CKNGLCVNTR	GSFKCQCPSG	MTLDATGRIC	LDIRLETCFL
970	980	990	1000	1010	1020	1030	1040
RYEDEECTLP	IAGRHRMDAC	CCSVGAAWGT	EECEECPMRN	TPEYEELCPR	GPGFATKEIT	NGKPFFKDIN	ECKMIPSLCT
1050	1060	1070	1080	1090	1100	1110	1120
HGKCRNTIGS	FKCRCDSGFA	LDSEERNCTD	IDECRISPDL	CGRGQCVNTP	GDFECKCDEG	YESGFMMMKN	CMDIDECQRD
1130	1140	1150	1160	1170	1180	1190	1200
PLLCRGGVCH	NTEGSYRCEC	PPGHQLSPNI	SACIDINECE	LSAHLCPNGR	CVNLIGKYQC	ACNPGYHSTP	DRLFCVDIDE
1210	1220	1230	1240	1250	1260	1270	1280
CSIMNGGCET	FCTNSEGSYE	CSCQPGFALM	PDQRSCTDID	ECEDNPNICD	GGQCTNIPGE	YRCLCYDGFM	ASEDMKTCVD
1290	1300	1310	1320	1330	1340	1350	1360
VNECDLNPNI	CLSGTCENTK	GSFICHCDMG	YSGKKGKTGC	TDINECEIGA	HNCGKHAVCT	NTAGSFKCSC	SPGWIGDGIK
1370	1380	1390	1400	1410	1420	1430	1440
CTDLDECSNG	THMCSQHADC	KNTMGSYRCL	CKEGYTGDGF	TCTDLDECSE	NLNLCGNGQC	LNAPGGYRCE	CDMGFVPSAD
1450	1460	1470	1480	1490	1500	1510	1520
GKACEDIDEC	SLPNICVFGT	CHNLPGLFRC	ECEIGYELDR	SGGNCTDVNE	CLDPTTCISG	NCVNTPGSYI	CDCPPDFELN
1530	1540	1550	1560	1570	1580	1590	1600
PTRVGCVDTR	SGNCYLDIRP	RGDNGDTACS	NEIGVGVSKA	SCCCSLGKAW	GTPCEMCPAV	NTSEYKILCP	GGEGFRPNPI
1610	1620	1630	1640	1650	1660	1670	1680
TVILEDIDEC	QELPGLCQGG	KCINTFGSFQ	CRCPTGYYLN	EDTRVCDDVN	ECETPGICGP	GTCYNTVGNY	TCICPPDYMQ
1690	1700	1710	1720	1730	1740	1750	1760
VNGGNNCMDM	RRSLCYRNYY	ADNQTCDGEL	LFNMTKKMCC	CSYNIGRAWN	KPCEQCPIPS	TDEFATLCGS	QRPGFVIDIY
1770	1780	1790	1800	1810	1820	1830	1840
TGLPVDIDEC	REIPGVCENG	VCINMVGSFR	CECPVGFFYN	DKLLVCEDID	ECQNGPVCQR	NAECINTAGS	YRCDCKPGYR
1850	1860	1870	1880	1890	1900	1910	1920
FTSTGQCNDR	NECQEIPNIC	SHGQCIDTVG	SFYCLCHTGF	KTNDDQTMCL	DINECERDAC	GNGTCRNTIG	SFNCRCNHGF
1930	1940	1950	1960	1970	1980	1990	2000
ILSHNNDCID	VDECASGNGN	LCRNGQCINT	VGSFQCQCNE	GYEVAPDGRT	CVDINECLLE	PRKCAPGTCQ	NLDGSYRCIC
2010	2020	2030	2040	2050	2060	2070	2080
PPGYSLQNEK	CEDIDECVEE	PEICALGTCS	NTEGSFKCLC	PEGFSLSSSG	RRCQDLRMSY	CYAKFEGGKC	SSPKSRNHSK
2090	2100	2110	2120	2130	2140	2150	2160
QECCCALKGE	GWGDPCELCP	TEPDEAFRQI	CPYGSGIIVG	PDDSAVDMDE	CKEPDVCKHG	QCINTDGSYR	CECPFGYILA
2170	2180	2190	2200	2210	2220	2230	2240

Detailed Protein Report

Cmpd.	No. of Cmpds.	m/z meas.	Δ m/z [ppm]	z	Rt [min]	Score	P	Range	Sequence	Modification
1872	1	1057.6895	-79.32	3	53.8	11.0	2	610-636	R.YCKDINECETPGICMNGRCVNTDGSYR.C	Carbamidomethyl: 2, 19; Oxidation: 15
748	8	635.3242	75.75	2	37.8	23.9	1	2482-2492	R.SCKDLDECATK.Q	Carbamidomethyl: 8
2864	1	703.3686	82.89	2	66.3	15.8	0	2681-2694	R.IGQGHCVSGMGMGR.G	Oxidation: 12

Detailed Protein Report

Protein 120: PREDICTED: nuclear receptor corepressor 1 isoform X13 [Homo sapiens]

**Accession:**	gi|578830118	**Score:**	50.4
**Database:**	refseq_human(refseq_human_20140103.fasta)	**MW [kDa]:**	277.7
**Database Date:**	2015-11-30	**pI:**	6.8
**Modification(s):**	Oxidation	**Sequence Coverage [%]:**	2.9
		**No. of unique Peptides:**	3

Quantitation

QU:MU	**Median:**	1.77	**CV:**	0.00 %	**No. of Peptides:**	1
WUP:QUP	**Median:**	0.67	**CV:**	0.00 %	**No. of Peptides:**	1

BRUKER

Detailed Protein Report

10	20	30	40	50	60	70	80
MSSSGYPPNQ	GAFSTEQSRY	PPHSVQYTFP	NTRHQQEFAV	PDYRSSHLEV	SQASQLLQQQ	QQQQLRRRPS	LLSEFHPGSD
90	100	110	120	130	140	150	160
RPQERRTSYE	PFHPGPSPVD	HDSLESKRPR	LEQVSDSHFQ	RVSAAVLPLV	HPLPEGLRAS	ADAKKDPAFG	GKHEAPSSPI
170	180	190	200	210	220	230	240
SGQPCGDDQN	ASPSKLSKEE	LIQSMDRVDR	EIAKVEQQIL	KLKKKQQQLE	EEAAKPPEPE	KPVSPPPVEQ	KHRSIVQIIY
250	260	270	280	290	300	310	320
DENRKKAEEA	HKIFEGLGPK	VELPLYNQPS	DTKVYHENIK	TGVPARRMMK	NQVMRKKLIL	FFKRRNHARK	QREQKICQRY
330	340	350	360	370	380	390	400
DQLMEAWEKK	VDRIENNPRR	KAKESKTREY	YEKQFPEIRK	QREQQERFQR	VGQRGAGLSA	TIARSEHEIS	EIIDGLSEQE
410	420	430	440	450	460	470	480
NNEKQMRQLS	VIPPMMFDAE	QRRVKFINMN	GLMEDPMKVY	KDRQFMNVWT	DHEKEIFKDK	FIQHPKNFGL	IASYLERKSV
490	500	510	520	530	540	550	560
PDCVLYYYLT	KKNENYKALV	RRNYGKRRGR	NQQQIARPSQ	EEKVEEKEED	KAEKTEKKEE	EKKDEEEKDE	KEDSKENTKE
570	580	590	600	610	620	630	640
KDKIDGTAEE	TEEREQATPR	GRKTANSQGR	RKGRITRSMT	NEAAAASAAA	AAATEEPPPP	LPPPPEPIST	EPVETSRWTE
650	660	670	680	690	700	710	720
EEMEVAKKGL	VEHGRNWAAI	AKMVGTKSEA	QCKNFYFNYK	RRHNLDNLLQ	QHKQKTSRKP	REERDVSQCE	SVASTVSAQE
730	740	750	760	770	780	790	800
DEDIEASNEE	ENPEDSEGAE	NSSDTESAPS	PSPVEAVKPS	EDSPENATSR	GNTEPAVELE	PTTETAPSTS	PSLAVPSTKP
810	820	830	840	850	860	870	880
AEDESVETQV	NDSISAETAE	QMDVDQQEHS	AEEGSVCDPP	PATKADSVDV	EVRVPENHAS	KVEGDNTKER	DLDRASEKVE
890	900	910	920	930	940	950	960
PRDEDLVVAQ	QINAQRPEPQ	SDNDSSATCS	ADEDVDGEPE	RQRMFPMDSK	PSLLNPTGSI	LVSSPLKPNP	LDLPQLQHRA
970	980	990	1000	1010	1020	1030	1040
AVIPPMVSCT	PCNIPIGTPV	SGYALYQRHI	KAMHESALLE	EQRQRQEQID	LECRSSTSPC	GTSKSPNREW	EGKSVAYMPY
1050	1060	1070	1080	1090	1100	1110	1120
AEVKRALEQE	AQMHNTAARS	ASPCRLSPRE	VSKAAPQPDM	SAARYSVPPV	LQPAPHQVIT	NLPEGVRLPT	TRPTRPPPPL
1130	1140	1150	1160	1170	1180	1190	1200
IPSSKTTVAS	EKPSFIMGGS	ISQGTPGTYL	TSHNQASYTQ	ETPKPSVGSI	SLGLPRQQES	AKSATLPYIK	QEEFSPRSQN
1210	1220	1230	1240	1250	1260	1270	1280
SQPEGLLVRA	QHEGVVRGTA	GAIQEGSITR	GTPTSKISVE	SIPSLRGSIT	QGTPALPQTG	IPTEALVKGS	ISRMPIEDSS
1290	1300	1310	1320	1330	1340	1350	1360
PEKGREEAAS	KGHVIYEGKS	GHILSYDNIK	NAREGTRSPR	TAHEISLKRS	YESVEGNIKQ	GMSMRESPVS	APLEGLICRA
1370	1380	1390	1400	1410	1420	1430	1440
LPRGSPHSDL	KERTVLSGSI	MQGTPRATTE	SFEDGLKYPK	QIKRESPPIR	AFEGAITKGK	PYDGITTIKE	MGRSIHEIPR
1450	1460	1470	1480	1490	1500	1510	1520
QDILTQESRK	TPEVVQSTRP	IIEGSISQGT	PIKFDNNSGQ	SAIKHNVKSL	ITGPSKLSRG	MPPLEIVPEN	IKVVERGKYE
1530	1540	1550	1560	1570	1580	1590	1600
DVKAGETVRS	RHTSVVSSGP	SVLRSTLHEA	PKAQLSPGIY	DDTSARRTPV	SYQNTMSRGS	PMMNRTSDVT	ISSNKSTNHE
1610	1620	1630	1640	1650	1660	1670	1680
RKSTLTPTQR	ESIPAKSPVP	GVDPVVSHSP	FDPHHRGSTA	GEVYRSHLPT	HLDPAMPFHR	ALDPAAAAYL	FQRQLSPTPG
1690	1700	1710	1720	1730	1740	1750	1760
YPSQYQLYAM	ENTRQTILND	YITSQQMQVN	LRPDVARGLS	PREQPLGLPY	PATRGIIDLT	NMPPTILVPH	PGGTSTPPMD
1770	1780	1790	1800	1810	1820	1830	1840
RITYIPGTQI	TFPPRPYNSA	SMSPGHPTHL	AAAASAERER	EREREKERER	ERIAAASSDL	YLRPGSEQPG	RPGSHGYVRS
1850	1860	1870	1880	1890	1900	1910	1920
PSPSVRTQET	MLQQRPSVFQ	GTNGTSVITP	LDPTAQLRIM	PLPAGGPSIS	QGLPASRYNT	AADALAALVD	AAASAPQMDV
1930	1940	1950	1960	1970	1980	1990	2000
SKTKESKHEA	ARLEENLRSR	SAAVSEQQQL	EQKTLEVEKR	SVQCLYTSSA	FPSGKPQPHS	SVVYSEAGKD	KGPPPKSRYE
2010	2020	2030	2040	2050	2060	2070	2080
EELRTRGKTT	ITAANFIDVI	ITRQIASDKD	ARERGSQSSD	SSSSLSSHRY	ETPSDAIEVI	SPASSPAPPQ	EKLQTYQPEV
2090	2100	2110	2120	2130	2140	2150	2160
VKANQAENDP	TRQYEGPLHH	YRPQQESPSP	QQQLPPSSQA	EGMGQVPRTH	RLITLADHIC	QIITQDFARN	QVSSQTPQQP
2170	2180	2190	2200	2210	2220	2230	2240

Detailed Protein Report

Cmpd.	No. of Cmpds.	m/z meas.	Δ m/z [ppm]	z	Rt [min]	Score	P	Range	Sequence	Modification	Ratios
2870	1	973.5098	46.41	2	64.6	11.5	1	426-441	K.FINMNGLMEDPMKVYK.D	Oxidation: 8	
47	3	1068.4733	-12.48	2	30.3	15.3	2	2030-2049	K.DARERGSQSSDSSSSLSSHR.Y		QU:MU 1.77<br>WUP:QUP 0.67
911	1	492.2027	-52.98	2	40.9	10.7	0	2341-2349	K.ALMGSFDDK.V		

Detailed Protein Report

Protein 121: tenascin-X isoform 1 precursor [Homo sapiens]

**Accession:**	gi|188528648	**Score:**	50.3
**Database:**	refseq_human(refseq_human_20140103.fasta)	**MW [kDa]:**	457.9
**Database Date:**	2015-11-30	**pI:**	4.9
**Modification(s):**	Carbamidomethyl	**Sequence Coverage [%]:**	1.5
		**No. of unique Peptides:**	4

Detailed Protein Report

10	20	30	40	50	60	70	80
MMPAQYALTS	SLVLLVLLST	ARAGPFSSRS	NVTLPAPRPP	PQPGGHTVGA	GVGSPSSQLY	EHTVEGGEKQ	VVFTHRINLP
90	100	110	120	130	140	150	160
PSTGCGCPPG	TEPPVLASEV	QALRVRLEIL	EELVKGLKEQ	CTGGCCPASA	QAGTGQTDVR	TLCSLHGVFD	LSRCTCSCEP
170	180	190	200	210	220	230	240
GWGGPTCSDP	TDAEIPPSSP	PSASGSCPDD	CNDQGRCVRG	RCVCFPGYTG	PSCGWPSCPG	DCQGRGRCVQ	GVCVCRAGFS
250	260	270	280	290	300	310	320
GPDCSQRSCP	RGCSQRGRCE	GGRCVCDPGY	TGDDCGMRSC	PRGCSQRGRC	ENGRCVCNPG	YTGEDCGVRS	CPRGCSQRGR
330	340	350	360	370	380	390	400
CKDGRCVCDP	GYTGEDCGTR	SCPWDCGEGG	RCVDGRCVCW	PGYTGEDCST	RTCPRDCRGR	GRCEDGECIC	DTGYSGDDCG
410	420	430	440	450	460	470	480
VRSCPGDCNQ	RGRCEDGRCV	CWPGYTGTDC	GSRACPRDCR	GRGRCENGVC	VCNAGYSGED	CGVRSCPGDC	RGRGRCESGR
490	500	510	520	530	540	550	560
CMCWPGYTGR	DCGTRACPGD	CRGRGRCVDG	RCVCNPGFTG	EDCGSRRCPG	DCRGHGLCED	GVCVCDAGYS	GEDCSTRSCP
570	580	590	600	610	620	630	640
GGCRGRGQCL	DGRCVCEDGY	SGEDCGVRQC	PNDCSQHGVC	QDGVCICWEG	YVSEDCSIRT	CPSNCHGRGR	CEEGRCLCDP
650	660	670	680	690	700	710	720
GYTGPTCATR	MCPADCRGRG	RCVQGVCLCH	VGYGGEDCGQ	EEPPASACPG	GCGPRELCRA	GQCVCVEGFR	GPDCAIQTCP
730	740	750	760	770	780	790	800
GDCRGRGECH	DGSCVCKDGY	AGEDCGEEVP	TIEGMRMHLL	EETTVRTEWT	PAPGPVDAYE	IQFIPTTEGA	SPPFTARVPS
810	820	830	840	850	860	870	880
SASAYDQRGL	APGQEYQVTV	RALRGTSWGL	PASKTITTMI	DGPQDLRVVA	VTPTTLELGW	LRPQAEVDRF	VVSYVSAGNQ
890	900	910	920	930	940	950	960
RVRLEVPPEA	DGTLLTDLMP	GVEYVVTVTA	ERGRAVSYPA	SVRANTGSSP	LGLLGTTDEP	PPSGPSTTQG	AQAPLLQQRP
970	980	990	1000	1010	1020	1030	1040
QELGELRVLG	RDETGRLRVV	WTAQPDTFAY	FQLRMRVPEG	PGAHEEVLPG	DVRQALVPPP	PPGTPYELSL	HGVPPGGKPS
1050	1060	1070	1080	1090	1100	1110	1120
DPIIYQGIMD	KDEEKPGKSS	GPPRLGELTV	TDRTSDSLLL	RWTVPEGEFD	SFVIQYKDRD	GQPQVVPVEG	PQRSAVITSL
1130	1140	1150	1160	1170	1180	1190	1200
DPGRKYKFVL	YGFVGKKRHG	PLVAEAKILP	QSDPSPGTPP	HLGNLWVTDP	TPDSLHLSWT	VPEGQFDTFM	VQYRDRDGRP
1210	1220	1230	1240	1250	1260	1270	1280
QVVPVEGPER	SFVVSSLDPD	HKYRFTLFGI	ANKKRYGPLT	ADGTTAPERK	EEPPRPEFLE	QPLLGELTVT	GVTPDSLRLS
1290	1300	1310	1320	1330	1340	1350	1360
WTVAQGPFDS	FMVQYKDAQG	QPQAVPVAGD	ENEVTVPGLD	PDRKYKMNLY	GLRGRQRVGP	ESVVAKTAPQ	EDVDETPSPT
1370	1380	1390	1400	1410	1420	1430	1440
ELGTEAPESP	EEPLLGELTV	TGSSPDSLSL	FWTVPQGSFD	SFTVQYKDRD	GRPRAVRVGG	KESEVTVGGL	EPGHKYKMHL
1450	1460	1470	1480	1490	1500	1510	1520
YGLHEGQRVG	PVSAVGVTAP	QQEETPPATE	SPLEPRLGEL	TVTDVTPNSV	GLSWTVPEGQ	FDSFIVQYKD	KDGQPQVVPV
1530	1540	1550	1560	1570	1580	1590	1600
AADQREVTVY	NLEPERKYKM	NMYGLHDGQR	MGPLSVVIVT	APLPPAPATE	ASKPPLEPRL	GELTVTDITP	DSVGLSWTVP
1610	1620	1630	1640	1650	1660	1670	1680
EGEFDSFVVQ	YKDRDGQPQV	VPVAADQREV	TIPDLEPSRK	YKFLLFGIQD	GKRRSPVSVE	AKTVARGDAS	PGAPPRLGEL
1690	1700	1710	1720	1730	1740	1750	1760
WVTDPTPDSL	RLSWTVPEGQ	FDSFVVQFKD	KDGPQVVPVE	GHERSVTVTP	LDAGRKYRFL	LYGLLGKKRH	GPLTADGTTE
1770	1780	1790	1800	1810	1820	1830	1840
ARSAMDDTGT	KRPPKPRLGE	ELQVTTVTQN	SVGLSWTVPE	GQFDSFVVQY	KDRDGQPQVV	PVEGSLREVS	VPGLDPAHRY
1850	1860	1870	1880	1890	1900	1910	1920
KLLLYGLHHG	KRVGPISAVA	ITAGREETET	ETTAPTPPAP	EPHLGELTVE	EATSHTLHLS	WMVTEGEFDS	FEIQYTDRDG
1930	1940	1950	1960	1970	1980	1990	2000
QLQMVRIGGD	RNDITLSGLE	SDHRYLVTLY	GFSDGKHVGP	VHVEALTVPE	EEKPSEPPTA	TPEPPIKPRL	GELTVTDATP
2010	2020	2030	2040	2050	2060	2070	2080
DSLSLSWTVP	EGQFDHFLVQ	YRNGDGQPKA	VRVPGHEEGV	TISGLEPDHK	YKMNLYGFHG	GQRMGPVSVV	GVTAAEEETP
2090	2100	2110	2120	2130	2140	2150	2160
SPTEPSMEAP	EPAEEPLLGE	LTVTGSSPDS	LSLSWTVPQG	RFDSFTVQYK	DRDGRPQVVR	VGGEESEVTV	GGLEPGRKYK
2170	2180	2190	2200	2210	2220	2230	2240

Detailed Protein Report

Cmpd.	No. of Cmpds.	m/z meas.	Δ m/z [ppm]	z	Rt [min]	Score	P	Range	Sequence	Modification
2748	1	1085.5225	-113.03	1	65.5	14.6	0	107-115	R.LEILEELVK.G	
15	1	1103.5160	91.05	2	29.8	14.6	1	264-282	R.CVCDPGYTGDDCGMRSCPR.G	Carbamidomethyl: 1, 3, 12
29	1	1103.5152	82.27	2	30.1	10.9	0	445-464	R.CENGVCVCNAGYSGEDCGVR.S	Carbamidomethyl: 6, 8, 17
2993	1	557.9657	38.29	3	66.2	10.2	0	2141-2157	R.VGGEESEVTVGGLEPGR.K	

Detailed Protein Report

Protein 122: PREDICTED: E3 ubiquitin-protein ligase HUWE1 isoform X8 [Homo sapiens]

**Accession:**	gi|530426358	**Score:**	50.2
**Database:**	refseq_human(refseq_human_20140103.fasta)	**MW [kDa]:**	466.2
**Database Date:**	2015-11-30	**pI:**	5.3
		**Sequence Coverage [%]:**	1.3
		**No. of unique Peptides:**	3

Quantitation

WUP:QUP	**Median:** 0.97	**CV:** 0.00 %	**No. of Peptides:** 1

Detailed Protein Report

10	20	30	40	50	60	70	80
MKVDRTKLKK	TPTEAPADCR	ALIDKLKVCN	DEQLLLELQQ	IKTWNIGKCE	LYHWVDLLDR	FDGILADAGQ	TVENMSWMLV
90	100	110	120	130	140	150	160
CDRPEREQLK	MLLLAVLNFT	ALLIEYSFSR	HLYSSIEHLT	TLLASSDMQV	VLAVLNLLYV	FSKRSNYITR	LGSDKRTPLL
170	180	190	200	210	220	230	240
TRLQHLAESW	GGKENGFGLA	ECCRDLHMMK	YPPSATTLHF	EFYADPGAEV	KIEKRTTSNT	LHYIHIEQLD	KISESPSEIM
250	260	270	280	290	300	310	320
ESLTKMYSIP	KDKQMLLFTH	IRLAHGFSNH	RKRLQAVQAR	LHAISILVYS	NALQESANSI	LYNGLIEELV	DVLQITDKQL
330	340	350	360	370	380	390	400
MEIKAASLRT	LTSIVHLERT	PKLSSIIDCT	GTASYHGFLP	VLVRNCIQAM	IDPSMDPYPH	QFATALFSFL	YHLASYDAGG
410	420	430	440	450	460	470	480
EALVSCGMME	ALLKVIKFLG	DEQDQITFVT	RAVRVVDLIT	NLDMAAFQSH	SGLSIFIYRL	EHEVDLCRKE	CPFVIKPKIQ
490	500	510	520	530	540	550	560
RPNTTQEGEE	METDMDGVQC	IPQRAALLKS	MLNFLKKAIQ	DPAFSDGIRH	VMDGSLPTSL	KHIISNAEYY	GPSLFLLATE
570	580	590	600	610	620	630	640
VVTVFVFQEP	SLLSSLQDNG	LTDVMLHALL	IKDVPATREV	LGSLPNVFSA	LCLNARGLQS	FVQCQPFERL	FKVLLSPDYL
650	660	670	680	690	700	710	720
PAMRRRRSSD	PLGDTASNLG	SAVDELMRHQ	PTLKTDATTA	IIKLLEEICN	LGRDPKYICQ	KPSIQKADGT	ATAPPPRSNH
730	740	750	760	770	780	790	800
AAEEASSEDE	EEEEVQAMQS	FNSTQQNETE	PNQQVVGTEE	RIPIPLMDYI	LNVMKFVESI	LSNNTTDDHC	QEFVNQKGLL
810	820	830	840	850	860	870	880
PLVTILGLPN	LPIDFPTSAA	CQAVAGVCKS	ILTLSHEPKV	LQEGLLQLDS	ILSSLEPLHR	PIESPGGSVL	LRELACAGNV
890	900	910	920	930	940	950	960
ADATLSAQAT	PLLHALTAAH	AYIMMFVHTC	RVGQSEIRSI	SVNQWGSQLG	LSVLSKLSQL	YCSLVWESTV	LLSLCTPNSL
970	980	990	1000	1010	1020	1030	1040
PSGCEFGQAD	MQKLVPKDEK	AGTTQGGKRS	DGEQDGAAGS	MDASTQGLLE	GIGLDGDTLA	PMETDEPTAS	DSKGKSKITP
1050	1060	1070	1080	1090	1100	1110	1120
AMAARIKQIK	PLLSASSRLG	RALAELFGLL	VKLCVGSPVR	QRRSHHAAST	TTAPTPAARS	TASALTKLLT	KGLSWQPPPY
1130	1140	1150	1160	1170	1180	1190	1200
TPTPRFRLTF	FICSVGFTSP	MLFDERKYPY	HLMLQKFLCS	GGHNALFETF	NWALSMGGKV	PVSEGLEHSD	LPDGTGEFLD
1210	1220	1230	1240	1250	1260	1270	1280
AWLMLVEKMV	NPTTVLESPH	SLPAKLPGGV	QNFPQFSALR	FLVVTQKAAF	TCIKNLWNRK	PLKVYGGRMA	ESMLAILCHI
1290	1300	1310	1320	1330	1340	1350	1360
LRGEPVIRER	LSKEKEGSRG	EEDTGQEEGG	SRREPQVNQQ	QLQQLMDMGF	TREHAMEALL	NTSTMEQATE	YLLTHPPPIM
1370	1380	1390	1400	1410	1420	1430	1440
GGVVRDLSMS	EEDQMMRAIA	MSLGQDIPMD	QRAESPEEVA	CRKEEEERKA	REKQEEEEAK	CLEKFQDADP	LEQDELHTFT
1450	1460	1470	1480	1490	1500	1510	1520
DTMLPGCFHL	LDELPDTVYR	VCDLIMTAIK	RNGADYRDMI	LKQVVNQVWE	AADVLIKAAL	PLTTSDTKTV	SEWISQMATL
1530	1540	1550	1560	1570	1580	1590	1600
PQASNLATRI	LLLTLLFEEL	KLPCAWVVES	SGILNVLIKL	LEVVQPCLQA	AKEQKEVQTP	KWITPVLLLI	DFYEKTAISS
1610	1620	1630	1640	1650	1660	1670	1680
KRRAQMTKYL	QSNSNNWRWF	DDRSGRWCSY	SASNNSTIDS	AWKSGETSVR	FTAGRRRYTV	QFTTMVQVNE	ETGNRRPVML
1690	1700	1710	1720	1730	1740	1750	1760
TLLRVPRLNK	NSKNSNGQEL	EKTLEESKEM	DIKRKENKGN	DTPLALESTN	TEKETSLEET	KIGEILIQGL	TEDMVTVLIR
1770	1780	1790	1800	1810	1820	1830	1840
ACVSMLGVPV	DPDTLHATLR	LCLRLTRDHK	YAMMFAELKS	TRMILNLTQS	SGFNGFTPLV	TLLLRHIIED	PCTLRHTMEK
1850	1860	1870	1880	1890	1900	1910	1920
VVRSAATSGA	GSTTSGVVSG	SLGSREINYI	LRVLGPAACR	NPDIFTEVAN	CCIRIALPAP	RGSGTASDDE	FENLRIKGPN
1930	1940	1950	1960	1970	1980	1990	2000
AVQLVKTTPL	KPSPLPVIPD	TIKEVIYDML	NALAAYHAPE	EADKSDPKPG	VMTQEVGQLL	QDMGDDVYQQ	YRSLTRQSSD
2010	2020	2030	2040	2050	2060	2070	2080
FDTQSGFSIN	SQVFAADGAS	TETSASGTSQ	GEASTPEESR	DGKKDKEGDR	ASEEGKQKGK	GSKPLMPTST	ILRLLAELVR
2090	2100	2110	2120	2130	2140	2150	2160
SYVGIATLIA	NYSYTVGQSE	LIKEDCSVLA	FVLDHLLPHT	QNAEDKDTPA	LARLFLASLA	AAGSGTDAQV	ALVNEVKAAL
2170	2180	2190	2200	2210	2220	2230	2240

Detailed Protein Report

Cmpd.	No. of Cmpds.	m/z meas.	Δ m/z [ppm]	z	Rt [min]	Score	P	Range	Sequence	Modification	Ratios
2725	2	1045.0312	6.89	2	64.4	11.5	2	1715-1733	R.KENKGNDTPLALESTNTEK.E		
2705	1	645.8441	-36.29	2	64.1	10.1	1	3005-3015	R.GVQYTRLAVQR.G		WUP:QUP 0.97
355	1	658.3846	-33.13	2	33.1	10.5	2	3311-3321	R.RSSLLTEKLLR.L		

Detailed Protein Report

Protein 123: pyruvate kinase PKM isoform a [Homo sapiens]

Accession: gi|33286418
Database: refseq_human(refseq_human_20140103.fasta)
Database Date: 2015-11-30

Score: 50.1
MW [kDa]: 57.9
pI: 9.0
Sequence Coverage [%]: 5.1
No. of unique Peptides: 2

Quantitation

QU:MU **Median:** 1.03 **CV:** 0.00 % **No. of Peptides:** 1

Alias proteins:

Accession	Name	Description
gi|530405979	refseq_human (refseq_human_20140103.fasta)	PREDICTED: pyruvate kinase PKM isoform X3 [Homo sapiens]

10	20	30	40	50	60	70	80
MSKPHSEAGT	AFIQTQQLHA	AMADTFLEHM	CRLDIDSPPI	TARNTGIICT	IGPASRSVET	LKEMIKSGMN	VARLNFSHGT
**90**	**100**	**110**	**120**	**130**	**140**	**150**	**160**
HEYHAETIKN	VRTATESFAS	DPILYRPVAV	ALDTKGPEIR	TGLIKGSGTA	EVELKKGATL	KITLDNAYME	KCDENILWLD
**170**	**180**	**190**	**200**	**210**	**220**	**230**	**240**
YKNICKVVEV	GSKIYVDDGL	ISLQVKQKGA	DFLVTEVENG	GSLGSKKGVN	LPGAAVDLPA	VSEKDIQDLK	FGVEQDVDMV
**250**	**260**	**270**	**280**	**290**	**300**	**310**	**320**
FASFIRKASD	VHEVRKVLGE	KGKNIKIISK	IENHEGVRRF	DEILEASDGI	MVARGDLGIE	IPAEKVFLAQ	KMMIGRCNRA
**330**	**340**	**350**	**360**	**370**	**380**	**390**	**400**
GKPVICATQM	LESMIKKPRP	TRAEGSDVAN	AVLDGADCIM	LSGETAKGDY	PLEAVRMQHL	IAREAEAAIY	HLQLFEELRR
**410**	**420**	**430**	**440**	**450**	**460**	**470**	**480**
LAPITSDPTE	ATAVGAVEAS	FKCCSGAIIV	LTKSGRSAHQ	VARYRPRAPI	IAVTRNPQTA	RQAHLYRGIF	PVLCKDPVQE
**490**	**500**	**510**	**520**	**530**	**540**		
AWAEDVDLRV	NFAMNVGKAR	GFFKKGDVVI	VLTGWRPGSG	FTNTMRVVPV	P		

Cmpd.	No. of Cmpds.	m/z meas.	Δ m/z [ppm]	z	Rt [min]	Score	P	Range	Sequence	Modification	Ratios
773	1	599.4299	171.21	2	39.7	39.3	0	33-43	R.LDIDSPPITAR.N		
183	1	628.6439	7.16	3	32.3	10.8	0	74-89	R.LNFSHGTHEYHAETIK.N		QU:MU 1.03

Detailed Protein Report

Protein 124: **PREDICTED: regulator of G-protein signaling 3 isoform X2 [Homo sapiens]**

**Accession:**	gi|578817733	**Score:**	50.1
**Database:**	refseq_human(refseq_human_20140103.fasta)	**MW [kDa]:**	120.1
**Database Date:**	2015-11-30	**pI:**	5.9
**Modification(s):**	Carbamidomethyl	**Sequence Coverage [%]:**	5.0
		**No. of unique Peptides:**	3

10	20	30	40	50	60	70	80
MERSLHRVSL	GSRRAHPDLS	FYLTTFGQLR	LSIDAQDRVL	LLHIIEGKGL	ISKQPGTCDP	YVKISLIPED	SRLRHQKTQT
**90**	**100**	**110**	**120**	**130**	**140**	**150**	**160**
VPDCRDPAFH	EHFFFPVQEE	DDQKRLLVTV	WNRASQSRQS	GLIGCMSFGV	KSLLTPDKEI	SGWYYLLGEH	LGRTKHLKVA
**170**	**180**	**190**	**200**	**210**	**220**	**230**	**240**
RRRLRPLRDP	LLRMPGGGDT	ENGKKLKITI	PRGKDGFGFT	ICCDSPVRVQ	AVDSGGPAER	AGLQQLDTVL	QLNERPVEHW
**250**	**260**	**270**	**280**	**290**	**300**	**310**	**320**
KCVELAHEIR	SCPSEIILLV	WRMVPQVKPG	PDGGVLRRAS	CKSTHDLQSP	PNKREKNCTH	GVQARPEQRH	SCHLVCDSSD
**330**	**340**	**350**	**360**	**370**	**380**	**390**	**400**
GLLLGGWERY	TEVAKRGGQH	TLPALSRATA	PTDPNYIILA	PLNPGSQLLR	PVYQEDTIPE	ESGSPSKGKS	YTGLGKKSRL
**410**	**420**	**430**	**440**	**450**	**460**	**470**	**480**
MKTVQTMKGH	GNYQNCPVVR	PHATHSSYGT	YVTLAPKVLV	FPVFVQPLDL	CNPARTLLLS	EELLLYEGRN	KAAEVTLFAY
**490**	**500**	**510**	**520**	**530**	**540**	**550**	**560**
SDLLLFTKED	EPGRCDVLRN	PLYLQSVKLQ	EGSSEDLKFC	VLYLAEKAEC	LFTLEAHSQE	QKKRVCWCLS	ENIAKQQQLA
**570**	**580**	**590**	**600**	**610**	**620**	**630**	**640**
ASPPDSKMFE	TEADEKREMA	LEEGKGPGAE	DSPPSKEPSP	GQELPPGQDL	PPNKDSPSGQ	EPAPSQEPLS	SKDSATSEGS
**650**	**660**	**670**	**680**	**690**	**700**	**710**	**720**
PPGPDAPPSK	DVPPCQEPPP	AQDLSPCQDL	PAGQEPLPHQ	DPLLTKDLPA	IQESPTRDLP	PCQDLPPSQV	SLPAKALTED
**730**	**740**	**750**	**760**	**770**	**780**	**790**	**800**
TMSSGDLLAA	TGDPPAAPRP	AFVIPEVRLD	STYSQKAGAE	QGCSGDEEDA	EEAEEVEEGE	EGEEDEDEDT	SDDNYGERSE
**810**	**820**	**830**	**840**	**850**	**860**	**870**	**880**
AKRSSMIETG	QGAEGGLSLR	VQNSLRRRTH	SEGSLLQEPR	GPCFASDTTL	HCSDGEGAAS	TWGMPSPSTL	KKELGRNGGS
**890**	**900**	**910**	**920**	**930**	**940**	**950**	**960**
MHHLSLFFTG	HRKMSGADTV	GDDDEASRKR	KSKNLAKDMK	NKLGIFRRRN	ESPGAPPAGK	ADKMMKSFKP	TSEEALKWGE
**970**	**980**	**990**	**1000**	**1010**	**1020**	**1030**	**1040**
SLEKLLVHKY	GLAVFQAFLR	TEFSEENLEF	WLACEDFKKV	KSQSKMASKA	KKIFAEYIAI	QACKEVNLDS	YTREHTKDNL
**1050**	**1060**	**1070**	**1080**	**1090**			
QSVTRGCFDL	AQKRIFGLME	KDSYPRFLRS	DLYLDLINQK	KMSPPL			

Cmpd.	No. of Cmpds.	m/z meas.	Δ m/z [ppm]	z	Rt [min]	Score	P	Range	Sequence	Modification
7	6	819.8312	-118.35	2	29.5	14.6	2	73-85	R.LRHQKTQTVPDCR.D	Carbamidomethyl: 12
512	1	593.1679	-216.84	2	36.4	10.4	0	209-220	R.VQAVDSGGPAER.A	
1949	1	599.9043	-89.98	3	52.7	12.8	0	877-892	R.NGGSMHHLSLFFTGHR.K	

Detailed Protein Report

Protein 125: PREDICTED: ubiquitin carboxyl-terminal hydrolase 24 isoform X1 [Homo sapiens]

**Accession:**	gi|530362258	**Score:**	50.0
**Database:**	refseq_human(refseq_human_20140103.fasta)	**MW [kDa]:**	275.5
**Database Date:**	2015-11-30	**pI:**	6.1
**Modification(s):**	Carbamidomethyl	**Sequence Coverage [%]:**	2.3
		**No. of unique Peptides:**	3

Quantitation

QU:MU	**Median:**	0.60	**CV:**	0.00 %	**No. of Peptides:**	1
WUP:QUP	**Median:**	2.27	**CV:**	0.00 %	**No. of Peptides:**	1

BRUKER

Detailed Protein Report

10	20	30	40	50	60	70	80
MDRCMPEAFK	KLLTSSAVHK	WGTEIHEGIY	NMLMLLIELV	AERIKQDPIP	TGLLGVLTMA	FNPDNEYHFK	NRMKVSQRNW
90	100	110	120	130	140	150	160
AEVFGEGNMF	AVSPVSTFQK	EPHGWVVDLV	NKFGELGGFA	AIQAKLHSED	IELGAVSALI	QPLGVCAEYL	NSSVVQPMLD
170	180	190	200	210	220	230	240
PVILTTIQDV	RSVEEKDLKD	KRLVSIPELL	SAVKLLCMRF	QPDLVTIVDD	LRLDILLRML	KSPHFSAKMN	SLKEVTKLIE
250	260	270	280	290	300	310	320
DSTLSKSVKN	AIDTDRLLDW	LVENSVLSIA	LEGNIDQAQY	CDRIKGIIEL	LGSKLSLDEL	TKIWKIQSGQ	SSTVIENIHT
330	340	350	360	370	380	390	400
IIAAAAVKFN	SDQLNHLFVL	IQKSWETESD	RVRQKLLSLI	GRIGREARFE	TTSGKVLDVL	WELAHLPTLP	SSLIQQALEE
410	420	430	440	450	460	470	480
HLTILSDAYA	VKEAIKRSYI	IKCIEDIKRP	GEWSGLEKNK	KDGFKSSQLN	NPQFVWVVPA	LRQLHEITRS	FIKQTYQKQD
490	500	510	520	530	540	550	560
KSIIQDLKKN	FEIVKLVTGS	LIACHRLAAA	VAGPGGLSGS	TLVDGRYTYR	EYLEAHLKFL	AFFLQEATLY	LGWNRAKEIW
570	580	590	600	610	620	630	640
ECLVTGQDVC	ELDREMCFEW	FTKGQHDLES	DVQQQLFKEK	ILKLESYEIT	MNGFNLFKTF	FENVNLCDHR	LKRQGAQLYV
650	660	670	680	690	700	710	720
EKLELIGMDF	IWKIAMESPD	EEIANEAIQL	IINYSYINLN	PRLKKDSVSL	HKKFIADCYT	RLEAASSALG	GPTLTHAVTR
730	740	750	760	770	780	790	800
ATKMLTATAM	PTVATSVQSP	YRSTKLVIIE	RLLLLAERYV	ITIEDFYSVP	RTILPHGASF	HGHLLTLNVT	YESTKDTFTV
810	820	830	840	850	860	870	880
EAHSNETIGS	VRWKIAKQLC	SPVDNIQIFT	NDSLLTVNKD	QKLLHQLGFS	DEQILTVKTS	GSGTPSGSSA	DSSTSSSSSS
890	900	910	920	930	940	950	960
SGVFSSSYAM	EQEKSLPGVV	MALVCNVFDM	LYQLANLEEP	RITLRVRKLL	LLIPTDPAIQ	EALDQLDSLG	RKKTLLSESS
970	980	990	1000	1010	1020	1030	1040
SQSSKSPSLS	SKQQHQPSAS	SILESLFRSF	APGMSTFRVL	YNLEVLSSKL	MPTADDDMAR	SCAKSFCENF	LKAGGLSLVV
1050	1060	1070	1080	1090	1100	1110	1120
NVMQRDSIPS	EVDYETRQGV	YSICLQLARF	LLVGQTMPTL	LDEDLTKDGI	EALSSRPFRN	VSRQTSRQMS	LCGTPEKSSY
1130	1140	1150	1160	1170	1180	1190	1200
RQLSVSDRSS	IRVEEIIPAA	RVAIQTMEVS	DFTSTVACFM	RLSWAAAAGR	LDLVGSSQPI	KESNSLCPAG	IRNRLSSSGS
1210	1220	1230	1240	1250	1260	1270	1280
NCSSGSEGEP	VALHAGICVR	QQSVSTKDSL	IAGEALSLLV	TCLQLRSQQL	ASFYNLPCVA	DFIIDILLGS	PSAEIRRVAC
1290	1300	1310	1320	1330	1340	1350	1360
DQLYTLSQTD	TSAHPDVQKP	NQFLLGVILT	AQLPLWSPTS	IMRGVNQRLL	SQCMEYFDLR	CQLLDDLTTS	EMEQLRISPA
1370	1380	1390	1400	1410	1420	1430	1440
TMLEDEITWL	DNFEPNRTAE	CETSEADNIL	LAGHLRLIKT	LLSLCGAEKE	MLGSSLIKPL	LDDFLFRASR	IILNSHSPAG
1450	1460	1470	1480	1490	1500	1510	1520
SAAISQQDFH	PKCSTANSRL	AAYEVLVMLA	DSSPSNLQII	IKELLSMHHQ	PDPALTKEFD	YLPPVDSRSS	SGFVGLRNGG
1530	1540	1550	1560	1570	1580	1590	1600
ATCYMNAVFQ	QLYMQPGLPE	SLLSVDDDTD	NPDDSVFYQV	QSLFGHLMES	KLQYYVPENF	WKIFKMWNKE	LYVREQQDAY
1610	1620	1630	1640	1650	1660	1670	1680
EFFTSLIDQM	DEYLKKMGRD	QIFKNTFQGI	YSDQKICKDC	PHRYEREEAF	MALNLGVTSC	QSLEISLDQF	VRGEVLEGSN
1690	1700	1710	1720	1730	1740	1750	1760
AYYCEKCKEK	RITVKRTCIK	SLPSVLVIHL	MRFGFDWESG	RSIKYDEQIR	FPWMLNMEPY	TVSGMARQDS	SSEVGENGRS
1770	1780	1790	1800	1810	1820	1830	1840
VDQGGGGSPR	KKVALTENYE	LVGVIVHSGQ	AHAGHYYSFI	KDRRGCGKGK	WYKFNDTVIE	EFDLNDETLE	YECFGGEYRP
1850	1860	1870	1880	1890	1900	1910	1920
KVYDQTNPYT	DVRRRYWNAY	MLFYQRVSDQ	NSPVLPKKSR	VSVVRQEAED	LSLSAPSSPE	ISPQSSPRPH	RPNNDRLSIL
1930	1940	1950	1960	1970	1980	1990	2000
TKLVKKGEKK	GLFVEKMPAR	IYQMVRDENL	KFMKNRDVYS	SDYFSFVLSL	ASLNATKLKH	PYYPCMAKVS	LQLAIQFLFQ
2010	2020	2030	2040	2050	2060	2070	2080
TYLRTKKKLR	VDTEEWIATI	EALLSKSFDA	CQWLVEYFIS	SEGRELIKIF	LLECNVREVR	VAVATILEKT	LDSALFYQDK
2090	2100	2110	2120	2130	2140	2150	2160
LKSLHQLLEV	LLALLDKDVP	ENCKNCAQYF	FLFNTFVQKQ	GIRAGDLLLR	HSALRHMISF	LLGASRQNNQ	IRRWSSAQAR
2170	2180	2190	2200	2210	2220	2230	2240

Detailed Protein Report

Cmpd.	No. of Cmpds.	m/z meas.	Δ m/z [ppm]	z	Rt [min]	Score	P	Range	Sequence	Modification	Ratios
2676	1	585.8808	7.47	2	61.8	11.9	1	284-294	R.IKGIIELLGSK.L		
2168	1	841.4140	51.38	2	57.5	11.5	1	1010-1024	K.LMPTADDDMARSCAK.S	Carbamidomethyl: 13	
1965	1	572.7269	-108.26	2	54.9	16.4	1	1760-1771	R.SVDQGGGGSPRK.K		WUP:QUP 2.27<br>QU:MU 0.60

BRUKER

Detailed Protein Report

Protein 126: apolipoprotein B-100 precursor [Homo sapiens]

**Accession:**	gi|105990532	**Score:**	49.8
**Database:**	refseq_human(refseq_human_20140103.fasta)	**MW [kDa]:**	515.2
**Database Date:**	2015-11-30	**pI:**	6.6
**Modification(s):**	Oxidation	**Sequence Coverage [%]:**	0.9
		**No. of unique Peptides:**	1

Quantitation

QU:MU	**Median:**	0.24	**CV:**	0.00 %	**No. of Peptides:**	1
WUP:QUP	**Median:**	0.38	**CV:**	0.00 %	**No. of Peptides:**	1

Detailed Protein Report

10	20	30	40	50	60	70	80
MDPPRPALLA	LLALPALLLL	LLAGARAEEE	MLENVSLVCP	KDATRFKHLR	KYTYNYEAES	SSGVPGTADS	RSATRINCKV
90	100	110	120	130	140	150	160
ELEVPQLCSF	ILKTSQCTLK	EVYGFNPEGK	ALLKKTKNSE	EFAAAMSRYE	LKLAIPEGKQ	VFLYPEKDEP	TYILNIKRGI
170	180	190	200	210	220	230	240
ISALLVPPET	EEAKQVLFLD	TVYGNCSTHF	TVKTRKGNVA	TEISTERDLG	QCDRFKPIRT	GISPLALIKG	MTRPLSTLIS
250	260	270	280	290	300	310	320
SSQSCQYTLD	AKRKHVAEAI	CKEQHLFLPF	SYKNKYGMVA	QVTQTLKLED	TPKINSRFFG	EGTKKMGLAF	ESTKSTSPPK
330	340	350	360	370	380	390	400
QAEAVLKTLQ	ELKKLTISEQ	NIQRANLFNK	LVTELRGLSD	EAVTSLLPQL	IEVSSPITLQ	ALVQCGQPQC	STHILQWLKR
410	420	430	440	450	460	470	480
VHANPLLIDV	VTYLVALIPE	PSAQQLREIF	NMARDQRSRA	TLYALSHAVN	NYHKTNPTGT	QELLDIANYL	MEQIQDDCTG
490	500	510	520	530	540	550	560
DEDYTYLILR	VIGNMGQTME	QLTPELKSSI	LKCVQSTKPS	LMIQKAAIQA	LRKMEPKDKD	QEVLLQTFLD	DASPGDKRLA
570	580	590	600	610	620	630	640
AYLMLMRSPS	QADINKIVQI	LPWEQNEQVK	NFVASHIANI	LNSEELDIQD	LKKLVKEALK	ESQLPTVMDF	RKFSRNYQLY
650	660	670	680	690	700	710	720
KSVSLPSLDP	ASAKIEGNLI	FDPNNYLPKE	SMLKTTLTAF	GFASADLIEI	GLEGKGFEPT	LEALFGKQGF	FPDSVNKALY
730	740	750	760	770	780	790	800
WVNGQVPDGV	SKVLVDHFGY	TKDDKHEQDM	VNGIMLSVEK	LIKDLKSKEV	PEARAYLRIL	GEELGFASLH	DLQLLGKLLL
810	820	830	840	850	860	870	880
MGARTLQGIP	QMIGEVIRKG	SKNDFFLHYI	FMENAFELPT	GAGLQLQISS	SGVIAPGAKA	GVKLEVANMQ	AELVAKPSVS
890	900	910	920	930	940	950	960
VEFVTNMGII	IPDFARSGVQ	MNTNFFHESG	LEAHVALKAG	KLKFIIPSPK	RPVKLLSGGN	TLHLVSTTKT	EVIPPLIENR
970	980	990	1000	1010	1020	1030	1040
QSWSVCKQVF	PGLNYCTSGA	YSNASSTDSA	SYYPLTGDTR	LELELRPTGE	IEQYSVSATY	ELQREDRALV	DTLKFVTQAE
1050	1060	1070	1080	1090	1100	1110	1120
GAKQTEATMT	FKYNRQSMTL	SSEVQIPDFD	VDLGTILRVN	DESTEGKTSY	RLTLDIQNKK	ITEVALMGHL	SCDTKEERKI
1130	1140	1150	1160	1170	1180	1190	1200
KGVISIPRLQ	AEARSEILAH	WSPAKLLLQM	DSSATAYGST	VSKRVAWHYD	EEKIEFEWNT	GTNVDTKKMT	SNFPVDLSDY
1210	1220	1230	1240	1250	1260	1270	1280
PKSLHMYANR	LLDHRVPQTD	MTFRHVGSKL	IVAMSSWLQK	ASGSLPYTQT	LQDHLNSLKE	FNLQNMGLPD	FHIPENLFLK
1290	1300	1310	1320	1330	1340	1350	1360
SDGRVKYTLN	KNSLKIEIPL	PFGGKSSRDL	KMLETVRTPA	LHFKSVGFHL	PSREFQVPTF	TIPKLYQLQV	PLLGVLDLST
1370	1380	1390	1400	1410	1420	1430	1440
NVYSNLYNWS	ASYSGGNTST	DHFSLRARYH	MKADSVVDLL	SYNVQGSGET	TYDHKNTFTL	SCDGSLRHKF	LDSNIKFSHV
1450	1460	1470	1480	1490	1500	1510	1520
EKLGNNPVSK	GLLIFDASSS	WGPQMSASVH	LDSKKKQHLF	VKEVKIDGQF	RVSSFYAKGT	YGLSCQRDPN	TGRLNGESNL
1530	1540	1550	1560	1570	1580	1590	1600
RFNSSYLQGT	NQITGRYEDG	TLSLTSTSDL	QSGIIKNTAS	LKYENYELTL	KSDTNGKYKN	FATSNKMDMT	FSKQNALLRS
1610	1620	1630	1640	1650	1660	1670	1680
EYQADYESLR	FFSLLSGSLN	SHGLELNADI	LGTDKINSGA	HKATLRIGQD	GISTSATTNL	KCSLLVLENE	LNAELGLSGA
1690	1700	1710	1720	1730	1740	1750	1760
SMKLTTNGRF	REHNAKFSLD	GKAALTELSL	GSAYQAMILG	VDSKNIFNFK	VSQEGLKLSN	DMMGSYAEMK	FDHTNSLNIA
1770	1780	1790	1800	1810	1820	1830	1840
GLSLDFSSKL	DNIYSSDKFY	KQTVNLQLQP	YSLVTTLNSD	LKYNALDLTN	NGKLRLEPLK	LHVAGNLKGA	YQNNEIKHIY
1850	1860	1870	1880	1890	1900	1910	1920
AISSAALSAS	YKADTVAKVQ	GVEFSHRLNT	DIAGLASAID	MSTNYNSDSL	HFSNVFRSVM	APFTMTIDAH	TNGNGKLALW
1930	1940	1950	1960	1970	1980	1990	2000
GEHTGQLYSK	FLLKAEPLAF	TFSHDYKGST	SHHLVSRKSI	SAALEHKVSA	LLTPAEQTGT	WKLKTQFNNN	EYSQDLDAYN
2010	2020	2030	2040	2050	2060	2070	2080
TKDKIGVELT	GRTLADLTLL	DSPIKVPLLL	SEPINIIDAL	EMRDAVEKPQ	EFTIVAFVKY	DKNQDVHSIN	LPFFETLQEY
2090	2100	2110	2120	2130	2140	2150	2160
FERNRQTIIV	VLENVQRNLK	HINIDQFVRK	YRAALGKLPQ	QANDYLNSFN	WERQVSHAKE	KLTALTKKYR	ITENDIQIAL
2170	2180	2190	2200	2210	2220	2230	2240

Detailed Protein Report

Cmpd.	No. of Cmpds.	m/z meas.	Δ m/z [ppm]	z	Rt [min]	Score	P	Range	Sequence	Modification	Ratios	
222	1	493.5794	-31.61	3	32.8	10.5	0	513-525	K.CVQSTKPSLMIQK.A	Oxidation: 10	WUP:QUP<br>QU:MU	0.38<br>0.24

Detailed Protein Report

Protein 127: transitional endoplasmic reticulum ATPase [Homo sapiens]

**Accession:**	gi|6005942	**Score:**	49.7
**Database:**	refseq_human(refseq_human_20140103.fasta)	**MW [kDa]:**	89.3
**Database Date:**	2015-11-30	**pI:**	5.0
**Modification(s):**	Carbamidomethyl	**Sequence Coverage [%]:**	5.5
		**No. of unique Peptides:**	1

10	20	30	40	50	60	70	80
MASGADSKGD	DLSTAILKQK	NRPNRLIVDE	AINEDNSVVS	LSQPKMDELQ	LFRGDTVLLK	GKKRREAVCI	VLSDDTCSDE
**90**	**100**	**110**	**120**	**130**	**140**	**150**	**160**
KIRMNRVVRN	NLRVRLGDVI	SIQPCPDVKY	GKRIHVLPID	DTVEGITGNL	FEVYLKPYFL	EAYRPIRKGD	IFLVRGGMRA
**170**	**180**	**190**	**200**	**210**	**220**	**230**	**240**
VEFKVVETDP	SPYCIVAPDT	VIHCEGEPIK	REDEEESLNE	VGYDDIGGCR	KQLAQIKEMV	ELPLRHPALF	KAIGVKPPRG
**250**	**260**	**270**	**280**	**290**	**300**	**310**	**320**
ILLYGPPGTG	KTLIARAVAN	ETGAFFFLIN	GPEIMSKLAG	ESESNLRKAF	EEAEKNAPAI	IFIDELDAIA	PKREKTHGEV
**330**	**340**	**350**	**360**	**370**	**380**	**390**	**400**
ERRIVSQLLT	LMDGLKQRAH	VIVMAATNRP	NSIDPALRRF	GRFDREVDIG	IPDATGRLEI	LQIHTKNMKL	ADDVDLEQVA
**410**	**420**	**430**	**440**	**450**	**460**	**470**	**480**
NETHGHVGAD	LAALCSEAAL	QAIRKKMDLI	DLEDETIDAE	VMNSLAVTMD	DFRWALSQSN	PSALRETVVE	VPQVTWEDIG
**490**	**500**	**510**	**520**	**530**	**540**	**550**	**560**
GLEDVKRELQ	ELVQYPVEHP	DKFLKFGMTP	SKGVLFYGPP	GCGKTLLAKA	IANECQANFI	SIKGPELLTM	WFGESEANVR
**570**	**580**	**590**	**600**	**610**	**620**	**630**	**640**
EIFDKARQAA	PCVLFFDELD	SIAKARGGNI	GDGGGAADRV	INQILTEMDG	MSTKKNVFII	GATNRPDIID	PAILRPGRLD
**650**	**660**	**670**	**680**	**690**	**700**	**710**	**720**
QLIYIPLPDE	KSRVAILKAN	LRKSPVAKDV	DLEFLAKMTN	GFSGADLTEI	CQRACKLAIR	ESIESEIRRE	RERQTNPSAM
**730**	**740**	**750**	**760**	**770**	**780**	**790**	**800**
EVEEDDPVPE	IRRDHFEEAM	RFARRSVSDN	DIRKYEMFAQ	TLQQSRGFGS	FRFPSGNQGG	AGPSQGSGGG	TGGSVYTEDN
**810**							
DDDLYG							

Cmpd.	No. of Cmpds.	m/z meas.	Δ m/z [ppm]	z	Rt [min]	Score	P	Range	Sequence	Modification
2857	1	1051.5637	82.66	2	66.2	14.5	1	678-696	K.MTNGFSGADLTEICQRACK.L	Carbamidomethyl: 18

Detailed Protein Report

Protein 128: **syntaxin-binding protein 5 isoform b [Homo sapiens]**

**Accession:**	gi|189217915	**Score:**	49.7
**Database:**	refseq_human(refseq_human_20140103.fasta)	**MW [kDa]:**	127.5
**Database Date:**	2015-11-30	**pI:**	7.1
		**Sequence Coverage [%]:**	5.0
		**No. of unique Peptides:**	3

10	20	30	40	50	60	70	80
MRKFNIRKVL	DGLTAGSSSA	SQQQQQQHPP	GNREPEIQET	LQSEHFQLCK	TVRHGFPYQP	SALAFDPVQK	ILAVGTQTGA
**90**	**100**	**110**	**120**	**130**	**140**	**150**	**160**
LRLFGRPGVE	CYCQHDSGAA	VIQLQFLINE	GALVSALADD	TLHLWNLRQK	RPAILHSLKF	CRERVTFCHL	PFQSKWLYVG
**170**	**180**	**190**	**200**	**210**	**220**	**230**	**240**
TERGNIHIVN	VESFTLSGYV	IMWNKAIELS	SKSHPGPVVH	ISDNPMDEGK	LLIGFESGTV	VLWDLKSKKA	DYRYTYDEAI
**250**	**260**	**270**	**280**	**290**	**300**	**310**	**320**
HSVAWHHEGK	QFICSHSDGT	LTIWNVRSPA	KPVQTITPHG	KQLKDGKKPE	PCKPILKVEF	KTTRSGEPFI	ILSGGLSYDT
**330**	**340**	**350**	**360**	**370**	**380**	**390**	**400**
VGRRPCLTVM	HGKSTAVLEM	DYSIVDFLTL	CETPYPNDFQ	EPYAVVVLLE	KDLVLIDLAQ	NGYPIFENPY	PLSIHESPVT
**410**	**420**	**430**	**440**	**450**	**460**	**470**	**480**
CCEYFADCPV	DLIPALYSVG	ARQKRQGYSK	KEWPINGGNW	GLGAQSYPEI	IITGHADGSV	KFWDASAITL	QVLYKLKTSK
**490**	**500**	**510**	**520**	**530**	**540**	**550**	**560**
VFEKSRNKDD	RPNTDIVDED	PYAIQIISWC	PESRMLCIAG	VSAHVIIYRF	SKQEVITEVI	PMLEVRLLYE	INDVETPEGE
**570**	**580**	**590**	**600**	**610**	**620**	**630**	**640**
QPPPLPTPVG	GSNPQPIPPQ	SHPSTSSSSS	DGLRDNVPCL	KVKNSPLKQS	PGYQTELVIQ	LVWVGGEPPQ	QITSLAVNSS
**650**	**660**	**670**	**680**	**690**	**700**	**710**	**720**
YGLVVFGNCN	GIAMVDYLQK	AVLLNLGTIE	LYGSNDPYRR	EPRSPRKSRQ	PSGAGLCDIS	EGTVVPEDRC	KSPTSGSSSP
**730**	**740**	**750**	**760**	**770**	**780**	**790**	**800**
HNSDDEQKMN	NFIEKVKTKS	RKFSKMVAND	IAKMSRKLSL	PTDLKPDLDV	KDNSFSRSRS	SSVTSIDKES	REAISALHFC
**810**	**820**	**830**	**840**	**850**	**860**	**870**	**880**
ETFTRKTDSS	PSPCLWVGTT	LGTVLVIALN	LPPGGEQRLL	QPVIVSPSGT	ILRLKGAILR	MAFLDTTGCL	IPPAYEPWRE
**890**	**900**	**910**	**920**	**930**	**940**	**950**	**960**
HNVPEEKDEK	EKLKKRRPVS	VSPSSSQEIS	ENQYAVICSE	KQAKVISLPT	QNCAYKQNIT	ETSFVLRGDI	VALSNSICLA
**970**	**980**	**990**	**1000**	**1010**	**1020**	**1030**	**1040**
CFCANGHIMT	FSLPSLRPLL	DVYYLPLTNM	RIARTFCFTN	NGQALYLVSP	TEIQRLTYSQ	ETCENLQEML	GELFTPVETP
**1050**	**1060**	**1070**	**1080**	**1090**	**1100**	**1110**	**1120**
EAPNRGFFKG	LFGGGAQSLD	REELFGESSS	GKASRSLAQH	IPGPGGIEGV	KGAASGVVGE	LARARLALDE	RGQKLGDLEE
**1130**	**1140**	**1150**	**1160**				
RTAAMLSSAE	SFSKHAHEIM	LKYKDKKWYQ	F				

Cmpd.	No. of Cmpds.	m/z meas.	Δ m/z [ppm]	z	Rt [min]	Score	P	Range	Sequence	Modification
1682	1	796.3351	-89.13	2	50.8	10.5	1	143-155	R.ERVTFCHLPFQSK.W	
1487	1	676.3270	-69.10	2	48.8	11.5	2	742-753	R.KFSKMVANDIAK.M	
1944	1	665.3207	-0.95	2	54.7	15.6	0	1122-1134	R.TAAMLSSAESFSK.H	

Detailed Protein Report

Protein 129: PREDICTED: rho GTPase-activating protein 26 isoform X6 [Homo sapiens]

**Accession:**	gi|530380462	**Score:**	49.3
**Database:**	refseq_human(refseq_human_20140103.fasta)	**MW [kDa]:**	69.9
**Database Date:**	2015-11-30	**pI:**	9.8
**Modification(s):**	Oxidation	**Sequence Coverage [%]:**	6.1
		**No. of unique Peptides:**	3

Quantitation

QU:MU	**Median:** 0.68	**CV:** 70.11 %	**No. of Peptides:**	2
WUP:QUP	**Median:** 0.77	**CV:** 223.20 %	**No. of Peptides:**	2

10	20	30	40	50	60	70	80
MKRETRRSAF	CVQGWSGGDP	ARVVLWGSAG	GRGRARHAGV	PAVGPECRAR	RSQRADPIVS	TAPAPLGAEL	LSLECPTVRM
**90**	**100**	**110**	**120**	**130**	**140**	**150**	**160**
ERALPRGRCL	PLGKDLSSAK	RKFADSLNEF	KFQCIGDAET	DDEMCIARSL	QEFATVLRNL	EDERIRMIEN	ASEVLITPLE
**170**	**180**	**190**	**200**	**210**	**220**	**230**	**240**
KFRKEQIGAA	KEAKKKYDKE	TEKYCGILEK	HLNLSSKKKE	SQLQEADSQV	DLVRQHFYEV	SLEYVFKVQE	VQERKMFEFV
**250**	**260**	**270**	**280**	**290**	**300**	**310**	**320**
EPLLAFLQGL	FTFYHHGYEL	AKDFGDFKTQ	LTISIQNTRN	RFEGTRSEVE	SLMKKMKENP	LEHKTISPYT	MEGYLYVQEK
**330**	**340**	**350**	**360**	**370**	**380**	**390**	**400**
RHFGTSWVKH	YCTYQRDSKQ	ITMVPFDQKS	GGKGGEDESV	ILKSCTRRKT	DSIEKRFCFD	VEAVDRPGVI	TMQALSEEDR
**410**	**420**	**430**	**440**	**450**	**460**	**470**	**480**
RLWMEAMDGR	EPVYNSNKDS	QSEGTAQLDS	IGFSIIRKCI	HAVETRGINE	QGLYRIVGVN	SRVQKLLSVL	MDPKTASETE
**490**	**500**	**510**	**520**	**530**	**540**	**550**	**560**
TDICAEWEIK	TITSALKTYL	RMLPGPLMMY	QFQRSFIKAA	KLENQESRVS	EIHSLVHRLP	EKNRQMLQLL	MNHLAKLIVP
**570**	**580**	**590**	**600**	**610**	**620**		
GTLKVTGSVD	MGKSSISLLF	KEMKLLKVRK	QQMLERMWRN	RNDFTLLVGV			

Cmpd.	No. of Cmpds.	m/z meas.	Δ m/z [ppm]	z	Rt [min]	Score	P	Range	Sequence	Modification	Ratios
1925	2	710.2851	-115.74	2	53.9	19.4	1	35-48	R.ARHAGVPAVGPECR.A		
47	3	768.2750	-160.02	2	30.4	15.4	1	340-353	K.QITMVPFDQKSGGK.G		WUP:QUP 0.20<br>QU:MU 1.27
1623	1	516.2455	-93.79	2	50.5	14.5	0	466-474	K.LLSVLMDPK.T	Oxidation: 6	WUP:QUP 2.95<br>QU:MU 0.36

Detailed Protein Report

Protein 130: **PREDICTED: autophagy-related protein 2 homolog B isoform X1 [Homo sapiens]**

**Accession:**	gi|578825925	**Score:**	49.3
**Database:**	refseq_human(refseq_human_20140103.fasta)	**MW [kDa]:**	232.5
**Database Date:**	2015-11-30	**pI:**	5.4
		**Sequence Coverage [%]:**	2.1
		**No. of unique Peptides:**	2

Detailed Protein Report

10	20	30	40	50	60	70	80
MPWPFSESIK	KRACRYLLQR	YLGHFLQEKL	SLEQLSLDLY	QGTGSLAQVP	LDKWCLNEIL	ESADAPLEVT	EGFIQSISLS
90	100	110	120	130	140	150	160
VPWGSLLQDN	CALEVRGLEM	VFRPRPRPAT	GSEPMYWSSF	MTSSMQLAKE	CLSQKLTDEQ	GEGSQPFEGL	EKFAETIETV
170	180	190	200	210	220	230	240
LRRVKVTFID	TVLRIEHVPE	NSKTGTALEI	RIERTVYCDE	TADESSGINV	HQPTAFAHKL	LQLSGVSLFW	DEFSASAKSS
250	260	270	280	290	300	310	320
PVCSTAPVET	EPKLSPSWNP	KIIYEPHPQL	TRNLPEIAPS	DPVQIGRLIG	RLELSLTLKQ	NEVLPGAKLD	VDGQIDSIHL
330	340	350	360	370	380	390	400
LLSPRQVHLL	LDMLAAIAGP	ENSSKIGLAN	KDRKNRPMQQ	EDEYRIQMEL	NRYYLRKDSL	SVGVSSEQSF	YETETARTPS
410	420	430	440	450	460	470	480
SREEVFFSMA	DMDMSHSLSS	LPPLGDPPNM	DLELSLTSTY	TNTPAGSPLS	ATVLQPTWGE	FLDHHKEQPV	RGSTFPSNLV
490	500	510	520	530	540	550	560
HPTPLQKTSL	PSRSVSVDES	RPELIFRLAV	GTFSISVLHI	DPLSPPETSQ	NLNPLTPMAV	AFFTCIEKID	PARFSTEDFK
570	580	590	600	610	620	630	640
SFRAVFAEAC	SHDHLRFIGT	GIKVSYEQRQ	RSASRYFSTD	MSIGQMEFLE	CLFPTDFHSV	PPHYTELLTF	HSKEETGSHS
650	660	670	680	690	700	710	720
PVCLQLHYKH	SENRGPQGNQ	ARLSSVPHKA	ELQIKLNPVC	CELDISIVDR	LNSLLQPQKL	ATVEMMASHM	YTSYNKHISL
730	740	750	760	770	780	790	800
HKAFTEVFLD	DSHSPANCRI	SVQVATPALN	LSVRFPIPDL	RSDQERGPWF	KKSLQKEILY	LAFTDLEFKT	EFIGGSTPEQ
810	820	830	840	850	860	870	880
IKLELTFREL	IGSFQEEKGD	PSIKFFHVSS	GVDGDTTSSD	DFDWPRIVLK	INPPAMHSIL	ERIAAEEEEE	NDGHYQEEEE
890	900	910	920	930	940	950	960
GGAHSLKDVC	DLRRPAPSPF	SSRRVMFENE	QMVMPGDPVE	MTEFQDKAIS	NSHYVLELTL	PNIYVTLPNK	SFYEKLYNRI
970	980	990	1000	1010	1020	1030	1040
FNDLLLWEPT	APSPVETFEN	ISYGIGLSVA	SQLINTFNKD	SFSAFKSAVH	YDEESGSEEE	TLQYFSTVDP	NYRSRRKKKL
1050	1060	1070	1080	1090	1100	1110	1120
DSQNKNSQSF	LSVLLNINHG	LIAVFTDVKQ	DNGDLLENKH	GEFWLEFNSG	SLFCVTKYEG	FDDKHYICLH	SSSFSLYHKG
1130	1140	1150	1160	1170	1180	1190	1200
IVNGVILPTE	TRLPSSTRPH	WLEPTIYSSE	EDGLSKTSSD	GVGGDSLNML	SVAVKILSDK	SESNTKEFLI	AVGLKGATLQ
1210	1220	1230	1240	1250	1260	1270	1280
HRMLPSGLSW	HEQILYFLNI	ADEPVLGYNP	PTSFTTFHVH	LWSCALDYRP	LYLPIRSLLT	VETFSVSSSV	ALDKSSSTLR
1290	1300	1310	1320	1330	1340	1350	1360
IILDEAALHL	SDKCNTVTIN	LSRDYVRVMD	MGLLELTITA	VKSDSDGEQT	EPRFELHCSS	DVVHIRTCSD	SCAALMNLIQ
1370	1380	1390	1400	1410	1420	1430	1440
YIASYGDLQT	PNKADMKPGA	FQRRSKVDSS	GRSSSRGPVL	PEADQQMLRD	LMSDAMEEID	MQQGTSSVKP	QANGVLDEKS
1450	1460	1470	1480	1490	1500	1510	1520
QIQEPCCSDL	FLFPDESGNV	SQESGPTYAS	FSHHFISDAM	TGVPTENDDF	CILFAPKAAM	QEKEEEPVIK	IMVDDAIVIR
1530	1540	1550	1560	1570	1580	1590	1600
DNYFSLPVNK	TDTSKAPLHF	PIPVIRYVVK	EVSLVWHLYG	GKDFGIVPPT	SPAKSYISPH	SSPSHTPTRH	GRNTVCGGKG
1610	1620	1630	1640	1650	1660	1670	1680
RNHDFLMEIQ	LSKVKFQHEV	YPPCKPDCDS	SLSEHPVSRQ	VFIVQDLEIR	DRLATSQMNK	FLYLYCSKEM	PRKAHSNMLT
1690	1700	1710	1720	1730	1740	1750	1760
VKALHVCPES	GRSPQECCLR	VSLMPLRLNI	DQDALFFLKD	FFTSLSAEVE	LQMTPDPEVK	KSPGADVTCS	LPRHLSTSKE
1770	1780	1790	1800	1810	1820	1830	1840
PNLVISFSGP	KQPSQNDSAN	SVEVVNGMEE	KNFSAEEASF	RDQPVFFREF	RFTSEVPIRL	DYHGKHVSMD	QGTLAGILIG
1850	1860	1870	1880	1890	1900	1910	1920
LAQLNCSELK	LKRLSYRHGL	LGVDKLFSYA	ITEWLNDIKK	NQLPGILGGV	GPMHSLVQLV	QGLKDLVWLP	IEQYRKDGRI
1930	1940	1950	1960	1970	1980	1990	2000
VRGFQRGAAS	FGTSTAMAAL	ELTNRMVQTI	QAAAETAYDM	VSPGTLSIEP	KKTKRFPHHR	LAHQPVDLRE	GVAKAYSVVK
2010	2020	2030	2040	2050	2060	2070	2080
EGITDTAQTI	YETAAREHES	RGVTGAVGEV	LRQIPPAVVK	PLIVATEATS	NVLGGMRNQI	RPDVRQDESQ	KWRHGDD

Cmpd.	No. of Cmpds.	m/z meas.	Δ m/z [ppm]	z	Rt [min]	Score	P	Range	Sequence	Modification

Detailed Protein Report

Cmpd.	No. of Cmpds.	m/z meas.	Δ m/z [ppm]	z	Rt [min]	Score	P	Range	Sequence	Modification
2278	1	713.2212	-186.29	2	56.8	10.0	1	549-560	K.IDPARFSTEDFK.S	
1611	1	621.3254	12.46	2	50.4	11.2	2	1590-1601	R.HGRNTVCGGKGR.N	

Detailed Protein Report

Protein 131: PREDICTED: probable E3 ubiquitin-protein ligase MYCBP2 isoform X2 [Homo sapiens]

**Accession:**	gi|530402184	**Score:**	49.2
**Database:**	refseq_human(refseq_human_20140103.fasta)	**MW [kDa]:**	504.8
**Database Date:**	2015-11-30	**pI:**	6.6
**Modification(s):**	Oxidation	**Sequence Coverage [%]:**	1.7
		**No. of unique Peptides:**	3

Quantitation

WUP:QUP	**Median:** 0.15	**CV:** 0.00 %	**No. of Peptides:** 1

Detailed Protein Report

10	20	30	40	50	60	70	80
MMMCAATASP	AAASSGLGGD	GFYPAATFSS	SPAPGALFMP	VPDGSVAAAG	LGLGLPAADS	RGHYQLLLSG	RALADRYRRI
90	100	110	120	130	140	150	160
YTAALNDRDQ	GGGSAGHPAS	RNKKILNKKK	LKRKQKSKSK	VKTRSKSENL	ENTVIIPDIK	LHSNPSAFNI	YCNVRHCVLE
170	180	190	200	210	220	230	240
WQKKEISLAA	ASKNSVQSGE	SDSDEEEESK	EPPIKLPKII	EVGLCEVFEL	IKETRFSHPS	LCLRSLQALL	NVLQGQQPEG
250	260	270	280	290	300	310	320
LQSEPPEVLE	SLFQLLLEIT	VRSTGMNDST	GQSLTALSCA	CLFSLVASWG	ETGRTLQAIS	AILTNNGSHA	CQTIQVPTIL
330	340	350	360	370	380	390	400
NSLQRSVQAV	LVGKIQIQDW	FSNGIKKAAL	MHKWPLKEIS	VDEDDQCLLQ	NDGFFLYLLC	KDGLYKIGSG	YSGTVRGHIY
410	420	430	440	450	460	470	480
NSTSRIRNRK	EKKSWLGYAQ	GYLLYRDVNN	HSMTAIRISP	ETLEQDGTVM	LPDCHTEGQN	ILFTDGEYIN	QIAASRDDGF
490	500	510	520	530	540	550	560
VVRIFATSTE	PVLQQELQLK	LARKCLHACG	ISLFDLEKDL	HIISTGFDEE	SAILGAGREF	ALMKTANGKI	YYTGKYQSLG
570	580	590	600	610	620	630	640
IKQGGPSAGK	WVELPITKSP	KIVHFSVGHD	GSHALLVAED	GSIFFTGSAS	KGEDGESTKS	RRQSKPYKPK	KIIKMEGKIV
650	660	670	680	690	700	710	720
VYTACNNGSS	SVISKDGELY	MFGKDAIYSD	SSSLVTDLKG	HFVTQVAMGK	AHTCVLMKNG	EVWTFGVNNK	GQCGRDTGAM
730	740	750	760	770	780	790	800
NQGGKGFGVE	NMATAMDEDL	EEELDEKDEK	SMMCPPGMHK	WKLEQCMVCT	VCGDCTGYGA	SCVSSGRPDR	VPGGICGCGS
810	820	830	840	850	860	870	880
GESGCAVCGC	CKACARELDG	QEARQRGILD	AVKEMIPLDL	LLAVPVPGVN	IEEHLQLRQE	EKRQRVIRRH	RLEEGRGPLV
890	900	910	920	930	940	950	960
FAGPIFMNHR	EQALARLRSH	PAQLKHKRDK	HKDGSGERGE	KDASKITTYP	PGSVRFDCEL	RAVQVSCGFH	HSVVLMENGD
970	980	990	1000	1010	1020	1030	1040
VYTFGYGQHG	QLGHGDVNSR	GCPTLVQALP	GPSTQVTAGS	NHTAVLLMDG	QVFTFGSFSK	GQLGRPILDV	PYWNAKPAPM
1050	1060	1070	1080	1090	1100	1110	1120
PNIGSKYGRK	ATWIGASGDQ	TFLRIDEALI	NSHVLATSEI	FASKHIIGLV	PASISEPPPF	KCLLINKVDG	SCKTFNDSEQ
1130	1140	1150	1160	1170	1180	1190	1200
EDLQGFGVCL	DPVYDVIWRF	RPNTRELWCY	NAVVADARLP	SAADMQSRCS	ILSPELALPT	GSRALTTRSH	AALHILGCLD
1210	1220	1230	1240	1250	1260	1270	1280
TLAAMQDLKM	GVASTEEETQ	AVMKVYSKED	YSVVNRFESH	GGGWGYSAHS	VEAIRFSADT	DILLGGLGLF	GGRGEYTAKI
1290	1300	1310	1320	1330	1340	1350	1360
KLFELGPDGG	DHETDGDLLA	ETDVLAYDCA	AREKYAMMFD	EPVLLQAGWW	YVAWARVSGP	SSDCGSHGQA	SITTDDGVVF
1370	1380	1390	1400	1410	1420	1430	1440
QFKSSKKSNN	GTDVNAGQIP	QLLYRLPTSD	GSASKGKQQT	SEPVHILKRS	FARTVSVECF	ESLLSILHWS	WTTLVLGVEE
1450	1460	1470	1480	1490	1500	1510	1520
LRGLKGFQFT	ATLLDLERLR	FVGTCCLRLL	RVYTCEIYPV	SATGKAVVEE	TSKLAECIGK	TRTLLRKILS	EGVDHCMVKL
1530	1540	1550	1560	1570	1580	1590	1600
DNDPQGYLSQ	PLSLLEAVLQ	ECHNTFTACF	HSFYPTPALQ	WACLCDLLNC	LDQDIQEANF	KTSSSRLLAA	VMSALCHTSV
1610	1620	1630	1640	1650	1660	1670	1680
KLTSIFPIAY	DGEVLLRSIV	KQVSTENDST	LVHRFPLLVA	HMEKLSQSEE	NISGMTSFRE	VLEKMLVIVV	LPVRNSLRRE
1690	1700	1710	1720	1730	1740	1750	1760
NELFSSHLVS	NTCGLLASIV	SELTASALGS	EVDGLNSLHS	VKASANRFTK	TSQGRSWNTG	NGSPDAICFS	VDKPGIVVVG
1770	1780	1790	1800	1810	1820	1830	1840
FSVYGGGGIH	EYELEVLVDD	SEHAGDSTHS	HRWTSLELVK	GTYTTDDSPS	DIAEIRLDKV	VPLKENVKYA	VRLRNYGSRT
1850	1860	1870	1880	1890	1900	1910	1920
ANGDGGMTTV	QCPDGVTFTF	STCSLSSNGT	NQTRGQIPQI	LYYRSEFDGD	LQSQLLSKAN	EEDKNCSRAL	SVVSTVVRAS
1930	1940	1950	1960	1970	1980	1990	2000
KDLLHRALAV	DADDIPELLS	SSSLFSMLLP	LIIAYIGPVA	AAIPKVAVEV	FGLVQQLLPS	VAILNQKYAP	PAFNPNQSTD
2010	2020	2030	2040	2050	2060	2070	2080
STTGNQPEQG	LSACTTSSHY	AVIESEHPYK	PACVMHYKVT	FPECVRWMTI	EFDPQCGTAQ	SEDVLRLLIP	VRTVQNSGYG
2090	2100	2110	2120	2130	2140	2150	2160
PKLTSVHENL	NSWIELKKFS	GSSGWPTMVL	VLPGNEALFS	LETASDYVKD	DKASFYGFKC	FAIGYEFSPG	PDEGVIQLEK
2170	2180	2190	2200	2210	2220	2230	2240

Detailed Protein Report

Cmpd.	No. of Cmpds.	m/z meas.	Δ m/z [ppm]	z	Rt [min]	Score	P	Range	Sequence	Modification	Ratios
1748	3	697.3510	1.93	3	50.2	15.7	1	680-698	K.GHFVTQVAMGKAHTCVLMK.N	Oxidation: 9, 18	
2398	1	1023.7079	112.56	2	59.9	13.0	2	2442-2460	K.VKDPPKGMIPPGTQLVKPK.S	Oxidation: 8	
2407	1	973.4149	-88.00	2	60.6	10.4	1	3799-3817	K.DGESTKIAGQISASVAQQR.N		WUP:QUP 0.15

Detailed Protein Report

Protein 132: **cytospin-B isoform 2 [Homo sapiens]**

**Accession:**	gi|75750474	**Score:**	49.0
**Database:**	refseq_human(refseq_human_20140103.fasta)	**MW [kDa]:**	110.2
**Database Date:**	2015-11-30	**pI:**	5.6
		**Sequence Coverage [%]:**	6.0
		**No. of unique Peptides:**	2

10	20	30	40	50	60	70	80
MGNHSGRPED	PEPGAFTTTK	RTGIPAPREF	SVTVSRERSV	PRGPSNPRKS	VSSPTSSNTP	TPTKHLRTPS	TKPKQENEGG
**90**	**100**	**110**	**120**	**130**	**140**	**150**	**160**
EKAALESQVR	ELLAEAKAKD	SEINRLRSEL	KKYKEKRTLN	AEGTDALGPN	VDGTSVSPGD	TEPMIRALEE	KNKNFQKELS
**170**	**180**	**190**	**200**	**210**	**220**	**230**	**240**
DLEEENRVLK	EKLIYLEHSP	NSEGAASHTG	DSSCPTSITQ	ESSFGSPTGN	QMSSDIDEYK	KNIHGNALRT	SGSSSSDVTK
**250**	**260**	**270**	**280**	**290**	**300**	**310**	**320**
ASLSPDASDF	EHITAETPSR	PLSSTSNPFK	SSKCSTAGSS	PNSVSELSLA	SLTEKIQKME	ENHHSTAEEL	QATLQELSDQ
**330**	**340**	**350**	**360**	**370**	**380**	**390**	**400**
QQMVQELTAE	NEKLVDEKTI	LETSFHQHRE	RAEQLSQENE	KLMNLLQERV	KNEEPTTQEG	KIIELEQKCT	GILEQGRFER
**410**	**420**	**430**	**440**	**450**	**460**	**470**	**480**
EKLLNIQQQL	TCSLRKVEEE	NQGALEMIKR	LKEENEKLNE	FLELERHNNN	MMAKTLEECR	VTLEGLKMEN	GSLKSHLQGE
**490**	**500**	**510**	**520**	**530**	**540**	**550**	**560**
KQKATEASAV	EQTAESCEVQ	EMLKVARAEK	DLLELSCNEL	RQELLKANGE	IKHVSSLLAK	VEKDYSYLKE	ICDHQAEQLS
**570**	**580**	**590**	**600**	**610**	**620**	**630**	**640**
RTSLKLQEKA	SESDAEIKDM	KETIFELEDQ	VEQHRAVKLH	NNQLISELES	SVIKLEEQKS	DLERQLKTLT	KQMKEETEEW
**650**	**660**	**670**	**680**	**690**	**700**	**710**	**720**
RRFQADLQTA	VVVANDIKCE	AQQELRTVKR	KLLEEEEKNA	RLQKELGDVQ	GHGRVVTSRA	APPPVDEEPE	SSEVDAAGRW
**730**	**740**	**750**	**760**	**770**	**780**	**790**	**800**
PGVCVSRTSP	TPPESATTVK	SLIKSFDLGR	PGGAGQNISV	HKTPRSPLSG	IPVRTAPAAA	VSPMQRHSTY	SSVRPASRGV
**810**	**820**	**830**	**840**	**850**	**860**	**870**	**880**
TQRLDLPDLP	LSDILKGRTE	TLKPDPHLRK	SPSLESLSRP	PSLGFGDTRL	LSASTRAWKP	QSKLSVERKD	PLAALAREYG
**890**	**900**	**910**	**920**	**930**	**940**	**950**	**960**
GSKRNALLKW	CQKKTQGYAN	IDITNFSSSW	SDGLAFCALL	HTYLPAHIPY	QELNSQEKKR	NLLLAFEAAE	SVGIKPSLEL
**970**	**980**	**990**					
SEMLYTDRPD	WQSVMQYVAQ	IYKYFET					

Cmpd.	No. of Cmpds.	m/z meas.	Δ m/z [ppm]	z	Rt [min]	Score	P	Range	Sequence	Modification
14	1	1103.5194	-101.41	2	29.8	16.5	2	799-818	R.GVTQRLDLPDLPLSDILKGR.T	
29	1	1001.9624	-50.81	2	30.0	16.6	0	831-849	K.SPSLESLSRPPSLGFGDTR.L	

Detailed Protein Report

Protein 133: myomesin-1 isoform b [Homo sapiens]

**Accession:**	gi|140560917	**Score:**	48.6
**Database:**	refseq_human(refseq_human_20140103.fasta)	**MW [kDa]:**	177.6
**Database Date:**	2015-11-30	**pI:**	6.4
		**Sequence Coverage [%]:**	2.1
		**No. of unique Peptides:**	2

10	20	30	40	50	60	70	80
MSLPFYQRCH	QHYDLSYRNK	DVRSTVSHYQ	REKKRSAVYT	QGSTAYSSRS	SAAHRRESEA	FRRASASSSQ	QQASQHALSS
90	100	110	120	130	140	150	160
EVSRKAASAY	DYGSSHGLTD	SSLLLDDYSS	KLSPKPKRAK	HSLLSGEEKE	NLPSDYMVPI	FSGRQKHVSG	ITDTEEERIK
170	180	190	200	210	220	230	240
EAAAYIAQRN	LLASEEGITT	SKQSTASKQT	TASKQSTASK	QSTASKQSTA	SRQSTASRQS	VVSKQATSAL	QQEETSEKKS
250	260	270	280	290	300	310	320
RKVVIREKAE	RLSLRKTLEE	TETYHAKLNE	DHLLHAPEFI	IKPRSHTVWE	KENVKLHCSI	AGWPEPRVTW	YKNQVPINVH
330	340	350	360	370	380	390	400
ANPGKYIIES	RYGMHTLEIN	GCDFEDTAQY	RASAMNVKGE	LSAYASVVVK	RYKGEFDETR	FHAGASTMPL	SFGVTPYGYA
410	420	430	440	450	460	470	480
SRFEIHFDDK	FDVSFGREGE	TMSLGCRVVI	TPEIKHFQPE	IQWYRNGVPL	SPSKWVQTLW	SGERATLTFS	HLNKEDEGLY
490	500	510	520	530	540	550	560
TIRVRMGEYY	EQYSAYVFVR	DADAEIEGAP	AAPLDVKCLE	ANKDYIIISW	KQPAVDGGSP	ILGYFIDKCE	VGTDSWSQCN
570	580	590	600	610	620	630	640
DTPVKFARFP	VTGLIEGRSY	IFRVRAVNKM	GIGFPSRVSE	PVAALDPAEK	ARLKSRPSAP	WTGQIIVTEE	EPSEGIVPGP
650	660	670	680	690	700	710	720
PTDLSVTEAT	RSYVVLSWKP	PGQRGHEGIM	YFVEKCEAGT	ENWQRVNTEL	PVKSPRFALF	DLAEGKSYCF	RVRCSNSAGV
730	740	750	760	770	780	790	800
GEPSEATEVT	VVGDKLDIPK	APGKIIPSRN	TDTSVVVSWE	ESKDAKELVG	YYIEASVAGS	GKWEPCNNNP	VKGSRFTCHG
810	820	830	840	850	860	870	880
LVTGQSYIFR	VRAVNAAGLS	EYSQDSEAIE	VKAAIAPPSP	PCDITCLESF	RDSMVLGWKQ	PDKIGGAEIT	GYYVNYREVI
890	900	910	920	930	940	950	960
DGVPGKWREA	NVKAVSEEAY	KISNLKENMV	YQFQVAAMNM	AGLGAPSAVS	ECFKCEEWTI	AVPGPPHSLK	CSEVRKDSLV
970	980	990	1000	1010	1020	1030	1040
LQWKPPVHSG	RTPVTGYFVD	LKEAKAKEDQ	WRGLNEAAIK	NVYLKVRGLK	EGVSYVFRVR	AINQAGVGKP	SDLAGPVVAE
1050	1060	1070	1080	1090	1100	1110	1120
TRPGTKEVVV	NVDDDGVISL	NFECDKMTPK	SEFSWSKDYV	STEDSPRLEV	ESKGNKTKMT	FKDLGMDDLG	IYSCDVTDTD
1130	1140	1150	1160	1170	1180	1190	1200
GIASSYLIDE	EELKRLLALS	HEHKFPTVPV	KSELAVEILE	KGQVRFWMQA	EKLSGNAKVN	YIFNEKEIFE	GPKYKMHIDR
1210	1220	1230	1240	1250	1260	1270	1280
NTGIIEMFME	KLQDEDEGTY	TFQLQDGKAT	NHSTVVLVGD	VFKKLQKEAE	FQRQEWIRKQ	GPHFVEYLSW	EVTGECNVLL
1290	1300	1310	1320	1330	1340	1350	1360
KCKVANIKKE	THIVWYKDER	EISVDEKHDF	KDGICTLLIT	EFSKKDAGIY	EVILKDDRGK	DKSRLKLVDE	AFKELMMEVC
1370	1380	1390	1400	1410	1420	1430	1440
KKIALSATDL	KIQSTAEGIQ	LYSFVTYYVE	DLKVNWSHNG	SAIRYSDRVK	TGVTGEQIWL	QINEPTPNDK	GKYVMELFDG
1450	1460	1470	1480	1490	1500	1510	1520
KTGHQKTVDL	SGQAYDEAYA	EFQRLKQAAI	AEKNRARVLG	GLPDVVTIQE	GKALNLTCNV	WGDPPPEVSW	LKNEKALASD
1530	1540	1550	1560	1570	1580	1590	
DHCNLKFEAG	RTAYFTINGV	STADSGKYGL	VVKNKYGSET	SDFTVSVFIP	EEEARMAALE	SLKGGKKAK	

Cmpd.	No. of Cmpds.	m/z meas.	Δ m/z [ppm]	z	Rt [min]	Score	P	Range	Sequence	Modification
792	1	555.7879	-13.08	2	38.3	14.3	0	697-706	R.FALFDLAEGK.S	
1871	2	621.3062	-102.44	2	53.7	17.1	2	1465-1475	R.LKQAAIAEKNR.A	

Detailed Protein Report

Protein 134: nucleolar transcription factor 1 isoform b [Homo sapiens]

**Accession:**	gi|115529449	**Score:**	48.5
**Database:**	refseq_human(refseq_human_20140103.fasta)	**MW [kDa]:**	84.9
**Database Date:**	2015-11-30	**pI:**	5.3
**Modification(s):**	Oxidation	**Sequence Coverage [%]:**	3.3
		**No. of unique Peptides:**	3

Alias proteins:

Accession	Name	Description
gi|115529451	refseq_human (refseq_human_20140103.fasta)	nucleolar transcription factor 1 isoform b [Homo sapiens]

10	20	30	40	50	60	70	80
MNGEADCPTD	LEMAAPKGQD	RWSQEDMLTL	LECMKNNLPS	NDSSKFKTTE	SHMDWEKVAF	KDFSGDMCKL	KWVEISNEVR
**90**	**100**	**110**	**120**	**130**	**140**	**150**	**160**
KFRTLTELIL	DAQEHVKNPY	KGKKLKKHPD	FPKKPLTPYF	RFFMEKRAKY	AKLHPEMSNL	DLTKILSKKY	KELPEKKKMK
**170**	**180**	**190**	**200**	**210**	**220**	**230**	**240**
YIQDFQREKQ	EFERNLARFR	EDHPDLIQNA	KKSDIPEKPK	TPQQLWYTHE	KKVYLKVRPD	EIMRDYIQKH	PELNISEEGI
**250**	**260**	**270**	**280**	**290**	**300**	**310**	**320**
TKSTLTKAER	QLKDKFDGRP	TKPPPNSYSL	YCAELMANMK	DVPSTERMVL	CSQQWKLLSQ	KEKDAYHKKC	DQKKKDYEVE
**330**	**340**	**350**	**360**	**370**	**380**	**390**	**400**
LLRFLESLPE	EEQQRVLGEE	KMLNINKKQA	TSPASKKPAQ	EGGKGGSEKP	KRPVSAMFIF	SEEKRRQLQE	ERPELSESEL
**410**	**420**	**430**	**440**	**450**	**460**	**470**	**480**
TRLLARMWND	LSEKKKAKYK	AREAALKAQS	ERKPGGEREE	RGKLPESPKR	AEEIWQQSVI	GDYLARFKND	RVKALKAMEM
**490**	**500**	**510**	**520**	**530**	**540**	**550**	**560**
TWNNMEKKEK	LMWIKKAAED	QKRYERELSE	MRAPPAATNS	SKKMKFQGEP	KKPPMNGYQK	FSQELLSNGE	LNHLPLKERM
**570**	**580**	**590**	**600**	**610**	**620**	**630**	**640**
VEIGSRWQRI	SQSQKEHYKK	LAEEQQKQYK	VHLDLWVKSL	SPQDRAAYKE	YISNKRKSMT	KLRGPNPKSS	RTTLQSKSES
**650**	**660**	**670**	**680**	**690**	**700**	**710**	**720**
EEDDEEDEDD	EDEDEEEEDD	ENGDSSEDGG	DSSESSSEDE	SEDGDENEED	DEDEDDDEDD	DEDEDNESEG	SSSSSSSSGD
**730**							
SSDSDSN							

Cmpd.	No. of Cmpds.	m/z meas.	Δ m/z [ppm]	z	Rt [min]	Score	P	Range	Sequence	Modification
2859	3	856.9262	37.72	2	66.2	13.0	1	474-487	K.ALKAMEMTWNNMEK.K	Oxidation: 5
2869	1	856.9447	59.29	2	66.4	17.0	1	474-487	K.ALKAMEMTWNNMEK.K	Oxidation: 7
1591	1	472.2351	-22.65	2	49.6	18.5	0	513-522	R.APPAATNSSK.K	

Detailed Protein Report

Protein 135: fibrinogen beta chain isoform 2 preproprotein [Homo sapiens]

Accession: gi|296080754
Database: refseq_human(refseq_human_20140103.fasta)
Database Date: 2015-11-30
Modification(s): Carbamidomethyl, Oxidation

Score: 48.5
MW [kDa]: 49.9
pI: 9.0
Sequence Coverage [%]: 8.6
No. of unique Peptides: 3

Quantitation

QU:MU	**Median:**	0.90	**CV:**	0.00 %	**No. of Peptides:**	1
WUP:QUP	**Median:**	0.69	**CV:**	0.00 %	**No. of Peptides:**	1

```
        10         20         30         40         50         60         70         80
MKRMVSWSFH KLKTMKHLLL LLLCVFLVKS QGVNDNEEGF FSARGHRPLD KKREEALLQQ ERPIRNSVDE LNNNVEAVSQ
        90        100        110        120        130        140        150        160
TSSSSFQYMY LLKDLWQKRQ KQVKDNENVV NEYSSELEKH QLYIDETVNS NIPTNLRVLR SILENLRSKI QKLESDVSAQ
       170        180        190        200        210        220        230        240
MEYCRTPCTV SCNIPVVSGK ECEEIIRKGG ETSEMYLIQP DSSVKPYRVY CDMNTENGGW TVIQNRQDGS VDFGRKWDPY
       250        260        270        280        290        300        310        320
KQGFGNVATN TDGKNYCGLP GEYWLGNDKI SQLTRMGPTE LLIEMEDWKG DKVKAHYGGF TVQNEANKYQ ISVNKYRGTA
       330        340        350        360        370        380        390        400
GNALMDGASQ LMGENRTMTI HNGMFFSTYD RDNDGWLTSD PRKQCSKEDG GGWWYNRCHA ANPNGRYYWG GQYTWDMAKH
       410        420        430        440
GTDDGVVWMN WKGSWYSMRK MSMKIRPFFP QQ
```

Cmpd.	No. of Cmpds.	m/z meas.	Δ m/z [ppm]	z	Rt [min]	Score	P	Range	Sequence	Modification	Ratios
590	2	809.8948	-6.44	2	37.4	25.6	0	166-180	R.TPCTVSCNIPVVSGK.E	Carbamidomethyl: 3, 7	QU:MU 0.90<br>WUP:QUP 0.69
1919	1	862.3067	-107.58	2	53.9	11.4	0	276-289	R.MGPTELLIEMEDWK.G	Oxidation: 1, 10	
747	1	516.6531	-255.12	2	37.8	11.5	0	425-432	K.IRPFFPQQ.-		

Detailed Protein Report

Protein 136: kinesin-like protein KIF20B isoform 2 [Homo sapiens]

Accession: gi|46049114
Database: refseq_human(refseq_human_20140103.fasta)
Database Date: 2015-11-30
Modification(s): Carbamidomethyl, Oxidation

Score: 48.5
MW [kDa]: 206.0
pI: 5.5
Sequence Coverage [%]: 3.8
No. of unique Peptides: 2

10	20	30	40	50	60	70	80
MESNFNQEGV	PRPSYVFSAD	PIARPSEINF	DGIKLDLSHE	FSLVAPNTEA	NSFESKDYLQ	VCLRIRPFTQ	SEKELESEGC
90	100	110	120	130	140	150	160
VHILDSQTVV	LKEPQCILGR	LSEKSSGQMA	QKFSFSKVFG	PATTQKEFFQ	GCIMQPVKDL	LKGQSRLIFT	YGLTNSGKTY
170	180	190	200	210	220	230	240
TFQGTEENIG	ILPRTLNVLF	DSLQERLYTK	MNLKPHRSRE	YLRLSSEQEK	EEIASKSALL	RQIKEVTVHN	DSDDTLYGSL
250	260	270	280	290	300	310	320
TNSLNISEFE	ESIKDYEQAN	LNMANSIKFS	VWVSFFEIYN	EYIYDLFVPV	SSKFQKRKML	RLSQDVKGYS	FIKDLQWIQV
330	340	350	360	370	380	390	400
SDSKEAYRLL	KLGIKHQSVA	FTKLNNASSR	SHSIFTVKIL	QIEDSEMSRV	IRVSELSLCD	LAGSERTMKT	QNEGERLRET
410	420	430	440	450	460	470	480
GNINTSLLTL	GKCINVLKNS	EKSKFQQHVP	FRESKLTHYF	QSFFNGKGKI	CMIVNISQCY	LAYDETLNVL	KFSAIAQKVC
490	500	510	520	530	540	550	560
VPDTLNSSQE	KLFGPVKSSQ	DVSLDSNSNS	KILNVKRATI	SWENSLEDLM	EDEDLVEELE	NAEETQNVET	KLLDEDLDKT
570	580	590	600	610	620	630	640
LEENKAFISH	EEKRKLLDLI	EDLKKKLINE	KKEKLTLEFK	IREEVTQEFT	QYWAQREADF	KETLLQEREI	LEENAERRLA
650	660	670	680	690	700	710	720
IFKDLVGKCD	TREEAAKDIC	ATKVETEEAT	ACLELKFNQI	KAELAKTKGE	LIKTKEELKK	RENESDSLIQ	ELETSNKKII
730	740	750	760	770	780	790	800
TQNQRIKELI	NIIDQKEDTI	NEFQNLKSHM	ENTFKCNDKA	DTSSLIINNK	LICNETVEVP	KDSKSKICSE	RKRVNENELQ
810	820	830	840	850	860	870	880
QDEPPAKKGS	IHVSSAITED	QKKSEEVRPN	IAEIEDIRVL	QENNEGLRAF	LLTIENELKN	EKEEKAELNK	QIVHFQQELS
890	900	910	920	930	940	950	960
LSEKKNLTLS	KEVQQIQSNY	DIAIAELHVQ	KSKNQEQEEK	IMKLSNEIET	ATRSITNNVS	QIKLMHTKID	ELRTLDSVSQ
970	980	990	1000	1010	1020	1030	1040
ISNIDLLNLR	DLSNGSEEDN	LPNTQLDLLG	NDYLVSKQVK	EYRIQEPNRE	NSFHSSIEAI	WEECKEIVKA	SSKKSHQIEE
1050	1060	1070	1080	1090	1100	1110	1120
LEQQIEKLQA	EVKGYKDENN	RLKEKEHKNQ	DDLLKEKETL	IQQLKEELQE	KNVTLDVQIQ	HVVEGKRALS	ELTQGVTCYK
1130	1140	1150	1160	1170	1180	1190	1200
AKIKELETIL	ETQKVECSHS	AKLEQDILEK	ESIILKLERN	LKEFQEHLQD	SVKNTKDLNV	KELKLKEEIT	QLTNNLQDMK
1210	1220	1230	1240	1250	1260	1270	1280
HLLQLKEEEE	ETNRQETEKL	KEELSASSAR	TQNLKADLQR	KEEDYADLKE	KLTDAKKQIK	QVQKEVSVMR	DEDKLLRIKI
1290	1300	1310	1320	1330	1340	1350	1360
NELEKKKNQC	SQELDMKQRT	IQQLKEQLNN	QKVEEAIQQY	ERACKDLNVK	EKIIEDMRMT	LEEQEQTQVE	QDQVLEAKLE
1370	1380	1390	1400	1410	1420	1430	1440
EVERLATELE	KWKEKCNDLE	TKNNQRSNKE	HENNTDVLGK	LTNLQDELQE	SEQKYNADRK	KWLEEKMMLI	TQAKEAENIR
1450	1460	1470	1480	1490	1500	1510	1520
NKEMKKYAED	RERFFKQQNE	MEILTAQLTE	KDSDLQKWRE	ERDQLVAALE	IQLKALISSN	VQKDNEIEQL	KRIISETSKI
1530	1540	1550	1560	1570	1580	1590	1600
ETQIMDIKPK	RISSADPDKL	QTEPLSTSFE	ISRNKIEDGS	VVLDSCEVST	ENDQSTRFPK	PELEIQFTPL	QPNKMAVKHP
1610	1620	1630	1640	1650	1660	1670	1680
GCTTPVTVKI	PKARKRKSNE	MEEDLVKCEN	KKNATPRTNL	KFPISDDRNS	SVKKEQKVAI	RPSSKKTYSL	RSQASIIGVN
1690	1700	1710	1720	1730	1740	1750	1760
LATKKKEGTL	QKFGDFLQHS	PSILQSKAKK	IIETMSSSKL	SNVEASKENV	SQPKRAKRKL	YTSEISSPID	ISGQVILMDQ
1770	1780	1790					
KMKESDHQII	KRRLRTKTAK						

Cmpd.	No. of	m/z meas.	Δ m/z	z	Rt	Score	P	Range	Sequence	Modification

	Cmpds.		[ppm]		[min]					
20	1	1103.5042	-42.73	2	29.9	14.1	2	93-112	K.EPQCILGRLSEKSSGQMAQK.F	Oxidation: 17
1683	1	954.5011	22.43	3	51.3	10.8	1	448-471	K.GKICMIVNISQCYLAYDETLNVLK.F	Carbamidomethyl: 4, 12; Oxidation: 5

Detailed Protein Report

Protein 137: PREDICTED: neurobeachin-like protein 1 isoform X8 [Homo sapiens]

**Accession:**	gi|578804777	**Score:**	48.3
**Database:**	refseq_human(refseq_human_20140103.fasta)	**MW [kDa]:**	305.4
**Database Date:**	2015-11-30	**pI:**	6.0
**Modification(s):**	Carbamidomethyl, Oxidation	**Sequence Coverage [%]:**	1.7
		**No. of unique Peptides:**	2

Detailed Protein Report

10	20	30	40	50	60	70	80
MASRERLFEL	WMLYCTKKDP	DYLKLWLDTF	VSSYEQFLDV	DFEKLPTRVD	DMPPGISLLP	DNILQVLRIQ	LLQCVQKMAD
90	100	110	120	130	140	150	160
GLEEQQQALS	ILLVKFFIIL	CRNLSNVEEI	GTCSYINYVI	TMTTLYIQQL	KSKKKEKEMA	DQTCIEEFVI	HALAFCESLY
170	180	190	200	210	220	230	240
DPYRNWRHRI	SGRILSTVEK	SRQKYKPASL	TVEFVPFFYQ	CFQESEHLKE	SLKCCLLHLF	GAIVAGGQRN	ALQAISPATM
250	260	270	280	290	300	310	320
EVLMRVLADC	DSWEDGDPEE	VGRKAELTLK	CLTEVVHILL	SSNSDQRQVE	TSTILENYFK	LLNSDHSALP	NQRRSRQWEN
330	340	350	360	370	380	390	400
RFIALQIKML	NTITAMLDCT	DRPVLQAIFL	NSNCFEHLIR	LLQNCKEVFK	ERIGYTHMLE	VLKSLGQPPL	ELLKELMNMA
410	420	430	440	450	460	470	480
VEGDHTSVGI	LGISNVQPLL	LLIQWLPELQ	SHDLQIFISD	WLKRICCINR	QSRTTCVNAN	MGIRIIETLD	LHSSLHQTCA
490	500	510	520	530	540	550	560
ENLIAIHGSL	GSQSVSSEEI	RRLLRLLRVD	ESESVHPYVT	PVTRAILTMA	RKLSLESALQ	YFNLSHSMAG	ISVPPIQKWP
570	580	590	600	610	620	630	640
GSAFSFSAWF	CLDQDQLTLG	IANKGGKRKQ	LYSFFTGSGM	GFEAFITHSG	MLVVAVCTKR	EYATVMLPDH	SFCDSLWHNI
650	660	670	680	690	700	710	720
TVVHMPGKRP	FGQSFVYIYD	NGQQKVSAPL	RFPAMNEPFT	SCCIGSAGQR	TTTPPPSQIP	DPPFSSPITP	HRTSFGGILS
730	740	750	760	770	780	790	800
SASWGGTIEK	SKLITKLISA	GTQDSEWGCP	TSLEGQLGSV	IIFYEPLQPP	QVKALYLAGP	NCLSPWKCQE	SDMADLPGNI
810	820	830	840	850	860	870	880
LLYYTAKACK	NSICLDLSTN	CLHGRLTGNK	VVNWDIKDII	NCIGGLNVLF	PLLEQISHFS	EGQIPEEKNE	STVPESVTPV
890	900	910	920	930	940	950	960
EGDWLVWTST	KASESRLERN	LVATFILIVK	HFIQRHPINQ	GNLIHSHGVA	TLGALLQKVP	STLMDVNVLM	AVQLLIEQVS
970	980	990	1000	1010	1020	1030	1040
LEKNMQLLQQ	MYQYLLFDFR	IWNRGDFPFR	IGHIQYLSTI	IKDSRRVFRK	KYGVQFLLDT	LRIYYGNGCK	YNELSLDDIR
1050	1060	1070	1080	1090	1100	1110	1120
TIRTSLYGLI	KYFLCKGGSH	EEIQSIMGYI	AATNEEEQLF	GILDVLFSLL	RTSPTRGQLF	LLLFEPGNAD	ILYALLLNQK
1130	1140	1150	1160	1170	1180	1190	1200
YSDRLREIIF	KIMEQMLKCT	NVYERSKQHI	RLREVGYSGL	GLLLNEALVN	TSLIKNLTHQ	IINTDPVINF	KDLLSVVYIS
1210	1220	1230	1240	1250	1260	1270	1280
HRAHINVRVA	ICRKVLQILQ	FQPDAAHQIS	QQVGWQDTLV	RLFLKAKFEN	GNTLHKHSRA	VLMKDNDKNM	STEDTKKNSD
1290	1300	1310	1320	1330	1340	1350	1360
EKTDEEKITS	FASANVSSDQ	WSLEDRHSLD	SNTPLFPEDS	SVGELSFKSE	NQEEFWHSNP	SHLSLDLSGI	DSCEMSDSGS
1370	1380	1390	1400	1410	1420	1430	1440
QVPDSLPSTP	SPVESTKSFS	VHSDRESSIT	NDMGFSDDFS	LLESQERCEE	ELLQLLTHIL	NYVMCKGLEK	SDDDTWIERG
1450	1460	1470	1480	1490	1500	1510	1520
QVFSALSKPG	ISSELLRPSD	EIKLTLLQKM	LEWAISENRE	AKTNPVTAEN	AFRLVLIIQD	FLQSEGLVNS	NMWTEKLLED
1530	1540	1550	1560	1570	1580	1590	1600
MMLLFDCLSV	CYSESPVWVK	LSQIQIQLLL	GFIGRGNLQV	CAMASAKLNT	LLQTKVIENQ	DEACYILGKL	EHVLSQSIKE
1610	1620	1630	1640	1650	1660	1670	1680
QTEIYSFLIP	LVRTLVSKIY	ELLFMNLHLP	SLPFTNGSSS	FFEDFQEYCN	SNEWQVYIEK	YIVPYMKQYE	AHTFYDGHEN
1690	1700	1710	1720	1730	1740	1750	1760
MALYWKDCYE	ALMVNMHKRD	REGGESKLKF	QELFVEPFNR	KARQENLRYN	NMLKQLSSQQ	LATLRRWKAI	QLYLTCERGP
1770	1780	1790	1800	1810	1820	1830	1840
WAKRKQNPIH	WKLANVENYS	RMRLKLVPNY	NFKTHEEASA	LRDNLGIQHS	QPSSDTLLLE	VVKQVKVSDM	VEDKLDLPEE
1850	1860	1870	1880	1890	1900	1910	1920
DITARVNVDE	KEEQDQKEKL	VLMEDCELIT	IIDVIPGRLE	ITTQHIYFYD	GSIEKEDGVG	FDFKWPHSQI	REIHLRRYNL
1930	1940	1950	1960	1970	1980	1990	2000
RRSALEIFHV	DQSNYFLNFK	KEVRNKIYSR	LLSLHSPNSY	YGSRSPQELF	KASGLTQKWV	NREISNFDYL	IQINTMAGRT
2010	2020	2030	2040	2050	2060	2070	2080
YNDLAQYPVF	PWILQDYTSE	ELDLNNPAVF	RDLSKPIGVV	NEKNAKAMRE	KYENFEDPMG	TIDKFHYGTH	YSNSAGVMHY
2090	2100	2110	2120	2130	2140	2150	2160
LIRVEPFTTL	HIQLQSGRFD	CADRQFHSIP	ATWQALMDNP	YDVKELIPEF	FYFPEFLENQ	NQFNLGRLQI	SKELVNDVIL
2170	2180	2190	2200	2210	2220	2230	2240

Detailed Protein Report

Cmpd.	No. of Cmpds.	m/z meas.	Δ m/z [ppm]	z	Rt [min]	Score	P	Range	Sequence	Modification
1799	1	1045.0496	94.89	2	50.8	15.5	0	672-690	R.FPAMNEPFTSCCIGSAGQR.T	Carbamidomethyl: 12; Oxidation: 4
2060	1	899.3817	-81.00	2	54.1	19.6	0	713-730	R.TSFGGILSSASWGGTIEK.S	

Detailed Protein Report

Protein 138: PREDICTED: protein transport protein Sec16A isoform X5 [Homo sapiens]

**Accession:**	gi|530426857	**Score:**	47.8
**Database:**	refseq_human(refseq_human_20140103.fasta)	**MW [kDa]:**	240.9
**Database Date:**	2015-11-30	**pI:**	5.7
**Modification(s):**	Oxidation	**Sequence Coverage [%]:**	2.1
		**No. of unique Peptides:**	3

Quantitation

QU:MU	**Median:**	1.63	**CV:**	0.00 %	**No. of Peptides:**	1
WUP:QUP	**Median:**	0.37	**CV:**	0.00 %	**No. of Peptides:**	1

Detailed Protein Report

10	20	30	40	50	60	70	80
MGAAGLGLGP	TTCSLTLFKQ	VRNLTPERQM	LAEQIGPGAT	SAGLKLSLHR	LRPCWRGTDI	TPSLTSRRPS	WGQGWLPPRE
90	100	110	120	130	140	150	160
DLSRCWELSL	PRCHRAGTPT	TAPALWKEQQ	RKAAALPCPC	QAGVQLQLRN	SYILLPSVSS	IDLCKGSVMQ	PPPQTVPSGM
170	180	190	200	210	220	230	240
AGPPPAGNPR	SVFWASSPYR	RRANNNAAVA	PTTCPLQPVT	DPFAFSRQAL	QSTPLGSSSK	SSPPVLQGPA	PAGFSQHPGL
250	260	270	280	290	300	310	320
LVPHTHARDS	SQGPCEPLPG	PLTQPRAHAS	PFSGALTPSA	PPGPEMNRSA	EVGPSSEPEV	QTLPYLPHYI	PGVDPETSHG
330	340	350	360	370	380	390	400
GHPHGNMPGL	DRPLSRQNPH	DGVVTPAASP	SLPQPGLQMP	GQWGPVQGGP	QPSGQHRSPC	PEGPVPSGVP	CATSVPHFPT
410	420	430	440	450	460	470	480
PSILHQGPGH	EQHSPLVAPP	AALPSDGRDE	VSHLQSGSHL	ANNSDPESTF	RQNPRIVNHW	ASPELRQNPG	VKNEHRPASA
490	500	510	520	530	540	550	560
LVNPLARGDS	PENRTHHPLG	AGAGSGCAPL	EADSGASGAL	AMFFQGGETE	NEENLSSEKA	GLSGQADFDD	FCSSPGLGRP
570	580	590	600	610	620	630	640
PAPTHVGAGS	LCQALLPGPS	NEAAGDVWGD	TASTGVPDAS	GSQYENVENL	EFVQNQEVLP	SEPLNLDPSS	PSDQFRYGPL
650	660	670	680	690	700	710	720
PGPAVPRHGA	VCHTGAPDAT	LHTVHPDSVS	SSYSSRSHGR	LSGSARPQEL	VGTFIQQEVG	KPEDEASGSF	FKQIDSSPVG
730	740	750	760	770	780	790	800
GETDETTVSQ	NYRGSVSQPS	TPSPPKPTGI	FQTSANSSFE	PVKSHLVGVK	PFEADRANVV	GEVRETCVRQ	KQCRPAAALP
810	820	830	840	850	860	870	880
DASPGNLEQP	PDNMETLCAP	QVCPLPLNST	TEAVHMLPHA	GAPPLDTVYP	APEKRPSART	QGPVKCESPA	TTLWAQSELP
890	900	910	920	930	940	950	960
DFGGNVLLAP	AAPALYVCAK	PQPPVVQPPE	EAMSGQQSRN	PSSAAPVQSR	GGIGASENLE	NPPKMGEEEA	LQSQVTKDAQ
970	980	990	1000	1010	1020	1030	1040
GQPGLERAQQ	ELVPPQQQAS	PPQLPKAMFS	ELSNPESLPA	QGQAQNSAQS	PASLVLVDAG	QQLPPRPPQS	SSVSLVSSGS
1050	1060	1070	1080	1090	1100	1110	1120
GQAAVPSEQP	WPQPVPALAP	GPPPQDLAAY	YYYRPLYDAY	QPQYSLPYPP	EPGAASLYYQ	DVYSLYEPRY	RPYDGAASAY
1130	1140	1150	1160	1170	1180	1190	1200
AQNYRYPEPE	RPSSRASHSS	ERPPPRQGYP	EGYYSSKSGW	SSQSDYYASY	YSSQYDYGDP	GHWDRYHYSA	RVRDPRTYDR
1210	1220	1230	1240	1250	1260	1270	1280
RYWCDAEYDA	YRREHSAFGD	RPEKRDNNWR	YDPRFTGSFD	DDPDPHRDPY	GEEVDRRSVH	SEHSARSLHS	AHSLASRRSS
1290	1300	1310	1320	1330	1340	1350	1360
LSSHSHQSQI	YRSHNVAAGS	YEAPLPPGSF	HGDFAYGTYR	SNFSSGPGFP	EYGYPADTVW	PAMEQVSSRP	TSPEKFSVPH
1370	1380	1390	1400	1410	1420	1430	1440
VCARFGPGGQ	LIKVIPNLPS	EGQPALVEVH	SMEALLQHTS	EQEEMRAFPG	PLAKDDTHKV	DVINFAQNKA	MKCLQNENLI
1450	1460	1470	1480	1490	1500	1510	1520
DKESASLLWN	FIVLLCRQNG	TVVGTDIAEL	LLRDHRTVWL	PGKSPNEANL	IDFTNEAVEQ	VEEEESGEAQ	LSFLTGGPAA
1530	1540	1550	1560	1570	1580	1590	1600
AASSLERETE	RFRELLLYGR	KKDALESAMK	NGLWGHALLL	ASKMDSRTHA	RVMTRFANSL	PINDPLQTVY	QLMSGRMPAA
1610	1620	1630	1640	1650	1660	1670	1680
STCCGDEKWG	DWRPHLAMVL	SNLNNNMDVE	SRTMATMGDT	LASRGLLDAA	HFCYLMAQAG	FGVYTKKTTK	LVLIGSNHSL
1690	1700	1710	1720	1730	1740	1750	1760
PFLKFATNEA	IQRTEAYEYA	QSLGAETCPL	PSFQVFKFIY	SCRLAEMGLA	TQAFHYCEAI	AKSILTQPHL	YSPVLISQLV
1770	1780	1790	1800	1810	1820	1830	1840
QMASQLRLFD	PQLKEKPEEE	SLAAPTWLVH	LQQVERQIKE	GAGVWHQDGA	LPQQCPGTPS	SEMEQLDRPG	LSQPGALGIA
1850	1860	1870	1880	1890	1900	1910	1920
NPLLAVPAPS	PEHSSPSVRL	LPSAPQTLPD	GPLASPARVP	MFPVPLPPGP	LEPGPGCVTP	GPALGFLEPS	GPGLPPGVPP
1930	1940	1950	1960	1970	1980	1990	2000
LQERRHLLQE	ARSPDPGIVP	QEAPVGNSLS	ELSEENFDGK	FANLTPSRTV	PDSEAPPGWD	RADSGPTQPP	LSLSPAPETK
2010	2020	2030	2040	2050	2060	2070	2080
RPGQAAKKET	KEPKKGESWF	FRWLPGKKKT	EAYLPDDKNK	SIVWDEKKNQ	WVNLNEPEEE	KKAPPPPPTS	MPKTVQAAPP
2090	2100	2110	2120	2130	2140	2150	2160
ALPGPPGAPV	NMYSRRAAGT	RARYVDVLNP	SGTQRSEPAL	APADFVAPLA	PLPIPSNLFV	PTPDAEEPQL	PDGTGREGPA
2170	2180	2190	2200	2210	2220	2230	2240

Detailed Protein Report

Cmpd.	No. of Cmpds.	m/z meas.	Δ m/z [ppm]	z	Rt [min]	Score	P	Range	Sequence	Modification	Ratios
118	1	635.6951	-152.31	2	30.4	10.0	0	1633-1644	R.TMATMGDTLASR.G	Oxidation: 2	
2722	2	607.3395	-24.29	2	62.5	12.3	2	2001-2011	K.RPGQAAKKETK.E		QU:MU 1.63<br>WUP:QUP 0.37
2457	1	647.2076	-173.21	2	61.2	14.3	1	2030-2040	K.TEAYLPDDKNK.S		

Detailed Protein Report

Protein 139: **PREDICTED: leucine-rich PPR motif-containing protein, mitochondrial isoform X2 [Homo sapiens]**

**Accession:**	gi|578802653	**Score:**	47.7
**Database:**	refseq_human(refseq_human_20140103.fasta)	**MW [kDa]:**	119.1
**Database Date:**	2015-11-30	**pI:**	5.9
		**Sequence Coverage [%]:**	3.2
		**No. of unique Peptides:**	2

Quantitation

QU:MU **Median:** 0.55 **CV:** 0.00 % **No. of Peptides:** 1

```
        10         20         30         40         50         60         70         80
MAALLRSARW LLRAGAAPRL PLSLRLLPGG PGRLHAASYL PAARAGPVAG GLLSPARLYA IAAKEKDIQE ESTFSSRKIS
        90        100        110        120        130        140        150        160
NQFDWALMRL DLSVRRTGRI PKKLLQKVFN DTCRSGGLGG SHALLLLRSC GSLLPELKLE ERTEFAHRIW DTLQKLGAVY
       170        180        190        200        210        220        230        240
DVSHYNALLK VYLQNEYKFS PTDFLAKMEE ANIQPNRVTY QRLIASYCNV GDIEGASKIL GFMKTKDLPV TEAVFSALVT
       250        260        270        280        290        300        310        320
GHARAGDMEN AENILTVMRD AGIEPGPDTY LALLNAYAEK GDIDHVKQTL EKVEKSELHL MDRDLLQIIF SFSKAGYPQY
       330        340        350        360        370        380        390        400
VSEILEKVTC ERRYIPDAMN LILLLVTEKL EDVALQILLA CPVSKEDGPS VFGSFFLQHC VTMNTPVEKL TDYCKKLKEV
       410        420        430        440        450        460        470        480
QMHSFPLQFT LHCALLANKT DLAKALMKAV KEEGFPIRPH YFWPLLVGRR KEKNVQGIIE ILKGMQELGV HPDQETYTDY
       490        500        510        520        530        540        550        560
VIPCFDSVNS ARAILQENGC LSDSDMFSQA GLRSEAANGN LDFVLSFLKS NTLPISLQSI RSSLLLGFRR SMNINLWSEI
       570        580        590        600        610        620        630        640
TELLYKDGRY CQEPRGPTEA VGYFLYNLID SMSDSEVQAK EEHLRQYFHQ LEKMNVKIPE NIYRGIRNLL ESYHVPELIK
       650        660        670        680        690        700        710        720
DAHLLVESKN LDFQKTVQLT SSELESTLET LKAENQPIRD VLKQLILVLC SEENMQKALE LKAKYESDMV TGGYAALINL
       730        740        750        760        770        780        790        800
CCRHDKVEDA LNLKEEFDRL DSSAVLDTGK YVGLVRVLAK HGKLQDAINI LKEMKEKDVL IKDTTALSFF HMLNGAALRG
       810        820        830        840        850        860        870        880
EIETVKQLHE AIVTLGLAEP STNISFPLVT VHLEKGDLST ALEVAIDCYE KYKVLPRIHD VLCKLVEKGE TDLIQKAMDF
       890        900        910        920        930        940        950        960
VSQEQGEMVM LYDLFFAFLQ TGNYKEAKKI IETPGIRARS ARLQWFCDRC VANNQVETLE KLVELTQKLF ECDRDQMYYN
       970        980        990       1000       1010       1020       1030       1040
LLKLYKINGD WQRADAVWNK IQEENVIPRE KTLRLLAEIL REGNQEVPFD VPELWYEDEK HSLNSSSAST TEPDFQKDIL
      1050       1060
IACRLNQKKR GI
```

Cmpd.	No. of Cmpds.	m/z meas.	Δ m/z [ppm]	z	Rt [min]	Score	P	Range	Sequence	Modification	Ratios
1368	1	428.2118	-70.40	3	47.2	12.4	1	673-683	K.AENQPIRDVLK.Q		
386	4	627.3328	-88.01	2	34.8	18.2	2	909-919	K.KIIETPGIRAR.S		QU:MU 0.55

Detailed Protein Report

Protein 140: cation-independent mannose-6-phosphate receptor precursor [Homo sapiens]

**Accession:**	gi|119964726	**Score:**	47.6
**Database:**	refseq_human(refseq_human_20140103.fasta)	**MW [kDa]:**	274.1
**Database Date:**	2015-11-30	**pI:**	5.5
**Modification(s):**	Carbamidomethyl	**Sequence Coverage [%]:**	2.6
		**No. of unique Peptides:**	2

Detailed Protein Report

10	20	30	40	50	60	70	80
MGAAAGRSPH	LGPAPARRPQ	RSLLLLQLLL	LVAAPGSTQA	QAAPFPELCS	YTWEAVDTKN	NVLYKINICG	SVDIVQCGPS
90	100	110	120	130	140	150	160
SAVCMHDLKT	RTYHSVGDSV	LRSATRSLLE	FNTTVSCDQQ	GTNHRVQSSI	AFLCGKTLGT	PEFVTATECV	HYFEWRTTAA
170	180	190	200	210	220	230	240
CKKDIFKANK	EVPCYVFDEE	LRKHDLNPLI	KLSGAYLVDD	SDPDTSLFIN	VCRDIDTLRD	PGSQLRACPP	GTAACLVRGH
250	260	270	280	290	300	310	320
QAFDVGQPRD	GLKLVRKDRL	VLSYVREEAG	KLDFCDGHSP	AVTITFVCPS	ERREGTIPKL	TAKSNCRYEI	EWITEYACHR
330	340	350	360	370	380	390	400
DYLESKTCSL	SGEQQDVSID	LTPLAQSGGS	SYISDGKEYL	FYLNVCGETE	IQFCNKKQAA	VCQVKKSDTS	QVKAAGRYHN
410	420	430	440	450	460	470	480
QTLRYSDGDL	TLIYFGGDEC	SSGFQRMSVI	NFECNKTAGN	DGKGTPVFTG	EVDCTYFFTW	DTEYACVKEK	EDLLCGATDG
490	500	510	520	530	540	550	560
KKRYDLSALV	RHAEPEQNWE	AVDGSQTETE	KKHFFINICH	RVLQEGKARG	CPEDAAVCAV	DKNGSKNLGK	FISSPMKEKG
570	580	590	600	610	620	630	640
NIQLSYSDGD	DCGHGKKIKT	NITLVCKPGD	LESAPVLRTS	GEGGCFYEFE	WHTAAACVLS	KTEGENCTVF	DSQAGFSFDL
650	660	670	680	690	700	710	720
SPLTKKNGAY	KVETKKYDFY	INVCGPVSVS	PCQPDSGACQ	VAKSDEKTWN	LGLSNAKLSY	YDGMIQLNYR	GGTPYNNERH
730	740	750	760	770	780	790	800
TPRATLITFL	CDRDAGVGFP	EYQEEDNSTY	NFRWYTSYAC	PEEPLECVVT	DPSTLEQYDL	SSLAKSEGGL	GGNWYAMDNS
810	820	830	840	850	860	870	880
GEHVTWRKYY	INVCRPLNPV	PGCNRYASAC	QMKYEKDQGS	FTEVVSISNL	GMAKTGPVVE	DSGSLLLEYV	NGSACTTSDG
890	900	910	920	930	940	950	960
RQTTYTTRIH	LVCSRGRLNS	HPIFSLNWEC	VVSFLWNTEA	ACPIQTTTDT	DQACSIRDPN	SGFVFNLNPL	NSSQGYNVSG
970	980	990	1000	1010	1020	1030	1040
IGKIFMFNVC	GTMPVCGTIL	GKPASGCEAE	TQTEELKNWK	PARPVGIEKS	LQLSTEGFIT	LTYKGPLSAK	GTADAFIVRF
1050	1060	1070	1080	1090	1100	1110	1120
VCNDDVYSGP	LKFLHQDIDS	GQGIRNTYFE	FETALACVPS	PVDCQVTDLA	GNEYDLTGLS	TVRKPWTAVD	TSVDGRKRTF
1130	1140	1150	1160	1170	1180	1190	1200
YLSVCNPLPY	IPGCQGSAVG	SCLVSEGNSW	NLGVVQMSPQ	AAANGSLSIM	YVNGDKCGNQ	RFSTRITFEC	AQISGSPAFQ
1210	1220	1230	1240	1250	1260	1270	1280
LQDGCEYVFI	WRTVEACPVV	RVEGDNCEVK	DPRHGNLYDL	KPLGLNDTIV	SAGEYTYYFR	VCGKLSSDVC	PTSDKSKVVS
1290	1300	1310	1320	1330	1340	1350	1360
SCQEKREPQG	FHKVAGLLTQ	KLTYENGLLK	MNFTGGDTCH	KVYQRSTAIF	FYCDRGTQRP	VFLKETSDCS	YLFEWRTQYA
1370	1380	1390	1400	1410	1420	1430	1440
CPPFDLTECS	FKDGAGNSFD	LSSLSRYSDN	WEAITGTGDP	EHYLINVCKS	LAPQAGTEPC	PPEAAACLLG	GSKPVNLGRV
1450	1460	1470	1480	1490	1500	1510	1520
RDGPQWRDGI	IVLKYVDGDL	CPDGIRKKST	TIRFTCSESQ	VNSRPMFISA	VEDCEYTFAW	PTATACPMKS	NEHDDCQVTN
1530	1540	1550	1560	1570	1580	1590	1600
PSTGHLFDLS	SLSGRAGFTA	AYSEKGLVYM	SICGENENCP	PGVGACFGQT	RISVGKANKR	LRYVDQVLQL	VYKDGSPCPS
1610	1620	1630	1640	1650	1660	1670	1680
KSGLSYKSVI	SFVCRPEAGP	TNRPMLISLD	KQTCTLFFSW	HTPLACEQAT	ECSVRNGSSI	VDLSPLIHRT	GGYEAYDESE
1690	1700	1710	1720	1730	1740	1750	1760
DDASDTNPDF	YINICQPLNP	MHGVPCPAGA	AVCKVPIDGP	PIDIGRVAGP	PILNPIANEI	YLNFESSTPC	LADKHFNYTS
1770	1780	1790	1800	1810	1820	1830	1840
LIAFHCKRGV	SMGTPKLLRT	SECDFVFEWE	TPVVCPDEVR	MDGCTLTDEQ	LLYSFNLSSL	STSTFKVTRD	SRTYSVGVCT
1850	1860	1870	1880	1890	1900	1910	1920
FAVGPEQGGC	KDGGVCLLSG	TKGASFGRLQ	SMKLDYRHQD	EAVVLSYVNG	DRCPPETDDG	VPCVFPFIFN	GKSYEECIIE
1930	1940	1950	1960	1970	1980	1990	2000
SRAKLWCSTT	ADYDRDHEWG	FCRHSNSYRT	SSIIFKCDED	EDIGRPQVFS	EVRGCDVTFE	WKTKVVCPPK	KLECKFVQKH
2010	2020	2030	2040	2050	2060	2070	2080
KTYDLRLLSS	LTGSWSLVHN	GVSYYINLCQ	KIYKGPLGCS	ERASICRRTT	TGDVQVLGLV	HTQKLGVIGD	KVVVTYSKGY
2090	2100	2110	2120	2130	2140	2150	2160
PCGGNKTASS	VIELTCTKTV	GRPAFKRFDI	DSCTYYFSWD	SRAACAVKPQ	EVQMVNGTIT	NPINGKSFSL	GDIYFKLFRA
2170	2180	2190	2200	2210	2220	2230	2240

Detailed Protein Report

Cmpd.	No. of Cmpds.	m/z meas.	Δ m/z [ppm]	z	Rt [min]	Score	P	Range	Sequence	Modification
1181	1	955.7839	6.62	3	44.9	12.0	1	267-292	R.EEAGKLDFCDGHSPAVTITFVCPSER.R	Carbamidomethyl: 22
2855	2	731.8691	-0.65	2	66.1	13.0	2	471-483	K.EDLLCGATDGKKR.Y	Carbamidomethyl: 5

Detailed Protein Report

Protein 141: **staphylococcal nuclease domain-containing protein 1 [Homo sapiens]**

**Accession:**	gi|77404397	**Score:**	47.5
**Database:**	refseq_human(refseq_human_20140103.fasta)	**MW [kDa]:**	101.9
**Database Date:**	2015-11-30	**pI:**	6.8
**Modification(s):**	Carbamidomethyl, Oxidation	**Sequence Coverage [%]:**	5.9
		**No. of unique Peptides:**	3

10	20	30	40	50	60	70	80
MASSAQSGGS	SGGPAVPTVQ	RGIIKMVLSG	CAIIVRGQPR	GGPPPERQIN	LSNIRAGNLA	RRAAATQPDA	KDTPDEPWAF
**90**	**100**	**110**	**120**	**130**	**140**	**150**	**160**
PAREFLRKKL	IGKEVCFTIE	NKTPQGREYG	MIYLGKDTNG	ENIAESLVAE	GLATRREGMR	ANNPEQNRLS	ECEEQAKAAK
**170**	**180**	**190**	**200**	**210**	**220**	**230**	**240**
KGMWSEGNGS	HTIRDLKYTI	ENPRHFVDSH	HQKPVNAIIE	HVRDGSVVRA	LLLPDYYLVT	VMLSGIKCPT	FRREADGSET
**250**	**260**	**270**	**280**	**290**	**300**	**310**	**320**
PEPFAAEAKF	FTESRLLQRD	VQIILESCHN	QNILGTILHP	NGNITELLLK	EGFARCVDWS	IAVYTRGAEK	LRAAERFAKE
**330**	**340**	**350**	**360**	**370**	**380**	**390**	**400**
RRLRIWRDYV	APTANLDQKD	KQFVAKVMQV	LNADAIVVKL	NSGDYKTIHL	SSIRPPRLEG	ENTQDKNKKL	RPLYDIPYMF
**410**	**420**	**430**	**440**	**450**	**460**	**470**	**480**
EAREFLRKKL	IGKKVNVTVD	YIRPASPATE	TVPAFSERTC	ATVTIGGINI	AEALVSKGLA	TVIRYRQDDD	QRSSHYDELL
**490**	**500**	**510**	**520**	**530**	**540**	**550**	**560**
AAEARAIKNG	KGLHSKKEVP	IHRVADISGD	TQKAKQFLPF	LQRAGRSEAV	VEYVFSGSRL	KLYLPKETCL	ITFLLAGIEC
**570**	**580**	**590**	**600**	**610**	**620**	**630**	**640**
PRGARNLPGL	VQEGEPFSEE	ATLFTKELVL	QREVEVEVES	MDKAGNFIGW	LHIDGANLSV	LLVEHALSKV	HFTAERSSYY
**650**	**660**	**670**	**680**	**690**	**700**	**710**	**720**
KSLLSAEEAA	KQKKEKVWAH	YEEQPVEEVM	PVLEEKERSA	SYKPVFVTEI	TDDLHFYVQD	VETGTQLEKL	MENMRNDIAS
**730**	**740**	**750**	**760**	**770**	**780**	**790**	**800**
HPPVEGSYAP	RRGEFCIAKF	VDGEWYRARV	EKVESPAKIH	VFYIDYGNRE	VLPSTRLGTL	SPAFSTRVLP	AQATEYAFAF
**810**	**820**	**830**	**840**	**850**	**860**	**870**	**880**
IQVPQDDDAR	TDAVDSVVRD	IQNTQCLLNV	EHLSAGCPHV	TLQFADSKGD	VGLGLVKEGL	VMVEVRKEKQ	FQKVITEYLN
**890**	**900**	**910**	**920**				
AQESAKSARL	NLWRYGDFRA	DDADEFGYSR					

Cmpd.	No. of Cmpds.	m/z meas.	Δ m/z [ppm]	z	Rt [min]	Score	P	Range	Sequence	Modification
2148	1	966.3470	-130.61	2	56.7	12.8	2	161-177	K.KGMWSEGNGSHTIRDLK.Y	Oxidation: 3
2741	1	656.8579	55.61	2	64.6	11.5	0	296-306	R.CVDWSIAVYTR.G	
297	2	626.2973	4.64	3	33.4	12.1	1	733-747	R.GEFCIAKFVDGEWYR.A	Carbamidomethyl: 4

Detailed Protein Report

Protein 142: PREDICTED: methylcytosine dioxygenase TET3 isoform X1 [Homo sapiens]

**Accession:**	gi|530367326	**Score:**	47.4
**Database:**	refseq_human(refseq_human_20140103.fasta)	**MW [kDa]:**	193.6
**Database Date:**	2015-11-30	**pI:**	9.0
		**Sequence Coverage [%]:**	1.8
		**No. of unique Peptides:**	2

Alias proteins:

Accession	Name	Description
gi|566559863	refseq_human (refseq_human_20140103.fasta)	methylcytosine dioxygenase TET3 [Homo sapiens]

Detailed Protein Report

10	20	30	40	50	60	70	80
MSQFQVPLAV	QPDLPGLYDF	PQRQVMVGSF	PGSGLSMAGS	ESQLRGGGDG	RKKRKRCGTC	EPCRRLENCG	ACTSCTNRRT
90	100	110	120	130	140	150	160
HQICKLRKCE	VLKKKVGLLK	EVEIKAGEGA	GPWGQGAAVK	TGSELSPVDG	PVPGQMDSGP	VYHGDSRQLS	ASGVPVNGAR
170	180	190	200	210	220	230	240
EPAGPSLLGT	GGPWRVDQKP	DWEAAPGPAH	TARLEDAHDL	VAFSAVAEAV	SSYGALSTRL	YETFNREMSR	EAGNNSRGPR
250	260	270	280	290	300	310	320
PGPEGCSAGS	EDLDTLQTAL	ALARHGMKPP	NCNCDGPECP	DYLEWLEGKI	KSVVMEGGEE	RPRLPGPLPP	GEAGLPAPST
330	340	350	360	370	380	390	400
RPLLSSEVPQ	ISPQEGLPLS	QSALSIAKEK	NISLQTAIAI	EALTQLSSAL	PQPSHSTPQA	SCPLPEALSP	PAPFRSPQSY
410	420	430	440	450	460	470	480
LRAPSWPVVP	PEEHSSFAPD	SSAFPPATPR	TEFPEAWGTD	TPPATPRSSW	PMPRPSPDPM	AELEQLLGSA	SDYIQSVFKR
490	500	510	520	530	540	550	560
PEALPTKPKV	KVEAPSSSPA	PAPSPVLQRE	APTPSSEPDT	HQKAQTALQQ	HLHHKRSLFL	EQVHDTSFPA	PSEPSAPGWW
570	580	590	600	610	620	630	640
PPPSSPVPRL	PDRPPKEKKK	KLPTPAGGPV	GTEKAAPGIK	PSVRKPIQIK	KSRPREAQPL	FPPVRQIVLE	GLRSPASQEV
650	660	670	680	690	700	710	720
QAHPPAPLPA	SQGSAVPLPP	EPSLALFAPS	PSRDSLLPPT	QEMRSPSPMT	ALQPGSTGPL	PPADDKLEEL	IRQFEAEFGD
730	740	750	760	770	780	790	800
SFGLPGPPSV	PIQDPENQQT	CLPAPESPFA	TRSPKQIKIE	SSGAVTVLST	TCFHSEEGGQ	EATPTKAENP	LTPTLSGFLE
810	820	830	840	850	860	870	880
SPLKYLDTPT	KSLLDTPAKR	AQAEFPTCDC	VEQIVEKDEG	PYYTHLGSGP	TVASIRELME	ERYGEKGKAI	RIEKVIYTGK
890	900	910	920	930	940	950	960
EGKSSRGCPI	AKWVIRRHTL	EEKLLCLVRH	RAGHHCQNAV	IVILILAWEG	IPRSLGDTLY	QELTDTLRKY	GNPTSRRCGL
970	980	990	1000	1010	1020	1030	1040
NDDRTCACQG	KDPNTCGASF	SFGCSWSMYF	NGCKYARSKT	PRKFRLAGDN	PKEEEVLRKS	FQDLATEVAP	LYKRLAPQAY
1050	1060	1070	1080	1090	1100	1110	1120
QNQVTNEEIA	IDCRLGLKEG	RPFAGVTACM	DFCAHAHKDQ	HNLYNGCTVV	CTLTKEDNRC	VGKIPEDEQL	HVLPLYKMAN
1130	1140	1150	1160	1170	1180	1190	1200
TDEFGSEENQ	NAKVGSGAIQ	VLTAFPREVR	RLPEPAKSCR	QRQLEARKAA	AEKKKIQKEK	LSTPEKIKQE	ALELAGITSD
1210	1220	1230	1240	1250	1260	1270	1280
PGLSLKGGLS	QQGLKPSLKV	EPQNHFSSFK	YSGNAVVESY	SVLGNCRPSD	PYSMNSVYSY	HSYYAQPSLT	SVNGFHSKYA
1290	1300	1310	1320	1330	1340	1350	1360
LPSFSYYGFP	SSNPVFPSQF	LGPGAWGHSG	SSGSFEKKPD	LHALHNSLSP	AYGGAEFAEL	PSQAVPTDAH	HPTPHHQQPA
1370	1380	1390	1400	1410	1420	1430	1440
YPGPKEYLLP	KAPLLHSVSR	DPSPFAQSSN	CYNRSIKQEP	VDPLTQAEPV	PRDAGKMGKT	PLSEVSQNGG	PSHLWGQYSG
1450	1460	1470	1480	1490	1500	1510	1520
GPSMSPKRTN	GVGGSWGVFS	SGESPAIVPD	KLSSFGASCL	APSHFTDGQW	GLFPGEGQQA	ASHSGGRLRG	KPWSPCKFGN
1530	1540	1550	1560	1570	1580	1590	1600
STSALAGPSL	TEKPWALGAG	DFNSALKGSP	GFQDKLWNPM	KGEEGRIPAA	GASQLDRAWQ	SFGLPLGSSE	KLFGALKSEE
1610	1620	1630	1640	1650	1660	1670	1680
KLWDPFSLEE	GPAEEPPSKG	AVKEEKGGGG	AEEEEEELWS	DSEHNFLDEN	IGGVAVAPAH	GSILIECARR	ELHATTPLKK
1690	1700	1710	1720	1730	1740	1750	1760
PNRCHPTRIS	LVFYQHKNLN	QPNHGLALWE	AKMKQLAERA	RARQEEAARL	GLGQQEAKLY	GKKRKWGGTV	VAEPQQKEKK
1770	1780	1790	1800				
GVVPTRQALA	VPTDSAVTVS	SYAYTKVTGP	YSRWI				

Cmpd.	No. of Cmpds.	m/z meas.	Δ m/z [ppm]	z	Rt [min]	Score	P	Range	Sequence	Modification
806	1	546.9707	-505.05	1	39.6	11.3	0	1562-1566	K.GEEGR.I	
1833	2	697.0345	-7.09	3	51.3	12.9	2	1724-1742	R.QEEAARLGLGQQEAKLYGK.K	

Detailed Protein Report

Protein 143: **PREDICTED: microtubule-actin cross-linking factor 1 isoform X29 [Homo sapiens]**

**Accession:**	gi|578798838	**Score:**	47.4
**Database:**	refseq_human(refseq_human_20140103.fasta)	**MW [kDa]:**	608.2
**Database Date:**	2015-11-30	**pI:**	5.2
**Modification(s):**	Carbamidomethyl, Oxidation	**Sequence Coverage [%]:**	0.8
		**No. of unique Peptides:**	3

Detailed Protein Report

10	20	30	40	50	60	70	80
MSSSDEETLS	ERSCRSERSC	RSERSYRSER	SGSLSPCPPG	DTLPWNLPLH	EQKKRKSQDS	VLDPAERAVV	RVADERDRVQ
90	100	110	120	130	140	150	160
KKTFTKWVNK	HLMKVRKHIN	DLYEDLRDGH	NLISLLEVLS	GIKLPREKGR	MRFHRLQNVQ	IALDFLKQRQ	VKLVNIRNDD
170	180	190	200	210	220	230	240
ITDGNPKLTL	GLIWTIILHF	QISDIYISGE	SGDMSAKEKL	LLWTQKVTAG	YTGIKCTNFS	SCWSDGKMFN	ALIHRYRPDL
250	260	270	280	290	300	310	320
VDMERVQIQS	NRENLEQAFE	VAERLGVTRL	LDAEDVDVPS	PDEKSVITYV	SSIYDAFPKV	PEGGEGISAT	EVDSRWQEYQ
330	340	350	360	370	380	390	400
SRVDSLIPWI	KQHTILMSDK	TFPQNPVELK	ALYNQYIHFK	ETEILAKERE	KGRIEELYKL	LEVWIEFGRI	KLPQGYHPND
410	420	430	440	450	460	470	480
VEEEWGKLII	EMLEREKSLR	PAVERLELLL	QIANKIQNGA	LNCEEKLTLA	KNTLQADAAH	LESGQPVQCE	SDVIMYIQEC
490	500	510	520	530	540	550	560
EGLIRQLQVD	LQILRDENYY	QLEELAFRVM	RLQDELVTLR	LECTNLYRKG	HFTSLELVPP	STLTTTHLKA	EPLTKATHSS
570	580	590	600	610	620	630	640
STSWFRKPMT	RAELVAISSS	EDEGNLRFVY	ELLSWVEEMQ	MKLERAEWGN	DLPSVELQLE	TQQHIHTSVE	ELGSSVKEAR
650	660	670	680	690	700	710	720
LYEGKMSQNF	HTSYAETLGK	LETQYCKLKE	TSSFRMRHLQ	SLHKFVSRAT	AELIWLNEKE	EEELAYDWSD	NNSNISAKRN
730	740	750	760	770	780	790	800
YFSELTMELE	EKQDVFRSLQ	DTAELLSLEN	HPAKQTVEAY	SAAVQSQLQW	MKQLCLCVEQ	HVKENTAYFQ	FFSDARELES
810	820	830	840	850	860	870	880
FLRNLQDSIK	RKYSCDHNTS	LSRLEDLLQD	SMDEKEQLIQ	SKSSVASLVG	RSKTIVQLKP	RSPDHVLKNT	ISVKAVCDYR
890	900	910	920	930	940	950	960
QIEITICKND	ECVLEDNSQR	TKWKVISPTG	NEAMVPSVCF	LIPPPNKDAI	EMASRVEQSY	QKVMALWHQL	HVNTKSLISW
970	980	990	1000	1010	1020	1030	1040
NYLRKDLDLV	QTWNLEKLRS	SAPGECHQIM	KNLQAHYEDF	LQDSRDSVLF	SVADRLRLEE	EVEACKARFQ	HLMKSMENED
1050	1060	1070	1080	1090	1100	1110	1120
KEETVAKMYI	SELKNIRLRL	EEYEQRVVKR	IQSLASSRTD	RDAWQDNALR	IAEQEHTQED	LQQLRSDLDA	VSMKCDSFLH
1130	1140	1150	1160	1170	1180	1190	1200
QSPSSSSVPT	LRSELNLLVE	KMDHVYGLST	VYLNKLKTVD	VIVRSIQDAE	LLVKGYEIKL	SQEEVVLADL	SALEAHWSTL
1210	1220	1230	1240	1250	1260	1270	1280
RHWLSDVKDK	NSVFSVLDEE	IAKAKVVAEQ	MSRLTPERNL	DLERYQEKGS	QLQERWHRVI	AQLEIRQSEL	ESIQEVLGDY
1290	1300	1310	1320	1330	1340	1350	1360
RACHGTLIKW	IEETTAQQEM	MKPGQAEDSR	VLSEQLSQQT	ALFAEIERNQ	TKLDQCQKFS	QQYSTIVKDY	ELQLMTYKAF
1370	1380	1390	1400	1410	1420	1430	1440
VESQQKSPGK	RRRMLSSSDA	ITQEFMDLRT	RYTALVTLTT	QHVKYISDAL	RRLEEEEKVV	EEEKQEHVEK	VKELLGWVST
1450	1460	1470	1480	1490	1500	1510	1520
LARNTQGKAT	SSETKESTDI	EKAILEQQVL	SEELTTKKEQ	VSEAIKTSQI	FLAKHGHKLS	EKEKKQISEQ	LNALNKAYHD
1530	1540	1550	1560	1570	1580	1590	1600
LCDGSANQLQ	QLQSQLAHQT	EQKTLQKQQN	TCHQQLEDLC	SWVGQAERAL	AGHQGRTTQQ	DLSALQKNQS	DLKDLQDDIQ
1610	1620	1630	1640	1650	1660	1670	1680
NRATSFATVV	KDIEGFMEEN	QTKLSPRELT	ALREKLHQAK	EQYEALQEET	RVAQKELEEA	VTSALQQETE	KSKAAKELAE
1690	1700	1710	1720	1730	1740	1750	1760
NKKKIDALLD	WVTSVGSSGG	QLLTNLPGME	QLSGASLEKG	ALDTTDGYMG	VNQAPEKLDK	QCEMMKARHQ	ELLSQQQNFI
1770	1780	1790	1800	1810	1820	1830	1840
LATQSAQAFL	DQHGHNLTPE	EQQMLQQKLG	ELKEQYSTSL	AQSEAELKQV	QTLQDELQKF	LQDHKEFESW	LERSEKELEN
1850	1860	1870	1880	1890	1900	1910	1920
MHKGGSSPET	LPSLLKRQGS	FSEDVISHKG	DLRFVTISGQ	KVLDMENSFK	EGKEPSEIGN	LVKDKLKDAT	ERYTALHSKC
1930	1940	1950	1960	1970	1980	1990	2000
TRLGSHLNML	LGQYHQFQNS	ADSLQAWMQA	CEANVEKLLS	DTVASDPGVL	QEQLATTKQL	QEELAEHQVP	VEKLQKVARD
2010	2020	2030	2040	2050	2060	2070	2080
IMEIEGEPAP	DHRHVQETTD	SILSHFQSLS	YSLAERSSLL	QKAIAQSQSV	QESLESLLQS	IGEVEQNLEG	KQVSSLSSGV
2090	2100	2110	2120	2130	2140	2150	2160
IQEALATNMK	LKQDIARQKS	SLEATREMVT	RFMETADSTT	AAVLQGKLAE	VSQRFEQLCL	QQQEKESSLK	KLLPQAEMFE
2170	2180	2190	2200	2210	2220	2230	2240

Detailed Protein Report

Cmpd.	No. of Cmpds.	m/z meas.	Δ m/z [ppm]	z	Rt [min]	Score	P	Range	Sequence	Modification
91	2	1240.0402	-66.65	2	31.1	23.4	2	1882-1903	K.VLDMENSFKEGKEPSEIGNLVK.D	Oxidation: 4
371	1	625.3885	104.40	2	34.3	11.3	2	2836-2845	R.LKDCMQKAQK.Y	Carbamidomethyl: 4
11	1	650.7452	-159.65	2	29.7	12.6	2	4482-4491	K.RAKQFHEAWK.K	

Detailed Protein Report

Protein 144: cadherin EGF LAG seven-pass G-type receptor 3 precursor [Homo sapiens]

**Accession:**	gi|145309304	**Score:**	47.3
**Database:**	refseq_human(refseq_human_20140103.fasta)	**MW [kDa]:**	358.0
**Database Date:**	2015-11-30	**pI:**	6.2
		**Sequence Coverage [%]:**	1.1
		**No. of unique Peptides:**	3

Quantitation

QU:MU	**Median:**	1.24	**CV:**	52.77 %	**No. of Peptides:**	2
WUP:QUP	**Median:**	0.72	**CV:**	0.00 %	**No. of Peptides:**	1

Detailed Protein Report

10	20	30	40	50	60	70	80
MMARRPPWRG	LGGRSTPILL	LLLLSLFPLS	QEELGGGGHQ	GWDPGLAATT	GPRAHIGGGA	LALCPESSGV	REDGGPGLGV
90	100	110	120	130	140	150	160
REPIFVGLRG	RRQSARNSRG	PPEQPNEELG	IEHGVQPLGS	RERETGQGPG	SVLYWRPEVS	SCGRTGPLQR	GSLSPGALSS
170	180	190	200	210	220	230	240
GVPGSGNSSP	LPSDFLIRHH	GPKPVSSQRN	AGTGSRKRVG	TARCCGELWA	TGSKGQGERA	TTSGAERTAP	RRNCLPGASG
250	260	270	280	290	300	310	320
SGPELDSAPR	TARTAPASGS	APRESRTAPE	PAPKRMRSRG	LFRCRFLPQR	PGPRPPGLPA	RPEARKVTSA	NRARFRRAAN
330	340	350	360	370	380	390	400
RHPQFPQYNY	QTLVPENEAA	GTAVLRVVAQ	DPDAGEAGRL	VYSLAALMNS	RSLELFSIDP	QSGLIRTAAA	LDRESMERHY
410	420	430	440	450	460	470	480
LRVTAQDHGS	PRLSATTMVA	VTVADRNDHS	PVFEQAQYRE	TLRENVEEGY	PILQLRATDG	DAPPNANLRY	RFVGPPAARA
490	500	510	520	530	540	550	560
AAAAAFEIDP	RSGLISTSGR	VDREHMESYE	LVVEASDQGQ	EPGPRSATVR	VHITVLDEND	NAPQFSEKRY	VAQVREDVRP
570	580	590	600	610	620	630	640
HTVVLRVTAT	DRDKDANGLV	HYNIISGNSR	GHFAIDSLTG	EIQVVAPLDF	EAEREYALRI	RAQDAGRPPL	SNNTGLASIQ
650	660	670	680	690	700	710	720
VVDINDHIPI	FVSTPFQVSV	LENAPLGHSV	IHIQAVDADH	GENARLEYSL	TGVAPDTPFV	INSATGWVSV	SGPLDRESVE
730	740	750	760	770	780	790	800
HYFFGVEARD	HGSPPLSASA	SVTVTVLDVN	DNRPEFTMKE	YHLRLNEDAA	VGTSVVSVTA	VDRDANSAIS	YQITGGNTRN
810	820	830	840	850	860	870	880
RFAISTQGGV	GLVTLALPLD	YKQERYFKLV	LTASDRALHD	HCYVHINITD	ANTHRPVFQS	AHYSVSVNED	RPMGSTIVVI
890	900	910	920	930	940	950	960
SASDDDVGEN	ARITYLLEDN	LPQFRIDADS	GAITLQAPLD	YEDQVTYTLA	ITARDNGIPQ	KADTTYVEVM	VNDVNDNAPQ
970	980	990	1000	1010	1020	1030	1040
FVASHYTGLV	SEDAPPFTSV	LQISATDRDA	HANGRVQYTF	QNGEDGDGDF	TIEPTSGIVR	TVRRLDREAV	SVYELTAYAV
1050	1060	1070	1080	1090	1100	1110	1120
DRGVPPLRTP	VSIQVMVQDV	NDNAPVFPAE	EFEVRVKENS	IVGSVVAQIT	AVDPDEGPNA	HIMYQIVEGN	IPELFQMDIF
1130	1140	1150	1160	1170	1180	1190	1200
SGELTALIDL	DYEARQEYVI	VVQATSAPLV	SRATVHVRLV	DQNDNSPVLN	NFQILFNNYV	SNRSDTFPSG	IIGRIPAYDP
1210	1220	1230	1240	1250	1260	1270	1280
DVSDHLFYSF	ERGNELQLLV	VNQTSGELRL	SRKLDNNRPL	VASMLVTVTD	GLHSVTAQCV	LRVVIITEEL	LANSLTVRLE
1290	1300	1310	1320	1330	1340	1350	1360
NMWQERFLSP	LLGRFLEGVA	AVLATPAEDV	FIFNIQNDTD	VGGTVLNVSF	SALAPRGAGA	GAAGPWFSSE	ELQEQLYVRR
1370	1380	1390	1400	1410	1420	1430	1440
AALAARSLLD	VLPFDDNVCL	REPCENYMKC	VSVLRFDSSA	PFLASASTLF	RPIQPIAGLR	CRCPPGFTGD	FCETELDLCY
1450	1460	1470	1480	1490	1500	1510	1520
SNPCRNGGAC	ARREGGYTCV	CRPRFTGEDC	ELDTEAGRCV	PGVCRNGGTC	TDAPNGGFRC	QCPAGGAFEG	PRCEVAARSF
1530	1540	1550	1560	1570	1580	1590	1600
PPSSFVMFRG	LRQRFHLTLS	LSFATVQQSG	LLFYNGRLNE	KHDFLALELV	AGQVRLTYST	GESNTVVSPT	VPGGLSDGQW
1610	1620	1630	1640	1650	1660	1670	1680
HTVHLRYYNK	PRTDALGGAQ	GPSKDKVAVL	SVDDCDVAVA	LQFGAEIGNY	SCAAAGVQTS	SKKSLDLTGP	LLLGGVPNLP
1690	1700	1710	1720	1730	1740	1750	1760
ENFPVSHKDF	IGCMRDLHID	GRRVDMAAFV	ANNGTMAGCQ	AKLHFCDSGP	CKNSGFCSER	WGSFSCDCPV	GFGGKDCQLT
1770	1780	1790	1800	1810	1820	1830	1840
MAHPHHFRGN	GTLSWNFGSD	MAVSVPWYLG	LAFRTRATQG	VLMQVQAGPH	STLLCQLDRG	LLSVTVTRGS	GRASHLLLDQ
1850	1860	1870	1880	1890	1900	1910	1920
VTVSDGRWHD	LRLELQEEPG	GRRGHHVLMV	SLDFSLFQDT	MAVGSELQGL	KVKQLHVGGL	PPGSAEEAPQ	GLVGCIQGVW
1930	1940	1950	1960	1970	1980	1990	2000
LGSTPSGSPA	LLPPSHRVNA	EPGCVVTNAC	ASGPCPPHAD	CRDLWQTFSC	TCQPGYYGPG	CVDACLLNPC	QNQGSCRHLP
2010	2020	2030	2040	2050	2060	2070	2080
GAPHGYTCDC	VGGYFGHHCE	HRMDQQCPRG	WWGSPTCGPC	NCDVHKGFDP	NCNKTNGQCH	CKEFHYRPRG	SDSCLPCDCY
2090	2100	2110	2120	2130	2140	2150	2160
PVGSTSRSCA	PHSGQCPCRP	GALGRQCNSC	DSPFAEVTAS	GCRVLYDACP	KSLRSGVWWP	QTKFGVLATV	PCPRGALGAA
2170	2180	2190	2200	2210	2220	2230	2240

Detailed Protein Report

Cmpd.	No. of Cmpds.	m/z meas.	Δ m/z [ppm]	z	Rt [min]	Score	P	Range	Sequence	Modification	Ratios	
1349	4	942.4987	46.29	2	46.5	16.8	1	232-250	R.RNCLPGASGSGPELDSAPR.T		QU:MU	0.75
1065	3	470.5947	-263.79	2	43.4	12.8	0	2331-2338	R.MEHPSSPR.G			
587	2	459.6056	-275.62	2	35.7	17.8	1	2636-2643	R.NVDRGAMR.F		WUP:QUP<br>QU:MU	0.72<br>2.03

Detailed Protein Report

Protein 145: PREDICTED: centlein isoform X2 [Homo sapiens]

**Accession:**	gi|578816553	**Score:**	46.9
**Database:**	refseq_human(refseq_human_20140103.fasta)	**MW [kDa]:**	155.6
**Database Date:**	2015-11-30	**pI:**	9.1
		**Sequence Coverage [%]:**	2.8
		**No. of unique Peptides:**	2

Quantitation

QU:MU **Median:** 0.85 **CV:** 0.00 % **No. of Peptides:** 1

10	20	30	40	50	60	70	80
MAARSPPSPH	PSPPARQLGP	RSPRVGRGAE	VHAMRSEASG	FAGAAREVVA	DESDKIWADK	EFVWSLWKRL	QVTNPDLTQV
90	100	110	120	130	140	150	160
VSLVVEREKQ	KSEAKDRKVL	EILQVKDAKI	QEFEQRESVL	KQEINDLVKR	KIAVDEENAF	LRKEFSDLEK	KFKDKSQEIK
170	180	190	200	210	220	230	240
DTKECVQNKE	EQNRLVIKNL	EEENKKLSTR	CTDLLNDLEK	LRKQEAHLRK	EKYSTDAKIK	TFEDNLIEAR	KEVEVSQSKY
250	260	270	280	290	300	310	320
NALSLQLSNK	QTELIQKDMD	ITLVRKELQE	LQNLYKQNST	HTAQQAELIQ	QLQVLNMDTQ	KVLRNQEDVH	TAESISYQKL
330	340	350	360	370	380	390	400
YNELHICFET	TKSNEAMLRQ	SVTNLQDQLL	QKEQENAKLK	EKLQESQGAP	LPLPQESDPD	YSAQVPHRPS	LSSLETLMVS
410	420	430	440	450	460	470	480
QKSEIEYLQE	KLKIANEKLS	ENISANKGFS	RKSIMTSAEG	KHKEPPVKRS	RSLSPKSSFT	DSEELQKLRK	AERKIENLEK
490	500	510	520	530	540	550	560
ALQLKSQEND	ELRDAHEKRK	ERLQMLQTNY	RAVKEQLKQW	EEGSGMTEIR	KIKRADPQQL	RQEDSDAVWN	ELAYFKRENQ
570	580	590	600	610	620	630	640
ELMIQKMNLE	EELDELKVHI	SIDKAAIQEL	NRCVAERREE	QLFRSGEDDE	VKRSTPEKNG	KEMLEQTLQK	VTELENRLKS
650	660	670	680	690	700	710	720
FEKRSRKLKE	GNKKLMKEND	FLKSLLKQQQ	EDTETREKEL	EQIIKGSKDV	EKENTELQVK	ISELETEVTS	LRRQVAEANA
730	740	750	760	770	780	790	800
LRNENEELIN	PMEKSHQSAD	RAKSEMATMK	VRSGRYDCKT	TMTKVKFKAA	KKNCSVGRHH	TVLNHSIKVM	SNVFENLSKD
810	820	830	840	850	860	870	880
GWEDVSESSS	DSEAQTSQTL	GTIIVETSQK	ISPTEDGKDQ	KESDPTEDSQ	TQGKEIVQTY	LNIDGKTPKD	YFHDKNAKKP
890	900	910	920	930	940	950	960
TFQKKNCKMQ	KSSHTAVPTR	VNREKYKNIT	AQKSSSNIIL	LRERIISLQQ	QNSVLQNAKK	TAELSVKEYK	EVNEKLLHQQ
970	980	990	1000	1010	1020	1030	1040
QVSDQRFQTS	RQTIKKLNLD	LAGLRKEKED	LLKKLESSSE	ITSLAEENSQ	VTFPRIQVTS	LSPSRSMDLE	MKQLQYKLKN
1050	1060	1070	1080	1090	1100	1110	1120
ATNELTKQSS	NVKTLKFELL	AKEEHIKEMH	EKISRMERDI	TMKRHLIEDL	KFRQKVNLES	NKSFSEMLQN	LDKKVKTLTE
1130	1140	1150	1160	1170	1180	1190	1200
ECSNKKVSID	SLKQRLNVAV	KEKSQYEQMY	QKSKEELEKK	DLKLTLLVSR	ISETESAMAE	IETAASKQLQ	ELALQSEQVL
1210	1220	1230	1240	1250	1260	1270	1280
EGAQKTLLLA	NEKVEEFTTF	VKALAKELQN	DVHVVRRQIR	ELKKMKKNRD	ACKTSTHKAQ	TLAASILNIS	RSDLEEILDT
1290	1300	1310	1320	1330	1340	1350	
EDQVEIEKTK	IDAENDKEWM	LYIQKLLEGQ	LPFASYLLEA	VLEKINEKKK	LVEGYFTIMK	DIR	

Cmpd.	No. of Cmpds.	m/z meas.	Δ m/z [ppm]	z	Rt [min]	Score	P	Range	Sequence	Modification	Ratios
848	1	512.1520	-290.70	2	40.7	15.4	1	1084-1091	K.RHLIEDLK.F		
562	1	884.4457	29.69	2	37.0	19.0	0	1171-1187	R.ISETESAMAEIETAASK.Q		QU:MU 0.85

Detailed Protein Report

Protein 146: **RNA polymerase II subunit A C-terminal domain phosphatase isoform 3 [Homo sapiens]**

Accession: gi|321267522
Database: refseq_human(refseq_human_20140103.fasta)
Database Date: 2015-11-30

Score: 46.6
MW [kDa]: 92.3
pI: 4.9
Sequence Coverage [%]: 7.1
No. of unique Peptides: 2

10	20	30	40	50	60	70	80
MKGLCAECGQ	DLTQLQSKNG	KQQVPLSTAT	VSMVHSVPEL	MVSSEQAEQL	GREDQQRLHR	NRKLVLMVDL	DQTLIHTTEQ
90	100	110	120	130	140	150	160
HCQQMSNKGI	FHFQLGRGEP	MLHTRLRPHC	KDFLEKIAKL	YELHVFTFGS	RLYAHTIAGF	LDPEKKLFSH	RILSRDECID
170	180	190	200	210	220	230	240
PFSKTGNLRN	LFPCGDSMVC	IIDDREDVWK	FAPNLITVKK	YVYFQGTGDM	NAPPGSRESQ	TRKKVNHSRG	TEVSEPSPPV
250	260	270	280	290	300	310	320
RDPEGVTQAP	GVEPSNGLEK	PARELNGSEA	ATPRDSPRPG	KPDERDIWPP	AQAPTSSQEL	AGAPEPQGSC	AQGGRVAPGQ
330	340	350	360	370	380	390	400
RPAQGATGTD	LDFDLSSDSE	SSSESEGTKS	SSSASDGESE	GKRGRQKPKA	APEGAGALAQ	GSSLEPGRPA	APSLPGEAEP
410	420	430	440	450	460	470	480
GAHAPDKEPE	LGGQEEGERD	GLCGLGNGCA	DRKEAETESQ	NSELSGVTAG	ESLDQSMEEE	EEEDTDEDDH	LIYLEEILVR
490	500	510	520	530	540	550	560
VHTDYYAKYD	RYLNKEIEEA	PDIRKIVPEL	KSKVLADVAI	IFSGLHPTNF	PIEKTREHYH	ATALGAKILT	RLVLSPDAPD
570	580	590	600	610	620	630	640
RATHLIAARA	GTEKVLQAQE	CGHLHVVNPD	WLWSCLERWD	KVEEQLFPLR	DDHTKAQREN	SPAAFPDREG	VPPTALFHPM
650	660	670	680	690	700	710	720
PVLPKAQPGP	EVRIYDSNTG	KLIRTGARGP	PAPSSSLPIR	QEPSSFRAVP	PPQPQMFGEE	LPDAQDGEQP	GPSRRKRQPS
730	740	750	760	770	780	790	800
MSETMPLYTL	CKEDLESMDK	EVDDILGEGS	DDSDSEKRRP	EEQEEEPQPR	KPGTRRERTL	GAPASSERSA	AGGRGPRGHK
810	820	830	840	850			
RKLNEEDAAS	ESSRESSNED	EGSSSEADEM	AKALEAELND	LM			

Cmpd.	No. of Cmpds.	m/z meas.	Δ m/z [ppm]	z	Rt [min]	Score	P	Range	Sequence	Modification
2677	1	973.4768	-94.08	2	63.6	15.5	2	662-680	K.LIRTGARGPPAPSSSLPIR.Q	
1965	3	899.3643	-116.81	2	52.9	16.3	2	779-797	R.TLGAPASSERSAAGGRGPR.G	

Detailed Protein Report

Protein 147: PREDICTED: G-protein coupled receptor 124 isoform X3 [Homo sapiens]

**Accession:**	gi|578815277	**Score:**	46.5
**Database:**	refseq_human(refseq_human_20140103.fasta)	**MW [kDa]:**	140.0
**Database Date:**	2015-11-30	**pI:**	9.9
**Modification(s):**	Carbamidomethyl	**Sequence Coverage [%]:**	2.6
		**No. of unique Peptides:**	3

10	20	30	40	50	60	70	80
MGAGGRRMRG	APARLLLPLL	PWLLLLLAPE	ARGAPGCPLS	IRSCKCSGER	PKGLSGGVPG	PARRRVVCSG	GDLPEPPEPG
90	100	110	120	130	140	150	160
LLPNGTVTLD	LRNNIISTVQ	PGAFLGLGEL	KRLDLSNNRI	GCLTSETFQG	LPRLLRLNIS	GNIFSSLQPG	VFDELPALKV
170	180	190	200	210	220	230	240
VDLGTEFLTC	DCHLRWLLPW	AQNRSLQLSE	HTLCAYPSAL	HAQALGSLQE	AQLCCEGALE	LHTHHLIPSL	RQVVFQGDRL
250	260	270	280	290	300	310	320
PFQCSASYLG	NDTRIRWYHN	RAPVEGDEQA	GILLAESLIH	DCTFITSELT	LSHIGVWASG	EWECTVSMAQ	GNASKKVEIV
330	340	350	360	370	380	390	400
VLETSASYCP	AERVANNRGD	FRWPRTLAGI	TAYQSCLQYP	FTSVPLGGGA	PGTRASRRCD	RAGRWEPGDY	SHCLYTNDIT
410	420	430	440	450	460	470	480
RVLYTFVLMP	INASNALTLA	HQLRVYTAEA	ASFSDMMDVV	YVAQMIQKFL	GYVDQIKELV	EVMVDMASNL	MLVDEHLLWL
490	500	510	520	530	540	550	560
AQREDKACSR	IVGALERIGG	AALSPHAQHI	SVNARNVALE	AYLIKPHSYV	GLTCTAFQRR	EGGVPGTRPG	SPGQNPPPEP
570	580	590	600	610	620	630	640
EPPADQQLRF	RCTTGRPNVS	LSSFHIKNSV	ALASIQLPPS	LFSSLPAALA	PPVPPDCTLQ	LLVFRNGRLF	HSHSNTSRPG
650	660	670	680	690	700	710	720
AAGPGKRRGV	ATPVIFAGTS	GCGVGNLTEP	VAVSLRHWAE	GAEPVAAWWS	QEGPGEAGGW	TSEGCQLRSS	QPNVSALHCQ
730	740	750	760	770	780	790	800
HLGNVAVLME	LSAFPREVGG	AGAGLHPVVY	PCTALLLLCL	FATIITYILN	HSSIRVSRKG	WHMLLNLCFH	IAMTSAVFAG
810	820	830	840	850	860	870	880
GITLTNYQMV	CQAVGITLHY	SSLSTLLWMG	VKARVLHKEL	TWRAPPPQEG	DPALPTPSPM	LRFYLIAGGI	PLIICGITAA
890	900	910	920	930	940	950	960
VNIHNYRDHS	PYCWLVWRPS	LGAFYIPVAL	ILLITWIYFL	CAGLRLRGPL	AQNPKAGNSR	ASLEAGEELR	GSTRLRGSGP
970	980	990	1000	1010	1020	1030	1040
LLSDSGSLLA	TGSARVGTPG	PPEDGDSLYS	PGVQLGALVT	THFLYLAMWA	CGALAVSQRW	LPRVVCSCLY	GVAASALGLF
1050	1060	1070	1080	1090	1100	1110	1120
VFTHHCARRR	DVRASWRACC	PPASPAAPHA	PPRALPAAAE	DGSPVFGEGP	PSLKSSPSGS	SGHPLALGPC	KLTNLQLAQS
1130	1140	1150	1160	1170	1180	1190	1200
QVCEAGAAAG	GEGEPEPAGT	RGNLAHRHPN	NVHHGRRAHK	SRAKGHRAGE	ACGKNRLKAL	RGGAAGALEL	LSSESGSLHN
1210	1220	1230	1240	1250	1260	1270	1280
SPTDSYLGSS	RNSPGAGLQL	EGEPMLTPSE	GSDTSAAPLS	EAGRAGQRRS	ASRDSLKGGG	ALEKESHRRS	YPLNAASLNG
1290	1300	1310	1320				
APKGGKYDDV	TLMGAEVASG	GCMKTGLWKS	ETTV				

Cmpd.	No. of Cmpds.	m/z meas.	Δ m/z [ppm]	z	Rt [min]	Score	P	Range	Sequence	Modification
2732	2	673.3595	29.93	2	64.5	15.8	1	33-45	R.GAPGCPLSIRSCK.C	Carbamidomethyl: 5
2870	9	673.3611	32.18	2	66.4	16.5	1	33-45	R.GAPGCPLSIRSCK.C	Carbamidomethyl: 12
1660	1	716.0007	12.17	3	50.5	14.1	1	1284-1304	K.GGKYDDVTLMGAEVASGGCMK.T	Carbamidomethyl: 19

Detailed Protein Report

Protein 148: **A disintegrin and metalloproteinase with thrombospondin motifs 19 preproprotein [Homo sapiens]**

**Accession:**	gi|112789555	**Score:**	46.3
**Database:**	refseq_human(refseq_human_20140103.fasta)	**MW [kDa]:**	134.0
**Database Date:**	2015-11-30	**pI:**	9.1
**Modification(s):**	Carbamidomethyl	**Sequence Coverage [%]:**	4.3
		**No. of unique Peptides:**	1

10	20	30	40	50	60	70	80
MRLTHICCCC	LLYQLGFLSN	GIVSELQFAP	DREEWEVVFP	ALWRREPVDP	AGGSGGSADP	GWVRGVGGGG	SARAQAAGSS
90	100	110	120	130	140	150	160
REVRSVAPVP	LEEPVEGRSE	SRLRPPPPSE	GEEDEELESQ	ELPRGSSGAA	ALSPGAPASW	QPPPPPQPPP	SPPPAQHAEP
170	180	190	200	210	220	230	240
DGDEVLLRIP	AFSRDLYLLL	RRDGRFLAPR	FAVEQRPNPG	PGPTGAASAP	QPPAPPDAGC	FYTGAVLRHP	GSLASFSTCG
250	260	270	280	290	300	310	320
GGLMGFIQLN	EDFIFIEPLN	DTMAITGHPH	RVYRQKRSME	EKVTEKSALH	SHYCGIISDK	GRPRSRKIAE	SGRGKRYSYK
330	340	350	360	370	380	390	400
LPQEYNIETV	VVADPAMVSY	HGADAARRFI	LTILNMVFNL	FQHKSLSVQV	NLRVIKLILL	HETPPELYIG	HHGEKMLESF
410	420	430	440	450	460	470	480
CKWQHEEFGK	KNDIHLEMST	NWGEDMTSVD	AAILITRKDF	CVHKDEPCDT	VGIAYLSGMC	SEKRKCIIAE	DNGLNLAFTI
490	500	510	520	530	540	550	560
AHEMGHNMGI	NHDNDHPSCA	DGLHIMSGEW	IKGQNLGDVS	WSRCSKEDLE	RFLRSKASNC	LLQTNPQSVN	SVMVPSKLPG
570	580	590	600	610	620	630	640
MTYTADEQCQ	ILFGPLASFC	QEMQHVICTG	LWCKVEGEKE	CRTKLDPPMD	GTDCDLGKWC	KAGECTSRTS	APEHLAGEWS
650	660	670	680	690	700	710	720
LWSPCSRTCS	AGISSRERKC	PGLDSEARDC	NGPRKQYRIC	ENPPCPAGLP	GFRDWQCQAY	SVRTSSPKHI	LQWQAVLDEE
730	740	750	760	770	780	790	800
KPCALFCSPV	GKEQPILLSE	KVMDGTSCGY	QGLDICANGR	CQKVGCDGLL	GSLAREDHCG	VCNGNGKSCK	IIKGDFNHTR
810	820	830	840	850	860	870	880
GAGYVEVLVI	PAGARRIKVV	EEKPAHSYLA	LRDAGKQSIN	SDWKIEHSGA	FNLAGTTVHY	VRRGLWEKIS	AKGPTTAPLH
890	900	910	920	930	940	950	960
LLVLLFQDQN	YGLHYEYTIP	SDPLPENQSS	KAPEPLFMWT	HTSWEDCDAT	CGGGERKTTV	SCTKIMSKNI	SIVDNEKCKY
970	980	990	1000	1010	1020	1030	1040
LTKPEPQIRK	CNEQPCQTRW	MMTEWTPCSR	TCGKGMQSRQ	VACTQQLSNG	TLIRARERDC	IGPKPASAQR	CEGQDCMTVW
1050	1060	1070	1080	1090	1100	1110	1120
EAGVWSECSV	KCGKGIRHRT	VRCTNPRKKC	VLSTRPREAE	DCEDYSKCYV	WRMGDWSKCS	ITCGKGMQSR	VIQCMHKITG
1130	1140	1150	1160	1170	1180	1190	1200
RHGNECFSSE	KPAAYRPCHL	QPCNEKINVN	TITSPRLAAL	TFKCLGDQWP	VYCRVIREKN	LCQDMRWYQR	CCETCRDFYA
1210							
QKLQQKS							

Cmpd.	No. of Cmpds.	m/z meas.	Δ m/z [ppm]	z	Rt [min]	Score	P	Range	Sequence	Modification
109	1	1098.0910	-64.88	3	31.1	12.4	2	619-647	K.WCKAGECTSRTSAPEHLAGEWSLWSPCSR.T	Carbamidomethyl: 2

Detailed Protein Report

Protein 149: treslin [Homo sapiens]

Accession: gi|118421085
Database: refseq_human(refseq_human_20140103.fasta)
Database Date: 2015-11-30

Score: 46.3
MW [kDa]: 210.7
pI: 9.8
Sequence Coverage [%]: 1.5
No. of unique Peptides: 1

10	20	30	40	50	60	70	80
MACCHKVMLL	LDTAGGAARH	SRVRRAALRL	LTYLSCRFGL	ARVHWAFKFF	DSQGARSRPS	RVSDFRELGS	RSWEDFEEEL
90	100	110	120	130	140	150	160
EARLEDRAHL	PGPAPRATHT	HGALMETLLD	YQWDRPEITS	PTKPILRSSG	RRLLDVESEA	KEAEAALGGL	VNAVFLLAPC
170	180	190	200	210	220	230	240
PHSQRELLQF	VSGCEAQAQR	LPPTPKQVME	KLLPKRVREV	MVARKITFYW	VDTTEWSKLW	ESPDHLGYWT	VCELLHHGGG
250	260	270	280	290	300	310	320
TVLPSESFSW	DFAQAGEMLL	RSGIKLSSEP	HLSPWISMLP	TDATLNRLLY	NSPEYEASFP	RMEGMLFLPV	EAGKEIQETW
330	340	350	360	370	380	390	400
TVTLEPLAMH	QRHFQKPVRI	FLKGSVAQWS	LPTSSTLGTD	SWMLGSPEES	TATQRLLFQQ	LVSRLTAEEL	HLVADVDPGE
410	420	430	440	450	460	470	480
GRPPITGVIS	PLSASAMILT	VCRTKEAEFQ	RHVLQTAVAD	SPRDTASLFS	DVVDSILNQT	HDSLADTASA	ASPVPEWAQQ
490	500	510	520	530	540	550	560
ELGHTTPWSP	AVVEKWFPFC	NISGASSDLM	ESFGLLQAAS	ANKEESSKTE	GELIHCLAEL	YQRKSREEST	IAHQEDSKKK
570	580	590	600	610	620	630	640
RGVPRTPVRQ	KMNTMCRSLK	MLNVARLNVK	AQKLHPDGSP	DVAGEKGIQK	IPSGRTVDKL	EDRGRTLRSS	KPKDFKTEEE
650	660	670	680	690	700	710	720
LLSYIRENYQ	KTVATGEIML	YACARNMIST	VKMFLKSKGT	KELEVNCLNQ	VKSSLLKTSK	SLRQNLGKKL	DKEDKVRECQ
730	740	750	760	770	780	790	800
LQVFLRLEMC	LQCPSINEST	DDMEQVVEEV	TDLLRMVCLT	EDSAYLAEFL	EEILRLYIDS	IPKTLGNLYN	SLGFVIPQKL
810	820	830	840	850	860	870	880
AGVLPTDFFS	DDSMTQENKS	PLLSVPFLSS	ARRSVSGSPE	SDELQELRTR	SAKKRRKNAL	IRHKSIAEVS	QNLRQIEIPK
890	900	910	920	930	940	950	960
VSKRATKKEN	SHPAPQQPSQ	PVKDTVQEVT	KVRRNLFNQE	LLSPSKRSLK	RGLPRSHSVS	AVDGLEDKLD	NFKKNKGYHK
970	980	990	1000	1010	1020	1030	1040
LLTKSVAETP	VHKQISKRLL	HRQIKGRSSD	PGPDIGVVEE	SPEKGDEISL	RRSPRIKQLS	FSRTHSASFY	SVSQPKSRSV
1050	1060	1070	1080	1090	1100	1110	1120
QRVHSFQQDK	SDQRENSPVQ	SIRSPKSLLF	GAMSEMISPS	EKGSARMKKR	SRNTLDSEVP	AAYQTPKKSH	QKSLSFSKTT
1130	1140	1150	1160	1170	1180	1190	1200
PRRISHTPQT	PLYTPERLQK	SPAKMTPTKQ	AAFKESLKDS	SSPGHDSPLD	SKITPQKRHT	QAGEGTSLET	KTPRTPKRQG
1210	1220	1230	1240	1250	1260	1270	1280
TQPPGFLPNC	TWPHSVNSSP	ESPSCPAPPT	SSTAQPRREC	LTPIRDPLRT	PPRAAAFMGT	PQNQTHQQPH	VLRAARAEEP
1290	1300	1310	1320	1330	1340	1350	1360
AQKLKDKAIK	TPKRPGNSTV	TSSPPVTPKK	LFTSPLCDVS	KKSPFRKSKI	ECPSPGELDQ	KEPQMSPSVA	ASLSCPVPST
1370	1380	1390	1400	1410	1420	1430	1440
PPELSQRATL	DTVPPPPPSK	VGKRCRKTSD	PRRSIVECQP	DASATPGVGT	ADSPAAPTDS	RDDQKGLSLS	PQSPPERRGY
1450	1460	1470	1480	1490	1500	1510	1520
PGPGLRSDWH	ASSPLLITSD	TEHVTLLSEA	EHHGIGDLKS	NVLSVEEGEG	LRTADAEKSS	LSHPGIPPSP	PSCGPGSPLM
1530	1540	1550	1560	1570	1580	1590	1600
PSRDVHCTTD	GRQCQASAQL	DNLPASAWHS	TDSASPQTYE	VELEMQASGL	PKLRIKKIDP	SSSLEAEPLS	KEESSLGEES
1610	1620	1630	1640	1650	1660	1670	1680
FLPALSMPRA	SRSLSKPEPT	YVSPPCPRLS	HSTPGKSRGQ	TYICQACTPT	HGPSSTPSPF	QTDGVPWTPS	PKHSGKTTPD
1690	1700	1710	1720	1730	1740	1750	1760
IIKDWPRRKR	AVGCGAGSSS	GRGEVGADLP	GSLSLLESEG	KDHGLELSIH	RTPILEDFEL	EGVCQLPDQS	PPRNSMPKAE
1770	1780	1790	1800	1810	1820	1830	1840
EASSWGQFGL	SSRKRVLLAK	EEADRGAKRI	CDLREDSEVS	KSKEGSPSWS	AWQLPSTGDE	EVFVSGSTPP	PSCAVRSCLS
1850	1860	1870	1880	1890	1900	1910	1920
ASALQALTQS	PLLFQGKTPS	SQSKDPRDED	VDVLPSTVED	SPFSRAFSRR	RPISRTYTRK	KLMGTWLEDL	

Cmpd.	No. of Cmpds.	m/z meas.	Δ m/z [ppm]	z	Rt [min]	Score	P	Range	Sequence	Modification

Detailed Protein Report

Cmpd.	No. of Cmpds.	m/z meas.	Δ m/z [ppm]	z	Rt [min]	Score	P	Range	Sequence	Modification
2455	1	669.2124	-220.96	2	61.2	12.2	1	1311-1322	K.LFTSPLCDVSKK.S	

Detailed Protein Report

Protein 150: PREDICTED: SH3 domain-containing protein 21 isoform X4 [Homo sapiens]

Accession: gi|578799774
Database: refseq_human(refseq_human_20140103.fasta)
Database Date: 2015-11-30

Score: 46.2
MW [kDa]: 84.7
pI: 5.6
Sequence Coverage [%]: 4.3
No. of unique Peptides: 2

Quantitation

WUP:QUP **Median:** 2.96 **CV:** 0.00 % **No. of Peptides:** 1

10	20	30	40	50	60	70	80
MEVLVLAGYR	AQKEDELSLA	PGDVVRQVRW	VPARGWLRGE	FGGRYGLFPE	RLVQEIPETL	RGSGEARRPR	CARRRGHPAK
90	100	110	120	130	140	150	160
HPRPQRWCKV	NFSYSPEQAD	ELKLQAGEIV	EMIKEIEDGW	WLGKKNGQLG	AFPSNFVELL	DSGPPSLGNP	DMPSVSPGPQ
170	180	190	200	210	220	230	240
RPPKLSSLAY	DSPPDYLQTV	SHPEVYRVLF	DYQPEAPDEL	ALRRGDVVKV	LSKTTEDKGW	WEGECQGRRG	VFPDNFVLPP
250	260	270	280	290	300	310	320
PPIKKLVPRK	VVSRESAPIK	EPKKLMPKTS	LPTVKKLATA	TTGPSKAKTS	RTPSRDSQKL	TSRDSGPNGG	FQSGGSYHPG
330	340	350	360	370	380	390	400
RKRSKTQTPQ	QRSVSSQEEE	HSSPVKAPSV	KRTPMPDKTA	TPERPPAPEN	APSSKKIPAP	DKVPSPEKTL	TLGDKASIPG
410	420	430	440	450	460	470	480
NSTSGKIPAP	DKVPTPEKMV	TPEDKASIPE	NSIIPEETLT	VDKPSTPERV	FSVEESPALE	APPMDKVPNP	KMAPLGDEAP
490	500	510	520	530	540	550	560
TLEKVLTPEL	SEEEVSTRDD	IQFHHFSSEE	ALQKVKYFVA	KEDPSSQEEA	HTPEAPPPQP	PSSERCLGEM	KCTLVRGDSS
570	580	590	600	610	620	630	640
PRQAELKSGP	ASRPALEKPH	PHEEATTLPE	EAPSNDERTP	EEEAPPNEQR	PLREEVLPKE	GVASKEEVTL	KEELPPKEEV
650	660	670	680	690	700	710	720
APKEEVPPIE	RAFAQKTRPI	KPPPDSQETL	ALPSLVPQNY	TENKNEGVDV	TSLRGEVESL	RRALELMEEE	ADRHLGGAEE
730	740	750	760	770	780		
REGAAPAAGG	PVYEPRALWG	QGDVCPGVCR	GLPWPSQARW	EKRQPCPGGF			

Cmpd.	No. of Cmpds.	m/z meas.	Δ m/z [ppm]	z	Rt [min]	Score	P	Range	Sequence	Modification	Ratios
328	1	658.3980	85.56	2	32.8	13.8	2	62-73	R.GSGEARRPRCAR.R		
2858	1	805.9291	-2.54	2	66.2	12.9	2	751-763	R.GLPWPSQARWEKR.Q		WUP:QUP 2.96

Detailed Protein Report

Protein 151: PREDICTED: PDZ domain-containing protein 2 isoform X8 [Homo sapiens]

**Accession:**	gi|578809880	**Score:**	46.1
**Database:**	refseq_human(refseq_human_20140103.fasta)	**MW [kDa]:**	289.4
**Database Date:**	2015-11-30	**pI:**	7.4
		**Sequence Coverage [%]:**	2.7
		**No. of unique Peptides:**	3

Detailed Protein Report

10	20	30	40	50	60	70	80
MPITQDNAVL	HLPLLYQWLQ	NSLQEGGDGP	EQRLCQAAIQ	KLQEYIQLNF	AVDESTVPPD	HSPPEMEICT	VYLTKELGDT
90	100	110	120	130	140	150	160
ETVGLSFGNI	PVFGDYGEKR	RGGKKRKTHQ	GPVLDVGCIW	VTELRKNSPA	GKSGKVRLRD	EILSLNGQLM	VGVDVSGASY
170	180	190	200	210	220	230	240
LAEQCWNGGF	IYLIMLRRFK	HKAHSTYNGN	SSNSSEPGET	PTLELGDRTA	KKGKRTRKFG	VISRPPANKA	PEESKGSAGC
250	260	270	280	290	300	310	320
EVSSDPSTEL	ENGPDPELGN	GHVFQLENGP	DSLKEVAGPH	LERSEVDRGT	EHRIPKTDAP	LTTSNDKRRF	SKGGKTDFQS
330	340	350	360	370	380	390	400
SDCLAREEVG	RIWKMELLKE	SDGLGIQVSG	GRGSKRSPHA	IVVTQVKEGG	AAHRDGRLSL	GDELLVINGH	LLVGLSHEEA
410	420	430	440	450	460	470	480
VAILRSATGM	VQLVVASKEN	SAEDLLRLTS	KSLPDLTSSV	EDVSSWTDNE	DQEADGEEDE	GTSSSVQRAM	PGTDEPQDVC
490	500	510	520	530	540	550	560
GAEESKGNLE	SPKQGSNKIK	LKSRLSGGVH	RLESVEEYNE	LMVRNGDPRI	RMLEVSRDGR	KHSLPQLLDS	SSASQEYHIV
570	580	590	600	610	620	630	640
KKSTRSLSTT	QVESPWRLIR	PSVISIIGLY	KEKGKGLGFS	IAGGRDCIRG	QMGIFVKTIF	PNGSAAEDGR	LKEGDEILDV
650	660	670	680	690	700	710	720
NGIPIKGLTF	QEAIHTFKQI	RSGLFVLTVR	TKLVSPSLTP	CSTPTHMSRS	ASPNFNTSGG	ASAGGSDEGS	SSSLGRKTPG
730	740	750	760	770	780	790	800
PKDRIVMEVT	LNKEPRVGLG	IGACCLALEN	SPPGIYIHSL	APGSVAKMES	NLSRGDQILE	VNSVNVRHAA	LSKVHAILSK
810	820	830	840	850	860	870	880
CPPGPVRLVI	GRHPNPKVSE	QEMDEVIARS	TYQESKEANS	SPGLGTPLKS	PSLAKKDSLI	SESELSQYFA	HDVPGPLSDF
890	900	910	920	930	940	950	960
MVAGSEDEDH	PGSGCSTSEE	GSLPPSTSTH	KEPGKPRANS	LVTLGSHRAS	GLFHKQVTVA	RQASLPGSPQ	ALRNPLLRQR
970	980	990	1000	1010	1020	1030	1040
KVGCYDANDA	SDEEEFDREG	DCISLPGALP	GPIRPLSEDD	PRRVSISSSK	GMDVHNQEER	PRKTLESLGK	LTTGDACVST
1050	1060	1070	1080	1090	1100	1110	1120
SCELASALSH	LDASHLTENL	PKAASELGQQ	PMTELDSSSD	LISSPGKKGA	AHPDPSKTSV	DTGQVSRPEN	PSQPASPRVT
1130	1140	1150	1160	1170	1180	1190	1200
KCKARSPVRL	PHEGSPSPGE	KAAAPPDYSK	TRSASETSTP	HNTRRVAALR	GAGPGAEGMT	PAGAVLPGDP	LTSQEQRQGA
1210	1220	1230	1240	1250	1260	1270	1280
PGNHSKALEM	TGIHAPESSQ	EPSLLEGADS	VSSRAPQASL	SMLPSTDNTK	EACGHVSGHC	CPGGSRESPV	TDIDSFIKEL
1290	1300	1310	1320	1330	1340	1350	1360
DASAARSPSS	QTGDSGSQEG	SAQGHPPAGA	GGGSSCRAEP	VPGGQTSSPR	RAWAAGAPAY	PQWASQPSVL	DSINPDKHFT
1370	1380	1390	1400	1410	1420	1430	1440
VNKNFLSNYS	RNFSSFHEDS	TSLSGLGDST	EPSLSSMYGD	AEDSSSDPES	LTEAPRASAR	DGWSPPRSRV	SLHKEDPSES
1450	1460	1470	1480	1490	1500	1510	1520
EEEQIEICST	RGCPNPPSSP	AHLPTQAAIC	PASAKVLSLK	YSTPRESVAS	PREKAACLPG	SYTSGPDSSQ	PSSLLEMSSQ
1530	1540	1550	1560	1570	1580	1590	1600
EHETHADIST	SQNHRPSCAE	ETTEVTSASS	AMENSPLSKV	ARHFHSPPII	LSSPNMVNGL	EHDLLDDETL	NQYETSINAA
1610	1620	1630	1640	1650	1660	1670	1680
ASLSSFSVDV	PKNGESVLEN	LHISESQDLD	DLLQKPKMIA	RRPIMAWFKE	INKHNQGTHL	RSKTEKEQPL	MPARSPDSKI
1690	1700	1710	1720	1730	1740	1750	1760
QMVSSSQKKG	VTVPHSPPQP	KTNLENKDLS	KKSPAEMLLT	NGQKAKCGPK	LKRLSLKGKA	KVNSEAPAAN	AVKAGGTDHR
1770	1780	1790	1800	1810	1820	1830	1840
KPLISPQTSH	KTLSKAVSQR	LHVADHEDPD	RNTTAAPRSP	QCVLESKPPL	ATSGPLKPSV	SDTSIRTFVS	PLTSPKPVPE
1850	1860	1870	1880	1890	1900	1910	1920
QGMWSRFHMA	VLSEPDRGCP	TTPKSPKCRA	EGRAPRADSG	PVSPAASRNG	MSVAGNRQSE	PRLASHVAAD	TAQPRPTGEK
1930	1940	1950	1960	1970	1980	1990	2000
GGNIMASDRL	ERTNQLKIVE	ISAEAVSETV	CGNKPAESDR	RGGCLAQGNC	QEKSEIRLYR	QVAESSTSHP	SSLPSHASQA
2010	2020	2030	2040	2050	2060	2070	2080
EQEMSRSFSM	AKLASSSSSL	QTAIRKAEYS	QGKSSLMSDS	RGVPRNSIPG	GPSGEDHLYF	TPRPATRTYS	MPAQFSSHFG
2090	2100	2110	2120	2130	2140	2150	2160
REGHPPHSLG	RSRDSQVPVT	SSVVPEAKAS	RGGLPSLANG	QGIYSVKPLL	DTSRNLPATD	EGDIISVQET	SCLVTDKIKV
2170	2180	2190	2200	2210	2220	2230	2240

Detailed Protein Report

Cmpd.	No. of Cmpds.	m/z meas.	Δ m/z [ppm]	z	Rt [min]	Score	P	Range	Sequence	Modification
892	1	675.8467	18.44	4	41.2	12.0	1	76-100	K.ELGDTETVGLSFGNIPVFGDYGEKR.R	
2006	1	917.8396	-134.11	2	53.5	11.6	1	2474-2492	R.KEGSGLGFSVAGGTDVEPK.S	
444	1	848.1390	136.74	2	35.2	10.0	1	2606-2621	R.IGRPTVLLGSSAVIRR.S	

Detailed Protein Report

Protein 152: leucine zipper protein 1 [Homo sapiens]

**Accession:**	gi|216548085	**Score:**	46.0
**Database:**	refseq_human(refseq_human_20140103.fasta)	**MW [kDa]:**	120.2
**Database Date:**	2015-11-30	**pI:**	9.4
		**Sequence Coverage [%]:**	5.9
		**No. of unique Peptides:**	2

Alias proteins:

Accession	Name	Description
gi|216548091	refseq_human (refseq_human_20140103.fasta)	leucine zipper protein 1 [Homo sapiens]

10	20	30	40	50	60	70	80
MAEFTSYKET	ASSRHLRFKL	QSLSRRLDEL	EEATKNLQKA	EDELLDLQDK	VIQAEGSNSS	MLAEIEVLRQ	RVLRIEGKDE
**90**	**100**	**110**	**120**	**130**	**140**	**150**	**160**
EIKRAEDLCR	LMKEKLEEEE	NLTRELKSEI	ERLQKRMAEL	EKLEEAFSRS	KNDCTQLCLS	LNEERNLTKK	ISSELEMLRV
**170**	**180**	**190**	**200**	**210**	**220**	**230**	**240**
KVKELESSED	RLDKTEQSLA	SELEKLKSLT	LSFVSERKYL	NEKEKENEKL	IKELTQKLEQ	NKKMNRDYTR	NASNLERNDL
**250**	**260**	**270**	**280**	**290**	**300**	**310**	**320**
RIEDGISSTL	PSKESRRKGG	LDYLKQVENE	TRNKSENEKN	RNQEDNKVKD	LNQEIEKLKT	QIKHFESLEE	ELKKMKSKNN
**330**	**340**	**350**	**360**	**370**	**380**	**390**	**400**
DLQDNYLSEQ	NKNKLLASQL	EEIKLQIKKQ	KELENGEVEG	EDAFLSSKGR	HERTKFRGHG	SEASVSKHTA	RELSPQHKRE
**410**	**420**	**430**	**440**	**450**	**460**	**470**	**480**
RLRNREFALN	NENYSLSNRQ	VSSPSFTNRR	AAKASHMGVS	TDSGTQETKK	TEDRFVPGSS	QSEGKKSREQ	PSVLSRYPPA
**490**	**500**	**510**	**520**	**530**	**540**	**550**	**560**
AQEHSKAWKG	TSKPGTESGL	KGKVEKTTRT	FSDTTHGSVP	SDPLGRADKA	SDTSSETVFG	KRGHVLGNGS	QVTQAANSGC
**570**	**580**	**590**	**600**	**610**	**620**	**630**	**640**
SKAIGALASS	RRSSSEGLSK	GKKAANGLEA	DNSCPNSKAP	VLSKYPYSCR	SQENILQGFS	TSHKEGVNQP	AAVVMEDSSP
**650**	**660**	**670**	**680**	**690**	**700**	**710**	**720**
HEALRCRVIK	SSGREKPDSD	DDLDIASLVT	AKLVNTTITP	EPEPKPQPNS	REKAKTRGAP	RTSLFENDKD	AGMENESVKS
**730**	**740**	**750**	**760**	**770**	**780**	**790**	**800**
VRASTNTMEL	PDTNGAGVKS	QRPFSPREAL	RSRAIIKPVI	VDKDVKKIMG	GSGTETTLEK	QKPVSKPGPN	KVTSSITIYP
**810**	**820**	**830**	**840**	**850**	**860**	**870**	**880**
SDSSSPRAAP	GEALRERHTS	TSNIQVGLAE	LTSVSNHVSS	PFELSIHKHD	ITLQLAEAER	MADGPLKDRP	ETVVSRSSII
**890**	**900**	**910**	**920**	**930**	**940**	**950**	**960**
IKPSDPVERN	SHAPPAETIR	WKSHSAPSEV	GFSDARHVTV	RNAWKSRRDL	KSLEDPPTRI	GKNVESTNSN	AYTQRSSTDF
**970**	**980**	**990**	**1000**	**1010**	**1020**	**1030**	**1040**
SELEQPRSCL	FEQGTRRVGP	SSGDAPEPSS	RRTQSSLTVS	EVLTRRNRVG	DTITVAAWNH	SASMEEEGED	CTLSVYRQLH
**1050**	**1060**	**1070**	**1080**				
NSLDPSELPG	KQGLPESGRV	RAEERLRPTR	PCAEEN				

Cmpd.	No. of Cmpds.	m/z meas.	Δ m/z [ppm]	z	Rt [min]	Score	P	Range	Sequence	Modification
2080	1	1044.4112	-88.13	2	55.9	13.0	1	510-529	R.TFSDTTHGSVPSDPLGRADK.A	
2108	1	698.2650	-143.11	2	56.2	11.7	0	849-860	K.HDITLQLAEAER.M	

Detailed Protein Report

Protein 153: cyclic AMP-dependent transcription factor ATF-5 [Homo sapiens]

**Accession:**	gi|12597625	**Score:**	45.7
**Database:**	refseq_human(refseq_human_20140103.fasta)	**MW [kDa]:**	30.7
**Database Date:**	2015-11-30	**pI:**	4.7
**Modification(s):**	Carbamidomethyl	**Sequence Coverage [%]:**	6.4
		**No. of unique Peptides:**	1

Alias proteins:

Accession	Name	Description
gi|302370956	refseq_human (refseq_human_20140103.fasta)	cyclic AMP-dependent transcription factor ATF-5 [Homo sapiens]

10	20	30	40	50	60	70	80
MSLLATLGLE	LDRALLPASG	LGWLVDYGKL	PPAPAPLAPY	EVLGGALEGG	LPVGGEPLAG	DGFSDWMTER	VDFTALLPLE
**90**	**100**	**110**	**120**	**130**	**140**	**150**	**160**
PPLPPGTLPQ	PSPTPPDLEA	MASLLKKELE	QMEDFFLDAP	PLPPPSPPPL	PPPPLPPAPS	LPLSLPSFDL	PQPPVLDTLD
**170**	**180**	**190**	**200**	**210**	**220**	**230**	**240**
LLAIYCRNEA	GQEEVGMPPL	PPPQQPPPPS	PPQPSRLAPY	PHPATTRGDR	KQKKRDQNKS	AALRYRQRKR	AEGEALEGEC
**250**	**260**	**270**	**280**	**290**			
QGLEARNREL	KERAESVERE	IQYVKDLLIE	VYKARSQRTR	SC			

Cmpd.	No. of Cmpds.	m/z meas.	Δ m/z [ppm]	z	Rt [min]	Score	P	Range	Sequence	Modification
533	1	625.6275	0.96	3	35.1	13.5	1	230-246	K.RAEGEALEGECQGLEAR.N	Carbamidomethyl: 11

Detailed Protein Report

Protein 154: **PREDICTED: fatty-acid amide hydrolase 2 isoform X4 [Homo sapiens]**

**Accession:**	gi|530426388	**Score:**	45.5
**Database:**	refseq_human(refseq_human_20140103.fasta)	**MW [kDa]:**	49.2
**Database Date:**	2015-11-30	**pI:**	9.3
**Modification(s):**	Oxidation	**Sequence Coverage [%]:**	8.5
		**No. of unique Peptides:**	2

10	20	30	40	50	60	70	80
MINGIVKYRF	EEAMKEAHAV	DQKLAEKQED	EATLENKWPF	LGVPLTVKEA	FQLQGMPNSS	GLMNRRDAIA	KTDATVVALL
**90**	**100**	**110**	**120**	**130**	**140**	**150**	**160**
KGAGAIPLGI	TNCSELCMWY	ESSNKIYGRS	NNPYDLQHIV	GGSSGGEGCT	LAAACSVIGV	GSDIGGSIRM	PAFFNGIFGH
**170**	**180**	**190**	**200**	**210**	**220**	**230**	**240**
KPSPGVVPNK	GQFPLAVGAQ	ELFLCTGPMC	RYAEDLAPML	KVMAGPGIKR	LKLDTKVHLK	DLKFYWMEHD	GGSFLMSKVD
**250**	**260**	**270**	**280**	**290**	**300**	**310**	**320**
QDLIMTQKKV	VVHLETILGA	SVQHVKLKKM	KYSFQLWIAM	MSAKGHDGKE	PVKFVDLLGD	HGKHVSPLWE	LIKWCLGLSV
**330**	**340**	**350**	**360**	**370**	**380**	**390**	**400**
YTIPSIGLAL	LEEKLRYSNE	KYQKFKAVEE	SLRKELVDML	GDDGVFLYPS	HPTVAPKHHV	PLTRPFNFAY	TGVFSALGLP
**410**	**420**	**430**	**440**	**450**			
VTQCPLGLNA	KGLPLGIQVV	AGPFNDHLTL	AVAQYLEKTF	GGWVCPGKF			

Cmpd.	No. of Cmpds.	m/z meas.	Δ m/z [ppm]	z	Rt [min]	Score	P	Range	Sequence	Modification
701	1	1099.6923	17.73	2	38.7	22.7	2	249-268	K.KVVVHLETILGASVQHVKLK.K	
1689	3	1051.5326	35.47	2	50.9	22.8	1	272-289	K.YSFQLWIAMMSAKGHDGK.E	Oxidation: 9, 10

Detailed Protein Report

Protein 155: CUB and sushi domain-containing protein 3 isoform 3 [Homo sapiens]

**Accession:**	gi|205277354	**Score:**	45.4
**Database:**	refseq_human(refseq_human_20140103.fasta)	**MW [kDa]:**	387.1
**Database Date:**	2015-11-30	**pI:**	5.5
**Modification(s):**	Carbamidomethyl	**Sequence Coverage [%]:**	1.3
		**No. of unique Peptides:**	2

Detailed Protein Report

10	20	30	40	50	60	70	80
MKGIRKGESR	AKESKPWEPG	KRRCAKCGRL	DFILMKKMGI	KSGFTFWNLV	FLLTVSCVKG	FIYTCGGTLK	GLNGTIESPG
90	100	110	120	130	140	150	160
FPYGYPNGAN	CTWVIIAEER	NRIQIVFQSF	ALEEEYDYLS	LYDGHPHPTN	FRTRLTGFHL	PPPVTSTKSV	FSLRLTSDFA
170	180	190	200	210	220	230	240
VSAHGFKVYY	EELQSSSCGN	PGVPPKGVLY	GTRFDVGDKI	RYSCVTGYIL	DGHPQLTCIA	NSVNTASWDF	PVPICRAEDA
250	260	270	280	290	300	310	320
CGGTMRGSSG	IISSPSFPNE	YHNNADCTWT	IVAEPGDTIS	LIFTDFQMEE	KYDYLEIEGS	EPPTIWLSGM	NIPPPIISNK
330	340	350	360	370	380	390	400
NWLRLHFVTD	SNHRYRGFSA	PYQVKKAIDF	KSRGFKLFPG	KDNSNKFSIL	NEGGIKTASN	LCPDPGEPEN	GKRIGSDFSL
410	420	430	440	450	460	470	480
GSTVQFSCDE	DYVLQGAKSI	TCQRIAEVFA	AWSDHRPVCK	VKTCGSNLQG	PSGTFTSPNF	PFQYDSNAQC	VWVITAVNTN
490	500	510	520	530	540	550	560
KVIQINFEEF	DLEIGYDTLT	IGDGGEVGDP	RTVLQVLTGS	FVPDLIVSMS	SQMWLHLQTD	ESVGSVGFKV	NYKEIEKESC
570	580	590	600	610	620	630	640
GDPGTPLYGI	REGDGFSNRD	VLRFECQFGF	ELIGEKSIVC	QENNQWSANI	PICIFPCLSN	FTAPMGTVLS	PDYPEGYGNN
650	660	670	680	690	700	710	720
LNCIWTIISD	PGSRIHLSFN	DFDLESQFDF	LAVKDGDSPE	SPILGTFTGA	EVPSHLTSNS	HILRLEFQAD	HSMSGRGFNI
730	740	750	760	770	780	790	800
TYNTFGHNEC	PDPGIPINAR	RFGDNFQLGS	SISVICEEGF	IKTQGTETIT	CILMDGKVMW	SGLIPKCGAP	CGGHFSAPSG
810	820	830	840	850	860	870	880
VILSPGWPGY	YKDSLNCEWV	IEAEPGHSIK	ITFERFQTEL	NYDVLEVHDG	PNLLSPLLGS	YNGTQVPQFL	FSSSNFIYLL
890	900	910	920	930	940	950	960
FTTDNSRSNN	GFKIHYESVT	VNTYSCLDPG	IPVHGRRYGH	DFSIGSTVSF	SCDSGYRLSH	EEPLLCEKNH	WWSHPLPTCD
970	980	990	1000	1010	1020	1030	1040
ALCGGDVRGP	SGTILSPGYP	EFYPNSLNCT	WTVDVTHGKG	VQFNFHTFHL	EDHHDYLLIT	ENGSFTQPLA	RLTGSDLPPT
1050	1060	1070	1080	1090	1100	1110	1120
INAGLYGNFR	AQLRFISDFS	ISYEGFNITF	SEYNLEPCED	PGIPQYGSRI	GFNFGIGDTL	TFSCSSGYRL	EGTSEIICLG
1130	1140	1150	1160	1170	1180	1190	1200
GGRRVWSAPL	PRCVAECGAS	ATNNEGILLS	PNYPLNYENN	HECIYSIQVQ	AGKGINISAR	TFHLAQGDVL	KIYDGKDKTT
1210	1220	1230	1240	1250	1260	1270	1280
HLLGAFTGAS	MRGLTLSSTS	NQLWLEFNSD	TEGTDEGFQL	VYTSFELSHC	EDPGIPQFGY	KISDQGHFAG	STIIYGCNPG
1290	1300	1310	1320	1330	1340	1350	1360
YTLHGSSLLK	CMTGERRAWD	YPLPSCIAEC	GGRFKGESSG	RILSPGYPFP	YDNNLRCMWM	IEVDPGNIVS	LQFLAFDTEA
1370	1380	1390	1400	1410	1420	1430	1440
SHDILRVWDG	PPENDMLLKE	ISGSLIPEGI	HSTLNIVTIQ	FDTDFYISKS	GFAIQFSSSV	ATACRDPGVP	MNGTRNGDGR
1450	1460	1470	1480	1490	1500	1510	1520
EPGDTVVFQC	DPGYELQGEE	RITCIQVENR	YFWQPSPPVC	IAPCGGNLTG	SSGFILSPNF	PHPYPHSRDC	DWTITVNADY
1530	1540	1550	1560	1570	1580	1590	1600
VISLAFISFS	IEPNYDFLYI	YDGPDSNSPL	IGSFQDSKLP	ERIESSSNTM	HLAFRSDGSV	SYTGFHLEYK	AKLRESCFDP
1610	1620	1630	1640	1650	1660	1670	1680
GNIMNGTRLG	MDYKLGSTVT	YYCDAGYVLQ	GYSTLTCIMG	DDGRPGWNRA	LPSCHAPCGS	RSTGSEGTVL	SPNYPKNYSV
1690	1700	1710	1720	1730	1740	1750	1760
GHNCVYSIAV	PKEFVVFGQF	VFFQTSLHDV	VEVYDGPTQQ	SSLLSSLSGS	HSGESLPLSS	GNQITIRFTS	VGPITAKGFH
1770	1780	1790	1800	1810	1820	1830	1840
FVYQAVPRTS	STQCSSVPEP	RFGRRIGNEF	AVGSSVLFDC	NPGYILHGSI	AIRCETVPNS	LAQWNDSLPT	CIVPCGGILT
1850	1860	1870	1880	1890	1900	1910	1920
KRKGTILSPG	YPEPYDNNLN	CVWKITVPEG	AGIQVQVVSF	ATEHNWDSLD	FYDGGDNNAP	RLGSYSGTTI	PHLLNSTSNN
1930	1940	1950	1960	1970	1980	1990	2000
LYLNFQSDIS	VSAAGFHLEY	TAIGLDSCPE	PQTPSSGIKI	GDRYMVGDVV	SFQCDQGYSL	QGHSHITCMP	GPVRRWNYPI
2010	2020	2030	2040	2050	2060	2070	2080
PICLAQCGGA	MSDFSGVILS	PGFPGNYPSS	LDCTWTINLP	IGFGVHLQFV	NFSTETIHDY	LEVRSGSSET	STVIGRLSGP
2090	2100	2110	2120	2130	2140	2150	2160
QIPSSLFSTT	HETSLYFHSD	YSQNKQGFHI	VYQAYQLQSC	PDPRPFRNGF	VIGNDFTVGQ	TISFECFPGY	TLIGNSALTC
2170	2180	2190	2200	2210	2220	2230	2240

Detailed Protein Report

Cmpd.	No. of Cmpds.	m/z meas.	Δ m/z [ppm]	z	Rt [min]	Score	P	Range	Sequence	Modification
1865	1	897.8571	-115.65	2	53.2	10.9	1	321-334	K.NWLRLHFVTDSNHR.Y	
1304	1	661.3930	144.57	1	46.4	11.2	0	2937-2941	R.QPECK.A	Carbamidomethyl: 4

Detailed Protein Report

Protein 156: rho GTPase-activating protein 36 isoform 1 precursor [Homo sapiens]

**Accession:**	gi|40255080	**Score:**	45.4
**Database:**	refseq_human(refseq_human_20140103.fasta)	**MW [kDa]:**	61.6
**Database Date:**	2015-11-30	**pI:**	10.2
		**Sequence Coverage [%]:**	5.9
		**No. of unique Peptides:**	2

10	20	30	40	50	60	70	80
MGGCIPFLKA	ARALCPRIMP	PLLLLSAFIF	LVSVLGGAPG	HNPDRRTKMV	SIHSLSELER	LKLQETAYHE	LVARHFLSEF
**90**	**100**	**110**	**120**	**130**	**140**	**150**	**160**
KPDRALPIDR	PNTLDKWFLI	LRGQQRAVSH	KTFGISLEEV	LVNEFTRRKH	LELTATMQVE	EATGQAAGRR	RGNVVRRVFG
**170**	**180**	**190**	**200**	**210**	**220**	**230**	**240**
RIRRFFSRRR	NEPTLPREFT	RRGRRGAVSV	DSLAELEDGA	LLLQTLQLSK	ISFPIGQRLL	GSKRKMSLNP	IAKQIPQVVE
**250**	**260**	**270**	**280**	**290**	**300**	**310**	**320**
ACCQFIEKHG	LSAVGIFTLE	YSVQRVRQLR	EEFDQGLDVV	LDDNQNVHDV	AALLKEFFRD	MKDSLLPDDL	YMSFLLTATL
**330**	**340**	**350**	**360**	**370**	**380**	**390**	**400**
KPQDQLSALQ	LLVYLMPPCH	SDTLERLLKA	LHKITENCED	SIGIDGQLVP	GNRMTSTNLA	LVFGSALLKK	GKFGKRESRK
**410**	**420**	**430**	**440**	**450**	**460**	**470**	**480**
TKLGIDHYVA	SVNVVRAMID	NWDVLFQVPP	HIQRQVAKRV	WKSSPEALDF	IRRRNLRKIQ	SARIKMEEDA	LLSDPVETSA
**490**	**500**	**510**	**520**	**530**	**540**	**550**	
EARAAVLAQS	KPSDEGSSEE	PAVPSGTARS	HDDEEGAGNP	PIPEQDRPLL	RVPREKEAKT	GVSYFFP	

Cmpd.	No. of Cmpds.	m/z meas.	Δ m/z [ppm]	z	Rt [min]	Score	P	Range	Sequence	Modification
1422	1	459.2842	44.39	2	47.9	16.9	0	211-218	K.ISFPIGQR.L	
2474	1	822.4025	-186.55	1	61.5	16.1	1	347-353	R.LLKALHK.I	

Detailed Protein Report

Protein 157: PREDICTED: uncharacterized protein KIAA1551 isoform X1 [Homo sapiens]

**Accession:**	gi|530399223	**Score:**	45.0
**Database:**	refseq_human(refseq_human_20140103.fasta)	**MW [kDa]:**	194.7
**Database Date:**	2015-11-30	**pI:**	9.7
**Modification(s):**	Carbamidomethyl	**Sequence Coverage [%]:**	2.3
		**No. of unique Peptides:**	2

10	20	30	40	50	60	70	80
MNWNEKPKSA	TLPPLYPKSQ	PPFLHQSLIN	QITTTSQSSF	SYPGSNQEAC	MYPGNSNPIS	QPLLNIQNYP	QQISVSDMHN
**90**	**100**	**110**	**120**	**130**	**140**	**150**	**160**
GTVVASHTSV	ERITYANVNG	PKQLTHNLQM	SSGVTQNVWL	NSPMRNPVHS	HIGATVSHQT	DFGANVPNMP	ALQSQLITSD
**170**	**180**	**190**	**200**	**210**	**220**	**230**	**240**
TYSMQMQMIP	SNSTRLPVAY	QGNQGLNQSF	SEQQVDWTQQ	CISKGLTYPD	YRPPPKLYRY	SPQSFLPDST	IQKQNFIPHT
**250**	**260**	**270**	**280**	**290**	**300**	**310**	**320**
SLQVKNSQLL	NSVLTLPSRQ	TSAVPSQQYA	TQTDKRPPPP	PYNCRYGSQP	LQSTQHITKH	LSMEVPQSRE	MLSSEIRTSF
**330**	**340**	**350**	**360**	**370**	**380**	**390**	**400**
QQQWQNPNEN	VSTIGNFTNL	KVNTNSKQPF	NSPIRSSVDG	VQTLAQTNEE	KIMDSCNPTS	NQVLDTSVAK	EKLVRDIKTL
**410**	**420**	**430**	**440**	**450**	**460**	**470**	**480**
VEIKQKFSEL	ARKIKINKDL	LMAAGCIKMT	NTSYSEPAQN	SKLSLKQTAK	IQSGPQITPV	MPENAERQTP	TVVESAETNK
**490**	**500**	**510**	**520**	**530**	**540**	**550**	**560**
TQCMLNSDIQ	EVNCRRFNQV	DSVLPNPVYS	EKRPMPDSSH	DVKVLTSKTS	AVEMTQAVLN	TQLSSENVTK	VEQNSPAVCE
**570**	**580**	**590**	**600**	**610**	**620**	**630**	**640**
TISVPKSMST	EEYKSKIQNE	NMLLLALLSQ	ARKTQKTVLK	DANQTIQDSK	PDSCEMNPNT	QMTGNQLNLK	NMETPSTSNV
**650**	**660**	**670**	**680**	**690**	**700**	**710**	**720**
SGRVLDNSFC	SGQESSTKGM	PAKSDSSCSM	EVLATCLSLW	KKQPSDTAKE	KECDKLRTNT	TAVGISKPAN	IHVKSPCSVV
**730**	**740**	**750**	**760**	**770**	**780**	**790**	**800**
GNSNSQNKIS	NPSQQTALSM	VMHNYESSGI	NITKGTELQI	AVVSPLVLSE	VKTLSVKGIT	PAVLPETVYP	VIKEGSVCSL
**810**	**820**	**830**	**840**	**850**	**860**	**870**	**880**
QNQLAENAKA	TAALKVDVSG	PVASTATSTK	IFPLTQKEKQ	NESTNGNSEV	TPNVNQGKHN	KLESAIHSPM	NDQQISQESR
**890**	**900**	**910**	**920**	**930**	**940**	**950**	**960**
NSTVVSSDTL	QIDNICSLVE	GDTSYNSQIA	KIFSSLPLKM	VEPQKPSLPN	QQGIGSREPE	KQLDNTTENK	DFGFQKDKPV
**970**	**980**	**990**	**1000**	**1010**	**1020**	**1030**	**1040**
QCTDVSHKIC	DQSKSEPPLE	SSFNNLETNR	VILEKSSLEH	ATEKSTANDT	CSSAAIQEDI	YPQEIDASSN	YTPQDPARNE
**1050**	**1060**	**1070**	**1080**	**1090**	**1100**	**1110**	**1120**
IHSDKAPVLY	LHDQLSELLK	EFPYGIEAVN	TREGSVGQQT	TYQTSEDQTA	DKTSSDSKDP	ADQIQITILS	SEQMKEIFPE
**1130**	**1140**	**1150**	**1160**	**1170**	**1180**	**1190**	**1200**
QDDQPYVVDK	LAEPQKEEPI	TEVVSQCDLQ	APAAGQSRDS	VILDSEKDDI	HCCALGWLSM	VYEGVPQCQC	NSIKNSSSEE
**1210**	**1220**	**1230**	**1240**	**1250**	**1260**	**1270**	**1280**
EKQKEQCSPL	DTNSCKQGER	TSDRDVTVVQ	FKSLVNNPKT	PPDGKSHFPE	LQDDSRKDTP	KTKHKSLPRT	EQELVAGQFS
**1290**	**1300**	**1310**	**1320**	**1330**	**1340**	**1350**	**1360**
SKCDKLNPLQ	NHKRKKLRFH	EVTFHSSNKM	TASYEQASQE	TRQKKHVTQN	SRPLKTKTAF	LPNKDVYKKH	SSLGQSLSPE
**1370**	**1380**	**1390**	**1400**	**1410**	**1420**	**1430**	**1440**
KIKLKLKSVS	FKQKRKLDQG	NVLDMEVKKK	KHDKQEQKGS	VGATFKLGDS	LSNPNERAIV	KEKMVSNTKS	VDTKASSSKF
**1450**	**1460**	**1470**	**1480**	**1490**	**1500**	**1510**	**1520**
SRILTPKEYL	QRQKHKEALS	NKASKKICVK	NVPCDSEHMR	PSKLAVQVES	CGKSNEKHSS	GVQTSKESLN	GLTSHGKNLK
**1530**	**1540**	**1550**	**1560**	**1570**	**1580**	**1590**	**1600**
IHHSQESKTY	NILRNVKEKV	GGKQPDKIWI	DKTKLDKLTN	ISNEAQFSQM	PPQVKDQKKL	YLNRVGFKCT	ERESISLTKL
**1610**	**1620**	**1630**	**1640**	**1650**	**1660**	**1670**	**1680**
ESSPRKLHKD	KRQENKHKTF	LPVKGNTEKS	NMLEFKLCPD	ILLKNTNSVE	ERKDVKPHPR	KEQAPLQVSG	IKSTKEDWLK
**1690**	**1700**	**1710**	**1720**	**1730**	**1740**	**1750**	
FVATKKRTQK	DSQERDNVNS	RLSKRSFSAD	GFEMLQNPVK	DSKEMFQTYK	QMYLEKRSRS	LGSSPVK	

Cmpd.	No. of Cmpds.	m/z meas.	Δ m/z [ppm]	z	Rt [min]	Score	P	Range	Sequence	Modification
2006	1	1044.4022	-89.89	2	55.0	16.6	1	957-974	K.DKPVQCTDVSHKICDQSK.S	Carbamidomethyl: 6
120	1	585.2911	-66.24	2	30.4	16.8	0	1662-	K.EQAPLQVSGIK.S	

Detailed Protein Report

Cmpd.	No. of Cmpds.	m/z meas.	Δ m/z [ppm]	z	Rt [min]	Score	P	Range	Sequence	Modification
								1672		

Detailed Protein Report

Protein 158: heterogeneous nuclear ribonucleoprotein U-like protein 2 [Homo sapiens]

Accession: gi|118601081
Database: refseq_human(refseq_human_20140103.fasta)
Database Date: 2015-11-30

Score: 45.0
MW [kDa]: 85.1
pI: 4.7
Sequence Coverage [%]: 5.0
No. of unique Peptides: 1

Quantitation

QU:MU	**Median:**	0.95	**CV:**	0.00 %	**No. of Peptides:**	1
WUP:QUP	**Median:**	1.52	**CV:**	0.00 %	**No. of Peptides:**	1

10	20	30	40	50	60	70	80
MEVKRLKVTE	LRSELQRRGL	DSRGLKVDLA	QRLQEALDAE	MLEDEAGGGG	AGPGGACKAE	PRPVAASGGG	PGGDEEEDEE
**90**	**100**	**110**	**120**	**130**	**140**	**150**	**160**
EEEEDEEALL	EDEDEEPPPA	QALGQAAQPP	PEPPEAAAME	AAAEPDASEK	PAEATAGSGG	VNGGEEQGLG	KREEDEPEER
**170**	**180**	**190**	**200**	**210**	**220**	**230**	**240**
SGDETPGSEV	PGDKAAEEQG	DDQDSEKSKP	AGSDGERRGV	KRQRDEKDEH	GRAYYEFREE	AYHSRSKSPL	PPEEEAKDEE
**250**	**260**	**270**	**280**	**290**	**300**	**310**	**320**
EDQTLVNLDT	YTSDLHFQVS	KDRYGGQPLF	SEKFPTLWSG	ARSTYGVTKG	KVCFEAKVTQ	NLPMKEGCTE	VSLLRVGWSV
**330**	**340**	**350**	**360**	**370**	**380**	**390**	**400**
DFSRPQLGED	EFSYGFDGRG	LKAENGQFEE	FGQTFGENDV	IGCFANFETE	EVELSFSKNG	EDLGVAFWIS	KDSLADRALL
**410**	**420**	**430**	**440**	**450**	**460**	**470**	**480**
PHVLCKNCVV	ELNFGQKEEP	FFPPPEEFVF	IHAVPVEERV	RTAVPPKTIE	ECEVILMVGL	PGSGKTQWAL	KYAKENPEKR
**490**	**500**	**510**	**520**	**530**	**540**	**550**	**560**
YNVLGAETVL	NQMRMKGLEE	PEMDPKSRDL	LVQQASQCLS	KLVQIASRTK	RNFILDQCNV	YNSGQRRKLL	LFKTFSRKVV
**570**	**580**	**590**	**600**	**610**	**620**	**630**	**640**
VVVPNEEDWK	KRLELRKEVE	GDDVPESIML	EMKANFSLPE	KCDYMDEVTY	GELEKEEAQP	IVTKYKEEAR	KLLPPSEKRT
**650**	**660**	**670**	**680**	**690**	**700**	**710**	**720**
NRRNNRNKRN	RQNRSRGQGY	VGGQRRGYDN	RAYGQQYWGQ	PGNRGGYRNF	YDRYRGDYDR	FYGRDYEYNR	YRDYYRQYNR
**730**	**740**	**750**					
DWQSYYYHHP	QDRDRYYRNY	YGYQGYR					

Cmpd.	No. of Cmpds.	m/z meas.	Δ m/z [ppm]	z	Rt [min]	Score	P	Range	Sequence	Modification	Ratios
196	1	838.3951	-57.68	2	32.2	16.3	1	507-521	K.SRDLLVQQASQCLSK.L		WUP:QUP 1.52<br>QU:MU 0.95

Detailed Protein Report

Protein 159: ADAMTS-like protein 1 isoform 4 precursor [Homo sapiens]

**Accession:**	gi|154275767	**Score:**	45.0
**Database:**	refseq_human(refseq_human_20140103.fasta)	**MW [kDa]:**	193.3
**Database Date:**	2015-11-30	**pI:**	9.5
**Modification(s):**	Carbamidomethyl	**Sequence Coverage [%]:**	2.4
		**No. of unique Peptides:**	1

10	20	30	40	50	60	70	80
MECCRRATPG	TLLLFLAFLL	LSSRTARSEE	DRDGLWDAWG	PWSECSRTCG	GGASYSLRRC	LSSKSCEGRN	IRYRTCSNVD
90	100	110	120	130	140	150	160
CPPEAGDFRA	QQCSAHNDVK	HHGQFYEWLP	VSNDPDNPCS	LKCQAKGTTL	VVELAPKVLD	GTRCYTESLD	MCISGLCQIV
170	180	190	200	210	220	230	240
GCDHQLGSTV	KEDNCGVCNG	DGSTCRLVRG	QYKSQLSATK	SDDTVVAIPY	GSRHIRLVLK	GPDHLYLETK	TLQGTKGENS
250	260	270	280	290	300	310	320
LSSTGTFLVD	NSSVDFQKFP	DKEILRMAGP	LTADFIVKIR	NSGSADSTVQ	FIFYQPIIHR	WRETDFFPCS	ATCGGGYQLT
330	340	350	360	370	380	390	400
SAECYDLRSN	RVVADQYCHY	YPENIKPKPK	LQECNLDPCP	ASDGYKQIMP	YDLYHPLPRW	EATPWTACSS	SCGGGIQSRA
410	420	430	440	450	460	470	480
VSCVEEDIQG	HVTSVEEWKC	MYTPKMPIAQ	PCNIFDCPKW	LAQEWSPCTV	TCGQGLRYRV	VLCIDHRGMH	TGGCSPKTKP
490	500	510	520	530	540	550	560
HIKEECIVPT	PCYKPKEKLP	VEAKLPWFKQ	AQELEEGAAV	SEEPSFIPEA	WSACTVTCGV	GTQVRIVRCQ	VLLSFSQSVA
570	580	590	600	610	620	630	640
DLPIDECEGP	KPASQRACYA	GPCSGEIPEF	NPDETDGLFG	GLQDFDELYD	WEYEGFTKCS	ESCGGGVQEA	VVSCLNKQTR
650	660	670	680	690	700	710	720
EPAEENLCVT	SRRPPQLLKS	CNLDPCPARW	EIGKWSPCSL	TCGVGLQTRD	VFCSHLLSRE	MNETVILADE	LCRQPKPSTV
730	740	750	760	770	780	790	800
QACNRFNCPP	AWYPAQWQPC	SRTCGGGVQK	REVLCKQRMA	DGSFLELPET	FCSASKPACQ	QACKKDDCPS	EWLLSDWTEC
810	820	830	840	850	860	870	880
STSCGEGTQT	RSAICRKMLK	TGLSTVVNST	LCPPLPFSSS	IRPCMLATCA	RPGRPSTKHS	PHIAAARKVY	IQTRRQRKLH
890	900	910	920	930	940	950	960
FVVGGFAYLL	PKTAVVLRCP	ARRVRKPLIT	WEKDGQHLIS	STHVTVAPFG	YLKIHRLKPS	DAGVYTCSAG	PAREHFVIKL
970	980	990	1000	1010	1020	1030	1040
IGGNRKLVAR	PLSPRSEEEV	LAGRKGGPKE	ALQTHKHQNG	IFSNGSKAEK	RGLAANPGSR	YDDLVSRLLE	QGGWPGELLA
1050	1060	1070	1080	1090	1100	1110	1120
SWEAQDSAER	NTTSEEDPGA	EQVLLHLPFT	MVTEQRRLDD	ILGNLSQQPE	ELRDLYSKHL	VAQLAQEIFR	SHLEHQDTLL
1130	1140	1150	1160	1170	1180	1190	1200
KPSERRTSPV	TLSPHKHVSG	FSSSLRTSST	GDAGGGSRRP	HRKPTILRKI	SAAQQLSASE	VVTHLGQTVA	LASGTLSVLL
1210	1220	1230	1240	1250	1260	1270	1280
HCEAIGHPRP	TISWARNGEE	VQFSDRILLQ	PDDSLQILAP	VEADVGFYTC	NATNALGYDS	VSIAVTLAGK	PLVKTSRMTV
1290	1300	1310	1320	1330	1340	1350	1360
INTEKPAVTV	DIGSTIKTVQ	GVNVTINCQV	AGVPEAEVTW	FRNKSKLGSP	HHLHEGSLLL	TNVSSSDQGL	YSCRAANLHG
1370	1380	1390	1400	1410	1420	1430	1440
ELTESTQLLI	LDPPQVPTQL	EDIRALLAAT	GPNLPSVLTS	PLGTQLVLDP	GNSALLGCPI	KGHPVPNITW	FHGGQPIVTA
1450	1460	1470	1480	1490	1500	1510	1520
TGLTHHILAA	GQILQVANLS	GGSQGEFSCL	AQNEAGVLMQ	KASLVIQDYW	WSVDRLATCS	ASCGNRGVQQ	PRLRCLLNST
1530	1540	1550	1560	1570	1580	1590	1600
EVNPAHCAGK	VRPAVQPIAC	NRRDCPSRWM	VTSWSACTRS	CGGGVQTRRV	TCQKLKASGI	STPVSNDMCT	QVAKRPVDTQ
1610	1620	1630	1640	1650	1660	1670	1680
ACNQQLCVEW	AFSSWGQCNG	PCIGPHLAVQ	HRQVFCQTRD	GITLPSEQCS	ALPRPVSTQN	CWSEACSVHW	RVSLWTLCTA
1690	1700	1710	1720	1730	1740	1750	1760
TCGNYGFQSR	RVECVHARTN	KAVPEHLCSW	GPRPANWQRC	NITPCENMEC	RDTTRYCEKV	KQLKLCQLSQ	FKSRCCGTCG
1770							
KA							

Cmpd.	No. of	m/z meas.	Δ m/z	z	Rt	Score	P	Range	Sequence	Modification

Detailed Protein Report

	Cmpds.		[ppm]		[min]					
1695	1	671.2184	-97.93	3	51.5	14.6	1	172-189	K.EDNCGVCNGDGSTCRLVR.G	Carbamidomethyl: 4, 7

Detailed Protein Report

Protein 160: voltage-dependent R-type calcium channel subunit alpha-1E isoform 2 [Homo sapiens]

**Accession:**	gi|329663531	**Score:**	44.8
**Database:**	refseq_human(refseq_human_20140103.fasta)	**MW [kDa]:**	254.4
**Database Date:**	2015-11-30	**pI:**	9.3
**Modification(s):**	Carbamidomethyl, Oxidation	**Sequence Coverage [%]:**	2.6
		**No. of unique Peptides:**	3

Quantitation

QU:MU	**Median:**	4.95	**CV:**	0.00 %	**No. of Peptides:**	1
WUP:QUP	**Median:**	0.60	**CV:**	0.00 %	**No. of Peptides:**	1

Detailed Protein Report

10	20	30	40	50	60	70	80
MARFGEAVVA	RPGSGDGDSD	QSRNRQGTPV	PASGQAAAYK	QTKAQRARTM	ALYNPIPVRQ	NCFTVNRSLF	IFGEDNIVRK
90	100	110	120	130	140	150	160
YAKKLIDWPP	FEYMILATII	ANCIVLALEQ	HLPEDDKTPM	SRRLEKTEPY	FIGIFCFEAG	IKIVALGFIF	HKGSYLRNGW
170	180	190	200	210	220	230	240
NVMDFIVVLS	GILATAGTHF	NTHVDLRTLR	AVRVLRPLKL	VSGIPSLQIV	LKSIMKAMVP	LLQIGLLLFF	AILMFAIIGL
250	260	270	280	290	300	310	320
EFYSGKLHRA	CFMNNSGILE	GFDPPHPCGV	QGCPAGYECK	DWIGPNDGIT	QFDNILFAVL	TVFQCITMEG	WTTVLYNTND
330	340	350	360	370	380	390	400
ALGATWNWLY	FIPLIIIGSF	FVLNLVLGVL	SGEFAKERER	VENRRAFMKL	RRQQQIEREL	NGYRAWIDKA	EEVMLAEENK
410	420	430	440	450	460	470	480
NAGTSALEVL	RRATIKRSRT	EAMTRDSSDE	HCVDISSVGT	PLARASIKSA	KVDGVSYFRH	KERLLRISIR	HMVKSQVFYW
490	500	510	520	530	540	550	560
IVLSLVALNT	ACVAIVHHNQ	PQWLTHLLYY	AEFLFLGLFL	LEMSLKMYGM	GPRLYFHSSF	NCFDFGVTVG	SIFEVVWAIF
570	580	590	600	610	620	630	640
RPGTSFGISV	LRALRLLRIF	KITKYWASLR	NLVVSLMSSM	KSIISLLFLL	FLFIVVFALL	GMQLFGGRFN	FNDGTPSANF
650	660	670	680	690	700	710	720
DTFPAAIMTV	FQILTGEDWN	EVMYNGIRSQ	GGVSSGMWSA	IYFIVLTLFG	NYTLLNVFLA	IAVDNLANAQ	ELTKDEQEEE
730	740	750	760	770	780	790	800
EAFNQKHALQ	KAKEVSPMSA	PNMPSIERER	RRRHHMSVWE	QRTSQLRKHM	QMSSQEALNR	EEAPTMNPLN	PLNPLSSLNP
810	820	830	840	850	860	870	880
LNAHPSLYRR	PRAIEGLALG	LALEKFEEER	ISRGGSLKGD	GGDRSSALDN	QRTPLSLGQR	EPPWLARPCH	GNCDPTQQEA
890	900	910	920	930	940	950	960
GGGEAVVTFE	DRARHRQSQR	RSRHRRVRTE	GKESSSASRS	RSASQERSLD	EAMPTEGEKD	HELRGNHGAK	EPTIQEERAQ
970	980	990	1000	1010	1020	1030	1040
DLRRTNSLMV	SRGSGLAGGL	DEADTPLVLP	HPELEVGKHV	VLTEQEPEGS	SEQALLGNVQ	LDMGRVISQS	EPDLSCITAN
1050	1060	1070	1080	1090	1100	1110	1120
TDKATTESTS	VTVAIPDVDP	LVDSTVVHIS	NKTDGEASPL	KEAEIREDEE	EVEKKKQKKE	KRETGKAMVP	HSSMFIFSTT
1130	1140	1150	1160	1170	1180	1190	1200
NPIRRACHYI	VNLRYFEMCI	LLVIAASSIA	LAAEDPVLTN	SERNKVLRYF	DYVFTGVFTF	EMVIKMIDQG	LILQDGSYFR
1210	1220	1230	1240	1250	1260	1270	1280
DLWNILDFVV	VVGALVAFAL	ANALGTNKGR	DIKTIKSLRV	LRVLRPLKTI	KRLPKLKAVF	DCVVTSLKNV	FNILIVYKLF
1290	1300	1310	1320	1330	1340	1350	1360
MFIFAVIAVQ	LFKGKFFYCT	DSSKDTEKEC	IGNYVDHEKN	KMEVKGREWK	RHEFHYDNII	WALLTLFTVS	TGEGWPQVLQ
1370	1380	1390	1400	1410	1420	1430	1440
HSVDVTEEDR	GPSRSNRMEM	SIFYVVYFVV	FPFFFVNIFV	ALIIITFQEQ	GDKMMEECSL	EKNERACIDF	AISAKPLTRY
1450	1460	1470	1480	1490	1500	1510	1520
MPQNRHTFQY	RVWHFVVSPS	FEYTIMAMIA	LNTVVLMMKY	YSAPCTYELA	LKYLNIAFTM	VFSLECVLKV	IAFGFLNYFR
1530	1540	1550	1560	1570	1580	1590	1600
DTWNIFDFIT	VIGSITEIIL	TDSKLVNTSG	FNMSFLKLFR	AARLIKLLRQ	GYTIRILLWT	FVQSFKALPY	VCLLIAMLFF
1610	1620	1630	1640	1650	1660	1670	1680
IYAIIGMQVF	GNIKLDEESH	INRHNNFRSF	FGSLMLLFRS	ATGEAWQEIM	LSCLGEKGCE	PDTTAPSGQN	ENERCGTDLA
1690	1700	1710	1720	1730	1740	1750	1760
YVYFVSFIFF	CSFLMLNLFV	AVIMDNFEYL	TRDSSILGPH	HLDEFVRVWA	EYDRAACGRI	HYTEMYEMLT	LMSPPLGLGK
1770	1780	1790	1800	1810	1820	1830	1840
RCPSKVAYKR	LVLMNMPVAE	DMTVHFTSTL	MALIRTALDI	KIAKGGADRQ	QLDSELQKET	LAIWPHLSQK	MLDLLVPMPK
1850	1860	1870	1880	1890	1900	1910	1920
ASDLTVGKIY	AAMMIMDYYK	QSKVKKQRQQ	LEEQKNAPMF	QRMEPSSLPQ	EIIANAKALP	YLQQDPVSGL	SGRSGYPSMS
1930	1940	1950	1960	1970	1980	1990	2000
PLSPQDIFQL	ACMDPADDGQ	FQERQSLVVT	DPSSMRRSFS	TIRDKRSNSS	WLEEFSMERS	SENTYKSRRR	SYHSSLRLSA
2010	2020	2030	2040	2050	2060	2070	2080
HRLNSDSGHK	SDTHRSGGRE	RGRSKERKHL	LSPDVSRCNS	EERGTQADWE	SPERRQSRSP	SEGRSQTPNR	QGTGSLSESS
2090	2100	2110	2120	2130	2140	2150	2160
IPSVSDTSTP	RRSRRQLPPV	PPKPRPLLSY	SSLIRHAGSI	SPPADGSEEG	SPLTSQALES	NNACLTESSN	SPHPQQSQHA
2170	2180	2190	2200	2210	2220	2230	2240

Detailed Protein Report

Cmpd.	No. of Cmpds.	m/z meas.	Δ m/z [ppm]	z	Rt [min]	Score	P	Range	Sequence	Modification	Ratios
2785	1	973.5222	64.95	2	63.4	10.8	1	734-750	K.EVSPMSAPNMPSIERER.R	Oxidation: 10	
183	1	823.9125	-35.10	2	31.1	10.2	2	959-972	R.AQDLRRTNSLMVSR.G		QU:MU 4.95<br>WUP:QUP 0.60
27	1	814.2746	-93.66	2	30.0	10.4	1	1296-1308	K.FFYCTDSSKDTEK.E	Carbamidomethyl: 4	

Detailed Protein Report

Protein 161: sortilin-related receptor preproprotein [Homo sapiens]

**Accession:**	gi|4507157	**Score:**	44.8
**Database:**	refseq_human(refseq_human_20140103.fasta)	**MW [kDa]:**	248.3
**Database Date:**	2015-11-30	**pI:**	5.2
**Modification(s):**	Carbamidomethyl	**Sequence Coverage [%]:**	0.7
		**No. of unique Peptides:**	2

Quantitation

QU:MU	**Median:**	0.72	**CV:**	0.00 %	**No. of Peptides:**	1
WUP:QUP	**Median:**	0.65	**CV:**	0.00 %	**No. of Peptides:**	1

Detailed Protein Report

10	20	30	40	50	60	70	80
MATRSSRRES	RLPFLFTLVA	LLPPGALCEV	WTQRLHGGSA	PLPQDRGFLV	VQGDPRELRL	WARGDARGAS	RADEKPLRRK
90	100	110	120	130	140	150	160
RSAALQPEPI	KVYGQVSLND	SHNQMVVHWA	GEKSNVIVAL	ARDSLALARP	KSSDVYVSYD	YGKSFKKISD	KLNFGLGNRS
170	180	190	200	210	220	230	240
EAVIAQFYHS	PADNKRYIFA	DAYAQYLWIT	FDFCNTLQGF	SIPFRAADLL	LHSKASNLLL	GFDRSHPNKQ	LWKSDDFGQT
250	260	270	280	290	300	310	320
WIMIQEHVKS	FSWGIDPYDK	PNTIYIERHE	PSGYSTVFRS	TDFFQSRENQ	EVILEEVRDF	QLRDKYMFAT	KVVHLLGSEQ
330	340	350	360	370	380	390	400
QSSVQLWVSF	GRKPMRAAQF	VTRHPINEYY	IADASEDQVF	VCVSHSNNRT	NLYISEAEGL	KFSLSLENVL	YYSPGGAGSD
410	420	430	440	450	460	470	480
TLVRYFANEP	FADFHRVEGL	QGVYIATLIN	GSMNEENMRS	VITFDKGGTW	EFLQAPAFTG	YGEKINCELS	QGCSLHLAQR
490	500	510	520	530	540	550	560
LSQLLNLQLR	RMPILSKESA	PGLIIATGSV	GKNLASKTNV	YISSSAGARW	REALPGPHYY	TWGDHGGIIT	AIAQGMETNE
570	580	590	600	610	620	630	640
LKYSTNEGET	WKTFIFSEKP	VFVYGLLTEP	GEKSTVFTIF	GSNKENVHSW	LILQVNATDA	LGVPCTENDY	KLWSPSDERG
650	660	670	680	690	700	710	720
NECLLGHKTV	FKRRTPHATC	FNGEDFDRPV	VVSNCSCTRE	DYECDFGFKM	SEDLSLEVCV	PDPEFSGKSY	SPPVPCPVGS
730	740	750	760	770	780	790	800
TYRRTRGYRK	ISGDTCSGGD	VEARLEGELV	PCPLAEENEF	ILYAVRKSIY	RYDLASGATE	QLPLTGLRAA	VALDFDYEHN
810	820	830	840	850	860	870	880
CLYWSDLALD	VIQRLCLNGS	TGQEVIINSG	LETVEALAFE	PLSQLLYWVD	AGFKKIEVAN	PDGDFRLTIV	NSSVLDRPRA
890	900	910	920	930	940	950	960
LVLVPQEGVM	FWTDWGDLKP	GIYRSNMDGS	AAYHLVSEDV	KWPNGISVDD	QWIYWTDAYL	ECIERITFSG	QQRSVILDNL
970	980	990	1000	1010	1020	1030	1040
PHPYAIAVFK	NEIYWDDWSQ	LSIFRASKYS	GSQMEILANQ	LTGLMDMKIF	YKGKNTGSNA	CVPRPCSLLC	LPKANNSRSC
1050	1060	1070	1080	1090	1100	1110	1120
RCPEDVSSSV	LPSGDLMCDC	PQGYQLKNNT	CVKEENTCLR	NQYRCSNGNC	INSIWWCDFD	NDCGDMSDER	NCPTTICDLD
1130	1140	1150	1160	1170	1180	1190	1200
TQFRCQESGT	CIPLSYKCDL	EDDCGDNSDE	SHCEMHQCRS	DEYNCSSGMC	IRSSWVCDGD	NDCRDWSDEA	NCTAIYHTCE
1210	1220	1230	1240	1250	1260	1270	1280
ASNFQCRNGH	CIPQRWACDG	DTDCQDGSDE	DPVNCEKKCN	GFRCPNGTCI	PSSKHCDGLR	DCSDGSDEQH	CEPLCTHFMD
1290	1300	1310	1320	1330	1340	1350	1360
FVCKNRQQCL	FHSMVCDGII	QCRDGSDEDA	AFAGCSQDPE	FHKVCDEFGF	QCQNGVCISL	IWKCDGMDDC	GDYSDEANCE
1370	1380	1390	1400	1410	1420	1430	1440
NPTEAPNCSR	YFQFRCENGH	CIPNRWKCDR	ENDCGDWSDE	KDCGDSHILP	FSTPGPSTCL	PNYYRCSSGT	CVMDTWVCDG
1450	1460	1470	1480	1490	1500	1510	1520
YRDCADGSDE	EACPLLANVT	AASTPTQLGR	CDRFEFECHQ	PKTCIPNWKR	CDGHQDCQDG	RDEANCPTHS	TLTCMSREFQ
1530	1540	1550	1560	1570	1580	1590	1600
CEDGEACIVL	SERCDGFLDC	SDESDEKACS	DELTVYKVQN	LQWTADFSGD	VTLTWMRPKK	MPSASCVYNV	YYRVVGESIW
1610	1620	1630	1640	1650	1660	1670	1680
KTLETHSNKT	NTVLKVLKPD	TTYQVKVQVQ	CLSKAHNTND	FVTLRTPEGL	PDAPRNLQLS	LPREAEGVIV	GHWAPPIHTH
1690	1700	1710	1720	1730	1740	1750	1760
GLIREYIVEY	SRSGSKMWAS	QRAASNFTEI	KNLLVNTLYT	VRVAAVTSRG	IGNWSDSKSI	TTIKGKVIPP	PDIHIDSYGE
1770	1780	1790	1800	1810	1820	1830	1840
NYLSFTLTME	SDIKVNGYVV	NLFWAFDTHK	QERRTLNFRG	SILSHKVGNL	TAHTSYEISA	WAKTDLGDSP	LAFEHVMTRG
1850	1860	1870	1880	1890	1900	1910	1920
VRPPAPSLKA	KAINQTAVEC	TWTGPRNVVY	GIFYATSFLD	LYRNPKSLTT	SLHNKTVIVS	KDEQYLFLVR	VVVPYQGPSS
1930	1940	1950	1960	1970	1980	1990	2000
DYVVVKMIPD	SRLPPRHLHV	VHTGKTSVVI	KWESPYDSPD	QDLLYAIAVK	DLIRKTDRSY	KVKSRNSTVE	YTLNKLEPGG
2010	2020	2030	2040	2050	2060	2070	2080
KYHIIVQLGN	MSKDSSIKIT	TVSLSAPDAL	KIITENDHVL	LFWKSLALKE	KHFNESRGYE	IHMFDSAMNI	TAYLGNTTDN
2090	2100	2110	2120	2130	2140	2150	2160
FFKISNLKMG	HNYTFTVQAR	CLFGNQICGE	PAILLYDELG	SGADASATQA	ARSTDVAAVV	VPILFLILLS	LGVGFAILYT
2170	2180	2190	2200	2210	2220		

Detailed Protein Report

Cmpd.	No. of Cmpds.	m/z meas.	Δ m/z [ppm]	z	Rt [min]	Score	P	Range	Sequence	Modification	Ratios
2231	1	599.9323	-0.25	3	57.8	14.4	1	1239-1254	K.CNGFRCPNGTCIPSSK.H	Carbamidomethyl: 1, 11	
1196	1	610.6448	-210.75	2	43.2	10.3	0	1244-1254	R.CPNGTCIPSSK.H	Carbamidomethyl: 1, 6	WUP:QUP 0.65<br>QU:MU 0.72

Detailed Protein Report

Protein 162: **PREDICTED: zinc finger protein 467 isoform X5 [Homo sapiens]**

**Accession:**	gi|578813954	**Score:**	44.8
**Database:**	refseq_human(refseq_human_20140103.fasta)	**MW [kDa]:**	66.0
**Database Date:**	2015-11-30	**pI:**	11.0
**Modification(s):**	Carbamidomethyl	**Sequence Coverage [%]:**	9.3
		**No. of unique Peptides:**	4

10	20	30	40	50	60	70	80
MKFPTPHPGL	PPSSLSPENR	ILPALSQVLW	PDPQEELLWV	TMRETLEALS	SLGFSVGQPE	MAPQSEPREG	SHNAQEQMSS
**90**	**100**	**110**	**120**	**130**	**140**	**150**	**160**
SREERALGVC	SGEEWMIRKV	KVEDEDQEAE	EEVEWPQHLS	LLPSPFPAPD	LGHLAAAYKL	EPGAPGALSG	LALSGWGPMP
**170**	**180**	**190**	**200**	**210**	**220**	**230**	**240**
EKPYGCGECE	RRFRDQLTLR	LHQRLHRGEG	PCACPDCGRS	FTQRAHMLLH	QRSHRGERPF	PCSECDKRFS	KKAHLTRHLR
**250**	**260**	**270**	**280**	**290**	**300**	**310**	**320**
THTGERPYPC	AECGKRFSQK	IHLGSHQKTH	TGERPFPCTE	CEKRFRKKTH	LIRHQRIHTG	ERPYQCAQCA	RSFTHKQHLV
**330**	**340**	**350**	**360**	**370**	**380**	**390**	**400**
RHQRVHQTAG	PARPSPDSSA	SPHSTAPSPT	PSFPGPKPFA	CSDCGLSFGW	KKNLATHQCL	HRSEGRPFGC	DECALGATVD
**410**	**420**	**430**	**440**	**450**	**460**	**470**	**480**
APAAKPLASA	PGGPGCGPGS	DPVVPQRAPS	GERSFFCPDC	GRGFSHGQHL	ARHPRVHTGE	RPFACTQCDR	RFGSRPNLVA
**490**	**500**	**510**	**520**	**530**	**540**	**550**	**560**
HSRAHSGARP	FACAQCGRRF	SRKSHLGRHQ	AVHTGSRPHA	CAVCARSFSS	KTNLVRHQAI	HTGSRPFSCP	QCGKSFSRKT
**570**	**580**	**590**	**600**				
HLVRHQLIHG	EAAHAAPDAA	LAAPAWSAPP	EVAPPPLFF				

Cmpd.	No. of Cmpds.	m/z meas.	Δ m/z [ppm]	z	Rt [min]	Score	P	Range	Sequence	Modification
1902	1	785.7933	-55.89	2	52.1	10.0	1	185-199	R.LHRGEGPCACPDCGR.S	
340	2	544.7855	123.91	2	32.9	10.7	0	434-442	R.SFFCPDCGR.G	Carbamidomethyl: 7
1119	2	555.1856	-176.56	2	44.1	13.3	0	443-452	R.GFSHGQHLAR.H	
2174	1	849.0086	-71.63	3	57.0	10.8	1	537-558	R.HQAIHTGSRPFSCPQCGKSFSR.K	Carbamidomethyl: 13, 16

Detailed Protein Report

Protein 163: ubiquitin carboxyl-terminal hydrolase 35 [Homo sapiens]

**Accession:**	gi|148746183	**Score:**	44.7
**Database:**	refseq_human(refseq_human_20140103.fasta)	**MW [kDa]:**	113.3
**Database Date:**	2015-11-30	**pI:**	5.3
**Modification(s):**	Carbamidomethyl	**Sequence Coverage [%]:**	6.2
		**No. of unique Peptides:**	1

Quantitation

QU:MU	**Median:**	0.49	**CV:**	0.00 %	**No. of Peptides:**	1
WUP:QUP	**Median:**	1.96	**CV:**	0.00 %	**No. of Peptides:**	1

10	20	30	40	50	60	70	80
MDKILEAVVT	SSYPVSVKQG	LVRRVLEAAR	QPLEREQCLA	LLALGARLYV	GGAEELPRRV	GCQLLHVAGR	HHPDVFAEFF
90	100	110	120	130	140	150	160
SARRVLRLLQ	GGAGPPGPRA	LACVQLGLQL	LPEGPAADEV	FALLRREVLR	TVCERPGPAA	CAQVARLLAR	HPRCVPDGPH
170	180	190	200	210	220	230	240
RLLFCQQLVR	CLGRFRCPAE	GEEGAVEFLE	QAQQVSGLLA	QLWRAQPAAI	LPCLKELFAV	ISCAEEEPPS	SALASVVQHL
250	260	270	280	290	300	310	320
PLELMDGVVR	NLSNDDSVTD	SQMLTAISRM	IDWVSWPLGK	NIDKWIIALL	KGLAAVKKFS	ILIEVSLTKI	EKVFSKLLYP
330	340	350	360	370	380	390	400
IVRGAALSVL	KYMLLTFQHS	HEAFHLLLPH	IPPMVASLVK	EDSNSGTSCL	EQLAELVHCM	VFRFPGFPDL	YEPVMEAIKD
410	420	430	440	450	460	470	480
LHVPNEDRIK	QLLGQDAWTS	QKSELAGFYP	RLMAKSDTGK	IGLINLGNTC	YVNSILQALF	MASDFRHCVL	RLTENNSQPL
490	500	510	520	530	540	550	560
MTKLQWLFGF	LEHSQRPAIS	PENFLSASWT	PWFSPGTQQD	CSEYLKYLLD	RLHEEEKTGT	RICQKLKQSS	SPSPPEEPPA
570	580	590	600	610	620	630	640
PSSTSVEKMF	GGKIVTRICC	LCCLNVSSRE	EAFTDLSLAF	PPPERCRRRR	LGSVMRPTED	ITARELPPPT	SAQGPGRVGP
650	660	670	680	690	700	710	720
RRQRKHCITE	DTPPTSLYIE	GLDSKEAGGQ	SSQEERIERE	EEGKEERTEK	EEVGEEEEST	RGEGEREKEE	EVEEEEEKVE
730	740	750	760	770	780	790	800
KETEKEAEQE	KEEDSLGAGT	HPDAAIPSGE	RTCGSEGSRS	VLDLVNYFLS	PEKLTAENRY	YCESCASLQD	AEKVVELSQG
810	820	830	840	850	860	870	880
PCYLILTLLR	FSFDLRTMRR	RKILDDVSIP	LLLRLPLAGG	RGQAYDLCSV	VVHSGVSSES	GHYYCYAREG	AARPAASLGT
890	900	910	920	930	940	950	960
ADRPEPENQW	YLFNDTRVSF	SSFESVSNVT	SFFPKDTAYV	LFYRQRPREG	PEAELGSSRV	RTEPTLHKDL	MEAISKDNIL
970	980	990	1000	1010	1020		
YLQEQEKEAR	SRAAYISALP	TSPHWGRGFD	EDKDEDEGSP	GGCNPAGGNG	GDFHRLVF		

Cmpd.	No. of Cmpds.	m/z meas.	Δ m/z [ppm]	z	Rt [min]	Score	P	Range	Sequence	Modification	Ratios
1261	1	1070.0005	-62.40	2	45.9	12.2	1	131-150	R.TVCERPGPAACAQVARLLAR.H	Carbamidomethyl: 3	QU:MU 0.49<br>WUP:QUP 1.96

Detailed Protein Report

Protein 164: PREDICTED: collagen alpha-2(I) chain-like [Homo sapiens]

**Accession:**	gi|530375734	**Score:**	44.7
**Database:**	refseq_human(refseq_human_20140103.fasta)	**MW [kDa]:**	28.8
**Database Date:**	2015-11-30	**pI:**	12.9
		**Sequence Coverage [%]:**	11.6
		**No. of unique Peptides:**	1

10	20	30	40	50	60	70	80
MAPFHLSHAP	AGARKVKLRH	PRKWSPSGSP	QPPAQLQPLE	APRLPGEVRT	SGRLATWFCR	LLARRSAAQR	KSKSRKDLNR
**90**	**100**	**110**	**120**	**130**	**140**	**150**	**160**
RVQSPLTPHS	FHYFSSQGIG	GGGGGRDKFA	GRLAWSNPRG	WDRPRGSGGA	LPRPEGDCGG	ARPPAGGAGR	WASPGAAGRW
**170**	**180**	**190**	**200**	**210**	**220**	**230**	**240**
SLPGRVCGAF	AQPAPCPHLT	SSPPRPLGGP	LRCAAQARSV	AARAVRPRSL	GQGPGAQGVG	RAGRERGRPS	TASGSARAGA
**250**	**260**	**270**	**280**				
ALPAAVRPRE	GPRGPSACAL	GRRRLCVGRP	GGPRGTD				

Cmpd.	No. of Cmpds.	m/z meas.	Δ m/z [ppm]	z	Rt [min]	Score	P	Range	Sequence	Modification
1759	1	881.8978	-107.69	2	50.4	14.1	1	204-221	R.AVRPRSLGQGPGAQGVGR.A	

Detailed Protein Report

Protein 165: **PREDICTED: lipopolysaccharide-responsive and beige-like anchor protein isoform X3 [Homo sapiens]**

**Accession:**	gi|530378475	**Score:**	44.6
**Database:**	refseq_human(refseq_human_20140103.fasta)	**MW [kDa]:**	318.2
**Database Date:**	2015-11-30	**pI:**	5.3
		**Sequence Coverage [%]:**	2.1
		**No. of unique Peptides:**	4

Quantitation

WUP:QUP	**Median:**	0.66	**CV:**	0.00 %	**No. of Peptides:**	1

Detailed Protein Report

10	20	30	40	50	60	70	80
MASEDNRVPS	PPPTGDDGGG	GGREETPTEG	GALSLKPGLP	IRGIRMKFAV	LTGLVEVGEV	SNRDIVETVF	NLLVGGQFDL
90	100	110	120	130	140	150	160
EMNFIIQEGE	SINCMVDLLE	KCDITCQAEV	WSMFTAILKK	SIRNLQVCTE	VGLVEKVLGK	IEKVDNMIAD	LLVDMLGVLA
170	180	190	200	210	220	230	240
SYNLTVRELK	LFFSKLQGDK	GRWPPHAGKL	LSVLKHMPQK	YGPDAFFNFP	GKSAAAIALP	PIAKWPYQNG	FTFHTWLRMD
250	260	270	280	290	300	310	320
PVNNINVDKD	KPYLYCFRTS	KGLGYSAHFV	GGCLIVTSIK	SKGKGFQHCV	KFDFKPQKWY	MVTIVHIYNR	WKNSELRCYV
330	340	350	360	370	380	390	400
NGELASYGEI	TWFVNTSDTF	DKCFLGSSET	ADANRVFCGQ	MTAVYLFSEA	LNAAQIFAIY	QLGLGYKGTF	KFKAESDLFL
410	420	430	440	450	460	470	480
AEHHKLLLYD	GKLSSAIAFT	YNPRATDAQL	CLESSPKDNP	SIFVHSPHAL	MLQDVKAVLT	HSIQSAMHSI	GGVQVLFPLF
490	500	510	520	530	540	550	560
AQLDYRQYLS	DEIDLTICST	LLAFIMELLK	NSIAMQEQML	ACKGFLVIGY	SLEKSSKSHV	SRAVLELCLA	FSKYLSNLQN
570	580	590	600	610	620	630	640
GMPLLKQLCD	HVLLNPAIWI	HTPAKVQLML	YTYLSTEFIG	TVNIYNTIRR	VGTVLLIMHT	LKYYYWAVNP	QDRSGITPKG
650	660	670	680	690	700	710	720
LDGPRPNQKE	MLSLRAFLLM	FIKQLVMKDS	GVKEDELQAI	LNYLLTMHED	DNLMDVLQLL	VALMSEHPNS	MIPAFDQRNG
730	740	750	760	770	780	790	800
LRVIYKLLAS	KSEGIRVQAL	KAMGYFLKHL	APKRKAEVML	GHGLFSLLAE	RLMLQTNLIT	MTTYNVLFEI	LIEQIGTQVI
810	820	830	840	850	860	870	880
HKQHPDPDSS	VKIQNPQILK	VIATLLRNSP	QCPESMEVRR	AFLSDMIKLF	NNSRENRRSL	LQCSVWQEWM	LSLCYFNPKN
890	900	910	920	930	940	950	960
SDEQKITEMV	YAIFRILLYH	AVKYEWGGWR	VWVDTLSITH	SKVTFEIHKE	NLANIFREQQ	GKVDEEIGLC	SSTSVQAASG
970	980	990	1000	1010	1020	1030	1040
IRRDINVSVG	SQQPDTKDSP	VCPHFTTNGN	ENSSIEKTSS	LESASNIELQ	TTNTSYEEMK	AEQENQELPD	EGTLEETLTN
1050	1060	1070	1080	1090	1100	1110	1120
ETRNADDLEV	SSDIIEAVAI	SSNSFITTGK	DSMTVSEVTA	SISSPSEEDA	SEMPEFLDKS	IVEEEEDDDY	VELKVEGSPT
1130	1140	1150	1160	1170	1180	1190	1200
EEANLPTELQ	DNSLSPAASE	AGEKLDMFGN	DDKLIFQEGK	PVTEKQTDTE	TQDSKDSGIQ	TMTASGSSAM	SPETTVSQIA
1210	1220	1230	1240	1250	1260	1270	1280
VESDLGQMLE	EGKKATNLTR	ETKLINDCHG	SVSEASSEQK	IAKLDVSNVA	TDTERLELKA	SPNVEAPQPH	RHVLEISRQH
1290	1300	1310	1320	1330	1340	1350	1360
EQPGQGIAPD	AVNGQRRDSR	STVFRIPEFN	WSQMHQRLLT	DLLFSIETDI	QMWRSHSTKT	VMDFVNSSDN	VIFVHNTIHL
1370	1380	1390	1400	1410	1420	1430	1440
ISQVMDNMVM	ACGGILPLLS	AATSATHELE	NIEPTQGLSI	EASVTFLQRL	ISLVDVLIFA	SSLGFTEIEA	EKSMSSGGIL
1450	1460	1470	1480	1490	1500	1510	1520
RQCLRLVCAV	AVRNCLECQQ	HSQLKTRGDK	ALKPMHSLIP	LGKSAAKSPV	DIVTGGISPV	RDLDRLLQDM	DINRLRAVVF
1530	1540	1550	1560	1570	1580	1590	1600
RDIEDSKQAQ	FLALAVVYFI	SVLMVSKYRD	ILEPQNERHS	QSCTETGSEN	ENVSLSEITP	AAFSTLTTAS	VEESESTSSA
1610	1620	1630	1640	1650	1660	1670	1680
RRRDSGIGEE	TATGLGSHVE	VTPHTAPPGV	SAGPDAISEV	LSTLSLEVNK	SPETKNDRGN	DLDTKATPSV	SVSKNVNVKD
1690	1700	1710	1720	1730	1740	1750	1760
ILRSLVNIPA	DGVTVDPALL	PPACLGALGD	LSVEQPVQFR	SFDRSVIVAA	KKSAVSPSTF	NTSIPTNAVS	VVSSVDSAQA
1770	1780	1790	1800	1810	1820	1830	1840
SDMGGESPGS	RSSNAKLPSV	PTVDSVSQDP	VSNMSITERL	EHALEKAAPL	LREIFVDFAP	FLSRTLLGSH	GQELLIEGTS
1850	1860	1870	1880	1890	1900	1910	1920
LVCMKSSSSV	VELVMLLCSQ	EWQNSIQKNA	GLAFIELVNE	GRLLSQTMKD	HLVRVANEAE	FILSRQRAED	IHRHAEFESL
1930	1940	1950	1960	1970	1980	1990	2000
CAQYSADKRE	DEKMCDHLIR	AAKYRDHVTA	TQLIQKIINI	LTDKHGAWGN	SAVSRPLEFW	RLDYWEDDLR	RRRRFVRNPL
2010	2020	2030	2040	2050	2060	2070	2080
GSTHPEATLK	TAVEHATDED	ILAKGKQSIR	SQALGNQNSE	NEILLEGDDD	TLSSVDEKDL	ENLAGPVSLS	TPAQLVAPSV
2090	2100	2110	2120	2130	2140	2150	2160
VVKGTLSVTS	SELYFEVDEE	DPNFKKIDPK	ILAYTEGLHG	KWLFTEIRSI	FSRRYLLQNT	ALEIFMANRV	AVMFNFPDPA
2170	2180	2190	2200	2210	2220	2230	2240

Detailed Protein Report

Cmpd.	No. of Cmpds.	m/z meas.	Δ m/z [ppm]	z	Rt [min]	Score	P	Range	Sequence	Modification	Ratios
1142	1	555.2290	-64.32	2	44.4	10.2	0	803-812	K.QHPDPDSSVK.I		
1230	1	715.1053	93.99	3	43.7	12.4	1	821-839	K.VIATLLRNSPQCPESMEVR.R		
1642	1	698.8555	-54.14	2	50.8	10.1	0	1488-1501	K.SPVDIVTGGISPVR.D		
2042	1	766.2799	-83.49	2	55.4	11.9	0	2669-2684	K.CSGIGDNPGSETAAPR.A		WUP:QUP 0.66

Detailed Protein Report

Protein 166: PREDICTED: lipoxygenase homology domain-containing protein 1 isoform X1 [Homo sapiens]

**Accession:**	gi|578832310	**Score:**	44.5
**Database:**	refseq_human(refseq_human_20140103.fasta)	**MW [kDa]:**	220.7
**Database Date:**	2015-11-30	**pI:**	5.2
**Modification(s):**	Carbamidomethyl	**Sequence Coverage [%]:**	2.0
		**No. of unique Peptides:**	3

Quantitation

QU:MU	**Median:** 1.31	**CV:** 0.00 %	**No. of Peptides:** 1

Detailed Protein Report

10	20	30	40	50	60	70	80
MMQLTLNTLF	PVVSTPAITY	IVTVFTGDVR	GAGTKSKIYL	VMYGARGNKN	SGKIFLEGGV	FDRGRTDIFH	IELAVLLSPL
90	100	110	120	130	140	150	160
SRVSVGHGNV	GVNRGWFCEK	VVILCPFTGI	QQTFPCSNWL	DEKKADGLIE	RQLYEMVSLR	KKRLKKFPWS	LWVWTTDLKK
170	180	190	200	210	220	230	240
AGTNSPIFIQ	IYGQKGRTDE	ILLNPNNKWF	KPGIIEKFRI	ELPDLGRFYK	IRVWHDKRSS	GSGWHLERMT	LMNTLNKDKY
250	260	270	280	290	300	310	320
NFNCNRWLDA	NEDDNEIVRE	MTAEGPTVRR	IMGMARYHVT	VCTGELEGAG	TDANVYLCLF	GDVGDTGERL	LYNCRNNTDL
330	340	350	360	370	380	390	400
FEKGNADEFT	IESVTMRNVR	RVRIRHDGKG	SGSGWYLDRV	LVREEGQPES	DNVEFPCLRW	LDKDKDDGQL	VRELLPSDSS
410	420	430	440	450	460	470	480
ATLKNFRYHI	SLKTGDVSGA	STDSRVYIKL	YGDKSDTIKQ	VLLVSDNNLK	DYFERGRVDE	FTLETLNIGN	INRLVIGHDS
490	500	510	520	530	540	550	560
TGMHASWFLG	SVQIRVPRQG	KQYTFPANRW	LDKNQADGRL	EVELYPSEVV	EIQKLVHYEV	EIWTGDVGGA	GTSARVYMQI
570	580	590	600	610	620	630	640
YGEKGKTEVL	FLSSRSKVFE	RASKDTFQLE	AADVGEVYKL	RLGHTGEGFG	PSWFVDTVWL	RHLVVREVDL	TPEEEARKKK
650	660	670	680	690	700	710	720
EKDKLRQLLK	KERLKAKLQR	KKKKRKGSDE	EDEGEEEESS	SSEESSSEEE	EMEEEEEEEE	FGPGMQEVIE	QHKFEAHRWL
730	740	750	760	770	780	790	800
ARGKEDNELV	VELVPAGKPG	PERNTYEVQV	VTGNVPKAGT	DANVYLTIYG	EEYGDTGERP	LKKSDKSNKF	EQGQTDTFTI
810	820	830	840	850	860	870	880
YAIDLGALTK	IRIRHDNTGN	RAGWFLDRID	ITDMNNEITY	YFPCQRWLAV	EEDDGQLSRE	LLPVDESYVL	PQSEEGRGGG
890	900	910	920	930	940	950	960
DNNPLDNLAL	EQKDKSTTFS	VTIKTGVKKN	AGTDANVFIT	LFGTQDDTGM	TLLKSSKTNS	DKFERDSIEI	FTVETLDLGD
970	980	990	1000	1010	1020	1030	1040
LWKVRLGHDN	TGKAPGWFVD	WVEVDAPSLG	KCMTFPCGRW	LAKNEDDGSI	IRDLFHAELQ	TRLYTPFVPY	EITLYTSDVF
1050	1060	1070	1080	1090	1100	1110	1120
AAGTDANIFI	IIYGCDAVCT	QQKYLCTNKR	EQKQFFERKS	ASRFIVELED	VGEIIEKIRI	GHNNTGMNPG	WHCSHVDIRR
1130	1140	1150	1160	1170	1180	1190	1200
LLPDKDGAET	LTFPCDRWLA	TSEDDKKTIR	ELVPYDIFTE	KYMKDGSLRQ	VYKEVEEPLD	IVLYSVQIFT	GNIPGAGTDA
1210	1220	1230	1240	1250	1260	1270	1280
KVYITIYGDL	GDTGERYLGK	SENRTNKFER	GTADTFIIEA	ADLGVIYKIK	LRHDNSKWCA	DWYVEKVEIW	NDTNEDEFLF
1290	1300	1310	1320	1330	1340	1350	1360
LCGRWLSLKK	EDGRLERLFY	EKEYTGDRSS	NCSSPADFWE	IALSSKMADV	DISTVTGPMA	DYVQEGPIIP	YYVSVTTGKH
1370	1380	1390	1400	1410	1420	1430	1440
KDAATDSRAF	IFLIGEDDER	SKRIWLDYPR	GKRGFSRGSV	EEFYVAGLDV	GIIKKIEVLY	EMTVWTGDVV	GGGTDSNIFM
1450	1460	1470	1480	1490	1500	1510	1520
TLYGINGSTE	EMQLDKKKAR	FEREQNDTFI	MEILDIAPFT	KMRIRIDGLG	SRPEWFLERI	LLKNMNTGDL	TMFYYGDWLS
1530	1540	1550	1560	1570	1580	1590	1600
QRKGKKTLVC	EMCAVIDEEE	MMEWTSYTVA	VKTSDILGAG	TDANVFIIIF	GENGDSGTLA	LKQSANWNKF	ERNNTDTFNF
1610	1620	1630	1640	1650	1660	1670	1680
PDMLSLGHLC	KLRVWHDNKG	IFPGWHLSYV	DVKDNSRDET	FHFQCDCWLS	KSEGDGQTVR	DFACANNKIC	DELEETTYEI
1690	1700	1710	1720	1730	1740	1750	1760
VIETGNGGET	RENVWLILEG	RKNRSKEFLM	ENSSRQRAFR	KGTTDTFEFD	SIYLGDIASL	CVGHLAREDR	FIPKRELAWH
1770	1780	1790	1800	1810	1820	1830	1840
VKTITITEME	YGNVYFFNCD	CLIPLKRKRK	YFKVFEVTKT	TESFASKVQS	LVPVKYEVIV	TTGYEPGAGT	DANVFVTIFG
1850	1860	1870	1880	1890	1900	1910	1920
ANGDTGKREL	KQKMRNLFER	GSTDRFFLET	LELGELRKVR	LEHDSSGYCS	GWLVEKVEVT	NTSTGVATIF	NCGRWLDKKR
1930	1940						
GDGLTWRDLF	PSV						

Cmpd.	No. of Cmpds.	m/z meas.	Δ m/z [ppm]	z	Rt [min]	Score	P	Range	Sequence	Modification	Ratios
2434	1	1045.2535	-261.87	1	60.9	17.6	1	384-392	K.DKDDGQLVR.E		
2071	1	1052.4367	-86.97	2	56.3	14.8	2	1120-1137	R.RLLPDKDGAETLTFPCDR.W	Carbamidomethyl: 16	

Detailed Protein Report

Cmpd.	No. of Cmpds.	m/z meas.	Δ m/z [ppm]	z	Rt [min]	Score	P	Range	Sequence	Modification	Ratios
1581	1	698.7063	-184.90	2	50.0	12.1	1	1707-1717	K.EFLMENSSRQR.A		QU:MU 1.31

Detailed Protein Report

Protein 167: PREDICTED: plexin-A1 isoform X1 [Homo sapiens]

**Accession:**	gi|578807485	**Score:**	44.5
**Database:**	refseq_human(refseq_human_20140103.fasta)	**MW [kDa]:**	212.5
**Database Date:**	2015-11-30	**pI:**	6.6
**Modification(s):**	Oxidation	**Sequence Coverage [%]:**	2.3
		**No. of unique Peptides:**	1

10	20	30	40	50	60	70	80
MMLTPAGPEH	RGPRPQPAMP	LPPRSLQVLL	LLLLLLLLLP	GMWAEAGLPR	AGGGSQPPFR	TFSASDWGLT	HLVVHEQTGE
90	100	110	120	130	140	150	160
VYVGAVNRIY	KLSGNLTLLR	AHVTGPVEDN	EKCYPPPSVQ	SCPHGLGSTD	NVNKLLLLDY	AANRLLACGS	ASQGICQFLR
170	180	190	200	210	220	230	240
LDDLFKLGEP	HHRKEHYLSS	VQEAGSMAGV	LIAGPPGQGQ	AKLFVGTPID	GKSEYFPTLS	SRRLMANEED	ADMFGFVYQD
250	260	270	280	290	300	310	320
EFVSSQLKIP	SDTLSKFPAF	DIYYVYSFRS	EQFVYYLTLQ	LDTQLTSPDA	AGEHFFTSKI	VRLCVDDPKF	YSYVEFPIGC
330	340	350	360	370	380	390	400
EQAGVEYRLV	QDAYLSRPGR	ALAHQLGLAE	DEDVLFTVFA	QGQKNRVKPP	KESALCLFTL	RAIKEKIKER	IQSCYRGEGK
410	420	430	440	450	460	470	480
LSLPWLLNKE	LGCINSPLQI	DDDFCGQDFN	QPLGGTVTIE	GTPLFVDKDD	GLTAVAAYDY	RGRTVVFAGT	RSGRIRKILV
490	500	510	520	530	540	550	560
DLSNPGGRPA	LAYESVVAQE	GSPILRDLVL	SPNHQYLYAM	TEKQVTRVPV	ESCVQYTSCE	LCLGSRDPHC	GWCVLHSICS
570	580	590	600	610	620	630	640
RRDACERADE	PQRFAADLLQ	CVQLTVQPRN	VSVTMSQVPL	VLQAWNVPDL	SAGVNCSFED	FTESESVLED	GRIHCRSPSA
650	660	670	680	690	700	710	720
REVAPITRGQ	GDQRVVKLYL	KSKETGKKFA	SVDFVFYNCS	VHQSCLSCVN	GSFPCHWCKY	RHVCTHNVAD	CAFLEGRVNV
730	740	750	760	770	780	790	800
SEDCPQILPS	TQIYVPVGVV	KPITLAARNL	PQPQSGQRGY	ECLFHIPGSP	ARVTALRFNS	SSLQCQNSSY	SYEGNDVSDL
810	820	830	840	850	860	870	880
PVNLSVVWNG	NFVIDNPQNI	QAHLYKCPAL	RESCGLCLKA	DPRFECGWCV	AERRCSLRHH	CAADTPASWM	HARHGSSRCT
890	900	910	920	930	940	950	960
DPKILKLSPE	TGPRQGGTRL	TITGENLGLR	FEDVRLGVRV	GKVLCSPVES	EYISAEQIVC	EIGDASSVRA	HDALVEVCVR
970	980	990	1000	1010	1020	1030	1040
DCSPHYRALS	PKRFTFVTPT	FYRVSPSRGP	LSGGTWIGIE	GSHLNAGSDV	AVSVGGRPCS	FSWRNSREIR	CLTPPGQSPG
1050	1060	1070	1080	1090	1100	1110	1120
SAPIIININR	AQLTNPEVKY	NYTEDPTILR	IDPEWSINSG	GTLLTVTGTN	LATVREPRIR	AKYGGIEREN	GCLVYNDTTM
1130	1140	1150	1160	1170	1180	1190	1200
VCRAPSVANP	VRSPPELGER	PDELGFVMDN	VRSLLVLNST	SFLYYPDPVL	EPLSPTGLLE	LKPSSPLILK	GRNLLPPAPG
1210	1220	1230	1240	1250	1260	1270	1280
NSRLNYTVLI	GSTPCTLTVS	ETQLLCEAPN	LTGQHKVTVR	AGGFEFSPGT	LQVYSDSLLT	LPAIVGIGGG	GGLLLLVIVA
1290	1300	1310	1320	1330	1340	1350	1360
VLIAYKRKSR	DADRTLKRLQ	LQMDNLESRV	ALECKEAFAE	LQTDIHELTN	DLDGAGIPFL	DYRTYAMRVL	FPGIEDHPVL
1370	1380	1390	1400	1410	1420	1430	1440
KEMEVQANVE	KSLTLFGQLL	TKKHFLLTFI	RTLEAQRSFS	MRDRGNVASL	IMTALQGEME	YATGVLKQLL	SDLIEKNLES
1450	1460	1470	1480	1490	1500	1510	1520
KNHPKLLLRR	TESVAEKMLT	NWFTFLLYKF	LKECAGEPLF	MLYCAIKQQM	EKGPIDAITG	EARYSLSEDK	LIRQQIDYKT
1530	1540	1550	1560	1570	1580	1590	1600
LTLNCVNPEN	ENAPEVPVKG	LDCDTVTQAK	EKLLDAAYKG	VPYSQRPKAA	DMDLEWRQGR	MARIILQDED	VTTKIDNDWK
1610	1620	1630	1640	1650	1660	1670	1680
RLNTLAHYQV	TDGSSVALVP	KQTSAYNISN	SSTFTKSLSR	YESMLRTASS	PDSLRSRTPM	ITPDLESGTK	LWHLVKNHDH
1690	1700	1710	1720	1730	1740	1750	1760
LDQREGDRGS	KMVSEIYLTR	LLATKKFVDD	LFETIFSTAH	RGSALPLAIK	YMFDFLDEQA	DKHQIHDADV	RHTWKSNCLP
1770	1780	1790	1800	1810	1820	1830	1840
LRFWVNVIKN	PQFVFDIHKN	SITDACLSVV	AQTFMDSCST	SEHKLGKDSP	SNKLLYAKDI	PNYKSWVERY	YADIAKMPAI
1850	1860	1870	1880	1890	1900	1910	1920
SDQDMSAYLA	EQSRLHLSQF	NSMSALHEIY	SYITKYKDEI	LAALEKDEQA	RRQRLRSKLE	QVVDTMALSS	

Cmpd.	No. of Cmpds.	m/z meas.	Δ m/z [ppm]	z	Rt [min]	Score	P	Range	Sequence	Modification

Detailed Protein Report

Cmpd.	No. of Cmpds.	m/z meas.	Δ m/z [ppm]	z	Rt [min]	Score	P	Range	Sequence	Modification
1881	1	628.2724	-43.47	2	53.9	15.1	0	1-11	-.MMLTPAGPEHR.G	Oxidation: 1

Detailed Protein Report

Protein 168: fibrillin-2 precursor [Homo sapiens]

**Accession:**	gi|66346695	**Score:**	44.4
**Database:**	refseq_human(refseq_human_20140103.fasta)	**MW [kDa]:**	314.6
**Database Date:**	2015-11-30	**pI:**	4.6
**Modification(s):**	Carbamidomethyl	**Sequence Coverage [%]:**	1.5
		**No. of unique Peptides:**	3

Quantitation

QU:MU	**Median:**	1.09	**CV:**	63.27 %	**No. of Peptides:**	2
WUP:QUP	**Median:**	1.03	**CV:**	41.37 %	**No. of Peptides:**	2

Detailed Protein Report

10	20	30	40	50	60	70	80
MGRRRRLCLQ	LYFLWLGCVV	LWAQGTAGQP	QPPPPKPPRP	QPPPQQVRSA	TAGSEGGFLA	PEYREEGAAV	ASRVRRRGQQ
90	100	110	120	130	140	150	160
DVLRGPNVCG	SRFHSYCCPG	WKTLPGGNQC	IVPICRNSCG	DGFCSRPNMC	TCSSGQISST	CGSKSIQQCS	VRCMNGGTCA
170	180	190	200	210	220	230	240
DDHCQCQKGY	IGTYCGQPVC	ENGCQNGGRC	IGPNRCACVY	GFTGPQCERD	YRTGPCFTQV	NNQMCQGQLT	GIVCTKTLCC
250	260	270	280	290	300	310	320
ATIGRAWGHP	CEMCPAQPQP	CRRGFIPNIR	TGACQDVDEC	QAIPGICQGG	NCINTVGSFE	CRCPAGHKQS	ETTQKCEDID
330	340	350	360	370	380	390	400
ECSIIPGICE	TGECSNTVGS	YFCVCPRGYV	TSTDGSRCID	QRTGMCFSGL	VNGRCAQELP	GRMTKMQCCC	EPGRCWGIGT
410	420	430	440	450	460	470	480
IPEACPVRGS	EEYRRLCMDG	LPMGGIPGSA	GSRPGGTGGN	GFAPSGNGNG	YGPGGTGFIP	IPGGNGFSPG	VGGAGVGAGG
490	500	510	520	530	540	550	560
QGPIITGLTI	LNQTIDICKH	HANLCLNGRC	IPTVSSYRCE	CNMGYKQDAN	GDCIDVDECT	SNPCTNGDCV	NTPGSYYCKC
570	580	590	600	610	620	630	640
HAGFQRTPTK	QACIDIDECI	QNGVLCKNGR	CVNTDGSFQC	ICNAGFELTT	DGKNCVDHDE	CTTTNMCLNG	MCINEDGSFK
650	660	670	680	690	700	710	720
CICKPGFVLA	PNGRYCTDVD	ECQTPGICMN	GHCINSEGSF	RCDCPPGLAV	GMDGRVCVDT	HMRSTCYGGI	KKGVCVRPFP
730	740	750	760	770	780	790	800
GAVTKSECCC	ANPDYGFGEP	CQPCPAKNSA	EFHGLCSSGV	GITVDGRDIN	ECALDPDICA	NGICENLRGS	YRCNCNSGYE
810	820	830	840	850	860	870	880
PDASGRNCID	IDECLVNRLL	CDNGLCRNTP	GSYSCTCPPG	YVFRTETETC	EDINECESNP	CVNGACRNNL	GSFNCECSPG
890	900	910	920	930	940	950	960
SKLSSTGLIC	IDSLKGTCWL	NIQDSRCEVN	INGATLKSEC	CATLGAAWGS	PCERCELDTA	CPRGLARIKG	VTCEDVNECE
970	980	990	1000	1010	1020	1030	1040
VFPGVCPNGR	CVNSKGSFHC	ECPEGLTLDG	TGRVCLDIRM	EQCYLKWDED	ECIHPVPGKF	RMDACCCAVG	AAWGTECEEC
1050	1060	1070	1080	1090	1100	1110	1120
PKPGTKEYET	LCPRGAGFAN	RGDVLTGRPF	YKDINECKAF	PGMCTYGKCR	NTIGSFKCRC	NSGFALDMEE	RNCTDIDECR
1130	1140	1150	1160	1170	1180	1190	1200
ISPDLCGSGI	CVNTPGSFEC	ECFEGYESGF	MMMKNCMDID	ECERNPLLCR	GGTCVNTEGS	FQCDCPLGHE	LSPSREDCVD
1210	1220	1230	1240	1250	1260	1270	1280
INECSLSDNL	CRNGKCVNMI	GTYQCSCNPG	YQATPDRQGC	TDIDECMIMN	GGCDTQCTNS	EGSYECSCSE	GYALMPDGRS
1290	1300	1310	1320	1330	1340	1350	1360
CADIDECENN	PDICDGGQCT	NIPGEYRCLC	YDGFMASMDM	KTCIDVNECD	LNSNICMFGE	CENTKGSFIC	HCQLGYSVKK
1370	1380	1390	1400	1410	1420	1430	1440
GTTGCTDVDE	CEIGAHNCDM	HASCLNIPGS	FKCSCREGWI	GNGIKCIDLD	ECSNGTHQCS	INAQCVNTPG	SYRCACSEGF
1450	1460	1470	1480	1490	1500	1510	1520
TGDGFTCSDV	DECAENINLC	ENGQCLNVPG	AYRCECEMGF	TPASDSRSCQ	DIDECSFQNI	CVFGTCNNLP	GMFHCICDDG
1530	1540	1550	1560	1570	1580	1590	1600
YELDRTGGNC	TDIDECADPI	NCVNGLCVNT	PGRYECNCPP	DFQLNPTGVG	CVDNRVGNCY	LKFGPRGDGS	LSCNTEIGVG
1610	1620	1630	1640	1650	1660	1670	1680
VSRSSCCCSL	GKAWGNPCET	CPPVNSTEYY	TLCPGGEGFR	PNPITIILED	IDECQELPGL	CQGGNCINTF	GSFQCECPQG
1690	1700	1710	1720	1730	1740	1750	1760
YYLSEDTRIC	EDIDECFAHP	GVCGPGTCYN	TLGNYTCICP	PEYMQVNGGH	NCMDMRKSFC	YRSYNGTTCE	NELPFNVTKR
1770	1780	1790	1800	1810	1820	1830	1840
MCCCTYNVGK	AWNKPCEPCP	TPGTADFKTI	CGNIPGFTFD	IHTGKAVDID	ECKEIPGICA	NGVCINQIGS	FRCECPTGFS
1850	1860	1870	1880	1890	1900	1910	1920
YNDLLLVCED	IDECSNGDNL	CQRNADCINS	PGSYRCECAA	GFKLSPNGAC	VDRNECLEIP	NVCSHGLCVD	LQGSYQCICH
1930	1940	1950	1960	1970	1980	1990	2000
NGFKASQDQT	MCMDVDECER	HPCGNGTCKN	TVGSYNCLCY	PGFELTHNND	CLDIDECSSF	FGQVCRNGRC	FNEIGSFKCL
2010	2020	2030	2040	2050	2060	2070	2080
CNEGYELTPD	GKNCIDTNEC	VALPGSCSPG	TCQNLEGSFR	CICPPGYEVK	SENCIDINEC	DEDPNICLFG	SCTNTPGGFQ
2090	2100	2110	2120	2130	2140	2150	2160
CLCPPGFVLS	DNGRRCFDTR	QSFCFTNFEN	GKCSVPKAFN	TTKAKCCCSK	MPGEGWGDPC	ELCPKDDEVA	FQDLCPYGHG
2170	2180	2190	2200	2210	2220	2230	2240

Detailed Protein Report

Cmpd.	No. of Cmpds.	m/z meas.	Δ m/z [ppm]	z	Rt [min]	Score	P	Range	Sequence	Modification	Ratios
186	1	806.9057	27.18	2	31.2	11.0	0	49-64	R.SATAGSEGGFLAPEYR.E		QU:MU 0.61<br>WUP:QUP 0.69
2112	1	600.2270	-134.67	3	54.8	15.6	2	77-92	R.RGQQDVLRGPNVCGSR.F	Carbamidomethyl: 13	
323	5	642.1545	-201.52	2	33.7	17.8	1	2529-2539	K.TCKDLDECQTK.Q		QU:MU 1.94<br>WUP:QUP 1.54

Detailed Protein Report

Protein 169: **sushi, nidogen and EGF-like domain-containing protein 1 precursor [Homo sapiens]**

**Accession:**	gi|122937283	**Score:**	44.1
**Database:**	refseq_human(refseq_human_20140103.fasta)	**MW [kDa]:**	152.1
**Database Date:**	2015-11-30	**pI:**	6.6
**Modification(s):**	Carbamidomethyl	**Sequence Coverage [%]:**	1.3
		**No. of unique Peptides:**	3

Alias proteins:

Accession	Name	Description
gi|578804032	refseq_human (refseq_human_20140103.fasta)	PREDICTED: sushi, nidogen and EGF-like domain-containing protein 1 isoform X2 [Homo sapiens]

10	20	30	40	50	60	70	80
MRHGVAWALL	VAAALGLGAR	GVRGAVALAD	FYPFGAERGD	AVTPKQDDGG	SGLRPLSVPF	PFFGAEHSGL	YVNNNGIISF
**90**	**100**	**110**	**120**	**130**	**140**	**150**	**160**
LKEVSQFTPV	AFPIAKDRCV	VAAFWADVDN	RRAGDVYYRE	ATDPAMLRRA	TEDVRHYFPE	LLDFNATWVF	VATWYRVTFF
**170**	**180**	**190**	**200**	**210**	**220**	**230**	**240**
GGSSSSPVNT	FQTVLITDGK	LSFTIFNYES	IVWTTGTHAS	SGGNATGLGG	IAAQAGFNAG	DGQRYFSIPG	SRTADMAEVE
**250**	**260**	**270**	**280**	**290**	**300**	**310**	**320**
TTTNVGVPGR	WAFRIDDAQV	RVGGCGHTTS	VCLALRPCLN	GGKCIDDCVT	GNPSYTCSCL	SGFTGRRCHL	DVNECASQPC
**330**	**340**	**350**	**360**	**370**	**380**	**390**	**400**
QNGGTCTHGI	NSFRCQCPAG	FGGPTCETAQ	SPCDTKECQH	GGQCQVENGS	AVCVCQAGYT	GAACEMDVDD	CSPDPCLNGG
**410**	**420**	**430**	**440**	**450**	**460**	**470**	**480**
SCVDLVGNYT	CLCAEPFKGL	RCETGDHPVP	DACLSAPCHN	GGTCVDADQG	YVCECPEGFM	GLDCRERVPD	DCECRNGGRC
**490**	**500**	**510**	**520**	**530**	**540**	**550**	**560**
LGANTTLCQC	PLGFFGLLCE	FEITAMPCNM	NTQCPDGGYC	MEHGGSYLCV	CHTDHNASHS	LPSPCDSDPC	FNGGSCDAHD
**570**	**580**	**590**	**600**	**610**	**620**	**630**	**640**
DSYTCECPRG	FHGKHCEKAR	PHLCSSGPCR	NGGTCKEAGG	EYHCSCPYRF	TGRHCEIGKP	DSCASGPCHN	GGTCFHYIGK
**650**	**660**	**670**	**680**	**690**	**700**	**710**	**720**
YKCDCPPGFS	GRHCEIAPSP	CFRSPCVNGG	TCEDRDTDFF	CHCQAGYMGR	RCQAEVDCGP	PEEVKHATLR	FNGTRLGAVA
**730**	**740**	**750**	**760**	**770**	**780**	**790**	**800**
LYACDRGYSL	SAPSRIRVCQ	PHGVWSEPPQ	CLEIDECRSQ	PCLHGGSCQD	RVAGYLCLCS	TGYEGAHCEL	ERDECRAHPC
**810**	**820**	**830**	**840**	**850**	**860**	**870**	**880**
RNGGSCRNLP	GAYVCRCPAG	FVGVHCETEV	DACDSSPCQH	GGRCESGGGA	YLCVCPESFF	GYHCETVSDP	CFSSPCGGRG
**890**	**900**	**910**	**920**	**930**	**940**	**950**	**960**
YCLASNGSHS	CTCKVGYTGE	DCAKELFPPT	ALKMERVEES	GVSISWNPPN	GPAARQMLDG	YAVTYVSSDG	SYRRTDFVDR
**970**	**980**	**990**	**1000**	**1010**	**1020**	**1030**	**1040**
TRSSHQLQAL	AAGRAYNISV	FSVKRNSNNK	NDISRPAVLL	ARTRPRPVEG	FEVTNVTAST	ISVQWALHRI	RHATVSGVRV
**1050**	**1060**	**1070**	**1080**	**1090**	**1100**	**1110**	**1120**
SIRHPEALRD	QATDVDRSVD	RFTFRALLPG	KRYTIQLTTL	SGLRGEEHPT	ESLATAPTHV	WTRPLPPANL	TAARVTATSA
**1130**	**1140**	**1150**	**1160**	**1170**	**1180**	**1190**	**1200**
HVVWDAPTPG	SLLEAYVINV	TTSQSTKSRY	VPNGKLASYT	VRDLLPGRRY	QLSVIAVQST	ELGPQHSEPA	HLYIITSPRD
**1210**	**1220**	**1230**	**1240**	**1250**	**1260**	**1270**	**1280**
GADRRWHQGG	HHPRVLKNRP	PPARLPELRL	LNDHSAPETP	TQPPRFSELV	DGRGRVSARF	GGSPSKAATV	RSQPTASAQL
**1290**	**1300**	**1310**	**1320**	**1330**	**1340**	**1350**	**1360**
ENMEEAPKRV	SLALQLPEHG	SKDIGNVPGN	CSENPCQNGG	TCVPGADAHS	CDCGPGFKGR	RCELACIKVS	RPCTRLFSET
**1370**	**1380**	**1390**	**1400**	**1410**	**1420**		
KAFPVWEGGV	CHHVYKRVYR	VHQDICFKES	CESTSLKKTP	NRKQSKSQTL	EKS		

Cmpd.	No. of Cmpds.	m/z meas.	Δ m/z [ppm]	z	Rt [min]	Score	P	Range	Sequence	Modification
2079	1	1044.9332	12.94	2	56.4	14.4	1	591-609	R.NGGTCKEAGGEYHCSCPYR.F	Carbamidomethyl: 5
1667	1	1073.2934	-127.64	2	49.2	19.6	1	591-609	R.NGGTCKEAGGEYHCSCPYR.F	Carbamidomethyl: 5, 16
2019	1	715.9639	11.03	3	53.6	10.1	1	591-609	R.NGGTCKEAGGEYHCSCPYR.F	Carbamidomethyl: 14, 16

Detailed Protein Report

Protein 170: PREDICTED: coiled-coil domain-containing protein 88B isoform X1 [Homo sapiens]

**Accession:**	gi|578821335	**Score:**	44.0
**Database:**	refseq_human(refseq_human_20140103.fasta)	**MW [kDa]:**	173.8
**Database Date:**	2015-11-30	**pI:**	5.0
		**Sequence Coverage [%]:**	2.8
		**No. of unique Peptides:**	3

Quantitation

WUP:QUP **Median:** 4.17 **CV:** 0.00 % **No. of Peptides:** 1

10	20	30	40	50	60	70	80
MEGGKGPRLR	DFLSGSLATW	ALGLAGLVGE	AEDSEGEEEE	EEEEPPLWLE	KRFLRLSDGA	LLLRVLGIIA	PSSRGGPRML
90	100	110	120	130	140	150	160
RGLDGPAAWR	VWNLNHLWGR	LRDFYQEELQ	LLILSPPPDL	QTLGFDPLSE	EAVEQLEGVL	RLLLGASVQC	EHRELFIRHI
170	180	190	200	210	220	230	240
QGLSLEVQSE	LAAAIQEVTQ	PGAGVVLALS	GPDPGELAPA	ELEMLSRSLM	GTLSKLARER	DLGAQRLAEL	LLEREPLCLR
250	260	270	280	290	300	310	320
PEAPSRAPAE	GPSHHLALQL	ANAKAQLRRL	RQELEEKAEL	LLDSQAEVQG	LEAEIRRLRQ	EAQALSGQAK	RAELYREEAE
330	340	350	360	370	380	390	400
ALRERAGRLP	RLQEELRRCR	ERLQAAEAYK	SQLEEERVLS	GVLEASKALL	EEQLEAARER	CARLHETQRE	NLLLRTRLGE
410	420	430	440	450	460	470	480
AHAELDSLRH	QVDQLAEENV	ELELELQRSL	EPPPGSPGEA	PLAGAAPSLQ	DEVREAEAGR	LRTLERENRE	LRGLLQVLQG
490	500	510	520	530	540	550	560
QPGGQHPLLE	APREDPVLPV	LEEAPQTPVA	FDHSPQGLVQ	KARDGGPQAL	DLAPPALDSV	LEASAECPQA	PDSDPQEAES
570	580	590	600	610	620	630	640
PLQAAAMDPQ	ASDWSPQESG	SPVETQESPE	KAGRRSSLQS	PASVAPPQGP	GTKIQAPQLL	GGETEGREAP	QGELVPEAWG
650	660	670	680	690	700	710	720
LRQEGPEHKP	GPSEPSSVQL	EEQEGPNQGL	DLATGQAEAR	EHDQRLEGTV	RDPAWQKPQQ	KSEGALEVQV	WEGPIPGESL
730	740	750	760	770	780	790	800
ASGVAEQEAL	REEVAQLRRK	AEALGDELEA	QARKLEAQNT	EAARLSKELA	QARRAEAEAH	REAEAQAWEQ	ARLREAVEAA
810	820	830	840	850	860	870	880
GQELESASQE	REALVEALAA	AGRERRQWER	EGSRLRAQSE	AAEERMQVLE	SEGRQHLEEA	ERERREKEAL	QAELEKAVVR
890	900	910	920	930	940	950	960
GKELGDRLEH	LQRELEQAAL	ERQEFLREKE	SQHQRYQGLE	QRLEAELQAA	ATSKEEALME	LKTRALQLEE	ELFQLRQGPA
970	980	990	1000	1010	1020	1030	1040
GLGPKKRAEP	QLVETQNVRL	IEVERSNAML	VAEKAALQGQ	LQHLEGQLGS	LQGRAQELLL	QSQRAQEHSS	RLQAEKSVLE
1050	1060	1070	1080	1090	1100	1110	1120
IQGQELHRKL	EVLEEEVRAA	RQSQEETRGQ	QQALLRDHKA	LAQLQRRQEA	ELEGLLVRHR	DLKANMRALE	LAHRELQGRH
1130	1140	1150	1160	1170	1180	1190	1200
EQLQAQRASV	EAQEVALLAE	RERLMQDGHR	QRGLEEELRR	LQSEHDRAQM	LLAELSRERG	ELQGERGELR	GRLARLELER
1210	1220	1230	1240	1250	1260	1270	1280
AQLEMQSQQL	RESNQQLDLS	ACRLTTQCEL	LTQLRSAQEE	ENRQLLAEVQ	ALSRENRELL	ERSLESRDHL	HREQREYLDQ
1290	1300	1310	1320	1330	1340	1350	1360
LNALRREKQK	LVEKIMDQYR	VLEPVPLPRT	KKGSWLADKG	PLPRHPCAGP	RAPSACGMRP	WQAGSGGNSA	QGSRWGEALS
1370	1380	1390	1400	1410	1420	1430	1440
HSALGTPLGN	DSDSAIQAPW	GRPSPTAKDL	VWDGRTPLRP	CRNTKQMPTE	RALRYRNRRN	VPSPHPSASD	TVGTAGLGVQ
1450	1460	1470	1480	1490	1500	1510	1520
PSRHWSVSGG	PRQPKSSGSQ	GPQGESLDKE	AWALRSSTVS	AGARRWSWDE	CVDRGDGWPP	RAAPGWSSGS	SRWLPLRQRS
1530	1540	1550	1560	1570			
LGDPPAEGGW	QELAREPPAL	SRWEAESQCW	GTVAWADLEP				

Cmpd.	No. of Cmpds.	m/z meas.	Δ m/z [ppm]	z	Rt [min]	Score	P	Range	Sequence	Modification	Ratios
245	1	715.7361	-205.90	2	33.1	13.1	1	755-767	K.LEAQNTEAARLSK.E		
2188	1	945.6234	129.84	2	57.8	19.6	2	1258-1272	R.ELLERSLESRDHLHR.E		
1580	1	781.3390	-87.96	2	49.5	11.3	1	1470-	K.EAWALRSSTVSAGAR.R		WUP:QUP 4.17

Detailed Protein Report

Cmpd.	No. of Cmpds.	m/z meas.	Δ m/z [ppm]	z	Rt [min]	Score	P	Range	Sequence	Modification	Ratios
								1484			

Detailed Protein Report

Protein 171: G-protein coupled receptor 98 precursor [Homo sapiens]

**Accession:**	gi|113722120	**Score:**	43.8
**Database:**	refseq_human(refseq_human_20140103.fasta)	**MW [kDa]:**	692.6
**Database Date:**	2015-11-30	**pI:**	4.4
		**Sequence Coverage [%]:**	0.8
		**No. of unique Peptides:**	1

BRUKER

Detailed Protein Report

10	20	30	40	50	60	70	80
MSVFLGPGMP	SASLLVNLLS	ALLILFVFGE	TEIRFTGQTE	FVVNETSTTV	IRLIIERIGE	PANVTAIVSL	YGEDAGDFFD
90	100	110	120	130	140	150	160
TYAAAFIPAG	ETNRTVYIAV	CDDDLPEPDE	TFIFHLTLQK	PSANVKLGWP	RTVTVTILSN	DNAFGIISFN	MLPSIAVSEP
170	180	190	200	210	220	230	240
KGRNESMPLT	LIREKGTYGM	VMVTFEVEGG	PNPPDEDLSP	VKGNITFPPG	RATVIYNLTV	LDDEVPENDE	IFLIQLKSVE
250	260	270	280	290	300	310	320
GGAEINTSRN	SIEIIIKKND	SPVRFLQSIY	LVPEEDHILI	IPVVRGKDNN	GNLIGSDEYE	VSISYAVTTG	NSTAHAQQNL
330	340	350	360	370	380	390	400
DFIDLQPNTT	VVFPPFIHES	HLKFQIVDDT	IPEIAESFHI	MLLKDTLQGD	AVLISPSVVQ	VTIKPNDKPY	GVLSFNSVLF
410	420	430	440	450	460	470	480
ERTVIIDEDR	ISRYEEITVV	RNGGTHGNVS	ANWVLTRNST	DPSPVTADIR	PSSGVLHFAQ	GQMLATIPLT	VVDDDLPEEA
490	500	510	520	530	540	550	560
EAYLLQILPH	TIRGGAEVSE	PAELLFYIQD	SDDVYGLITF	FPMENQKIES	SPGERYLSLS	FTRLGGTKGD	VRLLYSVLYI
570	580	590	600	610	620	630	640
PAGAVDPLQA	KEGILNISRR	NDLIFPEQKT	QVTTKLPIRN	DAFLQNGAHF	LVQLETVELL	NIIPLIPPIS	PRFGEICNIS
650	660	670	680	690	700	710	720
LLVTPAIANG	EIGFLSNLPI	ILHEPEDFAA	EVVYIPLHRD	GTDGQATVYW	SLKPSGFNSK	AVTPDDIGPF	NGSVLFLSGQ
730	740	750	760	770	780	790	800
SDTTINITIK	GDDIPEMNET	VTLSLDRVNV	ENQVLKSGYT	SRDLIILEND	DPGGVFEFSP	ASRGPYVIKE	GESVELHIIR
810	820	830	840	850	860	870	880
SRGSLVKQFL	HYRVEPRDSN	EFYGNTGVLE	FKPGEREIVI	TLLARLDGIP	ELDEHYWVVL	SSHGERESKL	GSATIVNITI
890	900	910	920	930	940	950	960
LKNDDPHGII	EFVSDGLIVM	INESKGDAIY	SAVYDVVRNR	GNFGDVSVSW	VVSPDFTQDV	FPVQGTVVFG	DQEFSKNITI
970	980	990	1000	1010	1020	1030	1040
YSLPDEIPEE	MEEFTVILLN	GTGGAKVGNR	TTATLRIRRN	DDPIYFAEPR	VVRVQEGETA	NFTVLRNGSV	DVTCMVQYAT
1050	1060	1070	1080	1090	1100	1110	1120
KDGKATARER	DFIPVEKGET	LIFEVGSRQQ	SISIFVNEDG	IPETDEPFYI	ILLNSTGDTV	VYQYGVATVI	IEANDDPNGI
1130	1140	1150	1160	1170	1180	1190	1200
FSLEPIDKAV	EEGKTNAFWI	LRHRGYFGSV	SVSWQLFQND	SALQPGQEFY	ETSGTVNFMD	GEEAKPIILH	AFPDKIPEFN
1210	1220	1230	1240	1250	1260	1270	1280
EFYFLKLVNI	SGGSPGPGGQ	LAETNLQVTV	MVPFNDDPFG	VFILDPECLE	REVAEDVLSE	DDMSYITNFT	ILRQQGVFGD
1290	1300	1310	1320	1330	1340	1350	1360
VQLGWEILSS	EFPAGLPPMI	DFLLVGIFPT	TVHLQQHMRR	HHSGTDALYF	TGLEGAFGTV	NPKYHPSRNN	TIANFTFSAW
1370	1380	1390	1400	1410	1420	1430	1440
VMPNANTNGF	IIAKDDGNGS	IYYGVKIQTN	ESHVTLSLHY	KTLGSNATYI	AKTTVMKYLE	ESVWLHLLII	LEDGIIEFYL
1450	1460	1470	1480	1490	1500	1510	1520
DGNAMPRGIK	SLKGEAITDG	PGILRIGAGI	NGNDRFTGLM	QDVRSYERKL	TLEEIYELHA	MPAKSDLHPI	SGYLEFRQGE
1530	1540	1550	1560	1570	1580	1590	1600
TNKSFIISAR	DDNDEEGEEL	FILKLVSVYG	GARISEENTT	ARLTIQKSDN	ANGLFGFTGA	CIPEIAEEGS	TISCVVERTR
1610	1620	1630	1640	1650	1660	1670	1680
GALDYVHVFY	TISQIETDGI	NYLVDDFANA	SGTITFLPWQ	RSEVLNIYVL	DDDIPELNEY	FRVTLVSAIP	GDGKLGSTPT
1690	1700	1710	1720	1730	1740	1750	1760
SGASIDPEKE	TTDITIKASD	HPYGLLQFST	GLPPQPKDAM	TLPASSVPHI	TVEEEDGEIR	LLVIRAQGLL	GRVTAEFRTV
1770	1780	1790	1800	1810	1820	1830	1840
SLTAFSPEDY	QNVAGTLEFQ	PGERYKYIFI	NITDNSIPEL	EKSFKVELLN	LEGGVAELFR	VDGSGSGDGD	MEFFLPTIHK
1850	1860	1870	1880	1890	1900	1910	1920
RASLGVASQI	LVTIAASDHA	HGVFEFSPES	LFVSGTEPED	GYSTVTLNVI	RHHGTLSPVT	LHWNIDSDPD	GDLAFTSGNI
1930	1940	1950	1960	1970	1980	1990	2000
TFEIGQTSAN	ITVEILPDED	PELDKAFSVS	VLSVSSGSLG	AHINATLTVL	ASDDPYGIFI	FSEKNRPVKV	EEATQNITLS
2010	2020	2030	2040	2050	2060	2070	2080
IIRLKGLMGK	VLVSYATLDD	MEKPPYFPPN	LARATQGRDY	IPASGFALFG	ANQSEATIAI	SILDDDEPER	SESVFIELLN
2090	2100	2110	2120	2130	2140	2150	2160
STLVAKVQSR	SIPNSPRLGP	KVETIAQLII	IANDDAFGTL	QLSAPIVRVA	ENHVGPIINV	TRTGGAFADV	SVKFKAVPIT
2170	2180	2190	2200	2210	2220	2230	2240
AIAGEDYSIA	SSDVVLLEGE	TSKAVPIYVI	NDIYPELEES	FLVQLMNETT	GGARLGALTE	AVIIIEASDD	PYGLFGFQIT
2250	2260	2270	2280	2290	2300	2310	2320
KLIVEEPEFN	SVKVNLPIIR	NSGTLGNVTV	QWVATINGQL	ATGDLRVVSG	NVTFAPGETI	QTLLLEVLAD	DVPEIEEVIQ
2330	2340	2350	2360	2370	2380	2390	2400

Detailed Protein Report

Cmpd.	No. of Cmpds.	m/z meas.	Δ m/z [ppm]	z	Rt [min]	Score	P	Range	Sequence	Modification
1940	2	643.2750	-74.26	2	54.6	11.3	0	4809-4819	K.EQPIVTENAER.Q	

Detailed Protein Report

Protein 172: FERM and PDZ domain-containing protein 1 [Homo sapiens]

**Accession:**	gi|239582741	**Score:**	43.6
**Database:**	refseq_human(refseq_human_20140103.fasta)	**MW [kDa]:**	173.3
**Database Date:**	2015-11-30	**pI:**	5.0
**Modification(s):**	Carbamidomethyl, Oxidation	**Sequence Coverage [%]:**	2.7
		**No. of unique Peptides:**	3

Quantitation

QU:MU	**Median:** 2.37	**CV:** 0.00 %	**No. of Peptides:**	1
WUP:QUP	**Median:** 1.06	**CV:** 0.00 %	**No. of Peptides:**	1

```
        10         20         30         40         50         60         70         80
MEELETSLFQ TRKAHRIEQM VARWLRRSRD SSARAKVAAA DGPARNPTQT LIPVRHTVKI DKDTLLQDYG FHISESLPLT
        90        100        110        120        130        140        150        160
VVAVTAGGSA HGKLFPGDQI LQMNNEPAED LSWERAVDIL REAEDSLSIT VVRCTSGVPK SSFLTEEKRA RLKTNPVKVH
       170        180        190        200        210        220        230        240
FAEEVLISGH SQGNSLLCMP NVLKLYLENG QTKAFKFEAN TTVKDIILTV KEKLSIRSIE YFALALEEQY SISRLHLLHE
       250        260        270        280        290        300        310        320
EELIQQVVER EESHDYRCLF RVCFVPKDPL DLLKEDPVAF EYLYLQSCSD VLQERFAVEM KCSSALRLAA LHIQERIYAC
       330        340        350        360        370        380        390        400
AQPQKISLKY IEKDWGIENF ISPTLLRNMK GKDIKKAISF HMKRNQNLLE PRQKQLISAA QLRLNYLQIL GELKTYGGRI
       410        420        430        440        450        460        470        480
FNATLMLQDR ESYIALLVGA KYGISQVINS KLNIMSTLAE FANISRVELT EESEKVSVVK VYLQDVKVLT LLLESNSAKD
       490        500        510        520        530        540        550        560
LACLIAGYYR LLVDPVTSIF LWPGNKQQAH RVSAEEGYES RACSDSEESS EVDCVLEPLS DRRLVKLAPC RSLIKEEQPP
       570        580        590        600        610        620        630        640
GNSPTPEVAR RGPSTCGASS TTDSAESEAS DSANTESRGY RTSGSSESMD ALEEDDLDTC SSSRSTFFHF GSPGLAESID
       650        660        670        680        690        700        710        720
SDSQEERSGI ETSGFLCLLD LAQRANPQCQ KTEFSESAAL ETFGWAPELS TVRLDPRLYE GSHADYYSLC SSVSPASYLS
       730        740        750        760        770        780        790        800
DSSESTASRQ GGAPPAWGQQ GWTEAQPSSM LEPLALHPPL AFEDGSSDEE YYDAADKLTP PGPPSGPRDV STAEPSATSL
       810        820        830        840        850        860        870        880
QNKASTSSPE NSLPCGPDGR QPSRRGGVKK YAKTLRKRRS FLQTDYTSQV SFPLVPSASL ESVDDVCYYD REPYLALGAP
       890        900        910        920        930        940        950        960
SPTVSSLQDM QGEPGLLETK ALGLLAPLRE TKSTNPASRV MEMEPETMET KSVIDSRVSS ISAIRFRIDP NNKENSGVVP
       970        980        990       1000       1010       1020       1030       1040
AASSSASTPH CSNPGSSGPD TAQARPSQIL PLSQDLDGIA PKEPTIEHGD SSFSLSSGDP NPDRACLASN PGLNNVSQGD
      1050       1060       1070       1080       1090       1100       1110       1120
TLELQLEPHV QLEMGLESFC TNHIQETAPK YTEPLLSPRD EPRSDECGIN PGEKIASIPT KEEPQGQLSL ERDREVTNKN
      1130       1140       1150       1160       1170       1180       1190       1200
GTNVFQEESR KDSGDSPGDV SNNVSQTLDI SSPAGKIVTS LSLDAPVTGT EQIPPHPPRD PQGQSREPPG QGCQAQEQKL
      1210       1220       1230       1240       1250       1260       1270       1280
FVELDLDPDF FLGKQTVSPA VPPEGIKAEA PNHVTGQDIA PRDSPEWVCF NPEPSLPEPL PCPQEDPHLE TSNHCLLSEG
      1290       1300       1310       1320       1330       1340       1350       1360
KSDSSSICLS AEKSFLCFAP ESHPEVSASL RVATSLGFAG MNEMVAPRIG MDQCSCQFSY ATCFRGPQPE TEEEDRDLEA
      1370       1380       1390       1400       1410       1420       1430       1440
HPMAPLTSPP SAGSPVVLPW RPARAHSCTT APLSRKSHIW PEYCSRALRQ LKATPASTPE GFIQLMESLL ELQDILETSW
      1450       1460       1470       1480       1490       1500       1510       1520
GVGNKHPPEK CTWHFTESRS RLCMGSQKLL SSCRHVIRMD QSPEEMQGAV RDTFQHLVQL AGLCFQFTDC SRCSARHREA
      1530       1540       1550       1560       1570       1580
AGNLRDVVYT YHQFIEAAKS TCERGYHDLS VKLLARQCTA LTAAVFCLTQ KFRASTAL
```

Cmpd.	No. of Cmpds.	m/z meas.	Δ m/z [ppm]	z	Rt [min]	Score	P	Range	Sequence	Modification	Ratios
2745	1	849.4011	-32.55	1	65.4	14.4	0	357-363	K.AISFHMK.R	Oxidation: 6	
2634	1	994.5127	-8.52	2	63.7	13.9	2	1462-1478	R.LCMGSQKLLSSCRHVIR.M	Carbamidomethyl: 2	

Detailed Protein Report

Cmpd.	No. of Cmpds.	m/z meas.	Δ m/z [ppm]	z	Rt [min]	Score	P	Range	Sequence	Modification	Ratios	
2077	1	1158.9066	-123.27	2	55.9	15.3	1	1526-1544	R.DVVYTYHQFIEAAKSTCER.G	Carbamidomethyl: 17	QU:MU<br>WUP:QUP	2.37<br>1.06

Detailed Protein Report

Protein 173: MAX gene-associated protein isoform 2 [Homo sapiens]

**Accession:**	gi|256017159	**Score:**	43.6
**Database:**	refseq_human(refseq_human_20140103.fasta)	**MW [kDa]:**	315.0
**Database Date:**	2015-11-30	**pI:**	6.4
**Modification(s):**	Carbamidomethyl	**Sequence Coverage [%]:**	0.8
		**No. of unique Peptides:**	2

Quantitation

WUP:QUP	**Median:** 5.06	**CV:** 0.00 %	**No. of Peptides:** 1

Detailed Protein Report

10	20	30	40	50	60	70	80
MEEKQQIILA	NQDGGTVAGA	APTFFVILKQ	PGNGKTDQGI	LVTNQDACAL	ASSVSSPVKS	KGKICLPADC	TVGGITVTLD
90	100	110	120	130	140	150	160
NNSMWNEFYH	RSTEMILTKQ	GRRMFPYCRY	WITGLDSNLK	YILVMDISPV	DNHRYKWNGR	WWEPSGKAEP	HVLGRVFIHP
170	180	190	200	210	220	230	240
ESPSTGHYWM	HQPVSFYKLK	LTNNTLDQEG	HIILHSMHRY	LPRLHLVPAE	KAVEVIQLNG	PGVHTFTFPQ	TEFFAVTAYQ
250	260	270	280	290	300	310	320
NIQITQLKID	YNPFAKGFRD	DGLNNKPQRD	GKQKNSSDQE	GNNISSSSGH	RVRLTEGQGS	EIQPGDLDPL	SRGHETSGKG
330	340	350	360	370	380	390	400
LEKTSLNIKR	DFLGFMDTDS	ALSEVPQLKQ	EISECLIASS	FEDDSRVASP	LDQNGSFNVV	IKEEPLDDYD	YELGECPEGV
410	420	430	440	450	460	470	480
TVKQEETDEE	TDVYSNSDDD	PILEKQLKRH	NKVDNPEADH	LSSKWLPSSP	SGVAKAKMFK	LDTGKMPVVY	LEPCAVTRST
490	500	510	520	530	540	550	560
VKISELPDNM	LSTSRKDKSS	MLAELEYLPT	YIENSNETAF	CLGKESENGL	RKHSPDLRVV	QKYPLLKEPQ	WKYPDISDSI
570	580	590	600	610	620	630	640
STERILDDSK	DSVGDSLSGK	EDLGRKRTTM	LKIATAAKVV	NANQNASPNV	PGKRGRPRKL	KLCKAGRPPK	NTGKSLISTK
650	660	670	680	690	700	710	720
NTPVSPGSTF	PDVKPDLEDV	DGVLFVSFES	KEALDIHAVD	GTTEESSSLQ	ASTTNDSGYR	ARISQLEKEL	IEDLKTLRHK
730	740	750	760	770	780	790	800
QVIHPGLQEV	GLKLNSVDPT	MSIDLKYLGV	QLPLAPATSF	PFWNLTGTNP	ASPDAGFPFV	SRTGKTNDFT	KIKGWRGKFH
810	820	830	840	850	860	870	880
SASASRNEGG	NSESSLKNRS	AFCSDKLDEY	LENEGKLMET	SMGFSSNAPT	SPVVYQLPTK	STSYVRTLDS	VLKKQSTISP
890	900	910	920	930	940	950	960
STSYSLKPHS	VPPVSRKAKS	QNRQATFSGR	TKSSYKSILP	YPVSPKQKYS	HVILGDKVTK	NSSGIISENQ	ANNFVVPTLD
970	980	990	1000	1010	1020	1030	1040
ENIFPKQISL	RQAQQQQQQQ	QGSRPPGLSK	SQVKLMDLED	CALWEGKPRT	YITEERADVS	LTTLLTAQAS	LKTKPIHTII
1050	1060	1070	1080	1090	1100	1110	1120
RKRAPPCNND	FCRLGCVCSS	LALEKRQPAH	CRRPDCMFGC	TCLKRKVVLV	KGGSKTKHFQ	RKAAHRDPVF	YDTLGEEARE
1130	1140	1150	1160	1170	1180	1190	1200
EEEGIREEEE	QLKEKKKRKK	LEYTICETEP	EQPVRHYPLW	VKVEGEVDPE	PVYIPTPSVI	EPMKPLLLPQ	PEVLSPTVKG
1210	1220	1230	1240	1250	1260	1270	1280
KLLTGIKSPR	SYTPKPNPVI	REEDKDPVYL	YFESMMTCAR	VRVYERKKED	QRQPSSSSSP	SPSFQQQTSC	HSSPENHNNA
1290	1300	1310	1320	1330	1340	1350	1360
KEPDSEQQPL	KQLTCDLEDD	SDKLQEKSWK	SSCNEGESSS	TSYMHQRSPG	GPTKLIEIIS	DCNWEEDRNK	ILSILSQHIN
1370	1380	1390	1400	1410	1420	1430	1440
SNMPQSLKVG	SFIIELASQR	KSRGEKNPPV	YSSRVKISMP	SCQDQDDMAE	KSGSETPDGP	LSPGKMEDIS	PVQTDALDSV
1450	1460	1470	1480	1490	1500	1510	1520
RERLHGGKGL	PFYAGLSPAG	KLVAYKRKPS	SSTSGLIQVA	SNAKVAASRK	PRTLLPSTSN	SKMASSSGTA	TNRPGKNLKA
1530	1540	1550	1560	1570	1580	1590	1600
FVPAKRPIEN	AAQIPVATPQ	VSPNTVKRAG	PRLLLIPVQQ	GSPTLRPVSN	TQLQGHRMVL	QPVRSPSGMN	LFRHPNGQIV
1610	1620	1630	1640	1650	1660	1670	1680
QLLPLHQLRG	SNTQPNLQPV	MFRNPGSVMG	IRLPAPSKPS	ETPPSSTSSS	AFSVMNPVIQ	AVGSSSAVNV	ITQAPSLLSS
1690	1700	1710	1720	1730	1740	1750	1760
GASFVSQAGT	LTLRISPPEP	QSFASKTGSE	TKITYSSGGQ	PVGTASLIPL	QSGSFALLQL	PGQKPVPSSI	LQHVASLQMK
1770	1780	1790	1800	1810	1820	1830	1840
RESQNPDQKD	ETNSIKREQE	TKKVLQSEGE	AVDPEANVIK	QNSGAATSEE	TLNDSLEDRG	DHLDEECLPE	EGCATVKPSE
1850	1860	1870	1880	1890	1900	1910	1920
HSCITGSHTD	QDYKDVNEEY	GARNRKSSKE	KVAVLEVRTI	SEKASNKTVQ	NLSKVQHQKL	GDVKVEQQKG	FDNPEENSSE
1930	1940	1950	1960	1970	1980	1990	2000
FPVTFKEESK	FELSGSKVME	QQSNLQPEAK	EKECGDSLEK	DRERWRKHLK	GPLTRKCVGA	SQECKKEADE	QLIKETKTCQ
2010	2020	2030	2040	2050	2060	2070	2080
ENSDVFQQEQ	GISDLLGKSG	ITEDARVLKT	ECDSWSRISN	PSAFSIVPRR	AAKSSRGNGH	FQGHLLLPGE	QIQPKQEKKG
2090	2100	2110	2120	2130	2140	2150	2160
GRSSADFTVL	DLEEDDEDDN	EKTDDSIDEI	VDVVSDYQSE	EVDDVEKNNC	VEYIEDDEEH	VDIETVEELS	EEINVAHLKT
2170	2180	2190	2200	2210	2220	2230	2240

Detailed Protein Report

Cmpd.	No. of Cmpds.	m/z meas.	Δ m/z [ppm]	z	Rt [min]	Score	P	Range	Sequence	Modification	Ratios
2438	1	611.7346	-126.77	2	60.9	12.8	0	1054-1065	R.LGCVCSSLALEK.R		
939	1	583.7035	-110.96	2	41.3	10.6	1	1976-1985	R.KCVGASQECK.K	Carbamidomethyl: 2, 9	WUP:QUP 5.06

Detailed Protein Report

Protein 174: PREDICTED: RING finger protein 17 isoform X10 [Homo sapiens]

**Accession:**	gi|578824967	**Score:**	43.6
**Database:**	refseq_human(refseq_human_20140103.fasta)	**MW [kDa]:**	168.9
**Database Date:**	2015-11-30	**pI:**	5.0
		**Sequence Coverage [%]:**	2.6
		**No. of unique Peptides:**	1

10	20	30	40	50	60	70	80
MLDTNTAEEI	DEALNTAHHS	FEQLSIAGKA	LEHMQKQTIE	ERERVIEVVE	KQFDQLLAFF	DSRKKNLCEE	FARTTDDYLS
90	100	110	120	130	140	150	160
NLIKAKSYIE	EKKNNLNAAM	NIARALQLSP	SLRTYCDLNQ	IIRTLQLTSD	SELAQVSSPQ	LRNPPRLSVN	CSEIICMFNN
170	180	190	200	210	220	230	240
MGKIEFRDST	KCYPQENEIR	QNVQKKYNNK	KELSCYDTYP	PLEKKKVDMS	VLTSEAPPPP	LQPETNDVHL	EAKNFQPQKD
250	260	270	280	290	300	310	320
VATASPKTIA	VLPQMGSSPD	VIIEEIIEDN	VESSAELVFV	SHVIDPCHFY	IRKYSQIKDA	KVLEKKVNEF	CNRSSHLDPS
330	340	350	360	370	380	390	400
DILELGARIF	VSSIKNGMWC	RGTITELIPI	EGRNTRKPCS	PTRLFVHEVA	LIQIFMVDFG	NSEVLIVTGV	VDTHVRPEHS
410	420	430	440	450	460	470	480
AKQHIALNDL	CLVLRKSEPY	TEGLLKDIQP	LAQPCSLKDI	VPQNSNEGWE	EEAKVEFLKM	VNNKAVSMKV	FREEDGVLIV
490	500	510	520	530	540	550	560
DLQKPPPNKI	SSDMPVSLRD	ALVFMELAKF	KSQSLRSHFE	KNTTLHYHPP	ILPKEMTDVS	VTVCHINSPG	DFYLQLIEGL
570	580	590	600	610	620	630	640
DILFLLKTIE	EFYKSEDGEN	LEILCPVQDQ	ACVAKFEDGI	WYRAKVIGLP	GHQEVEVKYV	DFGNTAKITI	KDVRKIKDEF
650	660	670	680	690	700	710	720
LNAPEKAIKC	KLAYIEPYKR	TMQWSKEAKE	KFEEKAQDKF	MTCSVIKILE	DNVLLVELFD	SLGAPEMTTT	SINDQLVKEG
730	740	750	760	770	780	790	800
LASYEIGYIL	KDNSQKHIEV	WDPSPEEIIS	NEVHNLNPVS	AKSLPNENFQ	SLYNKELPVH	ICNVISPEKI	YVQWLLTENL
810	820	830	840	850	860	870	880
LNSLEEKMIA	AYENSKWEPV	KWENDMHCAV	KIQDKNQWRR	GQIIRMVTDT	LVEVLLYDVG	VELVVNVDCL	RKLEENLKTM
890	900	910	920	930	940	950	960
GRLSLECSLV	DIRPAGGSDK	WTATACDCLS	LYLTGAVATI	ILQVDSEENN	TTWPLPVKIF	CRDEKGERVD	VSKYLIKKGL
970	980	990	1000	1010	1020	1030	1040
ALRERRINNL	DNSHSLSEKS	LEVPLEQEDS	VVTNCIKTNF	DPDKKTADII	SEQKVSEFQE	KILEPRTTRG	YKPPAIPNMN
1050	1060	1070	1080	1090	1100	1110	1120
VFEATVSCVG	DDGTIFVVPK	LSEFELIKMT	NEIQSNLKCL	GLLEPYFWKK	GEACAVRGSD	TLWYRGKVME	VVGGAVRVQY
1130	1140	1150	1160	1170	1180	1190	1200
LDHGFTEKIP	QCHLYPILLY	PDIPQFCIPC	QLHNTTPVGN	VWQPDAIEVL	QQLLSKRQVD	IHIMELPKNP	WEKLSIHLYF
1210	1220	1230	1240	1250	1260	1270	1280
DGMSLSYFMA	YYKYCTSEHT	EEMLKEKPRS	DHDKKYEEEQ	WEIRFEELLS	AETDTPLLPP	YLSSSLPSPG	ELYAVQVKHV
1290	1300	1310	1320	1330	1340	1350	1360
VSPNEVYICL	DSIETSNQSN	QHSDTDDSGV	SGESESESLD	EALQRVNKKV	EALPPLTDFR	TEMPCLAEYD	DGLWYRAKIV
1370	1380	1390	1400	1410	1420	1430	1440
AIKEFNPLSI	LVQFVDYGST	AKLTLNRLCQ	IPSHLMRYPA	RAIKVLLAGF	KPPLRDLGET	RIPYCPKWSM	EALWAMIDCL
1450	1460	1470	1480	1490			
QGKQLYAVSM	APAPEQIVTL	YDDEQHPVHM	PLVEMGLADK	DE			

Cmpd.	No. of Cmpds.	m/z meas.	Δ m/z [ppm]	z	Rt [min]	Score	P	Range	Sequence	Modification
200	1	642.8816	92.45	2	32.2	16.1	1	596-605	K.FEDGIWYRAK.V	

Detailed Protein Report

Protein 175: PREDICTED: uncharacterized protein LOC285556 isoform X1 [Homo sapiens]

**Accession:**	gi|578809699	**Score:**	43.5
**Database:**	refseq_human(refseq_human_20140103.fasta)	**MW [kDa]:**	190.0
**Database Date:**	2015-11-30	**pI:**	9.8
		**Sequence Coverage [%]:**	2.3
		**No. of unique Peptides:**	2

Quantitation

QU:MU	**Median:** 0.47	**CV:** 0.00 %	**No. of Peptides:**	1
WUP:QUP	**Median:** 2.21	**CV:** 0.00 %	**No. of Peptides:**	1

Alias proteins:

Accession	Name	Description
gi|578809701	refseq_human (refseq_human_20140103.fasta)	PREDICTED: uncharacterized protein LOC285556 isoform X2 [Homo sapiens]

Detailed Protein Report

10	20	30	40	50	60	70	80
MLSFHFWKSR	GQPTDAASSV	ADGIQTPRCC	RRCQANNWTG	QLSYRTLATV	SAGAAAPQPQ	TTSTASSRSL	PTSLRLAAAP
90	100	110	120	130	140	150	160
PQGLKNWEVV	AAVAAVPTAL	GPVQIRGTLL	RATLQPLRGQ	RRTQDFPSDH	HCLFLSLKPG	QGLIMEAAPP	ELNSKARQAE
170	180	190	200	210	220	230	240
VGDGVSSAQD	SQELKQQLWP	LPKPSASSQR	EAKYVDMCAS	AEVQRESPQT	MKLTLGHCPG	GQRASRSPKE	KAQDEPSSKT
250	260	270	280	290	300	310	320
PSPQNNPASS	QLSRSQHSAS	EEGGNFSSSS	SSSPMNKAEE	DGLSKMEDST	TSTGALATSS	SSLGFESESG	ESEGCQAVGG
330	340	350	360	370	380	390	400
EGEKISGGGG	GGKGGGGGGA	GDGTECRDII	AKSQGSRDPP	KVEEAHYITT	HEIQLSEVEQ	DMDFDVGLAS	RWDFEDNNVI
410	420	430	440	450	460	470	480
YSFVDYASFG	GSDETPGDIT	SLTEEDDDNS	CYLSTTPSTN	TTRTPSPTSS	DLARPNAGRS	GRDTSSTEVG	SGPSDSGPTP
490	500	510	520	530	540	550	560
PPTGPGTAPL	TEPLPETPEA	ASGAAAAAAS	SCGSAASQIL	LSIKPASRAI	NEPSNVRAKQ	NIIYAAKHEG	DMSLRVSTAA
570	580	590	600	610	620	630	640
EHNSSSLKQN	PAAAVAQDHA	KKFIAVPARL	QTRCGAIRAK	ELVDYSSGAS	SAVSELDDAD	KEVRNLTSRA	FRSLAYPYFE
650	660	670	680	690	700	710	720
ALNISSRESS	TTLSEVGFGR	WSTFLDLKCG	GVGARVEQSL	LRSSAASVAA	GLRKGSGARA	TADQLYIQSK	KSQTKALEFV
730	740	750	760	770	780	790	800
VSKVEGEIKH	VETPLCFQKQ	VQTGSRVVTL	LEPLNVRSES	KASSAPGPGR	ATKGPGKGPG	SAYTDDGSET	SEGSKPTSRA
810	820	830	840	850	860	870	880
DGPQKSKFAS	SLLKNVISKK	MQREHEFKME	RGEVMDTSHH	LSGTSKETEG	ARGSERQRER	GLQRQSSRHS	EAGSEYTVVS
890	900	910	920	930	940	950	960
MSDAGGEGSV	AGSKSPVFKA	STPRERNAGP	GRNFTDGHTE	VCEIKKSASE	TVKGIFLRSQ	NSAFRSWKEK	EAEKREEQAP
970	980	990	1000	1010	1020	1030	1040
IGKLKLPKGG	DWRADLGEIS	ASKNTIMSRL	FVPNIQQTPK	DKQPRKQATK	YPAAQATSTA	VIRPKAPEIK	IRLGSVQQPS
1050	1060	1070	1080	1090	1100	1110	1120
SDFNIAKLLT	PKLAGGSASN	LFKTIEDNSR	AQQKLFRGDN	LEKVPHFQVR	DIRDKSKAQG	PLHQVRDVRK	LIKGSGDSSD
1130	1140	1150	1160	1170	1180	1190	1200
KGSVTPEQGL	TGPKPRQLSA	AAGGSGSLSP	MVITCQAVVN	QREDSMDREP	RESMGKGGGS	RVLNSSSPEG	TVLVHRASGR
1210	1220	1230	1240	1250	1260	1270	1280
LPVATIAPNK	PEQGSYLPVL	KIVSKASTQK	TPEKLKEEEV	KEEGKATKPA	RNALEKLTAA	VRSMEELYSF	NRNEWKRKSD
1290	1300	1310	1320	1330	1340	1350	1360
PLPMMMDSHV	LSLIASEERE	GVVVADGDHD	KLSKRLGEVE	ERGTGNKAGV	VLRGAPIERL	QRRNSNPSAE	SVSARAAAFE
1370	1380	1390	1400	1410	1420	1430	1440
NLARERPRSL	YIPPVHKDVE	RTQPLQPLPP	LPSNRNVFTV	SASSIQKTGG	VAGKFPQGPS	PESPSAAKGI	KSQGLRSLKI
1450	1460	1470	1480	1490	1500	1510	1520
SPATRAPPDE	VTNRKSGSNL	EKSNSDCENY	LTIPLKGSSA	AGELLSRPGA	SREGPPNSSA	ATLCSLPPLS	ARSQVPSSSK
1530	1540	1550	1560	1570	1580	1590	1600
GSQVSGTSRP	AWRTKPDNPR	ETVAAPPGPQ	SPEHPPTTIY	HQPPLPFTLQ	GAQPQVLCFS	PPSMPAPAPA	ASAPVPTDPF
1610	1620	1630	1640	1650	1660	1670	1680
QQAQPQQTQR	KMLLDVTTGQ	YYLVDTPVQP	MTRRLFDPET	GQYVDVPMTS	QQQAVAPMSI	SVPPLALSPG	AYGPTYMIYP
1690	1700	1710	1720	1730	1740	1750	1760
GFLPTVLPTN	ALQPTPIARA	PRGSELSPMV	AEPSSKEAAA	TFTEAPYFMA	SGQSPASSTS	SAPAATSQLL	GAKAFAQLHG
1770	1780	1790	1800				
KPVISITSQP	LGPRIIAPPS	FDGTTMSFVV	EHR				

Cmpd.	No. of Cmpds.	m/z meas.	Δ m/z [ppm]	z	Rt [min]	Score	P	Range	Sequence	Modification	Ratios
886	1	575.7576	46.75	2	40.6	12.5	0	334-347	K.GGGGGGAGDGTECR.D		
1984	1	634.7974	-113.89	2	54.7	11.7	2	955-965	K.REEQAPIGKLK.L		WUP:QUP 2.21<br>QU:MU 0.47

Detailed Protein Report

Protein 176: tau-tubulin kinase 1 [Homo sapiens]

Accession: gi|58761548
Database: refseq_human(refseq_human_20140103.fasta)
Database Date: 2015-11-30

Score: 43.4
MW [kDa]: 142.6
pI: 5.4
Sequence Coverage [%]: 3.1
No. of unique Peptides: 2

Quantitation

QU:MU	**Median:**	0.66	**CV:**	0.00 %	**No. of Peptides:**	1
WUP:QUP	**Median:**	1.21	**CV:**	0.00 %	**No. of Peptides:**	1

10	20	30	40	50	60	70	80
MQCLAAALKD	ETNMSGGGEQ	ADILPANYVV	KDRWKVLKKI	GGGGFGEIYE	AMDLLTRENV	ALKVESAQQP	KQVLKMEVAV
90	100	110	120	130	140	150	160
LKKLQGKDHV	CRFIGCGRNE	KFNYVVMQLQ	GRNLADLRRS	QPRGTFTLST	TLRLGKQILE	SIEAIHSVGF	LHRDIKPSNF
170	180	190	200	210	220	230	240
AMGRLPSTYR	KCYMLDFGLA	RQYTNTTGDV	RPPRNVAGFR	GTVRYASVNA	HKNREMGRHD	DLWSLFYMLV	EFAVGQLPWR
250	260	270	280	290	300	310	320
KIKDKEQVGM	IKEKYEHRML	LKHMPSEFHL	FLDHIASLDY	FTKPDYQLIM	SVFENSMKER	GIAENEAFDW	EKAGTDALLS
330	340	350	360	370	380	390	400
TSTSTPPQQN	TRQTAAMFGV	VNVTPVPGDL	LRENTEDVLQ	GEHLSDQENA	PPILPGRPSE	GLGPSPHLVP	HPGGPEAEVW
410	420	430	440	450	460	470	480
EETDVNRNKL	RINIGKSPCV	EEEQSRGMGV	PSSPVRAPPD	SPTTPVRSLR	YRRVNSPESE	RLSTADGRVE	LPERRSRMDL
490	500	510	520	530	540	550	560
PGSPSRQACS	SQPAQMLSVD	TGHADRQASG	RMDVSASVEQ	EALSNAFRSV	PLAEEEDFDS	KEWVIIDKET	ELKDFPPGAE
570	580	590	600	610	620	630	640
PSTSGTTDEE	PEELRPLPEE	GEERRRLGAE	PTVRPRGRSM	QALAEEDLQH	LPPQPLPPQL	SQGDGRSETS	QPPTPGSPSH
650	660	670	680	690	700	710	720
SPLHSGPRPR	RRESDPTGPQ	RQVFSVAPPF	EVNGLPRAVP	LSLPYQDFKR	DLSDYRERAR	LLNRVRRVGF	SHMLLTTPQV
730	740	750	760	770	780	790	800
PLAPVQPQAN	GKEEEEEEEE	DEEEEEEDEE	EEEEEEEEEE	EEEEEEEEEE	EAAAAVALGE	VLGPRSGSSS	EGSERSTDRS
810	820	830	840	850	860	870	880
QEGAPSTLLA	DDQKESRGRA	SMADGDLEPE	EGSKTLVLVS	PGDMKKSPVT	AELAPDPDLG	TLAALTPQHE	RPQPTGSQLD
890	900	910	920	930	940	950	960
VSEPGTLSSV	LKSEPKPPGP	GAGLGAGTVT	TGVGGVAVTS	SPFTKVERTF	VHIAEKTHLN	VMSSGGQALR	SEEFSAGGEL
970	980	990	1000	1010	1020	1030	1040
GLELASDGGA	VEEGARAPLE	NGLALSGLNG	AEIEGSALSG	APRETPSEMA	TNSLPNGPAL	ADGPAPVSPL	EPSPEKVATI
1050	1060	1070	1080	1090	1100	1110	1120
SPRRHAMPGS	RPRSRIPVLL	SEEDTGSEPS	GSLSAKERWS	KRARPQQDLA	RLVMEKRQGR	LLLRLASGAS	SSSSEEQRRA
1130	1140	1150	1160	1170	1180	1190	1200
SETLSGTGSE	EDTPASEPAA	ALPRKSGRAA	ATRSRIPRPI	GLRMPMPVAA	QQPASRSHGA	APALDTAITS	RLQLQTPPGS
1210	1220	1230	1240	1250	1260	1270	1280
ATAADLRPKQ	PPGRGLGPGR	AQAGARPPAP	RSPRLPASTS	AARNASASPR	SQSLSRRESP	SPSHQARPGV	PPPRGVPPAR
1290	1300	1310	1320	1330			
AQPDGTPSPG	GSKKGPRGKL	QAQRATTKGR	AGGAEGRAGA	R			

Cmpd.	No. of Cmpds.	m/z meas.	Δ m/z [ppm]	z	Rt [min]	Score	P	Range	Sequence	Modification	Ratios
1658	1	697.8038	-143.18	2	50.5	11.5	1	124-136	R.GTFTLSTTLRLGK.Q		
1595	1	932.5310	13.94	2	50.2	20.1	0	1192-1209	R.LQLQTPPGSATAADLRPK.Q		QU:MU 0.66<br>WUP:QUP 1.21

Detailed Protein Report

Protein 177: PREDICTED: serine/threonine-protein kinase WNK2 isoform X12 [Homo sapiens]

**Accession:**	gi|578817784	**Score:**	43.3
**Database:**	refseq_human(refseq_human_20140103.fasta)	**MW [kDa]:**	227.1
**Database Date:**	2015-11-30	**pI:**	5.4
		**Sequence Coverage [%]:**	2.6
		**No. of unique Peptides:**	2

BRUKER

Detailed Protein Report

10	20	30	40	50	60	70	80
MDGDGGRRDV	PGTLMEPGRG	AGPAGMAEPR	AKAARPGPQR	FLRRSVVESD	QEEPPGLEAA	EAPGPQPPQP	LQRRVLLLCK
90	100	110	120	130	140	150	160
TRRLIAERAR	GRPAAPAPAA	LVAQPGAPGA	PADAGPEPVG	TQEPGPDPIA	AAVETAPAPD	GGPREEAAAT	VRKEDEGAAE
170	180	190	200	210	220	230	240
AKPEPGRTRR	DEPEEEEDDE	DDLKAVATSL	DGRFLKFDIE	LGRGSFKTVY	KGLDTETWVE	VAWCELQDRK	LTKLERQRFK
250	260	270	280	290	300	310	320
EEAEMLKGLQ	HPNIVRFYDF	WESSAKGKRC	IVLVTELMTS	GTLKTYLKRF	KVMKPKVLRS	WCRQILKGLL	FLHTRTPPII
330	340	350	360	370	380	390	400
HRDLKCDNIF	ITGPTGSVKI	GDLGLATLKR	ASFAKSVIGT	PEFMAPEMYE	EHYDESVDVY	AFGMCMLEMA	TSEYPYSECQ
410	420	430	440	450	460	470	480
NAAQIYRKVT	CGIKPASFEK	VHDPEIKEII	GECICKNKEE	RYEIKDLLSH	AFFAEDTGVR	VELAEEDHGR	KSTIALRLWV
490	500	510	520	530	540	550	560
EDPKKLKGKP	KDNGAIEFTF	DLEKETPDEV	AQEMIESGFF	HESDVKIVAK	SIRDRVALIQ	WRRERIWPAL	QPKEQQDVGS
570	580	590	600	610	620	630	640
PDKARGPPVP	LQVQVTYHAQ	AGQPGPPEPE	EPEADQHLLP	PTLPTSATSL	ASDSTFDSGQ	GSTVYSDSQS	SQQSVMLGSL
650	660	670	680	690	700	710	720
ADAAPSPAQC	VCSPPVSEGP	VLPQSLPSLG	AYQQPTAAPG	LPVGSVPAPA	CPPSLQQHFP	DPAMSFAPVL	PPPSTPMPTG
730	740	750	760	770	780	790	800
PGQPAPPGQQ	PPPLAQPTPL	PQVLAPQPVV	PLQPVPPHLP	PYLAPASQVG	APAQLKPLQM	PQAPLQPLAQ	VPPQMPPIPV
810	820	830	840	850	860	870	880
VPPITPLAGI	DGLPPALPDL	PTATVPPVPP	PQYFSPAVIL	PSLAAPLPPA	SPALPLQAVK	LPHPPGAPLA	MPCRTIVPNA
890	900	910	920	930	940	950	960
PATIPLLAVA	PPGVAALSIH	SAVAQLPGQP	VYPAAFPQMA	PTDVPPSPHH	TVQNMRATPP	QPALPPQPTL	PPQPVLPPQP
970	980	990	1000	1010	1020	1030	1040
TLPPQPVLPP	QPTRPPQPVL	PPQPMLPPQP	VLPPQPALPV	RPEPLQPHLP	EQAAPAATPG	SQILLGHPAP	YAVDVAAQVP
1050	1060	1070	1080	1090	1100	1110	1120
TVPVPPAAVL	SPPLPEVLLP	AAPELLPQFP	SSLATVSASV	QSVPTQTATL	LPPANPPLPG	GPGIASPCPT	VQLTVEPVQE
1130	1140	1150	1160	1170	1180	1190	1200
EQASQDKPPG	LPQSCESYGG	SDVTSGKELS	DSCEGAFGGG	RLEGRAARKH	HRRSTRARSR	QERASRPRLT	ILNVCNTGDK
1210	1220	1230	1240	1250	1260	1270	1280
MVECQLETHN	HKMVTFKFDL	DGDAPDEIAT	YMVEHDFILQ	AERETFIEQM	KDVMDKAEDM	LSEDTDADRG	SDPGTSPPHL
1290	1300	1310	1320	1330	1340	1350	1360
STCGLGTGEE	SRQSQANAPV	YQQNVLHTGK	RWFIICPVAE	HPAPEAPESS	PPLPLSSLPP	EASQDSAPYK	DQLSSKEQPS
1370	1380	1390	1400	1410	1420	1430	1440
FLASQQLLSQ	AGPSNPPGAP	PAPLAPSSPP	VTALPQDGAA	PATSTMPEPA	SGTASQAGGP	GTPQGLTSEL	ETSQPLAETH
1450	1460	1470	1480	1490	1500	1510	1520
EAPLAVQPLV	VGLAPCTPAP	EAASTRDASA	PREPLPPPAP	EPSPHSGTPQ	PALGQPAPLL	PAAVGAVSLA	TSQLPSPPLG
1530	1540	1550	1560	1570	1580	1590	1600
PTVPPQPPSA	LESDGEGPPP	RVGFVDSTIK	SLDEKLRTLL	YQEHVPTSSA	SAGTPVEVGD	RDFTLEPLRG	DQPRSEVCGG
1610	1620	1630	1640	1650	1660	1670	1680
DLALPPVPKE	AVSGRVQLPQ	PLVEKSELAP	TRGAVMEQGT	SSSMTAESSP	RSMLGYDRDG	RQVASDSHVV	PSVPQDVPAF
1690	1700	1710	1720	1730	1740	1750	1760
VRPARVEPTD	RDGGEAGESS	AEPPPSDMGT	VGGQASHPQT	LGARALGSPR	KRPEQQDVSS	PAKTVGRFSV	VSTQDEWTLA
1770	1780	1790	1800	1810	1820	1830	1840
SPHSLRYSAP	PDVYLDEAPS	SPDVKLAVRR	AQTASSIEVG	VGEPVSSDSG	DEGPRARPPV	QKQASLPVSG	SVAGDFVKKA
1850	1860	1870	1880	1890	1900	1910	1920
TAFLQRPSRA	GSLGPETPSR	VGMKVPTISV	TSFHSQSSYI	SSDNDSELED	ADIKKELQSL	REKHLKEISE	LQSQQKQEIE
1930	1940	1950	1960	1970	1980	1990	2000
ALYRRLGKPL	PPNVGFFHTA	PPTGRRRKTS	KSKLKAGKLL	NPLVRQLKVV	ASSTGSSTSS	LAPGPEPGPQ	PALHVQAQVN
2010	2020	2030	2040	2050	2060	2070	2080
NSNNKKGTFT	DDLHKLVDEW	TSKTVGAAQL	KPTLNQLKQT	QKLQDMEAQA	GWAAPGEARA	MTAPRAGVGM	PRLPPAPGPL
2090	2100	2110	2120	2130	2140	2150	
STTVIPGAAP	TLSVPTPDGA	LGTARRNQVW	FGLRVPPTAC	CGHSTQPRGG	QRVGSKTASF	AASDPVRS	

Detailed Protein Report

Cmpd.	No. of Cmpds.	m/z meas.	Δ m/z [ppm]	z	Rt [min]	Score	P	Range	Sequence	Modification
2405	1	904.4370	-32.64	2	60.5	15.1	1	323-339	R.DLKCDNIFITGPTGSVK.I	
1286	1	945.9319	-64.24	2	46.2	11.9	2	2129-2147	R.GGQRVGSKTASFAASDPVR.S	

Detailed Protein Report

Protein 178: probable G-protein coupled receptor 179 precursor [Homo sapiens]

**Accession:**	gi|93352554	**Score:**	43.3
**Database:**	refseq_human(refseq_human_20140103.fasta)	**MW [kDa]:**	257.2
**Database Date:**	2015-11-30	**pI:**	5.4
**Modification(s):**	Carbamidomethyl, Oxidation	**Sequence Coverage [%]:**	1.2
		**No. of unique Peptides:**	3

Quantitation

QU:MU	**Median:**	2.19	**CV:**	73.29 %	**No. of Peptides:**	2
WUP:QUP	**Median:**	0.17	**CV:**	0.00 %	**No. of Peptides:**	1

Detailed Protein Report

10	20	30	40	50	60	70	80
MGTRGAVMPP	PMWGLLGCCF	VCAWALGGPR	PIRSLPPLSS	QVKPGSVPMQ	VPLEGAEAAL	AYLYSGDAQQ	LSQVNCSERY
90	100	110	120	130	140	150	160
EARGAGAMPG	LPPSLQGAAG	TLAQAANFLN	MLLQANDIRE	SSVEEDVEWY	QALVRSVAEG	DPRVYRALLT	FNPPPGASHL
170	180	190	200	210	220	230	240
QLALQATRTG	EETILQDLSG	NWVQEENPPG	DLDTPALKKR	VLTNDLGSLG	SPKWPQADGY	VGDTQQVRLS	PPFLECQEGR
250	260	270	280	290	300	310	320
LRPGWLITLS	ATFYGLKPDL	SPEVRGQVQM	DVDLQSVDIN	QCASGPGWYS	NTHLCDLNST	QCVPLESQGF	VLGRYLCRCR
330	340	350	360	370	380	390	400
PGFYGASPSG	GLEESDFQTT	GQFGFPEGRS	GRLLQCLPCP	EGCTSCMDAT	PCLVEEAAVL	RAAVLACQAC	CMLAIFLSML
410	420	430	440	450	460	470	480
VSYRCRRNKR	IWASGVVLLE	TVLFGFLLLY	FPVFILYFKP	SVFRCIALRW	VRLLGFAIVY	GTIILKLYRV	LQLFLSRTAQ
490	500	510	520	530	540	550	560
RSALLSSGRL	LRRLGLLLLP	VLGFLAVWTV	GALERGIQHA	PLVIRGHTPS	GRHFYLCHHD	RWDYIMVVAE	LLLLCWGSFL
570	580	590	600	610	620	630	640
CYATRAVLSA	FHEPRYMGIA	LHNELLLSAA	FHTARFVLVP	SLHPDWTLLL	FFFHTHSTVT	TTLALIFIPK	FWKLGAPPRE
650	660	670	680	690	700	710	720
EMVDEVCEDE	LDLQHSGSYL	GSSIASAWSE	HSLDPGDIRD	ELKKLYAQLE	VHKTKEMAAN	NPHLPKKRGS	SCQGLGRSFM
730	740	750	760	770	780	790	800
RYLAEFPEAL	ARQHSRDSGS	PGHGSLPGSS	RRRLLSSSLQ	EPEGTPALHK	SRSTYDQRRE	QDPPLLDSLL	RRKLAKKASR
810	820	830	840	850	860	870	880
TESRESVEGP	PALGFRSASA	HNLTVGERLP	RARPASLQKS	LSVASSREKA	LLMASQAYLE	ETYRQAKERE	ERKKAKAAMA
890	900	910	920	930	940	950	960
SLVRRPSARR	LERPRGAPLS	APPSPAKSSS	VDSSHTSGRL	HEEARRRLPH	PPIRHQVSTP	ILALSGGLGE	PRMLSPTSTL
970	980	990	1000	1010	1020	1030	1040
APALLPALAP	TPAPALAPVP	VSPQSPNLLT	YICPWENAEL	PAKQENVPQE	GPSGPERGHH	SPAPARARLW	RALSVAVEKS
1050	1060	1070	1080	1090	1100	1110	1120
RAGENEMDAE	DAHHQREAND	VDEDRPKIFP	KSHSLKAPVQ	QGSMRSLGLA	IKALTRSRST	YREKESVEES	PEGQNSGTAG
1130	1140	1150	1160	1170	1180	1190	1200
ESMGAPSRSP	RLGRPKAVSK	QAALIPSDDK	ESLQNQQNAH	TSRMLQVCQR	EGSREQEDRG	RRMTQGLGER	KAERAGKTGL
1210	1220	1230	1240	1250	1260	1270	1280
AMLRQVSRDK	NIKQSKETPV	GWQELPKAGL	QSLGSADHRV	AEVCPWEVTE	SETRQPDSGN	KAEICPWETS	EGAPESRALR
1290	1300	1310	1320	1330	1340	1350	1360
QDPGDSQKKR	GEARGKSEPI	DVVPMMRKKP	ERLVREQEAV	CPWESADRGG	LSPGSAPQDP	GRIRDKSEAG	DSVEARKVEK
1370	1380	1390	1400	1410	1420	1430	1440
PGWEAAGPEA	HTPDITKAEP	CPWEASEGGE	DGKPAQEAVK	DLPQEKQKTR	KATFWKEQKP	GGDLESLCPW	ESTDFRGPSA
1450	1460	1470	1480	1490	1500	1510	1520
VSIQAPGSSE	CSGSLGSGIA	EVCLWEAGDA	PAIQKAEICP	WELDDNVMGQ	EMLSLGTGRE	SLQEKEKASR	KGSFGEMGEQ
1530	1540	1550	1560	1570	1580	1590	1600
TVKAVQKLSQ	QQESVCPRES	TVPGHSSPCL	DNSSSKAGSQ	FLCNGGSRAT	QVCPQEDLRP	EAQEATPAKT	EICPWEVNER
1610	1620	1630	1640	1650	1660	1670	1680
TREEWTSAQV	PRGGESQKDK	EKMPGKSEIE	DVTAWEKPEG	QIQKQEAVGP	WESVDPGSFS	PQPRPQDTER	PQTLLQMSGS
1690	1700	1710	1720	1730	1740	1750	1760
VGSKAADICP	LDVEENLTAG	KAEICPWEVG	AGAGEERALG	AEAIRKSPND	TGKVSADLGP	RERAVTAPEK	PQKPTPEWEV
1770	1780	1790	1800	1810	1820	1830	1840
ACPWGSVGPG	ACSQHPGTLD	ADGPKAGFQE	LDHMGCRPGE	VCPWEAQEAA	TSEKAKICPW	EVSEGTTGKG	LDQKAGSESA
1850	1860	1870	1880	1890	1900	1910	1920
EQREKALEKG	RLTSLGEDVS	KGMAKLCQQQ	ETICIWENKD	LRESPAQAPK	ISDLPSSMSS	EVAEGHSLEA	TEKGDLRQDP
1930	1940	1950	1960	1970	1980	1990	2000
KTGSFPEHIT	QEKAPAADTE	EFTTEDGEKT	SHELQSVCPW	ETTAPADSVS	HLDRQRPDQP	KASSQRLVST	GGRAADVCPW
2010	2020	2030	2040	2050	2060	2070	2080
DVPDAGVYKS	DSSAKAETCP	WEVTERIPVK	GVSRQDGKGD	SQEEKGRAPE	KSEPKGVPVQ	KKPEMADFRQ	QEAVCPWESQ
2090	2100	2110	2120	2130	2140	2150	2160
DGKGLSPQPA	PDASDRSRGS	SEAAGSVETR	VAEVCLWEVV	EAPSAKKAEI	CPWEAGGGAA	EEGEQERESQ	GQGEMFLQKA
2170	2180	2190	2200	2210	2220	2230	2240

Detailed Protein Report

Cmpd.	No. of Cmpds.	m/z meas.	Δ m/z [ppm]	z	Rt [min]	Score	P	Range	Sequence	Modification	Ratios	
671	1	648.3571	-64.53	2	36.8	12.2	2	1129-1140	R.SPRLGRPKAVSK.Q			
479	1	475.6002	-274.91	2	35.6	13.9	0	1164-1170	R.MLQVCQR.E	Carbamidomethyl: 5; Oxidation: 1	QU:MU	1.14
813	7	509.1525	-284.31	2	38.6	17.2	1	1195-1204	R.AGKTGLAMLR.Q		WUP:QUP<br>QU:MU	0.17<br>4.21

Detailed Protein Report

Protein 179: conserved oligomeric Golgi complex subunit 1 [Homo sapiens]

Accession: gi|21237783
Database: refseq_human(refseq_human_20140103.fasta)
Database Date: 2015-11-30
Modification(s): Oxidation

Score: 43.0
MW [kDa]: 108.9
pI: 7.1
Sequence Coverage [%]: 5.1
No. of unique Peptides: 3

Quantitation

WUP:QUP **Median:** 0.28 **CV:** 0.00 % **No. of Peptides:** 1

10	20	30	40	50	60	70	80
MATAATSPAL	KRLDLRDPAA	LFETHGAEEI	RGLERQVRAE	IEHKKEELRQ	MVGERYRDLI	EAADTIGQMR	RCAVGLVDAV
90	100	110	120	130	140	150	160
KATDQYCARL	RQAGSAAPRP	PRAQQPQQPS	QEKFYSMAAQ	IKLLLEIPEK	IWSSMEASQC	LHATQLYLLC	CHLHSLLQLD
170	180	190	200	210	220	230	240
SSSSRYSPVL	SRFPILIRQV	AAASHFRSTI	LHESKMLLKC	QGVSDQAVAE	ALCSIMLLEE	SSPRQALTDF	LLARKATIQK
250	260	270	280	290	300	310	320
LLNQPHHGAG	IKAQICSLVE	LLATTLKQAH	ALFYTLPEGL	LPDPALPCGL	LFSTLETITG	QHPAGKGTGV	LQEEMKLCSW
330	340	350	360	370	380	390	400
FKHLPASIVE	FQPTLRTLAH	PISQEYLKDT	LQKWIHMCNE	DIKNGITNLL	MYVKSMKGLA	GIRDAMWELL	TNESTNHSWD
410	420	430	440	450	460	470	480
VLCRRLLEKP	LLFWEDMMQQ	LFLDRLQTLT	KEGFDSISSS	SKELLVSALQ	ELESSTSNSP	SNKHIHFEYN	MSLFLWSESP
490	500	510	520	530	540	550	560
NDLPSDAAWV	SVANRGQFAS	SGLSMKAQAI	SPCVQNFCSA	LDSKLKVKLD	DLLAYLPSDD	SSLPKDVSPT	QAKSSAFDRY
570	580	590	600	610	620	630	640
ADAGTVQEML	RTQSVACIKH	IVDCIRAELQ	SIEEGVQGQQ	DALNSAKLHS	VLFMARLCQS	LGELCPHLKQ	CILGKSESSE
650	660	670	680	690	700	710	720
KPAREFRALR	KQGKVKTQEI	IPTQAKWQEV	KEVLLQQSVM	GYQVWSSAVV	KVLIHGFTQS	LLLDDAGSVL	ATATSWDELE
730	740	750	760	770	780	790	800
IQEEAESGSS	VTSKIRLPAQ	PSWYVQSFLF	SLCQEINRVG	GHALPKVTLQ	EMLKSCMVQV	VAAYEKLSEE	KQIKKEGAFP
810	820	830	840	850	860	870	880
VTQNRALQLL	YDLRYLNIVL	TAKGDEVKSG	RSKPDSRIEK	VTDHLEALID	PFDLDVFTPH	LNSNLHRLVQ	RTSVLFGLVT
890	900	910	920	930	940	950	960
GTENQLAPRS	STFNSQEPHN	ILPLASSQIR	FGLLPLSMTS	TRKAKSTRNI	ETKAQVVPPA	RSTAGDPTVP	GSLFRQLVSE
970	980	990					
EDNTSAPSLF	KLGWLSSMTK						

Cmpd.	No. of Cmpds.	m/z meas.	Δ m/z [ppm]	z	Rt [min]	Score	P	Range	Sequence	Modification	Ratios
2917	1	724.8590	4.01	2	65.2	12.8	0	58-70	R.DLIEAADTIGQMR.R	Oxidation: 12	WUP:QUP 0.28
2511	1	941.9690	8.39	2	62.0	16.9	1	72-89	R.CAVGLVDAVKATDQYCAR.L		
1712	1	1045.0032	-51.34	2	49.7	13.3	1	527-545	K.VKLDDLLAYLPSDDSSLPK.D		

Detailed Protein Report

Protein 180: **PREDICTED: uncharacterized aarF domain-containing protein kinase 1 isoform X2 [Homo sapiens]**

**Accession:**	gi|530404248	**Score:**	43.0
**Database:**	refseq_human(refseq_human_20140103.fasta)	**MW [kDa]:**	52.0
**Database Date:**	2015-11-30	**pI:**	9.7
**Modification(s):**	Carbamidomethyl	**Sequence Coverage [%]:**	9.8
		**No. of unique Peptides:**	2

Quantitation

WUP:QUP **Median:** 1.03 **CV:** 0.00 % **No. of Peptides:** 1

10	20	30	40	50	60	70	80
MARKALKLAS	WTSMALAASG	IYFYSNKYLD	PNDFGAVRVG	RAVATTAVIS	YDYLTSLKSV	PYGSEEYLQL	RSKVHLRSAR
**90**	**100**	**110**	**120**	**130**	**140**	**150**	**160**
RLCELCCANR	GTFIKVGQHL	GALDYLLPEE	YTSTLKVLHS	QAPQSSMQEI	RQVIREDLGK	EIHDLFQSFD	DTPLGTASLA
**170**	**180**	**190**	**200**	**210**	**220**	**230**	**240**
QVHKAVLHDG	RTVAVKVQHP	KVRAQSSKDI	LLMEVLVLAV	KQLFPEFEFM	WLVDEAKKNL	PLELDFLNEG	RNAEKVSQML
**250**	**260**	**270**	**280**	**290**	**300**	**310**	**320**
RHFDFLKVPR	IHWDLSTERV	LLMEFVDGGQ	VNDRDYMERN	KIDVNEISRH	LGKMYSEMIF	VNGFVHCDPH	PGNVLVRKHP
**330**	**340**	**350**	**360**	**370**	**380**	**390**	**400**
GTGKAEIVLL	DHGLYQDLEI	RNNAANYLPQ	ISHLLNHVPR	QMLLILKTND	LLRGIEAALG	TRASASSFLN	MSRCCIRALA
**410**	**420**	**430**	**440**	**450**	**460**		
EHKKKNTCSF	FRRTQISFSE	AFNLWQINLH	ELILRVKGLK	LADRVLALIC	WLFPAPL		

Cmpd.	No. of Cmpds.	m/z meas.	Δ m/z [ppm]	z	Rt [min]	Score	P	Range	Sequence	Modification	Ratios
2471	1	588.1664	-250.34	2	61.4	20.5	1	232-241	R.NAEKVSQMLR.H		
5	1	1205.0356	-39.18	2	29.0	11.7	2	383-403	R.ASASSFLNMSRCCIRALAEHK.K	Carbamidomethyl: 12, 13	WUP:QUP 1.03

Detailed Protein Report

Protein 181: probable G-protein coupled receptor 125 precursor [Homo sapiens]

**Accession:**	gi|59823631	**Score:**	42.9
**Database:**	refseq_human(refseq_human_20140103.fasta)	**MW [kDa]:**	146.1
**Database Date:**	2015-11-30	**pI:**	9.7
		**Sequence Coverage [%]:**	3.4
		**No. of unique Peptides:**	2

10	20	30	40	50	60	70	80
MEPPGRRRGR	AQPPLLLPLS	LLALLALLGG	GGGGGAAALP	AGCKHDGRPR	GAGRAAGAAE	GKVVCSSLEL	AQVLPPDTLP
90	100	110	120	130	140	150	160
NRTVTLILSN	NKISELKNGS	FSGLSLLERL	DLRNNLISSI	DPGAFWGLSS	LKRLDLTNNR	IGCLNADIFR	GLTNLVRLNL
170	180	190	200	210	220	230	240
SGNLFSSLSQ	GTFDYLASLR	SLEFQTEYLL	CDCNILWMHR	WVKEKNITVR	DTRCVYPKSL	QAQPVTGVKQ	ELLTCDPPLE
250	260	270	280	290	300	310	320
LPSFYMTPSH	RQVVFEGDSL	PFQCMASYID	QDMQVLWYQD	GRIVETDESQ	GIFVEKNMIH	NCSLIASALT	ISNIQAGSTG
330	340	350	360	370	380	390	400
NWGCHVQTKR	GNNTRTVDIV	VLESSAQYCP	PERVVNNKGD	FRWPRTLAGI	TAYLQCTRNT	HGSGIYPGNP	QDERKAWRRC
410	420	430	440	450	460	470	480
DRGGFWADDD	YSRCQYANDV	TRVLYMFNQM	PLNLTNAVAT	ARQLLAYTVE	AANFSDKMDV	IFVAEMIEKF	GRFTKEEKSK
490	500	510	520	530	540	550	560
ELGDVMVDIA	SNIMLADERV	LWLAQREAKA	CSRIVQCLQR	IATYRLAGGA	HVYSTYSPNI	ALEAYVIKST	GFTGMTCTVF
570	580	590	600	610	620	630	640
QKVAASDRTG	LSDYGRRDPE	GNLDKQLSFK	CNVSNTFSSL	ALKNTIVEAS	IQLPPSLFSP	KQKRELRPTD	DSLYKLQLIA
650	660	670	680	690	700	710	720
FRNGKLFPAT	GNSTNLADDG	KRRTVVTPVI	LTKIDGVNVD	THHIPVNVTL	RRIAHGADAV	AARWDFDLLN	GQGGWKSDGC
730	740	750	760	770	780	790	800
HILYSDENIT	TIQCYSLSNY	AVLMDLTGSE	LYTQAASLLH	PVVYTTAIIL	LLCLLAVIVS	YIYHHSLIRI	SLKSWHMLVN
810	820	830	840	850	860	870	880
LCFHIFLTCV	VFVGGITQTR	NASICQAVGI	ILHYSTLATV	LWVGVTARNI	YKQVTKKAKR	CQDPDEPPPP	PRPMLRFYLI
890	900	910	920	930	940	950	960
GGGIPIIVCG	ITAAANIKNY	GSRPNAPYCW	MAWEPSLGAF	YGPASFITFV	NCMYFLSIFI	QLKRHPERKY	ELKEPTEEQQ
970	980	990	1000	1010	1020	1030	1040
RLAANENGEI	NHQDSMSLSL	ISTSALENEH	TFHSQLLGAS	LTLLLYVALW	MFGALAVSLY	YPLDLVFSFV	FGATSLSFSA
1050	1060	1070	1080	1090	1100	1110	1120
FFVVHHCVNR	EDVRLAWIMT	CCPGRSSYSV	QVNVQPPNSN	GTNGEAPKCP	NSSAESSCTN	KSASSFKNSS	QGCKLTNLQA
1130	1140	1150	1160	1170	1180	1190	1200
AAAQCHANSL	PLNSTPQLDN	SLTEHSMDND	IKMHVAPLEV	QFRTNVHSSR	HHKNRSKGHR	ASRLTVLREY	AYDVPTSVEG
1210	1220	1230	1240	1250	1260	1270	1280
SVQNGLPKSR	LGNNEGHSRS	RRAYLAYRER	QYNPPQQDSS	DACSTLPKSS	RNFEKPVSTT	SKKDALRKPA	VVELENQQKS
1290	1300	1310	1320	1330			
YGLNLAIQNG	PIKSNGQEGP	LLGTDSTGNV	RTGLWKHETT	V			

Cmpd.	No. of Cmpds.	m/z meas.	Δ m/z [ppm]	z	Rt [min]	Score	P	Range	Sequence	Modification
2747	1	1045.5347	-21.10	2	65.5	16.6	2	211-229	R.DTRCVYPKSLQAQPVTGVK.Q	
430	1	706.0114	194.46	2	33.9	10.5	0	366-378	R.TLAGITAYLQCTR.N	

Detailed Protein Report

Protein 182: estrogen sulfotransferase [Homo sapiens]

**Accession:**	gi|4885617	**Score:**	42.8
**Database:**	refseq_human(refseq_human_20140103.fasta)	**MW [kDa]:**	35.1
**Database Date:**	2015-11-30	**pI:**	6.2
		**Sequence Coverage [%]:**	13.3
		**No. of unique Peptides:**	1

10	20	30	40	50	60	70	80
MNSELDYYEK	FEEVHGILMY	KDFVKYWDNV	EAFQARPDDL	VIATYPKSGT	TWVSEIVYMI	YKEGDVEKCK	EDVIFNRIPF
**90**	**100**	**110**	**120**	**130**	**140**	**150**	**160**
LECRKENLMN	GVKQLDEMNS	PRIVKTHLPP	ELLPASFWEK	DCKIIYLCRN	AKDVAVSFYY	FFLMVAGHPN	PGSFPEFVEK
**170**	**180**	**190**	**200**	**210**	**220**	**230**	**240**
FMQGQVPYGS	WYKHVKSWWE	KGKSPRVLFL	FYEDLKEDIR	KEVIKLIHFL	ERKPSEELVD	RIIHHTSFQE	MKNNPSTNYT
**250**	**260**	**270**	**280**	**290**	**300**		
TLPDEIMNQK	LSPFMRKGIT	GDWKNHFTVA	LNEKFDKHYE	QQMKESTLKF	RTEI		

Cmpd.	No. of Cmpds.	m/z meas.	Δ m/z [ppm]	z	Rt [min]	Score	P	Range	Sequence	Modification
2243	1	945.9550	-47.59	2	58.5	10.9	2	78-93	R.IPFLECRKENLMNGVK.Q	

Detailed Protein Report

Protein 183: synaptojanin-1 isoform d [Homo sapiens]

**Accession:**	gi|237757326	**Score:**	42.8
**Database:**	refseq_human(refseq_human_20140103.fasta)	**MW [kDa]:**	168.1
**Database Date:**	2015-11-30	**pI:**	6.9
**Modification(s):**	Carbamidomethyl	**Sequence Coverage [%]:**	3.4
		**No. of unique Peptides:**	3

10	20	30	40	50	60	70	80
MAFSKGFRIY	HKLDPPPFSL	IVETRHKEEC	LMFESGAVAV	LSSAEKEAIK	GTYSKVLDAY	GLLGVLRLNL	GDTMLHYLVL
90	100	110	120	130	140	150	160
VTGCMSVGKI	QESEVFRVTS	TEFISLRIDS	SDEDRISEVR	KVLNSGNFYF	AWSASGISLD	LSLNAHRSMQ	EQTTDNRFFW
170	180	190	200	210	220	230	240
NQSLHLHLKH	YGVNCDDWLL	RLMCGGVEIR	TIYAAHKQAK	ACLISRLSCE	RAGTRFNVRG	TNDDGHVANF	VETEQVVYLD
250	260	270	280	290	300	310	320
DSVSSFIQIR	GSVPLFWEQP	GLQVGSHRVR	MSRGFEANAP	AFDRHFRTLK	NLYGKQIIVN	LLGSKEGEHM	LSKAFQSHLK
330	340	350	360	370	380	390	400
ASEHAADIQM	VNFDYHQMVK	GGKAEKLHSV	LKPQVQKFLD	YGFFYFNGSE	VQRCQSGTVR	TNCLDCLDRT	NSVQAFLGLE
410	420	430	440	450	460	470	480
MLAKQLEALG	LAEKPQLVTR	FQEVFRSMWS	VNGDSISKIY	AGTGALEGKA	KAGKLKDGAR	SVTRTIQNNF	FDSSKQEAID
490	500	510	520	530	540	550	560
VLLLGNTLNS	DLADKARALL	TTGSLRASSK	VLKSMCENFY	KYSKPKKIRV	CVGTWNVNGG	KQFRSIAFKN	QTLTDWLLDA
570	580	590	600	610	620	630	640
PKLAGIQEFQ	DKRSKPTDIF	AIGFEEMVEL	NAGNIVSAST	TNQKLWAVEL	QKTISRDNKY	VLLASEQLVG	VCLFVFIRPQ
650	660	670	680	690	700	710	720
HAPFIRDVAV	DTVKTGMGGA	TGNKGAVAIR	MLFHTTSLCF	VCSHFAAGQS	QVKERNEDFI	EIARKLSFPM	GRMLFSHDYV
730	740	750	760	770	780	790	800
FWCGDFNYRI	DLPNEEVKEL	IRQQNWDSLI	AGDQLINQKN	AGQVFRGFLE	GKVTFAPTYK	YDLFSDDYDT	SEKCRTPAWT
810	820	830	840	850	860	870	880
DRVLWRRRKW	PFDRSAEDLD	LLNASFQDES	KILYTWTPGT	LLHYGRAELK	TSDHRPVVAL	IDIDIFEVEA	EERQNIYKEV
890	900	910	920	930	940	950	960
IAVQGPPDGT	VLVSIKSSLP	ENNFFDDALI	DELLQQFASF	GEVILIRFVE	DKMWVTFLEG	SSALNVLSLN	GKELLNRTIT
970	980	990	1000	1010	1020	1030	1040
IALKSPDWIK	NLEEEMSLEK	ISIALPSSTS	STLLGEDAEV	AADFDMEGDV	DDYSAEVEEL	LPQHLQPSSS	SGLGTSPSSS
1050	1060	1070	1080	1090	1100	1110	1120
PRTSPCQSPT	ISEGPVPSLP	IRPSRAPSRT	PGPPSAQSSP	IDAQPATPLP	QKDPAQPLEP	KRPPPPRPVA	PPTRPAPPQR
1130	1140	1150	1160	1170	1180	1190	1200
PPPPSGRSQP	SPQAGLAGPG	PAGYSTARPT	IPPRAGVISA	PQSHARASAG	RLTPESQSKT	SETSKGSTFL	PEPLKPQAAF
1210	1220	1230	1240	1250	1260	1270	1280
PPQSSLPPPA	QRLQEPLVPV	AAPMPQSGPQ	PNLETPPQPP	PRSRSSHSLP	SEASSQPQVK	TNGISDGKRE	SPLKIDPFED
1290	1300	1310	1320	1330	1340	1350	1360
LSFNLLAVSK	AQLSVQTSPV	PTPDPKRLIQ	LPSATQSNVL	SSVSCMPTMP	PIPARSQSQE	NMRSSPNPFI	TGLTRTNPFS
1370	1380	1390	1400	1410	1420	1430	1440
DRTAAPGNPF	RAKSEESEAT	SWFSKEEPVT	ISPFPSLQPL	GHNKSRASSS	LDGFKDSFDL	QGQSTLKISN	PKGWVTFEEE
1450	1460	1470	1480	1490	1500	1510	1520
EDFGVKGKSK	SACSDLLGNQ	PSSFSGSNLT	LNDDWNKGTN	VSFCVLPSRR	PPPPPVPLLP	PGTSPPVDPF	TTLASKASPT
1530							
LDFTER							

Cmpd.	No. of Cmpds.	m/z meas.	Δ m/z [ppm]	z	Rt [min]	Score	P	Range	Sequence	Modification
1994	1	1044.9084	-141.16	2	55.3	14.4	2	182-200	R.LMCGGVEIRTIYAAHKQAK.A	
2282	1	614.2542	-33.53	3	59.0	16.0	1	374-389	R.CQSGTVRTNCLDCLDR.T	Carbamidomethyl: 10
1652	1	626.6317	-42.50	3	50.4	12.3	1	1416-1432	K.DSFDLQGQSTLKISNPK.G	

Detailed Protein Report

Protein 184: PREDICTED: zinc finger protein 175 isoform X3 [Homo sapiens]

**Accession:**	gi|530417172	**Score:**	42.7
**Database:**	refseq_human(refseq_human_20140103.fasta)	**MW [kDa]:**	69.5
**Database Date:**	2015-11-30	**pI:**	10.1
**Modification(s):**	Carbamidomethyl	**Sequence Coverage [%]:**	3.5
		**No. of unique Peptides:**	2

Quantitation

WUP:QUP **Median:** 1.03 **CV:** 0.00 % **No. of Peptides:** 1

10	20	30	40	50	60	70	80
MPADVNLSQK	PQVLGPEKQD	GSCEASVSFE	DVTVDFSREE	WQQLDPAQRC	LYRDVMLELY	SHLFAVGYHI	PNPEVIFRML
**90**	**100**	**110**	**120**	**130**	**140**	**150**	**160**
KEKEPRVEEA	EVSHQRCQES	LKLNLEVNGQ	NESNDTEQLD	DVVGSGQLFS	HSSSDACSKN	IHTGETFCKG	NQCRKVCGHK
**170**	**180**	**190**	**200**	**210**	**220**	**230**	**240**
QSLKQHQIHT	QKKPDGCSEC	GGSFTQKSHL	FAQQRIHSVG	NLHECGKCGK	AFMPQLKLSV	YLTDHTGDIP	CICKECGKVF
**250**	**260**	**270**	**280**	**290**	**300**	**310**	**320**
IQRSELLTHQ	KTHTRKKPYK	CHDCGKAFFQ	MLSLFRHQRT	HSREKLYECS	ECGKGFSQNS	TLIIHQKIHT	GERQYACSEC
**330**	**340**	**350**	**360**	**370**	**380**	**390**	**400**
GKAFTQKSTL	SLHQRIHSGQ	KSYVCIECGQ	AFIQKAHLIV	HQRSHTGEKP	YQCHNCGKSF	ISKSQLDIHH	RIHTGEKPYE
**410**	**420**	**430**	**440**	**450**	**460**	**470**	**480**
CSDCGKTFTQ	KSHLNIHQKI	HTGERHHVCS	ECGKAFNQKS	ILSMHQRIHT	GEKPYKCSEC	GKAFTSKSQF	KEHQRIHTGE
**490**	**500**	**510**	**520**	**530**	**540**	**550**	**560**
KPYVCTECGK	AFNGRSNFHK	HQITHTRERP	FVCYKCGKAF	VQKSELITHQ	RTHMGEKPYE	CLDCGKSFSK	KPQLKVHQRI
**570**	**580**	**590**	**600**	**610**			
HTGERPYVCS	ECGKAFNNRS	NFNKHQTTHT	RDKSYKCSYS	VKGFTKQ			

Cmpd.	No. of Cmpds.	m/z meas.	Δ m/z [ppm]	z	Rt [min]	Score	P	Range	Sequence	Modification	Ratios
22	1	1205.0462	-16.45	2	30.0	19.7	1	218-238	K.LSVYLTDHTGDIPCICKECGK.V	Carbamidomethyl: 14, 16	WUP:QUP 1.03
6	2	1205.0350	-25.71	2	29.5	22.9	1	218-238	K.LSVYLTDHTGDIPCICKECGK.V	Carbamidomethyl: 14, 19	

Detailed Protein Report

Protein 185: ectodysplasin-A isoform 2 [Homo sapiens]

**Accession:**	gi|54112101	**Score:**	42.5
**Database:**	refseq_human(refseq_human_20140103.fasta)	**MW [kDa]:**	41.0
**Database Date:**	2015-11-30	**pI:**	9.6
		**Sequence Coverage [%]:**	8.7
		**No. of unique Peptides:**	2

10	20	30	40	50	60	70	80
MGYPEVERRE	LLPAAAPRER	GSQGCGCGGA	PARAGEGNSC	LLFLGFFGLS	LALHLLTLCC	YLELRSELRR	ERGAESRLGG
**90**	**100**	**110**	**120**	**130**	**140**	**150**	**160**
SGTPGTSGTL	SSLGGLDPDS	PITSHLGQPS	PKQQPLEPGE	AALHSDSQDG	HQMALLNFFF	PDEKPYSEEE	SRRVRRNKRS
**170**	**180**	**190**	**200**	**210**	**220**	**230**	**240**
KSNEGADGPV	KNKKKGKKAG	PPGPNGPPGP	PGPPGPQGPP	GIPGIPGIPG	TTVMGPPGPP	GPPGPQGPPG	LQGPSGAADK
**250**	**260**	**270**	**280**	**290**	**300**	**310**	**320**
AGTRENQPAV	VHLQGQGSAI	QVKNDLSGGV	LNDWSRITMN	PKVFKLHPRS	GELEVLVDGT	YFIYSQVYYI	NFTDFASYEV
**330**	**340**	**350**	**360**	**370**	**380**	**390**	
VVDEKPFLQC	TRSIETGKTN	YNTCYTAGVC	LLKARQKIAV	KMVHADISIN	MSKHTTFFGA	IRLGEAPAS	

Cmpd.	No. of Cmpds.	m/z meas.	Δ m/z [ppm]	z	Rt [min]	Score	P	Range	Sequence	Modification
1964	4	703.2668	-56.59	2	52.9	13.6	1	19-33	R.ERGSQGCGCGGAPAR.A	
1518	3	1059.0796	50.76	2	48.7	16.6	1	264-282	K.NDLSGGVLNDWSRITMNPK.V	

Detailed Protein Report

Protein 186: PREDICTED: talin-2 isoform X15 [Homo sapiens]

**Accession:**	gi|578827491	**Score:**	42.5
**Database:**	refseq_human(refseq_human_20140103.fasta)	**MW [kDa]:**	267.3
**Database Date:**	2015-11-30	**pI:**	5.4
		**Sequence Coverage [%]:**	1.9
		**No. of unique Peptides:**	1

Quantitation

QU:MU	**Median:** 1.45	**CV:** 0.00 %	**No. of Peptides:** 1

Detailed Protein Report

10	20	30	40	50	60	70	80
MVALSLKICV	RHCNVVKTMQ	FEPSTAVYDA	CRVIRERVPE	AQTGQASDYG	LFLSDEDPRK	GIWLEAGRTL	DYYMLRNGDI
90	100	110	120	130	140	150	160
LEYKKKQRPQ	KIRMLDGSVK	TVMVDDSKTV	GELLVTICSR	IGITNYEEYS	LIQETIEEKK	EEGTGTLKKD	RTLLRDERKM
170	180	190	200	210	220	230	240
EKLKAKLHTD	DDLNWLDHSR	TFREQGVDEN	ETLLLRRKFF	YSDQNVDSRD	PVQLNLLYVQ	ARDDILNGSH	PVSFEKACEF
250	260	270	280	290	300	310	320
GGFQAQIQFG	PHVEHKHKPG	FLDLKEFLPK	EYIKQRGAEK	RIFQEHKNCG	EMSEIEAKVK	YVKLARSLRT	YGVSFFLVKE
330	340	350	360	370	380	390	400
KMKGKNKLVP	RLLGITKDSV	MRVDEKTKEV	LQEWPLTTVK	RWAASPKSFT	LDFGEYQESY	YSVQTTEGEQ	ISQLIAGYID
410	420	430	440	450	460	470	480
IILKKKQSKD	RFGLEGDEES	TMLEESVSPK	KSTILQQQFN	RTGKAEHGSV	ALPAVMRSGS	SGPETFNVGS	MPSPQQQVMV
490	500	510	520	530	540	550	560
GQMHRGHMPP	LTSAQQALMG	TINTSMHAVQ	QAQDDLSELD	SLPPLGQDMA	SRVWVQNKVD	ESKHEIHSQV	DAITAGTASV
570	580	590	600	610	620	630	640
VNLTAGDPAD	TDYTAVGCAI	TTISSNLTEM	SKGVKLLAAL	MDDEVGSGED	LLRAARTLAG	AVSDLLKAVQ	PTSGEPRQTV
650	660	670	680	690	700	710	720
LTAAGSIGQA	SGDLLRQIGE	NETDERFQDV	LMSLAKAVAN	AAAMLVLKAK	NVAQVAEDTV	LQNRVIAAAT	QCALSTSQLV
730	740	750	760	770	780	790	800
ACAKVVSPTI	SSPVCQEQLI	EAGKLVDRSV	ENCVRACQAA	TTDSELLKQV	SAAASVVSQA	LHDLLQHVRQ	FASRGEPIGR
810	820	830	840	850	860	870	880
YDQATDTIMC	VTESIFSSMG	DAGEMVRQAR	VLAQATSDLV	NAMRSDAEAE	IDMENSKKLL	AAAKLLADST	ARMVEAAKGA
890	900	910	920	930	940	950	960
AANPENEDQQ	QRLREAAEGL	RVATNAAAQN	AIKKKIVNRL	EVAAKQAAAA	ATQTIAASQN	AAVSNKNPAA	QQQLVQSCKA
970	980	990	1000	1010	1020	1030	1040
VADHIPQLVQ	GVRGSQAQAE	DLSAQLALII	SSQNFLQPGS	KMVSSAKAAV	PTVSDQAAAM	QLSQCAKNLA	TSLAELRTAS
1050	1060	1070	1080	1090	1100	1110	1120
QKAHEACGPM	EIDSALNTVQ	TLKNELQDAK	MAAVESQLKP	LPGETLEKCA	QDLGSTSKAV	GSSMAQLLTC	AAQGNEHYTG
1130	1140	1150	1160	1170	1180	1190	1200
VAARETAQAL	KTLAQAARGV	AASTTDPAAA	HAMLDSARDV	MEGSAMLIQE	AKQALIAPGD	AERQQRLAQV	AKAVSHSLNN
1210	1220	1230	1240	1250	1260	1270	1280
CVNCLPGQKD	VDVALKSIGE	SSKKLLVDSL	PPSTKPFQEA	QSELNQAAAD	LNQSAGEVVH	ATRGQSGELA	AASGKFSDDF
1290	1300	1310	1320	1330	1340	1350	1360
DEFLDAGIEM	AGQAQTKEDQ	IQVIGNLKNI	SMASSKLLLA	AKSLSVDPGA	PNAKNLLAAA	ARAVTESINQ	LITLCTQQAP
1370	1380	1390	1400	1410	1420	1430	1440
GQKECDNALR	ELETVKGMLD	NPNEPVSDLS	YFDCIESVME	NSKVLGESMA	GISQNAKTGD	LPAFGECVGI	ASKALCGLTE
1450	1460	1470	1480	1490	1500	1510	1520
AAAQAAYLVG	ISDPNSQAGH	QGLVDPIQFA	RANQAIQMAC	QNLVDPGSSP	SQVLSAATIV	AKHTSALCNA	CRIASSKTAN
1530	1540	1550	1560	1570	1580	1590	1600
PVAKRHFVQS	AKEVANSTAN	LVKTIKALDG	DFSEDNRNKC	RIATAPLIEA	VENLTAFASN	PEFVSIPAQI	SSEGSQAQEP
1610	1620	1630	1640	1650	1660	1670	1680
ILVSAKTMLE	SSSYLIRTAR	SLAINPKDPP	TWSVLAGHSH	TVSDSIKSLI	TSIRDKAPGQ	RECDYSIDGI	NRCIRDIEQA
1690	1700	1710	1720	1730	1740	1750	1760
SLAAVSQSLA	TRDDISVEAL	QEQLTSVVQE	IGHLIDPIAT	AARGEAAQLG	HKVTQLASYF	EPLILAAVGV	ASKILDHQQQ
1770	1780	1790	1800	1810	1820	1830	1840
MTVLDQTKTL	AESALQMLYA	AKEGGGNPKA	QHTHDAITEA	AQLMKEAVDD	IMVTLNEAAS	EVGLVGGMVD	AIAEAMSKLD
1850	1860	1870	1880	1890	1900	1910	1920
EGTPPEPKGT	FVDYQTTVVK	YSKAIAVTAQ	EMIGFQIRTR	VQDLGHGCIF	LVQKAGALQV	CPTDSYTKRE	LIECARAVTE
1930	1940	1950	1960	1970	1980	1990	2000
KVSLVLSALQ	AGNKGTQACI	TAATAVSGII	ADLDTTIMFA	TAGTLNAENS	ETFADHRENI	LKTAKALVED	TKLLVSGAAS
2010	2020	2030	2040	2050	2060	2070	2080
TPDKLAQAAQ	SSAATITQLA	EVVKLGAASL	GSDDPETQVV	LINAIKDVAK	ALSDLISATK	GAASKPVDDP	SMYQLKGAAK
2090	2100	2110	2120	2130	2140	2150	2160
VMVTNVTSLL	KTVKAVEDEA	TRGTRALEAT	IECIKQELTV	FQSKDVPEKT	SSPEESIRMT	KGITMATAKA	VAAGNSCRQE
2170	2180	2190	2200	2210	2220	2230	2240
DVIATANLSR	KAVSDMLTAC	KQASFHPDVS	DEVRTRALRF	GTECTLGYLD	LLEHVLVILQ	KPTPEFKQQL	AAFSKRVAGA
2250	2260	2270	2280	2290	2300	2310	2320
VTELIQAAEA	MKGTEWVDPE	DPTVIAETEL	LGAAASIEAA	AKKLEQLKPR	AKPKQADETL	DFEEQILEAA	KSIAAATSAL
2330	2340	2350	2360	2370	2380	2390	2400

Detailed Protein Report

Cmpd.	No. of Cmpds.	m/z meas.	Δ m/z [ppm]	z	Rt [min]	Score	P	Range	Sequence	Modification	Ratios	
2049	1	812.3468	-109.11	2	56.0	14.1	1	442-457	R.TGKAEHGSVALPAVMR.S		QU:MU	1.45

Detailed Protein Report

Protein 187: **dematin isoform 3 [Homo sapiens]**

**Accession:**	gi|166706885	**Score:**	42.4
**Database:**	refseq_human(refseq_human_20140103.fasta)	**MW [kDa]:**	40.7
**Database Date:**	2015-11-30	**pI:**	9.4
		**Sequence Coverage [%]:**	5.6
		**No. of unique Peptides:**	1

10	20	30	40	50	60	70	80
MERLQKAKMD	NQVLGYKDLA	AIPKDKAILD	IERPDLMIYE	PHFTYSLLEH	VELPRSRERS	LSPKSTSPPP	SPEVWADSRS
**90**	**100**	**110**	**120**	**130**	**140**	**150**	**160**
PGIISQASAP	RTTGTPRTSL	PHFHHPETSR	PDSNIYKKPP	IYKQRESVGG	SPQTKHLIED	LIIESSKFPA	AQPPDPNQPA
**170**	**180**	**190**	**200**	**210**	**220**	**230**	**240**
KIETDYWPCP	PSLAVVETEW	RKRKASRRGA	EEEEEEEDDD	SGEEMKALRE	RQREELSKVT	SNLGKMILKE	EMEKSLPIRR
**250**	**260**	**270**	**280**	**290**	**300**	**310**	**320**
KTRSLPDRTP	FHTSLHQGTS	KSSSLPAYGR	TTLSRLQSTE	FSPSGSETGS	PGLQIYPYEM	LVVTNKGRTK	LPPGVDRMRL
**330**	**340**	**350**	**360**				
ERHLSAEDFS	RVFAMSPEEF	GKLALWKRNE	LKKKASLF				

Cmpd.	No. of Cmpds.	m/z meas.	Δ m/z [ppm]	z	Rt [min]	Score	P	Range	Sequence	Modification
953	1	531.2210	-62.44	2	40.3	19.5	0	323-331	R.HLSAEDFSR.V	

Detailed Protein Report

Protein 188: PREDICTED: partitioning defective 3 homolog isoform X4 [Homo sapiens]

**Accession:**	gi|530392319	**Score:**	42.3
**Database:**	refseq_human(refseq_human_20140103.fasta)	**MW [kDa]:**	149.4
**Database Date:**	2015-11-30	**pI:**	8.8
**Modification(s):**	Oxidation	**Sequence Coverage [%]:**	3.5
		**No. of unique Peptides:**	2

```
        10         20         30         40         50         60         70         80
MKVTVCFGRT RVVVPCGDGH MKVFSLIQQA VTRYRKAIAK DPNYWIQVHR LEHGDGGILD LDDILCDVAD DKDRLVAVFD
        90        100        110        120        130        140        150        160
EQDPHHGGDG TSASSTGTQS PEIFGSELGT NNVSAFQPYQ ATSEIEVTPS VLRANMPLHV RRSSDPALIG LSTSVSDSNF
       170        180        190        200        210        220        230        240
SSEEPSRKNP TRWSTTAGFL KQNTAGSPKT CDRKKDENYR SLPRDTSNWS NQFQRDNARS SLSASHPMVG KWLEKQEQDE
       250        260        270        280        290        300        310        320
DGTEEDNSRV EPVGHADTGL EHIPNFSLDD MVKLVEVPND GGPLGIHVVP FSARGGRTLG LLVKRLEKGG KAEHENLFRE
       330        340        350        360        370        380        390        400
NDCIVRINDG DLRNRRFEQA QHMFRQAMRT PIIWFHVVPA ANKEQYEQLS QSEKNNYYSS RFSPDSQYID NRSVNSAGLH
       410        420        430        440        450        460        470        480
TVQRAPRLNH PPEQIDSHSR LPHSAHPSGK PPSAPASAPQ NVFSTTVSSG YNTKKIGKRL NIQLKKGTEG LGFSITSRDV
       490        500        510        520        530        540        550        560
TIGGSAPIYV KNILPRGAAI QDGRLKAGDR LIEVNGVDLV GKSQEEVVSL LRSTKMEGTV SLLVFRQEDA FHPRELNAEP
       570        580        590        600        610        620        630        640
SQMQIPKETK AEDEDIVLTP DGTREFLTFE VPLNDSGSAG LGVSVKGNRS KENHADLGIF VKSIINGGAA SKDGRLRVND
       650        660        670        680        690        700        710        720
QLIAVNGESL LGKTNQDAME TLRRSMSTEG NKRGMIQLIV ARRISKCNEL KSPGSPPGPE LPIETALDDR ERRISHSLYS
       730        740        750        760        770        780        790        800
GIEGLDESPS RNAALSRIMG KYQLSPTVNM PQDDTVIIED DRLPVLPPHL SDQSSSSSHD DVGFVTADAG TWAKAAISDS
       810        820        830        840        850        860        870        880
ADCSLSPDVD PVLAFQREGF GRQSMSEKRT KQFSDASQLD FVKTRKSKSM DLGSSPSRDV GPSLGLKKSS SLESLQTAVA
       890        900        910        920        930        940        950        960
EVTLNGDIPF HRPRPRIIRG RGCNESFRAA IDKSYDKPAV DDDDEGMETL EEDTEESSRS GRESVSTASD QPSHSLERQM
       970        980        990       1000       1010       1020       1030       1040
NGNQEKGDKT DRKKDKTGKE KKKDRDKEKD KMKAKKGMLK GLGDMFRFGK HRKDDKIEKT GKIKIQESFT SEEERIRMKQ
      1050       1060       1070       1080       1090       1100       1110       1120
EQERIQAKTR EFRERQARER DYAEIQDFHR TFGCDDELMY GGVSSYEGSM ALNARPQSPR EGHMMDALYA QVKKPRNSKP
      1130       1140       1150       1160       1170       1180       1190       1200
SPVDSNRSTP SNHDRIQRLR QEFQQAKQDE DVEDRRRTYS FEQPWPNARP ATQSGRHSVS VEVQMQRQRQ EERESSQQAQ
      1210       1220       1230       1240       1250       1260       1270       1280
RQYSSLPRQS RKNASSVSQD SWEQNYSPGE GFQSAKENPR YSSYQGSRNG YLGGHGFNAR VMLETQELLR QEQRRKEQQM
      1290       1300       1310       1320       1330       1340
KKQPPSEGPS NYDSYKKVQD PSYAPPKGPF RQDVPPSPSQ VARLNRLQTP EKGRPFYS
```

Cmpd.	No. of Cmpds.	m/z meas.	Δ m/z [ppm]	z	Rt [min]	Score	P	Range	Sequence	Modification
2795	1	729.3498	-56.43	2	66.1	14.8	1	610-622	R.SKENHADLGIFVK.S	
2354	1	711.9476	-118.31	3	59.3	10.7	2	847-867	K.SKSMDLGSSPSRDVGPSLGLK.K	Oxidation: 4

Detailed Protein Report

Protein 189: endothelin-2 preproprotein [Homo sapiens]

**Accession:**	gi|4503463	**Score:**	42.3
**Database:**	refseq_human(refseq_human_20140103.fasta)	**MW [kDa]:**	19.9
**Database Date:**	2015-11-30	**pI:**	12.1
**Modification(s):**	Carbamidomethyl	**Sequence Coverage [%]:**	14.0
		**No. of unique Peptides:**	1

10	20	30	40	50	60	70	80
MVSVPTTWCS	VALALLVALH	EGKGQAAATL	EQPASSSHAQ	GTHLRLRRCS	CSSWLDKECV	YFCHLDIIWV	NTPEQTAPYG
**90**	**100**	**110**	**120**	**130**	**140**	**150**	**160**
LGNPPRRRRR	SLPRRCQCSS	ARDPACATFC	LRRPWTEAGA	VPSRKSPADV	FQTGKTGATT	GELLQRLRDI	STVKSLFAKR
**170**	**180**						
QQEAMREPRS	THSRWRKR						

Cmpd.	No. of Cmpds.	m/z meas.	Δ m/z [ppm]	z	Rt [min]	Score	P	Range	Sequence	Modification
2946	1	1051.5610	95.12	2	65.6	10.9	2	95-112	R.RCQCSSARDPACATFCLR.R	Carbamidomethyl: 4, 12

Detailed Protein Report

Protein 190: PREDICTED: protein FAM186A isoform X1 [Homo sapiens]

**Accession:**	gi|578823289	**Score:**	42.2
**Database:**	refseq_human(refseq_human_20140103.fasta)	**MW [kDa]:**	262.1
**Database Date:**	2015-11-30	**pI:**	9.1
**Modification(s):**	Oxidation	**Sequence Coverage [%]:**	1.1
		**No. of unique Peptides:**	1

Detailed Protein Report

10	20	30	40	50	60	70	80
MFFKMKNEID	NDPESEKCIK	DSTIMRREPQ	NILSPLMLPN	LEIPFSVKDI	ISRIERAQLH	RAREDIDMQL	SEIMNNVHRI
90	100	110	120	130	140	150	160
MTRYTLVFNS	SSERNVSLTE	HKKKQRTNFL	EKMATYAKTI	EIREKTLANI	LAWLEEWNDV	LSEMTLMDVD	EHHHWIAQME
170	180	190	200	210	220	230	240
LLPDTLKAIE	NNVKILSRFS	TSFLDEKKKQ	KKKILSRGTL	WKSWKERVIK	RPSTARALRP	DQMISDQLAT	NTKVSEIQGM
250	260	270	280	290	300	310	320
LQELIGTTMF	STLENNAIKY	ISSTIVNLST	ALSMLNDELK	CVNFQSSTVY	AHETSEAEKE	LSLKIIRDLS	NENEMLQQKL
330	340	350	360	370	380	390	400
QDAEEKCEQL	IRSKIVIEQL	YAKLSTSSTL	KVLPGPSPQS	SRAIIKVGDT	EDNMDNILDK	ELENIVDEVQ	RKETKDSGIK
410	420	430	440	450	460	470	480
WDSTISYTAQ	AERTPDLTEL	RQQPVASEDI	SEDSTKDNVS	LKKGDFYQED	ETDEYQSWKR	SHKKATYVYE	TSGPNLSDNK
490	500	510	520	530	540	550	560
SGQKVSEAKP	SQYYELQVLK	KKRKEMKSFS	EDKSKSPTEA	KRKHLSLTET	KSQGGKSGTS	MMMLEQFRKV	KRESPFDKRP
570	580	590	600	610	620	630	640
TAAEIKVEPT	TESLDKEGKG	EIRSLVEPLS	MIQFDDTAEP	QKGKIKGKKH	HISSGTITSK	EEKTEEKEEL	TKQVKSHQLV
650	660	670	680	690	700	710	720
KSLSRVAKET	SESTRVLESP	DGKSEQSNLE	EFQEAIMAFL	KQKIDNIGKA	FDKKTVPKEE	ELLKRAEAEK	LGIIKAKMEE
730	740	750	760	770	780	790	800
YFQKVAETVT	KILRKYKDTK	KEEQVGEKPI	KQKKVVSFMP	GLHFQKSPIS	AKSESSTLLS	YESTDPVINN	LIQMILAEIE
810	820	830	840	850	860	870	880
SERDIPTVST	VQKDHKEKEK	QRQEQYLQEG	QEQMSGMSLK	QQLLGERNLL	KEHYEKISEN	WEEKKAWLQM	KEGKQEQQSQ
890	900	910	920	930	940	950	960
KQWQEEEMWK	EEQKQATPKQ	AEQEEKQKQR	GQEEEELPKS	SLQRLEEGTQ	KMKTQGLLLE	KENGQMRQIQ	KEAKHLGPHR
970	980	990	1000	1010	1020	1030	1040
RREKGKEKQK	PERGLEDLER	QIKTKDQMQM	KETQPKELEK	MVIQTPMTLS	PRWKSVLKDV	QRSYEGKEFQ	RNLKTLENLP
1050	1060	1070	1080	1090	1100	1110	1120
DEKEPISITP	PPSLQYSLPG	ALPISGQPLT	KCIHLTPQQA	QEVGITLTPQ	QAQAQGITLT	LQQAQELGIP	LTPQQAQALE
1130	1140	1150	1160	1170	1180	1190	1200
ILFTPQQAQA	LGIPLTPQQT	QVQGITLTPQ	QDQAPGISLT	TQQAQKLGIP	LTPQQAQALG	IPLTPQQAQE	LGIPLTPQQA
1210	1220	1230	1240	1250	1260	1270	1280
QALRVSLTPQ	QAQELGIPLT	PQQAQALGIT	LTLQQAQQLG	IPLTPQQAQA	LGITLTPKQV	QELGIPLTPQ	QAQALGITLT
1290	1300	1310	1320	1330	1340	1350	1360
PKQAQELGIP	LNPQQAQTLG	IPLTPKQAQA	LGIPFTPQQA	QALGIPLTPQ	QAQTQEITLT	PQQAQALGMP	LTTQQAQELG
1370	1380	1390	1400	1410	1420	1430	1440
IPLTPQHAQA	LGMPLTTQQA	QELGIPLTPQ	QAQALGMPLT	TQQAQELGIP	LTPQQAQELG	IPFTPQQAQA	QEITLTPQQA
1450	1460	1470	1480	1490	1500	1510	1520
QALGMPLTAQ	QAQELGITLT	PQQAQELGIP	LTPQQAQALG	IPLIPPQAQE	LGIPLTPQQA	QALGILLIPP	QAQELGIPLT
1530	1540	1550	1560	1570	1580	1590	1600
PQQAQALGIP	LIPPQAQELG	IPLTPQQVQA	LGIPLIPPQA	QELEIPLTPQ	QAQALGIPLT	PQQAQELGIP	LTPQQAQELG
1610	1620	1630	1640	1650	1660	1670	1680
IPLTPQQAQA	QGIPLTPQQA	QALGISLTPQ	QAQAQGITLT	PQQAQALGVP	ITPVNAWVSA	VTLTSEQTHA	LESPMNLEQA
1690	1700	1710	1720	1730	1740	1750	1760
QEQLLKLGVP	LTLDKAHTLG	SPLTLKQVQW	SHRPFQKSKA	SLPTGQSIIS	RLSPSLRLSL	ASSAPTAEKS	SIFGVSSTPL
1770	1780	1790	1800	1810	1820	1830	1840
QISRVPLNQG	PFAPGKPLEM	GILSEPGKLG	APQTLRSSGQ	TLVYGGQSTS	AQFPAPQAPP	SPGQLPISRA	PPTPGQPFIA
1850	1860	1870	1880	1890	1900	1910	1920
GVPPTSGQIP	SLWAPLSPGQ	PLVPEASSIP	GDLLESGPLT	FSEQLQEFQP	PATAEQSPYL	QAPSTPGQHL	ATWTLPGRAS
1930	1940	1950	1960	1970	1980	1990	2000
SLWIPPTSRH	PPTLWPSPAP	GKPQKSWSPS	VAKKRLAIIS	SLKSKSVLIH	PSAPDFKVAQ	VPFTTKKFQM	SEVSDTSEET
2010	2020	2030	2040	2050	2060	2070	2080
QILRDTFAIE	SFRTFQSHFT	KYRTPVYQTP	YTDERALLTL	MKPTTSPSSL	TTLLRTSQIS	PLEWYQKSRF	PPIDKPWILS
2090	2100	2110	2120	2130	2140	2150	2160
SVSDTKKPKV	MVPPSSPQEL	EEKRYFVDVE	AQKKNLILLN	QAIKTCGLPS	QLHTMARTLI	IEILHMDTVQ	LGYLFRKYIA
2170	2180	2190	2200	2210	2220	2230	2240
YRLIQHARNN	IMKRLKAIQN	TGKGYEARNL	HMMLSRLDDY	GKKVMQVWTE	KQKSLGQKRN	QCLKKMIHVF	NQLKKIHELN
2250	2260	2270	2280	2290	2300	2310	2320
LSQPIPLIIE	EKQIPASTTF	VQKPFLKLLM	EEDRTSDICK	KFRQQEDQTE	AIWNVDLSTS	SYPIAEKTSM	HSLWAQLGGY
2330	2340	2350					

Detailed Protein Report

Cmpd.	No. of Cmpds.	m/z meas.	Δ m/z [ppm]	z	Rt [min]	Score	P	Range	Sequence	Modification
1615	1	781.3837	27.46	2	49.9	12.0	1	537-549	K.SGTSMMMLEQFRK.V	Oxidation: 6

Detailed Protein Report

Protein 191: PREDICTED: nesprin-1 isoform X19 [Homo sapiens]

**Accession:**	gi|578812604	**Score:**	42.2
**Database:**	refseq_human(refseq_human_20140103.fasta)	**MW [kDa]:**	1002.8
**Database Date:**	2015-11-30	**pI:**	5.3
**Modification(s):**	Carbamidomethyl, Oxidation	**Sequence Coverage [%]:**	0.7
		**No. of unique Peptides:**	3

Quantitation

QU:MU	**Median:**	0.70	**CV:**	0.00 %	**No. of Peptides:**	1	

Detailed Protein Report

10	20	30	40	50	60	70	80
MATSRGASRC	PRDIANVMQR	LQDEQEIVQK	RTFTKWINSH	LAKRKPPMVV	DDLFEDMKDG	VKLLALLEVL	SGQKLPCEQG
90	100	110	120	130	140	150	160
RRMKRIHAVA	NIGTALKFLE	GRKSMHRGSP	IKLVNINSTD	IADGRPSIVL	GLMWTIILYF	QIEELTSNLP	QLQSLSSSAS
170	180	190	200	210	220	230	240
SVDSIVSSET	PSPPSKRKVT	TKIQGNAKKA	LLKWVQYTAG	KQTGIEVKDF	GKSWRSGVAF	HSVIHAIRPE	LVDLETVKGR
250	260	270	280	290	300	310	320
SNRENLEDAF	TIAETELGIP	RLLDPEDVDV	DKPDEKSIMT	YVAQFLKHYP	DIHNASTDGQ	EDDEILPGFP	SFANSVQNFK
330	340	350	360	370	380	390	400
REDRVIFKEM	KVWIEQFERD	LTRAQMVESN	LQDKYQSFKH	FRVQYEMKRK	QIEHLIQPLH	RDGKLSLDQA	LVKQSWDRVT
410	420	430	440	450	460	470	480
SRLFDWHIQL	DKSLPAPLGT	IGAWLYRAEV	ALREEITVQQ	VHEETANTIQ	RKLEQHKDLL	QNTDAHKRAF	HEIYRTRSVN
490	500	510	520	530	540	550	560
GIPVPPDQLE	DMAERFHFVS	STSELHLMKM	EFLELKYRLL	SLLVLAESKL	KSWIIKYGRR	ESVEQLLQNY	VSFIENSKFF
570	580	590	600	610	620	630	640
EQYEVTYQIL	KQTAEMYVKA	DGSVEEAENV	MKFMNETTAQ	WRNLSVEVRS	VRSMLEEVIS	NWDRYGNTVA	SLQAWLEDAE
650	660	670	680	690	700	710	720
KMLNQSENAK	KDFFRNLPHW	IQQHTAMNDA	GNFLIETCDE	MVSRDLKQQL	LLLNGRWREL	FMEVKQYAQA	DEMDRMKKEY
730	740	750	760	770	780	790	800
TDCVVTLSAF	ATEAHKKLSE	PLEVSFMNVK	LLIQDLEDIE	QRVPVMDAQY	KIITKTAHLI	TKESPQEEGK	EMFATMSKLK
810	820	830	840	850	860	870	880
EQLTKVKECY	SPLLYESQQL	LIPLEELEKQ	MTSFYDSLGK	INEIITVLER	EAQSSALFKQ	KHQELLACQE	NCKKTLTLIE
890	900	910	920	930	940	950	960
KGSQSVQKFV	TLSNVLKHFD	QTRLQRQIAD	IHVAFQSMVK	KTGDWKKHVE	TNSRLMKKFE	ESRAELEKVL	RIAQEGLEEK
970	980	990	1000	1010	1020	1030	1040
GDPEELLRRH	TEFFSQLDQR	VLNAFLKACD	ELTDILPEQE	QQGLQEAVRK	LHKQWKDLQG	EAPYHLLHLK	IDVEKNRFLA
1050	1060	1070	1080	1090	1100	1110	1120
SVEECRTELD	RETKLMPQEG	SEKIIKEHRV	FFSDKGPHHL	CEKRLQLIEE	LCVKLPVRDP	VRDTPGTCHV	TLKELRAAID
1130	1140	1150	1160	1170	1180	1190	1200
STYRKLMEDP	DKWKDYTSRF	SEFSSWISTN	ETQLKGIKGE	AIDTANHGEV	KRAVEEIRNG	VTKRGETLSW	LKSRLKVLTE
1210	1220	1230	1240	1250	1260	1270	1280
VSSENEAQKQ	GDELAKLSSS	FKALVTLLSE	VEKMLSNFGD	CVQYKEIVKN	SLEELISGSK	EVQEQAEKIL	DTENLFEAQQ
1290	1300	1310	1320	1330	1340	1350	1360
LLLHHQQKTK	RISAKKRDVQ	QQIAQAQQGE	GGLPDRGHEE	LRKLESTLDG	LERSRERQER	RIQVTLRKWE	RFETNKETVV
1370	1380	1390	1400	1410	1420	1430	1440
RYLFQTGSSH	ERFLSFSSLE	SLSSELEQTK	EFSKRTESIA	VQAENLVKEA	SEIPLGPQNK	QLLQQQAKSI	KEQVKKLEDT
1450	1460	1470	1480	1490	1500	1510	1520
LEEDIKTMEM	VKTKWDHFGS	NFETLSVWIT	EKEKELNALE	TSSSAMDMQI	SQIKVTIQEI	ESKLSSIVGL	EEEAQSFAQF
1530	1540	1550	1560	1570	1580	1590	1600
VTTGESARIK	AKLTQIRRYG	EELREHAQCL	EGTILGHLSQ	QQKFEENLRK	IQQSVSEFED	KLAVPIKICS	SATETYKVLQ
1610	1620	1630	1640	1650	1660	1670	1680
EHMDLCQALE	SLSSAITAFS	ASARKVVNRD	SCVQEAAALQ	QQYEDILRRA	KERQTALENL	LAHWQRLEKE	LSSFLTWLER
1690	1700	1710	1720	1730	1740	1750	1760
GEAKASSPEM	DISADRVKVE	GELQLIQALQ	NEVVSQASFY	SKLLQLKESL	FSVASKDDVK	MMKLHLEQLD	ERWRDLPQII
1770	1780	1790	1800	1810	1820	1830	1840
NKRINFLQSV	VAEHQQFDEL	LLSFSVWIKL	FLSELQTTSE	ISIMDHQVAL	TRHKDHAAEV	ESKKGELQSL	QGHLAKLGSL
1850	1860	1870	1880	1890	1900	1910	1920
GRAEDLHLLQ	GKAEDCFQLF	EEASQVVERR	QLALSHLAEF	LQSHASLSGI	LRQLRQTVEA	TNSMNKNESD	LIEKDLNDAL
1930	1940	1950	1960	1970	1980	1990	2000
QNAKALESAA	VSLDGILSKA	QYHLKIGSSE	QRTSCRATAD	QLCGEVERIQ	NLLGTKQSEA	DALAVLKKAF	QDQKEELLKS
2010	2020	2030	2040	2050	2060	2070	2080
IEDIEERTDK	ERLKEPTRQA	LQQRLRVFNQ	LEDELNSHEH	ELCWLKDKAK	QIAQKDVAFA	PEVDREINRL	EVTWDDTKRL
2090	2100	2110	2120	2130	2140	2150	2160
IHENQGQCCG	LIDLMREYQN	LKSAVSKVLE	NASSVIVTRT	TIKDQEDLKW	AFSKHETAKN	KMNYKQKDLD	NFTSKGKHLL
2170	2180	2190	2200	2210	2220	2230	2240

Detailed Protein Report

Cmpd.	No. of Cmpds.	m/z meas.	Δ m/z [ppm]	z	Rt [min]	Score	P	Range	Sequence	Modification	Ratios	
280	1	607.8593	-49.80	2	33.5	10.5	1	3327-3337	R.TLKLEALLSVK.Q		QU:MU	0.70
2894	1	724.8707	49.22	2	64.9	10.6	1	4576-4586	K.EDFDKACHWLK.Q	Carbamidomethyl: 7		
2810	1	1024.4996	-15.58	3	65.5	10.3	2	6575-6600	K. LQDMYDELMMIIGSRRSGLNQNLT S	Oxidation: 9, 10		

Detailed Protein Report

Protein 192: utrophin [Homo sapiens]

**Accession:**	gi|110611228	**Score:**	42.1
**Database:**	refseq_human(refseq_human_20140103.fasta)	**MW [kDa]:**	394.2
**Database Date:**	2015-11-30	**pI:**	5.1
		**Sequence Coverage [%]:**	0.6
		**No. of unique Peptides:**	2

Quantitation

WUP:QUP	**Median:** 0.40	**CV:** 0.00 %	**No. of Peptides:** 1

Alias proteins:

Accession	Name	Description
gi|530384033	refseq_human (refseq_human_20140103.fasta)	PREDICTED: utrophin isoform X4 [Homo sapiens]

Detailed Protein Report

10	20	30	40	50	60	70	80
MAKYGEHEAS	PDNGQNEFSD	IIKSRSDEHN	DVQKKTFTKW	INARFSKSGK	PPINDMFTDL	KDGRKLLDLL	EGLTGTSLPK
90	100	110	120	130	140	150	160
ERGSTRVHAL	NNVNRVLQVL	HQNNVELVNI	GGTDIVDGNH	KLTLGLLWSI	ILHWQVKDVM	KDVMSDLQQT	NSEKILLSWV
170	180	190	200	210	220	230	240
RQTTRPYSQV	NVLNFTTSWT	DGLAFNAVLH	RHKPDLFSWD	KVVKMSPIER	LEHAFSKAQT	YLGIEKLLDP	EDVAVQLPDK
250	260	270	280	290	300	310	320
KSIIMYLTSL	FEVLPQQVTI	DAIREVETLP	RKYKKECEEE	AINIQSTAPE	EEHESPRAET	PSTVTEVDMD	LDSYQIALEE
330	340	350	360	370	380	390	400
VLTWLLSAED	TFQEQDDISD	DVEEVKDQFA	THEAFMMELT	AHQSSVGSVL	QAGNQLITQG	TLSDEEEFEI	QEQMTLLNAR
410	420	430	440	450	460	470	480
WEALRVESMD	RQSRLHDVLM	ELQKKQLQQL	SAWLTLTEER	IQKMETCPLD	DDVKSLQKLL	EEHKSLQSDL	EAEQVKVNSL
490	500	510	520	530	540	550	560
THMVVIVDEN	SGESATAILE	DQLQKLGERW	TAVCRWTEER	WNRLQEINIL	WQELLEEQCL	LKAWLTEKEE	ALNKVQTSNF
570	580	590	600	610	620	630	640
KDQKELSVSV	RRLAILKEDM	EMKRQTLDQL	SEIGQDVGQL	LDNSKASKKI	NSDSEELTQR	WDSLVQRLED	SSNQVTQAVA
650	660	670	680	690	700	710	720
KLGMSQIPQK	DLLETVRVRE	QAITKKSKQE	LPPPPPPKKR	QIHVDIEAKK	KFDAISAELL	NWILKWKTAI	QTTEIKEYMK
730	740	750	760	770	780	790	800
MQDTSEMKKK	LKALEKEQRE	RIPRADELNQ	TGQILVEQMG	KEGLPTEEIK	NVLEKVSSEW	KNVSQHLEDL	ERKIQLQEDI
810	820	830	840	850	860	870	880
NAYFKQLDEL	EKVIKTKEEW	VKHTSISESS	RQSLPSLKDS	CQRELTNLLG	LHPKIEMARA	SCSALMSQPS	APDFVQRGFD
890	900	910	920	930	940	950	960
SFLGRYQAVQ	EAVEDRQQHL	ENELKGQPGH	AYLETLKTLK	DVLNDSENKA	QVSLNVLNDL	AKVEKALQEK	KTLDEILENQ
970	980	990	1000	1010	1020	1030	1040
KPALHKLAEE	TKALEKNVHP	DVEKLYKQEF	DDVQGKWNKL	KVLVSKDLHL	LEEIALTLRA	FEADSTVIEK	WMDGVKDFLM
1050	1060	1070	1080	1090	1100	1110	1120
KQQAAQGDDA	GLQRQLDQCS	AFVNEIETIE	SSLKNMKEIE	TNLRSGPVAG	IKTWVQTRLG	DYQTQLEKLS	KEIATQKSRL
1130	1140	1150	1160	1170	1180	1190	1200
SESQEKAANL	KKDLAEMQEW	MTQAEEEYLE	RDFEYKSPEE	LESAVEEMKR	AKEDVLQKEV	RVKILKDNIK	LLAAKVPSGG
1210	1220	1230	1240	1250	1260	1270	1280
QELTSELNVV	LENYQLLCNR	IRGKCHTLEE	VWSCWIELLH	YLDLETTWLN	TLEERMKSTE	VLPEKTDAVN	EALESLESVL
1290	1300	1310	1320	1330	1340	1350	1360
RHPADNRTQI	RELGQTLIDG	GILDDIISEK	LEAFNSRYED	LSHLAESKQI	SLEKQLQVLR	ETDQMLQVLQ	ESLGELDKQL
1370	1380	1390	1400	1410	1420	1430	1440
TTYLTDRIDA	FQVPQEAQKI	QAEISAHELT	LEELRRNMRS	QPLTSPESRT	ARGGSQMDVL	QRKLREVSTK	FQLFQKPANF
1450	1460	1470	1480	1490	1500	1510	1520
EQRMLDCKRV	LDGVKAELHV	LDVKDVDPDV	IQTHLDKCMK	LYKTLSEVKL	EVETVIKTGR	HIVQKQQTDN	PKGMDEQLTS
1530	1540	1550	1560	1570	1580	1590	1600
LKVLYNDLGA	QVTEGKQDLE	RASQLARKMK	KEAASLSEWL	SATETELVQK	STSEGLLGDL	DTEISWAKNV	LKDLEKRKAD
1610	1620	1630	1640	1650	1660	1670	1680
LNTITESSAA	LQNLIEGSEP	ILEERLCVLN	AGWSRVRTWT	EDWCNTLMNH	QNQLEIFDGN	VAHISTWLYQ	AEALLDEIEK
1690	1700	1710	1720	1730	1740	1750	1760
KPTSKQEEIV	KRLVSELDDA	NLQVENVRDQ	ALILMNARGS	SSRELVEPKL	AELNRNFEKV	SQHIKSAKLL	IAQEPLYQCL
1770	1780	1790	1800	1810	1820	1830	1840
VTTETFETGV	PFSDLEKLEN	DIENMLKFVE	KHLESSDEDE	KMDEESAQIE	EVLQRGEEML	HQPMEDNKKE	KIRLQLLLLH
1850	1860	1870	1880	1890	1900	1910	1920
TRYNKIKAIP	IQQRKMGQLA	SGIRSSLLPT	DYLVEINKIL	LCMDDVELSL	NVPELNTAIY	EDFSFQEDSL	KNIKDQLDKL
1930	1940	1950	1960	1970	1980	1990	2000
GEQIAVIHEK	QPDVILEASG	PEAIQIRDTL	TQLNAKWDRI	NRMYSDRKGC	FDRAMEEWRQ	FHCDLNDLTQ	WITEAEELLV
2010	2020	2030	2040	2050	2060	2070	2080
DTCAPGGSLD	LEKARIHQQE	LEVGISSHQP	SFAALNRTGD	GIVQKLSQAD	GSFLKEKLAG	LNQRWDAIVA	EVKDRQPRLK
2090	2100	2110	2120	2130	2140	2150	2160
GESKQVMKYR	HQLDEIICWL	TKAEHAMQKR	STTELGENLQ	ELRDLTQEME	VHAEKLKWLN	RTELEMLSDK	SLSLPERDKI
2170	2180	2190	2200	2210	2220	2230	2240

Detailed Protein Report

Cmpd.	No. of Cmpds.	m/z meas.	Δ m/z [ppm]	z	Rt [min]	Score	P	Range	Sequence	Modification	Ratios
36	1	721.8221	-42.42	2	30.3	16.3	2	24-35	K.SRSDEHNDVQKK.T		
467	5	607.8289	-91.67	2	35.5	25.9	2	997-1006	K.WNKLKVLVSK.D		WUP:QUP 0.40

Detailed Protein Report

Protein 193: **A disintegrin and metalloproteinase with thrombospondin motifs 16 preproprotein [Homo sapiens]**

**Accession:**	gi|110735441	**Score:**	42.1
**Database:**	refseq_human(refseq_human_20140103.fasta)	**MW [kDa]:**	136.1
**Database Date:**	2015-11-30	**pI:**	10.2
**Modification(s):**	Carbamidomethyl, Oxidation	**Sequence Coverage [%]:**	3.8
		**No. of unique Peptides:**	3

10	20	30	40	50	60	70	80
MKPRARGWRG	LAALWMLLAQ	VAEQAPACAM	GPAAAAPGSP	SVPRPPPPAE	RPGWMEKGEY	DLVSAYEVDH	RGDYVSHEIM
**90**	**100**	**110**	**120**	**130**	**140**	**150**	**160**
HHQRRRRAVP	VSEVESLHLR	LKGSRHDFHM	DLRTSSSLVA	PGFIVQTLGK	TGTKSVQTLP	PEDFCFYQGS	LRSHRNSSVA
**170**	**180**	**190**	**200**	**210**	**220**	**230**	**240**
LSTCQGLSGM	IRTEEADYFL	RPLPSHLSWK	LGRAAQGSSP	SHVLYKRSTE	PHAPGASEVL	VTSRTWELAH	QPLHSSDLRL
**250**	**260**	**270**	**280**	**290**	**300**	**310**	**320**
GLPQKQHFCG	RRKKYMPQPP	KEDLFILPDE	YKSCLRHKRS	LLRSHRNEEL	NVETLVVVDK	KMMQNHGHEN	ITTYVLTILN
**330**	**340**	**350**	**360**	**370**	**380**	**390**	**400**
MVSALFKDGT	IGGNINIAIV	GLILLEDEQP	GLVISHHADH	TLSSFCQWQS	GLMGKDGTRH	DHAILLTGLD	ICSWKNEPCD
**410**	**420**	**430**	**440**	**450**	**460**	**470**	**480**
TLGFAPISGM	CSKYRSCTIN	EDTGLGLAFT	IAHESGHNFG	MIHDGEGNMC	KKSEGNIMSP	TLAGRNGVFS	WSPCSRQYLH
**490**	**500**	**510**	**520**	**530**	**540**	**550**	**560**
KFLSTAQAIC	LADQPKPVKE	YKYPEKLPGE	LYDANTQCKW	QFGEKAKLCM	LDFKKDICKA	LWCHRIGRKC	ETKFMPAAEG
**570**	**580**	**590**	**600**	**610**	**620**	**630**	**640**
TICGHDMWCR	GGQCVKYGDE	GPKPTHGHWS	DWSSWSPCSR	TCGGGVSHRS	RLCTNPKPSH	GGKFCEGSTR	TLKLCNSQKC
**650**	**660**	**670**	**680**	**690**	**700**	**710**	**720**
PRDSVDFRAA	QCAEHNSRRF	RGRHYKWKPY	TQVEDQDLCK	LYCIAEGFDF	FFSLSNKVKD	GTPCSEDSRN	VCIDGICERV
**730**	**740**	**750**	**760**	**770**	**780**	**790**	**800**
GCDNVLGSDA	VEDVCGVCNG	NNSACTIHRG	LYTKHHHTNQ	YYHMVTIPSG	ARSIRIYEMN	VSTSYISVRN	ALRRYYLNGH
**810**	**820**	**830**	**840**	**850**	**860**	**870**	**880**
WTVDWPGRYK	FSGTTFDYRR	SYNEPENLIA	TGPTNETLIV	ELLFQGRNPG	VAWEYSMPRL	GTEKQPPAQP	SYTWAIVRSE
**890**	**900**	**910**	**920**	**930**	**940**	**950**	**960**
CSVSCGGGQM	TVREGCYRDL	KFQVNMSFCN	PKTRPVTGLV	PCKVSACPPS	WSVGNWSACS	RTCGGGAQSR	PVQCTRRVHY
**970**	**980**	**990**	**1000**	**1010**	**1020**	**1030**	**1040**
DSEPVPASLC	PQPAPSSRQA	CNSQSCPPAW	SAGPWAECSH	TCGKGWRKRA	VACKSTNPSA	RAQLLPDAVC	TSEPKPRMHE
**1050**	**1060**	**1070**	**1080**	**1090**	**1100**	**1110**	**1120**
ACLLQRCHKP	KKLQWLVSAW	SQCSVTCERG	TQKRFLKCAE	KYVSGKYREL	ASKKCSHLPK	PSLELERACA	PLPCPRHPPF
**1130**	**1140**	**1150**	**1160**	**1170**	**1180**	**1190**	**1200**
AAAGPSRGSW	FASPWSQCTA	SCGGGVQTRS	VQCLAGGRPA	SGCLLHQKPS	ASLACNTHFC	PIAEKKDAFC	KDYFHWCYLV
**1210**	**1220**	**1230**					
PQHGMCSHKF	YGKQCCKTCS	KSNL					

Cmpd.	No. of Cmpds.	m/z meas.	Δ m/z [ppm]	z	Rt [min]	Score	P	Range	Sequence	Modification
1904	1	724.3383	-56.32	2	54.2	12.7	2	631-642	R.TLKLCNSQKCPR.D	Carbamidomethyl: 10
2449	1	1025.1077	-9.04	3	60.5	13.5	2	1186-1209	K.KDAFCKDYFHWCYLVPQHGMCSHK.F	Carbamidomethyl: 5, 12; Oxidation: 20
1728	1	636.2813	0.97	2	51.9	15.9	2	1214-1224	K.QCCKTCSKSNL.-	Carbamidomethyl: 2

Detailed Protein Report

Protein 194: PREDICTED: dedicator of cytokinesis protein 11 isoform X2 [Homo sapiens]

**Accession:**	gi|530422336	**Score:**	42.0
**Database:**	refseq_human(refseq_human_20140103.fasta)	**MW [kDa]:**	235.7
**Database Date:**	2015-11-30	**pI:**	8.1
**Modification(s):**	Oxidation	**Sequence Coverage [%]:**	1.7
		**No. of unique Peptides:**	3

Detailed Protein Report

10	20	30	40	50	60	70	80
MFKEKAKVVE	PLDYENVIAQ	RKTQIYSDPL	RDLLMFPMED	ISISVIGRQR	RTVQSTVPED	AEKRAQSLFV	KECIKTYSTD
90	100	110	120	130	140	150	160
WHVVNYKYED	FSGDFRMLPC	KSLRPEKIPN	HVFEIDEDCE	KDEDSSSLCS	QKGGVIKQGW	LHKANVNSTI	TVTMKVFKRR
170	180	190	200	210	220	230	240
YFYLTQLPDG	SYILNSYKDE	KNSKESKGCI	YLDACIDVVQ	CPKMRRHAFE	LKMLDKYSHY	LAAETEQEME	EWLITLKKII
250	260	270	280	290	300	310	320
QINTDSLVQE	KKETVETAQD	DETSSQGKAE	NIMASLERSM	HPELMKYGRE	TEQLNKLSRG	DGRQNLFSFD	SEVQRLDFSG
330	340	350	360	370	380	390	400
IEPDIKPFEE	KCNKRFLVNC	HDLTFNILGQ	IGDNAKGPPT	NVEPFFINLA	LFDVKNNCKI	SADFHVDLNP	PSVREMLWGS
410	420	430	440	450	460	470	480
STQLASDGSP	KGSSPESYIH	GIAESQLRYI	QQGIFSVTNP	HPEIFLVARI	EKVLQGNITH	CAEPYIKNSD	PVKTAQKVHR
490	500	510	520	530	540	550	560
TAKQVCSRLG	QYRMPFAWAA	RPIFKDTQGS	LDLDGRFSPL	YKQDSSKLSS	EDILKLLSEY	KKPEKTKLQI	IPGQLNITVE
570	580	590	600	610	620	630	640
CVPVDLSNCI	TSSYVPLKPF	EKNCQNITVE	VEEFVPEMTK	YCYPFTIYKN	HLYVYPLQLK	YDSQKTFAKA	RNIAVCVEFR
650	660	670	680	690	700	710	720
DSDESDASAL	KCIYGKPAGS	VFTTNAYAVV	SHHNQNPEFY	DEIKIELPIH	LHQKHHLLFT	FYHVSCEINT	KGTTKKQDTV
730	740	750	760	770	780	790	800
ETPVGFAWVP	LLKDGRIITF	EQQLPVSANL	PPGYLNLNDA	ESRRQCNVDI	KWVDGAKPLL	KIKSHLESTI	YTQDLHVHKF
810	820	830	840	850	860	870	880
FHHCQLIQSG	SKEVPGELIK	YLKCLHAMEI	QVMIQFLPVI	LMQLFRVLTN	MTHEDDVPIN	CTMVLLHIVS	KCHEEGLDSY
890	900	910	920	930	940	950	960
LRSFIKYSFR	PEKPSAPQAQ	LIHETLATTM	IAILKQSADF	LSINKLLKYS	WFFFEIIAKS	MATYLLEENK	IKLPRGQRFP
970	980	990	1000	1010	1020	1030	1040
ETYHHVLHSL	LLAIIPHVTI	RYAEIPDESR	NVNYSLASFL	KRCLTLMDRG	FIFNLINDYI	SGFSPKDPKV	LAEYKFEFLQ
1050	1060	1070	1080	1090	1100	1110	1120
TICNHEHYIP	LNLPMAFAKP	KLQRVQDFFS	FAVDRLTSVD	SNLEYSLSDE	YCKHHFLVGL	LLRETSIALQ	DNYEIRYTAI
1130	1140	1150	1160	1170	1180	1190	1200
SVIKNLLIKH	AFDTRYQHKN	QQAKIAQLYL	PFVGLLLENI	QRLAGRDTLY	SCAAMPNSAS	RDEFPCGFTS	PANRGSLSTD
1210	1220	1230	1240	1250	1260	1270	1280
KDTAYGSFQN	GHGIKREDSR	GSLIPEGATG	FPDQGNTGEN	TRQSSTRSSV	SQYNRLDQYE	IRSLLMCYLY	IVKMISEDTL
1290	1300	1310	1320	1330	1340	1350	1360
LTYWNKVSPQ	ELINILILLE	VCLFHFRYMG	KRNIARVHDA	WLSKHFGIDR	KSQTMPALRN	RSGVMQARLQ	HLSSLESSFT
1370	1380	1390	1400	1410	1420	1430	1440
LNHSSTTTEA	DIFHQALLEG	NTATEVSLTV	LDTISFFTQC	FKTQLLNNDG	HNPLMKKVFD	IHLAFLKNGQ	SEVSLKHVFA
1450	1460	1470	1480	1490	1500	1510	1520
SLRAFISKFP	SAFFKGRVNM	CAAFCYEVLK	CCTSKISSTR	NEASALLYLL	MRNNFEYTKR	KTFLRTHLQI	IIAVSQLIAD
1530	1540	1550	1560	1570	1580	1590	1600
VALSGGSRFQ	ESLFIINNFA	NSDRPMKATA	FPAEVKDLTK	RIRTVLMATA	QMKEHEKDPE	MLIDLQYSLA	KSYASTPELR
1610	1620	1630	1640	1650	1660	1670	1680
KTWLDSMAKI	HVKNGDFSEA	AMCYVHVAAL	VAEFLHRKKL	FPNGCSAFKK	ITPNIDEEGA	MKEDAGMMDV	HYSEEVLLEL
1690	1700	1710	1720	1730	1740	1750	1760
LEQCVDGLWK	AERYEIISEI	SKLIVPIYEK	RREFEKLTQV	YRTLHGAYTK	ILEVMHTKKR	LLGTFFRVAF	YGQSFFEEED
1770	1780	1790	1800	1810	1820	1830	1840
GKEYIYKEPK	LTGLSEISLR	LVKLYGEKFG	TENVKIIQDS	DKVNAKELDP	KYAHIQVTYV	KPYFDDKELT	ERKTEFERNH
1850	1860	1870	1880	1890	1900	1910	1920
NISRFVFEAP	YTLSGKKQGC	IEEQCKRRTI	LTTSNSFPYV	KKRIPINCEQ	QINLKPIDVA	TDEIKDKTAE	LQKLCSSTDV
1930	1940	1950	1960	1970	1980	1990	2000
DMIQLQLKLQ	GCVSVQVNAG	PLAYARAFLN	DSQASKYPPK	KVSELKDMFR	KFIQACSIAL	ELNERLIKED	QVEYHEGLKS
2010	2020	2030	2040	2050	2060		
NFRDMVKELS	DIIHEQILQE	DTMHSPWMSN	TLHVFCAISG	TSSDRGYGSP	RYAEV		

Cmpd.	No. of	m/z meas.	Δ m/z	z	Rt	Score	P	Range	Sequence	Modification

Detailed Protein Report

	Cmpds.		[ppm]		[min]					
180	1	1043.9250	-79.30	2	32.2	13.4	1	269-286	K.AENIMASLERSMHPELMK.Y	
2146	4	682.9130	127.21	2	57.2	18.0	1	279-289	R.SMHPELMKYGR.E	Oxidation: 7
2045	1	731.3221	-60.47	2	56.0	10.6	1	1650-1662	K.KITPNIDEEGAMK.E	Oxidation: 12

Detailed Protein Report

Protein 195: putative tyrosine-protein phosphatase auxilin isoform 3 [Homo sapiens]

**Accession:**	gi|379030617	**Score:**	41.9
**Database:**	refseq_human(refseq_human_20140103.fasta)	**MW [kDa]:**	98.6
**Database Date:**	2015-11-30	**pI:**	7.1
**Modification(s):**	Carbamidomethyl	**Sequence Coverage [%]:**	6.2
		**No. of unique Peptides:**	1

10	20	30	40	50	60	70	80
MEPSYGGGLF	DMVKGGAGRL	FSNLKDNLKD	TLKDTSSRVI	QSVTSYTKGD	LDFTYVTSRI	IVMSFPLDNV	DIGFRNQVDD
**90**	**100**	**110**	**120**	**130**	**140**	**150**	**160**
IRSFLDSRHL	DHYTVYNLSP	KSYRTAKFHS	RVSECSWPIR	QAPSLHNLFA	VCRNMYNWLL	QNPKNVCVVH	CLDGRAASSI
**170**	**180**	**190**	**200**	**210**	**220**	**230**	**240**
LVGAMFIFCN	LYSTPGPAIR	LLYAKRPGIG	LSPSHRRYLG	YMCDLLADKP	YRPHFKPLTI	KSITVSPIPF	FNKQRNGCRP
**250**	**260**	**270**	**280**	**290**	**300**	**310**	**320**
YCDVLIGETK	IYSTCTDFER	MKEYRVQDGK	IFIPLNITVQ	GDVVVSMYHL	RSTIGSRLQA	KVTNTQIFQL	QFHTGFIPLD
**330**	**340**	**350**	**360**	**370**	**380**	**390**	**400**
TTVLKFTKPE	LDACDVPEKY	PQLFQVTLDV	ELQPHDKVID	LTPPWEHYCT	KDVNPSILFS	SHQEHQDTLA	LGGQAPIDIP
**410**	**420**	**430**	**440**	**450**	**460**	**470**	**480**
PDNPRHYGQS	GFFASLCWQD	QKSEKSFCEE	DHAALVNQES	EQSDDELLTL	SSPHGNANGD	KPHGVKKPSK	KQQEPAAPPP
**490**	**500**	**510**	**520**	**530**	**540**	**550**	**560**
PEDVDLLGLE	GSAMSNSFSP	PAAPPTNSEL	LSDLFGGGGA	AGPTQAGQSG	VEDVFHPSGP	ASTQSTPRRS	ATSTSASPTL
**570**	**580**	**590**	**600**	**610**	**620**	**630**	**640**
RVGEGATFDP	FGAPSKPSGQ	DLLGSFLNTS	SASSDPFLQP	TRSPSPTVHA	SSTPAVNIQP	DVSGGWDWHA	KPGGFGMGSK
**650**	**660**	**670**	**680**	**690**	**700**	**710**	**720**
SAATSPTGSS	HGTPTHQSKP	QTLDPFADLG	TLGSSSFASK	PTTTPTGLGGG	FPPLSSPQKA	SPQPMGGGWQ	QGGAYNWQQP
**730**	**740**	**750**	**760**	**770**	**780**	**790**	**800**
QPKPQPSMPH	SSPQNRPNYN	VSFSAMPGGQ	NERGKGSSNL	EGKQKAADFE	DLLSGQGFNA	HKDKKGPRTI	AEMRKEEMAK
**810**	**820**	**830**	**840**	**850**	**860**	**870**	**880**
EMDPEKLKIL	EWIEGKERNI	RALLSTMHTV	LWAGETKWKP	VGMADLVTPE	QVKKVYRKAV	LVVHPDKATG	QPYEQYAKMI
**890**	**900**	**910**					
FMELNDAWSE	FENQGQKPLY						

Cmpd.	No. of Cmpds.	m/z meas.	Δ m/z [ppm]	z	Rt [min]	Score	P	Range	Sequence	Modification
1549	1	1052.1533	-35.54	3	49.1	11.3	2	108-133	K.FHSRVSECSWPIRQAPSLHNLFAVCR.N	Carbamidomethyl: 8, 25

Detailed Protein Report

Protein 196: PREDICTED: zinc finger protein 407 isoform X3 [Homo sapiens]

Accession: gi|578832590
Database: refseq_human(refseq_human_20140103.fasta)
Database Date: 2015-11-30
Modification(s): Oxidation

Score: 41.8
MW [kDa]: 180.5
pI: 6.5
Sequence Coverage [%]: 2.6
No. of unique Peptides: 1

10	20	30	40	50	60	70	80
MMDSENKPEN	DEDEKINKEA	QDLTKLSSHN	EDGGPVSDVI	ASFPENSMGK	RGFSESSNSD	SVVIGEDRNK	HASKRRKLDE
90	100	110	120	130	140	150	160
AEPLKSGKQG	ICRLETSESS	VTEGGIALDE	TGKETFLSDC	TVGGTCLPNA	LSPSCNFSTI	DVVSLKTDTE	KTSAQEMVSL
170	180	190	200	210	220	230	240
DLERESPFPP	KEISVSCTIG	NVDTVLKCSI	CGHLFSSCSD	LEKHAESHMQ	QPKEHTCCHC	SHKAESSSAL	HMHIKQAHGP
250	260	270	280	290	300	310	320
QKVFSCDLCG	FQCSEENLLN	AHYLGKTHLR	RQNLAARGGF	VQILTKQPFP	KKSRTMATKN	VHSKPRTSKS	IAKNSDSKGL
330	340	350	360	370	380	390	400
RNVGSTFKDF	RGSISKQSGS	SSELLVEMMP	SRNTLSQEVE	IVEEHVTSLG	LAQNPENQSR	KLDTLVTSEG	LLEKLESTKN
410	420	430	440	450	460	470	480
TLQAAHGNSV	TSRPRPERNI	LVLGNSFRRR	SSTFTLKGQA	KKRFNLLGIK	RGTSETQRMY	MKHLRTQMKT	HDAESVLKHL
490	500	510	520	530	540	550	560
EACSSVQRVC	VTTSETQEAE	QGQGSARPPD	SGLHSLTVKP	ASGSQTLCAC	TDCGQVATNR	TDLEIHVKRC	HAREMKFYCR
570	580	590	600	610	620	630	640
TCDFSSMSRR	DLDEHLHSNQ	HQQTASVLSC	QCCSFISLDE	INLRDHMKEK	HNMHFLCTPC	NLFFLSEKDV	EEHKATEKHI
650	660	670	680	690	700	710	720
NSLVQPKTLQ	SSNSDLVLQT	LPLSTLESEN	AKESMDDSGK	ASQEEPLKSR	VSHGNEVRHS	SKPQFQCKKC	FYKTRSSTVL
730	740	750	760	770	780	790	800
TRHIKLRHGQ	DYHFLCKACN	LYSLSKEGME	KHIKRSKHLE	NAKKNNIGLS	FEECIERVCI	GANDKKEEFD	VSGNGRIEGH
810	820	830	840	850	860	870	880
IGVQLQEHSY	LEKGMLASEE	LSQSGGSTKD	DELASTTTPK	RGRPKGNISR	TCSHCGLLAS	SITNLTVHIR	RKHSHQYSYL
890	900	910	920	930	940	950	960
CKVCKYYTVT	KGDMERHCAT	KKHKGRVEIE	ASGKHSSDII	VGPEGGSLEA	GKKNAGSAVT	MSDEHANKPA	ESPTSVLEKP
970	980	990	1000	1010	1020	1030	1040
DRGNSIEAEV	ENVFHSLDGE	VNSHLLDKKE	QISSEPEDFA	QPGDVYSQRD	VTGTGENKCL	HCEFSAHSSA	SLELHVKRKH
1050	1060	1070	1080	1090	1100	1110	1120
TKEFEFYCMA	CDYYAVTRRE	MTRHAATEKH	KMKRQSYLNS	ANVEAGSADM	SKNIIMPEEE	HQQNSEEFQI	ISGQPSDTLK
1130	1140	1150	1160	1170	1180	1190	1200
SRNAADCSIL	NENTNLDMSK	VLCAADSVEV	ETEEESNFNE	DHSFCETFQQ	APVKDKVRKP	EEMMSLTMSS	NYGSPSRFQN
1210	1220	1230	1240	1250	1260	1270	1280
ENSGSSALNC	ETAKKNHEIS	NDAGELRVHC	EGEGGNAGDG	GGVVPHRHLC	PVTLDGERSA	ESPVLVVTRI	TREQGNLESG
1290	1300	1310	1320	1330	1340	1350	1360
GQNRVARGHG	LEDLKGVQED	PVLGNKEILM	NSQHETEFIL	EEDGPASDST	VESSDVYETI	ISIDDKGQAM	YSFGRFDSSI
1370	1380	1390	1400	1410	1420	1430	1440
IRIKNPEDGE	LIDQSEEGLI	ATGVRISELP	LKDCAQGVKK	KKSEGSSIGE	STRIRCDDCG	FLADGLSGLN	VHIAMKHPTK
1450	1460	1470	1480	1490	1500	1510	1520
EKHFHCLLCG	KSFYTESNLH	QHLASAGHMR	NEQASVEELP	EGGATFKCVK	CTEPFDSEQN	LFLHIKGQHE	ELLREVNKYI
1530	1540	1550	1560	1570	1580	1590	1600
VEDTEQINRE	REENQGNVCK	YCGKMCRSSN	SMAFLAHIRT	HTGSKPFKCK	ICHFATAQLG	DARNHVKRHL	GMREYKCHVC
1610	1620	1630					
GVAFVMKKHL	NTHLLGKHGV	GTPKERQI					

Cmpd.	No. of Cmpds.	m/z meas.	Δ m/z [ppm]	z	Rt [min]	Score	P	Range	Sequence	Modification
2427	1	1103.9913	-10.11	2	58.6	12.6	2	1541-1559	K.YCGKMCRSSNSMAFLAHIR.T	Oxidation: 5, 12

Detailed Protein Report

Protein 197: DNA-directed RNA polymerase III subunit RPC5 isoform 4 [Homo sapiens]

**Accession:**	gi|384475537	**Score:**	41.8
**Database:**	refseq_human(refseq_human_20140103.fasta)	**MW [kDa]:**	75.7
**Database Date:**	2015-11-30	**pI:**	6.0
**Modification(s):**	Oxidation	**Sequence Coverage [%]:**	5.4
		**No. of unique Peptides:**	3

Quantitation

QU:MU	**Median:**	0.76	**CV:**	0.00 %	**No. of Peptides:**	1
WUP:QUP	**Median:**	2.02	**CV:**	0.00 %	**No. of Peptides:**	1

10	20	30	40	50	60	70	80
MANEEDDPVV	QEIKPKQQKV	ELEMAIDTLN	PNYCRSKGEQ	IALNVDGACA	DETSTYSSKL	MDKQTFCSSQ	TTSNTSRYAA
**90**	**100**	**110**	**120**	**130**	**140**	**150**	**160**
ALYRQGELHL	TPLHGILQLR	PSFSYLDKAD	AKHREREAAN	EAGDSSQDEA	EDDVKQITVR	FSRPESEQAR	QRRVQSYEFL
**170**	**180**	**190**	**200**	**210**	**220**	**230**	**240**
QKKHAEEPWV	HLHYYGLRDS	RSEHERQYLL	CPGSSGVENT	ELVKSPSEYL	MMLMPPSQEE	EKDKPVAPSN	VLSMAQLRTL
**250**	**260**	**270**	**280**	**290**	**300**	**310**	**320**
PLADQIKILM	KNVKVMPFAN	LMSLLGPSID	SVAVLRGIQK	VAMLVQGNWV	VKSDILYPKD	SSSPHSGVPA	EVLCRGRDFV
**330**	**340**	**350**	**360**	**370**	**380**	**390**	**400**
MWKFTQSRWV	VRKEVATVTK	LCAEDVKDFL	EHMAVVRINK	GWEFILPYDG	EFIKKHPDVV	QRQHMLWTGI	QAKLEKVYNL
**410**	**420**	**430**	**440**	**450**	**460**	**470**	**480**
VKETMPKKPD	AQSGPAGLVC	GDQRIQVAKT	KAQQNHALLE	RELQRRKEQL	RVPAVPPGVR	IKEEPVSEEG	EEDEEQEAEE
**490**	**500**	**510**	**520**	**530**	**540**	**550**	**560**
EPMDTSPSGL	HSKLANGLPL	GRAAGTDSFN	GHPPQGCAST	PVARELKAFV	EATFQRQFVL	TLSELKRLFN	LHLASLPPGH
**570**	**580**	**590**	**600**	**610**	**620**	**630**	**640**
TLFSGISDRM	LQDTVLAAGC	KQILVPFPPQ	TAASPDEQKV	FALWESGDMS	DQHRQVLLEI	FSKNYRVRRN	MIQSRLTQEC
**650**	**660**	**670**	**680**				
GEDLSKQEVD	KVLKDCCVSY	GGMWYLKGTV	QS				

Cmpd.	No. of Cmpds.	m/z meas.	Δ m/z [ppm]	z	Rt [min]	Score	P	Range	Sequence	Modification	Ratios
887	1	491.6754	-177.12	2	40.6	13.5	2	109-116	K.ADAKHRER.E		
2616	1	742.3664	-111.86	2	62.8	14.6	1	239-251	R.TLPLADQIKILMK.N		
828	1	897.4861	96.86	2	40.4	13.7	0	600-614	K.VFALWESGDMSDQHR.Q	Oxidation: 10	WUP:QUP 2.02<br>QU:MU 0.76

Detailed Protein Report

Protein 198: PREDICTED: uncharacterized protein KIAA1109 isoform X7 [Homo sapiens]

**Accession:**	gi|530378301	**Score:**	41.8
**Database:**	refseq_human(refseq_human_20140103.fasta)	**MW [kDa]:**	553.9
**Database Date:**	2015-11-30	**pI:**	6.1
		**Sequence Coverage [%]:**	0.9
		**No. of unique Peptides:**	2

Quantitation

QU:MU	**Median:** 2.25	**CV:** 0.00 %	**No. of Peptides:** 1

Detailed Protein Report

10	20	30	40	50	60	70	80
MDQRKNESIV	PSITQLEDFL	TEHNSNVVWL	LVATILSCGW	IIYLTYYNSR	NVGLILTLVL	NRLYKHGYIH	IGSFSFSVLS
90	100	110	120	130	140	150	160
GKVMVREIYY	ITEDMSIRIQ	DGFIIFRWWK	MYNPKQKQHD	PKAETRLYIT	VNDFEFHVYN	RSDLYGRLQE	LFGLEPTIIP
170	180	190	200	210	220	230	240
PKKDDDKTRE	IGRTRTQSKI	ERVKVKTESQ	DPTSSWRSLI	PVIKVNVSTG	RLAFGNHYQP	QTLCINFDDA	FLTYTTKPPS
250	260	270	280	290	300	310	320
SHLDQFMHIV	KGKLENVRVM	LVPSPRYVGL	QNDEPPRLMG	EGFVVMQSND	VDIYYYMDEP	GLVPEETEEN	IEGEMSSEDC
330	340	350	360	370	380	390	400
KLQDLPPCWG	LDIVCGKGTD	FNYGPWADRQ	RDCLWKFFFP	PDYQVLKVSE	IAQPGRPRQI	LAFELRMNII	ADATIDLLFT
410	420	430	440	450	460	470	480
KNRETNAVHV	NVGAGSYLEI	NIPMTVEENG	YTPAIKGQLL	HVDATTSMQY	RTLLEAEMLA	FHINASYPRI	WNMPQTWQCE
490	500	510	520	530	540	550	560
LEVYKATYHF	IFAQKNFFTD	LIQDWSSDSP	PDIFSFVPYT	WNFKIMFHQF	EMIWAANQHN	WIDCSTKQQE	NVYLAACGET
570	580	590	600	610	620	630	640
LNIDFSLPFT	DFVPATCNTK	FSLRGEDVDL	HLFLPDCHPS	KYSLFMLVKN	CHPNKMIHDT	GIPAECQSGQ	KTVKPKWRNV
650	660	670	680	690	700	710	720
TQEKSGWVEC	WTVPSVMLTI	DYTWHPIYPQ	KADEQLKQSL	SEMEETMLSV	LRPSQKTSDR	VVSSPSTSSR	PPIDPSELPP
730	740	750	760	770	780	790	800
DKLHVEMELS	PDSQITLYGP	LLNAFLCIKE	NYFGEDDMYM	DFEEVISSPV	LSLSTSSSSG	WTAVGMENDK	KENEGSAKSI
810	820	830	840	850	860	870	880
HPLALRPWDI	TVLVNLYKVH	GRLPVHGTTD	GPECPTAFLE	RLCFEMKKGF	RETMLQLILS	PLNVFVSDNY	QQRPPVDEVL
890	900	910	920	930	940	950	960
REGHINLSGL	QLRAHAMFSA	EGLPLGSDSL	EYAWLIDVQA	GSLTAKVTAP	QLACLLEWGQ	TFVFHVVCRE	YELERPKSVI
970	980	990	1000	1010	1020	1030	1040
ICQHGIDRRF	CESKLSCIPG	PCPTSDDLKY	TMIRLAVDGA	DIYIVEHGCA	TNIKMGAIRV	ANCNLHNQSV	GEGISAAIQD
1050	1060	1070	1080	1090	1100	1110	1120
FQVRQYIEQL	NNCRIGLQPA	VLRRAYWLEA	GSANLGLITV	DIALAADHHS	KHEAQRHFLE	THDARTKRLW	FLWPDDILKN
1130	1140	1150	1160	1170	1180	1190	1200
KRCRNKCGCL	GGCRFFGGTV	TGLDFFKLEE	LTPSSSSAFS	STSAESDMYY	GQSLLQPGEW	IITKEIPKII	DGNVNGMKRK
1210	1220	1230	1240	1250	1260	1270	1280
EWENKSVGIE	VERKTQHLSL	QVPLRSHSSS	SSSEENSSSS	AAQPLLAGEK	ESPSSVADDH	LVQKEFLHGT	KRDDGQASIP
1290	1300	1310	1320	1330	1340	1350	1360
TEISGNSPVS	PNTQDKSVGQ	SPLRSPLKRQ	ASVCSTRLGS	TKSLTAAFYG	DKQPVTVGVQ	FSSDVSRSDE	NVLDSPKQRR
1370	1380	1390	1400	1410	1420	1430	1440
SFGSFPYTPS	ADSNSFHQYR	SMDSSMSMAD	SEAYFSAAEE	FEPISSDEGP	GTYPGRKKKK	KQTQQIDYSR	GSIYHSVEGP
1450	1460	1470	1480	1490	1500	1510	1520
LTGHGESIQD	SRTLPFKTHP	SQASFVSALG	GEDDVIEHLY	IVEGEKTVES	EQITPQQPVM	NCYQTYLTQF	QVINWSVKHP
1530	1540	1550	1560	1570	1580	1590	1600
TNKRTSKSSL	HRPLDLDTPT	SEESSSSFEQ	LSVPTFKVIK	QGLTANSLLD	RGMQLSGSTS	NTPYTPLEKK	LADNTDDETL
1610	1620	1630	1640	1650	1660	1670	1680
TEEWTLDQPV	SQTRTTAIVE	VKGTVDIVLT	PLVAEALDRY	IEAMVHCAST	RHPAAIVDDL	HAKVLREAVQ	NSKTTFSENL
1690	1700	1710	1720	1730	1740	1750	1760
SSKQDIRGTK	TEQSTIGTTN	QGQAQTNLTM	KQDNVTIKGL	QTNVSIPKVN	LCLLQASVEE	SPTTAPSRSV	THVSLVALCF
1770	1780	1790	1800	1810	1820	1830	1840
DRIATQVRMN	RGVVEETSNN	AEPGRTSNFD	RYVHATKMQP	QSSGSLRSNA	GAEKGKEIAA	KLNIHRVHGQ	LRGLDTTDIG
1850	1860	1870	1880	1890	1900	1910	1920
TCAITAIPFE	KSKVLFTLEE	LDEFTFVDET	DQQAVPDVTR	IGPSQEKWGW	IMFECGLENL	TIKGGRQSGA	VLYNSFGIMG
1930	1940	1950	1960	1970	1980	1990	2000
KASDTERGGV	LTSNNSSDSP	TGSGYNTDVS	DDNLPCDRTS	PSSDLNGNSV	SDEQDEGVES	DDLKKDLPLM	PPPPDSCSMK
2010	2020	2030	2040	2050	2060	2070	2080
LTIKEIWFSF	AAPTNVRSHT	HAFSRQLNLL	STATPAVGAW	LVPIDQLKSS	LNKLETEGTL	RICAVMGCIM	TEALENKSVH
2090	2100	2110	2120	2130	2140	2150	2160
FPLRSKYNRL	TKVARFLQEN	PSCLLCNILH	HYLHQANYSI	IDDATMSDGL	PALVTLKKGL	VALARQWMKF	IVVTPAFKGV
2170	2180	2190	2200	2210	2220	2230	2240

Detailed Protein Report

Cmpd.	No. of Cmpds.	m/z meas.	Δ m/z [ppm]	z	Rt [min]	Score	P	Range	Sequence	Modification	Ratios
117	1	774.3916	-26.77	3	31.2	15.7	1	66-86	K.HGYIHIGSFSFSVLSGKVMVR.E		
1348	1	669.3232	-39.50	2	46.5	13.8	2	116-126	K.QKQHDPKAETR.L		QU:MU 2.25

Detailed Protein Report

Protein 199: ubiquitin carboxyl-terminal hydrolase 32 [Homo sapiens]

**Accession:**	gi|22550104	**Score:**	41.7
**Database:**	refseq_human(refseq_human_20140103.fasta)	**MW [kDa]:**	181.5
**Database Date:**	2015-11-30	**pI:**	6.0
		**Sequence Coverage [%]:**	2.1
		**No. of unique Peptides:**	1

Quantitation

QU:MU	**Median:**	0.46	**CV:**	0.00 %	**No. of Peptides:**	1
WUP:QUP	**Median:**	4.63	**CV:**	0.00 %	**No. of Peptides:**	1

10	20	30	40	50	60	70	80
MGAKESRIGF	LSYEEALRRV	TDVELKRLKD	AFKRTCGLSY	YMGQHCFIRE	VLGDGVPPKV	AEVIYCSFGG	TSKGLHFNNL
90	100	110	120	130	140	150	160
IVGLVLLTRG	KDEEKAKYIF	SLFSSESGNY	VIREEMERML	HVVDGKVPDT	LRKCFSEGEK	VNYEKFRNWL	FLNKDAFTFS
170	180	190	200	210	220	230	240
RWLLSGGVYV	TLTDDSDTPT	FYQTLAGVTH	LEESDIIDLE	KRYWLLKAQS	RTGRFDLETF	GPLVSPPIRP	SLSEGLFNAF
250	260	270	280	290	300	310	320
DENRDNHIDF	KEISCGLSAC	CRGPLAERQK	FCFKVFDVDR	DGVLSRVELR	DMVVALLEVW	KDNRTDDIPE	LHMDLSDIVE
330	340	350	360	370	380	390	400
GILNAHDTTK	MGHLTLEDYQ	IWSVKNVLAN	EFLNLLFQVC	HIVLGLRPAT	PEEEGQIIRG	WLERESRYGL	QAGHNWFIIS
410	420	430	440	450	460	470	480
MQWWQQWKEY	VKYDANPVVI	EPSSVLNGGK	YSFGTAAHPM	EQVEDRIGSS	LSYVNTTEEK	FSDNISTASE	ASETAGSGFL
490	500	510	520	530	540	550	560
YSATPGADVC	FARQHNTSDN	NNQCLLGANG	NILLHLNPQK	PGAIDNQPLV	TQEPVKATSL	TLEGGRLKRT	PQLIHGRDYE
570	580	590	600	610	620	630	640
MVPEPVWRAL	YHWYGANLAL	PRPVIKNSKT	DIPELELFPR	YLLFLRQQPA	TRTQQSNIWV	NMGNVPSPNA	PLKRVLAYTG
650	660	670	680	690	700	710	720
CFSRMQTIKE	IHEYLSQRLR	IKEEDMRLWL	YNSENYLTLL	DDEDHKLEYL	KIQDEQHLVI	EVRNKDMSWP	EEMSFIANSS
730	740	750	760	770	780	790	800
KIDRHKVPTE	KGATGLSNLG	NTCFMNSSIQ	CVSNTQPLTQ	YFISGRHLYE	LNRTNPIGMK	GHMAKCYGDL	VQELWSGTQK
810	820	830	840	850	860	870	880
NVAPLKLRWT	IAKYAPRFNG	FQQQDSQELL	AFLLDGLHED	LNRVHEKPYV	ELKDSDGRPD	WEVAAEAWDN	HLRRNRSIVV
890	900	910	920	930	940	950	960
DLFHGQLRSQ	VKCKTCGHIS	VRFDPFNFLS	LPLPMDSYMH	LEITVIKLDG	TTPVRYGLRL	NMDEKYTGLK	KQLSDLCGLN
970	980	990	1000	1010	1020	1030	1040
SEQILLAEVH	GSNIKNFPQD	NQKVRLSVSG	FLCAFEIPVP	VSPISASSPT	QTDFSSSPST	NEMFTLTTNG	DLPRPIFIPN
1050	1060	1070	1080	1090	1100	1110	1120
GMPNTVVPCG	TEKNFTNGMV	NGHMPSLPDS	PFTGYIIAVH	RKMMRTELYF	LSSQKNRPSL	FGMPLIVPCT	VHTRKKDLYD
1130	1140	1150	1160	1170	1180	1190	1200
AVWIQVSRLA	SPLPPQEASN	HAQDCDDSMG	YQYPFTLRVV	QKDGNSCAWC	PWYRFCRGCK	IDCGEDRAFI	GNAYIAVDWD
1210	1220	1230	1240	1250	1260	1270	1280
PTALHLRYQT	SQERVVDEHE	SVEQSRRAQA	EPINLDSCLR	AFTSEEELGE	NEMYYCSKCK	THCLATKKLD	LWRLPPILII
1290	1300	1310	1320	1330	1340	1350	1360
HLKRFQFVNG	RWIKSQKIVK	FPRESFDPSA	FLVPRDPALC	QHKPLTPQGD	ELSEPRILAR	EVKKVDAQSS	AGEEDVLLSK
1370	1380	1390	1400	1410	1420	1430	1440
SPSSLSANII	SSPKGSPSSS	RKSGTSCPSS	KNSSPNSSPR	TLGRSKGRLR	LPQIGSKNKL	SSSKENLDAS	KENGAGQICE
1450	1460	1470	1480	1490	1500	1510	1520
LADALSRGHV	LGGSQPELVT	PQDHEVALAN	GFLYEHEACG	NGYSNGQLGN	HSEEDSTDDQ	REDTRIKPIY	NLYAISCHSG
1530	1540	1550	1560	1570	1580	1590	1600
ILGGGHYVTY	AKNPNCKWYC	YNDSSCKELH	PDEIDTDSAY	ILFYEQQGID	YAQFLPKTDG	KKMADTSSMD	EDFESDYKKY
1610							
CVLQ							

Cmpd.	No. of Cmpds.	m/z meas.	Δ m/z [ppm]	z	Rt [min]	Score	P	Range	Sequence	Modification	Ratios

Detailed Protein Report

Cmpd.	No. of Cmpds.	m/z meas.	Δ m/z [ppm]	z	Rt [min]	Score	P	Range	Sequence	Modification	Ratios
598	1	823.8884	-6.53	2	37.5	15.8	0	1432-1447	K.ENGAGQICELADALSR.G		WUP:QUP 4.63<br>QU:MU 0.46

Detailed Protein Report

Protein 200: cGMP-specific 3',5'-cyclic phosphodiesterase isoform 1 [Homo sapiens]

**Accession:**	gi|61744435	**Score:**	41.7
**Database:**	refseq_human(refseq_human_20140103.fasta)	**MW [kDa]:**	99.9
**Database Date:**	2015-11-30	**pI:**	5.7
**Modification(s):**	Carbamidomethyl	**Sequence Coverage [%]:**	4.6
		**No. of unique Peptides:**	2

```
        10         20         30         40         50         60         70         80
MERAGPSFGQ QRQQQQPQQQ KQQQRDQDSV EAWLDDHWDF TFSYFVRKAT REMVNAWFAE RVHTIPVCKE GIRGHTESCS
        90        100        110        120        130        140        150        160
CPLQQSPRAD NSAPGTPTRK ISASEFDRPL RPIVVKDSEG TVSFLSDSEK KEQMPLTPPR FDHDEGDQCS RLLELVKDIS
       170        180        190        200        210        220        230        240
SHLDVTALCH KIFLHIHGLI SADRYSLFLV CEDSSNDKFL ISRLFDVAEG STLEEVSNNC IRLEWNKGIV GHVAALGEPL
       250        260        270        280        290        300        310        320
NIKDAYEDPR FNAEVDQITG YKTQSILCMP IKNHREEVVG VAQAINKKSG NGGTFTEKDE KDFAAYLAFC GIVLHNAQLY
       330        340        350        360        370        380        390        400
ETSLLENKRN QVLLDLASLI FEEQQSLEVI LKKIAATIIS FMQVQKCTIF IVDEDCSDSF SSVFHMECEE LEKSSDTLTR
       410        420        430        440        450        460        470        480
EHDANKINYM YAQYVKNTME PLNIPDVSKD KRFPWTTENT GNVNQQCIRS LLCTPIKNGK KNKVIGVCQL VNKMEENTGK
       490        500        510        520        530        540        550        560
VKPFNRNDEQ FLEAFVIFCG LGIQNTQMYE AVERAMAKQM VTLEVLSYHA SAAEEETREL QSLAAAVVPS AQTLKITDFS
       570        580        590        600        610        620        630        640
FSDFELSDLE TALCTIRMFT DLNLVQNFQM KHEVLCRWIL SVKKNYRKNV AYHNWRHAFN TAQCMFAALK AGKIQNKLTD
       650        660        670        680        690        700        710        720
LEILALLIAA LSHDLDHRGV NNSYIQRSEH PLAQLYCHSI MEHHHFDQCL MILNSPGNQI LSGLSIEEYK TTLKIIKQAI
       730        740        750        760        770        780        790        800
LATDLALYIK RRGEFFELIR KNQFNLEDPH QKELFLAMLM TACDLSAITK PWPIQQRIAE LVATEFFDQG DRERKELNIE
       810        820        830        840        850        860        870        880
PTDLMNREKK NKIPSMQVGF IDAICLQLYE ALTHVSEDCF PLLDGCRKNR QKWQALAEQQ EKMLINGESG QAKRN
```

Cmpd.	No. of Cmpds.	m/z meas.	Δ m/z [ppm]	z	Rt [min]	Score	P	Range	Sequence	Modification
1989	1	616.7715	-62.66	2	54.8	10.4	1	2-12	M.ERAGPSFGQQR.Q	
2072	1	1071.4033	-85.02	2	56.3	13.5	1	70-88	K.EGIRGHTESCSCPLQQSPR.A	Carbamidomethyl: 10

Detailed Protein Report

Protein 201: cingulin-like protein 1 [Homo sapiens]

**Accession:**	gi|31982906	**Score:**	41.7
**Database:**	refseq_human(refseq_human_20140103.fasta)	**MW [kDa]:**	149.0
**Database Date:**	2015-11-30	**pI:**	5.4
		**Sequence Coverage [%]:**	3.4
		**No. of unique Peptides:**	1

Alias proteins:

Accession	Name	Description
gi|356874770	refseq_human (refseq_human_20140103.fasta)	cingulin-like protein 1 [Homo sapiens]

```
        10         20         30         40         50         60         70         80
MELYFGEYQH VQQEYGVHLR LASDDTQKSR SSQNSKAGSY GVSIRVQGID GHPYIVLNNT ERCLAGTSFS ENGPPFPPPV
        90        100        110        120        130        140        150        160
INNLPLHSSN GSVPKENSEE LQLPENPYAQ PSPIRNLKQP LLHEGKNGVL DRKDGSVKPS HLLNFQRHPE LLQPYDPEKN
       170        180        190        200        210        220        230        240
ELNLQNHQPS ESNWLKTLTE EGINNKKPWT CFPKPSNSQP TSPSLEDPAK SGVTAIRLCS SVVIEDPKKQ TSVCVNVQSC
       250        260        270        280        290        300        310        320
TKERVGEEAL FTSGRPLTAH SPHAHPETKK TRPDVLPFRR QDSAGPVLDG ARSRRSSSSS TTPTSANSLY RFLLDDQECA
       330        340        350        360        370        380        390        400
IHADNVNRHE NRRYIPFLPG TGRDIDTGSI PGVDQLIEKF DQKPGLQRRG RSGKRNRINT DDRKRSRSVD SAFPFGLQGN
       410        420        430        440        450        460        470        480
SEYLIEFSRN LGKSSEHLLR PSQVCPQRPL SQERRGKQSV GRTFAKLQGA AHGASCAHSR PPQPNIDGKV LETEGSQEST
       490        500        510        520        530        540        550        560
VIRAPSLGAQ SKKEEEVKTA TATLMLQNRA TATSPDSGAK KISVKTFPSA SNTQATPDLL KGQQELTQQT NEETAKQILY
       570        580        590        600        610        620        630        640
NYLKEGSTDN DDATKRKVNL VFEKIQTLKS RAAGSAQGNN QACNSTSEVK DLLEQKSKLT IEVAELQRQL QLEVKNQQNI
       650        660        670        680        690        700        710        720
KEERERMRAN LEELRSQHNE KVEENSTLQQ RLEESEGELR KNLEELFQVK MEREQHQTEI RDLQDQLSEM HDELDSAKRS
       730        740        750        760        770        780        790        800
EDREKGALIE ELLQAKQDLQ DLLIAKEEQE DLLRKREREL TALKGALKEE VSSHDQEMDK LKEQYDAELQ ALRESVEEAT
       810        820        830        840        850        860        870        880
KNVEVLASRS NTSEQDQAGT EMRVKLLQEE NEKLQGRSEE LERRVAQLQR QIEDLKGDEA KAKETLKKYE GEIRQLEEAL
       890        900        910        920        930        940        950        960
VHARKEEKEA VSARRALENE LEAAQGNLSQ TTQEQKQLSE KLKEESEQKE QLRRLKNEME NERWHLGKTI EKLQKEMADI
       970        980        990       1000       1010       1020       1030       1040
VEASRTSTLE LQNQLDEYKE KNRRELAEMQ RQLKEKTLEA EKSRLTAMKM QDEMRLMEEE LRDYQRAQDE ALTKRQLLEQ
      1050       1060       1070       1080       1090       1100       1110       1120
TLKDLEYELE AKSHLKDDRS RLVKQMEDKV SQLEMELEEE RNNSDLLSER ISRSREQMEQ LRNELLQERA ARQDLECDKI
      1130       1140       1150       1160       1170       1180       1190       1200
SLERQNKDLK SRIIHLEGSY RSSKEGLVVQ MEARIAELED RLESEERDRA NLQLSNRRLE RKVKELVMQV DDEHLSLTDQ
      1210       1220       1230       1240       1250       1260       1270       1280
KDQLSLRLKA MKRQVEEAEE EIDRLESSKK KLQRELEEQM DMNEHLQGQL NSMKKDLRLK KLPSKVLDDM DDDDDLSTDG
      1290       1300       1310
GSLYEAPVSY TFSKDSTVAS QI
```

Cmpd.	No. of Cmpds.	m/z meas.	Δ m/z [ppm]	z	Rt [min]	Score	P	Range	Sequence	Modification
1287	1	611.9489	-57.31	3	46.2	10.8	1	494-509	K.EEEVKTATATLMLQNR.A	

Detailed Protein Report

Protein 202: **PREDICTED: neuroblastoma breakpoint family member 4 isoform X2 [Homo sapiens]**

**Accession:**	gi|578798443	**Score:**	41.6
**Database:**	refseq_human(refseq_human_20140103.fasta)	**MW [kDa]:**	70.3
**Database Date:**	2015-11-30	**pI:**	4.9
		**Sequence Coverage [%]:**	5.9
		**No. of unique Peptides:**	3

10	20	30	40	50	60	70	80
MTPPPSNSVI	ATADRYLVRW	LLPPSLLSES	ELQAVPSALS	SGIPVPVFAV	KDSKVLCRVH	QLFLNFPGST	SSATNVSMVV
**90**	**100**	**110**	**120**	**130**	**140**	**150**	**160**
SADPLSSERA	EMNILEINQE	LRSQLAESNQ	QFRDLKEKFL	ITQATAYSLA	NQLKKYKCEE	YKDIIDSVLR	DELQSMEKLA
**170**	**180**	**190**	**200**	**210**	**220**	**230**	**240**
EKLRQAEELR	QYKALVHSQA	KELTQLREKL	REGRDASRWL	NKHLKTLLTP	DDPDKSQGQD	LREQLAEGHR	LAEHLVHKLS
**250**	**260**	**270**	**280**	**290**	**300**	**310**	**320**
PENDEDEDED	EDDKDEEVEK	VQESPAPRHH	DKSNSYRHRE	VSFLALDEQK	VCSAQDVARD	YSNPKWDETS	LGFLEKQSDL
**330**	**340**	**350**	**360**	**370**	**380**	**390**	**400**
EEVKGQETVA	PRLSRGPLRV	DKHEIPQESL	DGCCLTPSIL	PDLTPSYHPY	WSTLYSFEDK	QVSLALVDKI	KKDQEEIEDQ
**410**	**420**	**430**	**440**	**450**	**460**	**470**	**480**
SPPCPRLSQE	LPEVKEQEVP	EDSVNEVYLT	PSVHHDVSDC	HQPYSSTLSS	LEDQLACSAL	DVASPTEAAC	PQGTWSGDLS
**490**	**500**	**510**	**520**	**530**	**540**	**550**	**560**
HHQSEVQVSQ	AQLEPSTLVP	SCLRLQLDQG	FHCGNGLAQR	GLSSTTCSFS	ANADSGNQWP	FQELVLEPSL	GMKNPPQLED
**570**	**580**	**590**	**600**	**610**	**620**	**630**	
DALEGSASNT	QGRQVTGRIR	ASLVLILKTI	RRRLPFSKWR	LAFRFAGPHA	ESAEIPNTAG	RTQRMAG	

Cmpd.	No. of Cmpds.	m/z meas.	Δ m/z [ppm]	z	Rt [min]	Score	P	Range	Sequence	Modification
2547	4	1044.5458	-3.00	2	62.5	15.8	1	1-19	-.MTPPPSNSVIATADRYLVR.W	
573	2	666.3169	-41.31	2	37.1	12.9	2	192-202	R.EGRDASRWLNK.H	
601	1	460.1164	-243.14	2	35.9	12.8	1	273-279	K.SNSYRHR.E	

Detailed Protein Report

Protein 203: DC-STAMP domain-containing protein 1 isoform 2 [Homo sapiens]

**Accession:**	gi|219521926	**Score:**	41.5
**Database:**	refseq_human(refseq_human_20140103.fasta)	**MW [kDa]:**	77.7
**Database Date:**	2015-11-30	**pI:**	10.3
**Modification(s):**	Carbamidomethyl, Oxidation	**Sequence Coverage [%]:**	10.0
		**No. of unique Peptides:**	3

10	20	30	40	50	60	70	80
MDIKHHQNGT	RGQRRKQPHT	TVQRLLTWGL	PVSCSWFLWR	QPGEFPVTAL	LLGAGAGGLL	AIGLGAMGWG	TSPHIRCASL
**90**	**100**	**110**	**120**	**130**	**140**	**150**	**160**
LLVPKMLGKE	GRLFVLGYAL	AAIYVGPVAN	LRHNLNNVIA	SLGCTVELQI	NNTRAAWRIS	TAPLRAMFKD	LLSSKELLRA
**170**	**180**	**190**	**200**	**210**	**220**	**230**	**240**
ETRNISATFE	DLDAQVNSET	GYTPEDTMDS	GETAQGREAR	QAPASRLHLS	TQKMYELKTK	LRCSYVVNQA	ILSCRRWFDR
**250**	**260**	**270**	**280**	**290**	**300**	**310**	**320**
KHEQCMKHIW	VPLLTHLLCL	PMKFKFFCGI	AKVMEVWCRN	RIPVEGNFGQ	TYDSLNQSIR	GLDGEFSANI	DFKEEKQAGV
**330**	**340**	**350**	**360**	**370**	**380**	**390**	**400**
LGLNTSWERV	STEVRDYVYR	QEARLEWALG	LLHVLLSCTF	LLVLHASFSY	MDSYNHDIRF	DNIYISTYFC	QIDDRRKKLG
**410**	**420**	**430**	**440**	**450**	**460**	**470**	**480**
KRTLLPLRKA	EEKTVIFPCK	PTIQASEMSN	VVRELLETLP	ILLLLVVLCG	LDWALYSIFD	TIRHHSFLQY	SFRSSHKLEV
**490**	**500**	**510**	**520**	**530**	**540**	**550**	**560**
KVGGDSMLAR	LLRKTIGALN	TSSETVMESN	NMPCLPQPVG	LDARAYWRAA	VPIGLLVCLC	LLQAFGYRLR	RVIAAFYFPK
**570**	**580**	**590**	**600**	**610**	**620**	**630**	**640**
REKKRILFLY	NDLLKKRAAF	TKLRRAAILR	RERQQKAPRH	PLADILHRGC	PLLRRWLCRR	CVVCQAPETP	ESYVCRTLDC
**650**	**660**	**670**	**680**	**690**			
EAVYCWSCWD	DMRQRCPVCT	PREELSSSAF	SDSNDDTAYA	G			

Cmpd.	No. of Cmpds.	m/z meas.	Δ m/z [ppm]	z	Rt [min]	Score	P	Range	Sequence	Modification
2414	1	964.4799	-71.72	3	60.6	15.7	2	86-112	K.MLGKEGRLFVLGYALAAIYVGPVANLR.H	
748	1	1010.1872	-33.67	3	39.3	12.9	2	248-272	K.HIWVPLLTHLLCLPMKFKFFCGIAK.V	Carbamidomethyl: 21; Oxidation: 15
2267	1	920.9067	1.92	2	58.8	12.9	0	621-636	R.CVVCQAPETPESYVCR.T	Carbamidomethyl: 1

Detailed Protein Report

Protein 204: E3 ubiquitin-protein ligase listerin [Homo sapiens]

**Accession:**	gi|231573214	**Score:**	41.5
**Database:**	refseq_human(refseq_human_20140103.fasta)	**MW [kDa]:**	205.0
**Database Date:**	2015-11-30	**pI:**	6.1
**Modification(s):**	Oxidation	**Sequence Coverage [%]:**	1.4
		**No. of unique Peptides:**	2

10	20	30	40	50	60	70	80
MDRVGGGKGP	GGSYYRVSSS	AARSRKLCPG	RVNRGLSAQS	TAATATMGGK	NKQRTKGNLR	PSNSGRAAEL	LAKEQGTVPG
90	100	110	120	130	140	150	160
FIGFGTSQSD	LGYVPAIQGA	EEIDSLVDSD	FRMVLRKLSK	KDVTTKLKAM	QEFGTMCTER	DTETVKGVLP	YWPRIFCKIS
170	180	190	200	210	220	230	240
LDHDRRVREA	TQQAFEKLIL	KVKKQLAPYL	KSLMGYWLMA	QCDTYTPAAF	AAKDAFEAAF	PPSKQPEAIA	FCKDEITSVL
250	260	270	280	290	300	310	320
QDHLIKETPD	TLSDPQTVPE	EEREAKFYRV	VTCSLLALKR	LLCLLPDNEL	DSLEEKFKSL	LSQNKFWKYG	KHSVPQIRSA
330	340	350	360	370	380	390	400
YFELVSALCQ	RIPQLMKEEA	SKVSPSVLLS	IDDSDPIVCP	ALWEAVLYTL	TTIEDCWLHV	NAKKSVFPKL	STVIREGGRG
410	420	430	440	450	460	470	480
LATVIYPYLL	PFISKLPQSI	TNPKLDFFKN	FLTSLVAGLS	TERTKTSSLE	SSAVISAFFE	CLRFIMQQNL	GEEEIEQMLV
490	500	510	520	530	540	550	560
NDQLIPFIDA	VLKDPGLQHG	QLFNHLAETL	SSWEAKADTE	KDEKTAHNLE	NVLIHFWERL	SEICVAKISE	PEADVESVLG
570	580	590	600	610	620	630	640
VSNLLQVLQK	PKSSLKSSKK	KNGKVRFADE	ILESNKENEK	CVSSEGEKIE	GWELTTEPSL	THNSSGLLSP	LRKKPLEDLV
650	660	670	680	690	700	710	720
CKLADISINY	VNERKSEQHL	RFLSTLLDSF	SSSRVFKMLL	GDEKQSIVQA	KPLEIAKLVQ	KNPAVQFLYQ	KLIGWLNEDQ
730	740	750	760	770	780	790	800
RKDFGFLVDI	LYSALRCCDN	DMERKKVLDD	LTKVDLKWNS	LLKIIEKACP	SSDKHALVTP	WLKGDILGEK	LVNLADCLCN
810	820	830	840	850	860	870	880
EDLESRVSSE	SHFSERWTLL	SLVLSQHVKN	DYLIGDVYVE	RIIVRLHETL	FKTKKLSEAE	SSDSSVSFIC	DVAYNYFSSA
890	900	910	920	930	940	950	960
KGCLLMPSSE	DLLLTLFQLC	AQSKEKTHLP	DFLICKLKNT	WLSGVNLLVH	QTDSSYKEST	FLHLSALWLK	NQVQASSLDI
970	980	990	1000	1010	1020	1030	1040
NSLQVLLSAV	DDLLNTLLES	EDSYLMGVYI	GSVMPNDSEW	EKMRQSLPMQ	WLHRPLLEGR	LSLNYECFKT	DFKEQDIKTL
1050	1060	1070	1080	1090	1100	1110	1120
PSHLCTSALL	SKMVLIALRK	ETVLENNELE	KIIAELLYSL	QWCEELDNPP	IFLIGFCEIL	QKMNITYDNL	RVLGNTSGLL
1130	1140	1150	1160	1170	1180	1190	1200
QLLFNRSREH	GTLWSLIIAK	LILSRSISSD	EVKPHYKRKE	SFFPLTEGNL	HTIQSLCPFL	SKEEKKEFSA	QCIPALLGWT
1210	1220	1230	1240	1250	1260	1270	1280
KKDLCSTNGG	FGHLAIFNSC	LQTKSIDDGE	LLHGILKIII	SWKKEHEDIF	LFSCNLSEAS	PEVLGVNIEI	IRFLSLFLKY
1290	1300	1310	1320	1330	1340	1350	1360
CSSPLAESEW	DFIMCSMLAW	LETTSENQAL	YSIPLVQLFA	CVSCDLACDL	SAFFDSTTLD	TIGNLPVNLI	SEWKEFFSQG
1370	1380	1390	1400	1410	1420	1430	1440
IHSLLLPILV	TVTGENKDVS	ETSFQNAMLK	PMCETLTYIS	KEQLLSHKLP	ARLVADQKTN	LPEYLQTLLN	TLAPLLLFRA
1450	1460	1470	1480	1490	1500	1510	1520
RPVQIAVYHM	LYKLMPELPQ	YDQDNLKSYG	DEEEEPALSP	PAALMSLLSI	QEDLLENVLG	CIPVGQIVTI	KPLSEDFCYV
1530	1540	1550	1560	1570	1580	1590	1600
LGYLLTWKLI	LTFFKAASSQ	LRALYSMYLR	KTKSLNKLLY	HLFRLMPENP	TYAETAVEVP	NKDPKTFFTE	ELQLSIRETT
1610	1620	1630	1640	1650	1660	1670	1680
MLPYHIPHLA	CSVYHMTLKD	LPAMVRLWWN	SSEKRVFNIV	DRFTSKYVSS	VLSFQEISSV	QTSTQLFNGM	TVKARATTRE
1690	1700	1710	1720	1730	1740	1750	1760
VMATYTIEDI	VIELIIQLPS	NYPLGSIIVE	SGKRVGVAVQ	QWRNWMLQLS	TYLTHQNGSI	MEGLALWKNN	VDKRFEGVED
1770	1780	1790	1800	1810	1820		
CMICFSVIHG	FNYSLPKKAC	RTCKKKFHSA	CLYKWFTSSN	KSTCPLCRET	FF		

Cmpd.	No. of	m/z meas.	Δ m/z	z	Rt	Score	P	Range	Sequence	Modification

Detailed Protein Report

	Cmpds.		[ppm]		[min]					
57	1	731.6721	-129.70	3	30.4	30.4	2	1127-1145	R.SREHGTLWSLIIAKLILSR.S	
2506	2	817.4892	80.15	1	61.9	11.1	0	1620-1626	K.DLPAMVR.L	Oxidation: 5

Detailed Protein Report

Protein 205: PREDICTED: palladin isoform X5 [Homo sapiens]

**Accession:**	gi|530377424	**Score:**	41.5
**Database:**	refseq_human(refseq_human_20140103.fasta)	**MW [kDa]:**	138.1
**Database Date:**	2015-11-30	**pI:**	8.0
		**Sequence Coverage [%]:**	3.1
		**No. of unique Peptides:**	2

Quantitation

QU:MU	**Median:**	3.61	**CV:**	0.00 %	**No. of Peptides:**	1
WUP:QUP	**Median:**	0.29	**CV:**	0.00 %	**No. of Peptides:**	1

10	20	30	40	50	60	70	80
MSGTSSHESF	YDSLSDMQEE	SKNTDFFPGL	SAFLSQEEIN	KSLDLARRAI	ADSETEDFDS	EKEISQIFST	SPASLCEHPS
90	100	110	120	130	140	150	160
HKETKLGEHA	SRRPQDNRST	PVQPLAEKQT	KSISSPVSKR	KPAMSPLLTR	PSYIRSLRKA	EKRGAKTPST	NVKPKTPHQR
170	180	190	200	210	220	230	240
KGGPQSQLCD	KAANLIEELT	SIFKAAKPRN	RSPNGESSSP	DSGYLSPKNQ	PSALLSASAS	QSPMEDQGEM	EREVKSPGAR
250	260	270	280	290	300	310	320
HCYQDNQDLA	VPHNRKSHPQ	PHSALHFPAA	PRFIQKLRSQ	EVAEGSRVYL	ECRVTGNPTP	RVRWFCEGKE	LHNTPDIQIH
330	340	350	360	370	380	390	400
CEGGDLHTLI	IAEAFEDDTG	RYTCLATNPS	GSDTTSAEVF	IEGASSTDSD	SESLAFKSRA	GAMPQAQKKT	TSVSLTIGSS
410	420	430	440	450	460	470	480
SPKTGVTTAV	IQPLSVPVQQ	VHSPTSYLCR	PDGTTTAYFP	PVFTKELQNT	AVAEGQVVVL	ECRVRGAPPL	QVQWFRQGSE
490	500	510	520	530	540	550	560
IQDSPDFRIL	QKKPRSTAEP	EEICTLVIAE	TFPEDAGIFT	CSARNDYGSA	TSTAQLVVTS	ANTENCSYES	MGESNNDHFQ
570	580	590	600	610	620	630	640
HFPPPPPILE	TSSLELASKK	PSEIQQVNNP	ELGLSRAALQ	MQFNAAERET	NGVHPSRGVN	GLINGKANSN	KSLPTPAVLL
650	660	670	680	690	700	710	720
SPTKEPPPLL	AKPKLDPLKL	QQLQNQIRLE	QEAGARQPPP	APRSAPPSPP	FPPPPAFPEL	AACTPPASPE	PMSALASRSA
730	740	750	760	770	780	790	800
PAMQSSGSFN	YARPKQFIAA	QNLGPASGHG	TPASSPSSSS	LPSPMSPTPR	QFGRAPVPPF	AQPFGAEPEA	PWGSSSPSPP
810	820	830	840	850	860	870	880
PPPPPVFSPT	AAFPVPDVFP	LPPPPPPLPS	PGQASHCSSP	ATRFGHSQTP	AAFLSALLPS	QPPPAAVNAL	GLPKGVTPAG
890	900	910	920	930	940	950	960
FPKKASRTAR	IASDEEIQGT	KDAVIQDLER	KLRFKEDLLN	NGQPIYWFKD	GKQISPKSDH	YTIQRDLDGT	CSLHTTASTL
970	980	990	1000	1010	1020	1030	1040
DDDGNYTIMA	ANPQGRISCT	GRLMVQAVNQ	RGRSPRSPSG	HPHVRRPRSR	SRDSGDENEP	IQERFFRPHF	LQAPGDLTVQ
1050	1060	1070	1080	1090	1100	1110	1120
EGKLCRMDCK	VSGLPTPDLS	WQLDGKPVRP	DSAHKMLVRE	NGVHSLIIEP	VTSRDAGIYT	CIATNRAGQN	SFSLELVVAA
1130	1140	1150	1160	1170	1180	1190	1200
KEAHKPPVFI	EKLQNTGVAD	GYPVRLECRV	LGVPPPQIFW	KKENESLTHS	TDRVSMHQDN	HGYICLLIQG	ATKEDAGWYT
1210	1220	1230	1240	1250	1260	1270	1280
VSAKNEAGIV	SCTARLDVYT	QWHQQSQSTK	PKKVRPSASR	YAALSDQGLD	IKAAFQPEAN	PSHLTLNTAL	VESEDL

Cmpd.	No. of Cmpds.	m/z meas.	Δ m/z [ppm]	z	Rt [min]	Score	P	Range	Sequence	Modification	Ratios
2009	2	945.8529	-132.43	2	55.0	12.3	1	1133-1149	K.LQNTGVADGYPVRLECR.V		
684	1	450.6291	-317.71	2	37.0	12.1	1	1233-1240	K.KVRPSASR.Y		WUP:QUP 0.29<br>QU:MU 3.61

Detailed Protein Report

Protein 206: PREDICTED: forkhead box protein P3 isoform X2 [Homo sapiens]

**Accession:**	gi|578838031	**Score:**	41.4
**Database:**	refseq_human(refseq_human_20140103.fasta)	**MW [kDa]:**	56.2
**Database Date:**	2015-11-30	**pI:**	10.7
		**Sequence Coverage [%]:**	9.5
		**No. of unique Peptides:**	1

10	20	30	40	50	60	70	80
MTRKGQRKKP	KISKFPFKSH	NQEKEKHRER	EKKNYENPSP	PRDYQRTHSS	KKIWIIRRER	SAASTPYSVV	FLLGIKAKLF
90	100	110	120	130	140	150	160
LIRDSFPQAR	LILFCQSTSP	SLPLDKDPMP	NPRPGKPSAP	SLALGPSPGA	SPSWRAAPKA	SDLLGARGPG	GTFQGRDLRG
170	180	190	200	210	220	230	240
GAHASSSSLN	PMPPSQLQLS	TVDAHARTPV	LQVHPLESPA	MISLTPPTTA	TGVFSLKARP	GLPPGINVAS	LEWVSREPAL
250	260	270	280	290	300	310	320
LCTFPNPSAP	RKDSTLSAVP	QSSYPLLANG	VCKWPGCEKV	FEEPEDFLKH	CQADHLLDEK	GRAQCLLQRE	MVQSLEQQLV
330	340	350	360	370	380	390	400
LEKEKLSAMQ	AHLAGKMALT	KASSVASSDK	GSCCIVAAGS	QGPVVPAWSG	PREAPDSLFA	VRRHLWGSHG	NSTFPEFLHN
410	420	430	440	450	460	470	480
MDYFKFHNMR	PPFTYATLIR	WAILEAPEKQ	RTLNEIYHWF	TRMFAFFRNH	PATWKNAIRH	NLSLHKCFVR	VESEKGAVWT
490	500	510					
VDELEFRKKR	SQRPSRCSNP	TPGP					

Cmpd.	No. of Cmpds.	m/z meas.	Δ m/z [ppm]	z	Rt [min]	Score	P	Range	Sequence	Modification
14	1	712.9876	-118.49	3	29.1	16.9	2	59-78	R.ERSAASTPYSVVFLLGIKAK.L	

Detailed Protein Report

Protein 207: **endonuclease 8-like 3 [Homo sapiens]**

**Accession:**	gi|157388969	**Score:**	41.3
**Database:**	refseq_human(refseq_human_20140103.fasta)	**MW [kDa]:**	67.8
**Database Date:**	2015-11-30	**pI:**	10.3
**Modification(s):**	Carbamidomethyl	**Sequence Coverage [%]:**	5.6
		**No. of unique Peptides:**	1

10	20	30	40	50	60	70	80
MVEGPGCTLN	GEKIRARVLP	GQAVTGVRGS	ALRSLQGRAL	RLAASTVVVS	PQAAALNNDS	SQNVLSLFNG	YVYSGVETLG
**90**	**100**	**110**	**120**	**130**	**140**	**150**	**160**
KELFMYFGPK	ALRIHFGMKG	FIMINPLEYK	YKNGASPVLE	VQLTKDLICF	FDSSVELRNS	MESQQRIRMM	KELDVCSPEF
**170**	**180**	**190**	**200**	**210**	**220**	**230**	**240**
SFLRAESEVK	KQKGRMLGDV	LMDQNVLPGV	GNIIKNEALF	DSGLHPAVKV	CQLTDEQIHH	LMKMIRDFSI	LFYRCRKAGL
**250**	**260**	**270**	**280**	**290**	**300**	**310**	**320**
ALSKHYKVYK	RPNCGQCHCR	ITVCRFGDNN	RMTYFCPHCQ	KENPQHVDIC	KLPTRNTIIS	WTSSRVDHVM	DSVARKSEEH
**330**	**340**	**350**	**360**	**370**	**380**	**390**	**400**
WTCVVCTLIN	KPSSKACDAC	LTSRPIDSVL	KSEENSTVFS	HLMKYPCNTF	GKPHTEVKIN	RKTAFGTTTL	VLTDFSNKSS
**410**	**420**	**430**	**440**	**450**	**460**	**470**	**480**
TLERKTKQNQ	ILDEEFQNSP	PASVCLNDIQ	HPSKKTTNDI	TQPSSKVNIS	PTISSESKLF	SPAHKKPKTA	QYSSPELKSC
**490**	**500**	**510**	**520**	**530**	**540**	**550**	**560**
NPGYSNSELQ	INMTDGPRTL	NPDSPRCSKH	NRLCILRVVR	KDGENKGRQF	YACPLPREAQ	CGFFEWADLS	FPFCNHGKRS
**570**	**580**	**590**	**600**	**610**			
TMKTVLKIGP	NNGKNFFVCP	LGKEKQCNFF	QWAENGPGIK	IIPGC			

Cmpd.	No. of Cmpds.	m/z meas.	Δ m/z [ppm]	z	Rt [min]	Score	P	Range	Sequence	Modification
1708	1	735.0267	-63.51	3	51.1	14.2	2	564-583	K.TVLKIGPNNGKNFFVCPLGK.E	Carbamidomethyl: 16

Detailed Protein Report

Protein 208: PREDICTED: insulin-like growth factor 1 receptor isoform X2 [Homo sapiens]

**Accession:**	gi|530407102	**Score:**	41.3
**Database:**	refseq_human(refseq_human_20140103.fasta)	**MW [kDa]:**	141.1
**Database Date:**	2015-11-30	**pI:**	5.4
**Modification(s):**	Carbamidomethyl, Oxidation	**Sequence Coverage [%]:**	2.4
		**No. of unique Peptides:**	3

10	20	30	40	50	60	70	80
MTNLKDIGLY	NLRNITRGAI	RIEKNADLCY	LSTVDWSLIL	DAVSNNYIVG	NKPPKECGDL	CPGTMEEKPM	CEKTTINNEY
90	100	110	120	130	140	150	160
NYRCWTTNRC	QKMCPSTCGK	RACTENNECC	HPECLGSCSA	PDNDTACVAC	RHYYYAGVCV	PACPPNTYRF	EGWRCVDRDF
170	180	190	200	210	220	230	240
CANILSAESS	DSEGFVIHDG	ECMQECPSGF	IRNGSQSMYC	IPCEGPCPKV	CEEEKKTKTI	DSVTSAQMLQ	GCTIFKGNLL
250	260	270	280	290	300	310	320
INIRRGNNIA	SELENFMGLI	EVVTGYVKIR	HSHALVSLSF	LKNLRLILGE	EQLEGNYSFY	VLDNQNLQQL	WDWDHRNLTI
330	340	350	360	370	380	390	400
KAGKMYFAFN	PKLCVSEIYR	MEEVTGTKGR	QSKGDINTRN	NGERASCESD	VLHFTSTTTS	KNRIIITWHR	YRPPDYRDLI
410	420	430	440	450	460	470	480
SFTVYYKEAP	FKNVTEYDGQ	DACGSNSWNM	VDVDLPPNKD	VEPGILLHGL	KPWTQYAVYV	KAVTLTMVEN	DHIRGAKSEI
490	500	510	520	530	540	550	560
LYIRTNASVP	SIPLDVLSAS	NSSSQLIVKW	NPPSLPNGNL	SYYIVRWQRQ	PQDGYLYRHN	YCSKDKIPIR	KYADGTIDIE
570	580	590	600	610	620	630	640
EVTENPKTEV	CGGEKGPCCA	CPKTEAEKQA	EKEEAEYRKV	FENFLHNSIF	VPRPERKRRD	VMQVANTTMS	SRSRNTTAAD
650	660	670	680	690	700	710	720
TYNITDPEEL	ETEYPFFESR	VDNKERTVIS	NLRPFTLYRI	DIHSCNHEAE	KLGCSASNFV	FARTMPAEGA	DDIPGPVTWE
730	740	750	760	770	780	790	800
PRPENSIFLK	WPEPENPNGL	ILMYEIKYGS	QVEDQRECVS	RQEYRKYGGA	KLNRLNPGNY	TARIQATSLS	GNGSWTDPVF
810	820	830	840	850	860	870	880
FYVQAKTGYE	NFIHLIIALP	VAVLLIVGGL	VIMLYVFHRK	RNNSRLGNGV	LYASVNPEYF	SAADVYVPDE	WEVAREKITM
890	900	910	920	930	940	950	960
SRELGQGSFG	MVYEGVAKGV	VKDEPETRVA	IKTVNEAASM	RERIEFLNEA	SVMKEFNCHH	VVRLLGVVSQ	GQPTLVIMEL
970	980	990	1000	1010	1020	1030	1040
MTRGDLKSYL	RSLRPEMENN	PVLAPPSLSK	MIQMAGEIAD	GMAYLNANKF	VHRDLAARNC	MVAEDFTVKI	GDFGMTRDIY
1050	1060	1070	1080	1090	1100	1110	1120
ETDYYRKGGK	GLLPVRWMSP	ESLKDGVFTT	YSDVWSFGVV	LWEIATLAEQ	PYQGLSNEQV	LRFVMEGGLL	DKPDNCPDML
1130	1140	1150	1160	1170	1180	1190	1200
FELMRMCWQY	NPKMRPSFLE	IISSIKEEME	PGFREVSFYY	SEENKLPEPE	ELDLEPENME	SVPLDPSASS	SSLPLPDRHS
1210	1220	1230	1240	1250			
GHKAENGPGP	GVLVLRASFD	ERQPYAHMNG	GRKNERALPL	PQSSTC			

Cmpd.	No. of Cmpds.	m/z meas.	Δ m/z [ppm]	z	Rt [min]	Score	P	Range	Sequence	Modification
1849	3	1045.0301	118.60	2	51.5	16.2	0	56-73	K.ECGDLCPGTMEEKPMCEK.T	Carbamidomethyl: 16; Oxidation: 10, 15
1628	1	716.0279	112.96	3	50.1	12.0	0	56-73	K.ECGDLCPGTMEEKPMCEK.T	Carbamidomethyl: 2, 16; Oxidation: 10, 15
1529	1	669.8325	-70.94	2	49.3	13.1	0	271-282	R.HSHALVSLSFLK.N	

Detailed Protein Report

Protein 209: PREDICTED: ubiquitin carboxyl-terminal hydrolase 40 isoform X4 [Homo sapiens]

**Accession:**	gi|530371419	**Score:**	41.3
**Database:**	refseq_human(refseq_human_20140103.fasta)	**MW [kDa]:**	125.8
**Database Date:**	2015-11-30	**pI:**	5.5
		**Sequence Coverage [%]:**	2.7
		**No. of unique Peptides:**	2

Quantitation

WUP:QUP **Median:** 1.84 **CV:** 0.00 % **No. of Peptides:** 1

10	20	30	40	50	60	70	80
MRQHDVQELN	RILFSALETS	LVGTSGHDLI	YRLYHGTIVN	QIVCKECKNV	SERQEDFLDL	TVAVKNVSGL	EDALWNMYVE
**90**	**100**	**110**	**120**	**130**	**140**	**150**	**160**
EEVFDCDNLY	HCGTCDRLVK	AAKSAKLRKL	PPFLTVSLLR	FNFDFVKCER	YKETSCYTFP	LRINLKPFCE	QSELDDLEYI
**170**	**180**	**190**	**200**	**210**	**220**	**230**	**240**
YDLFSVIIHK	GGCYGGHYHV	YIKDVDHLGN	WQFQEEKSKP	DVNLKDLQSE	EEIDHPLMIL	KAILLEEENN	LIPVDQLGQK
**250**	**260**	**270**	**280**	**290**	**300**	**310**	**320**
LLKKIGISWN	KKYRKQHGPL	RKFLQLHSQI	FLLSSDESTV	RLLKNSSLQA	ESDFQRNDQQ	IFKMLPPESP	GLNNSISCPH
**330**	**340**	**350**	**360**	**370**	**380**	**390**	**400**
WFDINDSKVQ	PIREKDIEQQ	FQGKESAYML	FYRKSQLQRP	PEARANPRYG	VPCHLLNEMD	AANIELQTKR	AECDSANNTF
**410**	**420**	**430**	**440**	**450**	**460**	**470**	**480**
ELHLHLGPQY	HFFNGALHPV	VSQTESVWDL	TFDKRKTLGD	LRQSIFQLLE	FWEGDMVLSV	AKLVPAGLHI	YQSLGGDELT
**490**	**500**	**510**	**520**	**530**	**540**	**550**	**560**
LCETEIADGE	DIFVWNGVEV	GGVHIQTGID	CEPLLLNVLH	LDTSSDGEKC	CQVIESPHVF	PANAEVGTVL	TALAIPAGVI
**570**	**580**	**590**	**600**	**610**	**620**	**630**	**640**
FINSAGCPGG	EGWTAIPKED	MRKTFREQGL	RNGSSILIQD	SHDDNSLLTK	EEKWVTSMNE	IDWLHVKNLC	QLESEEKQVK
**650**	**660**	**670**	**680**	**690**	**700**	**710**	**720**
ISATVNTMVF	DIRIKAIKEL	KLMKELADNS	CLRPIDRNGK	LLCPVPDSYT	LKEAELKMGS	SLGLCLGKAP	SSSQLFLFFA
**730**	**740**	**750**	**760**	**770**	**780**	**790**	**800**
MGSDVQPGTE	MEIVVEETIS	VRDCLKLMLK	KSGLQGDAWH	LRKMDWCYEA	GEPLCEEDAT	LKELLICSGD	TLLLIEGQLP
**810**	**820**	**830**	**840**	**850**	**860**	**870**	**880**
PLGFLKVPIW	WYQLQGPSGH	WESHQDQTNC	TSSWGRVWRA	TSSQGASGNE	PAQVSLLYLG	DIEISEDATL	AELKSQAMTL
**890**	**900**	**910**	**920**	**930**	**940**	**950**	**960**
PPFLEFGVPS	PAHLRAWTVE	RKRPGRLLRT	DRQPLREYKL	GRRIEICLEP	LQKGENLGPQ	DVLLRTQVRI	PGERTYAPAL
**970**	**980**	**990**	**1000**	**1010**	**1020**	**1030**	**1040**
DLVWNAAQGG	TAGSLRQRVA	DFYRLPVEKI	EIAKYFPEKF	EWLPISSWNQ	QITKRKKKKK	QDYLQGAPYY	LKDGDTIGVK
**1050**	**1060**	**1070**	**1080**	**1090**	**1100**	**1110**	
NLLIDDDDDF	STIRDDTGKE	KQKQRALGRR	KSQEALHEQS	SYILSSAETP	ARPRAPETSL	SIHVGSFR	

Cmpd.	No. of Cmpds.	m/z meas.	Δ m/z [ppm]	z	Rt [min]	Score	P	Range	Sequence	Modification	Ratios
2293	1	937.4457	-41.72	2	57.0	11.2	1	662-677	K.LMKELADNSCLRPIDR.N		WUP:QUP 1.84
2823	1	826.4280	41.02	2	65.7	30.1	0	1041-1054	K.NLLIDDDDDFSTIR.D		

Detailed Protein Report

Protein 210: histone H4 [Homo sapiens]

**Accession:**	gi|4504301	**Score:**	41.1
**Database:**	refseq_human(refseq_human_20140103.fasta)	**MW [kDa]:**	11.4
**Database Date:**	2015-11-30	**pI:**	11.8
		**Sequence Coverage [%]:**	7.8
		**No. of unique Peptides:**	1

Quantitation

QU:MU	**Median:** 1.36	**CV:** 0.00 %	**No. of Peptides:**	1
WUP:QUP	**Median:** 1.44	**CV:** 0.00 %	**No. of Peptides:**	1

Alias proteins:

Accession	Name	Description
gi|77539758	refseq_human (refseq_human_20140103.fasta)	histone H4 [Homo sapiens]
gi|28173560	refseq_human (refseq_human_20140103.fasta)	histone H4 [Homo sapiens]
gi|11415030	refseq_human (refseq_human_20140103.fasta)	histone H4 [Homo sapiens]
gi|4504323	refseq_human (refseq_human_20140103.fasta)	histone H4 [Homo sapiens]
gi|4504321	refseq_human (refseq_human_20140103.fasta)	histone H4 [Homo sapiens]
gi|4504317	refseq_human (refseq_human_20140103.fasta)	histone H4 [Homo sapiens]
gi|4504315	refseq_human (refseq_human_20140103.fasta)	histone H4 [Homo sapiens]
gi|4504313	refseq_human (refseq_human_20140103.fasta)	histone H4 [Homo sapiens]
gi|4504311	refseq_human (refseq_human_20140103.fasta)	histone H4 [Homo sapiens]
gi|4504309	refseq_human (refseq_human_20140103.fasta)	histone H4 [Homo sapiens]
gi|4504307	refseq_human (refseq_human_20140103.fasta)	histone H4 [Homo sapiens]
gi|4504305	refseq_human (refseq_human_20140103.fasta)	histone H4 [Homo sapiens]
gi|4504303	refseq_human (refseq_human_20140103.fasta)	histone H4 [Homo sapiens]

10	20	30	40	50	60	70	80
MSGRGKGGKG	LGKGGAKRHR	KVLRDNIQGI	TKPAIRRLAR	RGGVKRISGL	IYEETRGVLK	VFLENVIRDA	VTYTEHAKRK
**90**	**100**	**110**					
TVTAMDVVYA	LKRQGRTLYG	FGG					

Cmpd.	No. of Cmpds.	m/z meas.	Δ m/z [ppm]	z	Rt [min]	Score	P	Range	Sequence	Modification	Ratios
1293	9	495.1457	-296.53	2	45.8	41.1	0	61-68	K.VFLENVIR.D		QU:MU 1.36<br>WUP:QUP 1.44

Detailed Protein Report

Protein 211: uncharacterized protein C17orf85 [Homo sapiens]

**Accession:**	gi|166295179	**Score:**	40.8
**Database:**	refseq_human(refseq_human_20140103.fasta)	**MW [kDa]:**	70.5
**Database Date:**	2015-11-30	**pI:**	5.4
		**Sequence Coverage [%]:**	6.0
		**No. of unique Peptides:**	2

10	20	30	40	50	60	70	80
MAAVRGLRVS	VKAEAPAGPA	LGLPSPEAES	GVDRGEPEPM	EVEEGELEIV	PVRRSLKELI	PDTSRRYENK	AGSFITGIDV
**90**	**100**	**110**	**120**	**130**	**140**	**150**	**160**
TSKEAIEKKE	QRAKRFHFRS	EVNLAQRNVA	LDRDMMKKAI	PKVRLETIYI	CGVDEMSTQD	VFSYFKEYPP	AHIEWLDDTS
**170**	**180**	**190**	**200**	**210**	**220**	**230**	**240**
CNVVWLDEMT	ATRALINMSS	LPAQDKIRSR	DASEDKSAEK	RKKDKQEDSS	DDDEAEEGEV	EDENSSDVEL	DTLSQVEEES
**250**	**260**	**270**	**280**	**290**	**300**	**310**	**320**
LLRNDLRPAN	KLAKGNRLFM	RFATKDDKKE	LGAARRSQYY	MKYGNPNYGG	MKGILSNSWK	RRYHSRRIQR	DVIKKRALIG
**330**	**340**	**350**	**360**	**370**	**380**	**390**	**400**
DDVGLTSYKH	RHSGLVNVPE	EPIEEEEEEE	EEEEEEEEED	QDMDADDRVV	VEYHEELPAL	KQPRERSASR	RSSASSSDSD
**410**	**420**	**430**	**440**	**450**	**460**	**470**	**480**
EMDYDLELKM	ISTPSPKKSM	KMTMYADEVE	SQLKNIRNSM	RADSVSSSNI	KNRIGNKLPP	EKFADVRHLL	DEKRQHSRPR
**490**	**500**	**510**	**520**	**530**	**540**	**550**	**560**
PPVSSTKSDI	RQRLGKRPHS	PEKAFSSNPV	VRREPSSDVH	SRLGVPRQDS	KGLYADTREK	KSGNLWTRLG	SAPKTKEKNT
**570**	**580**	**590**	**600**	**610**	**620**	**630**	
KKVDHRAPGA	EEDDSELQRA	WGALIKEKEQ	SRQKKSRLDN	LPSLQIEVSR	ESSSGSEAES		

Cmpd.	No. of Cmpds.	m/z meas.	Δ m/z [ppm]	z	Rt [min]	Score	P	Range	Sequence	Modification
2859	1	648.3692	36.16	2	64.5	11.7	0	71-83	K.AGSFITGIDVTSK.E	
2116	1	945.7998	-143.91	2	56.8	14.7	1	419-434	K.SMKMTMYADEVESQLK.N	

Detailed Protein Report

Protein 212: **PREDICTED: peregrin isoform X6 [Homo sapiens]**

**Accession:**	gi|530373123	**Score:**	40.8
**Database:**	refseq_human(refseq_human_20140103.fasta)	**MW [kDa]:**	78.0
**Database Date:**	2015-11-30	**pI:**	6.9
		**Sequence Coverage [%]:**	4.4
		**No. of unique Peptides:**	2

Quantitation

QU:MU	**Median:**	1.67	**CV:**	0.00 %	**No. of Peptides:**	1
WUP:QUP	**Median:**	3.92	**CV:**	0.00 %	**No. of Peptides:**	1

10	20	30	40	50	60	70	80
MGVDFDVKTF	CHNLRATKPP	YECPVETCRK	VYKSYSGIEY	HLYHYDHDNP	PPPQQTPLRK	HKKKGRQSRP	ANKQSPSPSE
**90**	**100**	**110**	**120**	**130**	**140**	**150**	**160**
VSQSPGREVM	SYAQAQRMVE	VDLHGRVHRI	SIFDNLDVVS	EDEEAPEEAP	ENGSNKENTE	TPAATPKSGK	HKNKEKRKDS
**170**	**180**	**190**	**200**	**210**	**220**	**230**	**240**
NHHHHHNVSA	STTPKLPEVV	YRELEQDTPD	APPRPTSYYR	YIEKSAEELD	EEVEYDMDEE	DYIWLDIMNE	RRKTEGVSPI
**250**	**260**	**270**	**280**	**290**	**300**	**310**	**320**
PQEIFEYLMD	RLEKESYFES	HNKGDPNALV	DEDAVCCICN	DGECQNSNVI	LFCDMCNLAV	HQECYGVPYI	PEGQWLCRRC
**330**	**340**	**350**	**360**	**370**	**380**	**390**	**400**
LQSPSRAVDC	ALCPNKGGAF	KQTDDGRWAH	VVCALWIPEV	CFANTVFLEP	IDSIEHIPPA	RWKLTCYICK	QRGSGACIQC
**410**	**420**	**430**	**440**	**450**	**460**	**470**	**480**
HKANCYTAFH	VTCAQQAGLY	MKMEPVRETG	ANGTSFSVRK	TAYCDIHTPP	GSARRLPALS	HSEGEEDEDE	EEDEGKGWSS
**490**	**500**	**510**	**520**	**530**	**540**	**550**	**560**
EKVKKAKAKS	RIKMKKARKI	LAEKRAAAPV	VSVPCIPPHR	LSKITNRLTI	QRKSQFMQRL	HSYWTLKRQS	RNGVPLLRRL
**570**	**580**	**590**	**600**	**610**	**620**	**630**	**640**
QTHLQSQRNC	DQVGRDSEDK	NWALKEQLKS	WQRLRHDLER	ARLLVELIRK	REKLKRETIK	VQQIAMEMQL	TPFLILLRKT
**650**	**660**	**670**	**680**	**690**			
LEQLQEKDTG	NIFSEPVPLS	EGPEWEAVCT	PSPEAGTESA	K			

Cmpd.	No. of Cmpds.	m/z meas.	Δ m/z [ppm]	z	Rt [min]	Score	P	Range	Sequence	Modification	Ratios
256	1	715.8400	-27.55	2	33.2	17.3	1	137-150	K.ENTETPAATPKSGK.H		QU:MU 1.67<br>WUP:QUP 3.92
1974	1	757.2741	-191.30	2	55.1	10.7	0	506-520	R.AAAPVVSVPCIPPHR.L		

Detailed Protein Report

Protein 213: fibrocystin isoform 1 precursor [Homo sapiens]

**Accession:**	gi|126131102	**Score:**	40.7
**Database:**	refseq_human(refseq_human_20140103.fasta)	**MW [kDa]:**	446.4
**Database Date:**	2015-11-30	**pI:**	6.1
**Modification(s):**	Carbamidomethyl	**Sequence Coverage [%]:**	1.0
		**No. of unique Peptides:**	1

Detailed Protein Report

10	20	30	40	50	60	70	80
MTAWLISLMS	IEVLLLAVRH	LSLHIEPEEG	SLAGGTWITV	IFDGLELGVL	YPNNGSQLEI	HLVNVNMVVP	ALRSVPCDVF
90	100	110	120	130	140	150	160
PVFLDLPVVT	CRTRSVLSEA	HEGLYFLEAY	FGGQLVSSPN	PGPRDSCTFK	FSKAQTPIVH	QVYPPSGVPG	KLIHVYGWII
170	180	190	200	210	220	230	240
TGRLETFDFD	AEYIDSPVIL	EAQGDKWVTP	CSLINRQMGS	CYPIQEDHGL	GTLQCHVEGD	YIGSQNVSFS	VFNKGKSMVH
250	260	270	280	290	300	310	320
KKAWLISAKQ	DLFLYQTHSE	ILSVFPETGS	LGGRTNITIT	GDFFDNSAQV	TIAGIPCDIR	HVSPRKIECT	TRAPGKDVRL
330	340	350	360	370	380	390	400
TTPQPGNRGL	LFEVGDAVEG	LELTEATPGY	RWQIVPNASS	PFGFWSQEGQ	PFRARLSGFF	VAPETNNYTF	WIQADSQASL
410	420	430	440	450	460	470	480
HFSWSEEPRT	KVKVASISVG	TADWFDSWEQ	NRDEGTWQQK	TPKLELLGGA	MYYLEAEHHG	IAPSRGMRIG	VQIHNTWLNP
490	500	510	520	530	540	550	560
DVVTTYLREK	HQIRVRAQRL	PEVQVLNVSG	RGNFFLTWDN	VSSQPIPANA	TAHLIQTTIE	ELLAVKCKLE	PLWSNILLRL
570	580	590	600	610	620	630	640
GFERGPEVSN	SDGDLTSGTE	PFCGRFSLRQ	PRHLVLTPPA	AQKGYRLDQY	THLCLAYKGH	MNKILKMIVS	FTIGFQNMVK
650	660	670	680	690	700	710	720
NTTCDWSLTR	TSPESWQFDC	TDLWETCVRC	FGDLQPPPAN	SPVLVHQINL	LPLAQETGLF	YVDEIIIADT	NVTVSQADSG
730	740	750	760	770	780	790	800
TARPGGNLVE	SVSVVGSPPV	YSVTSWLAGC	GTELPLITAR	SVPTEGTEEG	SGLVLVTTQR	RQRTSPPLGG	HFRIQLPNTV
810	820	830	840	850	860	870	880
ISDVPVQISA	HHLHQLLQNN	ADDFTSRYLN	ASDFTVKEDL	YTCYEHVWTL	SWSTQIGDLP	NFIRVSDENL	TGVNPAAATR
890	900	910	920	930	940	950	960
VVYDGGVFLG	PIFGDMLATA	NQHTQVVVRV	NDVPAHCPGS	CSFQYLQGST	PCVHSVWYSI	DGDINLMIYI	TGTGFSGDSQ
970	980	990	1000	1010	1020	1030	1040
FLQVTVNKTS	CKVIFSNQTN	VVCQTDLLPV	GMHRILMLVR	PSGLAISATG	EDLFLNVKPR	LDMVEPSRAA	DIGGLWATIR
1050	1060	1070	1080	1090	1100	1110	1120
GSSLEGVSLI	LFGSYSCAIN	VATSNSSRIQ	CKVPPRGKDG	RIVNVTVIRG	DYSAVLPRAF	TYVSSLNPVI	VTLSRNISNI
1130	1140	1150	1160	1170	1180	1190	1200
AGGETLVIGV	ARLMNYTDLD	VEVHVQDALA	PVHTQSAWGL	EVALPPLPAG	LHRISVSING	VSIHSQGVDL	HIQYLTEVFS
1210	1220	1230	1240	1250	1260	1270	1280
IEPCCGSLLG	GTILSISGIG	FSRDPALVWV	LVGNRSCDIV	NLTEASIWCE	TLPAPQIPDA	GAPTVPAAVE	VWAGNRFFAR
1290	1300	1310	1320	1330	1340	1350	1360
GPSPSLVGKG	FTFMYEAAAT	PVVTAMQGEI	TNSSLSLHVG	GSNLSNSVIL	LGNLNCDVET	QSFQGNVSLS	GCSIPLHSLE
1370	1380	1390	1400	1410	1420	1430	1440
AGIYPLQVRQ	KQMGFANMSV	VLQQFAVMPR	IMAIFPSQGS	ACGGTILTVR	GLLLNSRRRS	VRVDLSGPFT	CVILSLGDHT
1450	1460	1470	1480	1490	1500	1510	1520
ILCQVSLEGD	PLPGASFSLN	VTVLVNGLTS	ECQGNCTLFI	REEASPVMDA	LSTNTSGSLT	TVLIRGQRLA	TTADEPMVFV
1530	1540	1550	1560	1570	1580	1590	1600
DDQLPCNVTF	FNASHVVCQT	RDLAPGPHYL	SVFYTRNGYA	CSGNVSRHFY	IMPQVFHYFP	KNFSLHGGSL	LTIEGTGLRG
1610	1620	1630	1640	1650	1660	1670	1680
QNTTSVYIDQ	QTCLTVNIGA	ELIRCIVPTG	NGSVALEIEV	DGLWYHIGVI	GYNKAFTPEL	ISISQSDDIL	TFAVAQISGA
1690	1700	1710	1720	1730	1740	1750	1760
ANIDIFIGMS	PCVGVSGNHT	VLQCVVPSLP	AGEYHVRGYD	CIRGWASSAL	VFTSRVIITA	VTENFGCLGG	RLVHVFGAGF
1770	1780	1790	1800	1810	1820	1830	1840
SPGNVSAAVC	GAPCRVLANA	TVSAFSCLVL	PLDVSLAFLC	GLKREEDSCE	AARHTYVQCD	LTVAMATEQL	LESWPYLYIC
1850	1860	1870	1880	1890	1900	1910	1920
EESSQCLFVP	DHWAESMFPS	FSGLFISPKL	ERDEVLIYNS	SCNITMETEA	EMECETPNQP	ITVKITEIRK	RWGQNTQGNF
1930	1940	1950	1960	1970	1980	1990	2000
SLQFCRRWSR	THSWFPERLP	QDGDNVTVEN	GQLLLLDTNT	SILNLLHIKG	GKLIFMAPGP	IELRAHAILV	SDGGELRIGS
2010	2020	2030	2040	2050	2060	2070	2080
EDKPFQGRAQ	ITLYGSSYST	PFFPYGVKFL	AVRNGTLSLH	GSLPEVIVTC	LRATAHALDT	VLALEDAVDW	NPGDEVVIIS
2090	2100	2110	2120	2130	2140	2150	2160
GTGVKGAKPM	EEIVTVETVQ	DTDLYLKSPL	RYSHNFTENW	VAGEHHILKA	TVALLSRSIT	IQGNLTNERE	KLLVSCQEAN
2170	2180	2190	2200	2210	2220	2230	2240

Detailed Protein Report

Cmpd.	No. of Cmpds.	m/z meas.	Δ m/z [ppm]	z	Rt [min]	Score	P	Range	Sequence	Modification
2756	1	1023.5380	40.37	2	63.0	11.3	1	1718-1735	R.GYDCIRGWASSALVFTSR.V	Carbamidomethyl: 4

Detailed Protein Report

Protein 214: myosin-IIIa [Homo sapiens]

**Accession:**	gi|145275208	**Score:**	40.7
**Database:**	refseq_human(refseq_human_20140103.fasta)	**MW [kDa]:**	186.1
**Database Date:**	2015-11-30	**pI:**	9.6
**Modification(s):**	Carbamidomethyl	**Sequence Coverage [%]:**	2.5
		**No. of unique Peptides:**	1

10	20	30	40	50	60	70	80
MFPLIGKTII	FDNFPDPSDT	WEITETIGKG	TYGKVFKVLN	KKNGQKAAVK	ILDPIHDIDE	EIEAEYNILK	ALSDHPNVVR
90	100	110	120	130	140	150	160
FYGIYFKKDK	VNGDKLWLVL	ELCSGGSVTD	LVKGFLKRGE	RMSEPLIAYI	LHEALMGLQH	LHNNKTIHRD	VKGNNILLTT
170	180	190	200	210	220	230	240
EGGVKLVDFG	VSAQLTSTRH	RRNTSVGTPF	WMAPEVIACE	QQLDTTYDAR	CDTWSLGITA	IELGDGDPPL	ADLHPMRALF
250	260	270	280	290	300	310	320
KIPRNPPPKL	RQPELWSAEF	NDFISKCLTK	DYEKRPTVSE	LLQHKFITQI	EGKDVMLQKQ	LTEFIGIHQC	MGGTEKARRE
330	340	350	360	370	380	390	400
RIHTKKGNFN	RPLISNLKDV	DDLATLEILD	ENTVSEQLEK	CYSRDQIYVY	VGDILIALNP	FQSLGLYSTK	HSKLYIGSKR
410	420	430	440	450	460	470	480
TASPPHIFAM	ADLGYQSMIT	YNSDQCIVIS	GESGAGKTEN	AHLLVQQLTV	LGKANNRTLQ	EKILQVNNLV	EAFGNACTII
490	500	510	520	530	540	550	560
NDNSSRFGKY	LEMKFTSSGA	VVGAQISEYL	LEKSRVIHQA	IGEKNFHIFY	YIYAGLAEKK	KLAHYKLPEN	KPPRYLQNDH
570	580	590	600	610	620	630	640
LRTVQDIMNN	SFYKSQYELI	EQCFKVIGFT	MEQLGSIYSI	LAAILNVGNI	EFSSVATEHQ	IDKSHISNHT	ALENCASLLC
650	660	670	680	690	700	710	720
IRADELQEAL	TSHCVVTRGE	TIIRPNTVEK	ATDVRDAMAK	TLYGRLFSWI	VNCINSLLKH	DSSPSGNGDE	LSIGILDIFG
730	740	750	760	770	780	790	800
FENFKKNSFE	QLCINIANEQ	IQYYYNQHVF	AWEQNEYLNE	DVDARVIEYE	DNWPLLDMFL	QKPMGLLSLL	DEESRFPKAT
810	820	830	840	850	860	870	880
DQTLVEKFEG	NLKSQYFWRP	KRMELSFGIH	HYAGKVLYNA	SGFLAKNRDT	LPTDIVLLLR	SSDNSVIRQL	VNHPLTKTGN
890	900	910	920	930	940	950	960
LPHSKTKNVI	NYQMRTSEKL	INLAKGDTGE	ATRHARETTN	MKTQTVASYF	RYSLMDLLSK	MVVGQPHFVR	CIKPNSERQA
970	980	990	1000	1010	1020	1030	1040
RKYDKEKVLL	QLRYTGILET	ARIRRLGFSH	RILFANFIKR	YYLLCYKSSE	EPRMSPDTCA	TILEKAGLDN	WALGKTKVFL
1050	1060	1070	1080	1090	1100	1110	1120
KYYHVEQLNL	MRKEAIDKLI	LIQACVRAFL	CSRRYQKIQE	KRKESAIIIQ	SAARGHLVRK	QRKEIVDMKN	TAVTTIQTSD
1130	1140	1150	1160	1170	1180	1190	1200
QEFDYKKNFE	NTRESFVKKQ	AENAISANER	FISAPNNKGS	VSVVKTSTFK	PEEETTNAVE	SNNRVYQTPK	KMNNVYEEEV
1210	1220	1230	1240	1250	1260	1270	1280
KQEFYLVGPE	VSPKQKSVKD	LEENSNLRKV	EKEEAMIQSY	YQRYTEERNC	EESKAAYLER	KAISERPSYP	VPWLAENETS
1290	1300	1310	1320	1330	1340	1350	1360
FKKTLEPTLS	QRSIYQNANS	MEKEKKTSVV	TQRAPICSQE	EGRGRLRHET	VKERQVEPVT	QAQEEEDKAA	VFIQSKYRGY
1370	1380	1390	1400	1410	1420	1430	1440
KRRQQLRKDK	MSSFKHQRIV	TTPTEVARNT	HNLYSYPTKH	EEINNIKKKD	NKDSKATSER	EACGLAIFSK	QISKLSEEYF
1450	1460	1470	1480	1490	1500	1510	1520
ILQKKLNEMI	LSQQLKSLYL	GVSHHKPINR	RVSSQQCLSG	VCKGEEPKIL	RPPRRPRKPK	TLNNPEDSTY	YYLLHKSIQE
1530	1540	1550	1560	1570	1580	1590	1600
EKRRPRKDSQ	GKLLDLEDFY	YKEFLPSRSG	PKEHSPSLRE	RRPQQELQNQ	CIKANERCWA	AESPEKEEER	EPAANPYDFR
1610	1620						
RLLRKTSQRR	RLVQQS						

Cmpd.	No. of Cmpds.	m/z meas.	Δ m/z [ppm]	z	Rt [min]	Score	P	Range	Sequence	Modification
875	1	648.3760	105.34	2	41.0	11.8	0	1472-1483	R.VSSQQCLSGVCK.G	Carbamidomethyl: 6

Detailed Protein Report

Protein 215: filamin-A isoform 1 [Homo sapiens]

**Accession:**	gi|116063573	**Score:**	40.7
**Database:**	refseq_human(refseq_human_20140103.fasta)	**MW [kDa]:**	279.8
**Database Date:**	2015-11-30	**pI:**	5.6
		**Sequence Coverage [%]:**	1.8
		**No. of unique Peptides:**	2

Detailed Protein Report

10	20	30	40	50	60	70	80
MSSSHSRAGQ	SAAGAAPGGG	VDTRDAEMPA	TEKDLAEDAP	WKKIQQNTFT	RWCNEHLKCV	SKRIANLQTD	LSDGLRLIAL
90	100	110	120	130	140	150	160
LEVLSQKKMH	RKHNQRPTFR	QMQLENVSVA	LEFLDRESIK	LVSIDSKAIV	DGNLKLILGL	IWTLILHYSI	SMPMWDEEED
170	180	190	200	210	220	230	240
EEAKKQTPKQ	RLLGWIQNKL	PQLPITNFSR	DWQSGRALGA	LVDSCAPGLC	PDWDSWDASK	PVTNAREAMQ	QADDWLGIPQ
250	260	270	280	290	300	310	320
VITPEEIVDP	NVDEHSVMTY	LSQFPKAKLK	PGAPLRPKLN	PKKARAYGPG	IEPTGNMVKK	RAEFTVETRS	AGQGEVLVYV
330	340	350	360	370	380	390	400
EDPAGHQEEA	KVTANNDKNR	TFSVWYVPEV	TGTHKVTVLF	AGQHIAKSPF	EVYVDKSQGD	ASKVTAQGPG	LEPSGNIANK
410	420	430	440	450	460	470	480
TTYFEIFTAG	AGTGEVEVVI	QDPMGQKGTV	EPQLEARGDS	TYRCSYQPTM	EGVHTVHVTF	AGVPIPRSPY	TVTVGQACNP
490	500	510	520	530	540	550	560
SACRAVGRGL	QPKGVRVKET	ADFKVYTKGA	GSGELKVTVK	GPKGEERVKQ	KDLGDGVYGF	EYYPMVPGTY	IVTITWGGQN
570	580	590	600	610	620	630	640
IGRSPFEVKV	GTECGNQKVR	AWGPGLEGGV	VGKSADFVVE	AIGDDVGTLG	FSVEGPSQAK	IECDDKGDGS	CDVRYWPQEA
650	660	670	680	690	700	710	720
GEYAVHVLCN	SEDIRLSPFM	ADIRDAPQDF	HPDRVKARGP	GLEKTGVAVN	KPAEFTVDAK	HGGKAPLRVQ	VQDNEGCPVE
730	740	750	760	770	780	790	800
ALVKDNGNGT	YSCSYVPRKP	VKHTAMVSWG	GVSIPNSPFR	VNVGAGSHPN	KVKVYGPGVA	KTGLKAHEPT	YFTVDCAEAG
810	820	830	840	850	860	870	880
QGDVSIGIKC	APGVVGPAEA	DIDFDIIRND	NDTFTVKYTP	RGAGSYTIMV	LFADQATPTS	PIRVKVEPSH	DASKVKAEGP
890	900	910	920	930	940	950	960
GLSRTGVELG	KPTHFTVNAK	AAGKGKLDVQ	FSGLTKGDAV	RDVDIIDHHD	NTYTVKYTPV	QQGPVGVNVT	YGGDPIPKSP
970	980	990	1000	1010	1020	1030	1040
FSVAVSPSLD	LSKIKVSGLG	EKVDVGKDQE	FTVKSKGAGG	QGKVASKIVG	PSGAAVPCKV	EPGLGADNSV	VRFLPREEGP
1050	1060	1070	1080	1090	1100	1110	1120
YEVEVTYDGV	PVPGSPFPLE	AVAPTKPSKV	KAFGPGLQGG	SAGSPARFTI	DTKGAGTGGL	GLTVEGPCEA	QLECLDNGDG
1130	1140	1150	1160	1170	1180	1190	1200
TCSVSYVPTE	PGDYNINILF	ADTHIPGSPF	KAHVVPCFDA	SKVKCSGPGL	ERATAGEVGQ	FQVDCSSAGS	AELTIEICSE
1210	1220	1230	1240	1250	1260	1270	1280
AGLPAEVYIQ	DHGDGTHTIT	YIPLCPGAYT	VTIKYGGQPV	PNFPSKLQVE	PAVDTSGVQC	YGPGIEGQGV	FREATTEFSV
1290	1300	1310	1320	1330	1340	1350	1360
DARALTQTGG	PHVKARVANP	SGNLTETYVQ	DRGDGMYKVE	YTPYEEGLHS	VDVTYDGSPV	PSSPFQVPVT	EGCDPSRVRV
1370	1380	1390	1400	1410	1420	1430	1440
HGPGIQSGTT	NKPNKFTVET	RGAGTGGLGL	AVEGPSEAKM	SCMDNKDGSC	SVEYIPYEAG	TYSLNVTYGG	HQVPGSPFKV
1450	1460	1470	1480	1490	1500	1510	1520
PVHDVTDASK	VKCSGPGLSP	GMVRANLPQS	FQVDTSKAGV	APLQVKVQGP	KGLVEPVDVV	DNADGTQTVN	YVPSREGPYS
1530	1540	1550	1560	1570	1580	1590	1600
ISVLYGDEEV	PRSPFKVKVL	PTHDASKVKA	SGPGLNTTGV	PASLPVEFTI	DAKDAGEGLL	AVQITDPEGK	PKKTHIQDNH
1610	1620	1630	1640	1650	1660	1670	1680
DGTYTVAYVP	DVTGRYTILI	KYGGDEIPFS	PYRVRAVPTG	DASKCTVTGA	GIGPTIQIGE	ETVITVDTKA	AGKGKVTCTV
1690	1700	1710	1720	1730	1740	1750	1760
CTPDGSEVDV	DVVENEDGTF	DIFYTAPQPG	KYVICVRFGG	EHVPNSPFQV	TALAGDQPSV	QPPLRSQQLA	PQYTYAQGGQ
1770	1780	1790	1800	1810	1820	1830	1840
QTWAPERPLV	GVNGLDVTSL	RPFDLVIPFT	IKKGEITGEV	RMPSGKVAQP	TITDNKDGTV	TVRYAPSEAG	LHEMDIRYDN
1850	1860	1870	1880	1890	1900	1910	1920
MHIPGSPLQF	YVDYVNCGHV	TAYGPGLTHG	VVNKPATFTV	NTKDAGEGGL	SLAIEGPSKA	EISCTDNQDG	TCSVSYLPVL
1930	1940	1950	1960	1970	1980	1990	2000
PGDYSILVKY	NEQHVPGSPF	TARVTGDDSM	RMSHLKVGSA	ADIPINISET	DLSLLTATVV	PPSGREEPCL	LKRLRNGHVG
2010	2020	2030	2040	2050	2060	2070	2080
ISFVPKETGE	HLVHVKKNGQ	HVASSPIPVV	ISQSEIGDAS	RVRVSGQGLH	EGHTFEPAEF	IIDTRDAGYG	GLSLSIEGPS
2090	2100	2110	2120	2130	2140	2150	2160
KVDINTEDLE	DGTCRVTYCP	TEPGNYIINI	KFADQHVPGS	PFSVKVTGEG	RVKESITRRR	RAPSVANVGS	HCDLSLKIPE
2170	2180	2190	2200	2210	2220	2230	2240

Detailed Protein Report

Cmpd.	No. of Cmpds.	m/z meas.	Δ m/z [ppm]	z	Rt [min]	Score	P	Range	Sequence	Modification
1673	1	694.3984	51.21	2	51.2	15.2	1	1451-1464	K.VKCSGPGLSPGMVR.A	
418	1	686.6239	-116.77	3	33.8	10.2	1	2339-2359	K.VNQPASFAVSLNGAKGAIDAK.V	

Detailed Protein Report

Protein 216: **PREDICTED: chromodomain-helicase-DNA-binding protein 4 isoform X6 [Homo sapiens]**

**Accession:**	gi|578822562	**Score:**	40.7
**Database:**	refseq_human(refseq_human_20140103.fasta)	**MW [kDa]:**	216.7
**Database Date:**	2015-11-30	**pI:**	5.7
**Modification(s):**	Carbamidomethyl, Oxidation	**Sequence Coverage [%]:**	2.5
		**No. of unique Peptides:**	2

Detailed Protein Report

10	20	30	40	50	60	70	80
MASGLGSPSP	CSAGSEEEDM	DALLNNSLPP	PHPENEEDPE	EDLSETETPK	LKKKKKPKKP	RDPKIPKSKR	QKKERMLLCR
90	100	110	120	130	140	150	160
QLGDSSGEGP	EFVEEEEEVA	LRSDSEGSDY	TPGKKKKKKL	GPKKEKKSKS	KRKEEEEEED	DDDDSKEPKS	SAQLLEDWGM
170	180	190	200	210	220	230	240
EDIDHVFSEE	DYRTLTNYKA	FSQFVRPLIA	AKNPKIAVSK	MMMVLGAKWR	EFSTNNPFKG	SSGASVAAAA	AAAVAVVESM
250	260	270	280	290	300	310	320
VTATEVAPPP	PPVEVPIRKA	KTKEGKGPNA	RRKPKGSPRV	PDAKKPKPKK	VAPLKIKLGG	FGSKRKRSSS	EDDDLDVESD
330	340	350	360	370	380	390	400
FDDASINSYS	VSDGSTSRSS	RSRKKLRTTK	KKKKDHQDYC	EVCQQGGEII	LCDTCPRAYH	MVCLDPDMEK	APEGKWSCPH
410	420	430	440	450	460	470	480
CEKEGIQWEA	KEDNSEGEEI	LEEVGGDLEE	EDDHHMEFCR	VCKDGGELLC	CDTCPSSYHI	HCLNPPLPEI	PNGEWLCPRC
490	500	510	520	530	540	550	560
TCPALKGKVQ	KILIWKWGQP	PSPTPVPRPP	DADPNTPSPK	PLEGRPERQF	FVKWQGMSYW	HCSWVSELQL	ELHCQVMFRN
570	580	590	600	610	620	630	640
YQRKNDMDEP	PSGDFGGDEE	KSRKRKNKDP	KFAEMEERFY	RYGIKPEWMM	IHRILNHSVD	KKGHVHYLIK	WRDLPYDQAS
650	660	670	680	690	700	710	720
WESEDVEIQD	YDLFKQSYWN	HRELMRGEEG	RPGKKLKKVK	LRKLERPPET	PTVDPTVKYE	RQPEYLDATG	GTLHPYQMEG
730	740	750	760	770	780	790	800
LNWLRFSWAQ	GTDTILADEM	GLGKTVQTAV	FLYSLYKEGH	SKGPFLVSAP	LSTIINWERE	FEMWAPDMYV	VTYVGDKDSR
810	820	830	840	850	860	870	880
AIIRENEFSF	EDNAIRGGKK	ASRMKKEASV	KFHVLLTSYE	LITIDMAILG	SIDWACLIVD	EAHRLKNNQS	KFFRVLNGYS
890	900	910	920	930	940	950	960
LQHKLLLTGT	PLQNNLEELF	HLLNFLTPER	FHNLEGFLEE	FADIAKEDQI	KKLHDMLGPH	MLRRLKADVF	KNMPSKTELI
970	980	990	1000	1010	1020	1030	1040
VRVELSPMQK	KYYKYILTRN	FEALNARGGG	NQVSLLNVVM	DLKKCCNHPY	LFPVAAMEAP	KMPNGMYDGS	ALIRASGKLL
1050	1060	1070	1080	1090	1100	1110	1120
LLQKMLKNLK	EGGHRVLIFS	QMTKMLDLLE	DFLEHEGYKY	ERIDGGITGN	MRQEAIDRFN	APGAQQFCFL	LSTRAGGLGI
1130	1140	1150	1160	1170	1180	1190	1200
NLATADTVII	YDSDWNPHND	IQAFSRAHRI	GQNKKVMIYR	FVTRASVEER	ITQVAKKKMM	LTHLVVRPGL	GSKTGSMSKQ
1210	1220	1230	1240	1250	1260	1270	1280
ELDDILKFGT	EELFKDEATD	GGGDNKEGED	SSVIHYDDKA	IERLLDRNQD	ETEDTELQGM	NEYLSSFKVA	QYVVREEEMG
1290	1300	1310	1320	1330	1340	1350	1360
EEEEVEREII	KQEESVDPDY	WEKLLRHHYE	QQQEDLARNL	GKGKRIRKQV	NYNDGSQEDR	DWQDDQSDNQ	SDYSVASEEG
1370	1380	1390	1400	1410	1420	1430	1440
DEDFDERSEA	PRRPSRKGLR	NDKDKPLPPL	LARVGGNIEV	LGFNARQRKA	FLNAIMRYGM	PPQDAFTTQW	LVRDLRGKSE
1450	1460	1470	1480	1490	1500	1510	1520
KEFKAYVSLF	MRHLCEPGAD	GAETFADGVP	REGLSRQHVL	TRIGVMSLIR	KKVQEFEHVN	GRWSMPELAE	VEENKKMSQP
1530	1540	1550	1560	1570	1580	1590	1600
GSPSPKTPTP	STPGDTQPNT	PAPVPPAEDG	IKIEENSLKE	EESIEGEKEV	KSTAPETAIE	CTQAPAPASE	DEKVVVEPPE
1610	1620	1630	1640	1650	1660	1670	1680
GEEKVEKAEV	KERTEEPMET	EPKGKGAADV	EKVEEKSAID	LTPIVVEDKE	EKKEEEEKKE	VMLQNGETPK	DLNDEKQKKN
1690	1700	1710	1720	1730	1740	1750	1760
IKQRFMFNIA	DGGFTELHSL	WQNEERAATV	TKKTYEIWHR	RHDYWLLAGI	INHGYARWQD	IQNDPRYAIL	NEPFKGEMNR
1770	1780	1790	1800	1810	1820	1830	1840
GNFLEIKNKF	LARRFKLLEQ	ALVIEEQLRR	AAYLNMSEDP	SHPSMALNTR	FAEVECLAES	HQHLSKESMA	GNKPANAVLH
1850	1860	1870	1880	1890	1900	1910	
KGILKQLEEL	LSDMKADVTR	LPATIARIPP	VAVRLQMSER	NILSRLANRA	PEPTPQQVAQ	QQ	

Cmpd.	No. of Cmpds.	m/z meas.	Δ m/z [ppm]	z	Rt [min]	Score	P	Range	Sequence	Modification
2366	1	649.8737	-146.01	3	59.4	13.6	0	1005-1021	K.CCNHPYLFPVAAMEAPK.M	Carbamidomethyl: 1
505	1	699.1373	103.36	3	35.9	10.8	2	1868-1885	R.IPPVAVRLQMSERNILSR.L	Oxidation: 10

Detailed Protein Report

Protein 217: PREDICTED: beta-enolase isoform X1 [Homo sapiens]

**Accession:**	gi|530410063	**Score:**	40.7
**Database:**	refseq_human(refseq_human_20140103.fasta)	**MW [kDa]:**	48.0
**Database Date:**	2015-11-30	**pI:**	9.1
**Modification(s):**	Oxidation	**Sequence Coverage [%]:**	10.8
		**No. of unique Peptides:**	1

10	20	30	40	50	60	70	80
MAVMRTLRAM	AMQKIFAREI	LDSRGNPTVE	VDLHTAKGRF	RAAVPSGAST	GIYEALELRD	GDKGRYLGKG	VLKAVENINN
**90**	**100**	**110**	**120**	**130**	**140**	**150**	**160**
TLGPALLQKK	LSVVDQEKVD	KFMIELDGTE	NKSKFGANAI	LGVSLAVCKA	GAAEKGVPLY	RHIADLAGNP	DLILPVPAFN
**170**	**180**	**190**	**200**	**210**	**220**	**230**	**240**
VINGGSHAGN	KLAMQEFMIL	PVGASSFKEA	MRIGAEVYHH	LKGVIKAKYG	KDATNVGDEG	GFAPNILENN	EALELLKTAI
**250**	**260**	**270**	**280**	**290**	**300**	**310**	**320**
QAAGYPDKVV	IGMDVAASEF	YRNGKYDLDF	KSPDDPARHI	TGEKLGELYK	SFIKNYPVVS	IEDPFDQDDW	ATWTSFLSGV
**330**	**340**	**350**	**360**	**370**	**380**	**390**	**400**
NIQIVGDDLT	VTNPKRIAQA	VEKKACNCLL	LKVNQIGSVT	ESIQACKLAQ	SNGWGVMVSH	RSGETEDTFI	ADLVVGLCTG
**410**	**420**	**430**	**440**	**450**			
QIKTGAPCRS	ERLAKYNQLM	RIEEALGDKA	IFAGRKFRNP	KAK			

Cmpd.	No. of Cmpds.	m/z meas.	Δ m/z [ppm]	z	Rt [min]	Score	P	Range	Sequence	Modification
2482	1	835.4239	2.22	2	61.6	10.7	2	1-14	-.MAVMRTLRAMAMQK.I	Oxidation: 4, 10

Detailed Protein Report

Protein 218: unconventional myosin-Ih [Homo sapiens]

Accession: gi|254028267
Database: refseq_human(refseq_human_20140103.fasta)
Database Date: 2015-11-30

Score: 40.6
MW [kDa]: 117.9
pI: 9.8
Sequence Coverage [%]: 4.4
No. of unique Peptides: 2

Quantitation

WUP:QUP **Median:** 6.53 **CV:** 0.00 % **No. of Peptides:** 1

10	20	30	40	50	60	70	80
MEGALTARDK	VGVQDFVLLD	AYTSESAFVD	NLRKRFSENL	IYTYIGTLLV	SVNPYQELGI	YTVSQMELYQ	GVNFFELPPH
90	100	110	120	130	140	150	160
VYAIADNAYR	MMCAELNNHF	ILISGESGAG	KTEASKKILE	YFAVTCPMTQ	SLQIARDRLL	FSNPVLEAFG	NARTLRNDNS
170	180	190	200	210	220	230	240
SRFGKYMDIQ	FDFQGIPVGG	HIISYLIEKS	RVVYQNEGER	NFHIFYQLLA	GGEEERLSYL	GLERDPQLYK	YLSQGHCAKE
250	260	270	280	290	300	310	320
SSISDKNDWK	TVSNAFSVID	FTEADLENLF	GIIASVLHLG	NIGFEEDDQG	CATIPDTHEI	KWIAKLLGVH	PSVLLEALTH
330	340	350	360	370	380	390	400
RKIEAKTEEV	ICPLTLELSV	YARDAMAKAV	YGRTFTWLVN	KINSSLVNKD	FTRKTVIGLL	DIYGFEVFDK	NGFEQFCINY
410	420	430	440	450	460	470	480
CNEKLQQLLI	ERTLKAEQAE	YEMEGIEWEP	IKYFNNKIIC	DLVEERHKGI	ISILDEECIR	PGPATDLSFL	EKLEEKVGKH
490	500	510	520	530	540	550	560
AHFETRKLAG	PKGRKRIGWM	EFRLLHYAGE	VTYCTKGFLE	KNNDLLYRHL	KEVLCKSKNI	ILRECFLLAE	LENRRRPPTV
570	580	590	600	610	620	630	640
GTQFKNSLSS	LLETLISKEP	SYIRCIKPND	RKEPSKFDDF	LIRHQIKYLG	LMEHLRVRRA	GFAYRRKYEH	FLQRYKSLCP
650	660	670	680	690	700	710	720
DTWPHWHGPP	AEGVERLIKY	IGYKPEEYKL	GKTKIFIRFP	RTLFATEDAF	EFSKHQLVAR	IQATYKRCLG	RREYVKKRQA
730	740	750	760	770	780	790	800
AIKLEAHWRG	ALARKAIQRR	KWAVRIIRKF	IKGFISRNKP	LCPDNEEFIV	FVRKNYILNL	RYHLPKTVLD	KSWLRPPGIL
810	820	830	840	850	860	870	880
ENASDLLRKM	CVRNLVQKYC	RGITAERKAM	MQQKVVTSEI	FRGRKDGYTE	SLNQPFVNSR	IDEGDINPKV	LQLISHEKIQ
890	900	910	920	930	940	950	960
YGVPVIKYDR	KGFKARQRQL	ILTQKAAYVV	ELAKIKQKIE	YSALKGVSTS	NLSDGILVIH	VSPEDSKQKG	DAVLQCGHVF
970	980	990	1000	1010	1020	1030	
EAVTKLVMLV	KKENIVNVVQ	GSLQFFISPG	KEGTIVFDTG	LEEQVYKNKN	GQLTVVSVRR	KS	

Cmpd.	No. of Cmpds.	m/z meas.	Δ m/z [ppm]	z	Rt [min]	Score	P	Range	Sequence	Modification	Ratios
354	1	1026.5219	53.51	2	34.4	10.0	0	416-432	K.AEQAEYEMEGIEWEPIK.Y		WUP:QUP 6.53
1186	1	863.9897	97.78	2	44.4	16.8	0	846-860	K.DGYTESLNQPFVNSR.I		

Detailed Protein Report

Protein 219: **PREDICTED: bromodomain adjacent to zinc finger domain protein 2A isoform X11 [Homo sapiens]**

**Accession:**	gi|578823227	**Score:**	40.5
**Database:**	refseq_human(refseq_human_20140103.fasta)	**MW [kDa]:**	170.8
**Database Date:**	2015-11-30	**pI:**	6.2
		**Sequence Coverage [%]:**	2.3
		**No. of unique Peptides:**	2

10	20	30	40	50	60	70	80
MEVACILHTR	GGVQKTGERR	EMEANDHFNF	TGLPPAPAAS	GLKPSPSSGE	GLYTNGSPMN	FPQQGKSLNG	DVNVNGLSTV
90	100	110	120	130	140	150	160
SHTTTSGILN	SAPHSSSTSH	LHHPSVAYDC	LWNYSQYPSA	NPGSNLKDPP	LLSQFSGGQY	PLNGILGGSR	QPSSPSHNTN
170	180	190	200	210	220	230	240
LRAGSQEFWA	NGTQSPMGLN	FDSQELYDSF	PDQNFEVMPN	GPPSFFTSPQ	TSPMLGSSIQ	TFAPSQEVGS	GIHPDEAAEK
250	260	270	280	290	300	310	320
EMTSVVAENG	TGLVGSLELE	EEQPELKMCG	YNGSVPSVES	LHQEVSVLVP	DPTVSCLDDP	SHLPDQLEDT	PILSEDSLEP
330	340	350	360	370	380	390	400
FNSLAPEPVS	GGLYGIDDTE	LMGAEDKLPL	EDSPVISALD	CPSLNNATAF	SLLADDSQTS	TSIFASPTSP	PVLGESVLQD
410	420	430	440	450	460	470	480
NSFDLNNGSD	AEQEEMETQS	SDFPPSLTQP	APDQSSTIQL	HPATSPAVSP	TTSPAVSLVV	SPAASPEISP	EVCPAASTVV
490	500	510	520	530	540	550	560
SPAVFSVVSP	ASSAVLPAVS	LEVPLTASVT	SPKASPVTSP	AAAFPTASPA	NKDVSSFLET	TADVEEITGE	GLTASGSGDV
570	580	590	600	610	620	630	640
MRRRIATPEE	VRLPLQHGWR	REVRIKKGSH	RWQGETWYYG	PCGKRMKQFP	EVIKYLSRNV	VHSVRREHFS	FSPRMPVGDF
650	660	670	680	690	700	710	720
FEERDTPEGL	QWVQLSAEEI	PSRIQAITGK	RGRPRNTEKA	KTKEVPKVKR	GRGRPPKVKI	TELLNKTDNR	PLKKLEAQET
730	740	750	760	770	780	790	800
LNEEDKAKIA	KSKKKMRQKV	QRGECQTTIQ	GQARNKRKQE	TKSLKQKEAK	KKSKAEKEKG	KTKQEKLKEK	VKREKKEKVK
810	820	830	840	850	860	870	880
MKEKEEVTKA	KPACKADKTL	ATQRRLEERQ	RQQMILEEMK	KPTEDMCLTD	HQPLPDFSRV	PGLTLPSGAF	SDCLTIVEFL
890	900	910	920	930	940	950	960
HSFGKVLGFD	PAKDVPSLGV	LQEGLLCQGD	SLGEVQDLLV	RLLKAALHDP	GFPSYCQSLK	ILGEKVSEIP	LTRDNVSEIL
970	980	990	1000	1010	1020	1030	1040
RCFLMAYGVE	PALCDRLRTQ	PFQAQPPQQK	AAVLAFLVHE	LNGSTLIINE	IDKTLESMSS	YRKNKWIVEG	RLRRLKTVLA
1050	1060	1070	1080	1090	1100	1110	1120
KRTGRSEVEM	EGPEECLGRR	RSSRIMEETS	GMEEEEEEES	IAAVPGRRGR	RDGEVDATAS	SIPELERQIE	KLSKRQLFFR
1130	1140	1150	1160	1170	1180	1190	1200
KKLLHSSQML	RAVSLGQDRY	RRRYWVLPYL	AGIFVEGTEG	NLVPEEVIKK	ETDSLKVAAH	ASLNPALFSM	KMELAGSNTT
1210	1220	1230	1240	1250	1260	1270	1280
ASSPARARGR	PRKTKPGSMQ	PRHLKSPVRG	QDSEQPQAQL	QPEAQLHAPA	QPQPQLQLQL	QSHKGFLEQE	GSPLSLGQSQ
1290	1300	1310	1320	1330	1340	1350	1360
HDLSQSAFLS	WLSQTQSHSS	LLSSSVLTPD	SSPGKLDPAP	SQPPEEPEPD	EAESSPDPQA	LWFNISAQMP	CNAAPTPPPA
1370	1380	1390	1400	1410	1420	1430	1440
VSEDQPTPSP	QQLASSKPMN	RPSAANPCSP	VQFSSTPLAG	LAPKRRAGDP	GEMPQSPTGL	GQPKRRGRPP	SKFFKQMEQR
1450	1460	1470	1480	1490	1500	1510	1520
YLTQLTAQPV	PPEMCSGWWW	IRDPEMLDAM	LKALHPRGIR	EKALHKHLNK	HRDFLQEVCL	RPSADPIFEP	RQLPAFQEGI
1530	1540	1550	1560				
MSWSPKEKTY	ETDLAVLQWV	EELEQRVIMS	DLQIR				

Cmpd.	No. of Cmpds.	m/z meas.	Δ m/z [ppm]	z	Rt [min]	Score	P	Range	Sequence	Modification
2577	1	941.5794	21.02	2	62.2	13.8	2	698-713	K.VKITELLNKTDNRPLK.K	
778	1	830.6256	153.36	1	39.7	11.3	0	700-706	K.ITELLNK.T	

Detailed Protein Report

Protein 220: **PREDICTED: plasma membrane calcium-transporting ATPase 3 isoform X5 [Homo sapiens]**

Accession: gi|530422822
Database: refseq_human(refseq_human_20140103.fasta)
Database Date: 2015-11-30
Modification(s): Oxidation

Score: 40.5
MW [kDa]: 127.0
pI: 5.6
Sequence Coverage [%]: 3.5
No. of unique Peptides: 2

10	20	30	40	50	60	70	80
MGDMANSSIE	FHPKPQQQRD	VPQAGGFGCT	LAELRTLMEL	RGAEALQKIE	EAYGDVSGLC	RRLKTSPTEG	LADNTNDLEK
**90**	**100**	**110**	**120**	**130**	**140**	**150**	**160**
RRQIYGQNFI	PPKQPKTFLQ	LVWEALQDVT	LIILEVAAIV	SLGLSFYAPP	GEESEACGNV	SGGAEDEGEA	EAGWIEGAAI
**170**	**180**	**190**	**200**	**210**	**220**	**230**	**240**
LLSVICVVLV	TAFNDWSKEK	QFRGLQSRIE	QEQKFTVIRN	GQLLQVPVAA	LVVGDIAQVK	YGDLLPADGV	LIQANDLKID
**250**	**260**	**270**	**280**	**290**	**300**	**310**	**320**
ESSLTGESDH	VRKSADKDPM	LLSGTHVMEG	SGRMVVTAVG	VNSQTGIIFT	LLGAGGEEEE	KKDKKAKKQD	GAVAMEMQPL
**330**	**340**	**350**	**360**	**370**	**380**	**390**	**400**
KSAEGGEMEE	REKKKANAPK	KEKSVLQGKL	TKLAVQIGKA	GLVMSAITVI	ILVLYFVIET	FVVEGRTWLA	ECTPVYVQYF
**410**	**420**	**430**	**440**	**450**	**460**	**470**	**480**
VKFFIIGVTV	LVVAVPEGLP	LAVTISLAYS	VKKMMKDNNL	VRHLDACETM	GNATAICSDK	TGTLTTNRMT	VVQSYLGDTH
**490**	**500**	**510**	**520**	**530**	**540**	**550**	**560**
YKEIPAPSAL	TPKILDLLVH	AISINSAYTT	KILPPEKEGA	LPRQVGNKTE	CALLGFVLDL	KRDFQPVREQ	IPEDKLYKVY
**570**	**580**	**590**	**600**	**610**	**620**	**630**	**640**
TFNSVRKSMS	TVIRMPDGGF	RLFSKGASEI	LLKKCTNILN	SNGELRGFRP	RDRDDMVRKI	IEPMACDGLR	TICIAYRDFS
**650**	**660**	**670**	**680**	**690**	**700**	**710**	**720**
AGQEPDWDNE	NEVVGDLTCI	AVVGIEDPVR	PEVPEAIRKC	QRAGITVRMV	TGDNINTARA	IAAKCGIIQP	GEDFLCLEGK
**730**	**740**	**750**	**760**	**770**	**780**	**790**	**800**
EFNRRIRNEK	GEIEQERLDK	VWPKLRVLAR	SSPTDKHTLV	KGIIDSTTGE	QRQVVAVTGD	GTNDGPALKK	ADVGFAMGIA
**810**	**820**	**830**	**840**	**850**	**860**	**870**	**880**
GTDVAKEASD	IILTDDNFTS	IVKAVMWGRN	VYDSISKFLQ	FQLTVNVVAV	IVAFTGACIT	QDSPLKAVQM	LWVNLIMDTF
**890**	**900**	**910**	**920**	**930**	**940**	**950**	**960**
ASLALATEPP	TESLLLRKPY	GRDKPLISRT	MMKNILGHAV	YQLAIIFTLL	FVGELFFDID	SGRNAPLHSP	PSEHYTIIFN
**970**	**980**	**990**	**1000**	**1010**	**1020**	**1030**	**1040**
TFVMMQLFNE	INARKIHGER	NVFDGIFSNP	IFCTIVLGTF	GIQIVIVQFG	GKPFSCSPLS	TEQWLWCLFV	GVGELVWGQV
**1050**	**1060**	**1070**	**1080**	**1090**	**1100**	**1110**	**1120**
IATIPTSQLK	CLKEAGHGPG	KDEMTDEELA	EGEEEIDHAE	RELRRGQILW	FRGLNRIQTQ	MEVVSTFKRS	GSVQGAVRRR
**1130**	**1140**	**1150**	**1160**				
SSVLSQLHDV	TNLSTPTHAI	LSAANPTSAA	GNPGGESVP				

Cmpd.	No. of Cmpds.	m/z meas.	Δ m/z [ppm]	z	Rt [min]	Score	P	Range	Sequence	Modification
2715	1	725.3796	55.65	2	65.1	12.1	0	309-321	K.QDGAVAMEMQPLK.S	Oxidation: 7, 9
70	1	877.8816	-104.57	2	30.6	11.7	1	757-772	K.HTLVKGIIDSTTGEQR.Q	

Detailed Protein Report

Protein 221: stAR-related lipid transfer protein 9 [Homo sapiens]

**Accession:**	gi|270265793	**Score:**	40.4
**Database:**	refseq_human(refseq_human_20140103.fasta)	**MW [kDa]:**	516.0
**Database Date:**	2015-11-30	**pI:**	5.9
**Modification(s):**	Carbamidomethyl	**Sequence Coverage [%]:**	0.8
		**No. of unique Peptides:**	1

Detailed Protein Report

10	20	30	40	50	60	70	80
MANVQVAVRV	RPLSKRETKE	GGRIIVEVDG	KVAKIRNLKV	DNRPDGFGDS	REKVMAFGFD	YCYWSVNPED	PQYASQDVVF
90	100	110	120	130	140	150	160
QDLGMEVLSG	VAKGYNICLF	AYGQTGSGKT	YTMLGTPASV	GLTPRICEGL	FVREKDCASL	PSSCRIKVSF	LEIYNERVRD
170	180	190	200	210	220	230	240
LLKQSGQKKS	YTLRVREHPE	MGPYVQGLSQ	HVVTNYKQVI	QLLEEGIANR	ITAATHVHEA	SSRSHAIFTI	HYTQAILENN
250	260	270	280	290	300	310	320
LPSEMASKIN	LVDLAGSERA	DPSYCKDRIA	EGANINKSLV	TLGIVISTLA	QNSQVFSSCQ	SLNSSVSNGG	DSGILSSPSG
330	340	350	360	370	380	390	400
TSSGGAPSRR	QSYIPYRDSV	LTWLLKDSLG	GNSKTIMVAT	VSPAHTSYSE	TMSTLRYASS	AKNIINKPRV	NEDANLKLIR
410	420	430	440	450	460	470	480
ELREEIERLK	ALLLSFELRN	FSSLSDENLK	ELVLQNELKI	DQLTKDWTQK	WNDWQALMEH	YSVDINRRRA	GVVIDSSLPH
490	500	510	520	530	540	550	560
LMALEDDVLS	TGVVLYHLKE	GTTKIGRIDS	DQEQDIVLQG	QWIERDHCTI	TSACGVVVLR	PARGARCTVN	GREVTASCRL
570	580	590	600	610	620	630	640
TQGAVITLGK	AQKFRFNHPA	EAAVLRQRRQ	VGEAAAGRGS	LEWLDLDGDL	AASRLGLSPL	LWKERRALEE	QCDEDHQTPR
650	660	670	680	690	700	710	720
DGETSHRAQI	QQQQSYVEDL	RHQILAEEIR	AAKELEFDQA	WISQQIKENQ	QCLLREETWL	ASLQQQQQED	QVAEKELEAS
730	740	750	760	770	780	790	800
VALDAWLQTD	PEIQPSPFVQ	SQKRVVHLQL	LRRHTLRAAE	RNVRRKKVSF	QLERIIKKQR	LLEAQKRLEK	LTTLCWLQDD
810	820	830	840	850	860	870	880
STQEPPYQVL	SPDATVPRPP	CRSKLTSCSS	LSPQRLCSKH	MPQLHSIFLS	WDPSTTLPPR	PDPTHQTSEK	TSSEEHLPQA
890	900	910	920	930	940	950	960
ASYPARTGCL	RKNGLHSSGH	GQPCTARAAL	ARKGASAPDA	CLTMSPNSVG	IQEMEMGVKQ	PHQMVSQGLA	SLRKSANKLK
970	980	990	1000	1010	1020	1030	1040
PRHEPKIFTS	TTQTRGAKGL	ADPSHTQAGW	RKEGNLGTHK	AAKGASCNSL	YPHGPRQTAG	HGKAVKTFWT	EYKPPSPSRA
1050	1060	1070	1080	1090	1100	1110	1120
SKRHQRVLAT	RVRNITKKSS	HLPLGSPLKR	QQNTRDPDTM	VPLTDFSPVM	DHSREKDNDL	SDTDSNYSLD	SLSCVYAKAL
1130	1140	1150	1160	1170	1180	1190	1200
IEPLKPEERK	WDFPEPENSE	SDDSQLSEDS	LAEKRYQSPK	NRLGGNRPTN	NRGQPRTRTR	ASVRGFTAAS	DSDLLAQTHR
1210	1220	1230	1240	1250	1260	1270	1280
SFSLDSLIDA	EEELGEDQQE	EPFPGSADEI	PTETFWHLED	SSLPVMDQEA	ICRLGPINYR	TAARLDAVLP	MSSSFYLDPQ
1290	1300	1310	1320	1330	1340	1350	1360
FQPHCELQPH	CELQPHCELQ	PHCEQAESQV	EPSYSEQADS	LQGMQLSRES	PLMSMDSWFS	CDSKINPSSP	PGIVGSLCPS
1370	1380	1390	1400	1410	1420	1430	1440
PDMQEFHSCK	GERPGYWPNT	EELKPSDAET	VLPYSSKLHQ	GSTELLCSAR	DEHTASAADT	SRLSLWGIQR	LIQPGADGTF
1450	1460	1470	1480	1490	1500	1510	1520
QGRCIPDMTQ	QGSSEASHNS	SVSNVLAASA	TTLTHVGSTH	ERDWSALQQK	YLLELSCPVL	EAIGAPKPAY	PYLEEDSGSL
1530	1540	1550	1560	1570	1580	1590	1600
AQASSKGGDT	LLPVGPRVSS	NLNLNNFPVH	LSRIRRLRAE	KEQDSLNAKL	EGVSDFFSTS	EKEASYDETY	SADLESLSAS
1610	1620	1630	1640	1650	1660	1670	1680
RSTNAQVFAT	ENAIPDSMTE	ACEVKQNNLE	ECLQSCRKPG	LMTSSDEDFF	QKNACHSNVT	TATKADHWSQ	GWAPLRKNSA
1690	1700	1710	1720	1730	1740	1750	1760
VQPGQLSPDS	HYPLEEEKTD	CQESSKEAVR	RHINVSFALP	SGPELYLHSA	PWNPLSSSLQ	PPLLETFYVT	KSRDALTETA
1770	1780	1790	1800	1810	1820	1830	1840
LEIPACREVR	VPSPPPREAW	GFGHNHQALQ	GAYLKNNLPV	LLQNQNSKIA	SSQQVTAEIP	VDLNTREVIR	ESGKCPGNIT
1850	1860	1870	1880	1890	1900	1910	1920
EESHDSVYSS	VTQNRHFLPS	TSTKVCEFEN	QVVILNKKHS	FPALEGGEVT	AQSCCGASSD	STESGKSLLF	RESEAREEEE
1930	1940	1950	1960	1970	1980	1990	2000
LDQNTVLRQT	INVSLEKDMP	GESAVSLKSR	SVDRRVSSPV	MVAQGGGPTP	KWEGKNETGL	LEKGLRPKDS	SEEFKLPGTK
2010	2020	2030	2040	2050	2060	2070	2080
PAYERFQLVA	CPQERNPSEC	KSQEMLNPNR	EPSGKKQNKR	VNNTDEMARL	IRSVMQLENG	ILEIESKQNK	QVHASHTPGT
2090	2100	2110	2120	2130	2140	2150	2160
DKELVFQDQK	EQEKTDHAFR	PDSSGNPLPS	KDQPSSPRQT	DDTVFRDSEA	GAMEVNSIGN	HPQVQKITPN	PFRSREGVRE
2170	2180	2190	2200	2210	2220	2230	2240
SEPVREHTHP	AGSDRPARDI	CDSLGKHTTC	REFTNTSLHP	QRMKALARAL	PLQPRLERSS	KNNGQFVKAS	ASLKGQPWGL
2250	2260	2270	2280	2290	2300	2310	2320
GSLEELETVK	GFQESQVAEH	VSSSNQEEPK	AQGKVEEMPM	QRGGSLQEEN	KVTQKFPSLS	QLCRDTFFRQ	ETVSPLLSRT
2330	2340	2350	2360	2370	2380	2390	2400

Detailed Protein Report

Cmpd.	No. of Cmpds.	m/z meas.	Δ m/z [ppm]	z	Rt [min]	Score	P	Range	Sequence	Modification
387	1	1294.0546	-62.74	2	34.8	12.0	2	136-157	K.DCASLPSSCRIKVSFLEIYNER.V	Carbamidomethyl: 2

Detailed Protein Report

Protein 222: MAP kinase-activating death domain protein isoform j [Homo sapiens]

Accession: gi|209863002
Database: refseq_human(refseq_human_20140103.fasta)
Database Date: 2015-11-30

Score: 40.4
MW [kDa]: 170.9
pI: 5.8
Sequence Coverage [%]: 2.5
No. of unique Peptides: 1

Alias proteins:

Accession	Name	Description
gi|530395946	refseq_human (refseq_human_20140103.fasta)	PREDICTED: MAP kinase-activating death domain protein isoform X18 [Homo sapiens]

10	20	30	40	50	60	70	80
MVQKKKFCPR	LLDYLVIVGA	RHPSSDSVAQ	TPELLRRYPL	EDHTEFPLPP	DVVFFCQPEG	CLSVRQRRMS	LRDDTSFVFT
90	100	110	120	130	140	150	160
LTDKDTGVTR	YGICVNFYRS	FQKRISKEKG	EGGAGSRGKE	GTHATCASEE	GGTESSESGS	SLQPLSADST	PDVNQSPRGK
170	180	190	200	210	220	230	240
RRAKAGSRSR	NSTLTSLCVL	SHYPFFSTFR	ECLYTLKRLV	DCCSERLLGK	KLGIPRGVQR	DTMWRIFTGS	LLVEEKSSAL
250	260	270	280	290	300	310	320
LHDLREIEAW	IYRLLRSPVP	VSGQKRVDIE	VLPQELQPAL	TFALPDPSRF	TLVDFPLHLP	LELLGVDACL	QVLTCILLEH
330	340	350	360	370	380	390	400
KVVLQSRDYN	ALSMSVMAFV	AMIYPLEYMF	PVIPLLPTCM	ASAEQLLLAP	TPYIIGVPAS	FFLYKLDFKM	PDDVWLVDLD
410	420	430	440	450	460	470	480
SNRVIAPTNA	EVLPILPEPE	SLELKKHLKQ	ALASMSLNTQ	PILNLEKFHE	GQEIPLLLGR	PSNDLQSTPS	TEFNPLIYGN
490	500	510	520	530	540	550	560
DVDSVDVATR	VAMVRFFNSA	NVLQGFQMHT	RTLRLFPRPV	VAFQAGSFLA	SRPRQTPFAE	KLARTQAVEY	FGEWILNPTN
570	580	590	600	610	620	630	640
YAFQRIHNNM	FDPALIGDKP	KWYAHQLQPI	HYRVYDSNSQ	LAEALSVPPE	RDSDSEPTDD	SGSDSMDYDD	SSSSYSSLGD
650	660	670	680	690	700	710	720
FVSEMMKCDI	NGDTPNVDPL	THAALGDASE	VEIDELQNQK	EAEEPGPDSE	NSQENPPLRS	SSSTTASSSP	STVIHGANSE
730	740	750	760	770	780	790	800
PADSTEMDDK	AAVGVSKPLP	SVPPSIGKSN	VDRRQAEIGE	GAQKLLRPNS	LRLASDSDAE	SDSRASSPNS	TVSNTSTEGF
810	820	830	840	850	860	870	880
GGIMSFASSL	YRNHSTSFSL	SNLTLPTKGA	REKATPFPSL	KGNRRALVDQ	KSSVIKHSPT	VKREPPSPQG	RSSNSSENQQ
890	900	910	920	930	940	950	960
FLKEVVHSVL	DGQGVGWLNM	KKVRRLLESE	QLRVFVLSKL	NRMVQSEDDA	RQDIIPDVEI	SRKVYKGMLD	LLKCTVLSLE
970	980	990	1000	1010	1020	1030	1040
QSYAHAGLGG	MASIFGLLEI	AQTHYYSKEP	DKRKRSPTES	VNTPVGKDPG	LAGRGDPKAM	AQLRVPQLGP	RAPSATGKGP
1050	1060	1070	1080	1090	1100	1110	1120
KELDTRSLKE	ENFIASIGPE	VIKPVFDLGE	TEEKKSQISA	DSGVSLTSSS	QRTDQDSVIG	VSPAVMIRSS	SQDSEVSNSS
1130	1140	1150	1160	1170	1180	1190	1200
GETLGADSDL	SSNAGDGPGG	EGSVHLASSR	GTLSDSEIET	NSATSTIFGK	AHSLKPSIKE	KLAGSPIRTS	EDVSQRVYLY
1210	1220	1230	1240	1250	1260	1270	1280
EGLLGKERST	LWDQMQFWED	AFLDAVMLER	EGMGMDQGPQ	EMIDRYLSLG	EHDRKRLEDD	EDRLLATLLH	NLISYMLLMK
1290	1300	1310	1320	1330	1340	1350	1360
VNKNDIRKKV	RRLMGKSHIG	LVYSQQINEV	LDQLANLNGR	DLSIWSSGSR	HMKKQTFVVH	AGTDTNGDIF	FMEVCDDCVV
1370	1380	1390	1400	1410	1420	1430	1440
LRSNIGTVYE	RWWYEKLINM	TYCPKTKVLC	LWRRNGSETQ	LNKFYTKKCR	ELYYCVKDSM	ERAAARQQSI	KPGPELGGEF
1450	1460	1470	1480	1490	1500	1510	1520
PVQDLKTGEG	GLLQVTLEGI	NLKFMHNQVF	IELNHIKKCN	TVRGVFVLEE	FVPEIKEVVS	HKYKTPMAHE	ICYSVLCLFS
1530	1540	1550					
YVAAVHSSEE	DLRTPPRPVS	S					

Cmpd.	No. of Cmpds.	m/z meas.	Δ m/z [ppm]	z	Rt [min]	Score	P	Range	Sequence	Modification
974	1	547.7654	-198.26	2	40.5	10.2	2	207-216	R.LLGKKLGIPR.G	

Detailed Protein Report

Protein 223: multicilin [Homo sapiens]

**Accession:**	gi|299782571	**Score:**	40.4
**Database:**	refseq_human(refseq_human_20140103.fasta)	**MW [kDa]:**	41.7
**Database Date:**	2015-11-30	**pI:**	5.5
**Modification(s):**	Carbamidomethyl, Oxidation	**Sequence Coverage [%]:**	6.5
		**No. of unique Peptides:**	2

10	20	30	40	50	60	70	80
MQACGGGAAG	RRAFDSICPN	RMLALPGRAL	LCKPGKPERK	FAPPRKFFPG	CTGGSPVSVY	EDPPDAEPTA	LPALTTIDLQ
**90**	**100**	**110**	**120**	**130**	**140**	**150**	**160**
DLADCSSLLG	SDAPPGGDLA	ASQNHSHQTE	ADFNLQDFRD	TVDDLISDSS	SMMSPTLASG	DFPFSPCDIS	PFGPCLSPPL
**170**	**180**	**190**	**200**	**210**	**220**	**230**	**240**
DPRALQSPPL	RPPDVPPPEQ	YWKEVADQNQ	RALGDALVEN	NQLHVTLTQK	QEEIASLKER	NVQLKELASR	TRHLASVLDK
**250**	**260**	**270**	**280**	**290**	**300**	**310**	**320**
LMITQSRDCG	AAAEPFLLKA	KAKRSLEELV	SAAGQDCAEV	DAILREISER	CDEALQSRDP	KRPRLLPEPA	NTDTRPGNLH
**330**	**340**	**350**	**360**	**370**	**380**	**390**	
GAFRGLRTDC	SRSALNLSHS	ELEEGGSFST	RIRSHSTIRT	LAFPQGNAFT	IRTANGGYKF	RWVPS	

Cmpd.	No. of Cmpds.	m/z meas.	Δ m/z [ppm]	z	Rt [min]	Score	P	Range	Sequence	Modification
809	1	526.2922	129.61	2	38.5	29.2	0	1-11	-.MQACGGGAAGR.R	Carbamidomethyl: 4; Oxidation: 1
1958	1	745.7963	-121.94	2	54.9	11.2	1	248-261	R.DCGAAAEPFLLKAK.A	Carbamidomethyl: 2

Detailed Protein Report

**Protein 224:**	**PREDICTED: neuroblast differentiation-associated protein AHNAK isoform X9 [Homo sapiens]**		
**Accession:**	gi|578821730	**Score:**	40.4
**Database:**	refseq_human(refseq_human_20140103.fasta)	**MW [kDa]:**	423.5
**Database Date:**	2015-11-30	**pI:**	5.9
		**Sequence Coverage [%]:**	1.1
		**No. of unique Peptides:**	2

Detailed Protein Report

10	20	30	40	50	60	70	80
MEKEETTREL	LLPNWQGSGS	HGLTIAQRDD	GVFVQEVTQN	SPAARTGVVK	EGDQIVGATI	YFDNLQSGEV	TQLLNTMGHH
90	100	110	120	130	140	150	160
TVGLKLHRKG	DRSPEPGQTW	TREVFSSCSS	EVVLSGDDEE	YQRIYTTKIK	PRLKSEDGVE	GDLGETQSRT	ITVTRRVTAY
170	180	190	200	210	220	230	240
TVDVTGREGA	KDIDISSPEF	KIKIPRHELT	EISNVDVETQ	SGKTVIRLPS	GSGAASPTGS	AVDIRAGAIS	ASGPELQGAG
250	260	270	280	290	300	310	320
HSKLQVTMPG	IKVGGSGVNV	NAKGLDLGGR	GGVQVPAVDI	SSSLGGRAVE	VQGPSLESGD	HGKIKFPTMK	VPKFGVSTGR
330	340	350	360	370	380	390	400
EGQTPKAGLR	VSAPEVSVGH	KGGKPGLTIQ	APQLEVSVPS	ANIEGLEGKL	KGPQITGPSL	EGDLGLKGAK	PQGHIGVDAS
410	420	430	440	450	460	470	480
APQIGGSITG	PSVEVQAPDI	DVQGPGSKLN	VPKMKVPKFS	VSGAKGEETG	IDVTLPTGEV	TVPGVSGDVS	LPEIATGGLE
490	500	510	520	530	540	550	560
GKMKGTKVKT	PEMIIQKPKI	SMQDVDLSLG	SPKLKGDIKV	SAPGVQGDVK	GPQVALKGSR	VDIETPNLEG	TLTGPRLGSP
570	580	590	600	610	620	630	640
SGKTGTCRIS	MSEVDLNVAA	PKVKGGVDVT	LPRVEGKVKV	PEVDVRGPKV	DVSAPDVEAH	GPEWNLKMPK	MKMPTFSTPG
650	660	670	680	690	700	710	720
AKGEGPDVHM	TLPKGDISIS	GPKVNVEAPD	VNLEGLGGKL	KGPDVKLPDM	SVKTPKISMP	DVDLHVKGTK	VKGEYDVTVP
730	740	750	760	770	780	790	800
KLEGELKGPK	VDIDAPDVDV	HGPDWHLKMP	KMKMPKFSVP	GFKAEGPEVD	VNLPKADIDV	SGPKVDIEGP	DVNIEGPEGK
810	820	830	840	850	860	870	880
LKGPKFKMPE	MNIKAPKISM	PDIDLNLKGP	KVKGDVDVSL	PKVEGDLKGP	EVDIKGPKVD	IDAPDVDVHG	PDWHLKMPKI
890	900	910	920	930	940	950	960
KMPKISMPGF	KGEGPDVDVN	LPKADIDVSG	PKVDVECPDV	NIEGPEGKWK	SPKFKMPEMH	FKTPKISMPD	IDLNLTGPKI
970	980	990	1000	1010	1020	1030	1040
KGDVDVTGPK	VEGDLKGPEV	DLKGPKVDID	VPDVNVQGPD	WHLKMPKMKM	PKFSMPGFKA	EGPEVDVNLP	KADVDVSGPK
1050	1060	1070	1080	1090	1100	1110	1120
VDVEGPDVNI	EGPEGKLKGP	KFKMPEMNIK	APKIPMPDFD	LHLKGPKVKG	DVDISLPKVE	GDLKGPEVDI	RGPQVDIDVP
1130	1140	1150	1160	1170	1180	1190	1200
DVGVQGPDWH	LKMPKVKMPK	FSMPGFKGEG	PDVDVNLPKA	DLDVSGPKVD	IDVPDVNIEG	PEGKLKGPKF	KMPEMNIKAP
1210	1220	1230	1240	1250	1260	1270	1280
KISMPDIDLN	LKGPKVKGDM	DVSLPKVEGD	MKVPDVDIKG	PKVDINAPDV	DVQGPDWHLK	MPKIKMPKIS	MPGFKGEGPE
1290	1300	1310	1320	1330	1340	1350	1360
VDVNLPKADL	DVSGPKVDVD	VPDVNIEGPD	AKLKGPKFKM	PEMNIKAPKI	SMPDLDLNLK	GPKMKGEVDV	SLANVEGDLK
1370	1380	1390	1400	1410	1420	1430	1440
GPALDIKGPK	IDVDAPDIDI	HGPDAKLKGP	KLKMPDMHVN	MPKISMPEID	LNLKGSKLKG	DVDVSGPKLE	GDIKAPSLDI
1450	1460	1470	1480	1490	1500	1510	1520
KGPEVDVSGP	KLNIEGKSKK	SRFKLPKFNF	SGSKVQTPEV	DVKGKKPDID	ITGPKVDINA	PDVEVQGKVK	GSKFKMPFLS
1530	1540	1550	1560	1570	1580	1590	1600
ISSPKVSMPD	VELNLKSPKV	KGDLDIAGPN	LEGDFKGPKV	DIKAPEVNLN	APDVDVHGPD	WNLKMPKMKM	PKFSVSGLKA
1610	1620	1630	1640	1650	1660	1670	1680
EGPDVAVDLP	KGDINIEGPS	MNIEGPDLNV	EGPEGGLKGP	KFKMPDMNIK	APKISMPDID	LNLKGPKVKG	DVDISLPKLE
1690	1700	1710	1720	1730	1740	1750	1760
GDLKGPEVDI	KGPKVDINAP	DVDVHGPDWH	LKMPKVKMPK	FSMPGFKGEG	PEVDVTLPKA	DIDISGPNVD	VDVPDVNIEG
1770	1780	1790	1800	1810	1820	1830	1840
PDAKLKGPKF	KMPEMNIKAP	KISMPDFDLN	LKGPKMKGDV	VVSLPKVEGD	LKGPEVDIKG	PKVDIDTPDI	NIEGSEGKFK
1850	1860	1870	1880	1890	1900	1910	1920
GPKFKIPEMH	LKAPKISMPD	IDLNLKGPKV	KGDVDVSLPK	MEGDLKGPEV	DIKGPKVDIN	APDVDVQGPD	WHLKMPKVKM
1930	1940	1950	1960	1970	1980	1990	2000
PKFSMPGFKG	EGPDVDVNLP	KADLDVSGPK	VDIDVPDVNI	EGPEGKLKGP	KFKMPEMNIK	APKISMPDID	LNLKGPKVKG
2010	2020	2030	2040	2050	2060	2070	2080
DMDVSLPKVE	GDMQVPDLDI	KGPKVDINAP	DVDVRGPDWH	LKMPKIKMPK	ISMPGFKGEG	PEVDVNLPKA	DLDVSGPKVD
2090	2100	2110	2120	2130	2140	2150	2160
VDVPDVNIEG	PDAKLKGPKF	KMPEMNIKAP	KISMPDFDLH	LKGPKVKGDV	DVSLPKMEGD	LKAPEVDIKG	PKVDIDAPDV
2170	2180	2190	2200	2210	2220	2230	2240

Detailed Protein Report

Cmpd.	No. of Cmpds.	m/z meas.	Δ m/z [ppm]	z	Rt [min]	Score	P	Range	Sequence	Modification
92	1	726.3490	-51.60	3	31.1	15.9	2	1041-1061	K.VDVEGPDVNIEGPEGKLKGPK.F	
1546	1	506.2878	21.02	2	47.6	11.8	1	2863-2872	K.GPEADIKGPK.V	

Detailed Protein Report

Protein 225: catenin alpha-1 [Homo sapiens]

**Accession:**	gi|55770844	**Score:**	40.3
**Database:**	refseq_human(refseq_human_20140103.fasta)	**MW [kDa]:**	100.0
**Database Date:**	2015-11-30	**pI:**	5.9
**Modification(s):**	Carbamidomethyl, Oxidation	**Sequence Coverage [%]:**	4.2
		**No. of unique Peptides:**	3

Alias proteins:

Accession	Name	Description
gi|578810148	refseq_human (refseq_human_20140103.fasta)	PREDICTED: catenin alpha-1 isoform X4 [Homo sapiens]

10	20	30	40	50	60	70	80
MTAVHAGNIN	FKWDPKSLEI	RTLAVERLLE	PLVTQVTTLV	NTNSKGPSNK	KRGRSKKAHV	LAASVEQATE	NFLEKGDKIA
90	100	110	120	130	140	150	160
KESQFLKEEL	VAAVEDVRKQ	GDLMKAAAGE	FADDPCSSVK	RGNMVRAARA	LLSAVTRLLI	LADMADVYKL	LVQLKVVEDG
170	180	190	200	210	220	230	240
ILKLRNAGNE	QDLGIQYKAL	KPEVDKLNIM	AAKRQQELKD	VGHRDQMAAA	RGILQKNVPI	LYTASQACLQ	HPDVAAYKAN
250	260	270	280	290	300	310	320
RDLIYKQLQQ	AVTGISNAAQ	ATASDDASQH	QGGGGGELAY	ALNNFDKQII	VDPLSFSEER	FRPSLEERLE	SIISGAALMA
330	340	350	360	370	380	390	400
DSSCTRDDRR	ERIVAECNAV	RQALQDLLSE	YMGNAGRKER	SDALNSAIDK	MTKKTRDLRR	QLRKAVMDHV	SDSFLETNVP
410	420	430	440	450	460	470	480
LLVLIEAAKN	GNEKEVKEYA	QVFREHANKL	IEVANLACSI	SNNEEGVKLV	RMSASQLEAL	CPQVINAALA	LAAKPQSKLA
490	500	510	520	530	540	550	560
QENMDLFKEQ	WEKQVRVLTD	AVDDITSIDD	FLAVSENHIL	EDVNKCVIAL	QEKDVDGLDR	TAGAIRGRAA	RVIHVVTSEM
570	580	590	600	610	620	630	640
DNYEPGVYTE	KVLEATKLLS	NTVMPRFTEQ	VEAAVEALSS	DPAQPMDENE	FIDASRLVYD	GIRDIRKAVL	MIRTPEELDD
650	660	670	680	690	700	710	720
SDFETEDFDV	RSRTSVQTED	DQLIAGQSAR	AIMAQLPQEQ	KAKIAEQVAS	FQEEKSKLDA	EVSKWDDSGN	DIIVLAKQMC
730	740	750	760	770	780	790	800
MIMMEMTDFT	RGKGPLKNTS	DVISAAKKIA	EAGSRMDKLG	RTIADHCPDS	ACKQDLLAYL	QRIALYCHQL	NICSKVKAEV
810	820	830	840	850	860	870	880
QNLGGELVVS	GVDSAMSLIQ	AAKNLMNAVV	QTVKASYVAS	TKYQKSQGMA	SLNLPAVSWK	MKAPEKKPLV	KREKQDETQT
890	900	910					
KIKRASQKKH	VNPVQALSEF	KAMDSI					

Cmpd.	No. of Cmpds.	m/z meas.	Δ m/z [ppm]	z	Rt [min]	Score	P	Range	Sequence	Modification
2291	2	682.7592	-174.39	2	59.1	12.9	0	138-149	R.LLILADMADVYK.L	
2280	1	920.8411	-26.04	2	58.9	13.1	0	718-731	K.QMCMIMMEMTDFTR.G	Carbamidomethyl: 3; Oxidation: 2
2652	3	659.3500	103.51	2	61.5	14.3	0	762-773	R.TIADHCPDSACK.Q	Carbamidomethyl: 11

Detailed Protein Report

Protein 226: PREDICTED: diacylglycerol kinase alpha isoform X2 [Homo sapiens]

**Accession:**	gi|530399854	**Score:**	40.3
**Database:**	refseq_human(refseq_human_20140103.fasta)	**MW [kDa]:**	69.9
**Database Date:**	2015-11-30	**pI:**	9.3
**Modification(s):**	Carbamidomethyl	**Sequence Coverage [%]:**	3.9
		**No. of unique Peptides:**	1

Alias proteins:

Accession	Name	Description
gi|530399856	refseq_human (refseq_human_20140103.fasta)	PREDICTED: diacylglycerol kinase alpha isoform X3 [Homo sapiens]

10	20	30	40	50	60	70	80
MVLRNRRGWG	ERLSPLGLNL	KEVDKIILQM	MRVAEYLDWD	VSELRPILQE	MMKEIDYDGS	GSVSQAEWVR	AGATTVPLLV
**90**	**100**	**110**	**120**	**130**	**140**	**150**	**160**
LLGLEMTLKD	DGQHMWRPKR	FPRPVYCNLC	ESSIGLGKQG	LSCNLCKYTV	HDQCAMKALP	CEVSTYAKSR	KDIGVQSHVW
**170**	**180**	**190**	**200**	**210**	**220**	**230**	**240**
VRGGCESGRC	DRCQKKIRIY	HSLTGLHCVW	CHLEIHDDCL	QAVGHECDCG	LLRDHILPPS	SIYPSVLASG	PDRKNSKTSQ
**250**	**260**	**270**	**280**	**290**	**300**	**310**	**320**
KTMDDLNLST	SEALRIDPVP	NTHPLLVFVN	PKSGGKQGQR	VLWKFQYILN	PRQVFNLLKD	GPEIGLRLFK	DVPDSRILVC
**330**	**340**	**350**	**360**	**370**	**380**	**390**	**400**
GGDGTVGWIL	ETIDKANLPV	LPPVAVLPLG	TGNDLARCLR	WGGGYEGQNL	AKILKDLEMS	KVVHMDRWSV	EVIPQQTEEK
**410**	**420**	**430**	**440**	**450**	**460**	**470**	**480**
SDPVPFQIIN	NYFSIGVDAS	IAHRFHIMRE	KYPEKFNSRM	KNKLWYFEFA	TSESIFSTCK	KLEESLTVEI	CGKPLDLSNL
**490**	**500**	**510**	**520**	**530**	**540**	**550**	**560**
SLEGIAVLNI	PSMHGGSNLW	GDTRRPHGDI	YGINQALGAT	AKVITDPDIL	KTCVPDLSDK	RLEVVGLEGA	IEMGQIYTKL
**570**	**580**	**590**	**600**	**610**	**620**	**630**	
KNAGRRLAKC	SEITFHTTKT	LPMQIDGEPW	MQTPCTIKIT	HKNQMPMLMG	PPPRSTNFFG	FLS	

Cmpd.	No. of Cmpds.	m/z meas.	Δ m/z [ppm]	z	Rt [min]	Score	P	Range	Sequence	Modification
1847	1	577.2815	93.89	2	52.9	16.5	1	163-172	R.GGCESGRCDR.C	Carbamidomethyl: 3, 8

Detailed Protein Report

Protein 227: PREDICTED: thyroglobulin isoform X9 [Homo sapiens]

**Accession:**	gi|530389281	**Score:**	40.2
**Database:**	refseq_human(refseq_human_20140103.fasta)	**MW [kDa]:**	232.1
**Database Date:**	2015-11-30	**pI:**	5.1
**Modification(s):**	Carbamidomethyl	**Sequence Coverage [%]:**	0.9
		**No. of unique Peptides:**	2

Quantitation

QU:MU	**Median:** 0.32	**CV:** 0.00 %	**No. of Peptides:** 1

BRUKER

Detailed Protein Report

10	20	30	40	50	60	70	80
MALVLEIFTL	LASICWVSAN	IFEYQVDAQP	LRPCELQRET	AFLKQADYVP	QCAEDGSFQT	VQCQNDGRSC	WCVGANGSEV
90	100	110	120	130	140	150	160
LGSRQPGRPV	ACLSFCQLQK	QQILLSGYIN	STDTSYLPQC	QDSGDYAPVQ	CDVQQVQCWC	VDAEGMEVYG	TRQLGRPKRC
170	180	190	200	210	220	230	240
PRSCEIRNRR	LLHGVGDKSP	PQCSAEGEFM	PVQCKFVNTT	DMMIFDLVHS	YNRFPDAFVT	FSSFQRRFPE	VSGYCHCADS
250	260	270	280	290	300	310	320
QGRELAETGL	ELLLDEIYDT	IFAGLDLPST	FTETTLYRIL	QRRFLAVQSV	ISGRFRCPTK	CEVERFTATS	FGHPYVPSCR
330	340	350	360	370	380	390	400
RNGDYQAVQC	QTEGPCWCVD	AQGKEMHGTR	QQGEPPSCAE	GQSCASERQQ	ALSRLYFGTS	GYFSQHDLFS	SPEKRWASPR
410	420	430	440	450	460	470	480
VARFATSCPP	TIKELFVDSG	LLRPMVEGQS	QQFSVSENLL	KEAIRAIFPS	RGLARLALQF	TTNPKRLQQN	LFGGKFLVNV
490	500	510	520	530	540	550	560
GQFNLSGALG	TRGTFNFSQF	FQQLGLASFL	NGGRQEDLAK	PLSVGLDSNS	STGTPEAAKK	DGTMNKPTVG	SFGFEINLQE
570	580	590	600	610	620	630	640
NQNALKFLAS	LLELPEFLLF	LQHAISVPED	VARDLGDVME	TVLSSQTCEQ	TPERLFVPSC	TTEGSYEDVQ	CFSGECWCVN
650	660	670	680	690	700	710	720
SWGKELPGSR	VRGGQPRCPT	DCEKQRARMQ	SLMGSQPAGS	TLFVPACTSE	GHFLPVQCFN	SECYCVDAEG	QAIPGTRSAI
730	740	750	760	770	780	790	800
GKPKKCPTPC	QLQSEQAFLR	TVQALLSNSS	MLPTLSDTYI	PQCSTDGQWR	QVQCNGPPEQ	VFELYQRWEA	QNKGQDLTPA
810	820	830	840	850	860	870	880
KLLVKIMSYR	EAASGNFSLF	IQSLYEAGQQ	DVFPVLSQYP	SLQDVPLAAL	EGKRPQPREN	ILLEPYLFWQ	ILNGQLSQYP
890	900	910	920	930	940	950	960
GSYSDFSTPL	AHFDLRNCWC	VDEAGQELEG	MRSEPSKLPT	CPGSCEEAKL	RVLQFIRETE	EIVSASNSSR	FPLGESFLVA
970	980	990	1000	1010	1020	1030	1040
KGIRLRNEDL	GLPPLFPPRE	AFAEQFLRGS	DYAIRLAAQS	TLSFYQRRRF	SPDDSAGASA	LLRSGPYMPQ	CDAFGSWEPV
1050	1060	1070	1080	1090	1100	1110	1120
QCHAGTGHCW	CVDEKGGFIP	GSLTARSLQI	PQCPTTCEKS	RTSGLLSSWK	QARSQENPSP	KDLFVPACLE	TGEYARLQAS
1130	1140	1150	1160	1170	1180	1190	1200
GAGTWCVDPA	SGEELRPGSS	SSAQCPSLCN	VLKSGVLSRR	VSPGYVPACR	AEDGGFSPVQ	CDQAQGSCWC	VMDSGEEVPG
1210	1220	1230	1240	1250	1260	1270	1280
TRVTGGQPAC	ESPRCPLPFN	ASEVVGGTIL	CETISGPTGS	AMQQCQLLCR	QGSWSVFPPG	PLICSLESGR	WESQLPQPRA
1290	1300	1310	1320	1330	1340	1350	1360
CQRPQLWQTI	QTQGHFQLQL	PPGKMCSADY	ADLLQTFQVF	ILDELTARGF	CQIQVKTFGT	LVSIPVCNNS	SVQVGCLTRE
1370	1380	1390	1400	1410	1420	1430	1440
RLGVNVTWKS	RLEDIPVASL	PDLHDIERAL	VGKDLLGRFT	DLIQSGSFQL	HLDSKTFPAE	TIRFLQGDHF	GTSPRTWFGC
1450	1460	1470	1480	1490	1500	1510	1520
SEGFYQVLTS	EASQDGLGCV	KCPEGSYSQD	EECIPCPVGF	YQEQAGSLAC	VPCPVGRTTI	SAGAFSQTHC	VTDCQRNEAG
1530	1540	1550	1560	1570	1580	1590	1600
LQCDQNGQYR	ASQKDRGSGK	AFCVDGEGRR	LPWWETEAPL	EDSQCLMMQK	FEKVPESKVI	FDANAPVAVR	SKVPDSEFPV
1610	1620	1630	1640	1650	1660	1670	1680
MQCLTDCTED	EACSFFTVST	TEPEISCDFY	AWTSDNVACM	TSDQKRDALG	NSKATSFGSL	RCQVKVRSHG	QDSPAVYLKK
1690	1700	1710	1720	1730	1740	1750	1760
GQGSTTTLQK	RFEPTGFQNM	LSGLYNPIVF	SASGANLTDA	HLFCLLACDR	DLCCDGFVLT	QVQGGAIICG	LLSSPSVLLC
1770	1780	1790	1800	1810	1820	1830	1840
NVKDWMDPSE	AWANATCPGV	TYDQESHQVI	LRLGDQEFIK	SLTPLEGTQD	TFTNFQQVYL	WKDSDMGSRP	ESMGCRKDTV
1850	1860	1870	1880	1890	1900	1910	1920
PRPASPTEAG	LTTELFSPVD	LNQVIVNGNQ	SLSSQKHWLF	KHLFSAQQAN	LWCLSRCVQE	HSFCQLAEIT	ESASLYFTCT
1930	1940	1950	1960	1970	1980	1990	2000
LYPEAQVCDD	IMESNAQGCR	LILPQMPKAL	FRKKVILEDK	VKNFYTRLPF	QKLMGISIRN	KVPMSEKSIS	NGFFECERRC
2010	2020	2030	2040	2050	2060	2070	2080
DADPCCTGFG	FLNVSQLKGG	EVTCLTLNSL	GIQMCSEENG	GAWRILDCGS	PDIEVHTYPF	GWYQKPIAQN	NAPSFCPLVV
2090	2100	2110					
LPSLTEKVFK	VREVRGTQLK	RME					

Detailed Protein Report

Cmpd.	No. of Cmpds.	m/z meas.	Δ m/z [ppm]	z	Rt [min]	Score	P	Range	Sequence	Modification	Ratios
2716	3	645.8279	25.63	2	64.3	20.3	2	160-169	R.CPRSCEIRNR.R	Carbamidomethyl: 1	
1580	1	502.6699	-218.64	2	50.0	19.9	0	467-475	R.LQQNLFGGK.F		QU:MU 0.32

Detailed Protein Report

Protein 228: pleckstrin homology domain-containing family A member 4 isoform 2 [Homo sapiens]

**Accession:**	gi|238859653	**Score:**	40.1
**Database:**	refseq_human(refseq_human_20140103.fasta)	**MW [kDa]:**	64.2
**Database Date:**	2015-11-30	**pI:**	10.7
		**Sequence Coverage [%]:**	5.3
		**No. of unique Peptides:**	2

Quantitation

QU:MU	**Median:**	0.67	**CV:**	0.00 %	**No. of Peptides:**	1
WUP:QUP	**Median:**	1.21	**CV:**	0.00 %	**No. of Peptides:**	1

```
        10         20         30         40         50         60         70         80
MEGSRPRSSL SLASSASTIS SLSSLSPKKP TRAVNKIHAF GKRGNALRRD PNLPVHIRGW LHKQDSSGLR LWKRRWFVLS
        90        100        110        120        130        140        150        160
GHCLFYYKDS REESVLGSVL LPSYNIRPDG PGAPRGRRFT FTAEHPGMRT YVLAADTLED LRGWLRALGR ASRAEGDDYG
       170        180        190        200        210        220        230        240
QPRSPARPQP GEGPGGPGGP PEVSRGEEGR ISESPEVTRL SRGRGRPRLL TPSPTTDLHS GLQMRRARSP DLFTPLSRPP
       250        260        270        280        290        300        310        320
SPLSLPRPRS APARRPPAPS GDTAPPARPH TPLSRIDVRP PLDWGPQRQT LSRPPTPRRG PPSEAGGGKP PRSPQHWSQE
       330        340        350        360        370        380        390        400
PRTQPGPPLE STFHQSLETD TLLTKLCGQD RLLRRLQEEI DQKQEEKEQL EAALELTRQQ LGQATREAGA PGRAWGRQRL
       410        420        430        440        450        460        470        480
LQDRLVSVRA TLCHLTQERE RVWDTYSGLE QELGTLRETL EYLLHLGSPQ DRVSAQQQLW MVEDTLAGLG GPQKPPPHTE
       490        500        510        520        530        540        550        560
PDSPSPVLQG EESSERESLP ESLELSSPRS PETDWGRPPG GDKDLASPHL GLGSPRVSRA SSPEGRHLPS PQLGTKSKEH
       570        580        590
HPLLADFRRS PGAGSQPLPS PGY
```

Cmpd.	No. of Cmpds.	m/z meas.	Δ m/z [ppm]	z	Rt [min]	Score	P	Range	Sequence	Modification	Ratios
489	3	603.8256	17.71	2	36.1	13.9	0	300-312	R.GPPSEAGGGKPPR.S		WUP:QUP 1.21<br>QU:MU 0.67
20	1	706.3988	42.59	2	29.2	15.0	2	387-399	R.EAGAPGRAWGRQR.L		

Detailed Protein Report

Protein 229: PREDICTED: nipped-B-like protein isoform X4 [Homo sapiens]

**Accession:**	gi|530378766	**Score:**	40.1
**Database:**	refseq_human(refseq_human_20140103.fasta)	**MW [kDa]:**	257.1
**Database Date:**	2015-11-30	**pI:**	9.1
**Modification(s):**	Oxidation	**Sequence Coverage [%]:**	2.0
		**No. of unique Peptides:**	2

Detailed Protein Report

10	20	30	40	50	60	70	80
MVSIDLHQAG	RVDSQASITQ	DSDSIKKPEE	IKQCNDAPVS	VLQEDIVGSL	KSTPENHPET	PKKKSDPELS	KSEMKQSESR
90	100	110	120	130	140	150	160
LAESKPNENR	LVETKSSENK	LETKVETQTE	ELKQNESRTT	ECKQNESTIV	EPKQNENRLS	DTKPNDNKQN	NGRSETTKSR
170	180	190	200	210	220	230	240
PETPKQKGES	RPETPKQKSD	GHPETPKQKG	DGRPETPKQK	GESRPETPKQ	KNEGRPETPK	HRHDNRRDSG	KPSTEKKPEV
250	260	270	280	290	300	310	320
SKHKQDTKSD	SPRLKSERAE	ALKQRPDGRS	VSESLRRDHD	NKQKSDDRGE	SERHRGDQSR	VRRPETLRSS	SRNEHGIKSD
330	340	350	360	370	380	390	400
SSKTDKLERK	HRHESGDSRE	RPSSGEQKSR	PDSPRVKQGD	SNKSRSDKLG	FKSPTSKDDK	RTEGNKSKVD	TNKAHPDNKA
410	420	430	440	450	460	470	480
EFPSYLLGGR	SGALKNFVIP	KIKRDKDGNV	TQETKKMEMK	GEPKDKVEKI	GLVEDLNKGA	KPVVVLQKLS	LDDVQKLIKD
490	500	510	520	530	540	550	560
REDKSRSSLK	PIKNKPSKSN	KGSIDQSVLK	ELPPELLAEI	ESTMPLCERV	KMNKRKRSTV	NEKPKYAEIS	SDEDNDSDEA
570	580	590	600	610	620	630	640
FESSRKRHKK	DDDKAWEYEE	RDRRSSGDHR	RSGHSHEGRR	SSGGGRYRNR	SPSDSDMEDY	SPPPSLSEVA	RKMKKKEKQK
650	660	670	680	690	700	710	720
KRKAYEPKLT	PEEMMDSSTF	KRFTASIENI	LDNLEDMDFT	AFGDDDEIPQ	ELLLGKHQLN	ELGSESAKIK	AMGIMDKLST
730	740	750	760	770	780	790	800
DKTVKVLNIL	EKNIQDGSKL	STLLNHNNDT	EEEERLWRDL	IMERVTKSAD	ACLTTINIMT	SPNMPKAVYI	EDVIERVIQY
810	820	830	840	850	860	870	880
TKFHLQNTLY	PQYDPVYRLD	PHGGGLLSSK	AKRAKCSTHK	QRVIVMLYNK	VCDIVSSLSE	LLEIQLLTDT	TILQVSSMGI
890	900	910	920	930	940	950	960
TPFFVENVSE	LQLCAIKLVT	AVFSRYEKHR	QLILEEIFTS	LARLPTSKRS	LRNFRLNSSD	MDGEPMYIQM	VTALVLQLIQ
970	980	990	1000	1010	1020	1030	1040
CVVHLPSSEK	DSNAEEDSNK	KIDQDVVITN	SYETAMRTAQ	NFLSIFLKKC	GSKQGEEDYR	PLFENFVQDL	LSTVNKPEWP
1050	1060	1070	1080	1090	1100	1110	1120
AAELLLSLLG	RLLVHQFSNK	STEMALRVAS	LDYLGTVAAR	LRKDAVTSKM	DQGSIERILK	QVSGGEDEIQ	QLQKALLDYL
1130	1140	1150	1160	1170	1180	1190	1200
DENTETDPSL	VFSRKFYIAQ	WFRDTTLETE	KAMKSQKDEE	SSEGTHHAKE	IETTGQIMHR	AENRKKFLRS	IIKTTPSQFS
1210	1220	1230	1240	1250	1260	1270	1280
TLKMNSDTVD	YDDACLIVRY	LASMRPFAQS	FDIYLTQILR	VLGENAIAVR	TKAMKCLSEV	VAVDPSILAR	LDMQRGVHGR
1290	1300	1310	1320	1330	1340	1350	1360
LMDNSTSVRE	AAVELLGRFV	LCRPQLAEQY	YDMLIERILD	TGISVRKRVI	KILRDICIEQ	PTFPKITEMC	VKMIRRVNDE
1370	1380	1390	1400	1410	1420	1430	1440
EGIKKLVNET	FQKLWFTPTP	HNDKEAMTRK	ILNITDVVAA	CRDTGYDWFE	QLLQNLLKSE	EDSSYKPVKK	ACTQLVDNLV
1450	1460	1470	1480	1490	1500	1510	1520
EHILKYEESL	ADSDNKGVNS	GRLVACITTL	FLFSKIRPQL	MVKHAMTMQP	YLTTKCSTQN	DFMVICNVAK	ILELVVPLME
1530	1540	1550	1560	1570	1580	1590	1600
HPSETFLATI	EEDLMKLIIK	YGMTVVQHCV	SCLGAVVNKV	TQNFKFVWAC	FNRYYGAISK	LKSQHQEDPN	NTSLLTNKPA
1610	1620	1630	1640	1650	1660	1670	1680
LLRSLFTVGA	LCRHFDFDLE	DFKGNSKVNI	KDKVLELLMY	FTKHSDEEVQ	TKAIIGLGFA	FIQHPSLMFE	QEVKNLYNNI
1690	1700	1710	1720	1730	1740	1750	1760
LSDKNSSVNL	KIQVLKNLQT	YLQEEDTRMQ	QADRDWKKVA	KQEDLKEMGD	VSSGMSSSIM	QLYLKQVLEA	FFHTQSSVRH
1770	1780	1790	1800	1810	1820	1830	1840
FALNVIALTL	NQGLIHPVQC	VPYLIAMGTD	PEPAMRNKAD	QQLVEIDKKY	AGFIHMKAVA	GMKMSYQVQQ	AINTCLKDPV
1850	1860	1870	1880	1890	1900	1910	1920
RGFRQDESSS	ALCSHLYSMI	RGNRQHRRAF	LISLLNLFDD	TAKTDVTMLL	YIADNLACFP	YQTQEEPLFI	MHHIDITLSV
1930	1940	1950	1960	1970	1980	1990	2000
SGSNLLQSFK	ESMVKDKRKE	RKSSPSKENE	SSDSEEEVSR	PRKSRKRVDS	DSDSDSEDDI	NSVMKCLPEN	SAPLIEFANV
2010	2020	2030	2040	2050	2060	2070	2080
SQGILLLLML	KQHLKNLCGF	SDSKIQKYSP	SESAKVYDKA	INRKTGVHFH	PKQTLDFLRS	DMANSKITEE	VKRSIVKQYL
2090	2100	2110	2120	2130	2140	2150	2160
DFKLLMEHLD	PDEEEEEGEV	SASTNARNKA	ITSLLGGGSP	KNNTAAETED	DESDGEDRGG	GTSGSLRRSK	RNSDSTELAA
2170	2180	2190	2200	2210	2220	2230	2240
QMNESVDVMD	VIAICCPKYK	DRPQIARVVQ	KTSSGFSVQW	MAGSYSGSWT	EAKRRDGRKL	VPWVDTIKES	DIIYKKIALT
2250	2260	2270					
SANKLTNKVV	QTLRSLYAAK	DGTSS					

Detailed Protein Report

Cmpd.	No. of Cmpds.	m/z meas.	Δ m/z [ppm]	z	Rt [min]	Score	P	Range	Sequence	Modification
2607	1	913.4633	-59.64	2	62.6	12.0	1	909-923	K.HRQLILEEIFTSLAR.L	
425	1	946.2023	58.66	3	35.0	14.6	2	1692-1714	K.IQVLKNLQTYLQEEDTRMQQADR.D	Oxidation: 18

Detailed Protein Report

Protein 230: ubiquitin carboxyl-terminal hydrolase 17 [Homo sapiens]

**Accession:**	gi|153792150	**Score:**	40.1
**Database:**	refseq_human(refseq_human_20140103.fasta)	**MW [kDa]:**	59.6
**Database Date:**	2015-11-30	**pI:**	9.5
**Modification(s):**	Carbamidomethyl	**Sequence Coverage [%]:**	8.5
		**No. of unique Peptides:**	2

Quantitation

QU:MU	**Median:**	2.48	**CV:**	0.00 %	**No. of Peptides:**	1
WUP:QUP	**Median:**	0.29	**CV:**	0.00 %	**No. of Peptides:**	1

10	20	30	40	50	60	70	80
MEDDSLYLGG	EWQFNHFSKL	TSSRPDAAFA	EIQRTSLPEK	SPLSSEARVD	LCDDLAPVAR	QLAPRKKLPL	SSRRPAAVGA
**90**	**100**	**110**	**120**	**130**	**140**	**150**	**160**
GLQNMGNTCY	ENASLQCLTY	TPPLANYMLS	REHSQTCQRP	KCCMLCTMQA	HITWALHSPG	HVIQPSQALA	AGFHRGKQED
**170**	**180**	**190**	**200**	**210**	**220**	**230**	**240**
AHEFLMFTVD	AMKKACLPGH	KQVDHHSKDT	TLIHQIFGGC	WRSQIKCLHC	HGISDTFDPY	LDIALDIQAA	QSVKQALEQL
**250**	**260**	**270**	**280**	**290**	**300**	**310**	**320**
VKPEELNGEN	AYHCGLCLQR	APASKTLTLH	TSAKVLILVL	KRFSDVTGNK	LAKNVQYPEC	LDMQPYMSQQ	NTGPLVYVLY
**330**	**340**	**350**	**360**	**370**	**380**	**390**	**400**
AVLVHAGWSC	HDGHYFSYVK	AQEGQWYKMD	DAKVTACSIT	SVLSQQAYVL	FYIQKSEWER	HSESVSRGRE	PRALGAEDTD
**410**	**420**	**430**	**440**	**450**	**460**	**470**	**480**
RRATQGELKR	DHPCLQAPEL	DERLVERATQ	ESTLDHWKFP	QEQNKTKPEF	NVRKVEGTLP	PNVLVIHQSK	YKCGMKNHHP
**490**	**500**	**510**	**520**	**530**	**540**		
EQQSSLLNLS	STTRTDQESV	NTGTLASLQG	RTRRSKGKNK	HSKRALLVCQ			

Cmpd.	No. of Cmpds.	m/z meas.	Δ m/z [ppm]	z	Rt [min]	Score	P	Range	Sequence	Modification	Ratios
1128	1	1019.1809	129.35	2	43.7	15.4	2	49-66	R.VDLCDDLAPVARQLAPRK.K	Carbamidomethyl: 4	
184	1	424.2193	37.35	3	32.0	12.9	0	112-121	R.EHSQTCQRPK.C	Carbamidomethyl: 6	WUP:QUP 0.29<br>QU:MU 2.48

Detailed Protein Report

Protein 231: zinc finger protein 714 [Homo sapiens]

Accession: gi|144953913
Database: refseq_human(refseq_human_20140103.fasta)
Database Date: 2015-11-30
Modification(s): Carbamidomethyl

Score: 39.9
MW [kDa]: 64.0
pI: 10.3
Sequence Coverage [%]: 5.0
No. of unique Peptides: 2

Quantitation

QU:MU	**Median:**	1.73	**CV:**	0.00 %	**No. of Peptides:**	1
WUP:QUP	**Median:**	5.59	**CV:**	0.00 %	**No. of Peptides:**	1

10	20	30	40	50	60	70	80
MNVMLENYKN	LVFLAGIAVS	KQDPITSLEQ	EKEPWNMKIC	EMVDESPAMC	SSFTRDLWPE	QDIKDSFQQV	ILRRHGKCEH
**90**	**100**	**110**	**120**	**130**	**140**	**150**	**160**
ENLQLRKGSA	NVVECKVYKK	GYNELNQCLT	TTQSKIFPCD	KYIKVFHKIF	NSNRHKTRHT	GEKPFKCKKC	DESFCMLLHL
**170**	**180**	**190**	**200**	**210**	**220**	**230**	**240**
HQHKRIHIRE	NSYQCEECDK	VFKRFSTLTR	HKRVHTGEKP	FKCEECGKAF	KHSSTLTTHK	MIHTGEKPYR	CEECGKAFYH
**250**	**260**	**270**	**280**	**290**	**300**	**310**	**320**
SSHLTTHKVI	HTGEKPFKCE	ECGKAFNHPS	ALTTHKFIHV	KEKPYKCEEC	DKAFNRFSYL	TKHKIIHSGE	KSYKCEQCGK
**330**	**340**	**350**	**360**	**370**	**380**	**390**	**400**
GFNWSSTLTK	HKRIHTGEKP	YKCEECGKAF	NVSSHLTTHK	MIHTGEKPYK	CEECGKAFNH	SSKLTIHKII	HTGEKPYKCE
**410**	**420**	**430**	**440**	**450**	**460**	**470**	**480**
ECGKAFNQSS	NLTKHKIIHT	GEKLYKCEEC	GKAFNRSSNL	TTHKRIHTGE	KPYKCEECGK	AFNRSSNLTK	HNIIHTGEKS
**490**	**500**	**510**	**520**	**530**	**540**	**550**	**560**
YKCEECGKAF	NQSSTLTKHR	KIQQGMVAHA	CNPNTLRGLG	EQIARSGVQD	QPGQHGKTPS	LLKIQKFAGC	GGRRL

Cmpd.	No. of Cmpds.	m/z meas.	Δ m/z [ppm]	z	Rt [min]	Score	P	Range	Sequence	Modification	Ratios
2246	1	953.4417	47.81	2	58.5	14.9	0	39-55	K.ICEMVDESPAMCSSFTR.D		WUP:QUP 5.59
2107	1	625.2545	-125.14	2	54.7	10.4	2	544-554	K.IQKFAGCGGRR.L	Carbamidomethyl: 7	QU:MU 1.73

Detailed Protein Report

Protein 232: von Willebrand factor preproprotein [Homo sapiens]

**Accession:**	gi|89191868	**Score:**	39.9
**Database:**	refseq_human(refseq_human_20140103.fasta)	**MW [kDa]:**	309.1
**Database Date:**	2015-11-30	**pI:**	5.2
**Modification(s):**	Carbamidomethyl	**Sequence Coverage [%]:**	1.3
		**No. of unique Peptides:**	3

Quantitation

QU:MU	**Median:**	2.03	**CV:**	152.70 %	**No. of Peptides:**	2
WUP:QUP	**Median:**	0.98	**CV:**	0.00 %	**No. of Peptides:**	1

BRUKER

Detailed Protein Report

10	20	30	40	50	60	70	80
MIPARFAGVL	LALALILPGT	LCAEGTRGRS	STARCSLFGS	DFVNTFDGSM	YSFAGYCSYL	LAGGCQKRSF	SIIGDFQNGK
90	100	110	120	130	140	150	160
RVSLSVYLGE	FFDIHLFVNG	TVTQGDQRVS	MPYASKGLYL	ETEAGYYKLS	GEAYGFVARI	DGSGNFQVLL	SDRYFNKTCG
170	180	190	200	210	220	230	240
LCGNFNIFAE	DDFMTQEGTL	TSDPYDFANS	WALSSGEQWC	ERASPPSSSC	NISSGEMQKG	LWEQCQLLKS	TSVFARCHPL
250	260	270	280	290	300	310	320
VDPEPFVALC	EKTLCECAGG	LECACPALLE	YARTCAQEGM	VLYGWTDHSA	CSPVCPAGME	YRQCVSPCAR	TCQSLHINEM
330	340	350	360	370	380	390	400
CQERCVDGCS	CPEGQLLDEG	LCVESTECPC	VHSGKRYPPG	TSLSRDCNTC	ICRNSQWICS	NEECPGECLV	TGQSHFKSFD
410	420	430	440	450	460	470	480
NRYFTFSGIC	QYLLARDCQD	HSFSIVIETV	QCADDRDAVC	TRSVTVRLPG	LHNSLVKLKH	GAGVAMDGQD	VQLPLLKGDL
490	500	510	520	530	540	550	560
RIQHTVTASV	RLSYGEDLQM	DWDGRGRLLV	KLSPVYAGKT	CGLCGNYNGN	QGDDFLTPSG	LAEPRVEDFG	NAWKLHGDCQ
570	580	590	600	610	620	630	640
DLQKQHSDPC	ALNPRMTRFS	EEACAVLTSP	TFEACHRAVS	PLPYLRNCRY	DVCSCSDGRE	CLCGALASYA	AACAGRGVRV
650	660	670	680	690	700	710	720
AWREPGRCEL	NCPKGQVYLQ	CGTPCNLTCR	SLSYPDEECN	EACLEGCFCP	PGLYMDERGD	CVPKAQCPCY	YDGEIFQPED
730	740	750	760	770	780	790	800
IFSDHHTMCY	CEDGFMHCTM	SGVPGSLLPD	AVLSSPLSHR	SKRSLSCRPP	MVKLVCPADN	LRAEGLECTK	TCQNYDLECM
810	820	830	840	850	860	870	880
SMGCVSGCLC	PPGMVRHENR	CVALERCPCF	HQGKEYAPGE	TVKIGCNTCV	CRDRKWNCTD	HVCDATCSTI	GMAHYLTFDG
890	900	910	920	930	940	950	960
LKYLFPGECQ	YVLVQDYCGS	NPGTFRILVG	NKGCSHPSVK	CKKRVTILVE	GGEIELFDGE	VNVKRPMKDE	THFEVVESGR
970	980	990	1000	1010	1020	1030	1040
YIILLLGKAL	SVVWDRHLSI	SVVLKQTYQE	KVCGLCGNFD	GIQNNDLTSS	NLQVEEDPVD	FGNSWKVSSQ	CADTRKVPLD
1050	1060	1070	1080	1090	1100	1110	1120
SSPATCHNNI	MKQTMVDSSC	RILTSDVFQD	CNKLVDPEPY	LDVCIYDTCS	CESIGDCACF	CDTIAAYAHV	CAQHGKVVTW
1130	1140	1150	1160	1170	1180	1190	1200
RTATLCPQSC	EERNLRENGY	ECEWRYNSCA	PACQVTCQHP	EPLACPVQCV	EGCHAHCPPG	KILDELLQTC	VDPEDCPVCE
1210	1220	1230	1240	1250	1260	1270	1280
VAGRRFASGK	KVTLNPSDPE	HCQICHCDVV	NLTCEACQEP	GGLVVPPTDA	PVSPTTLYVE	DISEPPLHDF	YCSRLLDLVF
1290	1300	1310	1320	1330	1340	1350	1360
LLDGSSRLSE	AEFEVLKAFV	VDMMERLRIS	QKWVRVAVVE	YHDGSHAYIG	LKDRKRPSEL	RRIASQVKYA	GSQVASTSEV
1370	1380	1390	1400	1410	1420	1430	1440
LKYTLFQIFS	KIDRPEASRI	TLLLMASQEP	QRMSRNFVRY	VQGLKKKKVI	VIPVGIGPHA	NLKQIRLIEK	QAPENKAFVL
1450	1460	1470	1480	1490	1500	1510	1520
SSVDELEQQR	DEIVSYLCDL	APEAPPPTLP	PDMAQVTVGP	GLLGVSTLGP	KRNSMVLDVA	FVLEGSDKIG	EADFNRSKEF
1530	1540	1550	1560	1570	1580	1590	1600
MEEVIQRMDV	GQDSIHVTVL	QYSYMVTVEY	PFSEAQSKGD	ILQRVREIRY	QGGNRTNTGL	ALRYLSDHSF	LVSQGDREQA
1610	1620	1630	1640	1650	1660	1670	1680
PNLVYMVTGN	PASDEIKRLP	GDIQVVPIGV	GPNANVQELE	RIGWPNAPIL	IQDFETLPRE	APDLVLQRCC	SGEGLQIPTL
1690	1700	1710	1720	1730	1740	1750	1760
SPAPDCSQPL	DVILLLDGSS	SFPASYFDEM	KSFAKAFISK	ANIGPRLTQV	SVLQYGSITT	IDVPWNVVPE	KAHLLSLVDV
1770	1780	1790	1800	1810	1820	1830	1840
MQREGGPSQI	GDALGFAVRY	LTSEMHGARP	GASKAVVILV	TDVSVDSVDA	AADAARSNRV	TVFPIGIGDR	YDAAQLRILA
1850	1860	1870	1880	1890	1900	1910	1920
GPAGDSNVVK	LQRIEDLPTM	VTLGNSFLHK	LCSGFVRICM	DEDGNEKRPG	DVWTLPDQCH	TVTCQPDGQT	LLKSHRVNCD
1930	1940	1950	1960	1970	1980	1990	2000
RGLRPSCPNS	QSPVKVEETC	GCRWTCPCVC	TGSSTRHIVT	FDGQNFKLTG	SCSYVLFQNK	EQDLEVILHN	GACSPGARQG
2010	2020	2030	2040	2050	2060	2070	2080
CMKSIEVKHS	ALSVELHSDM	EVTVNGRLVS	VPYVGGNMEV	NVYGAIMHEV	RFNHLGHIFT	FTPQNNEFQL	QLSPKTFASK
2090	2100	2110	2120	2130	2140	2150	2160
TYGLCGICDE	NGANDFMLRD	GTVTTDWKTL	VQEWTVQRPG	QTCQPILEEQ	CLVPDSSHCQ	VLLLPLFAEC	HKVLAPATFY
2170	2180	2190	2200	2210	2220	2230	2240

Detailed Protein Report

Cmpd.	No. of Cmpds.	m/z meas.	Δ m/z [ppm]	z	Rt [min]	Score	P	Range	Sequence	Modification	Ratios	
1145	2	729.1568	-199.23	2	42.6	14.5	0	1944-1956	R.WTCPCVCTGSSTR.H	Carbamidomethyl: 5	WUP:QUP<br>QU:MU	0.98<br>0.68
1133	2	729.1918	-151.33	2	42.4	15.1	0	1944-1956	R.WTCPCVCTGSSTR.H	Carbamidomethyl: 3		
99	1	845.3517	-47.80	3	31.0	10.4	1	2289-2311	K.VNCTTQPCPTAKAPTCGLCEVARL	Carbamidomethyl: 8, 16, 19	QU:MU	6.08

Detailed Protein Report

Protein 233: **PREDICTED: SPOC domain-containing protein 1 isoform X4 [Homo sapiens]**

**Accession:**	gi|578800076	**Score:**	39.8
**Database:**	refseq_human(refseq_human_20140103.fasta)	**MW [kDa]:**	100.9
**Database Date:**	2015-11-30	**pI:**	9.5
**Modification(s):**	Carbamidomethyl, Oxidation	**Sequence Coverage [%]:**	4.4
		**No. of unique Peptides:**	3

Quantitation

QU:MU	**Median:** 2.73	**CV:** 12.14 %	**No. of Peptides:**	2
WUP:QUP	**Median:** 0.59	**CV:** 55.09 %	**No. of Peptides:**	2

```
        10         20         30         40         50         60         70         80
MSQAGDVEGP STGDPVLSPQ HNCELLQNME GASSMPGLSP DGPGASSGPG VRAGSRRKIP RKEALRGGSS RAAGAAEVRP
        90        100        110        120        130        140        150        160
GVLELLAVVQ SRGSMLAPGL HMQLPSVPTQ GRALTSKRLQ VSLCDILDDS CPRKLCSRSA GLPERALACR ERLAGVEEVS
       170        180        190        200        210        220        230        240
CLRPREARDG GMSSPGCDRR SPTLSKEEPP GRPLTSSPDP VPVRLGSGPV IQLLGAISHG QAGGQLPPKL EVLEDLMEVS
       250        260        270        280        290        300        310        320
SPSPAQRLRR KKRPMVQGPA GCQVFQPSPS GGTAGDPGGL SDPFYPPRSG SLALGDPSSD PACSQSGPME AEEDSLPEQP
       330        340        350        360        370        380        390        400
EDSAQLQQEK PSLYIGVRGT VVRSMQEVLW TRLRELPDPV LSEEVVEGIA AGIEAALWDL TQGTNGRYKT KYRSLLFNLR
       410        420        430        440        450        460        470        480
DPRNLDLFLK VVHGDVTPYD LVRMSSMQLA PQELARWRDQ EEKRGLNIIE QQQKEPCRLP ASKMTHKGEV EIQRDMDQTL
       490        500        510        520        530        540        550        560
TLEDLVGPQM FMDCSPQALP IASEDTTGQH DHHFLDPNCH ICKDWEPSNE LLGSFEAAKS CGDNIFQKAL SQTPMPAPEM
       570        580        590        600        610        620        630        640
PKTRELSPTE PQDRVPPSGL HVPAAPTKAL PCLPPWEGVL DMFSIKRFRA RAQLVSGHSC RLVQALPTVI RSAGCIPSNI
       650        660        670        680        690        700        710        720
VWDLLASICP AKAKDVCVVR LCPHGARDTQ NCRLLYSYLN DRQRHGLASV EHMGMVLLPL PAFQPLPTRL RPLGGPGLEV
       730        740        750        760        770        780        790        800
THSSLLLAVL LPKEGLPDTA GSSPWLGKVQ KMVSFNSKVE KRYYQPDDRR PNVPLKGTPP PGGAWQQSQG RGSIAPRGIS
       810        820        830        840        850        860        870        880
AWQRPPRGRG RLWPEPENWQ HPGRGQWPPE PGLRQSQHPY SVAPAGHGFG RGQHFHRDSC PHQALLRHLE SLATMSHQLQ
       890        900        910        920        930        940
ALLCPQTKSS IPRPLQRLSS ALAAPEPPGP ARDSSLGPTD EAGSECPFPR KA
```

Cmpd.	No. of Cmpds.	m/z meas.	Δ m/z [ppm]	z	Rt [min]	Score	P	Range	Sequence	Modification	Ratios
815	1	647.6499	-180.41	2	38.6	10.3	1	169-180	R.DGGMSSPGCDRR.S	Carbamidomethyl: 9	QU:MU 2.42
1959	1	757.2595	-164.75	2	54.9	16.2	2	455-467	K.EPCRLPASKMTHK.G	Oxidation: 10	WUP:QUP 0.99
78	1	877.8658	-95.96	2	30.9	13.3	1	549-564	K.ALSQTPMPAPEMPKTR.E		QU:MU 3.08<br>WUP:QUP 0.35

Detailed Protein Report

Protein 234: putative hexokinase HKDC1 [Homo sapiens]

Accession: gi|156151420
Database: refseq_human(refseq_human_20140103.fasta)
Database Date: 2015-11-30
Modification(s): Carbamidomethyl, Oxidation

Score: 39.8
MW [kDa]: 102.4
pI: 7.2
Sequence Coverage [%]: 5.0
No. of unique Peptides: 3

Quantitation

WUP:QUP **Median:** 0.15 **CV:** 0.00 % **No. of Peptides:** 1

10	20	30	40	50	60	70	80
MFAVHLMAFY	FSKLKEDQIK	KVDRFLYHMR	LSDDTLLDIM	RRFRAEMEKG	LAKDTNPTAA	VKMLPTFVRA	IPDGSENGEF
**90**	**100**	**110**	**120**	**130**	**140**	**150**	**160**
LSLDLGGSKF	RVLKVQVAEE	GKRHVQMESQ	FYPTPNEIIR	GNGTELFEYV	ADCLADFMKT	KDLKHKKLPL	GLTFSFPCRQ
**170**	**180**	**190**	**200**	**210**	**220**	**230**	**240**
TKLEEGVLLS	WTKKFKARGV	QDTDVVSRLT	KAMRRHKDMD	VDILALVNDT	VGTMMTCAYD	DPYCEVGVII	GTGTNACYME
**250**	**260**	**270**	**280**	**290**	**300**	**310**	**320**
DMSNIDLVEG	DEGRMCINTE	WGAFGDDGAL	EDIRTEFDRE	LDLGSLNPGK	QLFEKMISGL	YLGELVRLIL	LKMAKAGLLF
**330**	**340**	**350**	**360**	**370**	**380**	**390**	**400**
GGEKSSALHT	KGKIETRHVA	AMEKYKEGLA	NTREILVDLG	LEPSEADCIA	VQHVCTIVSF	RSANLCAAAL	AAILTRLREN
**410**	**420**	**430**	**440**	**450**	**460**	**470**	**480**
KKVERLRTTV	GMDGTLYKIH	PQYPKRLHKV	VRKLVPSCDV	RFLLSESGST	KGAAMVTAVA	SRVQAQRKQI	DRVLALFQLT
**490**	**500**	**510**	**520**	**530**	**540**	**550**	**560**
REQLVDVQAK	MRAELEYGLK	KKSHGLATVR	MLPTYVCGLP	DGTEKGKFLA	LDLGGTNFRV	LLVKIRSGRR	SVRMYNKIFA
**570**	**580**	**590**	**600**	**610**	**620**	**630**	**640**
IPLEIMQGTG	EELFDHIVQC	IADFLDYMGL	KGASLPLGFT	FSFPCRQMSI	DKGTLIGWTK	GFKATDCEGE	DVVDMLREAI
**650**	**660**	**670**	**680**	**690**	**700**	**710**	**720**
KRRNEFDLDI	VAVVNDTVGT	MMTCGYEDPN	CEIGLIAGTG	SNMCYMEDMR	NIEMVEGGEG	KMCINTEWGG	FGDNGCIDDI
**730**	**740**	**750**	**760**	**770**	**780**	**790**	**800**
RTRYDTEVDE	GSLNPGKQRY	EKMTSGMYLG	EIVRQILIDL	TKQGLLFRGQ	ISERLRTRGI	FETKFLSQIE	SDRLALLQVR
**810**	**820**	**830**	**840**	**850**	**860**	**870**	**880**
RILQQLGLDS	TCEDSIVVKE	VCGAVSRRAA	QLCGAGLAAI	VEKRREDQGL	EHLRITVGVD	GTLYKLHPHF	SRILQETVKE
**890**	**900**	**910**	**920**				
LAPRCDVTFM	LSEDGSGKGA	ALITAVAKRL	QQAQKEN				

Cmpd.	No. of Cmpds.	m/z meas.	Δ m/z [ppm]	z	Rt [min]	Score	P	Range	Sequence	Modification	Ratios
2718	2	648.3573	22.96	2	62.4	12.9	2	43-53	R.FRAEMEKGLAK.D	Oxidation: 5	
1075	1	947.1189	99.83	2	43.5	13.9	2	147-162	K.KLPLGLTFSFPCRQTK.L	Carbamidomethyl: 12	
2984	5	973.5068	-23.83	2	66.1	13.0	2	313-331	K.MAKAGLLFGGEKSSALHTK.G		WUP:QUP 0.15

Detailed Protein Report

Protein 235: insulin receptor-related protein precursor [Homo sapiens]

**Accession:**	gi|31657140	**Score:**	39.8
**Database:**	refseq_human(refseq_human_20140103.fasta)	**MW [kDa]:**	143.6
**Database Date:**	2015-11-30	**pI:**	6.0
**Modification(s):**	Carbamidomethyl	**Sequence Coverage [%]:**	3.2
		**No. of unique Peptides:**	1

Quantitation

QU:MU **Median:** 1.52 **CV:** 0.00 % **No. of Peptides:** 1

10	20	30	40	50	60	70	80
MAVPSLWPWG	ACLPVIFLSL	GFGLDTVEVC	PSLDIRSEVA	ELRQLENCSV	VEGHLQILLM	FTATGEDFRG	LSFPRLTQVT
**90**	**100**	**110**	**120**	**130**	**140**	**150**	**160**
DYLLLFRVYG	LESLRDLFPN	LAVIRGTRLF	LGYALVIFEM	PHLRDVALPA	LGAVLRGAVR	VEKNQELCHL	STIDWGLLQP
**170**	**180**	**190**	**200**	**210**	**220**	**230**	**240**
APGANHIVGN	KLGEECADVC	PGVLGAAGEP	CAKTTFSGHT	DYRCWTSSHC	QRVCPCPHGM	ACTARGECCH	TECLGGCSQP
**250**	**260**	**270**	**280**	**290**	**300**	**310**	**320**
EDPRACVACR	HLYFQGACLW	ACPPGTYQYE	SWRCVTAERC	ASLHSVPGRA	STFGIHQGSC	LAQCPSGFTR	NSSSIFCHKC
**330**	**340**	**350**	**360**	**370**	**380**	**390**	**400**
EGLCPKECKV	GTKTIDSIQA	AQDLVGCTHV	EGSLILNLRQ	GYNLEPQLQH	SLGLVETITG	FLKIKHSFAL	VSLGFFKNLK
**410**	**420**	**430**	**440**	**450**	**460**	**470**	**480**
LIRGDAMVDG	NYTLYVLDNQ	NLQQLGSWVA	AGLTIPVGKI	YFAFNPRLCL	EHIYRLEEVT	GTRGRQNKAE	INPRTNGDRA
**490**	**500**	**510**	**520**	**530**	**540**	**550**	**560**
ACQTRTLRFV	SNVTEADRIL	LRWERYEPLE	ARDLLSFIVY	YKESPFQNAT	EHVGPDACGT	QSWNLLDVEL	PLSRTQEPGV
**570**	**580**	**590**	**600**	**610**	**620**	**630**	**640**
TLASLKPWTQ	YAVFVRAITL	TTEEDSPHQG	AQSPIVYLRT	LPAAPTVPQD	VISTSNSSSH	LLVRWKPPTQ	RNGNLTYYLV
**650**	**660**	**670**	**680**	**690**	**700**	**710**	**720**
LWQRLAEDGD	LYLNDYCHRG	LRLPTSNNDP	RFDGEDGDPE	AEMESDCCPC	QHPPPGQVLP	PLEAQEASFQ	KKFENFLHNA
**730**	**740**	**750**	**760**	**770**	**780**	**790**	**800**
ITIPISPWKV	TSINKSPQRD	SGRHRRAAGP	LRLGGNSSDF	EIQEDKVPRE	RAVLSGLRHF	TEYRIDIHAC	NHAAHTVGCS
**810**	**820**	**830**	**840**	**850**	**860**	**870**	**880**
AATFVFARTM	PHREADGIPG	KVAWEASSKN	SVLLRWLEPP	DPNGLILKYE	IKYRRLGEEA	TVLCVSRLRY	AKFGGVHLAL
**890**	**900**	**910**	**920**	**930**	**940**	**950**	**960**
LPPGNYSARV	RATSLAGNGS	WTDSVAFYIL	GPEEEDAGGL	HVLLTATPVG	LTLLIVLAAL	GFFYGKKRNR	TLYASVNPEY
**970**	**980**	**990**	**1000**	**1010**	**1020**	**1030**	**1040**
FSASDMYVPD	EWEVPREQIS	IIRELGQGSF	GMVYEGLARG	LEAGEESTPV	ALKTVNELAS	PRECIEFLKE	ASVMKAFKCH
**1050**	**1060**	**1070**	**1080**	**1090**	**1100**	**1110**	**1120**
HVVRLLGVVS	QGQPTLVIME	LMTRGDLKSH	LRSLRPEAEN	NPGLPQPALG	EMIQMAGEIA	DGMAYLAANK	FVHRDLAARN
**1130**	**1140**	**1150**	**1160**	**1170**	**1180**	**1190**	**1200**
CMVSQDFTVK	IGDFGMTRDV	YETDYYRKGG	KGLLPVRWMA	PESLKDGIFT	THSDVWSFGV	VLWEIVTLAE	QPYQGLSNEQ
**1210**	**1220**	**1230**	**1240**	**1250**	**1260**	**1270**	**1280**
VLKFVMDGGV	LEELEGCPLQ	LQELMSRCWQ	PNPRLRPSFT	HILDSIQEEL	RPSFRLLSFY	YSPECRGARG	SLPTTDAEPD
**1290**	**1300**						
SSPTPRDCSP	QNGGPGH						

Cmpd.	No. of Cmpds.	m/z meas.	Δ m/z [ppm]	z	Rt [min]	Score	P	Range	Sequence	Modification	Ratios	
608	1	730.3714	88.53	2	37.6	15.3	0	213-225	R.VCPCPHGMACTAR.G	Carbamidomethyl: 2, 4	QU:MU	1.52

Detailed Protein Report

Protein 236: PREDICTED: zinc finger protein 197 isoform X2 [Homo sapiens]

Accession: gi|578805510
Database: refseq_human(refseq_human_20140103.fasta)
Database Date: 2015-11-30
Modification(s): Carbamidomethyl, Oxidation

Score: 39.8
MW [kDa]: 96.1
pI: 10.1
Sequence Coverage [%]: 3.9
No. of unique Peptides: 3

10	20	30	40	50	60	70	80
MALMLLTAQP	QELVMFEEVS	VCFTSEEWAC	LGPIQRALYW	DVMLENYGNV	TSLEWETMTE	NEEVTSKPSS	SQRADSHKGT
**90**	**100**	**110**	**120**	**130**	**140**	**150**	**160**
SKRLQGSVPQ	VLDFEEECEW	QVLASQWGNE	TDERADTVKK	VSLCERDKKK	RTPPEKQGQK	WKELGDSLTF	GSAISESLIG
**170**	**180**	**190**	**200**	**210**	**220**	**230**	**240**
TEGKKFYKCD	MCCKHFNKIS	HLINHRRIHT	GEKPHKCKEC	GKGFIQRSSL	LMHLRNHSGE	KPYKCNECGK	AFSQSAYLLN
**250**	**260**	**270**	**280**	**290**	**300**	**310**	**320**
HQRIHTGEKP	YKCKECGKGF	YRHSGLIIHL	RRHSGERPYK	CNECGKVFSQ	NAYLIDHQRL	HKGEEPYKCN	KCQKAFILKK
**330**	**340**	**350**	**360**	**370**	**380**	**390**	**400**
SLILHQRIHS	GEKPYKCDEC	GKTFAQTTYL	IDHQRLHSAE	NPYKCKECGK	VFIRSKSLLL	HQRVHTEKKT	FGCKKCGKIF
**410**	**420**	**430**	**440**	**450**	**460**	**470**	**480**
SSKSNFIDHK	RMHSREKPYK	CTECGKAFTQ	SAYLFDHQRL	HNGEKPYECN	ECGKVFILKK	SLILHQRFHT	GENLYECKDC
**490**	**500**	**510**	**520**	**530**	**540**	**550**	**560**
GKVFGSNRNL	IDHERLHNGE	KPYECRECGK	TFIMSKSFMV	HQKLHTQEKA	YKCEDCGKAF	SYNSSLLVHR	RIHTGEKPFE
**570**	**580**	**590**	**600**	**610**	**620**	**630**	**640**
CSECGRAFSS	NRNLIEHKRI	HSGEKPYECD	ECGKCFILKK	SLIGHQRIHT	REKSYKCNDC	GKVFSYRSNL	IAHQRIHTGE
**650**	**660**	**670**	**680**	**690**	**700**	**710**	**720**
KPYACSECGK	GFTYNRNLIE	HQRIHSGEKT	YECHVCRKVL	TSSRNLMVHQ	RIHTGEKPYK	CNECGKDFSQ	NKNLVVHQRM
**730**	**740**	**750**	**760**	**770**	**780**	**790**	**800**
HTGEKPYECD	KCRKSFTSKR	NLVGHQRIHT	GEKPYGCNDC	SKVFRQRKNL	TVHQKIHTDE	KPCECDVSEK	EFSQTSNLHL
**810**	**820**	**830**					
QQKIHTIEEF	SWLQNTNESK	IEIQKI					

Cmpd.	No. of Cmpds.	m/z meas.	Δ m/z [ppm]	z	Rt [min]	Score	P	Range	Sequence	Modification
1461	1	671.4129	203.04	2	46.6	14.0	2	165-174	K.KFYKCDMCCK.H	Carbamidomethyl: 8; Oxidation: 7
926	1	539.7356	-166.84	2	41.1	10.3	2	312-320	K.CQKAFILKK.S	
2133	1	797.3986	-7.50	2	57.0	15.4	0	343-355	K.TFAQTTYLIDHQR.L	

Detailed Protein Report

Protein 237: **putative pre-mRNA-splicing factor ATP-dependent RNA helicase DHX16 isoform 1 [Homo sapiens]**

**Accession:**	gi|255982614	**Score:**	39.6
**Database:**	refseq_human(refseq_human_20140103.fasta)	**MW [kDa]:**	119.2
**Database Date:**	2015-11-30	**pI:**	6.4
**Modification(s):**	Oxidation	**Sequence Coverage [%]:**	3.1
		**No. of unique Peptides:**	1

10	20	30	40	50	60	70	80
MATPAGLERW	VQDELHSVLG	LSERHVAQFL	IGTAQRCTSA	EEFVQRLRDT	DTLDLSGPAR	DFALRLWNKV	PRKAVVEKPA
90	100	110	120	130	140	150	160
RAAEREARAL	LEKNRSYRLL	EDSEESSEET	VSRAGSSLQK	KRKKRKHLRK	KREEEEEEEA	SEKGKKKTGG	SKQQTEKPES
170	180	190	200	210	220	230	240
EDEWERTERE	RLQDLEERDA	FAERVRQRDK	DRTRNVLERS	DKKAYEEAQK	RLKMAEEDRK	AMVPELRKKS	RREYLAKRER
250	260	270	280	290	300	310	320
EKLEDLEAEL	ADEEFLFGDV	ELSRHERQEL	KYKRRVRDLA	REYRAAGEQE	KLEATNRYHM	PKETRGQPAR	AVDLVEEESG
330	340	350	360	370	380	390	400
APGEEQRRWE	EARLGAASLK	FGARDAASQE	PKYQLVLEEE	ETIEFVRATQ	LQGDEEPSAP	PTSTQAQQKE	SIQAVRRSLP
410	420	430	440	450	460	470	480
VFPFREELLA	AIANHQVLII	EGETGSGKTT	QIPQYLFEEG	YTNKGMKIAC	TQPRRVAAMS	VAARVAREMG	VKLGNEVGYS
490	500	510	520	530	540	550	560
IRFEDCTSER	TVLRYMTDGM	LLREFLSEPD	LASYSVVMVD	EAHERTLHTD	ILFGLIKDVA	RFRPELKVLV	ASATMDTARF
570	580	590	600	610	620	630	640
STFFDDAPVF	RIPGRRFPVD	IFYTKAPEAD	YLEACVVSVL	QIHVTQPPGD	ILVFLTGQEE	IEAACEMLQD	RCRRLGSKIR
650	660	670	680	690	700	710	720
ELLVLPIYAN	LPSDMQARIF	QPTPPGARKV	VVATNIAETS	LTIEGIIYVL	DPGFCKQKSY	NPRTGMESLT	VTPCSKASAN
730	740	750	760	770	780	790	800
QRAGRAGRVA	AGKCFRLYTA	WAYQHELEET	TVPEIQRTSL	GNVVLLLKSL	GIHDLMHFDF	LDPPPYETLL	LALEQLYALG
810	820	830	840	850	860	870	880
ALNHLGELTT	SGRKMAELPV	DPMLSKMILA	SEKYSCSEEI	LTVAAMLSVN	NSIFYRPKDK	VVHADNARVN	FFLPGGDHLV
890	900	910	920	930	940	950	960
LLNVYTQWAE	SGYSSQWCYE	NFVQFRSMRR	ARDVREQLEG	LLERVEVGLS	SCQGDYIRVR	KAITAGYFYH	TARLTRSGYR
970	980	990	1000	1010	1020	1030	1040
TVKQQQTVFI	HPNSSLFEQQ	PRWLLYHELV	LTTKEFMRQV	LEIESSWLLE	VAPHYYKAKE	LEDPHAKKMP	KKIGKTREEL
1050							
G							

Cmpd.	No. of Cmpds.	m/z meas.	Δ m/z [ppm]	z	Rt [min]	Score	P	Range	Sequence	Modification
1748	1	1044.9652	-43.04	2	51.7	25.1	2	285-302	R.AAGEQEKLEATNRYHMPK.E	Oxidation: 16

BRUKER

Detailed Protein Report

Protein 238: PREDICTED: zinc finger protein 41 isoform X5 [Homo sapiens]

Accession: gi|530421580
Database: refseq_human(refseq_human_20140103.fasta)
Database Date: 2015-11-30
Modification(s): Carbamidomethyl

Score: 39.6
MW [kDa]: 79.5
pI: 10.2
Sequence Coverage [%]: 6.2
No. of unique Peptides: 3

Quantitation

QU:MU	**Median:**	1.98	**CV:**	0.00 %	**No. of Peptides:**	1
WUP:QUP	**Median:**	1.01	**CV:**	0.00 %	**No. of Peptides:**	1

Alias proteins:

Accession	Name	Description
gi|578838101	refseq_human (refseq_human_20140103.fasta)	PREDICTED: zinc finger protein 41 isoform X15 [Homo sapiens]
gi|530421582	refseq_human (refseq_human_20140103.fasta)	PREDICTED: zinc finger protein 41 isoform X6 [Homo sapiens]

10	20	30	40	50	60	70	80
MLEGEAPHQS	CSGEAIGKMQ	QQGIPGGIFF	HCERFDQPIG	EDSLCSILEE	LWQDNDQLEQ	RQENQNNLLS	HVKVLIKERG
**90**	**100**	**110**	**120**	**130**	**140**	**150**	**160**
YEHKNIEKII	HVTTKLVPSI	KRLHNCDTIL	KHTLNSHNHN	RNSATKNLGK	IFGNGNNFPH	SPSSTKNENA	KTGANSCEHD
**170**	**180**	**190**	**200**	**210**	**220**	**230**	**240**
HYEKHLSHKQ	APTHHQKIHP	EEKLYVCTEC	VMGFTQKSHL	FEHQRIHAGE	KSRECDKSNK	VFPQKPQVDV	HPSVYTGEKP
**250**	**260**	**270**	**280**	**290**	**300**	**310**	**320**
YLCTQCGKVF	TLKSNLITHQ	KIHTGQKPYK	CSECGKAFFQ	RSDLFRHLRI	HTGEKPYECS	ECGKGFSQNS	DLSIHQKTHT
**330**	**340**	**350**	**360**	**370**	**380**	**390**	**400**
GEKHYECNEC	GKAFTRKSAL	RMHQRIHTGE	KPYVCADCGK	AFIQKSHFNT	HQRIHTGEKP	YECSDCGKSF	TKKSQLHVHQ
**410**	**420**	**430**	**440**	**450**	**460**	**470**	**480**
RIHTGEKPYI	CTECGKVFTH	RTNLTTHQKT	HTGEKPYMCA	ECGKAFTDQS	NLIKHQKTHT	GEKPYKCNGC	GKAFIWKSRL
**490**	**500**	**510**	**520**	**530**	**540**	**550**	**560**
KIHQKSHIGE	RHYECKDCGK	AFIQKSTLSV	HQRIHTGEKP	YVCPECGKAF	IQKSHFIAHH	RIHTGEKPYE	CSDCGKCFTK
**570**	**580**	**590**	**600**	**610**	**620**	**630**	**640**
KSQLRVHQKI	HTGEKPNICA	ECGKAFTDRS	NLITHQKIHT	REKPYECGDC	GKTFTWKSRL	NIHQKSHTGE	RHYECSKCGK
**650**	**660**	**670**	**680**	**690**	**700**		
AFIQKATLSM	HQIIHTGKKP	YACTECQKAF	TDRSNLIKHQ	KMHSGEKRYK	ASD		

Cmpd.	No. of Cmpds.	m/z meas.	Δ m/z [ppm]	z	Rt [min]	Score	P	Range	Sequence	Modification	Ratios
1222	1	955.6757	154.26	2	44.9	10.3	2	80-95	R.GYEHKNIEKIIHVTTK.L		
2726	1	884.9432	75.73	2	64.4	16.1	0	430-444	K.THTGEKPYMCAECGK.A	Carbamidomethyl: 10, 13	
1481	1	727.3860	6.97	2	48.7	13.2	1	455-466	K.HQKTHTGEKPYK.C		QU:MU 1.98<br>WUP:QUP 1.01

Detailed Protein Report

Protein 239: putative uncharacterized zinc finger protein 814 [Homo sapiens]

Accession: gi|222418585
Database: refseq_human(refseq_human_20140103.fasta)
Database Date: 2015-11-30
Modification(s): Carbamidomethyl

Score: 39.6
MW [kDa]: 97.3
pI: 10.2
Sequence Coverage [%]: 5.7
No. of unique Peptides: 2

Quantitation

QU:MU	**Median:** 0.80	**CV:** 23.03 %	**No. of Peptides:**	2
WUP:QUP	**Median:** 1.94	**CV:** 0.00 %	**No. of Peptides:**	1

10	20	30	40	50	60	70	80
MAAAATLRLS	AQGTVTFEDV	AVNFTWEEWN	LLSEAQRCLY	RDVTLENLAL	ISSLGCWCGV	EDEAAPSKQS	IYIQRETQVR
90	100	110	120	130	140	150	160
TPMAGVSPKK	AHPCEMCGPI	LGDILHVADH	QGTHHKQKLH	RCEAWGNKLY	DSGNFHQHQN	EHIGEKPYRG	SVEEALFAKR
170	180	190	200	210	220	230	240
CKLHVSGESS	VFSESGKDFL	PRSGLLQQEA	SHTGEKSNSK	TECVSPIQCG	GAHYSCGESM	KHFSTKHILS	QHQRLLTREE
250	260	270	280	290	300	310	320
CYVCCECGKS	FSKYASLSNH	QRVHTEKKHE	CGECGKSFSK	YVSFSNHQRV	HTEKKHECGE	CGKSFSKYVS	FSNHQRVHTG
330	340	350	360	370	380	390	400
KRPYECGECG	KSFSKYASFS	NHQRVHTEKK	HYECGECGKS	FSKYVSFSNH	QRVHTGKRPY	ECGECGKSFS	KYASFSNHQR
410	420	430	440	450	460	470	480
VHTDKKHYEC	GECGKSFSQK	SSLIQHQRFH	TGEKPYGCEE	CGKSFSSEGH	LRSHQRVHAG	ERPFKCGECV	KSFSHKRSLV
490	500	510	520	530	540	550	560
HHQRVHSGER	PYQCGECGKS	FSQKGNLVLH	QRVHTGARPY	ECGECGKSFS	SKGHLRNHQQ	IHTGDRLYEC	GECGKSFSHK
570	580	590	600	610	620	630	640
GTLILHQRVH	PRERSYGCGE	CGKSFSSIGH	LRSHQRVHTG	ERPYECGECG	KSFSHKRSLV	HHQRMHTGER	PYKCGDCGKS
650	660	670	680	690	700	710	720
FNEKGHLRNH	QRVHTTERPF	KCGECGKCFS	HKGNLILHQH	GHTGERPYVC	RECGKLFKKK	SHLLVHQRIH	NGEKPYACEA
730	740	750	760	770	780	790	800
CQKFFRNKYQ	LIAHQRVHTG	ERPYECNDCG	KSFTHSSTFC	VHKRIHTGEK	PYECSECGKS	FAESSSFTKH	KRVHTGEKPY
810	820	830	840	850	860		
ECSECGKSFA	ESSSLTKHKR	VHTGEKPYKC	EKCGKLFNKK	SHLLVHQSSH	WRKAI		

Cmpd.	No. of Cmpds.	m/z meas.	Δ m/z [ppm]	z	Rt [min]	Score	P	Range	Sequence	Modification	Ratios
2009	1	766.2777	-53.66	2	55.5	17.9	1	351-363	K.HYECGECGKSFSK.Y	Carbamidomethyl: 7	QU:MU 0.64
417	1	564.5160	-156.98	3	35.2	11.2	1	653-667	R.VHTTERPFKCGECGK.C		WUP:QUP 1.94<br>QU:MU 1.00

Detailed Protein Report

Protein 240: PREDICTED: uncharacterized protein LOC101928409 [Homo sapiens]

**Accession:**	gi|530358441	**Score:**	39.6
**Database:**	refseq_human(refseq_human_20140103.fasta)	**MW [kDa]:**	60.4
**Database Date:**	2015-11-30	**pI:**	11.3
**Modification(s):**	Carbamidomethyl, Oxidation	**Sequence Coverage [%]:**	7.0
		**No. of unique Peptides:**	3

Quantitation

QU:MU	**Median:** 1.04	**CV:** 0.00 %	**No. of Peptides:**	1
WUP:QUP	**Median:** 1.35	**CV:** 0.00 %	**No. of Peptides:**	1

10	20	30	40	50	60	70	80
MLGTVASPRP	GSLFSPPLAV	IKPGYEQGFK	SSLPHPQVYR	AAPELSDMLR	CGGQRRGGGI	ALCSLGVKSN	MNVKYCGKTE
**90**	**100**	**110**	**120**	**130**	**140**	**150**	**160**
SFQSRQRGPS	AGGPGRPESL	LPRVRLPEAL	LLAEDCRGNV	GEGEMDGGSR	AYKSDKYAHT	LTVTASHHAP	PPPTHMEGFE
**170**	**180**	**190**	**200**	**210**	**220**	**230**	**240**
LFHLPELCSP	SQDAQTTGRT	QMKPDHSPRP	SHRGRWVPKR	LLRLHPSSRV	VVRLVAEGAD	GARFGNTPHC	ILFHFTSTKE
**250**	**260**	**270**	**280**	**290**	**300**	**310**	**320**
ESRIGGRPPS	CFLMQITQHS	NRKSKLQGLL	LRGALEKEER	VVRARAGASE	AKSHSRGGWD	AWGLKANARG	RDGVGSGGRS
**330**	**340**	**350**	**360**	**370**	**380**	**390**	**400**
ALQSRPLPPA	SLEGGSGTCA	RSLSPGLCAS	AWRLPGKNLI	YFLPSRVSSL	PPRAAPFRRP	EPAPGPRGCS	GERGLGMSGG
**410**	**420**	**430**	**440**	**450**	**460**	**470**	**480**
VRAPPHPRAL	LGGDIQELKE	IPQEEVETSE	RRGNRLFCKK	TERRLEGLRK	AADGSQDNSV	RPVLQGNFNP	TRTQAKTIYI
**490**	**500**	**510**	**520**	**530**	**540**	**550**	**560**
YIYELTQNRA	TLVPTPPQVG	RHIQVALSED	GTWRLAGVAL	WRSRSPVSSY	EVSLLLQSSL	QRGDLATSLP	LGFPVNPL

Cmpd.	No. of Cmpds.	m/z meas.	Δ m/z [ppm]	z	Rt [min]	Score	P	Range	Sequence	Modification	Ratios
1973	1	902.8715	-113.53	2	55.1	11.5	1	57-74	R.GGGIALCSLGVKSNMNVK.Y	Carbamidomethyl: 7	WUP:QUP 1.35<br>QU:MU 1.04
1325	1	640.6479	-178.43	2	46.2	14.4	0	118-130	R.GNVGEGEMDGGSR.A	Oxidation: 8	
2625	1	885.4475	-93.73	1	62.9	13.6	0	515-522	R.LAGVALWR.S		

Detailed Protein Report

Protein 241: E3 ubiquitin-protein ligase TRIP12 isoform c [Homo sapiens]

**Accession:**	gi|10863903	**Score:**	39.5
**Database:**	refseq_human(refseq_human_20140103.fasta)	**MW [kDa]:**	220.3
**Database Date:**	2015-11-30	**pI:**	9.6
		**Sequence Coverage [%]:**	2.6
		**No. of unique Peptides:**	2

Detailed Protein Report

10	20	30	40	50	60	70	80
MSNRPNNNPG	GSLRRSQRNT	AGAQPQDDSI	GGRSCSSSSA	VIVPQPEDPD	RANTSERQKT	GQVPKKDNSR	GVKRSASPDY
90	100	110	120	130	140	150	160
NRTNSPSSAK	KPKALQHTES	PSETNKPHSK	SKKRHLDQEQ	QLKSAQSPST	SKAHTRKSGA	TGGSRSQKRK	RTESSCVKSG
170	180	190	200	210	220	230	240
SGSESTGAEE	RSAKPTKLAS	KSATSAKAGC	STITDSSSAA	STSSSSSAVA	SASSTVPPGA	RVKQGKDQNK	ARRSRSASSP
250	260	270	280	290	300	310	320
SPRRSSREKE	QSKTGGSSKF	DWAARFSPKV	SLPKTKLSLP	GSSKSETSKP	GPSGLQAKLA	SLRKSTKKRS	ESPPAELPSL
330	340	350	360	370	380	390	400
RRSTRQKTTG	SCASTSRRGS	GLGKRGAAEA	RRQEKMADPE	SNQEAVNSSA	ARTDEAPQGA	AGAVGMTTSG	ESESDDSEMG
410	420	430	440	450	460	470	480
RLQALLEARG	LPPHLFGPLG	PRMSQLFHRT	IGSGASSKAQ	QLLQGLQASD	ESQQLQAVIE	MCQLLVMGNE	ETLGGFPVKS
490	500	510	520	530	540	550	560
VVPALITLLQ	MEHNFDIMNH	ACRALTYMME	ALPRSSAVVV	DAIPVFLEKL	QVIQCIDVAE	QALTALEMLS	RRHSKAILQA
570	580	590	600	610	620	630	640
GGLADCLLYL	EFFSINAQRN	ALAIAANCCQ	SITPDEFHFV	ADSLPLLTQR	LTHQDKKSVE	STCLCFARLV	DNFQHEENLL
650	660	670	680	690	700	710	720
QQVASKDLLT	NVQQLLVVTP	PILSSGMFIM	VVRMFSLMCS	NCPTLAVQLM	KQNIAETLHF	LLCGASNGSC	QEQIDLVPRS
730	740	750	760	770	780	790	800
PQELYELTSL	ICELMPCLPK	EGIFAVDTML	KKGNAQNTDG	AIWQWRDDRG	LWHPYNRIDS	RIIEQINEDT	GTARAIQRKP
810	820	830	840	850	860	870	880
NPLANSNTSG	YSESKKDDAR	AQLMKEDPEL	AKSFIKTLFG	VLYEVYSSSA	GPAVRHKCLR	AILRIIYFAD	AELLKDVLKN
890	900	910	920	930	940	950	960
HAVSSHIASM	LSSQDLKIVV	GALQMAEILM	QKLPDIFSVY	FRREGVMHQV	KHLAESESLL	TSPPKACTNG	SGSMGSTTSV
970	980	990	1000	1010	1020	1030	1040
SSGTATAATH	AAADLGSPSL	QHSRDDSLDL	SPQGRLSDVL	KRKRLPKRGP	RRPKYSPPRD	DDKVDNQAKS	PTTTQSPKSS
1050	1060	1070	1080	1090	1100	1110	1120
FLASLNPKTW	GRLSTQSNSN	NIEPARTAGG	SGLARAASKD	TISNNREKIK	GWIKEQAHKF	VERYFSSENM	DGSNPALNVL
1130	1140	1150	1160	1170	1180	1190	1200
QRLCAATEQL	NLQVDGGAEC	LVEIRSIVSE	SDVSSFEIQH	SGFVKQLLLY	LTSKSEKDAV	SREIRLKRFL	HVFFSSPLPG
1210	1220	1230	1240	1250	1260	1270	1280
EEPIGRVEPV	GNAPLLALVH	KMNNCLSQME	QFPVKVHDFP	SGNGTGGSFS	LNRGSQALKF	FNTHQLKCQL	QRHPDCANVK
1290	1300	1310	1320	1330	1340	1350	1360
QWKGGPVKID	PLALVQAIER	YLVVRGYGRV	REDDEDSDDD	GSDEEIDESL	AAQFLNSGNV	RHRLQFYIGE	HLLPYNMTVY
1370	1380	1390	1400	1410	1420	1430	1440
QAVRQFSIQA	EDERESTDDE	SNPLGRAGIW	TKTHTIWYKP	VREDEESNKD	CVGGKRGRAQ	TAPTKTSPRN	AKKHDELWHD
1450	1460	1470	1480	1490	1500	1510	1520
GVCPSVSNPL	EVYLIPTPPE	NITFEDPSLD	VILLLRVLHA	ISRYWYYLYD	NAMCKEIIPT	SEFINSKLTA	KANRQLQDPL
1530	1540	1550	1560	1570	1580	1590	1600
VIMTGNIPTW	LTELGKTCPF	FFPFDTRQML	FYVTAFDRDR	AMQRLLDTNP	EINQSDSQDS	RVAPRLDRKK	RTVNREELLK
1610	1620	1630	1640	1650	1660	1670	1680
QAESVMQDLG	SSRAMLEIQY	ENEVGTGLGP	TLEFYALVSQ	ELQRADLGLW	RGEEVTLSNP	KGSQEGTKYI	QNLQGLFALP
1690	1700	1710	1720	1730	1740	1750	1760
FGRTAKPAHI	AKVKMKFRFL	GKLMAKAIMD	FRLVDLPLGL	PFYKWMLRQE	TSLTSHDLFD	IDPVVARSVY	HLEDIVRQKK
1770	1780	1790	1800	1810	1820	1830	1840
RLEQDKSQTK	ESLQYALETL	TMNGCSVEDL	GLDFTLPGFP	NIELKKGGKD	IPVTIHNLEE	YLRLVIFWAL	NEGVSRQFDS
1850	1860	1870	1880	1890	1900	1910	1920
FRDGFESVFP	LSHLQYFYPE	ELDQLLCGSK	ADTWDAKTLM	ECCRPDHGYT	HDSRAVKFLF	EILSSFDNEQ	QRLFLQFVTG
1930	1940	1950	1960	1970	1980	1990	2000
SPRLPVGGFR	SLNPPLTIVR	KTFESTENPD	DFLPSVMTCV	NYLKLPDYSS	IEIMREKLLI	AAREGQQSFH	LS

Cmpd.	No. of Cmpds.	m/z meas.	Δ m/z [ppm]	z	Rt [min]	Score	P	Range	Sequence	Modification
1453	1	929.4464	3.04	2	46.5	10.5	1	16-33	R.SQRNTAGAQPQDDSIGGR.S	
1915	1	643.3072	-50.38	2	54.3	12.5	2	133-145	K.AHTRKSGATGGSR.S	

Detailed Protein Report

Protein 242: PREDICTED: histone-lysine N-methyltransferase ASH1L isoform X2 [Homo sapiens]

**Accession:**	gi|530365070	**Score:**	39.4
**Database:**	refseq_human(refseq_human_20140103.fasta)	**MW [kDa]:**	296.8
**Database Date:**	2015-11-30	**pI:**	10.2
**Modification(s):**	Carbamidomethyl	**Sequence Coverage [%]:**	1.8
		**No. of unique Peptides:**	3

Detailed Protein Report

10	20	30	40	50	60	70	80
MDPRNTAMLG	LGSDSEGFSR	KSPSAISTGT	LVSKREVELE	KNTKEEEDLR	KRNRERNIEA	GKDDGLTDAQ	QQFSVKETNF
90	100	110	120	130	140	150	160
SEGNLKLKIG	LQAKRTKKPP	KNLENYVCRP	AIKTTIKHPR	KALKSGKMTD	EKNEHCPSKR	DPSKLYKKAD	DVAAIECQSE
170	180	190	200	210	220	230	240
EVIRLHSQGE	NNPLSKKLSP	VHSEMADYIN	ATPSTLLGSR	DPDLKDRALL	NGGTSVTEKL	AQLIATCPPS	KSSKTKPKKL
250	260	270	280	290	300	310	320
GTGTTAGLVS	KDLIRKAGVG	SVAGIIHKDL	IKKPTISTAV	GLVTKDPGKK	PVFNAAVGLV	NKDSVKKLGT	GTTAVFINKN
330	340	350	360	370	380	390	400
LGKKPGTITT	VGLLSKDSGK	KLGIGIVPGL	VHKESGKKLG	LGTVVGLVNK	DLGKKLGSTV	GLVAKDCAKK	IVASSAMGLV
410	420	430	440	450	460	470	480
NKDIGKKLMS	CPLAGLISKD	AINLKAEALL	PTQEPLKASC	STNINNQESQ	ELSESLKDSA	TSKTFEKNVV	RQNKESILEK
490	500	510	520	530	540	550	560
FSVRKEIINL	EKEMFNEGTC	IQQDSFSSSE	KGSYETSKHE	KQPPVYCTSP	DFKMGGASDV	STAKSPFSAV	GESNLPSPSP
570	580	590	600	610	620	630	640
TVSVNPLTRS	PPETSSQLAP	NPLLLSSTTE	LIEEISESVG	KNQFTSESTH	LNVGHRSVGH	SISIECKGID	KEVNDSKTTH
650	660	670	680	690	700	710	720
IDIPRISSSL	GKKPSLTSES	SIHTITPSVV	NFTSLFSNKP	FLKLGAVSAS	DKHCQVAESL	STSLQSKPLK	KRKGRKPRWT
730	740	750	760	770	780	790	800
KVVARSTCRS	PKGLELERSE	LFKNVSCSSL	SNSNSEPAKF	MKNIGPPSFV	DHDFLKRRLP	KLSKSTAPSL	ALLADSEKPS
810	820	830	840	850	860	870	880
HKSFATHKLS	SSMCVSSDLL	SDIYKPKRGR	PKSKEMPQLE	GPPKRTLKIP	ASKVFSLQSK	EEQEPPILQP	EIEIPSFKQG
890	900	910	920	930	940	950	960
LSVSPFPKKR	GRPKRQMRSP	VKMKPPVLSV	APFVATESPS	KLESESDNHR	SSSDFFESED	QLQDPDDLDD	SHRPSVCSMS
970	980	990	1000	1010	1020	1030	1040
DLEMEPDKKI	TKRNNGQLMK	TIIRKINKMK	TLKRKKLLNQ	ILSSSVESSN	KGKVQSKLHN	TVSSLAATFG	SKLGQQINVS
1050	1060	1070	1080	1090	1100	1110	1120
KKGTIYIGKR	RGRKPKTVLN	GILSGSPTSL	AVLEQTAQQA	AGSALGQILP	PLLPSSASSS	EILPSPICSQ	SSGTSGGQSP
1130	1140	1150	1160	1170	1180	1190	1200
VSSDAGFVEP	SSVPYLHLHS	RQGSMIQTLA	MKKASKGRRR	LSPPTLLPNS	PSHLSELTSL	KEATPSPISE	SHSDETIPSD
1210	1220	1230	1240	1250	1260	1270	1280
SGIGTDNNST	SDRAEKFCGQ	KKRRHSFEHV	SLIPPETSTV	LSSLKEKHKH	KCKRRNHDYL	SYDKMKRQKR	KRKKKYPQLR
1290	1300	1310	1320	1330	1340	1350	1360
NRQDPDFIAE	LEELISRLSE	IRITHRSHHF	IPRDLLPTIF	RINFNSFYTH	PSFPLDPLHY	IRKPDLKKKR	GRPPKMREAM
1370	1380	1390	1400	1410	1420	1430	1440
AEMPFMHSLS	FPLSSTGFYP	SYGMPYSPSP	LTAAPIGLGY	YGRYPPTLYP	PPPSPSFTTP	LPPPSYMHAG	HLLLNPAKYH
1450	1460	1470	1480	1490	1500	1510	1520
KKKHKLLRQE	AFLTTSRTPL	LSMSTYPSVP	PEMAYGWMVE	HKHRHRHKHR	EHRSSEQPQV	SMDTGSSRSV	LESLKRYRFG
1530	1540	1550	1560	1570	1580	1590	1600
KDAVGERYKH	KEKHRCHMSC	PHLSPSKSLI	NREEQWVHRE	PSESSPLALG	LQTPLQIDCS	ESSPSLSLGG	FTPNSEPASS
1610	1620	1630	1640	1650	1660	1670	1680
DEHTNLFTSA	IGSCRVSNPN	SSGRKKLTDS	PGLFSAQDTS	LNRLHRKESL	PSNERAVQTL	AGSQPTSDKP	SQRPSESTNC
1690	1700	1710	1720	1730	1740	1750	1760
SPTRKRSSSE	STSSTVNGVP	SRSPRLVASG	DDSVDSLLQR	MVQNEDQEPM	EKSIDAVIAT	ASAPPSSSPG	RSHSKDRTLG
1770	1780	1790	1800	1810	1820	1830	1840
KPDSLLVPAV	TSDSCNNSIS	LLSEKLTSSC	SPHHIKRSVV	EAMQRQARKM	CNYDKILATK	KNLDHVNKIL	KAKKLQRQAR
1850	1860	1870	1880	1890	1900	1910	1920
TGNNFVKRRP	GRPRKCPLQA	VVSMQAFQAA	QFVNPELNRD	EEGAALHLSP	DTVTDVIEAV	VQSVNLNPEH	KKGLKRKGWL
1930	1940	1950	1960	1970	1980	1990	2000
LEEQTRKKQK	PLPEEEEQEN	NKSFNEAPVE	IPSPSETPAK	PSEPESTLQP	VLSLIPREKK	PPRPPKKKYQ	KAGLYSDVYK
2010	2020	2030	2040	2050	2060	2070	2080
TTDPKSRLIQ	LKKEKLEYTP	GEHEYGLFPA	PIHVGKYLRQ	KRIDFQLPYD	ILWQWKHNQL	YKKPDVPLYK	KIRSNVYVDV
2090	2100	2110	2120	2130	2140	2150	2160
KPLSGYEATT	CNCKKPDDDT	RKGCVDDCLN	RMIFAECSPN	TCPCGEQCCN	QRIQRHEWVQ	CLERFRAEEK	GWGIRTKEPL
2170	2180	2190	2200	2210	2220	2230	2240

Detailed Protein Report

Cmpd.	No. of Cmpds.	m/z meas.	Δ m/z [ppm]	z	Rt [min]	Score	P	Range	Sequence	Modification
1736	1	853.3885	-153.79	2	50.1	10.2	2	256-272	R.KAGVGSVAGIIHKDLIK.K	
1687	1	696.2420	-115.78	3	49.4	11.7	1	744-762	K.NVSCSSLSNSNSEPAKFMK.N	Carbamidomethyl: 4
1905	1	673.3043	-162.57	2	54.2	17.5	0	2460-2472	R.QALAAPLLNLPPK.K	

Detailed Protein Report

Protein 243: probable exonuclease mut-7 homolog isoform a [Homo sapiens]

**Accession:**	gi|145279204	**Score:**	39.3
**Database:**	refseq_human(refseq_human_20140103.fasta)	**MW [kDa]:**	96.5
**Database Date:**	2015-11-30	**pI:**	9.9
**Modification(s):**	Carbamidomethyl	**Sequence Coverage [%]:**	4.2
		**No. of unique Peptides:**	1

10	20	30	40	50	60	70	80
MDPGDPAGDP	AAGERHRMGR	DPLLLLQALQ	TLWSTRERKQ	LREEAWRGFA	ALDDPLAGLL	DMLESCRGQR	GEGPSLAAWI
90	100	110	120	130	140	150	160
SHQLQCWLQA	QPCPSLAQHS	LRLKQLQARA	VKVLTESPPS	LAAPLASIFQ	LQDADRSCLL	AHVHRLHHEG	RFREAATLGA
170	180	190	200	210	220	230	240
TLKLQSELGV	EKMSIPLLLQ	DKVALVERYV	AGFPDLQRRL	LVLMDSWCQP	GFDIKDVARR	YPEVTSLSLE	KLSPKALSRQ
250	260	270	280	290	300	310	320
VLRLQERYGV	APALCPNAAI	QQRLAALRHL	CHKRFVEKSL	SQENWTDHVQ	GLVGQSPWLQ	EQLSQLLVSH	SDPVTAAQCA
330	340	350	360	370	380	390	400
MELLLPEERL	PAAVAVELRR	FRLQGRATEA	DSRLEVKDMK	DRYYQLPIPR	ENVHLLASWE	DLTRHEGALL	QCHQVVGVDV
410	420	430	440	450	460	470	480
EWTPVFVAGG	RPRPSLLQVA	VEGHVFLLDV	LALSQPPTGQ	GAQAFSRLVA	QLLSDPSITK	LGYGMVGDLQ	KLGTSCPALA
490	500	510	520	530	540	550	560
HVEKQILGGM	DLLLVHRQMR	VASVPAPAVD	RARELRGLSL	LVQQVLGTAL	DKTQQLSNWD	RRPLCEEQVI	YAAADAYCLL
570	580	590	600	610	620	630	640
EVHQALCREP	ARFHLSEDLA	GSRRPRHRER	PGARKPPGLQ	KASAPAAPRQ	VPVAVAVSEG	AAPQIPARAF	RVVCDNMLQG
650	660	670	680	690	700	710	720
LARSLRCLGV	DARMLGNGED	HRRAAEVARQ	EGRIILTSGQ	PFHKLRAQVG	AGRCLSVDCS	LKAQQQAKAV	LKHFNVRVTH
730	740	750	760	770	780	790	800
ADIFSRCQAC	NCDQYLKVSR	DMMKQLMWLS	SHQEGPRSSG	DEATQSQAVQ	EPGPAPDAAP	EGCTYDRPCR	WLQMADLRAE
810	820	830	840	850	860	870	880
TPDMLADGTR	LQLAGVPVGV	LRTPGLRCFY	CCTGCGKVFW	DGSHLGRVAT	HFRDMLESAP	SPCEPSPAPS	PASSPF

Cmpd.	No. of Cmpds.	m/z meas.	Δ m/z [ppm]	z	Rt [min]	Score	P	Range	Sequence	Modification
1319	1	571.1608	-69.20	2	46.1	14.7	0	828-837	R.CFYCCTGCGK.V	Carbamidomethyl: 5

Detailed Protein Report

Protein 244: vitamin D3 receptor isoform VDRA [Homo sapiens]

**Accession:**	gi|4507883	**Score:**	39.0
**Database:**	refseq_human(refseq_human_20140103.fasta)	**MW [kDa]:**	48.3
**Database Date:**	2015-11-30	**pI:**	6.1
**Modification(s):**	Carbamidomethyl, Oxidation	**Sequence Coverage [%]:**	2.6
		**No. of unique Peptides:**	2

Alias proteins:

Accession	Name	Description
gi|578824200	refseq_human (refseq_human_20140103.fasta)	PREDICTED: vitamin D3 receptor isoform X1 [Homo sapiens]
gi|63054845	refseq_human (refseq_human_20140103.fasta)	vitamin D3 receptor isoform VDRA [Homo sapiens]

10	20	30	40	50	60	70	80
MEAMAASTSL	PDPGDFDRNV	PRICGVCGDR	ATGFHFNAMT	CEGCKGFFRR	SMKRKALFTC	PFNGDCRITK	DNRRHCQACR
**90**	**100**	**110**	**120**	**130**	**140**	**150**	**160**
LKRCVDIGMM	KEFILTDEEV	QRKREMILKR	KEEEALKDSL	RPKLSEEQQR	IIAILLDAHH	KTYDPTYSDF	CQFRPPVRVN
**170**	**180**	**190**	**200**	**210**	**220**	**230**	**240**
DGGGSHPSRP	NSRHTPSFSG	DSSSSCSDHC	ITSSDMMDSS	SFSNLDLSEE	DSDDPSVTLE	LSQLSMLPHL	ADLVSYSIQK
**250**	**260**	**270**	**280**	**290**	**300**	**310**	**320**
VIGFAKMIPG	FRDLTSEDQI	VLLKSSAIEV	IMLRSNESFT	MDDMSWTCGN	QDYKYRVSDV	TKAGHSLELI	EPLIKFQVGL
**330**	**340**	**350**	**360**	**370**	**380**	**390**	**400**
KKLNLHEEEH	VLLMAICIVS	PDRPGVQDAA	LIEAIQDRLS	NTLQTYIRCR	HPPPGSHLLY	AKMIQKLADL	RSLNEEHSKQ
**410**	**420**	**430**					
YRCLSFQPEC	SMKLTPLVLE	VFGNEIS					

Cmpd.	No. of Cmpds.	m/z meas.	Δ m/z [ppm]	z	Rt [min]	Score	P	Range	Sequence	Modification
2332	3	665.1572	-192.83	2	59.6	12.9	0	403-413	R.CLSFQPECSMK.L	Carbamidomethyl: 1
2538	1	673.3328	74.17	2	61.7	14.1	0	403-413	R.CLSFQPECSMK.L	Carbamidomethyl: 8; Oxidation: 10

Detailed Protein Report

Protein 245: **PREDICTED: arf-GAP with Rho-GAP domain, ANK repeat and PH domain-containing protein 3 isoform X7 [Homo sapiens]**

**Accession:**	gi|578810904	**Score:**	39.0
**Database:**	refseq_human(refseq_human_20140103.fasta)	**MW [kDa]:**	149.1
**Database Date:**	2015-11-30	**pI:**	6.5
		**Sequence Coverage [%]:**	2.0
		**No. of unique Peptides:**	1

10	20	30	40	50	60	70	80
MDPGCLYYGV	QPVGTPGAPD	RRESRGVCQG	RAEHRLSRQD	LEAREDAGYA	SLELPGDSTL	LSPTLETEET	SDDLISPYAS
90	100	110	120	130	140	150	160
FSFTADRLTP	LLSGWLDKLS	PQGNYVFQRR	FVQFNGRSLM	YFGSDKDPFP	KGVIPLTAIE	MTRSSKDNKF	QVITGQRVFV
170	180	190	200	210	220	230	240
FRTESEAQRD	MWCSTLQSCL	KEQRLLGHPR	PPQPPRPLRT	GMLELRGHKA	KVFAALSPGE	LALYKSEQAF	SLGIGICFIE
250	260	270	280	290	300	310	320
LQGCSVRETK	SRSFDLLTPH	RCFSFTAESG	GARQSWAAAL	QEAVTETLSD	YEVAEKIWSN	RANRQCADCG	SSRPDWAAVN
330	340	350	360	370	380	390	400
LGVVICKQCA	GQHRALGSGI	SKVQSLKLDT	SVWSNEIVQL	FIVLGNDRAN	RFWAGTLPPG	EGLHPDATPG	PRGEFISRKY
410	420	430	440	450	460	470	480
RLGLFRKPHP	QYPDHSQLLQ	ALCAAVARPN	LLKNMTQLLC	VEAFEGEEPW	FPPAPDGSCP	GLLPSDPSPG	VYNEVVVRAT
490	500	510	520	530	540	550	560
YSGFLYCSPV	SNKAGPSPPR	RGRDAPPRLW	CVLGAALEMF	ASENSPEPLS	LIQPQDIVCL	GVSPPPTDPG	DRFPFSFELI
570	580	590	600	610	620	630	640
LAGGRIQHFG	TDGADSLEAW	TSAVGKWFSP	LSCHQLLGPG	LLRLGRLWLR	SPSHTAPAPG	LWLSGFGLLR	GDHLFLCSAP
650	660	670	680	690	700	710	720
GPGPPAPEDM	VHLRRLQEIS	VVSAADTPDK	KEHLVLVETG	RTLYLQGEGR	LDFTAWNAAI	GGAAGGGGTG	LQEQQMSRGD
730	740	750	760	770	780	790	800
IPIIVDACIS	FVTQHGLRLE	GVYRKGGARA	RSLRLLAEFR	RDARSVKLRP	GEHFVEDVTD	TLKRFFRELD	DPVTSARLLP
810	820	830	840	850	860	870	880
RWREAAELPQ	KNQRLEKYKD	VIGCLPRVNR	RTLATLIGHL	YRVQKCAALN	QMCTRNLALL	FAPSVFQTDG	RGEHEVRVLQ
890	900	910	920	930	940	950	960
ELIDGYISVF	DIDSDQVAQI	DLEVSLITTW	KDVQLSQAGD	LIMEVYIEQQ	LPDNCVTLKV	SPTLTAEELT	NQVLEMRGTA
970	980	990	1000	1010	1020	1030	1040
AGMDLWVTFE	IREHGELERP	LHPKEKVLEQ	ALQWCQLPEP	CSASLLLKKV	PLAQAGCLFT	GIRRESPRVG	LLRCREEPPR
1050	1060	1070	1080	1090	1100	1110	1120
LLGSRFQERF	FLLRGRCLLL	LKEKKSSKPE	REWPLEGAKV	YLGIRKKLKP	PTPWGFTLIL	EKMHLYLSCT	DEDEMWDWTT
1130	1140	1150	1160	1170	1180	1190	1200
SILKAQHDDQ	QPVVLRRHSS	SDLARQKFGT	MPLLPIRGDD	SGATLLSANQ	TLRRLHNRRT	LSMFFPMKSS	QGSVEEQEEL
1210	1220	1230	1240	1250	1260	1270	1280
EEPVYEEPVY	EEVGAFPELI	QDTSTSFSTT	REWTVKPENP	LTSQKSLDQP	FLSKSSTLGQ	EERPPEPPPG	PPSKSSPQAR
1290	1300	1310	1320	1330	1340	1350	
GSLEEQLLQE	LSSLILRKGE	TTAGLGSPSQ	PSSPQSPSPT	GLPTQTPGFP	TQPPCTSSPP	SSQPLT	

Cmpd.	No. of Cmpds.	m/z meas.	Δ m/z [ppm]	z	Rt [min]	Score	P	Range	Sequence	Modification
1500	1	850.8827	-87.14	2	48.9	14.3	1	656-671	R.LQEISVVSAADTPDKK.E	

Detailed Protein Report

Protein 246: **PREDICTED: coiled-coil domain-containing protein 136 isoform X4 [Homo sapiens]**

**Accession:**	gi|530386309	**Score:**	38.8
**Database:**	refseq_human(refseq_human_20140103.fasta)	**MW [kDa]:**	131.9
**Database Date:**	2015-11-30	**pI:**	4.5
		**Sequence Coverage [%]:**	2.7
		**No. of unique Peptides:**	1

10	20	30	40	50	60	70	80
MEAGAGAGAG	AAGWSCPGPG	PTVTTLGSYE	ASEGCERKKG	QRWGSLERRG	MQAMEGEVLL	PALYEEEEEE	EEEEEEVEEE
**90**	**100**	**110**	**120**	**130**	**140**	**150**	**160**
EEQVQKGGSV	GSLSVNKHRG	LSLTETELEE	LRAQVLQLVA	ELEETRELAG	QHEDDSLELQ	GLLEDERLAS	AQQAEVFTKQ
**170**	**180**	**190**	**200**	**210**	**220**	**230**	**240**
IQQLQGELRS	LREEISLLEH	EKESELKEIE	QELHLAQAEI	QSLRQAAEDS	ATEHESDIAS	LQEDLCRMQN	ELEDMERIRG
**250**	**260**	**270**	**280**	**290**	**300**	**310**	**320**
DYEMEIASLR	AEMEMKSSEP	SGSLGLSDYS	GLQEELQELR	ERYHFLNEEY	RALQESNSSL	TGQLADLESE	RTQRATERWL
**330**	**340**	**350**	**360**	**370**	**380**	**390**	**400**
QSQTLSMTSA	ESQTSEMDFL	EPDPEMQLLR	QQLRDAEEQM	HGMKNKCQEL	CCELEELQHH	RQVSEEEQRR	LQRELKCAQN
**410**	**420**	**430**	**440**	**450**	**460**	**470**	**480**
EVLRFQTSHS	VTQNEELKSR	LCTLQKKYDT	SQDEQNELLK	MQLQLQTELR	QLKVMKSTLV	ENQSEKELLC	RLQKLHLQHQ
**490**	**500**	**510**	**520**	**530**	**540**	**550**	**560**
NVTCEKEKLL	ERQQQLQEEL	QCHEAELQHL	RDTVASFKES	NEKDTETHAQ	LQEMKQLYQA	SKDELERQKH	MYDQLEQDLL
**570**	**580**	**590**	**600**	**610**	**620**	**630**	**640**
LCQLELKELK	ASHPIPEDKG	KCANKSQELL	TKLEDLCELQ	LLYQGMQEEQ	KKLIQNQDCV	LKEQLEIHEE	LRRFKESHFQ
**650**	**660**	**670**	**680**	**690**	**700**	**710**	**720**
EVLENPDDSK	LAKSSKCNRN	KQSKLLMEQM	QALQVMYDAG	QAKQELLQQE	QGRLLEERKR	LQADLQLCLE	EMQLLQVQSP
**730**	**740**	**750**	**760**	**770**	**780**	**790**	**800**
SIKMSLESYG	KSYGSMVPSN	ENCRKTYDTT	VDDNESYYKS	YTSTQTSSKS	FLKSYDSSTS	ASEAYGKSYC	TTSNSSITYK
**810**	**820**	**830**	**840**	**850**	**860**	**870**	**880**
KSYGSTSSSD	TCQKSFVSSC	TDEEPAEPED	MERFEEMVVK	VLIKLQAVQA	MYQISQEEHS	QLQEQMEKLL	AKQKDLKEEL
**890**	**900**	**910**	**920**	**930**	**940**	**950**	**960**
DACEREFKEC	MECLEKPMAP	QNDKNEIKEL	QTKLRELQLQ	YQASMDEQGR	LLVVQEQLEG	QLQCCQEELR	QLREKRPSVV
**970**	**980**	**990**	**1000**	**1010**	**1020**	**1030**	**1040**
KEARGKNANK	NMNKNANGVK	MKKVTKPCSD	TSESDLETRK	SLEVVLYYKA	SQRKLDGLAK	EEEKKEEMEE	EKKQVKEEAK
**1050**	**1060**	**1070**	**1080**	**1090**	**1100**	**1110**	**1120**
EQCGDELVAE	PADPEEAKST	EDQEENEEDK	EEEEKEEDSE	EEEDDADSSL	ESPEENNPLR	LSESKKNMFG	LWKPMVFLAI
**1130**	**1140**	**1150**					
AAVALYVLPN	MRQQESEFCL	ME					

Cmpd.	No. of Cmpds.	m/z meas.	Δ m/z [ppm]	z	Rt [min]	Score	P	Range	Sequence	Modification
488	1	686.3221	-115.45	2	36.1	10.5	2	905-915	K.NEIKELQTKLR.E	

Detailed Protein Report

Protein 247: **tripartite motif-containing protein 66 [Homo sapiens]**

**Accession:**	gi|209977097	**Score:**	38.8
**Database:**	refseq_human(refseq_human_20140103.fasta)	**MW [kDa]:**	134.6
**Database Date:**	2015-11-30	**pI:**	6.6
**Modification(s):**	Oxidation	**Sequence Coverage [%]:**	3.8
		**No. of unique Peptides:**	3

10	20	30	40	50	60	70	80
MARNCSECKE	KRAAHILCTY	CNRWLCSSCT	EEHRHSPVPG	GPFFPRAQKG	SPGVNGGPGD	FTLYCPLHTQ	EVLKLFCETC
**90**	**100**	**110**	**120**	**130**	**140**	**150**	**160**
DMLTCHSCLV	VEHKEHRCRH	VEEVLQNQRM	LLEGVTTQVA	HKKSSLQTSA	KQIEDRIFEV	KHQHRKVENQ	IKMAKMVLMN
**170**	**180**	**190**	**200**	**210**	**220**	**230**	**240**
ELNKQANGLI	EELEGITNER	KRKLEQQLQS	IMVLNRQFEH	VQNFINWAVC	SKTSVPFLFS	KELIVFQMQR	LLETSCNTDP
**250**	**260**	**270**	**280**	**290**	**300**	**310**	**320**
GSPWSIRFTW	EPNFWTKQLA	SLGCITTEGG	QMSRADAPAY	GGLQGSSPFY	QSHQSPVAQQ	EALSHPSHKF	QSPAVCSSSV
**330**	**340**	**350**	**360**	**370**	**380**	**390**	**400**
CCSHCSPVSP	SLKGQVPPPS	IHPAHSFRQP	PEMVPQQLGS	LQCSALLPRE	KELACSPHPP	KLLQPWLETQ	PPVEQESTSQ
**410**	**420**	**430**	**440**	**450**	**460**	**470**	**480**
RLGQQLTSQP	VCIVPPQDVQ	QGAHAQPTLQ	TPSIQVQFGH	HQKLKLSHFQ	QQPQQQLPPP	PPPLPHPPPP	LPPPPQQPHP
**490**	**500**	**510**	**520**	**530**	**540**	**550**	**560**
PLPPSQHLAS	SQHESPPGPA	CSQNMDIMHH	KFELEEMQKD	LELLLQAQQP	SLQLSQTKSP	QHLQQTIVGQ	INYIVRQPAP
**570**	**580**	**590**	**600**	**610**	**620**	**630**	**640**
VQSQSQEETL	QATDEPPASQ	GSKPALPLDK	NTAAALPQAS	GEETPLSVPP	VDSTIQHSSP	NVVRKHSTSL	SIMGFSNTLE
**650**	**660**	**670**	**680**	**690**	**700**	**710**	**720**
MELSSTRLER	PLEPQIQSVS	NLTAGAPQAV	PSLLSAPPKM	VSSLTSVQNQ	AMPSLTTSHL	QTVPSLVHST	FQSMPNLISD
**730**	**740**	**750**	**760**	**770**	**780**	**790**	**800**
SPQAMASLAS	DHPQAGPSLM	SGHTQAVPSL	ATCPLQSIPP	VSDMQPETGS	SSSSGRTSGS	LCPRDGADPS	LENALCKVKL
**810**	**820**	**830**	**840**	**850**	**860**	**870**	**880**
EEPINLSVKK	PPLAPVVSTS	TALQQYQNPK	ECENFEQGAL	ELDAKENQSI	RAFNSEHKIP	YVRLERLKIC	AASSGEMPVF
**890**	**900**	**910**	**920**	**930**	**940**	**950**	**960**
KLKPQKNDQD	GSFLLIIECG	TESSSMSIKV	SQDRLSEATQ	APGLEGRKVT	VTSLAGQRPP	EVEGTSPEEH	RLIPRTPGAK
**970**	**980**	**990**	**1000**	**1010**	**1020**	**1030**	**1040**
KGPPAPIENE	DFCAVCLNGG	ELLCCDRCPK	VFHLSCHVPA	LLSFPGGEWV	CTLCRSLTQP	EMEYDCENAC	YNQPGMRASP
**1050**	**1060**	**1070**	**1080**	**1090**	**1100**	**1110**	**1120**
GLSMYDQKKC	EKLVLSLCCN	NLSLPFHEPV	SPLARHYYQI	IKRPMDLSII	RRKLQKKDPA	HYTTPEEVVS	DVRLMFWNCA
**1130**	**1140**	**1150**	**1160**	**1170**	**1180**	**1190**	**1200**
KFNYPDSEVA	EAGRCLEVFF	EGWLKEIYPE	KRFAQPRQED	SDSEEVSSES	GCSTPQGFPW	PPYMQEGIQP	KRRRRHMENE
**1210**	**1220**						
RAKRMSFRLA	NSISQV						

Cmpd.	No. of Cmpds.	m/z meas.	Δ m/z [ppm]	z	Rt [min]	Score	P	Range	Sequence	Modification
1060	1	540.7960	-96.21	2	41.5	11.4	2	952-961	R.LIPRTPGAKK.G	
862	1	942.1586	54.56	3	40.3	12.8	1	1098-1121	K.DPAHYTTPEEVVSDVRLMFWNCAK.F	Oxidation: 18
2820	1	676.8699	24.74	2	63.9	14.6	1	1205-1216	R.MSFRLANSISQV.-	

Detailed Protein Report

Protein 248: frizzled-10 precursor [Homo sapiens]

Accession: gi|6005762
Database: refseq_human(refseq_human_20140103.fasta)
Database Date: 2015-11-30
Modification(s): Carbamidomethyl, Oxidation

Score: 38.7
MW [kDa]: 65.3
pI: 9.9
Sequence Coverage [%]: 6.4
No. of unique Peptides: 2

10	20	30	40	50	60	70	80
MQRPGPRLWL	VLQVMGSCAA	ISSMDMERPG	DGKCQPIEIP	MCKDIGYNMT	RMPNLMGHEN	QREAAIQLHE	FAPLVEYGCH
90	100	110	120	130	140	150	160
GHLRFFLCSL	YAPMCTEQVS	TPIPACRVMC	EQARLKCSPI	MEQFNFKWPD	SLDCRKLPNK	NDPNYLCMEA	PNNGSDEPTR
170	180	190	200	210	220	230	240
GSGLFPPLFR	PQRPHSAQEH	PLKDGGPGRG	GCDNPGKFHH	VEKSASCAPL	CTPGVDVYWS	REDKRFAVVW	LAIWAVLCFF
250	260	270	280	290	300	310	320
SSAFTVLTFL	IDPARFRYPE	RPIIFLSMCY	CVYSVGYLIR	LFAGAESIAC	DRDSGQLYVI	QEGLESTGCT	LVFLVLYYFG
330	340	350	360	370	380	390	400
MASSLWWVVL	TLTWFLAAGK	KWGHEAIEAN	SSYFHLAAWA	IPAVKTILIL	VMRRVAGDEL	TGVCYVGSMD	VNALTGFVLI
410	420	430	440	450	460	470	480
PLACYLVIGT	SFILSGFVAL	FHIRRVMKTG	GENTDKLEKL	MVRIGLFSVL	YTVPATCVIA	CYFYERLNMD	YWKILAAQHK
490	500	510	520	530	540	550	560
CKMNNQTKTL	DCLMAASIPA	VEIFMVKIFM	LLVVGITSGM	WIWTSKTLQS	WQQVCSRRLK	KKSRRKPASV	ITSGGIYKKA
570	580	590					
QHPQKTHHGK	YEIPAQSPTC	V					

Cmpd.	No. of Cmpds.	m/z meas.	Δ m/z [ppm]	z	Rt [min]	Score	P	Range	Sequence	Modification
1958	1	655.2730	-62.48	2	52.8	10.4	0	281-292	R.LFAGAESIACDR.D	Carbamidomethyl: 10
2186	1	928.7350	-68.15	3	55.7	17.1	1	483-507	K.MNNQTKTLDCLMAASIPAVEIFMVK.I	Oxidation: 23

Detailed Protein Report

Protein 249: PREDICTED: otoferlin isoform X1 [Homo sapiens]

**Accession:**	gi|530368284	**Score:**	38.7
**Database:**	refseq_human(refseq_human_20140103.fasta)	**MW [kDa]:**	225.9
**Database Date:**	2015-11-30	**pI:**	5.3
		**Sequence Coverage [%]:**	2.4
		**No. of unique Peptides:**	3

Quantitation

QU:MU	**Median:**	0.81	**CV:**	0.00 %	**No. of Peptides:**	1
WUP:QUP	**Median:**	1.12	**CV:**	0.00 %	**No. of Peptides:**	1

Detailed Protein Report

10	20	30	40	50	60	70	80
MALLIHLKTV	SELRGRGDRI	AKVTFRGQSF	YSRVLENCED	VADFDETFRW	PVASSIDRNE	MLEIQVFNYS	KVFSNKLIGT
90	100	110	120	130	140	150	160
FRMVLQKVVE	ESHVEVTDTL	IDDNNAIIKT	SLCVEVRYQA	TDGTVGSWDD	GDFLGDESLQ	EEEKDSQETD	GLLPGSRPSS
170	180	190	200	210	220	230	240
RPPGEKSFRS	KGREKTKGGR	DDEHKAGRSV	FSAMKLGKNR	SHKEEPQRPD	EPAVLEMEDL	DHLAIRLGDG	LDPDSVSLAS
250	260	270	280	290	300	310	320
VTALTTNVSN	KRSKPDIKME	PSAGRPMDYQ	VSITVIEARQ	LVGLNMDPVV	CVEVGDDKKY	TSMKESTNCP	YYNEYFVFDF
330	340	350	360	370	380	390	400
HVSPDVMFDK	IIKISVIHSK	NLLRSGTLVG	SFKMDVGTVY	SQPEHQFHHK	WAILSDPDDI	SSGLKGYVKC	DVAVVGKGDN
410	420	430	440	450	460	470	480
IKTPHKANET	DEDDIEGNLL	LPEGVPPERQ	WARFYVKIYR	AEGLPRMNTS	LMANVKKAFI	GENKDLVDPY	VQVFFAGQKG
490	500	510	520	530	540	550	560
KTSVQKSSYE	PLWNEQVVFT	DLFPPLCKRM	KVQIRDSDKV	NDVAIGTHFI	DLRKISNDGD	KGFLPTLGPA	WVNMYGSTRN
570	580	590	600	610	620	630	640
YTLLDEHQDL	NEGLGEGVSF	RARLLLGLAV	EIVDTSNPEL	TSSTEVQVEQ	ATPISESCAG	KMEEFFLFGA	FLEASMIDRR
650	660	670	680	690	700	710	720
NGDKPITFEV	TIGNYGNEVD	GLSRPQRPRP	RKEPGDEEEV	DLIQNASDDE	AGDAGDLASV	SSTPPMRPQV	TDRNYFHLPY
730	740	750	760	770	780	790	800
LERKPCIYIK	SWWPDQRRRL	YNANIMDHIA	DKLEEGLNDI	QEMIKTEKSY	PERRLRGVLE	ELSCGCCRFL	SLADKDQGHS
810	820	830	840	850	860	870	880
SRTRLDRERL	KSCMRELENM	GQQARMLRAQ	VKRHTVRDKL	RLCQNFLQKL	RFLADEPQHS	IPDIFIWMMS	NNKRVAYARV
890	900	910	920	930	940	950	960
PSKDLLFSIV	EEETGKDCAK	VKTLFLKLPG	KRGFGSAGWT	VQAKVELYLW	LGLSKQRKEF	LCGLPCGFQE	VKAAQGLGLH
970	980	990	1000	1010	1020	1030	1040
AFPPVSLVYT	KKQAFQLRAH	MYQARSLFAA	DSSGLSDPFA	RVFFINQSQC	TEVLNETLCP	TWDQMLVFDN	LELYGEAHEL
1050	1060	1070	1080	1090	1100	1110	1120
RDDPPIIVIE	IYDQDSMGKA	DFMGRTFAKP	LVKMADEAYC	PPRFPPQLEY	YQIYRGNATA	GDLLAAFELL	QIGPAGKADL
1130	1140	1150	1160	1170	1180	1190	1200
PPINGPVDVD	RGPIMPVPMG	IRPVLSKYRV	EVLFWGLRDL	KRVNLAQVDR	PRVDIECAGK	GVQSSLIHNY	KKNPNFNTLV
1210	1220	1230	1240	1250	1260	1270	1280
KWFEVDLPEN	ELLHPPLNIR	VVDCRAFGRY	TLVGSHAVSS	LRRFIYRPPD	RSAPSWNTTG	EVVVTMEPEV	PIKKLETMVK
1290	1300	1310	1320	1330	1340	1350	1360
LDATSEAVVK	VDVAEEEKEK	KKKKKGTAEE	PEEEEPDESM	LDWWSKYFAS	IDTMKEQLRQ	QEPSGIDLEE	KEEVDNTEGL
1370	1380	1390	1400	1410	1420	1430	1440
KGSMKGKEKA	RAAKEEKKKK	TQSSGSGQGS	EAPEKKKPKI	DELKVYPKEL	ESEFDNFEDW	LHTFNLLRGK	TGDDEDGSTE
1450	1460	1470	1480	1490	1500	1510	1520
EERIVGRFKG	SLCVYKVPLP	EDVSREAGYD	STYGMFQGIP	SNDPINVLVR	VYVVRATDLH	PADINGKADP	YIAIRLGKTD
1530	1540	1550	1560	1570	1580	1590	1600
IRDKENYISK	QLNPVFGKSF	DIEASFPMES	MLTVAVYDWD	LVGTDDLIGE	TKIDLENRFY	SKHRATCGIA	QTYSTHGYNI
1610	1620	1630	1640	1650	1660	1670	1680
WRDPMKPSQI	LTRLCKDGKV	DGPHFGPPGR	VKVANRVFTG	PSEIEDENGQ	RKPTDEHVAL	LALRHWEDIP	RAGCRLVPEH
1690	1700	1710	1720	1730	1740	1750	1760
VETRPLLNPD	KPGIEQGRLE	LWVDMFPMDM	PAPGTPLDIS	PRKPKKYELR	VIIWNTDEVV	LEDDDFFTGE	KSSDIFVRGW
1770	1780	1790	1800	1810	1820	1830	1840
LKGQQEDKQD	TDVHYHSLTG	EGNFNWRYLF	PFDYLAAEEK	IVISKKESMF	SWDETEYKIP	ARLTLQIWDA	DHFSADDFLG
1850	1860	1870	1880	1890	1900	1910	1920
AIELDLNRFP	RGAKTAKQCT	MEMATGEVDV	PLVSIFKQKR	VKGWWPLLAR	NENDEFELTG	KVEAELHLLT	AEEAEKNPVG
1930	1940	1950	1960	1970	1980	1990	2000
LARNEPDPLE	KPNRPDTAFV	WFLNPLKSIK	YLICTRYKWL	IIKIVLALLG	LLMLGLFLYS	LPGYMVKKLL	GA

Cmpd.	No. of Cmpds.	m/z meas.	Δ m/z [ppm]	z	Rt [min]	Score	P	Range	Sequence	Modification	Ratios
1211	2	839.3736	-36.62	2	45.3	13.7	2	181-195	R.DDEHKAGRSVFSAMK.L		WUP:QUP 1.12<br>QU:MU 0.81
1207	1	984.1444	151.03	2	43.4	10.7	0	542-559	K.GFLPTLGPAWVNMYGSTR.N		

Detailed Protein Report

Cmpd.	No. of Cmpds.	m/z meas.	Δ m/z [ppm]	z	Rt [min]	Score	P	Range	Sequence	Modification	Ratios
2705	1	558.6699	91.00	3	64.9	14.3	1	739-752	R.RLYNANIMDHIADK.L		

Detailed Protein Report

Protein 250: vasopressin-neurophysin 2-copeptin preproprotein [Homo sapiens]

Accession: gi|13259533
Database: refseq_human(refseq_human_20140103.fasta)
Database Date: 2015-11-30
Modification(s): Oxidation

Score: 38.5
MW [kDa]: 17.3
pI: 5.1
Sequence Coverage [%]: 11.0
No. of unique Peptides: 1

```
         10         20         30         40         50         60         70         80
MPDTMLPACF LGLLAFSSAC YFQNCPRGGK RAMSDLELRQ CLPCGPGGKG RCFGPSICCA DELGCFVGTA EALRCQEENY
         90        100        110        120        130        140        150        160
LPSPCQSGQK ACGSGGRCAA FGVCCNDESC VTEPECREGF HRRARASDRS NATQLDGPAG ALLLRLVQLA GAPEPFEPAQ
        170
PDAY
```

Cmpd.	No. of Cmpds.	m/z meas.	Δ m/z [ppm]	z	Rt [min]	Score	P	Range	Sequence	Modification
2048	2	945.8202	-134.22	2	56.0	14.9	1	32-49	R.AMSDLELRQCLPCGPGGK.G	Oxidation: 2

Detailed Protein Report

Protein 251: rho GTPase-activating protein 11A isoform 3 [Homo sapiens]

**Accession:**	gi|557128992	**Score:**	38.5
**Database:**	refseq_human(refseq_human_20140103.fasta)	**MW [kDa]:**	92.6
**Database Date:**	2015-11-30	**pI:**	9.9
		**Sequence Coverage [%]:**	5.8
		**No. of unique Peptides:**	3

Alias proteins:

Accession	Name	Description
gi|578827655	refseq_human (refseq_human_20140103.fasta)	PREDICTED: rho GTPase-activating protein 11A isoform X3 [Homo sapiens]
gi|557129007	refseq_human (refseq_human_20140103.fasta)	rho GTPase-activating protein 11A isoform 3 [Homo sapiens]

```
        10         20         30         40         50         60         70         80
MDSSNLAVIF APNLLQTSEG HEKMSSNTEK KLRLQAAVVQ TLIDYASDIG RVPDFILEKI PAMLGIDGLC ATPSLEGFEE
        90        100        110        120        130        140        150        160
GEYETPGEYK RKRRQSVGDF VSGALNKFKP NRTPSITPQE ERIAQLSESP VILTPNAKRT LPVDSSHGFS SKKRKSIKHN
       170        180        190        200        210        220        230        240
FNFELLPSNL FNSSSTPVSV HIDTSSEGSS QSSLSPVLIG GNHLITAGVP RRSKRIAGKK VCRVESGKAG CFSPKISHKE
       250        260        270        280        290        300        310        320
KVRRSLRLKF NLGKNGREVN GCSGVNRYES VGWRLANQQS LKNRIESVKT GLLFSPDVDE KLPKKGSEKI SKSEETLLTP
       330        340        350        360        370        380        390        400
ERLVGTNYRM SWTGPNNSSF QEVDANEASS MVENLEVENS LEPDIMVEKS PATSCELTPS NLNNKHNSNI TSSPLSGDEN
       410        420        430        440        450        460        470        480
NMTKETLVKV QKAFSESGSN LHALMNQRQS SVTNVGKVKL TEPSYLEDSP EENLFETNDL TIVESKEKYE HHTGKGEKCF
       490        500        510        520        530        540        550        560
SERDFSPLQT QTFNRETTIK CYSTQMKMEH EKDIHSNMPK DYLSKQEFSS DEEIKKQQSP KDKLNNKLKE NENMMEGNLP
       570        580        590        600        610        620        630        640
KCAAHSKDEA RSSFSQQSTC VVTNLSKPRP MRIAKQQSLE TCEKTVSESS QMTEHRKVSD HIQWFNKLSL NEPNRIKVKS
       650        660        670        680        690        700        710        720
PLKFQRTPVR QSVRRINSLL EYSRQPTGHK LASLGDTASP LVKSVSCDGA LSSCIESASK DSSVSCIKSG PKEQKSMSCE
       730        740        750        760        770        780        790        800
ESNIGAISKS SMELPSKSFL KMRKHPDSVN ASLRSTTVYK QKILSDGQVK VPLDDLTNHD IVKPVVNNNM GISSGINNRV
       810        820        830        840
LRRPSERGRA WYKGSPKHPI GKTQLLPTSK PVDL
```

Cmpd.	No. of Cmpds.	m/z meas.	Δ m/z [ppm]	z	Rt [min]	Score	P	Range	Sequence	Modification
1727	3	682.5948	-72.91	3	51.9	11.1	0	386-404	K.HNSNITSSPLSGDENNMTK.E	
2120	2	703.2439	-91.13	2	54.9	13.2	0	550-561	K.ENENMMEGNLPK.C	
2613	2	822.4595	113.47	2	63.4	14.2	0	684-700	K.SVSCDGALSSCIESASK.D	

Detailed Protein Report

Protein 252: PREDICTED: tetratricopeptide repeat protein 18 isoform X8 [Homo sapiens]

**Accession:**	gi|530392981	**Score:**	38.4
**Database:**	refseq_human(refseq_human_20140103.fasta)	**MW [kDa]:**	105.0
**Database Date:**	2015-11-30	**pI:**	5.6
**Modification(s):**	Carbamidomethyl	**Sequence Coverage [%]:**	4.3
		**No. of unique Peptides:**	2

Quantitation

QU:MU	**Median:** 0.36	**CV:** 0.00 %	**No. of Peptides:**	1
WUP:QUP	**Median:** 2.95	**CV:** 0.00 %	**No. of Peptides:**	1

Alias proteins:

Accession	Name	Description
gi|578818878	refseq_human (refseq_human_20140103.fasta)	PREDICTED: tetratricopeptide repeat protein 18 isoform X16 [Homo sapiens]
gi|578818876	refseq_human (refseq_human_20140103.fasta)	PREDICTED: tetratricopeptide repeat protein 18 isoform X15 [Homo sapiens]
gi|578818874	refseq_human (refseq_human_20140103.fasta)	PREDICTED: tetratricopeptide repeat protein 18 isoform X14 [Homo sapiens]

```
        10         20         30         40         50         60         70         80
MVGLQVPSLG EKDYPILFKN GTLKLGGERE PVPRPKKWPI ANILAPGANN IPDAFIVGGP YEEEEGELNH PEDSEFRNQA
        90        100        110        120        130        140        150        160
ECIKKRIIWD LESRCYLDPS AVVSFQKRIA DCRLWPVEIT RVPLVTIPKG KAGKTEKTDE EAQLSFHGVA YVNMVPLLYP
       170        180        190        200        210        220        230        240
GVKRIRGAFH VYPYLDSVVH EKTKCLLSLF RDIGHHLIHN NKIGGINSLL SKQAVSKNLK EDKPVKEKDI DGRPRPGDVQ
       250        260        270        280        290        300        310        320
APSIKSQSSD TPLEGEPPLS HNPEGQQYVE AGTYIVLEIQ LDKALVPKRM PEELARRVKE MIPPRPPLTR RTGGAQKAVS
       330        340        350        360        370        380        390        400
DYHIQIKNIS RAILDEYYRM FGKQVAKLES DMDSETLEEQ KCQLSYELNC SGKYFAFKEQ LKHAVVKIVR DKYLKTTSFE
       410        420        430        440        450        460        470        480
SQEELQTFIS ELYVFLVDQM HVALNQTMPD DVQGTVATIY TSSEQLQLFA FEAEVNENFE MAAAYYKERL VREPQNLDHW
       490        500        510        520        530        540        550        560
LDYGAFCLLT EDNIKAQECF QKALSLNQSH IHSLLLCGVL AVLLENYEQA EIFFEDATCL EPTNVVAWTL LGLYYEIQNN
       570        580        590        600        610        620        630        640
DIRMEMAFHE ASKQLQARML QAQVTKQKST GVEDTEERGK RESSLGPWGI TNGSATAIKV EAPAGPGAAL SILDKFLEES
       650        660        670        680        690        700        710        720
SKLQSDSQEP ILTTQTWDPS ISQKPSNTFI KEIPTKKEAS KCQDSSALLH PGLHYGVSQT TTIFMETIHF LMKVKAVQYV
       730        740        750        760        770        780        790        800
HRVLAHELLC PQGGPSCEYY LVLAQTHILK KNFAKAEEYL QQAAQMDYLN PNVWGLKGHL YFLSGNHSEA KACYERTISF
       810        820        830        840        850        860        870        880
VVDASEMHFI FLRLGLIYLE EKEYEKAKKT YMQACKRSPS CLTWLGLGIA CYRLEELTEA EDALSEANAL NNYNAEVWAY
       890        900        910        920        930
LALVCLKVGR QLEAEQAYKY MIKLKLKDEA LLAEIHTLQE TVGFGNPSF
```

Cmpd.	No. of Cmpds.	m/z meas.	Δ m/z [ppm]	z	Rt [min]	Score	P	Range	Sequence	Modification	Ratios
1779	1	516.2947	29.06	2	50.6	10.7	0	87-94	R.IIWDLESR.C		WUP:QUP 2.95<br>QU:MU 0.36
81	1	1120.1328	99.73	2	30.9	12.9	1	778-796	K.GHLYFLSGNHSEAKACYER.T	Carbamidomethyl: 16	

Detailed Protein Report

Protein 253: PREDICTED: serine-protein kinase ATM isoform X4 [Homo sapiens]

**Accession:**	gi|578822211	**Score:**	38.4
**Database:**	refseq_human(refseq_human_20140103.fasta)	**MW [kDa]:**	310.0
**Database Date:**	2015-11-30	**pI:**	5.9
		**Sequence Coverage [%]:**	1.3
		**No. of unique Peptides:**	1

Detailed Protein Report

10	20	30	40	50	60	70	80
MADICHQVFN	EDTRSLEISQ	SYTTTQRESS	DYSVPCKRKK	IELGWEVIKD	HLQKSQNDFD	LVPWLQIATQ	LISKYPASLP
90	100	110	120	130	140	150	160
NCELSPLLMI	LSQLLPQQRH	GERTPYVLRC	LTEVALCQDK	RSNLESSQKS	DLLKLWNKIW	CITFRGISSE	QIQAENFGLL
170	180	190	200	210	220	230	240
GAIIQGSLVE	VDREFWKLFT	GSACRPSCPA	VCCLTLALTT	SIVPGTVKMG	IEQNMCEVNR	SFSLKESIMK	WLLFYQLEGD
250	260	270	280	290	300	310	320
LENSTEVPPI	LHSNFPHLVL	EKILVSLTMK	NCKAAMNFFQ	SVPECEHHQK	DKEELSFSEV	EELFLQTTFD	KMDFLTIVRE
330	340	350	360	370	380	390	400
CGIEKHQSSI	GFSVHQNLKE	SLDRCLLGLS	EQLLNNYSSE	ITNSETLVRC	SRLLVGVLGC	YCYMGVIAEE	EAYKSELFQK
410	420	430	440	450	460	470	480
AKSLMQCAGE	SITLFKNKTN	EEFRIGSLRN	MMQLCTRCLS	NCTKKSPNKI	ASGFFLRLLT	SKLMNDIADI	CKSLASFIKK
490	500	510	520	530	540	550	560
PFDRGEVESM	EDDTNGNLME	VEDQSSMNLF	NDYPDSSVSD	ANEPGESQST	IGAINPLAEE	YLSKQDLLFL	DMLKFLCLCV
570	580	590	600	610	620	630	640
TTAQTNTVSF	RAADIRRKLL	MLIDSSTLEP	TKSLHLHMYL	MLLKELPGEE	YPLPMEDVLE	LLKPLSNVCS	LYRRDQDVCK
650	660	670	680	690	700	710	720
TILNHVLHVV	KNLGQSNMDS	ENTRDAQGQF	LTVIGAFWHL	TKERKYIFSV	RMALVNCLKT	LLEADPYSKW	AILNVMGKDF
730	740	750	760	770	780	790	800
PVNEVFTQFL	ADNHHQVRML	AAESINRLFQ	DTKGDSSRLL	KALPLKLQQT	AFENAYLKAQ	EGMREMSHSA	ENPETLDEIY
810	820	830	840	850	860	870	880
NRKSVLLTLI	AVVLSCSPIC	EKQALFALCK	SVKENGLEPH	LVKKVLEKVS	ETFGYRRLED	FMASHLDYLV	LEWLNLQDTE
890	900	910	920	930	940	950	960
YNLSSFPFIL	LNYTNIEDFY	RSCYKVLIPH	LVIRSHFDEV	KSIANQIQED	WKSLLTDCFP	KILVNILPYF	AYEGTRDSGM
970	980	990	1000	1010	1020	1030	1040
AQQRETATKV	YDMLKSENLL	GKQIDHLFIS	NLPEIVVELL	MTLHEPANSS	ASQSTDLCDF	SGDLDPAPNP	PHFPSHVIKA
1050	1060	1070	1080	1090	1100	1110	1120
TFAYISNCHK	TKLKSILEIL	SKSPDSYQKI	LLAICEQAAE	TNNVYKKHRI	LKIYHLFVSL	LLKDIKSGLG	GAWAFVLRDV
1130	1140	1150	1160	1170	1180	1190	1200
IYTLIHYINQ	RPSCIMDVSL	RSFSLCCDLL	SQVCQTAVTY	CKDALENHLH	VIVGTLIPLV	YEQVEVQKQV	LDLLKYLVID
1210	1220	1230	1240	1250	1260	1270	1280
NKDNENLYIT	IKLLDPFPDH	VVFKDLRITQ	QKIKYSRGPF	SLLEEINHFL	SVSVYDALPL	TRLEGLKDLR	RQLELHKDQM
1290	1300	1310	1320	1330	1340	1350	1360
VDIMRASQDN	PQDGIMVKLV	VNLLQLSKMA	INHTGEKEVL	EAVGSCLGEV	GPIDFSTIAI	QHSKDASYTK	ALKLFEDKEL
1370	1380	1390	1400	1410	1420	1430	1440
QWTFIMLTYL	NNTLVEDCVK	VRSAAVTCLK	NILATKTGHS	FWEIYKMTTD	PMLAYLQPFR	TSRKKFLEVP	RFDKENPFEG
1450	1460	1470	1480	1490	1500	1510	1520
LDDINLWIPL	SENHDIWIKT	LTCAFLDSGG	TKCEILQLLK	PMCEVKTDFC	QTVLPYLIHD	ILLQDTNESW	RNLLSTHVQG
1530	1540	1550	1560	1570	1580	1590	1600
FFTSCLRHFS	QTSRSTTPAN	LDSESEHFFR	CCLDKKSQRT	MLAVVDYMRR	QKRPSSGTIF	NDAFWLDLNY	LEVAKVAQSC
1610	1620	1630	1640	1650	1660	1670	1680
AAHFTALLYA	EIYADKKSMD	DQEKRSLAFE	EGSQSTTISS	LSEKSKEETG	ISLQDLLLEI	YRSIGEPDSL	YGCGGGKMLQ
1690	1700	1710	1720	1730	1740	1750	1760
PITRLRTYEH	EAMWGKALVT	YDLETAIPSS	TRQAGIIQAL	QNLGLCHILS	VYLKGLDYEN	KDWCPELEEL	HYQAAWRNMQ
1770	1780	1790	1800	1810	1820	1830	1840
WDHCTSVSKE	VEGTSYHESL	YNALQSLRDR	EFSTFYESLK	YARVKEVEEM	CKRSLESVYS	LYPTLSRLQA	IGELESIGEL
1850	1860	1870	1880	1890	1900	1910	1920
FSRSVTHRQL	SEVYIKWQKH	SQLLKDSDFS	FQEPIMALRT	VILEILMEKE	MDNSQRECIK	DILTKHLVEL	SILARTFKNT
1930	1940	1950	1960	1970	1980	1990	2000
QLPERAIFQI	KQYNSVSCGV	SEWQLEEAQV	FWAKKEQSLA	LSILKQMIKK	LDASCAANNP	SLKLTYTECL	RVCGNWLAET
2010	2020	2030	2040	2050	2060	2070	2080
CLENPAVIMQ	TYLEKAVEVA	GNYDGESSDE	LRNGKMKAFL	SLARFSDTQY	QRIENYMKSS	EFENKQALLK	RAKEEVGLLR
2090	2100	2110	2120	2130	2140	2150	2160
EHKIQTNRYT	VKVQRELELD	ELALRALKED	RKRFLCKAVE	NYINCLLSGE	EHDMWVFRLC	SLWLENSGVS	EVNGMMKRDG
2170	2180	2190	2200	2210	2220	2230	2240
MKIPTYKFLP	LMYQLAARMG	TKMMGGLGFH	EVLNNLISRI	SMDHPHHTLF	IILALANANR	DEFLTKPEVA	RRSRITKNVP
2250	2260	2270	2280	2290	2300	2310	2320
KQSSQLDEDR	TEAANRIICT	IRSRRPQMVR	SVEALCDAYI	ILANLDATQW	KTQRKGINIP	ADQPITKLKN	LEDVVVPTME
2330	2340	2350	2360	2370	2380	2390	2400

Detailed Protein Report

Cmpd.	No. of Cmpds.	m/z meas.	Δ m/z [ppm]	z	Rt [min]	Score	P	Range	Sequence	Modification
2544	1	885.4222	-31.19	2	61.8	14.9	1	403-418	K.SLMQCAGESITLFKNK.T	

BRUKER

Detailed Protein Report

Protein 254: **protein FAM104A isoform 3 [Homo sapiens]**

Accession: gi|574956994
Database: refseq_human(refseq_human_20140103.fasta)
Database Date: 2015-11-30

Score: 38.3
MW [kDa]: 9.8
pI: 12.7
Sequence Coverage [%]: 40.8
No. of unique Peptides: 1

```
        10         20         30         40         50         60         70         80
MGGRGADAGS SGGTGPTEGY SPPAASTRAA ARAKARGGGR GGRRNTTPSV PSLRGAAPRS FHPPAAMSER LRPSLQAVTV
        90        100        110
VGAAAAAAAA SIARTGPAGR KAA
```

Cmpd.	No. of Cmpds.	m/z meas.	Δ m/z [ppm]	z	Rt [min]	Score	P	Range	Sequence	Modification
2165	4	974.4048	-53.21	3	55.4	21.1	2	1-32	-.MGGRGADAGSSGGTGPTEGYSPPAASTRAAAR.A	

Detailed Protein Report

Protein 255: ATP-dependent DNA helicase Q1 [Homo sapiens]

**Accession:**	gi|14591902	**Score:**	38.3
**Database:**	refseq_human(refseq_human_20140103.fasta)	**MW [kDa]:**	73.4
**Database Date:**	2015-11-30	**pI:**	9.0
		**Sequence Coverage [%]:**	5.5
		**No. of unique Peptides:**	1

Alias proteins:

Accession	Name	Description
gi|578823000	refseq_human (refseq_human_20140103.fasta)	PREDICTED: ATP-dependent DNA helicase Q1 isoform X5 [Homo sapiens]
gi|530399347	refseq_human (refseq_human_20140103.fasta)	PREDICTED: ATP-dependent DNA helicase Q1 isoform X4 [Homo sapiens]
gi|530399345	refseq_human (refseq_human_20140103.fasta)	PREDICTED: ATP-dependent DNA helicase Q1 isoform X3 [Homo sapiens]
gi|530399343	refseq_human (refseq_human_20140103.fasta)	PREDICTED: ATP-dependent DNA helicase Q1 isoform X2 [Homo sapiens]
gi|530399341	refseq_human (refseq_human_20140103.fasta)	PREDICTED: ATP-dependent DNA helicase Q1 isoform X1 [Homo sapiens]
gi|14591904	refseq_human (refseq_human_20140103.fasta)	ATP-dependent DNA helicase Q1 [Homo sapiens]

10	20	30	40	50	60	70	80
MASVSALTEE	LDSITSELHA	VEIQIQELTE	RQQELIQKKK	VLTKKIKQCL	EDSDAGASNE	YDSSPAAWNK	EDFPWSGKVK
**90**	**100**	**110**	**120**	**130**	**140**	**150**	**160**
DILQNVFKLE	KFRPLQLETI	NVTMAGKEVF	LVMPTGGGKS	LCYQLPALCS	DGFTLVICPL	ISLMEDQLMV	LKQLGISATM
**170**	**180**	**190**	**200**	**210**	**220**	**230**	**240**
LNASSSKEHV	KWVHAEMVNK	NSELKLIYVT	PEKIAKSKMF	MSRLEKAYEA	RRFTRIAVDE	VHCCSQWGHD	FRPDYKALGI
**250**	**260**	**270**	**280**	**290**	**300**	**310**	**320**
LKRQFPNASL	IGLTATATNH	VLTDAQKILC	IEKCFTFTAS	FNRPNLYYEV	RQKPSNTEDF	IEDIVKLING	RYKGQSGIIY
**330**	**340**	**350**	**360**	**370**	**380**	**390**	**400**
CFSQKDSEQV	TVSLQNLGIH	AGAYHANLEP	EDKTTVHRKW	SANEIQVVVA	TVAFGMGIDK	PDVRFVIHHS	MSKSMENYYQ
**410**	**420**	**430**	**440**	**450**	**460**	**470**	**480**
ESGRAGRDDM	KADCILYYGF	GDIFRISSMV	VMENVGQQKL	YEMVSYCQNI	SKCRRVLMAQ	HFDEVWNSEA	CNKMCDNCCK
**490**	**500**	**510**	**520**	**530**	**540**	**550**	**560**
DSAFERKNIT	EYCRDLIKIL	KQAEELNEKL	TPLKLIDSWM	GKGAAKLRVA	GVVAPTLPRE	DLEKIIAHFL	IQQYLKEDYS
**570**	**580**	**590**	**600**	**610**	**620**	**630**	**640**
FTAYATISYL	KIGPKANLLN	NEAHAITMQV	TKSTQNSFRA	ESSQTCHSEQ	GDKKMEEKNS	GNFQKKAANM	LQQSGSKNTG
**650**							
AKKRKIDDA							

Cmpd.	No. of Cmpds.	m/z meas.	Δ m/z [ppm]	z	Rt [min]	Score	P	Range	Sequence	Modification
2269	1	701.1276	94.64	3	58.2	13.1	1	540-556	R.EDLEKIIAHFLIQQYLK.E	

Detailed Protein Report

Protein 256: uncharacterized protein LOC284677 [Homo sapiens]

Accession: gi|197116396
Database: refseq_human(refseq_human_20140103.fasta)
Database Date: 2015-11-30
Modification(s): Carbamidomethyl, Oxidation

Score: 38.3
MW [kDa]: 25.1
pI: 10.6
Sequence Coverage [%]: 8.1
No. of unique Peptides: 2

```
        10         20         30         40         50         60         70         80
MGMLAPGPLQ GRRPRKGHKG QEDAVAPGCK ASGRGSRVTH LLGYPTQNVS RSLRRKYAPP PCGGPEDVAL APCTAAAACE
        90        100        110        120        130        140        150        160
AGPSPVYVKV KSAEPADCAE GPVQCKNGLL VSSPHCEEPC AHSCAHPGLP PHLVHKLPLS YLQTQDTDAA SRRINAPLAA
       170        180        190        200        210        220        230        240
GWSWLRLWLV TLASGVDFPQ VSAWMRALPS PDCPGLRTTG EQMQKLLLKE NKVKTRKSKR RSGEGSHLTT SILEQ
```

Cmpd.	No. of Cmpds.	m/z meas.	Δ m/z [ppm]	z	Rt [min]	Score	P	Range	Sequence	Modification
2182	1	682.2713	-87.72	3	57.7	10.9	1	187-205	R.ALPSPDCPGLRTTGEQMQK.L	Oxidation: 17
2206	2	701.3221	-23.13	3	57.5	27.4	1	187-205	R.ALPSPDCPGLRTTGEQMQK.L	Carbamidomethyl: 7; Oxidation: 17

Detailed Protein Report

Protein 257: neuroblastoma breakpoint family member 6 isoform 2 [Homo sapiens]

**Accession:**	gi|221136848	**Score:**	38.3
**Database:**	refseq_human(refseq_human_20140103.fasta)	**MW [kDa]:**	72.2
**Database Date:**	2015-11-30	**pI:**	4.7
**Modification(s):**	Oxidation	**Sequence Coverage [%]:**	3.8
		**No. of unique Peptides:**	1

10	20	30	40	50	60	70	80
MVVSADPLSS	ERAEMNILEI	NQELRSQLAE	SNQQFRDLKE	KFLITQATAY	SLANQLKKYK	CEEYKDIIDS	VLRDELQSME
**90**	**100**	**110**	**120**	**130**	**140**	**150**	**160**
KLAEKLRQAE	ELRQYKALVH	SQAKELTQLR	EKLREGRDAS	RWLNKHLKTL	LTPDDPDKSQ	GQDLREQLAE	GHRLAEHLVH
**170**	**180**	**190**	**200**	**210**	**220**	**230**	**240**
KLSPENDEDE	DEDEDDKDEE	VEKVQESPAP	REVQKTEEKE	VPQDSLEECA	VTCSNSHNPS	NSNQPHRSTK	ITFKEHEVDS
**250**	**260**	**270**	**280**	**290**	**300**	**310**	**320**
ALVVESEHPH	DEEEEALNIP	PENQNDHEEE	EGKAPVPPRH	HDKSNSYRHR	EVSFLALDEQ	KVCSAQDVAR	DYSNPKWDET
**330**	**340**	**350**	**360**	**370**	**380**	**390**	**400**
SLGFLEKQSD	LEEVKGQETV	APRLSRGPLR	VDKHEIPQES	LDGCCLTPSI	LPDLTPSYHP	YWSTLYSFED	KQVSLALVDK
**410**	**420**	**430**	**440**	**450**	**460**	**470**	**480**
IKKDQEEIED	QSPPCPRLSQ	ELPEVKEQEV	PEDSVNEVYL	TPSVHHDVSD	CHQPYSSTLS	SLEDQLACSA	LDVASPTEAA
**490**	**500**	**510**	**520**	**530**	**540**	**550**	**560**
CPQGTWSGDL	SHHRSEVQIS	QAQLEPSTLV	PSCLRLQLDQ	GFHCGNGLAQ	RGLSSTTCSF	SANADSGNQW	PFQELVLEPS
**570**	**580**	**590**	**600**	**610**	**620**	**630**	**640**
LGMKNPPQLE	DDALEGSASN	TQGRQVTGRI	RASLVLILKT	IRRRLPFSKW	RLAFRFAGPH	AESAEIPNTA	ERMQRMIG

Cmpd.	No. of Cmpds.	m/z meas.	Δ m/z [ppm]	z	Rt [min]	Score	P	Range	Sequence	Modification
1180	1	384.1589	-56.37	2	44.9	12.5	1	633-638	R.MQRMIG.-	Oxidation: 1, 4

Detailed Protein Report

Protein 258: cadherin EGF LAG seven-pass G-type receptor 1 precursor [Homo sapiens]

**Accession:**	gi|7656967	**Score:**	38.2
**Database:**	refseq_human(refseq_human_20140103.fasta)	**MW [kDa]:**	329.3
**Database Date:**	2015-11-30	**pI:**	5.5
		**Sequence Coverage [%]:**	1.4
		**No. of unique Peptides:**	1

Detailed Protein Report

10	20	30	40	50	60	70	80
MAPPPPPVLP	VLLLLAAAAA	LPAMGLRAAA	WEPRVPGGTR	AFALRPGCTY	AVGAACTPRA	PRELLDVGRD	GRLAGRRRVS
90	100	110	120	130	140	150	160
GAGRPLPLQV	RLVARSAPTA	LSRRLRARTH	LPGCGARARL	CGTGARLCGA	LCFPVPGGCA	AAQHSALAAP	TTLPACRCPP
170	180	190	200	210	220	230	240
RPRPRCPGRP	ICLPPGGSVR	LRLLCALRRA	AGAVRVGLAL	EAATAGTPSA	SPSPSPPLPP	NLPEARAGPA	RRARRGTSGR
250	260	270	280	290	300	310	320
GSLKFPMPNY	QVALFENEPA	GTLILQLHAH	YTIEGEEERV	SYYMEGLFDE	RSRGYFRIDS	ATGAVSTDSV	LDRETKETHV
330	340	350	360	370	380	390	400
LRVKAVDYST	PPRSATTYIT	VLVKDTNDHS	PVFEQSEYRE	RVRENLEVGY	EVLTIRASDR	DSPINANLRY	RVLGGAWDVF
410	420	430	440	450	460	470	480
QLNESSGVVS	TRAVLDREEA	AEYQLLVEAN	DQGRNPGPLS	ATATVYIEVE	DENDNYPQFS	EQNYVVQVPE	DVGLNTAVLR
490	500	510	520	530	540	550	560
VQATDRDQGQ	NAAIHYSILS	GNVAGQFYLH	SLSGILDVIN	PLDFEDVQKY	SLSIKAQDGG	RPPLINSSGV	VSVQVLDVND
570	580	590	600	610	620	630	640
NEPIFVSSPF	QATVLENVPL	GYPVVHIQAV	DADSGENARL	HYRLVDTAST	FLGGGSAGPK	NPAPTPDFPF	QIHNSSGWIT
650	660	670	680	690	700	710	720
VCAELDREEV	EHYSFGVEAV	DHGSPPMSSS	TSVSITVLDV	NDNDPVFTQP	TYELRLNEDA	AVGSSVLTLQ	ARDRDANSVI
730	740	750	760	770	780	790	800
TYQLTGGNTR	NRFALSSQRG	GGLITLALPL	DYKQEQQYVL	AVTASDGTRS	HTAHVLINVT	DANTHRPVFQ	SSHYTVSVSE
810	820	830	840	850	860	870	880
DRPVGTSIAT	LSANDEDTGE	NARITYVIQD	PVPQFRIDPD	SGTMYTMMEL	DYENQVAYTL	TIMAQDNGIP	QKSDTTTLEI
890	900	910	920	930	940	950	960
LILDANDNAP	QFLWDFYQGS	IFEDAPPSTS	ILQVSATDRD	SGPNGRLLYT	FQGGDDGDGD	FYIEPTSGVI	RTQRRLDREN
970	980	990	1000	1010	1020	1030	1040
VAVYNLWALA	VDRGSPTPLS	ASVEIQVTIL	DINDNAPMFE	KDELELFVEE	NNPVGSVVAK	IRANDPDEGP	NAQIMYQIVE
1050	1060	1070	1080	1090	1100	1110	1120
GDMRHFFQLD	LLNGDLRAMV	ELDFEVRREY	VLVVQATSAP	LVSRATVHIL	LVDQNDNPPV	LPDFQILFNN	YVTNKSNSFP
1130	1140	1150	1160	1170	1180	1190	1200
TGVIGCIPAH	DPDVSDSLNY	TFVQGNELRL	LLLDPATGEL	QLSRDLDNNR	PLEALMEVSV	SDGIHSVTAF	CTLRVTIITD
1210	1220	1230	1240	1250	1260	1270	1280
DMLTNSITVR	LENMSQEKFL	SPLLALFVEG	VAAVLSTTKD	DVFVFNVQND	TDVSSNILNV	TFSALLPGGV	RGQFFPSEDL
1290	1300	1310	1320	1330	1340	1350	1360
QEQIYLNRTL	LTTISTQRVL	PFDDNICLRE	PCENYMKCVS	VLRFDSSAPF	LSSTTVLFRP	IHPINGLRCR	CPPGFTGDYC
1370	1380	1390	1400	1410	1420	1430	1440
ETEIDLCYSD	PCGANGRCRS	REGGYTCECF	EDFTGEHCEV	DARSGRCANG	VCKNGGTCVN	LLIGGFHCVC	PPGEYERPYC
1450	1460	1470	1480	1490	1500	1510	1520
EVTTRSFPPQ	SFVTFRGLRQ	RFHFTISLTF	ATQERNGLLL	YNGRFNEKHD	FIALEIVDEQ	VQLTFSAGET	TTTVAPKVPS
1530	1540	1550	1560	1570	1580	1590	1600
GVSDGRWHSV	QVQYYNKPNI	GHLGLPHGPS	GEKMAVVTVD	DCDTTMAVRF	GKDIGNYSCA	AQGTQTGSKK	SLDLTGPLLL
1610	1620	1630	1640	1650	1660	1670	1680
GGVPNLPEDF	PVHNRQFVGC	MRNLSVDGKN	VDMAGFIANN	GTREGCAARR	NFCDGRRCQN	GGTCVNRWNM	YLCECPLRFG
1690	1700	1710	1720	1730	1740	1750	1760
GKNCEQAMPH	PQLFSGESVV	SWSDLNIIIS	VPWYLGLMFR	TRKEDSVLME	ATSGGPTSFR	LQILNNYLQF	EVSHGPSDVE
1770	1780	1790	1800	1810	1820	1830	1840
SVMLSGLRVT	DGEWHHLLIE	LKNVKEDSEM	KHLVTMTLDY	GMDQNKADIG	GMLPGLTVRS	VVVGGASEDK	VSVRRGFRGC
1850	1860	1870	1880	1890	1900	1910	1920
MQGVRMGGTP	TNVATLNMNN	ALKVRVKDGC	DVDDPCTSSP	CPPNSRCHDA	WEDYSCVCDK	GYLGINCVDA	CHLNPCENMG
1930	1940	1950	1960	1970	1980	1990	2000
ACVRSPGSPQ	GYVCECGPSH	YGPYCENKLD	LPCPRGWWGN	PVCGPCHCAV	SKGFDPDCNK	TNGQCQCKEN	YYKLLAQDTC
2010	2020	2030	2040	2050	2060	2070	2080
LPCDCFPHGS	HSRTCDMATG	QCACKPGVIG	RQCNRCDNPF	AEVTTLGCEV	IYNGCPKAFE	AGIWWPQTKF	GQPAAVPCPK
2090	2100	2110	2120	2130	2140	2150	2160
GSVGNAVRHC	SGEKGWLPPE	LFNCTTISFV	DLRAMNEKLS	RNETQVDGAR	ALQLVRALRS	ATQHTGTLFG	NDVRTAYQLL
2170	2180	2190	2200	2210	2220	2230	2240

Detailed Protein Report

Cmpd.	No. of Cmpds.	m/z meas.	Δ m/z [ppm]	z	Rt [min]	Score	P	Range	Sequence	Modification
155	1	855.3273	-115.70	2	31.7	12.6	0	715-730	R.DANSVITYQLTGGNTR.N	

Detailed Protein Report

Protein 259: coiled-coil domain-containing protein 33 isoform 1 [Homo sapiens]

**Accession:**	gi|117168273	**Score:**	38.2
**Database:**	refseq_human(refseq_human_20140103.fasta)	**MW [kDa]:**	84.9
**Database Date:**	2015-11-30	**pI:**	6.8
		**Sequence Coverage [%]:**	5.8
		**No. of unique Peptides:**	1

10	20	30	40	50	60	70	80
MGLKNKKNTE	DPEEPLIASQ	STEPEIGHLS	PSKKETIMVT	LHGATNLPAC	KDGSEPWPYV	VVKSTSEEKN	NQSSKAVTSV
**90**	**100**	**110**	**120**	**130**	**140**	**150**	**160**
TSEPTRAPIW	GDTVNVEIQA	EDAGQEDVIL	KVVDNRKKQE	LLSYKIPIKY	LRVFHPYHFE	LVKPTESGKA	DEATAKTQLY
**170**	**180**	**190**	**200**	**210**	**220**	**230**	**240**
ATVVRKSSFI	PRYIGCNHMA	LEIFLRGVNE	PLANNPNPIV	VIARVVPNYK	EFKVSQANRD	LASVGLPITP	LSFPIPSMMN
**250**	**260**	**270**	**280**	**290**	**300**	**310**	**320**
FDVPRVSQNG	CPQLSKPGGP	PEQPLWNQSF	LFQGRDGATS	FSEDTALVLE	YYSSTSMKGS	QPWTLNQPLG	ISVLPLKSRL
**330**	**340**	**350**	**360**	**370**	**380**	**390**	**400**
YQKMLTGKGL	DGLHVERLPI	MDTSLKTIND	EAPTVALSFQ	LLSSERPENF	LTPNNSKALP	TLDPKILDKK	LRTIQESWSK
**410**	**420**	**430**	**440**	**450**	**460**	**470**	**480**
DTVSSTMDLS	TSTPREAEEE	PLVPEMSHDT	EMNNYRRAMQ	KMAEDILSLR	RQASILEGEN	RILRSRLAQQ	EEEEGQGKAS
**490**	**500**	**510**	**520**	**530**	**540**	**550**	**560**
EAQNTVSMKQ	KLLLSELDMK	KLRDRVQHLQ	NELIRKNDRE	KELLLLYQAQ	QPQAALLKQY	QGKLQKMKAL	EETVRHQEKV
**570**	**580**	**590**	**600**	**610**	**620**	**630**	**640**
IEKMERVLED	RLQDRSKPPP	LNRQQGKPYT	GFPMLSASGL	PLGSMGENLP	VELYSVLLAE	NAKLRTELDK	NRHQQAPIIL
**650**	**660**	**670**	**680**	**690**	**700**	**710**	**720**
QQQALPDLLS	GTSDKFNLLA	KLEHAQSRIL	SLESQLEDSA	RRWGREKQDL	ATRLQEQEKG	FRHPSNSIII	EQPSALTHSM
**730**	**740**	**750**	**760**				
DLKQPSELEP	LLPSSDSKLN	KPLSPQKETA	NSQQT				

Cmpd.	No. of Cmpds.	m/z meas.	Δ m/z [ppm]	z	Rt [min]	Score	P	Range	Sequence	Modification
1293	1	679.7520	127.71	3	46.3	12.9	1	52-69	K.DGSEPWPYVVVKSTSEEK.N	

Detailed Protein Report

Protein 260: PREDICTED: CLIP-associating protein 1 isoform X9 [Homo sapiens]

**Accession:**	gi|578803955	**Score:**	38.1
**Database:**	refseq_human(refseq_human_20140103.fasta)	**MW [kDa]:**	162.2
**Database Date:**	2015-11-30	**pI:**	9.4
**Modification(s):**	Oxidation	**Sequence Coverage [%]:**	2.3
		**No. of unique Peptides:**	2

10	20	30	40	50	60	70	80
MEPRMESCLA	QVLQKDVGKR	LQVGQELIDY	FSDKQKSADL	EHDQTMLDKL	VDGLATSWVN	SSNYKVVLLG	MDILSALVTR
90	100	110	120	130	140	150	160
LQDRFKAQIG	TVLPSLIDRL	GDAKDSVREQ	DQTLLLKIMD	QAANPQYVWD	RMLGGFKHKN	FRTREGICLC	LIATLNASGA
170	180	190	200	210	220	230	240
QTLTLSKIVP	HICNLLGDPN	SQVRDAAINS	LVEIYRHVGE	RVRADLSKKG	LPQSRLNVIF	TKFDEVQKSG	NMIQSANDKN
250	260	270	280	290	300	310	320
FDDEDSVDGN	RPSSASSTSS	KAPPSSRRNV	GMGTTRRLGS	STLGSKSSAA	KEGAGAVDEE	DFIKAFDDVP	VVQIYSSRDL
330	340	350	360	370	380	390	400
EESINKIREI	LSDDKHDWEQ	RVNALKKIRS	LLLAGAAEYD	NFFQHLRLLD	GAFKLSAKDL	RSQVVREACI	TLGHLSSVLG
410	420	430	440	450	460	470	480
NKFDHGAEAI	MPTIFNLIPN	SAKIMATSGV	VAVRLIIRHT	HIPRLIPVIT	SNCTSKSVAV	RRRCFEFLDL	LLQEWQTHSL
490	500	510	520	530	540	550	560
ERHISVLAET	IKKGIHDADS	EARIEARKCY	WGFHSHFSRE	AEHLYHTLES	SYQKALQSHL	KNSDSIVSLP	QSDRSSSSSQ
570	580	590	600	610	620	630	640
ESLNRPLSAK	RSPTGSTTSR	ASTVSTKSVS	TTGSLQRSRS	DIDVNAAASA	KSKVSSSSGT	TPFSSAAALP	PGSYASLESR
650	660	670	680	690	700	710	720
HMREDMEYIG	LDSGRIRTRR	QSSGSATNVA	STPDNRGRSR	AKVVSQSQPG	SRSSSPGKLL	GSGYGGLTGG	SSRGPPVTPS
730	740	750	760	770	780	790	800
SEKRSKIPRS	QGCSRETSPN	RIGLDRFGLG	QPGRIPGSVN	AMRVLSTSTD	LEAAVADALK	KPVRRRYEPY	GMYSDDDANS
810	820	830	840	850	860	870	880
DASSVCSERS	YGSRNGGIPH	YLRQTEDVAE	VLNHCASSNW	SERKEGLLGL	QNLLKSQRTL	SRVELKRLCE	IFTRMFADPH
890	900	910	920	930	940	950	960
SKRVFSMFLE	TLVDFIIIHK	DDLQDWLFVL	LTQLLKKMGA	DLLGSVQAKV	QKALDVTRDS	FPFDQQFNIL	MRFIVDQTQT
970	980	990	1000	1010	1020	1030	1040
PNLKVKVAIL	KYIESLARQM	DPTDFVNSSE	TRLAVSRIIT	WTTEPKSSDV	RKAAQIVLIS	LFELNTPEFT	MLLGALPKTF
1050	1060	1070	1080	1090	1100	1110	1120
QDGATKLLHN	HLKNSSNTSV	GSPSNTIGRT	PSRHTSSRTS	PLTSPTNCSH	GGLSPSMLDY	DTENLNSEEI	YSSLRGVTEA
1130	1140	1150	1160	1170	1180	1190	1200
IEKFSFRSQE	DLNEPIKRDG	KKECDIVSRD	GGAASPATEG	RGGSEVEGGR	TALDNKTSLL	NTQPPRAFPG	PRARDYNPYP
1210	1220	1230	1240	1250	1260	1270	1280
YSDAINTYDK	TALKEAVFDD	DMEQLRDVPI	DHSDLVADLL	KELSNHNERV	EERKGALLEL	LKITREDSLG	VWEEHFKTIL
1290	1300	1310	1320	1330	1340	1350	1360
LLLLETLGDK	DHSIRALALR	VLREILRNQP	ARFKNYAELT	IMKTLEAHKD	SHKEVVRAAE	EAASTLASSI	HPEQCIKVLC
1370	1380	1390	1400	1410	1420	1430	1440
PIIQTADYPI	NLAAIKMQTK	VVERIAKESL	LQLLVDIIPG	LLQGYDNTES	SVRKASVFCL	VAIYSVIGED	LKPHLAQLTG
1450	1460	1470	1480				
SKMKLLNLYI	KRAQTTNSNS	SSSSDVSTHS					

Cmpd.	No. of Cmpds.	m/z meas.	Δ m/z [ppm]	z	Rt [min]	Score	P	Range	Sequence	Modification
2270	1	614.2104	-96.89	3	58.8	10.1	1	641-655	R.HMREDMEYIGLDSGR.I	Oxidation: 2, 6
1389	1	816.1017	171.47	2	47.0	10.2	1	953-966	R.FIVDQTQTPNLKVK.V	

Detailed Protein Report

Protein 261: uncharacterized protein LOC100132994 [Homo sapiens]

**Accession:**	gi|223468690	**Score:**	38.1
**Database:**	refseq_human(refseq_human_20140103.fasta)	**MW [kDa]:**	54.4
**Database Date:**	2015-11-30	**pI:**	10.1
**Modification(s):**	Carbamidomethyl	**Sequence Coverage [%]:**	6.8
		**No. of unique Peptides:**	3

Quantitation

QU:MU	**Median:** 0.52	**CV:** 0.00 %	**No. of Peptides:**	1
WUP:QUP	**Median:** 1.31	**CV:** 0.00 %	**No. of Peptides:**	1

Alias proteins:

Accession	Name	Description
gi|223468693	refseq_human (refseq_human_20140103.fasta)	putative uncharacterized protein CXorf49 [Homo sapiens]

10	20	30	40	50	60	70	80
MSSPDKVSVC	GAGFDLEGGK	KAGSRTASPG	APGAHSHGLD	LGVPGSGDGK	SESGFTDPEG	FSFESESELI	EQGRVVLWGR
**90**	**100**	**110**	**120**	**130**	**140**	**150**	**160**
EGRPGTPVDD	QGDVVDYSFY	LADEPAAIVP	PPSVQGHPFP	EGAAAEGSAE	NWADAEVGPS	GRDVLGHSPG	KWQQASAGRL
**170**	**180**	**190**	**200**	**210**	**220**	**230**	**240**
HLCGPGPVRA	WKNPERGSKS	RWSLRVDPQQ	PSAKGPTRLP	THDSDSADES	SDLPLMKVGI	CRNEGSQAKP	GSPKKRADTS
**250**	**260**	**270**	**280**	**290**	**300**	**310**	**320**
RQASFHCKES	YLPVPGRFLT	SAPRGLTPVA	ERPAVGELED	SPQKKMQSRA	WGKVEVRPSC	SGAAAAGALP	QGLSRRKMAG
**330**	**340**	**350**	**360**	**370**	**380**	**390**	**400**
GKKSLGGASQ	LALGRGFPAC	GERLSAAPPE	PATFPPFSGV	RPQGMSKKPQ	KPKHSSPGKK	PAGRKTRESQ	AAAREDNDPN
**410**	**420**	**430**	**440**	**450**	**460**	**470**	**480**
RDEVPRAQLP	THRPGLPRLS	VRRGEFSSSD	PNIRAPQLPG	TSEPSAYSPG	GLVPRRHAPS	GNQQPPVHPP	RPERQQQPPG
**490**	**500**	**510**	**520**				
AQGCPRCIWL	QREIEDLTQQ	LAAMQFLTDK	FQDL				

Cmpd.	No. of Cmpds.	m/z meas.	Δ m/z [ppm]	z	Rt [min]	Score	P	Range	Sequence	Modification	Ratios
198	1	938.4465	-16.72	2	31.3	13.1	1	242-257	R.QASFHCKESYLPVPGR.F	Carbamidomethyl: 6	WUP:QUP 1.31<br>QU:MU 0.52
1392	1	895.6264	159.72	2	47.0	14.8	1	249-264	K.ESYLPVPGRFLTSAPR.G		
1182	1	662.2009	-171.93	2	44.9	10.2	0	475-486	R.QQQPPGAQGCPR.C	Carbamidomethyl: 10	

Detailed Protein Report

Protein 262: PREDICTED: ryanodine receptor 1 isoform X3 [Homo sapiens]

**Accession:**	gi|578834746	**Score:**	38.0
**Database:**	refseq_human(refseq_human_20140103.fasta)	**MW [kDa]:**	563.6
**Database Date:**	2015-11-30	**pI:**	5.1
**Modification(s):**	Carbamidomethyl, Oxidation	**Sequence Coverage [%]:**	0.9
		**No. of unique Peptides:**	3

Detailed Protein Report

10	20	30	40	50	60	70	80
MGDAEGEDEV	QFLRTDDEVV	LQCSATVLKE	QLKLCLAAEG	FGNRLCFLEP	TSNAQNVPPD	LAICCFVLEQ	SLSVRALQEM
90	100	110	120	130	140	150	160
LANTVEAGVE	SSQGGGHRTL	LYGHAILLRH	AHSRMYLSCL	TTSRSMTDKL	AFDVGLQEDA	TGEACWWTMH	PASKQRSEGE
170	180	190	200	210	220	230	240
KVRVGDDIIL	VSVSSERYLH	LSTASGELQV	DASFMQTLWN	MNPICSRCEE	GFVTGGHVLR	LFHGHMDECL	TISPADSDDQ
250	260	270	280	290	300	310	320
RRLVYYEGGA	VCTHARSLWR	LEPLRISWSG	SHLRWGQPLR	VRHVTTGQYL	ALTEDQGLVV	VDASKAHTKA	TSFCFRISKE
330	340	350	360	370	380	390	400
KLDVAPKRDV	EGMGPPEIKY	GESLCFVQHV	ASGLWLTYAA	PDPKALRLGV	LKKKAMLHQE	GHMDDALSLT	RCQQEESQAA
410	420	430	440	450	460	470	480
RMIHSTNGLY	NQFIKSLDSF	SGKPRGSGPP	AGTALPIEGV	ILSLQDLIIY	FEPPSEDLQH	EEKQSKLRSL	RNRQSLFQEE
490	500	510	520	530	540	550	560
GMLSMVLNCI	DRLNVYTTAA	HFAEFAGEEA	AESWKEIVNL	LYELLASLIR	GNRSNCALFS	TNLDWLVSKL	DRLEASSGIL
570	580	590	600	610	620	630	640
EVLYCVLIES	PEVLNIIQEN	HIKSIISLLD	KHGRNHKVLD	VLCSLCVCNG	VAVRSNQDLI	TENLLPGREL	LLQTNLINYV
650	660	670	680	690	700	710	720
TSIRPNIFVG	RAEGTTQYSK	WYFEVMVDEV	TPFLTAQATH	LRVGWALTEG	YTPYPGAGEG	WGGNGVGDDL	YSYGFDGLHL
730	740	750	760	770	780	790	800
WTGHVARPVT	SPGQHLLAPE	DVISCCLDLS	VPSISFRING	CPVQGVFESF	NLDGLFFPVV	SFSAGVKVRF	LLGGRHGEFK
810	820	830	840	850	860	870	880
FLPPPGYAPC	HEAVLPRERL	HLEPIKEYRR	EGPRGPHLVG	PSRCLSHTDF	VPCPVDTVQI	VLPPHLERIR	EKLAENIHEL
890	900	910	920	930	940	950	960
WALTRIEQGW	TYGPVRDDNK	RLHPCLVDFH	SLPEPERNYN	LQMSGETLKT	LLALGCHVGM	ADEKAEDNLK	KTKLPKTYMM
970	980	990	1000	1010	1020	1030	1040
SNGYKPAPLD	LSHVRLTPAQ	TTLVDRLAEN	GHNVWARDRV	GQGWSYSAVQ	DIPARRNPRL	VPYRLLDEAT	KRSNRDSLCQ
1050	1060	1070	1080	1090	1100	1110	1120
AVRTLLGYGY	NIEPPDQEPS	QVENQSRCDR	VRIFRAEKSY	TVQSGRWYFE	FEAVTTGEMR	VGWARPELRP	DVELGADELA
1130	1140	1150	1160	1170	1180	1190	1200
YVFNGHRGQR	WHLGSEPFGR	PWQPGDVVGC	MIDLTENTII	FTLNGEVLMS	DSGSETAFRE	IEIGDGFLPV	CSLGPGQVGH
1210	1220	1230	1240	1250	1260	1270	1280
LNLGQDVSSL	RFFAICGLQE	GFEPFAINMQ	RPVTTWFSKG	LPQFEPVPLE	HPHYEVSRVD	GTVDTPPCLR	LTHRTWGSQN
1290	1300	1310	1320	1330	1340	1350	1360
SLVEMLFLRL	SLPVQFHQHF	RCTAGATPLA	PPGLQPPAED	EARAAEPDPD	YENLRRSAGG	WSEAENGKEG	TAKEGAPGGT
1370	1380	1390	1400	1410	1420	1430	1440
PQAGGEAQPA	RAENEKDATT	EKNKKRGFLF	KAKKVAMMTQ	PPATPTLPRL	PHDVVPADNR	DDPEIILNTT	TYYYSVRVFA
1450	1460	1470	1480	1490	1500	1510	1520
GQEPSCVWAG	WVTPDYHQHD	MSFDLSKVRV	VTVTMGDEQG	NVHSSLKCSN	CYMVWGGDFV	SPGQQGRISH	TDLVIGCLVD
1530	1540	1550	1560	1570	1580	1590	1600
LATGLMTFTA	NGKESNTFFQ	VEPNTKLFPA	VFVLPTHQNV	IQFELGKQKN	IMPLSAAMFQ	SERKNPAPQC	PPRLEMQMLM
1610	1620	1630	1640	1650	1660	1670	1680
PVSWSRMPNH	FLQVETRRAG	ERLGWAVQCQ	EPLTMMALHI	PEENRCMDIL	ELSERLDLQR	FHSHTLRLYR	AVCALGNNRV
1690	1700	1710	1720	1730	1740	1750	1760
AHALCSHVDQ	AQLLHALEDA	HLPGPLRAGY	YDLLISIHLE	SACRSRRSML	SEYIVPLTPE	TRAITLFPPG	RSTENGHPRH
1770	1780	1790	1800	1810	1820	1830	1840
GLPGVGVTTS	LRPPHHFSPP	CFVAALPAAG	AAEAPARLSP	AIPLEALRDK	ALRMLGEAVR	DGGQHARDPV	GGSVEFQFVP
1850	1860	1870	1880	1890	1900	1910	1920
VLKLVSTLLV	MGIFGDEDVK	QILKMIEPEV	FTEEEEEEDE	EEEGEEEDEE	EKEEDEEETA	QEKEDEEKEE	EEAAEGEKEE
1930	1940	1950	1960	1970	1980	1990	2000
GLEEGLLQMK	LPESVKLQMC	HLLEYFCDQE	LQHRVESLAA	FAERYVDKLQ	ANQRSRYGLL	IKAFSMTAAE	TARRTREFRS
2010	2020	2030	2040	2050	2060	2070	2080
PPQEQINMLL	QFKDGTDEED	CPLPEEIRQD	LLDFHQDLLA	HCGIQLDGEE	EEPEEETTLG	SRLMSLLEKV	RLVKKKEEKP
2090	2100	2110	2120	2130	2140	2150	2160
EEERSAEESK	PRSLQELVSH	MVVRWAQEDF	VQSPELVRAM	FSLLHRQYDG	LGELLRALPR	AYTISPSSVE	DTMSLLECLG
2170	2180	2190	2200	2210	2220	2230	2240

Detailed Protein Report

Cmpd.	No. of Cmpds.	m/z meas.	Δ m/z [ppm]	z	Rt [min]	Score	P	Range	Sequence	Modification
2169	2	714.6084	-134.05	3	57.5	14.8	1	595-614	R.NHKVLDVLCSLCVCNGVAVR.S	
1512	1	727.4065	108.16	2	49.1	11.1	0	2601-2612	R.DVIEDCLMSLCR.Y	Carbamidomethyl: 11
2682	1	705.8622	37.53	2	64.6	12.1	2	3478-3490	K.MAKSGGSDQERTK.K	Oxidation: 1

Detailed Protein Report

Protein 263: symplekin [Homo sapiens]

**Accession:**	gi|124028529	**Score:**	37.7
**Database:**	refseq_human(refseq_human_20140103.fasta)	**MW [kDa]:**	141.1
**Database Date:**	2015-11-30	**pI:**	5.8
		**Sequence Coverage [%]:**	2.7
		**No. of unique Peptides:**	1

Alias proteins:

Accession	Name	Description
gi|530417312	refseq_human (refseq_human_20140103.fasta)	PREDICTED: symplekin isoform X1 [Homo sapiens]

10	20	30	40	50	60	70	80
MASGSGDSVT	RRSVASQFFT	QEEGPGIDGM	TTSERVVDLL	NQAALITNDS	KITVLKQVQE	LIINKDPTLL	DNFLDEIIAF
90	100	110	120	130	140	150	160
QADKSIEVRK	FVIGFIEEAC	KRDIELLLKL	IANLNMLLRD	ENVNVVKKAI	LTMTQLYKVA	LQWMVKSRVI	SELQEACWDM
170	180	190	200	210	220	230	240
VSAMAGDIIL	LLDSDNDGIR	THAIKFVEGL	IVTLSPRMAD	SEIPRRQEHD	ISLDRIPRDH	PYIQYNVLWE	EGKAALEQLL
250	260	270	280	290	300	310	320
KFMVHPAISS	INLTTALGSL	ANIARQRPMF	MSEVIQAYET	LHANLPPTLA	KSQVSSVRKN	LKLHLLSVLK	HPASLEFQAQ
330	340	350	360	370	380	390	400
ITTLLVDLGT	PQAEIARNMP	SSKDTRKRPR	DDSDSTLKKM	KLEPNLGEDD	EDKDLEPGPS	GTSKASAQIS	GQSDTDITAE
410	420	430	440	450	460	470	480
FLQPLLTPDN	VANLVLISMV	YLPEAMPASF	QAIYTPVESA	GTEAQIKHLA	RLMATQMTAA	GLGPGVEQTK	QCKEEPKEEK
490	500	510	520	530	540	550	560
VVKTESVLIK	RRLSAQGQAI	SVVGSLSSMS	PLEEEAPQAK	RRPEPIIPVT	QPRLAGAGGR	KKIFRLSDVL	KPLTDAQVEA
570	580	590	600	610	620	630	640
MKLGAVKRIL	RAEKAVACSG	AAQVRIKILA	SLVTQFNSGL	KAEVLSFILE	DVRARLDLAF	AWLYQEYNAY	LAAGASGSLD
650	660	670	680	690	700	710	720
KYEDCLIRLL	SGLQEKPDQK	DGIFTKVVLE	APLITESALE	VVRKYCEDES	RTYLGMSTLR	DLIFKRPSRQ	FQYLHVLLDL
730	740	750	760	770	780	790	800
SSHEKDKVRS	QALLFIKRMY	EKEQLREYVE	KFALNYLQLL	VHPNPPSVLF	GADKDTEVAA	PWTEETVKQC	LYLYLALLPQ
810	820	830	840	850	860	870	880
NHKLIHELAA	VYTEAIADIK	RTVLRVIEQP	IRGMGMNSPE	LLLLVENCPK	GAETLVTRCL	HSLTDKVPPS	PELVKRVRDL
890	900	910	920	930	940	950	960
YHKRLPDVRF	LIPVLNGLEK	KEVIQALPKL	IKLNPIVVKE	VFNRLLGTQH	GEGNSALSPL	NPGELLIALH	NIDSVKCDMK
970	980	990	1000	1010	1020	1030	1040
SIIKATNLCF	AERNVYTSEV	LAVVMQQLME	QSPLPMLLMR	TVIQSLTMYP	RLGGFVMNIL	SRLIMKQVWK	YPKVWEGFIK
1050	1060	1070	1080	1090	1100	1110	1120
CCQRTKPQSF	QVILQLPPQQ	LGAVFDKCPE	LREPLLAHVR	SFTPHQQAHI	PNSIMTILEA	SGKQEPEAKE	APAGPLEEDD
1130	1140	1150	1160	1170	1180	1190	1200
LEPLTLAPAP	APRPPQDLIG	LRLAQEKALK	RQLEEEQKLK	PGGVGAPSSS	SPSPSPSARP	GPPPSEEAMD	FREEGPECET
1210	1220	1230	1240	1250	1260	1270	1280
PGIFISMDDD	SGLTEAALLD	SSLEGPLPKE	TAAGGLTLKE	ERSPQTLAPV	GEDAMKTPSP	AAEDAREPEA	KGNS

Cmpd.	No. of Cmpds.	m/z meas.	Δ m/z [ppm]	z	Rt [min]	Score	P	Range	Sequence	Modification
2866	1	655.3693	-17.94	2	66.3	14.3	1	128-138	K.KAILTMTQLYK.V	

Detailed Protein Report

Protein 264: **PREDICTED: probable ATP-dependent RNA helicase DDX60-like isoform X6 [Homo sapiens]**

Accession: gi|578809622
Database: refseq_human(refseq_human_20140103.fasta)
Database Date: 2015-11-30
Modification(s): Carbamidomethyl, Oxidation

Score: 37.7
MW [kDa]: 193.4
pI: 9.2
Sequence Coverage [%]: 2.2
No. of unique Peptides: 3

Quantitation

QU:MU **Median:** 2.50 **CV:** 44.93 % **No. of Peptides:** 2

10	20	30	40	50	60	70	80
MGSKDHAVFF	REMTQLILNE	MPKAGYSSIL	NDFVESNFFV	IDGDSLLVTC	LGVKSFKWGQ	NLHFFYLVEC	YLVDLLSNGG
90	100	110	120	130	140	150	160
QFTIVFFKDA	EYAYFDFPEL	LSLRTALILH	LQHNTNIDVQ	TEFSGCLSQD	WKLFLEQHYP	YFLIVSEEGL	SDLQTYLFNF
170	180	190	200	210	220	230	240
LIIHSWGMKV	NVVLSSGHES	DTLRFYAYTM	ESTDRNQTFS	KENETVIQSA	YKSLIQHLEE	IRVLVLATHF	EHLKWNDMME
250	260	270	280	290	300	310	320
EAYQTLFLLQ	HLWSEGSDIQ	RVLCVTSCSL	SLRMYHRVLV	HSNCLSLQEV	EDFCRLRCLC	VAFQLHLPLS	QRACSRVITC
330	340	350	360	370	380	390	400
SWIRNSDSFL	KMNKWCEYFI	LSNLNVFGCW	NLNLNHVSDL	YDEQLLKNIA	FYYEFESTQE	PHLNLGDSIR	RDYEDLWNVV
410	420	430	440	450	460	470	480
SHLVKEFNVG	KSFPLRTTRR	HFLRQEKSVI	QEISLEKMPS	VGFIPMTSAV	IDEFVGDMMK	DLPILKSDDP	VVPSLFKQKT
490	500	510	520	530	540	550	560
SDELLHWHAQ	RLLSDDYDRI	KCHVDEQSRD	PHVLDFLKKI	QDYQQFYGKS	LESISTKVIV	TQTTRPKEDS	SGASGEILQN
570	580	590	600	610	620	630	640
TKPHQITKKS	KKKSFLKEDQ	NKAQQNDDLL	FSIEEEMKNN	LHSGIRKLED	YLTSCASNSV	KFGVEMLGLI	ACFKAWKKHC
650	660	670	680	690	700	710	720
RGEGKISKDL	SIAVQMMKRI	HSLLERYPEI	LEAEHHQYIA	KCLKYLGFND	LANSLDPTLI	GDDKNKKKYS	IDIGPARFQL
730	740	750	760	770	780	790	800
QYMGHYLIRD	ERKDRDPRVQ	DFIPNAWQQE	LLDVVDKNES	AVIVAPTSSG	KTYASYYCME	KVLRESDVGV	VVYVAPAKSL
810	820	830	840	850	860	870	880
VGQVAATVEN	RFTKTLPAGR	TLCGAFTRDY	CHNVLNCQVL	ITVPECFEIL	LLAPHRQKWV	ERIRYVIFDE	VHYLGREVGA
890	900	910	920	930	940	950	960
KFWELLLVII	RCPFLVLSAT	INNPNLLTKW	LQSVKQYWKQ	ADKIMEEKCI	SEKQADKCLN	FLQDHSYKNQ	SYEVRLVLCG
970	980	990	1000	1010	1020	1030	1040
ERYNDLEKHI	CSVKHDDVYF	DHFHPCAALT	TDIIEKYGFP	PDLTLTPQES	IQLYDTMAQV	WETWPRAQEL	CPEEFILFKN
1050	1060	1070	1080	1090	1100	1110	1120
KIVIKKLDAR	KYEENLKAEL	TNWIKNGQVK	KVKRVLKNLS	PDSLSSSKDM	VKMFPLLVEK	LRQMDKLPAI	FFLFKNDDVG
1130	1140	1150	1160	1170	1180	1190	1200
KRAGSVCTFL	EKTETKSHPH	TECHSYVFAI	DEVLEKVRKT	QKRISTKKNP	KKAEKLERKK	VYRAEYINFL	ENLKILEISE
1210	1220	1230	1240	1250	1260	1270	1280
DCTYADVKAL	HTEITRNKDS	TLERVLPRVR	FTRHGKELKA	LAQRGIGYHH	SSMYFKEKEF	VEILFVKGLI	RVVTATETLA
1290	1300	1310	1320	1330	1340	1350	1360
LGIHMPCKSV	VFAQDSVYLD	ALNYRQMSGR	AGRRGQDLLG	NVYFFDIPLP	KIKRLLASSV	PELRGQFPLS	ITLVLRLMLL
1370	1380	1390	1400	1410	1420	1430	1440
ASKGDDPEDA	KAKDYLNKKG	NPKKFAGLAS	YLHGHEPSNL	VFVNFLKRGL	FHNLCKPAWK	GSQQFSQDVM	EKLVLVLANL
1450	1460	1470	1480	1490	1500	1510	1520
FGRKYIPAKF	QNANLSFSQS	KVILAELPED	FKAALYEYNL	AVMKDFASFL	LIASKSVNMK	KEHQLPLSRI	KFTGKECEDS
1530	1540	1550	1560	1570	1580	1590	1600
QLVSHLMSCK	KGRVAISPFV	CLSGNTDNDL	LRPETINQVI	LRTVGVSGTQ	APLLWPWKLD	NRGRRMPLNA	YVLNFYKHNC
1610	1620	1630	1640	1650	1660	1670	1680
LTRLDQKNGM	RMGQLLKCLK	DFAFNIQAIS	DSLSELCENK	RDNVVLAFKQ	LSQTFYEKLQ	EMQIQMSQNH	LE

Cmpd.	No. of Cmpds.	m/z meas.	Δ m/z	z	Rt [min]	Score	P	Range	Sequence	Modification	Ratios

Detailed Protein Report

			[ppm]								
1058	1	586.1731	-222.69	2	42.8	12.2	2	639-648	K.HCRGEGKISK.D	Carbamidomethyl: 2	QU:MU 3.85
225	1	641.7812	-36.28	2	32.5	12.3	1	924-933	K.IMEEKCISEK.Q	Carbamidomethyl: 6; Oxidation: 2	
1639	1	926.8312	-90.99	2	50.2	13.2	1	1516-1531	K.ECEDSQLVSHLMSCKK.G	Oxidation: 12	QU:MU 1.63

Detailed Protein Report

Protein 265: tyrosine-protein kinase SYK isoform 2 [Homo sapiens]

**Accession:**	gi|205277435	**Score:**	37.6
**Database:**	refseq_human(refseq_human_20140103.fasta)	**MW [kDa]:**	69.5
**Database Date:**	2015-11-30	**pI:**	7.8
**Modification(s):**	Carbamidomethyl, Oxidation	**Sequence Coverage [%]:**	6.9
		**No. of unique Peptides:**	2

Alias proteins:

Accession	Name	Description
gi|293332611	refseq_human (refseq_human_20140103.fasta)	tyrosine-protein kinase SYK isoform 2 [Homo sapiens]

10	20	30	40	50	60	70	80
MASSGMADSA	NHLPFFFGNI	TREEAEDYLV	QGGMSDGLYL	LRQSRNYLGG	FALSVAHGRK	AHHYTIEREL	NGTYAIAGGR
**90**	**100**	**110**	**120**	**130**	**140**	**150**	**160**
THASPADLCH	YHSQESDGLV	CLLKKPFNRP	QGVQPKTGPF	EDLKENLIRE	YVKQTWNLQG	QALEQAIISQ	KPQLEKLIAT
**170**	**180**	**190**	**200**	**210**	**220**	**230**	**240**
TAHEKMPWFH	GKISREESEQ	IVLIGSKTNG	KFLIRARDNN	GSYALCLLHE	GKVLHYRIDK	DKTGKLSIPE	GKKFDTLWQL
**250**	**260**	**270**	**280**	**290**	**300**	**310**	**320**
VEHYSYKADG	LLRVLTVPCQ	KIGTQGNVNF	GGRPQLPGSH	PASSPAQGNR	QESTVSFNPY	EPELAPWAAD	KGPQREALPM
**330**	**340**	**350**	**360**	**370**	**380**	**390**	**400**
DTEVYESPYA	DPEEIRPKEV	YLDRKLLTLE	DKELGSGNFG	TVKKGYYQMK	KVVKTVAVKI	LKNEANDPAL	KDELLAEANV
**410**	**420**	**430**	**440**	**450**	**460**	**470**	**480**
MQQLDNPYIV	RMIGICEAES	WMLVMEMAEL	GPLNKYLQQN	RHVKDKNIIE	LVHQVSMGMK	YLEESNFVHR	DLAARNVLLV
**490**	**500**	**510**	**520**	**530**	**540**	**550**	**560**
TQHYAKISDF	GLSKALRADE	NYYKAQTHGK	WPVKWYAPEC	INYYKFSSKS	DVWSFGVLMW	EAFSYGQKPY	RGMKGSEVTA
**570**	**580**	**590**	**600**	**610**	**620**		
MLEKGERMGC	PAGCPREMYD	LMNLCWTYDV	ENRPGFAAVE	LRLRNYYYDV	VN		

Cmpd.	No. of Cmpds.	m/z meas.	Δ m/z [ppm]	z	Rt [min]	Score	P	Range	Sequence	Modification
2468	2	1044.5140	-52.13	2	61.4	15.4	2	173-191	K.ISREESEQIVLIGSKTNGK.F	
1105	1	653.6417	-205.60	2	42.1	10.9	1	565-576	K.GERMGCPAGCPR.E	Carbamidomethyl: 10; Oxidation: 4

Detailed Protein Report

Protein 266: eosinophil peroxidase preproprotein [Homo sapiens]

**Accession:**	gi|4503595	**Score:**	37.5
**Database:**	refseq_human(refseq_human_20140103.fasta)	**MW [kDa]:**	81.0
**Database Date:**	2015-11-30	**pI:**	11.5
**Modification(s):**	Carbamidomethyl	**Sequence Coverage [%]:**	3.6
		**No. of unique Peptides:**	2

Quantitation

WUP:QUP	**Median:** 0.65	**CV:** 0.00 %	**No. of Peptides:** 1

Alias proteins:

Accession	Name	Description
gi|578831504	refseq_human (refseq_human_20140103.fasta)	PREDICTED: eosinophil peroxidase isoform X1 [Homo sapiens]

10	20	30	40	50	60	70	80
MHLLPALAGV	LATLVLAQPC	EGTDPASPGA	VETSVLRDCI	AEAKLLVDAA	YNWTQKSIKQ	RLRSGSASPM	DLLSYFKQPV
**90**	**100**	**110**	**120**	**130**	**140**	**150**	**160**
AATRTVVRAA	DYMHVALGLL	EEKLQPQRSG	PFNVTDVLTE	PQLRLLSQAS	GCALRDQAER	CSDKYRTITG	RCNNKRRPLL
**170**	**180**	**190**	**200**	**210**	**220**	**230**	**240**
GASNQALARW	LPAEYEDGLS	LPFGWTPSRR	RNGFLLPLVR	AVSNQIVRFP	NERLTSDRGR	ALMFMQWGQF	IDHDLDFSPE
**250**	**260**	**270**	**280**	**290**	**300**	**310**	**320**
SPARVAFTAG	VDCERTCAQL	PPCFPIKIPP	NDPRIKNQRD	CIPFFRSAPS	CPQNKNRVRN	QINALTSFVD	ASMVYGSEVS
**330**	**340**	**350**	**360**	**370**	**380**	**390**	**400**
LSLRLRNRTN	YLGLLAINQR	FQDNGRALLP	FDNLHDDPCL	LTNRSARIPC	FLAGDTRSTE	TPKLAAMHTL	FMREHNRLAT
**410**	**420**	**430**	**440**	**450**	**460**	**470**	**480**
ELRRLNPRWN	GDKLYNEARK	IMGAMVQIIT	YRDFLPLVLG	KARARRTLGH	YRGYCSNVDP	RVANVFTLAF	RFGHTMLQPF
**490**	**500**	**510**	**520**	**530**	**540**	**550**	**560**
MFRLDSQYRA	SAPNSHVPLS	SAFFASWRIV	YEGGIDPILR	GLMATPAKLN	RQDAMLVDEL	RDRLFRQVRR	IGLDLAALNM
**570**	**580**	**590**	**600**	**610**	**620**	**630**	**640**
QRSRDHGLPG	YNAWRRFCGL	SQPRNLAQLS	RVLKNQDLAR	KFLNLYGTPD	NIDIWIGAIA	EPLLPGARVG	PLLACLFENQ
**650**	**660**	**670**	**680**	**690**	**700**	**710**	**720**
FRRARDGDRF	WWQKRGVFTK	RQRKALSRIS	LSRIICDNTG	ITTVSRDIFR	ANIYPRGFVN	CSRIPRLNLS	AWRGT

Cmpd.	No. of Cmpds.	m/z meas.	Δ m/z [ppm]	z	Rt [min]	Score	P	Range	Sequence	Modification	Ratios
873	1	575.3008	19.33	2	40.5	19.5	0	368-377	R.IPCFLAGDTR.S	Carbamidomethyl: 3	WUP:QUP 0.65
2217	1	927.3936	-145.10	2	56.1	18.0	2	547-562	R.QVRRIGLDLAALNMQR.S		

Detailed Protein Report

Protein 267: vacuolar protein sorting-associated protein 13B isoform 5 [Homo sapiens]

**Accession:**	gi|35493713	**Score:**	37.5
**Database:**	refseq_human(refseq_human_20140103.fasta)	**MW [kDa]:**	448.4
**Database Date:**	2015-11-30	**pI:**	6.0
**Modification(s):**	Carbamidomethyl, Oxidation	**Sequence Coverage [%]:**	1.4
		**No. of unique Peptides:**	3

Alias proteins:

Accession	Name	Description
gi|530388780	refseq_human (refseq_human_20140103.fasta)	PREDICTED: vacuolar protein sorting-associated protein 13B isoform X2 [Homo sapiens]
gi|530388778	refseq_human (refseq_human_20140103.fasta)	PREDICTED: vacuolar protein sorting-associated protein 13B isoform X1 [Homo sapiens]

Detailed Protein Report

10	20	30	40	50	60	70	80
MLESYVTPIL	MSYVNRYIKN	LKPSDLQLSL	WGGDVVLSKL	ELKLDVLEQE	LKLPFTFLSG	HIHELRIHVP	WTKLGSEPVV
90	100	110	120	130	140	150	160
ITINTMECIL	KLKDGIQDDH	ESCGSNSTNR	STAESTKSSI	KPRRMQQAAP	TDPDLPPGYV	QSLIRRVVNN	VNIVINNLIL
170	180	190	200	210	220	230	240
KYVEDDIVLS	VNITSAECYT	VGELWDRAFM	DISATDLVLR	KVINFSDCTV	CLDKRNASGK	IEFYQDPLLY	KCSFRTRLHF
250	260	270	280	290	300	310	320
TYENLNSKMP	SVIKIHTLVE	SLKLSITDQQ	LPMFIRIMQL	GIALYYGEIG	NFKEGEIEDL	TCHNKDMLGN	ITGSEDETRI
330	340	350	360	370	380	390	400
DMQYPAQHKG	QELYSQQDEE	QPQGWVSWAW	SFVPAIVSYD	DGEEDFVGND	PASTMHQQKA	QTLKDPIVSI	GFYCTKATVT
410	420	430	440	450	460	470	480
FKLTEMQVES	SYYSPQKVKS	KEVLCWEQEG	TTVEALMMGE	PFFDCQIGFV	GCRAMCLKGI	MGVKDFEENM	NRSETEACFF
490	500	510	520	530	540	550	560
ICGDNLSTKG	FTYLTNSLFD	YRSPENNGTR	AEFILDSTHH	KETYTEIAGM	QRFGAFYMDY	LYTMENTSGK	GSTNQQDFSS
570	580	590	600	610	620	630	640
GKSEDLGTVQ	EKSTKSLVIG	PLDFRLDSSA	VHRILKMIVC	ALEHEYEPYS	RLKSDIKDEN	ETILNPEEVA	LLEEYIPTRH
650	660	670	680	690	700	710	720
TSVTLLKCTC	TISMAEFNLL	DHLLPVIMGE	KNSSNFMNTT	NFQSLRPLPS	IRILVDKINL	EHSVPMYAEQ	LVHVVSSLTQ
730	740	750	760	770	780	790	800
PSDNLLHYCY	VHCYLKIFGF	QAGLTSLDCS	GSYCLPVPVI	PSFSTALYGK	LLKLPTCWTK	RSQIAITEGI	FELPNLTIQA
810	820	830	840	850	860	870	880
TRAQTLLLQA	IYQSWSHLGN	VSSSAVIEAL	INEIFLSIGV	KSKNPLPTLE	GSIQNVELKY	CSTSLVKCAS	GTMGSIKICA
890	900	910	920	930	940	950	960
KAPVDSGKEK	LIPLLQGPSD	TKDLHSTKWL	NESRKPESLL	APDLMAFTIQ	VPQYIDYCHN	SGAVLLCSIQ	GLAVNIDPIL
970	980	990	1000	1010	1020	1030	1040
YTWLIYQPQK	RTSRHMQQQP	VVAVPLVMPV	CRRKEDEVSI	GSAPLAKQQS	YQASEYASSP	VKTKTVTESR	PLSVPVKAML
1050	1060	1070	1080	1090	1100	1110	1120
NISESCRSPE	ERMKEFIGIV	WNAVKHLTLQ	LEVQSCCVFI	PNDSLPSPST	IVSGDIPGTV	RSWYHGQTSM	PGTLVLCLPQ
1130	1140	1150	1160	1170	1180	1190	1200
IKIISAGHKY	MEPLQEIPFV	IPRPILEEGD	AFPWTISLHN	FSIYTLLGKQ	VTLCLVEPMG	CTSTLAVTSQ	KLLATGPDTR
1210	1220	1230	1240	1250	1260	1270	1280
HSFVVCLHVD	LESLEIKCSN	PQVQLFYELT	DIMNKVWNKI	QKRGNLNLSP	TSPETMAGPV	PTSPVRSSIG	TAPPDTSTCS
1290	1300	1310	1320	1330	1340	1350	1360
PSADIGTTTE	GDSIQAGEES	PFSDSVTLEQ	TTSNIGGTSG	RVSLWMQWVL	PKITIKLFAP	DPENKGTEVC	MVSELEDLSA
1370	1380	1390	1400	1410	1420	1430	1440
SIDVQDVYTK	VKCKIESFNI	DHYRSSLGEE	CWSLGQCGGV	FLSCTDKLNR	RTLLVRPISK	QDPFSNCSGF	FPSTTTKLLD
1450	1460	1470	1480	1490	1500	1510	1520
GTHQQHGFLS	LTYTKAVTKN	VRHKLTSRNE	RRSFHKLSEG	LMDGSPHFLH	EILLSAQAFD	IVLYFPLLNA	IASIFQAKLP
1530	1540	1550	1560	1570	1580	1590	1600
KTQKEKRKSP	GQPMRTHTLT	SRNLPLIYVN	TSVIRIFIPK	TEEMQPTVEA	NQAAKEDTVV	LKIGSVAMAP	QADNPLGRSV
1610	1620	1630	1640	1650	1660	1670	1680
LRKDIYQRAL	NLGILRDPGS	EIEDRQYQID	LQSINIGTAQ	WHQLKPEKES	VSGGVVTETE	RNSQNPALEW	NMASSIRRHQ
1690	1700	1710	1720	1730	1740	1750	1760
ERRAILTPVL	TDFSVRITGA	PAVIFTKVVS	PENLHTEEIL	VCGHSLEVNI	TTNLDFFLSV	AQVQLLHQLI	VANMTGLEPS
1770	1780	1790	1800	1810	1820	1830	1840
NKAAEISKQE	QKKVDIFDGG	MAETSSRYSG	AQDSGIGSDS	VKIRIVQIEQ	HSGASQHRIA	RPSRQSSIVK	NLNFIPFDIF
1850	1860	1870	1880	1890	1900	1910	1920
ITASRISLMT	YSCMALSKSK	SQEQKNNEKT	DKSSLNLPEV	DSDVAKPNQA	CISTVTAEDL	LRSSISFPSG	KKIGVLSLES
1930	1940	1950	1960	1970	1980	1990	2000
LHASTRSSAR	QALGITIVRQ	PGRRGTGDLQ	LEPFLYFIVS	QPSLLLSCHH	RKQRVEVSIF	DAVLKGVASD	YKCIDPGKTL
2010	2020	2030	2040	2050	2060	2070	2080
PEALDYCTVW	LQTVPGEIDS	KSGIPPSFIT	LQIKDFLNGP	ADVNLDISKP	LKANLSFTKL	DQINLFLKKI	KNAHSLAHSE
2090	2100	2110	2120	2130	2140	2150	2160
ETSAMSNTMV	NKDDLPVSKY	YRGKLSKPKI	HGDGVQKISA	QENMWRAVSC	FQKISVQTTQ	IVISMETVPH	TSKPCLLASL
2170	2180	2190	2200	2210	2220	2230	2240

Detailed Protein Report

Cmpd.	No. of Cmpds.	m/z meas.	Δ m/z [ppm]	z	Rt [min]	Score	P	Range	Sequence	Modification
1794	2	1023.9088	-120.39	2	52.3	12.4	1	385-402	K.DPIVSIGFYCTKATVTFK.L	Carbamidomethyl: 10
1639	1	911.3413	-106.88	2	50.8	14.4	1	2218-2233	K.WCKHSGNPGPEQSIPK.I	Carbamidomethyl: 2
1842	1	1273.5438	-34.83	2	51.4	10.7	1	3135-3157	K.EYFRVPDSATFSICPGGEQPAMK.S	Oxidation: 22

Detailed Protein Report

Protein 268: triosephosphate isomerase isoform 3 [Homo sapiens]

Accession: gi|384475517
Database: refseq_human(refseq_human_20140103.fasta)
Database Date: 2015-11-30
Modification(s): Carbamidomethyl

Score: 37.4
MW [kDa]: 17.9
pI: 5.3
Sequence Coverage [%]: 7.8
No. of unique Peptides: 1

```
        10         20         30         40         50         60         70         80
MIKDCGATWV VLGHSERRHV FGESDELIGQ KVAHALAEGL GVIACIGEKL DEREAGITEK VVFEQTKVIA DNVKDWSKVV
        90        100        110        120        130        140        150        160
LAYEPVWAIG TGKTATPQQA QEVHEKLRGW LKSNVSDAVA QSTRIIYGGS VTGATCKELA SQPDVDGFLV GGASLKPEFV
       170
DIINAKQ
```

Cmpd.	No. of Cmpds.	m/z meas.	Δ m/z [ppm]	z	Rt [min]	Score	P	Range	Sequence	Modification
219	1	663.8152	-36.93	2	32.7	37.4	0	125-137	R.IIYGGSVTGATCK.E	Carbamidomethyl: 12

Detailed Protein Report

Protein 269: sortilin isoform 2 [Homo sapiens]

**Accession:**	gi|327315343	**Score:**	37.3
**Database:**	refseq_human(refseq_human_20140103.fasta)	**MW [kDa]:**	77.3
**Database Date:**	2015-11-30	**pI:**	5.1
**Modification(s):**	Carbamidomethyl	**Sequence Coverage [%]:**	3.9
		**No. of unique Peptides:**	1

10	20	30	40	50	60	70	80
MTFGQSKLYR	SEDYGKNFKD	ITDLINNTFI	RTEFGMAIGP	ENSGKVVLTA	EVSGGSRGGR	IFRSSDFAKN	FVQTDLPFHP
90	100	110	120	130	140	150	160
LTQMMYSPQN	SDYLLALSTE	NGLWVSKNFG	GKWEEIHKAV	CLAKWGSDNT	IFFTTYANGS	CTDLGALELW	RTSDLGKSFK
170	180	190	200	210	220	230	240
TIGVKIYSFG	LGGRFLFASV	MADKDTTRRI	HVSTDQGDTW	SMAQLPSVGQ	EQFYSILAAN	DDMVFMHVDE	PGDTGFGTIF
250	260	270	280	290	300	310	320
TSDDRGIVYS	KSLDRHLYTT	TGGETDFTNV	TSLRGVYITS	VLSEDNSIQT	MITFDQGGRW	THLRKPENSE	CDATAKNKNE
330	340	350	360	370	380	390	400
CSLHIHASYS	ISQKLNVPMA	PLSEPNAVGI	VIAHGSVGDA	ISVMVPDVYI	SDDGGYSWTK	MLEGPHYYTI	LDSGGIIVAI
410	420	430	440	450	460	470	480
EHSSRPINVI	KFSTDEGQCW	QTYTFTRDPI	YFTGLASEPG	ARSMNISIWG	FTESFLTSQW	VSYTIDFKDI	LERNCEEKDY
490	500	510	520	530	540	550	560
TIWLAHSTDP	EDYEDGCILG	YKEQFLRLRK	SSVCQNGRDY	VVTKQPSICL	CSLEDFLCDF	GYYRPENDSK	CVEQPELKGH
570	580	590	600	610	620	630	640
DLEFCLYGRE	EHLTTNGYRK	IPGDKCQGGV	NPVREVKDLK	KKCTSNFLSP	EKQNSKSNSV	PIILAIVGLM	LVTVVAGVLI
650	660	670	680	690	700		
VKKYVCGGRF	LVHRYSVLQQ	HAEANGVDGV	DALDTASHTN	KSGYHDDSDE	DLLE		

Cmpd.	No. of Cmpds.	m/z meas.	Δ m/z [ppm]	z	Rt [min]	Score	P	Range	Sequence	Modification
1682	1	1058.4833	-30.52	2	51.3	12.7	1	317-334	K.NKNECSLHIHASYSISQK.L	Carbamidomethyl: 5

Detailed Protein Report

Protein 270: zinc finger protein 573 isoform 4 [Homo sapiens]

**Accession:**	gi|289577063	**Score:**	37.2
**Database:**	refseq_human(refseq_human_20140103.fasta)	**MW [kDa]:**	77.9
**Database Date:**	2015-11-30	**pI:**	10.2
**Modification(s):**	Oxidation	**Sequence Coverage [%]:**	6.0
		**No. of unique Peptides:**	1

10	20	30	40	50	60	70	80
MFPVLEPHQV	GLIRSYNSKT	MTCELVTFRD	VAIDFSRQEW	EYLDPNQRDL	YRDVMLENYR	NLVSLGGHSI	SKPVVVDLLE
90	100	110	120	130	140	150	160
RGKEPWMILR	EETQFTDLDL	QCEIISYIEV	PTYETDISST	QLQSIYKREK	LYECKKCQKK	FSSGYQLILH	HRFHVIERPY
170	180	190	200	210	220	230	240
ECKECGKNFR	SGYQLTLHQR	FHTGEKPYEC	TECGKNFRSG	YQLTVHQRFH	TGEKTYECRQ	CGKAFIYASH	IVQHERIHTG
250	260	270	280	290	300	310	320
GKPYECQECG	RAFSQGGHLR	IHQRVHTGEK	PYKCKECGKT	FSRRSNLVEH	GQFHTDEKPY	ICEKCGKAFR	RGHQLTVHQR
330	340	350	360	370	380	390	400
VHTGKKPYEC	KECGKGYTTA	SYFLLHQRIH	KGGKPYECKE	CKKTFTLYRN	LTRHQNIHTG	EKLFECKQCG	KTYTTGSKLF
410	420	430	440	450	460	470	480
QHQKTHTGEK	PYECKECGKA	FSLYGYLKQH	QKIHTGMKHF	ECKECKKTFT	LYRNLTRHQN	IHTGKKLFEC	QECGKAYSTG
490	500	510	520	530	540	550	560
SNLIQHRKTH	TGEKPYKCKE	CGKTFSLHGY	LNQHQKIHTG	MKPYECKVCR	KTFTFYRNLT	LHQSIHTDEK	PFECKECGKT
570	580	590	600	610	620	630	640
FRRSSHLTAH	QSIHADKKPY	ECKECGKAFK	MYGYLTQHQK	IHTGGKPYEC	KECGKAFSRA	SNLVQHERIH	TGEKPYVCKQ
650	660	670					
CGKTFRYGSA	LKAHQRIHRS	IKV					

Cmpd.	No. of Cmpds.	m/z meas.	Δ m/z [ppm]	z	Rt [min]	Score	P	Range	Sequence	Modification
2167	1	900.3438	-97.52	3	55.5	11.8	2	15-37	R.SYNSKTMTCELVTFRDVAIDFSR.Q	Oxidation: 7

Detailed Protein Report

Protein 271: PERQ amino acid-rich with GYF domain-containing protein 2 isoform c [Homo sapiens]

**Accession:**	gi|156766047	**Score:**	37.1
**Database:**	refseq_human(refseq_human_20140103.fasta)	**MW [kDa]:**	149.4
**Database Date:**	2015-11-30	**pI:**	5.3
**Modification(s):**	Oxidation	**Sequence Coverage [%]:**	2.2
		**No. of unique Peptides:**	1

10	20	30	40	50	60	70	80
MAAETQTLNF	GPEWLRALSS	GGSITSPPLS	PALPKYKLAD	YRYGREEMLA	LFLKDNKIPS	DLLDKEFLPI	LQEEPLPPLA
**90**	**100**	**110**	**120**	**130**	**140**	**150**	**160**
LVPFTEEEQR	NFSMSVNSAA	VLRLTGRGGG	GTVVGAPRGR	SSSRGRGRGR	GECGFYQRSF	DEVEGVFGRG	GGREMHRSQS
**170**	**180**	**190**	**200**	**210**	**220**	**230**	**240**
WEERGDRRFE	KPGRKDVGRP	NFEEGGPTSV	GRKHEFIRSE	SENWRIFREE	QNGEDEDGGW	RLAGSRRDGE	RWRPHSPGWR
**250**	**260**	**270**	**280**	**290**	**300**	**310**	**320**
EHMERRRRFE	FDFRDRDDER	GYRRVRSGSG	SIDDDRDSLP	EWCLEDAEEE	MGTFDSSGAF	LSLKKVQKEP	IPEEQEMDFR
**330**	**340**	**350**	**360**	**370**	**380**	**390**	**400**
PVDEGEECSD	SEGSHNEEAK	EPDKTNKKEG	EKTDRVGVEA	SEETPQTSSS	SARPGTPSDH	QSQEASQFER	KDEPKTEQTE
**410**	**420**	**430**	**440**	**450**	**460**	**470**	**480**
KAEEETRMEN	SLPAKVPSRG	DEMVADVQQP	LSQIPSDTAS	PLLILPPPVP	NPSPTLRPVE	TPVVGAPGMG	SVSTEPDDEE
**490**	**500**	**510**	**520**	**530**	**540**	**550**	**560**
GLKHLEQQAE	KMVAYLQDSA	LDDERLASKL	QEHRAKGVSI	PLMHEAMQKW	YYKDPQGEIQ	GPFNNQEMAE	WFQAGYFTMS
**570**	**580**	**590**	**600**	**610**	**620**	**630**	**640**
LLVKRACDES	FQPLGDIMKM	WGRVPFSPGP	APPPHMGELD	QERLTRQQEL	TALYQMQHLQ	YQQFLIQQQY	AQVLAQQQKA
**650**	**660**	**670**	**680**	**690**	**700**	**710**	**720**
ALSSQQQQQL	ALLLQQFQTL	KMRISDQNII	PSVTRSVSVP	DTGSIWELQP	TASQPTVWEG	GSVWDLPLDT	TTPGPALEQL
**730**	**740**	**750**	**760**	**770**	**780**	**790**	**800**
QQLEKAKAAK	LEQERREAEM	RAKREEEERK	RQEELRRQQE	EILRRQQEEE	RKRREEEELA	RRKQEEALRR	QREQEIALRR
**810**	**820**	**830**	**840**	**850**	**860**	**870**	**880**
QREEEERQQQ	EEALRRLEER	RREEEERRKQ	EELLRKQEEE	AAKWAREEEE	AQRRLEENRL	RMEEEAARLR	HEEEERKRKE
**890**	**900**	**910**	**920**	**930**	**940**	**950**	**960**
LEVQRQKELM	RQRQQQEAL	RRLQQQQQQ	QLAQMKLPSS	STWGQQSNTT	ACQSQATLSL	AEIQKLEEER	ERQLREEQRR
**970**	**980**	**990**	**1000**	**1010**	**1020**	**1030**	**1040**
QQRELMKALQ	QQQQQQQKL	SGWGNVSKPS	GTTKSLLEIQ	QEEARQMQKQ	QQQQQHQQP	NRARNNTHSN	LHTSIGNSVW
**1050**	**1060**	**1070**	**1080**	**1090**	**1100**	**1110**	**1120**
GSINTGPPNQ	WASDLVSSIW	SNADTKNSNM	GFWDDAVKEV	GPRNSTNKNK	NNASLSKSVG	VSNRQNKKVE	EEEKLLKLFQ
**1130**	**1140**	**1150**	**1160**	**1170**	**1180**	**1190**	**1200**
GVNKAQDGFT	QWCEQMLHAL	NTANNLDVPT	FVSFLKEVES	PYEVHDYIRA	YLGDTSEAKE	FAKQFLERRA	KQKANQQRQQ
**1210**	**1220**	**1230**	**1240**	**1250**	**1260**	**1270**	**1280**
QQLPQQQQQQ	PPQQPPQQPQ	QQDSVWGMNH	STLHSVFQTN	QSNNQQSNFE	AVQSGKKKKK	QKMVRADPSL	LGFSVNASSE
**1290**	**1300**						
RLNMGEIETL	DDY						

Cmpd.	No. of Cmpds.	m/z meas.	Δ m/z [ppm]	z	Rt [min]	Score	P	Range	Sequence	Modification
1750	1	863.3936	-7.87	2	51.7	13.5	1	565-579	K.RACDESFQPLGDIMK.M	Oxidation: 14

Detailed Protein Report

Protein 272: probable global transcription activator SNF2L2 isoform c [Homo sapiens]

**Accession:**	gi|574957245	**Score:**	37.1
**Database:**	refseq_human(refseq_human_20140103.fasta)	**MW [kDa]:**	172.5
**Database Date:**	2015-11-30	**pI:**	6.7
**Modification(s):**	Oxidation	**Sequence Coverage [%]:**	2.4
		**No. of unique Peptides:**	2

10	20	30	40	50	60	70	80
MSTPTDPGAM	PHPGPSPGPG	PSPGPILGPS	PGPGPSPGSV	HSMMGPSPGP	PSVSHPMPTM	GSTDFPQEGM	HQMHKPIDGI
90	100	110	120	130	140	150	160
HDKGIVEDIH	CGSMKGTGMR	PPHPGMGPPQ	SPMDQHSQGY	MSPHPSPLGA	PEHVSSPMSG	GGPTPPQMPP	SQPGALIPGD
170	180	190	200	210	220	230	240
PQAMSQPNRG	PSPFSPVQLH	QLRAQILAYK	MLARGQPLPE	TLQLAVQGKR	TLPGLQQQQQ	QQQQQQQQQQ	QQQQQQQQPQ
250	260	270	280	290	300	310	320
QQPPQPQTQQ	QQQPALVNYN	RPSGPGPELS	GPSTPQKLPV	PAPGGRPSPA	PPAAAQPPAA	AVPGPSVPQP	APGQPSPVLQ
330	340	350	360	370	380	390	400
LQQKQSRISP	IQKPQGLDPV	EILQEREYRL	QARIAHRIQE	LENLPGSLPP	DLRTKATVEL	KALRLLNFQR	QLRQEVVACM
410	420	430	440	450	460	470	480
RRDTTLETAL	NSKAYKRSKR	QTLREARMTE	KLEKQQKIEQ	ERKRRQKHQE	YLNSILQHAK	DFKEYHRSVA	GKIQKLSKAV
490	500	510	520	530	540	550	560
ATWHANTERE	QKKETERIEK	ERMRRLMAED	EEGYRKLIDQ	KKDRRLAYLL	QQTDEYVANL	TNLVWEHKQA	QAAKEKKKRR
570	580	590	600	610	620	630	640
RRKKKAEENA	EGGESALGPD	GEPIDESSQM	SDLPVKVTHT	ETGKVLFGPE	APKASQLDAW	LEMNPGYEVA	PRSDSEESDS
650	660	670	680	690	700	710	720
DYEEEDEEEE	SSRQETEEKI	LLDPNSEEVS	EKDAKQIIET	AKQDVDDEYS	MQYSARGSQS	YYTVAHAISE	RVEKQSALLI
730	740	750	760	770	780	790	800
NGTLKHYQLQ	GLEWMVSLYN	NNLNGILADE	MGLGKTIQTI	ALITYLMEHK	RLNGPYLIIV	PLSTLSNWTY	EFDKWAPSVV
810	820	830	840	850	860	870	880
KISYKGTPAM	RRSLVPQLRS	GKFNVLLTTY	EYIIKDKHIL	AKIRWKYMIV	DEGHRMKNHH	CKLTQVDLNE	EETILIIRRL
890	900	910	920	930	940	950	960
HKVLRPFLLR	RLKKEVESQL	PEKVEYVIKC	DMSALQKILY	RHMQAKGILL	TDGSEKDKKG	KGGAKTLMNT	IMQLRKICNH
970	980	990	1000	1010	1020	1030	1040
PYMFQHIEES	FAEHLGYSNG	VINGAELYRA	SGKFELLDRI	LPKLRATNHR	VLLFCQMTSL	MTIMEDYFAF	RNFLYLRLDG
1050	1060	1070	1080	1090	1100	1110	1120
TTKSEDRAAL	LKKFNEPGSQ	YFIFLLSTRA	GGLGLNLQAA	DTVVIFDSDW	NPHQDLQAQD	RAHRIGQQNE	VRVLRLCTVN
1130	1140	1150	1160	1170	1180	1190	1200
SVEEKILAAA	KYKLNVDQKV	IQAGMFDQKS	SSHERRAFLQ	AILEHEEENE	EEDEVPDDET	LNQMIARREE	EFDLFMRMDM
1210	1220	1230	1240	1250	1260	1270	1280
DRRREDARNP	KRKPRLMEED	ELPSWIIKDD	AEVERLTCEE	EEEKIFGRGS	RQRRDVDYSD	ALTEKQWLRA	IEDGNLEEME
1290	1300	1310	1320	1330	1340	1350	1360
EEVRLKKRKR	RRNVDKDPAK	EDVEKAKKRR	GRPPAEKLSP	NPPKLTKQMN	AIIDTVINYK	DSSGRQLSEV	FIQLPSRKEL
1370	1380	1390	1400	1410	1420	1430	1440
PEYYELIRKP	VDFKKIKERI	RNHKYRSLGD	LEKDVMLLCH	NAQTFNLEGS	QIYEDSIVLQ	SVFKSARQKI	AKEEESEDES
1450	1460	1470	1480	1490	1500	1510	1520
NEEEEEEDEE	ESESEAKSVK	VKIKLNKKDD	KGRDKGKGKK	RPNRGKAKPV	VSDFDSDEEQ	DEREQSEGSG	TDDE

Cmpd.	No. of Cmpds.	m/z meas.	Δ m/z [ppm]	z	Rt [min]	Score	P	Range	Sequence	Modification
2593	1	648.3609	19.71	2	62.4	10.6	2	802-812	K.ISYKGTPAMRR.S	Oxidation: 9
2825	2	1024.0347	-1.30	2	65.7	15.7	1	1053-1069	K.KFNEPGSQYFIFLLSTR.A	

Detailed Protein Report

Protein 273: **polycystic kidney disease and receptor for egg jelly-related protein precursor [Homo sapiens]**

**Accession:**	gi|5174633	**Score:**	37.0
**Database:**	refseq_human(refseq_human_20140103.fasta)	**MW [kDa]:**	255.3
**Database Date:**	2015-11-30	**pI:**	10.0
**Modification(s):**	Carbamidomethyl	**Sequence Coverage [%]:**	1.5
		**No. of unique Peptides:**	2

Detailed Protein Report

10	20	30	40	50	60	70	80
MRPGPALLLL	GVGLSLSVGR	LPLPPVPRGA	QAAVSGAPGG	LLRGAPGLGV	RGGRALLSLR	PSAVRAGGAV	LSGRGSLCFP
90	100	110	120	130	140	150	160
HGGTGRRWYC	LDLRVLLSAQ	RLPWPAAPAL	ALVDLQLSAR	GGRLSLTWSV	RLPRSPGRLA	WAFRLRLLGP	GAARPASPAA
170	180	190	200	210	220	230	240
RVSPRSAAPG	PRPQQGFVAR	TECPTDGPAR	VMLQAVNSSS	HRAVESSVSC	QINACVIQRV	RINTDQKGAP	VRLSMQAEAT
250	260	270	280	290	300	310	320
INASVQLDCP	AARAIAQYWQ	VFSVPAVGQA	PDWTQPLDLP	QLEIRNSPLF	IHIPNNSLQW	GVYVFNFTVS	ITTGNPKMPE
330	340	350	360	370	380	390	400
VKDSDAVYVW	IVRSSLQAVM	LGDANITANF	TEQLILDGST	SSDPDADSPL	QGLQFFWYCT	TDPRNYGGDR	IILGSKEVCH
410	420	430	440	450	460	470	480
PEQANLKWPW	ASGPVLTLLP	ETLKGDHVYF	FRMVIRKDSR	TAFSDKRVHV	LQGPKAIAHI	TCIENCERNF	IVSDRFSLFL
490	500	510	520	530	540	550	560
NCTNCASRDF	YKWSILSSSG	GEMLFDWMGE	TVTGRNGAYL	SIKAFAFRHF	LEAEFSISLY	LACWSGVTSV	FRHSFIINHG
570	580	590	600	610	620	630	640
PQIGECKINP	AKGIALITKF	VVQCSNFRDK	HVPLTYKIIV	SDLHSVGEIS	SVKENTLGTI	LYLGPQSTVP	PSFLPVGMLA
650	660	670	680	690	700	710	720
SQYGLKIYAQ	VYDSLGAFSQ	VTLHATAQAP	TDKNSSKTVL	NQLLSFTVGP	SSLLSTLIQK	KDFLPAGYLL	YIVASVLNNM
730	740	750	760	770	780	790	800
KTELPLRDDR	VNLRKHLIDQ	SFLLPVSTLV	EIGQVVMTIT	KLTQKPSEFT	WDAQKRATMR	VWQANQALQE	YQQKDKRFRS
810	820	830	840	850	860	870	880
EQIEIVSTGI	LMSLSNILKM	TSPHQVVKDP	FYVIESLSDT	ILANKVPGNK	TTSMRTPNFN	MYVKKVEKWG	INQLFRNEKH
890	900	910	920	930	940	950	960
CRNCFYPTLN	VSSVPGLSAN	GPISTMFCDF	TNDLFPWLND	QENTSVEVSG	FRMTGVADNG	SVLEITPDVA	EVYLVRKNLT
970	980	990	1000	1010	1020	1030	1040
FAAFNLTVGP	NSEVDGSLKK	TTGGFSFQVD	STVLREVLVH	IVTEVMVLFT	VLVYTGSQIT	PTALVATFLV	PHDIPPFASQ
1050	1060	1070	1080	1090	1100	1110	1120
SALFDPACTV	KKARVVCLPV	SLLQLIAQHS	HSPHCTVSIV	LQAPRFVMKL	NDKLVRISIF	SVQCLDMYGI	QSEWREGYCI
1130	1140	1150	1160	1170	1180	1190	1200
LGEKTSWYEV	HCICKNVVRA	RRQLGTIGLT	GIHLHTHYVM	AKVIVIPNPV	DLRLNIIKSL	HQNPVTLFTV	LFIILLYVGL
1210	1220	1230	1240	1250	1260	1270	1280
AFWALYRDEM	DQHLRGHVIV	LPDNDPYDNL	CYLVTIFTGS	RWGSGTRANV	FVQLRGTVST	SDVHCLSHPH	FTTLYRGSIN
1290	1300	1310	1320	1330	1340	1350	1360
TFLLTTKSDL	GDIHSIRVWH	NNEGRSPSWY	LSRIKVENLF	SRHIWLFICQ	KWLSVDTTLD	RTFHVTHPDE	RLTRKDFFFI
1370	1380	1390	1400	1410	1420	1430	1440
DVSSNLRKNH	MWFSIFASVV	AKTFNRLQRL	SCCLAMLLSS	LLCNIMFFNL	NRQEQTESRE	RKYMRSMMIG	IESVLITIPV
1450	1460	1470	1480	1490	1500	1510	1520
QLLITFLFTC	SQRKPQADLK	EVSPQKHPLM	SEASEHWEEY	LRKWHAYETA	KVHPREVAKP	ASKGKPRLPK	ASPKATSKPK
1530	1540	1550	1560	1570	1580	1590	1600
HRHRKAQIKT	PETLGPNTNS	NNNIEDDQDV	HSEQHPSQKD	LQQLKKKPRI	VLPWWCVYVA	WFLVFATSSI	SSFFIVFYGL
1610	1620	1630	1640	1650	1660	1670	1680
TYGYDKSIEW	LFASFCSFCQ	SVLLVQPSKI	ILLSGFRTNK	PKYCKNLSWS	TKYKYTEIRL	DGMRMHPEEM	QRIHDQIVRI
1690	1700	1710	1720	1730	1740	1750	1760
RGTRMYQPLT	EDEIRIFKRK	KRIKRRALLF	LSYILTHFIF	LALLLILIVL	LRHTDCFYYN	QFIRDRFSMD	LATVTKLEDI
1770	1780	1790	1800	1810	1820	1830	1840
YRWLNSVLLP	LLHNDLNPTF	LPESSSKILG	LPLMRQVRAK	SSEKMCLPAE	KFVQNSIRRE	IHCHPKYGID	PEDTKNYSGF
1850	1860	1870	1880	1890	1900	1910	1920
WNEVDKQAID	ESTNGFTYKP	QGTQWLYYSY	GLLHTYGSGG	YALYFFPEQQ	RFNSTLRLKE	LQESNWLDEK	TWAVVLELTT
1930	1940	1950	1960	1970	1980	1990	2000
FNPDINLFCS	ISVIFEVSQL	GVVNTSISLH	SFSLADFDRK	ASAEIYLYVA	ILIFFLAYVV	DEGCIIMQER	ASYVRSVYNL
2010	2020	2030	2040	2050	2060	2070	2080
LNFALKCIFT	VLIVLFLRKH	FLATGIIRFY	LSNPEDFIPF	HAVSQVDHIM	RIILGFLLFL	TILKTLRYSR	FFYDVRLAQR
2090	2100	2110	2120	2130	2140	2150	2160
AIQAALPGIC	HMAFVVSVYF	FVYMAFGYLV	FGQHEWNYSN	LIHSTQTVFS	YCVSAFQNTE	FSNNRILGVL	FLSSFMLVMI
2170	2180	2190	2200	2210	2220	2230	2240

Detailed Protein Report

Cmpd.	No. of Cmpds.	m/z meas.	Δ m/z [ppm]	z	Rt [min]	Score	P	Range	Sequence	Modification
2763	1	741.8475	25.09	2	64.9	12.5	0	1125-1135	K.TSWYEVHCICK.N	Carbamidomethyl: 8, 10
2608	1	648.3616	-63.29	2	62.6	12.8	1	1788-1798	K.ILGLPLMRQVR.A	

Detailed Protein Report

Protein 274: PREDICTED: F-box/WD repeat-containing protein 11 isoform X5 [Homo sapiens]

**Accession:**	gi|530380941	**Score:**	36.9
**Database:**	refseq_human(refseq_human_20140103.fasta)	**MW [kDa]:**	56.8
**Database Date:**	2015-11-30	**pI:**	8.6
**Modification(s):**	Carbamidomethyl	**Sequence Coverage [%]:**	9.7
		**No. of unique Peptides:**	3

10	20	30	40	50	60	70	80
MISNGTSSVI	VSRKRPSEGN	YQKEKDLCIK	YFDQWSESDQ	VEFVEHLISR	MCHYQHGHIN	SYLKPMLQRD	FITALPEQGL
**90**	**100**	**110**	**120**	**130**	**140**	**150**	**160**
DHIAENILSY	LDARSLCAAE	LVCKEWQRVI	SEGMLWKKLI	ERMVRTDPLW	KGLSERRGWD	QYLFKNRPTD	GPPNSFYRSL
**170**	**180**	**190**	**200**	**210**	**220**	**230**	**240**
YPKIIQDIET	IESNWRCGRH	NLQRIQCRSE	NSKGVYCLQY	DDEKIISGLR	DNSIKIWDKT	SLECLKVLTG	HTGSVLCLQY
**250**	**260**	**270**	**280**	**290**	**300**	**310**	**320**
DERVIVTGSS	DSTVRVWDVN	TGEVLNTLIH	HNEAVLHLRF	SNGLMVTCSK	DRSIAVWDMA	SATDITLRRV	LVGHRAAVNV
**330**	**340**	**350**	**360**	**370**	**380**	**390**	**400**
VDFDDKYIVS	ASGDRTIKVW	STSTCEFVRT	LNGHKRGIAC	LQYRDRLVVS	GSSDNTIRLW	DIECGACLRV	LEGHEELVRC
**410**	**420**	**430**	**440**	**450**	**460**	**470**	**480**
IRFDNKRIVS	GAYDGKIKVW	DLQAALDPRA	PASTLCLRTL	VEHSGRVFRL	QFDEFQIISS	SHDDTILIWD	FLNVPPSAQN
**490**	**500**						
ETRSPSRTYT	YISR						

Cmpd.	No. of Cmpds.	m/z meas.	Δ m/z [ppm]	z	Rt [min]	Score	P	Range	Sequence	Modification
891	1	630.9500	-54.02	3	39.5	12.1	0	227-243	K.VLTGHTGSVLCLQYDER.V	
2270	3	596.7864	-15.83	2	56.7	13.1	0	316-326	R.AAVNVVDFDDK.Y	
1138	1	751.8302	130.90	3	42.5	11.7	1	419-438	K.VWDLQAALDPRAPASTLCLR.T	Carbamidomethyl: 18

Detailed Protein Report

Protein 275: metallothionein-4 [Homo sapiens]

Accession: gi|14269578
Database: refseq_human(refseq_human_20140103.fasta)
Database Date: 2015-11-30
Modification(s): Carbamidomethyl

Score: 36.8
MW [kDa]: 6.4
pI: 10.8
Sequence Coverage [%]: 53.2
No. of unique Peptides: 1

10	20	30	40	50	60	70
MDPRECVCMS	GGICMCGDNC	KCTTCNCKTC	RKSCCPCCPP	GCAKCARGCI	CKGGSDKCSC	CP

Cmpd.	No. of Cmpds.	m/z meas.	Δ m/z [ppm]	z	Rt [min]	Score	P	Range	Sequence	Modification
1709	1	892.3390	4.69	2	51.7	11.6	1	33-47	K.SCCPCCPPGCAKCAR.G	Carbamidomethyl: 2, 3, 6, 10, 13

Detailed Protein Report

Protein 276: tripartite motif-containing protein 65 isoform 2 [Homo sapiens]

Accession: gi|371122551
Database: refseq_human(refseq_human_20140103.fasta)
Database Date: 2015-11-30
Modification(s): Carbamidomethyl

Score: 36.8
MW [kDa]: 54.9
pI: 6.0
Sequence Coverage [%]: 8.1
No. of unique Peptides: 2

10	20	30	40	50	60	70	80
MAAQLLEEKL	TCAICLGLYQ	DPVTLPCGHN	FCGACIRDWW	DRCGKACPEC	REPFPDGAEL	RRNVALSGVL	EVVRAGPARD
90	100	110	120	130	140	150	160
PGPDPGPGPD	PAARCPRHGR	PLELFCRTEG	RCVCSVCTVR	ECRLHERALL	DAERLKREAQ	LRASLEVTQQ	QATQAEGQLL
170	180	190	200	210	220	230	240
ELRKQSSQIQ	NSACILASWV	SGKFSSLLQA	LEIQHTTALR	SIEVAKTQAL	AQARDEEQRL	RVHLEAVARH	GCRIRELLEQ
250	260	270	280	290	300	310	320
VDEQTFLQES	QLLQPPGPLG	PLTPLQWDED	QQLGDLKQLL	SRLCGLLLEE	GSHPGAPAKP	VDLAPVDYRN	LTFDPVSANR
330	340	350	360	370	380	390	400
HFYLSRQDQQ	VKHCRQSRGP	GGPGSFELWQ	VQCAQSFQAG	HHYWEVRASD	HSVTLGVSYP	QLPRCRLGPH	TDNIGRGPCS
410	420	430	440	450	460	470	480
WGLCVQEDSL	QAWHNGEAQR	LPGVSGRLLG	MDLDLASGCL	TFYSLEPQTQ	PLYTFHALFN	QPLTPVFWLL	EGRTLTLCHQ
490	500						
PGAVFPLGPQ	EEVLS						

Cmpd.	No. of Cmpds.	m/z meas.	Δ m/z [ppm]	z	Rt [min]	Score	P	Range	Sequence	Modification
347	1	764.8773	64.49	2	34.3	12.1	1	112-123	R.CVCSVCTVRECR.L	Carbamidomethyl: 1, 3, 6
40	1	661.7546	-162.80	2	30.3	10.4	1	124-134	R.LHERALLDAER.L	

Detailed Protein Report

Protein 277: PREDICTED: mucin-5AC [Homo sapiens]

**Accession:**	gi|578821001	**Score:**	36.8
**Database:**	refseq_human(refseq_human_20140103.fasta)	**MW [kDa]:**	520.3
**Database Date:**	2015-11-30	**pI:**	6.6
**Modification(s):**	Carbamidomethyl, Oxidation	**Sequence Coverage [%]:**	0.9
		**No. of unique Peptides:**	3

Detailed Protein Report

10	20	30	40	50	60	70	80
MSVGRRKLAL	LWALALALAC	TRHTGHAQDG	SSESSYKHHP	ALSPIARGPS	GVPLRGATVF	PSLRTIPVVR	ASNPAHNGRV
90	100	110	120	130	140	150	160
CSTWGSFHYK	TFDGDVFRFP	GLCNYVFSEH	CGAAYEDFNI	QLRRSQESAA	PTLSRVLMKV	DGVVIQLTKG	SVLVNGHPVL
170	180	190	200	210	220	230	240
LPFSQSGVLI	QQSSSYTKVE	ARLGLVLMWN	HDDSLLLELD	TKYANKTCGL	CGDFNGMPVV	SELLSHNTKL	TPMEFGNLQK
250	260	270	280	290	300	310	320
MDDPTDQCQD	PVPEPPRNCS	TGFGICEELL	HGQLFSGCVA	LVDVGSYLEA	CRQDLCFCED	TDLLSCVCHT	LAEYSRQCTH
330	340	350	360	370	380	390	400
AGGLPQDWRG	PDFCPQKCPN	NMQYHECRSP	CADTCSNQEH	SRACEDHCVA	GCFCPEGTVL	DDIGQTGCVP	VSKCACVYNG
410	420	430	440	450	460	470	480
AAYAPGATYS	TDCTNCTCSG	GRWSCQEVPC	PGTCSVLGGA	HFSTFDGKQY	TVHGDCSYVL	TKPCDSSAFT	VLAELRRCGL
490	500	510	520	530	540	550	560
TDSETCLKSV	TLSLDGAQTV	VVIKASGEVF	LNQIYTQLPI	SAANVTIFRP	STFFIIAQTS	LGLQLNLQLV	PTMQLFMQLA
570	580	590	600	610	620	630	640
PKLRGQTCGL	CGNFNSIQAD	DFRTLSGVVE	ATAAAFFNTF	KTQAACPNIR	NSFEDPCSLS	VENEKYAQHW	CSQLTDADGP
650	660	670	680	690	700	710	720
FGRCHAAVKP	GTYYSNCMFD	TCNCERSEDC	LCAALSSYVH	ACAAKGVQLG	GWRDGVCTKP	MTTCPKSMTY	HYHVSTCQPT
730	740	750	760	770	780	790	800
CRSLSEGDIT	CSVGFIPVDG	CICPKGTFLD	DTGKCVQASN	CPCYHRGSMI	PNGESVHDSG	AICTCTHGKL	SCIGGQAPAP
810	820	830	840	850	860	870	880
VCAAPMVFFD	CRNATPGDTG	AGCQKSCHTL	DMTCYSPQCV	PGCVCPDGLV	ADGEGGCITA	EDCPCVHNEA	SYRAGQTIRV
890	900	910	920	930	940	950	960
GCNTCTCDSR	MWRCTDDPCL	ATCAVYGDGH	YLTFDGQSYS	FNGDCEYTLV	QNHCGGKDST	QDSFRVVTEN	VPCGTTGTTC
970	980	990	1000	1010	1020	1030	1040
SKAIKIFLGG	FELKLSHGKV	EVIGTDESQE	VPYTIRQMGI	YLVVDTDIGL	VLLWDKKTSI	FINLSPEFKG	RVCGLCGNFD
1050	1060	1070	1080	1090	1100	1110	1120
DIAVNDFATR	SRSVVGDVLE	FGNSWKLSPS	CPDALAPKDP	CTANPFRKSW	AQKQCSILHG	PTFAACHAHV	EPARYYEACV
1130	1140	1150	1160	1170	1180	1190	1200
NDACACDSGG	DCECFCTAVA	AYAQACHEVG	LCVSWRTPSI	CPLFCDYYNP	EGQCEWHYQP	CGVPCLRTCR	NPRGDCLRDV
1210	1220	1230	1240	1250	1260	1270	1280
RGLEGCYPKC	PPEAPIFDED	KMQCVATCPT	PPLPPRCHVH	GKSYRPGAVV	PSDKNCQSCL	CTERGVECTY	KAEACVCTYN
1290	1300	1310	1320	1330	1340	1350	1360
GQRFHPGDVI	YHTTDGTGGC	ISARCGANGT	IERRVYPCSP	TTPVPPTTFS	FSTPPLVVSS	THTPSNGPSS	AHTGPPSSAW
1370	1380	1390	1400	1410	1420	1430	1440
PTTAGTSPRT	RLPTASASLP	PVCGEKCLWS	PWMDVSRPGR	GTDSGDFDTL	ENLRAHGYRV	CESPRSVECR	AEDAPGVPLR
1450	1460	1470	1480	1490	1500	1510	1520
ALGQRVQCSP	DVGLTCRNRE	QASGLCYNYQ	IRVQCCTPLP	CSTSSSPAQT	TPPTTSKTTE	TRASGSSAPS	STPGTVSLST
1530	1540	1550	1560	1570	1580	1590	1600
ARTTPAPGTA	TSVKKTFSTP	SPPPVPATST	SSMSTTAPGT	SVVSSKPTPT	EPSTSSCLQE	LCTWTEWIDG	SYPAPGINGG
1610	1620	1630	1640	1650	1660	1670	1680
DFDTFQNLRD	EGYTFCESPR	SVQCRAESFP	NTPLADLGQD	VICSHTEGLI	CLNKNQLPPI	CYNYEIRIQC	CETVNVCRDI
1690	1700	1710	1720	1730	1740	1750	1760
TRLPKTVATT	RPTPHPTGAQ	TQTTFTTHMP	SASTEQPTAT	SRGGPTATSV	TQGTHTTLVT	RNCHPRCTWT	KWFDVDFPSP
1770	1780	1790	1800	1810	1820	1830	1840
GPHGGDKETY	NNIIRSGEKI	CRRPEEITRL	QCRAKSHPEV	SIEHLGQVVQ	CSREEGLVCR	NQDQQGPFKM	CLNYEVRVLC
1850	1860	1870	1880	1890	1900	1910	1920
CETPRGCHMT	STPGSTSSSP	AQTTPSTTSK	TTETQASGSS	APSSTPGTVS	LSTARTTPAP	GTATSVKKTF	STPSPPPVPA
1930	1940	1950	1960	1970	1980	1990	2000
TSTSSMSTTA	PGTSVVSSKP	TPTEPSTSSC	LQELCTWTEW	IDGSYPAPGI	NGGDFDTFQN	LRDEGYTFCE	SPRSVQCRAE
2010	2020	2030	2040	2050	2060	2070	2080
SFPNTPLADL	GQDVICSHTE	GLICLNKNQL	PPICYNYEIR	IQCCETVNVC	RDITRPPKTV	ATTRPTPHPT	GAQTQTTFTT
2090	2100	2110	2120	2130	2140	2150	2160
HMPSASTEQP	TATSRGGPTA	TSVTQGTHTT	PVTRNCHPRC	TWTTWFDVDF	PSPGPHGGDK	ETYNNIIRSG	EKICRRPEEI
2170	2180	2190	2200	2210	2220	2230	2240

Detailed Protein Report

Cmpd.	No. of Cmpds.	m/z meas.	Δ m/z [ppm]	z	Rt [min]	Score	P	Range	Sequence	Modification
1356	1	863.9173	-123.12	2	46.6	12.1	1	7-22	R.KLALLWALALALACTR.H	
2649	2	734.8562	20.51	2	63.2	12.1	0	317-329	R.QCTHAGGLPQDWR.G	
2548	1	1044.5297	54.57	2	61.8	12.6	1	2203-2218	K.MCLNYEVRVLCCETPK.G	Carbamidomethyl: 2, 11, 12; Oxidation: 1

Detailed Protein Report

Protein 278: NF-X1-type zinc finger protein NFXL1 [Homo sapiens]

Accession: gi|89363020
Database: refseq_human(refseq_human_20140103.fasta)
Database Date: 2015-11-30
Modification(s): Carbamidomethyl

Score: 36.8
MW [kDa]: 101.3
pI: 10.5
Sequence Coverage [%]: 5.2
No. of unique Peptides: 2

Alias proteins:

Accession	Name	Description
gi|520261838	refseq_human (refseq_human_20140103.fasta)	NF-X1-type zinc finger protein NFXL1 [Homo sapiens]
gi|520261712	refseq_human (refseq_human_20140103.fasta)	NF-X1-type zinc finger protein NFXL1 [Homo sapiens]

10	20	30	40	50	60	70	80
MEASWRQVAG	GRGRSRGRAT	AAPSGNGVHL	RGAGGGREKG	SVGAVPSGTS	PGGVATTAAA	GSRHSPAGSQ	ALQTTAASEL
**90**	**100**	**110**	**120**	**130**	**140**	**150**	**160**
MSQKKFEEIK	KANQAAARKL	VEEQFSSSSE	EGDEDFEGKQ	GKILANTFIT	YTTQTDGDTR	ELERTKQYVN	EAFQAGAMTC
**170**	**180**	**190**	**200**	**210**	**220**	**230**	**240**
LICIASVKRN	QAVWSCSGCF	CIFHMPCIQK	WAKDSQFLVS	SVTDDDFGKK	DCPWPCPKCR	FEYKRSETPS	RYYCYCGKVE
**250**	**260**	**270**	**280**	**290**	**300**	**310**	**320**
DPPLDPWLVP	HSCGQVCERE	FKPPCGHKCL	LLCHPGPCPP	CPKMVTTTCY	CKKAKPIPRR	CSAKEWSCQL	PCGQKLLCGQ
**330**	**340**	**350**	**360**	**370**	**380**	**390**	**400**
HKCENPCHAG	SCQPCPRVSR	QKCVCGKKVA	ERSCASPLWH	CDQVCGKTLP	CGNHTCEQVC	HVGACGECPR	SGKRFCPCQK
**410**	**420**	**430**	**440**	**450**	**460**	**470**	**480**
SKFSLPCTED	VPTCGDSCDK	VLECGIHRCS	QRCHRGPCET	CRQEVEKHCR	CGKHTKRMPC	HKPYLCETKC	VKMRDCQKHQ
**490**	**500**	**510**	**520**	**530**	**540**	**550**	**560**
CRRKCCPGNC	PPCDQNCGRT	LGCRNHKCPS	VCHRGSCYPC	PETVDVKCNC	GNTKVTVPCG	RERTTRPPKC	KEQCSRPPTC
**570**	**580**	**590**	**600**	**610**	**620**	**630**	**640**
HHTSQEKHRC	HFGSCPPCHQ	PCQKVLEKCG	HLCPAPCHDQ	ALIKQTGRHQ	PTGPWEQPSE	PAFIQTALPC	PPCQVPIPME
**650**	**660**	**670**	**680**	**690**	**700**	**710**	**720**
CLGKHEVSPL	PCHAVGPYSC	KRVCGRILDC	QNHTCMKECH	KVTKTDGCTG	KNKAGPECLH	CEEGCSKSRP	LGCLHPCILR
**730**	**740**	**750**	**760**	**770**	**780**	**790**	**800**
CHPGECPPCV	QMLRIKCHCK	ITSLYVECRK	ITTADVNEKN	LLSCCKNQCP	KELPCGHRCK	EMCHPGECPF	NCNQKVKLRC
**810**	**820**	**830**	**840**	**850**	**860**	**870**	**880**
PCKRIKKELQ	CNKVRENQVS	IECDTTCKEM	KRKASEIKEA	EAKAALEEEK	RRQQAELEAF	ENRLKGRRKK	NRKRDEVAVE
**890**	**900**	**910**	**920**				
LSLWQKHKYY	LISVCGVVVV	VFAWYITHDV	N				

Cmpd.	No. of Cmpds.	m/z meas.	Δ m/z [ppm]	z	Rt [min]	Score	P	Range	Sequence	Modification
2093	1	732.3366	-79.70	2	56.6	10.4	1	17-31	R.GRATAAPSGNGVHLR.G	
1824	1	916.3237	-87.07	2	53.1	10.4	2	433-447	R.CHRGPCETCRQEVEK.H	Carbamidomethyl: 6

Detailed Protein Report

Protein 279: protein TANC1 isoform 2 [Homo sapiens]

**Accession:**	gi|225543463	**Score:**	36.4
**Database:**	refseq_human(refseq_human_20140103.fasta)	**MW [kDa]:**	152.1
**Database Date:**	2015-11-30	**pI:**	9.5
**Modification(s):**	Oxidation	**Sequence Coverage [%]:**	2.1
		**No. of unique Peptides:**	2

10	20	30	40	50	60	70	80
MLKAVLKKSR	EGGKGGKKEA	GSDFGPETSP	VLHLDHSADS	PVSSLPTAED	TYRVSLAKGV	SMSLPSSPLL	PRQSHLVQSR
**90**	**100**	**110**	**120**	**130**	**140**	**150**	**160**
VNKKSPGPVR	KPKYVESPRV	PGDAVIMPFR	EVAKPTEPDE	HAKADNEPSC	SPAAQELLTR	LGFLLGEGIP	SATHITIEDK
**170**	**180**	**190**	**200**	**210**	**220**	**230**	**240**
NETMCTALSQ	GISPCSTLTS	STASPSTDSP	CSTLNSCVSK	TAANKSPCET	ISSPSSTLES	KDSGIIATIT	SSSENDDRSG
**250**	**260**	**270**	**280**	**290**	**300**	**310**	**320**
SSLEWNKDGN	LRLGVQKGVL	HDRRADNCSP	VAEEETTGSA	ESTLPKAESS	AGDGPVPYSQ	GSSSLIMPRP	NSVAATSSTK
**330**	**340**	**350**	**360**	**370**	**380**	**390**	**400**
LEDLSYLDGQ	RNAPLRTSIR	LPWHNTAGAR	FAPYKPQDIL	LKPLLFEVPS	ITTDSVFVGR	DWLFHQIEEN	LRNTELAENR
**410**	**420**	**430**	**440**	**450**	**460**	**470**	**480**
GAVVVGNVGF	GKTAIISKLV	ALSCHGSRMR	QIASNSPGSS	PKTSDPTQDL	HFTPLLSPSS	STSASSTAKT	PLGSISAENQ
**490**	**500**	**510**	**520**	**530**	**540**	**550**	**560**
RPREDAVKYL	ASKVVAYHYC	QADNTYTCLV	PEFVHSIAAL	LCRSHQLAAY	RDLLIKEPQL	QSMLSLRSCV	QDPVAAFKRG
**570**	**580**	**590**	**600**	**610**	**620**	**630**	**640**
VLEPLTNLRN	EQKIPEEEYI	ILIDGLNEAE	FHKPDYGDTL	SSFITKIISK	FPAWLKLIVT	VRANFQEIIS	ALPFVKLSLD
**650**	**660**	**670**	**680**	**690**	**700**	**710**	**720**
DFPDNKDIHS	DLHAYVQHRV	HSSQDILSNI	SLNGKADATL	IGKVSSHLVL	RSLGSYLYLK	LTLDLFQRGH	LVIKSASYKV
**730**	**740**	**750**	**760**	**770**	**780**	**790**	**800**
VPVSLSELYL	LQCNMKFMTQ	SAFERALPIL	NVALASLHPM	TDEQIFQAIN	AGHIQGEQGW	EDFQQRMDAL	SCFLIKRRDK
**810**	**820**	**830**	**840**	**850**	**860**	**870**	**880**
TRMFCHPSFR	EWLVWRADGE	NTAFLCEPRN	GHALLAFMFS	RQEGKLNRQQ	TMELGHHILK	AHIFKGLSKK	TGISSSHLQA
**890**	**900**	**910**	**920**	**930**	**940**	**950**	**960**
LWIGYSTEGL	SAALASLRNL	YTPNVKVSRL	LILGGANVNY	RTEVLNNAPI	LCVQSHLGHE	EVVTLLLEFG	ACLDGTSENG
**970**	**980**	**990**	**1000**	**1010**	**1020**	**1030**	**1040**
MTALCYAAAA	GHMKLVCLLT	KKGVRVDHLD	KKGQCALVHS	ALRGHGDILQ	YLLTCEWSPG	PPQPGTLRKS	HALQQALTAA
**1050**	**1060**	**1070**	**1080**	**1090**	**1100**	**1110**	**1120**
ASMGHSSVVQ	CLLGMEKEHE	VEVNGTDTLW	GETALTAAAG	RGKLEVCELL	LGHGAAVSRT	NRRGVPPLFC	AARQGHWQIV
**1130**	**1140**	**1150**	**1160**	**1170**	**1180**	**1190**	**1200**
RLLLERGCDV	NLSDKQGRTP	LMVAACEGHL	STVEFLLSKG	AALSSLDKEG	LSALSWACLK	GHRAVVQYLV	EEGAAIDQTD
**1210**	**1220**	**1230**	**1240**	**1250**	**1260**	**1270**	**1280**
KNGRTPLDLA	AFYGDAETVL	YLVEKGAVIE	HVDHSGMRPL	DRAIGCRNTS	VVVALLRKGA	KLGNAAWAMA	TSKPDILIIL
**1290**	**1300**	**1310**	**1320**	**1330**	**1340**	**1350**	**1360**
LQKLMEEGNV	MYKKGKMKEA	AQRYQYALRK	FPREGFGEDM	RPFNELRVSL	YLNLSRCRRK	TNDFGMAEEF	ASKALELKPK
**1370**	**1380**	**1390**	**1400**				
SYEAFYARAR	AKRNSRYRNS	WQLWLTCKRL					

Cmpd.	No. of Cmpds.	m/z meas.	Δ m/z [ppm]	z	Rt [min]	Score	P	Range	Sequence	Modification
2703	5	673.3636	27.60	2	64.1	17.2	1	419-430	K.LVALSCHGSRMR.Q	Oxidation: 11
2221	1	953.0764	108.03	2	58.2	19.2	0	1226-1242	K.GAVIEHVDHSGMRPLDR.A	Oxidation: 12

Detailed Protein Report

Protein 280: PREDICTED: afadin isoform X15 [Homo sapiens]

**Accession:**	gi|578812779	**Score:**	36.4
**Database:**	refseq_human(refseq_human_20140103.fasta)	**MW [kDa]:**	185.3
**Database Date:**	2015-11-30	**pI:**	6.0
		**Sequence Coverage [%]:**	1.4
		**No. of unique Peptides:**	1

Quantitation

QU:MU	**Median:**	3.14	**CV:**	0.00 %	**No. of Peptides:**	1
WUP:QUP	**Median:**	0.05	**CV:**	0.00 %	**No. of Peptides:**	1

10	20	30	40	50	60	70	80
MSAGGRDEER	RKLADIIHHW	NANRLDLFEI	SQPTEDLEFH	GVMRFYFQDK	AAGNFATKCI	RVSSTATTQD	VIETLAEKFR
90	100	110	120	130	140	150	160
PDMRMLSSPK	YSLYEVHVSG	EERRLDIDEK	PLVVQLNWNK	DDREGRFVLK	NENDAIPPKK	AQSNGPEKQE	KEGVIQNFKR
170	180	190	200	210	220	230	240
TLSKKEKKEK	KKREKEALRQ	ASDKDDRPFQ	GEDVENSRLA	AEVYKDMPET	SFTRTISNPE	VVMKRRRQQK	LEKRMQEFRS
250	260	270	280	290	300	310	320
SDGRPDSGGT	LRIYADSLKP	NIPYKTILLS	TTDPADFAVA	EALEKYGLEK	ENPKDYCIAR	VMLPPGAQHS	DEKGAKEIIL
330	340	350	360	370	380	390	400
DDDECPLQIF	REWPSDKGIL	VFQLKRRPPD	HIPKKTKKHL	EGKTPKGKER	ADGSGYGSTL	PPEKLPYLVE	LSPGRRNHFA
410	420	430	440	450	460	470	480
YYNYHTYEDG	SDSRDKPKLY	RLQLSVTEVG	TEKLDDNSIQ	LFGPGIQPHH	CDLTNMDGVV	TVTPRSMDAE	TYVEGQRISE
490	500	510	520	530	540	550	560
TTMLQSGMKV	QFGASHVFKF	VDPSQDHALA	KRSVDGGLMV	KGPRHKPGIV	QETTFDLGGD	IHSGTALPTS	KSTTRLDSDR
570	580	590	600	610	620	630	640
VSSASSTAER	GMVKPMIRVE	QQPDYRRQES	RTQDASGPEL	ILPASIEFRE	SSEDSFLSAI	INYTNSSTVH	FKLSPTYVLY
650	660	670	680	690	700	710	720
MACRYVLSNQ	YRPDISPTER	THKVIAVVNK	MVSMMEGVIQ	EVDQVDQKQK	NIAGALAFWM	ANASELLNFI	KQDRDLSRIT
730	740	750	760	770	780	790	800
LDAQDVLAHL	VQMAFKYLVH	CLQSELNNYM	PAFLDDPEEN	SLQRPKIDDV	LHTLTGAMSL	LRRCRVNAAL	TIQLFSQLFH
810	820	830	840	850	860	870	880
FINMWLFNRL	VTDPDSGLCS	HYWGAIIRQQ	LGHIEAWAEK	QGLELAADCH	LSRIVQATTL	LTMDKYAPDD	IPNINSTCFK
890	900	910	920	930	940	950	960
LNSLQLQALL	QNYHCAPDEP	FIPTDLIENV	VTVAENTADE	LARSDGREVQ	LEEDPDLQLP	FLLPEDGYSC	DVVRNIPNGL
970	980	990	1000	1010	1020	1030	1040
QEFLDPLCQR	GFCRLIPHTR	SPGTWTIYFE	GADYESHLLR	ENTELAQPLR	KEPEIITVTL	KKQNGMGLSI	VAAKGAGQDK
1050	1060	1070	1080	1090	1100	1110	1120
LGIYVKSVVK	GGAADVDGRL	AAGDQLLSVD	GRSLVGLSQE	RAAELMTRTS	SVVTLEVAKQ	GAIYHGLATL	LNQPSPMMQR
1130	1140	1150	1160	1170	1180	1190	1200
ISDRRGSGKP	RPKSEGFELY	NNSTQNGSPE	SPQLPWAEYS	EPKKLPGDDR	LMKNRADHRS	SPNVANQPPS	PGGKSAYASG
1210	1220	1230	1240	1250	1260	1270	1280
TTAKITSVST	GNLCTEEQTP	PPRPEAYPIP	TQTYTREYFT	FPASKSQDRM	APPQNQWPNY	EEKPHMHTDS	NHSSIAIQRV
1290	1300	1310	1320	1330	1340	1350	1360
TRSQEELRED	KAYQLERHRI	EAAMDRKSDS	DMWINQSSSL	DSSTSSQEHL	NHSSKSVTPA	STLTKSGPGR	WKTPAAIPAT
1370	1380	1390	1400	1410	1420	1430	1440
PVAVSQPIRT	DLPPPPPPPP	VHYAGDFDGM	SMDLPLPPPP	SANQIGLPSA	QVAAAERRKR	EEHQRWYEKE	KARLEEERER
1450	1460	1470	1480	1490	1500	1510	1520
KRREQERKLG	QMRTQSLNPA	PFSPLTAQQM	KPEKPSTLQR	PQETVIRELQ	PQQQPRTIER	RDLQYITVSK	EELSSGDSLS
1530	1540	1550	1560	1570	1580	1590	1600
PDPWKRDAKE	KLEKQQQMHI	VDMLSKEIQE	LQSKPDRSAE	ESDRLRKLML	EWQFQKRLQE	SKQKDEDDEE	EEDDDVDTML
1610	1620	1630	1640				
IMQRLEAERR	ARQTAMPAIS	VLDLVCSCFF	PFGTFFLS				

Cmpd.	No. of Cmpds.	m/z meas.	Δ m/z [ppm]	z	Rt [min]	Score	P	Range	Sequence	Modification	Ratios

Detailed Protein Report

Cmpd.	No. of Cmpds.	m/z meas.	Δ m/z [ppm]	z	Rt [min]	Score	P	Range	Sequence	Modification	Ratios	
1199	1	533.6590	-250.21	2	44.6	12.3	0	1502-1510	R.DLQYITVSK.E		WUP:QUP<br>QU:MU	0.05<br>3.14

Detailed Protein Report

Protein 281: **PREDICTED: conserved oligomeric Golgi complex subunit 4 isoform X1 [Homo sapiens]**

**Accession:**	gi|578828932	**Score:**	36.3
**Database:**	refseq_human(refseq_human_20140103.fasta)	**MW [kDa]:**	73.7
**Database Date:**	2015-11-30	**pI:**	5.1
		**Sequence Coverage [%]:**	9.0
		**No. of unique Peptides:**	1

Quantitation

QU:MU **Median:** 0.74 **CV:** 0.00 % **No. of Peptides:** 1

10	20	30	40	50	60	70	80
MDGVQTALRS	EDYEQAAAHT	HRYLCLDKSV	IELSRQGKEG	SMIDANLKLL	QEAEQRLKAI	VAEKFAIATK	EGDLPQVERF
90	100	110	120	130	140	150	160
FKIFPLLGLH	EEGLRKFSEY	LCKQVASKAE	ENLLMVLGTD	MSDRRAAVIF	ADTLTLLFEG	IARIVETHQP	IVETYYGPGR
170	180	190	200	210	220	230	240
LYTLIKYLQV	ECDRQVEKVV	DKFIKQRDYH	QQFRHVQNNL	MRNSTTEKIE	PRELDPILTE	VTLMNARSEL	YLRFLKKRIS
250	260	270	280	290	300	310	320
SDFEVGDSMA	SEEVKQEHQK	CLDKLLNNCL	LSCTMQELIG	LYVTMEEYFM	RETVNKAVAL	DTYEKGQLTS	SMVDDVFYIV
330	340	350	360	370	380	390	400
KKCIGRALSS	SSIDCLCAMI	NLATTELESD	FRDVLCNKLR	MGFPATTFQD	IQRGVTSAVN	IMHSSLQQGK	FDTKGIESTD
410	420	430	440	450	460	470	480
EAKMSFLVTL	NNVEVCSENI	STLKKTLESD	CTKLFSQGIG	GEQAQAKFDS	CLSDLAAVSN	KFRDLLQEGL	TELNSTAIKP
490	500	510	520	530	540	550	560
QVQPWINSFF	SVSHNIEEEE	FNDYEANDPW	VQQFILNLEQ	QMAEFKASLS	PVIYDSLTGL	MTSLVAVELE	KVVLKSTFNR
570	580	590	600	610	620	630	640
LGGLQFDKEL	RSLIAYLTTV	TTWTIRDKFA	RLSQMATILN	LERVTEILDY	WGPNSGPLTW	RLTPAEVRQV	LALRIDFRSE
650							
DIKRLRL							

Cmpd.	No. of Cmpds.	m/z meas.	Δ m/z [ppm]	z	Rt [min]	Score	P	Range	Sequence	Modification	Ratios
1637	1	993.4172	-42.79	2	50.7	12.5	1	238-255	K.RISSDFEVGDSMASEEVK.Q		QU:MU 0.74

Detailed Protein Report

Protein 282: zinc finger protein castor homolog 1 isoform a [Homo sapiens]

**Accession:**	gi|119709834	**Score:**	36.1
**Database:**	refseq_human(refseq_human_20140103.fasta)	**MW [kDa]:**	189.9
**Database Date:**	2015-11-30	**pI:**	6.6
**Modification(s):**	Carbamidomethyl	**Sequence Coverage [%]:**	1.6
		**No. of unique Peptides:**	1

Alias proteins:

Accession	Name	Description
gi|578799307	refseq_human (refseq_human_20140103.fasta)	PREDICTED: zinc finger protein castor homolog 1 isoform X2 [Homo sapiens]

Detailed Protein Report

10	20	30	40	50	60	70	80
MDLGTAEGTR	CTDPPAGKPA	MAPKRKGGLK	LNAICAKLSR	QVVVEKRADA	GSHTEGSPSQ	PRDQERSGPE	SGAARAPRSE
90	100	110	120	130	140	150	160
EDKRRAVIEK	WVNGEYSEEP	APTPVLGRIA	REGLELPPEG	VYMVQPQGCS	DEEDHAEEPS	KDGGALEEKD	SDGAASKEDS
170	180	190	200	210	220	230	240
GPSTRQASGE	ASSLRDYAAS	TMTEFLGMFG	YDDQNTRDEL	ARKISFEKLH	AGSTPEAATS	SMLPTSEDTL	SKRARFSKYE
250	260	270	280	290	300	310	320
EYIRKLKAGE	QLSWPAPSTK	TEERVGKEVV	GTLPGLRLPS	STAHLETKAT	ILPLPSHSSV	QMQNLVARAS	KYDFFIQKLK
330	340	350	360	370	380	390	400
TGENLRPQNG	STYKKPSKYD	LENVKYLHLF	KPGEGSPDMG	GAIAFKTGKV	GRPSKYDVRG	IQKPGPAKVP	PTPSLAPAPL
410	420	430	440	450	460	470	480
ASVPSAPSAP	GPGPEPPASL	SFNTPEYLKS	TFSKTDSITT	GTVSTVKNGL	PTDKPAVTED	VNIYQKYIAR	FSGSQHCGHI
490	500	510	520	530	540	550	560
HCAYQYREHY	HCLDPECNYQ	RFTSKQDVIR	HYNMHKKRDN	SLQHGFMRFS	PLDDCSVYYH	GCHLNGKSTH	YHCMQVGCNK
570	580	590	600	610	620	630	640
VYTSTSDVMT	HENFHKKNTQ	LINDGFQRFR	ATEDCGTADC	QFYGQKTTHF	HCRRPGCTFT	FKNKCDIEKH	KSYHIKDDAY
650	660	670	680	690	700	710	720
AKDGFKKFYK	YEECKYEGCV	YSKATNHFHC	IRAGCGFTFT	STSQMTSHKR	KHERRHIRSS	GALGLPPSLL	GAKDTEHEES
730	740	750	760	770	780	790	800
SNDDLVDFSA	LSSKNSSLSA	SPTSQQSSAS	LAAATAATEA	GPSATKPPNS	KISGLLPQGL	PGSIPLALAL	SNSGLPTPTP
810	820	830	840	850	860	870	880
YFPILAGRGS	TSLPVGTPSL	LGAVSSGSAA	SATPDTPTLV	ASGAGDSAPV	AAASVPAPPA	SIMERISASK	GLISPMMARL
890	900	910	920	930	940	950	960
AAAALKPSAT	FDPGSGQQVT	PARFPPAQVK	PEPGESTGAP	GPHEASQDRS	LDLTVKEPSN	ESNGHAVPAN	SSLLSSLMNK
970	980	990	1000	1010	1020	1030	1040
MSQGNPGLGS	LLNIKAEAEG	SPAAEPSPFL	GKAVKALVQE	KLAEPWKVYL	RRFGTKDFCD	GQCDFLHKAH	FHCVVEECGA
1050	1060	1070	1080	1090	1100	1110	1120
LFSTLDGAIK	HANFHFRTEG	GAAKGNTEAA	FPASAAETKP	PMAPSSPPVP	PVTTATVSSL	EGPAPSPASV	PSTPTLLAWK
1130	1140	1150	1160	1170	1180	1190	1200
QLASTIPQMP	QIPASVPHLP	ASPLATTSLE	NAKPQVKPGF	LQFQENDPCL	ATDCKYANKF	HFHCLFGNCK	YVCKTSGKAE
1210	1220	1230	1240	1250	1260	1270	1280
SHCLDHINPN	NNLVNVRDQF	AYYSLQCLCP	NQHCEFRMRG	HYHCLRTGCY	FVTNITTKLP	WHIKKHEKAE	RRAANGFKYF
1290	1300	1310	1320	1330	1340	1350	1360
TKREECGRLG	CKYNQVNSHF	HCIREGCQFS	FLLKHQMTSH	ARKHMRRMLG	KNFDRVPPSQ	GPPGLMDAET	DECMDYTGCS
1370	1380	1390	1400	1410	1420	1430	1440
PGAMSSESST	MDRSCSSTPV	GNESTAAGNT	ISMPTASGAK	KRFWIIEDMS	PFGKRRKTAS	SRKMLDEGMM	LEGFRRFDLY
1450	1460	1470	1480	1490	1500	1510	1520
EDCKDAACQF	SLKVTHYHCT	RENCGYKFCG	RTHMYKHAQH	HDRVDNLVLD	DFKRFKASLS	CHFADCPFSG	TSTHFHCLRC
1530	1540	1550	1560	1570	1580	1590	1600
RFRCTDSTKV	TAHRKHHGKQ	DVISAAGFCQ	FSSSADCAVP	DCKYKLKCSH	FHCTFPGCRH	TVVGMSQMDS	HKRKHEKQER
1610	1620	1630	1640	1650	1660	1670	1680
GEPAAEGPAP	GPPISLDGSL	SLGAEPGSLL	FLQSAAAGLG	LALGDAGDPG	PPDAAAPGPR	EGAAAAAAAA	GESSQEDEEE
1690	1700	1710	1720	1730	1740	1750	1760
ELELPEEEAE	DDEDEDDDED	DDDEDDDEDD	DDEDLRTDSE	ESLPEAAAEA	AGAGARTPAL	AALAALGAPG	PAPTAASSP

Cmpd.	No. of Cmpds.	m/z meas.	Δ m/z [ppm]	z	Rt [min]	Score	P	Range	Sequence	Modification
1857	1	604.2655	-35.90	2	53.6	12.3	2	1283-1292	K.REECGRLGCK.Y	Carbamidomethyl: 9

Detailed Protein Report

Protein 283: PREDICTED: calcyphosin-like protein isoform X2 [Homo sapiens]

Accession: gi|578809847
Database: refseq_human(refseq_human_20140103.fasta)
Database Date: 2015-11-30
Modification(s): Oxidation

Score: 36.1
MW [kDa]: 23.1
pI: 8.9
Sequence Coverage [%]: 18.1
No. of unique Peptides: 2

10	20	30	40	50	60	70	80
MQETASIVSV	RSNWWQKMAG	TARHDREMAI	QAKKKLTTAT	DPIERLRLQC	LARGSAGIKG	LGRVFRIMDD	DNNRTLDFKE
**90**	**100**	**110**	**120**	**130**	**140**	**150**	**160**
FMKGLNDYAV	VMEKEEVEEL	FRRFDKDGNG	TIDFNEFLLT	LRPPMSRARK	EVIMQAFRKL	DKTGDGVITI	EDLREVYNAK
**170**	**180**	**190**	**200**				
HHPKYQNGEW	SEEQVFRKFL	DNFDSPYDKD	GLVHKVGAH				

Cmpd.	No. of Cmpds.	m/z meas.	Δ m/z [ppm]	z	Rt [min]	Score	P	Range	Sequence	Modification
2813	1	895.4559	30.47	2	63.8	12.6	1	80-94	K.EFMKGLNDYAVVMEK.E	Oxidation: 3
19	3	1205.0501	-38.96	2	29.8	23.5	0	107-127	K.DGNGTIDFNEFLLTLRPPMSR.A	Oxidation: 19

Detailed Protein Report

Protein 284: inhibitor of nuclear factor kappa-B kinase subunit alpha [Homo sapiens]

**Accession:**	gi|62241001	**Score:**	36.0
**Database:**	refseq_human(refseq_human_20140103.fasta)	**MW [kDa]:**	84.6
**Database Date:**	2015-11-30	**pI:**	6.3
**Modification(s):**	Carbamidomethyl, Oxidation	**Sequence Coverage [%]:**	2.7
		**No. of unique Peptides:**	2

Quantitation

QU:MU	**Median:**	1.11	**CV:**	0.00 %	**No. of Peptides:**	1
WUP:QUP	**Median:**	1.16	**CV:**	0.00 %	**No. of Peptides:**	1

10	20	30	40	50	60	70	80
MERPPGLRPG	AGGPWEMRER	LGTGGFGNVC	LYQHRELDLK	IAIKSCRLEL	STKNRERWCH	EIQIMKKLNH	ANVVKACDVP
**90**	**100**	**110**	**120**	**130**	**140**	**150**	**160**
EELNILIHDV	PLLAMEYCSG	GDLRKLLNKP	ENCCGLKESQ	ILSLLSDIGS	GIRYLHENKI	IHRDLKPENI	VLQDVGGKII
**170**	**180**	**190**	**200**	**210**	**220**	**230**	**240**
HKIIDLGYAK	DVDQGSLCTS	FVGTLQYLAP	ELFENKPYTA	TVDYWSFGTM	VFECIAGYRP	FLHHLQPFTW	HEKIKKKDPK
**250**	**260**	**270**	**280**	**290**	**300**	**310**	**320**
CIFACEEMSG	EVRFSSHLPQ	PNSLCSLVVE	PMENWLQLML	NWDPQQRGGP	VDLTLKQPRC	FVLMDHILNL	KIVHILNMTS
**330**	**340**	**350**	**360**	**370**	**380**	**390**	**400**
AKIISFLLPP	DESLHSLQSR	IERETGINTG	SQELLSETGI	SLDPRKPASQ	CVLDGVRGCD	SYMVYLFDKS	KTVYEGPFAS
**410**	**420**	**430**	**440**	**450**	**460**	**470**	**480**
RSLSDCVNYI	VQDSKIQLPI	IQLRKVWAEA	VHYVSGLKED	YSRLFQGQRA	AMLSLLRYNA	NLTKMKNTLI	SASQQLKAKL
**490**	**500**	**510**	**520**	**530**	**540**	**550**	**560**
EFFHKSIQLD	LERYSEQMTY	GISSEKMLKA	WKEMEEKAIH	YAEVGVIGYL	EDQIMSLHAE	IMELQKSPYG	RRQGDLMESL
**570**	**580**	**590**	**600**	**610**	**620**	**630**	**640**
EQRAIDLYKQ	LKHRPSDHSY	SDSTEMVKII	VHTVQSQDRV	LKELFGHLSK	LLGCKQKIID	LLPKVEVALS	NIKEADNTVM
**650**	**660**	**670**	**680**	**690**	**700**	**710**	**720**
FMQGKRQKEI	WHLLKIACTQ	SSARSLVGSS	LEGAVTPQTS	AWLPPTSAEH	DHSLSCVVTP	QDGETSAQMI	EENLNCLGHL
**730**	**740**	**750**					
STIIHEANEE	QGNSMMNLDW	SWLTE					

Cmpd.	No. of Cmpds.	m/z meas.	Δ m/z [ppm]	z	Rt [min]	Score	P	Range	Sequence	Modification	Ratios
2406	1	665.3146	-54.38	2	60.5	21.5	0	366-377	R.KPASQCVLDGVR.G	Carbamidomethyl: 6	
1405	1	533.6158	-246.47	2	45.9	14.5	1	510-517	K.AWKEMEEK.A	Oxidation: 5	QU:MU 1.11<br>WUP:QUP 1.16

Detailed Protein Report

Protein 285: thymidine phosphorylase isoform 1 proprotein [Homo sapiens]

Accession: gi|4503445
Database: refseq_human(refseq_human_20140103.fasta)
Database Date: 2015-11-30
Modification(s): Oxidation

Score: 36.0
MW [kDa]: 49.9
pI: 5.2
Sequence Coverage [%]: 9.5
No. of unique Peptides: 2

Alias proteins:

Accession	Name	Description
gi|384229049	refseq_human (refseq_human_20140103.fasta)	thymidine phosphorylase isoform 1 proprotein [Homo sapiens]
gi|166158925	refseq_human (refseq_human_20140103.fasta)	thymidine phosphorylase isoform 1 proprotein [Homo sapiens]
gi|166158922	refseq_human (refseq_human_20140103.fasta)	thymidine phosphorylase isoform 1 proprotein [Homo sapiens]

```
        10         20         30         40         50         60         70         80
MAALMTPGTG APPAPGDFSG EGSQGLPDPS PEPKQLPELI RMKRDGGRLS EADIRGFVAA VVNGSAQGAQ IGAMLMAIRL
        90        100        110        120        130        140        150        160
RGMDLEETSV LTQALAQSGQ QLEWPEAWRQ QLVDKHSTGG VGDKVSLVLA PALAACGCKV PMISGRGLGH TGGTLDKLES
       170        180        190        200        210        220        230        240
IPGFNVIQSP EQMQVLLDQA GCCIVGQSEQ LVPADGILYA ARDVTATVDS LPLITASILS KKLVEGLSAL VVDVKFGGAA
       250        260        270        280        290        300        310        320
VFPNQEQARE LAKTLVGVGA SLGLRVAAAL TAMDKPLGRC VGHALEVEEA LLCMDGAGPP DLRDLVTTLG GALLWLSGHA
       330        340        350        360        370        380        390        400
GTQAQGAARV AAALDDGSAL GRFERMLAAQ GVDPGLARAL CSGSPAERRQ LLPRAREQEE LLAPADGTVE LVRALPLALV
       410        420        430        440        450        460        470        480
LHELGAGRSR AGEPLRLGVG AELLVDVGQR LRRGTPWLRV HRDGPALSGP QSRALQEALV LSDRAPFAAP SPFAELVLPP
       490
QQ
```

Cmpd.	No. of Cmpds.	m/z meas.	Δ m/z [ppm]	z	Rt [min]	Score	P	Range	Sequence	Modification
41	1	906.3675	-118.89	2	29.5	10.9	1	140-157	K.VPMISGRGLGHTGGTLDK.L	Oxidation: 3
1488	1	671.3911	-17.76	2	47.0	12.0	0	223-235	K.LVEGLSALVVDVK.F	

Detailed Protein Report

Protein 286: **PREDICTED: centriolar coiled-coil protein of 110 kDa isoform X4 [Homo sapiens]**

**Accession:**	gi|578828704	**Score:**	35.9
**Database:**	refseq_human(refseq_human_20140103.fasta)	**MW [kDa]:**	109.0
**Database Date:**	2015-11-30	**pI:**	9.3
**Modification(s):**	Oxidation	**Sequence Coverage [%]:**	1.2
		**No. of unique Peptides:**	1

10	20	30	40	50	60	70	80
MEEYEKFCEK	SLARIQEASL	STESFLPAQS	ESISLIRFHG	VAILSPLLNI	EKRKEMQQEK	QKALDVEARK	QVNRKKALLT
**90**	**100**	**110**	**120**	**130**	**140**	**150**	**160**
RVQEILDNVQ	VRKAPNASDF	DQWEMETVYS	NSEVRNLNVP	ATFPNSFPSH	TEHSTAAKLD	KIAGILPLDN	EDQCKTDGID
**170**	**180**	**190**	**200**	**210**	**220**	**230**	**240**
LARDSEGFNS	PKQCDSSNIS	HVENEAFPKT	SSATPQETLI	SDGPFSVNEQ	QDLPLLAEVI	PDPYVMSLQN	LMKKSKEYIE
**250**	**260**	**270**	**280**	**290**	**300**	**310**	**320**
REQSRRSLRG	SINRIVNESH	LDKEHDAVEV	ADCVKEKGQL	TGKHCVSVIP	DKPSLNKSNV	LLQGASTQAS	SMSMPVLASF
**330**	**340**	**350**	**360**	**370**	**380**	**390**	**400**
SKVDIPIRTG	HPTVLESNSD	FKVIPTFVTE	NNVIKSLTGS	YAKLPSPEPS	MSPKMHRRRS	RTSSACHILI	NNPINACELS
**410**	**420**	**430**	**440**	**450**	**460**	**470**	**480**
PKGKEQAMDL	IIQDTDENTN	VPEIMPKLPT	DLAGVCSSKV	YVGKNTSEVK	EDVVLGKSNQ	VCQSSGNHLE	NKVTHGLVTV
**490**	**500**	**510**	**520**	**530**	**540**	**550**	**560**
EGQLTSDERG	AHIMNSTCAA	MPKLHEPYAS	SQCIASPNFG	TVSGLKPASM	LEKNCSLQTE	LNKSYDVKNP	SPLLMQNQNT
**570**	**580**	**590**	**600**	**610**	**620**	**630**	**640**
RQQMDTPMVS	CGNEQFLDNS	FEKVKRRLDL	DIDGLQKENC	PYVITSGITE	QERQHLPEKR	YPKGSGFVNK	NKMLGTSSKE
**650**	**660**	**670**	**680**	**690**	**700**	**710**	**720**
SEELLKSKML	AFEEMRKRLE	EQHAQQLSLL	IAEQEREQER	LQKEIEEQEK	MLKEKKAMTA	EASELDINNA	VELEWRKISD
**730**	**740**	**750**	**760**	**770**	**780**	**790**	**800**
SSLLETMLSQ	ADSLHTSNSN	SSGFTNSAMQ	YSFVSANEAP	FYLWGSSTSG	LTKLSVTRPF	GRAKTRWSQV	FSLEIQAKFN
**810**	**820**	**830**	**840**	**850**	**860**	**870**	**880**
KITAVAKGFL	TRRLMQTDKL	KQLRQTVKDT	MEFIRSFQSE	APLKRGIVSA	QDASLQERVL	AQLRAALYGI	HDIFFVMDAA
**890**	**900**	**910**	**920**	**930**	**940**	**950**	**960**
ERMSILHHDR	EVRKEKMLRQ	MDKMKSPRVA	LSAATQKSLD	RKKYMKAAEM	GMPNKKFLVK	QNPSETRSIC	RKNPKKAAKC
**970**	**980**						
CDNLRRQHSL	G						

Cmpd.	No. of Cmpds.	m/z meas.	Δ m/z [ppm]	z	Rt [min]	Score	P	Range	Sequence	Modification
2559	1	693.6709	-206.81	2	60.3	11.2	1	924-935	K.YMKAAEMGMPNK.K	Oxidation: 7

Detailed Protein Report

Protein 287: PREDICTED: multiple PDZ domain protein isoform X3 [Homo sapiens]

**Accession:**	gi|530390235	**Score:**	35.9
**Database:**	refseq_human(refseq_human_20140103.fasta)	**MW [kDa]:**	213.7
**Database Date:**	2015-11-30	**pI:**	4.8
		**Sequence Coverage [%]:**	2.3
		**No. of unique Peptides:**	3

Detailed Protein Report

10	20	30	40	50	60	70	80
MLEAIDKNRA	LHAAERLQTK	LRERGDVANE	DKLSLLKSVL	QSPLFSQILS	LQTSVQQLKD	QVNIATSATS	NIEYAHVPHL
90	100	110	120	130	140	150	160
SPAVIPTLQN	ESFLLSPNNG	NLEALTGPGI	PHINGKPACD	EFDQLIKNMA	QGRHVEVFEL	LKPPSGGLGF	SVVGLRSENR
170	180	190	200	210	220	230	240
GELGIFVQEI	QEGSVAHRDG	RLKETDQILA	INGQALDQTI	THQQAISILQ	KAKDTVQLVI	ARGSLPQLVS	PIVSRSPSAA
250	260	270	280	290	300	310	320
STISAHSNPV	HWQHMETIEL	VNDGSGLGFG	IIGGKATGVI	VKTILPGGVA	DQHGRLCSGD	HILKIGDTDL	AGMSSEQVAQ
330	340	350	360	370	380	390	400
VLRQCGNRVK	LMIARGAIEE	RTAPTALGIT	LSSSPTSTPE	LRVDASTQKG	EESETFDVEL	TKNVQGLGIT	IAGYIGDKKL
410	420	430	440	450	460	470	480
EPSGIFVKSI	TKSSAVEHDG	RIQIGDQIIA	VDGTNLQGFT	NQQAVEVLRH	TGQTVLLTLM	RRGMKQEAEL	MSREDVTKDA
490	500	510	520	530	540	550	560
DLSPVNASII	KENYEKDEDF	LSSTRNTNIL	PTEEEGYPLL	SAEIEEIEDA	QKQEAALLTK	WQRIMGINYE	IVVAHVSKFS
570	580	590	600	610	620	630	640
ENSGLGISLE	ATVGHHFIRS	VLPEGPVGHS	GKLFSGDELL	EVNGITLLGE	NHQDVVNILK	ELPIEVTMVC	CRRTVPPTTQ
650	660	670	680	690	700	710	720
SELDSLDLCD	IELTEKPHVD	LGEFIGSSET	EDPVLAMTDA	GQSTEEVQAP	LAMWEAGIQH	IELEKGSKGL	GFSILDYQDP
730	740	750	760	770	780	790	800
IDPASTVIII	RSLVPGGIAE	KDGRLLPGDR	LMFVNDVNLE	NSSLEEAVEA	LKGAPSGTVR	IGVAKPLPLS	PEEGYVSAKE
810	820	830	840	850	860	870	880
DSFLYPPHSC	EEAGLADKPL	FRADLALVGT	NDADLVDEST	FESPYSPEND	SIYSTQASIL	SLHGSSCGDG	LNYGSSLPSS
890	900	910	920	930	940	950	960
PPKDVIENSC	DPVLDLHMSL	EELYTQNLLQ	RQDENTPSVD	ISMGPASGFT	INDYTPANAI	EQQYECENTI	VWTESHLPSE
970	980	990	1000	1010	1020	1030	1040
VISSAELPSV	LPDSAGKGSE	YLLEQSSLAC	NAECVMLQNV	SKESFERTIN	IAKGNSSLGM	TVSANKDGLG	MIVRSIIHGG
1050	1060	1070	1080	1090	1100	1110	1120
AISRDGRIAI	GDCILSINEE	STISVTNAQA	RAMLRRHSLI	GPDIKITYVP	AEHLEEFKIS	LGQQSGRVMA	LDIFSSYTGR
1130	1140	1150	1160	1170	1180	1190	1200
DIPELPEREE	GEGEESELQN	TAYSNWNQPR	RVELWREPSK	SLGISIVGGR	GMGSRLSNGE	VMRGIFIKHV	LEDSPAGKNG
1210	1220	1230	1240	1250	1260	1270	1280
TLKPGDRIVE	APSQSESEPE	KAPLCSVPPP	PPSAFAEMGS	DHTQSSASKI	SQDVDKEDEF	GYSWKNIRER	YGTLTGELHM
1290	1300	1310	1320	1330	1340	1350	1360
IELEKGHSGL	GLSLAGNKDR	SRMSVFIVGI	DPNGAAGKDG	RLQIADELLE	INGQILYGRS	HQNASSIIKC	APSKVKIIFI
1370	1380	1390	1400	1410	1420	1430	1440
RNKDAVNQMA	VCPGNAVEPL	PSNSENLQNK	ETEPTVTTSD	AAVDLSSFKN	VQHLELPKDQ	GGLGIAISEE	DTLSGVIIKS
1450	1460	1470	1480	1490	1500	1510	1520
LTEHGVAATD	GRLKVGDQIL	AVDDEIVVGY	PIEKFISLLK	TAKMTVKLTI	HAENPDSQAV	PSAAGAASGE	KKNSSQSLMV
1530	1540	1550	1560	1570	1580	1590	1600
PQSGSPEPES	IRNTSRSSTP	AIFASDPATC	PIIPGCETTI	EISKGRTGLG	LSIVGGSDTL	LGAIIIHEVY	EEGAACKDGR
1610	1620	1630	1640	1650	1660	1670	1680
LWAGDQILEV	NGIDLRKATH	DEAINVLRQT	PQRVRLTLYR	DEAPYKEEEV	CDTLTIELQK	KPGKGLGLSI	VGKRNDTGVF
1690	1700	1710	1720	1730	1740	1750	1760
VSDIVKGGIA	DADGRLMQGD	QILMVNGEDV	RNATQEAVAA	LLKCSLGTVT	LEVGRIKAGP	FHSERRPSQS	SQVSEGSLSS
1770	1780	1790	1800	1810	1820	1830	1840
FTFPLSGSST	SESLESSSKK	NALASEIQGL	RTVEMKKGPT	DSLGISIAGG	VGSPLGDVPI	FIAMMHPTGV	AAQTQKLRVG
1850	1860	1870	1880	1890	1900	1910	1920
DRIVTICGTS	TEGMTHTQAV	NLLKNASGSI	EMQVVAGGDV	SVVTGHQQEP	ASSSLSFTGL	TSSSIFQDDL	GPPQCKSITL
1930	1940	1950	1960	1970	1980	1990	2000
ERGPDGLGFS	IVGGYGSPHG	DLPIYVKTVF	AKGAASEDGR	LKRGDQIIAV	NGQSLEGVTH	EEAVAILKRT	KGTVTLMVLS
2010							

Cmpd.	No. of Cmpds.	m/z meas.	Δ m/z [ppm]	z	Rt [min]	Score	P	Range	Sequence	Modification

Detailed Protein Report

Cmpd.	No. of Cmpds.	m/z meas.	Δ m/z [ppm]	z	Rt [min]	Score	P	Range	Sequence	Modification
2017	1	788.3019	-141.73	2	55.1	14.1	0	1303-1318	R.MSVFIVGIDPNGAAGK.D	
2930	1	899.4860	0.41	2	65.4	11.6	2	1340-1356	R.SHQNASSIIKCAPSKVK.I	
1435	1	627.2993	-161.59	2	47.6	10.2	1	1661-1673	K.KPGKGLGLSIVGK.R	

Detailed Protein Report

Protein 288: myosin light chain kinase, smooth muscle isoform 3B [Homo sapiens]

**Accession:**	gi|116008190	**Score:**	35.8
**Database:**	refseq_human(refseq_human_20140103.fasta)	**MW [kDa]:**	197.3
**Database Date:**	2015-11-30	**pI:**	6.1
**Modification(s):**	Carbamidomethyl	**Sequence Coverage [%]:**	2.3
		**No. of unique Peptides:**	3

Quantitation

QU:MU	**Median:**	1.66	**CV:**	43.99 %	**No. of Peptides:**	2
WUP:QUP	**Median:**	0.51	**CV:**	0.00 %	**No. of Peptides:**	1

Detailed Protein Report

10	20	30	40	50	60	70	80
MGDVKLVASS	HISKTSLSVD	PSRVDSMPLT	EAPAFILPPR	NLCIKEGATA	KFEGRVRGYP	EPQVTWHRNG	QPITSGGRFL
90	100	110	120	130	140	150	160
LDCGIRGTFS	LVIHAVHEED	RGKYTCEATN	GSGARQVTVE	LTVEGSFAKQ	LGQPVVSKTL	GDRFSAPAVE	TRPSIWGECP
170	180	190	200	210	220	230	240
PKFATKLGRV	VVKEGQMGRF	SCKITGRPQP	QVTWLKGNVP	LQPSARVSVS	EKNGMQVLEI	HGVNQDDVGV	YTCLVVNGSG
250	260	270	280	290	300	310	320
KASMSAELSI	QGLDSANRSF	VRETKATNSD	VRKEVTNVIS	KESKLDSLEA	AAKSKNCSSP	QRGGSPPWAA	NSQPQPPRES
330	340	350	360	370	380	390	400
KLESCKDSPR	TAPQTPVLQK	TSSSITLQAA	RVQPEPRAPG	LGVLSPSGEE	RKRPAPPRPA	TFPTRQPGLG	SQDVVSKAAN
410	420	430	440	450	460	470	480
RRIPMEGQRD	SAFPKFESKP	QSQEVKENQT	VKFRCEGLAV	MEVAPSFSSV	LKDCAVIEGQ	DFVLQCSVRG	TPVPRITWLL
490	500	510	520	530	540	550	560
NGQPIQYARS	TCEAGVAELH	IQDALPEDHG	TYTCLAENAL	GQVSCSAWVT	VHEKKSSRKS	EYLLPVAPSK	PTAPIFLQGL
570	580	590	600	610	620	630	640
SDLKVMDGSQ	VTMTVQVSGN	PPPEVIWLHN	GNEIQESEDF	HFEQRGTQHS	LCIQEVFPED	TGTYTCEAWN	SAGEVRTQAV
650	660	670	680	690	700	710	720
LTVQEPHDGT	QPWFISKPRS	VTASLGQSVL	ISCAIAGDPF	PTVHWLRDGK	ALCKDTGHFE	VLQNEDVFTL	VLKKVQPWHA
730	740	750	760	770	780	790	800
GQYEILLKNR	VGECSCQVSL	MLQNSSARAL	PRGREPASCE	DLCGGGVGAD	GGGSDRYGSL	RPGWPARGQG	WLEEEDGEDV
810	820	830	840	850	860	870	880
RGVLKRRVET	RQHTEEAIRQ	QEVEQLDFRD	LLGKKVSTKT	LSEDDLKEIP	AEQMDFRANL	QRQVKPKTVS	EEERKVHSPQ
890	900	910	920	930	940	950	960
QVDFRSVLAK	KGTSKTPVPE	KVPPPKPATP	DFRSVLGGKK	KLPAENGSSS	AETLNAKAVE	SSKPLSNAQP	SGPLKPVGNA
970	980	990	1000	1010	1020	1030	1040
KPAETLKPMG	NAKPAETLKP	MGNAKPDENL	KSASKEELKK	DVKNDVNCKR	GHAGTTDNEK	RSESQGTAPA	FKQKLQDVHV
1050	1060	1070	1080	1090	1100	1110	1120
AEGKKLLLQC	QVSSDPPATI	IWTLNGKTLK	TTKFIILSQE	GSLCSVSIEK	ALPEDRGLYK	CVAKNDAGQA	ECSCQVTVDD
1130	1140	1150	1160	1170	1180	1190	1200
APASENTKAP	EMKSRRPKSS	LPPVLGTESD	ATVKKKPAPK	TPPKAAMPPQ	IIQFPEDQKV	RAGESVELFG	KVTGTQPITC
1210	1220	1230	1240	1250	1260	1270	1280
TWMKFRKQIQ	ESEHMKVENS	ENGSKLTILA	ARQEHCGCYT	LLVENKLGSR	QAQVNLTVVD	KPDPPAGTPC	ASDIRSSSLT
1290	1300	1310	1320	1330	1340	1350	1360
LSWYGSSYDG	GSAVQSYSIE	IWDSANKTWK	ELATCRSTSF	NVQDLLPDHE	YKFRVRAINV	YGTSEPSQES	ELTTVGEKPE
1370	1380	1390	1400	1410	1420	1430	1440
EPKDEVEVSD	DDEKEPEVDY	RTVTINTEQK	VSDFYDIEER	LGSGKFGQVF	RLVEKKTRKV	WAGKFFKAYS	AKEKENIRQE
1450	1460	1470	1480	1490	1500	1510	1520
ISIMNCLHHP	KLVQCVDAFE	EKANIVMVLE	IVSGGELFER	IIDEDFELTE	RECIKYMRQI	SEGVEYIHKQ	GIVHLDLKPE
1530	1540	1550	1560	1570	1580	1590	1600
NIMCVNKTGT	RIKLIDFGLA	RRLENAGSLK	VLFGTPEFVA	PEVINYEPIG	YATDMWSIGV	ICYILNRLDC	TQCLQHPWLM
1610	1620	1630	1640	1650	1660	1670	1680
KDTKNMEAKK	LSKDRMKKYM	ARRKWQKTGN	AVRAIGRLSS	MAMISGLSGR	KSSTGSPTSP	LNAEKLESEE	DVSQAFLEAV
1690	1700	1710	1720	1730	1740	1750	1760
AEEKPHVKPY	FSKTIRDLEV	VEGSAARFDC	KIEGYPDPEV	VWFKDDQSIR	ESRHFQIDYD	EDGNCSLIIS	DVCGDDDAKY
1770	1780	1790	1800				
TCKAVNSLGE	ATCTAELIVE	TMEEGEGEGE	EEEE				

Cmpd.	No. of Cmpds.	m/z meas.	Δ m/z [ppm]	z	Rt [min]	Score	P	Range	Sequence	Modification	Ratios	
678	1	670.8078	-108.06	2	38.5	14.1	1	2-14	M.GDVKLVASSHISK.T		QU:MU	1.09
2055	1	643.7814	9.77	2	54.1	11.7	0	104-115	K.YTCEATNGSGAR.Q	Carbamidomethyl: 3		
2643	1	995.0088	-31.93	2	63.9	10.1	1	180-196	R.FSCKITGRPQPQVTWLK.G		WUP:QUP<br>QU:MU	0.51<br>2.52

Detailed Protein Report

Protein 289: PREDICTED: rho guanine nucleotide exchange factor 12 isoform X3 [Homo sapiens]

Accession: gi|530397943
Database: refseq_human(refseq_human_20140103.fasta)
Database Date: 2015-11-30

Score: 35.8
MW [kDa]: 161.8
pI: 5.3
Sequence Coverage [%]: 2.3
No. of unique Peptides: 2

10	20	30	40	50	60	70	80
MRAGVQTGDR	IIKVNGTLVT	HSNHLEVVKL	IKSGSYVALT	VQGRPPGSPQ	IPLADSEVEP	SVIGHMSPIM	TSPHSPGASG
**90**	**100**	**110**	**120**	**130**	**140**	**150**	**160**
NMERITSPVL	MGEENNVVHN	QKVEILRKML	QKEQERLQLL	QEDYNRTPAQ	RLLKEIQEAK	KHIPQLQEQL	SKATGSAQDG
**170**	**180**	**190**	**200**	**210**	**220**	**230**	**240**
AVVTPSRPLG	DTLTVSEAET	DPGDVLGRTD	CSSGDASRPS	SDNADSPKSG	PKERIYLEEN	PEKSETIQDT	DTQSLVGSPS
**250**	**260**	**270**	**280**	**290**	**300**	**310**	**320**
TRIAPHIIGA	EDDDFGTEHE	QINGQCSCFQ	SIELLKSRPA	HLAVFLHHVV	SQFDPATLLC	YLYSDLYKHT	NSKETRRIFL
**330**	**340**	**350**	**360**	**370**	**380**	**390**	**400**
EFHQFFLDRS	AHLKVSVPDE	MSADLEKRRP	ELIPEDLHRH	YIQTMQERVH	PEVQRHLEDF	RQKRSMGLTL	AESELTKLDA
**410**	**420**	**430**	**440**	**450**	**460**	**470**	**480**
ERDKDRLTLE	KERTCAEQIV	AKIEEVLMTA	QAVEEDKSST	MQYVILMYMK	HLGVKVKEPR	NLEHKRGRIG	FLPKIKQSMK
**490**	**500**	**510**	**520**	**530**	**540**	**550**	**560**
KDKEGEEKGK	RRGFPSILGP	PRRPSRHDNS	AIGRAMELQK	ARHPKHLSTP	SSVSPEPQDS	AKLRQSGLAN	EGTDAGYLPA
**570**	**580**	**590**	**600**	**610**	**620**	**630**	**640**
NSMSSVASGA	SFSQEGGKEN	DTGSKQVGET	SAPGDTLDGT	PRTLNTVFDF	PPPPLDQVQE	EECEVERVTE	HGTPKPFRKF
**650**	**660**	**670**	**680**	**690**	**700**	**710**	**720**
DSVAFGESQS	EDEQFENDLE	TDPPNWQQLV	SREVLLGLKP	CEIKRQEVIN	ELFYTERAHV	RTLKVLDQVF	YQRVSREGIL
**730**	**740**	**750**	**760**	**770**	**780**	**790**	**800**
SPSELRKIFS	NLEDILQLHI	GLNEQMKAVR	KRNETSVIDQ	IGEDLLTWFS	GPGEEKLKHA	AATFCSNQPF	ALEMIKSRQK
**810**	**820**	**830**	**840**	**850**	**860**	**870**	**880**
KDSRFQTFVQ	DAESNPLCRR	LQLKDIIPTQ	MQRLTKYPLL	LDNIAKYTEW	PTEREKVKKA	ADHCRQILNY	VNQAVKEAEN
**890**	**900**	**910**	**920**	**930**	**940**	**950**	**960**
KQRLEDYQRR	LDTSSLKLSE	YPNVEELRNL	DLTKRKMIHE	GPLVWKVNRD	KTIDLYTLLL	EDILVLLQKQ	DDRLVLRCHS
**970**	**980**	**990**	**1000**	**1010**	**1020**	**1030**	**1040**
KILASTADSK	HTFSPVIKLS	TVLVRQVATD	NKALFVISMS	DNGAQIYELV	AQTVSEKTVW	QDLICRMAAS	VKEQSTKPIP
**1050**	**1060**	**1070**	**1080**	**1090**	**1100**	**1110**	**1120**
LPQSTPGEGD	NDEEDPSKLK	EEQHGISVTG	LQSPDRDLGL	ESTLISSKPQ	SHSLSTSGKS	EVRDLFVAER	QFAKEQHTDG
**1130**	**1140**	**1150**	**1160**	**1170**	**1180**	**1190**	**1200**
TLKEVGEDYQ	IAIPDSHLPV	SEERWALDAL	RNLGLLKQLL	VQQLGLTEKS	VQEDWQHFPR	YRTASQGPQT	DSVIQNSENI
**1210**	**1220**	**1230**	**1240**	**1250**	**1260**	**1270**	**1280**
KAYHSGEGHM	PFRTGTGDIA	TCYSPRTSTE	SFAPRDSVGL	APQDSQASNI	LVMDHMIMTP	EMPTMEPEGG	LDDSGEHFFD
**1290**	**1300**	**1310**	**1320**	**1330**	**1340**	**1350**	**1360**
AREAHSDENP	SEGDGAVNKE	EKDVNLRISG	NYLILDGYDP	VQESSTDEEV	ASSLTLQPMT	GIPAVESTHQ	QQHSPQNTHS
**1370**	**1380**	**1390**	**1400**	**1410**	**1420**	**1430**	**1440**
DGAISPFTPE	FLVQQRWGAM	EYSCFEIQSP	SSCADSQSQI	MEYIHKIEAD	LEHLKKVEES	YTILCQRLAG	SALTDKHSDK
**1450**							
S							

Cmpd.	No. of Cmpds.	m/z meas.	Δ m/z [ppm]	z	Rt [min]	Score	P	Range	Sequence	Modification
85	1	710.2749	-87.79	2	30.8	12.0	0	335-347	K.VSVPDEMSADLEK.R	
1443	1	1022.6499	109.61	1	47.7	10.9	1	458-465	K.EPRNLEHK.R	

Detailed Protein Report

Protein 290: protein LAP2 isoform 7 [Homo sapiens]

**Accession:**	gi|55770895	**Score:**	35.7
**Database:**	refseq_human(refseq_human_20140103.fasta)	**MW [kDa]:**	146.0
**Database Date:**	2015-11-30	**pI:**	5.0
		**Sequence Coverage [%]:**	2.3
		**No. of unique Peptides:**	2

10	20	30	40	50	60	70	80
MTTKRSLFVR	LVPCRCLRGE	EETVTTLDYS	HCSLEQVPKE	IFTFEKTLEE	LYLDANQIEE	LPKQLFNCQS	LHKLSLPDND
90	100	110	120	130	140	150	160
LTTLPASIAN	LINLRELDVS	KNGIQEFPEN	IKNCKVLTIV	EASVNPISKL	PDGFSQLLNL	TQLYLNDAFL	EFLPANFGRL
170	180	190	200	210	220	230	240
TKLQILELRE	NQLKMLPKTM	NRLTQLERLD	LGSNEFTEVP	EVLEQLSGLK	EFWMDANRLT	FIPGFIGSLK	QLTYLDVSKN
250	260	270	280	290	300	310	320
NIEMVEEGIS	TCENLQDLLL	SSNSLQQLPE	TIGSLKNITT	LKIDENQLMY	LPDSIGGLIS	VEELDCSFNE	VEALPSSIGQ
330	340	350	360	370	380	390	400
LTNLRTFAAD	HNYLQQLPPE	IGSWKNITVL	FLHSNKLETL	PEEMGDMQKL	KVINLSDNRL	KNLPFSFTKL	QQLTAMWLSD
410	420	430	440	450	460	470	480
NQSKPLIPLQ	KETDSETQKM	VLTNYMFPQQ	PRTEDVMFIS	DNESFNPSLW	EEQRKQRAQV	AFECDEDKDE	REAPPREGNL
490	500	510	520	530	540	550	560
KRYPTPYPDE	LKNMVKTVQT	IVHRLKDEET	NEDSGRDLKP	HEDQQDINKD	VGVKTSESTT	TVKSKVDERE	KYMIGNSVQK
570	580	590	600	610	620	630	640
ISEPEAEISP	GSLPVTANMK	ASENLKHIVN	HDDVFEESEE	LSSDEEMKMA	EMRPPLIETS	INQPKVVALS	NNKKDDTKET
650	660	670	680	690	700	710	720
DSLSDEVTHN	SNQNNSNCSS	PSRMSDSVSL	NTDSSQDTSL	CSPVKQTHID	INSKIRQEDE	NFNSLLQNGD	ILNSSTEEKF
730	740	750	760	770	780	790	800
KAHDKKDFNL	PEYDLNVEER	LVLIEKSVDS	TATADDTHKL	DHINMNLNKL	ITNDTFQPEI	MERSKTQDIV	LGTSFLSINS
810	820	830	840	850	860	870	880
KEETEHLENG	NKYPNLESVN	KVNGHSEETS	QSPNRTEPHD	SDCSVDLGIS	KSTEDLSPQK	SGPVGSVVKS	HSITNMEIGG
890	900	910	920	930	940	950	960
LKIYDILSDN	GPQQPSTTVK	ITSAVDGKNI	VRSKSATLLY	DQPLQVFTGS	SSSSDLISGT	KAIFKFDSNH	NPEEPNIIRG
970	980	990	1000	1010	1020	1030	1040
PTSGPQSAPQ	IYGPPQYNIQ	YSSSAAVKDT	LWHSKQNPQI	DHASFPPQLL	PRSESTENQS	YAKHSANMNF	SNHNNVRANT
1050	1060	1070	1080	1090	1100	1110	1120
AYHLHQRLGP	ARHGEMWAIS	PNDRLIPAVT	RSTIQRQSSV	SSTASVNLGD	PGSTRRAQIP	EGDYLSYREF	HSAGRTPPMM
1130	1140	1150	1160	1170	1180	1190	1200
PGSQRPLSAR	TYSIDGPNAS	RPQSARPSIN	EIPERTMSVS	DFNYSRTSPS	KRPNARVGSE	HSLLDPPGKS	KVPRDWREQV
1210	1220	1230	1240	1250	1260	1270	1280
LRHIEAKKLE	KIRVRVEKDP	ELGFSISGGV	GGRGNPFRPD	DDGIFVTRVQ	PEGPASKLLQ	PGDKIIQANG	YSFINIEHGQ
1290	1300	1310					
AVSLLKTFQN	TVELIIVREV	SS					

Cmpd.	No. of Cmpds.	m/z meas.	Δ m/z [ppm]	z	Rt [min]	Score	P	Range	Sequence	Modification
486	1	668.2076	-211.28	2	34.5	10.8	0	1177-1189	R.VGSEHSLLDPPGK.S	
2597	1	913.4719	-133.82	1	63.1	13.2	2	1209-1215	K.LEKIRVR.V	

Detailed Protein Report

Protein 291: EP300-interacting inhibitor of differentiation 3 [Homo sapiens]

**Accession:**	gi|56605998	**Score:**	35.7
**Database:**	refseq_human(refseq_human_20140103.fasta)	**MW [kDa]:**	38.1
**Database Date:**	2015-11-30	**pI:**	4.9
**Modification(s):**	Oxidation	**Sequence Coverage [%]:**	14.7
		**No. of unique Peptides:**	3

Quantitation

WUP:QUP **Median:** 0.51 **CV:** 0.00 % **No. of Peptides:** 1

```
        10         20         30         40         50         60         70         80
MKMDVSVRAA GCSDDLSSGE ADVDPKLLEL TADEEKCRSI RRQYRQLMYC VRQNREDIVS SANNSLTEAL EEANVLFDGV
        90        100        110        120        130        140        150        160
SRTREAALDA RFLVMASDLG KEKAKQLNSD MNFFNQLAFC DFLFLFVGLN WMEGDPDKLS DCDDSIALSF WKAIEKEATS
       170        180        190        200        210        220        230        240
WMVKAETFHF VFGSFKLERS APKPRLEHQK KVRKMEENGN MPTKLQKLDL SSYPEATEKN VERILGLLQT YFRKYPDTPV
       250        260        270        280        290        300        310        320
SYFEFVIDPN SFSRTVENIF YVSFIVRDGF ARIRLDEDRL PILEPMNVNQ MGEGNDSSCH GRKQGVISLT LQEWKNIVAA
       330        340
FEISEAMITY SSY
```

Cmpd.	No. of Cmpds.	m/z meas.	Δ m/z [ppm]	z	Rt [min]	Score	P	Range	Sequence	Modification	Ratios
2097	1	900.3873	-17.91	3	54.6	12.2	2	1-26	-. MKMDVSVRAAGCSDDLSSGEAD\ L	Oxidation: 1	
936	1	647.6555	-210.59	2	41.8	10.9	1	194-204	R.KMEENGNMPTK.L	Oxidation: 8	WUP:QUP 0.51
399	1	701.3413	-70.15	2	35.0	12.6	0	304-315	K.QGVISLTLQEWK.N		

Detailed Protein Report

Protein 292: PREDICTED: pecanex-like protein 3 isoform X4 [Homo sapiens]

**Accession:**	gi|530396772	**Score:**	35.7
**Database:**	refseq_human(refseq_human_20140103.fasta)	**MW [kDa]:**	115.4
**Database Date:**	2015-11-30	**pI:**	6.0
		**Sequence Coverage [%]:**	4.0
		**No. of unique Peptides:**	1

10	20	30	40	50	60	70	80
MGSQVLQILR	QGVWASLTGG	WFFDPHQSTF	SNCFHLYVWI	FLLIFPFLLY	MVLPPSLMVA	GVYCLVVAVI	FATIKTVNYR
**90**	**100**	**110**	**120**	**130**	**140**	**150**	**160**
LHAMFDQGEI	VEKRSSTMGE	LEEEPAQGDS	NPPRDPGVEM	TVFRKVSSTP	PVRCSSQHSV	FGFNQVSELL	PRMEDSGPLR
**170**	**180**	**190**	**200**	**210**	**220**	**230**	**240**
DIKELVREQG	SNNVIVTSAD	REMLKLSSQE	KLIGDLPQTP	PGAVPDPSLA	STDSSEPSPL	AGDGAPWSGS	SMADTPMSPL
**250**	**260**	**270**	**280**	**290**	**300**	**310**	**320**
LKGSLSQELS	KSFLTLTQPD	RALVRTSSRR	EQRRGAGGYQ	PLDRRGSGEP	TPQKAGSSDS	CFSGTDRETL	SSFKSEKTNS
**330**	**340**	**350**	**360**	**370**	**380**	**390**	**400**
THLDSPPGGP	APEGSDTDPP	SEAELPASPD	AGVPSDDTLR	SFDTVIGAGT	PPGLAEPLLV	VRPKDLALLR	PSKRQPPLRR
**410**	**420**	**430**	**440**	**450**	**460**	**470**	**480**
HSPPGRAPRR	PLLEGGGFFE	DEDTSEGSEL	SPASSLRSQR	RYSTDSSSST	SCYSPESSRG	AAGGPRKRRA	PHGAEEGTAV
**490**	**500**	**510**	**520**	**530**	**540**	**550**	**560**
PPKRPYGTQR	TPSTASAKTH	ARVLSMDGAG	GDVLRPPLAG	CKAELEAQVG	VEQAASEPVV	LPAEARRGPA	ANQPGWRGEL
**570**	**580**	**590**	**600**	**610**	**620**	**630**	**640**
QEEGAVGGAA	EETGRRDRSS	SVRRTQAIRR	RHNAGSNPTP	PASVMGSPPS	SLQEAQRGRA	ASHSRALTLP	SALHFASSLL
**650**	**660**	**670**	**680**	**690**	**700**	**710**	**720**
LTRAGANVHE	ACTFDDTSEG	AVHYFYDESG	VRRSYTFGLA	GGGYENPVGQ	QGEQTANGAW	DRHSHSSSFH	SADVPEATGG
**730**	**740**	**750**	**760**	**770**	**780**	**790**	**800**
LNLLQPRPVV	LQGMQVRRVP	LEIPEFDLLD	QDSLHESQEQ	TLMEEAPPRA	QHSYKYWLLP	GRWTSVRYER	LALLALLDRT
**810**	**820**	**830**	**840**	**850**	**860**	**870**	**880**
RGVLENIFGV	GLSSLVAFLG	YLLLLKGFFT	DIWVFQFCLV	IASCQYSLLK	SVQPDAASPM	HGHNWVIAYS	RPVYFCICCL
**890**	**900**	**910**	**920**	**930**	**940**	**950**	**960**
LIWLLDALGS	AQPFPPVSLY	GLTLFSASFF	FCARDVATVF	TLCFPFVFLL	GLLPQVNTCL	MYLLEQIDMH	GFGGTAATSP
**970**	**980**	**990**	**1000**	**1010**	**1020**	**1030**	**1040**
LTAVFSLSRS	LLAAALLYGF	CLGAIKTPWP	EQHVPVLFSV	FCGLLVALSY	HLSRQSSDPT	VLWSLIRSKL	FPELEERSLE
**1050**	**1060**	**1070**					
TARAEPPDPL	PDKMRQSVLN	VSPGPAA					

Cmpd.	No. of Cmpds.	m/z meas.	Δ m/z [ppm]	z	Rt [min]	Score	P	Range	Sequence	Modification
2754	1	1086.0034	31.79	2	64.8	11.5	1	286-307	R.GSGEPTPQKAGSSDSCFSGTDR.E	

Detailed Protein Report

Protein 293: neurogenic locus notch homolog protein 1 preproprotein [Homo sapiens]

**Accession:**	gi|148833508	**Score:**	35.7
**Database:**	refseq_human(refseq_human_20140103.fasta)	**MW [kDa]:**	272.3
**Database Date:**	2015-11-30	**pI:**	4.8
		**Sequence Coverage [%]:**	1.3
		**No. of unique Peptides:**	1

Quantitation

QU:MU	**Median:**	2.27	**CV:**	0.00 %	**No. of Peptides:**	1
WUP:QUP	**Median:**	0.88	**CV:**	0.00 %	**No. of Peptides:**	1

Detailed Protein Report

10	20	30	40	50	60	70	80
MPPLLAPLLC	LALLPALAAR	GPRCSQPGET	CLNGGKCEAA	NGTEACVCGG	AFVGPRCQDP	NPCLSTPCKN	AGTCHVVDRR
90	100	110	120	130	140	150	160
GVADYACSCA	LGFSGPLCLT	PLDNACLTNP	CRNGGTCDLL	TLTEYKCRCP	PGWSGKSCQQ	ADPCASNPCA	NGGQCLPFEA
170	180	190	200	210	220	230	240
SYICHCPPSF	HGPTCRQDVN	ECGQKPGLCR	HGGTCHNEVG	SYRCVCRATH	TGPNCERPYV	PCSPSPCQNG	GTCRPTGDVT
250	260	270	280	290	300	310	320
HECACLPGFT	GQNCEENIDD	CPGNNCKNGG	ACVDGVNTYN	CRCPPEWTGQ	YCTEDVDECQ	LMPNACQNGG	TCHNTHGGYN
330	340	350	360	370	380	390	400
CVCVNGWTGE	DCSENIDDCA	SAACFHGATC	HDRVASFYCE	CPHGRTGLLC	HLNDACISNP	CNEGSNCDTN	PVNGKAICTC
410	420	430	440	450	460	470	480
PSGYTGPACS	QDVDECSLGA	NPCEHAGKCI	NTLGSFECQC	LQGYTGPRCE	IDVNECVSNP	CQNDATCLDQ	IGEFQCICMP
490	500	510	520	530	540	550	560
GYEGVHCEVN	TDECASSPCL	HNGRCLDKIN	EFQCECPTGF	TGHLCQYDVD	ECASTPCKNG	AKCLDGPNTY	TCVCTEGYTG
570	580	590	600	610	620	630	640
THCEVDIDEC	DPDPCHYGSC	KDGVATFTCL	CRPGYTGHHC	ETNINECSSQ	PCRHGGTCQD	RDNAYLCFCL	KGTTGPNCEI
650	660	670	680	690	700	710	720
NLDDCASSPC	DSGTCLDKID	GYECACEPGY	TGSMCNINID	ECAGNPCHNG	GTCEDGINGF	TCRCPEGYHD	PTCLSEVNEC
730	740	750	760	770	780	790	800
NSNPCVHGAC	RDSLNGYKCD	CDPGWSGTNC	DINNNECESN	PCVNGGTCKD	MTSGYVCTCR	EGFSGPNCQT	NINECASNPC
810	820	830	840	850	860	870	880
LNQGTCIDDV	AGYKCNCLLP	YTGATCEVVL	APCAPSPCRN	GGECRQSEDY	ESFSCVCPTG	WQGQTCEVDI	NECVLSPCRH
890	900	910	920	930	940	950	960
GASCQNTHGG	YRCHCQAGYS	GRNCETDIDD	CRPNPCHNGG	SCTDGINTAF	CDCLPGFRGT	FCEEDINECA	SDPCRNGANC
970	980	990	1000	1010	1020	1030	1040
TDCVDSYTCT	CPAGFSGIHC	ENNTPDCTES	SCFNGGTCVD	GINSFTCLCP	PGFTGSYCQH	DVNECDSQPC	LHGGTCQDGC
1050	1060	1070	1080	1090	1100	1110	1120
GSYRCTCPQG	YTGPNCQNLV	HWCDSSPCKN	GGKCWQTHTQ	YRCECPSGWT	GLYCDVPSVS	CEVAAQRQGV	DVARLCQHGG
1130	1140	1150	1160	1170	1180	1190	1200
LCVDAGNTHH	CRCQAGYTGS	YCEDLVDECS	PSPCQNGATC	TDYLGGYSCK	CVAGYHGVNC	SEEIDECLSH	PCQNGGTCLD
1210	1220	1230	1240	1250	1260	1270	1280
LPNTYKCSCP	RGTQGVHCEI	NVDDCNPPVD	PVSRSPKCFN	NGTCVDQVGG	YSCTCPPGFV	GERCEGDVNE	CLSNPCDARG
1290	1300	1310	1320	1330	1340	1350	1360
TQNCVQRVND	FHCECRAGHT	GRRCESVING	CKGKPCKNGG	TCAVASNTAR	GFICKCPAGF	EGATCENDAR	TCGSLRCLNG
1370	1380	1390	1400	1410	1420	1430	1440
GTCISGPRSP	TCLCLGPFTG	PECQFPASSP	CLGGNPCYNQ	GTCEPTSESP	FYRCLCPAKF	NGLLCHILDY	SFGGGAGRDI
1450	1460	1470	1480	1490	1500	1510	1520
PPPLIEEACE	LPECQEDAGN	KVCSLQCNNH	ACGWDGGDCS	LNFNDPWKNC	TQSLQCWKYF	SDGHCDSQCN	SAGCLFDGFD
1530	1540	1550	1560	1570	1580	1590	1600
CQRAEGQCNP	LYDQYCKDHF	SDGHCDQGCN	SAECEWDGLD	CAEHVPERLA	AGTLVVVVLM	PPEQLRNSSF	HFLRELSRVL
1610	1620	1630	1640	1650	1660	1670	1680
HTNVVFKRDA	HGQQMIFPYY	GREEELRKHP	IKRAAEGWAA	PDALLGQVKA	SLLPGGSEGG	RRRRELDPMD	VRGSIVYLEI
1690	1700	1710	1720	1730	1740	1750	1760
DNRQCVQASS	QCFQSATDVA	AFLGALASLG	SLNIPYKIEA	VQSETVEPPP	PAQLHFMYVA	AAAFVLLFFV	GCGVLLSRKR
1770	1780	1790	1800	1810	1820	1830	1840
RRQHGQLWFP	EGFKVSEASK	KKRREPLGED	SVGLKPLKNA	SDGALMDDNQ	NEWGDEDLET	KKFRFEEPVV	LPDLDDQTDH
1850	1860	1870	1880	1890	1900	1910	1920
RQWTQQHLDA	ADLRMSAMAP	TPPQGEVDAD	CMDVNVRGPD	GFTPLMIASC	SGGGLETGNS	EEEEDAPAVI	SDFIYQGASL
1930	1940	1950	1960	1970	1980	1990	2000
HNQTDRTGET	ALHLAARYSR	SDAAKRLLEA	SADANIQDNM	GRTPLHAAVS	ADAQGVFQIL	IRNRATDLDA	RMHDGTTPLI
2010	2020	2030	2040	2050	2060	2070	2080
LAARLAVEGM	LEDLINSHAD	VNAVDDLGKS	ALHWAAAVNN	VDAAVVLLKN	GANKDMQNNR	EETPLFLAAR	EGSYETAKVL
2090	2100	2110	2120	2130	2140	2150	2160
LDHFANRDIT	DHMDRLPRDI	AQERMHHDIV	RLLDEYNLVR	SPQLHGAPLG	GTPTLSPPLC	SPNGYLGSLK	PGVQGKKVRK
2170	2180	2190	2200	2210	2220	2230	2240
PSSKGLACGS	KEAKDLKARR	KKSQDGKGCL	LDSSGMLSPV	DSLESPHGYL	SDVASPPLLP	SPFQQSPSVP	LNHLPGMPDT
2250	2260	2270	2280	2290	2300	2310	2320
HLGIGHLNVA	AKPEMAALGG	GGRLAFETGP	PRLSHLPVAS	GTSTVLGSSS	GGALNFTVGG	STSLNGQCEW	LSRLQSGMVP
2330	2340	2350	2360	2370	2380	2390	2400

Detailed Protein Report

Cmpd.	No. of Cmpds.	m/z meas.	Δ m/z [ppm]	z	Rt [min]	Score	P	Range	Sequence	Modification	Ratios
2073	4	698.2445	-185.84	2	55.8	21.5	0	1992-2004	R.MHDGTTPLILAAR.L		WUP:QUP 0.88<br>QU:MU 2.27

Detailed Protein Report

Protein 294: **PREDICTED: proteasome-associated protein ECM29 homolog isoform X5 [Homo sapiens]**

**Accession:**	gi|530390837	**Score:**	35.7
**Database:**	refseq_human(refseq_human_20140103.fasta)	**MW [kDa]:**	203.7
**Database Date:**	2015-11-30	**pI:**	6.7
**Modification(s):**	Oxidation	**Sequence Coverage [%]:**	2.1
		**No. of unique Peptides:**	2

Quantitation

QU:MU	**Median:**	0.40	**CV:**	0.00 %	**No. of Peptides:**	1
WUP:QUP	**Median:**	3.27	**CV:**	0.00 %	**No. of Peptides:**	1

Detailed Protein Report

10	20	30	40	50	60	70	80
METGSDSDQL	ERVFLRLGHA	ETDEQLQNII	SKFLPPVLLK	LSSTQEGVRK	KVMELLVHLN	KRIKSRPKIQ	LPVETLLVQY
90	100	110	120	130	140	150	160
QDPAAVSFVT	NFTIIYVKMG	YPRLPVEKQC	ELAPTLLTAM	EGKPQPQQDS	LMHLLIPTLF	HMKYPVESSK	SASPFNLAEK
170	180	190	200	210	220	230	240
PKTVQLLLDF	MLDVLLMPYG	YVLNESQSRQ	NSSSAQGSSS	NSGGGSGIPQ	PPPGMSFYAA	KRVIGDNPWT	PEQLEQCKLG
250	260	270	280	290	300	310	320
IVKFIEAEQV	PELEAVLHLV	IASSDTRHSV	ATAADLELKS	KQSLIDWNNP	AIINKMYKVY	LGDIPLKTKE	GAVLKPELKR
330	340	350	360	370	380	390	400
DPVSTRVKLK	IVPHLLRSRQ	AAETFPANIQ	VVYDGLFGTN	TNSKLRTLSL	QFVHHICITC	PEIKIKPLGP	MLLNGLTKLI
410	420	430	440	450	460	470	480
NEYKEDPKLL	SMAYSAVGKL	SSRMPHLFTK	DIALVQQLFE	ALCKEEPETR	LAIQEALSMM	VGAYSTLEGA	QRTLMEALVA
490	500	510	520	530	540	550	560
SYLIKPEVQV	RQVAVKFAST	VFPSDHIPSR	YLLLLAAGDP	REEVHGEAQR	VLRCLPGRNR	KESTSEQMPS	FPEMVYYIQE
570	580	590	600	610	620	630	640
KASHRMKTPV	KYMTGTTVLP	FNPAAFGEIV	LYLRMCLAHS	AGVVPTSQSL	ADMQDHAPAI	GRYIRTLMSS	GQMAPSSSNK
650	660	670	680	690	700	710	720
SGETNPVQIY	IGLLQQLLAG	VGGLPVMYCL	LEAVSVYPEK	LATKFVDKTE	WIKSLMNNSK	EEMRELAALF	YSVVVSTVSG
730	740	750	760	770	780	790	800
NELKSMIEQL	IKTTKDNHSP	EIQHGSLLAL	GFTVGRYLAK	KKMRMSEQQD	LERNADTLPD	QEELIQSATE	TIGSFLDSTS
810	820	830	840	850	860	870	880
PLLAIAACTA	LGEIGRNGPL	PIPSEGSGFT	KLHLVESLLS	RIPSSKETNK	MKERAIQTLG	YFPVGDGDFP	HQKLLLQGLM
890	900	910	920	930	940	950	960
DSVEAKQIEL	QFTIGEAITS	AAIGTSSVAA	RDAWQMTEEE	YTPPAGAKVN	DVVPWVLDVI	LNKHIISPNP	HVRQAACIWL
970	980	990	1000	1010	1020	1030	1040
LSLVRKLSTH	KEVKSHLKEI	QSAFVSVLSE	NDELSQDVAS	KGLGLVYELG	NEQDQQELVS	TLVETLMTGK	RVKHEVSGET
1050	1060	1070	1080	1090	1100	1110	1120
VVFQGGALGK	TPDGQGLSTY	KELCSLASDL	SQPDLVYKFM	NLANHHAMWN	SRKGAAFGFN	VIATRAGEQL	APFLPQLVPR
1130	1140	1150	1160	1170	1180	1190	1200
LYRYQFDPNL	GIRQAMTSIW	NALVTDKSMV	DKYLKEILQD	LVKNLTSNMW	RVRESSCLAL	NDLLRGRPLD	DIIDKLPEIW
1210	1220	1230	1240	1250	1260	1270	1280
ETLFRVQDDI	KESVRKAAEL	ALKTLSKVCV	KMCDPAKGAA	GQRTIAALLP	CLLDKGMMST	VTEVRALSIN	TLVKISKSAG
1290	1300	1310	1320	1330	1340	1350	1360
AMLKPHAPKL	IPALLESLSV	LEPQVLNYLS	LRATEQEKAA	MDSARLSAAK	SSPMMETINM	CLQYLDVSVL	GELVPRLCEL
1370	1380	1390	1400	1410	1420	1430	1440
IRSGVGLGTK	GGCASVIVSL	TTQCPQDLTP	YSGKLMSALL	SGLTDRNSVI	QKSCAFAMGH	LVRTSRDSST	EKLLQKLNGW
1450	1460	1470	1480	1490	1500	1510	1520
YMEKEEPIYK	TSCALTIHAI	GRYSPDVLKN	HAKEVLPLAF	LGMHEIADEE	KSEKEECNLW	TEVWQENVPG	SFGGIRLYLQ
1530	1540	1550	1560	1570	1580	1590	1600
ELITITQKAL	QSQSWKMKAQ	GAIAMASIAK	QTSSLVPPYL	GMILTALLQG	LAGRTWAGKE	ELLKAIACVV	TACSAELEKS
1610	1620	1630	1640	1650	1660	1670	1680
VPNQPSTNEI	LQAVLKECSK	ENVKYKIVAI	SCAADILKAT	KEDRFQEFSN	IVIPLIKKNS	LESSGVRTTK	NEEENEKEKE
1690	1700	1710	1720	1730	1740	1750	1760
LQLEYLLGAF	ESLGKAWPRN	AETQRCYRQE	LCKLMCERLK	LSTWKVQLGV	LQSMNAFFQG	LMLLEEEHAD	PEALAEILLE
1770	1780	1790	1800	1810	1820	1830	1840
TCKSITYSLE	NKTYSSVRTE	ALSVIELLLK	KLEESKQWEC	LTSECRVLLI	ESLATMEPDS	RPELQEKAAL	LKKTLENLE

Cmpd.	No. of Cmpds.	m/z meas.	Δ m/z [ppm]	z	Rt [min]	Score	P	Range	Sequence	Modification	Ratios
681	1	625.0022	62.66	3	38.5	10.4	1	1079-1093	K.FMNLANHHAMWNSRK.G	Oxidation: 2	QU:MU 0.40<br>WUP:QUP 3.27
2061	1	703.7752	-146.50	2	54.1	12.0	1	1537-1550	K.MKAQGAIAMASIAK.Q	Oxidation: 1	

Detailed Protein Report

Protein 295: PREDICTED: histone-lysine N-methyltransferase 2B isoform X2 [Homo sapiens]

**Accession:**	gi|578835223	**Score:**	35.7
**Database:**	refseq_human(refseq_human_20140103.fasta)	**MW [kDa]:**	212.5
**Database Date:**	2015-11-30	**pI:**	6.8
		**Sequence Coverage [%]:**	2.3
		**No. of unique Peptides:**	1

Detailed Protein Report

10	20	30	40	50	60	70	80
MPPLEKARIA	GVGSLPLSGV	EEKMFSLLKR	AKVQLFKIDQ	QQQQKVAASM	PLSPGGQMEE	VAGAVKQISD	RGPVRSEDES
90	100	110	120	130	140	150	160
VEAKRERPSG	PESPVQGPRI	KHVCRHAAVA	LGQARAMVPE	DVPRLSALPL	RDRQDLATED	TSSASETESV	PSRSRRGKVE
170	180	190	200	210	220	230	240
AAGPGGESEP	TGSGGTLAHT	PRRSLPSHHG	KKMRMARCGH	CRGCLRVQDC	GSCVNCLDKP	KFGGPNTKKQ	CCVYRKCDKI
250	260	270	280	290	300	310	320
EARKMERLAK	KGRTIVKTLL	PWDSDESPEA	SPGPPGPRRG	AGAGGPREEV	VAHPGPEEQD	SLLQRKSARR	CVKQRPSYDI
330	340	350	360	370	380	390	400
FEDSDDSEPG	GPPAPRRRTP	RENELPLPEP	EEQSRPRKPT	LQPVLQLKAR	RRLDKDALAP	GPFASFPNGW	TGKQKSPDGV
410	420	430	440	450	460	470	480
HRVRVDFKED	CDLENVWLMG	GLSVLTSVPG	GPPMVCLLCA	SKGLHELVFC	QVCCDPFHPF	CLEEAERPLP	QHHDTWCCRR
490	500	510	520	530	540	550	560
CKFCHVCGRK	GRGSKHLLEC	ERCRHAYHPA	CLGPSYPTRA	TRKRRHWVRD	EICSACVRCK	SCGATPGKNW	DVEWSGDYSL
570	580	590	600	610	620	630	640
CPRCTQLYEK	GNYCPICTRC	YEDNDYESKM	MQCAQCDHWV	HAKCEGLSDE	DYEILSGLPD	SVLYTCGPCA	GAAQPRWREA
650	660	670	680	690	700	710	720
LSGALQGGLR	QVLQGLLSSK	VVGPLLLCTQ	CGPDGKQLHP	GPCGLQAVSQ	RFEDGHYKSV	HSFMEDMVGI	LMRHSEEGET
730	740	750	760	770	780	790	800
PDRRAGGQMK	GLLLKLLESA	FGWFDAHDPK	YWRRSTRLPN	GVLPNAVLPP	SLDHVYAQWR	QQEPETPESG	QPPGDPSAAF
810	820	830	840	850	860	870	880
QGKDPAAFSH	LEDPRQCALC	LKYGDADSKE	AGRLLYIGQN	EWTHVNCAIW	SAEVFEENDG	SLKNVHAAVA	RGRQMRCELC
890	900	910	920	930	940	950	960
LKPGATVGCC	LSSCLSNFHF	MCARASYCIF	QDDKKVFCQK	HTDLLDGKEI	VNPDGFDVLR	RVYVDFEGIN	FKRKFLTGLE
970	980	990	1000	1010	1020	1030	1040
PDAINVLIGS	IRIDSLGTLS	DLSDCEGRLF	PIGYQCSRLY	WSTVDARRRC	WYRCRILEYR	PWGPREEPAH	LEAAEENQTI
1050	1060	1070	1080	1090	1100	1110	1120
VHSPAPSSEP	PGGEDPPLDT	DVLVPGAPER	HSPIQNLDPP	LRPDSGSAPP	PAPRSFSGAR	IKVPNYSPSR	RPLGGVSFGP
1130	1140	1150	1160	1170	1180	1190	1200
LPSPGSPSSL	THHIPTVGDP	DFPAPPRRSR	RPSPLAPRPP	PSRWASPPLK	TSPQLRVPPP	TSVVTALTPT	SGELAPPGPA
1210	1220	1230	1240	1250	1260	1270	1280
PSPPPPEDLG	PDFEDMEVVS	GLSAADLDFA	ASLLGTEPFQ	EEIVAAGAMG	SSHGGPGDSS	EEESSPTSRY	IHFPVTVVSA
1290	1300	1310	1320	1330	1340	1350	1360
PGLAPSATPG	APRIEQLDGV	DDGTDSEAEA	VQQPRGQGTP	PSGPGVVRAG	VLGAAGDRAR	PPEDLPSEIV	DFVLKNLGGP
1370	1380	1390	1400	1410	1420	1430	1440
GDGGAGPREE	SLPPAPPLAN	GSQPSQGLTA	SPADPTRTFA	WLPGAPGVRV	LSLGPAPEPP	KPATSKIILV	NKLGQVFVKM
1450	1460	1470	1480	1490	1500	1510	1520
AGEGEPVPPP	VKQPPLPPTI	SPTAPTSWTL	PPGPLLGVLP	VVGVVRPAPP	PPPPPLTLVL	SSGPASPPRQ	AIRVKRVSTF
1530	1540	1550	1560	1570	1580	1590	1600
SGRSPPAPPP	YKAPRLDEDG	EASEDTPQVP	GLGSGGFSRV	RMKTPTVRGV	LDLDRPGEPA	GEESPGPLQE	RSPLLPLPED
1610	1620	1630	1640	1650	1660	1670	1680
GPPQVPDGPP	DLLLESQWHH	YSGEASSSEE	EPPSPDDKEN	QAPKRTGPHL	RFEISSEDGF	SVEAESLEGA	WRTLIEKVQE
1690	1700	1710	1720	1730	1740	1750	1760
ARGHARLRHL	SFSGMSGARL	LGIHHDAVIF	LAEQLPGAQR	CQHYKFRYHQ	QGEGQEEPPL	NPHGAARAEV	YLRKCTFDMF
1770	1780	1790	1800	1810	1820	1830	1840
NFLASQHRVL	PEGATCDEEE	DEVQLRSTRR	ATSLELPMAM	RFRHLKKTSK	EAVGVYRSAI	HGRGLFCKRN	IDAGEMVIEY
1850	1860	1870	1880	1890	1900	1910	1920
SGIVIRSVLT	DKREKFYDGK	GIGCYMFRMD	DFDVVDATMH	GNAARFINHS	CEPNCFSRVI	HVEGQKHIVI	FALRRILRGE
1930	1940	1950	1960				
ELTYDYKFPI	EDASNKLPCN	CGAKRCRRFL	N				

Cmpd.	No. of Cmpds.	m/z meas.	Δ m/z [ppm]	z	Rt [min]	Score	P	Range	Sequence	Modification
2760	1	841.9053	-83.22	2	63.0	12.2	1	7-23	K.ARIAGVGSLPLSGVEEK.M	

Detailed Protein Report

Protein 296: **PREDICTED: maestro heat-like repeat-containing protein family member 2A isoform X1 [Homo sapiens]**

**Accession:**	gi|578804228	**Score:**	35.7
**Database:**	refseq_human(refseq_human_20140103.fasta)	**MW [kDa]:**	189.4
**Database Date:**	2015-11-30	**pI:**	6.2
**Modification(s):**	Oxidation	**Sequence Coverage [%]:**	2.3
		**No. of unique Peptides:**	3

10	20	30	40	50	60	70	80
MTEAITEAAV	ASSEEVSEER	DDLGPLELHD	SGTFQQVVNL	LDIIDSESAK	TDTTGAGLDM	RKTLASVIIM	EKATTEPSVV
90	100	110	120	130	140	150	160
INTLIRCLQV	PEISTQRKVN	IYNILQDIIQ	QEGELEEQCV	QRLVAIASKE	MREIPEMEGY	MKAEVASDTL	VALSRNHFSL
170	180	190	200	210	220	230	240
VMYELQHHLK	PLNLTDEFVI	ITLAKLANGN	VFEFMPYMGI	TLATIFTMLR	LANEAKIRQA	ICSAMETFCE	TVQFYLKHLE
250	260	270	280	290	300	310	320
ESVYPVMTEE	EFALKVFPMY	RYFVTVWLRH	YNPEVKLGVI	KSLKPMLGLL	LPNDDLREQV	YDYIPLLLAE	YQGSLEVLFV
330	340	350	360	370	380	390	400
TQVLRQILEL	SVTTNTPVPQ	MQLHTIFTEL	HVQVCNKAPA	QHQYSSQNLM	EMVHCFVALA	RSYPKELMKF	FFSQMETNKE
410	420	430	440	450	460	470	480
AVRVGTLNLI	RAIVSADEPR	MSIRAIYLAI	RVVKNTISDT	RSKVRMAILH	IIGQLALCGY	QERIKGWGLK	YLSVQLTLST
490	500	510	520	530	540	550	560
YKLTNRREKF	YQRDLEERMV	HKVTMDTVKI	ITSSVSGMTT	EFWVRLLCYI	METDYVEALT	PICISLTNLA	EHQLHGQDVD
570	580	590	600	610	620	630	640
VSVAGKSRQV	DLPAPQKLLA	RLLVLMSSPY	KGEGRGIAML	NLLRTLSQSI	APSMADMWEL	EIALLVRYLE	EHTEFTWDQK
650	660	670	680	690	700	710	720
AWEDKLIQFL	RNSLKKTRGS	SWSLRLSKEL	NNQIASFDSP	SLEKGFLYRA	LGFTLATGLE	ASKVEVLLLE	LLYKTDYSND
730	740	750	760	770	780	790	800
FDSEGVIMCF	GLCARGQVKT	VLNVLHDFEE	RIQESEQSWQ	ISAWRKDHPW	RRETVKSALM	VMYSCVASYC	HPQLLLNLVD
810	820	830	840	850	860	870	880
SPITAKIIHH	YVSSCQDICL	KMAFMKSVVQ	VTKAINNIKD	LEDFHFAQKT	TLTSIIVAVI	KAEPTDNLVS	PVRALAMEAL
890	900	910	920	930	940	950	960
SHLSKLKPFY	STEENSELMD	ISIHSVISLQ	LPGEDNESIK	TLYANALSSL	EQLMESLLQR	QLDPKGLQEM	VQLLEKWILS
970	980	990	1000	1010	1020	1030	1040
EKEWEREKAV	SLHLYLMWIY	VHSTAVCIHL	KLGQFGTMVG	LIAPCTCDAH	QRTRMASMNV	LSSLLDLHAS	QTCSLWGPSK
1050	1060	1070	1080	1090	1100	1110	1120
QKELEKCKGD	LQSTDVEKIF	CASSRIAKVV	CMEFSCDEVV	SLIQKLCENT	GAMNLQHDKA	SVTWIAFFLQ	MRAKELEDKV
1130	1140	1150	1160	1170	1180	1190	1200
AEILSAILVH	LPVVDHPEVR	RLLIDGILLL	AHHHQETILT	SLLRQPLPME	SHLAEVWLAV	SENVPFARTM	LHSLMGRLQS
1210	1220	1230	1240	1250	1260	1270	1280
RLSPRISATS	KADIWRLAAV	DPLMTLCTIH	LLIQKLDEND	KLPDFLPDLI	YTLLLQLGSS	HRPEAAPPVL	KMWKLVHTTP
1290	1300	1310	1320	1330	1340	1350	1360
LPEEMNLQRV	TIKSMQLLFK	RVKSQHLAHT	LDEQAVWDLL	QDGGTFLEGV	SLLARLCMQH	VEGHRQRLAE	LVLRGMDSEV
1370	1380	1390	1400	1410	1420	1430	1440
LSCRISSTAV	CFMSGPVLYQ	EKLLKPAALL	LEKGADQEED	EALRVLSLRA	LGNMALGAPK	KVKQYRKVLL	EKCLGPLREP
1450	1460	1470	1480	1490	1500	1510	1520
VSNSVTAEGM	EALTKILAEL	REGDVGSSFD	AMSEQCRIFF	DNESELLRLK	AFILFGKLAR	VVGMSKKHFF	KGEVKKAWIP
1530	1540	1550	1560	1570	1580	1590	1600
LMLHSQDPCS	NAAQACMATM	FQCVHFWGWK	SLEHPSGPSD	TATDDKMTVF	QTTMCSILTR	KKPAVLYRFL	LETMAYVKNN
1610	1620	1630	1640	1650	1660	1670	1680
LSRIRIAACN	LAGIIMKQMS	THYLKKLDFP	ALRNSLQELQ	LDPDPGVRRA	ALETLTVLDS	CSQHGFLASP	QGMS

Cmpd.	No. of Cmpds.	m/z meas.	Δ m/z [ppm]	z	Rt [min]	Score	P	Range	Sequence	Modification
1832	2	577.2623	1.72	2	52.7	13.5	0	51-61	K.TDTTGAGLDMR.K	Oxidation: 10
2646	1	616.8461	-35.09	2	61.4	11.5	1	62-72	R.KTLASVIIMEK.A	
288	1	636.2301	-155.37	3	32.3	10.6	1	1348-	R.LAELVLRGMDSEVLSCR.I	Oxidation: 9

Detailed Protein Report

Cmpd.	No. of Cmpds.	m/z meas.	Δ m/z [ppm]	z	Rt [min]	Score	P	Range	Sequence	Modification
								1364		

Detailed Protein Report

Protein 297: PREDICTED: coiled-coil domain-containing protein 74B isoform X9 [Homo sapiens]

Accession: gi|578805131
Database: refseq_human(refseq_human_20140103.fasta)
Database Date: 2015-11-30
Modification(s): Carbamidomethyl

Score: 35.6
MW [kDa]: 36.8
pI: 12.0
Sequence Coverage [%]: 14.5
No. of unique Peptides: 2

10	20	30	40	50	60	70	80
MSGAGVAAGT	RPPSSPTPGS	RRRRQRPSVG	VQSLRPQSPQ	LRQSDPQKRN	LDLEKSLQFL	QQQHSEMLAK	LHEEIEHLKR
**90**	**100**	**110**	**120**	**130**	**140**	**150**	**160**
ENKGEPARGP	RPALPPQAHS	TLPLPQHRNT	AINSSTRLGS	GGTQDDLRYK	LIMNQTSQKK	DGPSGNHLSR	ASAPLGARWV
**170**	**180**	**190**	**200**	**210**	**220**	**230**	**240**
CINGVWVEPG	GPSPARLKEG	SSRTHRPGGK	HGRLAGGSAD	TVRSPADSLS	TSSFQSVKSI	SNSANSQGKA	RPQPGSFNKQ
**250**	**260**	**270**	**280**	**290**	**300**	**310**	**320**
DSKADVPQKA	DLEEEPLLHN	SKLDKVPGVQ	GQASPPHLGS	AKCSSASCGI	PTSCRPKSCS	TSSPSWKGAR	GPRQSRRKLA
**330**	**340**	**350**					
FPGTKKPRIS	PRSPPRASPR	NACF					

Cmpd.	No. of Cmpds.	m/z meas.	Δ m/z [ppm]	z	Rt [min]	Score	P	Range	Sequence	Modification
2681	1	945.9624	-37.93	2	61.9	11.2	2	140-158	K.KDGPSGNHLSRASAPLGAR.W	
1874	1	860.9002	-14.63	2	51.8	11.4	2	298-313	K.SCSTSSPSWKGARGPR.Q	Carbamidomethyl: 2

Detailed Protein Report

Protein 298: **protein FAM117B [Homo sapiens]**

Accession: gi|254910983
Database: refseq_human(refseq_human_20140103.fasta)
Database Date: 2015-11-30
Modification(s): Carbamidomethyl

Score: 35.6
MW [kDa]: 61.9
pI: 10.8
Sequence Coverage [%]: 7.1
No. of unique Peptides: 1

10	20	30	40	50	60	70	80
MSQRVRRNGS	PTPAGSLGGG	AVATAGGPGS	RLQPMRATVP	FQLKQQQQQQ	HGSPTRSGGG	GGGNNNGGCC	GGASGPAGGG
**90**	**100**	**110**	**120**	**130**	**140**	**150**	**160**
GGGGPRTASR	STSPTRGGGN	AAARTSPTVA	TQTGASATST	RGTSPTRSAA	PGARGSPPRP	PPPPPLLGTV	SSPSSSPTHL
**170**	**180**	**190**	**200**	**210**	**220**	**230**	**240**
WTGEVSAAPP	PARVRHRRRS	PEQSRSSPEK	RSPSAPVCKA	GDKTRQPSSS	PSSIIRRTSS	LDTLAAPYLA	GHWPRDSHGQ
**250**	**260**	**270**	**280**	**290**	**300**	**310**	**320**
AAPCMRDKAT	QTESAWAEEY	SEKKKGSHKR	SASWGSTDQL	KEIAKLRQQL	QRSKHSSRHH	RDKERQSPFH	GNHAAINQCQ
**330**	**340**	**350**	**360**	**370**	**380**	**390**	**400**
APVPKSALIP	VIPITKSTGS	RFRNSVEGLN	QEIEIIIKET	GEKEEQLIPQ	DIPDGHRAPP	PLVQRSSSTR	SIDTQTPGGA
**410**	**420**	**430**	**440**	**450**	**460**	**470**	**480**
DRGSNNSSRS	QSVSPTSFLT	ISNEGSEESP	CSADDLLVDP	RDKENGNNSP	LPKYATSPKP	NNSYMFKREP	PEGCERVKVF
**490**	**500**	**510**	**520**	**530**	**540**	**550**	**560**
EECSPKQLHE	IPAFYCPDKN	KVNFIPKSGS	AFCLVSILKP	LLPTPDLTLK	GSGHSLTVTT	GMTTTLLQPI	AVASLSTNTE
**570**	**580**	**590**					
QDRVSRGTST	VMPSASLLPP	PEPIEEAEG					

Cmpd.	No. of Cmpds.	m/z meas.	Δ m/z [ppm]	z	Rt [min]	Score	P	Range	Sequence	Modification
2079	1	772.9176	-77.41	3	55.9	13.4	0	57-86	R.SGGGGGGNNNGGCCGGASGPAGGGGGGGPR.T	Carbamidomethyl: 14

Detailed Protein Report

Protein 299: SKI family transcriptional corepressor 1 [Homo sapiens]

**Accession:**	gi|384407369	**Score:**	35.6
**Database:**	refseq_human(refseq_human_20140103.fasta)	**MW [kDa]:**	93.7
**Database Date:**	2015-11-30	**pI:**	8.5
		**Sequence Coverage [%]:**	6.2
		**No. of unique Peptides:**	2

Quantitation

WUP:QUP **Median:** 0.37 **CV:** 0.00 % **No. of Peptides:** 1

```
        10         20         30         40         50         60         70         80
MIPKSLVQAA ETQRGLCEPG GKGREGRSGG KGETRPRGVG TQEGKGTIPG SPREKRGERG EGDPALLPAE DLWRLPGSKD
        90        100        110        120        130        140        150        160
RLRSSLLPPP GPPSSDSGPG PPSSHSGKTA QGPRTLNWAR KQSARTSSNL CAWSLAMATK MAEIPSSPYE PGQRGMKDTQ
       170        180        190        200        210        220        230        240
RGDAPQRGSP EPRILQLARV GNVYLKEESS RSGRLEAKKW IPTKFLENLS VPHVDLGGNQ LWSGGMEALT TQLGPGREGS
       250        260        270        280        290        300        310        320
SSPNSKQELQ PYSGSSALKP NQVGETSLYG VPIVSLVIDG QERLCLAQIS NTLLKNYSYN EIHNRRVALG ITCVQCTPVQ
       330        340        350        360        370        380        390        400
LEILRRAGAM PISSRRCGMI TKREAERLCK SFLGEHKPPK LPENFAFDVV HECAWGSRGS FIPARYNSSR AKCIKCGYCS
       410        420        430        440        450        460        470        480
MYFSPNKFIF HSHRTPDAKY TQPDAANFNS WRRHLKLSDK SATDELSHAW EDRGLGLATG ASGPAGPGGP GGGAGVRSYP
       490        500        510        520        530        540        550        560
VIPVPSKGFG LLQKLPPPLF PHPYGFPTAF GLCPKKDDPV LGAGEPKGGS YVSAFRPVVK DTESIAKLYG SAREAYGAGP
       570        580        590        600        610        620        630        640
ARGPGPGAGS GGYVSPDFLS EGSSSYNSAS PDVDTADEPE VDVESNRFPD DEDAQEETEP SAPSAGGGPD AGRPAFGDLA
       650        660        670        680        690        700        710        720
AEDLVRRPER SPPSGGGGYE LREPCGPLGG PAPAKVFAPE RDEHVKSAAV ALGPAASYVC TPEAHEPDKE DNHSPADDLE
       730        740        750        760        770        780        790        800
TRKSYPDQRS ISQPSPANTD RGEDGLTLDV TGTHLVEKDI ENLAREELQK LLLEQMELRK KLEREFQSLK DNFQDQMKRE
       810        820        830        840        850        860        870
LAYREEMVQQ LQIVRDTLCN ELDQERKARY AIQQKLKEAH DALHHFSCKM LTPRHCTGNC SFKPPLLP
```

Cmpd.	No. of Cmpds.	m/z meas.	Δ m/z [ppm]	z	Rt [min]	Score	P	Range	Sequence	Modification	Ratios
1961	1	599.9124	-123.71	3	52.9	10.6	2	38-55	R.GVGTQEGKGTIPGSPREK.R		
2760	1	729.3797	-5.05	2	65.6	13.2	1	759-770	K.DIENLAREELQK.L		WUP:QUP 0.37

Detailed Protein Report

Protein 300: ectoderm-neural cortex protein 1 isoform 2 [Homo sapiens]

**Accession:**	gi|375268727	**Score:**	35.5
**Database:**	refseq_human(refseq_human_20140103.fasta)	**MW [kDa]:**	57.8
**Database Date:**	2015-11-30	**pI:**	5.7
**Modification(s):**	Carbamidomethyl	**Sequence Coverage [%]:**	6.8
		**No. of unique Peptides:**	2

Quantitation

WUP:QUP **Median:** 1.61	**CV:** 0.00 %	**No. of Peptides:**	1

10	20	30	40	50	60	70	80
MFSGGLKESQ	DSEVNFDNSI	HPEVLELLLD	YAYSSRVIIN	EENAESLLEA	GDMLEFQDIR	DACAEFLEKN	LHPTNCLGML
**90**	**100**	**110**	**120**	**130**	**140**	**150**	**160**
LLSDAHQCTK	LYELSWRMCL	SNFQTIRKNE	DFLQLPQDMV	VQLLSSEELE	TEDERLVYES	AINWISYDLK	KRYCYLPELL
**170**	**180**	**190**	**200**	**210**	**220**	**230**	**240**
QTVRLALLPA	IYLMENVAME	ELITKQRKSK	EIVEEAIRCK	LKILQNDGVV	TSLCARPRKT	GHALFLLGGQ	TFMCDKLYLV
**250**	**260**	**270**	**280**	**290**	**300**	**310**	**320**
DQKAKEIIPK	ADIPSPRKEF	SACAIGCKVY	ITGGRGSENG	VSKDVWVYDT	LHEEWSKAAP	MLVARFGHGS	AELKHCLYVV
**330**	**340**	**350**	**360**	**370**	**380**	**390**	**400**
GGHTAATGCL	PASPSVSLKQ	VEHYDPTINK	WTMVAPLREG	VSNAAVVSAK	LKLFAFGGTS	VSHDKLPKVQ	CYDQCENRWT
**410**	**420**	**430**	**440**	**450**	**460**	**470**	**480**
VPATCPQPWR	YTAAAVLGNQ	IFIMGGDTEF	SACSAYKFNS	ETYQWTKVGD	VTAKRMSCHA	VASGNKLYVV	GGYFGIQRCK
**490**	**500**	**510**	**520**				
TLDCYDPTLD	VWNSITTVPY	SLIPTAFVST	WKHLPS				

Cmpd.	No. of Cmpds.	m/z meas.	Δ m/z [ppm]	z	Rt [min]	Score	P	Range	Sequence	Modification	Ratios
1599	1	948.4653	4.77	2	50.2	12.3	0	220-236	K.TGHALFLLGGQTFMCDK.L	Carbamidomethyl: 15	WUP:QUP 1.61
1786	1	916.3119	-145.37	2	52.7	10.3	1	259-275	K.EFSACAIGCKVYITGGR.G	Carbamidomethyl: 5	

Detailed Protein Report

Protein 301: tenascin-N precursor [Homo sapiens]

Accession: gi|62988324
Database: refseq_human(refseq_human_20140103.fasta)
Database Date: 2015-11-30

Score: 35.4
MW [kDa]: 143.9
pI: 5.3
Sequence Coverage [%]: 1.5
No. of unique Peptides: 2

Quantitation

QU:MU **Median:** 2.27 **CV:** 0.00 % **No. of Peptides:** 1

10	20	30	40	50	60	70	80
MSLQEMFRFP	MGLLLGSVLL	VASAPATLEP	PGCSNKEQQV	TVSHTYKIDV	PKSALVQVDA	DPQPLSDDGA	SLLALGEARE
90	100	110	120	130	140	150	160
EQNIIFRHNI	RLQTPQKDCE	LAGSVQDLLA	RVKKLEEEMV	EMKEQCSAQR	CCQGVTDLSR	HCSGHGTFSL	ETCSCHCEEG
170	180	190	200	210	220	230	240
REGPACERLA	CPGACSGHGR	CVDGRCLCHE	PYVGADCGYP	ACPENCSGHG	ECVRGVCQCH	EDFMSEDCSE	KRCPGDCSGH
250	260	270	280	290	300	310	320
GFCDTGECYC	EEGFTGLDCA	QVVTPQGLQL	LKNTEDSLLV	SWEPSSQVDH	YLLSYYPLGK	ELSGKQIQVP	KEQHSYEILG
330	340	350	360	370	380	390	400
LLPGTKYIVT	LRNVKNEVSS	SPQHLLATTD	LAVLGTAWVT	DETENSLDVE	WENPSTEVDY	YKLRYGPMTG	QEVAEVTVPK
410	420	430	440	450	460	470	480
SSDPKSRYDI	TGLHPGTEYK	ITVVPMRGEL	EGKPILLNGR	TEIDSPTNVV	TDRVTEDTAT	VSWDPVQAVI	DKYVVRYTSA
490	500	510	520	530	540	550	560
DGDTKEMAVH	KDESSTVLTG	LKPGEAYKVY	VWAERGNQGS	KKADTNALTE	IDSPANLVTD	RVTENTATIS	WDPVQATIDK
570	580	590	600	610	620	630	640
YVVRYTSADD	QETREVLVGK	EQSSTVLTGL	RPGVEYTVHV	WAQKGDRESK	KADTNAPTDI	DSPKNLVTDR	VTENMATVSW
650	660	670	680	690	700	710	720
DPVQAAIDKY	VVRYTSAGGE	TREVPVGKEQ	SSTVLTGLRP	GMEYMVHVWA	QKGDQESKKA	DTKAQTDIDS	PQNLVTDRVT
730	740	750	760	770	780	790	800
ENMATVSWDP	VRATIDRYVV	RYTSAKDGET	REVPVGKEQS	STVLTGLRPG	VEYTVHVWAQ	KGAQESKKAD	TKAQTDIDSP
810	820	830	840	850	860	870	880
QNLVTDWVTE	NTATVSWDPV	QATIDRYVVH	YTSANGETRE	VPVGKEQSST	VLTGLRPGME	YTVHVWAQKG	NQESKKADTK
890	900	910	920	930	940	950	960
AQTEIDGPKN	LVTDWVTENM	ATVSWDPVQA	TIDKYMVRYT	SADGETREVP	VGKEHSSTVL	TGLRPGMEYM	VHVWAQKGAQ
970	980	990	1000	1010	1020	1030	1040
ESKKADTKAQ	TELDPPRNLR	PSAVTQSGGI	LTWTPPSAQI	HGYILTYQFP	DGTVKEMQLG	REDQRFALQG	LEQGATYPVS
1050	1060	1070	1080	1090	1100	1110	1120
LVAFKGGRRS	RNVSTTLSTV	GARFPHPSDC	SQVQQNSNAA	SGLYTIYLHG	DASRPLQVYC	DMETDGGGWI	VFQRRNTGQL
1130	1140	1150	1160	1170	1180	1190	1200
DFFKRWRSYV	EGFGDPMKEF	WLGLDKLHNL	TTGTPARYEV	RVDLQTANES	AYAIYDFFQV	ASSKERYKLT	VGKYRGTAGD
1210	1220	1230	1240	1250	1260	1270	1280
ALTYHNGWKF	TTFDRDNDIA	LSNCALTHHG	GWWYKNCHLA	NPNGRYGETK	HSEGVNWEPW	KGHEFSIPYV	ELKIRPHGYS
1290	1300						
REPVLGRKKR	TLRGRLRTF						

Cmpd.	No. of Cmpds.	m/z meas.	Δ m/z [ppm]	z	Rt [min]	Score	P	Range	Sequence	Modification	Ratios
2128	9	698.2808	-172.59	2	55.0	24.0	0	428-440	R.GELEGKPILLNGR.T		QU:MU 2.27
2573	1	590.4386	252.94	1	62.8	11.4	0	516-521	R.GNQGSK.K		

Detailed Protein Report

Protein 302: GH3 domain-containing protein isoform 2 precursor [Homo sapiens]

**Accession:**	gi|217330592	**Score:**	35.4
**Database:**	refseq_human(refseq_human_20140103.fasta)	**MW [kDa]:**	53.5
**Database Date:**	2015-11-30	**pI:**	10.0
		**Sequence Coverage [%]:**	6.5
		**No. of unique Peptides:**	3

Quantitation

QU:MU **Median:** 0.70 **CV:** 0.00 % **No. of Peptides:** 1

10	20	30	40	50	60	70	80
MLLWPLLLLL	LLLPTLALLR	QQRSQDARLS	WLAGLQHRVA	WGALVWAATW	QRRRLEQSTL	HVHQSQQQAL	RWCLQGAQRP
**90**	**100**	**110**	**120**	**130**	**140**	**150**	**160**
HCSLRRSTDI	STFRNHLPLT	KAYPEVLAQG	RTARVTLTSP	WPRPLPWPGN	TLGQVGTPGT	KDPRALLLDA	LRSPGLRALE
**170**	**180**	**190**	**200**	**210**	**220**	**230**	**240**
AGTAVELLDV	FLGLETDGEE	LAGAIAAGNP	GAPLRERAAE	LREALEQGPR	GLALRLWPKL	QVVVTLDAGG	QAEAVAALGA
**250**	**260**	**270**	**280**	**290**	**300**	**310**	**320**
LWCQGLAFFS	PAYAASGGVL	GLNLQPEQPH	GLYLLPPGAP	FIELLPVKEG	TQEEAASTLL	LAEAQQGKEY	ELVLTDRASL
**330**	**340**	**350**	**360**	**370**	**380**	**390**	**400**
TRCRLGDVVR	VVGAYNQCPV	VRFICRLDQT	LSVRGEDIGE	DLFSEALGRA	VGQWAGAKLL	DHGCVESSIL	DSSAGSAPHY
**410**	**420**	**430**	**440**	**450**	**460**	**470**	**480**
EVFVALRGLR	NLSEENRDKL	DHCLQEASPR	YKSLRFWGSV	GPARVHLVGQ	GAFRALRAAL	AACPSSPFPP	AMPRVLRHRH
**490**	**500**						
LAQCLQERVV	S						

Cmpd.	No. of Cmpds.	m/z meas.	Δ m/z [ppm]	z	Rt [min]	Score	P	Range	Sequence	Modification	Ratios
273	1	442.6382	-324.42	2	32.1	14.2	0	145-152	R.ALLLDALR.S		
2063	1	634.7944	-18.66	2	54.2	10.9	0	420-430	K.LDHCLQEASPR.Y		
734	1	712.3429	-104.91	2	39.2	10.3	1	445-457	R.VHLVGQGAFRALR.A		QU:MU 0.70

Detailed Protein Report

Protein 303: **PREDICTED: plasma membrane calcium-transporting ATPase 2 isoform X9 [Homo sapiens]**

**Accession:**	gi|578806173	**Score:**	35.3
**Database:**	refseq_human(refseq_human_20140103.fasta)	**MW [kDa]:**	129.4
**Database Date:**	2015-11-30	**pI:**	6.0
**Modification(s):**	Oxidation	**Sequence Coverage [%]:**	3.5
		**No. of unique Peptides:**	1

10	20	30	40	50	60	70	80
MGDMTNSDFY	SKNQRNESSH	GGEFGCTMEE	LRSLMELRGT	EAVVKIKETY	GDTEAICRRL	KTSPVEGLPG	TAPDLEKRKQ
**90**	**100**	**110**	**120**	**130**	**140**	**150**	**160**
IFGQNFIPPK	KPKTFLQLVW	EALQDVTLII	LEIAAIISLG	LSFYHPPGEG	NEGCATAQGG	AEDEGEAEAG	WIEGAAILLS
**170**	**180**	**190**	**200**	**210**	**220**	**230**	**240**
VICVVLVTAF	NDWSKEKQFR	GLQSRIEQEQ	KFTVVRAGQV	VQIPVAEIVV	GDIAQVKYGD	LLPADGLFIQ	GNDLKIDESS
**250**	**260**	**270**	**280**	**290**	**300**	**310**	**320**
LTGESDQVRK	SVDKDPMLLS	GTHVMEGSGR	MLVTAVGVNS	QTGIIFTLLG	AGGEEEEKKD	KKGKMQDGNV	DASQSKAKQQ
**330**	**340**	**350**	**360**	**370**	**380**	**390**	**400**
DGAAAMEMQP	LKSAEGGDAD	DRKKASMHKK	EKSVLQGKLT	KLAVQIGKAG	LVMSAITVII	LVLYFTVDTF	VVNKKPWLPE
**410**	**420**	**430**	**440**	**450**	**460**	**470**	**480**
CTPVYVQYFV	KFFIIGVTVL	VVAVPEGLPL	AVTISLAYSV	KKMMKDNNLV	RHLDACETMG	NATAICSDKT	GTLTTNRMTV
**490**	**500**	**510**	**520**	**530**	**540**	**550**	**560**
VQAYVGDVHY	KEIPDPSSIN	TKTMELLINA	IAINSAYTTK	ILPPEKEGAL	PRQVGNKTEC	GLLGFVLDLK	QDYEPVRSQM
**570**	**580**	**590**	**600**	**610**	**620**	**630**	**640**
PEEKLYKVYT	FNSVRKSMST	VIKLPDESFR	MYSKGASEIV	LKKCCKILNG	AGEPRVFRPR	DRDEMVKKVI	EPMACDGLRT
**650**	**660**	**670**	**680**	**690**	**700**	**710**	**720**
ICVAYRDFPS	SPEPDWDNEN	DILNELTCIC	VVGIEDPVRP	EVPEAIRKCQ	RAGITVRMVT	GDNINTARAI	AIKCGIIHPG
**730**	**740**	**750**	**760**	**770**	**780**	**790**	**800**
EDFLCLEGKE	FNRRIRNEKG	EIEQERIDKI	WPKLRVLARS	SPTDKHTLVK	GIIDSTHTEQ	RQVVAVTGDG	TNDGPALKKA
**810**	**820**	**830**	**840**	**850**	**860**	**870**	**880**
DVGFAMGIAG	TDVAKEASDI	ILTDDNFSSI	VKAVMWGRNV	YDSISKFLQF	QLTVNVVAVI	VAFTGACITQ	DSPLKAVQML
**890**	**900**	**910**	**920**	**930**	**940**	**950**	**960**
WVNLIMDTFA	SLALATEPPT	ETLLLRKPYG	RNKPLISRTM	MKNILGHAVY	QLALIFTLLF	VGEKMFQIDS	GRNAPLHSPP
**970**	**980**	**990**	**1000**	**1010**	**1020**	**1030**	**1040**
SEHYTIIFNT	FVMMQLFNEI	NARKIHGERN	VFDGIFRNPI	FCTIVLGTFA	IQIVIVQFGG	KPFSCSPLQL	DQWMWCIFIG
**1050**	**1060**	**1070**	**1080**	**1090**	**1100**	**1110**	**1120**
LGELVWGQVI	ATIPTSRLKF	LKEAGRLTQK	EEIPEEELNE	DVEEIDHAER	ELRRGQILWF	RGLNRIQTQI	EVVNTFKSGA
**1130**	**1140**	**1150**	**1160**	**1170**	**1180**		
SFQGALRRQS	SVTSQSQDVA	NLSSPSRVSL	SNALSSPTSL	PPAAAGHPRR	EGVP		

Cmpd.	No. of Cmpds.	m/z meas.	Δ m/z [ppm]	z	Rt [min]	Score	P	Range	Sequence	Modification
336	1	625.9844	-32.51	3	34.2	10.9	2	568-583	K.VYTFNSVRKSMSTVIK.L	Oxidation: 11

Detailed Protein Report

Protein 304: **A disintegrin and metalloproteinase with thrombospondin motifs 6 preproprotein [Homo sapiens]**

Accession: gi|64276808
Database: refseq_human(refseq_human_20140103.fasta)
Database Date: 2015-11-30
Modification(s): Carbamidomethyl, Oxidation

Score: 35.3
MW [kDa]: 125.2
pI: 6.3
Sequence Coverage [%]: 6.5
No. of unique Peptides: 3

Quantitation

QU:MU **Median:** 1.34 **CV:** 0.00 % **No. of Peptides:** 1

10	20	30	40	50	60	70	80
MEILWKTLTW	ILSLIMASSE	FHSDHRLSYS	SQEEFLTYLE	HYQLTIPIRV	DQNGAFLSFT	VKNDKHSRRR	RSMDPIDPQQ
90	100	110	120	130	140	150	160
AVSKLFFKLS	AYGKHFHLNL	TLNTDFVSKH	FTVEYWGKDG	PQWKHDFLDN	CHYTGYLQDQ	RSTTKVALSN	CVGLHGVIAT
170	180	190	200	210	220	230	240
EDEEYFIEPL	KNTTEDSKHF	SYENGHPHVI	YKKSALQQRH	LYDHSHCGVS	DFTRSGKPWW	LNDTSTVSYS	LPINNTHIHH
250	260	270	280	290	300	310	320
RQKRSVSIER	FVETLVVADK	MMVGYHGRKD	IEHYILSVMN	IVAKLYRDSS	LGNVVNIIVA	RLIVLTEDQP	NLEINHHADK
330	340	350	360	370	380	390	400
SLDSFCKWQK	SILSHQSDGN	TIPENGIAHH	DNAVLITRYD	ICTYKNKPCG	TLGLASVAGM	CEPERSCSIN	EDIGLGSAFT
410	420	430	440	450	460	470	480
IAHEIGHNFG	MNHDGIGNSC	GTKGHEAAKL	MAAHITANTN	PFSWSACSRD	YITSFLDSGR	GTCLDNEPPK	RDFLYPAVAP
490	500	510	520	530	540	550	560
GQVYDADEQC	RFQYGATSRQ	CKYGEVCREL	WCLSKSNRCV	TNSIPAAEGT	LCQTGNIEKG	WCYQGDCVPF	GTWPQSIDGG
570	580	590	600	610	620	630	640
WGPWSLWGEC	SRTCGGGVSS	SLRHCDSPAP	SGGGKYCLGE	RKRYRSCNTD	PCPLGSRDFR	EKQCADFDNM	PFRGKYYNWK
650	660	670	680	690	700	710	720
PYTGGGVKPC	ALNCLAEGYN	FYTERAPAVI	DGTQCNADSL	DICINGECKH	VGCDNILGSD	AREDRCRVCG	GDGSTCDAIE
730	740	750	760	770	780	790	800
GFFNDSLPRG	GYMEVVQIPR	GSVHIEVREV	AMSKNYIALK	SEGDDYYING	AWTIDWPRKF	DVAGTAFHYK	RPTDEPESLE
810	820	830	840	850	860	870	880
ALGPTSENLI	VMVLLQEQNL	GIRYKFNVPI	TRTGSGDNEV	GFTWNHQPWS	ECSATCAGGV	QRQEVVCKRL	DDNSIVQNNY
890	900	910	920	930	940	950	960
CDPDSKPPEN	QRACNTEPCP	PEWFIGDWLE	CSKTCDGGMR	TRAVLCIRKI	GPSEEETLDY	SGCLTHRPVE	KEPCNNQSCP
970	980	990	1000	1010	1020	1030	1040
PQWVALDWSE	CTPKCGPGFK	HRIVLCKSSD	LSKTFPAAQC	PEESKPPVRI	RCSLGRCPPP	RWVTGDWGQC	SAQCGLGQQM
1050	1060	1070	1080	1090	1100	1110	1120
RTVQCLSYTG	QASSDCLETV	RPPSMQQCES	KCDSTPISNT	EECKDVNKVA	YCPLVLKFKF	CSRAYFRQMC	CKTCQGH

Cmpd.	No. of Cmpds.	m/z meas.	Δ m/z [ppm]	z	Rt [min]	Score	P	Range	Sequence	Modification	Ratios
885	1	649.9950	65.11	3	39.5	10.1	1	584-601	R.HCDSPAPSGGGKYCLGER.K	Carbamidomethyl: 2, 14	
1669	1	1061.1845	59.73	3	50.6	11.4	0	833-862	R. TGSGDNEVGFTWNHQPWSECSA Q		
2995	1	964.4577	34.33	3	66.2	13.8	1	1017-1041	R. CPPPRWVTGDWGQCSAQCGLG[illegible] T	Carbamidomethyl: 14, 18; Oxidation: 24	QU:MU 1.34

Detailed Protein Report

Protein 305: protein-methionine sulfoxide oxidase MICAL3 isoform 1 [Homo sapiens]

**Accession:**	gi|209862789	**Score:**	35.1
**Database:**	refseq_human(refseq_human_20140103.fasta)	**MW [kDa]:**	224.2
**Database Date:**	2015-11-30	**pI:**	5.3
		**Sequence Coverage [%]:**	2.0
		**No. of unique Peptides:**	1

Quantitation

QU:MU	**Median:** 0.39	**CV:** 0.00 %	**No. of Peptides:** 1

Alias proteins:

Accession	Name	Description
gi|530426255	refseq_human (refseq_human_20140103.fasta)	PREDICTED: protein-methionine sulfoxide oxidase MICAL3 isoform X6 [Homo sapiens]

Detailed Protein Report

10	20	30	40	50	60	70	80
MEERKHETMN	PAHVLFDRFV	QATTCKGTLK	AFQELCDHLE	LKPKDYRSFY	HKLKSKLNYW	KAKALWAKLD	KRGSHKDYKK
90	100	110	120	130	140	150	160
GKACTNTKCL	IIGAGPCGLR	TAIDLSLLGA	KVVVIEKRDA	FSRNNVLHLW	PFTIHDLRGL	GAKKFYGKFC	AGAIDHISIR
170	180	190	200	210	220	230	240
QLQLILLKVA	LILGIEIHVN	VEFQGLIQPP	EDQENERIGW	RALVHPKTHP	VSEYEFEVII	GGDGRRNTLE	GFRRKEFRGK
250	260	270	280	290	300	310	320
LAIAITANFI	NRNTTAEAKV	EEISGVAFIF	NQKFFQELRE	ATGIDLENIV	YYKDDTHYFV	MTAKKQSLLD	KGVILHDYAD
330	340	350	360	370	380	390	400
TELLLSRENV	DQEALLSYAR	EAADFSTQQQ	LPSLDFAINH	YGQPDVAMFD	FTCMYASENA	ALVREQNGHQ	LLVALVGDSL
410	420	430	440	450	460	470	480
LEPFWPMGTG	IARGFLAAMD	SAWMVRSWSL	GTSPLEVLAE	RESIYRLLPQ	TTPENVSKNF	SQYSIDPVTR	YPNINVNFLR
490	500	510	520	530	540	550	560
PSQVRHLYDT	GETKDIHLEM	ESLVNSRTTP	KLTRNESVAR	SSKLLGWCQR	QTDGYAGVNV	TDLTMSWKSG	LALCAIIHRY
570	580	590	600	610	620	630	640
RPDLIDFDSL	DEQNVEKNNQ	LAFDIAEKEL	GISPIMTGKE	MASVGEPDKL	SMVMYLTQFY	EMFKDSLPSS	DTLDLNAEEK
650	660	670	680	690	700	710	720
AVLIASTRSP	ISFLSKLGQT	ISRKRSPKDK	KEKDLDGAGK	RRKTSQSEEE	EAPRGHRGER	PTLVSTLTDR	RMDVAVGNQN
730	740	750	760	770	780	790	800
KVKYMATQLL	AKFEENAPAQ	SIGIRRQGSM	KKEFPQNLGG	SDTCYFCQKR	VYVMERLSAE	GKFFHRSCFK	CEYCATTLRL
810	820	830	840	850	860	870	880
SAYAYDIEDG	KFYCKPHYCY	RLSGYAQRKR	PAVAPLSGKE	AKGPLQDGAT	TDANGRANAV	ASSTERTPGS	GVNGLEEPSI
890	900	910	920	930	940	950	960
AKRLRGTPER	IELENYRLSL	RQAEALQEVP	EETQAEHNLS	SVLDTGAEED	VASSSSESEM	EEEGEEEEEE	PRLPPSDLGG
970	980	990	1000	1010	1020	1030	1040
VPWKEAVRIH	ALLKGKSEEE	LEASKSFGPG	NEEEEEEEEE	YEEEEEEDYD	EEEEESSEAG	NQRLQQVMHA	ADPLEIQADV
1050	1060	1070	1080	1090	1100	1110	1120
HWTHIREREE	EERMAPASES	SASGAPLDEN	DLEEDVDSEP	AEIEGEAAED	GDPGDTGAEL	DDDQHWSDSP	SDADRELRLP
1130	1140	1150	1160	1170	1180	1190	1200
CPAEGEAELE	LRVSEDEEKL	PASPKHQERG	PSQATSPIRS	PQESALLFIP	VHSPSTEGPQ	LPPVPAATQE	KSPEERLFPE
1210	1220	1230	1240	1250	1260	1270	1280
PLLPKEKPKA	DAPSDLKAVH	SPIRSQPVTL	PEARTPVSPG	SPQPQPPVAA	STPPPSPLPI	CSQPQPSTEA	TVPSPTQSPI
1290	1300	1310	1320	1330	1340	1350	1360
RFQPAPAKTS	TPLAPLPVQS	QSDTKDRLGS	PLAVDEALRR	SDLVEEFWMK	SAEIRRSLGL	TPVDRSKGPE	PSFPTPAFRP
1370	1380	1390	1400	1410	1420	1430	1440
VSLKSYSVEK	SPQDEGLHLL	KPLSIPKRLG	LPKPEGEPLS	LPTPRSPSDR	ELRSAQEERR	ELSSSSGLGL	HGSSSNMKTL
1450	1460	1470	1480	1490	1500	1510	1520
GSQSFNTSDS	AMLTPPSSPP	PPPPPGEEPA	TLRRKLREAE	PNASVVPPPL	PATWMRPPRE	PAQPPREEVR	KSFVESVEEI
1530	1540	1550	1560	1570	1580	1590	1600
PFADDVEDTY	DDKTEDSSLQ	EKFFTPPSCW	PRPEKPRHPP	LAKENGRLPA	LEGTLQPQKR	GLPLVSAEAK	ELAEERMRAR
1610	1620	1630	1640	1650	1660	1670	1680
EKSVKSQALR	DAMARQLSRM	QQMELASGAP	RPRKASSAPS	QGKERRPDSP	TRPTLRGSEE	PTLKHEATSE	EVLSPPSDSG
1690	1700	1710	1720	1730	1740	1750	1760
GPDGSFTSSE	GSSGKSKKRS	SLFSPRRNKK	EKKSKGEGRP	PEKPSSNLLE	EAAAKPKSLW	KSVFSGYKKD	KKKKADDKSC
1770	1780	1790	1800	1810	1820	1830	1840
PSTPSSGATV	DSGKHRVLPV	VRAELQLRRQ	LSFSEDSDLS	SDDVLEKSSQ	KSRREPRTYT	EEELNAKLTR	RVQKAARRQA
1850	1860	1870	1880	1890	1900	1910	1920
KQEELKRLHR	AQIIQRQLQQ	VEERQRRLEE	RGVAVEKALR	GEAGMGKKDD	PKLMQEWFKL	VQEKNAMVRY	ESELMIFARE
1930	1940	1950	1960	1970	1980	1990	2000
LELEDRQSRL	QQELRERMAV	EDHLKTEEEL	SEEKQILNEM	LEVVEQRDSL	VALLEEQRLR	EREEDKDLEA	AMLSKGFSLN
2010							
WS							

Cmpd.	No. of Cmpds.	m/z meas.	Δ m/z [ppm]	z	Rt [min]	Score	P	Range	Sequence	Modification	Ratios	
1700	1	538.2445	-35.71	2	51.5	12.2	0	712-721	R.MDVAVGNQNK.V		QU:MU	0.39

Detailed Protein Report

Protein 306: NACHT, LRR and PYD domains-containing protein 2 isoform 3 [Homo sapiens]

**Accession:**	gi|291463280	**Score:**	35.1
**Database:**	refseq_human(refseq_human_20140103.fasta)	**MW [kDa]:**	117.8
**Database Date:**	2015-11-30	**pI:**	5.8
**Modification(s):**	Carbamidomethyl	**Sequence Coverage [%]:**	3.2
		**No. of unique Peptides:**	2

10	20	30	40	50	60	70	80
MVSSAQMGFN	LQALLEQLSQ	DELSKFKYLI	TTFSLAHELQ	KIPHKEMASL	QVFEKMHRMD	LSERAKDEVR	EAALKSFNKR
90	100	110	120	130	140	150	160
KPLSLGITRK	ERPPLDVDEM	LERFKTEAQA	FTETKGNVIC	LGKEVFKGKK	PDKDNRCRYI	LKTKFREMWK	SWPGDSKEVQ
170	180	190	200	210	220	230	240
VMAERYKMLI	PFSNPRVLPG	PFSYTVVLYG	PAGLGKTTLA	QKLMLDWAED	NLIHKFKYAF	YLSCRELSRL	GPCSFAELVF
250	260	270	280	290	300	310	320
RDWPELQDDI	PHILAQARKI	LFVIDGFDEL	GAAPGALIED	ICGDWEKKKP	VPVLLGSLLN	RVMLPKAALL	VTTRPRALRD
330	340	350	360	370	380	390	400
LRILAEEPIY	IRVEGFLEED	RRAYFLRHFG	DEDQAMRAFE	LMRSNAALFQ	LGSAPAVCWI	VCTTLKLQME	KGEDPVPTCL
410	420	430	440	450	460	470	480
TRTGLFLRFL	CSRFPQGAQL	RGALRTLSLL	AAQGLWAQTS	VLHREDLERL	GVQESDLRLF	LDGDILRQDR	VSKGCYSFIH
490	500	510	520	530	540	550	560
LSFQQFLTAL	FYTLEKEEEE	DRDGHTWDIG	DVQKLLSGVE	RLRNPDLIQA	GYYSFGLANE	KRAKELEATF	GCRMSPDIKQ
570	580	590	600	610	620	630	640
ELLRCDISCK	GGHSTVTDLQ	ELLGCLYESQ	EEELVKEVMA	QFKEISLHLN	AVDVVPSSFC	VKHCRNLQKM	SLQVIKENLP
650	660	670	680	690	700	710	720
ENVTASESDA	EVERSQDDQH	MLPFWTDLCS	IFGSNKDLMG	LAINDSFLSA	SLVRILCEQI	ASDTCHLQRV	VFKNISPADA
730	740	750	760	770	780	790	800
HRNLCLALRG	HKTVTYLTLQ	GNDQDDMFPA	LCEVLRHPEC	NLRYLGLVSC	SATTQQWADL	SLALEVNQSL	TCVNLSDNEL
810	820	830	840	850	860	870	880
LDEGAKLLYT	TLRHPKCFLQ	RLSLENCHLT	EANCKDLAAV	LVVSRELTHL	CLAKNPIGNT	GVKFLCEGLR	YPECKLQTLV
890	900	910	920	930	940	950	960
LWNCDITSDG	CCDLTKLLQE	KSSLLCLDLG	LNHIGVKGMK	FLCEALRKPL	CNLRCLWLWG	CSIPPFSCED	LCSALSCNQS
970	980	990	1000	1010	1020	1030	1040
LVTLDLGQNP	LGSSGVKMLF	ETLTCSSGTL	RTLRLKIDDF	NDELNKLLEE	IEEKNPQLII	DTEKHHPWAE	RPSSHDFMI

Cmpd.	No. of Cmpds.	m/z meas.	Δ m/z [ppm]	z	Rt [min]	Score	P	Range	Sequence	Modification
1922	1	893.8122	-134.43	2	53.9	11.2	0	695-709	R.ILCEQIASDTCHLQR.V	Carbamidomethyl: 3
223	1	636.6401	-68.84	3	32.8	23.9	1	846-863	R.ELTHLCLAKNPIGNTGVK.F	

BRUKER

Detailed Protein Report

Protein 307: **PREDICTED: bromodomain and WD repeat-containing protein 1 isoform X1 [Homo sapiens]**

**Accession:**	gi|530418950	**Score:**	35.1
**Database:**	refseq_human(refseq_human_20140103.fasta)	**MW [kDa]:**	253.3
**Database Date:**	2015-11-30	**pI:**	9.6
		**Sequence Coverage [%]:**	1.3
		**No. of unique Peptides:**	1

Quantitation

QU:MU	**Median:** 2.10	**CV:** 0.00 %	**No. of Peptides:** 1

Detailed Protein Report

10	20	30	40	50	60	70	80
MAEPSSARRP	VPLIESELYF	LIARYLSAGP	CRRAAQVLVQ	ELEQYQLLPK	RLDWEGNEHN	RSYEELVLSN	KHVAPDHLLQ
90	100	110	120	130	140	150	160
ICQRIGPMLD	KEIPPSISRV	TSLLGAGRQS	LLRTAKDCRH	TVWKGSAFAA	LHRGRPPEMP	VNYGSPPNLV	EIHRGKQLTG
170	180	190	200	210	220	230	240
CSTFSTAFPG	TMYQHIKMHR	RILGHLSAVY	CVAFDRTGHR	IFTGSDDCLV	KIWSTHNGRL	LSTLRGHSAE	ISDMAVNYEN
250	260	270	280	290	300	310	320
TMIAAGSCDK	IIRVWCLRTC	APVAVLQGHT	GSITSLQFSP	MAKGSQRYMV	STGADGTVCF	WQWDLESLKF	SPRPLKFTEK
330	340	350	360	370	380	390	400
PRPGVQMLCS	SFSVGGMFLA	TGSTDHVIRM	YFLGFEAPEK	IAELESHTDK	VDSIQFCNNG	DRFLSGSRDG	TARIWRFEQL
410	420	430	440	450	460	470	480
EWRSILLDMA	TRISGDLSSE	EERFMKPKVT	MIAWNQNDSI	VVTAVNDHVL	KVWNSYTGQL	LHNLMGHADE	VFVLETHPFD
490	500	510	520	530	540	550	560
SRIMLSAGHD	GSIFIWDITK	GTKMKHYFNM	IEGQGHGAVF	DCKFSQDGQH	FACTDSHGHL	LIFGFGCSKP	YEKIPDQMFF
570	580	590	600	610	620	630	640
HTDYRPLIRD	SNNYVLDEQT	QQAPHLMPPP	FLVDVDGNPH	PTKYQRLVPG	RENSADEHLI	PQLGYVATSD	GEVIEQIISL
650	660	670	680	690	700	710	720
QTNDNDERSP	ESSILDGMIR	QLQQQQDQRM	GADQDTIPRG	LSNGEETPRR	GFRRLSLDIQ	SPPNIGLRRS	GQVEGVRQMH
730	740	750	760	770	780	790	800
QNAPRSQIAT	ERDLQAWKRR	VVVPEVPLGI	FRKLEDFRLE	KGEEERNLYI	IGRKRKTLQL	SHKSDSVVLV	SQSRQRTCRR
810	820	830	840	850	860	870	880
KYPNYGRRNR	SWRELSSGNE	SSSSVRHETS	CDQSEGSGSS	EEDEWRSDRK	SESYSESSSD	SSSRYSDWTA	DAGINLQPPL
890	900	910	920	930	940	950	960
RTSCRRRITR	FCSSSEDEIS	TENLSPPKRR	RKRKKENKPK	KEDQELVKIV	GIRYEVGPPT	LCCLKLAFID	PATGKLMDKS
970	980	990	1000	1010	1020	1030	1040
FSIRYHDMPD	VIDFLVLRQF	YDEARQRNWQ	SCDRFRSIID	DAWWFGTVLS	QEPYQPQYPD	SHFQCYIVRW	DNTEIEKLSP
1050	1060	1070	1080	1090	1100	1110	1120
WDMEPIPDNV	DPPEELGASI	SVTTDELEKL	LYKPQAGEWG	QKSRDEECDR	IISGIDQLLN	LDIAAAFAGP	VDLCTYPKYC
1130	1140	1150	1160	1170	1180	1190	1200
TVVAYPTDLY	TIRMRLVNRF	YRRLSALVWE	VRYIEHNART	FNEPESVIAR	SAKKITDQLL	KFIKNQHCTN	ISELSNTSEN
1210	1220	1230	1240	1250	1260	1270	1280
DEQNAEDLDD	SDLPKTSSGR	RRVHDGKKSI	RATNYVESNW	KKQCKELVNL	IFQCEDSEPF	RQPVDLVEYP	DYRDIIDTPM
1290	1300	1310	1320	1330	1340	1350	1360
DFGTVRETLD	AGNYDSPLEF	CKDIRLIFSN	AKAYTPNKRS	KIYSMTLRLS	ALFEEKMKKI	SSDFKIGQKF	NEKLRRSQRF
1370	1380	1390	1400	1410	1420	1430	1440
KQRQNCKGDS	QPNKSIRNLK	PKRLKSQTKI	IPELVGSPTQ	STSSRTAYLG	THKTSAGISS	GVTSGDSSDS	AESSERRKRN
1450	1460	1470	1480	1490	1500	1510	1520
RPITNGSTLS	ESEVEDSLAT	SLSSSASSSS	EESKESSRAR	ESSSRSGLSR	SSNLRVTRTR	AAQRKTGPVS	LANGCGRKAT
1530	1540	1550	1560	1570	1580	1590	1600
RKRVYLSDSD	NNSLETGEIL	KARAGNNRKV	LRKCAAVAAN	KIKLMSDVEE	NSSSESVCSG	RKLPHRNASA	VARKKLLHNS
1610	1620	1630	1640	1650	1660	1670	1680
EDEQSLKSEI	EEEELKDENQ	LLPVSSSHTA	QSNVDESENR	DSESESDLRV	ARKNWHANGY	KSHTPAPSKT	KFLKIESSEE
1690	1700	1710	1720	1730	1740	1750	1760
DSKSHDSDHA	CNRTAGPSTS	VQKLKAESIS	EEADSEPGRS	GGRKYNTFHK	NASFFKKTKI	LSDSEDSESE	EQDREDGKCH
1770	1780	1790	1800	1810	1820	1830	1840
KMEMNPISGN	LNCDPIAMSQ	CSSDHGCETD	LDSDDDKIEK	PNNFMKDSAS	QDNGLSRKIS	RKRVCSSDSD	SSLQVVKKSS
1850	1860	1870	1880	1890	1900	1910	1920
KARTGLLRIT	RRCAATAANK	IKLMSDVEDV	SLENVHTRSK	NGRKKPLHLA	CTTAKKKLSD	CEGSVHCEVP	SEQYACEGKP
1930	1940	1950	1960	1970	1980	1990	2000
PDPDSEGSTK	VLSQALNGDS	DSEDMLNSEH	KHRHTNIHKI	DAPSKRKSSS	VTSSGEDSKS	HIPGSETDRT	FSSESTLAQK
2010	2020	2030	2040	2050	2060	2070	2080
ATAENNFEVE	LNYGLRRWNG	RRLRTYGKAP	FSKTKVIHDS	QETAEKEVKR	KRSHPELENV	KISETTGNSK	FRPDTSSKSS
2090	2100	2110	2120	2130	2140	2150	2160
DLGSVTESDI	DCTDNTKTKR	RKTKGKAKVV	RKEFVPRDRE	PNTKVRTCMH	NQKDAVQMPS	ETLKAKMVPE	KVPRRCATVA
2170	2180	2190	2200	2210	2220	2230	2240

Detailed Protein Report

Cmpd.	No. of Cmpds.	m/z meas.	Δ m/z [ppm]	z	Rt [min]	Score	P	Range	Sequence	Modification	Ratios	
127	1	724.8357	-58.74	2	31.3	12.0	1	762-773	K.GEEERNLYIIGR.K		QU:MU	2.10

Detailed Protein Report

Protein 308: TMF-regulated nuclear protein 1 [Homo sapiens]

Accession: gi|61966741
Database: refseq_human(refseq_human_20140103.fasta)
Database Date: 2015-11-30
Modification(s): Carbamidomethyl

Score: 35.0
MW [kDa]: 23.4
pI: 12.2
Sequence Coverage [%]: 14.1
No. of unique Peptides: 2

```
         10         20         30         40         50         60         70         80
MPGCRISACG PGAQEGTAEQ RSPPPPRDPM PSSQPPPPTP TLTPTPTPGQ SPPLPDAAGA SAGAAEDQEL QRWRQGASGI
         90        100        110        120        130        140        150        160
AGLAGPGGGS GAAAGAGGRA LELAEARRRL LEVEGRRRLV SELESRVLQL HRVFLAAELR LAHRAESLSR LSGGVAQAEL
        170        180        190        200        210        220        230
YLAAHGSRLK KGPRRGRRGR PPALLASALG LGGCVPWGAG RLRRGHGPEP DSPFRRSPPR GPASPQR
```

Cmpd.	No. of Cmpds.	m/z meas.	Δ m/z [ppm]	z	Rt [min]	Score	P	Range	Sequence	Modification
1625	2	682.3281	20.05	3	50.6	17.4	1	2-21	M.PGCRISACGPGAQEGTAEQR.S	Carbamidomethyl: 8
1148	1	676.2570	-106.06	2	44.5	17.6	1	205-216	R.GHGPEPDSPFRR.S	

Detailed Protein Report

Protein 309: **PREDICTED: GAS2-like protein 2 isoform X1 [Homo sapiens]**

**Accession:**	gi|578830659	**Score:**	35.0
**Database:**	refseq_human(refseq_human_20140103.fasta)	**MW [kDa]:**	92.5
**Database Date:**	2015-11-30	**pI:**	10.2
		**Sequence Coverage [%]:**	4.4
		**No. of unique Peptides:**	1

10	20	30	40	50	60	70	80
MSQPAGGRRK	PRTLGPPVCS	IRPFKSSEQY	LEAMKEDLAE	WLRDLYGLDI	DAANFLQVLE	TGLVLCQHAN	VVTDAALAFL
90	100	110	120	130	140	150	160
AEAPAQAQKI	PMPRVGVSCN	GAAQPGTFQA	RDNVSNFIQW	CRKEMGIQEV	LMFETEDLVL	RKNVKNVVLC	LLELGRRAWR
170	180	190	200	210	220	230	240
FGVAAPTLVQ	LEEEIEEEVR	RELALPPPDP	SPPAPPRRQP	CHFRNLDQMI	LRNHVMVRVG	GGWDTLGHYL	DKHDPCRCTS
250	260	270	280	290	300	310	320
LSHKPGSFLK	PPAPPVQHEV	RVQDGPSQTQ	PTMTISRSQS	PPPPVDWKTY	TSSDRRLRPP	TPSSPRPRRE	RGAGTGASRE
330	340	350	360	370	380	390	400
MAPFLRCQER	SLIPSWRQPT	AGDSPPSPQS	SSTQKGRDPQ	CTSSGKREER	YPPELPRGRI	PTSWVHEETD	SWGTDAGNPT
410	420	430	440	450	460	470	480
PQRLRAIEAT	TKGISARGPS	PLPRSFGPAE	CLGLRLPLRD	EAKGAFFQFR	EPESVRSPTP	VQGLTKIPIR	LPPARPPTPG
490	500	510	520	530	540	550	560
RSFPGATSGS	PRTELGRDPI	PLRAVTVDLA	GSTHGDCSVE	VRQEDQQLDI	QVMAEARESW	DLGLQEQEGR	YTPLPLGGNK
570	580	590	600	610	620	630	640
EQAIYCSLEE	EILGNMKLLE	VRSACPQGTR	SGVIPRSGVY	IPRLAGQWPE	PGGPYDKAIQ	ELAQGSPSLL	KVDLEAWKAA
650	660	670	680	690	700	710	720
PTGSPKPAVT	PGPGSLKGKL	GARQSGPRTK	ASLSAKGTHM	RKVPPQGGQD	CSASTVSASP	EAPTPSPLDP	NSDKAKACLS
730	740	750	760	770	780	790	800
KGRRTLRKPK	RVPSIYKLKL	RPRIRPRRDH	RPEKQPSRIP	RPLAYVFLGP	ARQPPKDRLL	RAVLGSKGGE	ASRVDGASVG
810	820	830	840	850			
EEEEEGKEEK	EPAAPLESSP	QPPEGLQPHW	LNQAPLPPEE	ESWV			

Cmpd.	No. of Cmpds.	m/z meas.	Δ m/z [ppm]	z	Rt [min]	Score	P	Range	Sequence	Modification
1978	1	643.7875	-102.56	2	54.6	14.5	2	671-682	K.ASLSAKGTHMRK.V	

Detailed Protein Report

Protein 310: **PREDICTED: putative uncharacterized protein CXorf49-like [Homo sapiens]**

**Accession:**	gi|410170282	**Score:**	34.9
**Database:**	refseq_human(refseq_human_20140103.fasta)	**MW [kDa]:**	53.2
**Database Date:**	2015-11-30	**pI:**	9.9
		**Sequence Coverage [%]:**	7.0
		**No. of unique Peptides:**	1

Quantitation

QU:MU	**Median:** 1.68	**CV:** 0.00 %	**No. of Peptides:**	1
WUP:QUP	**Median:** 2.35	**CV:** 0.00 %	**No. of Peptides:**	1

10	20	30	40	50	60	70	80
MSSTDEVSVC	RAGLGPEGGE	QAGAHTASPG	APRGDGHGRD	LNFGGQRSGK	GKGKGESGFT	DPESFSFESE	SELIEQGRVV
90	100	110	120	130	140	150	160
LWGQEGRPGT	PVDDQAGGGD	YSFYLADEPA	TIMPPSSVQG	HPSPEGATAK	GSTDIWADLE	VGPSWRGALS	PSPGEWQQAS
170	180	190	200	210	220	230	240
AGPLHLSVPG	PGPAWENPER	GSKSRLSFQV	DPQQPSAEGP	AGLNTDDSDS	ADESSDLPVI	RVIISTKEGS	QAKPGSPKKP
250	260	270	280	290	300	310	320
GDTCGRRSFH	RRESYLQVQG	PLLISPPRRL	TPVVERPAVG	ELDVPSLKKM	QSMVWGKRGV	RPSCSGAAVR	GPLPRGTLGR
330	340	350	360	370	380	390	400
KVAQEKKSLE	GAPELALRGA	FPAWGQRLSA	VPPDPASFPP	VSGVGLLGKS	VRPKEPKHSS	PGKKPAGRKT	RESQAAARED
410	420	430	440	450	460	470	480
NDPNRDEVPR	AQLPTQKPEL	ISLSVRRGEY	SSGDPNIRAP	QVLGTSQPSA	FTLRRLVPRC	HASSGDWQPP	VHPPRPERQQ
490	500	510					
QPPGAQGCPR	VMLCVAPRNR	GPH					

Cmpd.	No. of Cmpds.	m/z meas.	Δ m/z [ppm]	z	Rt [min]	Score	P	Range	Sequence	Modification	Ratios
674	1	512.2810	-49.78	2	38.0	12.2	1	311-320	R.GPLPRGTLGR.K		WUP:QUP 2.35<br>QU:MU 1.68

Detailed Protein Report

Protein 311: **PREDICTED: tau-tubulin kinase 2 isoform X5 [Homo sapiens]**

**Accession:**	gi|578826647	**Score:**	34.9
**Database:**	refseq_human(refseq_human_20140103.fasta)	**MW [kDa]:**	129.8
**Database Date:**	2015-11-30	**pI:**	6.5
**Modification(s):**	Oxidation	**Sequence Coverage [%]:**	3.0
		**No. of unique Peptides:**	2

10	20	30	40	50	60	70	80
MLPGKDHVCR	FIGCGRNDRF	NYVVMQLQGR	NLADLRRSQS	RGTFTISTTL	RLGRQILESI	ESIHSVGFLH	RDIKPSNFAM
**90**	**100**	**110**	**120**	**130**	**140**	**150**	**160**
GRFPSTCRKC	YMLDFGLARQ	FTNSCGDVRP	PRAVAGFRGT	VRYASINAHR	NREMGRHDDL	WSLFYMLVEF	VVGQLPWRKI
**170**	**180**	**190**	**200**	**210**	**220**	**230**	**240**
KDKEQVGSIK	ERYDHRLMLK	HLPPEFSIFL	DHISSLDYFT	KPDYQLLTSV	FDNSIKTFGV	IESDPFDWEK	TGNDGSLTTT
**250**	**260**	**270**	**280**	**290**	**300**	**310**	**320**
TTSTTPQLHT	RLTPAAIGIA	NATPIPGDLL	RENTDEVFPD	EQLSDGENGI	PVGVSPDKLP	GSLGHPRPQE	KDVWEEMDAN
**330**	**340**	**350**	**360**	**370**	**380**	**390**	**400**
KNKIKLGICK	AATEEENSHG	QANGLLNAPS	LGSPIRVRSE	ITQPDRDIPL	VRKLRSIHSF	ELEKRLTLEP	KPDTDKFLET
**410**	**420**	**430**	**440**	**450**	**460**	**470**	**480**
CLEKMQKDTS	AGKESILPAL	LHKPCVPAVS	RTDHIWHYDE	EYLPDASKPA	SANTPEQADG	GGSNGFIAVN	LSSCKQEIDS
**490**	**500**	**510**	**520**	**530**	**540**	**550**	**560**
KEWVIVDKEQ	DLQDFRTNEA	VGHKTTGSPS	DEEPEVLQVL	EASPQDEKLQ	LGPWAENDHL	KKETSGVVLA	LSAEGPPTAA
**570**	**580**	**590**	**600**	**610**	**620**	**630**	**640**
SEQYTDRLEL	QPGAASQFIA	ATPTSLMEAQ	AEGPLTAITI	PRPSVASTQS	TSGSFHCGQQ	PEKKDLQPME	PTVELYSPRE
**650**	**660**	**670**	**680**	**690**	**700**	**710**	**720**
NFSGLVVTEG	EPPSGGSRTD	LGLQIDHIGH	DMLPNIRESN	KSQDLGPKEL	PDHNRLVVRE	FENLPGETEE	KSILLESDNE
**730**	**740**	**750**	**760**	**770**	**780**	**790**	**800**
DEKLSRGQHC	IEISSLPGDL	VIVEKDHSAT	TEPLDVTKTQ	TFSVVPNQDK	NNEIMKLLTV	GTSEISSRDI	DPHVEGQIGQ
**810**	**820**	**830**	**840**	**850**	**860**	**870**	**880**
VAEMQKNKIS	KDDDIMSEDL	PGHQGDLSTF	LHQEGKREKI	TPRNGELFHC	VSENEHGAPT	RKDMVRSSFV	TRHSRIPVLA
**890**	**900**	**910**	**920**	**930**	**940**	**950**	**960**
QEIDSTLESS	SPVSAKEKLL	QKKAYQPDLV	KLLVEKRQFK	SFLGDLSSAS	DKLLEEKLAT	VPAPFCEEEV	LTPFSRLTVD
**970**	**980**	**990**	**1000**	**1010**	**1020**	**1030**	**1040**
SHLSRSAEDS	FLSPIISQSR	KSKIPRPVSW	VNTDQVNSST	SSQFFPRPPP	GKPPTRPGVE	ARLRRYKVLG	SSNSDSDLFS
**1050**	**1060**	**1070**	**1080**	**1090**	**1100**	**1110**	**1120**
RLAQILQNGS	QKPRSTTQCK	SPGSPHNPKT	PPKSPVVPRR	SPSASPRSSS	LPRTSSSSPS	RAGRPHHDQR	SSSPHLGRSK
**1130**	**1140**	**1150**	**1160**	**1170**	**1180**		
SPPSHSGSSS	SRRSCQQEHC	KPSKNGLKGS	GSLHHHSAST	KTPQGKSKPA	SKLSR		

Cmpd.	No. of Cmpds.	m/z meas.	Δ m/z [ppm]	z	Rt [min]	Score	P	Range	Sequence	Modification
1491	1	626.6231	-220.31	2	48.3	12.6	0	312-321	K.DVWEEMDANK.N	Oxidation: 6
2024	1	694.3465	21.44	2	55.7	10.8	1	1119-1132	R.SKSPPSHSGSSSSR.R	

Detailed Protein Report

Protein 312: myeloperoxidase precursor [Homo sapiens]

Accession: gi|4557759
Database: refseq_human(refseq_human_20140103.fasta)
Database Date: 2015-11-30

Score: 34.9
MW [kDa]: 83.8
pI: 10.1
Sequence Coverage [%]: 3.0
No. of unique Peptides: 1

10	20	30	40	50	60	70	80
MGVPFFSSLR	CMVDLGPCWA	GGLTAEMKLL	LALAGLLAIL	ATPQPSEGAA	PAVLGEVDTS	LVLSSMEEAK	QLVDKAYKER
**90**	**100**	**110**	**120**	**130**	**140**	**150**	**160**
RESIKQRLRS	GSASPMELLS	YFKQPVAATR	TAVRAADYLH	VALDLLERKL	RSLWRRPFNV	TDVLTPAQLN	VLSKSSGCAY
**170**	**180**	**190**	**200**	**210**	**220**	**230**	**240**
QDVGVTCPEQ	DKYRTITGMC	NNRRSPTLGA	SNRAFVRWLP	AEYEDGFSLP	YGWTPGVKRN	GFPVALARAV	SNEIVRFPTD
**250**	**260**	**270**	**280**	**290**	**300**	**310**	**320**
QLTPDQERSL	MFMQWGQLLD	HDLDFTPEPA	ARASFVTGVN	CETSCVQQPP	CFPLKIPPND	PRIKNQADCI	PFFRSCPACP
**330**	**340**	**350**	**360**	**370**	**380**	**390**	**400**
GSNITIRNQI	NALTSFVDAS	MVYGSEEPLA	RNLRNMSNQL	GLLAVNQRFQ	DNGRALLPFD	NLHDDPCLLT	NRSARIPCFL
**410**	**420**	**430**	**440**	**450**	**460**	**470**	**480**
AGDTRSSEMP	ELTSMHTLLL	REHNRLATEL	KSLNPRWDGE	RLYQEARKIV	GAMVQIITYR	DYLPLVLGPT	AMRKYLPTYR
**490**	**500**	**510**	**520**	**530**	**540**	**550**	**560**
SYNDSVDPRI	ANVFTNAFRY	GHTLIQPFMF	RLDNRYQPME	PNPRVPLSRV	FFASWRVVLE	GGIDPILRGL	MATPAKLNRQ
**570**	**580**	**590**	**600**	**610**	**620**	**630**	**640**
NQIAVDEIRE	RLFEQVMRIG	LDLPALNMQR	SRDHGLPGYN	AWRRFCGLPQ	PETVGQLGTV	LRNLKLARKL	MEQYGTPNNI
**650**	**660**	**670**	**680**	**690**	**700**	**710**	**720**
DIWMGGVSEP	LKRKGRVGPL	LACIIGTQFR	KLRDGDRFWW	ENEGVFSMQQ	RQALAQISLP	RIICDNTGIT	TVSKNNIFMS
**730**	**740**	**750**					
NSYPRDFVNC	STLPALNLAS	WREAS					

Cmpd.	No. of Cmpds.	m/z meas.	Δ m/z [ppm]	z	Rt [min]	Score	P	Range	Sequence	Modification
1893	1	640.7817	-156.88	2	54.0	15.3	0	537-548	R.VVLEGGIDPILR.G	

Detailed Protein Report

Protein 313: serine protease 58 precursor [Homo sapiens]

**Accession:**	gi|48255915	**Score:**	34.9
**Database:**	refseq_human(refseq_human_20140103.fasta)	**MW [kDa]:**	27.1
**Database Date:**	2015-11-30	**pI:**	6.4
**Modification(s):**	Carbamidomethyl, Oxidation	**Sequence Coverage [%]:**	20.7
		**No. of unique Peptides:**	2

```
        10         20         30         40         50         60         70         80
MKFILLWALL NLTVALAFNP DYTVSSTPPY LVYLKSDYLP CAGVLIHPLW VITAAHCNLP KLRVILGVTI PADSNEKHLQ
        90        100        110        120        130        140        150        160
VIGYEKMIHH PHFSVTSIDH DIMLIKLKTE AELNDYVKLA NLPYQTISEN TMCSVSTWSY NVCDIYKEPD SLQTVNISVI
       170        180        190        200        210        220        230        240
SKPQCRDAYK TYNITENMLC VGIVPGRRQP CKEVSAAPAI CNGMLQGILS FADGCVLRAD VGIYAKIFYY IPWIENVIQN
       250
N
```

Cmpd.	No. of Cmpds.	m/z meas.	Δ m/z [ppm]	z	Rt [min]	Score	P	Range	Sequence	Modification
1245	1	1072.1826	-52.32	3	43.9	19.6	1	36-63	K.SDYLPCAGVLIHPLWVITAAHCNLPKLR.V	Carbamidomethyl: 6, 22
2776	1	862.7428	-25.62	3	65.9	15.2	2	167-188	R.DAYKTYNITENMLCVGIVPGRR.Q	Carbamidomethyl: 14; Oxidation: 12

Detailed Protein Report

Protein 314: **PREDICTED: receptor-type tyrosine-protein phosphatase mu isoform X4 [Homo sapiens]**

**Accession:**	gi|578832170	**Score:**	34.8
**Database:**	refseq_human(refseq_human_20140103.fasta)	**MW [kDa]:**	161.1
**Database Date:**	2015-11-30	**pI:**	6.4
**Modification(s):**	Carbamidomethyl	**Sequence Coverage [%]:**	2.6
		**No. of unique Peptides:**	1

10	20	30	40	50	60	70	80
MPSGSFMLVN	ASGRPEGQRA	HLLLPQLKEN	DTHCIDFHYF	VSSKSNSPPG	LLNVYVKVNN	GPLGNPIWNI	SGDPTRTWNR
90	100	110	120	130	140	150	160
AELAISTFWP	NFYQVIFEVI	TSGHQGYLAI	DEVKVLGHPC	TRTPHFLRIQ	NVEVNAGQFA	TFQCSAIGRT	VAGDRLWLQG
170	180	190	200	210	220	230	240
IDVRDAPLKE	IKVTSSRRFI	ASFNVVNTTK	RDAGKYRCMI	RTEGGVGISN	YAELVVKEPP	VPIAPPQLAS	VGATYLWIQL
250	260	270	280	290	300	310	320
NANSINGDGP	IVAREVEYCT	ASGSWNDRQP	VDSTSYKIGH	LDPDTEYEIS	VLLTRPGEGG	TGSPGPALRT	RTKCADPMRG
330	340	350	360	370	380	390	400
PRKLEVVEVK	SRQITIRWEP	FGYNVTRCHS	YNLTVHYCYQ	VGGQEQVREE	VSWDTENSHP	QHTITNLSPY	TNVSVKLILM
410	420	430	440	450	460	470	480
NPEGRKESQE	LIVQTDEDLP	GAVPTESIQG	STFEEKIFLQ	WREPTQTYGV	ITLYEITYKA	VSSFDPEIDL	SNQSGRVSKL
490	500	510	520	530	540	550	560
GNETHFLFFG	LYPGTTYSFT	IRASTAKGFG	PPATNQFTTK	ISAPSMPAYE	LETPLNQTDN	TVTVMLKPAH	SRGAPVSVYQ
570	580	590	600	610	620	630	640
IVVEEERPRR	TKKTTEILKC	YPVPIHFQNA	SLLNSQYYFA	AEFPADSLQA	AQPFTIGDNK	TYNGYWNTPL	LPYKSYRIYF
650	660	670	680	690	700	710	720
QAASRANGET	KIDCVQVATK	AAIIVTQLTT	PYIRIAPAAG	DGQLTGAATP	KPVPEPEKQT	DHTVKIAGVI	AGILLFVIIF
730	740	750	760	770	780	790	800
LGVVLVMKKR	KLAKKRKETM	SSTRQEMTVM	VNSMDKSYAE	QGTNCDEAFS	FMDTHNLNGR	SVSSPSSFTM	KTNTLSTSVP
810	820	830	840	850	860	870	880
NSYYPDETHT	MASDTSSLVQ	SHTYKKREPA	DVPYQTGQLH	PAIRVADLLQ	HITQMKCAEG	YGFKEEYESF	FEGQSAPWDS
890	900	910	920	930	940	950	960
AKKDENRMKN	RYGNIIAYDH	SRVRLQTIEG	DTNSDYINGN	YIDGYHRPNH	YIATQGPMQE	TIYDFWRMVW	HENTASIIMV
970	980	990	1000	1010	1020	1030	1040
TNLVEVGRVK	CCKYWPDDTE	IYKDIKVTLI	ETELLAEYVI	RTFAVEKGGA	GGEANCSPSR	EVSQRGVHEI	REIRQFHFTG
1050	1060	1070	1080	1090	1100	1110	1120
WPDHGVPYHA	TGLLGFVRQV	KSKSPPSAGP	LVVHCSAGAG	RTGCFIVIDI	MLDMAEREGV	VDIYNCVREL	RSRRVNMVQT
1130	1140	1150	1160	1170	1180	1190	1200
EEQYVFIHDA	ILEACLCGDT	SVPASQVRSL	YYDMNKLDPQ	TNSSQIKEEF	RTLNMVTPTL	RVEDCSIALL	PRNHEKNRCM
1210	1220	1230	1240	1250	1260	1270	1280
DILPPDRCLP	FLITIDGESS	NYINAALMDS	YKQPSAFIVT	QHPLPNTVKD	FWRLVLDYHC	TSVVMLNDVD	PAQLCPQYWP
1290	1300	1310	1320	1330	1340	1350	1360
ENGVHRHGPI	QVEFVSADLE	EDIISRIFRI	YNAARPQDGY	RMVQQFQFLG	WPMYRDTPVS	KRSFLKLIRQ	VDKWQEEYNG
1370	1380	1390	1400	1410	1420	1430	1440
GEGRTVVHCL	NGGGRSGTFC	AISIVCEMLR	HQRTVDVFHA	VKTLRNNKPN	MVDLLDQYKF	CYEVALEYLN	SG

Cmpd.	No. of Cmpds.	m/z meas.	Δ m/z [ppm]	z	Rt [min]	Score	P	Range	Sequence	Modification
1436	1	927.4440	-10.35	3	47.6	22.6	1	1365-1390	R.TVVHCLNGGGRSGTFCAISIVCEMLR.H	Carbamidomethyl: 22

BRUKER

Detailed Protein Report

Protein 315: uncharacterized protein KIAA0408 [Homo sapiens]

**Accession:**	gi|59806363	**Score:**	34.8
**Database:**	refseq_human(refseq_human_20140103.fasta)	**MW [kDa]:**	79.1
**Database Date:**	2015-11-30	**pI:**	9.6
**Modification(s):**	Carbamidomethyl, Oxidation	**Sequence Coverage [%]:**	5.6
		**No. of unique Peptides:**	2

Quantitation

QU:MU	**Median:**	1.78	**CV:**	0.00 %	**No. of Peptides:**	1
WUP:QUP	**Median:**	0.50	**CV:**	0.00 %	**No. of Peptides:**	1

10	20	30	40	50	60	70	80
MDLHKQWENT	ETNWHKEKME	LLDQFDNERK	EWESQWKIMQ	KKIEELCREV	KLWRKININE	SAKIIDLYHE	KTIPEKVIES
**90**	**100**	**110**	**120**	**130**	**140**	**150**	**160**
SPNYPDLGQS	EFIRTNHKDG	LRKENKREQS	LVSGGNQMCK	EQKATKKSKV	GFLDPLATDN	QKECEAWPDL	RTSEEDSKSC
**170**	**180**	**190**	**200**	**210**	**220**	**230**	**240**
SGALSTALEE	LAKVSEELCS	FQEEIRKRSN	HRRMKSDSFL	QEMPNVTNIP	HGDPMINNDQ	CILPISLEKE	KQKNRKNLSC
**250**	**260**	**270**	**280**	**290**	**300**	**310**	**320**
TNVLQSNSTK	KCGIDTIDLK	RNETPPVPPP	RSTSRNFPSS	DSEQAYERWK	ERLDHNSWVP	HEGRSKRNYN	PHFPLRQQEM
**330**	**340**	**350**	**360**	**370**	**380**	**390**	**400**
SMLYPNEGKT	SKDGIIFSSL	VPEVKIDSKP	PSNEDVGLSM	WSCDIGIGAK	RSPSTSWFQK	TCSTPSNPKY	EMVIPDHPAK
**410**	**420**	**430**	**440**	**450**	**460**	**470**	**480**
SHPDLHVSND	CSSSVAESSS	PLRNFSCGFE	RTTRNEKLAA	KTDEFNRTVF	RTDRNCQAIQ	QNHSCSKSSE	DLKPCDTSST
**490**	**500**	**510**	**520**	**530**	**540**	**550**	**560**
HTGSISQSND	VSGIWKTNAH	MPVPMENVPD	NPTKKSTTGL	VRQMQGHLSP	RSYRNMLHEH	DWRPSNLSGR	PRSADPRSNY
**570**	**580**	**590**	**600**	**610**	**620**	**630**	**640**
GVVEKLLKTY	ETATESALQN	SKCFQDNWTK	CNSDVSGGAT	LSQHLEMLQM	EQQFQQKTAV	WGGQEVKQGI	DPKKITEESM
**650**	**660**	**670**	**680**	**690**	**700**		
SVNASHGKGF	SRPARPANRR	LPSRWASRSP	SAPPALRRTT	HNYTISLRSE	ALMV		

Cmpd.	No. of Cmpds.	m/z meas.	Δ m/z [ppm]	z	Rt [min]	Score	P	Range	Sequence	Modification	Ratios
2738	3	841.8972	10.76	2	62.7	13.5	1	17-29	K.EKMELLDQFDNER.K	Oxidation: 3	WUP:QUP 0.50<br>QU:MU 1.78
1324	1	727.1757	-204.08	2	46.2	10.1	0	108-120	R.EQSLVSGGNQMCK.E	Carbamidomethyl: 12; Oxidation: 11	

Detailed Protein Report

Protein 316: PREDICTED: zinc finger protein 554 isoform X2 [Homo sapiens]

**Accession:**	gi|578832959	**Score:**	34.8
**Database:**	refseq_human(refseq_human_20140103.fasta)	**MW [kDa]:**	43.7
**Database Date:**	2015-11-30	**pI:**	9.8
		**Sequence Coverage [%]:**	5.7
		**No. of unique Peptides:**	1

Quantitation

WUP:QUP **Median:** 2.44 **CV:** 0.00 % **No. of Peptides:** 1

10	20	30	40	50	60	70	80
MTVLRNQDST	YKKVALQEEP	ASGINMIKLI	REDGGWKQLE	DSHEDPQGLL	SQKASLHVVA	VPQEKATAWH	GFGENGNLSP
**90**	**100**	**110**	**120**	**130**	**140**	**150**	**160**
ALVLSQGSSK	GNHLCGSELD	ITSLASDSVL	NHHQLGYADR	RPCESNECGN	AIRQNSHFIQ	HGGKMFVYLE	NGQSLNHGMA
**170**	**180**	**190**	**200**	**210**	**220**	**230**	**240**
LTIHNKINTA	EKPFECHQCG	KVFNRRHSLS	EHQRIHTGEK	PYECQECGRA	FTHSSTLTRH	LRTHTGEKPY	GCGECGKAFN
**250**	**260**	**270**	**280**	**290**	**300**	**310**	**320**
RISSLTQHQR	IHTGEKPYKC	EDCGKSFCQS	SYLILHKRTH	TGEKPYECSE	CGKAFSDRSS	LNQHERTHTG	ENPYECKQCG
**330**	**340**	**350**	**360**	**370**	**380**	**390**	
RAFSQRSSLV	RHERTHTGEK	PYRCQECGKA	FSQSSSLVTH	QKTHSSQKTY	KIIDCGKAFY	QNRHLIGY	

Cmpd.	No. of Cmpds.	m/z meas.	Δ m/z [ppm]	z	Rt [min]	Score	P	Range	Sequence	Modification	Ratios
1743	1	560.7234	-120.44	2	52.1	14.2	0	210-219	R.AFTHSSTLTR.H		WUP:QUP 2.44

Detailed Protein Report

Protein 317: procollagen galactosyltransferase 1 precursor [Homo sapiens]

**Accession:**	gi|31377697	**Score:**	34.8
**Database:**	refseq_human(refseq_human_20140103.fasta)	**MW [kDa]:**	71.6
**Database Date:**	2015-11-30	**pI:**	7.0
		**Sequence Coverage [%]:**	4.2
		**No. of unique Peptides:**	1

10	20	30	40	50	60	70	80
MAAAPRAGRR	RGQPLLALLL	LLLAPLPPGA	PPGADAYFPE	ERWSPESPLQ	APRVLIALLA	RNAAHALPTT	LGALERLRHP
**90**	**100**	**110**	**120**	**130**	**140**	**150**	**160**
RERTALWVAT	DHNMDNTSTV	LREWLVAVKS	LYHSVEWRPA	EEPRSYPDEE	GPKHWSDSRY	EHVMKLRQAA	LKSARDMWAD
**170**	**180**	**190**	**200**	**210**	**220**	**230**	**240**
YILFVDADNL	ILNPDTLSLL	IAENKTVVAP	MLDSRAAYSN	FWCGMTSQGY	YKRTPAYIPI	RKRDRRGCFA	VPMVHSTFLI
**250**	**260**	**270**	**280**	**290**	**300**	**310**	**320**
DLRKAASRNL	AFYPPHPDYT	WSFDDIIVFA	FSCKQAEVQM	YVCNKEEYGF	LPVPLRAHST	LQDEAESFMH	VQLEVMVKHP
**330**	**340**	**350**	**360**	**370**	**380**	**390**	**400**
PAEPSRFISA	PTKTPDKMGF	DEVFMINLRR	RQDRRERMLR	ALQAQEIECR	LVEAVDGKAM	NTSQVEALGI	QMLPGYRDPY
**410**	**420**	**430**	**440**	**450**	**460**	**470**	**480**
HGRPLTKGEL	GCFLSHYNIW	KEVVDRGLQK	SLVFEDDLRF	EIFFKRRLMN	LMRDVEREGL	DWDLIYVGRK	RMQVEHPEKA
**490**	**500**	**510**	**520**	**530**	**540**	**550**	**560**
VPRVRNLVEA	DYSYWTLAYV	ISLQGARKLL	AAEPLSKMLP	VDEFLPVMFD	KHPVSEYKAH	FSLRNLHAFS	VEPLLIYPTH
**570**	**580**	**590**	**600**	**610**	**620**	**630**	
YTGDDGYVSD	TETSVVWNNE	HVKTDWDRAK	SQKMREQQAL	SREAKNSDVL	QSPLDSAARD	EL	

Cmpd.	No. of Cmpds.	m/z meas.	Δ m/z [ppm]	z	Rt [min]	Score	P	Range	Sequence	Modification
718	2	450.6336	-247.78	2	37.4	13.7	1	1-9	-.MAAAPRAGR.R	

Detailed Protein Report

Protein 318: metallothionein-1H [Homo sapiens]

Accession: gi|10835085
Database: refseq_human(refseq_human_20140103.fasta)
Database Date: 2015-11-30
Modification(s): Carbamidomethyl

Score: 34.7
MW [kDa]: 6.0
pI: 10.6
Sequence Coverage [%]: 49.2
No. of unique Peptides: 2

```
         10         20         30         40         50         60         70
MDPNCSCEAG GSCACAGSCK CKKCKCTSCK KSCCSCCPLG CAKCAQGCIC KGASEKCSCC A
```

Cmpd.	No. of Cmpds.	m/z meas.	Δ m/z [ppm]	z	Rt [min]	Score	P	Range	Sequence	Modification
2498	1	673.3379	138.11	2	61.8	10.5	0	32-43	K.SCCSCCPLGCAK.C	Carbamidomethyl: 5, 6, 10
1117	1	1019.4619	75.11	2	43.5	10.7	1	32-51	K.SCCSCCPLGCAKCAQGCICK.G	Carbamidomethyl: 2

Detailed Protein Report

Protein 319: **PREDICTED: zinc finger SWIM domain-containing protein 3 isoform X1 [Homo sapiens]**

**Accession:**	gi|578835782	**Score:**	34.6
**Database:**	refseq_human(refseq_human_20140103.fasta)	**MW [kDa]:**	78.3
**Database Date:**	2015-11-30	**pI:**	7.9
		**Sequence Coverage [%]:**	4.5
		**No. of unique Peptides:**	2

10	20	30	40	50	60	70	80
MKTRLERQAE	TTSGHPSLAS	LYYWTAPALP	GPELEMAPPP	HCAHNRYVQV	KFVCIRTQSN	RKRTREADMC	PAYLLLRYNE
**90**	**100**	**110**	**120**	**130**	**140**	**150**	**160**
RLDRLFISEL	NTQHIHGDSK	VASPGGDTTG	KSQKTMCLQR	LQPVQPTTKK	DLDTAEKSLV	EPSFCLDKVQ	VSSKPEQEGI
**170**	**180**	**190**	**200**	**210**	**220**	**230**	**240**
TPSDLAKIAK	VMKNFLKVDE	GSMASFSVGD	SQHLDRLSFQ	SSKMTDLFIR	FPENLLLHRV	ENTQGHILYA	FLVENKERES
**250**	**260**	**270**	**280**	**290**	**300**	**310**	**320**
RVVHFAVLKA	ETVTSVAKML	SIFTEFNSDW	PKVKVVFVDP	SFHYRAILQE	IFPAARILLS	IYHTTRLLEK	KLHRSSANPS
**330**	**340**	**350**	**360**	**370**	**380**	**390**	**400**
FKRLMKEALR	EAVFVTSEAS	LKNLCQMSQA	VLDEDLFNFL	QAHWFTCELL	WYMHVRKGLL	ACNTYMDSLD	IVTSKVSSLF
**410**	**420**	**430**	**440**	**450**	**460**	**470**	**480**
REQQSLLDCI	LCFVDYIDFF	NTKGLKNLPT	PPPKLKRARP	ASMPLKSKKA	FGICGESLTS	LPAEETKPDA	QQVQVQQQSQ
**490**	**500**	**510**	**520**	**530**	**540**	**550**	**560**
VPPSQVGMLD	TLHQSGSELA	YKLCHNEWEV	VQNSTHLVDM	AGSSVDVQLL	EDSHQVSKDG	CSCSCSFQQW	YHLPCRHILA
**570**	**580**	**590**	**600**	**610**	**620**	**630**	**640**
LLHTSQQPVG	EAMVCRRWQK	KYQYLLGPNG	ELQDRGMVPN	TGQPEKQGRN	DMIQDLSREL	ANLLMQTEGP	ELEERYSTLR
**650**	**660**	**670**	**680**	**690**	**700**		
KIVDIWAGPS	QPSELFQQPG	DFKDVGRLPF	LWGKQEEGEG	FPPATAVMHY			

Cmpd.	No. of Cmpds.	m/z meas.	Δ m/z [ppm]	z	Rt [min]	Score	P	Range	Sequence	Modification
2524	1	1044.0930	32.96	2	62.2	15.4	2	112-129	K.SQKTMCLQRLQPVQPTTK.K	
2543	2	701.8414	-144.22	2	61.8	19.2	2	424-436	K.GLKNLPTPPPKLK.R	

Detailed Protein Report

Protein 320: splicing factor, proline- and glutamine-rich [Homo sapiens]

**Accession:**	gi|4826998	**Score:**	34.5
**Database:**	refseq_human(refseq_human_20140103.fasta)	**MW [kDa]:**	76.1
**Database Date:**	2015-11-30	**pI:**	9.9
**Modification(s):**	Oxidation	**Sequence Coverage [%]:**	6.9
		**No. of unique Peptides:**	3

Quantitation

QU:MU	**Median:**	1.43	**CV:**	42.02 %	**No. of Peptides:**	2
WUP:QUP	**Median:**	0.84	**CV:**	25.78 %	**No. of Peptides:**	2

Alias proteins:

Accession	Name	Description
gi|530363144	refseq_human (refseq_human_20140103.fasta)	PREDICTED: splicing factor, proline- and glutamine-rich isoform X3 [Homo sapiens]
gi|530363142	refseq_human (refseq_human_20140103.fasta)	PREDICTED: splicing factor, proline- and glutamine-rich isoform X2 [Homo sapiens]
gi|530363140	refseq_human (refseq_human_20140103.fasta)	PREDICTED: splicing factor, proline- and glutamine-rich isoform X1 [Homo sapiens]

```
        10         20         30         40         50         60         70         80
MSRDRFRSRG GGGGGFHRRG GGGGRGGLHD FRSPPPGMGL NQNRGPMGPG PGQSGPKPPI PPPPPHQQQQ QPPPQQPPPQ
        90        100        110        120        130        140        150        160
QPPPHQPPPH PQPHQQQQPP PPPQDSSKPV VAQGPGPAPG VGSAPPASSS APPATPPTSG APPGSGPGPT PTPPPAVTSA
       170        180        190        200        210        220        230        240
PPGAPPPTPP SSGVPTTPPQ AGGPPPPPAA VPGPGPGPKQ GPGPGGPKGG KMPGGPKPGG GPGLSTPGGH PKPPHRGGGE
       250        260        270        280        290        300        310        320
PRGGRQHHPP YHQQHHQGPP PGGPGGRSEE KISDSEGFKA NLSLLRRPGE KTYTQRCRLF VGNLPADITE DEFKRLFAKY
       330        340        350        360        370        380        390        400
GEPGEVFINK GKGFGFIKLE SRALAEIAKA ELDDTPMRGR QLRVRFATHA AALSVRNLSP YVSNELLEEA FSQFGPIERA
       410        420        430        440        450        460        470        480
VVIVDDRGRS TGKGIVEFAS KPAARKAFER CSEGVFLLTT TPRPVIVEPL EQLDDEDGLP EKLAQKNPMY QKERETPPRF
       490        500        510        520        530        540        550        560
AQHGTFEYEY SQRWKSLDEM EKQQREQVEK NMKDAKDKLE SEMEDAYHEH QANLLRQDLM RRQEELRRME ELHNQEMQKR
       570        580        590        600        610        620        630        640
KEMQLRQEEE RRRREEEMMI RQREMEEQMR RQREESYSRM GYMDPRERDM RMGGGGAMNM GDPYGSGGQK FPPLGGGGGI
       650        660        670        680        690        700        710
GYEANPGVPP ATMSGSMMGS DMRTERFGQG GAGPVGGQGP RGMGPGTPAG YGRGREEYEG PNKKPRF
```

Cmpd.	No. of Cmpds.	m/z meas.	Δ m/z [ppm]	z	Rt [min]	Score	P	Range	Sequence	Modification	Ratios
406	1	678.2016	-174.41	2	34.7	11.5	1	268-279	R.SEEKISDSEGFK.A		WUP:QUP 1.08<br>QU:MU 0.96
6	1	736.0027	52.34	3	29.0	13.0	1	609-630	R.<br>DMRMGGGGAMNMGDPYGSGGQ<br>F	Oxidation: 4, 12	
284	1	671.3405	5.90	2	33.5	10.1	0	667-681	R.FGQGGAGPVGGQGPR.G		WUP:QUP 0.65<br>QU:MU 2.15

Detailed Protein Report

Protein 321: zinc finger protein 189 isoform 2 [Homo sapiens]

Accession: gi|37574608
Database: refseq_human(refseq_human_20140103.fasta)
Database Date: 2015-11-30

Score: 34.5
MW [kDa]: 68.1
pI: 10.2
Sequence Coverage [%]: 4.8
No. of unique Peptides: 1

Alias proteins:

Accession	Name	Description
gi|578817874	refseq_human (refseq_human_20140103.fasta)	PREDICTED: zinc finger protein 189 isoform X2 [Homo sapiens]
gi|578817872	refseq_human (refseq_human_20140103.fasta)	PREDICTED: zinc finger protein 189 isoform X1 [Homo sapiens]

10	20	30	40	50	60	70	80
MMENYGNLVS	LDVLNRDKDE	EPTVKQEIEE	IEEEVEPQGV	IVTRIKSEID	QDPMGRETFE	LVGRLDKQRG	IFLWEIPRES
**90**	**100**	**110**	**120**	**130**	**140**	**150**	**160**
LTQEQRMFRE	NTNIIRKRPN	SEEKCHKCEE	CGKGFVRKAH	FIQHQRVHTG	EKPFQCNECG	KSFSRSSFVI	EHQRIHTGER
**170**	**180**	**190**	**200**	**210**	**220**	**230**	**240**
PYECNYCGKT	FSVSSTLIRH	QRIHTGERPY	QCNQCKQSFS	QRRSLVKHQR	IHTGEKPHKC	SDCGKAFSWK	SHLIEHQRTH
**250**	**260**	**270**	**280**	**290**	**300**	**310**	**320**
TGEKPYHCTK	CKKSFSRNSL	LVEHQRIHTG	ERPHKCGECG	KAFRLSTYLI	QHQKIHTGEK	PFLCIECGKS	FSRSSFLIEH
**330**	**340**	**350**	**360**	**370**	**380**	**390**	**400**
QRIHTGERPY	QCKECGKSFS	QLCNLTRHQR	IHTGDKPHKC	EECGKAFSRS	SGLIQHQRIH	TREKTYPYNE	TKESFDPNCS
**410**	**420**	**430**	**440**	**450**	**460**	**470**	**480**
LVIQQEVYPK	EKSYKCDECG	KTFSVSAHLV	QHQRIHTGEK	PYLCTVCGKS	FSRSSFLIEH	QRIHTGERPY	LCRQCGKSFS
**490**	**500**	**510**	**520**	**530**	**540**	**550**	**560**
QLCNLIRHQG	VHTGNKPHKC	DECGKAFSRN	SGLIQHQRIH	TGEKPYKCEK	CDKSFSQQRS	LVNHQKIHAE	VKTQETHECD
**570**	**580**	**590**					
ACGEAFNCRI	SLIQHQKLHT	AWMQ					

Cmpd.	No. of Cmpds.	m/z meas.	Δ m/z [ppm]	z	Rt [min]	Score	P	Range	Sequence	Modification
1729	1	1051.5453	-9.31	2	51.4	14.5	1	70-86	R.GIFLWEIPRESLTQEQR.M	

Detailed Protein Report

Protein 322: G patch domain and KOW motifs-containing protein [Homo sapiens]

**Accession:**	gi|15811782	**Score:**	34.5
**Database:**	refseq_human(refseq_human_20140103.fasta)	**MW [kDa]:**	52.2
**Database Date:**	2015-11-30	**pI:**	5.8
		**Sequence Coverage [%]:**	6.1
		**No. of unique Peptides:**	1

Quantitation

QU:MU	**Median:**	0.76	**CV:**	0.00 %	**No. of Peptides:**	1
WUP:QUP	**Median:**	1.52	**CV:**	0.00 %	**No. of Peptides:**	1

10	20	30	40	50	60	70	80
MADSKEGVLP	LTAASTAPIS	FGFTRTSARR	RLADSGDGAG	PSPEEKDFLK	TVEGRELQSV	KPQEAPKELV	IPLIQNGHRR
**90**	**100**	**110**	**120**	**130**	**140**	**150**	**160**
QPPARPPGPS	TDTGALADGV	VSQAVKELIA	ESKKSLEERE	NAGVDPTLAI	PMIQKGCTPS	GEGADSEPRA	ETVPEEANYE
**170**	**180**	**190**	**200**	**210**	**220**	**230**	**240**
AVPVEAYGLA	MLRGMGWKPG	EGIGRTFNQV	VKPRVNSLRP	KGLGLGANLT	EAQALTPTGP	SRMPRPDEEQ	EKDKEDQPQG
**250**	**260**	**270**	**280**	**290**	**300**	**310**	**320**
LVPGGAVVVL	SGPHRGLYGK	VEGLDPDNVR	AMVRLAVGSR	VVTVSEYYLR	PVSQQEFDKN	TLDLRQQNGT	ASSRKTLWNQ
**330**	**340**	**350**	**360**	**370**	**380**	**390**	**400**
ELYIQQDNSE	RKRKHLPDRQ	DGPAAKSEKA	APRSQHWLHR	DLRVRFVDNM	YKGGQYYNTK	MIIEDVLSPD	TCVCRTDEGR
**410**	**420**	**430**	**440**	**450**	**460**	**470**	**480**
VLEGLREDML	ETLVPKAEGD	RVMVVLGPQT	GRVGHLLSRD	RARSRALVQL	PRENQVVELH	YDAICQYMGP	SDTDDD

Cmpd.	No. of Cmpds.	m/z meas.	Δ m/z [ppm]	z	Rt [min]	Score	P	Range	Sequence	Modification	Ratios
1518	1	816.3814	-50.98	2	49.2	16.7	1	256-270	R.GLYGKVEGLDPDNVR.A		QU:MU 0.76<br>WUP:QUP 1.52

Detailed Protein Report

Protein 323: **PREDICTED: ankyrin repeat domain-containing protein 30B isoform X1 [Homo sapiens]**

**Accession:**	gi|578832133	**Score:**	34.4
**Database:**	refseq_human(refseq_human_20140103.fasta)	**MW [kDa]:**	171.3
**Database Date:**	2015-11-30	**pI:**	5.9
		**Sequence Coverage [%]:**	2.1
		**No. of unique Peptides:**	1

10	20	30	40	50	60	70	80
MKRLLAAAGK	GVRGPEPPNP	FSERVYTEKD	YGTIYFGDLG	KIHTAASRGQ	VQKLEKMTVG	KKPVNLNKRD	MKKRTALHWA
**90**	**100**	**110**	**120**	**130**	**140**	**150**	**160**
CVNGHAEVVT	FLVDRKCQLN	VLDGEGRTPL	MKALQCEREA	CANILIDAGA	DLNYVDVYGN	TALHYAVYSE	NLLMVATLLS
**170**	**180**	**190**	**200**	**210**	**220**	**230**	**240**
YGAVIEVQNK	ASLTPLLLAI	QKRSKQTVEF	LLTKNANANA	FNESKCTALM	LAICEGSSEI	VGMLLQQNVD	VFAEDIHGIT
**250**	**260**	**270**	**280**	**290**	**300**	**310**	**320**
AERYAAACGV	NYIHQQLLEH	IRKLPKNPQN	TNPEGTSTGT	PDEAAPLAER	TPDTAESLLE	KTPDEAARLV	EGTSAKIQCL
**330**	**340**	**350**	**360**	**370**	**380**	**390**	**400**
GKATSGKFEQ	STEETPRKIL	RPTKETSEKF	SWPAKERSRK	ITWEEKETSV	KTECVAGVTP	NKTEVLEKGT	SNMIACPTKE
**410**	**420**	**430**	**440**	**450**	**460**	**470**	**480**
TSTKASTNVD	VSSVEPIFSL	FGTRTIENSQ	CTKVEEDFNL	ATKIISKSAA	QNYTCLPDAT	YQKDIKTINH	KIEDQMFPSE
**490**	**500**	**510**	**520**	**530**	**540**	**550**	**560**
SKREEDEEYS	WDSGSLFESS	AKTQVCIPES	MYQKVMEINR	EVEELPEKPS	AFKPAVEMQK	TVPNKAFELK	NEQTLRAAQM
**570**	**580**	**590**	**600**	**610**	**620**	**630**	**640**
FPSESKQKDD	EENSWDSESP	CETVSQKDVY	LPKATHQKEF	DTLSGKLEES	PVKDGLLKPT	CGRKVSLPNK	ALELKDRETF
**650**	**660**	**670**	**680**	**690**	**700**	**710**	**720**
KAESPDKDGL	LKPTCGRKVS	LPNKALELKD	RETLKAESPD	NDGLLKPTCG	RKVSLPNKAL	ELKDRETFKA	AQMFPSESKQ
**730**	**740**	**750**	**760**	**770**	**780**	**790**	**800**
KDDEENSWDF	ESFLETLLQN	DVCLPKATHQ	KEFDTLSGKL	EESPDKDGLL	KPTCGRKVSL	PNKALELKDR	ETLKAESPDK
**810**	**820**	**830**	**840**	**850**	**860**	**870**	**880**
DGLLKPTCVR	KVSLPNKALE	LKDRETLKAA	QMFPSESKQK	DDEENSWDFE	SFLEALLQND	GCLPKATHQK	EFDTLSGKLE
**890**	**900**	**910**	**920**	**930**	**940**	**950**	**960**
ESPDKDGLLK	PTCGMKISLP	NKALELKDRE	TFKAEDVSSV	ESTFSLFGKP	TTENSQSTKV	EEDFNLTTKE	GATKTVTGQQ
**970**	**980**	**990**	**1000**	**1010**	**1020**	**1030**	**1040**
ERDIGIIERA	PQDQTNKMPT	SELGRKEDTK	STSDSEIISV	SDTQNYECLP	EATYQKEIKT	TNGKIEESPE	KPSHFEPATE
**1050**	**1060**	**1070**	**1080**	**1090**	**1100**	**1110**	**1120**
MQNSVPNKGL	EWKNKQTLRA	DSTTLSKILD	ALPSCERGRE	LKKDNCEQIT	AKMEQTKNKF	CVLQKELSEA	KEIKSQLENQ
**1130**	**1140**	**1150**	**1160**	**1170**	**1180**	**1190**	**1200**
KAKWEQELCS	VRLTLNQEEE	KRRNVDILKE	KIRPEEQLRK	KLEVKQQLEQ	TLRIQDIELK	SVTSNLNQVS	HTHESENDLF
**1210**	**1220**	**1230**	**1240**	**1250**	**1260**	**1270**	**1280**
HENCMLKKEI	AMLKLEVATL	KHQHQVKENK	YFEDIKILQE	KNAELQMTLK	LKQKTVTKRA	SQYREQLKVL	TAENTMLTSK
**1290**	**1300**	**1310**	**1320**	**1330**	**1340**	**1350**	**1360**
LKEKQDKEIL	ETEIESHHPR	LASALQDHDQ	SVTSRKNQEL	AFHSAGDAPL	QGIMNVDVSN	TIYNNEVLHQ	PLYEAQRKSK
**1370**	**1380**	**1390**	**1400**	**1410**	**1420**	**1430**	**1440**
SPKINLNYAG	DDLRENALVS	EHAQRDRCET	QCQMKKAEHM	YQNEQDNVDK	HTEQQESLEQ	KLFQLESKNR	WLRQQLVYAH
**1450**	**1460**	**1470**	**1480**	**1490**	**1500**	**1510**	**1520**
KKVNKSKVTI	NIQFPEMKMQ	RHLNEKNEEV	FNYGNHLKER	IDQYEKEKAE	REVSIKKYKY	FSNFLKESGL	G

Cmpd.	No. of Cmpds.	m/z meas.	Δ m/z [ppm]	z	Rt [min]	Score	P	Range	Sequence	Modification
1677	1	619.9559	-45.33	3	51.2	22.1	1	760-776	K.LEESPDKDGLLKPTCGR.K	

Detailed Protein Report

Protein 324: proteoglycan 4 isoform D precursor [Homo sapiens]

**Accession:**	gi|189181724	**Score:**	34.4
**Database:**	refseq_human(refseq_human_20140103.fasta)	**MW [kDa]:**	136.5
**Database Date:**	2015-11-30	**pI:**	10.1
		**Sequence Coverage [%]:**	1.4
		**No. of unique Peptides:**	2

Quantitation

QU:MU	**Median:**	2.52	**CV:**	0.00 %	**No. of Peptides:**	1
WUP:QUP	**Median:**	0.51	**CV:**	0.00 %	**No. of Peptides:**	1

10	20	30	40	50	60	70	80
MAWKTLPIYL	LLLLSVFVIQ	QVSSQELSCK	GRCFESFERG	RECDCDAQCK	KYDKCCPDYE	SFCAEVKDNK	KNRTKKKPTP
90	100	110	120	130	140	150	160
KPPVVDEAGS	GLDNGDFKVT	TPDTSTTQHN	KVSTSPKITT	AKPINPRPSL	PPNSDTSKET	SLTVNKETTV	ETKETTTTNK
170	180	190	200	210	220	230	240
QTSTDGKEKT	TSAKETQSIE	KTSAKDLAPT	SKVLAKPTPK	AETTTKGPAL	TTPKEPTPTT	PKEPASTTPK	EPTPTTIKSA
250	260	270	280	290	300	310	320
PTTPKEPAPT	TTKSAPTTPK	EPAPTTTKEP	APTTPKEPAP	TTTKEPAPTT	TKSAPTTPKE	PAPTTPKKPA	PTTPKEPAPT
330	340	350	360	370	380	390	400
TPKEPTPTTP	KEPAPTTKEP	APTTPKEPAP	TAPKKPAPTT	PKEPAPTTPK	EPAPTTTKEP	SPTTPKEPAP	TTTKSAPTTT
410	420	430	440	450	460	470	480
KEPAPTTTKS	APTTPKEPSP	TTTKEPAPTT	PKEPAPTTPK	KPAPTTPKEP	APTTPKEPAP	TTTKKPAPTT	PKEPAPTTPK
490	500	510	520	530	540	550	560
ETAPTTPKKL	TPTTPEKLAP	TTPEKPAPTT	PEELAPTTPE	EPTPTTPEEP	APTTPKAAAP	NTPKEPAPTT	PKEPAPTTPK
570	580	590	600	610	620	630	640
EPAPTTPKET	APTTPKGTAP	TTLKEPAPTT	PKKPAPKELA	PTTTKEPTST	TCDKPAPTTP	KGTAPTTPKE	PAPTTPKEPA
650	660	670	680	690	700	710	720
PTTPKGTAPT	TLKEPAPTTP	KKPAPKELAP	TTTKGPTSTT	SDKPAPTTPK	ETAPTTPKEP	APTTPKKPAP	TTPETPPPTT
730	740	750	760	770	780	790	800
SEVSTPTTTK	EPTTIHKSPD	ESTPELSAEP	TPKALENSPK	EPGVPTTKTP	AATKPEMTTT	AKDKTTERDL	RTTPETTTAA
810	820	830	840	850	860	870	880
PKMTKETATT	TEKTTESKIT	ATTTQVTSTT	TQDTTPFKIT	TLKTTTLAPK	VTTTKKTITT	TEIMNKPEET	AKPKDRATNS
890	900	910	920	930	940	950	960
KATTPKPQKP	TKAPKKPTST	KKPKTMPRVR	KPKTTPTPRK	MTSTMPELNP	TSRIAEAMLQ	TTTRPNQTPN	SKLVEVNPKS
970	980	990	1000	1010	1020	1030	1040
EDAGGAEGET	PHMLLRPHVF	MPEVTPDMDY	LPRVPNQGII	INPMLSDETN	ICNGKPVDGL	TTLRNGTLVA	FRGHYFWMLS
1050	1060	1070	1080	1090	1100	1110	1120
PFSPPSPARR	ITEVWGIPSP	IDTVFTRCNC	EGKTFFFKDS	QYWRFTNDIK	DAGYPKPIFK	GFGGLTGQIV	AALSTAKYKN
1130	1140	1150	1160	1170	1180	1190	1200
WPESVYFFKR	GGSIQQYIYK	QEPVQKCPGR	RPALNYPVYG	ETTQVRRRRF	ERAIGPSQTH	TIRIQYSPAR	LAYQDKGVLH
1210	1220	1230	1240	1250	1260	1270	1280
NEVKVSILWR	GLPNVVTSAI	SLPNIRKPDG	YDYYAFSKDQ	YYNIDVPSRT	ARAITTRSGQ	TLSKVWYNCP	

Cmpd.	No. of Cmpds.	m/z meas.	Δ m/z [ppm]	z	Rt [min]	Score	P	Range	Sequence	Modification	Ratios
2237	1	994.8741	-142.64	2	57.8	23.9	2	785-802	K.TTERDLRTTPETTTAAPK.M		WUP:QUP 0.51<br>QU:MU 2.52
299	2	559.1629	-227.93	2	33.4	10.6	0	792-802	R.TTPETTTAAPK.M		

Detailed Protein Report

Protein 325: **SWI/SNF-related matrix-associated actin-dependent regulator of chromatin subfamily A member 5 [Homo sapiens]**

**Accession:**	gi|325651836	**Score:**	34.4
**Database:**	refseq_human(refseq_human_20140103.fasta)	**MW [kDa]:**	121.8
**Database Date:**	2015-11-30	**pI:**	9.0
**Modification(s):**	Oxidation	**Sequence Coverage [%]:**	2.1
		**No. of unique Peptides:**	1

10	20	30	40	50	60	70	80
MSSAAEPPPP	PPPESAPSKP	AASIASGGSN	SSNKGGPEGV	AAQAVASAAS	AGPADAEMEE	IFDDASPGKQ	KEIQEPDPTY
**90**	**100**	**110**	**120**	**130**	**140**	**150**	**160**
EEKMQTDRAN	RFEYLLKQTE	LFAHFIQPAA	QKTPTSPLKM	KPGRPRIKKD	EKQNLLSVGD	YRHRRTEQEE	DEELLTESSK
**170**	**180**	**190**	**200**	**210**	**220**	**230**	**240**
ATNVCTRFED	SPSYVKWGKL	RDYQVRGLNW	LISLYENGIN	GILADEMGLG	KTLQTISLLG	YMKHYRNIPG	PHMVLVPKST
**250**	**260**	**270**	**280**	**290**	**300**	**310**	**320**
LHNWMSEFKR	WVPTLRSVCL	IGDKEQRAAF	VRDVLLPGEW	DVCVTSYEML	IKEKSVFKKF	NWRYLVIDEA	HRIKNEKSKL
**330**	**340**	**350**	**360**	**370**	**380**	**390**	**400**
SEIVREFKTT	NRLLLTGTPL	QNNLHELWSL	LNFLLPDVFN	SADDFDSWFD	TNNCLGDQKL	VERLHMVLRP	FLLRRIKADV
**410**	**420**	**430**	**440**	**450**	**460**	**470**	**480**
EKSLPPKKEV	KIYVGLSKMQ	REWYTRILMK	DIDILNSAGK	MDKMRLLNIL	MQLRKCCNHP	YLFDGAEPGP	PYTTDMHLVT
**490**	**500**	**510**	**520**	**530**	**540**	**550**	**560**
NSGKMVVLDK	LLPKLKEQGS	RVLIFSQMTR	VLDILEDYCM	WRNYEYCRLD	GQTPHDERQD	SINAYNEPNS	TKFVFMLSTR
**570**	**580**	**590**	**600**	**610**	**620**	**630**	**640**
AGGLGINLAT	ADVVILYDSD	WNPQVDLQAM	DRAHRIGQTK	TVRVFRFITD	NTVEERIVER	AEMKLRLDSI	VIQQGRLVDQ
**650**	**660**	**670**	**680**	**690**	**700**	**710**	**720**
NLNKIGKDEM	LQMIRHGATH	VFASKESEIT	DEDIDGILER	GAKKTAEMNE	KLSKMGESSL	RNFTMDTESS	VYNFEGEDYR
**730**	**740**	**750**	**760**	**770**	**780**	**790**	**800**
EKQKIAFTEW	IEPPKRERKA	NYAVDAYFRE	ALRVSEPKAP	KAPRPPKQPN	VQDFQFFPPR	LFELLEKEIL	FYRKTIGYKV
**810**	**820**	**830**	**840**	**850**	**860**	**870**	**880**
PRNPELPNAA	QAQKEEQLKI	DEAESLNDEE	LEEKEKLLTQ	GFTNWNKRDF	NQFIKANEKW	GRDDIENIAR	EVEGKTPEEV
**890**	**900**	**910**	**920**	**930**	**940**	**950**	**960**
IEYSAVFWER	CNELQDIEKI	MAQIERGEAR	IQRRISIKKA	LDTKIGRYKA	PFHQLRISYG	TNKGKNYTEE	EDRFLICMLH
**970**	**980**	**990**	**1000**	**1010**	**1020**	**1030**	**1040**
KLGFDKENVY	DELRQCIRNS	PQFRFDWFLK	SRTAMELQRR	CNTLITLIER	ENMELEEKEK	AEKKKRGPKP	STQKRKMDGA
**1050**	**1060**						
PDGRGRKKKL	KL						

Cmpd.	No. of Cmpds.	m/z meas.	Δ m/z [ppm]	z	Rt [min]	Score	P	Range	Sequence	Modification
971	1	706.9594	14.52	2	42.2	20.7	2	485-496	K.MVVLDKLLPKLK.E	Oxidation: 1

Detailed Protein Report

Protein 326: PREDICTED: chromodomain-helicase-DNA-binding protein 9 isoform X9 [Homo sapiens]

**Accession:**	gi|530424378	**Score:**	34.4
**Database:**	refseq_human(refseq_human_20140103.fasta)	**MW [kDa]:**	269.5
**Database Date:**	2015-11-30	**pI:**	8.8
**Modification(s):**	Oxidation	**Sequence Coverage [%]:**	1.6
		**No. of unique Peptides:**	2

Quantitation

WUP:QUP	**Median:** 4.90	**CV:** 0.00 %	**No. of Peptides:** 1

Detailed Protein Report

10	20	30	40	50	60	70	80
MSEKKQRKKV	ESESKQEKAN	RIISEAIAKA	KERGERNIPR	VMSPENFPTA	SVEGKEEKKG	RRMKSKPKDK	DSKKTKTCSK
90	100	110	120	130	140	150	160
LKEKTKIGKL	IITLGKKQKR	KNESSDEISD	AEQMPQHTLK	DQDSQKRRSN	RQIKRKKYAE	DIEGKQSEEE	VKGSMKIKKN
170	180	190	200	210	220	230	240
SAPLPGEQPL	QLFVENPSEE	DAAIVDKILS	SRTVKKEISP	GVMIDTEEFF	VKYKNYSYLH	CEWATEEQLL	KDKRIQQKIK
250	260	270	280	290	300	310	320
RFKLRQAQRA	HFFADMEEEP	FNPDYVEVDR	VLEVSFCEDK	DTGEPVIYYL	VKWCSLPYED	STWELKEDVD	LAKIEEFEQL
330	340	350	360	370	380	390	400
QASRPDTRRL	DRPPSNIWKK	IDQSRDYKNG	NQLREYQLEG	LNWLLFNWYN	RRNCILADEM	GLGKTIQSIT	FLYEILLTGI
410	420	430	440	450	460	470	480
RGPFLIIAPL	STIANWEREF	RTWTDINVVV	YHGSLISRQM	IQQYEMYFRD	SQGRIIRGAY	RFQAIITTFE	MILGGCGELN
490	500	510	520	530	540	550	560
AIEWRCVIID	EAHRLKNKNC	KLLEGLKLMN	LEHKVLLTGT	PLQNTVEELF	SLLHFLEPLR	FPSESTFMQE	FGDLKTEEQV
570	580	590	600	610	620	630	640
QKLQAILKPM	MLRRLKEDVE	KKLAPKEETI	IEVELTNIQK	KYYRAILEKN	FSFLSKGAGQ	TNVPNLVNTM	MELRKCCNHP
650	660	670	680	690	700	710	720
YLIKGAEEKI	LGEFRDTYNP	AASDFHLQAM	IQSAGKLVLI	DKLLPKMKAG	GHKVLIFSQM	VRCLDILEDY	LIHKRYLYER
730	740	750	760	770	780	790	800
IDGRVRGNLR	QAAIDRFSKP	DSDRFVFLLC	TRAGGLGINL	TAADTCIIFD	SDWNPQNDLQ	AQARCHRIGQ	NKAVKVYRLV
810	820	830	840	850	860	870	880
TRNSYEREMF	DRASLKLGLD	KAVLQSMSGR	ESNVGGIQQL	SKKEIEDLLR	RGAYGAIMEE	EDEGSKFCEE	DIDQILLRRT
890	900	910	920	930	940	950	960
KTITIESEGR	GSTFAKASFV	ASGNRTDISL	DDPNFWQKWA	KKAEIDIEAI	SGRNSLVIDT	PRIRKQTRPF	SATKDELAEL
970	980	990	1000	1010	1020	1030	1040
SEAESEGDEK	PKLRRPCDRS	NGYGRTECFR	VEKNLLVYGW	GRWREILSHG	RFKRQLNEHD	VEIICRALLA	YCLVHYRGDE
1050	1060	1070	1080	1090	1100	1110	1120
KIKGFIWDLI	TPTEDGQTRE	LQNHLGLSAP	VPRGRKGKKV	KTQTSSFDIQ	KAEWLRKYNP	EQLLQDEGYK	KHIKHHCNKV
1130	1140	1150	1160	1170	1180	1190	1200
LLRVRMLYYL	KQEVIGNECQ	KVFDGVDASD	IDVWVPEPDH	SEVPAEWWDF	DADKSLLIGV	FKHGYEKYNT	IRADPALCFL
1210	1220	1230	1240	1250	1260	1270	1280
ERVGKPDEKA	VAAEQRANDY	MDGDVEDPEY	KPAPAIFKDD	IEDDVSSPGD	LVIADGDGQL	MEGDKVYWPT	QSALTTRLRR
1290	1300	1310	1320	1330	1340	1350	1360
LITAYQRTNK	NRQIQQIQPT	FSVPTSVMQP	IYEEATLNPK	MAAKIERQQR	WTRREEADFY	RVVSTFGVVF	DPDRGQFDWT
1370	1380	1390	1400	1410	1420	1430	1440
KFRAMARLHK	KTDDSLEKYL	YAFMSMCRRV	CRLPSKEELV	DPNIFIQPIT	EERASRTLYR	IELLRKVREQ	ALRHPQLFER
1450	1460	1470	1480	1490	1500	1510	1520
LKLCHPNPDL	PVWWECGPHD	RDLLIGAAKH	GVSRTDYHIL	RDPELSFMAA	QRNYSQSKMA	HSRTSTPLLQ	QYQVALSASP
1530	1540	1550	1560	1570	1580	1590	1600
LTSLPRLLDA	KGIILEEMKV	KSENLKEEPQ	SSEEESMSSV	ETRTLIKSEP	VSPKNGVLPQ	ATGDQKSGGK	CETDRRMVAA
1610	1620	1630	1640	1650	1660	1670	1680
RTEPLTPNPA	SKKPRVHKRG	SESSSDSDSD	SERSSCSSRS	SSSSSSSSCS	HSRSGSSSSS	SSSCSSASSS	SSSSTSSSSS
1690	1700	1710	1720	1730	1740	1750	1760
SSSSSSEESD	SDEEEAQKRA	ESTTHMKAYD	EESVASLSTT	QDETQDSFQM	NNGTPESAYI	LQGGYMLAAS	YWPKDRVMIN
1770	1780	1790	1800	1810	1820	1830	1840
RLDSICQTVL	KGKWPSARRS	YDANTVASFY	TTKLLDSPGA	ATEYSDPSVP	TPPGAGVKEE	HDQSTQMSKV	KKHVREKEFT
1850	1860	1870	1880	1890	1900	1910	1920
VKIKDEGGLK	LTFQKQGLAQ	KRPFDGEDGA	LGQQQYLTRL	RELQSASETS	LVNFPKSIPV	SGTSIQPTLG	ANGVILDNQP
1930	1940	1950	1960	1970	1980	1990	2000
IVKKRRGRRK	NVEGVDIFFF	NRNKPPNHVS	LGLTSSQIST	GINPALSYTQ	PQGIPDTESP	VPVINLKDGT	RLAGDDAPKR
2010	2020	2030	2040	2050	2060	2070	2080
KDLEKWLKEH	PGYVEDLGAF	IPRMQLHEGR	PKQKRHRCRN	PNKLDVNSLT	GEERVQLINR	RNARKVGGAF	APPLKDLCRF
2090	2100	2110	2120	2130	2140	2150	2160
LKENSEYGVA	PEWGDVVKQS	GFLPESMYER	ILTGPVVREE	VSRRGRRPKS	GIAKATAAAA	AASATSVSGN	PLLANGLLPG
2170	2180	2190	2200	2210	2220	2230	2240

Detailed Protein Report

Cmpd.	No. of Cmpds.	m/z meas.	Δ m/z [ppm]	z	Rt [min]	Score	P	Range	Sequence	Modification	Ratios
1309	1	863.9074	-143.00	2	46.0	10.4	2	563-576	K.LQAILKPMMLRRLK.E	Oxidation: 8	
2019	1	719.3122	-83.44	2	55.6	13.7	0	1342-1354	R.VVSTFGVVFDPDR.G		WUP:QUP 4.90

Detailed Protein Report

Protein 327: glutamine--tRNA ligase isoform b [Homo sapiens]

**Accession:**	gi|441478305	**Score:**	34.3
**Database:**	refseq_human(refseq_human_20140103.fasta)	**MW [kDa]:**	86.5
**Database Date:**	2015-11-30	**pI:**	6.6
**Modification(s):**	Oxidation	**Sequence Coverage [%]:**	5.6
		**No. of unique Peptides:**	1

10	20	30	40	50	60	70	80
MAALDSLSLF	TSLGLSEQKA	RETLKNSALS	AQLREAATQA	QQTLGSTIDK	ATGILLYGLA	SRLRDTRRLS	FLVSYIATAL
90	100	110	120	130	140	150	160
EYVRSHPLDP	IDTVDFEREC	GVGVIVTPEQ	IEEAVEAAIN	RHRPQLLVER	YHFNMGLLMG	EARAVLKWAD	GKMIKNEVDM
170	180	190	200	210	220	230	240
QVLHLLGPKL	EADLEKKFKV	AKARLEETDR	RTAKDVVENG	ETADQTLSLM	EQLRGEALKF	HKPGENYKTP	GYVVTPHTMN
250	260	270	280	290	300	310	320
LLKQHLEITG	GQVRTRFPPE	PNGILHIGHA	KAINFNFGYA	KANNGICFLR	FDDTNPEKEE	AKFFTAICDM	VAWLGYTPYK
330	340	350	360	370	380	390	400
VTYASDYFDQ	LYAWAVELIR	RGLAYVCHQR	GEELKGHNTL	PSPWRDRPME	ESLLLFEAMR	KGKFSEGEAT	LRMKLVMEDG
410	420	430	440	450	460	470	480
KMDPVAYRVK	YTPHHRTGDK	WCIYPTYDYT	HCLCDSIEHI	THSLCTKEFQ	ARRSSYFWLC	NALDVYCPVQ	WEYGRLNLHY
490	500	510	520	530	540	550	560
AVVSKRKILQ	LVATGAVRDW	DDPRLFTLTA	LRRRGFPPEA	INNFCARVGV	TVAQTTMEPH	LLEACVRDVL	NDTAPRAMAV
570	580	590	600	610	620	630	640
LESLRVIITN	FPAAKSLDIQ	VPNFPADETK	GFHQVPFAPI	VFIERTDFKE	EPEPGFKRLA	WGQPVGLRHT	GYVIELQHVV
650	660	670	680	690	700	710	720
KGPSGCVESL	EVTCRRADAG	EKPKAFIHWV	SQPLMCEVRL	YERLFQHKNP	EDPTEVPGGF	LSDLNLASLH	VVDAALVDCS
730	740	750	760	770			
VALAKPFDKF	QFERLGYFSV	DPDSHQGKLV	FNRTVTLKED	PGKV			

Cmpd.	No. of Cmpds.	m/z meas.	Δ m/z [ppm]	z	Rt [min]	Score	P	Range	Sequence	Modification
2780	1	841.6618	-115.85	3	65.2	10.5	2	131-152	R.YHFNMGLLMGEARAVLKWADGK.M	Oxidation: 9

Detailed Protein Report

Protein 328: sodium-driven chloride bicarbonate exchanger isoform 1 [Homo sapiens]

**Accession:**	gi|295821221	**Score:**	34.3
**Database:**	refseq_human(refseq_human_20140103.fasta)	**MW [kDa]:**	125.9
**Database Date:**	2015-11-30	**pI:**	6.0
		**Sequence Coverage [%]:**	3.3
		**No. of unique Peptides:**	3

Quantitation

QU:MU	**Median:** 2.10	**CV:** 0.00 %	**No. of Peptides:**	1
WUP:QUP	**Median:** 2.37	**CV:** 0.00 %	**No. of Peptides:**	1

10	20	30	40	50	60	70	80
MEIKDQGAQM	EPLLPTRNDE	EAVVDRGGTR	SILKTHFEKE	DLEGHRTLFI	GVHVPLGGRK	SHRRHRHRGH	KHRKRDRERD
**90**	**100**	**110**	**120**	**130**	**140**	**150**	**160**
SGLEDGRESP	SFDTPSQRVQ	FILGTEDDDE	EHIPHDLFTE	LDEICWREGE	DAEWRETARW	LKFEEDVEDG	GERWSKPYVA
**170**	**180**	**190**	**200**	**210**	**220**	**230**	**240**
TLSLHSLFEL	RSCILNGTVL	LDMHANTLEE	IADMVLDQQV	SSGQLNEDVR	HRVHEALMKQ	HHHQNQKKLT	NRIPIVRSFA
**250**	**260**	**270**	**280**	**290**	**300**	**310**	**320**
DIGKKQSEPN	SMDKNAGQVV	SPQSAPACVE	NKNDVSRENS	TVDFSKGLGG	QQKGHTSPCG	MKQRHEKGPP	HQQEREVDLH
**330**	**340**	**350**	**360**	**370**	**380**	**390**	**400**
FMKKIPPGAE	ASNILVGELE	FLDRTVVAFV	RLSPAVLLQG	LAEVPIPTRF	LFILLGPLGK	GQQYHEIGRS	IATLMTDEVF
**410**	**420**	**430**	**440**	**450**	**460**	**470**	**480**
HDVAYKAKDR	NDLVSGIDEF	LDQVTVLPPG	EWDPSIRIEP	PKNVPSQEKR	KIPAVPNGTA	AHGEAEPHGG	HSGPELQRTG
**490**	**500**	**510**	**520**	**530**	**540**	**550**	**560**
RIFGGLILDI	KRKAPYFWSD	FRDAFSLQCL	ASFLFLYCAC	MSPVITFGGL	LGEATEGRIS	AIESLFGASM	TGIAYSLFGG
**570**	**580**	**590**	**600**	**610**	**620**	**630**	**640**
QPLTILGSTG	PVLVFEKILF	KFCKEYGLSY	LSLRASIGLW	TATLCIILVA	TDASSLVCYI	TRFTEEAFAS	LICIIFIYEA
**650**	**660**	**670**	**680**	**690**	**700**	**710**	**720**
LEKLFELSEA	YPINMHNDLE	LLTQYSCNCV	EPHNPSNGTL	KEWRESNISA	SDIIWENLTV	SECKSLHGEY	VGRACGHDHP
**730**	**740**	**750**	**760**	**770**	**780**	**790**	**800**
YVPDVLFWSV	ILFFSTVTLS	ATLKQFKTSR	YFPTKVRSIV	SDFAVFLTIL	CMVLIDYAIG	IPSPKLQVPS	VFKPTRDDRG
**810**	**820**	**830**	**840**	**850**	**860**	**870**	**880**
WFVTPLGPNP	WWTVIAAIIP	ALLCTILIFM	DQQITAVIIN	RKEHKLKKGC	GYHLDLLMVA	VMLGVCSIMG	LPWFVAATVL
**890**	**900**	**910**	**920**	**930**	**940**	**950**	**960**
SITHVNSLKL	ESECSAPGEQ	PKFLGIREQR	VTGLMIFILM	GSSVFMTSIL	KFIPMPVLYG	VFLYMGASSL	KGIQFFDRIK
**970**	**980**	**990**	**1000**	**1010**	**1020**	**1030**	**1040**
LFWMPAKHQP	DFIYLRHVPL	RKVHLFTIIQ	MSCLGLLWII	KVSRAAIVFP	MMVLALVFVR	KLMDLLFTKR	ELSWLDDLMP
**1050**	**1060**	**1070**	**1080**	**1090**	**1100**	**1110**	**1120**
ESKKKKLEDA	EKEEEQSMLA	MEDEGTVQLP	LEGHYRDDPS	VINISDEMSK	TALWRNLLIT	ADNSKDKESS	FPSKSSPS

Cmpd.	No. of Cmpds.	m/z meas.	Δ m/z [ppm]	z	Rt [min]	Score	P	Range	Sequence	Modification	Ratios
63	1	724.6832	-193.81	2	29.7	11.3	1	128-139	R.EGEDAEWRETAR.W		QU:MU 2.10
1817	1	847.8355	-100.68	2	52.5	10.3	1	278-293	R.ENSTVDFSKGLGGQQK.G		
505	5	509.1120	-289.14	2	36.3	12.7	0	705-713	K.SLHGEYVGR.A		WUP:QUP 2.37

Detailed Protein Report

Protein 329: **monofunctional C1-tetrahydrofolate synthase, mitochondrial isoform 3 [Homo sapiens]**

**Accession:**	gi|337756507	**Score:**	34.3
**Database:**	refseq_human(refseq_human_20140103.fasta)	**MW [kDa]:**	99.2
**Database Date:**	2015-11-30	**pI:**	7.0
**Modification(s):**	Oxidation	**Sequence Coverage [%]:**	3.7
		**No. of unique Peptides:**	2

10	20	30	40	50	60	70	80
MQSRRARPRR	EVIQNSKEVL	SLLQEKNPAF	KPVLAIIQAG	DDNLMQEINQ	NLAEEAGLNI	THICLPPDSS	EAEIIDEILK
**90**	**100**	**110**	**120**	**130**	**140**	**150**	**160**
INEDTRVHGL	ALQISENLFS	NKVLNALKPE	KDVDGVTDIN	LGKLVRGDAH	ECFVSPVAKA	VIELLEKSVG	VNLDGKKILV
**170**	**180**	**190**	**200**	**210**	**220**	**230**	**240**
VGAHGSLEAA	LQCLFQRKGS	MTMSIQWKTR	QLQSKLHEAD	IVVLGSPKPE	EIPLTWIQPG	TTVLNCSHDF	LSGKVGCGSP
**250**	**260**	**270**	**280**	**290**	**300**	**310**	**320**
RIHFGGLIEE	DDVILLAAAL	RIQNMVSSGR	RWLREQQHRR	WRLHCLKLQP	LSPVPSDIEI	SRGQTPKAVD	VLAKEIGLLA
**330**	**340**	**350**	**360**	**370**	**380**	**390**	**400**
DEIEIYGKSK	AKVRLSVLER	LKDQADGKYV	LVAGITPTPL	GEGKSTVTIG	LVQALTAHLN	VNSFACLRQP	SQGPTFGVKG
**410**	**420**	**430**	**440**	**450**	**460**	**470**	**480**
GAAGGGYAQV	IPMEEFNLHL	TGDIHAITAA	NNLLAAAIDT	RILHENTQTD	KALYNRLVPL	VNGVREFSEI	QLARLKKLGI
**490**	**500**	**510**	**520**	**530**	**540**	**550**	**560**
NKTDPSTLTE	EEVSKFARLD	IDPSTITWQR	VLDTNDRFLR	KITIGQGNTE	KGHYRQAQFD	IAVASEIMAV	LALTDSLADM
**570**	**580**	**590**	**600**	**610**	**620**	**630**	**640**
KARLGRMVVA	SDKSGQPVTA	DDLGVTGALT	VLMKDAIKPN	LMQTLEGTPV	FVHAGPFANI	AHGNSSVLAD	KIALKLVGEE
**650**	**660**	**670**	**680**	**690**	**700**	**710**	**720**
GFVVTEAGFG	ADIGMEKFFN	IKCRASGLVP	NVVVLVATVR	ALKMHGGGPS	VTAGVPLKKE	YTEENIQLVA	DGCCNLQKQI
**730**	**740**	**750**	**760**	**770**	**780**	**790**	**800**
QITQLFGVPV	VVALNVFKTD	TRAEIDLVCE	LAKRAGAFDA	VPCYHWSVGG	KGSVDLARAV	REAASKRSRF	QFLYDVQVPI
**810**	**820**	**830**	**840**	**850**	**860**	**870**	**880**
VDKIRTIAQA	VYGAKDIELS	PEAQAKIDRY	TQQGFGNLPI	CMAKTHLSLS	HQPDKKGVPR	DFILPISDVR	ASIGAGFIYP
**890**	**900**	**910**	**920**				
LVGTMSTMPG	LPTRPCFYDI	DLDTETEQVK	GLF				

Cmpd.	No. of Cmpds.	m/z meas.	Δ m/z [ppm]	z	Rt [min]	Score	P	Range	Sequence	Modification
228	1	801.7963	-174.05	2	32.6	23.3	2	262-274	R.IQNMVSSGRRWLR.E	
1676	1	1045.0153	-29.27	2	50.7	10.9	0	574-594	K.SGQPVTADDLGVTGALTVLMK.D	Oxidation: 20

Detailed Protein Report

Protein 330: probable ATP-dependent RNA helicase DDX10 [Homo sapiens]

**Accession:**	gi|13514831	**Score:**	34.2
**Database:**	refseq_human(refseq_human_20140103.fasta)	**MW [kDa]:**	100.8
**Database Date:**	2015-11-30	**pI:**	9.3
		**Sequence Coverage [%]:**	4.6
		**No. of unique Peptides:**	2

10	20	30	40	50	60	70	80
MGKTANSPGS	GARPDPVRSF	NRWKKKHSHR	QNKKKQLRKQ	LKKPEWQVER	ESISRLMQNY	EKINVNEITR	FSDFPLSKKT
90	100	110	120	130	140	150	160
LKGLQEAQYR	LVTEIQKQTI	GLALQGKDVL	GAAKTGSGKT	LAFLVPVLEA	LYRLQWTSTD	GLGVLIISPT	RELAYQTFEV
170	180	190	200	210	220	230	240
LRKVGKNHDF	SAGLIIGGKD	LKHEAERINN	INILVCTPGR	LLQHMDETVS	FHATDLQMLV	LDEADRILDM	GFADTMNAVI
250	260	270	280	290	300	310	320
ENLPKKRQTL	LFSATQTKSV	KDLARLSLKN	PEYVWVHEKA	KYSTPATLEQ	NYIVCELQQK	ISVLYSFLRS	HLKKKSIVFF
330	340	350	360	370	380	390	400
SSCKEVQYLY	RVFCRLRPGV	SILALHGRQQ	QMRRMEVYNE	FVRKRAAVLF	ATDIAARGLD	FPAVNWVLQF	DCPEDANTYI
410	420	430	440	450	460	470	480
HRAGRTARYK	EDGEALLILL	PSEKAMVQQL	LQKKVPVKEI	KINPEKLIDV	QKKLESILAQ	DQDLKERAQR	CFVSYVRSVY
490	500	510	520	530	540	550	560
LMKDKEVFDV	SKLPIPEYAL	SLGLAVAPRV	RFLQKMQKQP	TKELVRSQAD	KVIEPRAPSL	TNDEVEEFRA	YFNEKMSILQ
570	580	590	600	610	620	630	640
KGGKRLEGTE	HRQDNDTGNE	EQEEEEDDEE	EMEEKLAKAK	GSQAPSLPNT	SEAQKIKEVP	TQFLDRDEEE	EDADFLKVKR
650	660	670	680	690	700	710	720
HNVFGLDLKD	EKTLQKKEPS	KSSIKKKMTK	VAEAKKVMKR	NFKVNKKITF	TDEGELVQQW	PQMQKSAIKD	AEEDDDTGGI
730	740	750	760	770	780	790	800
NLHKAKERLQ	EEDKFDKEEY	RKKIKAKHRE	KRLKEREARR	EANKRQAKAK	DEEEAFLDWS	DDDDDDDDGF	DPSTLPDPDK
810	820	830	840	850	860	870	880
YRSSEDSDSE	DMENKISDTK	KKQGMKKRSN	SEVEDVGPTS	HNRKKARWDT	LEPLDTGLSL	AEDEELVLHL	LRSQS

Cmpd.	No. of Cmpds.	m/z meas.	Δ m/z [ppm]	z	Rt [min]	Score	P	Range	Sequence	Modification
2045	3	599.9307	-69.34	3	53.9	11.2	1	1-18	-.MGKTANSPGSGARPDPVR.S	
2761	2	713.3401	-111.86	2	65.7	11.2	2	638-649	K.VKRHNVFGLDLK.D	

Detailed Protein Report

Protein 331: laminin subunit beta-2 precursor [Homo sapiens]

**Accession:**	gi|119703755	**Score:**	34.1
**Database:**	refseq_human(refseq_human_20140103.fasta)	**MW [kDa]:**	195.9
**Database Date:**	2015-11-30	**pI:**	6.1
		**Sequence Coverage [%]:**	2.6
		**No. of unique Peptides:**	1

Alias proteins:

Accession	Name	Description
gi|530372442	refseq_human (refseq_human_20140103.fasta)	PREDICTED: laminin subunit beta-2 isoform X1 [Homo sapiens]

Detailed Protein Report

10	20	30	40	50	60	70	80
MELTSRERGR	GQPLPWELRL	GLLLSVLAAT	LAQAPAPDVP	GCSRGSCYPA	TGDLLVGRAD	RLTASSTCGL	NGPQPYCIVS
90	100	110	120	130	140	150	160
HLQDEKKCFL	CDSRRPFSAR	DNPHSHRIQN	VVTSFAPQRR	AAWWQSENGI	PAVTIQLDLE	AEFHFTHLIM	TFKTFRPAAM
170	180	190	200	210	220	230	240
LVERSADFGR	TWHVYRYFSY	DCGADFPGVP	LAPPRHWDDV	VCESRYSEIE	PSTEGEVIYR	VLDPAIPIPD	PYSSRIQNLL
250	260	270	280	290	300	310	320
KITNLRVNLT	RLHTLGDNLL	DPRREIREKY	YYALYELVVR	GNCFCYGHAS	ECAPAPGAPA	HAEGMVHGAC	ICKHNTRGLN
330	340	350	360	370	380	390	400
CEQCQDFYRD	LPWRPAEDGH	SHACRKCECH	GHTHSCHFDM	AVYLASGNVS	GGVCDGCQHN	TAGRHCELCR	PFFYRDPTKD
410	420	430	440	450	460	470	480
LRDPAVCRSC	DCDPMGSQDG	GRCDSHDDPA	LGLVSGQCRC	KEHVVGTRCQ	QCRDGFFGLS	ISDRLGCRRC	QCNARGTVPG
490	500	510	520	530	540	550	560
STPCDPNSGS	CYCKRLVTGR	GCDRCLPGHW	GLSHDLLGCR	PCDCDVGGAL	DPQCDEGTGQ	CHCRQHMVGR	RCEQVQPGYF
570	580	590	600	610	620	630	640
RPFLDHLIWE	AEDTRGQVLD	VVERLVTPGE	TPSWTGSGFV	RLQEGQTLEF	LVASVPKAMD	YDLLLRLEPQ	VPEQWAELEL
650	660	670	680	690	700	710	720
IVQRPGPVPA	HSLCGHLVPK	DDRIQGTLQP	HARYLIFPNP	VCLEPGISYK	LHLKLVRTGG	SAQPETPYSG	PGLLIDSLVL
730	740	750	760	770	780	790	800
LPRVLVLEMF	SGGDAAALER	QATFERYQCH	EEGLVPSKTS	PSEACAPLLI	SLSTLIYNGA	LPCQCNPQGS	LSSECNPHGG
810	820	830	840	850	860	870	880
QCLCKPGVVG	RRCDLCAPGY	YGFGPTGCQA	CQCSHEGALS	SLCEKTSGQC	LCRTGAFGLR	CDRCQRGQWG	FPSCRPCVCN
890	900	910	920	930	940	950	960
GHADECNTHT	GACLGCRDHT	GGEHCERCIA	GFHGDPRLPY	GGQCRPCPCP	EGPGSQRHFA	TSCHQDEYSQ	QIVCHCRAGY
970	980	990	1000	1010	1020	1030	1040
TGLRCEACAP	GHFGDPSRPG	GRCQLCECSG	NIDPMDPDAC	DPHTGQCLRC	LHHTEGPHCA	HCKPGFHGQA	ARQSCHRCTC
1050	1060	1070	1080	1090	1100	1110	1120
NLLGTNPQQC	PSPDQCHCDP	SSGQCPCLPN	VQGPSCDRCA	PNFWNLTSGH	GCQPCACHPS	RARGPTCNEF	TGQCHCRAGF
1130	1140	1150	1160	1170	1180	1190	1200
GGRTCSECQE	LHWGDPGLQC	HACDCDSRGI	DTPQCHRFTG	HCSCRPGVSG	VRCDQCARGF	SGIFPACHPC	HACFGDWDRV
1210	1220	1230	1240	1250	1260	1270	1280
VQDLAARTQR	LEQRAQELQQ	TGVLGAFESS	FWHMQEKLGI	VQGIVGARNT	SAASTAQLVE	ATEELRREIG	EATEHLTQLE
1290	1300	1310	1320	1330	1340	1350	1360
ADLTDVQDEN	FNANHALSGL	ERDRLALNLT	LRQLDQHLDL	LKHSNFLGAY	DSIRHAHSQS	AEAERRANTS	ALAVPSPVSN
1370	1380	1390	1400	1410	1420	1430	1440
SASARHRTEA	LMDAQKEDFN	SKHMANQRAL	GKLSAHTHTL	SLTDINELVC	GAPGDAPCAT	SPCGGAGCRD	EDGQPRCGGL
1450	1460	1470	1480	1490	1500	1510	1520
SCNGAAATAD	LALGRARHTQ	AELQRALAEG	GSILSRVAET	RRQASEAQQR	AQAALDKANA	SRGQVEQANQ	ELQELIQSVK
1530	1540	1550	1560	1570	1580	1590	1600
DFLNQEGADP	DSIEMVATRV	LELSIPASAE	QIQHLAGAIA	ERVRSLADVD	AILARTVGDV	RRAEQLLQDA	RRARSWAEDE
1610	1620	1630	1640	1650	1660	1670	1680
KQKAETVQAA	LEEAQRAQGI	AQGAIRGAVA	DTRDTEQTLY	QVQERMAGAE	RALSSAGERA	RQLDALLEAL	KLKRAGNSLA
1690	1700	1710	1720	1730	1740	1750	1760
ASTAEETAGS	AQGRAQEAEQ	LLRGPLGDQY	QTVKALAERK	AQGVLAAQAR	AEQLRDEARD	LLQAAQDKLQ	RLQELEGTYE
1770	1780	1790	1800				
ENERALESKA	AQLDGLEARM	RSVLQAINLQ	VQIYNTCQ				

Cmpd.	No. of Cmpds.	m/z meas.	Δ m/z [ppm]	z	Rt [min]	Score	P	Range	Sequence	Modification
2931	1	1051.5566	80.57	2	65.4	10.0	0	918-937	R.LPYGGQCRPCPCPEGPGSQR.H	

Detailed Protein Report

Protein 332: sacsin isoform 2 [Homo sapiens]

**Accession:**	gi|491227401	**Score:**	34.1
**Database:**	refseq_human(refseq_human_20140103.fasta)	**MW [kDa]:**	504.2
**Database Date:**	2015-11-30	**pI:**	6.6
**Modification(s):**	Carbamidomethyl	**Sequence Coverage [%]:**	0.9
		**No. of unique Peptides:**	2

Detailed Protein Report

10	20	30	40	50	60	70	80
MAPYQGPALY	VYNNAVFTPE	DWHGIQEIAR	SRKKDDPLKV	GRFGIGFNSV	YHITDVPCIF	SGDQIGMLDP	HQTLFGPHES
90	100	110	120	130	140	150	160
GQCWNLKDDS	KEISELSDQF	APFVGIFGST	KETFINGNFP	GTFFRFPLRL	QPSQLSSNLY	NKQKVLELFE	SFRADADTVL
170	180	190	200	210	220	230	240
LFLKSVQDVS	LYVREADGTE	KLVFRVTSSE	SKALKHERPN	SIKILGTAIS	NYCKKTPSNN	ITCVTYHVNI	VLEEESTKDA
250	260	270	280	290	300	310	320
QKTSWLVCNS	VGGRGISSKL	DSLADELKFV	PIIGIAMPLS	SRDDEAKGAT	SDFSGKAFCF	LPLPPGEESS	TGLPVHISGF
330	340	350	360	370	380	390	400
FGLTDNRRSI	KWRELDQWRD	PAALWNEFLV	MNVVPKAYAT	LILDSIKRLE	MEKSSDFPLS	VDVIYKLWPE	ASKVKVHWQP
410	420	430	440	450	460	470	480
VLEPLFSELL	QNAVIYSISC	DWVRLEQVYF	SELDENLEYT	KTVLNYLQSS	GKQIAKVPGN	VDAAVQLTAA	SGTTPVRKVT
490	500	510	520	530	540	550	560
PAWVRQVLRK	CAHLGCAEEK	LHLLEFVLSD	QAYSELLGLE	LLPLQNGNFV	PFSSSVSDQD	VIYITSAEYP	RSLFPSLEGR
570	580	590	600	610	620	630	640
FILDNLKPHL	VAALKEAAQT	RGRPCTQLQL	LNPERFARLI	KEVMNTFWPG	RELIVQWYPF	DENRNHPSVS	WLKMVWKNLY
650	660	670	680	690	700	710	720
IHFSEDLTLF	DEMPLIPRTI	LEEGQTCVEL	IRLRIPSLVI	LDDESEAQLP	EFLADIVQKL	GGFVLKKLDA	SIQHPLIKKY
730	740	750	760	770	780	790	800
IHSPLPSAVL	QIMEKMPLQK	LCNQITSLLP	THKDALRKFL	ASLTDSSEKE	KRIIQELAIF	KRINHSSDQG	ISSYTKLKGC
810	820	830	840	850	860	870	880
KVLHHTAKLP	ADLRLSISVI	DSSDEATIRL	ANMLKIEQLK	TTSCLKLVLK	DIENAFYSHE	EVTQLMLWVL	ENLSSLKNEN
890	900	910	920	930	940	950	960
PNVLEWLTPL	KFIQISQEQM	VSAGELFDPD	IEVLKDLFCN	EEGTYFPPSV	FTSPDILHSL	RQIGLKNEAS	LKEKDVVQVA
970	980	990	1000	1010	1020	1030	1040
KKIEALQVGA	CPDQDVLLKK	AKTLLLVLNK	NHTLLQSSEG	KMTLKKIKWV	PACKERPPNY	PGSLVWKGDL	CNLCAPPDMC
1050	1060	1070	1080	1090	1100	1110	1120
DVGHAILIGS	SLPLVESIHV	NLEKALGIFT	KPSLSAVLKH	FKIVVDWYSS	KTFSDEDYYQ	FQHILLEIYG	FMHDHLNEGK
1130	1140	1150	1160	1170	1180	1190	1200
DSFRALKFPW	VWTGKKFCPL	AQAVIKPIHD	LDLQPYLHNV	PKTMAKFHQL	FKVCGSIEEL	TSDHISMVIQ	KIYLKSDQDL
1210	1220	1230	1240	1250	1260	1270	1280
SEQESKQNLH	LMLNIIRWLY	SNQIPASPNT	PVPIHHSKNP	SKLIMKPIHE	CCYCDIKVDD	LNDLLEDSVE	PIILVHEDIP
1290	1300	1310	1320	1330	1340	1350	1360
MKTAEWLKVP	CLSTRLINPE	NMGFEQSGQR	EPLTVRIKNI	LEEYPSVSDI	FKELLQNADD	ANATECSFLI	DMRRNMDIRE
1370	1380	1390	1400	1410	1420	1430	1440
NLLDPGMAAC	HGPALWSFNN	SQFSDSDFVN	ITRLGESLKR	GEVDKVGKFG	LGFNSVYHIT	DIPIIMSREF	MIMFDPNINH
1450	1460	1470	1480	1490	1500	1510	1520
ISKHIKDKSN	PGIKINWSKQ	QKRLRKFPNQ	FKPFIDVFGC	QLPLTVEAPY	SYNGTLFRLS	FRTQQEAKVS	EVSSTCYNTA
1530	1540	1550	1560	1570	1580	1590	1600
DIYSLVDEFS	LCGHRLIIFT	QSVKSMYLKY	LKIEETNPSL	AQDTVIIKKK	SCSSKALNTP	VLSVLKEAAK	LMKTCSSSNK
1610	1620	1630	1640	1650	1660	1670	1680
KLPSDEPKSS	CILQITVEEF	HHVFRRIADL	QSPLFRGPDD	DPAALFEMAK	SGQSKKPSDE	LSQKTVECTT	WLLCTCMDTG
1690	1700	1710	1720	1730	1740	1750	1760
EALKFSLSES	GRRLGLVPCG	AVGVQLSEIQ	DQKWTVKPHI	GEVFCYLPLR	IKTGLPVHIN	GCFAVTSNRK	EIWKTDTKGR
1770	1780	1790	1800	1810	1820	1830	1840
WNTTFMRHVI	VKAYLQVLSV	LRDLATSGEL	MDYTYYAVWP	DPDLVHDDFS	VICQGFYEDI	AHGKGKELTK	VFSDGSTWVS
1850	1860	1870	1880	1890	1900	1910	1920
MKNVRFLDDS	ILKRRDVGSA	AFKIFLKYLK	KTGSKNLCAV	ELPSSVKLGF	EEAGCKQILL	ENTFSEKQFF	SEVFFPNIQE
1930	1940	1950	1960	1970	1980	1990	2000
IEAELRDPLM	IFVLNEKVDE	FSGVLRVTPC	IPCSLEGHPL	VLPSRLIHPE	GRVAKLFDIK	DGRFPYGSTQ	DYLNPIILIK
2010	2020	2030	2040	2050	2060	2070	2080
LVQLGMAKDD	ILWDDMLERA	VSVAEINKSD	HVAACLRSSI	LLSLIDEKLK	IRDPRAKDFA	AKYQTIRFLP	FLTKPAGFSL
2090	2100	2110	2120	2130	2140	2150	2160
DWKGNSFKPE	TMFAATDLYT	AEHQDIVCLL	QPILNENSHS	FRGCGSVSLA	VKEFLGLLKK	PTVDLVINQL	KEVAKSVDDG
2170	2180	2190	2200	2210	2220	2230	2240

Detailed Protein Report

Cmpd.	No. of Cmpds.	m/z meas.	Δ m/z [ppm]	z	Rt [min]	Score	P	Range	Sequence	Modification
1798	1	916.4469	-78.82	2	50.8	10.5	2	1570-1586	K.KSCSSKALNTPVLSVLK.E	Carbamidomethyl: 3
362	1	529.2876	-26.74	3	34.5	12.9	2	4347-4361	R.GKSDKDVKPTALAQK.I	

Detailed Protein Report

Protein 333: leucine-, glutamate- and lysine-rich protein 1 isoform 1 [Homo sapiens]

**Accession:**	gi|262263420	**Score:**	34.1
**Database:**	refseq_human(refseq_human_20140103.fasta)	**MW [kDa]:**	81.2
**Database Date:**	2015-11-30	**pI:**	8.7
**Modification(s):**	Oxidation	**Sequence Coverage [%]:**	4.5
		**No. of unique Peptides:**	2

Alias proteins:

Accession	Name	Description
gi|530374591	refseq_human (refseq_human_20140103.fasta)	PREDICTED: leucine-, glutamate- and lysine-rich protein 1 isoform X1 [Homo sapiens]

10	20	30	40	50	60	70	80
MDHHIPMHAL	PEEIQKMLPE	EKVCKYCGVS	YLILHEFKAM	EEKVKAMEKE	MKFYQGSVDR	EKRLQEKLHS	LSQELEQYKI
**90**	**100**	**110**	**120**	**130**	**140**	**150**	**160**
DNKSKTERIY	DVGMQLKSQQ	NEFQKVKKQL	SHLQDELKIK	YRQSYIFSQR	LSEYKYFWNK	TLSLLTFTKR	ELTSIKNEVY
**170**	**180**	**190**	**200**	**210**	**220**	**230**	**240**
DNYQNWTSLK	GAVFLQIKSI	SETALTEIDI	LNKSLTVSQR	NKVCLEKEMK	NLKLLSDAAI	LRSQQIRTSR	QQEVNLQTRC
**250**	**260**	**270**	**280**	**290**	**300**	**310**	**320**
YDLQKEVLDL	QCLVEALGLK	LQKAVTEMDN	YKEMLMNKSN	EADDCQRELK	KLKFESIISE	SQHTMLLKEK	EDSLMTCQQI
**330**	**340**	**350**	**360**	**370**	**380**	**390**	**400**
YKALQEELTV	KEKQEEDIKR	RINLAENELE	ITKTLLNQTR	EEVLTLKNER	ELMLISHQKS	IEQLQETLRQ	KLLSDDNWKE
**410**	**420**	**430**	**440**	**450**	**460**	**470**	**480**
KIEAELAKER	AQHLVEFEEQ	ALLFKEETKL	QLDIEKEKHQ	DVIQKYKKEQ	EELQMKISDL	ITGATRDLRQ	EVTTLKEKLH
**490**	**500**	**510**	**520**	**530**	**540**	**550**	**560**
KSHIRYTEES	NSKEKEIENL	KNLVAEFESR	LKKEIDSNDS	VSENLRKEME	QKSDELKRVM	LAQTQLIEQF	NQSQEENTFL
**570**	**580**	**590**	**600**	**610**	**620**	**630**	**640**
QETVRRECEE	RFELTEALSQ	AREQLLELSK	LRGSLPFSPC	SLSKGSLTSP	AAAVSNHGER	SLARLNSEKG	IQIPNLRGVS
**650**	**660**	**670**	**680**	**690**	**700**		
KPTTFPTSDK	PKRVRSGVPI	LPQPHPPRGG	ASSANETRQR	LAAILRRRRS	QQ		

Cmpd.	No. of Cmpds.	m/z meas.	Δ m/z [ppm]	z	Rt [min]	Score	P	Range	Sequence	Modification
1884	1	734.7526	-169.10	2	53.9	10.2	1	86-97	K.TERIYDVGMQLK.S	Oxidation: 9
538	1	501.6964	-160.45	2	36.7	11.2	1	494-501	K.EKEIENLK.N	

Detailed Protein Report

Protein 334: dynein heavy chain domain-containing protein 1 isoform 1 [Homo sapiens]

**Accession:**	gi|222144249	**Score:**	34.1
**Database:**	refseq_human(refseq_human_20140103.fasta)	**MW [kDa]:**	533.3
**Database Date:**	2015-11-30	**pI:**	6.3
**Modification(s):**	Carbamidomethyl	**Sequence Coverage [%]:**	0.5
		**No. of unique Peptides:**	1

Quantitation

QU:MU	**Median:** 1.73	**CV:** 0.00 %	**No. of Peptides:** 1

Detailed Protein Report

10	20	30	40	50	60	70	80
MVPEERRVGL	SSDETSSDSL	KSWHSICVLD	SKEQPLACQQ	KQRQFVKPVT	ESEQPTVLEL	LLAELRTLFS	AVLQDSSPAA
90	100	110	120	130	140	150	160
WRYLHAVLGL	LPPYRELLVG	HLDLLPFLEQ	LYCWAPWVQT	HLHLDLLGAI	VQAFPPDSSL	LDSASHADCC	PQKRRLHHRP
170	180	190	200	210	220	230	240
PCPACPFVQA	QWSRQQVKEE	LATWLRPLTL	PELQRCLGIV	GAQVALEEAV	WLDGLSLLPL	ALAADIPVRY	ESSDTDNAEV
250	260	270	280	290	300	310	320
EPVGRKETRS	QLDYEVPREK	AFQKSSTGFS	PETSFLDSQV	MTALKMERYL	KKIHFLYLNV	APSRYFRPYS	LMVVPPDKVN
330	340	350	360	370	380	390	400
PEHYIFSPFG	ILHVHPVEGS	ETMTLGTWHH	HCVLWQQLQF	IPFFKYCLLR	KSFTCWKKNV	RLQGLHRLQK	FLENHLLLAV
410	420	430	440	450	460	470	480
PHFGAGLLHI	SRLLQELHSV	SWLPQELDRC	YELLDLQTAL	AEEKHKALRL	LHRCLNLCTS	ILRLVHEDTY	HMQQCLQERV
490	500	510	520	530	540	550	560
QNCDRIRTGQ	GSIYLQRVQH	KQLEQKLKQA	EAWWLQLGKF	ARLVDYMICQ	SLISVLEEQI	TSFVANILQA	PRQKPFLSSQ
570	580	590	600	610	620	630	640
LVFDDHGQLS	HVPCVENMIQ	TLTGGLQSVK	TSALQVVQSA	DLKTSSDSLY	SEEEDEEEDS	KDEFLMPKFQ	GQPSDAVSIF
650	660	670	680	690	700	710	720
CGPNVGLVWP	WKSHPIAGIL	EVRGCRLRGQ	YFPHNYKQLE	EDLDNNPKIQ	QALNIQQVLL	EGVLCKVQEF	CREHHWITGI
730	740	750	760	770	780	790	800
YEFLQSWGPQ	KLEDMRGGPI	KNYVTLVSRL	NVWQARVSSM	PIELLTKGGL	LLLSCHDVQA	EMESKLNSIR	KDILAHVQNE
810	820	830	840	850	860	870	880
CWNLSQQLMT	ELTDFMHIFR	TINSDIHAIA	QCTQKLNEAN	EQYVELEERM	EYVRALHELI	RNHFSLFSAE	NEALDISVRR
890	900	910	920	930	940	950	960
QFGESPIPPC	PPPPQPHLLH	CPLLAPQLLD	MWEAFQFEKS	QASEFLLSKR	HAIMPKLQQL	MAAALAELEG	LLAKALSGPF
970	980	990	1000	1010	1020	1030	1040
MDPTQDQRST	EHQLVSLERQ	FQNTVSDLSE	LHHAYAIFTE	DETPVPLPIC	GTRPIVQQQR	IWHLYRVISE	NISEWKCMAF
1050	1060	1070	1080	1090	1100	1110	1120
AKFSPAMAQE	KTEGWLTEAA	RMSTTLELHS	PVLQHCMRIL	GEFRSYLPLL	TKLGSLHPQS	LNCQCLLRAL	GLGSLQTIEL
1130	1140	1150	1160	1170	1180	1190	1200
LTLGQLLTYP	LLEFADRINQ	VWQNENERIH	AQETIRRLQR	YWEARQLRLL	NFILHVPYEP	PASERSKRQV	LRSPQWEVVD
1210	1220	1230	1240	1250	1260	1270	1280
KDSGTFILSD	YSNLQDSIQE	SLQVLSKILA	IEKSGDLNKI	ALEWVAIMHG	LGALLEVWLT	FQQKWIFLNK	VLHEMKIQFP
1290	1300	1310	1320	1330	1340	1350	1360
NADLNSRFKV	MDDQYRTLMR	ISVADPMVLS	LVVPSAERSP	YFQGQQLQQL	LQAGSVELEG	IIMSLESVLY	GVCAHFPRLF
1370	1380	1390	1400	1410	1420	1430	1440
FLSDSELVAL	LAARLESCEA	QLWVRRCFPH	VHAVSFRSCP	TGEKNTDDWE	SSPNTQTQVE	ALAVLGAGGE	EVKLQGPLPL
1450	1460	1470	1480	1490	1500	1510	1520
HPDLPKWLAS	LEKCLRLALV	HMLQGCVAAR	LARGPSLGEA	LKQLPKQNKL	YLQLYVQHWI	DLVQAFPWQC	VLVAEEVVWR
1530	1540	1550	1560	1570	1580	1590	1600
AEMEEALLEW	GTLAMVSMHM	RKLEVLVNFM	RAQRASQGGQ	SLPSVRQTSL	LSALLVMAVT	HRDIAQLLEQ	HQVSDLTDFH
1610	1620	1630	1640	1650	1660	1670	1680
WVRQLKYHLG	SPHIIPKSPL	QSLKTIASSE	PSLSPAACWI	DVLGRSFLYN	YEYLGPRLGP	LPSLLPERPA	LVLLLALEEV
1690	1700	1710	1720	1730	1740	1750	1760
ACGTVLGPNG	VGKRAIVNSL	AQALGRQLVM	LPCSPQIEAQ	CLSNYLNGAL	QGGAWLLLEK	VHQLPPGLLS	ALGQRLGELH
1770	1780	1790	1800	1810	1820	1830	1840
HLYAPLYQEA	SRNTSTIDPT	QPQLLGSSFF	EKHHVSVRLG	YGCLLVLRAL	SSAVPANLHL	LLRPVALALP	DLRQVAELTL
1850	1860	1870	1880	1890	1900	1910	1920
LGAGMRDAFQ	MATRLSKFFS	LERELVSGPL	PCRLPLLKQI	LEDTIRTLNV	TKEEPKCQKP	RSLAAIEEAA	LLRSPLFSIL
1930	1940	1950	1960	1970	1980	1990	2000
NGLHLHNLRG	LLCALFPSAS	QVLAEPMTYK	LMKPLVVEEL	QQVGLDPSPD	ILGSLEQLSQ	ALSRASGILL	LGPAGSGKTT
2010	2020	2030	2040	2050	2060	2070	2080
CWHSLFKIQN	RLAAMEDTST	QGCQPVEITH	LYPSGLSPQE	FLGWLEGSCW	HHGIFPKVLR	AAGQCNNMGQ	KRQTEESIGI
2090	2100	2110	2120	2130	2140	2150	2160
QHWIICDGAS	NGAWLDSITC	LLSELPQLSL	PSGQQIARPP	GTFLLMEVAD	TTGISPTVVG	CCALVWCGGE	QTWQCILSAL
2170	2180	2190	2200	2210	2220	2230	2240

Detailed Protein Report

Cmpd.	No. of Cmpds.	m/z meas.	Δ m/z [ppm]	z	Rt [min]	Score	P	Range	Sequence	Modification	Ratios	
2480	3	625.3103	-20.67	2	61.5	13.4	0	454-463	R.CLNLCTSILR.L	Carbamidomethyl: 1, 5	QU:MU	1.73

Detailed Protein Report

Protein 335: R-spondin-1 isoform 3 precursor [Homo sapiens]

Accession: gi|339276103
Database: refseq_human(refseq_human_20140103.fasta)
Database Date: 2015-11-30
Modification(s): Carbamidomethyl, Oxidation

Score: 34.0
MW [kDa]: 21.8
pI: 11.3
Sequence Coverage [%]: 26.5
No. of unique Peptides: 2

10	20	30	40	50	60	70	80
MRLGLCVVAL	VLSWTHLTIS	SRGIKGKRQR	RISAEGSQAC	AKGCELCSEV	NGCLKCSPKL	FILLERNDIR	QVGVCLPSCP
**90**	**100**	**110**	**120**	**130**	**140**	**150**	**160**
PGYFDARNPD	MNKCIKCKIE	HCEACFSHNF	CTKCKEGLYL	HKGRCYPACP	EGSSAANGTM	ECSSPGQKRR	KGGQGRRENA
**170**	**180**	**190**	**200**	**210**			
NRNLARKESK	EAGAGSRRRK	GQQQQQQQGT	VGPLTSAGPA				

Cmpd.	No. of Cmpds.	m/z meas.	Δ m/z [ppm]	z	Rt [min]	Score	P	Range	Sequence	Modification
2673	1	913.9847	93.98	2	63.5	12.9	1	43-59	K.GCELCSEVNGCLKCSPK.L	Carbamidomethyl: 2
1684	1	1273.9976	-19.18	2	50.8	10.8	1	125-149	R.CYPACPEGSSAANGTMECSSPGQKR.R	Oxidation: 16

Detailed Protein Report

Protein 336: myotubularin-related protein 8 [Homo sapiens]

**Accession:**	gi|21361759	**Score:**	34.0
**Database:**	refseq_human(refseq_human_20140103.fasta)	**MW [kDa]:**	78.9
**Database Date:**	2015-11-30	**pI:**	6.2
**Modification(s):**	Oxidation	**Sequence Coverage [%]:**	2.8
		**No. of unique Peptides:**	1

10	20	30	40	50	60	70	80
MDHITVPKVE	NVKLVDRYVS	KKPANGILYL	TATHLIYVEA	SGAARKETWI	ALHHIATVEK	LPITSLGCPL	TLRCKNFRVA
**90**	**100**	**110**	**120**	**130**	**140**	**150**	**160**
HFVLDSDLVC	HEVYISLLKL	SQPALPEDLY	AFSYNPKSSK	EMRESGWKLI	DPISDFGRMG	IPNRNWTITD	ANRNYEICST
**170**	**180**	**190**	**200**	**210**	**220**	**230**	**240**
YPPEIVVPKS	VTLGTVVGSS	KFRSKERVPV	LSYLYKENNA	AICRCSQPLS	GFYTRCVDDE	LLLEAISQTN	PGSQFMYVVD
**250**	**260**	**270**	**280**	**290**	**300**	**310**	**320**
TRPKLNAMAN	RAAGKGYENE	DNYANIRFRF	MGIENIHVMR	SSLQKLLEVC	ELKTPTMSEF	LSGLESSGWL	RHIKAIMDAG
**330**	**340**	**350**	**360**	**370**	**380**	**390**	**400**
IFITKAVKVE	KASVLVHCSD	GWDRTAQVCS	VASILLDPFY	RTFKGLMILI	EKEWISMGHK	FSQRCGHLDG	DSKEVSPIFT
**410**	**420**	**430**	**440**	**450**	**460**	**470**	**480**
QFLDCIWQLM	EQFPCAFEFN	ENFLLEIHDH	VFSCQFGNFL	GNCQKDREDL	RVYEKTHSVW	PFLVQRKPDF	RNPLYKGFTM
**490**	**500**	**510**	**520**	**530**	**540**	**550**	**560**
YGVLNPSTVP	YNIQFWCGMY	NRFDKGLQPK	QSMLESLLEI	KKQRAMLETD	VHELEKKLKV	RDEPPEEICT	CSQLGNILSQ
**570**	**580**	**590**	**600**	**610**	**620**	**630**	**640**
HLGSPLTNPL	GFMGINGDLN	TLMENGTLSR	EGGLRAQMDQ	VKSQGADLHH	NCCEIVGSLR	AINISGDVGI	SEAMGISGDM
**650**	**660**	**670**	**680**	**690**	**700**	**710**	
CTFEATGFSK	DLGICGAMDI	SEATGISGNL	GISEARGFSG	DMGILGDTGI	SKASTKEADY	SKHQ	

Cmpd.	No. of Cmpds.	m/z meas.	Δ m/z [ppm]	z	Rt [min]	Score	P	Range	Sequence	Modification
1315	8	956.5835	100.93	1	46.6	16.9	0	1-8	-.MDHITVPK.V	Oxidation: 1

Detailed Protein Report

Protein 337: PREDICTED: NEDD8 ultimate buster 1 isoform X3 [Homo sapiens]

**Accession:**	gi|530387034	**Score:**	33.9
**Database:**	refseq_human(refseq_human_20140103.fasta)	**MW [kDa]:**	67.7
**Database Date:**	2015-11-30	**pI:**	5.7
**Modification(s):**	Carbamidomethyl	**Sequence Coverage [%]:**	1.7
		**No. of unique Peptides:**	1

10	20	30	40	50	60	70	80
MKTNGGRCRI	RALCWSRREW	RGAGMAQKKY	LQAKLTQFLR	EDRIQLWKPP	YTDENKKVGL	ALKDLAKQYS	DRLECCENEV
**90**	**100**	**110**	**120**	**130**	**140**	**150**	**160**
EKVIEEIRCK	AIERGTGNDN	YRTTGIATIE	VFLPPRLKKD	RKNLLETRLH	ITGRELRSKI	AETFGLQENY	IKIVINKKQL
**170**	**180**	**190**	**200**	**210**	**220**	**230**	**240**
QLGKTLEEQG	VAHNVKAMVL	ELKQSEEDAR	KNFQLEEEEQ	NEAKLKEKQI	QRTKRGLEIL	AKRAAETVVD	PEMTPYLDIA
**250**	**260**	**270**	**280**	**290**	**300**	**310**	**320**
NQTGRSIRIP	PSERKALMLA	MGYHEKGRAF	LKRKEYGIAL	PCLLDADKYF	CECCRELLDT	VDNYAVLQLD	IVWCYFRLEQ
**330**	**340**	**350**	**360**	**370**	**380**	**390**	**400**
LECLDDAEKK	LNLAQKCFKN	CYGENHQRLV	HIKGNCGKEK	VLFLRLYLLQ	GIRNYHSGND	VEAYEYLNKA	RQLFKELYID
**410**	**420**	**430**	**440**	**450**	**460**	**470**	**480**
PSKVDNLLQL	GFTAQEARLG	LRACDGNVDH	AATHITNRRE	ILLSNPQMWW	LNDSNPETDN	RQESPSQENI	DRLVYMGFDA
**490**	**500**	**510**	**520**	**530**	**540**	**550**	**560**
LVAEAALRVF	RGNVQLAAQT	LAHNGGSLPP	ELPLSPEDSL	SPPATSPSDS	AGTSSASTDE	DMETEAVNEI	LEDIPEHEED
**570**	**580**	**590**	**600**				
YLDSTLEDEE	IIIAEYLSYV	ENRKSATKKN					

Cmpd.	No. of Cmpds.	m/z meas.	Δ m/z [ppm]	z	Rt [min]	Score	P	Range	Sequence	Modification
2092	1	625.2489	-174.33	2	54.5	12.9	2	330-339	K.KLNLAQKCFK.N	Carbamidomethyl: 8

Detailed Protein Report

Protein 338: EH domain-binding protein 1 isoform 3 [Homo sapiens]

**Accession:**	gi|217330572	**Score:**	33.9
**Database:**	refseq_human(refseq_human_20140103.fasta)	**MW [kDa]:**	132.2
**Database Date:**	2015-11-30	**pI:**	5.6
		**Sequence Coverage [%]:**	2.7
		**No. of unique Peptides:**	2

Quantitation

QU:MU	**Median:**	0.96	**CV:**	0.00 %	**No. of Peptides:**	1
WUP:QUP	**Median:**	1.29	**CV:**	0.00 %	**No. of Peptides:**	1

Alias proteins:

Accession	Name	Description
gi|217330574	refseq_human (refseq_human_20140103.fasta)	EH domain-binding protein 1 isoform 3 [Homo sapiens]

10	20	30	40	50	60	70	80
MASVWKRLQR	VGKHASKFQF	VASYQELMVE	CTKKWQPDKL	VVVWTRRSRR	KSSKAHSWQP	GIKNPYRGVV	VWPVPENIEI
**90**	**100**	**110**	**120**	**130**	**140**	**150**	**160**
TVTLFKDPHA	EEFEDKEWTF	VIENESPSGR	RKALATSSIN	MKQYASPMPT	QTDVKLKFKP	LSKKVVSAAL	QFSLSCIFLR
**170**	**180**	**190**	**200**	**210**	**220**	**230**	**240**
EGKATDEDMQ	SLASLMSMKQ	ADIGNLDDFE	EDNEDDDENR	VNQEEKAAKI	TELINKLNFL	DEAEKDLATV	NSNPFDDPDA
**250**	**260**	**270**	**280**	**290**	**300**	**310**	**320**
AELNPFGDPD	SEEPITETAS	PRKTEDSFYN	NSYNPFKEVQ	TPQYLNPFDE	PEAFVTIKDS	PPQSTKRKNI	RPVDMSKYLY
**330**	**340**	**350**	**360**	**370**	**380**	**390**	**400**
ADSSKTEEEE	LDESNPFYEP	KSTPPPNNLV	NPVQELETER	RVKRKAPAPP	VLSPKTGVLN	ENTVSAGKDL	STSPKPSPIP
**410**	**420**	**430**	**440**	**450**	**460**	**470**	**480**
SPVLGRKPNA	SQSLLVWCKE	VTKNYRGVKI	TNFTTSWRNG	LSFCAILHHF	RPDLIDYKSL	NPQDIKENNK	KAYDGFASIG
**490**	**500**	**510**	**520**	**530**	**540**	**550**	**560**
ISRLLEPSDM	VLLAIPDKLT	VMTYLYQIRA	HFSGQELNVV	QIEENSSKST	YKVGNYETDT	NSSVDQEKFY	AELSDLKREP
**570**	**580**	**590**	**600**	**610**	**620**	**630**	**640**
ELQQPISGAV	DFLSQDDSVF	VNDSGVGESE	SEHQTPDDHL	SPSTASPYCR	RTKSDTEPQK	SQQSSGRTSG	SDDPGICSNT
**650**	**660**	**670**	**680**	**690**	**700**	**710**	**720**
DSTQAQVLLG	KKRLLKAETL	ELSDLYVSDK	KKDMSPPFIC	EETDEQKLQT	LDIGSNLEKE	KLENSRSLEC	RSDPESPIKK
**730**	**740**	**750**	**760**	**770**	**780**	**790**	**800**
TSLSPTSKLG	YSYSRDLDLA	KKKHASLRQT	ESDPDADRTT	LNHADHSSKI	VQHRLLSRQE	ELKERARVLL	EQARRDAALK
**810**	**820**	**830**	**840**	**850**	**860**	**870**	**880**
AGNKHNTNTA	TPFCNRQLSD	QQDEERRRQL	RERARQLIAE	ARSGVKMSEL	PSYGEMAAEK	LKERSKASGE	QNSKLVDLKL
**890**	**900**	**910**	**920**	**930**	**940**	**950**	**960**
KKLLEVQPQV	ANSPSSAAQK	AVTESSEQDM	KSGTEDLRTE	RLQKTTERFR	NPVVFSKDST	VRKTQLQSFS	QYIENRPEMK
**970**	**980**	**990**	**1000**	**1010**	**1020**	**1030**	**1040**
RQRSIQEDTK	KGNEEKAAIT	ETQRKPSEDE	VLNKGFKDTS	QYVVGELAAL	ENEQKQIDTR	AALVEKRLRY	LMDTGRNTEE
**1050**	**1060**	**1070**	**1080**	**1090**	**1100**	**1110**	**1120**
EEAMMQEWFM	LVNKKNALIR	RMNQLSLLEK	EHDLERRYEL	LNRELRAMLA	IEDWQKTEAQ	KRREQLLLDE	LVALVNKRDA
**1130**	**1140**	**1150**	**1160**	**1170**			
LVRDLDAQEK	QAEEEDEHLE	RTLEQNKGKM	AKKEEKCVLQ				

Cmpd.	No. of Cmpds.	m/z meas.	Δ m/z [ppm]	z	Rt [min]	Score	P	Range	Sequence	Modification	Ratios
804	1	660.3468	-45.46	2	38.5	12.3	2	111-122	R.RKALATSSINMK.Q		
192	2	688.2999	-17.82	2	32.1	10.3	0	805-816	K.HNTNTATPFCNR.Q		WUP:QUP 1.29<br>QU:MU 0.96

Detailed Protein Report

Protein 339: **PREDICTED: uncharacterized protein C1orf159 isoform X8 [Homo sapiens]**

**Accession:**	gi|578799328	**Score:**	33.9
**Database:**	refseq_human(refseq_human_20140103.fasta)	**MW [kDa]:**	30.9
**Database Date:**	2015-11-30	**pI:**	11.7
		**Sequence Coverage [%]:**	8.9
		**No. of unique Peptides:**	1

```
         10         20         30         40         50         60         70         80
MSVVPILEGQ PHSISLAPHA PPCGRLCASA PGMALRHLAL LAGLLVGVAS KSMENTAQLP ECCVDVVGVN ASCPGASLCG
         90        100        110        120        130        140        150        160
PGCYRRWNAD GSASCVRCGN GTLPAYNGSE CRSFAGPGAP FPMNRSSGTP GRPHPGAPRV AASLFLGTFF ISSGLILSVA
        170        180        190        200        210        220        230        240
GFFYLKRSSK LPRACYRRNK APALQPGEAA AMIPPPQSSG PTLTSPVSDP FQYGSRATSG GSGPWTGPRI PLPSRGRPVS
        250        260        270        280        290        300        310
AMSDLEAETT PRTWRQGPGG RPLGGNQHKV LASPGARDSP GHSLGSESLR LPATDAKGSR AA
```

Cmpd.	No. of Cmpds.	m/z meas.	Δ m/z [ppm]	z	Rt [min]	Score	P	Range	Sequence	Modification
2555	1	701.8644	-5.90	2	61.9	10.1	0	256-269	R.QGPGGRPLGGNQHK.V	

Detailed Protein Report

Protein 340: **metabotropic glutamate receptor 4 isoform 5 [Homo sapiens]**

**Accession:**	gi|378548216	**Score:**	33.9
**Database:**	refseq_human(refseq_human_20140103.fasta)	**MW [kDa]:**	83.5
**Database Date:**	2015-11-30	**pI:**	9.9
		**Sequence Coverage [%]:**	6.3
		**No. of unique Peptides:**	2

10	20	30	40	50	60	70	80
MSCKIPQISY	ASTAPDLSDN	SRYDFFSRVV	PSDTYQAQAM	VDIVRALKWN	YVSTVASEGS	YGESGVEAFI	QKSREDGGVC
**90**	**100**	**110**	**120**	**130**	**140**	**150**	**160**
IAQSVKIPRE	PKAGEFDKII	RRLLETSNAR	AVIIFANEDD	IRRVLEAARR	ANQTGHFFWM	GSDSWGSKIA	PVLHLEEVAE
**170**	**180**	**190**	**200**	**210**	**220**	**230**	**240**
GAVTILPKRM	SVRGFDRYFS	SRTLDNNRRN	IWFAEFWEDN	FHCKLSRHAL	KKGSHVKKCT	NRERIGQDSA	YEQEGKVQFV
**250**	**260**	**270**	**280**	**290**	**300**	**310**	**320**
IDAVYAMGHA	LHAMHRDLCP	GRVGLCPRMD	PVDGTQLLKY	IRNVNFSGIA	GNPVTFNENG	DAPGRYDIYQ	YQLRNDSAEY
**330**	**340**	**350**	**360**	**370**	**380**	**390**	**400**
KVIGSWTDHL	HLRIERMHWP	GSGQQLPRSI	CSLPCQPGER	KKTVKGMPCC	WHCEPCTGYQ	YQVDRYTCKT	CPYDMRPTEN
**410**	**420**	**430**	**440**	**450**	**460**	**470**	**480**
RTGCRPIPII	KLEWGSPWAV	LPLFLAVVGI	AATLFVVITF	VRYNDTPIVK	ASGRELSYVL	LAGIFLCYAT	TFLMIAEPDL
**490**	**500**	**510**	**520**	**530**	**540**	**550**	**560**
GTCSLRRIFL	GLGMSISYAA	LLTKTNRIYR	IFEQGKRSVS	APRFISPASQ	LAITFSLISL	QLLGICVWFV	VDPSHSVVDF
**570**	**580**	**590**	**600**	**610**	**620**	**630**	**640**
QDQRTLDPRF	ARGVLKCDIS	DLSLICLLGY	SMLLMVTCTV	YAIKTRGVPE	TFNEAKPIGF	TMYTTCIVWL	AFIPIFFGTS
**650**	**660**	**670**	**680**	**690**	**700**	**710**	**720**
QSADKLYIQT	TTLTVSVSLS	ASVSLGMLYM	PKVYIILFHP	EQNVPKRKRS	LKAVVTAATM	SNKFTQKGNF	RPNGEAKSEL
**730**	**740**	**750**					
CENLEAPALA	TKQTYVTYTN	HAI					

Cmpd.	No. of Cmpds.	m/z meas.	Δ m/z [ppm]	z	Rt [min]	Score	P	Range	Sequence	Modification
2591	1	701.8584	-42.25	2	62.4	10.3	2	90-101	R.EPKAGEFDKIIR.R	
1390	1	977.5200	-44.32	2	47.5	12.4	1	487-504	R.RIFLGLGMSISYAALLTK.T	

Detailed Protein Report

Protein 341: PREDICTED: nuclear pore complex protein Nup214 isoform X4 [Homo sapiens]

**Accession:**	gi|530427224	**Score:**	33.9
**Database:**	refseq_human(refseq_human_20140103.fasta)	**MW [kDa]:**	209.0
**Database Date:**	2015-11-30	**pI:**	9.0
		**Sequence Coverage [%]:**	1.5
		**No. of unique Peptides:**	2

Detailed Protein Report

10	20	30	40	50	60	70	80
MRGQLRAAGA	EGRKFAVERP	GFRGQGRGRQ	RWLLRHTEGG	AMGDEMDAMI	PEREMKDFQF	RALKKVRIFD	SPEELPKERS
90	100	110	120	130	140	150	160
SLLAVSNKYG	LVFAGGASGL	QIFPTKNLLI	QNKPGDDPNK	IVDKVQGLLV	PMKFPIHHLA	LSCDNLTLSA	CMMSSEYGSI
170	180	190	200	210	220	230	240
IAFFDVRTFS	NEAKQQKRPF	AYHKLLKDAG	GMVIDMKWNP	TVPSMVAVCL	ADGSIAVLQV	TETVKVCATL	PSTVAVTSVC
250	260	270	280	290	300	310	320
WSPKGKQLAV	GKQNGTVVQY	LPTLQEKKVI	PCPPFYESDH	PVRVLDVLWI	GTYVFAIVYA	AADGTLETSP	DVVMALLPKK
330	340	350	360	370	380	390	400
EEKHPEIFVN	FMEPCYGSCT	ERQHHYYLSY	IEEWDLVLAA	SAASTEVSIL	ARQSDQINWE	SWLLEDSSRA	ELPVTDKSDD
410	420	430	440	450	460	470	480
SLPMGVVVDY	TNQVEITISD	EKTLPPAPVL	MLLSTDGVLC	PFYMINQNPG	VKSLIKTPER	LSLEGERQPK	SPASLAPTPA
490	500	510	520	530	540	550	560
ASPVAPSAAS	FSFGSSGFKP	TLESTPVPSV	SAPNIAMKPS	FPPSTSAVKV	NLSEKFTAAA	TSTPVSSSQS	APPMSPFSSA
570	580	590	600	610	620	630	640
SKPAASGPLS	HPTPLSAPPS	SVPLKSSVLP	SPSAGRSAQG	SSSPVPSMVQ	KSPRITPPAA	KPGSPQAKSL	QPAVAEKQGH
650	660	670	680	690	700	710	720
QWKDSDPVMA	GIGEEIAHFQ	KELEELKART	SKACFQVGTS	EEMKMLRTES	DDLHTFLLEI	KETTESLHGD	ISSLKTTLLE
730	740	750	760	770	780	790	800
GFAGVEEARE	QNERNRDSGY	LHLLYKRPLD	PKSEAQLQEI	RRLHQYVKFA	VQDVNDVLDL	EWDQHLEQKK	KQRHLLVPER
810	820	830	840	850	860	870	880
ETLFNTLANN	REIINQQRKR	LNHLVDSLQQ	LRLYKQTSLW	SLSSAVPSQS	SIHSFDSDLE	SLCNALLKTT	IESHTKSLPK
890	900	910	920	930	940	950	960
VPAKLSPMKQ	AQLRNFLAKR	KTPPVRSTAP	ASLSRSAFLS	QRYYEDLDEV	SSTSSVSQSL	ESEDARTSCK	DDEAVVQAPR
970	980	990	1000	1010	1020	1030	1040
HAPVVRTPSI	QPSLLPHAAP	FAKSHLVHGS	SPGVMGTSVA	TSASKIIPQG	ADSTMLATKT	VKHGAPSPSH	PISAPQAAAA
1050	1060	1070	1080	1090	1100	1110	1120
AALRRQMASQ	APAVNTLTES	TLKNVPQVVN	VQELKNNPAT	PSTAMGSSVP	YSTAKTPHPV	LTPVAANQAK	QGSLINSLKP
1130	1140	1150	1160	1170	1180	1190	1200
SGPTPASGQL	SSGDKASGTA	KIETAVTSTP	SASGQFSKPF	SFSPSGTGFN	FGIITPTPSS	NFTAAQGATP	STKESSQPDA
1210	1220	1230	1240	1250	1260	1270	1280
FSSGGGSKPS	YEAIPESSPP	SGITSASNTT	PGEPAASSSR	PVAPSGTALS	TTSSKLETPP	SKLGELLFPS	SLAGETLGSF
1290	1300	1310	1320	1330	1340	1350	1360
SGLRVGQADD	STKPTNKASS	TSLTSTQPTK	TSGVPSGFNF	TAPPVLGKHT	EPPVTSSATT	TSVAPPAATS	TSSTAVFGSL
1370	1380	1390	1400	1410	1420	1430	1440
PVTSAGSSGV	ISFGGTSLSA	GKTSFSFGSQ	QTNSTVPPSA	PPPTTAATPL	PTSFPTLSFG	SLLSSATTPS	LPMSAGRSTE
1450	1460	1470	1480	1490	1500	1510	1520
EATSSALPEK	PGDSEVSASA	ASLLEEQQSA	QLPQAPPQTS	DSVKKEPVLA	QPAVSNSGTA	ASSTSLVALS	AEATPATTGV
1530	1540	1550	1560	1570	1580	1590	1600
PDARTEAVPP	ASSFSVPGQT	AVTAAAISSA	GPVAVETSST	PIASSTTSIV	APGPSAEAAA	FGTVTSGSSV	FAQPPAASSS
1610	1620	1630	1640	1650	1660	1670	1680
SAFNQLTNNT	ATAPSATPVF	GQVAASTAPS	LFGQQTGSTA	STAAATPQVS	SSGFSSPAFG	TTAPGVFGQT	TFGQASVFGQ
1690	1700	1710	1720	1730	1740	1750	1760
SASSAASVFS	FSQPGFSSVP	AFGQPASSTP	TSTSGSVFGA	ASSTSSSSSF	SFGQSSPNTG	GGLFGQSNAP	AFGQSPGFGQ
1770	1780	1790	1800	1810	1820	1830	1840
GGSVFGGTSA	ATTTAATSGF	SFCQASGFGS	SNTGSVFGQA	ASTGGIVFGQ	QSSSSSGSVF	GSGNTGRGGG	FFSGLGGKPS
1850	1860	1870	1880	1890	1900	1910	1920
QDAANKNPFS	SASGGFGSTA	TSNTSNLFGN	SGAKTFGGFA	SSSFGEQKPT	GTFSSGGGSV	ASQGFGFSSP	NKTGGFGAAP
1930	1940	1950	1960	1970	1980	1990	2000
VFGSPPTFGG	SPGFGGVPAF	GSAPAFTSPL	GSTGGKVFGE	GTAAASAGGF	GFGSSSNTTS	FGTLASQNAP	TFGSLSQQTS
2010	2020	2030	2040				
GFGTQSSGFS	GFGSGTGGFS	FGSNNSSVQG	FGGWRS				

Cmpd.	No. of Cmpds.	m/z meas.	Δ m/z [ppm]	z	Rt [min]	Score	P	Range	Sequence	Modification

Detailed Protein Report

Cmpd.	No. of Cmpds.	m/z meas.	Δ m/z [ppm]	z	Rt [min]	Score	P	Range	Sequence	Modification
2741	1	715.3112	-107.27	2	62.7	17.4	1	66-77	K.VRIFDSPEELPK.E	
1945	4	1023.3932	-98.93	2	54.7	16.5	2	670-687	R.TSKACFQVGTSEEMKMLR.T	

Detailed Protein Report

Protein 342: PREDICTED: leucine-rich repeat serine/threonine-protein kinase 2 isoform X1 [Homo sapiens]

**Accession:**	gi|530399725	**Score:**	33.7
**Database:**	refseq_human(refseq_human_20140103.fasta)	**MW [kDa]:**	285.2
**Database Date:**	2015-11-30	**pI:**	6.3
**Modification(s):**	Oxidation	**Sequence Coverage [%]:**	1.6
		**No. of unique Peptides:**	2

Quantitation

QU:MU	**Median:**	0.46	**CV:**	0.00 %	**No. of Peptides:**	1
WUP:QUP	**Median:**	1.75	**CV:**	0.00 %	**No. of Peptides:**	1

Detailed Protein Report

10	20	30	40	50	60	70	80
MASGSCQGCE	EDEETLKKLI	VRLNNVQEGK	QIETLVQILE	DLLVFTYSER	ASKLFQGKNI	HVPLLIVLDS	YMRVASVQQV
90	100	110	120	130	140	150	160
GWSLLCKLIE	VCPGTMQSLM	GPQDVGNDWE	VLGVHQLILK	MLTVHNASVN	LSVIGLKTLD	LLLTSGKITL	LILDEESDIF
170	180	190	200	210	220	230	240
MLIFDAMHSF	PANDEVQKLG	CKALHVLFER	VSEEQLTEFV	ENKDYMILLS	ALTNFKDEEE	IVLHVLHCLH	SLAIPCNNVE
250	260	270	280	290	300	310	320
VLMSGNVRCY	NIVVEAMKAF	PMSERIQEVS	CCLLHRLTLG	NFFNILVLNE	VHEFVVKAVQ	QYPENAALQI	SALSCLALLT
330	340	350	360	370	380	390	400
ETIFLNQDLE	EKNENQENDD	EGEEDKLFWL	EACYKALTWH	RKNKHVQEAA	CWALNNLLMY	QNSLHEKIGD	EDGHFPAHRE
410	420	430	440	450	460	470	480
VMLSMLMHSS	SKEVFQASAN	ALSTLLEQNV	NFRKILLSKG	IHLNVLELMQ	KHIHSPEVAE	SGCKMLNHLF	EGSNTSLDIM
490	500	510	520	530	540	550	560
AAVVPKILTV	MKRHETSLPV	QLEALRAILH	FIVPGMPEES	REDTEFHHKL	NMVKKQCFKN	DIHKLVLAAL	NRFIGNPGIQ
570	580	590	600	610	620	630	640
KCGLKVISSI	VHFPDALEML	SLEGAMDSVL	HTLQMYPDDQ	EIQCLGLSLI	GYLITKKNVF	IGTGHLLAKI	LVSSLYRFKD
650	660	670	680	690	700	710	720
VAEIQTKGFQ	TILAILKLSA	SFSKLLVHHS	FDLVIFHQMS	SNIMEQKDQQ	FLNLCCKCFA	KVAMDDYLKN	VMLERACDQN
730	740	750	760	770	780	790	800
NSIMVECLLL	LGADANQAKE	GSSLICQVCE	KESSPKLVEL	LLNSGSREQD	VRKALTISIG	KGDSQIISLL	LRRLALDVAN
810	820	830	840	850	860	870	880
NSICLGGFCI	GKVEPSWLGP	LFPDKTSNLR	KQTNIASTLA	RMVIRYQMKS	AVEEGTASGS	DGNFSEDVLS	KFDEWTFIPD
890	900	910	920	930	940	950	960
SSMDSVFAQS	DDLDSEGSEG	SFLVKKKSNS	ISVGEFYRDA	VLQRCSPNLQ	RHSNSLGPIF	DHEDLLKRKR	KILSSDDSLR
970	980	990	1000	1010	1020	1030	1040
SSKLQSHMRH	SDSISSLASE	REYITSLDLS	ANELRDIDAL	SQKCCISVHL	EHLEKLELHQ	NALTSFPQQL	CETLKSLTHL
1050	1060	1070	1080	1090	1100	1110	1120
DLHSNKFTSF	PSYLLKMSCI	ANLDVSRNDI	GPSVVLDPTV	KCPTLKQFNL	SYNQLSFVPE	NLTDVVEKLE	QLILEGNKIS
1130	1140	1150	1160	1170	1180	1190	1200
GICSPLRLKE	LKILNLSKNH	ISSLSENFLE	ACPKVESFSA	RMNFLAAMPF	LPPSMTILKL	SQNKFSCIPE	AILNLPHLRS
1210	1220	1230	1240	1250	1260	1270	1280
LDMSSNDIQY	LPGPAHWKSL	NLRELLFSHN	QISILDLSEK	AYLWSRVEKL	HLSHNKLKEI	PPEIGCLENL	TSLDVSYNLE
1290	1300	1310	1320	1330	1340	1350	1360
LRSFPNEMGK	LSKIWDLPLD	ELHLNFDFKH	IGCKAKDIIR	FLQQRLKKAV	PYNRMKLMIV	GNTGSGKTTL	LQQLMKTKKS
1370	1380	1390	1400	1410	1420	1430	1440
DLGMQSATVG	IDVKDWPIQI	RDKRKRDLVL	NVWDFAGREE	FYSTHPHFMT	QRALYLAVYD	LSKGQAEVDA	MKPWLFNIKA
1450	1460	1470	1480	1490	1500	1510	1520
RASSSPVILV	GTHLDVSDEK	QRKACMSKIT	KELLNKRGFP	AIRDYHFVNA	TEESDALAKL	RKTIINESLN	FKIRDQLVVG
1530	1540	1550	1560	1570	1580	1590	1600
QLIPDCYVEL	EKIILSERKN	VPIEFPVIDR	KRLLQLVREN	QLQLDENELP	HAVHFLNESG	VLLHFQDPAL	QLSDLYFVEP
1610	1620	1630	1640	1650	1660	1670	1680
KWLCKIMAQI	LTVKVEGCPK	HPKGIISRRD	VEKFLSKKRK	FPKNYMSQYF	KLLEKFQIAL	PIGEEYLLVP	SSLSDHRPVI
1690	1700	1710	1720	1730	1740	1750	1760
ELPHCENSEI	IIRLYEMPYF	PMGFWSRLIN	RLLEISPYML	SGRERALRPN	RMYWRQGIYL	NWSPEAYCLV	GSEVLDNHPE
1770	1780	1790	1800	1810	1820	1830	1840
SFLKITVPSC	RKGCILLGQV	VDHIDSLMEE	WFPGLLEIDI	CGEGETLLKK	WALYSFNDGE	EHQKILLDDL	MKKAEEGDLL
1850	1860	1870	1880	1890	1900	1910	1920
VNPDQPRLTI	PISQIAPDLI	LADLPRNIML	NNDELEFEQA	PEFLLGDGSF	GSVYRAAYEG	EEVAVKIFNK	HTSLRLLRQE
1930	1940	1950	1960	1970	1980	1990	2000
LVVLCHLHHP	SLISLLAAGI	RPRMLVMELA	SKGSLDRLLQ	QDKASLTRTL	QHRIALHVAD	GLRYLHSAMI	IYRDLKPHNV
2010	2020	2030	2040	2050	2060	2070	2080
LLFTLYPNAA	IIAKIADYGI	AQYCCRMGIK	TSEGTPGFRA	PEVARGNVIY	NQQADVYSFG	LLLYDILTTG	GRIVEGLKFP
2090	2100	2110	2120	2130	2140	2150	2160
NEFDELEIQG	KLPDPVKEYG	CAPWPMVEKL	IKQCLKENPQ	ERPTSAQVFD	ILNSAELVCL	TRRILLPKNV	IVECMVATHH
2170	2180	2190	2200	2210	2220	2230	2240

Detailed Protein Report

Cmpd.	No. of Cmpds.	m/z meas.	Δ m/z [ppm]	z	Rt [min]	Score	P	Range	Sequence	Modification	Ratios
394	1	591.8037	-59.20	2	34.9	10.0	1	1964-1973	K.ASLTRTLQHR.I		QU:MU 0.46<br>WUP:QUP 1.75
1730	1	796.0646	-20.88	3	51.4	11.0	1	2457-2477	R.VMMTAQLGSLKNVMLVLGYNR.K	Oxidation: 2, 3, 14	

Detailed Protein Report

Protein 343: PREDICTED: zinc finger protein 708 isoform X1 [Homo sapiens]

Accession: gi|578833563
Database: refseq_human(refseq_human_20140103.fasta)
Database Date: 2015-11-30
Modification(s): Carbamidomethyl

Score: 33.6
MW [kDa]: 57.3
pI: 10.4
Sequence Coverage [%]: 4.0
No. of unique Peptides: 2

Quantitation

QU:MU **Median:** 2.98 **CV:** 0.00 % **No. of Peptides:** 1

10	20	30	40	50	60	70	80
MKRHEMAAKP	PAMCSHFAKD	LRPEQYIKNS	FQQVILRRYG	KCGYQKGCKS	VDEHKLHKGG	HKGLNRCVTT	TQSKIVQCDK
**90**	**100**	**110**	**120**	**130**	**140**	**150**	**160**
YVKVFHKYSN	AKRHKIRHTG	KNPFKCKECG	KSFCMLSQLT	QHEIIHTGEK	PYKCEECGKA	FKKSSNLTNH	KIIHTGEKPY
**170**	**180**	**190**	**200**	**210**	**220**	**230**	**240**
KCEECGKAFN	QSSTLTRHKI	IHTGEKLYKC	EECGKAFNRS	SNLTKHKIVH	TGEKPYKCEE	CGKAFKQSSN	LTNHKKIHTG
**250**	**260**	**270**	**280**	**290**	**300**	**310**	**320**
EKPYKCGECG	KAFTLSSHLT	THKRIHTGEK	PYKCEECGKA	FSVFSTLTKH	KIIHTEEKPY	KCEECGKAFN	RSSHLTNHKV
**330**	**340**	**350**	**360**	**370**	**380**	**390**	**400**
IHTGEKPYKC	EECGKAFTKS	STLTYHKVIH	TGKKPYKCEE	CGKAFSIFSI	LTKHKVIHTE	DKPYKCEECG	KTFNYSSNFT
**410**	**420**	**430**	**440**	**450**	**460**	**470**	**480**
NHKKIHTGEK	PYKCEECGKS	FILSSHLTTH	KIIHTGEKPY	KCKECGKAFN	QSSTLMKHKI	IHTGEKPYKC	EECGKAFNQS
**490**	**500**						
PNLTKHKRIH	TKEKPYKCK						

Cmpd.	No. of Cmpds.	m/z meas.	Δ m/z [ppm]	z	Rt [min]	Score	P	Range	Sequence	Modification	Ratios
252	1	605.6643	-205.83	2	32.9	12.7	2	102-111	K.NPFKCKECGK.S	Carbamidomethyl: 8	QU:MU 2.98
1258	2	586.7739	28.23	2	45.9	20.9	1	330-339	K.CEECGKAFTK.S	Carbamidomethyl: 4	

Detailed Protein Report

Protein 344: **PREDICTED: ras-specific guanine nucleotide-releasing factor 2 isoform X1 [Homo sapiens]**

**Accession:**	gi|530379425	**Score:**	33.5
**Database:**	refseq_human(refseq_human_20140103.fasta)	**MW [kDa]:**	133.5
**Database Date:**	2015-11-30	**pI:**	8.3
**Modification(s):**	Oxidation	**Sequence Coverage [%]:**	2.1
		**No. of unique Peptides:**	1

10	20	30	40	50	60	70	80
MQKSVRYNEG	HALYLAFLAR	KEGTKRGFLS	KKTAEASRWH	EKWFALYQNV	LFYFEGEQSC	RPAGMYLLEG	CSCERTPAPP
**90**	**100**	**110**	**120**	**130**	**140**	**150**	**160**
RAGAGQGGVR	DALDKQYYFT	VLFGHEGQKP	LELRCEEEQD	GKEWMEAIHQ	ASYADILIER	EVLMQKYIHL	VQIVETEKIA
**170**	**180**	**190**	**200**	**210**	**220**	**230**	**240**
ANQLRHQLED	QDTEIERLKS	EIIALNKTKE	RMRPYQSNQE	DEDPDIKKIK	KVQSFMRGWL	CRRKWKTIVQ	DYICSPHAES
**250**	**260**	**270**	**280**	**290**	**300**	**310**	**320**
MRKRNQIVFT	MVEAESEYVH	QLYILVNGFL	RPLRMAASSK	KPPISHDDVS	SIFLNSETIM	FLHEIFHQGL	KARIANWPTL
**330**	**340**	**350**	**360**	**370**	**380**	**390**	**400**
ILADLFDILL	PMLNIYQEFV	RNHQYSLQVL	ANCKQNRDFD	KLLKQYEANP	ACEGRMLETF	LTYPMFQIPR	YIITLHELLA
**410**	**420**	**430**	**440**	**450**	**460**	**470**	**480**
HTPHEHVERK	SLEFAKSKLE	ELSRVMHDEV	SDTENIRKNL	AIERMIVEGC	DILLDTSQTF	IRQGSLIQVP	SVERGKLSKV
**490**	**500**	**510**	**520**	**530**	**540**	**550**	**560**
RLGSLSLKKE	GERQCFLFTK	HFLICTRSSG	GKLHLLKTGG	VLSLIDCTLI	EEPDASDDDS	KGSGQVFGHL	DFKIVVEPPD
**570**	**580**	**590**	**600**	**610**	**620**	**630**	**640**
AAAFTVVLLA	PSRQEKAAWM	SDISQCVDNI	RCNGLMTIVF	EENSKVTVPH	MIKSDARLHK	DDTDICFSKT	LNSCKVPQIR
**650**	**660**	**670**	**680**	**690**	**700**	**710**	**720**
YASVERLLER	LTDLRFLSID	FLNTFLHTYR	IFTTAAVVLG	KLSDIYKRPF	TSIPVRSLEL	FFATSQNNRG	EHLVDGKSPR
**730**	**740**	**750**	**760**	**770**	**780**	**790**	**800**
LCRKFSSPPP	LAVSRTSSPV	RARKLSLTSP	LNSKIGALDL	TTSSSPTTTT	QSPAASPPPH	TGQIPLDLSR	GLSSPEQSPG
**810**	**820**	**830**	**840**	**850**	**860**	**870**	**880**
TVEENVDNPR	VDLCNKLKRS	IQKGFNNTER	TCDKEFIIRR	TATNRVLNVL	RHWVSKHAQD	FELNNELKMN	VLNLLEEVLR
**890**	**900**	**910**	**920**	**930**	**940**	**950**	**960**
DPDLLPQERK	AAANILRALS	QDDQDDIHLK	LEDIIQMTDC	MKAECFESLS	AMELAEQITL	LDHVIFRSIP	YEEFLGQGWM
**970**	**980**	**990**	**1000**	**1010**	**1020**	**1030**	**1040**
KLDKNERTPY	IMKTSQHFND	MSNLVASQIM	NYADVSSRAN	AIEKWVAVAD	ICRCLHNYNG	VLEITSALNR	SAIYRLKKTW
**1050**	**1060**	**1070**	**1080**	**1090**	**1100**	**1110**	**1120**
AKVSKQTKAL	MDKLQKTVSS	EGRFKNLRET	LKNCNPPAVP	YLGMYLTDLA	FIEEGTPNFT	EEGLVNFSKM	RMISHIIREI
**1130**	**1140**	**1150**	**1160**	**1170**			
RQFQQTSYRI	DHQPKVAQYL	LDKDLIIDED	TLYELSLKIE	PRLPA			

Cmpd.	No. of Cmpds.	m/z meas.	Δ m/z [ppm]	z	Rt [min]	Score	P	Range	Sequence	Modification
1027	1	984.1548	34.58	3	42.4	33.5	1	898-922	R.ALSQDDQDDIHLKLEDIIQMTDCMK.A	Oxidation: 20, 24

Detailed Protein Report

Protein 345: **PREDICTED: nascent polypeptide-associated complex subunit alpha isoform X6 [Homo sapiens]**

**Accession:**	gi|578823772	**Score:**	33.5
**Database:**	refseq_human(refseq_human_20140103.fasta)	**MW [kDa]:**	201.3
**Database Date:**	2015-11-30	**pI:**	10.2
		**Sequence Coverage [%]:**	1.8
		**No. of unique Peptides:**	2

Detailed Protein Report

10	20	30	40	50	60	70	80
MPGEATETVP	ATEQELPQPQ	AETAVLPMSS	ALSVTAALGQ	PGPTLPPPCS	PAPQQCPLSA	ANQASPFPSP	STIASTPLEV
90	100	110	120	130	140	150	160
PFPQSSSGTA	LPLGTAPEAP	TFLPNLIGPP	ISPAALALAS	PMIAPTLKGT	PSSSAPLALV	ALAPHSVQKS	SAFPPNLLTS
170	180	190	200	210	220	230	240
PPSVAVAESG	SVITLSAPIA	PSEPKTNLNK	VPSEVVPNPK	GTPSPPCIVS	TVPYHCVTPM	ASIQSGVASL	PQTTPTTTLA
250	260	270	280	290	300	310	320
IASPQVKDTT	ISSVLISPQN	PGSLSLKGPV	SPPAALSLST	QSLPVVTSSQ	KTAGPNTPPD	FPISLGSHLA	PLHQSSFGSV
330	340	350	360	370	380	390	400
QLLGQTGPSA	LSDPTVKTIS	VDHSSTGASY	PSQRSVIPPL	PSRNEVVPAT	VAAFPVVAPS	VDKGPSTISS	ITCSPSGSLN
410	420	430	440	450	460	470	480
VATSFSLSPT	TSLILKSSPN	ATYHYPLVAQ	MPVSSVGTTP	LVVTNPCTIA	AAPTTTFEVA	TCVSPPMSSG	PISNIEPTSP
490	500	510	520	530	540	550	560
AALVMAPVAP	KEPSTQVATT	LRIPVSPPLP	DPEDLKNLPS	SVLVKFPTQK	DLQTVPASLE	GAPFSPAQAG	LTTKKDPTVL
570	580	590	600	610	620	630	640
PLVQAAPKNS	PSFQSTSSSP	EIPLSPEATL	AKKSLGEPLP	IGKPASSMTS	PLGVNSSASV	IKTDSYAGPD	SAGPLLKSSL
650	660	670	680	690	700	710	720
ITPTVAAFPL	ESADPAGVAP	TTAKGTSTYT	TTASPFLEGT	VSLAPKNHPV	KEGTLTTLPL	VPTASENCPV	APSPQNTCAP
730	740	750	760	770	780	790	800
LATLVLAPEI	PKSVPSPSLP	PAGTPPGTKK	VDGISHTSAL	APVASSPKEC	PTEDSGASAT	ASSKGTLTYL	ADSPSPLGVS
810	820	830	840	850	860	870	880
VSPQTKRPPT	KKGSAGPDTP	IGNLSSPVSP	VEASFLPENS	LSFQGSKDSP	ATTHSPTPPS	PKGAPTPSAV	TPLSPKGVTL
890	900	910	920	930	940	950	960
PPKETPTPSV	VNLPFPKEGP	ATPAPKQAPA	LSMTSSSPKK	ARATPAPKGI	PASPSPKGAP	TPPAATPPSP	KGGPATPSPK
970	980	990	1000	1010	1020	1030	1040
WAPTPPAATP	PSPKGGPATP	SPKGAPTPPA	ATPPSPKGSP	AATPLPKGAP	TTPAATLPSP	KGGPATPSLK	GAPTPPAATP
1050	1060	1070	1080	1090	1100	1110	1120
PSPKGGPATP	SPKGAPMPPA	ATPPSPKGGL	ATPPHKGAPT	TPAATPPSPK	GGLATPPPKG	APTTPAATPP	SPKGGLATPP
1130	1140	1150	1160	1170	1180	1190	1200
PKGAPTTPAA	TPPSPKGGLA	TPSPKGAPTT	PAATPPSPKG	GLATPSPKGA	PTTPAATPPS	PKGGLATPSP	KGAPTTPAAT
1210	1220	1230	1240	1250	1260	1270	1280
PPSPKGGPAT	PPPKGAPTPP	AATPPSLKGG	LATPPHKGAP	NPAVVTPPSP	KGGPATSPPK	GAPTPPAATP	PSPKGSPGTP
1290	1300	1310	1320	1330	1340	1350	1360
PPKGAPTPPA	VTPPSPKGTP	TLPATTPSSK	GGPTTPSSKE	GPTPPAATPS	HKGGPAMTPP	SPKRGPAIPS	PKGDPTSPAV
1370	1380	1390	1400	1410	1420	1430	1440
IPLSPKKAPA	TPVTREGAAT	PSKGDLTPPA	VTPVSLKKAP	ATSAPKGGPA	TPSSKGDPTL	PAVTPPSPKE	PPAPKQVATS
1450	1460	1470	1480	1490	1500	1510	1520
SSPKKAPATP	APMGAPTLPA	VIPSSPKEVP	ATPSSRRDPI	APTATLLSKK	TPATLAPKEA	LIPPAMTVPS	PKKTPAIPTP
1530	1540	1550	1560	1570	1580	1590	1600
KEAPATPSSK	EASSPPAVTP	STYKGAPSPK	ELLIPPAVTS	PSPKEAPTPP	AVTPPSPEKG	PATPAPKGTP	TSPPVTPSSL
1610	1620	1630	1640	1650	1660	1670	1680
KDSPTSPASV	TCKMGATVPQ	ASKGLPAKKG	PTALKEVLVA	PAPESTPIIT	APTRKGPQTK	KSSATSPPIC	PDPSAKNGSK
1690	1700	1710	1720	1730	1740	1750	1760
GPLSTVAPAP	LLPVQKDSSK	TAKGKDASHS	PKGPLAPPES	KASTPLTAAA	FEKVLPKPES	ASVSAAPSPP	VSLPLAPSPV
1770	1780	1790	1800	1810	1820	1830	1840
PTLPPKQQFL	PSSPGLVLES	PSKPLAPADE	DELLPLIPPE	PISGGVPFQS	VLVNMPTPKS	AGIPVPTPSA	KQPVTKNNKG
1850	1860	1870	1880	1890	1900	1910	1920
SGTESDSDES	VPELEEQDST	QATTQQAQLA	AAAEIDEEPV	SKAKQSRSEK	KARKAMSKLG	LRQVTGVTRV	TIRKSKNILF
1930	1940	1950	1960	1970	1980	1990	2000
VITKPDVYKS	PASDTYIVFG	EAKIEDLSQQ	AQLAAAEKFK	VQGEAVSNIQ	ENTQTPTVQE	ESEEEEVDET	GVEVKDIELV
2010	2020	2030	2040				
MSQANVSRAK	AVRALKNNSN	DIVNAIMVSV	QAFVP				

Cmpd.	No. of Cmpds.	m/z meas.	Δ m/z [ppm]	z	Rt [min]	Score	P	Range	Sequence	Modification

Detailed Protein Report

Cmpd.	No. of Cmpds.	m/z meas.	Δ m/z [ppm]	z	Rt [min]	Score	P	Range	Sequence	Modification
732	1	472.2592	28.32	2	39.1	11.0	0	1468-1476	K.EVPATPSSR.R	
52	1	717.7555	-144.48	2	30.5	11.1	0	1531-1544	K.EASSPPAVTPSTYK.G	

Detailed Protein Report

Protein 346: **semaphorin-3A precursor [Homo sapiens]**

**Accession:**	gi|5174673	**Score:**	33.4
**Database:**	refseq_human(refseq_human_20140103.fasta)	**MW [kDa]:**	88.8
**Database Date:**	2015-11-30	**pI:**	7.2
		**Sequence Coverage [%]:**	2.6
		**No. of unique Peptides:**	2

Quantitation

QU:MU	**Median:**	0.64	**CV:**	0.00 %	**No. of Peptides:**	1
WUP:QUP	**Median:**	1.31	**CV:**	0.00 %	**No. of Peptides:**	1

Alias proteins:

Accession	Name	Description
gi|578813881	refseq_human (refseq_human_20140103.fasta)	PREDICTED: semaphorin-3A isoform X4 [Homo sapiens]
gi|578813879	refseq_human (refseq_human_20140103.fasta)	PREDICTED: semaphorin-3A isoform X3 [Homo sapiens]
gi|530385417	refseq_human (refseq_human_20140103.fasta)	PREDICTED: semaphorin-3A isoform X2 [Homo sapiens]
gi|530385415	refseq_human (refseq_human_20140103.fasta)	PREDICTED: semaphorin-3A isoform X1 [Homo sapiens]

10	20	30	40	50	60	70	80
MGWLTRIVCL	FWGVLLTARA	NYQNGKNNVP	RLKLSYKEML	ESNNVITFNG	LANSSSYHTF	LLDEERSRLY	VGAKDHIFSF
**90**	**100**	**110**	**120**	**130**	**140**	**150**	**160**
DLVNIKDFQK	IVWPVSYTRR	DECKWAGKDI	LKECANFIKV	LKAYNQTHLY	ACGTGAFHPI	CTYIEIGHHP	EDNIFKLENS
**170**	**180**	**190**	**200**	**210**	**220**	**230**	**240**
HFENGRGKSP	YDPKLLTASL	LIDGELYSGT	AADFMGRDFA	IFRTLGHHHP	IRTEQHDSRW	LNDPKFISAH	LISESDNPED
**250**	**260**	**270**	**280**	**290**	**300**	**310**	**320**
DKVYFFFREN	AIDGEHSGKA	THARIGQICK	NDFGGHRSLV	NKWTTFLKAR	LICSVPGPNG	IDTHFDELQD	VFLMNFKDPK
**330**	**340**	**350**	**360**	**370**	**380**	**390**	**400**
NPVVYGVFTT	SSNIFKGSAV	CMYSMSDVRR	VFLGPYAHRD	GPNYQWVPYQ	GRVPYPRPGT	CPSKTFGGFD	STKDLPDDVI
**410**	**420**	**430**	**440**	**450**	**460**	**470**	**480**
TFARSHPAMY	NPVFPMNNRP	IVIKTDVNYQ	FTQIVVDRVD	AEDGQYDVMF	IGTDVGTVLK	VVSIPKETWY	DLEEVLLEEM
**490**	**500**	**510**	**520**	**530**	**540**	**550**	**560**
TVFREPTAIS	AMELSTKQQQ	LYIGSTAGVA	QLPLHRCDIY	GKACAECCLA	RDPYCAWDGS	ACSRYFPTAK	RRTRRQDIRN
**570**	**580**	**590**	**600**	**610**	**620**	**630**	**640**
GDPLTHCSDL	HHDNHHGHSP	EERIIYGVEN	SSTFLECSPK	SQRALVYWQF	QRRNEERKEE	IRVDDHIIRT	DQGLLLRSLQ
**650**	**660**	**670**	**680**	**690**	**700**	**710**	**720**
QKDSGNYLCH	AVEHGFIQTL	LKVTLEVIDT	EHLEELLHKD	DDGDGSKTKE	MSNSMTPSQK	VWYRDFMQLI	NHPNLNTMDE
**730**	**740**	**750**	**760**	**770**	**780**		
FCEQVWKRDR	KQRRQRPGHT	PGNSNKWKHL	QENKKGRNRR	THEFERAPRS	V		

Cmpd.	No. of Cmpds.	m/z meas.	Δ m/z [ppm]	z	Rt [min]	Score	P	Range	Sequence	Modification	Ratios
1358	2	436.6061	-206.99	2	47.1	18.6	0	213-219	R.TEQHDSR.W		QU:MU 0.64<br>WUP:QUP 1.31
2058	1	722.8469	-17.26	2	56.1	14.8	1	265-277	R.IGQICKNDFGGHR.S		

Detailed Protein Report

Protein 347: transmembrane protein 169 [Homo sapiens]

**Accession:**	gi|19923913	**Score:**	33.3
**Database:**	refseq_human(refseq_human_20140103.fasta)	**MW [kDa]:**	33.6
**Database Date:**	2015-11-30	**pI:**	4.6
**Modification(s):**	Oxidation	**Sequence Coverage [%]:**	13.5
		**No. of unique Peptides:**	1

Alias proteins:

Accession	Name	Description
gi|578805184	refseq_human (refseq_human_20140103.fasta)	PREDICTED: transmembrane protein 169 isoform X1 [Homo sapiens]
gi|214831834	refseq_human (refseq_human_20140103.fasta)	transmembrane protein 169 [Homo sapiens]
gi|214831799	refseq_human (refseq_human_20140103.fasta)	transmembrane protein 169 [Homo sapiens]
gi|214831780	refseq_human (refseq_human_20140103.fasta)	transmembrane protein 169 [Homo sapiens]

10	20	30	40	50	60	70	80
MEEPTAVEGQ	VQLPSPHQGS	LRKAVAAALA	LDGESTMGHR	KKKRKESRPE	SIIIYRSDNE	KTDEEPGESE	GGDQPKEEEG
**90**	**100**	**110**	**120**	**130**	**140**	**150**	**160**
DDFLDYPVDD	DMWNLPLDSR	YVTLTGTITR	GKKKGQMVDI	HVTLTEKELQ	ELTKPKESSR	ETTPEGRMAC	QMGADRGPHV
**170**	**180**	**190**	**200**	**210**	**220**	**230**	**240**
VLWTLICLPV	VFILSFVVSF	YYGTITWYNI	FLVYNEERTF	WHKISYCPCL	VLFYPVLIMA	MASSLGLYAA	VVQLSWSWEA
**250**	**260**	**270**	**280**	**290**	**300**		
WWQAARDMEK	GFCGWLCSKL	GLEDCSPYSI	VELLESDNIS	STLSNKDPIQ	EVETSTV		

Cmpd.	No. of Cmpds.	m/z meas.	Δ m/z [ppm]	z	Rt [min]	Score	P	Range	Sequence	Modification
77	1	845.3458	-96.16	3	30.7	10.3	1	1-23	-.MEEPTAVEGQVQLPSPHQGSLRK.A	Oxidation: 1

Detailed Protein Report

Protein 348: heat shock 70 kDa protein 1A/1B [Homo sapiens]

**Accession:**	gi|167466173	**Score:**	33.3
**Database:**	refseq_human(refseq_human_20140103.fasta)	**MW [kDa]:**	70.0
**Database Date:**	2015-11-30	**pI:**	5.4
		**Sequence Coverage [%]:**	3.9
		**No. of unique Peptides:**	2

Quantitation

QU:MU	**Median:** 0.62	**CV:** 0.00 %	**No. of Peptides:** 1

Alias proteins:

Accession	Name	Description
gi|194248072	refseq_human (refseq_human_20140103.fasta)	heat shock 70 kDa protein 1A/1B [Homo sapiens]

10	20	30	40	50	60	70	80
MAKAAAIGID	LGTTYSCVGV	FQHGKVEIIA	NDQGNRTTPS	YVAFTDTERL	IGDAAKNQVA	LNPQNTVFDA	KRLIGRKFGD
**90**	**100**	**110**	**120**	**130**	**140**	**150**	**160**
PVVQSDMKHW	PFQVINDGDK	PKVQVSYKGE	TKAFYPEEIS	SMVLTKMKEI	AEAYLGYPVT	NAVITVPAYF	NDSQRQATKD
**170**	**180**	**190**	**200**	**210**	**220**	**230**	**240**
AGVIAGLNVL	RIINEPTAAA	IAYGLDRTGK	GERNVLIFDL	GGGTFDVSIL	TIDDGIFEVK	ATAGDTHLGG	EDFDNRLVNH
**250**	**260**	**270**	**280**	**290**	**300**	**310**	**320**
FVEEFKRKHK	KDISQNKRAV	RRLRTACERA	KRTLSSSTQA	SLEIDSLFEG	IDFYTSITRA	RFEELCSDLF	RSTLEPVEKA
**330**	**340**	**350**	**360**	**370**	**380**	**390**	**400**
LRDAKLDKAQ	IHDLVLVGGS	TRIPKVQKLL	QDFFNGRDLN	KSINPDEAVA	YGAAVQAAIL	MGDKSENVQD	LLLLDVAPLS
**410**	**420**	**430**	**440**	**450**	**460**	**470**	**480**
LGLETAGGVM	TALIKRNSTI	PTKQTQIFTT	YSDNQPGVLI	QVYEGERAMT	KDNNLLGRFE	LSGIPPAPRG	VPQIEVTFDI
**490**	**500**	**510**	**520**	**530**	**540**	**550**	**560**
DANGILNVTA	TDKSTGKANK	ITITNDKGRL	SKEEIERMVQ	EAEKYKAEDE	VQRERVSAKN	ALESYAFNMK	SAVEDEGLKG
**570**	**580**	**590**	**600**	**610**	**620**	**630**	**640**
KISEADKKKV	LDKCQEVISW	LDANTLAEKD	EFEHKRKELE	QVCNPIISGL	YQGAGGPGPG	GFGAQGPKGG	SGSGPTIEEV
**650**							
D							

Cmpd.	No. of Cmpds.	m/z meas.	Δ m/z [ppm]	z	Rt [min]	Score	P	Range	Sequence	Modification	Ratios
602	1	744.3677	18.07	2	37.5	10.9	0	37-49	R.TTPSYVAFTDTER.L		QU:MU 0.62
1835	2	599.2683	-138.16	2	53.3	22.4	0	160-171	K.DAGVIAGLNVLR.I		

Detailed Protein Report

Protein 349: PREDICTED: coiled-coil domain-containing protein 60 isoform X1 [Homo sapiens]

**Accession:**	gi|530401235	**Score:**	33.2
**Database:**	refseq_human(refseq_human_20140103.fasta)	**MW [kDa]:**	47.8
**Database Date:**	2015-11-30	**pI:**	10.4
**Modification(s):**	Oxidation	**Sequence Coverage [%]:**	7.4
		**No. of unique Peptides:**	2

10	20	30	40	50	60	70	80
MTKVPATKKL	QSSPNSGAVR	PFYASENLRQ	VPDKPMKSIK	YMDKEIINLK	KDLIRSRFLI	QSVKIGRGYF	AILREETAKK
**90**	**100**	**110**	**120**	**130**	**140**	**150**	**160**
KKQQQLQKLK	EEERNKFQPA	EKISEIHYGD	TLLSTYDDEK	LKTLGARVTR	RPFTPIHSCI	ISPSLTEAHV	EPLFRQLCAL
**170**	**180**	**190**	**200**	**210**	**220**	**230**	**240**
HWLLEALTID	HTHHTMKPVI	TCWNPKDPGG	SKSTIKKINK	DKSMGQKWEH	FITAPKTKKF	KIPTMRVTNR	KPSRRGSTLS
**250**	**260**	**270**	**280**	**290**	**300**	**310**	**320**
LSRASGGSSP	QSSMISVNPG	SDEPPSVNTQ	VTSSKDIEDN	ESSSTKPDEE	PLYMNLQKLL	EMVREDARRT	VTIENGMQRK
**330**	**340**	**350**	**360**	**370**	**380**	**390**	**400**
APSILSVLKQ	NKSNSAYKEM	QTTLKSSERS	SSTSAESHIQ	PVQKKSKNRT	NCDINIHYKS	GVCNTMRAKF	YSVAQEAGFC
**410**	**420**	**430**					
LQDKMEILMN	LLLDSRDHPS	R					

Cmpd.	No. of Cmpds.	m/z meas.	Δ m/z [ppm]	z	Rt [min]	Score	P	Range	Sequence	Modification
2869	1	724.8749	-14.66	2	64.6	19.1	2	309-320	R.RTVTIENGMQRK.A	Oxidation: 9
2374	1	1022.9281	-68.61	2	59.6	14.2	1	346-364	K.SSERSSSTSAESHIQPVQK.K	

Detailed Protein Report

Protein 350: PREDICTED: cylicin-1 isoform X1 [Homo sapiens]

**Accession:**	gi|530421958	**Score:**	33.2
**Database:**	refseq_human(refseq_human_20140103.fasta)	**MW [kDa]:**	74.1
**Database Date:**	2015-11-30	**pI:**	10.2
		**Sequence Coverage [%]:**	4.2
		**No. of unique Peptides:**	1

10	20	30	40	50	60	70	80
MSLPRLKVNI	RTYDNSIPIS	ESSRKSWNQK	HFALTFPKPL	QRGTNDKSRP	LKSQITVTRH	DKRKLEEGQK	PAHKWIRHSF
**90**	**100**	**110**	**120**	**130**	**140**	**150**	**160**
RKILQWPPIY	TAAREQTPFR	HLYTSKTHLK	KAEYKKSKDE	KGGTPLKKDS	KKKGGSYATN	PESKQIVEEK	TKRQNEADKT
**170**	**180**	**190**	**200**	**210**	**220**	**230**	**240**
PLKSSHENEQ	SKKSKSSSET	NPESQNSKTV	SKNCSQKDKK	DSKNSKKTNT	EFLHTKNNPK	KDLKRSKTSN	DPISEICSEN
**250**	**260**	**270**	**280**	**290**	**300**	**310**	**320**
SLNVDFLMLV	GQSDDESINF	DAWLRNYSQN	NSKNYSLKYT	KYTKKDTKKN	AKKSSDAESE	DSKDAKKDSK	KVKKNVKKDD
**330**	**340**	**350**	**360**	**370**	**380**	**390**	**400**
KKKDVKKDTE	STDAESGDSK	DERKDTKKDK	KKLKKDDKKK	DTKKYPESTD	TESGDAKDAR	NDSRNLKKAS	KNDDKKKDAK
**410**	**420**	**430**	**440**	**450**	**460**	**470**	**480**
KITFSTDSES	ELESKESQKD	EKKDKKDSKT	DNKKSVKNDE	ESTDADSEPK	GDSKKGKKDE	KKGKKDSKKD	DKKKDAKKNA
**490**	**500**	**510**	**520**	**530**	**540**	**550**	**560**
ESTEMESDLE	LKKDKKHSKE	KKGSKKDIKK	DARKDTESTD	AEFDESSKTG	FKTSTKIKGS	DTESEESLYK	PGAKKKIDES
**570**	**580**	**590**	**600**	**610**	**620**	**630**	**640**
DGTSANSKME	GLESKRGFRM	SSKKTTFNEK	GEKASTGRVP	PSREKPPLPA	CEPSLPSPKV	RRLCWCKMPP	PPPKPRYAPL
**650**	**660**						
PEAPWIHKLL							

Cmpd.	No. of Cmpds.	m/z meas.	Δ m/z [ppm]	z	Rt [min]	Score	P	Range	Sequence	Modification
547	1	472.6490	-226.33	2	35.3	16.3	1	119-127	K.DEKGGTPLK.K	

Detailed Protein Report

Protein 351: **PREDICTED: lysine-specific demethylase 2A isoform X3 [Homo sapiens]**

**Accession:**	gi|578821239	**Score:**	33.2
**Database:**	refseq_human(refseq_human_20140103.fasta)	**MW [kDa]:**	120.2
**Database Date:**	2015-11-30	**pI:**	9.2
		**Sequence Coverage [%]:**	2.1
		**No. of unique Peptides:**	2

10	20	30	40	50	60	70	80
MVDVMDVNTQ	KGIEMTMAQW	TRYYETPEEE	REKLYNVISL	EFSHTRLENM	VQRPSTVDFI	DWVDNMWPRH	LKESQTESTN
**90**	**100**	**110**	**120**	**130**	**140**	**150**	**160**
AILEMQYPKV	QKYCLMSVRG	CYTDFHVDFG	GTSVWYHIHQ	GGKVFWLIPP	TAHNLELYEN	WLLSGKQGDI	FLGDRVSDCQ
**170**	**180**	**190**	**200**	**210**	**220**	**230**	**240**
RIELKQGYTF	VIPSGWIHAV	YTPTDTLVFG	GNFLHSFNIP	MQLKIYNIED	RTRVPNKFRY	PFYYEMCWYV	LERYVYCITN
**250**	**260**	**270**	**280**	**290**	**300**	**310**	**320**
RSHLTKEFQK	ESLSMDLELN	GLESGNGDEE	AVDREPRRLS	SRRSVLTSPV	ANGVNLDYDG	LGKTCRSLPS	LKKTLAGDSS
**330**	**340**	**350**	**360**	**370**	**380**	**390**	**400**
SDCSRGSHNG	QVWDPQCAPR	KDRQVHLTHF	ELEGLRCLVD	KLESLPLHKK	CVPTGIEDED	ALIADVKILL	EELANSDPKL
**410**	**420**	**430**	**440**	**450**	**460**	**470**	**480**
ALTGVPIVQW	PKRDKLKFPT	RPKVRVPTIP	ITKPHTMKPA	PRLTPVRPAA	ASPIVSGARR	RRVRCRKCKA	CVQGECGVCH
**490**	**500**	**510**	**520**	**530**	**540**	**550**	**560**
YCRDMKKFGG	PGRMKQSCVL	RQCLAPRLPH	SVTCSLCGEV	DQNEETQDFE	KKLMECCICN	EIVHPGCLQM	DGEGLLNEEL
**570**	**580**	**590**	**600**	**610**	**620**	**630**	**640**
PNCWECPKCY	QEDSSEKAQK	RKMEESDEEA	VQAKVLRPLR	SCDEPLTPPP	HSPTSMLQLI	HDPVSPRGMV	TRSSPGAGPS
**650**	**660**	**670**	**680**	**690**	**700**	**710**	**720**
DHHSASRDER	FKRRQLLRLQ	ATERTMVREK	ENNPSGKKEL	SEVEKAKIRG	SYLTVTLQRP	TKELHGTSIV	PKLQAITASS
**730**	**740**	**750**	**760**	**770**	**780**	**790**	**800**
ANLRHSPRVL	VQHCPARTPQ	RGDEEGLGGE	EEEEEEEEEE	DDSAEEGGAA	RLNGRGSWAQ	DGDESWMQRE	VWMSVFRYLS
**810**	**820**	**830**	**840**	**850**	**860**	**870**	**880**
RRELCECMRV	CKTWYKWCCD	KRLWTKIDLS	RCKAIVPQAL	SGIIKRQPVS	LDLSWTNISK	KQLTWLVNRL	PGLKDLLLAG
**890**	**900**	**910**	**920**	**930**	**940**	**950**	**960**
CSWSAVSALS	TSSCPLLRTL	DLRWAVGIKD	PQIRDLLTPP	ADKPGQDNRS	KLRNMTDFRL	AGLDITDATL	RLIIRHMPLL
**970**	**980**	**990**	**1000**	**1010**	**1020**	**1030**	**1040**
SRLDLSHCSH	LTDQSSNLLT	AVGSSTRYSL	TELNMAGCNK	LTDQTLIYLR	RIANVTLIDL	RGCKQITRKA	CEHFISDLSI
**1050**	**1060**						
NSLYCLSDEK	LIQKIS						

Cmpd.	No. of Cmpds.	m/z meas.	Δ m/z [ppm]	z	Rt [min]	Score	P	Range	Sequence	Modification
846	1	663.6790	-184.30	2	40.6	10.3	1	313-325	K.KTLAGDSSSDCSR.G	
762	1	599.6213	-221.03	2	39.5	10.5	0	314-325	K.TLAGDSSSDCSR.G	

Detailed Protein Report

Protein 352: constitutive coactivator of peroxisome proliferator-activated receptor gamma isoform a [Homo sapiens]

Accession: gi|556503397
Database: refseq_human(refseq_human_20140103.fasta)
Database Date: 2015-11-30
Modification(s): Carbamidomethyl, Oxidation

Score: 33.2
MW [kDa]: 105.1
pI: 5.4
Sequence Coverage [%]: 5.3
No. of unique Peptides: 1

Quantitation

QU:MU	**Median:** 1.86	**CV:** 0.00 %	**No. of Peptides:**	1
WUP:QUP	**Median:** 0.76	**CV:** 0.00 %	**No. of Peptides:**	1

10	20	30	40	50	60	70	80
MDRQMILSRS	SVMGVRGLQG	FVGSTCPHIC	TVVNFKELAE	HHRSKYPGCT	PTIVVDAMCC	LRYWYTPESW	ICGGQWREYF
90	100	110	120	130	140	150	160
SALRDFVKTF	TAAGIKLIFF	FDGMVEQDKR	DEWVKRRLKN	NREISRIFHY	IKSHKEQPGR	NMFFIPSGLA	VFTRFALKTL
170	180	190	200	210	220	230	240
GQETLCSLQE	ADYEVASYGL	QHNCLGILGE	DTDYLIYDTC	PYFSISELCL	ESLDTVMLCR	EKLCESLGLC	VADLPLLACL
250	260	270	280	290	300	310	320
LGNDIIPEGM	FESFRYKCLS	SYTSVKENFD	KKGNIILAVS	DHISKVLYLY	QGEKKLEEIL	PLGPNKALFY	KGMASYLLPG
330	340	350	360	370	380	390	400
QKSPWFFQKP	KGVITLDKQV	ISTSSDAESR	EEVPMCSDAE	SRQEVPMCTG	PESRREVPVY	TDSEPRQEVP	MCSDPEPRQE
410	420	430	440	450	460	470	480
VPTCTGPESR	REVPMCSDPE	PRQEVPMCTG	PEARQEVPMY	TDSEPRQEVP	MYTDSEPRQE	VPMYTGSEPR	QEVPMYTGPE
490	500	510	520	530	540	550	560
SRQEVPMYTG	PESRQEVLIR	TDPESRQEIM	CTGHESKQEV	PICTDPISKQ	EDSMCTHAEI	NQKLPVATDF	EFKLEALMCT
570	580	590	600	610	620	630	640
NPEIKQEDPT	NVGPEVKQQV	TMVSDTEILK	VARTHHVQAE	SYLVYNIMSS	GEIECSNTLE	DELDQALPSQ	AFIYRPIRQR
650	660	670	680	690	700	710	720
VYSLLLEDCQ	DVTSTCLAVK	EWFVYPGNPL	RHPDLVRPLQ	MTIPGGTPSL	KILWLNQEPE	IQVRRLDTLL	ACFNLSSSRE
730	740	750	760	770	780	790	800
ELQAVESPFQ	ALCCLLIYLF	VQVDTLCLED	LHAFIAQALC	LQGKSTSQLV	NLQPDYINPR	AVQLGSLLVR	GLTTLVLVNS
810	820	830	840	850	860	870	880
ACGFPWKTSD	FMPWNVFDGK	LFHQKYLQSE	KGYAVEVLLE	QNRSRLTKFH	NLKAVVCKAC	MKENRRITGR	AHWGSHHAGR
890	900	910	920	930			
WGRQGSSYHR	TGSGYSRSSQ	GQPWRDQGPG	SRQYEHDQWR	RY			

Cmpd.	No. of Cmpds.	m/z meas.	Δ m/z [ppm]	z	Rt [min]	Score	P	Range	Sequence	Modification	Ratios
1242	1	666.6715	-165.15	2	45.6	11.1	0	412-422	R.EVPMCSDPEPR.Q	Carbamidomethyl: 5; Oxidation: 4	WUP:QUP 0.76<br>QU:MU 1.86

Detailed Protein Report

Protein 353: PREDICTED: acetyl-CoA acetyltransferase, cytosolic isoform X1 [Homo sapiens]

**Accession:**	gi|530383698	**Score:**	33.2
**Database:**	refseq_human(refseq_human_20140103.fasta)	**MW [kDa]:**	44.6
**Database Date:**	2015-11-30	**pI:**	9.9
		**Sequence Coverage [%]:**	9.4
		**No. of unique Peptides:**	1

10	20	30	40	50	60	70	80
MGSHPVLRIW	GNRRATAASL	GRSGGRLSSP	RLLRVVAPTL	TFAQTSRCSF	NGALAAVPVQ	DLGSTVIKEV	LKRATVAPED
**90**	**100**	**110**	**120**	**130**	**140**	**150**	**160**
VSEVIFGHVL	AAGCGQNPVR	QASVGAGIPY	SVPAWSCQMI	CGSGLKAVCL	AVQSIGIGDS	SIVVAGGMEN	MSKAPHLAYL
**170**	**180**	**190**	**200**	**210**	**220**	**230**	**240**
RTGVKIGEMP	LTDSILCDGL	TDAFHNCHMG	ITAENVAKKW	QVSREDQDKV	AVLSQNRTEN	AQKAGHFDKE	IVPVLVSTRK
**250**	**260**	**270**	**280**	**290**	**300**	**310**	**320**
GLIEVKTDEF	PRHGSNIEAM	SKLKPYFLTD	GTGTVTPANA	SGINDGAAAV	VLMKKSEADK	RGLTPLARIV	SWSQVGVEPS
**330**	**340**	**350**	**360**	**370**	**380**	**390**	**400**
IMGIGPIPAI	KQAVTKAGWS	LEDVDIFEIN	EAFAAVSAAI	VKELGLNPEK	VNIEGGAIAL	GHPLGASGCR	ILVTLLHTLE
**410**	**420**	**430**					
RMGRSRGVAA	LCIGGGMGIA	MCVQRE					

Cmpd.	No. of Cmpds.	m/z meas.	Δ m/z [ppm]	z	Rt [min]	Score	P	Range	Sequence	Modification
2173	1	1045.4885	-59.90	2	57.0	15.6	0	48-68	R.CSFNGALAAVPVQDLGSTVIK.E	

Detailed Protein Report

Protein 354: PREDICTED: latent-transforming growth factor beta-binding protein 1 isoform X5 [Homo sapiens]

**Accession:**	gi|530367595	**Score:**	33.1
**Database:**	refseq_human(refseq_human_20140103.fasta)	**MW [kDa]:**	134.0
**Database Date:**	2015-11-30	**pI:**	9.1
**Modification(s):**	Carbamidomethyl	**Sequence Coverage [%]:**	5.2
		**No. of unique Peptides:**	1

10	20	30	40	50	60	70	80
MAGAWLRWGL	LLWAGLLASS	AHGRLRRITY	VVHPGPGLAA	GALPLSGPPR	SRTFNVALNA	RYSRSSAAAG	APSRASPGVP
**90**	**100**	**110**	**120**	**130**	**140**	**150**	**160**
SERTRRTSKP	GGAALQGLRP	PPPPPPEPAR	PAVPGGQLHP	NPGGHPAAAP	FTKQGRQVVR	SKVPQETQSG	GGSRLQVHQK
**170**	**180**	**190**	**200**	**210**	**220**	**230**	**240**
QQLQGVNVCG	GRCCHGWSKA	PGSQRCTKPS	CVPPCQNGGM	CLRPQLCVCK	PGTKGKACET	IAAQDTSSPV	FGGQSPGAAS
**250**	**260**	**270**	**280**	**290**	**300**	**310**	**320**
SWGPPEQAAK	HTSSKKADTL	PRVSPVAQMT	LTLKPKPSVG	LPQQIHSQVT	PLSSQSVVIH	HGQTQEYVLK	PKYFPAQKGI
**330**	**340**	**350**	**360**	**370**	**380**	**390**	**400**
SGEQSTEGSF	PLRYVQDQVA	APFQLSNHTG	RIKVVFTPSI	CKVTCTKGSC	QNSCEKGNTT	TLISENGHAA	DTLTATNFRV
**410**	**420**	**430**	**440**	**450**	**460**	**470**	**480**
VICHLPCMNG	GQCSSRDKCQ	CPPNFTGKLC	QIPVHGASVP	KLYQHSQQPG	KALGTHVIHS	THTLPLTVTS	QQGVKVKFPP
**490**	**500**	**510**	**520**	**530**	**540**	**550**	**560**
NIVNIHVKHP	PEASVQIHQV	SRIDGPTGQK	TKEAQPGQSQ	VSYQGLPVQK	TQTIHSTYSH	QQVIPHVYPV	AAKTQLGRCF
**570**	**580**	**590**	**600**	**610**	**620**	**630**	**640**
QETIGSQCGK	ALPGLSKQED	CCGTVGTSWG	FNKCQKCPKK	PSYHGYNQMM	ECLPGYKRVN	NTFCQDINEC	QLQGVCPNGE
**650**	**660**	**670**	**680**	**690**	**700**	**710**	**720**
CLNTMGSYRC	TCKIGFGPDP	TFSSCVPDPP	VISEEKGPCY	RLVSSGRQCM	HPLSVHLTKQ	LCCCSVGKAW	GPHCEKCPLP
**730**	**740**	**750**	**760**	**770**	**780**	**790**	**800**
GTAAFKEICP	GGMGYTVSGV	HRRRPIHHHV	GKGPVFVKPK	NTQPVAKSTH	PPPLPAKEEP	VEALTFSREH	GPGVAEPEVA
**810**	**820**	**830**	**840**	**850**	**860**	**870**	**880**
TAPPEKEIPS	LDQEKTKLEP	GQPQLSPGIS	TIHLHPQFPV	VIEKTSPPVP	VEVAPEASTS	SASQVIAPTQ	VTEINECTVN
**890**	**900**	**910**	**920**	**930**	**940**	**950**	**960**
PDICGAGHCI	NLPVRYTCIC	YEGYRFSEQQ	RKCVDIDECT	QVQHLCSQGR	CENTEGSFLC	ICPAGFMASE	EGTNCIDVDE
**970**	**980**	**990**	**1000**	**1010**	**1020**	**1030**	**1040**
CLRPDVCGEG	HCVNTVGAFR	CEYCDSGYRM	TQRGRCEDID	ECLNPSTCPD	EQCVNSPGSY	QCVPCTEGFR	GWNGQCLDVD
**1050**	**1060**	**1070**	**1080**	**1090**	**1100**	**1110**	**1120**
ECLEPNVCAN	GDCSNLEGSY	MCSCHKGYTR	TPDHKHCRDI	DECQQGNLCV	NGQCKNTEGS	FRCTCGQGYQ	LSAAKDQCED
**1130**	**1140**	**1150**	**1160**	**1170**	**1180**	**1190**	**1200**
IDECQHRHLC	AHGQCRNTEG	SFQCVCDQGY	RASGLGDHCE	DINECLEDKS	VCQRGDCINT	AGSYDCTCPD	GFQLDDNKTC
**1210**	**1220**	**1230**	**1240**	**1250**			
QDINECEHPG	LCGPQGECLN	TEGSFHCVCQ	QGFSISADGR	TCEDVNE			

Cmpd.	No. of Cmpds.	m/z meas.	Δ m/z [ppm]	z	Rt [min]	Score	P	Range	Sequence	Modification
2143	1	1052.3999	-77.45	2	57.2	10.8	1	400-418	R.VVICHLPCMNGGQCSSRDK.C	Carbamidomethyl: 8

Detailed Protein Report

Protein 355: zinc finger protein 90 [Homo sapiens]

**Accession:**	gi|209862791	**Score:**	33.0
**Database:**	refseq_human(refseq_human_20140103.fasta)	**MW [kDa]:**	69.0
**Database Date:**	2015-11-30	**pI:**	10.3
**Modification(s):**	Carbamidomethyl	**Sequence Coverage [%]:**	4.3
		**No. of unique Peptides:**	1

Quantitation

WUP:QUP **Median:** 2.83 **CV:** 0.00 % **No. of Peptides:** 1

10	20	30	40	50	60	70	80
MGPLEFRDVA	IEFSLEEWHC	LDTAQQNLYR	DVMLENYRHL	VFLGIVVTKP	DLITCLEQGK	KPFTVKRHEM	IAKSPVMCFH
**90**	**100**	**110**	**120**	**130**	**140**	**150**	**160**
FAQDLCPEQS	LKDSFQKVIV	TRYEKREYGN	LELKKGCESV	DEGKVHKRGY	NGLNQCLTAT	QSKVFQCDTY	VKVSHIFSNS
**170**	**180**	**190**	**200**	**210**	**220**	**230**	**240**
NRHKIRDTGK	KPFKCIECGK	AFNQSSTLAT	HKKIHTGEIT	CKCEECGKAF	NRSSHLTSHK	RIHTGEKRYK	CEDCGKELKY
**250**	**260**	**270**	**280**	**290**	**300**	**310**	**320**
SSTLTAHKRI	HTGEKRYKCE	DCGKELKYSS	TLTAHKRIHT	GEKPYKCDKC	GRAFISSSIL	YVHKISHTEE	KPYKCEECGK
**330**	**340**	**350**	**360**	**370**	**380**	**390**	**400**
AFKLSSILST	HKRIHTGEKP	YKCEECGKAF	RRSLVLRTHK	RIHTGEKPYK	CDKCGKAFIS	SSLLYKHKIS	HSEKKPYKCE
**410**	**420**	**430**	**440**	**450**	**460**	**470**	**480**
ECGKAFKRSS	TLTIHKISHT	EEKPYKCQEC	DKVFKRSSAL	STHKIIHSGE	KPYKCEECGK	AFKRSSNLTT	HKISHTEEKL
**490**	**500**	**510**	**520**	**530**	**540**	**550**	**560**
YKCQECDKAF	KYSSALSTHK	IIHSGENPYK	CEECGKAFKR	SSVLSKHKII	HTGAKPYKCE	ECGKAFKRSS	QLTSHKISHT
**570**	**580**	**590**	**600**	**610**			
GEKPYKCEEC	GKAFNLSSDL	NTHKRIHIGQ	KAYIVKNMAN	L			

Cmpd.	No. of Cmpds.	m/z meas.	Δ m/z [ppm]	z	Rt [min]	Score	P	Range	Sequence	Modification	Ratios
1099	1	917.4452	-8.48	2	43.8	11.6	1	529-544	K.IIHTGAKPYKCEECGK.A	Carbamidomethyl: 11	WUP:QUP 2.83

Detailed Protein Report

Protein 356: E3 ubiquitin-protein ligase MARCH7 isoform c [Homo sapiens]

**Accession:**	gi|544346219	**Score:**	33.0
**Database:**	refseq_human(refseq_human_20140103.fasta)	**MW [kDa]:**	73.7
**Database Date:**	2015-11-30	**pI:**	6.0
**Modification(s):**	Oxidation	**Sequence Coverage [%]:**	2.7
		**No. of unique Peptides:**	1

10	20	30	40	50	60	70	80
MIGNYDHLMS	LVTSTSASAS	ASPFQSAWYS	ESEITQGARS	RSQNQQRDHD	SKRPKLSCTN	CTTSAGRNVG	NGLNTLSDSS
**90**	**100**	**110**	**120**	**130**	**140**	**150**	**160**
WRHSQVPRSS	SMVLGSFGTD	LMRERRDLER	RTDSSISNLM	DYSHRSGDFT	TSSYVQDRVP	SYSQGARPKE	NSMSTLQLNT
**170**	**180**	**190**	**200**	**210**	**220**	**230**	**240**
SSTNHQLPSE	HQTILSSRDS	RNSLRSNFSS	RESESSRSNT	QPGFSYSSSR	DEAPIISNSE	RVVSSQRPFQ	ESSDNEGRRT
**250**	**260**	**270**	**280**	**290**	**300**	**310**	**320**
TRRLLSRIAS	SMSSTFFSRR	SSQDSLNTRS	LNSENSYVSP	RILTASQSRS	NVPSASEVPD	NRASEASQGF	RFLRRRWGLS
**330**	**340**	**350**	**360**	**370**	**380**	**390**	**400**
SLSHNHSSES	DSENFNQESE	GRNTGPWLSS	SLRNRCTPLF	SRRRREGRDE	SSRIPTSDTS	SRSHIFRRES	NEVVHLEAQN
**410**	**420**	**430**	**440**	**450**	**460**	**470**	**480**
DPLGAAANRP	QASAASSSAT	TGGSTSDSAQ	GGRNTGISGI	LPGSLFRFAV	PPALGSNLTD	NVMITVDIIP	SGWNSADGKS
**490**	**500**	**510**	**520**	**530**	**540**	**550**	**560**
DKTKSAPSRD	PERLQKIKES	LLLEDSEEEE	GDLCRICQMA	AASSSNLLIE	PCKCTGSLQY	VHQDCMKKWL	QAKINSGSSL
**570**	**580**	**590**	**600**	**610**	**620**	**630**	**640**
EAVTTCELCK	EKLELNLEDF	DIHELHRAHA	NEQAEYEFIS	SGLYLVVLLH	LCEQSFSDMM	GNTNEPSTRV	RFINLARTLQ
**650**	**660**	**670**					
AHMEDLETSE	DDSEEDGDHN	RTFDIA					

Cmpd.	No. of Cmpds.	m/z meas.	Δ m/z [ppm]	z	Rt [min]	Score	P	Range	Sequence	Modification
1613	1	682.3429	11.62	3	50.4	22.6	2	89-106	R.SSSMVLGSFGTDLMRERR.D	Oxidation: 4

Detailed Protein Report

Protein 357: E3 ubiquitin-protein ligase RNF213 isoform 3 [Homo sapiens]

**Accession:**	gi|366039979	**Score:**	32.9
**Database:**	refseq_human(refseq_human_20140103.fasta)	**MW [kDa]:**	591.0
**Database Date:**	2015-11-30	**pI:**	6.0
		**Sequence Coverage [%]:**	0.6
		**No. of unique Peptides:**	1

Detailed Protein Report

10	20	30	40	50	60	70	80
MECPSCQHVS	KEETPKFCSQ	CGERLPPAAP	IADSENNNST	MASASEGEME	CGQELKEEGG	PCLFPGSDSW	QENPEEPCSK
90	100	110	120	130	140	150	160
ASWTVQESKK	KKRKKKKKGN	KSASSELASL	PLSPASPCHL	TLLSNPWPQD	TALPHSQAQQ	SGPTGQPSQP	PGTATTPLEG
170	180	190	200	210	220	230	240
DGLSAPTEVG	DSPLQAQALG	EAGVATGSEA	QSSPQFQDHT	EGEDQDASIP	SGGRGLSQEG	TGPPTSAGEG	HSRTEDAAQE
250	260	270	280	290	300	310	320
LLLPESKGGS	SEPGTELQTT	EQQAGASASM	AVDAVAEPAN	AVKGAGKEMK	EKTQRMKQPP	ATTPPFKTHC	QEAETKTKDE
330	340	350	360	370	380	390	400
MAAAEEKVGK	NEQGEPEDLK	KPEGKNRSAA	AVKNEKEQKN	QEADVQEVKA	STLSPGGGVT	VFFHAIISLH	FPFNPDLHKV
410	420	430	440	450	460	470	480
FIRGGEEFGE	SKWDSNICEL	HYTRDLGHDR	VLVEGIVCIS	KKHLDKYIPY	KYVIYNGESF	EYEFIYKHQQ	KKGEYVNRCL
490	500	510	520	530	540	550	560
FIKSSLLGSG	DWHQYYDIVY	MKPHGRLQKV	MNHITDGPRK	DLVKGKQIAA	ALMLDSTFSI	LQTWDTINLN	SFFTQFEQFC
570	580	590	600	610	620	630	640
FVLQQPMIYE	GQAQLWTDLQ	YREKEVKRYL	WQHLKKHVVP	LPDGKSTDFL	PVDCPVRSKL	KTGLIVLFVV	EKIELLLEGS
650	660	670	680	690	700	710	720
LDWLCHLLTS	DASSPDEFHR	DLSHILGIPQ	SWRLYLVNLC	QRCMDTRTYT	WLGALPVLHC	CMELAPRHKD	AWRQPEDTWA
730	740	750	760	770	780	790	800
ALEGLSFSPF	REQMLDTSSL	LQFMREKQHL	LSIDEPLFRS	WFSLLPLSHL	VMYMENFIEH	LGRFPAHILD	CLSGIYYRLP
810	820	830	840	850	860	870	880
GLEQVLNTQD	VQDVQNVQNI	LEMLLRLLDT	YRDKIPEEAL	SPSYLTVCLK	LHEAICSSTK	LLKFYELPAL	SAEIVCRMIR
890	900	910	920	930	940	950	960
LLSLVDSAGQ	RDETGNNSVQ	TVFQGTLAAT	KRWLREVFTK	NMLTSSGASF	TYVKEIEVWR	RLVEIQFPAE	HGWKESLLGD
970	980	990	1000	1010	1020	1030	1040
MEWRLTKEEP	LSQITAYCNS	CWDTKGLEDS	VAKTFEKCII	EAVSSACQSQ	TSILQGFSYS	DLRKFGIVLS	AVITKSWPRT
1050	1060	1070	1080	1090	1100	1110	1120
ADNFNDILKH	LLTLADVKHV	FRLCGTDEKI	LANVTEDAKR	LIAVADSVLT	KVVGDLLSGT	ILVGQLELII	KHKNQFLDIW
1130	1140	1150	1160	1170	1180	1190	1200
QLREKSLSPQ	DEQCAVEEAL	DWRREELLLL	KKEKRCVDSL	LKMCGNVKHL	IQVDFGVLAV	RHSQDLSSKR	LNDTVTVRLS
1210	1220	1230	1240	1250	1260	1270	1280
TSSNSQRATH	YHLSSQVQEM	AGKIDLLRDS	HIFQLFWREA	AEPLSEPKED	QEAAELLSEP	EEESERHILE	LEEVYDYLYQ
1290	1300	1310	1320	1330	1340	1350	1360
PSYRKFIKLH	QDLKSGEVTL	AEIDVIFKDF	VNKYTDLDSE	LKIMCTVDHQ	DQRDWIKDRV	EQIKEYHHLH	QAVHAAKVIL
1370	1380	1390	1400	1410	1420	1430	1440
QVKESLGLNG	DFSVLNTLLN	FTDNFDDFRR	ETLDQINQEL	IQAKKLLQDI	SEARCKGLQA	LSLRKEFICW	VREALGGINE
1450	1460	1470	1480	1490	1500	1510	1520
LKVFVDLASI	SAGENDIDVD	RVACFHDAVQ	GYASLLFKLD	PSVDFSAFMK	HLKKLWKALD	KDQYLPRKLC	DSARNLEWLK
1530	1540	1550	1560	1570	1580	1590	1600
TVNESHGSVE	RSSLTLATAI	NQRGIYVIQA	PKGGQKISPD	TVLHLILPES	PGSHEESREY	SLEEVKELLN	KLMLMSGKKD
1610	1620	1630	1640	1650	1660	1670	1680
RNNTEVERFS	EVFCSVQRLS	QAFIDLHSAG	NMLFRTWIAM	AYCSPKQGVS	LQMDFGLDLV	TELKEGGDVT	ELLAALCRQM
1690	1700	1710	1720	1730	1740	1750	1760
EHFLDSWKRF	VTQKRMEHFY	LNFYTAEQLV	YLSTELRKQP	PSDAALTMLS	FIKSNCTLRD	VLRASVGCGS	EAARYRMRRV
1770	1780	1790	1800	1810	1820	1830	1840
MEELPLMLLS	EFSLVDKLRI	IMEQSMRCLP	AFLPDCLDLE	TLGHCLAHLA	GMGGSPVERC	LPRGLQVGQP	NLVVCGHSEV
1850	1860	1870	1880	1890	1900	1910	1920
LPAALAVYMQ	TPSQPLPTYD	EVLLCTPATT	FEEVALLLRR	CLTLGSLGHK	VYSLLFADQL	SYEVARQAEE	LFHNLCTQQH
1930	1940	1950	1960	1970	1980	1990	2000
REDYQLVMVC	DGDWEHCYLP	SAFSQHKVFV	TPQAPLEAIQ	AYLAGHYRVP	KQTLSAAAVF	NDRLCVGIVA	SERAGVGKSL
2010	2020	2030	2040	2050	2060	2070	2080
YVKRLHDKMK	MQLNVKNVPL	KTIRLIDPQV	DESRVLGALL	PFLDAQYQKV	PVLFHLDVTS	SVQTGIWVFL	FKLLILQYLM
2090	2100	2110	2120	2130	2140	2150	2160
DINGKMWLRN	PCHLYIVEIL	ERRTSVPSRS	SSALRTRVPQ	FSFLDIFPKV	TCRPPKEVID	MELSALRSDT	EPGMDLWEFC
2170	2180	2190	2200	2210	2220	2230	2240

Detailed Protein Report

Cmpd.	No. of Cmpds.	m/z meas.	Δ m/z [ppm]	z	Rt [min]	Score	P	Range	Sequence	Modification
1737	1	863.0655	110.54	2	51.5	19.4	1	746-759	R.EKQHLLSIDEPLFR.S	

Detailed Protein Report

Protein 358: spectrin beta chain, non-erythrocytic 4 isoform sigma1 [Homo sapiens]

**Accession:**	gi|115430237	**Score:**	32.9
**Database:**	refseq_human(refseq_human_20140103.fasta)	**MW [kDa]:**	288.8
**Database Date:**	2015-11-30	**pI:**	5.7
		**Sequence Coverage [%]:**	1.1
		**No. of unique Peptides:**	1

Quantitation

QU:MU	**Median:**	4.03	**CV:**	0.00 %	**No. of Peptides:**	1
WUP:QUP	**Median:**	3.65	**CV:**	0.00 %	**No. of Peptides:**	1

Detailed Protein Report

10	20	30	40	50	60	70	80
MAQVPGEVDN	MEGLPAPNNN	PAARWESPDR	GWEREQPAAS	TAAASLFECS	RIKALADERE	AVQKKTFTKW	VNSHLARVGC
90	100	110	120	130	140	150	160
HIGDLYVDLR	DGFVLTRLLE	VLSGEQLPRP	TRGRMRIHSL	ENVDKALQFL	KEQRVHLENV	GSHDIVDGNH	RLTLGLVWTI
170	180	190	200	210	220	230	240
ILRFQIQVIK	IETEDNRETR	SAKDALLLWC	QMKTAGYPEV	NIQNFTTSWR	DGLAFNALIH	RHRPDLVDFS	KLTKSNANYN
250	260	270	280	290	300	310	320
LQRAFRTAEQ	HLGLARLLDP	EDVNMEAPDE	KSIITYVVSF	YHYFSKMKAL	AVEGKRIGKV	LDQVLEVGKI	IERYEELAAE
330	340	350	360	370	380	390	400
LLAWIHRTVG	LISNQKFANS	LSGVQQQLQA	FTAYCTLEKP	VKFQEKGNLE	VLLFSIQSKL	RACNRRLFVP	REGCGIWDID
410	420	430	440	450	460	470	480
KAWGELEKAE	HEREAALRAE	LIRQEKLELL	AQRFDHKVAM	RESWLNENQR	LVSQDNFGYE	LPAVEAAMKK	HEAIEADIAA
490	500	510	520	530	540	550	560
YEERVQGVAE	LAQALAAEGY	YDIRRVAAQR	DSVLRQWALL	TGLVGARRTR	LEQNLALQKV	FQEMVYMVDW	MEEMQAQLLS
570	580	590	600	610	620	630	640
RECGQHLVEA	DDLLQKHGLL	EGDIAAQSER	VEALNAAALR	FSQLQGYQPC	DPQVICNRVN	HVHGCLAELQ	EQAARRRAEL
650	660	670	680	690	700	710	720
EASRSLWALL	QELEEAESWA	RDKERLLEAA	GGGGAAGAAG	AAGTAGGAHD	LSSTARLLAQ	HKILQGELGG	RRALLQQALR
730	740	750	760	770	780	790	800
CGEELVAAGG	AVGPGADTVH	LVGLAERAAS	ARRRWQRLEE	AAARRERRLQ	EARALHQFGA	DLDGLLDWLR	DAYRLAAAGD
810	820	830	840	850	860	870	880
FGHDEASSRR	LARQHRALTG	EVEAHRGPVS	GLRRQLATLG	GASGAGPLVV	ALQVRVVEAE	QLFAEVTEVA	ALRRQWLRDA
890	900	910	920	930	940	950	960
LAVYRMFGEV	HACELWIGEK	EQWLLSMRVP	DSLDDVEVVQ	HRFESLDQEM	NSLMGRVLDV	NHTVQELVEG	GHPSSDEVRS
970	980	990	1000	1010	1020	1030	1040
CQDHLNSRWN	RIVELVEQRK	EEMSAVLLVE	NHVLEVAEVR	AQVREKRRAV	ESAPRAGGAL	QWRLSGLEAA	LQALEPRQAA
1050	1060	1070	1080	1090	1100	1110	1120
LLEEAALLAE	RFPAQAARLH	QGAEELGAEW	GALASAAQAC	GEAVAAAGRL	QRFLHDLDAF	LDWLVRAQEA	AGGSEGPLPN
1130	1140	1150	1160	1170	1180	1190	1200
SLEEADALLA	RHAALKEEVD	QREEDYARIV	AASEALLAAD	GAELGPGLAL	DEWLPHLELG	WHKLLGLWEA	RREALVQAHI
1210	1220	1230	1240	1250	1260	1270	1280
YQLFLRDLRQ	ALVVLRNQEM	ALSGAELPGT	VESVEEALKQ	HRDFLTTMEL	SQQKMQVAVQ	AAEGLLRQGN	IYGEQAQEAV
1290	1300	1310	1320	1330	1340	1350	1360
TRLLEKNQEN	QLRAQQWMQK	LHDQLELQHF	LRDCHELDGW	IHEKMLMARD	GTREDNHKLH	KRWLRHQAFM	AELAQNKEWL
1370	1380	1390	1400	1410	1420	1430	1440
EKIEREGQQL	MQEKPELAAS	VRKKLGEIRQ	CWAELESTTQ	AKARQLFEAS	KADQLVQSFA	ELDKKLLHME	SQLQDVDPGG
1450	1460	1470	1480	1490	1500	1510	1520
DLATVNSQLK	KLQSMESQVE	EWYREVGELQ	AQTAALPLEP	ASKELVGERQ	NAVGERLVRL	LEPLQERRRL	LLASKELHQV
1530	1540	1550	1560	1570	1580	1590	1600
AHDLDDELAW	VQERLPLAMQ	TERGNGLQAV	QQHIKKNQGL	RREIQAHGPR	LEEVLERAGA	LASLRSPEAE	AVRRGLEQLQ
1610	1620	1630	1640	1650	1660	1670	1680
SAWAGLREAA	ERRQQVLDAA	FQVEQYYFDV	AEVEAWLGEQ	ELLMMSEDKG	KDEQSTLQLL	KKHLQLEQGV	ENYEESIAQL
1690	1700	1710	1720	1730	1740	1750	1760
SRQCRALLEM	GHPDSEQISR	RQSQVDRLYV	ALKELGEERR	VALEQQYWLY	QLSRQVSELE	HWIAEKEVVA	GSPELGQDFE
1770	1780	1790	1800	1810	1820	1830	1840
HVSVLQEKFS	EFASETGMAG	RERLAAVNQM	VDELIECGHT	AAATMAEWKD	GLNEAWAELL	ELMGTRAQLL	AASRELHKFF
1850	1860	1870	1880	1890	1900	1910	1920
SDARELQGQI	EEKRRRLPRL	TTPPEPRPSA	SSMQRTLRAF	EHDLQLLVSQ	VRQLQEGAAQ	LRTVYAGEHA	EAIASREQEV
1930	1940	1950	1960	1970	1980	1990	2000
LQGWKELLSA	CEDARLHVSS	TADALRFHSQ	VRDLLSWMDG	IASQIGAADK	PRDVSSVEVL	MNYHQGLKTE	LEARVPELTT
2010	2020	2030	2040	2050	2060	2070	2080
CQELGRSLLL	NKSAMADEIQ	AQLDKLGTRK	EEVSEKWDRH	WEWLQQMLEV	HQFAQEAVVA	DAWLTAQEPL	LQSRELGSSV
2090	2100	2110	2120	2130	2140	2150	2160
DEVEQLIRRH	EAFRKAAAAW	EERFSSLRRL	TTIEKIKAEQ	SKQPPTPLLG	RKFFGDPTEL	AAKAAPLLRP	GGYERGLEPL
2170	2180	2190	2200	2210	2220	2230	2240

Detailed Protein Report

Cmpd.	No. of Cmpds.	m/z meas.	Δ m/z [ppm]	z	Rt [min]	Score	P	Range	Sequence	Modification	Ratios
1131	7	530.7679	3.99	2	44.3	18.5	2	2553-2562	R.EGGDRRASGR.R		WUP:QUP 3.65<br>QU:MU 4.03

Detailed Protein Report

Protein 359: PREDICTED: calcium-binding mitochondrial carrier protein SCaMC-3 isoform X1 [Homo sapiens]

**Accession:**	gi|578833593	**Score:**	32.9
**Database:**	refseq_human(refseq_human_20140103.fasta)	**MW [kDa]:**	45.9
**Database Date:**	2015-11-30	**pI:**	6.0
		**Sequence Coverage [%]:**	6.8
		**No. of unique Peptides:**	1

Quantitation

QU:MU	**Median:** 1.17	**CV:** 0.00 %	**No. of Peptides:**	1
WUP:QUP	**Median:** 1.48	**CV:** 0.00 %	**No. of Peptides:**	1

10	20	30	40	50	60	70	80
MRGSPGDAER	RQRWGRLFEE	LDSNKDGRVD	VHELRQGLAR	LGGGNPDPGA	QQGISSEGDA	DPDGGLDLEE	FSRYLQEREQ
**90**	**100**	**110**	**120**	**130**	**140**	**150**	**160**
RLLLMFHSLD	RNQDGHIDVS	EIQQSFRALG	ISISLEQAEK	ILHSMDRDGT	MTIDWQEWRD	HFLLHSLENV	EDVLYFWKHS
**170**	**180**	**190**	**200**	**210**	**220**	**230**	**240**
TVLDIGECLT	VPDEFSKQEK	LTGMWWKQLV	AGAVAGAVSR	TGTAPLDRLK	VFMQVHASKT	NRLNILGGLR	SMVLEGGIRS
**250**	**260**	**270**	**280**	**290**	**300**	**310**	**320**
LWRGNGINVL	KIAPESAIKF	MAYEQIKRAI	LGQQETLHVQ	ERFVAGSLAG	ATAQTIIYPM	ETLKNWWLQQ	YSHDSADPGI
**330**	**340**	**350**	**360**	**370**	**380**	**390**	**400**
LVLLACGTIS	STCGQIASYP	LALVRTRMQA	QASIEGGPQL	SMLGLLRHIL	SQEGMRGLYR	GIAPNFMKVI	PAVSISYVVY
**410**	**420**						
ENMKQALGVT	SR						

Cmpd.	No. of Cmpds.	m/z meas.	Δ m/z [ppm]	z	Rt [min]	Score	P	Range	Sequence	Modification	Ratios
1190	4	644.3159	-77.32	2	45.0	18.2	1	209-219	R.LKVFMQVHASK.T		QU:MU 1.17<br>WUP:QUP 1.48

Detailed Protein Report

Protein 360: PREDICTED: uncharacterized protein KIAA1210 isoform X1 [Homo sapiens]

**Accession:**	gi|578838720	**Score:**	32.8
**Database:**	refseq_human(refseq_human_20140103.fasta)	**MW [kDa]:**	167.9
**Database Date:**	2015-11-30	**pI:**	9.3
		**Sequence Coverage [%]:**	1.1
		**No. of unique Peptides:**	1

10	20	30	40	50	60	70	80
MAESLSEISD	SLDVLEAGDE	GKKKCKFKAL	KSFFVKKKEK	EAEDTQEEEM	LELSLSSSNI	NISSLQPVRE	NQPTKARAKS
**90**	**100**	**110**	**120**	**130**	**140**	**150**	**160**
SMGSKALSHD	SIFMLGPEPE	RSASKMFPSM	DPQRGRPQQR	SHISRTLPKP	RSKVPGVVSG	AMSGAVLQNV	PTSAVWVAGP
**170**	**180**	**190**	**200**	**210**	**220**	**230**	**240**
KITENPPSRR	RRLSIIPPVI	QPEIISKNLV	EISLDDESPK	NPQKKALPHK	SLTATQSFSE	LSSGPDCSQS	LTAFATLAST
**250**	**260**	**270**	**280**	**290**	**300**	**310**	**320**
SSTQLPIGFS	TPATTQGCLD	SSAARHKMTL	NPRKQKKNLQ	VIVEPKEEEP	NLPLVSEEEK	SITKPKEINE	KKLGMDSADS
**330**	**340**	**350**	**360**	**370**	**380**	**390**	**400**
SSQKQNNKTE	MYDKKTTDQA	PNTDASRSQG	YPMSAAYGRR	WRRKGASVSG	LSGCEFKGRS	LKQSSEGYGL	GDRAGSSPTN
**410**	**420**	**430**	**440**	**450**	**460**	**470**	**480**
KTARNVPFSH	LSLEKDNMEQ	PTTSQPETTT	PQGLLSDKDD	MGRRNAGIDF	GSRKASAAQP	IPENMDNSMV	SDPQPYHEDA
**490**	**500**	**510**	**520**	**530**	**540**	**550**	**560**
ASGAEKTEAR	ASLSLMVESL	STTQEEAILS	VAAEAQVFMN	PSHIQLEDQE	AFSFDLQKAQ	SKMESAQDVQ	TICKEKPSGN
**570**	**580**	**590**	**600**	**610**	**620**	**630**	**640**
VHQTFTASVL	GMTSTTAKGD	VYAKTLPPRS	LFQSSRKPDA	EEVSSDSENI	PEEGDGSEEL	AHGHSSQSLG	KFEDEQEVFS
**650**	**660**	**670**	**680**	**690**	**700**	**710**	**720**
ESKSFVEDLS	SSEEELDLRC	LSQALEEPED	AEVFTESSSY	VEKYNTSDDC	SSSEEDLPLR	HPAQALGKPK	NQQEVSSASN
**730**	**740**	**750**	**760**	**770**	**780**	**790**	**800**
NTPEEQNDFM	QQLPSRCPSQ	PIMNPTVQQQ	VPTSSVGTSI	KQSDSVEPIP	PRHPFQPWVN	PKVEQEVSSS	PKSMAVEESI
**810**	**820**	**830**	**840**	**850**	**860**	**870**	**880**
SMKPLPPKLL	CQPLMNPKVQ	QNMFSGSEDI	AVERVISVEP	LLPRYSPQSL	TDPQIRQISE	STAVEEGTYV	EPLPPRCLSQ
**890**	**900**	**910**	**920**	**930**	**940**	**950**	**960**
PSERPKFLDS	MSTSAEWSSP	VAPTPSKYTS	PPWVTPKFEE	LYQLSAHPES	TTVEEDISKE	QLLPRHLSQL	TVGNKVQQLS
**970**	**980**	**990**	**1000**	**1010**	**1020**	**1030**	**1040**
SNFERAAIEA	DISGSPLPPQ	YATQFLKRSK	VQEMTSRLEK	MAVEGTSNKS	PIPRRPTQSF	VKFMAQQIFS	ESSALKRGSD
**1050**	**1060**	**1070**	**1080**	**1090**	**1100**	**1110**	**1120**
VAPLPPNLPS	KSLSKPEVKH	QVFSDSGSAN	PKGGISSKML	PMKHPLQSLG	RPEDPQKVFS	YSERAPGKCS	SFKEQLSPRQ
**1130**	**1140**	**1150**	**1160**	**1170**	**1180**	**1190**	**1200**
LSQALRKPEY	EQKVSPVSAS	SPKEWRNSKK	QLPPKHSSQA	SDRSKFQPQM	SSKGPVNVPV	KQSSGEKHLP	SSSPFQQQVH
**1210**	**1220**	**1230**	**1240**	**1250**	**1260**	**1270**	**1280**
SSSVNAAARR	SVFESNSDNW	FLGRDEAFAI	KTKKFSQGSK	NPIKSIPAPA	TKPGKFTIAP	VRQTSTSGGI	YSKKEDLESG
**1290**	**1300**	**1310**	**1320**	**1330**	**1340**	**1350**	**1360**
DGNNNQHANL	SNQDDVEKLF	GVRLKRAPPS	QKYKSEKQDN	FTQLASVPSG	PISSSVGRGH	KIRSTSQGLL	DAAGNLTKIS
**1370**	**1380**	**1390**	**1400**	**1410**	**1420**	**1430**	**1440**
YVADKQQSRP	KSESMAKKQP	ACKTPGKPAG	QQSDYAVSEP	VWITMAKQKQ	KSFKAHISVK	ELKTKSNAGA	DAETKEPKYE
**1450**	**1460**	**1470**	**1480**	**1490**	**1500**	**1510**	**1520**
GAGSANENQP	KKMFTSSVHK	QEKTAQMKPP	KPTKSVGFEA	QKILQVPAME	KETKRSSTLP	AKFQNPVEPI	EPVWFSLARK
**1530**	**1540**						
KAKAWSHMAE	ITQ						

Cmpd.	No. of Cmpds.	m/z meas.	Δ m/z [ppm]	z	Rt [min]	Score	P	Range	Sequence	Modification
1818	1	582.2582	-176.44	1	53.1	16.6	0	1151-1155	K.QLPPK.H	

Detailed Protein Report

Protein 361: tyrosine-protein kinase Fgr [Homo sapiens]

**Accession:**	gi|4885235	**Score:**	32.8
**Database:**	refseq_human(refseq_human_20140103.fasta)	**MW [kDa]:**	59.4
**Database Date:**	2015-11-30	**pI:**	5.3
**Modification(s):**	Carbamidomethyl, Oxidation	**Sequence Coverage [%]:**	8.5
		**No. of unique Peptides:**	3

Alias proteins:

Accession	Name	Description
gi|578798632	refseq_human (refseq_human_20140103.fasta)	PREDICTED: tyrosine-protein kinase Fgr isoform X1 [Homo sapiens]
gi|112382244	refseq_human (refseq_human_20140103.fasta)	tyrosine-protein kinase Fgr [Homo sapiens]
gi|112382241	refseq_human (refseq_human_20140103.fasta)	tyrosine-protein kinase Fgr [Homo sapiens]

10	20	30	40	50	60	70	80
MGCVFCKKLE	PVATAKEDAG	LEGDFRSYGA	ADHYGPDPTK	ARPASSFAHI	PNYSNFSSQA	INPGFLDSGT	IRGVSGIGVT
**90**	**100**	**110**	**120**	**130**	**140**	**150**	**160**
LFIALYDYEA	RTEDDLTFTK	GEKFHILNNT	EGDWWEARSL	SSGKTGCIPS	NYVAPVDSIQ	AEEWYFGKIG	RKDAERQLLS
**170**	**180**	**190**	**200**	**210**	**220**	**230**	**240**
PGNPQGAFLI	RESETTKGAY	SLSIRDWDQT	RGDHVKHYKI	RKLDMGGYYI	TTRVQFNSVQ	ELVQHYMEVN	DGLCNLLIAP
**250**	**260**	**270**	**280**	**290**	**300**	**310**	**320**
CTIMKPQTLG	LAKDAWEISR	SSITLERRLG	TGCFGDVWLG	TWNGSTKVAV	KTLKPGTMSP	KAFLEEAQVM	KLLRHDKLVQ
**330**	**340**	**350**	**360**	**370**	**380**	**390**	**400**
LYAVVSEEPI	YIVTEFMCHG	SLLDFLKNPE	GQDLRLPQLV	DMAAQVAEGM	AYMERMNYIH	RDLRAANILV	GERLACKIAD
**410**	**420**	**430**	**440**	**450**	**460**	**470**	**480**
FGLARLIKDD	EYNPCQGSKF	PIKWTAPEAA	LFGRFTIKSD	VWSFGILLTE	LITKGRIPYP	GMNKREVLEQ	VEQGYHMPCP
**490**	**500**	**510**	**520**	**530**			
PGCPASLYEA	MEQTWRLDPE	ERPTFEYLQS	FLEDYFTSAE	PQYQPGDQT			

Cmpd.	No. of Cmpds.	m/z meas.	Δ m/z [ppm]	z	Rt [min]	Score	P	Range	Sequence	Modification
2000	1	899.3791	-86.20	2	53.4	11.8	2	1-16	-.MGCVFCKKLEPVATAK.E	Carbamidomethyl: 6; Oxidation: 1
2074	1	682.6660	-32.64	3	56.3	10.3	2	8-26	K.KLEPVATAKEDAGLEGDFR.S	
1458	1	1022.6515	107.92	2	46.6	10.7	0	73-91	R.GVSGIGVTLFIALYDYEAR.T	

Detailed Protein Report

Protein 362: **PREDICTED: NAD-dependent protein deacylase sirtuin-5, mitochondrial isoform X3 [Homo sapiens]**

**Accession:**	gi|530381706	**Score:**	32.7
**Database:**	refseq_human(refseq_human_20140103.fasta)	**MW [kDa]:**	31.7
**Database Date:**	2015-11-30	**pI:**	11.1
**Modification(s):**	Oxidation	**Sequence Coverage [%]:**	14.1
		**No. of unique Peptides:**	2

Quantitation

QU:MU	**Median:** 1.99	**CV:** 0.00 %	**No. of Peptides:**	1
WUP:QUP	**Median:** 1.07	**CV:** 115.25 %	**No. of Peptides:**	2

10	20	30	40	50	60	70	80
MRPLQIVPSR	LISQLYCGLK	PPASTRNQIC	LKMARPSSSM	ADFRKFFAKA	KHIVIISGAG	VSAESGVPTF	RGAGGYWRKW
**90**	**100**	**110**	**120**	**130**	**140**	**150**	**160**
QAQDLATPLA	FAHNPSRVWE	FYHYRREVMG	SKEPNAGHRA	IAECETRLGK	QGRRVVVITQ	NIDELHRKAG	TKNLLEIHGS
**170**	**180**	**190**	**200**	**210**	**220**	**230**	**240**
LFKTRCTSCG	VVAENYKSPI	CPALSGKGAP	EPGTQDASIP	VEKLPRWALP	LWCTQQPCLP	PRWLPGACQW	LNLTRRPPQL
**250**	**260**	**270**	**280**	**290**			
RTDSGFISRD	PVERLFLKPL	PVMKMKLFLK	CPGEERNYSI	SKN			

Cmpd.	No. of Cmpds.	m/z meas.	Δ m/z [ppm]	z	Rt [min]	Score	P	Range	Sequence	Modification	Ratios
324	1	686.3446	49.16	2	32.7	11.4	0	33-44	K.MARPSSSMADFR.K	Oxidation: 8	WUP:QUP 0.43<br>QU:MU 1.99
553	1	731.0325	126.18	2	36.5	10.4	1	255-266	R.LFLKPLPVMKMK.L	Oxidation: 9	WUP:QUP 2.69

Detailed Protein Report

Protein 363: adenomatous polyposis coli protein isoform a [Homo sapiens]

**Accession:**	gi|306922386	**Score:**	32.6
**Database:**	refseq_human(refseq_human_20140103.fasta)	**MW [kDa]:**	308.5
**Database Date:**	2015-11-30	**pI:**	9.0
		**Sequence Coverage [%]:**	1.4
		**No. of unique Peptides:**	2

Quantitation

WUP:QUP	**Median:** 0.63	**CV:** 0.00 %	**No. of Peptides:** 1

10	20	30	40	50	60	70	80
MYASLGSGPV	APLPASVPPS	VLGSWSTGGS	RSCVRQETKS	PGGARTSGHW	ASVWQEVLKQ	LQGSIEDEAM	ASSGQIDLLE
90	100	110	120	130	140	150	160
RLKELNLDSS	NFPGVKLRSK	MSLRSYGSRE	GSVSSRSGEC	SPVPMGSFPR	RGFVNGSRES	TGYLEELEKE	RSLLLADLDK
170	180	190	200	210	220	230	240
EEKEKDWYYA	QLQNLTKRID	SLPLTENFSL	QTDMTRRQLE	YEARQIRVAM	EEQLGTCQDM	EKRAQRSSQN	KHETGSHDAE
250	260	270	280	290	300	310	320
RQNEGQGVGE	INMATSGNGQ	GSTTRMDHET	ASVLSSSSTH	SAPRRLTSHL	GTKVEMVYSL	LSMLGTHDKD	DMSRTLLAMS
330	340	350	360	370	380	390	400
SSQDSCISMR	QSGCLPLLIQ	LLHGNDKDSV	LLGNSRGSKE	ARARASAALH	NIIHSQPDDK	RGRREIRVLH	LLEQIRAYCE
410	420	430	440	450	460	470	480
TCWEWQEAHE	PGMDQDKNPM	PAPVEHQICP	AVCVLMKLSF	DEEHRHAMNE	LGGLQAIAEL	LQVDCEMYGL	TNDHYSITLR
490	500	510	520	530	540	550	560
RYAGMALTNL	TFGDVANKAT	LCSMKGCMRA	LVAQLKSESE	DLQQVIASVL	RNLSWRADVN	SKKTLREVGS	VKALMECALE
570	580	590	600	610	620	630	640
VKKESTLKSV	LSALWNLSAH	CTENKADICA	VDGALAFLVG	TLTYRSQTNT	LAIIESGGGI	LRNVSSLIAT	NEDHRQILRE
650	660	670	680	690	700	710	720
NNCLQTLLQH	LKSHSLTIVS	NACGTLWNLS	ARNPKDQEAL	WDMGAVSMLK	NLIHSKHKMI	AMGSAAALRN	LMANRPAKYK
730	740	750	760	770	780	790	800
DANIMSPGSS	LPSLHVRKQK	ALEAELDAQH	LSETFDNIDN	LSPKASHRSK	QRHKQSLYGD	YVFDTNRHDD	NRSDNFNTGN
810	820	830	840	850	860	870	880
MTVLSPYLNT	TVLPSSSSSR	GSLDSSRSEK	DRSLERERGI	GLGNYHPATE	NPGTSSKRGL	QISTTAAQIA	KVMEEVSAIH
890	900	910	920	930	940	950	960
TSQEDRSSGS	TTELHCVTDE	RNALRRSSAA	HTHSNTYNFT	KSENSNRTCS	MPYAKLEYKR	SSNDSLNSVS	SSDGYGKRGQ
970	980	990	1000	1010	1020	1030	1040
MKPSIESYSE	DDESKFCSYG	QYPADLAHKI	HSANHMDDND	GELDTPINYS	LKYSDEQLNS	GRQSPSQNER	WARPKHIIED
1050	1060	1070	1080	1090	1100	1110	1120
EIKQSEQRQS	RNQSTTYPVY	TESTDDKHLK	FQPHFGQQEC	VSPYRSRGAN	GSETNRVGSN	HGINQNVSQS	LCQEDDYEDD
1130	1140	1150	1160	1170	1180	1190	1200
KPTNYSERYS	EEEQHEEEER	PTNYSIKYNE	EKRHVDQPID	YSLKYATDIP	SSQKQSFSFS	KSSSGQSSKT	EHMSSSSENT
1210	1220	1230	1240	1250	1260	1270	1280
STPSSNAKRQ	NQLHPSSAQS	RSGQPQKAAT	CKVSSINQET	IQTYCVEDTP	ICFSRCSSLS	SLSSAEDEIG	CNQTTQEADS
1290	1300	1310	1320	1330	1340	1350	1360
ANTLQIAEIK	EKIGTRSAED	PVSEVPAVSQ	HPRTKSSRLQ	GSSLSSESAR	HKAVEFSSGA	KSPSKSGAQT	PKSPPEHYVQ
1370	1380	1390	1400	1410	1420	1430	1440
ETPLMFSRCT	SVSSLDSFES	RSIASSVQSE	PCSGMVSGII	SPSDLPDSPG	QTMPPSRSKT	PPPPPQTAQT	KREVPKNKAP
1450	1460	1470	1480	1490	1500	1510	1520
TAEKRESGPK	QAAVNAAVQR	VQVLPDADTL	LHFATESTPD	GFSCSSSLSA	LSLDEPFIQK	DVELRIMPPV	QENDNGNETE
1530	1540	1550	1560	1570	1580	1590	1600
SEQPKESNEN	QEKEAEKTID	SEKDLLDDSD	DDDIEILEEC	IISAMPTKSS	RKAKKPAQTA	SKLPPPVARK	PSQLPVYKLL
1610	1620	1630	1640	1650	1660	1670	1680
PSQNRLQPQK	HVSFTPGDDM	PRVYCVEGTP	INFSTATSLS	DLTIESPPNE	LAAGEGVRGG	AQSGEFEKRD	TIPTEGRSTD
1690	1700	1710	1720	1730	1740	1750	1760
EAQGGKTSSV	TIPELDDNKA	EEGDILAECI	NSAMPKGKSH	KPFRVKKIMD	QVQQASASSS	APNKNQLDGK	KKKPTSPVKP
1770	1780	1790	1800	1810	1820	1830	1840
IPQNTEYRTR	VRKNADSKNN	LNAERVFSDN	KDSKKQNLKN	NSKVFNDKLP	NNEDRVRGSF	AFDSPHHYTP	IEGTPYCFSR
1850	1860	1870	1880	1890	1900	1910	1920
NDSLSSLDFD	DDDVDLSREK	AELRKAKENK	ESEAKVTSHT	ELTSNQQSAN	KTQAIAKQPI	NRGQPKPILQ	KQSTFPQSSK
1930	1940	1950	1960	1970	1980	1990	2000
DIPDRGAATD	EKLQNFAIEN	TPVCFSHNSS	LSSLSDIDQE	NNNKENEPIK	ETEPPDSQGE	PSKPQASGYA	PKSFHVEDTP
2010	2020	2030	2040	2050	2060	2070	2080
VCFSRNSSLS	SLSIDSEDDL	LQECISSAMP	KKKKPSRLKG	DNEKHSPRNM	GGILGEDLTL	DLKDIQRPDS	EHGLSPDSEN
2090	2100	2110	2120	2130	2140	2150	2160
FDWKAIQEGA	NSIVSSLHQA	AAAACLSRQA	SSDSDSILSL	KSGISLGSPF	HLTPDQEEKP	FTSNKGPRIL	KPGEKSTLET
2170	2180	2190	2200	2210	2220	2230	2240

Detailed Protein Report

Cmpd.	No. of Cmpds.	m/z meas.	Δ m/z [ppm]	z	Rt [min]	Score	P	Range	Sequence	Modification	Ratios
2988	1	619.0064	-1.40	3	66.1	10.6	0	1753-1768	K.KPTSPVKPIPQNTEYR.T		WUP:QUP 0.63
1774	1	620.2541	-173.92	2	52.0	10.5	1	1892-1902	K.TQAIAKQPINR.G		

Detailed Protein Report

Protein 364: PREDICTED: MAGUK p55 subfamily member 6 isoform X2 [Homo sapiens]

Accession: gi|530384825
Database: refseq_human(refseq_human_20140103.fasta)
Database Date: 2015-11-30
Modification(s): Oxidation

Score: 32.6
MW [kDa]: 48.7
pI: 9.5
Sequence Coverage [%]: 10.0
No. of unique Peptides: 2

10	20	30	40	50	60	70	80
MNNSSINNQL	LPVDAIRILG	IHKRAGEPLG	VTFRVENNDL	VIARILHGGM	IDRQGLLHVG	DIIKEVNGHE	VGNNPKELQE
**90**	**100**	**110**	**120**	**130**	**140**	**150**	**160**
LLKNISGSVT	LKILPSYRDT	ITPQQVFVKC	HFDYNPYNDN	LIPCKEAGLK	FSKGEILQIV	NREDPNWWQA	SHVKEGGSAG
**170**	**180**	**190**	**200**	**210**	**220**	**230**	**240**
LIPSQFLEEK	RKAFVRRDWD	NSGPFCGTIS	SKKKKKMMYL	TTRNAEFDRH	EIQIYEEVAK	MPPFQRKTLV	LIGAQGVGRR
**250**	**260**	**270**	**280**	**290**	**300**	**310**	**320**
SLKNRFIVLN	PTRFGTTVPF	TSRKPREDEK	DGQAYKFVSR	SEMEADIKAG	KYLEHGEYEG	NLYGTKIDSI	LEVVQTGRTC
**330**	**340**	**350**	**360**	**370**	**380**	**390**	**400**
ILDVNPQALK	VLRTSEFMPY	VVFIAAPELE	TLRAMHKAVV	DAGITTKLLT	DSDLKKTVDE	SARIQRAYNH	YFDLIIINDN
**410**	**420**	**430**					
LDKAFEKLQT	AIEKLRMEPQ	WVPISWVY					

Cmpd.	No. of Cmpds.	m/z meas.	Δ m/z [ppm]	z	Rt [min]	Score	P	Range	Sequence	Modification
2335	1	938.2188	84.20	3	57.5	10.4	1	334-357	R.TSEFMPYVVFIAAPELETLRAMHK.A	Oxidation: 5, 22
2850	1	994.4934	-74.35	2	64.3	22.2	2	358-376	K.AVVDAGITTKLLTDSDLKK.T	

Detailed Protein Report

Protein 365: PREDICTED: gamma-2-syntrophin isoform X1 [Homo sapiens]

**Accession:**	gi|578802555	**Score:**	32.6
**Database:**	refseq_human(refseq_human_20140103.fasta)	**MW [kDa]:**	53.7
**Database Date:**	2015-11-30	**pI:**	6.8
		**Sequence Coverage [%]:**	8.5
		**No. of unique Peptides:**	1

10	20	30	40	50	60	70	80
MLSVWAEATR	AGITVTLRRQ	PVGGLGLSIK	GGSEHNVPVV	ISKIFEDQAA	DQTGMLFVGD	AVLQVNGIHV	ENATHEEVVH
**90**	**100**	**110**	**120**	**130**	**140**	**150**	**160**
LLRNAGDEVT	ITVEYLREAP	AFLKLPLGSP	GPSSDHSSGA	SSPLFDSGLH	LNGNSSTTAP	SSPSSPIAKD	PRYEKRWLDT
**170**	**180**	**190**	**200**	**210**	**220**	**230**	**240**
LSVPLSMARI	SRYKAGTEKL	RWNAFEVLAL	DGVSSGILRF	YTAQDGTDWL	RAVSANIREL	TLQNMKMANK	CCSPSDQVVH
**250**	**260**	**270**	**280**	**290**	**300**	**310**	**320**
MGWVNEKLQG	ADSSQTFRPK	FLALKGPSFY	VFSTPPVSTF	DWVRAERTYH	LCEVLFKVHK	FWLTEDCWLQ	ANLYLGLQDF
**330**	**340**	**350**	**360**	**370**	**380**	**390**	**400**
DFEDQRPYCF	SIVAGHGKSH	VFNVELGSEL	AMWEKSFQRA	TFMEVQRTGS	RTYMCSWQGE	MLCFTVDFAL	GFTCFESKTK
**410**	**420**	**430**	**440**	**450**	**460**	**470**	**480**
NVLWRFKFSQ	LKGSSDDGKT	RVKLLFQNLD	TKQIETKELE	FQDLRAVLHC	IHSFIAAKVA	SVDPGFMDSQ	SLARKYMYSS
**490**							

Cmpd.	No. of Cmpds.	m/z meas.	Δ m/z [ppm]	z	Rt [min]	Score	P	Range	Sequence	Modification
2101	1	788.3311	-169.19	2	56.1	10.1	2	420-432	K.TRVKLLFQNLDTK.Q	

Detailed Protein Report

Protein 366: PREDICTED: CAS1 domain-containing protein 1 isoform X2 [Homo sapiens]

Accession: gi|578814529
Database: refseq_human(refseq_human_20140103.fasta)
Database Date: 2015-11-30
Modification(s): Carbamidomethyl

Score: 32.5
MW [kDa]: 72.0
pI: 9.6
Sequence Coverage [%]: 2.2
No. of unique Peptides: 2

Quantitation

QU:MU	**Median:**	1.55	**CV:**	0.00 %	**No. of Peptides:**	2
WUP:QUP	**Median:**	0.30	**CV:**	0.00 %	**No. of Peptides:**	2

```
        10         20         30         40         50         60         70         80
MGRCVLFSRN CSSIAPTWAQ VAASKLGDSP KARVRARRVP LVLSPHTPMA LPQPPPPAGA RDPVTPEHWA SGPPSQAQPL
        90        100        110        120        130        140        150        160
LRQEAKEEEE GEETGVQGAW GTGTAEQRRR GWGEAAESAA AEEGQAEVGG AAAAGSGSPA GGAGGGLGSW RPLLAWLQRR
       170        180        190        200        210        220        230        240
QPQCCPCAAP LSRSAAHCCH GGTKMAALAY NLGKREINHY FSVRSAKVLA LVAVLLLAAC HLASRRYRGN DSCEYLLSSG
       250        260        270        280        290        300        310        320
RFLGEKVWQP HSCMMHKYKI SEAKNCLVDK HIAFIGDSRI RQLFYSFVKI INPQFKEEGN KHENIPFEDK TASVKVDFLW
       330        340        350        360        370        380        390        400
HPEVNGSMKQ CIKVWTEDSI AKPHVIVAGA ATWSIKIHNG SSEALSQYKM NITSIAPLLE KLAKTSDVYW VLQDPVYEDL
       410        420        430        440        450        460        470        480
LSENRKMITN EKIDAYNEAA VSILNSSTRN SKSNVKMFSV SKLIAQETIM ESLDGLHLPE SSRETTAMIL MNVYCNKILK
       490        500        510        520        530        540        550        560
PVDGSCCQPR PPVTLIQKLA ACFFTLSIIG YLIFYIIHRN AHRKNKPCTD LESGEEKKNI INTPVSSLEI LLQSFCKLGL
       570        580        590        600        610        620        630        640
IMAYFYMCDR ANLFMKENKF YTHSSFFIPI IYILVLGVFY NENTKETKVL NREQTDEWKG WMQLVILIYH ISGASTIVGA
       650        660
IGTQEGEMTF I
```

Cmpd.	No. of Cmpds.	m/z meas.	Δ m/z [ppm]	z	Rt [min]	Score	P	Range	Sequence	Modification	Ratios
149	2	793.8480	-29.08	2	31.6	11.2	1	160-173	R.RQPQCCPCAAPLSR.S	Carbamidomethyl: 6	WUP:QUP 0.30<br>QU:MU 1.55
175	4	793.8315	-49.90	2	32.2	21.3	1	160-173	R.RQPQCCPCAAPLSR.S	Carbamidomethyl: 8	WUP:QUP 0.30<br>QU:MU 1.55

Detailed Protein Report

Protein 367: PREDICTED: PAX3- and PAX7-binding protein 1 isoform X2 [Homo sapiens]

**Accession:**	gi|578836733	**Score:**	32.5
**Database:**	refseq_human(refseq_human_20140103.fasta)	**MW [kDa]:**	86.2
**Database Date:**	2015-11-30	**pI:**	5.1
		**Sequence Coverage [%]:**	5.7
		**No. of unique Peptides:**	3

10	20	30	40	50	60	70	80
MFRKARRVNV	RKRNDSEEEE	RERDEEQEPP	PLLPPPGTGE	EAGPGGGDRA	PGGESLLGPG	PSPPSALTPG	LGAEAGGGFP
**90**	**100**	**110**	**120**	**130**	**140**	**150**	**160**
GGAEPGNGLK	PRKRPRENKE	VPRASLLSFQ	DEEEENEEVF	KVKKSSYSKK	IVKLLKKEYK	EDLEKSKIKT	ELNSSAESEQ
**170**	**180**	**190**	**200**	**210**	**220**	**230**	**240**
PLDKTGHVKD	TNQEDGVIIS	EHGEDEMDME	SEKEEEKPKT	GGAFSNALSS	LNVLRPGEIP	DAAFIHAARK	KRQMARELGD
**250**	**260**	**270**	**280**	**290**	**300**	**310**	**320**
FTPHDNEPGK	GRLVREDEND	ASDDEDDDEK	RRIVFSVKEK	SQRQKIAEEI	GIEGSDDDAL	VTGEQDEELS	RWEQEQIRKG
**330**	**340**	**350**	**360**	**370**	**380**	**390**	**400**
INIPQVQASQ	PAEVNMYYQN	TYQTMPYGSS	YGIPYSYTAY	GSSDAKSQKT	DNTVPFKTPS	NEMTPVTIDL	VKKQLKDRLD
**410**	**420**	**430**	**440**	**450**	**460**	**470**	**480**
SMKELHKTNR	QQHEKHLQSR	VDSTRAIERL	EGSSGGIGER	YKFLQEMRGY	VQDLLECFSE	KVPLINELES	AIHQLYKQRA
**490**	**500**	**510**	**520**	**530**	**540**	**550**	**560**
SRLVQRRQDD	IKDESSEFSS	HSNKALMAPN	LDSFGRDRAL	YQEHAKRRIA	EREARRTRRR	QAREQTGKMA	DHLEGLSSDD
**570**	**580**	**590**	**600**	**610**	**620**	**630**	**640**
EETSTDITNF	NLEKDRISKE	SGKVFEDVLE	SFYSIDCIKS	QFEAWRSKYY	TSYKDAYIGL	CLPKLFNPLI	RLQLLTWTPL
**650**	**660**	**670**	**680**	**690**	**700**	**710**	**720**
EAKCRDFENM	LWFESLLFYG	CEEREQEKDD	VDVALLPTIV	EKVILPKLTV	IAENMWDPFS	TTQTSRMVGI	TLKLINGYPS
**730**	**740**	**750**	**760**				
VVNAENKNTQ	VYLKALLLRM	RRTLDDDVFM	PLYPKNC				

Cmpd.	No. of Cmpds.	m/z meas.	Δ m/z [ppm]	z	Rt [min]	Score	P	Range	Sequence	Modification
584	1	824.2497	-169.01	2	36.9	10.9	0	150-164	K.TELNSSAESEQPLDK.T	
156	1	822.7809	-180.37	2	31.7	11.6	0	378-392	K.TPSNEMTPVTIDLVK.K	
2673	1	676.8390	-7.24	2	61.8	10.0	1	430-442	R.LEGSSGGIGERYK.F	

Detailed Protein Report

Protein 368: **kinesin-like protein KIF6 isoform 3 [Homo sapiens]**

**Accession:**	gi|573459724	**Score:**	32.5
**Database:**	refseq_human(refseq_human_20140103.fasta)	**MW [kDa]:**	86.2
**Database Date:**	2015-11-30	**pI:**	6.0
		**Sequence Coverage [%]:**	3.8
		**No. of unique Peptides:**	1

10	20	30	40	50	60	70	80
MVKQTIQIFA	RVKPPVRKHQ	QGIYSIDEDE	KLIPSLEIIL	PRDLADGFVN	NKRESYKFKF	QRIFDQDANQ	ETVFENIAKP
**90**	**100**	**110**	**120**	**130**	**140**	**150**	**160**
VAGSVLAGYN	GTIFAYGQTG	SGKTFTITGG	AERYSDRGII	PRTLSYIFEQ	LQKDSSKIYT	THISYLEIYN	ECGYDLLDPR
**170**	**180**	**190**	**200**	**210**	**220**	**230**	**240**
HEASSLEDLP	KVTILEDPDQ	NIHLKNLTLH	QATTEEEALN	LLFLGDTNRM	IAETPMNQAS	TRSHCIFTIH	LSSKEPGSAT
**250**	**260**	**270**	**280**	**290**	**300**	**310**	**320**
VRHAKLHLVD	LAGSERVAKT	GVGGHLLTEA	KYINLSLHYL	EQVIIALSEK	HRSHIPYRNS	MMTSVLRDSL	GGNCMTTMIA
**330**	**340**	**350**	**360**	**370**	**380**	**390**	**400**
TLSLEKRNLD	ESISTCRFAQ	RVALIKNEAV	LNEEINPRLV	IKRLQKEIQE	LKDELAMVTG	EQRTEALTEA	ELLQLEKLIT
**410**	**420**	**430**	**440**	**450**	**460**	**470**	**480**
SFLEDQDSDS	RLEVGADMRK	VHHCFHHLKK	LLNDKKILEN	NTVSSESKDQ	DCQEPLKEEE	YRKLRDILKQ	RDNEISILSE
**490**	**500**	**510**	**520**	**530**	**540**	**550**	**560**
VMKKLPLSWF	ERGMREEMSL	GCQEAFEIFK	RDHADSVTID	DNKQILKQRF	SEAKALGESI	NEARSKIGHL	KEEITQRHIQ
**570**	**580**	**590**	**600**	**610**	**620**	**630**	**640**
QVALGISENM	AVPLMPDQQE	EKLRSQLEEE	KRRYKTMFTR	LKALKVEIEH	LQLLMDKAKV	KLQKEFEVWW	AEEATNLQVN
**650**	**660**	**670**	**680**	**690**	**700**	**710**	**720**
SPAVNSLDHT	KPFLQTSDSQ	HEWSQLLSNK	SSGGWEVQDQ	GTGRFDVCDV	NARKILPSPC	PSPHSQKQSS	TSTPLEDSIP
**730**	**740**	**750**	**760**				
KRPVSSIPLT	GDSQTDSDII	AFIKARQSIL	QKQCLGSN				

Cmpd.	No. of Cmpds.	m/z meas.	Δ m/z [ppm]	z	Rt [min]	Score	P	Range	Sequence	Modification
2648	1	994.9726	-29.06	2	63.2	15.8	1	367-383	K.EIQELKDELAMVTGEQR.T	

Detailed Protein Report

Protein 369: nucleus accumbens-associated protein 1 [Homo sapiens]

**Accession:**	gi|16418383	**Score:**	32.5
**Database:**	refseq_human(refseq_human_20140103.fasta)	**MW [kDa]:**	57.2
**Database Date:**	2015-11-30	**pI:**	5.4
		**Sequence Coverage [%]:**	4.9
		**No. of unique Peptides:**	1

Quantitation

WUP:QUP **Median:** 1.25 **CV:** 0.00 % **No. of Peptides:** 1

Alias proteins:

Accession	Name	Description
gi|578832952	refseq_human (refseq_human_20140103.fasta)	PREDICTED: nucleus accumbens-associated protein 1 isoform X2 [Homo sapiens]
gi|530414464	refseq_human (refseq_human_20140103.fasta)	PREDICTED: nucleus accumbens-associated protein 1 isoform X1 [Homo sapiens]

```
        10         20         30         40         50         60         70         80
MAQTLQMEIP NFGNSILECL NEQRLQGLYC DVSVVVKGHA FKAHRAVLAA SSSYFRDLFN NSRSAVVELP AAVQPQSFQQ
        90        100        110        120        130        140        150        160
ILSFCYTGRL SMNVGDQFLL MYTAGFLQIQ EIMEKGTEFF LKVSSPSCDS QGLHAEEAPS SEPQSPVAQT SGWPACSTPL
       170        180        190        200        210        220        230        240
PLVSRVKTEQ QESDSVQCMP VAKRLWDSGQ KEAGGGGNGS RKMAKFSTPD LAANRPHQPP PPQQAPVVAA AQPAVAAGAG
       250        260        270        280        290        300        310        320
QPAGGVAAAG GVVSGPSTSE RTSPGTSSAY TSDSPGSYHN EEDEEEDGGE EGMDEQYRQI CNMYTMYSMM NVGQTAEKVE
       330        340        350        360        370        380        390        400
ALPEQVAPES RNRIRVRQDL ASLPAELINQ IGNRCHPKLY DEGDPSEKLE LVTGTNVYIT RAQLMNCHVS AGTRHKVLLR
       410        420        430        440        450        460        470        480
RLLASFFDRN TLANSCGTGI RSSTNDPRRK PLDSRVLHAV KYYCQNFAPN FKESEMNAIA ADMCTNARRV VRKSWMPKVK
       490        500        510        520        530
VLKAEDDAYT TFISETGKIE PDMMGVEHGF ETASHEGEAG PSAEALQ
```

Cmpd.	No. of Cmpds.	m/z meas.	Δ m/z [ppm]	z	Rt [min]	Score	P	Range	Sequence	Modification	Ratios
1731	6	916.4065	-40.21	2	51.4	20.9	2	184-201	K.RLWDSGQKEAGGGGNGSR.K		WUP:QUP 1.25

Detailed Protein Report

Protein 370: ubiquinone biosynthesis protein COQ7 homolog isoform 1 [Homo sapiens]

Accession: gi|25453484
Database: refseq_human(refseq_human_20140103.fasta)
Database Date: 2015-11-30
Modification(s): Oxidation

Score: 32.4
MW [kDa]: 24.3
pI: 9.5
Sequence Coverage [%]: 14.7
No. of unique Peptides: 1

10	20	30	40	50	60	70	80
MSCAGAAAAP	RLWRLRPGAR	RSLSAYGRRT	SVRFRSSGMT	LDNISRAAVD	RIIRVDHAGE	YGANRIYAGQ	MAVLGRTSVG
**90**	**100**	**110**	**120**	**130**	**140**	**150**	**160**
PVIQKMWDQE	KDHLKKFNEL	MVTFRVRPTV	LMPLWNVLGF	ALGAGTALLG	KEGAMACTVA	VEESIAHHYN	NQIRTLMEED
**170**	**180**	**190**	**200**	**210**	**220**		
PEKYEELLQL	IKKFRDEELE	HHDIGLDHDA	ELAPAYAVLK	SIIQAGCRVA	IYLSERL		

Cmpd.	No. of Cmpds.	m/z meas.	Δ m/z [ppm]	z	Rt [min]	Score	P	Range	Sequence	Modification
1373	1	1045.9338	-113.36	2	45.5	20.1	2	36-54	R.SSGMTLDNISRAAVDRIIR.V	Oxidation: 4

Detailed Protein Report

Protein 371: potassium channel subfamily K member 10 isoform 2 [Homo sapiens]

Accession: gi|20143944
Database: refseq_human(refseq_human_20140103.fasta)
Database Date: 2015-11-30
Modification(s): Oxidation

Score: 32.4
MW [kDa]: 60.3
pI: 9.8
Sequence Coverage [%]: 4.1
No. of unique Peptides: 2

Quantitation

QU:MU **Median:** 0.42 **CV:** 0.00 % **No. of Peptides:** 1

10	20	30	40	50	60	70	80
MKFPIETPRK	QVNWDPKVAV	PAAAPVCQPK	SATNGQPPAP	APTPTPRLSI	SSRATVVARM	EGTSQGGLQT	VMKWKTVVAI
**90**	**100**	**110**	**120**	**130**	**140**	**150**	**160**
FVVVVVYLVT	GGLVFRALEQ	PFESSQKNTI	ALEKAEFLRD	HVCVSPQELE	TLIQHALDAD	NAGVSPIGNS	SNNSSHWDLG
**170**	**180**	**190**	**200**	**210**	**220**	**230**	**240**
SAFFFAGTVI	TTIGYGNIAP	STEGGKIFCI	LYAIFGIPLF	GFLLAGIGDQ	LGTIFGKSIA	RVEKVFRKKQ	VSQTKIRVIS
**250**	**260**	**270**	**280**	**290**	**300**	**310**	**320**
TILFILAGCI	VFVTIPAVIF	KYIEGWTALE	SIYFVVVTLT	TVGFGDFVAG	GNAGINYREW	YKPLVWFWIL	VGLAYFAAVL
**330**	**340**	**350**	**360**	**370**	**380**	**390**	**400**
SMIGDWLRVL	SKKTKEEVGE	IKAHAAEWKA	NVTAEFRETR	RRLSVEIHDK	LQRAATIRSM	ERRRLGLDQR	AHSLDMLSPE
**410**	**420**	**430**	**440**	**450**	**460**	**470**	**480**
KRSVFAALDT	GRFKASSQES	INNRPNNLRL	KGPEQLNKHG	QGASEDNIIN	KFGSTSRLTK	RKNKDLKKTL	PEDVQKIYKT
**490**	**500**	**510**	**520**	**530**	**540**	**550**	
FRNYSLDEEK	KEEETEKMCN	SDNSSTAMLT	DCIQQHAELE	NGMIPTDTKD	REPENNSLLE	DRN	

Cmpd.	No. of Cmpds.	m/z meas.	Δ m/z [ppm]	z	Rt [min]	Score	P	Range	Sequence	Modification	Ratios
1557	2	494.2527	-64.89	2	49.7	17.6	1	3-10	K.FPIETPRK.Q		QU:MU 0.42
2750	1	741.8557	7.67	2	64.8	14.8	0	60-73	R.MEGTSQGGLQTVMK.W	Oxidation: 13	

Detailed Protein Report

Protein 372: PREDICTED: nardilysin isoform X1 [Homo sapiens]

**Accession:**	gi|530362704	**Score:**	32.4
**Database:**	refseq_human(refseq_human_20140103.fasta)	**MW [kDa]:**	105.5
**Database Date:**	2015-11-30	**pI:**	5.4
**Modification(s):**	Carbamidomethyl	**Sequence Coverage [%]:**	2.4
		**No. of unique Peptides:**	2

10	20	30	40	50	60	70	80
MVFMGSLKYP	DENGFDAFLK	KHGGSDNAST	DCERTVFQFD	VQRKYFKEAL	DRWAQFFIHP	LMIRDAIDRE	VEAVDSEYQL
**90**	**100**	**110**	**120**	**130**	**140**	**150**	**160**
ARPSDANRKE	MLFGSLARPG	HPMGKFFWGN	AETLKHEPRK	NNIDTHARLR	EFWMRYYSSH	YMTLVVQSKE	TLDTLEKWVT
**170**	**180**	**190**	**200**	**210**	**220**	**230**	**240**
EIFSQIPNNG	LPRPNFGHLT	DPFDTPAFNK	LYRVVPIRKI	HALTITWALP	PQQQHYRVKP	LHYISWLVGH	EGKGSILSFL
**250**	**260**	**270**	**280**	**290**	**300**	**310**	**320**
RKKCWALALF	GGNGETGFEQ	NSTYSVFSIS	ITLTDEGYEH	FYEVAYTVFQ	YLKMLQKLGP	EKRIFEEIRK	IEDNEFHYQE
**330**	**340**	**350**	**360**	**370**	**380**	**390**	**400**
QTDPVEYVEN	MCENMQLYPL	QDILTGDQLL	FEYKPEVIGE	ALNQLVPQKA	NLVLLSGANE	GKCDLKEKWF	GTQYSIEDIE
**410**	**420**	**430**	**440**	**450**	**460**	**470**	**480**
NSWAELWNSN	FELNPDLHLP	AENKYIATDF	TLKAFDCPET	EYPVKIVNTP	QGCLWYKKDN	KFKIPKAYIR	FHLISPLIQK
**490**	**500**	**510**	**520**	**530**	**540**	**550**	**560**
SAANVVLFDI	FVNILTHNLA	EPAYEADVAQ	LEYKLVAGEH	GLIIRVKGFN	HKLPLLFQLI	IDYLAEFNST	PAVFTMITEQ
**570**	**580**	**590**	**600**	**610**	**620**	**630**	**640**
LKKTYFNILI	KPETLAKDVR	LLILEYARWS	MIDKYQALMD	GLSLESLLSF	VKEFKSQLFV	EGLVQGNVTS	TESMDFLKYV
**650**	**660**	**670**	**680**	**690**	**700**	**710**	**720**
VDKLNFKPLE	QEMPVQFQVV	ELPSGHHLCK	VKALNKGDAN	SEVTVYYQSG	TRSLREYTLM	ELLVMHMEEP	CFDFLRTKQT
**730**	**740**	**750**	**760**	**770**	**780**	**790**	**800**
LGYHVYPTCR	NTSGILGFSV	TVGTQATKYN	SEVVDKKIEE	FLSSFEEKIE	NLTEEAFNTQ	VTALIKLKEC	EDTHLGEEVD
**810**	**820**	**830**	**840**	**850**	**860**	**870**	**880**
RNWNEVVTQQ	YLFDRLAHEI	EALKSFSKSD	LVNWFKAHRG	PGSKMLSVHV	VGYGKYELEE	DGTPSSEDSN	SSCEVMQLTY
**890**	**900**	**910**	**920**				
LPTSPLLADC	IIPITDIRAF	TTTLNLLPYH	KIVK				

Cmpd.	No. of Cmpds.	m/z meas.	Δ m/z [ppm]	z	Rt [min]	Score	P	Range	Sequence	Modification
2877	2	703.3682	134.70	2	66.7	17.6	0	22-34	K.HGGSDNASTDCER.T	Carbamidomethyl: 11
923	1	512.2392	-109.51	2	41.6	14.7	0	816-824	R.LAHEIEALK.S	

Detailed Protein Report

Protein 373: **protein scribble homolog isoform b [Homo sapiens]**

**Accession:**	gi|355390315	**Score:**	32.3
**Database:**	refseq_human(refseq_human_20140103.fasta)	**MW [kDa]:**	174.8
**Database Date:**	2015-11-30	**pI:**	4.9
**Modification(s):**	Carbamidomethyl	**Sequence Coverage [%]:**	1.7
		**No. of unique Peptides:**	1

10	20	30	40	50	60	70	80
MLKCIPLWRC	NRHVESVDKR	HCSLQAVPEE	IYRYSRSLEE	LLLDANQLRE	LPKPFFRLLN	LRKLGLSDNE	IQRLPPEVAN
**90**	**100**	**110**	**120**	**130**	**140**	**150**	**160**
FMQLVELDVS	RNDIPEIPES	IKFCKALEIA	DFSGNPLSRL	PDGFTQLRSL	AHLALNDVSL	QALPGDVGNL	ANLVTLELRE
**170**	**180**	**190**	**200**	**210**	**220**	**230**	**240**
NLLKSLPASL	SFLVKLEQLD	LGGNDLEVLP	DTLGALPNLR	ELWLDRNQLS	ALPPELGNLR	RLVCLDVSEN	RLEELPAELG
**250**	**260**	**270**	**280**	**290**	**300**	**310**	**320**
GLVLLTDLLL	SQNLLRRLPD	GIGQLKQLSI	LKVDQNRLCE	VTEAIGDCEN	LSELILTENL	LMALPRSLGK	LTKLTNLNVD
**330**	**340**	**350**	**360**	**370**	**380**	**390**	**400**
RNHLEALPPE	IGGCVALSVL	SLRDNRLAVL	PPELAHTTEL	HVLDVAGNRL	QSLPFALTHL	NLKALWLAEN	QAQPMLRFQT
**410**	**420**	**430**	**440**	**450**	**460**	**470**	**480**
EDDARTGEKV	LTCYLLPQQP	PPSLEDAGQQ	GSLSETWSDA	PPSRVSVIQF	LEAPIGDEDA	EEAAAEKRGL	QRRATPHPSE
**490**	**500**	**510**	**520**	**530**	**540**	**550**	**560**
LKVMKRSIEG	RRSEACPCQP	DSGSPLPAEE	EKRLSAESGL	SEDSRPSAST	VSEAEPEGPS	AEAQGGSQQE	ATTAGGEEDA
**570**	**580**	**590**	**600**	**610**	**620**	**630**	**640**
EEDYQEPTVH	FAEDALLPGD	DREIEEGQPE	APWTLPGGRQ	RLIRKDTPHY	KKHFKISKLP	QPEAVVALLQ	GMQPDGEGPV
**650**	**660**	**670**	**680**	**690**	**700**	**710**	**720**
APGGWHNGPH	APWAPRAQKE	EEEEEEGSPQ	EEEEEEEEEN	RAEEEEASTE	EEDKEGAVVS	APSVKGVSFD	QANNLLIEPA
**730**	**740**	**750**	**760**	**770**	**780**	**790**	**800**
RIEEEELTLT	ILRQTGGLGI	SIAGGKGSTP	YKGDDEGIFI	SRVSEEGPAA	RAGVRVGDKL	LEVNGVALQG	AEHHEAVEAL
**810**	**820**	**830**	**840**	**850**	**860**	**870**	**880**
RGAGTAVQMR	VWRERMVEPE	NAVTITPLRP	EDDYSPRERR	GGGLRLPLLP	PESPGPLRQR	HVACLARSER	GLGFSIAGGK
**890**	**900**	**910**	**920**	**930**	**940**	**950**	**960**
GSTPYRAGDA	GIFVSRIAEG	GAAHRAGTLQ	VGDRVLSING	VDVTEARHDH	AVSLLTAASP	TIALLLEREA	GGPLPPSPLP
**970**	**980**	**990**	**1000**	**1010**	**1020**	**1030**	**1040**
HSSPPTAAVA	TTSITTATPG	VPGLPSLAPS	LLAAALEGPY	PVEEIRLPRA	GGPLGLSIVG	GSDHSSHPFG	VQEPGVFISK
**1050**	**1060**	**1070**	**1080**	**1090**	**1100**	**1110**	**1120**
VLPRGLAARS	GLRVGDRILA	VNGQDVRDAT	HQEAVSALLR	PCLELSLLVR	RDPAPPGLRE	LCIQKAPGER	LGISIRGGAR
**1130**	**1140**	**1150**	**1160**	**1170**	**1180**	**1190**	**1200**
GHAGNPRDPT	DEGIFISKVS	PTGAAGRDGR	LRVGLRLLEV	NQQSLLGLTH	GEAVQLLRSV	GDTLTVLVCD	GFEASTDAAL
**1210**	**1220**	**1230**	**1240**	**1250**	**1260**	**1270**	**1280**
EVSPGVIANP	FAAGIGHRNS	LESISSIDRE	LSPEGPGKEK	ELPGQTLHWG	PEATEAAGRG	LQPLKLDYRA	LAAVPSAGSV
**1290**	**1300**	**1310**	**1320**	**1330**	**1340**	**1350**	**1360**
QRVPSGAAGG	KMAESPCSPS	GQQPPSPPSP	DELPANVKQA	YRAFAAVPTS	HPPEDAPAQP	PTPGPAASPE	QLSFRERQKY
**1370**	**1380**	**1390**	**1400**	**1410**	**1420**	**1430**	**1440**
FELEVRVPQA	EGPPKRVSLV	GADDLRKMQE	EEARKLQQKR	AQMLREAAEA	GAEARLALDG	ETLGEEEQED	EQPPWASPSP
**1450**	**1460**	**1470**	**1480**	**1490**	**1500**	**1510**	**1520**
TSRQSPASPP	PLGGGAPVRT	AKAERRHQER	LRVQSPEPPA	PERALSPAEL	RALEAEKRAL	WRAARMKSLE	QDALRAQMVL
**1530**	**1540**	**1550**	**1560**	**1570**	**1580**	**1590**	**1600**
SRSQEGRGTR	GPLERLAEAP	SPAPTPSPTP	VEDLGPQTST	SPGRLSPDFA	EELRSLEPSP	SPGPQEEDGE	VALVLLGRPS
**1610**	**1620**	**1630**	**1640**				
PGAVGPEDVA	LCSSRRPVRP	GRRGLGPVPS					

Cmpd.	No. of Cmpds.	m/z meas.	Δ m/z [ppm]	z	Rt [min]	Score	P	Range	Sequence	Modification
129	2	696.1281	130.45	3	31.3	16.1	2	861-880	R.HVACLARSERGLGFSIAGGK.G	Carbamidomethyl: 4

Detailed Protein Report

Protein 374: tumor suppressor candidate gene 1 protein [Homo sapiens]

Accession: gi|51944974
Database: refseq_human(refseq_human_20140103.fasta)
Database Date: 2015-11-30
Modification(s): Carbamidomethyl

Score: 32.3
MW [kDa]: 23.4
pI: 11.8
Sequence Coverage [%]: 8.5
No. of unique Peptides: 3

Quantitation

WUP:QUP **Median:** 1.49 **CV:** 0.00 % **No. of Peptides:** 2

10	20	30	40	50	60	70	80
MGPMWRMRGG	ATRRGSCCGG	DGAADGRGPG	RSGRARGGGS	PSGGGGGVGW	RGRADGARQQ	LEERFADLAA	SHLEAIRARD
90	100	110	120	130	140	150	160
EWDRQNARLR	QENARLRLEN	RRLKRENRSL	FRQALRLPGE	GGNGTPAEAR	RVPEEASTNR	RARDSGREDE	PGSPRALRAR
170	180	190	200	210	220		
LEKLEAMYRR	ALLQLHLEQR	GPRPSGDKEE	QPLQEPDSGL	RSRDSEPSGP	WL		

Cmpd.	No. of Cmpds.	m/z meas.	Δ m/z [ppm]	z	Rt [min]	Score	P	Range	Sequence	Modification	Ratios
1612	1	853.3687	3.55	2	49.9	10.3	2	14-31	R.RGSCCGGDGAADGRGPGR.S	Carbamidomethyl: 5	WUP:QUP 1.49
1610	1	853.3997	39.87	2	48.5	10.6	2	14-31	R.RGSCCGGDGAADGRGPGR.S	Carbamidomethyl: 4	WUP:QUP 1.49
474	1	775.3888	94.96	2	35.9	11.4	1	15-31	R.GSCCGGDGAADGRGPGR.S	Carbamidomethyl: 3	

Detailed Protein Report

Protein 375: **PREDICTED: protein Shroom4 isoform X2 [Homo sapiens]**

Accession: gi|578838225
Database: refseq_human(refseq_human_20140103.fasta)
Database Date: 2015-11-30
Modification(s): Carbamidomethyl

Score: 32.3
MW [kDa]: 147.5
pI: 6.0
Sequence Coverage [%]: 1.9
No. of unique Peptides: 1

Quantitation

QU:MU	**Median:**	4.76	**CV:**	0.00 %	**No. of Peptides:**	1
WUP:QUP	**Median:**	0.17	**CV:**	0.00 %	**No. of Peptides:**	1

10	20	30	40	50	60	70	80
MESLEQPGQA	TYESHLLPID	QNMYPNQRDS	AYSSFSASSN	ASDCALSLRP	EEPASTDCIM	QGPGPTKAPS	GRPNVAETSG
90	100	110	120	130	140	150	160
GSRRTNGGHL	TPSSQMSSRP	QEGYQSGPAK	AVRGPPQPPV	RRDSLQASRA	QLLNGEQRRA	SEPVVPLPQK	EKLSLEPVLP
170	180	190	200	210	220	230	240
ARNPNRFCCL	SGHDQVTSEG	HQNCEFSQPP	ESSQQGSEHL	LMQASTKAVG	SPKACDRASS	VDSNPLNEAS	AELAKASFGR
250	260	270	280	290	300	310	320
PPHLIGPTGH	RHSAPEQLLA	SHLQHVHLDT	RGSKGMELPP	VQDGHQWTLS	PLHSSHKGKK	SPCPPTGGTH	DQSSKERKTR
330	340	350	360	370	380	390	400
QVDDRSLVLG	HQSQSSPPHG	EADGHPSEKG	FLDPNRTSRA	ASELANQQPS	ASGSLVQQAT	DCSSTTKAAS	GTEAGEEGDS
410	420	430	440	450	460	470	480
EPKECSRMGG	RRSGGTRGRS	IQNRRKSERF	ATNLRNEIQR	RKAQLQKSKG	PLSQLCDTKE	PVEETQEPPE	SPPLTASNTS
490	500	510	520	530	540	550	560
LLSSCKKPPS	PRDKLFNKSM	MLRARSSECL	SQAPESHESR	TGLEGRISPG	QRPGQSSLGL	NTWWKAPDPS	SSDPEKAHAH
570	580	590	600	610	620	630	640
CGVRGGHWRW	SPEHNSQPLV	AAAMEGPSNP	GDNKELKAST	AQAGEDAILL	PFADRRKFFE	ESSKSLSTSH	LPGLTTHSNK
650	660	670	680	690	700	710	720
TFTQRPKPID	QNFQPMSSSC	RELRRHPMDQ	SYHSADQPYH	ATDQSYHSMS	PLQSETPTYS	ECFASKGLEN	SMCCKPLHCG
730	740	750	760	770	780	790	800
DFDYHRTCSY	SCSVQGALVH	DPCIYCSGEI	CPALLKRNMM	PNCYNCRCHH	HQCIRCSVCY	HNPQHSALED	SSLAPGNTWK
810	820	830	840	850	860	870	880
PRKLTVQEFP	GDKWNPITGN	RKTSQSGREM	AHSKTSFSWA	TPFHPCLENP	ALDLSSYRAI	SSLDLLGDFK	HALKKSEETS
890	900	910	920	930	940	950	960
VYEEGSSLAS	MPHPLRSRAF	SESHISLAPQ	STRAWGQHRR	ELFSKGDETQ	SDLLGARKKA	FPPPRPPPPN	WEKYRLFRAA
970	980	990	1000	1010	1020	1030	1040
QQQKQQQQQQ	KQQEEEEEEE	EEEEEEEEEE	EEEAEEEEEE	LPPQYFSSET	SGSCALNPEE	VLEQPQPLSF	GHLEGSRQGS
1050	1060	1070	1080	1090	1100	1110	1120
QSVPAEQESF	ALHSSDFLPP	IRGHLGSQPE	QAQPPCYYGI	GGLWRTSGQE	ATESAKQEFQ	HFSPPSGAPG	IPTSYSAYYN
1130	1140	1150	1160	1170	1180	1190	1200
ISVAKAELLN	KLKDQPEMAE	IGLGEEEVDH	ELAQKKIQLI	ESISRKLSVL	REAQRGLLED	INANSALGEE	VEANLKAVCK
1210	1220	1230	1240	1250	1260	1270	1280
SNEFEKYHLF	VGDLDKVVNL	LLSLSGRLAR	VENALNSIDS	EANQEKLVLI	EKKQQLTGQL	ADAKELKEHV	DRREKLVFGM
1290	1300	1310	1320	1330	1340		
VSRYLPQDQL	QDYQHFVKMK	SALIIEQREL	EEKIKLGEEQ	LKCLRESLLL	GPSNF		

Cmpd.	No. of Cmpds.	m/z meas.	Δ m/z [ppm]	z	Rt [min]	Score	P	Range	Sequence	Modification	Ratios
43	1	750.8112	-65.69	2	30.4	14.4	1	557-569	K.AHAHCGVRGGHWR.W	Carbamidomethyl: 5	WUP:QUP 0.17<br>QU:MU 4.76

Detailed Protein Report

Protein 376: PREDICTED: exosome complex exonuclease RRP44 isoform X1 [Homo sapiens]

**Accession:**	gi|530402172	**Score:**	32.2
**Database:**	refseq_human(refseq_human_20140103.fasta)	**MW [kDa]:**	90.5
**Database Date:**	2015-11-30	**pI:**	6.5
		**Sequence Coverage [%]:**	3.1
		**No. of unique Peptides:**	2

10	20	30	40	50	60	70	80
MSADNQLQVI	FITNDRRNKE	KAIEEGIPAF	TCEEYVKSLT	ANPELIDRLA	CLSEEGNEIE	SGKIIFSEHL	PLSKLQQGIK
**90**	**100**	**110**	**120**	**130**	**140**	**150**	**160**
SGTYLQGTFR	ASRENYLEAT	VWIHGDNEEN	KEIILQGLKH	LNRAVHEDIV	AVELLPKSQW	VAPSSVVLHD	EGQNEEDVEK
**170**	**180**	**190**	**200**	**210**	**220**	**230**	**240**
EEETERMLKT	AVSEKMLKPT	GRVVGIIKRN	WRPYCGMLSK	SDIKESRRHL	FTPADKRIPR	IRIETRQAST	LEGRRIIVAI
**250**	**260**	**270**	**280**	**290**	**300**	**310**	**320**
DGWPRNSRYP	NGHFVRNLGD	VGEKETETEV	LLLEHDVPHQ	PFSQAVLSFL	PKMPWSITEK	DMKNREDLRH	LCICSVDPPG
**330**	**340**	**350**	**360**	**370**	**380**	**390**	**400**
CTDIDDALHC	RELENGNLEV	GVHIADVSHF	IRPGNALDQE	SARRGTTVYL	CEKRIDMVPE	LLSSNLCSLK	CDVDRLAFSC
**410**	**420**	**430**	**440**	**450**	**460**	**470**	**480**
IWEMNHNAEI	LKTKFTKSVI	NSKASLTYAE	AQLRIDSANM	NDDITTSLRG	LNKLAKILKK	RRIEKGALTL	SSPEVRFHMD
**490**	**500**	**510**	**520**	**530**	**540**	**550**	**560**
SETHDPIDLQ	TKELRETNSM	VEEFMLLANI	SVAKKIHEEF	SEHALLRKHP	APPPSNYEIL	VKAARSRNLE	IKTDTAKSLA
**570**	**580**	**590**	**600**	**610**	**620**	**630**	**640**
ESLDQAESPT	FPYLNTLLRI	LATRCMMQAV	YFCSGMDNDF	HHYGLASPIY	THFTSPIRRY	ADVIVHRLLA	VAIGADCTYP
**650**	**660**	**670**	**680**	**690**	**700**	**710**	**720**
ELTDKHKLAD	ICKNLNFRHK	MAQYAQRASV	AFHTQLFFKS	KGIVSEEAYI	LFVRKNAIVV	LIPKYGLEGT	VFFEEKDKPN
**730**	**740**	**750**	**760**	**770**	**780**	**790**	**800**
PQLIYDDEIP	SLKIEDTVFH	VFDKVKVKIM	LDSSNLQHQK	IRMSLVEPQI	PGISIPTDTS	NMDLNGPKKK	KMKLGK

Cmpd.	No. of Cmpds.	m/z meas.	Δ m/z [ppm]	z	Rt [min]	Score	P	Range	Sequence	Modification
1167	1	977.5217	29.45	2	44.2	19.3	2	189-204	K.RNWRPYCGMLSKSDIK.E	
2612	1	588.6601	-226.00	2	60.9	13.0	2	301-309	K.DMKNREDLR.H	

Detailed Protein Report

Protein 377: PREDICTED: ankyrin-3 isoform X15 [Homo sapiens]

**Accession:**	gi|578819314	**Score:**	32.2
**Database:**	refseq_human(refseq_human_20140103.fasta)	**MW [kDa]:**	386.8
**Database Date:**	2015-11-30	**pI:**	5.6
		**Sequence Coverage [%]:**	0.8
		**No. of unique Peptides:**	1

Quantitation

QU:MU	**Median:**	1.57	**CV:**	0.00 %	**No. of Peptides:**	1
WUP:QUP	**Median:**	1.19	**CV:**	0.00 %	**No. of Peptides:**	1

10	20	30	40	50	60	70	80
MALPQSEDAM	TGDTDKYLGP	QDLKELGDDS	LPAEGYMGFS	LGARSASSDR	SYTLNRSSYA	RDSMMIEELL	VPSKEQHLTF
90	100	110	120	130	140	150	160
TREFDSDSLR	HYSWAADTLD	NVNLVSSPIH	SGFLVSFMVD	ARGGSMRGSR	HHGMRIIIPP	RKCTAPTRIT	CRLVKRHKLA
170	180	190	200	210	220	230	240
NPPPMVEGEG	LASRLVEMGP	AGAQFLGPVI	VEIPHFGSMR	GKERELIVLR	SENGETWKEH	QFDSKNEDLT	ELLNGMDEEL
250	260	270	280	290	300	310	320
DSPEELGKKR	ICRIITKDFP	QYFAVVSRIK	QESNQIGPEG	GILSSTTVPL	VQASFPEGAL	TKRIRVGLQA	QPVPDEIVKK
330	340	350	360	370	380	390	400
ILGNKATFSP	IVTVEPRRRK	FHKPITMTIP	VPPPSGEGVS	NGYKGDTTPN	LRLLCSITGG	TSPAQWEDIT	GTTPLTFIKD
410	420	430	440	450	460	470	480
CVSFTTNVSA	RFWLADCHQV	LETVGLATQL	YRELICVPYM	AKFVVFAKMN	DPVESSLRCF	CMTDDKVDKT	LEQQENFEEV
490	500	510	520	530	540	550	560
ARSKDIEVLE	GKPIYVDCYG	NLAPLTKGGQ	QLVFNFYSFK	ENRLPFSIKI	RDTSQEPCGR	LSFLKEPKTT	KGLPQTAVCN
570	580	590	600	610	620	630	640
LNITLPAHKK	ETESDQDDEI	EKTDRRQSFA	SLALRKRYSY	LTEPGMKTVE	RSTGATRSLP	TTYSYKPFFS	TRPYQSWTTA
650	660	670	680	690	700	710	720
PITVPGPAKS	GFTSLSSSSS	NTPSASPLKS	IWSVSTPSPI	KSTLGASTTS	SVKSISDVAS	PIRSFRTMSS	PIKTVVSQSP
730	740	750	760	770	780	790	800
YNIQVSSGTL	ARAPAVTEAT	PLKGLASNST	FSSRTSPVTT	AGSLLERSSI	TMTPPASPKS	NINMYSSSLP	FKSIITSAAP
810	820	830	840	850	860	870	880
LISSPLKSVV	SPVKSAVDVI	SSAKITMASS	LSSPVKQMPG	HAEVALVNGS	ISPLKYPSSS	TLINGCKATA	TLQEKISSAT
890	900	910	920	930	940	950	960
NSVSSVVSAA	TDTVEKVFST	TTAMPFSPLR	SYVSAAPSAF	QSLRTPSASA	LYTSLGSSIS	ATTSSVTSSI	ITVPVYSVVN
970	980	990	1000	1010	1020	1030	1040
VLPEPALKKL	PDSNSFTKSA	AALLSPIKTL	TTETHPQPHF	SRTSSPVKSS	LFLAPSALKL	STPSSLSSSQ	EILKDVAEMK
1050	1060	1070	1080	1090	1100	1110	1120
EDLMRMTAIL	QTDVPEEKPF	QPELPKEGRI	DDEEPFKIVE	KVKEDLVKVS	EILKKDVCVD	NKGSPKSPKS	DKGHSPEDDW
1130	1140	1150	1160	1170	1180	1190	1200
IEFSSEEIRE	ARQQAAASQS	PSLPERVQVK	AKAASEKDYN	LTKVIDYLTN	DIGSSSLTNL	KYKFEDAKKD	GEERQKRVLK
1210	1220	1230	1240	1250	1260	1270	1280
PAIALQEHKL	KMPPASMRTS	TSEKELCKMA	DSFFGTDTIL	ESPDDFSQHD	QDKSPLSDSG	FETRSEKTPS	APQSAESTGP
1290	1300	1310	1320	1330	1340	1350	1360
KPLFHEVPIP	PVITETRTEV	VHVIRSYDPS	AGDVPQTQPE	EPVSPKPSPT	FMELEPKPTT	SSIKEKVKAF	QMKASSEEDD
1370	1380	1390	1400	1410	1420	1430	1440
HNRVLSKGMR	VKEETHITTT	TRMVYHSPPG	GEGASERIEE	TMSVHDIMKA	FQSGRDPSKE	LAGLFEHKSA	VSPDVHKSAA
1450	1460	1470	1480	1490	1500	1510	1520
ETSAQHAEKD	NQMKPKLERI	IEVHIEKGNQ	AEPTEVIIRE	TKKHPEKEMY	VYQKDLSRGD	INLKDFLPEK	HDAFPCSEEQ
1530	1540	1550	1560	1570	1580	1590	1600
GQQEEEELTA	EESLPSYLES	SRVNTPVSQE	EDSRPSSAQL	ISDDSYKTLK	LLSQHSIEYH	DDELSELRGE	SYRFAEKMLL
1610	1620	1630	1640	1650	1660	1670	1680
SEKLDVSHSD	TEESVTDHAG	PPSSELQGSD	KRSREKIATA	PKKEILSKIY	KDVSENGVGK	VSKDEHFDKV	TVLHYSGNVS
1690	1700	1710	1720	1730	1740	1750	1760
SPKHAMWMRF	TEDRLDRGRE	KLIYEDRVDR	TVKEAEEKLT	EVSQFFRDKT	EKLNDELQSP	EKKARPKNGK	EYSSQSPTSS
1770	1780	1790	1800	1810	1820	1830	1840
SPEKVLLTEL	LASNDEWVKA	RQHGPDGQGF	PKAEEKAPSL	PSSPEKMVLS	QQTEDSKSTV	EAKGSISQSK	APDGPQSGFQ
1850	1860	1870	1880	1890	1900	1910	1920
LKQSKLSSIR	LKFEQGTHAK	SKDMSQEDRK	SDGQSRIPVK	KIQESKLPVY	QVFAREKQQK	AIDLPDESVS	VQKDFMVLKT
1930	1940	1950	1960	1970	1980	1990	2000
KDEHAQSNEI	VVNDSGSDNV	KKQRTEMSSK	AMPDSFSEQQ	AKDLACHITS	DLATRGPWDK	KVFRTWESSG	ATNNKSQKEK
2010	2020	2030	2040	2050	2060	2070	2080
LSHVLVHDVR	ENHIGHPESK	SVDQKNEFMS	VTERERKLLT	NGSLSEIKEM	TVKSPSKKVL	YREYVVKEGD	HPGGLLDQPS
2090	2100	2110	2120	2130	2140	2150	2160
RRSESSAVSH	IPVRVADERR	MLSSNIPDGF	CEQSAFPKHE	LSQKLSQSSM	SKETVETQHF	NSIEDEKVTY	SEISKVSKHQ
2170	2180	2190	2200	2210	2220	2230	2240

Detailed Protein Report

Cmpd.	No. of Cmpds.	m/z meas.	Δ m/z [ppm]	z	Rt [min]	Score	P	Range	Sequence	Modification	Ratios
534	1	676.8481	-14.81	2	35.1	20.1	1	3029-3041	K.TKALTTSSCVDVK.S		WUP:QUP 1.19<br>QU:MU 1.57

Detailed Protein Report

Protein 378: PREDICTED: alpha-actinin-4 isoform X1 [Homo sapiens]

**Accession:**	gi|530417302	**Score:**	32.1
**Database:**	refseq_human(refseq_human_20140103.fasta)	**MW [kDa]:**	104.3
**Database Date:**	2015-11-30	**pI:**	5.2
		**Sequence Coverage [%]:**	2.4
		**No. of unique Peptides:**	1

10	20	30	40	50	60	70	80
MVDYHAANQS	YQYGPSSAGN	GAGGGGSMGD	YMAQEDDWDR	DLLLDPAWEK	QQRKTFTAWC	NSHLRKAGTQ	IENIDEDFRD
**90**	**100**	**110**	**120**	**130**	**140**	**150**	**160**
GLKLMLLLEV	ISGERLPKPE	RGKMRVHKIN	NVNKALDFIA	SKGVKLVSIG	AEEIVDGNAK	MTLGMIWTII	LRFAIQDISV
**170**	**180**	**190**	**200**	**210**	**220**	**230**	**240**
EETSAKEGLL	LWCQRKTAPY	KNVNVQNFHI	SWKDGLAFNA	LIHRHRPELI	EYDKLRKDDP	VTNLNNAFEV	AEKYLDIPKM
**250**	**260**	**270**	**280**	**290**	**300**	**310**	**320**
LDAEDIVNTA	RPDEKAIMTY	VSSFYHAFSG	AQKAETAANR	ICKVLAVNQE	NEHLMEDYEK	LASDLLEWIR	RTIPWLEDRV
**330**	**340**	**350**	**360**	**370**	**380**	**390**	**400**
PQKTIQEMQQ	KLEDFRDYRR	VHKPPKVQEK	CQLEINFNTL	QTKLRLSNRP	AFMPSEGKMV	SDINNGWQHL	EQAEKGYEEW
**410**	**420**	**430**	**440**	**450**	**460**	**470**	**480**
LLNEIRRLER	LDHLAEKFRQ	KASIHEAWTD	GKEAMLKHRD	YETATLSDIK	ALIRKHEAFE	SDLAAHQDRV	EQIAAIAQEL
**490**	**500**	**510**	**520**	**530**	**540**	**550**	**560**
NELDYYDSHN	VNTRCQKICD	QWDALGSLTH	SRREALEKTE	KQLEAIDQLH	LEYAKRAAPF	NNWMESAMED	LQDMFIVHTI
**570**	**580**	**590**	**600**	**610**	**620**	**630**	**640**
EEIEGLISAH	DQFKSTLPDA	DREREAILAI	HKEAQRIAES	NHIKLSGSNP	YTTVTPQIIN	SKWEKVQQLV	PKRDHALLEE
**650**	**660**	**670**	**680**	**690**	**700**	**710**	**720**
QSKQQSNEHL	RRQFASQANV	VGPWIQTKME	EIGRISIEMN	GTLEDQLSHL	KQYERSIVDY	KPNLDLLEQQ	HQLIQEALIF
**730**	**740**	**750**	**760**	**770**	**780**	**790**	**800**
DNKHTNYTME	HIRVGWEQLL	TTIARTINEV	ENQILTRDAK	GISQEQMQEF	RASFNHFDKK	QTGSMDSDDF	RALLISTGYS
**810**	**820**	**830**	**840**	**850**	**860**	**870**	**880**
LGEAEFNRIM	SLVDPNHSGL	VTFQAFIDFM	SRETTDTDTA	DQVIASFKVL	AGDKNFITAE	ELRRELPPDQ	AEYCIARMAP
**890**	**900**	**910**					
YQGPDAVPGA	LDYKSFSTAL	YGESDL					

Cmpd.	No. of Cmpds.	m/z meas.	Δ m/z [ppm]	z	Rt [min]	Score	P	Range	Sequence	Modification
2706	1	693.8970	9.14	2	64.9	20.3	0	734-745	R.VGWEQLLTTIAR.T	

Detailed Protein Report

Protein 379: PREDICTED: centrosomal protein of 95 kDa isoform X10 [Homo sapiens]

**Accession:**	gi|578831608	**Score:**	32.1
**Database:**	refseq_human(refseq_human_20140103.fasta)	**MW [kDa]:**	71.1
**Database Date:**	2015-11-30	**pI:**	10.2
**Modification(s):**	Carbamidomethyl	**Sequence Coverage [%]:**	2.3
		**No. of unique Peptides:**	1

Alias proteins:

Accession	Name	Description
gi|578831610	refseq_human (refseq_human_20140103.fasta)	PREDICTED: centrosomal protein of 95 kDa isoform X11 [Homo sapiens]

10	20	30	40	50	60	70	80
MKICCTLLVF	CPRVGHPLLK	TVSCNGSETL	SVSGIPNARK	LGEPIRAAIP	LHPPYHPSEP	RAPCPIGKEY	LHSSHCSPAV
**90**	**100**	**110**	**120**	**130**	**140**	**150**	**160**
NSTGEHTEFS	GDLDDGLFLI	SKLPKGSKWE	VYPAQVQGPR	TRKPPKGKRN	ENRATASSCN	SPFPQRPRKR	LTEQELHDVS
**170**	**180**	**190**	**200**	**210**	**220**	**230**	**240**
EKLSQRLSEL	DWMLKSALGD	RIKEKTDHKE	ENTGNEEVED	GTEETLSQHS	DGIVEYGPKK	SRPGLSMRRK	PPYRSHSLSP
**250**	**260**	**270**	**280**	**290**	**300**	**310**	**320**
SPVNKHKQFH	LERKRQRKPR	ETDVRQFQAQ	AFTEAFEREL	RRHKVQENIG	PLRIHEKEEE	TEKIYRGEAV	RKGTPECSQP
**330**	**340**	**350**	**360**	**370**	**380**	**390**	**400**
WKIYSRKTTT	QSLRGGLPKP	NKAVPMKVSE	HSLLPLMLEQ	FPFLYVSGPT	LSKMWKQQIA	QVEQLKKEAC	RENRSKKKLQ
**410**	**420**	**430**	**440**	**450**	**460**	**470**	**480**
DEIEEALRRH	DLLTTLVKKE	YEHNKRLQDF	KDCIRRQRLT	QSKIKENRQQ	IVRARKYYDD	YRVQLCAKMM	RMRTREEMIF
**490**	**500**	**510**	**520**	**530**	**540**	**550**	**560**
KKLFEEGLNI	QKQRLRDLRN	YAKEKRDEQR	RRHQDELDSM	ENYYKDQFSL	LAEAISQEHQ	ELKAREKSQA	QVIIKIEAKS
**570**	**580**	**590**	**600**	**610**	**620**		
CTAWQCFFQQ	GTSYIGGWSN	RLYHIAPVCS	GLYSLGLCKY	TDILHNNKII			

Cmpd.	No. of Cmpds.	m/z meas.	Δ m/z [ppm]	z	Rt [min]	Score	P	Range	Sequence	Modification
1748	1	568.7494	-72.31	2	52.2	14.3	1	463-471	R.VQLCAKMMR.M	Carbamidomethyl: 4

Detailed Protein Report

Protein 380: **macrophage mannose receptor 1 precursor [Homo sapiens]**

Accession: gi|4505245
Database: refseq_human(refseq_human_20140103.fasta)
Database Date: 2015-11-30
Modification(s): Oxidation

Score: 32.1
MW [kDa]: 165.9
pI: 6.1
Sequence Coverage [%]: 1.6
No. of unique Peptides: 1

10	20	30	40	50	60	70	80
MRLPLLLVFA	SVIPGAVLLL	DTRQFLIYNE	DHKRCVDAVS	PSAVQTAACN	QDAESQKFRW	VSESQIMSVA	FKLCLGVPSK
90	100	110	120	130	140	150	160
TDWVAITLYA	CDSKSEFQKW	ECKNDTLLGI	KGEDLFFNYG	NRQEKNIMLY	KGSGLWSRWK	IYGTTDNLCS	RGYEAMYTLL
170	180	190	200	210	220	230	240
GNANGATCAF	PFKFENKWYA	DCTSAGRSDG	WLWCGTTTDY	DTDKLFGYCP	LKFEGSESLW	NKDPLTSVSY	QINSKSALTW
250	260	270	280	290	300	310	320
HQARKSCQQQ	NAELLSITEI	HEQTYLTGLT	SSLTSGLWIG	LNSLSFNSGW	QWSDRSPFRY	LNWLPGSPSA	EPGKSCVSLN
330	340	350	360	370	380	390	400
PGKNAKWENL	ECVQKLGYIC	KKGNTTLNSF	VIPSESDVPT	HCPSQWWPYA	GHCYKIHRDE	KKIQRDALTT	CRKEGGDLTS
410	420	430	440	450	460	470	480
IHTIEELDFI	ISQLGYEPND	ELWIGLNDIK	IQMYFEWSDG	TPVTFTKWLR	GEPSHENNRQ	EDCVVMKGKD	GYWADRGCEW
490	500	510	520	530	540	550	560
PLGYICKMKS	RSQGPEIVEV	EKGCRKGWKK	HHFYCYMIGH	TLSTFAEANQ	TCNNENAYLT	TIEDRYEQAF	LTSFVGLRPE
570	580	590	600	610	620	630	640
KYFWTGLSDI	QTKGTFQWTI	EEEVRFTHWN	SDMPGRKPGC	VAMRTGIAGG	LWDVLKCDEK	AKFVCKHWAE	GVTHPPKPTT
650	660	670	680	690	700	710	720
TPEPKCPEDW	GASSRTSLCF	KLYAKGKHEK	KTWFESRDFC	RALGGDLASI	NNKEEQQTIW	RLITASGSYH	KLFWLGLTYG
730	740	750	760	770	780	790	800
SPSEGFTWSD	GSPVSYENWA	YGEPNNYQNV	EYCGELKGDP	TMSWNDINCE	HLNNWICQIQ	KGQTPKPEPT	PAPQDNPPVT
810	820	830	840	850	860	870	880
EDGWVIYKDY	QYYFSKEKET	MDNARAFCKR	NFGDLVSIQS	ESEKKFLWKY	VNRNDAQSAY	FIGLLISLDK	KFAWMDGSKV
890	900	910	920	930	940	950	960
DYVSWATGEP	NFANEDENCV	TMYSNSGFWN	DINCGYPNAF	ICQRHNSSIN	ATTVMPTMPS	VPSGCKEGWN	FYSNKCFKIF
970	980	990	1000	1010	1020	1030	1040
GFMEEERKNW	QEARKACIGF	GGNLVSIQNE	KEQAFLTYHM	KDSTFSAWTG	LNDVNSEHTF	LWTDGRGVHY	TNWGKGYPGG
1050	1060	1070	1080	1090	1100	1110	1120
RRSSLSYEDA	DCVVIIGGAS	NEAGKWMDDT	CDSKRGYICQ	TRSDPSLTNP	PATIQTDGFV	KYGKSSYSLM	RQKFQWHEAE
1130	1140	1150	1160	1170	1180	1190	1200
TYCKLHNSLI	ASILDPYSNA	FAWLQMETSN	ERVWIALNSN	LTDNQYTWTD	KWRVRYTNWA	ADEPKLKSAC	VYLDLDGYWK
1210	1220	1230	1240	1250	1260	1270	1280
TAHCNESFYF	LCKRSDEIPA	TEPPQLPGRC	PESDHTAWIP	FHGHCYYIES	SYTRNWGQAS	LECLRMGSSL	VSIESAAESS
1290	1300	1310	1320	1330	1340	1350	1360
FLSYRVEPLK	SKTNFWIGLF	RNVEGTWLWI	NNSPVSFVNW	NTGDPSGERN	DCVALHASSG	FWSNIHCSSY	KGYICKRPKI
1370	1380	1390	1400	1410	1420	1430	1440
IDAKPTHELL	TTKADTRKMD	PSKPSSNVAG	VVIIVILLIL	TGAGLAAYFF	YKKRRVHLPQ	EGAFENTLYF	NSQSSPGTSD
1450	1460						
MKDLVGNIEQ	NEHSVI						

Cmpd.	No. of Cmpds.	m/z meas.	Δ m/z [ppm]	z	Rt [min]	Score	P	Range	Sequence	Modification
2976	1	928.0036	17.04	2	66.0	14.3	2	126-140	K.NIMLYKGSGLWSRWK.I	Oxidation: 3

Detailed Protein Report

Protein 381: desmoglein-2 preproprotein [Homo sapiens]

**Accession:**	gi|116534898	**Score:**	32.1
**Database:**	refseq_human(refseq_human_20140103.fasta)	**MW [kDa]:**	122.2
**Database Date:**	2015-11-30	**pI:**	5.0
		**Sequence Coverage [%]:**	1.7
		**No. of unique Peptides:**	1

10	20	30	40	50	60	70	80
MARSPGRAYA	LLLLLICFNV	GSGLHLQVLS	TRNENKLLPK	HPHLVRQKRA	WITAPVALRE	GEDLSKKNPI	AKIHSDLAEE
**90**	**100**	**110**	**120**	**130**	**140**	**150**	**160**
RGLKITYKYT	GKGITEPPFG	IFVFNKDTGE	LNVTSILDRE	ETPFFLLTGY	ALDARGNNVE	KPLELRIKVL	DINDNEPVFT
**170**	**180**	**190**	**200**	**210**	**220**	**230**	**240**
QDVFVGSVEE	LSAAHTLVMK	INATDADEPN	TLNSKISYRI	VSLEPAYPPV	FYLNKDTGEI	YTTSVTLDRE	EHSSYTLTVE
**250**	**260**	**270**	**280**	**290**	**300**	**310**	**320**
ARDGNGEVTD	KPVKQAQVQI	RILDVNDNIP	VVENKVLEGM	VEENQVNVEV	TRIKVFDADE	IGSDNWLANF	TFASGNEGGY
**330**	**340**	**350**	**360**	**370**	**380**	**390**	**400**
FHIETDAQTN	EGIVTLIKEV	DYEEMKNLDF	SVIVANKAAF	HKSIRSKYKP	TPIPIKVKVK	NVKEGIHFKS	SVISIYVSES
**410**	**420**	**430**	**440**	**450**	**460**	**470**	**480**
MDRSSKGQII	GNFQAFDEDT	GLPAHARYVK	LEDRDNWISV	DSVTSEIKLA	KLPDFESRYV	QNGTYTVKIV	AISEDYPRKT
**490**	**500**	**510**	**520**	**530**	**540**	**550**	**560**
ITGTVLINVE	DINDNCPTLI	EPVQTICHDA	EYVNVTAEDL	DGHPNSGPFS	FSVIDKPPGM	AEKWKIARQE	STSVLLQQSE
**570**	**580**	**590**	**600**	**610**	**620**	**630**	**640**
KKLGRSEIQF	LISDNQGFSC	PEKQVLTLTV	CECLHGSGCR	EAQHDSYVGL	GPAAIALMIL	AFLLLLLVPL	LLLMCHCGKG
**650**	**660**	**670**	**680**	**690**	**700**	**710**	**720**
AKGFTPIPGT	IEMLHPWNNE	GAPPEDKVVP	SFLPVDQGGS	LVGRNGVGGM	AKEATMKGSS	SASIVKGQHE	MSEMDGRWEE
**730**	**740**	**750**	**760**	**770**	**780**	**790**	**800**
HRSLLSGRAT	QFTGATGAIM	TTETTKTARA	TGASRDMAGA	QAAAVALNEE	FLRNYFTDKA	ASYTEEDENH	TAKDCLLVYS
**810**	**820**	**830**	**840**	**850**	**860**	**870**	**880**
QEETESLNAS	IGCCSFIEGE	LDDRFLDDLG	LKFKTLAEVC	LGQKIDINKE	IEQRQKPATE	TSMNTASHSL	CEQTMVNSEN
**890**	**900**	**910**	**920**	**930**	**940**	**950**	**960**
TYSSGSSFPV	PKSLQEANAE	KVTQEIVTER	SVSSRQAQKV	ATPLPDPMAS	RNVIATETSY	VTGSTMPPTT	VILGPSQPQS
**970**	**980**	**990**	**1000**	**1010**	**1020**	**1030**	**1040**
LIVTERVYAP	ASTLVDQPYA	NEGTVVVTER	VIQPHGGGSN	PLEGTQHLQD	VPYVMVRERE	SFLAPSSGVQ	PTLAMPNIAV
**1050**	**1060**	**1070**	**1080**	**1090**	**1100**	**1110**	**1120**
GQNVTVTERV	LAPASTLQSS	YQIPTENSMT	ARNTTVSGAG	VPGPLPDFGL	EESGHSNSTI	TTSSTRVTKH	STVQHSYS

Cmpd.	No. of Cmpds.	m/z meas.	Δ m/z [ppm]	z	Rt [min]	Score	P	Range	Sequence	Modification
1168	1	955.7541	206.12	1	44.8	19.8	1	33-40	R.NENKLLPK.H	

Detailed Protein Report

Protein 382: **PREDICTED: zinc finger protein 665 isoform X5 [Homo sapiens]**

**Accession:**	gi|578834915	**Score:**	32.1
**Database:**	refseq_human(refseq_human_20140103.fasta)	**MW [kDa]:**	76.3
**Database Date:**	2015-11-30	**pI:**	10.3
**Modification(s):**	Carbamidomethyl	**Sequence Coverage [%]:**	4.6
		**No. of unique Peptides:**	1

10	20	30	40	50	60	70	80
MVCPCSGLLL	AVGGMPPRCQ	GRTLEEEEEE	GKGVRDGSSS	DISCKCVNTD	LPPKGKNNMG	EAFYTVKLER	LESCDTVGLS
**90**	**100**	**110**	**120**	**130**	**140**	**150**	**160**
FQEVQKNTYD	FECQWKDDEG	NYKTVLMLQK	ENLPGRRAQR	DRRAAGNRHI	ENQLGVSFQS	HLPELQQFQH	EGKIYEYNQV
**170**	**180**	**190**	**200**	**210**	**220**	**230**	**240**
EKSPNNRGKH	YKCDECGKVF	SQNSRLTSHK	RIHTGEKPYQ	CNKCGKAFTV	RSNLTIHQVI	HTGEKPYKCN	ECGKVFSQPS
**250**	**260**	**270**	**280**	**290**	**300**	**310**	**320**
NLAGHQRIHT	GEKPYKCNEC	GKAFRAHSKL	TTHQVIHTGE	KPYKCKECGK	CFTQNSHLAS	HRRIHTGEKP	YKCNECGKAF
**330**	**340**	**350**	**360**	**370**	**380**	**390**	**400**
SVRSSLTTHQ	TIHTGEKPYK	CNECGKVFRH	NSYLAKHRRI	HTGEKPYKCN	ECGKAFSMHS	NLTKHQIIHT	GEKPFKCNEC
**410**	**420**	**430**	**440**	**450**	**460**	**470**	**480**
VKVFTQYSHL	ANHRRIHTGE	KPYRCDECGK	AFSVRSSLTT	HQAIHTGEKP	YKCNDCGKVF	TQNSHLASHR	GIHSGEKPYK
**490**	**500**	**510**	**520**	**530**	**540**	**550**	**560**
CDECGKAFSQ	TSQLARHWRV	HTGEKPYKCN	ECGKAFSVHS	SLTIHQTIHT	GQKPYKCNDC	GKVFRHNSYL	AIHQRIHTGE
**570**	**580**	**590**	**600**	**610**	**620**	**630**	**640**
KPYKCNECGK	AFSVHSNLAT	HQVIHTGEKP	YKCNECGKVF	TQNSHLANHR	RIHTGEKPYR	CNECGKAFSV	RSTLTTHMAV
**650**	**660**	**670**	**680**				
HTGDKPYKCN	QCGKVFTQNS	NLAKHRRIHS	G				

Cmpd.	No. of Cmpds.	m/z meas.	Δ m/z [ppm]	z	Rt [min]	Score	P	Range	Sequence	Modification
1354	1	684.3815	97.70	2	46.5	12.8	1	33-45	K.GVRDGSSSDISCK.C	Carbamidomethyl: 12

Detailed Protein Report

Protein 383: **unconventional myosin-Ie [Homo sapiens]**

**Accession:**	gi|55956916	**Score:**	32.1
**Database:**	refseq_human(refseq_human_20140103.fasta)	**MW [kDa]:**	127.0
**Database Date:**	2015-11-30	**pI:**	9.5
		**Sequence Coverage [%]:**	1.6
		**No. of unique Peptides:**	1

10	20	30	40	50	60	70	80
MGSKGVYQYH	WQSHNVKHSG	VDDMVLLSKI	TENSIVENLK	KRYMDDYIFT	YIGSVLISVN	PFKQMPYFGE	KEIEMYQGAA
90	100	110	120	130	140	150	160
QYENPPHIYA	LADNMYRNMI	IDRENQCVII	SGESGAGKTV	AAKYIMSYIS	RVSGGGTKVQ	HVKDIILQSN	PLLEAFGNAK
170	180	190	200	210	220	230	240
TVRNNNSSRF	GKYFEIQFSP	GGEPDGGKIS	NFLLEKSRVV	MRNPGERSFH	IFYQLIEGAS	AEQKHSLGIT	SMDYYYYLSL
250	260	270	280	290	300	310	320
SGSYKVDDID	DRREFQETLH	AMNVIGIFAE	EQTLVLQIVA	GILHLGNISF	KEVGNYAAVE	SEEFLAFPAY	LLGINQDRLK
330	340	350	360	370	380	390	400
EKLTSRQMDS	KWGGKSESIH	VTLNVEQACY	TRDALAKALH	ARVFDFLVDS	INKAMEKDHE	EYNIGVLDIY	GFEIFQKNGF
410	420	430	440	450	460	470	480
EQFCINFVNE	KLQQIFIELT	LKAEQEEYVQ	EGIRWTPIEY	FNNKIVCDLI	ENKVNPPGIM	SILDDVCATM	HAVGEGADQT
490	500	510	520	530	540	550	560
LLQKLQMQIG	SHEHFNSWNQ	GFIIHHYAGK	VSYDMDGFCE	RNRDVLFMDL	IELMQSSELP	FIKSLFPENL	QADKKGRPTT
570	580	590	600	610	620	630	640
AGSKIKKQAN	DLVSTLMKCT	PHYIRCIKPN	ETKKPRDWEE	SRVKHQVEYL	GLKENIRVRR	AGYAYRRIFQ	KFLQRYAILT
650	660	670	680	690	700	710	720
KATWPSWQGE	EKQGVLHLLQ	SVNMDSDQFQ	LGRSKVFIKA	PESLFLLEEM	RERKYDGYAR	VIQKSWRKFV	ARKKYVQMRE
730	740	750	760	770	780	790	800
EASDLLLNKK	ERRRNSINRN	FIGDYIGMEE	HPELQQFVGK	REKIDFADTV	TKYDRRFKGV	KRDLLLTPKC	LYLIGREKVK
810	820	830	840	850	860	870	880
QGPDKGLVKE	VLKRKIEIER	ILSVSLSTMQ	DDIFILHEQE	YDSLLESVFK	TEFLSLLAKR	YEEKTQKQLP	LKFSNTLELK
890	900	910	920	930	940	950	960
LKKENWGPWS	AGGSRQVQFH	QGFGDLAVLK	PSNKVLQVSI	GPGLPKNSRP	TRRNTTQNTG	YSSGTQNANY	PVRAAPPPPG
970	980	990	1000	1010	1020	1030	1040
YHQNGVIRNQ	YVPYPHAPGS	QRSNQKSLYT	SMARPPLPRQ	QSTSSDRVSQ	TPESLDFLKV	PDQGAAGVRR	QTTSRPPPAG
1050	1060	1070	1080	1090	1100	1110	
GRPKPQPKPK	PQVPQCKALY	AYDAQDTDEL	SFNANDIIDI	IKEDPSGWWT	GRLRGKQGLF	PNNYVTKI	

Cmpd.	No. of Cmpds.	m/z meas.	Δ m/z [ppm]	z	Rt [min]	Score	P	Range	Sequence	Modification
1182	1	478.1928	-225.01	2	43.0	13.4	1	782-789	K.RDLLLTPK.C	

Detailed Protein Report

Protein 384: cleavage and polyadenylation specificity factor subunit 2 [Homo sapiens]

**Accession:**	gi|34101288	**Score:**	32.1
**Database:**	refseq_human(refseq_human_20140103.fasta)	**MW [kDa]:**	88.4
**Database Date:**	2015-11-30	**pI:**	4.8
**Modification(s):**	Oxidation	**Sequence Coverage [%]:**	3.1
		**No. of unique Peptides:**	2

Alias proteins:

Accession	Name	Description
gi|530403998	refseq_human (refseq_human_20140103.fasta)	PREDICTED: cleavage and polyadenylation specificity factor subunit 2 isoform X1 [Homo sapiens]

10	20	30	40	50	60	70	80
MTSIIKLTTL	SGVQEESALC	YLLQVDEFRF	LLDCGWDEHF	SMDIIDSLRK	HVHQIDAVLL	SHPDPLHLGA	LPYAVGKLGL
**90**	**100**	**110**	**120**	**130**	**140**	**150**	**160**
NCAIYATIPV	YKMGQMFMYD	LYQSRHNTED	FTLFTLDDVD	AAFDKIQQLK	FSQIVNLKGK	GHGLSITPLP	AGHMIGGTIW
**170**	**180**	**190**	**200**	**210**	**220**	**230**	**240**
KIVKDGEEEI	VYAVDFNHKR	EIHLNGCSLE	MLSRPSLLIT	DSFNATYVQP	RRKQRDEQLL	TNVLETLRGD	GNVLIAVDTA
**250**	**260**	**270**	**280**	**290**	**300**	**310**	**320**
GRVLELAQLL	DQIWRTKDAG	LGVYSLALLN	NVSYNVVEFS	KSQVEWMSDK	LMRCFEDKRN	NPFQFRHLSL	CHGLSDLARV
**330**	**340**	**350**	**360**	**370**	**380**	**390**	**400**
PSPKVVLASQ	PDLECGFSRD	LFIQWCQDPK	NSIILTYRTT	PGTLARFLID	NPSEKITEIE	LRKRVKLEGK	ELEEYLEKEK
**410**	**420**	**430**	**440**	**450**	**460**	**470**	**480**
LKKEAAKKLE	QSKEADIDSS	DESDIEEDID	QPSAHKTKHD	LMMKGEGSRK	GSFFKQAKKS	YPMFPAPEER	IKWDEYGEII
**490**	**500**	**510**	**520**	**530**	**540**	**550**	**560**
KPEDFLVPEL	QATEEEKSKL	ESGLTNGDEP	MDQDLSDVPT	KCISTTESIE	IKARVTYIDY	EGRSDGDSIK	KIINQMKPRQ
**570**	**580**	**590**	**600**	**610**	**620**	**630**	**640**
LIIVHGPPEA	SQDLAECCRA	FGGKDIKVYM	PKLHETVDAT	SETHIYQVRL	KDSLVSSLQF	CKAKDAELAW	IDGVLDMRVS
**650**	**660**	**670**	**680**	**690**	**700**	**710**	**720**
KVDTGVILEE	GELKDDGEDS	EMQVEAPSDS	SVIAQQKAMK	SLFGDDEKET	GEESEIIPTL	EPLPPHEVPG	HQSVFMNEPR
**730**	**740**	**750**	**760**	**770**	**780**	**790**	
LSDFKQVLLR	EGIQAEFVGG	VLVCNNQVAV	RRTETGRIGL	EGCLCQDFYR	IRDLLYEQYA	IV	

Cmpd.	No. of Cmpds.	m/z meas.	Δ m/z [ppm]	z	Rt [min]	Score	P	Range	Sequence	Modification
303	1	1292.7280	120.56	1	33.5	21.9	1	439-449	K.HDLMMKGEGSR.K	Oxidation: 4, 5
1834	1	734.4148	23.62	2	51.3	10.2	1	610-622	R.LKDSLVSSLQFCK.A	

Detailed Protein Report

Protein 385: protein phosphatase 1 regulatory subunit 26 [Homo sapiens]

**Accession:**	gi|45387958	**Score:**	32.0
**Database:**	refseq_human(refseq_human_20140103.fasta)	**MW [kDa]:**	127.3
**Database Date:**	2015-11-30	**pI:**	9.8
**Modification(s):**	Carbamidomethyl	**Sequence Coverage [%]:**	2.4
		**No. of unique Peptides:**	2

Quantitation

QU:MU	**Median:**	0.43	**CV:**	0.00 %	**No. of Peptides:**	1
WUP:QUP	**Median:**	2.34	**CV:**	0.00 %	**No. of Peptides:**	1

Alias proteins:

Accession	Name	Description
gi|578818032	refseq_human (refseq_human_20140103.fasta)	PREDICTED: protein phosphatase 1 regulatory subunit 26 isoform X5 [Homo sapiens]
gi|578818030	refseq_human (refseq_human_20140103.fasta)	PREDICTED: protein phosphatase 1 regulatory subunit 26 isoform X4 [Homo sapiens]
gi|578818028	refseq_human (refseq_human_20140103.fasta)	PREDICTED: protein phosphatase 1 regulatory subunit 26 isoform X3 [Homo sapiens]
gi|530426639	refseq_human (refseq_human_20140103.fasta)	PREDICTED: protein phosphatase 1 regulatory subunit 26 isoform X2 [Homo sapiens]
gi|530426637	refseq_human (refseq_human_20140103.fasta)	PREDICTED: protein phosphatase 1 regulatory subunit 26 isoform X1 [Homo sapiens]

```
        10         20         30         40         50         60         70         80
MFLMNASPVV ALQSKWEAFG PPGSCRFPRC FSEADEGVES ASVSARVQML ISTLQRDGAA RGTSDERAAQ RGHRAEGCHD
        90        100        110        120        130        140        150        160
ARPAAKPTVH KEPPALAVCG LVADFDPMGE EETTDFGPLV LDSDSDDSVD RDIEEAIQEY LKAKSGAAQP GAGGAQPGAA
       170        180        190        200        210        220        230        240
QPSRAAGGGS RCKPEPAHGS APTALCPPKL VPGSGGGPGS QVGSSKDQGS ASPVSVSSDD SFEQSIRAEI EQFLNEKRQH
       250        260        270        280        290        300        310        320
ETQKCDGSVE KKPDTNENSA KSLLKSHQEP PTKVVHRQGL LGVQKEFAFR KPPRLAKMNV QPRSLRSKVT TTQENEGSTK
       330        340        350        360        370        380        390        400
PATPCRPSEA AQNKGGIKRS ASAARRGKRV MSAAQASEAS DSSSDDGIEE AIQLYQLQKT RKEADGDLPQ RVQLREERAP
       410        420        430        440        450        460        470        480
DPPAHSTSSA TKSALPETHR KTPSKKKLVA TKTMDPGPGG LDTDHAPKLL KETKAPPPAS PASRSEFVER SSCRADTSAE
       490        500        510        520        530        540        550        560
LMCAEAILDI SKTILPAPVE GSDGSLSASP LFYSPNVPSR SDGDSSSVDS DDSIEQEIRT FLALKAQSGS LLARGESCPQ
       570        580        590        600        610        620        630        640
AAQGPLLPPG LNSQTGGHKT PLSKTPDPLL GCKRKRRGGG HVRPSTPKKM QEVVKDGSQD ADHSQGRAEP GHERRDLPIQ
       650        660        670        680        690        700        710        720
GKASEALGGE GTARGPGDTR MSQGQGKTDE ARRLDEKESS EDKSSSLDSD EDLDTAIKDL LRSKRKLKKR CREPRAACRK
       730        740        750        760        770        780        790        800
KVRFSTAQTH FLEQLGGLRR DWKDRGPPVL KSCLSKSKRD SGEGPGKKPP SVFGSTAERM RQEGAASQDA ALAFRVRRPA
       810        820        830        840        850        860        870        880
SASASEGNPF PRESQGPAPS PGSLSDDSSS VDSNDSIELE IRKFLAEKAK ESVSSSEVQA EGPTALGTGG PARPEVLCRK
       890        900        910        920        930        940        950        960
EPAPPPGVCT RSQRARGVPH LAEGLRGTES AGAQGTAGLF SQGGKGLPAA PARGDPVPPR STSGGVSAKG LSVSRRNVYV
       970        980        990       1000       1010       1020       1030       1040
HKDQSPRGAE PAAKSAFGQL PSCATAGTEA GGARGTFHMG CGSPSFLTPS PGAERDAGAQ ADRTPPWSDF AHQSRLPSPW
      1050       1060       1070       1080       1090       1100       1110       1120
VLRSEGRDAV WRGGVGSERD KGSEGPARGL PSLPLAGFSP LLSTQLFHFG KGVSWGGRQA GLFSPHLGLP LQGPSFSAFR
      1130       1140       1150       1160       1170       1180       1190       1200
EAQAGPSPVF GSPHLLAKKD GGPWPTRKAQ AGLSLHDRRS SGSEESILDL RYRRRVNRDD QEQDALGSDA SDFSDTSTED
      1210
SGGSSVVKV
```

Detailed Protein Report

Cmpd.	No. of Cmpds.	m/z meas.	Δ m/z [ppm]	z	Rt [min]	Score	P	Range	Sequence	Modification	Ratios
1424	1	627.5982	-62.69	3	46.2	13.9	0	475-492	R.ADTSAELMCAEAILDISK.T		
1803	1	590.7798	-21.71	2	52.9	18.1	0	881-891	K.EPAPPPGVCTR.S	Carbamidomethyl: 9	QU:MU 0.43<br>WUP:QUP 2.34

Detailed Protein Report

Protein 386: pregnancy zone protein precursor [Homo sapiens]

**Accession:**	gi|162809334	**Score:**	31.9
**Database:**	refseq_human(refseq_human_20140103.fasta)	**MW [kDa]:**	163.8
**Database Date:**	2015-11-30	**pI:**	5.9
		**Sequence Coverage [%]:**	2.1
		**No. of unique Peptides:**	1

10	20	30	40	50	60	70	80
MRKDRLLHLC	LVLLLILLSA	SDSNSTEPQY	MVLVPSLLHT	EAPKKGCVLL	SHLNETVTVS	ASLESGRENR	SLFTDLVAEK
90	100	110	120	130	140	150	160
DLFHCVSFTL	PRISASSEVA	FLSIQIKGPT	QDFRKRNTVL	VLNTQSLVFV	QTDKPMYKPG	QTVRFRVVSV	DENFRPRNEL
170	180	190	200	210	220	230	240
IPLIYLENPR	RNRIAQWQSL	KLEAGINQLS	FPLSSEPIQG	SYRVVVQTES	GGRIQHPFTV	EEFVLPKFEV	KVQVPKIISI
250	260	270	280	290	300	310	320
MDEKVNITVC	GEYTYGKPVP	GLATVSLCRK	LSRVLNCDKQ	EVCEEFSQQL	NSNGCITQQV	HTKMLQITNT	GFEMKLRVEA
330	340	350	360	370	380	390	400
RIREEGTDLE	VTANRISEIT	NIVSKLKFVK	VDSHFRQGIP	FFAQVLLVDG	KGVPIPNKLF	FISVNDANYY	SNATTNEQGL
410	420	430	440	450	460	470	480
AQFSINTTSI	SVNKLFVRVF	TVHPNLCFHY	SWVAEDHQGA	QHTANRVFSL	SGSYIHLEPV	AGTLPCGHTE	TITAHYTLNR
490	500	510	520	530	540	550	560
QAMGELSELS	FHYLIMAKGV	IVRSGTHTLP	VESGDMKGSF	ALSFPVESDV	APIARMFIFA	ILPDGEVVGD	SEKFEIENCL
570	580	590	600	610	620	630	640
ANKVDLSFSP	AQSPPASHAH	LQVAAAPQSL	CALRAVDQSV	LLMKPEAELS	VSSVYNLLTV	KDLTNFPDNV	DQQEEEQGHC
650	660	670	680	690	700	710	720
PRPFFIHNGA	IYVPLSSNEA	DIYSFLKGMG	LKVFTNSKIR	KPKSCSVIPS	VSAGAVGQGY	YGAGLGVVER	PYVPQLGTYN
730	740	750	760	770	780	790	800
VIPLNNEQSS	GPVPETVRSY	FPETWIWELV	AVNSSGVAEV	GVTVPDTITE	WKAGAFCLSE	DAGLGISSTA	SLRAFQPFFV
810	820	830	840	850	860	870	880
ELTMPYSVIR	GEVFTLKATV	LNYLPKCIRV	SVQLKASPAF	LASQNTKGEE	SYCICGNERQ	TLSWTVTPKT	LGNVNFSVSA
890	900	910	920	930	940	950	960
EAMQSLELCG	NEVVEVPEIK	RKDTVIKTLL	VEAEGIEQEK	TFSSMTCASG	ANVSEQLSLK	LPSNVVKESA	RASFSVLGDI
970	980	990	1000	1010	1020	1030	1040
LGSAMQNIQN	LLQMPYGCGE	QNMVLFAPNI	YVLNYLNETQ	QLTQEIKAKA	VGYLITGYQR	QLNYKHQDGS	YSTFGERYGR
1050	1060	1070	1080	1090	1100	1110	1120
NQGNTWLTAF	VLKTFAQARS	YIFIDEAHIT	QSLTWLSQMQ	KDNGCFRSSG	SLLNNAIKGG	VEDEATLSAY	VTIALLEIPL
1130	1140	1150	1160	1170	1180	1190	1200
PVTNPIVRNA	LFCLESAWNV	AKEGTHGSHV	YTKALLAYAF	SLLGKQNQNR	EILNSLDKEA	VKEDNLVHWE	RPQRPKAPVG
1210	1220	1230	1240	1250	1260	1270	1280
HLYQTQAPSA	EVEMTSYVLL	AYLTAQPAPT	SGDLTSATNI	VKWIMKQQNA	QGGFSSTQDT	VVALHALSRY	GAATFTRTEK
1290	1300	1310	1320	1330	1340	1350	1360
TAQVTVQDSQ	TFSTNFQVDN	NNLLLLQQIS	LPELPGEYVI	TVTGERCVYL	QTSMKYNILP	EKEDSPFALK	VQTVPQTCDG
1370	1380	1390	1400	1410	1420	1430	1440
HKAHTSFQIS	LTISYTGNRP	ASNMVIVDVK	MVSGFIPLKP	TVKMLERSSS	VSRTEVSNNH	VLIYVEQVTN	QTLSFSFMVL
1450	1460	1470	1480	1490			
QDIPVGDLKP	AIVKVYDYYE	TDESVVAEYI	APCSTDTEHG	NV			

Cmpd.	No. of Cmpds.	m/z meas.	Δ m/z [ppm]	z	Rt [min]	Score	P	Range	Sequence	Modification
705	1	802.0356	159.48	2	38.8	16.0	1	322-335	R.IREEGTDLEVTANR.I	

Detailed Protein Report

Protein 387: cystatin-B [Homo sapiens]

Accession: gi|4503117
Database: refseq_human(refseq_human_20140103.fasta)
Database Date: 2015-11-30

Score: 31.9
MW [kDa]: 11.1
pI: 7.9
Sequence Coverage [%]: 12.2
No. of unique Peptides: 1

Quantitation

WUP:QUP **Median:** 4.54 **CV:** 0.00 % **No. of Peptides:** 1

```
         10         20         30         40         50         60         70         80
MMCGAPSATQ PATAETQHIA DQVRSQLEEK ENKKFPVFKA VSFKSQVVAG TNYFIKVHVG DEDFVHLRVF QSLPHENKPL
         90        100
TLSNYQTNKA KHDELTYF
```

Cmpd.	No. of Cmpds.	m/z meas.	Δ m/z [ppm]	z	Rt [min]	Score	P	Range	Sequence	Modification	Ratios
695	1	663.8458	-15.71	2	37.1	31.9	0	45-56	K.SQVVAGTNYFIK.V		WUP:QUP 4.54

Detailed Protein Report

Protein 388: peroxisome biogenesis factor 1 isoform 3 [Homo sapiens]

**Accession:**	gi|544186104	**Score:**	31.9
**Database:**	refseq_human(refseq_human_20140103.fasta)	**MW [kDa]:**	119.5
**Database Date:**	2015-11-30	**pI:**	5.7
		**Sequence Coverage [%]:**	0.9
		**No. of unique Peptides:**	1

```
        10         20         30         40         50         60         70         80
MMKELQTKQL QSNTVGITES NENESEIPVD SSSVASLWTM IGSIFSFQSE KKQETSWGLT EINAFKNMQS KVVPLDNIFR
        90        100        110        120        130        140        150        160
VCKSQPPSIY NASATSVFHK HCAIHVFPWD QEYFDVEPSF TVTYGKLVKL LSPKQQQSKT KQNVLSPEKE KQMSEPLDQK
       170        180        190        200        210        220        230        240
KIRSDHNEED EKACVLQVVW NGLEELNNAI KYTKNVEVLH LGKVWIPDDL RKRLNIEMHA VVRITPVEVT PKIPRSLKLQ
       250        260        270        280        290        300        310        320
PRENLPKDIS EEDIKTVFYS WLQQSTTTML PLVISEEEFI KLETKDGLKE FSLSIVHSWE KEKDKNIFLL SPNLLQKTTI
       330        340        350        360        370        380        390        400
QVLLDPMVKE ENSEEIDFIL PFLKLSSLGG VNSLGVSSLE HITHSLLGRP LSRQLMSLVA GLRNGALLLT GGKGSGKSTL
       410        420        430        440        450        460        470        480
AKAICKEAFD KLDAHVERVD CKALRGKRLE NIQKTLEVAF SEAVWMQPSV VLLDDLDLIA GLPAVPEHEH SPDAVQSQRL
       490        500        510        520        530        540        550        560
AHALNDMIKE FISMGSLVAL IATSQSQQSL HPLLVSAQGV HIFQCVQHIQ PPNQEQRCEI LCNVIKNKLD CDINKFTDLD
       570        580        590        600        610        620        630        640
LQHVAKETGG FVARDFTVLV DRAIHSRLSR QSISTREKLV LTTLDFQKAL RGFLPASLRS VNLHKPRDLG WDKIGGLHEV
       650        660        670        680        690        700        710        720
RQILMDTIQL PAKYPELFAN LPIRQRTGIL LYGPPGTGKT LLAGVIARES RMNFISVKGP ELLSKYIGAS EQAVRDIFIR
       730        740        750        760        770        780        790        800
AQAAKPCILF FDEFESIAPR RGHDNTGVTD RVVNQLLTQL DGVEGLQGVY VLAATSRPDL IDPALLRPGR LDKCVYCPPP
       810        820        830        840        850        860        870        880
DQVSRLEILN VLSDSLPLAD DVDLQHVASV TDSFTGADLK ALLYNAQLEA LHGMLLSSGL QDGSSSSDSD LSLSSMVFLN
       890        900        910        920        930        940        950        960
HSSGSDDSAG DGECGLDQSL VSLEMSEILP DESKFNMYRL YFGSSYESEL GNGTSSDLSS QCLSAPSSMT QDLPGVPGKD
       970        980        990       1000       1010       1020       1030       1040
QLFSQPPVLR TASQEGCQEL TQEQRDQLRA DISIIKGRYR SQSGEDESMN QPGPIKTRLA ISQSHLMTAL GHTRPSISED
      1050       1060       1070       1080
DWKNFAELYE SFQNPKRRKN QSGTMFRPGQ KVTLA
```

Cmpd.	No. of Cmpds.	m/z meas.	Δ m/z [ppm]	z	Rt [min]	Score	P	Range	Sequence	Modification
1073	4	1207.9210	130.55	1	43.0	31.9	2	233-242	K.IPRSLKLQPR.E	

Detailed Protein Report

Protein 389: PREDICTED: serine/threonine-protein kinase 31 isoform X3 [Homo sapiens]

**Accession:**	gi|578813577	**Score:**	31.7
**Database:**	refseq_human(refseq_human_20140103.fasta)	**MW [kDa]:**	95.7
**Database Date:**	2015-11-30	**pI:**	4.8
		**Sequence Coverage [%]:**	2.7
		**No. of unique Peptides:**	2

Quantitation

QU:MU	**Median:** 1.59	**CV:** 0.00 %	**No. of Peptides:**	1
WUP:QUP	**Median:** 0.20	**CV:** 0.00 %	**No. of Peptides:**	1

```
        10         20         30         40         50         60         70         80
MWVQGHSSRA SATESVSFSG IVQMDEDTHY DKVEDVVGSH IEDAVTFWAQ SINRNKDIMK IGCSLSEVCP QASSVLGNLD
        90        100        110        120        130        140        150        160
PNKIYGGLFS EDQCWYRCKV LKIISVEKCL VRYIDYGNTE ILNRSDIVEI PLELQFSSVA KKYKLWGLHI PSDQEVTQFD
       170        180        190        200        210        220        230        240
QQEKAAAVDL TNHLEYTLKT YIDTRMKNLA AKMEILKEMR HVDISVRFGK DLSDAIQVLD EGCFTTPASL NGLEIIWAEY
       250        260        270        280        290        300        310        320
SLAQENIKTC EYVSEGNILI AQRNEMQQKL YMSVEDFILE VDESSLNKRL KTLQDLSVSL EAVYGQAKEG ANSDEILKKF
       330        340        350        360        370        380        390        400
YDWKCDKREE FTSVRSETDA SLHRLVAWFQ RTLKVFDLSV EGSLISEDAM DNIDEILEKT ESSVCKELEI ALVDQGDADK
       410        420        430        440        450        460        470        480
EIISNTYSQV LQKIHSEERL IATVQAKYKD SIEFKKQLIE YLNKSPSVDH LLSIKKTLKS LKALLRWKLV EKSNLEESDD
       490        500        510        520        530        540        550        560
PDGSQIEKIK EEITQLRNNV FQEIYHEREE YEMLTSLAQK WFPELPLLHP EIGLLKYMNS GGLLTMSLER DLLDAEPMKE
       570        580        590        600        610        620        630        640
LSSKRPLVRS EVNGQIILLK GYSVDVDTEA KVIERAATYH RAWREAEGDS GLLPLIFLFL CKSDPMAYLM VPYYPRANLN
       650        660        670        680        690        700        710        720
AVQANMPLNS EETLKVMKGV AQGLHTLHKA DIIHGSLHQN NVFALNREQG IVGDFDFTKS VSQRASVNMM VGDLSLMSPE
       730        740        750        760        770        780        790        800
LKMGKPASPG SDLYAYGCLL LWLSVQNQEF EINKDGIPKV DQFHLDDKVK SLLCSLICYR SSMTAEQVLN AECFLMPKEQ
       810        820        830        840        850
SVPNPEKDTE YTLYKKEEEI KTENLDKCME KTRNGEANFD C
```

Cmpd.	No. of Cmpds.	m/z meas.	Δ m/z [ppm]	z	Rt [min]	Score	P	Range	Sequence	Modification	Ratios
181	8	592.2959	31.07	2	31.1	21.2	0	581-591	K.GYSVDVDTEAK.V		QU:MU 1.59<br>WUP:QUP 0.20
1907	1	734.2871	-85.93	2	52.2	10.5	2	822-833	K.TENLDKCMEKTR.N		

Detailed Protein Report

Protein 390: formin-1 isoform c [Homo sapiens]

Accession: gi|472235321
Database: refseq_human(refseq_human_20140103.fasta)
Database Date: 2015-11-30

Score: 31.7
MW [kDa]: 71.8
pI: 10.1
Sequence Coverage [%]: 5.7
No. of unique Peptides: 2

Quantitation

QU:MU **Median:** 2.49 **CV:** 0.00 % **No. of Peptides:** 1

10	20	30	40	50	60	70	80
MEGTHCTLQL	HKPITELCYI	SFCLPKGEVR	GFSYKGTVTL	DRSNKGFHNC	YQVREESDII	SLSQEPDEHP	GDIFFKQTPT
**90**	**100**	**110**	**120**	**130**	**140**	**150**	**160**
KDILTELYKL	TTERERLLTN	LLSSDHILGI	TMGNQEGKLQ	ELSVSLAPED	DCFQSAGDWQ	GELPVGPLNK	RSTHGNKKPR
**170**	**180**	**190**	**200**	**210**	**220**	**230**	**240**
RSSGRRESFG	ALPQKRTKRK	GRGGRESAPL	MGKDKICSSH	SLPLSRTRPN	LWVLEEKGNL	LPNGALACSL	QRRESCPPDI
**250**	**260**	**270**	**280**	**290**	**300**	**310**	**320**
PKTPDTDLGF	GSFETAFKDT	GLGREVLPPD	CSSTEAGGDG	IRRPPSGLEH	QQTGLSESHQ	DPEKHPEAEK	DEMEKPAKRT
**330**	**340**	**350**	**360**	**370**	**380**	**390**	**400**
CKQKPVSKVV	AKVQDLSSQV	QRVVKTHSKG	KETIAIRPAA	HAEFVPKADL	LTLPGAEAGA	HGSRRQGKER	QGDRSSQSPA
**410**	**420**	**430**	**440**	**450**	**460**	**470**	**480**
GETASISSVS	ASAEGAVNKV	PLKVIESEKL	DEAPEGKRLG	FPVHTSVPHT	RPETRNKRRA	GLPLGGHKSL	FLDLPHKVGP
**490**	**500**	**510**	**520**	**530**	**540**	**550**	**560**
DSSQPRGDKK	KPSPPAPAAL	GKVFNNSASQ	SSTHKQTSPV	PSPLSPRLPS	PQQHHRILRL	PALPGEREAA	LNDSPCRKSR
**570**	**580**	**590**	**600**	**610**	**620**	**630**	**640**
VFSGCVSADT	LEPPSSAKVT	ETKGASPAFL	RAGQPRLVPG	ETLEKSLGPG	KTTAEPQHQS	PPAFHWDLQQ	HFQEPVIRTV
**650**	**660**	**670**					
SISCASNLIK	EEAGKGKESR	SG					

Cmpd.	No. of Cmpds.	m/z meas.	Δ m/z [ppm]	z	Rt [min]	Score	P	Range	Sequence	Modification	Ratios
787	1	509.7081	-159.31	2	38.3	16.9	1	320-328	R.TCKQKPVSK.V		QU:MU 2.49
1018	1	920.4993	28.10	3	42.8	14.8	1	395-423	R. SSQSPAGETASISSVSASAEGAVN V		

Detailed Protein Report

Protein 391: **bifunctional UDP-N-acetylglucosamine 2-epimerase/N-acetylmannosamine kinase isoform 3 [Homo sapiens]**

**Accession:**	gi|298566325	**Score:**	31.6
**Database:**	refseq_human(refseq_human_20140103.fasta)	**MW [kDa]:**	78.5
**Database Date:**	2015-11-30	**pI:**	7.0
**Modification(s):**	Oxidation	**Sequence Coverage [%]:**	5.0
		**No. of unique Peptides:**	2

10	20	30	40	50	60	70	80
MPIGDCSVAA	KPRKQLLCSL	FQTTLGYRAR	ASGWKPMVIC	RGSHAFKDLI	NTYRMIEQDD	FDINTRLHTI	VRGEDEAAMV
**90**	**100**	**110**	**120**	**130**	**140**	**150**	**160**
ESVGLALVKL	PDVLNRLKPD	IMIVHGDRFD	ALALATSAAL	MNIRILHIEG	GEVSGTIDDS	IRHAITKLAH	YHVCCTRSAE
**170**	**180**	**190**	**200**	**210**	**220**	**230**	**240**
QHLISMCEDH	DRILLAGCPS	YDKLLSAKNK	DYMSIIRMWL	GDDVKSKDYI	VALQHPVTTD	IKHSIKMFEL	TLDALISFNK
**250**	**260**	**270**	**280**	**290**	**300**	**310**	**320**
RTLVLFPNID	AGSKEMVRVM	RKKGIEHHPN	FRAVKHVPFD	QFIQLVAHAG	CMIGNSSCGV	REVGAFGTPV	INLGTRQIGR
**330**	**340**	**350**	**360**	**370**	**380**	**390**	**400**
ETGENVLHVR	DADTQDKILQ	ALHLQFGKQY	PCSKIYGDGN	AVPRILKFLK	SIDLQEPLQK	KFCFPPVKEN	ISQDIDHILE
**410**	**420**	**430**	**440**	**450**	**460**	**470**	**480**
TLSALAVDLG	GTNLRVAIVS	MKGEIVKKYT	QFNPKTYEER	INLILQMCVE	AAAEAVKLNC	RILGVGISTG	GRVNPREGIV
**490**	**500**	**510**	**520**	**530**	**540**	**550**	**560**
LHSTKLIQEW	NSVDLRTPLS	DTLHLPVWVD	NDGNCAALAE	RKFGQGKGLE	NFVTLITGTG	IGGGIIHQHE	LIHGSSFCAA
**570**	**580**	**590**	**600**	**610**	**620**	**630**	**640**
ELGHLVVSLD	GPDCSCGSHG	CIEAYASGMA	LQREAKKLHD	EDLLLVEGMS	VPKDEAVGAL	HLIQAAKLGN	AKAQSILRTA
**650**	**660**	**670**	**680**	**690**	**700**	**710**	**720**
GTALGLGVVN	ILHTMNPSLV	ILSGVLASHY	IHIVKDVIRQ	QALSSVQDVD	VVVSDLVDPA	LLGAASMVLD	YTTRRIY

Cmpd.	No. of Cmpds.	m/z meas.	Δ m/z [ppm]	z	Rt [min]	Score	P	Range	Sequence	Modification
1574	1	1051.4917	-54.07	2	49.4	15.4	2	29-47	R.ARASGWKPMVICRGSHAFK.D	
2066	1	1044.9156	-88.64	2	56.2	16.2	2	191-207	K.DYMSIIRMWLGDDVKSK.D	Oxidation: 3, 8

Detailed Protein Report

Protein 392: **PDZ domain-containing protein MAGIX isoform d [Homo sapiens]**

**Accession:**	gi|532164745	**Score:**	31.6
**Database:**	refseq_human(refseq_human_20140103.fasta)	**MW [kDa]:**	26.7
**Database Date:**	2015-11-30	**pI:**	11.6
		**Sequence Coverage [%]:**	11.9
		**No. of unique Peptides:**	2

Quantitation

QU:MU **Median:** 1.32 **CV:** 0.00 % **No. of Peptides:** 1

10	20	30	40	50	60	70	80
MEPRTGDAAD	PRGSRGGRGP	SPLAGPSARQ	LLARLDARPL	AARAAVDVAA	LVRRAGATLR	LRRKEAVSVL	DSADIEVTDS
**90**	**100**	**110**	**120**	**130**	**140**	**150**	**160**
RLPHATIVDH	RPQVGDLVLH	INGESTQGLT	HAQAVERIRA	GGPQLHLVIR	RPLETHPGKP	RGVGEPRKGV	DRSPDPGGPE
**170**	**180**	**190**	**200**	**210**	**220**	**230**	**240**
VTGSRSSSTS	LVQHPPSRTT	LKKTRGSPEP	SPEAAADGPT	VSPPERRAED	PNDQIPGSPG	PWLVPSEERL	SRALGVRGAA
**250**	**260**						
QLAQEMAAGR	RRH						

Cmpd.	No. of Cmpds.	m/z meas.	Δ m/z [ppm]	z	Rt [min]	Score	P	Range	Sequence	Modification	Ratios
1080	1	841.9573	58.27	2	43.1	19.2	1	149-165	K.GVDRSPDPGGPEVTGSR.S		QU:MU 1.32
1707	2	1273.8380	161.91	1	51.6	12.4	0	238-250	R.GAAQLAQEMAAGR.R		

Detailed Protein Report

Protein 393: **serine/threonine-protein phosphatase 2A 55 kDa regulatory subunit B beta isoform isoform e [Homo sapiens]**

**Accession:**	gi|430727947	**Score:**	31.6
**Database:**	refseq_human(refseq_human_20140103.fasta)	**MW [kDa]:**	62.5
**Database Date:**	2015-11-30	**pI:**	6.4
		**Sequence Coverage [%]:**	6.6
		**No. of unique Peptides:**	1

10	20	30	40	50	60	70	80
MHQPPPASCS	SSSSSSSSSC	ECARVGVRVS	ALAPAAAPCP	APRQLPYPRL	PEPPSRGTST	LIPARLGPWC	SPTGSPAPLS
90	100	110	120	130	140	150	160
CETGCGEGSW	ILVCRLLVPT	QVSLLSMEED	IDTRKINNSF	LRDHSYATEA	DIISTVEFNH	TGELLATGDK	GGRVVIFQRE
170	180	190	200	210	220	230	240
QESKNQVHRR	GEYNVYSTFQ	SHEPEFDYLK	SLEIEEKINK	IRWLPQQNAA	YFLLSTNDKT	VKLWKVSERD	KRPEGYNLKD
250	260	270	280	290	300	310	320
EEGRLRDPAT	ITTLRVPVLR	PMDLMVEATP	RRVFANAHTY	HINSISVNSD	YETYMSADDL	RINLWNFEIT	NQSFNIVDIK
330	340	350	360	370	380	390	400
PANMEELTEV	ITAAEFHPHH	CNTFVYSSSK	GTIRLCDMRA	SALCDRHTKF	FEEPEDPSNR	SFFSEIISSI	SDVKFSHSGR
410	420	430	440	450	460	470	480
YIMTRDYLTV	KVWDLNMENR	PIETYQVHDY	LRSKLCSLYE	NDCIFDKFEC	VWNGSDSVIM	TGSYNNFFRM	FDRNTKRDVT
490	500	510	520	530	540	550	
LEASRENSKP	RAILKPRKVC	VGGKRRKDEI	SVDSLDFSKK	ILHTAWHPSE	NIIAVAATNN	LYIFQDKVN	

Cmpd.	No. of Cmpds.	m/z meas.	Δ m/z [ppm]	z	Rt [min]	Score	P	Range	Sequence	Modification
1632	1	1073.5401	-40.07	2	48.7	20.8	1	29-49	R.VSALAPAAAPCPAPRQLPYPR.L	

Detailed Protein Report

Protein 394: protein Hook homolog 1 [Homo sapiens]

**Accession:**	gi|7705415	**Score:**	31.5
**Database:**	refseq_human(refseq_human_20140103.fasta)	**MW [kDa]:**	84.6
**Database Date:**	2015-11-30	**pI:**	5.0
**Modification(s):**	Oxidation	**Sequence Coverage [%]:**	2.6
		**No. of unique Peptides:**	1

Alias proteins:

Accession	Name	Description
gi|530362743	refseq_human (refseq_human_20140103.fasta)	PREDICTED: protein Hook homolog 1 isoform X1 [Homo sapiens]

10	20	30	40	50	60	70	80
MEETQPPPQP	KLPLCDSLMI	WLQTFNTASP	CQDVKQLTSG	VAMAQVLHQI	DAAWFNESWL	SRIKEDVGDN	WRIKASNVKK
**90**	**100**	**110**	**120**	**130**	**140**	**150**	**160**
VLQGIMSYYH	EFLGQQISEA	LIPDLNQITE	CSDPVELGRL	LQLILGCAIN	CEKKQEHIQN	IMTLEESVQH	VVMTAIQELM
**170**	**180**	**190**	**200**	**210**	**220**	**230**	**240**
SKEILSSPPN	DAVGELEQQL	KRALEELQEA	LAEKEELRQR	CEELDMQVTT	LQDEKNSLVS	ENEMMNEKLD	QLDGSFDDPN
**250**	**260**	**270**	**280**	**290**	**300**	**310**	**320**
TVVAKKYFHA	QLQLEQLQEE	NFRLEAAKDD	YRVHCEELEK	QLIEFQHRND	ELTSLAEETR	ALKDEIDVLR	ATSDKANKLE
**330**	**340**	**350**	**360**	**370**	**380**	**390**	**400**
STVEIYRQKL	QDLNDLRKQV	KTLQETNMMY	MHNTVSLEEE	LKKANAARTQ	LETYKRQVQD	LHVKLSSESK	RADTLAFEMK
**410**	**420**	**430**	**440**	**450**	**460**	**470**	**480**
RLEEKHEALL	KEKERLIEQR	DTLKETNEEL	RCSQVQQDHL	NQTDASATKS	YENLAAEIMP	VEYREVFIRL	QHENKMLRLQ
**490**	**500**	**510**	**520**	**530**	**540**	**550**	**560**
QEGSENERIE	ELQEQLEQKH	RKMNELETEQ	RLSKERIREL	QQQIEDLQKS	LQEQGSKSEG	ESSSKLKQKL	EAHMEKLTEV
**570**	**580**	**590**	**600**	**610**	**620**	**630**	**640**
HEELQKKQEL	IEDLQPDINQ	NVQKINELEA	ALQKKDEDMK	AMEERYKMYL	EKARNVIKTL	DPKLNPASAE	IMLLRKQLAE
**650**	**660**	**670**	**680**	**690**	**700**	**710**	**720**
KERRIEILES	ECKVAKFRDY	EEKLIVSAWY	NKSLAFQKLG	MESRLVSGGG	ACSDTGACTP	ARSFLAQQRH	ITNTRRNLSV
**730**							
KVPATTSD							

Cmpd.	No. of Cmpds.	m/z meas.	Δ m/z [ppm]	z	Rt [min]	Score	P	Range	Sequence	Modification
1597	1	685.8641	-18.30	2	48.3	10.6	2	546-556	K.LKQKLEAHMEK.L	Oxidation: 9

Detailed Protein Report

Protein 395: **PREDICTED: ribosomal protein S6 kinase-like 1 isoform X5 [Homo sapiens]**

**Accession:**	gi|530404733	**Score:**	31.4
**Database:**	refseq_human(refseq_human_20140103.fasta)	**MW [kDa]:**	57.3
**Database Date:**	2015-11-30	**pI:**	7.0
**Modification(s):**	Carbamidomethyl	**Sequence Coverage [%]:**	10.3
		**No. of unique Peptides:**	2

Quantitation

QU:MU	**Median:** 0.47	**CV:** 0.00 %	**No. of Peptides:**	1
WUP:QUP	**Median:** 1.80	**CV:** 0.00 %	**No. of Peptides:**	1

```
        10         20         30         40         50         60         70         80
MSLVACECLP SPGLEPEPCS RARSQAHVYL EQIRNRVALG VPDMTKRDYL VDAATQIRLA LERDVSEDYE AAFNHYQNGV
        90        100        110        120        130        140        150        160
DVLLRGIHVD PNKERREAVK LKITKYLRRA EEIFNCHLQR PLSSGASPSA GFSSLRLRPI RTLSSAVEQL RGCRVVGVIE
       170        180        190        200        210        220        230        240
KVQLVQDPAT GGTFVVKSLP RCHMVSRERL TIIPHGVPYM TKLLRYFVSE DSIFLHLEHV QGGTLWSHLL SQAHSRHSGL
       250        260        270        280        290        300        310        320
SSGSTQERMK AQLNPHLNLL TPARLPSGHA PGQDRIALEP PRTSPNLLLA GEAPSTRPQR EAEGEPTART STSGSSDLPK
       330        340        350        360        370        380        390        400
APGGHLHLQA RRAGQNSDAG PPRGLTWVPE GAGPVLGGCG RGMDQSCLSA DGAGRGCGRA TWSVREEQVK QWAAEMLVAL
       410        420        430        440        450        460        470        480
EALHEQGVLC RDLHPGNLLL DQAGHIRLTY FGQWSEVEPQ CCGEAVDNLY SAPEVGGISE LTEACDWWSF GSLLYELLTG
       490        500        510        520        530
MALSQSHPSG IQAHTQLQLP EWLSRPAASL LTEVRCSPAR GSSCLA
```

Cmpd.	No. of Cmpds.	m/z meas.	Δ m/z [ppm]	z	Rt [min]	Score	P	Range	Sequence	Modification	Ratios
2112	1	1072.4083	-80.50	2	56.8	10.0	0	2-21	M.SLVACECLPSPGLEPEPCSR.A	Carbamidomethyl: 18	
2285	1	884.8843	-103.89	2	59.0	10.7	0	412-427	R.DLHPGNLLLDQAGHIR.L		WUP:QUP 1.80<br>QU:MU 0.47

Detailed Protein Report

Protein 396: **PREDICTED: sodium bicarbonate transporter-like protein 11 isoform X2 [Homo sapiens]**

**Accession:**	gi|530426013	**Score:**	31.4
**Database:**	refseq_human(refseq_human_20140103.fasta)	**MW [kDa]:**	96.0
**Database Date:**	2015-11-30	**pI:**	8.9
**Modification(s):**	Oxidation	**Sequence Coverage [%]:**	2.8
		**No. of unique Peptides:**	1

10	20	30	40	50	60	70	80
MSQNGYFEDS	SYYKCDTDDT	FEAREEILGD	EAFDTANSSI	VSGESIRFFV	NVNLEMQATN	TENEATSGGC	VLLHTSRKYL
**90**	**100**	**110**	**120**	**130**	**140**	**150**	**160**
KLKNFKEEIR	AHRDLDGFLA	QASIVLNETA	TSLDNVLRTM	LRRFARDPDN	NEPNCNLDLL	MAMLFTDAGA	PMRGKVHLLS
**170**	**180**	**190**	**200**	**210**	**220**	**230**	**240**
DTIQGVTATV	TGVRYQQSWL	CIICTMKALQ	KRHVCISRLV	RPQNWGENSC	EVRFVILVLA	PPKMKSTKTA	MEVARTFATM
**250**	**260**	**270**	**280**	**290**	**300**	**310**	**320**
FSDIAFRQKL	LETRTEEEFK	EALVHQRQLL	TMVSHGPVAP	RTKERSTVSL	PAHRHPEPPK	CKDFVPFGKG	IREDIARRFP
**330**	**340**	**350**	**360**	**370**	**380**	**390**	**400**
LYPLDFTDGI	IGKNKAVGKY	ITTTLFLYFA	CLLPTIAFGS	LNDENTDGAI	DVQKTIAGQS	IGGLLYALFS	GQPLVILLTT
**410**	**420**	**430**	**440**	**450**	**460**	**470**	**480**
APLALYIQVI	RVICDDYDLD	FNSFYAWTGL	WNSFFLALYA	FFNLSLVMSL	FKRSTEEIIA	LFISITFVLD	AVKGTVKIFW
**490**	**500**	**510**	**520**	**530**	**540**	**550**	**560**
KYYYGHYLDD	YHTKRTSSLV	SLSGLGASLN	ASLHTALNAS	FLASPTELPS	ATHSGQATAV	LSLLIMLGTL	WLGYTLYQFK
**570**	**580**	**590**	**600**	**610**	**620**	**630**	**640**
KSPYLHPCVR	EILSDCALPI	AVLAFSLISS	HGFREIEMSK	FRYNPSESPF	AMAQIQSLSL	RAVSGAMGLG	FLLSMLFFIE
**650**	**660**	**670**	**680**	**690**	**700**	**710**	**720**
QNLVAALVNA	PENRLVKGTA	YHWDLLLLAI	INTGLSLFGL	PWIHAAYPHS	PLHVRALALV	EERVENGHIY	DTIVNVKETR
**730**	**740**	**750**	**760**	**770**	**780**	**790**	**800**
LTSLGASVLV	GLSLLLLPVP	LQWIPKPVLY	GLFLYIALTS	LDGNQLVQRV	ALLLKEQTAY	PPTHYIRRVP	QRKIHYFTGL
**810**	**820**	**830**	**840**	**850**	**860**		
QVLQLLLLCA	FGMSSLPYMK	MIFPLIMIAM	IPIRYILLPR	IIEAKYLDVM	DAEHRP		

Cmpd.	No. of Cmpds.	m/z meas.	Δ m/z [ppm]	z	Rt [min]	Score	P	Range	Sequence	Modification
823	1	711.9737	188.22	2	38.7	20.7	0	236-247	R.TFATMFSDIAFR.Q	Oxidation: 5

Detailed Protein Report

Protein 397: **PREDICTED: myelin transcription factor 1-like protein isoform X7 [Homo sapiens]**

**Accession:**	gi|578802502	**Score:**	31.4
**Database:**	refseq_human(refseq_human_20140103.fasta)	**MW [kDa]:**	130.2
**Database Date:**	2015-11-30	**pI:**	4.7
		**Sequence Coverage [%]:**	1.9
		**No. of unique Peptides:**	2

10	20	30	40	50	60	70	80
MEVDTEEKRH	RTRSKGVRVP	VEPAIQELFS	CPTPGCDGSG	HVSGKYARHR	SVYGCPLAKK	RKTQDKQPQE	PAPKRKPFAV
90	100	110	120	130	140	150	160
KADSSSVDEC	DDSDGTEDMD	EKEEDEGEEY	SEDNDEPGDE	DEEDEEGDRE	EEEEIEEEDE	DDDEDGEDVE	DEEEEEEEEE
170	180	190	200	210	220	230	240
EEEEEEEEND	HQMNCHNTRI	MQDTEKDDNN	NDEYDNYDEL	VAKSLLNLGK	IAEDAAYRAR	TESEMNSNTS	NSLEDDSDKN
250	260	270	280	290	300	310	320
ENLGRKSELS	LDLDSDVVRE	TVDSLKLLAQ	GHGVVLSENM	NDRNYADSMS	QQDSRNMNYV	MLGKPMNNGL	MEKMVEESDE
330	340	350	360	370	380	390	400
EVCLSSLECL	RNQCFDLARK	LSETNPQERN	PQQNMNIRQH	VRPEEDFPGR	TPDRNYSDML	NLMRLEEQLS	PRSRVFASCA
410	420	430	440	450	460	470	480
KEDGCHERDD	DTTSVNSDRS	EEVFDMTKGN	LTLLEKAIAL	ETERAKAMRE	KMAMEAGRRD	NMRSYEDQSP	RQLPGEDRKP
490	500	510	520	530	540	550	560
KSSDSHVKKP	YYVLAMHESV	LKCPTPGCTG	RGHVNSNRNS	HRSLSGCPIA	AAEKLAKAQE	KHQSCDVSKS	SQASDRVLRP
570	580	590	600	610	620	630	640
MCFVKQLEIP	QYGYRNNVPT	TTPRSNLAKE	LEKYSKTSFE	YNSYDNHTYG	KRAIAPKVQT	RDISPKGYDD	AKRYCKDPSP
650	660	670	680	690	700	710	720
SSSSTSSYAP	SSSSNLSCGG	GSSASSTCSK	SSFDYTHDME	AAHMAATAIL	NLSTRCREMP	QNLSTKPQDL	CATRNPDMEV
730	740	750	760	770	780	790	800
DENGTLDLSM	NKQRPRDSCC	PILTPLEPMS	PQQQAVMNNR	CFQLGEGDCW	DLPVDYTKMK	PRRIDEDESK	DITPEDLDPF
810	820	830	840	850	860	870	880
QEALEERRYP	GEVTIPSPKP	KYPQCKESKK	DLITCPTPGC	DGSGHVTGNY	ASHRSLSGCP	LADKSIRSML	ATSSQELKCP
890	900	910	920	930	940	950	960
TPGCDGSGHI	TGNYASHRSL	SGCPRAKKSG	IRIAQSKEDK	EDQEPIRCPV	PGCDGQGHIT	GKYASHRSAS	GCPLAAKRQK
970	980	990	1000	1010	1020	1030	1040
DGYLNGSQFS	WKSVKTEGMS	CPTPGCDGSG	HVSGSFLTHR	SLSGCPRATS	AMKKAKLSGE	QMLTIKQRAS	NGIENDEEIK
1050	1060	1070	1080	1090	1100	1110	1120
QLDEEIKELN	ESNSQMEADM	IKLRTQVTIT	TMESNLKTIE	EENKVIEQQN	ESLLHELANL	SQSLIHSLAN	IQLPHMDPIN
1130	1140	1150	1160	1170			
EQNFDAYVTT	LTEMYTNQDR	YQSPENKALL	ENIKQAVRGI	QV			

Cmpd.	No. of Cmpds.	m/z meas.	Δ m/z [ppm]	z	Rt [min]	Score	P	Range	Sequence	Modification
1852	2	612.7877	57.61	2	53.5	15.8	0	409-419	R.DDDTTSVNSDR.S	
2754	2	639.3511	58.06	2	62.9	15.6	1	512-522	R.GHVNSNRNSHR.S	

Detailed Protein Report

Protein 398: **arf-GAP with coiled-coil, ANK repeat and PH domain-containing protein 1 [Homo sapiens]**

**Accession:**	gi|7661880	**Score:**	31.4
**Database:**	refseq_human(refseq_human_20140103.fasta)	**MW [kDa]:**	81.5
**Database Date:**	2015-11-30	**pI:**	8.7
**Modification(s):**	Carbamidomethyl, Oxidation	**Sequence Coverage [%]:**	4.2
		**No. of unique Peptides:**	2

10	20	30	40	50	60	70	80
MTVKLDFEEC	LKDSPRFRAS	IELVEAEVSE	LETRLEKLLK	LGTGLLESGR	HYLAASRAFV	VGICDLARLG	PPEPMMAECL
**90**	**100**	**110**	**120**	**130**	**140**	**150**	**160**
EKFTVSLNHK	LDSHAELLDA	TQHTLQQQIQ	TLVKEGLRGF	REARRDFWRG	AESLEAALTH	NAEVPRRRAQ	EAEEAGAALR
**170**	**180**	**190**	**200**	**210**	**220**	**230**	**240**
TARAGYRGRA	LDYALQINVI	EDKRKFDIME	FVLRLVEAQA	THFQQGHEEL	SRLSQYRKEL	GAQLHQLVLN	SAREKRDMEQ
**250**	**260**	**270**	**280**	**290**	**300**	**310**	**320**
RHVLLKQKEL	GGEEPEPSLR	EGPGGLVMEG	HLFKRASNAF	KTWSRRWFTI	QSNQLVYQKK	YKDPVTVVVD	DLRLCTVKLC
**330**	**340**	**350**	**360**	**370**	**380**	**390**	**400**
PDSERRFCFE	VVSTSKSCLL	QADSERLLQL	WVSAVQSSIA	SAFSQARLDD	SPRGPGQGSG	HLAIGSAATL	GSGGMARGRE
**410**	**420**	**430**	**440**	**450**	**460**	**470**	**480**
PGGVGHVVAQ	VQSVDGNAQC	CDCREPAPEW	ASINLGVTLC	IQCSGIHRSL	GVHFSKVRSL	TLDSWEPELV	KLMCELGNVI
**490**	**500**	**510**	**520**	**530**	**540**	**550**	**560**
INQIYEARVE	AMAVKKPGPS	CSRQEKEAWI	HAKYVEKKFL	TKLPEIRGRR	GGRGRPRGQP	PVPPKPSIRP	RPGSLRSKPE
**570**	**580**	**590**	**600**	**610**	**620**	**630**	**640**
PPSEDLGSLH	PGALLFRASG	HPPSLPTMAD	ALAHGADVNW	VNGGQDNATP	LIQATAANSL	LACEFLLQNG	ANVNQADSAG
**650**	**660**	**670**	**680**	**690**	**700**	**710**	**720**
RGPLHHATIL	GHTGLACLFL	KRGADLGARD	SEGRDPLTIA	METANADIVT	LLRLAKMREA	EAAQGQAGDE	TYLDIFRDFS
**730**	**740**	**750**					
LMASDDPEKL	SRRSHDLHTL						

Cmpd.	No. of Cmpds.	m/z meas.	Δ m/z [ppm]	z	Rt [min]	Score	P	Range	Sequence	Modification
1185	1	984.5174	36.04	2	44.4	11.5	2	1-16	-.MTVKLDFEECLKDSPR.F	Carbamidomethyl: 10
2485	1	821.9375	18.65	2	61.6	19.9	1	261-275	R.EGPGGLVMEGHLFKR.A	Oxidation: 8

Detailed Protein Report

Protein 399: calponin-3 isoform 2 [Homo sapiens]

Accession: gi|554506520
Database: refseq_human(refseq_human_20140103.fasta)
Database Date: 2015-11-30

Score: 31.3
MW [kDa]: 31.4
pI: 6.1
Sequence Coverage [%]: 8.1
No. of unique Peptides: 1

10	20	30	40	50	60	70	80
MTHFNKGPSY	GLSAEVKNKI	ASKYDHQAEE	DLRNWIEEVT	GMSIGPNFQL	GLKDGIILCE	LINKLQPGSV	KKVNESSLNW
**90**	**100**	**110**	**120**	**130**	**140**	**150**	**160**
PQAKTKGFHT	TIDIGVKYAE	KQTRRFDEGK	LKAGQSVIGL	QMGTNKCASQ	AGMTAYGTRR	HLYDPKMQTD	KPFDQTTISL
**170**	**180**	**190**	**200**	**210**	**220**	**230**	**240**
QMGTNKGASQ	AGMLAPGTRR	DIYDQKLTLQ	PVDNSTISLQ	MGTNKVASQK	GMSVYGLGRQ	VYDPKYCAAP	TEPVIHNGSQ
**250**	**260**	**270**	**280**	**290**			
GTGTNGSEIS	DSDYQAEYPD	EYHGEYQDDY	PRDYQYSDQG	IDY			

Cmpd.	No. of Cmpds.	m/z meas.	Δ m/z [ppm]	z	Rt [min]	Score	P	Range	Sequence	Modification
1867	1	702.3265	-60.80	2	53.7	14.0	0	113-126	K.AGQSVIGLQMGTNK.C	

Detailed Protein Report

Protein 400: ATP-binding cassette sub-family F member 2 isoform a [Homo sapiens]

**Accession:**	gi|27881506	**Score:**	31.3
**Database:**	refseq_human(refseq_human_20140103.fasta)	**MW [kDa]:**	71.2
**Database Date:**	2015-11-30	**pI:**	7.1
**Modification(s):**	Oxidation	**Sequence Coverage [%]:**	5.0
		**No. of unique Peptides:**	1

10	20	30	40	50	60	70	80
MPSDLAKKKA	AKKKEAAKAR	QRPRKGHEEN	GDVVTEPQVA	EKNEANGRET	TEVDLLTKEL	EDFEMKKAAA	RAVTGVLASH
**90**	**100**	**110**	**120**	**130**	**140**	**150**	**160**
PNSTDVHIIN	LSLTFHGQEL	LSDTKLELNS	GRRYGLIGLN	GIGKSMLLSA	IGKREVPIPE	HIDIYHLTRE	MPPSDKTPLH
**170**	**180**	**190**	**200**	**210**	**220**	**230**	**240**
CVMEVDTERA	MLEKEAERLA	HEDAECEKLM	ELYERLEELD	ADKAEMRASR	ILHGLGFTPA	MQRKKLKDFS	GGWRMRVALA
**250**	**260**	**270**	**280**	**290**	**300**	**310**	**320**
RALFIRPFML	LLDEPTNHLD	LDACVWLEEE	LKTFKRILVL	VSHSQDFLNG	VCTNIIHMHN	KKLKYYTGNY	DQYVKTRLEL
**330**	**340**	**350**	**360**	**370**	**380**	**390**	**400**
EENQMKRFHW	EQDQIAHMKN	YIARFGHGSA	KLARQAQSKE	KTLQKMMASG	LTERVVSDKT	LSFYFPPCGK	IPPPVIMVQN
**410**	**420**	**430**	**440**	**450**	**460**	**470**	**480**
VSFKYTKDGP	CIYNNLEFGI	DLDTRVALVG	PNGAGKSTLL	KLLTGELLPT	DGMIRKHSHV	KIGRYHQHLQ	EQLDLDLSPL
**490**	**500**	**510**	**520**	**530**	**540**	**550**	**560**
EYMMKCYPEI	KEKEEMRKII	GRYGLTGKQQ	VSPIRNLSDG	QKCRVCLAWL	AWQNPHMLFL	DEPTNHLDIE	TIDALADAIN
**570**	**580**	**590**	**600**	**610**	**620**	**630**	
EFEGGMMLVS	HDFRLIQQVA	QEIWVCEKQT	ITKWPGDILA	YKEHLKSKLV	DEEPQLTKRT	HNV	

Cmpd.	No. of Cmpds.	m/z meas.	Δ m/z [ppm]	z	Rt [min]	Score	P	Range	Sequence	Modification
2422	1	1044.2014	104.18	2	60.8	16.5	1	437-455	K.STLLKLLTGELLPTDGMIR.K	Oxidation: 17

Detailed Protein Report

Protein 401: **coilin [Homo sapiens]**

**Accession:**	gi|4758024	**Score:**	31.2
**Database:**	refseq_human(refseq_human_20140103.fasta)	**MW [kDa]:**	62.6
**Database Date:**	2015-11-30	**pI:**	10.0
		**Sequence Coverage [%]:**	5.2
		**No. of unique Peptides:**	1

10	20	30	40	50	60	70	80
MAASETVRLR	LQFDYPPPAT	PHCTAFWLLV	DLNRCRVVTD	LISLIRQRFG	FSSGAFLGLY	LEGGLLPPAE	SARLVRDNDC
**90**	**100**	**110**	**120**	**130**	**140**	**150**	**160**
LRVKLEERGV	AENSVVISNG	DINLSLRKAK	KRAFQLEEGE	ETEPDCKYSK	KHWKSRENNN	NNEKVLDLEP	KAVTDQTVSK
**170**	**180**	**190**	**200**	**210**	**220**	**230**	**240**
KNKRKNKATC	GTVGDDNEEA	KRKSPKKKEK	CEYKKKAKNP	KSPKVQAVKD	WANQRCSSPK	GSARNSLVKA	KRKGSVSVCS
**250**	**260**	**270**	**280**	**290**	**300**	**310**	**320**
KESPSSSSES	ESCDESISDG	PSKVTLEARN	SSEKLPTELS	KEEPSTKNTT	ADKLAIKLGF	SLTPSKGKTS	GTTSSSSDSS
**330**	**340**	**350**	**360**	**370**	**380**	**390**	**400**
AESDDQCLMS	SSTPECAAGF	LKTVGLFAGR	GRPGPGLSSQ	TAGAAGWRRS	GSNGGGQAPG	ASPSVSLPAS	LGRGWGREEN
**410**	**420**	**430**	**440**	**450**	**460**	**470**	**480**
LFSWKGAKGR	GMRGRGRGRG	HPVSCVVNRS	TDNQRQQQLN	DVVKNSSTII	QNPVETPKKD	YSLLPLLAAA	PQVGEKIAFK
**490**	**500**	**510**	**520**	**530**	**540**	**550**	**560**
LLELTSSYSP	DVSDYKEGRI	LSHNPETQQV	DIEILSSLPA	LREPGKFDLV	YHNENGAEVV	EYAVTQESKI	TVFWKELIDP
**570**	**580**						
RLIIESPSNT	SSTEPA						

Cmpd.	No. of Cmpds.	m/z meas.	Δ m/z [ppm]	z	Rt [min]	Score	P	Range	Sequence	Modification
1773	1	835.3568	-43.51	2	52.0	11.4	2	132-144	K.HWKSRENNNNNEK.V	

Detailed Protein Report

Protein 402: PREDICTED: oleosin-B4-like [Homo sapiens]

Accession: gi|410170263
Database: refseq_human(refseq_human_20140103.fasta)
Database Date: 2015-11-30
Modification(s): Carbamidomethyl

Score: 31.2
MW [kDa]: 49.8
pI: 12.2
Sequence Coverage [%]: 6.9
No. of unique Peptides: 2

10	20	30	40	50	60	70	80
MSSPVLEPSP	GPRSPYLHGP	YLLLGEVLGH	SALGAQHAQP	ANGDADELLE	LPALLQHPTG	CDPCASLRDS	RVTPATALLL
**90**	**100**	**110**	**120**	**130**	**140**	**150**	**160**
LSHRGRAQRR	SIQAAAAQEQ	STGRGCLGKE	PPSQESWTRS	DPRRCLSQDA	VRSGSRVCRS	RPQTRGGGGK	QPRRQKAAVA
**170**	**180**	**190**	**200**	**210**	**220**	**230**	**240**
GAKSRGGEGK	KPQRRGQKAA	TAGAKSRGGG	VRSLGGKKMR	RGQKGAAGAK	RCGGGKQPRR	RGQKASKSGG	VRCQTPQKAA
**250**	**260**	**270**	**280**	**290**	**300**	**310**	**320**
AQGVKSRKKL	RQRGQKAAAA	GQEAAAGKTC	GGGGEKHRGG	GDKKPLRKNS	RWRGQKAAAA	GGKKPQKAAA	AGGERRKKLR
**330**	**340**	**350**	**360**	**370**	**380**	**390**	**400**
RQKAAVARAK	SPKKPRWRGQ	NSGNGVEGQR	SLAFLEFDVE	GKVQRKTKIP	GGVVKQFVPA	DQGPLWQKAA	NLHKMSLPHQ
**410**	**420**	**430**	**440**	**450**	**460**	**470**	
LALKMEWNGI	IIEWNRMELS	SNGINRDHHR	MEPNGVIIEW	NRKASSCNGI	EWHHQRMDSK	GIIIE	

Cmpd.	No. of Cmpds.	m/z meas.	Δ m/z [ppm]	z	Rt [min]	Score	P	Range	Sequence	Modification
1321	1	1045.0547	21.73	2	44.8	13.5	2	90-109	R.RSIQAAAAQEQSTGRGCLGK.E	Carbamidomethyl: 17
928	1	573.2590	-111.53	2	41.2	17.7	2	176-187	R.GQKAATAGAKSR.G	

Detailed Protein Report

Protein 403: ATP-dependent RNA helicase DDX42 [Homo sapiens]

**Accession:**	gi|45446743	**Score:**	31.2
**Database:**	refseq_human(refseq_human_20140103.fasta)	**MW [kDa]:**	102.9
**Database Date:**	2015-11-30	**pI:**	6.6
		**Sequence Coverage [%]:**	3.0
		**No. of unique Peptides:**	1

Alias proteins:

Accession	Name	Description
gi|578830314	refseq_human (refseq_human_20140103.fasta)	PREDICTED: ATP-dependent RNA helicase DDX42 isoform X1 [Homo sapiens]
gi|45446747	refseq_human (refseq_human_20140103.fasta)	ATP-dependent RNA helicase DDX42 [Homo sapiens]

10	20	30	40	50	60	70	80
MNWNKGGPGT	KRGFGFGGFA	ISAGKKEEPK	LPQQSHSAFG	ATSSSSGFGK	SAPPQLPSFY	KIGSKRANFD	EENAYFEDEE
90	100	110	120	130	140	150	160
EDSSNVDLPY	IPAENSPTRQ	QFHSKPVDSD	SDDDPLEAFM	AEVEDQAARD	MKRLEEKDKE	RKNVKGIRDD	IEEEDDQEAY
170	180	190	200	210	220	230	240
FRYMAENPTA	GVVQEEEEDN	LEYDSDGNPI	APTKKIIDPL	PPIDHSEIDY	PPFEKNFYNE	HEEITNLTPQ	QLIDLRHKLN
250	260	270	280	290	300	310	320
LRVSGAAPPR	PGSSFAHFGF	DEQLMHQIRK	SEYTQPTPIQ	CQGVPVALSG	RDMIGIAKTG	SGKTAAFIWP	MLIHIMDQKE
330	340	350	360	370	380	390	400
LEPGDGPIAV	IVCPTRELCQ	QIHAECKRFG	KAYNLRSVAV	YGGGSMWEQA	KALQEGAEIV	VCTPGRLIDH	VKKKATNLQR
410	420	430	440	450	460	470	480
VSYLVFDEAD	RMFDMGFEYQ	VRSIASHVRP	DRQTLLFSAT	FRKKIEKLAR	DILIDPIRVV	QGDIGEANED	VTQIVEILHS
490	500	510	520	530	540	550	560
GPSKWNWLTR	RLVEFTSSGS	VLLFVTKKAN	AEELANNLKQ	EGHNLGLLHG	DMDQSERNKV	ISDFKKKDIP	VLVATDVAAR
570	580	590	600	610	620	630	640
GLDIPSIKTV	INYDVARDID	THTHRIGRTG	RAGEKGVAYT	LLTPKDSNFA	GDLVRNLEGA	NQHVSKELLD	LAMQNAWFRK
650	660	670	680	690	700	710	720
SRFKGGKGKK	LNIGGGGLGY	RERPGLGSEN	MDRGNNNVMS	NYEAYKPSTG	AMGDRLTAMK	AAFQSQYKSH	FVAASLSNQK
730	740	750	760	770	780	790	800
AGSSAAGASG	WTSAGSLNSV	PTNSAQQGHN	SPDSPVTSAA	KGIPGFGNTG	NISGAPVTYP	SAGAQGVNNT	ASGNNSREGT
810	820	830	840	850	860	870	880
GGSNGKRERY	TENRGSSRHS	HGETGNRHSD	SPRHGDGGRH	GDGYRHPESS	SRHTDGHRHG	ENRHGGSAGR	HGENRGANDG
890	900	910	920	930	940		
RNGESRKEAF	NRESKMEPKM	EPKVDSSKMD	KVDSKTDKTA	DGFAVPEPPK	RKKSRWDS		

Cmpd.	No. of Cmpds.	m/z meas.	Δ m/z [ppm]	z	Rt [min]	Score	P	Range	Sequence	Modification
2100	1	753.3769	8.39	2	56.6	15.7	2	896-908	K.MEPKMEPKVDSSK.M	

Detailed Protein Report

Protein 404: 28S ribosomal protein S31, mitochondrial [Homo sapiens]

**Accession:**	gi|186928854	**Score:**	31.1
**Database:**	refseq_human(refseq_human_20140103.fasta)	**MW [kDa]:**	45.3
**Database Date:**	2015-11-30	**pI:**	9.8
		**Sequence Coverage [%]:**	3.0
		**No. of unique Peptides:**	2

Quantitation

WUP:QUP **Median:** 0.38 **CV:** 33.11 % **No. of Peptides:** 2

10	20	30	40	50	60	70	80
MFPRVSTFLP	LRPLSRHPLS	SGSPETSAAA	IMLLTVRHGT	VRYRSSALLA	RTKNNIQRYF	GTNSVICSKK	DKQSVRTEET
**90**	**100**	**110**	**120**	**130**	**140**	**150**	**160**
SKETSESQDS	EKENTKKDLL	GIIKGMKVEL	STVNVRTTKP	PKRRPLKSLE	ATLGRLRRAT	EYAPKKRIEP	LSPELVAAAS
**170**	**180**	**190**	**200**	**210**	**220**	**230**	**240**
AVADSLPFDK	QTTKSELLSQ	LQQHEEESRA	QRDAKRPKIS	FSNIISDMKV	ARSATARVRS	RPELRIQFDE	GYDNYPGQEK
**250**	**260**	**270**	**280**	**290**	**300**	**310**	**320**
TDDLKKRKNI	FTGKRLNIFD	MMAVTKEAPE	TDTSPSLWDV	EFAKQLATVN	EQPLQNGFEE	LIQWTKEGKL	WEFPINNEAG
**330**	**340**	**350**	**360**	**370**	**380**	**390**	**400**
FDDDGSEFHE	HIFLEKHLES	FPKQGPIRHF	MELVTCGLSK	NPYLSVKQKV	EHIEWFRNYF	NEKKDILKES	NIQFN

Cmpd.	No. of Cmpds.	m/z meas.	Δ m/z [ppm]	z	Rt [min]	Score	P	Range	Sequence	Modification	Ratios	
2703	1	724.8604	-42.02	2	64.9	19.1	2	384-395	K.KDILKESNIQFN.-		WUP:QUP	0.28
1539	1	660.8309	-18.83	2	48.9	12.0	1	385-395	K.DILKESNIQFN.-		WUP:QUP	0.53

Detailed Protein Report

Protein 405: ubiquitin-conjugating enzyme E2 J2 isoform 3 [Homo sapiens]

**Accession:**	gi|37577130	**Score:**	31.1
**Database:**	refseq_human(refseq_human_20140103.fasta)	**MW [kDa]:**	22.9
**Database Date:**	2015-11-30	**pI:**	7.8
		**Sequence Coverage [%]:**	5.8
		**No. of unique Peptides:**	1

Alias proteins:

Accession	Name	Description
gi|37577132	refseq_human (refseq_human_20140103.fasta)	ubiquitin-conjugating enzyme E2 J2 isoform 3 [Homo sapiens]

```
        10         20         30         40         50         60         70         80
MTPYEGGYYH GKLIFPREFP FKPPSIYMIT PNGRFKCNTR LCLSITDFHP DTWNPAWSVS TILTGLLSFM VEKGPTLGSI
        90        100        110        120        130        140        150        160
ETSDFTKRQL AVQSLAFNLK DKVFCELFPE VVEEIKQKQK AQDELSSRPQ TLPLPDVVPD GETHLVQNGI QLLNGHAPGA
       170        180        190        200        210
VPNLAGLQQA NRHHGLLGGA LANLFVIVGF AAFAYTVKYV LRSIAQE
```

Cmpd.	No. of Cmpds.	m/z meas.	Δ m/z [ppm]	z	Rt [min]	Score	P	Range	Sequence	Modification
188	1	636.3948	167.51	2	32.1	12.9	0	2-12	M.TPYEGGYYHGK.L	

Detailed Protein Report

Protein 406: zinc finger protein 568 isoform 3 [Homo sapiens]

**Accession:**	gi|325651958	**Score:**	31.0
**Database:**	refseq_human(refseq_human_20140103.fasta)	**MW [kDa]:**	67.0
**Database Date:**	2015-11-30	**pI:**	10.0
		**Sequence Coverage [%]:**	1.6
		**No. of unique Peptides:**	1

Quantitation

QU:MU **Median:** 0.65 **CV:** 0.00 % **No. of Peptides:** 1

Alias proteins:

Accession	Name	Description
gi|325651960	refseq_human (refseq_human_20140103.fasta)	zinc finger protein 568 isoform 3 [Homo sapiens]

```
        10         20         30         40         50         60         70         80
MKPAQRNLYR DVMLENYSNL VTVGCQVTKP DVIFKLEQEE EPWVMEEEMF GRHCPEVWEV DEQIKKQQET LVRKVTSISK
        90        100        110        120        130        140        150        160
KILIKEKVIE CKKVAKIFPL SSDIVTSRQS FYDCDSLDKG LEHNLDLLRY EKGCVREKQS NEFGKPFYHC ASYVVTPFKC
       170        180        190        200        210        220        230        240
NQCGQDFSHK FDLIRHERIH AGEKPYECKE CGKAFSRKEN LITHQKIHTG EKPYKCNECG KAFIQMSNLI RHHRIHTGEK
       250        260        270        280        290        300        310        320
PYACKDCWKA FSQKSNLIEH ERIHTGEKPY ECKECGKSFS QKQNLIEHEK IHTGEKPYAC NECGRAFSRM SSVTLHMRSH
       330        340        350        360        370        380        390        400
TGEKPYKCNK CGKAFSQCSV FIIHMRSHTG EKPYVCSECG KAFSQSSSLT VHMRNHTAEK PYECKECGKA FSRKENLITH
       410        420        430        440        450        460        470        480
QKIHTGEKPY ECSECGKAFI QMSNLIRHQR IHTGEKPYAC TVCGKAFSQK SNLTEHEKIH TGEKPYHCNQ CGKAFSQRQN
       490        500        510        520        530        540        550        560
LLEHEKIHTG EKPFKCNECG KAFSRISSLT LHVRSHTGEK PYECNKCGKA FSQCSLLIIH MRSHTGEKPF ECNECGKAFS
       570        580        590
QRASLSIHKR GHTGERHQVY
```

Cmpd.	No. of Cmpds.	m/z meas.	Δ m/z [ppm]	z	Rt [min]	Score	P	Range	Sequence	Modification	Ratios	
858	1	501.2115	-157.63	2	39.1	12.8	1	66-73	K.KQQETLVR.K		QU:MU	0.65

Detailed Protein Report

Protein 407: cartilage intermediate layer protein 2 precursor [Homo sapiens]

**Accession:**	gi|145699139	**Score:**	30.9
**Database:**	refseq_human(refseq_human_20140103.fasta)	**MW [kDa]:**	126.2
**Database Date:**	2015-11-30	**pI:**	9.8
**Modification(s):**	Carbamidomethyl	**Sequence Coverage [%]:**	1.8
		**No. of unique Peptides:**	1

10	20	30	40	50	60	70	80
MASLLPLLCL	CVVAAHLAGA	RDATPTEEPM	ATALGLERRS	VYTGQPSPAL	EDWEEASEWT	SWFNVDHPGG	DGDFESLAAI
**90**	**100**	**110**	**120**	**130**	**140**	**150**	**160**
RFYYGPARVC	PRPLALEART	TDWALPSAVG	ERVHLNPTRG	FWCLNREQPR	GRRCSNYHVR	FRCPLEASWG	AWGPWGPCSG
**170**	**180**	**190**	**200**	**210**	**220**	**230**	**240**
SCGPGRRLRR	RHCPSPAGDA	CPGRPLEAQK	CVRPRCPGCS	LDTCECPDHI	LLGSVVTPSG	QPLLGARVSL	RDQPGTVATS
**250**	**260**	**270**	**280**	**290**	**300**	**310**	**320**
DAHGTFRVPG	VCADSRANIR	AQMDGFSAGE	AQAQANGSIS	VVTIILDKLE	KPYLVKHPES	RVREAGQNVT	FCCKASGTPM
**330**	**340**	**350**	**360**	**370**	**380**	**390**	**400**
PKKYSWFHNG	TLLDRRAHGY	GAHLELRGLR	PDQAGIYHCK	AWNEAGAVRS	GTARLTVLAP	GQPACDPRPR	EYLIKLPEDC
**410**	**420**	**430**	**440**	**450**	**460**	**470**	**480**
GQPGSGPAYL	DVGLCPDTRC	PSLAGSSPRC	GDASSRCCSV	RRLERREIHC	PGYVLPVKVV	AECGCQKCLP	PRGLVRGRVV
**490**	**500**	**510**	**520**	**530**	**540**	**550**	**560**
AADSGEPLRF	ARILLGQEPI	GFTAYQGDFT	IEVPPSTQRL	VVTFVDPSGE	FMDAVRVLPF	DPRGAGVYHE	VKAMRKKAPV
**570**	**580**	**590**	**600**	**610**	**620**	**630**	**640**
ILHTSQSNTI	PLGELEDEAP	LGELVLPSGA	FRRADGKPYS	GPVEARVTFV	DPRDLTSAAS	APSDLRFVDS	DGELAPLRTY
**650**	**660**	**670**	**680**	**690**	**700**	**710**	**720**
GMFSVDLRAP	GSAEQLQVGP	VAVRVAASQI	HMPGHVEALK	LWSLNPETGL	WEEESGFRRE	GSSGPRVRRE	ERVFLVGNVE
**730**	**740**	**750**	**760**	**770**	**780**	**790**	**800**
IRERRLFNLD	VPERRRCFVK	VRAYANDKFT	PSEQVEGVVV	TLVNLEPAPG	FSANPRAWGR	FDSAVTGPNG	ACLPAFCDAD
**810**	**820**	**830**	**840**	**850**	**860**	**870**	**880**
RPDAYTALVT	ATLGGEELEP	APSLPRPLPA	TVGVTQPYLD	RLGYRRTDHD	DPAFKRNGFR	INLAKPRPGD	PAEANGPVYP
**890**	**900**	**910**	**920**	**930**	**940**	**950**	**960**
WRSLRECQGA	PVTASHFRFA	RVEADKYEYN	VVPFREGTPA	SWTGDLLAWW	PNPQEFRACF	LKVKIQGPQE	YMVRSHNAGG
**970**	**980**	**990**	**1000**	**1010**	**1020**	**1030**	**1040**
SHPRTRGQLY	GLRDARSVRD	PERPGTSAAC	VEFKCSGMLF	DQRQVDRTLV	TIMPQGSCRR	VAVNGLLRDY	LTRHPPPVPA
**1050**	**1060**	**1070**	**1080**	**1090**	**1100**	**1110**	**1120**
EDPAAFSMLA	PLDPLGHNYG	VYTVTDQSPR	LAKEIAIGRC	FDGSSDGFSR	EMKADAGTAV	TFQCREPPAG	RPSLFQRLLE
**1130**	**1140**	**1150**	**1160**				
SPATALGDIR	REMSEAAQAQ	ARASGPLRTR	RGRVRQ				

Cmpd.	No. of Cmpds.	m/z meas.	Δ m/z [ppm]	z	Rt [min]	Score	P	Range	Sequence	Modification
1629	1	534.3438	61.87	2	50.6	13.1	1	468-476	K.CLPPRGLVR.G	Carbamidomethyl: 1

Detailed Protein Report

Protein 408: regulator of G-protein signaling 9 isoform 2 [Homo sapiens]

Accession: gi|126366080
Database: refseq_human(refseq_human_20140103.fasta)
Database Date: 2015-11-30

Score: 30.9
MW [kDa]: 76.6
pI: 10.0
Sequence Coverage [%]: 3.6
No. of unique Peptides: 2

Quantitation

QU:MU	**Median:**	1.13	**CV:**	0.00 %	**No. of Peptides:**	1
WUP:QUP	**Median:**	0.17	**CV:**	0.00 %	**No. of Peptides:**	1

10	20	30	40	50	60	70	80
MTIRHQGQQY	RPRMAFLQKI	EALVKDMQNP	ETGVRMQNQR	VLVTSVPHAM	TGSDVLQWIV	QRLWISSLEA	QNLGNFIVRY
**90**	**100**	**110**	**120**	**130**	**140**	**150**	**160**
GYIYPLQDPK	NLILKPDGSL	YRFQTPYFWP	TQQWPAEDTD	YAIYLAKRNI	KKKGILEEYE	KENYNFLNQK	MNYKWDFVIM
**170**	**180**	**190**	**200**	**210**	**220**	**230**	**240**
QAKEQYRAGK	ERNKADRYAL	DCQEKAYWLV	HRCPPGMDNV	LDYGLDRVTN	PNEVKKQTVV	AVKKEIMYYQ	QALMRSTVKS
**250**	**260**	**270**	**280**	**290**	**300**	**310**	**320**
SVSLGGIVKY	SEQFSSNDAI	MSGCLPSNPW	ITDDTQFWDL	NAKLVEIPTK	MRVERWAFNF	SELIRDPKGR	QSFQYFLKKE
**330**	**340**	**350**	**360**	**370**	**380**	**390**	**400**
FSGENLGFWE	ACEDLKYGDQ	SKVKEKAEEI	YKLFLAPGAR	RWINIDGKTM	DITVKGLKHP	HRYVLDAAQT	HIYMLMKKDS
**410**	**420**	**430**	**440**	**450**	**460**	**470**	**480**
YARYLKSPIY	KDMLAKAIEP	QETTKKSSTL	PFMRRHLRSS	PSPVILRQLE	EEAKAREAAN	TVDITQPGQH	MAPSPHLTVY
**490**	**500**	**510**	**520**	**530**	**540**	**550**	**560**
TGTCMPPSPS	SPFSSSCRSP	RKPFASPSRF	IRRPSTTICP	SPIRVALESS	SGLEQKGECS	GSMAPRGPSV	TESSEASLDT
**570**	**580**	**590**	**600**	**610**	**620**	**630**	**640**
SWPRSRPRAP	PKARMALSFS	RFLRRGCLAS	PVFARLSPKC	PAVSHGRVQP	LGDVGQQLPR	LKSKRVANFF	QIKMDVPTGS
**650**	**660**	**670**	**680**				
GTCLMDSEDA	GTGESGDRAT	EKEVICPWES	L				

Cmpd.	No. of Cmpds.	m/z meas.	Δ m/z [ppm]	z	Rt [min]	Score	P	Range	Sequence	Modification	Ratios
2782	1	681.4961	134.76	2	63.3	19.3	1	436-447	R.HLRSSPSPVILR.Q		
461	1	626.3269	-12.81	2	35.8	11.6	1	596-607	R.LSPKCPAVSHGR.V		WUP:QUP 0.17<br>QU:MU 1.13

Detailed Protein Report

Protein 409: steroid hormone receptor ERR2 [Homo sapiens]

**Accession:**	gi|238550159	**Score:**	30.9
**Database:**	refseq_human(refseq_human_20140103.fasta)	**MW [kDa]:**	56.2
**Database Date:**	2015-11-30	**pI:**	9.5
**Modification(s):**	Carbamidomethyl	**Sequence Coverage [%]:**	6.9
		**No. of unique Peptides:**	1

10	20	30	40	50	60	70	80
MSSDDRHLGS	SCGSFIKTEP	SSPSSGIDAL	SHHSPSGSSD	ASGGFGLALG	THANGLDSPP	MFAGAGLGGT	PCRKSYEDCA
**90**	**100**	**110**	**120**	**130**	**140**	**150**	**160**
SGIMEDSAIK	CEYMLNAIPK	RLCLVCGDIA	SGYHYGVASC	EACKAFFKRT	IQGNIEYSCP	ATNECEITKR	RRKSCQACRF
**170**	**180**	**190**	**200**	**210**	**220**	**230**	**240**
MKCLKVGMLK	EGVRLDRVRG	GRQKYKRRLD	SESSPYLSLQ	ISPPAKKPLT	KIVSYLLVAE	PDKLYAMPPP	GMPEGDIKAL
**250**	**260**	**270**	**280**	**290**	**300**	**310**	**320**
TTLCDLADRE	LVVIIGWAKH	IPGFSSLSLG	DQMSLLQSAW	MEILILGIVY	RSLPYDDKLV	YAEDYIMDEE	HSRLAGLLEL
**330**	**340**	**350**	**360**	**370**	**380**	**390**	**400**
YRAILQLVRR	YKKLKVEKEE	FVTLKALALA	NSDSMYIEDL	EAVQKLQDLL	HEALQDYELS	QRHEEPWRTG	KLLLTLPLLR
**410**	**420**	**430**	**440**	**450**	**460**	**470**	**480**
QTAAKAVQHF	YSVKLQGKVP	MHKLFLEMLE	AKVGQEQLRG	SPKDERMSSH	DGKCPFQSAA	FTSRDQSNSP	GIPNPRPSSP
**490**	**500**	**510**					
TPLNERGRQI	SPSTRTPGGQ	GKHLWLTM					

Cmpd.	No. of Cmpds.	m/z meas.	Δ m/z [ppm]	z	Rt [min]	Score	P	Range	Sequence	Modification
1383	2	1179.1487	0.19	2	47.4	17.9	1	239-259	K.ALTTLCDLADRELVVIIGWAK.H	Carbamidomethyl: 6

Detailed Protein Report

Protein 410: PREDICTED: rho GTPase-activating protein 23 isoform X2 [Homo sapiens]

**Accession:**	gi|578831178	**Score:**	30.9
**Database:**	refseq_human(refseq_human_20140103.fasta)	**MW [kDa]:**	153.2
**Database Date:**	2015-11-30	**pI:**	10.0
		**Sequence Coverage [%]:**	1.7
		**No. of unique Peptides:**	1

Alias proteins:

Accession	Name	Description
gi|578840369	refseq_human (refseq_human_20140103.fasta)	PREDICTED: rho GTPase-activating protein 23 isoform X2 [Homo sapiens]

10	20	30	40	50	60	70	80
MNGVAFCLVG	IPPRPEPRPP	QLPLGPRDGC	SPRRPFPWQG	PRTLLLYKSP	QDGFGFTLRH	FIVYPPESAV	HCSLKEEENG
90	100	110	120	130	140	150	160
GRGGGPSPRY	RLEPMDTIFV	KNVKEDGPAH	RAGLRTGDRL	VKVNGESVIG	KTYSQVIALI	QNSDDTLELS	IMPKDEDILQ
170	180	190	200	210	220	230	240
LAYSQDAYLK	GNEPYSGEAR	SIPEPPPICY	PRKTYAPPAR	ASTRATMVPE	PTSALPSDPR	SPAAWSDPGL	RVPPAARAHL
250	260	270	280	290	300	310	320
DNSSLGMSQP	RPSPGAFPHL	SSEPRTPRAF	PEPGSRVPPS	RLECQQALSH	WLSNQVPRRA	GERRCPAMAP	RARSASQDRL
330	340	350	360	370	380	390	400
EEVAAPRPWP	CSTSQDALSQ	LGQEGWHRAR	SDDYLSRATR	SAEALGPGAL	VSPRFERCGW	ASQRSSARTP	ACPTRDLPGP
410	420	430	440	450	460	470	480
QAPPPSGLQG	LDDLGYIGYR	SYSPSFQRRT	GLLHALSFRD	SPFGGLPTFN	LAQSPASFPP	EASEPPRVVR	PEPSTRALEP
490	500	510	520	530	540	550	560
PAEDRGDEVV	LRQKPPTGRK	VQLTPARQMN	LGFGDESPEP	EASGRGERLG	RKVAPLATTE	DSLASIPFID	EPTSPSIDLQ
570	580	590	600	610	620	630	640
AKHVPASAVV	SSAMNSAPVL	GTSPSSPTFT	FTLGRHYSQD	CSSIKAGRRS	SYLLAITTER	SKSCDDGLNT	FRDEGRVLRR
650	660	670	680	690	700	710	720
LPNRIPSLRM	LRSFFTDGSL	DSWGTSEDAD	APSKRHSTSD	LSDATFSDIR	REGWLYYKQI	LTKKGKAEDR	DDMLGWIRAI
730	740	750	760	770	780	790	800
RENSRAEGED	PGCANQALIS	KKLNDYRKVS	HSSGPKADSS	PKGSRGLGGL	KSEFLKQSAA	RGLRTQDLPA	GSKDDSAAAP
810	820	830	840	850	860	870	880
KTPWGINIIK	KNKKAAPRAF	GVRLEECQPA	TENQRVPLIV	AACCRIVEAR	GLESTGIYRV	PGNNAVVSSL	QEQLNRGPGD
890	900	910	920	930	940	950	960
INLQDERWQD	LNVISSLLKS	FFRKLPEPLF	TDDKYNDFIE	ANRIEDARER	MRTLRKLIRD	LPGHYYETLK	FLVGHLKTIA
970	980	990	1000	1010	1020	1030	1040
DHSEKNKMEP	RNLALVFGPT	LVRTSEDNMT	DMVTHMPDRY	KIVETLIQHS	DWFFSDEEDK	GERTPVGDKE	PQAVPNIEYL
1050	1060	1070	1080	1090	1100	1110	1120
LPNIGRTVPP	GDPGSDSTTC	SSAKSKGSWA	PKKEPYAREM	LAISFISAVN	RKRKKRREAR	GLGSSTDDDS	EQEAHKPGAG
1130	1140	1150	1160	1170	1180	1190	1200
ATAPGTQERP	QGPLPGAVAP	EAPGRLSPPA	APEERPAADT	RSIVSGYSTL	STMDRSVCSG	ASGRRAGAGD	EADDERSELS
1210	1220	1230	1240	1250	1260	1270	1280
HVETDTEGAA	GAGPGGRLTR	RPSFSSHHLM	PCDTLARRRL	ARGRPDGEGA	GRGGPRAPEP	PGSASSSSQE	SLRPPAAALA
1290	1300	1310	1320	1330	1340	1350	1360
SRPSRMEALR	LRLRGTADDM	LAVRLRRPLS	PETRRRRSSW	RRHTVVVQSP	LTDLNFNEWK	ELGGGGPPEP	AGARAHSDNK
1370	1380	1390	1400	1410			
DSGLSSLEST	KARAPSSAAS	QPPAPGDTGS	LQSQPPRRSA	ASRLHQCL			

Cmpd.	No. of Cmpds.	m/z meas.	Δ m/z [ppm]	z	Rt [min]	Score	P	Range	Sequence	Modification
2089	2	734.3401	-78.89	2	54.5	12.7	2	1239-1252	R.RLARGRPDGEGAGR.G	

Detailed Protein Report

Protein 411: **PREDICTED: maestro heat-like repeat-containing protein family member 1 isoform X10 [Homo sapiens]**

Accession: gi|578816157
Database: refseq_human(refseq_human_20140103.fasta)
Database Date: 2015-11-30

Score: 30.9
MW [kDa]: 163.0
pI: 6.3
Sequence Coverage [%]: 1.8
No. of unique Peptides: 1

10	20	30	40	50	60	70	80
MTESSMKKLA	STLLDAITDK	DPLVQEQVCS	ALCSLGEARP	VETLRACEEY	LRQHDKLAHP	YRAAVLRAME	RVLSSRASEL
90	100	110	120	130	140	150	160
DKDTASTIIL	LASSEMTKTK	DLVWDWQQAA	SGVLVAVGRQ	FISKVMEELL	RRLHPGTLPH	CAVLHTLASL	SVANAFGVVP
170	180	190	200	210	220	230	240
FLPSVLSSLL	PVLGVAKQDT	VRVAFCSALQ	RFSEGALEYL	ANLDRAPDPT	VRKDAFATDI	FSAYDVLFHQ	WLQSREAKLR
250	260	270	280	290	300	310	320
LAVVEALGPM	SHLLPSERLE	EQLPKLLPGI	LALYKKHAET	FYLSKSLGQI	LEAAVSVGSR	TLETQLDALL	AALHSQICVP
330	340	350	360	370	380	390	400
VESSSPLVMS	NQKEVLRCFT	VLACSSPDRL	LAFLLPRLDT	SNERTRVGTL	QVVRHVINSA	AAQMEDKKPF	ILSSMRLPLL
410	420	430	440	450	460	470	480
DTNSKVKRAV	VQVISAMAHH	GYLEQPGGEA	MIEYIVQQCA	LPPEQEPEKP	GPGSKDPKAD	SVRAISVRTL	YLVSTTVDRM
490	500	510	520	530	540	550	560
SHVLWPYLLQ	FLTPVRFTGA	LTPLCRSLVH	LAQKRQEAGA	DAFLIQYDAH	ASLPSPYAVT	GRLLVVSSSP	YLGDGRGAAA
570	580	590	600	610	620	630	640
LRLLSVLHPN	IHPLLGQHWE	TTVPLLLGYL	DEHTEETLPQ	EEWEEKLLMF	LRDTLAIISD	NAWICQLSLE	LCRQLPCYDE
650	660	670	680	690	700	710	720
APQEKNFLYK	CIGTTLGAAS	SKEVVRKHLQ	ELLETARYQE	EAEREGLACC	FGICAISHLE	DTLAQLEDFV	RSEVFRKSIG
730	740	750	760	770	780	790	800
ILNIFKDRSE	NEVEKVKSAL	ILCYGHVAAR	APRELVLAKV	ESDILRNICQ	HFSTKVLGIK	VETKDPALKL	CLVQSVCMVS
810	820	830	840	850	860	870	880
RAICSSTQAG	SFHFTRKAEL	VAQMMEFIRA	EPPDSLRTPI	RKKAMLTCTY	LVSVEPALDE	QARADVIHGC	LHSIMALLPE
890	900	910	920	930	940	950	960
PKEEDGGCQK	SLYLETLHAL	EDLLTSLLQR	NMTPQGLQIM	IEHLSPWIKS	PRGHERARAL	GLSALLLRYF	LEHLRVSALV
970	980	990	1000	1010	1020	1030	1040
PFHNLGLLIG	LFSPRCADLW	PATRQEAVDC	VYSLLYLQLG	YEGFSRDYRD	DVAERLLSLK	DGLVHPDPAI	LFHTCHSVGQ
1050	1060	1070	1080	1090	1100	1110	1120
IIAKRLPPDQ	LISLLLTMFE	ALGDPEKNCS	RAATVMINCL	LQERGGVLQE	KVPEIVSVLR	SKLQEAQGEH	VLPAAQHSVY
1130	1140	1150	1160	1170	1180	1190	1200
LLATQHCAAV	VSSLLGSPLP	LDSHTCMLWR	ALAVEPRLAA	QVLGLLLEKM	SRDVPFKESR	AFLLGRTPDR	VATLLPLSAT
1210	1220	1230	1240	1250	1260	1270	1280
CALFEVMSTP	AAGPAVLELY	PQLFVVLLLR	VSCTVGVQLP	RNLQAQERRG	ASPALATRNL	EPCSSAVDTL	RSMLLRSGSE
1290	1300	1310	1320	1330	1340	1350	1360
DVVQRMDLEG	GWELLRTSAG	HEEGATRLAR	AMAEHAGPRL	PLVLKTLACT	HSSAYENQRV	TTTAFLAELL	NSNVANDLML
1370	1380	1390	1400	1410	1420	1430	1440
LDSLLESLAA	RQKDTCASVR	RLVLRGLANL	ASGCPDKVRT	HGPQLLTAMI	GGLDDGDNPH	SPVALEAMLG	LARLVHLVES
1450	1460	1470	1480	1490			
WDLRSGLLHV	AIRIRPFFDS	VTVESADLQG	CCFAGPGSPH	P			

Cmpd.	No. of Cmpds.	m/z meas.	Δ m/z [ppm]	z	Rt [min]	Score	P	Range	Sequence	Modification
2554	1	821.4177	-87.55	2	61.9	12.2	1	1158-1172	R.LAAQVLGLLLEKMSR.D	

Detailed Protein Report

Protein 412: NADH-ubiquinone oxidoreductase 75 kDa subunit, mitochondrial isoform 5 [Homo sapiens]

Accession: gi|316983160
Database: refseq_human(refseq_human_20140103.fasta)
Database Date: 2015-11-30
Modification(s): Oxidation

Score: 30.8
MW [kDa]: 80.9
pI: 6.2
Sequence Coverage [%]: 2.7
No. of unique Peptides: 1

Quantitation

WUP:QUP **Median:** 2.24 **CV:** 0.00 % **No. of Peptides:** 1

10	20	30	40	50	60	70	80
MRIRGSSGTL	SRINMLRIPV	RKALVGLSKS	PKGCVRTTAT	AASNLIEVFV	DGQSVMVEPG	TTVLQACEKV	GMQIPRFCYH
90	100	110	120	130	140	150	160
ERLSVAGNCR	MCLVEIEKAP	KVVAACAMPV	MKGWNILTNS	EKSKKAREGV	MEFLLANHPL	DCPICDQGGE	CDLQDQSMMF
170	180	190	200	210	220	230	240
GNDRSRFLEG	KRAVEDKNIG	PLVKTIMTRC	IQCTRCIRFA	SEIAGVDDLG	TTGRGNDMQV	GTYIEKMFMS	ELSGNIIDIC
250	260	270	280	290	300	310	320
PVGALTSKPY	AFTARPWETR	KTESIDVMDA	VGSNIVVSTR	TGEVMRILPR	MHEDINEEWI	SDKTRFAYDG	LKRQRLTEPM
330	340	350	360	370	380	390	400
VRNEKGLLTY	TSWEDALSRV	AGMLQSFQGK	DVAAIAGGLV	DAEALVALKD	LLNRVDSDTL	CTEEVFPTAG	AGTDLRSNYL
410	420	430	440	450	460	470	480
LNTTIAGVEE	ADVVLLVGTN	PRFEAPLFNA	RIRKSWLHND	LKVALIGSPV	DLTYTYDHLG	DSPKILQDIA	SGSHPFSQVL
490	500	510	520	530	540	550	560
KEAKKPMVVL	GSSALQRNDG	AAILAAVSSI	AQKIRMTSGV	TGDWKVMNIL	HRIASQVAAL	DLGYKPGVEA	IRKNPPKVLF
570	580	590	600	610	620	630	640
LLGADGGCIT	RQDLPKDCFI	IYQGHHGDVG	APIADVILPG	AAYTEKSATY	VNTEGRAQQT	KVAVTPPGLA	REDWKIIRAL
650	660	670	680	690	700	710	720
SEIAGMTLPY	DTLDQVRNRL	EEVSPNLVRY	DDIEGANYFQ	QANELSKLVN	QQLLADPLVP	PQLTIKDFYM	TDSISRASQT
730	740	750					
MAKCVKAVTE	GAQAVEEPSI	C					

Cmpd.	No. of Cmpds.	m/z meas.	Δ m/z [ppm]	z	Rt [min]	Score	P	Range	Sequence	Modification	Ratios
2481	1	449.6389	-246.99	2	61.0	14.1	0	526-532	K.VMNILHR.I	Oxidation: 2	WUP:QUP 2.24

Detailed Protein Report

Protein 413: scaffold attachment factor B1 isoform 4 [Homo sapiens]

**Accession:**	gi|321267473	**Score:**	30.8
**Database:**	refseq_human(refseq_human_20140103.fasta)	**MW [kDa]:**	95.1
**Database Date:**	2015-11-30	**pI:**	6.0
		**Sequence Coverage [%]:**	2.6
		**No. of unique Peptides:**	1

Quantitation

QU:MU	**Median:**	4.03	**CV:**	0.00 %	**No. of Peptides:**	1
WUP:QUP	**Median:**	3.65	**CV:**	0.00 %	**No. of Peptides:**	1

10	20	30	40	50	60	70	80
MAETLSGLGD	SGAAGAAALS	SASSETGTRR	LSDLRVIDLR	AELRKRNVDS	SGNKSVLMER	LKKAIEDEGG	NPDEIEITSE
**90**	**100**	**110**	**120**	**130**	**140**	**150**	**160**
GNKKTSKRSS	KGRKPEEEGV	EDNGLEENSG	DGQIEDKETI	NNLDTSSSDF	TILQEIEEPS	LEPENEKILD	ILGETCKSEP
**170**	**180**	**190**	**200**	**210**	**220**	**230**	**240**
VKEESSELEQ	PFAQDTSSVG	PDRKLAEEED	LFDSAHPEEG	DLDLASESTA	HAQSSKADSL	LAVVKREPAE	QPGDGERTDC
**250**	**260**	**270**	**280**	**290**	**300**	**310**	**320**
EPVGLEPAVE	QSSAASELAE	ASSEELAEAP	TEAPSPEARD	SKEDGRKFDF	DACNEVPPAP	KESSTSEGAD	QKMSSPEDDS
**330**	**340**	**350**	**360**	**370**	**380**	**390**	**400**
DTKRLSKEEK	GRSSCGRNFW	VSGLSSTTRA	TDLKNLFSKY	GKVVGAKVVT	NARSPGARCY	GFVTMSTAEE	ATKCINHLHK
**410**	**420**	**430**	**440**	**450**	**460**	**470**	**480**
TELHGKMISV	EKAKNEPVGK	KTSDKRDSDG	KKEKSSNSDR	STNLKRDDKC	DRKDDAKKGD	DGSGEKSKDQ	DDQKPGPSER
**490**	**500**	**510**	**520**	**530**	**540**	**550**	**560**
SRATKSGSRG	TERTVVMDKS	KGVPVISVKT	SGSKERASKS	QDRKSASREK	RSVVSFDKVK	EPRKSRDSES	HSRVRERSER
**570**	**580**	**590**	**600**	**610**	**620**	**630**	**640**
EQRMQAQWER	EERERLEIAR	ERLAFQRQRL	ERERMERERL	ERERMHVEHE	RRREQERIHR	EREELRRQQE	LRYEQERRPA
**650**	**660**	**670**	**680**	**690**	**700**	**710**	**720**
VRRPYDLDRR	DDAYWPEAKR	AALDERYHSD	FNRQDRFHDF	DHRDRGRYPD	HSVDRREGSR	SMMGEREGQH	YPERHGGPER
**730**	**740**	**750**	**760**	**770**	**780**	**790**	**800**
HGRDSRDGWG	GYGSDKRMSE	GRGLPPPPRG	RRDWGDHGRR	EDDRSWQGTA	DGGMMDRDHK	RWQGGERSMS	GHSGPGHMMN
**810**	**820**	**830**	**840**	**850**			
RGGMSGRGSF	APGGASRGHP	IPHGGMQGGF	GGQSRGSRPS	DARFTRRY			

Cmpd.	No. of Cmpds.	m/z meas.	Δ m/z [ppm]	z	Rt [min]	Score	P	Range	Sequence	Modification	Ratios
980	2	530.6065	-255.21	2	40.6	14.1	1	545-553	K.SRDSESHSR.V		WUP:QUP 3.65<br>QU:MU 4.03

Detailed Protein Report

Protein 414: putative sodium-coupled neutral amino acid transporter 10 isoform a [Homo sapiens]

Accession: gi|83921602
Database: refseq_human(refseq_human_20140103.fasta)
Database Date: 2015-11-30
Modification(s): Oxidation

Score: 30.8
MW [kDa]: 119.7
pI: 5.4
Sequence Coverage [%]: 3.5
No. of unique Peptides: 2

Quantitation

QU:MU **Median:** 0.94 **CV:** 0.00 % **No. of Peptides:** 1

10	20	30	40	50	60	70	80
MTAAAASNWG	LITNIVNSIV	GVSVLTMPFC	FKQCGIVLGA	LLLVFCSWMT	HQSCMFLVKS	ASLSKRRTYA	GLAFHAYGKA
**90**	**100**	**110**	**120**	**130**	**140**	**150**	**160**
GKMLVETSMI	GLMLGTCIAF	YVVIGDLGSN	FFARLFGFQV	GGTFRMFLLF	AVSLCIVLPL	SLQRNMMASI	QSFSAMALLF
**170**	**180**	**190**	**200**	**210**	**220**	**230**	**240**
YTVFMFVIVL	SSLKHGLFSG	QWLRRVSYVR	WEGVFRCIPI	FGMSFACQSQ	VLPTYDSLDE	PSVKTMSSIF	ASSLNVVTTF
**250**	**260**	**270**	**280**	**290**	**300**	**310**	**320**
YVMVGFFGYV	SFTEATAGNV	LMHFPSNLVT	EMLRVGFMMS	VAVGFPMMIL	PCRQALSTLL	CEQQQKDGTF	AAGGYMPPLR
**330**	**340**	**350**	**360**	**370**	**380**	**390**	**400**
FKALTLSVVF	GTMVGGILIP	NVETILGLTG	ATMGSLICFI	CPALIYKKIH	KNALSSQVVL	WVGLGVLVVS	TVTTLSVSEE
**410**	**420**	**430**	**440**	**450**	**460**	**470**	**480**
VPEDLAEEAP	GGRLGEAEGL	MKVEAARLSA	QDPVVAVAED	GREKPKLPKE	REELEQAQIK	GPVDVPGRED	GKEAPEEAQL
**490**	**500**	**510**	**520**	**530**	**540**	**550**	**560**
DRPGQGIAVP	VGEAHRHEPP	VPHDKVVVDE	GQDREVPEEN	KPPSRHAGGK	APGVQGQMAP	PLPDSEREKQ	EPEQGEVGKR
**570**	**580**	**590**	**600**	**610**	**620**	**630**	**640**
PGQAQALEEA	GDLPEDPQKV	PEADGQPAVQ	PAKEDLGPGD	RGLHPRPQAV	LSEQQNGLAV	GGGEKAKGGP	PPGNAAGDTG
**650**	**660**	**670**	**680**	**690**	**700**	**710**	**720**
QPAEDSDHGG	KPPLPAEKPA	PGPGLPPEPR	EQRDVERAGG	NQAASQLEEA	GRAEMLDHAV	LLQVIKEQQV	QQKRLLDQQE
**730**	**740**	**750**	**760**	**770**	**780**	**790**	**800**
KLLAVIEEQH	KEIHQQRQED	EEDKPRQVEV	HQEPGAAVPR	GQEAPEGKAR	ETVENLPPLP	LDPVLRAPGG	RPAPSQDLNQ
**810**	**820**	**830**	**840**	**850**	**860**	**870**	**880**
RSLEHSEGPV	GRDPAGPPDG	GPDTEPRAAQ	AKLRDGQKDA	APRAAGTVKE	LPKGPEQVPV	PDPAREAGGP	EERLAEEFPG
**890**	**900**	**910**	**920**	**930**	**940**	**950**	**960**
QSQDVTGGSQ	DRKKPGKEVA	ATGTSILKEA	NWLVAGPGAE	TGDPRMKPKQ	VSRDLGLAAD	LPGGAEGAAA	QPQAVLRQPE
**970**	**980**	**990**	**1000**	**1010**	**1020**	**1030**	**1040**
LRVISDGEQG	GQQGHRLDHG	GHLEMRKARG	GDHVPVSHEQ	PRGGEDAAVQ	EPRQRPEPEL	GLKRAVPGGQ	RPDNAKPNRD
**1050**	**1060**	**1070**	**1080**	**1090**	**1100**	**1110**	**1120**
LKLQAGSDLR	RRRRDLGPHA	EGQLAPRDGV	IIGLNPLPDV	QVNDLRGALD	AQLRQAAGGA	LQVVHSRQLR	QAPGPPEES

Cmpd.	No. of Cmpds.	m/z meas.	Δ m/z [ppm]	z	Rt [min]	Score	P	Range	Sequence	Modification	Ratios
2229	1	701.2999	-60.69	3	57.7	11.7	0	275-293	R.VGFMMSVAVGFPMMILPCR.Q	Oxidation: 13	QU:MU 0.94
203	1	1043.9225	-84.97	2	32.5	19.1	1	854-873	K.GPEQVPVPDPAREAGGPEER.L		

Detailed Protein Report

Protein 415: **PREDICTED: nuclear envelope pore membrane protein POM 121C isoform X1 [Homo sapiens]**

Accession: gi|578813833
Database: refseq_human(refseq_human_20140103.fasta)
Database Date: 2015-11-30
Modification(s): Carbamidomethyl

Score: 30.8
MW [kDa]: 128.0
pI: 11.2
Sequence Coverage [%]: 2.7
No. of unique Peptides: 2

10	20	30	40	50	60	70	80
MTWRARDDAT	RGAVHAGIFK	SPPRRGAAMS	PAAAAAGAGE	RRRPIASVRD	GRGRGCGGPA	GAALLGLSLV	GLLLYLVPAA
90	100	110	120	130	140	150	160
AALAWLAVGT	TAAWWGLSRE	PRGSRPLSSF	VQKARHRRTL	FASPPAKSTA	NGNLLEPRTL	LEGPDPAELL	LMGSYLGKPG
170	180	190	200	210	220	230	240
PPQPAPAPEG	QDLRNRPGRR	PPARPAPRST	PPSQPTHRVH	HFYPSLPTPL	LRPSGRPSPR	DRGTLPDRFV	ITPRRRYPIH
250	260	270	280	290	300	310	320
QTQYSCPGVL	PTVCWNGYHK	KAVLSPRNSR	MVCSPVTVRI	APPDRRFSRS	AIPEQIISST	LSSPSSNAPD	PCAKETVLSA
330	340	350	360	370	380	390	400
LKEKKKKRTV	EEEDQIFLDG	QENKRRRHDS	SGSGHSAFEP	LVASGVPASF	VPKPGSLKRG	LNSQSSDDHL	NKRSRSSSMS
410	420	430	440	450	460	470	480
SLTGAYTSGI	PSSSRNAITS	SYSSTRGISQ	LWKRNGPSSS	PFSSPASSRS	QTPERPAKKI	REEELCHHSS	SSTPLAADKE
490	500	510	520	530	540	550	560
SQGEKAADTT	PRKKQNSNSQ	STPGSSGQRK	RKVQLLPSRR	GEQLTLPPPP	QLGYSITAED	LDLEKKASLQ	WFNQALEDKS
570	580	590	600	610	620	630	640
DAASNSVTET	PPTTQPSFTF	TLPAAATASP	PTSLLAPSTN	PLLESLKKMQ	TPPSLPPCPE	SAGAATTEAL	SPPKTPSLLP
650	660	670	680	690	700	710	720
PLGLSQSGPP	GLLPSPSFDS	KPPTTLLGLI	PAPSMVPATD	TKAPPTLQAE	TATKPQATSA	PSPAPKQSFL	FGTQNTSPSS
730	740	750	760	770	780	790	800
PAAPAASSAS	PMFKPIFTAP	PKSEKEGLTP	PGPSVSATAP	SSSSLPTTTS	TTAPTFQPVF	SSMGPPASVP	LPAPFFKQTT
810	820	830	840	850	860	870	880
TPATAPTTTA	PLFTGLASAT	SAVAPITSAS	PSTDSASKPA	FGFGINSVSS	SSVSTTTSTA	TAASQPFLFG	APQASAASFT
890	900	910	920	930	940	950	960
PAMGSIFQFG	KPPALPTTTT	VTTFSQSLPT	AVPTATSSSA	ADFSGFGSTL	ATSAPATSSQ	PTLTFSNTST	PTFNIPFGSS
970	980	990	1000	1010	1020	1030	1040
AKSPLPSYPG	ANPQPAFGAA	EGQPPGAAKP	ALTPSFGSSF	TFGNSAAPAP	ATAPTPAPAS	TIKIVPAHVP	TPIQPTFGGA
1050	1060	1070	1080	1090	1100	1110	1120
THSAFGLKAT	ASAFGAPASS	QPAFGGSTAV	FSFGAATSSG	FGATTQTASS	GSSSSVFGST	TPSPFTFGGS	AAPAGSGSFG
1130	1140	1150	1160	1170	1180	1190	1200
INVATPGSSA	TTGAFSFGAG	QSGSTATSTP	FTGGLGQNAL	GTTGQSTPFA	FNVGSTTESK	PVFGGTATPT	FGQNTPAPGV
1210	1220	1230	1240	1250	1260		
GTSGSSLSFG	ASSAPAQGFV	GVGPFGSAAP	SFSIGAGSKT	PGARQRLQAR	RQHTRKK		

Cmpd.	No. of Cmpds.	m/z meas.	Δ m/z [ppm]	z	Rt [min]	Score	P	Range	Sequence	Modification
2272	1	703.3240	-44.91	2	56.7	10.4	1	268-279	R.NSRMVCSPVTVR.I	Carbamidomethyl: 6
39	1	1103.4999	-21.74	2	30.2	20.4	1	394-415	R.SRSSSMSSLTGAYTSGIPSSSR.N	

Detailed Protein Report

Protein 416:	amyloid beta A4 precursor protein-binding family B member 1 isoform b [Homo sapiens]

**Accession:**	gi|22035554	**Score:**	30.7
**Database:**	refseq_human(refseq_human_20140103.fasta)	**MW [kDa]:**	76.9
**Database Date:**	2015-11-30	**pI:**	4.8
		**Sequence Coverage [%]:**	5.6
		**No. of unique Peptides:**	1

10	20	30	40	50	60	70	80
MSVPSSLSQS	AINANSHGGP	ALSLPLPLHA	AHNQLLNAKL	QATAVGPKDL	RSAMGEGGGP	EPGPANAKWL	KEGQNQLRRA
**90**	**100**	**110**	**120**	**130**	**140**	**150**	**160**
ATAHRDQNRN	VTLTLAEEAS	QEPEMAPLGP	KGLIHLYSEL	ELSAHNAANR	GLRGPGLIIS	TQEQGPDEGE	EKAAGEAEEE
**170**	**180**	**190**	**200**	**210**	**220**	**230**	**240**
EEDDDDEEEE	EDLSSPPGLP	EPLESVEAPP	RPQALTDGPR	EHSKSASLLF	GMRNSAASDE	DSSWATLSQG	SPSYGSPEDT
**250**	**260**	**270**	**280**	**290**	**300**	**310**	**320**
DSFWNPNAFE	TDSDLPAGWM	RVQDTSGTYY	WHIPTGTTQW	EPPGRASPSQ	GSSPQEESQL	TWTGFAHGEG	FEDGEFWKDE
**330**	**340**	**350**	**360**	**370**	**380**	**390**	**400**
PSDEAPMELG	LKEPEEGTLT	FPAQSLSPEP	LPQEEEKLPP	RNTNPGIKCF	AVRSLGWVEM	TEEELAPGRS	SVAVNNCIRQ
**410**	**420**	**430**	**440**	**450**	**460**	**470**	**480**
LSYHKNNLHD	PMSGGWGEGK	DLLLQLEDET	LKLVEPQSQA	LLHAQPIISI	RVWGVGRDSG	RDFAYVARDK	LTQMLKCHVF
**490**	**500**	**510**	**520**	**530**	**540**	**550**	**560**
RCEAPAKNIA	TSLHEICSKI	MAERRNARCL	VNGLSLDHSK	LVDVPFQVEF	PAPKNELVQK	FQVYYLGNVP	VAKPVGVDVI
**570**	**580**	**590**	**600**	**610**	**620**	**630**	**640**
NGALESVLSS	SSREQWTPSH	VSVAPATLTI	LHQQTEAVLG	ECRVRFLSFL	AVGRDVHTFA	FIMAAGPASF	CCHMFWCEPN
**650**	**660**	**670**	**680**	**690**	**700**	**710**	
AASLSEAVQA	ACMLRYQKCL	DARSQASTSC	LPAPPAESVA	RRVGWTVRRG	VQSLWGSLKP	KRLGAHTP	

Cmpd.	No. of Cmpds.	m/z meas.	Δ m/z [ppm]	z	Rt [min]	Score	P	Range	Sequence	Modification
2594	1	946.7123	-95.55	3	60.7	13.7	1	86-111	R.DQNRNVTLTLAEEASQEPEMAPLGPK.G	

Detailed Protein Report

Protein 417: ras/Rap GTPase-activating protein SynGAP [Homo sapiens]

Accession: gi|194248068
Database: refseq_human(refseq_human_20140103.fasta)
Database Date: 2015-11-30
Modification(s): Oxidation

Score: 30.6
MW [kDa]: 148.2
pI: 9.8
Sequence Coverage [%]: 1.6
No. of unique Peptides: 1

10	20	30	40	50	60	70	80
MSRSRASIHR	GSIPAMSYAP	FRDVRGPSMH	RTQYVHSPYD	RPGWNPRFCI	ISGNQLLMLD	EDEIHPLLIR	DRRSESSRNK
90	100	110	120	130	140	150	160
LLRRTVSVPV	EGRPHGEHEY	HLGRSRRKSV	PGGKQYSMEG	APAAPFRPSQ	GFLSRRLKSS	IKRTKSQPKL	DRTSSFRQIL
170	180	190	200	210	220	230	240
PRFRSADHDR	ARLMQSFKES	HSHESLLSPS	SAAEALELNL	DEDSIIKPVH	SSILGQEFCF	EVTTSSGTKC	FACRSAAERD
250	260	270	280	290	300	310	320
KWIENLQRAV	KPNKDNSRRV	DNVLKLWIIE	ARELPPKKRY	YCELCLDDML	YARTTSKPRS	ASGDTVFWGE	HFEFNNLPAV
330	340	350	360	370	380	390	400
RALRLHLYRD	SDKKRKKDKA	GYVGLVTVPV	ATLAGRHFTE	QWYPVTLPTG	SGGSGGMGSG	GGGGSGGGSG	GKGKGGCPAV
410	420	430	440	450	460	470	480
RLKARYQTMS	ILPMELYKEF	AEYVTNHYRM	LCAVLEPALN	VKGKEEVASA	LVHILQSTGK	AKDFLSDMAM	SEVDRFMERE
490	500	510	520	530	540	550	560
HLIFRENTLA	TKAIEEYMRL	IGQKYLKDAI	GEFIRALYES	EENCEVDPIK	CTASSLAEHQ	ANLRMCCELA	LCKVVNSHCV
570	580	590	600	610	620	630	640
FPRELKEVFA	SWRLRCAERG	REDIADRLIS	ASLFLRFLCP	AIMSPSLFGL	MQEYPDEQTS	RTLTLIAKVI	QNLANFSKFT
650	660	670	680	690	700	710	720
SKEDFLGFMN	EFLELEWGSM	QQFLYEISNL	DTLTNSSSFE	GYIDLGRELS	TLHALLWEVL	PQLSKEALLK	LGPLPRLLND
730	740	750	760	770	780	790	800
ISTALRNPNI	QRQPSRQSER	PRPQPVVLRG	PSAEMQGYMM	RDLNSSIDLQ	SFMARGLNSS	MDMARLPSPT	KEKPPPPPPG
810	820	830	840	850	860	870	880
GGKDLFYVSR	PPLARSSPAY	CTSSSDITEP	EQKMLSVNKS	VSMLDLQGDG	PGGRLNSSSV	SNLAAVGDLL	HSSQASLTAA
890	900	910	920	930	940	950	960
LGLRPAPAGR	LSQGSGSSIT	AAGMRLSQMG	VTTDGVPAQQ	LRIPLSFQNP	LFHMAADGPG	PPGGHGGGGG	HGPPSSHHHH
970	980	990	1000	1010	1020	1030	1040
HHHHHHRGGE	PPGDTFAPFH	GYSKSEDLSS	GVPKPPAASI	LHSHSYSDEF	GPSGTDFTRR	QLSLQDNLQH	MLSPPQITIG
1050	1060	1070	1080	1090	1100	1110	1120
PQRPAPSGPG	GGSGGGSGGG	GGGQPPPLQR	GKSQQLTVSA	AQKPRPSSGN	LLQSPEPSYG	PARPRQQSLS	KEGSIGGSGG
1130	1140	1150	1160	1170	1180	1190	1200
SGGGGGGGLK	PSITKQHSQT	PSTLNPTMPA	SERTVAWVSN	MPHLSADIES	AHIEREEYKL	KEYSKSMDES	RLDRVKEYEE
1210	1220	1230	1240	1250	1260	1270	1280
EIHSLKERLH	MSNRKLEEYE	RRLLSQEEQT	SKILMQYQAR	LEQSEKRLRQ	QQAEKDSQIK	SIIGRLMLVE	EELRRDHPAM
1290	1300	1310	1320	1330	1340	1350	
AEPLPEPKKR	LLDAQERQLP	PLGPTNPRVT	LAPPWNGLAP	PAPPPPPRLQ	ITENGEFRNT	ADH	

Cmpd.	No. of Cmpds.	m/z meas.	Δ m/z [ppm]	z	Rt [min]	Score	P	Range	Sequence	Modification
1986	1	687.3345	70.59	2	55.2	10.1	0	750-761	R.GPSAEMQGYMMR.D	Oxidation: 10

Detailed Protein Report

Protein 418: **PREDICTED: uncharacterized protein C4orf50 [Homo sapiens]**

**Accession:**	gi|530358255	**Score:**	30.6
**Database:**	refseq_human(refseq_human_20140103.fasta)	**MW [kDa]:**	97.6
**Database Date:**	2015-11-30	**pI:**	5.4
		**Sequence Coverage [%]:**	3.4
		**No. of unique Peptides:**	1

10	20	30	40	50	60	70	80
MPLLQGEASV	SKEGPESLSR	RERVEGYVWG	LRGGLSSENE	EVAPPATFSR	AHETKEPWPT	DSQLLAGKVR	ATGRTARGKE
**90**	**100**	**110**	**120**	**130**	**140**	**150**	**160**
ENQVWLGNAL	LLQGGSLEDK	GLEEEDEMPH	QEASGLGCRG	APEEPDSQEH	ESKEMLFFAG	ETGLPLFPRF	ALSVEGAEPT
**170**	**180**	**190**	**200**	**210**	**220**	**230**	**240**
DHGHPQAVSK	GHDRCALTID	ELAQDVEACF	QQLSTLQPGS	RGWQCSASAC	RGENWSFAQK	WHSGWERAHS	QQVWGNWGIC
**250**	**260**	**270**	**280**	**290**	**300**	**310**	**320**
SNEEAKSKES	GEGDKPGKTT	ALGTSEVPGN	PGTLPHWDEA	SPNPPQGPAE	PWGALERVRS	RFHQLISGLK	KQRSQILHDN
**330**	**340**	**350**	**360**	**370**	**380**	**390**	**400**
TKLHGDQERF	HERVCALERE	REREVTKISR	LERDNHRLVG	DISQLKKELD	QYLQAISDLE	DCNGKSYCKI	LELEEENETL
**410**	**420**	**430**	**440**	**450**	**460**	**470**	**480**
KGNLGQLQKA	TSESVRKSKD	TMEQVTLENW	KLQTLISELG	VSYKELIKDI	VLGIEDMIRA	LSGENEHLLR	RVHVLEREVT
**490**	**500**	**510**	**520**	**530**	**540**	**550**	**560**
LQRSTDQGRL	VRGREHLQGK	AKMHALDKEV	QVTPLTGQLL	SRACGPPLEE	EMSLAAGQTG	PSTGTGNSRR	GADSPPPSLV
**570**	**580**	**590**	**600**	**610**	**620**	**630**	**640**
WRNTGVANAL	QGNVSGAEVK	EAHLEKEEKR	PRCSVAQGQA	LSSLSNGPML	RDSEAEVTEE	DPRLRAQQLH	HRVLTLQCQL
**650**	**660**	**670**	**680**	**690**	**700**	**710**	**720**
RDQGAAHQAS	LDEATRLQEE	LQAKLEELQK	KQHEAKLAVT	PLKAKIASLV	RKCRERNRLI	THLLQELHRH	GLGNLLLSEL
**730**	**740**	**750**	**760**	**770**	**780**	**790**	**800**
AQNMLNDVAL	AEYTATFLAP	GVPETSHHLD	VKSEMTAALR	AQTYLLNPEM	DSVLQSSLSS	ESWPVPEPEW	PAQTAQLDSL
**810**	**820**	**830**	**840**	**850**	**860**	**870**	**880**
KLPLSLVSTL	DPGTCLAAVT	VEPGLPAQRL	QEKGGMPCPA	LQVDNVPAPS	ELLSPARILA	FHQELRQSIC	SNSQVHKSPL
**890**							
ELEM							

Cmpd.	No. of Cmpds.	m/z meas.	Δ m/z [ppm]	z	Rt [min]	Score	P	Range	Sequence	Modification
1506	1	1058.4824	21.34	2	48.5	10.4	1	202-220	R.GWQCSASACRGENWSFAQK.W	

Detailed Protein Report

Protein 419: hypoxia up-regulated protein 1 precursor [Homo sapiens]

**Accession:**	gi|5453832	**Score:**	30.5
**Database:**	refseq_human(refseq_human_20140103.fasta)	**MW [kDa]:**	111.3
**Database Date:**	2015-11-30	**pI:**	5.0
		**Sequence Coverage [%]:**	1.4
		**No. of unique Peptides:**	1

Alias proteins:

Accession	Name	Description
gi|195976805	refseq_human (refseq_human_20140103.fasta)	hypoxia up-regulated protein 1 precursor [Homo sapiens]

10	20	30	40	50	60	70	80
MADKVRRQRP	RRRVCWALVA	VLLADLLALS	DTLAVMSVDL	GSESMKVAIV	KPGVPMEIVL	NKESRRKTPV	IVTLKENERF
**90**	**100**	**110**	**120**	**130**	**140**	**150**	**160**
FGDSAASMAI	KNPKATLRYF	QHLLGKQADN	PHVALYQARF	PEHELTFDPQ	RQTVHFQISS	QLQFSPEEVL	GMVLNYSRSL
**170**	**180**	**190**	**200**	**210**	**220**	**230**	**240**
AEDFAEQPIK	DAVITVPVFF	NQAERRAVLQ	AARMAGLKVL	QLINDNTATA	LSYGVFRRKD	INTTAQNIMF	YDMGSGSTVC
**250**	**260**	**270**	**280**	**290**	**300**	**310**	**320**
TIVTYQMVKT	KEAGMQPQLQ	IRGVGFDRTL	GGLEMELRLR	ERLAGLFNEQ	RKGQRAKDVR	ENPRAMAKLL	REANRLKTVL
**330**	**340**	**350**	**360**	**370**	**380**	**390**	**400**
SANADHMAQI	EGLMDDVDFK	AKVTRVEFEE	LCADLFERVP	GPVQQALQSA	EMSLDEIEQV	ILVGGATRVP	RVQEVLLKAV
**410**	**420**	**430**	**440**	**450**	**460**	**470**	**480**
GKEELGKNIN	ADEAAAMGAV	YQAAALSKAF	KVKPFVVRDA	VVYPILVEFT	REVEEEPGIH	SLKHNKRVLF	SRMGPYPQRK
**490**	**500**	**510**	**520**	**530**	**540**	**550**	**560**
VITFNRYSHD	FNFHINYGDL	GFLGPEDLRV	FGSQNLTTVK	LKGVGDSFKK	YPDYESKGIK	AHFNLDESGV	LSLDRVESVF
**570**	**580**	**590**	**600**	**610**	**620**	**630**	**640**
ETLVEDSAEE	ESTLTKLGNT	ISSLFGGGTT	PDAKENGTDT	VQEEEESPAE	GSKDEPGEQV	ELKEEAEAPV	EDGSQPPPPE
**650**	**660**	**670**	**680**	**690**	**700**	**710**	**720**
PKGDATPEGE	KATEKENGDK	SEAQKPSEKA	EAGPEGVAPA	PEGEKKQKPA	RKRRMVEEIG	VELVVLDLPD	LPEDKLAQSV
**730**	**740**	**750**	**760**	**770**	**780**	**790**	**800**
QKLQDLTLRD	LEKQEREKAA	NSLEAFIFET	QDKLYQPEYQ	EVSTEEQREE	ISGKLSAAST	WLEDEGVGAT	TVMLKEKLAE
**810**	**820**	**830**	**840**	**850**	**860**	**870**	**880**
LRKLCQGLFF	RVEERKKWPE	RLSALDNLLN	HSSMFLKGAR	LIPEMDQIFT	EVEMTTLEKV	INETWAWKNA	TLAEQAKLPA
**890**	**900**	**910**	**920**	**930**	**940**	**950**	**960**
TEKPVLLSKD	IEAKMMALDR	EVQYLLNKAK	FTKPRPRPKD	KNGTRAEPPL	NASASDQGEK	VIPPAGQTED	AEPISEPEKV
**970**	**980**	**990**	**1000**				
ETGSEPGDTE	PLELGGPGAE	PEQKEQSTGQ	KRPLKNDEL				

Cmpd.	No. of Cmpds.	m/z meas.	Δ m/z [ppm]	z	Rt [min]	Score	P	Range	Sequence	Modification
419	1	643.8239	-17.73	2	35.2	10.7	2	298-308	K.DVRENPRAMAK.L	

Detailed Protein Report

Protein 420: **PREDICTED: myotubularin-related protein 12 isoform X2 [Homo sapiens]**

**Accession:**	gi|530378835	**Score:**	30.5
**Database:**	refseq_human(refseq_human_20140103.fasta)	**MW [kDa]:**	81.7
**Database Date:**	2015-11-30	**pI:**	6.4
**Modification(s):**	Carbamidomethyl, Oxidation	**Sequence Coverage [%]:**	3.0
		**No. of unique Peptides:**	2

10	20	30	40	50	60	70	80
MLGKGVVGGG	GGTKAPKPSF	VSYVRPEEIH	TNEKEVTEKE	VTLHLLPGEQ	LLCEASTVLK	YVQEDSCQHG	VYGRLVCTDF
**90**	**100**	**110**	**120**	**130**	**140**	**150**	**160**
KIAFLGDDES	ALDNDETQFK	NKVIGENDIT	LHCVDQIYGV	FDEKKKTLFG	QLKKYPEKLI	IHCKDLRVFQ	FCLRYTKEEE
**170**	**180**	**190**	**200**	**210**	**220**	**230**	**240**
VKRIVSGIIH	HTQAPKLLKR	LFLFSYATAA	QNNTVTDPKN	HTVMFDTLKD	WCWELERTKG	NMKYKAVSVN	EGYKVCERLP
**250**	**260**	**270**	**280**	**290**	**300**	**310**	**320**
AYFVVPTPLP	EENVQRFQGH	GIPIWCWSCH	NGSALLKMSA	LPKEQDDGIL	QIQKSFLDGI	YKTIHRPPYE	IVKTEDLSSN
**330**	**340**	**350**	**360**	**370**	**380**	**390**	**400**
FLSLQEIQTA	YSKFKQLFLI	DNSTEFWDTD	IKWFSLLESS	SWLDIIRRCL	KKAIEITECM	EAQNMNVLLL	EENASDLCCL
**410**	**420**	**430**	**440**	**450**	**460**	**470**	**480**
ISSLVQLMMD	PHCRTRIGFQ	SLIQKEWVMG	GHCFLDRCNH	LRQNDKEEGR	EGQDTQSKPL	NLLTVWDWSV	QFEPKAQTLL
**490**	**500**	**510**	**520**	**530**	**540**	**550**	**560**
KNPLYVEKPK	LDKGQRKGMR	FKLCLQAQNF	SPTESTKERA	VMHQRQLSLP	LTQSKSSPKR	GFFREETDHL	IKNLLGKRIS
**570**	**580**	**590**	**600**	**610**	**620**	**630**	**640**
KLINSSDELQ	DNFREFYDSW	HSKSTDYHGL	LLPHIEGPEI	KVWAQRYLRW	IPEAQILGGG	QVATLSKLLE	MMEEVQSLQE
**650**	**660**	**670**	**680**	**690**	**700**	**710**	**720**
KIDERHHSQQ	APQAEAPCLL	RNSARLSSLF	PFALLQRHSS	KPVLPTSGWK	ALGDEDDLAK	REDEFVDLGD	V

Cmpd.	No. of Cmpds.	m/z meas.	Δ m/z [ppm]	z	Rt [min]	Score	P	Range	Sequence	Modification
1197	1	662.2827	-107.94	1	45.1	19.7	0	278-283	K.MSALPK.E	Oxidation: 1
1995	1	862.3137	-122.84	2	54.8	10.8	0	503-517	K.LCLQAQNFSPTESTK.E	Carbamidomethyl: 2

Detailed Protein Report

Protein 421: **PREDICTED: bifunctional heparan sulfate N-deacetylase/N-sulfotransferase 2 isoform X2 [Homo sapiens]**

**Accession:**	gi|530394565	**Score:**	30.4
**Database:**	refseq_human(refseq_human_20140103.fasta)	**MW [kDa]:**	59.2
**Database Date:**	2015-11-30	**pI:**	9.8
		**Sequence Coverage [%]:**	6.3
		**No. of unique Peptides:**	2

```
        10         20         30         40         50         60         70         80
MLLKHRKEFW WFPHMWSHMQ PHLFHNRSVL ADQMRLNKQF ALEHGIPTDL GYAVAPHHSG VYPIHTQLYE AWKSVWGIQV
        90        100        110        120        130        140        150        160
TSTEEYPHLR PARYRRGFIH NGIMVLPRQT CGLFTHTIFY NEYPGGSREL DRSIRGGELF LTVLLNPISI FMTHLSNYGN
       170        180        190        200        210        220        230        240
DRLGLYTFES LVRFLQCWTR LRLQTLPPVP LAQKYFELFP QERSPLWQNP CDDKRHKDIW SKEKTCDRLP KFLIVGPQKT
       250        260        270        280        290        300        310        320
GTTAIHFFLS LHPAVTSSFP SPSTFEEIQF FNSPNYHKGI DWYMDFFPVP SNASTDFLFE KSATYFDSEV VPRRGAALLP
       330        340        350        360        370        380        390        400
RAKIITVLTN PADRAYSWYQ HQRAHGDPVA LNYTFYQVIS ASSQTPLALR SLQNRCLVPG YYSTHLQRWL TYYPSGQLLI
       410        420        430        440        450        460        470        480
VDGQELRTNP AASMESIQKF LGITPFLNYT RTLRFDDDKG FWCQGLEGGK TRCLGRSKGR RYPDMDTESR LFLTDFFRNH
       490        500        510
NLELSKLLSR LGQPVPSWLR EELQHSSLG
```

Cmpd.	No. of Cmpds.	m/z meas.	Δ m/z [ppm]	z	Rt [min]	Score	P	Range	Sequence	Modification
618	1	677.3937	25.26	2	37.3	10.3	0	97-108	R.GFIHNGIMVLPR.Q	
1699	1	1045.7064	77.05	2	51.0	20.0	2	315-334	R.GAALLPRAKIITVLTNPADR.A	

Detailed Protein Report

Protein 422: PREDICTED: serine/threonine-protein kinase ULK2 isoform X1 [Homo sapiens]

**Accession:**	gi|578830126	**Score:**	30.3
**Database:**	refseq_human(refseq_human_20140103.fasta)	**MW [kDa]:**	110.3
**Database Date:**	2015-11-30	**pI:**	9.8
**Modification(s):**	Carbamidomethyl, Oxidation	**Sequence Coverage [%]:**	2.7
		**No. of unique Peptides:**	2

Quantitation

WUP:QUP **Median:** 0.50 **CV:** 0.00 % **No. of Peptides:** 1

```
        10         20         30         40         50         60         70         80
MEVVGDFEYS KRDLVGHGAF AVVFRGRHRQ KTDWEVAIKS INKKNLSKSQ ILLGKEIKIL KELQHENIVA LYDVQELPNS
        90        100        110        120        130        140        150        160
VFLVMEYCNG GDLADYLQAK GTLSEDTIRV FLHQIAAAMR ILHSKGIIHR DLKPQNILLS YANRRKSSVS GIRIKIADFG
       170        180        190        200        210        220        230        240
FARYLHSNMM AATLCGSPMY MAPEVIMSQH YDAKADLWSI GTVIYQCLVG KPPFQANSPQ DLRMFYEKNR SLMPSIPRET
       250        260        270        280        290        300        310        320
SPYLANLLLG LLQRNQKDRM DFEAFFSHPF LEQGPVKKSC PVPVPMYSGS VSGSSCGSSP SCRFASPPSL PDMQHIQEEN
       330        340        350        360        370        380        390        400
LSSPPLGPPN YLQVSKDSAS TSSKNSSCDT DDFVLVPHNI SSDHSCDMPV GTAGRRASNE FLVCGGQCQP TVSPHSETAP
       410        420        430        440        450        460        470        480
IPVPTQIRNY QRIEQNLTST ASSGTNVHGS PRSAVVRRSN TSPMGFLRPG SCSPVPADTA QTVGRRLSTG SSRPYSPSPL
       490        500        510        520        530        540        550        560
VGTIPEQFSQ CCCGHPQGHD SRSRNSSGSP VPQAQSPQSL LSGARLQSAP TLTDIYQNKQ KLRKQHSDPV CPSHTGAGYS
       570        580        590        600        610        620        630        640
YSPQPSRPGS LGTSPTKHLG SSPRSSDWFF KTPLPTIIGS PTKTTAPFKI PKTQASSNLL ALVTRHGPAE EQSKDGNEPR
       650        660        670        680        690        700        710        720
ECAHCLLVQG SERQRAEQQS KAVFGRSVST GKLSDQQGKT PICRHQGSTD SLNTERPMDI GSPPHSAAAP TCTHMFLRTR
       730        740        750        760        770        780        790        800
TTSVGPSNSG GSLCAMSGRV CVGSPPGPGF GSSPPGAEAA PSLRYVPYGA SPPSLEGLIT FEAPELPEET LMEREHTDTL
       810        820        830        840        850        860        870        880
RHLNVMLMFT ECVLDLTAMR GGNPELCTSA VSLYQIQESV VVDQISQLSK DWGRVEQLVL YMKAAQLLAA SLHLAKAQIK
       890        900        910        920        930        940        950        960
SGKLSPSTAV KQVVKNLNER YKFCITMCKK LTEKLNRFFS DKQRFIDEIN SVTAEKLIYN CAVEMVQSAA LDEMFQQTED
       970        980        990       1000       1010
IVYRYHKAAL LLEGLSRILQ DPADIENVHK YKCSIERRLS ALCHSTATV
```

Cmpd.	No. of Cmpds.	m/z meas.	Δ m/z [ppm]	z	Rt [min]	Score	P	Range	Sequence	Modification	Ratios
1434	1	458.6103	-303.83	2	48.1	18.1	0	231-238	R.SLMPSIPR.E	Oxidation: 3	WUP:QUP 0.50
2561	2	913.4770	71.20	2	62.0	12.3	0	721-739	R.TTSVGPSNSGGSLCAMSGR.V	Carbamidomethyl: 14	

Detailed Protein Report

Protein 423: **PREDICTED: leukocyte immunoglobulin-like receptor subfamily B member 5 isoform X5 [Homo sapiens]**

Accession: gi|578833860
Database: refseq_human(refseq_human_20140103.fasta)
Database Date: 2015-11-30
Modification(s): Carbamidomethyl, Oxidation

Score: 30.3
MW [kDa]: 49.1
pI: 6.8
Sequence Coverage [%]: 6.2
No. of unique Peptides: 1

10	20	30	40	50	60	70	80
MTLTLSVLIC	LGLSVGPRTC	VQAGTLPKPT	LWAEPASVIA	RGKPVTLWCQ	GPLETEEYRL	DKEGLPWARK	RQNPLEPGAK
**90**	**100**	**110**	**120**	**130**	**140**	**150**	**160**
AKFHIPSTVY	DSAGRYRCYY	ETPAGWSEPS	DPLELVATGF	YAEPTLLALP	SPVVASGGNV	TLQCDTLDGL	LTFVLVEEEQ
**170**	**180**	**190**	**200**	**210**	**220**	**230**	**240**
KLPRTLYSQK	LPKGPSQALF	PVGPVTPSCR	WRFRCYYYYR	KNPQVWSNPS	DLLEILVPGV	SRKPSLLIPQ	GSVVARGGSL
**250**	**260**	**270**	**280**	**290**	**300**	**310**	**320**
TLQCRSDVGY	DIFVLYKEGE	HDLVQGSGQQ	PQAGLSQANF	TLGPVSRSHG	GQYRCYGAHN	LSPRWSAPSD	PLDILIAGLI
**330**	**340**	**350**	**360**	**370**	**380**	**390**	**400**
PDIPALSVQP	GPKVASGENV	TLLCQSWHQI	DTFFLTKEGA	AHPPLCLKSK	YQSYRHQAEF	SMSPVTSAQG	GTYRCYSAIR
**410**	**420**	**430**	**440**	**450**	**460**		
SYPYLLSSPS	YPQELVVSGP	SGDPSLSPTG	STPTPGPEDQ	PLTPTGLDPQ	SGE		

Cmpd.	No. of Cmpds.	m/z meas.	Δ m/z [ppm]	z	Rt [min]	Score	P	Range	Sequence	Modification
2477	1	973.4893	-56.45	2	61.5	15.4	0	1-18	-.MTLTLSVLICLGLSVGPR.T	Carbamidomethyl: 10; Oxidation: 1

Detailed Protein Report

Protein 424: **PREDICTED: tetratricopeptide repeat protein 13 isoform X7 [Homo sapiens]**

**Accession:**	gi|578802328	**Score:**	30.3
**Database:**	refseq_human(refseq_human_20140103.fasta)	**MW [kDa]:**	57.4
**Database Date:**	2015-11-30	**pI:**	9.3
		**Sequence Coverage [%]:**	4.4
		**No. of unique Peptides:**	1

10	20	30	40	50	60	70	80
MMLYHHGSLQ	EALKNFKRCL	QLEPYNEVCQ	YMKGLSHVAM	GQFYEGIKAQ	TKVMLNDPLP	GQKASPEYLK	VKYLREYSRY
**90**	**100**	**110**	**120**	**130**	**140**	**150**	**160**
LHAHLDTPLT	EYNIDVDLPG	SFKDHWAKNL	PFLIEDYEEQ	PGLQPHIKDV	LHQNFESYKP	EVQELICVAD	RLGSLMQYET
**170**	**180**	**190**	**200**	**210**	**220**	**230**	**240**
PGFLPNKRIH	RAMGLAALEV	MQAVQRTWTN	SKVRMNGKTR	LMQWRDMFDI	AVKWRRIADP	DQPVLWLDQM	PARSLSRGFN
**250**	**260**	**270**	**280**	**290**	**300**	**310**	**320**
NHINLIRGQV	INMRYLEYFE	KILHFIKDRI	LVYHGANNPK	GLLEVREALE	KVHKVEDLLP	IMKQFNTKTK	DGFTVNTKVP
**330**	**340**	**350**	**360**	**370**	**380**	**390**	**400**
SLKDQGKEYD	GFTITITGDK	VGNILFSVET	QTTEERTQLY	HAEIDALYKD	LTAKGKVLIL	SSEFGEADAV	CNLILSLVYY
**410**	**420**	**430**	**440**	**450**	**460**	**470**	**480**
FYNLMPLSRG	SSVIAYSVIV	GALMASGKEV	AGKIPKGKLV	DFEAMTAPGS	EAFSKVAKSW	MNLKSISPSY	KTLPSVSETF
**490**	**500**	**510**					
PTLRSMIEVL	NTDSSPRCLK	KL					

Cmpd.	No. of Cmpds.	m/z meas.	Δ m/z [ppm]	z	Rt [min]	Score	P	Range	Sequence	Modification
1682	4	820.8728	-97.18	2	49.4	15.9	1	234-247	R.SLSRGFNNHINLIR.G	

Detailed Protein Report

Protein 425: storkhead-box protein 1 isoform d [Homo sapiens]

**Accession:**	gi|194328693	**Score:**	30.2
**Database:**	refseq_human(refseq_human_20140103.fasta)	**MW [kDa]:**	98.7
**Database Date:**	2015-11-30	**pI:**	6.1
		**Sequence Coverage [%]:**	3.2
		**No. of unique Peptides:**	1

Alias proteins:

Accession	Name	Description
gi|578819078	refseq_human (refseq_human_20140103.fasta)	PREDICTED: storkhead-box protein 1 isoform X2 [Homo sapiens]
gi|578819076	refseq_human (refseq_human_20140103.fasta)	PREDICTED: storkhead-box protein 1 isoform X1 [Homo sapiens]

10	20	30	40	50	60	70	80
MNPITQSQFV	PLGEVLCCAI	SDMNTAQIVV	TQESLLERLM	KHYPGIAIPS	EDILYTTLGT	LIKERKIYHT	GEGYFIVTPQ
90	100	110	120	130	140	150	160
TYFITNTTTQ	ENKRMLPSDE	SRLMPASMTY	LVSMESCAES	AQENAAPISH	CQSCQCFRDM	HTQDVQEAPV	AAEVTRKSHR
170	180	190	200	210	220	230	240
GLGESVSWVQ	NGAVSVSAEH	HICESTKPLP	YTRDKEKGKK	FGFSLLWRSL	SRKEKPKTEH	SSFSAQFPPE	EWPVRDEDDL
250	260	270	280	290	300	310	320
DNIPRDVEHE	IIKRINPILT	VDNLIKHTVL	MQKYEEQKKY	NSQGTSTDML	TIGHKYPSKE	GVKKRQGLSA	KPQGQGHSRR
330	340	350	360	370	380	390	400
DRHKARNQGS	EFQPGSIRLE	KHPKLPATQP	IPRIKSPNEM	VGQKPLGEIT	TVLGSHLIYK	KRISNPFQGL	SHRGSTISKG
410	420	430	440	450	460	470	480
HKIQKTSDLK	PSQTGPKEKP	FQKPRSLDSS	RIFDGKAKEP	YAEQPNDKME	AESIYINDPT	VKPINDDFRG	HLFSHPQQSM
490	500	510	520	530	540	550	560
LQNDGKCCPF	MESMLRYEVY	GGENEVIPEV	LRKSHSHFDK	LGETKQTPHS	LPSRGASFSD	RTPSACRLVD	NTIHQFQNLG
570	580	590	600	610	620	630	640
LLDYPVGVNP	LRQAARQDKD	SEELLRKGFV	QDAETTSLEN	EQLSNDDQAL	YQNEVEDDDG	ACSSLYLEED	DISENDDLRQ
650	660	670	680	690	700	710	720
MLPGHSQYSF	TGGSQGNHLG	KQKVIERSLT	EYNSTMERVE	SQVLKRNECY	KPTGLHATPG	ESQEPNLSAE	SCGLNSGAQF
730	740	750	760	770	780	790	800
GFNYEEEPSV	AKCVQASAPA	DERIFDYYSA	RKASFEAEVI	QDTIGDTGKK	PASWSQSPQN	QEMRKHFPQK	FQLFNTSHMP
810	820	830	840	850	860	870	880
VLAQDVQYEH	SHLEGTENHS	MAGDSGIDSP	RTQSLGSNNS	VILDGLKRRQ	NFLQNVEGTK	SSQPLTSNSL	LPLTPVINV

Cmpd.	No. of Cmpds.	m/z meas.	Δ m/z [ppm]	z	Rt [min]	Score	P	Range	Sequence	Modification
1771	1	823.3088	-161.08	2	52.5	16.3	0	832-847	R.TQSLGSNNSVILDGLK.R	

Detailed Protein Report

Protein 426: homeobox protein Hox-A6 [Homo sapiens]

Accession: gi|13489077
Database: refseq_human(refseq_human_20140103.fasta)
Database Date: 2015-11-30
Modification(s): Carbamidomethyl

Score: 30.2
MW [kDa]: 26.3
pI: 9.8
Sequence Coverage [%]: 11.2
No. of unique Peptides: 1

10	20	30	40	50	60	70	80
MSSYFVNPTF	PGSLPSGQDS	FLGQLPLYQA	GYDALRPFPA	SYGASSLPDK	TYTSPCFYQQ	SNSVLACNRA	SYEYGASCFY
**90**	**100**	**110**	**120**	**130**	**140**	**150**	**160**
SDKDLSGASP	SGSGKQRGPG	DYLHFSPEQQ	YKPDSSSGQG	KALHDEGADR	KYTSPVYPWM	QRMNSCAGAV	YGSHGRRGRQ
**170**	**180**	**190**	**200**	**210**	**220**	**230**	**240**
TYTRYQTLEL	EKEFHFNRYL	TRRRRIEIAN	ALCLTERQIK	IWFQNRRMKW	KKENKLINST	QPSGEDSEAK	AGE

Cmpd.	No. of Cmpds.	m/z meas.	Δ m/z [ppm]	z	Rt [min]	Score	P	Range	Sequence	Modification
2519	1	701.8340	-53.57	2	61.5	17.0	0	186-197	R.IEIANALCLTER.Q	Carbamidomethyl: 8

Detailed Protein Report

Protein 427: PREDICTED: ubl carboxyl-terminal hydrolase 18 isoform X1 [Homo sapiens]

**Accession:**	gi|578836791	**Score:**	30.2
**Database:**	refseq_human(refseq_human_20140103.fasta)	**MW [kDa]:**	34.6
**Database Date:**	2015-11-30	**pI:**	9.4
**Modification(s):**	Oxidation	**Sequence Coverage [%]:**	8.1
		**No. of unique Peptides:**	1

Quantitation

QU:MU	**Median:**	2.31	**CV:**	0.00 %	**No. of Peptides:**	1
WUP:QUP	**Median:**	0.38	**CV:**	0.00 %	**No. of Peptides:**	1

```
        10         20         30         40         50         60         70         80
MNVDFTRILK RITVPRGADE QRRSVPFQML LLLEKMQDSR QKAVRPLELA YCLQKCNVPL FVQHDAAQLY LKLWNLIKDQ
        90        100        110        120        130        140        150        160
ITDVHLVERL QALYTIRVKD SLICVDCAME SSRNSSMLTL PLSLFDVDSK PLKTLEDALH CFFQPRELSS KSKCFCENCG
       170        180        190        200        210        220        230        240
KKTRGKQVLK LTHLPQTLTI HLMRFSIRNS QTRKICHSLY FPQSLDFSQI LPMKRESCDA EEQSGGQYEL FAVIAHVGMA
       250        260        270        280        290        300
DSGHYCVYIR NAVDGKWFCF NDSNICLVSW EDIQCTYGNP NYHWQETAYL LVYMKMEC
```

Cmpd.	No. of Cmpds.	m/z meas.	Δ m/z [ppm]	z	Rt [min]	Score	P	Range	Sequence	Modification	Ratios
374	1	795.4149	-53.97	2	34.7	14.0	1	23-35	R.RSVPFQMLLLLEK.M	Oxidation: 7	WUP:QUP 0.38<br>QU:MU 2.31

Detailed Protein Report

Protein 428: endothelial cell-specific molecule 1 isoform b precursor [Homo sapiens]

**Accession:**	gi|208022668	**Score:**	30.1
**Database:**	refseq_human(refseq_human_20140103.fasta)	**MW [kDa]:**	14.5
**Database Date:**	2015-11-30	**pI:**	6.4
		**Sequence Coverage [%]:**	16.4
		**No. of unique Peptides:**	1

```
        10         20         30         40         50         60         70         80
MKSVLLLTTL LVPAHLVAAW SNNYAVDCPQ HCDSSECKSS PRCKRTVLDD CGCCRVCAAG RGETCYRTVS GMDGMKCGPG
        90        100        110        120        130        140
LRCQPSNGED PFGEEFGICK EHDMASGDGN IVREEVVKEN AAGSPVMRKW LNPR
```

Cmpd.	No. of Cmpds.	m/z meas.	Δ m/z [ppm]	z	Rt [min]	Score	P	Range	Sequence	Modification
1903	1	643.3133	32.48	2	54.2	15.5	1	56-67	R.VCAAGRGETCYR.T	

Detailed Protein Report

Protein 429: kinesin light chain 4 isoform d [Homo sapiens]

**Accession:**	gi|573459761	**Score:**	30.1
**Database:**	refseq_human(refseq_human_20140103.fasta)	**MW [kDa]:**	59.6
**Database Date:**	2015-11-30	**pI:**	5.8
		**Sequence Coverage [%]:**	5.9
		**No. of unique Peptides:**	1

10	20	30	40	50	60	70	80
MSGLVLGQRD	EPAGHRLSQE	EILGSTRLVS	QGLEALRSEH	QAVLQSLSQT	IECLQQGGHE	EGLVHEKARQ	LRRSMENIEL
**90**	**100**	**110**	**120**	**130**	**140**	**150**	**160**
GLSEAQEEKE	GDATKDSLDD	LFPNEEEEDP	SNGLSRGQGA	TAAQQGGYEI	PARLRTLHNL	VIQYAAQGRY	EVAVPLCKQA
**170**	**180**	**190**	**200**	**210**	**220**	**230**	**240**
LEDLERTSGR	GHPDVATMLN	ILALVYRDQN	KYKEAAHLLN	DALSIRESTL	GPDHPAVAAT	LNNLAVLYGK	RGKYKEAEPL
**250**	**260**	**270**	**280**	**290**	**300**	**310**	**320**
CQRALEIREK	VLGTNHPDVA	KQLNNLALLC	QNQGKYEAVE	RYYQRALAIY	EGQLGPDNPN	VARTKNNLAS	CYLKQGKYAE
**330**	**340**	**350**	**360**	**370**	**380**	**390**	**400**
AETLYKEILT	RAHVQEFGSV	DDDHKPIWMH	AEEREEMSKS	RHHEGGTPYA	EYGGWYKACK	VSSPTVNTTL	RNLGALYRRQ
**410**	**420**	**430**	**440**	**450**	**460**	**470**	**480**
GKLEAAETLE	ECALRSRRQG	TDPISQTKVA	ELLGESDGRR	TSQEGPGDSV	KFEGGEDASV	AVEWSGDGSG	TLQRSGSLGK
**490**	**500**	**510**	**520**	**530**	**540**	**550**	
IRDVLRRSSE	LLVRKLQGTE	PRPSSSNMKR	AASLNYLNQP	SAAPLQVSRG	LSASTMDLSS	SS	

Cmpd.	No. of Cmpds.	m/z meas.	Δ m/z [ppm]	z	Rt [min]	Score	P	Range	Sequence	Modification
2084	1	900.3300	-81.09	3	54.4	16.9	2	355-377	R.EEMSKSRHHEGGTPYAEYGGWYK.A	

Detailed Protein Report

Protein 430: **PREDICTED: sprouty-related, EVH1 domain-containing protein 2 isoform X2 [Homo sapiens]**

**Accession:**	gi|530367354	**Score:**	30.1
**Database:**	refseq_human(refseq_human_20140103.fasta)	**MW [kDa]:**	25.4
**Database Date:**	2015-11-30	**pI:**	7.8
**Modification(s):**	Carbamidomethyl, Oxidation	**Sequence Coverage [%]:**	14.0
		**No. of unique Peptides:**	2

10	20	30	40	50	60	70	80
MPRPYRQVSF	PDDDEEIVRI	NPREKIWMTG	YEDYRHAPVR	GKYPDPSEDA	DSSYVRFAKG	EVPKHDYNYP	YVDSSDFGLG
**90**	**100**	**110**	**120**	**130**	**140**	**150**	**160**
EDPKGRGGSV	IKTQPSRGKS	RRRKEDGERS	RCVYCRDMFN	HEENRRGHCQ	DAPDSVRTCI	RRVSCMWCAD	SMLYHCMSDP
**170**	**180**	**190**	**200**	**210**	**220**	**230**	
EGDYTDPCSC	DTSDEKFCLR	WMALIALSFL	APCMCCYLPL	RACYHCGVMC	RCCGGKHKAA	A	

Cmpd.	No. of Cmpds.	m/z meas.	Δ m/z [ppm]	z	Rt [min]	Score	P	Range	Sequence	Modification
2553	3	973.5096	122.27	2	62.6	16.7	1	112-125	R.CVYCRDMFNHEENR.R	Carbamidomethyl: 1, 4; Oxidation: 7
1552	3	993.4662	70.96	2	49.6	13.4	2	202-218	R.ACYHCGVMCRCCGGKHK.A	Carbamidomethyl: 2, 5; Oxidation: 8

Detailed Protein Report

Protein 431: cytochrome b-245 heavy chain [Homo sapiens]

**Accession:**	gi|6996021	**Score:**	30.1
**Database:**	refseq_human(refseq_human_20140103.fasta)	**MW [kDa]:**	65.3
**Database Date:**	2015-11-30	**pI:**	9.8
		**Sequence Coverage [%]:**	4.9
		**No. of unique Peptides:**	1

Quantitation

QU:MU	**Median:**	0.65	**CV:**	0.00 %	**No. of Peptides:**	1
WUP:QUP	**Median:**	5.33	**CV:**	0.00 %	**No. of Peptides:**	1

10	20	30	40	50	60	70	80
MGNWAVNEGL	SIFVILVWLG	LNVFLFVWYY	RVYDIPPKFF	YTRKLLGSAL	ALARAPAACL	NFNCMLILLP	VCRNLLSFLR
**90**	**100**	**110**	**120**	**130**	**140**	**150**	**160**
GSSACCSTRV	RRQLDRNLTF	HKMVAWMIAL	HSAIHTIAHL	FNVEWCVNAR	VNNSDPYSVA	LSELGDRQNE	SYLNFARKRI
**170**	**180**	**190**	**200**	**210**	**220**	**230**	**240**
KNPEGGLYLA	VTLLAGITGV	VITLCLILII	TSSTKTIRRS	YFEVFWYTHH	LFVIFFIGLA	IHGAERIVRG	QTAESLAVHN
**250**	**260**	**270**	**280**	**290**	**300**	**310**	**320**
ITVCEQKISE	WGKIKECPIP	QFAGNPPMTW	KWIVGPMFLY	LCERLVRFWR	SQQKVVITKV	VTHPFKTIEL	QMKKKGFKME
**330**	**340**	**350**	**360**	**370**	**380**	**390**	**400**
VGQYIFVKCP	KVSKLEWHPF	TLTSAPEEDF	FSIHIRIVGD	WTEGLFNACG	CDKQEFQDAW	KLPKIAVDGP	FGTASEDVFS
**410**	**420**	**430**	**440**	**450**	**460**	**470**	**480**
YEVVMLVGAG	IGVTPFASIL	KSVWYKYCNN	ATNLKLKKIY	FYWLCRDTHA	FEWFADLLQL	LESQMQERNN	AGFLSYNIYL
**490**	**500**	**510**	**520**	**530**	**540**	**550**	**560**
TGWDESQANH	FAVHHDEEKD	VITGLKQKTL	YGRPNWDNEF	KTIASQHPNT	RIGVFLCGPE	ALAETLSKQS	ISNSESGPRG
**570**	**580**						
VHFIFNKENF							

Cmpd.	No. of Cmpds.	m/z meas.	Δ m/z [ppm]	z	Rt [min]	Score	P	Range	Sequence	Modification	Ratios
906	1	676.2230	-177.87	2	41.4	13.2	1	560-570	R.GVHFIFNKENF.-		QU:MU 0.65<br>WUP:QUP 5.33

Detailed Protein Report

Protein 432: zinc finger protein 831 [Homo sapiens]

**Accession:**	gi|123701326	**Score:**	30.1
**Database:**	refseq_human(refseq_human_20140103.fasta)	**MW [kDa]:**	177.8
**Database Date:**	2015-11-30	**pI:**	9.7
**Modification(s):**	Carbamidomethyl	**Sequence Coverage [%]:**	1.7
		**No. of unique Peptides:**	1

Alias proteins:

Accession	Name	Description
gi|578835748	refseq_human (refseq_human_20140103.fasta)	PREDICTED: zinc finger protein 831 isoform X5 [Homo sapiens]
gi|530417822	refseq_human (refseq_human_20140103.fasta)	PREDICTED: zinc finger protein 831 isoform X3 [Homo sapiens]
gi|530417820	refseq_human (refseq_human_20140103.fasta)	PREDICTED: zinc finger protein 831 isoform X2 [Homo sapiens]
gi|530417818	refseq_human (refseq_human_20140103.fasta)	PREDICTED: zinc finger protein 831 isoform X1 [Homo sapiens]

Detailed Protein Report

10	20	30	40	50	60	70	80
MEVPEPTCPA	PPARDQPAPT	PGPPGAPGGQ	ASPHLTLGPV	LLPPEQGLAP	PTVFLKALPI	PLYHTVPPGG	LQPRAPLVTG
90	100	110	120	130	140	150	160
SLDGGNVPFI	LSPVLQPEGP	GPTQVGKPAA	PTLTVNIVGT	LPVLSPGLGP	TLGSPGKVRN	AGKYLCPHCG	RDCLKPSVLE
170	180	190	200	210	220	230	240
KHIRSHTGER	PFPCATCGIA	FKTQSNLYKH	RRTQTHLNNS	RLSSESEGAG	GGLLEEGDKA	GEPPRPEGRG	ESRCQGMHEG
250	260	270	280	290	300	310	320
ASERPLSPGA	HVPLLAKNLD	VRTEAAPCPG	SAFADREAPW	DSAPMASPGL	PAASTQPWRK	LPEQKSPTAG	KPCALQRQQA
330	340	350	360	370	380	390	400
TAAEKPWDAK	APEGRLRKCE	STDSGYLSRS	DSAEQPHAPC	SPLHSLSEHS	AESEGEGGPG	PGPGVAGAEP	GAREAGLELE
410	420	430	440	450	460	470	480
KKRLEERIAQ	LISHNQAVVD	DAQLDNVRPR	KTGLSKQGSI	DLPTPYTYKD	SFHFDIRALE	PGRRRAPGPV	RSTWTPPDKS
490	500	510	520	530	540	550	560
RPLFFHSVPT	QLSTTVECVP	VTRSNSLPFV	EGSRTWLEPR	EPRDPWSRTQ	KPLSPRPGPA	RLGCRSGLSS	TDVPSGHPRA
570	580	590	600	610	620	630	640
LVRQAAVEDL	PGTPIGDALV	PAEDTDAKRT	AAREAMAGKG	RAGGRKCGQR	RLKMFSQEKW	QVYGDETFKR	IYQKMKASPH
650	660	670	680	690	700	710	720
GGKKAREVGM	GSGAELGFPL	QKEAAGSSGT	VPTQDRRTPV	HEDISAGATP	EPWGNPPALE	ASLVTEPTKH	GETVARRGDS
730	740	750	760	770	780	790	800
DRPRVEEAVS	SPALGGRDSP	CSGSRSPLVS	PNGRLELGWQ	MPPAPGPLKG	GDVEAPRPVW	PDPKLEGGAR	GVGDVQETCL
810	820	830	840	850	860	870	880
WAQTVLRWPS	RGSGEDKLPS	ERKKLKVEDL	HSWKQPEPVS	AETPGGPTQP	ASLSSQKQDA	DPGEVPGGSK	ESARQVGEPL
890	900	910	920	930	940	950	960
ESSGASLAAA	SVALKRVGPR	DKATPLHPAA	PAPAEHPSLA	TPPQAPRVLS	ALADNAFSPK	YLLRLPQAET	PLPLPIPWGP
970	980	990	1000	1010	1020	1030	1040
RHSQDSLCSS	GWPEERASFV	GSGLGTPLSP	SPASGPSPGE	ADSILEDPSC	SRPQDGRKGA	QLGGDKGDRM	ATSRPAAREL
1050	1060	1070	1080	1090	1100	1110	1120
PISAPGAPRE	ATSSPPTPTC	EAHLVQDMEG	DSHRIHRLCM	GSTLARARLS	GDVLNPWVPN	WELGEPPGNA	PEDPSSGPLV
1130	1140	1150	1160	1170	1180	1190	1200
GPDPCSPLQP	GSFLTALTRP	QGVPPGWPEL	ALSSHSGTSR	SHSTRSPHST	QNPFPSLKAE	PRLTWCCLSR	SVPLPAEQKA
1210	1220	1230	1240	1250	1260	1270	1280
KAASVYLAVH	FPGSSLRDEG	PNGPPGSNGG	WTWTSPGEGG	PAQMSKFSYP	TVPGVMPQHQ	VSEPEWKKGL	PWRAKMSRGN
1290	1300	1310	1320	1330	1340	1350	1360
SKQRKLKINP	KRYKGNFLQS	CVQLRASRLR	TPTWVRRRSR	HPPALEGLKP	CRTPGQTSSE	IAGLNLQEEP	SCATSESPPC
1370	1380	1390	1400	1410	1420	1430	1440
CGKEEKKEGD	CRQTLGTLSL	GTSSRIVREM	DKRTVKDISP	SAGEHGDCTT	HSTAATSGLS	LQSDTCLAVV	NDVPLPPGKG
1450	1460	1470	1480	1490	1500	1510	1520
LDLGLLETQL	LASQDSVSTD	PKPYIFSDAQ	RPSSFGSKGT	FPHHDIATSV	AAVCISLPVR	TDHIAQEIHS	AESRDHSQTA
1530	1540	1550	1560	1570	1580	1590	1600
GRTLTSSSPD	SKVTEEGRAQ	TLLPGRPSSG	QRISDSVPLE	STEKTHLEIP	ASGPSSASSH	HKEGRHKTFF	PSRGQYGCGE
1610	1620	1630	1640	1650	1660	1670	1680
MTVPCPSLGS	DGRKRQVSGL	ITRKDSVVPS	KPEQPIEIPE	APSKSLKKRS	LEGMRKQTRV	EFSDTSSDDE	DRLVIEI

Cmpd.	No. of Cmpds.	m/z meas.	Δ m/z [ppm]	z	Rt [min]	Score	P	Range	Sequence	Modification
2187	8	1023.8998	-84.84	2	57.2	18.3	1	258-276	K.NLDVRTEAAPCPGSAFADR.E	Carbamidomethyl: 11

Detailed Protein Report

Protein 433: transient receptor potential cation channel subfamily V member 5 [Homo sapiens]

Accession: gi|17505200
Database: refseq_human(refseq_human_20140103.fasta)
Database Date: 2015-11-30

Score: 30.0
MW [kDa]: 82.5
pI: 6.0
Sequence Coverage [%]: 3.0
No. of unique Peptides: 1

10	20	30	40	50	60	70	80
MGGFLPKAEG	PGSQLQKLLP	SFLVREQDWD	QHLDKLHMLQ	QKRILESPLL	RASKENDLSV	LRQLLLDCTC	DVRQRGALGE
**90**	**100**	**110**	**120**	**130**	**140**	**150**	**160**
TALHIAALYD	NLEAALVLME	AAPELVFEPT	TCEAFAGQTA	LHIAVVNQNV	NLVRALLTRR	ASVSARATGT	AFRRSPRNLI
**170**	**180**	**190**	**200**	**210**	**220**	**230**	**240**
YFGEHPLSFA	ACVNSEEIVR	LLIEHGADIR	AQDSLGNTVL	HILILQPNKT	FACQMYNLLL	SYDGHGDHLQ	PLDLVPNHQG
**250**	**260**	**270**	**280**	**290**	**300**	**310**	**320**
LTPFKLAGVE	GNTVMFQHLM	QKRRHIQWTY	GPLTSILYDL	TEIDSWGEEL	SFLELVVSSD	KREARQILEQ	TPVKELVSFK
**330**	**340**	**350**	**360**	**370**	**380**	**390**	**400**
WNKYGRPYFC	ILAALYLLYM	ICFTTCCVYR	PLKFRGGNRT	HSRDITILQQ	KLLQEAYETR	EDIIRLVGEL	VSIVGAVIIL
**410**	**420**	**430**	**440**	**450**	**460**	**470**	**480**
LLEIPDIFRV	GASRYFGKTI	LGGPFHVIII	TYASLVLVTM	VMRLTNTNGE	VVPMSFALVL	GWCSVMYFTR	GFQMLGPFTI
**490**	**500**	**510**	**520**	**530**	**540**	**550**	**560**
MIQKMIFGDL	MRFCWLMAVV	ILGFASAFYI	IFQTEDPTSL	GQFYDYPMAL	FTTFELFLTV	IDAPANYDVD	LPFMFSIVNF
**570**	**580**	**590**	**600**	**610**	**620**	**630**	**640**
AFAIIATLLM	LNLFIAMMGD	THWRVAQERD	ELWRAQVVAT	TVMLERKLPR	CLWPRSGICG	CEFGLGDRWF	LRVENHNDQN
**650**	**660**	**670**	**680**	**690**	**700**	**710**	**720**
PLRVLRYVEV	FKNSDKEDDQ	EHPSEKQPSG	AESGTLARAS	LALPTSSLSR	TASQSSSHRG	WEILRQNTLG	HLNLGLNLSE
**730**							
GDGEEVYHF							

Cmpd.	No. of Cmpds.	m/z meas.	Δ m/z [ppm]	z	Rt [min]	Score	P	Range	Sequence	Modification
87	1	829.3594	10.76	2	30.8	10.5	1	653-666	K.NSDKEDDQEHPSEK.Q	

Detailed Protein Report

Protein 434: PREDICTED: microtubule-associated protein 4 isoform X15 [Homo sapiens]

**Accession:**	gi|530372482	**Score:**	30.0
**Database:**	refseq_human(refseq_human_20140103.fasta)	**MW [kDa]:**	241.1
**Database Date:**	2015-11-30	**pI:**	6.0
		**Sequence Coverage [%]:**	1.7
		**No. of unique Peptides:**	1

Detailed Protein Report

10	20	30	40	50	60	70	80
MADLSLADAL	TEPSPDIEGE	IKRDFIATLE	AEAFDDVVGE	TVGKTDYIPL	LDVDEKTGNS	ESKKKPCSET	SQIEDTPSSK
90	100	110	120	130	140	150	160
PTLLANGGHG	VEGSDTTGSP	TEFLEEKMAY	QEYPNSQNWP	EDTNFCFQPE	QVVDPIQTDP	FKMYHDDDLA	DLVFPSSATA
170	180	190	200	210	220	230	240
DTSIFAGQND	PLKDSYVPLE	LAKEIEMASE	ERPPAQALEI	MMGLKTTDMA	PSKETEMALA	KDMALATKTE	VALAKDMESP
250	260	270	280	290	300	310	320
TKLDVTLAKD	MQPSMESDMA	LVKDMELPTE	KEVALVKDVR	WPTETDVSSA	KNVVLPTETE	VAPAKDVTLL	KETERASPIK
330	340	350	360	370	380	390	400
MDLAPSKDMG	PPKENKKETE	RASPIKMDLA	PSKDMGPPKE	NKIVPAKDLV	LLSEIEVAQA	NDIISSTEIS	SAEKVALSSE
410	420	430	440	450	460	470	480
TEVALARDMT	LPPETNVILT	KDKALPLEAE	VAPVKDMAQL	PETEIAPAKD	VAPSTVKEVG	LLKDMSPLSE	TEMALGKDVT
490	500	510	520	530	540	550	560
PPPETEVVLI	KNVCLPPEME	VALTEDQVPA	LKTEAPLAKD	GVLTLANNVT	PAKDVPPLSE	TEATPVPIKD	MEIAQTQKGI
570	580	590	600	610	620	630	640
SEDSHLESLQ	DVGQSAAPTF	MISPETVTGT	GKKCSLPAEE	DSVLEKLGER	KPCNSQPSEL	SSETSANFMY	CGTPPTQAKQ
650	660	670	680	690	700	710	720
VCRPSDRRST	RPKPARVPPE	LLGGSPPWKT	LDHRLGHCSL	SESGWVSGSS	SCGGPGNQRK	SIHVDSLEPQ	RDLGREAWDI
730	740	750	760	770	780	790	800
ESTPIMMKKK	KKKPKQKRYS	QPRAGGPSDD	DNADKPKGHP	FAADTQKSGV	LPSQPTTMGT	EYGLVSGENL	KRECLVNSSA
810	820	830	840	850	860	870	880
ARLVAENFVS	ESLRIPLYPS	EEAPKTAISS	QSKLRVEEES	KSNKSVLQNQ	DKKLLKQHEY	KPQPAPHLKT	PVDKSQSVGP
890	900	910	920	930	940	950	960
LNLKGPLAEV	SAYNVETPLD	IRLKEGCSPF	LDQEVMGVVS	KPTAAKEIPN	LVPTLIASNP	LECNLKEGNN	ESKMTKLQNV
970	980	990	1000	1010	1020	1030	1040
KLKEFPEGAE	EDKELKKEAF	PNERQEISIF	TSEQLQGQVL	VQVPGVENEP	FKRMAGDGKS	RKGRGSSGKM	RTDSGKVKAK
1050	1060	1070	1080	1090	1100	1110	1120
SELPFLLDSQ	KDGRAVLIPS	EPVSKTEGMT	TQDKSEELGL	NSSKQPGTKA	DLTEAVVMGE	PKEMTQPKVA	GTMQALIPLE
1130	1140	1150	1160	1170	1180	1190	1200
SGSGMTQTSG	VSTETGDVVK	DMGVNNQSKE	GRCPWKDHEA	APWISEKPKK	RGNEGKSKKF	KNNYSTQPAR	MERKEEILNP
1210	1220	1230	1240	1250	1260	1270	1280
PFEGKDGDTG	SIPHKSKEIG	FTFPKMHDSS	FSHTPDTPTV	EAVDRKGGNF	QVNFVELGTL	GENKISTVKA	STVTEPPAKV
1290	1300	1310	1320	1330	1340	1350	1360
TDVSCQEQIQ	GAGFVPSVVS	EENKTDAANR	YTAVADKPSK	RSNDGKSKKV	KNSSPEKHIL	ENKIDATKIH	VPMETTGDQG
1370	1380	1390	1400	1410	1420	1430	1440
IEGMAYMDEN	RNITFTCPRT	PSELINKSSP	LEVLESAACE	KLPTPTPQVV	KEGDSFPDTL	AKNGQEIAPA	QISKSLMVDN
1450	1460	1470	1480	1490	1500	1510	1520
YTKDGVPGQE	RPKGPSAVVP	STSTGGVALP	ITTAIETVNI	HGDHSLKNKA	ELADSMKNEA	GIDEGHVIGE	SESVHSGASK
1530	1540	1550	1560	1570	1580	1590	1600
HSVEKVTELA	KGHLLPGVPV	EDQSLPGEAR	ALEGYADRGN	FPAHPVNEEK	ETKEGSVAVQ	IPDLLEDKAQ	KLSFCEDQNA
1610	1620	1630	1640	1650	1660	1670	1680
QDRNSKGSDS	LNKKVDLTLL	SPKSENDKLK	EISLACKITE	LESVSLPTPE	IQSDFLHSKV	EAPPSEVADT	LVIMTASKGV
1690	1700	1710	1720	1730	1740	1750	1760
RLPEPKDKIL	ETPQKMTEKS	ESKTPGEGKK	EDKSRMAEPM	KGYMRPTKSR	GLTPLLPKST	IQEQERHKQL	KSAVCLSSST
1770	1780	1790	1800	1810	1820	1830	1840
VYQQLGMSVY	GIARPEEGRP	VVSGTGNDIT	TPPNKELPPS	PEKKTKPLAT	TQPAKTSTSK	AKTQPTSLPK	QPAPTTIGGL
1850	1860	1870	1880	1890	1900	1910	1920
NKKPMSLASG	LVPAAPPKRP	AVASARPSIL	PSKDVKPKPI	ADAKAPEKRA	SPSKPASAPA	SRSGSKSTQT	VAKTTTAAAV
1930	1940	1950	1960	1970	1980	1990	2000
ASTGPSSRSP	STLLPKKPTA	IKTEGKPAEV	KKMTAKSVPA	DLSRPKSTST	SSMKKTTTLS	GTAPAAGVVP	SRVKATPMPS
2010	2020	2030	2040	2050	2060	2070	2080
RPSTTPFIDK	KPTSAKPSST	TPRLSRLATN	TSAPDLKNVR	SKVGSTENIK	HQPGGGRAKV	EKKTEAAATT	RKPESNAVTK
2090	2100	2110	2120	2130	2140	2150	2160
TAGPIASAQK	QPAGKVQIVS	KKVSYSHIQS	KCGSKDNIKH	VPGGGNVQIQ	NKKVDISKVS	SKCGSKANIK	HKPGGGDVKI
2170	2180	2190	2200	2210	2220	2230	2240

Detailed Protein Report

Cmpd.	No. of Cmpds.	m/z meas.	Δ m/z [ppm]	z	Rt [min]	Score	P	Range	Sequence	Modification
1574	1	949.4919	-30.30	2	49.9	14.9	1	2072-2090	R.KPESNAVTKTAGPIASAQK.Q	

BRUKER

Detailed Protein Report

Protein 435: E3 ubiquitin-protein ligase Hakai isoform 2 [Homo sapiens]

**Accession:**	gi|546230945	**Score:**	29.9
**Database:**	refseq_human(refseq_human_20140103.fasta)	**MW [kDa]:**	54.4
**Database Date:**	2015-11-30	**pI:**	9.6
		**Sequence Coverage [%]:**	6.5
		**No. of unique Peptides:**	1

10	20	30	40	50	60	70	80
MDHTDNELQG	TNSSGSLGGL	DVRRRIPIKL	ISKQANKAKP	APRTQRTINR	MPAKAPPGDE	GFDYNEEERY	DCKGGELFAN
**90**	**100**	**110**	**120**	**130**	**140**	**150**	**160**
QRRFPGHLFW	DFQINILGEK	DDTPVHFCDK	CGLPIKIYGR	MIPCKHVFCY	DCAILHEKKG	DKMCPGCSDP	VQRIEQCTRG
**170**	**180**	**190**	**200**	**210**	**220**	**230**	**240**
SLFMCSIVQG	CKRTYLSQRD	LQAHINHRHM	RAGKPVTRAS	LENVHPPIAP	PPTEIPERFI	MPPDKHHMSH	IPPKQHIMMP
**250**	**260**	**270**	**280**	**290**	**300**	**310**	**320**
PPPLQHVPHE	HYNQPHEDIR	APPAELSMAP	PPPRSVSQET	FRISTRKHSN	LITVPIQDDS	NSGAREPPPP	APAPAHHHPE
**330**	**340**	**350**	**360**	**370**	**380**	**390**	**400**
YQGQPVVSHP	HHIMPPQQHY	APPPPPPPPI	SHPMPHPPQA	AGTPHLVYSQ	APPPPMTSAP	PPITPPPGHI	IAQMPPYMNH
**410**	**420**	**430**	**440**	**450**	**460**	**470**	**480**
PPPGPPPPQH	GGPPVTAPPP	HHYNPNSLPQ	FTEDQGTLSP	PFTQPGGMSP	GIWPAPRGPP	PPPRLQGPPS	QTPLPGPHHP
**490**	**500**						
DQTRYRPYYQ							

Cmpd.	No. of Cmpds.	m/z meas.	Δ m/z [ppm]	z	Rt [min]	Score	P	Range	Sequence	Modification
2201	1	753.7596	78.65	3	57.9	16.7	2	154-173	R.IEQCTRGSLFMCSIVQGCKR.T	

Detailed Protein Report

Protein 436: PREDICTED: zinc finger CCHC domain-containing protein 10 isoform X4 [Homo sapiens]

Accession: gi|530380033
Database: refseq_human(refseq_human_20140103.fasta)
Database Date: 2015-11-30

Score: 29.9
MW [kDa]: 13.8
pI: 11.3
Sequence Coverage [%]: 21.9
No. of unique Peptides: 2

Quantitation

QU:MU	**Median:**	0.79	**CV:**	15.24 %	**No. of Peptides:**	2
WUP:QUP	**Median:**	1.67	**CV:**	23.19 %	**No. of Peptides:**	2

```
        10         20         30         40         50         60         70         80
MATPMHRLIA RRQAFDTELQ PVKTFWILIQ PSIVISEANK QHVRCQKCLE FGHWTYECTG KRKYLHRPSR TAELKKALKE
        90        100        110        120
KENRLLLQQR SFFPPRVYQH WRNQCRKKGQ EKKV
```

Cmpd.	No. of Cmpds.	m/z meas.	Δ m/z [ppm]	z	Rt [min]	Score	P	Range	Sequence	Modification	Ratios
1467	1	1095.0485	53.51	2	48.5	15.5	2	45-62	R.CQKCLEFGHWTYECTGKR.K		QU:MU 0.92<br>WUP:QUP 2.10
380	1	464.6667	-199.17	2	34.4	14.4	0	64-70	K.YLHRPSR.T		QU:MU 0.68<br>WUP:QUP 1.33

Detailed Protein Report

Protein 437: zinc finger C4H2 domain-containing protein isoform 2 [Homo sapiens]

**Accession:**	gi|295842335	**Score:**	29.9
**Database:**	refseq_human(refseq_human_20140103.fasta)	**MW [kDa]:**	23.5
**Database Date:**	2015-11-30	**pI:**	7.9
		**Sequence Coverage [%]:**	13.9
		**No. of unique Peptides:**	1

Alias proteins:

Accession	Name	Description
gi|345091006	refseq_human (refseq_human_20140103.fasta)	zinc finger C4H2 domain-containing protein isoform 2 [Homo sapiens]

```
        10         20         30         40         50         60         70         80
MEKIKARLKA EFEALESEER HLKEYKQEMD LLLQEKMAHV EELRLIHADI NVMENTIKQS ENDLNKLLES TRRLHDEYKP
        90        100        110        120        130        140        150        160
LKEHVDALRM TLGLQRLPDL CEEEEKLSLD YFEKQKAEWQ TEPQEPPIPE SLAAAAAAAQ QLQVARKQDT RQTATFRQQP
       170        180        190        200        210
PPMKACLSCH QQIHRNAPIC PLCKAKSRSR NPKKPKRKQD E
```

Cmpd.	No. of Cmpds.	m/z meas.	Δ m/z [ppm]	z	Rt [min]	Score	P	Range	Sequence	Modification
2312	1	1051.3738	-133.19	2	58.8	13.3	1	158-175	R.QQPPPMKACLSCHQQIHR.N	

Detailed Protein Report

Protein 438: PREDICTED: coiled-coil domain-containing protein 108 isoform X2 [Homo sapiens]

**Accession:**	gi|578804012	**Score:**	29.9
**Database:**	refseq_human(refseq_human_20140103.fasta)	**MW [kDa]:**	87.3
**Database Date:**	2015-11-30	**pI:**	9.5
**Modification(s):**	Carbamidomethyl	**Sequence Coverage [%]:**	3.4
		**No. of unique Peptides:**	1

Quantitation

QU:MU	**Median:**	0.17	**CV:**	0.00 %	**No. of Peptides:**	1
WUP:QUP	**Median:**	1.74	**CV:**	0.00 %	**No. of Peptides:**	1

10	20	30	40	50	60	70	80
MDSLCVSSAY	ISLISSERPV	HNWRGKSVQK	KQAESKSQIK	LHTQSAPFGL	CPKDMMLTQA	PSSVVRSRNS	RNHTVNSGGS
**90**	**100**	**110**	**120**	**130**	**140**	**150**	**160**
CLSASTVAIP	AINDSSAAMS	ACSTISAQPA	SSMDTQMHSP	KKQERVNKRV	IWGIEVAEEL	HWKGWELGKE	TTRNLVLKNR
**170**	**180**	**190**	**200**	**210**	**220**	**230**	**240**
SLKLQKMKYR	PPKTKFFFTV	IPQPIFLSPG	ITLTLPIVFR	PLEAKEYMDQ	LWFEKAEGMF	CVGLRATLPC	HRLICRPPSL
**250**	**260**	**270**	**280**	**290**	**300**	**310**	**320**
QLPMCAVGDT	TEAFFCLDNV	GDLPTFFTWE	FSSPFQMLPA	TGLLEPGQAS	QIKVTFQPLT	AVIYEVQATC	WYGAGSRQRS
**330**	**340**	**350**	**360**	**370**	**380**	**390**	**400**
SIQLQAVAKC	AQLLVSIKHK	CPEDQDAEGF	QKLLYFGSVA	VGCTSERQIR	LHNPSAVNAP	FRIEISPDEL	AEDQAFSCPT
**410**	**420**	**430**	**440**	**450**	**460**	**470**	**480**
AHGIVLPGEK	KCVSVFFHPK	TLDTRTVDYC	SIMPSGCASK	TLLKVVGFCR	GPAVSLQHYC	VNFSWVNLGE	RSEQPLWIEN
**490**	**500**	**510**	**520**	**530**	**540**	**550**	**560**
QSDCTAHFQF	AIDCLESVFT	IRPAFGTLVG	KARMTLHCAF	QPTHPIICFR	RVACLIHHQD	PLFLDLMGTC	HSDSTKPAIL
**570**	**580**	**590**	**600**	**610**	**620**	**630**	**640**
KPQHLTWYRT	HLARGLTLYP	PDILDAMLKE	KKLAQDQNGA	LMIPIQDLED	MPAPQYPYIP	PMTEFFFDGT	SDITIFPPPI
**650**	**660**	**670**	**680**	**690**	**700**	**710**	**720**
SVEPVEVDFG	ACPGPEAPNP	VPLCLMNHTK	GKIMVVWTRR	SDCPFWVTPE	SCDVPPLKSM	AMRLHFQPPH	PNCLYTVELE
**730**	**740**	**750**	**760**	**770**	**780**	**790**	
AFAIYKVCAR	NEREECGVSA	RSLSGLVGWQ	EVTEGSFRLH	PLRARLSLGW	TVTPMSLSPP	KLLA	

Cmpd.	No. of Cmpds.	m/z meas.	Δ m/z [ppm]	z	Rt [min]	Score	P	Range	Sequence	Modification	Ratios
745	1	712.1831	-154.73	2	39.3	12.8	0	341-352	K.CPEDQDAEGFQK.L	Carbamidomethyl: 1	WUP:QUP 1.74<br>QU:MU 0.17

Detailed Protein Report

Protein 439: glycerol-3-phosphate dehydrogenase, mitochondrial precursor [Homo sapiens]

**Accession:**	gi|285002231	**Score:**	29.8
**Database:**	refseq_human(refseq_human_20140103.fasta)	**MW [kDa]:**	80.8
**Database Date:**	2015-11-30	**pI:**	8.4
**Modification(s):**	Oxidation	**Sequence Coverage [%]:**	2.8
		**No. of unique Peptides:**	1

Alias proteins:

Accession	Name	Description
gi|530370176	refseq_human (refseq_human_20140103.fasta)	PREDICTED: glycerol-3-phosphate dehydrogenase, mitochondrial isoform X1 [Homo sapiens]
gi|285002233	refseq_human (refseq_human_20140103.fasta)	glycerol-3-phosphate dehydrogenase, mitochondrial precursor [Homo sapiens]

10	20	30	40	50	60	70	80
MAFQKAVKGT	ILVGGGALAT	VLGLSQFAHY	RRKQMNLAYV	KAADCISEPV	NREPPSREAQ	LLTLQNTSEF	DILVIGGGAT
**90**	**100**	**110**	**120**	**130**	**140**	**150**	**160**
GSGCALDAVT	RGLKTALVER	DDFSSGTSSR	STKLIHGGVR	YLQKAIMKLD	IEQYRMVKEA	LHERANLLEI	APHLSAPLPI
**170**	**180**	**190**	**200**	**210**	**220**	**230**	**240**
MLPVYKWWQL	PYYWVGIKLY	DLVAGSNCLK	SSYVLSKSRA	LEHFPMLQKD	KLVGAIVYYD	GQHNDARMNL	AIALTAARYG
**250**	**260**	**270**	**280**	**290**	**300**	**310**	**320**
AATANYMEVV	SLLKKTDPQT	GKVRVSGARC	KDVLTGQEFD	VRAKCVINAT	GPFTDSVRKM	DDKDAAAICQ	PSAGVHIVMP
**330**	**340**	**350**	**360**	**370**	**380**	**390**	**400**
GYYSPESMGL	LDPATSDGRV	IFFLPWQKMT	IAGTTDTPTD	VTHHPIPSEE	DINFILNEVR	NYLSCDVEVR	RGDVLAAWSG
**410**	**420**	**430**	**440**	**450**	**460**	**470**	**480**
IRPLVTDPKS	ADTQSISRNH	VVDISESGLI	TIAGGKWTTY	RSMAEDTINA	AVKTHNLKAG	PSRTVGLFLQ	GGKDWSPTLY
**490**	**500**	**510**	**520**	**530**	**540**	**550**	**560**
IRLVQDYGLE	SEVAQHLAAT	YGDKAFEVAK	MASVTGKRWP	IVGVRLVSEF	PYIEAEVKYG	IKEYACTAVD	MISRRTRLAF
**570**	**580**	**590**	**600**	**610**	**620**	**630**	**640**
LNVQAAEEAL	PRIVELMGRE	LNWDDYKKQE	QLETARKFLY	YEMGYKSRSE	QLTDRSEISL	LPSDIDRYKK	RFHKFDADQK
**650**	**660**	**670**	**680**	**690**	**700**	**710**	**720**
GFITIVDVQR	VLESINVQMD	ENTLHEILNE	VDLNKNGQVE	LNEFLQLMSA	IQKGRVSGSR	LAILMKTAEE	NLDRRVPIPV
**730**							
DRSCGGL							

Cmpd.	No. of Cmpds.	m/z meas.	Δ m/z [ppm]	z	Rt [min]	Score	P	Range	Sequence	Modification
810	2	491.6440	-225.52	2	38.5	17.3	0	34-41	K.QMNLAYVK.A	Oxidation: 2

Detailed Protein Report

Protein 440: uncharacterized protein C9orf172 [Homo sapiens]

**Accession:**	gi|148762978	**Score:**	29.8
**Database:**	refseq_human(refseq_human_20140103.fasta)	**MW [kDa]:**	106.6
**Database Date:**	2015-11-30	**pI:**	10.2
		**Sequence Coverage [%]:**	3.6
		**No. of unique Peptides:**	1

10	20	30	40	50	60	70	80
MTRTDPPDLL	VSTVYQDIKV	ATPGPASKCS	PCERSVARPA	EPAPFNKRHC	RSFDFLEALD	GPAMETLPEP	PPPESAVPRA
90	100	110	120	130	140	150	160
RTREAEPRRR	ARSKSAPRAP	PGLTPAPASP	PVLPRRGREA	QRAARAEASP	RREPAYPALR	ALANELHPIK	LQPQRGGPGR
170	180	190	200	210	220	230	240
VAPLCAAAGR	CAPPEPPAGP	APHVRCRLDI	KPDDAVLQHA	TRGSRSCGPT	EAAHWARPAP	QFHGLTVPGP	RHMALSRTPT
250	260	270	280	290	300	310	320
PSDSYCADPR	AFYCDGPLPG	PRDYAERRSL	PFTTPPGPTQ	FFYTEEPQGF	RGSFAASPGP	TFDAYYPRPY	PSEELSGPSP
330	340	350	360	370	380	390	400
RRMGGYYAGE	VRTFPIQEPP	SRSYYGEAPR	AYGLPYGPRY	VPEEPRAHST	ARPFYTEDFG	RYRERDVLAR	TYPHPRSSPA
410	420	430	440	450	460	470	480
WADWGPRPYR	TLQVVPPSDP	DPLLASWHGG	TGTSPPRLAT	DSRHYSRSWD	NILAPGPRRE	DPLGRGRSYE	NLLGREVREP
490	500	510	520	530	540	550	560
RGVSPEGRRP	PVVVNLSTSP	RRYAALSLSE	TSLTEKGRAG	EGLGRNWYVT	PEITITDNDL	RATERPSARA	WELPGGRTRP
570	580	590	600	610	620	630	640
PPHAAPDGPT	SGRQRSLEQL	DELITDLVID	SRPTAGQASE	PAADCLGPQL	RRLLDSRPAG	SGAPALAPPR	SPPASAGSAE
650	660	670	680	690	700	710	720
EPAAPGEAAD	ASPEPSADED	DLMTCSNARC	RRTETMFNAC	LYFKSCHSCY	TYYCSRLCRR	EDWDAHKARC	VYGRVGSVCR
730	740	750	760	770	780	790	800
HVLQFCRDSG	PVHRAFSRIA	RVGFLSRGRG	VLFLGFPSPG	SADNFLRFGL	EGLLLSPTYL	SLRELATHAA	PLGSYARELA
810	820	830	840	850	860	870	880
AAGRLYEPAE	CFLLSVSVAV	GPGTAPPGTP	ALPAPAPRSH	GPTVRKFAKV	ALAAGSPARP	PPARSREPDM	ETLILTPPPG
890	900	910	920	930	940	950	960
TAGLDQDGEA	GRRAREVAFI	HIQRELRLRG	VFLRHEFPRV	YEQLCEFVEA	NRRFTPTTIY	PTDRRTGRPF	MCMIMAASEP
970	980						
RALDWVASAN	LLDDIM						

Cmpd.	No. of Cmpds.	m/z meas.	Δ m/z [ppm]	z	Rt [min]	Score	P	Range	Sequence	Modification
1901	1	599.9186	-47.48	3	53.6	13.3	0	946-961	R.TGRPFMCMIMAASEPR.A	

Detailed Protein Report

Protein 441: protein furry homolog [Homo sapiens]

**Accession:**	gi|117606355	**Score:**	29.7
**Database:**	refseq_human(refseq_human_20140103.fasta)	**MW [kDa]:**	338.7
**Database Date:**	2015-11-30	**pI:**	5.6
		**Sequence Coverage [%]:**	1.2
		**No. of unique Peptides:**	1

Detailed Protein Report

10	20	30	40	50	60	70	80
MASQQDSGFF	EISIKYLLKS	WSNTSPVGNG	YIKPPVPPAS	GTHREKGPPT	MLPINVDPDS	KPGEYVLKSL	FVNFTTQAER
90	100	110	120	130	140	150	160
KIRIIMAEPL	EKPLTKSLQR	GEDPQFDQVI	SSMSSLSEYC	LPSILRTLFD	WYKRQNGIED	ESHEYRPRTS	NKSKSDEQQR
170	180	190	200	210	220	230	240
DYLMERRDLA	IDFIFSLVLI	EVLKQIPLHP	VIDSLIHDVI	NLAFKHFKYK	EGYLGPNTGN	MHIVADLYAE	VIGVLAQAKF
250	260	270	280	290	300	310	320
PAVKKKFMAE	LKELRHKEQN	PYVVQSIISL	IMGMKFFRIK	MYPVEDFEAS	LQFMQECAHY	FLEVKDKDIK	HALAGLFVEI
330	340	350	360	370	380	390	400
LVPVAAAVKN	EVNVPCLRNF	VESLYDTTLE	LSSRKKHSLA	LYPLVTCLLC	VSQKQLFLNR	WHIFLNNCLS	NLKNKDPKMA
410	420	430	440	450	460	470	480
RVALESLYRL	LWVYMIRIKC	ESNTATQSRL	ITIITTLFPK	GSRGVVPRDM	PLNIFVKIIQ	FIAQERLDFA	MKEIIFDFLC
490	500	510	520	530	540	550	560
VGKPAKAFSL	NPERMNIGLR	AFLVIADSLQ	QKDGEPPMPV	TGAVLPSGNT	LRVKKTYLSK	TLTEEEAKMI	GMSLYYSQVR
570	580	590	600	610	620	630	640
KAVDNILRHL	DKEVGRCMML	TNVQMLNKEP	EDMITGERKP	KIDLFRTCVA	AIPRLLPDGM	SKLELIDLLA	RLSIHMDDEL
650	660	670	680	690	700	710	720
RHIAQNSLQG	LLVDFSDWRE	DVLFGFTNFL	LREVNDMHHT	LLDSSLKLLL	QLLTQWKLVI	QTQGKVYEQA	NKIRNSELIA
730	740	750	760	770	780	790	800
NGSSHRIQSE	RGPHCSVLHA	VEGFALVLLC	SFQVATRKLS	VLILKEIRAL	FIALGQPEDD	DRPMIDVMDQ	LSSSILESFI
810	820	830	840	850	860	870	880
HVAVSDSATL	PLTHNVDLQW	LVEWNAVLVN	SHYDVKSPSH	VWIFAQSVKD	PWVLCLFSFL	RQENLPKHCP	TALSYAWPYA
890	900	910	920	930	940	950	960
FTRLQSVMPL	VDPNSPINAK	KTSTAGSGDN	YVTLWRNYLI	LCFGVAKPSI	MSPGHLRAST	PEIMATTPDG	TVSYDNKAIG
970	980	990	1000	1010	1020	1030	1040
TPSVGVLLKQ	LVPLMRLESI	EITESLVLGF	GRTNSLVFRE	LVEELHPLMK	EALERRPENK	KRERRDLLR	LQLLRIFELL
1050	1060	1070	1080	1090	1100	1110	1120
ADAGVISDST	NGALERDTLA	LGALFLEYVD	LTRMLLEAEN	DKEVEILKDI	RAHFSAMVAN	LIQCVPVHHR	RFLFPQQSLR
1130	1140	1150	1160	1170	1180	1190	1200
HHLFILFSQW	AGPFSIMFTP	LDRYSDRNHQ	ITRYQYCALK	AMSAVLCCGP	VFDNVGLSPD	GYLYKWLDNI	LACQDLRVHQ
1210	1220	1230	1240	1250	1260	1270	1280
LGCEVVVLLL	ELNPDQINLF	NWAIDRCYTG	SYQLASGCFK	AIATVCGSRN	YPFDIVTLLN	LVLFKASDTN	REIYEISMQL
1290	1300	1310	1320	1330	1340	1350	1360
MQILEAKLFV	YSKKVAEQRP	GSILYGTHGP	LPPLYSVSLA	LLSCELARMY	PELTLPLFSE	VSQRFPTTHP	NGRQIMLTYL
1370	1380	1390	1400	1410	1420	1430	1440
LPWLHNIELV	DSRLLLPGSS	PSSPEDEVKD	REGDVTASHG	LRGNGWGSPE	ATSLVLNNLM	YMTAKYGDEV	PGPEMENAWN
1450	1460	1470	1480	1490	1500	1510	1520
ALANNEKWSN	NLRITLQFLI	SLCGVSSDTV	LLPYIKKVAI	YLCRNNTIQT	MEELLFELQQ	TEPVNPIVQH	CDNPPFYRFT
1530	1540	1550	1560	1570	1580	1590	1600
ASSKASAAAS	GTTSSSNTVV	AGQENFPDAE	ENKILKESDE	RFSNVIRAHT	RLESRYSNSS	GGSYDEDKND	PISPYTGWLL
1610	1620	1630	1640	1650	1660	1670	1680
TITETKQPQP	LPMPCTGGCW	APLVDYLPET	ITPRGPLHRC	NIAVIFMTEM	VVDHSVREDW	ALHLPLLLHA	VFLGLDHYRP
1690	1700	1710	1720	1730	1740	1750	1760
EVFEHSKKLL	LHLLIALSCN	SNFHSIASVL	LQTREMGEAK	TLTVQPAYQP	EYLYTGGFDF	LREDQSSPVP	DSGLSSSSTS
1770	1780	1790	1800	1810	1820	1830	1840
SSISLGGSSG	NLPQMTQEVE	DVDTAAETDE	KANKLIEFLT	TRAFGPLWCH	EDITPKNQNS	KSAEQLTNFL	RHVVSVFKDS
1850	1860	1870	1880	1890	1900	1910	1920
KSGFHLEHQL	SEVALQTALA	SSSRHYAGRS	FQIFRALKQP	LSAHALSDLL	SRLVEVIGEH	GDEIQGYVME	ALLTLEAAVD
1930	1940	1950	1960	1970	1980	1990	2000
NLSDCLKNSD	LLTVLSRSSS	PDLSSSSKLT	ASRKSTGQLN	MNPGTTSGNT	ATAERSRHQR	SFSVPKKFGV	IDRSSDPPRS
2010	2020	2030	2040	2050	2060	2070	2080
ATLDRIQACT	QQGLSSKTRS	SSSLKDSLTD	PSHINHPTNL	LATIFWVTVA	LMESDFEFEY	LMALRLLSRL	LAHMPLDKAE
2090	2100	2110	2120	2130	2140	2150	2160
NREKLEKLQA	QLKWADFSGL	QQLLLKGFTS	LTTTDLTLQL	FSLLTPVSKI	SMVDASHAIG	FPLNVLCLLP	QLIQHFENPN
2170	2180	2190	2200	2210	2220	2230	2240

Detailed Protein Report

Cmpd.	No. of Cmpds.	m/z meas.	Δ m/z [ppm]	z	Rt [min]	Score	P	Range	Sequence	Modification
2730	1	725.3873	9.20	3	65.2	13.7	2	589-606	K.EPEDMITGERKPKIDLFR.T	

Detailed Protein Report

Protein 442: collagen alpha-2(XI) chain isoform 3 preproprotein [Homo sapiens]

**Accession:**	gi|111118968	**Score:**	29.6
**Database:**	refseq_human(refseq_human_20140103.fasta)	**MW [kDa]:**	159.8
**Database Date:**	2015-11-30	**pI:**	9.5
		**Sequence Coverage [%]:**	1.5
		**No. of unique Peptides:**	1

10	20	30	40	50	60	70	80
MERCSRCHRL	LLLLPLVLGL	SAAPGWAGAP	PVDVLRALRF	PSLPDGVRRA	KGICPADVAY	RVARPAQLSA	PTRQLFPGGF
90	100	110	120	130	140	150	160
PKDFSLLTVV	RTRPGLQAPL	LTLYSAQGVR	QLGLELGRPV	RFLYEDQTGR	PQPPSQPVFR	GLSLADGKWH	RVAVAVKGQS
170	180	190	200	210	220	230	240
VTLIVDCKKR	VTRPLPRSAR	PVLDTHGVII	FGARILDEEV	FEGDVQELAI	VPGVQAAYES	CEQKELECEG	GQRERPQNQQ
250	260	270	280	290	300	310	320
PHRAQRSPQQ	QPSRLHRPQN	QEPQSQAAHG	PRGLKGEKGE	PAVLEPGMLV	EGPPGPEGPA	GLIGPPGIQG	NPGPVGDPGE
330	340	350	360	370	380	390	400
RGPPGRAGLP	GSDGAPGPPG	TSLMLPFRFG	SGGGDKGPVV	AAQEAQAQAI	LQQARLALRG	PPGPMGYTGR	PGPLGQPGSP
410	420	430	440	450	460	470	480
GLKGESGDLG	PQGPRGPQGL	TGPPGKAGRR	GRAGADGARG	MPGDPGVKGD	RGFDGLPGLP	GEKGHRGDTG	AQGLPGPPGE
490	500	510	520	530	540	550	560
DGERGDDGEI	GPRGLPGESG	PRGLLGPKGP	PGIPGPPGVR	GMDGPQGPKG	SLGPQGEPGP	PGQQGTPGTQ	GLPGPQGAIG
570	580	590	600	610	620	630	640
PHGEKGPQGK	PGLPGMPGSD	GPPGHPGKEG	PPGTKGNQGP	SGPQGPLGYP	GPRGVKGVDG	IRGLKGHKGE	KGEDGFPGFK
650	660	670	680	690	700	710	720
GDIGVKGDRG	EVGVPGSRGE	DGPEGPKGRT	GPTGDPGPPG	LMGEKGKLGV	PGLPGYPGRQ	GPKGSLGFPG	FPGASGEKGA
730	740	750	760	770	780	790	800
RGLSGKSGPR	GERGPTGPRG	QRGPRGATGK	SGAKGTSGGD	GPHGPPGERG	LPGPQGPNGF	PGPKGPPGPP	GKDGLPGHPG
810	820	830	840	850	860	870	880
QRGEVGFQGK	TGPPGPPGVV	GPQGAAGETG	PMGERGHPGP	PGPPGEQGLP	GTAGKEGTKG	DPGPPGAPGK	DGPAGLRGFP
890	900	910	920	930	940	950	960
GERGLPGTAG	GPGLKGNEGP	SGPPGPAGSP	GERGAAGSGG	PIGPPGRPGP	QGPPGAAGEK	GVPGEKGPIG	PTGRDGVQGP
970	980	990	1000	1010	1020	1030	1040
VGLPGPAGPP	GVAGEDGDKG	EVGDPGQKGT	KGNKGEHGPP	GPPGPIGPVG	QPGAAGADGE	PGARGPQGHF	GAKGDEGTRG
1050	1060	1070	1080	1090	1100	1110	1120
FNGPPGPIGL	QGLPGPSGEK	GETGDVGPMG	PPGPPGPRGP	AGPNGADGPQ	GPPGGVGNLG	PPGEKGEPGE	SGSPGIQGEP
1130	1140	1150	1160	1170	1180	1190	1200
GVKGPRGERG	EKGESGQPGE	PGPPGPKGPT	GDDGPKGNPG	PVGFPGDPGP	PGEGGPRGQD	GAKGDRGEDG	EPGQPGSPGP
1210	1220	1230	1240	1250	1260	1270	1280
TGENGPPGPL	GKRGPAGSPG	SEGRQGGKGA	KGDPGAIGAP	GKTGPVGPAG	PAGKPGPDGL	RGLPGSVGQQ	GRPGATGQAG
1290	1300	1310	1320	1330	1340	1350	1360
PPGPVGPPGL	PGLRGDAGAK	GEKGHPGLIG	LIGPPGEQGE	KGDRGLPGPQ	GSPGQKGEMG	IPGASGPIGP	GGPPGLPGPA
1370	1380	1390	1400	1410	1420	1430	1440
GPKGAKGATG	PGGPKGEKGV	QGPPGHPGPP	GEVIQPLPIQ	MPKKTRRSVD	GSRLMQEDEA	IPTGGAPGSP	GGLEEIFGSL
1450	1460	1470	1480	1490	1500	1510	1520
DSLREEIEQM	RRPTGTQDSP	ARTCQDLKLC	HPELPDGEYW	VDPNQGCARD	AFRVFCNFTA	GGETCVTPRD	DVTQFSYVDS
1530	1540	1550	1560	1570	1580	1590	1600
EGSPVGVVQL	TFLRLLSVSA	HQDVSYPCSG	AARDGPLRLR	GANEDELSPE	TSPYVKEFRD	GCQTQQGRTV	LEVRTPVLEQ
1610	1620	1630					
LPVLDASFSD	LGAPPRRGGV	LLGPVCFMG					

Cmpd.	No. of Cmpds.	m/z meas.	Δ m/z [ppm]	z	Rt [min]	Score	P	Range	Sequence	Modification
230	2	575.1791	-198.73	2	32.6	11.4	0	1618-1629	R.GGVLLGPVCFMG.-	

Detailed Protein Report

Protein 443: spindle and kinetochore-associated protein 1 [Homo sapiens]

**Accession:**	gi|21450832	**Score:**	29.6
**Database:**	refseq_human(refseq_human_20140103.fasta)	**MW [kDa]:**	29.5
**Database Date:**	2015-11-30	**pI:**	6.8
**Modification(s):**	Carbamidomethyl	**Sequence Coverage [%]:**	12.5
		**No. of unique Peptides:**	2

Alias proteins:

Accession	Name	Description
gi|88014625	refseq_human (refseq_human_20140103.fasta)	spindle and kinetochore-associated protein 1 [Homo sapiens]

```
        10         20         30         40         50         60         70         80
MASSDLEQLC SHVNEKIGNI KKTLSLRNCG QEPTLKTVLN KIGDEIIVIN ELLNKLELEI QYQEQTNNSL KELCESLEED
        90        100        110        120        130        140        150        160
YKDIEHLKEN VPSHLPQVTV TQSCVKGSDL DPEEPIKVEE PEPVKKPPKE QRSIKEMPFI TCDEFNGVPS YMKSRLTYNQ
       170        180        190        200        210        220        230        240
INDVIKEINK AVISKYKILH QPKKSMNSVT RNLYHRFIDE ETKDTKGRYF IVEADIKEFT TLKADKKFHV LLNILRHCRR
       250        260
LSEVRGGGLT RYVIT
```

Cmpd.	No. of Cmpds.	m/z meas.	Δ m/z [ppm]	z	Rt [min]	Score	P	Range	Sequence	Modification
72	1	1069.4912	-125.31	2	30.6	13.1	1	37-55	K.TVLNKIGDEIIVINELLNK.L	
1627	3	853.3841	-128.05	2	50.1	16.5	2	227-239	K.KFHVLLNILRHCR.R	Carbamidomethyl: 12

Detailed Protein Report

Protein 444: **PREDICTED: uveal autoantigen with coiled-coil domains and ankyrin repeats isoform X3 [Homo sapiens]**

**Accession:**	gi|530406156	**Score:**	29.6
**Database:**	refseq_human(refseq_human_20140103.fasta)	**MW [kDa]:**	160.2
**Database Date:**	2015-11-30	**pI:**	6.8
		**Sequence Coverage [%]:**	1.8
		**No. of unique Peptides:**	1

10	20	30	40	50	60	70	80
MMNCWFSCTP	KNRHAADWNK	YDDRLMKAAE	RGDVEKVTSI	LAKKGVNPGK	LDVEGRSVFH	VVTSKGNLEC	LNAILIHGVD
90	100	110	120	130	140	150	160
ITTSDTAGRN	ALHLAAKYGH	ALCLQKLLQY	NCPTEHADLQ	GRTALHDAAM	ADCPSSIQLL	CDHGASVNAK	DVDGRTPLVL
170	180	190	200	210	220	230	240
ATQMSRPTIC	QLLIDRGADV	NSRDKQNRTA	LMLGCEYGCR	DAVEVLIKNG	ADISLLDALG	HDSSYYARIG	DNLDILTLLK
250	260	270	280	290	300	310	320
TASENTNKGR	ELWKKGPSLQ	QRNLTHMQDE	VNVKSHQREH	QNIQDLEIEN	EDLKERLRKI	QQEQRILLDK	VNGLQLQLNE
330	340	350	360	370	380	390	400
REKLKSLLAA	KEKQHEESLR	TIEALKNRFK	YFESDHLGSG	SHFSNRKEDM	LLKQGQMYMA	DSQCTSPGIP	AHMQSRSMLR
410	420	430	440	450	460	470	480
PLELSLPSQT	SYSENEILKK	ELEAMRTFCE	SAKQDRLKLQ	NELAHKVAEC	KALALECERV	KEDSDEQIKQ	LEDALKDVQK
490	500	510	520	530	540	550	560
RMYESEGKVK	QMQTHFLALK	EHLTSEAASG	NHRLTEELKD	QLKDLKVKYE	GASAEVGKLR	NQIKQNEMIV	EEFKRDEGKL
570	580	590	600	610	620	630	640
IEENKRLQKE	LSMCEMEREK	KGRKVTEMEG	QAKELSAKLA	LSIPAEKFEN	MKSSLSNEVN	EKAKKLVEME	REHEKSLSEI
650	660	670	680	690	700	710	720
RQLKRELENV	KAKLAQHVKP	EEHEQVKSRL	EQKSGELGKK	ITELTLKNQT	LQKEIEKVYL	DNKLLKEQAH	NLTIEMKNHY
730	740	750	760	770	780	790	800
VPLKVSEDMK	KSHDAIIDDL	NRKLLDVTQK	YTEKKLEMEK	LLLENDSLSK	DVSRLETVFV	PPEKHEKEII	ALKSNIVELK
810	820	830	840	850	860	870	880
KQLSELKKKC	GEDQEKIHAL	TSENTNLKKM	MSNQYVPVKT	HEEVKMTLND	TLAKTNRELL	DVKKKFEDIN	QEFVKIKDKN
890	900	910	920	930	940	950	960
EILKRNLENT	QNQIKAEYIS	LAEHEAKMSS	LSQSMRKVQD	SNAEILANYR	KGQEEIVTLH	AEIKAQKKEL	DTIQECIKVK
970	980	990	1000	1010	1020	1030	1040
YAPIVSFEEC	ERKFKATEKE	LKDQLSEQTQ	KYSVSEEEVK	KNKQENDKLK	KEIFTLQKDL	RDKTVLIEKS	HEMERALSRK
1050	1060	1070	1080	1090	1100	1110	1120
TDELNKQLKD	LSQKYTEVKN	VKEKLVEENA	KQTSEILAVQ	NLLQKQHVPL	EQVEALKKSL	NGTIENLKEE	LKSMQRCYEK
1130	1140	1150	1160	1170	1180	1190	1200
EQQTVTKLHQ	LLENQKNSSV	PLAEHLQIKE	AFEKEVGIIK	ASLREKEEES	QNKMEEVSKL	QSEVQNTKQA	LKKLETREVV
1210	1220	1230	1240	1250	1260	1270	1280
DLSKYKATKS	DLETQISSLN	EKLANLNRKY	EEVCEEVLHA	KKKEISAKDE	KELLHFSIEQ	EIKDQKERCD	KSLTTITELQ
1290	1300	1310	1320	1330	1340	1350	1360
RRIQESAKQI	EAKDNKITEL	LNDVERLKQA	LNGLSQLTYT	SGNPTKRQSQ	LIDTLQHQVK	SLEQQLADAD	RQHQEVIAIY
1370	1380	1390	1400				
RTHLLSAAQG	HMDEDVQEAL	LQIIQMRQGL	VC				

Cmpd.	No. of Cmpds.	m/z meas.	Δ m/z [ppm]	z	Rt [min]	Score	P	Range	Sequence	Modification
1985	1	599.9122	-110.16	3	53.2	13.4	2	959-973	K.VKYAPIVSFEECERK.F	

Detailed Protein Report

Protein 445: cell cycle checkpoint control protein RAD9B isoform 1 [Homo sapiens]

**Accession:**	gi|169234617	**Score:**	29.6
**Database:**	refseq_human(refseq_human_20140103.fasta)	**MW [kDa]:**	48.1
**Database Date:**	2015-11-30	**pI:**	6.5
**Modification(s):**	Carbamidomethyl, Oxidation	**Sequence Coverage [%]:**	7.7
		**No. of unique Peptides:**	2

Quantitation

QU:MU **Median:** 0.92 **CV:** 0.00 % **No. of Peptides:** 1

10	20	30	40	50	60	70	80
MAAMLKCVMS	GSQVKVFGKA	VQALSRISDE	FWLDPSKKGL	ALRCVNSSRS	AYGCVLFSPV	FFQHYQWSAL	VKMSENELDT
**90**	**100**	**110**	**120**	**130**	**140**	**150**	**160**
TLHLKCKLGM	KSILPIFRCL	NSLERNIEKC	RIFTRSDKCK	VVIQFFYRHG	IKRTHNICFQ	ESQPLQVIFD	KNVCTNTLMI
**170**	**180**	**190**	**200**	**210**	**220**	**230**	**240**
QPRLLADAIV	LFTSSQEEVT	LAVTPLNFCL	KSSNEESMDL	SNAVHSEMFV	GSDEFDFFQI	GMDTEITFCF	KELKGILTFS
**250**	**260**	**270**	**280**	**290**	**300**	**310**	**320**
EATHAPISIY	FDFPGKPLAL	SIDDMLVEAN	FILATLADEQ	SRASSPQSLC	LSQKRKRSDL	IEKKAGKNVT	GQALECISKK
**330**	**340**	**350**	**360**	**370**	**380**	**390**	**400**
AAPRRLYPKE	TLTNISALEN	CGSPAMKRVD	GDVSEVSESS	VSNTEEVPGS	LCLRKFSCMF	FGAVSSDQQE	HFNHPFDSLA
**410**	**420**	**430**					
RASDSEEDMN	NVCCRKEFNG	SDAKYFCII					

Cmpd.	No. of Cmpds.	m/z meas.	Δ m/z [ppm]	z	Rt [min]	Score	P	Range	Sequence	Modification	Ratios
2570	8	1044.5429	14.94	2	62.8	15.9	2	1-19	-.MAAMLKCVMSGSQVKVFGK.A	Carbamidomethyl: 7; Oxidation: 4	
421	1	846.3629	-53.88	2	35.2	13.6	2	416-429	R.KEFNGSDAKYFCII.-	Carbamidomethyl: 12	QU:MU 0.92

Detailed Protein Report

**Protein 446:**	**PREDICTED: cadherin EGF LAG seven-pass G-type receptor 2 isoform X2 [Homo sapiens]**		
**Accession:**	gi|578798514	**Score:**	29.5
**Database:**	refseq_human(refseq_human_20140103.fasta)	**MW [kDa]:**	316.2
**Database Date:**	2015-11-30	**pI:**	5.0
		**Sequence Coverage [%]:**	0.9
		**No. of unique Peptides:**	2

Detailed Protein Report

10	20	30	40	50	60	70	80
MRSPATGVPL	PTPPPPLLLL	LLLLLPPPLL	GDQVGPCRSL	GSRGRGSSGA	CAPMGWLCPS	SASNLWLYTS	RCRDAGTELT
90	100	110	120	130	140	150	160
GHLVPHHDGL	RVWCPESEAH	IPLPPAPEGC	PWSCRLLGIG	GHLSPQGKLT	LPEEHPCLKA	PRLRCQSCKL	AQAPGLRAGE
170	180	190	200	210	220	230	240
RSPEESLGGR	RKRNVNTAPQ	FQPPSYQATV	PENQPAGTPV	ASLRAIDPDE	GEAGRLEYTM	DALFDSRSNQ	FFSLDPVTGA
250	260	270	280	290	300	310	320
VTTAEELDRE	TKSTHVFRVT	AQDHGMPRRS	ALATLTILVT	DTNDHDPVFE	QQEYKESLRE	NLEVGYEVLT	VRATDGDAPP
330	340	350	360	370	380	390	400
NANILYRLLE	GSGGSPSEVF	EIDPRSGVIR	TRGPVDREEV	ESYQLTVEAS	DQGRDPGPRS	TTAAVFLSVE	DDNDNAPQFS
410	420	430	440	450	460	470	480
EKRYVVQVRE	DVTPGAPVLR	VTASDRDKGS	NAVVHYSIMS	GNARGQFYLD	AQTGALDVVS	PLDYETTKEY	TLRVRAQDGG
490	500	510	520	530	540	550	560
RPPLSNVSGL	VTVQVLDIND	NAPIFVSTPF	QATVLESVPL	GYLVLHVQAI	DADAGDNARL	EYRLAGVGHD	FPFTINNGTG
570	580	590	600	610	620	630	640
WISVAAELDR	EEVDFYSFGV	EARDHGTPAL	TASASVSVTV	LDVNDNNPTF	TQPEYTVRLN	EDAAVGTSVV	TVSAVDRDAH
650	660	670	680	690	700	710	720
SVITYQITSG	NTRNRFSITS	QSGGGLVSLA	LPLDYKLERQ	YVLAVTASDG	TRQDTAQIVV	NVTDANTHRP	VFQSSHYTVN
730	740	750	760	770	780	790	800
VNEDRPAGTT	VVLISATDED	TGENARITYF	MEDSIPQFRI	DADTGAVTTQ	AELDYEDQVS	YTLAITARDN	GIPQKSDTTY
810	820	830	840	850	860	870	880
LEILVNDVND	NAPQFLRDSY	QGSVYEDVPP	FTSVLQISAT	DRDSGLNGRV	FYTFQGGDDG	DGDFIVESTS	GIVRTLRRLD
890	900	910	920	930	940	950	960
RENVAQYVLR	AYAVDKGMPP	ARTPMEVTVT	VLDVNDNPPV	FEQDEFDVFV	EENSPIGLAV	ARVTATDPDE	GTNAQIMYQI
970	980	990	1000	1010	1020	1030	1040
VEGNIPEVFQ	LDIFSGELTA	LVDLDYEDRP	EYVLVIQATS	APLVSRATVH	VRLLDRNDNP	PVLGNFEILF	NNYVTNRSSS
1050	1060	1070	1080	1090	1100	1110	1120
FPGGAIGRVP	AHDPDISDSL	TYSFERGNEL	SLVLLNASTG	ELKLSRALDN	NRPLEAIMSV	LVSDGVHSVT	AQCALRVTII
1130	1140	1150	1160	1170	1180	1190	1200
TDEMLTHSIT	LRLEDMSPER	FLSPLLGLFI	QAVAATLATP	PDHVVVFNVQ	RDTDAPGGHI	LNVSLSVGQP	PGPGGGPPFL
1210	1220	1230	1240	1250	1260	1270	1280
PSEDLQERLY	LNRSLLTAIS	AQRVLPFDDN	ICLREPCENY	MRCVSVLRFD	SSAPFIASSS	VLFRPIHPVG	GLRCRCPPGF
1290	1300	1310	1320	1330	1340	1350	1360
TGDYCETEVD	LCYSRPCGPH	GRCRSREGGY	TCLCRDGYTG	EHCEVSARSG	RCTPGVCKNG	GTCVNLLVGG	FKCDCPSGDF
1370	1380	1390	1400	1410	1420	1430	1440
EKPYCQVTTR	SFPAHSFITF	RGLRQRFHFT	LALSFATKER	DGLLLYNGRF	NEKHDFVALE	VIQEQVQLTF	SAGESTTTVS
1450	1460	1470	1480	1490	1500	1510	1520
PFVPGGVSDG	QWHTVQLKYY	NKPLLGQTGL	PQGPSEQKVA	VVTVDGCDTG	VALRFGSVLG	NYSCAAQGTQ	GGSKKSLDLT
1530	1540	1550	1560	1570	1580	1590	1600
GPLLLGGVPD	LPESFPVRMR	QFVGCMRNLQ	VDSRHIDMAD	FIANNGTVPG	CPAKKNVCDS	NTCHNGGTCV	NQWDAFSCEC
1610	1620	1630	1640	1650	1660	1670	1680
PLGFGGKSCA	QEMANPQHFL	GSSLVAWHGL	SLPISQPWYL	SLMFRTRQAD	GVLLQAITRG	RSTITLQLRE	GHVMLSVEGT
1690	1700	1710	1720	1730	1740	1750	1760
GLQASSLRLE	PGRANDGDWH	HAQLALGASG	GPGHAILSFD	YGQQRAEGNL	GPRLHGLHLS	NITVGGIPGP	AGGVARGFRG
1770	1780	1790	1800	1810	1820	1830	1840
CLQGVRVSDT	PEGVNSLDPS	HGESINVEQG	CSLPDPCDSN	PCPANSYCSN	DWDSYSCSCD	PGYYGDNCTN	VCDLNPCEHQ
1850	1860	1870	1880	1890	1900	1910	1920
SVCTRKPSAP	HGYTCECPPN	YLGPYCETRI	DQPCPRGWWG	HPTCGPCNCD	VSKGFDPDCN	KTSGECHCKE	NHYRPPGSPT
1930	1940	1950	1960	1970	1980	1990	2000
CLLCDCYPTG	SLSRVCDPED	GQCPCKPGVI	GRQCDRCDNP	FAEVTTNGCE	VNYDSCPRAI	EAGIWWPRTR	FGLPAAAPCP
2010	2020	2030	2040	2050	2060	2070	2080
KGSFGTAVRH	CDEHRGWLPP	NLFNCTSITF	SELKGFAERL	QRNESGLDSG	RSQQLALLLR	NATQHTAGYF	GSDVKVAYQL
2090	2100	2110	2120	2130	2140	2150	2160
ATRLLAHEST	QRGFGLSATQ	DVHFTENLLR	VGSALLDTAN	KRHWELIQQT	EGGTAWLLQH	YEAYASALAQ	NMRHTYLSPF
2170	2180	2190	2200	2210	2220	2230	2240

Detailed Protein Report

Cmpd.	No. of Cmpds.	m/z meas.	Δ m/z [ppm]	z	Rt [min]	Score	P	Range	Sequence	Modification
2164	1	937.1664	148.30	2	57.5	14.0	2	129-144	K.LTLPEEHPCLKAPRLR.C	
1989	3	572.7550	-154.40	2	55.2	15.5	1	1660-1669	R.GRSTITLQLR.E	

Detailed Protein Report

Protein 447: OTU domain-containing protein 7A [Homo sapiens]

**Accession:**	gi|18702331	**Score:**	29.5
**Database:**	refseq_human(refseq_human_20140103.fasta)	**MW [kDa]:**	100.6
**Database Date:**	2015-11-30	**pI:**	9.5
		**Sequence Coverage [%]:**	4.2
		**No. of unique Peptides:**	2

10	20	30	40	50	60	70	80
MVSSVLPNPT	SAECWAALLH	DPMTLDMDAV	LSDFVRSTGA	EPGLARDLLE	GKNWDLTAAL	SDYEQLRQVH	TANLPHVFNE
90	100	110	120	130	140	150	160
GRGPKQPERE	PQPGHKVERP	CLQRQDDIAQ	EKRLSRGISH	ASSAIVSLAR	SHVASECNNE	QFPLEMPIYT	FQLPDLSVYS
170	180	190	200	210	220	230	240
EDFRSFIERD	LIEQATMVAL	EQAGRLNWWS	TVCTSCKRLL	PLATTGDGNC	LLHAASLGMW	GFHDRDLVLR	KALYTMMRTG
250	260	270	280	290	300	310	320
AEREALKRRW	RWQQTQQNKE	EEWEREWTEL	LKLASSEPRT	HFSKNGGTGG	GVDNSEDPVY	ESLEEFHVFV	LAHILRRPIV
330	340	350	360	370	380	390	400
VVADTMLRDS	GGEAFAPIPF	GGIYLPLEVP	PNRCHCSPLV	LAYDQAHFSA	LVSMEQRDQQ	REQAVIPLTD	SEHKLLPLHF
410	420	430	440	450	460	470	480
AVDPGKDWEW	GKDDNDNARL	AHLILSLEAK	LNLLHSYMNV	TWIRIPSETR	APLAQPESPT	ASAGEDVQSL	ADSLDSDRDS
490	500	510	520	530	540	550	560
VCSNSNSNNG	KNGKDKEKEK	QRKEKDKTRA	DSVANKLGSF	SKTLGIKLKK	NMGGLGGLVH	GKMGRANSAN	GKNGDSAERG
570	580	590	600	610	620	630	640
KEKKAKSRKG	SKEESGASAS	TSPSEKTTPS	PTDKAAGASP	AEKGGGPRGD	AWKYSTDVKL	SLNILRAAMQ	GERKFIFAGL
650	660	670	680	690	700	710	720
LLTSHRHQFH	EEMIGYYLTS	AQERFSAEQE	QRRRDAATAA	AAAAAAAAAT	AKRPPRRPET	EGVPVPERAS	PGPPTQLVLK
730	740	750	760	770	780	790	800
LKERPSPGPA	AGRAARAAAG	GTASPGGGAR	RASASGPVPG	RSPPAPARQS	VIHVQASGAR	DEACAPAVGA	LRPCATYPQQ
810	820	830	840	850	860	870	880
NRSLSSQSYS	PARAAALRTV	NTVESLARAV	PGALPGAAGT	AGAAEHKSQT	YTNGFGALRD	GLEFADADAP	TARSNGECGR
890	900	910	920	930			
GGPGPVQRRC	QRENCAFYGR	AETEHYCSYC	YREELRRRRE	ARGARP			

Cmpd.	No. of Cmpds.	m/z meas.	Δ m/z [ppm]	z	Rt [min]	Score	P	Range	Sequence	Modification
2690	1	724.8511	-130.11	2	64.7	15.6	1	709-722	R.ASPGPPTQLVLKLK.E	
41	6	1068.4771	-72.42	2	30.4	13.9	2	737-761	R.AAAGGTASPGGGARRASASGPVPGR.S	

Detailed Protein Report

Protein 448: transcription elongation factor B polypeptide 2 isoform b [Homo sapiens]

**Accession:**	gi|46276893	**Score:**	29.5
**Database:**	refseq_human(refseq_human_20140103.fasta)	**MW [kDa]:**	17.9
**Database Date:**	2015-11-30	**pI:**	5.7
**Modification(s):**	Carbamidomethyl	**Sequence Coverage [%]:**	15.5
		**No. of unique Peptides:**	1

10	20	30	40	50	60	70	80
MDVFLMIRRH	KTTIFTDAKE	SSTVFELKRI	VEGILKRPPD	EQRLYKDDQL	LDDGKTLGEC	GFTSQTARPQ	APATVGLAFR
**90**	**100**	**110**	**120**	**130**	**140**	**150**	**160**
ADDTFEALCI	EPFSSPPELP	DVMKPQDSGS	SANEQAVHLH	VHSQTMAKSR	NTSWSQCPGL	TACSTREPQD	GPTQVHPRWG
**170**							
L							

Cmpd.	No. of Cmpds.	m/z meas.	Δ m/z [ppm]	z	Rt [min]	Score	P	Range	Sequence	Modification
2604	1	913.4894	96.33	2	63.2	15.9	0	131-146	R.NTSWSQCPGLTACSTR.E	Carbamidomethyl: 7, 13

Detailed Protein Report

Protein 449: PREDICTED: protein FAM214A isoform X6 [Homo sapiens]

**Accession:**	gi|578827201	**Score:**	29.5
**Database:**	refseq_human(refseq_human_20140103.fasta)	**MW [kDa]:**	111.5
**Database Date:**	2015-11-30	**pI:**	9.2
		**Sequence Coverage [%]:**	2.3
		**No. of unique Peptides:**	2

Alias proteins:

Accession	Name	Description
gi|578827203	refseq_human (refseq_human_20140103.fasta)	PREDICTED: protein FAM214A isoform X7 [Homo sapiens]

10	20	30	40	50	60	70	80
MVEMMLLPDC	CYSDDGPTTE	GIDLNDPAIK	QDALLLERWI	LEPVPRQNGD	RFIEEKTLLL	AVRSFVFFSQ	LSAWLSVSHG
**90**	**100**	**110**	**120**	**130**	**140**	**150**	**160**
AIPRNILYRI	SAADVDLQWN	FSQTPIEHVF	PVPNVSHNVA	LKVSVQSLPR	QSNYPVLTCS	IHTNIGLYEK	RIQQHKLKTH
**170**	**180**	**190**	**200**	**210**	**220**	**230**	**240**
QHHNPNEAEQ	CGTNSSQRLC	SKQTWTMAPE	SVLHAKSGPS	PEYTAAVKNI	KLYPGTGSKS	DHGTSQANIL	GFSGIGDIKS
**250**	**260**	**270**	**280**	**290**	**300**	**310**	**320**
QETSVRTLKS	FSMVDSSISN	RQSFWQSAGE	TNPLIGSLIQ	ERQEIIARIA	QHLIHCDPST	SHVSGRPFNT	QESSSLHSKL
**330**	**340**	**350**	**360**	**370**	**380**	**390**	**400**
FRVSQENENV	GKGKEAFSMT	FGSPEFSSPE	DTNEGKIRLK	PETPRSETCI	SNDFYSHMPV	GETNPLIGSL	LQERQDVIAR
**410**	**420**	**430**	**440**	**450**	**460**	**470**	**480**
IAQHLEHIDP	TASHIPRQSF	NMHDSSSVAS	KVFRSSYEDK	NLLKKNKDES	SVSISHTKCS	LLGDISDGKN	LVPNKCFTSF
**490**	**500**	**510**	**520**	**530**	**540**	**550**	**560**
KNNSKEKCSL	KHQTRNQCQN	NPSEIIQSTY	QETQNKSSSL	STSSILSQHK	ENNLDLTSRF	KEQEMSNGID	KQYSNCTTID
**570**	**580**	**590**	**600**	**610**	**620**	**630**	**640**
KQICTNKYKE	KIINENYNPK	FFGNLQSDDS	KKNDSKIKVT	VLEMSEYLNK	YESMSSNKDS	KRPKTCEQNT	QLNSIENYLN
**650**	**660**	**670**	**680**	**690**	**700**	**710**	**720**
KDNEGFKCKK	SDQLKNEQDK	QEDPTNEKSQ	NYSQRRSIKD	CLSTCEQPKN	TEVLRTTLKH	SNVWRKHNFH	SLDGTSTRAF
**730**	**740**	**750**	**760**	**770**	**780**	**790**	**800**
HPQTGLPLLS	SPVPQRKTQS	GCFDLDSSLL	HLKSFSSRSP	RPCLNIEDDP	DIHEKPFLSS	SAPPITSLSL	LGNFEESVLN
**810**	**820**	**830**	**840**	**850**	**860**	**870**	**880**
YRFDPLGIVD	GFTAEVGASG	AFCPTHLTLP	VEVSFYSVSD	DNAPSPYMGV	ITLESLGKRG	YRVPPSGTIQ	VTLFNPNKTV
**890**	**900**	**910**	**920**	**930**	**940**	**950**	**960**
VKMFVVIYDL	RDMPANHQTF	LRQRTFSVPV	KQEVKRSVNK	ENIRHTEERL	LRYLIHLRFQ	SSKSGKIYLH	RDVRLLFSRK
**970**	**980**	**990**					
SMEVDSGAAY	ELKSYTESPT	NPQFSPRC					

Cmpd.	No. of Cmpds.	m/z meas.	Δ m/z [ppm]	z	Rt [min]	Score	P	Range	Sequence	Modification
2952	1	557.9715	37.67	3	65.7	10.1	1	247-261	R.TLKSFSMVDSSISNR.Q	
1649	2	480.7758	122.24	2	48.9	19.4	0	661-668	K.QEDPTNEK.S	

Detailed Protein Report

Protein 450: coagulation factor VIII isoform a precursor [Homo sapiens]

**Accession:**	gi|4503647	**Score:**	29.5
**Database:**	refseq_human(refseq_human_20140103.fasta)	**MW [kDa]:**	266.8
**Database Date:**	2015-11-30	**pI:**	7.0
		**Sequence Coverage [%]:**	1.0
		**No. of unique Peptides:**	1

Detailed Protein Report

10	20	30	40	50	60	70	80
MQIELSTCFF	LCLLRFCFSA	TRRYYLGAVE	LSWDYMQSDL	GELPVDARFP	PRVPKSFPFN	TSVVYKKTLF	VEFTDHLFNI
90	100	110	120	130	140	150	160
AKPRPPWMGL	LGPTIQAEVY	DTVVITLKNM	ASHPVSLHAV	GVSYWKASEG	AEYDDQTSQR	EKEDDKVFPG	GSHTYVWQVL
170	180	190	200	210	220	230	240
KENGPMASDP	LCLTYSYLSH	VDLVKDLNSG	LIGALLVCRE	GSLAKEKTQT	LHKFILLFAV	FDEGKSWHSE	TKNSLMQDRD
250	260	270	280	290	300	310	320
AASARAWPKM	HTVNGYVNRS	LPGLIGCHRK	SVYWHVIGMG	TTPEVHSIFL	EGHTFLVRNH	RQASLEISPI	TFLTAQTLLM
330	340	350	360	370	380	390	400
DLGQFLLFCH	ISSHQHDGME	AYVKVDSCPE	EPQLRMKNNE	EAEDYDDDLT	DSEMDVVRFD	DDNSPSFIQI	RSVAKKHPKT
410	420	430	440	450	460	470	480
WVHYIAAEEE	DWDYAPLVLA	PDDRSYKSQY	LNNGPQRIGR	KYKKVRFMAY	TDETFKTREA	IQHESGILGP	LLYGEVGDTL
490	500	510	520	530	540	550	560
LIIFKNQASR	PYNIYPHGIT	DVRPLYSRRL	PKGVKHLKDF	PILPGEIFKY	KWTVTVEDGP	TKSDPRCLTR	YYSSFVNMER
570	580	590	600	610	620	630	640
DLASGLIGPL	LICYKESVDQ	RGNQIMSDKR	NVILFSVFDE	NRSWYLTENI	QRFLPNPAGV	QLEDPEFQAS	NIMHSINGYV
650	660	670	680	690	700	710	720
FDSLQLSVCL	HEVAYWYILS	IGAQTDFLSV	FFSGYTFKHK	MVYEDTLTLF	PFSGETVFMS	MENPGLWILG	CHNSDFRNRG
730	740	750	760	770	780	790	800
MTALLKVSSC	DKNTGDYYED	SYEDISAYLL	SKNNAIEPRS	FSQNSRHPST	RQKQFNATTI	PENDIEKTDP	WFAHRTPMPK
810	820	830	840	850	860	870	880
IQNVSSSDLL	MLLRQSPTPH	GLSLSDLQEA	KYETFSDDPS	PGAIDSNNSL	SEMTHFRPQL	HHSGDMVFTP	ESGLQLRLNE
890	900	910	920	930	940	950	960
KLGTTAATEL	KKLDFKVSST	SNNLISTIPS	DNLAAGTDNT	SSLGPPSMPV	HYDSQLDTTL	FGKKSSPLTE	SGGPLSLSEE
970	980	990	1000	1010	1020	1030	1040
NNDSKLLESG	LMNSQESSWG	KNVSSTESGR	LFKGKRAHGP	ALLTKDNALF	KVSISLLKTN	KTSNNSATNR	KTHIDGPSLL
1050	1060	1070	1080	1090	1100	1110	1120
IENSPSVWQN	ILESDTEFKK	VTPLIHDRML	MDKNATALRL	NHMSNKTTSS	KNMEMVQQKK	EGPIPPDAQN	PDMSFFKMLF
1130	1140	1150	1160	1170	1180	1190	1200
LPESARWIQR	THGKNSLNSG	QGPSPKQLVS	LGPEKSVEGQ	NFLSEKNKVV	VGKGEFTKDV	GLKEMVFPSS	RNLFLTNLDN
1210	1220	1230	1240	1250	1260	1270	1280
LHENNTHNQE	KKIQEEIEKK	ETLIQENVVL	PQIHTVTGTK	NFMKNLFLLS	TRQNVEGSYD	GAYAPVLQDF	RSLNDSTNRT
1290	1300	1310	1320	1330	1340	1350	1360
KKHTAHFSKK	GEEENLEGLG	NQTKQIVEKY	ACTTRISPNT	SQQNFVTQRS	KRALKQFRLP	LEETELEKRI	IVDDTSTQWS
1370	1380	1390	1400	1410	1420	1430	1440
KNMKHLTPST	LTQIDYNEKE	KGAITQSPLS	DCLTRSHSIP	QANRSPLPIA	KVSSFPSIRP	IYLTRVLFQD	NSSHLPAASY
1450	1460	1470	1480	1490	1500	1510	1520
RKKDSGVQES	SHFLQGAKKN	NLSLAILTLE	MTGDQREVGS	LGTSATNSVT	YKKVENTVLP	KPDLPKTSGK	VELLPKVHIY
1530	1540	1550	1560	1570	1580	1590	1600
QKDLFPTETS	NGSPGHLDLV	EGSLLQGTEG	AIKWNEANRP	GKVPFLRVAT	ESSAKTPSKL	LDPLAWDNHY	GTQIPKEEWK
1610	1620	1630	1640	1650	1660	1670	1680
SQEKSPEKTA	FKKKDTILSL	NACESNHAIA	AINEGQNKPE	IEVTWAKQGR	TERLCSQNPP	VLKRHQREIT	RTTLQSDQEE
1690	1700	1710	1720	1730	1740	1750	1760
IDYDDTISVE	MKKEDFDIYD	EDENQSPRSF	QKKTRHYFIA	AVERLWDYGM	SSSPHVLRNR	AQSGSVPQFK	KVVFQEFTDG
1770	1780	1790	1800	1810	1820	1830	1840
SFTQPLYRGE	LNEHLGLLGP	YIRAEVEDNI	MVTFRNQASR	PYSFYSSLIS	YEEDQRQGAE	PRKNFVKPNE	TKTYFWKVQH
1850	1860	1870	1880	1890	1900	1910	1920
HMAPTKDEFD	CKAWAYFSDV	DLEKDVHSGL	IGPLLVCHTN	TLNPAHGRQV	TVQEFALFFT	IFDETKSWYF	TENMERNCRA
1930	1940	1950	1960	1970	1980	1990	2000
PCNIQMEDPT	FKENYRFHAI	NGYIMDTLPG	LVMAQDQRIR	WYLLSMGSNE	NIHSIHFSGH	VFTVRKKEEY	KMALYNLYPG
2010	2020	2030	2040	2050	2060	2070	2080
VFETVEMLPS	KAGIWRVECL	IGEHLHAGMS	TLFLVYSNKC	QTPLGMASGH	IRDFQITASG	QYGQWAPKLA	RLHYSGSINA
2090	2100	2110	2120	2130	2140	2150	2160
WSTKEPFSWI	KVDLLAPMII	HGIKTQGARQ	KFSSLYISQF	IIMYSLDGKK	WQTYRGNSTG	TLMVFFGNVD	SSGIKHNIFN
2170	2180	2190	2200	2210	2220	2230	2240
PPIIARYIRL	HPTHYSIRST	LRMELMGCDL	NSCSMPLGME	SKAISDAQIT	ASSYFTNMFA	TWSPSKARLH	LQGRSNAWRP
2250	2260	2270	2280	2290	2300	2310	2320
QVNNPKEWLQ	VDFQKTMKVT	GVTTQGVKSL	LTSMYVKEFL	ISSSQDGHQW	TLFFQNGKVK	VFQGNQDSFT	PVVNSLDPPL
2330	2340	2350	2360				

Detailed Protein Report

Cmpd.	No. of Cmpds.	m/z meas.	Δ m/z [ppm]	z	Rt [min]	Score	P	Range	Sequence	Modification
843	1	653.3686	-17.24	2	40.6	10.7	2	1167-1178	K.NKVVVGKGEFTK.D	

Detailed Protein Report

Protein 451: PREDICTED: myosin-7B isoform X1 [Homo sapiens]

**Accession:**	gi|578836098	**Score:**	29.4
**Database:**	refseq_human(refseq_human_20140103.fasta)	**MW [kDa]:**	203.5
**Database Date:**	2015-11-30	**pI:**	5.7
		**Sequence Coverage [%]:**	1.5
		**No. of unique Peptides:**	1

10	20	30	40	50	60	70	80
MAPSLYGLRS	GCLQGKAPLR	FPAPYICGGG	QRLQRHAAQP	RQPVHADHQF	LATKTGGTLE	DQIIEANPAM	EAFGNAKTLR
**90**	**100**	**110**	**120**	**130**	**140**	**150**	**160**
NDNSSRFGKF	IRIHFGPSGK	LASADIDSYL	LEKSRVIFQL	PGERSYHVYY	QILSGRKPEL	QDMLLLSMNP	YDYHFCSQGV
**170**	**180**	**190**	**200**	**210**	**220**	**230**	**240**
ITVDNMNDGE	ELIATDHAMD	ILGFSVDEKC	ACYKIVGALL	HFGNMKFKQK	QREEQAEADG	TESADKAAYL	MGVSSGDLLK
**250**	**260**	**270**	**280**	**290**	**300**	**310**	**320**
GLLHPRVRVG	NEYVTKGQSV	EQVVFAVGAL	AKATYDRLFR	WLVSRINQTL	DTKLPRQFFI	GVLDIAGFEI	FEFNSFEQLC
**330**	**340**	**350**	**360**	**370**	**380**	**390**	**400**
INFTNEKLQQ	FFNQHMFVLE	QEEYKREGID	WVFIDFGLDL	QPCIDLIEKP	LGILSILEEE	CMFPKASDAS	FRAKLYDNHA
**410**	**420**	**430**	**440**	**450**	**460**	**470**	**480**
GKSPNFQQPR	PDKKRKYQAH	FEVVHYAGVV	PYSIVGWLEK	NKDPLNETVV	PIFQKSQNRL	LATLYENYAG	SCSTEPPKSG
**490**	**500**	**510**	**520**	**530**	**540**	**550**	**560**
VKEKRKKAAS	FQTVSQLHKE	NLNKLMTNLR	ATQPHFVRCI	VPNENKTPGV	MDAFLVLHQL	RCNGVLEGIR	ICRQGFPNRL
**570**	**580**	**590**	**600**	**610**	**620**	**630**	**640**
LYTDFRQRYR	ILNPSAIPDD	TFMDSRKATE	KLLGSLDLDH	TQYQFGHTKV	FFKAGLLGVL	EELRDQRLAK	VLTLLQARSR
**650**	**660**	**670**	**680**	**690**	**700**	**710**	**720**
GRLMRLEYQR	LLGGRDALFT	IQWNIRAFNA	VKNWSWMKLF	FKMKPLLRSA	QAEEELAALR	AELRGLRGAL	AAAEAKRQEL
**730**	**740**	**750**	**760**	**770**	**780**	**790**	**800**
EETHVSITQE	KNDLALQLQA	EQDNLADAEE	RCHLLIKSKV	QLEGKVKELS	ERLEDEEEVN	ADLAARRRKL	EDECTELKKD
**810**	**820**	**830**	**840**	**850**	**860**	**870**	**880**
IDDLELTLAK	AEKEKQATEN	KVKNLTEEMA	ALDESVARLT	KEKKALQEAH	QQALGDLQAE	EDRVSALTKA	KLRLEQQVED
**890**	**900**	**910**	**920**	**930**	**940**	**950**	**960**
LECSLEQEKK	LRMDTERAKR	KLEGDLKLTQ	ESVADAAQDK	QQLEEKLKKK	DSELSQLSLR	VEDEQLLGAQ	MQKKIKELQA
**970**	**980**	**990**	**1000**	**1010**	**1020**	**1030**	**1040**
RAEELEEELE	AERAARARVE	KQRAEAAREL	EELSERLEEA	GGASAGQREG	CRKREAELGR	LRRELEEAAL	RHEATVAALR
**1050**	**1060**	**1070**	**1080**	**1090**	**1100**	**1110**	**1120**
RKQAEGAAEL	GEQVDSLQRV	RQKLEKEKSE	LRMEVDDLAA	NVETLTRAKA	SAEKLCRTYE	DQLSEAKIKV	EELQRQLADA
**1130**	**1140**	**1150**	**1160**	**1170**	**1180**	**1190**	**1200**
STQRGRLQTE	SGELSRLLEE	KECLISQLSR	GKALAAQSLE	ELRRQLEEES	KAKSALAHAV	QALRHDCDLL	REQHEEEAEA
**1210**	**1220**	**1230**	**1240**	**1250**	**1260**	**1270**	**1280**
QAELQRLLSK	ANAEVAQWRS	KYEADAIQRT	EELEEAKKKL	ALRLQEAEEG	VEAANAKCSS	LEKAKLRLQT	ESEDVTLELE
**1290**	**1300**	**1310**	**1320**	**1330**	**1340**	**1350**	**1360**
RATSAAAALD	KKQRHLERAL	EERRRQEEEM	QRELEAAQRE	SRGLGTELFR	LRHGHEEALE	ALETLKRENK	NLQEEISDLT
**1370**	**1380**	**1390**	**1400**	**1410**	**1420**	**1430**	**1440**
DQVSLSGKSI	QELEKTKKAL	EGEKSEIQAA	LEEAEGALEL	EETKTLRIQL	ELSQVKAEVD	RKLAEKDEEC	ANLRRNHQRA
**1450**	**1460**	**1470**	**1480**	**1490**	**1500**	**1510**	**1520**
VESLQASLDA	ETRARNEALR	LKKKMEGDLN	DLELQLGHAT	RQATEAQAAT	RLMQAQLKEE	QAGRDEEQRL	AAELHEQAQA
**1530**	**1540**	**1550**	**1560**	**1570**	**1580**	**1590**	**1600**
LERRASLLAA	ELEELRAALE	QGERSRRLAE	QELLEATERL	NLLHSQNTGL	LNQKKKLEAD	LAQLSGEVEE	AAQERREAEE
**1610**	**1620**	**1630**	**1640**	**1650**	**1660**	**1670**	**1680**
KAKKAITDAA	MMAEELKKEQ	DTSAHLERMK	KTLEQTVREL	QARLEEAEQA	ALRGGKKQVQ	KLEAKVRELE	AELDAEQKKH
**1690**	**1700**	**1710**	**1720**	**1730**	**1740**	**1750**	**1760**
AEALKGVRKH	ERRVKELAYQ	AEEDRKNLAR	MQDLVDKLQS	KVKSYKRQFE	EAEQQANTNL	AKYRKAQHEL	DDAEERADMA
**1770**	**1780**	**1790**					
ETQANKLRAR	TRDALGPKLS	LSPQHKE					

Cmpd.	No. of	m/z meas.	Δ m/z	z	Rt	Score	P	Range	Sequence	Modification

Detailed Protein Report

	Cmpds.		[ppm]		[min]					
1310	1	939.8664	-147.69	2	46.0	16.1	1	1024-1040	R.ELEEAALRHEATVAALR.R	

Detailed Protein Report

Protein 452: regulator of G-protein signaling 10 isoform b [Homo sapiens]

Accession: gi|4506505
Database: refseq_human(refseq_human_20140103.fasta)
Database Date: 2015-11-30

Score: 29.4
MW [kDa]: 19.6
pI: 5.5
Sequence Coverage [%]: 16.8
No. of unique Peptides: 1

10	20	30	40	50	60	70	80
MEHIHDSDGS	SSSSHQSLKS	TAKWAASLEN	LLEDPEGVKR	FREFLKKEFS	EENVLFWLAC	EDFKKMQDKT	QMQEKAKEIY
**90**	**100**	**110**	**120**	**130**	**140**	**150**	**160**
MTFLSSKASS	QVNVEGQSRL	NEKILEEPHP	LMFQKLQDQI	FNLMKYDSYS	RFLKSDLFLK	HKRTEEEEED	LPDAQTAAKR
**170**							
ASRIYNT							

Cmpd.	No. of Cmpds.	m/z meas.	Δ m/z [ppm]	z	Rt [min]	Score	P	Range	Sequence	Modification
2594	2	1044.5480	50.13	2	63.1	15.6	2	143-160	K.RTEEEEEDLPDAQTAAKR.A	

Detailed Protein Report

Protein 453: PREDICTED: nuclear factor 1 X-type isoform X4 [Homo sapiens]

Accession: gi|530414877
Database: refseq_human(refseq_human_20140103.fasta)
Database Date: 2015-11-30
Modification(s): Oxidation

Score: 29.4
MW [kDa]: 54.1
pI: 9.7
Sequence Coverage [%]: 6.7
No. of unique Peptides: 1

10	20	30	40	50	60	70	80
MDEFHPFIEA	LLPHVRAFSY	TWFNLQARKR	KYFKKHEKRM	SKDEERAVKD	ELLGEKPEIK	QKWASRLLAK	LRKDIRPEFR
**90**	**100**	**110**	**120**	**130**	**140**	**150**	**160**
EDFVLTITGK	KPPCCVLSNP	DQKGKIRRID	CLRQADKVWR	LDLVMVILFK	GIPLESTDGE	RLYKSPQCSN	PGLCVQPHHI
**170**	**180**	**190**	**200**	**210**	**220**	**230**	**240**
GVTIKELDLY	LAYFVHTPES	GQSDSSNQQG	DADIKPLPNG	HLSFQDCFVT	SGVWNVTELV	RVSQTPVATA	SGPNFSLADL
**250**	**260**	**270**	**280**	**290**	**300**	**310**	**320**
ESPSYYNINQ	VTLGRRSITS	PPSTSTTKRP	KSIDDSEMES	PVDDVFYPGT	GRSPAAGSSQ	SSGWPNDVDA	GPASLKKSGK
**330**	**340**	**350**	**360**	**370**	**380**	**390**	**400**
LDFCSALSSQ	GSSPRMAFTH	HPLPVLAGVR	PGSPRATASA	LHFPSTSIIQ	QSSPYFTHPT	IRYHHHHGQD	SLKEFVQFVC
**410**	**420**	**430**	**440**	**450**	**460**	**470**	**480**
SDGSGQATGQ	PNGSGQGKVP	GSFLLPPPPP	VARPVPLPMP	DSKSTSTAPD	GAALTPPSPS	FATTGASSAN	RFVSIGPRDG
**490**	**500**						
NFLNIPQQSQ	SWFL						

Cmpd.	No. of Cmpds.	m/z meas.	Δ m/z [ppm]	z	Rt [min]	Score	P	Range	Sequence	Modification
392	1	874.3959	-106.40	3	34.9	13.0	0	419-443	K.VPGSFLLPPPPPVARPVPLPMPDSK.S	Oxidation: 21

Detailed Protein Report

Protein 454: PREDICTED: centrosomal protein KIAA1731 isoform X7 [Homo sapiens]

**Accession:**	gi|578822447	**Score:**	29.4
**Database:**	refseq_human(refseq_human_20140103.fasta)	**MW [kDa]:**	268.9
**Database Date:**	2015-11-30	**pI:**	5.4
		**Sequence Coverage [%]:**	0.5
		**No. of unique Peptides:**	1

Detailed Protein Report

10	20	30	40	50	60	70	80
MERFEKAHVR	GFQAMKKIHL	AQNQEKLMKE	LKQLQQEDLA	RRRQTVAQMP	PQLVELPYKR	SEMKEDWQRE	LEFAFEDMYN
90	100	110	120	130	140	150	160
ADRKVKGNLI	LHLEPEPLPT	VTNQIQDEEL	DLSMEQENLG	AAEDLPVTEA	EICSSETDVP	LVMKTQQIPS	KVLFKKLLNK
170	180	190	200	210	220	230	240
IRSQKSLWTI	KSMSEDESEM	ITTVSEIESK	APTVESGTIA	SKERTLSSGQ	EQVVESDTLT	IESGPLASED	KPLSCGTNSG
250	260	270	280	290	300	310	320
KEQEINETLP	ITTVAQSSVL	LHPQEAAARI	RMSARQKQIM	EIEEQKQKQL	ELLEQIEQQK	LRLETDCFRA	QLEEEKRKKT
330	340	350	360	370	380	390	400
QPTGVGCRKS	HHYINHLVGI	APASCPVISD	EDSHRQMIRN	YQHQLLQQNR	LHRQSVETAR	KQLLEYQTML	KGRCPSVSAP
410	420	430	440	450	460	470	480
SLITDSVISV	PSWKSERPTA	ISEHWDQGQR	LKLSPNKYQP	IQPIQTSKLE	QDHFQVARQN	HFPQRQVETT	ETLRASDILT
490	500	510	520	530	540	550	560
NQALESQEHL	RQFSQTETQQ	RDYKLVPKDS	ETLSRALSHD	RQLISQDARK	ISETFGATTF	QSLESQQLFS	ENSENISYHL
570	580	590	600	610	620	630	640
TEPSSFVPLV	PQHSFSSLPV	KVESGKIQEP	FSAMSKSTVS	TSHSIISQMH	DRPLLPSENI	TAQQGNMKAL	QEQLDLQKKV
650	660	670	680	690	700	710	720
LQATQEAQEQ	LLLCKQKEVE	QQTGLSVFLP	LVTPDSSALL	PSAKADLGRI	QESSPTKNNI	AVSSDHHVIS	QLQDKRLSLS
730	740	750	760	770	780	790	800
QPILSQQNNF	KFLQEQLNIQ	KDSLQARREA	QEVLYVHKQS	ELDRRVCSEQ	AEPSFPFQVA	QHTFTSLPSA	DTKSGKIQEQ
810	820	830	840	850	860	870	880
HSSKSEKGLV	SCQSDIPISQ	DGSLSFLQQF	LPLHDSLKLL	QEQLTKQRDT	LQARHEAQVE	LLLHRQRDLG	DSKSGLVSSS
890	900	910	920	930	940	950	960
SSPVVVQHSV	ASQASAKAEP	RRIQELYLSE	KENVGPSCHL	IIPTFQDKSL	SFPQHSLAQQ	ENLTILQEQS	QIQRVILGAK
970	980	990	1000	1010	1020	1030	1040
EGTQEFVHTE	SELEKRISSE	QTGTSSSLSQ	VDESERFQEC	ISIKSDSTIP	LSHPKIPRCQ	ERLLRVSQHM	LPLQDNLEEH
1050	1060	1070	1080	1090	1100	1110	1120
QAWLDTEKEA	FHFSQKTQEN	TSSEQTGSSS	FIPQLVQLSF	TSLASAESGT	ILEPLFTESE	SKIFSSHLQI	PQLQDRLLRI
1130	1140	1150	1160	1170	1180	1190	1200
SQLIQPQQDN	LKALQEQLAT	QREAIILARQ	EAREELLLHQ	SEWEGRISPE	QVDTSSLPLV	PQHSFASLPL	NESERNQEPC
1210	1220	1230	1240	1250	1260	1270	1280
SINSDNIVSS	GHSEIPTLPD	GLLGLSHLVL	PQQDNLIALE	EHLHAQTDFL	PSIEKTQKEL	VLSKPCKFEE	KVSSEHFIQS
1290	1300	1310	1320	1330	1340	1350	1360
HHGDLQALQQ	QLDTQKKAIR	SIQEVQEELL	LQRLSELEKR	VSSEQVCSSS	FVSQVPVADS	ERTQKSFPTK	SNDTLPSSHR
1370	1380	1390	1400	1410	1420	1430	1440
EIPRLQDRLL	SLSKPILPQQ	DNMTAQLDAQ	REVMYSYEKP	QEELSLNKQR	KLNKSESAEH	TIPSLFLPKE	TEHSFIPLPF
1450	1460	1470	1480	1490	1500	1510	1520
AEAKPKSTCE	LYSSQNEHAA	PPSNPVIPGF	QDRLLSFSQS	VLTQQDNLGL	QKQLDLQREV	LHYSQKAQEK	LLVQRQTALQ
1530	1540	1550	1560	1570	1580	1590	1600
QQIQKHEETL	KDFFKDSQIS	KPTVENDLKT	QKMGQLRDWF	PNTQDLAGND	QENIRHADRN	NSDDNHLASE	DTSAKQSGEH
1610	1620	1630	1640	1650	1660	1670	1680
LEKDLGRRSS	KPPVAKVKCG	LDLNQHELSA	IQEVESPAIG	RTSILGKPGI	YEDRDPLRVS	ISREQSFFGS	PLAHDPFSCL
1690	1700	1710	1720	1730	1740	1750	1760
QLVGQENVCG	DDYDEAVKLK	ESVVENHAVL	SYAVEEEHAY	LGPTVKPDDK	AKTLSYEPLS	SATVSTGSLL	SYENTDLSLT
1770	1780	1790	1800	1810	1820	1830	1840
DPESFSEHMD	DSKQESTTSK	EEETNIISSI	VPSTQDIYQR	QNSSDVHKSL	LPAVDETTCG	HTHFQQMIDK	YINEANLIPE
1850	1860	1870	1880	1890	1900	1910	1920
KTDLQELEHI	FPNLHHQLFK	PLEPHPDFDL	SSSSSGISPD	NRDFYQRSDS	SSESHCATGL	SKSTVYFTAL	RRTSMHSSLN
1930	1940	1950	1960	1970	1980	1990	2000
TSPNQQPDTN	LAHVGAHSFA	TENIIGGSEQ	CFEQLQPEYS	SQEESQHADL	PSIFSIEARD	SSQGMKNQNY	PSEEHTEILQ
2010	2020	2030	2040	2050	2060	2070	2080
NKKKIVHFQL	SIGNLSSVYS	SSDEANVFDQ	LNVQHSTPCG	SNSSECSTKH	QLESRKESMG	FEELSKRGVV	TMLQSQGLIE
2090	2100	2110	2120	2130	2140	2150	2160
DNKNETCRVL	DINPQVEETD	SRLCVRTVEM	GTSIQAPYSL	TTQNEKYFEN	SAETDIPKIT	KKLSQLGESE	LFASSGSFSL
2170	2180	2190	2200	2210	2220	2230	2240

Detailed Protein Report

Cmpd.	No. of Cmpds.	m/z meas.	Δ m/z [ppm]	z	Rt [min]	Score	P	Range	Sequence	Modification
97	1	608.3115	-17.30	2	30.2	16.4	0	749-758	R.EAQEVLYVHK.Q	

Detailed Protein Report

Protein 455: PREDICTED: nucleobindin-2 isoform X9 [Homo sapiens]

Accession: gi|578820568
Database: refseq_human(refseq_human_20140103.fasta)
Database Date: 2015-11-30

Score: 29.3
MW [kDa]: 40.3
pI: 4.9
Sequence Coverage [%]: 3.8
No. of unique Peptides: 2

Quantitation

QU:MU	**Median:** 1.46	**CV:** 0.00 %	**No. of Peptides:** 1
WUP:QUP	**Median:** 2.06	**CV:** 0.00 %	**No. of Peptides:** 1

```
        10         20         30         40         50         60         70         80
MRWRTILLQY CFLLITCLLT ALEAVPIDID KTKVQNIHPV ESAKIEPPDT GLYYDEYLKQ VIDVLETDKH FREKLQKADI
        90        100        110        120        130        140        150        160
EEIKSGRLSK ELDLVSHHVR TKLDELKRQE VGRLRMLIKA KLDSLQDIGM DHQALLKQFD HLNHLNPDKF ESTDLDMLIK
       170        180        190        200        210        220        230        240
AATSDLEHYD KTRHEEFKKY EMMKEHERRE YLKTLNEEKR KEEESKFEEM KKKHENHPKV NHPGSKDQLK EVWEETDGLD
       250        260        270        280        290        300        310        320
PNDFDPKTFF KLHDVNSDGF LDEQELEALF TKELEKVYDP KNEEDDMVEM EEERLRMREH VMNEVDTNKD RLVTLEEFLK
       330        340
ATEKKEFLEP DSWESPSI
```

Cmpd.	No. of Cmpds.	m/z meas.	Δ m/z [ppm]	z	Rt [min]	Score	P	Range	Sequence	Modification	Ratios
585	1	537.7782	-18.47	2	37.3	11.4	2	172-179	K.TRHEEFKK.Y		QU:MU 1.46<br>WUP:QUP 2.06
1116	1	701.1743	-178.79	1	44.1	17.9	0	180-184	K.YEMMK.E		

Detailed Protein Report

Protein 456: **PREDICTED: peptide chain release factor 1-like, mitochondrial isoform X1 [Homo sapiens]**

Accession: gi|530383831
Database: refseq_human(refseq_human_20140103.fasta)
Database Date: 2015-11-30

Score: 29.3
MW [kDa]: 39.1
pI: 9.8
Sequence Coverage [%]: 8.7
No. of unique Peptides: 1

10	20	30	40	50	60	70	80
MRSRVLWGAA	RWLWPRRAVG	PARRPLSSGS	PPLEELFTRG	GPLRTFLERQ	AGSEAHLKVR	RPELLAVIKL	LNEKERELRE
**90**	**100**	**110**	**120**	**130**	**140**	**150**	**160**
TEHLLHDENE	DLRKLAENEI	TLCQKEITQL	KHQIILLLVP	SEETDENDLI	LEVTAGVGGG	LRHASASIGG	SEAYRHMKFE
**170**	**180**	**190**	**200**	**210**	**220**	**230**	**240**
GGVHRVQRVP	KTEKQGRVHT	STMTVAILPQ	PTEINLVINP	KDLRIDTKRA	SGAGGQHVNT	TDSAVRIVHL	PTGVVSECQQ
**250**	**260**	**270**	**280**	**290**	**300**	**310**	**320**
ERSQLKNKEL	AMTKLRAKLY	SMHLEEEINK	RQNARKIQIG	SKGRSEKIRT	YNFPQNRVTD	HRINKTLHDL	ETFMQGDYLL
**330**	**340**	**350**					
DELVQSLKEY	ADYESLVEII	SQKV					

Cmpd.	No. of Cmpds.	m/z meas.	Δ m/z [ppm]	z	Rt [min]	Score	P	Range	Sequence	Modification
1562	1	1051.5516	-26.89	2	49.2	16.6	2	94-111	R.KLAENEITLCQKEITQLK.H	

Detailed Protein Report

Protein 457: PREDICTED: phosphatidylinositol 4-phosphate 3-kinase C2 domain-containing subunit beta isoform X2 [Homo sapiens]

**Accession:**	gi|530364909	**Score:**	29.3
**Database:**	refseq_human(refseq_human_20140103.fasta)	**MW [kDa]:**	181.7
**Database Date:**	2015-11-30	**pI:**	7.2
**Modification(s):**	Carbamidomethyl	**Sequence Coverage [%]:**	2.1
		**No. of unique Peptides:**	1

10	20	30	40	50	60	70	80
MSSTQGNGEH	WKSLESVGIS	RKELAMAEAL	QMEYDALSRL	RHDKEENRAK	QNADPSLISW	DEPGVDFYSK	PAGRRTDLKL
**90**	**100**	**110**	**120**	**130**	**140**	**150**	**160**
LRGLSGSDPT	LNYNSLSPQE	GPPNHSTSQG	PQPGSDPWPK	GSLSGDYLYI	FDGSDGGVSS	SPGPGDIEGS	CKKLSPPPLP
**170**	**180**	**190**	**200**	**210**	**220**	**230**	**240**
PRASIWDTPP	LPPRKGSPSS	SKISQPSDIN	TFSLVEQLPG	KLLEHRILEE	EEVLGGGGQG	RLLGSVDYDG	INDAITRLNL
**250**	**260**	**270**	**280**	**290**	**300**	**310**	**320**
KSTYDAEMLR	DATRGWKEGR	GPLDFSKDTS	GKPVARSKTM	PPQVPPRTYA	SRYGNRKNAT	PGKNRRISAA	PVGSRPHTVA
**330**	**340**	**350**	**360**	**370**	**380**	**390**	**400**
NGHELFEVSE	ERDEEVAAFC	HMLDILRSGS	DIQDYFLTGY	VWSAVTPSPE	HLGDEVNLKV	TVLCDRLQEA	LTFTCNCSST
**410**	**420**	**430**	**440**	**450**	**460**	**470**	**480**
VDLLIYQTLC	YTHDDLRNVD	VGDFVLKPCG	LEEFLQNKHA	LGSHEYIQYC	RKFDIDIRLQ	LMEQKVVRSD	LARTVNDDQS
**490**	**500**	**510**	**520**	**530**	**540**	**550**	**560**
PSTLNYLVHL	QERPVKQTIS	RQALSLLFDT	YHNEVDAFLL	ADGDFPLKAD	RVVQSVKAIC	NALAAVETPE	ITSALNQLPP
**570**	**580**	**590**	**600**	**610**	**620**	**630**	**640**
CPSRMQPKIQ	KDPSVLAVRE	NREKVVEALT	AAILDLVELY	CNTFNADFQT	AVPGSRKHDL	VQEACHFARS	LAFTVYATHR
**650**	**660**	**670**	**680**	**690**	**700**	**710**	**720**
IPIIWATSYE	DFYLSCSLSH	GGKELCSPLQ	TRRAHFSKYL	FHLIVWDQQI	CFPVQVNRLP	RETLLCATLY	ALPIPPPGSS
**730**	**740**	**750**	**760**	**770**	**780**	**790**	**800**
SEANKQRRVP	EALGWVTTPL	FNFRQVLTCG	RKLLGLWPAT	QENPSARWSA	PNFHQPDSVI	LQIDFPTSAF	DIKFTSPPGD
**810**	**820**	**830**	**840**	**850**	**860**	**870**	**880**
KFSPRYEFGS	LREEDQRKLK	DIMQKESLYW	LTDADKKRLW	EKRYYCHSEQ	WTHMNHQDAL	GLLHATFPDQ	EVRRMAVQWI
**890**	**900**	**910**	**920**	**930**	**940**	**950**	**960**
GSLSDAELLD	YLPQLVQALK	YECYLDSPLV	RFLLKRAVSD	LRVTHYFFWL	LKDGLKDSQF	SIRYQYLLAA	LLCCCGKGLR
**970**	**980**	**990**	**1000**	**1010**	**1020**	**1030**	**1040**
EEFNRQCWLV	NALAKLAQQV	REAAPSARQG	ILRTGLEEVK	QFFALNGSCR	LPLSPSLLVK	GIVPRDCSYF	NSNAVPLKLS
**1050**	**1060**	**1070**	**1080**	**1090**	**1100**	**1110**	**1120**
FQNVDPLGEN	IRVIFKCGDD	LRQDMLTLQM	IRIMSKIWVQ	EGLDMRMVIF	RCFSTGRGRG	MVEMIPNAET	LRKIQVEHGV
**1130**	**1140**	**1150**	**1160**	**1170**	**1180**	**1190**	**1200**
TGSFKDRPLA	DWLQKHNPGE	DEYEKAVENF	IYSCAGCCVA	TYVLGICDRH	NDNIMLKTTG	HMFHIDFGRF	LGHAQMFGNI
**1210**	**1220**	**1230**	**1240**	**1250**	**1260**	**1270**	**1280**
KRDRAPFVFT	SDMAYVINGG	DKPSSRFHDF	VDLCCQAYNL	IRKHTHLFLN	LLGLMLSCGI	PELSDLEDLK	YVYDALRPQD
**1290**	**1300**	**1310**	**1320**	**1330**	**1340**	**1350**	**1360**
TEANATTYFT	RLIESSLGSV	ATKLNFFIHN	LAQMKFTGSD	DRLTLSFASR	THTLKSSGRI	SDVFLCRHEK	IFHPNKGYIY
**1370**	**1380**	**1390**	**1400**	**1410**	**1420**	**1430**	**1440**
VVKVMRENTH	EATYIQRTFE	EFQELHNKLR	LLFPSSHLPS	FPSRFVIGRS	RGEAVAERRR	EELNGYIWHL	IHAPPEVAEC
**1450**	**1460**	**1470**	**1480**	**1490**	**1500**	**1510**	**1520**
DLVYTFFHPL	PRDEKAMGTS	PAPKSSDGTW	ARPVGKVGGE	VKLSISYKNN	KLFIMVMHIR	GLQLLQDGND	PDPYVKIYLL
**1530**	**1540**	**1550**	**1560**	**1570**	**1580**	**1590**	**1600**
PDPQKTTKRK	TKVARKTCNP	TYNEMLVYDG	IPKGDLQQRE	LQLSVLSEQG	FWENVLLGEV	NIRLRELDLA	QEKTGWFALG
**1610**							
SRSHGTL							

Cmpd.	No. of Cmpds.	m/z meas.	Δ m/z [ppm]	z	Rt [min]	Score	P	Range	Sequence	Modification
2067	1	714.5942	-120.32	3	56.2	13.5	1	1227-1243	R.FHDFVDLCCQAYNLIRK.H	Carbamidomethyl: 8

Detailed Protein Report

Protein 458: protein FAM107A isoform a [Homo sapiens]

**Accession:**	gi|6005924	**Score:**	29.3
**Database:**	refseq_human(refseq_human_20140103.fasta)	**MW [kDa]:**	17.4
**Database Date:**	2015-11-30	**pI:**	10.3
		**Sequence Coverage [%]:**	18.1
		**No. of unique Peptides:**	1

Alias proteins:

Accession	Name	Description
gi|530371819	refseq_human (refseq_human_20140103.fasta)	PREDICTED: protein FAM107A isoform X3 [Homo sapiens]
gi|115583665	refseq_human (refseq_human_20140103.fasta)	protein FAM107A isoform a [Homo sapiens]

```
        10         20         30         40         50         60         70         80
MYSEIQRERA DIGGLMARPE YREWNPELIK PKKLLNPVKA SRSHQELHRE LLMNHRRGLG VDSKPELQRV LEHRRRNQLI
        90        100        110        120        130        140        150
KKKKEELEAK RLQCPFEQEL LRRQQRLNQL EKPPEKEEDH APEFIKVREN LRRIATLTSE EREL
```

Cmpd.	No. of Cmpds.	m/z meas.	Δ m/z [ppm]	z	Rt [min]	Score	P	Range	Sequence	Modification
2090	1	696.9638	-136.58	3	54.5	18.1	2	57-74	R.RGLGVDSKPELQRVLEHR.R	

Detailed Protein Report

Protein 459: zinc finger protein 469 [Homo sapiens]

**Accession:**	gi|188536004	**Score:**	29.3
**Database:**	refseq_human(refseq_human_20140103.fasta)	**MW [kDa]:**	409.9
**Database Date:**	2015-11-30	**pI:**	8.9
		**Sequence Coverage [%]:**	0.6
		**No. of unique Peptides:**	1

Detailed Protein Report

10	20	30	40	50	60	70	80
MPGERPRGAP	PPTMTGDLQP	RQVASSPGHP	SQPPLEDNTP	ATRTTKGARE	AGGQAQAMEL	PEAQPRQARD	GELKPPSLRG
90	100	110	120	130	140	150	160
QAPSSTPGKR	GSPQTPPGRS	PLQAPSRLAG	RAEGSPPQRY	ILGIASSRTK	PTLDETPENP	QLEAAQLPEV	DTPQGPGTGA
170	180	190	200	210	220	230	240
PLRPGLPRTE	AQPAAEELGF	HRCFQEPPSS	FTSTNYTSPS	ATPRPPAPGP	PQSRGTSPLQ	PGSYPEYQAS	GADSWPPAAE
250	260	270	280	290	300	310	320
NSFPGANFGV	PPAEPEPIPK	GSRPGGSPRG	VSFQFPFPAL	HGASTKPFPA	DVAGHAFTNG	PLVFAFHQPQ	GAWPEEAVGT
330	340	350	360	370	380	390	400
GPAYPLPTQP	APSPLPCYQG	QPGGLNRHSD	LSGALSSPGA	AHSAPRPFSD	SLHKSLTKIL	PERPPSAQDG	LGSTRGPPSS
410	420	430	440	450	460	470	480
LPQRHFPGQA	YRASGVDTSP	GPPDTELAAP	GPPPARLPQL	WDPTAAPYPT	PPGGPLAATR	SMFFNGQPSP	GQRLCLPQSA
490	500	510	520	530	540	550	560
PLPWPQVLPT	ARPSPHGMEM	LSRLPFPAGG	PEWQGGSQGA	LGTAGKTPGP	REKLPAVRSS	QGGSPALFTY	NGMTDPGAQP
570	580	590	600	610	620	630	640
LFFGVAQPQV	SPHGTPSLPP	PRVVGASPSE	SPLPSPATNT	AGSTCSSLSP	MSSSPANPSS	EESQLPGPLG	PSAFFHPPTH
650	660	670	680	690	700	710	720
PQETGSPFPS	PEPPHSLPTH	YQPEPAKAFP	FPADGLGAEG	AFQCLEETPF	PHEGPEVGRG	GLQGFPRAPP	PYPTHHFSLS
730	740	750	760	770	780	790	800
SASLDQLDVL	LTCRQCDRNY	SSLAAFLAHR	QFCGLLLARA	KDGHQRSPGP	PGLPSPPAAP	RVPADAHAGL	LSHAKTFLLA
810	820	830	840	850	860	870	880
GDAQAEGKDD	PLRTGFLPSL	AATPFPLPAS	DLDMEDDAKL	DSLITEALNG	MEYQSDNPEI	DSSFIDVFAD	EEPSGPRGPS
890	900	910	920	930	940	950	960
SGHPLKSKAG	VTPESKAPPP	LPAATPDPQT	PRPGDRGCPA	RGRPKTRSLG	LAPTEADAPS	QGRQQRRGKQ	LKLFRKDLDS
970	980	990	1000	1010	1020	1030	1040
GGAAEGSGSG	GGGRASGLRP	RRNDGLGERP	PPRPRRPRTQ	APGSRADPAP	RVPRAAALPE	ETRSSRRRRL	PPRKDPRKRK
1050	1060	1070	1080	1090	1100	1110	1120
ARGGAWGKEL	ILKIVQQKNR	LREYDFASES	EEDEQPPPRG	PGFRGRRGRG	EKRKEVELTQ	GPREDEPQKP	RKAARQEAGG
1130	1140	1150	1160	1170	1180	1190	1200
DGAPANPEEP	GGSRPGPGRS	PQARGPSRSL	ETGAAAREGG	PKCADRPSVA	PKDPLQVPTN	TETSEETRPS	LDFPQEAKEP
1210	1220	1230	1240	1250	1260	1270	1280
ETAEESAPDS	TEFTEALRSP	PAACAGEMGA	SPGLLIPEQP	PPSRHDTGTP	KPSGSLANTA	PHGSSPTPGV	GSLLGGPGGT
1290	1300	1310	1320	1330	1340	1350	1360
QAPVSHNSKD	PPARQPGEFL	APVANPSSTA	CPKPSVLSSK	ISSFGCDPAG	FNRDPLGVPV	AKKGPQPYSS	PHSELFLGPK
1370	1380	1390	1400	1410	1420	1430	1440
DLAGCFLEEL	HPKPSARDAP	PASSSCLCQD	GEDAGSLEPQ	LPRSPPGTAE	TEPGRAASPP	TLESSSLFPD	LPVDRFDPPL
1450	1460	1470	1480	1490	1500	1510	1520
YGSLSANRDS	GLPFACADPP	QKTVPSDPPY	PSFLLLEEVS	PMLPSHFPDL	SGGKVLSKTC	PPERTVVPGA	APSLPGKGSG
1530	1540	1550	1560	1570	1580	1590	1600
CSVALMSHLS	EDELEIQKLV	TELESQLQRS	KDTRGAPREL	AEAESVGRVE	LGTGTEPPSQ	RRTCQATVPH	EDTFSAADLT
1610	1620	1630	1640	1650	1660	1670	1680
RVGESTAHRE	GAESAVATVE	AVQGRPGGTW	PCPASFHPGH	AALLPCAQED	LVSGAPFSPR	GANFHFQPVQ	KAGASKTGLC
1690	1700	1710	1720	1730	1740	1750	1760
QAEGDSRPPQ	DVCLPEPSKQ	PGPQLDAGSL	AKCSPDQELS	FPKNKEAASS	QESEDSLRLL	PCEQRGGFLP	EPGTADQPHR
1770	1780	1790	1800	1810	1820	1830	1840
GAPAPEAFGS	PAVHLAPDLA	FQGDGAPPLD	ATWPFGASPS	HAAQGHSAGR	AGGHLPHPTAG	RPGFEGNEFA	PAGASSLTAP
1850	1860	1870	1880	1890	1900	1910	1920
RGREAWLVPV	PSPACVSNTH	PSRRSQDPAL	SPPIRQLQLP	GPGVAKSKDG	ILGLQELTPA	AQSPPRVNPS	GLEGGTVEGG
1930	1940	1950	1960	1970	1980	1990	2000
KVACGPAQGS	PGGVQVTTLP	AVAGHQLGLE	ADGHWGLLGQ	AEKTQGQGTA	NQLQPENGVS	PGGTDNHASV	NASPKTALTG
2010	2020	2030	2040	2050	2060	2070	2080
PTEGAVLLEK	CKGSRAAMSL	QEEAEPTPSP	PSPNRESLAL	ALTAAHSRSG	SEGRTPERAS	SPGLNKPLLA	TGDSPAPSVG
2090	2100	2110	2120	2130	2140	2150	2160
DLAACAPSPT	SAAHMPCSLG	PLPREDPLTS	PSRAQGGLGG	QLPASPSCRD	PPGPQQLLAC	SPAWAPLEEA	DGVQATTDTG
2170	2180	2190	2200	2210	2220	2230	2240

Detailed Protein Report

Cmpd.	No. of Cmpds.	m/z meas.	Δ m/z [ppm]	z	Rt [min]	Score	P	Range	Sequence	Modification
2716	2	715.3661	-0.43	2	62.4	12.8	2	3063-3076	R.RTEEAAGAGRAQGR.G	

Detailed Protein Report

Protein 460: SCO-spondin precursor [Homo sapiens]

**Accession:**	gi|134031945	**Score:**	29.3
**Database:**	refseq_human(refseq_human_20140103.fasta)	**MW [kDa]:**	547.1
**Database Date:**	2015-11-30	**pI:**	5.6
**Modification(s):**	Carbamidomethyl	**Sequence Coverage [%]:**	1.0
		**No. of unique Peptides:**	2

Detailed Protein Report

10	20	30	40	50	60	70	80
MLLPALLFGM	AWALADGRWC	EWTETIRVEE	EVAPRQEDLV	PCASLDHYSR	LGWRLDLPWS	GRSGLTRSPA	PGLCPIYKPP
90	100	110	120	130	140	150	160
ETRPAKWNRT	VRTCCPGWGG	AHCTEALAKA	SPEGHCFAMW	QCQLQAGSAN	ASAGSLEECC	ARPWGQSWWD	GSSQACRSCS
170	180	190	200	210	220	230	240
SRHLPGSASS	PALLQPLAGA	VGQLWSQHQR	PSATCASWSG	FHYRTFDGRH	YHFLGRCTYL	LAGAADSTWA	VHLTPGDRCP
250	260	270	280	290	300	310	320
QPGHCQRVTM	GPEEVLIQAG	NVSVKGQLVP	EGQSWLLHGL	SLQWLGDWLV	LSGGLGVVVR	LDRTGSISIS	VDHELWGQTQ
330	340	350	360	370	380	390	400
GLCGLYNGWP	EDDFMEPGGG	LAMLAATFGN	SWRLPGSESG	CLDAVEVAQG	CDPLGLIDAD	VEPGHLRAEA	QDVCHQLLEG
410	420	430	440	450	460	470	480
PFGQCHAQVS	PAEYHEACLF	AYCAGAMAGS	GQEGRQQAVC	ATFASYVQAC	ARRHIHIRWR	KPGFCERLCP	GGQLYSDCVS
490	500	510	520	530	540	550	560
LCPPSCEAVG	QGEEESCREE	CVSGCECPRG	LFWNGTLCVP	AAHCPCYYCR	QRYVPGDTVR	QLCNPCVCRD	GRWHCAQALC
570	580	590	600	610	620	630	640
PAECAVGGDG	HYLTFDGRSY	SFWGGQGCRY	SLVQDYVKGQ	LLILLEHGAC	DAGSCLHAIS	VSLEDTHIQL	RDSGAVLVNG
650	660	670	680	690	700	710	720
QDVGLPWIGA	EGLSVRRASS	AFLLLRWPGA	QVLWGLSDPV	AYITLDPRHA	HQVQGLCGTF	TQNQQDDFLT	PAGDVETSIA
730	740	750	760	770	780	790	800
AFASKFQVAG	KGRCPSEDSA	LLSPCTTHSQ	RHAFAEAACA	ILHSSVFQEC	HRLVDKEPFY	LRCLAAVCGC	DPGSDCLCPV
810	820	830	840	850	860	870	880
LSAYARRCAQ	EGASPPWRNQ	TLCPVMCPGG	QEYRECAPAC	GQHCGKPEDC	GELGSCVAGC	NCPLGLLWDP	EGQCVPPSLC
890	900	910	920	930	940	950	960
PCQLGARRYA	PGSATMKECN	RCICQERGLW	NCTARHCPSQ	AFCPRELVYA	PGACLLTCDS	PSANHSCPAG	STDGCVCPPG
970	980	990	1000	1010	1020	1030	1040
TVLLDERCVP	PDLCPCRHSG	QWYLPNATIQ	EDCNVCVCRG	RQWHCTGQRR	SGRCQASGAP	HYVTFDGLAF	TYPGACEYLL
1050	1060	1070	1080	1090	1100	1110	1120
VREASGLFTV	SAQNLPCGAS	GLTCTKALAV	RLEGTVVHML	RGRAVTVNGV	SVTPPKVYTG	PGLSLRRAGL	FLLLSTHLGL
1130	1140	1150	1160	1170	1180	1190	1200
TLLWDGGTRV	LVQLSPQFRG	RVAGLCGDFD	GDASNDLRSR	QGVLEPTAEL	AAHSWRLSPL	CPEPGDLPHP	CTMNTHRAGW
1210	1220	1230	1240	1250	1260	1270	1280
ARARCGALLQ	PLFTLCHAEV	PPQQHYEWCL	YDACGCDSGG	DCECLCSAIA	TYADECARHG	HHVRWRSQEL	CSLQCEGGQV
1290	1300	1310	1320	1330	1340	1350	1360
YEACGPTCPP	TCHEQHPEPG	WHCQVVACVE	GCFCPEGTLL	HGGACLEPAS	CPCEWGRNSF	PPGSVLQKDC	GNCTCQEGQW
1370	1380	1390	1400	1410	1420	1430	1440
HCGGDGGHCE	ELVPACAEGE	ALCQENGHCV	PHGWLCDNQD	DCGDGSDEEG	CAAPGCGEGQ	MTCSSGHCLP	LALLCDRQDD
1450	1460	1470	1480	1490	1500	1510	1520
CGDGTDEPSY	PCPQGLLACA	DGRCLPPALL	CDGHPDCLDA	ADEESCLGQV	TCVPGEVSCV	DGTCLGAIQL	CDGVWDCPDG
1530	1540	1550	1560	1570	1580	1590	1600
ADEGPGHCPL	PSLPTPPAST	LPGPSPGSLD	TASSPLASAS	PAPPCGPFEF	RCGSGECTPR	GWRCDQEEDC	ADGSDERGCG
1610	1620	1630	1640	1650	1660	1670	1680
GPCAPHHAPC	ARGPHCVSPE	QLCDGVRQCP	DGSDEGPDAC	GGLPALGGPN	RTGLPCPEYT	CPNGTCIGFQ	LVCDGQPDCG
1690	1700	1710	1720	1730	1740	1750	1760
RPGQVGPSPE	EQGCGAWGPW	SPWGPCSRTC	GPWGQGRSRR	CSPLGLLVLQ	NCPGPEHQSQ	ACFTAACPVD	GEWSTWSPWS
1770	1780	1790	1800	1810	1820	1830	1840
VCSEPCRGTM	TRQRQCHSPQ	NGGRTCAALP	GGLHSTRQTK	PCPQDGCPNA	TCSGELMFQP	CAPCPLTCDD	ISGQVTCPPD
1850	1860	1870	1880	1890	1900	1910	1920
WPCGSPGCWC	PEGQVLGSEG	WCVWPRQCPC	LVDGARYWPG	QRIKADCQLC	ICQDGRPRRC	RLNPDCAVDC	GWSSWSPWAK
1930	1940	1950	1960	1970	1980	1990	2000
CLGPCGSQSI	QWSFRSSNNP	RPSGRGRQCR	GIHRKARRCQ	TEPCEGCEHQ	GQVHRVGERW	HGGPCRVCQC	LHNLTAHCSP
2010	2020	2030	2040	2050	2060	2070	2080
YCPLGSCPQG	WVLVEGTGES	CCHCALPGEN	QTVQPMATPA	AAPAPSPQIR	FPLATYILPP	SGDPCYSPLG	LAGLAEGSLH
2090	2100	2110	2120	2130	2140	2150	2160
ASSQQLEHPT	QAALLGAPTQ	GPSPQGWHAG	GDAYAKWHTR	PHYLQLDLLQ	PRNLTGILVP	ETGSSNAYAS	SFSLQFSSNG
2170	2180	2190	2200	2210	2220	2230	2240

Detailed Protein Report

Cmpd.	No. of Cmpds.	m/z meas.	Δ m/z [ppm]	z	Rt [min]	Score	P	Range	Sequence	Modification
1387	1	927.1161	66.38	3	47.5	19.2	0	2250-2277	R.GGPCDGVLDCEDGSDEEGCVLLPEGTGR.F	
1393	1	927.1317	74.51	3	47.0	10.1	1	4052-4077	R.LCNPMPCEAGCPAGMEVVTCANRCPR.R	Carbamidomethyl: 2

Detailed Protein Report

Protein 461: leukocyte receptor cluster member 9 [Homo sapiens]

**Accession:**	gi|206725452	**Score:**	29.2
**Database:**	refseq_human(refseq_human_20140103.fasta)	**MW [kDa]:**	53.1
**Database Date:**	2015-11-30	**pI:**	9.5
		**Sequence Coverage [%]:**	8.2
		**No. of unique Peptides:**	1

10	20	30	40	50	60	70	80
MGSRPPCGAT	SSARRACQFP	APMAAAREPE	LPQEAPATEP	APPPACRFFL	EGRCRFGARC	RQPHPGAPAP	PGREAQPEAG
**90**	**100**	**110**	**120**	**130**	**140**	**150**	**160**
AKKPPLRTAA	DVIQRIRWDP	RLDPADFSVG	YVDRFLGVRE	EPFSAFCWDQ	PLAALGPGVL	AVPQHRVRFF	RFHGRLVWDR
**170**	**180**	**190**	**200**	**210**	**220**	**230**	**240**
ASRTDLVFGS	GSAAGRGPTI	LDAPNTEGAH	GAEGAEWTLA	GTGQEAQAAP	KRGSTRPLCT	GHQEPGVEEP	GELEAAQERA
**250**	**260**	**270**	**280**	**290**	**300**	**310**	**320**
LGTAADLGTL	APRGRLAGVT	EEALKPTAAT	RTTLLGGKEA	QALGVPGGSA	ETTEAEWGPA	AWPEDKRARL	SVAAPCQPRP
**330**	**340**	**350**	**360**	**370**	**380**	**390**	**400**
THFVALMVTE	PGLQAEVTKA	QEYLVHVAPH	CANFLVPSQN	LHLTLALLRL	AGAGEEAAAI	GALRRALLAP	GLNAPPRLSF
**410**	**420**	**430**	**440**	**450**	**460**	**470**	**480**
RKLVLLGPHV	LCAPPSPTLE	SMAQVLSQRL	EAEGLSTLQS	PGQLHPHLTV	AKVPHGSQVH	LPKLEFTLSQ	EVGCQPLQTL
**490**	**500**	**510**					
WLCRIGRTGG	PFQPLAEIRL	E					

Cmpd.	No. of Cmpds.	m/z meas.	Δ m/z [ppm]	z	Rt [min]	Score	P	Range	Sequence	Modification
1675	1	1011.7711	-47.99	3	51.2	10.4	1	212-239	K.RGSTRPLCTGHQEPGVEEPGELEAAQER.A	

Detailed Protein Report

Protein 462: rho GTPase-activating protein 42 [Homo sapiens]

**Accession:**	gi|221307575	**Score:**	29.2
**Database:**	refseq_human(refseq_human_20140103.fasta)	**MW [kDa]:**	98.5
**Database Date:**	2015-11-30	**pI:**	8.9
**Modification(s):**	Carbamidomethyl, Oxidation	**Sequence Coverage [%]:**	2.9
		**No. of unique Peptides:**	2

Quantitation

QU:MU	**Median:** 1.65	**CV:** 0.00 %	**No. of Peptides:** 1
WUP:QUP	**Median:** 0.71	**CV:** 0.00 %	**No. of Peptides:** 1

10	20	30	40	50	60	70	80
MGLPTLEFSD	SYLDSPDFRE	RLQCHEIELE	RTNKFIKELI	KDGSLLIGAL	RNLSMAVQKF	SQSLQDFQFE	CIGDAETDDE
**90**	**100**	**110**	**120**	**130**	**140**	**150**	**160**
ISIAQSLKEF	ARLLIAVEEE	RRRLIQNAND	VLIAPLEKFR	KEQIGAAKDG	KKKFDKESEK	YYSILEKHLN	LSAKKKESHL
**170**	**180**	**190**	**200**	**210**	**220**	**230**	**240**
QEADTQIDRE	HQNFYEASLE	YVFKIQEVQE	KKKFEFVEPL	LSFLQGLFTF	YHEGYELAQE	FAPYKQQLQF	NLQNTRNNFE
**250**	**260**	**270**	**280**	**290**	**300**	**310**	**320**
STRQEVERLM	QRMKSANQDY	RPPSQWTMEG	YLYVQEKRPL	GFTWIKHYCT	YDKGSKTFTM	SVSEMKSSGK	MNGLVTSSPE
**330**	**340**	**350**	**360**	**370**	**380**	**390**	**400**
MFKLKSCIRR	KTDSIDKRFC	FDIEVVERHG	IITLQAFSEA	NRKLWLEAMD	GKEPIYTLPA	IISKKEEMYL	NEAGFNFVRK
**410**	**420**	**430**	**440**	**450**	**460**	**470**	**480**
CIQAVETRGI	TILGLYRIGG	VNSKVQKLMN	TTFSPKSPPD	IDIDIELWDN	KTITSGLKNY	LRCLAEPLMT	YKLHKDFIIA
**490**	**500**	**510**	**520**	**530**	**540**	**550**	**560**
VKSDDQNYRV	EAVHALVHKL	PEKNREMLDI	LIKHLVKVSL	HSQQNLMTVS	NLGVIFGPTL	MRAQEETVAA	MMNIKFQNIV
**570**	**580**	**590**	**600**	**610**	**620**	**630**	**640**
VEILIEHYEK	IFHTAPDPSI	PLPQPQSRSG	SRRTRAICLS	TGSRKPRGRY	TPCLAEPDSD	SYSSSPDSTP	MGSIESLSSH
**650**	**660**	**670**	**680**	**690**	**700**	**710**	**720**
SSEQNSTTKS	ASCQPREKSG	GIPWIATPSS	SNGQKSLGLW	TTSPESSSRE	DATKTDAESD	CQSVASVTSP	GDVSPPIDLV
**730**	**740**	**750**	**760**	**770**	**780**	**790**	**800**
KKEPYGLSGL	KRASASSLRS	ISAAEGNKSY	SGSIQSLTSV	GSKETPKASP	NPDLPPKMCR	RLRLDTASSN	GYQRPGSVVA
**810**	**820**	**830**	**840**	**850**	**860**	**870**	**880**
AKAQLFENVG	SPKPVSSGRQ	AKAMYSCKAE	HSHELSFPQG	AIFSNVYPSV	EPGWLKATYE	GKTGLVPENY	VVFL

Cmpd.	No. of Cmpds.	m/z meas.	Δ m/z [ppm]	z	Rt [min]	Score	P	Range	Sequence	Modification	Ratios
150	1	849.3532	-94.77	2	31.6	11.8	1	311-325	K.MNGLVTSSPEMFKLK.S	Oxidation: 11	WUP:QUP 0.71<br>QU:MU 1.65
1883	2	621.2961	-4.47	2	53.9	17.5	0	463-472	R.CLAEPLMTYK.L	Carbamidomethyl: 1;<br>Oxidation: 7	

Detailed Protein Report

Protein 463: unconventional myosin-Id [Homo sapiens]

Accession: gi|51100974
Database: refseq_human(refseq_human_20140103.fasta)
Database Date: 2015-11-30

Score: 29.2
MW [kDa]: 116.1
pI: 10.0
Sequence Coverage [%]: 1.8
No. of unique Peptides: 1

Quantitation

WUP:QUP **Median:** 0.37 **CV:** 0.00 % **No. of Peptides:** 1

10	20	30	40	50	60	70	80
MAEQESLEFG	KADFVLMDTV	SMPEFMANLR	LRFEKGRIYT	FIGEVVVSVN	PYKLLNIYGR	DTIEQYKGRE	LYERPPHLFA
90	100	110	120	130	140	150	160
IADAAYKAMK	RRSKDTCIVI	SGESGAGKTE	ASKYIMQYIA	AITNPSQRAE	VERVKNMLLK	SNCVLEAFGN	AKTNRNDNSS
170	180	190	200	210	220	230	240
RFGKYMDINF	DFKGDPIGGH	INNYLLEKSR	VIVQQPGERS	FHSFYQLLQG	GSEQMLRSLH	LQKSLSSYNY	IHVGAQLKSS
250	260	270	280	290	300	310	320
INDAAEFRVV	ADAMKVIGFK	PEEIQTVYKI	LAAILHLGNL	KFVVDGDTPL	IENGKVVSII	AELLSTKTDM	VEKALLYRTV
330	340	350	360	370	380	390	400
ATGRDIIDKQ	HTEQEASYGR	DAFAKAIYER	LFCWIVTRIN	DIIEVKNYDT	TIHGKNTVIG	VLDIYGFEIF	DNNSFEQFCI
410	420	430	440	450	460	470	480
NYCNEKLQQL	FIQLVLKQEQ	EEYQREGIPW	KHIDYFNNQI	IVDLVEQQHK	GIIAILDDAC	MNVGKVTDEM	FLEALNSKLG
490	500	510	520	530	540	550	560
KHAHFSSRKL	CASDKILEFD	RDFRIRHYAG	DVVYSVIGFI	DKNKDTLFQD	FKRLMYNSSN	PVLKNMWPEG	KLSITEVTKR
570	580	590	600	610	620	630	640
PLTAATLFKN	SMIALVDNLA	SKEPYYVRCI	KPNDKKSPQI	FDDERCRHQV	EYLGLLENVR	VRRAGFAFRQ	TYEKFLHRYK
650	660	670	680	690	700	710	720
MISEFTWPNH	DLPSDKEAVK	KLIERCGFQD	DVAYGKTKIF	IRTPRTLFTL	EELRAQMLIR	IVLFLQKVWR	GTLARMRYKR
730	740	750	760	770	780	790	800
TKAALTIIRY	YRRYKVKSYI	HEVARRFHGV	KTMRDYGKHV	KWPSPPKVLR	RFEEALQTIF	NRWRASQLIK	SIPASDLPQV
810	820	830	840	850	860	870	880
RAKVAAVEML	KGQRADLGLQ	RAWEGNYLAS	KPDTPQTSGT	FVPVANELKR	KDKYMNVLFS	CHVRKVNRFS	KVEDRAIFVT
890	900	910	920	930	940	950	960
DRHLYKMDPT	KQYKVMKTIP	LYNLTGLSVS	NGKDQLVVFH	TKDNKDLIVC	LFSKQPTHES	RIGELVGVLV	NHFKSEKRHL
970	980	990	1000	1010			
QVNVTNPVQC	SLHGKKCTVS	VETRLNQPQP	DFTKNRSGFI	LSVPGN			

Cmpd.	No. of Cmpds.	m/z meas.	Δ m/z [ppm]	z	Rt [min]	Score	P	Range	Sequence	Modification	Ratios
2982	3	729.3741	-5.85	2	66.0	18.2	1	985-996	R.LNQPQPDFTKNR.S		WUP:QUP 0.37

Detailed Protein Report

Protein 464: plakophilin-1 isoform 1a [Homo sapiens]

Accession: gi|53729344
Database: refseq_human(refseq_human_20140103.fasta)
Database Date: 2015-11-30
Modification(s): Carbamidomethyl

Score: 29.1
MW [kDa]: 80.4
pI: 10.0
Sequence Coverage [%]: 3.3
No. of unique Peptides: 1

10	20	30	40	50	60	70	80
MNHSPLKTAL	AYECFQDQDN	STLALPSDQK	MKTGTSGRQR	VQEQVMMTVK	RQKSKSSQSS	TLSHSNRGSM	YDGLADNYNY
90	100	110	120	130	140	150	160
GTTSRSSYYS	KFQAGNGSWG	YPIYNGTLKR	EPDNRRFSSY	SQMENWSRHY	PRGSCNTTGA	GSDICFMQKI	KASRSEPDLY
170	180	190	200	210	220	230	240
CDPRGTLRKG	TLGSKGQKTT	QNRYSFYSTC	SGQKAIKKCP	VRPPSCASKQ	DPVYIPPISC	NKDLSFGHSR	ASSKICSEDI
250	260	270	280	290	300	310	320
ECSGLTIPKA	VQYLSSQDEK	YQAIGAYYIQ	HTCFQDESAK	QQVYQLGGIC	KLVDLLRSPN	QNVQQAAAGA	LRNLVFRSTT
330	340	350	360	370	380	390	400
NKLETRRQNG	IREAVSLLRR	TGNAEIQKQL	TGLLWNLSST	DELKEELIAD	ALPVLADRVI	IPFSGWCDGN	SNMSREVVDP
410	420	430	440	450	460	470	480
EVFFNATGCL	RNLSSADAGR	QTMRNYSGLI	DSLMAYVQNC	VAASRCDDKS	VENCMCVLHN	LSYRLDAEVP	TRYRQLEYNA
490	500	510	520	530	540	550	560
RNAYTEKSST	GCFSNKSDKM	MNNNYDCPLP	EEETNPKGSG	WLYHSDAIRT	YLNLMGKSKK	DATLEACAGA	LQNLTASKGL
570	580	590	600	610	620	630	640
MSSGMSQLIG	LKEKGLPQIA	RLLQSGNSDV	VRSGASLLSN	MSRHPLLHRV	MGNQVFPEVT	RLLTSHTGNT	SNSEDILSSA
650	660	670	680	690	700	710	720
CYTVRNLMAS	QPQLAKQYFS	SSMLNNIINL	CRSSASPKAA	EAARLLLSDM	WSSKELQGVL	RQQGFDRNML	GTLAGANSLR
730							
NFTSRF							

Cmpd.	No. of Cmpds.	m/z meas.	Δ m/z [ppm]	z	Rt [min]	Score	P	Range	Sequence	Modification
1592	1	783.4244	89.54	2	50.1	14.5	1	152-164	K.ASRSEPDLYCDPR.G	Carbamidomethyl: 10

Detailed Protein Report

Protein 465: interferon regulatory factor 2-binding protein 2 isoform B [Homo sapiens]

Accession: gi|116734706
Database: refseq_human(refseq_human_20140103.fasta)
Database Date: 2015-11-30
Modification(s): Carbamidomethyl

Score: 29.1
MW [kDa]: 59.4
pI: 10.1
Sequence Coverage [%]: 4.4
No. of unique Peptides: 2

10	20	30	40	50	60	70	80
MAAAVAVAAA	SRRQSCYLCD	LPRMPWAMIW	DFTEPVCRGC	VNYEGADRVE	FVIETARQLK	RAHGCFPEGR	SPPGAAASAA
**90**	**100**	**110**	**120**	**130**	**140**	**150**	**160**
AKPPPLSAKD	ILLQQQQQLG	HGGPEAAPRA	PQALERYPLA	AAAERPPRLG	SDFGSSRPAA	SLAQPPTPQP	PPVNGILVPN
**170**	**180**	**190**	**200**	**210**	**220**	**230**	**240**
GFSKLEEPPE	LNRQSPNPRR	GHAVPPTLVP	LMNGSATPLP	TALGLGGRAA	ASLAAVSGTA	AASLGSAQPT	DLGAHKRPAS
**250**	**260**	**270**	**280**	**290**	**300**	**310**	**320**
VSSSAAVEHE	QREAAAKEKQ	PPPPAHRGPA	DSLSTAAGAA	ELSAEGAGKS	RGSGEQDWVN	RPKTVRDTLL	ALHQHGHSGP
**330**	**340**	**350**	**360**	**370**	**380**	**390**	**400**
FESKFKKEPA	LTAVARTARK	RKPSPEPEGE	VGPPKINGEA	QPWLSTSTEG	LKIPMTPTSS	FVSPPPPTAS	PHSNRTTPPE
**410**	**420**	**430**	**440**	**450**	**460**	**470**	**480**
AAQNGQSPMA	ALILVADNAG	GSHASKDANQ	VHSTTRRNSN	SPPSPSSMNQ	RRLGPREVGG	QGAGNTGGLE	PVHPASLPDS
**490**	**500**	**510**	**520**	**530**	**540**	**550**	**560**
SLATSAPLCC	TLCHERLEDT	HFVQCPSVPS	HKFCFPCSRQ	SIKQQGASGE	VYCPSGEKCP	LVGSNVPWAF	MQGEIATILA
**570**	**580**						
GDVKVKKERD	S						

Cmpd.	No. of Cmpds.	m/z meas.	Δ m/z [ppm]	z	Rt [min]	Score	P	Range	Sequence	Modification
1850	1	724.2703	-144.49	2	53.0	18.8	0	342-355	R.KPSPEPEGEVGPPK.I	
2516	1	715.2357	-144.98	2	61.4	10.3	1	513-523	K.FCFPCSRQSIK.Q	Carbamidomethyl: 2, 5

Detailed Protein Report

Protein 466: PREDICTED: YLP motif-containing protein 1 isoform X1 [Homo sapiens]

**Accession:**	gi|530404193	**Score:**	29.1
**Database:**	refseq_human(refseq_human_20140103.fasta)	**MW [kDa]:**	231.6
**Database Date:**	2015-11-30	**pI:**	6.0
**Modification(s):**	Oxidation	**Sequence Coverage [%]:**	0.9
		**No. of unique Peptides:**	2

Quantitation

QU:MU	**Median:**	1.46	**CV:**	0.00 %	**No. of Peptides:**	1
WUP:QUP	**Median:**	0.74	**CV:**	0.00 %	**No. of Peptides:**	1

Detailed Protein Report

10	20	30	40	50	60	70	80
MYPNWGRYGG	SSHYPPPPVP	PPPPVALPEA	SPGPGYSSST	TPAAPSSSGF	MSFREQHLAQ	LQQLQQMHQK	QMQCVLQPHH
90	100	110	120	130	140	150	160
LPPPPLPPPP	VMPGGGYGDW	QPPPPPMPPP	PGPALSYQKQ	QQYKHQMLHH	QRDGPPGLVP	MELESPPESP	PVPPGSYMPP
170	180	190	200	210	220	230	240
SQSYMPPPQP	PPSYYPPTSS	QPYLPPAQPS	PSQSPPSQSY	LAPTPSYSSS	SSSSQSYLSH	SQSYLPSSQA	SPSRPSQGHS
250	260	270	280	290	300	310	320
KSQLLAPPPP	SAPPGNKTTV	QQEPLESGAK	NKSTEQQQAA	PEPDPSTMTP	QEQQQYWYRQ	HLLSLQQRTK	VHLPGHKKGP
330	340	350	360	370	380	390	400
VVAKDTPEPV	KEEVTVPATS	QVPESPSSEE	PPLPPPNEEV	PPPLPPEEPQ	SEDPEEDARL	KQLQAAAAHW	QQHQQHRVGF
410	420	430	440	450	460	470	480
QYQGIMQKHT	QLQQILQQYQ	QIIQPPPHIQ	TMSVDMQLRH	YEMQQQQFQH	LYQEWEREFQ	LWEEQLHSYP	HKDQLQEYEK
490	500	510	520	530	540	550	560
QWKTWQGHMK	ATQSYLQEKV	NSFQNMKNQY	MGNMSMPPPF	VPYSQMPPPL	PTMPPPVLPP	SLPPPVMPPA	LPATVPPPGM
570	580	590	600	610	620	630	640
PPPVMPPSLP	TSVPPPGMPP	SLSSAGPPPV	LPPPSLSSAG	PPPVLPPPSL	SSTAPPPVMP	LPPLSSATPP	PGIPPPGVPQ
650	660	670	680	690	700	710	720
GIPPQLTAAP	VPPASSSQSS	QVPEKPRPAL	LPTPVSFGSA	PPTTYHPPLQ	SAGPSEQVNS	KAPLSKSALP	YSSFSSDQGL
730	740	750	760	770	780	790	800
GESSAAPSQP	ITAVKDMPVR	SGGLLPDPPR	SSYLESPRGP	RFDGPRRFED	LGSRCEGPRP	KGPRFEGNRP	DGPRPRYEGH
810	820	830	840	850	860	870	880
PAEGTKSKWG	MIPRGPASQF	YITPSTSLSP	RQSGPQWKGP	KPAFGQQHQQ	QPKSQAEPLS	GNKEPLADTS	SNQQKNFKMQ
890	900	910	920	930	940	950	960
SAAFSIAADV	KDVKAAQSNE	NLSDSQQEPP	KSEVSEGPVE	PSNWDQNVQS	METQIDKAQA	VTQPVPLANK	PVPAQSTFPS
970	980	990	1000	1010	1020	1030	1040
KTGGMEGGTA	VATSSLTADN	DFKPVGIGLP	HSENNQDKGL	PRPDNRDNRL	EGNRGNSSSY	RGPGQSRMED	TRDKGLVNRG
1050	1060	1070	1080	1090	1100	1110	1120
RGQAISRGPG	LVKQEDFRDK	MMGRREDSRE	KMNRGEGSRD	RGLVRPGSSR	EKVPGGLQGS	QDRGAAGSRE	RGPPRRAGSQ
1130	1140	1150	1160	1170	1180	1190	1200
ERGPLRRAGS	RERIPPRRAG	SRERGPPRGP	GSRERGLGRS	DFGRDRGPFR	PEPGDGGEKM	YPYHRDEPPR	APWNHGEERG
1210	1220	1230	1240	1250	1260	1270	1280
HEEFPLDGRN	APMERERLDD	WDRERYWREC	ERDYQDDTLE	LYNREDRFSA	PPSRSHDGDR	RGPWWDDWER	DQDMDEDYNR
1290	1300	1310	1320	1330	1340	1350	1360
EMERDMDRDV	DRISRPMDMY	DRSLDNEWDR	DYGRPLDEQE	SQFRERDIPS	LPPLPPLPPL	PPLDRYRDDR	WREERNREHG
1370	1380	1390	1400	1410	1420	1430	1440
YDRDFRDRGE	LRIREYPERG	DTWREKRDYV	PDRMDWERER	LSDRWYPSDV	DRHSPMAEHM	PSSHHSSEMM	GSDASLDSDQ
1450	1460	1470	1480	1490	1500	1510	1520
GLGGVMVLSQ	RQHEIILKAA	QELKMLREQK	EQLQKMKDFG	SEPQMADHLP	PQESRLQNTS	SRPGMYPPPG	SYRPPPPMGK
1530	1540	1550	1560	1570	1580	1590	1600
PPGSIVRPSA	PPARSSVPVT	RPPVPIPPPP	PPPPLPPPPP	VIKPQTSAVE	QERWDEDSFY	GLWDTNDEQG	LNSEFKSETA
1610	1620	1630	1640	1650	1660	1670	1680
AIPSAPVLPP	PPVHSSIPPP	GPVPMGMPPM	SKPPPVQQTV	DYGHGRDIST	NKVEQIPYGE	RITLRPDPLP	ERSTFETEHA
1690	1700	1710	1720	1730	1740	1750	1760
GQRDRYDRER	DREPYFDRQS	NVIADHRDFK	RDRETHRDRD	RDRGVIDYDR	DRFDRERRPR	DDRAQSYRDK	KDHSSSRRGG
1770	1780	1790	1800	1810	1820	1830	1840
FDRPSYDRKS	DRPVYEGPSM	FGGERRTYPE	ERMPLPAPSL	SHQPPPAPRV	EKKPESKNVD	DILKPPGRES	RPERIVVIMR
1850	1860	1870	1880	1890	1900	1910	1920
GLPGSGKTHV	AKLIRDKEVE	FGGPAPRVLS	LDDYFITEVE	KEEKDPDSGK	KVKKKVMEYE	YEAEMEETYR	TSMFKTFKKT
1930	1940	1950	1960	1970	1980	1990	2000
LDDGFFPFII	LDAINDRVRH	FDQFWSAAKT	KGFEVYLAEM	SADNQTCGKR	NIHGRKLKEI	NKMADHWETA	PRHMMRLDIR
2010	2020	2030	2040	2050	2060	2070	
SLLQDAAIEE	VEMEDFDANI	EEQKEEKKDA	EEEESELGYI	PKSKWEMDTS	EAKLVCHVEE	V	

Cmpd.	No. of Cmpds.	m/z meas.	Δ m/z [ppm]	z	Rt [min]	Score	P	Range	Sequence	Modification	Ratios

Detailed Protein Report

Cmpd.	No. of Cmpds.	m/z meas.	Δ m/z [ppm]	z	Rt [min]	Score	P	Range	Sequence	Modification	Ratios
44	1	767.2360	-155.99	2	30.4	11.8	1	1673-1685	R.STFETEHAGQRDR.Y		
2835	5	746.4789	26.21	1	64.1	17.2	0	1835-1840	R.IVVIMR.G	Oxidation: 5	WUP:QUP 0.74<br>QU:MU 1.46

Detailed Protein Report

Protein 467: multidrug resistance protein 3 isoform C [Homo sapiens]

Accession: gi|9961252
Database: refseq_human(refseq_human_20140103.fasta)
Database Date: 2015-11-30

Score: 29.0
MW [kDa]: 135.2
pI: 9.2
Sequence Coverage [%]: 1.9
No. of unique Peptides: 2

10	20	30	40	50	60	70	80
MDLEAAKNGT	AWRPTSAEGD	FELGISSKQK	RKKTKTVKMI	GVLTLFRYSD	WQDKLFMSLG	TIMAIAHGSG	LPLMMIVFGE
90	100	110	120	130	140	150	160
MTDKFVDTAG	NFSFPVNFSL	SLLNPGKILE	EEMTRYAYYY	SGLGAGVLVA	AYIQVSFWTL	AAGRQIRKIR	QKFFHAILRQ
170	180	190	200	210	220	230	240
EIGWFDINDT	TELNTRLTDD	ISKISEGIGD	KVGMFFQAVA	TFFAGFIVGF	IRGWKLTLVI	MAISPILGLS	AAVWAKILSA
250	260	270	280	290	300	310	320
FSDKELAAYA	KAGAVAEEAL	GAIRTVIAFG	GQNKELERYQ	KHLENAKEIG	IKKAISANIS	MGIAFLLIYA	SYALAFWYGS
330	340	350	360	370	380	390	400
TLVISKEYTI	GNAMTVFFSI	LIGAFSVGQA	APCIDAFANA	RGAAYVIFDI	IDNNPKIDSF	SERGHKPDSI	KGNLEFNDVH
410	420	430	440	450	460	470	480
FSYPSRANVK	ILKGLNLKVQ	SGQTVALVGS	SGCGKSTTVQ	LIQRLYDPDE	GTINIDGQDI	RNFNVNYLRE	IIGVVSQEPV
490	500	510	520	530	540	550	560
LFSTTIAENI	CYGRGNVTMD	EIKKAVKEAN	AYEFIMKLPQ	KFDTLVGERG	AQLSGGQKQR	IAIARALVRN	PKILLLDEAT
570	580	590	600	610	620	630	640
SALDTESEAE	VQAALDKARE	GRTTIVIAHR	LSTVRNADVI	AGFEDGVIVE	QGSHSELMKK	EGVYFKLVNM	QTSGSQIQSE
650	660	670	680	690	700	710	720
EFELNDEKAA	TRMAPNGWKS	RLFRHSTQKN	LKNSQMCQKS	LDVETDGLEA	NVPPVSFLKV	LKLNKTEWPY	FVVGTVCAIA
730	740	750	760	770	780	790	800
NGGLQPAFSV	IFSEIIAIFG	PGDDAVKQQK	CNIFSLIFLF	LGIISFFTFF	LQGFTFGKAG	EILTRRLRSM	AFKAMLRQDM
810	820	830	840	850	860	870	880
SWFDDHKNST	GALSTRLATD	AAQVQGATGT	RLALIAQNIA	NLGTGIIISF	IYGWQLTLLL	LAVVPIIAVS	GIVEMKLLAG
890	900	910	920	930	940	950	960
NAKRDKKELE	AAGKIATEAI	ENIRTVVSLT	QERKFESMYV	EKLYGPYRVF	SAIVFGAVAL	GHASSFAPDY	AKAKLSAAHL
970	980	990	1000	1010	1020	1030	1040
FMLFERQPLI	DSYSEEGLKP	DKFEGNITFN	EVVFNYPTRA	NVPVLQGLSL	EVKKGQTLAL	VGSSGCGKST	VVQLLERFYD
1050	1060	1070	1080	1090	1100	1110	1120
PLAGTVLLDG	QEAKKLNVQW	LRAQLGIVSQ	EPILFDCSIA	ENIAYGDNSR	VVSQDEIVSA	AKAANIHPFI	ETLPHKYETR
1130	1140	1150	1160	1170	1180	1190	1200
VGDKGTQLSG	GQKQRIAIAR	ALIRQPQILL	LDEATSALDT	ESEKVVQEAL	DKAREGRTCI	VIAHRLSTIQ	NADLIVVFQN
1210	1220	1230	1240				
GRVKEHGTHQ	QLLAQKGIYF	SMVSVQAGTQ	NL				

Cmpd.	No. of Cmpds.	m/z meas.	Δ m/z [ppm]	z	Rt [min]	Score	P	Range	Sequence	Modification
408	1	655.8392	-137.81	2	35.1	15.6	2	407-418	R.ANVKILKGLNLK.V	
439	1	565.3076	0.55	2	35.1	13.4	1	530-540	R.GAQLSGGQKQR.I	

Detailed Protein Report

Protein 468: **PREDICTED: replication initiator 1 isoform X5 [Homo sapiens]**

**Accession:**	gi|578814165	**Score:**	29.0
**Database:**	refseq_human(refseq_human_20140103.fasta)	**MW [kDa]:**	69.8
**Database Date:**	2015-11-30	**pI:**	12.1
**Modification(s):**	Carbamidomethyl	**Sequence Coverage [%]:**	3.9
		**No. of unique Peptides:**	1

Alias proteins:

Accession	Name	Description
gi|578814167	refseq_human (refseq_human_20140103.fasta)	PREDICTED: replication initiator 1 isoform X6 [Homo sapiens]

10	20	30	40	50	60	70	80
MGIGVSLLLQ	FSLTPGGYRS	VGRSRRCSRG	SIPRNIPKRS	WKKPHPQLCS	LQEEEPMLER	RCRGPLAMGL	AQPRLLSGPS
90	100	110	120	130	140	150	160
QESPQTLGKE	SRGLRQQGTS	VAQSGAQAPG	RAHRCAHCRR	HFPGWVALWL	HTRRCQARLP	LPCPECGRRF	RHAPFLALHR
170	180	190	200	210	220	230	240
QVHAAATPDL	GFACHLCGQS	FRGWVALVLH	LRAHSAAKRP	IACPKCERRF	WRRKQLRAHL	RRCHPPAPEA	RPFICGNCGR
250	260	270	280	290	300	310	320
SFAQWDQLVA	HKRVHVAEAL	EEAAAKALGP	RPRGRPAVTA	PRPGGDAVDR	PFQCACCGKR	FRHKPNLIAH	RRVHTGERPH
330	340	350	360	370	380	390	400
QCPECGKRFT	NKPYLTSHRR	IHTGEKPYPC	KECGRRFRHK	PNLLSHSKIH	KRSEGSAQAA	PGPGSPQLPA	GPQESAAEPT
410	420	430	440	450	460	470	480
PAVPLKPAQE	PPPGAPPEHP	QDPIEAPPSL	YSCDDCGRSF	RLERFLRAHQ	RQHTGERPFT	CAECGKNFGK	KTHLVAHSRV
490	500	510	520	530	540	550	560
HSGERPFACE	ECGRRFSQGS	HLAAHRRDHA	PDRPFVCPDC	GKAFRHKPYL	AAHRRIHTGE	KPYVCPDCGK	AFSQKSNLVS
570	580	590	600	610	620	630	
HRRIHTGERP	YACPDCDRSF	SQKSNLITHR	KSHIRDGAFC	CAICGQTFDD	EERLLAHQKK	HDV	

Cmpd.	No. of Cmpds.	m/z meas.	Δ m/z [ppm]	z	Rt [min]	Score	P	Range	Sequence	Modification
1852	1	897.8615	-114.49	2	53.0	11.7	2	193-208	R.AHSAAKRPIACPKCER.R	Carbamidomethyl: 11

Detailed Protein Report

Protein 469: ectonucleoside triphosphate diphosphohydrolase 4 isoform b [Homo sapiens]

**Accession:**	gi|193083168	**Score:**	29.0
**Database:**	refseq_human(refseq_human_20140103.fasta)	**MW [kDa]:**	69.4
**Database Date:**	2015-11-30	**pI:**	9.3
**Modification(s):**	Oxidation	**Sequence Coverage [%]:**	5.8
		**No. of unique Peptides:**	1

10	20	30	40	50	60	70	80
MGRIGISCLF	PASWHFSISP	VGCPRILNTN	LRQIMVISVL	AAAVSLLYFS	VVIIRNKYGR	LTRDKKFQRY	LARVTDIEAT
90	100	110	120	130	140	150	160
DTNNPNVNYG	IVVDCGSSGS	RVFVYCWPRH	NGNPHDLLDI	RQMRDKNRKP	VVMKIKPGIS	EFATSPEKVS	DYISPLLNFA
170	180	190	200	210	220	230	240
AEHVPRAKHK	ETPLYILCTA	GMRILPESQQ	KAILEDLLTD	IPVHFDFLFS	DSHAEVISGK	QEGVYAWIGI	NFVLGRFEHI
250	260	270	280	290	300	310	320
EDDDEAVVEV	NIPGSESSEA	IVRKRTAGIL	DMGGVSTQIA	YEVPKTEEVA	KNLLAEFNLG	CDVHQTEHVY	RVYVATFLGF
330	340	350	360	370	380	390	400
GGNAARQRYE	DRIFANTIQK	NRLLGKQTGL	TPDMPYLDPC	LPLDIKDEIQ	QNGQTIYLRG	TGDFDLCRET	IQPFMNKTNE
410	420	430	440	450	460	470	480
TQTSLNGVYQ	PPIHFQNSEF	YGFSEFYYCT	EDVLRMGGDY	NAAKFTKAAK	DYCATKWSIL	RERFDRGLYA	SHADLHRLKY
490	500	510	520	530	540	550	560
QCFKSAWMFE	VFHRGFSFPV	NYKSLKTALQ	VYDKEVQWTL	GAILYRTRFL	PLRDIQQEAF	RASHTHWRGV	SFVYNHYLFS
570	580	590	600	610			
GCFLVVLLAI	LLYLLRLRRI	HRRTPRSSSA	AALWMEEGLP	AQNAPGTL			

Cmpd.	No. of Cmpds.	m/z meas.	Δ m/z [ppm]	z	Rt [min]	Score	P	Range	Sequence	Modification
2772	1	696.6903	49.74	3	65.0	14.8	1	380-397	R.GTGDFDLCRETIQPFMNK.T	Oxidation: 16

Detailed Protein Report

Protein 470: **PREDICTED: MDS1 and EVI1 complex locus protein EVI1 isoform X16 [Homo sapiens]**

Accession: gi|578807160
Database: refseq_human(refseq_human_20140103.fasta)
Database Date: 2015-11-30
Modification(s): Carbamidomethyl

Score: 28.9
MW [kDa]: 93.9
pI: 6.2
Sequence Coverage [%]: 3.2
No. of unique Peptides: 2

10	20	30	40	50	60	70	80
MRSKGRARKL	ATNNECVYGN	YPEIPLEEMP	DADGVASTPS	LNIQEPCSPA	TSSEAFTPKE	GSPYKAPIYI	PDDIPIPAEF
**90**	**100**	**110**	**120**	**130**	**140**	**150**	**160**
ELRESNMPGA	GLGIWTKRKI	EVGEKFGPYV	GEQRSNLKDP	SYGWEILDEF	YNVKFCIDAS	QPDVGSWLKY	IRFAGCYDQH
**170**	**180**	**190**	**200**	**210**	**220**	**230**	**240**
NLVACQINDQ	IFYRVVADIA	PGEELLLFMK	SEDYPHETMA	PDIHEERQYR	CEDCDQLFES	KAELADHQKF	PCSTPHSAFS
**250**	**260**	**270**	**280**	**290**	**300**	**310**	**320**
MVEEDFQQKL	ESENDLQEIH	TIQECKECDQ	VFPDLQSLEK	HMLSHTEERE	YKCDQCPKAF	NWKSNLIRHQ	MSHDSGKHYE
**330**	**340**	**350**	**360**	**370**	**380**	**390**	**400**
CENCAKQVFT	DPSNLQRHIR	SQHVGARAHA	CPECGKTFAT	SSGLKQHKHI	HSSVKPFICE	VCHKSYTQFS	NLCRHKRMHA
**410**	**420**	**430**	**440**	**450**	**460**	**470**	**480**
DCRTQIKCKD	CGQMFSTTSS	LNKHRRFCEG	KNHFAAGGFF	GQGISLPGTP	AMDKTSMVNM	SHANPGLADY	FGANRHPAGL
**490**	**500**	**510**	**520**	**530**	**540**	**550**	**560**
TFPTAPGFSF	SFPGLFPSGL	YHRPPLIPAS	SPVKGLSSTE	QTNKSQSPLM	THPQILPATQ	DILKALSKHP	SVGDNKPVEL
**570**	**580**	**590**	**600**	**610**	**620**	**630**	**640**
QPERSSEERP	FEKISDQSES	SDLDDVSTPS	GSDLETTSGS	DLESDIESDK	EKFKENGKMF	KDKVSPLQNL	ASINNKKEYS
**650**	**660**	**670**	**680**	**690**	**700**	**710**	**720**
NHSIFSPSLE	EQTAVSGAVN	DSIKAIASIA	EKYFGSTGLV	GLQDKKVGAL	PYPSMFPLPF	FPAFSQSMYP	FPDRDLRSLP
**730**	**740**	**750**	**760**	**770**	**780**	**790**	**800**
LKMEPQSPGE	VKKLQKGSSE	SPFDLTTKRK	DEKPLTPVPS	KPPVTPATSQ	DQPLDLSMGS	RSRASGTKLT	EPRKNHVFGG
**810**	**820**	**830**	**840**	**850**			
KKGSNVESRP	ASDGSLQHAR	PTPFFMDPIY	RCQLLKTWQK	S			

Cmpd.	No. of Cmpds.	m/z meas.	Δ m/z [ppm]	z	Rt [min]	Score	P	Range	Sequence	Modification
2185	1	1052.4145	-10.10	2	57.2	11.8	1	309-326	R.HQMSHDSGKHYECENCAK.Q	
668	2	602.8050	-39.80	2	37.9	17.1	1	832-840	R.CQLLKTWQK.S	Carbamidomethyl: 1

Detailed Protein Report

Protein 471: PREDICTED: E3 ubiquitin-protein ligase RING2 isoform X2 [Homo sapiens]

**Accession:**	gi|530365226	**Score:**	28.9
**Database:**	refseq_human(refseq_human_20140103.fasta)	**MW [kDa]:**	32.2
**Database Date:**	2015-11-30	**pI:**	7.8
		**Sequence Coverage [%]:**	4.2
		**No. of unique Peptides:**	1

10	20	30	40	50	60	70	80
MCPICLDMLK	NTMTTKECLH	RFCADCIITA	LRSGNKECPT	CRKKLVSKRS	LRPDPNFDAL	ISKIYPSRDE	YEAHQERVLA
**90**	**100**	**110**	**120**	**130**	**140**	**150**	**160**
RINKHNNQQA	LSHSIEEGLK	IQAMNRLQRG	KKQQIENGSG	AEDNGDSSHC	SNASTHSNQE	AGPSNKRTKT	SDDSGLELDN
**170**	**180**	**190**	**200**	**210**	**220**	**230**	**240**
NNAAMAIDPV	MDGASEIELV	FRPHPTLMEK	DDSAQTRYIK	TSGNATVDHL	SKYLAVRLAL	EELRSKGESN	QMNLDTASEK
**250**	**260**	**270**	**280**	**290**			
QYTIYIATAS	GQFTVLNGSF	SLELVSEKYW	KVNKPMELYY	APTKEHK			

Cmpd.	No. of Cmpds.	m/z meas.	Δ m/z [ppm]	z	Rt [min]	Score	P	Range	Sequence	Modification
1896	1	621.2840	-141.50	1	54.1	16.2	0	213-217	K.YLAVR.L	

Detailed Protein Report

Protein 472: peptidyl-prolyl cis-trans isomerase FKBP5 isoform 1 [Homo sapiens]

**Accession:**	gi|4758384	**Score:**	28.9
**Database:**	refseq_human(refseq_human_20140103.fasta)	**MW [kDa]:**	51.2
**Database Date:**	2015-11-30	**pI:**	5.6
**Modification(s):**	Carbamidomethyl	**Sequence Coverage [%]:**	5.9
		**No. of unique Peptides:**	1

Quantitation

WUP:QUP	**Median:** 0.99	**CV:** 0.00 %	**No. of Peptides:** 1

Alias proteins:

Accession	Name	Description
gi|224809329	refseq_human (refseq_human_20140103.fasta)	peptidyl-prolyl cis-trans isomerase FKBP5 isoform 1 [Homo sapiens]
gi|224809327	refseq_human (refseq_human_20140103.fasta)	peptidyl-prolyl cis-trans isomerase FKBP5 isoform 1 [Homo sapiens]

10	20	30	40	50	60	70	80
MTTDEGAKNN	EESPTATVAE	QGEDITSKKD	RGVLKIVKRV	GNGEETPMIG	DKVYVHYKGK	LSNGKKFDSS	HDRNEPFVFS
90	100	110	120	130	140	150	160
LGKGQVIKAW	DIGVATMKKG	EICHLLCKPE	YAYGSAGSLP	KIPSNATLFF	EIELLDFKGE	DLFEDGGIIR	RTKRKGEGYS
170	180	190	200	210	220	230	240
NPNEGATVEI	HLEGRCGGRM	FDCRDVAFTV	GEGEDHDIPI	GIDKALEKMQ	REEQCILYLG	PRYGFGEAGK	PKFGIEPNAE
250	260	270	280	290	300	310	320
LIYEVTLKSF	EKAKESWEMD	TKEKLEQAAI	VKEKGTVYFK	GGKYMQAVIQ	YGKIVSWLEM	EYGLSEKESK	ASESFLLAAF
330	340	350	360	370	380	390	400
LNLAMCYLKL	REYTKAVECC	DKALGLDSAN	EKGLYRRGEA	QLLMNEFESA	KGDFEKVLEV	NPQNKAARLQ	ISMCQKKAKE
410	420	430	440	450	460		
HNERDRRIYA	NMFKKFAEQD	AKEEANKAMG	KKTSEGVTNE	KGTDSQAMEE	EKPEGHV		

Cmpd.	No. of Cmpds.	m/z meas.	Δ m/z [ppm]	z	Rt [min]	Score	P	Range	Sequence	Modification	Ratios
2372	1	911.8056	-124.45	2	57.9	13.0	1	336-352	K.AVECCDKALGLDSANEK.G	Carbamidomethyl: 4	WUP:QUP 0.99

Detailed Protein Report

Protein 473: NKAP-like protein [Homo sapiens]

Accession: gi|56090620
Database: refseq_human(refseq_human_20140103.fasta)
Database Date: 2015-11-30

Score: 28.9
MW [kDa]: 46.3
pI: 10.3
Sequence Coverage [%]: 5.7
No. of unique Peptides: 1

10	20	30	40	50	60	70	80
MPPVSRSSYS	EDIVGSRRRR	RSSSGSPPSP	QSRCSSWDGC	SRSHSRGREG	LRPPWSELDV	GALYPFSRSG	SRGRLPRFRN
**90**	**100**	**110**	**120**	**130**	**140**	**150**	**160**
YAFASSWSTS	YSGYRYHRHC	YAEERQSAED	YEKEESHRQR	RLKERERIGE	LGAPEVWGPS	PKFPQLDSDE	HTPVEDEEEV
**170**	**180**	**190**	**200**	**210**	**220**	**230**	**240**
THQKSSSSDS	NSEEHRKKKT	SRSRNKKKRK	NKSSKRKHRK	YSDSDSNSES	DTNSDSDDDK	KRVKAKKKKK	KKKHKTKKKK
**250**	**260**	**270**	**280**	**290**	**300**	**310**	**320**
NKKTKKESSD	SSCKDSEEDL	SEATWMEQPN	VADTMDLIGP	EAPIIHTSQD	EKPLKYGHAL	LPGEGAAMAE	YVKAGKRIPR
**330**	**340**	**350**	**360**	**370**	**380**	**390**	**400**
RGEIGLTSEE	IGSFECSGYV	MSGSRHRRME	AVRLRKENQI	YSADEKRALA	SFNQEERRKR	ESKILASFRE	MVHKKTKEKD
**410**							
DK							

Cmpd.	No. of Cmpds.	m/z meas.	Δ m/z [ppm]	z	Rt [min]	Score	P	Range	Sequence	Modification
1782	1	938.9355	-33.28	2	50.6	12.7	0	296-313	K.YGHALLPGEGAAMAEYVK.A	

Detailed Protein Report

Protein 474: relA-associated inhibitor [Homo sapiens]

**Accession:**	gi|63003907	**Score:**	28.9
**Database:**	refseq_human(refseq_human_20140103.fasta)	**MW [kDa]:**	89.0
**Database Date:**	2015-11-30	**pI:**	6.4
**Modification(s):**	Oxidation	**Sequence Coverage [%]:**	4.1
		**No. of unique Peptides:**	1

Alias proteins:

Accession	Name	Description
gi|215820635	refseq_human (refseq_human_20140103.fasta)	relA-associated inhibitor [Homo sapiens]

10	20	30	40	50	60	70	80
MDSEAFQSAR	DFLDMNFQSL	AMKHMDLKQM	ELDTAAAKVD	ELTKQLESLW	SDSPAPPGPQ	AGPPSRPPRY	SSSSIPEPFG
**90**	**100**	**110**	**120**	**130**	**140**	**150**	**160**
SRGSPRKAAT	DGADTPFGRS	ESAPTLHPYS	PLSPKGRPSS	PRTPLYLQPD	AYGSLDRATS	PRPRAFDGAG	SSLGRAPSPR
**170**	**180**	**190**	**200**	**210**	**220**	**230**	**240**
PGPGPLRQQG	PPTPFDFLGR	AGSPRGSPLA	EGPQAFFPER	GPSPRPPATA	YDAPASAFGS	SLLGSGGSAF	APPLRAQDDL
**250**	**260**	**270**	**280**	**290**	**300**	**310**	**320**
TLRRRPPKAW	NESDLDVAYE	KKPSQTASYE	RLDVFARPAS	PSLQLLPWRE	SSLDGLGGTG	KDNLTSATLP	RNYKVSPLAS
**330**	**340**	**350**	**360**	**370**	**380**	**390**	**400**
DRRSDAGSYR	RSLGSAGPSG	TLPRSWQPVS	RIPMPPSSPQ	PRGAPRQRPI	PLSMIFKLQN	AFWEHGASRA	MLPGSPLFTR
**410**	**420**	**430**	**440**	**450**	**460**	**470**	**480**
APPPKLQPQP	QPQPQPQSQP	QPQLPPQPQT	QPQTPTPAPQ	HPQQTWPPVN	EGPPKPPTEL	EPEPEIEGLL	TPVLEAGDVD
**490**	**500**	**510**	**520**	**530**	**540**	**550**	**560**
EGPVARPLSP	TRLQPALPPE	AQSVPELEEV	ARVLAEIPRP	LKRRGSMEQA	PAVALPPTHK	KQYQQIISRL	FHRHGGPGPG
**570**	**580**	**590**	**600**	**610**	**620**	**630**	**640**
GPEPELSPIT	EGSEARAGPP	APAPPAPIPP	PAPSQSSPPE	QPQSMEMRSV	LRKAGSPRKA	RRARLNPLVL	LLDAALTGEL
**650**	**660**	**670**	**680**	**690**	**700**	**710**	**720**
EVVQQAVKEM	NDPSQPNEEG	ITALHNAICG	ANYSIVDFLI	TAGANVNSPD	SHGWTPLHCA	ASCNDTVICM	ALVQHGAAIF
**730**	**740**	**750**	**760**	**770**	**780**	**790**	**800**
ATTLSDGATA	FEKCDPYREG	YADCATYLAD	VEQSMGLMNS	GAVYALWDYS	AEFGDELSFR	EGESVTVLRR	DGPEETDWWW
**810**	**820**	**830**					
AALHGQEGYV	PRNYFGLFPR	VKPQRSKV					

Cmpd.	No. of Cmpds.	m/z meas.	Δ m/z [ppm]	z	Rt [min]	Score	P	Range	Sequence	Modification
100	1	1301.5284	-95.57	2	31.0	12.3	1	378-400	K.LQNAFWEHGASRAMLPGSPLFTR.A	Oxidation: 14

Detailed Protein Report

Protein 475: rho GTPase-activating protein 20 isoform 4 [Homo sapiens]

**Accession:**	gi|385862212	**Score:**	28.9
**Database:**	refseq_human(refseq_human_20140103.fasta)	**MW [kDa]:**	128.9
**Database Date:**	2015-11-30	**pI:**	8.9
**Modification(s):**	Carbamidomethyl	**Sequence Coverage [%]:**	2.2
		**No. of unique Peptides:**	1

Alias proteins:

Accession	Name	Description
gi|385862214	refseq_human (refseq_human_20140103.fasta)	rho GTPase-activating protein 20 isoform 4 [Homo sapiens]

10	20	30	40	50	60	70	80
MKTLAERRRS	APSLILDKAL	QKRPTTRDSP	SASVDTCTFL	SSLVCSNRTL	LIDGRAELKR	GLQRQERHLF	LFNDLFVVAK
**90**	**100**	**110**	**120**	**130**	**140**	**150**	**160**
IKYNNNFKIK	NKIKLTDMWT	ASCVDEVGEG	NTNAMKSFVL	GWPTVNFVAT	FSSPEQKDKW	LSLLQRYINL	EKEKDYPKSI
**170**	**180**	**190**	**200**	**210**	**220**	**230**	**240**
PLKIFAKDIG	NCAYSKTITV	MNSDTANEVI	NMSLPMLGIT	GSERDYQLWV	NSGKEEAPYP	LIGHEYPYGI	KMSHLRDSAL
**250**	**260**	**270**	**280**	**290**	**300**	**310**	**320**
LTPGSKDSTT	PFNLQEPFLM	EQLPREMQCQ	FILKPSRLAA	AQQLSDSGHK	TFKRRRSIIN	WAFWRGSSTH	LDNLPSSPTS
**330**	**340**	**350**	**360**	**370**	**380**	**390**	**400**
PMPGQLFGIS	LPNICENDNL	PKPVLDMLFF	LNQKGPLTKG	IFRQSANVKS	CRELKEKLNS	GVEVHLDCES	IFVIASVLKD
**410**	**420**	**430**	**440**	**450**	**460**	**470**	**480**
FLRNIPGSIF	SSDLYDHWVS	VMDQGNDEEK	INTVQRLLDQ	LPRANVVLLR	YLFGVLHNIE	QHSSSNQMTA	FNLAVCVAPS
**490**	**500**	**510**	**520**	**530**	**540**	**550**	**560**
ILWPPASSSP	ELENEFTKKV	SLLIQFLIEN	CLRIFGEEIT	SLFREVSVRC	DTRENASDIS	CFQLNDSSYD	SLENELNEDV
**570**	**580**	**590**	**600**	**610**	**620**	**630**	**640**
DAPCSDLVKK	LGQGSRSMDS	VLTLSDYDLD	QPEVEGLLTL	SDFDLAHSKD	EDVQMKRPLE	SKPVNILVYT	KIPLRDHARA
**650**	**660**	**670**	**680**	**690**	**700**	**710**	**720**
PSAMCTPSYL	STAAANAAKS	LRRHRRCSEP	SIDYLDSKLS	YLREFYQKKL	RKSSCDAILS	QKDEDYLKQN	QPLQEEGKTC
**730**	**740**	**750**	**760**	**770**	**780**	**790**	**800**
FKQSLVTGTD	VSKKNATTQN	TKKKSLSGSE	GNHVKLFPKS	KPVAISVASY	SPMSSQDHSK	NQPFDVNTSG	YSPPHTADAL
**810**	**820**	**830**	**840**	**850**	**860**	**870**	**880**
KGPRTHRRCS	EPNIEDQNRK	LTYLRGIYSK	KQHKTSCEAG	LLHGEEDYLK	RHKSLQMEGQ	KLINQSLVMG	IEVGKSSATN
**890**	**900**	**910**	**920**	**930**	**940**	**950**	**960**
QNTEKVLPPR	LNLCPRTSYS	SLSSPGTSPS	GSSVSSQDSA	FSQISEHSVF	TPTETSSPID	CTFQAQRKRE	DLSPDFSNAS
**970**	**980**	**990**	**1000**	**1010**	**1020**	**1030**	**1040**
HVSGMPGPSS	GQACSRPAYT	KKDTMEWHSQ	MHSVTLHPST	WLRNGVASLK	NWSLKKKAKA	ARPEEEKIAS	PKGPLEPPPH
**1050**	**1060**	**1070**	**1080**	**1090**	**1100**	**1110**	**1120**
ASGVPEANSL	QEEQKDLPLR	AAEGLSPVQS	AQRCSSSPFQ	DSERHCSSPF	SLVESRLKLC	MKSHEEIEPG	SQSSSGSLPW
**1130**	**1140**	**1150**	**1160**				
ERASASSWTL	EDATSPDSGP	TVVCDIEDRY	LTKDI				

Cmpd.	No. of Cmpds.	m/z meas.	Δ m/z [ppm]	z	Rt [min]	Score	P	Range	Sequence	Modification
1952	3	703.2693	-86.35	2	52.8	11.2	0	1085-1096	R.HCSSPFSLVESR.L	Carbamidomethyl: 2

Detailed Protein Report

Protein 476: PREDICTED: ninein isoform X5 [Homo sapiens]

**Accession:**	gi|578825856	**Score:**	28.9
**Database:**	refseq_human(refseq_human_20140103.fasta)	**MW [kDa]:**	231.1
**Database Date:**	2015-11-30	**pI:**	4.8
		**Sequence Coverage [%]:**	1.4
		**No. of unique Peptides:**	1

Detailed Protein Report

10	20	30	40	50	60	70	80
MDEVEQDQHE	ARLKELFDSF	DTTGTGSLGQ	EELTDLCHML	SLEEVAPVLQ	QTLLQDNLLG	RVHFDQFKEA	LILILSRTLS
90	100	110	120	130	140	150	160
NEEHFQEPDC	SLEAQPKYVR	GGKRYGRRSL	PEFQESVEEF	PEVTVIEPLD	EEARPSHIPA	GDCSEHWKTQ	RSEEYEAEGQ
170	180	190	200	210	220	230	240
LRFWNPDDLN	ASQSGSSPPQ	DWIEEKLQEV	CEDLGITRDG	HLNRKKLVSI	CEQYGLQNVD	GEMLEEVFHN	LDPDGTMSVE
250	260	270	280	290	300	310	320
DFFYGLFKNG	KSLTPSASTP	YRQLKRHLSM	QSFDESGRRT	TTSSAMTSTI	GFRVFSCLDD	GMGHASVERI	LDTWQEEGIE
330	340	350	360	370	380	390	400
NSQEILKALD	FSLDGNINLT	ELTLALENEL	LVTKNSIHQA	ALASFKAEIR	HLLERVDQVV	REKEKLRSDL	DKAEKLKSLM
410	420	430	440	450	460	470	480
ASEVDDHHAA	IERRNEYNLR	KLDEEYKERI	AALKNELRKE	REQILQQAGK	QRLELEQEIE	KAKTEENYIR	DRLALSLKEN
490	500	510	520	530	540	550	560
SRLENELLEN	AEKLAEYENL	TNKLQRNLEN	VLAEKFGDLD	PSSAEFFLQE	ERLTQMRNEY	ERQCRVLQDQ	VDELQSELEE
570	580	590	600	610	620	630	640
YRAQGRVLRL	PLKNSPSEEV	EANSGGIEPE	HGLGSEECNP	LNMSIEAELV	IEQMKEQHHR	DICCLRLELE	DKVRHYEKQL
650	660	670	680	690	700	710	720
DETVVSCKKA	QENMKQRHEN	ETHTLEKQIS	DLKNEIAELQ	GQAAVLKEAH	HEATCRHEEE	KKQLQVKLEE	EKTHLQEKLR
730	740	750	760	770	780	790	800
LQHEMELKAR	LTQAQASFER	EREGLQSSAW	TEEKVRGLTQ	ELEQFHQEQL	TSLVEKHTLE	KEELRKELLE	KHQRELQEGR
810	820	830	840	850	860	870	880
EKMETECNRR	TSQIEAQFQS	DCQKVTERCE	SALQSLEGRY	RQELKDLQEQ	QREEKSQWEF	EKDELTQECA	EAQELLKETL
890	900	910	920	930	940	950	960
KREKTTSLVL	TQEREMLEKT	YKEHLNSMVV	ERQQLLQDLE	DLRNVSETQQ	SLLSDQILEL	KSSHKRELRE	REEVLCQAGA
970	980	990	1000	1010	1020	1030	1040
SEQLASQRLE	RLEMEHDQER	QEMMSKLLAM	ENIHKATCET	ADRERAEMST	EISRLQSKIK	EMQQATSPLS	MLQSGCQVIG
1050	1060	1070	1080	1090	1100	1110	1120
EEEVEGDGAL	SLLQQGEQLL	EENGDVLLSL	QRAHEQAVKE	NVKMATEISR	LQQRLQKLEP	GLVMSSCLDE	PATEFFGNTA
1130	1140	1150	1160	1170	1180	1190	1200
EQTEQFLQQN	RTKQVEGVTR	RHVLSDLEDD	EVRDLGSTGT	SSVQRQEVKI	EESEASVEGF	SELENSEETR	TESWELKNQI
1210	1220	1230	1240	1250	1260	1270	1280
SQLQEQLMML	CADCDRASEK	KQDLLFDVSV	LKKKLKMLER	IPEASPKYKL	LYEDVSREND	CLQEELRMME	TRYDEALENN
1290	1300	1310	1320	1330	1340	1350	1360
KELTAEVFRL	QDELKKMEEV	TETFLSLEKS	YDEVKIENEG	LNVLVLRLQG	KIEKLQESVV	QRCDCCLWEA	SLENLEIEPD
1370	1380	1390	1400	1410	1420	1430	1440
GNILQLNQTL	EECVPRVRSV	HHVIEECKQE	NQYLEGNTQL	LEKVKAHEIA	WLHGTIQTHQ	ERPRVQNQVI	LEENTTLLGF
1450	1460	1470	1480	1490	1500	1510	1520
QDKHFQHQAT	IAELELEKTK	LQELTRKLKE	RVTILVKQKD	VLSHGEKEEE	LKAMMHDLQI	TCSEMQQKVE	LLRYESEKLQ
1530	1540	1550	1560	1570	1580	1590	1600
QENSILRNEI	TTLNEEDSIS	NLKLGTLNGS	QEEMWQKTET	VKQENAAVQK	MVENLKKQIS	ELKIKNQQLD	LENTELSQKN
1610	1620	1630	1640	1650	1660	1670	1680
SQNQEKLQEL	NQRLTEMLCQ	KEKEPGNSAL	EEREQEKFNL	KEELERCKVQ	SSTLVSSLEA	ELSEVKIQTH	IVQQENHLLK
1690	1700	1710	1720	1730	1740	1750	1760
DELEKMKQLH	RCPDLSDFQQ	KISSVLSYNE	KLLKEKEALS	EELNSCVDKL	AKSSLLEHRI	ATMKQEQKSW	EHQSASLKSQ
1770	1780	1790	1800	1810	1820	1830	1840
LVASQEKVQN	LEDTVQNVNL	QMSRMKSDLR	VTQQEKEALK	QEVMSLHKQL	QNAGGKSWAP	EIATHPSGLH	NQQKRLSWDK
1850	1860	1870	1880	1890	1900	1910	1920
LDHLMNEEQQ	LLWQENERLQ	TMVQNTKAEL	THSREKVRQL	ESNLLPKHQK	HLNPSGTMNP	TEQEKLSLKR	ECDQFQKEQS
1930	1940	1950	1960	1970	1980	1990	
PANRKVSQMN	SLEQELETIH	LENEGLKKKQ	VKLDEQLMEN	SVVGSSREGC	SSLPEIVCEG	RTFSFNELS	

Cmpd.	No. of Cmpds.	m/z meas.	Δ m/z [ppm]	z	Rt [min]	Score	P	Range	Sequence	Modification
1578	1	685.8860	27.18	2	48.0	10.3	0	913-923	R.QQLLQDLEDLR.N	

Detailed Protein Report

Protein 477: beta-1,4 N-acetylgalactosaminyltransferase 2 isoform c [Homo sapiens]

**Accession:**	gi|227497758	**Score:**	28.8
**Database:**	refseq_human(refseq_human_20140103.fasta)	**MW [kDa]:**	54.2
**Database Date:**	2015-11-30	**pI:**	8.9
		**Sequence Coverage [%]:**	4.6
		**No. of unique Peptides:**	1

Quantitation

QU:MU	**Median:** 0.80	**CV:** 0.00 %	**No. of Peptides:**	1
WUP:QUP	**Median:** 0.31	**CV:** 0.00 %	**No. of Peptides:**	1

10	20	30	40	50	60	70	80
MFGSMFLQAV	FSSPKPELPS	PAPGVQKLKL	LPEERLRNLF	SYDGIWLFPK	NQCKCEANKE	QGGYNFQDAY	GQSDLPAVKA
**90**	**100**	**110**	**120**	**130**	**140**	**150**	**160**
RRQAEFEHFQ	RREGLPRPLP	LLVQPNLPFG	YPVHGVEVMP	LHTVPIPGLQ	FEGPDAPVYE	VTLTASLGTL	NTLADVPDSV
**170**	**180**	**190**	**200**	**210**	**220**	**230**	**240**
VQGRGQKQLI	ISTSDRKLLK	FILQHVTYTS	TGYQHQKVDI	VSLESRSSVA	KFPVTIRHPV	IPKLYDPGPE	RKLRNLVTIA
**250**	**260**	**270**	**280**	**290**	**300**	**310**	**320**
TKTFLRPHKL	MIMLRSIREY	YPDLTVIVAD	DSQKPLEIKD	NHVEYYTMPF	GKGWFAGRNL	AISQVTTKYV	LWVDDDFLFN
**330**	**340**	**350**	**360**	**370**	**380**	**390**	**400**
EETKIEVLVD	VLEKTELDVV	GGSVLGNVFQ	FKLLLEQSEN	GACLHKRMGF	FQPLDGFPSC	VVTSGVVNFF	LAHTERLQRV
**410**	**420**	**430**	**440**	**450**	**460**	**470**	**480**
GFDPRLQRVA	HSEFFIDGLG	TLLVGSCPEV	IIGHQSRSPV	VDSELAALEK	TYNTYRSNTL	TRVQFKLALH	YFKNHLQCAA
**490**							

Cmpd.	No. of Cmpds.	m/z meas.	Δ m/z [ppm]	z	Rt [min]	Score	P	Range	Sequence	Modification	Ratios
26	1	537.7695	-77.75	2	30.0	13.9	0	299-308	R.NLAISQVTTK.Y		QU:MU 0.80<br>WUP:QUP 0.31

Detailed Protein Report

Protein 478: **PREDICTED: T-complex protein 11-like protein 1 isoform X2 [Homo sapiens]**

**Accession:**	gi|578820625	**Score:**	28.8
**Database:**	refseq_human(refseq_human_20140103.fasta)	**MW [kDa]:**	36.6
**Database Date:**	2015-11-30	**pI:**	6.2
**Modification(s):**	Oxidation	**Sequence Coverage [%]:**	9.3
		**No. of unique Peptides:**	1

10	20	30	40	50	60	70	80
MMGTLCAPAR	DEEVKKLKDI	KEIVPLFREI	FSVLDLMKVD	MANFAISSIR	PHLMQQSVEY	ERKKFQEILE	RQPNSLDFVT
**90**	**100**	**110**	**120**	**130**	**140**	**150**	**160**
QWLEEASEDL	MTQKYKHALP	VGGMAAGSGD	MPRLSPVAVQ	NYAYLKLLKW	DHLQRPFPET	VLMDQSRFHE	LQLQLEQLTI
**170**	**180**	**190**	**200**	**210**	**220**	**230**	**240**
LGAVLLVTFS	MAAPGISSQA	DFAEKLKMIV	KILLTDMHLP	SFHLKDVLTT	IGEKVCLEVS	SCLSLCGSSP	FTTDKETVLK
**250**	**260**	**270**	**280**	**290**	**300**	**310**	**320**
GQIQAVASPD	DPIRRIMESR	ILTFLETYLA	SGHQKPLPTV	PGGLSPVQRE	LEEVAIKFAR	LVNYNKMVFC	PYYDAILSKI
**330**							
LVRS							

Cmpd.	No. of Cmpds.	m/z meas.	Δ m/z [ppm]	z	Rt [min]	Score	P	Range	Sequence	Modification
780	1	541.6146	-237.37	2	39.7	10.1	0	1-10	-.MMGTLCAPAR.D	Oxidation: 1, 2

Detailed Protein Report

Protein 479: angiopoietin-related protein 1 precursor [Homo sapiens]

**Accession:**	gi|4757752	**Score:**	28.8
**Database:**	refseq_human(refseq_human_20140103.fasta)	**MW [kDa]:**	56.7
**Database Date:**	2015-11-30	**pI:**	9.1
		**Sequence Coverage [%]:**	7.5
		**No. of unique Peptides:**	1

Alias proteins:

Accession	Name	Description
gi|530365564	refseq_human (refseq_human_20140103.fasta)	PREDICTED: angiopoietin-related protein 1 isoform X1 [Homo sapiens]

10	20	30	40	50	60	70	80
MKTFTWTLGV	LFFLLVDTGH	CRGGQFKIKK	INQRRYPRAT	DGKEEAKKCA	YTFLVPEQRI	TGPICVNTKG	QDASTIKDMI
**90**	**100**	**110**	**120**	**130**	**140**	**150**	**160**
TRMDLENLKD	VLSRQKREID	VLQLVVDVDG	NIVNEVKLLR	KESRNMNSRV	TQLYMQLLHE	IIRKRDNSLE	LSQLENKILN
**170**	**180**	**190**	**200**	**210**	**220**	**230**	**240**
VTTEMLKMAT	RYRELEVKYA	SLTDLVNNQS	VMITLLEEQC	LRIFSRQDTH	VSPPLVQVVP	QHIPNSQQYT	PGLLGGNEIQ
**250**	**260**	**270**	**280**	**290**	**300**	**310**	**320**
RDPGYPRDLM	PPPDLATSPT	KSPFKIPPVT	FINEGPFKDC	QQAKEAGHSV	SGIYMIKPEN	SNGPMQLWCE	NSLDPGGWTV
**330**	**340**	**350**	**360**	**370**	**380**	**390**	**400**
IQKRTDGSVN	FFRNWENYKK	GFGNIDGEYW	LGLENIYMLS	NQDNYKLLIE	LEDWSDKKVY	AEYSSFRLEP	ESEFYRLRLG
**410**	**420**	**430**	**440**	**450**	**460**	**470**	**480**
TYQGNAGDSM	MWHNGKQFTT	LDRDKDMYAG	NCAHFHKGGW	WYNACAHSNL	NGVWYRGGHY	RSKHQDGIFW	AEYRGGSYSL
**490**	**500**						
RAVQMMIKPI	D						

Cmpd.	No. of Cmpds.	m/z meas.	Δ m/z [ppm]	z	Rt [min]	Score	P	Range	Sequence	Modification
2041	1	1018.3938	-75.83	3	55.4	17.0	0	341-366	K.GFGNIDGEYWLGLENIYMLSNQDNYK.L	

Detailed Protein Report

Protein 480: PREDICTED: protein DBF4 homolog B isoform X7 [Homo sapiens]

**Accession:**	gi|578831452	**Score:**	28.8
**Database:**	refseq_human(refseq_human_20140103.fasta)	**MW [kDa]:**	48.4
**Database Date:**	2015-11-30	**pI:**	10.4
**Modification(s):**	Carbamidomethyl	**Sequence Coverage [%]:**	4.7
		**No. of unique Peptides:**	1

10	20	30	40	50	60	70	80
MSEPGKGDDC	LELESSMAES	RLRAPDLGVS	RCLGKCQKNS	PGARKHPFSG	KSFYLDLPAG	KNLQFLTGAI	QQLGGVIEGF
90	100	110	120	130	140	150	160
LSKEVSYIVS	SRREVKAESS	GKSHRGCPSP	SPSEVRVETS	AMVDPKGSHP	RPSRKPVDSV	PLSRGKELLQ	KAIRNQGSIS
170	180	190	200	210	220	230	240
GGGSGGSSSL	LTNARSWGVR	ILHVDEMMMH	VQQLSLASLC	VKKQQPKKPE	GTCPAAESRT	RKVARLKAPF	LKIEDESRKF
250	260	270	280	290	300	310	320
RPFHHQFKSF	PEISFLGPKD	ASPFEAPTTL	GSMHHTRESK	DGEPSPRSAA	HTMPRRKKGY	CECCQEAFEE	LHVHLQSAQH
330	340	350	360	370	380	390	400
RSFALEAHLY	AEVDRIIAQL	SHSFADIPFQ	AGLPSDPRQG	CGPAAMDRIT	RWCDGTSCKS	HMCECHNPPA	GLAQGLQGAG
410	420	430	440	450			
LPLSLPSLLY	PVSSGHFLGS	AAWGVVACRG	HAPPSLPRKL	LCPGGHSC			

Cmpd.	No. of Cmpds.	m/z meas.	Δ m/z [ppm]	z	Rt [min]	Score	P	Range	Sequence	Modification
1527	2	472.2297	42.07	2	49.3	18.1	0	440-448	K.LLCPGGHSC.-	Carbamidomethyl: 9

Detailed Protein Report

Protein 481: PREDICTED: zinc finger protein 675 isoform X4 [Homo sapiens]

**Accession:**	gi|530414624	**Score:**	28.8
**Database:**	refseq_human(refseq_human_20140103.fasta)	**MW [kDa]:**	57.4
**Database Date:**	2015-11-30	**pI:**	10.2
**Modification(s):**	Carbamidomethyl	**Sequence Coverage [%]:**	3.7
		**No. of unique Peptides:**	1

Alias proteins:

Accession	Name	Description
gi|578833063	refseq_human (refseq_human_20140103.fasta)	PREDICTED: zinc finger protein 675 isoform X6 [Homo sapiens]

10	20	30	40	50	60	70	80
MCSHFAQEFW	PEQNIKDSFE	KVTLRRYEKC	GNDNFQLKGC	KSVDECKLHK	GGYNGLNQCL	PTMQSKMFQC	DKYVKVFNKF
**90**	**100**	**110**	**120**	**130**	**140**	**150**	**160**
SHSDRHKIKH	MENKPFKCKE	CGRSFCMLSH	LTRHERNYTK	VNFCKCEECE	KAVNQSSKLT	KHKRIYTCEK	LYKCQECDRT
**170**	**180**	**190**	**200**	**210**	**220**	**230**	**240**
FNQFSNLTEY	KKDYAREKPY	KCEECGKAFN	QSSHLTTHKI	IHTGEKPYKC	EECGKAFNQF	SNLTTHKKIH	TGEQPYICEE
**250**	**260**	**270**	**280**	**290**	**300**	**310**	**320**
CGKAFTQSST	LTTHKRIHTG	EKPYKCEECG	KAFNRSSKLT	EHKNIHTGEQ	PYKCEECGKA	FNRSSNLTEH	RKIHTEEKPY
**330**	**340**	**350**	**360**	**370**	**380**	**390**	**400**
KCKECGKAFK	HSSALTTHKR	IHTGEKPYKC	EECGKAFNRS	SKLTEHKKLH	TGKKPYKCEE	CGKAFIQSSK	LTEHKKIHSG
**410**	**420**	**430**	**440**	**450**	**460**	**470**	**480**
EIPYKCEECG	KAFKHSSSLT	THKRIHTGEK	PYKCEECGKA	FSRSSKLTEH	KIIHTGEKPY	KCERCDKAFN	QSANLTKHKK
**490**	**500**						
IHTGEKLQNW	NV						

Cmpd.	No. of Cmpds.	m/z meas.	Δ m/z [ppm]	z	Rt [min]	Score	P	Range	Sequence	Modification
2266	1	682.3051	5.92	3	58.8	17.1	2	30-47	K.CGNDNFQLKGCKSVDECK.L	Carbamidomethyl: 1

Detailed Protein Report

Protein 482: PREDICTED: zinc finger protein 646 isoform X3 [Homo sapiens]

**Accession:**	gi|530409439	**Score:**	28.8
**Database:**	refseq_human(refseq_human_20140103.fasta)	**MW [kDa]:**	198.5
**Database Date:**	2015-11-30	**pI:**	6.8
**Modification(s):**	Carbamidomethyl	**Sequence Coverage [%]:**	1.2
		**No. of unique Peptides:**	1

10	20	30	40	50	60	70	80
MEDTPPSLSC	SDCQRHFPSL	PELSRHRELL	HPSPNQDSEE	ADSIPRPYRC	QQCGRGYRHP	GSLVNHRRTH	ETGLFPCTTC
90	100	110	120	130	140	150	160
GKDFSNPMAL	KSHMRTHAPE	GRRRHRPPRP	KEATPHLQGE	TVSTDSWGQR	LGSSEGWENQ	TKHTEETPDC	ESVPDPRAAS
170	180	190	200	210	220	230	240
GTWEDLPTRQ	REGLASHPGP	EDGADGWGPS	TNSARAPPLP	IPASSLLSNL	EQYLAESVVN	FTGGQEPTQS	PPAEEERRYK
250	260	270	280	290	300	310	320
CSQCGKTYKH	AGSLTNHRQS	HTLGIYPCAI	CFKEFSNLMA	LKNHSRLHAQ	YRPYHCPHCP	RVFRLPRELL	EHQQSHEGER
330	340	350	360	370	380	390	400
QEPRWEEKGM	PTTNGHTDES	SQDQLPSAQM	LNGSAELSTS	GELEDSGLEE	YRPFRCGDCG	RTYRHAGSLI	NHRKSHQTGV
410	420	430	440	450	460	470	480
YPCSLCSKQL	FNAAALKNHV	RAHHRPRQGV	GENGQPSVPP	APLLLAETTH	KEEEDPTTTL	DHRPYKCSEC	GRAYRHRGSL
490	500	510	520	530	540	550	560
VNHRHSHRTG	EYQCSLCPRK	YPNLMALRNH	VRVHCKAARR	SADIGAEGAP	SHLKVELPPD	PVEAEAAPHT	DQDHVCKHEE
570	580	590	600	610	620	630	640
EATDITPAAD	KTAAHICSIC	GLLFEDAESL	ERHGLTHGAG	EKENSRTETT	MSPPRAFACR	DCGKSYRHSG	SLINHRQTHQ
650	660	670	680	690	700	710	720
TGDFSCGACA	KHFHTMAAMK	NHLRRHSRRR	SRRHRKRAGG	ASGGREAKLL	AAESWTRELE	DNEGLESPQD	PSGESPHGAE
730	740	750	760	770	780	790	800
GNLESDGDCL	QAESEGDKCG	LERDETHFQG	DKESGGTGEG	LERKDASLLD	NLDIPGEEGG	GTHFCDSLTG	VDEDQKPATG
810	820	830	840	850	860	870	880
QPNSSSHSAN	AVTGWQAGAA	HTCSDCGHSF	PHATGLLSHR	PCHPPGIYQC	SLCPKEFDSL	PALRSHFQNH	RPGEATSAQP
890	900	910	920	930	940	950	960
FLCCLCGMIF	PGRAGYRLHR	RQAHSSSGMT	EGSEEEGEEE	GVAEAAPARS	PPLQLSEAEL	LNQLQREVEA	LDSAGYGHIC
970	980	990	1000	1010	1020	1030	1040
GCCGQTYDDL	GSLERHHQSQ	SSGTTADKAP	SPLGVAGDAM	EMVVDSVLED	IVNSVSGEGG	DAKSQEGAGT	PLGDSLCIQG
1050	1060	1070	1080	1090	1100	1110	1120
GESLLEAQPR	PFRCNQCGKT	YRHGGSLVNH	RKIHQTGDFL	CPVCSRCYPN	LAAYRNHLRN	HPRCKGSEPQ	VGPIPEAAGS
1130	1140	1150	1160	1170	1180	1190	1200
SELQVGPIPE	GGSNKPQHMA	EEGPGQAEVE	KLQEELKVEP	LEEVARVKEE	VWEETTVKGE	EIEPRLETAE	KGCQTEASSE
1210	1220	1230	1240	1250	1260	1270	1280
RPFSCEVCGR	SYKHAGSLIN	HRQSHQTGHF	GCQACSKGFS	NLMSLKNHRR	IHADPRRFRC	SECGKAFRLR	KQLASHQRVH
1290	1300	1310	1320	1330	1340	1350	1360
MERRGGGGTR	KATREDRPFR	CGQCGRTYRH	AGSLLNHRRS	HETGQYSCPT	CPKTYSNRMA	LKDHQRLHSE	NRRRRAGRSR
1370	1380	1390	1400	1410	1420	1430	1440
RTAVRCALCG	RSFPGRGSLE	RHLREHEETE	REPANGQGGL	DGTAASEANL	TGSQGLETQL	GGAEPVPHLE	DGVPRPGERS
1450	1460	1470	1480	1490	1500	1510	1520
QSPIRAASSE	APEPLSWGAG	KAGGWPVGGG	LGNHSGGWVP	QFLTRSEEPE	DSVHRSPCHA	GDCQLNGPTL	SHMDSWDNRD
1530	1540	1550	1560	1570	1580	1590	1600
NSSQLQPGSH	SSCSQCGKTY	CQSGSLLNHN	TNKTDRHYCL	LCSKEFLNPV	ATKSHSHNHI	DAQTFACPDC	GKAFESHQEL
1610	1620	1630	1640	1650	1660	1670	1680
ASHLQAHARG	HSQVPAQMEE	ARDPKAGTGE	DQVVLPGQGK	AQEAPSETPR	GPGESVERAR	GGQAVTSMAA	EDKERPFRCT
1690	1700	1710	1720	1730	1740	1750	1760
QCGRSYRHAG	SLLNHQKAHT	TGLYPCSLCP	KLLPNLLSLK	NHSRTHTDPK	RHCCSICGKA	FRTAARLEGH	GRVHAPREGP
1770	1780	1790	1800	1810			
FTCPHCPRHF	RRRISFVQHQ	QQHQEEWTVA	GSGRGHEGSQ	EEEPQWHQ			

Cmpd.	No. of	m/z meas.	Δ m/z	z	Rt	Score	P	Range	Sequence	Modification

Detailed Protein Report

	Cmpds.		[ppm]		[min]					
1417	7	1023.6578	178.56	1	47.9	16.5	0	1557-1564	R.HYCLLCSK.E	Carbamidomethyl: 3

Detailed Protein Report

Protein 483: 3-hydroxyisobutyryl-CoA hydrolase, mitochondrial isoform 2 precursor [Homo sapiens]

Accession: gi|37594469
Database: refseq_human(refseq_human_20140103.fasta)
Database Date: 2015-11-30
Modification(s): Oxidation

Score: 28.8
MW [kDa]: 38.0
pI: 9.5
Sequence Coverage [%]: 11.8
No. of unique Peptides: 1

10	20	30	40	50	60	70	80
MGQREMWRLM	SRFNAFKRTN	TILHHLRMSK	HTDAAEEVLL	EKKGCTGVIT	LNRPKFLNAL	TLNMIRQIYP	QLKKWEQDPE
**90**	**100**	**110**	**120**	**130**	**140**	**150**	**160**
TFLIIIKGAG	GKAFCAGGDI	RVISEAEKAK	QKIAPVFFRE	EYMLNNAVGS	CQKPYVALIH	GITMGGGVGL	SVHGQFRVAT
**170**	**180**	**190**	**200**	**210**	**220**	**230**	**240**
EKCLFAMPET	AIGLFPDVGG	GYFLPRLQGK	LGYFLALTGF	RLKGRDVYRA	GIATHFVDSE	KLAMLEEDLL	ALKSPSKENI
**250**	**260**	**270**	**280**	**290**	**300**	**310**	**320**
ASVLENYHTE	SKIDRDKSFI	LEEHMDKINS	CFSANTVEEI	IENLQQDGSS	FALEQLKVIN	KMSPTSLKIT	LRQLMEGSSK
**330**	**340**						
TLQEVLTMEY	RLSQACMF						

Cmpd.	No. of Cmpds.	m/z meas.	Δ m/z [ppm]	z	Rt [min]	Score	P	Range	Sequence	Modification
2789	1	863.0890	-7.69	3	66.0	12.9	0	163-186	K.CLFAMPETAIGLFPDVGGGYFLPR.L	Oxidation: 5

Detailed Protein Report

Protein 484: leucine-rich repeat-containing protein 16A isoform 2 [Homo sapiens]

**Accession:**	gi|291190783	**Score:**	28.8
**Database:**	refseq_human(refseq_human_20140103.fasta)	**MW [kDa]:**	150.8
**Database Date:**	2015-11-30	**pI:**	8.8
**Modification(s):**	Oxidation	**Sequence Coverage [%]:**	3.2
		**No. of unique Peptides:**	1

10	20	30	40	50	60	70	80
MTEESSDVPR	ELIESIKDVI	GRKIKISVKK	KVKLEVKGDK	VENKVLVLTS	CRAFLVTARI	PTKLELTFSY	LEIHGVVCSK
**90**	**100**	**110**	**120**	**130**	**140**	**150**	**160**
SAQMIVETEK	CSISMKMASP	EDVSEVLAHI	GTCLRKIFPG	LSPVRIMKKV	SMEPSERLAS	LQALWDSQTV	AEQGPCGGFS
**170**	**180**	**190**	**200**	**210**	**220**	**230**	**240**
QMYACVCDWL	GFSYREEVQW	DVDTIYLTQD	TRELNLQDFS	HLDHRDLIPI	IAALEYNQWF	TKLSSKDLKL	STDVCEQILR
**250**	**260**	**270**	**280**	**290**	**300**	**310**	**320**
VVSRSNRLEE	LVLENAGLRT	DFAQKLASAL	AHNPNSGLHT	INLAGNPLED	RGVSSLSIQF	AKLPKGLKHL	NLSKTSLSPK
**330**	**340**	**350**	**360**	**370**	**380**	**390**	**400**
GVNSLSQSLS	ANPLTASTLV	HLDLSGNVLR	GDDLSHMYNF	LAQPNAIVHL	DLSNTECSLD	MVCGALLRGC	LQYLAVLNLS
**410**	**420**	**430**	**440**	**450**	**460**	**470**	**480**
RTVFSHRKGK	EVPPSFKQFF	SSSLALMHIN	LSGTKLSPEP	LKALLLGLAC	NHNLKGVSLD	LSNCELRSGG	AQVLEGCIAE
**490**	**500**	**510**	**520**	**530**	**540**	**550**	**560**
IHNITSLDIS	DNGLESDLST	LIVWLSKNRS	IQHLALGKNF	NNMKSKNLTP	VLDNLVQMIQ	DEESPLQSLS	LADSKLKTEV
**570**	**580**	**590**	**600**	**610**	**620**	**630**	**640**
TIIINALGSN	TSLTKVDISG	NGMGDMGAKM	LAKALQINTK	LRTVIWDKNN	ITAQGFQDIA	VAMEKNYTLR	FMPIPMYDAS
**650**	**660**	**670**	**680**	**690**	**700**	**710**	**720**
QALKTNPEKT	EDALQKIENY	LLRNHETRKY	LQEQAYRLQQ	GIVTSTTQQM	IDRICVKVQD	HLNSLRNCGG	DAIQEDLKSA
**730**	**740**	**750**	**760**	**770**	**780**	**790**	**800**
ERLMRDAKNS	KTLLPNLYHV	GGASWAGASG	LLSSPIQETL	ESMAGEVTRV	VDEQLKALLE	SMVDAAENLC	PNVMKKAHIR
**810**	**820**	**830**	**840**	**850**	**860**	**870**	**880**
QDLIHASTEK	ISIPRTFVKN	VLLEQSGIDI	LNKISEVKLT	VASFLSDRIV	DEILDALSHC	HHKLADHFSR	RGKTLPQQES
**890**	**900**	**910**	**920**	**930**	**940**	**950**	**960**
LEIELAEEKP	VKRSIITVEE	LTEIERLEDL	DTCMMTPKSK	RKSIHSRMLR	PVSRAFEMEF	DLDKALEEVP	IHIEDPPFPS
**970**	**980**	**990**	**1000**	**1010**	**1020**	**1030**	**1040**
LRQEKRSSGF	ISELPSEEGK	KLEHFTKLRP	KRNKKQQPTQ	AAVCAANIVS	QDGEQNGLMG	RVDEGVDEFF	TKKVTKMDSK
**1050**	**1060**	**1070**	**1080**	**1090**	**1100**	**1110**	**1120**
KWSTRGSESH	ELNEGGDEKK	KRDSRKSSGF	LNLIKSRSKS	ERPPTILMTE	EPSSPKGAVR	SPPVDCPRKD	TKAAEHNGNS
**1130**	**1140**	**1150**	**1160**	**1170**	**1180**	**1190**	**1200**
ERIEEIKTPD	SFEESQGEEI	GKVERSDSKS	SPQAGRRYGV	QVMGSGLLAE	MKAKQEKRAA	CAQKKLGNDA	VSQDSSSPAL
**1210**	**1220**	**1230**	**1240**	**1250**	**1260**	**1270**	**1280**
SGVERSDGGG	AGLPENRFGL	GTPEKNTKAE	PKAEAGSRSR	SSSSTPTSPK	PLLQSPKPSL	AARPVIPQKP	RTASRPDDIP
**1290**	**1300**	**1310**	**1320**	**1330**	**1340**	**1350**	**1360**
DSPSSPKVAL	LPPVLKKVPS	DKERDGQSSP	QPSPRTFSQE	VSRRSWGQQA	QEYQEQKQRS	SSKDGHQGSK	SNDSGEEAEK
**1370**							
EFIFV							

Cmpd.	No. of Cmpds.	m/z meas.	Δ m/z [ppm]	z	Rt [min]	Score	P	Range	Sequence	Modification
843	1	946.5224	65.61	3	40.1	18.7	2	91-116	K.CSISMKMASPEDVSEVLAHIGTCLRK.I	Oxidation: 5, 7

Detailed Protein Report

Protein 485: PREDICTED: 1,5-anhydro-D-fructose reductase isoform X4 [Homo sapiens]

**Accession:**	gi|578818599	**Score:**	28.7
**Database:**	refseq_human(refseq_human_20140103.fasta)	**MW [kDa]:**	22.1
**Database Date:**	2015-11-30	**pI:**	6.4
**Modification(s):**	Carbamidomethyl	**Sequence Coverage [%]:**	10.7
		**No. of unique Peptides:**	1

10	20	30	40	50	60	70	80
MGDIPAVGLS	SWKASPGKVT	EAVKEAIDAG	YRHFDCAYFY	HNEREVGAGI	RCKIKEGAVR	REDLFIATKP	PHPEWIMSCS
**90**	**100**	**110**	**120**	**130**	**140**	**150**	**160**
ELSFCLSHPR	VQDLPLDESN	MVIPSDTDFL	DTWEAMEDLV	ITGLVKNIGV	SNFNHEQLER	LLNKPGLRFK	PLTNQILIRF
**170**	**180**	**190**	**200**				
QIQRNVIVIP	GSITPSHIKE	NIQVCYTLRG	NAILRQ				

Cmpd.	No. of Cmpds.	m/z meas.	Δ m/z [ppm]	z	Rt [min]	Score	P	Range	Sequence	Modification
1457	1	960.4393	-73.19	2	47.9	10.9	1	180-195	K.ENIQVCYTLRGNAILR.Q	Carbamidomethyl: 6

Detailed Protein Report

Protein 486: PREDICTED: protein FAM45A isoform X1 [Homo sapiens]

Accession: gi|530393695
Database: refseq_human(refseq_human_20140103.fasta)
Database Date: 2015-11-30
Modification(s): Carbamidomethyl

Score: 28.7
MW [kDa]: 39.8
pI: 6.3
Sequence Coverage [%]: 7.2
No. of unique Peptides: 2

10	20	30	40	50	60	70	80
MLSSDHLSTV	EKDTNGEVLW	VWCYPSTTAT	LRNLLLRKCC	LTDENKLLHP	FVFGQYRRTW	FYITTIEVPD	SSILKKVTHF
**90**	**100**	**110**	**120**	**130**	**140**	**150**	**160**
SIVLTAKDFN	PEKYAAFTRI	LCRMYLKHGS	PVKMMESYIA	VLTKGICQSE	ENGSFLSKDF	DARKAYLAGS	IKDIVSQFGM
**170**	**180**	**190**	**200**	**210**	**220**	**230**	**240**
ETVILHTALM	LKKRIVVYHP	KIEAVQEFTR	TLPALVWHRQ	DWTILHSYVH	LNADELEALQ	MCTGYVAGFV	DLEVSNRPDL
**250**	**260**	**270**	**280**	**290**	**300**	**310**	**320**
YDVFVNLAES	EITIAPLAKE	AMAMGKLHKE	MGQLIVQSAE	DPEKSESHVI	QDIALKTREI	FTNLAPFSEV	SADGEKRVLN
**330**	**340**	**350**					
LEALKQKRFP	PATENFLYHL	AAAEQMLKI					

Cmpd.	No. of Cmpds.	m/z meas.	Δ m/z [ppm]	z	Rt [min]	Score	P	Range	Sequence	Modification
837	1	491.6643	-76.81	2	38.9	11.9	0	39-46	K.CCLTDENK.L	Carbamidomethyl: 1
2666	1	973.5093	-13.80	2	63.4	16.7	1	77-93	K.VTHFSIVLTAKDFNPEK.Y	

Detailed Protein Report

Protein 487: PREDICTED: FERM and PDZ domain-containing protein 4 isoform X2 [Homo sapiens]

Accession: gi|578837988
Database: refseq_human(refseq_human_20140103.fasta)
Database Date: 2015-11-30
Modification(s): Carbamidomethyl

Score: 28.6
MW [kDa]: 189.7
pI: 5.2
Sequence Coverage [%]: 0.9
No. of unique Peptides: 1

10	20	30	40	50	60	70	80
MDTVHPGSHS	WDFCHRTKSS	GWPPPSGTWG	LSQVPPYGWE	MTANRDGRDY	FINHMTQAIP	FDDPRLESCQ	IIPPAPRKVE
90	100	110	120	130	140	150	160
MRRDPVLGFG	FVAGSEKPVV	VRSVTPGGPS	EGKLIPGDQI	VMINDEPVSA	APRERVIDLV	RSCKESILLT	VIQPYPSPKS
170	180	190	200	210	220	230	240
AFISAAKKAR	LKSNPVKVRF	SEEVIINGQV	SETVKDNSLL	FMPNVLKVYL	ENGQTKSFRF	DCSTSIKDVI	LTLQEKLSIK
250	260	270	280	290	300	310	320
GIEHFSLMLE	QRTEGAGTKL	LLLHEQETLT	QVTQRPSSHK	MRCLFRISFV	PKDPIDLLRR	DPVAFEYLYV	QSCNDVVQER
330	340	350	360	370	380	390	400
FGPELKYDIA	LRLAALQMYI	ATVTTKQTQK	ISLKYIEKEW	GLETFLPSAV	LQSMKEKNIK	KALSHLVKAN	QNLVPPGKKQ
410	420	430	440	450	460	470	480
AEKRSEVTLL	VGPRYGISHV	INTKTNLVAL	LADFSHVNRI	EMFSEEESLV	RVELHVLDVK	PITLLMESSD	AMNLACLTAG
490	500	510	520	530	540	550	560
YYRLLVDSRR	SIFNMANKKN	TATQETGPEN	KGKHNLLGPD	WNCIPQMTTF	IGEGEQEAQI	TYIDSKQKTV	EITDSTMCPK
570	580	590	600	610	620	630	640
EHRHLYIDNA	YSSDGLNQQL	SQPGEAPCEA	DYRSLAQRSL	LTLSGPETLK	KAQESPRGAK	VSFIFGDFAL	DDGISPPTLG
650	660	670	680	690	700	710	720
YETLLDEGPE	MLEKQRNLYI	GSANDMKGLD	LTPEAEGIQF	VENSVYANIG	DVKSFQAAEG	IEEPLLHDIC	YAENTDDAED
730	740	750	760	770	780	790	800
EDEVSCEEDL	VVGEMNQPAI	LNLSGSSDDI	IDLTSLPPPE	GDDNEDDFLL	RSLNMAIAAP	PPGFRDSSDE	EDSQSQAASF
810	820	830	840	850	860	870	880
PEDKEKGSSL	QNDEIPVSLI	DAVPTSAEGK	CEKGLDNAVV	STLGALEALS	VSEEQQTSDN	SGVAILRAYS	PESSSDSGNE
890	900	910	920	930	940	950	960
TNSSEMTESS	ELATAQKQSE	NLSRMFLATH	EGYHPLAEEQ	TEFPASKTPA	GGLPPKSSHA	LAARPATDLP	PKVVPSKQLL
970	980	990	1000	1010	1020	1030	1040
HSDHMEMEPE	TMETKSVTDY	FSKLHMGSVA	YSCTSKRKSK	LADGEGKAPP	NGNTTGKKQQ	GTKTAEMEEE	ASGKFGTVSS
1050	1060	1070	1080	1090	1100	1110	1120
RDSQHLSTFN	LERTAFRKDS	QRWYVATEGG	MAEKSGLEAA	TGKTFPRASG	LGAREAEGKE	EGAPDGETSD	GSGLGQGDRF
1130	1140	1150	1160	1170	1180	1190	1200
LTDVTCASSA	KDLDNPEDAD	SSTCDHPSKL	PEADESVARL	CDYHLAKRMS	SLQSEGHFSL	QSSQGSSVDA	GCGTGSSGSA
1210	1220	1230	1240	1250	1260	1270	1280
CATPVESPLC	PSLGKHLIPD	ASGKGVNYIP	SEERAPGLPN	HGATFKELHP	QTEGMCPRMT	VPALHTAINT	EPLFGTLRDG
1290	1300	1310	1320	1330	1340	1350	1360
CHRLPKIKET	TALTEPGKER	RGGMPSAWSQ	HPEADPILLP	SNIHSESKVP	IPNQDPNDFS	QANQAYGEAV	SWRPPDLRGG
1370	1380	1390	1400	1410	1420	1430	1440
SLRTPPSQKA	LRHSSSILSG	SVDLETFRER	TKGAVSLKCP	GITEAQEASS	ERRAELPLGR	KLTKSFSQSS	MHLSSEGRFH
1450	1460	1470	1480	1490	1500	1510	1520
KRSPVAHKDS	KLYRTLPLRK	LEGSNWRCRG	PFSYCFLNRG	QDEDGEEEEE	RGEATVQVSC	LYRPQMTQAM	PEPSSPCLAV
1530	1540	1550	1560	1570	1580	1590	1600
AIQKQRGELS	RGSVLKVWAE	DLRDPDDLDF	SNLAFDARIA	RINALKESTY	AMPDGFLAAQ	NDANELLCLV	RATKEKREES
1610	1620	1630	1640	1650	1660	1670	1680
RPEAYDLTLS	QYKQLLSIES	RQLGSACRKM	AMAEKSPEEM	LLAMTSSFQV	LCCLTEACMR	LVKVVNSETQ	RQEIVGKIDE
1690	1700	1710	1720	1730	1740		
VVINYICLLK	AAEAATGKNP	GDPNVGLSAR	HSTTMAALVS	TLTRSLKRLL	NK		

Cmpd.	No. of Cmpds.	m/z meas.	Δ m/z [ppm]	z	Rt [min]	Score	P	Range	Sequence	Modification
684	1	460.1264	-252.38	2	38.1	14.1	1	1622-1629	R.QLGSACRK.M	Carbamidomethyl: 6

Detailed Protein Report

Protein 488: ankyrin repeat and zinc finger domain-containing protein 1 isoform 1 [Homo sapiens]

**Accession:**	gi|109150425	**Score:**	28.6
**Database:**	refseq_human(refseq_human_20140103.fasta)	**MW [kDa]:**	80.9
**Database Date:**	2015-11-30	**pI:**	9.6
		**Sequence Coverage [%]:**	2.9
		**No. of unique Peptides:**	1

Alias proteins:

Accession	Name	Description
gi|109150435	refseq_human (refseq_human_20140103.fasta)	ankyrin repeat and zinc finger domain-containing protein 1 isoform 1 [Homo sapiens]

10	20	30	40	50	60	70	80
MSPAPDAAPA	PASISLFDLS	ADAPVFQGLS	LVSHAPGEAL	ARAPRTSCSG	SGERESPERK	LLQGPMDISE	KLFCSTCDQT
90	100	110	120	130	140	150	160
FQNHQEQREH	YKLDWHRFNL	KQRLKDKPLL	SALDFEKQSS	TGDLSSISGS	EDSDSASEED	LQTLDRERAT	FEKLSRPPGF
170	180	190	200	210	220	230	240
YPHRVLFQNA	QGQFLYAYRC	VLGPHQDPPE	EAELLLQNLQ	SRGPRDCVVL	MAAAGHFAGA	IFQGREVVTH	KTFHRYTVRA
250	260	270	280	290	300	310	320
KRGTAQGLRD	ARGGPSHSAG	ANLRRYNEAT	LYKDVRDLLA	GPSWAKALEE	AGTILLRAPR	SGRSLFFGGK	GAPLQRGDPR
330	340	350	360	370	380	390	400
LWDIPLATRR	PTFQELQRVL	HKLTTLHVYE	EDPREAVRLH	SPQTHWKTVR	EERKKPTEEE	IRKICRDEKE	ALGQNEESPK
410	420	430	440	450	460	470	480
QGSGSEGEDG	FQVELELVEL	TVGTLDLCES	EVLPKRRRRK	RNKKEKSRDQ	EAGAHRTLLQ	QTQEEEPSTQ	SSQAVAAPLG
490	500	510	520	530	540	550	560
PLLDEAKAPG	QPELWNALLA	ACRAGDVGVL	KLQLAPSPAD	PRVLSLLSAP	LGSGGFTLLH	AAAAAGRGSV	VRLLLEAGAD
570	580	590	600	610	620	630	640
PTVQDSRARP	PYTVAADKST	RNEFRRFMEK	NPDAYDYNKA	QVPGPLTPEM	EARQATRKRE	QKAARRQEE	QQQRQQEQEE
650	660	670	680	690	700	710	720
REREEQRRFA	ALSDREKRAL	AAERRLAAQL	GAPTSPIPDS	AIVNTRRCWS	CGASLQGLTP	FHYLDFSFCS	TRCLQDHRRQ
730							
AGRPSS							

Cmpd.	No. of Cmpds.	m/z meas.	Δ m/z [ppm]	z	Rt [min]	Score	P	Range	Sequence	Modification
883	3	529.1891	-185.79	2	39.4	16.5	0	277-286	R.DLLAGPSWAK.A	

Detailed Protein Report

Protein 489: prolow-density lipoprotein receptor-related protein 1 precursor [Homo sapiens]

**Accession:**	gi|126012562	**Score:**	28.6
**Database:**	refseq_human(refseq_human_20140103.fasta)	**MW [kDa]:**	504.3
**Database Date:**	2015-11-30	**pI:**	5.0
**Modification(s):**	Carbamidomethyl, Oxidation	**Sequence Coverage [%]:**	0.6
		**No. of unique Peptides:**	2

Detailed Protein Report

10	20	30	40	50	60	70	80
MLTPPLLLLL	PLLSALVAAA	IDAPKTCSPK	QFACRDQITC	ISKGWRCDGE	RDCPDGSDEA	PEICPQSKAQ	RCQPNEHNCL
90	100	110	120	130	140	150	160
GTELCVPMSR	LCNGVQDCMD	GSDEGPHCRE	LQGNCSRLGC	QHHCVPTLDG	PTCYCNSSFQ	LQADGKTCKD	FDECSVYGTC
170	180	190	200	210	220	230	240
SQLCTNTDGS	FICGCVEGYL	LQPDNRSCKA	KNEPVDRPPV	LLIANSQNIL	ATYLSGAQVS	TITPTSTRQT	TAMDFSYANE
250	260	270	280	290	300	310	320
TVCWVHVGDS	AAQTQLKCAR	MPGLKGFVDE	HTINISLSLH	HVEQMAIDWL	TGNFYFVDDI	DDRIFVCNRN	GDTCVTLLDL
330	340	350	360	370	380	390	400
ELYNPKGIAL	DPAMGKVFFT	DYGQIPKVER	CDMDGQNRTK	LVDSKIVFPH	GITLDLVSRL	VYWADAYLDY	IEVVDYEGKG
410	420	430	440	450	460	470	480
RQTIIQGILI	EHLYGLTVFE	NYLYATNSDN	ANAQQKTSVI	RVNRFNSTEY	QVVTRVDKGG	ALHIYHQRRQ	PRVRSHACEN
490	500	510	520	530	540	550	560
DQYGKPGGCS	DICLLANSHK	ARTCRCRSGF	SLGSDGKSCK	KPEHELFLVY	GKGRPGIIRG	MDMGAKVPDE	HMIPIENLMN
570	580	590	600	610	620	630	640
PRALDFHAET	GFIYFADTTS	YLIGRQKIDG	TERETILKDG	IHNVEGVAVD	WMGDNLYWTD	DGPKKTISVA	RLEKAAQTRK
650	660	670	680	690	700	710	720
TLIEGKMTHP	RAIVVDPLNG	WMYWTDWEED	PKDSRRGRLE	RAWMDGSHRD	IFVTSKTVLW	PNGLSLDIPA	GRLYWVDAFY
730	740	750	760	770	780	790	800
DRIETILLNG	TDRKIVYEGP	ELNHAFGLCH	HGNYLFWTEY	RSGSVYRLER	GVGGAPPTVT	LLRSERPPIF	EIRMYDAQQQ
810	820	830	840	850	860	870	880
QVGTNKCRVN	NGGCSSLCLA	TPGSRQCACA	EDQVLDADGV	TCLANPSYVP	PPQCQPGEFA	CANSRCIQER	WKCDGDNDCL
890	900	910	920	930	940	950	960
DNSDEAPALC	HQHTCPSDRF	KCENNRCIPN	RWLCDGDNDC	GNSEDESNAT	CSARTCPPNQ	FSCASGRCIP	ISWTCDLDDD
970	980	990	1000	1010	1020	1030	1040
CGDRSDESAS	CAYPTCFPLT	QFTCNNGRCI	NINWRCDNDN	DCGDNSDEAG	CSHSCSSTQF	KCNSGRCIPE	HWTCDGDNDC
1050	1060	1070	1080	1090	1100	1110	1120
GDYSDETHAN	CTNQATRPPG	GCHTDEFQCR	LDGLCIPLRW	RCDGDTDCMD	SSDEKSCEGV	THVCDPSVKF	GCKDSARCIS
1130	1140	1150	1160	1170	1180	1190	1200
KAWVCDGDND	CEDNSDEENC	ESLACRPPSH	PCANNTSVCL	PPDKLCDGND	DCGDGSDEGE	LCDQCSLNNG	GCSHNCSVAP
1210	1220	1230	1240	1250	1260	1270	1280
GEGIVCSCPL	GMELGPDNHT	CQIQSYCAKH	LKCSQKCDQN	KFSVKCSCYE	GWVLEPDGES	CRSLDPFKPF	IIFSNRHEIR
1290	1300	1310	1320	1330	1340	1350	1360
RIDLHKGDYS	VLVPGLRNTI	ALDFHLSQSA	LYWTDVVEDK	IYRGKLLDNG	ALTSFEVVIQ	YGLATPEGLA	VDWIAGNIYW
1370	1380	1390	1400	1410	1420	1430	1440
VESNLDQIEV	AKLDGTLRTT	LLAGDIEHPR	AIALDPRDGI	LFWTDWDASL	PRIEAASMSG	AGRRTVHRET	GSGGWPNGLT
1450	1460	1470	1480	1490	1500	1510	1520
VDYLEKRILW	IDARSDAIYS	ARYDGSGHME	VLRGHEFLSH	PFAVTLYGGE	VYWTDWRTNT	LAKANKWTGH	NVTVVQRTNT
1530	1540	1550	1560	1570	1580	1590	1600
QPFDLQVYHP	SRQPMAPNPC	EANGGQGPCS	HLCLINYNRT	VSCACPHLMK	LHKDNTTCYE	FKKFLLYARQ	MEIRGVDLDA
1610	1620	1630	1640	1650	1660	1670	1680
PYYNYIISFT	VPDIDNVTVL	DYDAREQRVY	WSDVRTQAIK	RAFINGTGVE	TVVSADLPNA	HGLAVDWVSR	NLFWTSYDTN
1690	1700	1710	1720	1730	1740	1750	1760
KKQINVARLD	GSFKNAVVQG	LEQPHGLVVH	PLRGKLYWTD	GDNISMANMD	GSNRTLLFSG	QKGPVGLAID	FPESKLYWIS
1770	1780	1790	1800	1810	1820	1830	1840
SGNHTINRCN	LDGSGLEVID	AMRSQLGKAT	ALAIMGDKLW	WADQVSEKMG	TCSKADGSGS	VVLRNSTTLV	MHMKVYDESI
1850	1860	1870	1880	1890	1900	1910	1920
QLDHKGTNPC	SVNNGDCSQL	CLPTSETTRS	CMCTAGYSLR	SGQQACEGVG	SFLLYSVHEG	IRGIPLDPND	KSDALVPVSG
1930	1940	1950	1960	1970	1980	1990	2000
TSLAVGIDFH	AENDTIYWVD	MGLSTISRAK	RDQTWREDVV	TNGIGRVEGI	AVDWIAGNIY	WTDQGFDVIE	VARLNGSFRY
2010	2020	2030	2040	2050	2060	2070	2080
VVISQGLDKP	RAITVHPEKG	YLFWTEWGQY	PRIERSRLDG	TERVVLVNVS	ISWPNGISVD	YQDGKLYWCD	ARTDKIERID
2090	2100	2110	2120	2130	2140	2150	2160
LETGENREVV	LSSNNMDMFS	VSVFEDFIYW	SDRTHANGSI	KRGSKDNATD	SVPLRTGIGV	QLKDIKVFNR	DRQKGTNVCA
2170	2180	2190	2200	2210	2220	2230	2240

Detailed Protein Report

Cmpd.	No. of Cmpds.	m/z meas.	Δ m/z [ppm]	z	Rt [min]	Score	P	Range	Sequence	Modification
87	2	849.3639	-63.47	2	30.0	12.9	1	1560-1573	R.TVSCACPHLMKLHK.D	Carbamidomethyl: 4, 6; Oxidation: 10
2236	2	873.3305	-116.11	2	57.8	15.6	1	1952-1966	R.DQTWREDVVTNGIGR.V	

Detailed Protein Report

Protein 490: **PREDICTED: zinc finger and SCAN domain-containing protein 12 isoform X3 [Homo sapiens]**

**Accession:**	gi|578812153	**Score:**	28.6
**Database:**	refseq_human(refseq_human_20140103.fasta)	**MW [kDa]:**	65.7
**Database Date:**	2015-11-30	**pI:**	6.2
		**Sequence Coverage [%]:**	5.0
		**No. of unique Peptides:**	1

Quantitation

QU:MU	**Median:**	0.27	**CV:**	0.00 %	**No. of Peptides:**	1
WUP:QUP	**Median:**	0.85	**CV:**	0.00 %	**No. of Peptides:**	1

10	20	30	40	50	60	70	80
MASTWAIQAH	MDQDEPLEVK	IEEEKYTTRQ	DWDLRKNNTH	SREVFRQYFR	QFCYQETSGP	REALSRLREL	CHQWLRPETH
90	100	110	120	130	140	150	160
TKEQILELLV	LEQFLTILPE	ELQAWVQEQH	PESGEEVVTV	LEDLERELDE	PGEQVSVHTG	EQEMFLQETV	RLRKEGEPSM
170	180	190	200	210	220	230	240
SLQSMKAQPK	YESPELESQQ	EQVLDVETGN	EYGNLKQEVS	EEMEPHGKTS	SKFENDMSKS	ARCGETREPE	EITEEPSACS
250	260	270	280	290	300	310	320
REDKQPTCDE	NGVSLTENSD	HTEHQRICPG	EESYGCDDCG	KAFSQHSHLI	EHQRIHTGDR	PYKCEECGKA	FRGRTVLIRH
330	340	350	360	370	380	390	400
KIIHTGEKPY	KCNECGKAFG	RWSALNQHQR	LHTGEKHYHC	NDCGKAFSQK	AGLFHHIKIH	TRDKPYQCTQ	CNKSFSRRSI
410	420	430	440	450	460	470	480
LTQHQGVHTG	AKPYECNECG	KAFVYNSSLV	SHQEIHHKEK	CYQCKECGKS	FSQSGLIQHQ	RIHTGEKPYK	CDVCEKAFIQ
490	500	510	520	530	540	550	560
RTSLTEHQRI	HTGERPYKCD	KCGKAFTQRS	VLTEHQRIHT	GERPYKCDEC	GNAFRGITSL	IQHQRIHTGE	KPYQCDECGK
570							
AFRQR							

Cmpd.	No. of Cmpds.	m/z meas.	Δ m/z [ppm]	z	Rt [min]	Score	P	Range	Sequence	Modification	Ratios
224	1	666.0908	127.27	3	32.5	10.0	0	422-438	K.AFVYNSSLVSHQEIHHK.E		WUP:QUP 0.85<br>QU:MU 0.27

Detailed Protein Report

Protein 491: **PREDICTED: KN motif and ankyrin repeat domain-containing protein 1 isoform X16 [Homo sapiens]**

**Accession:**	gi|578816444	**Score:**	28.5
**Database:**	refseq_human(refseq_human_20140103.fasta)	**MW [kDa]:**	122.8
**Database Date:**	2015-11-30	**pI:**	5.0
**Modification(s):**	Oxidation	**Sequence Coverage [%]:**	3.2
		**No. of unique Peptides:**	1

Quantitation

QU:MU	**Median:**	1.65	**CV:**	0.00 %	**No. of Peptides:**	1
WUP:QUP	**Median:**	0.44	**CV:**	0.00 %	**No. of Peptides:**	1

10	20	30	40	50	60	70	80
MAHTTKVNGS	ASGKAGDILS	GDQDKEQKDP	YFVETPYGYQ	LDLDFLKYVD	DIQKGNTIKR	LNIQKRRKPS	VPCPEPRTTS
90	100	110	120	130	140	150	160
GQQGIWTSTE	SLSSSNSDDN	KQCPNFLIAR	SQVTSTPISK	PPPPLETSLP	FLTIPENRQL	PPPSPQLPKH	NLHVTKTLME
170	180	190	200	210	220	230	240
TRRRLEQERA	TMQMTPGEFR	RPRLASFGGM	GTTSSLPSFV	GSGNHNPAKH	QLQNGYQGNG	DYGSYAPAAP	TTSSMGSSIR
250	260	270	280	290	300	310	320
HSPLSSGIST	PVTNVSPMHL	QHIREQMAIA	LKRLKELEEQ	VRTIPVLQVK	ISVLQEEKRQ	LVSQLKNQRA	ASQINVCGVR
330	340	350	360	370	380	390	400
KRSYSAGNAS	QLEQLSRARR	SGGELYIDYE	EEEMETVEQS	TQRIKEFRQL	TADMQALEQK	IQDSSCEASS	ELRENGECRS
410	420	430	440	450	460	470	480
VAVGAEENMN	DIVVYHRGSR	SCKDAAVGTL	VEMRNCGVSV	TEAMLGVMTE	ADKEIELQQQ	TIESLKEKIY	RLEVQLRETT
490	500	510	520	530	540	550	560
HDREMTKLKQ	ELQAAGSRKK	VDKATMAQPL	VFSKVVEAVV	QTRDQMVGSH	MDLVDTCVGT	SVETNSVGIS	CQPECKNKVV
570	580	590	600	610	620	630	640
GPELPMNWWI	VKERVEMHDR	CAGRSVEMCD	KSVSVEVSVC	ETGSNTEESV	NDLTLLKTNL	NLKEVRSIGC	GDCSVDVTVC
650	660	670	680	690	700	710	720
SPKECASRGV	NTEAVSQVEA	AVMAVPRTAD	QDTSTDLEQV	HQFTNTETAT	LIESCTNTCL	STLDKQTSTQ	TVETRTVAVG
730	740	750	760	770	780	790	800
EGRVKDINSS	TKTRSIGVGT	LLSGHSGFDR	PSAVKTKESG	VGQININDNY	LVGLKMRTIA	CGPPQLTVGL	TASRRSVGVG
810	820	830	840	850	860	870	880
DDPVGESLEN	PQPQAPLGMM	TGLDHYIERI	QKLLAEQQTL	LAENYSELAE	AFGEPHSQMG	SLNSQLISTL	SSINSVMKSA
890	900	910	920	930	940	950	960
STEELRNPDF	QKTSLGKITG	NYLGYTCKCG	GLQSGSPLSS	QTSQPEQEVG	TSEGKPISSL	DAFPTQEGTL	SPVNLTDDQI
970	980	990	1000	1010	1020	1030	1040
AAGLYACTNN	ESTLKSIMKK	KDGNKDSNGA	KKNLQFVGIN	GGYETTSSDD	SSSDESSSSE	SDDECDVIEY	PLEEEEEEED
1050	1060	1070	1080	1090	1100	1110	1120
EDTRGMAEGH	HAVNIEGLKS	ARVEDEMQVQ	ECEPEKVEIR	ERYELSEKML	SACNLLKNTI	NDPKALTSKD	MWLNLWKWNL
1130							

Cmpd.	No. of Cmpds.	m/z meas.	Δ m/z [ppm]	z	Rt [min]	Score	P	Range	Sequence	Modification	Ratios	
23	1	736.0076	3.59	3	30.0	11.9	1	1063-1080	R.VEDEMQVQECEPEKVEIR.E	Oxidation: 5	WUP:QUP<br>QU:MU	0.44<br>1.65

Detailed Protein Report

Protein 492: **PREDICTED: probable threonine--tRNA ligase 2, cytoplasmic isoform X3 [Homo sapiens]**

**Accession:**	gi|578826598	**Score:**	28.5
**Database:**	refseq_human(refseq_human_20140103.fasta)	**MW [kDa]:**	82.5
**Database Date:**	2015-11-30	**pI:**	5.7
**Modification(s):**	Carbamidomethyl, Oxidation	**Sequence Coverage [%]:**	6.5
		**No. of unique Peptides:**	2

10	20	30	40	50	60	70	80
MAAEALAAEA	VASRLERQEE	DIRWLWSEVE	RLRDEQLNAP	YSCQAEGPCL	TREVAQLRAE	NCDLRHRLCS	LRLCLAEERS
**90**	**100**	**110**	**120**	**130**	**140**	**150**	**160**
RQATLESAEL	EAAQEAGAQP	PPSQSQDKDM	KKKKMKESEA	DSEVKHQPIF	IKERLKLFEI	LKKDHQLLLA	IYGKKGDTSN
**170**	**180**	**190**	**200**	**210**	**220**	**230**	**240**
IITVRVADGQ	TVQGEVWKTT	PYQVAAEISQ	ELAESTVIAK	VNGELWDLDR	PLEGDSSLEL	LTFDNEEAQA	VYWHSSAHIL
**250**	**260**	**270**	**280**	**290**	**300**	**310**	**320**
GEAMELYYGG	HLCYGPPIEN	GFYYDMFIED	RAVSSTELSA	LENICKAIIK	EKQPFERLEV	SKEILLEMFK	YNKFKCRILN
**330**	**340**	**350**	**360**	**370**	**380**	**390**	**400**
EKVNTATTTV	YRCGPLIDLC	KGPHVRHTGK	IKTIKIFKNS	STYWEGNPEM	ETLQRIYGIS	FPDNKMMRDW	EKFQEEAKNR
**410**	**420**	**430**	**440**	**450**	**460**	**470**	**480**
DHRKIGKEQE	LFFFHDLSPG	SCFFLPRGAF	IYNTLTDFIR	EEYHKRDFTE	VLSPNMYNSK	LWEASGHWQH	YSENMFTFEI
**490**	**500**	**510**	**520**	**530**	**540**	**550**	**560**
EKDTFALKPM	NCPGHCLMFA	HRPRSWREMP	IRFADFGVLH	RNELSGTLSG	LTRVRRFQQD	DAHIFCTVEQ	IEEEIKGCLQ
**570**	**580**	**590**	**600**	**610**	**620**	**630**	**640**
FLQSVYSTFG	FSFQLNLSTR	PENFLGEIEM	WNEAEKQLQN	SLMDFGEPWK	MNPGDGAFYG	PKIDIKIKDA	IGRYHQCATI
**650**	**660**	**670**	**680**	**690**	**700**	**710**	**720**
QLDFQLPIRF	NLTYVSKDGD	DKKRPVIIHR	AILGSVERMI	AILSENYGGK	WYPVNFLKKD	LWLTLTWITV	VH

Cmpd.	No. of Cmpds.	m/z meas.	Δ m/z [ppm]	z	Rt [min]	Score	P	Range	Sequence	Modification
2780	1	849.0600	-40.11	3	65.9	10.9	2	1-23	-.MAAEALAAEAVASRLERQEEDIR.W	Oxidation: 1
569	1	689.8555	-24.02	4	37.1	17.6	2	627-649	K.IKDAIGRYHQCATIQLDFQLPIR.F	Carbamidomethyl: 11

Detailed Protein Report

Protein 493: **PREDICTED: keratin-associated protein 9-8 [Homo sapiens]**

Accession: gi|578846434
Database: refseq_human(refseq_human_20140103.fasta)
Database Date: 2015-11-30
Modification(s): Carbamidomethyl

Score: 28.5
MW [kDa]: 16.8
pI: 11.9
Sequence Coverage [%]: 20.1
No. of unique Peptides: 2

10	20	30	40	50	60	70	80
MTHCCSPCCQ	PTCCRTTCWK	PTTVTTCSST	PCCQPSCCVS	SCCQPCCRPT	CCQNTCCQPI	CVTSCCQPSC	CSTPCCQPTC
**90**	**100**	**110**	**120**	**130**	**140**	**150**	**160**
CGQTSCGSSC	GQSSSCAPVY	CRRTCYHPTT	VCLPGCLNQS	CGSSCCQPCC	RPACCETTCC	RTTCFQPTCV	YSCCQPSCC

Cmpd.	No. of Cmpds.	m/z meas.	Δ m/z [ppm]	z	Rt [min]	Score	P	Range	Sequence	Modification
2540	1	856.9279	151.49	2	62.4	10.7	0	2-15	M.THCCSPCCQPTCCR.T	Carbamidomethyl: 3, 4, 13
2119	1	1044.4110	33.02	2	56.4	17.8	0	142-159	R.TTCFQPTCVYSCCQPSCC.-	Carbamidomethyl: 3, 8

Detailed Protein Report

Protein 494: **ectonucleotide pyrophosphatase/phosphodiesterase family member 2 isoform 3 preproprotein [Homo sapiens]**

Accession: gi|195947389
Database: refseq_human(refseq_human_20140103.fasta)
Database Date: 2015-11-30
Modification(s): Carbamidomethyl

Score: 28.5
MW [kDa]: 101.9
pI: 8.6
Sequence Coverage [%]: 2.1
No. of unique Peptides: 1

10	20	30	40	50	60	70	80
MARRSSFQSC	QIISLFTFAV	GVNICLGFTA	HRIKRAEGWE	EGPPTVLSDS	PWTNISGSCK	GRCFELQEAG	PPDCRCDNLC
**90**	**100**	**110**	**120**	**130**	**140**	**150**	**160**
KSYTSCCHDF	DELCLKTARG	WECTKDRCGE	VRNEENACHC	SEDCLARGDC	CTNYQVVCKG	ESHWVDDDCE	EIKAAECPAG
**170**	**180**	**190**	**200**	**210**	**220**	**230**	**240**
FVRPPLIIFS	VDGFRASYMK	KGSKVMPNIE	KLRSCGTHSP	YMRPVYPTKT	FPNLYTLATG	LYPESHGIVG	NSMYDPVFDA
**250**	**260**	**270**	**280**	**290**	**300**	**310**	**320**
TFHLRGREKF	NHRWWGGQPL	WITATKQGVK	AGTFFWSVVI	PHERRILTIL	QWLTLPDHER	PSVYAFYSEQ	PDFSGHKYGP
**330**	**340**	**350**	**360**	**370**	**380**	**390**	**400**
FGPEMTNPLR	EIDKIVGQLM	DGLKQLKLHR	CVNVIFVGDH	GMEDVTCDRT	EFLSNYLTNV	DDITLVPGTL	GRIRSKFSNN
**410**	**420**	**430**	**440**	**450**	**460**	**470**	**480**
AKYDPKAIIA	NLTCKKPDQH	FKPYLKQHLP	KRLHYANNRR	IEDIHLLVER	RWHVARKPLD	VYKKPSGKCF	FQGDHGFDNK
**490**	**500**	**510**	**520**	**530**	**540**	**550**	**560**
VNSMQTVFVG	YGSTFKYKTK	VPPFENIELY	NVMCDLLGLK	PAPNNGTHGS	LNHLLRTNTF	RPTMPEEVTR	PNYPGIMYLQ
**570**	**580**	**590**	**600**	**610**	**620**	**630**	**640**
SDFDLGCTCD	DKVEPKNKLD	ELNKRLHTKG	STEAETRKFR	GSRNENKENI	NGNFEPRKER	HLLYGRPAVL	YRTRYDILYH
**650**	**660**	**670**	**680**	**690**	**700**	**710**	**720**
TDFESGYSEI	FLMPLWTSYT	VSKQAEVSSV	PDHLTSCVRP	DVRVSPSFSQ	NCLAYKNDKQ	MSYGFLFPPY	LSSSPEAKYD
**730**	**740**	**750**	**760**	**770**	**780**	**790**	**800**
AFLVTNMVPM	YPAFKRVWNY	FQRVLVKKYA	SERNGVNVIS	GPIFDYDYDG	LHDTEDKIKQ	YVEGSSIPVP	THYYSIITSC
**810**	**820**	**830**	**840**	**850**	**860**	**870**	**880**
LDFTQPADKC	DGPLSVSSFI	LPHRPDNEES	CNSSEDESKW	VEELMKMHTA	RVRDIEHLTS	LDFFRKTSRS	YPEILTLKTY
**890**							
LHTYESEI							

Cmpd.	No. of Cmpds.	m/z meas.	Δ m/z [ppm]	z	Rt [min]	Score	P	Range	Sequence	Modification
1449	1	405.2085	100.81	2	48.3	16.6	0	76-81	R.CDNLCK.S	Carbamidomethyl: 1, 5

Detailed Protein Report

Protein 495: putative ATP-dependent RNA helicase DHX57 [Homo sapiens]

**Accession:**	gi|39777586	**Score:**	28.5
**Database:**	refseq_human(refseq_human_20140103.fasta)	**MW [kDa]:**	155.5
**Database Date:**	2015-11-30	**pI:**	8.7
		**Sequence Coverage [%]:**	2.2
		**No. of unique Peptides:**	1

10	20	30	40	50	60	70	80
MSSSVRRKGK	PGKGGGKGSS	RGGRGGRSHA	SKSHGSGGGG	GGGGGGGGGN	RKASSRIWDD	GDDFCIFSES	RRPSRPSNSN
**90**	**100**	**110**	**120**	**130**	**140**	**150**	**160**
ISKGESRPKW	KPKAKVPLQT	LHMTSENQEK	VKALLRDLQE	QDADAGSERG	LSGEEEDDEP	DCCNDERYWP	AGQEPSLVPD
**170**	**180**	**190**	**200**	**210**	**220**	**230**	**240**
LDPLEYAGLA	SVEPYVPEFT	VSPFAVQKLS	RYGFNTERCQ	AVLRMCDGDV	GASLEHLLTQ	CFSETFGERM	KISEAVNQIS
**250**	**260**	**270**	**280**	**290**	**300**	**310**	**320**
LDECMEQRQE	EAFALKSICG	EKFIERIQNR	VWTIGLELEY	LTSRFRKSKP	KESTKNVQEN	SLEICKFYLK	GNCKFGSKCR
**330**	**340**	**350**	**360**	**370**	**380**	**390**	**400**
FKHEVPPNQI	VGRIERSVDD	SHLNAIEDAS	FLYELEIRFS	KDHKYPYQAP	LVAFYSTNEN	LPLACRLHIS	EFLYDKALTF
**410**	**420**	**430**	**440**	**450**	**460**	**470**	**480**
AETSEPVVYS	LITLLEEESE	IVKLLTNTHH	KYSDPPVNFL	PVPSRTRINN	PACHKTVIPN	NSFVSNQIPE	VEKASESEES
**490**	**500**	**510**	**520**	**530**	**540**	**550**	**560**
DEDDGPAPVI	VENESYVNLK	KKISKRYDWQ	AKSVHAENGK	ICKQFRMKQA	SRQFQSILQE	RQSLPAWEER	ETILNLLRKH
**570**	**580**	**590**	**600**	**610**	**620**	**630**	**640**
QVVVISGMTG	CGKTTQIPQF	ILDDSLNGPP	EKVANIICTQ	PRRISAISVA	ERVAKERAER	VGLTVGYQIR	LESVKSSATR
**650**	**660**	**670**	**680**	**690**	**700**	**710**	**720**
LLYCTTGVLL	RRLEGDTALQ	GVSHIIVDEV	HERTEESDFL	LLVLKDIVSQ	RPGLQVILMS	ATLNAELFSD	YFNSCPVITI
**730**	**740**	**750**	**760**	**770**	**780**	**790**	**800**
PGRTFPVDQF	FLEDAIAVTR	YVLQDGSPYM	RSMKQISKEK	LKARRNRTAF	EEVEEDLRLS	LHLQDQDSVK	DAVPDQQLDF
**810**	**820**	**830**	**840**	**850**	**860**	**870**	**880**
KQLLARYKGV	SKSVIKTMSI	MDFEKVNLEL	IEALLEWIVD	GKHSYPPGAI	LVFLPGLAEI	KMLYEQLQSN	SLFNNRRSNR
**890**	**900**	**910**	**920**	**930**	**940**	**950**	**960**
CVIHPLHSSL	SSEEQQAVFV	KPPAGVTKII	ISTNIAETSI	TIDDVVYVID	SGKMKEKRYD	ASKGMESLED	TFVSQANALQ
**970**	**980**	**990**	**1000**	**1010**	**1020**	**1030**	**1040**
RKGRAGRVAS	GVCFHLFTSH	HYNHQLLKQQ	LPEIQRVPLE	QLCLRIKILE	MFSAHNLQSV	FSRLIEPPHT	DSLRASKIRL
**1050**	**1060**	**1070**	**1080**	**1090**	**1100**	**1110**	**1120**
RDLGALTPDE	RLTPLGYHLA	SLPVDVRIGK	LMLFGSIFRC	LDPALTIAAS	LAFKSPFVSP	WDKKEEANQK	KLEFAFANSD
**1130**	**1140**	**1150**	**1160**	**1170**	**1180**	**1190**	**1200**
YLALLQAYKG	WQLSTKEGVR	ASYNYCRQNF	LSGRVLQEMA	SLKRQFTELL	SDIGFAREGL	RAREIEKRAQ	GGDGVLDATG
**1210**	**1220**	**1230**	**1240**	**1250**	**1260**	**1270**	**1280**
EEANSNAENP	KLISAMLCAA	LYPNVVQVKS	PEGKFQKTST	GAVRMQPKSA	ELKFVTKNDG	YVHIHPSSVN	YQVRHFDSPY
**1290**	**1300**	**1310**	**1320**	**1330**	**1340**	**1350**	**1360**
LLYHEKIKTS	RVFIRDCSMV	SVYPLVLFGG	GQVNVQLQRG	EFVVSLDDGW	IRFVAASHQV	AELVKELRCE	LDQLLQDKIK
**1370**	**1380**	**1390**					
NPSIDLCTCP	RGSRIISTIV	KLVTTQ					

Cmpd.	No. of Cmpds.	m/z meas.	Δ m/z [ppm]	z	Rt [min]	Score	P	Range	Sequence	Modification
1483	2	699.6611	-193.23	2	48.2	18.4	0	33-51	K.SHGSGGGGGGGGGGGGNR.K	

Detailed Protein Report

Protein 496: PREDICTED: DNA mismatch repair protein Mlh3 isoform X4 [Homo sapiens]

**Accession:**	gi|530403519	**Score:**	28.5
**Database:**	refseq_human(refseq_human_20140103.fasta)	**MW [kDa]:**	143.6
**Database Date:**	2015-11-30	**pI:**	6.4
		**Sequence Coverage [%]:**	1.3
		**No. of unique Peptides:**	1

10	20	30	40	50	60	70	80
MIKCLSVEVQ	AKLRSGLAIS	SLGQCVEELA	LNSIDAEAKC	VAVRVNMETF	QVQVIDNGFG	MGSDDVEKVG	NRYFTSKCHS
**90**	**100**	**110**	**120**	**130**	**140**	**150**	**160**
VQDLENPRFY	GFRGEALANI	ADMASAVEIS	SKKNRTMKTF	VKLFQSGKAL	KACEADVTRA	SAGTTVTVYN	LFYQLPVRRK
**170**	**180**	**190**	**200**	**210**	**220**	**230**	**240**
CMDPRLEFEK	VRQRIEALSL	MHPSISFSLR	NDVSGSMVLQ	LPKTKDVCSR	FCQIYGLGKS	QKLREISFKY	KEFELSGYIS
**250**	**260**	**270**	**280**	**290**	**300**	**310**	**320**
SEAHYNKNMQ	FLFVNKRLVL	RTKLHKLIDF	LLRKESIICK	PKNGPTSRQM	NSSLRHRSTP	ELYGIYVINV	QCQFCEYDVC
**330**	**340**	**350**	**360**	**370**	**380**	**390**	**400**
MEPAKTLIEF	QNWDTLLFCI	QEGVKMFLKQ	EKLFVELSGE	DIKEFSEDNG	FSLFDATLQK	RVTSDERSNF	QEACNNILDS
**410**	**420**	**430**	**440**	**450**	**460**	**470**	**480**
YEMFNLQSKA	VKRKTTAENV	NTQSSRDSEA	TRKNTNDAFL	YIYESGGPGH	SKMTEPSLQN	KDSSCSESKM	LEQETIVASE
**490**	**500**	**510**	**520**	**530**	**540**	**550**	**560**
AGENEKHKKS	FLEHSSLENP	CGTSLEMFLS	PFQTPCHFEE	SGQDLEIWKE	STTVNGMAAN	ILKNNRIQNQ	PKRFKDATEV
**570**	**580**	**590**	**600**	**610**	**620**	**630**	**640**
GCQPLPFATT	LWGVHSAQTE	KEKKKESSNC	GRRNVFSYGR	VKLCSTGFIT	HVVQNEKTKS	TETEHSFKNY	VRPGPTRAQE
**650**	**660**	**670**	**680**	**690**	**700**	**710**	**720**
TFGNRTRHSV	ETPDIKDLAS	TLSKESGQLP	NKKNCRTNIS	YGLENEPTAT	YTMFSAFQEG	SKKSQTDCIL	SDTSPSFPWY
**730**	**740**	**750**	**760**	**770**	**780**	**790**	**800**
RHVSNDSRKT	DKLIGFSKPI	VRKKLSLSSQ	LGSLEKFKRQ	YGKVENPLDT	EVEESNGVTT	NLSLQVEPDI	LLKDKNRLEN
**810**	**820**	**830**	**840**	**850**	**860**	**870**	**880**
SDVCKITTME	HSDSDSSCQP	ASHILNSEKF	PFSKDEDCLE	QQMPSLRESP	MTLKELSLFN	RKPLDLEKSS	ESLASKLSRL
**890**	**900**	**910**	**920**	**930**	**940**	**950**	**960**
KGSERETQTM	GMMSRFNELP	NSDSSRKDSK	LCSVLTQDFC	MLFNNKHEKT	ENGVIPTSDS	ATQDNSFNKN	SKTHSNSNTT
**970**	**980**	**990**	**1000**	**1010**	**1020**	**1030**	**1040**
ENCVISETPL	VLPYNNSKVT	GKDSDVLIRA	SEQQIGSLDS	PSGMLMNPVE	DATGDQNGIC	FQSEESKARA	CSETEESNTC
**1050**	**1060**	**1070**	**1080**	**1090**	**1100**	**1110**	**1120**
CSDWQRHFDV	ALGRMVYVNK	MTGLSTFIAP	TEDIQAACTK	DLTTVAVDVV	LENGSQYRCQ	PFRSDLVLPF	LPRARAERTV
**1130**	**1140**	**1150**	**1160**	**1170**	**1180**	**1190**	**1200**
MRQDNRDTVD	DTVSSESLQS	LFSEWDNPVF	ARYPEVAVDV	SSGQAESLAV	KIHNILYPYR	FTKGMIHSMQ	VLQQVDNKFI
**1210**	**1220**	**1230**	**1240**	**1250**	**1260**	**1270**	**1280**
ACLMSTKTEE	NGEAGGNLLV	LVDQHAAHER	IRLEQLIIDS	YEKQQAQGSG	RKKLLSSTLI	PPLEITVTEE	QRRLL

Cmpd.	No. of Cmpds.	m/z meas.	Δ m/z [ppm]	z	Rt [min]	Score	P	Range	Sequence	Modification
964	1	523.2194	-171.68	2	40.4	11.9	2	877-885	K.LSRLKGSER.E	

Detailed Protein Report

Protein 497: phosphatidylinositol phosphatase PTPRQ precursor [Homo sapiens]

**Accession:**	gi|222537743	**Score:**	28.5
**Database:**	refseq_human(refseq_human_20140103.fasta)	**MW [kDa]:**	257.1
**Database Date:**	2015-11-30	**pI:**	5.4
		**Sequence Coverage [%]:**	1.2
		**No. of unique Peptides:**	1

Detailed Protein Report

10	20	30	40	50	60	70	80
MDFLIIFLLL	FIGTSETQVD	VSNVVPGTRY	DITISSISTT	YTSPVTRIVT	TNVTKPGPPV	FLAGERVGSA	GILLSWNTPP
90	100	110	120	130	140	150	160
NPNGRIISYI	VKYKEVCPWM	QTVYTQVRSK	PDSLEVLLTN	LNPGTTYEIK	VAAENSAGIG	VFSDPFLFQT	AESAPGKVVN
170	180	190	200	210	220	230	240
LTVEAYNASA	VKLIWYLPRQ	PNGKITSFKI	SVKHARSGIV	VKDVSIRVED	ILTGKLPECN	ENSESFLWST	ASPSPTLGRV
250	260	270	280	290	300	310	320
TPPSRTTHSS	STLTQNEISS	VWKEPISFVV	THLRPYTTYL	FEVSAATTEA	GYIDSTIVRT	PESVPEGPPQ	NCVTGNITGK
330	340	350	360	370	380	390	400
SFSILWDPPT	IVTGKFSYRV	ELYGPSGRIL	DNSTKDLKFA	FTNLTPFTMY	DVYIAAETSA	GTGPKSNISV	FTPPDVPGAV
410	420	430	440	450	460	470	480
FDLQLAEVES	TQVRITWKKP	RQPNGIINQY	RVKVLVPETG	IILENTLLTG	NNEYINDPMA	PEIVNIVEPM	VGLYEGSAEM
490	500	510	520	530	540	550	560
SSDLHSLATF	IYNSHPDKNF	PARNRAEDQT	SPVVTTRNQY	ITDIAAEQLS	YVIRRLVPFT	EHMISVSAFT	IMGEGPPTVL
570	580	590	600	610	620	630	640
SVRTRQQVPS	SIKIINYKNI	SSSSILLYWD	PPEYPNGKIT	HYTIYAMELD	TNRAFQITTI	DNSFLITGLK	KYTKYKMRVA
650	660	670	680	690	700	710	720
ASTHVGESSL	SEENDIFVRT	SEDEPESSPQ	DVEVIDVTAD	EIRLKWSPPE	KPNGIIIAYE	VLYKNIDTLY	MKNTSTTDII
730	740	750	760	770	780	790	800
LRNLRPHTLY	NISVRSYTRF	GHGNQVSSLL	SVRTSETVPD	SAPENITYKN	ISSGEIELSF	LPPSSPNGII	QKYTIYLKRS
810	820	830	840	850	860	870	880
NGNEERTINT	TSLTQNIKVL	KKYTQYIIEV	SASTLKGEGV	RSAPISILTE	EDAPDSPPQD	FSVKQLSGVT	VKLSWQPPLE
890	900	910	920	930	940	950	960
PNGIILYYTV	YVWNRSSLKT	INVTETSLEL	SDLDYNVEYS	AYVTASTRFG	DGKTRSNIIS	FQTPEGAPSD	PPKDVYYANL
970	980	990	1000	1010	1020	1030	1040
SSSSIILFWT	PPSKPNGIIQ	YYSVYYRNTS	GTFMQNFTLH	EVTNDFDNMT	VSTIIDKLTI	FSYYTFWLTA	STSVGNGNKS
1050	1060	1070	1080	1090	1100	1110	1120
SDIIEVYTDQ	DIPEGFVGNL	TYESISSTAI	NVSWVPPAQP	NGLVFYYVSL	ILQQTPRHVR	PPLVTYERSI	YFDNLEKYTD
1130	1140	1150	1160	1170	1180	1190	1200
YILKITPSTE	KGFSDTYTAQ	LYIKTEEDVP	ETSPIINTFK	NLSSTSVLLS	WDPPVKPNGA	IISYDLTLQG	PNENYSFITS
1210	1220	1230	1240	1250	1260	1270	1280
DNYIILEELS	PFTLYSFFAA	ARTRKGLGPS	SILFFYTDES	VPLAPPQNLT	LINCTSDFVW	LKWSPSPLPG	GIVKVYSFKI
1290	1300	1310	1320	1330	1340	1350	1360
HEHETDTIYY	KNISGFKTEA	KLVGLEPVST	YSIRVSAFTK	VGNGNQFSNV	VKFTTQESVP	DVVQNMQCMA	TSWQSVLVKW
1370	1380	1390	1400	1410	1420	1430	1440
DPPKKANGII	TQYMVTVERN	STKVSPQDHM	YTFIKLLANT	SYVFKVRAST	SAGEGDESTC	HVSTLPETVP	SVPTNIAFSD
1450	1460	1470	1480	1490	1500	1510	1520
VQSTSATLTW	IRPDTILGYF	QNYKITTQLR	AQKCKEWESE	ECVEYQKIQY	LYEAHLTEET	VYGLKKFRWY	RFQVAASTNA
1530	1540	1550	1560	1570	1580	1590	1600
GYGNASNWIS	TKTLPGPPDG	PPENVHVVAT	SPFSISISWS	EPAVITGPTC	YLIDVKSVDN	DEFNISFIKS	NEENKTIEIK
1610	1620	1630	1640	1650	1660	1670	1680
DLEIFTRYSV	VITAFTGNIS	AAYVEGKSSA	EMIVTTLESA	PKDPPNNMTF	QKIPDEVTKF	QLTFLPPSQP	NGNIQVYQAL
1690	1700	1710	1720	1730	1740	1750	1760
VYREDDPTAV	QIHNLSIIQK	TNTFVIAMLE	GLKGGHTYNI	SVYAVNSAGA	GPKVPMRITM	DIKAPARPKT	KPTPIYDATG
1770	1780	1790	1800	1810	1820	1830	1840
KLLVTSTTIT	IRMPICYYSD	DHGPIKNVQV	LVTETGAQHD	GNVTKWYDAY	FNKARPYFTN	EGFPNPPCTE	GKTKFSGNEE
1850	1860	1870	1880	1890	1900	1910	1920
IYIIGADNAC	MIPGNEDKIC	NGPLKPKKQY	LFKFRATNIM	GQFTDSDYSD	PVKTLGEGLS	ERTVEIILSV	TLCILSIILL
1930	1940	1950	1960	1970	1980	1990	2000
GTAIFAFARI	RQKQKEGGTY	SPQDAEIIDT	KLKLDQLITV	ADLELKDERL	TRLLSYRKSI	KPISKKSFLQ	HVEELCTNNN
2010	2020	2030	2040	2050	2060	2070	2080
LKFQEEFSEL	PKFLQDLSST	DADLPWNRAK	NRFPNIKPYN	NNRVKLIADA	SVPGSDYINA	SYISGYLCPN	EFIATQGPLP
2090	2100	2110	2120	2130	2140	2150	2160
GTVGDFWRMV	WETRAKTLVM	LTQCFEKGRI	RCHQYWPEDN	KPVTVFGDIV	ITKLMEDVQI	DWTIRDLKIE	RHGDCMTVRQ
2170	2180	2190	2200	2210	2220	2230	2240

Detailed Protein Report

Cmpd.	No. of Cmpds.	m/z meas.	Δ m/z [ppm]	z	Rt [min]	Score	P	Range	Sequence	Modification
13	1	1002.9641	-45.61	2	29.1	11.9	0	30-47	R.YDITISSISTTYTSPVTR.I	

Detailed Protein Report

Protein 498: zinc finger and SCAN domain-containing protein 32 isoform b [Homo sapiens]

**Accession:**	gi|32129212	**Score:**	28.4
**Database:**	refseq_human(refseq_human_20140103.fasta)	**MW [kDa]:**	54.9
**Database Date:**	2015-11-30	**pI:**	10.1
		**Sequence Coverage [%]:**	6.2
		**No. of unique Peptides:**	1

Quantitation

QU:MU	**Median:**	2.42	**CV:**	0.00 %	**No. of Peptides:**	1
WUP:QUP	**Median:**	0.51	**CV:**	0.00 %	**No. of Peptides:**	1

Alias proteins:

Accession	Name	Description
gi|578828138	refseq_human (refseq_human_20140103.fasta)	PREDICTED: zinc finger and SCAN domain-containing protein 32 isoform X7 [Homo sapiens]
gi|530408311	refseq_human (refseq_human_20140103.fasta)	PREDICTED: zinc finger and SCAN domain-containing protein 32 isoform X6 [Homo sapiens]

10	20	30	40	50	60	70	80
MRDNRAVSLC	QQEWMCPGPA	QRALYRGATQ	RKDSHVSLAT	GVPWGYEETK	TLLAILSSSQ	FYGKLQTCQQ	NSQIYRAMAE
**90**	**100**	**110**	**120**	**130**	**140**	**150**	**160**
GLWEQGFLRT	PEQCRTKFKS	LQLSYRKVRR	GRVPEPCIFY	EEMNALSGSW	ASAPPMASDA	VPGQEGSDIE	AGELNHQNGE
**170**	**180**	**190**	**200**	**210**	**220**	**230**	**240**
PTEVEDGTVD	GADRDEKDFR	NPGQEVRKLD	LPVLFPNRLG	FEFKNEIKKE	NLKWDDSEEV	EINKALQRKS	RGVYWHSELQ
**250**	**260**	**270**	**280**	**290**	**300**	**310**	**320**
KGLESEPTSR	RQCRNSPGES	EEKTPSQEKM	SHQSFCARDK	ACTHILCGKN	CSQSVHSPHK	PALKLEKVSQ	CPECGKTFSR
**330**	**340**	**350**	**360**	**370**	**380**	**390**	**400**
SSYLVRHQRI	HTGEKPHKCS	ECGKGFSERS	NLTAHLRTHT	GERPYQCGQC	GKSFNQSSSL	IVHQRTHTGE	KPYQCIVCGK
**410**	**420**	**430**	**440**	**450**	**460**	**470**	**480**
RFNNSSQFSA	HRRIHTGESP	YKCAVCGKIF	NNSSHFSAHR	KTHTGEKPYR	CSHCERGFTK	NSALTRHQTV	HMKAVLSSQE
**490**							
GRDAL							

Cmpd.	No. of Cmpds.	m/z meas.	Δ m/z [ppm]	z	Rt [min]	Score	P	Range	Sequence	Modification	Ratios
707	1	647.6537	-225.58	2	38.4	15.1	0	402-412	R.FNNSSQFSAHR.R		WUP:QUP 0.51<br>QU:MU 2.42

Detailed Protein Report

Protein 499: PREDICTED: peroxisomal biogenesis factor 5 isoform X9 [Homo sapiens]

**Accession:**	gi|578822993	**Score:**	28.4
**Database:**	refseq_human(refseq_human_20140103.fasta)	**MW [kDa]:**	63.0
**Database Date:**	2015-11-30	**pI:**	4.2
		**Sequence Coverage [%]:**	4.8
		**No. of unique Peptides:**	1

10	20	30	40	50	60	70	80
MDDLLAEMQQ	IEQSNFRQAP	QRAPGVADLA	LSENWAQEFL	AAGDAVDVTQ	DYNETDWSQE	FISEVTDPLS	VSPARWAEEY
**90**	**100**	**110**	**120**	**130**	**140**	**150**	**160**
LEQSEEKLWL	GEPEGTATDR	WYDEYHPEED	LQHTASDFVA	KVDDPKLANS	EWPVCVSVPQ	FLKFVRQIGE	GQVSLESGAG
**170**	**180**	**190**	**200**	**210**	**220**	**230**	**240**
SGRAQAEQWA	AEFIQQQGTS	DAWVDQFTRP	VNTSALDMEF	ERAKSAIESD	VDFWDKLQAE	LEEMAKRDAE	AHPWLSDYDD
**250**	**260**	**270**	**280**	**290**	**300**	**310**	**320**
LTSATYDKGY	QFEEENPLRD	HPQPFEEGLR	RLQEGDLPNA	VLLFEAAVQQ	DPKHMEAWQY	LGTTQAENEQ	ELLAISALRR
**330**	**340**	**350**	**360**	**370**	**380**	**390**	**400**
CLELKPDNQT	ALMALAVSFT	NESLQRQACE	TLRDWLRYTP	AYAHLVTPAE	EGAGGAGLGP	SKRILGSLLS	DSLFLEVKEL
**410**	**420**	**430**	**440**	**450**	**460**	**470**	**480**
FLAAVRLDPT	SIDPDVQCGL	GVLFNLSGEY	DKAVDCFTAA	LSVRPNDYLL	WNKLGATLAN	GNQSEEAVAA	YRRALELQPG
**490**	**500**	**510**	**520**	**530**	**540**	**550**	**560**
YIRSRYNLGI	SCINLGAHRE	AVEHFLEALN	MQRKSRGPRG	EGGAMSENIW	STLRLALSML	GQSDAYGAAD	ARDLSTLLTM
**570**							
FGLPQ							

Cmpd.	No. of Cmpds.	m/z meas.	Δ m/z [ppm]	z	Rt [min]	Score	P	Range	Sequence	Modification
438	1	718.3578	-29.07	2	34.0	11.2	0	553-565	R.DLSTLLTMFGLPQ.-	

Detailed Protein Report

Protein 500: proto-oncogene vav isoform 3 [Homo sapiens]

**Accession:**	gi|384551649	**Score:**	28.3
**Database:**	refseq_human(refseq_human_20140103.fasta)	**MW [kDa]:**	94.4
**Database Date:**	2015-11-30	**pI:**	6.3
		**Sequence Coverage [%]:**	3.3
		**No. of unique Peptides:**	1

10	20	30	40	50	60	70	80
MELWRQCTHW	LIQCRVLPPS	HRVTWDGAQV	CELAQALRDG	VLLCQLLNNL	LPHAINLREV	NLRPQMSQFL	CLKNIRTFLS
90	100	110	120	130	140	150	160
TCCEKFGLKR	SELFEAFDLF	DVQDFGKVIY	TLSALSWTPI	AQNRGIMPFP	TEEESVGDED	IYSGLSDQID	DTVEEDEDLY
170	180	190	200	210	220	230	240
DCVENEEAEG	DEIYEDLMRS	EPVSMPHFLK	PLQRFLKPQD	IEIIFINIED	LLRVHTHFLK	EMKEALGTPG	AANLYQVFIK
250	260	270	280	290	300	310	320
YKERFLVYGR	YCSQVESASK	HLDRVAAARE	DVQMKLEECS	QRANNGRFTL	RDLLMVPMQR	VLKYHLLLQE	LVKHTQEAME
330	340	350	360	370	380	390	400
KENLRLALDA	MRDLAQCVNE	VKRDNETLRQ	ITNFQLSIEN	LDQSLAHYGR	PKIDGELKIT	SVERRSKMDR	YAFLLDKALL
410	420	430	440	450	460	470	480
ICKRRGDSYD	LKDFVNLHSF	QVRDDSSGDR	DNKKWSHMFL	LIEDQGAQGY	ELFFKTRELK	KKWMEQFEMA	ISNIYPENAT
490	500	510	520	530	540	550	560
ANGHDFQMFS	FEETTSCKAC	QMLLRGTFYQ	GYRCHRCRAS	AHKECLGRVP	PCGRHGQDFP	GTMKKDKLHR	RAQDKKRNEL
570	580	590	600	610	620	630	640
GLPKMEVFQE	YYGLPPPPGA	IGPFLRLNPG	DIVELTKAEA	EQNWWEGRNT	STNEIGWFPC	NRVKPYVHGP	PQDLSVHLWY
650	660	670	680	690	700	710	720
AGPMERAGAE	SILANRSDGT	FLVRQRVKDA	AEFAISIKYN	VEVKHIKIMT	AEGLYRITEK	KAFRGLTELV	EFYQQNSLKD
730	740	750	760	770	780	790	800
CFKSLDTTLQ	FPFKEPEKRT	ISRPAVGSTK	YFGTAKARYD	FCARDRSELS	LKEGDIIKIL	NKKGQQGWWR	GEIYGRVGWF
810	820						
PANYVEEDYS	EYC						

Cmpd.	No. of Cmpds.	m/z meas.	Δ m/z [ppm]	z	Rt [min]	Score	P	Range	Sequence	Modification
2147	1	788.3156	-165.74	2	56.7	13.2	2	665-678	R.QRVKDAAEFAISIK.Y	

Detailed Protein Report

Protein 501: sushi, von Willebrand factor type A, EGF and pentraxin domain-containing protein 1 precursor [Homo sapiens]

**Accession:**	gi|148886654	**Score:**	28.2
**Database:**	refseq_human(refseq_human_20140103.fasta)	**MW [kDa]:**	389.9
**Database Date:**	2015-11-30	**pI:**	5.2
		**Sequence Coverage [%]:**	0.7
		**No. of unique Peptides:**	2

Quantitation

QU:MU	**Median:** 2.04	**CV:** 0.00 %	**No. of Peptides:** 1

BRUKER

Detailed Protein Report

10	20	30	40	50	60	70	80
MWPRLAFCCW	GLALVSGWAT	FQQMSPSRNF	SFRLFPETAP	GAPGSIPAPP	APGDEAAGSR	VERLGQAFRR	RVRLLRELSE
90	100	110	120	130	140	150	160
RLELVFLVDD	SSSVGEVNFR	SELMFVRKLL	SDFPVVPTAT	RVAIVTFSSK	NYVVPRVDYI	STRRARQHKC	ALLLQEIPAI
170	180	190	200	210	220	230	240
SYRGGGTYTK	GAFQQAAQIL	LHARENSTKV	VFLITDGYSN	GGDPRPIAAS	LRDSGVEIFT	FGIWQGNIRE	LNDMASTPKE
250	260	270	280	290	300	310	320
EHCYLLHSFE	EFEALARRAL	HEDLPSGSFI	QDDMVHCSYL	CDEGKDCCDR	MGSCKCGTHT	GHFECICEKG	YYGKGLQYEC
330	340	350	360	370	380	390	400
TACPSGTYKP	EGSPGGISSC	IPCPDENHTS	PPGSTSPEDC	VCREGYRASG	QTCELVHCPA	LKPPENGYFI	QNTCNNHFNA
410	420	430	440	450	460	470	480
ACGVRCHPGF	DLVGSSIILC	LPNGLWSGSE	SYCRVRTCPH	LRQPKHGHIS	CSTREMLYKT	TCLVACDEGY	RLEGSDKLTC
490	500	510	520	530	540	550	560
QGNSQWDGPE	PRCVERHCST	FQMPKDVIIS	PHNCGKQPAK	FGTICYVSCR	QGFILSGVKE	MLRCTTSGKW	NVGVQAAVCK
570	580	590	600	610	620	630	640
DVEAPQINCP	KDIEAKTLEQ	QDSANVTWQI	PTAKDNSGEK	VSVHVHPAFT	PPYLFPIGDV	AIVYTATDLS	GNQASCIFHI
650	660	670	680	690	700	710	720
KVIDAEPPVI	DWCRSPPPVQ	VSEKVHAASW	DEPQFSDNSG	AELVITRSHT	QGDLFPQGET	IVQYTATDPS	GNNRTCDIHI
730	740	750	760	770	780	790	800
VIKGSPCEIP	FTPVNGDFIC	TPDNTGVNCT	LTCLEGYDFT	EGSTDKYYCA	YEDGVWKPTY	TTEWPDCAKK	RFANHGFKSF
810	820	830	840	850	860	870	880
EMFYKAARCD	DTDLMKKFSE	AFETTLGKMV	PSFCSDAEDI	DCRLEENLTK	KYCLEYNYDY	ENGFAIGPGG	WGAANRLDYS
890	900	910	920	930	940	950	960
YDDFLDTVQE	TATSIGNAKS	SRIKRSAPLS	DYKIKLIFNI	TASVPLPDER	NDTLEWENQQ	RLLQTLETIT	NKLKRTLNKD
970	980	990	1000	1010	1020	1030	1040
PMYSFQLASE	ILIADSNSLE	TKKASPFCRP	GSVLRGRMCV	NCPLGTYYNL	EHFTCESCRI	GSYQDEEGQL	ECKLCPSGMY
1050	1060	1070	1080	1090	1100	1110	1120
TEYIHSRNIS	DCKAQCKQGT	YSYSGLETCE	SCPLGTYQPK	FGSRSCLSCP	ENTSTVKRGA	VNISACGVPC	PEGKFSRSGL
1130	1140	1150	1160	1170	1180	1190	1200
MPCHPCPRDY	YQPNAGKAFC	LACPFYGTTP	FAGSRSITEC	SSFSSTFSAA	EESVVPPASL	GHIKKRHEIS	SQVFHECFFN
1210	1220	1230	1240	1250	1260	1270	1280
PCHNSGTCQQ	LGRGYVCLCP	LGYTGLKCET	DIDECSPLPC	LNNGVCKDLV	GEFICECPSG	YTGQRCEENI	NECSSSPCLN
1290	1300	1310	1320	1330	1340	1350	1360
KGICVDGVAG	YRCTCVKGFV	GLHCETEVNE	CQSNPCLNNA	VCEDQVGGFL	CKCPPGFLGT	RCGKNVDECL	SQPCKNGATC
1370	1380	1390	1400	1410	1420	1430	1440
KDGANSFRCL	CAAGFTGSHC	ELNINECQSN	PCRNQATCVD	ELNSYSCKCQ	PGFSGKRCET	EQSTGFNLDF	EVSGIYGYVM
1450	1460	1470	1480	1490	1500	1510	1520
LDGMLPSLHA	LTCTFWMKSS	DDMNYGTPIS	YAVDNGSDNT	LLLTDYNGWV	LYVNGREKIT	NCPSVNDGRW	HHIAITWTSA
1530	1540	1550	1560	1570	1580	1590	1600
NGIWKVYIDG	KLSDGGAGLS	VGLPIPGGGA	LVLGQEQDKK	GEGFSPAESF	VGSISQLNLW	DYVLSPQQVK	SLATSCPEEL
1610	1620	1630	1640	1650	1660	1670	1680
SKGNVLAWPD	FLSGIVGKVK	IDSKSIFCSD	CPRLGGSVPH	LRTASEDLKP	GSKVNLFCDP	GFQLVGNPVQ	YCLNQGQWTQ
1690	1700	1710	1720	1730	1740	1750	1760
PLPHCERISC	GVPPPLENGF	HSADDFYAGS	TVTYQCNNGY	YLLGDSRMFC	TDNGSWNGVS	PSCLDVDECA	VGSDCSEHAS
1770	1780	1790	1800	1810	1820	1830	1840
CLNVDGSYIC	SCVPPYTGDG	KNCAEPIKCK	APGNPENGHS	SGEIYTVGAE	VTFSCQEGYQ	LMGVTKITCL	ESGEWNHLIP
1850	1860	1870	1880	1890	1900	1910	1920
YCKAVSCGKP	AIPENGCIEE	LAFTFGSKVT	YRCNKGYTLA	GDKESSCLAN	SSWSHSPPVC	EPVKCSSPEN	INNGKYILSG
1930	1940	1950	1960	1970	1980	1990	2000
LTYLSTASYS	CDTGYSLQGP	SIIECTASGI	WDRAPPACHL	VFCGEPPAIK	DAVITGNNFT	FRNTVTYTCK	EGYTLAGLDT
2010	2020	2030	2040	2050	2060	2070	2080
IECLADGKWS	RSDQQCLAVS	CDEPPIVDHA	SPETAHRLFG	DIAFYYCSDG	YSLADNSQLL	CNAQGKWVPP	EGQDMPRCIA
2090	2100	2110	2120	2130	2140	2150	2160
HFCEKPPSVS	YSILESVSKA	KFAAGSVVSF	KCMEGFVLNT	SAKIECMRGG	QWNPSPMSIQ	CIPVRCGEPP	SIMNGYASGS
2170	2180	2190	2200	2210	2220	2230	2240

Detailed Protein Report

Cmpd.	No. of Cmpds.	m/z meas.	Δ m/z [ppm]	z	Rt [min]	Score	P	Range	Sequence	Modification	Ratios
2764	2	639.3642	-0.12	2	63.1	17.4	2	903-913	R.IKRSAPLSDYK.I		
281	1	743.8595	-21.15	2	33.5	10.8	0	1214-1227	R.GYVCLCPLGYTGLK.C		QU:MU 2.04

Detailed Protein Report

Protein 502: PREDICTED: protein NLRC5 isoform X13 [Homo sapiens]

**Accession:**	gi|578829278	**Score:**	28.2
**Database:**	refseq_human(refseq_human_20140103.fasta)	**MW [kDa]:**	152.2
**Database Date:**	2015-11-30	**pI:**	6.4
**Modification(s):**	Carbamidomethyl	**Sequence Coverage [%]:**	2.9
		**No. of unique Peptides:**	1

10	20	30	40	50	60	70	80
MDPVGLQLGN	KNLWSCLVRL	LTKDPEWLNA	KMKFFLPNTD	LDSRNETLDP	EQRVILQLNK	LHVQGSDTWQ	SFIHCVCMQL
90	100	110	120	130	140	150	160
EVPLDLEVLL	LSTFGYDDGF	TSQLGAEGKS	QPESQLHHGL	KRPHQSCGSS	PRRKQCKKQQ	LELAKKYLQL	LRTSAQQRYR
170	180	190	200	210	220	230	240
SQIPGSGQPH	AFHQVYVPPI	LRRATASLDT	PEGAIMGDVK	VEDGADVSIS	DLFNTRVNKG	PRVTVLLGKA	GMGKTTLAHR
250	260	270	280	290	300	310	320
LCQKWAEGHL	NCFQALFLFE	FRQLNLITRF	LTPSELLFDL	YLSPESDHDT	VFQYLEKNAD	QVLLIFDGLD	EALQPMGPDG
330	340	350	360	370	380	390	400
PGPVLTLFSH	LCNGTLLPGC	RVMATSRPGK	LPACLPAEAA	MVHMLGFDGP	RVEEYVNHFF	SAQPSREGAL	VELQTNGRLR
410	420	430	440	450	460	470	480
SLCAVPALCQ	VACLCLHHLL	PDHAPGQSVA	LLPNMTQLYM	QMVLALSPPG	HLPTSSLLDL	GEVALRGLET	GKVIFYAKDI
490	500	510	520	530	540	550	560
APPLIAFGAT	HSLLTSFCVC	TGPGHQQTGY	AFTHLSLQEF	LAALHLMASP	KVNKDTLTQY	VTLHSRWVQR	TKARLGLSDH
570	580	590	600	610	620	630	640
LPTFLAGLAS	CTCRPFLSHL	AQGNEDCVGA	KQAAVVQVLK	KLATRKLTGP	KVVELCHCVD	ETQEPELASL	TAQSLPYQLP
650	660	670	680	690	700	710	720
FHNFPLTCTD	LATLTNILEH	REAPIHLDFD	GCPLEPHCPE	ALVGCGQIEN	LSFKSRKCGD	AFAEALSRSL	PTMGRLQMLG
730	740	750	760	770	780	790	800
LAGSKITARG	ISHLVKALPL	CPQLKEVSFR	DNQLSDQVVL	NIVEVLPHLP	RLRKLDLSSN	SICVSTLLCL	ARVAVTCPTV
810	820	830	840	850	860	870	880
RMLQAREADL	IFLLSPPTET	TAELQRAPDL	QESDGQRKGA	QSRSLTLRLQ	KCQLQVHDAE	ALIALLQEGP	HLEEVDLSGN
890	900	910	920	930	940	950	960
QLEDEGCRLM	AEAASQLHIA	RKLDLSNNGL	SVAGVHCVLR	AVSACWTLAE	LHISLQHKTV	IFMFAQEPEE	QKGPQERAAF
970	980	990	1000	1010	1020	1030	1040
LDSLMLQMPS	ELPLSSRRMR	LTHCGLQEKH	LEQLCKALGG	SCHLGHLHLD	FSGNALGDEG	AARLAQLLPG	LGALQSLNLS
1050	1060	1070	1080	1090	1100	1110	1120
ENGLSLDAVL	GLVRCFSTLQ	WLFRLDISFE	SQHILLRGDK	TSRDMWATGS	LPDFPAAAKF	LGFRQRCIPR	SLCLSECPLE
1130	1140	1150	1160	1170	1180	1190	1200
PPSLTRLCAT	LKDCPGPLEL	QLSCEFLSDQ	SLETLLDCLP	QLPQLSLLQL	SQTGLSPKSP	FLLANTLSLC	PRVKKVDLRS
1210	1220	1230	1240	1250	1260	1270	1280
LHHATLHFRS	NEEEEGVCCG	RFTGCSLSQE	HVESLCWLLS	KCKDLSQVDL	SANLLGDSGL	RCLLECLPQV	PISGLLDLSH
1290	1300	1310	1320	1330	1340	1350	1360
NSISQESALY	LLETLPSCPR	VREASVNLGS	EQSFRIHFSR	EDQAGKTLRL	SECSFRPEHV	SRLATGLSKS	LQLTELTLTQ
1370	1380	1390					
CCLGQKQLAI	LLSLVGRPAG	LFSLS					

Cmpd.	No. of Cmpds.	m/z meas.	Δ m/z [ppm]	z	Rt [min]	Score	P	Range	Sequence	Modification
1152	1	983.1542	145.88	2	44.5	13.7	0	775-792	K.LDLSSNSICVSTLLCLAR.V	Carbamidomethyl: 15

Detailed Protein Report

Protein 503: DNA repair and recombination protein RAD54B isoform 1 [Homo sapiens]

**Accession:**	gi|6912622	**Score:**	28.1
**Database:**	refseq_human(refseq_human_20140103.fasta)	**MW [kDa]:**	102.9
**Database Date:**	2015-11-30	**pI:**	9.4
**Modification(s):**	Oxidation	**Sequence Coverage [%]:**	2.9
		**No. of unique Peptides:**	1

10	20	30	40	50	60	70	80
MRRSAAPSQL	QGNSFKKPKF	IPPGRSNPGL	NEEITKLNPD	IKLFEGVAIN	NTFLPSQNDL	RICSLNLPSE	ESTREINNRD
90	100	110	120	130	140	150	160
NCSGKYCFEA	PTLATLDPPH	TVHSAPKEVA	VSKEQEEKSD	SLVKYFSVVW	CKPSKKKHKK	WEGDAVLIVK	GKSFILKNLE
170	180	190	200	210	220	230	240
GKDIGRGIGY	KFKELEKIEE	GQTLMICGKE	IEVMGVISPD	DFSSGRCFQL	GGGSTAISHS	SQVARKCFSN	PFKSVCKPSS
250	260	270	280	290	300	310	320
KENRQNDFQN	CKPRHDPYTP	NSLVMPRPDK	NHQWVFNKNC	FPLVDVVIDP	YLVYHLRPHQ	KEGIIFLYEC	VMGMRMNGRC
330	340	350	360	370	380	390	400
GAILADEMGL	GKTLQCISLI	WTLQCQGPYG	GKPVIKKTLI	VTPGSLVNNW	KKEFQKWLGS	ERIKIFTVDQ	DHKVEEFIKS
410	420	430	440	450	460	470	480
IFYSVLIISY	EMLLRSLDQI	KNIKFDLLIC	DEGHRLKNSA	IKTTTALISL	SCEKRIILTG	TPIQNDLQEF	FALIDFVNPG
490	500	510	520	530	540	550	560
ILGSLSSYRK	IYEEPIILSR	EPSASEEEKE	LGERRAAELT	CLTGLFILRR	TQEIINKYLP	PKIENVVFCR	PGALQIELYR
570	580	590	600	610	620	630	640
KLLNSQVVRF	CLQGLLENSP	HLICIGALKK	LCNHPCLLFN	SIKEKECSST	CDKNEEKSLY	KGLLSVFPAD	YNPLLFTEKE
650	660	670	680	690	700	710	720
SGKLQVLSKL	LAVIHELRPT	EKVVLVSNYT	QTLNILQEVC	KRHGYAYTRL	DGQTPISQRQ	QIVDGFNSQH	SSFFIFLLSS
730	740	750	760	770	780	790	800
KAGGVGLNLI	GGSHLILYDI	DWNPATDIQA	MSRVWRDGQK	YPVHIYRLLT	TGTIEEKIYQ	RQISKQGLCG	AVVDLTKTSE
810	820	830	840	850	860	870	880
HIQFSVEELK	NLFTLHESSD	CVTHDLLDCE	CTGEEVHTGD	SLEKFIVSRD	CQLGPHHQKS	NSLKPLSMSQ	LKQWKHFSGD
890	900	910	920				
HLNLTDPFLE	RITENVSFIF	QNITTQATGT					

Cmpd.	No. of Cmpds.	m/z meas.	Δ m/z [ppm]	z	Rt [min]	Score	P	Range	Sequence	Modification
920	1	945.9532	-58.91	2	39.9	10.3	1	860-875	K.SNSLKPLSMSQLKQWK.H	Oxidation: 9

Detailed Protein Report

Protein 504: **PREDICTED: serine/threonine-protein kinase MRCK beta isoform X4 [Homo sapiens]**

**Accession:**	gi|530404963	**Score:**	28.1
**Database:**	refseq_human(refseq_human_20140103.fasta)	**MW [kDa]:**	169.5
**Database Date:**	2015-11-30	**pI:**	5.8
**Modification(s):**	Carbamidomethyl	**Sequence Coverage [%]:**	1.8
		**No. of unique Peptides:**	1

10	20	30	40	50	60	70	80
MSAKVRLKKL	EQLLLDGPWR	NESALSVETL	LDVLVCLYTE	CSHSALRRDK	YVAEFLEWAK	PFTQLVKEMQ	LHREDFEIIK
90	100	110	120	130	140	150	160
VIGRGAFGEV	AVVKMKNTER	IYAMKILNKW	EMLKRAETAC	FREERDVLVN	GDCQWITALH	YAFQDENHLY	LVMDYYVGGD
170	180	190	200	210	220	230	240
LLTLLSKFED	KLPEDMARFY	IGEMVLAIDS	IHQLHYVHRD	IKPDNVLLDV	NGHIRLADFG	SCLKMNDDGT	VQSSVAVGTP
250	260	270	280	290	300	310	320
DYISPEILQA	MEDGMGKYGP	ECDWWSLGVC	MYEMLYGETP	FYAESLVETY	GKIMNHEERF	QFPSHVTDVS	EEAKDLIQRL
330	340	350	360	370	380	390	400
ICSRERRLGQ	NGIEDFKKHA	FFEGLNWENI	RNLEAPYIPD	VSSPSDTSNF	DVDDDVLRNT	EILPPGSHTG	FSGLHLPFIG
410	420	430	440	450	460	470	480
FTFTTESCFS	DRGSLKSIMQ	SNTLTKDEDV	QRDLEHSLQM	EAYERRIRRL	EQEKLELSRK	LQESTQTVQS	LHGSSRALSN
490	500	510	520	530	540	550	560
SNRDKEIKKL	NEEIERLKNK	IADSNRLERQ	LEDTVALRQE	REDSTQRLRG	LEKQHRVVRQ	EKEELHKQLV	EASERLKSQA
570	580	590	600	610	620	630	640
KELKDAHQQR	KLALQEFSEL	NERMAELRAQ	KQKVSRQLRD	KEEEMEVATQ	KVDAMRQEMR	RAEKLRKELE	AQLDDAVAEA
650	660	670	680	690	700	710	720
SKERKLREHS	ENFCKQMESE	LEALKVKQGG	RGAGATLEHQ	QEISKIKSEL	EKKVLFYEEE	LVRREASHVL	EVKNVKKEVH
730	740	750	760	770	780	790	800
DSESHQLALQ	KEILMLKDKL	EKSKRERHNE	MEEAVGTIKD	KYERERAMLF	DENKKLTAEN	EKLCSFVDKL	TAQNRQLEDE
810	820	830	840	850	860	870	880
LQDLAAKKES	VAHWEAQIAE	IIQWVSDEKD	ARGYLQALAS	KMTEELEALR	SSSLGSRTLD	PLWKVRRSQK	LDMSARLELQ
890	900	910	920	930	940	950	960
SALEAEIRAK	QLVQEELRKV	KDANLTLESK	LKDSEAKNRE	LLEEMEILKK	KMEEKFRADT	GLKLPDFQDS	IFEYFNTAPL
970	980	990	1000	1010	1020	1030	1040
AHDLTFRDSL	SSSSASSLLA	FWEETSSASE	QETQAPKPEA	SPSMSVAASE	QQEDMARPPQ	RPSAVPLPTT	QALALAGPKP
1050	1060	1070	1080	1090	1100	1110	1120
KAHQFSIKSF	SSPTQCSHCT	SLMVGLIRQG	YACEVCSFAC	HVSCKDGAPQ	VCPIPPEQSK	RPLGVDVQRG	IGTAYKGHVK
1130	1140	1150	1160	1170	1180	1190	1200
VPKPTGVKKG	WQRAYAVVCD	CKLFLYDLPE	GKSTQPGVIA	SQVLDLRDDE	FSVSSVLASD	VIHATRRDIP	CIFRVTASLL
1210	1220	1230	1240	1250	1260	1270	1280
GAPSKTSSLL	ILTENENEKR	KWVGILEGLQ	SILHKNRLRN	QVVHVPLEAY	DSSLPLIKAI	LTAAIVDADR	IAVGLEEGLY
1290	1300	1310	1320	1330	1340	1350	1360
VIEVTRDVIV	RAADCKKVHQ	IELAPREKIV	ILLCGRNHHV	HLYPWSSLDG	AEGSFDIKLP	ETKGCQLMAT	ATLKRNSGTC
1370	1380	1390	1400	1410	1420	1430	1440
LFVAVKRLIL	CYEIQRTKPF	HRKFNEIVAP	GSVQCLAVLR	DRLCVGYPSG	FCLLSIQGDG	QPLNLVNPND	PSLAFLSQQS
1450	1460	1470	1480	1490	1500		
FDALCAVELE	SEEYLLCFSH	MGLYVDPQGR	RARAQELMWP	AAPVACKEKS			

Cmpd.	No. of Cmpds.	m/z meas.	Δ m/z [ppm]	z	Rt [min]	Score	P	Range	Sequence	Modification
413	1	946.2338	-157.99	2	34.8	16.0	0	1069-1085	R.QGYACEVCSFACHVSCK.D	Carbamidomethyl: 16

Detailed Protein Report

Protein 505: sodium/hydrogen exchanger 9B1 isoform 2 [Homo sapiens]

**Accession:**	gi|154937338	**Score:**	28.1
**Database:**	refseq_human(refseq_human_20140103.fasta)	**MW [kDa]:**	52.2
**Database Date:**	2015-11-30	**pI:**	9.5
**Modification(s):**	Carbamidomethyl	**Sequence Coverage [%]:**	4.4
		**No. of unique Peptides:**	2

10	20	30	40	50	60	70	80
MHTTESKNEH	LEDENFQTST	TPQSLIDPNN	TAHEETKTVL	SDTEEIKPQT	KKETYISCPL	RGVLNVIITN	GVILFVIWCM
**90**	**100**	**110**	**120**	**130**	**140**	**150**	**160**
TWSILGSEAL	PGGNLFGLFI	IFYSAIIGGK	ILQLIRIPLV	PPLPPLLGML	LAGFTIRNVP	FINEHVHVPN	TWSSILRSIA
**170**	**180**	**190**	**200**	**210**	**220**	**230**	**240**
LTIILIRAGL	GLDPQALRHL	KVVCFRLAVG	PCLMEASAAA	VFSHFIMKFP	WQWAFLLGFV	LGAVSPAVVV	PYMMVLQENG
**250**	**260**	**270**	**280**	**290**	**300**	**310**	**320**
YGVEEGIPTL	LMAASSMDDI	LAITGFNTCL	SIVFSSGGIL	NNAIASIRNV	CISLLAGIVL	GFFVRYFPSE	DQKKLTLKRG
**330**	**340**	**350**	**360**	**370**	**380**	**390**	**400**
FLVLTMCVSA	VLGSQRIGLH	GSGGLCTLVL	SFIAGTKWSQ	EKMKVQKIIT	TVWDIFQPLL	FGLVGAEVSV	SSLESNIVGI
**410**	**420**	**430**	**440**	**450**	**460**	**470**	**480**
SVATLSLALC	VRILTTYLLM	CFAGFSFKEK	IFIALAWMPK	ATVQINQAIL	LLFLLREEWT	NCKVAKKCEY	TKERQ

Cmpd.	No. of Cmpds.	m/z meas.	Δ m/z [ppm]	z	Rt [min]	Score	P	Range	Sequence	Modification
2206	1	682.1436	141.92	3	58.0	11.6	0	337-357	R.IGLHGSGGLCTLVLSFIAGTK.W	
2202	1	701.1391	121.39	3	57.9	16.6	0	337-357	R.IGLHGSGGLCTLVLSFIAGTK.W	Carbamidomethyl: 10

Detailed Protein Report

Protein 506: centrosomal protein of 170 kDa protein B isoform 2 [Homo sapiens]

Accession: gi|326319990
Database: refseq_human(refseq_human_20140103.fasta)
Database Date: 2015-11-30

Score: 28.1
MW [kDa]: 163.5
pI: 6.5
Sequence Coverage [%]: 2.4
No. of unique Peptides: 1

Quantitation

QU:MU	**Median:** 1.10	**CV:** 0.00 %	**No. of Peptides:** 1
WUP:QUP	**Median:** 0.87	**CV:** 0.00 %	**No. of Peptides:** 1

10	20	30	40	50	60	70	80
MRIPDQKYVT	LKLNDVIRFG	YDSNMYVLER	VQHRVPEEAL	KHEKYTSQLQ	VSVKGLAPKR	SEALPEHTPY	CEASNPRPEK
90	100	110	120	130	140	150	160
GDRRPGTEAA	SYRTPLYGQP	SWWGEDDGST	LPDAQRQGEP	YPERPKGPVQ	QDGELHGFRA	PAEPQGCSFR	REPSYFEIPT
170	180	190	200	210	220	230	240
KETPQPSQPP	EVPAHEMPTK	DAEAGGGGAA	PVVQSHASFT	IEFDDCSPGK	MKIKDHITKF	SLRQRRPPGK	EATPGEMVSA
250	260	270	280	290	300	310	320
ETKVADWLVQ	NDPSLLHRVG	PGDDRHSTKS	DLPVHTRTLK	GHKHEDGTQS	DSEDPLAKAA	SAAGVPLEAS	GEQVRLQRQI
330	340	350	360	370	380	390	400
KRDPQELLHN	QQAFVIEFFD	EDTPRKKRSQ	SFTHSPSGDP	KADKRRGPTP	ADRDRPSVPA	PVQAGGRSSG	PQRAGSLKRE
410	420	430	440	450	460	470	480
KTEERLGSPS	PASRTPARPF	GSVGRRSRLA	QDFMAQCLRE	SSPAARPSPE	KVPPVLPAPL	TPHGTSPVGP	PTPPPAPTDP
490	500	510	520	530	540	550	560
QLTKARKQEE	DDSLSDAGTY	TIETEAQDTE	VEEARKMIDQ	VFGVLESPEL	SRASSATFRP	VIRGDRDESD	DGGVAQRMAL
570	580	590	600	610	620	630	640
LQEFASRPLG	AAPQAEHQGL	PVPGSPGGQK	WVSRWASLAD	SYSDPGLTED	GLGRRGGEPE	GSLPVRMRRR	LPQLPSERAD
650	660	670	680	690	700	710	720
SPAGPESSRR	SGPGPPELDS	EQPSRLFGQE	ELDPDSLSDA	SGSDGGRGPE	PGVEPQDSRR	RSPQEGPTWS	RGRRSPRAPG
730	740	750	760	770	780	790	800
EPTPASFFIG	DQNGDAVLSR	KPLAAPGDGE	GLGQTAQPSP	PARDGVYVSA	NGRMVIQLRP	GRSPEPDGPA	PAFLRQESFT
810	820	830	840	850	860	870	880
KEPASGPPAP	GKPPHISSHP	LLQDLAATRA	ARMDFHSQDT	HLILKETETA	LAALEARLLS	NSVDAECEGG	STPRPPEDAL
890	900	910	920	930	940	950	960
SGDSDVDTAS	TVSLRSGKSG	PSPTTPQPLR	AQKEMSPSPP	AAQDPGGTAL	VSAREQSSER	QHHPLGPTDM	GRGEPVRRSA
970	980	990	1000	1010	1020	1030	1040
IRRGHRPRGS	LDWPSEERGP	VLAHLPSSDV	MASNHETPEA	TGAGRLGSRR	KPAAPPPSPA	AREEQSRSSA	SSQKGPQALT
1050	1060	1070	1080	1090	1100	1110	1120
RSNSLSTPRP	TRASRLRRAR	LGDASDTEAA	DGERGSLGNP	EPVGRPAAEQ	AKKLSRLDIL	AMPRKRAGSF	TGTSDPEAAP
1130	1140	1150	1160	1170	1180	1190	1200
ARTSFSGRSV	ELCCASRKPT	MAEARAVSRK	AANTATTTGP	RQPFSRARSG	SARYTSNTRR	RQQGSDYTST	SEEEYGSRHG
1210	1220	1230	1240	1250	1260	1270	1280
SPKHTRSHTS	TATQTPRAGS	SSRARSRAPG	PRDTDDDEEE	PDPYGFIVQT	AEIAEIARLS	QTLVKDVAIL	AQEIHDVAGD
1290	1300	1310	1320	1330	1340	1350	1360
GDTLGSSEPA	HSASLSNMPS	TPASTISARE	ELVQRIPEAS	LNFQKVPPGS	LNSRDFDQNM	NDSCEDALAN	KTRPRNREEV
1370	1380	1390	1400	1410	1420	1430	1440
IFDNLMLNPV	SQLSQAIREN	TEHLAEKMKI	LFQNTGRAWE	DLEARINAEN	EVPILKTSNK	EISSILKELR	RVQKQLEVIN
1450	1460	1470	1480	1490	1500	1510	1520
AIVDPSGSLD	LLTGNRSLAS	SAQPGLGKGR	VAAQSPPSPA	SAEALLPALP	LRNFPQRASC	GPPSLPDPTF	LPDAERFLI

Cmpd.	No. of Cmpds.	m/z meas.	Δ m/z [ppm]	z	Rt [min]	Score	P	Range	Sequence	Modification	Ratios
1914	1	909.8535	-115.14	2	54.3	13.0	2	1105-1122	R.KRAGSFTGTSDPEAAPAR.T		QU:MU 1.10<br>WUP:QUP 0.87

Detailed Protein Report

Protein 507: zinc finger protein Aiolos isoform 2 [Homo sapiens]

**Accession:**	gi|38045959	**Score:**	28.1
**Database:**	refseq_human(refseq_human_20140103.fasta)	**MW [kDa]:**	51.6
**Database Date:**	2015-11-30	**pI:**	5.5
**Modification(s):**	Carbamidomethyl	**Sequence Coverage [%]:**	5.5
		**No. of unique Peptides:**	1

10	20	30	40	50	60	70	80
MEDIQTNAEL	KSTQEQSVPA	ESAAVLNDYS	LTKSHEMENV	DSGEGPANED	EDIGDDSMKV	KDEYSERDEN	VLKSEPMGNA
**90**	**100**	**110**	**120**	**130**	**140**	**150**	**160**
EEPEIPYSYS	REYNEYENIK	LERHVVSFDS	SRPTSGKMNC	DVCGLSCISF	NVLMVHKRSH	TVEKPYKCEF	CGRSYKQRSS
**170**	**180**	**190**	**200**	**210**	**220**	**230**	**240**
LEEHKERCRT	FLQSTDPGDT	ASAEARHIKA	EMGSERALVL	DRLASNVAKR	KSSMPQKFIG	EKRHCFDVNY	NSSYMYEKES
**250**	**260**	**270**	**280**	**290**	**300**	**310**	**320**
ELIQTRMMDQ	AINNAISYLG	AEALRPLVQT	PPAPTSEMVP	VISSMYPIAL	TRAEMSNGAP	QELEKKSIHL	PEKSVPSERG
**330**	**340**	**350**	**360**	**370**	**380**	**390**	**400**
LSPNNSGHDS	TDTDSNHEER	QNHIYQQNHM	VLSRARNGMP	LLKEVPRSYE	LLKPPPICPR	DSVKVINKEG	EVMDVYRCDH
**410**	**420**	**430**	**440**	**450**	**460**		
CRVLFLDYVM	FTIHMGCHGF	RDPFECNMCG	YRSHDRYEFS	SHIARGEHRA	LLK		

Cmpd.	No. of Cmpds.	m/z meas.	Δ m/z [ppm]	z	Rt [min]	Score	P	Range	Sequence	Modification
278	1	998.9505	-27.17	2	33.5	14.8	2	138-153	K.RSHTVEKPYKCEFCGR.S	Carbamidomethyl: 11

Detailed Protein Report

Protein 508: PREDICTED: SNW domain-containing protein 1 isoform X2 [Homo sapiens]

**Accession:**	gi|530403266	**Score:**	28.1
**Database:**	refseq_human(refseq_human_20140103.fasta)	**MW [kDa]:**	65.4
**Database Date:**	2015-11-30	**pI:**	10.2
		**Sequence Coverage [%]:**	6.0
		**No. of unique Peptides:**	1

Quantitation

QU:MU	**Median:** 1.04	**CV:** 0.00 %	**No. of Peptides:**	1
WUP:QUP	**Median:** 0.32	**CV:** 0.00 %	**No. of Peptides:**	1

10	20	30	40	50	60	70	80
MALTSFLPAP	TQLSQDQLEA	EEKARSQRSR	QTSLVSSRRE	PPPYGYRKGW	IPRLLEDFGD	GGAFPEIHVA	QYPLDMGRKK
**90**	**100**	**110**	**120**	**130**	**140**	**150**	**160**
KMSNALAIQV	DSEGKIKYDA	IARQGQSKDK	VIYSKYTDLV	PKEVMNADDP	DLQRPDEEAI	KEITEKTRVA	LEKSVSQKVA
**170**	**180**	**190**	**200**	**210**	**220**	**230**	**240**
AAMPVRAADK	LAPAQYIRYT	PSQQGVAFNS	GAKQRVIRMV	EMQKDPMEPP	RFKINKKIPR	GPPSPPAPVM	HSPSRKMTVK
**250**	**260**	**270**	**280**	**290**	**300**	**310**	**320**
EQQEWKIPPC	ISNWKNAKGY	TIPLDKRLAA	DGRGLQTVHI	NENFAKLAEA	LYIADRKARE	AVEMRAQVER	KMAQKEKEKH
**330**	**340**	**350**	**360**	**370**	**380**	**390**	**400**
EEKLREMAQK	ARERRAGIKT	HVEKEDGEAR	ERDEIRHDRR	KERQHDRNLS	RAAPDKRSKL	QRNENRDISE	VIALGVPNPR
**410**	**420**	**430**	**440**	**450**	**460**	**470**	**480**
TSNEVQYDQR	LFNQSKGMDS	GFAGGEDEIY	NVYDQAWRGG	KDMAQSIYRP	SKNLDKDMYG	DDLEARIKTN	RCQAIQLNFS
**490**	**500**	**510**	**520**	**530**	**540**	**550**	**560**
VYTGESKVVH	SFFSFSLDLF	PTRSFLVQTV	DREAEKDQCS	LRKILLVWTS	FWKKPNSMVA	LKDPQIAAAP	RNTSMKARRG
**570**	**580**						
GRNRHRSLQS	E						

Cmpd.	No. of Cmpds.	m/z meas.	Δ m/z [ppm]	z	Rt [min]	Score	P	Range	Sequence	Modification	Ratios
2282	1	863.8185	-157.42	2	56.9	13.4	2	543-558	K.DPQIAAAPRNTSMKAR.R		QU:MU 1.04<br>WUP:QUP 0.32

BRUKER

Detailed Protein Report

Protein 509: tetratricopeptide repeat protein 37 [Homo sapiens]

**Accession:**	gi|7662078	**Score:**	28.1
**Database:**	refseq_human(refseq_human_20140103.fasta)	**MW [kDa]:**	175.4
**Database Date:**	2015-11-30	**pI:**	8.1
		**Sequence Coverage [%]:**	1.9
		**No. of unique Peptides:**	2

10	20	30	40	50	60	70	80
MSSKEVKTAL	KSARDAIRNK	EYKEALKHCK	TVLKQEKNNY	NAWVFIGVAA	AELEQPDQAQ	SAYKKAAELE	PDQLLAWQGL
90	100	110	120	130	140	150	160
ANLYEKYNHI	NAKDDLPGVY	QKLLDLYESV	DKQKWCDVCK	KLVDLYYQEK	KHLEVARTWH	KLIKTRQEQG	AENEELHQLW
170	180	190	200	210	220	230	240
RKLTQFLAES	TEDQNNETQQ	LLFTAFENAL	GLSDKIPSED	HQVLYRHFIQ	SLSKFPHESA	RLKKACEGMI	NIYPTVQYPL
250	260	270	280	290	300	310	320
EVLCLHLIES	GNLTDEGQQY	CCRLVEMDSK	SGPGLIGLGI	KALQDKKYED	AVRNLTEGLK	ESPVCTSGWY	HLAEAQVKMH
330	340	350	360	370	380	390	400
RPKEAVLSCS	QALKIVDNLG	ASGNSLYQRN	LCLHLKAEAL	IKLSDYDSSE	EAIRTLDQIS	DADNIPGLLV	LKSLAYRNKG
410	420	430	440	450	460	470	480
SFDEAAKIME	DLLSSYPDLA	EVHALEALIH	FTKKDYLQAE	KCFQRALEKD	TEVAEYHYQL	GLTYWFMGEE	TRKDKTKALT
490	500	510	520	530	540	550	560
HFLKAARLDT	YMGKVFCYLG	HYYRDVVGDK	NRARGCYRKA	FELDDTDAES	GAAAVDLSVE	LEDMEMALAI	LTTVTQKASA
570	580	590	600	610	620	630	640
GTAKWAWLRR	GLYYLKAGQH	SQAVADLQAA	LRADPKDFNC	WESLGEAYLS	RGGYTTALKS	FTKASELNPE	SIYSVFKVAA
650	660	670	680	690	700	710	720
IQQILGKYKE	AVAQYQMIIK	KKEDYVPALK	GLGECHLMMA	KAALVDYLDG	KAVDYIEKAL	EYFTCALQHR	ADVSCLWKLA
730	740	750	760	770	780	790	800
GDACTCLYAV	APSKVNVHVL	GVLLGQKEGK	QVLKKNELLH	LGGRCYGRAL	KLMSTSNTWC	DLGINYYRQA	QHLAETGSNM
810	820	830	840	850	860	870	880
NDLKELLEKS	LHCLKKAVRL	DSNNHLYWNA	LGVVACYSGI	GNYALAQHCF	IKSIQSEQIN	AVAWTNLGVL	YLTNENIEQA
890	900	910	920	930	940	950	960
HEAFKMAQSL	DPSYLMCWIG	QALIAEAVGS	YDTMDLFRHT	TELNMHTEGA	LGYAYWVCTT	LQDKSNRETE	LYQYNILQMN
970	980	990	1000	1010	1020	1030	1040
AIPAAQVILN	KYVERIQNYA	PAFTMLGYLN	EHLQLKKEAA	NAYQRAILLL	QTAEDQDTYN	VAIRNYGRLL	CSTGEYDKAI
1050	1060	1070	1080	1090	1100	1110	1120
QAFKSTPLEV	LEDIIGFALA	LFMKGLYKES	SKAYERALSI	VESEQDKAHI	LTALAITEYK	QGKTDVAKTL	LFKCSILKEP
1130	1140	1150	1160	1170	1180	1190	1200
TTESLQALCA	LGLAMQDATL	SKAALNELLK	HIKHKDSNYQ	RCLLTSAIYA	LQGRSVAVQK	QISKAVHSNP	GDPALWSLLS
1210	1220	1230	1240	1250	1260	1270	1280
RVVAQYAQRN	AKGGVVAGNV	AHILDSNHGK	KALLYTAVNQ	LAMGSSSAED	EKNTALKTIQ	KAALLSPGDP	AIWAGLMAAC
1290	1300	1310	1320	1330	1340	1350	1360
HADDKLALVN	NTQPKRIDLY	LALLSAVSAS	IKDEKFFENY	NQSLEKWSLS	QAVTGLIDTG	RISEAETLCT	KNLKSNPDQP
1370	1380	1390	1400	1410	1420	1430	1440
AVILLLRQVQ	CKPLLESQKP	LPDAVLEELQ	KTVMSNSTSV	PAWQWLAHVY	QSQGMMRAAE	MCYRKSLQLA	SQRGSWSGKL
1450	1460	1470	1480	1490	1500	1510	1520
SSLLRLALLA	LKVCMANISN	DHWPSLVQEA	TTEALKLCFC	PLAVLLQALL	QFKRKMGARE	TRRLLERVVY	QPGYPKSIAS
1530	1540	1550	1560	1570			
TARWYLLRHL	YAKDDYELID	VLVNNAKTHG	DTRALELNQR	LSSQ			

Cmpd.	No. of Cmpds.	m/z meas.	Δ m/z [ppm]	z	Rt [min]	Score	P	Range	Sequence	Modification
2200	1	899.4081	-76.29	2	55.9	13.7	1	319-334	K.MHRPKEAVLSCSQALK.I	
728	1	705.9560	-25.50	2	37.6	14.3	1	1440-1452	K.LSSLLRLALLALK.V	

Detailed Protein Report

Protein 510: **PREDICTED: huntingtin-interacting protein 1-related protein isoform X2 [Homo sapiens]**

**Accession:**	gi|530401786	**Score:**	28.0
**Database:**	refseq_human(refseq_human_20140103.fasta)	**MW [kDa]:**	118.6
**Database Date:**	2015-11-30	**pI:**	6.2
		**Sequence Coverage [%]:**	2.6
		**No. of unique Peptides:**	1

Quantitation

QU:MU **Median:** 1.07 **CV:** 0.00 % **No. of Peptides:** 1

10	20	30	40	50	60	70	80
MEARFSPINQ	ILPWCRQDLA	ISISKAINTQ	EAPVKEKHAR	RIILGTHHEK	GAFTFWSYAI	GLPLPSSSIL	SWKFCHVLHK
90	100	110	120	130	140	150	160
VLRDGHPNVL	HDCQRYRSNI	REIGDLWGHL	HDRYGQLVNV	YTKLLLTKIS	FHLKHPQFPA	GLEVTDEVLE	KAAGTDVNNI
170	180	190	200	210	220	230	240
FQLTVEMFDY	MDCELKLSES	VFRQLNTAIA	VSQMSSGQCR	LAPLIQVIQD	CSHLYHYTVK	LLFKLHSCLP	ADTLQGHRDR
250	260	270	280	290	300	310	320
FHEQFHSLRN	FFRRASDMLY	FKRLIQIPRL	PEGPPNFLRA	SALAEHIKPV	VVIPEEAPED	EEPENLIEIS	TGPPAGEPVV
330	340	350	360	370	380	390	400
VADLFDQTFG	PPNGSVKDDR	TSVPRDLQIE	SLKREVEMLR	SELEKIKLEA	QRYIAQLKSQ	VNALEGELEE	QRKQKQKALV
410	420	430	440	450	460	470	480
DNEQLRHELA	QLRAAQLEGE	RSQGLREEAE	RKASATEARY	NKLKEKHSEL	VHVHAELLRK	NADTAKQLTV	TQQSQEEVAR
490	500	510	520	530	540	550	560
VKEQLAFQVE	QVKRESELKL	EEKSDQLEKL	KRELEAKAGE	LARAQEALSH	TEQSKSELSS	RLDTLSAEKD	ALSGAVRQRE
570	580	590	600	610	620	630	640
ADLLAAQSLV	RETEAALSRE	QQRSSQEQGE	LQGRLAERES	QEQGLRQRLL	DEQFAVLRGA	AAEAAGILQD	AVSKLDDPLH
650	660	670	680	690	700	710	720
LRCTSSPDYL	VSRAQEALDA	VSTLEEGHAQ	YLTSLADASA	LVAALTRFSH	LAADTIINGG	ATSHLAPTDP	ADRLIDTCRE
730	740	750	760	770	780	790	800
CGARALELMG	QLQDQQALRH	MQASLVRTPL	QGILQLGQEL	KPKSLDVRQE	ELGAVVDKEM	AATSAAIEDA	VRRIEDMMNQ
810	820	830	840	850	860	870	880
ARHASSGVKL	EVNERILNSC	TDLMKAIRLL	VTTSTSLQKE	IVESGRGAAT	QQEFYAKNSR	WTEGLISASK	AVGWGATQLV
890	900	910	920	930	940	950	960
EAADKVVLHT	GKYEELIVCS	HEIAASTAQL	VAASKVKANK	HSPHLSRLQE	CSRTVNERAA	NVVASTKSGQ	EQIEDRDTMD
970	980	990	1000	1010	1020	1030	1040
FSGLSLIKLK	KQEMETQVRV	LELEKTLEAE	RMRLGELRKQ	HYVLAGASGS	PGEEVAIRPS	TAPRSVTTKK	PPLAQKPSVA
1050	1060	1070					
PRQDHQLDKK	DGIYPAQLVN	Y					

Cmpd.	No. of Cmpds.	m/z meas.	Δ m/z [ppm]	z	Rt [min]	Score	P	Range	Sequence	Modification	Ratios
662	1	593.6995	-211.24	2	38.3	11.3	1	513-523	R.ELEAKAGELAR.A		QU:MU 1.07

Detailed Protein Report

Protein 511: contactin-associated protein-like 4 isoform 2 [Homo sapiens]

**Accession:**	gi|148664242	**Score:**	28.0
**Database:**	refseq_human(refseq_human_20140103.fasta)	**MW [kDa]:**	137.4
**Database Date:**	2015-11-30	**pI:**	6.1
**Modification(s):**	Oxidation	**Sequence Coverage [%]:**	3.1
		**No. of unique Peptides:**	2

10	20	30	40	50	60	70	80
MWNYDCDDPL	VSALPQASFS	SSSELSSSHG	PGFARLNRRD	GAGGWSPLVS	NKYQWLQIDL	GERMEVTAVA	TQGGYGSSNW
90	100	110	120	130	140	150	160
VTSYLLMFSD	SGWNWKQYRQ	EDSIWGFSGN	ANADSVVYYR	LQPSIKARFL	RFIPLEWNPK	GRIGMRIEVF	GCAYRSEVVD
170	180	190	200	210	220	230	240
LDGKSSLLYR	FDQKSLSPIK	DIISLKFKTM	QSDGILLHRE	GPNGDHITLQ	LRRARLFLLI	NSGEAKLPST	STLVNLTLGS
250	260	270	280	290	300	310	320
LLDDQHWHSV	LIQRLGKQVN	FTVDEHRHHF	HARGEFNLMN	LDYEGNVSFS	CSQPQSMPVT	FLSSRSYLAL	PDFSGEEEVS
330	340	350	360	370	380	390	400
ATFQFRTWNK	AGLLLFSELQ	LISGGILLFL	SDGKLKSNLY	QPGKLPSDIT	AGVELNDGQW	HSVSLSAKKN	HLSVAVDGQM
410	420	430	440	450	460	470	480
ASAAPLLGPE	QIYSGGTYYF	GGCPDKSFGS	KCKSPLGGFQ	GCMRLISISG	KVVDLISVQQ	GSLGNFSDLQ	IDSCGISDRC
490	500	510	520	530	540	550	560
LPNYCEHGGE	CSQSWSTFHC	NCTNTGYRGA	TCHNSIYEQS	CEAYKHRGNT	SGFYYIDSDG	SGPLEPFLLY	CNMTETAWTI
570	580	590	600	610	620	630	640
IQHNGSDLTR	VRNTNPENPY	AGFFEYVASM	EQLQATINRA	EHCEQEFTYY	CKKSRLVNKQ	DGTPLSWWVG	RTNETQTYWG
650	660	670	680	690	700	710	720
GSSPDLQKCT	CGLEGNCIDS	QYYCNCDADR	NEWTNDTGLL	AYKEHLPVTK	IVITDTGRLH	SEAAYKLGPL	LCQGDRSFWN
730	740	750	760	770	780	790	800
SASFDTEASY	LHFPTFHGEL	SADVSFFFKT	TASSGVFLEN	LGIADFIRIE	LRSPTVVTFS	FDVGNGPFEI	SVQSPTHFND
810	820	830	840	850	860	870	880
NQWHHVRVER	NMKEASLQVD	QLTPKTQPAP	ADGHVLLQLN	SQLFVGGTAT	RQRGFLGCIR	SLQLNGMTLD	LEERAQVTPE
890	900	910	920	930	940	950	960
VQPGCRGHCS	SYGKLCRNGG	KCRERPIGFF	CDCTFSAYTG	PFCSNEISAY	FGSGSSVIYN	FQENYLLSKN	SSSHAASFHG
970	980	990	1000	1010	1020	1030	1040
DMKLSREMIK	FSFRTTRTPS	LLLFVSSFYK	EYLSVIIAKN	GSLQIRYKLN	KYQEPDVVNF	DFKNMADGQL	HHIMINREEG
1050	1060	1070	1080	1090	1100	1110	1120
VVFIEIDDNR	RRQVHLSSGT	EFSAVKSLVL	GRILEHSDVD	QDTALAGAQG	FTGCLSAVQL	SHVAPLKAAL	HPSHPDPVTV
1130	1140	1150	1160	1170	1180	1190	1200
TGHVTESSCM	AQPGTDATSR	ERTHSFADHS	GTIDDREPLA	NAIKSDSAVI	GGLIAVVIFI	LLCITAIAVR	IYQQKRLYKR
1210	1220	1230	1240				
SEAKRSENVD	SAEAVLKSEL	NIQNAVNENQ	KEYFF				

Cmpd.	No. of Cmpds.	m/z meas.	Δ m/z [ppm]	z	Rt [min]	Score	P	Range	Sequence	Modification
2542	1	715.3361	-67.84	2	61.7	15.1	2	964-974	K.LSREMIKFSFR.T	Oxidation: 5
1571	1	1061.1650	-10.98	3	49.3	12.9	1	1024-1050	K.NMADGQLHHIMINREEGVVFIEIDDNR.R	Oxidation: 2

Detailed Protein Report

Protein 512: PREDICTED: synaptojanin-2 isoform X3 [Homo sapiens]

**Accession:**	gi|530384175	**Score:**	27.9
**Database:**	refseq_human(refseq_human_20140103.fasta)	**MW [kDa]:**	122.5
**Database Date:**	2015-11-30	**pI:**	6.9
		**Sequence Coverage [%]:**	2.4
		**No. of unique Peptides:**	1

10	20	30	40	50	60	70	80
MNCLDCLDRT	NTVQSFIALE	VLHLQLKTLG	LSSKPIVDRF	VESFKAMWSL	NGHSLSKVFT	GSRALEGKAK	VGKLKDGARS
90	100	110	120	130	140	150	160
MSRTIQSNFF	DGVKQEAIKL	LLVGDVYGEE	VADKGGMLLD	STALLVTPRI	LKAMTERQSE	FTNFKRIRIA	MGTWNVNGGK
170	180	190	200	210	220	230	240
QFRSNVLRTA	ELTDWLLDSP	QLSGATDSQD	DSSPADIFAV	GFEEMVELSA	GNIVNASTTN	KKMWGEQLQK	AISRSHRYIL
250	260	270	280	290	300	310	320
LTSAQLVGVC	LYIFVRPYHV	PFIRDVAIDT	VKTGMGGKAG	NKGAVGIRFQ	FHSTSFCFIC	SHLTAGQSQV	KERNEDYKEI
330	340	350	360	370	380	390	400
TQKLCFPMGR	NVFSHDYVFW	CGDFNYRIDL	TYEEVFYFVK	RQDWKKLLEF	DQLQLQKSSG	KIFKDFHEGA	INFGPTYKYD
410	420	430	440	450	460	470	480
VGSAAYDTSD	KCRTPAWTDR	VLWWRKKHPF	DKTAGELNLL	DSDLDVDTKV	RHTWSPGALQ	YYGRAELQAS	DHRPVLAIVE
490	500	510	520	530	540	550	560
VEVQEVDVGA	RERVFQEVSS	FQGPLDATVV	VNLQSPTLEE	KNEFPEDLRT	ELMQTLGSYG	TIVLVRINQG	QMLVTFADSH
570	580	590	600	610	620	630	640
SALSVLDVDG	MKVKGRAVKI	RPKTKDWLKG	LREEIIRKRD	SMAPVSPTAN	SCLLEENFDF	TSLDYESEGD	ILEDDEDYLV
650	660	670	680	690	700	710	720
DEFNQPGVSD	SELGGDDLSD	VPGPTALAPP	SKSPALTKKK	QHPTYKDDAD	LVELKRELEA	VGEFRHRSPS	RSLSVPNRPR
730	740	750	760	770	780	790	800
PPQPPQRPPP	PTGLMVKKSA	SDASISSGTH	GQYSILQTAR	LLPGAPQQPP	KARTGISKPY	NVKQIKTTNA	QEAEAAIRCL
810	820	830	840	850	860	870	880
LEARGGASEE	ALSAVAPRDL	EASSEPEPTP	GAAKPETPQA	PPLLPRRPPP	RVPAIKKPTL	RRTGKPLSPE	EQFEQQTVHF
890	900	910	920	930	940	950	960
TIGPPETSVE	APPVVTAPRV	PPVPKPRTFQ	PGKAAERPSH	RKPASDEAPP	GAGASVPPPL	EAPPLVPKVP	PRRKKSAPAA
970	980	990	1000	1010	1020	1030	1040
FHLQVLQSNS	QLLQGLTYNS	SDSPSGHPPA	AGTVFPQGDF	LSTSSATSPD	SDGTKAMKPE	AAPLLGDYQD	PFWNLLHHPK
1050	1060	1070	1080	1090	1100	1110	1120
LLNNTWLSKS	SDPLDSGTRS	PKRDPIDPVS	AGASAAKAEL	PPDHEHKTLG	HWVTISDQEK	RTALQVFDPL	AKT

Cmpd.	No. of Cmpds.	m/z meas.	Δ m/z [ppm]	z	Rt [min]	Score	P	Range	Sequence	Modification
1895	1	643.2981	-123.24	2	54.1	16.3	0	28-39	K.TLGLSSKPIVDR.F	

Detailed Protein Report

Protein 513: myosin-2 [Homo sapiens]

**Accession:**	gi|153791586	**Score:**	27.9
**Database:**	refseq_human(refseq_human_20140103.fasta)	**MW [kDa]:**	222.9
**Database Date:**	2015-11-30	**pI:**	5.5
**Modification(s):**	Oxidation	**Sequence Coverage [%]:**	1.6
		**No. of unique Peptides:**	1

Alias proteins:

Accession	Name	Description
gi|153792663	refseq_human (refseq_human_20140103.fasta)	myosin-2 [Homo sapiens]

Detailed Protein Report

10	20	30	40	50	60	70	80
MSSDSELAVF	GEAAPFLRKS	ERERIEAQNR	PFDAKTSVFV	AEPKESFVKG	TIQSREGGKV	TVKTEGGATL	TVKDDQVFPM
90	100	110	120	130	140	150	160
NPPKYDKIED	MAMMTHLHEP	AVLYNLKERY	AAWMIYTYSG	LFCVTVNPYK	WLPVYKPEVV	TAYRGKKRQE	APPHIFSISD
170	180	190	200	210	220	230	240
NAYQFMLTDR	ENQSILITGE	SGAGKTVNTK	RVIQYFATIA	VTGEKKKEEI	TSGKIQGTLE	DQIISANPLL	EAFGNAKTVR
250	260	270	280	290	300	310	320
NDNSSRFGKF	IRIHFGTTGK	LASADIETYL	LEKSRVVFQL	KAERSYHIFY	QITSNKKPEL	IEMLLITTNP	YDYPFVSQGE
330	340	350	360	370	380	390	400
ISVASIDDQE	ELMATDSAID	ILGFTNEEKV	SIYKLTGAVM	HYGNLKFKQK	QREEQAEPDG	TEVADKAAYL	QSLNSADLLK
410	420	430	440	450	460	470	480
ALCYPRVKVG	NEYVTKGQTV	EQVSNAVGAL	AKAVYEKMFL	WMVARINQQL	DTKQPRQYFI	GVLDIAGFEI	FDFNSLEQLC
490	500	510	520	530	540	550	560
INFTNEKLQQ	FFNHHMFVLE	QEEYKKEGIE	WTFIDFGMDL	AACIELIEKP	MGIFSILEEE	CMFPKATDTS	FKNKLYDQHL
570	580	590	600	610	620	630	640
GKSANFQKPK	VVKGKAEAHF	ALIHYAGVVD	YNITGWLEKN	KDPLNETVVG	LYQKSAMKTL	AQLFSGAQTA	EGEGAGGGAK
650	660	670	680	690	700	710	720
KGGKKKGSSF	QTVSALFREN	LNKLMTNLRS	THPHFVRCII	PNETKTPGAM	EHELVLHQLR	CNGVLEGIRI	CRKGFPSRIL
730	740	750	760	770	780	790	800
YADFKQRYKV	LNASAIPEGQ	FIDSKKASEK	LLASIDIDHT	QYKFGHTKVF	FKAGLLGLLE	EMRDDKLAQL	ITRTQARCRG
810	820	830	840	850	860	870	880
FLARVEYQRM	VERREAIFCI	QYNIRSFMNV	KHWPWMKLFF	KIKPLLKSAE	TEKEMATMKE	EFQKIKDELA	KSEAKRKELE
890	900	910	920	930	940	950	960
EKMVTLLKEK	NDLQLQVQAE	AEGLADAEER	CDQLIKTKIQ	LEAKIKEVTE	RAEDEEEINA	ELTAKKRKLE	DECSELKKDI
970	980	990	1000	1010	1020	1030	1040
DDLELTLAKV	EKEKHATENK	VKNLTEEMAG	LDETIAKLTK	EKKALQEAHQ	QTLDDLQAEE	DKVNTLTKAK	IKLEQQVDDL
1050	1060	1070	1080	1090	1100	1110	1120
EGSLEQEKKL	RMDLERAKRK	LEGDLKLAQE	SIMDIENEKQ	QLDEKLKKKE	FEISNLQSKI	EDEQALGIQL	QKKIKELQAR
1130	1140	1150	1160	1170	1180	1190	1200
IEELEEEIEA	ERASRAKAEK	QRSDLSRELE	EISERLEEAG	GATSAQIEMN	KKREAEFQKM	RRDLEEATLQ	HEATAATLRK
1210	1220	1230	1240	1250	1260	1270	1280
KHADSVAELG	EQIDNLQRVK	QKLEKEKSEM	KMEIDDLASN	VETVSKAKGN	LEKMCRTLED	QLSELKSKEE	EQQRLINDLT
1290	1300	1310	1320	1330	1340	1350	1360
AQRGRLQTES	GEFSRQLDEK	EALVSQLSRG	KQAFTQQIEE	LKRQLEEEIK	AKNALAHALQ	SSRHDCDLLR	EQYEEEQESK
1370	1380	1390	1400	1410	1420	1430	1440
AELQRALSKA	NTEVAQWRTK	YETDAIQRTE	ELEEAKKKLA	QRLQAAEEHV	EAVNAKCASL	EKTKQRLQNE	VEDLMLDVER
1450	1460	1470	1480	1490	1500	1510	1520
TNAACAALDK	KQRNFDKILA	EWKQKCEETH	AELEASQKEA	RSLGTELFKI	KNAYEESLDQ	LETLKRENKN	LQQEISDLTE
1530	1540	1550	1560	1570	1580	1590	1600
QIAEGGKRIH	ELEKIKKQVE	QEKCELQAAL	EEAEASLEHE	EGKILRIQLE	LNQVKSEVDR	KIAEKDEEID	QLKRNHIRIV
1610	1620	1630	1640	1650	1660	1670	1680
ESMQSTLDAE	IRSRNDAIRL	KKKMEGDLNE	MEIQLNHANR	MAAEALRNYR	NTQGILKDTQ	IHLDDALRSQ	EDLKEQLAMV
1690	1700	1710	1720	1730	1740	1750	1760
ERRANLLQAE	IEELRATLEQ	TERSRKIAEQ	ELLDASERVQ	LLHTQNTSLI	NTKKKLETDI	SQMQGEMEDI	LQEARNAEEK
1770	1780	1790	1800	1810	1820	1830	1840
AKKAITDAAM	MAEELKKEQD	TSAHLERMKK	NMEQTVKDLQ	LRLDEAEQLA	LKGGKKQIQK	LEARVRELEG	EVESEQKRNA
1850	1860	1870	1880	1890	1900	1910	1920
EAVKGLRKHE	RRVKELTYQT	EEDRKNILRL	QDLVDKLQAK	VKSYKRQAEE	AEEQSNTNLA	KFRKLQHELE	EAEERADIAE
1930	1940	1950					
SQVNKLRVKS	REVHTKVISE	E					

Cmpd.	No. of Cmpds.	m/z meas.	Δ m/z [ppm]	z	Rt [min]	Score	P	Range	Sequence	Modification
2807	1	714.6776	11.25	3	66.4	12.9	1	1228-1246	K.SEMKMEIDDLASNVETVSK.A	Oxidation: 3

Detailed Protein Report

Protein 514: **PREDICTED: nuclear receptor subfamily 1 group D member 2 isoform X1 [Homo sapiens]**

Accession: gi|530373510
Database: refseq_human(refseq_human_20140103.fasta)
Database Date: 2015-11-30
Modification(s): Oxidation

Score: 27.9
MW [kDa]: 45.6
pI: 9.9
Sequence Coverage [%]: 6.1
No. of unique Peptides: 1

Quantitation

WUP:QUP **Median:** 0.35 **CV:** 0.00 % **No. of Peptides:** 1

10	20	30	40	50	60	70	80
MEVNAGGVIA	YISSSSSASS	PASCHSEGSE	NSFQSSSSSV	PSSPNSSNSD	TNGNPKNGDL	ANIEGILKND	RIDCSMKTSK
**90**	**100**	**110**	**120**	**130**	**140**	**150**	**160**
SSAPGMTKSH	SGVTKFSGMV	LLCKVCGDVA	SGFHYGVHAC	EGCKGFFRRS	IQQNIQYKKC	LKNENCSIMR	MNRNRCQQCR
**170**	**180**	**190**	**200**	**210**	**220**	**230**	**240**
FKKCLSVGMS	RDAVRFGRIP	KREKQRMLIE	MQSAMKTMMN	SQFSGHLQND	TLVEHHEQTA	LPAQEQLRPK	PQLEQENIKS
**250**	**260**	**270**	**280**	**290**	**300**	**310**	**320**
SSPPSSDFAK	EEVIGMVTRA	HKDTFMYNQE	QQENSAESMQ	PQRGERIPKN	MEQYNLNHDH	CGNGLSSHFP	CSESQQHLNG
**330**	**340**	**350**	**360**	**370**	**380**	**390**	**400**
QFKGRNIMHY	PNGHAICIAN	GHCMNFSNAY	TQRVCDRVPI	DGFSQNENKN	SYLCNTGGRM	HLYSVHIQPK	FHQKQQYHTI
**410**							
SSLNHEVL							

Cmpd.	No. of Cmpds.	m/z meas.	Δ m/z [ppm]	z	Rt [min]	Score	P	Range	Sequence	Modification	Ratios
80	1	877.8666	-76.26	2	30.7	12.1	2	183-196	R.EKQRMLIEMQSAMK.T	Oxidation: 9, 13	WUP:QUP 0.35

Detailed Protein Report

Protein 515: ephrin type-A receptor 7 isoform 2 precursor [Homo sapiens]

**Accession:**	gi|568599847	**Score:**	27.9
**Database:**	refseq_human(refseq_human_20140103.fasta)	**MW [kDa]:**	111.4
**Database Date:**	2015-11-30	**pI:**	5.5
		**Sequence Coverage [%]:**	3.5
		**No. of unique Peptides:**	1

10	20	30	40	50	60	70	80
MVFQTRYPSW	IILCYIWLLR	FAHTGEAQAA	KEVLLLDSKA	QQTELEWISS	PPNGWEEISG	LDENYTPIRT	YQVCQVMEPN
90	100	110	120	130	140	150	160
QNNWLRTNWI	SKGNAQRIFV	ELKFTLRDCN	SLPGVLGTCK	ETFNLYYYET	DYDTGRNIRE	NLYVKIDTIA	ADESFTQGDL
170	180	190	200	210	220	230	240
GERKMKLNTE	VREIGPLSKK	GFYLAFQDVG	ACIALVSVKV	YYKKCWSIIE	NLAIFPDTVT	GSEFSSLVEV	RGTCVSSAEE
250	260	270	280	290	300	310	320
EAENAPRMHC	SAEGEWLVPI	GKCICKAGYQ	QKGDTCEPCG	RGFYKSSSQD	LQCSRCPTHS	FSDKEGSSRC	ECEDGYYRAP
330	340	350	360	370	380	390	400
SDPPYVACTR	PPSAPQNLIF	NINQTTVSLE	WSPPADNGGR	NDVTYRILCK	RCSWEQGECV	PCGSNIGYMP	QQTGLEDNYV
410	420	430	440	450	460	470	480
TVMDLLAHAN	YTFEVEAVNG	VSDLSRSQRL	FAAVSITTGQ	AAPSQVSGVM	KERVLQRSVE	LSWQEPEHPN	GVITEYEIKY
490	500	510	520	530	540	550	560
YEKDQRERTY	STVKTKSTSA	SINNLKPGTV	YVFQIRAFTA	AGYGNYSPRL	DVATLEEATA	TAVSSEQNPV	IIIAVVAVAG
570	580	590	600	610	620	630	640
TIILVFMVFG	FIIGRRHCGY	SKADQEGDEE	LYFHFKFPGT	KTYIDPETYE	DPNRAVHQFA	KELDASCIKI	ERVIGAGEFG
650	660	670	680	690	700	710	720
EVCSGRLKLP	GKRDVAVAIK	TLKVGYTEKQ	RRDFLCEASI	MGQFDHPNVV	HLEGVVTRGK	PVMIVIEFME	NGALDAFLRK
730	740	750	760	770	780	790	800
HDGQFTVIQL	VGMLRGIAAG	MRYLADMGYV	HRDLAARNIL	VNSNLVCKVS	DFGLSRVIED	DPEAVYTTTG	GKIPVRWTAP
810	820	830	840	850	860	870	880
EAIQYRKFTS	ASDVWSYGIV	MWEVMSYGER	PYWDMSNQDV	IKAIEEGYRL	PAPMDCPAGL	HQLMLDCWQK	ERAERPKFEQ
890	900	910	920	930	940	950	960
IVGILDKMIR	NPNSLKTPLG	TCSRPISPLL	DQNTPDFTTF	CSVGEWLQAI	KMERYKDNFT	AAGYNSLESV	ARMTIEDVMS
970	980	990	1000				
LGITLVGHQK	KIMSSIQTMR	AQMLHLHGTG	IQV				

Cmpd.	No. of Cmpds.	m/z meas.	Δ m/z [ppm]	z	Rt [min]	Score	P	Range	Sequence	Modification
1992	1	857.8267	-91.44	2	55.3	11.3	0	937-952	K.DNFTAAGYNSLESVAR.M	

Detailed Protein Report

Protein 516: **PREDICTED: protein 4.1 isoform X29 [Homo sapiens]**

**Accession:**	gi|578798602	**Score:**	27.8
**Database:**	refseq_human(refseq_human_20140103.fasta)	**MW [kDa]:**	90.9
**Database Date:**	2015-11-30	**pI:**	6.1
**Modification(s):**	Oxidation	**Sequence Coverage [%]:**	3.9
		**No. of unique Peptides:**	2

10	20	30	40	50	60	70	80
MHCKVSLLDD	TVYECVVEKH	AKGQDLLKRV	CEHLNLLEED	YFGLAIWDNA	TSKTWLDSAK	EIKKQVRGVP	WNFTFNVKFY
**90**	**100**	**110**	**120**	**130**	**140**	**150**	**160**
PPDPAQLTED	ITRYYLCLQL	RQDIVAGRLP	CSFATLALLG	SYTIQSELGD	YDPELHGVDY	VSDFKLAPNQ	TKELEEKVME
**170**	**180**	**190**	**200**	**210**	**220**	**230**	**240**
LHKSYRSMTP	AQADLEFLEN	AKKLSMYGVD	LHKAKDLEGV	DIILGVCSSG	LLVYKDKLRI	NRFPWPKVLK	ISYKRSSFFI
**250**	**260**	**270**	**280**	**290**	**300**	**310**	**320**
KIRPGEQEQY	ESTIGFKLPS	YRAAKKLWKV	CVEHHTFFRL	TSTDTIPKSK	FLALGSKFRY	SGRTQAQTRQ	ASALIDRPAP
**330**	**340**	**350**	**360**	**370**	**380**	**390**	**400**
HFERTASKRA	SRSLDGAAAV	DSADRSPRPT	SAPAITQGQV	AEGGVLDASA	KKTVVPKAQK	ETVKAEVKKE	DEPPEQAEPE
**410**	**420**	**430**	**440**	**450**	**460**	**470**	**480**
PTEAWKVEKT	HIEVTVPTSN	GDQTQKLAEK	TEDLIRMRKK	KRERLDGENI	YIRHSNLMLE	DLDKSQEEIK	KHHASISELK
**490**	**500**	**510**	**520**	**530**	**540**	**550**	**560**
KNFMESVPEP	RPSEWDKRLS	THSPFRTLNI	NGQIPTGEGV	KKTSVLPSER	KSINGIRTEE	VAVVTKGPST	NPDSEWEGPK
**570**	**580**	**590**	**600**	**610**	**620**	**630**	**640**
HSVVPSKSQM	TTSSESLQSF	AFGSLSISSK	ETEEKEEGAA	GYLDIKEMPR	GPTGGCIGVE	EQASALKFSV	TPASCQLQPG
**650**	**660**	**670**	**680**	**690**	**700**	**710**	**720**
VKKAESSEEH	VTPGEPPGKQ	NGSFLDFHVG	NQFPTLIRSF	QPPLVKTQTV	TISDNANAVK	SEIPTKDVPI	VHTETKTITY
**730**	**740**	**750**	**760**	**770**	**780**	**790**	**800**
EAAQTDDNSG	DLDPGVLLTA	QTITSETPSS	TTTTQITKTV	KGGISETRIE	KRIVITGDAD	IDHDQVLVQA	IKEAKEQHPD
**810**	**820**						
MSVTKVVVHQ	ETEIADE						

Cmpd.	No. of Cmpds.	m/z meas.	Δ m/z [ppm]	z	Rt [min]	Score	P	Range	Sequence	Modification
1704	1	682.2585	-109.76	3	51.6	12.4	1	454-470	R.HSNLMLEDLDKSQEEIK.K	Oxidation: 5
100	1	565.6045	-3.81	3	31.2	15.5	1	596-610	K.EEGAAGYLDIKEMPR.G	Oxidation: 13

Detailed Protein Report

Protein 517: mucin-2 precursor [Homo sapiens]

**Accession:**	gi|557129045	**Score:**	27.7
**Database:**	refseq_human(refseq_human_20140103.fasta)	**MW [kDa]:**	539.8
**Database Date:**	2015-11-30	**pI:**	5.4
**Modification(s):**	Oxidation	**Sequence Coverage [%]:**	0.6
		**No. of unique Peptides:**	1

Quantitation

QU:MU	**Median:**	0.72	**CV:**	0.00 %	**No. of Peptides:**	1
WUP:QUP	**Median:**	1.63	**CV:**	0.00 %	**No. of Peptides:**	1

Detailed Protein Report

10	20	30	40	50	60	70	80
MGLPLARLAA	VCLALSLAGG	SELQTEGRTR	NHGHNVCSTW	GNFHYKTFDG	DVFRFPGPCD	YNFASDCRGS	YKEFAVHLKR
90	100	110	120	130	140	150	160
GPGQAEAPAG	VESILLTIKD	DTIYLTRHLA	VLNGAVVSTP	HYSPGLLIEK	SDAYTKVYSR	AGLTLMWNRE	DALMLELDTK
170	180	190	200	210	220	230	240
FRNHTCGLCG	DYNGLQSYSE	FLSDGVLFSP	LEFGNMQKIN	QPDVVCEDPE	EEVAPASCSE	HRAECERLLT	AEAFADCQDL
250	260	270	280	290	300	310	320
VPLEPYLRAC	QQDRCRCPGG	DTCVCSTVAE	FSRQCSHAGG	RPGNWRTATL	CPKTCPGNLV	YLESGSPCMD	TCSHLEVSSL
330	340	350	360	370	380	390	400
CEEHRMDGCF	CPEGTVYDDI	GDSGCVPVSQ	CHCRLHGHLY	TPGQEITNDC	EQCVCNAGRW	VCKDLPCPGT	CALEGGSHIT
410	420	430	440	450	460	470	480
TFDGKTYTFH	GDCYYVLAKG	DHNDSYALLG	ELAPCGSTDK	QTCLKTVVLL	ADKKKNVVVF	KSDGSVLLNE	LQVNLPHVTA
490	500	510	520	530	540	550	560
SFSVFRPSSY	HIMVSMAIGV	RLQVQLAPVM	QLFVTLDQAS	QGQVQGLCGN	FNGLEGDDFK	TASGLVEATG	AGFANTWKAQ
570	580	590	600	610	620	630	640
STCHDKLDWL	DDPCSLNIES	ANYAEHWCSL	LKKTETPFGR	CHSAVDPAEY	YKRCKYDTCN	CQNNEDCLCA	ALSSYARACT
650	660	670	680	690	700	710	720
AKGVMLWGWR	EHVCNKDVGS	CPNSQVFLYN	LTTCQQTCRS	LSEADSHCLE	GFAPVDGCGC	PDHTFLDEKG	RCVPLAKCSC
730	740	750	760	770	780	790	800
YHRGLYLEAG	DVVVRQEERC	VCRDGRLHCR	QIRLIGQSCT	APKIHMDCSN	LTALATSKPR	ALSCQTLAAG	YYHTECVSGC
810	820	830	840	850	860	870	880
VCPDGLMDDG	RGGCVVEKEC	PCVHNNDLYS	SGAKIKVDCN	TCTCKRGRWV	CTQAVCHGTC	SIYGSGHYIT	FDGKYYDFDG
890	900	910	920	930	940	950	960
HCSYVAVQDY	CGQNSSLGSF	SIITENVPCG	TTGVTCSKAI	KIFMGRTELK	LEDKHRVVIQ	RDEGHHVAYT	TREVGQYLVV
970	980	990	1000	1010	1020	1030	1040
ESSTGIIVIW	DKRTTVFIKL	APSYKGTVCG	LCGNFDHRSN	NDFTTRDHMV	VSSELDFGNS	WKEAPTCPDV	STNPEPCSLN
1050	1060	1070	1080	1090	1100	1110	1120
PHRRSWAEKQ	CSILKSSVFS	ICHSKVDPKP	FYEACVHDSC	SCDTGGDCEC	FCSAVASYAQ	ECTKEGACVF	WRTPDLCPIF
1130	1140	1150	1160	1170	1180	1190	1200
CDYYNPPHEC	EWHYEPCGNR	SFETCRTING	IHSNISVSYL	EGCYPRCPKD	RPIYEEDLKK	CVTADKCGCY	VEDTHYPPGA
1210	1220	1230	1240	1250	1260	1270	1280
SVPTEETCKS	CVCTNSSQVV	CRPEEGKILN	QTQDGAFCYW	EICGPNGTVE	KHFNICSITT	RPSTLTTFTT	ITLPTTPTTF
1290	1300	1310	1320	1330	1340	1350	1360
TTTTTTTTPT	SSTVLSTTPK	LCCLWSDWIN	EDHPSSGSDD	GDRETFDGVC	GAPEDIECRS	VKDPHLSLEQ	LGQKVQCDVS
1370	1380	1390	1400	1410	1420	1430	1440
VGFICKNEDQ	FGNGPFGLCY	DYKIRVNCCW	PMDKCITTPS	PPTTTPSPPP	TSTTTLPPTT	TPSPPTTTTT	TPPPTTTPSP
1450	1460	1470	1480	1490	1500	1510	1520
PITTTTTPPP	TTTPSPPIST	TTTPPPTTTP	SPPTTTPSPP	TTTPSPPTTT	TTTPPPTTTP	SPPTTTPITP	PASTTTLPPT
1530	1540	1550	1560	1570	1580	1590	1600
TTPSPPTTTT	TTPPPTTTPS	PPTTTPITPP	TSTTTLPPTT	TPSPPPTTTT	TPPPTTTPSP	PTTTTPSPPT	ITTTTPPPTT
1610	1620	1630	1640	1650	1660	1670	1680
TPSPPTTTTT	TPPPTTTPSP	PTTTPITPPT	STTTLPPTTT	PSPPPTTTTT	PPPTTTPSPP	TTTTPSPPIT	TTTTPPPTTT
1690	1700	1710	1720	1730	1740	1750	1760
PSSPITTTPS	PPTTTMTTPS	PTTTPSSPIT	TTTTPSSTTT	PSPPPTTMTT	PSPTTTPSPP	TTTMTTLPPT	TTSSPLTTTP
1770	1780	1790	1800	1810	1820	1830	1840
LPPSITPPTF	SPFSTTTPTT	PCVPLCNWTG	WLDSGKPNFH	KPGGDTELIG	DVCGPGWAAN	ISCRATMYPD	VPIGQLGQTV
1850	1860	1870	1880	1890	1900	1910	1920
VCDVSVGLIC	KNEDQKPGGV	IPMAFCLNYE	INVQCCECVT	QPTTMTTTTT	ENPTPPTTTP	ITTTTTVTPT	PTPTGTQTPT
1930	1940	1950	1960	1970	1980	1990	2000
TTPITTTTTV	TPTPTPTGTQ	TPTTTPITTT	TTVTPTPTPT	GTQTPTTTPI	TTTTTVTPTP	TPTGTQTPTT	TPITTTTTVT
2010	2020	2030	2040	2050	2060	2070	2080
PTPTPTGTQT	PTTTPITTTT	TVTPTPTPTG	TQTPTTTPIT	TTTTVTPTPT	PTGTQTPTTT	PITTTTTVTP	TPTPTGTQTP
2090	2100	2110	2120	2130	2140	2150	2160
TTTPITTTTT	VTPTPTPTGT	QTPTTTPITT	TTTVTPTPTP	TGTQTPTTTP	ITTTTTVTPT	PTPTGTQTPT	TTPITTTTTV
2170	2180	2190	2200	2210	2220	2230	2240
TPTPTPTGTQ	TPTTTPITTT	TTVTPTPTPT	GTQTPTTTPI	TTTTTVTPTP	TPTGTQTPTT	TPITTTTTVT	PTPTPTGTQT
2250	2260	2270	2280	2290	2300	2310	2320
PTTTPITTTT	TVTPTPTPTG	TQTPTTTPIT	TTTTVTPTPT	PTGTQTPTTT	PITTTTTVTP	TPTPTGTQTP	TTTPITTTTT
2330	2340	2350	2360	2370	2380	2390	2400

Detailed Protein Report

Cmpd.	No. of Cmpds.	m/z meas.	Δ m/z [ppm]	z	Rt [min]	Score	P	Range	Sequence	Modification	Ratios	
2127	3	937.3845	-87.61	2	56.5	16.1	0	764-780	K.IHMDCSNLTALATSKPR.A	Oxidation: 3	WUP:QUP<br>QU:MU	1.63<br>0.72

Detailed Protein Report

Protein 518: **PREDICTED: von Willebrand factor A domain-containing protein 5B1 isoform X1 [Homo sapiens]**

**Accession:**	gi|578798398	**Score:**	27.6
**Database:**	refseq_human(refseq_human_20140103.fasta)	**MW [kDa]:**	102.9
**Database Date:**	2015-11-30	**pI:**	9.8
		**Sequence Coverage [%]:**	3.1
		**No. of unique Peptides:**	1

10	20	30	40	50	60	70	80
MPHVLIEKGD	MTLGEFDQHL	KGRTDFIKGM	KKKSRAERKT	EIIRKRLHKD	IPHHSVIMLN	FCPDLQSVQP	CLRKAHGEFI
**90**	**100**	**110**	**120**	**130**	**140**	**150**	**160**
FLIDRSSSMS	GISMHRVKDA	MLVALKSLMP	ACLFNIIGFG	STFKSLFPSS	QTYSEDSLAM	ACDDIQRMKA	DMGGTNILSP
**170**	**180**	**190**	**200**	**210**	**220**	**230**	**240**
LKWVIRQPVH	RGHPRLLFVI	TDGAVNNTGK	VLELVRNHAF	STRCYSFGIG	PNVCHRLVKG	LASVSEGSAE	LLMEGERLQP
**250**	**260**	**270**	**280**	**290**	**300**	**310**	**320**
KMVKSLKKAM	APVLSDVTVE	WIFPETTEVL	VSPVSASSLF	PGERLVGYGI	VCDASLHISN	PRSDKRRRYS	MLHSQESGSS
**330**	**340**	**350**	**360**	**370**	**380**	**390**	**400**
VFYHSQDDGP	GLEGGDCAKN	SGAPFILGQA	KNARLASGDS	TTKHDLNLSQ	RRRAYSTNQI	TNHKPLPRAT	MASDPMPAAK
**410**	**420**	**430**	**440**	**450**	**460**	**470**	**480**
RYPLRKARLQ	DLTNQTSLDV	QRWQIDLQPL	LNSGQDLNQG	PKLRGPGARR	PSLLPQGCQP	FLPWGQETQA	WSPVRERTSD
**490**	**500**	**510**	**520**	**530**	**540**	**550**	**560**
SRSPGDLEPS	HHPSAFETET	SSDWDPPAES	QERASPSRPA	TPAPVLGKAL	VKGLHDSQRL	QWEVSFELGT	PGPERGGAQD
**570**	**580**	**590**	**600**	**610**	**620**	**630**	**640**
ADLWSETFHH	LAARAIIRDF	EQLAEREGEI	EQGSNRRYQV	SALHTSKACN	IISKYTAFVP	VDVSKSRYLP	TVVEYPNSGA
**650**	**660**	**670**	**680**	**690**	**700**	**710**	**720**
ALRMLGSRAL	AQQWRGTSSG	FGRPQTMLGE	DSAPGNDMEA	SPTALFSEAR	SPGREKHGAS	EGPQRSLATN	TLSSMKASEN
**730**	**740**	**750**	**760**	**770**	**780**	**790**	**800**
LFGSWLNLNK	SRLLTRAAKG	FLSKPLIKAV	ESTSGNQSFD	YIPLVSLQLA	SGAFLLNEAF	CEATHIPMEK	LKWTSPFTCH
**810**	**820**	**830**	**840**	**850**	**860**	**870**	**880**
RVSLTTRPSE	SKTPSPQLCT	SSPPRHPSCD	SFSLEPLAKG	KLGLEPRAVV	EHTGKLWATV	VGLAWLEHSS	ASYFTEWELV
**890**	**900**	**910**	**920**	**930**	**940**		
AAKANSWLEQ	QEVPEGRTQG	TLKAAARQLF	VLLRHWDENL	EFNMLCYNPN	YV		

Cmpd.	No. of Cmpds.	m/z meas.	Δ m/z [ppm]	z	Rt [min]	Score	P	Range	Sequence	Modification
1946	1	745.7827	-91.37	2	54.7	12.0	0	9-21	K.GDMTLGEFDQHLK.G	

Detailed Protein Report

Protein 519: glyoxalase domain-containing protein 4 [Homo sapiens]

**Accession:**	gi|217330598	**Score:**	27.6
**Database:**	refseq_human(refseq_human_20140103.fasta)	**MW [kDa]:**	33.2
**Database Date:**	2015-11-30	**pI:**	5.3
		**Sequence Coverage [%]:**	8.4
		**No. of unique Peptides:**	1

Quantitation

QU:MU	**Median:**	0.22	**CV:**	0.00 %	**No. of Peptides:**	1
WUP:QUP	**Median:**	1.40	**CV:**	0.00 %	**No. of Peptides:**	1

10	20	30	40	50	60	70	80
MAARRALHFV	FKVGNRFQTA	RFYRDVLGMK	VLRHEEFEEG	CKAACNGPYD	GKWSKTMVGF	GPEDDHFVAE	LTYNYGVGDY
**90**	**100**	**110**	**120**	**130**	**140**	**150**	**160**
KLGNDFMGIT	LASSQAVSNA	RKLEWPLTEV	AEGVFETEAP	GGYKFYLQNR	SLPQSDPVLK	VTLAVSDLQK	SLNYWCNLLG
**170**	**180**	**190**	**200**	**210**	**220**	**230**	**240**
MKIYEKDEEK	QRALLGYADN	QCKLELQGVK	GGVDHAAAFG	RIAFSCPQKE	LPDLEDLMKR	ENQKILTPLV	SLDTPGKATV
**250**	**260**	**270**	**280**	**290**	**300**		
QVVILADPDG	HEICFVGDEA	FRELSKMDPE	GSKLLDDAMA	ADKSDEWFAK	HNKPKASG		

Cmpd.	No. of Cmpds.	m/z meas.	Δ m/z [ppm]	z	Rt [min]	Score	P	Range	Sequence	Modification	Ratios
1097	1	631.1089	141.12	3	42.0	17.0	2	6-21	R.ALHFVFKVGNRFQTAR.F		WUP:QUP 1.40<br>QU:MU 0.22

Detailed Protein Report

Protein 520: **PREDICTED: T-lymphoma invasion and metastasis-inducing protein 1 isoform X4 [Homo sapiens]**

**Accession:**	gi|530419050	**Score:**	27.6
**Database:**	refseq_human(refseq_human_20140103.fasta)	**MW [kDa]:**	161.7
**Database Date:**	2015-11-30	**pI:**	6.4
		**Sequence Coverage [%]:**	2.1
		**No. of unique Peptides:**	1

Quantitation

QU:MU	**Median:**	4.76	**CV:**	0.00 %	**No. of Peptides:**	1
WUP:QUP	**Median:**	0.17	**CV:**	0.00 %	**No. of Peptides:**	1

10	20	30	40	50	60	70	80
MGNAESQHVE	HEFYGEKHAS	LGRKHTSRSL	RLSHKTRRTR	HASSGKVIHR	NSEVSTRSSS	TPSIPQSLAE	NGLEPFSQDG
**90**	**100**	**110**	**120**	**130**	**140**	**150**	**160**
TLEDFGSPIW	VDRVDMGLRP	VSYTDSSVTP	SVDSSIVLTA	ASVQSMPDTE	ESRLYGDDAT	YLAEGGRRQH	SYTSNGPTFM
**170**	**180**	**190**	**200**	**210**	**220**	**230**	**240**
ETASFKKKRS	KSADIWREDS	LEFSLSDLSQ	EHLTSNEEIL	GSAEEKDCEE	ARGMETRASP	RQLSTCQRAN	SLGDLYAQKN
**250**	**260**	**270**	**280**	**290**	**300**	**310**	**320**
SGVTANGGPG	SKFAGYCRNL	VSDIPNLANH	KMPPAAAEET	PPYSNYNTLP	CRKSHCLSEG	ATNPQISHSN	SMQGRRAKTT
**330**	**340**	**350**	**360**	**370**	**380**	**390**	**400**
QDVNAGEGSE	FADSGIEGAT	TDTDLLSRRS	NATNSSYSPT	TGRAFVGSDS	GSSSTGDAAR	QGVYENFRRE	LEMSTTNSES
**410**	**420**	**430**	**440**	**450**	**460**	**470**	**480**
LEEAGSAHSD	EQSSGTLSSP	GQSDILLTAA	QGTVRKAGAL	AVKNFLVHKK	NKKVESATRR	KWKHYWVSLK	GCTLFFYESD
**490**	**500**	**510**	**520**	**530**	**540**	**550**	**560**
GRSGIDHNSI	PKHAVWVENS	IVQAVPEHPK	KDFVFCLSNS	LGDAFLFQTT	SQTELENWIT	AIHSACATAV	ARHHHKEDTL
**570**	**580**	**590**	**600**	**610**	**620**	**630**	**640**
RLLKSEIKKL	EQKIDMDEKM	KKMGEMQLSS	VTDSKKKKTI	LDQIFVWEQN	LEQFQMDLFR	FRCYLASLQG	GELPNPKRLL
**650**	**660**	**670**	**680**	**690**	**700**	**710**	**720**
AFASRPTKVA	MGRLGIFSVS	SFHALVAART	GETGVRRRTQ	AMSRSASKRR	SRFSSLWGLD	TTSKKKQGRP	SINQVFGEGT
**730**	**740**	**750**	**760**	**770**	**780**	**790**	**800**
EAVKKSLEGI	FDDIVPDGKR	EKEVVLPNVH	QHNPDCDIWV	HEYFTPSWFC	LPNNQPALTV	VRPGDTARDT	LELICKTHQL
**810**	**820**	**830**	**840**	**850**	**860**	**870**	**880**
DHSAHYLRLK	FLIENKMQLY	VPQPEEDIYE	LLYKEIEICP	KVTQSIHIEK	SDTAADTYGF	SLSSVEEDGI	RRLYVNSVKE
**890**	**900**	**910**	**920**	**930**	**940**	**950**	**960**
TGLASKKGLK	AGDEILEINN	RAADALNSSM	LKDFLSQPSL	GLLVRTYPEL	EEGVELLESP	PHRVDGPADL	GESPLAFLTS
**970**	**980**	**990**	**1000**	**1010**	**1020**	**1030**	**1040**
NPGHSLCSEQ	GSSAETAPEE	TEGPDLESSD	ETDHSSKSTE	QVAAFCRSLH	EMNPSDQSPS	PQDSTGPQLA	TMRQLSDADK
**1050**	**1060**	**1070**	**1080**	**1090**	**1100**	**1110**	**1120**
LRKVICELLE	TERTYVKDLN	CLMERYLKPL	QKETFLTQDE	LDVLFGNLTE	MVEFQVEFLK	TLEDGVRLVP	DLEKLEKVDQ
**1130**	**1140**	**1150**	**1160**	**1170**	**1180**	**1190**	**1200**
FKKVLFSLGG	SFLYYADRFK	LYSAFCASHT	KVPKVLVKAK	TDTAFKAFLD	AQNPKQQHSS	TLESYLIKPI	QRILKYPLLL
**1210**	**1220**	**1230**	**1240**	**1250**	**1260**	**1270**	**1280**
RELFALTDAE	SEEHYHLDVA	IKTMNKVASH	INEMQKIHEE	FGAVFDQLIA	EQTGEKKEVA	DLSMGDLLLH	TTVIWLNPPA
**1290**	**1300**	**1310**	**1320**	**1330**	**1340**	**1350**	**1360**
SLGKWKKEPE	LAAFVFKTAV	VLVYKDGSKQ	KKKLVGSHRL	SIYEDWDPFR	FRHMIPTEAL	QVRALASADA	EANAVCEIVH
**1370**	**1380**	**1390**	**1400**	**1410**	**1420**	**1430**	**1440**
VKSESEGRPE	RVFHLCCSSP	ESRKDFLKAV	HSILRDKHRR	QLLKTESLPS	SQQYVPFGGK	RLCALKGARP	AMSRAGTVGI
**1450**	**1460**						
RTFKIPVTPT	PQ						

Cmpd.	No. of Cmpds.	m/z meas.	Δ m/z [ppm]	z	Rt [min]	Score	P	Range	Sequence	Modification	Ratios
44	2	750.8174	-45.94	2	30.3	15.2	0	134-147	R.LYGDDATYLAEGGR.R		WUP:QUP 0.17<br>QU:MU 4.76

Detailed Protein Report

Protein 521: ataxin-7-like protein 3 isoform b [Homo sapiens]

**Accession:**	gi|149588534	**Score:**	27.6
**Database:**	refseq_human(refseq_human_20140103.fasta)	**MW [kDa]:**	38.6
**Database Date:**	2015-11-30	**pI:**	6.8
**Modification(s):**	Carbamidomethyl, Oxidation	**Sequence Coverage [%]:**	6.1
		**No. of unique Peptides:**	1

10	20	30	40	50	60	70	80
MKMEEMSLSG	LDNSKLEAIA	QEIYADLVED	SCLGFCFEVH	RAVKCGYFFL	DDTDPDSMKD	FEIVDQPGLD	IFGQVFNQWK
**90**	**100**	**110**	**120**	**130**	**140**	**150**	**160**
SKECVCPNCS	RSIAASRFAP	HLEKCLGMGR	NSSRIANRRI	ANSNNMNKSE	SDQEDNDDIN	DNDWSYGSEK	KAKKRKSDKN
**170**	**180**	**190**	**200**	**210**	**220**	**230**	**240**
PNSPRRSKSL	KHKNGELSNS	DPFKYNNSTG	ISYETLGPEE	LRSLLTTQCG	VISEHTKKMC	TRSLRCPQHT	DEQRRTVRIY
**250**	**260**	**270**	**280**	**290**	**300**	**310**	**320**
FLGPSAVLPE	VESSLDNDSF	DMTDSQALIS	RLQWDGSSDL	SPSDSGSSKT	SENQGWGLGT	NSSESRKTKK	KKSHLSLVGT
**330**	**340**	**350**					
ASGLGSNKKK	KPKPPAPPTP	SIYDDIN					

Cmpd.	No. of Cmpds.	m/z meas.	Δ m/z [ppm]	z	Rt [min]	Score	P	Range	Sequence	Modification
631	1	766.4842	137.27	2	37.4	10.7	1	98-110	R.FAPHLEKCLGMGR.N	Carbamidomethyl: 8; Oxidation: 11

Detailed Protein Report

Protein 522: **PREDICTED: E3 ubiquitin-protein ligase UBR5 isoform X7 [Homo sapiens]**

**Accession:**	gi|530389115	**Score:**	27.6
**Database:**	refseq_human(refseq_human_20140103.fasta)	**MW [kDa]:**	200.9
**Database Date:**	2015-11-30	**pI:**	5.2
**Modification(s):**	Oxidation	**Sequence Coverage [%]:**	1.4
		**No. of unique Peptides:**	1

10	20	30	40	50	60	70	80
MMRRSLRAAG	LGRHEAGASS	SDHQDPVSPP	IAPPSWVPDP	PAMDPDGDID	FILAPAVGSL	TTAATGTGQG	PSTSTIPGPS
90	100	110	120	130	140	150	160
TEPSVVESKD	RKANAHFILK	LLCDSVVLQP	YLRELLSAKD	ARGMTPFMSA	VSGRAYPAAI	TILETAQKIA	KAEISSSEKE
170	180	190	200	210	220	230	240
EDVFMGMVCP	SGTNPDDSPL	YVLCCNDTCS	FTWTGAEHIN	QDIFECRTCG	LLESLCCCTE	CARVCHKGHD	CKLKRTSPTA
250	260	270	280	290	300	310	320
YCDCWEKCKC	KTLIAGQKSA	RLDLLYRLLT	ATNLVTLPNS	RGEHLLLFLV	QTVARQTVEH	CQYRPPRIRE	DRNRKTASPE
330	340	350	360	370	380	390	400
DSDMPDHDLE	PPRFAQLALE	RVLQDWNALK	SMIMFGSQEN	KDPLSASSRI	GHLLPEEQVY	LNQQSGTIRL	DCFTHCLIVK
410	420	430	440	450	460	470	480
CTADILLLDT	LLGTLVKELQ	NKYTPGRREE	AIAVTMRFLR	SVARVFVILS	VEMASSKKKN	NFIPQPIGKC	KRVFQALLPY
490	500	510	520	530	540	550	560
AVEELCNVAE	SLIVPVRMGI	ARPTAPFTLA	STSIDAMQGS	EELFSVEPLP	PRPSSDQSSS	SSQSQSSYII	RNPQQRRISQ
570	580	590	600	610	620	630	640
SQPVRGRDEE	QDDIVSADVE	EVEVVEGVAG	EEDHHDEQEE	HGEENAEAEG	QHDEHDEDGS	DMELDLLAAA	ETESDSESNH
650	660	670	680	690	700	710	720
SNQDNASGRR	SVVTAATAGS	EAGASSVPAF	FSEDDSQSND	SSDSDSSSSQ	SDDIEQETFM	LDEPLERTTN	SSHANGAAQA
730	740	750	760	770	780	790	800
PRSMQWAVRN	TQHQRAASTA	PSSTSTPAAS	SAGLIYIDPS	NLRRSGTIST	SAAAAAAALE	ASNASSYLTS	ASSLARAYSI
810	820	830	840	850	860	870	880
VIRQISDLMG	LIPKYNHLVY	SQIPAAVKLT	YQDAVNLQNY	VEEKLIPTWN	WMVSIMDSTE	AQLRYGSALA	SAGDPGHPNH
890	900	910	920	930	940	950	960
PLHASQNSAR	RERMTAREEA	SLRTLEGRRR	ATLLSARQGM	MSARGDFLNY	ALSLMRSHND	EHSDVLPVLD	VCSLKHVAYV
970	980	990	1000	1010	1020	1030	1040
FQALIYWIKA	MNQQTTLDTP	QLERKRTREL	LELGIDNEDS	EHENDDDTNQ	SATLNDKDDD	SLPAETGQNH	PFFRRSDSMT
1050	1060	1070	1080	1090	1100	1110	1120
FLGCIPPNPF	EVPLAEAIPL	ADQPHLLQPN	ARKEDLFGRP	SQGLYSSSAS	SGKCLMEVTV	DRNCLEVLPT	KMSYAANLKN
1130	1140	1150	1160	1170	1180	1190	1200
VMNMQNRQKK	EGEEQPVLPE	ETESSKPGPS	AHDLAAQLKS	SLLAEIGLTE	SEGPPLTSFR	PQCSFMGMVI	SHDMLLGRWR
1210	1220	1230	1240	1250	1260	1270	1280
LSLELFGRVF	MEDVGAEPGS	ILTELGGFEV	KESKFRREME	KLRNQQSRDL	SLEVKVDRDR	DLLIQQTMRQ	LNNHFGRRCA
1290	1300	1310	1320	1330	1340	1350	1360
TTPMAVHRVK	VTFKDEPGEG	SGVARSFYTA	IAQAFLSNEK	LPNLECIQNA	NKGTHTSLMQ	RLRNRGERDR	ERERERERMRR
1370	1380	1390	1400	1410	1420	1430	1440
SSGLRAGSRR	DRDRDFRRQL	SIDTRPFRPA	SEGNPSDDPE	PLPAHRQALG	ERLYPRVQAM	QPAFASKITG	MLLELSPAQL
1450	1460	1470	1480	1490	1500	1510	1520
LLLLASEDSL	RARVDEAMEL	IIAHGRENGA	DSILDLGLVD	SSEKVQQENR	KRHGSSRSVV	DMDLDDTDDG	DDNAPLFYQP
1530	1540	1550	1560	1570	1580	1590	1600
GKRGFYTPRP	GKNTEARLNC	FRNIGRILGL	CLLQNELCPI	TLNRHVIKVL	LGRKVNWHDF	AFFDPVMYES	LRQLILASQS
1610	1620	1630	1640	1650	1660	1670	1680
SDADAVFSAM	DLAFAIDLCK	EEGGGQVELI	PNGVNIPVTP	QNVYEYVRKY	AEHRMLVVAE	QPLHAMRKGL	LDVLPKNSLE
1690	1700	1710	1720	1730	1740	1750	1760
DLTAEDFRLL	VNGCGEVNVQ	MLISFTSFND	ESGENAEKLL	QFKRWFWSIV	EKMSMTERQD	LVYFWTSSPS	LPASEEGFQP
1770	1780	1790	1800	1810	1820		
MPSITIRPPD	DQHLPTANTC	ISRLYVPLYS	SKQILKQKLL	LAIKTKNFGF	V		

Cmpd.	No. of	m/z meas.	Δ m/z	z	Rt	Score	P	Range	Sequence	Modification

Detailed Protein Report

	Cmpds.		[ppm]		[min]					
2795	1	958.9896	49.22	2	65.4	17.4	1	1112-1127	K.MSYAANLKNVMNMQNR.Q	Oxidation: 11, 13

Detailed Protein Report

Protein 523: **EGF domain-specific O-linked N-acetylglucosamine transferase isoform a precursor [Homo sapiens]**

**Accession:**	gi|522838254	**Score:**	27.6
**Database:**	refseq_human(refseq_human_20140103.fasta)	**MW [kDa]:**	62.0
**Database Date:**	2015-11-30	**pI:**	6.6
**Modification(s):**	Carbamidomethyl	**Sequence Coverage [%]:**	4.7
		**No. of unique Peptides:**	1

Alias proteins:

Accession	Name	Description
gi|578805996	refseq_human (refseq_human_20140103.fasta)	PREDICTED: EGF domain-specific O-linked N-acetylglucosamine transferase isoform X3 [Homo sapiens]
gi|530373685	refseq_human (refseq_human_20140103.fasta)	PREDICTED: EGF domain-specific O-linked N-acetylglucosamine transferase isoform X2 [Homo sapiens]
gi|530373683	refseq_human (refseq_human_20140103.fasta)	PREDICTED: EGF domain-specific O-linked N-acetylglucosamine transferase isoform X1 [Homo sapiens]

10	20	30	40	50	60	70	80
MLMLFVFGVL	LHEVSLSGQN	EAPPNTHSIP	GEPLYNYASI	RLPEEHIPFF	LHNNRHIATV	CRKDSLCPYK	KHLEKLKYCW
**90**	**100**	**110**	**120**	**130**	**140**	**150**	**160**
GYEKSCKPEF	RFGYPVCSYV	DMGWTDTLES	AEDIFWKQAD	FGYARERLEE	MHVLCQPKET	SDSSLVCSRY	LQYCRATNLY
**170**	**180**	**190**	**200**	**210**	**220**	**230**	**240**
LDLRNIKRNH	DRFKEDFFQS	GEIGGHCKLD	IRTLTSEGQR	KSPLQSWFAE	LQSYTQLNFR	PIEDAKCDIV	IEKPTYFMKL
**250**	**260**	**270**	**280**	**290**	**300**	**310**	**320**
DAGVNMYHHF	CDFINLYITQ	HVNNSFSTDV	YIVMWDTSSY	GYGDLFSDTW	NAFTDYDVIH	LKTYDSKRVC	FKEAVFSLLP
**330**	**340**	**350**	**360**	**370**	**380**	**390**	**400**
RMRYGLFYNT	PLISGCQNTG	LFRAFAQHVL	HRLNITQEGP	KDGKIRVTIL	ARSTEYRKIL	NQNELVNALK	TVSTFEVQIV
**410**	**420**	**430**	**440**	**450**	**460**	**470**	**480**
DYKYRELGFL	DQLRITHNTD	IFIGMHGAGL	THLLFLPDWA	AVFELYNCED	ERCYLDLARL	RGVHYITWRR	QNKVFPQDKG
**490**	**500**	**510**	**520**	**530**			
HHPTLGEHPK	FTNYSFDVEE	FMYLVLQAAD	HVLQHPKWPF	KKKHDEL			

Cmpd.	No. of Cmpds.	m/z meas.	Δ m/z [ppm]	z	Rt [min]	Score	P	Range	Sequence	Modification
674	1	698.6888	27.74	3	38.4	13.0	2	76-91	K.LKYCWGYEKSCKPEFR.F	Carbamidomethyl: 11

Detailed Protein Report

Protein 524: cytoskeleton-associated protein 2 isoform 3 [Homo sapiens]

**Accession:**	gi|557878731	**Score:**	27.6
**Database:**	refseq_human(refseq_human_20140103.fasta)	**MW [kDa]:**	71.2
**Database Date:**	2015-11-30	**pI:**	10.0
		**Sequence Coverage [%]:**	3.2
		**No. of unique Peptides:**	1

10	20	30	40	50	60	70	80
MLSSRDQRVV	TSEDQVQEGT	KVLKLKTKMA	DKENMKRPAE	SKNNTVVGKH	CIPLKPSNEL	TNSTVVIDTH	KPKDSNQTPH
**90**	**100**	**110**	**120**	**130**	**140**	**150**	**160**
LLLTEDDPQS	QHMTLSQAFH	LKNNSKKKQM	TTEKQKQDAN	MPKKPVLGSY	RGQIVQSKIN	SFRKPLQVKD	ESSAATKKLS
**170**	**180**	**190**	**200**	**210**	**220**	**230**	**240**
ATIPKATKPQ	PVNTSSVTVK	SNRSSNMTAT	TKFVSTTSQN	TQLVRPPIRS	HHSNTRDTVK	QGISRTSANV	TIRKGPHEKE
**250**	**260**	**270**	**280**	**290**	**300**	**310**	**320**
LLQSKTALSS	VKTSSSQGII	RNKTLSRSIA	SEVIARPASL	SNDKLMEKSE	PVDQRRHTAG	KAIVDSRSAQ	PKETSEERKA
**330**	**340**	**350**	**360**	**370**	**380**	**390**	**400**
RLSEWKAGKG	RVLKRPPNSV	VTQHEPAGQN	EKPVGSFWTT	MAEEDEQRLF	TEKVNNTFSE	CLNLINEGCP	KEDILVTLND
**410**	**420**	**430**	**440**	**450**	**460**	**470**	**480**
LIKNIPDAKK	LVKYWICLAL	IEPITSPIEN	IIAIYEKAIL	AGAQPIEEMR	HTIVDILTMK	SQEKANLGEN	MEKSCASKEE
**490**	**500**	**510**	**520**	**530**	**540**	**550**	**560**
VKEVSIEDTG	VDVDPEKLEM	ESKLHRNLLF	QDCEKEQDNK	TKDPTHDVKT	PNTETRTSCL	IKYNVSTTPY	LQSVKKKVQF
**570**	**580**	**590**	**600**	**610**	**620**	**630**	**640**
DGTNSAFKEL	KFLTPVRRSR	RLQEKTSKLP	DMLKDHYPCV	SSLEQLTELG	RETDAFVCRP	NAALCRVYYE	ADTT

Cmpd.	No. of Cmpds.	m/z meas.	Δ m/z [ppm]	z	Rt [min]	Score	P	Range	Sequence	Modification
2617	1	732.5346	128.71	1	63.5	10.7	0	572-577	K.FLTPVR.R	

Detailed Protein Report

Protein 525: long-chain-fatty-acid--CoA ligase 1 isoform d [Homo sapiens]

**Accession:**	gi|557878742	**Score:**	27.6
**Database:**	refseq_human(refseq_human_20140103.fasta)	**MW [kDa]:**	58.5
**Database Date:**	2015-11-30	**pI:**	8.6
		**Sequence Coverage [%]:**	5.1
		**No. of unique Peptides:**	2

10	20	30	40	50	60	70	80
MVIVPLYDTL	GNEAITYIVN	KAELSLVFVD	KPEKAKLLLE	GVENKLIPGL	KIIVVMDAYG	SELVERGQRC	GVEVTSMKAM
90	100	110	120	130	140	150	160
EDLGRANRRK	PKPPAPEDLA	VICFTSGTTG	NPKGAMVTHR	NIVSDCSAFV	KATENTVNPC	PDDTLISFLP	LAHMFERVVE
170	180	190	200	210	220	230	240
CVMLCHGAKI	GFFQGDIRLL	MDDLKVLQPT	VFPVVPRLLN	RMFDRIFGQA	NTTLKRWLLD	FASKRKEAEL	RSGIIRNNSL
250	260	270	280	290	300	310	320
WDRLIFHKVQ	SSLGGRVRLM	VTGAAPVSAT	VLTFLRAALG	CQFYEGYGQT	ECTAGCCLTM	PGDWTAGHVG	APMPCNLIKL
330	340	350	360	370	380	390	400
VDVEEMNYMA	AEGEGEVCVK	GPNVFQGYLK	DPAKTAEALD	KDGWLHTGDI	GKWLPNGTLK	IIDRKKHIFK	LAQGEYIAPE
410	420	430	440	450	460	470	480
KIENIYMRSE	PVAQVFVHGE	SLQAFLIAIV	VPDVETLCSW	AQKRGFEGSF	EELCRNKDVK	KAILEDMVRL	GKDSGLKPFE
490	500	510	520	530			
QVKGITLHPE	LFSIDNGLLT	PTMKAKRPEL	RNYFRSQIDD	LYSTIKV			

Cmpd.	No. of Cmpds.	m/z meas.	Δ m/z [ppm]	z	Rt [min]	Score	P	Range	Sequence	Modification
2760	1	848.9242	-91.80	2	64.9	14.2	2	244-258	R.LIFHKVQSSLGGRVR.L	
2516	1	715.3738	61.15	2	62.0	13.4	1	444-455	K.RGFEGSFEELCR.N	

Detailed Protein Report

Protein 526: PREDICTED: nucleoprotein TPR isoform X1 [Homo sapiens]

**Accession:**	gi|530365345	**Score:**	27.5
**Database:**	refseq_human(refseq_human_20140103.fasta)	**MW [kDa]:**	274.8
**Database Date:**	2015-11-30	**pI:**	4.9
		**Sequence Coverage [%]:**	0.8
		**No. of unique Peptides:**	1

Quantitation

QU:MU	**Median:** 0.29	**CV:** 0.00 %	**No. of Peptides:** 1

Detailed Protein Report

10	20	30	40	50	60	70	80
MPSSGSVSRS	RQRGRSMASR	GFDGSRRGSL	RRAGAREPPS	EVIEAAAQAS	GSCWSSPFFS	ASTPSAAATG	VPGPTDMAAV
90	100	110	120	130	140	150	160
LQQVLERTEL	NKLPKSVQNK	LEKFLADQQS	EIDGLKGRHE	KFKVESEQQY	FEIEKRLSHS	QERLVNETRE	CQSLRLELEK
170	180	190	200	210	220	230	240
LNNQLKALTE	KNKELEIAQD	RNIAIQSQFT	RTKEELEAEK	RDLIRTNERL	SQELEYLTED	VKRLNEKLKE	SNTTKGELQL
250	260	270	280	290	300	310	320
KLDELQASDV	SVKYREKRLE	QEKELLHSQN	TWLNTELKTK	TDELLALGRE	KGNEILELKC	NLENKKEEVS	RLEEQMNGLK
330	340	350	360	370	380	390	400
TSNEHLQKHV	EDLLTKLKEA	KEQQASMEEK	FHNELNAHIK	LSNLYKSAAD	DSEAKSNELT	RAVEELHKLL	KEAGEANKAI
410	420	430	440	450	460	470	480
QDHLLEVEQS	KDQMEKEMLE	KIGRLEKELE	NANDLLSATK	RKGAILSEEE	LAAMSPTAAA	VAKIVKPGMK	LTELYNAYVE
490	500	510	520	530	540	550	560
TQDQLLLEKL	ENKRINKYLD	EIVKEVEAKA	PILKRQREEY	ERAQKAVASL	SVKLEQAMKE	IQRLQEDTDK	ANKQSSVLER
570	580	590	600	610	620	630	640
DNRRMEIQVK	DLSQQIRVLL	MELEEARGNH	VIRDEEVSSA	DISSSSEVIS	QHLVSYRNIE	ELQQQNQRLL	VALRELGETR
650	660	670	680	690	700	710	720
EREEQETTSS	KITELQLKLE	SALTELEQLR	KSRQHQMQLV	DSIVRQRDMY	RILLSQTTGV	AIPLHASSLD	DVSLASTPKR
730	740	750	760	770	780	790	800
PSTSQTVSTP	APVPVIESTE	AIEAKAALKQ	LQEIFENYKK	EKAENEKIQN	EQLEKLQEQV	TDLRSQNTKI	STQLDFASKR
810	820	830	840	850	860	870	880
YEMLQDNVEG	YRREITSLHE	RNQKLTATTQ	KQEQIINTMT	QDLRGANEKL	AVAEVRAENL	KKEKEMLKLS	EVRLSQQRES
890	900	910	920	930	940	950	960
LLAEQRGQNL	LLTNLQTIQG	ILERSETETK	QRLSSQIEKL	EHEISHLKKK	LENEVEQRHT	LTRNLDVQLL	DTKRQLDTET
970	980	990	1000	1010	1020	1030	1040
NLHLNTKELL	KNAQKEIATL	KQHLSNMEVQ	VASQSSQRTG	KGQPSNKEDV	DDLVSQLRQT	EEQVNDLKER	LKTSTSNVEQ
1050	1060	1070	1080	1090	1100	1110	1120
YQAMVTSLEE	SLNKEKQVTE	EVRKNIEVRL	KESAEFQTQL	EKKLMEVEKE	KQELQDDKRR	AIESMEQQLS	ELKKTLSSVQ
1130	1140	1150	1160	1170	1180	1190	1200
NEVQEALQRA	STALSNEQQA	RRDCQEQAKI	AVEAQNKYER	ELMLHAADVE	ALQAAKEQVS	KMASVRQHLE	ETTQKAESQL
1210	1220	1230	1240	1250	1260	1270	1280
LECKASWEER	ERMLKDEVSK	CVCRCEDLEK	QNRLLHDQIE	KLSDKVVASV	KEGVQGPLNV	SLSEEGKSQE	QILEILRFIR
1290	1300	1310	1320	1330	1340	1350	1360
REKEIAETRF	EVAQVESLRY	RQRVELLERE	LQELQDSLNA	EREKVQVTAK	TMAQHEELMK	KTETMNVVME	TNKMLREEKE
1370	1380	1390	1400	1410	1420	1430	1440
RLEQDLQQMQ	AKVRKLELDI	LPLQEANAEL	SEKSGMLQAE	KKLLEEDVKR	WKARNQHLVS	QQKDPDTEEY	RKLLSEKEVH
1450	1460	1470	1480	1490	1500	1510	1520
TKRIQQLTEE	IGRLKAEIAR	SNASLTNNQN	LIQSLKEDLN	KVRTEKETIQ	KDLDAKIIDI	QEKVKTITQV	KKIGRRYKTQ
1530	1540	1550	1560	1570	1580	1590	1600
YEELKAQQDK	VMETSAQSSG	DHQEQHVSVQ	EMQELKETLN	QAETKSKSLE	SQVENLQKTL	SEKETEARNL	QEQTVQLQSE
1610	1620	1630	1640	1650	1660	1670	1680
LSRLRQDLQD	RTTQEEQLRQ	QITEKEEKTR	KAIVAAKSKI	AHLAGVKDQL	TKENEELKQR	NGALDQQKDE	LDVRITALKS
1690	1700	1710	1720	1730	1740	1750	1760
QYEGRISRLE	RELREHQERH	LEQRDEPQEP	SNKVPEQQRQ	ITLKTTPASG	ERGIASTSDP	PTANIKPTPV	VSTPSKVTAA
1770	1780	1790	1800	1810	1820	1830	1840
AMAGNKSTPR	ASIRPMVTPA	TVTNPTTTPT	ATVMPTTQVE	SQEAMQSEGP	VEHVPVFGST	SGSVRSTSPN	VQPSISQPIL
1850	1860	1870	1880	1890	1900	1910	1920
TVQQQTQATA	FVQPTQQSHP	QIEPANQELS	SNIVEVVQSS	PVERPSTSTA	VFGTVSATPS	SSLPKRTREE	EEDSTIEASD
1930	1940	1950	1960	1970	1980	1990	2000
QVSDDTVEMP	LPKKLKSVTP	VGTEEEVMAE	ESTDGEVETQ	VYNQDSQDSI	GEGVTQGDYT	PMEDSEETSQ	SLQIDLGPLQ
2010	2020	2030	2040	2050	2060	2070	2080
SDQQTTTSSQ	DGQGKGDDVI	VIDSDDEEED	DDENDGEHED	YEEDEEDDDD	DEDDTGMGDE	GEDSNEGTGS	ADGNDGYEAD
2090	2100	2110	2120	2130	2140	2150	2160
DAEGGDGTDP	GTETEESMGG	GEGNHRAADS	QNSGEGNTGA	AESSFSQEVS	REQQPSSASE	RQAPRAPQSP	RRPPHPLPPR
2170	2180	2190	2200	2210	2220	2230	2240
LTIHAPPQEL	GPPVQRIQMT	RRQSVGRGLQ	LTPGIGGMQQ	HFFDDEDRTV	PSTPTLVVPH	RTDGFAEAIH	SPQVAGVPRF
2250	2260	2270	2280	2290	2300	2310	2320
RFGPPEDMPQ	TSSSHSDLGQ	LASQGGLGMY	ETPLFLAHEE	ESGGRSVPTT	PLQVAAPVTV	FTESTTSDAS	EHASQSVPMV
2330	2340	2350	2360	2370	2380	2390	2400

Detailed Protein Report

Cmpd.	No. of Cmpds.	m/z meas.	Δ m/z [ppm]	z	Rt [min]	Score	P	Range	Sequence	Modification	Ratios
1647	1	524.8144	75.03	2	50.8	12.8	2	12-20	R.QRGRSMASR.G		QU:MU 0.29

Detailed Protein Report

Protein 527: **PREDICTED: pre-mRNA-splicing factor ATP-dependent RNA helicase PRP16 isoform X2 [Homo sapiens]**

**Accession:**	gi|530424571	**Score:**	27.5
**Database:**	refseq_human(refseq_human_20140103.fasta)	**MW [kDa]:**	136.2
**Database Date:**	2015-11-30	**pI:**	5.9
**Modification(s):**	Carbamidomethyl	**Sequence Coverage [%]:**	3.1
		**No. of unique Peptides:**	2

Quantitation

QU:MU	**Median:** 2.31	**CV:** 0.00 %	**No. of Peptides:**	1
WUP:QUP	**Median:** 0.51	**CV:** 37.25 %	**No. of Peptides:**	2

10	20	30	40	50	60	70	80
MGDTSEDASI	HRLEGTDLDC	QVGGLICKSK	SAASEQHVFK	APAPRPSLLG	LDLLASLKRR	EREEKDDGED	KKKSKVSSYK
90	100	110	120	130	140	150	160
DWEESKDDQK	DAEEEGGDQA	GQNIRKDRHY	RSARVETPSH	PGGVSEEFWE	RSRQRERERR	EHGVYASSKE	EKDWKKEKSR
170	180	190	200	210	220	230	240
DRDYDRKRDR	DAATPSRSTW	EEEDSGYGSS	RRSQWESPSP	TPSYRDSERS	HRLSTRDRDR	SVRGKYSDDT	PLPTPSYKYN
250	260	270	280	290	300	310	320
EWADDRRHLG	STPRLSRGRG	RREEGEEGIS	FDTEEERQQW	EDDQRQADRD	WYMMDEGYDE	FHNPLAYSSE	DYVRRREQHL
330	340	350	360	370	380	390	400
HKQKQKRISA	QRRQINEDNE	RWETNRMLTS	GVVHRLEVDE	DFEEDNAAKV	HLMVHNLVPP	FLDGRIVFTK	QPEPVIPVKD
410	420	430	440	450	460	470	480
ATSDLAIIAR	KGSQTVRKHR	EQKERKKAQH	KHWELAGTKL	GDIMGVKKEE	EPDKAVTEDG	KVDYRTEQKF	ADHMKRKSEA
490	500	510	520	530	540	550	560
SSEFAKKKSI	LEQRQYLPIF	AVQQELLTII	RDNSIVIVVG	ETGSGKTTQL	TQYLHEDGYT	DYGMIGCTQP	RRVAAMSVAK
570	580	590	600	610	620	630	640
RVSEEMGGNL	GEEVGYAIRF	EDCTSENTLI	KYMTDGILLR	ESLREADLDH	YSAIIMDEAH	ERSLNTDVLF	GLLREVVARR
650	660	670	680	690	700	710	720
SDLKLIVTSA	TMDAEKFAAF	FGNVPIFHIP	GRTFPVDILF	SKTPQEDYVE	AAVKQSLQVH	LSGAPGDILI	FMPGQEDIEV
730	740	750	760	770	780	790	800
TSDQIVEHLE	ELENAPALAV	LPIYSQLPSD	LQAKIFQKAP	DGVRKCIVAT	NIAETSLTVD	GIMFVIDSGY	CKLKVFNPRI
810	820	830	840	850	860	870	880
GMDALQIYPI	SQANANQRSG	RAGRTGPGQC	FRLYTQSAYK	NELLTTTVPE	IQRTNLANVV	LLLKSLGVQD	LLQFHFMDPP
890	900	910	920	930	940	950	960
PEDNMLNSMY	QLWILGALDN	TGGLTSTGRL	MVEFPLDPAL	SKMLIVSCDM	GCSSEILLIV	SMLSVPAIFY	RPKGREEESD
970	980	990	1000	1010	1020	1030	1040
QIREKFAVPE	SDHLTYLNVY	LQWKNNNYST	IWCNDHFIHA	KAMRKVREVR	AQLKDIMVQQ	RMSLASCGTD	WDIVRKCICA
1050	1060	1070	1080	1090	1100	1110	1120
AYFHQAAKLK	GIGEYVNIRT	GMPCHLHPTS	SLFGMGYTPD	YIVYHELVMT	TKEYMQCVTA	VDGEWLAELG	PMFYSVKQAG
1130	1140	1150	1160	1170	1180	1190	1200
KSRQENRRRA	KEEASAMEEE	MALAEEQLRA	RRQEQEKRSP	LGSVRSTKIY	TPGRKEQGEP	MTPRRTPARF	GL

Cmpd.	No. of Cmpds.	m/z meas.	Δ m/z [ppm]	z	Rt [min]	Score	P	Range	Sequence	Modification	Ratios	
145	1	896.4035	-68.20	3	31.5	16.8	2	800-824	R. IGMDALQIYPISQANANQRSGRAG T		QU:MU<br>WUP:QUP	2.31<br>0.73
1651	1	720.3147	-23.44	2	50.9	10.7	0	1037-1048	K.CICAAYFHQAAK.L	Carbamidomethyl: 1, 3	WUP:QUP	0.35

Detailed Protein Report

Protein 528: **retinoblastoma-associated protein [Homo sapiens]**

**Accession:**	gi|108773787	**Score:**	27.5
**Database:**	refseq_human(refseq_human_20140103.fasta)	**MW [kDa]:**	106.1
**Database Date:**	2015-11-30	**pI:**	8.9
**Modification(s):**	Carbamidomethyl, Oxidation	**Sequence Coverage [%]:**	2.0
		**No. of unique Peptides:**	1

10	20	30	40	50	60	70	80
MPPKTPRKTA	ATAAAAAAEP	PAPPPPPPPE	EDPEQDSGPE	DLPLVRLEFE	ETEEPDFTAL	CQKLKIPDHV	RERAWLTWEK
**90**	**100**	**110**	**120**	**130**	**140**	**150**	**160**
VSSVDGVLGG	YIQKKKELWG	ICIFIAAVDL	DEMSFTFTEL	QKNIEISVHK	FFNLLKEIDT	STKVDNAMSR	LLKKYDVLFA
**170**	**180**	**190**	**200**	**210**	**220**	**230**	**240**
LFSKLERTCE	LIYLTQPSSS	ISTEINSALV	LKVSWITFLL	AKGEVLQMED	DLVISFQLML	CVLDYFIKLS	PPMLLKEPYK
**250**	**260**	**270**	**280**	**290**	**300**	**310**	**320**
TAVIPINGSP	RTPRRGQNRS	ARIAKQLEND	TRIIEVLCKE	HECNIDEVKN	VYFKNFIPFM	NSLGLVTSNG	LPEVENLSKR
**330**	**340**	**350**	**360**	**370**	**380**	**390**	**400**
YEEIYLKNKD	LDARLFLDHD	KTLQTDSIDS	FETQRTPRKS	NLDEEVNVIP	PHTPVRTVMN	TIQQLMMILN	SASDQPSENL
**410**	**420**	**430**	**440**	**450**	**460**	**470**	**480**
ISYFNNCTVN	PKESILKRVK	DIGYIFKEKF	AKAVGQGCVE	IGSQRYKLGV	RLYYRVMESM	LKSEEERLSI	QNFSKLLNDN
**490**	**500**	**510**	**520**	**530**	**540**	**550**	**560**
IFHMSLLACA	LEVVMATYSR	STSQNLDSGT	DLSFPWILNV	LNLKAFDFYK	VIESFIKAEG	NLTREMIKHL	ERCEHRIMES
**570**	**580**	**590**	**600**	**610**	**620**	**630**	**640**
LAWLSDSPLF	DLIKQSKDRE	GPTDHLESAC	PLNLPLQNNH	TAADMYLSPV	RSPKKKGSTT	RVNSTANAET	QATSAFQTQK
**650**	**660**	**670**	**680**	**690**	**700**	**710**	**720**
PLKSTSLSLF	YKKVYRLAYL	RLNTLCERLL	SEHPELEHII	WTLFQHTLQN	EYELMRDRHL	DQIMMCSMYG	ICKVKNIDLK
**730**	**740**	**750**	**760**	**770**	**780**	**790**	**800**
FKIIVTAYKD	LPHAVQETFK	RVLIKEEEYD	SIIVFYNSVF	MQRLKTNILQ	YASTRPPTLS	PIPHIPRSPY	KFPSSPLRIP
**810**	**820**	**830**	**840**	**850**	**860**	**870**	**880**
GGNIYISPLK	SPYKISEGLP	TPTKMTPRSR	ILVSIGESFG	TSEKFQKINQ	MVCNSDRVLK	RSAEGSNPPK	PLKKLRFDIE
**890**	**900**	**910**	**920**	**930**			
GSDEADGSKH	LPGESKFQQK	LAEMTSTRTR	MQKQKMNDSM	DTSNKEEK			

Cmpd.	No. of Cmpds.	m/z meas.	Δ m/z [ppm]	z	Rt [min]	Score	P	Range	Sequence	Modification
2018	1	1044.9422	-30.44	2	55.6	16.6	1	699-715	R.HLDQIMMCSMYGICKVK.N	Carbamidomethyl: 14; Oxidation: 6, 10

Detailed Protein Report

Protein 529: metastasis-associated in colon cancer protein 1 [Homo sapiens]

**Accession:**	gi|157502191	**Score:**	27.5
**Database:**	refseq_human(refseq_human_20140103.fasta)	**MW [kDa]:**	96.6
**Database Date:**	2015-11-30	**pI:**	6.5
		**Sequence Coverage [%]:**	3.2
		**No. of unique Peptides:**	1

Quantitation

WUP:QUP **Median:** 0.50 **CV:** 0.00 % **No. of Peptides:** 1

10	20	30	40	50	60	70	80
MLITERKHFR	SGRIAQSMSE	ANLIDMEAGK	LSKSCNITEC	QDPDLLHNWP	DAFTLRGNNA	SKVANPFWNQ	LSASNPFLDD
90	100	110	120	130	140	150	160
ITQLRNNRKR	NNISILKEDP	FLFCREIENG	NSFDSSGDEL	DVHQLLRQTS	SRNSGRSKSV	SELLDILDDT	AHAHQSIHNS
170	180	190	200	210	220	230	240
DQILLHDLEW	LKNDREAYKM	AWLSQRQLAR	SCLDLNTISQ	SPGWAQTQLA	EVTIACKVNH	QGGSVQLPES	DITVHVPQGH
250	260	270	280	290	300	310	320
VAVGEFQEVS	LRAFLDPPHM	LNHDLSCTVS	PLLEIMLGNL	NTMEALLLEM	KIGAEVRKDP	FSQVMTEMVC	LHSLGKEGPF
330	340	350	360	370	380	390	400
KVLSNCYIYK	DTIQVKLIDL	SQVMYLVVAA	QAKALPSPAA	TIWDYIHKTT	SIGIYGPKYI	HPSFTVVLTV	CGHNYMPGQL
410	420	430	440	450	460	470	480
TISDIKKGGK	NISPVVFQLW	GKQSFLLDKP	QDLSISIFSC	DPDFEVKTEG	ERKEIKQKQL	EAGEVVHQQF	LFSLVEHREM
490	500	510	520	530	540	550	560
HLFDFCVQVE	PPNGEPVAQF	SITTPDPTPN	LKRLSNLPGY	LQKKEEIKSA	PLSPKILVKY	PTFQDKTLNF	SNYGVTLKAV
570	580	590	600	610	620	630	640
LRQSKIDYFL	EYFKGDTIAL	LGEGKVKAIG	QSKVKEWYVG	VLRGKIGLVH	CKNVKVISKE	QVMFMSDSVF	TTRNLLEQIV
650	660	670	680	690	700	710	720
LPLKKLTYIY	SVVLTLVSEK	VYDWKVLADV	LGYSHLSLED	FDQIQADKES	EKVSYVIKKL	KEDCHTERNT	RKFLYELIVA
730	740	750	760	770	780	790	800
LLKMDCQELV	ARLIQEAAVL	TSAVKLGKGW	RELAEKLVRL	TKQQMEAYEI	PHRGNTGDVA	VEMMWKPAYD	FLYTWSAHYG
810	820	830	840	850	860		
NNYRDVLQDL	QSALDRMKNP	VTKHWRELTG	VLILVNSLEV	LRVTAFSTSE	EV		

Cmpd.	No. of Cmpds.	m/z meas.	Δ m/z [ppm]	z	Rt [min]	Score	P	Range	Sequence	Modification	Ratios
2511	1	841.8162	-157.28	2	61.3	16.7	0	354-368	K.ALPSPAATIWDYIHK.T		WUP:QUP 0.50

Detailed Protein Report

Protein 530: ephrin type-A receptor 3 isoform b precursor [Homo sapiens]

**Accession:**	gi|32967314	**Score:**	27.5
**Database:**	refseq_human(refseq_human_20140103.fasta)	**MW [kDa]:**	60.9
**Database Date:**	2015-11-30	**pI:**	5.5
**Modification(s):**	Carbamidomethyl	**Sequence Coverage [%]:**	3.0
		**No. of unique Peptides:**	1

10	20	30	40	50	60	70	80
MDCQLSILLL	LSCSVLDSFG	ELIPQPSNEV	NLLDSKTIQG	ELGWISYPSH	GWEEISGVDE	HYTPIRTYQV	CNVMDHSQNN
**90**	**100**	**110**	**120**	**130**	**140**	**150**	**160**
WLRTNWVPRN	SAQKIYVELK	FTLRDCNSIP	LVLGTCKETF	NLYYMESDDD	HGVKFREHQF	TKIDTIAADE	SFTQMDLGDR
**170**	**180**	**190**	**200**	**210**	**220**	**230**	**240**
ILKLNTEIRE	VGPVNKKGFY	LAFQDVGACV	ALVSVRVYFK	KCPFTVKNLA	MFPDTVPMDS	QSLVEVRGSC	VNNSKEEDPP
**250**	**260**	**270**	**280**	**290**	**300**	**310**	**320**
RMYCSTEGEW	LVPIGKCSCN	AGYEERGFMC	QACRPGFYKA	LDGNMKCAKC	PPHSSTQEDG	SMNCRCENNY	FRADKDPPSM
**330**	**340**	**350**	**360**	**370**	**380**	**390**	**400**
ACTRPPSSPR	NVISNINETS	VILDWSWPLD	TGGRKDVTFN	IICKKCGWNI	KQCEPCSPNV	RFLPRQFGLT	NTTVTVTDLL
**410**	**420**	**430**	**440**	**450**	**460**	**470**	**480**
AHTNYTFEID	AVNGVSELSS	PPRQFAAVSI	TTNQAAPSPV	LTIKKDRTSR	NSISLSWQEP	EHPNGIILDY	EVKYYEKQEQ
**490**	**500**	**510**	**520**	**530**	**540**		
ETSYTILRAR	GTNVTISSLK	PDTIYVFQIR	ARTAAGYGTN	SRKFEFETSP	DCMYYFNAV		

Cmpd.	No. of Cmpds.	m/z meas.	Δ m/z [ppm]	z	Rt [min]	Score	P	Range	Sequence	Modification
2832	2	945.9883	67.92	2	64.0	16.1	1	366-381	K.CGWNIKQCEPCSPNVR.F	Carbamidomethyl: 8

Detailed Protein Report

Protein 531: **PREDICTED: delta and Notch-like epidermal growth factor-related receptor isoform X1 [Homo sapiens]**

**Accession:**	gi|530371157	**Score:**	27.5
**Database:**	refseq_human(refseq_human_20140103.fasta)	**MW [kDa]:**	73.8
**Database Date:**	2015-11-30	**pI:**	5.0
**Modification(s):**	Carbamidomethyl	**Sequence Coverage [%]:**	6.9
		**No. of unique Peptides:**	2

10	20	30	40	50	60	70	80
MQPRRAQAPG	AQLLPALALL	LLLLGAGPRG	SSLANPVPAA	PLSAPGPCAA	QPCRNGGVCT	SRPEPDPQHP	APAGEPGYSC
**90**	**100**	**110**	**120**	**130**	**140**	**150**	**160**
TCPAGISGAN	CQLVADPCAS	NPCHHGNCSS	SSSSSSDGYL	CICNEGYEGP	NCEQALPSLP	ATGWTESMAP	RQLQPVPATQ
**170**	**180**	**190**	**200**	**210**	**220**	**230**	**240**
EPDKILPRSQ	ATVTLPTWQP	KTGQKVVEMK	WDQVEVIPDI	ACGNASSNSS	AGGRLVSFEV	PQNTSVKIRQ	DATASLILLW
**250**	**260**	**270**	**280**	**290**	**300**	**310**	**320**
KVTATGFQQC	SLIDGRSVTP	LQASGGLVLL	EEMLALGNNH	FIGFVNDSVT	KSIVALRLTL	VVKVSTCVPG	ESHANDLECS
**330**	**340**	**350**	**360**	**370**	**380**	**390**	**400**
GKGKCTTKPS	EATFSCTCEE	QYVGTFCEEY	DACQRKPCQN	NASCIDANEK	QDGSNFTCVC	LPGYTGELCQ	SKIDYCILDP
**410**	**420**	**430**	**440**	**450**	**460**	**470**	**480**
CRNGATCISS	LSGFTCQCPE	GYFGSACEEK	VDPCASSPCQ	NNGTCYVDGV	HFTCNCSPGF	TGPTCAQLID	FCALSPCAHG
**490**	**500**	**510**	**520**	**530**	**540**	**550**	**560**
TCRSVGTSYK	CLCDPGTHCE	LYKDPCANVS	CLNGATCDSD	GLNGTCICAP	GFTGEECDID	INECDSNPCH	HGGSCLDQPN
**570**	**580**	**590**	**600**	**610**	**620**	**630**	**640**
GYNCHCPHGW	VGANCEIHLQ	WKSGHMAESL	TNMPRHSLYI	IIGALCVAFI	LMLIILIVGI	CRISRIEYQG	SSRPAYEEFY
**650**	**660**	**670**	**680**	**690**	**700**		
NCRSIDSEFS	NAIASIRHAR	FGKKSRPAMY	DVSPIAYEDY	SPDDKPLVTL	IKTKDL		

Cmpd.	No. of Cmpds.	m/z meas.	Δ m/z [ppm]	z	Rt [min]	Score	P	Range	Sequence	Modification
2308	1	1002.6780	75.60	3	59.3	12.4	2	1-29	-.MQPRRAQAPGAQLLPALALLLLLLGAGPR.G	
1231	1	1023.8326	-111.10	2	45.5	15.1	0	304-322	K.VSTCVPGESHANDLECSGK.G	Carbamidomethyl: 4, 16

Detailed Protein Report

Protein 532: protein dopey-2 [Homo sapiens]

**Accession:**	gi|45827701	**Score:**	27.4
**Database:**	refseq_human(refseq_human_20140103.fasta)	**MW [kDa]:**	258.1
**Database Date:**	2015-11-30	**pI:**	5.9
		**Sequence Coverage [%]:**	1.0
		**No. of unique Peptides:**	1

Alias proteins:

Accession	Name	Description
gi|578836735	refseq_human (refseq_human_20140103.fasta)	PREDICTED: protein dopey-2 isoform X1 [Homo sapiens]

Detailed Protein Report

10	20	30	40	50	60	70	80
MDPEEQELLN	DYRYRSYSSV	IEKALRNFES	SSEWADLISS	LGKLNKALQS	NLRYSLLPRR	LLISKRLAQC	LHPALPSGVH
90	100	110	120	130	140	150	160
LKALETYEII	FKIVGTKWLA	KDLFLYSCGL	FPLLAHAAVS	VRPVLLTLYE	KYFLPLQKLL	LPSLQAFIVG	LLPGLEEGSE
170	180	190	200	210	220	230	240
ISDRTDALLL	RLSLVVGKEV	FYTALWGSVL	ASPSIRLPAS	VFVVGHINRD	APGREQKYML	GTNHQLTVKS	LRASLLDSNV
250	260	270	280	290	300	310	320
LVQRNNLEIV	LFFFPFYTCL	DSNERAIPLL	RSDIVRILSA	ATQTLLRRDM	SLNRRLYAWL	LGSDIKGNTV	VPESEISNSY
330	340	350	360	370	380	390	400
EDQSSYFFEK	YSKDLLVEGL	AEILHQKFID	ADVEERHHAY	LKPFRVLISL	LDKPEIGPQV	VGNLFLEVIR	AFYSYCRDAL
410	420	430	440	450	460	470	480
GSDLKLSYTQ	SGNSLISAIK	ENRNASEIVK	TVNLLITSLS	TDFLWDYMTR	CFEECFRPVK	QRYSVRNSVS	PPPTVSELCA
490	500	510	520	530	540	550	560
LLVFLLDVIP	LELYSEVQTQ	YLPQVLGCLV	QPLAEDMEAL	SLPELTHALK	TCFKVLSKVQ	MPPSYLDTES	TSGTSSPVKG
570	580	590	600	610	620	630	640
ENGKIILETK	AVIPGDEDAS	FPPLKSEDSG	IGLSASSPEL	SEHLRVPRVS	LERDDVWKKG	GSMQRTFLCI	QELIANFASK
650	660	670	680	690	700	710	720
NIFGVQLTAS	GEESKSEEPA	GKRDRDGTQS	LAANDSSRKN	SWEPKPITVP	QFKQMLSDLF	TARGSPFKTK	SSESPSSSPS
730	740	750	760	770	780	790	800
SPARKNGGEW	DVEKVVIDLG	GSREERREAF	AAACHLLLDC	ATFPVYLSEE	ETEQLCATLF	QLPGAGDSSF	PSWLKSLMTI
810	820	830	840	850	860	870	880
CCCVTDCYLQ	NVAISTLLEV	INHSQSLALV	IEDKMKRYKS	SGHNPFFGKL	QMVTVPPIAP	GILKVIAEKT	DFYQRVARVL
890	900	910	920	930	940	950	960
WNQLNKETRE	HHVTCVELFY	RLHCLAPTAN	ICEDIICHAL	LDPDKGTRLE	ALFRFSVIWH	LTREIQGSRV	TSHNRSFDRS
970	980	990	1000	1010	1020	1030	1040
LFVVLDSLAC	TDGAIGAAAQ	GWLVRALSLG	DVARILEPVL	LLLLQPKTQR	TSIHCLKQEN	SADDLHRWFN	RKKTSFREAC
1050	1060	1070	1080	1090	1100	1110	1120
AVPEPQESGS	EEHLPLSQFT	TVDREAIWAE	VEKEPEKYPL	RGELSEEELP	YYVELPDRTA	HGAPDSSEHT	ESADTSSCHT
1130	1140	1150	1160	1170	1180	1190	1200
DSENTSSFSS	PSHDLQELSN	EENCCAPIPM	GGRAYPKRSA	LLAAFQSESF	KAGAKLSLVR	VDSDKTQASE	SFSSDEEADL
1210	1220	1230	1240	1250	1260	1270	1280
ELQALTTSRL	LKQQRERQEA	VEALFKHILL	YLQPYDSRRV	LYAFSVLEAV	LKTNPKEFIE	AVSRTSMDTS	STAHLNLISN
1290	1300	1310	1320	1330	1340	1350	1360
LLARHQEALI	GQSFYGKLQT	QVPNVCPHSL	LLELLTYLCL	SFLRSYYPCY	LKVSHRDILG	NRDVQVKSVE	VLIRIMMQLV
1370	1380	1390	1400	1410	1420	1430	1440
SVAKSSEGKN	VEFIHSLLQR	CKVQEFVLLS	LSASMYTSQK	RYGLATAHHG	RALPEDSLFE	ESLINLGQDQ	IWSEHPLQIE
1450	1460	1470	1480	1490	1500	1510	1520
LLKLLQVLIV	LEHHLGRAHE	EAENQPDLSR	EWQRALNFQQ	AISALQYVQP	HPLTSQGLLV	SAVVRGLQPA	YGYGMHPAWV
1530	1540	1550	1560	1570	1580	1590	1600
SLVTHSLPYF	GKSLGWTVTP	FVVQICKNLD	DLVKQYESES	VKLSVSTTSK	RENISPDYPL	TLLEGLTTIS	HFCLLEQANQ
1610	1620	1630	1640	1650	1660	1670	1680
NKKTMAAGDP	ANLRNARNAI	LEELPRTVNT	MALLWNVLRK	EETQKRPVDL	LGATKGSSSV	YFKTTKTIRQ	KILDFLNPLT
1690	1700	1710	1720	1730	1740	1750	1760
AHLGVQLTAA	VAAVWSRKKA	QRHSKMKIIP	TASASQLTLV	DLVCALSTLQ	TDTLLHLVKE	VVKRPPQVKG	GDEKSPLVDI
1770	1780	1790	1800	1810	1820	1830	1840
PVLQFCYAFL	QRLPVPALQE	NFSSLLGVLK	ESVQLNLAPP	GYFLLLSMLN	DFVTRTPNLE	NKKDQKDLQE	ITQKILEAVG
1850	1860	1870	1880	1890	1900	1910	1920
NIAGSSLEQT	SWLSRNLEVK	AQPQASLEES	DAEEDLYDAA	AASAMVSSSA	PSVYSVQALS	LLAEVLASLL	DMVYRSDEKE
1930	1940	1950	1960	1970	1980	1990	2000
KAVPLISRLL	YYVFPYLRNH	SAYNAPSFRA	GAQLLSSLSG	YAYTKRAWRK	EVLELFLDPA	FFQMDTSCVH	WKSIIDHLLT
2010	2020	2030	2040	2050	2060	2070	2080
HEKTMFKDLM	NMQSSSLKLF	SSFEQKAMLL	KRQAFAVFSG	ELDQYHLYLP	LIQERLTDNL	RVGQTSIVAA	QMFLFFRVLL
2090	2100	2110	2120	2130	2140	2150	2160
LRISPQHLTS	LWPIMVSELI	QTFTQLEEDL	KDEDESLRST	NKVNRTKVSV	PDANGPSVGE	IPQSELILYL	SACKFLDTAL
2170	2180	2190	2200	2210	2220	2230	2240
SFPPDKMPLF	QIYRWAFIPE	VDTEGPAFLS	DVEENHQECK	PHTVRILELL	KLKFGEISSS	DEITMKSEFP	LLRQHSVSSI
2250	2260	2270	2280	2290	2300		
RQLMPFFMTL	NGAFKTQRQL	PADSPGTPFL	DFPVTDSPRI	LKQLEECIEY	DFLEHPEC		

Detailed Protein Report

Cmpd.	No. of Cmpds.	m/z meas.	Δ m/z [ppm]	z	Rt [min]	Score	P	Range	Sequence	Modification
1889	1	673.7777	-150.29	2	54.0	16.1	0	1929-1938	R.LLYYVFPYLR.N	

Detailed Protein Report

Protein 533: PREDICTED: peroxidasin-like protein isoform X2 [Homo sapiens]

**Accession:**	gi|530388290	**Score:**	27.4
**Database:**	refseq_human(refseq_human_20140103.fasta)	**MW [kDa]:**	95.0
**Database Date:**	2015-11-30	**pI:**	9.5
**Modification(s):**	Carbamidomethyl	**Sequence Coverage [%]:**	1.2
		**No. of unique Peptides:**	1

10	20	30	40	50	60	70	80
MCGSEFFWPC	CDQHVSYSHG	KPHTSSDLLA	QFHYPRDPLI	VEMARAGEIF	EHTLQLIRER	VKQGLTVDLE	GKEFRYNDLV
90	100	110	120	130	140	150	160
SPRSLSLIAN	LSGCTARRPL	PNCSNRCFHA	KYRAHDGTCN	NLQQPTWGAA	LTAFARLLQP	AYRDGIRAPR	GLGLPVGSRQ
170	180	190	200	210	220	230	240
PLPPPRLVAT	VWARAAAVTP	DHSYTRMLMH	WGWFLEHDLD	HTVPALSTAR	FSDGRPCSSV	CTNDPPCFPM	NTRHADPRGT
250	260	270	280	290	300	310	320
HAPCMLFARS	SPACASGRPS	ATVDSVYARE	QINQQTAYID	GSNVYGSSER	ESQALRDPSV	PRGLLKTGFP	WPPSGKPLLP
330	340	350	360	370	380	390	400
FSTGPPTECA	RQEQESPCFL	AGDHRANEHL	ALAAMHTLWF	REHNRMATEL	SALNPHWEGN	TVYQEARKIV	GAELQHITYS
410	420	430	440	450	460	470	480
HWLPKVLGDP	GTRMLRGYRG	YNPNVNAGII	NSFATAAFRF	GHTLINPILY	RLNATLGEIS	EGHLPFHKAL	FSPSRIIKEG
490	500	510	520	530	540	550	560
GIDPVLRGLF	GVAAKWRAPS	YLLSPELTQR	LFSAAYSAAV	DSAATIIQRG	RDHGIPPYVD	FRVFCNLTSV	KNFEDLQNEI
570	580	590	600	610	620	630	640
KDSEIRQKLR	KLYGSPGDID	LWPALMVEDL	IPGTRVGPTL	MCLFVTQFQR	LRDGDRFWYE	NPGVFTPAQL	TQLKQASLSR
650	660	670	680	690	700	710	720
VLCDNGDSIQ	QVQADVFVKA	EYPQDYLNCS	EIPKVDLRVW	QDCCADCRSR	GQFRAVTQES	QKKRSAQYSY	PVDKDMELSH
730	740	750	760	770	780	790	800
LRSRQQDKIY	VGEDARNVTV	LAKTKFSQDF	STFAAEIQET	ITALREQINK	LEARLRQAGC	TDVRGVPRKA	EERWMKEDCT
810	820	830	840	850			
HCICESGQVT	CVVEICPPAP	CPSPELVKGT	CCPVCRDRGM	PSDSPEKR			

Cmpd.	No. of Cmpds.	m/z meas.	Δ m/z [ppm]	z	Rt [min]	Score	P	Range	Sequence	Modification
1949	1	656.8239	117.11	2	54.8	14.5	0	679-688	R.VWQDCCADCR.S	Carbamidomethyl: 5, 9

Detailed Protein Report

Protein 534: PREDICTED: zinc finger protein 449 isoform X1 [Homo sapiens]

Accession: gi|530422388
Database: refseq_human(refseq_human_20140103.fasta)
Database Date: 2015-11-30
Modification(s): Carbamidomethyl

Score: 27.4
MW [kDa]: 32.2
pI: 10.5
Sequence Coverage [%]: 6.5
No. of unique Peptides: 1

Quantitation

WUP:QUP **Median:** 1.24 **CV:** 0.00 % **No. of Peptides:** 1

10	20	30	40	50	60	70	80
METMYPFIVT	LEGNALQGPI	LQKDYVQLEN	QWETPPEDLQ	TDLAKLVDQQ	NPTLGETPEN	SNLEEPLNPK	PHKKKSPGEK
**90**	**100**	**110**	**120**	**130**	**140**	**150**	**160**
PHRCPQCGKC	FARKSQLTGH	QRIHSGEEPH	KCPECGKRFL	RSSDLYRHQR	LHTGERPYEC	TVCKKRFTRR	SHLIGHQRTH
**170**	**180**	**190**	**200**	**210**	**220**	**230**	**240**
SEEETYKCLE	CGKSFCHGSS	LKRHLKTHTG	EKPHRCHNCG	KSFSRLTALT	LHQRTHTEER	PFKCNYCGKS	FRQRPSLVIH
**250**	**260**	**270**	**280**				
LRIHTGEKPY	KCTHCSKSFR	QRAGLIMHQV	THFRGLI				

Cmpd.	No. of Cmpds.	m/z meas.	Δ m/z [ppm]	z	Rt [min]	Score	P	Range	Sequence	Modification	Ratios
2275	1	1130.5320	8.03	2	58.9	12.4	2	215-232	R.THTEERPFKCNYCGKSFR.Q	Carbamidomethyl: 10	WUP:QUP 1.24

Detailed Protein Report

Protein 535: PREDICTED: AP-3 complex subunit beta-2 isoform X2 [Homo sapiens]

**Accession:**	gi|578827454	**Score:**	27.4
**Database:**	refseq_human(refseq_human_20140103.fasta)	**MW [kDa]:**	114.4
**Database Date:**	2015-11-30	**pI:**	5.4
		**Sequence Coverage [%]:**	2.0
		**No. of unique Peptides:**	1

Alias proteins:

Accession	Name	Description
gi|578827456	refseq_human (refseq_human_20140103.fasta)	PREDICTED: AP-3 complex subunit beta-2 isoform X3 [Homo sapiens]

10	20	30	40	50	60	70	80
MLDTNKDSLK	LEAMKRIVAM	IARGKNASDL	FPAVVKNVAC	KNIEVKKLVY	VYLVRYAEEQ	QDLALLSIST	FQRGLKDPNQ
**90**	**100**	**110**	**120**	**130**	**140**	**150**	**160**
LIRASALRVL	SSIRVPIIVP	IMMLAIKEAA	SDMSPYVRKT	AAHAIPKLYS	LDSDQKDQLI	EVIEKLLADK	TTLVAGSVVM
**170**	**180**	**190**	**200**	**210**	**220**	**230**	**240**
AFEEVCPERI	DLIHKNYRKL	CNLLIDVEEW	GQVVIISMLT	RYARTQFLSP	TQNESLLEEN	AEKAFYGSEE	DEAKGAGSEE
**250**	**260**	**270**	**280**	**290**	**300**	**310**	**320**
TAAAAAPSRK	PYVMDPDHRL	LLRNTKPLLQ	SRSAAVVMAV	AQLYFHLAPK	AEVGVIAKAL	VRLLRSHSEV	QYVVLQNVAT
**330**	**340**	**350**	**360**	**370**	**380**	**390**	**400**
MSIKRRGMFE	PYLKSFYIRS	TDPTQIKILK	LEVLTNLANE	TNIPTVLREF	QTYIRSMDKD	FVAATIQAIG	RCATNIGRVR
**410**	**420**	**430**	**440**	**450**	**460**	**470**	**480**
DTCLNGLVQL	LSNRDELVVA	ESVVVIKKLL	QMQPAQHGEI	IKHLAKLTDN	IQVPMARASI	LWLIGEYCEH	VPRIAPDVLR
**490**	**500**	**510**	**520**	**530**	**540**	**550**	**560**
KMAKSFTAEE	DIVKLQVINL	AAKLYLTNSK	QTKLLTQYVL	SLAKYDQNYD	IRDRARFTRQ	LIVPSEQGGA	LSRHAKKLFL
**570**	**580**	**590**	**600**	**610**	**620**	**630**	**640**
APKPAPVLES	SFKDRDHFQL	GSLSHLLNAK	ATGYQELPDW	PEEAPDPSVR	NVEVPEWTKC	SNREKRKEKE	KPFYSDSEGE
**650**	**660**	**670**	**680**	**690**	**700**	**710**	**720**
SGPTESADSD	PESESESDSK	SSSESGSGES	SSESDNEDQD	EDEEKGRGSE	SEQSEEDGKR	KTKKKVPERK	GEASSSDEGS
**730**	**740**	**750**	**760**	**770**	**780**	**790**	**800**
DSSSSSSESE	MTSESEEEQL	EPASWSRKTP	PSSKSAPATK	EISLLDLEDF	TPPSVQPVSP	PAIVSTSLAA	DLEGLTLTDS
**810**	**820**	**830**	**840**	**850**	**860**	**870**	**880**
TLVPSLLSPV	SGVGRQELLH	RVAGEGLAVD	YTFSRQPFSG	DPHMVSVHIH	FSNSSDTPIK	GLHVGTPKLP	AGISIQEFPE
**890**	**900**	**910**	**920**	**930**	**940**	**950**	**960**
IESLAPGESA	TAVMGINFCD	STQAANFQLC	TQTRQFYVSI	QPPVGELMAP	VFMSENEFKK	EQGKLMGMNE	ITEKLMLPDT
**970**	**980**	**990**	**1000**	**1010**	**1020**	**1030**	**1040**
CRSDHIVVQK	VTATANLGRV	PCGTSDEYRF	AGRTLTGGSL	VLLTLDARPA	GAAQLTVNSE	KMVIGTMLVK	DVIQALTQ

Cmpd.	No. of Cmpds.	m/z meas.	Δ m/z [ppm]	z	Rt [min]	Score	P	Range	Sequence	Modification
1596	1	677.3556	42.21	2	50.2	10.6	1	108-119	K.EAASDMSPYVRK.T	

Detailed Protein Report

Protein 536: serine/threonine-protein kinase MRCK gamma [Homo sapiens]

Accession: gi|156766068
Database: refseq_human(refseq_human_20140103.fasta)
Database Date: 2015-11-30
Modification(s): Oxidation

Score: 27.4
MW [kDa]: 172.4
pI: 5.9
Sequence Coverage [%]: 1.9
No. of unique Peptides: 1

10	20	30	40	50	60	70	80
MERRLRALEQ	LARGEAGGCP	GLDGLLDLLL	ALHHELSSGP	LRRERSVAQF	LSWASPFVSK	VKELRLQRDD	FEILKVIGRG
90	100	110	120	130	140	150	160
AFGEVTVVRQ	RDTGQIFAMK	MLHKWEMLKR	AETACFREER	DVLVKGDSRW	VTTLHYAFQD	EEYLYLVMDY	YAGGDLLTLL
170	180	190	200	210	220	230	240
SRFEDRLPPE	LAQFYLAEMV	LAIHSLHQLG	YVHRDVKPDN	VLLDVNGHIR	LADFGSCLRL	NTNGMVDSSV	AVGTPDYISP
250	260	270	280	290	300	310	320
EILQAMEEGK	GHYGPQCDWW	SLGVCAYELL	FGETPFYAES	LVETYGKIMN	HEDHLQFPPD	VPDVPASAQD	LIRQLLCRQE
330	340	350	360	370	380	390	400
ERLGRGGLDD	FRNHPFFEGV	DWERLASSTA	PYIPELRGPM	DTSNFDVDDD	TLNHPGTLPP	PSHGAFSGHH	LPFVGFTYTS
410	420	430	440	450	460	470	480
GSHSPESSSE	AWAALERKLQ	CLEQEKVELS	RKHQEALHAP	TDHRELEQLR	KEVQTLRDRL	PEMLRDKASL	SQTDGPPAGS
490	500	510	520	530	540	550	560
PGQDSDLRQE	LDRLHRELAE	GRAGLQAQEQ	ELCRAQGQQE	ELLQRLQEAQ	EREAATASQT	RALSSQLEEA	RAAQRELEAQ
570	580	590	600	610	620	630	640
VSSLSRQVTQ	LQGQWEQRLE	ESSQAKTIHT	ASETNGMGPP	EGGPQEAQLR	KEVAALREQL	EQAHSHRPSG	KEEALCQLQE
650	660	670	680	690	700	710	720
ENRRLSREQE	RLEAELAQEQ	ESKQRLEGER	RETESNWEAQ	LADILSWVND	EKVSRGYLQA	LATKMAEELE	SLRNVGTQTL
730	740	750	760	770	780	790	800
PARPLDHQWK	ARRLQKMEAS	ARLELQSALE	AEIRAKQGLQ	ERLTQVQEAQ	LQAERRLQEA	EKQSQALQQE	LAMLREELRA
810	820	830	840	850	860	870	880
RGPVDTKPSN	SLIPFLSFRS	SEKDSAKDPG	ISGEATRHGG	EPDLRPEGRR	SLRMGAVFPR	APTANTASTE	GLPAKPGSHT
890	900	910	920	930	940	950	960
LRPRSFPSPT	KCLRCTSLML	GLGRQGLGCD	ACGYFCHTTC	APQAPPCPVP	PDLLRTALGV	HPETGTGTAY	EGFLSVPRPS
970	980	990	1000	1010	1020	1030	1040
GVRRGWQRVF	AALSDSRLLL	FDAPDLRLSP	PSGALLQVLD	LRDPQFSATP	VLASDVIHAQ	SRDLPRIFRV	TTSQLAVPPT
1050	1060	1070	1080	1090	1100	1110	1120
TCTVLLLAES	EGERERWLQV	LGELQRLLLD	ARPRPRPVYT	LKEAYDNGLP	LLPHTLCAAI	LDQDRLALGT	EEGLFVIHLR
1130	1140	1150	1160	1170	1180	1190	1200
SNDIFQVGEC	RRVQQLTLSP	SAGLLVVLCG	RGPSVRLFAL	AELENIEVAG	AKIPESRGCQ	VLAAGSILQA	RTPVLCVAVK
1210	1220	1230	1240	1250	1260	1270	1280
RQVLCYQLGP	GPGPWQRRIR	ELQAPATVQS	LGLLGDRLCV	GAAGGFALYP	LLNEAAPLAL	GAGLVPEELP	PSRGGLGEAL
1290	1300	1310	1320	1330	1340	1350	1360
GAVELSLSEF	LLLFTTAGIY	VDGAGRKSRG	HELLWPAAPM	GWGYAAPYLT	VFSENSIDVF	DVRRAEWVQT	VPLKKVRPLN
1370	1380	1390	1400	1410	1420	1430	1440
PEGSLFLYGT	EKVRLTYLRN	QLAEKDEFDI	PDLTDNSRRQ	LFRTKSKRRF	FFRVSEEQQK	QQRREMLKDP	FVRSKLISPP
1450	1460	1470	1480	1490	1500	1510	1520
TNFNHLVHVG	PANGRPGARD	KSPAPEEKGR	VARGSGPQRP	HSFSEALRRP	ASMGSEGLGG	DADPMKRKPW	TSLSSESVSC
1530	1540	1550	1560				
PQGSLSPATS	LMQVSERPRS	LPLSPELESS	P				

Cmpd.	No. of Cmpds.	m/z meas.	Δ m/z [ppm]	z	Rt [min]	Score	P	Range	Sequence	Modification
2153	1	974.3330	-124.42	2	55.3	15.2	1	1489-1507	R.RPASMGSEGLGGDADPMKR.K	Oxidation: 5

Detailed Protein Report

Protein 537: tubulin-specific chaperone E isoform b [Homo sapiens]

**Accession:**	gi|568215736	**Score:**	27.3
**Database:**	refseq_human(refseq_human_20140103.fasta)	**MW [kDa]:**	64.8
**Database Date:**	2015-11-30	**pI:**	7.1
		**Sequence Coverage [%]:**	5.4
		**No. of unique Peptides:**	1

10	20	30	40	50	60	70	80
MSDTLTADVI	GRRVEVNGEH	ATVRFAGVVP	PVAGPWLGVE	WDNPERGKHD	GSHEGTVYFK	CRHPTGGSFI	RPNKVNFGTD
**90**	**100**	**110**	**120**	**130**	**140**	**150**	**160**
FLTAIKNRYV	LEDGPEEDRK	EQIVTIGNKP	VETIGFDSIM	KQQSQLSKLQ	EVSLRNCAVS	CAGEKGGVAE	ACPNIRKVDL
**170**	**180**	**190**	**200**	**210**	**220**	**230**	**240**
SKNLLSSWDE	VIHIADQLRH	LEVLNVSENK	LKFPSGSVLT	GTLSVLKVLV	LNQTGITWAE	AHAQCGGSRH	GLDMQKDASK
**250**	**260**	**270**	**280**	**290**	**300**	**310**	**320**
FVDLCVLQKC	STSNCIISAK	DHTSMRMNVA	KVLRCVAGCP	GLEELYLESN	NIFISERPTD	VLQTVKLLDL	SSNQLIDENQ
**330**	**340**	**350**	**360**	**370**	**380**	**390**	**400**
LYLIAHLPRL	EQLILSDTGI	SSLHFPDAGI	GCKTSMFPSL	KYLVVNDNQI	SQWSFFNELE	KLPSLRALSC	LRNPLTKEDK
**410**	**420**	**430**	**440**	**450**	**460**	**470**	**480**
EAETARLLII	ASIGQLKTLN	KCEILPEERR	RAELDYRKAF	GNEWKQAGGH	KDPEKNRLSE	EFLTAHPRYQ	FLCLKYGAPE
**490**	**500**	**510**	**520**	**530**	**540**	**550**	**560**
DWELKTQQPL	MLKNQLLTLK	IKYPHQLDQK	VLEKQLPGSM	TIQKVKGLLS	RLLKVPVSDL	LLSYESPKKP	GREIELENDL
**570**	**580**						
KSLQFYSVEN	GDCLLVRW						

Cmpd.	No. of Cmpds.	m/z meas.	Δ m/z [ppm]	z	Rt [min]	Score	P	Range	Sequence	Modification
1805	1	781.2970	-97.72	2	52.9	10.5	1	47-60	R.GKHDGSHEGTVYFK.C	

Detailed Protein Report

Protein 538: PREDICTED: synaptotagmin-7 isoform X3 [Homo sapiens]

**Accession:**	gi|530397573	**Score:**	27.3
**Database:**	refseq_human(refseq_human_20140103.fasta)	**MW [kDa]:**	68.4
**Database Date:**	2015-11-30	**pI:**	9.9
		**Sequence Coverage [%]:**	3.9
		**No. of unique Peptides:**	2

Quantitation

QU:MU	**Median:**	1.55	**CV:**	0.00 %	**No. of Peptides:**	1
WUP:QUP	**Median:**	1.74	**CV:**	0.00 %	**No. of Peptides:**	1

10	20	30	40	50	60	70	80
MYRDPEAASP	GAPSRDVLLV	SAIITVSLSV	TVVLCGLCHW	CQRKLGKRYK	NSLETVGTPD	SGRGRSEKKA	INDLDRDFWN
**90**	**100**	**110**	**120**	**130**	**140**	**150**	**160**
NNESTVQQKW	SSYPPKEFIL	NISPYAPYGD	PRLSLNFEDS	TLSTATTLES	IPSSTGEPKC	QRPRTLMRQQ	SLQQPLSQHQ
**170**	**180**	**190**	**200**	**210**	**220**	**230**	**240**
RGRQPSQPTT	SQSLGQLQAH	MASAPGPNPR	AYGRGQARQG	TSAGSKYRAA	GGRSRSNPGS	WDHVVGQIRN	RGLDMKSFLE
**250**	**260**	**270**	**280**	**290**	**300**	**310**	**320**
GRMVVLSLVL	GLSEQDDFAN	IPDLQNPGTQ	QNQNAQGDKR	LPAGGKAVNT	APVPGQTPHD	ESDRRTEPRS	SVSDLVNSLT
**330**	**340**	**350**	**360**	**370**	**380**	**390**	**400**
SEMLMLSPGS	EEDEAHEGCS	RENLGRIQFS	VGYNFQESTL	TVKIMKAQEL	PAKDFSGTSD	PFVKIYLLPD	KKHKLETKVK
**410**	**420**	**430**	**440**	**450**	**460**	**470**	**480**
RKNLNPHWNE	TFLFEGFPYE	KVVQRILYLQ	VLDYDRFSRN	DPIGEVSIPL	NKVDLTQMQT	FWKDLKPCSD	GSGSRGELLL
**490**	**500**	**510**	**520**	**530**	**540**	**550**	**560**
SLCYNPSANS	IIVNIIKARN	LKAMDIGGTS	DPYVKVWLMY	KDKRVEKKKT	VTMKRNLNPI	FNESFAFDIP	TEKLRETTII
**570**	**580**	**590**	**600**	**610**	**620**		
ITVMDKDKLS	RNDVIGKIYL	SWKSGPGEVK	HWKDMIARPR	QPVAQWHQLK	A		

Cmpd.	No. of Cmpds.	m/z meas.	Δ m/z [ppm]	z	Rt [min]	Score	P	Range	Sequence	Modification	Ratios
285	1	733.8676	-12.20	2	33.5	11.8	2	195-208	R.GQARQGTSAGSKYR.A		QU:MU 1.55<br>WUP:QUP 1.74
2844	1	655.3688	27.19	2	66.0	15.5	1	591-600	K.HWKDMIARPR.Q		

Detailed Protein Report

Protein 539: pyrroline-5-carboxylate reductase 2 isoform 2 [Homo sapiens]

**Accession:**	gi|410991931	**Score:**	27.3
**Database:**	refseq_human(refseq_human_20140103.fasta)	**MW [kDa]:**	25.9
**Database Date:**	2015-11-30	**pI:**	10.2
		**Sequence Coverage [%]:**	8.5
		**No. of unique Peptides:**	1

```
        10         20         30         40         50         60         70         80
MSVGFIGAGQ LAYALARGFT AAGILSAHKI IASSPEMNLP TVSALRKMGV NLTRSNKETV KHSDVLFLAV KPHIIPFILD
        90        100        110        120        130        140        150        160
EIGADVQARH IVVSCAAGVT ISSVEKAFMA LDALADGGVK MGLPRRLAIQ LGAQALLGAA KMLLDSEQHP CQLKDNVCSP
       170        180        190        200        210        220        230        240
GGATIHALHF LESGGFRSLL INAVEASCIR TRELQSMADQ EKISPAALKK TLLDRVKLES PTVSTLTPSS PGKLLTRSLA
       250
LGGKKD
```

Cmpd.	No. of Cmpds.	m/z meas.	Δ m/z [ppm]	z	Rt [min]	Score	P	Range	Sequence	Modification
325	1	1074.4954	-137.82	2	34.1	27.3	2	121-141	K.MGLPRRLAIQLGAQALLGAAK.M	

Detailed Protein Report

Protein 540: chloride anion exchanger [Homo sapiens]

**Accession:**	gi|4557535	**Score:**	27.3
**Database:**	refseq_human(refseq_human_20140103.fasta)	**MW [kDa]:**	84.5
**Database Date:**	2015-11-30	**pI:**	9.5
**Modification(s):**	Oxidation	**Sequence Coverage [%]:**	3.7
		**No. of unique Peptides:**	1

Alias proteins:

Accession	Name	Description
gi|578813973	refseq_human (refseq_human_20140103.fasta)	PREDICTED: chloride anion exchanger isoform X1 [Homo sapiens]

10	20	30	40	50	60	70	80
MIEPFGNQYI	VARPVYSTNA	FEENHKKTGR	HHKTFLDHLK	VCCSCSPQKA	KRIVLSLFPI	ASWLPAYRLK	EWLLSDIVSG
**90**	**100**	**110**	**120**	**130**	**140**	**150**	**160**
ISTGIVAVLQ	GLAFALLVDI	PPVYGLYASF	FPAIIYLFFG	TSRHISVGPF	PILSMMVGLA	VSGAVSKAVP	DRNATTLGLP
**170**	**180**	**190**	**200**	**210**	**220**	**230**	**240**
NNSNNSSLLD	DERVRVAAAA	SVTVLSGIIQ	LAFGILRIGF	VVIYLSESLI	SGFTTAAAVH	VLVSQLKFIF	QLTVPSHTDP
**250**	**260**	**270**	**280**	**290**	**300**	**310**	**320**
VSIFKVLYSV	FSQIEKTNIA	DLVTALIVLL	VVSIVKEINQ	RFKDKLPVPI	PIEFIMTVIA	AGVSYGCDFK	NRFKVAVVGD
**330**	**340**	**350**	**360**	**370**	**380**	**390**	**400**
MNPGFQPPIT	PDVETFQNTV	GDCFGIAMVA	FAVAFSVASV	YSLKYDYPLD	GNQELIALGL	GNIVCGVFRG	FAGSTALSRS
**410**	**420**	**430**	**440**	**450**	**460**	**470**	**480**
AVQESTGGKT	QIAGLIGAII	VLIVVLAIGF	LLAPLQKSVL	AALALGNLKG	MLMQFAEIGR	LWRKDKYDCL	IWIMTFIFTI
**490**	**500**	**510**	**520**	**530**	**540**	**550**	**560**
VLGLGLGLAA	SVAFQLLTIV	FRTQFPKCST	LANIGRTNIY	KNKKDYYDMY	EPEGVKIFRC	PSPIYFANIG	FFRRKLIDAV
**570**	**580**	**590**	**600**	**610**	**620**	**630**	**640**
GFSPLRILRK	RNKALRKIRK	LQKQGLLQVT	PKGFICTVDT	IKDSDEELDN	NQIEVLDQPI	NTTDLPFHID	WNDDLPLNIE
**650**	**660**	**670**	**680**	**690**	**700**	**710**	**720**
VPKISLHSLI	LDFSAVSFLD	VSSVRGLKSI	LQEFIRIKVD	VYIVGTDDDF	IEKLNRYEFF	DGEVKSSIFF	LTIHDAVLHI
**730**	**740**	**750**	**760**	**770**			
LMKKDYSTSK	FNPSQEKDGK	IDFTINTNGG	LRNRVYEVPV	ETKF			

Cmpd.	No. of Cmpds.	m/z meas.	Δ m/z [ppm]	z	Rt [min]	Score	P	Range	Sequence	Modification
2677	1	862.3965	-52.92	2	64.6	13.5	1	450-463	K.GMLMQFAEIGRLWR.K	Oxidation: 4

Detailed Protein Report

Protein 541: TBC1 domain family member 22A isoform b [Homo sapiens]

Accession: gi|546231127
Database: refseq_human(refseq_human_20140103.fasta)
Database Date: 2015-11-30

Score: 27.3
MW [kDa]: 50.9
pI: 5.6
Sequence Coverage [%]: 5.9
No. of unique Peptides: 1

10	20	30	40	50	60	70	80
MASDGARKQF	WKRSNSKLPG	SLLRSTAKMP	TTPVKAKRVS	TFQEFESNTS	DAWDAGEDDD	ELLAMAAESL	NSEVVMETAN
**90**	**100**	**110**	**120**	**130**	**140**	**150**	**160**
RVLRNHSQRQ	GRPTLQEGPG	LQQKPRPEAE	PPSPPSGDLR	LVKSVSESHT	SCPAEELRRL	SWSGIPKPVR	PMTWKLLSGY
**170**	**180**	**190**	**200**	**210**	**220**	**230**	**240**
LPANVDRRPA	TLQRKQKEYF	AFIEHYYDSR	NDEVHQDTYR	QIHIDIPRMS	PEALILQPKV	TEIFERILFI	WAIRHPASGY
**250**	**260**	**270**	**280**	**290**	**300**	**310**	**320**
VQGINDLVTP	FFVVFICEYI	EAEEVDTVDV	SGVPAEVLCN	IEADTYWCMS	KLLDGIQDNY	TFAQPGIQMK	VKMLEELVSR
**330**	**340**	**350**	**360**	**370**	**380**	**390**	**400**
IDEQVHRHLD	QHEVRYLQFA	FRWMNNLLMR	EVPLRCTIRL	WDTYQSEPDG	FSHFHLYVCA	AFLVRWRKEI	LEEKDFQELL
**410**	**420**	**430**	**440**				
LFLQNLPTAH	WDDEDISLLL	AEAYRLKFAF	ADAPNHYKK				

Cmpd.	No. of Cmpds.	m/z meas.	Δ m/z [ppm]	z	Rt [min]	Score	P	Range	Sequence	Modification
1413	1	816.3918	26.50	2	47.3	12.4	0	124-138	K.SVSESHTSCPAEELR.R	

Detailed Protein Report

Protein 542: dynein heavy chain 12, axonemal isoform 1 [Homo sapiens]

**Accession:**	gi|194440727	**Score:**	27.2
**Database:**	refseq_human(refseq_human_20140103.fasta)	**MW [kDa]:**	356.7
**Database Date:**	2015-11-30	**pI:**	5.8
**Modification(s):**	Oxidation	**Sequence Coverage [%]:**	1.3
		**No. of unique Peptides:**	2

Quantitation

WUP:QUP	**Median:** 1.30	**CV:** 0.00 %	**No. of Peptides:** 1

Detailed Protein Report

10	20	30	40	50	60	70	80
MSDANKAAIA	AEKEALNLKL	PPIVHLPENI	GVDTPTQSKL	LKYRRSKEQQ	QKINQLVIDG	AKRNLDRTLG	KRTPLLPPPD
90	100	110	120	130	140	150	160
YPQTMTSEMK	KKGFNYIYMK	QCVESSPLVP	IQQEWLDHML	RLIPESLKEG	KEREELLESL	INEVSSDFEN	SMKRYLVQSV
170	180	190	200	210	220	230	240
LVKPPVKSLE	DEGGPLPESP	VGLDYSNPWH	SSYVQARNQI	FSNLHIIHPT	MKMLLDLGYT	TFADTVLLDF	TGIRAKGPID
250	260	270	280	290	300	310	320
CESLKTDLSI	QTRNAEEKIM	NTWYPKVINL	FTKKEALEGV	KPEKLDAFYS	CVSTLMSNQL	KDLLRRTVEG	FVKLFDPKDQ
330	340	350	360	370	380	390	400
QRLPIFKIEL	TFDDDKMEFY	PTFQDLEDNV	LSLVERIAEA	LQNVQTIPSW	LSGTSTPVNL	DTELPEHVLH	WAVDTLKAAV
410	420	430	440	450	460	470	480
HRNLEGARKH	YETYVEKYNW	LLDGTAVENI	ETFQTEDHTF	DEYTEFIEKF	LSLASEIMLL	PQWIHYTMVR	LDCEDLKTGL
490	500	510	520	530	540	550	560
TNKAKAFANI	LLNDIASKYR	KENECICSEF	EAIKEHALKV	PETTEEMMDL	ISYVEKARTV	GIEELILRIQ	ESKRQMSYFL
570	580	590	600	610	620	630	640
DVFLFPQEDL	ALNATVLMWP	RKINPIFDEN	DELIENAKHK	KENELMAKRE	KLILEIEKES	RRMEEFTEFA	ELERMQQYVT
650	660	670	680	690	700	710	720
DVRQLQKRIQ	ESEEAVQFIN	KEEELFKWEL	TKYPELDKLK	VNIEPYQKFF	NFVLKWQRSE	KRWMDGGFLD	LNGESMEADV
730	740	750	760	770	780	790	800
EEFSREIFKT	LKFFQTKLKK	ELQEKRKAAR	KRSLEEEKIE	EEPKDNATIT	MCRMRARHWK	QISEIVGYDL	TPDSGTTLRK
810	820	830	840	850	860	870	880
VLKLNLTPYL	EQFEVISAGA	SKEFSLEKAM	NTMIGTWEDI	AFHISLYRDT	GVCILSSVDE	IQAILDDQII	KTQTMRGSPF
890	900	910	920	930	940	950	960
IKPFEHEIKA	WEDRLIRIQE	TIDEWLKVQA	QWLYLEPIFC	SEDIMQQMPE	EGRQFQTVDR	HWRDIMKFCA	KDPKVLAATS
970	980	990	1000	1010	1020	1030	1040
LTGLLEKLQN	CNELLEKIMK	GLNAYLEKKR	LFFPRFFFLS	NDEMLEILSE	TKDPLRVQPH	LKKCFEGIAK	LEFLPNLDIK
1050	1060	1070	1080	1090	1100	1110	1120
AMYSSEGERV	ELIALISTSA	ARGAVEKWLI	QVEDLMLRSV	HDVIAAARLA	YPESARRDWV	REWPGQVVLC	ISQMFWTSET
1130	1140	1150	1160	1170	1180	1190	1200
QEVISGGTEG	LKKYYKELQN	QLNEIVELVR	GKLSKQTRTT	LGALVTIDVH	ARDVVMDMIK	MGVSHDTDFL	WLAQLRYYWE
1210	1220	1230	1240	1250	1260	1270	1280
NENARVRIIN	CNVKYAYEYL	GNSPRLVITP	LTDRCYRTLI	GAFYLNLGGA	PEGPAGTGKT	ETTKDLAKAL	AVQCVVFNCS
1290	1300	1310	1320	1330	1340	1350	1360
DGLDYLAMGK	FFKGLASSGA	WACFDEFNRI	ELEVLSVVAQ	QILCIQRAIQ	QKLVVFVFEG	TELKLNPNCF	VAITMNPGYA
1370	1380	1390	1400	1410	1420	1430	1440
GRSELPDNLK	VLFRTVAMMV	PNYALIAEIS	LYSYGFLNAR	PLSVKIVMTY	RLCSEQLSSQ	FHYDYGMRAV	KAVLVAAGNL
1450	1460	1470	1480	1490	1500	1510	1520
KLKYPNENED	ILLLRSIKDV	NEPKFLSHDI	PLFNGITSDL	FPGIKLPEAD	YHEFLECAHE	ACNVHNLQPV	KFFLEKIIQT
1530	1540	1550	1560	1570	1580	1590	1600
YEMMIVRHGF	MLVGEPFAAK	TKVLHVLADT	LTLMNEHGYG	EEEKVIYRTV	NPKSITMGQL	FGQFDPVSHE	WTDGIVANTF
1610	1620	1630	1640	1650	1660	1670	1680
REFALSETPD	RKWVVFDGPI	DTLWIESMNT	VLDDNKKLCL	MSGEIIQMSP	QMSLIFETMD	LSQASPATVS	RCGMIYLEPS
1690	1700	1710	1720	1730	1740	1750	1760
QLGWEPLVSS	WLNSLKGPLC	EPEYQALLRG	LFAWLIPPSL	NQRVELFQLN	YLYTTIVSKI	LKILITFRIS	NYFKYVPLKT
1770	1780	1790	1800	1810	1820	1830	1840
QCTFIKFFLH	QQACFIFSLI	WSIGGSCDTD	GRRVFDTFIR	LIILGKDDEN	PVPDSVGKWE	CPFDEKGLVY	DYMYELKNKG
1850	1860	1870	1880	1890	1900	1910	1920
RWVHWNELIK	NTNLGDKQIK	IQDIIVPTMD	TIRYTFLMDL	SITYAKPLLF	VGPTGTGKSV	YVKDKLMNHL	EKDQYFPFYI
1930	1940	1950	1960	1970	1980	1990	2000
NLSARTSANQ	VQNIIMARLD	KRRKGVFGPP	MGKKCIIFID	DMNMPALEKY	GAQPPIELLR	QFFDCGHWYD	LKDTSKITLV
2010	2020	2030	2040	2050	2060	2070	2080
DIELIAAMGP	PGGGRNPVTP	RCIRHFNICS	INSFSDETMV	RIFSSIVAFY	LRTHEFPPEY	FVIGNQIVNG	TMEIYKQSVE
2090	2100	2110	2120	2130	2140	2150	2160
NLLPTPTKSH	YTFNLRDFSR	VIRGCLLIER	DAVANKHTMI	RLFVHEVLRV	FYDRLINDDD	RRWLFQLTKT	VIKDHFKESF
2170	2180	2190	2200	2210	2220	2230	2240

Detailed Protein Report

Cmpd.	No. of Cmpds.	m/z meas.	Δ m/z [ppm]	z	Rt [min]	Score	P	Range	Sequence	Modification	Ratios
2090	1	1159.8674	-54.47	3	56.0	10.4	2	1512-1540	K.FFLEKIIQTYEMMIVRHGFMLVGEF T	Oxidation: 12, 20	
1721	1	646.7566	-109.88	2	51.8	16.9	1	1602-1612	R.EFALSETPDRK.W		WUP:QUP 1.30

Detailed Protein Report

Protein 543: kalirin isoform 2 [Homo sapiens]

**Accession:**	gi|4504335	**Score:**	27.2
**Database:**	refseq_human(refseq_human_20140103.fasta)	**MW [kDa]:**	192.1
**Database Date:**	2015-11-30	**pI:**	5.7
		**Sequence Coverage [%]:**	1.3
		**No. of unique Peptides:**	1

Quantitation

QU:MU	**Median:**	1.55	**CV:**	0.00 %	**No. of Peptides:**	1
WUP:QUP	**Median:**	1.02	**CV:**	0.00 %	**No. of Peptides:**	1

10	20	30	40	50	60	70	80
MTDRFWDQWY	LWYLRLLRLL	DRGSFRNDGL	KASDVLPILK	EKVAFVSGGR	DKRGGPILTF	PARSNHDRIR	QEDLRKLVTY
90	100	110	120	130	140	150	160
LASVPSEDVC	KRGFTVIIDM	RGSKWDLIKP	LLKTLQEAFP	AEIHVALIIK	PDNFWQKQKT	NFGSSKFIFE	TSMVSVEGLT
170	180	190	200	210	220	230	240
KLVDPSQLTE	EFDGSLDYNH	EEWIELRLSL	EEFFNSAVHL	LSRLEDLQEM	LARKEFPVDV	EGSRRLIDEH	TQLKKKVLKA
250	260	270	280	290	300	310	320
PVEELDREGQ	RLLQCIRCSD	GFSGRNCIPG	SADFQSLVPK	ITSLLDKLHS	TRQHLHQMWH	VRKLKLDQCF	QLRLFEQDAE
330	340	350	360	370	380	390	400
KMFDWISHNK	ELFLQSHTEI	GVSYQYALDL	QTQHNHFAMN	SMNAYVNINR	IMSVASRLSE	AGHYASQQIK	QISTQLDQEW
410	420	430	440	450	460	470	480
KSFAAALDER	STILAMSAVF	HQKAEQFLSG	VDAWCKMCSE	GGLPSEMQDL	ELAIHHHQTL	YEQVTQAYTE	VSQDGKALLD
490	500	510	520	530	540	550	560
VLQRPLSPGN	SESLTATANY	SKAVHQVLDV	VHEVLHHQRR	LESIWQHRKV	RLHQRLQLCV	FQQDVQQVLD	WIENHGEAFL
570	580	590	600	610	620	630	640
SKHTGVGKSL	HRARALQKRH	DDFEEVAQNT	YTNADKLLEA	AEQLAQTGEC	DPEEIYKAAR	HLEVRIQDFV	RRVEQRKLLL
650	660	670	680	690	700	710	720
DMSVSFHTHT	KELWTWMEDL	QKEMLEDVCA	DSVDAVQELI	KQFQQQQTAT	LDATLNVIKE	GEDLIQQLRS	APPSLGEPSE
730	740	750	760	770	780	790	800
ARDSAVSNNK	TPHSSSISHI	ESVLQQLDDA	QVQMEELFHE	RKIKLDIFLQ	LRIFEQYTIE	VTAELDAWNE	DLLRQMNDFN
810	820	830	840	850	860	870	880
TEDLTLAEQR	LQRHTERKLA	MNNMTFEVIQ	QGQDLHQYIT	EVQASGIELI	CEKDIDLAAQ	VQELLEFLHE	KQHELELNAE
890	900	910	920	930	940	950	960
QTHKRLEQCL	QLRHLQAEVK	QVLGWIRNGE	SMLNASLVNA	SSLSEAEQLQ	REHEQFQLAI	ESLFHATSLQ	KTHQSALQVQ
970	980	990	1000	1010	1020	1030	1040
QKAEVLLQAG	HYDADAIREC	AEKVALHWQQ	LMLKMEDRLK	LVNASVAFYK	TSEQVCSVLE	SLEQEYRRDE	DWCGGRDKLG
1050	1060	1070	1080	1090	1100	1110	1120
PAAEIDHVIP	LISKHLEQKE	AFLKACTLAR	RNAEVFLKYI	HRNNVSMPSV	ASHTRGPEQQ	VKAILSELLQ	RENRVLHFWT
1130	1140	1150	1160	1170	1180	1190	1200
LKKRRLDQCQ	QYVVFERSAK	QALDWIQETG	EFYLSTHTST	GETTEETQEL	LKEYGEFRVP	AKQTKEKVKL	LIQLADSFVE
1210	1220	1230	1240	1250	1260	1270	1280
KGHIHATEIR	KWVTTVDKHY	RDFSLRMGKY	RYSLEKALGV	NTEDNKDLEL	DIIPASLSDR	EVKLRDANHE	VNEEKRKSAR
1290	1300	1310	1320	1330	1340	1350	1360
KKEFIMAELL	QTEKAYVRDL	HECLETYLWE	MTSGVEEIPP	GILNKEHIIF	GNIQEIYDFH	NNIFLKELEK	YEQLPEDVGH
1370	1380	1390	1400	1410	1420	1430	1440
CFVTWADKFQ	MYVTYCKNKP	DSNQLILEHA	GTFFDEIQQR	HGLANSISSY	LIKPVQRITK	YQLLLKELLT	CCEEGKGELK
1450	1460	1470	1480	1490	1500	1510	1520
DGLEVMLSVP	KKANDAMHVS	MLEGFDENLD	VQGELILQDA	FQVWDPKSLI	RKGRERHLFL	FEISLVFSKE	IKDSSGHTKY
1530	1540	1550	1560	1570	1580	1590	1600
VYKNKLLTSE	LGVTEHVEGD	PCKFALWSGR	TPSSDNKTVL	KASNIETKQE	WIKNIREVIQ	ERIIHLKGAL	KEPLQLPKTP
1610	1620	1630	1640	1650	1660	1670	
AKQRNNSKRD	GVEDIDSQGD	GSSQPDTISI	ASRTSQNTVD	SDKDGNLVPR	WHLGPGDPFS	TYV	

Cmpd.	No. of Cmpds.	m/z meas.	Δ m/z [ppm]	z	Rt [min]	Score	P	Range	Sequence	Modification	Ratios
599	1	562.7645	22.71	2	37.5	10.4	0	1427-1436	K.ELLTCCEEGK.G		QU:MU 1.55<br>WUP:QUP 1.02

Detailed Protein Report

Protein 544: Krueppel-like factor 12 [Homo sapiens]

Accession: gi|21071074
Database: refseq_human(refseq_human_20140103.fasta)
Database Date: 2015-11-30

Score: 27.2
MW [kDa]: 44.2
pI: 10.6
Sequence Coverage [%]: 8.5
No. of unique Peptides: 1

Alias proteins:

Accession	Name	Description
gi|578824739	refseq_human (refseq_human_20140103.fasta)	PREDICTED: Krueppel-like factor 12 isoform X3 [Homo sapiens]
gi|530402087	refseq_human (refseq_human_20140103.fasta)	PREDICTED: Krueppel-like factor 12 isoform X2 [Homo sapiens]
gi|530402085	refseq_human (refseq_human_20140103.fasta)	PREDICTED: Krueppel-like factor 12 isoform X1 [Homo sapiens]

10	20	30	40	50	60	70	80
MNIHMKRKTI	KNINTFENRM	LMLDGMPAVR	VKTELLESEQ	GSPNVHNYPD	MEAVPLLLNN	VKGEPPEDSL	SVDHFQTQTE
**90**	**100**	**110**	**120**	**130**	**140**	**150**	**160**
PVDLSINKAR	TSPTAVSSSP	VSMTASASSP	SSTSTSSSSS	SRLASSPTVI	TSVSSASSSS	TVLTPGPLVA	SASGVGGQQF
**170**	**180**	**190**	**200**	**210**	**220**	**230**	**240**
LHIIHPVPPS	SPMNLQSNKL	SHVHRIPVVV	QSVPVVYTAV	RSPGNVNNTI	VVPLLEDGRG	HGKAQMDPRG	LSPRQSKSDS
**250**	**260**	**270**	**280**	**290**	**300**	**310**	**320**
DDDDLPNVTL	DSVNETGSTA	LSIARAVQEV	HPSPVSRVRG	NRMNNQKFPC	SISPFSIEST	RRQRRSESPD	SRKRRIHRCD
**330**	**340**	**350**	**360**	**370**	**380**	**390**	**400**
FEGCNKVYTK	SSHLKAHRRT	HTGEKPYKCT	WEGCTWKFAR	SDELTRHYRK	HTGVKPFKCA	DCDRSFSRSD	HLALHRRRHM
**410**							
LV							

Cmpd.	No. of Cmpds.	m/z meas.	Δ m/z [ppm]	z	Rt [min]	Score	P	Range	Sequence	Modification
1218	1	758.4479	56.96	3	44.8	16.1	1	202-223	R.SPGNVNNTIVVPLLEDGRGHGK.A	

Detailed Protein Report

Protein 545: **very long-chain specific acyl-CoA dehydrogenase, mitochondrial isoform 2 precursor [Homo sapiens]**

**Accession:**	gi|76496475	**Score:**	27.2
**Database:**	refseq_human(refseq_human_20140103.fasta)	**MW [kDa]:**	68.0
**Database Date:**	2015-11-30	**pI:**	9.5
		**Sequence Coverage [%]:**	6.3
		**No. of unique Peptides:**	1

10	20	30	40	50	60	70	80
MQAARMAASL	GRQLLRLGGG	SSRLTALLGQ	PRPGPARRPY	AGGAAQESKS	FAVGMFKGQL	TTDQVFPYPS	VLNEEQTQFL
**90**	**100**	**110**	**120**	**130**	**140**	**150**	**160**
KELVEPVSRF	FEEVNDPAKN	DALEMVEETT	WQGLKELGAF	GLQVPSELGG	VGLCNTQYAR	LVEIVGMHDL	GVGITLGAHQ
**170**	**180**	**190**	**200**	**210**	**220**	**230**	**240**
SIGFKGILLF	GTKAQKEKYL	PKLASGETVA	AFCLTEPSSG	SDAASIRTSA	VPSPCGKYYT	LNGSKLWISN	GGLADIFTVF
**250**	**260**	**270**	**280**	**290**	**300**	**310**	**320**
AKTPVTDPAT	GAVKEKITAF	VVERGFGGIT	HGPPEKKMGI	KASNTAEVFF	DGVRVPSENV	LGEVGSGFKV	AMHILNNGRF
**330**	**340**	**350**	**360**	**370**	**380**	**390**	**400**
GMAAALAGTM	RGIIAKAVDH	ATNRTQFGEK	IHNFGLIQEK	LARMVMLQYV	TESMAYMVSA	NMDQGATDFQ	IEAAISKIFG
**410**	**420**	**430**	**440**	**450**	**460**	**470**	**480**
SEAAWKVTDE	CIQIMGGMGF	MKEPGVERVL	RDLRIFRIFE	GTNDILRLFV	ALQGCMDKGK	ELSGLGSALK	NPFGNAGLLL
**490**	**500**	**510**	**520**	**530**	**540**	**550**	**560**
GEAGKQLRRR	AGLGSGLSLS	GLVHPELSRS	GELAVRALEQ	FATVVEAKLI	KHKKGIVNEQ	FLLQRLADGA	IDLYAMVVVL
**570**	**580**	**590**	**600**	**610**	**620**	**630**	**640**
SRASRSLSEG	HPTAQHEKML	CDTWCIEAAA	RIREGMAALQ	SDPWQQELYR	NFKSISKALV	ERGGVVTSNP	LGF

Cmpd.	No. of Cmpds.	m/z meas.	Δ m/z [ppm]	z	Rt [min]	Score	P	Range	Sequence	Modification
1617	2	1051.5420	13.25	2	50.0	13.5	1	38-57	R.RPYAGGAAQESKSFAVGMFK.G	

Detailed Protein Report

Protein 546: **transient receptor potential cation channel subfamily M member 3 isoform d [Homo sapiens]**

**Accession:**	gi|154091318	**Score:**	27.2
**Database:**	refseq_human(refseq_human_20140103.fasta)	**MW [kDa]:**	176.1
**Database Date:**	2015-11-30	**pI:**	6.4
**Modification(s):**	Oxidation	**Sequence Coverage [%]:**	1.6
		**No. of unique Peptides:**	1

10	20	30	40	50	60	70	80
MYVRVSFDTK	PDLLLHLMTK	EWQLELPKLL	ISVHGGLQNF	ELQPKLKQVF	GKGLIKAAMT	TGAWIFTGGV	NTGVIRHVGD
90	100	110	120	130	140	150	160
ALKDHASKSR	GKICTIGIAP	WGIVENQEDL	IGRDVVRPYQ	TMSNPMSKLT	VLNSMHSHFI	LADNGTTGKY	GAEVKLRRQL
170	180	190	200	210	220	230	240
EKHISLQKIN	TRIGQGVPVV	ALIVEGGPNV	ISIVLEYLRD	TPPVPVVVCD	GSGRASDILA	FGHKYSEEGG	LINESLRDQL
250	260	270	280	290	300	310	320
LVTIQKTFTY	TRTQAQHLFI	ILMECMKKKE	LITVFRMGSE	GHQDIDLAIL	TALLKGANAS	APDQLSLALA	WNRVDIARSQ
330	340	350	360	370	380	390	400
IFIYGQQWPV	GSLEQAMLDA	LVLDRVDFVK	LLIENGVSMH	RFLTISRLEE	LYNTRHGPSN	TLYHLVRDVK	KGNLPPDYRI
410	420	430	440	450	460	470	480
SLIDIGLVIE	YLMGGAYRCN	YTRKRFRTLY	HNLFGPKRDD	IPLRRGRKTT	KKREEEVDID	LDDPEINHFP	FPFHELMVWA
490	500	510	520	530	540	550	560
VLMKRQKMAL	FFWQHGEEAM	AKALVACKLC	KAMAHEASEN	DMVDDISQEL	NHNSRDFGQL	AVELLDQSYK	QDEQLAMKLL
570	580	590	600	610	620	630	640
TYELKNWSNA	TCLQLAVAAK	HRDFIAHTCS	QMLLTDMWMG	RLRMRKNSGL	KVILGILLPP	SILSLEFKNK	DDMPYMSQAQ
650	660	670	680	690	700	710	720
EIHLQEKEAE	EPEKPTKEKE	EEDMELTAML	GRNNGESSRK	KDEEEVQSKH	RLIPLGRKIY	EFYNAPIVKF	WFYTLAYIGY
730	740	750	760	770	780	790	800
LMLFNYIVLV	KMERWPSTQE	WIVISYIFTL	GIEKMREILM	SEPGKLLQKV	KVWLQEYWNV	TDLIAILLFS	VGMILRLQDQ
810	820	830	840	850	860	870	880
PFRSDGRVIY	CVNIIYWYIR	LLDIFGVNKY	LGPYVMMIGK	MMIDMMYFVI	IMLVVLMSFG	VARQAILFPN	EEPSWKLAKN
890	900	910	920	930	940	950	960
IFYMPYWMIY	GEVFADQIDP	PCGQNETRED	GKIIQLPPCK	TGAWIVPAIM	ACYLLVANIL	LVNLLIAVFN	NTFFEVKSIS
970	980	990	1000	1010	1020	1030	1040
NQVWKFQRYQ	LIMTFHERPV	LPPPLIIFSH	MTMIFQHLCC	RWRKHESDPD	ERDYGLKLFI	TDDELKKVHD	FEEQCIEEYF
1050	1060	1070	1080	1090	1100	1110	1120
REKDDRFNSS	NDERIRVTSE	RVENMSMRLE	EVNEREHSMK	ASLQTVDIRL	AQLEDLIGRM	ATALERLTGL	ERAESNKIRS
1130	1140	1150	1160	1170	1180	1190	1200
RTSSDCTDAA	YIVRQSSFNS	QEGNTFKLQE	SIDPAGEETM	SPTSPTLMPR	MRSHSFYSVN	MKDKGGIEKL	ESIFKERSLS
1210	1220	1230	1240	1250	1260	1270	1280
LHRATSSHSV	AKEPKAPAAP	ANTLAIVPDS	RRPSSCIDIY	VSAMDELHCD	IDPLDNSVNI	LGLGEPSFST	PVPSTAPSSS
1290	1300	1310	1320	1330	1340	1350	1360
AYATLAPTDR	PPSRSIDFED	ITSMDTRSFS	SDYTHLPECQ	NPWDSEPPMY	HTIERSKSSR	YLATTPFLLE	EAPIVKSHSF
1370	1380	1390	1400	1410	1420	1430	1440
MFSPSRSYYA	NFGVPVKTAE	YTSITDCIDT	RCVNAPQAIA	DRAAFPGGLG	DKVEDLTCCH	PEREAELSHP	SSDSEENEAK
1450	1460	1470	1480	1490	1500	1510	1520
GRRATIAISS	QEGDNSERTL	SNNITVPKIE	RANSYSAEEP	SAPYAHTRKS	FSISDKLDRQ	RNTASLRNPF	QRSKSSKPEG
1530	1540	1550					
RGDSLSMRRL	SRTSAFQSFE	SKHN					

Cmpd.	No. of Cmpds.	m/z meas.	Δ m/z [ppm]	z	Rt [min]	Score	P	Range	Sequence	Modification
2053	1	575.2161	-129.75	3	55.6	15.5	2	1055-1068	R.IRVTSERVENMSMR.L	Oxidation: 11

Detailed Protein Report

Protein 547: deoxyribonuclease gamma isoform 2 precursor [Homo sapiens]

**Accession:**	gi|375151549	**Score:**	27.2
**Database:**	refseq_human(refseq_human_20140103.fasta)	**MW [kDa]:**	31.8
**Database Date:**	2015-11-30	**pI:**	9.7
**Modification(s):**	Oxidation	**Sequence Coverage [%]:**	9.1
		**No. of unique Peptides:**	1

Quantitation

QU:MU	**Median:**	1.28	**CV:**	0.00 %	**No. of Peptides:**	1
WUP:QUP	**Median:**	1.55	**CV:**	0.00 %	**No. of Peptides:**	1

10	20	30	40	50	60	70	80
MSRELAPLLL	LLLSIHSALA	MRICSFNVRS	FGESKQEDKN	AMDVIVKVIK	RCDIILVMEI	KDSNNRICPI	LMEKLNREKL
**90**	**100**	**110**	**120**	**130**	**140**	**150**	**160**
VSVKRSYHYH	DYQDGDADVF	SREPFVVWFQ	SPHTAVKDFV	IIPLHTTPET	SVKEIDELVE	VYTDVKHRWK	AENFIFMGDF
**170**	**180**	**190**	**200**	**210**	**220**	**230**	**240**
NAGCSYVPKK	AWKNIRLRTD	PRFVWLIGDQ	EDTTVKKSTN	CAYDRIVLRG	QEIVSSVVPK	SNSVFDFQKA	YKLTEEEALD
**250**	**260**	**270**	**280**				
VSDHFPVEFK	LQSSRAFTNS	KKSVTLRKKT	KSKRS				

Cmpd.	No. of Cmpds.	m/z meas.	Δ m/z [ppm]	z	Rt [min]	Score	P	Range	Sequence	Modification	Ratios
2460	1	873.4565	-23.34	2	59.0	11.5	2	36-50	K.QEDKNAMDVIVKVIK.R	Oxidation: 7	QU:MU 1.28<br>WUP:QUP 1.55

Detailed Protein Report

Protein 548: **PREDICTED: rab11 family-interacting protein 4 isoform X1 [Homo sapiens]**

**Accession:**	gi|530411171	**Score:**	27.1
**Database:**	refseq_human(refseq_human_20140103.fasta)	**MW [kDa]:**	67.7
**Database Date:**	2015-11-30	**pI:**	4.8
**Modification(s):**	Carbamidomethyl	**Sequence Coverage [%]:**	5.6
		**No. of unique Peptides:**	1

10	20	30	40	50	60	70	80
MSPQLDTKSP	SAHAGLPATG	GGGAAPSTGA	QKCERQRRGR	DCSCASGLPA	ASPRAADPRP	LASAPLPGSG	PRMRTPPALG
**90**	**100**	**110**	**120**	**130**	**140**	**150**	**160**
SQGSEVTGPT	FADGELIPRE	PGFFPEDEEE	AMTLAPPEGP	QELYTDSPME	STQSLEGSVG	SPAEKDGGLG	GLFLPEDKSL
**170**	**180**	**190**	**200**	**210**	**220**	**230**	**240**
VHTPSMTTSD	LSTHSTTSLI	SNEEQFEDYG	EGDDVDCAPS	SPCPDDETRT	NVYSDLGSSV	SSSAGQTPRK	MRHVYNSELL
**250**	**260**	**270**	**280**	**290**	**300**	**310**	**320**
DVYCSQCCKK	INLLNDLEAR	LKNLKANSPN	RKISSTAFGR	QLMHSSNFSS	SNGSTEDLFR	DSIDSCDNDI	TEKVSFLEKK
**330**	**340**	**350**	**360**	**370**	**380**	**390**	**400**
VTELENDSLT	NGDLKSKLKQ	ENTQLVHRVH	ELEEMVKDQE	TTAEQALEEE	ARRHREAYGK	LEREKATEVE	LLNARVQQLE
**410**	**420**	**430**	**440**	**450**	**460**	**470**	**480**
EENTELRTTV	TRLKSQTEKL	DEERQRMSDR	LEDTSLRLKD	EMDLYKRMMD	KLRQNRLEFQ	KEREATQELI	EDLRKELEHL
**490**	**500**	**510**	**520**	**530**	**540**	**550**	**560**
QMYKLDCERP	GRGRSASSGL	GEFNARAREV	ELEHEVKRLK	QENYKLRDQN	DDLNGQILSL	SLYEAKNLFA	AQTKAQSLAA
**570**	**580**	**590**	**600**	**610**			
EIDTASRDEL	MEALKEQEEI	NFRLRQYMDK	IILAILDHNP	SILEIKH			

Cmpd.	No. of Cmpds.	m/z meas.	Δ m/z [ppm]	z	Rt [min]	Score	P	Range	Sequence	Modification
1981	1	696.2626	-62.02	2	54.7	16.3	0	41-54	R.DCSCASGLPAASPR.A	Carbamidomethyl: 2

Detailed Protein Report

Protein 549: sterol 26-hydroxylase, mitochondrial precursor [Homo sapiens]

Accession: gi|4503211
Database: refseq_human(refseq_human_20140103.fasta)
Database Date: 2015-11-30

Score: 27.1
MW [kDa]: 60.2
pI: 9.7
Sequence Coverage [%]: 5.3
No. of unique Peptides: 1

10	20	30	40	50	60	70	80
MAALGCARLR	WALRGAGRGL	CPHGARAKAA	IPAALPSDKA	TGAPGAGPGV	RRRQRSLEEI	PRLGQLRFFF	QLFVQGYALQ
90	100	110	120	130	140	150	160
LHQLQVLYKA	KYGPMWMSYL	GPQMHVNLAS	APLLEQVMRQ	EGKYPVRNDM	ELWKEHRDQH	DLTYGPFTTE	GHHWYQLRQA
170	180	190	200	210	220	230	240
LNQRLLKPAE	AALYTDAFNE	VIDDFMTRLD	QLRAESASGN	QVSDMAQLFY	YFALEAICYI	LFEKRIGCLQ	RSIPEDTVTF
250	260	270	280	290	300	310	320
VRSIGLMFQN	SLYATFLPKW	TRPVLPFWKR	YLDGWNAIFS	FGKKLIDEKL	EDMEAQLQAA	GPDGIQVSGY	LHFLLASGQL
330	340	350	360	370	380	390	400
SPREAMGSLP	ELLMAGVDTT	SNTLTWALYH	LSKDPEIQEA	LHEEVVGVVP	AGQVPQHKDF	AHMPLLKAVL	KETLRLYPVV
410	420	430	440	450	460	470	480
PTNSRIIEKE	IEVDGFLFPK	NTQFVFCHYV	VSRDPTAFSE	PESFQPHRWL	RNSQPATPRI	QHPFGSVPFG	YGVRACLGRR
490	500	510	520	530	540		
IAELEMQLLL	ARLIQKYKVV	LAPETGELKS	VARIVLVPNK	KVGLQFLQRQ	C		

Cmpd.	No. of Cmpds.	m/z meas.	Δ m/z [ppm]	z	Rt [min]	Score	P	Range	Sequence	Modification
2189	1	937.1073	154.72	2	57.8	12.8	2	124-137	K.YPVRNDMELWKEHR.D	

Detailed Protein Report

Protein 550: proline dehydrogenase 1, mitochondrial isoform 1 precursor [Homo sapiens]

**Accession:**	gi|304766736	**Score:**	27.0
**Database:**	refseq_human(refseq_human_20140103.fasta)	**MW [kDa]:**	67.9
**Database Date:**	2015-11-30	**pI:**	8.8
		**Sequence Coverage [%]:**	4.5
		**No. of unique Peptides:**	1

10	20	30	40	50	60	70	80
MALRRALPAL	RPCIPRFVPL	STAPASREQP	AAGPAAVPGG	GSATAVRPPV	PAVDFGNAQE	AYRSRRTWEL	ARSLLVLRLC
**90**	**100**	**110**	**120**	**130**	**140**	**150**	**160**
AWPALLARHE	QLLYVSRKLL	GQRLFNKLMK	MTFYGHFVAG	EDQESIQPLL	RHYRAFGVSA	ILDYGVEEDL	SPEEAEHKEM
**170**	**180**	**190**	**200**	**210**	**220**	**230**	**240**
ESCTSAAERD	GSGTNKRDKQ	YQAHRAFGDR	RNGVISARTY	FYANEAKCDS	HMETFLRCIE	ASGRVSDDGF	IAIKLTALGR
**250**	**260**	**270**	**280**	**290**	**300**	**310**	**320**
PQFLLQFSEV	LAKWRCFFHQ	MAVEQGQAGL	AAMDTKLEVA	VLQESVAKLG	IASRAEIEDW	FTAETLGVSG	TMDLLDWSSL
**330**	**340**	**350**	**360**	**370**	**380**	**390**	**400**
IDSRTKLSKH	LVVPNAQTGQ	LEPLLSRFTE	EEELQMTRML	QRMDVLAKKA	TEMGVRLMVD	AEQTYFQPAI	SRLTLEMQRK
**410**	**420**	**430**	**440**	**450**	**460**	**470**	**480**
FNVEKPLIFN	TYQCYLKDAY	DNVTLDVELA	RREGWCFGAK	LVRGAYLAQE	RARAAEIGYE	DPINPTYEAT	NAMYHRCLDY
**490**	**500**	**510**	**520**	**530**	**540**	**550**	**560**
VLEELKHNAK	AKVMVASHNE	DTVRFALRRM	EELGLHPADH	QVYFGQLLGM	CDQISFPLGQ	AGYPVYKYVP	YGPVMEVLPY
**570**	**580**	**590**	**600**	**610**			
LSRRALENSS	LMKGTHRERQ	LLWLELLRRL	RTGNLFHRPA				

Cmpd.	No. of Cmpds.	m/z meas.	Δ m/z [ppm]	z	Rt [min]	Score	P	Range	Sequence	Modification
1081	1	851.8806	-166.32	2	43.6	11.8	2	2-16	M.ALRRALPALRPCIPR.F	

Detailed Protein Report

Protein 551: tyrosine-protein phosphatase non-receptor type 4 [Homo sapiens]

**Accession:**	gi|4506295	**Score:**	27.0
**Database:**	refseq_human(refseq_human_20140103.fasta)	**MW [kDa]:**	105.8
**Database Date:**	2015-11-30	**pI:**	7.4
**Modification(s):**	Oxidation	**Sequence Coverage [%]:**	3.3
		**No. of unique Peptides:**	2

10	20	30	40	50	60	70	80
MTSRFRLPAG	RTYNVRASEL	ARDRQHTEVV	CNILLLDNTV	QAFKVNKHDQ	GQVLLDVVFK	HLDLTEQDYF	GLQLADDSTD
**90**	**100**	**110**	**120**	**130**	**140**	**150**	**160**
NPRWLDPNKP	IRKQLKRGSP	YSLNFRVKFF	VSDPNKLQEE	YTRYQYFLQI	KQDILTGRLP	CPSNTAALLA	SFAVQSELGD
**170**	**180**	**190**	**200**	**210**	**220**	**230**	**240**
YDQSENLSGY	LSDYSFIPNQ	PQDFEKEIAK	LHQQHIGLSP	AEAEFNYLNT	ARTLELYGVE	FHYARDQSNN	EIMIGVMSGG
**250**	**260**	**270**	**280**	**290**	**300**	**310**	**320**
ILIYKNRVRM	NTFPWLKIVK	ISFKCKQFFI	QLRKELHESR	ETLLGFNMVN	YRACKNLWKA	CVEHHTFFRL	DRPLPPQKNF
**330**	**340**	**350**	**360**	**370**	**380**	**390**	**400**
FAHYFTLGSK	FRYCGRTEVQ	SVQYGKEKAN	KDRVFARSPS	KPLARKLMDW	EVVSRNSISD	DRLETQSLPS	RSPPGTPNHR
**410**	**420**	**430**	**440**	**450**	**460**	**470**	**480**
NSTFTQEGTR	LRPSSVGHLV	DHMVHTSPSE	VFVNQRSPSS	TQANSIVLES	SPSQETPGDG	KPPALPPKQS	KKNSWNQIHY
**490**	**500**	**510**	**520**	**530**	**540**	**550**	**560**
SHSQQDLESH	INETFDIPSS	PEKPTPNGGI	PHDNLVLIRM	KPDENGRFGF	NVKGGYDQKM	PVIVSRVAPG	TPADLCVPRL
**570**	**580**	**590**	**600**	**610**	**620**	**630**	**640**
NEGDQVVLIN	GRDIAEHTHD	QVVLFIKASC	ERHSGELMLL	VRPNAVYDVV	EEKLENEPDF	QYIPEKAPLD	SVHQDDHSLR
**650**	**660**	**670**	**680**	**690**	**700**	**710**	**720**
ESMIQLAEGL	ITGTVLTQFD	QLYRKKPGMT	MSCAKLPQNI	SKNRYRDISP	YDATRVILKG	NEDYINANYI	NMEIPSSSII
**730**	**740**	**750**	**760**	**770**	**780**	**790**	**800**
NQYIACQGPL	PHTCTDFWQM	TWEQGSSMVV	MLTTQVERGR	VKCHQYWPEP	TGSSSYGCYQ	VTCHSEEGNT	AYIFRKMTLF
**810**	**820**	**830**	**840**	**850**	**860**	**870**	**880**
NQEKNESRPL	TQIQYIAWPD	HGVPDDSSDF	LDFVCHVRNK	RAGKEEPVVV	HCSAGIGRTG	VLITMETAMC	LIECNQPVYP
**890**	**900**	**910**	**920**	**930**			
LDIVRTMRDQ	RAMMIQTPSQ	YRFVCEAILK	VYEEGFVKPL	TTSTNK			

Cmpd.	No. of Cmpds.	m/z meas.	Δ m/z [ppm]	z	Rt [min]	Score	P	Range	Sequence	Modification
702	1	733.4640	133.70	3	38.8	14.9	0	226-245	R.DQSNNEIMIGVMSGGILIYK.N	Oxidation: 12
2736	1	694.8753	-23.24	2	64.6	12.1	2	676-686	K.LPQNISKNRYR.D	

Detailed Protein Report

Protein 552: coiled-coil domain-containing protein lobo homolog isoform b [Homo sapiens]

**Accession:**	gi|574584573	**Score:**	27.0
**Database:**	refseq_human(refseq_human_20140103.fasta)	**MW [kDa]:**	96.2
**Database Date:**	2015-11-30	**pI:**	5.4
**Modification(s):**	Oxidation	**Sequence Coverage [%]:**	2.1
		**No. of unique Peptides:**	1

10	20	30	40	50	60	70	80
MEVLREKVEE	EEEAEREEAA	EWAEWARMEK	MMRPVEVRKE	EITLKQETLR	DLEKKLSEIQ	ITVSAELPAF	TKDTIDISKL
**90**	**100**	**110**	**120**	**130**	**140**	**150**	**160**
PISYKTNTPK	EEHLLQVADN	FSRQYSHLCP	DRVPLFLHPL	NECEVPKFVS	TTLRPTLMPY	PELYNWDSCA	QFVSDFLTMV
**170**	**180**	**190**	**200**	**210**	**220**	**230**	**240**
PLPDPLKPTI	KKEEKVLPKK	YTIKPPRDLC	SRFEQEQEVK	KQQEIRAQEK	KRLREEEERL	MEAEKAKPDA	LHGLRVHSWV
**250**	**260**	**270**	**280**	**290**	**300**	**310**	**320**
LVLSGKREVP	ENFFIDPFTG	HSYSTQDEHF	LGIESLWNHK	NYWINMQDCW	NCCKDLIFDL	GDPVRWEYML	LGTDKSQLSL
**330**	**340**	**350**	**360**	**370**	**380**	**390**	**400**
TEEDDSGIND	EDDVENLGKE	DEDKSFDMPH	SWVEQIEISP	EAFETRCPNG	KKVIQYKRAK	LEKWAPYLNS	NGLVSRLTTY
**410**	**420**	**430**	**440**	**450**	**460**	**470**	**480**
EDLQCTNILE	IKEWYQNRED	MLELKHINKT	TDLKTDYFKP	GHPQALRVHS	YKSMQPEMDR	VIEFYETARV	DGLMKREETP
**490**	**500**	**510**	**520**	**530**	**540**	**550**	**560**
RTMTEYYQGR	PDFLSYRHAS	FGPRVKKLTL	SSAESNPRPI	VKITERFFRN	PAKPAEEDVA	ERVFLVAEER	IQLRYHCRED
**570**	**580**	**590**	**600**	**610**	**620**	**630**	**640**
HITASKREFL	RRTEVDSKGN	KIIMTPDMCI	SFEVEPMEHT	KKLLYQYEAM	MHLKREEKLS	RHQVWESELE	VLEILKLREE
**650**	**660**	**670**	**680**	**690**	**700**	**710**	**720**
EEAAHTLTIS	IYDTKRNEKS	KEYREAMERM	MHEEHLRQVE	TQLDYLAPFL	AQLPPGEKLT	CWQAVRLKDE	CLSDFKQRLI
**730**	**740**	**750**	**760**	**770**	**780**	**790**	**800**
NKANLIQARF	EKETQELQKK	QQWYQENQVT	LTPEDEDLYL	SYCSQAMFRI	RILEQRLNRH	KELAPLKYLA	LEEKLYKDPR
**810**							
LGELQKIFA							

Cmpd.	No. of Cmpds.	m/z meas.	Δ m/z [ppm]	z	Rt [min]	Score	P	Range	Sequence	Modification
1516	1	513.1182	-169.11	2	48.6	13.7	0	453-460	K.SMQPEMDR.V	Oxidation: 2, 6

Detailed Protein Report

Protein 553: tubby-related protein 4 isoform 1 [Homo sapiens]

**Accession:**	gi|55953100	**Score:**	27.0
**Database:**	refseq_human(refseq_human_20140103.fasta)	**MW [kDa]:**	168.9
**Database Date:**	2015-11-30	**pI:**	8.3
		**Sequence Coverage [%]:**	1.9
		**No. of unique Peptides:**	1

Quantitation

WUP:QUP	**Median:** 0.72	**CV:** 0.00 %	**No. of Peptides:** 1

Alias proteins:

Accession	Name	Description
gi|530383880	refseq_human (refseq_human_20140103.fasta)	PREDICTED: tubby-related protein 4 isoform X1 [Homo sapiens]

10	20	30	40	50	60	70	80
MYAAVEHGPV	LCSDSNILCL	SWKGRVPKSE	KEKPVCRRRY	YEEGWLATGN	GRGVVGVTFT	SSHCRRDRST	PQRINFNLRG
90	100	110	120	130	140	150	160
HNSEVVLVRW	NEPYQKLATC	DADGGIFVWI	QYEGRWSVEL	VNDRGAQVSD	FTWSHDGTQA	LISYRDGFVL	VGSVSGQRHW
170	180	190	200	210	220	230	240
SSEINLESQI	TCGIWTPDDQ	QVLFGTADGQ	VIVMDCHGRM	LAHVLLHESD	GVLGMSWNYP	IFLVEDSSES	DTDSDDYAPP
250	260	270	280	290	300	310	320
QDGPAAYPIP	VQNIKPLLTV	SFTSGDISLM	NNYDDLSPTV	IRSGLKEVVA	QWCTQGDLLA	VAGMERQTQL	GELPNGPLLK
330	340	350	360	370	380	390	400
SAMVKFYNVR	GEHIFTLDTL	VQRPIISICW	GHRDSRLLMA	SGPALYVVRV	EHRVSSLQLL	CQQAIASTLR	EDKDVSKLTL
410	420	430	440	450	460	470	480
PPRLCSYLST	AFIPTIKPPI	PDPNNMRDFV	SYPSAGNERL	HCTMKRTEDD	PEVGGPCYTL	YLEYLGGLVP	ILKGRRISKL
490	500	510	520	530	540	550	560
RPEFVIMDPR	TDSKPDEIYG	NSLISTVIDS	CNCSDSSDIE	LSDDWAAKKS	PKISRASKSP	KLPRISIEAR	KSPKLPRAAQ
570	580	590	600	610	620	630	640
ELSRSPRLPL	RKPSVGSPSL	TRREFPFEDI	TQHNYLAQVT	SNIWGTKFKI	VGLAAFLPTN	LGAVIYKTSL	LHLQPRQMTI
650	660	670	680	690	700	710	720
YLPEVRKISM	DYINLPVFNP	NVFSEDEDDL	PVTGASGVPE	NSPPCTVNIP	IAPIHSSAQA	MSPTQSIGLV	QSLLANQNVQ
730	740	750	760	770	780	790	800
LDVLTNQTTA	VGTAEHAGDS	ATQYPVSNRY	SNPGQVIFGS	VEMGRIIQNP	PPLSLPPPPQ	GPMQLSTVGH	GDRDHEHLQK
810	820	830	840	850	860	870	880
SAKALRPTPQ	LAAEGDAVVF	SAPQEVQVTK	INPPPPYPGT	IPAAPTTAAP	PPPLPPPQPP	VDVCLKKGDF	SLYPTSVHYQ
890	900	910	920	930	940	950	960
TPLGYERITT	FDSSGNVEEV	CRPRTRMLCS	QNTYTLPGPG	SSATLRLTAT	EKKVPQPCSS	ATLNRLTVPR	YSIPTGDPPP
970	980	990	1000	1010	1020	1030	1040
YPEIASQLAQ	GRGAAQRSDN	SLIHATLRRN	NREATLKMAQ	LADSPRAPLQ	PLAKSKGGPG	GVVTQLPARP	PPALYTCSQC
1050	1060	1070	1080	1090	1100	1110	1120
SGTGPSSQPG	ASLAHTASAS	PLASQSSYSL	LSPPDSARDR	TDYVNSAFTE	DEALSQHCQL	EKPLRHPPLP	EAAVTLKRPP
1130	1140	1150	1160	1170	1180	1190	1200
PYQWDPMLGE	DVWVPQERTA	QTSGPNPLKL	SSLMLSQGQH	LDVSRLPFIS	PKSPASPTAT	FQTGYGMGVP	YPGSYNNPPL
1210	1220	1230	1240	1250	1260	1270	1280
PGVQAPCSPK	DALSPTQFAQ	QEPAVVLQPL	YPPSLSYCTL	PPMYPGSSTC	SSLQLPPVAL	HPWSSYSACP	PMQNPQGTLP
1290	1300	1310	1320	1330	1340	1350	1360
PKPHLVVEKP	LVSPPPADLQ	SHLGTEVMVE	TADNFQEVLS	LTESPVPQRT	EKFGKKNRKR	LDSRAEEGSV	QAITEGKVKK
1370	1380	1390	1400	1410	1420	1430	1440
EARTLSDFNS	LISSPHLGRE	KKKVKSQKDQ	LKSKKLNKTN	EFQDSSESEP	ELFISGDELM	NQSQGSRKGW	KSKRSPRAAG
1450	1460	1470	1480	1490	1500	1510	1520
ELEEAKCRRA	SEKEDGRLGS	QGFVYVMANK	QPLWNEATQV	YQLDFGGRVT	QESAKNFQIE	LEGRQVMQFG	RIDGSAYILD
1530	1540	1550					
FQYPFSAVQA	FAVALANVTQ	RLK					

Detailed Protein Report

Cmpd.	No. of Cmpds.	m/z meas.	Δ m/z [ppm]	z	Rt [min]	Score	P	Range	Sequence	Modification	Ratios	
2366	1	649.2906	-70.12	3	60.0	16.8	2	1340-1357	K.RLDSRAEEGSVQAITEGK.V		WUP:QUP	0.72

Detailed Protein Report

Protein 554: PREDICTED: transmembrane channel-like protein 8 isoform X4 [Homo sapiens]

Accession: gi|578830481
Database: refseq_human(refseq_human_20140103.fasta)
Database Date: 2015-11-30
Modification(s): Oxidation

Score: 26.9
MW [kDa]: 45.7
pI: 10.7
Sequence Coverage [%]: 2.2
No. of unique Peptides: 1

10	20	30	40	50	60	70	80
MLLPRSVSSE	RAPGVPEPEE	LWEAEMERLR	GSGTPVRGLP	YAMMDKRLIW	QLREPAGVQT	LRWQRWQRRR	QTVERRLREA
**90**	**100**	**110**	**120**	**130**	**140**	**150**	**160**
AQRLARGLGL	WEGALYEIGG	LFGTGIRSYF	TFLRFLLLLN	LLSLLLTASF	VLLPLVWLRP	PDPGPTLNLT	LQCPGSRQSP
**170**	**180**	**190**	**200**	**210**	**220**	**230**	**240**
PGVLRFHNQL	WHVLTGRAFT	NTYLFYGAYR	VGPESSSVYS	IRLAYLLSPL	ACLLLCFCGT	LRRMVKGLPQ	KTLLGQGYQA
**250**	**260**	**270**	**280**	**290**	**300**	**310**	**320**
PLSAKVFSSW	DFCIRVQEAA	TIKKHEISNE	FKVELEEGRR	FQLMQQQTRA	QTACRLLSYL	RVNVLNGLLV	VGAISAIFWA
**330**	**340**	**350**	**360**	**370**	**380**	**390**	**400**
TKYSQDNKEV	SGVPVSAAPV	PAPWGHRPGQ	LPGSPAVHIS	GPAGELPSQH	GGQPHSDLVR	GAEAGQLGDV	LRLPGSDHTV
**410**							
HWQRQEQL							

Cmpd.	No. of Cmpds.	m/z meas.	Δ m/z [ppm]	z	Rt [min]	Score	P	Range	Sequence	Modification
1031	1	598.1512	-245.53	2	43.0	26.9	0	281-289	R.FQLMQQQTR.A	Oxidation: 4

Detailed Protein Report

Protein 555: E3 ubiquitin-protein ligase UHRF1 isoform 1 [Homo sapiens]

**Accession:**	gi|115430235	**Score:**	26.9
**Database:**	refseq_human(refseq_human_20140103.fasta)	**MW [kDa]:**	89.8
**Database Date:**	2015-11-30	**pI:**	8.7
**Modification(s):**	Carbamidomethyl, Oxidation	**Sequence Coverage [%]:**	3.5
		**No. of unique Peptides:**	2

10	20	30	40	50	60	70	80
MWIQVRTMDG	RQTHTVDSLS	RLTKVEELRR	KIQELFHVEP	GLQRLFYRGK	QMEDGHTLFD	YEVRLNDTIQ	LLVRQSLVLP
**90**	**100**	**110**	**120**	**130**	**140**	**150**	**160**
HSTKERDSEL	SDTDSGCCLG	QSESDKSSTH	GEAAAETDSR	PADEDMWDET	ELGLYKVNEY	VDARDTNMGA	WFEAQVVRVT
**170**	**180**	**190**	**200**	**210**	**220**	**230**	**240**
RKAPSRDEPC	SSTSRPALEE	DVIYHVKYDD	YPENGVVQMN	SRDVRARART	IIKWQDLEVG	QVVMLNYNPD	NPKERGFWYD
**250**	**260**	**270**	**280**	**290**	**300**	**310**	**320**
AEISRKRETR	TARELYANVV	LGDDSLNDCR	IIFVDEVFKI	ERPGEGSPMV	DNPMRRKSGP	SCKHCKDDVN	RLCRVCACHL
**330**	**340**	**350**	**360**	**370**	**380**	**390**	**400**
CGGRQDPDKQ	LMCDECDMAF	HIYCLDPPLS	SVPSEDEWYC	PECRNDASEV	VLAGERLRES	KKKAKMASAT	SSSQRDWGKG
**410**	**420**	**430**	**440**	**450**	**460**	**470**	**480**
MACVGRTKEC	TIVPSNHYGP	IPGIPVGTMW	RFRVQVSESG	VHRPHVAGIH	GRSNDGAYSL	VLAGGYEDDV	DHGNFFTYTG
**490**	**500**	**510**	**520**	**530**	**540**	**550**	**560**
SGGRDLSGNK	RTAEQSCDQK	LTNTNRALAL	NCFAPINDQE	GAEAKDWRSG	KPVRVVRNVK	GGKNSKYAPA	EGNRYDGIYK
**570**	**580**	**590**	**600**	**610**	**620**	**630**	**640**
VVKYWPEKGK	SGFLVWRYLL	RRDDDEPGPW	TKEGKDRIKK	LGLTMQYPEG	YLEALANRER	EKENSKREEE	EQQEGGFASP
**650**	**660**	**670**	**680**	**690**	**700**	**710**	**720**
RTGKGKWKRK	SAGGGPSRAG	SPRRTSKKTK	VEPYSLTAQQ	SSLIREDKSN	AKLWNEVLAS	LKDRPASGSP	FQLFLSKVEE
**730**	**740**	**750**	**760**	**770**	**780**	**790**	**800**
TFQCICCQEL	VFRPITTVCQ	HNVCKDCLDR	SFRAQVFSCP	ACRYDLGRSY	AMQVNQPLQT	VLNQLFPGYG	NGR

Cmpd.	No. of Cmpds.	m/z meas.	Δ m/z [ppm]	z	Rt [min]	Score	P	Range	Sequence	Modification
2815	2	626.8578	121.32	2	63.8	11.9	1	396-406	R.DWGKGMACVGR.T	Carbamidomethyl: 8; Oxidation: 6
2699	1	973.4997	26.66	2	64.8	15.0	2	544-560	K.NSKYAPAEGNRYDGIYK.V	

Detailed Protein Report

Protein 556: PREDICTED: core histone macro-H2A.1 isoform X3 [Homo sapiens]

Accession: gi|530380257
Database: refseq_human(refseq_human_20140103.fasta)
Database Date: 2015-11-30

Score: 26.9
MW [kDa]: 39.0
pI: 10.3
Sequence Coverage [%]: 9.0
No. of unique Peptides: 2

10	20	30	40	50	60	70	80
MSSRGGKKKS	TKTSRSAKAG	VIFPVGRMLR	YIKKGHPKYR	IGVGAPVYMA	AVLEYLTAEI	LELAGNAARD	NKKGRVTPRH
90	100	110	120	130	140	150	160
ILLAVANDEE	LNQLLKGVTI	ASGGVLPNIH	PELLAKKRGS	KGKLEAIITP	PPAKKAKSPS	QKKPVSKKAG	GKKGARKSKK
170	180	190	200	210	220	230	240
QGEVSKAASA	DSTTEGTPAD	GFTVLSTKSL	FLGQKLQVVQ	ADIASIDSDA	VVHPTNTDFY	IGGEVGNTLE	KKGGKEFVEA
250	260	270	280	290	300	310	320
VLELRKKNGP	LEVAGAAVSA	GHGLPAKFVI	HCNSPVWGAD	KCEELLEKTV	KNCLALADDK	KLKSIAFPSI	GSGRNGFPKQ
330	340	350	360	370			
TAAQLILKAI	SSYFVSTMSS	SIKTVYFVLF	DSESIGIYVQ	EMAKLDAN			

Cmpd.	No. of Cmpds.	m/z meas.	Δ m/z [ppm]	z	Rt [min]	Score	P	Range	Sequence	Modification
2553	1	639.3415	-83.77	2	61.9	15.2	1	124-135	K.LEAIITPPPAKK.A	
2834	1	806.4289	45.18	3	65.9	11.7	1	268-288	K.FVIHCNSPVWGADKCEELLEK.T	

Detailed Protein Report

Protein 557: PREDICTED: LIM domain only protein 7 isoform X22 [Homo sapiens]

**Accession:**	gi|530402379	**Score:**	26.9
**Database:**	refseq_human(refseq_human_20140103.fasta)	**MW [kDa]:**	147.1
**Database Date:**	2015-11-30	**pI:**	6.1
**Modification(s):**	Carbamidomethyl	**Sequence Coverage [%]:**	2.5
		**No. of unique Peptides:**	2

Quantitation

QU:MU **Median:** 2.64 **CV:** 0.00 % **No. of Peptides:** 1

10	20	30	40	50	60	70	80
MEGLEEAEAN	CSVAFAEAQR	WVEAVTEKNF	ETKDFRASLE	NGVLLCDLIN	KLKPGVIKKI	NRLSTPIAGL	DNINVFLKAC
**90**	**100**	**110**	**120**	**130**	**140**	**150**	**160**
EQIGLKEAQL	FHPGDLQDLS	NRVTVKQEET	DRRVKNVLIT	LYWLGRKAQS	NPYYNGPHLN	LKAFENLLGQ	ALTKALEDSS
**170**	**180**	**190**	**200**	**210**	**220**	**230**	**240**
FLKRSGRDSG	YGDIWCPERG	EFLAPPRHHK	REDSFESLDS	LGSRSLTSCS	SDITLRGGRE	GFESDTDSEF	TFKMQDYNKD
**250**	**260**	**270**	**280**	**290**	**300**	**310**	**320**
DMSYRRISAV	EPKTALPFNR	FLPNKSRQPS	YVPAPLRKKK	PDKHEDNRRS	WASPVYTEAD	GTFSRLFQKI	YGENGSKSMS
**330**	**340**	**350**	**360**	**370**	**380**	**390**	**400**
DVSAEDVQNL	RQLRYEEMQK	IKSQLKEQDQ	KWQDDLAKWK	DRRKSYTSDL	QKKKEEREEI	EKQALEKSKR	SSKTFKEMLQ
**410**	**420**	**430**	**440**	**450**	**460**	**470**	**480**
DRESQNQKST	VPSRRRMYSF	DDVLEEGKRP	PTMTVSEASY	QSERVEEKGA	TYPSEIPKED	STTFAKREDR	VTTEIQLPSQ
**490**	**500**	**510**	**520**	**530**	**540**	**550**	**560**
SPVEEQSPAS	LSSLRSRSTQ	MESTRVSASL	PRSYRKTDTV	RLTSVVTPRP	FGSQTRGISS	LPRSYTMDDA	WKYNGDVEDI
**570**	**580**	**590**	**600**	**610**	**620**	**630**	**640**
KRTPNNVVST	PAPSPDASQL	ASSLSSQKEV	AATEEDVTRL	PSPTSPFSSL	SQDQAATSKA	TLSSTSGLDL	MSESGEGEIS
**650**	**660**	**670**	**680**	**690**	**700**	**710**	**720**
PQREVSRSQD	QFSDMRISIN	QTPGKSLDFG	FTIKWDIPGI	FVASVEAGSP	AEFSQLQVDD	EIIAINNTKF	SYNDSKEWEE
**730**	**740**	**750**	**760**	**770**	**780**	**790**	**800**
AMAKAQETGH	LVMDVRRYGK	AGSPETKWID	ATSGIYNSEK	SSNLSVTTDF	SESLQSSNIE	SKEINGIHDE	SNAFESKASE
**810**	**820**	**830**	**840**	**850**	**860**	**870**	**880**
SISLKNLKRR	SQFFEQGSSD	SVVPDLPVPT	ISAPSRWVWD	QEEERKRQER	WQKEQDRLLQ	EKYQREQEKL	REEWQRAKQE
**890**	**900**	**910**	**920**	**930**	**940**	**950**	**960**
AERENSKYLD	EELMVLSSNS	MSLTTREPSL	ATWEATWSEG	SKSSDREGTR	AGEEERRQPQ	EEVVHEDQGK	KPQDQLVIER
**970**	**980**	**990**	**1000**	**1010**	**1020**	**1030**	**1040**
ERKWEQQLQE	EQEQKRLQAE	AEEQKRPAEE	QKRQAEIERE	TSVRIYQYRR	PVDSYDIPKT	EEASSGFLPG	DRNKSRSTTE
**1050**	**1060**	**1070**	**1080**	**1090**	**1100**	**1110**	**1120**
LDDYSTNKNG	NNKYLDQIGN	MTSSQRRSKK	EQVPSGAELE	RQQILQEMRK	RTPLHNDNSW	IRQRSASVNK	EPVSLPGIMR
**1130**	**1140**	**1150**	**1160**	**1170**	**1180**	**1190**	**1200**
RGESLDNLDS	PRSNSWRQPP	WLNQPTGFYA	SSSVQDFSRP	PPQLVSTSNR	AYMRNPSSSV	PPPSAGSVKT	STTGVATTQS
**1210**	**1220**	**1230**	**1240**	**1250**	**1260**	**1270**	**1280**
PTPRSHSPSA	SQSGSQLRNR	SVSGKRICSY	CNNILGKGAA	MIIESLGLCY	HLHCFKCVAC	ECDLGGSSSG	AEVRIRNHQL
**1290**	**1300**						
YCNDCYLRFK	SGRPTAM						

Cmpd.	No. of Cmpds.	m/z meas.	Δ m/z [ppm]	z	Rt [min]	Score	P	Range	Sequence	Modification	Ratios
51	1	857.9018	-103.42	2	29.6	11.0	0	63-78	R.LSTPIAGLDNINVFLK.A		
1358	1	955.9298	-2.00	2	46.6	15.9	2	164-179	K.RSGRDSGYGDIWCPER.G	Carbamidomethyl: 13	QU:MU 2.64

Detailed Protein Report

Protein 558: PREDICTED: dedicator of cytokinesis protein 7 isoform X2 [Homo sapiens]

Accession: gi|530363514
Database: refseq_human(refseq_human_20140103.fasta)
Database Date: 2015-11-30
Modification(s): Oxidation

Score: 26.9
MW [kDa]: 209.0
pI: 6.1
Sequence Coverage [%]: 2.0
No. of unique Peptides: 1

10	20	30	40	50	60	70	80
MAERRAFAQK	ISRTVAAEVR	KQISGQYSGS	PQLLKNLNIV	GNISHHTTVP	LTEAVDPVDL	EDYLITHPLA	VDSGPLRDLI
90	100	110	120	130	140	150	160
EFPPDDIEVV	YSPRDCRTLV	SAVPEESEMD	PHVRDCIRSY	TEDWAIVIRK	YHKLGTGFNP	NTLDKQKERQ	KGLPKQVFES
170	180	190	200	210	220	230	240
DEAPDGNSYQ	DDQDDLKRRS	MSIDDTPRGS	WACSIFDLKN	SLPDALLPNL	LDRTPNEEID	RQNDDQRKSN	RHKELFALHP
250	260	270	280	290	300	310	320
SPDEEEPIER	LSVPDIPKEH	FGQRLLVKCL	SLKFEIEIEP	IFASLALYDV	KEKKKISENF	YFDLNSEQMK	GLLRPHVPPA
330	340	350	360	370	380	390	400
AITTLARSAI	FSITYPSQDV	FLVIKLEKVL	QQGDIGECAE	PYMIFKEADA	TKNKEKLEKL	KSQADQFCQR	LGKYRMPFAW
410	420	430	440	450	460	470	480
TAIHLMNIVS	SAGSLERDST	EVEISTGERK	GSWSERRNSS	IVGRRSLERT	TSGDDACNLT	SFRPATLTVT	NFFKQEGDRL
490	500	510	520	530	540	550	560
SDEDLYKFLA	DMRRPSSVLR	RLRPITAQLK	IDISPAPENP	HYCLTPELLQ	VKLYPDSRVR	PTREILEFPA	RDVYVPNTTY
570	580	590	600	610	620	630	640
RNLLYIYPQS	LNFANRQGSA	RNITVKVQFM	YGEDPSNAMP	VIFGKSSCSE	FSKEAYTAVV	YHNRSPDFHE	EIKVKLPATL
650	660	670	680	690	700	710	720
TDHHHLLFTF	YHVSCQQKQN	TPLETPVGYT	WIPMLQNGRL	KTGQFCLPVS	LEKPPQAYSV	LSPEVPLPGM	KWVDNHKGVF
730	740	750	760	770	780	790	800
NVEVVAVSSI	HTQDPYLDKF	FALVNALDEH	LFPVRIGDMR	IMENNLENEL	KSSISALNSS	QLEPVVRFLH	LLLDKLILLV
810	820	830	840	850	860	870	880
IRPPVIAGQI	VNLGQASFEA	MASIINRLHK	NLEGNHDQHG	RNSLLASYIH	YVFRLPNTYP	NSSSPGPGGL	GGSVHYATMA
890	900	910	920	930	940	950	960
RSAVRPASLN	LNRSRSLSNS	NPDISGTPTS	PDDEVRSIIG	SKGLDRSNSW	VNTGGPKAAP	WGSNPSPSAE	STQAMDRSCN
970	980	990	1000	1010	1020	1030	1040
RMSSHTETSS	FLQTLTGRLP	TKKLFHEELA	LQWVVCSGSV	RESALQQAWF	FFELMVKSMV	HHLYFNDKLE	APRKSRFPER
1050	1060	1070	1080	1090	1100	1110	1120
FMDDIAALVS	TIASDIVSRF	QKDTEMVERL	NTSLAFFLND	LLSVMDRGFV	FSLIKSCYKQ	VSSKLYSLPN	PSVLVSLRLD
1130	1140	1150	1160	1170	1180	1190	1200
FLRIICSHEH	YVTLNLPCSL	LTPPASPSPS	VSSATSQSSG	FSTNVQDQKI	ANMFELSVPF	RQQHYLAGLV	LTELAVILDP
1210	1220	1230	1240	1250	1260	1270	1280
DAEGLFGLHK	KVINMVHNLL	SSHDSDPRYS	DPQIKARVAM	LYLPLIGIIM	ETVPQLYDFT	ETHNQRGRPI	CIATDDYESE
1290	1300	1310	1320	1330	1340	1350	1360
SGSMISQTVA	MAIAGTSVPQ	LTRPGSFLLT	STSGRQHTTF	SAESSRSLLI	CLLWVLKNAD	ETVLQKWFTD	LSVLQLNRLL
1370	1380	1390	1400	1410	1420	1430	1440
DLLYLCVSCF	EYKGKKVFER	MNSLTFKKSK	DMRAKLEEAI	LGSIGARQEM	VRRSRGQLER	SPSGSAFGSQ	ENLRWRKDMT
1450	1460	1470	1480	1490	1500	1510	1520
HWRQNTEKLD	KSRAEIEHEA	LIDGNLATEA	NLIILDTLEI	VVQTVSVTES	KESILGGVLK	VLLHSMACNQ	SAVYLQHCFA
1530	1540	1550	1560	1570	1580	1590	1600
TQRALVSKFP	ELLFEEETEQ	CADLCLRLLR	HCSSSIGTIR	SHASASLYLL	MRQNFEIGNN	FARVKMQVTM	SLSSLVGTSQ
1610	1620	1630	1640	1650	1660	1670	1680
NFNEEFLRRS	LKTILTYAEE	DLELRETTFP	DQVQDLVFNL	HMILSDTVKM	KEHQEDPEML	IDLMYRIAKG	YQTSPDLRLT
1690	1700	1710	1720	1730	1740	1750	1760
WLQNMAGKHS	ERSNHAEAAQ	CLVHSAALVA	EYLSMLEDRK	YLPVGCVTFQ	NISSNVLEES	AVSDDVVSPD	EEGICSGKYF
1770	1780	1790	1800	1810	1820	1830	1840
TESGLVGLLE	QAAASFSMAG	MYEAVNEVYK	VLIPIHEANR	DAKKLSTIHG	KLQEAFSKIV	HQSTGWEFYL	VYIFLFDSMK
1850	1860						
TSVILFFLKA	MFPS						

Cmpd.	No. of Cmpds.	m/z meas.	Δ m/z [ppm]	z	Rt [min]	Score	P	Range	Sequence	Modification

Detailed Protein Report

Cmpd.	No. of Cmpds.	m/z meas.	Δ m/z [ppm]	z	Rt [min]	Score	P	Range	Sequence	Modification
122	1	879.4101	-14.16	3	30.5	16.1	0	1501-1523	K.VLLHSMACNQSAVYLQHCFATQR.A	Oxidation: 6

Detailed Protein Report

Protein 559: cation channel sperm-associated protein subunit beta precursor [Homo sapiens]

Accession: gi|51339295
Database: refseq_human(refseq_human_20140103.fasta)
Database Date: 2015-11-30
Modification(s): Oxidation

Score: 26.8
MW [kDa]: 126.8
pI: 6.8
Sequence Coverage [%]: 2.7
No. of unique Peptides: 1

Quantitation

QU:MU **Median:** 0.92 **CV:** 0.00 % **No. of Peptides:** 1

10	20	30	40	50	60	70	80
MESPLIYVSV	LLLNIFEFSS	GIVYNKDDTE	KRFACSNKGF	PQENEIIKLY	LFLENLKIQC	FFQTENEIAS	KAMLSVFTSG
90	100	110	120	130	140	150	160
GLAPSLGIMN	STYNGIFHFN	LTLFSDRILW	LVDIPRENIT	QSTDIAAVEE	WLVRITLHHG	LNIYATEGTL	LDVIREPILQ
170	180	190	200	210	220	230	240
WTPGDVIPES	EISKLYPHVV	DLKVTKCPCA	NDVALLGFIV	DTIVDGVYIG	ITFGGFWHDY	DTTWFNMTQT	IYSQLQEEYE
250	260	270	280	290	300	310	320
DLSLVDMVLT	NHFLVILTSL	GLFVSEDLRY	PSRHSLSFSR	ADFCGFERVD	YVKGKLWYNE	RCFANREHFE	VDYVTVTFER
330	340	350	360	370	380	390	400
NRTLSESSSC	FYSQEPFLEW	VPCLPHIFKG	IKIFPTVLTF	LVDQERGTGV	YLFYNKVRKT	AIASVSTLRN	NEPNSQSKFP
410	420	430	440	450	460	470	480
IFRFPSSFSS	PVGMVFHPRS	HFLYAYGNQI	WLSVDGGNTF	QLIANFHDDI	IKKTFHSFYT	SAITFVSQRG	KVYSTKAGMG
490	500	510	520	530	540	550	560
RYSAVGSVTE	RIFTLYYDHL	GFLHKLTLGR	FEASGPPTAF	GNSRNLFGQP	PDMGFETALA	PQHTSLDEII	FFAYVPENEP
570	580	590	600	610	620	630	640
QETIYSKKFG	NIHYGKVIHS	GKTGRAYIRK	VLQHTTPKGF	LSSVIAEMKE	PFGLEEVNES	SCLSSSLLIN	KAGNVYKLTL
650	660	670	680	690	700	710	720
DSQVVQALFE	DTDIEKTVVL	PGYSSFLITS	ILDNKNALAI	ATMPESAPNN	MTFLKSTWFL	YNFGQRNGRT	WKIYSKPCNY
730	740	750	760	770	780	790	800
WFQHDDSPSL	NIVKYIDLGN	SYVLKAKVIR	NAKGFRMLEI	PLLTVFVGNP	NLLEVTAEVT	FDDTDSYVIT	ISAASKVLHQ
810	820	830	840	850	860	870	880
GSTSLAFIMW	SASTECFVTT	MVPTLKSSCS	YLRSMHHIPS	KFIPFEDWIS	GVHKDSQGFN	LIKTLPINYR	PPSNMGIAIP
890	900	910	920	930	940	950	960
LTDNFYHADP	SKPIPRNMFH	MSKKTGKFKQ	CANVSTREEC	NCTKDQKFSH	AVAFSDCREK	VPRFKFPITQ	YPVSLEIINE
970	980	990	1000	1010	1020	1030	1040
DGRVPLQSPY	LVTVTEVNMR	HNWKLKHTVP	ENIKRMKQLV	EPILGAAVYN	PSGLNLSIKG	SELFHFRVTV	ISGVTFCNLI
1050	1060	1070	1080	1090	1100	1110	1120
EEFQIYVDEA	PLPFPGHTLI	AVATAVVLGG	LIFIAFMFQL	QGIHPWRTFQ	RWIRRNQEKF	SSISLSELIH	RSKSEE

Cmpd.	No. of Cmpds.	m/z meas.	Δ m/z [ppm]	z	Rt [min]	Score	P	Range	Sequence	Modification	Ratios
2126	1	1051.4446	-118.59	2	56.4	11.5	1	591-609	K.VLQHTTPKGFLSSVIAEMK.E	Oxidation: 18	QU:MU 0.92

Detailed Protein Report

Protein 560: coiled-coil domain-containing protein 80 precursor [Homo sapiens]

**Accession:**	gi|41152074	**Score:**	26.8
**Database:**	refseq_human(refseq_human_20140103.fasta)	**MW [kDa]:**	108.1
**Database Date:**	2015-11-30	**pI:**	10.2
**Modification(s):**	Oxidation	**Sequence Coverage [%]:**	4.2
		**No. of unique Peptides:**	2

Quantitation

QU:MU	**Median:** 1.45	**CV:** 0.00 %	**No. of Peptides:**	1
WUP:QUP	**Median:** 1.23	**CV:** 0.00 %	**No. of Peptides:**	1

Alias proteins:

Accession	Name	Description
gi|41152076	refseq_human (refseq_human_20140103.fasta)	coiled-coil domain-containing protein 80 precursor [Homo sapiens]

10	20	30	40	50	60	70	80
MTWRMGPRFT	MLLAMWLVCG	SEPHPHATIR	GSHGGRKVPL	VSPDSSRPAR	FLRHTGRSRG	IERSTLEEPN	LQPLQRRRSV
**90**	**100**	**110**	**120**	**130**	**140**	**150**	**160**
PVLRLARPTE	PPARSDINGA	AVRPEQRPAA	RGSPREMIRD	EGSSARSRML	RFPSGSSSPN	ILASFAGKNR	VWVISAPHAS
**170**	**180**	**190**	**200**	**210**	**220**	**230**	**240**
EGYYRLMMSL	LKDDVYCELA	ERHIQQIVLF	HQAGEEGGKV	RRITSEGQIL	EQPLDPSLIP	KLMSFLKLEK	GKFGMVLLKK
**250**	**260**	**270**	**280**	**290**	**300**	**310**	**320**
TLQVEERYPY	PVRLEAMYEV	IDQGPIRRIE	KIRQKGFVQK	CKASGVEGQV	VAEGNDGGGG	AGRPSLGSEK	KKEDPRRAQV
**330**	**340**	**350**	**360**	**370**	**380**	**390**	**400**
PPTRESRVKV	LRKLAATAPA	LPQPPSTPRA	TTLPPAPATT	VTRSTSRAVT	VAARPMTTTA	FPTTQRPWTP	SPSHRPPTTT
**410**	**420**	**430**	**440**	**450**	**460**	**470**	**480**
EVITARRPSV	SENLYPPSRK	DQHRERPQTT	RRPSKATSLE	SFTNAPPTTI	SEPSTRAAGP	GRFRDNRMDR	REHGHRDPNV
**490**	**500**	**510**	**520**	**530**	**540**	**550**	**560**
VPGPPKPAKE	KPPKKKAQDK	ILSNEYEEKY	DLSRPTASQL	EDELQVGNVP	LKKAKESKKH	EKLEKPEKEK	KKKMKNENAD
**570**	**580**	**590**	**600**	**610**	**620**	**630**	**640**
KLLKSEKQMK	KSEKKSKQEK	EKSKKKKGGK	TEQDGYQKPT	NKHFTQSPKK	SVADLLGSFE	GKRRLLLITA	PKAENNMYVQ
**650**	**660**	**670**	**680**	**690**	**700**	**710**	**720**
QRDEYLESFC	KMATRKISVI	TIFGPVNNST	MKIDHFQLDN	EKPMRVVDDE	DLVDQRLISE	LRKEYGMTYN	DFFMVLTDVD
**730**	**740**	**750**	**760**	**770**	**780**	**790**	**800**
LRVKQYYEVP	ITMKSVFDLI	DTFQSRIKDM	EKQKKEGIVC	KEDKKQSLEN	FLSRFRWRRR	LLVISAPNDE	DWAYSQQLSA
**810**	**820**	**830**	**840**	**850**	**860**	**870**	**880**
LSGQACNFGL	RHITILKLLG	VGEEVGGVLE	LFPINGSSVV	EREDVPAHLV	KDIRNYFQVS	PEYFSMLLVG	KDGNVKSWYP
**890**	**900**	**910**	**920**	**930**	**940**	**950**	**960**
SPMWSMVIVY	DLIDSMQLRR	QEMAIQQSLG	MRCPEDEYAG	YGYHSYHQGY	QDGYQDDYRH	HESYHHGYPY	

Cmpd.	No. of Cmpds.	m/z meas.	Δ m/z [ppm]	z	Rt [min]	Score	P	Range	Sequence	Modification	Ratios
1362	5	698.8981	6.72	2	46.7	13.9	0	350-363	R.ATTLPPAPATTVTR.S		WUP:QUP 1.23<br>QU:MU 1.45
1645	1	1062.1080	-81.53	3	50.3	12.9	2	697-722	R. LISELRKEYGMTYNDFFMVLTDVD V	Oxidation: 18	

Detailed Protein Report

Protein 561: pleckstrin homology domain-containing family G member 1 [Homo sapiens]

**Accession:**	gi|71274148	**Score:**	26.8
**Database:**	refseq_human(refseq_human_20140103.fasta)	**MW [kDa]:**	155.3
**Database Date:**	2015-11-30	**pI:**	5.8
**Modification(s):**	Oxidation	**Sequence Coverage [%]:**	2.7
		**No. of unique Peptides:**	1

Alias proteins:

Accession	Name	Description
gi|578812862	refseq_human (refseq_human_20140103.fasta)	PREDICTED: pleckstrin homology domain-containing family G member 1 isoform X7 [Homo sapiens]
gi|578812859	refseq_human (refseq_human_20140103.fasta)	PREDICTED: pleckstrin homology domain-containing family G member 1 isoform X6 [Homo sapiens]
gi|530383900	refseq_human (refseq_human_20140103.fasta)	PREDICTED: pleckstrin homology domain-containing family G member 1 isoform X4 [Homo sapiens]
gi|530383898	refseq_human (refseq_human_20140103.fasta)	PREDICTED: pleckstrin homology domain-containing family G member 1 isoform X3 [Homo sapiens]
gi|530383896	refseq_human (refseq_human_20140103.fasta)	PREDICTED: pleckstrin homology domain-containing family G member 1 isoform X2 [Homo sapiens]

10	20	30	40	50	60	70	80
MELSDSDRPV	SFGSTSSSAS	SRDSHGSFGS	RMTLVSNSHM	GLFNQDKEVG	AIKLELIPAR	PFSSSELQRD	NPATGQQNAD
90	100	110	120	130	140	150	160
EGSERPPRAQ	WRVDSNGAPK	TIADSATSPK	LLYVDRVVQE	ILETERTYVQ	DLKSIVEDYL	DCIRDQTKLP	LGTEERSALF
170	180	190	200	210	220	230	240
GNIQDIYHFN	SELLQDLENC	ENDPVAIAEC	FVSKSEEFHI	YTQYCTNYPR	SVAVLTECMR	NKILAKFFRE	RQETLKHSLP
250	260	270	280	290	300	310	320
LGSYLLKPVQ	RILKYHLLLH	EIENHLDKDT	EGYDVVLDAI	DTMQRVAWHI	NDMKRKHEHA	VRLQEIQSLL	TNWKGPDLTS
330	340	350	360	370	380	390	400
YGELVLEGTF	RIQRAKNERT	LFLFDKLLLI	TKKRDDTFTY	KAHILCGNLM	LVEVIPKEPL	SFSVFHYKNP	KLQHTVQAKS
410	420	430	440	450	460	470	480
QQDKRLWVLH	LKRLILENHA	AKIPAKAKQA	ILEMDAIHHP	GFCYSPEGGT	KALFGSKEGS	APYRLRRKSE	PSSRSHKVLK
490	500	510	520	530	540	550	560
TSETAQDIQK	VSREEGSPQL	SSARPSPAQR	NSQPSSSTMI	SVLRAGGALR	NIWTDHQIRQ	ALFPSRRSPQ	ENEDDEDDYQ
570	580	590	600	610	620	630	640
MFVPSFSSSD	LNSTRLCEDS	TSSRPCSWHM	GQMESTETSS	SGHRIVRRAS	SAGESNTCPP	EIGTSDRTRE	LQNSPKTEGQ
650	660	670	680	690	700	710	720
EEMTPFGSSI	ELTIDDIDHV	YDNISYEDLK	LMVAKREEAE	STPSKSARDS	VRPKSTPELA	FTKRQAGHSK	GSLYAQTDGT
730	740	750	760	770	780	790	800
LSGGEASSQS	THELQAVEEN	IYDTIGLPDP	PSLGFKCSSL	KRAKRSTFLG	LEADFVCCDS	LRPFVSQDSL	QLSEDEAPYH
810	820	830	840	850	860	870	880
QATPDHGYLS	LLYDSPSGNL	SMPHKPVSDK	LSEEVDEIWN	DLENYIKKNE	DKARDRLLAA	FPVSKDDVPD	RLHAESTPEL
890	900	910	920	930	940	950	960
SRDVGRSVST	LSLPESQALL	TPVKSRAGRA	SRANCPFEED	LISKEGSFMS	LNRLSLASEM	PLMDNPYDLA	NSGLSQTDPE
970	980	990	1000	1010	1020	1030	1040
NPDLGMEATD	KTKSRVFMMA	RQYSQKIKKA	NQLLKVKSLE	LEQPPASQHQ	KSMHKDLAAI	LEEKKQGGPA	IGARIAEYSQ
1050	1060	1070	1080	1090	1100	1110	1120
LYDQIVFRES	PLKIQKDGWA	SPQESSLLRS	VSPSQVHHGS	GDWLLHSTYS	NGELADFCLP	PEQDLRSRYP	TFEINTKSTP
1130	1140	1150	1160	1170	1180	1190	1200
RQLSAACSVP	SLQTSDPLPG	SVQRCSVVVS	QPNKENWCQD	HLYNSLGRKG	ISAKSQPYHR	SQSSSSVLIN	KSMDSINYPS
1210	1220	1230	1240	1250	1260	1270	1280
DVGKQQLLSL	HRSSRCESHQ	DLLPDIADSH	QQGTEKLSDL	TLQDSQKVVV	VNRNLPLNAQ	IATQNYFSNF	KETDGDEDDY
1290	1300	1310	1320	1330	1340	1350	1360
VEIKSEEDES	ELELSHNRRR	KSDSKFVDAD	FSDNVCSGNT	LHSLNSPRTP	KKPVNSKLGL	SPYLTPYNDS	DKLNDYLWRG
1370	1380	1390					
PSPNQQNIVQ	SLREKFQCLS	SSSFA					

Cmpd.	No. of	m/z meas.	Δ m/z	z	Rt	Score	P	Range	Sequence	Modification

Detailed Protein Report

	Cmpds.		[ppm]		[min]					
2063	1	697.5833	-140.16	3	56.2	11.8	1	998-1015	K.SLELEQPPASQHQKSMHK.D	Oxidation: 16

Detailed Protein Report

Protein 562: PREDICTED: collagen alpha-2(I) chain-like [Homo sapiens]

**Accession:**	gi|578803403	**Score:**	26.8
**Database:**	refseq_human(refseq_human_20140103.fasta)	**MW [kDa]:**	69.1
**Database Date:**	2015-11-30	**pI:**	12.4
		**Sequence Coverage [%]:**	3.6
		**No. of unique Peptides:**	1

10	20	30	40	50	60	70	80
MKNRRPGTPS	HRDSWVPFRP	TPHTPYTLWR	CALARAILSQ	CMYFLAGFLT	ENREGGELGD	VRVWSKSLPP	TTSCTSRRED
**90**	**100**	**110**	**120**	**130**	**140**	**150**	**160**
ELSAPLQAAG	AASPPRWLSG	SGLRESGRRA	RRLPTFPKHN	PGKFRLREGD	GAVGRGGDEN	RGRRFQPGSS	SHTQRLPLTA
**170**	**180**	**190**	**200**	**210**	**220**	**230**	**240**
RGGGVNGGIP	TASRGRRLLG	EAAPRRSRRS	GGARSPESHI	RAGSESGRPP	IRLGGAGQHR	AVRSAAAGQG	LRLSAAPPPG
**250**	**260**	**270**	**280**	**290**	**300**	**310**	**320**
RRRPPSALPA	SPLSAAARSP	PASPRSPAVS	SPSVVCVCAR	ARAHGSVWGN	RKFCRLGEQY	GKSEPRRQRW	RAGEAARGRS
**330**	**340**	**350**	**360**	**370**	**380**	**390**	**400**
GGRVSRGAGT	PRGTPGCGGA	GLDARAGGRG	WPWMGPAGPR	ISRGPCGQHC	LLEPGMGEWR	GPCCPRGLGT	LPLPTPTRAA
**410**	**420**	**430**	**440**	**450**	**460**	**470**	**480**
AGRSSLRSSG	PSVTLSRDAV	IHGGGGGGRG	RDKHGSGEVL	NSVLLSCLSL	GTFSPLGSVL	DFAGCENLLT	SESSVEPRLG
**490**	**500**	**510**	**520**	**530**	**540**	**550**	**560**
LWSAYFLQSD	CSCLRWSLSV	PLWGTSALIS	ALTAAVRAAG	KWGAPSGGRV	AVSLLISSVT	FKLSLEATAA	AGHVRRALGC
**570**	**580**	**590**	**600**	**610**	**620**	**630**	**640**
WGKISSAGSR	DRAGGRRGTA	PRPRLGTWEG	RAGALGPGAR	PSWDTREDGG	GEEVGSAAGE	LGIMQMGKEN	LRPRPSAKSS
**650**	**660**	**670**					
LGICRYCGHV	MEGSGKYGLI	F					

Cmpd.	No. of Cmpds.	m/z meas.	Δ m/z [ppm]	z	Rt [min]	Score	P	Range	Sequence	Modification
195	1	572.8054	9.14	2	32.4	16.7	2	312-323	R.AGEAARGRSGGR.V	

Detailed Protein Report

Protein 563: PREDICTED: dynamin-binding protein isoform X1 [Homo sapiens]

**Accession:**	gi|578819186	**Score:**	26.8
**Database:**	refseq_human(refseq_human_20140103.fasta)	**MW [kDa]:**	172.2
**Database Date:**	2015-11-30	**pI:**	5.1
		**Sequence Coverage [%]:**	2.3
		**No. of unique Peptides:**	1

10	20	30	40	50	60	70	80
MEAGSVVRAI	FDFCPSVSEE	LPLFVGDIIE	VLAVVDEFWL	LGKKEDVTGQ	FPSSFVEIVT	IPSLKEGERL	FVCICEFTSQ
90	100	110	120	130	140	150	160
ELDNLPLHRG	DLVILDGIPT	AGWLQGRSCW	GARGFFPSSC	VRELCLSSQS	RQWHSQSALF	QIPEYSMGQA	RALMGLSAQL
170	180	190	200	210	220	230	240
DEELDFREGD	VITIIGVPEP	GWFEGELEGR	RGIFPEGFVE	LLGPLRTVDE	SVSSGNQDDC	IVNGEVDTPV	GEEEIGPDED
250	260	270	280	290	300	310	320
EEEPGTYGVA	LYRFQALEPN	ELDFEVGDKI	RILATLEDGW	LEGSLKGRTG	IFPYRFVKLC	PDTRVEETMA	LPQEGSLARI
330	340	350	360	370	380	390	400
PETSLDCLEN	TLGVEEQRHE	TSDHEAEEPD	CIISEAPTSP	LGHLTSEYDT	DRNSYQDEDT	AGGPPRSPGV	EWEMPLATDS
410	420	430	440	450	460	470	480
PTSDPTEVVN	GISSQPQVPF	HPNLQKSQYY	STVGGSHPHS	EQYPDLLPLE	ARTRDYASLP	PKRMYSQLKT	LQKPVLPLYR
490	500	510	520	530	540	550	560
GSSVSASRVV	KPRQSSPQLH	NLASYTKKHH	TSSVYSISER	LEMKPGPQAQ	GLVMEAATHS	QGDGSTDLDS	KLTQQLIEFE
570	580	590	600	610	620	630	640
KSLAGPGTEP	DKILRHFSIM	DFNSEKDIVR	GSSKLITEQE	LPERRKALRP	PPPRPCTPVS	TSPHLLVDQN	LKPAPPLVVR
650	660	670	680	690	700	710	720
PSRPAPLPPS	AQQRTNAVSP	KLLSRHRPTC	ETLEKEGPGH	MGRSLDQTSP	CPLVLVRIEE	MERDLDMYSR	AQEELNLMLE
730	740	750	760	770	780	790	800
EKQDESSRAE	TLEDLKFCES	NIESLNMELQ	QLREMTLLSS	QSSSLVAPSG	SVSAENPEQR	MLEKRAKVIE	ELLQTERDYI
810	820	830	840	850	860	870	880
RDLEMCIERI	MVPMQQAQVP	NIDFEGLFGN	MQMVIKVSKQ	LLAALEISDA	VGPVFLGHRD	ELEGTYKIYC	QNHDEAIALL
890	900	910	920	930	940	950	960
EIYEKDEKIQ	KHLQDSLADL	KSLYNEWGCT	NYINLGSFLI	KPVQRVMRYP	LLLMELLNST	PESHPDKVPL	TNAVLAVKEI
970	980	990	1000	1010	1020	1030	1040
NVNINEYKRR	KDLVLKYRKG	DEDSLMEKIS	KLNIHSIIKK	SNRVSSHLKH	LTGFAPQIKD	EVFEETEKNF	RMQERLIKSF
1050	1060	1070	1080	1090	1100	1110	1120
IRDLSLYLQH	IRKERTERLV	ISPLNQLLSM	FTGPHKLVQK	RFDKLLDFYN	CTERAEKLKD	KKTLEELQSA	RNNYEALNAQ
1130	1140	1150	1160	1170	1180	1190	1200
LLDELPKFHQ	YAQGLFTNCV	HGYAEAHCDF	VHQALEQLKP	LLSLLKVAGR	EGNLIAIFHE	EHSRVLQQLQ	VFTFFPESLP
1210	1220	1230	1240	1250	1260	1270	1280
ATKKPFERKT	IDRQSARKPL	LGLPSYMLQS	EELRASLLAR	YPPEKLFQAE	RNFNAAQDLD	VSLLEGDLVG	VIKKKDPMGS
1290	1300	1310	1320	1330	1340	1350	1360
QNRWLIDNGV	TKGFVYSSFL	KPYNPRRSHS	DASVGSHSST	ESEHGSSSPR	FPRQNSGSTL	TFNPSSMAVS	FTSGSCQKQP
1370	1380	1390	1400	1410	1420	1430	1440
QDASPPPKEC	DQGTLSASLN	PSNSESSPSR	CPSDPDSTSQ	PRSGDSADVA	RDVKQPTATP	RSYRNFRHPE	IVGYSVPGRN
1450	1460	1470	1480	1490	1500	1510	1520
GQSQDLVKGC	ARTAQAPEDR	STEPDGSEAE	GNQVYFAVYT	FKARNPNELS	VSANQKLKIL	EFKDVTGNTE	WWLAEVNGKK
1530	1540						
GYVPSNYIRK	TEYT						

Cmpd.	No. of Cmpds.	m/z meas.	Δ m/z [ppm]	z	Rt [min]	Score	P	Range	Sequence	Modification
2669	1	995.0095	-40.18	2	63.5	15.5	2	1235-1251	R.ASLLARYPPEKLFQAER.N	

Detailed Protein Report

Protein 564: sialate O-acetylesterase isoform 2 [Homo sapiens]

**Accession:**	gi|321267600	**Score:**	26.8
**Database:**	refseq_human(refseq_human_20140103.fasta)	**MW [kDa]:**	54.5
**Database Date:**	2015-11-30	**pI:**	7.1
**Modification(s):**	Carbamidomethyl	**Sequence Coverage [%]:**	3.5
		**No. of unique Peptides:**	1

10	20	30	40	50	60	70	80
MVLQKEPAGA	VIWGFGTPGA	TVTVTLRQGQ	ETIMKKVTSV	KAHSDTWMVV	LDPMKPGGPF	EVMAQQTLEK	INFTLRVHDV
**90**	**100**	**110**	**120**	**130**	**140**	**150**	**160**
LFGDVWLCSG	QSNMQMTVLQ	IFNATRELSN	TAAYQSVRIL	SVSPIQAEQE	LEDLVAVDLQ	WSKPTSENLG	HGYFKYMSAV
**170**	**180**	**190**	**200**	**210**	**220**	**230**	**240**
CWLFGRHLYD	TLQYPIGLIA	SSWGGTPIEA	WSSGRSLKAC	GVPKQGSIPY	DSVTGPSKHS	VLWNAMIHPL	CNMTLKGVVW
**250**	**260**	**270**	**280**	**290**	**300**	**310**	**320**
YQGESNINYN	TDLYNCTFPA	LIEDWRETFH	RGSQGQTERF	FPFGLVQLSS	DLSKKSSDDG	FPQIRWHQTA	DFGYVPNPKM
**330**	**340**	**350**	**360**	**370**	**380**	**390**	**400**
PNTFMAVAMD	LCDRDSPFGS	IHPRDKQTVA	YRLHLGARAL	AYGEKNLTFE	GPLPEKIELL	AHKGLLNLTY	YQQIQVQKKD
**410**	**420**	**430**	**440**	**450**	**460**	**470**	**480**
NKIFEISCCS	DHRCKWLPAS	MNTVSTQSLT	LAIDSCHGTV	VALRYAWTTW	PCEYKQCPLY	HPSSALPAPP	FIAFITDQGP
**490**							
GHQSNVAK							

Cmpd.	No. of Cmpds.	m/z meas.	Δ m/z [ppm]	z	Rt [min]	Score	P	Range	Sequence	Modification
693	1	1035.0409	84.42	2	38.6	14.9	2	400-415	K.DNKIFEISCCSDHRCK.W	Carbamidomethyl: 9, 10, 15

Detailed Protein Report

Protein 565: PREDICTED: RAD51-associated protein 2 isoform X1 [Homo sapiens]

**Accession:**	gi|530367071	**Score:**	26.7
**Database:**	refseq_human(refseq_human_20140103.fasta)	**MW [kDa]:**	132.8
**Database Date:**	2015-11-30	**pI:**	7.1
		**Sequence Coverage [%]:**	2.7
		**No. of unique Peptides:**	1

10	20	30	40	50	60	70	80
MAELRKPTSS	LTPPEDPDSQ	PPSSKRLCLE	EPGGVFKAGW	RLPLVPRLSE	AEKVWELSPR	PFKGLLVSTN	AIFDNSTDSC
**90**	**100**	**110**	**120**	**130**	**140**	**150**	**160**
VEKSVSGKQI	CNLKCSNLKF	QMSSCLQSPP	SQSPDSDLRA	SGRSEAGLHD	REAFSVHRSN	SSKAGVSQLL	PSTSIHDIHG
**170**	**180**	**190**	**200**	**210**	**220**	**230**	**240**
IRNENRKQQF	VQGRDNVHKE	NPFLDVTFYK	ETKSPFHEIK	NRCKANSVVP	SNKRENNISS	SVLKISKSQN	QPSLEIAKPS
**250**	**260**	**270**	**280**	**290**	**300**	**310**	**320**
YFRDSGTISV	PQFPMDLNSK	MSSVYLKEIA	KKKNDKKEAY	VRDFTNIYWS	QNRPDVKKQK	LQNDKKTVEA	ENIFSKCYEN
**330**	**340**	**350**	**360**	**370**	**380**	**390**	**400**
DYPSLSSQNT	CKRKDLISSN	YCNCSSIQCN	VRDSRKNFAI	LENANWEEAE	CLDSYVLTRL	EKSQNWDCNV	RHILRRNRGN
**410**	**420**	**430**	**440**	**450**	**460**	**470**	**480**
CWIINNCKTK	CENMKKTEEK	WNWLLLLEID	LLSKEDYHCA	KVINAYEEQS	KLLVREILGS	QTALITTVWL	NGKGENDNTL
**490**	**500**	**510**	**520**	**530**	**540**	**550**	**560**
QLRYNTTQKV	FHVNNPFESF	IIEIFYFHKS	ISGNKKDNSI	LTCCNILKCK	KQIGIIGIQN	LITRNMNTNI	KNGILSIYLQ
**570**	**580**	**590**	**600**	**610**	**620**	**630**	**640**
DSVSEPLDIL	LKTNIAFLLN	NFDSLTRIEN	DFELEEECIF	KCMLYLKYPK	NIVENHTAYL	VKILTSSRLL	EDNMKPMLKK
**650**	**660**	**670**	**680**	**690**	**700**	**710**	**720**
RKLFRTEQVF	EKSKKKLINS	FSMTTQNTGF	PIFETYEKIP	LLMDFDDMDE	ISLIREITCQ	NMSCPQQVVN	VENWAHYNSS
**730**	**740**	**750**	**760**	**770**	**780**	**790**	**800**
TVKAHGNSCP	QFIQNNRGYI	NENFYEVNMH	SQDLNMERKQ	GHNKISNFDC	EHIFEDLCNV	RQQAIPASHN	IIHNEETHTT
**810**	**820**	**830**	**840**	**850**	**860**	**870**	**880**
SITQVLNFWN	LLSEIEEKKY	DLILKEEVKV	TAESLTNSCQ	VHKDTKIEKE	EKDSFFPMDD	MFSVQSVSLI	SKEVNVEENK
**890**	**900**	**910**	**920**	**930**	**940**	**950**	**960**
YVNQNYVTNT	NEYESILPER	EIANSKDFHR	KNDSALYINH	QFETGLSEGN	DECFQDLAAK	YLSTEALTIV	KDFEMKRKFD
**970**	**980**	**990**	**1000**	**1010**	**1020**	**1030**	**1040**
LVLEELRMFH	EISRENELLS	TVETNNGQEN	YFGENDAEKV	KMEIEKDLKM	VVVNKIRASS	SFHDTIAGPN	MGKSHQSLFK
**1050**	**1060**	**1070**	**1080**	**1090**	**1100**	**1110**	**1120**
WKTVPNNGEQ	EVPNESCYPS	RSEEELLYST	SEKDCETPLP	KRPAFLPDEC	KEEFNYLLRG	GSHFPHGISR	VRPLKTCSRP
**1130**	**1140**	**1150**	**1160**				
IRIGLSRKAR	IKQLHPYLKQ	MCYGNLKENF					

Cmpd.	No. of Cmpds.	m/z meas.	Δ m/z [ppm]	z	Rt [min]	Score	P	Range	Sequence	Modification
1446	1	698.8871	-14.31	2	47.7	15.2	2	261-272	K.MSSVYLKEIAKK.K	

Detailed Protein Report

Protein 566: complexin-3 precursor [Homo sapiens]

**Accession:**	gi|71725345	**Score:**	26.7
**Database:**	refseq_human(refseq_human_20140103.fasta)	**MW [kDa]:**	17.5
**Database Date:**	2015-11-30	**pI:**	4.7
**Modification(s):**	Oxidation	**Sequence Coverage [%]:**	19.6
		**No. of unique Peptides:**	1

```
         10         20         30         40         50         60         70         80
MAFMVKTMVG GQLKNLTGSL GGGEDKGDGD KSAAEAQGMS REEYEEYQKQ LVEEKMERDA QFTQRKAERA TLRSHFRDKY
         90        100        110        120        130        140        150        160
RLPKNETDES QIQMAGGDVE LPRELAKMIE EDTEEEEEKA SVLGQLASLP GLNLGSLKDK AQATLGDLKQ SAEKCHVM
```

Cmpd.	No. of Cmpds.	m/z meas.	Δ m/z [ppm]	z	Rt [min]	Score	P	Range	Sequence	Modification
1306	1	721.2739	-148.07	2	45.9	11.1	1	2-14	M.AFMVKTMVGGQLK.N	Oxidation: 3, 7

Detailed Protein Report

Protein 567: metallothionein-1G [Homo sapiens]

**Accession:**	gi|10835230	**Score:**	26.7
**Database:**	refseq_human(refseq_human_20140103.fasta)	**MW [kDa]:**	6.1
**Database Date:**	2015-11-30	**pI:**	10.5
**Modification(s):**	Carbamidomethyl	**Sequence Coverage [%]:**	70.5
		**No. of unique Peptides:**	1

```
         10         20         30         40         50         60         70
MDPNCSCAAG VSCTCASSCK CKECKCTSCK KSCCSCCPVG CAKCAQGCIC KGASEKCSCC A
```

Cmpd.	No. of Cmpds.	m/z meas.	Δ m/z [ppm]	z	Rt [min]	Score	P	Range	Sequence	Modification
1987	2	862.6346	-31.70	3	53.2	13.2	2	1-25	-.MDPNCSCAAGVSCTCASSCKCKECK.C	Carbamidomethyl: 7

Detailed Protein Report

Protein 568: death-inducer obliterator 1 isoform c [Homo sapiens]

**Accession:**	gi|301129165	**Score:**	26.7
**Database:**	refseq_human(refseq_human_20140103.fasta)	**MW [kDa]:**	243.7
**Database Date:**	2015-11-30	**pI:**	8.9
		**Sequence Coverage [%]:**	1.2
		**No. of unique Peptides:**	1

Quantitation

QU:MU	**Median:** 0.74	**CV:** 0.00 %	**No. of Peptides:** 1

Alias proteins:

Accession	Name	Description
gi|301129170	refseq_huma (refseq_human_20140103.fasta)	death-inducer obliterator 1 isoform c [Homo sapiens]

Detailed Protein Report

10	20	30	40	50	60	70	80
MDDKGDPSNE	EAPKAIKPTS	KEFRKTWGFR	RTTIAKREGA	GDAEADPLEP	PPPQQQLGLS	LRRSGRQPKR	TERVEQFLTI
90	100	110	120	130	140	150	160
ARRRGRRSMP	VSLEDSGEPT	SCPATDAETA	SEGSVESASE	TRSGPQSAST	AVKERPASSE	KVKGGDDHDD	TSDSDSDGLT
170	180	190	200	210	220	230	240
LKELQNRLRR	KREQEPTERP	LKGIQSRLRK	KRREEGPAET	VGSEASDTVE	GVLPSKQEPE	NDQGVVSQAG	KDDRESKLEG
250	260	270	280	290	300	310	320
KAAQDIKDEE	PGDLGRPKPE	CEGYDPNALY	CICRQPHNNR	FMICCDRCEE	WFHGDCVGIS	EARGRLLERN	GEDYICPNCT
330	340	350	360	370	380	390	400
ILQVQDETHS	ETADQQEAKW	RPGDADGTDC	TSIGTIEQKS	SEDQGIKGRI	EKAANPSGKK	KLKIFQPVIE	APGASKCIGP
410	420	430	440	450	460	470	480
GCCHVAQPDS	VYCSNDCILK	HAAATMKFLS	SGKEQKPKPK	EKMKMKPEKP	SLPKCGAQAG	IKISSVHKRP	APEKKETTVK
490	500	510	520	530	540	550	560
KAVVVPARSE	ALGKEAACES	STPSWASDHN	YNAVKPEKTA	APSPSLLYKS	TKEDRRSEEK	AAAMAASKKT	APPGSAVGKQ
570	580	590	600	610	620	630	640
PAPRNLVPKK	SSFANVAAAT	PAIKKPPSGF	KGTIPKRPWL	SATPSSGASA	ARQAGPAPAA	ATAASKKFPG	SAALVGAVRK
650	660	670	680	690	700	710	720
PVVPSVPMAS	PAPGRLGAMS	AAPSQPNSQI	RQNIRRSLKE	ILWKRVNDSD	DLIMTENEVG	KIALHIEKEM	FNLFQVTDNR
730	740	750	760	770	780	790	800
YKSKYRSIMF	NLKDPKNQGL	FHRVLREEIS	LAKLVRLKPE	ELVSKELSTW	KERPARSVME	SRTKLHNESK	KTAPRQEAIP
810	820	830	840	850	860	870	880
DLEDSPPVSD	SEEQQESARA	VPEKSTAPLL	DVFSSMLKDT	TSQHRAHLFD	LNCKICTGQV	PSAEDEPAPK	KQKLSASVKK
890	900	910	920	930	940	950	960
EDLKSKHDSS	APDPAPDSAD	EVMPEAVPEV	ASEPGLESAS	HPNVDRTYFP	GPPGDGHPEP	SPLEDLSPCP	ASCGSGVVTT
970	980	990	1000	1010	1020	1030	1040
VTVSGRDPRT	APSSSCTAVA	SAASRPDSTH	MVEARQDVPK	PVLTSVMVPK	SILAKPSSSP	DPRYLSVPPS	PNISTSESRS
1050	1060	1070	1080	1090	1100	1110	1120
PPEGDTTLFL	SRLSTIWKGF	INMQSVAKFV	TKAYPVSGCF	DYLSEDLPDT	IHIGGRIAPK	TVWDYVGKLK	SSVSKELCLI
1130	1140	1150	1160	1170	1180	1190	1200
RFHPATEEEE	VAYISLYSYF	SSRGRFGVVA	NNNRHVKDLY	LIPLSAQDPV	PSKLLPFEGP	GLESPRPNII	LGLVICQKIK
1210	1220	1230	1240	1250	1260	1270	1280
RPANSGELDK	MDEKRTRLQP	EEADVPAYPK	VATVPQSEKK	PSKYPLCSAD	AAVSTTPPGS	PPPPPPLPEP	PVLKVLSSLK
1290	1300	1310	1320	1330	1340	1350	1360
PAAPSPATAA	TTAAAASTAA	SSTASSASKT	ASPLEHILQT	LFGKKKSFDP	SAREPPGSTA	GLPQEPKTTA	EDGVPAPPLL
1370	1380	1390	1400	1410	1420	1430	1440
DPIVQQFGQF	SKDKALEEEE	DDRPYDPEEE	YDPERAFDTQ	LVERGRRHEV	ERAPEAAAAE	REEVAYDPED	ETILEEAKVT
1450	1460	1470	1480	1490	1500	1510	1520
VDDLPNRMCA	DVRRNSVERP	AEPVAGAATP	SLVEQQKMLE	ELNKQIEEQK	RQLEEQEEAL	RQQRAAVGVS	MAHFSVSDAL
1530	1540	1550	1560	1570	1580	1590	1600
MSPPPKSSLP	KAELFQQEQQ	SADKPASLPP	ASQASNHRDP	RQARRLATET	GEGEGEPLSR	LSARGAQGAL	PERDASRGGL
1610	1620	1630	1640	1650	1660	1670	1680
VGQAPMPVPE	EKEPASSPWA	SGEKPPAGSE	QDGWKAEPGE	GTRPATVGDS	SARPARRVLL	PTPPCGALQP	GFPLQHDGER
1690	1700	1710	1720	1730	1740	1750	1760
DPFTCPGFAS	QDKALGSAQY	EDPRNLHSAG	RSSSPAGETE	GDREPQARPG	EGTAPLPPPG	QKVGGSQPPF	QGQREPGPHA
1770	1780	1790	1800	1810	1820	1830	1840
LGMSGLHGPN	FPGPRGPAPP	FPEENIASND	GPRGPPPARF	GAQKGPIPSL	FSGQHGPPPY	GDSRGPSPSY	LGGPRGVAPS
1850	1860	1870	1880	1890	1900	1910	1920
QFEERKDPHG	EKREFQDAPY	NEVTGAPAQF	EGTEQAPFLG	SRGGAPFQFG	GQRRPLLSQL	KGPRGGPPPS	QFGGQRGPPP
1930	1940	1950	1960	1970	1980	1990	2000
GHFVGPRGPH	PSQFETARGP	HPNQFEGPRG	QAPNFMPGPR	GIQPQQFEDQ	RVHSPPRFTN	QRAPAPLQFG	GLRGSAPFSE
2010	2020	2030	2040	2050	2060	2070	2080
KNEQTPSRFH	FQGQAPQVMK	PGPRPLLELP	SHPPQHRKDR	WEEAGPPSAL	SSSAPGQGPE	ADGQWASADF	REGKGHEYRN
2090	2100	2110	2120	2130	2140	2150	2160
QTFEGRQRER	FDVGPKEKPL	EEPDAQGRAS	EDRRRERERG	RNWSRERDWD	RPREWDRHRD	KDSSRDWDRN	RERSANRDRE
2170	2180	2190	2200	2210	2220	2230	2240

Detailed Protein Report

Cmpd.	No. of Cmpds.	m/z meas.	Δ m/z [ppm]	z	Rt [min]	Score	P	Range	Sequence	Modification	Ratios	
411	1	701.3611	72.76	2	35.1	11.7	1	2-14	M.DDKGDPSNEEAPK.A		QU:MU	0.74

Detailed Protein Report

Protein 569: **cullin-associated NEDD8-dissociated protein 2 isoform 2 [Homo sapiens]**

**Accession:**	gi|112420977	**Score:**	26.7
**Database:**	refseq_human(refseq_human_20140103.fasta)	**MW [kDa]:**	122.6
**Database Date:**	2015-11-30	**pI:**	5.5
**Modification(s):**	Carbamidomethyl, Oxidation	**Sequence Coverage [%]:**	3.3
		**No. of unique Peptides:**	2

10	20	30	40	50	60	70	80
MSTAAFHISS	LLEKMTSSDK	DFRFMATSDL	MSELQKDSIQ	LDEDSERKVV	KMLLRLLEDK	NGEVQNLAVK	WLGVPLGAFH
**90**	**100**	**110**	**120**	**130**	**140**	**150**	**160**
ASLLHCLLPQ	LSSPRLAVRK	RAVGALGHLA	AACSTDLFVE	LADHLLDRLP	GPRVPTSPTA	IRTLIQCLGS	VGRQAGHRLG
**170**	**180**	**190**	**200**	**210**	**220**	**230**	**240**
AHLDRLVPLV	EDFCNLDDDE	LRESCLQAFE	AFLRKCPKEM	GPHVPNVTSL	CLQYIKHDPN	YNYDSDEDEE	QMETEDSEFS
**250**	**260**	**270**	**280**	**290**	**300**	**310**	**320**
EQESEDEYSD	DDDMSWKVRR	AAAKCIAALI	SSRPDLLPDF	HCTLAPVLIR	RFKEREENVK	ADVFTAYIVL	LRQTQPPKGW
**330**	**340**	**350**	**360**	**370**	**380**	**390**	**400**
LEAMEEPTQT	GSNLHMLRGQ	VPLVVKALQR	QLKDRSVRAR	QGCFSLLTEL	AGVLPGSLAE	HMPVLVSGII	FSLADRSSSS
**410**	**420**	**430**	**440**	**450**	**460**	**470**	**480**
TIRMDALAFL	QGLLGTEPAE	AFHPHLPILL	PPVMACVADS	FYKIAAEALV	VLQELVRALW	PLHRPRMLDP	EPYVGEMSAV
**490**	**500**	**510**	**520**	**530**	**540**	**550**	**560**
TLARLRATDL	DQEVKERAIS	CMGHLVGHLG	DRLGDDLEPT	LLLLLDRLRN	EITRLPAIKA	LTLVAVSPLQ	LDLQPILAEA
**570**	**580**	**590**	**600**	**610**	**620**	**630**	**640**
LHILASFLRK	NQRALRLATL	AALDALAQSQ	GLSLPPSAVQ	AVLAELPALV	NESDMHVAQL	AVDFLATVTQ	AQPASLVEVS
**650**	**660**	**670**	**680**	**690**	**700**	**710**	**720**
GPVLSELLRL	LRSPLLPAGV	LAAAEGFLQA	LVGTRPPCVD	YAKLISLLTA	PVYEQAVDGG	PGLHKQVFHS	LARCVAALSA
**730**	**740**	**750**	**760**	**770**	**780**	**790**	**800**
ACPQEAASTA	SRLVCDARSP	HSSTGVKVLA	FLSLAEVGQV	AGPGHQRELK	AVLLEALGSP	SEDVRAAASY	ALGRVGAGSL
**810**	**820**	**830**	**840**	**850**	**860**	**870**	**880**
PDFLPFLLEQ	IEAEPRRQYL	LLHSLREALG	AAQPDSLKPY	AEDIWALLFQ	RCEGAEEGTR	GVVAECIGKL	VLVNPSFLLP
**890**	**900**	**910**	**920**	**930**	**940**	**950**	**960**
RLRKQLAAGR	PHTRSTVITA	VKFLISDQPH	PIDPLLKSFI	AVHNKPSLVR	DLLDDILPLL	YQETKIRRDL	IREVEMGPFK
**970**	**980**	**990**	**1000**	**1010**	**1020**	**1030**	**1040**
HTVDDGLDVR	KAAFECMYSL	LESCLGQLDI	CEFLNHVEDG	LKDHYDIRML	TFIMVARLAT	LCPAPVLQRV	DRLIEPLRAT
**1050**	**1060**	**1070**	**1080**	**1090**	**1100**	**1110**	**1120**
CTAKVKAGSV	KQEFEKQDEL	KRSAMRAVAA	LLTIPEVGKS	PIMADFSSQI	RSNPELAALF	ESIQKDSASA	PSTDSMELS

Cmpd.	No. of Cmpds.	m/z meas.	Δ m/z [ppm]	z	Rt [min]	Score	P	Range	Sequence	Modification
1132	1	701.3400	-16.93	3	44.3	12.1	0	199-216	K.EMGPHVPNVTSLCLQYIK.H	Carbamidomethyl: 13; Oxidation: 2
2247	1	1017.5903	-9.72	2	58.5	14.5	2	884-902	R.KQLAAGRPHTRSTVITAVK.F	

Detailed Protein Report

Protein 570: **1-phosphatidylinositol 4,5-bisphosphate phosphodiesterase eta-1 isoform c [Homo sapiens]**

**Accession:**	gi|195972873	**Score:**	26.6
**Database:**	refseq_human(refseq_human_20140103.fasta)	**MW [kDa]:**	114.4
**Database Date:**	2015-11-30	**pI:**	8.3
**Modification(s):**	Carbamidomethyl, Oxidation	**Sequence Coverage [%]:**	2.4
		**No. of unique Peptides:**	1

Quantitation

WUP:QUP **Median:** 0.72 **CV:** 0.00 % **No. of Peptides:** 1

10	20	30	40	50	60	70	80
MADLEVYKNL	SPEKVERCMS	VMQSGTQMIK	LKRGTKGLVR	LFYLDEHRTR	LRWRPSRKSE	KAKILIDSIY	KVTEGRQSEI
90	100	110	120	130	140	150	160
FHRQAEGNFD	PSCCFTIYHG	NHMESLDLIT	SNPEEARTWI	TGLKYLMAGI	SDEDSLAKRQ	RTHDQWVKQT	FEEADKNGDG
170	180	190	200	210	220	230	240
LLNIEEIHQL	MHKLNVNLPR	RKVRQMFQEA	DTDENQGTLT	FEEFCVFYKM	MSLRRDLYLL	LLSYSDKKDH	LTVEELAQFL
250	260	270	280	290	300	310	320
KVEQKMNNVT	TDYCLDIIKK	FEVSEENKVK	NVLGIEGFTN	FMRSPACDIF	NPLHHEVYQD	MDQPLCNYYI	ASSHNTYLTG
330	340	350	360	370	380	390	400
DQLLSQSKVD	MYARVLQEGC	RCVEVDCWDG	PDGEPVVHHG	YTLTSKILFR	DVVETINKHA	FVKNEFPVIL	SIENHCSIQQ
410	420	430	440	450	460	470	480
QRKIAQYLKG	IFGDKLDLSS	VDTGECKQLP	SPQSLKGKIL	VKGKKLPYHL	GDDAEEGEVS	DEDSADEIED	ECKFKLHYSN
490	500	510	520	530	540	550	560
GTTEHQVESF	IRKKLESLLK	ESQIRDKEDP	DSFTVRALLK	ATHEGLNAHL	KQSPDVKESG	KKSHGRSLMT	NFGKHKKTTK
570	580	590	600	610	620	630	640
SRSKSYSTDD	EEDTQQSTGK	EGGQLYRLGR	RRKTMKLCRE	LSDLVVYTNS	VAAQDIVDDG	TTGNVLSFSE	TRAHQVVQQK
650	660	670	680	690	700	710	720
SEQFMIYNQK	QLTRIYPSAY	RIDSSNFNPL	PYWNAGCQLV	ALNYQSEGRM	MQLNRAKFKA	NGNCGYVLKP	QQMCKGTFNP
730	740	750	760	770	780	790	800
FSGDPLPANP	KKQLILKVIS	GQQLPKPPDS	MFGDRGEIID	PFVEVEIIGL	PVDCCKDQTR	VVDDNGFNPV	WEETLTFTVH
810	820	830	840	850	860	870	880
MPEIALVRFL	VWDHDPIGRD	FVGQRTVTFS	SLVPGYRHVY	LEGLTEASIF	VHITINEIYG	KWSPLILNPS	YTILHFLGAT
890	900	910	920	930	940	950	960
KNRQLQGLKG	LFNKNPRHSS	SENNSHYVRK	RSIGDRILRR	TASAPAKGRK	KSKMGFQEMV	EIKDSVSEAT	RDQDGVLRRT
970	980	990	1000	1010			
TRSLQARPVS	MPVDRNLLGA	LSLPVSETAK	DIEGKENSLV	QI			

Cmpd.	No. of Cmpds.	m/z meas.	Δ m/z [ppm]	z	Rt [min]	Score	P	Range	Sequence	Modification	Ratios
2076	1	649.2852	-47.07	3	54.3	10.8	2	18-33	R.CMSVMQSGTQMIKLKR.G	Carbamidomethyl: 1; Oxidation: 2, 5, 11	WUP:QUP 0.72

Detailed Protein Report

Protein 571: teashirt homolog 2 isoform 2 [Homo sapiens]

**Accession:**	gi|301171536	**Score:**	26.6
**Database:**	refseq_human(refseq_human_20140103.fasta)	**MW [kDa]:**	114.5
**Database Date:**	2015-11-30	**pI:**	7.5
		**Sequence Coverage [%]:**	2.2
		**No. of unique Peptides:**	1

Quantitation

QU:MU **Median:** 1.12 **CV:** 0.00 % **No. of Peptides:** 1

10	20	30	40	50	60	70	80
MMAAALLHYT	GYAQEEQLKE	EEEIKEEEEE	EDSGSVAQLQ	GGNDTGTDEE	LETGPEQKGC	FSYQNSPGSH	LSNQDAENES
**90**	**100**	**110**	**120**	**130**	**140**	**150**	**160**
LLSDASDQVS	DIKSVCGRDA	SDKKAHTHVR	LPNEAHNCMD	KMTAVYANIL	SDSYWSGLGL	GFKLSNSERR	NCDTRNGSNK
**170**	**180**	**190**	**200**	**210**	**220**	**230**	**240**
SDFDWHQDAL	SKSLQQNLPS	RSVSKPSLFS	SVQLYRQSSK	MCGTVFTGAS	RFRCRQCSAA	YDTLVELTVH	MNETGHYQDD
**250**	**260**	**270**	**280**	**290**	**300**	**310**	**320**
NRKKDKLRPT	SYSKPRKRAF	QDMDKEDAQK	VLKCMFCGDS	FDSLQDLSVH	MIKTKHYQKV	PLKEPVPTIS	SKMVTPAKKR
**330**	**340**	**350**	**360**	**370**	**380**	**390**	**400**
VFDVNRPCSP	DSTTGSFADS	FSSQKNANLQ	LSSNNRYGYQ	NGASYTWQFE	ACKSQILKCM	ECGSSHDTLQ	QLTTHMMVTG
**410**	**420**	**430**	**440**	**450**	**460**	**470**	**480**
HFLKVTSSAS	KKGKQLVLDP	LAVEKMQSLS	EAPNSDSLAP	KPSSNSASDC	TASTTELKKE	SKKERPEETS	KDEKVVKSED
**490**	**500**	**510**	**520**	**530**	**540**	**550**	**560**
YEDPLQKPLD	PTIKYQYLRE	EDLEDGSKGG	GDILKSLENT	VTTAINKAQN	GAPSWSAYPS	IHAAYQLSEG	TKPPLPMGSQ
**570**	**580**	**590**	**600**	**610**	**620**	**630**	**640**
VLQIRPNLTN	KLRPIAPKWK	VMPLVSMPTH	LAPYTQVKKE	SEDKDEAVKE	CGKESPHEEA	SSFSHSEGDS	FRKSETPPEA
**650**	**660**	**670**	**680**	**690**	**700**	**710**	**720**
KKTELGPLKE	EEKLMKEGSE	KEKPQPLEPT	SALSNGCALA	NHAPALPCIN	PLSALQSVLN	NHLGKATEPL	RSPSCSSPSS
**730**	**740**	**750**	**760**	**770**	**780**	**790**	**800**
STISMFHKSN	LNVMDKPVLS	PASTRSASVS	RRYLFENSDQ	PIDLTKSKSK	KAESSQAQSC	MSPPQKHALS	DIADMVKVLP
**810**	**820**	**830**	**840**	**850**	**860**	**870**	**880**
KATTPKPASS	SRVPPMKLEM	DVRRFEDVSS	EVSTLHKRKG	RQSNWNPQHL	LILQAQFASS	LFQTSEGKYL	LSDLGPQERM
**890**	**900**	**910**	**920**	**930**	**940**	**950**	**960**
QISKFTGLSM	TTISHWLANV	KYQLRKTGGT	KFLKNMDKGH	PIFYCSDCAS	QFRTPSTYIS	HLESHLGFQM	KDMTRLSVDQ
**970**	**980**	**990**	**1000**	**1010**	**1020**	**1030**	**1040**
QSKVEQEISR	VSSAQRSPET	IAAEEDTDSK	FKCKLCCRTF	VSKHAVKLHL	SKTHSKSPEH	HSQFVTDVDE	E

Cmpd.	No. of Cmpds.	m/z meas.	Δ m/z [ppm]	z	Rt [min]	Score	P	Range	Sequence	Modification	Ratios
50	1	645.8133	-54.63	2	29.6	11.6	0	516-527	K.SLENTVTTAINK.A		QU:MU 1.12

Detailed Protein Report

Protein 572: von Willebrand factor A domain-containing protein 8 isoform b precursor [Homo sapiens]

Accession: gi|57863273
Database: refseq_human(refseq_human_20140103.fasta)
Database Date: 2015-11-30

Score: 26.6
MW [kDa]: 116.9
pI: 9.0
Sequence Coverage [%]: 3.6
No. of unique Peptides: 1

10	20	30	40	50	60	70	80
MQSRLLLLGA	PGGHGGPASR	RMRLLLRQVV	QRRPGGDRQR	PEVRLLHAGS	GADTGDTVNI	GDVSYKLKIP	KNPELVPQNY
90	100	110	120	130	140	150	160
ISDSLAQSVV	QHLRWIMQKD	LLGQDVFLIG	PPGPLRRSIA	MQYLELTKRE	VEYIALSRDT	TETDLKQRRE	IRAGTAFYID
170	180	190	200	210	220	230	240
QCAVRAATEG	RTLILEGLEK	AERNVLPVLN	NLLENREMQL	EDGRFLMSAE	RYDKLLRDHT	KKELDSWKIV	RVSENFRVIA
250	260	270	280	290	300	310	320
LGLPVPRYSG	NPLDPPLRSR	FQARDIYYLP	FKDQLKLLYS	IGANVSAEKV	SQLLSFATTL	CSQESSTLGL	PDFPLDSLAA
330	340	350	360	370	380	390	400
AVQILDSFPM	MPIKHAIQWL	YPYSILLGHE	GKMAVEGVLK	RFELQDSGSS	LLPKEIVKVE	KMMENHVSQA	SVTIRIADKE
410	420	430	440	450	460	470	480
VTIKVPAGTR	LLSQPCASDR	FIQTLSHKQL	QAEMMQSHMV	KDICLIGGKG	CGKTVIAKNF	ADTLGYNIEP	IMLYQDMTAR
490	500	510	520	530	540	550	560
DLLQQRYTLP	NGDTAWRSSP	LVNAALEGKL	VLLDGIHRVN	AGTLAVLQRL	IHDRELSLYD	GSRLLREDRY	MRLKEELQLS
570	580	590	600	610	620	630	640
DEQLQKRSIF	PIHPSFRIIA	LAEPPVIGST	AHQWLGPEFL	TMFFFHYMKP	LVKSEEIQVI	KEKVPNVPQE	ALDKLLSFTH
650	660	670	680	690	700	710	720
KLRETQDPTA	QSLAASLSTR	QLLRISRRLS	QYPNENLHSA	VTKACLSRFL	PSLARSALEK	NLADATIEIN	TDDNLEPELK
730	740	750	760	770	780	790	800
DYKCEVTSGT	LRIGAVSAPI	YNAHEKMKVP	DVLFYDNIQH	VIVMEDMLKD	FLLGEHLLLV	GNQGVGKNKI	VDRFLHLLNR
810	820	830	840	850	860	870	880
PREYIQLHRD	TTVQTLTLQP	SVKDGLIVYE	DSPLVKAVKL	GHILVVDEAD	KAPTNVTCIL	KTLVENGEMI	LADGRRIVAN
890	900	910	920	930	940	950	960
SANVNGRENV	VVIHPDFRMI	VLANRPGFPF	LGNDFFGTLG	DIFSCHAVDN	PKPHSELEML	RQYGPNVPEP	ILQKLVAAFG
970	980	990	1000	1010	1020	1030	1040
ELRSLADQGI	INYPYSTREV	VNIVKHLQKF	PTEGLSSVVR	NVFDFDSYNN	DMREILINTL	HKYGIPIGAK	PTSVQLAKE

Cmpd.	No. of Cmpds.	m/z meas.	Δ m/z [ppm]	z	Rt [min]	Score	P	Range	Sequence	Modification
2188	1	958.6176	79.28	2	55.7	10.0	2	5-23	R.LLLLGAPGGHGGPASRRMR.L	

Detailed Protein Report

Protein 573: **PREDICTED: microtubule-associated serine/threonine-protein kinase 4 isoform X12 [Homo sapiens]**

**Accession:**	gi|578810348	**Score:**	26.6
**Database:**	refseq_human(refseq_human_20140103.fasta)	**MW [kDa]:**	247.6
**Database Date:**	2015-11-30	**pI:**	9.5
		**Sequence Coverage [%]:**	1.3
		**No. of unique Peptides:**	1

Detailed Protein Report

10	20	30	40	50	60	70	80
MRPRSRSLSP	GRSPACCDHE	IIMMNHVYKE	RFPKATAQME	ERLKEIITSY	SPDNVLPLAD	GVLSFTHHQI	IELARDCLDK
90	100	110	120	130	140	150	160
SHQGLITSRY	FLELQHKLDK	LLQEAHDRSE	SGELAFIKQL	VRKILIVIAR	PARLLECLEF	DPEEFYYLLE	AAEGHAKEGQ
170	180	190	200	210	220	230	240
GIKTDIPRYI	ISQLGLNKDP	LEEMAHLGNY	DSGTAETPET	DESVSSSNAS	LKLRRKPRES	DFETIKLISN	GAYGAVYFVR
250	260	270	280	290	300	310	320
HKESRQRFAM	KKINKQNLIL	RNQIQQAFVE	RDILTFAENP	FVVSMYCSFE	TRRHLCMVME	YVEGGDCATL	MKNMGPLPVD
330	340	350	360	370	380	390	400
MARMYFAETV	LALEYLHNYG	IVHRDLKPDN	LLVTSMGHIK	LTDFGLSKVG	LMSMTTNLYE	GHIEKDAREF	LDKQVCGTPE
410	420	430	440	450	460	470	480
YIAPEVILRQ	GYGKPVDWWA	MGIILYEFLV	GCVPFFGDTP	EELFGQVISD	EINWPEKDEA	PPPDAQDLIT	LLLRQNPLER
490	500	510	520	530	540	550	560
LGTGGAYEVK	QHRFFRSLDW	NSLLRQKAEF	IPQLESEDDT	SYFDTRSEKY	HHMETEEEDD	TNDEDFNVEI	RQFSSCSHRF
570	580	590	600	610	620	630	640
SKVFSSIDRI	TQNSAEEKED	SVDKTKSTTL	PSTETLSWSS	EYSEMQQLST	SNSSDTESNR	HKLSSGLLPK	LAISTEGEQD
650	660	670	680	690	700	710	720
EAASCPGDPH	EEPGKPALPP	EECAQEEPEV	TTPASTISSS	TLSVGSFSEH	LDQINGRSEC	VDSTDNSSKP	SSEPASHMAR
730	740	750	760	770	780	790	800
QRLESTEKKK	ISGKVTKSLS	ASALSLMIPG	DMFAVSPLGS	PMSPHSLSSD	PSSSRDSSPS	RDSSAASASP	HQPIVIHSSG
810	820	830	840	850	860	870	880
KNYGFTIRAI	RVYVGDSDIY	TVHHIVWNVE	EGSPACQAGL	KAGDLITHIN	GEPVHGLVHT	EVIELLLKSG	NKVSITTTPF
890	900	910	920	930	940	950	960
ENTSIKTGPA	RRNSYKSRMV	RRSKKSKKKE	SLERRRSLFK	KLAKQPSPLL	HTSRSFSCLN	RSLSSGESLP	GSPTHSLSPR
970	980	990	1000	1010	1020	1030	1040
SPTPSYRSTP	DFPSGTNSSQ	SSSPSSSAPN	SPAGSGHIRP	STLHGLAPKL	GGQRYRSGRR	KSAGNIPLSP	LARTPSPTPQ
1050	1060	1070	1080	1090	1100	1110	1120
PTSPQRSPSP	LLGHSLGNSK	IAQAFPSKMH	SPPTIVRHIV	RPKSAEPPRS	PLLKRVQSEE	KLSPSYGSDK	KHLCSRKHSL
1130	1140	1150	1160	1170	1180	1190	1200
EVTQEEVQRE	QSQREAPLQS	LDENVCDVPP	LSRARPVEQG	CLKRPVSRKV	GRQESVDDLD	RDKLKAKVVV	KKADGFPEKQ
1210	1220	1230	1240	1250	1260	1270	1280
ESHQKSHGPG	SDLENFALFK	LEEREKKVYP	KAVERSSTFE	NKASMQEAPP	LGSLLKDALH	KQASVRASEG	AMSDGRVPAE
1290	1300	1310	1320	1330	1340	1350	1360
HRQGGGDFRR	APAPGTLQDG	LCHSLDRGIS	GKGEGTEKSS	QAKELLRCEK	LDSKLANIDY	LRKKMSLEDK	EDNLCPVLKP
1370	1380	1390	1400	1410	1420	1430	1440
KMTAGSHECL	PGNPVRPTGG	QQEPPPASES	RAFVSSTHAA	QMSAVSFVPL	KALTGRVDSG	TEKPGLVAPE	SPVRKSPSEY
1450	1460	1470	1480	1490	1500	1510	1520
KLEGRSVSCL	KPIEGTLDIA	LLSGPQASKT	ELPSPESAQS	PSPSGDVRAS	VPPVLPSSSG	KKNDTTSARE	LSPSSLKMNK
1530	1540	1550	1560	1570	1580	1590	1600
SYLLEPWFLP	PSRGLQNSPA	VSLPDPEFKR	DRKGPHPTAR	SPGTVMESNP	QQREGSSPKH	QDHTTDPKLL	TCLGQNLHSP
1610	1620	1630	1640	1650	1660	1670	1680
DLARPRCPLP	PEASPSREKP	GLRESSERGP	PTARSERSAA	RADTCREPSM	ELCFPETAKT	SDNSKNLLSV	GRTHPDFYTQ
1690	1700	1710	1720	1730	1740	1750	1760
TQAMEKAWAP	GGKTNHKDGP	GEARPPPRDN	SSLHSAGIPC	EKELGKVRRG	VEPKPEALLA	RRSLQPPGIE	SEKSEKLSSF
1770	1780	1790	1800	1810	1820	1830	1840
PSLQKDGAKE	PERKEQPLQR	HPSSIPPPPL	TAKDLSSPAA	RQHCSSPSHA	SGREPGAKPS	TAEPSSSPQD	PPKPVAAHSE
1850	1860	1870	1880	1890	1900	1910	1920
SSSHKPRPGP	DPGPPKTKHP	DRSLSSQKPS	VGATKGKEPA	TQSLGGSSRE	GKGHSKSGPD	VFPATPGSQN	KASDGIGQGE
1930	1940	1950	1960	1970	1980	1990	2000
GGPSVPLHTD	RAPLDAKPQP	TSGGRPLEVL	EKPVHLPRPG	HPGPSEPADQ	KLSAVGEKQT	LSPKHPKPST	VKDCPTLCKQ
2010	2020	2030	2040	2050	2060	2070	2080
TDNRQTDKSP	SQPAANTDRR	AEGKKCTEAL	YAPAEGDKLE	AGLSFVHSEN	RLKGAERPAA	GVGKGFPEAR	GKGPGPQKPP
2090	2100	2110	2120	2130	2140	2150	2160
TEADKPNGMK	RSPSATGQSS	FRSTALPEKS	LSCSSSFPET	RAGVREASAA	SSDTSSAKAA	GGMLELPAPS	NRDHRKAQPA
2170	2180	2190	2200	2210	2220	2230	2240

Detailed Protein Report

Cmpd.	No. of Cmpds.	m/z meas.	Δ m/z [ppm]	z	Rt [min]	Score	P	Range	Sequence	Modification
2513	1	694.6522	-36.12	3	59.7	10.7	0	1135-1153	R.EAPLQSLDENVCDVPPLSR.A	

Detailed Protein Report

Protein 574: sushi domain-containing protein 1 isoform 3 precursor [Homo sapiens]

**Accession:**	gi|544063419	**Score:**	26.6
**Database:**	refseq_human(refseq_human_20140103.fasta)	**MW [kDa]:**	80.4
**Database Date:**	2015-11-30	**pI:**	6.0
**Modification(s):**	Carbamidomethyl	**Sequence Coverage [%]:**	4.0
		**No. of unique Peptides:**	1

10	20	30	40	50	60	70	80
MGRGPWDAGP	SRRLLPLLLL	LGLARGAAGA	PGPDGLDVCA	TCHEHATCQQ	REGKKICICN	YGFVGNGRTQ	CVDKNECQFG
**90**	**100**	**110**	**120**	**130**	**140**	**150**	**160**
ATLVCGNHTS	CHNTPGGFYC	ICLEGYRATN	NNKTFIPNDG	TFCTDIDECE	VSGLCRHGGR	CVNTHGSFEC	YCMDGYLPRN
**170**	**180**	**190**	**200**	**210**	**220**	**230**	**240**
GPEPFHPTTD	ATSCTEIDCG	TPPEVPDGYI	IGNYTSSLGS	QVRYACREGF	FSVPEDTVSS	CTGLGTWESP	KLHCQEINCG
**250**	**260**	**270**	**280**	**290**	**300**	**310**	**320**
NPPEMRHAIL	VGNHSSRLGG	VARYVCQEGF	ESPGGKITSV	CTEKGTWRES	TLTCTEILTK	INDVSLFNDT	CVRWQINSRR
**330**	**340**	**350**	**360**	**370**	**380**	**390**	**400**
INPKISYVIS	IKGQRLDPME	SVREETVNLT	TDSRTPEVCL	ALYPGTNYTV	NISTAPPRRS	MPAVIGFQTA	EVDLLEDDGS
**410**	**420**	**430**	**440**	**450**	**460**	**470**	**480**
FNISIFNETC	LKLNRRSRKV	GSEHMYQFTV	LGQRWYLANF	SHATSFNFTT	REQVPVVCLD	LYPTTDYTVN	VTLLRSPKRH
**490**	**500**	**510**	**520**	**530**	**540**	**550**	**560**
SVQITIATPP	AVKQTISNIS	GFNETCLRWR	SIKTADMEEM	YLFHIWGQRW	YQKEFAQEMT	FNISSSSRDP	EVCLDLRPGT
**570**	**580**	**590**	**600**	**610**	**620**	**630**	**640**
NYNVSLRALS	SELPVVISLT	TQITEPPLPE	VEFFTVHRGP	LPRLRLRKAK	EKNGPISSYQ	VLVLPLALQS	TFSCDSEGAS
**650**	**660**	**670**	**680**	**690**	**700**	**710**	**720**
SFFSNASDAD	GYVAAELLAK	DVPDDAMEIP	IGDRLYYGEY	YNAPLKRGSD	YCIILRITSE	WNKIRHSCCC	RWRVLDWVPW
**730**							
LL							

Cmpd.	No. of Cmpds.	m/z meas.	Δ m/z [ppm]	z	Rt [min]	Score	P	Range	Sequence	Modification
893	1	736.8280	-19.37	2	41.2	11.4	0	56-68	K.ICICNYGFVGNGR.T	Carbamidomethyl: 4

Detailed Protein Report

Protein 575: PREDICTED: coiled-coil domain-containing protein 111 isoform X3 [Homo sapiens]

**Accession:**	gi|578808887	**Score:**	26.6
**Database:**	refseq_human(refseq_human_20140103.fasta)	**MW [kDa]:**	58.1
**Database Date:**	2015-11-30	**pI:**	8.8
		**Sequence Coverage [%]:**	2.2
		**No. of unique Peptides:**	1

Quantitation

QU:MU	**Median:**	2.36	**CV:**	0.00 %	**No. of Peptides:**	1
WUP:QUP	**Median:**	0.06	**CV:**	0.00 %	**No. of Peptides:**	1

10	20	30	40	50	60	70	80
MNRKWEAKLK	QIEERASHYE	RKPLSSVYRP	RLSKPEEPPS	IWRLFHRQAQ	AFNFVKSCKE	DVHVFALECK	VGDGQRIYLV
**90**	**100**	**110**	**120**	**130**	**140**	**150**	**160**
TTYAEFWFYY	KSRKNLLHCY	EVIPENAVCK	LYFDLEFNKP	ANPGADGKKM	VALLIEYVCK	ALQELYGVNC	SAEDVLNLDS
**170**	**180**	**190**	**200**	**210**	**220**	**230**	**240**
STDEKFSRHL	IFQLHDVAFK	DNIHVGNFLR	KILQPALDLL	GSEDDDSAPE	TTGHGFPHFS	EAPARQGFSF	NKMFTEKATE
**250**	**260**	**270**	**280**	**290**	**300**	**310**	**320**
ESWTSNSKKL	ERLGSAEQSS	PDLSFLVVKN	NMGEKHLFVD	LGVYTRNRNF	RLYKSSKIGK	RVALEVTEDN	KFFPIQSKDV
**330**	**340**	**350**	**360**	**370**	**380**	**390**	**400**
SDEYQYFLSS	LVSNVRFSDT	LRILTCEPSQ	NKQKGVGYFN	SIGTSVETIE	GFQCSPYPEV	DHFVLSLVNK	DGIKGGIRRW
**410**	**420**	**430**	**440**	**450**	**460**	**470**	**480**
NYFFPEELLV	YDICKYRWCE	NIGRAHKSNN	IMILVDLKNE	VWYQKCHDPV	CKAENFKSDC	FPLPAEVCLL	FLFKELDCPL
**490**	**500**	**510**					
VKKLNPPLLF	RRGVLKSMCT	EL					

Cmpd.	No. of Cmpds.	m/z meas.	Δ m/z [ppm]	z	Rt [min]	Score	P	Range	Sequence	Modification	Ratios
1088	1	540.6458	-238.97	2	43.2	12.3	1	493-502	R.GVLKSMCTEL.-		QU:MU 2.36<br>WUP:QUP 0.06

Detailed Protein Report

Protein 576: ubiquitin carboxyl-terminal hydrolase 12 [Homo sapiens]

Accession: gi|301500675
Database: refseq_human(refseq_human_20140103.fasta)
Database Date: 2015-11-30
Modification(s): Carbamidomethyl

Score: 26.6
MW [kDa]: 42.8
pI: 6.2
Sequence Coverage [%]: 7.0
No. of unique Peptides: 1

10	20	30	40	50	60	70	80
MEILMTVSKF	ASICTMGANA	SALEKEIGPE	QFPVNEHYFG	LVNFGNTCYC	NSVLQALYFC	RPFREKVLAY	KSQPRKKESL
**90**	**100**	**110**	**120**	**130**	**140**	**150**	**160**
LTCLADLFHS	IATQKKKVGV	IPPKKFITRL	RKENELFDNY	MQQDAHEFLN	YLLNTIADIL	QEERKQEKQN	GRLPNGNIDN
**170**	**180**	**190**	**200**	**210**	**220**	**230**	**240**
ENNNSTPDPT	WVHEIFQGTL	TNETRCLTCE	TISSKDEDFL	DLSVDVEQNT	SITHCLRGFS	NTETLCSEYK	YYCEECRSKQ
**250**	**260**	**270**	**280**	**290**	**300**	**310**	**320**
EAHKRMKVKK	LPMILALHLK	RFKYMDQLHR	YTKLSYRVVF	PLELRLFNTS	GDATNPDRMY	DLVAVVVHCG	SGPNRGHYIA
**330**	**340**	**350**	**360**	**370**	**380**		
IVKSHDFWLL	FDDDIVEKID	AQAIEEFYGL	TSDISKNSES	GYILFYQSRD			

Cmpd.	No. of Cmpds.	m/z meas.	Δ m/z [ppm]	z	Rt [min]	Score	P	Range	Sequence	Modification
620	1	647.6390	-191.68	2	36.2	14.2	1	231-239	K.YYCEECRSK.Q	Carbamidomethyl: 3, 6

Detailed Protein Report

Protein 577: PREDICTED: RING finger protein 32 isoform X5 [Homo sapiens]

Accession: gi|578813915
Database: refseq_human(refseq_human_20140103.fasta)
Database Date: 2015-11-30
Modification(s): Carbamidomethyl

Score: 26.6
MW [kDa]: 27.1
pI: 10.6
Sequence Coverage [%]: 9.8
No. of unique Peptides: 1

10	20	30	40	50	60	70	80
MLKNKGHSSK	KDNLAVNAVA	LQDHILHDLQ	LRNLSVADHS	KTQVQKKENK	SLKRDTKAII	DTGLKKTTQC	PKLEDSEKEY
**90**	**100**	**110**	**120**	**130**	**140**	**150**	**160**
VLDPKPPPLT	LAQKLGLIGP	PPPPLSSDEW	EKVKQRSLLQ	GDSVQPCPIC	KEEFELRPQV	LLSCSHVFHK	ACLQAFEKFT
**170**	**180**	**190**	**200**	**210**	**220**	**230**	**240**
NKKTCPLCRK	NQYQTRVIHD	GARLFRIKCV	TRIQAYWRGC	VVRKWYRNLR	KTVPPTDAKL	RKKFFEKKTQ	DWKPA

Cmpd.	No. of Cmpds.	m/z meas.	Δ m/z [ppm]	z	Rt [min]	Score	P	Range	Sequence	Modification
428	1	862.8357	-97.27	2	35.3	14.1	2	164-176	K.TCPLCRKNQYQTR.V	Carbamidomethyl: 2, 5

Detailed Protein Report

Protein 578: PREDICTED: putative Polycomb group protein ASXL2 isoform X2 [Homo sapiens]

**Accession:**	gi|578802980	**Score:**	26.6
**Database:**	refseq_human(refseq_human_20140103.fasta)	**MW [kDa]:**	125.4
**Database Date:**	2015-11-30	**pI:**	8.8
		**Sequence Coverage [%]:**	2.5
		**No. of unique Peptides:**	1

10	20	30	40	50	60	70	80
MKRTKCADID	VETPDSILVN	TNLRALINKH	TFSVLPGDCQ	QRLLLLLPEV	DRQVGPDGLM	KLNGSALNNE	FFTSAAQGWK
**90**	**100**	**110**	**120**	**130**	**140**	**150**	**160**
ERLSEGEFTP	EMQVRIRQEI	EKEKKVEPWK	EQFFESYYGQ	SSGLSLEDSK	KLTASPSDPK	VKKTPAEQPK	SMPVSEASLI
**170**	**180**	**190**	**200**	**210**	**220**	**230**	**240**
RIVPVVSQSE	CKEEALQMSS	PGRKEECESQ	GEVQPNFSTS	SEPLLSSALN	THELSSILPI	KCPKDEDLLE	QKPVTSAEQE
**250**	**260**	**270**	**280**	**290**	**300**	**310**	**320**
SEKNHLTTAS	NYNKSESQES	LVTSPSKPKS	PGVEKPIVKP	TAGAGPQETN	MKEPLATLVD	QSPESLKRKS	SLTQEEAPVS
**330**	**340**	**350**	**360**	**370**	**380**	**390**	**400**
WEKRPRVTEN	RQHQQPFQVS	PQPFLNRGDR	IQVRKVPPLK	IPVSRISPMP	FHPSQVSPRA	RFPVSITSPN	RTGARTLADI
**410**	**420**	**430**	**440**	**450**	**460**	**470**	**480**
KAKAQLVKAQ	RAAAAAAAAA	AAAASVGGTI	PGPGPGGGQG	PGEGGEGQTA	RGGSPGSDRV	SETGKGPTLE	LAGTGSRGGT
**490**	**500**	**510**	**520**	**530**	**540**	**550**	**560**
RELLPCGPET	QPQSETKTTP	SQAQPHSVSG	AQLQQTPPVP	PTPAVSGACT	SVPSPAHIEK	LDNEKLNPTR	ATATVASVSH
**570**	**580**	**590**	**600**	**610**	**620**	**630**	**640**
PQGPSSCRQE	KAPSPTGPAL	ISGASPVHCA	ADGTVELKAG	PSKNIPNPSA	SSKTDASVPV	AVTPSPLTSL	LTTATLEKLP
**650**	**660**	**670**	**680**	**690**	**700**	**710**	**720**
VPQVSATTAP	AGSAPPSSTL	PAASSLKTPG	TSLNMNGPTL	RPTSSIPANN	PLVTQLLQGK	DVPMEQILPK	PLTKVEMKTV
**730**	**740**	**750**	**760**	**770**	**780**	**790**	**800**
PLTAKEERGM	GALIATNTTE	NSTREEVNER	QSHPATQQQL	GKTLQSKQLP	QVPRPLQLFS	AKELRDSSID	THQYHEGLSK
**810**	**820**	**830**	**840**	**850**	**860**	**870**	**880**
ATQDQILQTL	IQRVRRQNLL	SVVPPSQFNF	AHSGFQLEDI	STSQRFMLGF	AGRRTSKPAM	AGHYLLNIST	YGRGSESFRR
**890**	**900**	**910**	**920**	**930**	**940**	**950**	**960**
THSVNPEDRF	CLSSPTEALK	MGYTDCKNAT	GESSSSKEDD	TDEESTGDEQ	ESVTVKEEPQ	VSQSAGKGDT	SSGPHSRETL
**970**	**980**	**990**	**1000**	**1010**	**1020**	**1030**	**1040**
STSDCLASKN	VKAEIPLNEQ	TTLSKENYLF	TRGQTFDEKT	LARDLIQAAQ	KQMAHAVRGK	AIRSSPELFS	STVLPLPADS
**1050**	**1060**	**1070**	**1080**	**1090**	**1100**	**1110**	**1120**
PTHQPLLLPP	LQTPKLYGSP	TQIGPSYRGM	INVSTSSDMD	HNSAVPGSQV	SSNVGDVMSF	SVTVTTIPAS	QAMNPSSHGQ
**1130**	**1140**	**1150**	**1160**	**1170**	**1180**		
TIPVQAFSEE	NSIEGTPSKC	YCRLKAMIMC	KGCGAFCHDD	CIGPSKLCVS	CLVVR		

Cmpd.	No. of Cmpds.	m/z meas.	Δ m/z [ppm]	z	Rt [min]	Score	P	Range	Sequence	Modification
326	1	761.7364	-167.46	2	34.1	10.4	0	83-95	R.LSEGEFTPEMQVR.I	

Detailed Protein Report

Protein 579: PREDICTED: inositol 1,4,5-trisphosphate receptor type 2 isoform X1 [Homo sapiens]

**Accession:**	gi|578822836	**Score:**	26.6
**Database:**	refseq_human(refseq_human_20140103.fasta)	**MW [kDa]:**	212.9
**Database Date:**	2015-11-30	**pI:**	6.3
		**Sequence Coverage [%]:**	0.9
		**No. of unique Peptides:**	2

10	20	30	40	50	60	70	80
MTEKMSSFLY	IGDIVSLYAE	GSVNGFISTL	GLVDDRCVVH	PEAGDLANPP	KKFRDCLFKV	CPMNRYSAQK	QYWKAKQAKQ
**90**	**100**	**110**	**120**	**130**	**140**	**150**	**160**
GNHTEAALLK	KLQHAAELEQ	KQNESENKKL	LGEIVKYSNV	IQLLHIKSNK	YLTVNKRLPA	LLEKNAMRVS	LDAAGNEGSW
**170**	**180**	**190**	**200**	**210**	**220**	**230**	**240**
FYIHPFWKLR	SEGDNIVVGD	KVVLMPVNAG	QPLHASNIEL	LDNPGCKEVN	AVNCNTSWKI	TLFMKYSSYR	EDVLKGGDVV
**250**	**260**	**270**	**280**	**290**	**300**	**310**	**320**
RLFHAEQEKF	LTCDEYEKKQ	HIFLRTTLRQ	SATSATSSKA	LWEIEVVHHD	PCRGGAGQWN	SLFRFKHLAT	GNYLAAELNP
**330**	**340**	**350**	**360**	**370**	**380**	**390**	**400**
DYRDAQNEGK	NVRDGVPPTS	KKKRQAGEKI	MYTLVSVPHG	NDIASLFELD	ATTLQRADCL	VPRNSYVRLR	HLCTNTWVTS
**410**	**420**	**430**	**440**	**450**	**460**	**470**	**480**
TSIPIDTDEE	RPVMLKIGTC	QTKEDKEAFA	IVSVPLSEVR	DLDFANDANK	VLATTVKKLE	NGTITQNERR	FVTKLLEDLI
**490**	**500**	**510**	**520**	**530**	**540**	**550**	**560**
FFVADVPNNG	QEVLDVVITK	PNRERQKLMR	EQNILAQVFG	ILKAPFKEKA	GEGSMLRLED	LGDQRYAPYK	YMLRLCYRVL
**570**	**580**	**590**	**600**	**610**	**620**	**630**	**640**
RHSQQDYRKN	QEYIAKNFCV	MQSQIGYDIL	AEDTITALLH	NNRKLLEKHI	TAKEIETFVS	LLRRNREPRF	LDYLSDLCVS
**650**	**660**	**670**	**680**	**690**	**700**	**710**	**720**
NTTAIPVTQE	LICKFMLSPG	NADILIQTKV	VSMQADNPME	SSILSDDIDD	EEVWLYWIDS	NKEPHGKAIR	HLAQEAKEGT
**730**	**740**	**750**	**760**	**770**	**780**	**790**	**800**
KADLEVLTYY	RYQLNLFARM	CLDRQYLAIN	QISTQLSVDL	ILRCVSDESL	PFDLRASFCR	LMLHMHVDRD	PQESVVPVRY
**810**	**820**	**830**	**840**	**850**	**860**	**870**	**880**
ARLWTEIPTK	ITIHEYDSIT	DSSRNDMKRK	FALTMEFVEE	YLKEVVNQPF	PFGDKEKNKL	TFEVVHLARN	LIYFGFYSFS
**890**	**900**	**910**	**920**	**930**	**940**	**950**	**960**
ELLRLTRTLL	AILDIVQAPM	SSYFERLSKF	QDGGNNVMRT	IHGVGEMMTQ	MVLSRGSIFP	MSVPDVPPSI	HPSKQGSPTE
**970**	**980**	**990**	**1000**	**1010**	**1020**	**1030**	**1040**
HEDVTVMDTK	LKIIEILQFI	LSVRLDYRIS	YMLSIYKKEF	GEDNDNAETS	ASGSPDTLLP	SAIVPDIDEI	AAQAETMFAG
**1050**	**1060**	**1070**	**1080**	**1090**	**1100**	**1110**	**1120**
RKEKNPVQLD	DEGGRTFLRV	LIHLIMHDYP	PLLSGALQLL	FKHFSQRAEV	LQAFKQVQLL	VSNQDVDNYK	QIKADLDQLR
**1130**	**1140**	**1150**	**1160**	**1170**	**1180**	**1190**	**1200**
LTVEKSELWV	EKSSNYENGE	IGESQVKGGE	EPIEESNILS	PVQDGTKKPQ	IDSNKSNNYR	IVKEILIRLS	KLCVQNKKCR
**1210**	**1220**	**1230**	**1240**	**1250**	**1260**	**1270**	**1280**
NQHQRLLKNM	GAHSVVLDLL	QIPYEKNDEK	MNEVMNLAHT	FLQNFCRGNP	QNQVLLHKHL	NLFLTPGLLE	AETMRHIFMN
**1290**	**1300**	**1310**	**1320**	**1330**	**1340**	**1350**	**1360**
NYHLCNEISE	RVVQHFVHCI	ETHGRHVEYL	RFLQTIVKAD	GKYVKKCQDM	VMTELINGGE	DVLIFYNDRA	SFPILLHMMC
**1370**	**1380**	**1390**	**1400**	**1410**	**1420**	**1430**	**1440**
SERDRGDESG	PLAYHITLVE	LLAACTEGKN	VYTEIKCNSL	LPLDDIVRVV	THDDCIPEVK	IAYVNFVNHC	YVDTEVEMKE
**1450**	**1460**	**1470**	**1480**	**1490**	**1500**	**1510**	**1520**
IYTSNHIWKL	FENFLVDMAR	VCNTTTDRKH	ADIFLEKCVT	ESIMNIVSGF	FNSPFSDNST	SLQTHQPVFI	QLLQSAFRIY
**1530**	**1540**	**1550**	**1560**	**1570**	**1580**	**1590**	**1600**
NCTWPNPAQK	ASVESCIRTL	AEVAKNRGIA	IPVDLDSQVN	TLFMKSHSNM	VQRAAMGWRL	SARSGPRFKE	ALGGPAWDYR
**1610**	**1620**	**1630**	**1640**	**1650**	**1660**	**1670**	**1680**
NIIEKLQDVV	ASLEHQFSPM	MQAEFSVLVD	VLYSPELLFP	EGSDARIRCG	AFMSKLINHT	KKLMEKEEKL	CIKILQTLRE
**1690**	**1700**	**1710**	**1720**	**1730**	**1740**	**1750**	**1760**
MLEKKDSFVE	EGNTLRKILL	NRYFKGDYSI	GVNGHLSGAY	SKTAQVGGSF	SGQDSDKMGI	SMSDIQCLLD	KEGASELVID
**1770**	**1780**	**1790**	**1800**	**1810**	**1820**	**1830**	**1840**
VIVNTKNDRI	FSEGIFLGIA	LLEGGNTQTQ	YSFYQQLHEQ	KKSEKFFKVL	YDRMKAAQKE	IRSTVTVNTI	DLGNKKRDDD
**1850**	**1860**	**1870**					
NELMTSGPRM	REFWSWGDPK	TALQD					

Detailed Protein Report

Cmpd.	No. of Cmpds.	m/z meas.	Δ m/z [ppm]	z	Rt [min]	Score	P	Range	Sequence	Modification
725	1	771.2336	-342.03	1	39.0	11.8	0	110-116	K.LLGEIVK.Y	
1690	4	648.2368	-188.91	2	51.4	14.8	2	1199-1208	K.CRNQHQRLLK.N	

Detailed Protein Report

Protein 580: zinc finger protein 570 [Homo sapiens]

**Accession:**	gi|21389599	**Score:**	26.5
**Database:**	refseq_human(refseq_human_20140103.fasta)	**MW [kDa]:**	62.3
**Database Date:**	2015-11-30	**pI:**	9.7
		**Sequence Coverage [%]:**	7.5
		**No. of unique Peptides:**	1

10	20	30	40	50	60	70	80
MAVGLLKAMY	QELVTFRDVA	VDFSQEEWDC	LDSSQRHLYS	NVMLENYRIL	VSLGLCFSKP	SVILLLEQGK	APWMVKRELT
**90**	**100**	**110**	**120**	**130**	**140**	**150**	**160**
KGLCSGWEPI	CETEELTPKQ	DFYEEHQSQK	IIETLTSYNL	EYSSLREEWK	CEGYFERQPG	NQKACFKEEI	ITHEEPLFDE
**170**	**180**	**190**	**200**	**210**	**220**	**230**	**240**
REQEYKSWGS	FHQNPLLCTQ	KIIPKEEKVH	KHDTQKRSFK	KNLMAIKPKS	VCAEKKLLKC	NDCEKVFSQS	SSLTLHQRIH
**250**	**260**	**270**	**280**	**290**	**300**	**310**	**320**
TGEKPYKCIE	CGKAFSQRSN	LVQHQRIHTG	EKPYECKECR	KAFSQNAHLV	QHLRVHTGEK	PYECKVCRKA	FSQFAYLAQH
**330**	**340**	**350**	**360**	**370**	**380**	**390**	**400**
QRVHTGEKPY	ECIECGKAFS	NRSSIAQHQR	VHTGEKPYEC	NVCGKAFSLR	AYLTVHQRIH	TGERPYECKE	CGKAFSQNSH
**410**	**420**	**430**	**440**	**450**	**460**	**470**	**480**
LAQHQRIHTG	EKPYKCQECR	KAFSQIAYLA	QHQRVHTGEK	PYECIECGKA	FSNDSSLTQH	QRVHTGEKPY	ECTVCGKAFS
**490**	**500**	**510**	**520**	**530**	**540**		
YCGSLAQHQR	IHTGERPYEC	KECKKTFRQH	AHLAHHQRIH	IGESLSPPNP	VNHQVL		

Cmpd.	No. of Cmpds.	m/z meas.	Δ m/z [ppm]	z	Rt [min]	Score	P	Range	Sequence	Modification
17	1	1103.4962	-28.67	2	29.9	11.4	1	144-161	K.ACFKEEIITHEEPLFDER.E	

Detailed Protein Report

Protein 581: cyclic nucleotide-gated cation channel beta-1 isoform c [Homo sapiens]

Accession: gi|554790418
Database: refseq_human(refseq_human_20140103.fasta)
Database Date: 2015-11-30

Score: 26.5
MW [kDa]: 139.1
pI: 4.6
Sequence Coverage [%]: 1.6
No. of unique Peptides: 2

Quantitation

QU:MU	**Median:**	0.40	**CV:**	0.00 %	**No. of Peptides:**	1
WUP:QUP	**Median:**	2.56	**CV:**	0.00 %	**No. of Peptides:**	1

```
        10         20         30         40         50         60         70         80
MLGWVQRVLP QPPGTPRKTK MQEEEEVEPE PEMEAEVEPE PNPEEAETES ESMPPEESFK EEEVAVADPS PQETKEAALT
        90        100        110        120        130        140        150        160
STISLRAQGA EISEMNSPSR RVLTWLMKGV EKVIPQPVHS ITEDPAQILG HGSTGDTGCT DEPNEALEAQ DTRPGLRLLL
       170        180        190        200        210        220        230        240
WLEQNLERVL PQPPKSSEVW RDEPAVATAP PGRPQEMGPK LQARETPSLP TPIPLQPKEE PKEAPAPEPQ PGSQAQTSSL
       250        260        270        280        290        300        310        320
PPTRDPARLV AWVLHRLEMA LPQPVLHGKI GEQEPDSPGI CDVQTISILP GGQVEPDLVL EEVEPPWEDA HQDVSTSPQG
       330        340        350        360        370        380        390        400
TEVVPAYEEE NKAVEKMPRE LSRIEEEKED EEEEEEEEEE EEEEEVTEVL LDSCVVSQVG VGQSEEDGTR PQSTSDQKLW
       410        420        430        440        450        460        470        480
EEVGEEAKKE AEEKAKEEAE EVAEEEAEKE PQDWAETKEE PEAEAEAASS GVPATKQHPE VQVEDTDADS CPLMAEENPP
       490        500        510        520        530        540        550        560
STVLPPPSPA KSDTLIVPSS ASGTHRKKLP SEDDEAEELK ALSPAESPVV AWSDPTTPKD TDGQDRAAST ASTNSAIIND
       570        580        590        600        610        620        630        640
RLQELVKLFK ERTEKVKEKL IDPDVTSDEE SPKPSPAKKA PEPAPDTKPA EAEPVEEEHY CDMLCCKFKH RPWKKYQFPQ
       650        660        670        680        690        700        710        720
SIDPLTNLMY VLWLFFVVMA WNWNCWLIPV RWAFPYQTPD NIHHWLLMDY LCDLIYFLDI TVFQTRLQFV RGGDIITDKK
       730        740        750        760        770        780        790        800
DMRNNYLKSR RFKMDLLSLL PLDFLYLKVG VNPLLRLPRC LKYMAFFEFN SRLESILSKA YVYRVIRTTA YLLYSLHLNS
       810        820        830        840        850        860        870        880
CLYYWASAYQ GLGSTHWVYD GVGNSYIRCY YFAVKTLITI GGLPDPKTLF EIVFQLLNYF TGVFAFSVMI GQMRDVVGAA
       890        900        910        920        930        940        950        960
TAGQTYYRSC MDSTVKYMNF YKIPKSVQNR VKTWYEYTWH SQGMLDESEL MVQLPDKMRL DLAIDVNYNI VSKVALFQGC
       970        980        990       1000       1010       1020       1030       1040
DRQMIFDMLK RLRSVVYLPN DYVCKKGEIG REMYIIQAGQ VQVLGGPDGK SVLVTLKAGS VFGEISLLAV GGGNRRTANV
      1050       1060       1070       1080       1090       1100       1110       1120
VAHGFTNLFI LDKKDLNEIL VHYPESQKLL RKKARRMLRS NNKPKEEKSV LILPPRAGTP KLFNAALAMT GKMGGKGAKG
      1130       1140       1150       1160       1170       1180       1190       1200
GKLAHLRARL KELAALEAAA KQQELVEQAK SSQDVKGEEG SAAPDQHTHP KEAATDPPAP RTPPEPPGSP PSSPPPASLG
      1210       1220       1230       1240       1250
RPEGEEEGPA EPEEHSVRIC MSPGPEPGEQ ILSVKMPEER EEKAE
```

Cmpd.	No. of Cmpds.	m/z meas.	Δ m/z [ppm]	z	Rt [min]	Score	P	Range	Sequence	Modification	Ratios
1888	3	612.7759	-142.99	2	54.0	15.4	0	836-847	K.TLITIGGLPDPK.T		QU:MU 0.40<br>WUP:QUP 2.56
1512	1	851.5801	69.44	1	48.6	11.1	1	1120-1127	K.GGKLAHLR.A		

Detailed Protein Report

Protein 582: **PREDICTED: alpha-N-acetylneuraminide alpha-2,8-sialyltransferase isoform X2 [Homo sapiens]**

**Accession:**	gi|530399363	**Score:**	26.5
**Database:**	refseq_human(refseq_human_20140103.fasta)	**MW [kDa]:**	23.1
**Database Date:**	2015-11-30	**pI:**	10.8
		**Sequence Coverage [%]:**	13.2
		**No. of unique Peptides:**	1

10	20	30	40	50	60	70	80
MSPCGRARRQ	TSRGAMAVLA	WKFPRTRLPM	GASALCVVVL	CWLYIFPVYR	LPNEKEIVQG	VLQQGTAWRR	NQTAARAFRK
90	100	110	120	130	140	150	160
QMEDCCDPAH	LFAMTKMNSP	MGKSMWYDGE	FLYSFTIDNS	TYSLFPQATP	FQLPLKKCAV	VGNGGILKKS	GCGRQIDEAN
170	180	190	200	210			
FVMRCNLPPL	SSEYTKDVGS	KSQLVTANPS	IIRQRDFTAV	ERVKK			

Cmpd.	No. of Cmpds.	m/z meas.	Δ m/z [ppm]	z	Rt [min]	Score	P	Range	Sequence	Modification
550	1	581.8032	-38.57	2	36.9	10.8	2	71-80	R.NQTAARAFRK.Q	

Detailed Protein Report

Protein 583: RNA 3'-terminal phosphate cyclase isoform a [Homo sapiens]

**Accession:**	gi|195927044	**Score:**	26.5
**Database:**	refseq_human(refseq_human_20140103.fasta)	**MW [kDa]:**	40.7
**Database Date:**	2015-11-30	**pI:**	9.6
**Modification(s):**	Oxidation	**Sequence Coverage [%]:**	7.1
		**No. of unique Peptides:**	1

Quantitation

WUP:QUP **Median:** 1.59 **CV:** 0.00 % **No. of Peptides:** 1

10	20	30	40	50	60	70	80
MAGPRVEVDG	SIMEGGGQIL	RVSTALSCLL	GLPLRVQKIR	AGRSTPGLSS	GGWKSKIKVL	TRPQHLSGLE	MIRDLCDGQL
**90**	**100**	**110**	**120**	**130**	**140**	**150**	**160**
EGAEIGSTEI	TFTPEKIKGG	IHTADTKTAG	SVCLLMQVSM	PCVLFAASPS	ELHLKGGTNA	EMAPQIDYTV	MVFKPIVEKF
**170**	**180**	**190**	**200**	**210**	**220**	**230**	**240**
GFIFNCDIKT	RGYYPKGGGE	VIVRMSPVKQ	LNPINLTERG	CVTKIYGRAF	VAGVLPFKVA	KDMAAAAVRC	IRKEIRDLYV
**250**	**260**	**270**	**280**	**290**	**300**	**310**	**320**
NIQPVQEPKD	QAFGNGNGII	IIAETSTGCL	FAGSSLGKRG	VNADKVGIEA	AEMLLANLRH	GGTVDEYLQD	QLIVFMALAN
**330**	**340**	**350**	**360**	**370**	**380**		
GVSRIKTGPV	TLHTQTAIHF	AEQIAKAKFI	VKKSEDEEDA	AKDTYIIECQ	GIGMTNPNL		

Cmpd.	No. of Cmpds.	m/z meas.	Δ m/z [ppm]	z	Rt [min]	Score	P	Range	Sequence	Modification	Ratios
2681	1	559.7841	-33.68	2	64.6	11.9	1	219-229	K.VAKDMAAAAVR.C	Oxidation: 5	WUP:QUP 1.59

Detailed Protein Report

Protein 584: PREDICTED: DNA polymerase zeta catalytic subunit isoform X4 [Homo sapiens]

**Accession:**	gi|530383948	**Score:**	26.5
**Database:**	refseq_human(refseq_human_20140103.fasta)	**MW [kDa]:**	301.0
**Database Date:**	2015-11-30	**pI:**	9.0
**Modification(s):**	Carbamidomethyl, Oxidation	**Sequence Coverage [%]:**	0.7
		**No. of unique Peptides:**	1

Detailed Protein Report

10	20	30	40	50	60	70	80
MFSVRIVTAD	YYMASPLQGL	DTCQSPLTQA	PVKKVPVVRV	FGATPAGQKT	CLHLHGIFPY	LYVPYDGYGQ	QPESYLSQMA
90	100	110	120	130	140	150	160
FSIDRALNVA	LGNPSSTAQH	VFKVSLVSGM	PFYGYHEKER	HFMKIYLYNP	TMVKRICELL	QSGAIMNKFY	QPHEAHIPYL
170	180	190	200	210	220	230	240
LQLFIDYNLY	GMNLINLAAV	KFRKARRKSN	TLHATGSCKN	HLSGNSLADT	LFRWEQDEIP	SSLILEGVEP	QSTCELEVDA
250	260	270	280	290	300	310	320
VAADILNRLD	IEAQIGGNPG	LQAIWEDEKQ	RRRNRNETSQ	MSQPESQDHR	FVPATESEKK	FQKRLQEILK	QNDFSVTLSG
330	340	350	360	370	380	390	400
SVDYSDGSQE	FSAELTLHSE	VLSPEMLQCT	PANMVEVHKD	KESSKGHTRH	KVEEALINEE	AILNLMENSQ	TFQPLTQRLS
410	420	430	440	450	460	470	480
ESPVFMDSSP	DEALVHLLAG	LESDGYRGER	NRMPSPCRSF	GNNKYPQNSD	DEENEPQIEK	EEMELSLVMS	QRWDSNIEEH
490	500	510	520	530	540	550	560
CAKKRSLCRN	THRSSTEDDD	SSSGEEMEWS	DNSLLLASLS	IPQLDGTADE	NSDNPLNNEN	SRTHSSVIAT	SKLSVKPSIF
570	580	590	600	610	620	630	640
HKDAATLEPS	SSAKITFQCK	HTSALSSHVL	NKEDLIEDLS	QTNKNTEKGL	DNSVTSFTNE	STYSMKYPGS	LSSTVHSENS
650	660	670	680	690	700	710	720
HKENSKKEIL	PVSSCESSIF	DYEEDIPSVT	RQVPSRKYTN	IRKIEKDSPF	IHMHRHPNEN	TLGKNSFNFS	DLNHSKNKVS
730	740	750	760	770	780	790	800
SEGNEKGNST	ALSSLFPSSF	TENCELLSCS	GENRTMVHSL	NSTADESGLN	KLKIRYEEFQ	EHKTEKPSLS	QQAAHYMFFP
810	820	830	840	850	860	870	880
SVVLSNCLTR	PQKLSPVTYK	LQPGNKPSRL	KLNKRKLAGH	QETSTKSSET	GSTKDNFIQN	NPCNSNPEKD	NALASDLTKT
890	900	910	920	930	940	950	960
TRGAFENKTP	TDGFIDCHFG	DGTLETEQSF	GLYGNKYTLR	AKRKVNYETE	DSESSFVTHN	SKISLPHPME	IGESLDGTLK
970	980	990	1000	1010	1020	1030	1040
SRKRRKMSKK	LPPVIIKYII	INRFRGRKNM	LVKLGKIDSK	EKQVILTEEK	MELYKKLAPL	KDFWPKVPDS	PATKYPIYPL
1050	1060	1070	1080	1090	1100	1110	1120
TPKKSHRRKS	KHKSAKKKTG	KQQRTNNENI	KRTLSFRKKR	SHAILSPPSP	SYNAETEDCD	LNYSDVMSKL	GFLSERSTSP
1130	1140	1150	1160	1170	1180	1190	1200
INSSPPRCWS	PTDPRAEEIM	AAAEKEAMLF	KGPNVYKKTV	NSRIGKTSRA	RAQIKKSKAK	LANPSIVTKK	RNKRNQTNKL
1210	1220	1230	1240	1250	1260	1270	1280
VDDGKKKPRA	KQKTNEKGTS	RKHTTLKDEK	IKSQSGAEVK	FVLKHQNVSE	FASSSGGSQL	LFKQKDMPLM	GSAVDHPLSA
1290	1300	1310	1320	1330	1340	1350	1360
SLPTGINAQQ	KLSGCFSSFL	ESKKSVDLQT	FPSSRDDLHP	SVVCNSIGPG	VSKINVQRPH	NQSAMFTLKE	STLIQKNIFD
1370	1380	1390	1400	1410	1420	1430	1440
LSNHLSQVAQ	NTQISSGMSS	KIEDNANNIQ	RNYLSSIGKL	SEYRNSLESK	LDQAYTPNFL	HCKDSQQQIV	CIAEQSKHSE
1450	1460	1470	1480	1490	1500	1510	1520
TCSPGNTASE	ESQMPNNCFV	TSLRSPIKQI	AWEQKQRGFI	LDMSNFKPER	VKPRSLSEAI	SQTKALSQCK	NRNVSTPSAF
1530	1540	1550	1560	1570	1580	1590	1600
GEGQSGLAVL	KELLQKRQQK	AQNANTTQDP	LSNKHQPNKN	ISGSLEHNKA	NKRTRSVTSP	RKPRTPRSTK	QKEKIPKLLK
1610	1620	1630	1640	1650	1660	1670	1680
VDSLNLQNSS	QLDNSVSDDS	PIFFSDPGFE	SCYSLEDSLS	PEHNYNFDIN	TIGQTGFCSF	YSGSQFVPAD	QNLPQKFLSD
1690	1700	1710	1720	1730	1740	1750	1760
AVQDLFPGQA	IEKNEFLSHD	NQKCDEDKHH	TTDSASWIRS	GTLSPEIFEK	STIDSNENRR	HNQWKNSFHP	LTTRSNSIMD
1770	1780	1790	1800	1810	1820	1830	1840
SFCVQQAEDC	LSEKSRLNRS	SVSKEVFLSL	PQPNNSDWIQ	GHTRKEMGQS	LDSANTSFTA	ILSSPDGELV	DVACEDLELY
1850	1860	1870	1880	1890	1900	1910	1920
VSRNNDMLTP	TPDSSPRSTS	SPSQSKNGSF	TPRTANILKP	LMSPPSREEI	MATLLDHDLS	ETIYQEPFCS	NPSDVPEKPR
1930	1940	1950	1960	1970	1980	1990	2000
EIGGRLLMVE	TRLANDLAEF	EGDFSLEGLR	LWKTAFSAMT	QNPRPGSPLR	SGQGVVNKGS	SNSPKMVEDK	KIVIMPCKCA
2010	2020	2030	2040	2050	2060	2070	2080
PSRQLVQVWL	QAKEEYERSK	KLPKTKPTGV	VKSAENFSSS	VNPDDKPVVP	PKMDVSPCIL	PTTAHTKEDV	DNSQIALQAP
2090	2100	2110	2120	2130	2140	2150	2160
TTGCSQTASE	SQMLPPVASA	SDPEKDEDDD	DNYYISYSSP	DSPVIPPWQQ	PISPDSKALN	GDDRPSSPVE	ELPSLAFENF
2170	2180	2190	2200	2210	2220	2230	2240

Detailed Protein Report

Cmpd.	No. of Cmpds.	m/z meas.	Δ m/z [ppm]	z	Rt [min]	Score	P	Range	Sequence	Modification
727	2	517.1500	-168.10	2	37.5	14.5	1	431-438	R.NRMPSPCR.S	Carbamidomethyl: 7; Oxidation: 3

Detailed Protein Report

Protein 585: zinc finger protein 865 [Homo sapiens]

Accession: gi|307219238
Database: refseq_human(refseq_human_20140103.fasta)
Database Date: 2015-11-30

Score: 26.4
MW [kDa]: 111.0
pI: 10.6
Sequence Coverage [%]: 2.7
No. of unique Peptides: 2

Quantitation

QU:MU **Median:** 1.21 **CV:** 0.00 % **No. of Peptides:** 1

10	20	30	40	50	60	70	80
MEANPAGSGA	GGGGSSGIGG	EDGVHFQSYP	FDFLEFLNHQ	RFEPMELYGE	HAKAVAALPC	APGPPPQPPP	QPPPPQYDYP
**90**	**100**	**110**	**120**	**130**	**140**	**150**	**160**
PQSTFKPKAE	VPSSSSSSSS	SSSSSSSSSS	SSSSSSSQAK	KPDPPLPPAF	GAPPPPLFDA	AFPTPQWGIV	DLSGHQHLFG
**170**	**180**	**190**	**200**	**210**	**220**	**230**	**240**
NLKRGGPASG	PGVTPGLGAP	AGAPGPLPAP	SQTPPGPPAA	AACDPTKDDK	GYFRRLKYLM	ERRFPCGVCQ	KSFKQSSHLV
**250**	**260**	**270**	**280**	**290**	**300**	**310**	**320**
QHMLVHSGER	PYECGVCGRT	YNHVSSLIRH	RRCHKDVPPA	AGGPPQPGPH	LPPLGLPAPA	ASAATAAAPS	TVSSGPPATP
**330**	**340**	**350**	**360**	**370**	**380**	**390**	**400**
VAPAPSADGS	AAPAGVGVPP	PATGGGDGPF	ACPLCWKVFK	KPSHLHQHQI	IHTGEKPFSC	SVCSKSFNRR	ESLKRHVKTH
**410**	**420**	**430**	**440**	**450**	**460**	**470**	**480**
SADLLRLPCG	ICGKAFRDAS	YLLKHQAAHA	GAGAGGPRPV	YPCDLCGKSY	SAPQSLLRHK	AAHAPPAAAA	EAPKDGAASA
**490**	**500**	**510**	**520**	**530**	**540**	**550**	**560**
PQPPPTFPPG	PYLLPPDPPT	TDSEKAAAAA	AAVVYGAVPV	PLLGAHPLLL	GGAGTSGAGG	SGASVPGKTF	CCGICGRGFG
**570**	**580**	**590**	**600**	**610**	**620**	**630**	**640**
RRETLKRHER	IHTGEKPHQC	PVCGKRFRES	FHLSKHHVVH	TRERPYKCEL	CGKVFGYPQS	LTRHRQVHRL	QLPCALAGAA
**650**	**660**	**670**	**680**	**690**	**700**	**710**	**720**
GLPSTQGTPG	ACGPGASGTS	AGPTDGLSYA	CSDCGEHFPD	LFHVMSHKEV	HMAEKPYGCD	ACGKTFGFIE	NLMWHKLVHQ
**730**	**740**	**750**	**760**	**770**	**780**	**790**	**800**
AAPERLLPPA	PGGLQPPDGS	SGTDAASVLD	NGLAGEVGAA	VAALAGVSGG	EDAGGAAVAG	AGGGASSGPE	RFSCATCGQS
**810**	**820**	**830**	**840**	**850**	**860**	**870**	**880**
FKHFLGLVTH	KYVHLVRRTL	GCGLCGQSFA	GAYDLLLHRR	SHRQKRGFRC	PVCGKRFWEA	ALLMRHQRCH	TEQRPYRCGV
**890**	**900**	**910**	**920**	**930**	**940**	**950**	**960**
CGRGFLRSWY	LRQHRVVHTG	ERAFKCGVCA	KRFAQSSSLA	EHRRLHAVAR	PQRCSACGKT	FRYRSNLLEH	QRLHLGERAY
**970**	**980**	**990**	**1000**	**1010**	**1020**	**1030**	**1040**
RCEHCGKGFF	YLSSVLRHQR	AHEPPRPELR	CPACLKAFKD	PGYFRKHLAA	HQGGRPFRCS	SCGEGFANTY	GLKKHRLAHK
**1050**	**1060**						
AENLGGPGAG	AGTLAGKDA						

Cmpd.	No. of Cmpds.	m/z meas.	Δ m/z [ppm]	z	Rt [min]	Score	P	Range	Sequence	Modification	Ratios
10	2	819.8432	-130.81	2	29.6	16.4	2	260-272	R.TYNHVSSLIRHRR.C		
2279	1	869.2738	-114.47	2	58.9	10.1	0	689-704	K.EVHMAEKPYGCDACGK.T		QU:MU 1.21

Detailed Protein Report

Protein 586: zinc finger protein 20 isoform 2 [Homo sapiens]

**Accession:**	gi|322303718	**Score:**	26.4
**Database:**	refseq_human(refseq_human_20140103.fasta)	**MW [kDa]:**	61.1
**Database Date:**	2015-11-30	**pI:**	10.0
**Modification(s):**	Carbamidomethyl	**Sequence Coverage [%]:**	6.8
		**No. of unique Peptides:**	1

10	20	30	40	50	60	70	80
MDSVAFEDVA	VSFTQEEWAL	LDPSQKNLYR	DVMQETFKNL	TSVGKTWKVQ	NIEDEYKNPR	RNLSLMREKL	CESKESHHCG
**90**	**100**	**110**	**120**	**130**	**140**	**150**	**160**
ESFNQIADDM	LNRKTLPGIT	PCESSVCGEV	GTGHSSLNTH	IRADTGHKSS	EYQEYGENPY	RNKECKKAFS	YLDSFQSHDK
**170**	**180**	**190**	**200**	**210**	**220**	**230**	**240**
ACTKEKPYDG	KECTETFISH	SCIQRHRVMH	SGDGPYKCKF	CGKAFYFLNL	CLIHERIHTG	VKPYKCKQCG	KAFTRSTTLP
**250**	**260**	**270**	**280**	**290**	**300**	**310**	**320**
VHERTHTGVN	ADECKECGNA	FSFPSEIRRH	KRSHTGEKPY	ECKQCGKVFI	SFSSIQYHKM	THTGEKPYEC	KQCGKAFRCG
**330**	**340**	**350**	**360**	**370**	**380**	**390**	**400**
SHLQKHGRTH	TGEKPYECRQ	CGKAFRCTSD	LQRHEKTHTE	DKPYGCKQCG	KGFRCASQLQ	IHERTHSGEK	PHECKECGKV
**410**	**420**	**430**	**440**	**450**	**460**	**470**	**480**
FKYFSSLRIH	ERTHTGEKPH	ECKQCGKAFR	YFSSLHIHER	THTGDKPYEC	KVCGKAFTCS	SSIRYHERTH	TGEKPYECKH
**490**	**500**	**510**	**520**	**530**			
CGKAFISNYI	RYHERTHTGE	KPYQCKQCGK	AFIRASSCRE	HERTHTINR			

Cmpd.	No. of Cmpds.	m/z meas.	Δ m/z [ppm]	z	Rt [min]	Score	P	Range	Sequence	Modification
2376	1	1130.4062	-64.30	2	58.0	12.0	0	75-93	K.ESHHCGESFNQIADDMLNR.K	Carbamidomethyl: 5

Detailed Protein Report

Protein 587: ral GTPase-activating protein subunit alpha-1 isoform 1 [Homo sapiens]

**Accession:**	gi|51226124	**Score:**	26.4
**Database:**	refseq_human(refseq_human_20140103.fasta)	**MW [kDa]:**	229.7
**Database Date:**	2015-11-30	**pI:**	5.8
**Modification(s):**	Oxidation	**Sequence Coverage [%]:**	1.6
		**No. of unique Peptides:**	1

Detailed Protein Report

10	20	30	40	50	60	70	80
MFSKKPHGDV	KKSTQKVLDT	KKDALTRLKH	LRIVIENAES	IDLKQFFDQH	FSHIYYVFFE	NFVTIEASLK	QKGHKSQREE
90	100	110	120	130	140	150	160
LDAILFIFEK	ILQLLPERIH	QRWQFHSIGL	ILKKLLHTGN	SLKIRREGVR	LFLLWLQALQ	NNCSKEQLWM	FSCLIPGFSA
170	180	190	200	210	220	230	240
PQSEHGPRTL	DNLINPPLNL	QETQVTIEEI	TPLVPPQSGD	KGQEDLTSYF	LEALLKYIVI	QVKSLEWKNK	ENQERGFSFL
250	260	270	280	290	300	310	320
FSHFKKYYLP	YIFPNICKEN	SLYHPILDIP	QMRPKPHYVV	IKKDAETNEA	IYCTKEPFIK	ARVIVIRWLV	SFWLEPKPHT
330	340	350	360	370	380	390	400
GPHIPGMEGE	VLPKNIQRAA	ASLVSREESK	NDNADKTDRT	TEPEQSHSNT	STLTEREPSS	SSLCSIDEEH	LTDIEIVRRV
410	420	430	440	450	460	470	480
FSSKRSNVNF	VTEIFRQAFL	LPICEAAAMR	KVVKVYQEWI	QQEEKPLFMQ	EPEEIVITSS	DLPCIENVTD	HDISMEEGEK
490	500	510	520	530	540	550	560
REEENGTNTA	DHVRNSSWAK	NGSYQGALHN	ASEEATEQNI	RAGTQAVLQV	FIINSSNIFL	LEPANEIKNL	LDEHTDMCKR
570	580	590	600	610	620	630	640
ILNIYRYMVV	QVSMDKKTWE	QMLLVLLRVT	ESVLKMPSQA	FLQFQGKKNM	TLAGRLAGPL	FQTLIVAWIK	ANLNVYISRE
650	660	670	680	690	700	710	720
LWDDLLSVLS	SLTYWEELAT	EWSLTMETLT	KVLARNLYSL	DLSDLPLDKL	SEQKQKKHKG	KGVGHEFQKV	SVDKSFSRGW
730	740	750	760	770	780	790	800
SRDQPGQAPM	RQRSATTTGS	PGTEKARSIV	RQKTVDIDDA	QILPRSTRVR	HFSQSEETGN	EVFGALNEEQ	PLPRSSSTSD
810	820	830	840	850	860	870	880
ILEPFTVERA	KVNKEDMSQK	LPPLNSDIGG	SSANVPDLMD	EFIAERLRSG	NASTMTRRGS	SPGSLEIPKD	LPDILNKQNQ
890	900	910	920	930	940	950	960
MRPIDDPGVP	SEWTSPASAG	SSDLISSDSH	SDSFSAFQYD	GRKFDNFGFG	TDTGVTSSAD	VDSGSGHHQS	AEEQEVASLT
970	980	990	1000	1010	1020	1030	1040
TLHIDSETSS	LNQQAFSAEV	ATITGSESAS	PVHSPLGSRS	QTPSPSTLNI	DHMEQKDLQL	DEKLHHSVLQ	TPDDLEISEF
1050	1060	1070	1080	1090	1100	1110	1120
PSECCSVMAG	GTLTGWHADV	ATVMWRRMLG	ILGDVNSIMD	PEIHAQVFDY	LCELWQNLAK	IRDNLGISTD	NLTSPSPPVL
1130	1140	1150	1160	1170	1180	1190	1200
IPPLRILTPW	LFKATMLTDK	YKQGKLHAYK	LICNTMKRRQ	DVSPNRDFLT	HFYNIMHCGL	LHIDQDIVNT	IIKHCSPQFF
1210	1220	1230	1240	1250	1260	1270	1280
SLGLPGATML	IMDFIVAAGR	VASSAFLNAP	RVEAQVLLGS	LVCFPNLYCE	LPSLHPNIPD	VAVSQFTDVK	ELIIKTVLSS
1290	1300	1310	1320	1330	1340	1350	1360
ARDEPSGPAR	CVALCSLGIW	ICEELVHESH	HPQIKEALNV	ICVSLKFTNK	TVAHVACNML	HMLVHYVPRL	QIYQPDSPLK
1370	1380	1390	1400	1410	1420	1430	1440
IIQILIATIT	HLLPSTEASS	YEMDKRLVVS	LLLCLLDWIM	ALPLKTLLQP	FHATGAESDK	TEKSVLNCIY	KVLHGCVYGA
1450	1460	1470	1480	1490	1500	1510	1520
QCFSNPRYFP	MSLSDLASVD	YDPFMHLESL	KEPEPLHSPD	SERSSKLQPV	TEVKTQMQHG	LISIAARTVI	THLVNHLGHY
1530	1540	1550	1560	1570	1580	1590	1600
PMSGGPAMLT	SQVCENHDNH	YSESTELSPE	LFESPNIQFF	VLNNTTLVSC	IQIRSEENMP	GGGLSAGLAS	ANSNVRIIVR
1610	1620	1630	1640	1650	1660	1670	1680
DLSGKYSWDS	AILYGPPPVS	GLSEPTSFML	SLSHQEKPEE	PPTSNECLED	ITVKDGLSLQ	FKRFRETVPT	WDTIRDEEDV
1690	1700	1710	1720	1730	1740	1750	1760
LDELLQYLGV	TSPECLQRTG	ISLNIPAPQP	VCISEKQEND	VINAILKQHT	EEKEFVEKHF	NDLNMKAVEQ	DEPIPQKPQS
1770	1780	1790	1800	1810	1820	1830	1840
AFYYCRLLLS	ILGMNSWDKR	RSFHLLKKNE	KLLRELRNLD	SRQCRETHKI	AVFYVAEGQE	DKHSILTNTG	GSQAYEDFVA
1850	1860	1870	1880	1890	1900	1910	1920
GLGWEVNLTN	HCGFMGGLQK	NKSTGLTTPY	FATSTVEVIF	HVSTRMPSDS	DDSLTKKLRH	LGNDEVHIVW	SEHTRDYRRG
1930	1940	1950	1960	1970	1980	1990	2000
IIPTEFGDVL	IVIYPMKNHM	FSIQIMKKPE	VPFFGPLFDG	AIVNGKVLPI	MVRATAINAS	RALKSLIPLY	QNFYEEARY
2010	2020	2030	2040				
LQTIVQHHLE	PTTFEDFAAQ	VFSPAPYHHL	PSDADH				

Cmpd.	No. of Cmpds.	m/z meas.	Δ m/z [ppm]	z	Rt [min]	Score	P	Range	Sequence	Modification

Detailed Protein Report

Cmpd.	No. of Cmpds.	m/z meas.	Δ m/z [ppm]	z	Rt [min]	Score	P	Range	Sequence	Modification
800	1	709.3960	15.98	2	39.5	11.8	2	1-12	-.MFSKKPHGDVKK.S	Oxidation: 1

Detailed Protein Report

Protein 588: **PREDICTED: poly [ADP-ribose] polymerase 14 isoform X1 [Homo sapiens]**

**Accession:**	gi|530374806	**Score:**	26.4
**Database:**	refseq_human(refseq_human_20140103.fasta)	**MW [kDa]:**	187.2
**Database Date:**	2015-11-30	**pI:**	8.1
		**Sequence Coverage [%]:**	1.2
		**No. of unique Peptides:**	2

Quantitation

WUP:QUP **Median:** 0.04 **CV:** 0.00 % **No. of Peptides:** 1

10	20	30	40	50	60	70	80
MEDIPEECEN	ISSLVAFENL	KANVTDIMLI	LLVENISGLS	NDDFQVEIIR	DFDVAVVTFQ	KHIDTIRFVD	DCTKHHSIKQ
90	100	110	120	130	140	150	160
LQLSPRLLEV	TNTIRVENLP	PGADDYSLKL	FFENPYNGGG	RVANVEYFPE	ESSALIEFFD	RKVLDTIMAT	KLDFNKMPLS
170	180	190	200	210	220	230	240
VFPYYASLGT	ALYGKEKPLI	KLPAPFEESL	DLPLWKFLQK	KNHLIEEIND	EMRRCHCELT	WSQLSGKVTI	RPAATLVNEG
250	260	270	280	290	300	310	320
RPRIKTWQAD	TSTTLSSIRS	KYKVNPIKVD	PTMWDTIKND	VKDDRILIEF	DTLKEMVILA	GKSEDVQSIE	VQVRELIEST
330	340	350	360	370	380	390	400
TQKIKREEQS	LKEKMIISPG	RYFLLCHSSL	LDHLLTECPE	IEICYDRVTQ	HLCLKGPSAD	VYKAKCEIQE	KVYTMAQKNI
410	420	430	440	450	460	470	480
QVSPEIFQFL	QQVNWKEFSK	CLFIAQKILA	LYELEGTTVL	LTSCSSEALL	EAEKQMLSAL	NYKRIEVENK	EVLHGKKWKG
490	500	510	520	530	540	550	560
LTHNLLKKQN	SSPNTVIINE	LTSETTAEVI	ITGCVKEVNE	TYKLLFNFVE	QNMKIERLVE	VKPSLVIDYL	KTEKKLFWPK
570	580	590	600	610	620	630	640
IKKVNVQVSF	NPENKQKGIL	LTGSKTEVLK	AVDIVKQVWD	SVCVKSVHTD	KPGAKQFFQD	KARFYQSEIK	RLFGCYIELQ
650	660	670	680	690	700	710	720
ENEVMKEGGS	PAGQKCFSRT	VLAPGVVLIV	QQGDLARLPV	DVVVNASNED	LKHYGGLAAA	LSKAAGPELQ	ADCDQIVKRE
730	740	750	760	770	780	790	800
GRLLPGNATI	SKAGKLPYHH	VIHAVGPRWS	GYEAPRCVYL	LRRAVQLSLC	LAEKYKYRSI	AIPAISSGVF	GFPLGRCVET
810	820	830	840	850	860	870	880
IVSAIKENFQ	FKKDGHCLKE	IYLVDVSEKT	VEAFAEAVKT	VFKATLPDTA	APPGLPPAAA	GPGKTSWEKG	SLVSPGGLQM
890	900	910	920	930	940	950	960
LLVKEGVQNA	KTDVVVNSVP	LDLVLSRGPL	SKSLLEKAGP	ELQEELDTVG	QGVAVSMGTV	LKTSSWNLDC	RYVLHVVAPE
970	980	990	1000	1010	1020	1030	1040
WRNGSTSSLK	IMEDIIRECM	EITESLSLKS	IAFPAIGTGN	LGFPKNIFAE	LIISEVFKFS	SKNQLKTLQE	VHFLLHPSDH
1050	1060	1070	1080	1090	1100	1110	1120
ENIQAFSDEF	ARRANGNLVS	DKIPKAKDTQ	GFYGTVSSPD	SGVYEMKIGS	IIFQVASGDI	TKEEADVIVN	STSNSFNLKA
1130	1140	1150	1160	1170	1180	1190	1200
GVSKAILECA	GQNVERECSQ	QAQQRKNDYI	ITGGGFLRCK	NIIHVIGGND	VKSSVSSVLQ	ECEKKNYSSI	CLPAIGTGNA
1210	1220	1230	1240	1250	1260	1270	1280
KQHPDKVAEA	IIDAIEDFVQ	KGSAQSVKKV	KVVIFLPQVL	DVFYANMKKR	EGTQLSSQQS	VMSKLASFLG	FSKQSPQKKN
1290	1300	1310	1320	1330	1340	1350	1360
HLVLEKKTES	ATFRVCGENV	TCVEYAISWL	QDLIEKEQCP	YTSEDECIKD	FDEKEYQELN	ELQKKLNINI	SLDHKRPLIK
1370	1380	1390	1400	1410	1420	1430	1440
VLGISRDVMQ	ARDEIEAMIK	RVRLAKEQES	RADCISEFIE	WQYNDNNTSH	CFNKMTNLKL	EDARREKKKT	VDVKINHRHY
1450	1460	1470	1480	1490	1500	1510	1520
TVNLNTYTAT	DTKGHSLSVQ	RLTKSKVDIP	AHWSDMKQQN	FCVVELLPSD	PEYNTVASKF	NQTCSHFRIE	KIERIQNPDL
1530	1540	1550	1560	1570	1580	1590	1600
WNSYQAKKKT	MDAKNGQTMN	EKQLFHGTDA	GSVPHVNRNG	FNRSYAGKNA	VAYGKGTYFA	VNANYSANDT	YSRPDANGRK
1610	1620	1630	1640	1650	1660	1670	
HVYYVRVLTG	IYTHGNHSLI	VPPSKNPQNP	TDLYDTVTDN	VHHPSLFVAF	YDYQAYPEYL	ITFRK	

Cmpd.	No. of Cmpds.	m/z meas.	Δ m/z [ppm]	z	Rt [min]	Score	P	Range	Sequence	Modification	Ratios
554	1	476.1400	-265.76	2	36.9	12.8	1	1019-	K.FSSKNQLK.T		

Detailed Protein Report

Cmpd.	No. of Cmpds.	m/z meas.	Δ m/z [ppm]	z	Rt [min]	Score	P	Range	Sequence	Modification	Ratios
								1026			
2539	1	648.3564	69.94	2	62.4	13.6	0	1173-1184	K.SSVSSVLQECEK.K		WUP:QUP 0.04

Detailed Protein Report

Protein 589: huntingtin [Homo sapiens]

**Accession:**	gi|90903231	**Score:**	26.3
**Database:**	refseq_human(refseq_human_20140103.fasta)	**MW [kDa]:**	347.6
**Database Date:**	2015-11-30	**pI:**	5.8
		**Sequence Coverage [%]:**	0.9
		**No. of unique Peptides:**	1

Detailed Protein Report

10	20	30	40	50	60	70	80
MATLEKLMKA	FESLKSFQQQ	QQQQQQQQQQ	QQQQQQQQQQ	PPPPPPPPPP	PQLPQPPPQA	QPLLPQPQPP	PPPPPPPPGP
90	100	110	120	130	140	150	160
AVAEEPLHRP	KKELSATKKD	RVNHCLTICE	NIVAQSVRNS	PEFQKLLGIA	MELFLLCSDD	AESDVRMVAD	ECLNKVIKAL
170	180	190	200	210	220	230	240
MDSNLPRLQL	ELYKEIKKNG	APRSLRAALW	RFAELAHLVR	PQKCRPYLVN	LLPCLTRTSK	RPEESVQETL	AAAVPKIMAS
250	260	270	280	290	300	310	320
FGNFANDNEI	KVLLKAFIAN	LKSSSPTIRR	TAAGSAVSIC	QHSRRTQYFY	SWLLNVLLGL	LVPVEDEHST	LLILGVLLTL
330	340	350	360	370	380	390	400
RYLVPLLQQQ	VKDTSLKGSF	GVTRKEMEVS	PSAEQLVQVY	ELTLHHTQHQ	DHNVVTGALE	LLQQLFRTPP	PELLQTLTAV
410	420	430	440	450	460	470	480
GGIGQLTAAK	EESGGRSRSG	SIVELIAGGG	SSCSPVLSRK	QKGKVLLGEE	EALEDDSESR	SDVSSSALTA	SVKDEISGEL
490	500	510	520	530	540	550	560
AASSGVSTPG	SAGHDIITEQ	PRSQHTLQAD	SVDLASCDLT	SSATDGDEED	ILSHSSSQVS	AVPSDPAMDL	NDGTQASSPI
570	580	590	600	610	620	630	640
SDSSQTTTEG	PDSAVTPSDS	SEIVLDGTDN	QYLGLQIGQP	QDEDEEATGI	LPDEASEAFR	NSSMALQQAH	LLKNMSHCRQ
650	660	670	680	690	700	710	720
PSDSSVDKFV	LRDEATEPGD	QENKPCRIKG	DIGQSTDDDS	APLVHCVRLL	SASFLLTGGK	NVLVPDRDVR	VSVKALALSC
730	740	750	760	770	780	790	800
VGAAVALHPE	SFFSKLYKVP	LDTTEYPEEQ	YVSDILNYID	HGDPQVRGAT	AILCGTLICS	ILSRSRFHVG	DWMGTIRTLT
810	820	830	840	850	860	870	880
GNTFSLADCI	PLLRKTLKDE	SSVTCKLACT	AVRNCVMSLC	SSSYSELGLQ	LIIDVLTLRN	SSYWLVRTEL	LETLAEIDFR
890	900	910	920	930	940	950	960
LVSFLEAKAE	NLHRGAHHYT	GLLKLQERVL	NNVVIHLLGD	EDPRVRHVAA	ASLIRLVPKL	FYKCDQGQAD	PVVAVARDQS
970	980	990	1000	1010	1020	1030	1040
SVYLKLLMHE	TQPPSHFSVS	TITRIYRGYN	LLPSITDVTM	ENNLSRVIAA	VSHELITSTT	RALTFGCCEA	LCLLSTAFPV
1050	1060	1070	1080	1090	1100	1110	1120
CIWSLGWHCG	VPPLSASDES	RKSCTVGMAT	MILTLLSSAW	FPLDLSAHQD	ALILAGNLLA	ASAPKSLRSS	WASEEEANPA
1130	1140	1150	1160	1170	1180	1190	1200
ATKQEEVWPA	LGDRALVPMV	EQLFSHLLKV	INICAHVLDD	VAPGPAIKAA	LPSLTNPPSL	SPIRRKGKEK	EPGEQASVPL
1210	1220	1230	1240	1250	1260	1270	1280
SPKKGSEASA	ASRQSDTSGP	VTTSKSSSLG	SFYHLPSYLK	LHDVLKATHA	NYKVTLDLQN	STEKFGGFLR	SALDVLSQIL
1290	1300	1310	1320	1330	1340	1350	1360
ELATLQDIGK	CVEEILGYLK	SCFSREPMMA	TVCVQQLLKT	LFGTNLASQF	DGLSSNPSKS	QGRAQRLGSS	SVRPGLYHYC
1370	1380	1390	1400	1410	1420	1430	1440
FMAPYTHFTQ	ALADASLRNM	VQAEQENDTS	GWFDVLQKVS	TQLKTNLTSV	TKNRADKNAI	HNHIRLFEPL	VIKALKQYTT
1450	1460	1470	1480	1490	1500	1510	1520
TTCVQLQKQV	LDLLAQLVQL	RVNYCLLDSD	QVFIGFVLKQ	FEYIEVGQFR	ESEAIIPNIF	FFLVLLSYER	YHSKQIIGIP
1530	1540	1550	1560	1570	1580	1590	1600
KIIQLCDGIM	ASGRKAVTHA	IPALQPIVHD	LFVLRGTNKA	DAGKELETQK	EVVVSMLLRL	IQYHQVLEMF	ILVLQQCHKE
1610	1620	1630	1640	1650	1660	1670	1680
NEDKWKRLSR	QIADIILPML	AKQQMHIDSH	EALGVLNTLF	EILAPSSLRP	VDMLLRSMFV	TPNTMASVST	VQLWISGILA
1690	1700	1710	1720	1730	1740	1750	1760
ILRVLISQST	EDIVLSRIQE	LSFSPYLISC	TVINRLRDGD	STSTLEEHSE	GKQIKNLPEE	TFSRFLLQLV	GILLEDIVTK
1770	1780	1790	1800	1810	1820	1830	1840
QLKVEMSEQQ	HTFYCQELGT	LLMCLIHIFK	SGMFRRITAA	ATRLFRSDGC	GGSFYTLDSL	NLRARSMITT	HPALVLLWCQ
1850	1860	1870	1880	1890	1900	1910	1920
ILLLVNHTDY	RWWAEVQQTP	KRHSLSSTKL	LSPQMSGEEE	DSDLAAKLGM	CNREIVRRGA	LILFCDYVCQ	NLHDSEHLTW
1930	1940	1950	1960	1970	1980	1990	2000
LIVNHIQDLI	SLSHEPPVQD	FISAVHRNSA	ASGLFIQAIQ	SRCENLSTPT	MLKKTLQCLE	GIHLSQSGAV	LTLYVDRLLC
2010	2020	2030	2040	2050	2060	2070	2080
TPFRVLARMV	DILACRRVEM	LLAANLQSSM	AQLPMEELNR	IQEYLQSSGL	AQRHQRLYSL	LDRFRLSTMQ	DSLSPSPPVS
2090	2100	2110	2120	2130	2140	2150	2160
SHPLDGDGHV	SLETVSPDKD	WYVHLVKSQC	WTRSDSALLE	GAELVNRIPA	EDMNAFMMNS	EFNLSLLAPC	LSLGMSEISG
2170	2180	2190	2200	2210	2220	2230	2240

Detailed Protein Report

Cmpd.	No. of Cmpds.	m/z meas.	Δ m/z [ppm]	z	Rt [min]	Score	P	Range	Sequence	Modification
347	1	524.3773	160.20	2	33.0	15.3	2	93-101	K.ELSATKKDR.V	

Detailed Protein Report

Protein 590: PREDICTED: zinc finger protein 841 isoform X1 [Homo sapiens]

**Accession:**	gi|578834341	**Score:**	26.3
**Database:**	refseq_human(refseq_human_20140103.fasta)	**MW [kDa]:**	99.6
**Database Date:**	2015-11-30	**pI:**	10.4
**Modification(s):**	Carbamidomethyl	**Sequence Coverage [%]:**	5.1
		**No. of unique Peptides:**	2

10	20	30	40	50	60	70	80
MALPQGSLTF	RDVAVEFSQE	EWKCLDPVQK	ALYRDVMLEN	YRNLGFLGLC	LPDLNIISML	EQGKEPWTVV	SQVKIARNPN
**90**	**100**	**110**	**120**	**130**	**140**	**150**	**160**
CGECMKGVIT	GISPKCVIKE	LPPIQNSNTG	EKFQAVMLEG	HESYDTENFY	FREIRKNLQE	VDFQWKDGEI	NYKEGPMTHK
**170**	**180**	**190**	**200**	**210**	**220**	**230**	**240**
NNLTGQRVRH	SQGDVENKHM	ENQLILRFQS	GLGELQKFQT	AEKIYGCNQI	ERTVNNCFLA	SPLQRIFPGV	QTNISRKYGN
**250**	**260**	**270**	**280**	**290**	**300**	**310**	**320**
DFLQLSLPTQ	DEKTHIREKP	YIGNECGKAF	RVSSSLINHQ	MIHTTEKPYR	CNESGKAFHR	GSLLTVHQIV	HTRGKPYQCD
**330**	**340**	**350**	**360**	**370**	**380**	**390**	**400**
VCGRIFRQNS	DLVNHRRSHT	GDKPYICNEC	GKSFSKSSHL	AVHQRIHTGE	KPYKCNRCGK	CFSQSSSLAT	HQTVHTGDKP
**410**	**420**	**430**	**440**	**450**	**460**	**470**	**480**
YKCNECGKTF	KRNSSLTAHH	IIHAGKKPYT	CDVCGKVFYQ	NSQLVRHQII	HTGETPYKCN	ECGKVFFQRS	RLAGHRRIHT
**490**	**500**	**510**	**520**	**530**	**540**	**550**	**560**
GEKPYKCNEC	GKVFSQHSHL	AVHQRVHTGE	KPYKCNECGK	AFNWGSLLTV	HQRIHTGEKP	YKCNVCGKVF	IDSGNLSIHR
**570**	**580**	**590**	**600**	**610**	**620**	**630**	**640**
RSHTGEKPFQ	CNECGKVFSY	YSCLARHRKI	HTGEKPYKCN	DCGKAYTQRS	SLTKHLVIHT	GENPYHCNEF	GEAFIQSSKL
**650**	**660**	**670**	**680**	**690**	**700**	**710**	**720**
ARYHRNPTGE	KPHKCSECGR	TFSHKTSLVY	HQRRHTGEMP	YKCIECGKVF	NSTTTLARHR	RIHTGEKPYK	CNECGKVFRY
**730**	**740**	**750**	**760**	**770**	**780**	**790**	**800**
RSGLARHWSI	HTGEKPYKCN	ECGKAFRVRS	ILLNHQMMHT	GEKPYKCNEC	GKAFIERSNL	VYHQRNHTGE	KPYKCMECGK
**810**	**820**	**830**	**840**	**850**	**860**	**870**	
AFGRRSCLTK	HQRIHSSEKP	YKCNECGKSY	ISRSGLTKHQ	IKHAGENLTT	KLNVERPLDV	VLTSGIPK	

Cmpd.	No. of Cmpds.	m/z meas.	Δ m/z [ppm]	z	Rt [min]	Score	P	Range	Sequence	Modification
1732	1	1061.1490	-2.43	3	51.5	11.3	2	561-586	R.RSHTGEKPFQCNECGKVFSYYSCLAR.H	Carbamidomethyl: 11, 14, 23
1286	1	738.4255	114.34	3	45.7	15.0	2	643-660	R.YHRNPTGEKPHKCSECGR.T	Carbamidomethyl: 13, 16

Detailed Protein Report

Protein 591: integrator complex subunit 1 [Homo sapiens]

**Accession:**	gi|160948599	**Score:**	26.2
**Database:**	refseq_human(refseq_human_20140103.fasta)	**MW [kDa]:**	244.1
**Database Date:**	2015-11-30	**pI:**	5.7
**Modification(s):**	Carbamidomethyl	**Sequence Coverage [%]:**	1.7
		**No. of unique Peptides:**	2

BRUKER

Detailed Protein Report

10	20	30	40	50	60	70	80
MNRAKPTTVR	RPSAAAKPSG	HPPPGDFIAL	GSKGQANESK	TASTLLKPAP	SGLPSERKRD	AAAALSSASA	LTGLTKRPKL
90	100	110	120	130	140	150	160
SSTPPLSALG	RLAEAAVAEK	RAISPSIKEP	SVVPIEVLPT	VLLDEIEAAE	LEGNDDRIEG	VLCGAVKQLK	VTRAKPDSTL
170	180	190	200	210	220	230	240
YLSLMYLAKI	KPNIFATEGV	IEALCSLLRR	DASINFKAKG	NSLVSVLACN	LLMAAYEEDE	NWPEIFVKVY	IEDSLGERIW
250	260	270	280	290	300	310	320
VDSPHCKTFV	DNIQTAFNTR	MPPRSVLLQG	EAGRVAGDLG	AGSSPHPSLT	EEEDSQTELL	IAEEKLSPEQ	EGQLMPRYEE
330	340	350	360	370	380	390	400
LAESVEEYVL	DMLRDQLNRR	QPIDNVSRNL	LRLLTSTCGY	KEVRLLAVQK	LEMWLQNPKL	TRPAQDLLMS	VCMNCNTHGS
410	420	430	440	450	460	470	480
EDMDVISHLI	KIRLKPKVLL	NHFMLCIREL	LSAHKDNLGT	TIKLVIFNEL	SSARNPNNMQ	VLYTALQHSS	ELAPKFLAMV
490	500	510	520	530	540	550	560
FQDLLTNKDD	YLRASRALLR	EIIKQTKHEI	NFQAFCLGLM	QERKEPQYLE	MEFKERFVVH	ITDVLAVSMM	LGITAQVKEA
570	580	590	600	610	620	630	640
GIAWDKGEKR	NLEVLRSFQN	QIAAIQRDAV	WWLHTVVPSI	SKLAPKDYVH	CLHKVLFTEQ	PETYYKWDNW	PPESDRNFFL
650	660	670	680	690	700	710	720
RLCSEVPILE	DTLMRILVIG	LSRELPLGPA	DAMELADHLV	KRAAAVQADD	VEVLKVGRTQ	LIDAVLNLCT	YHHPENIQLP
730	740	750	760	770	780	790	800
PGYQPPNLAI	STLYWKAWPL	LLVVAAFNPE	NIGLAAWEEY	PTLKMLMEMV	MTNNYSYPPC	TLTDEETRTE	MLNRELQTAQ
810	820	830	840	850	860	870	880
REKQEILAFE	GHLAAASTKQ	TITESSSLLL	SQLTSLDPQG	PPRRPPPHIL	DQVKSLNQSL	RLGHLLCRSR	NPDFLLHIIQ
890	900	910	920	930	940	950	960
RQASSQSMPW	LADLVQSSEG	SLDVLPVQCL	CEFLLHDAVD	DAASGEEDDE	GESKEQKAKK	RQRQQKQRQL	LGRLQDLLLG
970	980	990	1000	1010	1020	1030	1040
PKADEQTTCE	VLDYFLRRLG	SSQVASRVLA	MKGLSLVLSE	GSLRDGEEKE	PPMEEDVGDT	DVLQGYQWLL	RDLPRLPLFD
1050	1060	1070	1080	1090	1100	1110	1120
SVRSTTALAL	QQAIHMETDP	QTISAYLIYL	SQHTPVEEQA	QHSDLALDVA	RLVVERSTIM	SHLFSKLSPS	AASDAVLSAL
1130	1140	1150	1160	1170	1180	1190	1200
LSIFSRYVRR	MRQSKEGEEV	YSWSESQDQV	FLRWSSGETA	TMHILVVHAM	VILLTLGPPR	ADDSEFQALL	DIWFPEEKPL
1210	1220	1230	1240	1250	1260	1270	1280
PTAFLVDTSE	EALLLPDWLK	LRMIRSEVLR	LVDAALQDLE	PQQLLLFVQS	FGIPVSSMSK	LLQFLDQAVA	HDPQTLEQNI
1290	1300	1310	1320	1330	1340	1350	1360
MDKNYMAHLV	EVQHERGASG	GQTFHSLLTA	SLPPRRDSTE	APKPKSSPEQ	PIGQGRIRVG	TQLRVLGPED	DLAGMFLQIF
1370	1380	1390	1400	1410	1420	1430	1440
PLSPDPRWQS	SSPRPVALAL	QQALGQELAR	VVQGSPEVPG	ITVRVLQALA	TLLSSPHGGA	LVMSMHRSHF	LACPLLRQLC
1450	1460	1470	1480	1490	1500	1510	1520
QYQRCVPQDT	GFSSLFLKVL	LQMLQWLDSP	GVEGGPLRAQ	LRMLASQASA	GRRLSDVRGG	LLRLAEALAF	RQDLEVVSST
1530	1540	1550	1560	1570	1580	1590	1600
VRAVIATLRS	GEQCSVEPDL	ISKVLQGLIE	VRSPHLEELL	TAFFSATADA	ASPFPACKPV	VVVSSLLLQE	EEPLAGGKPG
1610	1620	1630	1640	1650	1660	1670	1680
ADGGSLEAVR	LGPSSGLLVD	WLEMLDPEVV	SSCPDLQLRL	LFSRRKGKGQ	AQVPSFRPYL	LTLFTHQSSW	PTLHQCIRVL
1690	1700	1710	1720	1730	1740	1750	1760
LGKSREQRFD	PSASLDFLWA	CIHVPRIWQG	RDQRTPQKRR	EELVLRVQGP	ELISLVELIL	AEAETRSQDG	DTAACSLIQA
1770	1780	1790	1800	1810	1820	1830	1840
RLPLLLSCCC	GDDESVRKVT	EHLSGCIQQW	GDSVLGRRCR	DLLLQLYLQR	PELRVPVPEV	LLHSEGAASS	SVCKLDGLIH
1850	1860	1870	1880	1890	1900	1910	1920
RFITLLADTS	DSRALENRGA	DASMACRKLA	VAHPLLLLRH	LPMIAALLHG	RTHLNFQEFR	QQNHLSCFLH	VLGLLELLQP
1930	1940	1950	1960	1970	1980	1990	2000
HVFRSEHQGA	LWDCLLSFIR	LLLNYRKSSR	HLAAFINKFV	QFIHKYITYN	APAAISFLQK	HADPLHDLSF	DNSDLVMLKS
2010	2020	2030	2040	2050	2060	2070	2080
LLAGLSLPSR	DDRTDRGLDE	EGEEESSAGS	LPLVSVSLFT	PLTAAEMAPY	MKRLSRGQTV	EDLLEVLSDI	DEMSRRRPEI
2090	2100	2110	2120	2130	2140	2150	2160
LSFFSTNLQR	LMSSAEECCR	NLAFSLALRS	MQNSPSIAAA	FLPTFMYCLG	SQDFEVVQTA	LRNLPEYALL	CQEHAAVLLH
2170	2180	2190	2200				

Detailed Protein Report

Cmpd.	No. of Cmpds.	m/z meas.	Δ m/z [ppm]	z	Rt [min]	Score	P	Range	Sequence	Modification
1586	1	948.9589	-43.25	2	50.1	10.1	0	1297-1315	R.GASGGQTFHSLLTASLPPR.R	
464	1	724.3818	41.56	3	35.8	16.2	1	2091-2109	R.LMSSAEECCRNLAFSLALR.S	Carbamidomethyl: 8

Detailed Protein Report

Protein 592: uncharacterized membrane protein C3orf80 precursor [Homo sapiens]

Accession: gi|270265902
Database: refseq_human(refseq_human_20140103.fasta)
Database Date: 2015-11-30
Modification(s): Carbamidomethyl

Score: 26.2
MW [kDa]: 25.7
pI: 5.4
Sequence Coverage [%]: 13.4
No. of unique Peptides: 2

Quantitation

QU:MU	**Median:**	1.05	**CV:**	0.00 %	**No. of Peptides:**	1
WUP:QUP	**Median:**	0.30	**CV:**	0.00 %	**No. of Peptides:**	1

```
        10         20         30         40         50         60         70         80
MWGPGVTAEG LSVAPAPPPL LPLLLLLALA LVAPSRGGGG CAELACGERE RCCDATNATA VRCCKLPLHA FLDNVGWFVR
        90        100        110        120        130        140        150        160
KLSGLLILLV LFAIGYFLQR IICPSPRRYP RGQARPGQRP GPPGGAGPLG GAGPPDDDDD SPALLRDEAA AGSQDSLLDS
       170        180        190        200        210        220        230        240
GGGGRGRGGG GRSDPSCASE HEMRVVSPVF LQLPSYEEVK YLPTYEESMR LQQLSPGEVV LPVSVLGRPR GGVAAEPDGG
       250
EGRYPLI
```

Cmpd.	No. of Cmpds.	m/z meas.	Δ m/z [ppm]	z	Rt [min]	Score	P	Range	Sequence	Modification	Ratios
536	2	786.8751	66.36	2	36.7	12.7	1	52-65	R.CCDATNATAVRCCK.L	Carbamidomethyl: 1, 2	WUP:QUP 0.30<br>QU:MU 1.05
2758	1	973.5021	83.13	2	64.9	13.5	2	166-184	R.GRGGGGRSDPSCASEHEMR.V		

Detailed Protein Report

Protein 593: **PREDICTED: sarcolemmal membrane-associated protein isoform X16 [Homo sapiens]**

**Accession:**	gi|530373158	**Score:**	26.2
**Database:**	refseq_human(refseq_human_20140103.fasta)	**MW [kDa]:**	90.8
**Database Date:**	2015-11-30	**pI:**	5.1
		**Sequence Coverage [%]:**	3.3
		**No. of unique Peptides:**	1

Quantitation

QU:MU **Median:** 3.85 **CV:** 0.00 % **No. of Peptides:** 1

10	20	30	40	50	60	70	80
MPSALAIFTC	RPNSHPFQER	HVYLDEPIKI	GRSVARCRPA	QNNATFDCKV	LSRNHALVWF	DHKTGKFYLQ	DTKSSNGTFI
90	100	110	120	130	140	150	160
NSQRLSRGSE	ESPPCEILSG	DIIQFGVDVT	ENTRKVTHGC	IVSTIKLFLP	DGMEARLRSD	VIHAPLPSPV	DKVAANTPSM
170	180	190	200	210	220	230	240
YSQELFQLSQ	YLQEALHREQ	MLEQKLATLQ	RLLAITQEAS	DTSWQALIDE	DRLLSRLEVM	GNQLQACSKN	QTEDSLRKEL
250	260	270	280	290	300	310	320
IALQEDKHNY	ETTAKESLRR	VLQEKIEVVR	KLSEVERSLS	NTEDECTHLK	EMNERTQEEL	RELANKYNGA	VNEIKDLSDK
330	340	350	360	370	380	390	400
LKVAEGKQEE	IQQKGQAEKK	ELQHKIDEME	EKEQELQAKI	EALQADNDFT	NERLTALQEK	LIVEGHLTKA	VEETKLSKEN
410	420	430	440	450	460	470	480
QTRAKESDFS	DTLSPSKEKS	SDDTTDAQMD	EQDLNEPLAK	VSLLKDDLQG	AQSEIEAKQE	IQHLRKELIE	AQELARTSKQ
490	500	510	520	530	540	550	560
KCFELQALLE	EERKAYRNQV	EESTKQIQVL	QAQLQRLHID	TENLREEKDS	EITSTRDELL	SARDEILLLH	QAAAKVASER
570	580	590	600	610	620	630	640
DTDIASLQEE	LKKVRAELER	WRKAASEYEK	EITSLQNSFQ	LRCQQCEDQQ	REEATRLQGE	LEKLRKEWNA	LETECHSLKR
650	660	670	680	690	700	710	720
ENVLLSSELQ	RQEKELHNSQ	KQSLELTSDL	SILQMSRKEL	ENQVGSLKEQ	HLRDSADLKT	LLSKAENQAK	DVQKEYEKTQ
730	740	750	760	770	780	790	800
TVLSELKLKF	EMTEQEKQSI	TDELKQCKNN	LKLLREKGNN	KPWPWMPMLA	ALVAVTAIVL	YVPGLARASP	

Cmpd.	No. of Cmpds.	m/z meas.	Δ m/z [ppm]	z	Rt [min]	Score	P	Range	Sequence	Modification	Ratios
1042	1	586.1730	-249.89	2	41.3	10.5	0	467-476	K.ELIEAQELAR.T		QU:MU 3.85

Detailed Protein Report

Protein 594: **PREDICTED: suppressor of tumorigenicity 7 protein-like isoform X10 [Homo sapiens]**

Accession: gi|530362849
Database: refseq_human(refseq_human_20140103.fasta)
Database Date: 2015-11-30
Modification(s): Oxidation

Score: 26.1
MW [kDa]: 35.9
pI: 9.9
Sequence Coverage [%]: 5.6
No. of unique Peptides: 2

10	20	30	40	50	60	70	80
MADRGGVGEA	AAVGASPASV	PGLNPTLGWR	ERLRAGLAGT	GASLWFVAGL	GLLYALRIPL	RLCENLAAVT	VFLNSLTPKF
**90**	**100**	**110**	**120**	**130**	**140**	**150**	**160**
YVALTGTSSL	ISGLIFIFEW	WYFHKHGTSF	IEQVSVSHLQ	PLMGGTESSI	SEPGSPSRNR	ENETSRQNLS	ECKVWRNPLN
**170**	**180**	**190**	**200**	**210**	**220**	**230**	**240**
LFRGAEYRRY	TWVTGKEPLT	YYDMNLSAQD	HQTFFTCDTD	FLRPSDTVMQ	KAWRERNPPA	RIKAAYQALE	LNNDCATAYV
**250**	**260**	**270**	**280**	**290**	**300**	**310**	**320**
LLAEEEATTI	VDAERLFKQA	LKAGETIYRQ	SQQCQHQSPQ	HEAQLRRDTN	VLVYIKRRLA	MCARKLGRIR	EAVKIMRDI

Cmpd.	No. of Cmpds.	m/z meas.	Δ m/z [ppm]	z	Rt [min]	Score	P	Range	Sequence	Modification
2033	4	625.2626	-137.48	2	55.3	14.2	1	259-269	K.QALKAGETIYR.Q	
1102	2	836.1988	-267.93	1	43.4	12.0	1	298-304	R.RLAMCAR.K	Oxidation: 4

Detailed Protein Report

Protein 595: PREDICTED: sodium/myo-inositol cotransporter 2 isoform X3 [Homo sapiens]

**Accession:**	gi|530407547	**Score:**	26.1
**Database:**	refseq_human(refseq_human_20140103.fasta)	**MW [kDa]:**	56.6
**Database Date:**	2015-11-30	**pI:**	6.3
**Modification(s):**	Carbamidomethyl	**Sequence Coverage [%]:**	6.6
		**No. of unique Peptides:**	2

Quantitation

QU:MU	**Median:**	0.66	**CV:**	0.00 %	**No. of Peptides:**	1
WUP:QUP	**Median:**	1.92	**CV:**	0.00 %	**No. of Peptides:**	1

10	20	30	40	50	60	70	80
MYAGAIFIQQ	SLHLDLYLAI	VGLLAITAVY	TVAGGLAAVI	YTDALQTLIM	LIGALTLMGY	SFAAVGGMEG	LKEKYFLALA
**90**	**100**	**110**	**120**	**130**	**140**	**150**	**160**
SNRSENSSCG	LPREDAFHIF	RDPLTSDLPW	PGVLFGMSIP	SLWYWCTDQV	IVQRTLAAKN	LSHAKGGALM	AAYLKVLPLF
**170**	**180**	**190**	**200**	**210**	**220**	**230**	**240**
IMVFPGMVSR	ILFPDQVACA	DPEICQKICS	NPSGCSDIAY	PKLVLELLPT	GLRGLMMAVM	VAALMSSLTS	IFNSASTIFT
**250**	**260**	**270**	**280**	**290**	**300**	**310**	**320**
MDLWNHLRPR	ASEKELMIVG	RVFVLLLVLV	SILWIPVVQA	SQGGQLFIYI	QSISSYLQPP	VAVVFIMGCF	WKRTNEKGAF
**330**	**340**	**350**	**360**	**370**	**380**	**390**	**400**
WGLISGLLLG	LVRLVLDFIY	VQPRCDQPDE	RPVLVKSIHY	LYFSMILSTV	TLITVSTVSW	FTEPPSKEMV	SHLTWFTRHD
**410**	**420**	**430**	**440**	**450**	**460**	**470**	**480**
PVVQKEQAPP	AAPLSLTLSQ	NGMPEASSSS	SVQFEMVQEN	TSKTHSCDMT	PKQSKVVKAI	LWLCGIQEKG	KEELPARAEA
**490**	**500**	**510**	**520**				
IIVSLEENPL	VKTLLDVNLI	FCVSCAIFIW	GYFA				

Cmpd.	No. of Cmpds.	m/z meas.	Δ m/z [ppm]	z	Rt [min]	Score	P	Range	Sequence	Modification	Ratios
2244	1	714.6704	-20.12	3	58.5	12.0	1	75-93	K.YFLALASNRSENSSCGLPR.E	Carbamidomethyl: 15	
816	1	812.9497	-14.41	2	40.2	14.1	0	478-492	R.AEAIIVSLEENPLVK.T		QU:MU 0.66<br>WUP:QUP 1.92

Detailed Protein Report

Protein 596: glutamate decarboxylase 2 [Homo sapiens]

Accession: gi|4503875
Database: refseq_human(refseq_human_20140103.fasta)
Database Date: 2015-11-30
Modification(s): Oxidation

Score: 26.1
MW [kDa]: 65.4
pI: 6.5
Sequence Coverage [%]: 4.1
No. of unique Peptides: 1

Alias proteins:

Accession	Name	Description
gi|197276620	refseq_human (refseq_human_20140103.fasta)	glutamate decarboxylase 2 [Homo sapiens]

10	20	30	40	50	60	70	80
MASPGSGFWS	FGSEDGSGDS	ENPGTARAWC	QVAQKFTGGI	GNKLCALLYG	DAEKPAESGG	SQPPRAAARK	AACACDQKPC
**90**	**100**	**110**	**120**	**130**	**140**	**150**	**160**
SCSKVDVNYA	FLHATDLLPA	CDGERPTLAF	LQDVMNILLQ	YVVKSFDRST	KVIDFHYPNE	LLQEYNWELA	DQPQNLEEIL
**170**	**180**	**190**	**200**	**210**	**220**	**230**	**240**
MHCQTTLKYA	IKTGHPRYFN	QLSTGLDMVG	LAADWLTSTA	NTNMFTYEIA	PVFVLLEYVT	LKKMREIIGW	PGGSGDGIFS
**250**	**260**	**270**	**280**	**290**	**300**	**310**	**320**
PGGAISNMYA	MMIARFKMFP	EVKEKGMAAL	PRLIAFTSEH	SHFSLKKGAA	ALGIGTDSVI	LIKCDERGKM	IPSDLERRIL
**330**	**340**	**350**	**360**	**370**	**380**	**390**	**400**
EAKQKGFVPF	LVSATAGTTV	YGAFDPLLAV	ADICKKYKIW	MHVDAAWGGG	LLMSRKHKWK	LSGVERANSV	TWNPHKMMGV
**410**	**420**	**430**	**440**	**450**	**460**	**470**	**480**
PLQCSALLVR	EEGLMQNCNQ	MHASYLFQQD	KHYDLSYDTG	DKALQCGRHV	DVFKLWLMWR	AKGTTGFEAH	VDKCLELAEY
**490**	**500**	**510**	**520**	**530**	**540**	**550**	**560**
LYNIIKNREG	YEMVFDGKPQ	HTNVCFWYIP	PSLRTLEDNE	ERMSRLSKVA	PVIKARMMEY	GTTMVSYQPL	GDKVNFFRMV
**570**	**580**	**590**					
ISNPAATHQD	IDFLIEEIER	LGQDL					

Cmpd.	No. of Cmpds.	m/z meas.	Δ m/z [ppm]	z	Rt [min]	Score	P	Range	Sequence	Modification
2204	1	767.2614	-182.11	2	57.4	12.9	0	397-410	K.MMGVPLQCSALLVR.E	Oxidation: 2

Detailed Protein Report

Protein 597: PREDICTED: zinc finger protein 40 isoform X2 [Homo sapiens]

**Accession:**	gi|578811634	**Score:**	26.0
**Database:**	refseq_human(refseq_human_20140103.fasta)	**MW [kDa]:**	222.1
**Database Date:**	2015-11-30	**pI:**	9.5
**Modification(s):**	Oxidation	**Sequence Coverage [%]:**	1.3
		**No. of unique Peptides:**	2

Quantitation

QU:MU	**Median:**	0.91	**CV:**	0.00 %	**No. of Peptides:**	1
WUP:QUP	**Median:**	2.10	**CV:**	0.00 %	**No. of Peptides:**	1

Detailed Protein Report

10	20	30	40	50	60	70	80
MPRTKQIHPR	NLRDKIEEAQ	KELNGAEVSK	KEILQAGVKG	TSESLKGVKR	KKIVAENHLK	KIPKSPLRNP	LQAKHKQNTE
90	100	110	120	130	140	150	160
ESSFAVLHSA	SESHKKQNYI	PVKNGKQFTK	QNGETPGIIA	EASKSEESVS	PKKPLFLQQP	SELRRWRSEG	ADPAKFSDLD
170	180	190	200	210	220	230	240
EQCDSSSLSS	KTRTDNSECI	SSHCGTTSPS	YTNTAFDVLL	KAMEPELSTL	SQKGSPCAIK	TEKLRPNKTA	RSPPKLKNSS
250	260	270	280	290	300	310	320
MDAPNQTSQE	LVAESQSSCT	SYTVHMSAAQ	KNEQGAMQSA	SHLYHQHEHF	VPKSNQHNQQ	LPGCSGFTGS	LTNLQNQENA
330	340	350	360	370	380	390	400
KLEQVYNIAV	TSSVGLTSPS	SRSQVTPQNQ	QMDSASPLSI	SPANSTQSPP	MPIYNSTHVA	SVVNQSVEQM	CNLLLKDQKP
410	420	430	440	450	460	470	480
KKQGKYICEY	CNRACAKPSV	LLKHIRSHTG	ERPYPCVTCG	FSFKTKSNLY	KHKKSHAHTI	KLGLVLQPDA	GGLFLSHESP
490	500	510	520	530	540	550	560
KALSIHSDVE	DSGESEEEGA	TDERQHDLGA	MELQPVHIIK	RMSNAETLLK	SSFTPSSPEN	VIGDFLLQDR	SAESQAVTEL
570	580	590	600	610	620	630	640
PKVVVHHVTV	SPLRTDSPKA	MDPKPELSSA	QKQKDLQVTN	VQPLSANMSQ	GGVSRLETNE	NSHQKGDMNP	LEGKQDSHVG
650	660	670	680	690	700	710	720
TVHAQLQRQQ	ATDYSQEQQG	KLLSPRSLGS	TDSGYFSRSE	SADQTVSPPT	PFARRLPSTE	QDSGRSNGPS	AALVTTSTPS
730	740	750	760	770	780	790	800
ALPTGEKALL	LPGQMRPPLA	TKTLEERISK	LISDNEALVD	DKQLDSVKPR	RTSLSRRGSI	DSPKSYIFKD	SFQFDLKPVG
810	820	830	840	850	860	870	880
RRTSSSSDIP	KSPFTPTEKS	KQVFLLSVPS	LDCLPITRSN	SMPTTGYSAV	PANIIPPPHP	LRGSQSFDDK	IGAFYDDVFV
890	900	910	920	930	940	950	960
SGPNAPVPQS	GHPRTLVRQA	AIEDSSANES	HVLGTGQSLD	ESHQGCHAAG	EAMSVRSKAL	AQGPHIEKKK	SHQGRGTMFE
970	980	990	1000	1010	1020	1030	1040
CETCRNRYRK	LENFENHKKF	YCSELHGPKT	KVAMREPEHS	PVPGGLPQPI	LHYRVAGSSG	IWEQTPQIRK	RRKMKSVGDD
1050	1060	1070	1080	1090	1100	1110	1120
EELQQNESGT	SPKSSEGLQF	QNALGCNPSL	PKHNVTIRSD	QQHKNIQLQN	SHIHLVARGP	EQTMDPKLST	IMEQQISSAA
1130	1140	1150	1160	1170	1180	1190	1200
QDKIELQRHG	TGISVIQHTN	SLSRPNSFDK	PEPFERASPV	SFQELNRTGK	SGSLKVIGIS	QEESHPSRDG	SHPHQLALSD
1210	1220	1230	1240	1250	1260	1270	1280
ALRGELQESS	RKSPSERHVL	GQPSRLVRQH	NIQVPEILVT	EEPDRDLEAQ	CHDQEKSEKF	SWPQRSETLS	KLPTEKLPPK
1290	1300	1310	1320	1330	1340	1350	1360
KKRLRLAEIE	HSSTESSFDS	TLSRSLSRES	SLSHTSSFSA	SLDIEDVSKT	EASPKIDFLN	KAEFLMIPAG	LNTLNVPGCH
1370	1380	1390	1400	1410	1420	1430	1440
REMRRTASEQ	INCTQTSMEV	SDLRSKSFDC	GSITPPQTTP	LTELQPPSSP	SRVGVTGHVP	LLERRRGPLV	RQISLNIAPD
1450	1460	1470	1480	1490	1500	1510	1520
SHLSPVHPTS	FQNTALPSVN	AVPYQGPQLT	STSLAEFSAN	TLHSQTQVKD	LQAETSNSSS	TNVFPVQQLC	DINLLNQIHA
1530	1540	1550	1560	1570	1580	1590	1600
PPSHQSTQLS	LQVSTQGSKP	DKNSVLSGSS	KSEDCFAPKY	QLHCQVFTSG	PSCSSNPVHS	LPNQVISDPV	GTDHCVTSAT
1610	1620	1630	1640	1650	1660	1670	1680
LPTKLIDSMS	NSHPLLPPEL	RPLGSQVQKV	PSSFMLPIRL	QSSVPAYCFA	TLTSLPQILV	TQDLPNQPIC	QTNHSVVPIS
1690	1700	1710	1720	1730	1740	1750	1760
EEQNSVPTLQ	KGHQNALPNP	EKEFLCENVF	SEMSQNSSLS	ESLPITQKIS	VGRLSPQQES	SASSKRMLSP	ANSLDIAMEK
1770	1780	1790	1800	1810	1820	1830	1840
HQKRAKDENG	AVCATDVRPL	EALSSRVNEA	SKQKKPILVR	QVCTTEPLDG	VMLEKDVFSQ	PEISNEAVNL	TNVLPADNSS
1850	1860	1870	1880	1890	1900	1910	1920
TGCSKFVVIE	PISELQEFEN	IKSSTSLTLT	VRSSPAPSEN	THISPLKCTD	NNQERKSPGV	KNQGDKVNIQ	EQSQQPVTSL
1930	1940	1950	1960	1970	1980	1990	2000
SLFNIKDTQQ	LAFPSLKTTT	NFTWCYLLRQ	KSLHLPQKDQ	KTSAYTDWTV	SASNPNPLGL	PTKVALALLN	SKQNTGKSLY
2010	2020	2030					
CQAITTHSKS	DLLVYSSKWK	SSLSKI					

Cmpd.	No. of Cmpds.	m/z meas.	Δ m/z [ppm]	z	Rt [min]	Score	P	Range	Sequence	Modification	Ratios

Detailed Protein Report

Cmpd.	No. of Cmpds.	m/z meas.	Δ m/z [ppm]	z	Rt [min]	Score	P	Range	Sequence	Modification	Ratios	
1515	1	719.8406	-37.88	2	49.1	13.1	0	1176-1188	K.VIGISQEESHPSR.D		WUP:QUP<br>QU:MU	2.10<br>0.91
52	1	776.2895	-107.95	2	30.3	12.9	0	1747-1760	R.MLSPANSLDIAMEK.H	Oxidation: 1, 12		

Detailed Protein Report

Protein 598: lengsin isoform a [Homo sapiens]

Accession: gi|221307485
Database: refseq_human(refseq_human_20140103.fasta)
Database Date: 2015-11-30
Modification(s): Oxidation

Score: 26.0
MW [kDa]: 57.2
pI: 5.9
Sequence Coverage [%]: 3.5
No. of unique Peptides: 1

Quantitation

QU:MU	**Median:**	4.45	**CV:**	0.00 %	**No. of Peptides:**	1
WUP:QUP	**Median:**	0.60	**CV:**	0.00 %	**No. of Peptides:**	1

```
        10         20         30         40         50         60         70         80
MNNEEDLLQE DSTRDEGNET EANSMNTLRR TRKKVTKPYV CSTEVGETDM SNSNDCMRDS SQILTPPQLS SRMKHIRQAM
        90        100        110        120        130        140        150        160
AKNRLQFVRF EATDLHGVSR SKTIPAHFFQ EKVSHGVCMP RGYLEVIPNP KDNEMNNIRA TCFNSDIVLM PELSTFRVLP
       170        180        190        200        210        220        230        240
WADRTARVIC DTFTVTGEPL LTSPRYIAKR QLSHLQASGF SLLSAFIYDF CIFGVPEILN SKIISFPALT FLNNHDQPFM
       250        260        270        280        290        300        310        320
QELVDGLYHT GANVESFSSS TRPGQMEISF LPEFGISSAD NAFTLRTGVK EVARKYNYIA SFFIETGFCD SGILSHSLWD
       330        340        350        360        370        380        390        400
VDRKKNMFCS TSGTEQLTIT GKKWLAGLLK HSAALSCLMA PSVSCRKRYS KDRKDLKKSV PTTWGYNDNS CIFNIKCHGE
       410        420        430        440        450        460        470        480
KGTRIENKLG SATANPYLVL AATVAAGLDG LHSSNEVLAG PDESTDFYQV EPSEIPLKLE DALVALEEDQ CLRQALGETF
       490        500        510
IRYFVAMKKY ELENEEIAAE RNKFLEYFI
```

Cmpd.	No. of Cmpds.	m/z meas.	Δ m/z [ppm]	z	Rt [min]	Score	P	Range	Sequence	Modification	Ratios
109	1	981.4823	13.57	2	30.3	10.6	1	326-343	K.NMFCSTSGTEQLTITGKK.W	Oxidation: 2	QU:MU 4.45<br>WUP:QUP 0.60

Detailed Protein Report

Protein 599: PREDICTED: mediator of RNA polymerase II transcription subunit 1 isoform X1 [Homo sapiens]

Accession: gi|530412429
Database: refseq_human(refseq_human_20140103.fasta)
Database Date: 2015-11-30
Modification(s): Carbamidomethyl

Score: 26.0
MW [kDa]: 149.1
pI: 9.6
Sequence Coverage [%]: 2.8
No. of unique Peptides: 2

Quantitation

QU:MU	**Median:**	1.02	**CV:**	0.00 %	**No. of Peptides:**	1
WUP:QUP	**Median:**	1.04	**CV:**	0.00 %	**No. of Peptides:**	1

10	20	30	40	50	60	70	80
MYLALQSLEQ	DLSKMAIMYW	KATNAGPLDK	ILHGSVGYLT	PRSGGHLMNL	KYYVSPSDLL	DDKTASPIIL	HENNVSRSLG
90	100	110	120	130	140	150	160
MNASVTIEGT	SAVYKLPIAP	LIMGSHPVDN	KWTPSFSSIT	SANSVDLPAC	FFLKFPQPIP	VSRAFVQKLQ	NCTGIPLFET
170	180	190	200	210	220	230	240
QPTYAPLYEL	ITQFELSKDP	DPIPLNHNMR	FYAALPGQQH	CYFLNKDAPL	PDGRSLQGTL	VSKITFQHPG	RVPLILNLIR
250	260	270	280	290	300	310	320
HQVAYNTLIG	SCVKRTILKE	DSPGLLQFEV	CPLSESRFSV	SFQHPVNDSL	VCVVMDVQDS	THVSCKLYKG	LSDALICTDD
330	340	350	360	370	380	390	400
FIAKVVQRCM	SIPVTMRAIR	RKAETIQADT	PALSLIAETV	EDMVKKNLPP	ASSPGYGMTT	GNNPMSGTTT	PTNTFPGGPI
410	420	430	440	450	460	470	480
TTLFNMSMSI	KDRHESVGHG	EDFSKVSQNP	ILTSLLQITG	NGGSTIGSSP	TPPHHTPPPV	SSMAGNTKNH	PMLMNLLKDN
490	500	510	520	530	540	550	560
PAQDFSTLYG	SSPLERQNSS	SGSPRMEICS	GSNKTKKKKS	SRLPPEKPKH	QTEDDFQREL	FSMDVDSQNP	IFDVNMTADT
570	580	590	600	610	620	630	640
LDTPHITPAP	SQCSTPPTTY	PQPVPHPQPS	IQRMVRLSSS	DSIGPDVTDI	LSDIAEEASK	LPSTSDDCPA	IGTPLRDSSS
650	660	670	680	690	700	710	720
SGHSQSTLFD	SDVFQTNNNE	NPYTDPADLI	ADAAGSPSSD	SPTNHFFHDG	VDFNPDLLNS	QSQSGFGEEY	FDESSQSGDN
730	740	750	760	770	780	790	800
DDFKGFASQA	LNTLGVPMLG	GDNGETKFKG	NNQADTVDFS	IISVAGKALA	PADLMEHHSG	SQGPLLTTGD	LGKEKTQKRV
810	820	830	840	850	860	870	880
KEGNGTSNST	LSGPGLDSKP	GKRSRTPSND	GKSKDKPPKR	KKADTEGKSP	SHSSSNRPFT	PPTSTGGSKS	PGSAGRSQTP
890	900	910	920	930	940	950	960
PGVATPPIPK	ITIQIPKGTV	MVGKPSSHSQ	YTSSGSVSSS	GSKSHHSHSS	SSSSSASTSG	KMKSSKSEGS	SSSKLSSSMY
970	980	990	1000	1010	1020	1030	1040
SSQGSSGSSQ	SKNSSQSGGK	PGSSPITKHG	LSSGSSSTKM	KPQGKPSSLM	NPSLSKPNIS	PSHSRPPGGS	DKLASPMKPV
1050	1060	1070	1080	1090	1100	1110	1120
PGTPPSSKAK	SPISSGSGGS	HMSGTSSSSG	MKSSSGLGSS	GSLSQKTPPS	SNSCTASSSS	FSSSGSSMSS	SQNQHGSSKG
1130	1140	1150	1160	1170	1180	1190	1200
KSPSRNKKPS	LTAVIDKLKH	GVVTSGPGGE	DPLDGQMGVS	TNSSSHPMSS	KHNMSGGEFQ	GKREKSDKDK	SKVSTSGSSV
1210	1220	1230	1240	1250	1260	1270	1280
DSSKKTSESK	NVGSTGVAKI	IISKHDGGSP	SIKAKVTLQK	PGESSGEGLR	PQMASSKNYG	SPLISGSTPK	HERGSPSHSK
1290	1300	1310	1320	1330	1340	1350	1360
SPAYTPQNLD	SESESGSSIA	EKSYQNSPSS	DDGIRPLPEY	STEKHKKHKK	EKKKVKDKDR	DRDRDKDRDK	KKSHSIKPES
1370	1380	1390	1400	1410			
WSKSPISSDQ	SLSMTSNTIL	SADRPSRLSP	DFMIGEEDDD	LMDVALIGN			

Cmpd.	No. of Cmpds.	m/z meas.	Δ m/z [ppm]	z	Rt [min]	Score	P	Range	Sequence	Modification	Ratios
2702	1	842.5321	94.33	3	62.2	11.9	2	307-328	K.LYKGLSDALICTDDFIAKVVQR.C	Carbamidomethyl: 11	
1993	1	918.3565	-113.08	2	53.3	14.1	0	750-767	K.GNNQADTVDFSIISVAGK.A		WUP:QUP 1.04<br>QU:MU 1.02

Detailed Protein Report

Protein 600: PREDICTED: pulmonary surfactant-associated protein C isoform X1 [Homo sapiens]

**Accession:**	gi|530387966	**Score:**	26.0
**Database:**	refseq_human(refseq_human_20140103.fasta)	**MW [kDa]:**	28.6
**Database Date:**	2015-11-30	**pI:**	10.8
		**Sequence Coverage [%]:**	14.4
		**No. of unique Peptides:**	1

10	20	30	40	50	60	70	80
MAPRDAHRDP	EMPAVLGSPA	GQLPTWLRPS	THPGHTWERR	RGEHSTCSKM	DVGSKEVLME	SPPDYSAAPR	GRFGIPCCPV
**90**	**100**	**110**	**120**	**130**	**140**	**150**	**160**
HLKRLLIVVV	VVVLIVVVIV	GALLMGLHMS	QKHTEMVLEM	SIGAPEAQQR	LALSEHLVTT	ATFSIGSTGL	VVYDYQQLLI
**170**	**180**	**190**	**200**	**210**	**220**	**230**	**240**
AYKPAPGTCC	YIMKIAPESI	PSLEALTRKV	HNFQGQWKPQ	RERKRPGQRV	FCSFCRRARS	CFCPHRRDKP	WRNGSLGRGW
**250**	**260**						
EWAEVAPRGP	GTPATTE						

Cmpd.	No. of Cmpds.	m/z meas.	Δ m/z [ppm]	z	Rt [min]	Score	P	Range	Sequence	Modification
1479	1	1006.6165	136.56	2	46.9	11.4	1	239-257	R.GWEWAEVAPRGPGTPATTE.-	

Detailed Protein Report

Protein 601: PREDICTED: microtubule-associated protein 2 isoform X12 [Homo sapiens]

**Accession:**	gi|530370373	**Score:**	25.9
**Database:**	refseq_human(refseq_human_20140103.fasta)	**MW [kDa]:**	192.1
**Database Date:**	2015-11-30	**pI:**	4.8
		**Sequence Coverage [%]:**	1.7
		**No. of unique Peptides:**	1

Alias proteins:

Accession	Name	Description
gi|530370375	refseq_human (refseq_human_20140103.fasta)	PREDICTED: microtubule-associated protein 2 isoform X13 [Homo sapiens]

Detailed Protein Report

10	20	30	40	50	60	70	80
MEFHDQQELT	PSTAEPSDQK	EKESEKQSKP	GEDLKHAALV	SQPETTKTYP	DKKDMQGTEE	EKAPLALFGH	TLVASLEDMK
90	100	110	120	130	140	150	160
QKTEPSLVVP	GIDLPKEPPT	PKEQKDWFIE	MPTEAKKDEW	GLVAPISPGP	LTPMREKDVF	DDIPKWEGKQ	FDSPMPSPFQ
170	180	190	200	210	220	230	240
GGSFTLPLDV	MKNEIVTETS	PFAPAFLQPD	DKKSLQQTSG	PATAKDSFKI	EEPHEAKPDK	MAEAPPSEAM	TLPKDAHIPV
250	260	270	280	290	300	310	320
VEEHVMGKVL	EEEKEAINQE	TVQQRDTFTP	SGQEPILTEK	ETELKLEEKT	TISDKEAVPK	ESKPPKPADE	EIGIIQTSTE
330	340	350	360	370	380	390	400
HTFSEQKDQE	PTTDMLKQDS	FPVSLEQAVT	DSAMTSKTLE	KAMTEPSALI	EKSSIQELFE	MRVDDKDKIE	GVGAATSAEL
410	420	430	440	450	460	470	480
DMPFYEDKSG	MSKYFETSAL	KEEATKSIEP	GSDYYELSDT	RESVHESIDT	MSPMHKNGDK	EFQTGKESQP	SPPAQEAGYS
490	500	510	520	530	540	550	560
TLAQSYPSDL	PEEPSSPQER	MFTIDPKVYG	EKRDLHSKNK	DDLTLSRSLG	LGGRSAIEQR	SMSINLPMSC	LDSIALGFNF
570	580	590	600	610	620	630	640
GRGHDLSPLA	SDILTNTSGS	MDEGDDYLPA	TTPALEKAPC	FPVESKEEEQ	IEKVKATGEE	STQAEISCES	PFLAKDFYKN
650	660	670	680	690	700	710	720
GTVMAPDLPE	MLDLAGTRSR	LASVSADAEV	ARRKSVPSET	VVEDSRTGLP	PVTDENHVIV	KTDSQLEDLG	YCVFNKYTVP
730	740	750	760	770	780	790	800
LPSPVQDSEN	LSGESGTFYE	GTDDKVRRDL	ATDLSLIEVK	LAAAGRVKDE	FSVDKEASAH	ISGDKSGLSK	EFDQEKKAND
810	820	830	840	850	860	870	880
RLDTVLEKSE	EHADSKEHAK	KTEEAGDEIE	TFGLGVTYEQ	ALAKDLSIPT	DASSEKAEKG	LSSVPEIAEV	EPSKKVEQGL
890	900	910	920	930	940	950	960
DFAVQGQLDV	KISDFGQMAS	GLNIDDRRAT	ELKLEATQDM	TPSSKAPQEA	DAFMGVESGH	MKEGTKVSET	EVKEKVAKPD
970	980	990	1000	1010	1020	1030	1040
LVHQEAVDKE	ESYESSGEHE	SLTMESLKAD	EGKKETSPES	SLIQDEIAVK	LSVEIPCPPA	VSEADLATDE	RADVQMEFIQ
1050	1060	1070	1080	1090	1100	1110	1120
GPKEESKETP	DISITPSDVA	EPLHETIVSE	PAEIQSEEEE	IEAQGEYDKL	LFRSDTLQIT	DLGVSGAREE	FVETCPSEHK
1130	1140	1150	1160	1170	1180	1190	1200
GVIESVVTIE	DDFITVVQTT	TDEGESGSHS	VRFAALEQPE	VERRPSPHDE	EEFEVEEAAE	AQAEPKDGSP	EAPASPEREE
1210	1220	1230	1240	1250	1260	1270	1280
VALSEYKTET	YDDYKDETTI	DDSIMDADSL	WVDTQDDDRS	IMTEQLETIP	KEEKAEKEAR	RSSLEKHRKE	KPFKTGRGRI
1290	1300	1310	1320	1330	1340	1350	1360
STPERKVAKK	EPSTVSRDEV	RRKKAVYKKA	ELAKKTEVQA	HSPSRKFILK	PAIKYTRPTH	LSCVKRKTTA	AGGESALAPS
1370	1380	1390	1400	1410	1420	1430	1440
VFKQAKDKVS	NSTLSKIPAL	QGSTKSPRYS	SACPSTTKRA	TFSDSLLIQP	TSAGSTDRLP	YSKSGNKDGV	TKSPEKRSSL
1450	1460	1470	1480	1490	1500	1510	1520
PRPSSILPPR	RGVSGDRDEN	SFSLNSSISS	SARRTTRSEP	IRRAGKSGTS	TPTTPGSTAI	TPGTPPSYSS	RTPGTPGTPS
1530	1540	1550	1560	1570	1580	1590	1600
YPRTPHTPGT	PKSAILVPSE	KKVAIIRTPP	KSPATPKQLR	LINQPLPDLK	NVKSKIGSTD	NIKYQPKGGQ	VRILNKKIDF
1610	1620	1630	1640	1650	1660	1670	1680
SKVQSRCGSK	DNIKHSAGGG	NVQIVTKKID	LSHVTSKCGS	LKNIRHRPGG	GRVKIESVKL	DFKEKAQAKV	GSLDNAHHVP
1690	1700	1710	1720	1730	1740	1750	1760
GGGNVKIDSQ	KLNFREHAKA	RVDHGAEIIT	QSPGRSSVAS	PRRLSNVSSS	GSINLLESPQ	LATLAEDVTA	ALAKQGL

Cmpd.	No. of Cmpds.	m/z meas.	Δ m/z [ppm]	z	Rt [min]	Score	P	Range	Sequence	Modification
2357	1	994.6851	123.33	2	57.7	11.8	2	1524-1542	R.TPHTPGTPKSAILVPSEKK.V	

Detailed Protein Report

Protein 602: collagen alpha-1(IV) chain preproprotein [Homo sapiens]

**Accession:**	gi|148536825	**Score:**	25.9
**Database:**	refseq_human(refseq_human_20140103.fasta)	**MW [kDa]:**	160.5
**Database Date:**	2015-11-30	**pI:**	9.4
**Modification(s):**	Oxidation	**Sequence Coverage [%]:**	2.5
		**No. of unique Peptides:**	2

10	20	30	40	50	60	70	80
MGPRLSVWLL	LLPAALLLHE	EHSRAAAKGG	CAGSGCGKCD	CHGVKGQKGE	RGLPGLQGVI	GFPGMQGPEG	PQGPPGQKGD
90	100	110	120	130	140	150	160
TGEPGLPGTK	GTRGPPGASG	YPGNPGLPGI	PGQDGPPGPP	GIPGCNGTKG	ERGPLGPPGL	PGFAGNPGPP	GLPGMKGDPG
170	180	190	200	210	220	230	240
EILGHVPGML	LKGERGFPGI	PGTPGPPGLP	GLQGPVGPPG	FTGPPGPPGP	PGPPGEKGQM	GLSFQGPKGD	KGDQGVSGPP
250	260	270	280	290	300	310	320
GVPGQAQVQE	KGDFATKGEK	GQKGEPGFQG	MPGVGEKGEP	GKPGPRGKPG	KDGDKGEKGS	PGFPGEPGYP	GLIGRQGPQG
330	340	350	360	370	380	390	400
EKGEAGPPGP	PGIVIGTGPL	GEKGERGYPG	TPGPRGEPGP	KGFPGLPGQP	GPPGLPVPGQ	AGAPGFPGER	GEKGDRGFPG
410	420	430	440	450	460	470	480
TSLPGPSGRD	GLPGPPGSPG	PPGQPGYTNG	IVECQPGPPG	DQGPPGIPGQ	PGFIGEIGEK	GQKGESCLIC	DIDGYRGPPG
490	500	510	520	530	540	550	560
PQGPPGEIGF	PGQPGAKGDR	GLPGRDGVAG	VPGPQGTPGL	IGQPGAKGEP	GEFYFDLRLK	GDKGDPGFPG	QPGMTGRAGS
570	580	590	600	610	620	630	640
PGRDGHPGLP	GPKGSPGSVG	LKGERGPPGG	VGFPGSRGDT	GPPGPPGYGP	AGPIGDKGQA	GFPGGPGSPG	LPGPKGEPGK
650	660	670	680	690	700	710	720
IVPLPGPPGA	EGLPGSPGFP	GPQGDRGFPG	TPGRPGLPGE	KGAVGQPGIG	FPGPPGPKGV	DGLPGDMGPP	GTPGRPGFNG
730	740	750	760	770	780	790	800
LPGNPGVQGQ	KGEPGVGLPG	LKGLPGLPGI	PGTPGEKGSI	GVPGVPGEHG	AIGPPGLQGI	RGEPGPPGLP	GSVGSPGVPG
810	820	830	840	850	860	870	880
IGPPGARGPP	GGQGPPGLSG	PPGIKGEKGF	PGFPGLDMPG	PKGDKGAQGL	PGITGQSGLP	GLPGQQGAPG	IPGFPGSKGE
890	900	910	920	930	940	950	960
MGVMGTPGQP	GSPGPVGAPG	LPGEKGDHGF	PGSSGPRGDP	GLKGDKGDVG	LPGKPGSMDK	VDMGSMKGQK	GDQGEKGQIG
970	980	990	1000	1010	1020	1030	1040
PIGEKGSRGD	PGTPGVPGKD	GQAGQPGQPG	PKGDPGISGT	PGAPGLPGPK	GSVGGMGLPG	TPGEKGVPGI	PGPQGSPGLP
1050	1060	1070	1080	1090	1100	1110	1120
GDKGAKGEKG	QAGPPGIGIP	GLRGEKGDQG	IAGFPGSPGE	KGEKGSIGIP	GMPGSPGLKG	SPGSVGYPGS	PGLPGEKGDK
1130	1140	1150	1160	1170	1180	1190	1200
GLPGLDGIPG	VKGEAGLPGT	PGPTGPAGQK	GEPGSDGIPG	SAGEKGEPGL	PGRGFPGFPG	AKGDKGSKGE	VGFPGLAGSP
1210	1220	1230	1240	1250	1260	1270	1280
GIPGSKGEQG	FMGPPGPQGQ	PGLPGSPGHA	TEGPKGDRGP	QGQPGLPGLP	GPMGPPGLPG	IDGVKGDKGN	PGWPGAPGVP
1290	1300	1310	1320	1330	1340	1350	1360
GPKGDPGFQG	MPGIGGSPGI	TGSKGDMGPP	GVPGFQGPKG	LPGLQGIKGD	QGDQGVPGAK	GLPGPPGPPG	PYDIIKGEPG
1370	1380	1390	1400	1410	1420	1430	1440
LPGPEGPPGL	KGLQGLPGPK	GQQGVTGLVG	IPGPPGIPGF	DGAPGQKGEM	GPAGPTGPRG	FPGPPGPDGL	PGSMGPPGTP
1450	1460	1470	1480	1490	1500	1510	1520
SVDHGFLVTR	HSQTIDDPQC	PSGTKILYHG	YSLLYVQGNE	RAHGQDLGTA	GSCLRKFSTM	PFLFCNINNV	CNFASRNDYS
1530	1540	1550	1560	1570	1580	1590	1600
YWLSTPEPMP	MSMAPITGEN	IRPFISRCAV	CEAPAMVMAV	HSQTIQIPPC	PSGWSSLWIG	YSFVMHTSAG	AEGSGQALAS
1610	1620	1630	1640	1650	1660	1670	
PGSCLEEFRS	APFIECHGRG	TCNYYANAYS	FWLATIERSE	MFKKPTPSTL	KAGELRTHVS	RCQVCMRRT	

Cmpd.	No. of Cmpds.	m/z meas.	Δ m/z [ppm]	z	Rt [min]	Score	P	Range	Sequence	Modification
177	1	942.3962	-112.61	2	32.2	12.0	1	808-828	R.GPPGGQGPPGLSGPPGIKGEK.G	
62	1	713.3305	-7.33	3	30.5	13.9	1	927-947	K.GDVGLPGKPGSMDKVDMGSMK.G	Oxidation: 12, 17

Detailed Protein Report

Protein 603: endoplasmic reticulum-Golgi intermediate compartment protein 2 [Homo sapiens]

**Accession:**	gi|50959176	**Score:**	25.9
**Database:**	refseq_human(refseq_human_20140103.fasta)	**MW [kDa]:**	42.5
**Database Date:**	2015-11-30	**pI:**	6.3
		**Sequence Coverage [%]:**	5.6
		**No. of unique Peptides:**	1

10	20	30	40	50	60	70	80
MRRLNRKKTL	SLVKELDAFP	KVPESYVETS	ASGGTVSLIA	FTTMALLTIM	EFSVYQDTWM	KYEYEVDKDF	SSKLRINIDI
**90**	**100**	**110**	**120**	**130**	**140**	**150**	**160**
TVAMKCQYVG	ADVLDLAETM	VASADGLVYE	PTVFDLSPQQ	KEWQRMLQLI	QSRLQEEHSL	QDVIFKSAFK	STSTALPPRE
**170**	**180**	**190**	**200**	**210**	**220**	**230**	**240**
DDSSQSPNAC	RIHGHLYVNK	VAGNFHITVG	KAIPHPRGHA	HLAALVNHES	YNFSHRIDHL	SFGELVPAII	NPLDGTEKIA
**250**	**260**	**270**	**280**	**290**	**300**	**310**	**320**
IDHNQMFQYF	ITVVPTKLHT	YKISADTHQF	SVTERERIIN	HAAGSHGVSG	IFMKYDLSSL	MVTVTEEHMP	FWQFFVRLCG
**330**	**340**	**350**	**360**	**370**	**380**		
IVGGIFSTTG	MLHGIGKFIV	EIICCRFRLG	SYKPVNSVPF	EDGHTDNHLP	LLENNTH		

Cmpd.	No. of Cmpds.	m/z meas.	Δ m/z [ppm]	z	Rt [min]	Score	P	Range	Sequence	Modification
1411	1	1095.6964	116.34	1	47.3	11.8	0	338-346	K.FIVEIICCR.F	

Detailed Protein Report

Protein 604: centrosomal protein of 162 kDa isoform b [Homo sapiens]

**Accession:**	gi|555289992	**Score:**	25.9
**Database:**	refseq_human(refseq_human_20140103.fasta)	**MW [kDa]:**	153.0
**Database Date:**	2015-11-30	**pI:**	5.3
**Modification(s):**	Oxidation	**Sequence Coverage [%]:**	1.5
		**No. of unique Peptides:**	1

Alias proteins:

Accession	Name	Description
gi|578812505	refseq_human (refseq_human_20140103.fasta)	PREDICTED: centrosomal protein of 162 kDa isoform X7 [Homo sapiens]

10	20	30	40	50	60	70	80
MEIEEESAEK	IQFLKSSGTS	LLSTDSLETN	ELVVSELNHS	SLGVGLDTLE	EQEEKEQFFA	RLEKGLTSSI	DYSRLNKELD
**90**	**100**	**110**	**120**	**130**	**140**	**150**	**160**
SNDSTHFKAL	HSNQANAELT	DDEHENESKH	EELAENYSDD	FEDEYVGAPL	TTKDEEMPSK	ENSKSEKISV	PKQEEEKTGM
**170**	**180**	**190**	**200**	**210**	**220**	**230**	**240**
LANVVLLDSL	DSVAEVNLDE	QDKITPKPRC	LPEMTENEMT	GTGVSYGQSS	SDVEALHQAY	CHIAHSLGDE	DKQKIESNTV
**250**	**260**	**270**	**280**	**290**	**300**	**310**	**320**
EDIKSSVKGH	PQENEENSKN	ISTMESDLPT	VEELMKPIRI	DSFGISGFDL	QPVSSEKVAE	RKETEFFSSL	PLKMNPNILS
**330**	**340**	**350**	**360**	**370**	**380**	**390**	**400**
QDSQHVNLFF	DKNDENVILQ	KTTNESMENS	CPQVTEVTAT	EEHVDKMYLN	ILRKKITVNS	SSLSQDDKIN	KTYRSQLSSE
**410**	**420**	**430**	**440**	**450**	**460**	**470**	**480**
EEGAVMGKQV	PYKKARSAPP	LLKRKPQSGL	YASVRSSGYG	KPSSPLKMFS	TLEKKTSEDI	IKSKNLRSIS	TSNQPRKKEI
**490**	**500**	**510**	**520**	**530**	**540**	**550**	**560**
LSGTKLIKPA	ALDKPAHKTE	SCLSTRKKSE	NPTETDSCIQ	FQTDSLGYCG	ENKEKKLLMF	KRVQEAEDKW	RGAQALIEQI
**570**	**580**	**590**	**600**	**610**	**620**	**630**	**640**
KATFSEKEKE	LENKLEELKK	QQEKELFKLN	QDNYILQAKL	SSFEETNKKQ	RWLHFGEAAD	PVTGEKLKQI	QKEIQEQETL
**650**	**660**	**670**	**680**	**690**	**700**	**710**	**720**
LQGYQQENER	LYNQVKDLQE	QNKKNEERMF	KENQSLFSEV	ASLKEQMHKS	RFLSQVVEDS	EPTRNQNFTD	LLAELRMAQK
**730**	**740**	**750**	**760**	**770**	**780**	**790**	**800**
EKDSLLEDIK	RLKQDKQALE	VDFEKMKKER	DQAKDQIAYV	TGEKLYEIKI	LEETHKQEIS	RLQKRLQWYA	ENQELLDKDA
**810**	**820**	**830**	**840**	**850**	**860**	**870**	**880**
LRLREANEEI	EKLKLEIEKL	KAESGNPSIR	QKIRLKDKAA	DAKKIQDLER	QVKEMEGILK	RRYPNSLPAL	ILAASAAGDT
**890**	**900**	**910**	**920**	**930**	**940**	**950**	**960**
VDKNTVEFME	KRIKKLEADL	EGKDEDAKKS	LRTMEQQFQK	MKIQYEQRLE	QQEQLLACKL	NQHDSPRIKA	LEKELDDIKE
**970**	**980**	**990**	**1000**	**1010**	**1020**	**1030**	**1040**
AHQITVRNLE	AEIDVLKHQN	AELDVKKNDK	DDEDFQSIEF	QVEQAHAKAK	LVRLNEELAA	KKREIQDLSK	TVERLQKDRR
**1050**	**1060**	**1070**	**1080**	**1090**	**1100**	**1110**	**1120**
MMLSNQNSKG	REEMSAKRAK	KDVLHSSKGN	ANSFPGTLDS	KLYQPHTFTD	SHVSEVLQEN	YRLKNELEGL	ISEKNELKMK
**1130**	**1140**	**1150**	**1160**	**1170**	**1180**	**1190**	**1200**
SEAVMNQFEN	SMRRVKEDTA	AHIASLKASH	QREIEKLLCQ	NAVENSSSKV	AELNRKIATQ	EVLIRHFQSQ	VNELQSKQES
**1210**	**1220**	**1230**	**1240**	**1250**	**1260**	**1270**	**1280**
LVVSEVREEI	LQKEITKLLE	ELREAKENHT	PEMKHFVGLE	KKIKQMEMRH	AQREQELQQI	IQQTHQVVET	EQNKEVEKWK
**1290**	**1300**	**1310**	**1320**	**1330**			
RLAQLKNREL	EKFRTELDSI	LDVLRELHRQ	GVVVPVAFAD	EMNAPEY			

Cmpd.	No. of Cmpds.	m/z meas.	Δ m/z [ppm]	z	Rt [min]	Score	P	Range	Sequence	Modification
377	1	657.6657	-223.40	2	34.7	10.5	1	913-922	R.TMEQQFQKMK.I	Oxidation: 2

Detailed Protein Report

Protein 605: **PREDICTED: tolloid-like protein 1 isoform X2 [Homo sapiens]**

**Accession:**	gi|530378097	**Score:**	25.9
**Database:**	refseq_human(refseq_human_20140103.fasta)	**MW [kDa]:**	82.0
**Database Date:**	2015-11-30	**pI:**	5.5
**Modification(s):**	Carbamidomethyl	**Sequence Coverage [%]:**	2.2
		**No. of unique Peptides:**	1

10	20	30	40	50	60	70	80
MISTVSCTMP	GTPSQGWSLR	GMFLDTILPS	RDDNGIRPAI	GQRTRLSKGD	IAQARKLYRC	PACGETLQES	NGNLSSPGFP
**90**	**100**	**110**	**120**	**130**	**140**	**150**	**160**
NGYPSYTHCI	WRVSVTPGEK	IVLNFTTMDL	YKSSLCWYDY	IEVRDGYWRK	SPLLGRFCGD	KLPEVLTSTD	SRMWIEFRSS
**170**	**180**	**190**	**200**	**210**	**220**	**230**	**240**
SNWVGKGFAA	VYEAICGGEI	RKNEGQIQSP	NYPDDYRPMK	ECVWKITVSE	SYHVGLTFQS	FEIERHDNCA	YDYLEVRDGT
**250**	**260**	**270**	**280**	**290**	**300**	**310**	**320**
SENSPLIGRF	CGYDKPEDIR	STSNTLWMKF	VSDGTVNKAG	FAANFFKEED	ECAKPDRGGC	EQRCLNTLGS	YQCACEPGYE
**330**	**340**	**350**	**360**	**370**	**380**	**390**	**400**
LGPDRRSCEA	ACGGLLTKLN	GTITTPGWPK	EYPPNKNCVW	QVVAPTQYRI	SVKFEFFELE	GNEVCKYDYV	EIWSGLSSES
**410**	**420**	**430**	**440**	**450**	**460**	**470**	**480**
KLHGKFCGAE	VPEVITSQFN	NMRIEFKSDN	TVSKKGFKAH	FFSDKDECSK	DNGGCQHECV	NTMGSYMCQC	RNGFVLHDNK
**490**	**500**	**510**	**520**	**530**	**540**	**550**	**560**
HDCKEAECEQ	KIHSPSGLIT	SPNWPDKYPS	RKECTWEISA	TPGHRIKLAF	SEFEIEQHQE	CAYDHLEVFD	GETEKSPILG
**570**	**580**	**590**	**600**	**610**	**620**	**630**	**640**
RLCGNKIPDP	LVATGNKMFV	RFVSDASVQR	KGFQATHSTE	CGGRLKAESK	PRDLYSHAQF	GDNNYPGQVD	CEWLLVSERG
**650**	**660**	**670**	**680**	**690**	**700**	**710**	**720**
SRLELSFQTF	EVEEEADCGY	DYVELFDGLD	STAVGLGRFC	GSGPPEEIYS	IGDSVLIHFH	TDDTINKKGF	HIRYKSIRYP
**730**							
DTTHTKK							

Cmpd.	No. of Cmpds.	m/z meas.	Δ m/z [ppm]	z	Rt [min]	Score	P	Range	Sequence	Modification
2771	1	848.8935	-73.79	2	65.0	12.1	1	562-577	R.LCGNKIPDPLVATGNK.M	Carbamidomethyl: 2

Detailed Protein Report

Protein 606: **PREDICTED: chondroadherin-like protein isoform X2 [Homo sapiens]**

**Accession:**	gi|530419599	**Score:**	25.9
**Database:**	refseq_human(refseq_human_20140103.fasta)	**MW [kDa]:**	81.7
**Database Date:**	2015-11-30	**pI:**	10.9
**Modification(s):**	Carbamidomethyl	**Sequence Coverage [%]:**	4.4
		**No. of unique Peptides:**	2

Quantitation

QU:MU **Median:** 1.45 **CV:** 0.00 % **No. of Peptides:** 1

10	20	30	40	50	60	70	80
MEGPRSSTHV	PLVLPLLVLL	LLAPARQAAA	QRCPQACICD	NSRRHVACRY	QNLTEVPDAI	PELTQRLDLQ	GNLLKVIPAA
**90**	**100**	**110**	**120**	**130**	**140**	**150**	**160**
AFQGVPHLTH	LDLRHCEVEL	VAEGAFRGLG	RLLLLNLASN	HLRELPQEAL	DGLGSLRRLE	LEGNALEELR	PGTFGALGAL
**170**	**180**	**190**	**200**	**210**	**220**	**230**	**240**
ATLNLAHNAL	VYLPAMAFQG	LLRVRWLRLS	HNALSVLAPE	ALAGLPALRR	LSLHHNELQA	LPGPVLSQAR	GLARLELGHN
**250**	**260**	**270**	**280**	**290**	**300**	**310**	**320**
PLTYAGEEDG	LALPGLRELL	LDGGALQALG	PRAFAHCPRL	HTLDLRGNQL	DTLPPLQGPG	QLRRLRLQGN	PLWCGCQARP
**330**	**340**	**350**	**360**	**370**	**380**	**390**	**400**
LLEWLARARV	RSDGACQGPR	RLRGEALDAL	RPWDLRCPGD	AAQEEEELEE	RAVAGPRAPP	RGPPRGPGEE	RAVAPCPRAC
**410**	**420**	**430**	**440**	**450**	**460**	**470**	**480**
VCVPESRHSS	CEGCGLQAVP	RGFPSDTQLL	DLRRNHFPSV	PRAAFPGLGH	LVSLHLQHCG	IAELEAGALA	GLGRLIYLYL
**490**	**500**	**510**	**520**	**530**	**540**	**550**	**560**
SDNQLAGLSA	AALEGAPRLG	YLYLERNRFL	QVPGAALRAL	PSLFSLHLQD	NAVDRLAPGD	LGRTRALRWV	YLSGNRITEV
**570**	**580**	**590**	**600**	**610**	**620**	**630**	**640**
SLGALGPARE	LEKLHLDRNQ	LREVPTGALE	GLPALLELQL	SGNPLRALRD	GAFQPVGRSL	QHLFLNSSGL	EQICPGAFSG
**650**	**660**	**670**	**680**	**690**	**700**	**710**	**720**
LGPGLQSLHL	QKNQLRALPA	LPSLSQLELI	DLSSNPFHCD	CQLLPLHRWL	TGLNLRVGAT	CATPPNARGQ	RVKAAAAVFE
**730**	**740**	**750**	**760**				
DCPGWAARKA	KRTPASRPSA	RRTPIKGRQC	GADKGTL				

Cmpd.	No. of Cmpds.	m/z meas.	Δ m/z [ppm]	z	Rt [min]	Score	P	Range	Sequence	Modification	Ratios
173	1	679.8611	32.65	2	32.1	14.2	2	330-341	R.VRSDGACQGPRR.L	Carbamidomethyl: 7	QU:MU 1.45
2204	1	1130.1280	10.71	2	58.0	11.7	1	549-569	R.WVYLSGNRITEVSLGALGPAR.E		

Detailed Protein Report

Protein 607: E3 SUMO-protein ligase PIAS3 [Homo sapiens]

**Accession:**	gi|115298686	**Score:**	25.8
**Database:**	refseq_human(refseq_human_20140103.fasta)	**MW [kDa]:**	68.0
**Database Date:**	2015-11-30	**pI:**	8.9
		**Sequence Coverage [%]:**	5.4
		**No. of unique Peptides:**	2

10	20	30	40	50	60	70	80
MAELGELKHM	VMSFRVSELQ	VLLGFAGRNK	SGRKHELLAK	ALHLLKSSCA	PSVQMKIKEL	YRRRFPRKTL	GPSDLSLLSL
**90**	**100**	**110**	**120**	**130**	**140**	**150**	**160**
PPGTSPVGSP	GPLAPIPPTL	LAPGTLLGPK	REVDMHPPLP	QPVHPDVTMK	PLPFYEVYGE	LIRPTTLAST	SSQRFEEAHF
**170**	**180**	**190**	**200**	**210**	**220**	**230**	**240**
TFALTPQQVQ	QILTSREVLP	GAKCDYTIQV	QLRFCLCETS	CPQEDYFPPN	LFVKVNGKLC	PLPGYLPPTK	NGAEPKRPSR
**250**	**260**	**270**	**280**	**290**	**300**	**310**	**320**
PINITPLARL	SATVPNTIVV	NWSSEFGRNY	SLSVYLVRQL	TAGTLLQKLR	AKGIRNPDHS	RALIKEKLTA	DPDSEVATTS
**330**	**340**	**350**	**360**	**370**	**380**	**390**	**400**
LRVSLMCPLG	KMRLTVPCRA	LTCAHLQSFD	AALYLQMNEK	KPTWTCPVCD	KKAPYESLII	DGLFMEILSS	CSDCDEIQFM
**410**	**420**	**430**	**440**	**450**	**460**	**470**	**480**
EDGSWCPMKP	KKEASEVCPP	PGYGLDGLQY	SPVQGGDPSE	NKKKVEVIDL	TIESSSDEED	LPPTKKHCSV	TSAAIPALPG
**490**	**500**	**510**	**520**	**530**	**540**	**550**	**560**
SKGVLTSGHQ	PSSVLRSPAM	GTLGGDFLSS	LPLHEYPPAF	PLGADIQGLD	LFSFLQTESQ	HYGPSVITSL	DEQDALGHFF
**570**	**580**	**590**	**600**	**610**	**620**	**630**	
QYRGTPSHFL	GPLAPTLGSS	HCSATPAPPP	GRVSSIVAPG	GALREGHGGP	LPSGPSLTGC	RSDIISLD	

Cmpd.	No. of Cmpds.	m/z meas.	Δ m/z [ppm]	z	Rt [min]	Score	P	Range	Sequence	Modification
2323	1	965.9652	-82.27	2	57.3	14.9	2	16-33	R.VSELQVLLGFAGRNKSGR.K	
2512	1	856.9301	-38.31	2	62.0	11.0	1	41-56	K.ALHLLKSSCAPSVQMK.I	

Detailed Protein Report

Protein 608: PREDICTED: LIM/homeobox protein Lhx3 isoform X1 [Homo sapiens]

**Accession:**	gi|530426634	**Score:**	25.8
**Database:**	refseq_human(refseq_human_20140103.fasta)	**MW [kDa]:**	42.3
**Database Date:**	2015-11-30	**pI:**	9.5
**Modification(s):**	Carbamidomethyl	**Sequence Coverage [%]:**	8.5
		**No. of unique Peptides:**	2

10	20	30	40	50	60	70	80
MTLNMKETRW	GAPAPEIPLC	AGCDQHILDR	FILKALDRHW	HSKCLKCSDC	HTPLAERCFS	RGESVYCKDD	FFKRFGTKCA
**90**	**100**	**110**	**120**	**130**	**140**	**150**	**160**
ACQLGIPPTQ	VVRRAQDFVY	HLHCFACVVC	KRQLATGDEF	YLMEDSRLVC	KADYETAKQR	EAEATAKRPR	TTITAKQLET
**170**	**180**	**190**	**200**	**210**	**220**	**230**	**240**
LKSAYNTSPK	PARHVREQLS	SETGLDMRVV	QVWFQNRRAK	EKRLKKDAGR	QRWGQYFRNM	KRSRGGSKSD	KDSVQEGQDS
**250**	**260**	**270**	**280**	**290**	**300**	**310**	**320**
DAEVSFPDEP	SLAEMGPANG	LYGSLGEPTQ	ALGRPSGALG	NFSLEHGGLA	GPEQYRELRP	GSPYGVPPSP	AAPQSLPGPQ
**330**	**340**	**350**	**360**	**370**	**380**	**390**	
PLLSSLVYPD	TSLGLVPSGA	PGGPPPMRVL	AGNGPSSDLS	TGSSGGYPDF	PASPASWLDE	VDHAQF	

Cmpd.	No. of Cmpds.	m/z meas.	Δ m/z [ppm]	z	Rt [min]	Score	P	Range	Sequence	Modification
2005	1	788.2933	-81.32	2	55.0	10.3	1	44-57	K.CLKCSDCHTPLAER.C	
2172	1	1051.9909	-51.91	2	57.0	15.5	1	75-93	R.FGTKCAACQLGIPPTQVVR.R	Carbamidomethyl: 5, 8

Detailed Protein Report

Protein 609: **rap guanine nucleotide exchange factor 1 isoform a [Homo sapiens]**

**Accession:**	gi|38373675	**Score:**	25.8
**Database:**	refseq_human(refseq_human_20140103.fasta)	**MW [kDa]:**	120.5
**Database Date:**	2015-11-30	**pI:**	5.6
**Modification(s):**	Oxidation	**Sequence Coverage [%]:**	2.9
		**No. of unique Peptides:**	2

10	20	30	40	50	60	70	80
MDTDSQRSHL	SSFTMKLMDK	FHSPKIKRTP	SKKGKPAEVS	VKIPEKPVNK	EATDRFLPEG	YPLPLDLEQQ	AVEFMSTSAV
**90**	**100**	**110**	**120**	**130**	**140**	**150**	**160**
ASRSQRQKNL	SWLEEKEKEV	VSALRYFKTI	VDKMAIDKKV	LEMLPGSASK	VLEAILPLVQ	NDPRIQHSSA	LSSCYSRVYQ
**170**	**180**	**190**	**200**	**210**	**220**	**230**	**240**
SLANLIRWSD	QVMLEGVNSE	DKEMVTTVKG	VIKAVLDGVK	ELVRLTIEKQ	GRPSPTSPVK	PSSPASKPDG	PAELPLTDRE
**250**	**260**	**270**	**280**	**290**	**300**	**310**	**320**
VEILNKTTGM	SQSTELLPDA	TDEEVAPPKP	PLPGIRVVDN	SPPPALPPKK	RQSAPSPTRV	AVVAPMSRAT	SGSSLPVGIN
**330**	**340**	**350**	**360**	**370**	**380**	**390**	**400**
RQDFDVDCYA	QRRLSGGSHS	YGGESPRLSP	CSSIGKLSKS	DEQLSSLDRD	SGQCSRNTSC	ETLDHYDPDY	EFLQQDLSNA
**410**	**420**	**430**	**440**	**450**	**460**	**470**	**480**
DQIPQQTAWN	LSPLPESLGE	SGSPFLGPPF	QLPLGGHPQP	DGPLAPGQQT	DTPPALPEKK	RRSAASQTAD	GSGCRVSYER
**490**	**500**	**510**	**520**	**530**	**540**	**550**	**560**
HPSQYDNISG	EDLQSTAPIP	SVPYAPFAAI	LPFQHGGSSA	PVEFVGDFTA	PESTGDPEKP	PPLPEKKNKH	MLAYMQLLED
**570**	**580**	**590**	**600**	**610**	**620**	**630**	**640**
YSEPQPSMFY	QTPQNEHIYQ	QKNKLLMEVY	GFSDSFSGVD	SVQELAPPPA	LPPKQRQLEP	PAGKDGHPRD	PSAVSGVPGK
**650**	**660**	**670**	**680**	**690**	**700**	**710**	**720**
DSRDGSERAP	KSPDALESAQ	SEEEVDELSL	IDHNEIMSRL	TLKQEGDDGP	DVRGGSGDIL	LVHATETDRK	DLVLYCEAFL
**730**	**740**	**750**	**760**	**770**	**780**	**790**	**800**
TTYRTFISPE	ELIKKLQYRY	EKFSPFADTF	KKRVSKNTFF	VLVRVVDELC	LVELTEEILK	LLMELVFRLV	CNGELSLARV
**810**	**820**	**830**	**840**	**850**	**860**	**870**	**880**
LRKNILDKVD	QKKLLRCATS	SQPLAARGVA	ARPGTLHDFH	SHEIAEQLTL	LDAELFYKIE	IPEVLLWAKE	QNEEKSPNLT
**890**	**900**	**910**	**920**	**930**	**940**	**950**	**960**
QFTEHFNNMS	YWVRSIIMLQ	EKAQDRERLL	LKFIKIMKHL	RKLNNFNSYL	AILSALDSAP	IRRLEWQKQT	SEGLAEYCTL
**970**	**980**	**990**	**1000**	**1010**	**1020**	**1030**	**1040**
IDSSSSFRAY	RAALSEVEPP	CIPYLGLILQ	DLTFVHLGNP	DYIDGKVNFS	KRWQQFNILD	SMRCFQQAHY	DMRRNDDIIN
**1050**	**1060**	**1070**	**1080**				
FFNDFSDHLA	EEALWELSLK	IKPRNITRRK	TDREEKT				

Cmpd.	No. of Cmpds.	m/z meas.	Δ m/z [ppm]	z	Rt [min]	Score	P	Range	Sequence	Modification
2613	3	885.4451	-28.15	2	62.7	13.1	1	292-308	R.QSAPSPTRVAVVAPMSR.A	Oxidation: 15
1627	1	686.4490	148.95	2	48.7	12.7	1	630-643	R.DPSAVSGVPGKDSR.D	

Detailed Protein Report

Protein 610: protein kinase C theta type isoform 3 [Homo sapiens]

Accession: gi|544063437
Database: refseq_human(refseq_human_20140103.fasta)
Database Date: 2015-11-30
Modification(s): Oxidation

Score: 25.8
MW [kDa]: 77.9
pI: 9.2
Sequence Coverage [%]: 4.3
No. of unique Peptides: 1

Quantitation

[truncated: 353,640 more chars]
