# Supplementary material for: Exploration of key factors in Gingival Crevicular fluids from patients undergoing Periodontally Accelerated Osteogenic Orthodontics (PAOO) using proteome analysis
Source: BMC Oral Health. 2023 Nov 27;23:934. doi: 10.1186/s12903-023-03606-7 (PMC10683118; doi:10.1186/s12903-023-03606-7)
Supplement: Supplementary file 6 — Supplementary Material 6 [file 12903_2023_3606_MOESM6_ESM.pdf]

Detailed Protein Report

Project Info

Name: teeth-151208 **Date:** Dec 8, 2015

Note:

Sample Info & Protocols

Name: id-ALL **Date:** Dec 10, 2015

Fraction:

Volume: **Concentration:**

Note:

Buffer: **Organism:**

Search Result Info

Search Result	Location	Search Engine	Database
ZT_human_PE_2015-12-11 21:24:46	/teeth-151208/id-ALL/"Combined MS/MS spectra"	Mascot	refseq_human

Protein 1: serum albumin preproprotein [Homo sapiens]

Accession: gi\|4502027
Database: refseq_human(refseq_human_20140103.fasta)
Database Date: 2015-11-30
Modification(s): Carbamidomethyl, Oxidation

Score: 1677.2
MW [kDa]: 69.3
pI: 5.9
Sequence Coverage [%]: 56.2
No. of unique Peptides: 35

Quantitation

Q:W	**Median:** 1.04	**CV:** 76.74 %	**No. of Peptides:** 22
M:Q	**Median:** 0.77	**CV:** 54.66 %	**No. of Peptides:** 27

```
        10         20         30         40         50         60         70         80
MKWVTFISLL FLFSSAYSRG VFRRDAHKSE VAHRFKDLGE ENFKALVLIA FAQYLQQCPF EDHVKLVNEV TEFAKTCVAD
        90        100        110        120        130        140        150        160
ESAENCDKSL HTLFGDKLCT VATLRETYGE MADCCAKQEP ERNECFLQHK DDNPNLPRLV RPEVDVMCTA FHDNEETFLK
       170        180        190        200        210        220        230        240
KYLYEIARRH PYFYAPELLF FAKRYKAAFT ECCQAADKAA CLLPKLDELR DEGKASSAKQ RLKCASLQKF GERAFKAWAV
       250        260        270        280        290        300        310        320
ARLSQRFPKA EFAEVSKLVT DLTKVHTECC HGDLLECADD RADLAKYICE NQDSISSKLK ECCEKPLLEK SHCIAEVEND
       330        340        350        360        370        380        390        400
EMPADLPSLA ADFVESKDVC KNYAEAKDVF LGMFLYEYAR RHPDYSVVLL LRLAKTYETT LEKCCAAADP HECYAKVFDE
       410        420        430        440        450        460        470        480
FKPLVEEPQN LIKQNCELFE QLGEYKFQNA LLVRYTKKVP QVSTPTLVEV SRNLGKVGSK CCKHPEAKRM PCAEDYLSVV
       490        500        510        520        530        540        550        560
LNQLCVLHEK TPVSDRVTKC CTESLVNRRP CFSALEVDET YVPKEFNAET FTFHADICTL SEKERQIKKQ TALVELVKHK
       570        580        590        600        610
PKATKEQLKA VMDDFAAFVE KCCKADDKET CFAEEGKKLV AASQAALGL
```

Cmpd.	No. of Cmpds.	m/z meas.	Δ m/z [ppm]	z	Rt [min]	Score	P	Range	Sequence	Modification	Ratios
298	24	613.8004	-9.46	2	32.4	71.1	1	35-44	R.FKDLGEENFK.A		M:Q 1.05<br>Q:W 0.78
514	32	575.2390	-125.46	2	34.9	63.4	0	66-75	K.LVNEVTEFAK.T		M:Q 0.70<br>Q:W 1.13
529	18	509.2667	-10.16	2	36.6	49.5	0	89-97	K.SLHTLFGDK.L		M:Q 1.98<br>Q:W 0.24
299	2	467.2609	-4.27	2	33.7	52.0	0	98-105	K.LCTVATLR.E	Carbamidomethyl: 2	M:Q 1.15
171	1	659.8150	4.22	4	31.4	26.5	2	118-138	K.QEPERNECFLQHKDDNPNLPR.L	Carbamidomethyl: 8	M:Q 0.57<br>Q:W 1.05

Cmpd.	No. of Cmpds.	m/z meas.	Δ m/z [ppm]	z	Rt [min]	Score	P	Range	Sequence	Modification	Ratios	
328	1	509.7430	1.26	2	33.5	11.8	0	123-130	R.NECFLQHK.D			
213	3	666.3221	11.20	3	32.0	43.3	1	123-138	R.NECFLQHKDDNPNLPR.L	Carbamidomethyl: 3	M:Q<br>Q:W	0.76<br>1.21
350	3	528.2959	-3.73	2	34.4	39.6	1	161-168	K.KYLYEIAR.R		M:Q<br>Q:W	1.24<br>0.52
532	20	464.1198	-281.27	2	35.3	30.2	0	162-168	K.YLYEIAR.R		M:Q<br>Q:W	0.91<br>1.20
33	5	686.2794	-11.06	2	29.6	70.0	0	187-198	K.AAFTECCQAADK.A	Carbamidomethyl: 6, 7		
1590	1	977.4188	-47.42	2	50.1	11.8	1	187-205	K.AAFTECCQAADKAACLLPK.L			
31	9	537.7767	3.24	2	30.1	45.4	1	206-214	K.LDELRDEGK.A		M:Q<br>Q:W	0.57<br>2.49
235	7	696.2743	-13.94	3	31.7	57.7	0	265-281	K.VHTECCHGDLLECADDR.A	Carbamidomethyl: 5, 6, 13	M:Q<br>Q:W	0.76<br>0.88
45	14	722.3225	-3.01	2	30.4	77.4	0	287-298	K.YICENQDSISSK.L	Carbamidomethyl: 3	Q:W<br>M:Q	2.14<br>0.70
75	3	773.8989	-4.03	2	30.2	28.9	1	299-310	K.LKECCEKPLLEK.S	Carbamidomethyl: 4, 5	M:Q<br>Q:W	0.75<br>1.61
2459	1	997.4538	2.48	3	59.0	27.7	0	311-337	K.<br>SHCIAEVENDEMPADLPSLAADFV<br>D	Carbamidomethyl: 3;<br>Oxidation: 12		
2988	1	992.1219	2.21	3	66.1	32.9	0	311-337	K.<br>SHCIAEVENDEMPADLPSLAADFV<br>D	Carbamidomethyl: 3	M:Q	0.37
2841	5	812.4044	8.52	2	65.9	42.2	0	348-360	K.DVFLGMFLYEYAR.R			
2516	13	820.4094	17.73	2	59.7	54.5	0	348-360	K.DVFLGMFLYEYAR.R	Oxidation: 6	M:Q<br>Q:W	1.26<br>0.82
42	1	492.7365	-23.11	2	29.7	28.1	0	376-383	K.TYETTLEK.C		Q:W	1.81
8	10	776.7890	-17.37	2	29.1	42.3	0	384-396	K.CCAAADPHECYAK.V	Carbamidomethyl: 1, 2, 10	Q:W<br>M:Q	0.35<br>1.78
2246	92	682.3456	-35.78	3	56.6	62.3	0	397-413	K.VFDEFKPLVEEPQNLIK.Q			
1582	43	829.3539	-31.44	2	48.3	77.6	0	414-426	K.QNCELFEQLGEYK.F	Carbamidomethyl: 3	Q:W<br>M:Q	2.08<br>0.38
742	34	480.7783	-13.77	2	39.3	53.1	0	427-434	K.FQNALLVR.Y		M:Q	0.96
783	12	820.4638	-10.68	2	38.6	67.6	1	438-452	K.KVPQVSTPTLVEVSR.N		Q:W<br>M:Q	2.19<br>0.69
1019	3	756.4151	-13.09	2	41.5	37.1	0	439-452	K.VPQVSTPTLVEVSR.N		M:Q<br>Q:W	0.95<br>2.13
78	8	569.7455	-12.45	2	30.7	62.5	0	500-508	K.CCTESLVNR.R	Carbamidomethyl: 1, 2	M:Q<br>Q:W	0.36<br>2.77
1753	7	637.6399	-13.90	3	50.5	67.0	0	509-524	R.RPCFSALEVDETYVPK.E	Carbamidomethyl: 3	M:Q	0.34
2419	18	753.9221	-119.82	3	58.5	64.2	0	525-543	K.EFNAETFTFHADICTLSEK.E	Carbamidomethyl: 14	M:Q<br>Q:W	0.80<br>0.88
2154	1	848.9827	-91.45	3	55.4	11.2	1	525-545	K.EFNAETFTFHADICTLSEKER.<br>Q	Carbamidomethyl: 14		
421	3	564.7074	-257.77	2	34.6	52.0	1	549-558	K.KQTALVELVK.H		M:Q<br>Q:W	0.96<br>0.88
860	27	500.8124	13.72	2	39.6	52.8	0	550-558	K.QTALVELVK.H		M:Q	0.61
2105	69	671.7288	-137.21	2	54.9	98.5	0	570-581	K.AVMDDFAAFVEK.C		M:Q<br>Q:W	0.85<br>0.32
21	3	543.2367	-19.25	3	29.6	43.1	2	585-598	K.ADDKETCFAEEGKK.L	Carbamidomethyl: 7	Q:W<br>M:Q	0.54<br>1.85
895	1	571.3207	-52.32	2	39.6	22.1	1	598-609	K.KLVAASQAALGL.-		M:Q	0.26

Detailed Protein Report

Protein 2: hemoglobin subunit beta [Homo sapiens]

**Accession:**	gi|4504349	**Score:**	811.9
**Database:**	refseq_human(refseq_human_20140103.fasta)	**MW [kDa]:**	16.0
**Database Date:**	2015-11-30	**pI:**	6.9
**Modification(s):**	Carbamidomethyl, Oxidation	**Sequence Coverage [%]:**	84.4
		**No. of unique Peptides:**	13

Quantitation

Q:W	**Median:**	1.98	**CV:**	66.22 %	**No. of Peptides:**	10
M:Q	**Median:**	1.29	**CV:**	77.56 %	**No. of Peptides:**	9

```
        10         20         30         40         50         60         70         80
MVHLTPEEKS AVTALWGKVN VDEVGGEALG RLLVVYPWTQ RFFESFGDLS TPDAVMGNPK VKAHGKKVLG AFSDGLAHLD
        90        100        110        120        130        140        150
NLKGTFATLS ELHCDKLHVD PENFRLLGNV LVCVLAHHFG KEFTPPVQAA YQKVVAGVAN ALAHKYH
```

Cmpd.	No. of Cmpds.	m/z meas.	Δ m/z [ppm]	z	Rt [min]	Score	P	Range	Sequence	Modification	Ratios
63	5	476.7531	-11.35	2	30.5	38.7	0	2-9	M.VHLTPEEK.S		M:Q 0.70<br>Q:W 3.02
629	18	466.7591	-9.74	2	36.3	58.8	0	10-18	K.SAVTALWGK.V		Q:W 1.05<br>M:Q 3.04
496	16	657.8496	20.65	2	36.2	81.3	0	19-31	K.VNVDEVGGEALGR.L		M:Q 0.96<br>Q:W 2.10
2284	42	637.8143	-81.79	2	56.9	61.8	0	32-41	R.LLVVYPWTQR.F		Q:W 1.78
1677	1	1037.9101	-62.48	2	49.6	60.1	0	42-60	R.FFESFGDLSTPDAVMGNPK.V	Oxidation: 15	
2107	19	1029.8517	-122.16	2	56.7	64.9	0	42-60	R.FFESFGDLSTPDAVMGNPK.V		M:Q 4.42<br>Q:W 0.70
2072	40	599.9066	-155.81	3	54.5	102.5	1	67-83	K.KVLGAFSDGLAHLDNLK.G		M:Q 0.72
2939	23	557.3045	4.87	3	65.5	94.6	0	68-83	K.VLGAFSDGLAHLDNLK.G		
595	5	739.7263	-168.37	2	36.3	53.2	0	84-96	K.GTFATLSELHCDK.L	Carbamidomethyl: 11	M:Q 0.97<br>Q:W 4.67
187	24	563.6579	-226.49	2	31.2	45.5	0	97-105	K.LHVDPENFR.L		Q:W 3.40<br>M:Q 0.81
563	3	689.7251	-186.44	2	35.9	45.0	0	122-133	K.EFTPPVQAAYQK.V		Q:W 4.20<br>M:Q 0.73
213	10	575.2065	-233.10	2	31.9	88.0	0	134-145	K.VVAGVANALAHK.Y		Q:W 1.18
430	1	725.3587	-59.21	2	35.4	17.7	1	134-147	K.VVAGVANALAHKYH.-		M:Q 2.71<br>Q:W 1.45

Detailed Protein Report

Protein 3: keratin, type I cytoskeletal 13 isoform a [Homo sapiens]

Accession: gi|131412225
Database: refseq_human(refseq_human_20140103.fasta)
Database Date: 2015-11-30
Modification(s): Carbamidomethyl

Score: 753.3
MW [kDa]: 49.5
pI: 4.8
Sequence Coverage [%]: 39.7
No. of unique Peptides: 16

Quantitation

Q:W	**Median:**	0.42	**CV:**	37.76 %	**No. of Peptides:**	10
M:Q	**Median:**	0.76	**CV:**	30.01 %	**No. of Peptides:**	12

10	20	30	40	50	60	70	80
MSLRLQSSSA	SYGGGFGGGS	CQLGGGRGVS	TCSTRFVSGG	SAGGYGGGVS	CGFGGGAGSG	FGGGYGGGLG	GGYGGGLGGG
90	100	110	120	130	140	150	160
FGGGFAGGFV	DFGACDGGLL	TGNEKITMQN	LNDRLASYLE	KVRALEEANA	DLEVKIRDWH	LKQSPASPER	DYSPYYKTIE
170	180	190	200	210	220	230	240
ELRDKILTAT	IENNRVILEI	DNARLAADDF	RLKYENELAL	RQSVEADING	LRRVLDELTL	SKTDLEMQIE	SLNEELAYMK
250	260	270	280	290	300	310	320
KNHEEEMKEF	SNQVVGQVNV	EMDATPGIDL	TRVLAEMREQ	YEAMAERNRR	DAEEWFHAKS	AELNKEVSTN	TAMIQTSKTE
330	340	350	360	370	380	390	400
ITELRRTLQG	LEIELQSQLS	MKAGLENTVA	ETECRYALQL	QQIQGLISSI	EAQLSELRSE	MECQNQEYKM	LLDIKTRLEQ
410	420	430	440	450	460		
EIATYRSLLE	GQDAKMIGFP	SSAGSVSPRS	TSVTTTSSAS	VTTTSNASGR	RTSDVRRP		

Cmpd.	No. of Cmpds.	m/z meas.	Δ m/z [ppm]	z	Rt [min]	Score	P	Range	Sequence	Modification	Ratios
337	2	1073.9554	-24.90	2	34.2	51.4	0	5-27	R.LQSSSASYGGGFGGGSCQLGGGG	Carbamidomethyl: 17	M:Q 0.56<br>Q:W 0.55
323	6	651.3296	-4.86	2	34.0	78.4	0	124-135	R.ALEEANADLEVK.I		
264	5	572.8011	-32.35	2	33.3	62.0	0	166-175	K.ILTATIENNR.V		
528	8	521.6763	-233.65	2	35.9	61.6	0	176-184	R.VILEIDNAR.L		M:Q 0.64<br>Q:W 0.36
342	5	624.7261	-199.84	2	33.4	46.8	1	192-201	R.LKYENELALR.Q		M:Q 0.44<br>Q:W 0.65
379	5	601.3190	11.24	2	34.1	54.4	0	202-212	R.QSVEADINGLR.R		Q:W 0.17<br>M:Q 0.93
235	2	679.3515	-16.64	2	32.3	26.4	1	202-213	R.QSVEADINGLRR.V		Q:W 0.54<br>M:Q 0.73
686	1	587.3207	-42.26	2	37.1	41.5	1	213-222	R.RVLDELTLSK.T		
727	5	509.2783	-32.74	2	39.1	53.0	0	214-222	R.VLDELTLSK.T		
434	3	566.7484	-14.34	2	35.4	31.5	0	291-299	R.DAEEWFHAK.S		Q:W 0.35<br>M:Q 1.23
173	1	705.3260	-34.92	2	30.9	13.5	0	306-318	K.EVSTNTAMIQTSK.T		M:Q 1.24<br>Q:W 0.34
2903	1	909.4716	-17.49	2	65.4	28.3	0	327-342	R.TLQGLEIELQSQLSMK.A		M:Q 0.90<br>Q:W 0.57
142	6	725.3217	-19.13	2	31.2	69.4	0	343-355	K.AGLENTVAETECR.Y	Carbamidomethyl: 12	Q:W 0.42<br>M:Q 0.77
269	6	690.3609	-9.58	2	33.1	55.3	1	396-406	K.TRLEQEIATYR.S		M:Q 0.73<br>Q:W 0.48
768	4	696.8079	-61.40	2	39.0	28.8	0	416-429	K.MIGFPSSAGSVSPR.S		M:Q 0.77
33	5	1001.9745	-5.27	2	29.8	50.9	0	430-450	R.STSVTTTSSASVTTTSNASGR.R		M:Q 0.58

Detailed Protein Report

Protein 4: apolipoprotein A-I preproprotein [Homo sapiens]

**Accession:**	gi|4557321	**Score:**	601.3
**Database:**	refseq_human(refseq_human_20140103.fasta)	**MW [kDa]:**	30.8
**Database Date:**	2015-11-30	**pI:**	5.5
		**Sequence Coverage [%]:**	48.3
		**No. of unique Peptides:**	15

Quantitation

Q:W	**Median:**	1.21	**CV:**	63.01 %	**No. of Peptides:**	8
M:Q	**Median:**	0.91	**CV:**	31.98 %	**No. of Peptides:**	6

Alias proteins:

Accession	Name	Description
gi|530398069	refseq_human (refseq_human_20140103.fasta)	PREDICTED: apolipoprotein A-I isoform X2 [Homo sapiens]
gi|530398067	refseq_human (refseq_human_20140103.fasta)	PREDICTED: apolipoprotein A-I isoform X1 [Homo sapiens]

```
        10         20         30         40         50         60         70         80
MKAAVLTLAV LFLTGSQARH FWQQDEPPQS PWDRVKDLAT VYVDVLKDSG RDYVSQFEGS ALGKQLNLKL LDNWDSVTST
        90        100        110        120        130        140        150        160
FSKLREQLGP VTQEFWDNLE KETEGLRQEM SKDLEEVKAK VQPYLDDFQK KWQEEMELYR QKVEPLRAEL QEGARQKLHE
       170        180        190        200        210        220        230        240
LQEKLSPLGE EMRDRARAHV DALRTHLAPY SDELRQRLAA RLEALKENGG ARLAEYHAKA TEHLSTLSEK AKPALEDLRQ
       250        260        270
GLLPVLESFK VSFLSALEEY TKKLNTQ
```

Cmpd.	No. of Cmpds.	m/z meas.	Δ m/z [ppm]	z	Rt [min]	Score	P	Range	Sequence	Modification	Ratios
1873	12	731.8942	-48.16	2	52.1	53.6	1	35-47	R.VKDLATVYVDVLK.D		
2093	4	618.2734	-120.22	2	54.7	58.3	0	37-47	K.DLATVYVDVLK.D		Q:W 2.19
805	1	908.4284	-0.73	2	38.9	11.7	1	48-64	K.DSGRDYVSQFEGSALGK.Q		M:Q 0.87<br>Q:W 1.38
893	5	700.8159	-31.87	2	39.6	45.2	0	52-64	R.DYVSQFEGSALGK.Q		Q:W 2.00
1807	26	806.8737	-27.99	2	51.3	94.1	0	70-83	K.LLDNWDSVTSTFSK.L		Q:W 1.62<br>M:Q 1.00
2784	1	734.7111	-0.04	3	66.2	16.8	1	84-101	K.LREQLGPVTQEFWDNLEK.E		
2426	1	966.9435	-27.92	2	58.6	21.1	0	86-101	R.EQLGPVTQEFWDNLEK.E		
2011	2	704.2707	-123.01	1	55.5	10.6	0	102-107	K.ETEGLR.Q		
497	7	626.6875	-201.93	2	34.7	29.3	0	121-130	K.VQPYLDDFQK.K		M:Q 0.56<br>Q:W 1.62
257	4	690.8471	-20.89	2	32.0	53.5	1	121-131	K.VQPYLDDFQKK.W		M:Q 0.72<br>Q:W 1.38
860	2	642.2972	11.37	2	39.2	28.3	0	132-140	K.WQEEMELYR.Q		M:Q 1.53<br>Q:W 0.45
1475	4	896.3833	-111.83	1	48.6	11.9	0	158-164	K.LHELQEK.L		Q:W 0.47
283	5	651.3276	-0.37	2	33.5	58.6	0	185-195	R.THLAPYSDELR.Q		M:Q 1.05
2876	27	615.8555	-4.42	2	64.7	47.2	0	240-250	R.QGLLPVLESFK.V		
2300	25	693.8302	-44.61	2	57.2	61.0	0	251-262	K.VSFLSALEEYTK.K		

Detailed Protein Report

Protein 5: keratin, type II cytoskeletal 6A [Homo sapiens]

**Accession:**	gi|5031839	**Score:**	566.1
**Database:**	refseq_human(refseq_human_20140103.fasta)	**MW [kDa]:**	60.0
**Database Date:**	2015-11-30	**pI:**	8.9
**Modification(s):**	Carbamidomethyl	**Sequence Coverage [%]:**	27.1
		**No. of unique Peptides:**	13

Quantitation

Q:W	**Median:** 0.58	**CV:** 111.72 %	**No. of Peptides:** 8
M:Q	**Median:** 0.71	**CV:** 109.19 %	**No. of Peptides:** 9

10	20	30	40	50	60	70	80
MASTSTTIRS	HSSSRRGFSA	NSARLPGVSR	SGFSSVSVSR	SRGSGGLGGA	CGGAGFGSRS	LYGLGGSKRI	SIGGGSCAIS
**90**	**100**	**110**	**120**	**130**	**140**	**150**	**160**
GGYGSRAGGS	YGFGGAGSGF	GFGGGAGIGF	GLGGGAGLAG	GFGGPGFPVC	PPGGIQEVTV	NQSLLTPLNL	QIDPTIQRVR
**170**	**180**	**190**	**200**	**210**	**220**	**230**	**240**
AEEREQIKTL	NNKFASFIDK	VRFLEQQNKV	LETKWTLLQE	QGTKTVRQNL	EPLFEQYINN	LRRQLDSIVG	ERGRLDSELR
**250**	**260**	**270**	**280**	**290**	**300**	**310**	**320**
GMQDLVEDFK	NKYEDEINKR	TAAENEFVTL	KKDVDAAYMN	KVELQAKADT	LTDEINFLRA	LYDAELSQMQ	THISDTSVVL
**330**	**340**	**350**	**360**	**370**	**380**	**390**	**400**
SMDNNRNLDL	DSIIAEVKAQ	YEEIAQRSRA	EAESWYQTKY	EELQVTAGRH	GDDLRNTKQE	IAEINRMIQR	LRSEIDHVKK
**410**	**420**	**430**	**440**	**450**	**460**	**470**	**480**
QCANLQAAIA	DAEQRGEMAL	KDAKNKLEGL	EDALQKAKQD	LARLLKEYQE	LMNVKLALDV	EIATYRKLLE	GEECRLNGEG
**490**	**500**	**510**	**520**	**530**	**540**	**550**	**560**
VGQVNISVVQ	STVSSGYGGA	SGVGSGLGLG	GGSSYSYGSG	LGVGGGFSSS	SGRAIGGGLS	SVGGGSSTIK	YTTTSSSSRK
**570**							
SYKH							

Cmpd.	No. of Cmpds.	m/z meas.	Δ m/z [ppm]	z	Rt [min]	Score	P	Range	Sequence	Modification	Ratios	
152	2	712.8348	20.14	2	31.9	14.7	0	43-59	R.GSGGLGGACGGAGFGSR.S	Carbamidomethyl: 9	Q:W	0.10
470	1	799.8881	6.12	2	35.9	18.2	0	70-86	R.ISIGGGSCAISGGYGSR.A	Carbamidomethyl: 8	Q:W<br>M:Q	0.57<br>0.92
910	12	602.3097	-20.53	2	39.9	44.2	0	195-204	K.WTLLQEQGTK.T		Q:W<br>M:Q	0.85<br>0.72
2908	9	945.9990	10.55	2	65.1	35.8	0	208-222	R.QNLEPLFEQYINNLR.R			
2813	2	682.9825	-67.79	3	65.6	26.1	1	208-223	R.QNLEPLFEQYINNLRR.Q			
1215	2	591.1296	-252.65	2	44.9	47.4	0	241-250	R.GMQDLVEDFK.N		M:Q	2.16
328	3	675.8743	11.03	2	34.1	67.2	1	261-272	R.TAAENEFVTLKK.D		M:Q<br>Q:W	1.09<br>0.30
1975	19	704.2826	-108.99	2	55.1	74.7	0	288-299	K.ADTLTDEINFLR.A		Q:W	0.45
2923	33	665.3592	-11.18	2	65.3	86.6	0	327-338	R.NLDLDSIIAEVK.A		Q:W<br>M:Q	1.26<br>1.09
145	7	583.2816	-24.68	2	31.1	63.8	0	360-369	K.YEELQVTAGR.H		Q:W<br>M:Q	0.62<br>0.90
303	1	577.1338	-255.87	2	33.1	22.3	0	447-455	K.EYQELMNVK.L		M:Q	1.02
2191	15	632.2117	-219.91	2	56.0	48.4	0	456-466	K.LALDVEIATYR.K		M:Q<br>Q:W	0.34<br>2.61
359	1	724.3637	-37.90	2	33.6	16.6	0	534-550	R.AIGGGLSSVGGGSSTIK.Y		M:Q	0.08

Detailed Protein Report

Protein 6: hemoglobin subunit delta [Homo sapiens]

Accession: gi|4504351
Database: refseq_human(refseq_human_20140103.fasta)
Database Date: 2015-11-30
Modification(s): Carbamidomethyl, Oxidation

Score: 556.3
MW [kDa]: 16.0
pI: 9.1
Sequence Coverage [%]: 70.1
No. of unique Peptides: 3

Quantitation

Q:W	**Median:**	5.16	**CV:**	0.00 %	**No. of Peptides:**	1
M:Q	**Median:**	0.88	**CV:**	0.00 %	**No. of Peptides:**	1

```
        10         20         30         40         50         60         70         80
MVHLTPEEKT AVNALWGKVN VDAVGGEALG RLLVVYPWTQ RFFESFGDLS SPDAVMGNPK VKAHGKKVLG AFSDGLAHLD
        90        100        110        120        130        140        150
NLKGTFSQLS ELHCDKLHVD PENFRLLGNV LVCVLARNFG KEFTPQMQAA YQKVVAGVAN ALAHKYH
```

Cmpd.	No. of Cmpds.	m/z meas.	Δ m/z [ppm]	z	Rt [min]	Score	P	Range	Sequence	Modification	Ratios
467	1	628.8213	-19.13	2	35.8	42.8	0	19-31	K.VNVDAVGGEALGR.L		M:Q 0.88<br>Q:W 5.16
2232	1	1030.8450	-118.44	2	56.4	33.3	0	42-60	R.FFESFGDLSSPDAVMGNPK.V	Oxidation: 15	
1086	2	761.3593	7.32	2	42.3	31.4	0	84-96	K.GTFSQLSELHCDK.L	Carbamidomethyl: 11	

Detailed Protein Report

Protein 7: serotransferrin precursor [Homo sapiens]

Accession: gi|4557871
Database: refseq_human(refseq_human_20140103.fasta)
Database Date: 2015-11-30
Modification(s): Carbamidomethyl

Score: 547.8
MW [kDa]: 77.0
pI: 7.0
Sequence Coverage [%]: 26.8
No. of unique Peptides: 15

Quantitation

Q:W	**Median:**	1.14	**CV:**	64.30 %	**No. of Peptides:**	11
M:Q	**Median:**	0.79	**CV:**	31.49 %	**No. of Peptides:**	11

10	20	30	40	50	60	70	80
MRLAVGALLV	CAVLGLCLAV	PDKTVRWCAV	SEHEATKCQS	FRDHMKSVIP	SDGPSVACVK	KASYLDCIRA	IAANEADAVT
90	100	110	120	130	140	150	160
LDAGLVYDAY	LAPNNLKPVV	AEFYGSKEDP	QTFYYAVAVV	KKDSGFQMNQ	LRGKKSCHTG	LGRSAGWNIP	IGLLYCDLPE
170	180	190	200	210	220	230	240
PRKPLEKAVA	NFFSGSCAPC	ADGTDFPQLC	QLCPGCGCST	LNQYFGYSGA	FKCLKDGAGD	VAFVKHSTIF	ENLANKADRD
250	260	270	280	290	300	310	320
QYELLCLDNT	RKPVDEYKDC	HLAQVPSHTV	VARSMGGKED	LIWELLNQAQ	EHFGKDKSKE	FQLFSSPHGK	DLLFKDSAHG
330	340	350	360	370	380	390	400
FLKVPPRMDA	KMYLGYEYVT	AIRNLREGTC	PEAPTDECKP	VKWCALSHHE	RLKCDEWSVN	SVGKIECVSA	ETTEDCIAKI
410	420	430	440	450	460	470	480
MNGEADAMSL	DGGFVYIAGK	CGLVPVLAEN	YNKSDNCEDT	PEAGYFAVAV	VKKSASDLTW	DNLKGKKSCH	TAVGRTAGWN
490	500	510	520	530	540	550	560
IPMGLLYNKI	NHCRFDEFFS	EGCAPGSKKD	SSLCKLCMGS	GLNLCEPNNK	EGYYGYTGAF	RCLVEKGDVA	FVKHQTVPQN
570	580	590	600	610	620	630	640
TGGKNPDPWA	KNLNEKDYEL	LCLDGTRKPV	EEYANCHLAR	APNHAVVTRK	DKEACVHKIL	RQQQHLFGSN	VTDCSGNFCL
650	660	670	680	690	700		
FRSETKDLLF	RDDTVCLAKL	HDRNTYEKYL	GEEYVKAVGN	LRKCSTSSLL	EACTFRRP		

Cmpd.	No. of Cmpds.	m/z meas.	Δ m/z [ppm]	z	Rt [min]	Score	P	Range	Sequence	Modification	Ratios
610	3	708.3500	-19.16	2	36.2	66.2	0	47-60	K.SVIPSDGPSVACVK.K	Carbamidomethyl: 12	M:Q 0.81<br>Q:W 0.97
1759	1	815.3781	-41.02	2	50.7	25.1	0	108-121	K.EDPQTFYYAVAVVK.K		
431	3	637.3141	-25.64	2	35.0	51.8	0	226-236	K.HSTIFENLANK.A		M:Q 0.89<br>Q:W 0.97
302	1	850.4388	3.54	3	33.8	10.5	1	252-273	R.<br>KPVDEYKDCHLAQVPSHTVVAR.<br>S	Carbamidomethyl: 9	Q:W 1.85
498	1	746.3968	18.25	2	36.2	42.7	1	298-310	K.SKEFQLFSSPHGK.D		
678	4	638.8051	-22.74	2	38.0	31.2	0	300-310	K.EFQLFSSPHGK.D		Q:W 2.38<br>M:Q 1.01
172	4	761.3492	-29.85	2	31.0	78.7	1	372-384	R.LKCDEWSVNSVGK.I	Carbamidomethyl: 3	M:Q 0.63<br>Q:W 1.43
255	1	863.4074	23.48	2	33.2	14.5	0	385-399	K.IECVSAETTEDCIAK.I	Carbamidomethyl: 3, 12	M:Q 0.81<br>Q:W 0.94
708	2	625.2611	-72.75	2	37.7	48.9	0	454-464	K.SASDLTWDNLK.G		Q:W 2.05<br>M:Q 0.61
817	2	789.3225	-12.58	2	39.0	49.5	0	495-508	R.FDEFFSEGCAPGSK.K	Carbamidomethyl: 9	M:Q 0.99<br>Q:W 0.79
840	1	853.8816	-5.85	2	40.5	22.5	0	516-530	K.LCMGSGLNLCEPNNK.E	Carbamidomethyl: 2, 10	M:Q 0.75<br>Q:W 0.43
448	1	642.1562	-205.50	2	34.1	34.3	0	531-541	K.EGYYGYTGAFR.C		M:Q 1.53<br>Q:W 0.45
1439	1	783.3662	-47.33	2	46.8	10.9	1	647-659	K.DLLFRDDTVCLAK.L	Carbamidomethyl: 10	
232	3	500.6172	-271.08	2	32.1	36.9	0	669-676	K.YLGEEYVK.A		Q:W 2.44<br>M:Q 0.45
915	2	766.3408	-8.88	2	40.2	24.0	0	684-696	K.CSTSSLLEACTFR.R	Carbamidomethyl: 1, 10	M:Q 0.66

Detailed Protein Report

Protein 8: PREDICTED: titin isoform X6 [Homo sapiens]

**Accession:**	gi|578804834	**Score:**	475.7
**Database:**	refseq_human(refseq_human_20140103.fasta)	**MW [kDa]:**	3773.1
**Database Date:**	2015-11-30	**pI:**	6.0
**Modification(s):**	Carbamidomethyl, Oxidation	**Sequence Coverage [%]:**	1.4
		**No. of unique Peptides:**	35

Quantitation

Q:W	**Median:**	0.90	**CV:**	62.64 %	**No. of Peptides:**	5
M:Q	**Median:**	0.75	**CV:**	44.89 %	**No. of Peptides:**	8

Detailed Protein Report

10	20	30	40	50	60	70	80
MTTQAPTFTQ	PLQSVVVLEG	STATFEAHIS	GFPVPEVSWF	RDGQVISTST	LPGVQISFSD	GRAKLTIPAV	TKANSGRYSL
90	100	110	120	130	140	150	160
KATNGSGQAT	STAELLVKAE	TAPPNFVQRL	QSMTVRQGSQ	VRLQVRVTGI	PTPVVKFYRD	GAEIQSSLDF	QISQEGDLYS
170	180	190	200	210	220	230	240
LLIAEAYPED	SGTYSVNATN	SVGRATSTAE	LLVQGEEEVP	AKKTKTIVST	AQISESRQTR	IEKKIEAHFD	ARSIATVEMV
250	260	270	280	290	300	310	320
IDGAAGQQLP	HKTPPRIPPK	PKSRSPTPPS	IAAKAQLARQ	QSPSPIRHSP	SPVRHVRAPT	PSPVRSVSPA	ARISTSPIRS
330	340	350	360	370	380	390	400
VRSPLLMRKT	QASTVATGPE	VPPPWKQEGY	VASSSEAEMR	ETTLTTSTQI	RTEERWEGRY	GVQEQVTISG	AAGAAASVSA
410	420	430	440	450	460	470	480
SASYAAEAVA	TGAKEVKQDA	DKSAAVATVV	AAVDMARVRE	PVISAVEQTA	QRTTTTAVHI	QPAQEQQVRK	EAEKTAVTKV
490	500	510	520	530	540	550	560
VVAADKAKEQ	ELKSRTKEVI	TTKQEQMHVT	HEQIRKETEK	TFVPKVVISA	AKAKEQETRI	SEEITKKQKQ	VTQEAIRQET
570	580	590	600	610	620	630	640
EITAASMVVV	ATAKSTKLET	VPGAQEETTT	QQDQMHLSYE	KIMKETRKTV	VPKVIVATPK	VKEQDLVSRG	REGITTKREQ
650	660	670	680	690	700	710	720
VQITQEKMRK	EAEKTALSTI	AVATAKAKEQ	ETILRTRETM	ATRQEQIQVT	HGKVDVGKKA	EAVATVVAAV	DQARVREPRE
730	740	750	760	770	780	790	800
PGHLEESYAQ	QTTLEYGYKE	RISAAKVAEP	PQRPASEPHV	VPKAVKPRVI	QAPSETHIKT	TDQKGMHISS	QIKKTTDLTT
810	820	830	840	850	860	870	880
ERLVHVDKRP	RTASPHFTVS	KISVPKTEHG	YEASIAGSAI	ATLQKELSAT	SSAQKITKSV	KAPTVKPSET	RVRAEPTPLP
890	900	910	920	930	940	950	960
QFPFADTPDT	YKSEAGVEVK	KEVGVSITGT	TVREERFEVL	HGREAKVTET	ARVPAPVEIP	VTPPTLVSGL	KNVTVIEGES
970	980	990	1000	1010	1020	1030	1040
VTLECHISGY	PSPTVTWYRE	DYQIESSIDF	QITFQSGIAR	LMIREAFAED	SGRFTCSAVN	EAGTVSTSCY	LAVQVSEEFE
1050	1060	1070	1080	1090	1100	1110	1120
KETTAVTEKF	TTEEKRFVES	RDVVMTDTSL	TEEQAGPGEP	AAPYFITKPV	VQKLVEGGSV	VFGCQVGGNP	KPHVYWKKSG
1130	1140	1150	1160	1170	1180	1190	1200
VPLTTGYRYK	VSYNKQTGEC	KLVISMTFAD	DAGEYTIVVR	NKHGETSASA	SLLEEADYEL	LMKSQQEMLY	QTQVTAFVQE
1210	1220	1230	1240	1250	1260	1270	1280
PKVGETAPGF	VYSEYEKEYE	KEQALIRKKM	AKDTVVVRTY	VEDQEFHISS	FEERLIKEIE	YRIIKTTLEE	LLEEDGEEKM
1290	1300	1310	1320	1330	1340	1350	1360
AVDISESEAV	ESGFDSRIKN	YRILEGMGVT	FHCKMSGYPL	PKIAWYKDGK	RIKHGERYQM	DFLQDGRASL	RIPVVLPEDE
1370	1380	1390	1400	1410	1420	1430	1440
GIYTAFASNI	KGNAICSGKL	YVEPAAPLGA	PTYIPTLEPV	SRIRSLSPRS	VSRSPIRMSP	ARMSPARMSP	ARMSPARMSP
1450	1460	1470	1480	1490	1500	1510	1520
GRRLEETDES	QLERLYKPVF	VLKPVSFKCL	EGQTARFDLK	VVGRPMPETF	WFHDGQQIVN	DYTHKVVIKE	DGTQSLIIVP
1530	1540	1550	1560	1570	1580	1590	1600
ATPSDSGEWT	VVAQNRAGRS	SISVILTVEA	VEHQVKPMFV	EKLKNVNIKE	GSRLEMKVRA	TGNPNPDIVW	LKNSDIIVPH
1610	1620	1630	1640	1650	1660	1670	1680
KYPKIRIEGT	KGEAALKIDS	TVSQDSAWYT	ATAINKAGRD	TTRCKVNVEV	EFAEPEPERK	LIIPRGTYRA	KEIAAPELEP
1690	1700	1710	1720	1730	1740	1750	1760
LHLRYGQEQW	EEGDLYDKEK	QQKPFFKKKL	TSLRLKRFGP	AHFECRLTPI	GDPTMVVEWL	HDGKPLEAAN	RLRMINEFGY
1770	1780	1790	1800	1810	1820	1830	1840
CSLDYGVAYS	RDSGIITCRA	TNKYGTDHTS	ATLIVKDEKS	LVEESQLPEG	RKGLQRIEEL	ERMAHEGALT	GVTTDQKEKQ
1850	1860	1870	1880	1890	1900	1910	1920
KPDIVLYPEP	VRVLEGETAR	FRCRVTGYPQ	PKVNWYLNGQ	LIRKSKRFRV	RYDGIHYLDI	VDCKSYDTGE	VKVTAENPEG
1930	1940	1950	1960	1970	1980	1990	2000
VIEHKVKLEI	QQREDFRSVL	RRAPEPRPEF	HVHEPGKLQF	EVQKVDRPVD	TTETKEVVKL	KRAERITHEK	VPEESEELRS
2010	2020	2030	2040	2050	2060	2070	2080
KFKRRTEEGY	YEAITAVELK	SRKKDESYEE	LLRKTKDELL	HWTKELTEEE	KKALAEEGKI	TIPTFKPDKI	ELSPSMEAPK
2090	2100	2110	2120	2130	2140	2150	2160
IFERIQSQTV	GQGSDAHFRV	RVVGKPDPEC	EWYKNGVKIE	RSDRIYWYWP	EDNVCELVIR	DVTAEDSASI	MVKAINIAGE
2170	2180	2190	2200	2210	2220	2230	2240

Detailed Protein Report

Cmpd.	No. of Cmpds.	m/z meas.	Δ m/z [ppm]	z	Rt [min]	Score	P	Range	Sequence	Modification	Ratios
1676	1	839.3937	-10.73	2	49.5	10.3	2	1418-1432	R.MSPARMSPARMSPAR.M	Oxidation: 6, 11	
619	1	690.3860	77.00	2	37.3	10.5	0	5395-5407	R.VTNEVGSSICSAR.V	Carbamidomethyl: 10	M:Q 0.78
2868	1	805.9212	74.64	2	66.3	17.2	0	6519-6533	K.AQNEVGSDTCVCTVK.L	Carbamidomethyl: 10	
1459	1	851.5485	120.85	2	48.0	14.8	0	7111-7125	K.DIEQTVGLPVTLTCR.L	Carbamidomethyl: 14	
2735	1	1045.0652	51.04	2	64.6	11.1	1	7556-7574	K.NDAGMRECSAVLTVLEPAR.I	Carbamidomethyl: 8	
1761	1	785.8716	-131.54	2	51.8	13.2	2	10202-10215	K.IHVAISKRVEPPPK.V		Q:W 1.20
967	1	752.8772	-83.84	2	40.6	12.6	2	10536-10548	K.VPEEPKKPVPEKK.V		
1296	2	640.2561	-89.77	2	45.9	12.9	1	10647-10656	K.EVTIMEEKER.A	Oxidation: 5	Q:W 0.94
2926	1	713.8984	-28.68	2	65.7	11.1	1	11136-11148	K.VEPPPPPKVPEIK.K		
1495	1	620.4184	75.49	3	48.4	11.8	2	11427-11443	K.GPEISEKIIPPKKPPTK.V		
1106	15	555.2011	-153.95	2	43.9	16.9	0	11876-11886	K.GTAIFACDIAK.D		
2424	1	822.3339	-55.35	2	60.7	10.9	2	12942-12954	R.EKEMARFECELSR.E	Oxidation: 4	
2695	1	805.4194	19.35	2	64.9	15.3	2	12948-12960	R.FECELSRENAKVK.W	Carbamidomethyl: 3	
1925	1	661.3041	-146.74	2	54.4	13.5	1	13185-13196	R.AAAHLTVIEKLR.I		M:Q 0.86<br>Q:W 0.89
958	15	481.1097	-309.72	2	41.5	15.0	0	13690-13697	K.TWVLATDR.A		M:Q 0.52
1842	1	740.3191	-71.84	2	53.4	15.5	2	14176-14188	K.GYIVERCPRGSDK.W		M:Q 1.17
1108	1	540.7821	22.96	2	42.5	15.6	1	15620-15628	K.EIREGADYK.L		M:Q 0.71
2888	1	900.4707	-22.65	2	64.8	13.7	0	15990-16005	K.NETVIEKPTDALQITK.E		
2737	1	885.4690	-162.81	1	64.6	11.5	1	16573-16580	K.LKIGLITK.N		
2512	6	813.3358	-60.53	2	61.3	13.8	0	17628-17640	R.VPDLLEGCQYEFR.V	Carbamidomethyl: 8	
2371	2	805.2633	-115.33	2	60.0	12.7	0	26736-26748	R.EEEPLFDIDSEMR.K		
1812	1	658.3022	-97.61	2	52.7	11.3	0	27840-27851	K.AGEDVQVLIPFK.G		
2869	1	710.3695	-29.92	2	65.0	10.9	0	28132-28143	R.IGHNVHLELPYK.G		
2745	1	656.8669	67.42	2	62.7	10.9	0	28981-28992	K.YTLTVENNSGSK.S		
69	1	731.7932	-168.95	2	30.8	10.2	1	29155-29166	K.QLTFTVERLVEK.T		
2485	1	862.3731	-106.42	2	61.5	10.8	0	29616-29633	R.AGSDLVLDAAVGGKPEPK.I		
1065	4	515.1664	-172.63	2	41.8	12.4	0	29692-29701	K.VLDSPGPCGK.L	Carbamidomethyl: 8	M:Q 0.75<br>Q:W 0.32
560	1	607.8375	45.47	2	37.0	10.1	1	30266-30276	R.AQNEKGVSEPR.E		M:Q 1.43
98	1	529.8207	-183.86	3	30.5	11.1	1	30910-30923	K.DDGGSRVTGYYIER.K		
2686	1	937.4503	-21.14	2	62.5	12.9	0	31044-31059	K.QFTIGGLLEATEYEFR.V		
658	2	677.6643	-16.40	3	38.2	11.8	2	31650-31667	K.DAGFYVVCAKNRFGIDQK.T		
2593	1	616.1731	-223.16	2	60.7	11.0	1	32525-32534	R.MPYDVPEPRK.Y		
2811	1	1052.5183	-55.98	2	65.6	16.6	0	32829-32846	R.SLSPTYIELMRPVSELIR.S		
27	2	731.6483	-99.39	3	29.3	11.9	2	33357-33375	K.TNLQFMGQAFKSIHEKVSK.I		M:Q 0.32<br>Q:W 1.80
1561	1	745.7829	-73.36	2	49.4	15.1	0	33469-33482	K.TDTSDSGLYTCTVK.N		

Detailed Protein Report

Protein 9: hemoglobin subunit alpha [Homo sapiens]

Accession: gi|4504345
Database: refseq_human(refseq_human_20140103.fasta)
Database Date: 2015-11-30
Modification(s): Oxidation

Score: 472.8
MW [kDa]: 15.2
pI: 9.4
Sequence Coverage [%]: 50.7
No. of unique Peptides: 8

Quantitation

Q:W	**Median:** 2.26	**CV:** 109.95 %	**No. of Peptides:**	7
M:Q	**Median:** 1.12	**CV:** 16.19 %	**No. of Peptides:**	4

Alias proteins:

Accession	Name	Description
gi|4504347	refseq_human (refseq_human_20140103.fasta)	hemoglobin subunit alpha [Homo sapiens]

```
        10         20         30         40         50         60         70         80
MVLSPADKTN VKAAWGKVGA HAGEYGAEAL ERMFLSFPTT KTYFPHFDLS HGSAQVKGHG KKVADALTNA VAHVDDMPNA
        90        100        110        120        130        140        150
LSALSDLHAH KLRVDPVNFK LLSHCLLVTL AAHLPAEFTP AVHASLDKFL ASVSTVLTSK YR
```

Cmpd.	No. of Cmpds.	m/z meas.	Δ m/z [ppm]	z	Rt [min]	Score	P	Range	Sequence	Modification	Ratios	
4	1	729.4098	-5.99	1	29.0	28.6	0	2-8	M.VLSPADK.T			
39	3	586.3399	3.78	2	29.7	55.0	1	2-12	M.VLSPADKTNVK.A		Q:W	0.98
111	20	765.3757	6.39	2	30.7	80.1	0	18-32	K.VGAHAGEYGAEALER.M		Q:W<br>M:Q	2.13<br>0.92
1126	3	544.1344	-264.30	2	42.3	39.7	0	33-41	R.MFLSFPTTK.T	Oxidation: 1	M:Q<br>Q:W	1.23<br>4.22
1845	62	536.1590	-227.09	2	51.4	57.9	0	33-41	R.MFLSFPTTK.T		Q:W	0.41
1032	16	917.4300	-21.34	2	41.7	79.5	0	42-57	K.TYFPHFDLSHGSAQVK.G		M:Q<br>Q:W	1.01<br>3.01
592	6	544.3095	-13.04	2	37.4	51.7	1	92-100	K.LRVDPVNFK.L		Q:W	4.58
1648	52	626.8403	-33.03	2	48.9	80.4	0	129-140	K.FLASVSTVLTSK.Y		M:Q<br>Q:W	1.38<br>6.08

Detailed Protein Report

Protein 10: alpha-2-macroglobulin precursor [Homo sapiens]

Accession: gi|66932947
Database: refseq_human(refseq_human_20140103.fasta)
Database Date: 2015-11-30
Modification(s): Carbamidomethyl

Score: 452.3
MW [kDa]: 163.2
pI: 6.0
Sequence Coverage [%]: 13.8
No. of unique Peptides: 15

Quantitation

Q:W	**Median:**	1.35	**CV:**	22.47 %	**No. of Peptides:**	7
M:Q	**Median:**	0.77	**CV:**	33.36 %	**No. of Peptides:**	9

10	20	30	40	50	60	70	80
MGKNKLLHPS	LVLLLLVLLP	TDASVSGKPQ	YMVLVPSLLH	TETTEKGCVL	LSYLNETVTV	SASLESVRGN	RSLFTDLEAE
90	100	110	120	130	140	150	160
NDVLHCVAFA	VPKSSSNEEV	MFLTVQVKGP	TQEFKKRTTV	MVKNEDSLVF	VQTDKSIYKP	GQTVKFRVVS	MDENFHPLNE
170	180	190	200	210	220	230	240
LIPLVYIQDP	KGNRIAQWQS	FQLEGGLKQF	SFPLSSEPFQ	GSYKVVVQKK	SGGRTEHPFT	VEEFVLPKFE	VQVTVPKIIT
250	260	270	280	290	300	310	320
ILEEEMNVSV	CGLYTYGKPV	PGHVTVSICR	KYSDASDCHG	EDSQAFCEKF	SGQLNSHGCF	YQQVKTKVFQ	LKRKEYEMKL
330	340	350	360	370	380	390	400
HTEAQIQEEG	TVVELTGRQS	SEITRTITKL	SFVKVDSHFR	QGIPFFGQVR	LVDGKGVPIP	NKVIFIRGNE	ANYYSNATTD
410	420	430	440	450	460	470	480
EHGLVQFSIN	TTNVMGTSLT	VRVNYKDRSP	CYGYQWVSEE	HEEAHHTAYL	VFSPSKSFVH	LEPMSHELPC	GHTQTVQAHY
490	500	510	520	530	540	550	560
ILNGGTLLGL	KKLSFYYLIM	AKGGIVRTGT	HGLLVKQEDM	KGHFSISIPV	KSDIAPVARL	LIYAVLPTGD	VIGDSAKYDV
570	580	590	600	610	620	630	640
ENCLANKVDL	SFSPSQSLPA	SHAHLRVTAA	PQSVCALRAV	DQSVLLMKPD	AELSASSVYN	LLPEKDLTGF	PGPLNDQDDE
650	660	670	680	690	700	710	720
DCINRHNVYI	NGITYTPVSS	TNEKDMYSFL	EDMGLKAFTN	SKIRKPKMCP	QLQQYEMHGP	EGLRVGFYES	DVMGRGHARL
730	740	750	760	770	780	790	800
VHVEEPHTET	VRKYFPETWI	WDLVVVNSAG	VAEVGVTVPD	TITEWKAGAF	CLSEDAGLGI	SSTASLRAFQ	PFFVELTMPY
810	820	830	840	850	860	870	880
SVIRGEAFTL	KATVLNYLPK	CIRVSVQLEA	SPAFLAVPVE	KEQAPHCICA	NGRQTVSWAV	TPKSLGNVNF	TVSAEALESQ
890	900	910	920	930	940	950	960
ELCGTEVPSV	PEHGRKDTVI	KPLLVEPEGL	EKETTFNSLL	CPSGGEVSEE	LSLKLPPNVV	EESARASVSV	LGDILGSAMQ
970	980	990	1000	1010	1020	1030	1040
NTQNLLQMPY	GCGEQNMVLF	APNIYVLDYL	NETQQLTPEI	KSKAIGYLNT	GYQRQLNYKH	YDGSYSTFGE	RYGRNQGNTW
1050	1060	1070	1080	1090	1100	1110	1120
LTAFVLKTFA	QARAYIFIDE	AHITQALIWL	SQRQKDNGCF	RSSGSLLNNA	IKGGVEDEVT	LSAYITIALL	EIPLTVTHPV
1130	1140	1150	1160	1170	1180	1190	1200
VRNALFCLES	AWKTAQEGDH	GSHVYTKALL	AYAFALAGNQ	DKRKEVLKSL	NEEAVKKDNS	VHWERPQKPK	APVGHFYEPQ
1210	1220	1230	1240	1250	1260	1270	1280
APSAEVEMTS	YVLLAYLTAQ	PAPTSEDLTS	ATNIVKWITK	QQNAQGGFSS	TQDTVVALHA	LSKYGAATFT	RTGKAAQVTI
1290	1300	1310	1320	1330	1340	1350	1360
QSSGTFSSKF	QVDNNNRLLL	QQVSLPELPG	EYSMKVTGEG	CVYLQTSLKY	NILPEKEEFP	FALGVQTLPQ	TCDEPKAHTS
1370	1380	1390	1400	1410	1420	1430	1440
FQISLSVSYT	GSRSASNMAI	VDVKMVSGFI	PLKPTVKMLE	RSNHVSRTEV	SSNHVLIYLD	KVSNQTLSLF	FTVLQDVPVR
1450	1460	1470	1480				
DLKPAIVKVY	DYYETDEFAI	AEYNAPCSKD	LGNA				

Cmpd.	No. of Cmpds.	m/z meas.	Δ m/z [ppm]	z	Rt [min]	Score	P	Range	Sequence	Modification	Ratios
743	1	697.8370	-9.33	2	39.3	19.9	0	124-135	K.NEDSLVFVQTDK.S		
1683	8	802.8311	-117.18	2	49.7	41.2	0	175-188	R.IAQWQSFQLEGGLK.Q		Q:W 1.08<br>M:Q 0.99
2299	2	924.9110	-35.46	2	57.0	20.1	0	189-204	K.QFSFPLSSEPFQGSYK.V		Q:W 1.46<br>M:Q 0.77
2052	5	574.7391	-130.69	2	54.0	26.2	0	361-370	R.QGIPFFGQVR.L		M:Q 0.61

Detailed Protein Report

Cmpd.	No. of Cmpds.	m/z meas.	Δ m/z [ppm]	z	Rt [min]	Score	P	Range	Sequence	Modification	Ratios	
											Q:W	1.47
898	2	542.6752	-250.41	2	39.6	25.9	0	522-531	K.GHFSISIPVK.S		M:Q	0.90
2454	1	922.9898	-34.93	2	59.0	33.4	0	540-557	R.LLIYAVLPTGDVIGDSAK.Y			
276	2	636.8299	-16.03	2	33.4	30.1	0	587-598	R.VTAAPQSVCALR.A	Carbamidomethyl: 9	M:Q	0.90
1177	1	509.7828	-34.15	2	43.4	37.2	0	812-820	K.ATVLNYLPK.C		Q:W<br>M:Q	1.85<br>0.40
2461	3	942.5232	-4.52	2	59.1	21.9	0	824-841	R.VSVQLEASPAFLAVPVEK.E			
305	2	558.6600	-261.38	2	32.5	48.3	0	854-863	R.QTVSWAVTPK.S		Q:W<br>M:Q	1.74<br>0.57
234	2	605.7801	-74.07	2	32.3	35.4	0	935-945	K.LPPNVVEESAR.A		M:Q<br>Q:W	0.96<br>1.09
2462	1	669.8332	5.93	2	59.1	43.0	0	1123-1133	R.NALFCLESAWK.T	Carbamidomethyl: 5		
2729	1	783.4212	1.90	2	65.2	28.1	0	1148-1162	K.ALLAYAFALAGNQDK.R		M:Q<br>Q:W	1.23<br>1.02
1151	1	796.4102	8.78	3	43.1	23.4	0	1241-1263	K.QQNAQGGFSSTQDTVVALHALSKY			
891	2	809.4242	-4.39	2	39.9	18.3	0	1408-1421	R.TEVSSNHVLIYLDK.V			

Detailed Protein Report

Protein 11: keratin, type II cytoskeletal 5 [Homo sapiens]

Accession: gi|119395754
Database: refseq_human(refseq_human_20140103.fasta)
Database Date: 2015-11-30
Modification(s): Carbamidomethyl

Score: 433.7
MW [kDa]: 62.3
pI: 8.6
Sequence Coverage [%]: 17.5
No. of unique Peptides: 4

Quantitation

Q:W	**Median:** 0.41	**CV:** 48.83 %	**No. of Peptides:** 3
M:Q	**Median:** 0.66	**CV:** 17.74 %	**No. of Peptides:** 4

```
        10         20         30         40         50         60         70         80
MSRQSSVSFR SGGSRSFSTA SAITPSVSRT SFTSVSRSGG GGGGGFGRVS LAGACGVGGY GSRSLYNLGG SKRISISTSG
        90        100        110        120        130        140        150        160
GSFRNRFGAG AGGGYGFGGG AGSGFGFGGG AGGGFGLGGG AGFGGGFGGP GFPVCPPGGI QEVTVNQSLL TPLNLQIDPS
       170        180        190        200        210        220        230        240
IQRVRTEERE QIKTLNNKFA SFIDKVRFLE QQNKVLDTKW TLLQEQGTKT VRQNLEPLFE QYINNLRRQL DSIVGERGRL
       250        260        270        280        290        300        310        320
DSELRNMQDL VEDFKNKYED EINKRTTAEN EFVMLKKDVD AAYMNKVELE AKVDALMDEI NFMKMFFDAE LSQMQTHVSD
       330        340        350        360        370        380        390        400
TSVVLSMDNN RNLDLDSIIA EVKAQYEEIA NRSRTEAESW YQTKYEELQQ TAGRHGDDLR NTKHEISEMN RMIQRLRAEI
       410        420        430        440        450        460        470        480
DNVKKQCANL QNAIADAEQR GELALKDARN KLAELEEALQ KAKQDMARLL REYQELMNTK LALDVEIATY RKLLEGEECR
       490        500        510        520        530        540        550        560
LSGEGVGPVN ISVVTSSVSS GYGSGSGYGG GLGGGLGGGL GGGLAGGSSG SYYSSSSGGV GLGGGLSVGG SGFSASSGRG
       570        580        590        600
LGVGFGSGGG SSSSVKFVST TSSSRKSFKS
```

Cmpd.	No. of Cmpds.	m/z meas.	Δ m/z [ppm]	z	Rt [min]	Score	P	Range	Sequence	Modification	Ratios
419	1	705.9605	130.39	2	34.6	27.4	1	266-277	R.TTAENEFVMLKK.D		M:Q 0.89
553	2	851.4187	19.30	2	36.2	57.4	0	406-420	K.QCANLQNAIADAEQR.G	Carbamidomethyl: 2	Q:W 0.48<br>M:Q 0.64
804	1	572.1668	-261.37	2	38.5	48.3	0	432-441	K.LAELEEALQK.A		M:Q 0.56<br>Q:W 0.22
334	4	720.3453	-20.22	2	33.5	59.4	0	560-576	R.GLGVGFGSGGGSSSSVK.F		Q:W 0.66<br>M:Q 0.61

Detailed Protein Report

Protein 12: keratin, type I cytoskeletal 14 [Homo sapiens]

**Accession:**	gi|15431310	**Score:**	405.3
**Database:**	refseq_human(refseq_human_20140103.fasta)	**MW [kDa]:**	51.6
**Database Date:**	2015-11-30	**pI:**	4.9
**Modification(s):**	Oxidation	**Sequence Coverage [%]:**	22.0
		**No. of unique Peptides:**	7

Quantitation

Q:W	**Median:**	0.66	**CV:**	73.93 %	**No. of Peptides:**	2
M:Q	**Median:**	1.31	**CV:**	77.01 %	**No. of Peptides:**	5

```
        10         20         30         40         50         60         70         80
MTTCSRQFTS SSSMKGSCGI GGGIGGGSSR ISSVLAGGSC RAPSTYGGGL SVSSSRFSSG GAYGLGGGYG GGFSSSSSSF
        90        100        110        120        130        140        150        160
GSGFGGGYGG GLGAGLGGGF GGGFAGGDGL LVGSEKVTMQ NLNDRLASYL DKVRALEEAN ADLEVKIRDW YQRQRPAEIK
       170        180        190        200        210        220        230        240
DYSPYFKTIE DLRNKILTAT VDNANVLLQI DNARLAADDF RTKYETELNL RMSVEADING LRRVLDELTL ARADLEMQIE
       250        260        270        280        290        300        310        320
SLKEELAYLK KNHEEEMNAL RGQVGGDVNV EMDAAPGVDL SRILNEMRDQ YEKMAEKNRK DAEEWFFTKT EELNREVATN
       330        340        350        360        370        380        390        400
SELVQSGKSE ISELRRTMQN LEIELQSQLS MKASLENSLE ETKGRYCMQL AQIQEMIGSV EEQLAQLRCE MEQQNQEYKI
       410        420        430        440        450        460        470        480
LLDVKTRLEQ EIATYRRLLE GEDAHLSSSQ FSSGSQSSRD VTSSSRQIRT KVMDVHDGKV VSTHEQVLRT KN
```

Cmpd.	No. of Cmpds.	m/z meas.	Δ m/z [ppm]	z	Rt [min]	Score	P	Range	Sequence	Modification	Ratios	
121	3	713.3373	-20.71	2	30.8	66.0	0	42-56	R.APSTYGGGLSVSSSR.F		M:Q<br>Q:W	1.86<br>0.34
296	1	532.8004	-15.38	2	33.7	58.7	1	126-134	R.LASYLDKVR.A		M:Q	3.67
282	1	633.8578	31.13	2	33.5	20.5	1	202-211	R.TKYETELNLR.M			
639	1	680.8203	-49.04	2	36.6	15.5	1	212-223	R.MSVEADINGLRR.V		M:Q	0.84
1899	3	586.6929	-125.55	2	52.2	33.2	0	301-309	K.DAEEWFFTK.T		M:Q	1.36
1027	2	636.9803	-7.37	3	41.6	10.2	0	337-352	R.TMQNLEIELQSQLSMK.A	Oxidation: 15	Q:W<br>M:Q	1.28<br>0.49
246	2	512.5147	-194.60	3	33.1	22.6	2	406-417	K.TRLEQEIATYRR.L			

Detailed Protein Report

Protein 13: annexin A1 [Homo sapiens]

Accession: gi|4502101
Database: refseq_human(refseq_human_20140103.fasta)
Database Date: 2015-11-30
Modification(s): Carbamidomethyl, Oxidation

Score: 346.8
MW [kDa]: 38.7
pI: 6.7
Sequence Coverage [%]: 35.5
No. of unique Peptides: 9

Quantitation

Q:W	**Median:**	0.47	**CV:**	50.31 %	**No. of Peptides:**	5
M:Q	**Median:**	1.20	**CV:**	24.24 %	**No. of Peptides:**	4

```
        10         20         30         40         50         60         70         80
MAMVSEFLKQ AWFIENEEQE YVQTVKSSKG GPGSAVSPYP TFNPSSDVAA LHKAIMVKGV DEATIIDILT KRNNAQRQQI
        90        100        110        120        130        140        150        160
KAAYLQETGK PLDETLKKAL TGHLEEVVLA LLKTPAQFDA DELRAAMKGL GTDEDTLIEI LASRTNKEIR DINRVYREEL
       170        180        190        200        210        220        230        240
KRDLAKDITS DTSGDFRNAL LSLAKGDRSE DFGVNEDLAD SDARALYEAG ERRKGTDVNV FNTILTTRSY PQLRRVFQKY
       250        260        270        280        290        300        310        320
TKYSKHDMNK VLDLELKGDI EKCLTAIVKC ATSKPAFFAE KLHQAMKGVG TRHKALIRIM VSRSEIDMND IKAFYQKMYG
       330        340        350
ISLCQAILDE TKGDYEKILV ALCGGN
```

Cmpd.	No. of Cmpds.	m/z meas.	Δ m/z [ppm]	z	Rt [min]	Score	P	Range	Sequence	Modification	Ratios	
1254	2	786.0316	-32.49	3	45.3	18.2	0	30-53	K.GGPGSAVSPYPTFNPSSDVAALHA		M:Q<br>Q:W	0.93<br>0.53
2994	1	973.5095	-39.14	2	66.2	10.2	1	54-71	K.AIMVKGVDEATIIDILTK.R	Oxidation: 3		
2228	1	694.3075	-115.27	2	56.3	17.4	0	59-71	K.GVDEATIIDILTK.R		M:Q	1.76
537	1	635.6517	-48.42	3	35.3	22.1	1	82-98	K.AAYLQETGKPLDETLKK.A			
422	2	631.7779	-41.67	2	35.3	82.8	0	114-124	K.TPAQFDADELR.A		M:Q<br>Q:W	1.21<br>0.55
2959	6	851.9284	-21.25	2	65.8	81.3	0	129-144	K.GLGTDEDTLIEILASR.T			
174	2	607.2652	-8.28	2	30.9	68.2	0	167-177	K.DITSDTSGDFR.N		Q:W	0.21
2388	2	775.8834	-37.22	2	58.2	36.2	0	215-228	K.GTDVNVFNTILTTR.S		Q:W<br>M:Q	0.89<br>1.05
2020	1	678.7710	-93.49	2	55.4	10.5	0	270-281	K.CATSKPAFFAEK.L	Carbamidomethyl: 1	Q:W	0.42

Detailed Protein Report

Protein 14: alpha-1-antitrypsin precursor [Homo sapiens]

Accession: gi|50363217
Database: refseq_human(refseq_human_20140103.fasta)
Database Date: 2015-11-30

Score: 268.7
MW [kDa]: 46.7
pI: 5.3
Sequence Coverage [%]: 26.3
No. of unique Peptides: 6

Quantitation

Q:W	**Median:** 1.63	**CV:** 46.80 %	**No. of Peptides:**	3
M:Q	**Median:** 1.08	**CV:** 23.67 %	**No. of Peptides:**	4

Alias proteins:

Accession	Name	Description
gi|189163542	refseq_human (refseq_human_20140103.fasta)	alpha-1-antitrypsin precursor [Homo sapiens]
gi|189163540	refseq_human (refseq_human_20140103.fasta)	alpha-1-antitrypsin precursor [Homo sapiens]
gi|189163538	refseq_human (refseq_human_20140103.fasta)	alpha-1-antitrypsin precursor [Homo sapiens]
gi|189163536	refseq_human (refseq_human_20140103.fasta)	alpha-1-antitrypsin precursor [Homo sapiens]
gi|189163534	refseq_human (refseq_human_20140103.fasta)	alpha-1-antitrypsin precursor [Homo sapiens]
gi|189163532	refseq_human (refseq_human_20140103.fasta)	alpha-1-antitrypsin precursor [Homo sapiens]
gi|189163530	refseq_human (refseq_human_20140103.fasta)	alpha-1-antitrypsin precursor [Homo sapiens]
gi|189163528	refseq_human (refseq_human_20140103.fasta)	alpha-1-antitrypsin precursor [Homo sapiens]
gi|50363221	refseq_human (refseq_human_20140103.fasta)	alpha-1-antitrypsin precursor [Homo sapiens]
gi|50363219	refseq_human (refseq_human_20140103.fasta)	alpha-1-antitrypsin precursor [Homo sapiens]

```
        10         20         30         40         50         60         70         80
MPSSVSWGIL LLAGLCCLVP VSLAEDPQGD AAQKTDTSHH DQDHPTFNKI TPNLAEFAFS LYRQLAHQSN STNIFFSPVS
        90        100        110        120        130        140        150        160
IATAFAMLSL GTKADTHDEI LEGLNFNLTE IPEAQIHEGF QELLRTLNQP DSQLQLTTGN GLFLSEGLKL VDKFLEDVKK
       170        180        190        200        210        220        230        240
LYHSEAFTVN FGDTEEAKKQ INDYVEKGTQ GKIVDLVKEL DRDTVFALVN YIFFKGKWER PFEVKDTEEE DFHVDQVTTV
       250        260        270        280        290        300        310        320
KVPMMKRLGM FNIQHCKKLS SWVLLMKYLG NATAIFFLPD EGKLQHLENE LTHDIITKFL ENEDRRSASL HLPKLSITGT
       330        340        350        360        370        380        390        400
YDLKSVLGQL GITKVFSNGA DLSGVTEEAP LKLSKAVHKA VLTIDEKGTE AAGAMFLEAI PMSIPPEVKF NKPFVFLMIE
       410        420
QNTKSPLFMG KVVNPTQK
```

Cmpd.	No. of Cmpds.	m/z meas.	Δ m/z [ppm]	z	Rt [min]	Score	P	Range	Sequence	Modification	Ratios	
2521	1	821.3964	-47.50	2	61.5	24.3	0	50-63	K.ITPNLAEFAFSLYR.Q			
2910	3	858.7649	-23.64	3	65.6	35.3	0	126-149	R. TLNQPDSQLQLTTGNGLFLSEGLK L			
722	1	729.3434	-11.09	3	37.9	16.3	1	161-179	K.LYHSEAFTVNFGDTEEAKK.Q		M:Q<br>Q:W	1.41<br>1.18
762	1	601.9916	0.24	3	38.4	25.3	0	284-298	K.LQHLENELTHDIITK.F		Q:W<br>M:Q	3.06<br>1.05
904	7	555.7989	-12.32	2	39.8	70.1	0	315-324	K.LSITGTYDLK.S		M:Q	0.76
1068	4	508.1796	-258.32	2	42.1	41.2	0	325-334	K.SVLGQLGITK.V		M:Q<br>Q:W	1.24<br>1.21

Detailed Protein Report

Protein 15: actin, cytoplasmic 1 [Homo sapiens]

Accession: gi|4501885
Database: refseq_human(refseq_human_20140103.fasta)
Database Date: 2015-11-30

Score: 267.4
MW [kDa]: 41.7
pI: 5.2
Sequence Coverage [%]: 24.0
No. of unique Peptides: 6

Quantitation

Q:W	**Median:**	0.25	**CV:**	86.94 %	**No. of Peptides:**	5
M:Q	**Median:**	1.66	**CV:**	25.39 %	**No. of Peptides:**	4

10	20	30	40	50	60	70	80
MDDDIAALVV	DNGSGMCKAG	FAGDDAPRAV	FPSIVGRPRH	QGVMVGMGQK	DSYVGDEAQS	KRGILTLKYP	IEHGIVTNWD
**90**	**100**	**110**	**120**	**130**	**140**	**150**	**160**
DMEKIWHHTF	YNELRVAPEE	HPVLLTEAPL	NPKANREKMT	QIMFETFNTP	AMYVAIQAVL	SLYASGRTTG	IVMDSGDGVT
**170**	**180**	**190**	**200**	**210**	**220**	**230**	**240**
HTVPIYEGYA	LPHAILRLDL	AGRDLTDYLM	KILTERGYSF	TTTAEREIVR	DIKEKLCYVA	LDFEQEMATA	ASSSSLEKSY
**250**	**260**	**270**	**280**	**290**	**300**	**310**	**320**
ELPDGQVITI	GNERFRCPEA	LFQPSFLGME	SCGIHETTFN	SIMKCDVDIR	KDLYANTVLS	GGTTMYPGIA	DRMQKEITAL
**330**	**340**	**350**	**360**	**370**	**380**		
APSTMKIKII	APPERKYSVW	IGGSILASLS	TFQQMWISKQ	EYDESGPSIV	HRKCF		

Cmpd.	No. of Cmpds.	m/z meas.	Δ m/z [ppm]	z	Rt [min]	Score	P	Range	Sequence	Modification	Ratios	
659	5	599.7070	-249.11	2	36.8	51.4	0	29-39	R.AVFPSIVGRPR.H		M:Q<br>Q:W	1.63<br>0.30
1159	5	651.9980	-43.48	3	44.1	29.5	0	96-113	R.VAPEEHPVLLTEAPLNPK.A			
1678	9	895.8924	-63.80	2	49.5	48.9	0	239-254	K.SYELPDGQVITIGNER.F		M:Q<br>Q:W	1.12<br>0.51
2043	1	1107.8969	-127.87	2	54.1	33.7	0	292-312	K.DLYANTVLSGGTTMYPGIADR.M		M:Q<br>Q:W	1.89<br>0.15
563	1	581.2929	-34.36	2	37.0	51.0	0	316-326	K.EITALAPSTMK.I		Q:W	0.08
74	5	758.8545	-0.57	2	30.1	53.0	0	360-372	K.QEYDESGPSIVHR.K		Q:W<br>M:Q	0.55<br>2.20

Detailed Protein Report

Protein 16: keratin, type II cytoskeletal 75 [Homo sapiens]

Accession: gi|153791158
Database: refseq_human(refseq_human_20140103.fasta)
Database Date: 2015-11-30

Score: 251.8
MW [kDa]: 59.5
pI: 8.7
Sequence Coverage [%]: 8.2
No. of unique Peptides: 1

10	20	30	40	50	60	70	80
MSRQSSITFQ	SGSRRGFSTT	SAITPAAGRS	RFSSVSVARS	AAGSGGLGRI	SSAGASFGSR	SLYNLGGAKR	VSINGCGSSC
**90**	**100**	**110**	**120**	**130**	**140**	**150**	**160**
RSGFGGRASN	RFGVNSGFGY	GGGVGGGFSG	PSFPVCPPGG	IQEVTVNQSL	LTPLHLQIDP	TIQRVRAEER	EQIKTLNNKF
**170**	**180**	**190**	**200**	**210**	**220**	**230**	**240**
ASFIDKVRFL	EQQNKVLETK	WALLQEQGSR	TVRQNLEPLF	DSYTSELRRQ	LESITTERGR	LEAELRNMQD	VVEDFKVRYE
**250**	**260**	**270**	**280**	**290**	**300**	**310**	**320**
DEINKRTAAE	NEFVALKKDV	DAAYMNKVEL	EAKVKSLPEE	INFIHSVFDA	ELSQLQTQVG	DTSVVLSMDN	NRNLDLDSII
**330**	**340**	**350**	**360**	**370**	**380**	**390**	**400**
AEVKAQYEDI	ANRSRAEAES	WYQTKYEELQ	VTAGRHGDDL	RNTKQEISEM	NRMIQRLRAE	IDSVKKQCSS	LQTAIADAEQ
**410**	**420**	**430**	**440**	**450**	**460**	**470**	**480**
RGELALKDAR	AKLVDLEEAL	QKAKQDMARL	LREYQELMNI	KLALDVEIAT	YRKLLEGEEC	RLSGEGVSPV	NISVVTSTLS
**490**	**500**	**510**	**520**	**530**	**540**	**550**	**560**
SGYGSGSSIG	GGNLGLGGGS	GYSFTTSGGH	SLGAGLGGSG	FSATSNRGLG	GSGSSVKFVS	TTSSSQKSYT	H

Cmpd.	No. of Cmpds.	m/z meas.	Δ m/z [ppm]	z	Rt [min]	Score	P	Range	Sequence	Modification
566	3	678.9555	96.02	2	35.7	53.0	1	413-424	K.LVDLEEALQKAK.Q	

Detailed Protein Report

Protein 17: lactotransferrin isoform 2 [Homo sapiens]

**Accession:**	gi|312433998	**Score:**	243.7
**Database:**	refseq_human(refseq_human_20140103.fasta)	**MW [kDa]:**	73.1
**Database Date:**	2015-11-30	**pI:**	9.3
**Modification(s):**	Carbamidomethyl	**Sequence Coverage [%]:**	15.2
		**No. of unique Peptides:**	8

Quantitation

Q:W	**Median:** 0.31	**CV:** 27.32 %	**No. of Peptides:**	2
M:Q	**Median:** 1.25	**CV:** 64.74 %	**No. of Peptides:**	6

10	20	30	40	50	60	70	80
MRKVRGPPVS	CIKRDSPIQC	IQAIAENRAD	AVTLDGGFIY	EAGLAPYKLR	PVAAEVYGTE	RQPRTHYYAV	AVVKKGGSFQ
**90**	**100**	**110**	**120**	**130**	**140**	**150**	**160**
LNELQGLKSC	HTGLRRTAGW	NVPIGTLRPF	LNWTGPPEPI	EAAVARFFSA	SCVPGADKGQ	FPNLCRLCAG	TGENKCAFSS
**170**	**180**	**190**	**200**	**210**	**220**	**230**	**240**
QEPYFSYSGA	FKCLRDGAGD	VAFIRESTVF	EDLSDEAERD	EYELLCPDNT	RKPVDKFKDC	HLARVPSHAV	VARSVNGKED
**250**	**260**	**270**	**280**	**290**	**300**	**310**	**320**
AIWNLLRQAQ	EKFGKDKSPK	FQLFGSPSGQ	KDLLFKDSAI	GFSRVPPRID	SGLYLGSGYF	TAIQNLRKSE	EEVAARRARV
**330**	**340**	**350**	**360**	**370**	**380**	**390**	**400**
VWCAVGEQEL	RKCNQWSGLS	EGSVTCSSAS	TTEDCIALVL	KGEADAMSLD	GGYVYTAGKC	GLVPVLAENY	KSQQSSDPDP
**410**	**420**	**430**	**440**	**450**	**460**	**470**	**480**
NCVDRPVEGY	LAVAVVRRSD	TSLTWNSVKG	KKSCHTAVDR	TAGWNIPMGL	LFNQTGSCKF	DEYFSQSCAP	GSDPRSNLCA
**490**	**500**	**510**	**520**	**530**	**540**	**550**	**560**
LCIGDEQGEN	KCVPNSNERY	YGYTGAFRCL	AENAGDVAFV	KDVTVLQNTD	GNNNEAWAKD	LKLADFALLC	LDGKRKPVTE
**570**	**580**	**590**	**600**	**610**	**620**	**630**	**640**
ARSCHLAMAP	NHAVVSRMDK	VERLKQVLLH	QQAKFGRNGS	DCPDKFCLFQ	SETKNLLFND	NTECLARLHG	KTTYEKYLGP
**650**	**660**	**670**					
QYVAGITNLK	KCSTSPLLEA	CEFLRK					

Cmpd.	No. of Cmpds.	m/z meas.	Δ m/z [ppm]	z	Rt [min]	Score	P	Range	Sequence	Modification	Ratios	
1629	2	695.8567	-18.94	2	48.9	36.9	0	76-88	K.GGSFQLNELQGLK.S		M:Q	0.95
852	1	598.3199	18.32	2	40.1	36.0	0	261-271	K.FQLFGSPSGQK.D		M:Q	0.52
739	1	787.3768	-43.94	2	39.2	13.3	1	320-332	R.VVWCAVGEQELRK.C	Carbamidomethyl: 4	M:Q	2.68
1575	3	681.8469	-16.13	2	49.9	23.1	0	380-391	K.CGLVPVLAENYK.S	Carbamidomethyl: 1		
458	1	619.2938	-20.74	2	35.7	45.8	0	419-429	R.SDTSLTWNSVK.G		M:Q	0.74
593	1	904.3991	-2.21	2	37.4	38.9	0	476-491	R.SNLCALCIGDEQGENK.C	Carbamidomethyl: 4, 7		
2333	5	668.3646	18.05	2	59.0	28.6	0	543-554	K.LADFALLCLDGK.R	Carbamidomethyl: 8	M:Q<br>Q:W	1.97<br>0.41
1229	1	790.3991	23.83	2	45.5	20.9	0	615-627	K.NLLFNDNTECLAR.L	Carbamidomethyl: 10	M:Q<br>Q:W	1.93<br>0.24

Detailed Protein Report

Protein 18: haptoglobin isoform 1 preproprotein [Homo sapiens]

Accession: gi|4826762
Database: refseq_human(refseq_human_20140103.fasta)
Database Date: 2015-11-30
Modification(s): Carbamidomethyl

Score: 217.0
MW [kDa]: 45.2
pI: 6.1
Sequence Coverage [%]: 13.3
No. of unique Peptides: 4

Quantitation

Q:W	**Median:** 1.53	**CV:** 45.35 %	**No. of Peptides:**	4
M:Q	**Median:** 0.81	**CV:** 8.16 %	**No. of Peptides:**	4

10	20	30	40	50	60	70	80
MSALGAVIAL	LLWGQLFAVD	SGNDVTDIAD	DGCPKPPEIA	HGYVEHSVRY	QCKNYYKLRT	EGDGVYTLND	KKQWINKAVG
**90**	**100**	**110**	**120**	**130**	**140**	**150**	**160**
DKLPECEADD	GCPKPPEIAH	GYVEHSVRYQ	CKNYYKLRTE	GDGVYTLNNE	KQWINKAVGD	KLPECEAVCG	KPKNPANPVQ
**170**	**180**	**190**	**200**	**210**	**220**	**230**	**240**
RILGGHLDAK	GSFPWQAKMV	SHHNLTTGAT	LINEQWLLTT	AKNLFLNHSE	NATAKDIAPT	LTLYVGKKQL	VEIEKVVLHP
**250**	**260**	**270**	**280**	**290**	**300**	**310**	**320**
NYSQVDIGLI	KLKQKVSVNE	RVMPICLPSK	DYAEVGRVGY	VSGWGRNANF	KFTDHLKYVM	LPVADQDQCI	RHYEGSTVPE
**330**	**340**	**350**	**360**	**370**	**380**	**390**	**400**
KKTPKSPVGV	QPILNEHTFC	AGMSKYQEDT	CYGDAGSAFA	VHDLEEDTWY	ATGILSFDKS	CAVAEYGVYV	KVTSIQDWVQ
**410**							
KTIAEN							

Cmpd.	No. of Cmpds.	m/z meas.	Δ m/z [ppm]	z	Rt [min]	Score	P	Range	Sequence	Modification	Ratios	
136	3	854.8916	-43.33	2	31.0	42.7	1	117-131	K.LRTEGDGVYTLNNEK.Q		M:Q<br>Q:W	0.79<br>1.41
73	4	619.9745	-5.86	3	29.9	61.3	1	137-153	K.AVGDKLPECEAVCGKPK.N	Carbamidomethyl: 9, 13	Q:W<br>M:Q	1.60<br>0.83
1754	7	645.8334	-54.84	2	50.3	65.3	0	216-227	K.DIAPTLTLYVGK.K		M:Q<br>Q:W	0.90<br>2.87
741	3	602.2951	-44.79	2	39.2	47.7	0	392-401	K.VTSIQDWVQK.T		Q:W<br>M:Q	0.85<br>0.72

Detailed Protein Report

Protein 19: keratin, type II cytoskeletal 2 epidermal [Homo sapiens]

**Accession:**	gi|47132620	**Score:**	205.4
**Database:**	refseq_human(refseq_human_20140103.fasta)	**MW [kDa]:**	65.4
**Database Date:**	2015-11-30	**pI:**	8.9
		**Sequence Coverage [%]:**	5.5
		**No. of unique Peptides:**	1

Quantitation

Q:W	**Median:**	1.09	**CV:**	0.00 %	**No. of Peptides:**	1
M:Q	**Median:**	1.42	**CV:**	0.00 %	**No. of Peptides:**	1

10	20	30	40	50	60	70	80
MSCQISCKSR	GRGGGGGGFR	GFSSGSAVVS	GGSRRSTSSF	SCLSRHGGGG	GGFGGGGFGS	RSLVGLGGTK	SISISVAGGG
**90**	**100**	**110**	**120**	**130**	**140**	**150**	**160**
GGFGAAGGFG	GRGGGFGGGS	SFGGGSGFSG	GGFGGGGFGG	GRFGGFGGPG	GVGGLGGPGG	FGPGGYPGGI	HEVSVNQSLL
**170**	**180**	**190**	**200**	**210**	**220**	**230**	**240**
QPLNVKVDPE	IQNVKAQERE	QIKTLNNKFA	SFIDKVRFLE	QQNQVLQTKW	ELLQQMNVGT	RPINLEPIFQ	GYIDSLKRYL
**250**	**260**	**270**	**280**	**290**	**300**	**310**	**320**
DGLTAERTSQ	NSELNNMQDL	VEDYKKKYED	EINKRTAAEN	DFVTLKKDVD	NAYMIKVELQ	SKVDLLNQEI	EFLKVLYDAE
**330**	**340**	**350**	**360**	**370**	**380**	**390**	**400**
ISQIHQSVTD	TNVILSMDNS	RNLDLDSIIA	EVKAQYEEIA	QRSKEEAEAL	YHSKYEELQV	TVGRHGDSLK	EIKIEISELN
**410**	**420**	**430**	**440**	**450**	**460**	**470**	**480**
RVIQRLQGEI	AHVKKQCKNV	QDAIADAEQR	GEHALKDARN	KLNDLEEALQ	QAKEDLARLL	RDYQELMNVK	LALDVEIATY
**490**	**500**	**510**	**520**	**530**	**540**	**550**	**560**
RKLLEGEECR	MSGDLSSNVT	VSVTSSTISS	NVASKAAFGG	SGGRGSSSGG	GYSSGSSSYG	SGGRQSGSRG	GSGGGGSISG
**570**	**580**	**590**	**600**	**610**	**620**	**630**	**640**
GGYGSGGGSG	GRYGSGGGSK	GGSISGGGYG	SGGGKHSSGG	GSRGGSSSGG	GYGSGGGGSS	SVKGSSGEAF	GSSVTFSFR

Cmpd.	No. of Cmpds.	m/z meas.	Δ m/z [ppm]	z	Rt [min]	Score	P	Range	Sequence	Modification	Ratios
412	2	738.3807	-21.14	2	35.1	70.4	0	198-209	R.FLEQQNQVLQTK.W		M:Q 1.42<br>Q:W 1.09

Detailed Protein Report

Protein 20: **keratin, type II cytoskeletal 1 [Homo sapiens]**

Accession: gi|119395750
Database: refseq_human(refseq_human_20140103.fasta)
Database Date: 2015-11-30

Score: 204.4
MW [kDa]: 66.0
pI: 8.8
Sequence Coverage [%]: 11.8
No. of unique Peptides: 5

Quantitation

Q:W **Median:** 1.31 **CV:** 0.00 % **No. of Peptides:** 1

10	20	30	40	50	60	70	80
MSRQFSSRSG	YRSGGGFSSG	SAGIINYQRR	TTSSSTRRSG	GGGGRFSSCG	GGGGSFGAGG	GFGSRSLVNL	GGSKSISISV
90	100	110	120	130	140	150	160
ARGGGRGSGF	GGGYGGGGFG	GGGFGGGGFG	GGGIGGGGFG	GFGSGGGGFG	GGGFGGGGYG	GGYGPVCPPG	GIQEVTINQS
170	180	190	200	210	220	230	240
LLQPLNVEID	PEIQKVKSRE	REQIKSLNNQ	FASFIDKVRF	LEQQNQVLQT	KWELLQQVDT	STRTHNLEPY	FESFINNLRR
250	260	270	280	290	300	310	320
RVDQLKSDQS	RLDSELKNMQ	DMVEDYRNKY	EDEINKRTNA	ENEFVTIKKD	VDGAYMTKVD	LQAKLDNLQQ	EIDFLTALYQ
330	340	350	360	370	380	390	400
AELSQMQTQI	SETNVILSMD	NNRSLDLDSI	IAEVKAQYED	IAQKSKAEAE	SLYQSKYEEL	QITAGRHGDS	VRNSKIEISE
410	420	430	440	450	460	470	480
LNRVIQRLRS	EIDNVKKQIS	NLQQSISDAE	QRGENALKDA	KNKLNDLEDA	LQQAKEDLAR	LLRDYQELMN	TKLALDLEIA
490	500	510	520	530	540	550	560
TYRTLLEGEE	SRMSGECAPN	VSVSVSTSHT	TISGGGSRGG	GGGGYGSGGS	SYGSGGGSYG	SGGGGGGGRG	SYGSGGSSYG
570	580	590	600	610	620	630	640
SGGGSYGSGG	GGGGHGSYGS	GSSSGGYRGG	SGGGGGGSSG	GRGSGGGSSG	GSIGGRGSSS	GGVKSSGGSS	SVKFVSTTYS
650							
GVTR							

Cmpd.	No. of Cmpds.	m/z meas.	Δ m/z [ppm]	z	Rt [min]	Score	P	Range	Sequence	Modification	Ratios
1554	1	692.3258	-33.18	2	49.6	24.5	0	186-197	K.SLNNQFASFIDK.V		Q:W 1.31
1307	1	738.3615	-22.47	2	45.1	53.5	0	212-223	K.WELLQQVDTSTR.T		
2843	2	651.8509	-15.86	2	64.1	16.0	0	344-355	R.SLDLDSIIAEVK.A		
1168	1	922.9918	16.42	2	44.2	12.8	1	417-432	K.KQISNLQQSISDAEQR.G		
906	2	679.3419	-14.12	2	39.8	27.2	0	444-455	K.LNDLEDALQQAK.E		

Detailed Protein Report

Protein 21: actin, aortic smooth muscle [Homo sapiens]

Accession: gi|4501883
Database: refseq_human(refseq_human_20140103.fasta)
Database Date: 2015-11-30
Modification(s): Carbamidomethyl

Score: 199.2
MW [kDa]: 42.0
pI: 5.1
Sequence Coverage [%]: 23.3
No. of unique Peptides: 3

Quantitation

Q:W	**Median:** 2.40	**CV:** 0.00 %	**No. of Peptides:**	1
M:Q	**Median:** 0.81	**CV:** 0.00 %	**No. of Peptides:**	1

Alias proteins:

Accession	Name	Description
gi|213688375	r e f s e q _ h u m a n (refseq_human_20140103.fasta)	actin, aortic smooth muscle [Homo sapiens]

```
        10         20         30         40         50         60         70         80
MCEEEDSTAL VCDNGSGLCK AGFAGDDAPR AVFPSIVGRP RHQGVMVGMG QKDSYVGDEA QSKRGILTLK YPIEHGIITN
        90        100        110        120        130        140        150        160
WDDMEKIWHH SFYNELRVAP EEHPTLLTEA PLNPKANREK MTQIMFETFN VPAMYVAIQA VLSLYASGRT TGIVLDSGDG
       170        180        190        200        210        220        230        240
VTHNVPIYEG YALPHAIMRL DLAGRDLTDY LMKILTERGY SFVTTAEREI VRDIKEKLCY VALDFENEMA TAASSSSLEK
       250        260        270        280        290        300        310        320
SYELPDGQVI TIGNERFRCP ETLFQPSFIG MESAGIHETT YNSIMKCDID IRKDLYANNV LSGGTTMYPG IADRMQKEIT
       330        340        350        360        370        380
ALAPSTMKIK IIAPPERKYS VWIGGSILAS LSTFQQMWIS KQEYDEAGPS IVHRKCF
```

Cmpd.	No. of Cmpds.	m/z meas.	Δ m/z [ppm]	z	Rt [min]	Score	P	Range	Sequence	Modification	Ratios
2365	1	1015.4376	26.15	2	60.0	17.3	0	2-20	M.CEEEDSTALVCDNGSGLCK.A	Carbamidomethyl: 11	
2088	2	652.6205	-100.50	3	54.7	15.4	0	98-115	R.VAPEEHPTLLTEAPLNPK.A		
267	1	750.8467	-14.36	2	32.1	15.2	0	362-374	K.QEYDEAGPSIVHR.K		Q:W 2.40<br>M:Q 0.81

Detailed Protein Report

Protein 22: keratin, type II cytoskeletal 4 [Homo sapiens]

Accession: gi|331999954
Database: refseq_human(refseq_human_20140103.fasta)
Database Date: 2015-11-30

Score: 179.5
MW [kDa]: 56.1
pI: 6.2
Sequence Coverage [%]: 10.2
No. of unique Peptides: 5

Quantitation

Q:W	**Median:** 0.76	**CV:** 30.81 %	**No. of Peptides:**	3
M:Q	**Median:** 0.61	**CV:** 5.54 %	**No. of Peptides:**	3

10	20	30	40	50	60	70	80
MIARQQCVRG	GPRGFSCGSA	IVGGGKRGAF	SSVSMSGGAG	RCSSGGFGSR	SLYNLRGNKS	ISMSVAGSRQ	GACFGGAGGF
**90**	**100**	**110**	**120**	**130**	**140**	**150**	**160**
GTGGFGGGFG	GSFSGKGGPG	FPVCPAGGIQ	EVTINQSLLT	PLHVEIDPEI	QKVRTEEREQ	IKLLNNKFAS	FIDKVQFLEQ
**170**	**180**	**190**	**200**	**210**	**220**	**230**	**240**
QNKVLETKWN	LLQQQTTTTS	SKNLEPLFET	YLSVLRKQLD	TLGNDKGRLQ	SELKTMQDSV	EDFKTKYEEE	INKRTAAEND
**250**	**260**	**270**	**280**	**290**	**300**	**310**	**320**
FVVLKKDVDA	AYLNKVELEA	KVDSLNDEIN	FLKVLYDAEL	SQMQTHVSDT	SVVLSMDNNR	NLDLDSIIAE	VRAQYEEIAQ
**330**	**340**	**350**	**360**	**370**	**380**	**390**	**400**
RSKAEAEALY	QTKVQQLQIS	VDQHGDNLKN	TKSEIAELNR	MIQRLRAEIE	NIKKQCQTLQ	VSVADAEQRG	ENALKDAHSK
**410**	**420**	**430**	**440**	**450**	**460**	**470**	**480**
RVELEAALQQ	AKEELARMLR	EYQELMSVKL	ALDIEIATYR	KLLEGEEYRM	SGECQSAVSI	SVVSGSTSTG	GISGGLGSGS
**490**	**500**	**510**	**520**	**530**			
GFGLSSGFGS	GSGSGFGFGG	SVSGSSSSKI	ISTTTLNKRR				

Cmpd.	No. of Cmpds.	m/z meas.	Δ m/z [ppm]	z	Rt [min]	Score	P	Range	Sequence	Modification	Ratios
950	1	603.8017	-33.37	2	40.4	11.5	0	235-245	R.TAAENDFVVLK.K		M:Q 0.59
530	2	667.8552	-21.26	2	35.2	56.2	1	235-246	R.TAAENDFVVLKK.D		M:Q 0.59<br>Q:W 0.62
1586	2	703.8014	-85.66	2	49.7	20.9	0	262-273	K.VDSLNDEINFLK.V		Q:W 1.15
365	2	678.3483	-55.17	2	33.6	39.9	1	401-412	K.RVELEAALQQAK.E		M:Q 0.66<br>Q:W 0.60
1796	1	599.9815	-22.51	3	51.1	17.3	1	402-417	R.VELEAALQQAKEELAR.M		

Detailed Protein Report

Protein 23: **POTE ankyrin domain family member E [Homo sapiens]**

**Accession:**	gi|134133226	**Score:**	175.4
**Database:**	refseq_human(refseq_human_20140103.fasta)	**MW [kDa]:**	121.3
**Database Date:**	2015-11-30	**pI:**	5.8
		**Sequence Coverage [%]:**	6.4
		**No. of unique Peptides:**	2

10	20	30	40	50	60	70	80
MVVEVDSMPA	ASSVKKPFGL	RSKMGKWCCR	CFPCYRESGK	SNVGTSGDHD	DSAMKTLRSK	MGKWCHHCFP	CCRGSGKSNV
**90**	**100**	**110**	**120**	**130**	**140**	**150**	**160**
GASGDHDDSA	MKTLRNKMGK	WCCHCFPCCR	GSGKSKVGAW	GDYDDSAFME	PRYHVRGEDL	DKLHRAAWWG	KVPRKDLIVM
**170**	**180**	**190**	**200**	**210**	**220**	**230**	**240**
LRDTDVNKKD	KQKRTALHLA	SANGNSEVVK	LLLDRRCQLN	VLDNKKRTAL	IKAVQCQEDE	CALMLLEHGT	DPNIPDEYGN
**250**	**260**	**270**	**280**	**290**	**300**	**310**	**320**
TTLHYAIYNE	DKLMAKALLL	YGADIESKNK	HGLTPLLLGV	HEQKQQVVKF	LIKKKANLNA	LDRYGRTALI	LAVCCGSASI
**330**	**340**	**350**	**360**	**370**	**380**	**390**	**400**
VSLLLEQNID	VSSQDLSGQT	AREYAVSSHH	HVICQLLSDY	KEKQMLKISS	ENSNPEQELK	LTSEEESQRF	KGSENSQPEK
**410**	**420**	**430**	**440**	**450**	**460**	**470**	**480**
MSQELEINKD	GDREVEEEMK	KHESNNVGLL	ENLTNGVTAG	NGDNGLIPQR	KSRTPENQQF	PDNESEEYHR	ICELLSDYKE
**490**	**500**	**510**	**520**	**530**	**540**	**550**	**560**
KQMPKYSSEN	SNPEQDLKLT	SEEESQRLKG	SENGQPEKRS	QEPEINKDGD	RELENFMAIE	EMKKHGSTHV	GFPENLTNGA
**570**	**580**	**590**	**600**	**610**	**620**	**630**	**640**
TAGNGDDGLI	PPRKSRTPES	QQFPDTENEE	YHSDEQNDTQ	KQFCEEQNTG	ILHDEILIHE	EKQIEVVEKM	NSELSLSCKK
**650**	**660**	**670**	**680**	**690**	**700**	**710**	**720**
EKDVLHENST	LREEIAMLRL	ELDTMKHQSQ	LREKKYLEDI	ESVKKKNDNL	LKALQLNELT	MDDDTAVLVI	DNGSGMCKAG
**730**	**740**	**750**	**760**	**770**	**780**	**790**	**800**
FAGDDAPRAV	FPSIVGRPRQ	QGMMGGMHQK	ESYVGKEAQS	KRGILTLKYP	MEHGIITNWD	DMEKIWHHTF	YNELRVAPEE
**810**	**820**	**830**	**840**	**850**	**860**	**870**	**880**
HPILLTEAPL	NPKANREKMT	QIMFETFNTP	AMYVAIQAVP	SLYTSGRTTG	IVMDSGDGVT	HTVPIYEGNA	LPHATLRLDL
**890**	**900**	**910**	**920**	**930**	**940**	**950**	**960**
AGRELPDYLM	KILTERGYRF	TTMAEREIVR	DIKEKLCYVA	LDFEQEMATA	ASSSLEKSY	ELPDGQVITI	GNERFRCPEA
**970**	**980**	**990**	**1000**	**1010**	**1020**	**1030**	**1040**
LFQPCFLGME	SCGIHETTFN	SIMKSDVDIR	KDLYTNTVLS	GGTTMYPGMA	HRMQKEIAAL	APSMMKIRII	APPKRKYSVW
**1050**	**1060**	**1070**	**1080**				
VGGSILASLS	TFQQMWISKQ	EYDESGPSIV	HRKCF				

Cmpd.	No. of Cmpds.	m/z meas.	Δ m/z [ppm]	z	Rt [min]	Score	P	Range	Sequence	Modification
1070	1	670.5538	205.94	2	43.5	10.8	2	152-162	K.VPRKDLIVMLR.D	
1146	1	984.4853	-59.25	2	43.0	11.3	0	796-813	R.VAPEEHPILLTEAPLNPK.A	

Detailed Protein Report

Protein 24: alpha-enolase isoform 1 [Homo sapiens]

**Accession:**	gi|4503571	**Score:**	161.6
**Database:**	refseq_human(refseq_human_20140103.fasta)	**MW [kDa]:**	47.1
**Database Date:**	2015-11-30	**pI:**	7.7
**Modification(s):**	Carbamidomethyl	**Sequence Coverage [%]:**	16.6
		**No. of unique Peptides:**	5

Quantitation

Q:W	**Median:** 0.89	**CV:** 19.41 %	**No. of Peptides:**	5
M:Q	**Median:** 0.93	**CV:** 29.08 %	**No. of Peptides:**	4

```
        10         20         30         40         50         60         70         80
MSILKIHARE IFDSRGNPTV EVDLFTSKGL FRAAVPSGAS TGIYEALELR DNDKTRYMGK GVSKAVEHIN KTIAPALVSK
        90        100        110        120        130        140        150        160
KLNVTEQEKI DKLMIEMDGT ENKSKFGANA ILGVSLAVCK AGAVEKGVPL YRHIADLAGN SEVILPVPAF NVINGGSHAG
       170        180        190        200        210        220        230        240
NKLAMQEFMI LPVGAANFRE AMRIGAEVYH NLKNVIKEKY GKDATNVGDE GGFAPNILEN KEGLELLKTA IGKAGYTDKV
       250        260        270        280        290        300        310        320
VIGMDVAASE FFRSGKYDLD FKSPDDPSRY ISPDQLADLY KSFIKDYPVV SIEDPFDQDD WGAWQKFTAS AGIQVVGDDL
       330        340        350        360        370        380        390        400
TVTNPKRIAK AVNEKSCNCL LLKVNQIGSV TESLQACKLA QANGWGVMVS HRSGETEDTF IADLVVGLCT GQIKTGAPCR
       410        420        430        440
SERLAKYNQL LRIEEELGSK AKFAGRNFRN PLAK
```

Cmpd.	No. of Cmpds.	m/z meas.	Δ m/z [ppm]	z	Rt [min]	Score	P	Range	Sequence	Modification	Ratios	
1541	1	703.8713	13.64	2	47.8	10.1	0	16-28	R.GNPTVEVDLFTSK.G		Q:W	1.15
2401	3	902.9549	-22.96	2	58.7	34.1	0	33-50	R.AAVPSGASTGIYEALELR.D		M:Q<br>Q:W	0.99<br>0.84
1618	10	713.3563	-14.47	2	50.5	67.0	0	270-281	R.YISPDQLADLYK.S		M:Q<br>Q:W	1.45<br>0.67
584	1	817.3947	-24.01	2	36.2	33.0	0	344-358	K.VNQIGSVTESLQACK.L	Carbamidomethyl: 14	Q:W<br>M:Q	0.81<br>0.69
307	1	846.3647	-102.88	2	33.2	17.4	1	407-420	K.YNQLLRIEEELGSK.A		M:Q<br>Q:W	0.77<br>1.04

Detailed Protein Report

Protein 25: keratin, type I cytoskeletal 19 [Homo sapiens]

Accession: gi|24234699
Database: refseq_human(refseq_human_20140103.fasta)
Database Date: 2015-11-30
Modification(s): Oxidation

Score: 158.8
MW [kDa]: 44.1
pI: 4.9
Sequence Coverage [%]: 15.8
No. of unique Peptides: 1

10	20	30	40	50	60	70	80
MTSYSYRQSS	ATSSFGGLGG	GSVRFGPGVA	FRAPSIHGGS	GGRGVSVSSA	RFVSSSSSGA	YGGGYGGVLT	ASDGLLAGNE
**90**	**100**	**110**	**120**	**130**	**140**	**150**	**160**
KLTMQNLNDR	LASYLDKVRA	LEAANGELEV	KIRDWYQKQG	PGPSRDYSHY	YTTIQDLRDK	ILGATIENSR	IVLQIDNARL
**170**	**180**	**190**	**200**	**210**	**220**	**230**	**240**
AADDFRTKFE	TEQALRMSVE	ADINGLRRVL	DELTLARTDL	EMQIEGLKEE	LAYLKKNHEE	EISTLRGQVG	GQVSVEVDSA
**250**	**260**	**270**	**280**	**290**	**300**	**310**	**320**
PGTDLAKILS	DMRSQYEVMA	EQNRKDAEAW	FTSRTEELNR	EVAGHTEQLQ	MSRSEVTDLR	RTLQGLEIEL	QSQLSMKAAL
**330**	**340**	**350**	**360**	**370**	**380**	**390**	**400**
EDTLAETEAR	FGAQLAHIQA	LISGIEAQLG	DVRADSERQN	QEYQRLMDIK	SRLEQEIATY	RSLLEGQEDH	YNNLSASKVL
**410**							

Cmpd.	No. of Cmpds.	m/z meas.	Δ m/z [ppm]	z	Rt [min]	Score	P	Range	Sequence	Modification
1981	1	1051.4041	-77.63	2	53.4	11.4	1	248-264	K.ILSDMRSQYEVMAEQNR.K	Oxidation: 5, 12

Detailed Protein Report

Protein 26: keratin, type I cytoskeletal 10 [Homo sapiens]

**Accession:**	gi|195972866	**Score:**	150.8
**Database:**	refseq_human(refseq_human_20140103.fasta)	**MW [kDa]:**	58.8
**Database Date:**	2015-11-30	**pI:**	5.0
		**Sequence Coverage [%]:**	6.0
		**No. of unique Peptides:**	1

10	20	30	40	50	60	70	80
MSVRYSSSKH	YSSSRSGGGG	GGGGCGGGGG	VSSLRISSSK	GSLGGGFSSG	GFSGGSFSRG	SSGGGCFGGS	SGGYGGLGGF
**90**	**100**	**110**	**120**	**130**	**140**	**150**	**160**
GGGSFRGSYG	SSSFGGSYGG	SFGGGSFGGG	SFGGGSFGGG	GFGGGGFGGG	FGGGFGGDGG	LLSGNEKVTM	QNLNDRLASY
**170**	**180**	**190**	**200**	**210**	**220**	**230**	**240**
LDKVRALEES	NYELEGKIKE	WYEKHGNSHQ	GEPRDYSKYY	KTIDDLKNQI	LNLTTDNANI	LLQIDNARLA	ADDFRLKYEN
**250**	**260**	**270**	**280**	**290**	**300**	**310**	**320**
EVALRQSVEA	DINGLRRVLD	ELTLTKADLE	MQIESLTEEL	AYLKKNHEEE	MKDLRNVSTG	DVNVEMNAAP	GVDLTQLLNN
**330**	**340**	**350**	**360**	**370**	**380**	**390**	**400**
MRSQYEQLAE	QNRKDAEAWF	NEKSKELTTE	IDNNIEQISS	YKSEITELRR	NVQALEIELQ	SQLALKQSLE	ASLAETEGRY
**410**	**420**	**430**	**440**	**450**	**460**	**470**	**480**
CVQLSQIQAQ	ISALEEQLQQ	IRAETECQNT	EYQQLLDIKI	RLENEIQTYR	SLLEGEGSSG	GGGRGGGSFG	GGYGGGSSGG
**490**	**500**	**510**	**520**	**530**	**540**	**550**	**560**
GSSGGGHGGG	HGGSSGGGYG	GGSSGGGSSG	GGYGGGSSSG	GHGGSSSGGY	GGGSSGGGGG	GYGGGSSGGG	SSSGGGYGGG
**570**	**580**	**590**					
SSSGGHKSSS	SGSVGESSSK	GPRY					

Cmpd.	No. of Cmpds.	m/z meas.	Δ m/z [ppm]	z	Rt [min]	Score	P	Range	Sequence	Modification
348	1	806.4041	46.58	2	34.3	11.3	1	185-198	K.HGNSHQGEPRDYSK.Y	

Detailed Protein Report

Protein 27: keratin, type II cytoskeletal 79 [Homo sapiens]

Accession: gi|32567786
Database: refseq_human(refseq_human_20140103.fasta)
Database Date: 2015-11-30
Modification(s): Oxidation

Score: 145.7
MW [kDa]: 57.8
pI: 6.9
Sequence Coverage [%]: 6.7
No. of unique Peptides: 1

10	20	30	40	50	60	70	80
MRSSVSRQTY	STKGGFSSNS	ASGGSGSQAR	TSFSSVTVSR	SSGSGGGAHC	GPGTGGFGSR	SLYNLGGHKS	ISVSVAGGAL
90	100	110	120	130	140	150	160
LGRALGGFGF	GSRAFMGQGA	GRQTFGPACP	PGGIQEVTVN	QSLLTPLHVE	IDPEIQRVRT	QEREQIKTLN	NKFASFIDKV
170	180	190	200	210	220	230	240
RFLEQQNKVL	ETKWALLQEQ	GQNLGVTRNN	LEPLFEAYLG	SMRSTLDRLQ	SERGRLDSEL	RNVQDLVEDF	KNKYEDEINK
250	260	270	280	290	300	310	320
HTAAENEFVV	LKKDVDAAYM	GRMDLHGKVG	TLTQEIDFLQ	QLYEMELSQV	QTHVSNTNVV	LSMDNNRNLD	LDSIIAEVKA
330	340	350	360	370	380	390	400
QYELIAQRSR	AEAEAWYQTK	YEELQVTAGK	HGDNLRDTKN	EIAELTRTIQ	RLQGEADAAK	KQCQQLQTAI	AEAEQRGELA
410	420	430	440	450	460	470	480
LKDAQKKLGD	LDVALHQAKE	DLTRLLRDYQ	ELMNVKLALD	VEIATYRKLL	ESEESRMSGE	CPSAVSISVT	GNSTTVCGGG
490	500	510	520	530	540		
AASFGGGISL	GGSGGATKGG	FSTNVGYSTV	KGGPVSAGTS	ILRKTTTVKT	SSQRY		

Cmpd.	No. of Cmpds.	m/z meas.	Δ m/z [ppm]	z	Rt [min]	Score	P	Range	Sequence	Modification
140	1	773.9092	30.36	2	31.7	10.7	2	1-13	-.MRSSVSRQTYSTK.G	Oxidation: 1

Detailed Protein Report

Protein 28: fibrinogen gamma chain isoform gamma-A precursor [Homo sapiens]

**Accession:**	gi|70906437	**Score:**	139.8
**Database:**	refseq_human(refseq_human_20140103.fasta)	**MW [kDa]:**	49.5
**Database Date:**	2015-11-30	**pI:**	5.7
		**Sequence Coverage [%]:**	10.8
		**No. of unique Peptides:**	4

Quantitation

Q:W	**Median:** 1.40	**CV:** 38.93 %	**No. of Peptides:**	2
M:Q	**Median:** 0.85	**CV:** 0.00 %	**No. of Peptides:**	1

```
        10         20         30         40         50         60         70         80
MSWSLHPRNL ILYFYALLFL SSTCVAYVAT RDNCCILDER FGSYCPTTCG IADFLSTYQT KVDKDLQSLE DILHQVENKT
        90        100        110        120        130        140        150        160
SEVKQLIKAI QLTYNPDESS KPNMIDAATL KSRKMLEEIM KYEASILTHD SSIRYLQEIY NSNNQKIVNL KEKVAQLEAQ
       170        180        190        200        210        220        230        240
CQEPCKDTVQ IHDITGKDCQ DIANKGAKQS GLYFIKPLKA NQQFLVYCEI DGSGNGWTVF QKRLDGSVDF KKNWIQYKEG
       250        260        270        280        290        300        310        320
FGHLSPTGTT EFWLGNEKIH LISTQSAIPY ALRVELEDWN GRTSTADYAM FKVGPEADKY RLTYAYFAGG DAGDAFDGFD
       330        340        350        360        370        380        390        400
FGDDPSDKFF TSHNGMQFST WDNDNDKFEG NCAEQDGSGW WMNKCHAGHL NGVYYQGGTY SKASTPNGYD NGIIWATWKT
       410        420        430        440
RWYSMKKTTM KIIPFNRLTI GEGQQHHLGG AKQAGDV
```

Cmpd.	No. of Cmpds.	m/z meas.	Δ m/z [ppm]	z	Rt [min]	Score	P	Range	Sequence	Modification	Ratios
127	2	757.2233	-190.62	2	30.5	61.4	0	135-146	R.YLQEIYNSNNQK.I		M:Q 0.85<br>Q:W 2.04
1191	1	647.3732	-13.52	2	43.1	26.2	0	189-199	K.QSGLYFIKPLK.A		
2104	2	841.8525	-155.05	2	54.7	23.4	0	259-273	K.IHLISTQSAIPYALR.V		
329	2	559.1487	-212.08	2	33.2	28.9	0	274-282	R.VELEDWNGR.T		Q:W 0.96

Detailed Protein Report

Protein 29: nesprin-2 isoform 5 [Homo sapiens]

**Accession:**	gi|118918407	**Score:**	131.1
**Database:**	refseq_human(refseq_human_20140103.fasta)	**MW [kDa]:**	798.4
**Database Date:**	2015-11-30	**pI:**	5.1
**Modification(s):**	Oxidation	**Sequence Coverage [%]:**	1.9
		**No. of unique Peptides:**	10

Quantitation

Q:W	**Median:**	0.65	**CV:**	0.00 %	**No. of Peptides:**	1
M:Q	**Median:**	1.86	**CV:**	72.29 %	**No. of Peptides:**	2

Detailed Protein Report

10	20	30	40	50	60	70	80
MASSPELPTE	DEQGSWGIDD	LHISLQAEQE	DTQKKAFTCW	INSQLARHTS	PSVISDLFTD	IKKGHVLLDL	LEVLSGQQLP
90	100	110	120	130	140	150	160
RDKGSNTFQC	RINIEHALTF	LRNRSIKLIN	IHVTDIIDGN	PSIILGLIWT	IILHFHIEKL	AQTLSCNYNQ	PSLDDVSVVD
170	180	190	200	210	220	230	240
SSPASSPPAK	KCSKVQARWQ	MSARKALLLW	AQEQCATYES	VNVTDFKSSW	RNGMAFLAII	HALRPDLIDM	KSVKHRSNKD
250	260	270	280	290	300	310	320
NLREAFRIAE	QELKIPRLLE	PEDVDVVDPD	EKSIMTYVAQ	FLQYSKDAPG	TGEEAQGKVK	DAMGWLTLQK	EKLQKLLKDS
330	340	350	360	370	380	390	400
ENDTYFKKYN	SLLSFMESFN	EEKKSFLDVL	SIKRDLDELD	KDHLQLREAW	DGLDHQINAW	KIKLNYALPP	PLHQTEAWLQ
410	420	430	440	450	460	470	480
EVEELMDEDL	SASQDHSQAV	TLIQEKMTLF	KSLMDRFEHH	SNILLTFENK	DENHLPLVPP	NKLEEMKRRI	NNILEKKFIL
490	500	510	520	530	540	550	560
LLEFHYYKCL	VLGLVDEVKS	KLDIWNIKYG	SRESVELLLE	DWHKFIEEKE	FLARLDTSFQ	KCGEIYKNLA	GECQNINKQY
570	580	590	600	610	620	630	640
MMVKSDVCMY	RKNIYNVKST	LQKVLACWAT	YVENLRLLRA	CFEETKKEEI	KEVPFETLAQ	WNLEHATLNE	AGNFLVEVSN
650	660	670	680	690	700	710	720
DVVGSSISKE	LRRLNKRWRK	LVSKTQLEMN	LPLMIKKQDQ	PTFDNSGNIL	SKEEKATVEF	STDMSVELPE	NYNQNIKAGE
730	740	750	760	770	780	790	800
KHEKENEEFT	GQLKVAKDVE	KLIGQVEIWE	AEAKSVLDQD	DVDTSMEESL	KHLIAKGSMF	DELMARSEDM	LQMDIQNISS
810	820	830	840	850	860	870	880
QESFQHVLTT	GLQAKIQEAK	EKVQINVVKL	IAALKNLTDV	SPDLDIRLKM	EESQKELESY	MMRAQQLLGQ	RESPGELISK
890	900	910	920	930	940	950	960
HKEALIISNT	KSLAKYLKAV	EELKNNVTED	IKMSLEEKSR	DVCAKWESLH	HELSLYVQQL	KIDIEKGKLS	DNILKLEKQI
970	980	990	1000	1010	1020	1030	1040
NKEKKLIRRG	RTKGLIKEHE	ACFSEEGCLY	QLNHHMEVLR	ELCEELPSQK	SQQEVKRLLK	DYEQKIERLL	KCASEIHMTL
1050	1060	1070	1080	1090	1100	1110	1120
QPTAGGTSKN	EGTITTSENR	GGDPHSEAPF	AKSDNQPSTE	KAMEPTMKFS	LASVLRPLQE	ESIMEKDYSA	SINSLLERYD
1130	1140	1150	1160	1170	1180	1190	1200
TYRDILEHHL	QNNKFRITSD	FSSEEDRSSS	CLQAKLTDLQ	VIKNETDARW	KEFEIISLKL	ENHVNDIKKP	FVIKERDTLK
1210	1220	1230	1240	1250	1260	1270	1280
ERERELQMTL	NTRMESLETA	LRLVLPVEKA	SLLLCGSDLP	LHKMAIQGFH	LIDADRIYQH	LRNIQDSIAK	QIEICNRLEE
1290	1300	1310	1320	1330	1340	1350	1360
PGNFVLKELH	PFDLHAMQNI	ILKYKTQFEG	MNHRVQRSED	TLKALEDFLA	SLRTAKLSAE	PVTDLSASDT	QVAQENTLTV
1370	1380	1390	1400	1410	1420	1430	1440
KNKEGEIHLM	KDKAKHLDKC	LKMLDMSFKD	AERGDDTSCE	NLLDAFSIKL	SETHGYGVQE	EFTEENKLLE	ACIFKNNELL
1450	1460	1470	1480	1490	1500	1510	1520
KNIQDVQSQI	SKIGLKDPTV	PAVKHRKKSL	IRLDKVLDEY	EEEKRHLQEM	ANSLPHFKDG	REKTVNQQCQ	NTVVLWENTK
1530	1540	1550	1560	1570	1580	1590	1600
ALVTECLEQC	GRVLELLKQY	QNFKSILTTL	IQKEESVISL	QASYMGKENL	KKRIAEIEIV	KEEFNEHLEV	VDKINQVCKN
1610	1620	1630	1640	1650	1660	1670	1680
LQFYLNKMKT	FEEPPFEKEA	NIIVDRWLDI	NEKTEDYYEN	LGRALALWDK	LFNLKNVIDE	WTEKALQKME	LHQLTEEDRE
1690	1700	1710	1720	1730	1740	1750	1760
RLKEELQVHE	QKTSEFSRRV	AEIQFLLQSS	EIPLELQVME	SSILNKMEHV	QKCLTGESNC	HALSGSTAEL	REDLDQAKTQ
1770	1780	1790	1800	1810	1820	1830	1840
IGMTESLLKA	LSPSDSLEIF	TKLEEIQQQI	LQQKHSMILL	ENQIGCLTPE	LSELKKQYES	VSDLFNTKKS	VLQDHFSKLL
1850	1860	1870	1880	1890	1900	1910	1920
NDQCKNFNDW	FSNIKVNLKE	CFESSETKKS	VEQKLQKLSD	FLTLEGRNSK	IKQVDSVLKH	VKKHLPKAHV	KELISWLVGQ
1930	1940	1950	1960	1970	1980	1990	2000
EFELEKMESI	CQARAKELED	SLQQLLRLQD	DHRNLRKWLT	NQEEKWKGME	EPGEKTELFC	QALARKREQF	ESVAQLNNSL
2010	2020	2030	2040	2050	2060	2070	2080
KEYGFTEEEE	IIMEATCLMD	RYQTLLRQLS	EIEEEDKLLP	TEDQSFNDLA	HDVIHWIKEI	KESLMVLNSS	EGKMPLEERI
2090	2100	2110	2120	2130	2140	2150	2160
QKIKEIILLK	PEGDARIETI	MKQAESSEAP	LVQKTLTDIS	NQWDNTLHLA	STYLSHQEKL	LLEGEKYLQS	KEDLRLMLIE
2170	2180	2190	2200	2210	2220	2230	2240

Detailed Protein Report

Cmpd.	No. of Cmpds.	m/z meas.	Δ m/z [ppm]	z	Rt [min]	Score	P	Range	Sequence	Modification	Ratios	
2639	1	686.8883	34.43	2	63.0	14.6	0	451-462	K.DENHLPLVPPNK.L			
2757	1	849.0563	-8.59	3	65.6	10.4	0	696-717	K.<br>ATVEFSTDMSVELPENYNQNIK.<br>A	Oxidation: 9		
2961	1	927.4882	-20.92	2	65.8	15.8	1	864-880	R.AQQLLGQRESPGELISK.H			
289	1	715.8764	45.86	2	32.3	10.0	0	1817-<br>1828	K.QYESVSDLFNTK.K			
133	1	679.8336	-94.89	2	31.4	13.6	1	2192-<br>2203	K.AQDLTSLLKELK.S		M:Q<br>Q:W	0.97<br>0.65
1892	1	628.2831	-31.74	2	54.0	13.1	0	2204-<br>2214	K.SQGNYLLECTK.N			
2908	1	922.9773	-1.53	2	65.5	11.3	1	3576-<br>3590	K.ELVQTEIQERHSFTK.E		M:Q	3.55
1662	2	882.4096	-80.38	2	51.0	13.3	2	3618-<br>3632	K.SEQFEELQSILKKGK.L			
1911	1	568.7711	-62.35	2	52.2	11.0	1	4627-<br>4636	K.SLKAGLDYNR.S			
2841	2	648.3701	-36.81	1	64.1	18.0	1	6824-<br>6828	K.FRAVR.T			

Detailed Protein Report

Protein 30: laminin subunit alpha-2 isoform a precursor [Homo sapiens]

**Accession:**	gi|28559088	**Score:**	127.6
**Database:**	refseq_human(refseq_human_20140103.fasta)	**MW [kDa]:**	343.7
**Database Date:**	2015-11-30	**pI:**	6.0
**Modification(s):**	Carbamidomethyl, Oxidation	**Sequence Coverage [%]:**	4.5
		**No. of unique Peptides:**	10

Quantitation

Q:W	**Median:**	0.71	**CV:**	89.66 %	**No. of Peptides:**	3
M:Q	**Median:**	1.07	**CV:**	37.41 %	**No. of Peptides:**	2

Detailed Protein Report

10	20	30	40	50	60	70	80
MPGAAGVLLL	LLLSGGLGGV	QAQRPQQQRQ	SQAHQQRGLF	PAVLNLASNA	LITTNATCGE	KGPEMYCKLV	EHVPGQPVRN
90	100	110	120	130	140	150	160
PQCRICNQNS	SNPNQRHPIT	NAIDGKNTWW	QSPSIKNGIE	YHYVTITLDL	QQVFQIAYVI	VKAANSPRPG	NWILERSLDD
170	180	190	200	210	220	230	240
VEYKPWQYHA	VTDTECLTLY	NIYPRTGPPS	YAKDDEVICT	SFYSKIHPLE	NGEIHISLIN	GRPSADDPSP	ELLEFTSARY
250	260	270	280	290	300	310	320
IRLRFQRIRT	LNADLMMFAH	KDPREIDPIV	TRRYYYSVKD	ISVGGMCICY	GHARACPLDP	ATNKSRCECE	HNTCGDSCDQ
330	340	350	360	370	380	390	400
CCPGFHQKPW	RAGTFLTKTE	CEACNCHGKA	EECYYDENVA	RRNLSLNIRG	KYIGGGVCIN	CTQNTAGINC	ETCTDGFFRP
410	420	430	440	450	460	470	480
KGVSPNYPRP	CQPCHCDPIG	SLNEVCVKDE	KHARRGLAPG	SCHCKTGFGG	VSCDRCARGY	TGYPDCKACN	CSGLGSKNED
490	500	510	520	530	540	550	560
PCFGPCICKE	NVEGGDCSRC	KSGFFNLQED	NWKGCDECFC	SGVSNRCQSS	YWTYGKIQDM	SGWYLTDLPG	RIRVAPQQDD
570	580	590	600	610	620	630	640
LDSPQQISIS	NAEARQALPH	SYYWSAPAPY	LGNKLPAVGG	QLTFTISYDL	EEEEEDTERV	LQLMIILEGN	DLSISTAQDE
650	660	670	680	690	700	710	720
VYLHPSEEHT	NVLLLKEESF	TIHGTHFPVR	RKEFMTVLAN	LKRVLLQITY	SFGMDAIFRL	SSVNLESAVS	YPTDGSIAAA
730	740	750	760	770	780	790	800
VEVCQCPPGY	TGSSCESCWP	RHRRVNGTIF	GGICEPCQCF	GHAESCDDVT	GECLNCKDHT	GGPYCDKCLP	GFYGEPTKGT
810	820	830	840	850	860	870	880
SEDCQPCACP	LNIPSNNFSP	TCHLDRSLGL	ICDGCPVGYT	GPRCERCAEG	YFGQPSVPGG	SCQPCQCNDN	LDFSIPGSCD
890	900	910	920	930	940	950	960
SLSGSCLICK	PGTTGRYCEL	CADGYFGDAV	DAKNCQPCRC	NAGGSFSEVC	HSQTGQCECR	ANVQGQRCDK	CKAGTFGLQS
970	980	990	1000	1010	1020	1030	1040
ARGCVPCNCN	SFGSKSFDCE	ESGQCWCQPG	VTGKKCDRCA	HGYFNFQEGG	CTACECSHLG	NNCDPKTGRC	ICPPNTIGEK
1050	1060	1070	1080	1090	1100	1110	1120
CSKCAPNTWG	HSITTGCKAC	NCSTVGSLDF	QCNVNTGQCN	CHPKFSGAKC	TECSRGHWNY	PRCNLCDCFL	PGTDATTCDS
1130	1140	1150	1160	1170	1180	1190	1200
ETKKCSCSDQ	TGQCTCKVNV	EGIHCDRCRP	GKFGLDAKNP	LGCSSCYCFG	TTTQCSEAKG	LIRTWVTLKA	EQTILPLVDE
1210	1220	1230	1240	1250	1260	1270	1280
ALQHTTTKGI	VFQHPEIVAH	MDLMREDLHL	EPFYWKLPEQ	FEGKKLMAYG	GKLKYAIYFE	AREETGFSTY	NPQVIIRGGT
1290	1300	1310	1320	1330	1340	1350	1360
PTHARIIVRH	MAAPLIGQLT	RHEIEMTEKE	WKYYGDDPRV	HRTVTREDFL	DILYDIHYIL	IKATYGNFMR	QSRISEISME
1370	1380	1390	1400	1410	1420	1430	1440
VAEQGRGTTM	TPPADLIEKC	DCPLGYSGLS	CEACLPGFYR	LRSQPGGRTP	GPTLGTCVPC	QCNGHSSLCD	PETSICQNCQ
1450	1460	1470	1480	1490	1500	1510	1520
HHTAGDFCER	CALGYYGIVK	GLPNDCQQCA	CPLISSSNNF	SPSCVAEGLD	DYRCTACPRG	YEGQYCERCA	PGYTGSPGNP
1530	1540	1550	1560	1570	1580	1590	1600
GGSCQECECD	PYGSLPVPCD	PVTGFCTCRP	GATGRKCDGC	KHWHAREGWE	CVFCGDECTG	LLLGDLARLE	QMVMSINLTG
1610	1620	1630	1640	1650	1660	1670	1680
PLPAPYKMLY	GLENMTQELK	HLLSPQRAPE	RLIQLAEGNL	NTLVTEMNEL	LTRATKVTAD	GEQTGQDAER	TNTRAKSLGE
1690	1700	1710	1720	1730	1740	1750	1760
FIKELARDAE	AVNEKAIKLN	ETLGTRDEAF	ERNLEGLQKE	IDQMIKELRR	KNLETQKEIA	EDELVAAEAL	LKKVKKLFGE
1770	1780	1790	1800	1810	1820	1830	1840
SRGENEEMEK	DLREKLADYK	NKVDDAWDLL	REATDKIREA	NRLFAVNQKN	MTALEKKKEA	VESGKRQIEN	TLKEGNDILD
1850	1860	1870	1880	1890	1900	1910	1920
EANRLADEIN	SIIDYVEDIQ	TKLPPMSEEL	NDKIDDLSQE	IKDRKLAEKV	SQAESHAAQL	NDSSAVLDGI	LDEAKNISFN
1930	1940	1950	1960	1970	1980	1990	2000
ATAAFKAYSN	IKDYIDEAEK	VAKEAKDLAH	EATKLATGPR	GLLKEDAKGC	LQKSFRILNE	AKKLANDVKE	NEDHLNGLKT
2010	2020	2030	2040	2050	2060	2070	2080
RIENADARNG	DLLRTLNDTL	GKLSAIPNDT	AAKLQAVKDK	ARQANDTAKD	VLAQITELHQ	NLDGLKKNYN	KLADSVAKTN
2090	2100	2110	2120	2130	2140	2150	2160
AVVKDPSKNK	IIADADATVK	NLEQEADRLI	DKLKPIKELE	DNLKKNISEI	KELINQARKQ	ANSIKVSVSS	GGDCIRTYKP
2170	2180	2190	2200	2210	2220	2230	2240

Detailed Protein Report

Cmpd.	No. of Cmpds.	m/z meas.	Δ m/z [ppm]	z	Rt [min]	Score	P	Range	Sequence	Modification	Ratios	
2885	1	1023.0269	2.26	2	64.8	14.8	2	248-264	R.IRTLNADLMMFAHKDPR.E	Oxidation: 9	Q:W	2.00
2021	1	665.3031	-44.41	2	55.2	14.0	1	295-306	R.ACPLDPATNKSR.C	Carbamidomethyl: 2		
1220	1	1018.6209	-90.98	3	44.9	11.4	0	307-331	R. CECEHNTCGDSCDQCCPGFHQK A	Carbamidomethyl: 12, 15, 16		
892	1	739.3524	121.81	2	39.5	10.1	0	1125-1137	K.CSCSDQTGQCTCK.V	Carbamidomethyl: 1, 10		
2360	1	1073.7380	-80.56	3	59.8	11.8	2	1380-1408	K. CDCPLGYSGLSCEACLPGFYRLR T	Carbamidomethyl: 1, 12		
1065	1	836.1966	-317.81	1	42.9	11.8	1	1756-1762	K.KLFGESR.G			
445	1	475.6547	-205.40	2	34.1	10.7	1	1810-1817	K.NMTALEKK.K	Oxidation: 2	Q:W<br>M:Q	0.55<br>0.75
1656	1	803.8745	-81.91	2	50.5	10.4	2	1969-1982	K.GCLQKSFRILNEAK.K			
1760	1	568.7537	-31.99	2	52.3	14.8	0	2146-2156	K.VSVSSGGDCIR.T	Carbamidomethyl: 9		
2430	1	531.0471	-454.49	1	60.8	17.8	0	2291-2295	K.ADAVR.V		M:Q<br>Q:W	1.54<br>0.32

Detailed Protein Report

Protein 31: PREDICTED: dystonin isoform X18 [Homo sapiens]

**Accession:**	gi|578811869	**Score:**	125.3
**Database:**	refseq_human(refseq_human_20140103.fasta)	**MW [kDa]:**	851.5
**Database Date:**	2015-11-30	**pI:**	5.0
**Modification(s):**	Carbamidomethyl, Oxidation	**Sequence Coverage [%]:**	1.7
		**No. of unique Peptides:**	8

Quantitation

Q:W	**Median:**	1.00	**CV:**	21.27 %	**No. of Peptides:**	2
M:Q	**Median:**	0.86	**CV:**	41.32 %	**No. of Peptides:**	3

Detailed Protein Report

10	20	30	40	50	60	70	80
MAGYLSPAAY	LYVEEQEYLQ	AYEDVLERYK	DERDKVQKKT	FTKWINQHLM	KVRKHVNDLY	EDLRDGHNLI	SLLEVLSGDT
90	100	110	120	130	140	150	160
LPREKGRMRF	HRLQNVQIAL	DYLKRRQVKL	VNIRNDDITD	GNPKLTLGLI	WTIILHFQIS	DIHVTGESED	MSAKERLLLW
170	180	190	200	210	220	230	240
TQQATEGYAG	IRCENFTTCW	RDGKLFNAII	HKYRPDLIDM	NTVAVQSNLA	NLEHAFYVAE	KIGVIRLLDP	EDVDVSSPDE
250	260	270	280	290	300	310	320
KSVITYVSSL	YDAFPKVPEG	GEGIGANDVE	VKWIEYQNMV	NYLIQWIRHH	VTTMSERTFP	NNPVELKALY	NQYLQFKETE
330	340	350	360	370	380	390	400
IPPKETEKSK	IKRLYKLLEI	WIEFGRIKLL	QGYHPNDIEK	EWGKLIIAML	EREKALRPEV	ERLEMLQQIA	NRVQRDSVIC
410	420	430	440	450	460	470	480
EDKLILAGNA	LQSDSKRLES	GVQFQNEAEI	AGYILECENL	LRQHVIDVQI	LIDGKYYQAD	QLVQRVAKLR	DEIMALRNEC
490	500	510	520	530	540	550	560
SSVYSKGRIL	TTEQTKLMIS	GITQSLNSGF	AQTLHPSLTS	GLTQSLTPSL	TSSSMTSGLS	SGMTSRLTPS	VTPAYTPGFP
570	580	590	600	610	620	630	640
SGLVPNFSSG	VEPNSLQTLK	LMQIRKPLLK	SSLLDQNLTE	EEINMKFVQD	LLNWVDEMQV	QLDRTEWGSD	LPSVESHLEN
650	660	670	680	690	700	710	720
HKNVHRAIEE	FESSLKEAKI	SEIQMTAPLK	LTYAEKLHRL	ESQYAKLLNT	SRNQERHLDT	LHNFVSRATN	ELIWLNEKEE
730	740	750	760	770	780	790	800
EEVAYDWSER	NTNIARKKDY	HAELMRELDQ	KEENIKSVQE	IAEQLLLENH	PARLTIEAYR	AAMQTQWSWI	LQLCQCVEQH
810	820	830	840	850	860	870	880
IKENTAYFEF	FNDAKEATDY	LRNLKDAIQR	KYSCDRSSSI	HKLEDLVQES	MEEKEELLQY	KSTIANLMGK	AKTIIQLKPR
890	900	910	920	930	940	950	960
NSDCPLKTSI	PIKAICDYRQ	IEITIYKDDE	CVLANNSHRA	KWKVISPTGN	EAMVPSVCFT	VPPPNKEAVD	LANRIEQQYQ
970	980	990	1000	1010	1020	1030	1040
NVLTLWHESH	INMKSVVSWH	YLINEIDRIR	ASNVASIKTM	LPGEHQQVLS	NLQSRFEDFL	EDSQESQVFS	GSDITQLEKE
1050	1060	1070	1080	1090	1100	1110	1120
VNVCKQYYQE	LLKSAEREEQ	EESVYNLYIS	EVRNIRLRLE	NCEDRLIRQI	RTPLERDDLH	ESVFRITEQE	KLKKELERLK
1130	1140	1150	1160	1170	1180	1190	1200
DDLGTITNKC	EEFFSQAAAS	SSVPTLRSEL	NVVLQNMNQV	YSMSSTYIDK	LKTVNLVLKN	TQAAEALVKL	YETKLCEEEA
1210	1220	1230	1240	1250	1260	1270	1280
VIADKNNIEN	LISTLKQWRS	EVDEKRQVFH	ALEDELQKAK	AISDEMFKTY	KERDLDFDWH	KEKADQLVER	WQNVHVQIDN
1290	1300	1310	1320	1330	1340	1350	1360
RLRDLEGIGK	SLKYYRDTYH	PLDDWIQQVE	TTQRKIQENQ	PENSKTLATQ	LNQQKMLVSE	IEMKQSKMDE	CQKYAEQYSA
1370	1380	1390	1400	1410	1420	1430	1440
TVKDYELQTM	TYRAMVDSQQ	KSPVKRRRMQ	SSADLIIQEF	MDLRTRYTAL	VTLMTQYIKF	AGDSLKRLEE	EEKSLEEEKK
1450	1460	1470	1480	1490	1500	1510	1520
EHVEKAKELQ	KWVSNISKTL	KDAEKAGKPP	FSKQKISSEE	ISTKKEQLSE	ALQTIQLFLA	KHGDKMTDEE	RNELEKQVKT
1530	1540	1550	1560	1570	1580	1590	1600
LQESYNLLFS	ESLKQLQESQ	TSGDVKVEEK	LDKVIAGTID	QTTGEVLSVF	QAVLRGLIDY	DTGIRLLETQ	LMISGLISPE
1610	1620	1630	1640	1650	1660	1670	1680
LRKCFDLKDA	KSHGLIDEQI	LCQLKELSKA	KEIISAASPT	TIPVLDALAQ	SMITESMAIK	VLEILLSTGS	LVIPATGEQL
1690	1700	1710	1720	1730	1740	1750	1760
TLQKAFQQNL	VSSALFSKVL	ERQNMCKDLI	DPCTSEKVSL	IDMVQRSTLQ	ENTGMWLLPV	RPQEGGRITL	KCGRNISILR
1770	1780	1790	1800	1810	1820	1830	1840
AAHEGLIDRE	TMFRLLSAQL	LSGGLINSNS	GQRMTVEEAV	REGVIDRDTA	SSILTYQVQT	GGIIQSNPAK	RLTVDEAVQC
1850	1860	1870	1880	1890	1900	1910	1920
DLITSSSALL	VLEAQRGYVG	LIWPHSGEIF	PTSSSLQQEL	ITNELAYKIL	NGRQKIAALY	IPESSQVIGL	DAAKQLGIID
1930	1940	1950	1960	1970	1980	1990	2000
NNTASILKNI	TLPDKMPDLG	DLEACKNARR	WLSFCKFQPS	TVHDYRQEED	VFDGEEPVTT	QTSEETKKLF	LSYLMINSYM
2010	2020	2030	2040	2050	2060	2070	2080
DANTGQRLLL	YDGDLDEAVG	MLLEGCHAEF	DGNTAIKECL	DVLSSSGVFL	NNASGREKDE	CTATPSSFNK	CHCGEPEHEE
2090	2100	2110	2120	2130	2140	2150	2160
TPENRKCAID	EEFNEMRNTV	INSEFSQSGK	LASTISIDPK	VNSSPSVCVP	SLISYLTQTE	LADISMLRSD	SENILTNYEN
2170	2180	2190	2200	2210	2220	2230	2240

Detailed Protein Report

Cmpd.	No. of Cmpds.	m/z meas.	Δ m/z [ppm]	z	Rt [min]	Score	P	Range	Sequence	Modification	Ratios	
585	1	558.7340	-142.02	2	36.6	17.2	1	469-477	K.LRDEIMALR.N		M:Q<br>Q:W	0.57<br>1.23
1982	1	927.8655	-42.70	2	53.4	10.5	1	1703-1717	R.QNMCKDLIDPCTSEK.V	Carbamidomethyl: 4, 11; Oxidation: 3		
1902	1	805.7986	-138.23	2	54.1	11.0	1	3497-3510	R.TKQIMLAIDSEMSK.L	Oxidation: 12	M:Q<br>Q:W	0.77<br>0.81
103	1	822.3979	-76.79	2	30.5	11.5	2	3730-3743	K.LNEAKIKCEQLNLK.A			
779	1	1105.2011	129.15	2	38.2	12.7	2	4130-4148	K.TLDDIVGRYEDLSKSVNER.N			
2450	1	684.2042	-212.00	2	61.0	10.9	1	4867-4878	K.EEQNKSHPISAK.L			
2575	1	588.6984	-194.01	2	60.9	10.5	0	6044-6053	K.LLDVMELAEK.F	Oxidation: 5	M:Q	1.47
1793	6	781.2979	-163.01	2	52.8	12.6	2	6661-6674	R.SLKEKTSLADDNLK.L			

Detailed Protein Report

Protein 32: beta-actin-like protein 2 [Homo sapiens]

**Accession:**	gi|63055057	**Score:**	123.5
**Database:**	refseq_human(refseq_human_20140103.fasta)	**MW [kDa]:**	42.0
**Database Date:**	2015-11-30	**pI:**	5.3
**Modification(s):**	Oxidation	**Sequence Coverage [%]:**	14.6
		**No. of unique Peptides:**	1

10	20	30	40	50	60	70	80
MTDNELSALV	VDNGSGMCKA	GFGGDDAPRA	VFPSMIGRPR	HQGVMVGMGQ	KDCYVGDEAQ	SKRGVLTLKY	PIEHGVVTNW
**90**	**100**	**110**	**120**	**130**	**140**	**150**	**160**
DDMEKIWYHT	FYNELRVAPD	EHPILLTEAP	LNPKINREKM	TQIMFEAFNT	PAMYVAIQAV	LSLYASGRTT	GIVMDSGDGV
**170**	**180**	**190**	**200**	**210**	**220**	**230**	**240**
THIVPIYEGY	ALPHAILRLD	LAGRDLTDYL	MKILTERGYN	FTTTAERPIV	RDVKEKLCYV	ALDFEQEMVR	AAASSSPERS
**250**	**260**	**270**	**280**	**290**	**300**	**310**	**320**
YELPDGQVIT	IGNERFRCPE	AIFQPSFLGI	ESSGIHETTF	NSIMKCDVDI	RKDLYANTVL	SGGSTMYPGI	ADRMQKEIIT
**330**	**340**	**350**	**360**	**370**	**380**		
LAPSTMKIKI	IAPPERKYSV	WIGGSILASL	STFQQMWISK	QEYDEAGPPI	VHRKCF		

Cmpd.	No. of Cmpds.	m/z meas.	Δ m/z [ppm]	z	Rt [min]	Score	P	Range	Sequence	Modification
2065	1	1108.9201	-97.52	2	54.4	45.1	0	293-313	K.DLYANTVLSGGSTMYPGIADR.M	Oxidation: 14

Detailed Protein Report

Protein 33: transthyretin precursor [Homo sapiens]

**Accession:**	gi|4507725	**Score:**	120.5
**Database:**	refseq_human(refseq_human_20140103.fasta)	**MW [kDa]:**	15.9
**Database Date:**	2015-11-30	**pI:**	5.4
		**Sequence Coverage [%]:**	18.4
		**No. of unique Peptides:**	3

Quantitation

Q:W	**Median:** 0.80	**CV:** 0.00 %	**No. of Peptides:**	1
M:Q	**Median:** 0.88	**CV:** 0.00 %	**No. of Peptides:**	1

```
        10         20         30         40         50         60         70         80
MASHRLLLLC LAGLVFVSEA GPTGTGESKC PLMVKVLDAV RGSPAINVAV HVFRKAADDT WEPFASGKTS ESGELHGLTT
        90        100        110        120        130        140        150
EEEFVEGIYK VEIDTKSYWK ALGISPFHEH AEVVFTANDS GPRRYTIAAL LSPYSYSTTA VVTNPKE
```

Cmpd.	No. of Cmpds.	m/z meas.	Δ m/z [ppm]	z	Rt [min]	Score	P	Range	Sequence	Modification	Ratios
1401	2	683.8722	-15.95	2	45.9	76.1	0	42-54	R.GSPAINVAVHVFR.K		
594	2	761.8596	-3.46	2	37.4	15.4	1	55-68	R.KAADDTWEPFASGK.T		M:Q 0.88<br>Q:W 0.80
810	2	697.8221	10.50	2	40.1	29.0	0	56-68	K.AADDTWEPFASGK.T		

Detailed Protein Report

Protein 34: pyruvate kinase PKM isoform f [Homo sapiens]

**Accession:**	gi|332164781	**Score:**	119.1
**Database:**	refseq_human(refseq_human_20140103.fasta)	**MW [kDa]:**	58.5
**Database Date:**	2015-11-30	**pI:**	8.7
**Modification(s):**	Carbamidomethyl	**Sequence Coverage [%]:**	9.9
		**No. of unique Peptides:**	4

Quantitation

Q:W	**Median:** 0.49	**CV:** 3.89 %	**No. of Peptides:**	3
M:Q	**Median:** 0.80	**CV:** 40.92 %	**No. of Peptides:**	3

```
        10         20         30         40         50         60         70         80
MTSAAMSKPH SEAGTAFIQT QQLHAAMADT FLEHMCRLDI DSPPITARNT GIICTIGPAS RSVETLKEMI KSGMNVARLN
        90        100        110        120        130        140        150        160
FSHGTHEYHA ETIKNVRTAT ESFASDPILY RPVAVALDTK GPEIRTGLIK GSGTAEVELK KGATLKITLD NAYMEKCDEN
       170        180        190        200        210        220        230        240
ILWLDYKNIC KVVEVGSKIY VDDGLISLQV KQKGADFLVT EVENGGSLGS KKGVNLPGAA VDLPAVSEKD IQDLKFGVEQ
       250        260        270        280        290        300        310        320
DVDMVFASFI RKASDVHEVR KVLGEKGKNI KIISKIENHE GVRRFDEILE ASDGIMVARG DLGIEIPAEK VFLAQKMMIG
       330        340        350        360        370        380        390        400
RCNRAGKPVI CATQMLESMI KKPRPTRAEG SDVANAVLDG ADCIMLSGET AKGDYPLEAV RMQHLIAREA EAAMFHRKLF
       410        420        430        440        450        460        470        480
EELVRASSHS TDLMEAMAMG SVEASYKCLA AALIVLTESG RSAHQVARYR PRAPIIAVTR NPQTARQAHL YRGIFPVLCK
       490        500        510        520        530        540
DPVQEAWAED VDLRVNFAMN VGKARGFFKK GDVVIVLTGW RPGSGFTNTM RVVPVP
```

Cmpd.	No. of Cmpds.	m/z meas.	Δ m/z [ppm]	z	Rt [min]	Score	P	Range	Sequence	Modification	Ratios	
859	3	599.3167	-17.70	2	39.2	51.2	0	38-48	R.LDIDSPPITAR.N		M:Q<br>Q:W	0.54<br>0.47
566	1	680.2094	-215.66	2	36.4	14.7	0	49-61	R.NTGIICTIGPASR.S	Carbamidomethyl: 6	M:Q<br>Q:W	1.37<br>0.49
111	2	628.6466	11.60	3	30.8	41.8	0	79-94	R.LNFSHGTHEYHAETIK.N		M:Q<br>Q:W	0.69<br>0.51
2063	1	731.7933	-161.16	2	54.4	11.5	0	179-191	K.IYVDDGLISLQVK.Q			

Detailed Protein Report

Protein 35: PREDICTED: DNA-directed RNA polymerase, mitochondrial isoform X1 [Homo sapiens]

**Accession:**	gi|530425287	**Score:**	116.5
**Database:**	refseq_human(refseq_human_20140103.fasta)	**MW [kDa]:**	145.0
**Database Date:**	2015-11-30	**pI:**	10.3
**Modification(s):**	Carbamidomethyl, Oxidation	**Sequence Coverage [%]:**	5.9
		**No. of unique Peptides:**	6

Quantitation

Q:W	**Median:**	1.93	**CV:**	116.30 %	**No. of Peptides:**	2
M:Q	**Median:**	0.65	**CV:**	0.47 %	**No. of Peptides:**	2

10	20	30	40	50	60	70	80
MPSRLRCILG	VYFRLGFPFA	ASVDHETTTP	GRRRGRMRRR	GPVGWPGAAC	VMSALCWGRG	AAGLKRALRP	CGRPGLPGKE
90	100	110	120	130	140	150	160
GTAGGVCGPR	RSSSASPQEQ	DQDRRKDWGH	VELLEVLQAR	VRQLQAESVS	EVVVNRVDVA	RLPECGSGDG	SLQPPRKVQM
170	180	190	200	210	220	230	240
GAKDATPVPC	GRWAKILEKD	KRTQQMRMQR	LKAKLQMPFQ	SGEFKALTRR	LQVEPRLLSK	QMAGCLEDCT	RQAPESPWEE
250	260	270	280	290	300	310	320
QLARLLQEAP	GKLSLDVEQA	PSGQHSQAQL	SGQQQRLLAF	FKCCLLTDQL	PLAHHLLVVH	HGQRQKRKLL	TLDMYNAVML
330	340	350	360	370	380	390	400
GWARQGAFKE	LVYVLFMVKD	AGLTPDLLSY	AAALQCMGRQ	DQDAGTIERC	LEQMSQEGLK	LQALFTAVLL	SEEDRATVLK
410	420	430	440	450	460	470	480
AVHKVKPTFS	LPPQLPPPVN	TSKLLRDVYA	KDGRVSYPKL	HLPLKTLQCL	FEKQLHMELA	SRVCVVSVEK	PTLPSKEVKH
490	500	510	520	530	540	550	560
ARKTLKTLRD	QWEKALCRAL	RETKNRLERE	VYEGRFSLYP	FLCLLDEREV	VRMLLQVLQA	LPAQGESFTT	LARELSARTF
570	580	590	600	610	620	630	640
SRHVVQRQRV	SGQVQALQNH	YRKYLCLLAS	DAEVPEPCLP	RQYWEELGAP	EALREQPWPL	PVQMELGKLL	AEMLVQATQM
650	660	670	680	690	700	710	720
PCSLDKPHRS	SRLVPVLYHV	YSFRNVQQIG	ILKPHPAYVQ	LLEKAAEPTL	TFEAVDVPML	CPPLPWTSPH	SGAFLLSPTK
730	740	750	760	770	780	790	800
LMRTVEGATQ	HQELLETCPP	TALHGALDAL	TQLGNCAWRV	NGRVLDLVLQ	LFQAKGCPQL	GVPAPPSEAP	QPPEAHLPHS
810	820	830	840	850	860	870	880
AAPARKAELR	RELAHCQKVA	REMHSLRAEA	LYRLSLAQHL	RDRVFWLPHN	MDFRGRTYPC	PPHFNHLGSD	VARALLEFAQ
890	900	910	920	930	940	950	960
GRPLGPHGLD	WLKIHLVNLT	GLKKREPLRK	RLAFAEEVMD	DILDSADQPL	TGRKWWMGAE	EPWQTLACCM	EVANAVRASD
970	980	990	1000	1010	1020	1030	1040
PAAYVSHLPV	HQDGSCNGLQ	HYAALGRDSV	GAASVNLEPS	DVPQDVYSGV	AAQVEVFRRQ	DAQRGMRVAQ	VLEGFITRKV
1050	1060	1070	1080	1090	1100	1110	1120
VKQTVMTVVY	GVTRYGGRLQ	IEKRLRELSD	FPQEFVWEAS	HYLVRQVFKS	LQEMFSGTRA	IQHWLTESAR	LISHMGSVVE
1130	1140	1150	1160	1170	1180	1190	1200
WVTPLGVPVI	QPYRLDSKVK	QIGGIQSIT	YTHNGDISRK	PNTRKQKNGF	PPNFIHSLDS	SHMMLTALHC	YRSPPLCHRK
1210	1220	1230	1240	1250	1260	1270	1280
GLTFVSVHDC	YWTHAADVSV	MNQVCREQFV	RLHSEPILQD	LSRFLVKRFC	SEPQKILEAS	QLKETLQAVP	KPGAFDLEQV
1290							
KRSTYFFS							

Cmpd.	No. of Cmpds.	m/z meas.	Δ m/z [ppm]	z	Rt [min]	Score	P	Range	Sequence	Modification	Ratios
2442	1	857.6864	-95.07	3	60.9	18.7	2	40-65	R. RGPVGWPGAACVMSALCWGRG/ R		
3	1	1244.4602	-117.14	2	28.9	10.3	1	41-65	R. GPVGWPGAACVMSALCWGRGA/ R	Carbamidomethyl: 10; Oxidation: 12	
125	2	793.8591	-42.14	2	30.8	18.8	1	158-172	K.VQMGAKDATPVPCGR.W	Carbamidomethyl: 13	M:Q 0.65<br>Q:W 4.87
2861	1	682.6750	3.46	3	66.2	16.6	0	584-601	K.YLCLLASDAEVPEPCLPR.Q	Carbamidomethyl: 3	
390	1	685.3636	4.04	2	34.8	10.5	0	1043-	K.QTVMTVVYGVTR.Y	Oxidation: 4	M:Q 0.65

Detailed Protein Report

Cmpd.	No. of Cmpds.	m/z meas.	Δ m/z [ppm]	z	Rt [min]	Score	P	Range	Sequence	Modification	Ratios
								1054			Q:W 0.77
2603	1	678.4838	186.95	1	63.1	15.6	0	1227-1231	R.EQFVR.L		

Detailed Protein Report

Protein 36: ankyrin repeat domain-containing protein 11 [Homo sapiens]

**Accession:**	gi|56676397	**Score:**	116.2
**Database:**	refseq_human(refseq_human_20140103.fasta)	**MW [kDa]:**	297.7
**Database Date:**	2015-11-30	**pI:**	6.7
**Modification(s):**	Oxidation	**Sequence Coverage [%]:**	4.4
		**No. of unique Peptides:**	9

Quantitation

Q:W	**Median:**	0.78	**CV:**	56.06 %	**No. of Peptides:**	2
M:Q	**Median:**	0.93	**CV:**	66.82 %	**No. of Peptides:**	4

Alias proteins:

Accession	Name	Description
gi|371874367	refseq_human (refseq_human_20140103.fasta)	ankyrin repeat domain-containing protein 11 [Homo sapiens]
gi|371874306	refseq_human (refseq_human_20140103.fasta)	ankyrin repeat domain-containing protein 11 [Homo sapiens]

Detailed Protein Report

10	20	30	40	50	60	70	80
MPKGGCPKAP	QQEELPLSSD	MVEKQTGKKD	KDKVSLTKTP	KLERGDGGKE	VRERASKRKL	PFTAGANGEQ	KDSDTEKQGP
90	100	110	120	130	140	150	160
ERKRIKKEPV	TRKAGLLFGM	GLSGIRAGYP	LSERQQVALL	MQMTAEESAN	SPVDTTPKHP	SQSTVCQKGT	PNSASKTKDK
170	180	190	200	210	220	230	240
VNKRNERGET	RLHRAAIRGD	ARRIKELISE	GADVNVKDFA	GWTALHEACN	RGYYDVAKQL	LAAGAEVNTK	GLDDDTPLHD
250	260	270	280	290	300	310	320
AANNGHYKVV	KLLLRYGGNP	QQSNRKGETP	LKVANSPTMV	NLLLGKGTYT	SSEESSTESS	EEEDAPSFAP	SSSVDGNNTD
330	340	350	360	370	380	390	400
SEFEKGLKHK	AKNPEPQKAT	APVKDEYEFD	EDDEQDRVPP	VDDKHLLKKD	YRKETKSNSF	ISIPKMEVKS	YTKNNTIAPK
410	420	430	440	450	460	470	480
KASHRILSDT	SDEEDASVTV	GTGEKLRLSA	HTILPGSKTR	EPSNAKQQKE	KNKVKKKRKK	ETKGREVRFG	KRSDKFCSSE
490	500	510	520	530	540	550	560
SESESSESGE	DDRDSLGSSG	CLKGSPLVLK	DPSLFSSLSA	SSTSSHGSSA	AQKQNPSHTD	QHTKHWRTDN	WKTISSPAWS
570	580	590	600	610	620	630	640
EVSSLSDSTR	TRLTSESDYS	SEGSSVESLK	PVRKRQEHRK	RASLSEKKSP	FLSSAEGAVP	KLDKEGKVVK	KHKTKHKHKN
650	660	670	680	690	700	710	720
KEKGQCSISQ	ELKLKSFTYE	YEDSKQKSDK	AILLENDLST	ENKLKVLKHD	RDHFKKEEKL	SKMKLEEKEW	LFKDEKSLKR
730	740	750	760	770	780	790	800
IKDTNKDISR	SFREEKDRSN	KAEKERSLKE	KSPKEEKLRL	YKEERKKKSK	DRPSKLEKKN	DLKEDKISKE	KEKIFKEDKE
810	820	830	840	850	860	870	880
KLKKEKVYRE	DSAFDEYCNK	NQFLENEDTK	FSLSDDQRDR	WFSDLSDSSF	DFKGEDSWDS	PVTDYRDMKS	DSVAKLILET
890	900	910	920	930	940	950	960
VKEDSKERRR	DSRAREKRDY	REPFFRKKDR	DYLDKNSEKR	KEQTEKHKSV	PGYLSEKDKK	RRESAEAGRD	RKDALESCKE
970	980	990	1000	1010	1020	1030	1040
RRDGRAKPEE	AHREELKECG	CESGFKDKSD	GDFGKGLEPW	ERHHPAREKE	KKDGPDKERK	EKTKPERYKE	KSSDKDKSEK
1050	1060	1070	1080	1090	1100	1110	1120
SILEKCQKDK	EFDKCFKEKK	DTKEKHKDTH	GKDKERKASL	DQGKEKKEKA	FPGIISEDFS	EKKDDKKGKE	KSWYIADIFT
1130	1140	1150	1160	1170	1180	1190	1200
DESEDDRDSC	MGSGFKMGEA	SDLPRTDGLQ	EKEEGREAYA	SDRHRKSSDK	QHPERQKDKE	PRDRRKDRGA	ADAGRDKKEK
1210	1220	1230	1240	1250	1260	1270	1280
VFEKHKEKKD	KESTEKYKDR	KDRASVDSTQ	DKKNKQKLPE	KAEKKHAAED	KAKSKHKEKS	DKEHSKERKS	SRSADAEKSL
1290	1300	1310	1320	1330	1340	1350	1360
LEKLEEEALH	EYREDSNDKI	SEVSSDSFTD	RGQEPGLTAF	LEVSFTEPPG	DDKPRESACL	PEKLKEKERH	RHSSSSSKKS
1370	1380	1390	1400	1410	1420	1430	1440
HDRERAKKEK	AEKKEKGEDY	KEGGSRKDSG	QYEKDFLEAD	AYGVSYNMKA	DIEDELDKTI	ELFSTEKKDK	NDSEREPSKK
1450	1460	1470	1480	1490	1500	1510	1520
IEKELKPYGS	SAINILKEKK	KREKHREKWR	DEKERHRDRH	ADGLLRHHRD	ELLRHHRDEQ	KPATRDKDSP	PRVLKDKSRD
1530	1540	1550	1560	1570	1580	1590	1600
EGPRLGDAKL	KEKFKDGAEK	EKGDPVKMSN	GNDKVAPSKD	PGKKDARPRE	KLLGDGDLMM	TSFERMLSQK	DLEIEERHKR
1610	1620	1630	1640	1650	1660	1670	1680
HKERMKQMEK	LRHRSGDPKL	KEKAKPADDG	RKKGLDIPAK	KPPGLDPPFK	DKKLKESTPI	PPAAENKLHP	ASGADSKDWL
1690	1700	1710	1720	1730	1740	1750	1760
AGPHMKEVLP	ASPRPDQSRP	TGVPTPTSVL	SCPSYEEVMH	TPRTPSCSAD	DYADLVFDCA	DSQHSTPVPT	APTSACSPSF
1770	1780	1790	1800	1810	1820	1830	1840
FDRFSVASSG	LSENASQAPA	RPLSTNLYRS	VSVDIRRTPE	EEFSVGDKLF	RQQSVPAASS	YDSPMPPSME	DRAPLPPVPA
1850	1860	1870	1880	1890	1900	1910	1920
EKFACLSPGY	YSPDYGLPSP	KVDALHCPPA	AVVTVTPSPE	GVFSSLQAKP	SPSPRAELLV	PSLEGALPPD	LDTSEDQQAT
1930	1940	1950	1960	1970	1980	1990	2000
AAIIPPEPSY	LEPLDEGPFS	AVITEEPVEW	AHPSEQALAS	SLIGGTSENP	VSWPVGSDLL	LKSPQRFPES	PKRFCPADPL
2010	2020	2030	2040	2050	2060	2070	2080
HSAAPGPFSA	SEAPYPAPPA	SPAPYALPVA	EPGLEDVKDG	VDAVPAAIST	SEAAPYAPPS	GLESFFSNCK	SLPEAPLDVA
2090	2100	2110	2120	2130	2140	2150	2160
PEPACVAAVA	QVEALGPLEN	SFLDGSRGLS	HLGQVEPVPW	ADAFAGPEDD	LDLGPFSLPE	LPLQTKDAAD	GEAEPVEESL
2170	2180	2190	2200	2210	2220	2230	2240

Detailed Protein Report

Cmpd.	No. of Cmpds.	m/z meas.	Δ m/z [ppm]	z	Rt [min]	Score	P	Range	Sequence	Modification	Ratios
2918	1	1155.0468	-67.97	2	65.2	13.4	2	93-114	R. KAGLLFGMGLSGIRAGYPLSER. Q	Oxidation: 8	
793	1	1105.5255	-96.76	2	39.9	11.9	2	266-286	R.KGETPLKVANSPTMVNLLLGK. G		
1383	2	637.4051	150.05	3	45.8	14.2	0	553-570	K.TISSPAWSEVSSLSDSTR.T		M:Q 2.07<br>Q:W 0.46
462	1	475.6124	-248.29	2	34.3	11.4	1	1270-1278	K.SSRSADAEK.S		M:Q 0.91
2600	1	741.8765	-22.04	2	62.5	14.8	2	1478-1489	R.DRHADGLLRHHR.D		
313	3	501.1733	-259.03	2	33.0	13.5	2	1525-1533	R.LGDAKLKEK.F		M:Q 1.05<br>Q:W 1.31
2267	1	937.3359	-108.02	2	56.7	10.6	1	1570-1585	R.EKLLGDGDLMMTSFER.M	Oxidation: 10, 11	
1507	1	619.9684	9.93	3	48.5	11.3	1	1570-1585	R.EKLLGDGDLMMTSFER.M	Oxidation: 11	M:Q 0.38
2584	1	658.2319	-130.00	2	61.0	15.1	0	2424-2434	K.LDAIEPYHSDR.A		

Detailed Protein Report

Protein 37: protein AHNAK2 [Homo sapiens]

**Accession:**	gi|156766050	**Score:**	116.0
**Database:**	refseq_human(refseq_human_20140103.fasta)	**MW [kDa]:**	616.2
**Database Date:**	2015-11-30	**pI:**	5.1
**Modification(s):**	Oxidation	**Sequence Coverage [%]:**	2.3
		**No. of unique Peptides:**	8

Quantitation

Q:W	**Median:**	1.96	**CV:**	1141.42 %	**No. of Peptides:**	2
M:Q	**Median:**	1.22	**CV:**	79.66 %	**No. of Peptides:**	4

Detailed Protein Report

10	20	30	40	50	60	70	80
MCDCFHMVLP	TWPGTPGSVS	GRQLQPGEPG	AETEDDHSVT	EGPADEGIRP	RPQGSSPVYE	YTTEAADFGL	QEDAPGRQGS
90	100	110	120	130	140	150	160
AGRRRSWWKR	DSGDSRTFFR	MSRPEAVQEA	TEVTLKTEVE	AGASGYSVTG	GGDQGIFVKQ	VLKDSSAAKL	FNLREGDQLL
170	180	190	200	210	220	230	240
STTVFFENIK	YEDALKILQY	SEPYKVQFKI	RRQLPAPQDE	EWASSDAQHG	PQGKEKEDTD	VADGCRETPT	KTLEGDGDQE
250	260	270	280	290	300	310	320
RLISKPRVGR	GRQSQRERLS	WPKFQSIKSK	RGPGPQRSHS	SSEAYEPRDA	HDVSPTSTDT	EAQLTVERQE	QKAGPGSQRR
330	340	350	360	370	380	390	400
RKFLNLRFRT	GSGQGPSSTG	QPGRGFQSGV	GRAGVLEELG	PWGDSLEETG	AATGSRREER	AEQDREVMPA	QSMPLPTELG
410	420	430	440	450	460	470	480
DPRLCEGTPQ	EGGLRAARLH	GKTLEGQAQE	TAVAQRKPRA	QPTPGMSREG	EGEGLQSLEI	GIARLSLRDT	TEGGTQIGPP
490	500	510	520	530	540	550	560
EIRVRVHDLK	TPKFAFSTEK	EPERERRLST	PQRGKRQDAS	SKAGTGLKGE	EVEGAGWMPG	REPTTHAEAQ	GDEGDGEEGL
570	580	590	600	610	620	630	640
QRTRITEEQD	KGREDTEGQI	RMPKFKIPSL	GWSPSKHTKT	GREKATEDTE	QGREGEATAT	ADRREQRRTE	EGLKDKEDSD
650	660	670	680	690	700	710	720
SMTNTTKIQL	IHDEKRLKKE	QILTEKEVAT	KDSKFKMPKF	KMPLFGASAP	GKSMEASVDV	SAPKVEADVS	LLSMQGDLKT
730	740	750	760	770	780	790	800
TDLSVQTPSA	DLEVQDGQVD	VKLPEGPLPE	GASLKGHLPK	VQRPSLKMPK	VDLKGPKLDL	KGPKAEVTAP	DVKMSLSSME
810	820	830	840	850	860	870	880
VDVQAPRAKL	DGARLEGDLS	LADKEVTAKD	SKFKMPKFKM	PSFGVSAPGK	SMEDSVDVSA	PKVEADVSLS	SMQGDLKATD
890	900	910	920	930	940	950	960
LSIQPPSADL	EVQAGQVDVK	LPEGPVPEGA	GPKVHLPKVE	MPSFKMPKVD	LKGPQIDVKG	PKLDLKGPKA	EVTAPDGEVS
970	980	990	1000	1010	1020	1030	1040
LPSMEVDVQA	QKAKLDGAWL	EGDLSLADKD	VTAKDSKFKM	PKFKMPSFGV	SAPGKSIKAL	VDVSAPKVEA	DLSLPSMQGD
1050	1060	1070	1080	1090	1100	1110	1120
LKTTDLSIQP	ASTDLKVQAD	QVDVKLPEGH	LPEGAGLKGH	LPKVEMPSFK	MPKVALKGPQ	VDVKGPKLDL	KSPKAEVTAP
1130	1140	1150	1160	1170	1180	1190	1200
DVEVSLPSVE	VDVEAPGAKL	DSARLEGELS	LADKDVTAKD	SRFKMPKFKM	PSFGASAPGK	SIEASVDVSA	PKVEADVSLP
1210	1220	1230	1240	1250	1260	1270	1280
SMQGDLKTTD	LSIQPPSADL	EVHAGQVDVK	LLEGHVPEGA	GFKGHLPKVQ	MPSLKMPKVD	LKGPQVEVRG	PKLDLKGHKA
1290	1300	1310	1320	1330	1340	1350	1360
EVTAHEVAVS	LPSVEVDMQA	PGAKLDGAQL	DGDLSLADKD	VTAKDSKFKM	PKFKMPSFGV	SAPGKSIEAS	VDLSAPKVEA
1370	1380	1390	1400	1410	1420	1430	1440
DMSLPSMQGD	LKTTDLSIQP	PSTDLELQAG	QLDVKLPEGP	VPEGAGLKGH	LPKLQMPSFK	VPKVDLKGPE	IDIKGPKLDL
1450	1460	1470	1480	1490	1500	1510	1520
KDPKVEVTAP	DVEVSLPSVE	VDVEAPGAKL	DGGRLEEDMS	LADKDLTTKD	SKFKMPKFKM	PSFGVSAPGK	SIEASVDVSA
1530	1540	1550	1560	1570	1580	1590	1600
PKVEADVSLP	SMQGDLKATD	LSIQPPSADL	EVQAGQVDVK	LPEGPVSEGA	GLKGHLPKVQ	MPSFKMPKVD	LKGPQIDVKG
1610	1620	1630	1640	1650	1660	1670	1680
PKLDLKGPKV	EVTAPDVKMS	LSSMEVDVQA	PRAKLDGAQL	EGDLSLADKA	VTAKDSKFKM	PKFKMPSFGV	SAPGKSIEAS
1690	1700	1710	1720	1730	1740	1750	1760
VDVSEPKVEA	DVSLPSMQGD	LKTTDLSIQS	PSADLEVQAG	QVNVKLPEGP	LPEGAGFKGH	LPKVQMPSLK	MPKVALKGPQ
1770	1780	1790	1800	1810	1820	1830	1840
MDVKGPKLDL	KGPKAEVMAP	DVEVSLPSVE	VDVEAPGAKL	DSVRLEGDLS	LADKDVTAKD	SKFKMPKFKM	PSFGVSAPGK
1850	1860	1870	1880	1890	1900	1910	1920
SIEASVDVSA	PKVEAEVSLP	SMQGDLKTTD	LCIPLPSADL	VVQAGQVDMK	LPEGQVPEGA	GLKGHLPKVD	MPSFKMPKVD
1930	1940	1950	1960	1970	1980	1990	2000
LKGPQTDVKG	AKLDLKGPKA	EVTAPDVEVS	LPSMEVDVQA	QKAKLDGARL	EGDLSLADKD	MTAKDSKFKM	PKFKMPSFGV
2010	2020	2030	2040	2050	2060	2070	2080
SAPGRSIEAS	VDVPAPKVEA	DVSLPSMQGD	LKTTDLSIQP	PSADLKVQTG	QVDVKLPEGH	VPEGAGLKGH	LPKVEMPSLK
2090	2100	2110	2120	2130	2140	2150	2160
MPKVDLKGPQ	VDIKGPKLDL	KDPKVEMRVP	DVEVSLPSME	VDVQAPRAKL	DSAHLQGDLT	LANKDLTTKD	SKFKMPKFKM
2170	2180	2190	2200	2210	2220	2230	2240

Detailed Protein Report

Cmpd.	No. of Cmpds.	m/z meas.	Δ m/z [ppm]	z	Rt [min]	Score	P	Range	Sequence	Modification	Ratios	
2566	1	913.4598	-15.26	2	62.5	16.5	1	469-485	R.DTTEGGTQIGPPEIRVR.V			
2046	2	596.7306	-219.53	2	56.0	16.0	2	484-493	R.VRVHDLKTPK.F		M:Q	1.68
597	1	1230.6275	14.93	2	36.8	18.6	1	785-807	K.<br>AEVTAPDVKMSLSSMEVDVQAPR<br>A			
267	1	862.8828	-39.08	2	33.3	11.9	1	1475-<br>1489	R.LEEDMSLADKDLTTK.D	Oxidation: 5		
738	1	769.3703	-105.01	2	39.2	11.2	2	3408-<br>3421	K.SPQVDIKGPKLDLK.V		M:Q<br>Q:W	0.67<br>0.22
1307	2	926.0984	-40.13	3	46.5	19.2	1	3491-<br>3517	R.<br>SIEASLDVSAPKVEADVSLSSMQG<br>A			
2190	3	648.2929	-93.26	2	56.0	10.4	1	5699-<br>5710	R.FPKLGFSSSPTK.K		M:Q<br>Q:W	0.59<br>17.81
1331	2	1023.0328	-5.50	2	45.4	12.2	1	5762-<br>5779	R.VMVTSAARTELILPEQDR.K	Oxidation: 2	M:Q	3.28

Detailed Protein Report

Protein 38: Alstrom syndrome protein 1 [Homo sapiens]

**Accession:**	gi|110349786	**Score:**	115.7
**Database:**	refseq_human(refseq_human_20140103.fasta)	**MW [kDa]:**	460.9
**Database Date:**	2015-11-30	**pI:**	5.8
**Modification(s):**	Carbamidomethyl, Oxidation	**Sequence Coverage [%]:**	2.6
		**No. of unique Peptides:**	5

Quantitation

Q:W	**Median:**	1.92	**CV:**	0.00 %	**No. of Peptides:**	1
M:Q	**Median:**	2.18	**CV:**	144.52 %	**No. of Peptides:**	2

Detailed Protein Report

10	20	30	40	50	60	70	80
MEPEDLPWPG	ELEEEEEEEE	EEEEEEEEEA	AAAAAAANVDD	VVVVEEVEEE	AGRELDSDSH	YGPQHLESID	DEEDEEAKAW
90	100	110	120	130	140	150	160
LQAHPGRILP	PLSPPQHRYS	EGERTSLEKI	VPLTCHVWQQ	IVYQGNSRTQ	ISDTNVVCLE	TTAQRGSGDD	QKTESWHCLP
170	180	190	200	210	220	230	240
QEMDSSQTLD	TSQTRFNVRT	EDTEVTDFPS	LEEGILTQSE	NQVKEPNRDL	FCSPLLVIQD	SFASPDLPLL	TCLTQDQEFA
250	260	270	280	290	300	310	320
PDSLFHQSEL	SFAPLRGIPD	KSEDTEWSSR	PSEVSEALFQ	ATAEVASDLA	SSRFSVSQHP	LIGSTAVGSQ	CPFLPSEQGN
330	340	350	360	370	380	390	400
NEETISSVDE	LKIPKDCDRY	DDLCSYMSWK	TRKDTQWPEN	NLADKDQVSV	ATSFDITDEN	IATKRSDHFD	AARSYGQYWT
410	420	430	440	450	460	470	480
QEDSSKQAET	YLTKGLQGKV	ESDVITLDGL	NENAVVCSER	VAELQRKPTR	ESEYHSSDLR	MLRMSPDTVP	KAPKHLKAGD
490	500	510	520	530	540	550	560
TSKGGIAKVT	QSNLKSGITT	TPVDSDIGSH	LSLSLEDLSQ	LAVSSPLETT	TGQHTDTLNQ	KTLADTHLTE	ETLKVTAIPE
570	580	590	600	610	620	630	640
PADQKTATPT	VLSSSHSHRG	KPSIFYQQGL	PDSHLTEEAL	KVSAAPGLAD	QTTGMSTLTS	TSYSHREKPG	TFYQQELPES
650	660	670	680	690	700	710	720
NLTEEPLEVS	AAPGPVEQKT	GIPTVSSTSH	SHVEDLLFFY	RQTLPDGHLT	DQALKVSAVS	GPADQKTGTA	TVLSTPHSHR
730	740	750	760	770	780	790	800
EKPGIFYQQE	FADSHQTEET	LTKVSATPGP	ADQKTEIPAV	QSSSYSQREK	PSILYPQDLA	DSHLPEEGLK	VSAVAGPADQ
810	820	830	840	850	860	870	880
KTGLPTVPSS	AYSHREKLLV	FYQQALLDSH	LPEEALKVSA	VSGPADGKTG	TPAVTSTSSA	SSSLGEKPSA	FYQQTLPNSH
890	900	910	920	930	940	950	960
LTEEALKVSI	VPGPGDQKTG	IPSAPSSFYS	HREKPIIFSQ	QTLPDFLFPE	EALKVSAVSV	LAAQKTGTPT	VSSNSHSHSE
970	980	990	1000	1010	1020	1030	1040
KSSVFYQQEL	PDSDLPRESL	KMSAIPGLTD	QKTVPTPTVP	SGSFSHREKP	SIFYQQEWPD	SYATEKALKV	STGPGPADQK
1050	1060	1070	1080	1090	1100	1110	1120
TEIPAVQSSS	YPQREKPSVL	YPQVLSDSHL	PEESLKVSAF	PGPADQMTDT	PAVPSTFYSQ	REKPGIFYQQ	TLPESHLPKE
1130	1140	1150	1160	1170	1180	1190	1200
ALKISVAPGL	ADQKTGTPTV	TSTSYSQHRE	KPSIFHQQAL	PGTHIPEEAQ	KVSAVTGPGN	QKTWIPRVLS	TFYSQREKPG
1210	1220	1230	1240	1250	1260	1270	1280
IFYQQTLPGS	HIPEEAQKVS	PVLGPADQKT	GTPTPTSASY	SHTEKPGIFY	QQVLPDNHPT	EEALKISVAS	EPVDQTTGTP
1290	1300	1310	1320	1330	1340	1350	1360
AVTSTSYSQY	REKPSIFYQQ	SLPSSHLTEE	AKNVSAVPGP	ADQKTVIPIL	PSTFYSHTEK	PGVFYQQVLP	HSHPTEEALK
1370	1380	1390	1400	1410	1420	1430	1440
ISVASEPVDQ	TTGTPTVTST	SYSQHTEKPS	IFYQQSLPGS	HLTEEAKNVS	AVPGPGDRKT	GIPTLPSTFY	SHTEKPGSFY
1450	1460	1470	1480	1490	1500	1510	1520
QQVLPHSHLP	EEALEVSVAP	GPVDQTIGTP	TVTSPSSSFG	EKPIVIYKQA	FPEGHLPEES	LKVSVAPGPV	GQTTGAPTIT
1530	1540	1550	1560	1570	1580	1590	1600
SPSYSQHRAK	SGSFYQLALL	GSQIPEEALR	VSSAPGPADQ	TTGIPTITST	SYSFGEKPIV	NYKQAFPDGH	LPEEALKVSI
1610	1620	1630	1640	1650	1660	1670	1680
VSGPTEKKTD	IPAGPLGSSA	LGEKPITFYR	QALLDSPLNK	EVVKVSAAPG	PADQKTETLP	VHSTSYSNRG	KPVIFYQQTL
1690	1700	1710	1720	1730	1740	1750	1760
SDSHLPEEAL	KVPPVPGPDA	QKTETPSVSS	SLYSYREKPI	VFYQQALPDS	ELTQEALKVS	AVPQPADQKT	GLSTVTSSFY
1770	1780	1790	1800	1810	1820	1830	1840
SHTEKPNISY	QQELPDSHLT	EEALKVSNVP	GPADQKTGVS	TVTSTSYSHR	EKPIVSYQRE	LPHFTEAGLK	ILRVPGPADQ
1850	1860	1870	1880	1890	1900	1910	1920
KTGINILPSN	SYPQREHSVI	SYEQELPDLT	EVTLKAIGVP	GPADQKTGIQ	IASSSSYSNR	EKASIFHQQE	LPDVTEEALN
1930	1940	1950	1960	1970	1980	1990	2000
VFVVPGQGDR	KTEIPTVPLS	YYSRREKPSV	ISQQELPDSH	LTEEALKVSP	VSIPAEQKTG	IPIGLSSSYS	HSHKEKLKIS
2010	2020	2030	2040	2050	2060	2070	2080
TVHIPDDQKT	EFPAATLSSY	SQIEKPKIST	VIGPNDQKTP	SQTAFHSSYS	QTVKPNILFQ	QQLPDRDQSK	GILKISAVPE
2090	2100	2110	2120	2130	2140	2150	2160
LTDVNTGKPV	SLSSSYFHRE	KSNIFSPQEL	PGSHVTEDVL	KVSTIPGPAG	QKTVLPTALP	SSFSHREKPD	IFYQKDLPDR
2170	2180	2190	2200	2210	2220	2230	2240

Detailed Protein Report

Cmpd.	No. of Cmpds.	m/z meas.	Δ m/z [ppm]	z	Rt [min]	Score	P	Range	Sequence	Modification	Ratios
312	1	862.8548	-22.20	2	33.0	10.7	1	340-352	R.YDDLCSYMSWKTR.K	Carbamidomethyl: 5	
881	1	555.8212	17.82	2	40.6	26.2	0	1219-1229	K.VSPVLGPADQK.T		Q:W 1.92<br>M:Q 0.76
1890	1	1036.8846	-93.94	2	54.0	16.6	2	2503-2519	R.NAEEEESRVRAHAWNMK.F	Oxidation: 16	
160	1	896.7503	-5.56	3	31.7	21.2	1	2566-2589	R. GMGCKPEAVCSHIIIESHEKGCFR T	Carbamidomethyl: 4	M:Q 6.32
1836	3	863.4125	-60.24	2	51.6	18.6	1	3791-3805	R.LIQAFGHERVCLSPR.R		

Detailed Protein Report

Protein 39: plectin isoform 1d [Homo sapiens]

**Accession:**	gi|41322910	**Score:**	115.3
**Database:**	refseq_human(refseq_human_20140103.fasta)	**MW [kDa]:**	512.3
**Database Date:**	2015-11-30	**pI:**	5.5
**Modification(s):**	Carbamidomethyl	**Sequence Coverage [%]:**	2.6
		**No. of unique Peptides:**	5

Quantitation

Q:W	**Median:**	1.10	**CV:**	0.00 %	**No. of Peptides:**	1
M:Q	**Median:**	0.94	**CV:**	9.14 %	**No. of Peptides:**	2

Detailed Protein Report

10	20	30	40	50	60	70	80
MKIVPDERDR	VQKKTFTKWV	NKHLIKAQRH	ISDLYEDLRD	GHNLISLLEV	LSGDSLPREK	GRMRFHKLQN	VQIALDYLRH
90	100	110	120	130	140	150	160
RQVKLVNIRN	DDIADGNPKL	TLGLIWTIIL	HFQISDIQVS	GQSEDMTAKE	KLLLWSQRMV	EGYQGLRCDN	FTSSWRDGRL
170	180	190	200	210	220	230	240
FNAIIHRHKP	LLIDMNKVYR	QTNLENLDQA	FSVAERDLGV	TRLLDPEDVD	VPQPDEKSII	TYVSSLYDAM	PRVPDVQDGV
250	260	270	280	290	300	310	320
RANELQLRWQ	EYRELVLLLL	QWMRHHTAAF	EERRFPSSFE	EIEILWSQFL	KFKEMELPAK	EADKNRSKGI	YQSLEGAVQA
330	340	350	360	370	380	390	400
GQLKVPPGYH	PLDVEKEWGK	LHVAILEREK	QLRSEFERLE	CLQRIVTKLQ	MEAGLCEEQL	NQADALLQSD	VRLLAAGKVP
410	420	430	440	450	460	470	480
QRAGEVERDL	DKADSMIRLL	FNDVQTLKDG	RHPQGEQMYR	RVYRLHERLV	AIRTEYNLRL	KAGVAAPATQ	VAQVTLQSVQ
490	500	510	520	530	540	550	560
RRPELEDSTL	RYLQDLLAWV	EENQHRVDGA	EWGVDLPSVE	AQLGSHRGLH	QSIEEFRAKI	ERARSDEGQL	SPATRGAYRD
570	580	590	600	610	620	630	640
CLGRLDLQYA	KLLNSSKARL	RSLESLHSFV	AAATKELMWL	NEKEEEEVGF	DWSDRNTNMT	AKKESYSALM	RELELKEKKI
650	660	670	680	690	700	710	720
KELQNAGDRL	LREDHPARPT	VESFQAALQT	QWSWMLQLCC	CIEAHLKENA	AYFQFFSDVR	EAEGQLQKLQ	EALRRKYSCD
730	740	750	760	770	780	790	800
RSATVTRLED	LLQDAQDEKE	QLNEYKGHLS	GLAKRAKAVV	QLKPRHPAHP	MRGRLPLLAV	CDYKQVEVTV	HKGDECQLVG
810	820	830	840	850	860	870	880
PAQPSHWKVL	SSSGSEAAVP	SVCFLVPPPN	QEAQEAVTRL	EAQHQALVTL	WHQLHVDMKS	LLAWQSLRRD	VQLIRSWSLA
890	900	910	920	930	940	950	960
TFRTLKPEEQ	RQALHSLELH	YQAFLRDSQD	AGGFGPEDRL	MAEREYGSCS	HHYQQLLQSL	EQGAQEESRC	QRCISELKDI
970	980	990	1000	1010	1020	1030	1040
RLQLEACETR	TVHRLRLPLD	KEPARECAQR	IAEQQKAQAE	VEGLGKGVAR	LSAEAEKVLA	LPEPSPAAPT	LRSELELTLG
1050	1060	1070	1080	1090	1100	1110	1120
KLEQVRSLSA	IYLEKLKTIS	LVIRGTQGAE	EVLRAHEEQL	KEAQAVPATL	PELEATKASL	KKLRAQAEAQ	QPTFDALRDE
1130	1140	1150	1160	1170	1180	1190	1200
LRGAQEVGER	LQQRHGERDV	EVERWRERVA	QLLERWQAVL	AQTDVRQREL	EQLGRQLRYY	RESADPLGAW	LQDARRRQEQ
1210	1220	1230	1240	1250	1260	1270	1280
IQAMPLADSQ	AVREQLRQEQ	ALLEEIERHG	EKVEECQRFA	KQYINAIKDY	ELQLVTYKAQ	LEPVASPAKK	PKVQSGSESV
1290	1300	1310	1320	1330	1340	1350	1360
IQEYVDLRTH	YSELTTLTSQ	YIKFISETLR	RMEEEERLAE	QQRAEEERERL	AEVEAALEKQ	RQLAEAHAQA	KAQAEREAKE
1370	1380	1390	1400	1410	1420	1430	1440
LQQRMQEEVV	RREEAAVDAQ	QQKRSIQEEL	QQLRQSSEAE	IQAKARQAEA	AERSRLRIEE	EIRVVRLQLE	ATERQRGGAE
1450	1460	1470	1480	1490	1500	1510	1520
GELQALRARA	EEAEAQKRQA	QEEAERLRRQ	VQDESQRKRQ	AEVELASRVK	AEAEAAREKQ	RALQALEELR	LQAEEAERRL
1530	1540	1550	1560	1570	1580	1590	1600
RQAEVERARQ	VQVALETAQR	SAEAELQSKR	ASFAEKTAQL	ERSLQEEHVA	VAQLREEAER	RAQQQAEAER	AREEAERELE
1610	1620	1630	1640	1650	1660	1670	1680
RWQLKANEAL	RLRLQAEEVA	QQKSLAQAEA	EKQKEEAERE	ARRRGKAEEQ	AVRQRELAEQ	ELEKQRQLAE	GTAQQRLAAE
1690	1700	1710	1720	1730	1740	1750	1760
QELIRLRAET	EQGEQQRQLL	EEELARLQRE	AAAATQKRQE	LEAELAKVRA	EMEVLLASKA	RAEEESRSTS	EKSKQRLEAE
1770	1780	1790	1800	1810	1820	1830	1840
AGRFRELAEE	AARLRALAEE	AKRQRQLAEE	DAARQRAEAE	RVLAEKLAAI	GEATRLKTEA	EIALKEKEAE	NERLRRLAED
1850	1860	1870	1880	1890	1900	1910	1920
EAFQRRRLEE	QAAQHKADIE	ERLAQLRKAS	DSELERQKGL	VEDTLRQRRQ	VEEEILALKA	SFEKAAAGKA	ELELELGRIR
1930	1940	1950	1960	1970	1980	1990	2000
SNAEDTLRSK	EQAELEAARQ	RQLAAEEERR	RREAEERVQK	SLAAEEEAAR	QRKAALEEVE	RLKAKVEEAR	RLRERAEQES
2010	2020	2030	2040	2050	2060	2070	2080
ARQLQLAQEA	AQKRLQAEEK	AHAFAVQQKE	QELQQTLQQE	QSVLDQLRGE	AEAARRAAEE	AEEARVQAER	EAAQSRRQVE
2090	2100	2110	2120	2130	2140	2150	2160
EAERLKQSAE	EQAQARAQAQ	AAAEKLRKEA	EQEAARRAQA	EQAALRQKQA	ADAEMEKHKK	FAEQTLRQKA	QVEQELTTLR
2170	2180	2190	2200	2210	2220	2230	2240

Detailed Protein Report

Cmpd.	No. of Cmpds.	m/z meas.	Δ m/z [ppm]	z	Rt [min]	Score	P	Range	Sequence	Modification	Ratios
2471	1	588.1664	-172.67	2	61.4	13.5	0	1688-1697	R.AETEQGEQQR.Q		M:Q 1.03
1213	1	713.6809	-62.89	3	43.8	10.9	2	2243-2262	K.QVAEEAARLSVAAQEAARLR.Q		
1692	4	582.1401	-225.95	2	51.1	12.2	0	2343-2352	R.QLEMSAEAER.L		
42	1	768.2950	-141.23	2	29.9	18.7	1	3570-3583	R.AQLMADFQAGRVTK.E		M:Q 0.86<br>Q:W 1.10
1932	1	1021.8810	-139.60	2	52.6	12.9	2	3832-3849	R.CRRDDGTGQLLLPLSDAR.K	Carbamidomethyl: 1	

Detailed Protein Report

Protein 40: heat shock protein beta-1 [Homo sapiens]

Accession: gi|4504517
Database: refseq_human(refseq_human_20140103.fasta)
Database Date: 2015-11-30

Score: 113.8
MW [kDa]: 22.8
pI: 6.0
Sequence Coverage [%]: 21.5
No. of unique Peptides: 2

Quantitation

Q:W	**Median:**	0.64	**CV:**	0.00 %	**No. of Peptides:**	1
M:Q	**Median:**	1.22	**CV:**	0.00 %	**No. of Peptides:**	1

```
        10         20         30         40         50         60         70         80
MTERRVPFSL LRGPSWDPFR DWYPHSRLFD QAFGLPRLPE EWSQWLGGSS WPGYVRPLPP AAIESPAVAA PAYSRALSRQ
        90        100        110        120        130        140        150        160
LSSGVSEIRH TADRWRVSLD VNHFAPDELT VKTKDGVVEI TGKHEERQDE HGYISRCFTR KYTLPPGVDP TQVSSSLSPE
       170        180        190        200        210
GTLTVEAPMP KLATQSNEIT IPVTFESRAQ LGGPEAAKSD ETAAK
```

Cmpd.	No. of Cmpds.	m/z meas.	Δ m/z [ppm]	z	Rt [min]	Score	P	Range	Sequence	Modification	Ratios
1666	4	953.4317	-71.06	2	49.5	35.6	0	172-188	K.LATQSNEITIPVTFESR.A		M:Q 1.22<br>Q:W 0.64
2886	1	822.4678	63.76	2	64.8	15.6	1	189-205	R.AQLGGPEAAKSDETAAK.-		

Detailed Protein Report

Protein 41: trinucleotide repeat-containing gene 18 protein [Homo sapiens]

**Accession:**	gi|169658378	**Score:**	113.7
**Database:**	refseq_human(refseq_human_20140103.fasta)	**MW [kDa]:**	314.3
**Database Date:**	2015-11-30	**pI:**	9.6
**Modification(s):**	Carbamidomethyl, Oxidation	**Sequence Coverage [%]:**	4.0
		**No. of unique Peptides:**	5

Alias proteins:

Accession	Name	Description
gi|578813684	refseq_human (refseq_human_20140103.fasta)	PREDICTED: trinucleotide repeat-containing gene 18 protein isoform X1 [Homo sapiens]

Detailed Protein Report

10	20	30	40	50	60	70	80
MDGRDFGPQR	SVHGPPPPLL	SGLAMDSHRV	GAATAGRLPA	SGLPGPLPPG	KYMAGLNLHP	HPGEAFLGSF	VASGMGPSAS
90	100	110	120	130	140	150	160
SHGSPVPLPS	DLSFRSPTPS	NLPMVQLWAA	HAHEGFSHLP	SGLYPSYLHL	NHLEPPSSGS	PLLSQLGQPS	IFDTQKGQGP
170	180	190	200	210	220	230	240
GGDGFYLPTA	GAPGSLHSHA	PSARTPGGGH	SSGAPAKGSS	SRDGPAKERA	GRGGEPPPLF	GKKDPRARGE	EASGPRGVVD
250	260	270	280	290	300	310	320
LTQEARAEGR	QDRGPPRLAE	RLSPFLAESK	TKNAALQPSV	LTMCNGGAGD	VGLPALVAEA	GRGGAKEAAR	QDEGARLLRR
330	340	350	360	370	380	390	400
TETLLPGPRP	CPSPLPPPPA	PPKGPPAPPA	ATPAGVYTVF	REQGREHRVV	APTFVPSVEA	FDERPGPIQI	ASQARDARAR
410	420	430	440	450	460	470	480
EREAGRPGVL	QAPPGSPRPL	DRPEGLREKN	SVIRSLKRPP	PADAPTVRAT	RASPDPRAYV	PAKELLKPEA	DPRPCERAPR
490	500	510	520	530	540	550	560
GPAGPAAQQA	AKLFGLEPGR	PPPTGPEHKW	KPFELGNFAA	TQMAVLAAQH	HHSRAEEEAA	VVAASSSKKA	YLDPGAVLPR
570	580	590	600	610	620	630	640
SAATCGRPVA	DMHSAAHGSG	EASAMQSLIK	YSGSFARDAV	AVRPGGCGKK	SPFGGLGTMK	PEPAPTSAGA	SRAQARLPHS
650	660	670	680	690	700	710	720
GGPAAGGGRQ	LKRDPERPES	AKAFGREGSG	AQGEAEVRHP	PVGIAVAVAR	QKDSGGSGRL	GPGLVDQERS	LSLSNVKGHG
730	740	750	760	770	780	790	800
RADEDCVDDR	ARHREERLLG	ARLDRDQEKL	LRESKELADL	ARLHPTSCAP	NGLNPNLMVT	GGPALAGSGR	WSADPAAHLA
810	820	830	840	850	860	870	880
THPWLPRSGN	ASMWLAGHPY	GLGPPSLHQG	MAPAFPPGLG	GSLPSAYQFV	RDPQSGQLVV	IPSDHLPHFA	ELMERATVPP
890	900	910	920	930	940	950	960
LWPALYPPGR	SPLHHAQQLQ	LFSQQHFLRQ	QEFLYLQQQA	AQALELQRSA	QLVQERLKAQ	EHRAEMEEKG	SKRGLEAAGK
970	980	990	1000	1010	1020	1030	1040
AGLATAGPGL	LPRKPPGLAA	GPAGTYGKAV	SPPPSPRASP	VAALKAKVIQ	KLEDVSKPPA	YAYPATPSSH	PTSPPPASPP
1050	1060	1070	1080	1090	1100	1110	1120
PTPGITRKEE	APENVVEKKD	LELEKEAPSP	FQALFSDIPP	RYPFQALPPH	YGRPYPFLLQ	PTAAADADGL	APDVPLPADG
1130	1140	1150	1160	1170	1180	1190	1200
PERLALSPED	KPIRLSPSKI	TEPLREGPEE	EPLAEREVKA	EVEDMDEGPT	ELPPLESPLP	LPAAEAMATP	SPAGGCGGGL
1210	1220	1230	1240	1250	1260	1270	1280
LEAQALSATG	QSCAEPSECP	DFVEGPEPRV	DSPGRTEPCT	AALDLGVQLT	PETLVEAKEE	PVEVPVAVPV	VEAVPEEGLA
1290	1300	1310	1320	1330	1340	1350	1360
QVAPSESQPT	LEMSDCDVPA	GEGQCPSLEP	QEAVPVLGST	CFLEEASSDQ	FLPSLEDPLA	GMNALAAAAE	LPQARPLPSP
1370	1380	1390	1400	1410	1420	1430	1440
GAAGAQALEK	LEAAESLVLE	QSFLHGITLL	SEIAELELER	RSQEMGGAER	ALVARPSLES	LLAAGSHMLR	EVLDGPVVDP
1450	1460	1470	1480	1490	1500	1510	1520
LKNLRLPREL	KPNKKYSWMR	KKEERMYAMK	SSLEDMDALE	LDFRMRLAEV	QRQYKEKQRE	LVKLQRRRDS	EDRREEPHRS
1530	1540	1550	1560	1570	1580	1590	1600
LARRGPGRPR	KRTHAPSALS	PPRKRGKSGH	SSGKLSSKSL	LTSDDYELGA	GIRKRHKGSE	EEHDALIGMG	KARGRNQTWD
1610	1620	1630	1640	1650	1660	1670	1680
EHEASSDFIS	QLKIKKKKMA	SDQEQLASKL	DKALSLTKQD	KLKSPFKFSD	SAGGKSKTSG	GCGRYLTPYD	SLLGKNRKAL
1690	1700	1710	1720	1730	1740	1750	1760
AKGLGLSLKS	SREGKHKRAA	KTRKMEVGFK	ARGQPKSAHS	PFASEVSSYS	YNTDSEEDEE	FLKDEWPAQG	PSSSKLTPSL
1770	1780	1790	1800	1810	1820	1830	1840
LCSMVAKNSK	AAGGPKLTKR	GLAAPRTLKP	KPATSRKQPF	CLLLREAEAR	SSFSDSSEES	FDQDESSEEE	DEEEELEEED
1850	1860	1870	1880	1890	1900	1910	1920
EASGGGYRLG	ARERALSPGL	EESGLGLLAR	FAASALPSPT	VGPSLSVVQL	EAKQKARKKE	ERQSLLGTEF	EYTDSESEVK
1930	1940	1950	1960	1970	1980	1990	2000
VRKRSPAGLL	RPKKGLGEPG	PSLAAPTPGA	RGPDPSSPDK	AKLAVEKGRK	ARKLRGPKEP	GFEAGPEASD	DDLWTRRRSE
2010	2020	2030	2040	2050	2060	2070	2080
RIFLHDASAA	APAPVSTAPA	TKTSRCAKGG	PLSPRKDAGR	AKDRKDPRKK	KKGKEAGPGA	GLPPPRAPAL	PSEARAPHAS
2090	2100	2110	2120	2130	2140	2150	2160
SLTAAKRSKA	KAKGKEVKKE	NRGKGGAVSK	LMESMAAEED	FEPNQDSSFS	EDEHLPRGGA	VERPLTPAPR	SCIIDKDELK
2170	2180	2190	2200	2210	2220	2230	2240

Detailed Protein Report

Cmpd.	No. of Cmpds.	m/z meas.	Δ m/z [ppm]	z	Rt [min]	Score	P	Range	Sequence	Modification
1629	1	1052.2731	65.37	3	50.1	21.1	1	271-302	K.TKNAALQPSVLTMCNGGAGDVGLPALVAEAGR.G	Carbamidomethyl: 14; Oxidation: 13
1053	2	500.6425	-250.20	2	42.8	13.1	2	1545-1554	K.RGKSGHSSGK.L	
2682	1	734.8888	-6.21	2	63.7	11.3	2	1642-1655	K.LKSPFKFSDSAGGK.S	
177	1	613.7776	-171.05	2	31.9	22.8	1	1787-1797	R.TLKPKPATSRK.Q	
1643	1	713.3402	-67.63	2	50.8	15.8	2	2464-2476	K.LDHEGVTSPKSKK.A	

Detailed Protein Report

Protein 42: PREDICTED: dynein heavy chain 5, axonemal isoform X1 [Homo sapiens]

**Accession:**	gi|530378724	**Score:**	112.2
**Database:**	refseq_human(refseq_human_20140103.fasta)	**MW [kDa]:**	526.8
**Database Date:**	2015-11-30	**pI:**	5.7
**Modification(s):**	Carbamidomethyl, Oxidation	**Sequence Coverage [%]:**	2.6
		**No. of unique Peptides:**	6

Quantitation

Q:W	**Median:**	0.86	**CV:**	0.00 %	**No. of Peptides:**	1
M:Q	**Median:**	1.04	**CV:**	0.00 %	**No. of Peptides:**	1

BRUKER

Detailed Protein Report

10	20	30	40	50	60	70	80
MDKLQRLKGE	KEAKRALLDA	RHNYLFAIVA	SCLDLNKTEV	EDAILEGNQI	ERIDQLFAVG	GLRHLMFYYQ	DVEEAETGQL
90	100	110	120	130	140	150	160
GSLGGVNLVS	GKIKKPKVFV	TEGNDVALTG	VCVFFIRTDP	SKAITPDNIH	QEVSFNMLDA	ADGGLLNSVR	RLLSDIFIPA
170	180	190	200	210	220	230	240
LRATSHGWGE	LEGLQDAANI	RQEFLSSLEG	FVNVLSGAQE	SLKEKVNLRK	CDILELKTLK	EPTDYLTLAN	NPETLGKIED
250	260	270	280	290	300	310	320
CMKVWIKQTE	QVLAENNQLL	KEADDVGPRA	ELEHWKKRLS	KFNYLLEQLK	SPDVKAVLAV	LAAAKSKLLK	TWREMDIRIT
330	340	350	360	370	380	390	400
DATNEAKDNV	KYLYTLEKCC	DPLYSSDPLS	MMDAIPTLIN	AIKMIYSISH	YYNTSEKITS	LFVKVTNQII	SACKAYITNN
410	420	430	440	450	460	470	480
GTASIWNQPQ	DVVEEKILSA	IKLKQEYQLC	FHKTKQKLKQ	NPNAKQFDFS	EMYIFGKFET	FHRRLAKIID	IFTTLKTYSV
490	500	510	520	530	540	550	560
LQDSTIEGLE	DMATKYQGIV	ATIKKKEYNF	LDQRKMDFDQ	DYEEFCKQTN	DLHNELRKFM	DVTFAKIQNT	NQALRMLKKF
570	580	590	600	610	620	630	640
ERLNIPNLGI	DDKYQLILEN	YGADIDMISK	LYTKQKYDPP	LARNQPPIAG	KILWARQLFH	RIQQPMQLFQ	QHPAVLSTAE
650	660	670	680	690	700	710	720
AKPIIRSYNR	MAKVLLEFEV	LFHRAWLRQI	EEIHVGLEAS	LLVKAPGTGE	LFVNFDPQIL	ILFRETECMA	QMGLEVSPLA
730	740	750	760	770	780	790	800
TSLFQKRDRY	KRNFSNMKMM	LAEYQRVKSK	IPAAIEQLIV	PHLAKVDEAL	QPGLAALTWT	SLNIEAYLEN	TFAKIKDLEL
810	820	830	840	850	860	870	880
LLDRVNDLIE	FRIDAILEEM	SSTPLCQLPQ	EEPLTCEEFL	QMTKDLCVNG	AQILHFKSSL	VEEAVNELVN	MLLDVEVLSE
890	900	910	920	930	940	950	960
EESEKISNEN	SVNYKNESSA	KREEGNFDTL	TSSINARANA	LLLTTVTRKK	KETEMLGEEA	RELLSHFNHQ	NMDALLKVTR
970	980	990	1000	1010	1020	1030	1040
NTLEAIRKRI	HSSHTINFRD	SNSASNMKQN	SLPIFRASVT	LAIPNIVMAP	ALEDVQQTLN	KAVECIISVP	KGVRQWSSEL
1050	1060	1070	1080	1090	1100	1110	1120
LSKKKIQERK	MAALQSNEDS	DSDVEMGENE	LQDTLEIASV	NLPIPVQTKN	YYKNVSENKE	IVKLVSVLST	IINSTKKEVI
1130	1140	1150	1160	1170	1180	1190	1200
TSMDCFKRYN	HIWQKGKEEA	IKTFITQSPL	LSEFESQILY	FQNLEQEINA	EPEYVCVGSI	ALYTADLKFA	LTAETKAWMV
1210	1220	1230	1240	1250	1260	1270	1280
VIGRHCNKKY	RSEMENIFML	IEEFNKKLNR	PIKDLDDIRI	AMAALKEIRE	EQISIDFQVG	PIEESYALLN	RYGLLIAREE
1290	1300	1310	1320	1330	1340	1350	1360
IDKVDTLHYA	WEKLLARAGE	VQNKLVSLQP	SFKKELISAV	EVFLQDCHQF	YLDYDLNGPM	ASGLKPQEAS	DRLIMFQNQF
1370	1380	1390	1400	1410	1420	1430	1440
DNIYRKYITY	TGGEELFGLP	ATQYPQLLEI	KKQLNLLQKI	YTLYNSVIET	VNSYYDILWS	EVNIEKINNE	LLEFQNRCRK
1450	1460	1470	1480	1490	1500	1510	1520
LPRALKDWQA	FLDLKKIIDD	FSECCPLLEY	MASKAMMERH	WERITTLTGH	SLDVGNESFK	LRNIMEAPLL	KYKEEIEDIC
1530	1540	1550	1560	1570	1580	1590	1600
ISAVKERDIE	QKLKQVINEW	DNKTFTFGSF	KTRGELLLRG	DSTSEIIANM	EDSLMLLGSL	LSNRYNMPFK	AQIQKWVQYL
1610	1620	1630	1640	1650	1660	1670	1680
SNSTDIIESW	MTVQNLWIYL	EAVFVGGDIA	KQLPKEAKRF	SNIDKSWVKI	MTRAHEVPSV	VQCCVGDETL	GQLLPHLLDQ
1690	1700	1710	1720	1730	1740	1750	1760
LEICQKSLTG	YLEKKRLCFP	RFFFVSDPAL	LEILGQASDS	HTIQAHLLNV	FDNIKSVKFH	EKIYDRILSI	SSQEGETIEL
1770	1780	1790	1800	1810	1820	1830	1840
DKPVMAEGNV	EVWLNSLLEE	SQSSLHLVIR	QAAANIQETG	FQLTEFLSSF	PAQVGLLGIQ	MIWTRDSEEA	LRNAKFDKKI
1850	1860	1870	1880	1890	1900	1910	1920
MQKTNQAFLE	LLNTLIDVTT	RDLSSTERVK	YETLITIHVH	QRDIFDDLCH	MHIKSPMDFE	WLKQCRFYFN	EDSDKMMIHI
1930	1940	1950	1960	1970	1980	1990	2000
TDVAFIYQNE	FLGCTDRLVI	TPLTDRCYIT	LAQALGMSMG	GAPAGPAGTG	KTETTKDMGR	CLGKYVVVFN	CSDQMDFRGL
2010	2020	2030	2040	2050	2060	2070	2080
GRIFKGLAQS	GSWGCFDEFN	RIDLPVLSVA	AQQISIILTC	KKEHKKSFIF	TDGDNVTMNP	EFGLFLTMNP	GYAGRQELPE
2090	2100	2110	2120	2130	2140	2150	2160
NLKINFRSVA	MMVPDRQIII	RVKLASCGFI	DNVVLARKFF	TLYKLCEEQL	SKQVHYDFGL	RNILSVLRTL	GAAKRANPMD
2170	2180	2190	2200	2210	2220	2230	2240

Detailed Protein Report

Cmpd.	No. of Cmpds.	m/z meas.	Δ m/z [ppm]	z	Rt [min]	Score	P	Range	Sequence	Modification	Ratios
1349	1	991.5669	90.75	3	47.0	28.2	1	1947-1976	R.CYITLAQALGMSMGGAPAGPAGTD	Carbamidomethyl: 1; Oxidation: 11, 13	
2802	1	849.0671	-21.12	3	66.2	12.2	2	1999-2021	R.GLGRIFKGLAQSGSWGCFDEFNRI		
78	1	529.5845	-42.73	3	29.9	11.4	2	3729-3741	R.THLMEDVTANKRR.M	Oxidation: 4	
2750	1	905.4797	-0.39	2	65.5	16.2	2	3993-4008	R.TIAQARKYIVDSMGEK.Y		
1942	2	709.7806	-178.71	2	52.9	13.6	0	4122-4133	K.QFPITLLQMSIK.F		
592	2	607.1554	-159.80	2	36.0	18.7	0	4415-4424	R.DALDCMFDAR.I	Carbamidomethyl: 5	M:Q 1.04<br>Q:W 0.86

Detailed Protein Report

Protein 43: PREDICTED: probable E3 ubiquitin-protein ligase MYCBP2 isoform X12 [Homo sapiens]

**Accession:**	gi|578824802	**Score:**	111.7
**Database:**	refseq_human(refseq_human_20140103.fasta)	**MW [kDa]:**	514.4
**Database Date:**	2015-11-30	**pI:**	6.7
**Modification(s):**	Carbamidomethyl, Oxidation	**Sequence Coverage [%]:**	3.5
		**No. of unique Peptides:**	6

Quantitation

Q:W	**Median:**	1.23	**CV:**	0.00 %	**No. of Peptides:**	1
M:Q	**Median:**	1.93	**CV:**	0.00 %	**No. of Peptides:**	1

Detailed Protein Report

10	20	30	40	50	60	70	80
MMMCAATASP	AAASSGLGGD	GFYPAATFSS	SPAPGALFMP	VPDGSVAAAG	LGLGLPAADS	RGHYQLLLSG	RALADRYRRI
90	100	110	120	130	140	150	160
YTAALNDRDQ	GGGSAGHPAS	RNKKILNKKK	LKRKQKSKSK	VKTRSKSENL	ENTVIIPDIK	LHSNPSAFNI	YCNVRHCVLE
170	180	190	200	210	220	230	240
WQKKEISLAA	ASKNSVQSGE	SDSDEEEESK	EPPIKLPKII	EVGLCEVFEL	IKETRFSHPS	LCLRSLQALL	NVLQGQQPEG
250	260	270	280	290	300	310	320
LQSEPPEVLE	SLFQLLLEIT	VRSTGMNDST	GQSLTALSCA	CLFSLVASWG	ETGRTLQAIS	AILTNNGSHA	CQTIQVPTIL
330	340	350	360	370	380	390	400
NSLQRSVQAV	LVGKIQIQDW	FSNGIKKAAL	MHKWPLKEIS	VDEDDQCLLQ	NDGFFLYLLC	KDGLYKIGSG	YSGTVRGHIY
410	420	430	440	450	460	470	480
NSTSRIRNRK	EKKSWLGYAQ	GYLLYRDVNN	HSMTAIRISP	ETLEQDGTVM	LPDCHTEGQN	ILFTDGEYIN	QIAASRDDGF
490	500	510	520	530	540	550	560
VVRIFATSTE	PVLQQELQLK	LARKCLHACG	ISLFDLEKDL	HIISTGFDEE	SAILGAGREF	ALMKTANGKI	YYTGKYQSLG
570	580	590	600	610	620	630	640
IKQGGPSAGK	WVELPITKSP	KIVHFSVGHD	GSHALLVAED	GSIFFTGSAS	KGEDGESTKS	RRQSKPYKPK	KIIKMEGKIV
650	660	670	680	690	700	710	720
VYTACNNGSS	SVISKDGELY	MFGKDAIYSD	SSSLVTDLKG	HFVTQVAMGK	AHTCVLMKNG	EVWTFGVNNK	GQCGRDTGAM
730	740	750	760	770	780	790	800
NQGGKGFGVE	NMATAMDEDL	EEELDEKDEK	SMMCPPGMHK	WKLEQCMVCT	VCGDCTGYGA	SCVSSGRPDR	VPGGICGCGS
810	820	830	840	850	860	870	880
GESGCAVCGC	CKACARELDG	QEARQRGILD	AVKEMIPLDL	LLAVPVPGVN	IEEHLQLRQE	EKRQRVIRRH	RLEEGRGPLV
890	900	910	920	930	940	950	960
FAGPIFMNHR	EQALARLRSH	PAQLKHKRDK	HKDGSGERGE	KDASKITTYP	PGSVRFDCEL	RAVQVSCGFH	HSVVLMENGD
970	980	990	1000	1010	1020	1030	1040
VYTFGYGQHG	QLGHGDVNSR	GCPTLVQALP	GPSTQVTAGS	NHTAVLLMDG	QVFTFGSFSK	GQLGRPILDV	PYWNAKPAPM
1050	1060	1070	1080	1090	1100	1110	1120
PNIGSKYGRK	ATWIGASGDQ	TFLRIDEALI	NSHVLATSEI	FASKHIIGLV	PASISEPPPF	KCLLINKVDG	SCKTFNDSEQ
1130	1140	1150	1160	1170	1180	1190	1200
EDLQGFGVCL	DPVYDVIWRF	RPNTRELWCY	NAVVADARLP	SAADMQSRCS	ILSPELALPT	GSRALTTRSH	AALHILGCLD
1210	1220	1230	1240	1250	1260	1270	1280
TLAAMQDLKM	GVASTEEETQ	AVMKVYSKED	YSVVNRFESH	GGGWGYSAHS	VEAIRFSADT	DILLGGLGLF	GGRGEYTAKI
1290	1300	1310	1320	1330	1340	1350	1360
KLFELGPDGG	DHETDGDLLA	ETDVLAYDCA	AREKYAMMFD	EPVLLQAGWW	YVAWARVSGP	SSDCGSHGQA	SITTDDGVVF
1370	1380	1390	1400	1410	1420	1430	1440
QFKSSKKSNN	GTDVNAGQIP	QLLYRLPTSD	GSASKGKQQT	SEPVHILKRS	FARTVSVECF	ESLLSILHWS	WTTLVLGVEE
1450	1460	1470	1480	1490	1500	1510	1520
LRGLKGFQFT	ATLLDLERLR	FVGTCCLRLL	RVYTCEIYPV	SATGKAVVEE	TSKLAECIGK	TRTLLRKILS	EGVDHCMVKL
1530	1540	1550	1560	1570	1580	1590	1600
DNDPQGYLSQ	PLSLLEAVLQ	ECHNTFTACF	HSFYPTPALQ	WACLCDLLNC	LDQDIQEANF	KTSSSRLLAA	VMSALCHTSV
1610	1620	1630	1640	1650	1660	1670	1680
KLTSIFPIAY	DGEVLLRSIV	KQVSTENDST	LVHRFPLLVA	HMEKLSQSEE	NISGMTSFRE	VLEKMLVIVV	LPVRNSLRRE
1690	1700	1710	1720	1730	1740	1750	1760
NELFSSHLVS	NTCGLLASIV	SELTASALGS	EVDGLNSLHS	VKASANRFTK	TSQGRSWNTG	NGSPDAICFS	VDKPGIVVVG
1770	1780	1790	1800	1810	1820	1830	1840
FSVYGGGGIH	EYELEVLVDD	SEHAGDSTHS	HRWTSLELVK	GTYTTDDSPS	DIAEIRLDKV	VPLKENVKYA	VRLRNYGSRT
1850	1860	1870	1880	1890	1900	1910	1920
ANGDGGMTTV	QCPDGVTFTF	STCSLSSNGT	NQTRGQIPQI	LYYRSEFDGD	LQSQLLSKAN	EEDKNCSRAL	SVVSTVVRAS
1930	1940	1950	1960	1970	1980	1990	2000
KDLLHRALAV	DADDIPELLS	SSSLFSMLLP	LIIAYIGPVA	AAIPKVAVEV	FGLVQQLLPS	VAILNQKYAP	PAFNPNQSTD
2010	2020	2030	2040	2050	2060	2070	2080
STTGNQPEQG	LSACTTSSHY	AVIESEHPYK	PACVMHYKVT	FPECVRWMTI	EFDPQCGTAQ	SEDVLRLLIP	VRTVQNSGYG
2090	2100	2110	2120	2130	2140	2150	2160
PKLTSVHENL	NSWIELKKFS	GSSGWPTMVL	VLPGNEALFS	LETASDYVKD	DKASFYGFKC	FAIGYEFSPG	PDEGVIQLEK
2170	2180	2190	2200	2210	2220	2230	2240

Detailed Protein Report

Cmpd.	No. of Cmpds.	m/z meas.	Δ m/z [ppm]	z	Rt [min]	Score	P	Range	Sequence	Modification	Ratios
1748	3	697.3510	1.93	3	50.2	15.7	1	680-698	K.GHFVTQVAMGKAHTCVLMK.N	Oxidation: 9, 18	
1222	1	927.2551	104.81	3	44.9	11.3	1	1184-1209	R.ALTTRSHAALHILGCLDTLAAMQDM	Oxidation: 22	
1800	1	658.7973	-153.52	2	52.5	13.4	1	1909-1921	R.ALSVVSTVVRASK.D		
2398	1	1023.7079	112.56	2	59.9	13.0	2	2442-2460	K.VKDPPKGMIPPGTQLVKPK.S	Oxidation: 8	
2407	1	973.4149	-88.00	2	60.6	10.4	1	3885-3903	K.DGESTKIAGQISASVAQQR.N		M:Q 1.93<br>Q:W 1.23
1461	1	799.6263	-94.96	3	48.0	13.1	1	4348-4368	K.AMVEFREHTGKPTTSSSEACR.F	Carbamidomethyl: 20; Oxidation: 2	

Detailed Protein Report

Protein 44: cadherin EGF LAG seven-pass G-type receptor 3 precursor [Homo sapiens]

**Accession:**	gi|145309304	**Score:**	110.7
**Database:**	refseq_human(refseq_human_20140103.fasta)	**MW [kDa]:**	358.0
**Database Date:**	2015-11-30	**pI:**	6.2
**Modification(s):**	Carbamidomethyl, Oxidation	**Sequence Coverage [%]:**	2.8
		**No. of unique Peptides:**	6

Quantitation

Q:W	**Median:** 1.03	**CV:**	58.31 %	**No. of Peptides:**	2
M:Q	**Median:** 1.45	**CV:**	0.00 %	**No. of Peptides:**	1

Detailed Protein Report

10	20	30	40	50	60	70	80
MMARRPPWRG	LGGRSTPILL	LLLLSLFPLS	QEELGGGGHQ	GWDPGLAATT	GPRAHIGGGA	LALCPESSGV	REDGGPGLGV
90	100	110	120	130	140	150	160
REPIFVGLRG	RRQSARNSRG	PPEQPNEELG	IEHGVQPLGS	RERETGQGPG	SVLYWRPEVS	SCGRTGPLQR	GSLSPGALSS
170	180	190	200	210	220	230	240
GVPGSGNSSP	LPSDFLIRHH	GPKPVSSQRN	AGTGSRKRVG	TARCCGELWA	TGSKGQGERA	TTSGAERTAP	RRNCLPGASG
250	260	270	280	290	300	310	320
SGPELDSAPR	TARTAPASGS	APRESRTAPE	PAPKRMRSRG	LFRCRFLPQR	PGPRPPGLPA	RPEARKVTSA	NRARFRRAAN
330	340	350	360	370	380	390	400
RHPQFPQYNY	QTLVPENEAA	GTAVLRVVAQ	DPDAGEAGRL	VYSLAALMNS	RSLELFSIDP	QSGLIRTAAA	LDRESMERHY
410	420	430	440	450	460	470	480
LRVTAQDHGS	PRLSATTMVA	VTVADRNDHS	PVFEQAQYRE	TLRENVEEGY	PILQLRATDG	DAPPNANLRY	RFVGPPAARA
490	500	510	520	530	540	550	560
AAAAAFEIDP	RSGLISTSGR	VDREHMESYE	LVVEASDQGQ	EPGPRSATVR	VHITVLDEND	NAPQFSEKRY	VAQVREDVRP
570	580	590	600	610	620	630	640
HTVVLRVTAT	DRDKDANGLV	HYNIISGNSR	GHFAIDSLTG	EIQVVAPLDF	EAEREYALRI	RAQDAGRPPL	SNNTGLASIQ
650	660	670	680	690	700	710	720
VVDINDHIPI	FVSTPFQVSV	LENAPLGHSV	IHIQAVDADH	GENARLEYSL	TGVAPDTPFV	INSATGWVSV	SGPLDRESVE
730	740	750	760	770	780	790	800
HYFFGVEARD	HGSPPLSASA	SVTVTVLDVN	DNRPEFTMKE	YHLRLNEDAA	VGTSVVSVTA	VDRDANSAIS	YQITGGNTRN
810	820	830	840	850	860	870	880
RFAISTQGGV	GLVTLALPLD	YKQERYFKLV	LTASDRALHD	HCYVHINITD	ANTHRPVFQS	AHYSVSVNED	RPMGSTIVVI
890	900	910	920	930	940	950	960
SASDDDVGEN	ARITYLLEDN	LPQFRIDADS	GAITLQAPLD	YEDQVTYTLA	ITARDNGIPQ	KADTTYVEVM	VNDVNDNAPQ
970	980	990	1000	1010	1020	1030	1040
FVASHYTGLV	SEDAPPFTSV	LQISATDRDA	HANGRVQYTF	QNGEDGDGDF	TIEPTSGIVR	TVRRLDREAV	SVYELTAYAV
1050	1060	1070	1080	1090	1100	1110	1120
DRGVPPLRTP	VSIQVMVQDV	NDNAPVFPAE	EFEVRVKENS	IVGSVVAQIT	AVDPDEGPNA	HIMYQIVEGN	IPELFQMDIF
1130	1140	1150	1160	1170	1180	1190	1200
SGELTALIDL	DYEARQEYVI	VVQATSAPLV	SRATVHVRLV	DQNDNSPVLN	NFQILFNNYV	SNRSDTFPSG	IIGRIPAYDP
1210	1220	1230	1240	1250	1260	1270	1280
DVSDHLFYSF	ERGNELQLLV	VNQTSGELRL	SRKLDNNRPL	VASMLVTVTD	GLHSVTAQCV	LRVVIITEEL	LANSLTVRLE
1290	1300	1310	1320	1330	1340	1350	1360
NMWQERFLSP	LLGRFLEGVA	AVLATPAEDV	FIFNIQNDTD	VGGTVLNVSF	SALAPRGAGA	GAAGPWFSSE	ELQEQLYVRR
1370	1380	1390	1400	1410	1420	1430	1440
AALAARSLLD	VLPFDDNVCL	REPCENYMKC	VSVLRFDSSA	PFLASASTLF	RPIQPIAGLR	CRCPPGFTGD	FCETELDLCY
1450	1460	1470	1480	1490	1500	1510	1520
SNPCRNGGAC	ARREGGYTCV	CRPRFTGEDC	ELDTEAGRCV	PGVCRNGGTC	TDAPNGGFRC	QCPAGGAFEG	PRCEVAARSF
1530	1540	1550	1560	1570	1580	1590	1600
PPSSFVMFRG	LRQRFHLTLS	LSFATVQQSG	LLFYNGRLNE	KHDFLALELV	AGQVRLTYST	GESNTVVSPT	VPGGLSDGQW
1610	1620	1630	1640	1650	1660	1670	1680
HTVHLRYYNK	PRTDALGGAQ	GPSKDKVAVL	SVDDCDVAVA	LQFGAEIGNY	SCAAAGVQTS	SKKSLDLTGP	LLLGGVPNLP
1690	1700	1710	1720	1730	1740	1750	1760
ENFPVSHKDF	IGCMRDLHID	GRRVDMAAFV	ANNGTMAGCQ	AKLHFCDSGP	CKNSGFCSER	WGSFSCDCPV	GFGGKDCQLT
1770	1780	1790	1800	1810	1820	1830	1840
MAHPHHFRGN	GTLSWNFGSD	MAVSVPWYLG	LAFRTRATQG	VLMQVQAGPH	STLLCQLDRG	LLSVTVTRGS	GRASHLLLDQ
1850	1860	1870	1880	1890	1900	1910	1920
VTVSDGRWHD	LRLELQEEPG	GRRGHHVLMV	SLDFSLFQDT	MAVGSELQGL	KVKQLHVGGL	PPGSAEEAPQ	GLVGCIQGVW
1930	1940	1950	1960	1970	1980	1990	2000
LGSTPSGSPA	LLPPSHRVNA	EPGCVVTNAC	ASGPCPPHAD	CRDLWQTFSC	TCQPGYYGPG	CVDACLLNPC	QNQGSCRHLP
2010	2020	2030	2040	2050	2060	2070	2080
GAPHGYTCDC	VGGYFGHHCE	HRMDQQCPRG	WWGSPTCGPC	NCDVHKGFDP	NCNKTNGQCH	CKEFHYRPRG	SDSCLPCDCY
2090	2100	2110	2120	2130	2140	2150	2160
PVGSTSRSCA	PHSGQCPCRP	GALGRQCNSC	DSPFAEVTAS	GCRVLYDACP	KSLRSGVWWP	QTKFGVLATV	PCPRGALGAA
2170	2180	2190	2200	2210	2220	2230	2240

Detailed Protein Report

Cmpd.	No. of Cmpds.	m/z meas.	Δ m/z [ppm]	z	Rt [min]	Score	P	Range	Sequence	Modification	Ratios
1647	9	942.4685	14.29	2	49.1	22.8	1	232-250	R.RNCLPGASGSGPELDSAPR.T		Q:W 0.60<br>M:Q 1.45
2229	1	956.4132	-125.14	2	56.5	12.5	1	2144-2162	K.FGVLATVPCPRGALGAAVR.L	Carbamidomethyl: 9	
1065	4	470.5947	-263.79	2	43.4	12.8	0	2331-2338	R.MEHPSSPR.G		
2129	1	698.8200	-38.23	2	55.2	13.9	2	2331-2342	R.MEHPSSPRGARR.Y	Oxidation: 1	
587	2	459.6056	-275.62	2	35.7	17.8	1	2636-2643	R.NVDRGAMR.F		Q:W 1.77
118	1	1069.1641	-33.12	3	30.2	17.4	1	3083-3111	R.ERLEEAPAPVLRPLSRPGSQECM L	Carbamidomethyl: 22; Oxidation: 23	

Detailed Protein Report

Protein 45: fibrillin-2 precursor [Homo sapiens]

**Accession:**	gi|66346695	**Score:**	110.4
**Database:**	refseq_human(refseq_human_20140103.fasta)	**MW [kDa]:**	314.6
**Database Date:**	2015-11-30	**pI:**	4.6
**Modification(s):**	Carbamidomethyl, Oxidation	**Sequence Coverage [%]:**	3.1
		**No. of unique Peptides:**	7

Quantitation

Q:W	**Median:**	0.57	**CV:**	77.12 %	**No. of Peptides:**	3
M:Q	**Median:**	0.96	**CV:**	58.37 %	**No. of Peptides:**	2

Detailed Protein Report

10	20	30	40	50	60	70	80
MGRRRRLCLQ	LYFLWLGCVV	LWAQGTAGQP	QPPPPKPPRP	QPPPQQVRSA	TAGSEGGFLA	PEYREEGAAV	ASRVRRRGQQ
90	100	110	120	130	140	150	160
DVLRGPNVCG	SRFHSYCCPG	WKTLPGGNQC	IVPICRNSCG	DGFCSRPNMC	TCSSGQISST	CGSKSIQQCS	VRCMNGGTCA
170	180	190	200	210	220	230	240
DDHCQCQKGY	IGTYCGQPVC	ENGCQNGGRC	IGPNRCACVY	GFTGPQCERD	YRTGPCFTQV	NNQMCQGQLT	GIVCTKTLCC
250	260	270	280	290	300	310	320
ATIGRAWGHP	CEMCPAQPQP	CRRGFIPNIR	TGACQDVDEC	QAIPGICQGG	NCINTVGSFE	CRCPAGHKQS	ETTQKCEDID
330	340	350	360	370	380	390	400
ECSIIPGICE	TGECSNTVGS	YFCVCPRGYV	TSTDGSRCID	QRTGMCFSGL	VNGRCAQELP	GRMTKMQCCC	EPGRCWGIGT
410	420	430	440	450	460	470	480
IPEACPVRGS	EEYRRLCMDG	LPMGGIPGSA	GSRPGGTGGN	GFAPSGNGNG	YGPGGTGFIP	IPGGNGFSPG	VGGAGVGAGG
490	500	510	520	530	540	550	560
QGPIITGLTI	LNQTIDICKH	HANLCLNGRC	IPTVSSYRCE	CNMGYKQDAN	GDCIDVDECT	SNPCTNGDCV	NTPGSYYCKC
570	580	590	600	610	620	630	640
HAGFQRTPTK	QACIDIDECI	QNGVLCKNGR	CVNTDGSFQC	ICNAGFELTT	DGKNCVDHDE	CTTTNMCLNG	MCINEDGSFK
650	660	670	680	690	700	710	720
CICKPGFVLA	PNGRYCTDVD	ECQTPGICMN	GHCINSEGSF	RCDCPPGLAV	GMDGRVCVDT	HMRSTCYGGI	KKGVCVRPFP
730	740	750	760	770	780	790	800
GAVTKSECCC	ANPDYGFGEP	CQPCPAKNSA	EFHGLCSSGV	GITVDGRDIN	ECALDPDICA	NGICENLRGS	YRCNCNSGYE
810	820	830	840	850	860	870	880
PDASGRNCID	IDECLVNRLL	CDNGLCRNTP	GSYSCTCPPG	YVFRTETETC	EDINECESNP	CVNGACRNNL	GSFNCECSPG
890	900	910	920	930	940	950	960
SKLSSTGLIC	IDSLKGTCWL	NIQDSRCEVN	INGATLKSEC	CATLGAAWGS	PCERCELDTA	CPRGLARIKG	VTCEDVNECE
970	980	990	1000	1010	1020	1030	1040
VFPGVCPNGR	CVNSKGSFHC	ECPEGLTLDG	TGRVCLDIRM	EQCYLKWDED	ECIHPVPGKF	RMDACCCAVG	AAWGTECEEC
1050	1060	1070	1080	1090	1100	1110	1120
PKPGTKEYET	LCPRGAGFAN	RGDVLTGRPF	YKDINECKAF	PGMCTYGKCR	NTIGSFKCRC	NSGFALDMEE	RNCTDIDECR
1130	1140	1150	1160	1170	1180	1190	1200
ISPDLCGSGI	CVNTPGSFEC	ECFEGYESGF	MMMKNCMDID	ECERNPLLCR	GGTCVNTEGS	FQCDCPLGHE	LSPSREDCVD
1210	1220	1230	1240	1250	1260	1270	1280
INECSLSDNL	CRNGKCVNMI	GTYQCSCNPG	YQATPDRQGC	TDIDECMIMN	GGCDTQCTNS	EGSYECSCSE	GYALMPDGRS
1290	1300	1310	1320	1330	1340	1350	1360
CADIDECENN	PDICDGGQCT	NIPGEYRCLC	YDGFMASMDM	KTCIDVNECD	LNSNICMFGE	CENTKGSFIC	HCQLGYSVKK
1370	1380	1390	1400	1410	1420	1430	1440
GTTGCTDVDE	CEIGAHNCDM	HASCLNIPGS	FKCSCREGWI	GNGIKCIDLD	ECSNGTHQCS	INAQCVNTPG	SYRCACSEGF
1450	1460	1470	1480	1490	1500	1510	1520
TGDGFTCSDV	DECAENINLC	ENGQCLNVPG	AYRCECEMGF	TPASDSRSCQ	DIDECSFQNI	CVFGTCNNLP	GMFHCICDDG
1530	1540	1550	1560	1570	1580	1590	1600
YELDRTGGNC	TDIDECADPI	NCVNGLCVNT	PGRYECNCPP	DFQLNPTGVG	CVDNRVGNCY	LKFGPRGDGS	LSCNTEIGVG
1610	1620	1630	1640	1650	1660	1670	1680
VSRSSCCCSL	GKAWGNPCET	CPPVNSTEYY	TLCPGGEGFR	PNPITIILED	IDECQELPGL	CQGGNCINTF	GSFQCECPQG
1690	1700	1710	1720	1730	1740	1750	1760
YYLSEDTRIC	EDIDECFAHP	GVCGPGTCYN	TLGNYTCICP	PEYMQVNGGH	NCMDMRKSFC	YRSYNGTTCE	NELPFNVTKR
1770	1780	1790	1800	1810	1820	1830	1840
MCCCTYNVGK	AWNKPCEPCP	TPGTADFKTI	CGNIPGFTFD	IHTGKAVDID	ECKEIPGICA	NGVCINQIGS	FRCECPTGFS
1850	1860	1870	1880	1890	1900	1910	1920
YNDLLLVCED	IDECSNGDNL	CQRNADCINS	PGSYRCECAA	GFKLSPNGAC	VDRNECLEIP	NVCSHGLCVD	LQGSYQCICH
1930	1940	1950	1960	1970	1980	1990	2000
NGFKASQDQT	MCMDVDECER	HPCGNGTCKN	TVGSYNCLCY	PGFELTHNND	CLDIDECSSF	FGQVCRNGRC	FNEIGSFKCL
2010	2020	2030	2040	2050	2060	2070	2080
CNEGYELTPD	GKNCIDTNEC	VALPGSCSPG	TCQNLEGSFR	CICPPGYEVK	SENCIDINEC	DEDPNICLFG	SCTNTPGGFQ
2090	2100	2110	2120	2130	2140	2150	2160
CLCPPGFVLS	DNGRRCFDTR	QSFCFTNFEN	GKCSVPKAFN	TTKAKCCCSK	MPGEGWGDPC	ELCPKDDEVA	FQDLCPYGHG
2170	2180	2190	2200	2210	2220	2230	2240

Detailed Protein Report

Cmpd.	No. of Cmpds.	m/z meas.	Δ m/z [ppm]	z	Rt [min]	Score	P	Range	Sequence	Modification	Ratios
186	1	806.9057	27.18	2	31.2	11.0	0	49-64	R.SATAGSEGGFLAPEYR.E		M:Q 1.64<br>Q:W 1.45
2112	1	600.2270	-134.67	3	54.8	15.6	2	77-92	R.RGQQDVLRGPNVCGSR.F	Carbamidomethyl: 13	
534	2	730.1886	-131.49	2	36.0	10.1	1	383-394	R.MTKMQCCCEPGR.C	Carbamidomethyl: 6; Oxidation: 4	
522	1	730.1851	-136.21	2	35.9	15.8	1	383-394	R.MTKMQCCCEPGR.C	Carbamidomethyl: 8; Oxidation: 4	
472	1	516.1120	-268.37	2	34.5	12.2	0	1884-1893	K.LSPNGACVDR.N		Q:W 0.29
281	1	477.4617	-193.67	3	32.2	10.8	0	2436-2448	K.ICPHGPGYTTDGR.D	Carbamidomethyl: 2	
304	9	642.1738	-171.43	2	33.2	24.4	1	2529-2539	K.TCKDLDECQTK.Q		M:Q 0.56<br>Q:W 0.45

Detailed Protein Report

Protein 46: centromere protein F [Homo sapiens]

**Accession:**	gi|55770834	**Score:**	109.1
**Database:**	refseq_human(refseq_human_20140103.fasta)	**MW [kDa]:**	357.3
**Database Date:**	2015-11-30	**pI:**	4.9
**Modification(s):**	Oxidation	**Sequence Coverage [%]:**	3.2
		**No. of unique Peptides:**	6

Quantitation

M:Q **Median:** 2.43 **CV:** 0.00 % **No. of Peptides:** 1

Detailed Protein Report

10	20	30	40	50	60	70	80
MSWALEEWKE	GLPTRALQKI	QELEGQLDKL	KKEKQQRQFQ	LDSLEAALQK	QKQKVENEKT	EGTNLKRENQ	RLMEICESLE
90	100	110	120	130	140	150	160
KTKQKISHEL	QVKESQVNFQ	EGQLNSGKKQ	IEKLEQELKR	CKSELERSQQ	AAQSADVSLN	PCNTPQKIFT	TPLTPSQYYS
170	180	190	200	210	220	230	240
GSKYEDLKEK	YNKEVEERKR	LEAEVKALQA	KKASQTLPQA	TMNHRDIARH	QASSSVFSWQ	QEKTPSHLSS	NSQRTPIRRD
250	260	270	280	290	300	310	320
FSASYFSGEQ	EVTPSRSTLQ	IGKRDANSSF	FDNSSSPHLL	DQLKAQNQEL	RNKINELELR	LQGHEKEMKG	QVNKFQELQL
330	340	350	360	370	380	390	400
QLEKAKVELI	EKEKVLNKCR	DELVRTTAQY	DQASTKYTAL	EQKLKKLTED	LSCQRQNAES	ARCSLEQKIK	EKEKEFQEEL
410	420	430	440	450	460	470	480
SRQQRSFQTL	DQECIQMKAR	LTQELQQAKN	MHNVLQAELD	KLTSVKQQLE	NNLEEFKQKL	CRAEQAFQAS	QIKENELRRS
490	500	510	520	530	540	550	560
MEEMKKENNL	LKSHSEQKAR	EVCHLEAELK	NIKQCLNQSQ	NFAEEMKAKN	TSQETMLRDL	QEKINQQENS	LTLEKLKLAV
570	580	590	600	610	620	630	640
ADLEKQRDCS	QDLLKKREHH	IEQLNDKLSK	TEKESKALLS	ALELKKKEYE	ELKEEKTLFS	CWKSENEKLL	TQMESEKENL
650	660	670	680	690	700	710	720
QSKINHLETC	LKTQQIKSHE	YNERVRTLEM	DRENLSVEIR	NLHNVLDSKS	VEVETQKLAY	MELQQKAEFS	DQKHQKEIEN
730	740	750	760	770	780	790	800
MCLKTSQLTG	QVEDLEHKLQ	LLSNEIMDKD	RCYQDLHAEY	ESLRDLLKSK	DASLVTNEDH	QRSLLAFDQQ	PAMHHSFANI
810	820	830	840	850	860	870	880
IGEQGSMPSE	RSECRLEADQ	SPKNSAILQN	RVDSLEFSLE	SQKQMNSDLQ	KQCEELVQIK	GEIEENLMKA	EQMHQSFVAE
890	900	910	920	930	940	950	960
TSQRISKLQE	DTSAHQNVVA	ETLSALENKE	KELQLLNDKV	ETEQAEIQEL	KKSNHLLEDS	LKELQLLSET	LSLEKKEMSS
970	980	990	1000	1010	1020	1030	1040
IISLNKREIE	ELTQENGTLK	EINASLNQEK	MNLIQKSESF	ANYIDEREKS	ISELSDQYKQ	EKLILLQRCE	ETGNAYEDLS
1050	1060	1070	1080	1090	1100	1110	1120
QKYKAAQEKN	SKLECLLNEC	TSLCENRKNE	LEQLKEAFAK	EHQEFLTKLA	FAEERNQNLM	LELETVQQAL	RSEMTDNQNN
1130	1140	1150	1160	1170	1180	1190	1200
SKSEAGGLKQ	EIMTLKEEQN	KMQKEVNDLL	QENEQLMKVM	KTKHECQNLE	SEPIRNSVKE	RESERNQCNF	KPQMDLEVKE
1210	1220	1230	1240	1250	1260	1270	1280
ISLDSYNAQL	VQLEAMLRNK	ELKLQESEKE	KECLQHELQT	IRGDLETSNL	QDMQSQEISG	LKDCEIDAEE	KYISGPHELS
1290	1300	1310	1320	1330	1340	1350	1360
TSQNDNAHLQ	CSLQTTMNKL	NELEKICEIL	QAEKYELVTE	LNDSRSECIT	ATRKMAEEVG	KLLNEVKILN	DDSGLLHGEL
1370	1380	1390	1400	1410	1420	1430	1440
VEDIPGGEFG	EQPNEQHPVS	LAPLDESNSY	EHLTLSDKEV	QMHFAELQEK	FLSLQSEHKI	LHDQHCQMSS	KMSELQTYVD
1450	1460	1470	1480	1490	1500	1510	1520
SLKAENLVLS	TNLRNFQGDL	VKEMQLGLEE	GLVPSLSSSC	VPDSSSLSSL	GDSSFYRALL	EQTGDMSLLS	NLEGAVSANQ
1530	1540	1550	1560	1570	1580	1590	1600
CSVDEVFCSS	LQEENLTRKE	TPSAPAKGVE	ELESLCEVYR	QSLEKLEEKM	ESQGIMKNKE	IQELEQLLSS	ERQELDCLRK
1610	1620	1630	1640	1650	1660	1670	1680
QYLSENEQWQ	QKLTSVTLEM	ESKLAAEKKQ	TEQLSLELEV	ARLQLQGLDL	SSRSLLGIDT	EDAIQGRNES	CDISKEHTSE
1690	1700	1710	1720	1730	1740	1750	1760
TTERTPKHDV	HQICDKDAQQ	DLNLDIEKIT	ETGAVKPTGE	CSGEQSPDTN	YEPPGEDKTQ	GSSECISELS	FSGPNALVPM
1770	1780	1790	1800	1810	1820	1830	1840
DFLGNQEDIH	NLQLRVKETS	NENLRLLHVI	EDRDRKVESL	LNEMKELDSK	LHLQEVQLMT	KIEACIELEK	IVGELKKENS
1850	1860	1870	1880	1890	1900	1910	1920
DLSEKLEYFS	CDHQELLQRV	ETSEGLNSDL	EMHADKSSRE	DIGDNVAKVN	DSWKERFLDV	ENELSRIRSE	KASIEHEALY
1930	1940	1950	1960	1970	1980	1990	2000
LEADLEVVQT	EKLCLEKDNE	NKQKVIVCLE	EELSVVTSER	NQLRGELDTM	SKKTTALDQL	SEKMKEKTQE	LESHQSECLH
2010	2020	2030	2040	2050	2060	2070	2080
CIQVAEAEVK	EKTELLQTLS	SDVSELLKDK	THLQEKLQSL	EKDSQALSLT	KCELENQIAQ	LNKEKELLVK	ESESLQARLS
2090	2100	2110	2120	2130	2140	2150	2160
ESDYEKLNVS	KALEAALVEK	GEFALRLSST	QEEVHQLRRG	IEKLRVRIEA	DEKKQLHIAE	KLKERERERND	SLKDKVENLE
2170	2180	2190	2200	2210	2220	2230	2240

Detailed Protein Report

Cmpd.	No. of Cmpds.	m/z meas.	Δ m/z [ppm]	z	Rt [min]	Score	P	Range	Sequence	Modification	Ratios
104	2	1043.8924	-98.05	2	30.5	15.3	0	128-147	R.SQQAAQSADVSLNPCNTPQK.I		
2707	1	821.4735	62.12	2	64.1	11.9	2	301-314	R.LQGHEKEMKGQVNK.F	Oxidation: 8	
1209	1	573.1437	-261.24	2	43.6	14.6	0	981-990	K.EINASLNQEK.M		M:Q 2.43
1276	1	917.4679	30.80	2	46.1	11.7	0	2267-2283	K.ELNEAVAALCGDQEIMK.A		
552	1	600.8199	-67.28	2	35.3	13.6	1	2316-2325	K.KQLCVLQQLK.E		
2721	2	974.5198	32.44	2	63.0	14.2	1	2466-2482	K.AKEQNLSSQVECLELEK.A		

Detailed Protein Report

Protein 47: neutrophil defensin 1 preproprotein [Homo sapiens]

Accession: gi|4758146
Database: refseq_human(refseq_human_20140103.fasta)
Database Date: 2015-11-30
Modification(s): Carbamidomethyl

Score: 108.8
MW [kDa]: 10.2
pI: 7.6
Sequence Coverage [%]: 19.1
No. of unique Peptides: 2

Quantitation

Q:W	**Median:**	0.47	**CV:**	0.00 %	**No. of Peptides:**	1
M:Q	**Median:**	0.98	**CV:**	25.99 %	**No. of Peptides:**	2

Alias proteins:

Accession	Name	Description
gi|124248516	refseq_human (refseq_human_20140103.fasta)	neutrophil defensin 1 precursor [Homo sapiens]

```
        10         20         30         40         50         60         70         80
MRTLAILAAI LLVALQAQAE PLQARADEVA AAPEQIAADI PEVVVSLAWD ESLAPKHPGS RKNMACYCRI PACIAGERRY
        90        100
GTCIYQGRLW AFCC
```

Cmpd.	No. of Cmpds.	m/z meas.	Δ m/z [ppm]	z	Rt [min]	Score	P	Range	Sequence	Modification	Ratios	
147	3	493.7604	4.77	2	31.8	52.9	0	70-78	R.IPACIAGER.R	Carbamidomethyl: 4	M:Q	1.26
108	3	559.2630	8.21	2	31.3	55.9	0	80-88	R.YGTCIYQGR.L	Carbamidomethyl: 4	Q:W<br>M:Q	0.47<br>0.76

Detailed Protein Report

Protein 48: PREDICTED: dynein heavy chain 3, axonemal isoform X1 [Homo sapiens]

**Accession:**	gi|578828592	**Score:**	108.2
**Database:**	refseq_human(refseq_human_20140103.fasta)	**MW [kDa]:**	466.0
**Database Date:**	2015-11-30	**pI:**	6.0
**Modification(s):**	Carbamidomethyl, Oxidation	**Sequence Coverage [%]:**	2.7
		**No. of unique Peptides:**	6

Quantitation

Q:W	**Median:**	0.38	**CV:**	0.00 %	**No. of Peptides:**	1
M:Q	**Median:**	1.17	**CV:**	46.90 %	**No. of Peptides:**	2

Detailed Protein Report

10	20	30	40	50	60	70	80
MGDMDCSSQK	IAKSDSIHHM	SHSQGQPELP	PLPASANEEP	SGLYQTVMSH	SFYPPLMQRT	SWTLAAPFKE	QHHHRGPSDS
90	100	110	120	130	140	150	160
IANNYSLMAQ	DLKLKDLLKV	YQPATISVPR	DRTGQGLPSS	GNRSSSEPMR	KKTKFSSRNK	EDSTRIKLAF	KTSIFSPMKK
170	180	190	200	210	220	230	240
EVKTSLTFPG	SRPMSPEQQL	DVMLQQEMEM	ESKEKKPSES	DLERYYYYLT	NGIRKDMIAP	EEGEVMVRIS	KLISNTLLTS
250	260	270	280	290	300	310	320
PFLEPLMVVL	VQEKENDYYC	SLMKSIVDYI	LMDPMERKRL	FIESIPRLFP	QRVIRAPVPW	HSVYRSAKKW	NEEHLHTVNP
330	340	350	360	370	380	390	400
MMLRLKELWF	AEFRDLRFVR	TAEILAGKLP	LQPQEFWDVI	QKHCLEAHQT	LLNKWIPTCA	QLFTSRKEHW	IHFAPKSNYD
410	420	430	440	450	460	470	480
SSRNIEEYFA	SVASFMSLQL	RELVIKSLED	LVSLFMIHKD	GNDFKEPYQE	MKFFIPQLIM	IKLEVSEPII	VFNPSFDGCW
490	500	510	520	530	540	550	560
ELIRDSFLEI	IKNSNGIPKV	ESVLFPELKG	YNLLLGTVNA	EEKLVSDFLI	QTFKVFQKNQ	VGPCKYLNVY	KKYVDLLDNT
570	580	590	600	610	620	630	640
AEQNIAAFLK	ENHDIDDFVT	KINAIKKRRN	EIASMNITVP	LAMFCLDATA	LNHDLCERAQ	NLKDHLIQFQ	VDVNRDTNTS
650	660	670	680	690	700	710	720
ICNQYSHIAD	KVSEVPANTK	ELVSLIEFLK	KSSAVTVFKL	RRQLRDASER	LEFLMDYADL	PYEDIKLNST	LFLWPDQIED
730	740	750	760	770	780	790	800
IFDNSRNLLL	HKRDQAEMDL	IKRCSEFELR	LEGYHRELES	FRKREVMTTE	EMKHNVEKLN	ELSKNLNRAF	AEFELINKEE
810	820	830	840	850	860	870	880
ELLEKEKSTY	PLLQAMLKNK	VPYEQLWSTA	YEFSIKSEEW	MNGPLFLLNA	EQIAEEIGNM	WRTTYKLIKT	LSDVPAPRRL
890	900	910	920	930	940	950	960
AENVKIKIDK	FKQYIPILSI	SCNPGMKDRH	WQQISEIVGY	EIKPTETTCL	SNMLEFGFGK	FVEKLEPIGA	AASKEYSLEK
970	980	990	1000	1010	1020	1030	1040
NLDRMKLDWV	NVTFSFVKYR	DTDTNILCAI	DDIQMLLDDH	VIKTQTMCGS	PFIKPIEAEC	RKWEEKLIRI	QDNLDAWLKC
1050	1060	1070	1080	1090	1100	1110	1120
QATWLYLEPI	FSSEDIIAQM	PEEGRKFGIV	DSYWKSLMSQ	AVKDNRILVA	ADQPRMAEKL	QEANFLLEDI	QKGLNDYLEK
1130	1140	1150	1160	1170	1180	1190	1200
KRLFFPRFFF	LSNDELLEIL	SETKDPLRVQ	PHLKKCFEGI	AKLEFTDNLE	IVGMISSEKE	TVPFIQKIYP	ANAKGMVEKW
1210	1220	1230	1240	1250	1260	1270	1280
LQQVEQMMLA	SMREVIGLGI	EAYVKVPRNH	WVLQWPGQVV	ICVSSIFWTQ	EVSQALAENT	LLDFLKKSND	QIAQIVQLVR
1290	1300	1310	1320	1330	1340	1350	1360
GKLSSGARLT	LGALTVIDVH	ARDVVAKLSE	DRVSDLNDFQ	WISQLRYYWV	AKDVQVQIIT	TEALYGYEYL	GNSPRLVITP
1370	1380	1390	1400	1410	1420	1430	1440
LTDRCYRTLM	GALKLNLGGA	PEGPAGTGKT	ETTKDLAKAL	AKQCVVFNCS	DGLDYKAMGK	FFKGLAQAGA	WACFDEFNRI
1450	1460	1470	1480	1490	1500	1510	1520
EVEVLSVVAQ	QILSIQQAII	RKLKTFIFEG	TELSLNPTCA	VFITMNPGYA	GRAELPDNLK	ALFRTVAMMV	PDYALIGEIS
1530	1540	1550	1560	1570	1580	1590	1600
LYSMGFLDSR	SLAQKIVATY	RLCSEQLSSQ	HHYDYGMRAV	KSVLTAAGNL	KLKYPEENES	VLLLRALLDV	NLAKFLAQDV
1610	1620	1630	1640	1650	1660	1670	1680
PLFQGIISDL	FPGVVLPKPD	YEVFLKVLND	NIKKMKLQPV	PWFIGKIIQI	YEMMLVRHGY	MIVGDPMGGK	TSAYKVLAAA
1690	1700	1710	1720	1730	1740	1750	1760
LGDLHAANQM	EEFAVEYKII	NPKAITMGQL	YGCFDQVSHE	WMDGVLANAF	REQASSLSDD	RKWIIFDGPV	DAIWIENMNT
1770	1780	1790	1800	1810	1820	1830	1840
VLDDNKKLCL	MSGEIIQMNS	KMSLIFEPAD	LEQASPATVS	RCGMIYMEPH	QLGWKPLKDS	YMDTLPSSLT	KEHKELVNDM
1850	1860	1870	1880	1890	1900	1910	1920
FMWLVQPCLE	FGRLHCKFVV	QTSPIHLAFS	MMRLYSSLLD	EIRAVEEEEM	ELGEGLSSQQ	IFLWLQGLFL	FSLVWTVAGT
1930	1940	1950	1960	1970	1980	1990	2000
INADSRKKFD	VFFRNLIMGM	DDNHPRPKSV	KLTKNNIFPE	RGSIYDFYFI	KQASGHWETW	TQYITKEEEK	VPAGAKVSEL
2010	2020	2030	2040	2050	2060	2070	2080
IIPTMETARQ	SFFLKTYLDH	EIPMLFVGPT	GTGKSAITNN	FLLHLPKNTY	LPNCINFSAR	TSANQTQDII	MSKLDRRRKG
2090	2100	2110	2120	2130	2140	2150	2160
LFGPPIGKKA	VVFVDDLNMP	AKEVYGAQPP	IELLRQWIDH	GYWFDKKDTT	RLDIVDMLLV	TAMGPPGGGR	NDITGRFTRH
2170	2180	2190	2200	2210	2220	2230	2240

Detailed Protein Report

Cmpd.	No. of Cmpds.	m/z meas.	Δ m/z [ppm]	z	Rt [min]	Score	P	Range	Sequence	Modification	Ratios
301	1	874.1753	76.12	3	33.1	22.7	0	463-484	K.LEVSEPIIVFNPSFDGCWELIR.D	Carbamidomethyl: 17	
565	1	607.8337	-71.29	2	35.7	10.9	1	1562-1573	K.SVLTAAGNLKLK.Y		M:Q 1.83
2111	30	835.2916	-121.21	2	54.8	20.6	0	1935-1948	R.NLIMGMDDNHPRPK.S	Oxidation: 4, 6	
315	1	1201.1883	61.60	2	32.6	16.3	1	2128-2150	K.DTTRLDIVDMLLVTAMGPPGGGR.N	Oxidation: 10	
1563	1	949.5033	8.02	2	49.7	12.9	2	2269-2283	R.LIDKEDRQVFFNMVK.E	Oxidation: 13	
1965	2	572.7269	-161.97	2	54.9	12.8	1	2924-2934	K.LISGLGGEKDR.W		M:Q 0.75<br>Q:W 0.38

Detailed Protein Report

Protein 49: PREDICTED: histone-lysine N-methyltransferase 2A isoform X1 [Homo sapiens]

**Accession:**	gi|578822195	**Score:**	107.6
**Database:**	refseq_human(refseq_human_20140103.fasta)	**MW [kDa]:**	323.9
**Database Date:**	2015-11-30	**pI:**	9.2
**Modification(s):**	Carbamidomethyl, Oxidation	**Sequence Coverage [%]:**	3.7
		**No. of unique Peptides:**	6

Quantitation

Q:W	**Median:** 1.43	**CV:** 37.29 %	**No. of Peptides:** 2

Detailed Protein Report

10	20	30	40	50	60	70	80
MLAQADKLPM	TDKRVASLLK	KAKAQLCKIE	KSKSLKQTDQ	PKAQGQESDS	SETSVRGPRI	KHVCRRAAVA	LGRKRAVFPD
90	100	110	120	130	140	150	160
DMPTLSALPW	EEREKILSSM	GNDDKSSIAG	SEDAEPLAPP	IKPIKPVTRN	KAPQEPPVKK	GRRSRRCGQC	PGCQVPEDCG
170	180	190	200	210	220	230	240
VCTNCLDKPK	FGGRNIKKQC	CKMRKCQNLQ	WMPSKAYLQK	QAKAVKKKEK	KSKTSEKKDS	KESSVVKNVV	DSSQKPTPSA
250	260	270	280	290	300	310	320
REDPAPKKSS	SEPPPRKPVE	EKSEEGNVSA	PGPESKQATT	PASRKSSKQV	SQPALVIPPQ	PPTTGPPRKE	VPKTTPSEPK
330	340	350	360	370	380	390	400
KKQPPPPESG	PEQSKQKKVA	PRPSIPVKQK	PKEKEKPPPV	NKQENAGTLN	ILSTLSNGNS	SKQKIPADGV	HRIRVDFKED
410	420	430	440	450	460	470	480
CEAENVWEMG	GLGILTSVPI	TPRVVCFLCA	SSGHVEFVYC	QVCCEPFHKF	CLEENERPLE	DQLENWCCRR	CKFCHVCGRQ
490	500	510	520	530	540	550	560
HQATKQLLEC	NKCRNSYHPE	CLGPNYPTKP	TKKKKVWICT	KCVRCKSCGS	TTPGKGWDAQ	WSHDFSLCHD	CAKLFAKGNF
570	580	590	600	610	620	630	640
CPLCDKCYDD	DDYESKMMQC	GKCDRWVHSK	CENLSGTEDE	MYEILSNLPE	SVAYTCVNCT	ERHPAEWRLA	LEKELQISLK
650	660	670	680	690	700	710	720
QVLTALLNSR	TTSHLLRYRQ	AAKPPDLNPE	TEESIPSRSS	PEGPDPPVLT	EVSKQDDQQP	LDLEGVKRKM	DQGNYTSVLE
730	740	750	760	770	780	790	800
FSDDIVKIIQ	AAINSDGGQP	EIKKANSMVK	SFFIRQMERV	FPWFSVKKSR	FWEPNKVSSN	SGMLPNAVLP	PSLDHNYAQW
810	820	830	840	850	860	870	880
QEREENSHTE	QPPLMKKIIP	APKPKGPGEP	DSPTPLHPPT	PPILSTDRSR	EDSPELNPPP	GIEDNRQCAL	CLTYGDDSAN
890	900	910	920	930	940	950	960
DAGRLLYIGQ	NEWTHVNCAL	WSAEVFEDDD	GSLKNVHMAV	IRGKQLRCEF	CQKPGATVGC	CLTSCTSNYH	FMCSRAKNCV
970	980	990	1000	1010	1020	1030	1040
FLDDKKVYCQ	RHRDLIKGEV	VPENGFEVFR	RVFVDFEGIS	LRRKFLNGLE	PENIHMMIGS	MTIDCLGILN	DLSDCEDKLF
1050	1060	1070	1080	1090	1100	1110	1120
PIGYQCSRVY	WSTTDARKRC	VYTCKIVECR	PPVVEPDINS	TVEHDENRTI	AHSPTSFTES	SSKESQNTAE	IISPPSPDRP
1130	1140	1150	1160	1170	1180	1190	1200
PHSQTSGSCY	YHVISKVPRI	RTPSYSPTQR	SPGCRPLPSA	GSPTPTTHEI	VTVGDPLLSS	GLRSIGSRRH	STSSLSPQRS
1210	1220	1230	1240	1250	1260	1270	1280
KLRIMSPMRT	GNTYSRNNVS	SVSTTGTATD	LESSAKVVDH	VLGPLNSSTS	LGQNTSTSSN	LQRTVVTVGN	KNSHLDGSSS
1290	1300	1310	1320	1330	1340	1350	1360
SEMKQSSASD	LVSKSSSLKG	EKTKVLSSKS	SEGSAHNVAY	PGIPKLAPQV	HNTTSRELNV	SKIGSFAEPS	SVSFSSKEAL
1370	1380	1390	1400	1410	1420	1430	1440
SFPHLHLRGQ	RNDRDQHTDS	TQSANSSPDE	DTEVKTLKLS	GMSNRSSIIN	EHMGSSSRDR	RQKGKKSCKE	TFKEKHSSKS
1450	1460	1470	1480	1490	1500	1510	1520
FLEPGQVTTG	EEGNLKPEFM	DEVLTPEYMG	QRPCNNVSSD	KIGDKGLSMP	GVPKAPPMQV	EGSAKELQAP	RKRTVKVTLT
1530	1540	1550	1560	1570	1580	1590	1600
PLKMENESQS	KNALKESSPA	SPLQIESTSP	TEPISASENP	GDGPVAQPSP	NNTSCQDSQS	NNYQNLPVQD	RNLMLPDGPK
1610	1620	1630	1640	1650	1660	1670	1680
PQEDGSFKRR	YPRRSARARS	NMFFGLTPLY	GVRSYGEEDI	PFYSSSTGKK	RGKRSAEGQV	DGADDLSTSD	EDDLYYYNFT
1690	1700	1710	1720	1730	1740	1750	1760
RTVISSGGEE	RLASHNLFRE	EEQCDLPKIS	QLDGVDDGTE	SDTSVTATTR	KSSQIPKRNG	KENGTENLKI	DRPEDAGEKE
1770	1780	1790	1800	1810	1820	1830	1840
HVTKSSVGHK	NEPKMDNCHS	VSRVKTQGQD	SLEAQLSSLE	SSRRVHTSTP	SDKNLLDTYN	TELLKSDSDN	NNSDDCGNIL
1850	1860	1870	1880	1890	1900	1910	1920
PSDIMDFVLK	NTPSMQALGE	SPESSSSELL	NLGEGLGLDS	NREKDMGLFE	VFSQQLPTTE	PVDSSVSSSI	SAEEQFELPL
1930	1940	1950	1960	1970	1980	1990	2000
ELPSDLSVLT	TRSPTVPSQN	PSRLAVISDS	GEKRVTITEK	SVASSESDPA	LLSPGVDPTP	EGHMTPDHFI	QGHMDADHIS
2010	2020	2030	2040	2050	2060	2070	2080
SPPCGSVEQG	HGNNQDLTRN	SSTPGLQVPV	SPTVPIQNQK	YVPNSTDSPG	PSQISNAAVQ	TTPPHLKPAT	EKLIVVNQNM
2090	2100	2110	2120	2130	2140	2150	2160
QPLYVLQTLP	NGVTQKIQLT	SSVSSTPSVM	ETNTSVLGPM	GGGLTLTTGL	NPSLPTSQSL	FPSASKGLLP	MSHHQHLHSF
2170	2180	2190	2200	2210	2220	2230	2240

Detailed Protein Report

Cmpd.	No. of Cmpds.	m/z meas.	Δ m/z [ppm]	z	Rt [min]	Score	P	Range	Sequence	Modification	Ratios	
1751	1	1061.1573	33.03	3	51.7	15.9	2	144-170	R. SRRCGQCPGCQVPEDCGVCTNC F	Carbamidomethyl: 4, 10, 16, 19, 22		
908	1	541.3264	90.29	3	41.4	12.3	2	242-256	R.EDPAPKKSSSEPPPR.K			
53	1	1096.9888	-34.10	2	30.4	17.5	1	1264-1284	R. TVVTVGNKNSHLDGSSSSEMK. Q	Oxidation: 20		
11	1	820.3475	-49.73	1	29.3	12.9	1	2734-2739	K.HCRNYK.F		Q:W	1.00
2967	2	753.3836	9.44	2	66.3	16.1	1	2767-2779	R.KSAFDMFNFLASK.H			
395	1	625.3909	142.58	2	34.6	10.9	2	2950-2960	K.LPCNCGAKKCR.K	Carbamidomethyl: 5	Q:W	2.05

Detailed Protein Report

Protein 50: **fibrinogen beta chain isoform 2 preproprotein [Homo sapiens]**

**Accession:**	gi|296080754	**Score:**	107.6
**Database:**	refseq_human(refseq_human_20140103.fasta)	**MW [kDa]:**	49.9
**Database Date:**	2015-11-30	**pI:**	9.0
**Modification(s):**	Carbamidomethyl, Oxidation	**Sequence Coverage [%]:**	14.4
		**No. of unique Peptides:**	5

Quantitation

Q:W	**Median:**	1.52	**CV:**	17.59 %	**No. of Peptides:**	2
M:Q	**Median:**	0.53	**CV:**	42.01 %	**No. of Peptides:**	2

10	20	30	40	50	60	70	80
MKRMVSWSFH	KLKTMKHLLL	LLLCVFLVKS	QGVNDNEEGF	FSARGHRPLD	KKREEALLQQ	ERPIRNSVDE	LNNNVEAVSQ
**90**	**100**	**110**	**120**	**130**	**140**	**150**	**160**
TSSSSFQYMY	LLKDLWQKRQ	KQVKDNENVV	NEYSSELEKH	QLYIDETVNS	NIPTNLRVLR	SILENLRSKI	QKLESDVSAQ
**170**	**180**	**190**	**200**	**210**	**220**	**230**	**240**
MEYCRTPCTV	SCNIPVVSGK	ECEEIIRKGG	ETSEMYLIQP	DSSVKPYRVY	CDMNTENGGW	TVIQNRQDGS	VDFGRKWDPY
**250**	**260**	**270**	**280**	**290**	**300**	**310**	**320**
KQGFGNVATN	TDGKNYCGLP	GEYWLGNDKI	SQLTRMGPTE	LLIEMEDWKG	DKVKAHYGGF	TVQNEANKYQ	ISVNKYRGTA
**330**	**340**	**350**	**360**	**370**	**380**	**390**	**400**
GNALMDGASQ	LMGENRTMTI	HNGMFFSTYD	RDNDGWLTSD	PRKQCSKEDG	GGWWYNRCHA	ANPNGRYYWG	GQYTWDMAKH
**410**	**420**	**430**	**440**				
GTDDGVVWMN	WKGSWYSMRK	MSMKIRPFFP	QQ				

Cmpd.	No. of Cmpds.	m/z meas.	Δ m/z [ppm]	z	Rt [min]	Score	P	Range	Sequence	Modification	Ratios
590	2	809.8948	-6.44	2	37.4	25.6	0	166-180	R.TPCTVSCNIPVVSGK.E	Carbamidomethyl: 3, 7	Q:W 1.28<br>M:Q 0.80
1961	3	893.2648	-149.91	2	53.0	20.5	0	255-269	K.NYCGLPGEYWLGNDK.I	Carbamidomethyl: 3	
1919	1	862.3067	-107.58	2	53.9	11.4	0	276-289	R.MGPTELLIEMEDWK.G	Oxidation: 1, 10	
656	1	620.1188	-231.67	2	36.8	38.6	0	368-377	K.EDGGGWWYNR.C		
747	1	516.6531	-255.12	2	37.8	11.5	0	425-432	K.IRPFFPQQ.-		M:Q 0.36<br>Q:W 1.81

Detailed Protein Report

Protein 51: dynein heavy chain 11, axonemal [Homo sapiens]

**Accession:**	gi|459642365	**Score:**	107.5
**Database:**	refseq_human(refseq_human_20140103.fasta)	**MW [kDa]:**	520.0
**Database Date:**	2015-11-30	**pI:**	6.0
**Modification(s):**	Carbamidomethyl	**Sequence Coverage [%]:**	2.2
		**No. of unique Peptides:**	7

Quantitation

Q:W	**Median:**	1.38	**CV:**	0.00 %	**No. of Peptides:**	1
M:Q	**Median:**	0.83	**CV:**	0.00 %	**No. of Peptides:**	1

Detailed Protein Report

10	20	30	40	50	60	70	80
MAAQVAAREA	RDFREAPTLR	LTSGAGLEAV	GAVELEEEEE	NEEEAAARRA	RSFAQDARVR	FLGGRLAMML	GFTEEKWSQY
90	100	110	120	130	140	150	160
LESEDNRQVL	GEFLESTSPA	CLVFSFAASG	RLAASQEIPR	DANHKLVFIS	KKITESIGVN	DFSQVVLFGE	LPALSLGHVS
170	180	190	200	210	220	230	240
AFLDEILVPV	LSNKNNHKSW	SCFTSQDMEY	HIEVMKKKMY	IFRGKMSRRT	LLPIPTVAGK	MDLDQNCSEN	KPPSNERIIL
250	260	270	280	290	300	310	320
HAIESVVIEW	SHQIQEIIER	DSVQRLLNGL	HLSPQAELDF	WMMRRENLSC	IYDQLQAPVV	LKMVKILTTK	QSSYFPTLKD
330	340	350	360	370	380	390	400
IFLAVENALL	EAQDVELYLR	PLRRHIQCLQ	ETEFPQTRIL	IAPLFHTICL	IWSHSKFYNT	PARVIVLLQE	FCNLFINQAT
410	420	430	440	450	460	470	480
AYLSPEDLLR	GEIEESLEKV	QVAVNILKTF	KNSFFNYRKK	LASYFMGRKL	RPWDFQSHLV	FCRFDKFLDR	LIKIEDIFAT
490	500	510	520	530	540	550	560
TLEFEKLERL	EFGGTKGAIL	NGQVHEMSEE	LMELCKLFKQ	STYDPSDCTN	MEFESDYVAF	KSKTLEFDRR	LGTIICEAFF
570	580	590	600	610	620	630	640
NCNGLEAAFK	LLTIFGNFLE	KPVVMEIFSL	HYSTLVHMFN	TELDVCKQLY	NEHMKQIECG	HVVLNKNMPF	TSGNMKWAQQ
650	660	670	680	690	700	710	720
VLQRLQMFWS	NFASLRYLFL	GNPDHALVYQ	KYVEMTTLLD	QFESRIYNEW	KSNVDEICEF	NLNQPLVKFS	AINGLLCVNF
730	740	750	760	770	780	790	800
DPKLVAVLRE	VKYLLMLKKQ	DIPDSALAIF	KKRNTILKYI	GNLDLLVQGY	NKLKQTLLEV	EYPLIEDELR	AIDEQLTAAT
810	820	830	840	850	860	870	880
TWLTWQDDCW	GYIERVRAAT	SELEHRVERT	QKNVKVIQQT	MRGWARCVLP	PRREHRREAA	FTLEDKGDLF	TKKYKLIQGD
890	900	910	920	930	940	950	960
GCKIHNLVEE	NRKLFKANPS	LDTWKIYVEF	IDDIVVEGFF	QAIMHDLDFF	LKNTEKQLKP	APFFQAQMIL	LPPEIVFKPS
970	980	990	1000	1010	1020	1030	1040
LDREAGDGFY	DLVEEMLCNS	FRMSAQMNRI	ATHLEIKNYQ	NDMDNMLGLA	EVRQEIMNRV	VNVINKVLDF	RNTLETHTYL
1050	1060	1070	1080	1090	1100	1110	1120
WVDDRAEFMK	HFLLYGHAVS	SDEMDAHANE	EIPEQPPTLE	QFKEQIDIYE	ALYVQMSKFE	DFRVFDSWFK	VDMKPFKVSL
1130	1140	1150	1160	1170	1180	1190	1200
LTIIKKWSWM	FQEHLLRFVI	DSLNELQEFI	KETDSGLQRE	LNEGDHDGLV	DIMVHLLAVR	SRQRATDELF	EPLKETITLL
1210	1220	1230	1240	1250	1260	1270	1280
ESYGQKMPEQ	VYIQLEELPE	RWETTKKIAA	TVRHEVSPLH	NAEVTLIRKK	CILFDAKQAE	FRERFRHYAP	LGFNAENPYT
1290	1300	1310	1320	1330	1340	1350	1360
ALDKANEELE	ALEEEMLQMQ	ESTRLFEVAL	PEYKQMKQCR	KEIKLLKGLW	DVIIYVRRSI	DNWTKTQWRQ	IHVEQMDVEL
1370	1380	1390	1400	1410	1420	1430	1440
RRFAKEIWSL	NKEVRVWDAY	TGLEGTVKDM	TASLRAITEL	QSPALRDRHW	HQLMKAIGVK	FLINEATTLA	DLLALRLHRV
1450	1460	1470	1480	1490	1500	1510	1520
EDDVRRIVDK	AVKELGTEKV	ITEISQTWAT	MKFSYEVHYR	TGIPLLKSDE	QLFETLEHNQ	VQLQTLLQSK	YVEYFIEQVL
1530	1540	1550	1560	1570	1580	1590	1600
SWQNKLNIAD	LVIFTWMEVQ	RTWSHLESIF	VCSEDIRIQL	VKDARRFDGV	DAEFKELMFK	TAKVENVLEA	TCRPNLYEKL
1610	1620	1630	1640	1650	1660	1670	1680
KDLQSRLSLC	EKALAEYLET	KRIAFPRFYF	VSSADLLDIL	SKGAQPKQVT	CHLAKLFDSI	ADLQFEDNQD	VSAHRAVGMY
1690	1700	1710	1720	1730	1740	1750	1760
SKEKEYVPFQ	AECECVGHVE	TWLLQLEQTM	QETVRHSITE	AIVAYEEKPR	ELWIFDFPAQ	VALTSSQIWW	TTDVGIAFSR
1770	1780	1790	1800	1810	1820	1830	1840
LEEGYETALK	DFHKKQISQL	NTLITLLLGE	LPPGDRQKIM	TICTIDVHAR	DVVAKLISQK	VVSPQAFTWL	SQLRHRWEDT
1850	1860	1870	1880	1890	1900	1910	1920
QKHCFVNICD	AQFQYFYEYL	GNSPRLVITP	LTDRCYITLT	QSLHLTMSGA	PAGPAGTGKT	ETTKDLGRAL	GMMVYVFNCS
1930	1940	1950	1960	1970	1980	1990	2000
EQMDYKSIGN	IYKGLVQTGA	WGCFDEFNRI	SVEVLSVVAV	QVKMIHDAIR	NRKKRFVFLG	EAITLKPSVG	IFITMNPGYA
2010	2020	2030	2040	2050	2060	2070	2080
GRTELPENLK	ALFRPCAMVA	PDIELICEIL	LVAEGFVDAR	ALARKFITLY	TLCKELLSKQ	DHYDWGLRAI	KSVLVVAGSL
2090	2100	2110	2120	2130	2140	2150	2160
KRGDKNRPED	QVLMRALRDF	NMPKIVTDDI	PVFLGLVGDL	FPALDVPRRR	KLHFEQMVRQ	STLELRLQPE	ESFILKVVQL
2170	2180	2190	2200	2210	2220	2230	2240

Cmpd.	No. of Cmpds.	m/z meas.	Δ m/z [ppm]	z	Rt [min]	Score	P	Range	Sequence	Modification	Ratios
2700	1	920.8814	-102.30	2	65.0	16.0	2	857-872	R.REAAFTLEDKGDLFTK.K		
882	1	647.8007	-79.74	2	39.8	12.8	2	1437-1446	R.LHRVEDDVRR.I		
36	1	779.3158	-100.01	2	29.7	10.1	1	2083-2095	R.GDKNRPEDQVLMR.A		
1912	2	634.8166	-103.44	2	54.3	17.9	0	2157-2167	K.VVQLEELLAVR.H		
2478	1	589.2111	-228.33	2	60.9	14.1	0	2296-2304	R.LLFEIHHLR.S		
2496	1	822.4302	-68.99	2	61.8	12.6	1	2977-2990	R.LQLKIILCFSPVGR.T	Carbamidomethyl: 8	Q:W 1.38<br>M:Q 0.83
1863	1	965.8216	-153.66	2	53.3	12.4	2	4470-4484	R.QETKQTYECPVYRTK.L	Carbamidomethyl: 9	

Detailed Protein Report

Protein 52: dynein heavy chain 1, axonemal [Homo sapiens]

**Accession:**	gi|197927452	**Score:**	106.5
**Database:**	refseq_human(refseq_human_20140103.fasta)	**MW [kDa]:**	487.2
**Database Date:**	2015-11-30	**pI:**	5.5
**Modification(s):**	Carbamidomethyl, Oxidation	**Sequence Coverage [%]:**	2.3
		**No. of unique Peptides:**	7

Quantitation

Q:W	**Median:**	0.98	**CV:**	0.00 %	**No. of Peptides:**	1
M:Q	**Median:**	1.33	**CV:**	45.24 %	**No. of Peptides:**	3

Alias proteins:

Accession	Name	Description
gi|578805960	refseq_human (refseq_human_20140103.fasta)	PREDICTED: dynein heavy chain 1, axonemal isoform X1 [Homo sapiens]

BRUKER

Detailed Protein Report

10	20	30	40	50	60	70	80
MEQPNSKGYS	LGRTPQGPEC	SSAPAVQVGT	HRGLEYNPGK	ILPGSDYGLG	NPPALDPKLP	HLPLPPAPPT	LSDLGQPRKS
90	100	110	120	130	140	150	160
PLTGTDKKYP	LMKQRGFYSD	ILSPGTLDQL	GEVCRGPRMS	QNLLRQADLD	KFTPRVGSFE	VPEDFQERME	QQCIGSTTRL
170	180	190	200	210	220	230	240
LAQTDFPLQA	YEPKMQVPFQ	VLPGQHPRKI	EIERRKQQYL	SLDIEQLLFS	QGIDSNKLMP	RHLDHQHPQT	IEQGHDPIFP
250	260	270	280	290	300	310	320
IYLPLKVFDN	EDFDCRTPRE	WINMGLEPGS	LDRKPVPGKA	LLPTDDFLGH	EDPKSQKLKY	KWCEVGVLDY	DEEKKLYLVH
330	340	350	360	370	380	390	400
KTDEKGLVRD	EMGRPILNAG	VTTEGRPPLQ	VCQYWVPRIQ	LLFCAEDPCM	FAQRVVQANA	LRKNTEALLL	YNLYVDCMPS
410	420	430	440	450	460	470	480
DGQHVISEQS	LSKIKQWALS	TPRMRKGPSV	LEHLSSLARE	VSLDYERSMN	KINFDHVVSS	KPETFSYVTL	PKKEEEQVPE
490	500	510	520	530	540	550	560
RGLVSVPKYH	FWEQKEDFTF	VSLLTRPEVI	TALSKVRAEC	NKVTAMSLFH	SSLSKYSHLE	EFEQIQSQTF	SQVQMFLKDS
570	580	590	600	610	620	630	640
WISSLKVAMR	SSLRDMSKGW	YNLYETNWEV	YLMSKLRKLM	ELVKYMLQDT	LRFLVQDSLA	SFSQFISDTC	CSVLNCTDDM
650	660	670	680	690	700	710	720
VWGDDLINSP	YRPRKNPLFI	MDLVLDSSGV	HYSTPLEQFE	ASLLNLFDKG	ILATHAVPQL	EKLVMEDIFI	SGDPLLESVG
730	740	750	760	770	780	790	800
LHEPLVEELR	ATIASAVSKA	MIPLQAYAKE	YRKYLELNNN	DIASFLKTYQ	TQGLLAQEVR	EVVLTHLREK	EILDSSLPSS
810	820	830	840	850	860	870	880
IIIGPFYINT	DNVKQSLSKK	RKALATSVLD	ILAKNLHKEV	DSICEEFRSI	SRKIYEKPNS	IEELAELREW	MKGIPERLVG
890	900	910	920	930	940	950	960
LEERIVKVMD	DYQVMDEFLY	NLSSDDFNDK	WIASNWPSKI	LGQIELVQQQ	HVEDEEKFRK	IQIMDQNNFQ	EKLEGLQLVV
970	980	990	1000	1010	1020	1030	1040
AGFSIHVEIS	RAHEIANEVR	RVKKQLKDCQ	QLAMLYNNRE	RIFSLPITNY	DKLSRMVKEF	QPYLDLWTTA	SDWLRWSESW
1050	1060	1070	1080	1090	1100	1110	1120
MNDPLSAIDA	EQLEKNVVEA	FKTMHKCVKQ	FKDMPACQEV	ALDIRARIEE	FKPYIPLIQG	LRNPGMRIRH	WETLSNQINI
1130	1140	1150	1160	1170	1180	1190	1200
NVRPKANLTF	ARCLEMNLQD	HIESISKVAE	VAGKEYAIEQ	ALDKMEKEWS	TILFNVLPYK	ATDTYILKSP	DEASQLLDDH
1210	1220	1230	1240	1250	1260	1270	1280
IVMTQNMSFS	PYKKPFEQRI	NSWENKLKLT	QEVLEEWLNC	QRSWLYLEPI	FSSEDINQQL	PVESKRYQTM	ERIWKKIMKN
1290	1300	1310	1320	1330	1340	1350	1360
AYENREVINV	CSDLRMLDSL	RDCNKILDLV	QKGLSEYLET	KRSAFPRFYF	LSDDELLEIL	SQTKDPTAVQ	PHLRKCFENI
1370	1380	1390	1400	1410	1420	1430	1440
ARLLFQEDLE	ITHMYSAEGE	EVQLCFSIYP	SSNVEDWLRE	VERSMKASVH	DIIEKAIRAY	PTMPRTQWVL	NWPGQVTIAG
1450	1460	1470	1480	1490	1500	1510	1520
CQTYWTMEVA	EALEAGNLRS	QLFPQLCQQL	SDLVALVRGK	LSRMQRAVLS	ALIVIEVHAK	DVVSKLIQEN	VVSVNDFQWI
1530	1540	1550	1560	1570	1580	1590	1600
SQLRYYWTNN	DLYIRAVNAE	FIYGYEYLGN	SGRLVITPLT	DRCYLTLTGA	LHLKFGGAPA	GPAGTGKTET	TKDLGKALAI
1610	1620	1630	1640	1650	1660	1670	1680
QTVVFNCSDQ	LDFMAMGKFF	KGLASAGAWA	CFDEFNRIDI	EVLSVVAQQI	TTIQKAQQQR	VERFMFEGVE	IPLVPSCAVF
1690	1700	1710	1720	1730	1740	1750	1760
ITMNPGYAGR	TELPDNLKAL	FRPVAMMVPD	YAMITEISLY	SFGFNEASVL	AKKITTTFKL	SSEQLSSQDH	YDFGMRAVKT
1770	1780	1790	1800	1810	1820	1830	1840
VISAAGNLKR	ENPSMNEELI	CLRAIRDVNV	PKFLQEDLKL	FSGIVSDLFP	TIKEEDTDYG	ILDEAIREAC	RNSNLKDVEG
1850	1860	1870	1880	1890	1900	1910	1920
FLTKCIQLYE	TTVVRHGLML	VGPTGSGKST	CYRVLAAAMT	SLKGQPSISG	GMYEAVNYYV	LNPKSITMGQ	LYGEFDLLTH
1930	1940	1950	1960	1970	1980	1990	2000
EWTDGIFSSF	IRAGAITSDT	NKKWYMFDGP	VDAIWIENMN	TVLDDNKKLC	LSSGEIIKLT	EAMTMMFEVQ	DLAVASPATV
2010	2020	2030	2040	2050	2060	2070	2080
SRCGMVYLEP	SILGLMPFIE	CWLRKLPPLL	KPYEEHFKAL	FVSFLEESIS	FVRSSVKEVI	ASTNCNLTMS	LLKLLDCFFK
2090	2100	2110	2120	2130	2140	2150	2160
PFLPREGLKK	IPSEKLSRIV	ELIEPWFIFS	LIWSVGATGD	SSGRTSFSHW	LRLKMENEQL	TLLFPEEGLV	FDYRLEDAGI
2170	2180	2190	2200	2210	2220	2230	2240

Detailed Protein Report

Cmpd.	No. of Cmpds.	m/z meas.	Δ m/z [ppm]	z	Rt [min]	Score	P	Range	Sequence	Modification	Ratios	
1891	2	918.3580	-147.46	2	53.5	15.4	2	559-574	K.DSWISSLKVAMRSSLR.D		M:Q<br>Q:W	0.93<br>0.98
449	4	565.3223	-66.47	2	35.3	17.9	2	596-604	K.LRKLMELVK.Y			
2181	1	990.9291	-65.49	2	55.9	12.3	2	1828-1844	R.EACRNSNLKDVEGFLTK.C	Carbamidomethyl: 3		
870	1	573.1350	-254.46	2	40.3	10.7	1	2478-2486	R.DRLVNEEDR.S		M:Q	2.43
178	2	715.8622	76.35	2	31.9	12.3	0	2496-2506	R.CMEQWEVTFNK.V	Oxidation: 2	M:Q	1.03
972	1	730.6935	389.04	1	40.6	12.0	0	3422-3428	K.AAEIQAK.V			
51	2	706.3163	-75.95	3	30.3	15.4	2	3699-3718	K.KLSAISLGQGQGPRAEAMMR.S	Oxidation: 18		

Detailed Protein Report

Protein 53: coiled-coil domain-containing protein 168 [Homo sapiens]

**Accession:**	gi|226246554	**Score:**	103.7
**Database:**	refseq_human(refseq_human_20140103.fasta)	**MW [kDa]:**	801.4
**Database Date:**	2015-11-30	**pI:**	9.6
**Modification(s):**	Carbamidomethyl, Oxidation	**Sequence Coverage [%]:**	1.8
		**No. of unique Peptides:**	7

Quantitation

Q:W	**Median:**	1.27	**CV:**	15.77 %	**No. of Peptides:**	2
M:Q	**Median:**	0.56	**CV:**	1.18 %	**No. of Peptides:**	2

Detailed Protein Report

10	20	30	40	50	60	70	80
MSKQYYSFKK	GVGSGLEDNT	FMTLWDFLES	WIIQNDWVAI	FFIILLGIIF	EIILMKACAS	FWKKPTLPEK	GSSDVQETED
90	100	110	120	130	140	150	160
SCPKSRKLAP	ENWSVINSSS	GERVGTFLEK	RITSSLTSEE	KECNFEDRIL	FSREILWSGT	SESEDQVSPS	SESHVPSSNG
170	180	190	200	210	220	230	240
ISSSLPLFYS	EVEETCLSHT	EHPDREYETI	QFSSKKLFSM	MKTNKNKNSG	FSSDLSFSAS	RFTVENEDLD	VAPCPLAHLF
250	260	270	280	290	300	310	320
LSRDQVRLLE	ENVRNQIPSK	PKTKLGSRTT	YQCSRSQESL	NQNQPSVGMV	ISVQAQDSFP	GQNAFQNQGL	YEVQFTSQAQ
330	340	350	360	370	380	390	400
YINHNQESIK	SQPESKASNF	AQPEDVMKKP	FSSSTQDSFQ	SQDLDRNQHF	VEVPSIVEAK	YSVKGLESDE	HLGEDQHCVW
410	420	430	440	450	460	470	480
FIDSNKVKYS	IKGQDTIFKN	AEFLVLTLNP	NLVTEDMPQL	RSVKAQGQQQ	IVSSELNQDS	VYSSVPLLST	IKGQKNRRKT
490	500	510	520	530	540	550	560
PDSKSKLSLN	VPSLKAKKTP	TSQVFQITVC	HTLKNRNELG	CKNNTEKKEL	HERKDISDIA	LHLISVSKLI	LPYVKNYSRK
570	580	590	600	610	620	630	640
QLVKVMPGLI	KCGHFLQKQN	KSPDTEKINY	AGPLEETGIS	DITKKEKEYD	KENKRLKNIS	PKMLPQLEQS	FMVNTVQLKA
650	660	670	680	690	700	710	720
PCLLVETNGK	SKESLKDSIT	QAKGIGITEF	HVLNSKKPFD	LHIPKHKTSL	EEAISKPMQK	LVSSPEMESN	NRMKIQEDLQ
730	740	750	760	770	780	790	800
SSENSHLQLS	NGEELPTSTP	KTQRCFPREN	TQKQKDFLEL	VLELSNVGLL	ISPGSKMHKS	SEELEAIKIQ	VNTESVNLKE
810	820	830	840	850	860	870	880
SKPLILNVTE	DSDLRESEEL	ECNTGSNITN	MHQDKETSDA	FHSATYTTIS	QLPDTETHSI	SKAKADTLRI	IRLSHSASKQ
890	900	910	920	930	940	950	960
EKLPDEKETQ	NAEYIDKSCT	FKKPQQCDRK	EQEKEANSEL	TQGFRFSIHL	KQKPKYVKFQ	MEQISSGSSK	APNKEQEVQP
970	980	990	1000	1010	1020	1030	1040
QTLSTQTILE	NSPCPMMDPF	QVEKVKQSTD	RPTDRESAGD	PKNPLTMPEN	LPVGELLIET	TEYSVPFGGN	LQKTTDSHIA
1050	1060	1070	1080	1090	1100	1110	1120
EEKEDVKRYL	PAVALGSFNN	HLLTLPYFKR	QEIKKKLSET	KSVLSVKYVI	MKVKKPAISL	MPYINICGTS	NHRKKMGGNF
1130	1140	1150	1160	1170	1180	1190	1200
EIIIKQILQD	KIAAGMLLNV	IYPPMSILPN	TRMYSRLNAE	NHSHIKLVQE	ESQIEREEKY	PYFINEGNES	QNTLDAKLQD
1210	1220	1230	1240	1250	1260	1270	1280
EVKGVKETLP	KAVLHDSCNL	GLDAHLEKEI	KTEKEMHQPI	PFTETIIESV	VSPIMELSHA	ENVKSTQKTQ	TDCKCTADSE
1290	1300	1310	1320	1330	1340	1350	1360
TPSPISGKSL	IGDPLNQTRE	SYIPSNGSDT	REMGYCFAEE	KTEIPKDLPA	TSPETFNYCT	PVLSCSKVMK	KRVTFALTTS
1370	1380	1390	1400	1410	1420	1430	1440
TAKPKCVNTK	AVKPSISETV	SVTSHRKKSE	LDFKTKFKKI	NQTKGLVPEC	LNTLCSPMHS	RLQREFCLPA	SQLKQGETAD
1450	1460	1470	1480	1490	1500	1510	1520
KTYTDVFAKN	SISHDREEKL	QDGKEEEHKV	LLEAAPQLSQ	HLGSEAGQMK	EIHLESDPVL	NCLTLELHIN	GQRLQHQTGF
1530	1540	1550	1560	1570	1580	1590	1600
EQTTLETSLQ	MGPLEAEELQ	KANETENDIK	VLGGPKIPPP	KALQALENSD	GLILNAYQKD	NELVKSDEEL	NQPGSTNIQV
1610	1620	1630	1640	1650	1660	1670	1680
QPQTHFTQTI	LKSTSCPTLD	QFPFEKVESH	VRFSPLKSGE	AKVDEIIFYA	REGGISSDSS	HQKEQAGGTE	KKETAIFGSC
1690	1700	1710	1720	1730	1740	1750	1760
MPALSTPKTT	RNLKQFSDMK	TLVNPKCGII	KAKKPSISYM	LNIRAGAGPK	RRKELSCNLT	TKMKELHQGK	KGVDETYAFL
1770	1780	1790	1800	1810	1820	1830	1840
TMTPDINKYS	KVETEKDTLR	EKRLSSTQVK	QDTSPHEDSI	TSRDIKETLL	QDEEQEERKQ	EALLKVIPQH	LQHFMFRSGQ
1850	1860	1870	1880	1890	1900	1910	1920
GKDLDFHKLE	NQGSRKILFV	TKQDVPQQLQ	PAEPIQREET	KKCLQTQNGT	ICTVNSKLLP	LKSEDSVNGE	VLTGAIKRGV
1930	1940	1950	1960	1970	1980	1990	2000
PTDRKCMGEQ	HNSGKGEKAE	FNKDLQATVL	ELQKSPHGGE	AQKANLTDME	SGSSNAMNMN	VQHEREDKNI	QKMLTESVPC
2010	2020	2030	2040	2050	2060	2070	2080
YSQHLRFSTH	QMKDPDPCKS	GSEPKSPEGR	SWNLSHIVQK	TKQETHFRET	VLEPISGYMM	KQSPHMQEGI	KCMEGLKTSF
2090	2100	2110	2120	2130	2140	2150	2160
PKTGKSKIGS	IPRDTPWDEN	PRRKWDSSIS	EKTAWNQKNL	QTVLKPLDFS	SLMSSEYESR	SYTLEFIGKK	SMSPKCVTLK
2170	2180	2190	2200	2210	2220	2230	2240

Detailed Protein Report

Cmpd.	No. of Cmpds.	m/z meas.	Δ m/z [ppm]	z	Rt [min]	Score	P	Range	Sequence	Modification	Ratios
2367	1	805.3600	-33.80	2	60.0	10.6	0	1883-1897	K.CLQTQNGTICTVNSK.L		
270	1	642.3049	-15.58	2	33.4	11.3	0	2241-2251	R.QNLDGHITEEK.E		M:Q 0.56<br>Q:W 1.49
2490	1	610.2540	-89.47	2	61.1	10.6	2	3706-3716	K.QEGKMQEGKGK.S		
1634	1	803.8641	-92.41	2	50.2	10.9	0	5427-5440	K.MCILFSKPLPSNLK.L	Oxidation: 1	
2371	9	974.2361	79.07	3	60.1	12.7	2	5658-5682	R.DEDIYFTGFGTIRSGKRPEWLFTGK		
2107	1	698.3422	-40.67	2	54.9	13.3	1	6680-6691	K.KQSPHENSSLIR.K		M:Q 0.57<br>Q:W 1.09
1685	1	916.4063	-73.78	2	50.8	10.7	2	6846-6860	R.ECRKETLIITPPSCK.S	Carbamidomethyl: 2, 14	

Detailed Protein Report

Protein 54: obscurin isoform b [Homo sapiens]

**Accession:**	gi|403501446	**Score:**	101.9
**Database:**	refseq_human(refseq_human_20140103.fasta)	**MW [kDa]:**	867.9
**Database Date:**	2015-11-30	**pI:**	5.6
**Modification(s):**	Carbamidomethyl	**Sequence Coverage [%]:**	1.0
		**No. of unique Peptides:**	7

Quantitation

M:Q	**Median:** 0.81	**CV:** 0.00 %	**No. of Peptides:** 1

Detailed Protein Report

10	20	30	40	50	60	70	80
MDQPQFSGAP	RFLTRPKAFV	VSVGKDATLS	CQIVGNPTPQ	VSWEKDQQPV	AAGARFRLAQ	DGDLYRLTIL	DLALGDSGQY
90	100	110	120	130	140	150	160
VCRARNAIGE	AFAAVGLQVD	AEAACAEQAP	HFLLRPTSIR	VREGSEATFR	CRVGGSPRPA	VSWSKDGRRL	GEPDGPRVRV
170	180	190	200	210	220	230	240
EELGEASALR	IRAARPRDGG	TYEVRAENPL	GAASAAAALV	VDSDAADTAS	RPGTSTAALL	AHLQRRREAM	RAEGAPASPP
250	260	270	280	290	300	310	320
STGTRTCTVT	EGKHARLSCY	VTGEPKPETV	WKKDGQLVTE	GRRHVVYEDA	QENFVLKILF	CKQSDRGLYT	CTASNLVGQT
330	340	350	360	370	380	390	400
YSSVLVVVRE	PAVPFKKRLQ	DLEVREKESA	TFLCEVPQPS	TEAAWFKEET	RLWASAKYGI	EEEGTERRLT	VRNVSADDDA
410	420	430	440	450	460	470	480
VYICETPEGS	RTVAELAVQG	NLLRKLPRKT	AVRVGDTAMF	CVELAVPVGP	VHWLRNQEEV	VAGGRVAISA	EGTRHTLTIS
490	500	510	520	530	540	550	560
QCCLEDVGQV	AFMAGDCQTS	TQFCVSAPRK	PPLQPPVDPV	VKARMESSVI	LSWSPPPHGE	RPVTIDGYLV	EKKKLGTYTW
570	580	590	600	610	620	630	640
IRCHEAEWVA	TPELTVADVA	EEGNFQFRVS	ALNSFGQSPY	LEFPGTVHLA	PKLAVRTPLK	AVQAVEGGEV	TFSVDLTVAS
650	660	670	680	690	700	710	720
AGEWFLDGQA	LKASSVYEIH	CDRTRHTLTI	REVPASLHGA	QLKFVANGIE	SSIRMEVRAA	PGLTANKPPA	AAAREVLARL
730	740	750	760	770	780	790	800
HEEAQLLAEL	SDQAAAVTWL	KDGRTLSPGP	KYEVQASAGR	RVLLVRDVAR	DDAGLYECVS	RGGRIAYQLS	VQGLARFLHK
810	820	830	840	850	860	870	880
DMAGSCVDAV	AGGPAQFECE	TSEAHVHVHW	YKDGMELGHS	GERFLQEDVG	TRHRLVAATV	TRQDEGTYSC	RVGEDSVDFR
890	900	910	920	930	940	950	960
LRVSEPKVVF	AKEQLARRKL	QAEAGASATL	SCEVAQAQTE	VTWYKDGKKL	SSSSKVCMEA	TGCTRRLVVQ	QAGQADAGEY
970	980	990	1000	1010	1020	1030	1040
SCEAGGQRLS	FHLDVKEPKV	VFAKDQVAHS	EVQAEAGASA	TLSCEVAQAQ	TEVMWYKDGK	KLSSSLKVHV	EAKGCRRRLV
1050	1060	1070	1080	1090	1100	1110	1120
VQQAGKTDAG	DYSCEARGQR	VSFRLHITEP	KMMFAKEQSV	HNEVQAEAGA	SAMLSCEVAQ	AQTEVTWYKD	GKKLSSSSKV
1130	1140	1150	1160	1170	1180	1190	1200
GMEVKGCTRR	LVLPQAGKAD	AGEYSCEAGG	QRVSFHLHIT	EPKGVFAKEQ	SVHNEVQAEA	GTTAMLSCEV	AQPQTEVTWY
1210	1220	1230	1240	1250	1260	1270	1280
KDGKKLSSSS	KVRMEVKGCT	RRLVVQQVGK	ADAGEYSCEA	GGQRVSFQLH	ITEPKAVFAK	EQLVHNEVRT	EAGASATLSC
1290	1300	1310	1320	1330	1340	1350	1360
EVAQAQTEVT	WYKDGKKLSS	SSKVRIEAAG	CMRQLVVQQA	GQADAGEYTC	EAGGQRLSFH	LDVSEPKAVF	AKEQLAHRKV
1370	1380	1390	1400	1410	1420	1430	1440
QAEAGAIATL	SCEVAQAQTE	VTWYKDGKKL	SSSSKVRMEA	VGCTRRLVVQ	QACQADTGEY	SCEAGGQRLS	FSLDVAEPKV
1450	1460	1470	1480	1490	1500	1510	1520
VFAKEQPVHR	EVQAQAGAST	TLSCEVAQAQ	TEVMWYKDGK	KLSFSSKVRM	EAVGCTRRLV	VQQAGQAVAG	EYSCEAGSQR
1530	1540	1550	1560	1570	1580	1590	1600
LSFHLHVAEP	KAVFAKEQPA	SREVQAEAGT	SATLSCEVAQ	AQTEVTWYKD	GKKLSSSSKV	RMEAVGCTRR	LVVQEAGQAD
1610	1620	1630	1640	1650	1660	1670	1680
AGEYSCKAGD	QRLSFHLHVA	EPKVVFAKEQ	PAHREVQAEA	GASATLSCEV	AQAQTEVTWY	KDGKKLSSSS	KVRVEAVGCT
1690	1700	1710	1720	1730	1740	1750	1760
RRLVVQQAGQ	AEAGEYSCEA	GGQQLSFRLQ	VAELEPQISE	RPCRREPLVV	KEHEDIILTA	TLATPSAATV	TWLKDGVEIR
1770	1780	1790	1800	1810	1820	1830	1840
RSKRHETASQ	GDTHTLTVHG	AQVLDSAIYS	CRVGAEGQDF	PVQVEEVAAK	FCRLLEPVCG	ELGGTVTLAC	ELSPACAEVV
1850	1860	1870	1880	1890	1900	1910	1920
WRCGNTQLRV	GKRFQMVAEG	PVRSLTVLGL	RAEDAGEYVC	ESRDDHTSAQ	LTVSVPRVVK	FMSGLSTVVA	EEGGEATFQC
1930	1940	1950	1960	1970	1980	1990	2000
VVSPSDVAVV	WFRDGALLQP	SEKFAISQSG	ASHSLTISDL	VLEDAGQITV	EAEGASSSAA	LRVREAPVLF	KKKLEPQTVE
2010	2020	2030	2040	2050	2060	2070	2080
ERSSVTLEVE	LTRPWPELRW	TRNATALAPG	KNVEIHAEGA	RHRLVLHNVG	FADRGFFGCE	TPDDKTQAKL	TVEMRQVRLV
2090	2100	2110	2120	2130	2140	2150	2160
RGLQAVEARE	QGTATMEVQL	SHADVDGSWT	RDGLRFQQGP	TCHLAVRGPM	HTLTLSGLRP	EDSGLMVFKA	EGVHTSARLV
2170	2180	2190	2200	2210	2220	2230	2240

Detailed Protein Report

Cmpd.	No. of Cmpds.	m/z meas.	Δ m/z [ppm]	z	Rt [min]	Score	P	Range	Sequence	Modification	Ratios
1991	1	643.3110	-83.65	2	54.8	19.9	2	338-347	K.RLQDLEVREK.E		
961	1	512.1292	-217.77	2	42.1	10.5	0	872-880	R.VGEDSVDFR.L		M:Q 0.81
2661	2	686.8980	-30.78	2	63.3	13.1	2	881-892	R.LRVSEPKVVFAK.E		
2890	1	922.9785	45.19	2	65.3	10.6	2	929-945	K.KLSSSSKVCMEATGCTR.R	Carbamidomethyl: 15	
2923	1	951.4595	12.61	2	65.7	13.9	2	929-945	K.KLSSSSKVCMEATGCTR.R	Carbamidomethyl: 9, 15	
2091	1	709.3658	-116.08	2	56.0	20.8	2	5981-5992	R.KYLLQARTAIIK.S		
2727	1	846.9286	-83.14	2	65.2	13.0	2	7693-7708	K.ASGRALAAKIIPYHPK.D		

Detailed Protein Report

Protein 55: immunoglobulin lambda-like polypeptide 5 isoform 1 [Homo sapiens]

Accession: gi|295986608
Database: refseq_human(refseq_human_20140103.fasta)
Database Date: 2015-11-30
Modification(s): Carbamidomethyl

Score: 100.4
MW [kDa]: 23.0
pI: 10.0
Sequence Coverage [%]: 23.8
No. of unique Peptides: 3

Quantitation

Q:W	**Median:** 1.29	**CV:** 0.00 %	**No. of Peptides:**	1
M:Q	**Median:** 1.41	**CV:** 0.00 %	**No. of Peptides:**	1

```
        10         20         30         40         50         60         70         80
MRPKTGQVGC ETPEELGPGP RQRWPLLLLG LAMVAHGLLR PMVAPQSGDP DPGASVGSSR SSLRSLWGRL LLQPSPQRAD
        90        100        110        120        130        140        150        160
PRCWPRGFWS EPQSLCYVFG TGTKVTVLGQ PKANPTVTLF PPSSEELQAN KATLVCLISD FYPGAVTVAW KADGSPVKAG
       170        180        190        200        210        220
VETTKPSKQS NNKYAASSYL SLTPEQWKSH RSYSCQVTHE GSTVEKTVAP TECS
```

Cmpd.	No. of Cmpds.	m/z meas.	Δ m/z [ppm]	z	Rt [min]	Score	P	Range	Sequence	Modification	Ratios
2042	1	1045.4503	-102.49	2	55.9	14.4	2	70-86	R.LLLQPSPQRADPRCWPR.G	Carbamidomethyl: 14	
1891	5	1021.8855	-134.81	2	52.1	22.0	0	113-131	K.ANPTVTLFPPSSEELQANK.A		
1789	23	872.4180	-17.25	2	50.7	64.0	0	174-188	K.YAASSYLSLTPEQWK.S		M:Q 1.41<br>Q:W 1.29

Detailed Protein Report

Protein 56: keratin, type II cytoskeletal 1b [Homo sapiens]

Accession: gi|194018511
Database: refseq_human(refseq_human_20140103.fasta)
Database Date: 2015-11-30

Score: 100.2
MW [kDa]: 61.9
pI: 5.6
Sequence Coverage [%]: 9.0
No. of unique Peptides: 2

10	20	30	40	50	60	70	80
MSHQFSSQSA	FSSMSRRVYS	TSSSAGSGGG	SPAVGSVCYA	RGRCGGGGYG	IHGRGFGSRS	LYNLGGSRSI	SINLMGRSTS
90	100	110	120	130	140	150	160
GFCQGGGVGG	FGGGRGFGVG	STGAGGFGGG	GFGGAGFGTS	NFGLGGFGPY	CPPGGIQEVT	INQSLLEPLH	LEVDPEIQRI
170	180	190	200	210	220	230	240
KTQEREQIMV	LNNKFASFID	KVRFLEQQNQ	VLQTKWELLQ	QVNTSTGTNN	LEPLLENYIG	DLRRQVDLLS	AEQMRQNAEV
250	260	270	280	290	300	310	320
RSMQDVVEDY	KSKYEDEINK	RTGSENDFVV	LKKDVDAAYV	SKVDLESRVD	TLTGEVNFLK	YLFLTELSQV	QTHISDTNVI
330	340	350	360	370	380	390	400
LSMDNNRSLD	LDSIIDAVRT	QYELIAQRSK	DEAEALYQTK	YQELQITAGR	HGDDLKNSKM	EIAELNRTVQ	RLQAEISNVK
410	420	430	440	450	460	470	480
KQIEQMQSLI	SDAEERGEQA	LQDAWQKLQD	LEEALQQSKE	ELARLLRDYQ	AMLGVKLSLD	VEIATYRQLL	EGEESRMSGE
490	500	510	520	530	540	550	560
LQSHVSISVQ	NSQVSVNGGA	GGGGSYGSGG	YGGGSGGGYG	GGRSYRGGGA	RGRSGGGYGS	GCGGGGGSYG	GSGRSGRGSS
570	580						
RVQIIQTSTN	TSHRRILE						

Cmpd.	No. of Cmpds.	m/z meas.	Δ m/z [ppm]	z	Rt [min]	Score	P	Range	Sequence	Modification
2213	1	1103.9435	-56.00	2	56.2	13.0	0	18-41	R.VYSTSSSAGSGGGSPAVGSVCYAR.G	
2780	1	922.4553	-14.26	2	66.1	16.9	1	361-376	K.YQELQITAGRHGDDLK.N	

Detailed Protein Report

Protein 57: glyceraldehyde-3-phosphate dehydrogenase isoform 1 [Homo sapiens]

**Accession:**	gi|7669492	**Score:**	99.9
**Database:**	refseq_human(refseq_human_20140103.fasta)	**MW [kDa]:**	36.0
**Database Date:**	2015-11-30	**pI:**	9.3
**Modification(s):**	Carbamidomethyl	**Sequence Coverage [%]:**	13.7
		**No. of unique Peptides:**	3

Quantitation

Q:W	**Median:**	0.66	**CV:**	28.83 %	**No. of Peptides:**	2
M:Q	**Median:**	1.28	**CV:**	20.51 %	**No. of Peptides:**	3

```
        10         20         30         40         50         60         70         80
MGKVKVGVNG FGRIGRLVTR AAFNSGKVDI VAINDPFIDL NYMVYMFQYD STHGKFHGTV KAENGKLVIN GNPITIFQER
        90        100        110        120        130        140        150        160
DPSKIKWGDA GAEYVVESTG VFTTMEKAGA HLQGGAKRVI ISAPSADAPM FVMGVNHEKY DNSLKIISNA SCTTNCLAPL
       170        180        190        200        210        220        230        240
AKVIHDNFGI VEGLMTTVHA ITATQKTVDG PSGKLWRDGR GALQNIIPAS TGAAKAVGKV IPELNGKLTG MAFRVPTANV
       250        260        270        280        290        300        310        320
SVVDLTCRLE KPAKYDDIKK VVKQASEGPL KGILGYTEHQ VVSSDFNSDT HSSTFDAGAG IALNDHFVKL ISWYDNEFGY
       330        340
SNRVVDLMAH MASKE
```

Cmpd.	No. of Cmpds.	m/z meas.	Δ m/z [ppm]	z	Rt [min]	Score	P	Range	Sequence	Modification	Ratios	
676	4	917.4657	2.44	2	38.0	53.0	0	146-162	K.IISNASCTTNCLAPLAK.V	Carbamidomethyl: 7, 11	M:Q<br>Q:W	1.25<br>0.50
904	2	706.3818	-24.09	2	40.1	26.3	0	201-215	R.GALQNIIPASTGAAK.A		Q:W<br>M:Q	0.87<br>1.00
1648	5	882.4064	1.76	2	50.9	20.6	0	310-323	K.LISWYDNEFGYSNR.V		M:Q	1.65

Detailed Protein Report

Protein 58: PREDICTED: nebulin isoform X22 [Homo sapiens]

**Accession:**	gi|530370465	**Score:**	98.9
**Database:**	refseq_human(refseq_human_20140103.fasta)	**MW [kDa]:**	961.5
**Database Date:**	2015-11-30	**pI:**	9.5
**Modification(s):**	Oxidation	**Sequence Coverage [%]:**	1.1
		**No. of unique Peptides:**	6

Quantitation

Q:W	**Median:**	1.12	**CV:**	20.66 %	**No. of Peptides:**	3
M:Q	**Median:**	1.12	**CV:**	41.82 %	**No. of Peptides:**	3

10	20	30	40	50	60	70	80
MADDEDYEEV	VEYYTEEVVY	EEVPGETITK	IYETTTTRTS	DYEQSETSKP	ALAQPALAQP	ASAKPVERRK	VIRKKVDPSK
90	100	110	120	130	140	150	160
FMTPYIAHSQ	KMQDLFSPNK	YKEKFEKTKG	QPYASTTDTP	ELRRIKKVQD	QLSEVKYRMD	GDVAKTICHV	DEKAKDIEHA
170	180	190	200	210	220	230	240
KKVSQQVSKV	LYKQNWEDTK	DKYLLPPDAP	ELVQAVKNTA	MFSKKLYTED	WEADKSLFYP	YNDSPELRRV	AQAQKALSDV
250	260	270	280	290	300	310	320
AYKKGLAEQQ	AQFTPLADPP	DIEFAKKVTN	QVSKQKYKED	YENKIKGKWS	ETPCFEVANA	RMNADNISTR	KYQEDFENMK
330	340	350	360	370	380	390	400
DQIYFMQTET	PEYKMNKKAG	VAASKVKYKE	DYEKNKGKAD	YNVLPASENP	QLRQLKAAGD	ALSDKLYKEN	YEKTKAKSIN
410	420	430	440	450	460	470	480
YCETPKFKLD	TVLQNFSSDK	KYKDSYLKDI	LGHYVGSFED	PYHSHCMKVT	AQNSDKNYKA	EYEEDRGKGF	FPQTITQEYE
490	500	510	520	530	540	550	560
AIKKLDQCKD	HTYKVHPDKT	KFTQVTDSPV	LLQAQVNSKQ	LSDLNYKAKH	ESEKFKCHIP	PDTPAFIQHK	VNAYNLSDNL
570	580	590	600	610	620	630	640
YKQDWEKSKA	KKFDIKVDAI	PLLAAKANTK	NTSDVMYKKD	YEKNKGKMIG	VLSINDDPKM	LHSLKVAKNQ	SDRLYKENYE
650	660	670	680	690	700	710	720
KTKAKSMNYC	ETPKYQLDTQ	LKNFSEARYK	DLYVKDVLGH	YVGSMEDPYH	THCMKVAAQN	SDKSYKAEYE	EDKGKCYFPQ
730	740	750	760	770	780	790	800
TITQEYEAIK	KLDQCKDHTY	KVHPDKTKFT	AVTDSPVLLQ	AQLNTKQLSD	LNYKAKHEGE	KFKCHIPADA	PQFIQHRVNA
810	820	830	840	850	860	870	880
YNLSDNVYKQ	DWEKSKAKKF	DIKVDAIPLL	AAKANTKNTS	DVMYKKDYEK	SKGKMIGALS	INDDPKMLHS	LKTAKNQSDR
890	900	910	920	930	940	950	960
EYRKDYEKSK	TIYTAPLDML	QVTQAKKSQA	IASDVDYKHI	LHSYSYPPDS	INVDLAKKAY	ALQSDVEYKA	DYNSWMKGCG
970	980	990	1000	1010	1020	1030	1040
WVPFGSLEME	KAKRASDILN	EKKYRQHPDT	LKFTSIEDAP	ITVQSKINQA	QRSDIAYKAK	GEEIIHKYNL	PPDLPQFIQA
1050	1060	1070	1080	1090	1100	1110	1120
KVNAYNISEN	MYKADLKDLS	KKGYDLRTDA	IPIRAAKAAR	QAASDVQYKK	DYEKAKGKMV	GFQSLQDDPK	LVHYMNVAKI
1130	1140	1150	1160	1170	1180	1190	1200
QSDREYKKDY	EKTKSKYNTP	HDMFNVVAAK	KAQDVVSNVN	YKHSLHHYTY	LPDAMDLELS	KNMMQIQSDN	VYKEDYNNWM
1210	1220	1230	1240	1250	1260	1270	1280
KGIGWIPIGS	LDVEKVKKAG	DALNEKKYRQ	HPDTLKFTSI	VDSPVMVQAK	QNTKQVSDIL	YKAKGEDVKH	KYTMSPDLPQ
1290	1300	1310	1320	1330	1340	1350	1360
FLQAKCNAYN	ISDVCYKRDW	YDLIAKGNNV	LGDAIPITAA	KASRNIASDY	KYKEAYEKSK	GKHVGFRSLQ	DDPKLVHYMN
1370	1380	1390	1400	1410	1420	1430	1440
VAKLQSDREY	KKNYENTKTS	YHTPGDMVSI	TAAKMAQDVA	TNVNYKQPLH	HYTYLPDAMS	LEHTRNVNQI	QSDNVYKDEY
1450	1460	1470	1480	1490	1500	1510	1520
NSFLKGIGWI	PIGSLEVEKV	KKAGDALNER	KYRQHPDTVK	FTSVPDSMGM	VLAQHNTKQL	SDLNYKVEGE	KLKHKYTIDP
1530	1540	1550	1560	1570	1580	1590	1600
ELPQFIQAKV	NALNMSDAHY	KADWKKTIAK	GYDLRPDAIP	IVAAKSSRNI	ASDCKYKEAY	EKAKGKQVGF	LSLQDDPKLV
1610	1620	1630	1640	1650	1660	1670	1680
HYMNVAKIQS	DREYKKGYEA	SKTKYHTPLD	MVSVTAAKKS	QEVATNANYR	QSYHHYTLLP	DALNVEHSRN	AMQIQSDNLY
1690	1700	1710	1720	1730	1740	1750	1760
KSDFTNWMKG	IGWVPIESLE	VEKAKKAGEI	LSEKKYRQHP	EKLKFTYAMD	TMEQALNKSN	KLNMDKRLYT	EKWNKDKTTI
1770	1780	1790	1800	1810	1820	1830	1840
HVMPDTPDIL	LSRVNQITMS	DKLYKAGWEE	EKKKGYDLRP	DAIAIKAARA	SRDIASDYKY	KKAYEQAKGK	HIGFRSLEDD
1850	1860	1870	1880	1890	1900	1910	1920
PKLVHFMQVA	KMQSDREYKK	GYEKSKTSFH	TPVDMLSVVA	AKKSQEVATN	ANYRNVIHTY	NMLPDAMSFE	LAKNMMQIQS
1930	1940	1950	1960	1970	1980	1990	2000
DNQYKADYAD	FMKGIGWLPL	GSLEAEKNKK	AMEIISEKKY	RQHPDTLKYS	TLMDSMNMVL	AQNNAKIMNE	HLYKQAWEAD
2010	2020	2030	2040	2050	2060	2070	2080
KTKVHIMPDI	PQIILAKANA	INMSDKLYKL	SLEESKKKGY	DLRPDAIPIK	AAKASRDIAS	DYKYKYNYEK	GKGKMVGFRS
2090	2100	2110	2120	2130	2140	2150	2160
LEDDPKLVHS	MQVAKMQSDR	EYKKNYENTK	TSYHTPADML	SVTAAKDAQA	NITNTNYKHL	IHKYILLPDA	MNIELTRNMN
2170	2180	2190	2200	2210	2220	2230	2240

Detailed Protein Report

Cmpd.	No. of Cmpds.	m/z meas.	Δ m/z [ppm]	z	Rt [min]	Score	P	Range	Sequence	Modification	Ratios
457	5	544.7651	77.28	2	35.3	13.1	0	646-654	K.SMNYCETPK.Y	Oxidation: 2	
1248	1	796.3930	45.30	2	45.3	13.7	1	2813-2825	R.DDPKMMWSMHVAK.I	Oxidation: 5	
1176	2	729.3599	-9.93	2	43.4	18.3	1	3617-3628	R.KAYDLQSDNLYK.S		M:Q 1.55<br>Q:W 1.27
993	1	662.3199	-62.62	2	41.2	12.9	2	7204-7214	K.VKDEVSDLKYK.E		M:Q 0.64<br>Q:W 0.84
624	2	607.8333	-21.30	2	36.4	10.4	2	7714-7723	K.RDLELEVKGR.G		M:Q 1.43<br>Q:W 1.33
2801	2	994.5013	20.67	2	63.5	13.2	1	8000-8015	K.LNQENFSSVLYKENMR.K	Oxidation: 15	

Detailed Protein Report

Protein 59: tenascin-X isoform 1 precursor [Homo sapiens]

**Accession:**	gi|188528648	**Score:**	98.2
**Database:**	refseq_human(refseq_human_20140103.fasta)	**MW [kDa]:**	457.9
**Database Date:**	2015-11-30	**pI:**	4.9
**Modification(s):**	Carbamidomethyl	**Sequence Coverage [%]:**	2.7
		**No. of unique Peptides:**	7

Quantitation

Q:W	**Median:**	0.89	**CV:**	0.00 %	**No. of Peptides:**	1
M:Q	**Median:**	1.40	**CV:**	0.00 %	**No. of Peptides:**	1

Detailed Protein Report

10	20	30	40	50	60	70	80
MMPAQYALTS	SLVLLVLLST	ARAGPFSSRS	NVTLPAPRPP	PQPGGHTVGA	GVGSPSSQLY	EHTVEGGEKQ	VVFTHRINLP
90	100	110	120	130	140	150	160
PSTGCGCPPG	TEPPVLASEV	QALRVRLEIL	EELVKGLKEQ	CTGGCCPASA	QAGTGQTDVR	TLCSLHGVFD	LSRCTCSCEP
170	180	190	200	210	220	230	240
GWGGPTCSDP	TDAEIPPSSP	PSASGSCPDD	CNDQGRCVRG	RCVCFPGYTG	PSCGWPSCPG	DCQGRGRCVQ	GVCVCRAGFS
250	260	270	280	290	300	310	320
GPDCSQRSCP	RGCSQRGRCE	GGRCVCDPGY	TGDDCGMRSC	PRGCSQRGRC	ENGRCVCNPG	YTGEDCGVRS	CPRGCSQRGR
330	340	350	360	370	380	390	400
CKDGRCVCDP	GYTGEDCGTR	SCPWDCGEGG	RCVDGRCVCW	PGYTGEDCST	RTCPRDCRGR	GRCEDGECIC	DTGYSGDDCG
410	420	430	440	450	460	470	480
VRSCPGDCNQ	RGRCEDGRCV	CWPGYTGTDC	GSRACPRDCR	GRGRCENGVC	VCNAGYSGED	CGVRSCPGDC	RGRGRCESGR
490	500	510	520	530	540	550	560
CMCWPGYTGR	DCGTRACPGD	CRGRGRCVDG	RCVCNPGFTG	EDCGSRRCPG	DCRGHGLCED	GVCVCDAGYS	GEDCSTRSCP
570	580	590	600	610	620	630	640
GGCRGRGQCL	DGRCVCEDGY	SGEDCGVRQC	PNDCSQHGVC	QDGVCICWEG	YVSEDCSIRT	CPSNCHGRGR	CEEGRCLCDP
650	660	670	680	690	700	710	720
GYTGPTCATR	MCPADCRGRG	RCVQGVCLCH	VGYGGEDCGQ	EEPPASACPG	GCGPRELCRA	GQCVCVEGFR	GPDCAIQTCP
730	740	750	760	770	780	790	800
GDCRGRGECH	DGSCVCKDGY	AGEDCGEEVP	TIEGMRMHLL	EETTVRTEWT	PAPGPVDAYE	IQFIPTTEGA	SPPFTARVPS
810	820	830	840	850	860	870	880
SASAYDQRGL	APGQEYQVTV	RALRGTSWGL	PASKTITTMI	DGPQDLRVVA	VTPTTLELGW	LRPQAEVDRF	VVSYVSAGNQ
890	900	910	920	930	940	950	960
RVRLEVPPEA	DGTLLTDLMP	GVEYVVTVTA	ERGRAVSYPA	SVRANTGSSP	LGLLGTTDEP	PPSGPSTTQG	AQAPLLQQRP
970	980	990	1000	1010	1020	1030	1040
QELGELRVLG	RDETGRLRVV	WTAQPDTFAY	FQLRMRVPEG	PGAHEEVLPG	DVRQALVPPP	PPGTPYELSL	HGVPPGGKPS
1050	1060	1070	1080	1090	1100	1110	1120
DPIIYQGIMD	KDEEKPGKSS	GPPRLGELTV	TDRTSDSLLL	RWTVPEGEFD	SFVIQYKDRD	GQPQVVPVEG	PQRSAVITSL
1130	1140	1150	1160	1170	1180	1190	1200
DPGRKYKFVL	YGFVGKKRHG	PLVAEAKILP	QSDPSPGTPP	HLGNLWVTDP	TPDSLHLSWT	VPEGQFDTFM	VQYRDRDGRP
1210	1220	1230	1240	1250	1260	1270	1280
QVVPVEGPER	SFVVSSLDPD	HKYRFTLFGI	ANKKRYGPLT	ADGTTAPERK	EEPPRPEFLE	QPLLGELTVT	GVTPDSLRLS
1290	1300	1310	1320	1330	1340	1350	1360
WTVAQGPFDS	FMVQYKDAQG	QPQAVPVAGD	ENEVTVPGLD	PDRKYKMNLY	GLRGRQRVGP	ESVVAKTAPQ	EDVDETPSPT
1370	1380	1390	1400	1410	1420	1430	1440
ELGTEAPESP	EEPLLGELTV	TGSSPDSLSL	FWTVPQGSFD	SFTVQYKDRD	GRPRAVRVGG	KESEVTVGGL	EPGHKYKMHL
1450	1460	1470	1480	1490	1500	1510	1520
YGLHEGQRVG	PVSAVGVTAP	QQEETPPATE	SPLEPRLGEL	TVTDVTPNSV	GLSWTVPEGQ	FDSFIVQYKD	KDGQPQVVPV
1530	1540	1550	1560	1570	1580	1590	1600
AADQREVTVY	NLEPERKYKM	NMYGLHDGQR	MGPLSVVIVT	APLPPAPATE	ASKPPLEPRL	GELTVTDITP	DSVGLSWTVP
1610	1620	1630	1640	1650	1660	1670	1680
EGEFDSFVVQ	YKDRDGQPQV	VPVAADQREV	TIPDLEPSRK	YKFLLFGIQD	GKRRSPVSVE	AKTVARGDAS	PGAPPRLGEL
1690	1700	1710	1720	1730	1740	1750	1760
WVTDPTPDSL	RLSWTVPEGQ	FDSFVVQFKD	KDGPQVVPVE	GHERSVTVTP	LDAGRKYRFL	LYGLLGKKRH	GPLTADGTTE
1770	1780	1790	1800	1810	1820	1830	1840
ARSAMDDTGT	KRPPKPRLGE	ELQVTTVTQN	SVGLSWTVPE	GQFDSFVVQY	KDRDGQPQVV	PVEGSLREVS	VPGLDPAHRY
1850	1860	1870	1880	1890	1900	1910	1920
KLLLYGLHHG	KRVGPISAVA	ITAGREETET	ETTAPTPPAP	EPHLGELTVE	EATSHTLHLS	WMVTEGEFDS	FEIQYTDRDG
1930	1940	1950	1960	1970	1980	1990	2000
QLQMVRIGGD	RNDITLSGLE	SDHRYLVTLY	GFSDGKHVGP	VHVEALTVPE	EEKPSEPPTA	TPEPPIKPRL	GELTVTDATP
2010	2020	2030	2040	2050	2060	2070	2080
DSLSLSWTVP	EGQFDHFLVQ	YRNGDGQPKA	VRVPGHEEGV	TISGLEPDHK	YKMNLYGFHG	GQRMGPVSVV	GVTAAEEETP
2090	2100	2110	2120	2130	2140	2150	2160
SPTEPSMEAP	EPAEEPLLGE	LTVTGSSPDS	LSLSWTVPQG	RFDSFTVQYK	DRDGRPQVVR	VGGEESEVTV	GGLEPGRKYK
2170	2180	2190	2200	2210	2220	2230	2240

Detailed Protein Report

Cmpd.	No. of Cmpds.	m/z meas.	Δ m/z [ppm]	z	Rt [min]	Score	P	Range	Sequence	Modification	Ratios
2748	1	1085.5225	-113.03	1	65.5	14.6	0	107-115	R.LEILEELVK.G		
15	1	1103.5160	91.05	2	29.8	14.6	1	264-282	R.CVCDPGYTGDDCGMRSCPR.G	Carbamidomethyl: 1, 3, 12	
29	1	1103.5152	82.27	2	30.1	10.9	0	445-464	R.CENGVCVCNAGYSGEDCGVR.S	Carbamidomethyl: 6, 8, 17	
2799	1	732.8911	134.58	2	64.1	13.0	1	725-737	R.GRGECHDGSCVCK.D	Carbamidomethyl: 10, 12	
1929	1	890.3591	-119.35	2	52.8	13.5	1	1418-1435	R.VGGKESEVTVGGLEPGHK.Y		M:Q 1.40<br>Q:W 0.89
2906	2	557.9675	41.54	3	65.1	15.9	0	2141-2157	R.VGGEESEVTVGGLEPGR.K		
2882	1	981.9684	-29.71	2	65.2	15.6	1	2349-2366	R.VPGHEDRVTISGLEPDNK.Y		

Detailed Protein Report

Protein 60: macrophage mannose receptor 1 precursor [Homo sapiens]

**Accession:**	gi|4505245	**Score:**	98.1
**Database:**	refseq_human(refseq_human_20140103.fasta)	**MW [kDa]:**	165.9
**Database Date:**	2015-11-30	**pI:**	6.1
**Modification(s):**	Carbamidomethyl, Oxidation	**Sequence Coverage [%]:**	6.0
		**No. of unique Peptides:**	6

Quantitation

Q:W	**Median:** 1.02	**CV:** 24.87 %	**No. of Peptides:**	2
M:Q	**Median:** 1.04	**CV:** 5.09 %	**No. of Peptides:**	2

10	20	30	40	50	60	70	80
MRLPLLLVFA	SVIPGAVLLL	DTRQFLIYNE	DHKRCVDAVS	PSAVQTAACN	QDAESQKFRW	VSESQIMSVA	FKLCLGVPSK
90	100	110	120	130	140	150	160
TDWVAITLYA	CDSKSEFQKW	ECKNDTLLGI	KGEDLFFNYG	NRQEKNIMLY	KGSGLWSRWK	IYGTTDNLCS	RGYEAMYTLL
170	180	190	200	210	220	230	240
GNANGATCAF	PFKFENKWYA	DCTSAGRSDG	WLWCGTTTDY	DTDKLFGYCP	LKFEGSESLW	NKDPLTSVSY	QINSKSALTW
250	260	270	280	290	300	310	320
HQARKSCQQQ	NAELLSITEI	HEQTYLTGLT	SSLTSGLWIG	LNSLSFNSGW	QWSDRSPFRY	LNWLPGSPSA	EPGKSCVSLN
330	340	350	360	370	380	390	400
PGKNAKWENL	ECVQKLGYIC	KKGNTTLNSF	VIPSESDVPT	HCPSQWWPYA	GHCYKIHRDE	KKIQRDALTT	CRKEGGDLTS
410	420	430	440	450	460	470	480
IHTIEELDFI	ISQLGYEPND	ELWIGLNDIK	IQMYFEWSDG	TPVTFTKWLR	GEPSHENNRQ	EDCVVMKGKD	GYWADRGCEW
490	500	510	520	530	540	550	560
PLGYICKMKS	RSQGPEIVEV	EKGCRKGWKK	HHFYCYMIGH	TLSTFAEANQ	TCNNENAYLT	TIEDRYEQAF	LTSFVGLRPE
570	580	590	600	610	620	630	640
KYFWTGLSDI	QTKGTFQWTI	EEEVRFTHWN	SDMPGRKPGC	VAMRTGIAGG	LWDVLKCDEK	AKFVCKHWAE	GVTHPPKPTT
650	660	670	680	690	700	710	720
TPEPKCPEDW	GASSRTSLCF	KLYAKGKHEK	KTWFESRDFC	RALGGDLASI	NNKEEQQTIW	RLITASGSYH	KLFWLGLTYG
730	740	750	760	770	780	790	800
SPSEGFTWSD	GSPVSYENWA	YGEPNNYQNV	EYCGELKGDP	TMSWNDINCE	HLNNWICQIQ	KGQTPKPEPT	PAPQDNPPVT
810	820	830	840	850	860	870	880
EDGWVIYKDY	QYYFSKEKET	MDNARAFCKR	NFGDLVSIQS	ESEKKFLWKY	VNRNDAQSAY	FIGLLISLDK	KFAWMDGSKV
890	900	910	920	930	940	950	960
DYVSWATGEP	NFANEDENCV	TMYSNSGFWN	DINCGYPNAF	ICQRHNSSIN	ATTVMPTMPS	VPSGCKEGWN	FYSNKCFKIF
970	980	990	1000	1010	1020	1030	1040
GFMEEERKNW	QEARKACIGF	GGNLVSIQNE	KEQAFLTYHM	KDSTFSAWTG	LNDVNSEHTF	LWTDGRGVHY	TNWGKGYPGG
1050	1060	1070	1080	1090	1100	1110	1120
RRSSLSYEDA	DCVVIIGGAS	NEAGKWMDDT	CDSKRGYICQ	TRSDPSLTNP	PATIQTDGFV	KYGKSSYSLM	RQKFQWHEAE
1130	1140	1150	1160	1170	1180	1190	1200
TYCKLHNSLI	ASILDPYSNA	FAWLQMETSN	ERVWIALNSN	LTDNQYTWTD	KWRVRYTNWA	ADEPKLKSAC	VYLDLDGYWK
1210	1220	1230	1240	1250	1260	1270	1280
TAHCNESFYF	LCKRSDEIPA	TEPPQLPGRC	PESDHTAWIP	FHGHCYYIES	SYTRNWGQAS	LECLRMGSSL	VSIESAAESS
1290	1300	1310	1320	1330	1340	1350	1360
FLSYRVEPLK	SKTNFWIGLF	RNVEGTWLWI	NNSPVSFVNW	NTGDPSGERN	DCVALHASSG	FWSNIHCSSY	KGYICKRPKI
1370	1380	1390	1400	1410	1420	1430	1440
IDAKPTHELL	TTKADTRKMD	PSKPSSNVAG	VVIIVILLIL	TGAGLAAYFF	YKKRRVHLPQ	EGAFENTLYF	NSQSSPGTSD
1450	1460						
MKDLVGNIEQ	NEHSVI						

Cmpd.	No. of Cmpds.	m/z meas.	Δ m/z [ppm]	z	Rt [min]	Score	P	Range	Sequence	Modification	Ratios
1884	1	621.2922	12.19	2	53.6	15.7	1	95-103	K.SEFQKWECK.N	Carbamidomethyl: 8	
2976	1	928.0036	17.04	2	66.0	14.3	2	126-140	K.NIMLYKGSGLWSRWK.I	Oxidation: 3	
1638	1	1069.9824	-53.73	2	49.0	18.2	2	324-341	K.NAKWENLECVQKLGYICK.K		M:Q 1.09<br>Q:W 1.30
1876	1	914.3217	-127.99	2	53.5	10.7	2	477-491	R.GCEWPLGYICKMKSR.S	Carbamidomethyl: 10	
											M:Q 0.98

Detailed Protein Report

Cmpd.	No. of Cmpds.	m/z meas.	Δ m/z [ppm]	z	Rt [min]	Score	P	Range	Sequence	Modification	Ratios
2392	1	736.0727	87.47	3	60.2	10.6	1	586-604	R.FTHWNSDMPGRKPGCVAMR.T	Oxidation: 8	
196	1	651.1714	-173.27	2	31.8	10.9	1	819-829	K.ETMDNARAFCK.R	Oxidation: 3	Q:W 0.80

Detailed Protein Report

Protein 61: desmoplakin isoform I [Homo sapiens]

**Accession:**	gi|58530840	**Score:**	97.7
**Database:**	refseq_human(refseq_human_20140103.fasta)	**MW [kDa]:**	331.6
**Database Date:**	2015-11-30	**pI:**	6.4
**Modification(s):**	Carbamidomethyl	**Sequence Coverage [%]:**	3.6
		**No. of unique Peptides:**	6

Quantitation

Q:W	**Median:**	1.27	**CV:**	0.00 %	**No. of Peptides:**	1
M:Q	**Median:**	0.86	**CV:**	50.55 %	**No. of Peptides:**	2

Detailed Protein Report

10	20	30	40	50	60	70	80
MSCNGGSHPR	INTLGRMIRA	ESGPDLRYEV	TSGGGGTSRM	YYSRRGVITD	QNSDGYCQTG	TMSRHQNQNT	IQELLQNCSD
90	100	110	120	130	140	150	160
CLMRAELIVQ	PELKYGDGIQ	LTRSRELDEC	FAQANDQMEI	LDSLIREMRQ	MGQPCDAYQK	RLLQLQEQMR	ALYKAISVPR
170	180	190	200	210	220	230	240
VRRASSKGGG	GYTCQSGSGW	DEFTKHVTSE	CLGWMRQQRA	EMDMVAWGVD	LASVEQHINS	HRGIHNSIGD	YRWQLDKIKA
250	260	270	280	290	300	310	320
DLREKSAIYQ	LEEEYENLLK	ASFERMDHLR	QLQNIIQATS	REIMWINDCE	EEELLYDWSD	KNTNIAQKQE	AFSIRMSQLE
330	340	350	360	370	380	390	400
VKEKELNKLK	QESDQLVLNQ	HPASDKIEAY	MDTLQTQWSW	ILQITKCIDV	HLKENAAYFQ	FFEEAQSTEA	YLKGLQDSIR
410	420	430	440	450	460	470	480
KKYPCDKNMP	LQHLLEQIKE	LEKEREKILE	YKRQVQNLVN	KSKKIVQLKP	RNPDYRSNKP	IILRALCDYK	QDQKIVHKGD
490	500	510	520	530	540	550	560
ECILKDNNER	SKWYVTGPGG	VDMLVPSVGL	IIPPPNPLAV	DLSCKIEQYY	EAILALWNQL	YINMKSLVSW	HYCMIDIEKI
570	580	590	600	610	620	630	640
RAMTIAKLKT	MRQEDYMKTI	ADLELHYQEF	IRNSQGSEMF	GDDDKRKIQS	QFTDAQKHYQ	TLVIQLPGYP	QHQTVTTTEI
650	660	670	680	690	700	710	720
THHGTCQDVN	HNKVIETNRE	NDKQETWMLM	ELQKIRRQIE	HCEGRMTLKN	LPLADQGSSH	HITVKINELK	SVQNDSQAIA
730	740	750	760	770	780	790	800
EVLNQLKDML	ANFRGSEKYC	YLQNEVFGLF	QKLENINGVT	DGYLNSLCTV	RALLQAILQT	EDMLKVYEAR	LTEEETVCLD
810	820	830	840	850	860	870	880
LDKVEAYRCG	LKKIKNDLNL	KKSLLATMKT	ELQKAQQIHS	QTSQQYPLYD	LDLGKFGEKV	TQLTDRWQRI	DKQIDFRLWD
890	900	910	920	930	940	950	960
LEKQIKQLRN	YRDNYQAFCK	WLYDAKRRQD	SLESMKFGDS	NTVMRFLNEQ	KNLHSEISGK	RDKSEEVQKI	AELCANSIKD
970	980	990	1000	1010	1020	1030	1040
YELQLASYTS	GLETLLNIPI	KRTMIQSPSG	VILQEAADVH	ARYIELLTRS	GDYYRFLSEM	LKSLEDLKLK	NTKIEVLEEE
1050	1060	1070	1080	1090	1100	1110	1120
LRLARDANSE	NCNKNKFLDQ	NLQKYQAECS	QFKAKLASLE	ELKRQAELDG	KSAKQNLDKC	YGQIKELNEK	ITRLTYEIED
1130	1140	1150	1160	1170	1180	1190	1200
EKRRRKSVED	RFDQQKNDYD	QLQKARQCEK	ENLGWQKLES	EKAIKEKEYE	IERLRVLLQE	EGTRKREYEN	ELAKVRNHYN
1210	1220	1230	1240	1250	1260	1270	1280
EEMSNLRNKY	ETEINITKTT	IKEISMQKED	DSKNLRNQLD	RLSRENRDLK	DEIVRLNDSI	LQATEQRRRA	EENALQQKAC
1290	1300	1310	1320	1330	1340	1350	1360
GSEIMQKKQH	LEIELKQVMQ	QRSEDNARHK	QSLEEAAKTI	QDKNKEIERL	KAEFQEEAKR	RWEYENELSK	VRNNYDEEII
1370	1380	1390	1400	1410	1420	1430	1440
SLKNQFETEI	NITKTTIHQL	TMQKEEDTSG	YRAQIDNLTR	ENRSLSEEIK	RLKNTLTQTT	ENLRRVEEDI	QQQKATGSEV
1450	1460	1470	1480	1490	1500	1510	1520
SQRKQQLEVE	LRQVTQMRTE	ESVRYKQSLD	DAAKTIQDKN	KEIERLKQLI	DKETNDRKCL	EDENARLQRV	QYDLQKANSS
1530	1540	1550	1560	1570	1580	1590	1600
ATETINKLKV	QEQELTRLRI	DYERVSQERT	VKDQDITRFQ	NSLKELQLQK	QKVEEELNRL	KRTASEDSCK	RKKLEEEELEG
1610	1620	1630	1640	1650	1660	1670	1680
MRRSLKEQAI	KITNLTQQLE	QASIVKKRSE	DDLRQQRDVL	DGHLREKQRT	QEELRRLSSE	VEALRRQLLQ	EQESVKQAHL
1690	1700	1710	1720	1730	1740	1750	1760
RNEHFQKAIE	DKSRSLNESK	IEIERLQSLT	ENLTKEHLML	EEELRNLRLE	YDDLRRGRSE	ADSDKNATIL	ELRSQLQISN
1770	1780	1790	1800	1810	1820	1830	1840
NRTLELQGLI	NDLQRERENL	RQEIEKFQKQ	ALEASNRIQE	SKNQCTQVVQ	ERESLLVKIK	VLEQDKARLQ	RLEDELNRAK
1850	1860	1870	1880	1890	1900	1910	1920
STLEAETRVK	QRLECEKQQI	QNDLNQWKTQ	YSRKEEAIRK	IESEREKSER	EKNSLRSEIE	RLQAEIKRIE	ERCRRKLEDS
1930	1940	1950	1960	1970	1980	1990	2000
TRETQSQLET	ERSRYQREID	KLRQRPYGSH	RETQTECEWT	VDTSKLVFDG	LRKKVTAMQL	YECQLIDKTT	LDKLLKGKKS
2010	2020	2030	2040	2050	2060	2070	2080
VEEVASEIQP	FLRGAGSIAG	ASASPKEKYS	LVEAKRKKLI	SPESTVMLLE	AQAATGGIID	PHRNEKLTVD	SAIARDLIDF
2090	2100	2110	2120	2130	2140	2150	2160
DDRQQIYAAE	KAITGFDDPF	SGKTVSVSEA	IKKNLIDRET	GMRLLEAQIA	SGGVVDPVNS	VFLPKDVALA	RGLIDRDLYR
2170	2180	2190	2200	2210	2220	2230	2240

Detailed Protein Report

Cmpd.	No. of Cmpds.	m/z meas.	Δ m/z [ppm]	z	Rt [min]	Score	P	Range	Sequence	Modification	Ratios
2092	1	1045.4569	7.22	2	56.0	12.2	0	46-64	R.GVITDQNSDGYCQTGTMSR.H	Carbamidomethyl: 12	
2178	4	596.7430	-119.04	2	57.6	11.1	1	1330-1339	R.LKAEFQEEAK.R		M:Q 0.53<br>Q:W 1.27
35	1	796.7935	-155.66	2	29.4	12.2	1	1351-1363	K.VRNNYDEEIISLK.N		
1858	1	616.2551	-101.28	2	53.1	10.2	1	1488-1497	K.QLIDKETNDR.K		
2423	1	837.8362	-126.41	2	60.6	16.4	2	1545-1558	R.VSQERTVKDQDITR.F		
1485	1	600.4356	127.57	2	47.3	10.5	2	1819-1828	K.IKVLEQDKAR.L		M:Q 1.38

Detailed Protein Report

**Protein 62:**	**PREDICTED: neuroblast differentiation-associated protein AHNAK isoform X3 [Homo sapiens]**		
**Accession:**	gi|530397283	**Score:**	97.5
**Database:**	refseq_human(refseq_human_20140103.fasta)	**MW [kDa]:**	614.7
**Database Date:**	2015-11-30	**pI:**	5.8
**Modification(s):**	Oxidation	**Sequence Coverage [%]:**	1.6
		**No. of unique Peptides:**	6

Quantitation

Q:W	**Median:**	0.38	**CV:**	0.00 %	**No. of Peptides:**	1
M:Q	**Median:**	2.20	**CV:**	31.55 %	**No. of Peptides:**	2

Detailed Protein Report

10	20	30	40	50	60	70	80
MEKEETTREL	LLPNWQGSGS	HGLTIAQRDD	GVFVQEVTQN	SPAARTGVVK	EGDQIVGATI	YFDNLQSGEV	TQLLNTMGHH
90	100	110	120	130	140	150	160
TVGLKLHRKG	DRSPEPGQTW	TREVFSSCSS	EVVLSGDDEE	YQRIYTTKIK	PRLKSEDGVE	GDLGETQSRT	ITVTRRVTAY
170	180	190	200	210	220	230	240
TVDVTGREGA	KDIDISSPEF	KIKIPRHELT	EISNVDVETQ	SGKTVIRLPS	GSGAASPTGS	AVDIRAGAIS	ASGPELQGAG
250	260	270	280	290	300	310	320
HSKLQVTMPG	IKVGGSGVNV	NAKGLDLGGR	GGVQVPAVDI	SSSLGGRAVE	VQGPSLESGD	HGKIKFPTMK	VPKFGVSTGR
330	340	350	360	370	380	390	400
EGQTPKAGLR	VSAPEVSVGH	KGGKPGLTIQ	APQLEVSVPS	ANIEGLEGKL	KGPQITGPSL	EGDLGLKGAK	PQGHIGVDAS
410	420	430	440	450	460	470	480
APQIGGSITG	PSVEVQAPDI	DVQGPGSKLN	VPKMKVPKFS	VSGAKGEETG	IDVTLPTGEV	TVPGVSGDVS	LPEIATGGLE
490	500	510	520	530	540	550	560
GKMKGTKVKT	PEMIIQKPKI	SMQDVDLSLG	SPKLKGDIKV	SAPGVQGDVK	GPQVALKGSR	VDIETPNLEG	TLTGPRLGSP
570	580	590	600	610	620	630	640
SGKTGTCRIS	MSEVDLNVAA	PKVKGGVDVT	LPRVEGKVKV	PEVDVRGPKV	DVSAPDVEAH	GPEWNLKMPK	MKMPTFSTPG
650	660	670	680	690	700	710	720
AKGEGPDVHM	TLPKGDISIS	GPKVNVEAPD	VNLEGLGGKL	KGPDVKLPDM	SVKTPKISMP	DVDLHVKGTK	VKGEYDVTVP
730	740	750	760	770	780	790	800
KLEGELKGPK	VDIDAPDVDV	HGPDWHLKMP	KMKMPKFSVP	GFKAEGPEVD	VNLPKADVDI	SGPKIDVTAP	DVSIEEPEGK
810	820	830	840	850	860	870	880
LKGPKFKMPE	MNIKVPKISM	PDVDLHLKGP	NVKGEYDVTM	PKVESEIKVP	DVELKSAKMD	IDVPDVEVQG	PDWHLKMPKM
890	900	910	920	930	940	950	960
KMPKFSMPGF	KAEGPEVDVN	LPKADVDISG	PKVGVEVPDV	NIEGPEGKLK	GPKFKMPEMN	IKAPKISMPD	VDLHMKGPKV
970	980	990	1000	1010	1020	1030	1040
KGEYDMTVPK	LEGDLKGPKV	DVSAPDVEMQ	GPDWNLKMPK	IKMPKFSMPS	LKGEGPEFDV	NLSKANVDIS	APKVDTNAPD
1050	1060	1070	1080	1090	1100	1110	1120
LSLEGPEGKL	KGPKFKMPEM	HFRAPKMSLP	DVDLDLKGPK	MKGNVDISAP	KIEGEMQVPD	VDIRGPKVDI	KAPDVEGQGL
1130	1140	1150	1160	1170	1180	1190	1200
DWSLKIPKMK	MPKFSMPSLK	GEGPEVDVNL	PKADVVVSGP	KVDIEAPDVS	LEGPEGKLKG	PKFKMPEMHF	KTPKISMPDV
1210	1220	1230	1240	1250	1260	1270	1280
DLHLKGPKVK	GDVDVSVPKV	EGEMKVPDVD	IKGPKVDISA	PDVDVHGPDW	HLKMPKVKMP	KFSMPGFKGE	GPEVDVKLPK
1290	1300	1310	1320	1330	1340	1350	1360
ADVDVSGPKM	DAEVPDVNIE	GPDAKLKGPK	FKMPEMSIKP	QKISIPDVGL	HLKGPKMKGD	YDVTVPKVEG	EIKAPDVDIK
1370	1380	1390	1400	1410	1420	1430	1440
GPKVDINAPD	VEVHGPDWHL	KMPKVKMPKF	SMPGFKGEGP	EVDMNLPKAD	LGVSGPKVDI	DVPDVNLEAP	EGKLKGPKFK
1450	1460	1470	1480	1490	1500	1510	1520
MPSMNIQTHK	ISMPDVGLNL	KAPKLKTDVD	VSLPKVEGDL	KGPEIDVKAP	KMDVNVGDID	IEGPEGKLKG	PKFKMPEMHF
1530	1540	1550	1560	1570	1580	1590	1600
KAPKISMPDV	DLHLKGPKVK	GDMDVSVPKV	EGEMKVPDVD	IKGPKVDIDA	PDVEVHDPDW	HLKMPKMKMP	KFSMPGFKAE
1610	1620	1630	1640	1650	1660	1670	1680
GPEVDVNLPK	ADIDVSGPSV	DTDAPDLDIE	GPEGKLKGSK	FKMPKLNIKA	PKVSMPDVDL	NLKGPKLKGE	IDASVPELEG
1690	1700	1710	1720	1730	1740	1750	1760
DLRGPQVDVK	GPFVEAEVPD	VDLECPDAKL	KGPKFKMPEM	HFKAPKISMP	DVDLHLKGPK	VKGDADVSVP	KLEGDLTGPS
1770	1780	1790	1800	1810	1820	1830	1840
VGVEVPDVEL	ECPDAKLKGP	KFKMPDMHFK	APKISMPDVD	LHLKGPKVKG	DVDVSVPKLE	GDLTGPSVGV	EVPDVELECP
1850	1860	1870	1880	1890	1900	1910	1920
DAKLKGPKFK	MPEMHFKTPK	ISMPDVDLHL	KGPKVKGDMD	VSVPKVEGEM	KVPDVDIKGP	KMDIDAPDVD	VHGPDWHLKM
1930	1940	1950	1960	1970	1980	1990	2000
PKMKMPKFSM	PGFKAEGPEV	DVNLPKADVV	VSGPKVDVEV	PDVSLEGPEG	KLKGPKLKMP	EMHFKAPKIS	MPDVDLHLKG
2010	2020	2030	2040	2050	2060	2070	2080
PKVKGDVDVS	LPKLEGDLTG	PSVDVEVPDV	ELECPDAKLK	GPKFKMPEMH	FKTPKISMPD	VNLNLKGPKV	KGDMDVSVPK
2090	2100	2110	2120	2130	2140	2150	2160
VEGEMKVPDV	DIRGPKVDID	APDVDVHGPD	WHLKMPKMKM	PKFSMPGFKG	EGPEVDVNLP	KADVDVSGPK	VDVEVPDVSL
2170	2180	2190	2200	2210	2220	2230	2240

Detailed Protein Report

Cmpd.	No. of Cmpds.	m/z meas.	Δ m/z [ppm]	z	Rt [min]	Score	P	Range	Sequence	Modification	Ratios
2716	1	849.3583	-170.01	2	65.2	15.5	2	485-499	K.GTKVKTPEMIIQKPK.I		
863	1	578.2806	-119.29	2	40.2	10.1	2	1643-1652	K.MPKLNIKAPK.V	Oxidation: 1	
1213	1	764.7682	-167.48	2	44.8	12.8	1	2723-2737	K.GDVDVTGPKVEGDLK.G		
92	1	726.3490	-51.60	3	31.1	15.9	2	2802-2822	K.VDVEGPDVNIEGPEGKLKGPK.F		
1557	1	472.2329	-53.94	2	49.3	18.8	0	2851-2859	K.GDVDISLPK.V		M:Q 1.61<br>Q:W 0.38
1546	1	506.2878	21.02	2	47.6	11.8	1	4624-4633	K.GPEADIKGPK.V		M:Q 2.99

Detailed Protein Report

Protein 63: apolipoprotein A-II preproprotein [Homo sapiens]

Accession: gi|4502149
Database: refseq_human(refseq_human_20140103.fasta)
Database Date: 2015-11-30

Score: 97.3
MW [kDa]: 11.2
pI: 7.1
Sequence Coverage [%]: 32.0
No. of unique Peptides: 2

10	20	30	40	50	60	70	80
MKLLAATVLL	LTICSLEGAL	VRRQAKEPCV	ESLVSQYFQT	VTDYGKDLME	KVKSPELQAE	AKSYFEKSKE	QLTPLIKKAG
90	100	110					
TELVNFLSYF	VELGTQPATQ						

Cmpd.	No. of Cmpds.	m/z meas.	Δ m/z [ppm]	z	Rt [min]	Score	P	Range	Sequence	Modification
457	3	578.8364	-24.29	2	35.7	60.5	1	68-77	K.SKEQLTPLIK.K	
2890	2	1193.0548	-39.81	2	64.9	36.7	0	79-100	K.AGTELVNFLSYFVELGTQPATQ.-	

Detailed Protein Report

Protein 64: dynein heavy chain 2, axonemal [Homo sapiens]

**Accession:**	gi|75677365	**Score:**	97.3
**Database:**	refseq_human(refseq_human_20140103.fasta)	**MW [kDa]:**	507.4
**Database Date:**	2015-11-30	**pI:**	5.9
**Modification(s):**	Carbamidomethyl, Oxidation	**Sequence Coverage [%]:**	2.3
		**No. of unique Peptides:**	8

Quantitation

M:Q	**Median:** 1.19	**CV:** 0.00 %	**No. of Peptides:** 1

Alias proteins:

Accession	Name	Description
gi|530409951	refseq_human (refseq_human_20140103.fasta)	PREDICTED: dynein heavy chain 2, axonemal isoform X1 [Homo sapiens]

Detailed Protein Report

10	20	30	40	50	60	70	80
MSSKAEKKQR	LSGRGSSQAS	WSGRATRAAV	ATQEQGNAPA	VSEPELQAEL	PKEEPEPRLE	GPQAQSEESV	EPEADVKPLF
90	100	110	120	130	140	150	160
LSRAALTGLA	DAVWTQEHDA	ILEHFAQDPT	ESILTIFIDP	CFGLKLELGM	PVQTQNQLVY	FIRQAPVPIT	WENFEATVQF
170	180	190	200	210	220	230	240
GTVRGPYIPA	LLRLLGGVFA	PQIFANTGWP	ESIRNHFASH	LHKFLACLTD	TRYKLEGHTV	LYIPAEAMNM	KPEMVIKDKE
250	260	270	280	290	300	310	320
LVQRLETSMI	HWTRQIKEML	SAQETVETGE	NLGPLEEIEF	WRNRCMDLSG	ISKQLVKKGV	KHVESILHLA	KSSYLAPFMK
330	340	350	360	370	380	390	400
LAQQIQDGSR	QAQSNLTFLS	ILKEPYQELA	FMKPKDISSK	LPKLISLIRI	IWVNSPHYNT	RERLTSLFRK	VCDCQYHFAR
410	420	430	440	450	460	470	480
WEDGKQGPLP	CFFGAQGPQI	TRNLLEIEDI	FHKNLHTLRA	VRGGILDVKN	TCWHEDYNKF	RAGIKDLEVM	TQNLITSAFE
490	500	510	520	530	540	550	560
LVRDVPHGVL	LLDTFHRLAS	REAIKRTYDK	KAVDLYMLFN	SELALVNRER	NKKWPDLEPY	VAQYSGKARW	VHILRRRIDR
570	580	590	600	610	620	630	640
VMTCLAGAHF	LPRIGTGKES	VHTYQQMVQA	IDELVRKTFQ	EWTSSLDKDC	IRRLDTPLLR	ISQEKAGMLD	VNFDKSLLIL
650	660	670	680	690	700	710	720
FAEIDYWERL	LFETPHYVVN	VAERAEDLRI	LRENLLLVAR	DYNRIIAMLS	PDEQALFKER	IRLLDKKIHP	GLKKLHWALK
730	740	750	760	770	780	790	800
GASAFFITEC	RIHASKVQMI	VNEFKASTLT	IGWRAQEMSE	KLLVRISGKR	VYRDLEFEED	QREHRAAVQQ	KLMNLHQDVV
810	820	830	840	850	860	870	880
TIMTNSYEVF	KNDGPEIQQQ	WMLYMIRLDR	MMEDALRLNV	KWSLLELSKA	INGDGKTSPN	PLFQVLVILK	NDLQGSVAQV
890	900	910	920	930	940	950	960
EFSPTLQTLA	GVVNDIGNHL	FSTISVFCHL	PDILTKRKLH	REPIQTVVEQ	DEDIKKIQTQ	ISSGMTNNAS	LLQNYLKTWD
970	980	990	1000	1010	1020	1030	1040
MYREIWEINK	DSFIHRYQRL	NPPVSSFVAD	IARYTEVANN	VQKEETVTNI	QFVLLDCSHL	KFSLVQHCNE	WQNKFATLLR
1050	1060	1070	1080	1090	1100	1110	1120
EMAAGRLLEL	HTYLKENAEK	ISRPPQTLEE	LGVSLQLVDA	LKHDLANVET	QIPPIHEQFA	ILEKYEVPVE	DSVLEMLDSL
1130	1140	1150	1160	1170	1180	1190	1200
NGEWVVFQQT	LLDSKQMLKK	HKEKFKTGLI	HSADDFKKKA	HTLLEDFEFK	GHFTSNVGYM	SALDQITQVR	AMLMAMREEE
1210	1220	1230	1240	1250	1260	1270	1280
NSLRANLGIF	KIEQPPSKDL	QNLEKELDAL	QQIWEIARDW	EENWNEWKTG	RFLILQTETM	ETTAHGLFRR	LTKLAKEYKD
1290	1300	1310	1320	1330	1340	1350	1360
RNWEIIETTR	SKIEQFKRTM	PLISDLRNPA	LRERHWDQVR	DEIQREFDQE	SESFTLEQIV	ELGMDQHVEK	IGEISASATK
1370	1380	1390	1400	1410	1420	1430	1440
ELAIEVALQN	IAKTWDVTQL	DIVPYKDKGH	HRLRGTEEVF	QALEDNQVAL	STMKASRFVK	AFEKDVDHWE	RCLSLILEVI
1450	1460	1470	1480	1490	1500	1510	1520
EMILTVQRQW	MYLENIFLGE	DIRKQLPNES	TLFDQVNSNW	KAIMDRMNKD	NNALRSTHHP	GLLDTLIEMN	TILEDIQKSL
1530	1540	1550	1560	1570	1580	1590	1600
DMYLETKRHI	FPRFYFLSND	DLLEILGQSR	NPEAVQPHLK	KCFDNIKLLR	IQKVGGPSSK	WEAVGMFSGD	GEYIDFLHSV
1610	1620	1630	1640	1650	1660	1670	1680
FLEGPVESWL	GDVEQTMRVT	LRDLLRNCHL	ALRKFLNKRD	KWVKEWAGQV	VITASQIQWT	ADVTKCLLTA	KERADKKILK
1690	1700	1710	1720	1730	1740	1750	1760
VMKKNQVSIL	NKYSEAIRGN	LTKIMRLKIV	ALVTIEIHAR	DVLEKLYKSG	LMDVNSFDWL	SQLRFYWEKD	LDDCVIRQTN
1770	1780	1790	1800	1810	1820	1830	1840
TQFQYNYEYL	GNSGRLVITP	LTDRCYMTLT	TALHLHRGGS	PKGPAGTGKT	ETVKDLGKAL	GIYVIVVNCS	EGLDYKSMGR
1850	1860	1870	1880	1890	1900	1910	1920
MYSGLAQTGA	WGCFDEFNRI	NIEVLSVVAH	QILCILSALA	AGLTHFHFDG	FEINLVWSCG	IFITMNPGYA	GRTELPENLK
1930	1940	1950	1960	1970	1980	1990	2000
SMFRPIAMVV	PDSTLIAEII	LFGEGFGNCK	ILAKKVYTLY	SLAVQQLSRQ	DHYDFGLRAL	TSLLRYAGKK	RRLQPDLTDE
2010	2020	2030	2040	2050	2060	2070	2080
EVLLLSMRDM	NIAKLTSVDA	PLFNAIVQDL	FPNIELPVID	YGKLRETVEQ	EIRDMGLQST	PFTLTKVFQL	YETKNSRHST
2090	2100	2110	2120	2130	2140	2150	2160
MIVGCTGSGK	TASWRILQAS	LSSLCRAGDP	NFNIVREFPL	NPKALSLGEL	YGEYDLSTNE	WTDGILSSVM	RTACADEKPD
2170	2180	2190	2200	2210	2220	2230	2240

Detailed Protein Report

Cmpd.	No. of Cmpds.	m/z meas.	Δ m/z [ppm]	z	Rt [min]	Score	P	Range	Sequence	Modification	Ratios
2498	1	464.1929	-197.80	2	59.9	11.5	0	673-680	R.ENLLLVAR.D		
58	1	827.0195	-82.92	3	30.1	13.2	1	1393-1414	R.LRGTEEVFQALEDNQVALSTMK.A		
271	1	701.3595	-80.46	2	33.1	11.1	2	1681-1692	K.VMKKNQVSILNK.Y		M:Q 1.19
2974	1	826.4375	-16.40	2	65.9	10.8	2	1693-1706	K.YSEAIRGNLTKIMR.L		
1377	1	976.4425	-17.17	2	46.8	10.3	1	2078-2095	R.HSTMIVGCTGSGKTASWR.I	Carbamidomethyl: 8; Oxidation: 4	
312	1	712.8533	-32.33	2	33.2	10.2	2	3248-3258	K.LEMLKKQYDEK.L		
2410	1	1023.8441	-100.67	2	60.0	14.9	2	4373-4389	K.KSAKGMYSCPCYYYPNR.A	Oxidation: 6	
2695	1	1044.5484	84.19	2	63.9	15.3	2	4373-4389	K.KSAKGMYSCPCYYYPNR.A	Carbamidomethyl: 9	

Detailed Protein Report

Protein 65: usherin isoform B [Homo sapiens]

**Accession:**	gi|219842266	**Score:**	97.2
**Database:**	refseq_human(refseq_human_20140103.fasta)	**MW [kDa]:**	575.2
**Database Date:**	2015-11-30	**pI:**	6.4
**Modification(s):**	Carbamidomethyl	**Sequence Coverage [%]:**	2.1
		**No. of unique Peptides:**	5

Quantitation

Q:W	**Median:**	1.50	**CV:**	0.00 %	**No. of Peptides:**	1
M:Q	**Median:**	0.57	**CV:**	0.00 %	**No. of Peptides:**	1

BRUKER

Detailed Protein Report

10	20	30	40	50	60	70	80
MNCPVLSLGS	GFLFQVIEML	IFAYFASISL	TESRGLFPRL	ENVGAFKKVS	IVPTQAVCGL	PDRSTFCHSS	AAAESIQFCT
90	100	110	120	130	140	150	160
QRFCIQDCPY	RSSHPTYTAL	FSAGLSSCIT	PDKNDLHPNA	HSNSASFIFG	NHKSCFSSPP	SPKLMASFTL	AVWLKPEQQG
170	180	190	200	210	220	230	240
VMCVIEKTVD	GQIVFKLTIS	EKETMFYYRT	VNGLQPPIKV	MTLGRILVKK	WIHLSVQVHQ	TKISFFINGV	EKDHTPFNAR
250	260	270	280	290	300	310	320
TLSGSITDFA	SGTVQIGQSL	NGLEQFVGRM	QDFRLYQVAL	TNREILEVFS	GDLLRLHAQS	HCRCPGSHPR	VHPLAQRYCI
330	340	350	360	370	380	390	400
PNDAGDTADN	RVSRLNPEAH	PLSFVNDNDV	GTSWVSNVFT	NITQLNQGVT	ISVDLENGQY	QVFYIIIQFF	SPQPTEIRIQ
410	420	430	440	450	460	470	480
RKKENSLDWE	DWQYFARNCG	AFGMKNNGDL	EKPDSVNCLQ	LSNFTPYSRG	NVTFSILTPG	PNYRPGYNNF	YNTPSLQEFV
490	500	510	520	530	540	550	560
KATQIRFHFH	GQYYTTETAV	NLRHRYYAVD	EITISGRCQC	HGHADNCDTT	SQPYRCLCSQ	ESFTEGLHCD	RCLPLYNDKP
570	580	590	600	610	620	630	640
FRQGDQVYAF	NCKPCQCNSH	SKSCHYNISV	DPFPFEHFRG	GGGVCDDCEH	NTTGRNCELC	KDYFFRQVGA	DPSAIDVCKP
650	660	670	680	690	700	710	720
CDCDTVGTRN	GSILCDQIGG	QCNCKRHVSG	RQCNQCQNGF	YNLQELDPDG	CSPCNCNTSG	TVDGDITCHQ	NSGQCKCKAN
730	740	750	760	770	780	790	800
VIGLRCDHCN	FGFKFLRSFN	DVGCEPCQCN	LHGSVNKFCN	PHSGQCECKK	EAKGLQCDTC	RENFYGLDVT	NCKACDCDTA
810	820	830	840	850	860	870	880
GSLPGTVCNA	KTGQCICKPN	VEGRQCNKCL	EGNFYLRQNN	SFLCLPCNCD	KTGTINGSLL	CNKSTGQCPC	KLGVTGLRCN
890	900	910	920	930	940	950	960
QCEPHRYNLT	IDNFQHCQMC	ECDSLGTLPG	TICDPISGQC	LCVPNRQGRR	CNQCQPGFYI	SPGNATGCLP	CSCHTTGAVN
970	980	990	1000	1010	1020	1030	1040
HICNSLTGQC	VCQDASIAGQ	RCDQCKDHYF	GFDPQTGRCQ	PCNCHLSGAL	NETCHLVTGQ	CFCKQFVTGS	KCDACVPSAS
1050	1060	1070	1080	1090	1100	1110	1120
HLDVNNLLGC	SKTPFQQPPP	RGQVQSSSAI	NLSWSPPDSP	NAHWLTYSLL	RDGFEIYTTE	DQYPYSIQYF	LDTDLLPYTK
1130	1140	1150	1160	1170	1180	1190	1200
YSYYIETTNV	HGSTRSVAVT	YKTKPGVPEG	NLTLSYIIPI	GSDSVTLTWT	TLSNQSGPIE	KYILSCAPLA	GGQPCVSYEG
1210	1220	1230	1240	1250	1260	1270	1280
HETSATIWNL	VPFAKYDFSV	QACTSGGCLH	SLPITVTTAQ	APPQRLSPPK	MQKISSTELH	VEWSPPAELN	GIIIRYELYM
1290	1300	1310	1320	1330	1340	1350	1360
RRLRSTKETT	SEESRVFQSS	GWLSPHSFVE	SANENALKPP	QTMTTITGLE	PYTKYEFRVL	AVNMAGSVSS	AWVSERTGES
1370	1380	1390	1400	1410	1420	1430	1440
APVFMIPPSV	FPLSSYSLNI	SWEKPADNVT	RGKVVGYDIN	MLSEQSPQQS	IPMAFSQLLH	TAKSQELSYT	VEGLKPYRIY
1450	1460	1470	1480	1490	1500	1510	1520
EFTITLCNSV	GCVTSASGAG	QTLAAAPAQL	RPPLVKGINS	TTIHLKWFPP	EELNGPSPIY	QLERRESSLP	ALMTTMMKGI
1530	1540	1550	1560	1570	1580	1590	1600
RFIGNGYCKF	PSSTHPVNTD	FTGIKASFRT	KVPEGLIVFA	ASPGNQEEYF	ALQLKKGRLY	FLFDPQGSPV	EVTTTNDHGK
1610	1620	1630	1640	1650	1660	1670	1680
QYSDGKWHEI	IAIRHQAFGQ	ITLDGIYTGS	SAILNGSTVI	GDNTGVFLGG	LPRSYTILRK	DPEIIQKGFV	GCLKDVHFMK
1690	1700	1710	1720	1730	1740	1750	1760
NYNPSAIWEP	LDWQSSEEQI	NVYNSWEGCP	ASLNEGAQFL	GAGFLELHPY	MFHGGMNFEI	SFKFRTDQLN	GLLLFVYNKD
1770	1780	1790	1800	1810	1820	1830	1840
GPDFLAMELK	SGILTFRLNT	SLAFTQVDLL	LGLSYCNGKW	NKVIIKKEGS	FISASVNGLM	KHASESGDQP	LVVNSPVYVG
1850	1860	1870	1880	1890	1900	1910	1920
GIPQELLNSY	QHLCLEQGFG	GCMKDVKFTR	GAVVNLASVS	SGAVRVNLDG	CLSTDSAVNC	RGNDSILVYQ	GKEQSVYEGG
1930	1940	1950	1960	1970	1980	1990	2000
LQPFTEYLYR	VIASHEGGSV	YSDWSRGRTT	GAAPQSVPTP	SRVRSLNGYS	IEVTWDEPVV	RGVIEKYILK	AYSEDSTRPP
2010	2020	2030	2040	2050	2060	2070	2080
RMPSASAEFV	NTSNLTGILT	GLLPFKNYAV	TLTACTLAGC	TESSHALNIS	TPQEAPQEVQ	PPVAKSLPSS	LLLSWNPPKK
2090	2100	2110	2120	2130	2140	2150	2160
ANGIITQYCL	YMDGRLIYSG	SEENYTVTDL	AVFTPHQFLL	SACTHVGCTN	SSWVLLYTAQ	LPPEHVDSPV	LTVLDSRTIH
2170	2180	2190	2200	2210	2220	2230	2240

Detailed Protein Report

Cmpd.	No. of Cmpds.	m/z meas.	Δ m/z [ppm]	z	Rt [min]	Score	P	Range	Sequence	Modification	Ratios
2191	1	863.3484	-20.01	2	55.8	14.2	0	794-811	K.ACDCDTAGSLPGTVCNAK.T		
2688	2	885.4613	98.77	2	63.8	13.3	0	838-851	R.QNNSFLCLPCNCDK.T	Carbamidomethyl: 7, 10, 12	
1593	1	1172.1148	23.38	2	50.1	15.7	0	3098-3119	K.SNGTQITTVEDTPSDIPTPTIR.G		
864	1	471.1407	-194.52	2	39.2	18.2	0	3546-3554	R.GTSLSFSDK.E		
206	1	898.4111	-54.24	3	32.3	10.4	2	5085-5108	K.RMSPLNVYPPGENHMGLADTKIPI S		M:Q 0.57<br>Q:W 1.50

Detailed Protein Report

Protein 66: PREDICTED: MAX gene-associated protein isoform X16 [Homo sapiens]

**Accession:**	gi|578826753	**Score:**	96.7
**Database:**	refseq_human(refseq_human_20140103.fasta)	**MW [kDa]:**	290.2
**Database Date:**	2015-11-30	**pI:**	8.7
**Modification(s):**	Carbamidomethyl, Oxidation	**Sequence Coverage [%]:**	3.4
		**No. of unique Peptides:**	6

Quantitation

Q:W	**Median:**	0.52	**CV:**	0.00 %	**No. of Peptides:**	1
M:Q	**Median:**	0.86	**CV:**	0.00 %	**No. of Peptides:**	1

Detailed Protein Report

10	20	30	40	50	60	70	80
MFKLDTGKMP	VVYLEPCAVT	RSTVKISELP	DNMLSTSRKD	KSSMLAELEY	LPTYIENSNE	TAFCLGKESE	NGLRKHSPDL
90	100	110	120	130	140	150	160
RVVQKYPLLK	EPQWKYPDIS	DSISTERILD	DSKDSVGDSL	SGKEDLGRKR	TTMLKIATAA	KVVNANQNAS	PNVPGKRGRP
170	180	190	200	210	220	230	240
RKLKLCKAGR	PPKNTGKSLI	STKNTPVSPG	STFPDVKPDL	EDVDGVLFVS	FESKEALDIH	AVDGTTEESS	SLQASTTNDS
250	260	270	280	290	300	310	320
GYRARISQLE	KELIEDLKTL	RHKQVIHPGL	QEVGLKLNSV	DPTMSIDLKY	LGVQLPLAPA	TSFPFWNLTG	TNPASPDAGF
330	340	350	360	370	380	390	400
PFVSRTGKTN	DFTKIKGWRG	KFHSASASRN	EGGNSESSLK	NRSAFCSDKL	DEYLENEGKL	METSMGFSSN	APTSPVVYQL
410	420	430	440	450	460	470	480
PTKSTSYVRT	LDSVLKKQST	ISPSTSYSLK	PHSVPPVSRK	AKSQNRQATF	SGRTKSSYKS	ILPYPVSPKQ	KYSHVILGDK
490	500	510	520	530	540	550	560
VTKNSSGIIS	ENQANNFVVP	TLDENIFPKQ	ISLRQAQQQQ	QQQQGSRPPG	LSKSQVKLMD	LEDCALWEGK	PRTYITEERA
570	580	590	600	610	620	630	640
DVSLTTLLTA	QASLKTKPIH	TIIRKRAPPC	NNDFCRLGCV	CSSLALEKRQ	PAHCRRPDCM	FGCTCLKRKV	VLVKGGSKTK
650	660	670	680	690	700	710	720
HFQRKAAHRD	PVFYDTLGEE	AREEEEGIRE	EEEQLKEKKK	RKKLEYTICE	TEPEQPVRHY	PLWVKVEGEV	DPEPVYIPTP
730	740	750	760	770	780	790	800
SVIEPMKPLL	LPQPEVLSPT	VKGKLLTGIK	SPRSYTPKPN	PVIREEDKDP	VYLYFESMMT	CARVRVYERK	KEDQRQPSSS
810	820	830	840	850	860	870	880
SSPSPSFQQQ	TSCHSSPENH	NNAKEPDSEQ	QPLKQLTCDL	EDDSDKLQEK	SWKSSCNEGE	SSSTSYMHQR	SPGGPTKLIE
890	900	910	920	930	940	950	960
IISDCNWEED	RNKILSILSQ	HINSNMPQSL	KVGSFIIELA	SQRKSRGEKN	PPVYSSRVKI	SMPSCQDQDD	MAEKSGSETP
970	980	990	1000	1010	1020	1030	1040
DGPLSPGKME	DISPVQTDAL	DSVRERLHGG	KGLPFYAGLS	PAGKLVAYKR	KPSSSTSGLI	QVNGKSYPQA	KLLLGQMGAL
1050	1060	1070	1080	1090	1100	1110	1120
HPANRLAAYI	TGRLRPSVLD	LSTLSTVISK	VASNAKVAAS	RKPRTLLPST	SNSKMASSSG	TATNRPGKNL	KAFVPAKRPI
1130	1140	1150	1160	1170	1180	1190	1200
AARPSPGGVF	TQFVMSKVGA	LQQKIPGVST	PQTLAGTQKF	SIRPSPVMVV	TPVVSSEPVQ	VCSPVTAAVT	TTTPQVFLEN
1210	1220	1230	1240	1250	1260	1270	1280
TTAVTPMTAI	SDVETKETTY	SSGATTTGVV	EVSETNTSTS	VTSTQSTATV	NLTKTTGITT	PVASVAFPKS	LVASPSTITL
1290	1300	1310	1320	1330	1340	1350	1360
PVASTASTSL	VVVTAAASSS	MVTTPTSSLG	SVPIILSGIN	GSPPVSQRPE	NAAQIPVATP	QVSPNTVKRA	GPRLLLIPVQ
1370	1380	1390	1400	1410	1420	1430	1440
QGSPTLRPVS	NTQLQGHRMV	LQPVRSPSGM	NLFRHPNGQI	VQLLPLHQLR	GSNTQPNLQP	VMFRNPGSVM	GIRLPAPSKP
1450	1460	1470	1480	1490	1500	1510	1520
SETPPSSTSS	SAFSVMNPVI	QAVGSSSAVN	VITQAPSLLS	SGASFVSQAG	TLTLRISPPE	PQSFASKTGS	ETKITYSSGG
1530	1540	1550	1560	1570	1580	1590	1600
QPVGTASLIP	LQSGSFALLQ	LPGQKPVPSS	ILQHVASLQM	KRESQNPDQK	DETNSIKREQ	ETKKVLQSEG	EAVDPEANVI
1610	1620	1630	1640	1650	1660	1670	1680
KQNSGAATSE	ETLNDSLEDR	GDHLDEECLP	EEGCATVKPS	EHSCITGSHT	DQDYKDVNEE	YGARNRKSSK	EKVAVLEVRT
1690	1700	1710	1720	1730	1740	1750	1760
ISEKASNKTV	QNLSKVQHQK	LGDVKVEQQK	GFDNPEENSS	EFPVTFKEES	KFELSGSKVM	EQQSNLQPEA	KEKECGDSLE
1770	1780	1790	1800	1810	1820	1830	1840
KDRERWRKHL	KGPLTRKCVG	ASQECKKEAD	EQLIKETKTC	QENSDVFQQE	QGISDLLGKS	GITEDARVLK	TECDSWSRIS
1850	1860	1870	1880	1890	1900	1910	1920
NPSAFSIVPR	RAAKSSRGNG	HFQGHLLLPG	EQIQPKQEKK	GGRSSADFTV	LDLEEDDEDD	NEKTDDSIDE	IVDVVSDYQS
1930	1940	1950	1960	1970	1980	1990	2000
EEVDDVEKVN	NCVEYIEDDE	EHVDIETVEE	LSEEINVAHL	KTTAAHTQSF	KQPSCTHISA	DEKAAERSRK	APPIPLKLKP
2010	2020	2030	2040	2050	2060	2070	2080
DYWSDKLQKE	AEAFAYYRRT	HTANERRRRG	EMRDLFEKLK	ITLGLLHSSK	VSKSLILTRA	FSEIQGLTDQ	ADKLIGQKNL
2090	2100	2110	2120	2130	2140	2150	2160
LTRKRNILIR	KVSSLSGKTE	EVVLKKLEYI	YAKQQALEAQ	KRKKKMGSDE	FDISPRISKQ	QEGSSASSVD	LGQMFINNRR
2170	2180	2190	2200	2210	2220	2230	2240

Detailed Protein Report

Cmpd.	No. of Cmpds.	m/z meas.	Δ m/z [ppm]	z	Rt [min]	Score	P	Range	Sequence	Modification	Ratios
2438	1	611.7346	-126.77	2	60.9	12.8	0	597-608	R.LGCVCSSLALEK.R		
1755	1	636.2889	-110.13	2	51.9	13.3	0	754-764	R.SYTPKPNPVIR.E		
1840	1	899.4842	21.23	2	51.7	12.0	0	1585-1601	K.VLQSEGEAVDPEANVIK.Q		
2040	1	841.7905	46.48	3	55.6	10.9	2	1732-1753	K.FELSGSKVMEQQSNLQPEAKEK.E	Oxidation: 9	
939	1	583.7035	-110.96	2	41.3	10.6	1	1777-1786	R.KCVGASQECK.K	Carbamidomethyl: 2, 9	
238	1	897.4097	-91.61	2	31.7	17.0	1	2603-2620	K.VMPCLAPIAAKVGSVGHK.M	Oxidation: 2	M:Q 0.86<br>Q:W 0.52

Detailed Protein Report

Protein 67: stAR-related lipid transfer protein 9 [Homo sapiens]

**Accession:**	gi|270265793	**Score:**	95.2
**Database:**	refseq_human(refseq_human_20140103.fasta)	**MW [kDa]:**	516.0
**Database Date:**	2015-11-30	**pI:**	5.9
**Modification(s):**	Carbamidomethyl	**Sequence Coverage [%]:**	1.8
		**No. of unique Peptides:**	4

10	20	30	40	50	60	70	80
MANVQVAVRV	RPLSKRETKE	GGRIIVEVDG	KVAKIRNLKV	DNRPDGFGDS	REKVMAFGFD	YCYWSVNPED	PQYASQDVVF
90	100	110	120	130	140	150	160
QDLGMEVLSG	VAKGYNICLF	AYGQTGSGKT	YTMLGTPASV	GLTPRICEGL	FVREKDCASL	PSSCRIKVSF	LEIYNERVRD
170	180	190	200	210	220	230	240
LLKQSGQKKS	YTLRVREHPE	MGPYVQGLSQ	HVVTNYKQVI	QLLEEGIANR	ITAATHVHEA	SSRSHAIFTI	HYTQAILENN
250	260	270	280	290	300	310	320
LPSEMASKIN	LVDLAGSERA	DPSYCKDRIA	EGANINKSLV	TLGIVISTLA	QNSQVFSSCQ	SLNSSVSNGG	DSGILSSPSG
330	340	350	360	370	380	390	400
TSSGGAPSRR	QSYIPYRDSV	LTWLLKDSLG	GNSKTIMVAT	VSPAHTSYSE	TMSTLRYASS	AKNIINKPRV	NEDANLKLIR
410	420	430	440	450	460	470	480
ELREEIERLK	ALLLSFELRN	FSSLSDENLK	ELVLQNELKI	DQLTKDWTQK	WNDWQALMEH	YSVDINRRRA	GVVIDSSLPH
490	500	510	520	530	540	550	560
LMALEDDVLS	TGVVLYHLKE	GTTKIGRIDS	DQEQDIVLQG	QWIERDHCTI	TSACGVVVLR	PARGARCTVN	GREVTASCRL
570	580	590	600	610	620	630	640
TQGAVITLGK	AQKFRFNHPA	EAAVLRQRRQ	VGEAAAGRGS	LEWLDLDGDL	AASRLGLSPL	LWKERRALEE	QCDEDHQTPR
650	660	670	680	690	700	710	720
DGETSHRAQI	QQQQSYVEDL	RHQILAEEIR	AAKELEFDQA	WISQQIKENQ	QCLLREETWL	ASLQQQQQED	QVAEKELEAS
730	740	750	760	770	780	790	800
VALDAWLQTD	PEIQPSPFVQ	SQKRVVHLQL	LRRHTLRAAE	RNVRRKKVSF	QLERIIKKQR	LLEAQKRLEK	LTTLCWLQDD
810	820	830	840	850	860	870	880
STQEPPYQVL	SPDATVPRPP	CRSKLTSCSS	LSPQRLCSKH	MPQLHSIFLS	WDPSTTLPPR	PDPTHQTSEK	TSSEEHLPQA
890	900	910	920	930	940	950	960
ASYPARTGCL	RKNGLHSSGH	GQPCTARAAL	ARKGASAPDA	CLTMSPNSVG	IQEMEMGVKQ	PHQMVSQGLA	SLRKSANKLK
970	980	990	1000	1010	1020	1030	1040
PRHEPKIFTS	TTQTRGAKGL	ADPSHTQAGW	RKEGNLGTHK	AAKGASCNSL	YPHGPRQTAG	HGKAVKTFWT	EYKPPSPSRA
1050	1060	1070	1080	1090	1100	1110	1120
SKRHQRVLAT	RVRNITKKSS	HLPLGSPLKR	QQNTRDPDTM	VPLTDFSPVM	DHSREKDNDL	SDTDSNYSLD	SLSCVYAKAL
1130	1140	1150	1160	1170	1180	1190	1200
IEPLKPEERK	WDFPEPENSE	SDDSQLSEDS	LAEKRYQSPK	NRLGGNRPTN	NRGQPRTRTR	ASVRGFTAAS	DSDLLAQTHR
1210	1220	1230	1240	1250	1260	1270	1280
SFSLDSLIDA	EEELGEDQQE	EPFPGSADEI	PTETFWHLED	SSLPVMDQEA	ICRLGPINYR	TAARLDAVLP	MSSSFYLDPQ
1290	1300	1310	1320	1330	1340	1350	1360
FQPHCELQPH	CELQPHCELQ	PHCEQAESQV	EPSYSEQADS	LQGMQLSRES	PLMSMDSWFS	CDSKINPSSP	PGIVGSLCPS
1370	1380	1390	1400	1410	1420	1430	1440
PDMQEFHSCK	GERPGYWPNT	EELKPSDAET	VLPYSSKLHQ	GSTELLCSAR	DEHTASAADT	SRLSLWGIQR	LIQPGADGTF
1450	1460	1470	1480	1490	1500	1510	1520
QGRCIPDMTQ	QGSSEASHNS	SVSNVLAASA	TTLTHVGSTH	ERDWSALQQK	YLLELSCPVL	EAIGAPKPAY	PYLEEDSGSL
1530	1540	1550	1560	1570	1580	1590	1600
AQASSKGGDT	LLPVGPRVSS	NLNLNNFPVH	LSRIRRLRAE	KEQDSLNAKL	EGVSDFFSTS	EKEASYDETY	SADLESLSAS
1610	1620	1630	1640	1650	1660	1670	1680
RSTNAQVFAT	ENAIPDSMTE	ACEVKQNNLE	ECLQSCRKPG	LMTSSDEDFF	QKNACHSNVT	TATKADHWSQ	GWAPLRKNSA
1690	1700	1710	1720	1730	1740	1750	1760
VQPGQLSPDS	HYPLEEEKTD	CQESSKEAVR	RHINVSFALP	SGPELYLHSA	PWNPLSSSLQ	PPLLETFYVT	KSRDALTETA
1770	1780	1790	1800	1810	1820	1830	1840
LEIPACREVR	VPSPPPREAW	GFGHNHQALQ	GAYLKNNLPV	LLQNQNSKIA	SSQQVTAEIP	VDLNTREVIR	ESGKCPGNIT
1850	1860	1870	1880	1890	1900	1910	1920
EESHDSVYSS	VTQNRHFLPS	TSTKVCEFEN	QVVILNKKHS	FPALEGGEVT	AQSCCGASSD	STESGKSLLF	RESEAREEEE
1930	1940	1950	1960	1970	1980	1990	2000
LDQNTVLRQT	INVSLEKDMP	GESAVSLKSR	SVDRRVSSPV	MVAQGGGPTP	KWEGKNETGL	LEKGLRPKDS	SEEFKLPGTK
2010	2020	2030	2040	2050	2060	2070	2080
PAYERFQLVA	CPQERNPSEC	KSQEMLNPNR	EPSGKKQNKR	VNNTDEMARL	IRSVMQLENG	ILEIESKQNK	QVHASHTPGT
2090	2100	2110	2120	2130	2140	2150	2160
DKELVFQDQK	EQEKTDHAFR	PDSSGNPLPS	KDQPSSPRQT	DDTVFRDSEA	GAMEVNSIGN	HPQVQKITPN	PFRSREGVRE
2170	2180	2190	2200	2210	2220	2230	2240

Detailed Protein Report

Cmpd.	No. of Cmpds.	m/z meas.	Δ m/z [ppm]	z	Rt [min]	Score	P	Range	Sequence	Modification
2859	3	648.3692	122.78	2	64.5	15.6	1	134-145	R.EKDCASLPSSCR.I	
387	1	1294.0546	-62.74	2	34.8	12.0	2	136-157	K.DCASLPSSCRIKVSFLEIYNER.V	Carbamidomethyl: 2
2847	1	665.3691	57.41	2	64.1	17.6	0	2192-2202	R.EFTNTSLHPQR.M	
1886	1	863.9273	-37.56	2	52.2	12.3	1	2739-2754	K.KVVAALPSQAPYDDPR.V	

Detailed Protein Report

Protein 68: plastin-2 [Homo sapiens]

Accession: gi|167614506
Database: refseq_human(refseq_human_20140103.fasta)
Database Date: 2015-11-30
Modification(s): Carbamidomethyl, Oxidation

Score: 93.8
MW [kDa]: 70.2
pI: 5.2
Sequence Coverage [%]: 14.5
No. of unique Peptides: 6

Quantitation

Q:W	**Median:** 0.89	**CV:** 8.51 %	**No. of Peptides:**	2
M:Q	**Median:** 1.04	**CV:** 0.00 %	**No. of Peptides:**	1

Alias proteins:

Accession	Name	Description
gi|530402335	refseq_human (refseq_human_20140103.fasta)	PREDICTED: plastin-2 isoform X2 [Homo sapiens]

```
        10         20         30         40         50         60         70         80
MARGSVSDEE MMELREAFAK VDTDGNGYIS FNELNDLFKA ACLPLPGYRV REITENLMAT GDLDQDGRIS FDEFIKIFHG
        90        100        110        120        130        140        150        160
LKSTDVAKTF RKAINKKEGI CAIGGTSEQS SVGTQHSYSE EEKYAFVNWI NKALENDPDC RHVIPMNPNT NDLFNAVGDG
       170        180        190        200        210        220        230        240
IVLCKMINLS VPDTIDERTI NKKKLTPFTI QENLNLALNS ASAIGCHVVN IGAEDLKEGK PYLVLGLLWQ VIKIGLFADI
       250        260        270        280        290        300        310        320
ELSRNEALIA LLREGESLED LMKLSPEELL LRWANYHLEN AGCNKIGNFS TDIKDSKAYY HLLEQVAPKG DEEGVPAVVI
       330        340        350        360        370        380        390        400
DMSGLREKDD IQRAECMLQQ AERLGCRQFV TATDVVRGNP KLNLAFIANL FNRYPALHKP ENQDIDWGAL EGETREERTF
       410        420        430        440        450        460        470        480
RNWMNSLGVN PRVNHLYSDL SDALVIFQLY EKIKVPVDWN RVNKPPYPKL GGNMKKLENC NYAVELGKNQ AKFSLVGIGG
       490        500        510        520        530        540        550        560
QDLNEGNRTL TLALIWQLMR RYTLNILEEI GGGQKVNDDI IVNWVNETLR EAKKSSSISS FKDPKISTSL PVLDLIDAIQ
       570        580        590        600        610        620        630
PGSINYDLLK TENLNDDEKL NNAKYAISMA RKIGARVYAL PEDLVEVNPK MVMTVFACLM GKGMKRV
```

Cmpd.	No. of Cmpds.	m/z meas.	Δ m/z [ppm]	z	Rt [min]	Score	P	Range	Sequence	Modification	Ratios
2609	1	658.3945	34.02	2	61.3	14.5	1	77-88	K.IFHGLKSTDVAK.T		
545	1	652.1661	-227.16	2	36.1	14.5	0	402-412	R.NWMNSLGVNPR.V	Oxidation: 3	M:Q 1.04<br>Q:W 0.82
2352	1	836.1191	173.33	2	59.3	10.6	2	442-456	R.VNKPPYPKLGGNMKK.L		
1806	1	649.7882	-96.92	2	52.6	11.2	2	531-542	R.EAKKSSSISSFK.D		
2231	1	793.3805	-58.93	2	56.5	14.2	0	597-610	R.VYALPEDLVEVNPK.M		Q:W 0.97
2744	1	710.3515	31.99	2	65.6	10.2	0	611-622	K.MVMTVFACLMGK.G	Carbamidomethyl: 8;<br>Oxidation: 1, 3	

Detailed Protein Report

Protein 69: myosin-9 [Homo sapiens]

**Accession:**	gi|12667788	**Score:**	93.3
**Database:**	refseq_human(refseq_human_20140103.fasta)	**MW [kDa]:**	226.4
**Database Date:**	2015-11-30	**pI:**	5.4
**Modification(s):**	Oxidation	**Sequence Coverage [%]:**	3.5
		**No. of unique Peptides:**	5

Quantitation

Q:W	**Median:**	1.13	**CV:**	250.26 %	**No. of Peptides:**	2
M:Q	**Median:**	0.69	**CV:**	29.61 %	**No. of Peptides:**	2

Detailed Protein Report

10	20	30	40	50	60	70	80
MAQQAADKYL	YVDKNFINNP	LAQADWAAKK	LVWVPSDKSG	FEPASLKEEV	GEEAIVELVE	NGKKVKVNKD	DIQKMNPPKF
90	100	110	120	130	140	150	160
SKVEDMAELT	CLNEASVLHN	LKERYYSGLI	YTYSGLFCVV	INPYKNLPIY	SEEIVEMYKG	KKRHEMPPHI	YAITDTAYRS
170	180	190	200	210	220	230	240
MMQDREDQSI	LCTGESGAGK	TENTKKVIQY	LAYVASSHKS	KKDQGELERQ	LLQANPILEA	FGNAKTVKND	NSSRFGKFIR
250	260	270	280	290	300	310	320
INFDVNGYIV	GANIETYLLE	KSRAIRQAKE	ERTFHIFYYL	LSGAGEHLKT	DLLLEPYNKY	RFLSNGHVTI	PGQQDKDMFQ
330	340	350	360	370	380	390	400
ETMEAMRIMG	IPEEEQMGLL	RVISGVLQLG	NIVFKKERNT	DQASMPDNTA	AQKVSHLLGI	NVTDFTRGIL	TPRIKVGRDY
410	420	430	440	450	460	470	480
VQKAQTKEQA	DFAIEALAKA	TYERMFRWLV	LRINKALDKT	KRQGASFIGI	LDIAGFEIFD	LNSFEQLCIN	YTNEKLQQLF
490	500	510	520	530	540	550	560
NHTMFILEQE	EYQREGIEWN	FIDFGLDLQP	CIDLIEKPAG	PPGILALLDE	ECWFPKATDK	SFVEKVMQEQ	GTHPKFQKPK
570	580	590	600	610	620	630	640
QLKDKADFCI	IHYAGKVDYK	ADEWLMKNMD	PLNDNIATLL	HQSSDKFVSE	LWKDVDRIIG	LDQVAGMSET	ALPGAFKTRK
650	660	670	680	690	700	710	720
GMFRTVGQLY	KEQLAKLMAT	LRNTNPNFVR	CIIPNHEKKA	GKLDPHLVLD	QLRCNGVLEG	IRICRQGFPN	RVVFQEFRQR
730	740	750	760	770	780	790	800
YEILTPNSIP	KGFMDGKQAC	VLMIKALELD	SNLYRIGQSK	VFFRAGVLAH	LEEERDLKIT	DVIIGFQACC	RGYLARKAFA
810	820	830	840	850	860	870	880
KRQQQLTAMK	VLQRNCAAYL	KLRNWQWWRL	FTKVKPLLQV	SRQEEEMMAK	EEELVKVREK	QLAAENRLTE	METLQSQLMA
890	900	910	920	930	940	950	960
EKLQLQEQLQ	AETELCAEAE	ELRARLTAKK	QELEEICHDL	EARVEEEEER	CQHLQAEKKK	MQQNIQELEE	QLEEEESARQ
970	980	990	1000	1010	1020	1030	1040
KLQLEKVTTE	AKLKKLEEEQ	IILEDQNCKL	AKEKKLLEDR	IAEFTTNLTE	EEEKSKSLAK	LKNKHEAMIT	DLEERLRREE
1050	1060	1070	1080	1090	1100	1110	1120
KQRQELEKTR	RKLEGDSTDL	SDQIAELQAQ	IAELKMQLAK	KEEELQAALA	RVEEEAAQKN	MALKKIRELE	SQISELQEDL
1130	1140	1150	1160	1170	1180	1190	1200
ESERASRNKA	EKQKRDLGEE	LEALKTELED	TLDSTAAQQE	LRSKREQEVN	ILKKTLEEEA	KTHEAQIQEM	RQKHSQAVEE
1210	1220	1230	1240	1250	1260	1270	1280
LAEQLEQTKR	VKANLEKAKQ	TLENERGELA	NEVKVLLQGK	GDSEHKRKKV	EAQLQELQVK	FNEGERVRTE	LADKVTKLQV
1290	1300	1310	1320	1330	1340	1350	1360
ELDNVTGLLS	QSDSKSSKLT	KDFSALESQL	QDTQELLQEE	NRQKLSLSTK	LKQVEDEKNS	FREQLEEEEE	AKHNLEKQIA
1370	1380	1390	1400	1410	1420	1430	1440
TLHAQVADMK	KKMEDSVGCL	ETAEEVKRKL	QKDLEGLSQR	HEEKVAAYDK	LEKTKTRLQQ	ELDDLLVDLD	HQRQSACNLE
1450	1460	1470	1480	1490	1500	1510	1520
KKQKKFDQLL	AEEKTISAKY	AEERDRAEAE	AREKETKALS	LARALEEAME	QKAELERLNK	QFRTEMEDLM	SSKDDVGKSV
1530	1540	1550	1560	1570	1580	1590	1600
HELEKSKRAL	EQQVEEMKTQ	LEELEDELQA	TEDAKLRLEV	NLQAMKAQFE	RDLQGRDEQS	EEKKKQLVRQ	VREMEAELED
1610	1620	1630	1640	1650	1660	1670	1680
ERKQRSMAVA	ARKKLEMDLK	DLEAHIDSAN	KNRDEAIKQL	RKLQAQMKDC	MRELDDTRAS	REEILAQAKE	NEKKLKSMEA
1690	1700	1710	1720	1730	1740	1750	1760
EMIQLQEELA	AAERAKRQAQ	QERDELADEI	ANSSGKGALA	LEEKRRLEAR	IAQLEEELEE	EQGNTELIND	RLKKANLQID
1770	1780	1790	1800	1810	1820	1830	1840
QINTDLNLER	SHAQKNENAR	QQLERQNKEL	KVKLQEMEGT	VKSKYKASIT	ALEAKIAQLE	EQLDNETKER	QAACKQVRRT
1850	1860	1870	1880	1890	1900	1910	1920
EKKLKDVLLQ	VDDERRNAEQ	YKDQADKAST	RLKQLKRQLE	EAEEEAQRAN	ASRRKLQREL	EDATETADAM	NREVSSLKNK
1930	1940	1950	1960	1970			
LRRGDLPFVV	PRRMARKGAG	DGSDEEVDGK	ADGAEAKPAE				

Cmpd.	No. of Cmpds.	m/z meas.	Δ m/z [ppm]	z	Rt [min]	Score	P	Range	Sequence	Modification	Ratios
573	1	472.1766	-189.39	2	36.0	12.4	0	31-38	K.LVWVPSDK.S		Q:W 4.62<br>M:Q 0.52
1950	1	856.8003	-136.41	2	54.5	12.5	1	910-923	K.KQELEEICHDLEAR.V		

Detailed Protein Report

Cmpd.	No. of Cmpds.	m/z meas.	Δ m/z [ppm]	z	Rt [min]	Score	P	Range	Sequence	Modification	Ratios
187	1	849.4195	-61.73	2	32.3	21.9	2	1358-1372	K.QIATLHAQVADMKKK.M	Oxidation: 12	
2426	1	625.6164	-235.71	2	60.8	13.5	0	1593-1602	R.EMEAELEDER.K		
502	2	567.1670	-259.21	2	35.6	22.1	2	1604-1613	K.QRSMAVAARK.K	Oxidation: 4	M:Q 0.92<br>Q:W 0.28

Detailed Protein Report

Protein 70: PREDICTED: uncharacterized protein KIAA1551 isoform X1 [Homo sapiens]

**Accession:**	gi|530399223	**Score:**	93.3
**Database:**	refseq_human(refseq_human_20140103.fasta)	**MW [kDa]:**	194.7
**Database Date:**	2015-11-30	**pI:**	9.7
**Modification(s):**	Carbamidomethyl, Oxidation	**Sequence Coverage [%]:**	4.2
		**No. of unique Peptides:**	6

Quantitation

Q:W **Median:** 0.20 **CV:** 54.75 % **No. of Peptides:** 2

10	20	30	40	50	60	70	80
MNWNEKPKSA	TLPPLYPKSQ	PPFLHQSLIN	QITTTSQSSF	SYPGSNQEAC	MYPGNSNPIS	QPLLNIQNYP	QQISVSDMHN
90	100	110	120	130	140	150	160
GTVVASHTSV	ERITYANVNG	PKQLTHNLQM	SSGVTQNVWL	NSPMRNPVHS	HIGATVSHQT	DFGANVPNMP	ALQSQLITSD
170	180	190	200	210	220	230	240
TYSMQMQMIP	SNSTRLPVAY	QGNQGLNQSF	SEQQVDWTQQ	CISKGLTYPD	YRPPPKLYRY	SPQSFLPDST	IQKQNFIPHT
250	260	270	280	290	300	310	320
SLQVKNSQLL	NSVLTLPSRQ	TSAVPSQQYA	TQTDKRPPPP	PYNCRYGSQP	LQSTQHITKH	LSMEVPQSRE	MLSSEIRTSF
330	340	350	360	370	380	390	400
QQQWQNPNEN	VSTIGNFTNL	KVNTNSKQPF	NSPIRSSVDG	VQTLAQTNEE	KIMDSCNPTS	NQVLDTSVAK	EKLVRDIKTL
410	420	430	440	450	460	470	480
VEIKQKFSEL	ARKIKINKDL	LMAAGCIKMT	NTSYSEPAQN	SKLSLKQTAK	IQSGPQITPV	MPENAERQTP	TVVESAETNK
490	500	510	520	530	540	550	560
TQCMLNSDIQ	EVNCRRFNQV	DSVLPNPVYS	EKRPMPDSSH	DVKVLTSKTS	AVEMTQAVLN	TQLSSENVTK	VEQNSPAVCE
570	580	590	600	610	620	630	640
TISVPKSMST	EEYKSKIQNE	NMLLLALLSQ	ARKTQKTVLK	DANQTIQDSK	PDSCEMNPNT	QMTGNQLNLK	NMETPSTSNV
650	660	670	680	690	700	710	720
SGRVLDNSFC	SGQESSTKGM	PAKSDSSCSM	EVLATCLSLW	KKQPSDTAKE	KECDKLRTNT	TAVGISKPAN	IHVKSPCSVV
730	740	750	760	770	780	790	800
GNSNSQNKIS	NPSQQTALSM	VMHNYESSGI	NITKGTELQI	AVVSPLVLSE	VKTLSVKGIT	PAVLPETVYP	VIKEGSVCSL
810	820	830	840	850	860	870	880
QNQLAENAKA	TAALKVDVSG	PVASTATSTK	IFPLTQKEKQ	NESTNGNSEV	TPNVNQGKHN	KLESAIHSPM	NDQQISQESR
890	900	910	920	930	940	950	960
NSTVVSSDTL	QIDNICSLVE	GDTSYNSQIA	KIFSSLPLKM	VEPQKPSLPN	QQGIGSREPE	KQLDNTTENK	DFGFQKDKPV
970	980	990	1000	1010	1020	1030	1040
QCTDVSHKIC	DQSKSEPPLE	SSFNNLETNR	VILEKSSLEH	ATEKSTANDT	CSSAAIQEDI	YPQEIDASSN	YTPQDPARNE
1050	1060	1070	1080	1090	1100	1110	1120
IHSDKAPVLY	LHDQLSELLK	EFPYGIEAVN	TREGSVGQQT	TYQTSEDQTA	DKTSSDSKDP	ADQIQITILS	SEQMKEIFPE
1130	1140	1150	1160	1170	1180	1190	1200
QDDQPYVVDK	LAEPQKEEPI	TEVVSQCDLQ	APAAGQSRDS	VILDSEKDDI	HCCALGWLSM	VYEGVPQCQC	NSIKNSSSEE
1210	1220	1230	1240	1250	1260	1270	1280
EKQKEQCSPL	DTNSCKQGER	TSDRDVTVVQ	FKSLVNNPKT	PPDGKSHFPE	LQDDSRKDTP	KTKHKSLPRT	EQELVAGQFS
1290	1300	1310	1320	1330	1340	1350	1360
SKCDKLNPLQ	NHKRKKLRFH	EVTFHSSNKM	TASYEQASQE	TRQKKHVTQN	SRPLKTKTAF	LPNKDVYKKH	SSLGQSLSPE
1370	1380	1390	1400	1410	1420	1430	1440
KIKLKLKSVS	FKQKRKLDQG	NVLDMEVKKK	KHDKQEQKGS	VGATFKLGDS	LSNPNERAIV	KEKMVSNTKS	VDTKASSSKF
1450	1460	1470	1480	1490	1500	1510	1520
SRILTPKEYL	QRQKHKEALS	NKASKKICVK	NVPCDSEHMR	PSKLAVQVES	CGKSNEKHSS	GVQTSKESLN	GLTSHGKNLK
1530	1540	1550	1560	1570	1580	1590	1600
IHHSQESKTY	NILRNVKEKV	GGKQPDKIWI	DKTKLDKLTN	ISNEAQFSQM	PPQVKDQKKL	YLNRVGFKCT	ERESISLTKL
1610	1620	1630	1640	1650	1660	1670	1680
ESSPRKLHKD	KRQENKHKTF	LPVKGNTEKS	NMLEFKLCPD	ILLKNTNSVE	ERKDVKPHPR	KEQAPLQVSG	IKSTKEDWLK
1690	1700	1710	1720	1730	1740	1750	
FVATKKRTQK	DSQERDNVNS	RLSKRSFSAD	GFEMLQNPVK	DSKEMFQTYK	QMYLEKRSRS	LGSSPVK	

Detailed Protein Report

Cmpd.	No. of Cmpds.	m/z meas.	Δ m/z [ppm]	z	Rt [min]	Score	P	Range	Sequence	Modification	Ratios	
2628	1	644.4984	138.82	1	63.6	19.8	1	391-395	K.EKLVR.D		Q:W	0.12
2689	1	731.8632	-38.63	2	64.8	14.6	1	416-428	K.INKDLLMAAGCIK.M	Carbamidomethyl: 11; Oxidation: 7		
2776	1	710.3755	7.84	2	63.8	11.0	0	816-830	K.VDVSGPVASTATSTK.I			
2006	1	1044.4022	-89.89	2	55.0	16.6	1	957-974	K.DKPVQCTDVSHKICDQSK.S	Carbamidomethyl: 6		
2741	1	654.3586	-33.86	2	65.4	11.6	1	1325-1335	K.KHVTQNSRPLK.T		Q:W	0.34
258	2	585.2801	-84.98	2	32.0	19.7	0	1662-1672	K.EQAPLQVSGIK.S			

Detailed Protein Report

Protein 71: coagulation factor VIII isoform a precursor [Homo sapiens]

**Accession:**	gi|4503647	**Score:**	92.5
**Database:**	refseq_human(refseq_human_20140103.fasta)	**MW [kDa]:**	266.8
**Database Date:**	2015-11-30	**pI:**	7.0
**Modification(s):**	Carbamidomethyl, Oxidation	**Sequence Coverage [%]:**	4.8
		**No. of unique Peptides:**	6

Quantitation

Q:W	**Median:**	0.60	**CV:**	0.00 %	**No. of Peptides:**	1
M:Q	**Median:**	0.26	**CV:**	0.00 %	**No. of Peptides:**	1

BRUKER

Detailed Protein Report

10	20	30	40	50	60	70	80
MQIELSTCFF	LCLLRFCFSA	TRRYYLGAVE	LSWDYMQSDL	GELPVDARFP	PRVPKSFPFN	TSVVYKKTLF	VEFTDHLFNI
90	100	110	120	130	140	150	160
AKPRPPWMGL	LGPTIQAEVY	DTVVITLKNM	ASHPVSLHAV	GVSYWKASEG	AEYDDQTSQR	EKEDDKVFPG	GSHTYVWQVL
170	180	190	200	210	220	230	240
KENGPMASDP	LCLTYSYLSH	VDLVKDLNSG	LIGALLVCRE	GSLAKEKTQT	LHKFILLFAV	FDEGKSWHSE	TKNSLMQDRD
250	260	270	280	290	300	310	320
AASARAWPKM	HTVNGYVNRS	LPGLIGCHRK	SVYWHVIGMG	TTPEVHSIFL	EGHTFLVRNH	RQASLEISPI	TFLTAQTLLM
330	340	350	360	370	380	390	400
DLGQFLLFCH	ISSHQHDGME	AYVKVDSCPE	EPQLRMKNNE	EAEDYDDDLT	DSEMDVVRFD	DDNSPSFIQI	RSVAKKHPKT
410	420	430	440	450	460	470	480
WVHYIAAEEE	DWDYAPLVLA	PDDRSYKSQY	LNNGPQRIGR	KYKKVRFMAY	TDETFKTREA	IQHESGILGP	LLYGEVGDTL
490	500	510	520	530	540	550	560
LIIFKNQASR	PYNIYPHGIT	DVRPLYSRRL	PKGVKHLKDF	PILPGEIFKY	KWTVTVEDGP	TKSDPRCLTR	YYSSFVNMER
570	580	590	600	610	620	630	640
DLASGLIGPL	LICYKESVDQ	RGNQIMSDKR	NVILFSVFDE	NRSWYLTENI	QRFLPNPAGV	QLEDPEFQAS	NIMHSINGYV
650	660	670	680	690	700	710	720
FDSLQLSVCL	HEVAYWYILS	IGAQTDFLSV	FFSGYTFKHK	MVYEDTLTLF	PFSGETVFMS	MENPGLWILG	CHNSDFRNRG
730	740	750	760	770	780	790	800
MTALLKVSSC	DKNTGDYYED	SYEDISAYLL	SKNNAIEPRS	FSQNSRHPST	RQKQFNATTI	PENDIEKTDP	WFAHRTPMPK
810	820	830	840	850	860	870	880
IQNVSSSDLL	MLLRQSPTPH	GLSLSDLQEA	KYETFSDDPS	PGAIDSNNSL	SEMTHFRPQL	HHSGDMVFTP	ESGLQLRLNE
890	900	910	920	930	940	950	960
KLGTTAATEL	KKLDFKVSST	SNNLISTIPS	DNLAAGTDNT	SSLGPPSMPV	HYDSQLDTTL	FGKKSSPLTE	SGGPLSLSEE
970	980	990	1000	1010	1020	1030	1040
NNDSKLLESG	LMNSQESSWG	KNVSSTESGR	LFKGKRAHGP	ALLTKDNALF	KVSISLLKTN	KTSNNSATNR	KTHIDGPSLL
1050	1060	1070	1080	1090	1100	1110	1120
IENSPSVWQN	ILESDTEFKK	VTPLIHDRML	MDKNATALRL	NHMSNKTTSS	KNMEMVQQKK	EGPIPPDAQN	PDMSFFKMLF
1130	1140	1150	1160	1170	1180	1190	1200
LPESARWIQR	THGKNSLNSG	QGPSPKQLVS	LGPEKSVEGQ	NFLSEKNKVV	VGKGEFTKDV	GLKEMVFPSS	RNLFLTNLDN
1210	1220	1230	1240	1250	1260	1270	1280
LHENNTHNQE	KKIQEEIEKK	ETLIQENVVL	PQIHTVTGTK	NFMKNLFLLS	TRQNVEGSYD	GAYAPVLQDF	RSLNDSTNRT
1290	1300	1310	1320	1330	1340	1350	1360
KKHTAHFSKK	GEEENLEGLG	NQTKQIVEKY	ACTTRISPNT	SQQNFVTQRS	KRALKQFRLP	LEETELEKRI	IVDDTSTQWS
1370	1380	1390	1400	1410	1420	1430	1440
KNMKHLTPST	LTQIDYNEKE	KGAITQSPLS	DCLTRSHSIP	QANRSPLPIA	KVSSFPSIRP	IYLTRVLFQD	NSSHLPAASY
1450	1460	1470	1480	1490	1500	1510	1520
RKKDSGVQES	SHFLQGAKKN	NLSLAILTLE	MTGDQREVGS	LGTSATNSVT	YKKVENTVLP	KPDLPKTSGK	VELLPKVHIY
1530	1540	1550	1560	1570	1580	1590	1600
QKDLFPTETS	NGSPGHLDLV	EGSLLQGTEG	AIKWNEANRP	GKVPFLRVAT	ESSAKTPSKL	LDPLAWDNHY	GTQIPKEEWK
1610	1620	1630	1640	1650	1660	1670	1680
SQEKSPEKTA	FKKKDTILSL	NACESNHAIA	AINEGQNKPE	IEVTWAKQGR	TERLCSQNPP	VLKRHQREIT	RTTLQSDQEE
1690	1700	1710	1720	1730	1740	1750	1760
IDYDDTISVE	MKKEDFDIYD	EDENQSPRSF	QKKTRHYFIA	AVERLWDYGM	SSSPHVLRNR	AQSGSVPQFK	KVVFQEFTDG
1770	1780	1790	1800	1810	1820	1830	1840
SFTQPLYRGE	LNEHLGLLGP	YIRAEVEDNI	MVTFRNQASR	PYSFYSSLIS	YEEDQRQGAE	PRKNFVKPNE	TKTYFWKVQH
1850	1860	1870	1880	1890	1900	1910	1920
HMAPTKDEFD	CKAWAYFSDV	DLEKDVHSGL	IGPLLVCHTN	TLNPAHGRQV	TVQEFALFFT	IFDETKSWYF	TENMERNCRA
1930	1940	1950	1960	1970	1980	1990	2000
PCNIQMEDPT	FKENYRFHAI	NGYIMDTLPG	LVMAQDQRIR	WYLLSMGSNE	NIHSIHFSGH	VFTVRKKEEY	KMALYNLYPG
2010	2020	2030	2040	2050	2060	2070	2080
VFETVEMLPS	KAGIWRVECL	IGEHLHAGMS	TLFLVYSNKC	QTPLGMASGH	IRDFQITASG	QYGQWAPKLA	RLHYSGSINA
2090	2100	2110	2120	2130	2140	2150	2160
WSTKEPFSWI	KVDLLAPMII	HGIKTQGARQ	KFSSLYISQF	IIMYSLDGKK	WQTYRGNSTG	TLMVFFGNVD	SSGIKHNIFN
2170	2180	2190	2200	2210	2220	2230	2240

Detailed Protein Report

Cmpd.	No. of Cmpds.	m/z meas.	Δ m/z [ppm]	z	Rt [min]	Score	P	Range	Sequence	Modification	Ratios
2170	1	937.3499	-130.54	2	55.7	11.5	0	1-15	-.MQIELSTCFFLCLLR.F	Carbamidomethyl: 12	Q:W 0.60<br>M:Q 0.26
2041	1	856.8157	-171.77	2	55.6	11.8	2	1060-1073	K.KVTPLIHDRMLMDK.N	Oxidation: 12	
2966	2	953.4397	1.72	2	66.3	11.8	0	1101-1117	K.EGPIPPDAQNPDMSFFK.M	Oxidation: 13	
843	1	653.3686	-17.24	2	40.6	10.7	2	1167-1178	K.NKVVVGKGEFTK.D		
2184	1	1061.5230	-9.68	3	57.5	13.4	0	1523-1553	K.<br>DLFPTETSNGSPGHLDLVEGSLLQ<br>W		
2720	1	710.3296	-131.82	2	65.3	14.5	0	2092-2104	K.VDLLAPMIIHGIK.T		

Detailed Protein Report

Protein 72: splicing factor, proline- and glutamine-rich [Homo sapiens]

**Accession:**	gi|4826998	**Score:**	91.8
**Database:**	refseq_human(refseq_human_20140103.fasta)	**MW [kDa]:**	76.1
**Database Date:**	2015-11-30	**pI:**	9.9
**Modification(s):**	Oxidation	**Sequence Coverage [%]:**	6.9
		**No. of unique Peptides:**	5

Quantitation

Q:W	**Median:**	1.32	**CV:**	26.15 %	**No. of Peptides:**	2
M:Q	**Median:**	0.53	**CV:**	20.95 %	**No. of Peptides:**	2

Alias proteins:

Accession	Name	Description
gi|530363144	refseq_human (refseq_human_20140103.fasta)	PREDICTED: splicing factor, proline- and glutamine-rich isoform X3 [Homo sapiens]
gi|530363142	refseq_human (refseq_human_20140103.fasta)	PREDICTED: splicing factor, proline- and glutamine-rich isoform X2 [Homo sapiens]
gi|530363140	refseq_human (refseq_human_20140103.fasta)	PREDICTED: splicing factor, proline- and glutamine-rich isoform X1 [Homo sapiens]

10	20	30	40	50	60	70	80
MSRDRFRSRG	GGGGGFHRRG	GGGGRGGLHD	FRSPPPGMGL	NQNRGPMGPG	PGQSGPKPPI	PPPPPHQQQQ	QPPPQQPPPQ
90	100	110	120	130	140	150	160
QPPPHQPPPH	PQPHQQQQPP	PPPQDSSKPV	VAQGPGPAPG	VGSAPPASSS	APPATPPTSG	APPGSGPGPT	PTPPPAVTSA
170	180	190	200	210	220	230	240
PPGAPPPTPP	SSGVPTTPPQ	AGGPPPPPAA	VPGPGPGPKQ	GPGPGGPKGG	KMPGGPKPGG	GPGLSTPGGH	PKPPHRGGGE
250	260	270	280	290	300	310	320
PRGGRQHHPP	YHQQHHQGPP	PGGPGGRSEE	KISDSEGFKA	NLSLLRRPGE	KTYTQRCRLF	VGNLPADITE	DEFKRLFAKY
330	340	350	360	370	380	390	400
GEPGEVFINK	GKGFGFIKLE	SRALAEIAKA	ELDDTPMRGR	QLRVRFATHA	AALSVRNLSP	YVSNELLEEA	FSQFGPIERA
410	420	430	440	450	460	470	480
VVIVDDRGRS	TGKGIVEFAS	KPAARKAFER	CSEGVFLLTT	TPRPVIVEPL	EQLDDEDGLP	EKLAQKNPMY	QKERETPPRF
490	500	510	520	530	540	550	560
AQHGTFEYEY	SQRWKSLDEM	EKQQREQVEK	NMKDAKDKLE	SEMEDAYHEH	QANLLRQDLM	RRQEELRRME	ELHNQEMQKR
570	580	590	600	610	620	630	640
KEMQLRQEEE	RRRREEEMMI	RQREMEEQMR	RQREESYSRM	GYMDPRERDM	RMGGGGAMNM	GDPYGSGGQK	FPPLGGGGGI
650	660	670	680	690	700	710	
GYEANPGVPP	ATMSGSMMGS	DMRTERFGQG	GAGPVGGQGP	RGMGPGTPAG	YGRGREEYEG	PNKKPRF	

Cmpd.	No. of Cmpds.	m/z meas.	Δ m/z [ppm]	z	Rt [min]	Score	P	Range	Sequence	Modification	Ratios
14	1	1103.5206	70.68	2	29.5	14.3	1	609-630	R.<br>DMRMGGGGAMNMGDPYGSGGQ<br>F	Oxidation: 2, 12	
18	3	736.0042	54.34	3	29.4	11.7	1	609-630	R.<br>DMRMGGGGAMNMGDPYGSGGQ<br>F	Oxidation: 2, 10	M:Q 0.43<br>Q:W 1.71
20	3	735.9950	41.81	3	29.6	11.7	1	609-630	R.<br>DMRMGGGGAMNMGDPYGSGGQ<br>F	Oxidation: 4, 10	
8	3	736.0117	64.51	3	29.3	14.9	1	609-630	R.<br>DMRMGGGGAMNMGDPYGSGGQ<br>F	Oxidation: 4, 12	
284	1	671.3405	5.90	2	33.5	10.1	0	667-681	R.FGQGGAGPVGGQGPR.G		Q:W 1.02<br>M:Q 0.65

Detailed Protein Report

Protein 73: **PREDICTED: beta-enolase isoform X1 [Homo sapiens]**

Accession: gi|530410063
Database: refseq_human(refseq_human_20140103.fasta)
Database Date: 2015-11-30
Modification(s): Oxidation

Score: 89.5
MW [kDa]: 48.0
pI: 9.1
Sequence Coverage [%]: 14.2
No. of unique Peptides: 1

10	20	30	40	50	60	70	80
MAVMRTLRAM	AMQKIFAREI	LDSRGNPTVE	VDLHTAKGRF	RAAVPSGAST	GIYEALELRD	GDKGRYLGKG	VLKAVENINN
**90**	**100**	**110**	**120**	**130**	**140**	**150**	**160**
TLGPALLQKK	LSVVDQEKVD	KFMIELDGTE	NKSKFGANAI	LGVSLAVCKA	GAAEKGVPLY	RHIADLAGNP	DLILPVPAFN
**170**	**180**	**190**	**200**	**210**	**220**	**230**	**240**
VINGGSHAGN	KLAMQEFMIL	PVGASSFKEA	MRIGAEVYHH	LKGVIKAKYG	KDATNVGDEG	GFAPNILENN	EALELLKTAI
**250**	**260**	**270**	**280**	**290**	**300**	**310**	**320**
QAAGYPDKVV	IGMDVAASEF	YRNGKYDLDF	KSPDDPARHI	TGEKLGELYK	SFIKNYPVVS	IEDPFDQDDW	ATWTSFLSGV
**330**	**340**	**350**	**360**	**370**	**380**	**390**	**400**
NIQIVGDDLT	VTNPKRIAQA	VEKKACNCLL	LKVNQIGSVT	ESIQACKLAQ	SNGWGVMVSH	RSGETEDTFI	ADLVVGLCTG
**410**	**420**	**430**	**440**	**450**			
QIKTGAPCRS	ERLAKYNQLM	RIEEALGDKA	IFAGRKFRNP	KAK			

Cmpd.	No. of Cmpds.	m/z meas.	Δ m/z [ppm]	z	Rt [min]	Score	P	Range	Sequence	Modification
2482	1	835.4239	2.22	2	61.6	10.7	2	1-14	-.MAVMRTLRAMAMQK.I	Oxidation: 4, 10

Detailed Protein Report

Protein 74: PREDICTED: microtubule-actin cross-linking factor 1 isoform X23 [Homo sapiens]

**Accession:**	gi|578798826	**Score:**	89.4
**Database:**	refseq_human(refseq_human_20140103.fasta)	**MW [kDa]:**	844.7
**Database Date:**	2015-11-30	**pI:**	5.2
**Modification(s):**	Carbamidomethyl, Oxidation	**Sequence Coverage [%]:**	1.0
		**No. of unique Peptides:**	6

Quantitation

Q:W	**Median:** 0.67	**CV:** 0.00 %	**No. of Peptides:** 1

10	20	30	40	50	60	70	80
MSSSDEETLS	ERSCRSERSC	RSERSYRSER	SGSLSPCPPG	DTLPWNLPLH	EQKKRKSQDS	VLDPAERAVV	RVADERDRVQ
90	100	110	120	130	140	150	160
KKTFTKWVNK	HLMKVRKHIN	DLYEDLRDGH	NLISLLEVLS	GIKLPREKGR	MRFHRLQNVQ	IALDFLKQRQ	VKLVNIRNDD
170	180	190	200	210	220	230	240
ITDGNPKLTL	GLIWTIILHF	QISDIYISGE	SGDMSAKEKL	LLWTQKVTAG	YTGIKCTNFS	SCWSDGKMFN	ALIHRYRPDL
250	260	270	280	290	300	310	320
VDMERVQIQS	NRENLEQAFE	VAERLGVTRL	LDAEDVDVPS	PDEKSVITYV	SSIYDAFPKV	PEGGEGISAT	EVDSRWQEYQ
330	340	350	360	370	380	390	400
SRVDSLIPWI	KQHTILMSDK	TFPQNPVELK	ALYNQYIHFK	ETEILAKERE	KGRIEELYKL	LEVWIEFGRI	KLPQGYHPND
410	420	430	440	450	460	470	480
VEEEWGKLII	EMLEREKSLR	PAVERLELLL	QIANKIQNGA	LNCEEKLTLA	KNTLQADAAH	LESGQPVQCE	SDVIMYIQEC
490	500	510	520	530	540	550	560
EGLIRQLQVD	LQILRDENYY	QLEELAFRVM	RLQDELVTLR	LECTNLYRKG	HFTSLELVPP	STLTTTHLKA	EPLTKATHSS
570	580	590	600	610	620	630	640
STSWFRKPMT	RAELVAISSS	EDEGNLRFVY	ELLSWVEEMQ	MKLERAEWGN	DLPSVELQLE	TQQHIHTSVE	ELGSSVKEAR
650	660	670	680	690	700	710	720
LYEGKMSQNF	HTSYAETLGK	LETQYCKLKE	TSSFRMRHLQ	SLHKFVSRAT	AELIWLNEKE	EEELAYDWSD	NNSNISAKRN
730	740	750	760	770	780	790	800
YFSELTMELE	EKQDVFRSLQ	DTAELLSLEN	HPAKQTVEAY	SAAVQSQLQW	MKQLCLCVEQ	HVKENTAYFQ	FFSDARELES
810	820	830	840	850	860	870	880
FLRNLQDSIK	RKYSCDHNTS	LSRLEDLLQD	SMDEKEQLIQ	SKSSVASLVG	RSKTIVQLKP	RSPDHVLKNT	ISVKAVCDYR
890	900	910	920	930	940	950	960
QIEITICKND	ECVLEDNSQR	TKWKVISPTG	NEAMVPSVCF	LIPPPNKDAI	EMASRVEQSY	QKVMALWHQL	HVNTKSLISW
970	980	990	1000	1010	1020	1030	1040
NYLRKDLDLV	QTWNLEKLRS	SAPGECHQIM	KNLQAHYEDF	LQDSRDSVLF	SVADRLRLEE	EVEACKARFQ	HLMKSMENED
1050	1060	1070	1080	1090	1100	1110	1120
KEETVAKMYI	SELKNIRLRL	EEYEQRVVKR	IQSLASSRTD	RDAWQDNALR	IAEQEHTQED	LQQLRSDLDA	VSMKCDSFLH
1130	1140	1150	1160	1170	1180	1190	1200
QSPSSSSVPT	LRSELNLLVE	KMDHVYGLST	VYLNKLKTVD	VIVRSIQDAE	LLVKGYEIKL	SQEEVVLADL	SALEAHWSTL
1210	1220	1230	1240	1250	1260	1270	1280
RHWLSDVKDK	NSVFSVLDEE	IAKAKVVAEQ	MSRLTPERNL	DLERYQEKGS	QLQERWHRVI	AQLEIRQSEL	ESIQEVLGDY
1290	1300	1310	1320	1330	1340	1350	1360
RACHGTLIKW	IEETTAQQEM	MKPGQAEDSR	VLSEQLSQQT	ALFAEIERNQ	TKLDQCQKFS	QQYSTIVKDY	ELQLMTYKAF
1370	1380	1390	1400	1410	1420	1430	1440
VESQQKSPGK	RRRMLSSSDA	ITQEFMDLRT	RYTALVTLTT	QHVKYISDAL	RRLEEEEKVV	EEEKQEHVEK	VKELLGWVST
1450	1460	1470	1480	1490	1500	1510	1520
LARNTQGKAT	SSETKESTDI	EKAILEQQVL	SEELTTKKEQ	VSEAIKTSQI	FLAKHGHKLS	EKEKKQISEQ	LNALNKAYHD
1530	1540	1550	1560	1570	1580	1590	1600
LCDGSANQLQ	QLQSQLAHQT	EQKECRAVAG	VIDLGTVEIF	PIFKAMQKGL	LDQDTGLVLL	ESQVIMSGLI	APETGENLSL
1610	1620	1630	1640	1650	1660	1670	1680
EEGIARNLIN	PQMYQQLREL	QDALALISRL	TESRGPLSVV	EAIEKRIISE	TVGLKILEAH	LATGGFSLSP	SENCINLEEA
1690	1700	1710	1720	1730	1740	1750	1760
FHQGLISAWL	HSVLESYLRT	SKNLIDPNTA	EKIGLLDLMQ	RCIVHQESGF	KLLPVKQLAG	GMVSLKSGRK	VSIFRAVQEG
1770	1780	1790	1800	1810	1820	1830	1840
LIDRQVTVRL	LEAQLFAGGI	VDPRTGHRLT	VEEAVRHNLI	DQDMACAILI	RQLQTGGIID	TVTGQRLTID	EAVSNDLVAA
1850	1860	1870	1880	1890	1900	1910	1920
KIALVILESL	WSFMGLLWPE	SGEILPITDA	LEQGIVSTEL	AHKILSNRQH	IKALFLPATT	EILSWKKAIE	SGILDRDLAN
1930	1940	1950	1960	1970	1980	1990	2000
NLKSICIPDV	MPHMQLADSA	EQNINPGAAV	LPCSKSHPKA	TASQSENLLF	QLMTHSYINV	QNGQRLLLLD	KELMETLTSR
2010	2020	2030	2040	2050	2060	2070	2080
DEYQTSPPKV	VEIGHQRQKT	PEGLQESANV	KISGTFSSGW	TVRLPEFQFS	SQNKEYPDRE	DCTTEKGKKT	TVETEDSSVE
2090	2100	2110	2120	2130	2140	2150	2160
NPEQDLFVEQ	KERNPNIDAL	KVINKVKLEV	QRQLIGTQRE	DQTAVSVREN	ASRGHLLTIP	PAEAEGVPLV	VDKDVFSVET
2170	2180	2190	2200	2210	2220	2230	2240

Detailed Protein Report

Cmpd.	No. of Cmpds.	m/z meas.	Δ m/z [ppm]	z	Rt [min]	Score	P	Range	Sequence	Modification	Ratios
324	1	755.2309	-145.08	2	33.4	13.7	1	1035-1047	K.SMENEDKEETVAK.M		Q:W 0.67
1103	1	1355.1276	184.19	1	42.5	16.9	2	2204-2215	K.LQVQVKKTLGIK.L		
91	2	1240.0402	-66.65	2	31.1	23.4	2	3949-3970	K.VLDMENSFKEGKEPSEIGNLVK.D	Oxidation: 4	
371	1	625.3885	104.40	2	34.3	11.3	2	4903-4912	R.LKDCMQKAQK.Y	Carbamidomethyl: 4	
1272	1	673.3365	1.16	2	45.6	11.4	1	5818-5827	R.QQQEEMRQLR.E		
11	1	650.7452	-159.65	2	29.7	12.6	2	6549-6558	K.RAKQFHEAWK.K		

Detailed Protein Report

Protein 75: DNA-dependent protein kinase catalytic subunit isoform 1 [Homo sapiens]

**Accession:**	gi|13654237	**Score:**	89.2
**Database:**	refseq_human(refseq_human_20140103.fasta)	**MW [kDa]:**	468.8
**Database Date:**	2015-11-30	**pI:**	6.8
**Modification(s):**	Carbamidomethyl, Oxidation	**Sequence Coverage [%]:**	2.1
		**No. of unique Peptides:**	6

Quantitation

Q:W	**Median:**	0.92	**CV:**	494.79 %	**No. of Peptides:**	2
M:Q	**Median:**	0.86	**CV:**	0.03 %	**No. of Peptides:**	2

Detailed Protein Report

10	20	30	40	50	60	70	80
MAGSGAGVRC	SLLRLQETLS	AADRCGAALA	GHQLIRGLGQ	ECVLSSSPAV	LALQTSLVFS	RDFGLLVFVR	KSLNSIEFRE
90	100	110	120	130	140	150	160
CREEILKFLC	IFLEKMGQKI	APYSVEIKNT	CTSVYTKDRA	AKCKIPALDL	LIKLLQTFRS	SRLMDEFKIG	ELFSKFYGEL
170	180	190	200	210	220	230	240
ALKKKIPDTV	LEKVYELLGL	LGEVHPSEMI	NNAENLFRAF	LGELKTQMTS	AVREPKLPVL	AGCLKGLSSL	LCNFTKSMEE
250	260	270	280	290	300	310	320
DPQTSREIFN	FVLKAIRPQI	DLKRYAVPSA	GLRLFALHAS	QFSTCLLDNY	VSLFEVLLKW	CAHTNVELKK	AALSALESFL
330	340	350	360	370	380	390	400
KQVSNMVAKN	AEMHKNKLQY	FMEQFYGIIR	NVDSNNKELS	IAIRGYGLFA	GPCKVINAKD	VDFMYVELIQ	RCKQMFLTQT
410	420	430	440	450	460	470	480
DTGDDRVYQM	PSFLQSVASV	LLYLDTVPEV	YTPVLEHLVV	MQIDSFPQYS	PKMQLVCCRA	IVKVFLALAA	KGPVLRNCIS
490	500	510	520	530	540	550	560
TVVHQGLIRI	CSKPVVLPKG	PESESEDHRA	SGEVRTGKWK	VPTYKDYVDL	FRHLLSSDQM	MDSILADEAF	FSVNSSSESL
570	580	590	600	610	620	630	640
NHLLYDEFVK	SVLKIVEKLD	LTLEIQTVGE	QENGDEAPGV	WMIPTSDPAA	NLHPAKPKDF	SAFINLVEFC	REILPEKQAE
650	660	670	680	690	700	710	720
FFEPWVYSFS	YELILQSTRL	PLISGFYKLL	SITVRNAKKI	KYFEGVSPKS	LKHSPEDPEK	YSCFALFVKF	GKEVAVKMKQ
730	740	750	760	770	780	790	800
YKDELLASCL	TFLLSLPHNI	IELDVRAYVP	ALQMAFKLGL	SYTPLAEVGL	NALEEWSIYI	DRHVMQPYYK	DILPCLDGYL
810	820	830	840	850	860	870	880
KTSALSDETK	NNWEVSALSR	AAQKGFNKVV	LKHLKKTKNL	SSNEAISLEE	IRIRVVQMLG	SLGGQINKNL	LTVTSSDEMM
890	900	910	920	930	940	950	960
KSYVAWDREK	RLSFAVPFRE	MKPVIFLDVF	LPRVTELALT	ASDRQTKVAA	CELLHSMVMF	MLGKATQMPE	GGQGAPPMYQ
970	980	990	1000	1010	1020	1030	1040
LYKRTFPVLL	RLACDVDQVT	RQLYEPLVMQ	LIHWFTNNKK	FESQDTVALL	EAILDGIVDP	VDSTLRDFCG	RCIREFLKWS
1050	1060	1070	1080	1090	1100	1110	1120
IKQITPQQQE	KSPVNTKSLF	KRLYSLALHP	NAFKRLGASL	AFNNIYREFR	EEESLVEQFV	FEALVIYMES	LALAHADEKS
1130	1140	1150	1160	1170	1180	1190	1200
LGTIQQCCDA	IDHLCRIIEK	KHVSLNKAKK	RRLPRGFPPS	ASLCLLDLVK	WLLAHCGRPQ	TECRHKSIEL	FYKFVPLLPG
1210	1220	1230	1240	1250	1260	1270	1280
NRSPNLWLKD	VLKEEGVSFL	INTFEGGGCG	QPSGILAQPT	LLYLRGPFSL	QATLCWLDLL	LAALECYNTF	IGERTVGALQ
1290	1300	1310	1320	1330	1340	1350	1360
VLGTEAQSSL	LKAVAFFLES	IAMHDIIAAE	KCFGTGAAGN	RTSPQEGERY	NYSKCTVVVR	IMEFTTTLLN	TSPEGWKLLK
1370	1380	1390	1400	1410	1420	1430	1440
KDLCNTHLMR	VLVQTLCEPA	SIGFNIGDVQ	VMAHLPDVCV	NLMKALKMSP	YKDILETHLR	EKITAQSIEE	LCAVNLYGPD
1450	1460	1470	1480	1490	1500	1510	1520
AQVDRSRLAA	VVSACKQLHR	AGLLHNILPS	QSTDLHHSVG	TELLSLVYKG	IAPGDERQCL	PSLDLSCKQL	ASGLLELAFA
1530	1540	1550	1560	1570	1580	1590	1600
FGGLCERLVS	LLLNPAVLST	ASLGSSQGSV	IHFSHGEYFY	SLFSETINTE	LLKNLDLAVL	ELMQSSVDNT	KMVSAVLNGM
1610	1620	1630	1640	1650	1660	1670	1680
LDQSFRERAN	QKHQGLKLAT	TILQHWKKCD	SWWAKDSPLE	TKMAVLALLA	KILQIDSSVS	FNTSHGSFPE	VFTTYISLLA
1690	1700	1710	1720	1730	1740	1750	1760
DTKLDLHLKG	QAVTLLPFFT	SLTGGSLEEL	RRVLEQLIVA	HFPMQSREFP	PGTPRFNNYV	DCMKKFLDAL	ELSQSPMLLE
1770	1780	1790	1800	1810	1820	1830	1840
LMTEVLCREQ	QHVMEELFQS	SFRRIARRGS	CVTQVGLLES	VYEMFRKDDP	RLSFTRQSFV	DRSLLTLLWH	CSLDALREFF
1850	1860	1870	1880	1890	1900	1910	1920
STIVVDAIDV	LKSRFTKLNE	STFDTQITKK	MGYYKILDVM	YSRLPKDDVH	AKESKINQVF	HGSCITEGNE	LTKTLIKLCY
1930	1940	1950	1960	1970	1980	1990	2000
DAFTENMAGE	NQLLERRRLY	HCAAYNCAIS	VICCVFNELK	FYQGFLFSEK	PEKNLLIFEN	LIDLKRRYNF	PVEVEVPMER
2010	2020	2030	2040	2050	2060	2070	2080
KKKYIEIRKE	AREAANGDSD	GPSYMSSLSY	LADSTLSEEM	SQFDFSTGVQ	SYSYSSQDPR	PATGRFRRRE	QRDPTVHDDV
2090	2100	2110	2120	2130	2140	2150	2160
LELEMDELNR	HECMAPLTAL	VKHMHRSLGP	PQGEEDSVPR	DLPSWMKFLH	GKLGNPIVPL	NIRLFLAKLV	INTEEVFRPY
2170	2180	2190	2200	2210	2220	2230	2240

Detailed Protein Report

Cmpd.	No. of Cmpds.	m/z meas.	Δ m/z [ppm]	z	Rt [min]	Score	P	Range	Sequence	Modification	Ratios
1135	2	473.1745	-251.80	2	44.3	15.1	0	1643-1651	K.MAVLALLAK.I	Oxidation: 1	Q:W 0.15<br>M:Q 0.86
9	1	798.7834	5.94	3	29.1	17.8	2	2314-2334	K.EVYAAAAEVLGLILRYVMERK.N		
698	2	512.2692	-22.73	2	37.6	10.8	0	2434-2441	K.VCLDIIYK.M	Carbamidomethyl: 2	M:Q 0.86<br>Q:W 5.54
272	1	898.1750	46.75	3	33.4	12.1	2	3030-3050	K.IWSEPFYQETYLPYMIRSKLK.L		
2214	1	722.7106	-205.39	2	56.0	11.5	2	3236-3247	K.FSMKMKMIDSAR.K		
2232	1	937.4206	-14.54	2	56.5	22.1	1	3682-3696	K.ECSPWMSDFKVEFLR.N		

Detailed Protein Report

Protein 76: uncharacterized protein C4orf21 [Homo sapiens]

**Accession:**	gi|254826809	**Score:**	88.5
**Database:**	refseq_human(refseq_human_20140103.fasta)	**MW [kDa]:**	236.5
**Database Date:**	2015-11-30	**pI:**	5.8
**Modification(s):**	Carbamidomethyl	**Sequence Coverage [%]:**	4.7
		**No. of unique Peptides:**	4

Quantitation

Q:W	**Median:** 1.27	**CV:** 0.00 %	**No. of Peptides:** 1

Alias proteins:

Accession	Name	Description
gi|530377937	refseq_human (refseq_human_20140103.fasta)	PREDICTED: uncharacterized protein C4orf21 isoform X1 [Homo sapiens]

Detailed Protein Report

10	20	30	40	50	60	70	80
MESQEFIVLY	THQKMKKSKV	WQDGILKITH	LGNKAILYDD	KGACLESLFL	KCLEVKPGDD	LESDRYLITV	EEVKVAGAIG
90	100	110	120	130	140	150	160
IVKQNVNKEA	PELNSRTFIS	SGRSLGCQPS	GLKRKFTGFQ	GPRQVPKKMV	IMESGESAAS	HEAKKTGPTI	FSPFCSMPPL
170	180	190	200	210	220	230	240
FPTVGKKDVN	NILADPENIV	TYKNRERNAM	DFSSVFSPSF	QINPEVLCEE	NYFCSPVNSG	NKLSDSLLTN	EPVKRDSLAS
250	260	270	280	290	300	310	320
HYSGVSQNIR	SKAQILALLK	SESSSSCEEL	NSEMTEHFPQ	KQPQGSLKIA	TKPKYLIQQE	ECAEMKSTEN	LYYQHQSENT
330	340	350	360	370	380	390	400
MRNKSRWAMY	LSSQSSPIHS	STVDGNDTER	KPKAQEDDVN	SNLKDLSLQK	IIQFVETYAE	ERKKYNVDQS	VGNNDPSWNQ
410	420	430	440	450	460	470	480
EVKLEIPSFN	ESSSLQVTCS	SAENDGILSE	SDIQEDNKIP	FNQNDKGCIK	GSVLIKENAQ	EVNTCGTLEK	EYEQSESSLP
490	500	510	520	530	540	550	560
ELKHLQIESS	NNSRISDDIT	DMISESKMDN	ESLNSIHESL	SNVTQPFLEV	TFNLNNFETS	DTEEESQESN	KISQDSESWV
570	580	590	600	610	620	630	640
KDILVNDGNS	CFQKRSENTN	CEEIEGEHLP	FLTSVSDKPT	VTFPVKETLP	SQFCDKTYVG	FDMGICKTEN	TGKEIEEYSD
650	660	670	680	690	700	710	720
TLSNFESFKW	TDAVYGDNKE	DANKPIQEVR	INYDFALPPN	KSKGINMNLH	IPHIQNQIAE	NSNLFSEDAQ	PQPFILGSDL
730	740	750	760	770	780	790	800
DKNDEHVLPS	TSSSDNSVQL	LNTNQNHYEC	IALDKSNTHI	SNSLFYPLGK	KHLISKDTEA	HISEPEDLGK	IRSPPPDHVE
810	820	830	840	850	860	870	880
VETAREGKQY	WNPRNSSELS	GLVNTISILK	SLCEHSTALD	SLEILKKKNT	VFQQGTQQTY	EPDSPPEVRK	PFITVVSPKS
890	900	910	920	930	940	950	960
PHLHKDSQQI	LKEDEVELSE	PLQSVQFSSS	GSKEETAFQA	VIPKQIERKT	CDPKPVEFQG	HQVKGSATSG	VMVRGHSSQL
970	980	990	1000	1010	1020	1030	1040
GCSQFPDSTE	YENFMTETPE	LPSTCMQIDF	LQVTSPEENI	STLSPVSTFS	LNSRDEDFMV	EFSETSLKAR	TLPDDLHFLN
1050	1060	1070	1080	1090	1100	1110	1120
LEGMKKSRSL	ENENLQRLSL	LSRTQVPLIT	LPRTDGPPDL	DSHSYMINSN	TYESSGSPML	NLCEKSAVLS	FSIEPEDQNE
1130	1140	1150	1160	1170	1180	1190	1200
TFFSEESREV	NPGDVSLNNI	STQSKWLKYQ	NTSQCNVATP	NRVDKRITDG	FFAEAVSGMH	FRDTSERQSD	AVNESSLDSV
1210	1220	1230	1240	1250	1260	1270	1280
HLQMIKGMLY	QQRQDFSSQD	SVSRKKVLSL	NLKQTSKTEE	IKNVLGGSTC	YNYSVKDLQE	ISGSELCFPS	GQKIKSAYLP
1290	1300	1310	1320	1330	1340	1350	1360
QRQIHIPAVF	QSPAHYKQTF	TSCLIEHLNI	LLFGLAQNLQ	KALSKVDISF	YTSLKGEKLK	NAENNVPSCH	HSQPAKLVMV
1370	1380	1390	1400	1410	1420	1430	1440
KKEGPNKGRL	FYTCDGPKAD	RCKFFKWLED	VTPGYSTQEG	ARPGMVLSDI	KSIGLYLRSQ	KIPLYEECQL	LVRKGFDFQR
1450	1460	1470	1480	1490	1500	1510	1520
KQYGKLKKFT	TVNPEFYNEP	KTKLYLKLSR	KERSSAYSKN	DLWVVSKTLD	FELDTFIACS	AFFGPSSINE	IEILPLKGYF
1530	1540	1550	1560	1570	1580	1590	1600
PSNWPTNMVV	HALLVCNAST	ELTTLKNIQD	YFNPATLPLT	QYLLTTSSPT	IVSNKRVSKR	KFIPPAFTNV	STKFELLSLG
1610	1620	1630	1640	1650	1660	1670	1680
ATLKLASELI	QVHKLNKDQA	TALIQIAQMM	ASHESIEEVK	ELQTHTFPIT	IIHGVFGAGK	SYLLAVVILF	FVQLFEKSEA
1690	1700	1710	1720	1730	1740	1750	1760
PTIGNARPWK	LLISSSTNVA	VDRVLLGLLS	LGFENFIRVG	SVRKIAKPIL	PYSLHAGSEN	ESEQLKELHA	LMKEDLTPTE
1770	1780	1790	1800	1810	1820	1830	1840
RVYVRKSIEQ	HKLGTNRTLL	KQVRVVGVTC	AACPFPCMND	LKFPVVVLDE	CSQITEPASL	LPIARFECEK	LILVGDPKQL
1850	1860	1870	1880	1890	1900	1910	1920
PPTIQGSDAA	HENGLEQTLF	DRLCLMGHKP	ILLRTQYRCH	PAISAIANDL	FYKGALMNGV	TEIERSPLLE	WLPTLCFYNV
1930	1940	1950	1960	1970	1980	1990	2000
KGLEQIERDN	SFHNVAEATF	TLKLIQSLIA	SGIAGSMIGV	ITLYKSQMYK	LCHLLSAVDF	HHPDIKTVQV	STVDAFQGAE
2010	2020	2030	2040	2050	2060	2070	2080
KEIIILSCVR	TRQVGFIDSE	KRMNVALTRG	KRHLLIVGNL	ACLRKNQLWG	RVIQHCEGRE	DGLQHANQYE	PQLNHLLKDY
2090	2100	2110					
FEKQVEEKQK	KKSEKEKSKD	KSHS					

Detailed Protein Report

Cmpd.	No. of Cmpds.	m/z meas.	Δ m/z [ppm]	z	Rt [min]	Score	P	Range	Sequence	Modification	Ratios
2045	1	788.3012	-86.79	2	55.5	10.9	0	52-65	K.CLEVKPGDDLESDR.Y		
1998	1	902.8620	-77.52	2	55.4	13.3	1	128-144	K.KMVIMESGESAASHEAK.K		
2940	2	973.5071	-8.25	2	65.5	10.8	1	167-183	K.KDVNNILADPENIVTYK.N		Q:W 1.27
2882	1	938.5029	-0.62	2	64.8	14.3	1	1419-1433	R.SQKIPLYEECQLLVR.K	Carbamidomethyl: 10	

Detailed Protein Report

Protein 77: probable helicase senataxin [Homo sapiens]

**Accession:**	gi|113722133	**Score:**	87.2
**Database:**	refseq_human(refseq_human_20140103.fasta)	**MW [kDa]:**	302.7
**Database Date:**	2015-11-30	**pI:**	6.9
**Modification(s):**	Carbamidomethyl, Oxidation	**Sequence Coverage [%]:**	3.7
		**No. of unique Peptides:**	5

Quantitation

M:Q	**Median:**	0.47	**CV:**	0.00 %	**No. of Peptides:**	1

Detailed Protein Report

10	20	30	40	50	60	70	80
MSTCCWCTPG	GASTIDFLKR	YASNTPSGEF	QTADEDLCYC	LECVAEYHKA	RDELPFLHEV	LWELETLRLI	NHFEKSMKAE
90	100	110	120	130	140	150	160
IGDDDELYIV	DNNGEMPLFD	ITGQDFENKL	RVPLLEILKY	PYLLLHERVN	ELCVEALCRM	EQANCSFQVF	DKHPGIYLFL
170	180	190	200	210	220	230	240
VHPNEMVRRW	AILTARNLGK	VDRDDYYDLQ	EVLLCLFKVI	ELGLLESPDI	YTSSVLEKGK	LILLPSHMYD	TTNYKSYWLG
250	260	270	280	290	300	310	320
ICMLLTILEE	QAMDSLLLGS	DKQNDFMQSI	LHTMEREADD	DSVDPFWPAL	HCFMVILDRL	GSKVWGQLMD	PIVAFQTIIN
330	340	350	360	370	380	390	400
NASYNREIRH	IRNSSVRTKL	EPESYLDDMV	TCSQIVYNYN	PEKTKKDSGW	RTAICPDYCP	NMYEEMETLA	SVLQSDIGQD
410	420	430	440	450	460	470	480
MRVHNSTFLW	FIPFVQSLMD	LKDLGVAYIA	QVVNHLYSEV	KEVLNQTDAV	CDKVTEFFLL	ILVSVIELHR	NKKCLHLLWV
490	500	510	520	530	540	550	560
SSQQWVEAVV	KCAKLPTTAF	TRSSEKSSGN	CSKGTAMISS	LSLHSMPSNS	VQLAYVQLIR	SLLKEGYQLG	QQSLCKRFWD
570	580	590	600	610	620	630	640
KLNLFLRGNL	SLGWQLTSQE	THELQSCLKQ	IIRNIKFKAP	PCNTFVDLTS	ACKISPASYN	KEESEQMGKT	SRKDMHCLEA
650	660	670	680	690	700	710	720
SSPTFSKEPM	KVQDSVLIKA	DNTIEGDNNE	QNYIKDVKLE	DHLLAGSCLK	QSSKNIFTER	AEDQIKISTR	KQKSVKEISS
730	740	750	760	770	780	790	800
YTPKDCTSRN	GPERGCDRGI	IVSTRLLTDS	STDALEKVST	SNEDFSLKDD	ALAKTSKRKT	KVQKDEICAK	LSHVIKKQHR
810	820	830	840	850	860	870	880
KSTLVDNTIN	LDENLTVSNI	ESFYSRKDTG	VQKGDGFIHN	LSLDPSGVLD	DKNGEQKSQN	NVLPKEKQLK	NEELVIFSFH
890	900	910	920	930	940	950	960
ENNCKIQEFH	VDGKELIPFT	EMTNASEKKS	SPFKDLMTVP	ESRDEEMSNS	TSVIYSNLTR	EQAPDISPKS	DTLTDSQIDR
970	980	990	1000	1010	1020	1030	1040
DLHKLSLLAQ	ASVITFPSDS	PQNSSQLQRK	VKEDKRCFTA	NQNNVGDTSR	GQVIIISDSD	DDDDERILSL	EKLTKQDKIC
1050	1060	1070	1080	1090	1100	1110	1120
LEREHPEQHV	STVNSKEEKN	PVKEEKTETL	FQFEESDSQC	FEFESSSEVF	SVWQDHPDDN	NSVQDGEKKC	LAPIANTTNG
1130	1140	1150	1160	1170	1180	1190	1200
QGCTDYVSEV	VKKGAEGIEE	HTRPRSISVE	EFCEIEVKKP	KRKRSEKPMA	EDPVRPSSSV	RNEGQSDTNK	RDLVGNDFKS
1210	1220	1230	1240	1250	1260	1270	1280
IDRRTSTPNS	RIQRATTVSQ	KKSSKLCTCT	EPIRKVPVSK	TPKKTHSDAK	KGQNRSSNYL	SCRTTPAIVP	PKKFRQCPEP
1290	1300	1310	1320	1330	1340	1350	1360
TSTAEKLGLK	KGPRKAYELS	QRSLDYVAQL	RDHGKTVGVV	DTRKKTKLIS	PQNLSVRNNK	KLLTSQELQM	QRQIRPKSQK
1370	1380	1390	1400	1410	1420	1430	1440
NRRRLSDCES	TDVKRAGSHT	AQNSDIFVPE	SDRSDYNCTG	GTEVLANSNR	KQLIKCMPSE	PETIKAKHGS	PATDDACPLN
1450	1460	1470	1480	1490	1500	1510	1520
QCDSVVLNGT	VPTNEVIVST	SEDPLGGGDP	TARHIEMAAL	KEGEPDSSSD	AEEDNLFLTQ	NDPEDMDLCS	QMENDNYKLI
1530	1540	1550	1560	1570	1580	1590	1600
ELIHGKDTVE	VEEDSVSRPQ	LESLSGTKCK	YKDCLETTKN	QGEYCPKHSE	VKAADEDVFR	KPGLPPPASK	PLRPTTKIFS
1610	1620	1630	1640	1650	1660	1670	1680
SKSTSRIAGL	SKSLETSSAL	SPSLKNKSKG	IQSILKVPQP	VPLIAQKPVG	EMKNSCNVLH	PQSPNNSNRQ	GCKVPFGESK
1690	1700	1710	1720	1730	1740	1750	1760
YFPSSSPVNI	LLSSQSVSDT	FVKEVLKWKY	EMFLNFGQCG	PPASLCQSIS	RPVPVRFHNY	GDYFNVFFPL	MVLNTFETVA
1770	1780	1790	1800	1810	1820	1830	1840
QEWLNSPNRE	NFYQLQVRKF	PADYIKYWEF	AVYLEECELA	KQLYPKENDL	VFLAPERINE	EKKDTERNDI	QDLHEYHSGY
1850	1860	1870	1880	1890	1900	1910	1920
VHKFRRTSVM	RNGKTECYLS	IQTQENFPAN	LNELVNCIVI	SSLVTTQRKL	KAMSLLGSRN	QLARAVLNPN	PMDFCTKDLL
1930	1940	1950	1960	1970	1980	1990	2000
TTTSERIIAY	LRDFNEDQKK	AIETAYAMVK	HSPSVAKICL	IHGPPGTGKS	KTIVGLLYRL	LTENQRKGHS	DENSNAKIKQ
2010	2020	2030	2040	2050	2060	2070	2080
NRVLVCAPSN	AAVDELMKKI	ILEFKEKCKD	KKNPLGNCGD	INLVRLGPEK	SINSEVLKFS	LDSQVNHRMK	KELPSHVQAM
2090	2100	2110	2120	2130	2140	2150	2160
HKRKEFLDYQ	LDELSRQRAL	CRGGREIQRQ	ELDENISKVS	KERQELASKI	KEVQGRPQKT	QSIIILESHI	ICCTLSTSGG
2170	2180	2190	2200	2210	2220	2230	2240

Detailed Protein Report

Cmpd.	No. of Cmpds.	m/z meas.	Δ m/z [ppm]	z	Rt [min]	Score	P	Range	Sequence	Modification	Ratios
2508	3	812.3607	-22.41	2	61.8	14.0	0	599-613	K.APPCNTFVDLTSACK.I	Carbamidomethyl: 14	M:Q 0.47
896	1	576.9688	-1.76	3	41.3	12.0	1	679-694	K.LEDHLLAGSCLKQSSK.N		
1617	1	781.3954	-38.20	2	48.5	10.2	1	1613-1627	K.SLETSSALSPSLKNK.S		
399	1	658.3859	48.11	2	34.0	10.8	2	2099-2109	R.ALCRGGREIQR.Q	Carbamidomethyl: 3	
2158	1	682.2802	-2.49	3	57.4	22.7	1	2220-2235	K.AQEYGYDQSMMARFCR.L	Carbamidomethyl: 15; Oxidation: 10, 11	

Detailed Protein Report

Protein 78: dynein heavy chain 9, axonemal isoform 2 [Homo sapiens]

**Accession:**	gi|114155133	**Score:**	87.1
**Database:**	refseq_human(refseq_human_20140103.fasta)	**MW [kDa]:**	511.6
**Database Date:**	2015-11-30	**pI:**	5.6
**Modification(s):**	Oxidation	**Sequence Coverage [%]:**	1.5
		**No. of unique Peptides:**	4

Detailed Protein Report

10	20	30	40	50	60	70	80
MRLAEERAAL	AAENADGEPG	ADRRLRLLGT	YVAMSLRPAA	GAWERCAGSA	EAEQLLQAFL	GRDAAEGPRP	LLVVRPGPRG
90	100	110	120	130	140	150	160
LAIRPGLEVG	PESGLAGAKA	LFFLRTGPEP	PGPDSFRGAV	VCGDLPAAPL	EHLAALFSEV	VLPVLANEKN	RLNWPHMICE
170	180	190	200	210	220	230	240
DVRRHAHSLQ	CDLSVILEQV	KGKTLLPLPA	GSEKMEFADS	KSETVLDSID	KSVIYAIESA	VIKWSYQVQV	VLKRESSQPL
250	260	270	280	290	300	310	320
LQGENPTPKV	ELEFWKSRYE	DLKYIYNQLR	TITVRGMAKL	LDKLQSSYFP	AFKAMYRDVV	AALAEAQDIH	VHLIPLQRHL
330	340	350	360	370	380	390	400
EALENAEFPE	VKPQLRPLLH	VVCLIWATCK	SYRSPGRLTV	LLQEICNLLI	QQASNYLSPE	DLLRSEVEES	QRKLQVVSDT
410	420	430	440	450	460	470	480
LSFFKQEFQD	RRENLHTYFK	ENQEVKEWDF	QSSLVFVRLD	GFLGQLHVVE	GLLKTALDFH	KLGKVEFSGV	RGNALSQQVQ
490	500	510	520	530	540	550	560
QMHEEFQEMY	RLLSGSSSDC	LYLQSTDFEN	DVSEFNQKVE	DLDRRLGTIF	IQAFDDAPGL	EHAFKLLDIA	GNLLERPLVA
570	580	590	600	610	620	630	640
RDTSDKYLVL	IQMFNKDLDA	VRMIYSQHVQ	EEAELGFSPV	HKNMPTVAGG	LRWAQELRQR	IQGPFSNFGR	ITHPCMESAE
650	660	670	680	690	700	710	720
GKRMQQKYED	MLSLLEKYET	RLYEDWCRTV	SEKSQYNLSQ	PLLKRDPETK	EITINFNPQL	ISVLKEMSYL	EPREMKHMPE
730	740	750	760	770	780	790	800
TAAAMFSSRD	FYRQLVANLE	LMANWYNKVM	KTLLEVEFPL	VEEELQNIDL	RLRAAEETLN	WKTEGICDYV	TEITSSIHDL
810	820	830	840	850	860	870	880
EQRIQKTKDN	VEEIQNIMKT	WVTPIFKTKD	GKRESLLSLD	DRHDRMEKYY	NLIKESGLKI	HALVQENLGL	FSADPTSNIW
890	900	910	920	930	940	950	960
KTYVNSIDNL	LLNGFFLAIE	CSLKYLLENT	ECKAGLTPIF	EAQLSLAIPE	LVFYPSLESG	VKGGFCDIVE	GLITSIFRIP
970	980	990	1000	1010	1020	1030	1040
SLVPRLSPQN	GSPHYQVDLD	GIPDLANMRR	TLMERVQRMM	GLCCGYQSTF	SQYSYLYVED	RKEVLGQFLL	YGHILTPEEI
1050	1060	1070	1080	1090	1100	1110	1120
EDHVEDGIPE	NPPLLSQFKV	QIDSYETLYE	EVCRLEPIKV	FDGWMKIDIR	PFKASLLNII	KRWSLLFKQH	LVDHVTHSLA
1130	1140	1150	1160	1170	1180	1190	1200
NLDAFIKKSE	SGLLKKVEKG	DFQGLVEIMG	HLMAVKERQS	NTDEMFEPLK	QTIELLKTYE	QELPETVFKQ	LEELPEKWNN
1210	1220	1230	1240	1250	1260	1270	1280
IKKVAITVKQ	QVAPLQANEV	TLLRQRCTAF	DAEQQQFWEQ	FHKEAPFRFD	SIHPHQMLDA	RHIEIQQMES	TMASISESAS
1290	1300	1310	1320	1330	1340	1350	1360
LFEVNVPDYK	QLRQCRKEVC	QLKELWDTIG	MVTSSIHAWE	TTPWRNINVE	AMELECKQFA	RHIRNLDKEV	RAWDAFTGLE
1370	1380	1390	1400	1410	1420	1430	1440
STVWNTLSSL	RAVAELQNPA	IRERHWRQLM	QATGVSFTMD	QDTTLAHLLQ	LQLHHYEDEV	RGIVDKAAKE	MGMEKTLKEL
1450	1460	1470	1480	1490	1500	1510	1520
QTTWAGMEFQ	YEPHPRTNVP	LLCSDEDLIE	VLEDNQVQLQ	NLVMSKYVAF	FLEEVSGWQK	KLSTVDAVIS	IWFEVQRTWT
1530	1540	1550	1560	1570	1580	1590	1600
HLESIFTGSE	DIRAQLPQDS	KRFEGIDIDF	KELAYDAQKI	PNVVQTTNKP	GLYEKLEDIQ	GRLCLCEKAL	AEYLDTKRLA
1610	1620	1630	1640	1650	1660	1670	1680
FPRFYFLSSS	DLLDILSNGT	APQQVQRHLS	KLFDNMAKMR	FQLDASGEPT	KTSLGMYSKE	EEYVAFSEPC	DCSGQVEIWL
1690	1700	1710	1720	1730	1740	1750	1760
NHVLGHMKAT	VRHEMTEGVT	AYEEKPREQW	LFDHPAQVAL	TCTQIWWTTE	VGMAFARLEE	GYESAMKDYY	KKQVAQLKTL
1770	1780	1790	1800	1810	1820	1830	1840
ITMLIGQLSK	GDRQKIMTIC	TIDVHARDVV	AKMIAQKVDN	AQAFLWLSQL	RHRWDDEVKH	CFANICDAQF	LYSYEYLGNT
1850	1860	1870	1880	1890	1900	1910	1920
PRLVITPLTD	RCYITLTQSL	HLTMSGAPAG	PAGTGKTETT	KDLGRALGIL	VYVFNCSEQM	DYKSCGNIYK	GLAQTGAWGC
1930	1940	1950	1960	1970	1980	1990	2000
FDEFNRISVE	VLSVVAVQVK	SIQDAIRDKK	QWFSFLGEEI	SLNPSVGIFI	TMNPGYAGRT	ELPENLKSLF	RPCAMVVPDF
2010	2020	2030	2040	2050	2060	2070	2080
ELICEIMLVA	EGFIEAQSLA	RKFITLYQLC	KELLSKQDHY	DWGLRAIKSV	LVVAGSLKRG	DPDRPEDQVL	MRSLRDFNIP
2090	2100	2110	2120	2130	2140	2150	2160
KIVTDDMPIF	MGLIGDLFPA	LDVPRRRDPN	FEALVRKAIV	DLKLQAEDNF	VLKVVQLEEL	LAVRHSVFVV	GGAGTGKSQV
2170	2180	2190	2200	2210	2220	2230	2240

Detailed Protein Report

Cmpd.	No. of Cmpds.	m/z meas.	Δ m/z [ppm]	z	Rt [min]	Score	P	Range	Sequence	Modification
106	1	1068.1367	75.32	2	30.1	13.8	2	182-201	K.GKTLLPLPAGSEKMEFADSK.S	Oxidation: 14
2873	2	731.8707	-28.75	2	66.4	25.7	1	1075-1086	R.LEPIKVFDGWMK.I	
54	1	796.7965	-120.13	2	30.4	11.9	0	3448-3461	R.MSVENATILINCER.W	
1679	7	648.2912	-180.05	2	51.3	17.9	0	4475-4486	K.WVLAGVALLLQI.-	

Detailed Protein Report

Protein 79: centlein isoform 1 [Homo sapiens]

Accession: gi|110815815
Database: refseq_human(refseq_human_20140103.fasta)
Database Date: 2015-11-30

Score: 84.8
MW [kDa]: 162.1
pI: 8.8
Sequence Coverage [%]: 5.2
No. of unique Peptides: 5

Quantitation

Q:W	**Median:**	3.20	**CV:**	59.19 %	**No. of Peptides:**	2
M:Q	**Median:**	0.70	**CV:**	27.62 %	**No. of Peptides:**	3

```
        10         20         30         40         50         60         70         80
MAARSPPSPH PSPPARQLGP RSPRVGRGAE VHAMRSEASG FAGAAREVVA DESDKIWVGE EGSGGRRGPG GAAPAHAPLL
        90        100        110        120        130        140        150        160
SAPMGSRRLE GISVEEAMVT RTQLLEEELS SLKEELALCQ ADKEFVWSLW KRLQVTNPDL TQVVSLVVER EKQKSEAKDR
       170        180        190        200        210        220        230        240
KVLEILQVKD AKIQEFEQRE SVLKQEINDL VKRKIAVDEE NAFLRKEFSD LEKKFKDKSQ EIKDTKECVQ NKEEQNRLVI
       250        260        270        280        290        300        310        320
KNLEEENKKL STRCTDLLND LEKLRKQEAH LRKEKYSTDA KIKTFEDNLI EARKEVEVSQ SKYNALSLQL SNKQTELIQK
       330        340        350        360        370        380        390        400
DMDITLVRKE LQELQNLYKQ NSTHTAQQAE LIQQLQVLNM DTQKVLRNQE DVHTAESISY QKLYNELHIC FETTKSNEAM
       410        420        430        440        450        460        470        480
LRQSVTNLQD QLLQKEQENA KLKEKLQESQ GAPLPLPQES DPDYSAQVPH RPSLSSLETL MVSQKSEIEY LQEKLKIANE
       490        500        510        520        530        540        550        560
KLSENISANK GFSRKSIMTS AEGKHKEPPV KRSRSLSPKS SFTDSEELQK LRKAERKIEN LEKALQLKSQ ENDELRDAHE
       570        580        590        600        610        620        630        640
KRKERLQMLQ TNYRAVKEQL KQWEEGSGMT EIRKIKRADP QQLRQEDSDA VWNELAYFKR ENQELMIQKM NLEEELDELK
       650        660        670        680        690        700        710        720
VHISIDKAAI QELNRCVAER REEQLFRSGE DDEVKRSTPE KNGKEMLEQT LQKVTELENR LKSFEKRSRK LKEGNKKLMK
       730        740        750        760        770        780        790        800
ENDFLKSLLK QQQEDTETRE KELEQIIKGS KDVEKENTEL QVKISELETE VTSLRRQVAE ANALRNENEE LINPMEKSHQ
       810        820        830        840        850        860        870        880
SADRAKSEMA TMKVRSGRYD CKTTMTKVKF KAAKKNCSVG RHHTVLNHSI KVMSNVFENL SKDGWEDVSE SSSDSEAQTS
       890        900        910        920        930        940        950        960
QTLGTIIVET SQKISPTEDG KDQKESDPTE DSQTQGKEIV QTYLNIDGKT PKDYFHDKNA KKPTFQKKNC KMQKSSHTAV
       970        980        990       1000       1010       1020       1030       1040
PTRVNREKYK NITAQKSSSN IILLRERIIS LQQQNSVLQN AKKTAELSVK EYKEVNEKLL HQQQVSDQRF QTSRQTIKKL
      1050       1060       1070       1080       1090       1100       1110       1120
NLDLAGLRKE KEDLLKKLES SSEITSLAEE NSQVTFPRIQ VTSLSPSRSM DLEMKQLQYK LKNATNELTK QSSNVKTLKF
      1130       1140       1150       1160       1170       1180       1190       1200
ELLAKEEHIK EMHEKISRME RDITMKRHLI EDLKFRQKVN LESNKSFSEM LQNLDKKVKT LTEECSNKKV SIDSLKQRLN
      1210       1220       1230       1240       1250       1260       1270       1280
VAVKEKSQYE QMYQKSKEEL EKKDLKLTLL VSRISETESA MAEIETAASK QLQELALQSE QVLEGAQKTL LLANEKVEEF
      1290       1300       1310       1320       1330       1340       1350       1360
TTFVKALAKE LQNDVHVVRR QIRELKKMKK NRDACKTSTH KAQTLAASIL NISRSDLEEI LDTEDQVEIE KTKIDAENDK
      1370       1380       1390       1400       1410
EWMLYIQKLL EGQLPFASYL LEAVLEKINE KKKLVEGYFT IMKDIR
```

Cmpd.	No. of Cmpds.	m/z meas.	Δ m/z [ppm]	z	Rt [min]	Score	P	Range	Sequence	Modification	Ratios
255	1	795.3881	-39.33	2	32.4	10.8	1	88-101	R.RLEGISVEEAMVTR.T		M:Q 0.48<br>Q:W 1.85
848	1	512.1520	-290.70	2	40.7	15.4	1	1147-1154	K.RHLIEDLK.F		M:Q 0.86<br>Q:W 5.54
1953	1	643.3047	-169.45	1	54.5	11.9	0	1199-1204	R.LNVAVK.E		
2736	1	683.3656	57.65	3	65.5	15.2	2	1207-1222	K.SQYEQMYQKSKEELEK.K		
562	1	884.4457	29.69	2	37.0	19.0	0	1234-	R.ISETESAMAEIETAASK.Q		M:Q 0.84

Detailed Protein Report

Cmpd.	No. of Cmpds.	m/z meas.	Δ m/z [ppm]	z	Rt [min]	Score	P	Range	Sequence	Modification	Ratios
								1250			

Detailed Protein Report

Protein 80: sacsin isoform 1 [Homo sapiens]

**Accession:**	gi|163659918	**Score:**	84.7
**Database:**	refseq_human(refseq_human_20140103.fasta)	**MW [kDa]:**	520.8
**Database Date:**	2015-11-30	**pI:**	6.7
**Modification(s):**	Carbamidomethyl	**Sequence Coverage [%]:**	2.2
		**No. of unique Peptides:**	6

Quantitation

Q:W	**Median:**	0.53	**CV:**	46.51 %	**No. of Peptides:**	2
M:Q	**Median:**	2.74	**CV:**	0.00 %	**No. of Peptides:**	1

Detailed Protein Report

10	20	30	40	50	60	70	80
METKENRWVP	VTVLPGCVGC	RTVAALASWT	VRDVKERIFA	ETGFPVSEQR	LWRGGRELSD	WIKIGDLTSK	NCHLFVNLQS
90	100	110	120	130	140	150	160
KGLKGGGRFG	QTTPPLVDFL	KDILRRYPEG	GQILKELIQN	AEDAGATEVK	FLYDETQYGT	ETLWSKDMAP	YQGPALYVYN
170	180	190	200	210	220	230	240
NAVFTPEDWH	GIQEIARSRK	KDDPLKVGRF	GIGFNSVYHI	TDVPCIFSGD	QIGMLDPHQT	LFGPHESGQC	WNLKDDSKEI
250	260	270	280	290	300	310	320
SELSDQFAPF	VGIFGSTKET	FINGNFPGTF	FRFPLRLQPS	QLSSNLYNKQ	KVLELFESFR	ADADTVLLFL	KSVQDVSLYV
330	340	350	360	370	380	390	400
READGTEKLV	FRVTSSESKA	LKHERPNSIK	ILGTAISNYC	KKTPSNNITC	VTYHVNIVLE	EESTKDAQKT	SWLVCNSVGG
410	420	430	440	450	460	470	480
RGISSKLDSL	ADELKFVPII	GIAMPLSSRD	DEAKGATSDF	SGKAFCFLPL	PPGEESSTGL	PVHISGFFGL	TDNRRSIKWR
490	500	510	520	530	540	550	560
ELDQWRDPAA	LWNEFLVMNV	VPKAYATLIL	DSIKRLEMEK	SSDFPLSVDV	IYKLWPEASK	VKVHWQPVLE	PLFSELLQNA
570	580	590	600	610	620	630	640
VIYSISCDWV	RLEQVYFSEL	DENLEYTKTV	LNYLQSSGKQ	IAKVPGNVDA	AVQLTAASGT	TPVRKVTPAW	VRQVLRKCAH
650	660	670	680	690	700	710	720
LGCAEEKLHL	LEFVLSDQAY	SELLGLELLP	LQNGNFVPFS	SSVSDQDVIY	ITSAEYPRSL	FPSLEGRFIL	DNLKPHLVAA
730	740	750	760	770	780	790	800
LKEAAQTRGR	PCTQLQLLNP	ERFARLIKEV	MNTFWPGREL	IVQWYPFDEN	RNHPSVSWLK	MVWKNLYIHF	SEDLTLFDEM
810	820	830	840	850	860	870	880
PLIPRTILEE	GQTCVELIRL	RIPSLVILDD	ESEAQLPEFL	ADIVQKLGGF	VLKKLDASIQ	HPLIKKYIHS	PLPSAVLQIM
890	900	910	920	930	940	950	960
EKMPLQKLCN	QITSLLPTHK	DALRKFLASL	TDSSEKEKRI	IQELAIFKRI	NHSSDQGISS	YTKLKGCKVL	HHTAKLPADL
970	980	990	1000	1010	1020	1030	1040
RLSISVIDSS	DEATIRLANM	LKIEQLKTTS	CLKLVLKDIE	NAFYSHEEVT	QLMLWVLENL	SSLKNENPNV	LEWLTPLKFI
1050	1060	1070	1080	1090	1100	1110	1120
QISQEQMVSA	GELFDPDIEV	LKDLFCNEEG	TYFPPSVFTS	PDILHSLRQI	GLKNEASLKE	KDVVQVAKKI	EALQVGACPD
1130	1140	1150	1160	1170	1180	1190	1200
QDVLLKKAKT	LLLVLNKNHT	LLQSSEGKMT	LKKIKWVPAC	KERPPNYPGS	LVWKGDLCNL	CAPPDMCDVG	HAILIGSSLP
1210	1220	1230	1240	1250	1260	1270	1280
LVESIHVNLE	KALGIFTKPS	LSAVLKHFKI	VVDWYSSKTF	SDEDYYQFQH	ILLEIYGFMH	DHLNEGKDSF	RALKFPWVWT
1290	1300	1310	1320	1330	1340	1350	1360
GKKFCPLAQA	VIKPIHDLDL	QPYLHNVPKT	MAKFHQLFKV	CGSIEELTSD	HISMVIQKIY	LKSDQDLSEQ	ESKQNLHLML
1370	1380	1390	1400	1410	1420	1430	1440
NIIRWLYSNQ	IPASPNTPVP	IHHSKNPSKL	IMKPIHECCY	CDIKVDDLND	LLEDSVEPII	LVHEDIPMKT	AEWLKVPCLS
1450	1460	1470	1480	1490	1500	1510	1520
TRLINPENMG	FEQSGQREPL	TVRIKNILEE	YPSVSDIFKE	LLQNADDANA	TECSFLIDMR	RNMDIRENLL	DPGMAACHGP
1530	1540	1550	1560	1570	1580	1590	1600
ALWSFNNSQF	SDSDFVNITR	LGESLKRGEV	DKVGKFGLGF	NSVYHITDIP	IIMSREFMIM	FDPNINHISK	HIKDKSNPGI
1610	1620	1630	1640	1650	1660	1670	1680
KINWSKQQKR	LRKFPNQFKP	FIDVFGCQLP	LTVEAPYSYN	GTLFRLSFRT	QQEAKVSEVS	STCYNTADIY	SLVDEFSLCG
1690	1700	1710	1720	1730	1740	1750	1760
HRLIIFTQSV	KSMYLKYLKI	EETNPSLAQD	TVIIKKKSCS	SKALNTPVLS	VLKEAAKLMK	TCSSSNKKLP	SDEPKSSCIL
1770	1780	1790	1800	1810	1820	1830	1840
QITVEEFHHV	FRRIADLQSP	LFRGPDDDPA	ALFEMAKSGQ	SKKPSDELSQ	KTVECTTWLL	CTCMDTGEAL	KFSLSESGRR
1850	1860	1870	1880	1890	1900	1910	1920
LGLVPCGAVG	VQLSEIQDQK	WTVKPHIGEV	FCYLPLRIKT	GLPVHINGCF	AVTSNRKEIW	KTDTKGRWNT	TFMRHVIVKA
1930	1940	1950	1960	1970	1980	1990	2000
YLQVLSVLRD	LATSGELMDY	TYYAVWPDPD	LVHDDFSVIC	QGFYEDIAHG	KGKELTKVFS	DGSTWVSMKN	VRFLDDSILK
2010	2020	2030	2040	2050	2060	2070	2080
RRDVGSAAFK	IFLKYLKKTG	SKNLCAVELP	SSVKLGFEEA	GCKQILLENT	FSEKQFFSEV	FFPNIQEIEA	ELRDPLMIFV
2090	2100	2110	2120	2130	2140	2150	2160
LNEKVDEFSG	VLRVTPCIPC	SLEGHPLVLP	SRLIHPEGRV	AKLFDIKDGR	FPYGSTQDYL	NPIILIKLVQ	LGMAKDDILW
2170	2180	2190	2200	2210	2220	2230	2240

Detailed Protein Report

Cmpd.	No. of Cmpds.	m/z meas.	Δ m/z [ppm]	z	Rt [min]	Score	P	Range	Sequence	Modification	Ratios
1798	1	916.4469	-78.82	2	50.8	10.5	2	1717-1733	K.KSCSSKALNTPVLSVLK.E	Carbamidomethyl: 3	
2078	1	1029.6855	128.38	2	56.1	10.5	0	1861-1877	K.WTVKPHIGEVFCYLPLR.I		
71	1	845.3572	-104.63	2	30.3	12.1	1	2019-2034	K.TGSKNLCAVELPSSVK.L	Carbamidomethyl: 7	
2171	1	1022.8841	-140.49	2	57.3	14.3	2	2478-2495	K.DTTVKYCHADIPREVAVK.L		
1779	1	516.2947	105.86	2	50.6	10.7	0	2483-2490	K.YCHADIPR.E	Carbamidomethyl: 2	M:Q 2.74<br>Q:W 0.34
362	1	529.2876	-26.74	3	34.5	12.9	2	4494-4508	R.GKSDKDVKPTALAQK.I		Q:W 0.82

Detailed Protein Report

Protein 81: histone-lysine N-methyltransferase, H3 lysine-36 and H4 lysine-20 specific isoform b [Homo sapiens]

**Accession:**	gi|19923586	**Score:**	84.6
**Database:**	refseq_human(refseq_human_20140103.fasta)	**MW [kDa]:**	296.5
**Database Date:**	2015-11-30	**pI:**	9.5
**Modification(s):**	Carbamidomethyl	**Sequence Coverage [%]:**	1.8
		**No. of unique Peptides:**	4

Quantitation

Q:W	**Median:**	1.43	**CV:**	34.59 %	**No. of Peptides:**	3
M:Q	**Median:**	0.49	**CV:**	0.00 %	**No. of Peptides:**	1

Alias proteins:

Accession	Name	Description
gi|578811198	refseq_human (refseq_human_20140103.fasta)	PREDICTED: histone-lysine N-methyltransferase, H3 lysine-36 and H4 lysine-20 specific isoform X5 [Homo sapiens]
gi|530381144	refseq_human (refseq_human_20140103.fasta)	PREDICTED: histone-lysine N-methyltransferase, H3 lysine-36 and H4 lysine-20 specific isoform X1 [Homo sapiens]

Detailed Protein Report

10	20	30	40	50	60	70	80
MDQTCELPRR	NCLLPFSNPV	NLDAPEDKDS	PFGNGQSNFS	EPLNGCTMQL	STVSGTSQNA	YGQDSPSCYI	PLRRLQDLAS
90	100	110	120	130	140	150	160
MINVEYLNGS	ADGSESFQDP	EKSDSRAQTP	IVCTSLSPGG	PTALAMKQEP	SCNNSPELQV	KVTKTIKNGF	LHFENFTCVD
170	180	190	200	210	220	230	240
DADVDSEMDP	EQPVTEDESI	EEIFEETQTN	ATCNYETKSE	NGVKVAMGSE	QDSTPESRHG	AVKSPFLPLA	PQTETQKNKQ
250	260	270	280	290	300	310	320
RNEVDGSNEK	AALLPAPFSL	GDTNITIEEQ	LNSINLSFQD	DPDSSTSTLG	NMLELPGTSS	SSTSQELPFC	QPKKKSTPLK
330	340	350	360	370	380	390	400
YEVGDLIWAK	FKRRPWWPCR	ICSDPLINTH	SKMKVSNRRP	YRQYYVEAFG	DPSERAWVAG	KAIVMFEGRH	QFEELPVLRR
410	420	430	440	450	460	470	480
RGKQKEKGYR	HKVPQKILSK	WEASVGLAEQ	YDVPKGSKNR	KCIPGSIKLD	SEEDMPFEDC	TNDPESEHDL	LLNGCLKSLA
490	500	510	520	530	540	550	560
FDSEHSADEK	EKPCAKSRAR	KSSDNPKRTS	VKKGHIQFEA	HKDERRGKIP	ENLGLNFISG	DISDTQASNE	LSRIANSLTG
570	580	590	600	610	620	630	640
SNTAPGSFLF	SSCGKNTAKK	EFETSNGDSL	LGLPEGALIS	KCSREKNKPQ	RSLVCGSKVK	LCYIGAGDEE	KRSDSISICT
650	660	670	680	690	700	710	720
TSDDGSSDLD	PIEHSSESDN	SVLEIPDAFD	RTENMLSMQK	NEKIKYSRFA	ATNTRVKAKQ	KPLISNSHTD	HLMGCTKSAE
730	740	750	760	770	780	790	800
PGTETSQVNL	SDLKASTLVH	KPQSDFTNDA	LSPKFNLSSS	ISSENSLIKG	GAANQALLHS	KSKQPKFRSI	KCKHKENPVM
810	820	830	840	850	860	870	880
AEPPVINEEC	SLKCCSSDTK	GSPLASISKS	GKVDGLKLLN	NMHEKTRDSS	DIETAVVKHV	LSELKELSYR	SLGEDVSDSG
890	900	910	920	930	940	950	960
TSKPSKPLLF	SSASSQNHIP	IEPDYKFSTL	LMMLKDMHDS	KTKEQRLMTA	QNLVSYRSPG	RGDCSTNSPV	GVSKVLVSGG
970	980	990	1000	1010	1020	1030	1040
STHNSEKKGD	GTQNSANPSP	SGGDSALSGE	LSASLPGLLS	DKRDLPASGK	SRSDCVTRRN	CGRSKPSSKL	RDAFSAQMVK
1050	1060	1070	1080	1090	1100	1110	1120
NTVNRKALKT	ERKRKLNQLP	SVTLDAVLQG	DRERGGSLRG	GAEDPSKEDP	LQIMGHLTSE	DGDHFSDVHF	DSKVKQSDPG
1130	1140	1150	1160	1170	1180	1190	1200
KISEKGLSFE	NGKGPELDSV	MNSENDELNG	VNQVVPKKRW	QRLNQRRTKP	RKRMNRFKEK	ENSECAFRVL	LPSDPVQEGR
1210	1220	1230	1240	1250	1260	1270	1280
DEFPEHRTPS	ASILEEPLTE	QNHADCLDSA	GPRLNVCDKS	SASIGDMEKE	PGIPSLTPQA	ELPEPAVRSE	KKRLRKPSKW
1290	1300	1310	1320	1330	1340	1350	1360
LLEYTEEYDQ	IFAPKKKQKK	VQEQVHKVSS	RCEEESLLAR	GRSSAQNKQV	DENSLISTKE	EPPVLEREAP	FLEGPLAQSE
1370	1380	1390	1400	1410	1420	1430	1440
LGGGHAELPQ	LTLSVPVAPE	VSPRPALESE	ELLVKTPGNY	ESKRQRKPTK	KLLESNDLDP	GFMPKKGDLG	LSKKCYEAGH
1450	1460	1470	1480	1490	1500	1510	1520
LENGITESCA	TSYSKDFGGG	TTKIFDKPRK	RKRQRHAAAK	MQCKKVKNDD	SSKEIPGSEG	ELMPHRTATS	PKETVEEGVE
1530	1540	1550	1560	1570	1580	1590	1600
HDPGMPASKK	MQGERGGGAA	LKENVCQNCE	KLGELLLCEA	QCCGAFHLEC	LGLTEMPRGK	FICNECRTGI	HTCFVCKQSG
1610	1620	1630	1640	1650	1660	1670	1680
EDVKRCLLPL	CGKFYHEECV	QKYPPTVMQN	KGFRCSLHIC	ITCHAANPAN	VSASKGRLMR	CVRCPVAYHA	NDFCLAAGSK
1690	1700	1710	1720	1730	1740	1750	1760
ILASNSIICP	NHFTPRRGCR	NHEHVNVSWC	FVCSEGGSLL	CCDSCPAAFH	RECLNIDIPE	GNWYCNDCKA	GKKPHYREIV
1770	1780	1790	1800	1810	1820	1830	1840
WVKVGRYRWW	PAEICHPRAV	PSNIDKMRHD	VGEFPVLFFG	SNDYLWTHQA	RVFPYMEGDV	SSKDKMGKGV	DGTYKKALQE
1850	1860	1870	1880	1890	1900	1910	1920
AAARFEELKA	QKELRQLQED	RKNDKKPPPY	KHIKVNRPIG	RVQIFTADLS	EIPRCNCKAT	DENPCGIDSE	CINRMLLYEC
1930	1940	1950	1960	1970	1980	1990	2000
HPTVCPAGGR	CQNQCFSKRQ	YPEVEIFRTL	QRGWGLRTKT	DIKKGEFVNE	YVGELIDEEE	CRARIRYAQE	HDITNFYMLT
2010	2020	2030	2040	2050	2060	2070	2080
LDKDRIIDAG	PKGNYARFMN	HCCQPNCETQ	KWSVNGDTRV	GLFALSDIKA	GTELTFNYNL	ECLGNGKTVC	KCGAPNCSGF
2090	2100	2110	2120	2130	2140	2150	2160
LGVRPKNQPI	ATEEKSKKFK	KKQQGKRRTQ	GEITKEREDE	CFSCGDAGQL	VSCKKPGCPK	VYHADCLNLT	KRPAGKWECP
2170	2180	2190	2200	2210	2220	2230	2240

Detailed Protein Report

Cmpd.	No. of Cmpds.	m/z meas.	Δ m/z [ppm]	z	Rt [min]	Score	P	Range	Sequence	Modification	Ratios
1121	3	509.6492	-167.81	2	42.3	11.4	0	2-9	M.DQTCELPR.R	Carbamidomethyl: 4	Q:W 1.85
2073	1	902.2674	-271.88	1	56.1	16.1	1	441-448	R.KCIPGSIK.L	Carbamidomethyl: 2	Q:W 0.89
617	1	459.6161	-280.24	2	37.0	16.5	1	491-498	K.EKPCAKSR.A		M:Q 0.49<br>Q:W 1.77
330	1	539.2345	-72.41	2	32.8	12.9	0	1623-1631	K.YPPTVMQNK.G		

Detailed Protein Report

Protein 82: complement C3 precursor [Homo sapiens]

Accession: gi|115298678
Database: refseq_human(refseq_human_20140103.fasta)
Database Date: 2015-11-30
Modification(s): Carbamidomethyl

Score: 84.4
MW [kDa]: 187.0
pI: 6.0
Sequence Coverage [%]: 3.5
No. of unique Peptides: 4

Quantitation

Q:W	**Median:** 2.35	**CV:** 0.00 %	**No. of Peptides:**	1
M:Q	**Median:** 0.57	**CV:** 3.65 %	**No. of Peptides:**	2

10	20	30	40	50	60	70	80
MGPTSGPSLL	LLLLTHLPLA	LGSPMYSIIT	PNILRLESEE	TMVLEAHDAQ	GDVPVTVTVH	DFPGKKLVLS	SEKTVLTPAT
90	100	110	120	130	140	150	160
NHMGNVTFTI	PANREFKSEK	GRNKFVTVQA	TFGTQVVEKV	VLVSLQSGYL	FIQTDKTIYT	PGSTVLYRIF	TVNHKLLPVG
170	180	190	200	210	220	230	240
RTVMVNIENP	EGIPVKQDSL	SSQNQLGVLP	LSWDIPELVN	MGQWKIRAYY	ENSPQQVFST	EFEVKEYVLP	SFEVIVEPTE
250	260	270	280	290	300	310	320
KFYYIYNEKG	LEVTITARFL	YGKKVEGTAF	VIFGIQDGEQ	RISLPESLKR	IPIEDGSGEV	VLSRKVLLDG	VQNPRAEDLV
330	340	350	360	370	380	390	400
GKSLYVSATV	ILHSGSDMVQ	AERSGIPIVT	SPYQIHFTKT	PKYFKPGMPF	DLMVFVTNPD	GSPAYRVPVA	VQGEDTVQSL
410	420	430	440	450	460	470	480
TQGDGVAKLS	INTHPSQKPL	SITVRTKKQE	LSEAEQATRT	MQALPYSTVG	NSNNYLHLSV	LRTELRPGET	LNVNFLLRMD
490	500	510	520	530	540	550	560
RAHEAKIRYY	TYLIMNKGRL	LKAGRQVREP	GQDLVVLPLS	ITTDFIPSFR	LVAYYTLIGA	SGQREVVADS	VWVDVKDSCV
570	580	590	600	610	620	630	640
GSLVVKSGQS	EDRQPVPGQQ	MTLKIEGDHG	ARVVLVAVDK	GVFVLNKKNK	LTQSKIWDVV	EKADIGCTPG	SGKDYAGVFS
650	660	670	680	690	700	710	720
DAGLTFTSSS	GQQTAQRAEL	QCPQPAARRR	RSVQLTEKRM	DKVGKYPKEL	RKCCEDGMRE	NPMRFSCQRR	TRFISLGEAC
730	740	750	760	770	780	790	800
KKVFLDCCNY	ITELRRQHAR	ASHLGLARSN	LDEDIIAEEN	IVSRSEFPES	WLWNVEDLKE	PPKNGISTKL	MNIFLKDSIT
810	820	830	840	850	860	870	880
TWEILAVSMS	DKKGICVADP	FEVTVMQDFF	IDLRLPYSVV	RNEQVEIRAV	LYNYRQNQEL	KVRVELLHNP	AFCSLATTKR
890	900	910	920	930	940	950	960
RHQQTVTIPP	KSSLSVPYVI	VPLKTGLQEV	EVKAAVYHHF	ISDGVRKSLK	VVPEGIRMNK	TVAVRTLDPE	RLGREGVQKE
970	980	990	1000	1010	1020	1030	1040
DIPPADLSDQ	VPDTESETRI	LLQGTPVAQM	TEDAVDAERL	KHLIVTPSGC	GEQNMIGMTP	TVIAVHYLDE	TEQWEKFGLE
1050	1060	1070	1080	1090	1100	1110	1120
KRQGALELIK	KGYTQQLAFR	QPSSAFAAFV	KRAPSTWLTA	YVVKVFSLAV	NLIAIDSQVL	CGAVKWLILE	KQKPDGVFQE
1130	1140	1150	1160	1170	1180	1190	1200
DAPVIHQEMI	GGLRNNNEKD	MALTAFVLIS	LQEAKDICEE	QVNSLPGSIT	KAGDFLEANY	MNLQRSYTVA	IAGYALAQMG
1210	1220	1230	1240	1250	1260	1270	1280
RLKGPLLNKF	LTTAKDKNRW	EDPGKQLYNV	EATSYALLAL	LQLKDFDFVP	PVVRWLNEQR	YYGGGYGSTQ	ATFMVFQALA
1290	1300	1310	1320	1330	1340	1350	1360
QYQKDAPDHQ	ELNLDVSLQL	PSRSSKITHR	IHWESASLLR	SEETKENEGF	TVTAEGKGQG	TLSVVTMYHA	KAKDQLTCNK
1370	1380	1390	1400	1410	1420	1430	1440
FDLKVTIKPA	PETEKRPQDA	KNTMILEICT	RYRGDQDATM	SILDISMMTG	FAPDTDDLKQ	LANGVDRYIS	KYELDKAFSD
1450	1460	1470	1480	1490	1500	1510	1520
RNTLIIYLDK	VSHSEDDCLA	FKVHQYFNVE	LIQPGAVKVY	AYYNLEESCT	RFYHPEKEDG	KLNKLCRDEL	CRCAEENCFI
1530	1540	1550	1560	1570	1580	1590	1600
QKSDDKVTLE	ERLDKACEPG	VDYVYKTRLV	KVQLSNDFDE	YIMAIEQTIK	SGSDEVQVGQ	QRTFISPIKC	REALKLEEKK
1610	1620	1630	1640	1650	1660	1670	
HYLMWGLSSD	FWGEKPNLSY	IIGKDTWVEH	WPEEDECQDE	ENQKQCQDLG	AFTESMVVFG	CPN	

Cmpd.	No. of Cmpds.	m/z meas.	Δ m/z [ppm]	z	Rt [min]	Score	P	Range	Sequence	Modification	Ratios

Detailed Protein Report

Cmpd.	No. of Cmpds.	m/z meas.	Δ m/z [ppm]	z	Rt [min]	Score	P	Range	Sequence	Modification	Ratios
2933	1	940.0026	13.02	2	65.4	22.8	0	226-241	K.EYVLPSFEVIVEPTEK.F		M:Q 0.55<br>Q:W 2.35
2396	6	701.3860	-50.24	2	59.8	21.6	0	892-904	K.SSLSVPYVIVPLK.T		M:Q 0.59
1782	5	1086.9427	-40.31	2	50.9	20.1	2	1505-1522	K.LCRDELCRCAEENCFIQK.S		
192	1	650.8121	22.36	2	31.2	19.9	0	1536-1546	K.ACEPGVDYVYK.T	Carbamidomethyl: 2	

Detailed Protein Report

Protein 83: PREDICTED: ceruloplasmin isoform X3 [Homo sapiens]

**Accession:**	gi|578807065	**Score:**	84.1
**Database:**	refseq_human(refseq_human_20140103.fasta)	**MW [kDa]:**	122.1
**Database Date:**	2015-11-30	**pI:**	5.4
**Modification(s):**	Carbamidomethyl	**Sequence Coverage [%]:**	2.6
		**No. of unique Peptides:**	2

Quantitation

Q:W	**Median:**	1.27	**CV:**	0.00 %	**No. of Peptides:**	1
M:Q	**Median:**	1.50	**CV:**	0.00 %	**No. of Peptides:**	1

10	20	30	40	50	60	70	80
MKILILGIFL	FLCSTPAWAK	EKHYYIGIIE	TTWDYASDHG	EKKLISVDTE	HSNIYLQNGP	DRIGRLYKKA	LYLQYTDETF
90	100	110	120	130	140	150	160
RTTIEKPVWL	GFLGPIIKAE	TGDKVYVHLK	NLASRPYTFH	SHGITYYKEH	EGAIYPDNTT	DFQRADDKVY	PGEQYTYMLL
170	180	190	200	210	220	230	240
ATEEQSPGEG	DGNCVTRIYH	SHIDAPKDIA	SGLIGPLIIC	KKDSLDKEKE	KHIDREFVVM	FSVVDENFSW	YLEDNIKTYC
250	260	270	280	290	300	310	320
SEPEKVDKDN	EDFQESNRMY	SVNGYTFGSL	PGLSMCAEDR	VKWYLFGMGN	EVDVHAAFFH	GQALTNKNYR	IDTINLFPAT
330	340	350	360	370	380	390	400
LFDAYMVAQN	PGEWMLSCQN	LNHLKAGLQA	FFQVQECNKS	SSKDNIRGKH	VRHYYIAAEE	IIWNYAPSGI	DIFTKENLTA
410	420	430	440	450	460	470	480
PGSDSAVFFE	QGTTRIGGSY	KKLVYREYTD	ASFTNRKERG	PEEEHLGILG	PVIWAEVGDT	IRVTFHNKGA	YPLSIEPIGV
490	500	510	520	530	540	550	560
RFNKNNEGTY	YSPNYNPQSR	SVPPSASHVA	PTETFTYEWT	VPKEVGPTNA	DPVCLAKMYY	SAVEPTKDIF	TGLIGPMKIC
570	580	590	600	610	620	630	640
KKGSLHANGR	QKDVDKEFYL	FPTVFDENES	LLLEDNIRMF	TTAPDQVDKE	DEDFQESNKM	HSMNGFMYGN	QPGLTMCKGD
650	660	670	680	690	700	710	720
SVVWYLFSAG	NEADVHGIYF	SGNTYLWRGE	RRDTANLFPQ	TSLTLHMWPD	TEGTFNVECL	TTDHYTGGMK	QKYTVNQCRR
730	740	750	760	770	780	790	800
QSEDSTFYLG	ERTYYIAAVE	VEWDYSPQRE	WEKELHHLQE	QNVSNAFLDK	GEFYIGSKYK	KVVYRQYTDS	TFRVPVERKA
810	820	830	840	850	860	870	880
EEEHLGILGP	QLHADVGDKV	KIIFKNMATR	PYSIHAHGVQ	TESSTVTPTL	PGETLTYVWK	IPERSGAGTE	DSACIPWAYY
890	900	910	920	930	940	950	960
STVDQVKDLY	SGLIGPLIVC	RRPYLKVFNP	RRKLEFALLF	LVFDENESWY	LDDNIKTYSD	HPEKVNKDDE	EFIESNKMHA
970	980	990	1000	1010	1020	1030	1040
INGRMFGNLQ	GLTMHVGDEV	NWYLMGMGNE	IDLHTVHFHG	HSFQYKHRGV	YSSDVFDIFP	GTYQTLEMFP	RTPGIWLLHC
1050	1060	1070					
HVTDHIHAGM	ETTYTVLQNE	GGTSM					

Cmpd.	No. of Cmpds.	m/z meas.	Δ m/z [ppm]	z	Rt [min]	Score	P	Range	Sequence	Modification	Ratios
2949	6	735.4039	-26.64	2	65.6	54.0	0	188-201	K.DIASGLIGPLIICK.K	Carbamidomethyl: 13	M:Q 1.50<br>Q:W 1.27
2789	2	788.4137	-22.96	2	65.3	30.2	0	888-901	K.DLYSGLIGPLIVCR.R	Carbamidomethyl: 13	

Detailed Protein Report

Protein 84: PREDICTED: zinc finger protein 737 isoform X3 [Homo sapiens]

**Accession:**	gi|530414417	**Score:**	83.8
**Database:**	refseq_human(refseq_human_20140103.fasta)	**MW [kDa]:**	60.4
**Database Date:**	2015-11-30	**pI:**	10.4
**Modification(s):**	Carbamidomethyl	**Sequence Coverage [%]:**	9.6
		**No. of unique Peptides:**	3

10	20	30	40	50	60	70	80
MGPLQFRDVA	IEFSLEEWHC	LDTAQRNLYR	NVMLENYRNL	VFLGIVVSKP	DLITCLEQGK	KPLTMKKHEM	VANPSVTCSH
**90**	**100**	**110**	**120**	**130**	**140**	**150**	**160**
FARDLWPEQS	IKDSFQKVTL	RRYENYGHDN	LQFKKGCESV	DECKVHKRGY	NGLNQYLTTT	QSKIFQCDKY	VKVIHKFSNS
**170**	**180**	**190**	**200**	**210**	**220**	**230**	**240**
NRHKIRHTGK	KPFKCIECGK	AFNQSSTLTT	HKKIHTGEKP	FKCEECGKAF	NWSSHLTTHK	RIHTGEKRYK	CEDCGKAFSR
**250**	**260**	**270**	**280**	**290**	**300**	**310**	**320**
FSYLTAHKII	HSGEKPYKCE	ECGKAFKRSS	ILTAHKIIHS	GEKPYKCEEC	GKAFKHPSVL	TTHKRIHTGE	KPYKCEECGR
**330**	**340**	**350**	**360**	**370**	**380**	**390**	**400**
AFKYFSSLTT	HKIIHSGEKP	YKCEECGKAF	NWSSHLTTHK	RIHTGEKPYK	CEECGEAFKY	SSSLTTHKII	HTGQQPFKCE
**410**	**420**	**430**	**440**	**450**	**460**	**470**	**480**
ECGKAFKCFS	ILTTHKRIHT	GEKPYKCEEC	GKAFNSSSHL	TAHKRIHTGE	KPYKCERCGK	AFKRSFILTR	HKRIHTGEKP
**490**	**500**	**510**	**520**	**530**			
YKCEECGKGF	KCPSTLTTHK	RSGCPTRKLL	LSSVSTLNNS	EWK			

Cmpd.	No. of Cmpds.	m/z meas.	Δ m/z [ppm]	z	Rt [min]	Score	P	Range	Sequence	Modification
2919	1	981.9597	-7.66	2	65.7	10.8	2	305-320	K.RIHTGEKPYKCEECGR.A	Carbamidomethyl: 11
2022	1	1051.4074	-85.21	2	53.9	10.6	2	399-416	K.CEECGKAFKCFSILTTHK.R	Carbamidomethyl: 10
2081	4	1051.4297	-64.07	2	54.6	15.9	2	399-416	K.CEECGKAFKCFSILTTHK.R	Carbamidomethyl: 1

Detailed Protein Report

Protein 85: PREDICTED: dynein heavy chain 10, axonemal isoform X1 [Homo sapiens]

**Accession:**	gi|530401632	**Score:**	83.7
**Database:**	refseq_human(refseq_human_20140103.fasta)	**MW [kDa]:**	528.4
**Database Date:**	2015-11-30	**pI:**	5.5
**Modification(s):**	Oxidation	**Sequence Coverage [%]:**	1.7
		**No. of unique Peptides:**	6

Quantitation

Q:W	**Median:**	1.06	**CV:**	52.16 %	**No. of Peptides:**	2
M:Q	**Median:**	0.97	**CV:**	0.00 %	**No. of Peptides:**	1

Detailed Protein Report

10	20	30	40	50	60	70	80
MDDLRVLWMR	DRVYAAFGIT	DPQLFEDLLN	RDDGQGEDLI	LHFLNQASEE	EGPSALFIYR	TMVPEEVEVE	IDEIPVLSEE
90	100	110	120	130	140	150	160
GEEEEETYSQ	KVESVDKVRA	KRVSLRTESL	GQPLNREDEE	MDKEISEKLP	SKRTAKHIME	KMHLHMLCTP	LPEEFLDQNV
170	180	190	200	210	220	230	240
VFFLRNTKEA	ISEATDMKEA	MEIMPETLEY	GIINANVLHF	LKNIICQVFL	PALSFNQHRT	STTVGVTSGE	VSNSSEHESD
250	260	270	280	290	300	310	320
LPPMPGEAVE	YHSIQLIRDE	FLMNVQKFAS	NIQRTMQQLE	GEIKLEMPII	SVEGEVSDLA	ADPETVDILE	QCVINWLNQI
330	340	350	360	370	380	390	400
STAVEAQLKK	TPQGKGPLAE	IEFWRERNAT	LSALHEQTKL	PIVRKVLDVI	KESDSMLVAN	LQPVFTELFK	FHTEASDNVR
410	420	430	440	450	460	470	480
FLSTVERYFK	NITHGSGFHV	VLDTIPAMMS	ALRMVWIISR	HYNKDERMIP	LMERIAWEIA	ERVCRVVNLR	TLFKENRASA
490	500	510	520	530	540	550	560
QSKTLEARNT	LRLWKKAYFD	TRAKIEASGR	EDRWEFDRKR	LFERTDYMAT	ICQDLSDVLQ	ILEEFYNIFG	PELKAVTGDP
570	580	590	600	610	620	630	640
KRIDDVLCRV	DGLVTPMENL	TFDPFSIKSS	QFWKYVMDEF	KIEVLVIEKE	AKHFIDESFK	TLRSAEAAFD	MLLKFKHIRS
650	660	670	680	690	700	710	720
REAVNRQMMM	KFNDILAQYC	KEIDIINKIF	VQNLENPPLY	KNHPPVAGAI	YWERSLFFRI	KHTILRFQEV	QEILDSDRGQ
730	740	750	760	770	780	790	800
EVKQKYLEVG	RTMKEYEDRK	YEQWMEVTEQ	VLPALMKKSL	LTKSSIATEE	PSTLERGAVF	AINFSPALRE	IINETKYLEQ
810	820	830	840	850	860	870	880
LGFTVPELAR	NVALQEDKFL	RYTAGIQRML	DHYHMLIGTL	NDAESVLLKD	HSQELLRVFR	SGYKRLNWNS	LGIGDYITGC
890	900	910	920	930	940	950	960
KQAIGKFESL	VHQIHKNADD	ISSRLTLIEA	INLFKYPAAK	SEEELPGVKE	FFEHIERERA	SDVDHMVRWY	LAIGPLLTKV
970	980	990	1000	1010	1020	1030	1040
EGLVVHTNTG	KAPKLASYYK	YWEKKIYEVL	TKLILKNLQS	FNSLILGNVP	LFHTETILTA	PEIILHPNTN	EIDKMCFHCV
1050	1060	1070	1080	1090	1100	1110	1120
RNCVEITKHF	VRWMNGSCIE	CPPQKGEEEE	VVIINFYNDI	SLNPQIIEQA	VMIPQNVHRI	LINLMKYLQK	WKRYRPLWKL
1130	1140	1150	1160	1170	1180	1190	1200
DKAIVMEKFA	AKKPPCVAYD	EKLQFYSKIA	YEVMRHPLIK	DEHCIRLQLR	HLANTVQENA	KSWVISLGKL	LNESAKEELY
1210	1220	1230	1240	1250	1260	1270	1280
NLHEEMEHLA	KNLRKIPNTL	EDLKFVLATI	AEIRSKSLVM	ELRYRDVQER	YRTMAMYNLF	PPDAEKELVD	KIESIWSNLF
1290	1300	1310	1320	1330	1340	1350	1360
NDSVNVEHAL	GDIKRTFTEL	TRGEIMNYRV	QIEEFAKRFY	SEGPGSVGDD	LDKGVELLGV	YERELARHEK	SRQELANAEK
1370	1380	1390	1400	1410	1420	1430	1440
LFDLPITMYP	ELLKVQKEMS	GLRMIYELYE	GLKVAKEEWS	QTLWINLNVQ	ILQEGIEGFL	RALRKLPRPV	RGLSVTYYLE
1450	1460	1470	1480	1490	1500	1510	1520
AKMKAFKDSI	PLLLDLKNEA	LRDRHWKELM	EKTSVFFEMT	ETFTLENMFA	MELHKHTDVL	NEIVTAAIKE	VAIEKAVKEI
1530	1540	1550	1560	1570	1580	1590	1600
LDTWENMKFT	VVKYCKGTQE	RGYILGSVDE	IIQSLDDNTF	NLQSISGSRF	VGPFLQTVHK	WEKTLSLIGE	VIEIWMLVQR
1610	1620	1630	1640	1650	1660	1670	1680
KWMYLESIFI	GGDIRSQLPE	EAKKFDNIDK	VFKRIMGETL	KDPVIKRCCE	APNRLSDLQN	VSEGLEKCQK	SLNDYLDSKR
1690	1700	1710	1720	1730	1740	1750	1760
NAFPRFFFIS	DDELLSILGS	SDPLCVQEHM	IKMYDNIASL	RFNDGDSGEK	LVSAMISAEG	EVMEFRKILR	AEGRVEDWMT
1770	1780	1790	1800	1810	1820	1830	1840
AVLNEMRRTN	RLITKEAIFR	YCEDRSRVDW	MLLYQGMVVL	AASQVWWTWE	VEDVFHKAQK	GEKQAMKNYG	RKMHRQIDEL
1850	1860	1870	1880	1890	1900	1910	1920
VTRITMPLSK	NDRKKYNTVL	IIDVHARDIV	DSFIRGSILE	AREFDWESQL	RFYWDREPDE	LNIRQCTGTF	GYGYEYMGLN
1930	1940	1950	1960	1970	1980	1990	2000
GRLVITPLTD	RIYLTLTQAL	SMYLGGAPAG	PAGTGKTETT	KDLAKALGLL	CVVTNCGEGM	DYRAVGKIFS	GLAQCGAWGC
2010	2020	2030	2040	2050	2060	2070	2080
FDEFNRIDAS	VLSVISSQIQ	TIRNALIHQL	TTFQFEGQEI	SLDSRMGIFI	TMNPGYAGRT	ELPESVKALF	RPVVVIVPDL
2090	2100	2110	2120	2130	2140	2150	2160
QQICEIMLFS	EGFLEAKTLA	KKMTVLYKLA	REQLSKQYHY	DFGLRALKSV	LVMAGELKRG	SSDLREDVVL	MRALRDMNLP
2170	2180	2190	2200	2210	2220	2230	2240

Detailed Protein Report

Cmpd.	No. of Cmpds.	m/z meas.	Δ m/z [ppm]	z	Rt [min]	Score	P	Range	Sequence	Modification	Ratios
1643	1	938.9588	-53.24	2	50.3	11.9	2	624-639	R.SAEAAFDMLLKFKHIR.S		
1629	1	711.6687	-76.59	3	49.1	10.5	1	897-915	K.NADDISSRLTLIEAINLFK.Y		
2459	1	1045.2266	-235.83	1	61.2	14.2	0	940-948	R.ASDVDHMVR.W	Oxidation: 7	
2662	1	835.4217	30.04	2	61.6	11.7	1	2634-2646	K.RLLVFMDDMNMPR.V	Oxidation: 6, 9	Q:W 1.73
115	2	679.8485	-38.99	2	30.8	24.0	0	2780-2791	R.VFNGLVLTNPER.F		M:Q 0.97<br>Q:W 0.65
1556	1	502.7809	-7.71	2	49.7	11.3	1	3742-3750	K.ATEVSEKLK.L		

Detailed Protein Report

Protein 86: intersectin-1 isoform ITSN-I [Homo sapiens]

**Accession:**	gi|47717123	**Score:**	83.4
**Database:**	refseq_human(refseq_human_20140103.fasta)	**MW [kDa]:**	195.3
**Database Date:**	2015-11-30	**pI:**	8.5
**Modification(s):**	Oxidation	**Sequence Coverage [%]:**	4.8
		**No. of unique Peptides:**	4

Quantitation

M:Q	**Median:** 0.61	**CV:** 14.02 %	**No. of Peptides:** 2

10	20	30	40	50	60	70	80
MAQFPTPFGG	SLDIWAITVE	ERAKHDQQFH	SLKPISGFIT	GDQARNFFFQ	SGLPQPVLAQ	IWALADMNND	GRMDQVEFSI
90	100	110	120	130	140	150	160
AMKLIKLKLQ	GYQLPSALPP	VMKQQPVAIS	SAPAFGMGGI	ASMPPLTAVA	PVPMGSIPVV	GMSPTLVSSV	PTAAVPPLAN
170	180	190	200	210	220	230	240
GAPPVIQPLP	AFAHPAATLP	KSSSFSRSGP	GSQLNTKLQK	AQSFDVASVP	PVAEWAVPQS	SRLKYRQLFN	SHDKTMSGHL
250	260	270	280	290	300	310	320
TGPQARTILM	QSSLPQAQLA	SIWNLSDIDQ	DGKLTAEEFI	LAMHLIDVAM	SGQPLPPVLP	PEYIPPSFRR	VRSGSGISVI
330	340	350	360	370	380	390	400
SSTSVDQRLP	EEPVLEDEQQ	QLEKKLPVTF	EDKKRENFER	GNLELEKRRQ	ALLEQQRKEQ	ERLAQLERAE	QERKERERQE
410	420	430	440	450	460	470	480
QERKRQLELE	KQLEKQRELE	RQREEERRKE	IERREAAKRE	LERQRQLEWE	RNRRQELLNQ	RNKEQEDIVV	LKAKKKTLEF
490	500	510	520	530	540	550	560
ELEALNDKKH	QLEGKLQDIR	CRLTTQRQEI	ESTNKSRELR	IAEITHLQQQ	LQESQQMLGR	LIPEKQILND	QLKQVQQNSL
570	580	590	600	610	620	630	640
HRDSLVTLKR	ALEAKELARQ	HLRDQLDEVE	KETRSKLQEI	DIFNNQLKEL	REIHNKQQLQ	KQKSMEAERL	KQKEQERKII
650	660	670	680	690	700	710	720
ELEKQKEEAQ	RRAQERDKQW	LEHVQQEDEH	QRPRKLHEEE	KLKREESVKK	KDGEEKGKQE	AQDKLGRLFH	QHQEPAKPAV
730	740	750	760	770	780	790	800
QAPWSTAEKG	PLTISAQENV	KVVYYRALYP	FESRSHDEIT	IQPGDIVMVK	GEWVDESQTG	EPGWLGGELK	GKTGWFPANY
810	820	830	840	850	860	870	880
AEKIPENEVP	APVKPVTDST	SAPAPKLALR	ETPAPLAVTS	SEPSTTPNNW	ADFSSTWPTS	TNEKPETDNW	DAWAAQPSLT
890	900	910	920	930	940	950	960
VPSAGQLRQR	SAFTPATATG	SSPSPVLGQG	EKVEGLQAQA	LYPWRAKKDN	HLNFNKNDVI	TVLEQQDMWW	FGEVQGQKGW
970	980	990	1000	1010	1020	1030	1040
FPKSYVKLIS	GPIRKSTSMD	SGSSESPASL	KRVASPAAKP	VVSGEEFIAM	YTYESSEQGD	LTFQQGDVIL	VTKKDGDWWT
1050	1060	1070	1080	1090	1100	1110	1120
GTVGDKAGVF	PSNYVRLKDS	EGSGTAGKTG	SLGKKPEIAQ	VIASYTATGP	EQLTLAPGQL	ILIRKKNPGG	WWEGELQARG
1130	1140	1150	1160	1170	1180	1190	1200
KKRQIGWFPA	NYVKLLSPGT	SKITPTEPPK	STALAAVCQV	IGMYDYTAQN	DDELAFNKGQ	IINVLNKEDP	DWWKGEVNGQ
1210	1220	1230	1240	1250	1260	1270	1280
VGLFPSNYVK	LTTDMDPSQQ	WCSDLHLLDM	LTPTERKRQG	YIHELIVTEE	NYVNDLQLVT	EIFQKPLMES	ELLTEKEVAM
1290	1300	1310	1320	1330	1340	1350	1360
IFVNWKELIM	CNIKLLKALR	VRKKMSGEKM	PVKMIGDILS	AQLPHMQPYI	RFCSRQLNGA	ALIQQKTDEA	PDFKEFVKRL
1370	1380	1390	1400	1410	1420	1430	1440
AMDPRCKGMP	LSSFILKPMQ	RVTRYPLIIK	NILENTPENH	PDHSHLKHAL	EKAEELCSQV	NEGVREKENS	DRLEWIQAHV
1450	1460	1470	1480	1490	1500	1510	1520
QCEGLSEQLV	FNSVTNCLGP	RKFLHSGKLY	KAKSNKELYG	FLFNDFLLLT	QITKPLGSSG	TDKVFSPKSN	LQYKMYKTPI
1530	1540	1550	1560	1570	1580	1590	1600
FLNEVLVKLP	TDPSGDEPIF	HISHIDRVYT	LRAESINERT	AWVQKIKAAS	ELYIETEKKK	REKAYLVRSQ	RATGIGRLMV
1610	1620	1630	1640	1650	1660	1670	1680
NVVEGIELKP	CRSHGKSNPY	CEVTMGSQCH	ITKTIQDTLN	PKWNSNCQFF	IRDLEQEVLC	ITVFERDQFS	PDDFLGRTEI
1690	1700	1710	1720	1730			
RVADIKKDQG	SKGPVTKCLL	LHEVPTGEIV	VRLDLQLFDE	P			

Detailed Protein Report

Cmpd.	No. of Cmpds.	m/z meas.	Δ m/z [ppm]	z	Rt [min]	Score	P	Range	Sequence	Modification	Ratios	
85	1	827.0046	76.69	2	31.0	15.0	1	73-86	R.MDQVEFSIAMKLIK.L			
1569	1	788.7801	-144.71	2	49.5	15.7	2	584-596	R.DQLDEVEKETRSK.L		M:Q	0.53
2747	1	1045.5347	10.58	2	65.5	12.9	0	891-912	R. SAFTPATATGSSPSPVLGQGEK. V			
2785	1	874.5334	88.10	1	63.9	14.2	1	1359-1365	K.RLAMDPR.C	Oxidation: 4	M:Q	0.70

Detailed Protein Report

Protein 87: replication factor C subunit 1 isoform 2 [Homo sapiens]

**Accession:**	gi|325296984	**Score:**	83.4
**Database:**	refseq_human(refseq_human_20140103.fasta)	**MW [kDa]:**	128.2
**Database Date:**	2015-11-30	**pI:**	9.9
**Modification(s):**	Carbamidomethyl	**Sequence Coverage [%]:**	10.2
		**No. of unique Peptides:**	5

10	20	30	40	50	60	70	80
MDIRKFFGVI	PSGKKLVSET	VKKNEKTKSD	EETLKAKKGI	KEIKVNSSRK	EDDFKQKQPS	KKKRIIYDSD	SESEETLQVK
90	100	110	120	130	140	150	160
NAKKPPEKLP	VSSKPGKISR	QDPVTYISET	DEEDDFMCKK	AASKSKENGR	STNSHLGTSN	MKKNEENTKT	KNKPLSPIKL
170	180	190	200	210	220	230	240
TPTSVLDYFG	TGSVQRSNKK	MVASKRKELS	QNTDESGLND	EAIAKQLQLD	EDAELERQLH	EDEEFARTLA	MLDEEPKTKK
250	260	270	280	290	300	310	320
ARKDTEAGET	FSSVQANLSK	AEKHKYPHKV	KTAQVSDERK	SYSPRKQSKY	ESSKESQQHS	KSSADKIGEV	SSPKASSKLA
330	340	350	360	370	380	390	400
IMKRKEESSY	KEIEPVASKR	KENAIKLKGE	TKTPKKTKSS	PAKKESVSPE	DSEKKRTNYQ	AYRSYLNREG	PKALGSKEIP
410	420	430	440	450	460	470	480
KGAENCLEGL	IFVITGVLES	IERDEAKSLI	ERYGGKVTGN	VSKKTNYLVM	GRDSGQSKSD	KAAALGTKII	DEDGLLNLIR
490	500	510	520	530	540	550	560
TMPGKKSKYE	IAVETEMKKE	SKLERTPQKN	VQGKRKISPS	KKESESKKSR	PTSKRDSLAK	TIKKETDVFW	KSLDFKEQVA
570	580	590	600	610	620	630	640
EETSGDSKAR	NLADDSSENK	VENLLWVDKY	KPTSLKTIIG	QQGDQSCANK	LLRWLRNWQK	SSSEDKKHAA	KFGKFSGKDD
650	660	670	680	690	700	710	720
GSSFKAALLS	GPPGVGKTTT	ASLVCQELGY	SYVELNASDT	RSKSSLKAIV	AESLNNTSIK	GFYSNGAASS	VSTKHALIMD
730	740	750	760	770	780	790	800
EVDGMAGNED	RGGIQELIGL	IKHTKIPIIC	MCNDRNHPKI	RSLVHYCFDL	RFQRPRVEQI	KGAMMSIAFK	EGLKIPPPAM
810	820	830	840	850	860	870	880
NEIILGANQD	IRQVLHNLSM	WCARSKALTY	DQAKADSHRA	KKDIKMGPFD	VARKVFAAGE	ETAHMSLVDK	SDLFFHDYSI
890	900	910	920	930	940	950	960
APLFVQENYI	HVKPVAAGGD	MKKHLMLLSR	AADSICDGDL	VDSQIRSKQN	WSLLPAQAIY	ASVLPGELMR	GYMTQFPTFP
970	980	990	1000	1010	1020	1030	1040
SWLGKHSSTG	KHDRIVQDLA	LHMSLRTYSS	KRTVNMDYLS	LLRDALVQPL	TSQGVDGVQD	VVALMDTYYL	MKEDFENIME
1050	1060	1070	1080	1090	1100	1110	1120
ISSWGGKPSP	FSKLDPKVKA	AFTRAYNKEA	HLTPYSLQAI	KASRHSTSPS	LDSEYNEELN	EDDSQSDEKD	QDAIETDAMI
1130	1140	1150					
KKKTKSSKPS	KPEKDKEPRK	GKGKSSKK					

Cmpd.	No. of Cmpds.	m/z meas.	Δ m/z [ppm]	z	Rt [min]	Score	P	Range	Sequence	Modification
967	2	530.7788	-57.17	2	40.4	11.1	2	42-50	K.EIKVNSSRK.E	
1884	1	974.4729	4.34	2	51.9	11.6	2	125-142	K.SKENGRSTNSHLGTSNMK.K	
2929	1	965.1417	7.65	3	65.4	10.9	1	658-683	K.TTTASLVCQELGYSYVELNASDTRSK.S	Carbamidomethyl: 8
1265	1	990.4490	-59.66	2	45.4	11.3	2	746-761	K.IPIICMCNDRNHPKIR.S	Carbamidomethyl: 5
6	1	776.7890	-162.05	2	28.8	17.4	1	777-790	R.VEQIKGAMMSIAFK.E	

Detailed Protein Report

Protein 88: low-density lipoprotein receptor-related protein 2 precursor [Homo sapiens]

**Accession:**	gi|126012573	**Score:**	82.9
**Database:**	refseq_human(refseq_human_20140103.fasta)	**MW [kDa]:**	521.6
**Database Date:**	2015-11-30	**pI:**	4.8
**Modification(s):**	Carbamidomethyl	**Sequence Coverage [%]:**	1.9
		**No. of unique Peptides:**	6

Quantitation

Q:W	**Median:**	2.22	**CV:**	0.00 %	**No. of Peptides:**	1
M:Q	**Median:**	1.40	**CV:**	0.00 %	**No. of Peptides:**	1

Detailed Protein Report

10	20	30	40	50	60	70	80
MDRGPAAVAC	TLLLALVACL	APASGQECDS	AHFRCGSGHC	IPADWRCDGT	KDCSDDADEI	GCAVVTCQQG	YFKCQSEGQC
90	100	110	120	130	140	150	160
IPNSWVCDQD	QDCDDGSDER	QDCSQSTCSS	HQITCSNGQC	IPSEYRCDHV	RDCPDGADEN	DCQYPTCEQL	TCDNGACYNT
170	180	190	200	210	220	230	240
SQKCDWKVDC	RDSSDEINCT	EICLHNEFSC	GNGECIPRAY	VCDHDNDCQD	GSDEHACNYP	TCGGYQFTCP	SGRCIYQNWV
250	260	270	280	290	300	310	320
CDGEDDCKDN	GDEDGCESGP	HDVHKCSPRE	WSCPESGRCI	SIYKVCDGIL	DCPGREDENN	TSTGKYCSMT	LCSALNCQYQ
330	340	350	360	370	380	390	400
CHETPYGGAC	FCPPGYIINH	NDSRTCVEFD	DCQIWGICDQ	KCESRPGRHL	CHCEEGYILE	RGQYCKANDS	FGEASIIFSN
410	420	430	440	450	460	470	480
GRDLLIGDIH	GRSFRILVES	QNRGVAVGVA	FHYHLQRVFW	TDTVQNKVFS	VDINGLNIQE	VLNVSVETPE	NLAVDWVNNK
490	500	510	520	530	540	550	560
IYLVETKVNR	IDMVNLDGSY	RVTLITENLG	HPRGIAVDPT	VGYLFFSDWE	SLSGEPKLER	AFMDGSNRKD	LVKTKLGWPA
570	580	590	600	610	620	630	640
GVTLDMISKR	VYWVDSRFDY	IETVTYDGIQ	RKTVVHGGSL	IPHPFGVSLF	EGQVFFTDWT	KMAVLKANKF	TETNPQVYYQ
650	660	670	680	690	700	710	720
ASLRPYGVTV	YHSLRQPYAT	NPCKDNNGGC	EQVCVLSHRT	DNDGLGFRCK	CTFGFQLDTD	ERHCIAVQNF	LIFSSQVAIR
730	740	750	760	770	780	790	800
GIPFTLSTQE	DVMVPVSGNP	SFFVGIDFDA	QDSTIFFSDM	SKHMIFKQKI	DGTGREILAA	NRVENVESLA	FDWISKNLYW
810	820	830	840	850	860	870	880
TDSHYKSISV	MRLADKTRRT	VVQYLNNPRS	VVVHPFAGYL	FFTDWFRPAK	IMRAWSDGSH	LLPVINTTLG	WPNGLAIDWA
890	900	910	920	930	940	950	960
ASRLYWVDAY	FDKIEHSTFD	GLDRRRLGHI	EQMTHPFGLA	IFGEHLFFTD	WRLGAIIRVR	KADGGEMTVI	RSGIAYILHL
970	980	990	1000	1010	1020	1030	1040
KSYDVNIQTG	SNACNQPTHP	NGDCSHFCFP	VPNFQRVCGC	PYGMRLASNH	LTCEGDPTNE	PPTEQCGLFS	FPCKNGRCVP
1050	1060	1070	1080	1090	1100	1110	1120
NYYLCDGVDD	CHDNSDEQLC	GTLNNTCSSS	AFTCGHGECI	PAHWRCDKRN	DCVDGSDEHN	CPTHAPASCL	DTQYTCDNHQ
1130	1140	1150	1160	1170	1180	1190	1200
CISKNWVCDT	DNDCGDGSDE	KNCNSTETCQ	PSQFNCPNHR	CIDLSFVCDG	DKDCVDGSDE	VGCVLNCTAS	QFKCASGDKC
1210	1220	1230	1240	1250	1260	1270	1280
IGVTNRCDGV	FDCSDNSDEA	GCPTRPPGMC	HSDEFQCQED	GICIPNFWEC	DGHPDCLYGS	DEHNACVPKT	CPSSYFHCDN
1290	1300	1310	1320	1330	1340	1350	1360
GNCIHRAWLC	DRDNDCGDMS	DEKDCPTQPF	RCPSWQWQCL	GHNICVNLSV	VCDGIFDCPN	GTDESPLCNG	NSCSDFNGGC
1370	1380	1390	1400	1410	1420	1430	1440
THECVQEPFG	AKCLCPLGFL	LANDSKTCED	IDECDILGSC	SQHCYNMRGS	FRCSCDTGYM	LESDGRTCKV	TASESLLLLV
1450	1460	1470	1480	1490	1500	1510	1520
ASQNKIIADS	VTSQVHNIYS	LVENGSYIVA	VDFDSISGRI	FWSDATQGKT	WSAFQNGTDR	RVVFDSSIIL	TETIAIDWVG
1530	1540	1550	1560	1570	1580	1590	1600
RNLYWTDYAL	ETIEVSKIDG	SHRTVLISKN	LTNPRGLALD	PRMNEHLLFW	SDWGHHPRIE	RASMDGSMRT	VIVQDKIFWP
1610	1620	1630	1640	1650	1660	1670	1680
CGLTIDYPNR	LLYFMDSYLD	YMDFCDYNGH	HRRQVIASDL	IIRHPYALTL	FEDSVYWTDR	ATRRVMRANK	WHGGNQSVVM
1690	1700	1710	1720	1730	1740	1750	1760
YNIQWPLGIV	AVHPSKQPNS	VNPCAFSRCS	HLCLLSSQGP	HFYSCVCPSG	WSLSPDLLNC	LRDDQPFLIT	VRQHIIFGIS
1770	1780	1790	1800	1810	1820	1830	1840
LNPEVKSNDA	MVPIAGIQNG	LDVEFDDAEQ	YIYWVENPGE	IHRVKTDGTN	RTVFASISMV	GPSMNLALDW	ISRNLYSTNP
1850	1860	1870	1880	1890	1900	1910	1920
RTQSIEVLTL	HGDIRYRKTL	IANDGTALGV	GFPIGITVDP	ARGKLYWSDQ	GTDSGVPAKI	ASANMDGTSV	KTLFTGNLEH
1930	1940	1950	1960	1970	1980	1990	2000
LECVTLDIEE	QKLYWAVTGR	GVIERGNVDG	TDRMILVHQL	SHPWGIAVHD	SFLYYTDEQY	EVIERVDKAT	GANKIVLRDN
2010	2020	2030	2040	2050	2060	2070	2080
VPNLRGLQVY	HRRNAAESSN	GCSNNMNACQ	QICLPVPGGL	FSCACATGFK	LNPDNRSCSP	YNSFIVVSML	SAIRGFSLEL
2090	2100	2110	2120	2130	2140	2150	2160
SDHSETMVPV	AGQGRNALHV	DVDVSSGFIY	WCDFSSSVAS	DNAIRRIKPD	GSSLMNIVTH	GIGENGVRGI	AVDWVAGNLY
2170	2180	2190	2200	2210	2220	2230	2240

Detailed Protein Report

Cmpd.	No. of Cmpds.	m/z meas.	Δ m/z [ppm]	z	Rt [min]	Score	P	Range	Sequence	Modification	Ratios
573	1	656.8903	-140.87	3	35.8	15.5	1	279-295	R.CISIYKVCDGILDCPGR.E	Carbamidomethyl: 1, 8	
1173	1	786.4695	28.78	1	44.3	12.4	0	776-782	R.EILAANR.V		
1012	1	974.7041	-35.28	3	41.2	13.1	1	1173-1199	K.DCVDGSDEVGCVLNCTASQFKCA C	Carbamidomethyl: 11, 15, 22	
936	1	647.6555	-174.87	2	41.8	10.3	0	3696-3706	K.WAVCNGVDDCR.D	Carbamidomethyl: 4	
23	1	788.3643	33.00	3	29.6	20.9	0	3797-3816	R.TCHPEYFQCTSGHCVHSELK. C	Carbamidomethyl: 2	
1208	1	441.6040	-232.73	2	44.7	10.7	1	4259-4265	K.YDGTDRR.V		M:Q 1.40<br>Q:W 2.22

Detailed Protein Report

Protein 89: PREDICTED: dynein heavy chain 12, axonemal isoform X1 [Homo sapiens]

**Accession:**	gi|578805765	**Score:**	82.3
**Database:**	refseq_human(refseq_human_20140103.fasta)	**MW [kDa]:**	454.9
**Database Date:**	2015-11-30	**pI:**	5.5
**Modification(s):**	Oxidation	**Sequence Coverage [%]:**	2.6
		**No. of unique Peptides:**	5

Quantitation

Q:W	**Median:**	1.30	**CV:**	0.00 %	**No. of Peptides:**	1
M:Q	**Median:**	1.09	**CV:**	0.00 %	**No. of Peptides:**	1

Detailed Protein Report

10	20	30	40	50	60	70	80
MSDANKAAIA	AEKEALNLKL	PPIVHLPENI	GVDTPTQSKL	LKYRRSKEQQ	QKINQLVIDG	AKRNLDRTLG	KRTPLLPPPD
90	100	110	120	130	140	150	160
YPQTMTSEMK	KKGFNYIYMK	QCVESSPLVP	IQQEWLDHML	RLIPESLKEG	KEREELLESL	INEVSSDFEN	SMKRYLVQSV
170	180	190	200	210	220	230	240
LVKPPVKSLE	DEGGPLPESP	VGLDYSNPWH	SSYVQARNQI	FSNLHIIHPT	MKMLLDLGYT	TFADTVLLDF	TGIRAKGPID
250	260	270	280	290	300	310	320
CESLKTDLSI	QTRNAEEKIM	NTWYPKVINL	FTKKEALEGV	KPEKLDAFYS	CVSTLMSNQL	KDLLRRTVEG	FVKLFDPKDQ
330	340	350	360	370	380	390	400
QRLPIFKIEL	TFDDDKMEFY	PTFQDLEDNV	LSLVERIAEA	LQNVQTIPSW	LSGTSTPVNL	DTELPEHVLH	WAVDTLKAAV
410	420	430	440	450	460	470	480
HRNLEGARKH	YETYVEKYNW	LLDGTAVENI	ETFQTEDHTF	DEYTEFIEKF	LSLASEIMLL	PQWIHYTMVR	LDCEDLKTGL
490	500	510	520	530	540	550	560
TNKAKAFANI	LLNDIASKYR	KENECICSEF	EAIKEHALKV	PETTEEMMDL	ISYVEKARTV	GIEELILRIQ	ESKRQMSYFL
570	580	590	600	610	620	630	640
DVFLFPQEDL	ALNATVLMWP	RKINPIFDEN	DELIENAKHK	KENELMAKRE	KLILEIEKES	RRMEEFTEFA	ELERMQQYVT
650	660	670	680	690	700	710	720
DVRQLQKRIQ	ESEEAVQFIN	KEEELFKWEL	TKYPELDKLK	VNIEPYQKFF	NFVLKWQRSE	KRWMDGGFLD	LNGESMEADV
730	740	750	760	770	780	790	800
EEFSREIFKT	LKFFQTKLKK	ELQEKRKAAR	KRSLEEEKIE	EEPKDNATIT	MCSTVMEQIK	AFKEYIPTVS	ILCNPGMRAR
810	820	830	840	850	860	870	880
HWKQISEIVG	YDLTPDSGTT	LRKVLKLNLT	PYLEQFEVIS	AGASKEFSLE	KAMNTMIGTW	EDIAFHISLY	RDTGVCILSS
890	900	910	920	930	940	950	960
VDEIQAILDD	QIIKTQTMRG	SPFIKPFEHE	IKAWEDRLIR	IQETIDEWLK	VQAQWLYLEP	IFCSEDIMQQ	MPEEGRQFQT
970	980	990	1000	1010	1020	1030	1040
VDRHWRDIMK	FCAKDPKVLA	ATSLTGLLEK	LQNCNELLEK	IMKGLNAYLE	KKRLFFPRFF	FLSNDEMLEI	LSETKDPLRV
1050	1060	1070	1080	1090	1100	1110	1120
QPHLKKCFEG	IAKLEFLPNL	DIKAMYSSEG	ERVELIALIS	TSAARGAVEK	WLIQVEDLML	RSVHDVIAAA	RLAYPESARR
1130	1140	1150	1160	1170	1180	1190	1200
DWVREWPGQV	VLCISQMFWT	SETQEVISGG	TEGLKKYYKE	LQNQLNEIVE	LVRGKLSKQT	RTTLGALVTI	DVHARDVVMD
1210	1220	1230	1240	1250	1260	1270	1280
MIKMGVSHDT	DFLWLAQLRY	YWENENARVR	IINCNVKYAY	EYLGNSPRLV	ITPLTDRCYR	TLIGAFYLNL	GGAPEGPAGT
1290	1300	1310	1320	1330	1340	1350	1360
GKTETTKDLA	KALAVQCVVF	NCSDGLDYLA	MGKFFKGLAS	SGAWACFDEF	NRIELEVLSV	VAQQILCIQR	AIQQKLVVFV
1370	1380	1390	1400	1410	1420	1430	1440
FEGTELKLNP	NCFVAITMNP	GYAGRSELPD	NLKVLFRTVA	MMVPNYALIA	EISLYSYGFL	NARPLSVKIV	MTYRLCSEQL
1450	1460	1470	1480	1490	1500	1510	1520
SSQFHYDYGM	RAVKAVLVAA	GNLKLKYPNE	NEDILLLRSI	KDVNEPKFLS	HDIPLFNGIT	SDLFPGIKLP	EADYHEFLEC
1530	1540	1550	1560	1570	1580	1590	1600
AHEACNVHNL	QPVKFFLEKI	IQTYEMMIVR	HGFMLVGEPF	AAKTKVLHVL	ADTLTLMNEH	GYGEEEKVIY	RTVNPKSITM
1610	1620	1630	1640	1650	1660	1670	1680
GQLFGQFDPV	SHEWTDGIVA	NTFREFALSE	TPDRKWVVFD	GPIDTLWIES	MNTVLDDNKK	LCLMSGEIIQ	MSPQMSLIFE
1690	1700	1710	1720	1730	1740	1750	1760
TMDLSQASPA	TVSRCGMIYL	EPSQLGWEPL	VSSWLNSLKG	PLCEPEYQAL	LRGLFAWLIP	PSLNQRVELF	QLNYLYTTIV
1770	1780	1790	1800	1810	1820	1830	1840
SKILKILITF	RISNYFKYVP	LKTQCTFIKF	FLHQQACFIF	SLIWSIGGSC	DTDGRRVFDT	FIRLIILGKD	DENPVPDSVG
1850	1860	1870	1880	1890	1900	1910	1920
KWECPFDEKG	LVYDYMYELK	NKGRWVHWNE	LIKNTNLGDK	QIKIQDIIVP	TMDTIRYTFL	MDLSITYAKP	LLFVGPTGTG
1930	1940	1950	1960	1970	1980	1990	2000
KSVYVKDKLM	NHLEKDQYFP	FYINLSARTS	ANQVQNIIMA	RLDKRRKGVF	GPPMGKKCII	FIDDMNMPAL	EKYGAQPPIE
2010	2020	2030	2040	2050	2060	2070	2080
LLRQFFDCGH	WYDLKDTSKI	TLVDIELIAA	MGPPGGGRNP	VTPRCIRHFN	ICSINSFSDE	TMVRIFSSIV	AFYLRTHEFP
2090	2100	2110	2120	2130	2140	2150	2160
PEYFVIGNQI	VNGTMEIYKQ	SVENLLPTPT	KSHYTFNLRD	FSRVIRGCLL	IERDAVANKH	TMIRLFVHEV	LRVFYDRLIN
2170	2180	2190	2200	2210	2220	2230	2240

Detailed Protein Report

Cmpd.	No. of Cmpds.	m/z meas.	Δ m/z [ppm]	z	Rt [min]	Score	P	Range	Sequence	Modification	Ratios
47	1	1103.4984	-39.62	2	29.9	12.9	1	73-91	R.TPLLPPPDYPQTMTSEMKK.K	Oxidation: 13, 17	
1482	1	681.7771	64.45	3	47.3	18.6	2	267-284	K.VINLFTKKEALEGVKPEK.L		
1201	1	1070.0173	-20.70	2	43.7	12.3	1	583-600	K.INPIFDENDELIENAKHK.K		M:Q 1.09<br>Q:W 1.30
2090	1	1159.8674	-54.47	3	56.0	10.4	2	1535-1563	K.<br>FFLEKIIQTYEMMIVRHGFMLVGEF<br>T	Oxidation: 12, 20	
1721	1	646.7566	-109.88	2	51.8	16.9	1	1625-1635	R.EFALSETPDRK.W		

Detailed Protein Report

Protein 90: protocadherin Fat 2 precursor [Homo sapiens]

**Accession:**	gi|13787217	**Score:**	81.4
**Database:**	refseq_human(refseq_human_20140103.fasta)	**MW [kDa]:**	479.0
**Database Date:**	2015-11-30	**pI:**	4.9
		**Sequence Coverage [%]:**	2.0
		**No. of unique Peptides:**	3

Alias proteins:

Accession	Name	Description
gi|578810809	refseq_human (refseq_human_20140103.fasta)	PREDICTED: protocadherin Fat 2 isoform X1 [Homo sapiens]

Detailed Protein Report

10	20	30	40	50	60	70	80
MTIALLGFAI	FLLHCATCEK	PLEGILSSSA	WHFTHSHYNA	TIYENSSPKT	YVESFEKMGI	YLAEPQWAVR	YRIISGDVAN
90	100	110	120	130	140	150	160
VFKTEEYVVG	NFCFLRIRTK	SSNTALLNRE	VRDSYTLIIQ	ATEKTLELEA	LTRVVVHILD	QNDLKPLFSP	PSYRVTISED
170	180	190	200	210	220	230	240
MPLKSPICKV	TATDADLGQN	AEFYYAFNTR	SEMFAIHPTS	GVVTVAGKLN	VTWRGKHELQ	VLAVDRMRKI	SEGNGFGSLA
250	260	270	280	290	300	310	320
ALVVHVEPAL	RKPPAIASVV	VTPPDSNDGT	TYATVLVDAN	SSGAEVESVE	VVGGDPGKHF	KAIKSYARSN	EFSLVSVKDI
330	340	350	360	370	380	390	400
NWMEYLHGFN	LSLQARSGSG	PYFYSQIRGF	HLPPSKLSSL	KFEKAVYRVQ	LSEFSPPGSR	VVMVRVTPAF	PNLQYVLKPS
410	420	430	440	450	460	470	480
SENVGFKLNA	RTGLITTTKL	MDFHDRAHYQ	LHIRTSPGQA	STVVVIDIVD	CNNHAPLFNR	SSYDGTLDEN	IPPGTSVLAV
490	500	510	520	530	540	550	560
TATDRDHGEN	GYVTYSIAGP	KALPFSIDPY	LGIISTSKPM	DYELMKRIYT	FRVRASDWGS	PFRREKEVSI	FLQLRNLNDN
570	580	590	600	610	620	630	640
QPMFEEVNCT	GSIRQDWPVG	KSIMTMSAID	VDELQNLKYE	IVSGNELEYF	DLNHFSGVIS	LKRPFINLTA	GQPTSYSLKI
650	660	670	680	690	700	710	720
TASDGKNYAS	PTTLNITVVK	DPHFEVPVTC	DKTGVLTQFT	KTILHFIGLQ	NQESSDEEFT	SLSTYQINHY	TPQFEDHFPQ
730	740	750	760	770	780	790	800
SIDVLESVPI	NTPLARLAAT	DPDAGFNGKL	VYVIADGNEE	GCFDIELETG	LLTVAAPLDY	EATNFYILNV	TVYDLGTPQK
810	820	830	840	850	860	870	880
SSWKLLTVNV	KDWNDNAPRF	PPGGYQLTIS	EDTEVGTTIA	ELTTKDADSE	DNGRVRYTLL	SPTEKFSLHP	LTGELVVTGH
890	900	910	920	930	940	950	960
LDRESEPRYI	LKVEARDQPS	KGHQLFSVTD	LIITLEDVND	NSPQCITEHN	RLKVPEDLPP	GTVLTFLDAS	DPDLGPAGEV
970	980	990	1000	1010	1020	1030	1040
RYVLMDGAHG	TFRVDLMTGA	LILERELDFE	RRAGYNLSLW	ASDGGRPLAR	RTLCHVEVIV	LDVNENLHPP	HFASFVHQGQ
1050	1060	1070	1080	1090	1100	1110	1120
VQENSPSGTQ	VIVVAAQDDD	SGLDGELQYF	LRAGTGLAAF	SINQDTGMIQ	TLAPLDREFA	SYYWLTVLAV	DRGSVPLSSV
1130	1140	1150	1160	1170	1180	1190	1200
TEVYIEVTDA	NDNPPQMSQA	VFYPSIQEDA	PVGTSVLQLD	AWDPDSSSKG	KLTFNITSGN	YMGFFMIHPV	TGLLSTAQQL
1210	1220	1230	1240	1250	1260	1270	1280
DRENKDEHIL	EVTVLDNGEP	SLKSTSRVVV	GILDVNDNPP	IFSHKLFNVR	LPERLSPVSP	GPVYRLVASD	LDEGLNGRVT
1290	1300	1310	1320	1330	1340	1350	1360
YSIEDSDEEA	FSIDLVTGVV	SSSSTFTAGE	YNILTIKATD	SGQPPLSASV	RLHIEWIPWP	RPSSIPLAFD	ETYYSFTVME
1370	1380	1390	1400	1410	1420	1430	1440
TDPVNHMVGV	ISVEGRPGLF	WFNISGGDKD	MDFDIEKTTG	SIVIARPLDT	RRRSNYNLTV	EVTDGSRTIA	TQVHIFMIAN
1450	1460	1470	1480	1490	1500	1510	1520
INHHRPQFLE	TRYEVRVPQD	TVPGVELLRV	QAIDQDKGKS	LIYTIHGSQD	PGSASLFQLD	PSSGVLVTVG	KLDLGSGPSQ
1530	1540	1550	1560	1570	1580	1590	1600
HTLTVMVRDQ	EIPIKRNFVW	VTIHVEDGNL	HPPRFTQLHY	EASVPDTIAP	GTELLQVRAM	DADRGVNAEV	HYSLLKGNSE
1610	1620	1630	1640	1650	1660	1670	1680
GFFNINALLG	IITLAQKLDQ	ANHAPHTLTV	KAEDQGSPQW	HDLATVIIHV	YPSDRSAPIF	SKSEYFVEIP	ESIPVGSPIL
1690	1700	1710	1720	1730	1740	1750	1760
LVSAMSPSEV	TYELREGNKD	GVFSMNSYSG	LISTQKKLDH	EKISSYQLKI	RGSNMAGAFT	DVMVVVDIID	ENDNAPMFLK
1770	1780	1790	1800	1810	1820	1830	1840
STFVGQISEA	APLYSMIMDK	NNNPFVIHAS	DSDKEANSLL	VYKILEPEAL	KFFKIDPSMG	TLTIVSEMDY	ESMPSFQFCV
1850	1860	1870	1880	1890	1900	1910	1920
YVHDQGSPVL	FAPRPAQVII	HVRDVNDSPP	RFSEQIYEVA	IVGPIHPGME	LLMVRASDED	SEVNYSIKTG	NADEAVTIHP
1930	1940	1950	1960	1970	1980	1990	2000
VTGSISVLNP	AFLGLSRKLT	IRASDGLYQD	TALVKISLTQ	VLDKSLQFDQ	DVYWAAVKEN	LQDRKALVIL	GAQGNHLNDT
2010	2020	2030	2040	2050	2060	2070	2080
LSYFLLNGTD	MFHMVQSAGV	LQTRGVAFDR	EQQDTHELAV	EVRDNRTPQR	VAQGLVRVSI	EDVNDNPPKF	KHLPYYTIIQ
2090	2100	2110	2120	2130	2140	2150	2160
DGTEPGDVLF	QVSATDEDLG	TNGAVTYEFA	EDYTYFRIDP	YLGDISLKKP	FDYQALNKYH	LKVIARDGGT	PSLQSEEEVL
2170	2180	2190	2200	2210	2220	2230	2240
VTVRNKSNPL	FQSPYYKVRV	PENITLYTPI	LHTQARSPEG	LRLIYNIVEE	EPLMLFTTDF	KTGVLTVTGP	LDYESKTKHV
2250	2260	2270	2280	2290	2300	2310	2320
FTVRATDTAL	GSFSEATVEV	LVEDVNDNPP	TFSQLVYTTS	ISEGLPAQTP	VIQLLASDQD	SGRNRDVSYQ	IVEDGSDVSK
2330	2340	2350	2360	2370	2380	2390	2400

Detailed Protein Report

Cmpd.	No. of Cmpds.	m/z meas.	Δ m/z [ppm]	z	Rt [min]	Score	P	Range	Sequence	Modification
2633	1	741.3552	-72.85	2	63.0	15.7	1	71-83	R.YRIISGDVANVFK.T	
1721	1	1030.6326	87.08	2	51.3	16.6	1	993-1011	R.AGYNLSLWASDGGRPLARR.T	
65	2	1096.9820	-88.55	2	30.5	12.9	2	3376-3395	K.GELQVAKALDREQASSYSLK.L	

Detailed Protein Report

Protein 91: **PREDICTED: A-kinase anchor protein 9 isoform X3 [Homo sapiens]**

**Accession:**	gi|578813854	**Score:**	81.2
**Database:**	refseq_human(refseq_human_20140103.fasta)	**MW [kDa]:**	447.3
**Database Date:**	2015-11-30	**pI:**	4.8
**Modification(s):**	Oxidation	**Sequence Coverage [%]:**	1.4
		**No. of unique Peptides:**	3

Detailed Protein Report

10	20	30	40	50	60	70	80
MEDEERQKKL	EAGKAKLAQF	RQRKAQSDGQ	SPSKKQKKKR	KTSSSKHDVS	AHHDLNIDQS	QCNEMYINSS	QRVESTVIPE
90	100	110	120	130	140	150	160
STIMRTLHSG	EITSHEQGFS	VELESEISTT	ADDCSSEVNG	CSFVMRTGKP	TNLLREEEFG	VDDSYSEQGA	QDSPTHLEMM
170	180	190	200	210	220	230	240
ESELAGKQHE	IEELNRELEE	MRVTYGTEGL	QQLQEFEAAI	KQRDGIITQL	TANLQQARRE	KDETMREFLE	LTEQSQKLQI
250	260	270	280	290	300	310	320
QFQQLQASET	LRNSTHSSTA	ADLLQAKQQI	LTHQQQLEEQ	DHLLEDYQKK	KEDFTMQISF	LQEKIKVYEM	EQDKKVENSN
330	340	350	360	370	380	390	400
KEEIQEKETI	IEELNTKIIE	EEKKTLELKD	KLTTADKLLG	ELQEQIVQKN	QEIKNMKLEL	TNSKQKERQS	SEEIKQLMGT
410	420	430	440	450	460	470	480
VEELQKRNHK	DSQFETDIVQ	RMEQETQRKL	EQLRAELDEM	YGQQIVQMKQ	ELIRQHMAQM	EEMKTRHKGE	MENALRSYSN
490	500	510	520	530	540	550	560
ITVNEDQIKL	MNVAINELNI	KLQDTNSQKE	KLKEELGLIL	EEKCALQRQL	EDLVEELSFS	REQIQRARQT	IAEQESKLNE
570	580	590	600	610	620	630	640
AHKSLSTVED	LKAEIVSASE	SRKELELKHE	AEVTNYKIKL	EMLEKEKNAV	LDRMAESQEA	ELERLRTQLL	FSHEEELSKL
650	660	670	680	690	700	710	720
KEDLEIEHRI	NIEKLKDNLG	IHYKQQIDGL	QNEMSQKIET	MQFEKDNLIT	KQNQLILEIS	KLKDLQQSLV	NSKSEEMTLQ
730	740	750	760	770	780	790	800
INELQKEIEI	LRQEEKEKGT	LEQEVQELQL	KTELLEKQMK	EKENDLQEKF	AQLEAENSIL	KDEKKTLEDM	LKIHTPVSQE
810	820	830	840	850	860	870	880
ERLIFLDSIK	SKSKDSVWEK	EIEILIEENE	DLKQQCIQLN	EEIEKQRNTF	SFAEKNFEVN	YQELQEEYAC	LLKVKDDLED
890	900	910	920	930	940	950	960
SKNKQELEYK	SKLKALNEEL	HLQRINPTTV	KMKSSVFDED	KTFVAETLEM	GEVVEKDTTE	LMEKLEVTKR	EKLELSQRLS
970	980	990	1000	1010	1020	1030	1040
DLSEQLKQKH	GEISFLNEEV	KSLKQEKEQV	SLRCRELEII	INHNRAENVQ	SCDTQVSSLL	DGVVTMTSRG	AEGSVSKVNK
1050	1060	1070	1080	1090	1100	1110	1120
SFGEESKIMV	EDKVSFENMT	VGEESKQEQL	ILDHLPSVTK	ESSLRATQPS	ENDKLQKELN	VLKSEQNDLR	LQMEAQRICL
1130	1140	1150	1160	1170	1180	1190	1200
SLVYSTHVDQ	VREYMENEKD	KALCSLKEEL	IFAQEEKIKE	LQKIHQLELQ	TMKTQETGDE	GKPLHLLIGK	LQKAVSEECS
1210	1220	1230	1240	1250	1260	1270	1280
YFLQTLCSVL	GEYYTPALKC	EVNAEDKENS	GDYISENEDP	ELQDYRYEVQ	DFQENMHTLL	NKVTEEYNKL	LVLQTRLSKI
1290	1300	1310	1320	1330	1340	1350	1360
WGQQTDGMKL	EFGEENLPKE	ETEFLSIHSQ	MTNLEDIDVN	HKSKLSSLQD	LEKTKLEEQV	QELESLISSL	QQQLKETEQN
1370	1380	1390	1400	1410	1420	1430	1440
YEAEIHCLQK	RLQAVSESTV	PPSLPVDSVV	ITESDAQRTM	YPGSCVKKNI	DGTIEFSGEF	GVKEETNIVK	LLEKQYQEQL
1450	1460	1470	1480	1490	1500	1510	1520
EEEVAKVIVS	MSIAFAQQTE	LSRISGGKEN	TASSKQAHAV	CQQEQHYFNE	MKLSQDQIGF	QTFETVDVKF	KEEFKPLSKE
1530	1540	1550	1560	1570	1580	1590	1600
LGEHGKEILL	SNSDPHDIPE	SKDCVLTISE	EMFSKDKTFI	VRQSIHDEIS	VSSMDASRQL	MLNEEQLEDM	RQELVRQYQE
1610	1620	1630	1640	1650	1660	1670	1680
HQQATELLRQ	AHMRQMERQR	EDQEQLQEEI	KRLNRQLAQN	GNENQGEVEE	QTFKEKELDR	KPEDVPPEIL	SNERYALQKA
1690	1700	1710	1720	1730	1740	1750	1760
NNRLLKILLE	VVKTTAAVEE	TIGRHVLGIL	DRSSKSQSSA	SLIWRSEAEA	SVKSCVHEEH	TRVTDESIPS	YSGSDMPRND
1770	1780	1790	1800	1810	1820	1830	1840
INMWSKVTEE	GTELSQRLVR	SGFAGTEIDP	ENEELMLNIS	SRLQAAVEKL	LEAISETSSQ	LEHAKVTQTE	LMRESFRQKQ
1850	1860	1870	1880	1890	1900	1910	1920
EATESLKCQE	ELRERLHEES	RAREQLAVEL	SKAEGVIDGY	ADEKTLFERQ	IQEKTDIIDR	LEQELLCASN	RLQELEAEQQ
1930	1940	1950	1960	1970	1980	1990	2000
QIQEERELLS	RQKEAMKAEA	GPVEQQLLQE	TEKLMKEKLE	VQCQAEKVRD	DLQKQVKALE	IDVEEQVSRF	IELEQEKNTE
2010	2020	2030	2040	2050	2060	2070	2080
LMDLRQQNQA	LEKQLEKMRK	FLDEQAIDRE	HERDVFQQEI	QKLEQQLKVV	PRFQPISEHQ	TREVEQLANH	LKEKTDKCSE
2090	2100	2110	2120	2130	2140	2150	2160
LLLSKEQLQR	DIQERNEEIE	KLEFRVRELE	QALLVSADTF	QKVEDRKHFG	AVEAKPELSL	EVQLQAERDA	IDRKEKEITN
2170	2180	2190	2200	2210	2220	2230	2240

Detailed Protein Report

Cmpd.	No. of Cmpds.	m/z meas.	Δ m/z [ppm]	z	Rt [min]	Score	P	Range	Sequence	Modification
2830	1	648.3562	-0.14	1	63.9	11.7	0	352-357	K.LTTADK.L	
27	10	768.2815	-117.99	2	29.6	16.0	2	758-769	K.QMKEKENDLQEK.F	Oxidation: 2
49	1	819.8727	-87.95	2	29.3	12.5	1	3738-3751	R.WHRVTGSVSININR.D	

Detailed Protein Report

Protein 92: **basement membrane-specific heparan sulfate proteoglycan core protein precursor [Homo sapiens]**

**Accession:**	gi|126012571	**Score:**	80.3
**Database:**	refseq_human(refseq_human_20140103.fasta)	**MW [kDa]:**	468.5
**Database Date:**	2015-11-30	**pI:**	6.1
**Modification(s):**	Oxidation	**Sequence Coverage [%]:**	2.3
		**No. of unique Peptides:**	4

Detailed Protein Report

10	20	30	40	50	60	70	80
MGWRAAGALL	LALLLHGRLL	AVTHGLRAYD	GLSLPEDIET	VTASQMRWTH	SYLSDDEDML	ADSISGDDLG	SGDLGSGDFQ
90	100	110	120	130	140	150	160
MVYFRALVNF	TRSIEYSPQL	EDAGSREFRE	VSEAVVDTLE	SEYLKIPGDQ	VVSVVFIKEL	DGWVFVELDV	GSEGNADGAQ
170	180	190	200	210	220	230	240
IQEMLLRVIS	SGSVASYVTS	PQGFQFRRLG	TVPQFPRACT	EAEFACHSYN	ECVALEYRCD	RRPDCRDMSD	ELNCEEPVLG
250	260	270	280	290	300	310	320
ISPTFSLLVE	TTSLPPRPET	TIMRQPPVTH	APQPLLPGSV	RPLPCGPQEA	ACRNGHCIPR	DYLCDGQEDC	EDGSDELDCG
330	340	350	360	370	380	390	400
PPPPCEPNEF	PCGNGHCALK	LWRCDGDFDC	EDRTDEANCP	TKRPEEVCGP	TQFRCVSTNM	CIPASFHCDE	ESDCPDRSDE
410	420	430	440	450	460	470	480
FGCMPPQVVT	PPRESIQASR	GQTVTFTCVA	IGVPTPIINW	RLNWGHIPSH	PRVTVTSEGG	RGTLIIRDVK	ESDQGAYTCE
490	500	510	520	530	540	550	560
AMNARGMVFG	IPDGVLELVP	QRGPCPDGHF	YLEHSAACLP	CFCFGITSVC	QSTRRFRDQI	RLRFDQPDDF	KGVNVTMPAQ
570	580	590	600	610	620	630	640
PGTPPLSSTQ	LQIDPSLHEF	QLVDLSRRFL	VHDSFWALPE	QFLGNKVDSY	GGSLRYNVRY	ELARGMLEPV	QRPDVVLMGA
650	660	670	680	690	700	710	720
GYRLLSRGHT	PTQPGALNQR	QVQFSEEHWV	HESGRPVQRA	ELLQVLQSLE	AVLIQTVYNT	KMASVGLSDI	AMDTTVTHAT
730	740	750	760	770	780	790	800
SHGRAHSVEE	CRCPIGYSGL	SCESCDAHFT	RVPGGPYLGT	CSGCNCNGHA	SSCDPVYGHC	LNCQHNTEGP	QCNKCKAGFF
810	820	830	840	850	860	870	880
GDAMKATATS	CRPCPCPYID	ASRRFSDTCF	LDTDGQATCD	ACAPGYTGRR	CESCAPGYEG	NPIQPGGKCR	PVNQEIVRCD
890	900	910	920	930	940	950	960
ERGSMGTSGE	ACRCKNNVVG	RLCNECADGS	FHLSTRNPDG	CLKCFCMGVS	RHCTSSSWSR	AQLHGASEEP	GHFSLTNAAS
970	980	990	1000	1010	1020	1030	1040
THTTNEGIFS	PTPGELGFSS	FHRLLSGPYF	WSLPSRFLGD	KVTSYGGELR	FTVTQRSQPG	STPLHGQPLV	VLQGNNIILE
1050	1060	1070	1080	1090	1100	1110	1120
HHVAQEPSPG	QPSTFIVPFR	EQAWQRPDGQ	PATREHLLMA	LAGIDTLLIR	ASYAQQPAES	RVSGISMDVA	VPEETGQDPA
1130	1140	1150	1160	1170	1180	1190	1200
LEVEQCSCPP	GYRGPSCQDC	DTGYTRTPSG	LYLGTCERCS	CHGHSEACEP	ETGACQGCQH	HTEGPRCEQC	QPGYYGDAQR
1210	1220	1230	1240	1250	1260	1270	1280
GTPQDCQLCP	CYGDPAAGQA	AHTCFLDTDG	HPTCDACSPG	HSGRHCERCA	PGYYGNPSQG	QPCQRDSQVP	GPIGCNCDPQ
1290	1300	1310	1320	1330	1340	1350	1360
GSVSSQCDAA	GQCQCKAQVE	GLTCSHCRPH	HFHLSASNPD	GCLPCFCMGI	TQQCASSAYT	RHLISTHFAP	GDFQGFALVN
1370	1380	1390	1400	1410	1420	1430	1440
PQRNSRLTGE	FTVEPVPEGA	QLSFGNFAQL	GHESFYWQLP	ETYQGDKVAA	YGGKLRYTLS	YTAGPQGSPL	SDPDVQITGN
1450	1460	1470	1480	1490	1500	1510	1520
NIMLVASQPA	LQGPERRSYE	IMFREEFWRR	PDGQPATREH	LLMALADLDE	LLIRATFSSV	PLAASISAVS	LEVAQPGPSN
1530	1540	1550	1560	1570	1580	1590	1600
RPRALEVEEC	RCPPGYIGLS	CQDCAPGYTR	TGSGLYLGHC	ELCECNGHSD	LCHPETGACS	QCQHNAAGEF	CELCAPGYYG
1610	1620	1630	1640	1650	1660	1670	1680
DATAGTPEDC	QPCACPLTNP	ENMFSRTCES	LGAGGYRCTA	CEPGYTGQYC	EQCGPGYVGN	PSVQGGQCLP	ETNQAPLVVE
1690	1700	1710	1720	1730	1740	1750	1760
VHPARSIVPQ	GGSHSLRCQV	SGSPPHYFYW	SREDGRPVPS	GTQQRHQGSE	LHFPSVQPSD	AGVYICTCRN	LHQSNTSRAE
1770	1780	1790	1800	1810	1820	1830	1840
LLVTEAPSKP	ITVTVEEQRS	QSVRPGADVT	FICTAKSKSP	AYTLVWTRLH	NGKLPTRAMD	FNGILTIRNV	QLSDAGTYVC
1850	1860	1870	1880	1890	1900	1910	1920
TGSNMFAMDQ	GTATLHVQAS	GTLSAPVVSI	HPPQLTVQPG	QLAEFRCSAT	GSPTPTLEWT	GGPGGQLPAK	AQIHGGILRL
1930	1940	1950	1960	1970	1980	1990	2000
PAVEPTDQAQ	YLCRAHSSAG	QQVARAVLHV	HGGGGPRVQV	SPERTQVHAG	RTVRLYCRAA	GVPSATITWR	KEGGSLPPQA
2010	2020	2030	2040	2050	2060	2070	2080
RSERTDIATL	LIPAITTADA	GFYLCVATSP	AGTAQARIQV	VVLSASDASP	PPVKIESSSP	SVTEGQTLDL	NCVVAGSAHA
2090	2100	2110	2120	2130	2140	2150	2160
QVTWYRRGGS	LPPHTQVHGS	RLRLPQVSPA	DSGEYVCRVE	NGSGPKEASI	TVSVLHGTHS	GPSYTPVPGS	TRPIRIEPSS
2170	2180	2190	2200	2210	2220	2230	2240

Detailed Protein Report

Cmpd.	No. of Cmpds.	m/z meas.	Δ m/z [ppm]	z	Rt [min]	Score	P	Range	Sequence	Modification
2417	1	1175.2100	-20.32	2	60.1	10.2	1	5-27	R.AAGALLLALLLHGRLLAVTHGLR.A	
2083	1	1052.3950	-135.43	2	56.4	13.6	0	625-643	R.GMLEPVQRPDVVLMGAGYR.L	Oxidation: 2
1714	1	1011.6020	66.80	3	51.7	12.9	1	3737-3764	R.FDAGSGMATIRHPTPLALGHFHTVTLLR.S	Oxidation: 7
362	1	457.1238	-295.03	2	33.9	17.6	0	4004-4012	R.SAEPLALGR.W	

Detailed Protein Report

Protein 93: smoothelin isoform e [Homo sapiens]

Accession: gi|333360860
Database: refseq_human(refseq_human_20140103.fasta)
Database Date: 2015-11-30
Modification(s): Carbamidomethyl, Oxidation

Score: 79.9
MW [kDa]: 104.8
pI: 9.9
Sequence Coverage [%]: 4.0
No. of unique Peptides: 3

Quantitation

Q:W **Median:** 0.20 **CV:** 0.00 % **No. of Peptides:** 1

10	20	30	40	50	60	70	80
MGGMGTPGDL	HAACPSTTRR	DALCLGPEFE	FDLRHSAGCP	GDLGFFPLDP	DTEAGGSGCL	PTWLPLRRLA	RGRLEVTADL
**90**	**100**	**110**	**120**	**130**	**140**	**150**	**160**
AERRRIRSAI	RELQRQELER	EEEALASKRF	RAERQDNKEN	WLHSQQREAE	QRAALARLAG	QLESMNDVEE	LTALLRSAGE
**170**	**180**	**190**	**200**	**210**	**220**	**230**	**240**
YEERKLIRAA	IRRVRAQEIE	AATLAGRLYS	GRPNSGSRED	SKGLAAHRLE	QCEVPEREEQ	EQQAEVSKPT	PTPEGTSQDV
**250**	**260**	**270**	**280**	**290**	**300**	**310**	**320**
TTVTLLLRAP	PGSTSSSPAS	PSSSPTPASP	EPPLEPAEAQ	CLTAEVPGSP	EPPPSPPKTT	SPEPQESPTL	PSTEGQVVNK
**330**	**340**	**350**	**360**	**370**	**380**	**390**	**400**
LLSGPKETPA	AQSPTRGPSD	TKRADVAGPR	PCQRSLSVLS	PRQPAQNRES	TPLASGPSSF	QRAGSVRDRV	HKFTSDSPMA
**410**	**420**	**430**	**440**	**450**	**460**	**470**	**480**
ARLQDGTPQA	ALSPLTPARL	LGPSLTSTTP	ASSSSGSSSR	GPSDTSSRFS	KEQRGVAQPL	AQLRSCPQEE	GPRGRGLAAR
**490**	**500**	**510**	**520**	**530**	**540**	**550**	**560**
PLENRAGGPV	ARSEEPGAPL	PVAVGTAEPG	GSMKTTFTIE	IKDGRGQAST	GRVLLPTGNQ	RAELTLGLRA	PPTLLSTSSG
**570**	**580**	**590**	**600**	**610**	**620**	**630**	**640**
GKSTITRVNS	PGTLARLGSV	THVTSFSHAP	PSSRGGCSIK	MEAEPAEPLA	AAVEAANGAE	QTRVNKAPEG	RSPLSAEELM
**650**	**660**	**670**	**680**	**690**	**700**	**710**	**720**
TIEDEGVLDK	MLDQSTDFEE	RKLIRAALRE	LRQRKRDQRD	KERERRLQEA	RGRPGEGRGN	TATETTTRHS	QRAADGSAVS
**730**	**740**	**750**	**760**	**770**	**780**	**790**	**800**
TVTKTERLVH	SNDGTRTART	TTVESSFVRR	SENGSGSTMM	QTKTFSSSSS	SKKMGSIFDR	EDQASPRAGS	LAALEKRQAE
**810**	**820**	**830**	**840**	**850**	**860**	**870**	**880**
KKKELMKAQS	LPKTSASQAR	KAMIEKLEKE	GAAGSPGGPR	AAVQRSTSFG	VPNANSIKQM	LLDWCRAKTR	GYEHVDIQNF
**890**	**900**	**910**	**920**	**930**	**940**	**950**	**960**
SSSWSDGMAF	CALVHNFFPE	AFDYGQLSPQ	NRRQNFEVAF	SSAETHADCP	QLLDTEDMVR	LREPDWKCVY	TYIQEFYRCL
**970**	**980**						
VQKGLVKTKK	S						

Cmpd.	No. of Cmpds.	m/z meas.	Δ m/z [ppm]	z	Rt [min]	Score	P	Range	Sequence	Modification	Ratios
2098	2	938.2855	-133.64	2	54.8	13.7	0	1-19	-.MGGMGTPGDLHAACPSTTR.R	Oxidation: 1	
1663	1	1044.8761	-92.00	2	50.6	17.6	1	1-20	-.MGGMGTPGDLHAACPSTTRR.D	Carbamidomethyl: 14; Oxidation: 1	
1621	1	502.6927	-108.16	2	50.1	15.3	1	523-532	K.DGRGQASTGR.V		Q:W 0.20

Detailed Protein Report

Protein 94: PREDICTED: collagen alpha-1(XIV) chain isoform X1 [Homo sapiens]

**Accession:**	gi|530389313	**Score:**	79.9
**Database:**	refseq_human(refseq_human_20140103.fasta)	**MW [kDa]:**	191.8
**Database Date:**	2015-11-30	**pI:**	5.1
		**Sequence Coverage [%]:**	4.0
		**No. of unique Peptides:**	4

Quantitation

Q:W	**Median:** 2.29	**CV:** 0.00 %	**No. of Peptides:**	1
M:Q	**Median:** 0.55	**CV:** 0.00 %	**No. of Peptides:**	1

Detailed Protein Report

10	20	30	40	50	60	70	80
MKIFQRKMRY	WLLPPFLAIV	YFCTIVQGQV	APPTRLRYNV	ISHDSIQISW	KAPRGKFGGY	KLLVTPTSGG	KTNQLNLQNT
90	100	110	120	130	140	150	160
ATKAIIQGLM	PDQNYTVQII	AYNKDKESKP	AQGQFRIKDL	EKRKDPKPRV	KVVDRGNGSR	PSSPEEVKFV	CQTPAIADIV
170	180	190	200	210	220	230	240
ILVDGSWSIG	RFNFRLVRHF	LENLVTAFDV	GSEKTRIGLA	QYSGDPRIEW	HLNAFSTKDE	VIEAVRNLPY	KGGNTLTGLA
250	260	270	280	290	300	310	320
LNYIFENSFK	PEAGSRTGVS	KIGILITDGK	SQDDIIPPSR	NLRESGVELF	AIGVKNADVN	ELQEIASEPD	STHVYNVAEF
330	340	350	360	370	380	390	400
DLMHTVVESL	TRTLCSRVEE	QDREIKASAH	AITGPPTELI	TSEVTARSFM	VNWTHAPGNV	EKYRVVYYPT	RGGKPDEVVV
410	420	430	440	450	460	470	480
DGTVSSTVLK	NLMSLTEYQI	AVFAIYAHTA	SEGLRGTETT	LALPMASDLL	LYDVTENSMR	VKWDAVPGAS	GYLILYAPLT
490	500	510	520	530	540	550	560
EGLAGDEKEM	KIGETHTDIE	LSGLLPNTEY	TVTVYAMFGE	EASDPVTGQE	TTLALSPPRN	LRISNVGSNS	ARLTWDPTSR
570	580	590	600	610	620	630	640
QINGYRIVYN	NADGTEINEV	EVDPITTFPL	KGLTPLTEYT	IAIFSIYDEG	QSEPLTGVFT	TEEVPAQQYL	EIDEVTTDSF
650	660	670	680	690	700	710	720
RVTWHPLSAD	EGLHKLMWIP	VYGGKTEEVV	LKEEQDSHVI	EGLEPGTEYE	VSLLAVLDDG	SESEVVTAVG	TTLDSFWTEP
730	740	750	760	770	780	790	800
ATTIVPTTSV	TSVFQTGIRN	LVVGDETTSS	LRVKWDISDS	DVQQFRVTYM	TAQGDPEEEV	IGTVMVPGSQ	NNLLLKPLLP
810	820	830	840	850	860	870	880
DTEYKVTVTP	IYTDGEGVSV	SAPGKTLPSS	GPQNLRVSEE	WYNRLRITWD	PPSSPVKGYR	IVYKPVSVPG	PTLETFVGAD
890	900	910	920	930	940	950	960
INTILITNLL	SGMDYNVKIF	ASQASGFSDA	LTGMVKTLFL	GVTNLQAKHV	EMTSLCAHWQ	VHRHATAYRV	VIESLQDRQK
970	980	990	1000	1010	1020	1030	1040
QESTVGGGTT	RHCFYGLQPD	SEYKISVYTK	LQEIEGPSVS	IMEKTQSLPT	RPPTFPPTIP	PAKEVCKAAK	ADLVFMVDGS
1050	1060	1070	1080	1090	1100	1110	1120
WSIGDENFNK	IISFLYSTVG	ALNKIGTDGT	QVAMVQFTDD	PRTEFKLNAY	KTKETLLDAI	KHISYKGGNT	KTGKAIKYVR
1130	1140	1150	1160	1170	1180	1190	1200
DTLFTAESGT	RRGIPKVIVV	ITDGRSQDDV	NKISREMQLD	GYSIFAIGVA	DADYSELVSI	GSKPSARHVF	FVDDFDAFKK
1210	1220	1230	1240	1250	1260	1270	1280
IEDELITFVC	ETASATCPVV	HKDGIDLAGF	KMMEMFGLVE	KDFSSVEGVS	MEPGTFNVFP	CYQLHKDALV	SQPTRYLHPE
1290	1300	1310	1320	1330	1340	1350	1360
GLPSDYTISF	LFRILPDTPQ	EPFALWEILN	KNSDPLVGVI	LDNGGKTLTY	FNYDQSGDFQ	TVTFEGPEIR	KIFYGSFHKL
1370	1380	1390	1400	1410	1420	1430	1440
HIVVSETLVK	VVIDCKQVGE	KAMNASANIT	SDGVEVLGKM	VRSRGPGGNS	APFQLQMFDI	VCSTSWANTD	KCCELPGLRD
1450	1460	1470	1480	1490	1500	1510	1520
DESCPDLPHS	CSCSETNEVA	LGPAGPPGGP	GLRGPKGQQG	EPGPKGPDGP	RGEIGLPGPQ	GPPGPQGPSG	LSIQGMPGMP
1530	1540	1550	1560	1570	1580	1590	1600
GEKGEKGDTG	LPGPQGIPGG	VGSPGRDGSP	GQRGLPGKDG	SSGPPGPPGP	IGIPGTPGVP	GITGSMGPQG	ALGPPGVPGA
1610	1620	1630	1640	1650	1660	1670	1680
KGERGERGDL	QSQAMVRSVA	RQVCEQLIQS	HMARYTAILN	QIPSHSSSIR	TVQGPPGEPG	RPGSPGAPGE	QGPPGTPGFP
1690	1700	1710	1720	1730	1740	1750	1760
GNAGVPGTPG	ERGLTGIKGE	KGNPGVGTQG	PRGPPGPAGP	SGESRPGSPG	PPGSPGPRGP	PGHLGVPGPQ	GPSGQPGYCD
1770	1780	1790					
PSSCSAYGVR	DLIPYNDYQH						

Cmpd.	No. of Cmpds.	m/z meas.	Δ m/z [ppm]	z	Rt [min]	Score	P	Range	Sequence	Modification	Ratios
2684	15	673.3700	31.83	2	63.7	19.1	0	208-218	R.IEWHLNAFSTK.D		
74	2	809.3972	-57.78	2	30.6	10.2	1	740-754	R.NLVVGDETTSSLRVK.W		Q:W 2.29<br>M:Q 0.55
2197	1	585.1748	-243.43	2	57.7	10.6	0	826-836	K.TLPSSGPQNLR.V		
834	1	683.9031	-31.20	2	40.0	13.1	1	1133-1145	R.GIPKVIVVITDGR.S		

BRUKER Detailed Protein Report

Protein 95: zinc finger protein 492 [Homo sapiens]

Accession: gi|148806865
Database: refseq_human(refseq_human_20140103.fasta)
Database Date: 2015-11-30
Modification(s): Carbamidomethyl, Oxidation

Score: 79.6
MW [kDa]: 61.1
pI: 10.3
Sequence Coverage [%]: 11.3
No. of unique Peptides: 4

Quantitation

Q:W	**Median:**	1.05	**CV:**	0.00 %	**No. of Peptides:**	1
M:Q	**Median:**	0.46	**CV:**	22.43 %	**No. of Peptides:**	2

Alias proteins:

Accession	Name	Description
gi|578833408	refseq_human (refseq_human_20140103.fasta)	PREDICTED: zinc finger protein 492 isoform X1 [Homo sapiens]

```
        10         20         30         40         50         60         70         80
MLENYRNLVF VGIAASKPDL ITCLEQGKEP WNVKRHEMVA EPPVVCSYFA RDLWPKQGKK NYFQKVILRR YKKCGCENLQ
        90        100        110        120        130        140        150        160
LRKYCKSMDE CKVHKECYNG LNQCLTTTQN KIFQCDKYVK VFHKFSNSNR HTIRHTGKKS FKCKECEKSF CMLSHLAQHK
       170        180        190        200        210        220        230        240
RIHSGEKPYK CKECGKAYNE TSNLSTHKRI HTGKKPYKCE ECGKAFNRLS HLTTHKIIHT GKKPYKCEEC GKAFNQSANL
       250        260        270        280        290        300        310        320
TTHKRIHTGE KPYKCEECGR AFSQSSTLTA HKIIHAGEKP YKCEECGKAF SQSSTLTTHK IIHTGEKFYK CEECGKAFSQ
       330        340        350        360        370        380        390        400
LSHLTTHKRI HSGEKPYKCE ECGKAFKQSS TLTTHKRIHA GEKFYKCEVC SKAFSRFSHL TTHKRIHTGE KPYKCEECGK
       410        420        430        440        450        460        470        480
AFNLSSQLTT HKIIHTGEKP YKCEECGKAF NQSSTLSKHK VIHTGEKPYK YEECGKAFNQ SSHLTTHKMI HTGEKPYKCE
       490        500        510        520        530        540
ECGKAFNNSS ILNRHKMIHT GEKLYKPESC NNACDNIAKI SKYKRNCAGE K
```

Cmpd.	No. of Cmpds.	m/z meas.	Δ m/z [ppm]	z	Rt [min]	Score	P	Range	Sequence	Modification	Ratios	
1839	1	582.2374	12.53	2	53.0	13.4	1	84-92	K.YCKSMDECK.V	Carbamidomethyl: 8		
1912	1	621.2738	-20.17	2	54.0	13.3	1	195-204	K.KPYKCEECGK.A	Carbamidomethyl: 5		
2688	1	992.0980	-39.40	3	64.8	18.1	2	495-519	R.HKMIHTGEKLYKPESCNNACDNIAK.I	Carbamidomethyl: 16, 20; Oxidation: 3	M:Q	0.37
122	1	879.4101	-4.12	3	30.5	10.1	1	497-519	K.MIHTGEKLYKPESCNNACDNIAK.I	Carbamidomethyl: 14	M:Q<br>Q:W	0.57<br>1.05

Detailed Protein Report

Protein 96: Golgin subfamily A member 4 isoform 2 [Homo sapiens]

**Accession:**	gi|6715600	**Score:**	78.5
**Database:**	refseq_human(refseq_human_20140103.fasta)	**MW [kDa]:**	261.0
**Database Date:**	2015-11-30	**pI:**	5.2
**Modification(s):**	Oxidation	**Sequence Coverage [%]:**	3.3
		**No. of unique Peptides:**	6

Quantitation

M:Q	**Median:** 0.85	**CV:** 0.00 %	**No. of Peptides:** 1

Detailed Protein Report

10	20	30	40	50	60	70	80
MFKKLKQKIS	EEQQQLQQAL	APAQASSNSS	TPTRMRSRTS	SFTEQLDEGT	PNRESGDTQS	FAQKLQLRVP	SVESLFRSPI
90	100	110	120	130	140	150	160
KESLFRSSSK	ESLVRTSSRE	SLNRLDLDSS	TASFDPPSDM	DSEAEDLVGN	SDSLNKEQLI	QRLRRMERSL	SSYRGKYSEL
170	180	190	200	210	220	230	240
VTAYQMLQRE	KKKLQGILSQ	SQDKSLRRIA	ELREELQMDQ	QAKKHLQEEF	DASLEEKDQY	ISVLQTQVSL	LKQRLRNGPM
250	260	270	280	290	300	310	320
NVDVLKPLPQ	LEPQAEVFTK	EENPESDGEP	VVEDGTSVKT	LETLQQRVKR	QENLLKRCKE	TIQSHKEQCT	LLTSEKEALQ
330	340	350	360	370	380	390	400
EQLDERLQEL	EKIKDLHMAE	KTKLITQLRD	AKNLIEQLEQ	DKGMVIAETK	RQMHETLEMK	EEEIAQLRSR	IKQMTTQGEE
410	420	430	440	450	460	470	480
LREQKEKSER	AAFEELEKAL	STAQKTEEAR	RKLKAEMDEQ	IKTIEKTSEE	ERISLQQELS	RVKQEVVDVM	KKSSEEQIAK
490	500	510	520	530	540	550	560
LQKLHEKELA	RKEQELTKKL	QTREREFQEQ	MKVALEKSQS	EYLKISQEKE	QQESLALEEL	ELQKKAILTE	SENKLRDLQQ
570	580	590	600	610	620	630	640
EAETYRTRIL	ELESSLEKSL	QENKNQSKDL	AVHLEAEKNK	HNKEITVMVE	KHKTELESLK	HQQDALWTEK	LQVLKQQYQT
650	660	670	680	690	700	710	720
EMEKLREKCE	QEKETLLKDK	EIIFQAHIEE	MNEKTLEKLD	VKQTELESLS	SELSEVLKAR	HKLEEELSVL	KDQTDKMKQE
730	740	750	760	770	780	790	800
LEAKMDEQKN	HHQQQVDSII	KEHEVSIQRT	EKALKDQINQ	LELLLKERDK	HLKEHQAHVE	NLEADIKRSE	GELQQASAKL
810	820	830	840	850	860	870	880
DVFQSYQSAT	HEQTKAYEEQ	LAQLQQKLLD	LETERILLTK	QVAEVEAQKK	DVCTELDAHK	IQVQDLMQQL	EKQNSEMEQK
890	900	910	920	930	940	950	960
VKSLTQVYES	KLEDGNKEQE	QTKQILVEKE	NMILQMREGQ	KKEIEILTQK	LSAKEDSIHI	LNEEYETKFK	NQEKKMEKVK
970	980	990	1000	1010	1020	1030	1040
QKAKEMQETL	KKKLLDQEAK	LKKELENTAL	ELSQKEKQFN	AKMLEMAQAN	SAGISDAVSR	LETNQKEQIE	SLTEVHRREL
1050	1060	1070	1080	1090	1100	1110	1120
NDVISIWEKK	LNQQAEELQE	IHEIQLQEKE	QEVAELKQKI	LLFGCEKEEM	NKEITWLKEE	GVKQDTTLNE	LQEQLKQKSA
1130	1140	1150	1160	1170	1180	1190	1200
HVNSLAQDET	KLKAHLEKLE	VDLNKSLKEN	TFLQEQLVEL	KMLAEEDKRK	VSELTSKLKT	TDEEFQSLKS	SHEKSNKSLE
1210	1220	1230	1240	1250	1260	1270	1280
DKSLEFKKLS	EELAIQLDIC	CKKTEALLEA	KTNELINISS	SKTNAILSRI	SHCQHRTTKV	KEALLIKTCT	VSELEAQLRQ
1290	1300	1310	1320	1330	1340	1350	1360
LTEEQNTLNI	SFQQATHQLE	EKENQIKSMK	ADIESLVTEK	EALQKEGGNQ	QQAASEKESC	ITQLKKELSE	NINAVTLMKE
1370	1380	1390	1400	1410	1420	1430	1440
ELKEKKVEIS	SLSKQLTDLN	VQLQNSISLS	EKEAAISSLR	KQYDEEKCEL	LDQVQDLSFK	VDTLSKEKIS	ALEQVDDWSN
1450	1460	1470	1480	1490	1500	1510	1520
KFSEWKKKAQ	SRFTQHQNTV	KELQIQLELK	SKEAYEKDEQ	INLLKEELDQ	QNKRFDCLKG	EMEDDKSKME	KKESNLETEL
1530	1540	1550	1560	1570	1580	1590	1600
KSQTARIMEL	EDHITQKTIE	IESLNEVLKN	YNQQKDIEHK	ELVQKLQHFQ	ELGEEKDNRV	KEAEEKILTL	ENQVYSMKAE
1610	1620	1630	1640	1650	1660	1670	1680
LETKKKELEH	VNLSVKSKEE	ELKALEDRLE	SESAAKLAEL	KRKAEQKIAA	IKKQLLSQME	EKEEQYKKGT	ESHLSELNTK
1690	1700	1710	1720	1730	1740	1750	1760
LQEREREVHI	LEEKLKSVES	SQSETLIVPR	SAKNVAAYTE	QEEADSQGCV	QKTYEEKISV	LQRNLTEKEK	LLQRVGQEKE
1770	1780	1790	1800	1810	1820	1830	1840
ETVSSHFEMR	CQYQERLIKL	EHAEAKQHED	QSMIGHLQEE	LEEKNKKYSL	IVAQHVEKEG	GKNNIQAKQN	LENVFDDVQK
1850	1860	1870	1880	1890	1900	1910	1920
TLQEKELTCQ	ILEQKIKELD	SCLVRQKEVH	RVEMEELTSK	YEKLQALQQM	DGRNKPTELL	EENTEEKSKS	HLVQPKLLSN
1930	1940	1950	1960	1970	1980	1990	2000
MEAQHNDLEF	KLAGAEREKQ	KLGKEIVRLQ	KDLRMLRKEH	QQELEILKKE	YDQEREEKIK	QEQEDLELKH	NSTLKQLMRE
2010	2020	2030	2040	2050	2060	2070	2080
FNTQLAQKEQ	ELEMTIKETI	NKAQEVEAEL	LESHQEETNQ	LLKKIAEKDD	DLKRTAKRYE	EILDAREEEM	TAKVRDLQTQ
2090	2100	2110	2120	2130	2140	2150	2160
LEELQKKYQQ	KLEQEENPGN	DNVTIMELQT	QLAQKTTLIS	DSKLKEQEFR	EQIHNLEDRL	KKYEKNVYAT	TVGTPYKGGN
2170	2180	2190	2200	2210	2220	2230	2240

Detailed Protein Report

Cmpd.	No. of Cmpds.	m/z meas.	Δ m/z [ppm]	z	Rt [min]	Score	P	Range	Sequence	Modification	Ratios
2067	1	672.2171	-202.08	2	56.0	14.5	1	601-611	K.HNKEITVMVEK.H	Oxidation: 8	
362	2	539.2298	-100.57	2	33.2	13.3	1	963-971	K.AKEMQETLK.K		M:Q 0.85
2557	1	715.3634	85.34	2	61.9	10.2	1	1495-1506	R.FDCLKGEMEDDK.S		
2790	2	683.3645	24.15	3	65.3	12.8	1	1829-1845	K.QNLENVFDDVQKTLQEK.E		
190	1	716.5084	147.04	2	32.1	12.0	2	1856-1867	K.IKELDSCLVRQK.E		
1522	1	620.4115	154.27	3	48.7	15.7	2	1866-1880	R.QKEVHRVEMEELTSK.Y	Oxidation: 9	

Detailed Protein Report

Protein 97: PREDICTED: ubiquitin carboxyl-terminal hydrolase 24 isoform X1 [Homo sapiens]

**Accession:**	gi|530362258	**Score:**	78.2
**Database:**	refseq_human(refseq_human_20140103.fasta)	**MW [kDa]:**	275.5
**Database Date:**	2015-11-30	**pI:**	6.1
**Modification(s):**	Carbamidomethyl, Oxidation	**Sequence Coverage [%]:**	2.7
		**No. of unique Peptides:**	4

Quantitation

Q:W	**Median:**	0.99	**CV:**	0.00 %	**No. of Peptides:**	1
M:Q	**Median:**	0.87	**CV:**	0.00 %	**No. of Peptides:**	1

Detailed Protein Report

10	20	30	40	50	60	70	80
MDRCMPEAFK	KLLTSSAVHK	WGTEIHEGIY	NMLMLLIELV	AERIKQDPIP	TGLLGVLTMA	FNPDNEYHFK	NRMKVSQRNW
90	100	110	120	130	140	150	160
AEVFGEGNMF	AVSPVSTFQK	EPHGWVVDLV	NKFGELGGFA	AIQAKLHSED	IELGAVSALI	QPLGVCAEYL	NSSVVQPMLD
170	180	190	200	210	220	230	240
PVILTTIQDV	RSVEEKDLKD	KRLVSIPELL	SAVKLLCMRF	QPDLVTIVDD	LRLDILLRML	KSPHFSAKMN	SLKEVTKLIE
250	260	270	280	290	300	310	320
DSTLSKSVKN	AIDTDRLLDW	LVENSVLSIA	LEGNIDQAQY	CDRIKGIIEL	LGSKLSLDEL	TKIWKIQSGQ	SSTVIENIHT
330	340	350	360	370	380	390	400
IIAAAAVKFN	SDQLNHLFVL	IQKSWETESD	RVRQKLLSLI	GRIGREARFE	TTSGKVLDVL	WELAHLPTLP	SSLIQQALEE
410	420	430	440	450	460	470	480
HLTILSDAYA	VKEAIKRSYI	IKCIEDIKRP	GEWSGLEKNK	KDGFKSSQLN	NPQFVWVVPA	LRQLHEITRS	FIKQTYQKQD
490	500	510	520	530	540	550	560
KSIIQDLKKN	FEIVKLVTGS	LIACHRLAAA	VAGPGGLSGS	TLVDGRYTYR	EYLEAHLKFL	AFFLQEATLY	LGWNRAKEIW
570	580	590	600	610	620	630	640
ECLVTGQDVC	ELDREMCFEW	FTKGQHDLES	DVQQQLFKEK	ILKLESYEIT	MNGFNLFKTF	FENVNLCDHR	LKRQGAQLYV
650	660	670	680	690	700	710	720
EKLELIGMDF	IWKIAMESPD	EEIANEAIQL	IINYSYINLN	PRLKKDSVSL	HKKFIADCYT	RLEAASSALG	GPTLTHAVTR
730	740	750	760	770	780	790	800
ATKMLTATAM	PTVATSVQSP	YRSTKLVIIE	RLLLLAERYV	ITIEDFYSVP	RTILPHGASF	HGHLLTLNVT	YESTKDTFTV
810	820	830	840	850	860	870	880
EAHSNETIGS	VRWKIAKQLC	SPVDNIQIFT	NDSLLTVNKD	QKLLHQLGFS	DEQILTVKTS	GSGTPSGSSA	DSSTSSSSSS
890	900	910	920	930	940	950	960
SGVFSSSYAM	EQEKSLPGVV	MALVCNVFDM	LYQLANLEEP	RITLRVRKLL	LLIPTDPAIQ	EALDQLDSLG	RKKTLLSESS
970	980	990	1000	1010	1020	1030	1040
SQSSKSPSLS	SKQQHQPSAS	SILESLFRSF	APGMSTFRVL	YNLEVLSSKL	MPTADDDMAR	SCAKSFCENF	LKAGGLSLVV
1050	1060	1070	1080	1090	1100	1110	1120
NVMQRDSIPS	EVDYETRQGV	YSICLQLARF	LLVGQTMPTL	LDEDLTKDGI	EALSSRPFRN	VSRQTSRQMS	LCGTPEKSSY
1130	1140	1150	1160	1170	1180	1190	1200
RQLSVSDRSS	IRVEEIIPAA	RVAIQTMEVS	DFTSTVACFM	RLSWAAAAGR	LDLVGSSQPI	KESNSLCPAG	IRNRLSSSGS
1210	1220	1230	1240	1250	1260	1270	1280
NCSSGSEGEP	VALHAGICVR	QQSVSTKDSL	IAGEALSLLV	TCLQLRSQQL	ASFYNLPCVA	DFIIDILLGS	PSAEIRRVAC
1290	1300	1310	1320	1330	1340	1350	1360
DQLYTLSQTD	TSAHPDVQKP	NQFLLGVILT	AQLPLWSPTS	IMRGVNQRLL	SQCMEYFDLR	CQLLDDLTTS	EMEQLRISPA
1370	1380	1390	1400	1410	1420	1430	1440
TMLEDEITWL	DNFEPNRTAE	CETSEADNIL	LAGHLRLIKT	LLSLCGAEKE	MLGSSLIKPL	LDDFLFRASR	IILNSHSPAG
1450	1460	1470	1480	1490	1500	1510	1520
SAAISQQDFH	PKCSTANSRL	AAYEVLVMLA	DSSPSNLQII	IKELLSMHHQ	PDPALTKEFD	YLPPVDSRSS	SGFVGLRNGG
1530	1540	1550	1560	1570	1580	1590	1600
ATCYMNAVFQ	QLYMQPGLPE	SLLSVDDDTD	NPDDSVFYQV	QSLFGHLMES	KLQYYVPENF	WKIFKMWNKE	LYVREQQDAY
1610	1620	1630	1640	1650	1660	1670	1680
EFFTSLIDQM	DEYLKKMGRD	QIFKNTFQGI	YSDQKICKDC	PHRYEREEAF	MALNLGVTSC	QSLEISLDQF	VRGEVLEGSN
1690	1700	1710	1720	1730	1740	1750	1760
AYYCEKCKEK	RITVKRTCIK	SLPSVLVIHL	MRFGFDWESG	RSIKYDEQIR	FPWMLNMEPY	TVSGMARQDS	SSEVGENGRS
1770	1780	1790	1800	1810	1820	1830	1840
VDQGGGGSPR	KKVALTENYE	LVGVIVHSGQ	AHAGHYYSFI	KDRRGCGKGK	WYKFNDTVIE	EFDLNDETLE	YECFGGEYRP
1850	1860	1870	1880	1890	1900	1910	1920
KVYDQTNPYT	DVRRRYWNAY	MLFYQRVSDQ	NSPVLPKKSR	VSVVRQEAED	LSLSAPSSPE	ISPQSSPRPH	RPNNDRLSIL
1930	1940	1950	1960	1970	1980	1990	2000
TKLVKKGEKK	GLFVEKMPAR	IYQMVRDENL	KFMKNRDVYS	SDYFSFVLSL	ASLNATKLKH	PYYPCMAKVS	LQLAIQFLFQ
2010	2020	2030	2040	2050	2060	2070	2080
TYLRTKKKLR	VDTEEWIATI	EALLSKSFDA	CQWLVEYFIS	SEGRELIKIF	LLECNVREVR	VAVATILEKT	LDSALFYQDK
2090	2100	2110	2120	2130	2140	2150	2160
LKSLHQLLEV	LLALLDKDVP	ENCKNCAQYF	FLFNTFVQKQ	GIRAGDLLLR	HSALRHMISF	LLGASRQNNQ	IRRWSSAQAR
2170	2180	2190	2200	2210	2220	2230	2240

Detailed Protein Report

Cmpd.	No. of Cmpds.	m/z meas.	Δ m/z [ppm]	z	Rt [min]	Score	P	Range	Sequence	Modification	Ratios
9	1	650.7714	-9.80	2	29.3	16.5	1	1-10	-.MDRCMPEAFK.K	Carbamidomethyl: 4; Oxidation: 1	
2676	1	585.8808	7.47	2	61.8	11.9	1	284-294	R.IKGIIELLGSK.L		
2168	1	841.4140	51.38	2	57.5	11.5	1	1010-1024	K.LMPTADDDMARSCAK.S	Carbamidomethyl: 13	
143	2	849.3662	-2.35	2	31.0	11.7	1	1010-1024	K.LMPTADDDMARSCAK.S	Carbamidomethyl: 13; Oxidation: 2	M:Q 0.87<br>Q:W 0.99

Detailed Protein Report

Protein 98: syntaxin-binding protein 5 isoform b [Homo sapiens]

Accession: gi|189217915
Database: refseq_human(refseq_human_20140103.fasta)
Database Date: 2015-11-30
Modification(s): Oxidation

Score: 77.7
MW [kDa]: 127.5
pI: 7.1
Sequence Coverage [%]: 5.3
No. of unique Peptides: 4

Quantitation

M:Q **Median:** 1.51 **CV:** 0.00 % **No. of Peptides:** 1

10	20	30	40	50	60	70	80
MRKFNIRKVL	DGLTAGSSSA	SQQQQQQHPP	GNREPEIQET	LQSEHFQLCK	TVRHGFPYQP	SALAFDPVQK	ILAVGTQTGA
90	100	110	120	130	140	150	160
LRLFGRPGVE	CYCQHDSGAA	VIQLQFLINE	GALVSALADD	TLHLWNLRQK	RPAILHSLKF	CRERVTFCHL	PFQSKWLYVG
170	180	190	200	210	220	230	240
TERGNIHIVN	VESFTLSGYV	IMWNKAIELS	SKSHPGPVVH	ISDNPMDEGK	LLIGFESGTV	VLWDLKSKKA	DYRYTYDEAI
250	260	270	280	290	300	310	320
HSVAWHHEGK	QFICSHSDGT	LTIWNVRSPA	KPVQTITPHG	KQLKDGKKPE	PCKPILKVEF	KTTRSGEPFI	ILSGGLSYDT
330	340	350	360	370	380	390	400
VGRRPCLTVM	HGKSTAVLEM	DYSIVDFLTL	CETPYPNDFQ	EPYAVVVLLE	KDLVLIDLAQ	NGYPIFENPY	PLSIHESPVT
410	420	430	440	450	460	470	480
CCEYFADCPV	DLIPALYSVG	ARQKRQGYSK	KEWPINGGNW	GLGAQSYPEI	IITGHADGSV	KFWDASAITL	QVLYKLKTSK
490	500	510	520	530	540	550	560
VFEKSRNKDD	RPNTDIVDED	PYAIQIISWC	PESRMLCIAG	VSAHVIIYRF	SKQEVITEVI	PMLEVRLLYE	INDVETPEGE
570	580	590	600	610	620	630	640
QPPPLPTPVG	GSNPQPIPPQ	SHPSTSSSSS	DGLRDNVPCL	KVKNSPLKQS	PGYQTELVIQ	LVWVGGEPPQ	QITSLAVNSS
650	660	670	680	690	700	710	720
YGLVVFGNCN	GIAMVDYLQK	AVLLNLGTIE	LYGSNDPYRR	EPRSPRKSRQ	PSGAGLCDIS	EGTVVPEDRC	KSPTSGSSSP
730	740	750	760	770	780	790	800
HNSDDEQKMN	NFIEKVKTKS	RKFSKMVAND	IAKMSRKLSL	PTDLKPDLDV	KDNSFSRSRS	SSVTSIDKES	REAISALHFC
810	820	830	840	850	860	870	880
ETFTRKTDSS	PSPCLWVGTT	LGTVLVIALN	LPPGGEQRLL	QPVIVSPSGT	ILRLKGAILR	MAFLDTTGCL	IPPAYEPWRE
890	900	910	920	930	940	950	960
HNVPEEKDEK	EKLKKRRPVS	VSPSSSQEIS	ENQYAVICSE	KQAKVISLPT	QNCAYKQNIT	ETSFVLRGDI	VALSNSICLA
970	980	990	1000	1010	1020	1030	1040
CFCANGHIMT	FSLPSLRPLL	DVYYLPLTNM	RIARTFCFTN	NGQALYLVSP	TEIQRLTYSQ	ETCENLQEML	GELFTPVETP
1050	1060	1070	1080	1090	1100	1110	1120
EAPNRGFFKG	LFGGGAQSLD	REELFGESSS	GKASRSLAQH	IPGPGGIEGV	KGAASGVVGE	LARARLALDE	RGQKLGDLEE
1130	1140	1150	1160				
RTAAMLSSAE	SFSKHAHEIM	LKYKDKKWYQ	F				

Cmpd.	No. of Cmpds.	m/z meas.	Δ m/z [ppm]	z	Rt [min]	Score	P	Range	Sequence	Modification	Ratios
1682	1	796.3351	-89.13	2	50.8	10.5	1	143-155	R.ERVTFCHLPFQSK.W		
1487	1	676.3270	-69.10	2	48.8	11.5	2	742-753	R.KFSKMVANDIAK.M		M:Q 1.51
2747	1	807.3929	-21.85	2	65.6	15.2	2	743-756	K.FSKMVANDIAKMSR.K	Oxidation: 4	
1944	1	665.3207	-0.95	2	54.7	15.6	0	1122-1134	R.TAAMLSSAESFSK.H		

Detailed Protein Report

Protein 99: PREDICTED: E3 ubiquitin-protein ligase UBR4 isoform X11 [Homo sapiens]

**Accession:**	gi|578798772	**Score:**	77.4
**Database:**	refseq_human(refseq_human_20140103.fasta)	**MW [kDa]:**	574.7
**Database Date:**	2015-11-30	**pI:**	5.6
**Modification(s):**	Carbamidomethyl, Oxidation	**Sequence Coverage [%]:**	1.9
		**No. of unique Peptides:**	5

Detailed Protein Report

10	20	30	40	50	60	70	80
MATSGGEEAA	AAAPAPGTPA	TGADTTPGWE	VAVRPLLSAS	YSAFEMKELP	QLVASVIESE	SEILHHEKQY	EPFYSSFVAL
90	100	110	120	130	140	150	160
STHYITTVCS	LIPRNQLQSV	AAACKVLIEF	SLLRLENPDE	ACAVSQKHLI	LLIKGLCTGC	SRLDRTEIIT	FTAMMKSAKL
170	180	190	200	210	220	230	240
PQTVKTLSDV	EDQKELASPV	SPELRQKEVQ	MNFLNQLTSV	FNPRTVASQP	ISTQTLVEGE	NDEQSSTDQA	SAIKTKNVFI
250	260	270	280	290	300	310	320
AQNVASLQEL	GGSEKLLRVC	LNLPYFLRYI	NRFQDAVLAN	SFFIMPATVA	DATAVRNGFH	SLVIDVTMAL	DTLSLPVLEP
330	340	350	360	370	380	390	400
LNPSRLQDVT	VLSLSCLYAG	VSVATCMAIL	HVGSAQQVRT	GSTSSKEDDY	ESDAATIVQK	CLEIYDMIGQ	AISSSRRAGG
410	420	430	440	450	460	470	480
EHYQNFQLLG	AWCLLNSLFL	ILNLSPTALA	DKGKEKDPLA	ALRVRDILSR	TKEGVGSPKL	GPGKGHQGFG	VLSVILANHA
490	500	510	520	530	540	550	560
IKLLTSLFQD	LQVEALHKGW	ETDGPPAALS	IMAQSTSIQR	IQRLIDSVPL	MNLLLTLLST	SYRKACVLQR	QRKGSMSSDA
570	580	590	600	610	620	630	640
SASTDSNTYY	EDDFSSTEED	SSQDDDSEPI	LGQWFEETIS	PSKEKAAPPP	PPPPPPLESS	PRVKSPSKQA	PGEKGNILAS
650	660	670	680	690	700	710	720
RKDPELFLGL	ASNILNFITS	SMLNSRNNFI	RNYLSVSLSE	HHMATLASII	KEVDKDGLKG	SSDEEFAAAL	YHFNHSLVTS
730	740	750	760	770	780	790	800
DLQSPNLQNT	LLQQLGVAPF	SEGPWPLYIH	PQSLSVLSRL	LLIWQHKASA	QGDPDVPECL	KVWDRFLSTM	KQNALQGVVP
810	820	830	840	850	860	870	880
SETEDLNVEH	LQMLLLIFHN	FTETGRRAIL	SLFVQIIQEL	SVNMDAQMRF	VPLILARLLL	IFDYLLHQYS	KAPVYLFEQV
890	900	910	920	930	940	950	960
QHNLLSPPFG	WASGSQDSNS	RRATTPLYHG	FKEVEENWSK	HFSSDAVPHP	RFYCVLSPEA	SEDDLNRLDS	VACDVLFSKL
970	980	990	1000	1010	1020	1030	1040
VKYDELYAAL	TALLAAGSQL	DTVRRKENKN	VTALEACALQ	YYFLILWRIL	GILPPSKTYI	NQLSMNSPEM	SECDILHTLR
1050	1060	1070	1080	1090	1100	1110	1120
WSSRLRISSY	VNWIKDHLIK	QGMKAEHASS	LLELASTTKC	SSVKYDVEIV	EEYFARQISS	FCSIDCTTIL	QLHEIPSLQS
1130	1140	1150	1160	1170	1180	1190	1200
IYTLDAAISK	VQVSLDEHFS	KMAAETDPHK	SSEITKNLLP	ATLQLIDTYA	SFTRAYLLQN	FNEEGTTEKP	SKEKLQGFAA
1210	1220	1230	1240	1250	1260	1270	1280
VLAIGSSRCK	ANTLGPTLVQ	NLPSSVQTVC	ESWNNINTNE	FPNIGSWRNA	FANDTIPSES	YISAVQAAHL	GTLCSQSLPL
1290	1300	1310	1320	1330	1340	1350	1360
AASLKHTLLS	LVRLTGDLIV	WSDEMNPPQV	IRTLLPLLLE	SSTESVAEIS	SNSLERILGP	AESDEFLARV	YEKLITGCYN
1370	1380	1390	1400	1410	1420	1430	1440
ILANHADPNS	GLDESILEEC	LQYLEKQLES	SQARKAMEEF	FSDSGELVQI	MMATANENLS	AKFCNRVLKF	FTKLFQLTEK
1450	1460	1470	1480	1490	1500	1510	1520
SPNPSLLHLC	GSLAQLACVE	PVRLQAWLTR	MTTSPPKDSD	QLDVIQENRQ	LLQLLTTYIV	RENSQVGEGV	CAVLLGTLTP
1530	1540	1550	1560	1570	1580	1590	1600
MATEMLANGD	GTGFPELMVV	MATLASAGQG	AGHLQLHNAA	VDWLSRCKKY	LSQKNVVEKL	NANVMHGKHV	MILECTCHIM
1610	1620	1630	1640	1650	1660	1670	1680
SYLADVTNAL	SQSNGQGPSH	LSVDGEERAI	EVDSDWVEEL	AVEEEDSQAE	DSDEDSLCNK	LCTFTITQKE	FMNQHWYHCH
1690	1700	1710	1720	1730	1740	1750	1760
TCKMVDGVGV	CTVCAKVCHK	DHEISYAKYG	SFFCDCGAKE	DGSCLALVKR	TPSSGMSSTM	KESAFQSEPR	ISESLVRHAS
1770	1780	1790	1800	1810	1820	1830	1840
TSSPADKAKV	TISDGKVADE	EKPKKSSLCR	TVEGCREELQ	NQANFSFAPL	VLDMLNFLMD	AIQTNFQQAS	AVGSSSRAQQ
1850	1860	1870	1880	1890	1900	1910	1920
ALSELHTVEK	AVEMTDQLMV	PTLGSQEGAF	ENVRMNYSGD	QGQTIRQLIS	AHVLRRVAMC	VLSSPHGRRQ	HLAVSHEKGK
1930	1940	1950	1960	1970	1980	1990	2000
ITVLQLSALL	KQADSSKRKL	TLTRLASAPV	PFTVLSLTGN	PCKEDYLAVC	GLKDCHVLTF	SSSGSVSDHL	VLHPQLATGN
2010	2020	2030	2040	2050	2060	2070	2080
FIIKAVWLPG	SQTELAIVTA	DFVKIYDLCV	DALSPTFYFL	LPSSKIRDVT	FLFNEEGKNI	IVIMSSAGYI	YTQLMEEASS
2090	2100	2110	2120	2130	2140	2150	2160
AQQGPFYVTN	VLEINHEDLK	QDSNSQVAGG	GVSVYYSHVL	QMLFFSYCQG	KSFAATISRT	TLEVLQLFPI	NIKSSNGGSK
2170	2180	2190	2200	2210	2220	2230	2240

Detailed Protein Report

Cmpd.	No. of Cmpds.	m/z meas.	Δ m/z [ppm]	z	Rt [min]	Score	P	Range	Sequence	Modification
270	1	530.5999	-29.12	3	33.1	13.6	2	1777-1790	K.VADEEKPKKSSLCR.T	
1928	1	917.3279	-102.08	2	54.5	15.2	1	2608-2623	K.LPQMETGMDEGKEPQK.Q	Oxidation: 4
1934	1	745.7866	-161.90	2	54.6	10.5	1	3240-3252	R.DLHTLDSHVRGIK.K	
1759	2	920.8290	-80.75	2	52.0	12.7	0	4486-4502	K.MAGVMAQCGGLECMLNR.L	Carbamidomethyl: 13
375	1	878.7841	3.73	3	34.7	12.0	2	4668-4690	K.VFLDCFCKIAAGIKNNSNGHQLK.D	Carbamidomethyl: 5, 7

Detailed Protein Report

Protein 100: TNF receptor-associated factor 2 [Homo sapiens]

Accession: gi|22027612
Database: refseq_human(refseq_human_20140103.fasta)
Database Date: 2015-11-30
Modification(s): Carbamidomethyl

Score: 77.4
MW [kDa]: 55.8
pI: 9.0
Sequence Coverage [%]: 9.2
No. of unique Peptides: 2

10	20	30	40	50	60	70	80
MAAASVTPPG	SLELLQPGFS	KTLLGTKLEA	KYLCSACRNV	LRRPFQAQCG	HRYCSFCLAS	ILSSGPQNCA	ACVHEGIYEE
**90**	**100**	**110**	**120**	**130**	**140**	**150**	**160**
GISILESSSA	FPDNAARREV	ESLPAVCPSD	GCTWKGTLKE	YESCHEGRCP	LMLTECPACK	GLVRLGEKER	HLEHECPERS
**170**	**180**	**190**	**200**	**210**	**220**	**230**	**240**
LSCRHCRAPC	CGADVKAHHE	VCPKFPLTCD	GCGKKKIPRE	KFQDHVKTCG	KCRVPCRFHA	IGCLETVEGE	KQQEHEVQWL
**250**	**260**	**270**	**280**	**290**	**300**	**310**	**320**
REHLAMLLSS	VLEAKPLLGD	QSHAGSELLQ	RCESLEKKTA	TFENIVCVLN	REVERVAMTA	EACSRQHRLD	QDKIEALSSK
**330**	**340**	**350**	**360**	**370**	**380**	**390**	**400**
VQQLERSIGL	KDLAMADLEQ	KVLEMEASTY	DGVFIWKISD	FARKRQEAVA	GRIPAIFSPA	FYTSRYGYKM	CLRIYLNGDG
**410**	**420**	**430**	**440**	**450**	**460**	**470**	**480**
TGRGTHLSLF	FVVMKGPNDA	LLRWPFNQKV	TLMLLDQNNR	EHVIDAFRPD	VTSSSFQRPV	NDMNIASGCP	LFCPVSKMEA
**490**	**500**	**510**					
KNSYVRDDAI	FIKAIVDLTG	L					

Cmpd.	No. of Cmpds.	m/z meas.	Δ m/z [ppm]	z	Rt [min]	Score	P	Range	Sequence	Modification
2037	1	1051.4158	-90.99	2	54.1	10.8	1	214-231	R.VPCRFHAIGCLETVEGEK.Q	Carbamidomethyl: 3, 10
2147	1	1022.9076	-91.14	2	57.0	15.3	1	214-231	R.VPCRFHAIGCLETVEGEK.Q	Carbamidomethyl: 10

Detailed Protein Report

Protein 101: fibrillin-1 precursor [Homo sapiens]

**Accession:**	gi|281485550	**Score:**	77.3
**Database:**	refseq_human(refseq_human_20140103.fasta)	**MW [kDa]:**	312.1
**Database Date:**	2015-11-30	**pI:**	4.7
**Modification(s):**	Carbamidomethyl, Oxidation	**Sequence Coverage [%]:**	3.2
		**No. of unique Peptides:**	5

Quantitation

Q:W	**Median:**	2.32	**CV:**	34.06 %	**No. of Peptides:**	2
M:Q	**Median:**	0.41	**CV:**	1.01 %	**No. of Peptides:**	2

Detailed Protein Report

10	20	30	40	50	60	70	80
MRRGRLLEIA	LGFTVLLASY	TSHGADANLE	AGNVKETRAS	RAKRRGGGGH	DALKGPNVCG	SRYNAYCCPG	WKTLPGGNQC
90	100	110	120	130	140	150	160
IVPICRHSCG	DGFCSRPNMC	TCPSGQIAPS	CGSRSIQHCN	IRCMNGGSCS	DDHCLCQKGY	IGTHCGQPVC	ESGCLNGGRC
170	180	190	200	210	220	230	240
VAPNRCACTY	GFTGPQCERD	YRTGPCFTVI	SNQMCQGQLS	GIVCTKTLCC	ATVGRAWGHP	CEMCPAQPHP	CRRGFIPNIR
250	260	270	280	290	300	310	320
TGACQDVDEC	QAIPGLCQGG	NCINTVGSFE	CKCPAGHKLN	EVSQKCEDID	ECSTIPGICE	GGECTNTVSS	YFCKCPPGFY
330	340	350	360	370	380	390	400
TSPDGTRCID	VRPGYCYTAL	TNGRCSNQLP	QSITKMQCCC	DAGRCWSPGV	TVAPEMCPIR	ATEDFNKLCS	VPMVIPGRPE
410	420	430	440	450	460	470	480
YPPPPLGPIP	PVLPVPPGFP	PGPQIPVPRP	PVEYLYPSRE	PPRVLPVNVT	DYCQLVRYLC	QNGRCIPTPG	SYRCECNKGF
490	500	510	520	530	540	550	560
QLDLRGECID	VDECEKNPCA	GGECINNQGS	YTCQCRAGYQ	STLTRTECRD	IDECLQNGRI	CNNGRCINTD	GSFHCVCNAG
570	580	590	600	610	620	630	640
FHVTRDGKNC	EDMDECSIRN	MCLNGMCINE	DGSFKCICKP	GFQLASDGRY	CKDINECETP	GICMNGRCVN	TDGSYRCECF
650	660	670	680	690	700	710	720
PGLAVGLDGR	VCVDTHMRST	CYGGYKRGQC	IKPLFGAVTK	SECCCASTEY	AFGEPCQPCP	AQNSAEYQAL	CSSGPGMTSA
730	740	750	760	770	780	790	800
GSDINECALD	PDICPNGICE	NLRGTYKCIC	NSGYEVDSTG	KNCVDINECV	LNSLLCDNGQ	CRNTPGSFVC	TCPKGFIYKP
810	820	830	840	850	860	870	880
DLKTCEDIDE	CESSPCINGV	CKNSPGSFIC	ECSSESTLDP	TKTICIETIK	GTCWQTVIDG	RCEININGAT	LKSQCCSSLG
890	900	910	920	930	940	950	960
AAWGSPCTLC	QVDPICGKGY	SRIKGTQCED	IDECEVFPGV	CKNGLCVNTR	GSFKCQCPSG	MTLDATGRIC	LDIRLETCFL
970	980	990	1000	1010	1020	1030	1040
RYEDEECTLP	IAGRHRMDAC	CCSVGAAWGT	EECEECPMRN	TPEYEELCPR	GPGFATKEIT	NGKPFFKDIN	ECKMIPSLCT
1050	1060	1070	1080	1090	1100	1110	1120
HGKCRNTIGS	FKCRCDSGFA	LDSEERNCTD	IDECRISPDL	CGRGQCVNTP	GDFECKCDEG	YESGFMMMKN	CMDIDECQRD
1130	1140	1150	1160	1170	1180	1190	1200
PLLCRGGVCH	NTEGSYRCEC	PPGHQLSPNI	SACIDINECE	LSAHLCPNGR	CVNLIGKYQC	ACNPGYHSTP	DRLFCVDIDE
1210	1220	1230	1240	1250	1260	1270	1280
CSIMNGGCET	FCTNSEGSYE	CSCQPGFALM	PDQRSCTDID	ECEDNPNICD	GGQCTNIPGE	YRCLCYDGFM	ASEDMKTCVD
1290	1300	1310	1320	1330	1340	1350	1360
VNECDLNPNI	CLSGTCENTK	GSFICHCDMG	YSGKKGKTGC	TDINECEIGA	HNCGKHAVCT	NTAGSFKCSC	SPGWIGDGIK
1370	1380	1390	1400	1410	1420	1430	1440
CTDLDECSNG	THMCSQHADC	KNTMGSYRCL	CKEGYTGDGF	TCTDLDECSE	NLNLCGNGQC	LNAPGGYRCE	CDMGFVPSAD
1450	1460	1470	1480	1490	1500	1510	1520
GKACEDIDEC	SLPNICVFGT	CHNLPGLFRC	ECEIGYELDR	SGGNCTDVNE	CLDPTTCISG	NCVNTPGSYI	CDCPPDFELN
1530	1540	1550	1560	1570	1580	1590	1600
PTRVGCVDTR	SGNCYLDIRP	RGDNGDTACS	NEIGVGVSKA	SCCCSLGKAW	GTPCEMCPAV	NTSEYKILCP	GGEGFRPNPI
1610	1620	1630	1640	1650	1660	1670	1680
TVILEDIDEC	QELPGLCQGG	KCINTFGSFQ	CRCPTGYYLN	EDTRVCDDVN	ECETPGICGP	GTCYNTVGNY	TCICPPDYMQ
1690	1700	1710	1720	1730	1740	1750	1760
VNGGNNCMDM	RRSLCYRNYY	ADNQTCDGEL	LFNMTKKMCC	CSYNIGRAWN	KPCEQCPIPS	TDEFATLCGS	QRPGFVIDIY
1770	1780	1790	1800	1810	1820	1830	1840
TGLPVDIDEC	REIPGVCENG	VCINMVGSFR	CECPVGFFYN	DKLLVCEDID	ECQNGPVCQR	NAECINTAGS	YRCDCKPGYR
1850	1860	1870	1880	1890	1900	1910	1920
FTSTGQCNDR	NECQEIPNIC	SHGQCIDTVG	SFYCLCHTGF	KTNDDQTMCL	DINECERDAC	GNGTCRNTIG	SFNCRCNHGF
1930	1940	1950	1960	1970	1980	1990	2000
ILSHNNDCID	VDECASGNGN	LCRNGQCINT	VGSFQCQCNE	GYEVAPDGRT	CVDINECLLE	PRKCAPGTCQ	NLDGSYRCIC
2010	2020	2030	2040	2050	2060	2070	2080
PPGYSLQNEK	CEDIDECVEE	PEICALGTCS	NTEGSFKCLC	PEGFSLSSSG	RRCQDLRMSY	CYAKFEGGKC	SSPKSRNHSK
2090	2100	2110	2120	2130	2140	2150	2160
QECCCALKGE	GWGDPCELCP	TEPDEAFRQI	CPYGSGIIVG	PDDSAVDMDE	CKEPDVCKHG	QCINTDGSYR	CECPFGYILA
2170	2180	2190	2200	2210	2220	2230	2240

Detailed Protein Report

Cmpd.	No. of Cmpds.	m/z meas.	Δ m/z [ppm]	z	Rt [min]	Score	P	Range	Sequence	Modification	Ratios
25	1	796.8073	-13.58	2	29.5	12.2	0	166-179	R.CACTYGFTGPQCER.D	Carbamidomethyl: 1	M:Q 0.42<br>Q:W 3.24
1152	1	991.7003	-40.24	3	44.0	14.6	1	356-380	K.<br>MQCCCDAGRCWSPGVTVAPEM(<br>A	Carbamidomethyl: 3, 4, 5, 22; Oxidation: 1, 21	
1872	1	1057.6895	-79.32	3	53.8	11.0	2	610-636	R.<br>YCKDINECETPGICMNGRCVNTD(<br>C	Carbamidomethyl: 2, 19; Oxidation: 15	
748	17	635.3242	75.75	2	37.8	23.9	1	2482-2492	R.SCKDLDECATK.Q	Carbamidomethyl: 8	M:Q 0.41<br>Q:W 1.67
2864	1	703.3686	82.89	2	66.3	15.8	0	2681-2694	R.IGQGHCVSGMGMGR.G	Oxidation: 12	

Detailed Protein Report

Protein 102: NF-X1-type zinc finger protein NFXL1 [Homo sapiens]

**Accession:**	gi|89363020	**Score:**	77.2
**Database:**	refseq_human(refseq_human_20140103.fasta)	**MW [kDa]:**	101.3
**Database Date:**	2015-11-30	**pI:**	10.5
**Modification(s):**	Carbamidomethyl	**Sequence Coverage [%]:**	10.1
		**No. of unique Peptides:**	5

Quantitation

Q:W	**Median:**	0.67	**CV:**	0.00 %	**No. of Peptides:**	1
M:Q	**Median:**	0.08	**CV:**	0.00 %	**No. of Peptides:**	1

Alias proteins:

Accession	Name	Description
gi|520261838	refseq_human (refseq_human_20140103.fasta)	NF-X1-type zinc finger protein NFXL1 [Homo sapiens]
gi|520261712	refseq_human (refseq_human_20140103.fasta)	NF-X1-type zinc finger protein NFXL1 [Homo sapiens]

10	20	30	40	50	60	70	80
MEASWRQVAG	GRGRSRGRAT	AAPSGNGVHL	RGAGGGREKG	SVGAVPSGTS	PGGVATTAAA	GSRHSPAGSQ	ALQTTAASEL
90	100	110	120	130	140	150	160
MSQKKFEEIK	KANQAAARKL	VEEQFSSSSE	EGDEDFEGKQ	GKILANTFIT	YTTQTDGDTR	ELERTKQYVN	EAFQAGAMTC
170	180	190	200	210	220	230	240
LICIASVKRN	QAVWSCSGCF	CIFHMPCIQK	WAKDSQFLVS	SVTDDDFGKK	DCPWPCPKCR	FEYKRSETPS	RYYCYCGKVE
250	260	270	280	290	300	310	320
DPPLDPWLVP	HSCGQVCERE	FKPPCGHKCL	LLCHPGPCPP	CPKMVTTTCY	CKKAKPIPRR	CSAKEWSCQL	PCGQKLLCGQ
330	340	350	360	370	380	390	400
HKCENPCHAG	SCQPCPRVSR	QKCVCGKKVA	ERSCASPLWH	CDQVCGKTLP	CGNHTCEQVC	HVGACGECPR	SGKRFCPCQK
410	420	430	440	450	460	470	480
SKFSLPCTED	VPTCGDSCDK	VLECGIHRCS	QRCHRGPCET	CRQEVEKHCR	CGKHTKRMPC	HKPYLCETKC	VKMRDCQKHQ
490	500	510	520	530	540	550	560
CRRKCCPGNC	PPCDQNCGRT	LGCRNHKCPS	VCHRGSCYPC	PETVDVKCNC	GNTKVTVPCG	RERTTRPPKC	KEQCSRPPTC
570	580	590	600	610	620	630	640
HHTSQEKHRC	HFGSCPPCHQ	PCQKVLEKCG	HLCPAPCHDQ	ALIKQTGRHQ	PTGPWEQPSE	PAFIQTALPC	PPCQVPIPME
650	660	670	680	690	700	710	720
CLGKHEVSPL	PCHAVGPYSC	KRVCGRILDC	QNHTCMKECH	KVTKTDGCTG	KNKAGPECLH	CEEGCSKSRP	LGCLHPCILR
730	740	750	760	770	780	790	800
CHPGECPPCV	QMLRIKCHCK	ITSLYVECRK	ITTADVNEKN	LLSCCKNQCP	KELPCGHRCK	EMCHPGECPF	NCNQKVKLRC
810	820	830	840	850	860	870	880
PCKRIKKELQ	CNKVRENQVS	IECDTTCKEM	KRKASEIKEA	EAKAALEEEK	RRQQAELEAF	ENRLKGRRKK	NRKRDEVAVE
890	900	910	920				
LSLWQKHKYY	LISVCGVVVV	VFAWYITHDV	N				

Cmpd.	No. of Cmpds.	m/z meas.	Δ m/z [ppm]	z	Rt [min]	Score	P	Range	Sequence	Modification	Ratios	
2093	1	732.3366	-79.70	2	56.6	10.4	1	17-31	R.GRATAAPSGNGVHLR.G			
2885	1	1129.0588	55.84	2	64.8	16.4	2	323-342	K.CENPCHAGSCQPCPRVSRQK.C	Carbamidomethyl: 1		
1824	1	916.3237	-87.07	2	53.1	10.4	2	433-447	R.CHRGPCETCRQEVEK.H	Carbamidomethyl: 6	Q:W	0.67
490	1	587.1540	-200.57	2	35.5	10.2	2	475-483	R.DCQKHQCRR.K		M:Q	0.08
425	1	626.3269	55.74	3	35.3	13.9	0	589-604	K.CGHLCPAPCHDQALIK.Q	Carbamidomethyl: 1, 5, 9		

Detailed Protein Report

Protein 103: PREDICTED: bromodomain-containing protein 1 isoform X2 [Homo sapiens]

**Accession:**	gi|530419805	**Score:**	75.9
**Database:**	refseq_human(refseq_human_20140103.fasta)	**MW [kDa]:**	133.2
**Database Date:**	2015-11-30	**pI:**	9.9
**Modification(s):**	Carbamidomethyl	**Sequence Coverage [%]:**	6.3
		**No. of unique Peptides:**	4

Alias proteins:

Accession	Name	Description
gi|578837119	refseq_human (refseq_human_20140103.fasta)	PREDICTED: bromodomain-containing protein 1 isoform X8 [Homo sapiens]
gi|578837117	refseq_human (refseq_human_20140103.fasta)	PREDICTED: bromodomain-containing protein 1 isoform X7 [Homo sapiens]

10	20	30	40	50	60	70	80
MRRKGRCHRG	SAARHPSSPC	SVKHSPTRET	LTYAQAQRMV	EIEIEGRLHR	ISIFDPLEII	LEDDLTAQEM	SECNSNKENS
90	100	110	120	130	140	150	160
ERPPVCLRTK	RHKNNRVKKK	NEALPSAHGT	PASASALPEP	KVRIVEYSPP	SAPRRPPVYY	KFIEKSAEEL	DNEVEYDMDE
170	180	190	200	210	220	230	240
EDYAWLEIVN	EKRKGDCVPA	VSQSMFEFLM	DRFEKESHCE	NQKQGEQQSL	IDEDAVCCIC	MDGECQNSNV	ILFCDMCNLA
250	260	270	280	290	300	310	320
VHQECYGVPY	IPEGQWLCRH	CLQSRARPAD	CVLCPNKGGA	FKKTDDDRWG	HVVCALWIPE	VGFANTVFIE	PIDGVRNIPP
330	340	350	360	370	380	390	400
ARWKLTCYLC	KQKGVGACIQ	CHKANCYTAF	HVTCAQKAGL	YMKMEPVKEL	TGGGTTFSVR	KTAYCDVHTP	PGCTRRPLNI
410	420	430	440	450	460	470	480
YGDVEMKNGV	CRKESSVKTV	RSTSKVRKKA	KKAKKALAEP	CAVLPTVCAP	YIPPQRLNRI	ANQVAIQRKK	QFVERAHSYW
490	500	510	520	530	540	550	560
LLKRLSRNGA	PLLRRLQSSL	QSQRSSQQRE	NDEEMKAAKE	KLKYWQRLRH	DLERARLLIE	LLRKREKLKR	EQVKVEQVAM
570	580	590	600	610	620	630	640
ELRLTPLTVL	LRSVLDQLQD	KDPARIFAQP	VSLKEVPDYL	DHIKHPMDFA	TMRKRLEAQG	YKNLHEFEED	FDLIIDNCMK
650	660	670	680	690	700	710	720
YNARDTVFYR	AAVRLRDQGG	VVLRQARREV	DSIGLEEASG	MHLPERPAAA	PRRPFSWEDV	DRLLDPANRA	HLGLEEQLRE
730	740	750	760	770	780	790	800
LLDMLDLTCA	MKSSGSRSKR	AKLLKKEIAL	LRNKLSQQHS	QPLPTGPGLE	GFEEDGAALG	PEAGEEGDKS	PPKLEPSDAL
810	820	830	840	850	860	870	880
PLPSNSETNS	EPPTLKPVEL	NPEQSKLFKR	VTFDNESHSA	CTQSALVSGR	PPEPTRASSG	DVPAAAASAV	AEPASDVNRR
890	900	910	920	930	940	950	960
TSVLFCKSKS	VSPPKSAKNT	ETQPTSPQLG	TKTFLSVVLP	RLETLLQPRK	RSRSTCGDSE	VEEESPGKRL	DAGLTNGFGG
970	980	990	1000	1010	1020	1030	1040
ARSEQEPGGG	LGRKATPRRR	CASESSISSS	NSPLCDSSFN	APKCGRGKPA	LVRRHTLEDR	SELISCIENG	NYAKAARIAA
1050	1060	1070	1080	1090	1100	1110	1120
EVGQSSMWIS	TDAAASVLEP	LKVVWAKCSG	YPSYPALIID	PKMPRVPGHH	NGVTIPAPPL	DVLKIGEHMQ	TKSDEKLFLV
1130	1140	1150	1160	1170	1180	1190	
LFFDNKRSWQ	WLPKSKMVPL	GIDETIDKLK	MMEGRNSSIR	KAVRIAFDRA	MNHLSRVHGE	PTSDLSDID	

Cmpd.	No. of Cmpds.	m/z meas.	Δ m/z [ppm]	z	Rt [min]	Score	P	Range	Sequence	Modification
832	1	902.3468	-117.98	2	39.2	13.7	1	266-282	R.ARPADCVLCPNKGGAFK.K	Carbamidomethyl: 6
1578	2	873.9352	-97.55	2	48.2	14.4	2	530-543	R.HDLERARLLIELLR.K	
63	1	1097.5505	-106.80	1	30.7	16.6	1	922-930	R.LETLLQPRK.R	
2766	1	702.8107	-85.42	2	65.0	18.8	1	949-962	K.RLDAGLTNGFGGAR.S	

Detailed Protein Report

Protein 104: chromodomain-helicase-DNA-binding protein 2 isoform 1 [Homo sapiens]

**Accession:**	gi|118421089	**Score:**	75.8
**Database:**	refseq_human(refseq_human_20140103.fasta)	**MW [kDa]:**	211.2
**Database Date:**	2015-11-30	**pI:**	8.8
**Modification(s):**	Oxidation	**Sequence Coverage [%]:**	4.3
		**No. of unique Peptides:**	6

Quantitation

Q:W	**Median:**	0.84	**CV:**	0.00 %	**No. of Peptides:**	1
M:Q	**Median:**	0.70	**CV:**	0.00 %	**No. of Peptides:**	1

BRUKER

Detailed Protein Report

10	20	30	40	50	60	70	80
MMRNKDKSQE	EDSSLHSNAS	SHSASEEASG	SDSGSQSESE	QGSDPGSGHG	SESNSSSESS	ESQSESESES	AGSKSQPVLP
90	100	110	120	130	140	150	160
EAKEKPASKK	ERIADVKKMW	EEYPDVYGVR	RSNRSRQEPS	RFNIKEEASS	GSESGSPKRR	GQRQLKKQEK	WKQEPSEDEQ
170	180	190	200	210	220	230	240
EQGTSAESEP	EQKKVKARRP	VPRRTVPKPR	VKKQPKTQRG	KRKKQDSSDE	DDDDDEAPKR	QTRRRAAKNV	SYKEDDDFET
250	260	270	280	290	300	310	320
DSDDLIEMTG	EGVDEQQDNS	ETIEKVLDSR	LGKKGATGAS	TTVYAIEANG	DPSGDFDTEK	DEGEIQYLIK	WKGWSYIHST
330	340	350	360	370	380	390	400
WESEESLQQQ	KVKGLKKLEN	FKKKEDEIKQ	WLGKVSPEDV	EYFNCQQELA	SELNKQYQIV	ERVIAVKTSK	STLGQTDFPA
410	420	430	440	450	460	470	480
HSRKPAPSNE	PEYLCKWMGL	PYSECSWEDE	ALIGKKFQNC	IDSFHSRNNS	KTIPTRECKA	LKQRPRFVAL	KKQPAYLGGE
490	500	510	520	530	540	550	560
NLELRDYQLE	GLNWLAHSWC	KNNSVILADE	MGLGKTIQTI	SFLSYLFHQH	QLYGPFLIVV	PLSTLTSWQR	EFEIWAPEIN
570	580	590	600	610	620	630	640
VVVYIGDLMS	RNTIREYEWI	HSQTKRLKFN	ALITTYEILL	KDKTVLGSIN	WAFLGVDEAH	RLKNDDSLLY	KTLIDFKSNH
650	660	670	680	690	700	710	720
RLLITGTPLQ	NSLKELWSLL	HFIMPEKFEF	WEDFEEDHGK	GRENGYQSLH	KVLEPFLLRR	VKKDVEKSLP	AKVEQILRVE
730	740	750	760	770	780	790	800
MSALQKQYYK	WILTRNYKAL	AKGTRGSTSG	FLNIVMELKK	CCNHCYLIKP	PEENERENGQ	EILLSLIRSS	GKLILLDKLL
810	820	830	840	850	860	870	880
TRLRERGNRV	LIFSQMVRML	DILAEYLTIK	HYPFQRLDGS	IKGEIRKQAL	DHFNADGSED	FCFLLSTRAG	GLGINLASAD
890	900	910	920	930	940	950	960
TVVIFDSDWN	PQNDLQAQAR	AHRIGQKKQV	NIYRLVTKGT	VEEEIIERAK	KKMVLDHLVI	QRMDTTGRTI	LENNSGRSNS
970	980	990	1000	1010	1020	1030	1040
NPFNKEELTA	ILKFGAEDLF	KELEGEESEP	QEMDIDEILR	LAETRENEVS	TSATDELLSQ	FKVANFATME	DEEELEERPH
1050	1060	1070	1080	1090	1100	1110	1120
KDWDEIIPEE	QRKKVEEEER	QKELEEIYML	PRIRSSTKKA	QTNDSDSDTE	SKRQAQRSSA	SESETEDSDD	DKKPKRRGRP
1130	1140	1150	1160	1170	1180	1190	1200
RSVRKDLVEG	FTDAEIRRFI	KAYKKFGLPL	ERLECIARDA	ELVDKSVADL	KRLGELIHNS	CVSAMQEYEE	QLKENASEGK
1210	1220	1230	1240	1250	1260	1270	1280
GPGKRRGPTI	KISGVQVNVK	SIIQHEEEFE	MLHKSIPVDP	EEKKKYCLTC	RVKAAHFDVE	WGVEDDSRLL	LGIYEHGYGN
1290	1300	1310	1320	1330	1340	1350	1360
WELIKTDPEL	KLTDKILPVE	TDKKPQGKQL	QTRADYLLKL	LRKGLEKKGA	VTGGEEAKLK	KRKPRVKKEN	KVPRLKEEHG
1370	1380	1390	1400	1410	1420	1430	1440
IELSSPRHSD	NPSEEGEVKD	DGLEKSPMKK	KQKKKENKEN	KEKQMSSRKD	KEGDKERKKS	KDKKEKPKSG	DAKSSSKSKR
1450	1460	1470	1480	1490	1500	1510	1520
SQGPVHITAG	SEPVPIGEDE	DDDLDQETFS	ICKERMRPVK	KALKQLDKPD	KGLNVQEQLE	HTRNCLLKIG	DRIAECLKAY
1530	1540	1550	1560	1570	1580	1590	1600
SDQEHIKLWR	RNLWIFVSKF	TEFDARKLHK	LYKMAHKKRS	QEEEEQKKKD	DVTGGKKPFR	PEASGSSRDS	LISQSHTSHN
1610	1620	1630	1640	1650	1660	1670	1680
LHPQKPHLPA	SHGPQMHGHP	RDNYNHPNKR	HFSNADRGDW	QRERKFNYGG	GNNNPPWGSD	RHHQYEQHWY	KDHHYGDRRH
1690	1700	1710	1720	1730	1740	1750	1760
MDAHRSGSYR	PNNMSRKRPY	DQYSSDRDHR	GHRDYYDRHH	HDSKRRRSDE	FRPQNYHQQD	FRRMSDHRPA	MGYHGQGPSD
1770	1780	1790	1800	1810	1820	1830	
HYRSFHTDKL	GEYKQPLPPL	HPAVSDPRSP	PSQKSPHDSK	SPLDHRSPLE	RSLEQKNNPD	YNWNVRKT	

Cmpd.	No. of Cmpds.	m/z meas.	Δ m/z [ppm]	z	Rt [min]	Score	P	Range	Sequence	Modification	Ratios
1421	1	846.6046	143.39	1	47.5	10.1	2	147-152	K.KQEKWK.Q		
2644	1	913.4624	-16.46	2	63.1	14.1	1	743-759	K.GTRGSTSGFLNIVMELK.K	Oxidation: 14	
1777	2	1022.8922	-139.78	2	52.6	14.7	2	932-948	K.KMVLDHLVIQRMDTTGR.T	Oxidation: 2, 12	
1792	1	898.7912	-12.20	3	52.2	11.6	1	1212-1234	K.ISGVQVNVKSIIQHEEEFEMLHK.S		
2192	1	885.2814	-167.85	2	57.8	12.6	0	1221-1234	K.SIIQHEEEFEMLHK.S		

Detailed Protein Report

Cmpd.	No. of Cmpds.	m/z meas.	Δ m/z [ppm]	z	Rt [min]	Score	P	Range	Sequence	Modification	Ratios
1619	1	1015.0002	-35.61	2	50.0	12.7	2	1531-1546	R.RNLWIFVSKFTEFDAR.K		Q:W 0.84<br>M:Q 0.70

Detailed Protein Report

Protein 105: ubiquitin carboxyl-terminal hydrolase 34 [Homo sapiens]

**Accession:**	gi|110347427	**Score:**	75.1
**Database:**	refseq_human(refseq_human_20140103.fasta)	**MW [kDa]:**	404.0
**Database Date:**	2015-11-30	**pI:**	5.4
**Modification(s):**	Oxidation	**Sequence Coverage [%]:**	2.2
		**No. of unique Peptides:**	3

Detailed Protein Report

10	20	30	40	50	60	70	80
MCENCADLVE	VLNEISDVEG	GDGLQLRKEH	TLKIFTYINS	WTQRQCLCCF	KEYKHLEIFN	QVVCALINLV	IAQVQVLRDQ
90	100	110	120	130	140	150	160
LCKHCTTINI	DSTWQDESNQ	AEEPLNIDRE	CNEGSTERQK	SIEKKSNSTR	ICNLTEEESS	KSSDPFSLWS	TDEKEKLLLC
170	180	190	200	210	220	230	240
VAKIFQIQFP	LYTAYKHNTH	PTIEDISTQE	SNILGAFCDM	NDVEVPLHLL	RYVCLFCGKN	GLSLMKDCFE	YGTPETLPFL
250	260	270	280	290	300	310	320
IAHAFITVVS	NIRIWLHIPA	VMQHIIPFRT	YVIRYLCKLS	DQELRQSAAR	NMADLMWSTV	KEPLDTTLCF	DKESLDLAFK
330	340	350	360	370	380	390	400
YFMSPTLTMR	LAGLSQITNQ	LHTFNDVCNN	ESLVSDTETS	IAKELADWLI	SNNVVEHIFG	PNLHIEIIKQ	CQVILNFLAA
410	420	430	440	450	460	470	480
EGRLSTQHID	CIWAAAQLKH	CSRYIHDLFP	SLIKNLDPVP	LRHLLNLVSA	LEPSVHTEQT	LYLASMLIKA	LWNNALAAKA
490	500	510	520	530	540	550	560
QLSKQSSFAS	LLNTNIPIGN	KKEEEELRRT	APSPWSPAAS	PQSSDNSDTH	QSGGSDIEMD	EQLINRTKHV	QQRLSDTEES
570	580	590	600	610	620	630	640
MQGSSDETAN	SGEDGSSGPG	SSSGHSDGSS	NEVNSSHASQ	SAGSPGSEVQ	SEDIADIEAL	KEEDEDDDHG	HNPPKSSCGT
650	660	670	680	690	700	710	720
DLRNRKLESQ	AGICLGDSQG	MSERNGTSSG	TGKDLVFNTE	SLPSVDNRMR	MLDACSHSED	PEHDISGEMN	ATHIAQGSQE
730	740	750	760	770	780	790	800
SCITRTGDFL	GETIGNELFN	CRQFIGPQHH	HHHHHHHHHH	DGHMVDDMLS	ADDVSCSSSQ	VSAKSEKNMA	DFDGEESGCE
810	820	830	840	850	860	870	880
EELVQINSHA	ELTSHLQQHL	PNLASIYHEH	LSQGPVVHKH	QFNSNAVTDI	NLDNVCKKGN	TLLWDIVQDE	DAVNLSEGLI
890	900	910	920	930	940	950	960
NEAEKLLCSL	VCWFTDRQIR	MRFIEGCLEN	LGNNRSVVIS	LRLLPKLFGT	FQQFGSSYDT	HWITMWAEKE	LNMMKLFFDN
970	980	990	1000	1010	1020	1030	1040
LVYYIQTVRE	GRQKHALYSH	SAEVQVRLQF	LTCVFSTLGS	PDHFRLSLEQ	VDILWHCLVE	DSECYDDALH	WFLNQVRSKD
1050	1060	1070	1080	1090	1100	1110	1120
QHAMGMETYK	HLFLEKMPQL	KPETISMTGL	NLFQHLCNLA	RLATSAYDGC	SNSELCGMDQ	FWGIALRAQS	GDVSRAAIQY
1130	1140	1150	1160	1170	1180	1190	1200
INSYYINGKT	GLEKEQEFIS	KCMESLMIAS	SSLEQESHSS	LMVIERGLLM	LKTHLEAFRR	RFAYHLRQWQ	IEGTGISSHL
1210	1220	1230	1240	1250	1260	1270	1280
KALSDKQSLP	LRVVCQPAGL	PDKMTIEMYP	SDQVADLRAE	VTHWYENLQK	EQINQQAQLQ	EFGQSNRKGE	FPGGLMGPVR
1290	1300	1310	1320	1330	1340	1350	1360
MISSGHELTT	DYDEKALHEL	GFKDMQMVFV	SLGAPRRERK	GEGVQLPASC	LPPPQKDNIP	MLLLLQEPHL	TTLFDLLEML
1370	1380	1390	1400	1410	1420	1430	1440
ASFKPPSGKV	AVDDSESLRC	EELHLHAENL	SRRVWELLML	LPTCPNMLMA	FQNISDEQSN	DGFNWKELLK	IKSAHKLLYA
1450	1460	1470	1480	1490	1500	1510	1520
LEIIEALGKP	NRRIRRESTG	SYSDLYPDSD	DSSEDQVENS	KNSWSCKFVA	AGGLQQLLEI	FNSGILEPKE	QESWTVWQLD
1530	1540	1550	1560	1570	1580	1590	1600
CLACLLKLIC	QFAVDPSDLD	LAYHDVFAWS	GIAESHRKRT	WPGKSRKAAG	DHAKGLHIPR	LTEVFLVLVQ	GTSLIQRLMS
1610	1620	1630	1640	1650	1660	1670	1680
VAYTYDNLAP	RVLKAQSDHR	SRHEVSHYSM	WLLVSWAHCC	SLVKSSLADS	DHLQDWLKKL	TLLIPETAVR	HESCSGLYKL
1690	1700	1710	1720	1730	1740	1750	1760
SLSGLDGGDS	INRSFLLLAA	STLLKFLPDA	QALKPIRIDD	YEEEPILKPG	CKEYFWLLCK	LVDNIHIKDA	SQTTLLDLDA
1770	1780	1790	1800	1810	1820	1830	1840
LARHLADCIR	SREILDHQDG	NVEDDGLTGL	LRLATSVVKH	KPPFKFSREG	QEFLRDIFNL	LFLLPSLKDR	QQPKCKSHSS
1850	1860	1870	1880	1890	1900	1910	1920
RAAAYDLLVE	MVKGSVENYR	LIHNWVMAQH	MQSHAPYKWD	YWPHEDVRAE	CRFVGLTNLG	ATCYLASTIQ	QLYMIPEARQ
1930	1940	1950	1960	1970	1980	1990	2000
AVFTAKYSED	MKHKTTLLEL	QKMFTYLMES	ECKAYNPRPF	CKTYTMDKQP	LNTGEQKDMT	EFFTDLITKI	EEMSPELKNT
2010	2020	2030	2040	2050	2060	2070	2080
VKSLFGGVIT	NNVVSLDCEH	VSQTAEEFYT	VRCQVADMKN	IYESLDEVTI	KDTLEGDNMY	TCSHCGKKVR	AEKRACFKKL
2090	2100	2110	2120	2130	2140	2150	2160
PRILSFNTMR	YTFNMVTMMK	EKVNTHFSFP	LRLDMTPYTE	DFLMGKSERK	EGFKEVSDHS	KDSESYEYDL	IGVTVHTGTA
2170	2180	2190	2200	2210	2220	2230	2240

Detailed Protein Report

Cmpd.	No. of Cmpds.	m/z meas.	Δ m/z [ppm]	z	Rt [min]	Score	P	Range	Sequence	Modification
1545	1	948.9999	79.00	2	47.6	10.2	0	647-664	K.LESQAGICLGDSQGMSER.N	Oxidation: 15
2681	1	1079.0243	41.33	2	64.7	12.9	2	2052-2070	K.DTLEGDNMYTCSHCGKKVR.A	
597	2	642.8360	18.78	2	36.1	18.4	0	2940-2950	R.SCWTTLISAFR.I	

Detailed Protein Report

Protein 106: PREDICTED: rap guanine nucleotide exchange factor 3 isoform X2 [Homo sapiens]

Accession: gi|530399600
Database: refseq_human(refseq_human_20140103.fasta)
Database Date: 2015-11-30
Modification(s): Carbamidomethyl, Oxidation

Score: 74.6
MW [kDa]: 87.5
pI: 7.2
Sequence Coverage [%]: 4.9
No. of unique Peptides: 4

Quantitation

Q:W	**Median:**	1.36	**CV:**	0.00 %	**No. of Peptides:**	1
M:Q	**Median:**	1.01	**CV:**	0.00 %	**No. of Peptides:**	1

Alias proteins:

Accession	Name	Description
gi|530399602	refseq_human (refseq_human_20140103.fasta)	PREDICTED: rap guanine nucleotide exchange factor 3 isoform X3 [Homo sapiens]

```
        10         20         30         40         50         60         70         80
MGSWPWDLGS IPGAKLWESA RCCWMKVPSA MDRDAQFYRF PGPEPEPVRT HEMEEELAEA VALLSQRGPD ALLTVALRKP
        90        100        110        120        130        140        150        160
PGQRTDEELD LIFEELLHIK AVAHLSNSVK RELAAVLLFE PHSKAGTVLF SQGDKGTSWY IIWKGSVNVV THGKGLVTTL
       170        180        190        200        210        220        230        240
HEGDDFGQLA LVNDAPRAAT IILREDNCHF LRVDKQDFNR IIKDVEAKTM RLEEHGKVVL VLERASQGAG PSRPPTPGRN
       250        260        270        280        290        300        310        320
RYTVMSGTPE KILELLLEAM GPDSSAHDPT ETFLSDFLLT HRVFMPSAQL CAALLHHFHV EPAGGSEQER STYVCNKRQQ
       330        340        350        360        370        380        390        400
ILRLVSQWVA LYGSMLHTDP VATSFLQKLS DLVGRDTRLS NLLREQWPER RRCHRLENGC GNASPQMKAR NLPVWLPNQD
       410        420        430        440        450        460        470        480
EPLPGSSCAI QVGDKVPYDI CRPDHSVLTL QLPVTASVRE VMAALAQEDG WTKGQVLVKV NSAGDAIGLQ PDARGVATSL
       490        500        510        520        530        540        550        560
GLNERLFVVN PQEVHELIPH PDQLGPTVGS AEGLDLVSAK DLAGQLTDHD WSLFNSIHQV ELIHYVLGPQ HLRDVTTANL
       570        580        590        600        610        620        630        640
ERFMRRFNEL QYWVATELCL CPVPGPRAQL LRKFIKLAAH LKEQKNLNSF FAVMFGLSNS AISRLAHTWE RLPHKVRKLY
       650        660        670        680        690        700        710        720
SALERLLDPS WNHRVYRLAL AKLSPPVIPF MPLLLKDMTF IHEGNHTLVE NLINFEKMRM MARAARMLHH CRSHNPVPLS
       730        740        750        760        770        780
PLRSRVSHLH EDSQVARIST CSEQSLSTRS PASTWAYVQQ LKVIDNQREL SRLSRELEP
```

Cmpd.	No. of Cmpds.	m/z meas.	Δ m/z [ppm]	z	Rt [min]	Score	P	Range	Sequence	Modification	Ratios
1402	1	727.3976	-11.71	2	45.9	15.2	0	112-124	R.ELAAVLLFEPHSK.A		Q:W 1.36<br>M:Q 1.01
1805	1	937.7928	-121.06	2	52.4	13.0	1	373-388	R.CHRLENGCGNASPQMK.A	Carbamidomethyl: 1, 8; Oxidation: 15	
870	1	711.3337	31.93	2	39.7	13.9	0	376-388	R.LENGCGNASPQMK.A	Carbamidomethyl: 5; Oxidation: 12	
997	5	555.1971	-145.78	2	41.0	15.0	2	698-706	K.MRMMARAAR.M	Oxidation: 1	

Detailed Protein Report

Protein 107: PREDICTED: nesprin-1 isoform X19 [Homo sapiens]

**Accession:**	gi|578812604	**Score:**	74.6
**Database:**	refseq_human(refseq_human_20140103.fasta)	**MW [kDa]:**	1002.8
**Database Date:**	2015-11-30	**pI:**	5.3
**Modification(s):**	Carbamidomethyl, Oxidation	**Sequence Coverage [%]:**	1.1
		**No. of unique Peptides:**	5

Quantitation

Q:W	**Median:** 1.33	**CV:** 0.00 %	**No. of Peptides:** 1

Detailed Protein Report

10	20	30	40	50	60	70	80
MATSRGASRC	PRDIANVMQR	LQDEQEIVQK	RTFTKWINSH	LAKRKPPMVV	DDLFEDMKDG	VKLLALLEVL	SGQKLPCEQG
90	100	110	120	130	140	150	160
RRMKRIHAVA	NIGTALKFLE	GRKSMHRGSP	IKLVNINSTD	IADGRPSIVL	GLMWTIILYF	QIEELTSNLP	QLQSLSSSAS
170	180	190	200	210	220	230	240
SVDSIVSSET	PSPPSKRKVT	TKIQGNAKKA	LLKWVQYTAG	KQTGIEVKDF	GKSWRSGVAF	HSVIHAIRPE	LVDLETVKGR
250	260	270	280	290	300	310	320
SNRENLEDAF	TIAETELGIP	RLLDPEDVDV	DKPDEKSIMT	YVAQFLKHYP	DIHNASTDGQ	EDDEILPGFP	SFANSVQNFK
330	340	350	360	370	380	390	400
REDRVIFKEM	KVWIEQFERD	LTRAQMVESN	LQDKYQSFKH	FRVQYEMKRK	QIEHLIQPLH	RDGKLSLDQA	LVKQSWDRVT
410	420	430	440	450	460	470	480
SRLFDWHIQL	DKSLPAPLGT	IGAWLYRAEV	ALREEITVQQ	VHEETANTIQ	RKLEQHKDLL	QNTDAHKRAF	HEIYRTRSVN
490	500	510	520	530	540	550	560
GIPVPPDQLE	DMAERFHFVS	STSELHLMKM	EFLELKYRLL	SLLVLAESKL	KSWIIKYGRR	ESVEQLLQNY	VSFIENSKFF
570	580	590	600	610	620	630	640
EQYEVTYQIL	KQTAEMYVKA	DGSVEEAENV	MKFMNETTAQ	WRNLSVEVRS	VRSMLEEVIS	NWDRYGNTVA	SLQAWLEDAE
650	660	670	680	690	700	710	720
KMLNQSENAK	KDFFRNLPHW	IQQHTAMNDA	GNFLIETCDE	MVSRDLKQQL	LLLNGRWREL	FMEVKQYAQA	DEMDRMKKEY
730	740	750	760	770	780	790	800
TDCVVTLSAF	ATEAHKKLSE	PLEVSFMNVK	LLIQDLEDIE	QRVPVMDAQY	KIITKTAHLI	TKESPQEEGK	EMFATMSKLK
810	820	830	840	850	860	870	880
EQLTKVKECY	SPLLYESQQL	LIPLEELEKQ	MTSFYDSLGK	INEIITVLER	EAQSSALFKQ	KHQELLACQE	NCKKTLTLIE
890	900	910	920	930	940	950	960
KGSQSVQKFV	TLSNVLKHFD	QTRLQRQIAD	IHVAFQSMVK	KTGDWKKHVE	TNSRLMKKFE	ESRAELEKVL	RIAQEGLEEK
970	980	990	1000	1010	1020	1030	1040
GDPEELLRRH	TEFFSQLDQR	VLNAFLKACD	ELTDILPEQE	QQGLQEAVRK	LHKQWKDLQG	EAPYHLLHLK	IDVEKNRFLA
1050	1060	1070	1080	1090	1100	1110	1120
SVEECRTELD	RETKLMPQEG	SEKIIKEHRV	FFSDKGPHHL	CEKRLQLIEE	LCVKLPVRDP	VRDTPGTCHV	TLKELRAAID
1130	1140	1150	1160	1170	1180	1190	1200
STYRKLMEDP	DKWKDYTSRF	SEFSSWISTN	ETQLKGIKGE	AIDTANHGEV	KRAVEEIRNG	VTKRGETLSW	LKSRLKVLTE
1210	1220	1230	1240	1250	1260	1270	1280
VSSENEAQKQ	GDELAKLSSS	FKALVTLLSE	VEKMLSNFGD	CVQYKEIVKN	SLEELISGSK	EVQEQAEKIL	DTENLFEAQQ
1290	1300	1310	1320	1330	1340	1350	1360
LLLHHQQKTK	RISAKKRDVQ	QQIAQAQQGE	GGLPDRGHEE	LRKLESTLDG	LERSRERQER	RIQVTLRKWE	RFETNKETVV
1370	1380	1390	1400	1410	1420	1430	1440
RYLFQTGSSH	ERFLSFSSLE	SLSSELEQTK	EFSKRTESIA	VQAENLVKEA	SEIPLGPQNK	QLLQQQAKSI	KEQVKKLEDT
1450	1460	1470	1480	1490	1500	1510	1520
LEEDIKTMEM	VKTKWDHFGS	NFETLSVWIT	EKEKELNALE	TSSSAMDMQI	SQIKVTIQEI	ESKLSSIVGL	EEEAQSFAQF
1530	1540	1550	1560	1570	1580	1590	1600
VTTGESARIK	AKLTQIRRYG	EELREHAQCL	EGTILGHLSQ	QQKFEENLRK	IQQSVSEFED	KLAVPIKICS	SATETYKVLQ
1610	1620	1630	1640	1650	1660	1670	1680
EHMDLCQALE	SLSSAITAFS	ASARKVVNRD	SCVQEAAALQ	QQYEDILRRA	KERQTALENL	LAHWQRLEKE	LSSFLTWLER
1690	1700	1710	1720	1730	1740	1750	1760
GEAKASSPEM	DISADRVKVE	GELQLIQALQ	NEVVSQASFY	SKLLQLKESL	FSVASKDDVK	MMKLHLEQLD	ERWRDLPQII
1770	1780	1790	1800	1810	1820	1830	1840
NKRINFLQSV	VAEHQQFDEL	LLSFSVWIKL	FLSELQTTSE	ISIMDHQVAL	TRHKDHAAEV	ESKKGELQSL	QGHLAKLGSL
1850	1860	1870	1880	1890	1900	1910	1920
GRAEDLHLLQ	GKAEDCFQLF	EEASQVVERR	QLALSHLAEF	LQSHASLSGI	LRQLRQTVEA	TNSMNKNESD	LIEKDLNDAL
1930	1940	1950	1960	1970	1980	1990	2000
QNAKALESAA	VSLDGILSKA	QYHLKIGSSE	QRTSCRATAD	QLCGEVERIQ	NLLGTKQSEA	DALAVLKKAF	QDQKEELLKS
2010	2020	2030	2040	2050	2060	2070	2080
IEDIEERTDK	ERLKEPTRQA	LQQRLRVFNQ	LEDELNSHEH	ELCWLKDKAK	QIAQKDVAFA	PEVDREINRL	EVTWDDTKRL
2090	2100	2110	2120	2130	2140	2150	2160
IHENQGQCCG	LIDLMREYQN	LKSAVSKVLE	NASSVIVTRT	TIKDQEDLKW	AFSKHETAKN	KMNYKQKDLD	NFTSKGKHLL
2170	2180	2190	2200	2210	2220	2230	2240

Detailed Protein Report

Cmpd.	No. of Cmpds.	m/z meas.	Δ m/z [ppm]	z	Rt [min]	Score	P	Range	Sequence	Modification	Ratios
1807	1	945.8341	-138.33	2	51.1	10.6	2	1681-1698	R.GEAKASSPEMDISADRVK.V		
742	1	524.3579	134.01	2	37.8	10.5	1	2443-2452	R.TGDSKVLEAK.L		
280	1	607.8593	-49.80	2	33.5	10.5	1	3327-3337	R.TLKLEALLSVK.Q		Q:W 1.33
2894	1	724.8707	49.22	2	64.9	10.6	1	4576-4586	K.EDFDKACHWLK.Q	Carbamidomethyl: 7	
2810	1	1024.4996	-15.58	3	65.5	10.3	2	6575-6600	K. LQDMYDELMMIIGSRRSGLNQNLT S	Oxidation: 9, 10	

Detailed Protein Report

Protein 108: myosin-6 [Homo sapiens]

**Accession:**	gi|156104908	**Score:**	74.2
**Database:**	refseq_human(refseq_human_20140103.fasta)	**MW [kDa]:**	223.6
**Database Date:**	2015-11-30	**pI:**	5.5
**Modification(s):**	Carbamidomethyl	**Sequence Coverage [%]:**	3.5
		**No. of unique Peptides:**	3

Quantitation

Q:W	**Median:**	0.24	**CV:**	0.00 %	**No. of Peptides:**	1
M:Q	**Median:**	1.23	**CV:**	113.96 %	**No. of Peptides:**	2

Detailed Protein Report

10	20	30	40	50	60	70	80
MTDAQMADFG	AAAQYLRKSE	KERLEAQTRP	FDIRTECFVP	DDKEEFVKAK	ILSREGGKVI	AETENGKTVT	VKEDQVLQQN
90	100	110	120	130	140	150	160
PPKFDKIEDM	AMLTFLHEPA	VLFNLKERYA	AWMIYTYSGL	FCVTVNPYKW	LPVYNAEVVA	AYRGKKRSEA	PPHIFSISDN
170	180	190	200	210	220	230	240
AYQYMLTDRE	NQSILITGES	GAGKTVNTKR	VIQYFASIAA	IGDRGKKDNA	NANKGTLEDQ	IIQANPALEA	FGNAKTVRND
250	260	270	280	290	300	310	320
NSSRFGKFIR	IHFGATGKLA	SADIETYLLE	KSRVIFQLKA	ERNYHIFYQI	LSNKKPELLD	MLLVTNNPYD	YAFVSQGEVS
330	340	350	360	370	380	390	400
VASIDDSEEL	MATDSAFDVL	GFTSEEKAGV	YKLTGAIMHY	GNMKFKQKQR	EEQAEPDGTE	DADKSAYLMG	LNSADLLKGL
410	420	430	440	450	460	470	480
CHPRVKVGNE	YVTKGQSVQQ	VYYSIGALAK	AVYEKMFNWM	VTRINATLET	KQPRQYFIGV	LDIAGFEIFD	FNSFEQLCIN
490	500	510	520	530	540	550	560
FTNEKLQQFF	NHHMFVLEQE	EYKKEGIEWT	FIDFGMDLQA	CIDLIEKPMG	IMSILEEECM	FPKATDMTFK	AKLYDNHLGK
570	580	590	600	610	620	630	640
SNNFQKPRNI	KGKQEAHFSL	IHYAGTVDYN	ILGWLEKNKD	PLNETVVALY	QKSSLKLMAT	LFSSYATADT	GDSGKSKGGK
650	660	670	680	690	700	710	720
KKGSSFQTVS	ALHRENLNKL	MTNLRTTHPH	FVRCIIPNER	KAPGVMDNPL	VMHQLRCNGV	LEGIRICRKG	FPNRILYGDF
730	740	750	760	770	780	790	800
RQRYRILNPV	AIPEGQFIDS	RKGTEKLLSS	LDIDHNQYKF	GHTKVFFKAG	LLGLLEEMRD	ERLSRIITRM	QAQARGQLMR
810	820	830	840	850	860	870	880
IEFKKIVERR	DALLVIQWNI	RAFMGVKNWP	WMKLYFKIKP	LLKSAETEKE	MATMKEEFGR	IKETLEKSEA	RRKELEEKMV
890	900	910	920	930	940	950	960
SLLQEKNDLQ	LQVQAEQDNL	NDAEERCDQL	IKNKIQLEAK	VKEMNERLED	EEEMNAELTA	KKRKLEDECS	ELKKDIDDLE
970	980	990	1000	1010	1020	1030	1040
LTLAKVEKEK	HATENKVKNL	TEEMAGLDEI	IAKLTKEKKA	LQEAHQQALD	DLQVEEDKVN	SLSKSKVKLE	QQVDDLEGSL
1050	1060	1070	1080	1090	1100	1110	1120
EQEKKVRMDL	ERAKRKLEGD	LKLTQESIMD	LENDKLQLEE	KLKKKEFDIN	QQNSKIEDEQ	VLALQLQKKL	KENQARIEEL
1130	1140	1150	1160	1170	1180	1190	1200
EEELEAERTA	RAKVEKLRSD	LSRELEEISE	RLEEAGGATS	VQIEMNKKRE	AEFQKMRRDL	EEATLQHEAT	AAALRKKHAD
1210	1220	1230	1240	1250	1260	1270	1280
SVAELGEQID	NLQRVKQKLE	KEKSEFKLEL	DDVTSNMEQI	IKAKANLEKV	SRTLEDQANE	YRVKLEEAQR	SLNDFTTQRA
1290	1300	1310	1320	1330	1340	1350	1360
KLQTENGELA	RQLEEKEALI	SQLTRGKLSY	TQQMEDLKRQ	LEEEGKAKNA	LAHALQSARH	DCDLLREQYE	EETEAKAELQ
1370	1380	1390	1400	1410	1420	1430	1440
RVLSKANSEV	AQWRTKYETD	AIQRTEELEE	AKKKLAQRLQ	DAEEAVEAVN	AKCSSLEKTK	HRLQNEIEDL	MVDVERSNAA
1450	1460	1470	1480	1490	1500	1510	1520
AAALDKKQRN	FDKILAEWKQ	KYEESQSELE	SSQKEARSLS	TELFKLKNAY	EESLEHLETF	KRENKNLQEE	ISDLTEQLGE
1530	1540	1550	1560	1570	1580	1590	1600
GGKNVHELEK	VRKQLEVEKL	ELQSALEEAE	ASLEHEEGKI	LRAQLEFNQI	KAEIERKLAE	KDEEMEQAKR	NHQRVVDSLQ
1610	1620	1630	1640	1650	1660	1670	1680
TSLDAETRSR	NEVLRVKKKM	EGDLNEMEIQ	LSHANRMAAE	AQKQVKSLQS	LLKDTQIQLD	DAVRANDDLK	ENIAIVERRN
1690	1700	1710	1720	1730	1740	1750	1760
NLLQAELEEL	RAVVEQTERS	RKLAEQELIE	TSERVQLLHS	QNTSLINQKK	KMESDLTQLQ	SEVEEAVQEC	RNAEEKAKKA
1770	1780	1790	1800	1810	1820	1830	1840
ITDAAMMAEE	LKKEQDTSAH	LERMKKNMEQ	TIKDLQHRLD	EAEQIALKGG	KKQLQKLEAR	VRELEGELEA	EQKRNAESVK
1850	1860	1870	1880	1890	1900	1910	1920
GMRKSERRIK	ELTYQTEEDK	KNLLRLQDLV	DKLQLKVKAY	KRQAEEAEEQ	ANTNLSKFRK	VQHELDEAEE	RADIAESQVN
1930	1940						
KLRAKSRDIG	AKQKMHDEE						

Cmpd.	No. of Cmpds.	m/z meas.	Δ m/z [ppm]	z	Rt [min]	Score	P	Range	Sequence	Modification	Ratios	
1830	1	938.9541	-31.45	2	51.2	10.3	1	666-680	R.TTHPHFVRCIIPNER.K	Carbamidomethyl: 9	M:Q	0.49
2955	1	953.4514	-13.99	2	66.1	12.0	2	928-943	R.LEDEEEMNAELTAKKR.K		Q:W<br>M:Q	0.24<br>3.05

Detailed Protein Report

Cmpd.	No. of Cmpds.	m/z meas.	Δ m/z [ppm]	z	Rt [min]	Score	P	Range	Sequence	Modification	Ratios
202	1	561.6237	-229.75	2	31.3	12.3	0	945-953	K.LEDECSELK.K	Carbamidomethyl: 5	

Detailed Protein Report

Protein 109: **PREDICTED: sp110 nuclear body protein isoform X3 [Homo sapiens]**

**Accession:**	gi|530370295	**Score:**	74.1
**Database:**	refseq_human(refseq_human_20140103.fasta)	**MW [kDa]:**	58.5
**Database Date:**	2015-11-30	**pI:**	10.2
**Modification(s):**	Oxidation	**Sequence Coverage [%]:**	16.9
		**No. of unique Peptides:**	5

Quantitation

M:Q **Median:** 0.94 **CV:** 0.00 % **No. of Peptides:** 1

10	20	30	40	50	60	70	80
MNAEEDSEEM	PSLLTSTVQV	ASDNLIPQIR	DKEDPQEMPH	SPLGSMPEIR	DNSPEPNDPE	EPQEVSSTPS	DKKGKKRKRC
**90**	**100**	**110**	**120**	**130**	**140**	**150**	**160**
IWSTPKRRHK	KKSLPGGTAS	SRHGIQKKLK	RVDQVPQKKD	DSTCNSTVET	RAQKARTECA	RKSRSEEIID	GTSEMNEGKR
**170**	**180**	**190**	**200**	**210**	**220**	**230**	**240**
SQKTPSTPRR	VTQGAASPGH	GIQEKLQVVD	KVTQRKDDST	WNSEVMMRVQ	KARTKCARKS	RLKEKKKEKD	ICSSSKRRFQ
**250**	**260**	**270**	**280**	**290**	**300**	**310**	**320**
KNIHRRGKPK	SDTVDFHCSK	LPVTCGEAKG	ILYKKKMKHG	SSVKCIRNED	GTWLTPNEFE	VEGKGRNAKN	WKRNIRCEGM
**330**	**340**	**350**	**360**	**370**	**380**	**390**	**400**
TLGELLKRKN	SDECEVCCQG	GQLLCCGTCP	RVFHEDCHIP	PVEAKRMLWS	CTFCRMKRSS	GSQQCHHVSK	TLERQMQPQD
**410**	**420**	**430**	**440**	**450**	**460**	**470**	**480**
QLKCEFLLLK	AYCHPQSSFF	TGIPFNIRDY	GEPFQEAMWL	DLVKERLITE	MYTVAWFVRD	MRLMFRNHKT	FYKASDFGQV
**490**	**500**	**510**	**520**				
GLDLEAEFEK	DLKDVLGFHE	ANDGGFWTLP					

Cmpd.	No. of Cmpds.	m/z meas.	Δ m/z [ppm]	z	Rt [min]	Score	P	Range	Sequence	Modification	Ratios
1618	1	882.8999	95.52	3	48.5	11.3	2	171-195	R. VTQGAASPGHGIQEKLQVVDKVT[illegible] K		
220	1	978.1311	-3.65	3	32.7	10.8	2	279-304	K. HGSSVKCIRNEDGTWLTPNEFEV[illegible] G		
1829	1	781.3008	-84.17	2	53.2	11.5	2	366-377	K.RMLWSCTFCRMK.R		
2818	1	989.4652	-45.70	2	64.4	15.5	1	395-410	R.QMQPQDQLKCEFLLLK.A	Oxidation: 2	
2660	2	484.7692	52.33	2	61.6	14.6	1	460-466	R.DMRLMFR.N		M:Q 0.94

Detailed Protein Report

Protein 110: PREDICTED: rho GTPase-activating protein 26 isoform X13 [Homo sapiens]

**Accession:**	gi|578810840	**Score:**	73.4
**Database:**	refseq_human(refseq_human_20140103.fasta)	**MW [kDa]:**	71.6
**Database Date:**	2015-11-30	**pI:**	9.8
**Modification(s):**	Oxidation	**Sequence Coverage [%]:**	9.9
		**No. of unique Peptides:**	3

Quantitation

Q:W	**Median:** 0.34	**CV:** 0.00 %	**No. of Peptides:**	1
M:Q	**Median:** 2.74	**CV:** 0.00 %	**No. of Peptides:**	1

```
        10         20         30         40         50         60         70         80
MKRETRRSAF CVQGWSGGDP ARVVLWGSAG GRGRARHAGV PAVGPECRAR RSQRADPIVS TAPAPLGAEL LSLECPTVRM
        90        100        110        120        130        140        150        160
ERALPRGRCL PLGKDLSSAK RKFADSLNEF KFQCIGDAET DDEMCIARSL QEFATVLRNL EDERIRMIEN ASEVLITPLE
       170        180        190        200        210        220        230        240
KFRKEQIGAA KEAKKKYDKE TEKYCGILEK HLNLSSKKKE SQLQEADSQV DLVRQHFYEV SLEYVFKVQE VQERKMFEFV
       250        260        270        280        290        300        310        320
EPLLAFLQGL FTFYHHGYEL AKDFGDFKTQ LTISIQNTRN RFEGTRSEVE SLMKKMKENP LEHKTISPYT MEGYLYVQEK
       330        340        350        360        370        380        390        400
RHFGTSWVKH YCTYQRDSKQ ITMVPFDQKS GGKGGEDESV ILKSCTRRKT DSIEKRFCFD VEAVDRPGVI TMQALSEEDR
       410        420        430        440        450        460        470        480
RLWMEAMDGR EPVYNSNKDS QSEGTAQLDS IGFSIIRKCI HAVETRGINE QGLYRIVGVN SRVQKLLSVL MDPKTASETE
       490        500        510        520        530        540        550        560
TDICAEWEIK TITSALKTYL RMLPGPLMMY QFQRSFIKAA KLENQESRVS EIHSLVHRLP EKNRQMLQLL MNHLANSAFV
       570        580        590        600        610        620        630
SLSHYCIRYA GGLGKKCVKC SLQWRTAANA RWPSQRMLGK CSCNQATKQS FQGDFTYGDQ GQIGVCCP
```

Cmpd.	No. of Cmpds.	m/z meas.	Δ m/z [ppm]	z	Rt [min]	Score	P	Range	Sequence	Modification	Ratios
2004	1	710.2849	-115.96	2	55.4	11.7	1	35-48	R.ARHAGVPAVGPECR.A		
286	1	716.6484	355.53	1	32.9	20.0	0	165-171	K.EQIGAAK.E		
1623	1	516.2455	-93.79	2	50.5	14.5	0	466-474	K.LLSVLMDPK.T	Oxidation: 6	M:Q 2.74<br>Q:W 0.34

Detailed Protein Report

Protein 111: neurogenic locus notch homolog protein 1 preproprotein [Homo sapiens]

**Accession:**	gi|148833508	**Score:**	73.3
**Database:**	refseq_human(refseq_human_20140103.fasta)	**MW [kDa]:**	272.3
**Database Date:**	2015-11-30	**pI:**	4.8
**Modification(s):**	Carbamidomethyl	**Sequence Coverage [%]:**	2.2
		**No. of unique Peptides:**	4

Quantitation

Q:W	**Median:**	1.39	**CV:**	24.91 %	**No. of Peptides:**	2
M:Q	**Median:**	0.57	**CV:**	0.00 %	**No. of Peptides:**	1

Detailed Protein Report

10	20	30	40	50	60	70	80
MPPLLAPLLC	LALLPALAAR	GPRCSQPGET	CLNGGKCEAA	NGTEACVCGG	AFVGPRCQDP	NPCLSTPCKN	AGTCHVVDRR
90	100	110	120	130	140	150	160
GVADYACSCA	LGFSGPLCLT	PLDNACLTNP	CRNGGTCDLL	TLTEYKCRCP	PGWSGKSCQQ	ADPCASNPCA	NGGQCLPFEA
170	180	190	200	210	220	230	240
SYICHCPPSF	HGPTCRQDVN	ECGQKPGLCR	HGGTCHNEVG	SYRCVCRATH	TGPNCERPYV	PCSPSPCQNG	GTCRPTGDVT
250	260	270	280	290	300	310	320
HECACLPGFT	GQNCEENIDD	CPGNNCKNGG	ACVDGVNTYN	CRCPPEWTGQ	YCTEDVDECQ	LMPNACQNGG	TCHNTHGGYN
330	340	350	360	370	380	390	400
CVCVNGWTGE	DCSENIDDCA	SAACFHGATC	HDRVASFYCE	CPHGRTGLLC	HLNDACISNP	CNEGSNCDTN	PVNGKAICTC
410	420	430	440	450	460	470	480
PSGYTGPACS	QDVDECSLGA	NPCEHAGKCI	NTLGSFECQC	LQGYTGPRCE	IDVNECVSNP	CQNDATCLDQ	IGEFQCICMP
490	500	510	520	530	540	550	560
GYEGVHCEVN	TDECASSPCL	HNGRCLDKIN	EFQCECPTGF	TGHLCQYDVD	ECASTPCKNG	AKCLDGPNTY	TCVCTEGYTG
570	580	590	600	610	620	630	640
THCEVDIDEC	DPDPCHYGSC	KDGVATFTCL	CRPGYTGHHC	ETNINECSSQ	PCRHGGTCQD	RDNAYLCFCL	KGTTGPNCEI
650	660	670	680	690	700	710	720
NLDDCASSPC	DSGTCLDKID	GYECACEPGY	TGSMCNINID	ECAGNPCHNG	GTCEDGINGF	TCRCPEGYHD	PTCLSEVNEC
730	740	750	760	770	780	790	800
NSNPCVHGAC	RDSLNGYKCD	CDPGWSGTNC	DINNNECESN	PCVNGGTCKD	MTSGYVCTCR	EGFSGPNCQT	NINECASNPC
810	820	830	840	850	860	870	880
LNQGTCIDDV	AGYKCNCLLP	YTGATCEVVL	APCAPSPCRN	GGECRQSEDY	ESFSCVCPTG	WQGQTCEVDI	NECVLSPCRH
890	900	910	920	930	940	950	960
GASCQNTHGG	YRCHCQAGYS	GRNCETDIDD	CRPNPCHNGG	SCTDGINTAF	CDCLPGFRGT	FCEEDINECA	SDPCRNGANC
970	980	990	1000	1010	1020	1030	1040
TDCVDSYTCT	CPAGFSGIHC	ENNTPDCTES	SCFNGGTCVD	GINSFTCLCP	PGFTGSYCQH	DVNECDSQPC	LHGGTCQDGC
1050	1060	1070	1080	1090	1100	1110	1120
GSYRCTCPQG	YTGPNCQNLV	HWCDSSPCKN	GGKCWQTHTQ	YRCECPSGWT	GLYCDVPSVS	CEVAAQRQGV	DVARLCQHGG
1130	1140	1150	1160	1170	1180	1190	1200
LCVDAGNTHH	CRCQAGYTGS	YCEDLVDECS	PSPCQNGATC	TDYLGGYSCK	CVAGYHGVNC	SEEIDECLSH	PCQNGGTCLD
1210	1220	1230	1240	1250	1260	1270	1280
LPNTYKCSCP	RGTQGVHCEI	NVDDCNPPVD	PVSRSPKCFN	NGTCVDQVGG	YSCTCPPGFV	GERCEGDVNE	CLSNPCDARG
1290	1300	1310	1320	1330	1340	1350	1360
TQNCVQRVND	FHCECRAGHT	GRRCESVING	CKGKPCKNGG	TCAVASNTAR	GFICKCPAGF	EGATCENDAR	TCGSLRCLNG
1370	1380	1390	1400	1410	1420	1430	1440
GTCISGPRSP	TCLCLGPFTG	PECQFPASSP	CLGGNPCYNQ	GTCEPTSESP	FYRCLCPAKF	NGLLCHILDY	SFGGGAGRDI
1450	1460	1470	1480	1490	1500	1510	1520
PPPLIEEACE	LPECQEDAGN	KVCSLQCNNH	ACGWDGGDCS	LNFNDPWKNC	TQSLQCWKYF	SDGHCDSQCN	SAGCLFDGFD
1530	1540	1550	1560	1570	1580	1590	1600
CQRAEGQCNP	LYDQYCKDHF	SDGHCDQGCN	SAECEWDGLD	CAEHVPERLA	AGTLVVVVLM	PPEQLRNSSF	HFLRELSRVL
1610	1620	1630	1640	1650	1660	1670	1680
HTNVVFKRDA	HGQQMIFPYY	GREEELRKHP	IKRAAEGWAA	PDALLGQVKA	SLLPGGSEGG	RRRRELDPMD	VRGSIVYLEI
1690	1700	1710	1720	1730	1740	1750	1760
DNRQCVQASS	QCFQSATDVA	AFLGALASLG	SLNIPYKIEA	VQSETVEPPP	PAQLHFMYVA	AAAFVLLFFV	GCGVLLSRKR
1770	1780	1790	1800	1810	1820	1830	1840
RRQHGQLWFP	EGFKVSEASK	KKRREPLGED	SVGLKPLKNA	SDGALMDDNQ	NEWGDEDLET	KKFRFEEPVV	LPDLDDQTDH
1850	1860	1870	1880	1890	1900	1910	1920
RQWTQQHLDA	ADLRMSAMAP	TPPQGEVDAD	CMDVNVRGPD	GFTPLMIASC	SGGGLETGNS	EEEEDAPAVI	SDFIYQGASL
1930	1940	1950	1960	1970	1980	1990	2000
HNQTDRTGET	ALHLAARYSR	SDAAKRLLEA	SADANIQDNM	GRTPLHAAVS	ADAQGVFQIL	IRNRATDLDA	RMHDGTTPLI
2010	2020	2030	2040	2050	2060	2070	2080
LAARLAVEGM	LEDLINSHAD	VNAVDDLGKS	ALHWAAAVNN	VDAAVLLLKN	GANKDMQNNR	EETPLFLAAR	EGSYETAKVL
2090	2100	2110	2120	2130	2140	2150	2160
LDHFANRDIT	DHMDRLPRDI	AQERMHHDIV	RLLDEYNLVR	SPQLHGAPLG	GTPTLSPPLC	SPNGYLGSLK	PGVQGKKVRK
[illegible]	[illegible]	[illegible]	[illegible]	[illegible]	[illegible]	[illegible]	[illegible]

Detailed Protein Report

Cmpd.	No. of Cmpds.	m/z meas.	Δ m/z [ppm]	z	Rt [min]	Score	P	Range	Sequence	Modification	Ratios
1878	2	716.0566	111.85	3	52.1	10.3	1	1331-1350	R.GFICKCPAGFEGATCENDAR.T	Carbamidomethyl: 6	Q:W 1.77
1825	2	715.9982	30.20	3	51.5	11.6	1	1331-1350	R.GFICKCPAGFEGATCENDAR.T	Carbamidomethyl: 4	
1618	6	697.0380	98.52	3	48.9	15.1	1	1331-1350	R.GFICKCPAGFEGATCENDAR.T		
2073	10	698.2445	-185.84	2	55.8	21.5	0	1992-2004	R.MHDGTTPLILAAR.L		M:Q 0.57<br>Q:W 1.09

Detailed Protein Report

Protein 112: rapamycin-insensitive companion of mTOR isoform 3 [Homo sapiens]

**Accession:**	gi|550544215	**Score:**	73.3
**Database:**	refseq_human(refseq_human_20140103.fasta)	**MW [kDa]:**	155.8
**Database Date:**	2015-11-30	**pI:**	6.8
		**Sequence Coverage [%]:**	3.9
		**No. of unique Peptides:**	3

Quantitation

Q:W	**Median:**	0.96	**CV:**	73.60 %	**No. of Peptides:**	2
M:Q	**Median:**	0.58	**CV:**	19.84 %	**No. of Peptides:**	2

10	20	30	40	50	60	70	80
MGIIATFRSW	AGIINLCKPG	NSGIQSLIGV	LCIPNMEIRR	GLLEVLYDIF	RLPLPVVTEE	FIEALLSVDP	GRFQDSWRLS
90	100	110	120	130	140	150	160
DGFVAAEAKT	ILPHRARSRP	DLMDNYLALI	LSAFIRNGLL	EGLVEVITNS	DDHISVRATI	LLGELLHMAN	TILPHSHSHH
170	180	190	200	210	220	230	240
LHCLPTLMNM	AASFDIPKEK	RLRASAALNC	LKRFHEMKKR	GPKPYSLHLD	HIIQKAIATH	QKRDQYLRVQ	KDIFILKDTE
250	260	270	280	290	300	310	320
EALLINLRDS	QVLQHKENLE	WNWNLIGTIL	KWPNVNLRNY	KDEQLHRFVR	RLLYFYKPSS	KLYANLDLDF	AKAKQLTVVG
330	340	350	360	370	380	390	400
CQFTEFLLES	EEDGQGYLED	LVKDIVQWLN	ASSGMKPERS	LQNNGLLTTL	SQHYFLFIGT	LSCHPHGVKM	LEKCSVFQCL
410	420	430	440	450	460	470	480
LNLCSLKNQD	HLLKLTVSSL	DYSRDGLARV	ILSKILTAAT	DACRLYATKH	LRVLLRANVE	FFNNWGIELL	VTQLHDKNKT
490	500	510	520	530	540	550	560
ISSEALDILD	EACEDKANLH	ALIQMKPALS	HLGDKGLLLL	LRFLSIPKGF	SYLNERGYVA	KQLEKWHREY	NSKYVDLIEE
570	580	590	600	610	620	630	640
QLNEALTTYR	KPVDGDNYVR	RSNQRLQRPH	VYLPIHLYGQ	LVHHKTGCHL	LEVQNIITEL	CRNVRTPDLD	KWEEIKKLKA
650	660	670	680	690	700	710	720
SLWALGNIGS	SNWGLNLLQE	ENVIPDILKL	AKQCEVLSIR	GTCVYVLGLI	AKTKQGCDIL	KCHNWDAVRH	SRKHLWPVVP
730	740	750	760	770	780	790	800
DDVEQLCNEL	SSIPSTLSLN	SESTSSRHNS	ESESVPSSMF	ILEDDRFGSS	STSTFFLDIN	EDTEPTFYDR	SGPIKDKNSF
810	820	830	840	850	860	870	880
PFFASSKLVK	NRILNSLTLP	NKKHRSSSDP	KGGKLSSESK	TSNRRIRTLT	EPSVDFNHSD	DFTPISTVQK	TLQLETSFMG
890	900	910	920	930	940	950	960
NKHIEDTGST	PSIGENDLKF	TKNFGTENHR	ENTSRERLVV	ESSTSSHMKI	RSQSFNTDTT	TSGISSMSSS	PSRETVGVDA
970	980	990	1000	1010	1020	1030	1040
TTMDTDCGSM	STVVSTKTIK	TSHYLTPQSN	HLSLSKSNSV	SLVPPGSSHT	LPRRAQSLKA	PSIATIKSLA	DCNFSYTSSR
1050	1060	1070	1080	1090	1100	1110	1120
DAFGYATLKR	LQQQRMHPSL	SHSEALASPA	KDVLFTDTIT	MKANSFESRL	TPSRFMKALS	YASLDKEDLL	SPINQNTLQR
1130	1140	1150	1160	1170	1180	1190	1200
SSSVRSMVSS	ATYGGSDDYI	GLALPVDIND	IFQVKDIPYF	QTKNIPPHDD	RGARAFAHDA	GESTEDTGLQ	EHTDDNCLYC
1210	1220	1230	1240	1250	1260	1270	1280
VCIEILGFQP	SNQLSAICSH	SDFQDIPYSD	WCEQTIHNPL	EVVPSKFSGI	SGCSDGVSQE	GSASSTKSTE	LLLGVKTIPD
1290	1300	1310	1320	1330	1340	1350	1360
DTPMCRILLR	KEVLRLVINL	SSSVSTKCHE	TGLLTIKEKY	PQTFDDICLY	SEVSHLLSHC	TFRLPCRRFI	QELFQDVQFL
1370	1380	1390					
QMHEEAEAVL	ATPPKQPIVD	TSAES					

Cmpd.	No. of Cmpds.	m/z meas.	Δ m/z [ppm]	z	Rt [min]	Score	P	Range	Sequence	Modification	Ratios
2622	1	658.3808	-6.32	2	61.5	16.9	2	182-193	R.LRASAALNCLKR.F		
2751	1	805.9143	-22.12	2	65.5	23.4	1	435-449	K.ILTAATDACRLYATK.H		Q:W 0.50<br>M:Q 0.71
275	1	795.2750	-158.71	2	33.1	12.4	1	916-929	R.ERLVVESSTSSHMK.I		M:Q 0.48<br>Q:W 1.85

Detailed Protein Report

Protein 113: dynein heavy chain 17, axonemal [Homo sapiens]

**Accession:**	gi|256542310	**Score:**	73.0
**Database:**	refseq_human(refseq_human_20140103.fasta)	**MW [kDa]:**	509.0
**Database Date:**	2015-11-30	**pI:**	5.4
**Modification(s):**	Carbamidomethyl, Oxidation	**Sequence Coverage [%]:**	1.6
		**No. of unique Peptides:**	3

Quantitation

Q:W	**Median:**	0.45	**CV:**	0.00 %	**No. of Peptides:**	1
M:Q	**Median:**	0.96	**CV:**	0.00 %	**No. of Peptides:**	1

10	20	30	40	50	60	70	80
MTMAPDVRLE	YLEEVASIVL	KFKPDKWSKL	IGAEENVALF	TEFFEKPDVQ	VLVLTLNAAG	MIIPCLGFPQ	SLKSKGVYFI
90	100	110	120	130	140	150	160
KTKSENINKD	NYRARLLYGD	ISPTPVDQLI	AVVEEVLSSL	LNQSENMAGW	PQVVSEDIVK	QVHRLKNEMF	VMSGKIKGKT
170	180	190	200	210	220	230	240
LLPIPEHLGS	LDGTLESMER	IPSSLDNLLL	HAIETTIIDW	SHQIRDVLSK	DSAQALLDGL	HPLPQVEFEF	WDTRLLNLKC
250	260	270	280	290	300	310	320
IHEQLNRPKV	NKIVEILEKA	KSCYWPALQN	VYTNVTEGLK	EANDIVLYLK	PLRILLEEME	QADFTMLPTF	IAKVLDTICF
330	340	350	360	370	380	390	400
IWATSEYYNT	PARIIVILQE	FCNQIIEMTR	TFLSPEEVLK	GLQGEIEEVL	SGISLAVNVL	KELYQTYDFC	CVNMKLFFKD
410	420	430	440	450	460	470	480
KEPVPWEFPS	SLAFSRINSF	FQRIQTIEEL	YKTAIEFLKL	EKIELGGVRG	NLLGSLVTRI	YDEVFELVKV	FADCKYDPLD
490	500	510	520	530	540	550	560
PGDSNFDRDY	ADFEIKIQDL	DRRLATIFCQ	GFDDCSCIKS	SAKLLYMCGG	LMERPLILAE	VAPRYSVMLE	LFDAELDNAK
570	580	590	600	610	620	630	640
ILYDAQMAAS	EEGNIPLIHK	NMPPVAGQLK	WSLELQERLE	VSMKHLKHVE	HPVMSGAEAK	LTYQKYDEMM	ELLRCHREKI
650	660	670	680	690	700	710	720
YQQWVAGVDQ	DCHFNLGQPL	ILRDAASNLI	HVNFSKALVA	VLREVKYLNF	QQQKEIPDSA	ESLFSENETF	RKFVGNLELI
730	740	750	760	770	780	790	800
VGWYNEIKTI	VKAVEFLLIK	SELEAIDVKL	LSAETTLFWN	GEGVFQYIQE	VREILHNLQN	RMQKAKQNIE	GISQAMKDWS
810	820	830	840	850	860	870	880
ANPLFERKDN	KKEALLDLDG	RIANLNKRYA	AVRDAGVKIQ	AMVAENAELF	RADTLSLPWK	DYVIYIDDMV	LDEFDQFIRK
890	900	910	920	930	940	950	960
SLSFLMDNMV	IDESIAPLFE	IRMELDEDGL	TFNPTLEVGS	DRGFLALIEG	LVNDIYNVAR	LIPRLAKDRM	NYKMDLEDNT
970	980	990	1000	1010	1020	1030	1040
DLIEMREEVS	SLVINAMKEA	EEYQDSFERY	SYLWTDNLQE	FMKNFLIYGC	AVTAEDLDTW	TDDTIPKTPP	TLAQFQEQID
1050	1060	1070	1080	1090	1100	1110	1120
SYEKLYEEVS	KCENTKVFHG	WLQCDCRPFK	QALLSTIRRW	GFMFKRHLSN	HVTNSLADLE	AFMKVARMGL	TKPLKEGDYD
1130	1140	1150	1160	1170	1180	1190	1200
GLVEVMGHLM	KVKERQAATD	NMFEPLKQTI	ELLKTYGEEM	PEEIHLKLQE	LPEHWANTKK	LAIQVKLTVA	PLQANEVSIL
1210	1220	1230	1240	1250	1260	1270	1280
RRKCQQFELK	QHEFRERFRR	EAPFSFSDPN	PYKSLNKQQK	SISAMEGIME	ALSKSGGLFE	VPVPDYKQLK	ACHREVRLLK
1290	1300	1310	1320	1330	1340	1350	1360
ELWDMVVVVN	TSIEDWKTTK	WKDINVEQMD	IDCKKFAKDM	RSLDKEMKTW	DAFVGLDNTV	KNVITSLRAV	SELQNPAIRE
1370	1380	1390	1400	1410	1420	1430	1440
RHWQQLMQAT	QVKFKMSEET	TLADLLQLNL	HSYEDEVRNI	VDKAVKESGM	EKVLKALDST	WSMMEFQHEP	HPRTGTMMLK
1450	1460	1470	1480	1490	1500	1510	1520
SSEVLVETLE	DNQVQLQNLM	MSKYLAHFLK	EVTSWQQKLS	TADSVISIWF	EVQRTWSHLE	SIFIGSEDIR	TQLPGDSQRF
1530	1540	1550	1560	1570	1580	1590	1600
DDINQEFKAL	MEDAVKTPNV	VEATSKPGLY	NKLEALKKSL	AICEKALAEY	LETKRLAFPR	FYFVSSADLL	DILSNGNDPV
1610	1620	1630	1640	1650	1660	1670	1680
EVSRHLSKLF	DSLCKLKFRL	DASDKPLKVG	LGMYSKEDEY	MVFDQECDLS	GQVEVWLNRV	LDRMCSTLRH	EIPEAVVTYE
1690	1700	1710	1720	1730	1740	1750	1760
EKPREQWILD	YPAQVALTCT	QIWWTTEVGL	AFARLEEGYE	NAIRDYNKKQ	ISQLNVLITL	LMGNLNAGDR	MKIMTICTID
1770	1780	1790	1800	1810	1820	1830	1840
VHARDVVAKM	IVAKVESSQA	FTWQAQLRHR	WDEEKRHCFA	NICDAQIQYS	YEYLGNTPRL	VITPLTDRCY	ITLTQSLHLI
1850	1860	1870	1880	1890	1900	1910	1920
MGGAPAGPAG	TGKTETTKDL	GRALGTMVYV	FNCSEQMDYK	SCGNIYKGLA	QTGAWGCFDE	FNRISVEVLS	VIAVQVKCVQ
1930	1940	1950	1960	1970	1980	1990	2000
DAIRAKKKAF	NFLGEIIGLI	PTVGIFITMN	PGYAGRAELP	ENLKALFRPC	AMVVPDFELI	CEIMLMAEGF	LEARLLARKF
2010	2020	2030	2040	2050	2060	2070	2080
ITLYTLCKEL	LSKQDHYDWG	LRAIKSVLVV	AGSLKRGDPS	RAEDQVLMRA	LRDFNIPKIV	TDDLPVFMGL	IGDLFPALDV
2090	2100	2110	2120	2130	2140	2150	2160
PRKRDLNFEK	IIKQSIVELK	LQAEDSFVLK	VVQLEELLQV	RHSVFIVGNA	GSGKSQVLKS	LNKTYQNLKR	KPVAVDLDPK
2170	2180	2190	2200	2210	2220	2230	2240

Detailed Protein Report

Cmpd.	No. of Cmpds.	m/z meas.	Δ m/z [ppm]	z	Rt [min]	Score	P	Range	Sequence	Modification	Ratios
1870	1	704.2582	-146.54	2	53.4	13.7	0	1255-1267	K.SGGLFEVPVPDYK.Q		M:Q 0.96<br>Q:W 0.45
1689	1	1051.5326	21.27	2	50.9	16.9	1	4025-4041	K.CMLFALCYFHAVVAERR.K	Carbamidomethyl: 1; Oxidation: 2	
637	1	635.8011	-148.78	2	36.5	10.8	2	4247-4257	R.RSLKELNLGLK.G		

Detailed Protein Report

Protein 114: filaggrin [Homo sapiens]

**Accession:**	gi|60097902	**Score:**	72.6
**Database:**	refseq_human(refseq_human_20140103.fasta)	**MW [kDa]:**	434.9
**Database Date:**	2015-11-30	**pI:**	9.6
		**Sequence Coverage [%]:**	1.8
		**No. of unique Peptides:**	3

Detailed Protein Report

10	20	30	40	50	60	70	80
MSTLLENIFA	IINLFKQYSK	KDKNTDTLSK	KELKELLEKE	FRQILKNPDD	PDMVDVFMDH	LDIDHNKKID	FTEFLLMVFK
90	100	110	120	130	140	150	160
LAQAYYESTR	KENLPISGHK	HRKHSHHDKH	EDNKQEENKE	NRKRPSSLER	RNNRKGNKGR	SKSPRETGGK	RHESSSEKKE
170	180	190	200	210	220	230	240
RKGYSPTHRE	EEYGKNHHNS	SKKEKNKTEN	TRLGDNRKRL	SERLEEKEDN	EEGVYDYENT	GRMTQKWIQS	GHIATYYTIQ
250	260	270	280	290	300	310	320
DEAYDTTDSL	LEENKIYERS	RSSDGKSSSQ	VNRSRHENTS	QVPLQESRTR	KRRGSRVSQD	RDSEGHSEDS	ERHSGSASRN
330	340	350	360	370	380	390	400
HHGSAWEQSR	DGSRHPRSHD	EDRASHGHSA	DSSRQSGTRH	AETSSRGQTA	SSHEQARSSP	GERHGSGHQQ	SADSSRHSAT
410	420	430	440	450	460	470	480
GRGQASSAVS	DRGHRGSSGS	QASDSEGHSE	NSDTQSVSGH	GKAGLRQQSH	QESTRGRSGE	RSGRSGSSLY	QVSTHEQPDS
490	500	510	520	530	540	550	560
AHGRTGTSTG	GRQGSHHEQA	RDSSRHSASQ	EGQDTIRGHP	GSSRGGRQGS	HHEQSVNRSG	HSGSHHSHTT	SQGRSDASHG
570	580	590	600	610	620	630	640
QSGSRSASRQ	TRNEEQSGDG	TRHSGSRHHE	ASSQADSSRH	SQVGQGQSSG	PRTSRNQGSS	VSQDSDSQGH	SEDSERWSGS
650	660	670	680	690	700	710	720
ASRNHHGSAQ	EQSRDGSRHP	RSHHEDRAGH	GHSADSSRKS	GTRHTQNSSS	GQAASSHEQA	RSSAGERHGS	RHQLQSADSS
730	740	750	760	770	780	790	800
RHSGTGHGQA	SSAVRDSGHR	GSSGSQATDS	EGHSEDSDTQ	SVSGHGQAGH	HQQSHQESAR	DRSGERSRRS	GSFLYQVSTH
810	820	830	840	850	860	870	880
KQSESSHGWT	GPSTGVRQGS	HHEQARDNSR	HSASQDGQDT	IRGHPGSSRR	GRQGSHHEQS	VDRSGHSGSH	HSHTTSQGRS
890	900	910	920	930	940	950	960
DASRGQSGSR	SASRTTRNEE	QSRDGSRHSG	SRHHEASSHA	DISRHSQAGQ	GQSEGSRTSR	RQGSSVSQDS	DSEGHSEDSE
970	980	990	1000	1010	1020	1030	1040
RWSGSASRNH	RGSAQEQSRH	GSRHPRSHHE	DRAGHGHSAD	SSRQSGTPHA	ETSSGGQAAS	SHEQARSSPG	ERHGSRHQQS
1050	1060	1070	1080	1090	1100	1110	1120
ADSSRHSGIP	RRQASSAVRD	SGHWGSSGSQ	ASDSEGHSEE	SDTQSVSGHG	QDGPHQQSHQ	ESARDWSGGR	SGRSGSFIYQ
1130	1140	1150	1160	1170	1180	1190	1200
VSTHEQSESA	HGRTRTSTGR	RQGSHHEQAR	DSSRHSASQE	GQDTIRAHPG	SRRGGRQGSH	HEQSVDRSGH	SGSHHSHTTS
1210	1220	1230	1240	1250	1260	1270	1280
QGRSDASHGQ	SGSRSASRQT	RKDKQSGDGS	RHSGSRHHEA	ASWADSSRHS	QVGQEQSSGS	RTSRHQGSSV	SQDSDSERHS
1290	1300	1310	1320	1330	1340	1350	1360
DDSERLSGSA	SRNHHGSSRE	QSRDGSRHPG	FHQEDRASHG	HSADSSRQSG	THHTESSSHG	QAVSSHEQAR	SSPGERHGSR
1370	1380	1390	1400	1410	1420	1430	1440
HQQSADSSRH	SGIGHRQASS	AVRDSGHRGS	SGSQVTNSEG	HSEDSDTQSV	SAHGQAGPHQ	QSHKESARGQ	SGESSGRSRS
1450	1460	1470	1480	1490	1500	1510	1520
FLYQVSSHEQ	SESTHGQTAP	STGGRQGSRH	EQARNSSRHS	ASQDGQDTIR	GHPGSSRGGR	QGSYHEQSVD	RSGHSGYHHS
1530	1540	1550	1560	1570	1580	1590	1600
HTTPQGRSDA	SHGQSGPRSA	SRQTRNEEQS	GDGSRHSGSR	HHEPSTRAGS	SRHSQVGQGE	SAGSKTSRRQ	GSSVSQDRDS
1610	1620	1630	1640	1650	1660	1670	1680
EGHSEDSERR	SESASRNHYG	SAREQSRHGS	RNPRSHQEDR	ASHGHSAESS	RQSGTRHAET	SSGGQAASSQ	EQARSSPGER
1690	1700	1710	1720	1730	1740	1750	1760
HGSRHQQSAD	SSTDSGTGRR	QDSSVVGDSG	NRGSSGSQAS	DSEGHSEESD	TQSVSAHGQA	GPHQQSHQES	TRGQSGERSG
1770	1780	1790	1800	1810	1820	1830	1840
RSGSFLYQVS	THEQSESAHG	RTGPSTGGRQ	RSRHEQARDS	SRHSASQEGQ	DTIRGHPGSS	RGGRQGSHYE	QSVDSSGHSG
1850	1860	1870	1880	1890	1900	1910	1920
SHHSHTTSQE	RSDVSRGQSG	SRSVSRQTRN	EKQSGDGSRH	SGSRHHEASS	RADSSRHSQV	GQGQSSGPRT	SRNQGSSVSQ
1930	1940	1950	1960	1970	1980	1990	2000
DSDSQGHSED	SERWSGSASR	NHLGSAWEQS	RDGSRHPGSH	HEDRAGHGHS	ADSSRQSGTR	HTESSSRGQA	ASSHEQARSS
2010	2020	2030	2040	2050	2060	2070	2080
AGERHGSHHQ	LQSADSSRHS	GIGHGQASSA	VRDSGHRGYS	GSQASDSEGH	SEDSDTQSVS	AQGKAGPHQQ	SHKESARGQS
2090	2100	2110	2120	2130	2140	2150	2160
GESSGRSGSF	LYQVSTHEQS	ESTHGQSAPS	TGGRQGSHYD	QAQDSSRHSA	SQEGQDTIRG	HPGPSRGGRQ	GSHQEQSVDR
2170	2180	2190	2200	2210	2220	2230	2240

Detailed Protein Report

Cmpd.	No. of Cmpds.	m/z meas.	Δ m/z [ppm]	z	Rt [min]	Score	P	Range	Sequence	Modification
1715	1	867.8788	-56.88	2	50.0	14.2	2	3238-3252	R.NHRGSVQEQSRHGSR.H	
46	1	731.3139	-20.71	3	30.3	16.4	1	3577-3596	R.HPTSHHEDRAGHGHSAESSR.Q	
371	1	938.4376	1.41	2	34.6	15.1	2	3797-3814	R.SDASHGQSGSRSASRETR.N	

Detailed Protein Report

Protein 115: **biorientation of chromosomes in cell division protein 1-like 1 [Homo sapiens]**

**Accession:**	gi|87299628	**Score:**	71.9
**Database:**	refseq_human(refseq_human_20140103.fasta)	**MW [kDa]:**	330.3
**Database Date:**	2015-11-30	**pI:**	4.9
**Modification(s):**	Oxidation	**Sequence Coverage [%]:**	1.9
		**No. of unique Peptides:**	3

Detailed Protein Report

10	20	30	40	50	60	70	80
MATNPQPQPP	PPAPPPPPPQ	PQPQPPPPPP	GPGAGPGAGG	AGGAGAGAGD	PQLVAMIVNH	LKSQGLFDQF	RRDCLADVDT
90	100	110	120	130	140	150	160
KPAYQNLRQR	VDNFVANHLA	THTWSPHLNK	NQLRNNIRQQ	VLKSGMLESG	IDRIISQVVD	PKINHTFRPQ	VEKAVHEFLA
170	180	190	200	210	220	230	240
TLNHKEEGSG	NTAPDDEKPD	TSLITQGVPT	PGPSANVAND	AMSILETITS	LNQEASAARA	STETSNAKTS	ERASKKLPSQ
250	260	270	280	290	300	310	320
PTTDTSTDKE	RTSEDMADKE	KSTADSGGEG	LETAPKSEEF	SDLPCPVEEI	KNYTKEHNNL	ILLNKDVQQE	SSEQKNKSTD
330	340	350	360	370	380	390	400
KGEKKPDSNE	KGERKKEKKE	KTEKKFDHSK	KSEDTQKVKD	EKQAKEKEVE	SLKLPSEKNS	NKAKTVEGTK	EDFSLIDSDV
410	420	430	440	450	460	470	480
DGLTDITVSS	VHTSDLSSFE	EDTEEEVVTS	DSMEEGEITS	DDEEKNKQNK	TKTQTSDSSE	GKTKSVRHAY	VHKPYLYSKY
490	500	510	520	530	540	550	560
YSDSDDELTV	EQRRQSIAKE	KEERLLRRQI	NREKLEEKRK	QKAEKTKSSK	TKGQGRSSVD	LEESSTKSLE	PKAARIKEVL
570	580	590	600	610	620	630	640
KERKVLEKKV	ALSKKRKKDS	RNVEENSKKK	QQYEEDSKET	LKTSEHCEKE	KISSSKELKH	VHAKSEPSKP	ARRLSESLHV
650	660	670	680	690	700	710	720
VDENKNESKL	EREHKRRTST	PVIMEGVQEE	TDTRDVKRQV	ERSEICTEEP	QKQKSTLKNE	KHLKKDDSET	PHLKSLLKKE
730	740	750	760	770	780	790	800
VKSSKEKPER	EKTPSEDKLS	VKHKYKGDCM	HKTGDETELH	SSEKGLKVEE	NIQKQSQQTK	LSSDDKTERK	SKHRNERKLS
810	820	830	840	850	860	870	880
VLGKDGKPVS	EYIIKTDENV	RKENNKKERR	LSAEKTKAEH	KSRRSSDSKI	QKDSLGSKQH	GITLQRRSES	YSEDKCDMDS
890	900	910	920	930	940	950	960
TNMDSNLKPE	EVVHKEKRRT	KSLLEEKLVL	KSKSKTQGKQ	VKVVETELQE	GATKQATTPK	PDKEKNTEEN	DSEKQRKSKV
970	980	990	1000	1010	1020	1030	1040
EDKPFEETGV	EPVLETASSS	AHSTQKDSSH	RAKLPLAKEK	YKSDKDSTST	RLERKLSDGH	KSRSLKHSSK	DIKKKDENKS
1050	1060	1070	1080	1090	1100	1110	1120
DDKDGKEVDS	SHEKARGNSS	LMEKKLSRRL	CENRRGSLSQ	EMAKGEEKLA	ANTLSTPSGS	SLQRPKKSGD	MTLIPEQEPM
1130	1140	1150	1160	1170	1180	1190	1200
EIDSEPGVEN	VFEVSKTQDN	RNNNSQQDID	SENMKQKTSA	TVQKDELRTC	TADSKATAPA	YKPGRGTGVN	SNSEKHADHR
1210	1220	1230	1240	1250	1260	1270	1280
STLTKKMHIQ	SAVSKMNPGE	KEPIHRGTTE	VNIDSETVHR	MLLSAPSEND	RVQKNLKNTA	AEEHVAQGDA	TLEHSTNLDS
1290	1300	1310	1320	1330	1340	1350	1360
SPSLSSVTVV	PLRESYDPDV	IPLFDKRTVL	EGSTASTSPA	DHSALPNQSL	TVRESEVLKT	SDSKEGGEGF	TVDTPAKASI
1370	1380	1390	1400	1410	1420	1430	1440
TSKRHIPEAH	QATLLDGKQG	KVIMPLGSKL	TGVIVENENI	TKEGGLVDMA	KKENDLNAEP	NLKQTIKATV	ENGKKDGIAV
1450	1460	1470	1480	1490	1500	1510	1520
DHVVGLNTEK	YAETVKLKHK	RSPGKVKDIS	IDVERRNENS	EVDTSAGSGS	APSVLHQRNG	QTEDVATGPR	RAEKTSVATS
1530	1540	1550	1560	1570	1580	1590	1600
TEGKDKDVTL	SPVKAGPATT	TSSETRQSEV	ALPCTSIEAD	EGLIIGTHSR	NNPLHVGAEA	SECTVFAAAE	EGGAVVTEGF
1610	1620	1630	1640	1650	1660	1670	1680
AESETFLTST	KEGESGECAV	AESEDRAADL	LAVHAVKIEA	NVNSVVTEEK	DDAVTSAGSE	EKCDGSLSRD	SEIVEGTITF
1690	1700	1710	1720	1730	1740	1750	1760
ISEVESDGAV	TSAGTEIRAG	SISSEEVDGS	QGNMMRMGPK	KETEGTVTCT	GAEGRSDNFV	ICSVTGAGPR	EERMVTGAGV
1770	1780	1790	1800	1810	1820	1830	1840
VLGDNDAPPG	TSASQEGDGS	VNDGTEGESA	VTSTGITEDG	EGPASCTGSE	DSSEGFAISS	ESEENGESAM	DSTVAKEGTN
1850	1860	1870	1880	1890	1900	1910	1920
VPLVAAGPCD	DEGIVTSTGA	KEEDEEGEDV	VTSTGRGNEI	GHASTCTGLG	EESEGVLICE	SAEGDSQIGT	VVEHVEAEAG
1930	1940	1950	1960	1970	1980	1990	2000
AAIMNANENN	VDSMSGTEKG	SKDTDICSSA	KGIVESSVTS	AVSGKDEVTP	VPGGCEGPMT	SAASDQSDSQ	LEKVEDTTIS
2010	2020	2030	2040	2050	2060	2070	2080
TGLVGGSYDV	LVSGEVPECE	VAHTSPSEKE	DEDIITSVEN	EECDGLMATT	ASGDITNQNS	LAGGKNQGKV	LIISTSTTND
2090	2100	2110	2120	2130	2140	2150	2160
YTPQVSAITD	VEGGLSDALR	TEENMEGTRV	TTEEFEAPMP	SAVSGDDSQL	TASRSEEKDE	CAMISTSIGE	EFELPISSAT
2170	2180	2190	2200	2210	2220	2230	2240

Detailed Protein Report

Cmpd.	No. of Cmpds.	m/z meas.	Δ m/z [ppm]	z	Rt [min]	Score	P	Range	Sequence	Modification
1228	1	954.9820	37.30	2	45.5	23.8	0	658-674	R.TSTPVIMEGVQEETDTR.D	Oxidation: 7
582	1	848.5905	146.58	2	36.6	10.8	2	853-867	K.DSLGSKQHGITLQRR.S	
2965	1	557.6376	40.39	3	65.8	10.0	2	2881-2894	K.YPVETTLKMKDDSK.T	Oxidation: 9

Detailed Protein Report

Protein 116: **PREDICTED: RANBP2-like and GRIP domain-containing protein 5/6 isoform X6 [Homo sapiens]**

**Accession:**	gi|578805009	**Score:**	71.9
**Database:**	refseq_human(refseq_human_20140103.fasta)	**MW [kDa]:**	196.8
**Database Date:**	2015-11-30	**pI:**	5.9
		**Sequence Coverage [%]:**	4.6
		**No. of unique Peptides:**	3

Quantitation

Q:W **Median:** 1.49 **CV:** 8.81 % **No. of Peptides:** 2

10	20	30	40	50	60	70	80
MGPRVTVKSM	KGFYFAKLYY	EAKEYDLAKK	YICTYINVQE	RDPKAHRFLG	LLYELEENTE	KAVECYRRSV	ELNPTQKDLV
90	100	110	120	130	140	150	160
LKIAELLCKN	DVTDGRAKYW	VERAAKLFPG	SPAIYKLKEQ	LLDCEGEDGW	NKLFDLIQSE	LYVRPDDVHV	NIRLVELYRS
170	180	190	200	210	220	230	240
TKRLKDAVAH	CHEAERNIAL	RSSLEWNSCV	VQTLKEYLES	LQCLESDKSD	WQATNTDLLL	AYANLMLLTL	STRDVQENRE
250	260	270	280	290	300	310	320
LLESFDSALQ	SAKSSLGGND	ELSATFLEMK	GHFYMYAGSL	LLKMGQHGNN	VQWRALSELA	ALCYLIAFQV	PRPKIKLREG
330	340	350	360	370	380	390	400
KAGQNLLEMM	ACDRLSQSGH	MLLSLSRGKQ	DFLKEVVETF	ANKIGQSALY	DALFSSQSPK	DTSFLGSDDI	GKIDVQEPEL
410	420	430	440	450	460	470	480
EDLARYDVGA	IRAHNGSLQH	LTWLGLQWNS	LPALPGIRKW	LKQLFHRLPH	ETSRLETNAP	ESICILDLEV	FLLGVVYTSH
490	500	510	520	530	540	550	560
LQLKEKCNSH	HSSYQPLCLP	FPVCKQLCTE	RQKSWWDAVC	TLIHRKAVPG	NLAKLRLLVQ	HEINTLRAQE	KHGLQPALLV
570	580	590	600	610	620	630	640
HWAKYLQKTG	SGLNSFYGQL	EYIGRSVHYW	KKVLPLLKII	KKNSIPEPID	PLFKHFHSVD	IQASEIVEYE	EDAHITFAML
650	660	670	680	690	700	710	720
DAVNGNIEDA	VTAFESIKSV	VSYWNLALIF	HRKAEDIEND	ALSPEEQEEC	RNYLTKTRDY	LIKIIDDGDS	NLSVVKKLPV
730	740	750	760	770	780	790	800
PLESVKQMLN	SVMQELEDYS	EGGPLYKNGS	LRNADSEIKH	STPSPTKYSL	SPSKSYKYSP	ETPPRWTEDR	NSLLNMICQQ
810	820	830	840	850	860	870	880
VEAIKKEMQE	LKLNSSKSAS	RHRWPTENYG	PDSVPDGYQG	SQTFHGAPLT	VATTGPSVYY	SQSPAYNSQY	LLRPAANVTP
890	900	910	920	930	940	950	960
TKGSSNTEFK	STKEGFSIPV	SADGFKFGIS	EPGNQEKKRE	KPLENDTGFQ	AQDISGRKKG	RGVIFGQTSS	TFTFADVAKS
970	980	990	1000	1010	1020	1030	1040
TSGEGFQFGK	KDLNFKGFSG	AGEKLFSSRY	GKMANKANTS	GDFEKDDDAY	KTEDSDDIHF	EPVVQMPEKV	ELVTGEEGEK
1050	1060	1070	1080	1090	1100	1110	1120
VLYSQGVKLF	RFDAEVRQWK	ERGLGNLKIL	KNEVNGKLRM	LMRREQVLKV	CANHWITTTM	NLKPLSGSDR	AWMWSASDFS
1130	1140	1150	1160	1170	1180	1190	1200
DGDAKLERLA	AKFKTPELAE	EFKQKFEECQ	RLLLDIPLQT	PHKLVDTGRA	AKLIQRAEEM	KSGLKDFKTF	LTNDQTKVTE
1210	1220	1230	1240	1250	1260	1270	1280
EENKGSGTGA	AGASDTTIKP	NAENTGPTLE	WDNYDLREDA	LDDSVSSSSV	HASPLASSPV	RKNLFRFDES	TTGSNFSFKS
1290	1300	1310	1320	1330	1340	1350	1360
ALSLSKSPAK	LNQSGTSVGT	DEESVVTQEE	ERDGQYFEPV	VPLPDLVEVS	SGEENEQVVF	SHRAEIYRYD	KDVGQWKERG
1370	1380	1390	1400	1410	1420	1430	1440
IGDIKILQNY	DNKQVRIVMR	RDQVLKLCAN	HRITPDMSLQ	NMKGTERVWV	WTACDFADGE	RKVEHLAVRF	KLQDVADSFK
1450	1460	1470	1480	1490	1500	1510	1520
KIFDEAKTAQ	EKDSLITPHV	SRSSTPRESP	CGKIAVAILE	ETTRERTDVI	QGDDVADAAS	EVEVSSTSET	TTKAVVSPPK
1530	1540	1550	1560	1570	1580	1590	1600
FVFVSESVKR	IFSSEKSKPF	VFGNSSATGS	LFGFSFNAPL	KSNNSETSSV	AQSGSESKVE	PKKCELSKNS	DIEQSSDSKV
1610	1620	1630	1640	1650	1660	1670	1680
KNLSASFPTE	ESSINYTFKT	PEKEPPLWHA	EFTKEELVQK	LRSTTKSADH	LNGLLREIEA	TNAVLMEQIK	LLKSEIRRLE
1690	1700	1710	1720	1730	1740	1750	
RNQEREKSAA	NLEYLKNVLL	QFIFLKPGSE	RERLLPVINT	MLQLSPEEKG	KLAAVAQDEE	ENASRSSG	

Detailed Protein Report

Cmpd.	No. of Cmpds.	m/z meas.	Δ m/z [ppm]	z	Rt [min]	Score	P	Range	Sequence	Modification	Ratios
2775	1	557.2986	22.82	3	63.1	14.9	1	891-906	K.STKEGFSIPVSADGFK.F		Q:W 1.63
1997	1	648.7895	-86.66	2	53.5	11.3	1	1382-1392	R.DQVLKLCANHR.I		Q:W 1.37
1535	1	1023.6824	101.59	2	49.0	12.2	1	1697-1713	K.NVLLQFIFLKPGSERER.L		

Detailed Protein Report

Protein 117: PREDICTED: regulator of G-protein signaling 3 isoform X1 [Homo sapiens]

Accession: gi|578817731
Database: refseq_human(refseq_human_20140103.fasta)
Database Date: 2015-11-30
Modification(s): Carbamidomethyl, Oxidation

Score: 71.5
MW [kDa]: 120.9
pI: 5.9
Sequence Coverage [%]: 7.6
No. of unique Peptides: 5

Quantitation

M:Q **Median:** 0.81 **CV:** 16.90 % **No. of Peptides:** 2

10	20	30	40	50	60	70	80
MEWLSPDIAL	PRRDEWTQTS	PARKRITHAK	VQGAGQLRLS	IDAQDRVLLL	HIIEGKGLIS	KQPGTCDPYV	KISLIPEDSR
**90**	**100**	**110**	**120**	**130**	**140**	**150**	**160**
LRHQKTQTVP	DCRDPAFHEH	FFFPVQEEDD	QKRLLVTVWN	RASQSRQSGL	IGCMSFGVKS	LLTPDKEISG	WYYLLGEHLG
**170**	**180**	**190**	**200**	**210**	**220**	**230**	**240**
RTKHLKVARR	RLRPLRDPLL	RMPGGGDTEN	GKKLKITIPR	GKDGFGFTIC	CDSPVRVQAV	DSGGPAERAG	LQQLDTVLQL
**250**	**260**	**270**	**280**	**290**	**300**	**310**	**320**
NERPVEHWKC	VELAHEIRSC	PSEIILLVWR	MVPQVKPGPD	GGVLRRASCK	STHDLQSPPN	KREKNCTHGV	QARPEQRHSC
**330**	**340**	**350**	**360**	**370**	**380**	**390**	**400**
HLVCDSSDGL	LLGGWERYTE	VAKRGGQHTL	PALSRATAPT	DPNYIILAPL	NPGSQLLRPV	YQEDTIPEES	GSPSKGKSYT
**410**	**420**	**430**	**440**	**450**	**460**	**470**	**480**
GLGKKSRLMK	TVQTMKGHGN	YQNCPVVRPH	ATHSSYGTYV	TLAPKVLVFP	VFVQPLDLCN	PARTLLLSEE	LLLYEGRNKA
**490**	**500**	**510**	**520**	**530**	**540**	**550**	**560**
AEVTLFAYSD	LLLFTKEDEP	GRCDVLRNPL	YLQSVKLQEG	SSEDLKFCVL	YLAEKAECLF	TLEAHSQEQK	KRVCWCLSEN
**570**	**580**	**590**	**600**	**610**	**620**	**630**	**640**
IAKQQQLAAS	PPDSKMFETE	ADEKREMALE	EGKGPGAEDS	PPSKEPSPGQ	ELPPGQDLPP	NKDSPSGQEP	APSQEPLSSK
**650**	**660**	**670**	**680**	**690**	**700**	**710**	**720**
DSATSEGSPP	GPDAPPSKDV	PPCQEPPPAQ	DLSPCQDLPA	GQEPLPHQDP	LLTKDLPAIQ	ESPTRDLPPC	QDLPPSQVSL
**730**	**740**	**750**	**760**	**770**	**780**	**790**	**800**
PAKALTEDTM	SSGDLLAATG	DPPAAPRPAF	VIPEVRLDST	YSQKAGAEQG	CSGDEEDAEE	AEEVEEGEEG	EEDEDEDTSD
**810**	**820**	**830**	**840**	**850**	**860**	**870**	**880**
DNYGERSEAK	RSSMIETGQG	AEGGLSLRVQ	NSLRRRTHSE	GSLLQEPRGP	CFASDTTLHC	SDGEGAASTW	GMPSPSTLKK
**890**	**900**	**910**	**920**	**930**	**940**	**950**	**960**
ELGRNGGSMH	HLSLFFTGHR	KMSGADTVGD	DDEASRKRKS	KNLAKDMKNK	LGIFRRRNES	PGAPPAGKAD	KMMKSFKPTS
**970**	**980**	**990**	**1000**	**1010**	**1020**	**1030**	**1040**
EEALKWGESL	EKLLVHKYGL	AVFQAFLRTE	FSEENLEFWL	ACEDFKKVKS	QSKMASKAKK	IFAEYIAIQA	CKEVNLDSYT
**1050**	**1060**	**1070**	**1080**	**1090**	**1100**		
REHTKDNLQS	VTRGCFDLAQ	KRIFGLMEKD	SYPRFLRSDL	YLDLINQKKM	SPPL		

Cmpd.	No. of Cmpds.	m/z meas.	Δ m/z [ppm]	z	Rt [min]	Score	P	Range	Sequence	Modification	Ratios	
2171	1	576.2313	-138.85	3	55.7	10.6	1	31-46	K.VQGAGQLRLSIDAQDR.V		M:Q	0.96
7	10	819.8312	-118.35	2	29.5	14.6	2	81-93	R.LRHQKTQTVPDCR.D	Carbamidomethyl: 12		
1945	1	700.2356	-147.65	2	54.4	10.8	0	127-139	R.QSGLIGCMSFGVK.S	Carbamidomethyl: 7; Oxidation: 8	M:Q	0.69
512	1	593.1679	-216.84	2	36.4	10.4	0	217-228	R.VQAVDSGGPAER.A			
1949	1	599.9043	-89.98	3	52.7	12.8	0	885-900	R.NGGSMHHLSLFFTGHR.K			

Detailed Protein Report

Protein 118: endothelin-2 preproprotein [Homo sapiens]

Accession: gi|4503463
Database: refseq_human(refseq_human_20140103.fasta)
Database Date: 2015-11-30
Modification(s): Carbamidomethyl

Score: 71.1
MW [kDa]: 19.9
pI: 12.1
Sequence Coverage [%]: 14.0
No. of unique Peptides: 2

10	20	30	40	50	60	70	80
MVSVPTTWCS	VALALLVALH	EGKGQAAATL	EQPASSSHAQ	GTHLRLRRCS	CSSWLDKECV	YFCHLDIIWV	NTPEQTAPYG
**90**	**100**	**110**	**120**	**130**	**140**	**150**	**160**
LGNPPRRRRR	SLPRRCQCSS	ARDPACATFC	LRRPWTEAGA	VPSRKSPADV	FQTGKTGATT	GELLQRLRDI	STVKSLFAKR
**170**	**180**						
QQEAMREPRS	THSRWRKR						

Cmpd.	No. of Cmpds.	m/z meas.	Δ m/z [ppm]	z	Rt [min]	Score	P	Range	Sequence	Modification
2946	1	1051.5610	95.12	2	65.6	10.9	2	95-112	R.RCQCSSARDPACATFCLR.R	Carbamidomethyl: 4, 12
2155	1	1051.4608	-0.17	2	55.5	16.2	2	95-112	R.RCQCSSARDPACATFCLR.R	Carbamidomethyl: 12, 16

Detailed Protein Report

Protein 119: **PREDICTED: beta/gamma crystallin domain-containing protein 3 isoform X1 [Homo sapiens]**

**Accession:**	gi|530373918	**Score:**	70.8
**Database:**	refseq_human(refseq_human_20140103.fasta)	**MW [kDa]:**	298.2
**Database Date:**	2015-11-30	**pI:**	5.0
**Modification(s):**	Oxidation	**Sequence Coverage [%]:**	2.8
		**No. of unique Peptides:**	4

Quantitation

Q:W	**Median:**	0.98	**CV:**	0.00 %	**No. of Peptides:**	1
M:Q	**Median:**	0.74	**CV:**	23.23 %	**No. of Peptides:**	2

Detailed Protein Report

10	20	30	40	50	60	70	80
MKGNLLEGPL	EDSDCSKTSF	NKENSLTNNP	ELQNIASSNN	LLNKNAWGSI	ERNRSSPSSV	TNSSYDGESD	SQHHLSCEPV
90	100	110	120	130	140	150	160
SQTNRNLVCS	ALLTGSNHRK	VPCSPDFQRV	TTTENTIKEN	STVMSNRTLV	QREELVEPQG	PAISDFSCSK	SDGSDTTEQE
170	180	190	200	210	220	230	240
STNLPSPNKS	IRHEHLQLPE	SECSDKQTID	SSSKQAATHT	NIIALQRHAV	TDTEFVNEGK	RLSAQDSQKN	VAVREIRRET
250	260	270	280	290	300	310	320
ESASAGESIA	SSHVKAPEDK	IESLPKDTDQ	YFETKAKKLD	FRSHDKIPHI	RMNKKDLASL	NYISESAVVA	SLGNENAPEL
330	340	350	360	370	380	390	400
KFELNRSHIS	ETPLDSESPQ	QAEVSPDAKT	SLSLDCKKLN	FSISPPTFVS	GVGMLSKLDI	PDLMNEGSPV	PIETGNVNIV
410	420	430	440	450	460	470	480
GISYQPRKCK	EENVKNHVEA	AGRKSPPPSF	CLEYTSAIFE	FKEVLSNSEK	CQVLPGSEAS	GPHLTGLELL	SFDSGNLSKD
490	500	510	520	530	540	550	560
CSSILSQDPN	RVELVSSNTK	ANMSIIEKSD	SLSLEAKTAN	IVSKAEIDGQ	NNVLVESHSG	RGKTISLSKV	SLSKVEPRNI
570	580	590	600	610	620	630	640
SQDKMSSFPL	KITHVPEKPI	LSELTFLEVE	QGKRFQSINH	NEIGEKCSDA	GLKENCQAEL	SPAASKYEDK	PEPEVDALGS
650	660	670	680	690	700	710	720
PPALLKSNIS	WILPPIHDEK	ISRQMAQNCE	AHTCVFHQSL	DICGTKKISG	HSEMAELSLT	NISPKFQETG	SMKVNSPFLD
730	740	750	760	770	780	790	800
SDSSLEKNSS	ASEDSSFLKV	PSVLKLEKKS	SSYRKKENIH	FLNGGIDSVS	SSSSYPEEVS	MIVNSHKPQN	NLDSIQVTKD
810	820	830	840	850	860	870	880
LTHEGTSVTN	LLYPTTSYLE	FETSVSIGTE	VTPFQEHFGI	YTGKISIDFP	TAAQFDNLVE	AETGAVAGPA	ASVNSSGQQC
890	900	910	920	930	940	950	960
SEASAEHIEA	RRRAHDQLLD	LKSSLLKKAD	TLIGEIFNSV	REELKFKHTV	STCQEHIAIE	GIMNLGTLKE	DISEKNPSEV
970	980	990	1000	1010	1020	1030	1040
TLTEIQQTEG	LEEQGMENMS	EVKEKPCVSP	TVGEKNLLVD	PNSMNVSCLL	EDKARELVNE	IIYVAQEKLR	NDTFEDTEDT
1050	1060	1070	1080	1090	1100	1110	1120
WDSELQANTS	KILNSDSVKP	HDVVREFLVS	EQPVNQSTQI	SENKVLNEFF	SLSNLASGTE	SIKGGEIVLY	QKSLFSGNGS
1130	1140	1150	1160	1170	1180	1190	1200
GLSDSINLQE	SDTVLLAEDM	SHKRLDDRVK	THLFRSEDCN	ETMEIENVDN	NKTETEDRRT	LVLNFKWPPL	VNDDIHAPGT
1210	1220	1230	1240	1250	1260	1270	1280
SKSSLSDSLV	CISEKNLPGH	SKNTPLAMSD	VGKVHKKDNE	INIGKIELIP	SMLETGKTNK	KDAELNILKY	EAVPPMIEMG
1290	1300	1310	1320	1330	1340	1350	1360
RIHKMDAELN	VTKTEPKANV	FKMGEVYQMD	AESCIEKTEG	SAVILGMEKA	YKMKDTEGDI	GKIEVIPMMP	EVKNIHQKDA
1370	1380	1390	1400	1410	1420	1430	1440
EGDIVKTEMT	PVTVDMENIY	QTHAEGDIGK	TGTIALSEVE	NIHQKGGEGI	SEKAEVIPVT	LAMENTYQKD	AEGDIGKAEV
1450	1460	1470	1480	1490	1500	1510	1520
MPVRLEMENT	YPKDTERDGG	KTEVMPLALE	VVNTYQKNAK	GFTGNTEGSV	LKMEATYRKT	AEEVIKNTEI	VPCVLKVKEA
1530	1540	1550	1560	1570	1580	1590	1600
HETAPAPLEM	EKACKRDVKE	TIGATVSTPS	VIEMEKISPE	DRGENIGKHK	VLPAVVDIEK	IHGTGLELTT	KQGEAMLPAF
1610	1620	1630	1640	1650	1660	1670	1680
ESKTPQEYAE	GSVEETKEEP	TEIKEGLIAH	ENRLPTYFRG	YESPTLSKDY	EGYPAPAMPD	FQPGDTTVRL	DKRMSLTAIY
1690	1700	1710	1720	1730	1740	1750	1760
DKRRETDYSD	KGYNLAFVSQ	DEQENSSFTI	LYEEPLQEED	KYASAEARQT	QSVLFHDTSA	DSMPVLACER	SESRTDLVHH
1770	1780	1790	1800	1810	1820	1830	1840
FEKGTKLGET	FDSDSSEMFL	SVEAKRYKIY	PLALSPIYED	DSSQEDILSS	EVSPGHHGPR	KSRDSENQSS	SVLSLLQSVS
1850	1860	1870	1880	1890	1900	1910	1920
ERLKMNFDED	DREAADEEEE	EEEAAVLHKG	DLRAGSGERV	TFQLPDPSIT	FYPDDQESVG	ISKNSYVMPN	EPTTSNLQVG
1930	1940	1950	1960	1970	1980	1990	2000
LWPEKTSFLQ	KSDLTSKLHS	SLKSAYHQYL	QTSQSHSSEK	GARFGGIFQE	PVSKYFRVQD	SPGRLSPFIE	NVDKQTLRCN
2010	2020	2030	2040	2050	2060	2070	2080
PRPGKMVIYD	LHESTYKQEV	YCNIPDATSW	SFPNGVLIKV	VRGCWILYEK	PHFRGQKCVL	EEGEKVLNRD	WILQNRRHPQ
2090	2100	2110	2120	2130	2140	2150	2160
RNFILGSLKR	VLKDCSIPEI	ELFPQSDPAC	CPVYIQRAVP	NLEELNISKS	VSFTVKSGVW	LAYPDINFKG	QATVLEEDHG
2170	2180	2190	2200	2210	2220	2230	2240

Detailed Protein Report

Cmpd.	No. of Cmpds.	m/z meas.	Δ m/z [ppm]	z	Rt [min]	Score	P	Range	Sequence	Modification	Ratios
1963	1	918.3504	-135.25	2	54.9	12.1	1	501-517	K.ANMSIIEKSDSLSLEAK.T		M:Q 0.93<br>Q:W 0.98
2896	1	1012.4872	-151.04	1	65.4	13.7	1	740-748	K.VPSVLKLEK.K		M:Q 0.59
137	1	793.8712	-30.92	2	31.4	10.2	1	1430-1444	K.DAEGDIGKAEVMPVR.L		
1726	1	900.4572	47.30	3	49.9	14.8	1	1649-1672	K.DYEGYPAPAMPDFQPGDTTVRLDR	Oxidation: 10	

Detailed Protein Report

Protein 120: transcription factor HIVEP3 isoform b [Homo sapiens]

**Accession:**	gi|189181750	**Score:**	70.7
**Database:**	refseq_human(refseq_human_20140103.fasta)	**MW [kDa]:**	259.2
**Database Date:**	2015-11-30	**pI:**	8.6
**Modification(s):**	Carbamidomethyl	**Sequence Coverage [%]:**	3.0
		**No. of unique Peptides:**	2

Quantitation

Q:W	**Median:**	2.06	**CV:**	0.00 %	**No. of Peptides:**	1
M:Q	**Median:**	0.62	**CV:**	0.00 %	**No. of Peptides:**	1

Detailed Protein Report

10	20	30	40	50	60	70	80
MDPEQSVKGT	KKAEGSPRKR	LTKGEAIQTS	VSSSVPYPGS	GTAATQESPA	QELLAPQPFP	GPSSVLREGS	QEKTGQQQKP
90	100	110	120	130	140	150	160
PKRPPIEASV	HISQLPQHPL	TPAFMSPGKP	EHLLEGSTWQ	LVDPMRPGPS	GSFVAPGLHP	QSQLLPSHAS	IIPPEDLPGV
170	180	190	200	210	220	230	240
PKVFVPRPSQ	VSLKPTEEAH	KKERKPQKPG	KYICQYCSRP	CAKPSVLQKH	IRSHTGERPY	PCGPCGFSFK	TKSNLYKHRK
250	260	270	280	290	300	310	320
SHAHRIKAGL	ASGMGGEMYP	HGLEMERIPG	EEFEEPTEGE	STDSEEETSA	TSGHPAELSP	RPKQPLLSSG	LYSSGSHSSS
330	340	350	360	370	380	390	400
HERCSLSQSS	TAQSLEDPPP	FVEPSSEHPL	SHKPEDTHTI	KQKLALRLSE	RKKVIDEQAF	LSPGSKGSTE	SGYFSRSESA
410	420	430	440	450	460	470	480
EQQVSPPNTN	AKSYAEIIFG	KCGRIGQRTA	MLTATSTQPL	LPLSTEDKPS	LVPLSVPRTQ	VIEHITKLIT	INEAVVDTSE
490	500	510	520	530	540	550	560
IDSVKPRRSS	LSRRSSMESP	KSSLYREPLS	SHSEKTKPEQ	SLLSLQHPPS	TAPPVPLLRS	HSMPSAACTI	STPHHPFRGS
570	580	590	600	610	620	630	640
YSFDDHITDS	EALSHSSHVF	TSHPRMLKRQ	PAIELPLGGE	YSSEEPGPSS	KDTASKPSDE	VEPKESELTK	KTKKGLKTKG
650	660	670	680	690	700	710	720
VIYECNICGA	RYKKRDNYEA	HKKYYCSELQ	IAKPISAGTH	TSPEAEKSQI	EHEPWSQMMH	YKLGTTLELT	PLRKRRKEKS
730	740	750	760	770	780	790	800
LGDEEEPPAF	ESTKSQFGSP	GPSDAARNLP	LESTKSPAEP	SKSVPSLEGP	TGFQPRTPKP	GSGSESGKER	RTTSKEISVI
810	820	830	840	850	860	870	880
QHTSSFEKSD	SLEQPSGLEG	EDKPLAQFPS	PPPAPHGRSA	HSLQPKLVRQ	PNIQVPEILV	TEEPDRPDTE	PEPPPKEPEK
890	900	910	920	930	940	950	960
TEEFQWPQRS	QTLAQLPAEK	LPPKKKRLRL	AEMAQSSGES	SFESSVPLSR	SPSQESNVSL	SGSSRSASFE	RDDHGKAEAP
970	980	990	1000	1010	1020	1030	1040
SPSSDMRPKP	LGTHMLTVPS	HHPHAREMRR	SASEQSPNVS	HSAHMTETRS	KSFDYGSLSL	TGPSAPAPVA	PPARVAPPER
1050	1060	1070	1080	1090	1100	1110	1120
RKCFLVRQAS	LSRPPESELE	VAPKGRQESE	EPQPSSSKPS	AKSSLSQISS	AATSHGGPPG	GKGPGQDRPP	LGPTVPYTEA
1130	1140	1150	1160	1170	1180	1190	1200
LQVFHHPVAQ	TPLHEKPYLP	PPVSLFSFQH	LVQHEPGQSP	EFFSTQAMSS	LLSSPYSMPP	LPPSLFQAPP	LPLQPTVLHP
1210	1220	1230	1240	1250	1260	1270	1280
GQLHLPQLMP	HPANIPFRQP	PSFLPMPYPT	SSALSSGFFL	PLQSQFALQL	PGDVESHLPQ	IKTSLAPLAT	GSAGLSPSTE
1290	1300	1310	1320	1330	1340	1350	1360
YSSDIRLPPV	APPASSSAPT	SAPPLALPAC	PDTMVSLVVP	VRVQTNMPSY	GSAMYTTLSQ	ILVTQSQGSS	ATVALPKFEE
1370	1380	1390	1400	1410	1420	1430	1440
PPSKGTTVCG	ADVHEVGPGP	SGLSEEQSRA	FPTPYLRVPV	TLPERKGTSL	SSESILSLEG	SSSTAGGSKR	VLSPAGSLEL
1450	1460	1470	1480	1490	1500	1510	1520
TMETQQQKRV	KEEEASKADE	KLELVKPCSV	VLTSTEDGKR	PEKSHLGNQG	QGRRELEMLS	SLSSDPSDTK	EIPPLPHPAL
1530	1540	1550	1560	1570	1580	1590	1600
SHGTAPGSEA	LKEYPQPSGK	PHRRGLTPLS	VKKEDSKEQP	DLPSLAPPSS	LPLSETSSRP	AKSQEGTDSK	KVLQFPSLHT
1610	1620	1630	1640	1650	1660	1670	1680
TTNVSWCYLN	YIKPNHIQHA	DRRSSVYAGW	CISLYNPNLP	GVSTKAALSL	LRSKQKVSKE	TYTMATAPHP	EAGRLVPSSS
1690	1700	1710	1720	1730	1740	1750	1760
RKPRMTEVHL	PSLVSPEGQK	DLARVEKEEE	RRGEPEEDAP	ASQRGEPARI	KIFEGGYKSN	EEYVYVRGRG	RGKYVCEECG
1770	1780	1790	1800	1810	1820	1830	1840
IRCKKPSMLK	KHIRTHTDVR	PYVCKHCHFA	FKTKGNLTKH	MKSKAHSKKC	QETGVLEELE	AEEGTSDDLF	QDSEGREGSE
1850	1860	1870	1880	1890	1900	1910	1920
AVEEHQFSDL	EDSDSDSDLD	EDEDEDEEES	QDELSRPSSE	APPPGPPHAL	RADSSPILGP	QPPDAPASGT	EATRGSSVSE
1930	1940	1950	1960	1970	1980	1990	2000
AERLTASSCS	MSSQSMPGLP	WLGPAPLGSV	EKDTGSALSY	KPVSPRRPWS	PSKEAGSRPP	LARKHSLTKN	DSSPQRCSPA
2010	2020	2030	2040	2050	2060	2070	2080
REPQASAPSP	PGLHVDPGRG	MGALPCGSPR	LQLSPLTLCP	LGRELAPRAH	VLSKLEGTTD	PGLPRYSPTR	RWSPGQAESP
2090	2100	2110	2120	2130	2140	2150	2160
PRSAPPGKWA	LAGPGSPSAG	EHGPGLGLDP	RVLFPPAPLP	HKLLSRSPET	CASPWKAESR	SPSCSPGPAH	PLSSRPFSAL
2170	2180	2190	2200	2210	2220	2230	2240

Detailed Protein Report

Cmpd.	No. of Cmpds.	m/z meas.	Δ m/z [ppm]	z	Rt [min]	Score	P	Range	Sequence	Modification	Ratios
2516	1	941.4560	-33.22	2	61.9	13.2	2	635-651	K.GLKTKGVIYECNICGAR.Y	Carbamidomethyl: 11	
22	1	657.3901	133.33	2	28.9	15.8	1	1752-1762	R.GKYVCEECGIR.C	Carbamidomethyl: 5	M:Q 0.62<br>Q:W 2.06

Detailed Protein Report

Protein 121: E3 ubiquitin-protein ligase RNF213 isoform 3 [Homo sapiens]

**Accession:**	gi|366039979	**Score:**	70.0
**Database:**	refseq_human(refseq_human_20140103.fasta)	**MW [kDa]:**	591.0
**Database Date:**	2015-11-30	**pI:**	6.0
**Modification(s):**	Oxidation	**Sequence Coverage [%]:**	1.3
		**No. of unique Peptides:**	4

Detailed Protein Report

10	20	30	40	50	60	70	80
MECPSCQHVS	KEETPKFCSQ	CGERLPPAAP	IADSENNNST	MASASEGEME	CGQELKEEGG	PCLFPGSDSW	QENPEEPCSK
90	100	110	120	130	140	150	160
ASWTVQESKK	KKRKKKKKGN	KSASSELASL	PLSPASPCHL	TLLSNPWPQD	TALPHSQAQQ	SGPTGQPSQP	PGTATTPLEG
170	180	190	200	210	220	230	240
DGLSAPTEVG	DSPLQAQALG	EAGVATGSEA	QSSPQFQDHT	EGEDQDASIP	SGGRGLSQEG	TGPPTSAGEG	HSRTEDAAQE
250	260	270	280	290	300	310	320
LLLPESKGGS	SEPGTELQTT	EQQAGASASM	AVDAVAEPAN	AVKGAGKEMK	EKTQRMKQPP	ATTPPFKTHC	QEAETKTKDE
330	340	350	360	370	380	390	400
MAAAEEKVGK	NEQGEPEDLK	KPEGKNRSAA	AVKNEKEQKN	QEADVQEVKA	STLSPGGGVT	VFFHAIISLH	FPFNPDLHKV
410	420	430	440	450	460	470	480
FIRGGEEFGE	SKWDSNICEL	HYTRDLGHDR	VLVEGIVCIS	KKHLDKYIPY	KYVIYNGESF	EYEFIYKHQQ	KKGEYVNRCL
490	500	510	520	530	540	550	560
FIKSSLLGSG	DWHQYYDIVY	MKPHGRLQKV	MNHITDGPRK	DLVKGKQIAA	ALMLDSTFSI	LQTWDTINLN	SFFTQFEQFC
570	580	590	600	610	620	630	640
FVLQQPMIYE	GQAQLWTDLQ	YREKEVKRYL	WQHLKKHVVP	LPDGKSTDFL	PVDCPVRSKL	KTGLIVLFVV	EKIELLLEGS
650	660	670	680	690	700	710	720
LDWLCHLLTS	DASSPDEFHR	DLSHILGIPQ	SWRLYLVNLC	QRCMDTRTYT	WLGALPVLHC	CMELAPRHKD	AWRQPEDTWA
730	740	750	760	770	780	790	800
ALEGLSFSPF	REQMLDTSSL	LQFMREKQHL	LSIDEPLFRS	WFSLLPLSHL	VMYMENFIEH	LGRFPAHILD	CLSGIYYRLP
810	820	830	840	850	860	870	880
GLEQVLNTQD	VQDVQNVQNI	LEMLLRLLDT	YRDKIPEEAL	SPSYLTVCLK	LHEAICSSTK	LLKFYELPAL	SAEIVCRMIR
890	900	910	920	930	940	950	960
LLSLVDSAGQ	RDETGNNSVQ	TVFQGTLAAT	KRWLREVFTK	NMLTSSGASF	TYVKEIEVWR	RLVEIQFPAE	HGWKESLLGD
970	980	990	1000	1010	1020	1030	1040
MEWRLTKEEP	LSQITAYCNS	CWDTKGLEDS	VAKTFEKCII	EAVSSACQSQ	TSILQGFSYS	DLRKFGIVLS	AVITKSWPRT
1050	1060	1070	1080	1090	1100	1110	1120
ADNFNDILKH	LLTLADVKHV	FRLCGTDEKI	LANVTEDAKR	LIAVADSVLT	KVVGDLLSGT	ILVGQLELII	KHKNQFLDIW
1130	1140	1150	1160	1170	1180	1190	1200
QLREKSLSPQ	DEQCAVEEAL	DWRREELLLL	KKEKRCVDSL	LKMCGNVKHL	IQVDFGVLAV	RHSQDLSSKR	LNDTVTVRLS
1210	1220	1230	1240	1250	1260	1270	1280
TSSNSQRATH	YHLSSQVQEM	AGKIDLLRDS	HIFQLFWREA	AEPLSEPKED	QEAAELLSEP	EEESERHILE	LEEVYDYLYQ
1290	1300	1310	1320	1330	1340	1350	1360
PSYRKFIKLH	QDLKSGEVTL	AEIDVIFKDF	VNKYTDLDSE	LKIMCTVDHQ	DQRDWIKDRV	EQIKEYHHLH	QAVHAAKVIL
1370	1380	1390	1400	1410	1420	1430	1440
QVKESLGLNG	DFSVLNTLLN	FTDNFDDFRR	ETLDQINQEL	IQAKKLLQDI	SEARCKGLQA	LSLRKEFICW	VREALGGINE
1450	1460	1470	1480	1490	1500	1510	1520
LKVFVDLASI	SAGENDIDVD	RVACFHDAVQ	GYASLLFKLD	PSVDFSAFMK	HLKKLWKALD	KDQYLPRKLC	DSARNLEWLK
1530	1540	1550	1560	1570	1580	1590	1600
TVNESHGSVE	RSSLTLATAI	NQRGIYVIQA	PKGGQKISPD	TVLHLILPES	PGSHEESREY	SLEEVKELLN	KLMLMSGKKD
1610	1620	1630	1640	1650	1660	1670	1680
RNNTEVERFS	EVFCSVQRLS	QAFIDLHSAG	NMLFRTWIAM	AYCSPKQGVS	LQMDFGLDLV	TELKEGGDVT	ELLAALCRQM
1690	1700	1710	1720	1730	1740	1750	1760
EHFLDSWKRF	VTQKRMEHFY	LNFYTAEQLV	YLSTELRKQP	PSDAALTMLS	FIKSNCTLRD	VLRASVGCGS	EAARYRMRRV
1770	1780	1790	1800	1810	1820	1830	1840
MEELPLMLLS	EFSLVDKLRI	IMEQSMRCLP	AFLPDCLDLE	TLGHCLAHLA	GMGGSPVERC	LPRGLQVGQP	NLVVCGHSEV
1850	1860	1870	1880	1890	1900	1910	1920
LPAALAVYMQ	TPSQPLPTYD	EVLLCTPATT	FEEVALLLRR	CLTLGSLGHK	VYSLLFADQL	SYEVARQAEE	LFHNLCTQQH
1930	1940	1950	1960	1970	1980	1990	2000
REDYQLVMVC	DGDWEHCYLP	SAFSQHKVFV	TPQAPLEAIQ	AYLAGHYRVP	KQTLSAAAVF	NDRLCVGIVA	SERAGVGKSL
2010	2020	2030	2040	2050	2060	2070	2080
YVKRLHDKMK	MQLNVKNVPL	KTIRLIDPQV	DESRVLGALL	PFLDAQYQKV	PVLFHLDVTS	SVQTGIWVFL	FKLLILQYLM
2090	2100	2110	2120	2130	2140	2150	2160
DINGKMWLRN	PCHLYIVEIL	ERRTSVPSRS	SSALRTRVPQ	FSFLDIFPKV	TCRPPKEVID	MELSALRSDT	EPGMDLWEFC
2170	2180	2190	2200	2210	2220	2230	2240

Detailed Protein Report

Cmpd.	No. of Cmpds.	m/z meas.	Δ m/z [ppm]	z	Rt [min]	Score	P	Range	Sequence	Modification
1737	1	863.0655	110.54	2	51.5	19.4	1	746-759	R.EKQHLLSIDEPLFR.S	
887	1	646.8226	-33.57	2	39.6	12.6	1	1778-1787	K.LRIIMEQSMR.C	Oxidation: 5
398	1	646.4124	124.84	2	34.0	11.1	0	2137-2147	K.EVIDMELSALR.S	Oxidation: 5
2481	1	856.9085	-24.89	2	61.4	13.6	1	4661-4674	R.LLNFDTELSTKEMR.N	Oxidation: 13

Detailed Protein Report

Protein 122: DNA-directed RNA polymerase II subunit RPB1 [Homo sapiens]

**Accession:**	gi|4505939	**Score:**	69.9
**Database:**	refseq_human(refseq_human_20140103.fasta)	**MW [kDa]:**	217.1
**Database Date:**	2015-11-30	**pI:**	7.1
**Modification(s):**	Oxidation	**Sequence Coverage [%]:**	4.5
		**No. of unique Peptides:**	5

Detailed Protein Report

10	20	30	40	50	60	70	80
MHGGGPPSGD	SACPLRTIKR	VQFGVLSPDE	LKRMSVTEGG	IKYPETTEGG	RPKLGGLMDP	RQGVIERTGR	CQTCAGNMTE
90	100	110	120	130	140	150	160
CPGHFGHIEL	AKPVFHVGFL	VKTMKVLRCV	CFFCSKLLVD	SNNPKIKDIL	AKSKGQPKKR	LTHVYDLCKG	KNICEGGEEM
170	180	190	200	210	220	230	240
DNKFGVEQPE	GDEDLTKEKG	HGGCGRYQPR	IRRSGLELYA	EWKHVNEDSQ	EKKILLSPER	VHEIFKRISD	EECFVLGMEP
250	260	270	280	290	300	310	320
RYARPEWMIV	TVLPVPPLSV	RPAVVMQGSA	RNQDDLTHKL	ADIVKINNQL	RRNEQNGAAA	HVIAEDVKLL	QFHVATMVDN
330	340	350	360	370	380	390	400
ELPGLPRAMQ	KSGRPLKSLK	QRLKGKEGRV	RGNLMGKRVD	FSARTVITPD	PNLSIDQVGV	PRSIAANMTF	AEIVTPFNID
410	420	430	440	450	460	470	480
RLQELVRRGN	SQYPGAKYII	RDNGDRIDLR	FHPKPSDLHL	QTGYKVERHM	CDGDIVIFNR	QPTLHKMSMM	GHRVRILPWS
490	500	510	520	530	540	550	560
TFRLNLSVTT	PYNADFDGDE	MNLHLPQSLE	TRAEIQELAM	VPRMIVTPQS	NRPVMGIVQD	TLTAVRKFTK	RDVFLERGEV
570	580	590	600	610	620	630	640
MNLLMFLSTW	DGKVPQPAIL	KPRPLWTGKQ	IFSLIIPGHI	NCIRTHSTHP	DDEDSGPYKH	ISPGDTKVVV	ENGELIMGIL
650	660	670	680	690	700	710	720
CKKSLGTSAG	SLVHISYLEM	GHDITRLFYS	NIQTVINNWL	LIEGHTIGIG	DSIADSKTYQ	DIQNTIKKAK	QDVIEVIEKA
730	740	750	760	770	780	790	800
HNNELEPTPG	NTLRQTFENQ	VNRILNDARD	KTGSSAQKSL	SEYNNFKSMV	VSGAKGSKIN	ISQVIAVVGQ	QNVEGKRIPF
810	820	830	840	850	860	870	880
GFKHRTLPHF	IKDDYGPESR	GFVENSYLAG	LTPTEFFFHA	MGGREGLIDT	AVKTAETGYI	QRRLIKSMES	VMVKYDATVR
890	900	910	920	930	940	950	960
NSINQVVQLR	YGEDGLAGES	VEFQNLATLK	PSNKAFEKKF	RFDYTNERAL	RRTLQEDLVK	DVLSNAHIQN	ELEREFERMR
970	980	990	1000	1010	1020	1030	1040
EDREVLRVIF	PTGDSKVVLP	CNLLRMIWNA	QKIFHINPRL	PSDLHPIKVV	EGVKELSKKL	VIVNGDDPLS	RQAQENATLL
1050	1060	1070	1080	1090	1100	1110	1120
FNIHLRSTLC	SRRMAEEFRL	SGEAFDWLLG	EIESKFNQAI	AHPGEMVGAL	AAQSLGEPAT	QMTLNTFHYA	GVSAKNVTLG
1130	1140	1150	1160	1170	1180	1190	1200
VPRLKELINI	SKKPKTPSLT	VFLLGQSARD	AERAKDILCR	LEHTTLRKVT	ANTAIYYDPN	PQSTVVAEDQ	EWVNVYYEMP
1210	1220	1230	1240	1250	1260	1270	1280
DFDVARISPW	LLRVELDRKH	MTDRKLTMEQ	IAEKINAGFG	DDLNCIFNDD	NAEKLVLRIR	IMNSDENKMQ	EEEEVVDKMD
1290	1300	1310	1320	1330	1340	1350	1360
DDVFLRCIES	NMLTDMTLQG	IEQISKVYMH	LPQTDNKKKI	IITEDGEFKA	LQEWILETDG	VSLMRVLSEK	DVDPVRTTSN
1370	1380	1390	1400	1410	1420	1430	1440
DIVEIFTVLG	IEAVRKALER	ELYHVISFDG	SYVNYRHLAL	LCDTMTCRGH	LMAITRHGVN	RQDTGPLMKC	SFEETVDVLM
1450	1460	1470	1480	1490	1500	1510	1520
EAAAHGESDP	MKGVSENIML	GQLAPAGTGC	FDLLLDAEKC	KYGMEIPTNI	PGLGAAGPTG	MFFGSAPSPM	GGISPAMTPW
1530	1540	1550	1560	1570	1580	1590	1600
NQGATPAYGA	WSPSVGSGMT	PGAAGFSPSA	ASDASGFSPG	YSPAWSPTPG	SPGSPGPSSP	YIPSPGGAMS	PSYSPTSPAY
1610	1620	1630	1640	1650	1660	1670	1680
EPRSPGGYTP	QSPSYSPTSP	SYSPTSPSYS	PTSPNYSPTS	PSYSPTSPSY	SPTSPSYSPT	SPSYSPTSPS	YSPTSPSYSP
1690	1700	1710	1720	1730	1740	1750	1760
TSPSYSPTSP	SYSPTSPSYS	PTSPSYSPTS	PSYSPTSPSY	SPTSPSYSPT	SPSYSPTSPS	YSPTSPNYSP	TSPNYTPTSP
1770	1780	1790	1800	1810	1820	1830	1840
SYSPTSPSYS	PTSPNYTPTS	PNYSPTSPSY	SPTSPSYSPT	SPSYSPSSPR	YTPQSPTYTP	SSPSYSPSSP	SYSPTSPKYT
1850	1860	1870	1880	1890	1900	1910	1920
PTSPSYSPSS	PEYTPTSPKY	SPTSPKYSPT	SPKYSPTSPT	YSPTTPKYSP	TSPTYSPTSP	VYTPTSPKYS	PTSPTYSPTS
1930	1940	1950	1960	1970	1980		
PKYSPTSPTY	SPTSPKGSTY	SPTSPGYSPT	SPTYSLTSPA	ISPDDSDEEN			

Cmpd.	No. of Cmpds.	m/z meas.	Δ m/z [ppm]	z	Rt [min]	Score	P	Range	Sequence	Modification
2144	1	953.3984	-95.27	2	57.0	16.6	2	2-20	M.HGGGPPSGDSACPLRTIKR.V	
96	1	822.4073	-70.22	2	30.1	11.5	2	20-33	K.RVQFGVLSPDELKR.M	

Detailed Protein Report

Cmpd.	No. of Cmpds.	m/z meas.	Δ m/z [ppm]	z	Rt [min]	Score	P	Range	Sequence	Modification
652	1	918.1693	7.00	3	37.5	17.7	2	358-382	K.RVDFSARTVITPDPNLSIDQVGVPR.S	
2282	1	614.2542	-87.39	3	59.0	11.8	2	461-475	R.QPTLHKMSMMGHRVR.I	Oxidation: 9, 10
349	1	626.3032	-27.82	3	34.0	12.3	0	1330-1345	K.ALQEWILETDGVSLMR.V	Oxidation: 15

Detailed Protein Report

Protein 123: PREDICTED: neurobeachin-like protein 1 isoform X7 [Homo sapiens]

**Accession:**	gi|578804775	**Score:**	69.9
**Database:**	refseq_human(refseq_human_20140103.fasta)	**MW [kDa]:**	307.1
**Database Date:**	2015-11-30	**pI:**	6.0
**Modification(s):**	Carbamidomethyl, Oxidation	**Sequence Coverage [%]:**	2.1
		**No. of unique Peptides:**	4

Quantitation

Q:W	**Median:**	3.00	**CV:**	0.00 %	**No. of Peptides:**	1	

Detailed Protein Report

10	20	30	40	50	60	70	80
MASRERLFEL	WMLYCTKKDP	DYLKLWLDTF	VSSYEQFLDV	DFEKLPTRVD	DMPPGISLLP	DNILQVLRIQ	LLQCVQKMAD
90	100	110	120	130	140	150	160
GLEEQQQALS	ILLVKFFIIL	CRNLSNVEEI	GTCSYINYVI	TMTTLYIQQL	KSKKKEKEMA	DQTCIEEFVI	HALAFCESLY
170	180	190	200	210	220	230	240
DPYRNWRHRI	SGRILSTVEK	SRQKYKPASL	TVEFVPFFYQ	CFQESEHLKE	SLKCCLLHLF	GAIVAGGQRN	ALQAISPATM
250	260	270	280	290	300	310	320
EVLMRVLADC	DSWEDGDPEE	VGRKAELTLK	CLTEVVHILL	SSNSDQRQVE	TSTILENYFK	LLNSDHSALP	NQRRSRQWEN
330	340	350	360	370	380	390	400
RFIALQIKML	NTITAMLDCT	DRPVLQAIFL	NSNCFEHLIR	LLQNCKLFLN	ANNKVADKNE	KDLANKLLTE	MNEDQVFQGQ
410	420	430	440	450	460	470	480
LDCLAISTIQ	ALTAVMNKSP	AAKEVFKERI	GYTHMLEVLK	SLGQPPLELL	KELMNMAVEG	DHTSVGILGI	SNVQPLLLLI
490	500	510	520	530	540	550	560
QWLPELQSHD	LQIFISDWLK	RICCINRQSR	TTCVNANMGI	RIIETLDLHS	SLHQTCAENL	IAIHGSLGSQ	SVSSEEIRRL
570	580	590	600	610	620	630	640
LRLLRVDESE	SVHPYVTPVT	RAILTMARKL	SLESALQYFN	LSHSMAGISV	PPIQKWPGSA	FSFSAWFCLD	QDQLTLGIAN
650	660	670	680	690	700	710	720
KGGKRKQLYS	FFTGSGMGFE	AFITHSGMLV	VAVCTKREYA	TVMLPDHSFC	DSLWHNITVV	HMPGKRPFGQ	SFVYIYDNGQ
730	740	750	760	770	780	790	800
QKVSAPLRFP	AMNEPFTSCC	IGSAGQRTTT	PPPSQIPDPP	FSSPITPHRT	SFGGILSSAS	WGGTIEKSKL	ITKLISAGTQ
810	820	830	840	850	860	870	880
DSEWGCPTSL	EGQLGSVIIF	YEPLQPPQVK	ALYLAGPNCL	SPWKCQESDM	ADLPGNILLY	YTAKACKNSI	CLDLSTNCLH
890	900	910	920	930	940	950	960
GRLTGNKVVN	WDIKDIINCI	GGLNVLFPLL	EQISHFSEGQ	IPEEKNESTV	PESVTPVEGD	WLVWTSTKAS	ESRLERNLVA
970	980	990	1000	1010	1020	1030	1040
TFILIVKHFI	QRHPINQGNL	IHSHGVATLG	ALLQKVPSTL	MDVNVLMAVQ	LLIEQVSLEK	NMQLLQQMYQ	YLLFDFRIWN
1050	1060	1070	1080	1090	1100	1110	1120
RGDFPFRIGH	IQYLSTIIKD	SRRVFRKKYG	VQFLLDTLRI	YYGNGCKYNE	LSLDDIRTIR	TSLYGLIKYF	LCKGGSHEEI
1130	1140	1150	1160	1170	1180	1190	1200
QSIMGYIAAT	NEEEQLFGIL	DVLFSLLRTS	PTRGQLFLLL	FEPGNADILY	ALLLNQKYSD	RLREIIFKIM	EQMLKCTNVY
1210	1220	1230	1240	1250	1260	1270	1280
ERSKQHIRLR	EVGYSGLGLL	LNEALVNTSL	IKNLTHQIIN	TDPVINFKDL	LSVVYISHRA	HINVRVAICR	KVLQILQFQP
1290	1300	1310	1320	1330	1340	1350	1360
DAAHQISQQV	GWQDTLVRLF	LKAKFENGNT	LHKHSRAVLM	KDNDKNMSTE	DTKKNSDEKT	DEEKITSFAS	ANVSSDQWSL
1370	1380	1390	1400	1410	1420	1430	1440
EDRHSLDSNT	PLFPEDSSVG	ELSFKSENQE	EFWHSNPSHL	SLDLSGIDSC	EMSDSGSQVP	DSLPSTPSPV	ESTKSFSVHS
1450	1460	1470	1480	1490	1500	1510	1520
DRESSITNDM	GFSDDFSLLE	SQERCEEELL	QLLTHILNYV	MCKGLEKSDD	DTWIERGQVF	SALSKPGISS	ELLRPSDEIK
1530	1540	1550	1560	1570	1580	1590	1600
LTLLQKMLEW	AISENREAKT	NPVTAENAFR	LVLIIQDFLQ	SEGLVNSNMW	TEKLLEDMML	LFDCLSVCYS	ESPVWVKLSQ
1610	1620	1630	1640	1650	1660	1670	1680
IQIQLLLGFI	GRGNLQVCAM	ASAKLNTLLQ	TKVIENQDEA	CYILGKLEHV	LSQSIKEQTE	IYSFLIPLVR	TLVSKIYELL
1690	1700	1710	1720	1730	1740	1750	1760
FMNLHLPSLP	FTNGSSSFFE	DFQEYCNSNE	WQVYIEKYIV	PYMKQYEAHT	FYDGHENMAL	YWKDCYEALM	VNMHKRDREG
1770	1780	1790	1800	1810	1820	1830	1840
GESKLKFQEL	FVEPFNRKAR	QENLRYNNML	KQLSSQQLAT	LRRWKAIQLY	LTCERGPWAK	RKQNPIHWKL	ANVENYSRMR
1850	1860	1870	1880	1890	1900	1910	1920
LKLVPNYNFK	THEEASALRD	NLGIQHSQPS	SDTLLLEVVK	QVKVSDMVED	KLDLPEEDIT	ARVNVDEKEE	QDQKEKLVLM
1930	1940	1950	1960	1970	1980	1990	2000
EDCELITIID	VIPGRLEITT	QHIYFYDGSI	EKEDGVGFDF	KWPHSQIREI	HLRRYNLRRS	ALEIFHVDQS	NYFLNFKKEV
2010	2020	2030	2040	2050	2060	2070	2080
RNKIYSRLLS	LHSPNSYYGS	RSPQELFKAS	GLTQKWVNRE	ISNFDYLIQI	NTMAGRTYND	LAQYPVFPWI	LQDYTSEELD
2090	2100	2110	2120	2130	2140	2150	2160
LNNPAVFRDL	SKPIGVVNEK	NAKAMREKYE	NFEDPMGTID	KFHYGTHYSN	SAGVMHYLIR	VEPFTTLHIQ	LQSGRFDCAD
2170	2180	2190	2200	2210	2220	2230	2240

Detailed Protein Report

Cmpd.	No. of Cmpds.	m/z meas.	Δ m/z [ppm]	z	Rt [min]	Score	P	Range	Sequence	Modification	Ratios	
2277	1	816.2926	-155.86	2	58.7	11.5	1	7-18	R.LFELWMLYCTKK.D	Carbamidomethyl: 9		
1799	1	1045.0496	94.89	2	50.8	15.5	0	729-747	R.FPAMNEPFTSCCIGSAGQR.T	Carbamidomethyl: 12; Oxidation: 4		
2060	1	899.3817	-81.00	2	54.1	19.6	0	770-787	R.TSFGGILSSASWGGTIEK.S		Q:W	3.00
2089	1	925.3261	-72.47	1	56.3	10.1	0	1326-1333	K.NMSTEDTK.K			

Detailed Protein Report

Protein 124: **PREDICTED: chromodomain-helicase-DNA-binding protein 7 isoform X2 [Homo sapiens]**

**Accession:**	gi|530388491	**Score:**	69.9
**Database:**	refseq_human(refseq_human_20140103.fasta)	**MW [kDa]:**	335.6
**Database Date:**	2015-11-30	**pI:**	5.9
**Modification(s):**	Carbamidomethyl, Oxidation	**Sequence Coverage [%]:**	2.5
		**No. of unique Peptides:**	4

Detailed Protein Report

10	20	30	40	50	60	70	80
MADPGMMSLF	GEDGNIFSEG	LEGLGECGYP	ENPVNPMGQQ	MPIDQGFASL	QPSLHHPSTN	QNQTKLTHFD	HYNQYEQQKM
90	100	110	120	130	140	150	160
HLMDQPNRMM	SNTPGNGLAS	PHSQYHTPPV	PQVPHGGSGG	GQMGVYPGMQ	NERHGQSFVD	SSSMWGPRAV	QVPDQIRAPY
170	180	190	200	210	220	230	240
QQQQPQPQPP	QPAPSGPPAQ	GHPQHMQQMG	SYMARGDFSM	QQHGQPQQRM	SQFSQGQEGL	NQGNPFIATS	GPGHLSHVPQ
250	260	270	280	290	300	310	320
QSPSMAPSLR	HSVQQFHHHP	STALHGESVA	HSPRFSPNPP	QQGAVRPQTL	NFSSRSQTVP	SPTINNSGQY	SRYPYSNLNQ
330	340	350	360	370	380	390	400
GLVNNTGMNQ	NLGLTNNTPM	NQSVPRYPNA	VGFPSNSGQG	LMHQQPIHPS	GSLNQMNTQT	MHPSQPQGTY	ASPPPMSPMK
410	420	430	440	450	460	470	480
AMSNPAGTPP	PQVRPGSAGI	PMEVGSYPNM	PHPQPSHQPP	GAMGIGQRNM	GPRNMQQSRP	FIGMSSAPRE	LTGHMRPNGC
490	500	510	520	530	540	550	560
PGVGLGDPQA	IQERLIPGQQ	HPGQQPSFQQ	LPTCPPLQPH	PGLHHQSSPP	HPHHQPWAQL	HPSPQNTPQK	VPVHQHSPSE
570	580	590	600	610	620	630	640
PFLEKPVPDM	TQVSGPNAQL	VKSDDYLPSI	EQQPQQKKKK	KKNNHIVAED	PSKGFGKDDF	PGGVDNQELN	RNSLDGSQEE
650	660	670	680	690	700	710	720
KKKKKRSKAK	KDPKEPKEPK	EKKEPKEPKT	PKAPKIPKEP	KEKKAKTATP	KPKSSKKSSN	KKPDSEASAL	KKKVNKGKTE
730	740	750	760	770	780	790	800
GSENSDLDKT	PPPSPPPEED	EDPGVQKRRS	SRQVKRKRYT	EDLEFKISDE	EADDADAAGR	DSPSNTSQSE	QQESVDAEGP
810	820	830	840	850	860	870	880
VVEKIMSSRS	VKKQKESGEE	VEIEEFYVKY	KNFSYLHCQW	ASIEDLEKDK	RIQQKIKRFK	AKQGQNKFLS	EIEDELFNPD
890	900	910	920	930	940	950	960
YVEVDRIMDF	ARSTDDRGEP	VTHYLVKWCS	LPYEDSTWER	RQDIDQAKIE	EFEKLMSREP	ETERVERPPA	DDWKKSESSR
970	980	990	1000	1010	1020	1030	1040
EYKNNNKLRE	YQLEGVNWLL	FNWYNMRNCI	LADEMGLGKT	IQSITFLYEI	YLKGIHGPFL	VIAPLSTIPN	WEREFRTWTE
1050	1060	1070	1080	1090	1100	1110	1120
LNVVVYHGSQ	ASRRTIQLYE	MYFKDPQGRV	IKGSYKFHAI	ITTFEMILTD	CPELRNIPWR	CVVIDEAHRL	KNRNCKLLEG
1130	1140	1150	1160	1170	1180	1190	1200
LKMMDLEHKV	LLTGTPLQNT	VEELFSLLHF	LEPSRFPSET	TFMQEFGDLK	TEEQVQKLQA	ILKPMMLRRL	KEDVEKNLAP
1210	1220	1230	1240	1250	1260	1270	1280
KEETIIEVEL	TNIQKKYYRA	ILEKNFTFLS	KGGGQANVPN	LLNTMMELRK	CCNHPYLING	AEEKILEEFK	ETHNAESPDF
1290	1300	1310	1320	1330	1340	1350	1360
QLQAMIQAAG	KLVLIDKLLP	KLKAGGHRVL	IFSQMVRCLD	ILEDYLIQRR	YPYERIDGRV	RGNLRQAAID	RFSKPDSDRF
1370	1380	1390	1400	1410	1420	1430	1440
VFLLCTRAGG	LGINLTAADT	CIIFDSDWNP	QNDLQAQARC	HRIGQSKSVK	IYRLITRNSY	EREMFDKASL	KLGLDKAVLQ
1450	1460	1470	1480	1490	1500	1510	1520
SMSGRENATN	GVQQLSKKEI	EDLLRKGAYG	ALMDEEDEGS	KFCEEDIDQI	LLRRTHTITI	ESEGKGSTFA	KASFVASGNR
1530	1540	1550	1560	1570	1580	1590	1600
TDISLDDPNF	WQKWAKKAEL	DIDALNGRNN	LVIDTPRVRK	QTRLYSAVKE	DELMEFSDLE	SDSEEKPCAK	PRRPQDKSQG
1610	1620	1630	1640	1650	1660	1670	1680
YARSECFRVE	KNLLVYGWGR	WTDILSHGRY	KRQLTEQDVE	TICRTILVYC	LNHYKGDENI	KSFIWDLITP	TADGQTRALV
1690	1700	1710	1720	1730	1740	1750	1760
NHSGLSAPVP	RGRKGKKVKA	QSTQPVVQDA	DWLASCNPDA	LFQEDSYKKH	LKHHCNKVLL	RVRMLYYLRQ	EVIGDQADKI
1770	1780	1790	1800	1810	1820	1830	1840
LEGADSSEAD	VWIPEPFHAE	VPADWWDKEA	DKSLLIGVFK	HGYEKYNSMR	ADPALCFLER	VGMPDAKAIA	AEQRGTDMLA
1850	1860	1870	1880	1890	1900	1910	1920
DGGDGGEFDR	EDEDPEYKPT	RTPFKDEIDE	FANSPSEDKE	ESMEIHATGK	HSESNAELGQ	LYWPNTSTLT	TRLRRLITAY
1930	1940	1950	1960	1970	1980	1990	2000
QRSYKRQQMR	QEALMKTDRR	RRRPREEVRA	LEAEREAIIS	EKRQKWTRRE	EADFYRVVST	FGVIFDPVKQ	QFDWNQFRAF
2010	2020	2030	2040	2050	2060	2070	2080
ARLDKKSDES	LEKYFSCFVA	MCRRVCRMPV	KPDDEPPDLS	SIIEPITEER	ASRTLYRIEL	LRKIREQVLH	HPQLGERLKL
2090	2100	2110	2120	2130	2140	2150	2160
CQPSLDLPEW	WECGRHDRDL	LVGAAKHGVS	RTDYHILNDP	ELSFLDAHKN	FAQNRGAGNT	SSLNPLAVGF	VQTPPVISSA
2170	2180	2190	2200	2210	2220	2230	2240

Detailed Protein Report

Cmpd.	No. of Cmpds.	m/z meas.	Δ m/z [ppm]	z	Rt [min]	Score	P	Range	Sequence	Modification
1309	1	863.9074	-143.00	2	46.0	10.4	2	1178-1191	K.LQAILKPMMLRRLK.E	Oxidation: 8
2753	2	937.4586	-76.18	2	63.4	14.1	2	1428-1445	K.ASLKLGLDKAVLQSMSGR.E	
1713	1	806.6994	-162.85	2	51.4	15.5	0	1835-1850	R.GTDMLADGGDGGEFDR.E	
2811	1	995.0043	51.30	2	63.6	17.4	0	2080-2095	K.LCQPSLDLPEWWECGR.H	Carbamidomethyl: 14

Detailed Protein Report

Protein 125: ubiquitin carboxyl-terminal hydrolase 35 [Homo sapiens]

**Accession:**	gi|148746183	**Score:**	69.5
**Database:**	refseq_human(refseq_human_20140103.fasta)	**MW [kDa]:**	113.3
**Database Date:**	2015-11-30	**pI:**	5.3
**Modification(s):**	Carbamidomethyl	**Sequence Coverage [%]:**	8.3
		**No. of unique Peptides:**	4

Quantitation

Q:W	**Median:** 1.30	**CV:** 0.00 %	**No. of Peptides:**	1
M:Q	**Median:** 0.39	**CV:** 134.28 %	**No. of Peptides:**	2

```
        10         20         30         40         50         60         70         80
MDKILEAVVT SSYPVSVKQG LVRRVLEAAR QPLEREQCLA LLALGARLYV GGAEELPRRV GCQLLHVAGR HHPDVFAEFF
        90        100        110        120        130        140        150        160
SARRVLRLLQ GGAGPPGPRA LACVQLGLQL LPEGPAADEV FALLRREVLR TVCERPGPAA CAQVARLLAR HPRCVPDGPH
       170        180        190        200        210        220        230        240
RLLFCQQLVR CLGRFCRPAE GEEGAVEFLE QAQQVSGLLA QLWRAQPAAI LPCLKELFAV ISCAEEEPPS SALASVVQHL
       250        260        270        280        290        300        310        320
PLELMDGVVR NLSNDDSVTD SQMLTAISRM IDWVSWPLGK NIDKWIIALL KGLAAVKKFS ILIEVSLTKI EKVFSKLLYP
       330        340        350        360        370        380        390        400
IVRGAALSVL KYMLLTFQHS HEAFHLLLPH IPPMVASLVK EDSNSGTSCL EQLAELVHCM VFRFPGFPDL YEPVMEAIKD
       410        420        430        440        450        460        470        480
LHVPNEDRIK QLLGQDAWTS QKSELAGFYP RLMAKSDTGK IGLINLGNTC YVNSILQALF MASDFRHCVL RLTENNSQPL
       490        500        510        520        530        540        550        560
MTKLQWLFGF LEHSQRPAIS PENFLSASWT PWFSPGTQQD CSEYLKYLLD RLHEEEKTGT RICQKLKQSS SPSPPEEPPA
       570        580        590        600        610        620        630        640
PSSTSVEKMF GGKIVTRICC LCCLNVSSRE EAFTDLSLAF PPPERCRRRR LGSVMRPTED ITARELPPPT SAQGPGRVGP
       650        660        670        680        690        700        710        720
RRQRKHCITE DTPPTSLYIE GLDSKEAGGQ SSQEERIERE EEGKEERTEK EEVGEEEEST RGEGEREKEE EVEEEEEKVE
       730        740        750        760        770        780        790        800
KETEKEAEQE KEEDSLGAGT HPDAAIPSGE RTCGSEGSRS VLDLVNYFLS PEKLTAENRY YCESCASLQD AEKVVELSQG
       810        820        830        840        850        860        870        880
PCYLILTLLR FSFDLRTMRR RKILDDVSIP LLLRLPLAGG RGQAYDLCSV VVHSGVSSES GHYYCYAREG AARPAASLGT
       890        900        910        920        930        940        950        960
ADRPEPENQW YLFNDTRVSF SSFESVSNVT SFFPKDTAYV LFYRQRPREG PEAELGSSRV RTEPTLHKDL MEAISKDNIL
       970        980        990       1000       1010       1020
YLQEQEKEAR SRAAYISALP TSPHWGRGFD EDKDEDEGSP GGCNPAGGNG GDFHRLVF
```

Cmpd.	No. of Cmpds.	m/z meas.	Δ m/z [ppm]	z	Rt [min]	Score	P	Range	Sequence	Modification	Ratios
1261	1	1070.0005	-62.40	2	45.9	12.2	1	131-150	R.TVCERPGPAACAQVARLLAR.H	Carbamidomethyl: 3	M:Q 1.09<br>Q:W 1.30
574	1	469.1409	-166.57	2	36.0	10.2	0	154-161	R.CVPDGPHR.L	Carbamidomethyl: 1	M:Q 0.14
2059	1	698.3372	-172.92	2	54.3	14.6	1	285-297	K.WIIALLKGLAAVK.K		
806	1	546.9707	-505.05	1	39.6	11.3	0	702-706	R.GEGER.E		

Detailed Protein Report

Protein 126: centrosomal protein of 290 kDa [Homo sapiens]

**Accession:**	gi|109255234	**Score:**	69.1
**Database:**	refseq_human(refseq_human_20140103.fasta)	**MW [kDa]:**	290.2
**Database Date:**	2015-11-30	**pI:**	5.7
**Modification(s):**	Oxidation	**Sequence Coverage [%]:**	2.5
		**No. of unique Peptides:**	5

Quantitation

Q:W	**Median:** 0.39	**CV:** 0.00 %	**No. of Peptides:** 1

Detailed Protein Report

10	20	30	40	50	60	70	80
MPPNINWKEI	MKVDPDDLPR	QEELADNLLI	SLSKVEVNEL	KSEKQENVIH	LFRITQSLMK	MKAQEVELAL	EEVEKAGEEQ
90	100	110	120	130	140	150	160
AKFENQLKTK	VMKLENELEM	AQQSAGGRDT	RFLRNEICQL	EKQLEQKDRE	LEDMEKELEK	EKKVNEQLAL	RNEEAENENS
170	180	190	200	210	220	230	240
KLRRENKRLK	KKNEQLCQDI	IDYQKQIDSQ	KETLLSRRGE	DSDYRSQLSK	KNYELIQYLD	EIQTLTEANE	KIEVQNQEMR
250	260	270	280	290	300	310	320
KNLEESVQEM	EKMTDEYNRM	KAIVHQTDNV	IDQLKKENDH	YQLQVQELTD	LLKSKNEEDD	PIMVAVNAKV	EEWKLILSSK
330	340	350	360	370	380	390	400
DDEIIEYQQM	LHNLREKLKN	AQLDADKSNV	MALQQGIQER	DSQIKMLTEQ	VEQYTKEMEK	NTCIIEDLKN	ELQRNKGAST
410	420	430	440	450	460	470	480
LSQQTHMKIQ	STLDILKEKT	KEAERTAELA	EADAREKDKE	LVEALKRLKD	YESGVYGLED	AVVEIKNCKN	QIKIRDREIE
490	500	510	520	530	540	550	560
ILTKEINKLE	LKISDFLDEN	EALRERVGLE	PKTMIDLTEF	RNSKHLKQQQ	YRAENQILLK	EIESLEEERL	DLKKKIRQMA
570	580	590	600	610	620	630	640
QERGKRSATS	GLTTEDLNLT	ENISQGDRIS	ERKLDLLSLK	NMSEAQSKNE	FLSRELIEKE	RDLERSRTVI	AKFQNKLKEL
650	660	670	680	690	700	710	720
VEENKQLEEG	MKEILQAIKE	MQKDPDVKGG	ETSLIIPSLE	RLVNAIESKN	AEGIFDASLH	LKAQVDQLTG	RNEELRQELR
730	740	750	760	770	780	790	800
ESRKEAINYS	QQLAKANLKI	DHLEKETSLL	RQSEGSNVVF	KGIDLPDGIA	PSSASIINSQ	NEYLIHLLQE	LENKEKKLKN
810	820	830	840	850	860	870	880
LEDSLEDYNR	KFAVIRHQQS	LLYKEYLSEK	ETWKTESKTI	KEEKRKLEDQ	VQQDAIKVKE	YNNLLNALQM	DSDEMKKILA
890	900	910	920	930	940	950	960
ENSRKITVLQ	VNEKSLIRQY	TTLVELERQL	RKENEKQKNE	LLSMEAEVCE	KIGCLQRFKE	MAIFKIAALQ	KVVDNSVSLS
970	980	990	1000	1010	1020	1030	1040
ELELANKQYN	ELTAKYRDIL	QKDNMLVQRT	SNLEHLECEN	ISLKEQVESI	NKELEITKEK	LHTIEQAWEQ	ETKLGNESSM
1050	1060	1070	1080	1090	1100	1110	1120
DKAKKSITNS	DIVSISKKIT	MLEMKELNER	QRAEHCQKMY	EHLRTSLKQM	EERNFELETK	FAELTKINLD	AQKVEQMLRD
1130	1140	1150	1160	1170	1180	1190	1200
ELADSVSKAV	SDADRQRILE	LEKNEMELKV	EVSKLREISD	IARRQVEILN	AQQQSRDKEV	ESLRMQLLDY	QAQSDEKSLI
1210	1220	1230	1240	1250	1260	1270	1280
AKLHQHNVSL	QLSEATALGK	LESITSKLQK	MEAYNLRLEQ	KLDEKEQALY	YARLEGRNRA	KHLRQTIQSL	RRQFSGALPL
1290	1300	1310	1320	1330	1340	1350	1360
AQQEKFSKTM	IQLQNDKLKI	MQEMKNSQQE	HRNMENKTLE	MELKLKGLEE	LISTLKDTKG	AQKVINWHMK	IEELRLQELK
1370	1380	1390	1400	1410	1420	1430	1440
LNRELVKDKE	EIKYLNNIIS	EYERTISSLE	EEIVQQNKFH	EERQMAWDQR	EVDLERQLDI	FDRQQNEILN	AAQKFEEATG
1450	1460	1470	1480	1490	1500	1510	1520
SIPDPSLPLP	NQLEIALRKI	KENIRIILET	RATCKSLEEK	LKEKESALRL	AEQNILSRDK	VINELRLRLP	ATAEREKLIA
1530	1540	1550	1560	1570	1580	1590	1600
ELGRKEMEPK	SHHTLKIAHQ	TIANMQARLN	QKEEVLKKYQ	RLLEKAREEQ	REIVKKHEED	LHILHHRLEL	QADSSLNKFK
1610	1620	1630	1640	1650	1660	1670	1680
QTAWDLMKQS	PTPVPTNKHF	IRLAEMEQTV	AEQDDSLSSL	LVKLKKVSQD	LERQREITEL	KVKEFENIKL	QLQENHEDEV
1690	1700	1710	1720	1730	1740	1750	1760
KKVKAEVEDL	KYLLDQSQKE	SQCLKSELQA	QKEANSRAPT	TTMRNLVERL	KSQLALKEKQ	QKALSRALLE	LRAEMTAAAE
1770	1780	1790	1800	1810	1820	1830	1840
ERIISATSQK	EAHLNVQQIV	DRHTRELKTQ	VEDLNENLLK	LKEALKTSKN	RENSLTDNLN	DLNNELQKKQ	KAYNKILREK
1850	1860	1870	1880	1890	1900	1910	1920
EEIDQENDEL	KRQIKRLTSG	LQGKPLTDNK	QSLIEELQRK	VKKLENQLEG	KVEEVDLKPM	KEKNAKEELI	RWEEGKKWQA
1930	1940	1950	1960	1970	1980	1990	2000
KIEGIRNKLK	EKEGEVFTLT	KQLNTLKDLF	AKADKEKLTL	QRKLKTTGMT	VDQVLGIRAL	ESEKELEELK	KRNLDLENDI
2010	2020	2030	2040	2050	2060	2070	2080
LYMRAHQALP	RDSVVEDLHL	QNRYLQEKLH	ALEKQFSKDT	YSKPSISGIE	SDDHCQREQE	LQKENLKLSS	ENIELKFQLE
2090	2100	2110	2120	2130	2140	2150	2160
QANKDLPRLK	NQVRDLKEMC	EFLKKEKAEV	QRKLGHVRGS	GRSGKTIPEL	EKTIGLMKKV	VEKVQRENEQ	LKKASGILTS
2170	2180	2190	2200	2210	2220	2230	2240

Detailed Protein Report

Cmpd.	No. of Cmpds.	m/z meas.	Δ m/z [ppm]	z	Rt [min]	Score	P	Range	Sequence	Modification	Ratios
2146	2	937.4291	-49.48	2	55.4	15.4	2	115-129	R.NEICQLEKQLEQKDR.E		
1675	1	745.8585	-74.75	2	50.7	11.8	1	1101-1113	K.FAELTKINLDAQK.V		
1969	1	703.7594	-155.73	2	54.7	10.7	0	1966-1978	K.TTGMTVDQVLGIR.A	Oxidation: 4	
1615	1	494.1461	-276.33	2	50.1	10.7	2	2106-2113	K.EKAEVQRK.L		Q:W 0.39
1648	1	858.4049	-102.70	1	49.1	10.3	0	2438-2444	K.NILLEEK.V		

Detailed Protein Report

Protein 127: PREDICTED: striated muscle preferentially expressed protein kinase isoform X4 [Homo sapiens]

**Accession:**	gi|530369685	**Score:**	69.0
**Database:**	refseq_human(refseq_human_20140103.fasta)	**MW [kDa]:**	339.0
**Database Date:**	2015-11-30	**pI:**	9.7
**Modification(s):**	Carbamidomethyl	**Sequence Coverage [%]:**	1.7
		**No. of unique Peptides:**	2

Quantitation

Q:W	**Median:**	0.34	**CV:**	0.00 %	**No. of Peptides:**	1
M:Q	**Median:**	1.08	**CV:**	0.00 %	**No. of Peptides:**	1

Detailed Protein Report

10	20	30	40	50	60	70	80
MKKLWVKKRF	QKTGHSRRAF	GRLTHVFRSC	RKQSYDSETA	EDDISDVQGT	QRLELRDDGA	FSTPTGGSDT	LVGTSLDTPP
90	100	110	120	130	140	150	160
TSVTGTSEEQ	VSWWGSGQTV	LEQEAGSGGG	TRRLPGSPSS	VPQSGLRREE	PDLQPQLASE	APRRPAQPPP	SKSALLPPPS
170	180	190	200	210	220	230	240
PRVGKRSPPG	PPAQPAATPT	SPHRRTQEPV	LPEDTTTEEK	RGKKSKSSGP	SLAGTAESRP	QTPLSEASGR	LSALGRSPRL
250	260	270	280	290	300	310	320
VRAGSRILDK	LQFFEERRRS	LERSDSPPAP	LRPWVPLRKA	RSLEQPKSER	GAPWGTPGAS	QEELRAPGSV	AERRRLFQQK
330	340	350	360	370	380	390	400
AASLDERTRQ	RSPASDLELR	FAQELGRIRR	STSREELVRS	HESLRATLQR	APSPREPGEP	PLFSRPSTPK	TSRAVSPAAA
410	420	430	440	450	460	470	480
QPPSPSSAEK	PGDEPGRPRS	RGPAGRTEPG	EGPQQEVRRR	DQFPLTRSRA	IQECRSPVPP	PAADPPEART	KAPPGRKREP
490	500	510	520	530	540	550	560
PAQAVRFLPW	ATPGLEGAAV	PQTLEKNRAG	PEAEKRLRRG	PEEDGPWGPW	DRRGARSQGK	GRRARPTSPE	LESSDDSYVS
570	580	590	600	610	620	630	640
AGEEPLEAPV	FEIPLQNVVV	APGADVLLKC	IITANPPPQV	SWHKDGSALR	SEGRLLLRAE	GERHTLLLRE	ARAADAGSYM
650	660	670	680	690	700	710	720
ATATNELGQA	TCAASLTVRP	GGSTSPFSSP	ITSDEEYLSP	PEEFPEPGET	WPRTPTMKPS	PSQNRRSSDT	GSKAPPTFKV
730	740	750	760	770	780	790	800
SLMDQSVREG	QDVIMSIRVQ	GEPKPVVSWL	RNRQPVRPDQ	RRFAEEAEGG	LCRLRILAAE	RGDAGFYTCK	AVNEYGARQC
810	820	830	840	850	860	870	880
EARLEVRAHP	ESRSLAVLAP	LQDVDVGAGE	MALFECLVAG	PTDVEVDWLC	RGRLLQPALL	KCKMHFDGRK	CKLLLTSVHE
890	900	910	920	930	940	950	960
DDSGVYTCKL	STAKDELTCS	ARLTVRPSLA	PLFTRLLEDV	EVLEGRAARF	DCKISGTPPP	VVTWTHFGCP	MEESENLRLR
970	980	990	1000	1010	1020	1030	1040
QDGGLHSLHI	AHVGSEDEGL	YAVSAVNTHG	QAHCSAQLYV	EEPRTAASGP	SSKLEKMPSI	PEEPEQGELE	RLSIPDFLRP
1050	1060	1070	1080	1090	1100	1110	1120
LQDLEVGLAK	EAMLECQVTG	LPYPTISWFH	NGHRIQSSDD	RRMTQYRDVH	RLVFPAVGPQ	HAGVYKSVIA	NKLGKAACYA
1130	1140	1150	1160	1170	1180	1190	1200
HLYVTDVVPG	PPDGAPQVVA	VTGRMVTLTW	NPPRSLDMAI	DPDSLTYTVQ	HQVLGSDQWT	ALVTGLREPG	WAATGLRKGV
1210	1220	1230	1240	1250	1260	1270	1280
QHIFRVLSTT	VKSSSKPSPP	SEPVQLLEHG	PTLEEAPAML	DKPDIVYVVE	GQPASVTVTF	NHVEAQVVWR	SCRGALLEAR
1290	1300	1310	1320	1330	1340	1350	1360
AGVYELSQPD	DDQYCLRICR	VSRRDMGALT	CTARNRHGTQ	TCSVTLELAE	APRFESIMED	VEVGAGETAR	FAVVVEGKPL
1370	1380	1390	1400	1410	1420	1430	1440
PDIMWYKDEV	LLTESSHVSF	VYEENECSLV	VLSTGAQDGG	VYTCTAQNLA	GEVSCKAELA	VHSAQTAMEV	EGVGEDEDHR
1450	1460	1470	1480	1490	1500	1510	1520
GRRLSDFYDI	HQEIGRGAFS	YLRRIVERSS	GLEFAAKFIP	SQAKPKASAR	REARLLARLQ	HDCVLYFHEA	FERRRGLVIV
1530	1540	1550	1560	1570	1580	1590	1600
TELCTEELLE	RIARKPTVCE	SEIRAYMRQV	LEGIHYLHQS	HVLHLDVKPE	NLLVWDGAAG	EQQVRICDFG	NAQELTPGEP
1610	1620	1630	1640	1650	1660	1670	1680
QYCQYGTPEF	VAPEIVNQSP	VSGVTDIWPV	GVVAFLCLTG	ISPFVGENDR	TTLMNIRNYN	VAFEETTFLS	LSREARGFLI
1690	1700	1710	1720	1730	1740	1750	1760
KVLVQDRLRP	TAEETLEHPW	FKTQAKGAEV	STDHLKLFLS	RRRWQRSQIS	YKCHLVLRPI	PELLRAPPER	VWVTMPRRPP
1770	1780	1790	1800	1810	1820	1830	1840
PSGGLSSSSD	SEEEELEELP	SVPRPLQPEF	SGSRVSLTDI	PTEDEALGTP	ETGAATPMDW	QEQGRAPSQD	QEAPSPEALP
1850	1860	1870	1880	1890	1900	1910	1920
SPGQEPAAGA	SPRRGELRRG	SSAESALPRA	GPRELGRGLH	KAASVELPQR	RSPSPGATRL	ARGGLGEGEY	AQRLQALRQR
1930	1940	1950	1960	1970	1980	1990	2000
LLRGGPEDGK	VSGLRGPLLE	SLGGRARDPR	MARAASSEAA	PHHQPPLENR	GLQKSSSFSQ	GEAEPRGRHR	RAGAPLEIPV
2010	2020	2030	2040	2050	2060	2070	2080
ARLGARRLQE	SPSLSALSEA	QPSSPARPSA	PKPSTPKSAE	PSATTPSDAP	QPPAPQPAQD	KAPEPRPEPV	RASKPAPPPQ
2090	2100	2110	2120	2130	2140	2150	2160
ALQTLALPLT	PYAQIIQSLQ	LSGHAQGPSQ	GPAAPPSEPK	PHAAVFARVA	SPPPGAPEKR	VPSAGGPPVL	AEKARVPTVP
2170	2180	2190	2200	2210	2220	2230	2240

Detailed Protein Report

Cmpd.	No. of Cmpds.	m/z meas.	Δ m/z [ppm]	z	Rt [min]	Score	P	Range	Sequence	Modification	Ratios
146	2	531.9457	-37.14	3	31.8	25.5	2	1936-1950	R.GPLLESLGGRARDPR.M		M:Q 1.08<br>Q:W 0.34
43	1	796.8047	-128.81	2	30.2	14.7	2	2824-2837	R.FGVVRACRENATGR.T	Carbamidomethyl: 7	

Detailed Protein Report

Protein 128: fanconi-associated nuclease 1 isoform a [Homo sapiens]

**Accession:**	gi|226246523	**Score:**	68.8
**Database:**	refseq_human(refseq_human_20140103.fasta)	**MW [kDa]:**	114.2
**Database Date:**	2015-11-30	**pI:**	7.4
**Modification(s):**	Carbamidomethyl, Oxidation	**Sequence Coverage [%]:**	7.2
		**No. of unique Peptides:**	3

Quantitation

Q:W	**Median:** 0.69	**CV:** 0.00 %	**No. of Peptides:**	1
M:Q	**Median:** 0.90	**CV:** 0.00 %	**No. of Peptides:**	1

Alias proteins:

Accession	Name	Description
gi|578840975	refseq_human (refseq_human_20140103.fasta)	PREDICTED: fanconi-associated nuclease 1 isoform X4 [Homo sapiens]
gi|578840973	refseq_human (refseq_human_20140103.fasta)	PREDICTED: fanconi-associated nuclease 1 isoform X3 [Homo sapiens]
gi|578840971	refseq_human (refseq_human_20140103.fasta)	PREDICTED: fanconi-associated nuclease 1 isoform X2 [Homo sapiens]
gi|578840969	refseq_human (refseq_human_20140103.fasta)	PREDICTED: fanconi-associated nuclease 1 isoform X1 [Homo sapiens]
gi|530405539	refseq_human (refseq_human_20140103.fasta)	PREDICTED: fanconi-associated nuclease 1 isoform X4 [Homo sapiens]
gi|530405537	refseq_human (refseq_human_20140103.fasta)	PREDICTED: fanconi-associated nuclease 1 isoform X3 [Homo sapiens]
gi|530405535	refseq_human (refseq_human_20140103.fasta)	PREDICTED: fanconi-associated nuclease 1 isoform X2 [Homo sapiens]
gi|530405533	refseq_human (refseq_human_20140103.fasta)	PREDICTED: fanconi-associated nuclease 1 isoform X1 [Homo sapiens]

10	20	30	40	50	60	70	80
MMSEGKPPDK	KRPRRSLSIS	KNKKKASNSI	ISCFNNAPPA	KLACPVCSKM	VPRYDLNRHL	DEMCANNDFV	QVDPGQVGLI
**90**	**100**	**110**	**120**	**130**	**140**	**150**	**160**
NSNVSMVDLT	SVTLEDVTPK	KSPPPKTNLT	PGQSDSAKRE	VKQKISPYFK	SNDVVCKNQD	ELRNRSVKVI	CLGSLASKLS
**170**	**180**	**190**	**200**	**210**	**220**	**230**	**240**
RKYVKAKKSI	DKDEEFAGSS	PQSSKSTVVK	SLIDNSSEIE	DEDQILENSS	QKENVFKCDS	LKEECIPEHM	VRGSKIMEAE
**250**	**260**	**270**	**280**	**290**	**300**	**310**	**320**
SQKATRECEK	SALTPGFSDN	AIMLFSPDFT	LRNTLKSTSE	DSLVKQECIK	EVVEKREACH	CEEVKMTVAS	EAKIQLSDSE
**330**	**340**	**350**	**360**	**370**	**380**	**390**	**400**
AKSHSSADDA	SAWSNIQEAP	LQDDSCLNND	IPHSIPLEQG	SSCNGPGQTT	GHPYYLRSFL	VVLKTVLENE	DDMLLFDEQE
**410**	**420**	**430**	**440**	**450**	**460**	**470**	**480**
KGIVTKFYQL	SATGQKLYVR	LFQRKLSWIK	MTKLEYEEIA	LDLTPVIEEL	TNAGFLQTES	ELQELSEVLE	LLSAPELKSL
**490**	**500**	**510**	**520**	**530**	**540**	**550**	**560**
AKTFHLVNPN	GQKQQLVDAF	LKLAKQRSVC	TWGKNKPGIG	AVILKRAKAL	AGQSVRICKG	PRAVFSRILL	LFSLTDSMED
**570**	**580**	**590**	**600**	**610**	**620**	**630**	**640**
EDAACGGQGQ	LSTVLLVNLG	RMEFPSYTIN	RKTHIFQDRD	DLIRYAAATH	MLSDISSAMA	NGNWEEAKEL	AQCAKRDWNR
**650**	**660**	**670**	**680**	**690**	**700**	**710**	**720**
LKNHPSLRCH	EDLPLFLRCF	TVGWIYTRIL	SRFVEILQRL	HMYEEAVREL	ESLLSQRIYC	PDSRGRWWDR	LALNLHQHLK
**730**	**740**	**750**	**760**	**770**	**780**	**790**	**800**
RLEPTIKCIT	EGLADPEVRT	GHRLSLYQRA	VRLRESPSCK	KFKHLFQQLP	EMAVQDVKHV	TITGRLCPQR	GMCKSVFVME
**810**	**820**	**830**	**840**	**850**	**860**	**870**	**880**
AGEAADPTTV	LCSVEELALA	HYRRSGFDQG	IHGEGSTFST	LYGLLLWDII	FMDGIPDVFR	NACQAFPLDL	CTDSFFTSRR
**890**	**900**	**910**	**920**	**930**	**940**	**950**	**960**
PALEARLQLI	HDAPEESLRA	WVAATWHEQE	GRVASLVSWD	RFTSLQQAQD	LVSCLGGPVL	SGVCRHLAAD	FRHCRGGLPD
**970**	**980**	**990**	**1000**	**1010**	**1020**		
LVVWNSQSRH	FKLVEVKGPN	DRLSHKQMIW	LAELQKLGAE	VEVCHVVAVG	AKSQSLS		

Detailed Protein Report

Cmpd.	No. of Cmpds.	m/z meas.	Δ m/z [ppm]	z	Rt [min]	Score	P	Range	Sequence	Modification	Ratios
443	1	874.1657	67.87	3	34.2	13.4	2	25-49	K. KASNSIISCFNNAPPAKLACPVCSI M	Carbamidomethyl: 20	
691	1	765.8997	57.54	2	38.6	18.5	1	223-235	K.EECIPEHMVRGSK.I	Oxidation: 8	M:Q 0.90<br>Q:W 0.69
97	2	826.6883	-79.67	3	31.1	17.4	0	922-945	R. FTSLQQAQDLVSCLGGPVLSGVC H		

Detailed Protein Report

Protein 129: **PREDICTED: sushi, nidogen and EGF-like domain-containing protein 1 isoform X4 [Homo sapiens]**

**Accession:**	gi|578804038	**Score:**	68.7
**Database:**	refseq_human(refseq_human_20140103.fasta)	**MW [kDa]:**	142.7
**Database Date:**	2015-11-30	**pI:**	6.4
**Modification(s):**	Carbamidomethyl	**Sequence Coverage [%]:**	4.4
		**No. of unique Peptides:**	5

10	20	30	40	50	60	70	80
MRHGVAWALL	VAAALGLGAR	GVRGAVALAD	FYPFGAERGD	AVTPKQDDGG	SGLRPLSVPF	PFFGAEHSGL	YVNNNGIISF
**90**	**100**	**110**	**120**	**130**	**140**	**150**	**160**
LKEVSQFTPV	AFPIAKDRCV	VAAFWADVDN	RRAGDVYYRE	ATDPAMLRRA	TEDVRHYFPE	LLDFNATWVF	VATWYRVTFF
**170**	**180**	**190**	**200**	**210**	**220**	**230**	**240**
GGSSSSPVNT	FQTVLITDGK	LSFTIFNYES	IVWTTGTHAS	SGGNATGLGG	IAAQAGFNAG	DGQRYFSIPG	SRTADMAEVE
**250**	**260**	**270**	**280**	**290**	**300**	**310**	**320**
TTTNVGVPGR	WAFRIDDAQV	RVGGCGHTTS	VCLALRPCLN	GGKCIDDCVT	GNPSYTCSCL	SGFTGRRCHL	DVNECASQPC
**330**	**340**	**350**	**360**	**370**	**380**	**390**	**400**
QNGGTCTHGI	NSFRCQCPAG	FGGPTCETAQ	SPCDTKECQH	GGQCQVENGS	AVCVCQAGYT	GAACEMDVDD	CSPDPCLNGG
**410**	**420**	**430**	**440**	**450**	**460**	**470**	**480**
SCVDLVGNYT	CLCAEPFKGL	RCETGDHPVP	DACLSAPCHN	GGTCVDADQG	YVCECPEGFM	GLDCRERVPD	DCECRNGGRC
**490**	**500**	**510**	**520**	**530**	**540**	**550**	**560**
LGANTTLCQC	PLGFFGLLCE	FEITAMPCNM	NTQCPDGGYC	MEHGGSYLCV	CHTDHNASHS	LPSPCDSDPC	FNGGSCDAHD
**570**	**580**	**590**	**600**	**610**	**620**	**630**	**640**
DSYTCECPRG	FHGKHCEKAR	PHLCSSGPCR	NGGTCKEAGG	EYHCSCPYRF	TGRHCEIGKP	DSCASGPCHN	GGTCFHYIGK
**650**	**660**	**670**	**680**	**690**	**700**	**710**	**720**
YKCDCPPGFS	GRHCEIAPSP	CFRSPCVNGG	TCEDRDTDFF	CHCQAGYMGR	RCQAEVDCGP	PEEVKHATLR	FNGTRLGAVA
**730**	**740**	**750**	**760**	**770**	**780**	**790**	**800**
LYACDRGYSL	SAPSRIRVCQ	PHGVWSEPPQ	CLEIDECRSQ	PCLHGGSCQD	RVAGYLCLCS	TGYEGAHCEL	ERDECRAHPC
**810**	**820**	**830**	**840**	**850**	**860**	**870**	**880**
RNGGSCRNLP	GAYVCRCPAG	FVGVHCETEV	DACDSSPCQH	GGRCESGGGA	YLCVCPESFF	GYHCETVSDP	CFSSPCGGRG
**890**	**900**	**910**	**920**	**930**	**940**	**950**	**960**
YCLASNGSHS	CTCKVGYTGE	DCAKELFPPT	ALKMERVEES	GVSISWNPPN	GPAARQMLDG	YAVTYVSSDG	SYRRTDFVDR
**970**	**980**	**990**	**1000**	**1010**	**1020**	**1030**	**1040**
TRSSHQLQAL	AAGRAYNISV	FSVKRNSNNK	NDISRPAVLL	ARTRPRPVEG	FEVTNVTAST	ISVQWALHRI	RHATVSGVRV
**1050**	**1060**	**1070**	**1080**	**1090**	**1100**	**1110**	**1120**
SIRHPEALRD	QATDVDRSVD	RFTFRALLPG	KRYTIQLTTL	SGLRGEEHPT	ESLATAPTHV	WTRPLPPANL	TAARVTATSA
**1130**	**1140**	**1150**	**1160**	**1170**	**1180**	**1190**	**1200**
HVVWDAPTPG	SLLEAYVINV	TTSQSTKSRY	VPNGKLASYT	VRDLLPGRRY	QLSVIAVQST	ELGPQHSEPA	HLYIITSPRD
**1210**	**1220**	**1230**	**1240**	**1250**	**1260**	**1270**	**1280**
GADRRWHQGG	HHPRVLKNRP	PPARLPELRL	LNDHSAPETP	TQPPRFSELV	DGRGRVSARF	GGSPSKAATV	RSQPTASAQL
**1290**	**1300**	**1310**	**1320**	**1330**			
ENMEEAPKRV	SLALQLPEHG	SKDIGSLDLL	LAPVSTRRTG	LCIRRE			

Cmpd.	No. of Cmpds.	m/z meas.	Δ m/z [ppm]	z	Rt [min]	Score	P	Range	Sequence	Modification
1017	1	788.6695	-45.57	3	41.2	10.3	2	570-590	R.GFHGKHCEKARPHLCSSGPCR.N	Carbamidomethyl: 15
2079	1	1044.9332	12.94	2	56.4	14.4	1	591-609	R.NGGTCKEAGGEYHCSCPYR.F	Carbamidomethyl: 5
1667	1	1073.2934	-127.64	2	49.2	19.6	1	591-609	R.NGGTCKEAGGEYHCSCPYR.F	Carbamidomethyl: 5, 16
2019	1	715.9639	11.03	3	53.6	10.1	1	591-609	R.NGGTCKEAGGEYHCSCPYR.F	Carbamidomethyl: 14, 16
2962	1	1019.9724	-38.21	2	66.2	14.4	1	895-913	K.VGYTGEDCAKELFPPTALK.M	

Detailed Protein Report

Protein 130: PREDICTED: E3 ubiquitin-protein ligase HUWE1 isoform X8 [Homo sapiens]

**Accession:**	gi|530426358	**Score:**	68.6
**Database:**	refseq_human(refseq_human_20140103.fasta)	**MW [kDa]:**	466.2
**Database Date:**	2015-11-30	**pI:**	5.3
		**Sequence Coverage [%]:**	1.4
		**No. of unique Peptides:**	3

Quantitation

Q:W	**Median:**	1.04	**CV:**	0.00 %	**No. of Peptides:**	1
M:Q	**Median:**	1.01	**CV:**	49.57 %	**No. of Peptides:**	2

Detailed Protein Report

10	20	30	40	50	60	70	80
MKVDRTKLKK	TPTEAPADCR	ALIDKLKVCN	DEQLLLELQQ	IKTWNIGKCE	LYHWVDLLDR	FDGILADAGQ	TVENMSWMLV
90	100	110	120	130	140	150	160
CDRPEREQLK	MLLLAVLNFT	ALLIEYSFSR	HLYSSIEHLT	TLLASSDMQV	VLAVLNLLYV	FSKRSNYITR	LGSDKRTPLL
170	180	190	200	210	220	230	240
TRLQHLAESW	GGKENGFGLA	ECCRDLHMMK	YPPSATTLHF	EFYADPGAEV	KIEKRTTSNT	LHYIHIEQLD	KISESPSEIM
250	260	270	280	290	300	310	320
ESLTKMYSIP	KDKQMLLFTH	IRLAHGFSNH	RKRLQAVQAR	LHAISILVYS	NALQESANSI	LYNGLIEELV	DVLQITDKQL
330	340	350	360	370	380	390	400
MEIKAASLRT	LTSIVHLERT	PKLSSIIDCT	GTASYHGFLP	VLVRNCIQAM	IDPSMDPYPH	QFATALFSFL	YHLASYDAGG
410	420	430	440	450	460	470	480
EALVSCGMME	ALLKVIKFLG	DEQDQITFVT	RAVRVVDLIT	NLDMAAFQSH	SGLSIFIYRL	EHEVDLCRKE	CPFVIKPKIQ
490	500	510	520	530	540	550	560
RPNTTQEGEE	METDMDGVQC	IPQRAALLKS	MLNFLKKAIQ	DPAFSDGIRH	VMDGSLPTSL	KHIISNAEYY	GPSLFLLATE
570	580	590	600	610	620	630	640
VVTVFVFQEP	SLLSSLQDNG	LTDVMLHALL	IKDVPATREV	LGSLPNVFSA	LCLNARGLQS	FVQCQPFERL	FKVLLSPDYL
650	660	670	680	690	700	710	720
PAMRRRRSSD	PLGDTASNLG	SAVDELMRHQ	PTLKTDATTA	IIKLLEEICN	LGRDPKYICQ	KPSIQKADGT	ATAPPPRSNH
730	740	750	760	770	780	790	800
AAEEASSEDE	EEEEVQAMQS	FNSTQQNETE	PNQQVVGTEE	RIPIPLMDYI	LNVMKFVESI	LSNNTTDDHC	QEFVNQKGLL
810	820	830	840	850	860	870	880
PLVTILGLPN	LPIDFPTSAA	CQAVAGVCKS	ILTLSHEPKV	LQEGLLQLDS	ILSSLEPLHR	PIESPGGSVL	LRELACAGNV
890	900	910	920	930	940	950	960
ADATLSAQAT	PLLHALTAAH	AYIMMFVHTC	RVGQSEIRSI	SVNQWGSQLG	LSVLSKLSQL	YCSLVWESTV	LLSLCTPNSL
970	980	990	1000	1010	1020	1030	1040
PSGCEFGQAD	MQKLVPKDEK	AGTTQGGKRS	DGEQDGAAGS	MDASTQGLLE	GIGLDGDTLA	PMETDEPTAS	DSKGKSKITP
1050	1060	1070	1080	1090	1100	1110	1120
AMAARIKQIK	PLLSASSRLG	RALAELFGLL	VKLCVGSPVR	QRRSHHAAST	TTAPTPAARS	TASALTKLLT	KGLSWQPPPY
1130	1140	1150	1160	1170	1180	1190	1200
TPTPRFRLTF	FICSVGFTSP	MLFDERKYPY	HLMLQKFLCS	GGHNALFETF	NWALSMGGKV	PVSEGLEHSD	LPDGTGEFLD
1210	1220	1230	1240	1250	1260	1270	1280
AWLMLVEKMV	NPTTVLESPH	SLPAKLPGGV	QNFPQFSALR	FLVVTQKAAF	TCIKNLWNRK	PLKVYGGRMA	ESMLAILCHI
1290	1300	1310	1320	1330	1340	1350	1360
LRGEPVIRER	LSKEKEGSRG	EEDTGQEEGG	SRREPQVNQQ	QLQQLMDMGF	TREHAMEALL	NTSTMEQATE	YLLTHPPPIM
1370	1380	1390	1400	1410	1420	1430	1440
GGVVRDLSMS	EEDQMMRAIA	MSLGQDIPMD	QRAESPEEVA	CRKEEEERKA	REKQEEEEAK	CLEKFQDADP	LEQDELHTFT
1450	1460	1470	1480	1490	1500	1510	1520
DTMLPGCFHL	LDELPDTVYR	VCDLIMTAIK	RNGADYRDMI	LKQVVNQVWE	AADVLIKAAL	PLTTSDTKTV	SEWISQMATL
1530	1540	1550	1560	1570	1580	1590	1600
PQASNLATRI	LLLTLLFEEL	KLPCAWVVES	SGILNVLIKL	LEVVQPCLQA	AKEQKEVQTP	KWITPVLLLI	DFYEKTAISS
1610	1620	1630	1640	1650	1660	1670	1680
KRRAQMTKYL	QSNSNNWRWF	DDRSGRWCSY	SASNNSTIDS	AWKSGETSVR	FTAGRRRYTV	QFTTMVQVNE	ETGNRRPVML
1690	1700	1710	1720	1730	1740	1750	1760
TLLRVPRLNK	NSKNSNGQEL	EKTLEESKEM	DIKRKENKGN	DTPLALESTN	TEKETSLEET	KIGEILIQGL	TEDMVTVLIR
1770	1780	1790	1800	1810	1820	1830	1840
ACVSMLGVPV	DPDTLHATLR	LCLRLTRDHK	YAMMFAELKS	TRMILNLTQS	SGFNGFTPLV	TLLLRHIIED	PCTLRHTMEK
1850	1860	1870	1880	1890	1900	1910	1920
VVRSAATSGA	GSTTSGVVSG	SLGSREINYI	LRVLGPAACR	NPDIFTEVAN	CCIRIALPAP	RGSGTASDDE	FENLRIKGPN
1930	1940	1950	1960	1970	1980	1990	2000
AVQLVKTTPL	KPSPLPVIPD	TIKEVIYDML	NALAAYHAPE	EADKSDPKPG	VMTQEVGQLL	QDMGDDVYQQ	YRSLTRQSSD
2010	2020	2030	2040	2050	2060	2070	2080
FDTQSGFSIN	SQVFAADGAS	TETSASGTSQ	GEASTPEESR	DGKKDKEGDR	ASEEGKQKGK	GSKPLMPTST	ILRLLAELVR
2090	2100	2110	2120	2130	2140	2150	2160
SYVGIATLIA	NYSYTVGQSE	LIKEDCSVLA	FVLDHLLPHT	QNAEDKDTPA	LARLFLASLA	AAGSGTDAQV	ALVNEVKAAL
2170	2180	2190	2200	2210	2220	2230	2240

Detailed Protein Report

Cmpd.	No. of Cmpds.	m/z meas.	Δ m/z [ppm]	z	Rt [min]	Score	P	Range	Sequence	Modification	Ratios
2739	1	701.4113	40.63	1	62.6	10.5	0	1576-1581	K.EVQTPK.W		
2862	4	645.8328	-53.66	2	64.5	18.4	1	3005-3015	R.GVQYTRLAVQR.G		Q:W 1.04<br>M:Q 1.61
355	1	658.3846	-33.13	2	33.1	10.5	2	3311-3321	R.RSSLLTEKLLR.L		M:Q 0.63

Detailed Protein Report

Protein 131: PREDICTED: protein FAM186A isoform X1 [Homo sapiens]

**Accession:**	gi|578823289	**Score:**	68.5
**Database:**	refseq_human(refseq_human_20140103.fasta)	**MW [kDa]:**	262.1
**Database Date:**	2015-11-30	**pI:**	9.1
		**Sequence Coverage [%]:**	2.2
		**No. of unique Peptides:**	3

Quantitation

Q:W	**Median:**	5.98	**CV:**	151.36 %	**No. of Peptides:**	2
M:Q	**Median:**	1.22	**CV:**	82.57 %	**No. of Peptides:**	2

Detailed Protein Report

10	20	30	40	50	60	70	80
MFFKMKNEID	NDPESEKCIK	DSTIMRREPQ	NILSPLMLPN	LEIPFSVKDI	ISRIERAQLH	RAREDIDMQL	SEIMNNVHRI
90	100	110	120	130	140	150	160
MTRYTLVFNS	SSERNVSLTE	HKKKQRTNFL	EKMATYAKTI	EIREKTLANI	LAWLEEWNDV	LSEMTLMDVD	EHHHWIAQME
170	180	190	200	210	220	230	240
LLPDTLKAIE	NNVKILSRFS	TSFLDEKKKQ	KKKILSRGTL	WKSWKERVIK	RPSTARALRP	DQMISDQLAT	NTKVSEIQGM
250	260	270	280	290	300	310	320
LQELIGTTMF	STLENNAIKY	ISSTIVNLST	ALSMLNDELK	CVNFQSSTVY	AHETSEAEKE	LSLKIIRDLS	NENEMLQQKL
330	340	350	360	370	380	390	400
QDAEEKCEQL	IRSKIVIEQL	YAKLSTSSTL	KVLPGPSPQS	SRAIIKVGDT	EDNMDNILDK	ELENIVDEVQ	RKETKDSGIK
410	420	430	440	450	460	470	480
WDSTISYTAQ	AERTPDLTEL	RQQPVASEDI	SEDSTKDNVS	LKKGDFYQED	ETDEYQSWKR	SHKKATYVYE	TSGPNLSDNK
490	500	510	520	530	540	550	560
SGQKVSEAKP	SQYYELQVLK	KKRKEMKSFS	EDKSKSPTEA	KRKHLSLTET	KSQGGKSGTS	MMMLEQFRKV	KRESPFDKRP
570	580	590	600	610	620	630	640
TAAEIKVEPT	TESLDKEGKG	EIRSLVEPLS	MIQFDDTAEP	QKGKIKGKKH	HISSGTITSK	EEKTEEKEEL	TKQVKSHQLV
650	660	670	680	690	700	710	720
KSLSRVAKET	SESTRVLESP	DGKSEQSNLE	EFQEAIMAFL	KQKIDNIGKA	FDKKTVPKEE	ELLKRAEAEK	LGIIKAKMEE
730	740	750	760	770	780	790	800
YFQKVAETVT	KILRKYKDTK	KEEQVGEKPI	KQKKVVSFMP	GLHFQKSPIS	AKSESSTLLS	YESTDPVINN	LIQMILAEIE
810	820	830	840	850	860	870	880
SERDIPTVST	VQKDHKEKEK	QRQEQYLQEG	QEQMSGMSLK	QQLLGERNLL	KEHYEKISEN	WEEKKAWLQM	KEGKQEQQSQ
890	900	910	920	930	940	950	960
KQWQEEEMWK	EEQKQATPKQ	AEQEEKQKQR	GQEEEELPKS	SLQRLEEGTQ	KMKTQGLLLE	KENGQMRQIQ	KEAKHLGPHR
970	980	990	1000	1010	1020	1030	1040
RREKGKEKQK	PERGLEDLER	QIKTKDQMQM	KETQPKELEK	MVIQTPMTLS	PRWKSVLKDV	QRSYEGKEFQ	RNLKTLENLP
1050	1060	1070	1080	1090	1100	1110	1120
DEKEPISITP	PPSLQYSLPG	ALPISGQPLT	KCIHLTPQQA	QEVGITLTPQ	QAQAQGITLT	LQQAQELGIP	LTPQQAQALE
1130	1140	1150	1160	1170	1180	1190	1200
ILFTPQQAQA	LGIPLTPQQT	QVQGITLTPQ	QDQAPGISLT	TQQAQKLGIP	LTPQQAQALG	IPLTPQQAQE	LGIPLTPQQA
1210	1220	1230	1240	1250	1260	1270	1280
QALRVSLTPQ	QAQELGIPLT	PQQAQALGIT	LTLQQAQQLG	IPLTPQQAQA	LGITLTPKQV	QELGIPLTPQ	QAQALGITLT
1290	1300	1310	1320	1330	1340	1350	1360
PKQAQELGIP	LNPQQAQTLG	IPLTPKQAQA	LGIPFTPQQA	QALGIPLTPQ	QAQTQEITLT	PQQAQALGMP	LTTQQAQELG
1370	1380	1390	1400	1410	1420	1430	1440
IPLTPQHAQA	LGMPLTTQQA	QELGIPLTPQ	QAQALGMPLT	TQQAQELGIP	LTPQQAQELG	IPFTPQQAQA	QEITLTPQQA
1450	1460	1470	1480	1490	1500	1510	1520
QALGMPLTAQ	QAQELGITLT	PQQAQELGIP	LTPQQAQALG	IPLIPPQAQE	LGIPLTPQQA	QALGILLIPP	QAQELGIPLT
1530	1540	1550	1560	1570	1580	1590	1600
PQQAQALGIP	LIPPQAQELG	IPLTPQQVQA	LGIPLIPPQA	QELEIPLTPQ	QAQALGIPLT	PQQAQELGIP	LTPQQAQELG
1610	1620	1630	1640	1650	1660	1670	1680
IPLTPQQAQA	QGIPLTPQQA	QALGISLTPQ	QAQAQGITLT	PQQAQALGVP	ITPVNAWVSA	VTLTSEQTHA	LESPMNLEQA
1690	1700	1710	1720	1730	1740	1750	1760
QEQLLKLGVP	LTLDKAHTLG	SPLTLKQVQW	SHRPFQKSKA	SLPTGQSIIS	RLSPSLRLSL	ASSAPTAEKS	SIFGVSSTPL
1770	1780	1790	1800	1810	1820	1830	1840
QISRVPLNQG	PFAPGKPLEM	GILSEPGKLG	APQTLRSSGQ	TLVYGGQSTS	AQFPAPQAPP	SPGQLPISRA	PPTPGQPFIA
1850	1860	1870	1880	1890	1900	1910	1920
GVPPTSGQIP	SLWAPLSPGQ	PLVPEASSIP	GDLLESGPLT	FSEQLQEFQP	PATAEQSPYL	QAPSTPGQHL	ATWTLPGRAS
1930	1940	1950	1960	1970	1980	1990	2000
SLWIPPTSRH	PPTLWPSPAP	GKPQKSWSPS	VAKKRLAIIS	SLKSKSVLIH	PSAPDFKVAQ	VPFTTKKFQM	SEVSDTSEET
2010	2020	2030	2040	2050	2060	2070	2080
QILRDTFAIE	SFRTFQSHFT	KYRTPVYQTP	YTDERALLTL	MKPTTSPSSL	TTLLRTSQIS	PLEWYQKSRF	PPIDKPWILS
2090	2100	2110	2120	2130	2140	2150	2160
SVSDTKKPKV	MVPPSSPQEL	EEKRYFVDVE	AQKKNLILLN	QAIKTCGLPS	QLHTMARTLI	IEILHMDTVQ	LGYLFRKYIA
2170	2180	2190	2200	2210	2220	2230	2240

Detailed Protein Report

Cmpd.	No. of Cmpds.	m/z meas.	Δ m/z [ppm]	z	Rt [min]	Score	P	Range	Sequence	Modification	Ratios
82	1	724.8160	-30.23	2	30.3	10.0	0	308-319	R.DLSNENEMLQQK.L		
2521	1	648.3547	-3.42	2	62.0	14.3	1	609-620	K.KHHISSGTITSK.E		M:Q 0.59<br>Q:W 17.81
768	3	495.2668	-47.89	2	38.4	14.6	1	2337-2345	R.KSLASLQSR.S		M:Q 2.50<br>Q:W 2.01

Detailed Protein Report

Protein 132: lysine-specific demethylase 3A [Homo sapiens]

**Accession:**	gi|20357522	**Score:**	68.4
**Database:**	refseq_human(refseq_human_20140103.fasta)	**MW [kDa]:**	147.2
**Database Date:**	2015-11-30	**pI:**	9.4
**Modification(s):**	Carbamidomethyl, Oxidation	**Sequence Coverage [%]:**	3.3
		**No. of unique Peptides:**	3

Quantitation

Q:W	**Median:**	4.62	**CV:**	0.00 %	**No. of Peptides:**	1
M:Q	**Median:**	0.52	**CV:**	0.00 %	**No. of Peptides:**	1

Alias proteins:

Accession	Name	Description
gi|226442779	r e f s e q _ h u m a (refseq_human_20140103.fasta)	lysine-specific demethylase 3A [Homo sapiens]

10	20	30	40	50	60	70	80
MVLTLGESWP	VLVGRRFLSL	SAADGSDGSH	DSWDVERVAE	WPWLSGTIRA	VSHTDVTKKD	LKVCVEFDGE	SWRKRRWIEV
90	100	110	120	130	140	150	160
YSLLRRAFLV	EHNLVLAERK	SPEISERIVQ	WPAITYKPLL	DKAGLGSITS	VRFLGDQQRV	FLSKDLLKPI	QDVNSLRLSL
170	180	190	200	210	220	230	240
TDNQIVSKEF	QALIVKHLDE	SHLLKGDKNL	VGSEVKIYSL	DPSTQWFSAT	VINGNPASKT	LQVNCEEIPA	LKIVDPSLIH
250	260	270	280	290	300	310	320
VEVVHDNLVT	CGNSARIGAV	KRKSSENNGT	LVSKQAKSCS	EASPSMCPVQ	SVPTTVFKEI	LLGCTAATPP	SKDPRQQSTP
330	340	350	360	370	380	390	400
QAANSPPNLG	AKIPQGCHKQ	SLPEEISSCL	NTKSEALRTK	PDVCKAGLLS	KSSQIGTGDL	KILTEPKGSC	TQPKTNTDQE
410	420	430	440	450	460	470	480
NRLESVPQAL	TGLPKECLPT	KASSKAELEI	ANPPELQKHL	EHAPSPSDVS	NAPEVKAGVN	SDSPNNCSGK	KVEPSALACR
490	500	510	520	530	540	550	560
SQNLKESSVK	VDNESCCSRS	NNKIQNAPSR	KSVLTDPAKL	KKLQQSGEAF	VQDDSCVNIV	AQLPKCRECR	LDSLRKDKEQ
570	580	590	600	610	620	630	640
QKDSPVFCRF	FHFRRLQFNK	HGVLRVEGFL	TPNKYDNEAI	GLWLPLTKNV	VGIDLDTAKY	ILANIGDHFC	QMVISEKEAM
650	660	670	680	690	700	710	720
STIEPHRQVA	WKRAVKGVRE	MCDVCDTTIF	NLHWVCPRCG	FGVCVDCYRM	KRKNCQQGAA	YKTFSWLKCV	KSQIHEPENL
730	740	750	760	770	780	790	800
MPTQIIPGKA	LYDVGDIVHS	VRAKWGIKAN	CPCSNRQFKL	FSKPASKEDL	KQTSLAGEKP	TLGAVLQQNP	SVLEPAAVGG
810	820	830	840	850	860	870	880
EAASKPAGSM	KPACPASTSP	LNWLADLTSG	NVNKENKEKQ	PTMPILKNEI	KCLPPLPPLS	KSSTVLHTFN	STILTPVSNN
890	900	910	920	930	940	950	960
NSGFLRNLLN	SSTGKTENGL	KNTPKILDDI	FASLVQNKTT	SDLSKRPQGL	TIKPSILGFD	TPHYWLCDNR	LLCLQDPNNK
970	980	990	1000	1010	1020	1030	1040
SNWNVFRECW	KQGQPVMVSG	VHHKLNSELW	KPESFRKEFG	EQEVDLVNCR	TNEIITGATV	GDFWDGFEDV	PNRLKNEKEP
1050	1060	1070	1080	1090	1100	1110	1120
MVLKLKDWPP	GEDFRDMMPS	RFDDLMANIP	LPEYTRRDGK	LNLASRLPNY	FVRPDLGPKM	YNAYGLITPE	DRKYGTTNLH
1130	1140	1150	1160	1170	1180	1190	1200
LDVSDAANVM	VYVGIPKGQC	EQEEEVLKTI	QDGDSDELTI	KRFIEGKEKP	GALWHIYAAK	DTEKIREFLK	KVSEEQGQEN
1210	1220	1230	1240	1250	1260	1270	1280
PADHDPIHDQ	SWYLDRSLRK	RLHQEYGVQG	WAIVQFLGDV	VFIPAGAPHQ	VHNLYSCIKV	AEDFVSPEHV	KHCFWLTQEF
1290	1300	1310	1320	1330			
RYLSQTHTNH	EDKLQVKNVI	YHAVKDAVAM	LKASESSFGK	P			

Cmpd.	No. of Cmpds.	m/z meas.	Δ m/z [ppm]	z	Rt [min]	Score	P	Range	Sequence	Modification	Ratios
62	1	675.3854	139.53	2	29.5	11.1	0	457-470	K.AGVNSDSPNNCSGK.K		
81	1	821.0339	-27.87	3	30.4	17.2	2	457-480	K. AGVNSDSPNNCSGKKVEPSALAC S	Carbamidomethyl: 23	
755	2	472.1298	-292.09	2	39.4	27.4	0	840-847	K.QPTMPILK.N	Oxidation: 4	Q:W 4.62

Detailed Protein Report

Cmpd.	No. of Cmpds.	m/z meas.	Δ m/z [ppm]	z	Rt [min]	Score	P	Range	Sequence	Modification	Ratios	
											M:Q	0.52

Detailed Protein Report

Protein 133: zymogen granule protein 16 homolog B precursor [Homo sapiens]

**Accession:**	gi|94536866	**Score:**	68.1
**Database:**	refseq_human(refseq_human_20140103.fasta)	**MW [kDa]:**	22.7
**Database Date:**	2015-11-30	**pI:**	7.6
		**Sequence Coverage [%]:**	14.4
		**No. of unique Peptides:**	2

Quantitation

Q:W	**Median:** 0.39	**CV:** 0.00 %	**No. of Peptides:** 1
M:Q	**Median:** 1.80	**CV:** 0.00 %	**No. of Peptides:** 1

```
        10         20         30         40         50         60         70         80
MGAQGAQESI KAMWRVPGTT RRPVTGESPG MHRPEAMLLL LTLALLGGPT WAGKMYGPGG GKYFSTTEDY DHEITGLRVS
        90        100        110        120        130        140        150        160
VGLLLVKSVQ VKLGDSWDVK LGALGGNTQE VTLQPGEYIT KVFVAFQAFL RGMVMYTSKD RYFYFGKLDG QISSAYPSQE
       170        180        190        200        210
GQVLVGIYGQ YQLLGIKSIG FEWNYPLEEP TTEPPVNLTY SANSPVGR
```

Cmpd.	No. of Cmpds.	m/z meas.	Δ m/z [ppm]	z	Rt [min]	Score	P	Range	Sequence	Modification	Ratios
2510	2	464.1779	-296.31	2	60.1	35.2	0	79-87	R.VSVGLLLVK.S		
1743	6	1095.0646	-10.46	2	50.3	32.9	0	101-121	K.LGALGGNTQEVTLQPGEYITK.V		M:Q 1.80<br>Q:W 0.39

Detailed Protein Report

Protein 134: **PREDICTED: latent-transforming growth factor beta-binding protein 1 isoform X4 [Homo sapiens]**

**Accession:**	gi|530367593	**Score:**	67.8
**Database:**	refseq_human(refseq_human_20140103.fasta)	**MW [kDa]:**	176.5
**Database Date:**	2015-11-30	**pI:**	5.4
**Modification(s):**	Carbamidomethyl, Oxidation	**Sequence Coverage [%]:**	5.5
		**No. of unique Peptides:**	4

```
        10         20         30         40         50         60         70         80
MAGAWLRWGL LLWAGLLASS AHGRLRRITY VVHPGPGLAA GALPLSGPPR SRTFNVALNA RYSRSSAAAG APSRASPGVP
        90        100        110        120        130        140        150        160
SERTRRTSKP GGAALQGLRP PPPPPPEPAR PAVPGGQLHP NPGGHPAAAP FTKQGRQVVR SKVPQETQSG GGSRLQVHQK
       170        180        190        200        210        220        230        240
QQLQGVNVCG GRCCHGWSKA PGSQRCTKPS CVPPCQNGGM CLRPQLCVCK PGTKGKACET IAAQDTSSPV FGGQSPGAAS
       250        260        270        280        290        300        310        320
SWGPPEQAAK HTSSKKADTL PRVSPVAQMT LTLKPKPSVG LPQQIHSQVT PLSSQSVVIH HGQTQEYVLK PKYFPAQKGI
       330        340        350        360        370        380        390        400
SGEQSTEGSF PLRYVQDQVA APFQLSNHTG RIKVVFTPSI CKVTCTKGSC QNSCEKGNTT TLISENGHAA DTLTATNFRV
       410        420        430        440        450        460        470        480
VICHLPCMNG GQCSSRDKCQ CPPNFTGKLC QIPVHGASVP KLYQHSQQPG KALGTHVIHS THTLPLTVTS QQGVKVKFPP
       490        500        510        520        530        540        550        560
NIVNIHVKHP PEASVQIHQV SRIDGPTGQK TKEAQPGQSQ VSYQGLPVQK TQTIHSTYSH QQVIPHVYPV AAKTQLGRCF
       570        580        590        600        610        620        630        640
QETIGSQCGK ALPGLSKQED CCGTVGTSWG FNKCQKCPKK PSYHGYNQMM ECLPGYKRVN NTFCQDINEC QLQGVCPNGE
       650        660        670        680        690        700        710        720
CLNTMGSYRC TCKIGFGPDP TFSSCVPDPP VISEEKGPCY RLVSSGRQCM HPLSVHLTKQ LCCCSVGKAW GPHCEKCPLP
       730        740        750        760        770        780        790        800
GTAKEEPVEA LTFSREHGPG VAEPEVATAP PEKEIPSLDQ EKTKLEPGQP QLSPGISTIH LHPQFPVVIE KTSPPVPVEV
       810        820        830        840        850        860        870        880
APEASTSSAS QVIAPTQVTE INECTVNPDI CGAGHCINLP VRYTCICYEG YRFSEQQRKC VDIDECTQVQ HLCSQGRCEN
       890        900        910        920        930        940        950        960
TEGSFLCICP AGFMASEEGT NCIDVDECLR PDVCGEGHCV NTVGAFRCEY CDSGYRMTQR GRCEDIDECL NPSTCPDEQC
       970        980        990       1000       1010       1020       1030       1040
VNSPGSYQCV PCTEGFRGWN GQCLDVDECL EPNVCANGDC SNLEGSYMCS CHKGYTRTPD HKHCRDIDEC QQGNLCVNGQ
      1050       1060       1070       1080       1090       1100       1110       1120
CKNTEGSFRC TCGQGYQLSA AKDQCEDIDE CQHRHLCAHG QCRNTEGSFQ CVCDQGYRAS GLGDHCEDIN ECLEDKSVCQ
      1130       1140       1150       1160       1170       1180       1190       1200
RGDCINTAGS YDCTCPDGFQ LDDNKTCQDI NECEHPGLCG PQGECLNTEG SFHCVCQQGF SISADGRTCE DVNECELLSG
      1210       1220       1230       1240       1250       1260       1270       1280
VCGEAFCENV EGSFLCVCAD ENQEYSPMTG QCRSRTSTDL DVDVDQPKEE KKECYYNLND ASLCDNVLAP NVTKQECCCT
      1290       1300       1310       1320       1330       1340       1350       1360
SGVGWGDNCE IFPCPVLGTA EFTEMCPKGK GFVPAGESSS EAGGENYKDA DECLLFGQEI CKNGFCLNTR PGYECYCKQG
      1370       1380       1390       1400       1410       1420       1430       1440
TYYDPVKLQC FDMDECQDPS SCIDGQCVNT EGSYNCFCTH PMVLDASEKR CIRPAESNEQ IEETDVYQDL CWEHLSDEYV
      1450       1460       1470       1480       1490       1500       1510       1520
CSRPLVGKQT TYTECCCLYG EAWGMQCALC PLKDSDDYAQ LCNIPVTGRR QPYGRDALVD FSEQYTPEAD PYFIQDRFLN
      1530       1540       1550       1560       1570       1580       1590       1600
SFEELQAEEC GILNGCENGR CVRVQEGYTC DCFDGYHLDT AKMTCVDVNE CDELNNRMSL CKNAKCINTD GSYKCLCLPG
      1610       1620       1630
YVPSDKPNYC TPLNTALNLE KDSDLE
```

Cmpd.	No. of Cmpds.	m/z meas.	Δ m/z [ppm]	z	Rt [min]	Score	P	Range	Sequence	Modification
65	1	1240.0095	73.75	3	30.2	22.4	1	180-214	K.APGSQRCTKPSCVPPCQNGGMCLRPQLCVCKPGTK.G	Carbamidomethyl: 7; Oxidation: 21
109	1	1098.0910	-73.60	3	31.1	10.3	0	186-214	R.CTKPSCVPPCQNGGMCLRPQLCVCKPGTK.G	Carbamidomethyl: 1, 6, 10,

Detailed Protein Report

Cmpd.	No. of Cmpds.	m/z meas.	Δ m/z [ppm]	z	Rt [min]	Score	P	Range	Sequence	Modification
										16; Oxidation: 15
2143	1	1052.3999	-77.45	2	57.2	10.8	1	400-418	R.VVICHLPCMNGGQCSSRDK.C	Carbamidomethyl: 8
1842	1	1074.5505	92.79	2	51.7	12.2	1	578-596	K.QEDCCGTVGTSWGFNKCQK.C	Carbamidomethyl: 4

Detailed Protein Report

Protein 135: PREDICTED: centrosomal protein of 290 kDa isoform X4 [Homo sapiens]

**Accession:**	gi|530400856	**Score:**	67.7
**Database:**	refseq_human(refseq_human_20140103.fasta)	**MW [kDa]:**	234.7
**Database Date:**	2015-11-30	**pI:**	6.1
		**Sequence Coverage [%]:**	2.8
		**No. of unique Peptides:**	1

Detailed Protein Report

10	20	30	40	50	60	70	80
MQKLSLRQKS	AIFCQQIHEN	RADMDKSQVA	TLEEEQVHSQ	VKYADINLKE	DIIKSEVPLQ	TEILKNKLKV	NLPDPVSITA
90	100	110	120	130	140	150	160
QSKLSQINSL	ENLIEQLRRE	LVFLRSQNEI	IAQEFLIKEA	ECRNADIELE	HHRSQAEQNE	FLSRELIEKE	RDLERSRTVI
170	180	190	200	210	220	230	240
AKFQNKLKEL	VEENKQLEEG	MKEILQAIKE	MQKDPDVKGG	ETSLIIPSLE	RLVNAIESKN	AEGIFDASLH	LKAQVDQLTG
250	260	270	280	290	300	310	320
RNEELRQELR	ESRKEAINYS	QQLAKANLKI	DHLEKETSLL	RQSEGSNVVF	KGIDLPDGIA	PSSASIINSQ	NEYLIHLLQE
330	340	350	360	370	380	390	400
LENKEKKLKN	LEDSLEDYNR	KFAVIRHQQS	LLYKEYLSEK	ETWKTESKTI	KEEKRKLEDQ	VQQDAIKVKE	YNNLLNALQM
410	420	430	440	450	460	470	480
DSDEMKKILA	ENSRKITVLQ	VNEKSLIRQY	TTLVELERQL	RKENEKQKNE	LLSMEAEVCE	KIGCLQRFKE	MAIFKIAALQ
490	500	510	520	530	540	550	560
KVVDNSVSLS	ELELANKQYN	ELTAKYRDIL	QKDNMLVQRT	SNLEHLECEN	ISLKEQVESI	NKELEITKEK	LHTIEQAWEQ
570	580	590	600	610	620	630	640
ETKLGNESSM	DKAKKSITNS	DIVSISKKIT	MLEMKELNER	QRAEHCQKMY	EHLRTSLKQM	EERNFELETK	FAELTKINLD
650	660	670	680	690	700	710	720
AQKVEQMLRD	ELADSVSKAV	SDADRQRILE	LEKNEMELKV	EVSKLREISD	IARRQVEILN	AQQQSRDKEV	ESLRMQLLDY
730	740	750	760	770	780	790	800
QAQSDEKSLI	AKLHQHNVSL	QLSEATALGK	LESITSKLQK	MEAYNLRLEQ	KLDEKEQALY	YARLEGRNRA	KHLRQTIQSL
810	820	830	840	850	860	870	880
RRQFSGALPL	AQQEKFSKTM	IQLQNDKLKI	MQEMKNSQQE	HRNMENKTLE	MELKLKGLEE	LISTLKDTKG	AQKVINWHMK
890	900	910	920	930	940	950	960
IEELRLQELK	LNRELVKDKE	EIKYLNNIIS	EYERTISSLE	EEIVQQNKFH	EERQMAWDQR	EVDLERQLDI	FDRQQNEILN
970	980	990	1000	1010	1020	1030	1040
AAQKFEEATG	SIPDPSLPLP	NQLEIALRKI	KENIRIILET	RATCKSLEEK	LKEKESALRL	AEQNILSRDK	VINELRLRLP
1050	1060	1070	1080	1090	1100	1110	1120
ATAEREKLIA	ELGRKEMEPK	SHHTLKIAHQ	TIANMQARLN	QKEEVLKKYQ	RLLEKAREEQ	REIVKKHEED	LHILHHRLEL
1130	1140	1150	1160	1170	1180	1190	1200
QADSSLNKFK	QTAWDLMKQS	PTPVPTNKHF	IRLAEMEQTV	AEQDDSLSSL	LVKLKKVSQD	LERQREITEL	KVKEFENIKL
1210	1220	1230	1240	1250	1260	1270	1280
QLQENHEDEV	KKVKAEVEDL	KYLLDQSQKE	SQCLKSELQA	QKEANSRAPT	TTMRNLVERL	KSQLALKEKQ	QKALSRALLE
1290	1300	1310	1320	1330	1340	1350	1360
LRAEMTAAAE	ERIISATSQK	EAHLNVQQIV	DRHTRELKTQ	VEDLNENLLK	LKEALKTSKN	RENSLTDNLN	DLNNELQKKQ
1370	1380	1390	1400	1410	1420	1430	1440
KAYNKILREK	EEIDQENDEL	KRQIKRLTSG	LQGKPLTDNK	QSLIEELQRK	VKKLENQLEG	KVEEVDLKPM	KEKNAKEELI
1450	1460	1470	1480	1490	1500	1510	1520
RWEEGKKWQA	KIEGIRNKLK	EKEGEVFTLT	KQLNTLKDLF	AKADKEKLTL	QRKLKTTGMT	VDQVLGIRAL	ESEKELEELK
1530	1540	1550	1560	1570	1580	1590	1600
KRNLDLENDI	LYMRAHQALP	RDSVVEDLHL	QNRYLQEKLH	ALEKQFSKDT	YSKPSISGIE	SDDHCQREQE	LQKENLKLSS
1610	1620	1630	1640	1650	1660	1670	1680
ENIELKFQLE	QANKDLPRLK	NQVRDLKEMC	EFLKKEKAEV	QRKLGHVRGS	GRSGKTIPEL	EKTIGLMKKV	VEKVQRENEQ
1690	1700	1710	1720	1730	1740	1750	1760
LKKASGILTS	EKMANIEQEN	EKLKAELEKL	KAHLGHQLSM	HYESKTKGTE	KIIAENERLR	KELKKETDAA	EKLRIAKNNL
1770	1780	1790	1800	1810	1820	1830	1840
EILNEKMTVQ	LEETGKRLQF	AESRGPQLEG	ADSKSWKSIV	VTRMYETKLK	ELETDIAKKN	QSITDLKQLV	KEATEREQKV
1850	1860	1870	1880	1890	1900	1910	1920
NKYNEDLEQQ	IKILKHVPEG	AETEQGLKRE	LQVLRLANHQ	LDKEKAELIH	QIEANKDQSG	AESTIPDADQ	LKEKIKDLET
1930	1940	1950	1960	1970	1980	1990	2000
QLKMSDLEKQ	HLKEEIKKLK	KELENFDPSF	FEEIEDLKYN	YKEEVKKNIL	LEEKVKKLSE	QLGVELTSPV	AASEEFEDEE
2010							
ESPVNFPIY							

Cmpd.	No. of Cmpds.	m/z meas.	Δ m/z [ppm]	z	Rt [min]	Score	P	Range	Sequence	Modification

Detailed Protein Report

Cmpd.	No. of Cmpds.	m/z meas.	Δ m/z [ppm]	z	Rt [min]	Score	P	Range	Sequence	Modification
1190	5	500.7991	-35.31	2	43.5	14.1	2	2-9	M.QKLSLRQK.S	

Detailed Protein Report

Protein 136: PREDICTED: myosin-7B isoform X1 [Homo sapiens]

**Accession:**	gi|578836098	**Score:**	67.7
**Database:**	refseq_human(refseq_human_20140103.fasta)	**MW [kDa]:**	203.5
**Database Date:**	2015-11-30	**pI:**	5.7
**Modification(s):**	Oxidation	**Sequence Coverage [%]:**	3.9
		**No. of unique Peptides:**	4

Quantitation

Q:W	**Median:**	0.28	**CV:**	0.00 %	**No. of Peptides:**	1
M:Q	**Median:**	1.00	**CV:**	0.00 %	**No. of Peptides:**	1

Detailed Protein Report

10	20	30	40	50	60	70	80
MAPSLYGLRS	GCLQGKAPLR	FPAPYICGGG	QRLQRHAAQP	RQPVHADHQF	LATKTGGTLE	DQIIEANPAM	EAFGNAKTLR
90	100	110	120	130	140	150	160
NDNSSRFGKF	IRIHFGPSGK	LASADIDSYL	LEKSRVIFQL	PGERSYHVYY	QILSGRKPEL	QDMLLLSMNP	YDYHFCSQGV
170	180	190	200	210	220	230	240
ITVDNMNDGE	ELIATDHAMD	ILGFSVDEKC	ACYKIVGALL	HFGNMKFKQK	QREEQAEADG	TESADKAAYL	MGVSSGDLLK
250	260	270	280	290	300	310	320
GLLHPRVRVG	NEYVTKGQSV	EQVVFAVGAL	AKATYDRLFR	WLVSRINQTL	DTKLPRQFFI	GVLDIAGFEI	FEFNSFEQLC
330	340	350	360	370	380	390	400
INFTNEKLQQ	FFNQHMFVLE	QEEYKREGID	WVFIDFGLDL	QPCIDLIEKP	LGILSILEEE	CMFPKASDAS	FRAKLYDNHA
410	420	430	440	450	460	470	480
GKSPNFQQPR	PDKKRKYQAH	FEVVHYAGVV	PYSIVGWLEK	NKDPLNETVV	PIFQKSQNRL	LATLYENYAG	SCSTEPPKSG
490	500	510	520	530	540	550	560
VKEKRKKAAS	FQTVSQLHKE	NLNKLMTNLR	ATQPHFVRCI	VPNENKTPGV	MDAFLVLHQL	RCNGVLEGIR	ICRQGFPNRL
570	580	590	600	610	620	630	640
LYTDFRQRYR	ILNPSAIPDD	TFMDSRKATE	KLLGSLDLDH	TQYQFGHTKV	FFKAGLLGVL	EELRDQRLAK	VLTLLQARSR
650	660	670	680	690	700	710	720
GRLMRLEYQR	LLGGRDALFT	IQWNIRAFNA	VKNWSWMKLF	FKMKPLLRSA	QAEEELAALR	AELRGLRGAL	AAAEAKRQEL
730	740	750	760	770	780	790	800
EETHVSITQE	KNDLALQLQA	EQDNLADAEE	RCHLLIKSKV	QLEGKVKELS	ERLEDEEEVN	ADLAARRRKL	EDECTELKKD
810	820	830	840	850	860	870	880
IDDLELTLAK	AEKEKQATEN	KVKNLTEEMA	ALDESVARLT	KEKKALQEAH	QQALGDLQAE	EDRVSALTKA	KLRLEQQVED
890	900	910	920	930	940	950	960
LECSLEQEKK	LRMDTERAKR	KLEGDLKLTQ	ESVADAAQDK	QQLEEKLKKK	DSELSQLSLR	VEDEQLLGAQ	MQKKIKELQA
970	980	990	1000	1010	1020	1030	1040
RAEELEEELE	AERAARARVE	KQRAEAAREL	EELSERLEEA	GGASAGQREG	CRKREAELGR	LRRELEEAAL	RHEATVAALR
1050	1060	1070	1080	1090	1100	1110	1120
RKQAEGAAEL	GEQVDSLQRV	RQKLEKEKSE	LRMEVDDLAA	NVETLTRAKA	SAEKLCRTYE	DQLSEAKIKV	EELQRQLADA
1130	1140	1150	1160	1170	1180	1190	1200
STQRGRLQTE	SGELSRLLEE	KECLISQLSR	GKALAAQSLE	ELRRQLEEES	KAKSALAHAV	QALRHDCDLL	REQHEEEAEA
1210	1220	1230	1240	1250	1260	1270	1280
QAELQRLLSK	ANAEVAQWRS	KYEADAIQRT	EELEEAKKKL	ALRLQEAEEG	VEAANAKCSS	LEKAKLRLQT	ESEDVTLELE
1290	1300	1310	1320	1330	1340	1350	1360
RATSAAAALD	KKQRHLERAL	EERRRQEEEM	QRELEAAQRE	SRGLGTELFR	LRHGHEEALE	ALETLKRENK	NLQEEISDLT
1370	1380	1390	1400	1410	1420	1430	1440
DQVSLSGKSI	QELEKTKKAL	EGEKSEIQAA	LEEAEGALEL	EETKTLRIQL	ELSQVKAEVD	RKLAEKDEEC	ANLRRNHQRA
1450	1460	1470	1480	1490	1500	1510	1520
VESLQASLDA	ETRARNEALR	LKKKMEGDLN	DLELQLGHAT	RQATEAQAAT	RLMQAQLKEE	QAGRDEEQRL	AAELHEQAQA
1530	1540	1550	1560	1570	1580	1590	1600
LERRASLLAA	ELEELRAALE	QGERSRRLAE	QELLEATERL	NLLHSQNTGL	LNQKKKLEAD	LAQLSGEVEE	AAQERREAEE
1610	1620	1630	1640	1650	1660	1670	1680
KAKKAITDAA	MMAEELKKEQ	DTSAHLERMK	KTLEQTVREL	QARLEEAEQA	ALRGGKKQVQ	KLEAKVRELE	AELDAEQKKH
1690	1700	1710	1720	1730	1740	1750	1760
AEALKGVRKH	ERRVKELAYQ	AEEDRKNLAR	MQDLVDKLQS	KVKSYKRQFE	EAEQQANTNL	AKYRKAQHEL	DDAEERADMA
1770	1780	1790					
ETQANKLRAR	TRDALGPKLS	LSPQHKE					

Cmpd.	No. of Cmpds.	m/z meas.	Δ m/z [ppm]	z	Rt [min]	Score	P	Range	Sequence	Modification	Ratios
987	1	574.1653	-236.77	2	40.8	12.3	0	931-940	K.DSELSQLSLR.V		M:Q 1.00<br>Q:W 0.28
1310	1	939.8664	-147.69	2	46.0	16.1	1	1024-1040	R.ELEEAALRHEATVAALR.R		
2175	1	1031.4134	-120.21	2	55.8	11.8	2	1313-1330	R.ELEAAQRESRGLGTELFR.L		
2480	3	625.3103	-63.96	2	61.5	13.4	2	1629-1638	R.MKKTLEQTVR.E	Oxidation: 1	

Detailed Protein Report

Protein 137: PREDICTED: keratin, type I cytoskeletal 24 isoform X1 [Homo sapiens]

Accession: gi|578830527
Database: refseq_human(refseq_human_20140103.fasta)
Database Date: 2015-11-30
Modification(s): Oxidation

Score: 67.2
MW [kDa]: 46.1
pI: 4.7
Sequence Coverage [%]: 4.9
No. of unique Peptides: 1

Quantitation

Q:W **Median:** 0.39 **CV:** 0.00 % **No. of Peptides:** 1

10	20	30	40	50	60	70	80
MSCSSRASSS	RAGGSSSARV	SAGGSSFSSG	SRCGLGGSSA	QGFRGGASSC	SLSGGSSDKV	RALEEANTDL	ENKIKEWYDK
**90**	**100**	**110**	**120**	**130**	**140**	**150**	**160**
YGPGSGDGGS	GRDYSKYYSI	IEDLRNQIIA	ATVENAGIIL	HIDNARLAAD	DFRLKYENEL	CLRQSVEADI	NGLRKVLDDL
**170**	**180**	**190**	**200**	**210**	**220**	**230**	**240**
TMTRSDLEMQ	IESFTEELAY	LRKNHEEEMK	NMQGSSGGEV	TVEMNAAPGT	DLTKLLNDMR	AQYEELAEQN	RREAEERFNK
**250**	**260**	**270**	**280**	**290**	**300**	**310**	**320**
QSASLQAQIS	TDAGAATSAK	NEITELKRTL	QALEIELQSQ	LAMKSSLEGT	LADTEAGYVA	QLSEIQTQIS	ALEEEICQIW
**330**	**340**	**350**	**360**	**370**	**380**	**390**	**400**
GETKCQNAEY	KQLLDIKTRL	EVEIETYRRL	LDGEGGGSSF	AEFGGRNSGS	VNMGSRDLVS	GDSRSGSCSG	QGRDSSKTRV
**410**	**420**	**430**					
TKTIVEELVD	GKVVSSQVSS	ISEVKVK					

Cmpd.	No. of Cmpds.	m/z meas.	Δ m/z [ppm]	z	Rt [min]	Score	P	Range	Sequence	Modification	Ratios	
587	1	512.7149	-24.47	2	35.9	12.9	0	367-376	R.NSGSVNMGSR.D	Oxidation: 7	Q:W	0.39

Detailed Protein Report

Protein 138: PREDICTED: integrin beta-4 isoform X9 [Homo sapiens]

**Accession:**	gi|578830859	**Score:**	67.2
**Database:**	refseq_human(refseq_human_20140103.fasta)	**MW [kDa]:**	198.8
**Database Date:**	2015-11-30	**pI:**	5.8
**Modification(s):**	Carbamidomethyl, Oxidation	**Sequence Coverage [%]:**	2.3
		**No. of unique Peptides:**	3

Quantitation

Q:W	**Median:**	0.54	**CV:**	0.00 %	**No. of Peptides:**	1
M:Q	**Median:**	1.70	**CV:**	0.00 %	**No. of Peptides:**	1

Detailed Protein Report

10	20	30	40	50	60	70	80
MVVAVRNRAG	SEGLESPSWG	LREGQQEGQE	GRRKRMAGPR	PSPWARLLLA	ALISVSLSGT	LANRCKKAPV	KSCTECVRVD
**90**	**100**	**110**	**120**	**130**	**140**	**150**	**160**
KDCAYCTDEM	FRDRRCNTQA	ELLAAGCQRE	SIVVMESSFQ	ITEETQIDTT	LRRSQMSPQG	LRVRLRPGEE	RHFELEVFEP
**170**	**180**	**190**	**200**	**210**	**220**	**230**	**240**
LESPVDLYIL	MDFSNSMSDD	LDNLKKMGQN	LARVLSQLTS	DYTIGFGKFV	DKVSVPQTDM	RPEKLKEPWP	NSDPPFSFKN
**250**	**260**	**270**	**280**	**290**	**300**	**310**	**320**
VISLTEDVDE	FRNKLQGERI	SGNLDAPEGG	FDAILQTAVC	TRDIGWRPDS	THLLVFSTES	AFHYEADGAN	VLAGIMSRND
**330**	**340**	**350**	**360**	**370**	**380**	**390**	**400**
ERCHLDTTGT	YTQYRTQDYP	SVPTLVRLLA	KHNIIPIFAV	TNYSYSYYEK	LHTYFPVSSL	GVLQEDSSNI	VELLEEAFNR
**410**	**420**	**430**	**440**	**450**	**460**	**470**	**480**
IRSNLDIRAL	DSPRGLRTEV	TSKMFQKTRT	GSFHIRRGEV	GIYQVQLRAL	EHVDGTHVCQ	LPEDQKGNIH	LKPSFSDGLK
**490**	**500**	**510**	**520**	**530**	**540**	**550**	**560**
MDAGIICDVC	TCELQKEVRS	ARCSFNGDFV	CGQCVCSEGW	SGQTCNCSTG	SLSDIQPCLR	EGEDKPCSGR	GECQCGHCVC
**570**	**580**	**590**	**600**	**610**	**620**	**630**	**640**
YGEGRYEGQF	CEYDNFQCPR	TSGFLCNDRG	RCSMGQCVCE	PGWTGPSCDC	PLSNATCIDS	NGGICNGRGH	CECGRCHCHQ
**650**	**660**	**670**	**680**	**690**	**700**	**710**	**720**
QSLYTDTICE	INYSAIHPGL	CEDLRSCVQC	QAWGTGEKKG	RTCEECNFKV	KMVDELKRAE	EVVVRCSFRD	EDDDCTYSYT
**730**	**740**	**750**	**760**	**770**	**780**	**790**	**800**
MEGDGAPGPN	STVLVHKKKD	CPPGSFWWLI	PLLLLLLPLL	ALLLLLCWKY	CACCKACLAL	LPCCNRGHMV	GFKEDHYMLR
**810**	**820**	**830**	**840**	**850**	**860**	**870**	**880**
ENLMASDHLD	TPMLRSGNLK	GRDVVRWKVT	NNMQRPGFAT	HAASINPTEL	VPYGLSLRLA	RLCTENLLKP	DTRECAQLRQ
**890**	**900**	**910**	**920**	**930**	**940**	**950**	**960**
EVEENLNEVY	RQISGVHKLQ	QTKFRQQPNA	GKKQDHTIVD	TVLMAPRSAK	PALLKLTEKQ	VEQRAFHDLK	VAPGYYTLTA
**970**	**980**	**990**	**1000**	**1010**	**1020**	**1030**	**1040**
DQDARGMVEF	QEGVELVDVR	VPLFIRPEDD	DEKQLLVEAI	DVPAGTATLG	RRLVNITIIK	EQARDVVSFE	QPEFSVSRGD
**1050**	**1060**	**1070**	**1080**	**1090**	**1100**	**1110**	**1120**
QVARIPVIRR	VLDGGKSQVS	YRTQDGTAQG	NRDYIPVEGE	LLFQPGEAWK	ELQVKLLELQ	EVDSLLRGRQ	VRRFHVQLSN
**1130**	**1140**	**1150**	**1160**	**1170**	**1180**	**1190**	**1200**
PKFGAHLGQP	HSTTIIIRDP	DELDRSFTSQ	MLSSQPPPHG	DLGAPQNPNA	KAAGSRKIHF	NWLPPSGKPM	GYRVKYWIQG
**1210**	**1220**	**1230**	**1240**	**1250**	**1260**	**1270**	**1280**
DSESEAHLLD	SKVPSVELTN	LYPYCDYEMK	VCAYGAQGEG	PYSSLVSCRT	HQEVPSEPGR	LAFNVVSSTV	TQLSWAEPAE
**1290**	**1300**	**1310**	**1320**	**1330**	**1340**	**1350**	**1360**
TNGEITAYEV	CYGLVNDDNR	PIGPMKKVLV	DNPKNRMLLI	ENLRESQPYR	YTVKARNGAG	WGPEREAIIN	LATQPKRPMS
**1370**	**1380**	**1390**	**1400**	**1410**	**1420**	**1430**	**1440**
IPIIPDIPIV	DAQSGEDYDS	FLMYSDDVLR	SPSGSQRPSV	SDDTEHLVNG	RMDFAFPGST	NSLHRMTTTS	AAAYGTHLSP
**1450**	**1460**	**1470**	**1480**	**1490**	**1500**	**1510**	**1520**
HVPHRVLSTS	STLTRDYNSL	TRSEHSHSTT	LPRDYSTLTS	VSSHDSRLTA	GVPDTPTRLV	FSALGPTSLR	VSWQEPRCER
**1530**	**1540**	**1550**	**1560**	**1570**	**1580**	**1590**	**1600**
PLQGYSVEYQ	LLNGGELHRL	NIPNPAQTSV	VVEDLLPNHS	YVFRVRAQSQ	EGWGREREGV	ITIESQVHPQ	SPLCPLPGSA
**1610**	**1620**	**1630**	**1640**	**1650**	**1660**	**1670**	**1680**
FTLSTPSAPG	PLVFTALSPD	SLQLSWERPR	RPNGDIVGYL	VTCEMAQGGG	PATAFRVDGD	SPESRLTVPG	LSENVPYKFK
**1690**	**1700**	**1710**	**1720**	**1730**	**1740**	**1750**	**1760**
VQARTTEGFG	PEREGIITIE	SQDGGPFPQL	GSRAGLFQHP	LQSEYSSITT	THTSATEPFL	VDGLTLGAQH	LEAGGSLTRH
**1770**	**1780**	**1790**					
VTQEFVSRTL	TTSGTLSTHM	DQQFFQT					

Cmpd.	No. of Cmpds.	m/z meas.	Δ m/z [ppm]	z	Rt [min]	Score	P	Range	Sequence	Modification	Ratios
1596	1	480.7905	34.21	2	49.7	13.2	1	1-8	-.MVVAVRNR.A	Oxidation: 1	M:Q 1.70<br>Q:W 0.54
2157	1	841.3003	-27.93	2	57.2	16.5	1	82-94	K.DCAYCTDEMFRDR.R	Carbamidomethyl: 2	
522	1	678.9701	154.68	2	36.5	13.0	1	403-414	R.SNLDIRALDSPR.G		

Detailed Protein Report

Protein 139: NACHT, LRR and PYD domains-containing protein 2 isoform 3 [Homo sapiens]

Accession: gi|291463280
Database: refseq_human(refseq_human_20140103.fasta)
Database Date: 2015-11-30
Modification(s): Carbamidomethyl

Score: 67.2
MW [kDa]: 117.8
pI: 5.8
Sequence Coverage [%]: 5.6
No. of unique Peptides: 5

Quantitation

Q:W	**Median:**	1.10	**CV:**	21.14 %	**No. of Peptides:**	2
M:Q	**Median:**	1.01	**CV:**	0.00 %	**No. of Peptides:**	1

```
        10         20         30         40         50         60         70         80
MVSSAQMGFN LQALLEQLSQ DELSKFKYLI TTFSLAHELQ KIPHKEMASL QVFEKMHRMD LSERAKDEVR EAALKSFNKR
        90        100        110        120        130        140        150        160
KPLSLGITRK ERPPLDVDEM LERFKTEAQA FTETKGNVIC LGKEVFKGKK PDKDNRCRYI LKTKFREMWK SWPGDSKEVQ
       170        180        190        200        210        220        230        240
VMAERYKMLI PFSNPRVLPG PFSYTVVLYG PAGLGKTTLA QKLMLDWAED NLIHKFKYAF YLSCRELSRL GPCSFAELVF
       250        260        270        280        290        300        310        320
RDWPELQDDI PHILAQARKI LFVIDGFDEL GAAPGALIED ICGDWEKKKP VPVLLGSLLN RVMLPKAALL VTTRPRALRD
       330        340        350        360        370        380        390        400
LRILAEEPIY IRVEGFLEED RRAYFLRHFG DEDQAMRAFE LMRSNAALFQ LGSAPAVCWI VCTTLKLQME KGEDPVPTCL
       410        420        430        440        450        460        470        480
TRTGLFLRFL CSRFPQGAQL RGALRTLSLL AAQGLWAQTS VLHREDLERL GVQESDLRLF LDGDILRQDR VSKGCYSFIH
       490        500        510        520        530        540        550        560
LSFQQFLTAL FYTLEKEEEE DRDGHTWDIG DVQKLLSGVE RLRNPDLIQA GYYSFGLANE KRAKELEATF GCRMSPDIKQ
       570        580        590        600        610        620        630        640
ELLRCDISCK GGHSTVTDLQ ELLGCLYESQ EEELVKEVMA QFKEISLHLN AVDVVPSSFC VKHCRNLQKM SLQVIKENLP
       650        660        670        680        690        700        710        720
ENVTASESDA EVERSQDDQH MLPFWTDLCS IFGSNKDLMG LAINDSFLSA SLVRILCEQI ASDTCHLQRV VFKNISPADA
       730        740        750        760        770        780        790        800
HRNLCLALRG HKTVTYLTLQ GNDQDDMFPA LCEVLRHPEC NLRYLGLVSC SATTQQWADL SLALEVNQSL TCVNLSDNEL
       810        820        830        840        850        860        870        880
LDEGAKLLYT TLRHPKCFLQ RLSLENCHLT EANCKDLAAV LVVSRELTHL CLAKNPIGNT GVKFLCEGLR YPECKLQTLV
       890        900        910        920        930        940        950        960
LWNCDITSDG CCDLTKLLQE KSSLLCLDLG LNHIGVKGMK FLCEALRKPL CNLRCLWLWG CSIPPFSCED LCSALSCNQS
       970        980        990       1000       1010       1020       1030       1040
LVTLDLGQNP LGSSGVKMLF ETLTCSSGTL RTLRLKIDDF NDELNKLLEE IEEKNPQLII DTEKHHPWAE RPSSHDFMI
```

Cmpd.	No. of Cmpds.	m/z meas.	Δ m/z [ppm]	z	Rt [min]	Score	P	Range	Sequence	Modification	Ratios
1922	1	893.8122	-134.43	2	53.9	11.2	0	695-709	R.ILCEQIASDTCHLQR.V	Carbamidomethyl: 3	
1358	1	727.3750	-33.15	2	45.7	10.3	1	710-722	R.VVFKNISPADAHR.N		Q:W 1.36<br>M:Q 1.01
223	1	636.6401	-68.84	3	32.8	23.9	1	846-863	R.ELTHLCLAKNPIGNTGVK.F		
1270	1	419.0769	-336.69	2	44.6	11.0	0	864-870	K.FLCEGLR.Y		Q:W 0.89
1197	1	786.4909	152.63	2	44.6	10.8	1	864-875	K.FLCEGLRYPECK.L	Carbamidomethyl: 3, 11	

Detailed Protein Report

Protein 140: phosphatidylinositol 3,4,5-trisphosphate 5-phosphatase 1 isoform b [Homo sapiens]

Accession: gi|40254823
Database: refseq_human(refseq_human_20140103.fasta)
Database Date: 2015-11-30
Modification(s): Oxidation

Score: 67.1
MW [kDa]: 133.1
pI: 7.9
Sequence Coverage [%]: 5.2
No. of unique Peptides: 3

Quantitation

Q:W **Median:** 1.12 **CV:** 0.00 % **No. of Peptides:** 1

10	20	30	40	50	60	70	80
MVPCWNHGNI	TRSKAEELLS	RTGKDGSFLV	RASESISRAY	ALCVLYRNCV	YTYRILPNED	DKFTVQASEG	VSMRFFTKLD
**90**	**100**	**110**	**120**	**130**	**140**	**150**	**160**
QLIEFYKKEN	MGLVTHLQYP	VPLEEEDTGD	DPEEDTESVV	SPPELPPRNI	PLTASSCEAK	EVPFSNENPR	ATETSRPSLS
**170**	**180**	**190**	**200**	**210**	**220**	**230**	**240**
ETLFQRLQSM	DTSGLPEEHL	KAIQDYLSTQ	LAQDSEFVKT	GSSSLPHLKK	LTTLLCKELY	GEVIRTLPSL	ESLQRLFDQQ
**250**	**260**	**270**	**280**	**290**	**300**	**310**	**320**
LSPGLRPRPQ	VPGEANPINM	VSKLSQLTSL	LSSIEDKVKA	LLHEGPESPH	RPSLIPPVTF	EVKAESLGIP	QKMQLKVDVE
**330**	**340**	**350**	**360**	**370**	**380**	**390**	**400**
SGKLIIKKSK	DGSEDKFYSH	KKILQLIKSQ	KFLNKLVILV	ETEKEKILRK	EYVFADSKKR	EGFCQLLQQM	KNKHSEQPEP
**410**	**420**	**430**	**440**	**450**	**460**	**470**	**480**
DMITIFIGTW	NMGNAPPPKK	ITSWFLSKGQ	GKTRDDSADY	IPHDIYVIGT	QEDPLSEKEW	LEILKHSLQE	ITSVTFKTVA
**490**	**500**	**510**	**520**	**530**	**540**	**550**	**560**
IHTLWNIRIV	VLAKPEHENR	ISHICTDNVK	TGIANTLGNK	GAVGVSFMFN	GTSLGFVNSH	LTSGSEKKLR	RNQNYMNILR
**570**	**580**	**590**	**600**	**610**	**620**	**630**	**640**
FLALGDKKLS	PFNITHRFTH	LFWFGDLNYR	VDLPTWEAET	IIQKIKQQQY	ADLLSHDQLL	TERREQKVFL	HFEEEEITFA
**650**	**660**	**670**	**680**	**690**	**700**	**710**	**720**
PTYRFERLTR	DKYAYTKQKA	TGMKYNLPSW	CDRVLWKSYP	LVHVVCQSYG	STSDIMTSDH	SPVFATFEAG	VTSQFVSKNG
**730**	**740**	**750**	**760**	**770**	**780**	**790**	**800**
PGTVDSQGQI	EFLRCYATLK	TKSQTKFYLE	FHSSCLESFV	KSQEGENEEG	SEGELVVKFG	ETLPKLKPII	SDPEYLLDQH
**810**	**820**	**830**	**840**	**850**	**860**	**870**	**880**
ILISIKSSDS	DESYGEGCIA	LRLEATETQL	PIYTPLTHHG	ELTGHFQGEI	KLQTSQGKTR	EKLYDFVKTE	RDESSGPKTL
**890**	**900**	**910**	**920**	**930**	**940**	**950**	**960**
KSLTSHDPMK	QWEVTSRAPP	CSGSSITEII	NPNYMGVGPF	GPPMPLHVKQ	TLSPDQQPTA	WSYDQPPKDS	PLGPCRGESP
**970**	**980**	**990**	**1000**	**1010**	**1020**	**1030**	**1040**
PTPPGQPPIS	PKKFLPSTAN	RGLPPRTQES	RPSDLGKNAG	DTLPQEDLPL	TKPEMFENPL	YGSLSSFPKP	APRKDQESPK
**1050**	**1060**	**1070**	**1080**	**1090**	**1100**	**1110**	**1120**
MPRKEPPPCP	EPGILSPSIV	LTKAQEADRG	EGPGKQVPAP	RLRSFTCSSS	AEGRAAGGDK	SQGKPKTPVS	SQAPVPAKRP
**1130**	**1140**	**1150**	**1160**	**1170**	**1180**	**1190**	
IKPSRSEINQ	QTPPTPTPRP	PLPVKSPAVL	HLQHSKGRDY	RDNTELPHHG	KHRPEEGPPG	PLGRTAMQ	

Cmpd.	No. of Cmpds.	m/z meas.	Δ m/z [ppm]	z	Rt [min]	Score	P	Range	Sequence	Modification	Ratios
499	3	584.8345	31.59	2	35.6	27.2	0	79-87	K.LDQLIEFYK.K		Q:W 1.12
234	1	850.8764	-41.35	2	32.6	12.8	0	167-181	R.LQSMDTSGLPEEHLK.A	Oxidation: 4	
1543	1	1008.6543	64.47	2	49.1	16.6	1	1107-1125	K.TPVSSQAPVPAKRPIKPSR.S		

Detailed Protein Report

Protein 141: ryanodine receptor 1 isoform 2 [Homo sapiens]

**Accession:**	gi|113204617	**Score:**	66.9
**Database:**	refseq_human(refseq_human_20140103.fasta)	**MW [kDa]:**	564.3
**Database Date:**	2015-11-30	**pI:**	5.1
**Modification(s):**	Carbamidomethyl, Oxidation	**Sequence Coverage [%]:**	1.5
		**No. of unique Peptides:**	4

10	20	30	40	50	60	70	80
MGDAEGEDEV	QFLRTDDEVV	LQCSATVLKE	QLKLCLAAEG	FGNRLCFLEP	TSNAQNVPPD	LAICCFVLEQ	SLSVRALQEM
90	100	110	120	130	140	150	160
LANTVEAGVE	SSQGGGHRTL	LYGHAILLRH	AHSRMYLSCL	TTSRSMTDKL	AFDVGLQEDA	TGEACWWTMH	PASKQRSEGE
170	180	190	200	210	220	230	240
KVRVGDDIIL	VSVSSERYLH	LSTASGELQV	DASFMQTLWN	MNPICSRCEE	GFVTGGHVLR	LFHGHMDECL	TISPADSDDQ
250	260	270	280	290	300	310	320
RRLVYYEGGA	VCTHARSLWR	LEPLRISWSG	SHLRWGQPLR	VRHVTTGQYL	ALTEDQGLVV	VDASKAHTKA	TSFCFRISKE
330	340	350	360	370	380	390	400
KLDVAPKRDV	EGMGPPEIKY	GESLCFVQHV	ASGLWLTYAA	PDPKALRLGV	LKKKAMLHQE	GHMDDALSLT	RCQQEESQAA
410	420	430	440	450	460	470	480
RMIHSTNGLY	NQFIKSLDSF	SGKPRGSGPP	AGTALPIEGV	ILSLQDLIIY	FEPPSEDLQH	EEKQSKLRSL	RNRQSLFQEE
490	500	510	520	530	540	550	560
GMLSMVLNCI	DRLNVYTTAA	HFAEFAGEEA	AESWKEIVNL	LYELLASLIR	GNRSNCALFS	TNLDWLVSKL	DRLEASSGIL
570	580	590	600	610	620	630	640
EVLYCVLIES	PEVLNIIQEN	HIKSIISLLD	KHGRNHKVLD	VLCSLCVCNG	VAVRSNQDLI	TENLLPGREL	LLQTNLINYV
650	660	670	680	690	700	710	720
TSIRPNIFVG	RAEGTTQYSK	WYFEVMVDEV	TPFLTAQATH	LRVGWALTEG	YTPYPGAGEG	WGGNGVGDDL	YSYGFDGLHL
730	740	750	760	770	780	790	800
WTGHVARPVT	SPGQHLLAPE	DVISCCLDLS	VPSISFRING	CPVQGVFESF	NLDGLFFPVV	SFSAGVKVRF	LLGGRHGEFK
810	820	830	840	850	860	870	880
FLPPPGYAPC	HEAVLPRERL	HLEPIKEYRR	EGPRGPHLVG	PSRCLSHTDF	VPCPVDTVQI	VLPPHLERIR	EKLAENIHEL
890	900	910	920	930	940	950	960
WALTRIEQGW	TYGPVRDDNK	RLHPCLVDFH	SLPEPERNYN	LQMSGETLKT	LLALGCHVGM	ADEKAEDNLK	KTKLPKTYMM
970	980	990	1000	1010	1020	1030	1040
SNGYKPAPLD	LSHVRLTPAQ	TTLVDRLAEN	GHNVWARDRV	GQGWSYSAVQ	DIPARRNPRL	VPYRLLDEAT	KRSNRDSLCQ
1050	1060	1070	1080	1090	1100	1110	1120
AVRTLLGYGY	NIEPPDQEPS	QVENQSRCDR	VRIFRAEKSY	TVQSGRWYFE	FEAVTTGEMR	VGWARPELRP	DVELGADELA
1130	1140	1150	1160	1170	1180	1190	1200
YVFNGHRGQR	WHLGSEPFGR	PWQPGDVVGC	MIDLTENTII	FTLNGEVLMS	DSGSETAFRE	IEIGDGFLPV	CSLGPGQVGH
1210	1220	1230	1240	1250	1260	1270	1280
LNLGQDVSSL	RFFAICGLQE	GFEPFAINMQ	RPVTTWFSKG	LPQFEPVPLE	HPHYEVSRVD	GTVDTPPCLR	LTHRTWGSQN
1290	1300	1310	1320	1330	1340	1350	1360
SLVEMLFLRL	SLPVQFHQHF	RCTAGATPLA	PPGLQPPAED	EARAAEPDPD	YENLRRSAGG	WSEAENGKEG	TAKEGAPGGT
1370	1380	1390	1400	1410	1420	1430	1440
PQAGGEAQPA	RAENEKDATT	EKNKKRGFLF	KAKKVAMMTQ	PPATPTLPRL	PHDVVPADNR	DDPEIILNTT	TYYYSVRVFA
1450	1460	1470	1480	1490	1500	1510	1520
GQEPSCVWAG	WVTPDYHQHD	MSFDLSKVRV	VTVTMGDEQG	NVHSSLKCSN	CYMVWGGDFV	SPGQQGRISH	TDLVIGCLVD
1530	1540	1550	1560	1570	1580	1590	1600
LATGLMTFTA	NGKESNTFFQ	VEPNTKLFPA	VFVLPTHQNV	IQFELGKQKN	IMPLSAAMFQ	SERKNPAPQC	PPRLEMQMLM
1610	1620	1630	1640	1650	1660	1670	1680
PVSWSRMPNH	FLQVETRRAG	ERLGWAVQCQ	EPLTMMALHI	PEENRCMDIL	ELSERLDLQR	FHSHTLRLYR	AVCALGNNRV
1690	1700	1710	1720	1730	1740	1750	1760
AHALCSHVDQ	AQLLHALEDA	HLPGPLRAGY	YDLLISIHLE	SACRSRRSML	SEYIVPLTPE	TRAITLFPPG	RSTENGHPRH
1770	1780	1790	1800	1810	1820	1830	1840
GLPGVGVTTS	LRPPHHFSPP	CFVAALPAAG	AAEAPARLSP	AIPLEALRDK	ALRMLGEAVR	DGGQHARDPV	GGSVEFQFVP
1850	1860	1870	1880	1890	1900	1910	1920
VLKLVSTLLV	MGIFGDEDVK	QILKMIEPEV	FTEEEEEEDE	EEEGEEEDEE	EKEEDEEETA	QEKEDEEKEE	EEAAEGEKEE
1930	1940	1950	1960	1970	1980	1990	2000
GLEEGLLQMK	LPESVKLQMC	HLLEYFCDQE	LQHRVESLAA	FAERYVDKLQ	ANQRSRYGLL	IKAFSMTAAE	TARRTREFRS
2010	2020	2030	2040	2050	2060	2070	2080
PPQEQINMLL	QFKDGTDEED	CPLPEEIRQD	LLDFHQDLLA	HCGIQLDGEE	EEPEEETTLG	SRLMSLLEKV	RLVKKKEEKP
2090	2100	2110	2120	2130	2140	2150	2160
EEERSAEESK	PRSLQELVSH	MVVRWAQEDF	VQSPELVRAM	FSLLHRQYDG	LGELLRALPR	AYTISPSSVE	DTMSLLECLG
2170	2180	2190	2200	2210	2220	2230	2240

Detailed Protein Report

Cmpd.	No. of Cmpds.	m/z meas.	Δ m/z [ppm]	z	Rt [min]	Score	P	Range	Sequence	Modification
2169	2	714.6084	-134.05	3	57.5	14.8	1	595-614	R.NHKVLDVLCSLCVCNGVAVR.S	
2128	1	1051.4436	-85.59	2	55.2	12.7	1	901-917	K.RLHPCLVDFHSLPEPER.N	Carbamidomethyl: 5
1512	1	727.4065	108.16	2	49.1	11.1	0	2601-2612	R.DVIEDCLMSLCR.Y	Carbamidomethyl: 11
2682	1	705.8622	37.53	2	64.6	12.1	2	3478-3490	K.MAKSGGSDQERTK.K	Oxidation: 1

Detailed Protein Report

Protein 142: zinc finger protein 622 [Homo sapiens]

Accession: gi|15529978
Database: refseq_human(refseq_human_20140103.fasta)
Database Date: 2015-11-30
Modification(s): Carbamidomethyl

Score: 66.9
MW [kDa]: 54.2
pI: 5.8
Sequence Coverage [%]: 14.3
No. of unique Peptides: 4

10	20	30	40	50	60	70	80
MATYTCITCR	VAFRDADMQR	AHYKTDWHRY	NLRRKVASMA	PVTAEGFQER	VRAQRAVAEE	ESKGSATYCT	VCSKKFASFN
**90**	**100**	**110**	**120**	**130**	**140**	**150**	**160**
AYENHLKSRR	HVELEKKAVQ	AVNRKVEMMN	EKNLEKGLGV	DSVDKDAMNA	AIQQAIKAQP	SMSPKKAPPA	PAKEARNVVA
**170**	**180**	**190**	**200**	**210**	**220**	**230**	**240**
VGTGGRGTHD	RDPSEKPPRL	QWFEQQAKKL	AKQQEEDSEE	EEEDLDGDDW	EDIDSDEELE	CEDTEAMDDV	VEQDAEEEEA
**250**	**260**	**270**	**280**	**290**	**300**	**310**	**320**
EEGPPLGAIP	ITDCLFCSHH	SSSLMKNVAH	MTKDHSFFIP	DIEYLSDIKG	LIKYLGEKVG	VGKICLWCNE	KGKSFYSTEA
**330**	**340**	**350**	**360**	**370**	**380**	**390**	**400**
VQAHMNDKSH	CKLFTDGDAA	LEFADFYDFR	SSYPDHKEGE	DPNKAEELPS	EKNLEYDDET	MELILPSGAR	VGHRSLMRYY
**410**	**420**	**430**	**440**	**450**	**460**	**470**	**480**
KQRFGLSRAV	AVAKNRKAVG	RVLQQYRALG	WTGSTGAALM	RERDMQYVQR	MKSKWMLKTG	MKNNATKQMH	FRVQVRF

Cmpd.	No. of Cmpds.	m/z meas.	Δ m/z [ppm]	z	Rt [min]	Score	P	Range	Sequence	Modification
2592	1	822.7237	16.97	3	62.4	10.6	2	1-20	-.MATYTCITCRVAFRDADMQR.A	Carbamidomethyl: 6, 9
1757	1	560.2452	1.92	2	52.3	12.6	0	64-74	K.GSATYCTVCSK.K	
2237	1	753.3462	-49.90	2	56.3	10.4	1	299-311	K.VGVGKICLWCNEK.G	Carbamidomethyl: 7
1586	1	480.6461	-220.76	2	49.5	14.0	1	395-401	R.SLMRYYK.Q	

Detailed Protein Report

Protein 143: zinc finger protein 281 isoform 1 [Homo sapiens]

**Accession:**	gi|6912752	**Score:**	66.8
**Database:**	refseq_human(refseq_human_20140103.fasta)	**MW [kDa]:**	96.9
**Database Date:**	2015-11-30	**pI:**	9.5
**Modification(s):**	Carbamidomethyl	**Sequence Coverage [%]:**	2.5
		**No. of unique Peptides:**	2

Alias proteins:

Accession	Name	Description
gi|526253064	refseq_human (refseq_human_20140103.fasta)	zinc finger protein 281 isoform 1 [Homo sapiens]

10	20	30	40	50	60	70	80
MKIGSGFLSG	GGGTGSSGGS	GSGGGGSGGG	GGGGSSGRRA	EMEPTFPQGM	VMFNHRLPPV	TSFTRPAGSA	APPPQCVLSS
**90**	**100**	**110**	**120**	**130**	**140**	**150**	**160**
STSAAPAAEP	PPPPAPDMTF	KKEPAASAAA	FPSQRTSWGF	LQSLVSIKQE	KPADPEEQQS	HHHHHHHHYG	GLFAGAEERS
**170**	**180**	**190**	**200**	**210**	**220**	**230**	**240**
PGLGGGEGGS	HGVIQDLSIL	HQHVQQQPAQ	HHRDVLLSSS	SRTDDHHGTE	EPKQDTNVKK	AKRPKPESQG	IKAKRKPSAS
**250**	**260**	**270**	**280**	**290**	**300**	**310**	**320**
SKPSLVGDGE	GAILSPSQKP	HICDHCSAAF	RSSYHLRRHV	LIHTGERPFQ	CSQCSMGFIQ	KYLLQRHEKI	HSREKPFGCD
**330**	**340**	**350**	**360**	**370**	**380**	**390**	**400**
QCSMKFIQKY	HMERHKRTHS	GEKPYKCDTC	QQYFSRTDRL	LKHRRTCGEV	IVKGATSAEP	GSSNHTNMGN	LAVLSQGNTS
**410**	**420**	**430**	**440**	**450**	**460**	**470**	**480**
SSRRKTKSKS	IAIENKEQKT	GKTNESQISN	NINMQSYSVE	MPTVSSSGGI	IGTGIDELQK	RVPKLIFKKG	SRKNTDKNYL
**490**	**500**	**510**	**520**	**530**	**540**	**550**	**560**
NFVSPLPDIV	GQKSLSGKPS	GSLGIVSNNS	VETIGLLQST	SGKQGQISSN	YDDAMQFSKK	RRYLPTASSN	SAFSINVGHM
**570**	**580**	**590**	**600**	**610**	**620**	**630**	**640**
VSQQSVIQSA	GVSVLDNEAP	LSLIDSSALN	AEIKSCHDKS	GIPDEVLQSI	LDQYSNKSES	QKEDPFNIAE	PRVDLHTSGE
**650**	**660**	**670**	**680**	**690**	**700**	**710**	**720**
HSELVQEENL	SPGTQTPSND	KASMLQEYSK	YLQQAFEKST	NASFTLGHGF	QFVSLSSPLH	NHTLFPEKQI	YTTSPLECGF
**730**	**740**	**750**	**760**	**770**	**780**	**790**	**800**
GQSVTSVLPS	SLPKPPFGML	FGSQPGLYLS	ALDATHQQLT	PSQELDDLID	SQKNLETSSA	FQSSSQKLTS	QKEQKNLESS
**810**	**820**	**830**	**840**	**850**	**860**	**870**	**880**
TGFQIPSQEL	ASQIDPQKDI	EPRTTYQIEN	FAQAFGSQFK	SGSRVPMTFI	TNSNGEVDHR	VRTSVSDFSG	YTNMMSDVSE
**890**	**900**						
PCSTRVKTPT	SQSYR						

Cmpd.	No. of Cmpds.	m/z meas.	Δ m/z [ppm]	z	Rt [min]	Score	P	Range	Sequence	Modification
2793	1	840.4111	74.23	2	65.3	14.1	1	347-359	K.CDTCQQYFSRTDR.L	Carbamidomethyl: 4
2653	1	811.8976	73.50	2	64.2	24.6	1	347-359	K.CDTCQQYFSRTDR.L	

Detailed Protein Report

Protein 144: PREDICTED: histone-lysine N-methyltransferase ASH1L isoform X2 [Homo sapiens]

**Accession:**	gi|530365070	**Score:**	66.6
**Database:**	refseq_human(refseq_human_20140103.fasta)	**MW [kDa]:**	296.8
**Database Date:**	2015-11-30	**pI:**	10.2
**Modification(s):**	Carbamidomethyl	**Sequence Coverage [%]:**	3.0
		**No. of unique Peptides:**	4

Detailed Protein Report

10	20	30	40	50	60	70	80
MDPRNTAMLG	LGSDSEGFSR	KSPSAISTGT	LVSKREVELE	KNTKEEEDLR	KRNRERNIEA	GKDDGLTDAQ	QQFSVKETNF
90	100	110	120	130	140	150	160
SEGNLKLKIG	LQAKRTKKPP	KNLENYVCRP	AIKTTIKHPR	KALKSGKMTD	EKNEHCPSKR	DPSKLYKKAD	DVAAIECQSE
170	180	190	200	210	220	230	240
EVIRLHSQGE	NNPLSKKLSP	VHSEMADYIN	ATPSTLLGSR	DPDLKDRALL	NGGTSVTEKL	AQLIATCPPS	KSSKTKPKKL
250	260	270	280	290	300	310	320
GTGTTAGLVS	KDLIRKAGVG	SVAGIIHKDL	IKKPTISTAV	GLVTKDPGKK	PVFNAAVGLV	NKDSVKKLGT	GTTAVFINKN
330	340	350	360	370	380	390	400
LGKKPGTITT	VGLLSKDSGK	KLGIGIVPGL	VHKESGKKLG	LGTVVGLVNK	DLGKKLGSTV	GLVAKDCAKK	IVASSAMGLV
410	420	430	440	450	460	470	480
NKDIGKKLMS	CPLAGLISKD	AINLKAEALL	PTQEPLKASC	STNINNQESQ	ELSESLKDSA	TSKTFEKNVV	RQNKESILEK
490	500	510	520	530	540	550	560
FSVRKEIINL	EKEMFNEGTC	IQQDSFSSSE	KGSYETSKHE	KQPPVYCTSP	DFKMGGASDV	STAKSPFSAV	GESNLPSPSP
570	580	590	600	610	620	630	640
TVSVNPLTRS	PPETSSQLAP	NPLLLSSTTE	LIEEISESVG	KNQFTSESTH	LNVGHRSVGH	SISIECKGID	KEVNDSKTTH
650	660	670	680	690	700	710	720
IDIPRISSSL	GKKPSLTSES	SIHTITPSVV	NFTSLFSNKP	FLKLGAVSAS	DKHCQVAESL	STSLQSKPLK	KRKGRKPRWT
730	740	750	760	770	780	790	800
KVVARSTCRS	PKGLELERSE	LFKNVSCSSL	SNSNSEPAKF	MKNIGPPSFV	DHDFLKRRLP	KLSKSTAPSL	ALLADSEKPS
810	820	830	840	850	860	870	880
HKSFATHKLS	SSMCVSSDLL	SDIYKPKRGR	PKSKEMPQLE	GPPKRTLKIP	ASKVFSLQSK	EEQEPPILQP	EIEIPSFKQG
890	900	910	920	930	940	950	960
LSVSPFPKKR	GRPKRQMRSP	VKMKPPVLSV	APFVATESPS	KLESESDNHR	SSSDFFESED	QLQDPDDLDD	SHRPSVCSMS
970	980	990	1000	1010	1020	1030	1040
DLEMEPDKKI	TKRNNGQLMK	TIIRKINKMK	TLKRKKLLNQ	ILSSSVESSN	KGKVQSKLHN	TVSSLAATFG	SKLGQQINVS
1050	1060	1070	1080	1090	1100	1110	1120
KKGTIYIGKR	RGRKPKTVLN	GILSGSPTSL	AVLEQTAQQA	AGSALGQILP	PLLPSSASSS	EILPSPICSQ	SSGTSGGQSP
1130	1140	1150	1160	1170	1180	1190	1200
VSSDAGFVEP	SSVPYLHLHS	RQGSMIQTLA	MKKASKGRRR	LSPPTLLPNS	PSHLSELTSL	KEATPSPISE	SHSDETIPSD
1210	1220	1230	1240	1250	1260	1270	1280
SGIGTDNNST	SDRAEKFCGQ	KKRRHSFEHV	SLIPPETSTV	LSSLKEKHKH	KCKRRNHDYL	SYDKMKRQKR	KRKKKYPQLR
1290	1300	1310	1320	1330	1340	1350	1360
NRQDPDFIAE	LEELISRLSE	IRITHRSHHF	IPRDLLPTIF	RINFNSFYTH	PSFPLDPLHY	IRKPDLKKKR	GRPPKMREAM
1370	1380	1390	1400	1410	1420	1430	1440
AEMPFMHSLS	FPLSSTGFYP	SYGMPYSPSP	LTAAPIGLGY	YGRYPPTLYP	PPPSPSFTTP	LPPPSYMHAG	HLLLNPAKYH
1450	1460	1470	1480	1490	1500	1510	1520
KKKHKLLRQE	AFLTTSRTPL	LSMSTYPSVP	PEMAYGWMVE	HKHRHRHKHR	EHRSSEQPQV	SMDTGSSRSV	LESLKRYRFG
1530	1540	1550	1560	1570	1580	1590	1600
KDAVGERYKH	KEKHRCHMSC	PHLSPSKSLI	NREEQWVHRE	PSESSPLALG	LQTPLQIDCS	ESSPSLSLGG	FTPNSEPASS
1610	1620	1630	1640	1650	1660	1670	1680
DEHTNLFTSA	IGSCRVSNPN	SSGRKKLTDS	PGLFSAQDTS	LNRLHRKESL	PSNERAVQTL	AGSQPTSDKP	SQRPSESTNC
1690	1700	1710	1720	1730	1740	1750	1760
SPTRKRSSSE	STSSTVNGVP	SRSPRLVASG	DDSVDSLLQR	MVQNEDQEPM	EKSIDAVIAT	ASAPPSSSPG	RSHSKDRTLG
1770	1780	1790	1800	1810	1820	1830	1840
KPDSLLVPAV	TSDSCNNSIS	LLSEKLTSSC	SPHHIKRSVV	EAMQRQARKM	CNYDKILATK	KNLDHVNKIL	KAKKLQRQAR
1850	1860	1870	1880	1890	1900	1910	1920
TGNNFVKRRP	GRPRKCPLQA	VVSMQAFQAA	QFVNPELNRD	EEGAALHLSP	DTVTDVIEAV	VQSVNLNPEH	KKGLKRKGWL
1930	1940	1950	1960	1970	1980	1990	2000
LEEQTRKKQK	PLPEEEEQEN	NKSFNEAPVE	IPSPSETPAK	PSEPESTLQP	VLSLIPREKK	PPRPPKKKYQ	KAGLYSDVYK
2010	2020	2030	2040	2050	2060	2070	2080
TTDPKSRLIQ	LKKEKLEYTP	GEHEYGLFPA	PIHVGKYLRQ	KRIDFQLPYD	ILWQWKHNQL	YKKPDVPLYK	KIRSNVYVDV
2090	2100	2110	2120	2130	2140	2150	2160
KPLSGYEATT	CNCKKPDDDT	RKGCVDDCLN	RMIFAECSPN	TCPCGEQCCN	QRIQRHEWVQ	CLERFRAEEK	GWGIRTKEPL
2170	2180	2190	2200	2210	2220	2230	2240

Detailed Protein Report

Cmpd.	No. of Cmpds.	m/z meas.	Δ m/z [ppm]	z	Rt [min]	Score	P	Range	Sequence	Modification
1736	1	853.3885	-153.79	2	50.1	10.2	2	256-272	R.KAGVGSVAGIIHKDLIK.K	
2429	1	856.6272	147.06	2	60.7	15.4	1	273-289	K.KPTISTAVGLVTKDPGK.K	
1687	1	696.2420	-115.78	3	49.4	11.7	1	744-762	K.NVSCSSLSNSNSEPAKFMK.N	Carbamidomethyl: 4
1905	1	673.3043	-162.57	2	54.2	17.5	0	2460-2472	R.QALAAPLLNLPPK.K	

Detailed Protein Report

Protein 145: PREDICTED: zinc finger protein 845 isoform X3 [Homo sapiens]

**Accession:**	gi|578835119	**Score:**	66.6
**Database:**	refseq_human(refseq_human_20140103.fasta)	**MW [kDa]:**	89.9
**Database Date:**	2015-11-30	**pI:**	10.5
**Modification(s):**	Carbamidomethyl, Oxidation	**Sequence Coverage [%]:**	10.1
		**No. of unique Peptides:**	2

10	20	30	40	50	60	70	80
MALSQGLLTF	RDVAIEFSQE	EWKCLDPAQR	TLYRDVMLEN	YRNLVSLDIS	SKCMMKEFSS	TAQGNTEVIH	TGTLQRHERH
**90**	**100**	**110**	**120**	**130**	**140**	**150**	**160**
HIGDFCFQEM	EKDIHDFEFQ	WKEDERNSHE	APMTEIKQLT	GSTNRHDQRH	AGNKPIKDQL	GSSFHSHLPE	LHMFQTEGKI
**170**	**180**	**190**	**200**	**210**	**220**	**230**	**240**
GNQVEKSINS	ASLVSTSQRI	SCRPKTHISK	NYGNNFLNSS	LLTQKQEVHM	REKSFQCNES	GKAFNYSSVL	RKHQIIHLGA
**250**	**260**	**270**	**280**	**290**	**300**	**310**	**320**
KQYKCDVCGK	VFNQKRYLAC	HRRCHTGKKP	YKCNDCGKTF	SQELTLTCHH	RLHTGEKHYK	CSECGKTFSR	NSALVIHKAI
**330**	**340**	**350**	**360**	**370**	**380**	**390**	**400**
HTGEKSYKCN	ECGKTFSQTS	YLVYHRRLHT	GEKPYKCEEC	DKAFSFKSNL	ERHRKIHTGE	KPYKCNECSR	TFSRKSSLTR
**410**	**420**	**430**	**440**	**450**	**460**	**470**	**480**
HRRLHTGEKP	YKCNDCGKTF	SQMSSLVYHR	RLHTGEKPYK	CEECDEAFSF	KSNLQRHRRI	HTGEKPYRCN	ECGKTFSRKS
**490**	**500**	**510**	**520**	**530**	**540**	**550**	**560**
YLTCHRRLHT	GEKPYKCNEC	GKTFGRNSAL	IIHKAIHTGE	KPYKCNECGK	AFSQKSSLTC	HLRLHTGEKP	YKCEECDKVF
**570**	**580**	**590**	**600**	**610**	**620**	**630**	**640**
SRKSSLEKHR	RIHTGEKPYK	CKVCDKAFGR	DSHLAQHTRI	HTGEKPYKCN	ECGKNFRHNS	ALVIHKAIHS	GEKPYKCNEC
**650**	**660**	**670**	**680**	**690**	**700**	**710**	**720**
GKTFRHNSAL	EIHKAIHTGE	KPYKCSECGK	VFNRKANLSR	HHRLHTGEKP	YKCNKCGKVF	NQQAHLACHH	RIHTGEKPYK
**730**	**740**	**750**	**760**	**770**	**780**		
CNECGKTFRH	NSVLVIHKTI	HTGEKPYKCN	ECGKVFNRKA	KLARHHRIHT	GKKH		

Cmpd.	No. of Cmpds.	m/z meas.	Δ m/z [ppm]	z	Rt [min]	Score	P	Range	Sequence	Modification
2753	1	920.1206	31.91	3	65.6	17.8	1	53-76	K.CMMKEFSSTAQGNTEVIHTGTLQR.H	Carbamidomethyl: 1; Oxidation: 2, 3
1972	1	665.2897	-23.77	2	54.8	11.9	2	469-479	R.CNECGKTFSRK.S	Carbamidomethyl: 4

Detailed Protein Report

Protein 146: spectrin beta chain, non-erythrocytic 1 isoform 2 [Homo sapiens]

**Accession:**	gi|112382252	**Score:**	66.5
**Database:**	refseq_human(refseq_human_20140103.fasta)	**MW [kDa]:**	251.2
**Database Date:**	2015-11-30	**pI:**	5.3
		**Sequence Coverage [%]:**	2.5
		**No. of unique Peptides:**	3

Quantitation

Q:W	**Median:**	0.47	**CV:**	0.00 %	**No. of Peptides:**	1
M:Q	**Median:**	1.09	**CV:**	27.85 %	**No. of Peptides:**	2

Detailed Protein Report

10	20	30	40	50	60	70	80
MELQRTSSIS	GPLSPAYTGQ	VPYNYNQLEG	RFKQLQDERE	AVQKKTFTKW	VNSHLARVSC	RITDLYTDLR	DGRMLIKLLE
90	100	110	120	130	140	150	160
VLSGERLPKP	TKGRMRIHCL	ENVDKALQFL	KEQRVHLENM	GSHDIVDGNH	RLTLGLIWTI	ILRFQIQDIS	VETEDNKEKK
170	180	190	200	210	220	230	240
SAKDALLLWC	QMKTAGYPNV	NIHNFTTSWR	DGMAFNALIH	KHRPDLIDFD	KLKKSNAHYN	LQNAFNLAEQ	HLGLTKLLDP
250	260	270	280	290	300	310	320
EDISVDHPDE	KSIITYVVTY	YHYFSKMKAL	AVEGKRIGKV	LDNAIETEKM	IEKYESLASD	LLEWIEQTII	ILNNRKFANS
330	340	350	360	370	380	390	400
LVGVQQQLQA	FNTYRTVEKP	PKFTEKGNLE	VLLFTIQSKM	RANNQKVYMP	REGKLISDIN	KAWERLEKAE	HERELALRNE
410	420	430	440	450	460	470	480
LIRQEKLEQL	ARRFDRKAAM	RETWLSENQR	LVSQDNFGFD	LPAVEAATKK	HEAIETDIAA	YEERVQAVVA	VARELEAENY
490	500	510	520	530	540	550	560
HDIKRITARK	DNVIRLWEYL	LELLRARRQR	LEMNLGLQKI	FQEMLYIMDW	MDEMKVLVLS	QDYGKHLLGV	EDLLQKHTLV
570	580	590	600	610	620	630	640
EADIGIQAER	VRGVNASAQK	FATDGEGYKP	CDPQVIRDRV	AHMEFCYQEL	CQLAAERRAR	LEESRRLWKF	FWEMAEEEGW
650	660	670	680	690	700	710	720
IREKEKILSS	DDYGKDLTSV	MRLLSKHRAF	EDEMSGRSGH	FEQAIKEGED	MIAEEHFGSE	KIRERIIYIR	EQWANLEQLS
730	740	750	760	770	780	790	800
AIRKKRLEEA	SLLHQFQADA	DDIDAWMLDI	LKIVSSSDVG	HDEYSTQSLV	KKHKDVAEEI	ANYRPTLDTL	HEQASALPQE
810	820	830	840	850	860	870	880
HAESPDVRGR	LSGIEERYKE	VAELTRLRKQ	ALQDTLALYK	MFSEADACEL	WIDEKEQWLN	NMQIPEKLED	LEVIQHRFES
890	900	910	920	930	940	950	960
LEPEMNNQAS	RVAVVNQIAR	QLMHSGHPSE	KEIKAQQDKL	NTRWSQFREL	VDRKKDALLS	ALSIQNYHLE	CNETKSWIRE
970	980	990	1000	1010	1020	1030	1040
KTKVIESTQD	LGNDLAGVMA	LQRKLTGMER	DLVAIEAKLS	DLQKEAEKLE	SEHPDQAQAI	LSRLAEISDV	WEEMKTTLKN
1050	1060	1070	1080	1090	1100	1110	1120
REASLGEASK	LQQFLRDLDD	FQSWLSRTQT	AIASEDMPNT	LTEAEKLLTQ	HENIKNEIDN	YEEDYQKMRD	MGEMVTQGQT
1130	1140	1150	1160	1170	1180	1190	1200
DAQYMFLRQR	LQALDTGWNE	LHKMWENRQN	LLSQSHAYQQ	FLRDTKQAEA	FLNNQEYVLA	HTEMPTTLEG	AEAAIKKQED
1210	1220	1230	1240	1250	1260	1270	1280
FMTTMDANEE	KINAVVETGR	RLVSDGNINS	DRIQEKVDSI	DDRHRKNRET	ASELLMRLKD	NRDLQKFLQD	CQELSLWINE
1290	1300	1310	1320	1330	1340	1350	1360
KMLTAQDMSY	DEARNLHSKW	LKHQAFMAEL	ASNKEWLDKI	EKEGMQLISE	KPETEAVVKE	KLTGLHKMWE	VLESTTQTKA
1370	1380	1390	1400	1410	1420	1430	1440
QRLFDANKAE	LFTQSCADLD	KWLHGLESQI	QSDDYGKDLT	SVNILLKKQQ	MLENQMEVRK	KEIEELQSQA	QALSQEGKST
1450	1460	1470	1480	1490	1500	1510	1520
DEVDSKRLTV	QTKFMELLEP	LNERKHNLLA	SKEIHQFNRD	VEDEILWVGE	RMPLATSTDH	GHNLQTVQLL	IKKNQTLQKE
1530	1540	1550	1560	1570	1580	1590	1600
IQGHQPRIDD	IFERSQNIVT	DSSSLSAEAI	RQRLADLKQL	WGLLIEETEK	RHRRLEEAHR	AQQYYFDAAE	AEAWMSEQEL
1610	1620	1630	1640	1650	1660	1670	1680
YMMSEEKAKD	EQSAVSMLKK	HQILEQAVED	YAETVHQLSK	TSRALVADSH	PESERISMRQ	SKVDKLYAGL	KDLAEERRGK
1690	1700	1710	1720	1730	1740	1750	1760
LDERHRLFQL	NREVDDLEQW	IAEREVVAGS	HELGQDYEHV	TMLQERFREF	ARDTGNIGQE	RVDTVNHLAD	ELINSGHSDA
1770	1780	1790	1800	1810	1820	1830	1840
ATIAEWKDGL	NEAWADLLEL	IDTRTQILAA	SYELHKFYHD	AKEIFGRIQD	KHKKLPEELG	RDQNTVETLQ	RMHTTFEHDI
1850	1860	1870	1880	1890	1900	1910	1920
QALGTQVRQL	QEDAARLQAA	YAGDKADDIQ	KRENEVLEAW	KSLLDACESR	RVRLVDTGDK	FRFFSMVRDL	MLWMEDVIRQ
1930	1940	1950	1960	1970	1980	1990	2000
IEAQEKPRDV	SSVELLMNNH	QGIKAEIDAR	NDSFTTCIEL	GKSLLARKHY	ASEEIKEKLL	QLTEKRKEMI	DKWEDRWEWL
2010	2020	2030	2040	2050	2060	2070	2080
RLILEVHQFS	RDASVAEAWL	LGQEPYLSSR	EIGQSVDEVE	KLIKRHEAFE	KSAATWDERF	SALERLTTLE	LLEVRRQQEE
2090	2100	2110	2120	2130	2140	2150	2160
EERKRRPPSP	EPSTKVSEEA	ESQQQWDTSK	GEQVSQNGLP	AEQGSPRVSY	RSQTYQNYKN	FNSRRTASDQ	PWSGL

Detailed Protein Report

Cmpd.	No. of Cmpds.	m/z meas.	Δ m/z [ppm]	z	Rt [min]	Score	P	Range	Sequence	Modification	Ratios
418	10	607.8310	-55.05	2	34.3	14.1	2	820-829	K.EVAELTRLRK.Q		M:Q 1.43
601	2	548.1743	-250.21	2	36.1	12.2	0	1087-1095	K.LLTQHENIK.N		M:Q 0.83<br>Q:W 0.47
7	1	689.3232	-33.69	1	29.3	10.0	0	1866-1871	K.ADDIQK.R		

BRUKER

Detailed Protein Report

Protein 147: **PREDICTED: T-lymphoma invasion and metastasis-inducing protein 1 isoform X4 [Homo sapiens]**

**Accession:**	gi|530419050	**Score:**	66.3
**Database:**	refseq_human(refseq_human_20140103.fasta)	**MW [kDa]:**	161.7
**Database Date:**	2015-11-30	**pI:**	6.4
		**Sequence Coverage [%]:**	3.9
		**No. of unique Peptides:**	3

Quantitation

Q:W	**Median:** 2.40	**CV:** 0.00 %	**No. of Peptides:**	1
M:Q	**Median:** 1.63	**CV:** 79.73 %	**No. of Peptides:**	2

10	20	30	40	50	60	70	80
MGNAESQHVE	HEFYGEKHAS	LGRKHTSRSL	RLSHKTRRTR	HASSGKVIHR	NSEVSTRSSS	TPSIPQSLAE	NGLEPFSQDG
**90**	**100**	**110**	**120**	**130**	**140**	**150**	**160**
TLEDFGSPIW	VDRVDMGLRP	VSYTDSSVTP	SVDSSIVLTA	ASVQSMPDTE	ESRLYGDDAT	YLAEGGRRQH	SYTSNGPTFM
**170**	**180**	**190**	**200**	**210**	**220**	**230**	**240**
ETASFKKKRS	KSADIWREDS	LEFSLSDLSQ	EHLTSNEEIL	GSAEEKDCEE	ARGMETRASP	RQLSTCQRAN	SLGDLYAQKN
**250**	**260**	**270**	**280**	**290**	**300**	**310**	**320**
SGVTANGGPG	SKFAGYCRNL	VSDIPNLANH	KMPPAAAEET	PPYSNYNTLP	CRKSHCLSEG	ATNPQISHSN	SMQGRRAKTT
**330**	**340**	**350**	**360**	**370**	**380**	**390**	**400**
QDVNAGEGSE	FADSGIEGAT	TDTDLLSRRS	NATNSSYSPT	TGRAFVGSDS	GSSSTGDAAR	QGVYENFRRE	LEMSTTNSES
**410**	**420**	**430**	**440**	**450**	**460**	**470**	**480**
LEEAGSAHSD	EQSSGTLSSP	GQSDILLTAA	QGTVRKAGAL	AVKNFLVHKK	NKKVESATRR	KWKHYWVSLK	GCTLFFYESD
**490**	**500**	**510**	**520**	**530**	**540**	**550**	**560**
GRSGIDHNSI	PKHAVWVENS	IVQAVPEHPK	KDFVFCLSNS	LGDAFLFQTT	SQTELENWIT	AIHSACATAV	ARHHHKEDTL
**570**	**580**	**590**	**600**	**610**	**620**	**630**	**640**
RLLKSEIKKL	EQKIDMDEKM	KKMGEMQLSS	VTDSKKKKTI	LDQIFVWEQN	LEQFQMDLFR	FRCYLASLQG	GELPNPKRLL
**650**	**660**	**670**	**680**	**690**	**700**	**710**	**720**
AFASRPTKVA	MGRLGIFSVS	SFHALVAART	GETGVRRRTQ	AMSRSASKRR	SRFSSLWGLD	TTSKKKQGRP	SINQVFGEGT
**730**	**740**	**750**	**760**	**770**	**780**	**790**	**800**
EAVKKSLEGI	FDDIVPDGKR	EKEVVLPNVH	QHNPDCDIWV	HEYFTPSWFC	LPNNQPALTV	VRPGDTARDT	LELICKTHQL
**810**	**820**	**830**	**840**	**850**	**860**	**870**	**880**
DHSAHYLRLK	FLIENKMQLY	VPQPEEDIYE	LLYKEIEICP	KVTQSIHIEK	SDTAADTYGF	SLSSVEEDGI	RRLYVNSVKE
**890**	**900**	**910**	**920**	**930**	**940**	**950**	**960**
TGLASKKGLK	AGDEILEINN	RAADALNSSM	LKDFLSQPSL	GLLVRTYPEL	EEGVELLESP	PHRVDGPADL	GESPLAFLTS
**970**	**980**	**990**	**1000**	**1010**	**1020**	**1030**	**1040**
NPGHSLCSEQ	GSSAETAPEE	TEGPDLESSD	ETDHSSKSTE	QVAAFCRSLH	EMNPSDQSPS	PQDSTGPQLA	TMRQLSDADK
**1050**	**1060**	**1070**	**1080**	**1090**	**1100**	**1110**	**1120**
LRKVICELLE	TERTYVKDLN	CLMERYLKPL	QKETFLTQDE	LDVLFGNLTE	MVEFQVEFLK	TLEDGVRLVP	DLEKLEKVDQ
**1130**	**1140**	**1150**	**1160**	**1170**	**1180**	**1190**	**1200**
FKKVLFSLGG	SFLYYADRFK	LYSAFCASHT	KVPKVLVKAK	TDTAFKAFLD	AQNPKQQHSS	TLESYLIKPI	QRILKYPLLL
**1210**	**1220**	**1230**	**1240**	**1250**	**1260**	**1270**	**1280**
RELFALTDAE	SEEHYHLDVA	IKTMNKVASH	INEMQKIHEE	FGAVFDQLIA	EQTGEKKEVA	DLSMGDLLLH	TTVIWLNPPA
**1290**	**1300**	**1310**	**1320**	**1330**	**1340**	**1350**	**1360**
SLGKWKKEPE	LAAFVFKTAV	VLVYKDGSKQ	KKKLVGSHRL	SIYEDWDPFR	FRHMIPTEAL	QVRALASADA	EANAVCEIVH
**1370**	**1380**	**1390**	**1400**	**1410**	**1420**	**1430**	**1440**
VKSESEGRPE	RVFHLCCSSP	ESRKDFLKAV	HSILRDKHRR	QLLKTESLPS	SQQYVPFGGK	RLCALKGARP	AMSRAGTVGI
**1450**	**1460**						
RTFKIPVTPT	PQ						

Cmpd.	No. of Cmpds.	m/z meas.	Δ m/z [ppm]	z	Rt [min]	Score	P	Range	Sequence	Modification	Ratios
44	2	750.8174	-45.94	2	30.3	15.2	0	134-147	R.LYGDDATYLAEGGR.R		Q:W 2.40<br>M:Q 0.81
1584	1	1022.9716	-69.17	2	49.7	16.6	1	707-725	K.QGRPSINQVFGEGTEAVKK.S		M:Q 3.28
2671	10	822.4677	-5.20	1	64.5	22.1	0	873-879	R.LYVNSVK.E		

Detailed Protein Report

Protein 148: small proline-rich protein 2A [Homo sapiens]

Accession: gi|5174693
Database: refseq_human(refseq_human_20140103.fasta)
Database Date: 2015-11-30
Modification(s): Carbamidomethyl

Score: 66.3
MW [kDa]: 8.0
pI: 10.3
Sequence Coverage [%]: 36.1
No. of unique Peptides: 3

Quantitation

Q:W	**Median:** 0.86	**CV:** 33.96 %	**No. of Peptides:**	2
M:Q	**Median:** 0.87	**CV:** 0.00 %	**No. of Peptides:**	1

```
        10         20         30         40         50         60         70         80
MSYQQQQCKQ PCQPPPVCPT PKCPEPCPPP KCPEPCPPPK CPQPCPPQQC QQKYPPVTPS PPCQSKYPPK SK
```

Cmpd.	No. of Cmpds.	m/z meas.	Δ m/z [ppm]	z	Rt [min]	Score	P	Range	Sequence	Modification	Ratios
2928	1	724.8814	36.56	2	65.4	11.5	0	10-22	K.QPCQPPPVCPTPK.C	Carbamidomethyl: 3	
85	6	753.3519	-18.26	2	30.5	41.0	0	10-22	K.QPCQPPPVCPTPK.C	Carbamidomethyl: 3, 9	M:Q 0.87<br>Q:W 0.62
171	1	729.5012	195.91	2	31.9	13.8	0	54-66	K.YPPVTPSPPCQSK.Y	Carbamidomethyl: 10	Q:W 1.20

Detailed Protein Report

Protein 149: PREDICTED: uncharacterized protein KIAA1109 isoform X14 [Homo sapiens]

**Accession:**	gi|578809471	**Score:**	66.3
**Database:**	refseq_human(refseq_human_20140103.fasta)	**MW [kDa]:**	555.0
**Database Date:**	2015-11-30	**pI:**	6.1
		**Sequence Coverage [%]:**	1.6
		**No. of unique Peptides:**	4

Quantitation

Q:W	**Median:**	2.74	**CV:**	0.00 %	**No. of Peptides:**	1
M:Q	**Median:**	0.44	**CV:**	0.00 %	**No. of Peptides:**	1

Detailed Protein Report

10	20	30	40	50	60	70	80
MDQRKNESIV	PSITQLEDFL	TEHNSNVVWL	LVATILSCGW	IIYLTYYNSR	NVGLILTLVL	NRLYKHGYIH	IGSFSFSVLS
90	100	110	120	130	140	150	160
GKVMVREIYY	ITEDMSIRIQ	DGFIIFRWWK	MYNPKQKQHD	PKAETRLYIT	VNDFEFHVYN	RSDLYGRLQE	LFGLEPTIIP
170	180	190	200	210	220	230	240
PKKDDDKTRE	IGRTRTQSKI	ERVKVKTESQ	DPTSSWRSLI	PVIKVNVSTG	RLAFGNHYQP	QTLCINFDDA	FLTYTTKPPS
250	260	270	280	290	300	310	320
SHLDQFMHIV	KGKLENVRVM	LVPSPRYVGL	QNDEPPRLMG	EGFVVMQSND	VDIYYYMDEP	GLVPEETEEN	IEGEMSSEDC
330	340	350	360	370	380	390	400
KLQDLPPCWG	LDIVCGKGTD	FNYGPWADRQ	RDCLWKFFFP	PDYQVLKVSE	IAQPGRPRQI	LAFELRMNII	ADATIDLLFT
410	420	430	440	450	460	470	480
KNRETNAVHV	NVGAGSYLEI	NIPMTVEENG	YTPAIKGQLL	HVDATTSMQY	RTLLEAEMLA	FHINASYPRI	WNMPQTWQCE
490	500	510	520	530	540	550	560
LEVYKATYHF	IFAQKNFFTD	LIQDWSSDSP	PDIFSFVPYT	WNFKIMFHQF	EMIWAANQHN	WIDCSTKQQE	NVYLAACGET
570	580	590	600	610	620	630	640
LNIDFSLPFT	DFVPATCNTK	FSLRGEDVDL	HLFLPDCHPS	KYSLFMLVKN	CHPNKMIHDT	GIPAECQSGQ	KTVKPKWRNV
650	660	670	680	690	700	710	720
TQEKSGWVEC	WTVPSVMLTI	DYTWHPIYPQ	KADEQLKQSL	SEMEETMLSV	LRPSQKTSDR	VVSSPSTSSR	PPIDPSELPP
730	740	750	760	770	780	790	800
DKLHVEMELS	PDSQITLYGP	LLNAFLCIKE	NYFGEDDMYM	DFEEVISSPV	LSLSTSSSSG	WTAVGMENDK	KENEGSAKSI
810	820	830	840	850	860	870	880
HPLALRPWDI	TVLVNLYKVH	GRLPVHGTTD	GPECPTAFLE	RLCFEMKKGF	RETMLQLILS	PLNVFVSDNY	QRPPVDEVLR
890	900	910	920	930	940	950	960
EGHINLSGLQ	LRAHAMFSAE	GLPLGSDSLE	YAWLIDVQAG	SLTAKVTAPQ	LACLLEWGQT	FVFHVVCREY	ELERPKSVII
970	980	990	1000	1010	1020	1030	1040
CQHGIDRRFC	ESKLSCIPGP	CPTSDDLKYT	MIRLAVDGAD	IYIVEHGCAT	NIKMGAIRVA	NCNLHNQSVG	EGISAAIQDF
1050	1060	1070	1080	1090	1100	1110	1120
QVRQYIEQLN	NCRIGLQPAV	LRRAYWLEAG	SANLGLITVD	IALAADHHSK	HEAQRHFLET	HDARTKRLWF	LWPDDILKNK
1130	1140	1150	1160	1170	1180	1190	1200
RCRNKCGCLG	GCRFFGGTVT	GLDFFKLEEL	TPSSSSAFSS	TSAESDMYYG	QSLLQPGEWI	ITKEIPKIID	GNVNGMKRKE
1210	1220	1230	1240	1250	1260	1270	1280
WENKSVGIEV	ERKTQHLSLQ	VPLRSHSSSS	SSEENSSSSA	AQPLLAGEKE	SPSSVADDHL	VQKEFLHGTK	RDDGQASIPT
1290	1300	1310	1320	1330	1340	1350	1360
EISGNSPVSP	NTQDKSVGQS	PLRSPLKRQA	SVCSTRLGST	KSLTAAFYGD	KQPVTVGVQF	SSDVSRSDEN	VLDSPKQRRS
1370	1380	1390	1400	1410	1420	1430	1440
FGSFPYTPSA	DSNSFHQYRS	MDSSMSMADS	EAYFSAAEEF	EPISSDEGPG	TYPGRKKKKK	QTQQIDYSRG	SIYHSVEGPL
1450	1460	1470	1480	1490	1500	1510	1520
TGHGESIQDS	RTLPFKTHPS	QASFVSALGG	EDDVIEHLYI	VEGEKTVESE	QITPQQPVMN	CYQTYLTQFQ	VINWSVKHPT
1530	1540	1550	1560	1570	1580	1590	1600
NKRTSKSSLH	RPLDLDTPTS	EESSSSFEQL	SVPTFKVIKQ	GLTANSLLDR	GMQLSGSTSN	TPYTPLEKKL	ADNTDDETLT
1610	1620	1630	1640	1650	1660	1670	1680
EEWTLDQPVS	QTRTTAIVEV	KGTVDIVLTP	LVAEALDRYI	EAMVHCASTR	HPAAIVDDLH	AKVLREAVQN	SKTTFSENLS
1690	1700	1710	1720	1730	1740	1750	1760
SKQDIRGTKT	EQSTIGTTNQ	GQAQTNLTMK	QDNVTIKGLQ	TNVSIPKVNL	CLLQASVEES	PTTAPSRSVT	HVSLVALCFD
1770	1780	1790	1800	1810	1820	1830	1840
RIATQVRMNR	GVVEETSNNA	EPGRTSNFDR	YVHATKMQPQ	SSGSLRSNAG	AEKGKEIAAK	LNIHRVHGQL	RGLDTTDIGT
1850	1860	1870	1880	1890	1900	1910	1920
CAITAIPFEK	SKVLFTLEEL	DEFTFVDETD	QQAVPDVTRI	GPSQEKWGWI	MFECGLENLT	IKGGRQSGAV	LYNSFGIMGK
1930	1940	1950	1960	1970	1980	1990	2000
ASDTERGGVL	TSNNSSDSPT	GSGYNTDVSD	DNLPCDRTSP	SSDLNGNSVS	DEQDEGVESD	DLKKDLPLMP	PPPDSCSMKL
2010	2020	2030	2040	2050	2060	2070	2080
TIKEIWFSFA	APTNVRSHTH	AFSRQLNLLS	TATPAVGAWL	VPIDQLKSSL	NKLETEGTLR	ICAVMGCIMT	EALENKSVHF
2090	2100	2110	2120	2130	2140	2150	2160
PLRSKYNRLT	KVARFLQENP	SCLLCNILHH	YLHQANYSII	DDATMSDGLP	ALVTLKKGLV	ALARQWMKFI	VVTPAFKGVS
2170	2180	2190	2200	2210	2220	2230	2240

Detailed Protein Report

Cmpd.	No. of Cmpds.	m/z meas.	Δ m/z [ppm]	z	Rt [min]	Score	P	Range	Sequence	Modification	Ratios
117	1	774.3916	-26.77	3	31.2	15.7	1	66-86	K.HGYIHIGSFSFSVLSGKVMVR.E		
1348	1	669.3232	-39.50	2	46.5	13.8	2	116-126	K.QKQHDPKAETR.L		M:Q 0.44<br>Q:W 2.74
2455	1	669.2124	-199.17	2	61.2	12.2	1	1309-1321	R.QASVCSTRLGSTK.S		
2265	1	618.9864	-97.88	3	56.8	10.2	2	4978-4994	K.VLSVLIKKLGTALQDEK.E		

Detailed Protein Report

Protein 150: PREDICTED: centriolin isoform X3 [Homo sapiens]

**Accession:**	gi|578817016	**Score:**	66.2
**Database:**	refseq_human(refseq_human_20140103.fasta)	**MW [kDa]:**	258.5
**Database Date:**	2015-11-30	**pI:**	5.3
**Modification(s):**	Carbamidomethyl, Oxidation	**Sequence Coverage [%]:**	3.0
		**No. of unique Peptides:**	5

BRUKER

Detailed Protein Report

10	20	30	40	50	60	70	80
MKKGSQQKIF	SKAKIPSSSH	SPIPSSMSNM	RSRSLSPLIG	SETLPFHSGG	QWCEQVEIAD	ENNMLLDYQD	HKGADSHAGV
90	100	110	120	130	140	150	160
RYITEALIKK	LTKQDNLALI	KSLNLSLSKD	GGKKFKYIEN	LEKCVKLEVL	NLSYNLIGKI	EKLDKLLKLR	ELNLSYNKIS
170	180	190	200	210	220	230	240
KIEGIENMCN	LQKLNLAGNE	IEHIPVWLGK	KLKSLRVLNL	KGNKISSLQD	ISKLKPLQDL	ISLILVENPV	VTLPHYLQFT
250	260	270	280	290	300	310	320
IFHLRSLESL	EGQPVTTQDR	QEAFERFSLE	EVERLERDLE	KKMIETEELK	SKQTRFLEEI	KNQDKLNKSL	KEEAMLQKQS
330	340	350	360	370	380	390	400
CEELKSDLNT	KNELLKQKTI	ELTRACQKQY	ELEQELAFYK	IDAKFEPLNY	YPSEYAEIDK	APDESPYIGK	SRYKRNMFAT
410	420	430	440	450	460	470	480
ESYIIDSAQA	VQIKKMEPDE	QLRNDHMNLR	GHTPLDTQLE	DKEKKISAAQ	TRLSELHDEI	EKAEQQILRA	TEEFKQLEEA
490	500	510	520	530	540	550	560
IQLKKISEAG	KDLLYKQLSG	RLQLVNKLRQ	EALDLELQME	KQKQEIAGKQ	KEIKDLQIAI	DSLDSKDPKH	SHMKAQKSGK
570	580	590	600	610	620	630	640
EQQLDIMNKQ	YQQLESRLDE	ILSRIAKETE	EIKDLEEQLT	EGQIAANEAL	KKDLEGVISG	LQEYLGTIKG	QATQAQNECR
650	660	670	680	690	700	710	720
KLRDEKETLL	QRLTEVEQER	DQLEIVAMDA	ENMRKELAEL	ESALQEQHEV	NASLQQTQGD	LSAYEAELEA	RLNLRDAEAN
730	740	750	760	770	780	790	800
QLKEELEKVT	RLTQLEQSAL	QAELEKERQA	LKNALGKAQF	SEEKEQENSE	LHAKLKHLQD	DNNLLKQQLK	DFQNHLNHVV
810	820	830	840	850	860	870	880
DGLVRPEEVA	ARVDELRRKL	KLGTGEMNIH	SPSDVLGKSL	ADLQKQFSEI	LARSKWERDE	AQVRERKLQE	EMALQQEKLA
890	900	910	920	930	940	950	960
TGQEEFRQAC	ERALEARMNF	DKRQHEARIQ	QMENEIHYLQ	ENLKSMEEIQ	GLTDLQLQEA	DEEKERILAQ	LRELEKKKKL
970	980	990	1000	1010	1020	1030	1040
EDAKSQEQVF	GLDKELKKLK	KAVATSDKLA	TAELTIAKDQ	LKSLHGTVMK	INQERAEELQ	EAERFSRKAA	QAARDLTRAE
1050	1060	1070	1080	1090	1100	1110	1120
AEIELLQNLL	RQKGEQFRLE	MEKTGVGTGA	NSQVLEIEKL	NETMERQRTE	IARLQNVLDL	TGSDNKGGFE	NVLEEIAELR
1130	1140	1150	1160	1170	1180	1190	1200
REVSYQNDYI	SSMADPFKRR	GYWYFMPPPP	SSKVSSHSSQ	ATKDSGVGLK	YSASTPVRKP	RPGQQDGKEG	SQPPPASGYW
1210	1220	1230	1240	1250	1260	1270	1280
VYSPIRSGLH	KLFPSRDADS	GGDSQEESEL	DDQEEPPFVP	PPGYMMYTVL	PDGSPVPQGM	ALYAPPPPLP	NNSRPLTPGT
1290	1300	1310	1320	1330	1340	1350	1360
VVYGPPPAGA	PMVYGPPPPN	FSIPFIPMGV	LHCNVPEHHN	LENEVSRLED	IMQHLKSKKR	EERWMRASKR	QSEKEMEELH
1370	1380	1390	1400	1410	1420	1430	1440
HNIDDLLQEK	KSLECEVEEL	HRTVQKRQQQ	KDFIDGNVES	LMTELEIEKS	LKHHEDIVDE	IECIEKTLLK	RRSELREADR
1450	1460	1470	1480	1490	1500	1510	1520
LLAEAESELS	CTKEKTKNAV	EKFTDAKRSL	LQTESDAEEL	ERRAQETAVN	LVKADQQLRS	LQADAKDLEQ	HKIKQEEILK
1530	1540	1550	1560	1570	1580	1590	1600
EINKIVAAKD	SDFQCLSKKK	EKLTEELQKL	QKDIEMAERN	EDHHLQVLKE	SEVLLQAKRA	ELEKLKSQVT	SQQQEMAVLD
1610	1620	1630	1640	1650	1660	1670	1680
RQLGHKKEEL	HLLQGSMVQA	KADLQEALRL	GETEVTEKCN	HIREVKSLLE	ELSFQKGELN	VQISERKTQL	TLIKQEIEKE
1690	1700	1710	1720	1730	1740	1750	1760
EENLQVVLRQ	MSKHKTELKN	ILDMLQLENH	ELQGLKLQHD	QRVSELEKTQ	VAVLEEKLEL	ENLQQISQQQ	KGEIEWQKQL
1770	1780	1790	1800	1810	1820	1830	1840
LERDKREIER	MTAESRALQS	CVECLSKEKE	DLQEKCDIWE	KKLAQTKRVL	AAAEENSKME	QSNLEKLELN	VRKLQQELDQ
1850	1860	1870	1880	1890	1900	1910	1920
LNRDKLSLHN	DISAMQQQLQ	EKREAVNSLQ	EELANVQDHL	NLAKQDLLHT	TKHQDVLLSE	QTRLQKDISE	WANRFEDCQK
1930	1940	1950	1960	1970	1980	1990	2000
EEETKQQQLQ	VLQNEIEENK	LKLVQQEMMF	QRLQKERESE	ESKLETSKVT	LKEQQHQLEK	ELTDQKSKLD	QVLSKVLAAE
2010	2020	2030	2040	2050	2060	2070	2080
ERVRTLQEEE	RWCESLEKTL	SQTKRQLSER	EQQLVEKSGE	LLALQKEADS	MRADFSLLRN	QFLTERKKAE	KQVASLKEAL
2090	2100	2110	2120	2130	2140	2150	2160
KIQRSQLEKN	LLFLPELPAD	LEAILERNEN	LEGELESLKE	NLPFTMNEGP	FEEKLNFSQV	HIMDEHWRGE	ALREKLRHRE
2170	2180	2190	2200	2210	2220	2230	2240

Detailed Protein Report

Cmpd.	No. of Cmpds.	m/z meas.	Δ m/z [ppm]	z	Rt [min]	Score	P	Range	Sequence	Modification
951	1	677.3400	12.76	3	40.4	10.0	1	13-31	K.AKIPSSSHSPIPSSMSNMR.S	Oxidation: 18
2056	1	1029.5727	91.82	2	55.8	13.5	2	415-430	K.KMEPDEQLRNDHMNLR.G	Oxidation: 2, 13
1818	1	582.2582	-133.26	1	53.1	16.6	0	1602-1606	R.QLGHK.K	
1797	1	638.2204	-128.45	3	51.0	10.7	1	1771-1787	R.MTAESRALQSCVECLSK.E	Carbamidomethyl: 14
36	1	721.8221	18.43	2	30.3	15.4	1	1915-1925	R.FEDCQKEEETK.Q	Carbamidomethyl: 4

Detailed Protein Report

Protein 151: neuralized-like protein 4 isoform 2 [Homo sapiens]

**Accession:**	gi|53829368	**Score:**	66.0
**Database:**	refseq_human(refseq_human_20140103.fasta)	**MW [kDa]:**	166.6
**Database Date:**	2015-11-30	**pI:**	5.5
		**Sequence Coverage [%]:**	3.3
		**No. of unique Peptides:**	5

Quantitation

Q:W	**Median:** 0.46	**CV:** 0.00 %	**No. of Peptides:**	1
M:Q	**Median:** 2.22	**CV:** 0.00 %	**No. of Peptides:**	1

10	20	30	40	50	60	70	80
MAAGSGGSGG	SGGGPGPGPG	GGGGPSGSGS	GPGSNGGLGS	GGELHPRTGR	LVSLSACGRT	ARRQQPGQEF	NHGLVLSREP
90	100	110	120	130	140	150	160
LRDGRVFTVR	IDRKVNSWSG	SIEIGVTALD	PSVLDFPSSA	TGLKGGSWVV	SGCSVLRDGR	SVLEEYGQDL	DQLGEGDRVG
170	180	190	200	210	220	230	240
VERTVAGELR	LWVNGRDCGV	AATGLPPRVW	AVVDLYGKCT	QITVLPPEPG	FSPPTPIPTP	PLEPLAPTED	SALAEQGTSA
250	260	270	280	290	300	310	320
DEAFMVSPAQ	ARPETFPNSL	ESHNDFANME	LSEVVSNTIL	SAYNGGLLNV	NLSSPPAGEG	LGSSGAATSP	ILTSNDALLF
330	340	350	360	370	380	390	400
HEKCGTLIKL	SNNNKTAERR	RPLDEFNNGV	VMTNRPLRDN	EMFEIRIDKL	VDKWSGSIEI	GVTTHNPNSL	EYPATMTNLQ
410	420	430	440	450	460	470	480
SGTIMMSGCG	ILTNGKGTRR	EYCEFSLDEL	QEGDHIGLTR	KSNSALHFFI	NGIDQGVATP	LTPPVVYGVV	DLYGMAVKVT
490	500	510	520	530	540	550	560
IVHNNNHSDR	LRRNNAILRA	LSPEGALRRA	APAAQAEPER	LLFHPNCGQK	AAITHEGRTA	LRPHATDDFN	HGVVLSSRAL
570	580	590	600	610	620	630	640
RDGEVFQVRI	DKMVDKWAGS	IEIGVTTHNP	AYLQLPSTMT	NLRSGTWMMT	GNGVMHNGTT	ILDEYGHNLD	RLKAGDTVGV
650	660	670	680	690	700	710	720
VRREDGTLHF	FVNGMTQGPA	AWNVPPGVYA	VVDLYGQAAQ	ATIVDDVEVA	PVPEPLPEGN	NQVSPSSPSS	GAGGSDLRFH
730	740	750	760	770	780	790	800
QLHGSNAVIT	NGGRTALRHN	CRSEFNDAIV	ISNRALRDGE	LFEIVIQKMV	DRWSGSIEAG	VTAIRPEDLE	FPNTMTDIDY
810	820	830	840	850	860	870	880
DTWMLSGTAI	MQDGNTMRNN	YGCDLDALGT	GARIGMMRTA	KGDLHYFING	QDQGAACSGL	PPEVYAVVDL	YGQCVQVSIT
890	900	910	920	930	940	950	960
NATGPMDNSL	ATSNTATEKS	FPLHSPVAGV	AHRFHSTCGK	NVTLEEDGTR	AVRAAGYAHG	LVFSTKELRA	EEVFEVKVEE
970	980	990	1000	1010	1020	1030	1040
LDEKWAGSLR	LGLTTLAPGE	MGPGAGGGGP	GLPPSLPELR	TKTTWMVSSC	EVRRDGQLQR	MNYGRNLERL	GVGSRVGVRR
1050	1060	1070	1080	1090	1100	1110	1120
GADDTMHILV	DGEDMGPAAT	GIAKNVWAVL	DLYGPVRGVS	IVSSTRLEES	EGTQPPSPSS	DTGSEGEEDD	EGEEHGLGGQ
1130	1140	1150	1160	1170	1180	1190	1200
NEVGIIPTTL	EFLENHGKNI	LLSNGNRTAT	RVASYNQGIV	VINQPLVPQL	LVQVRIDFLN	RQWTSSLVLG	VITCAPERLN
1210	1220	1230	1240	1250	1260	1270	1280
FPASACALKR	AAWLLRGRGV	FHNGLKICEK	FGPNLDTCPE	GTILGLRLDS	SGGLHLHVNG	VDQGVAVPDV	PQPCHALVDL
1290	1300	1310	1320	1330	1340	1350	1360
YGQCEQVTIV	NPEPGAASGK	SAGTQGDMEK	ADMVDGIKES	VCWGPPPAAS	PLKSCEYHAL	CSRFQELLLL	PEDYFMPPPK
1370	1380	1390	1400	1410	1420	1430	1440
RSLCYCESCR	KLRGDEAHRR	RGEPPREYAL	PFGWCRFNLR	VNPRLEAGTL	TKKWHMAYHG	SNVAAVRRVL	DRGELGAGTA
1450	1460	1470	1480	1490	1500	1510	1520
SILSCRPLKG	EPGVGFEEPG	ENCAPPREEQ	PPPVLLSPSL	QYAGAETLAS	KVQFRDPKSQ	RTHQAQVAFQ	VCVRPGSYTP
1530	1540	1550	1560	1570			
GPPSAALGEP	PDPHFSPAEL	EWVTKEKGAT	LLCALLVRVE				

Cmpd.	No. of Cmpds.	m/z meas.	Δ m/z [ppm]	z	Rt [min]	Score	P	Range	Sequence	Modification	Ratios
760	1	856.2336	-324.05	1	38.9	11.7	1	493-499	R.RNNAILR.A		
512	1	457.1269	-288.36	2	35.0	10.5	0	500-508	R.ALSPEGALR.R		
2675	1	715.3668	-42.44	2	61.8	10.6	1	1139-	K.NILLSNGNRTATR.V		

Detailed Protein Report

Cmpd.	No. of Cmpds.	m/z meas.	Δ m/z [ppm]	z	Rt [min]	Score	P	Range	Sequence	Modification	Ratios
								1151			
1492	1	555.2127	-156.80	2	47.2	20.0	2	1372-1380	K.LRGDEAHRR.R		Q:W 0.46<br>M:Q 2.22
2678	1	693.8667	-63.13	2	64.6	13.3	1	1546-1558	K.EKGATLLCALLVR.V		

Detailed Protein Report

Protein 152: E3 ubiquitin-protein ligase BRE1A [Homo sapiens]

**Accession:**	gi|34878777	**Score:**	65.8
**Database:**	refseq_human(refseq_human_20140103.fasta)	**MW [kDa]:**	113.6
**Database Date:**	2015-11-30	**pI:**	5.6
**Modification(s):**	Carbamidomethyl, Oxidation	**Sequence Coverage [%]:**	6.4
		**No. of unique Peptides:**	4

Quantitation

Q:W	**Median:**	0.98	**CV:**	0.00 %	**No. of Peptides:**	1
M:Q	**Median:**	0.75	**CV:**	0.00 %	**No. of Peptides:**	1

10	20	30	40	50	60	70	80
MSGIGNKRAA	GEPGTSMPPE	KKAAVEDSGT	TVETIKLGGV	SSTEELDIRT	LQTKNRKLAE	MLDQRQAIED	ELREHIEKLE
90	100	110	120	130	140	150	160
RRQATDDASL	LIVNRYWSQF	DENIRIILKR	YDLEQGLGDL	LTERKALVVP	EPEPDSDSNQ	ERKDDRERGE	GQEPAFSFLA
170	180	190	200	210	220	230	240
TLASSSSEEM	ESQLQERVES	SRRAVSQIVT	VYDKLQEKVE	LLSRKLNSGD	NLIVEEAVQE	LNSFLAQENM	RLQELTDLLQ
250	260	270	280	290	300	310	320
EKHRTMSQEF	SKLQSKVETA	ESRVSVLESM	IDDLQWDIDK	IRKREQRLNR	HLAEVLERVN	SKGYKVYGAG	SSLYGGTITI
330	340	350	360	370	380	390	400
NARKFEEMNA	ELEENKELAQ	NRLCELEKLR	QDFEEVTTQN	EKLKVELRSA	VEQVVKETPE	YRCMQSQFSV	LYNESLQLKA
410	420	430	440	450	460	470	480
HLDEARTLLH	GTRGTHQHQV	ELIERDEVSL	HKKLRTEVIQ	LEDTLAQVRK	EYEMLRIEFE	QTLAANEQAG	PINREMRHLI
490	500	510	520	530	540	550	560
SSLQNHNHQL	KGEVLRYKRK	LREAQSDLNK	TRLRSGSALL	QSQSSTEDPK	DEPAELKPDS	EDLSSQSSAS	KASQEDANEI
570	580	590	600	610	620	630	640
KSKRDEEERE	RERREKERER	EREREKEKER	EREKQKLKES	EKERDSAKDK	EKGKHDDGRK	KEAEIIKQLK	IELKKAQESQ
650	660	670	680	690	700	710	720
KEMKLLLDMY	RSAPKEQRDK	VQLMAAEKKS	KAELEDLRQR	LKDLEDKEKK	ENKKMADEDA	LRKIRAVEEQ	IEYLQKKLAM
730	740	750	760	770	780	790	800
AKQEEEALLS	EMDVTGQAFE	DMQEQNIRLM	QQLREKDDAN	FKLMSERIKS	NQIHKLLKEE	KEELADQVLT	LKTQVDAQLQ
810	820	830	840	850	860	870	880
VVRKLEEKEH	LLQSNIGTGE	KELGLRTQAL	EMNKRKAMEA	AQLADDLKAQ	LELAQKKLHD	FQDEIVENSV	TKEKDMFNFK
890	900	910	920	930	940	950	960
RAQEDISRLR	RKLETTKKPD	NVPKCDEILM	EEIKDYKARL	TCPCCNMRKK	DAVLTKCFHV	FCFECVKTRY	DTRQRKCPKC
970	980						
NAAFGANDFH	RIYIG						

Cmpd.	No. of Cmpds.	m/z meas.	Δ m/z [ppm]	z	Rt [min]	Score	P	Range	Sequence	Modification	Ratios	
1337	1	926.7938	6.01	3	45.5	12.7	1	257-280	K.VETAESRVSVLESMIDDLQWDIDK.I			
463	1	475.6020	-277.84	2	35.1	11.6	0	827-834	R.TQALEMNK.R	Oxidation: 6	M:Q	0.75
2961	1	788.3914	-25.63	2	65.8	15.7	2	835-848	K.RKAMEAAQLADDLK.A	Oxidation: 4		
1957	2	918.3405	-92.02	2	52.8	13.9	2	956-971	R.KCPKCNAAFGANDFHR.I	Carbamidomethyl: 5	Q:W	0.98

Detailed Protein Report

Protein 153: PREDICTED: focal adhesion kinase 1 isoform X5 [Homo sapiens]

**Accession:**	gi|530389208	**Score:**	65.8
**Database:**	refseq_human(refseq_human_20140103.fasta)	**MW [kDa]:**	125.2
**Database Date:**	2015-11-30	**pI:**	6.2
**Modification(s):**	Carbamidomethyl, Oxidation	**Sequence Coverage [%]:**	6.5
		**No. of unique Peptides:**	4

Quantitation

Q:W	**Median:** 1.16	**CV:** 0.00 %	**No. of Peptides:**	1
M:Q	**Median:** 0.26	**CV:** 0.00 %	**No. of Peptides:**	1

```
        10         20         30         40         50         60         70         80
MLLELAGQEA LKPAGAIYME KSGCSPFPVC WAKEYDRYLA SSKIMAAAYL DPNLNHTPNS STKTHLGTGM ERSPGAMERV
        90        100        110        120        130        140        150        160
LKVFHYFESN SEPTTWASII RHGDATDVRG IIQKIVDSHK VKHVACYGFR LSHLRSEEVH WLHVDMGVSS VREKYELAHP
       170        180        190        200        210        220        230        240
PEEWKYELRI RYLPKGFLNQ FTEDKPTLNF FYQQVKSDYM LEIADQVDQE IALKLGCLEI RRSYWEMRGN ALEKKSNYEV
       250        260        270        280        290        300        310        320
LEKDVGLKRF FPKSLLDSVK AKTLRKLIQQ TFRQFANLNR EESILKFFEI LSPVYRFDKE CFKCALGSSW IISVELAIGP
       330        340        350        360        370        380        390        400
EEGISYLTDK GCNPTHLADF TQVQTIQYSN SEDKDRKGML QLKIAGAPEP LTVTAPSLTI AENMADLIDG YCRLVNGTSQ
       410        420        430        440        450        460        470        480
SFIIRPQKEG ERALPSIPKL ANSEKQGMRT HAVSVSETDD YAEIIDEEDT YTMPSKSYGI DEARDYEIQR ERIELGRCIG
       490        500        510        520        530        540        550        560
EGQFGDVHQG IYMSPENPAL AVAIKTCKNC TSDSVREKFL QEALTMRQFD HPHIVKLIGV ITENPVWIIM ELCTLGELRS
       570        580        590        600        610        620        630        640
FLQVRKYSLD LASLILYAYQ LSTALAYLES KRFVHRDIAA RNVLVSSNDC VKLGDFGLSR YMEDSTYYKA SKGKLPIKWM
       650        660        670        680        690        700        710        720
APESINFRRF TSASDVWMFG VCMWEILMHG VKPFQGVKNN DVIGRIENGE RLPMPPNCPP TLYSLMTKCW AYDPSRRPRF
       730        740        750        760        770        780        790        800
TELKAQLSTI LEEEKAQQEE RMRMESRRQA TVSWDSGGSD EAPPKPSRPG YPSPRSSEGF YPSPQHMVQT NHYQVSGYPG
       810        820        830        840        850        860        870        880
SHGITAMAGS IYPGQASLLD QTDSWNHRPQ EIAMWQPNVE DSTVLDLRGI GQVLPTHLME ERLIRQQQEM EEDQRWLEKE
       890        900        910        920        930        940        950        960
ERFLKPDVRL SRGSIDREDG SLQGPIGNQH IYQPVGKPDP AAPPKKPPRP GAPGHLGSLA SLSSPADSYN EGVKPWRLQP
       970        980        990       1000       1010       1020       1030       1040
QEISPPPTAN LDRSNDKVYE NVTGLVKAVI EMSSKIQPAP PEEYVPMVKE VGLALRTLLA TVDETIPLLP ASTHREIEMA
      1050       1060       1070       1080       1090       1100       1110
QKLLNSDLGE LINKMKLAQQ YVMTSLQQEY KKQMLTAAHA LAVDAKNLLD VIDQARLKML GQTRPH
```

Cmpd.	No. of Cmpds.	m/z meas.	Δ m/z [ppm]	z	Rt [min]	Score	P	Range	Sequence	Modification	Ratios
2292	1	920.6271	128.21	2	59.1	13.3	2	413-429	R.ALPSIPKLANSEKQGMR.T		
153	3	635.8187	61.87	2	31.1	14.9	1	506-516	K.TCKNCTSDSVR.E	Carbamidomethyl: 2	
2249	1	937.4207	-94.13	2	56.6	13.7	2	635-649	K.LPIKWMAPESINFRR.F	Oxidation: 6	M:Q 0.26<br>Q:W 1.16
2062	1	720.2626	-173.22	2	54.3	10.7	0	1073-1086	K.QMLTAAHALAVDAK.N		

Detailed Protein Report

Protein 154: PREDICTED: dystrophin isoform X3 [Homo sapiens]

**Accession:**	gi|578837834	**Score:**	65.7
**Database:**	refseq_human(refseq_human_20140103.fasta)	**MW [kDa]:**	426.9
**Database Date:**	2015-11-30	**pI:**	5.5
**Modification(s):**	Carbamidomethyl	**Sequence Coverage [%]:**	1.5
		**No. of unique Peptides:**	3

Quantitation

Q:W	**Median:**	0.55	**CV:**	92.92 %	**No. of Peptides:**	2
M:Q	**Median:**	0.43	**CV:**	0.00 %	**No. of Peptides:**	1

Detailed Protein Report

10	20	30	40	50	60	70	80
MLWWEEVEDC	YEREDVQKKT	FTKWVNAQFS	KFGKQHIENL	FSDLQDGRRL	LDLLEGLTGQ	KLPKEKGSTR	VHALNNVNKA
90	100	110	120	130	140	150	160
LRVLQNNNVD	LVNIGSTDIV	DGNHKLTLGL	IWNIILHWQV	KNVMKNIMAG	LQQTNSEKIL	LSWVRQSTRN	YPQVNVINFT
170	180	190	200	210	220	230	240
TSWSDGLALN	ALIHSHRPDL	FDWNSVVCQQ	SATQRLEHAF	NIARYQLGIE	KLLDPEDVDT	TYPDKKSILM	YITSLFQVLP
250	260	270	280	290	300	310	320
QQVSIEAIQE	VEMLPRPPKV	TKEEHFQLHH	QMHYSQQITV	SLAQGYERTS	SPKPRFKSYA	YTQAAYVTTS	DPTRSPFPSQ
330	340	350	360	370	380	390	400
HLEAPEDKSF	GSSLMESEVN	LDRYQTALEE	VLSWLLSAED	TLQAQGEISN	DVEVVKDQFH	THEGYMMDLT	AHQGRVGNIL
410	420	430	440	450	460	470	480
QLGSKLIGTG	KLSEDEETEV	QEQMNLLNSR	WECLRVASME	KQSNLHRVLM	DLQNQKLKEL	NDWLTKTEER	TRKMEEEPLG
490	500	510	520	530	540	550	560
PDLEDLKRQV	QQHKVLQEDL	EQEQVRVNSL	THMVVVVDES	SGDHATAALE	EQLKVLGDRW	ANICRWTEDR	WVLLQDILLK
570	580	590	600	610	620	630	640
WQRLTEEQCL	FSAWLSEKED	AVNKIHTTGF	KDQNEMLSSL	QKLAVLKADL	EKKKQSMGKL	YSLKQDLLST	LKNKSVTQKT
650	660	670	680	690	700	710	720
EAWLDNFARC	WDNLVQKLEK	STAQISQAVT	TTQPSLTQTT	VMETVTTVTT	REQILVKHAQ	EELPPPPPQK	KRQITVDSEI
730	740	750	760	770	780	790	800
RKRLDVDITE	LHSWITRSEA	VLQSPEFAIF	RKEGNFSDLK	EKVNAIEREK	AEKFRKLQDA	SRSAQALVEQ	MVNEGVNADS
810	820	830	840	850	860	870	880
IKQASEQLNS	RWIEFCQLLS	ERLNWLEYQN	NIIAFYNQLQ	QLEQMTTTAE	NWLKIQPTTP	SEPTAIKSQL	KICKDEVNRL
890	900	910	920	930	940	950	960
SDLQPQIERL	KIQSIALKEK	GQGPMFLDAD	FVAFTNHFKQ	VFSDVQAREK	ELQTIFDTLP	PMRYQETMSA	IRTWVQQSET
970	980	990	1000	1010	1020	1030	1040
KLSIPQLSVT	DYEIMEQRLG	ELQALQSSLQ	EQQSGLYYLS	TTVKEMSKKA	PSEISRKYQS	EFEEIEGRWK	KLSSQLVEHC
1050	1060	1070	1080	1090	1100	1110	1120
QKLEEQMNKL	RKIQNHIQTL	KKWMAEVDVF	LKEEWPALGD	SEILKKQLKQ	CRLLVSDIQT	IQPSLNSVNE	GGQKIKNEAE
1130	1140	1150	1160	1170	1180	1190	1200
PEFASRLETE	LKELNTQWDH	MCQQVYARKE	ALKGGLEKTV	SLQKDLSEMH	EWMTQAEEEY	LERDFEYKTP	DELQKAVEEM
1210	1220	1230	1240	1250	1260	1270	1280
KRAKEEAQQK	EAKVKLLTES	VNSVIAQAPP	VAQEALKKEL	ETLTTNYQWL	CTRLNGKCKT	LEEVWACWHE	LLSYLEKANK
1290	1300	1310	1320	1330	1340	1350	1360
WLNEVEFKLK	TTENIPGGAE	EISEVLDSLE	NLMRHSEDNP	NQIRILAQTL	TDGGVMDELI	NEELETFNSR	WRELHEEAVR
1370	1380	1390	1400	1410	1420	1430	1440
RQKLLEQSIQ	SAQETEKSLH	LIQESLTFID	KQLAAYIADK	VDAAQMPQEA	QKIQSDLTSH	EISLEEMKKH	NQGKEAAQRV
1450	1460	1470	1480	1490	1500	1510	1520
LSQIDVAQKK	LQDVSMKFRL	FQKPANFEQR	LQESKMILDE	VKMHLPALET	KSVEQEVVQS	QLNHCVNLYK	SLSEVKSEVE
1530	1540	1550	1560	1570	1580	1590	1600
MVIKTGRQIV	QKKQTENPKE	LDERVTALKL	HYNELGAKVT	ERKQQLEKCL	KLSRKMRKEM	NVLTEWLAAT	DMELTKRSAV
1610	1620	1630	1640	1650	1660	1670	1680
EGMPSNLDSE	VAWGKATQKE	IEKQKVHLKS	ITEVGEALKT	VLGKKETLVE	DKLSLLNSNW	IAVTSRAEEW	LNLLLEYQKH
1690	1700	1710	1720	1730	1740	1750	1760
METFDQNVDH	ITKWIIQADT	LLDESEKKKP	QQKEDVLKRL	KAELNDIRPK	VDSTRDQAAN	LMANRGDHCR	KLVEPQISEL
1770	1780	1790	1800	1810	1820	1830	1840
NHRFAAISHR	IKTGKASIPL	KELEQFNSDI	QKLLEPLEAE	IQQGVNLKEE	DFNKDMNEDN	EGTVKELLQR	GDNLQQRITD
1850	1860	1870	1880	1890	1900	1910	1920
ERKREEIKIK	QQLLQTKHNA	LKDLRSQRRK	KALEISHQWY	QYKRQADDLL	KCLDDIEKKL	ASLPEPRDER	KIKEIDRELQ
1930	1940	1950	1960	1970	1980	1990	2000
KKKEELNAVR	RQAEGLSEDG	AAMAVEPTQI	QLSKRWREIE	SKFAQFRRLN	FAQIHTVREE	TMMVMTEDMP	LEISYVPSTY
2010	2020	2030	2040	2050	2060	2070	2080
LTEITHVSQA	LLEVEQLLNA	PDLCAKDFED	LFKQEESLKN	IKDSLQQSSG	RIDIIHSKKT	AALQSATPVE	RVKLQEALSQ
2090	2100	2110	2120	2130	2140	2150	2160
LDFQWEKVNK	MYKDRQGRFD	RSVEKWRRFH	YDIKIFNQWL	TEAEQFLRKT	QIPENWEHAK	YKWYLKELQD	GIGQRQTVVR
2170	2180	2190	2200	2210	2220	2230	2240

Detailed Protein Report

Cmpd.	No. of Cmpds.	m/z meas.	Δ m/z [ppm]	z	Rt [min]	Score	P	Range	Sequence	Modification	Ratios	
129	1	619.6434	29.86	3	30.9	11.2	2	1736-1751	R.DQAANLMANRGDHCRK.L	Carbamidomethyl: 14	Q:W<br>M:Q	0.25<br>0.43
982	1	712.1299	-301.49	1	41.8	17.8	1	2091-2095	K.MYKDR.Q			
1813	1	785.8684	-31.80	2	51.3	11.2	0	2659-2671	K.VHMITENINASWR.S		Q:W	1.20

Detailed Protein Report

Protein 155: short transient receptor potential channel 4 isoform zeta [Homo sapiens]

**Accession:**	gi|209863032	**Score:**	65.6
**Database:**	refseq_human(refseq_human_20140103.fasta)	**MW [kDa]:**	92.2
**Database Date:**	2015-11-30	**pI:**	9.3
**Modification(s):**	Oxidation	**Sequence Coverage [%]:**	6.0
		**No. of unique Peptides:**	1

Quantitation

Q:W	**Median:** 0.99	**CV:** 0.00 %	**No. of Peptides:**	1
M:Q	**Median:** 0.87	**CV:** 0.00 %	**No. of Peptides:**	1

```
        10         20         30         40         50         60         70         80
MAQFYYKRNV NAPYRDRIPL RIVRAESELS PSEKAYLNAV EKGDYASVKK SLEEAEIYFK ININCIDPLG RTALLIAIEN
        90        100        110        120        130        140        150        160
ENLELIELLL SFNVYVGDAL LHAIRKEVVG AVELLLNHKK PSGEKQFVAQ PNCQQLLASR WYDEFPGWRR RHWAVKMVTC
       170        180        190        200        210        220        230        240
FIIGLLFPVF SVCYLIAPKS PLGLFIRKPF IKFICHTASY LTFLFLLLLA SQHIDRSDLN RQGPPPTIVE WMILPWVLGF
       250        260        270        280        290        300        310        320
IWGEIKQMWD GGLQDYIHDW WNLMDFVMNS LYLATISLKI VAFVKYSALN PRESWDMWHP TLVAEALFAI ANIFSSLRLI
       330        340        350        360        370        380        390        400
SLFTANSHLG PLQISLGRML LDILKFLFIY CLVLLAFANG LNQLYFYYEE TKGLTCKGIR CEKQNNAFST LFETLQSLFW
       410        420        430        440        450        460        470        480
SIFGLINLYV TNVKAQHEFT EFVGATMFGT YNVISLVVLL NMLIAMMNNS YQLIADHADI EWKFARTKLW MSYFEEGGTL
       490        500        510        520        530        540        550        560
PTPFNVIPSP KSLWYLIKWI WTHLCKKKMR RKPESFGTIG RRAADNLRRH HQYQEVMRNL VKRYVAAMIR DAKTEEGLTE
       570        580        590        600        610        620        630        640
ENFKELKQDI SSFRFEVLGL LRGSKLSTIQ SANASKESSN SADSDEKSDS EGNSKDKKKN FSLFDLTTLI HPRSAAIASE
       650        660        670        680        690        700        710        720
RHNISNGSAL VVQEPPREKQ RKVNFVTDIK NFGLFHRRSK QNAAEQNANQ IFSVSEEVAR QQAAGPLERN IQLESRGLAS
       730        740        750        760        770        780        790        800
RGDLSIPGLS EQCVLVDHRE RNTDTLGLQV GKRVCPFKSE KVVVEDTVPI IPKEKHAKEE DSSIDYDLNL PDTVTHEDYV
       810
TTRL
```

Cmpd.	No. of Cmpds.	m/z meas.	Δ m/z [ppm]	z	Rt [min]	Score	P	Range	Sequence	Modification	Ratios	
117	1	849.3965	-40.26	2	30.9	18.1	1	530-542	R.HHQYQEVMRNLVK.R	Oxidation: 8	M:Q<br>Q:W	0.87<br>0.99

Detailed Protein Report

Protein 156: PREDICTED: CDK5 regulatory subunit-associated protein 2 isoform X4 [Homo sapiens]

**Accession:**	gi|578817652	**Score:**	65.3
**Database:**	refseq_human(refseq_human_20140103.fasta)	**MW [kDa]:**	211.3
**Database Date:**	2015-11-30	**pI:**	5.4
**Modification(s):**	Oxidation	**Sequence Coverage [%]:**	3.3
		**No. of unique Peptides:**	4

Quantitation

Q:W	**Median:**	4.30	**CV:**	12.53 %	**No. of Peptides:**	2
M:Q	**Median:**	0.54	**CV:**	54.26 %	**No. of Peptides:**	4

Detailed Protein Report

10	20	30	40	50	60	70	80
MMDLVLEEDV	TVPGTLSGCS	GLVPSVPDDL	DGINPNAGLG	NGLLPNVSEE	TVSPTRARNM	KDFENQITEL	KKENFNLKLR
90	100	110	120	130	140	150	160
IYFLEERMQQ	EFHGPTEHIY	KTNIELKVEV	ESLKRELQER	EQLLIKASKA	VESLAEAGGS	EIQRVKEDAR	KKVQQVEDLL
170	180	190	200	210	220	230	240
TKRILLLEKD	VTAAQAELEK	AFAGTETEKA	LRLRLESKLS	EMKKMHEGDL	AMALVLDEKD	RLIEELKLSL	KSKEALIQCL
250	260	270	280	290	300	310	320
KEEKSQMACP	DENVSSGELR	GLCAAPREEK	ERETEAAQME	HQKERNSFEE	RIQALEEDLR	EKEREIATEK	KNSLKRDKAI
330	340	350	360	370	380	390	400
QGLTMALKSK	EKKVEELNSE	IEKLSAAFAK	AREALQKAQT	QEFQGSEDYE	TALSGKEALS	AALRSQNLTK	STENHRLRRS
410	420	430	440	450	460	470	480
IKKITQELSD	LQQERERLEK	DLEEAHREKS	KGDCTIRDLR	NEVEKLRNEV	NEREKAMENR	YKSLLSESNK	KLHNQEQVIK
490	500	510	520	530	540	550	560
HLTESTNQKD	VLLQKFNEKD	LEVIQQNCYL	MAAEDLELRS	EGLITEKCSS	QQPPGSKTIF	SKEKKQSSDY	EELIQVLKKE
570	580	590	600	610	620	630	640
QDIYTHLVKS	LQESDSINNL	QAELNKIFAL	RKQLEQDVLS	YQNLRKTLEE	QISEIRRREE	ESFSLYSDQT	SYLSICLEEN
650	660	670	680	690	700	710	720
NRFQVEHFSQ	EELKKKVSDL	IQLVKELYTD	NQHLKKTIFD	LSCMGFQGNG	FPDRLASTEQ	TEIMKDLSKG	GCKNGYLRHT
730	740	750	760	770	780	790	800
ESKISDCDGA	HAPGCLEEGA	FINLLAPLFN	EKATLLLESR	PDLLKVVREL	LLGQLFLTEQ	EVSGEHLDGK	TEKTPKQKGE
810	820	830	840	850	860	870	880
LVHFVQTNSF	SKPHDELKLS	CEAQLVKAGE	VPKVGLKDAS	VQTVATEGDL	LRFKHEATRE	AWEEKPINTA	LSAEHRPENL
890	900	910	920	930	940	950	960
HGVPGWQAAL	LSLPGITNRE	AKKSRLPILI	KPSRSLGNMY	RLPATQEVVT	QLQSQILELQ	GELKEFKTCN	KQLHQKLILA
970	980	990	1000	1010	1020	1030	1040
EAVMEGRPTP	DKTLLNAQPP	VGAAYQDSPG	EQKGIKTTSS	VWRDKEMDSD	QQRSYEIDSE	ICPPDDLASL	PSCKENPEDV
1050	1060	1070	1080	1090	1100	1110	1120
LSPTSVATYL	SSKSQPSAKV	SVMGTDQSES	INTSNETEYL	KQKIHDLETE	LEGYQNFIFQ	LQKHSQCSEA	IITVLCGTEG
1130	1140	1150	1160	1170	1180	1190	1200
AQDGLSKPKN	GSDGEEMTFS	SLHQVRYVKH	VKILGPLAPE	MIDSRVLENL	KQQLEEQEYK	LQKEQNLNMQ	LFSEIHNLQN
1210	1220	1230	1240	1250	1260	1270	1280
KFRDLSPPRY	DSLVQSQARE	LSLQRQQIKD	GHGICVISRQ	HMNTMIKAFE	ELLQASDVDY	CVAEGFQEQL	NQCAELLEKL
1290	1300	1310	1320	1330	1340	1350	1360
EKLFLNGKSV	GVEMNTQNEL	MERIEEDNLT	YQHLLPESPE	PSASHALSDY	ETSEKSFFSR	DQKQDNETEK	TSVMVNSFSQ
1370	1380	1390	1400	1410	1420	1430	1440
DLLMEHIQEI	RTLRKRLEES	IKTNEKLRKQ	LERQGSEFVQ	GSTSIFASGS	ELHSSLTSEI	HFLRKQNQAL	NAMLIKGSRD
1450	1460	1470	1480	1490	1500	1510	1520
KQKENDKLRE	SLSRKTVSLE	HLQREYASVK	EENERLQKEG	SEKERHNQQL	IQEVRCSGQE	LSRVQEEVKL	RQQLLSQNDK
1530	1540	1550	1560	1570	1580	1590	1600
LLQSLRVELK	AYEKLDEEHR	RLREASGEGW	KGQDPFRDLH	SLLMEIQALR	LQLERSIETS	STLQSRLKEQ	LARGAEKAQE
1610	1620	1630	1640	1650	1660	1670	1680
GALTLAVQAV	SIPEVPLQPD	KHDGDKYPME	SDNSFDLFDS	SQAVTPKSVS	ETPPLSGNDT	DSLSCDSGSS	ATSTPCVSRL
1690	1700	1710	1720	1730	1740	1750	1760
VTGHHLWASK	NGRHVLGLIE	DYEALLKQIS	QGQRLLAEMD	IQTQEAPSST	SQELGTKGPH	PAPLSKFVSS	VSTAKLTLEE
1770	1780	1790	1800	1810	1820	1830	1840
AYRRLKLLWR	VSLPEDGQCP	LHCEQIGEMK	AEVTKLHKKL	FEQEKKLQNT	MKLLQLSKRQ	EKVIFDQLVV	THKILRKARG
1850	1860	1870					
NLELRPGGAH	PGTCSPSRPG	S					

Cmpd.	No. of Cmpds.	m/z meas.	Δ m/z [ppm]	z	Rt [min]	Score	P	Range	Sequence	Modification	Ratios	
93	1	529.5810	3.65	3	30.1	13.6	1	271-283	K.ERETEAAQMEHQK.E		M:Q<br>Q:W	0.65<br>4.87
2305	1	937.4652	-77.24	2	57.1	11.9	1	906-921	R.LPILIKPSRSLGNMYR.L	Oxidation: 14	M:Q	0.26
268	3	501.1673	-209.52	2	32.1	13.8	1	1589-1597	K.EQLARGAEK.A		M:Q<br>Q:W	1.05<br>3.80
578	2	575.8071	-12.69	2	36.1	15.6	1	1756-	K.LTLEEAYRR.L		M:Q	0.48

Detailed Protein Report

Cmpd.	No. of Cmpds.	m/z meas.	Δ m/z [ppm]	z	Rt [min]	Score	P	Range	Sequence	Modification	Ratios
								1764			

Detailed Protein Report

Protein 157: PREDICTED: methylcytosine dioxygenase TET3 isoform X1 [Homo sapiens]

**Accession:**	gi|530367326	**Score:**	65.2
**Database:**	refseq_human(refseq_human_20140103.fasta)	**MW [kDa]:**	193.6
**Database Date:**	2015-11-30	**pI:**	9.0
		**Sequence Coverage [%]:**	3.1
		**No. of unique Peptides:**	1

Alias proteins:

Accession	Name	Description
gi|566559863	refseq_huma (refseq_human_20140103.fasta)	methylcytosine dioxygenase TET3 [Homo sapiens]

10	20	30	40	50	60	70	80
MSQFQVPLAV	QPDLPGLYDF	PQRQVMVGSF	PGSGLSMAGS	ESQLRGGGDG	RKKRKRCGTC	EPCRRLENCG	ACTSCTNRRT
90	100	110	120	130	140	150	160
HQICKLRKCE	VLKKKVGLLK	EVEIKAGEGA	GPWGQGAAVK	TGSELSPVDG	PVPGQMDSGP	VYHGDSRQLS	ASGVPVNGAR
170	180	190	200	210	220	230	240
EPAGPSLLGT	GGPWRVDQKP	DWEAAPGPAH	TARLEDAHDL	VAFSAVAEAV	SSYGALSTRL	YETFNREMSR	EAGNNSRGPR
250	260	270	280	290	300	310	320
PGPEGCSAGS	EDLDTLQTAL	ALARHGMKPP	NCNCDGPECP	DYLEWLEGKI	KSVVMEGGEE	RPRLPGPLPP	GEAGLPAPST
330	340	350	360	370	380	390	400
RPLLSSEVPQ	ISPQEGLPLS	QSALSIAKEK	NISLQTAIAI	EALTQLSSAL	PQPSHSTPQA	SCPLPEALSP	PAPFRSPQSY
410	420	430	440	450	460	470	480
LRAPSWPVVP	PEEHSSFAPD	SSAFPPATPR	TEFPEAWGTD	TPPATPRSSW	PMPRPSPDPM	AELEQLLGSA	SDYIQSVFKR
490	500	510	520	530	540	550	560
PEALPTKPKV	KVEAPSSSPA	PAPSPVLQRE	APTPSSEPDT	HQKAQTALQQ	HLHHKRSLFL	EQVHDTSFPA	PSEPSAPGWW
570	580	590	600	610	620	630	640
PPPSSPVPRL	PDRPPKEKKK	KLPTPAGGPV	GTEKAAPGIK	PSVRKPIQIK	KSRPREAQPL	FPPVRQIVLE	GLRSPASQEV
650	660	670	680	690	700	710	720
QAHPPAPLPA	SQGSAVPLPP	EPSLALFAPS	PSRDSLLPPT	QEMRSPSPMT	ALQPGSTGPL	PPADDKLEEL	IRQFEAEFGD
730	740	750	760	770	780	790	800
SFGLPGPPSV	PIQDPENQQT	CLPAPESPFA	TRSPKQIKIE	SSGAVTVLST	TCFHSEEGGQ	EATPTKAENP	LTPTLSGFLE
810	820	830	840	850	860	870	880
SPLKYLDTPT	KSLLDTPAKR	AQAEFPTCDC	VEQIVEKDEG	PYYTHLGSGP	TVASIRELME	ERYGEKGKAI	RIEKVIYTGK
890	900	910	920	930	940	950	960
EGKSSRGCPI	AKWVIRRHTL	EEKLLCLVRH	RAGHHCQNAV	IVILILAWEG	IPRSLGDTLY	QELTDTLRKY	GNPTSRRCGL
970	980	990	1000	1010	1020	1030	1040
NDDRTCACQG	KDPNTCGASF	SFGCSWSMYF	NGCKYARSKT	PRKFRLAGDN	PKEEEVLRKS	FQDLATEVAP	LYKRLAPQAY
1050	1060	1070	1080	1090	1100	1110	1120
QNQVTNEEIA	IDCRLGLKEG	RPFAGVTACM	DFCAHAHKDQ	HNLYNGCTVV	CTLTKEDNRC	VGKIPEDEQL	HVLPLYKMAN
1130	1140	1150	1160	1170	1180	1190	1200
TDEFGSEENQ	NAKVGSGAIQ	VLTAFPREVR	RLPEPAKSCR	QRQLEARKAA	AEKKKIQKEK	LSTPEKIKQE	ALELAGITSD
1210	1220	1230	1240	1250	1260	1270	1280
PGLSLKGGLS	QQGLKPSLKV	EPQNHFSSFK	YSGNAVVESY	SVLGNCRPSD	PYSMNSVYSY	HSYYAQPSLT	SVNGFHSKYA
1290	1300	1310	1320	1330	1340	1350	1360
LPSFSYYGFP	SSNPVFPSQF	LGPGAWGHSG	SSGSFEKKPD	LHALHNSLSP	AYGGAEFAEL	PSQAVPTDAH	HPTPHHQQPA
1370	1380	1390	1400	1410	1420	1430	1440
YPGPKEYLLP	KAPLLHSVSR	DPSPFAQSSN	CYNRSIKQEP	VDPLTQAEPV	PRDAGKMGKT	PLSEVSQNGG	PSHLWGQYSG
1450	1460	1470	1480	1490	1500	1510	1520
GPSMSPKRTN	GVGGSWGVFS	SGESPAIVPD	KLSSFGASCL	APSHFTDGQW	GLFPGEGQQA	ASHSGGRLRG	KPWSPCKFGN
1530	1540	1550	1560	1570	1580	1590	1600
STSALAGPSL	TEKPWALGAG	DFNSALKGSP	GFQDKLWNPM	KGEEGRIPAA	GASQLDRAWQ	SFGLPLGSSE	KLFGALKSEE
1610	1620	1630	1640	1650	1660	1670	1680
KLWDPFSLEE	GPAEEPPSKG	AVKEEKGGGG	AEEEEEELWS	DSEHNFLDEN	IGGVAVAPAH	GSILIECARR	ELHATTPLKK
1690	1700	1710	1720	1730	1740	1750	1760
PNRCHPTRIS	LVFYQHKNLN	QPNHGLALWE	AKMKQLAERA	RARQEEAARL	GLGQQEAKLY	GKKRKWGGTV	VAEPQQKEKK
1770	1780	1790	1800				
GVVPTRQALA	VPTDSAVTVS	SYAYTKVTGP	YSRWI				

Detailed Protein Report

Cmpd.	No. of Cmpds.	m/z meas.	Δ m/z [ppm]	z	Rt [min]	Score	P	Range	Sequence	Modification
1833	2	697.0345	-7.09	3	51.3	12.9	2	1724-1742	R.QEEAARLGLGQQEAKLYGK.K	

Detailed Protein Report

Protein 158: zinc finger protein 574 [Homo sapiens]

**Accession:**	gi|154744870	**Score:**	65.2
**Database:**	refseq_human(refseq_human_20140103.fasta)	**MW [kDa]:**	98.8
**Database Date:**	2015-11-30	**pI:**	9.7
		**Sequence Coverage [%]:**	2.2
		**No. of unique Peptides:**	1

Quantitation

Q:W	**Median:**	17.81	**CV:**	0.00 %	**No. of Peptides:**	1
M:Q	**Median:**	0.59	**CV:**	0.00 %	**No. of Peptides:**	1

Alias proteins:

Accession	Name	Description
gi|530417050	refseq_human (refseq_human_20140103.fasta)	PREDICTED: zinc finger protein 574 isoform X1 [Homo sapiens]

10	20	30	40	50	60	70	80
MTEESEETVL	YIEHRYVCSE	CNQLYGSLEE	VLMHQNSHVP	QQHFELVGVA	DPGVTVATDT	ASGTGLYQTL	VQESQYQCLE
90	100	110	120	130	140	150	160
CGQLLMSPSQ	LLEHQELHLK	MMAPQEAVPA	EPSPKAPPLS	SSTIHYECVD	CKALFASQEL	WLNHRQTHLR	ATPTKAPAPV
170	180	190	200	210	220	230	240
VLGSPVVLGP	PVGQARVAVE	HSYRKAEEGG	EGATVPSAAA	TTTEVVTEVE	LLLYKCSECS	QLFQLPADFL	EHQATHFPAP
250	260	270	280	290	300	310	320
VPESQEPALQ	QEVQASSPAE	VPVSQPDPLP	ASDHSYELRN	GEAIGRDRRG	RRARRNNSGE	AGGAATQELF	CSACDQLFLS
330	340	350	360	370	380	390	400
PHQLQQHLRS	HREGVFKCPL	CSRVFPSPSS	LDQHLGDHSS	ESHFLCVDCG	LAFGTEALLL	AHRRAHTPNP	LHSCPCGKTF
410	420	430	440	450	460	470	480
VNLTKFLYHR	RTHGVGGVPL	PTTPVPPEEP	VIGFPEPAPA	ETGEPEAPEP	PVSEETSAGP	AAPGTYRCLL	CSREFGKALQ
490	500	510	520	530	540	550	560
LTRHQRFVHR	LERRHKCSIC	GKMFKKKSHV	RNHLRTHTGE	RPFPCPDCSK	PFNSPANLAR	HRLTHTGERP	YRCGDCGKAF
570	580	590	600	610	620	630	640
TQSSTLRQHR	LVHAQHFPYR	CQECGVRFHR	PYRLLMHRYH	HTGEYPYKCR	ECPRSFLLRR	LLEVHQLVVH	AGRQPHRCPS
650	660	670	680	690	700	710	720
CGAAFPSSLR	LREHRCAAAA	AQAPRRFECG	TCGKKVGSAA	RLQAHEAAHA	AAGPGEVLAK	EPPAPRAPRA	TRAPVASPAA
730	740	750	760	770	780	790	800
LGSTATASPA	APARRRGLEC	SECKKLFSTE	TSLQVHRRIH	TGERPYPCPD	CGKAFRQSTH	LKDHRRLHTG	ERPFACEVCG
810	820	830	840	850	860	870	880
KAFAISMRLA	EHRRIHTGER	PYSCPDCGKS	YRSFSNLWKH	RKTHQQQHQA	AVRQQLAEAE	AAVGLAVMET	AVEALPLVEA
890	900						
IEIYPLAEAE	GVQISG						

Cmpd.	No. of Cmpds.	m/z meas.	Δ m/z [ppm]	z	Rt [min]	Score	P	Range	Sequence	Modification	Ratios
2725	1	648.3400	66.02	2	62.5	12.4	0	638-650	R.CPSCGAAFPSSLR.L		M:Q 0.59<br>Q:W 17.81

Detailed Protein Report

Protein 159: latent-transforming growth factor beta-binding protein 4 isoform b [Homo sapiens]

**Accession:**	gi|110347412	**Score:**	65.1
**Database:**	refseq_human(refseq_human_20140103.fasta)	**MW [kDa]:**	169.3
**Database Date:**	2015-11-30	**pI:**	5.0
**Modification(s):**	Carbamidomethyl	**Sequence Coverage [%]:**	4.3
		**No. of unique Peptides:**	4

Quantitation

Q:W	**Median:**	0.42	**CV:**	0.00 %	**No. of Peptides:**	1
M:Q	**Median:**	0.59	**CV:**	196.24 %	**No. of Peptides:**	3

10	20	30	40	50	60	70	80
MGDVKALLFV	AAARARRLGG	AAASESLAVS	EAFCRVRSCQ	PKKCAGPQRC	LNPVPAVPSP	SPSVRKRQVS	LNWQPLTLQE
90	100	110	120	130	140	150	160
ARALLKRRRP	RGPGGRGLLR	RRPPQRAPAG	KAPVLCPLIC	HNGGVCVKPD	RCLCPPDFAG	KFCQLHSSGA	RPPAPAVPGL
170	180	190	200	210	220	230	240
TRSVYTMPLA	NHRDDEHGVA	SMVSVHVEHP	QEASVVVHQV	ERVSGPWEEA	DAEAVARAEA	AARAEAAAPY	TVLAQSAPRE
250	260	270	280	290	300	310	320
DGYSDASGFG	YCFRELRGGE	CASPLPGLRT	QEVCCRGAGL	AWGVHDCQLC	SERLGNSERV	SAPDGPCPTG	FERVNGSCED
330	340	350	360	370	380	390	400
VDECATGGRC	QHGECANTRG	GYTCVCPDGF	LLDSSRSSCI	SQHVISEAKG	PCFRVLRDGG	CSLPILRNIT	KQICCCSRVG
410	420	430	440	450	460	470	480
KAWGRGCQLC	PPFGSEGFRE	ICPAGPGYHY	SASDLRYNTR	PLGQEPPRVS	LSQPRTLPAT	SRPSAGFLPT	HRLEPRPEPR
490	500	510	520	530	540	550	560
PDPRPGPELP	LPSIPAWTGP	EIPESGPSSG	MCQRNPQVCG	PGRCISRPSG	YTCACDSGFR	LSPQGTRCID	VDECRRVPPP
570	580	590	600	610	620	630	640
CAPGRCENSP	GSFRCVCGPG	FRAGPRAAEC	LDVDECHRVP	PPCDLGRCEN	TPGSFLCVCP	AGYQAAPHGA	SCQDVDECTQ
650	660	670	680	690	700	710	720
SPGLCGRGAC	KNLPGSFRCV	CPAGFRGSAC	EEDVDECAQE	PPPCGPGRCD	NTAGSFHCAC	PAGFRSRGPG	APCQDVDECA
730	740	750	760	770	780	790	800
RSPPPCTYGR	CENTEGSFQC	VCPMGFQPNT	AGSECEDVDE	CENHLACPGQ	ECVNSPGSFQ	CRTCPSGHHL	HRGRCTDVDE
810	820	830	840	850	860	870	880
CSSGAPPCGP	HGHCTNTEGS	FRCSCAPGYR	APSGRPGPCA	DVNECLEGDF	CFPHGECLNT	DGSFACTCAP	GYRPGPRGAS
890	900	910	920	930	940	950	960
CLDVDECSEE	DLCQSGICTN	TDGSFECICP	PGHRAGPDLA	SCLDVDECRE	RGPALCGSQR	CENSPGSYRC	VRDCDPGYHA
970	980	990	1000	1010	1020	1030	1040
GPEGTCDDVD	ECQEYGPEIC	GAQRCENTPG	SYRCTPACDP	GYQPTPGGGC	QDVDECRNRS	FCGAHAVCQN	LPGSFQCLCD
1050	1060	1070	1080	1090	1100	1110	1120
QGYEGARDGR	HCVDVNECET	LQGVCGAALC	ENVEGSFLCV	CPNSPEEFDP	MTGRCVPPRT	SAGTFPGSQP	QAPASPVLPA
1130	1140	1150	1160	1170	1180	1190	1200
RPPPPPLPRR	PSTPRQGPVG	SGRRECYFDT	AAPDACDNIL	ARNVTWQECC	CTVGEGWGSG	CRIQQCPGTE	TAEYQSLCPH
1210	1220	1230	1240	1250	1260	1270	1280
GRGYLAPSGD	LSLRRDVDEC	QLFRDQVCKS	GVCVNTAPGY	SCYCSNGYYY	HTQRLECIDN	DECADEEPAC	EGGRCVNTVG
1290	1300	1310	1320	1330	1340	1350	1360
SYHCTCEPPL	VLDGSQRRCV	SNESQSLDDN	LGVCWQEVGA	DLVCSHPRLD	RQATYTECCC	LYGEAWGMDC	ALCPAQDSDD
1370	1380	1390	1400	1410	1420	1430	1440
FEALCNVLRP	PAYSPPRPGG	FGLPYEYGPD	LGPPYQGLPY	GPELYPPPAL	PYDPYPPPPG	PFARREAPYG	APRFDMPDFE
1450	1460	1470	1480	1490	1500	1510	1520
DDGGPYGESE	APAPPGPGTR	WPYRSRDTRR	SFPEPEEPPE	GGSYAGSLAE	PYEELEAEEC	GILDGCTNGR	CVRVPEGFTC
1530	1540	1550	1560	1570	1580	1590	
RCFDGYRLDM	TRMACVDINE	CDEAEAASPL	CVNARCLNTD	GSFRCICRPG	FAPTHQPHHC	APARPRA	

Cmpd.	No. of Cmpds.	m/z meas.	Δ m/z [ppm]	z	Rt [min]	Score	P	Range	Sequence	Modification	Ratios
407	1	846.5265	154.58	2	33.7	10.2	1	541-555	R.LSPQGTRCIDVDECR.R		M:Q 0.77<br>Q:W 0.42
1055	1	941.2810	-147.80	2	42.8	15.8	1	932-949	R.GPALCGSQRCENSPGSYR.C		M:Q 2.35

Detailed Protein Report

Cmpd.	No. of Cmpds.	m/z meas.	Δ m/z [ppm]	z	Rt [min]	Score	P	Range	Sequence	Modification	Ratios	
373	1	457.1205	-287.06	2	34.0	13.0	1	1136-1144	R.QGPVGSGRR.E		M:Q	0.11
1279	1	955.9599	14.50	2	46.1	15.5	2	1215-1229	R.RDVDECQLFRDQVCK.S	Carbamidomethyl: 6		

Detailed Protein Report

Protein 160: zinc finger protein 841 [Homo sapiens]

**Accession:**	gi|211057416	**Score:**	65.0
**Database:**	refseq_human(refseq_human_20140103.fasta)	**MW [kDa]:**	106.0
**Database Date:**	2015-11-30	**pI:**	10.4
**Modification(s):**	Carbamidomethyl	**Sequence Coverage [%]:**	10.8
		**No. of unique Peptides:**	3

10	20	30	40	50	60	70	80
MALPQGSLTF	RDVAVEFSQE	EWKCLDPVQK	ALYRDVMLEN	YRNLGFLGLC	LPDLNIISML	EQGKEPWTVV	SQVKIARNPN
**90**	**100**	**110**	**120**	**130**	**140**	**150**	**160**
CGECMKGVIT	GISPKCVIKE	LPPIQNSNTG	EKFQAVMLEG	HESYDTENFY	FREIRKNLQE	VDFQWKDGEI	NYKEGPMTHK
**170**	**180**	**190**	**200**	**210**	**220**	**230**	**240**
NNLTGQRVRH	SQGDVENKHM	ENQLILRFQS	GLGELQKFQT	AEKIYGCNQI	ERTVNNCFLA	SPLQRIFPGV	QTNISRKYGN
**250**	**260**	**270**	**280**	**290**	**300**	**310**	**320**
DFLQLSLPTQ	DEKTHIREKP	YIGNECGKAF	RVSSSLINHQ	MIHTTEKPYR	CNESGKAFHR	GSLLTVHQIV	HTRGKPYQCD
**330**	**340**	**350**	**360**	**370**	**380**	**390**	**400**
VCGRIFRQNS	DLVNHRRSHT	GDKPYICNEC	GKSFSKSSHL	AVHQRIHTGE	KPYKCNRCGK	CFSQSSSLAT	HQTVHTGDKP
**410**	**420**	**430**	**440**	**450**	**460**	**470**	**480**
YKCNECGKTF	KRNSSLTAHH	IIHAGKKPYT	CDVCGKVFYQ	NSQLVRHQII	HTGETPYKCN	ECGKVFFQRS	RLAGHRRIHT
**490**	**500**	**510**	**520**	**530**	**540**	**550**	**560**
GEKPYKCNEC	GKVFSQHSHL	AVHQRVHTGE	KPYKCNECGK	AFNWGSLLTV	HQRIHTGEKP	YKCNVCGKVF	NYGGYLSVHM
**570**	**580**	**590**	**600**	**610**	**620**	**630**	**640**
RCHTGEKPLH	CNKCGMVFTY	YSCLARHQRM	HTGEKPYKCN	VCGKVFIDSG	NLSIHRRSHT	GEKPFQCNEC	GKVFSYYSCL
**650**	**660**	**670**	**680**	**690**	**700**	**710**	**720**
ARHRKIHTGE	KPYKCNDCGK	AYTQRSSLTK	HLVIHTGENP	YHCNEFGEAF	IQSSKLARYH	RNPTGEKPHK	CSECGRTFSH
**730**	**740**	**750**	**760**	**770**	**780**	**790**	**800**
KTSLVYHQRR	HTGEMPYKCI	ECGKVFNSTT	TLARHRRIHT	GEKPYKCNEC	GKVFRYRSGL	ARHWSIHTGE	KPYKCNECGK
**810**	**820**	**830**	**840**	**850**	**860**	**870**	**880**
AFRVRSILLN	HQMMHTGEKP	YKCNECGKAF	IERSNLVYHQ	RNHTGEKPYK	CMECGKAFGR	RSCLTKHQRI	HSSEKPYKCN
**890**	**900**	**910**	**920**	**930**			
ECGKSYISRS	GLTKHQIKHA	GENLTTKLNV	ERPLDVVLTS	GIPK			

Cmpd.	No. of Cmpds.	m/z meas.	Δ m/z [ppm]	z	Rt [min]	Score	P	Range	Sequence	Modification
1460	1	1011.7530	-79.39	3	48.0	16.8	2	590-616	R.MHTGEKPYKCNVCGKVFIDSGNLSIHR.R	
1732	1	1061.1490	-2.43	3	51.5	11.3	2	617-642	R.RSHTGEKPFQCNECGKVFSYYSCLAR.H	Carbamidomethyl: 11, 14, 23
1286	1	738.4255	114.34	3	45.7	15.0	2	699-716	R.YHRNPTGEKPHKCSECGR.T	Carbamidomethyl: 13, 16

Detailed Protein Report

Protein 161: PREDICTED: serine/threonine-protein kinase 31 isoform X3 [Homo sapiens]

**Accession:**	gi|578813577	**Score:**	64.6
**Database:**	refseq_human(refseq_human_20140103.fasta)	**MW [kDa]:**	95.7
**Database Date:**	2015-11-30	**pI:**	4.8
**Modification(s):**	Carbamidomethyl, Oxidation	**Sequence Coverage [%]:**	4.4
		**No. of unique Peptides:**	4

Quantitation

Q:W	**Median:** 5.37	**CV:** 0.00 %	**No. of Peptides:** 1
M:Q	**Median:** 0.96	**CV:** 0.00 %	**No. of Peptides:** 1

10	20	30	40	50	60	70	80
MWVQGHSSRA	SATESVSFSG	IVQMDEDTHY	DKVEDVVGSH	IEDAVTFWAQ	SINRNKDIMK	IGCSLSEVCP	QASSVLGNLD
90	100	110	120	130	140	150	160
PNKIYGGLFS	EDQCWYRCKV	LKIISVEKCL	VRYIDYGNTE	ILNRSDIVEI	PLELQFSSVA	KKYKLWGLHI	PSDQEVTQFD
170	180	190	200	210	220	230	240
QQEKAAAVDL	TNHLEYTLKT	YIDTRMKNLA	AKMEILKEMR	HVDISVRFGK	DLSDAIQVLD	EGCFTTPASL	NGLEIIWAEY
250	260	270	280	290	300	310	320
SLAQENIKTC	EYVSEGNILI	AQRNEMQQKL	YMSVEDFILE	VDESSLNKRL	KTLQDLSVSL	EAVYGQAKEG	ANSDEILKKF
330	340	350	360	370	380	390	400
YDWKCDKREE	FTSVRSETDA	SLHRLVAWFQ	RTLKVFDLSV	EGSLISEDAM	DNIDEILEKT	ESSVCKELEI	ALVDQGDADK
410	420	430	440	450	460	470	480
EIISNTYSQV	LQKIHSEERL	IATVQAKYKD	SIEFKKQLIE	YLNKSPSVDH	LLSIKKTLKS	LKALLRWKLV	EKSNLEESDD
490	500	510	520	530	540	550	560
PDGSQIEKIK	EEITQLRNNV	FQEIYHEREE	YEMLTSLAQK	WFPELPLLHP	EIGLLKYMNS	GGLLTMSLER	DLLDAEPMKE
570	580	590	600	610	620	630	640
LSSKRPLVRS	EVNGQIILLK	GYSVDVDTEA	KVIERAATYH	RAWREAEGDS	GLLPLIFLFL	CKSDPMAYLM	VPYYPRANLN
650	660	670	680	690	700	710	720
AVQANMPLNS	EETLKVMKGV	AQGLHTLHKA	DIIHGSLHQN	NVFALNREQG	IVGDFDFTKS	VSQRASVNMM	VGDLSLMSPE
730	740	750	760	770	780	790	800
LKMGKPASPG	SDLYAYGCLL	LWLSVQNQEF	EINKDGIPKV	DQFHLDDKVK	SLLCSLICYR	SSMTAEQVLN	AECFLMPKEQ
810	820	830	840	850			
SVPNPEKDTE	YTLYKKEEEI	KTENLDKCME	KTRNGEANFD	C			

Cmpd.	No. of Cmpds.	m/z meas.	Δ m/z [ppm]	z	Rt [min]	Score	P	Range	Sequence	Modification	Ratios
452	1	524.1225	-252.65	2	34.6	17.8	0	551-559	R.DLLDAEPMK.E	Oxidation: 8	
181	11	592.2959	31.07	2	31.1	21.2	0	581-591	K.GYSVDVDTEAK.V		Q:W 5.37<br>M:Q 0.96
2669	1	948.3641	-79.89	2	64.6	15.1	2	817-831	K.EEEIKTENLDKCMEK.T	Carbamidomethyl: 12	
1907	1	734.2871	-85.93	2	52.2	10.5	2	822-833	K.TENLDKCMEKTR.N		

Detailed Protein Report

Protein 162: **PREDICTED: extracellular matrix protein FRAS1 isoform X2 [Homo sapiens]**

**Accession:**	gi|578809381	**Score:**	64.6
**Database:**	refseq_human(refseq_human_20140103.fasta)	**MW [kDa]:**	438.5
**Database Date:**	2015-11-30	**pI:**	5.2
**Modification(s):**	Carbamidomethyl	**Sequence Coverage [%]:**	1.7
		**No. of unique Peptides:**	2

Detailed Protein Report

10	20	30	40	50	60	70	80
MGVLKVWLGL	ALALAEFAVL	PHHSEGACVY	QDSLLADATI	WKPDSCQSCR	CHGDIVICKP	AVCRNPQCAF	EKGEVLQIAA
90	100	110	120	130	140	150	160
NQCCPECVLR	TPGSCHHEKK	IHEHGTEWAS	SPCSVCSCNH	GEVRCTPQPC	PPLSCGHQEL	AFIPEGSCCP	VCVGLGKPCS
170	180	190	200	210	220	230	240
YEGHVFQDGE	DWRLSRCAKC	LCRNGVAQCF	TAQCQPLFCN	QDETVVRVPG	KCCPQCSARS	CSAAGQVYEH	GEQWSENACT
250	260	270	280	290	300	310	320
TCICDRGEVR	CHKQACLPLR	CGKGQSRARR	HGQCCEECVS	PAGSCSYDGV	VRYQDEMWKG	SACEFCMCDH	GQVTCQTGEC
330	340	350	360	370	380	390	400
AKVECARDEE	LIHLDGKCCP	ECISRNGYCV	YEETGEFMSS	NASEVKRIPE	GEKWEDGPCK	VCECRGAQVT	CYEPSCPPCP
410	420	430	440	450	460	470	480
VGTLALEVKG	QCCPDCTSVH	CHPDCLTCSQ	SPDHCDLCQD	PTKLLQNGWC	VHSCGLGFYQ	AGSLCLACQP	QCSTCTSGLE
490	500	510	520	530	540	550	560
CSSCQPPLLM	RHGQCVPTCG	DGFYQDRHSC	AVCHESCAGC	WGPTEKHCLA	CRDPLHVLRD	GGCESSCGKG	FYNRQGTCSA
570	580	590	600	610	620	630	640
CDQSCDSCGP	SSPRCLTCTE	KTVLHDGKCM	SECPGGYYAD	ATGRCKACHS	SCLACMGPAP	SHCTGCKKPE	EGLQVEQLSD
650	660	670	680	690	700	710	720
VGIPSGECLA	QCRAHFYLES	TGICEACHQS	CFRCAGKSPH	NCTDCGPSHV	LLDGQCLSQC	PDGYFHQEGS	CTECHPTCRQ
730	740	750	760	770	780	790	800
CHGPLESDCI	SCYPHISLTN	GNCRTSCREE	QFLNLVGYCA	DCHHLCQHCA	ADLHNTGSIC	LRCQNAHYLL	LGDHCVPDCP
810	820	830	840	850	860	870	880
SGYYAERGAC	KKCHSSCRTC	QGRGPFSCSS	CDTNLVLSHT	GTCSTTCFPG	HYLDDNHVCQ	PCNTHCGSCD	SQASCTSCRD
890	900	910	920	930	940	950	960
PNKVLLFGEC	QYESCAPQYY	LDFSTNTCKE	CDWSCSACSG	PLKTDCLQCM	DGYVLQDGAC	VEQCLSSFYQ	DSGLCKNCDS
970	980	990	1000	1010	1020	1030	1040
YCLQCQGPHE	CTRCKGPFLL	LEAQCVQECG	KGYFADHAKH	KCTACPQGCL	QCSHRDRCHL	CDHGFFLKSG	LCVYNCVPGF
1050	1060	1070	1080	1090	1100	1110	1120
SVHTSNETCS	GKIHTPSLHV	NGSLILPIGS	IKPLDFSLLN	VQDQEGRVED	LLFHVVSTPT	NGQLVLSRNG	KEVQLDKAGR
1130	1140	1150	1160	1170	1180	1190	1200
FSWKDVNEKK	VRFVHSKEKL	RKGYLFLKIS	DQQFFSEPQL	INIQAFSTQA	PYVLRNEVLH	ISRGERATIT	TQMLDIRDDD
1210	1220	1230	1240	1250	1260	1270	1280
NPQDVVIEII	DPPLHGQLLQ	TLQSPATPIY	QFQLDELSRG	LLHYAHDGSD	STSDVAVLQA	NDGHSFHNIL	FQVKTVPQND
1290	1300	1310	1320	1330	1340	1350	1360
RGLQLVANSM	VWVPEGGMLQ	ITNRILQAEA	PGASAEEIIY	KITQDYPQFG	EVVLLVNMPA	DSPADEGQHL	PDGRTATPTS
1370	1380	1390	1400	1410	1420	1430	1440
TFTQQDINEG	IVWYRHSGAP	AQSDSFRFEV	SSASNAQTRL	ESHMFNIAIL	PQTPEAPKVS	LEASLHMTAR	EDGLTVIQPH
1450	1460	1470	1480	1490	1500	1510	1520
SLSFINSEKP	SGKIVYNITL	PLHPNQGIIE	HRDHPHSPIR	YFTQEDINQG	KVMYRPPPAA	PHLQELMAFS	FAGLPESVKF
1530	1540	1550	1560	1570	1580	1590	1600
HFTVSDGEHT	SPEMVLTIHL	LPSDQQLPVF	QVTAPRLAVS	PGGSTSVGLQ	VVVRDAETAP	KELFFELRRP	PQHGVLLKHT
1610	1620	1630	1640	1650	1660	1670	1680
AEFRRPMATG	DTFTYEDVEK	NALQYIHDGS	STREDSMEIS	VTDGLTVTML	EVRVEVSLSE	DRGPRLAAGS	SLSITVASKS
1690	1700	1710	1720	1730	1740	1750	1760
TAIITRSHLA	YVDDSSPDPE	IWIQLNYLPS	YGTLLRISGS	EVEELSEVSN	FTMEDINNKK	IRYSAVFETD	GHLVTDSFYF
1770	1780	1790	1800	1810	1820	1830	1840
SVSDMDHNHL	DNQIFTIMIT	PAENPPPVIA	FADLITVDEG	GRAPLSFHHF	FATDDDDNLQ	RDAIIKLSAL	PKYGCIENTG
1850	1860	1870	1880	1890	1900	1910	1920
TGDRFGPETA	SDLEASFPIQ	DVLENYIYYF	QSVHESIEPT	HDIFSFYVSD	GTSRSEIHSI	NITIERKNDE	PPRMTLQPLR
1930	1940	1950	1960	1970	1980	1990	2000
VQLSSGVVIS	NSSLSLQDLD	TPDNELIFVL	TKKPDHGHVL	WRQTASEPLE	NGRVLVQGST	FTYQDILAGL	VGYVPSVPGM
2010	2020	2030	2040	2050	2060	2070	2080
VVDEFQFSLT	DGLHVDTGRM	KIYTELPASD	TPHLAINQGL	QLSAGSVARI	TEQHLKVTDI	DSDDHQVMYI	MKEDPGAGRL
2090	2100	2110	2120	2130	2140	2150	2160
QMMKHGNLEQ	ISIKGPIRSF	TQADISQGHV	EYSHGTGEPG	GSFAFKFDVV	DGEGNRLIDK	SFSISILEDK	SPPVITTNKG
2170	2180	2190	2200	2210	2220	2230	2240
LVLDENSVKK	ITTLQLSATD	QDSGPTELIY	RITRQPQLGH	LEHAASPGIQ	ISSFTQADLT	SRNVQYVHSS	EAEKHSDAFS
2250	2260	2270	2280	2290	2300	2310	2320
FTLSDGVSEV	TQTFHITLHP	VDDSLPVVQN	LGMRVQEGMR	KTITEFELKA	VDADTEAESV	TFTIVQPPRH	GTIERTSNGQ
2330	2340	2350	2360	2370	2380	2390	2400

Detailed Protein Report

Cmpd.	No. of Cmpds.	m/z meas.	Δ m/z [ppm]	z	Rt [min]	Score	P	Range	Sequence	Modification
2609	1	1176.3241	-122.97	2	60.9	16.9	1	605-627	R.CKACHSSCLACMGPAPSHCTGCK.K	Carbamidomethyl: 1
2457	1	647.2076	-153.78	2	61.2	15.1	0	2338-2349	R.VSYSHDGSNSLK.D	

Detailed Protein Report

Protein 163: **keratin, type II cytoskeletal 73 [Homo sapiens]**

**Accession:**	gi|28173564	**Score:**	64.4
**Database:**	refseq_human(refseq_human_20140103.fasta)	**MW [kDa]:**	58.9
**Database Date:**	2015-11-30	**pI:**	7.6
		**Sequence Coverage [%]:**	6.9
		**No. of unique Peptides:**	1

10	20	30	40	50	60	70	80
MSRQFTYKSG	AAAKGGFSGC	SAVLSGGSSS	SYRAGGKGLS	GGFSSRSLYS	LGGARSISFN	VASGSGWAGG	YGFGRGRASG
90	100	110	120	130	140	150	160
FAGSMFGSVA	LGSVCPSLCP	PGGIHQVTIN	KSLLAPLNVE	LDPEIQKVRA	QEREQIKVLN	NKFASFIDKV	RFLEQQNQVL
170	180	190	200	210	220	230	240
ETKWELLQQL	DLNNCKNNLE	PILEGYISNL	RKQLETLSGD	RVRLDSELRS	VREVVEDYKK	RYEEEINKRT	TAENEFVVLK
250	260	270	280	290	300	310	320
KDVDAAYTSK	VELQAKVDAL	DGEIKFFKCL	YEGETAQIQS	HISDTSIILS	MDNNRNLDLD	SIIAEVRAQY	EEIARKSKAE
330	340	350	360	370	380	390	400
AEALYQTKFQ	ELQLAAGRHG	DDLKHTKNEI	SELTRLIQRL	RSEIESVKKQ	CANLETAIAD	AEQRGDCALK	DARAKLDELE
410	420	430	440	450	460	470	480
GALQQAKEEL	ARMLREYQEL	LSVKLSLDIE	IATYRKLLEG	EECRMSGEYT	NSVSISVINS	SMAGMAGTGA	GFGFSNAGTY
490	500	510	520	530	540	550	
GYWPSSVSGG	YSMLPGGCVT	GSGNCSPRGE	ARTRLGSASE	FRDSQGKTLA	LSSPTKKTMR		

Cmpd.	No. of Cmpds.	m/z meas.	Δ m/z [ppm]	z	Rt [min]	Score	P	Range	Sequence	Modification
2930	1	899.4860	22.51	2	65.4	12.0	1	329-344	K.FQELQLAAGRHGDDLK.H	

Detailed Protein Report

Protein 164: PREDICTED: dmX-like protein 2 isoform X3 [Homo sapiens]

**Accession:**	gi|530405585	**Score:**	64.2
**Database:**	refseq_human(refseq_human_20140103.fasta)	**MW [kDa]:**	291.5
**Database Date:**	2015-11-30	**pI:**	5.9
**Modification(s):**	Carbamidomethyl, Oxidation	**Sequence Coverage [%]:**	2.2
		**No. of unique Peptides:**	3

Detailed Protein Report

10	20	30	40	50	60	70	80
MHLHQVLTGA	VNPGDNCYSV	GSVGDVPFTA	YGSGCDIVIL	ANDFECVQII	PGAKHGNIQV	SCVECSNQQG	RIAASYGNAV
90	100	110	120	130	140	150	160
CIFEPLGINS	HKRNCQLKCQ	WLKTGQFFLS	SVTYNLAWDP	QDNRLLTATD	SIQLWAPPGD	DILEEEEEID	NTVPPVLNDW
170	180	190	200	210	220	230	240
KCVWQCKTSV	SVHLMEWSPD	GEYFATAGKD	DCLLKVWYPM	TGWKSSIIPQ	DHHEVKRRQS	STQFSFVYLA	HPRAVTGFSW
250	260	270	280	290	300	310	320
RKTSKYMPRG	SVCNVLLTSC	HDGVCRLWAE	TLLPEDCLLG	EQICETTTSS	IASSLSHAGR	HKDRIQHALE	TIHHLKNLRK
330	340	350	360	370	380	390	400
GQRRSSVLVT	HAELMPDQTA	MHEVQRHISH	HANALCHFHI	AASINPATDI	PNVLVGTAFN	VDDGNGGFVV	HWLNNKEFHF
410	420	430	440	450	460	470	480
TSSTEVFMHQ	LRKLSDKQVD	HENDDADRED	EEHSQEDRER	GLHMKLDHDL	SLDRESEAGT	GSSEHEDGER	EGSPRTYSRL
490	500	510	520	530	540	550	560
SVPMPLPTVL	LDRKIETLLT	EWNKNPDMLF	TIHPVDGTFL	VWHVKYLDEY	NPGIFRQVQV	SFSSRIPVAF	PSGDASSLSK
570	580	590	600	610	620	630	640
NIMMYACINA	TKDSHHTLLH	QEGMSVGSPH	GSQPHSRSHS	THMNILAPTV	MMISKHIDGS	LNQWAVTFAD	KSAFTTVLTV
650	660	670	680	690	700	710	720
SHKFRYCGHR	FHLNDLACHS	VLPLLLTSSH	HNALLTPELD	CQWDSDNKLS	RLMDPVKHIK	GSSKQPLRNA	ATRTFHDPNA
730	740	750	760	770	780	790	800
IYSELILWRV	DPIGPLSYTG	GVSELARINS	LHTSAFSNVA	WLPTLIPSYC	LGTYCNSASA	CFVASDGKNL	RLYQAVVDAR
810	820	830	840	850	860	870	880
KLLDELSDPE	SSKLIGEVFN	IVSQQSTARP	GCIIELDAIT	NQCGSNTQLL	HVFQEDFIIG	YKPHKEDMEK	KETEIFFQPS
890	900	910	920	930	940	950	960
QGYRPPPFSE	KFFLVVIEKD	SNNNSILHMW	HLHLKSVQAC	LAKASEGASS	ESLLSVPGQK	NVDSSPETSP	SVSPMPHSSS
970	980	990	1000	1010	1020	1030	1040
IANLQTASKL	ILSSRLVYSQ	PLDLPESVEV	IRATPSAGHL	SSSSIYPVCL	APYLVVTTCS	DNKVRFWKCC	MEANPECNKS
1050	1060	1070	1080	1090	1100	1110	1120
DEKEIYHWKR	WPLMNDEGED	NSSTVSIVGR	PVAVSCSYTG	RLAVAYKQPI	HHNGFVSKEF	SMHVCIFECE	STGGSEWVLE
1130	1140	1150	1160	1170	1180	1190	1200
QTIHLDDLVK	VGSVLDSRVS	VDSNLFVYSK	SDALLSKDRY	LIPNIKHLVH	LDWVSKEDGS	HILTVGVGAN	IFMYGRLSGI
1210	1220	1230	1240	1250	1260	1270	1280
VTEQTNSKDG	VAVITLPLGG	SIKQGVKSRW	VLLRSIDLVS	SVDGTPSLPV	SLSWVRDGIL	VVGMDCEMHV	YAQWKHAVKF
1290	1300	1310	1320	1330	1340	1350	1360
GDTEADSSNA	EEAAMQDHST	FKSNMLARKS	VVEGTAISDD	VFCSPTVIQD	GGLFEAAHVL	SPTLPQYHPT	QLLELMDLGK
1370	1380	1390	1400	1410	1420	1430	1440
VRRAKAILSH	LVKCIAGEVA	IVRDPDAGEG	TKRHLSRTIS	VSGSTAKETV	TVGKDGTRDY	TEIDSIPPLP	LYALLAADQD
1450	1460	1470	1480	1490	1500	1510	1520
TSYRISEEST	KIPQSYEDQT	VSQPEDQYSE	LFQIQDIPTD	DIDLEPEKRE	NKSKVINLSQ	YGPAYFGQEH	ARVLSSHLMH
1530	1540	1550	1560	1570	1580	1590	1600
SSLPGLTRLE	QMFLVALADT	VATTSTELDE	SRDKSCSGRD	TLDECGLRYL	LAMRLHTCLL	TSLPPLYRVQ	LLHQGVSTCH
1610	1620	1630	1640	1650	1660	1670	1680
FAWAFHSEAE	EELINMIPAI	QRGDPQWSEL	RAMGIGWWVR	NINTLRRCIE	KVAKASFQRN	NDALDAALFY	LSMKKKAVVW
1690	1700	1710	1720	1730	1740	1750	1760
GLFRSQHDEK	MTTFFSHNFN	EDRWRKAALK	NAFSLLGKQR	FEQSAAFFLL	AGSLKDAIEV	CLEKMEDIQL	AMVIARLYES
1770	1780	1790	1800	1810	1820	1830	1840
EFETSSTYIS	ILNQKILGCQ	KDGSGFSCKR	LHPDPFLRSL	AYWVMKDYTR	ALDTLLEQTP	KEDDEHQVII	KSCNPVAFSF
1850	1860	1870	1880	1890	1900	1910	1920
YNYLRTHPLL	IRRNLASPEG	TLATLGLKTE	KNFVDKINLI	ERKLFFTTAN	AHFKVGCPVL	ALEVLSKIPK	VTKTSALSAK
1930	1940	1950	1960	1970	1980	1990	2000
KDQPDFISHR	MDDVPSHSKA	LSDGNGSSGI	EWSNVTSSQY	DWSQPIVKVD	EEPLNLDWGE	DHDSALDEEE	DDAVGLVMKS
2010	2020	2030	2040	2050	2060	2070	2080
TDAREKDKQS	DQKASDPNML	LTPQEEDDPE	GDTEVDVIAE	QLKFRACLKI	LMTELRTLAT	GYEVDGGKLR	FQLYNWLEKE
2090	2100	2110	2120	2130	2140	2150	2160
IAALHEICNH	ESVIKEYSSK	TYSKVESDLL	DQEEMVDKPD	IGSYERHQIE	RRRLQAKREH	AERRKSWLQK	NQDLLRVFLS
2170	2180	2190	2200	2210	2220	2230	2240

Detailed Protein Report

Cmpd.	No. of Cmpds.	m/z meas.	Δ m/z [ppm]	z	Rt [min]	Score	P	Range	Sequence	Modification
2579	1	731.3629	44.45	2	62.7	16.3	0	561-572	K.NIMMYACINATK.D	Carbamidomethyl: 7; Oxidation: 3, 4
1518	1	816.3814	-111.76	2	49.2	16.9	2	2557-2569	R.SFKRHLLDIFSLR.K	
1839	1	780.2904	-91.58	2	51.5	14.1	1	2574-2588	R.VSCSSHRGVPGGMGR.K	Carbamidomethyl: 3; Oxidation: 13

Detailed Protein Report

Protein 165: PREDICTED: thyroglobulin isoform X9 [Homo sapiens]

**Accession:**	gi|530389281	**Score:**	64.1
**Database:**	refseq_human(refseq_human_20140103.fasta)	**MW [kDa]:**	232.1
**Database Date:**	2015-11-30	**pI:**	5.1
**Modification(s):**	Carbamidomethyl	**Sequence Coverage [%]:**	2.3
		**No. of unique Peptides:**	4

Quantitation

Q:W	**Median:**	0.20	**CV:**	0.00 %	**No. of Peptides:**	1
M:Q	**Median:**	1.34	**CV:**	16.34 %	**No. of Peptides:**	3

Detailed Protein Report

10	20	30	40	50	60	70	80
MALVLEIFTL	LASICWVSAN	IFEYQVDAQP	LRPCELQRET	AFLKQADYVP	QCAEDGSFQT	VQCQNDGRSC	WCVGANGSEV
90	100	110	120	130	140	150	160
LGSRQPGRPV	ACLSFCQLQK	QQILLSGYIN	STDTSYLPQC	QDSGDYAPVQ	CDVQQVQCWC	VDAEGMEVYG	TRQLGRPKRC
170	180	190	200	210	220	230	240
PRSCEIRNRR	LLHGVGDKSP	PQCSAEGEFM	PVQCKFVNTT	DMMIFDLVHS	YNRFPDAFVT	FSSFQRRFPE	VSGYCHCADS
250	260	270	280	290	300	310	320
QGRELAETGL	ELLLDEIYDT	IFAGLDLPST	FTETTLYRIL	QRRFLAVQSV	ISGRFRCPTK	CEVERFTATS	FGHPYVPSCR
330	340	350	360	370	380	390	400
RNGDYQAVQC	QTEGPCWCVD	AQGKEMHGTR	QQGEPPSCAE	GQSCASERQQ	ALSRLYFGTS	GYFSQHDLFS	SPEKRWASPR
410	420	430	440	450	460	470	480
VARFATSCPP	TIKELFVDSG	LLRPMVEGQS	QQFSVSENLL	KEAIRAIFPS	RGLARLALQF	TTNPKRLQQN	LFGGKFLVNV
490	500	510	520	530	540	550	560
GQFNLSGALG	TRGTFNFSQF	FQQLGLASFL	NGGRQEDLAK	PLSVGLDSNS	STGTPEAAKK	DGTMNKPTVG	SFGFEINLQE
570	580	590	600	610	620	630	640
NQNALKFLAS	LLELPEFLLF	LQHAISVPED	VARDLGDVME	TVLSSQTCEQ	TPERLFVPSC	TTEGSYEDVQ	CFSGECWCVN
650	660	670	680	690	700	710	720
SWGKELPGSR	VRGGQPRCPT	DCEKQRARMQ	SLMGSQPAGS	TLFVPACTSE	GHFLPVQCFN	SECYCVDAEG	QAIPGTRSAI
730	740	750	760	770	780	790	800
GKPKKCPTPC	QLQSEQAFLR	TVQALLSNSS	MLPTLSDTYI	PQCSTDGQWR	QVQCNGPPEQ	VFELYQRWEA	QNKGQDLTPA
810	820	830	840	850	860	870	880
KLLVKIMSYR	EAASGNFSLF	IQSLYEAGQQ	DVFPVLSQYP	SLQDVPLAAL	EGKRPQPREN	ILLEPYLFWQ	ILNGQLSQYP
890	900	910	920	930	940	950	960
GSYSDFSTPL	AHFDLRNCWC	VDEAGQELEG	MRSEPSKLPT	CPGSCEEAKL	RVLQFIRETE	EIVSASNSSR	FPLGESFLVA
970	980	990	1000	1010	1020	1030	1040
KGIRLRNEDL	GLPPLFPPRE	AFAEQFLRGS	DYAIRLAAQS	TLSFYQRRRF	SPDDSAGASA	LLRSGPYMPQ	CDAFGSWEPV
1050	1060	1070	1080	1090	1100	1110	1120
QCHAGTGHCW	CVDEKGGFIP	GSLTARSLQI	PQCPTTCEKS	RTSGLLSSWK	QARSQENPSP	KDLFVPACLE	TGEYARLQAS
1130	1140	1150	1160	1170	1180	1190	1200
GAGTWCVDPA	SGEELRPGSS	SSAQCPSLCN	VLKSGVLSRR	VSPGYVPACR	AEDGGFSPVQ	CDQAQGSCWC	VMDSGEEVPG
1210	1220	1230	1240	1250	1260	1270	1280
TRVTGGQPAC	ESPRCPLPFN	ASEVVGGTIL	CETISGPTGS	AMQQCQLLCR	QGSWSVFPPG	PLICSLESGR	WESQLPQPRA
1290	1300	1310	1320	1330	1340	1350	1360
CQRPQLWQTI	QTQGHFQLQL	PPGKMCSADY	ADLLQTFQVF	ILDELTARGF	CQIQVKTFGT	LVSIPVCNNS	SVQVGCLTRE
1370	1380	1390	1400	1410	1420	1430	1440
RLGVNVTWKS	RLEDIPVASL	PDLHDIERAL	VGKDLLGRFT	DLIQSGSFQL	HLDSKTFPAE	TIRFLQGDHF	GTSPRTWFGC
1450	1460	1470	1480	1490	1500	1510	1520
SEGFYQVLTS	EASQDGLGCV	KCPEGSYSQD	EECIPCPVGF	YQEQAGSLAC	VPCPVGRTTI	SAGAFSQTHC	VTDCQRNEAG
1530	1540	1550	1560	1570	1580	1590	1600
LQCDQNGQYR	ASQKDRGSGK	AFCVDGEGRR	LPWWETEAPL	EDSQCLMMQK	FEKVPESKVI	FDANAPVAVR	SKVPDSEFPV
1610	1620	1630	1640	1650	1660	1670	1680
MQCLTDCTED	EACSFFTVST	TEPEISCDFY	AWTSDNVACM	TSDQKRDALG	NSKATSFGSL	RCQVKVRSHG	QDSPAVYLKK
1690	1700	1710	1720	1730	1740	1750	1760
GQGSTTTLQK	RFEPTGFQNM	LSGLYNPIVF	SASGANLTDA	HLFCLLACDR	DLCCDGFVLT	QVQGGAIICG	LLSSPSVLLC
1770	1780	1790	1800	1810	1820	1830	1840
NVKDWMDPSE	AWANATCPGV	TYDQESHQVI	LRLGDQEFIK	SLTPLEGTQD	TFTNFQQVYL	WKDSDMGSRP	ESMGCRKDTV
1850	1860	1870	1880	1890	1900	1910	1920
PRPASPTEAG	LTTELFSPVD	LNQVIVNGNQ	SLSSQKHWLF	KHLFSAQQAN	LWCLSRCVQE	HSFCQLAEIT	ESASLYFTCT
1930	1940	1950	1960	1970	1980	1990	2000
LYPEAQVCDD	IMESNAQGCR	LILPQMPKAL	FRKKVILEDK	VKNFYTRLPF	QKLMGISIRN	KVPMSEKSIS	NGFFECERRC
2010	2020	2030	2040	2050	2060	2070	2080
DADPCCTGFG	FLNVSQLKGG	EVTCLTLNSL	GIQMCSEENG	GAWRILDCGS	PDIEVHTYPF	GWYQKPIAQN	NAPSFCPLVV
2090	2100	2110					
LPSLTEKVFK	VREVRGTQLK	RME					

Detailed Protein Report

Cmpd.	No. of Cmpds.	m/z meas.	Δ m/z [ppm]	z	Rt [min]	Score	P	Range	Sequence	Modification	Ratios	
2716	7	645.8279	25.63	2	64.3	20.3	2	160-169	R.CPRSCEIRNR.R	Carbamidomethyl: 1	M:Q	1.61
865	1	623.9049	-34.22	3	40.3	10.8	0	228-243	R.FPEVSGYCHCADSQGR.E	Carbamidomethyl: 8, 10	M:Q	1.09
1580	1	502.6699	-218.64	2	50.0	19.9	0	467-475	R.LQQNLFGGK.F		Q:W	0.20
1861	1	719.7564	-125.05	2	51.8	13.2	2	1537-1550	R.GSGKAFCVDGEGRR.L		M:Q	1.36

Detailed Protein Report

Protein 166: zinc finger protein 92 isoform 1 [Homo sapiens]

**Accession:**	gi|37537684	**Score:**	64.1
**Database:**	refseq_human(refseq_human_20140103.fasta)	**MW [kDa]:**	60.2
**Database Date:**	2015-11-30	**pI:**	10.3
		**Sequence Coverage [%]:**	11.4
		**No. of unique Peptides:**	3

10	20	30	40	50	60	70	80
MVDKTPVMCS	HFAQDVWPEH	SIKDSFQKVI	LRTYGKYGHE	NLQLRKDHKS	VDACKVYKGG	YNGLNQCLTT	TDSKIFQCDK
**90**	**100**	**110**	**120**	**130**	**140**	**150**	**160**
YVKVFHKFPN	VNRNKIRHTG	KKPFKCKNRG	KSFCMLSQLT	QHKKIHTREY	SYKCEECGKA	FNWSSTLTKH	KIIHTGEKPY
**170**	**180**	**190**	**200**	**210**	**220**	**230**	**240**
KCEECGKAFN	RSSNLTKHKI	IHTGEKPYKC	EECGKAFNRS	STLTKHKRIH	TEEKPYKCEE	CGKAFNQFSI	LNKHKRIHME
**250**	**260**	**270**	**280**	**290**	**300**	**310**	**320**
DKPYKCEECG	KAFRVFSILK	KHKIIHTGEK	PYKCEECGKA	FNQFSNLTKH	KIIHTGEKPY	KCDECGKAFN	QSSTLTKHKR
**330**	**340**	**350**	**360**	**370**	**380**	**390**	**400**
IHTGEKPYKC	EECGKAFKQS	STLTEHKIIH	TGEKPYKCEK	CGKAFSWSSA	FTKHKRNHME	DKPYKCEECG	KAFSVFSTLT
**410**	**420**	**430**	**440**	**450**	**460**	**470**	**480**
KHKIIHTREK	PYKCEECGKA	FNQSSIFTKH	KIIHTEGKSY	KCEKCGNAFN	QSSNLTARKI	IYTGEKPYKY	EECDKAFNKF
**490**	**500**	**510**	**520**				
STLITHQIIY	TGEKPCKHEC	GRAFNKSSNY	TKEKLQT				

Cmpd.	No. of Cmpds.	m/z meas.	Δ m/z [ppm]	z	Rt [min]	Score	P	Range	Sequence	Modification
1095	1	540.7909	-75.68	2	41.9	25.1	1	252-260	K.AFRVFSILK.K	
2650	3	710.3764	51.40	2	61.9	12.6	1	361-373	K.CGKAFSWSSAFTK.H	
2728	6	741.8636	30.01	2	64.4	16.2	0	445-458	K.CGNAFNQSSNLTAR.K	

Detailed Protein Report

Protein 167: fibroblast growth factor receptor substrate 2 [Homo sapiens]

**Accession:**	gi|110347408	**Score:**	63.8
**Database:**	refseq_human(refseq_human_20140103.fasta)	**MW [kDa]:**	57.0
**Database Date:**	2015-11-30	**pI:**	5.6
**Modification(s):**	Carbamidomethyl	**Sequence Coverage [%]:**	12.6
		**No. of unique Peptides:**	5

Alias proteins:

Accession	Name	Description
gi|507834075	refseq_human (refseq_human_20140103.fasta)	fibroblast growth factor receptor substrate 2 [Homo sapiens]
gi|507834073	refseq_human (refseq_human_20140103.fasta)	fibroblast growth factor receptor substrate 2 [Homo sapiens]
gi|507834071	refseq_human (refseq_human_20140103.fasta)	fibroblast growth factor receptor substrate 2 [Homo sapiens]
gi|507834069	refseq_human (refseq_human_20140103.fasta)	fibroblast growth factor receptor substrate 2 [Homo sapiens]
gi|507834066	refseq_human (refseq_human_20140103.fasta)	fibroblast growth factor receptor substrate 2 [Homo sapiens]
gi|507834061	refseq_human (refseq_human_20140103.fasta)	fibroblast growth factor receptor substrate 2 [Homo sapiens]
gi|110347414	refseq_human (refseq_human_20140103.fasta)	fibroblast growth factor receptor substrate 2 [Homo sapiens]

```
        10         20         30         40         50         60         70         80
MGSCCSCPDK DTVPDNHRNK FKVINVDDDG NELGSGIMEL TDTELILYTR KRDSVKWHYL CLRRYGYDSN LFSFESGRRC
        90        100        110        120        130        140        150        160
QTGQGIFAFK CARAEELFNM LQEIMQNNSI NVVEEPVVER NNHQTELEVP RTPRTPTTPG FAAQNLPNGY PRYPSFGDAS
       170        180        190        200        210        220        230        240
SHPSSRHPSV GSARLPSVGE ESTHPLLVAE EQVHTYVNTT GVQEERKNRT SVHVPLEARV SNAESSTPKE EPSSIEDRDP
       250        260        270        280        290        300        310        320
QILLEPEGVK FVLGPTPVQK QLMEKEKLEQ LGRDQVSGSG ANNTEWDTGY DSDERRDAPS VNKLVYENIN GLSIPSASGV
       330        340        350        360        370        380        390        400
RRGRLTSTST SDTQNINNSA QRRTALLNYE NLPSLPPVWE ARKLSRDEDD NLGPKTPSLN GYHNNLDPMH NYVNTENVTV
       410        420        430        440        450        460        470        480
PASAHKIEYS RRRDCTPTVF NFDIRRPSLE HRQLNYIQVD LEGGSDSDNP QTPKTPTTPL PQTPTRRTEL YAVIDIERTA
       490        500        510
AMSNLQKALP RDDGTSRKTR HNSTDLPM
```

Cmpd.	No. of Cmpds.	m/z meas.	Δ m/z [ppm]	z	Rt [min]	Score	P	Range	Sequence	Modification
1454	1	450.0854	-157.84	2	48.4	13.2	0	2-10	M.GSCCSCPDK.D	
390	2	524.3566	177.97	2	33.5	14.0	0	57-63	K.WHYLCLR.R	Carbamidomethyl: 5
2113	1	899.3324	-89.81	2	56.6	12.8	1	64-78	R.RYGYDSNLFSFESGR.R	
2420	1	822.3542	-46.73	2	60.7	12.7	1	80-93	R.CQTGQGIFAFKCAR.A	Carbamidomethyl: 1, 12
1513	1	1023.0018	-57.43	2	49.1	11.1	1	304-322	K.LVYENINGLSIPSASGVRR.G	

Detailed Protein Report

Protein 168: dynein heavy chain 6, axonemal [Homo sapiens]

**Accession:**	gi|194353966	**Score:**	63.7
**Database:**	refseq_human(refseq_human_20140103.fasta)	**MW [kDa]:**	475.7
**Database Date:**	2015-11-30	**pI:**	5.6
**Modification(s):**	Oxidation	**Sequence Coverage [%]:**	1.7
		**No. of unique Peptides:**	4

Alias proteins:

Accession	Name	Description
gi|578802753	refseq_human (refseq_human_20140103.fasta)	PREDICTED: dynein heavy chain 6, axonemal isoform X1 [Homo sapiens]

Detailed Protein Report

10	20	30	40	50	60	70	80
MTFRATDSEF	DLTNIEEYAE	NSALSRLNNI	KAKQRVSYVT	STENESDTQI	LTFRHITKAQ	EKTRKRQQPI	KLEPLPVLKV
90	100	110	120	130	140	150	160
YQDHKQPEYI	HEQNRFQLMT	AGIIKRPVSI	AKKSFATSST	QFLEHQDAVK	KMQIHRPYVE	VFSPSPPKLP	HTGIGKRGLF
170	180	190	200	210	220	230	240
GTRSSAYPKY	TFHDREEVVK	ANIRDPLQII	KIIRENEHLG	FLYMIPAVPR	SSIEYDTYNL	KVVSYENINK	NDYYTISQRA
250	260	270	280	290	300	310	320
VTHIYNEDIE	FIEIDRWEQE	YLYHRELTKI	PIFSLFRKWK	AFSVWRKNVR	SKKITGCQKS	LQKNLFIVNP	HLRPALLKIN
330	340	350	360	370	380	390	400
ELCYHLSFMG	LCYIEKCHTY	TLQEFKAAQV	IRLAEVTERL	GEFRNEAKYV	VRRACRFALR	AAGFVPDDCA	FGPFEDYHKV
410	420	430	440	450	460	470	480
QSSGSFINTP	HELPTYGDSE	KMTYTEQASK	RHYCMRLTCF	IRLNDYLIEN	TMHILTVNAV	NSLLNHLTDK	LKRTPSADVI
490	500	510	520	530	540	550	560
QKWITEEKPE	VPDKKGTLMV	EKQEEDESLI	PMFLTELMLT	VQSLLFEPSL	EDFLDGILGA	VNHCQNTVLS	VPNLVPDSYF
570	580	590	600	610	620	630	640
DAFTSPYINN	KLEGKTCGTG	PSLAAVFEDD	KNFHTIISQI	KETIQAAFES	ARIYAATFEK	FQIFFKENES	LDLQALKLQE
650	660	670	680	690	700	710	720
PDINFFSEQL	EKYHKQHKDA	VALRPTRNVG	LLLIDTRLLR	EKLIPSPLRC	LEVLNFMLPR	QSKKKVDAII	FEAQDAEYKL
730	740	750	760	770	780	790	800
EFVPTTTTEY	VHSLLFLDEI	QERIESLEDE	GNIVTQMYKL	MEQYQVPTPP	EDFAVFATMK	PSIVAVRNAI	DKSVGDRESS
810	820	830	840	850	860	870	880
IKQFCVHLGS	DLEELNNEVN	EVKLQAQDPQ	ILDISADQDK	IRLILNNLQS	VLADLQKRAF	QYKSYQKNFK	VEVSKFEALE
890	900	910	920	930	940	950	960
EVSAELKLKQ	LLWDSFSEWD	KLQQEWLKSK	FDCLDPEVLN	GQVSKYAKFV	TQLEKGLPPN	SVVPQLKYKV	EKMKEKLPVI
970	980	990	1000	1010	1020	1030	1040
IDLRNPTLKA	RHWAAIEQTV	DATLVDAEIP	LTLERLSQLH	VFDFGQEIQD	ISGQASGEAA	LEAILKKVED	SWKTTEFVIL
1050	1060	1070	1080	1090	1100	1110	1120
PHRDSKDVFI	LGGTDDIQVL	LDDSTINVAT	LASSRYLGPL	KTRVDEWQKQ	LALFNQTLEE	WLTCQRNWLY	LESIFNAPDI
1130	1140	1150	1160	1170	1180	1190	1200
QRQLPAEAKM	FLQVDKSWKE	IMRKVNRLPN	ALRAATQPGL	LETFQNNNAL	LDQIQKCLEA	YLESKRVIFP	RFYFLSNDEL
1210	1220	1230	1240	1250	1260	1270	1280
LEILAQTRNP	QAVQPHLRKC	FDSISKLEFA	LMPPAEGKIP	GIDGEPEKVY	TNDILAMLSP	EGERVSLGKG	LKARGNVEEW
1290	1300	1310	1320	1330	1340	1350	1360
LGKVEEAMFT	SLRRLCKAAI	ADYQGKLRTD	WVVAGHPSQV	ILTVSQIMWC	RDLTECLETE	HSNHIQALKN	FEKVNFERLN
1370	1380	1390	1400	1410	1420	1430	1440
ALAAIVQGSL	PKLHRNILTA	LITIDVHARD	IVTELVQSKV	ETVESFDWQR	QLRYYWDIDL	DNCVARMALS	QYTYGYEYLG
1450	1460	1470	1480	1490	1500	1510	1520
ACPRLVITPL	TDRCYLCLMG	ALQLDLGGAP	AGPAGTGKTE	TTKDLAKALA	IQCVVFNCSD	GLDYKMMGRF	FSGLAQSGAW
1530	1540	1550	1560	1570	1580	1590	1600
CCFDEFNRID	IEVLSVIAQQ	LITIRNAKAA	KLSRFMFEGR	EIKLVMTCAA	FITMNPGYAG	RTELPDNLKA	LFRPFAMMVP
1610	1620	1630	1640	1650	1660	1670	1680
NYALIAEVIL	YSEGFESSKI	LARKMTQMYK	LCSEQLSQQD	HYDFGMRAVK	SVLVMAGSLK	RENPDLNEDV	VLIRALQDSN
1690	1700	1710	1720	1730	1740	1750	1760
LPKFLTDDAL	LFSGIISDLF	PGVQIPEHDY	GILQSTIVDV	MNRQNLQPEM	CMVRKVIQFY	ETMLVRHGVM	LVGPTGGGKT
1770	1780	1790	1800	1810	1820	1830	1840
TVYRILAETL	GNLQKLGIEN	SFYQAVKTYV	LNPKSITMGE	LYGEVNNLTL	EWKDGLMALS	VRAAVNDTSE	DHKWIISDGP
1850	1860	1870	1880	1890	1900	1910	1920
VDALWIENMN	TVLDDNKMLC	LANSERIKLT	PQIHMLFEVQ	DLRVASPATV	SRCGMVFVDP	EELKWMPYVK	TWMKGISKKL
1930	1940	1950	1960	1970	1980	1990	2000
TEETQEYILN	LFQRYVDEGL	HFINKKCSQA	IPQVDISKVT	TLCCLLESLI	LGKDGVNLAM	EQTKLNTILC	QTFVFCYLWS
2010	2020	2030	2040	2050	2060	2070	2080
LGGNLTENYY	DSFDTFIRTQ	FDDNPDARLP	NSGDLWSIHM	DFDTKRLDPW	ERIIPTFKYN	RDVPFFEMLV	PTTDTVRYGY
2090	2100	2110	2120	2130	2140	2150	2160
LMEKLLAVKH	SVLFTGITGV	GKSVIAKGLL	NKIQESAGYV	PVYLNFSAQT	SSARTQEIIE	SKLERKRKNI	LGAPGNKRIV
2170	2180	2190	2200	2210	2220	2230	2240

Detailed Protein Report

Cmpd.	No. of Cmpds.	m/z meas.	Δ m/z [ppm]	z	Rt [min]	Score	P	Range	Sequence	Modification
2662	1	698.0710	213.61	2	62.1	10.4	0	1359-1372	R.LNALAAIVQGSLPK.L	
2264	1	1011.7667	170.21	1	56.8	10.2	1	2148-2157	R.KNILGAPGNK.R	
1872	1	973.5013	-53.30	2	51.7	13.0	2	2694-2710	K.NGLTKLLETNILVDKMK.L	Oxidation: 16
2924	1	1015.5128	-43.32	2	65.3	18.0	2	2905-2922	K.LRAAQAELDITMATLREK.Q	

Detailed Protein Report

Protein 169: **PREDICTED: uncharacterized protein LOC101927628 [Homo sapiens]**

Accession: gi|578827891
Database: refseq_human(refseq_human_20140103.fasta)
Database Date: 2015-11-30
Modification(s): Carbamidomethyl
Score: 63.5
MW [kDa]: 109.0
pI: 12.3
Sequence Coverage [%]: 7.8
No. of unique Peptides: 5

Quantitation

M:Q **Median:** 1.17 **CV:** 0.00 % **No. of Peptides:** 1

10	20	30	40	50	60	70	80
MEESAEQAQR	RDETRVGRRR	HSWRSRQSRQ	TKEFIFSELL	SNLYSRRNLQ	TLVEESAEQA	QRCDEMLLLR	AAVQPALAWE
**90**	**100**	**110**	**120**	**130**	**140**	**150**	**160**
REDTRGGVGR	AGRPRSSSSQ	SCCPTCTHVG	TSRHSWRRRQ	SRQTKEFIFS	ELLSNLHSRG	NEKTLVKASA	EKADQGVHLL
**170**	**180**	**190**	**200**	**210**	**220**	**230**	**240**
RAAVQPALAW	EREDTRGGVG	RAGRPRSSSS	QSCCPNCTRV	GTRRHSWRSR	QSRQTKEFIF	SELLSNLYSR	RNLQTLVEES
**250**	**260**	**270**	**280**	**290**	**300**	**310**	**320**
AEQAQRCDEM	LLLRAAVQPA	LAWEREDTRG	GVGRAGRPRS	SSSQSCCPTC	TRVGTRRHSW	RRRQSRQTKE	FIFSELLSNL
**330**	**340**	**350**	**360**	**370**	**380**	**390**	**400**
HSRGNEKTLM	EESAEQAQRR	DETRVGRRRH	SWRSRQSRQT	KEFIFSELLS	NLYSHRNLQT	LVEESAEQAQ	RCDEMLLLRA
**410**	**420**	**430**	**440**	**450**	**460**	**470**	**480**
AVQPALAWER	EDTRGGVGRA	GRPRSSSSQS	CCPTCTHVGT	SRHSWRRRQS	RQTKEFIFSE	LLSNLHSRGN	EKTLVKASAE
**490**	**500**	**510**	**520**	**530**	**540**	**550**	**560**
KADQGVHLLR	AAVQPALAWE	REDTRGGVGR	AGRPRSSSSQ	SCCPNCTRVG	TRRHSWRSRQ	SRHRGVTRLA	WEEEDTPGGV
**570**	**580**	**590**	**600**	**610**	**620**	**630**	**640**
GRAGRPRSSS	SRSCCPTCTR	VGTCRHLWRS	RQSRHSGATR	CCFSELLSNL	HSRGNKKTLV	EALAEQADQG	VHLLRAAVQP
**650**	**660**	**670**	**680**	**690**	**700**	**710**	**720**
VLAWEREDTR	GGVGRAGRPR	SSSSQSCCPT	CTRVGTRRHS	CRSRQSRHSG	VTRLAWEEED	IPGGVGRAGR	ARSSSSRSCC
**730**	**740**	**750**	**760**	**770**	**780**	**790**	**800**
PTCTRVGTCR	HLWRSRQSRH	SGATRCCFSE	LLSNLHSRGN	QKTLVEASAE	QADQGVHLLR	AAVQPVLAWE	REDTRGGVGR
**810**	**820**	**830**	**840**	**850**	**860**	**870**	**880**
AGRPRSSSSQ	SCCPTCTRVG	TRRHSWRSRP	SRHSGVTRLA	WEEEDTPGGV	GRAGRPRSSS	SRSCCPTCTR	VGTCRHLWRS
**890**	**900**	**910**	**920**	**930**	**940**	**950**	**960**
RQSRHSGATR	CCFSELLSNL	HWRGNEKTLV	EASAEQADQG	VHLLRAAVQP	VLAWERADTR	GGVGRAGRPR	NSSSQSCCPT
**970**	**980**						
CTRVGMRRHS							

Cmpd.	No. of Cmpds.	m/z meas.	Δ m/z [ppm]	z	Rt [min]	Score	P	Range	Sequence	Modification	Ratios
2702	1	856.8935	-53.50	2	65.0	10.9	1	71-85	R.AAVQPALAWEREDTR.G		
254	1	977.7698	-10.85	3	31.9	11.3	2	91-117	R. AGRPRSSSSQSCCPTCTHVGTSR R		
2075	3	937.3867	-15.36	2	54.5	13.9	1	280-296	R.SSSSQSCCPTCTRVGTR.R	Carbamidomethyl: 7, 8	
2276	2	937.4372	47.94	2	56.9	12.7	1	951-967	R.NSSSQSCCPTCTRVGMR.R	Carbamidomethyl: 8	
2316	1	937.2798	-119.93	2	57.4	14.7	1	951-967	R.NSSSQSCCPTCTRVGMR.R	Carbamidomethyl: 7	M:Q 1.17

Detailed Protein Report

Protein 170: vacuolar protein sorting-associated protein 13B isoform 1 [Homo sapiens]

**Accession:**	gi|35493701	**Score:**	63.5
**Database:**	refseq_human(refseq_human_20140103.fasta)	**MW [kDa]:**	445.7
**Database Date:**	2015-11-30	**pI:**	6.0
**Modification(s):**	Carbamidomethyl, Oxidation	**Sequence Coverage [%]:**	2.0
		**No. of unique Peptides:**	4

Quantitation

M:Q	**Median:** 1.51	**CV:** 0.00 %	**No. of Peptides:** 1

Detailed Protein Report

10	20	30	40	50	60	70	80
MLESYVTPIL	MSYVNRYIKN	LKPSDLQLSL	WGGDVVLSKL	ELKLDVLEQE	LKLPFTFLSG	HIHELRIHVP	WTKLGSEPVV
90	100	110	120	130	140	150	160
ITINTMECIL	KLKDGIQDDH	ESCGSNSTNR	STAESTKSSI	KPRRMQQAAP	TDPDLPPGYV	QSLIRRVVNN	VNIVINNLIL
170	180	190	200	210	220	230	240
KYVEDDIVLS	VNITSAECYT	VGELWDRAFM	DISATDLVLR	KVINFSDCTV	CLDKRNASGK	IEFYQDPLLY	KCSFRTRLHF
250	260	270	280	290	300	310	320
TYENLNSKMP	SVIKIHTLVE	SLKLSITDQQ	LPMFIRIMQL	GIALYYGEIG	NFKEGEIEDL	TCHNKDMLGN	ITGSEDETRI
330	340	350	360	370	380	390	400
DMQYPAQHKG	QELYSQQDEE	QPQGWVSWAW	SFVPAIVSYD	DGEEDFVGND	PASTMHQQKA	QTLKDPIVSI	GFYCTKATVT
410	420	430	440	450	460	470	480
FKLTEMQVES	SYYSPQKVKS	KEVLCWEQEG	TTVEALMMGE	PFFDCQIGFV	GCRAMCLKGI	MGVKDFEENM	NRSETEACFF
490	500	510	520	530	540	550	560
ICGDNLSTKG	FTYLTNSLFD	YRSPENNGTR	AEFILDSTHH	KETYTEIAGM	QRFGAFYMDY	LYTMENTSGK	GSTNQQDFSS
570	580	590	600	610	620	630	640
GKSEDLGTVQ	EKSTKSLVIG	PLDFRLDSSA	VHRILKMIVC	ALEHEYEPYS	RLKSDIKDEN	ETILNPEEVA	LLEEYIPTRH
650	660	670	680	690	700	710	720
TSVTLLKCTC	TISMAEFNLL	DHLLPVIMGE	KNSSNFMNTT	NFQSLRPLPS	IRILVDKINL	EHSVPMYAEQ	LVHVVSSLTQ
730	740	750	760	770	780	790	800
PSDNLLHYCY	VHCYLKIFGF	QAGLTSLDCS	GSYCLPVPVI	PSFSTALYGK	LLKLPTCWTK	RSQIAITEGI	FELPNLTIQA
810	820	830	840	850	860	870	880
TRAQTLLLQA	IYQSWSHLGN	VSSSAVIEAL	INEIFLSIGV	KSKNPLPTLE	GSIQNVELKY	CSTSLVKCAS	GTMGSIKICA
890	900	910	920	930	940	950	960
KAPVDSGKEK	LIPLLQGPSD	TKDLHSTKWL	NESRKPESLL	APDLMAFTIQ	VPQYIDYCHN	SGAVLLCSIQ	GLAVNIDPIL
970	980	990	1000	1010	1020	1030	1040
YTWLIYQPQK	RTSRHMQQQP	VVAVPLVMPV	CRRKEDEVSI	GSAPLAKQQS	YQASEYASSP	VKTKTVTESR	PLSVPVKAML
1050	1060	1070	1080	1090	1100	1110	1120
NISESCRSPE	ERMKEFIGIV	WNAVKHLTLQ	LEVQSCCVFI	PNDSLPSPST	IVSGDIPGTV	RSWYHGQTSM	PGTLVLCLPQ
1130	1140	1150	1160	1170	1180	1190	1200
IKIISAGHKY	MEPLQEIPFV	IPRPILEEGD	AFPWTISLHN	FSIYTLLGKQ	VTLCLVEPMG	CTSTLAVTSQ	KLLATGPDTR
1210	1220	1230	1240	1250	1260	1270	1280
HSFVVCLHVD	LESLEIKCSN	PQVQLFYELT	DIMNKVWNKI	QKRGNLNLSP	TSPETMAGPV	PTSPVRSSIG	TAPPDTSTCS
1290	1300	1310	1320	1330	1340	1350	1360
PSADIGTTTE	GDSIQAGEES	PFSDSVTLEQ	TTSNIGGTSG	RVSLWMQWVL	PKITIKLFAP	DPENKGTEVC	MVSELEDLSA
1370	1380	1390	1400	1410	1420	1430	1440
SIDVQDVYTK	VKCKIESFNI	DHYRSRPGEG	WQSGHFEGVF	LQCKEKSVTT	TKLLDGTHQQ	HGFLSLTYTK	AVTKNVRHKL
1450	1460	1470	1480	1490	1500	1510	1520
TSRNERRSFH	KLSEGLMDGS	PHFLHEILLS	AQAFDIVLYF	PLLNAIASIF	QAKLPKTQKE	KRKSPGQPMR	THTLTSRNLP
1530	1540	1550	1560	1570	1580	1590	1600
LIYVNTSVIR	IFIPKTEEMQ	PTVEANQAAK	EDTVVLKIGS	VAMAPQADNP	LGRSVLRKDI	YQRALNLGIL	RDPGSEIEDR
1610	1620	1630	1640	1650	1660	1670	1680
QYQIDLQSIN	IGTAQWHQLK	PEKESVSGGV	VTETERNSQN	PALEWNMASS	IRRHQERRAI	LTPVLTDFSV	RITGAPAVIF
1690	1700	1710	1720	1730	1740	1750	1760
TKVVSPENLH	TEEILVCGHS	LEVNITTNLD	FFLSVAQVQL	LHQLIVANMT	GLEPSNKAAE	ISKQEQKKVD	IFDGGMAETS
1770	1780	1790	1800	1810	1820	1830	1840
SRYSGAQDSG	IGSDSVKIRI	VQIEQHSGAS	QHRIARPSRQ	SSIVKNLNFI	PFDIFITASR	ISLMTYSCMA	LSKSKSQEQK
1850	1860	1870	1880	1890	1900	1910	1920
NNEKTDKSSL	NLPEVDSDVA	KPNQACISTV	TAEDLLRSSI	SFPSGKKIGV	LSLESLHAST	RSSARQALGI	TIVRQPGRRG
1930	1940	1950	1960	1970	1980	1990	2000
TGDLQLEPFL	YFIVSQPSLL	LSCHHRKQRV	EVSIFDAVLK	GVASDYKCID	PGKTLPEALD	YCTVWLQTVP	GEIDSKSGIP
2010	2020	2030	2040	2050	2060	2070	2080
PSFITLQIKD	FLNGPADVNL	DISKPLKANL	SFTKLDQINL	FLKKIKNAHS	LAHSEETSAM	SNTMVNKDDL	PVSKYYRGKL
2090	2100	2110	2120	2130	2140	2150	2160
SKPKIHGDGV	QKISAQENMW	RAVSCFQKIS	VQTTQIVISM	ETVPHTSKPC	LLASLSNLNG	SLSVKATQKV	PGIILGSSFL
2170	2180	2190	2200	2210	2220	2230	2240

Detailed Protein Report

Cmpd.	No. of Cmpds.	m/z meas.	Δ m/z [ppm]	z	Rt [min]	Score	P	Range	Sequence	Modification	Ratios
1794	2	1023.9088	-120.39	2	52.3	12.4	1	385-402	K.DPIVSIGFYCTKATVTFK.L	Carbamidomethyl: 10	
1639	1	911.3413	-106.88	2	50.8	14.4	1	2193-2208	K.WCKHSGNPGPEQSIPK.I	Carbamidomethyl: 2	M:Q 1.51
1721	1	682.3288	-51.59	3	51.5	13.2	1	2196-2214	K.HSGNPGPEQSIPKISIDLR.G		
1842	1	1273.5438	-34.83	2	51.4	10.7	1	3110-3132	K.EYFRVPDSATFSICPGGEQPAMK.S	Oxidation: 22	

Detailed Protein Report

Protein 171: glycerol-3-phosphate dehydrogenase, mitochondrial precursor [Homo sapiens]

**Accession:**	gi|285002231	**Score:**	63.4
**Database:**	refseq_human(refseq_human_20140103.fasta)	**MW [kDa]:**	80.8
**Database Date:**	2015-11-30	**pI:**	8.4
**Modification(s):**	Carbamidomethyl, Oxidation	**Sequence Coverage [%]:**	7.0
		**No. of unique Peptides:**	5

Quantitation

Q:W	**Median:** 1.46	**CV:** 0.00 %	**No. of Peptides:**	1
M:Q	**Median:** 1.24	**CV:** 0.00 %	**No. of Peptides:**	1

Alias proteins:

Accession	Name	Description
gi|530370176	refseq_human (refseq_human_20140103.fasta)	PREDICTED: glycerol-3-phosphate dehydrogenase, mitochondrial isoform X1 [Homo sapiens]
gi|285002233	refseq_human (refseq_human_20140103.fasta)	glycerol-3-phosphate dehydrogenase, mitochondrial precursor [Homo sapiens]

```
        10         20         30         40         50         60         70         80
MAFQKAVKGT ILVGGGALAT VLGLSQFAHY RRKQMNLAYV KAADCISEPV NREPPSREAQ LLTLQNTSEF DILVIGGGAT
        90        100        110        120        130        140        150        160
GSGCALDAVT RGLKTALVER DDFSSGTSSR STKLIHGGVR YLQKAIMKLD IEQYRMVKEA LHERANLLEI APHLSAPLPI
       170        180        190        200        210        220        230        240
MLPVYKWWQL PYYWVGIKLY DLVAGSNCLK SSYVLSKSRA LEHFPMLQKD KLVGAIVYYD GQHNDARMNL AIALTAARYG
       250        260        270        280        290        300        310        320
AATANYMEVV SLLKKTDPQT GKVRVSGARC KDVLTGQEFD VRAKCVINAT GPFTDSVRKM DDKDAAICQ PSAGVHIVMP
       330        340        350        360        370        380        390        400
GYYSPESMGL LDPATSDGRV IFFLPWQKMT IAGTTDTPTD VTHHPIPSEE DINFILNEVR NYLSCDVEVR RGDVLAAWSG
       410        420        430        440        450        460        470        480
IRPLVTDPKS ADTQSISRNH VVDISESGLI TIAGGKWTTY RSMAEDTINA AVKTHNLKAG PSRTVGLFLQ GGKDWSPTLY
       490        500        510        520        530        540        550        560
IRLVQDYGLE SEVAQHLAAT YGDKAFEVAK MASVTGKRWP IVGVRLVSEF PYIEAEVKYG IKEYACTAVD MISRRTRLAF
       570        580        590        600        610        620        630        640
LNVQAAEEAL PRIVELMGRE LNWDDYKKQE QLETARKFLY YEMGYKSRSE QLTDRSEISL LPSDIDRYKK RFHKFDADQK
       650        660        670        680        690        700        710        720
GFITIVDVQR VLESINVQMD ENTLHEILNE VDLNKNGQVE LNEFLQLMSA IQKGRVSGSR LAILMKTAEE NLDRRVPIPV
       730
DRSCGGL
```

Cmpd.	No. of Cmpds.	m/z meas.	Δ m/z [ppm]	z	Rt [min]	Score	P	Range	Sequence	Modification	Ratios
810	2	491.6440	-225.52	2	38.5	17.3	0	34-41	K.QMNLAYVK.A	Oxidation: 2	
1028	1	863.9268	-97.49	2	41.6	10.0	2	114-128	K.LIHGGVRYLQKAIMK.L		
2820	1	676.8699	33.04	2	63.9	12.5	0	179-190	K.LYDLVAGSNCLK.S	Carbamidomethyl: 10	
29	1	706.3122	-81.53	3	29.6	11.9	1	179-197	K.LYDLVAGSNCLKSSYVLSK.S	Carbamidomethyl: 10	
2822	1	607.3358	86.08	2	64.4	11.7	0	598-606	K.FLYYEMGYK.S		M:Q 1.24<br>Q:W 1.46

Detailed Protein Report

Protein 172: PREDICTED: 14-3-3 protein epsilon isoform X1 [Homo sapiens]

**Accession:**	gi|530410617	**Score:**	63.4
**Database:**	refseq_human(refseq_human_20140103.fasta)	**MW [kDa]:**	27.4
**Database Date:**	2015-11-30	**pI:**	4.6
**Modification(s):**	Oxidation	**Sequence Coverage [%]:**	11.3
		**No. of unique Peptides:**	2

Quantitation

Q:W **Median:** 0.40 **CV:** 0.00 % **No. of Peptides:** 1

Alias proteins:

Accession	Name	Description
gi|578840292	refseq_human (refseq_human_20140103.fasta)	PREDICTED: 14-3-3 protein epsilon isoform X1 [Homo sapiens]

```
        10         20         30         40         50         60         70         80
MDDREDLVYQ AKLAEQAERY DEMVESMKKV AGMDVELTVE ERNLLSVAYK NVIGARRASW RIISSIEQKE ENKGGEDKLK
        90        100        110        120        130        140        150        160
MIREYRQMVE TELKLICCDI LDVLDKHLIP AANTGESKVF YYKMKGDYHR YLAEFATGND RKEAAENSLV AYKAASDIAM
       170        180        190        200        210        220        230        240
TELPPTHPIR LGLALNFSVF YYEILNSPDR ACRLAKAAFD DAIAELDTLS EESYKDSTLI MQLLRDNLTL WTSDMQGDDS
       250
```

Cmpd.	No. of Cmpds.	m/z meas.	Δ m/z [ppm]	z	Rt [min]	Score	P	Range	Sequence	Modification	Ratios
2589	1	1045.0814	94.73	2	62.4	16.1	2	13-29	K.LAEQAERYDEMVESMKK.V	Oxidation: 11, 15	
2725	7	595.3333	-1.39	2	65.2	47.3	0	216-225	K.DSTLIMQLLR.D		Q:W 0.40

Detailed Protein Report

Protein 173: PREDICTED: autophagy-related protein 2 homolog B isoform X1 [Homo sapiens]

**Accession:**	gi|578825925	**Score:**	63.3
**Database:**	refseq_human(refseq_human_20140103.fasta)	**MW [kDa]:**	232.5
**Database Date:**	2015-11-30	**pI:**	5.4
		**Sequence Coverage [%]:**	2.8
		**No. of unique Peptides:**	3

Quantitation

Q:W	**Median:**	2.74	**CV:**	0.00 %	**No. of Peptides:**	1
M:Q	**Median:**	1.38	**CV:**	0.00 %	**No. of Peptides:**	1

Detailed Protein Report

10	20	30	40	50	60	70	80
MPWPFSESIK	KRACRYLLQR	YLGHFLQEKL	SLEQLSLDLY	QGTGSLAQVP	LDKWCLNEIL	ESADAPLEVT	EGFIQSISLS
90	100	110	120	130	140	150	160
VPWGSLLQDN	CALEVRGLEM	VFRPRPRPAT	GSEPMYWSSF	MTSSMQLAKE	CLSQKLTDEQ	GEGSQPFEGL	EKFAETIETV
170	180	190	200	210	220	230	240
LRRVKVTFID	TVLRIEHVPE	NSKTGTALEI	RIERTVYCDE	TADESSGINV	HQPTAFAHKL	LQLSGVSLFW	DEFSASAKSS
250	260	270	280	290	300	310	320
PVCSTAPVET	EPKLSPSWNP	KIIYEPHPQL	TRNLPEIAPS	DPVQIGRLIG	RLELSLTLKQ	NEVLPGAKLD	VDGQIDSIHL
330	340	350	360	370	380	390	400
LLSPRQVHLL	LDMLAAIAGP	ENSSKIGLAN	KDRKNRPMQQ	EDEYRIQMEL	NRYYLRKDSL	SVGVSSEQSF	YETETARTPS
410	420	430	440	450	460	470	480
SREEVFFSMA	DMDMSHSLSS	LPPLGDPPNM	DLELSLTSTY	TNTPAGSPLS	ATVLQPTWGE	FLDHHKEQPV	RGSTFPSNLV
490	500	510	520	530	540	550	560
HPTPLQKTSL	PSRSVSVDES	RPELIFRLAV	GTFSISVLHI	DPLSPPETSQ	NLNPLTPMAV	AFFTCIEKID	PARFSTEDFK
570	580	590	600	610	620	630	640
SFRAVFAEAC	SHDHLRFIGT	GIKVSYEQRQ	RSASRYFSTD	MSIGQMEFLE	CLFPTDFHSV	PPHYTELLTF	HSKEETGSHS
650	660	670	680	690	700	710	720
PVCLQLHYKH	SENRGPQGNQ	ARLSSVPHKA	ELQIKLNPVC	CELDISIVDR	LNSLLQPQKL	ATVEMMASHM	YTSYNKHISL
730	740	750	760	770	780	790	800
HKAFTEVFLD	DSHSPANCRI	SVQVATPALN	LSVRFPIPDL	RSDQERGPWF	KKSLQKEILY	LAFTDLEFKT	EFIGGSTPEQ
810	820	830	840	850	860	870	880
IKLELTFREL	IGSFQEEKGD	PSIKFFHVSS	GVDGDTTSSD	DFDWPRIVLK	INPPAMHSIL	ERIAAEEEEE	NDGHYQEEEE
890	900	910	920	930	940	950	960
GGAHSLKDVC	DLRRPAPSPF	SSRRVMFENE	QMVMPGDPVE	MTEFQDKAIS	NSHYVLELTL	PNIYVTLPNK	SFYEKLYNRI
970	980	990	1000	1010	1020	1030	1040
FNDLLLWEPT	APSPVETFEN	ISYGIGLSVA	SQLINTFNKD	SFSAFKSAVH	YDEESGSEEE	TLQYFSTVDP	NYRSRRKKKL
1050	1060	1070	1080	1090	1100	1110	1120
DSQNKNSQSF	LSVLLNINHG	LIAVFTDVKQ	DNGDLLENKH	GEFWLEFNSG	SLFCVTKYEG	FDDKHYICLH	SSSFSLYHKG
1130	1140	1150	1160	1170	1180	1190	1200
IVNGVILPTE	TRLPSSTRPH	WLEPTIYSSE	EDGLSKTSSD	GVGGDSLNML	SVAVKILSDK	SESNTKEFLI	AVGLKGATLQ
1210	1220	1230	1240	1250	1260	1270	1280
HRMLPSGLSW	HEQILYFLNI	ADEPVLGYNP	PTSFTTFHVH	LWSCALDYRP	LYLPIRSLLT	VETFSVSSSV	ALDKSSSTLR
1290	1300	1310	1320	1330	1340	1350	1360
IILDEAALHL	SDKCNTVTIN	LSRDYVRVMD	MGLLELTITA	VKSDSDGEQT	EPRFELHCSS	DVVHIRTCSD	SCAALMNLIQ
1370	1380	1390	1400	1410	1420	1430	1440
YIASYGDLQT	PNKADMKPGA	FQRRSKVDSS	GRSSSRGPVL	PEADQQMLRD	LMSDAMEEID	MQQGTSSVKP	QANGVLDEKS
1450	1460	1470	1480	1490	1500	1510	1520
QIQEPCCSDL	FLFPDESGNV	SQESGPTYAS	FSHHFISDAM	TGVPTENDDF	CILFAPKAAM	QEKEEEPVIK	IMVDDAIVIR
1530	1540	1550	1560	1570	1580	1590	1600
DNYFSLPVNK	TDTSKAPLHF	PIPVIRYVVK	EVSLVWHLYG	GKDFGIVPPT	SPAKSYISPH	SSPSHTPTRH	GRNTVCGGKG
1610	1620	1630	1640	1650	1660	1670	1680
RNHDFLMEIQ	LSKVKFQHEV	YPPCKPDCDS	SLSEHPVSRQ	VFIVQDLEIR	DRLATSQMNK	FLYLYCSKEM	PRKAHSNMLT
1690	1700	1710	1720	1730	1740	1750	1760
VKALHVCPES	GRSPQECCLR	VSLMPLRLNI	DQDALFFLKD	FFTSLSAEVE	LQMTPDPEVK	KSPGADVTCS	LPRHLSTSKE
1770	1780	1790	1800	1810	1820	1830	1840
PNLVISFSGP	KQPSQNDSAN	SVEVVNGMEE	KNFSAEEASF	RDQPVFFREF	RFTSEVPIRL	DYHGKHVSMD	QGTLAGILIG
1850	1860	1870	1880	1890	1900	1910	1920
LAQLNCSELK	LKRLSYRHGL	LGVDKLFSYA	ITEWLNDIKK	NQLPGILGGV	GPMHSLVQLV	QGLKDLVWLP	IEQYRKDGRI
1930	1940	1950	1960	1970	1980	1990	2000
VRGFQRGAAS	FGTSTAMAAL	ELTNRMVQTI	QAAAETAYDM	VSPGTLSIEP	KKTKRFPHHR	LAHQPVDLRE	GVAKAYSVVK
2010	2020	2030	2040	2050	2060	2070	2080
EGITDTAQTI	YETAAREHES	RGVTGAVGEV	LRQIPPAVVK	PLIVATEATS	NVLGGMRNQI	RPDVRQDESQ	KWRHGDD

Cmpd.	No. of Cmpds.	m/z meas.	Δ m/z [ppm]	z	Rt [min]	Score	P	Range	Sequence	Modification	Ratios

Detailed Protein Report

Cmpd.	No. of Cmpds.	m/z meas.	Δ m/z [ppm]	z	Rt [min]	Score	P	Range	Sequence	Modification	Ratios	
2179	1	766.3372	-41.60	2	55.9	12.0	0	239-253	K.SSPVCSTAPVETEPK.L		M:Q	1.38
2278	1	713.2212	-186.29	2	56.8	10.0	1	549-560	K.IDPARFSTEDFK.S		Q:W	2.74
1611	1	621.3254	12.46	2	50.4	11.2	2	1590-1601	R.HGRNTVCGGKGR.N			

Detailed Protein Report

Protein 174: numb-like protein [Homo sapiens]

Accession: gi|10863899
Database: refseq_human(refseq_human_20140103.fasta)
Database Date: 2015-11-30

Score: 63.3
MW [kDa]: 64.9
pI: 10.1
Sequence Coverage [%]: 12.8
No. of unique Peptides: 3

Quantitation

M:Q **Median:** 0.52 **CV:** 3.09 % **No. of Peptides:** 2

10	20	30	40	50	60	70	80
MSRSAAASGG	PRRPERHLPP	APCGAPGPPE	TCRTEPDGAG	TMNKLRQSLR	RRKPAYVPEA	SRPHQWQADE	DAVRKGTCSF
**90**	**100**	**110**	**120**	**130**	**140**	**150**	**160**
PVRYLGHVEV	EESRGMHVCE	DAVKKLKAMG	RKSVKSVLWV	SADGLRVVDD	KTKDLLVDQT	IEKVSFCAPD	RNLDKAFSYI
**170**	**180**	**190**	**200**	**210**	**220**	**230**	**240**
CRDGTTRRWI	CHCFLALKDS	GERLSHAVGC	AFAACLERKQ	RREKECGVTA	AFDASRTSFA	REGSFRLSGG	GRPAEREAPD
**250**	**260**	**270**	**280**	**290**	**300**	**310**	**320**
KKKAEAAAAP	TVAPGPAQPG	HVSPTPATTS	PGEKGEAGTP	VAAGTTAAAI	PRRHAPLEQL	VRQGSFRGFP	ALSQKNSPFK
**330**	**340**	**350**	**360**	**370**	**380**	**390**	**400**
RQLSLRLNEL	PSTLQRRTDF	QVKGTVPEME	PPGAGDSDSI	NALCTQISSS	FASAGAPAPG	PPPATTGTSA	WGEPSVPPAA
**410**	**420**	**430**	**440**	**450**	**460**	**470**	**480**
AFQPGHKRTP	SEAERWLEEV	SQVAKAQQQQ	QQQQQQQQQQ	QQQQQQAASV	APVPTMPPAL	QPFPAPVGPF	DAAPAQVAVF
**490**	**500**	**510**	**520**	**530**	**540**	**550**	**560**
LPPPHMQPPF	VPAYPGLGYP	PMPRVPVVGI	TPSQMVANAF	CSAAQLQPQP	ATLLGKAGAF	PPPAIPSAPG	SQARPRPNGA
**570**	**580**	**590**	**600**	**610**			
PWPPEPAPAP	APELDPFEAQ	WAALEGKATV	EKPSNPFSGD	LQKTFEIEL			

Cmpd.	No. of Cmpds.	m/z meas.	Δ m/z [ppm]	z	Rt [min]	Score	P	Range	Sequence	Modification	Ratios
2693	1	1023.5273	37.29	2	64.9	10.3	2	203-221	R.EKECGVTAAFDASRTSFAR.E		M:Q 0.50
2525	1	834.4332	-13.93	2	62.0	11.3	2	227-242	R.LSGGGRPAEREAPDKK.K		
2024	2	788.7867	-185.87	2	53.8	13.7	2	308-321	R.GFPALSQKNSPFKR.Q		M:Q 0.53

Detailed Protein Report

Protein 175: PREDICTED: E3 ubiquitin-protein ligase UBR5 isoform X4 [Homo sapiens]

**Accession:**	gi|530389108	**Score:**	63.3
**Database:**	refseq_human(refseq_human_20140103.fasta)	**MW [kDa]:**	308.7
**Database Date:**	2015-11-30	**pI:**	5.5
**Modification(s):**	Oxidation	**Sequence Coverage [%]:**	2.7
		**No. of unique Peptides:**	3

Quantitation

M:Q	**Median:** 0.43	**CV:** 0.00 %	**No. of Peptides:** 1

Detailed Protein Report

10	20	30	40	50	60	70	80
MTSIHFVVHP	LPGTEDQLND	RLREVSEKLN	KYNLNSHPPL	NVLEQATIKQ	CVVGPNHAAF	LLEDGRVCRI	GFSVQPDRLE
90	100	110	120	130	140	150	160
LGKPDNNDGS	KLNSNSGAGR	TSRPGRTSDS	PWFLSGSETL	GRLAGNTLGS	RWSSGVGGSG	GGSSGRSSAG	ARDSRRQTRV
170	180	190	200	210	220	230	240
IRTGRDRGSG	LLGSQPQPVI	PASVIPEELI	SQAQVVLQGK	SRSVIIRELQ	RTNLDVNLAV	NNLLSRDDED	GDDGDDTASE
250	260	270	280	290	300	310	320
SYLPGEDLMS	LLDADIHSAH	PSVIIDADAM	FSEDISYFGY	PSFRRSSLSR	LGSSRERDSE	LLRERESVLR	LRERRWLDGA
330	340	350	360	370	380	390	400
SFDNERGSTS	KEGEPNLDKK	NTPVQSPVSL	GEDLQWWPDK	DGTKFICIGA	LYSELLAVSS	KGELYQWKWS	ESEPYRNAQN
410	420	430	440	450	460	470	480
PSLHHPRATF	LGLTNEKIVL	LSANSIRATV	ATENNKVATW	VDETLSSVAS	KLEHTAQTYS	ELQGERIVSL	HCCALYTCAQ
490	500	510	520	530	540	550	560
LENSLYWWGV	VPFSQRKKML	EKARAKNKKP	KSSAGISSMP	NITVGTQVCL	RNNPLYHAGA	VAFSISAGIP	KVGVLMESVW
570	580	590	600	610	620	630	640
NMNDSCRFQL	RSPESLKNME	KASKTTEAKP	ESKQEPVKTE	MGPPPSPAST	CSDASSIASS	ASMPYKRRRS	TPAPKEEEKV
650	660	670	680	690	700	710	720
NEEQWSLREV	VFVEDVKNVP	VGKVLKVDGA	YVAVKFPGTS	SNTNCQNSSG	PDADPSSLLQ	DCRLLRIDEL	QVVKTGGTPK
730	740	750	760	770	780	790	800
VPDCFQRTPK	KLCIPEKTEI	LAVNVDSKGV	HAVLKTGNWV	RYCIFDLATG	KAEQENNFPT	SSIAFLGQNE	RNVAIFTAGQ
810	820	830	840	850	860	870	880
ESPIILRDGN	GTIYPMAKDC	MGGIRDPDWL	DLPPISSLGM	GVHSLINLPA	NSTIKKKAAV	IIMAVEKQTL	MQHILRCDYE
890	900	910	920	930	940	950	960
ACRQYLMNLE	QAVVLEQNLQ	MLQTFISHRC	DGNRNILHAC	VSVCFPTSNK	ETKEEEEAER	SERNTFAERL	SAVEAIANAI
970	980	990	1000	1010	1020	1030	1040
SVVSSNGPGN	RAGSSSSRSL	RLREMMRRSL	RAAGLGRHEA	GASSSDHQDP	VSPPIAPPSW	VPDPPAMDPD	GDIDFILAPA
1050	1060	1070	1080	1090	1100	1110	1120
VGSLTTAATG	TGQGPSTSTI	PGPSTEPSVV	ESKDRKANAH	FILKLLCDSV	VLQPYLRELL	SAKDARGMTP	FMSAVSGRAY
1130	1140	1150	1160	1170	1180	1190	1200
PAAITILETA	QKIAKAEISS	SEKEEDVFMG	MVCPSGTNPD	DSPLYVLCCN	DTCSFTWTGA	EHINQDIFEC	RTCGLLESLC
1210	1220	1230	1240	1250	1260	1270	1280
CCTECARVCH	KGHDCKLKRT	SPTAYCDCWE	KCKCKTLIAG	QKSARLDLLY	RLLTATNLVT	LPNSRGEHLL	LFLVQTVARQ
1290	1300	1310	1320	1330	1340	1350	1360
TVEHCQYRPP	RIREDRNRKT	ASPEDSDMPD	HDLEPPRFAQ	LALERVLQDW	NALKSMIMFG	SQENKDPLSA	SSRIGHLLPE
1370	1380	1390	1400	1410	1420	1430	1440
EQVYLNQQSG	TIRLDCFTHC	LIVKCTADIL	LLDTLLGTLV	KELQNKYTPG	RREEAIAVTM	RFLRSVARVF	VILSVEMASS
1450	1460	1470	1480	1490	1500	1510	1520
KKKNNFIPQP	IGKCKRVFQA	LLPYAVEELC	NVAESLIVPV	RMGIARPTAP	FTLASTSIDA	MQGSEELFSV	EPLPPRPSSD
1530	1540	1550	1560	1570	1580	1590	1600
QSSSSSQSQS	SYIIRNPQQR	RISQSQPVRG	RDEEQDDIVS	ADVEEVEVVE	GVAGEEDHHD	EQEEHGEENA	EAEGQHDEHD
1610	1620	1630	1640	1650	1660	1670	1680
EDGSDMELDL	LAAAETESDS	ESNHSNQDNA	SGRRSVVTAA	TAGSEAGASS	VPAFFSEDDS	QSNDSSDSDS	SSSQSDDIEQ
1690	1700	1710	1720	1730	1740	1750	1760
ETFMLDEPLE	RTTNSSHANG	AAQAPRSMQW	AVRNTQHQRA	ASTAPSSTST	PAASSAGLIY	IDPSNLRRSG	TISTSAAAAA
1770	1780	1790	1800	1810	1820	1830	1840
AALEASNASS	YLTSASSLAR	AYSIVIRQIS	DLMGLIPKYN	HLVYSQIPAA	VKLTYQDAVN	LQNYVEEKLI	PTWNWMVSIM
1850	1860	1870	1880	1890	1900	1910	1920
DSTEAQLRYG	SALASAGDPG	HPNHPLHASQ	NSARRERMTA	REEASLRTLE	GRRRATLLSA	RQGMMSARGD	FLNYALSLMR
1930	1940	1950	1960	1970	1980	1990	2000
SHNDEHSDVL	PVLDVCSLKH	VAYVFQALIY	WIKAMNQQTT	LDTPQLERKR	TRELLELGID	NEDSEHENDD	DTNQSATLND
2010	2020	2030	2040	2050	2060	2070	2080
KDDDSLPAET	GQNHPFFRRS	DSMTFLGCIP	PNPFEVPLAE	AIPLADQPHL	LQPNARKEDL	FGRPSQGLYS	SSASSGKCLM
2090	2100	2110	2120	2130	2140	2150	2160
EVTVDRNCLE	VLPTKMSYAA	NLKNVMNMQN	RQKKEGEEQP	VLPEETESSK	PGPSAHDLAA	QLKSSLLAEI	GLTESEGPPL
2170	2180	2190	2200	2210	2220	2230	2240

Detailed Protein Report

Cmpd.	No. of Cmpds.	m/z meas.	Δ m/z [ppm]	z	Rt [min]	Score	P	Range	Sequence	Modification	Ratios
1093	1	647.6752	-180.54	2	42.2	14.8	0	132-146	R.WSSGVGGSGGGSSGR.S		M:Q 0.43
31	1	712.9884	0.22	3	29.6	11.4	1	1299-1317	R.KTASPEDSDMPDHDLEPPR.F		
2778	1	958.9830	42.28	2	66.1	16.7	1	2096-2111	K.MSYAANLKNVMNMQNR.Q	Oxidation: 11, 13	

Detailed Protein Report

Protein 176: PREDICTED: zinc finger protein DZIP1 isoform X6 [Homo sapiens]

**Accession:**	gi|578825196	**Score:**	63.2
**Database:**	refseq_human(refseq_human_20140103.fasta)	**MW [kDa]:**	101.2
**Database Date:**	2015-11-30	**pI:**	6.2
**Modification(s):**	Oxidation	**Sequence Coverage [%]:**	5.8
		**No. of unique Peptides:**	3

10	20	30	40	50	60	70	80
MQAEAADWFS	SMPFQKHVYY	PLASGPEGPD	VAVAAAAAGA	ASMACAPPSA	ASGPLPFFQF	RPRLESVDWR	RLSAIDVDKV
90	100	110	120	130	140	150	160
AGAVDVLTLQ	ENIMNITFCK	LEDEKCPHCQ	SGVDPVLLKL	IRLAQFTIEY	LLHSQEFLTS	QLHTLEERLR	LSHCDGEQSK
170	180	190	200	210	220	230	240
KLLTKQAGEI	KTLKEECKRR	KKMISTQQLM	IEAKANYYQC	HFCDKAFMNQ	AFLQSHIQRR	HTEENSHFEY	QKNAQIEKLR
250	260	270	280	290	300	310	320
SEIVVLKEEL	QLTRSELEAA	HHASAVRFSK	EYEMQKTKEE	DFLKLFDRWK	EEEKEKLVDE	MEKVKEMFMK	EFKELTSKNS
330	340	350	360	370	380	390	400
ALEYQLSEIQ	KSNMQIKSNI	GTLKDAHEFK	EDRSPYPQDF	HNVMQLLDSQ	ESKWTARVQA	IHQEHKKEKG	RLLSHIEKLR
410	420	430	440	450	460	470	480
TSMIDDLNAS	NVFYKKRIEE	LGQRLQEQNE	LIITQRQQIK	DFTCNPLNSI	SEPKGNPLAW	QAFESQPAAP	AVPMNAPALH
490	500	510	520	530	540	550	560
TLETKSSLPM	VHEQAFSSHI	LEPIEELSEE	EKGRENEQKL	NNNKMHLRKA	LKSNSSLTKG	LRTMVEQNLM	EKLETLGINA
570	580	590	600	610	620	630	640
DIRGISSDQL	HRVLKSVESE	RHKQEREIPN	FHQIREFLEH	QVSCKIEEKA	LLSSDQCSVS	QMDTLSTGEV	PKMIQLPSKN
650	660	670	680	690	700	710	720
RQLIRQKAVS	TDRTSVPKIK	KNVMEDPFPR	KSSTITTPPF	SSEEEQEDDD	LIRAYASPGP	LPVPPPQNKG	SFGKNTVKSD
730	740	750	760	770	780	790	800
ADGTEGSEIE	DTDDSPKPAG	VAVKTPTEKV	EKMFPHRKNV	NKPVGGTNVP	EMFIKKEELQ	ELKCADVEDE	DWDISSLEEE
810	820	830	840	850	860	870	880
ISLGKKSGKE	QKEPPPAKNE	PHFAHVLNAW	GAFNPKGPKG	EGLLLSPLIQ	ARSIFLLVVF	HLYKYLINLC	WYIIRQKSCQ
890							
CFLYVI							

Cmpd.	No. of Cmpds.	m/z meas.	Δ m/z [ppm]	z	Rt [min]	Score	P	Range	Sequence	Modification
1895	3	704.7670	-135.29	2	52.2	17.1	0	183-194	K.MISTQQLMIEAK.A	Oxidation: 8
2012	1	616.7504	-93.75	2	55.5	10.3	1	662-671	K.NVMEDPFPRK.S	
1639	1	1023.6248	76.10	1	49.2	11.8	1	810-818	K.EQKEPPPAK.N	

Detailed Protein Report

Protein 177: PREDICTED: chromodomain-helicase-DNA-binding protein 9 isoform X9 [Homo sapiens]

**Accession:**	gi|530424378	**Score:**	63.1
**Database:**	refseq_human(refseq_human_20140103.fasta)	**MW [kDa]:**	269.5
**Database Date:**	2015-11-30	**pI:**	8.8
		**Sequence Coverage [%]:**	2.6
		**No. of unique Peptides:**	3

Quantitation

Q:W	**Median:**	0.45	**CV:**	18.30 %	**No. of Peptides:**	2
M:Q	**Median:**	1.55	**CV:**	0.00 %	**No. of Peptides:**	1

Detailed Protein Report

10	20	30	40	50	60	70	80
MSEKKQRKKV	ESESKQEKAN	RIISEAIAKA	KERGERNIPR	VMSPENFPTA	SVEGKEEKKG	RRMKSKPKDK	DSKKTKTCSK
90	100	110	120	130	140	150	160
LKEKTKIGKL	IITLGKKQKR	KNESSDEISD	AEQMPQHTLK	DQDSQKRRSN	RQIKRKKYAE	DIEGKQSEEE	VKGSMKIKKN
170	180	190	200	210	220	230	240
SAPLPGEQPL	QLFVENPSEE	DAAIVDKILS	SRTVKKEISP	GVMIDTEEFF	VKYKNYSYLH	CEWATEEQLL	KDKRIQQKIK
250	260	270	280	290	300	310	320
RFKLRQAQRA	HFFADMEEEP	FNPDYVEVDR	VLEVSFCEDK	DTGEPVIYYL	VKWCSLPYED	STWELKEDVD	LAKIEEFEQL
330	340	350	360	370	380	390	400
QASRPDTRRL	DRPPSNIWKK	IDQSRDYKNG	NQLREYQLEG	LNWLLFNWYN	RRNCILADEM	GLGKTIQSIT	FLYEILLTGI
410	420	430	440	450	460	470	480
RGPFLIIAPL	STIANWEREF	RTWTDINVVV	YHGSLISRQM	IQQYEMYFRD	SQGRIIRGAY	RFQAIITTFE	MILGGCGELN
490	500	510	520	530	540	550	560
AIEWRCVIID	EAHRLKNKNC	KLLEGLKLMN	LEHKVLLTGT	PLQNTVEELF	SLLHFLEPLR	FPSESTFMQE	FGDLKTEEQV
570	580	590	600	610	620	630	640
QKLQAILKPM	MLRRLKEDVE	KKLAPKEETI	IEVELTNIQK	KYYRAILEKN	FSFLSKGAGQ	TNVPNLVNTM	MELRKCCNHP
650	660	670	680	690	700	710	720
YLIKGAEEKI	LGEFRDTYNP	AASDFHLQAM	IQSAGKLVLI	DKLLPKMKAG	GHKVLIFSQM	VRCLDILEDY	LIHKRYLYER
730	740	750	760	770	780	790	800
IDGRVRGNLR	QAAIDRFSKP	DSDRFVFLLC	TRAGGLGINL	TAADTCIIFD	SDWNPQNDLQ	AQARCHRIGQ	NKAVKVYRLV
810	820	830	840	850	860	870	880
TRNSYEREMF	DRASLKLGLD	KAVLQSMSGR	ESNVGGIQQL	SKKEIEDLLR	RGAYGAIMEE	EDEGSKFCEE	DIDQILLRRT
890	900	910	920	930	940	950	960
KTITIESEGR	GSTFAKASFV	ASGNRTDISL	DDPNFWQKWA	KKAEIDIEAI	SGRNSLVIDT	PRIRKQTRPF	SATKDELAEL
970	980	990	1000	1010	1020	1030	1040
SEAESEGDEK	PKLRRPCDRS	NGYGRTECFR	VEKNLLVYGW	GRWREILSHG	RFKRQLNEHD	VEIICRALLA	YCLVHYRGDE
1050	1060	1070	1080	1090	1100	1110	1120
KIKGFIWDLI	TPTEDGQTRE	LQNHLGLSAP	VPRGRKGKKV	KTQTSSFDIQ	KAEWLRKYNP	EQLLQDEGYK	KHIKHHCNKV
1130	1140	1150	1160	1170	1180	1190	1200
LLRVRMLYYL	KQEVIGNECQ	KVFDGVDASD	IDVWVPEPDH	SEVPAEWWDF	DADKSLLIGV	FKHGYEKYNT	IRADPALCFL
1210	1220	1230	1240	1250	1260	1270	1280
ERVGKPDEKA	VAAEQRANDY	MDGDVEDPEY	KPAPAIFKDD	IEDDVSSPGD	LVIADGDGQL	MEGDKVYWPT	QSALTTRLRR
1290	1300	1310	1320	1330	1340	1350	1360
LITAYQRTNK	NRQIQQIQPT	FSVPTSVMQP	IYEEATLNPK	MAAKIERQQR	WTRREEADFY	RVVSTFGVVF	DPDRGQFDWT
1370	1380	1390	1400	1410	1420	1430	1440
KFRAMARLHK	KTDDSLEKYL	YAFMSMCRRV	CRLPSKEELV	DPNIFIQPIT	EERASRTLYR	IELLRKVREQ	ALRHPQLFER
1450	1460	1470	1480	1490	1500	1510	1520
LKLCHPNPDL	PVWWECGPHD	RDLLIGAAKH	GVSRTDYHIL	RDPELSFMAA	QRNYSQSKMA	HSRTSTPLLQ	QYQVALSASP
1530	1540	1550	1560	1570	1580	1590	1600
LTSLPRLLDA	KGIILEEMKV	KSENLKEEPQ	SSEEESMSSV	ETRTLIKSEP	VSPKNGVLPQ	ATGDQKSGGK	CETDRRMVAA
1610	1620	1630	1640	1650	1660	1670	1680
RTEPLTPNPA	SKKPRVHKRG	SESSSDSDSD	SERSSCSSRS	SSSSSSSSCS	HSRSGSSSSS	SSSCSSASSS	SSSSTSSSSS
1690	1700	1710	1720	1730	1740	1750	1760
SSSSSSEESD	SDEEEAQKRA	ESTTHMKAYD	EESVASLSTT	QDETQDSFQM	NNGTPESAYI	LQGGYMLAAS	YWPKDRVMIN
1770	1780	1790	1800	1810	1820	1830	1840
RLDSICQTVL	KGKWPSARRS	YDANTVASFY	TTKLLDSPGA	ATEYSDPSVP	TPPGAGVKEE	HDQSTQMSKV	KKHVREKEFT
1850	1860	1870	1880	1890	1900	1910	1920
VKIKDEGGLK	LTFQKQGLAQ	KRPFDGEDGA	LGQQQYLTRL	RELQSASETS	LVNFPKSIPV	SGTSIQPTLG	ANGVILDNQP
1930	1940	1950	1960	1970	1980	1990	2000
IVKKRRGRRK	NVEGVDIFFF	NRNKPPNHVS	LGLTSSQIST	GINPALSYTQ	PQGIPDTESP	VPVINLKDGT	RLAGDDAPKR
2010	2020	2030	2040	2050	2060	2070	2080
KDLEKWLKEH	PGYVEDLGAF	IPRMQLHEGR	PKQKRHRCRN	PNKLDVNSLT	GEERVQLINR	RNARKVGGAF	APPLKDLCRF
2090	2100	2110	2120	2130	2140	2150	2160
LKENSEYGVA	PEWGDVVKQS	GFLPESMYER	ILTGPVVREE	VSRRGRRPKS	GIAKATAAAA	AASATSVSGN	PLLANGLLPG
2170	2180	2190	2200	2210	2220	2230	2240

Detailed Protein Report

Cmpd.	No. of Cmpds.	m/z meas.	Δ m/z [ppm]	z	Rt [min]	Score	P	Range	Sequence	Modification	Ratios
85	1	710.2749	-156.22	2	30.8	10.2	1	994-1004	K.NLLVYGWGRWR.E		
2019	1	719.3122	-83.44	2	55.6	13.7	0	1342-1354	R.VVSTFGVVFDPDR.G		Q:W 0.54
2610	2	449.6838	-219.89	2	60.9	14.6	1	2055-2061	R.VQLINRR.N		M:Q 1.55<br>Q:W 0.37

Detailed Protein Report

Protein 178: **PREDICTED: arf-GAP with Rho-GAP domain, ANK repeat and PH domain-containing protein 3 isoform X1 [Homo sapiens]**

**Accession:**	gi|530380655	**Score:**	63.0
**Database:**	refseq_human(refseq_human_20140103.fasta)	**MW [kDa]:**	168.0
**Database Date:**	2015-11-30	**pI:**	6.4
**Modification(s):**	Oxidation	**Sequence Coverage [%]:**	2.5
		**No. of unique Peptides:**	2

10	20	30	40	50	60	70	80
MAAPQDLDIA	VWLATVHLEQ	YADTFRRHGL	ATAGAARGLG	HEELKQLGIS	ATGHRKRILR	LLQTGTEEGS	LDPKSDSAME
90	100	110	120	130	140	150	160
PSPSPAPQAQ	PPKPVPKPRT	VFGGLSGPAT	TQRPGLSPAL	GGPGVSRSPE	PSPRPPPLPT	SSSEQSSALN	TVEMMPNSIY
170	180	190	200	210	220	230	240
FGLDSRGRAQ	AAQDKAPDSS	QISAPTPALR	PTTGTVHIMD	PGCLYYGVQP	VGTPGAPDRR	ESRGVCQGRA	EHRLSRQDLE
250	260	270	280	290	300	310	320
AREDAGYASL	ELPGDSTLLS	PTLETEETSD	DLISPYASFS	FTADRLTPLL	SGWLDKLSPQ	GNYVFQRRFV	QFNGRSLMYF
330	340	350	360	370	380	390	400
GSDKDPFPKG	VIPLTAIEMT	RSSKDNKFQV	ITGQRVFVFR	TESEAQRDMW	CSTLQSCLKE	QRLLGHPRPP	QPPRPLRTGM
410	420	430	440	450	460	470	480
LELRGHKAKV	FAALSPGELA	LYKSEQAFSL	GIGICFIELQ	GCSVRETKSR	SFDLLTPHRC	FSFTAESGGA	RQSWAAALQE
490	500	510	520	530	540	550	560
AVTETLSDYE	VAEKIWSNRA	NRQCADCGSS	RPDWAAVNLG	VVICKQCAGQ	HRALGSGISK	VQSLKLDTSV	WSNEIVQLFI
570	580	590	600	610	620	630	640
VLGNDRANRF	WAGTLPPGEG	LHPDATPGPR	GEFISRKYRL	GLFRKPHPQY	PDHSQLLQAL	CAAVARPNLL	KNMTQLLCVE
650	660	670	680	690	700	710	720
AFEGEEPWFP	PAPDGSCPGL	LPSDPSPGVY	NEVVVRATYS	GFLYCSPVSN	KAGPSPPRRG	RDAPPRLWCV	LGAALEMFAS
730	740	750	760	770	780	790	800
ENSPEPLSLI	QPQDIVCLGV	SPPPTDPGDR	FPFSFELILA	GGRIQHFGTD	GADSLEAWTS	AVGKWFSPLS	CHQLLGPGLL
810	820	830	840	850	860	870	880
RLGRLWLRSP	SHTAPAPGLW	LSGFGLLRGD	HLFLCSAPGP	GPPAPEDMVH	LRRLQEISVV	SAADTPDKKE	HLVLVETGRT
890	900	910	920	930	940	950	960
LYLQGEGRLD	FTAWNAAIGG	AAGGGGTGLQ	EQQMSRGDIP	IIVDACISFV	TQHGLRLEGV	YRKGGARARS	LRLLAEFRRD
970	980	990	1000	1010	1020	1030	1040
ARSVKLRPGE	HFVEDVTDTL	KRFFRELDDP	VTSARLLPRW	REAAELPQKN	QRLEKYKDVI	GCLPRVNRRT	LATLIGHLYR
1050	1060	1070	1080	1090	1100	1110	1120
VQKCAALNQM	CTRNLALLFA	PSVFQTDGRG	EHEVRVLQEL	IDGYISVFDI	DSDQVAQIDL	EVSLITTWKD	VQLSQAGDLI
1130	1140	1150	1160	1170	1180	1190	1200
MEVYIEQQLP	DNCVTLKVSP	TLTAEELTNQ	VLEMRGTAAG	MDLWVTFEIR	EHGELERPLH	PKEKVLEQAL	QWCQLPEPCS
1210	1220	1230	1240	1250	1260	1270	1280
ASLLLKKVPL	AQAGCLFTGI	RRESPRVGLL	RCREEPPRLL	GSRFQERFFL	LRGRCLLLLK	EKKSSKPERE	WPLEGAKVYL
1290	1300	1310	1320	1330	1340	1350	1360
GIRKKLKPPT	PWGFTLILEK	MHLYLSCTDE	DEMWDWTTSI	LKAQHDDQQP	VVLRRHSSSD	LARQKFGTMP	LLPIRGDDSG
1370	1380	1390	1400	1410	1420	1430	1440
ATLLSANQTL	PMKSSQGSVE	EQEELEEPVY	EEPVYEEVGA	FPELIQDTST	SFSTTREWTV	KPENPLTSQK	SLDQPFLSKS
1450	1460	1470	1480	1490	1500	1510	1520
STLGQEERPP	EPPPGPPSKS	SPQARGSLEE	QLLQELSSLI	LRKGETTAGL	GSPSQPSSPQ	SPSPTGLPTQ	TPGFPTQPPC
1530	1540						
TSSPPSSQPL	T						

Cmpd.	No. of Cmpds.	m/z meas.	Δ m/z [ppm]	z	Rt [min]	Score	P	Range	Sequence	Modification
164	1	715.8153	8.42	2	31.4	24.0	0	368-379	R.DMWCSTLQSCLK.E	Oxidation: 2
1500	1	850.8827	-87.14	2	48.9	14.3	1	854-869	R.LQEISVVSAADTPDKK.E	

Detailed Protein Report

Protein 179: **PREDICTED: UDP-glucose:glycoprotein glucosyltransferase 1 isoform X3 [Homo sapiens]**

Accession: gi|578804605
Database: refseq_human(refseq_human_20140103.fasta)
Database Date: 2015-11-30
Modification(s): Carbamidomethyl, Oxidation

Score: 63.0
MW [kDa]: 176.2
pI: 5.3
Sequence Coverage [%]: 5.6
No. of unique Peptides: 5

10	20	30	40	50	60	70	80
MGCKGDASGV	CYKMGVLVVL	TVLWLFSSVK	ADSKAITTSL	TTKWFSTPLL	LEASEFLAED	SQEKFWNFVE	ASQNIGSSDH
90	100	110	120	130	140	150	160
DGTDYSYYHA	ILEAAFQFLS	PLQQNLFKFC	LSLRSYSATI	QAFQQIAADE	PPPEGCNSFF	SVHGKKTCES	DTLEALLLTA
170	180	190	200	210	220	230	240
SERPKPLLFK	GDHRYPSSNP	ESPVVIFYSE	IGSEEFSNFH	RQLISKSNAG	KINYVFRHYI	FNPRKEPVYL	SGYGVELAIK
250	260	270	280	290	300	310	320
STEYKAKDDT	QVKGTEVNTT	VIGENDPIDE	VQGFLFGKLR	DLHPDLEGQL	KELRKHLVES	TNEMAPLKVW	QLQDLSFQTA
330	340	350	360	370	380	390	400
ARILASPVEL	ALVVMKDLSQ	NFPTKARAIT	KTAVSSELRT	EVEENQKYFK	GTLGLQPGDS	ALFINGLHMD	LDTQDIFSLF
410	420	430	440	450	460	470	480
DVLRNEARVM	EGLHRLGIEG	LSLHNVLKLN	IQPSEADYAV	DIRSPAISWV	NNLEVDSRYN	SWPSSLQELL	RPTFPGVIRQ
490	500	510	520	530	540	550	560
IRKNLHNMVF	IVDPAHETTA	ELMNTAEMFL	SNHIPLRIGF	IFVVNDSEDV	DGMQDAGVAV	LRAYNYVAQE	VDDYHAFQTL
570	580	590	600	610	620	630	640
THIYNKVRTG	EKVKVEHVVS	VLEKKYPYVE	VNSILGIDSA	YDRNRKEARG	YYEQTGVGPL	PVVLFNGMPF	EREQLDPDEL
650	660	670	680	690	700	710	720
ETITMHKILE	TTTFFQRAVY	LGELPHDQDV	VEYIMNQPNV	VPRINSRILT	AERDYLDLTA	SNNFFVDDYA	RFTILDSQGK
730	740	750	760	770	780	790	800
TAAVANSMNY	LTKKGMSSKE	IYDDSFIRPV	TFWIVGDFDS	PSGRQLLYDA	IKHQKSSNNV	RISMINNPAK	EISYENTQIS
810	820	830	840	850	860	870	880
RAIWAALQTQ	TSNAAKNFIT	KMAKEGAAEA	LAAGADIAEF	SVGGMDFSLF	KEVFESSKMD	FILSHAVYCR	DVLKLKKGQR
890	900	910	920	930	940	950	960
AVISNGRIIG	PLEDSELFNQ	DDFHLLENII	LKTSGQKIKS	HIQQLRVEED	VASDLVMKVD	ALLSAQPKGD	PRIEYQFFED
970	980	990	1000	1010	1020	1030	1040
RHSAIKLRPK	EGETYFDVVA	VVDPVTREAQ	RLAPLLLVLA	QLINMNLRVF	MNCQSKLSDM	PLKSFYRYVL	EPEISFTSDN
1050	1060	1070	1080	1090	1100	1110	1120
SFAKGPIAKF	LDMPQSPLFT	LNLNTPESWM	VESVRTPYDL	DNIYLEEVDS	VVAAEYELEY	LLLEGHCYDI	TTGQPPRGLQ
1130	1140	1150	1160	1170	1180	1190	1200
FTLGTSANPV	IVDTIVMANL	GYFQLKANPG	AWILRLRKGR	SEDIYRIYSH	DGTDSPPDAD	EVVIVLNNFK	SKIIKVKVQK
1210	1220	1230	1240	1250	1260	1270	1280
KADMVNEDLL	SDGTSENESG	FWDSFKWGFT	GQKTEEVKQD	KDDIINIFSV	ASGHLYERFL	RIMMLSVLKN	TKTPVKFWFL
1290	1300	1310	1320	1330	1340	1350	1360
KNYLSPTFKE	FIPYMANEYN	FQYELVQYKW	PRWLHQQTEK	QRIIWGYKIL	FLDVLFPLVV	DKFLFVDADQ	IVRTDLKELR
1370	1380	1390	1400	1410	1420	1430	1440
DFNLDGAPYG	YTPFCDSRRE	MDGYRFWKSG	YWASHLAGRK	YHISALYVVD	LKKFRKIAAG	DRLRGQYQGL	SQDPNSLSNL
1450	1460	1470	1480	1490	1500	1510	1520
DQDLPNNMIH	QVPIKSLPQE	WLWCETWCDD	ASKKRAKTID	LCNNPMTKEP	KLEAAVRIVP	EWQDYDQEIK	QLQIRFQKEK
1530	1540	1550					
ETGALYKEKT	KEPSREGPQK	REEL					

Cmpd.	No. of Cmpds.	m/z meas.	Δ m/z [ppm]	z	Rt [min]	Score	P	Range	Sequence	Modification
1963	1	696.2530	-54.87	2	54.6	12.7	1	1-13	-.MGCKGDASGVCYK.M	Carbamidomethyl: 11; Oxidation: 1
1565	1	1018.8365	-18.21	3	49.3	12.6	2	606-632	R.KEARGYYEQTGVGPLPVVLFNGMPFER.E	
1888	1	600.2525	-61.03	3	53.5	13.2	0	633-647	R.EQLDPDELETITMHK.I	
2009	1	599.9210	-67.30	3	53.5	11.7	1	1009-1023	R.VFMNCQSKLSDMPLK.S	Carbamidomethyl: 5

Detailed Protein Report

Cmpd.	No. of Cmpds.	m/z meas.	Δ m/z [ppm]	z	Rt [min]	Score	P	Range	Sequence	Modification
2056	1	973.8963	-78.49	2	54.1	12.8	0	1028-1044	R.YVLEPEISFTSDNSFAK.G	

Detailed Protein Report

Protein 180: 14-3-3 protein sigma [Homo sapiens]

Accession: gi|5454052
Database: refseq_human(refseq_human_20140103.fasta)
Database Date: 2015-11-30

Score: 62.7
MW [kDa]: 27.8
pI: 4.5
Sequence Coverage [%]: 11.3
No. of unique Peptides: 1

10	20	30	40	50	60	70	80
MERASLIQKA	KLAEQAERYE	DMAAFMKGAV	EKGEELSCEE	RNLLSVAYKN	VVGGQRAAWR	VLSSIEQKSN	EEGSEEKGPE
**90**	**100**	**110**	**120**	**130**	**140**	**150**	**160**
VREYREKVET	ELQGVCDTVL	GLLDSHLIKE	AGDAESRVFY	LKMKGDYYRY	LAEVATGDDK	KRIIDSARSA	YQEAMDISKK
**170**	**180**	**190**	**200**	**210**	**220**	**230**	**240**
EMPPTNPIRL	GLALNFSVFH	YEIANSPEEA	ISLAKTTFDE	AMADLHTLSE	DSYKDSTLIM	QLLRDNLTLW	TADNAGEEGG
**250**							
EAPQEPQS							

Cmpd.	No. of Cmpds.	m/z meas.	Δ m/z [ppm]	z	Rt [min]	Score	P	Range	Sequence	Modification
2035	3	1051.3758	-123.72	2	55.8	15.4	2	10-27	K.AKLAEQAERYEDMAAFMK.G	

Detailed Protein Report

Protein 181: prolow-density lipoprotein receptor-related protein 1 precursor [Homo sapiens]

**Accession:**	gi|126012562	**Score:**	62.7
**Database:**	refseq_human(refseq_human_20140103.fasta)	**MW [kDa]:**	504.3
**Database Date:**	2015-11-30	**pI:**	5.0
**Modification(s):**	Carbamidomethyl, Oxidation	**Sequence Coverage [%]:**	1.6
		**No. of unique Peptides:**	3

Detailed Protein Report

10	20	30	40	50	60	70	80
MLTPPLLLLL	PLLSALVAAA	IDAPKTCSPK	QFACRDQITC	ISKGWRCDGE	RDCPDGSDEA	PEICPQSKAQ	RCQPNEHNCL
90	100	110	120	130	140	150	160
GTELCVPMSR	LCNGVQDCMD	GSDEGPHCRE	LQGNCSRLGC	QHHCVPTLDG	PTCYCNSSFQ	LQADGKTCKD	FDECSVYGTC
170	180	190	200	210	220	230	240
SQLCTNTDGS	FICGCVEGYL	LQPDNRSCKA	KNEPVDRPPV	LLIANSQNIL	ATYLSGAQVS	TITPTSTRQT	TAMDFSYANE
250	260	270	280	290	300	310	320
TVCWVHVGDS	AAQTQLKCAR	MPGLKGFVDE	HTINISLSLH	HVEQMAIDWL	TGNFYFVDDI	DDRIFVCNRN	GDTCVTLLDL
330	340	350	360	370	380	390	400
ELYNPKGIAL	DPAMGKVFFT	DYGQIPKVER	CDMDGQNRTK	LVDSKIVFPH	GITLDLVSRL	VYWADAYLDY	IEVVDYEGKG
410	420	430	440	450	460	470	480
RQTIIQGILI	EHLYGLTVFE	NYLYATNSDN	ANAQQKTSVI	RVNRFNSTEY	QVVTRVDKGG	ALHIYHQRRQ	PRVRSHACEN
490	500	510	520	530	540	550	560
DQYGKPGGCS	DICLLANSHK	ARTCRCRSGF	SLGSDGKSCK	KPEHELFLVY	GKGRPGIIRG	MDMGAKVPDE	HMIPIENLMN
570	580	590	600	610	620	630	640
PRALDFHAET	GFIYFADTTS	YLIGRQKIDG	TERETILKDG	IHNVEGVAVD	WMGDNLYWTD	DGPKKTISVA	RLEKAAQTRK
650	660	670	680	690	700	710	720
TLIEGKMTHP	RAIVVDPLNG	WMYWTDWEED	PKDSRRGRLE	RAWMDGSHRD	IFVTSKTVLW	PNGLSLDIPA	GRLYWVDAFY
730	740	750	760	770	780	790	800
DRIETILLNG	TDRKIVYEGP	ELNHAFGLCH	HGNYLFWTEY	RSGSVYRLER	GVGGAPPTVT	LLRSERPPIF	EIRMYDAQQQ
810	820	830	840	850	860	870	880
QVGTNKCRVN	NGGCSSLCLA	TPGSRQCACA	EDQVLDADGV	TCLANPSYVP	PPQCQPGEFA	CANSRCIQER	WKCDGDNDCL
890	900	910	920	930	940	950	960
DNSDEAPALC	HQHTCPSDRF	KCENNRCIPN	RWLCDGDNDC	GNSEDESNAT	CSARTCPPNQ	FSCASGRCIP	ISWTCDLDDD
970	980	990	1000	1010	1020	1030	1040
CGDRSDESAS	CAYPTCFPLT	QFTCNNGRCI	NINWRCDNDN	DCGDNSDEAG	CSHSCSSTQF	KCNSGRCIPE	HWTCDGDNDC
1050	1060	1070	1080	1090	1100	1110	1120
GDYSDETHAN	CTNQATRPPG	GCHTDEFQCR	LDGLCIPLRW	RCDGDTDCMD	SSDEKSCEGV	THVCDPSVKF	GCKDSARCIS
1130	1140	1150	1160	1170	1180	1190	1200
KAWVCDGDND	CEDNSDEENC	ESLACRPPSH	PCANNTSVCL	PPDKLCDGND	DCGDGSDEGE	LCDQCSLNNG	GCSHNCSVAP
1210	1220	1230	1240	1250	1260	1270	1280
GEGIVCSCPL	GMELGPDNHT	CQIQSYCAKH	LKCSQKCDQN	KFSVKCSCYE	GWVLEPDGES	CRSLDPFKPF	IIFSNRHEIR
1290	1300	1310	1320	1330	1340	1350	1360
RIDLHKGDYS	VLVPGLRNTI	ALDFHLSQSA	LYWTDVVEDK	IYRGKLLDNG	ALTSFEVVIQ	YGLATPEGLA	VDWIAGNIYW
1370	1380	1390	1400	1410	1420	1430	1440
VESNLDQIEV	AKLDGTLRTT	LLAGDIEHPR	AIALDPRDGI	LFWTDWDASL	PRIEAASMSG	AGRRTVHRET	GSGGWPNGLT
1450	1460	1470	1480	1490	1500	1510	1520
VDYLEKRILW	IDARSDAIYS	ARYDGSGHME	VLRGHEFLSH	PFAVTLYGGE	VYWTDWRTNT	LAKANKWTGH	NVTVVQRTNT
1530	1540	1550	1560	1570	1580	1590	1600
QPFDLQVYHP	SRQPMAPNPC	EANGGQGPCS	HLCLINYNRT	VSCACPHLMK	LHKDNTTCYE	FKKFLLYARQ	MEIRGVDLDA
1610	1620	1630	1640	1650	1660	1670	1680
PYYNYIISFT	VPDIDNVTVL	DYDAREQRVY	WSDVRTQAIK	RAFINGTGVE	TVVSADLPNA	HGLAVDWVSR	NLFWTSYDTN
1690	1700	1710	1720	1730	1740	1750	1760
KKQINVARLD	GSFKNAVVQG	LEQPHGLVVH	PLRGKLYWTD	GDNISMANMD	GSNRTLLFSG	QKGPVGLAID	FPESKLYWIS
1770	1780	1790	1800	1810	1820	1830	1840
SGNHTINRCN	LDGSGLEVID	AMRSQLGKAT	ALAIMGDKLW	WADQVSEKMG	TCSKADGSGS	VVLRNSTTLV	MHMKVYDESI
1850	1860	1870	1880	1890	1900	1910	1920
QLDHKGTNPC	SVNNGDCSQL	CLPTSETTRS	CMCTAGYSLR	SGQQACEGVG	SFLLYSVHEG	IRGIPLDPND	KSDALVPVSG
1930	1940	1950	1960	1970	1980	1990	2000
TSLAVGIDFH	AENDTIYWVD	MGLSTISRAK	RDQTWREDVV	TNGIGRVEGI	AVDWIAGNIY	WTDQGFDVIE	VARLNGSFRY
2010	2020	2030	2040	2050	2060	2070	2080
VVISQGLDKP	RAITVHPEKG	YLFWTEWGQY	PRIERSRLDG	TERVVLVNVS	ISWPNGISVD	YQDGKLYWCD	ARTDKIERID
2090	2100	2110	2120	2130	2140	2150	2160
LETGENREVV	LSSNNMDMFS	VSVFEDFIYW	SDRTHANGSI	KRGSKDNATD	SVPLRTGIGV	QLKDIKVFNR	DRQKGTNVCA
2170	2180	2190	2200	2210	2220	2230	2240

Detailed Protein Report

Cmpd.	No. of Cmpds.	m/z meas.	Δ m/z [ppm]	z	Rt [min]	Score	P	Range	Sequence	Modification
127	3	849.3809	-43.54	2	30.9	15.1	1	1560-1573	R.TVSCACPHLMKLHK.D	Carbamidomethyl: 4, 6; Oxidation: 10
2236	2	873.3305	-116.11	2	57.8	15.6	1	1952-1966	R.DQTWREDVVTNGIGR.V	
2647	1	822.4641	61.16	1	64.0	11.2	0	3754-3760	R.CLSSSLR.C	Carbamidomethyl: 1

Detailed Protein Report

Protein 182: profilin-1 [Homo sapiens]

**Accession:**	gi|4826898	**Score:**	62.6
**Database:**	refseq_human(refseq_human_20140103.fasta)	**MW [kDa]:**	15.0
**Database Date:**	2015-11-30	**pI:**	9.4
**Modification(s):**	Carbamidomethyl	**Sequence Coverage [%]:**	20.7
		**No. of unique Peptides:**	2

Quantitation

Q:W	**Median:**	1.45	**CV:**	4.87 %	**No. of Peptides:**	2
M:Q	**Median:**	0.54	**CV:**	0.00 %	**No. of Peptides:**	1

```
         10         20         30         40         50         60         70         80
MAGWNAYIDN LMADGTCQDA AIVGYKDSPS VWAAVPGKTF VNITPAEVGV LVGKDRSSFY VNGLTLGGQK CSVIRDSLLQ
         90        100        110        120        130        140        150
DGEFSMDLRT KSTGGAPTFN VTVTKTDKTL VLLMGKEGVH GGLINKKCYE MASHLRRSQY
```

Cmpd.	No. of Cmpds.	m/z meas.	Δ m/z [ppm]	z	Rt [min]	Score	P	Range	Sequence	Modification	Ratios	
2934	10	822.4733	1.65	2	65.5	46.8	0	39-54	K.TFVNITPAEVGVLVGK.D		Q:W	1.38
1495	1	850.7379	-173.16	2	48.5	15.9	2	128-140	K.CYEMASHLRRSQY.-	Carbamidomethyl: 1	M:Q<br>Q:W	0.54<br>1.52

Detailed Protein Report

Protein 183: protein SOGA1 isoform 1 [Homo sapiens]

**Accession:**	gi|257467639	**Score:**	62.0
**Database:**	refseq_human(refseq_human_20140103.fasta)	**MW [kDa]:**	183.7
**Database Date:**	2015-11-30	**pI:**	6.1
**Modification(s):**	Carbamidomethyl	**Sequence Coverage [%]:**	3.0
		**No. of unique Peptides:**	3

Quantitation

M:Q **Median:** 0.37 **CV:** 0.00 % **No. of Peptides:** 1

10	20	30	40	50	60	70	80
MEAPAAEPPV	RGCGPQPAPA	PAPAPERKKS	HRAPSPARPK	DVAGWSLAKG	RRGPGPGSAV	ACSAAFSSRP	DKKGRAVAPG
90	100	110	120	130	140	150	160
ARGAGVRVAG	VRTGVRAKGR	PRSGAGPRPP	PPPPSLTDSS	SEVSDCASEE	ARLLGLELAL	SSDAESAAGG	PAGVRTGQPA
170	180	190	200	210	220	230	240
QPAPSAQQPP	RPPASPDEPS	VAASSVGSSR	LPLSASLAFS	DLTEEMLDCG	PSGLVRELEE	LRSENDYLKD	EIEELRAEML
250	260	270	280	290	300	310	320
EMRDVYMEED	VYQLQELRQQ	LDQASKTCRI	LQYRLRKAER	RSLRAAQTGQ	VDGELIRGLE	QDVKVSKDIS	MRLHKELEVV
330	340	350	360	370	380	390	400
EKKRARLEEE	NEELRQRLIE	TELAKQVLQT	ELERPREHSL	KKRGTRSLGK	ADKKTLVQED	SADLKCQLHF	AKEESALMCK
410	420	430	440	450	460	470	480
KLTKLAKEND	SMKEELLKYR	SLYGDLDSAL	SAEELADAPH	SRETELKVHL	KLVEEEANLL	SRRIVELEVE	NRGLRAEMDD
490	500	510	520	530	540	550	560
MKDHGGGCGG	PEARLAFSAL	GGGECGESLA	ELRRHLQFVE	EEAELLRRSS	AELEDQNKLL	LNELAKFRSE	HELDVALSED
570	580	590	600	610	620	630	640
SCSVLSEPSQ	EELAAAKLQI	GELSGKVKKL	QYENRVLLSN	LQRCDLASCQ	STRPMLETDA	EAGDSAQCVP	APLGETHESH
650	660	670	680	690	700	710	720
AVRLCRAREA	EVLPGLREQA	ALVSKAIDVL	VADANGFTAG	LRLCLDNECA	DFRLHEAPDN	SEGPRDTKLI	HAILVRLSVL
730	740	750	760	770	780	790	800
QQELNAFTRK	ADAVLGCSVK	EQQESFSSLP	PLGSQGLSKE	ILLAKDLGSD	FQPPDFRDLP	EWEPRIREAF	RTGDLDSKPD
810	820	830	840	850	860	870	880
PSRSFRPYRA	EDNDSYASEI	KELQLVLAEA	HDSLRGLQEQ	LSQERQLRKE	EADNFNQKMV	QLKEDQQRAL	LRREFELQSL
890	900	910	920	930	940	950	960
SLQRRLEQKF	WSQEKNMLVQ	ESQQFKHNFL	LLFMKLRWFL	KRWRQGKVLP	SEGDDFLEVN	SMKELYLLME	EEEINAQHSD
970	980	990	1000	1010	1020	1030	1040
NKACTGDSWT	QNTPNEYIKT	LADMKVTLKE	LCWLLRDERR	GLTELQQQFA	KAKATWETER	AELKGHTSQM	ELKTGKGAGE
1050	1060	1070	1080	1090	1100	1110	1120
RAGPDWKAAL	QREREEQQHL	LAESYSAVME	LTRQLQISER	NWSQEKLQLV	ERLQGEKQQV	EQQVKELQNR	LSQLQKAADP
1130	1140	1150	1160	1170	1180	1190	1200
WVLKHSELEK	QDNSWKETRS	EKIHDKEAVS	EVELGGNGLK	RTKSVSSMSE	FESLLDCSPY	LAGGDARGKK	LPNNPAFGFV
1210	1220	1230	1240	1250	1260	1270	1280
SSEPGDPEKD	TKEKPGLSSR	DCNHLGALAC	QDPPGRQMQR	SYTAPDKTGI	RVYYSPPVAR	RLGVPVVHDK	EGKIIIEPGF
1290	1300	1310	1320	1330	1340	1350	1360
LFTTAKPKES	AEADGLAESS	YGRWLCNFSR	QRLDGGSAGS	PSAAGPGFPA	ALHDFEMSGN	MSDDMKEITN	CVRQAMRSGS
1370	1380	1390	1400	1410	1420	1430	1440
LERKVKSTSS	QTVGLASVGT	QTIRTVSVGL	QTDPPRSSLH	GKAWSPRSSS	LVSVRSKQIS	SSLDKVHSRI	ERPCCSPKYG
1450	1460	1470	1480	1490	1500	1510	1520
SPKLQRRSVS	KLDSSKDRSL	WNLHQGKQNG	SAWARSTTTR	DSPVLRNIND	GLSSLFSVVE	HSGSTESVWK	LGMSETRAKP
1530	1540	1550	1560	1570	1580	1590	1600
EPPKYGIVQE	FFRNVCGRAP	SPTSSAGEEG	TKKPEPLSPA	SYHQPEGVAR	ILNKKAAKLG	SSEEVRLTML	PQVGKDGVLR
1610	1620	1630	1640	1650	1660	1670	
DGDGAVVLPN	EDAVCDCSTQ	SLTSCFARSS	RSAIRHSPSK	CRLHPSESSW	GGEERALPPS	E	

Cmpd.	No. of Cmpds.	m/z meas.	Δ m/z [ppm]	z	Rt [min]	Score	P	Range	Sequence	Modification	Ratios
861	1	491.8136	29.86	2	39.6	10.2	2	73-82	K.KGRAVAPGAR.G		M:Q 0.37
2190	1	701.1048	122.16	3	57.8	13.2	2	1426-1443	K.VHSRIERPCCSPKYGSPK.L	Carbamidomethyl: 9	
2282	1	701.1125	133.17	3	58.4	14.5	2	1426-	K.VHSRIERPCCSPKYGSPK.L	Carbamidomethyl: 10	

Detailed Protein Report

Cmpd.	No. of Cmpds.	m/z meas.	Δ m/z [ppm]	z	Rt [min]	Score	P	Range	Sequence	Modification	Ratios
								1443			

Detailed Protein Report

Protein 184: PREDICTED: protein KIAA1199 isoform X3 [Homo sapiens]

**Accession:**	gi|578827243	**Score:**	61.9
**Database:**	refseq_human(refseq_human_20140103.fasta)	**MW [kDa]:**	90.7
**Database Date:**	2015-11-30	**pI:**	9.6
		**Sequence Coverage [%]:**	5.8
		**No. of unique Peptides:**	2

10	20	30	40	50	60	70	80
MYSHGNLEIP	WSLPVLITPC	TRQKSSRCFP	ADPAPPTRSK	WQIKDVVGYN	SLGHCFFTED	GPEERNTFDH	CLGLLVKSGT
**90**	**100**	**110**	**120**	**130**	**140**	**150**	**160**
LLPSDRDSKM	CKMITEDSYP	GYIPKPRQDC	NAVSTFWMAN	PNNNLINCAA	AGSEETGFWF	IFHHVPTGPS	VGMYSPGYSE
**170**	**180**	**190**	**200**	**210**	**220**	**230**	**240**
HIPLGKFYNN	RAHSNYRAGM	IIDNGVKTTE	ASAKDKRPFL	SIISARYSPH	QDADPLKPRE	PAIIRHFIAY	KNQDHGAWLR
**250**	**260**	**270**	**280**	**290**	**300**	**310**	**320**
GGDVWLDSCR	FADNGIGLTL	ASGGTFPYDD	GSKQEIKNSL	FVGESGNVGT	EMMDNRIWGP	GGLDHSGRTL	PIGQNFPIRG
**330**	**340**	**350**	**360**	**370**	**380**	**390**	**400**
IQLYDGPINI	QNCTFRKFVA	LEGRHTSALA	FRLNNAWQSC	PHNNVTGIAF	EDVPITSRVF	FGEPGPWFNQ	LDMDGDKTSV
**410**	**420**	**430**	**440**	**450**	**460**	**470**	**480**
FHDVDGSVSE	YPGSYLTKND	NWLVRHPDCI	NVPDWRGAIC	SGCYAQMYIQ	AYKTSNLRMK	IIKNDFPSHP	LYLEGALTRS
**490**	**500**	**510**	**520**	**530**	**540**	**550**	**560**
THYQQYQPVV	TLQKGYTIHW	DQTAPAELAI	WLINFNKGDW	IRVGLCYPRG	TTFSILSDVH	NRLLKQTSKT	GVFVRTLQMD
**570**	**580**	**590**	**600**	**610**	**620**	**630**	**640**
KVEQSYPGRS	HYYWDEDSGL	LFLKLKAQNE	REKFAFCSMK	GCERIKIKAL	IPKNAGVSDC	TATAYPKFTE	RAVVDVPMPK
**650**	**660**	**670**	**680**	**690**	**700**	**710**	**720**
KLFGSQLKTK	DHFLEVKMES	SKQHFFHLWN	DFAYIEVDGK	KYPSSEDGIQ	VVVIDGNQGR	VVSHTSFRNS	ILQGIPWQLF
**730**	**740**	**750**	**760**	**770**	**780**	**790**	**800**
NYVATIPDNS	IVLMASKGRY	VSRGPWTRVL	EKLGADRGLK	LKEQMAFVGF	KGSFRPIWVT	LDTEDHKAKI	FQVVPIPVVK
**810**							
KKKL							

Cmpd.	No. of Cmpds.	m/z meas.	Δ m/z [ppm]	z	Rt [min]	Score	P	Range	Sequence	Modification
737	1	936.9549	11.85	2	39.2	10.8	0	570-584	R.SHYYWDEDSGLLFLK.L	
143	1	796.0835	178.80	2	30.7	10.0	2	740-752	R.YVSRGPWTRVLEK.L	

Detailed Protein Report

Protein 185: protein unc-45 homolog A isoform 3 [Homo sapiens]

**Accession:**	gi|89179321	**Score:**	61.8
**Database:**	refseq_human(refseq_human_20140103.fasta)	**MW [kDa]:**	101.6
**Database Date:**	2015-11-30	**pI:**	5.7
**Modification(s):**	Carbamidomethyl, Oxidation	**Sequence Coverage [%]:**	5.5
		**No. of unique Peptides:**	4

Quantitation

Q:W	**Median:**	0.97	**CV:**	0.00 %	**No. of Peptides:**	1	

10	20	30	40	50	60	70	80
MTASSVEQLR	KEGNELFKCG	DYGGALAAYT	QALGLDATPQ	DQAVLHRNRA	ACHLKLEDYD	KAETEASKAI	EKDGGDVKAL
**90**	**100**	**110**	**120**	**130**	**140**	**150**	**160**
YRRSQALEKL	GRLDQAVLDL	QRCVSLEPKN	KVFQEALRNI	GGQIQEKVRY	MSSTDAKVEQ	MFQILLDPEE	KGTEKKQKAS
**170**	**180**	**190**	**200**	**210**	**220**	**230**	**240**
QNLVVLARED	AGAEKIFRSN	GVQLLQRLLD	MGETDLMLAA	LRTLVGICSE	HQSRTVATLS	ILGTRRVVSI	LGVESQAVSL
**250**	**260**	**270**	**280**	**290**	**300**	**310**	**320**
AACHLLQVMF	DALKEGVKKG	FRGKEGAIIV	DPARELKVLI	SNLLDLLTEV	GVSGQGRDNA	LTLLIKAVPR	KSLKDPNNSL
**330**	**340**	**350**	**360**	**370**	**380**	**390**	**400**
TLWVIDQGLK	KILEVGGSLQ	DPPGELAVTA	NSRMSASILL	SKLFDDLKCD	AERENFHRLC	ENYIKSWFEG	QGLAGKLRAI
**410**	**420**	**430**	**440**	**450**	**460**	**470**	**480**
QTVSCLLQGP	CDAGNRALEL	SGVMESVIAL	CASEQEEEQL	VAVEALIHAA	GKAKRASFIT	ANGVSLLKDL	YKCSEKDSIR
**490**	**500**	**510**	**520**	**530**	**540**	**550**	**560**
IRALVGLCKL	GSAGGTDFSM	KQFAEGSTLK	LAKQCRKWLC	NDQIDAGTRR	WAVEGLAYLT	FDADVKEEFV	EDAAALKALF
**570**	**580**	**590**	**600**	**610**	**620**	**630**	**640**
QLSRLEERSV	LFAVASALVN	CTNSYDYEEP	DPKMVELAKY	AKQHVPEQHP	KDKPSFVRAR	VKKLLAAGVV	SAMVCMVKTE
**650**	**660**	**670**	**680**	**690**	**700**	**710**	**720**
SPVLTSSCRE	LLSRVFLALV	EEVEDRGTVV	AQGGGRALIP	LALEGTDVGQ	TKAAQALAKL	TITSNPEMTF	PGERIYEVVR
**730**	**740**	**750**	**760**	**770**	**780**	**790**	**800**
PLVSLLHLNC	SGLQNFEALM	ALTNLAGISE	RLRQKILKEK	AVPMIEGYMF	EEHEMIRRAA	TECMCNLAMS	KEVQDLFEAQ
**810**	**820**	**830**	**840**	**850**	**860**	**870**	**880**
GNDRLKLLVL	YSGEDDELLQ	RAAAGGLAML	TSMRPTLCSR	IPQVTTHWLE	ILQALLLSSN	QELQHRGAVV	VLNMVEASRE
**890**	**900**	**910**	**920**	**930**			
IASTLMESEM	MEILSVLAKG	DHSPVTRAAA	ACLDKAVEYG	LIQPNQDGE			

Cmpd.	No. of Cmpds.	m/z meas.	Δ m/z [ppm]	z	Rt [min]	Score	P	Range	Sequence	Modification	Ratios
2777	1	703.4893	208.47	2	63.3	10.5	1	50-61	R.AACHLKLEDYDK.A		
1687	1	793.3789	34.81	2	51.0	14.1	1	778-791	R.RAATECMCNLAMSK.E	Carbamidomethyl: 6	Q:W 0.97
2677	1	731.3736	106.62	2	64.7	11.8	0	779-791	R.AATECMCNLAMSK.E	Carbamidomethyl: 5; Oxidation: 6, 11	
150	1	977.7995	37.53	3	31.3	12.2	1	779-804	R. AATECMCNLAMSKEVQDLFEAQG L	Carbamidomethyl: 5	

Detailed Protein Report

Protein 186: nucleolar transcription factor 1 isoform a [Homo sapiens]

**Accession:**	gi|7657671	**Score:**	61.6
**Database:**	refseq_human(refseq_human_20140103.fasta)	**MW [kDa]:**	89.4
**Database Date:**	2015-11-30	**pI:**	5.5
**Modification(s):**	Oxidation	**Sequence Coverage [%]:**	5.0
		**No. of unique Peptides:**	3

Quantitation

Q:W	**Median:**	0.38	**CV:**	0.00 %	**No. of Peptides:**	1
M:Q	**Median:**	1.01	**CV:**	49.66 %	**No. of Peptides:**	2

Alias proteins:

Accession	Name	Description
gi|578831354	refseq_human (refseq_human_20140103.fasta)	PREDICTED: nucleolar transcription factor 1 isoform X3 [Homo sapiens]
gi|578831352	refseq_human (refseq_human_20140103.fasta)	PREDICTED: nucleolar transcription factor 1 isoform X2 [Homo sapiens]
gi|578831350	refseq_human (refseq_human_20140103.fasta)	PREDICTED: nucleolar transcription factor 1 isoform X1 [Homo sapiens]

10	20	30	40	50	60	70	80
MNGEADCPTD	LEMAAPKGQD	RWSQEDMLTL	LECMKNNLPS	NDSSKFKTTE	SHMDWEKVAF	KDFSGDMCKL	KWVEISNEVR
90	100	110	120	130	140	150	160
KFRTLTELIL	DAQEHVKNPY	KGKKLKKHPD	FPKKPLTPYF	RFFMEKRAKY	AKLHPEMSNL	DLTKILSKKY	KELPEKKKMK
170	180	190	200	210	220	230	240
YIQDFQREKQ	EFERNLARFR	EDHPDLIQNA	KKSDIPEKPK	TPQQLWYTHE	KKVYLKVRPD	ATTKEVKDSL	GKQWSQLSDK
250	260	270	280	290	300	310	320
KRLKWIHKAL	EQRKEYEEIM	RDYIQKHPEL	NISEEGITKS	TLTKAERQLK	DKFDGRPTKP	PPNSYSLYCA	ELMANMKDVP
330	340	350	360	370	380	390	400
STERMVLCSQ	QWKLLSQKEK	DAYHKKCDQK	KKDYEVELLR	FLESLPEEEQ	QRVLGEEKML	NINKKQATSP	ASKKPAQEGG
410	420	430	440	450	460	470	480
KGGSEKPKRP	VSAMFIFSEE	KRRQLQEERP	ELSESELTRL	LARMWNDLSE	KKKAKYKARE	AALKAQSERK	PGGEREERGK
490	500	510	520	530	540	550	560
LPESPKRAEE	IWQQSVIGDY	LARFKNDRVK	ALKAMEMTWN	NMEKKEKLMW	IKKAAEDQKR	YERELSEMRA	PPAATNSSKK
570	580	590	600	610	620	630	640
MKFQGEPKKP	PMNGYQKFSQ	ELLSNGELNH	LPLKERMVEI	GSRWQRISQS	QKEHYKKLAE	EQQKQYKVHL	DLWVKSLSPQ
650	660	670	680	690	700	710	720
DRAAYKEYIS	NKRKSMTKLR	GPNPKSSRTT	LQSKSESEED	DEEDEDDEDE	DEEEEDDENG	DSSEDGGDSS	ESSSEDESED
730	740	750	760	770			
GDENEEDDED	EDDDEDDDED	EDNESEGSSS	SSSSSGDSSD	SDSN			

Cmpd.	No. of Cmpds.	m/z meas.	Δ m/z [ppm]	z	Rt [min]	Score	P	Range	Sequence	Modification	Ratios
1966	1	871.8504	-139.97	2	54.7	11.1	1	108-121	K.HPDFPKKPLTPYFR.F		M:Q 0.63
2869	1	856.9447	59.29	2	66.4	17.0	1	511-524	K.ALKAMEMTWNNMEK.K	Oxidation: 7	
1591	1	472.2351	-22.65	2	49.6	18.5	0	550-559	R.APPAATNSSK.K		M:Q 1.61<br>Q:W 0.38

Detailed Protein Report

Protein 187: **PREDICTED: pleckstrin homology domain-containing family A member 4 isoform X3 [Homo sapiens]**

**Accession:**	gi|530416942	**Score:**	61.5
**Database:**	refseq_human(refseq_human_20140103.fasta)	**MW [kDa]:**	65.5
**Database Date:**	2015-11-30	**pI:**	10.7
		**Sequence Coverage [%]:**	10.4
		**No. of unique Peptides:**	4

Quantitation

Q:W	**Median:**	0.54	**CV:**	37.77 %	**No. of Peptides:**	2
M:Q	**Median:**	0.54	**CV:**	8.15 %	**No. of Peptides:**	2

```
        10         20         30         40         50         60         70         80
MEGSRPRSSL SLASSASTIS SLSSLSPKKP TRAVNKIHAF GKRGNALRRD PNLPVHIRGW LHKQDSSGLR LWKRRWFVLS
        90        100        110        120        130        140        150        160
GHCLFYYKDS REESVLGSVL LPSYNIRPDG PGAPRGRRFT FTAEHPGMRT YVLAADTLED LRGWLRALGR ASRAEGDDYG
       170        180        190        200        210        220        230        240
QPRSPARPQP GEGPGGPGGP PEVSRGEEGR ISESPEVTRL SRGRGRPRLL TPSPTTDLHS GLQMRRARSP DLFTPLSRPP
       250        260        270        280        290        300        310        320
SPLSLPRPRS APARRPPAPS GDTAPPARPH TPLSRIDVRP PLDWGPQRQT LSRPPTPRRG PPSEAGGGKP PRSPQHWSQE
       330        340        350        360        370        380        390        400
PRTQAHSGSP TYLQLPPRPP GTRASMVLLP GPPLESTFHQ SLETDTLLTK LCGQDRLLRR LQEEIDQKQE EKEQLEAALE
       410        420        430        440        450        460        470        480
LTRQQLGQAT REAGAPGRAW GRQRLLQDRL VSVRATLCHL TQERERVWDT YSGLEQELGT LRETLEYLLH LGSPQDRVSA
       490        500        510        520        530        540        550        560
QQQLWMVEDT LAGLGGPQKP PPHTEPDSPS PVLQGEESSE RESLPESLEL SSPRSPETDW GRPPGGDKDL ASPHLGLGSP
       570        580        590        600
RVSRASSPEG RHLPSPQLGT KKYGSITDWP ALRSM
```

Cmpd.	No. of Cmpds.	m/z meas.	Δ m/z [ppm]	z	Rt [min]	Score	P	Range	Sequence	Modification	Ratios
361	2	711.1755	-208.54	2	33.6	11.6	1	151-163	R.ASRAEGDDYGQPR.S		M:Q 0.50<br>Q:W 0.37
411	3	603.6782	-226.42	2	34.8	12.5	0	300-312	R.GPPSEAGGGKPPR.S		Q:W 0.78<br>M:Q 0.59
20	1	706.3988	42.59	2	29.2	15.0	2	412-424	R.EAGAPGRAWGRQR.L		
1037	1	945.4829	-32.03	2	41.7	11.1	2	565-582	R.ASSPEGRHLPSPQLGTKK.Y		

Detailed Protein Report

**Protein 188:**	**PREDICTED: cadherin EGF LAG seven-pass G-type receptor 2 isoform X2 [Homo sapiens]**

**Accession:**	gi|578798514	**Score:**	61.4
**Database:**	refseq_human(refseq_human_20140103.fasta)	**MW [kDa]:**	316.2
**Database Date:**	2015-11-30	**pI:**	5.0
		**Sequence Coverage [%]:**	1.5
		**No. of unique Peptides:**	1

Detailed Protein Report

10	20	30	40	50	60	70	80
MRSPATGVPL	PTPPPPLLLL	LLLLLPPPLL	GDQVGPCRSL	GSRGRGSSGA	CAPMGWLCPS	SASNLWLYTS	RCRDAGTELT
90	100	110	120	130	140	150	160
GHLVPHHDGL	RVWCPESEAH	IPLPPAPEGC	PWSCRLLGIG	GHLSPQGKLT	LPEEHPCLKA	PRLRCQSCKL	AQAPGLRAGE
170	180	190	200	210	220	230	240
RSPEESLGGR	RKRNVNTAPQ	FQPPSYQATV	PENQPAGTPV	ASLRAIDPDE	GEAGRLEYTM	DALFDSRSNQ	FFSLDPVTGA
250	260	270	280	290	300	310	320
VTTAEELDRE	TKSTHVFRVT	AQDHGMPRRS	ALATLTILVT	DTNDHDPVFE	QQEYKESLRE	NLEVGYEVLT	VRATDGDAPP
330	340	350	360	370	380	390	400
NANILYRLLE	GSGGSPSEVF	EIDPRSGVIR	TRGPVDREEV	ESYQLTVEAS	DQGRDPGPRS	TTAAVFLSVE	DDNDNAPQFS
410	420	430	440	450	460	470	480
EKRYVVQVRE	DVTPGAPVLR	VTASDRDKGS	NAVVHYSIMS	GNARGQFYLD	AQTGALDVVS	PLDYETTKEY	TLRVRAQDGG
490	500	510	520	530	540	550	560
RPPLSNVSGL	VTVQVLDIND	NAPIFVSTPF	QATVLESVPL	GYLVLHVQAI	DADAGDNARL	EYRLAGVGHD	FPFTINNGTG
570	580	590	600	610	620	630	640
WISVAAELDR	EEVDFYSFGV	EARDHGTPAL	TASASVSVTV	LDVNDNNPTF	TQPEYTVRLN	EDAAVGTSVV	TVSAVDRDAH
650	660	670	680	690	700	710	720
SVITYQITSG	NTRNRFSITS	QSGGGLVSLA	LPLDYKLERQ	YVLAVTASDG	TRQDTAQIVV	NVTDANTHRP	VFQSSHYTVN
730	740	750	760	770	780	790	800
VNEDRPAGTT	VVLISATDED	TGENARITYF	MEDSIPQFRI	DADTGAVTTQ	AELDYEDQVS	YTLAITARDN	GIPQKSDTTY
810	820	830	840	850	860	870	880
LEILVNDVND	NAPQFLRDSY	QGSVYEDVPP	FTSVLQISAT	DRDSGLNGRV	FYTFQGGDDG	DGDFIVESTS	GIVRTLRRLD
890	900	910	920	930	940	950	960
RENVAQYVLR	AYAVDKGMPP	ARTPMEVTVT	VLDVNDNPPV	FEQDEFDVFV	EENSPIGLAV	ARVTATDPDE	GTNAQIMYQI
970	980	990	1000	1010	1020	1030	1040
VEGNIPEVFQ	LDIFSGELTA	LVDLDYEDRP	EYVLVIQATS	APLVSRATVH	VRLLDRNDNP	PVLGNFEILF	NNYVTNRSSS
1050	1060	1070	1080	1090	1100	1110	1120
FPGGAIGRVP	AHDPDISDSL	TYSFERGNEL	SLVLLNASTG	ELKLSRALDN	NRPLEAIMSV	LVSDGVHSVT	AQCALRVTII
1130	1140	1150	1160	1170	1180	1190	1200
TDEMLTHSIT	LRLEDMSPER	FLSPLLGLFI	QAVAATLATP	PDHVVVFNVQ	RDTDAPGGHI	LNVSLSVGQP	PGPGGGPPFL
1210	1220	1230	1240	1250	1260	1270	1280
PSEDLQERLY	LNRSLLTAIS	AQRVLPFDDN	ICLREPCENY	MRCVSVLRFD	SSAPFIASSS	VLFRPIHPVG	GLRCRCPPGF
1290	1300	1310	1320	1330	1340	1350	1360
TGDYCETEVD	LCYSRPCGPH	GRCRSREGGY	TCLCRDGYTG	EHCEVSARSG	RCTPGVCKNG	GTCVNLLVGG	FKCDCPSGDF
1370	1380	1390	1400	1410	1420	1430	1440
EKPYCQVTTR	SFPAHSFITF	RGLRQRFHFT	LALSFATKER	DGLLLYNGRF	NEKHDFVALE	VIQEQVQLTF	SAGESTTTVS
1450	1460	1470	1480	1490	1500	1510	1520
PFVPGGVSDG	QWHTVQLKYY	NKPLLGQTGL	PQGPSEQKVA	VVTVDGCDTG	VALRFGSVLG	NYSCAAQGTQ	GGSKKSLDLT
1530	1540	1550	1560	1570	1580	1590	1600
GPLLLGGVPD	LPESFPVRMR	QFVGCMRNLQ	VDSRHIDMAD	FIANNGTVPG	CPAKKNVCDS	NTCHNGGTCV	NQWDAFSCEC
1610	1620	1630	1640	1650	1660	1670	1680
PLGFGGKSCA	QEMANPQHFL	GSSLVAWHGL	SLPISQPWYL	SLMFRTRQAD	GVLLQAITRG	RSTITLQLRE	GHVMLSVEGT
1690	1700	1710	1720	1730	1740	1750	1760
GLQASSLRLE	PGRANDGDWH	HAQLALGASG	GPGHAILSFD	YGQQRAEGNL	GPRLHGLHLS	NITVGGIPGP	AGGVARGFRG
1770	1780	1790	1800	1810	1820	1830	1840
CLQGVRVSDT	PEGVNSLDPS	HGESINVEQG	CSLPDPCDSN	PCPANSYCSN	DWDSYSCSCD	PGYYGDNCTN	VCDLNPCEHQ
1850	1860	1870	1880	1890	1900	1910	1920
SVCTRKPSAP	HGYTCECPPN	YLGPYCETRI	DQPCPRGWWG	HPTCGPCNCD	VSKGFDPDCN	KTSGECHCKE	NHYRPPGSPT
1930	1940	1950	1960	1970	1980	1990	2000
CLLCDCYPTG	SLSRVCDPED	GQCPCKPGVI	GRQCDRCDNP	FAEVTTNGCE	VNYDSCPRAI	EAGIWWPRTR	FGLPAAAPCP
2010	2020	2030	2040	2050	2060	2070	2080
KGSFGTAVRH	CDEHRGWLPP	NLFNCTSITF	SELKGFAERL	QRNESGLDSG	RSQQLALLLR	NATQHTAGYF	GSDVKVAYQL
2090	2100	2110	2120	2130	2140	2150	2160
ATRLLAHEST	QRGFGLSATQ	DVHFTENLLR	VGSALLDTAN	KRHWELIQQT	EGGTAWLLQH	YEAYASALAQ	NMRHTYLSPF
2170	2180	2190	2200	2210	2220	2230	2240
TIVTPNIVIS	VVRLDKGNFA	GAKLPRYEAL	RGEQPPDLET	TVILPESVFR	ETPPVVRPAG	PGEAQEPEEL	ARRQRRHPEL
2250	2260	2270	2280	2290	2300	2310	2320
SQGEAVASVI	IYRTLAGLLP	HNYDPDKRSL	RVPKRPIINT	PVVSISVHDD	EELLPRALDK	PVTVQFRLLE	TEERTKPICV
2330	2340	2350	2360	2370	2380	2390	2400

Detailed Protein Report

Cmpd.	No. of Cmpds.	m/z meas.	Δ m/z [ppm]	z	Rt [min]	Score	P	Range	Sequence	Modification
2164	1	937.1664	148.30	2	57.5	14.0	2	129-144	K.LTLPEEHPCLKAPRLR.C	

Detailed Protein Report

Protein 189: voltage-dependent R-type calcium channel subunit alpha-1E isoform 2 [Homo sapiens]

**Accession:**	gi|329663531	**Score:**	61.3
**Database:**	refseq_human(refseq_human_20140103.fasta)	**MW [kDa]:**	254.4
**Database Date:**	2015-11-30	**pI:**	9.3
**Modification(s):**	Carbamidomethyl, Oxidation	**Sequence Coverage [%]:**	3.1
		**No. of unique Peptides:**	5

Quantitation

Q:W	**Median:**	0.86	**CV:**	50.67 %	**No. of Peptides:**	2
M:Q	**Median:**	0.58	**CV:**	167.04 %	**No. of Peptides:**	3

Detailed Protein Report

10	20	30	40	50	60	70	80
MARFGEAVVA	RPGSGDGDSD	QSRNRQGTPV	PASGQAAAYK	QTKAQRARTM	ALYNPIPVRQ	NCFTVNRSLF	IFGEDNIVRK
90	100	110	120	130	140	150	160
YAKKLIDWPP	FEYMILATII	ANCIVLALEQ	HLPEDDKTPM	SRRLEKTEPY	FIGIFCFEAG	IKIVALGFIF	HKGSYLRNGW
170	180	190	200	210	220	230	240
NVMDFIVVLS	GILATAGTHF	NTHVDLRTLR	AVRVLRPLKL	VSGIPSLQIV	LKSIMKAMVP	LLQIGLLLFF	AILMFAIIGL
250	260	270	280	290	300	310	320
EFYSGKLHRA	CFMNNSGILE	GFDPPHPCGV	QGCPAGYECK	DWIGPNDGIT	QFDNILFAVL	TVFQCITMEG	WTTVLYNTND
330	340	350	360	370	380	390	400
ALGATWNWLY	FIPLIIIGSF	FVLNLVLGVL	SGEFAKERER	VENRRAFMKL	RRQQQIEREL	NGYRAWIDKA	EEVMLAEENK
410	420	430	440	450	460	470	480
NAGTSALEVL	RRATIKRSRT	EAMTRDSSDE	HCVDISSVGT	PLARASIKSA	KVDGVSYFRH	KERLLRISIR	HMVKSQVFYW
490	500	510	520	530	540	550	560
IVLSLVALNT	ACVAIVHHNQ	PQWLTHLLYY	AEFLFLGLFL	LEMSLKMYGM	GPRLYFHSSF	NCFDFGVTVG	SIFEVVWAIF
570	580	590	600	610	620	630	640
RPGTSFGISV	LRALRLLRIF	KITKYWASLR	NLVVSLMSSM	KSIISLLFLL	FLFIVVFALL	GMQLFGGRFN	FNDGTPSANF
650	660	670	680	690	700	710	720
DTFPAAIMTV	FQILTGEDWN	EVMYNGIRSQ	GGVSSGMWSA	IYFIVLTLFG	NYTLLNVFLA	IAVDNLANAQ	ELTKDEQEEE
730	740	750	760	770	780	790	800
EAFNQKHALQ	KAKEVSPMSA	PNMPSIERER	RRRHHMSVWE	QRTSQLRKHM	QMSSQEALNR	EEAPTMNPLN	PLNPLSSLNP
810	820	830	840	850	860	870	880
LNAHPSLYRR	PRAIEGLALG	LALEKFEEER	ISRGGSLKGD	GGDRSSALDN	QRTPLSLGQR	EPPWLARPCH	GNCDPTQQEA
890	900	910	920	930	940	950	960
GGGEAVVTFE	DRARHRQSQR	RSRHRRVRTE	GKESSSASRS	RSASQERSLD	EAMPTEGEKD	HELRGNHGAK	EPTIQEERAQ
970	980	990	1000	1010	1020	1030	1040
DLRRTNSLMV	SRGSGLAGGL	DEADTPLVLP	HPELEVGKHV	VLTEQEPEGS	SEQALLGNVQ	LDMGRVISQS	EPDLSCITAN
1050	1060	1070	1080	1090	1100	1110	1120
TDKATTESTS	VTVAIPDVDP	LVDSTVVHIS	NKTDGEASPL	KEAEIREDEE	EVEKKKQKKE	KRETGKAMVP	HSSMFIFSTT
1130	1140	1150	1160	1170	1180	1190	1200
NPIRRACHYI	VNLRYFEMCI	LLVIAASSIA	LAAEDPVLTN	SERNKVLRYF	DYVFTGVFTF	EMVIKMIDQG	LILQDGSYFR
1210	1220	1230	1240	1250	1260	1270	1280
DLWNILDFVV	VVGALVAFAL	ANALGTNKGR	DIKTIKSLRV	LRVLRPLKTI	KRLPKLKAVF	DCVVTSLKNV	FNILIVYKLF
1290	1300	1310	1320	1330	1340	1350	1360
MFIFAVIAVQ	LFKGKFFYCT	DSSKDTEKEC	IGNYVDHEKN	KMEVKGREWK	RHEFHYDNII	WALLTLFTVS	TGEGWPQVLQ
1370	1380	1390	1400	1410	1420	1430	1440
HSVDVTEEDR	GPSRSNRMEM	SIFYVVYFVV	FPFFFVNIFV	ALIIITFQEQ	GDKMMEECSL	EKNERACIDF	AISAKPLTRY
1450	1460	1470	1480	1490	1500	1510	1520
MPQNRHTFQY	RVWHFVVSPS	FEYTIMAMIA	LNTVVLMMKY	YSAPCTYELA	LKYLNIAFTM	VFSLECVLKV	IAFGFLNYFR
1530	1540	1550	1560	1570	1580	1590	1600
DTWNIFDFIT	VIGSITEIIL	TDSKLVNTSG	FNMSFLKLFR	AARLIKLLRQ	GYTIRILLWT	FVQSFKALPY	VCLLIAMLFF
1610	1620	1630	1640	1650	1660	1670	1680
IYAIIGMQVF	GNIKLDEESH	INRHNNFRSF	FGSLMLLFRS	ATGEAWQEIM	LSCLGEKGCE	PDTTAPSGQN	ENERCGTDLA
1690	1700	1710	1720	1730	1740	1750	1760
YVYFVSFIFF	CSFLMLNLFV	AVIMDNFEYL	TRDSSILGPH	HLDEFVRVWA	EYDRAACGRI	HYTEMYEMLT	LMSPPLGLGK
1770	1780	1790	1800	1810	1820	1830	1840
RCPSKVAYKR	LVLMNMPVAE	DMTVHFTSTL	MALIRTALDI	KIAKGGADRQ	QLDSELQKET	LAIWPHLSQK	MLDLLVPMPK
1850	1860	1870	1880	1890	1900	1910	1920
ASDLTVGKIY	AAMMIMDYYK	QSKVKKQRQQ	LEEQKNAPMF	QRMEPSSLPQ	EIIANAKALP	YLQQDPVSGL	SGRSGYPSMS
1930	1940	1950	1960	1970	1980	1990	2000
PLSPQDIFQL	ACMDPADDGQ	FQERQSLVVT	DPSSMRRSFS	TIRDKRSNSS	WLEEFSMERS	SENTYKSRRR	SYHSSLRLSA
2010	2020	2030	2040	2050	2060	2070	2080
HRLNSDSGHK	SDTHRSGGRE	RGRSKERKHL	LSPDVSRCNS	EERGTQADWE	SPERRQSRSP	SEGRSQTPNR	QGTGSLSESS
2090	2100	2110	2120	2130	2140	2150	2160
IPSVSDTSTP	RRSRRQLPPV	PPKPRPLLSY	SSLIRHAGSI	SPPADGSEEG	SPLTSQALES	NNACLTESSN	SPHPQQSQHA
2170	2180	2190	2200	2210	2220	2230	2240

Detailed Protein Report

Cmpd.	No. of Cmpds.	m/z meas.	Δ m/z [ppm]	z	Rt [min]	Score	P	Range	Sequence	Modification	Ratios	
2194	2	973.4542	-4.91	2	57.6	16.3	1	734-750	K.EVSPMSAPNMPSIERER.R	Oxidation: 10	Q:W	1.39
183	1	823.9125	-35.10	2	31.1	10.2	2	959-972	R.AQDLRRTNSLMVSR.G		M:Q	0.12
27	1	814.2746	-93.66	2	30.0	10.4	1	1296-1308	K.FFYCTDSSKDTEK.E	Carbamidomethyl: 4	Q:W<br>M:Q	0.54<br>1.85
118	1	753.3697	-54.70	2	31.4	13.4	0	1426-1439	R.ACIDFAISAKPLTR.Y		M:Q	0.87
1976	1	698.7954	-42.34	2	53.2	11.0	1	1728-1739	R.VWAEYDRAACGR.I			

Detailed Protein Report

Protein 190: PREDICTED: glutamine-rich protein 2 isoform X2 [Homo sapiens]

Accession: gi|578831523
Database: refseq_human(refseq_human_20140103.fasta)
Database Date: 2015-11-30
Modification(s): Oxidation

Score: 61.2
MW [kDa]: 72.2
pI: 9.9
Sequence Coverage [%]: 8.1
No. of unique Peptides: 3

10	20	30	40	50	60	70	80
MRSRPAKPTW	RRSSSCWHRW	VERLQRILEG	EGNQEAGKEL	KAGELRLQLG	VLRVTVADIE	KELAELRESQ	DRGKAAMENS
**90**	**100**	**110**	**120**	**130**	**140**	**150**	**160**
VSEASLYLQD	QLDKLRMIIE	SMLTSSSTLL	SMSMAPHKAH	TLAPGQIDPE	ATCPACSLDV	SHQVSTLVRR	YEQLQDMVNS
**170**	**180**	**190**	**200**	**210**	**220**	**230**	**240**
LAVSRPSKKA	KLQRQDEELL	GRVQSAILQV	QGDCEKLNIT	TSNLIEDHRQ	KQKDIAMLYQ	GLEKLEKEKA	NREHLEMEID
**250**	**260**	**270**	**280**	**290**	**300**	**310**	**320**
VKADKSALAT	KVSRVQFDAT	TEQLNHMMQE	LVAKMSGQEQ	DWQKMLDRLL	TEMDNKLDRL	ELDPVKQLLE	DRWKSLRQQL
**330**	**340**	**350**	**360**	**370**	**380**	**390**	**400**
RERPPLYQAD	EAAAMRRQLL	AHFHCLSCDR	PLETPVTGHA	IPVTPAGPGL	PGHHSIRPYT	VFELEQVRQH	SRNLKLGSAF
**410**	**420**	**430**	**440**	**450**	**460**	**470**	**480**
PRGDLAQMEQ	SVGRLRSMHS	KMLMNIEKVQ	IHFGGSTKAS	SQIIRELLHA	QCLGSPCYKR	VTDMADYTYS	TVPRRCGGSH
**490**	**500**	**510**	**520**	**530**	**540**	**550**	**560**
TLTYPYHRSR	PQHLPRGLYP	TEEIQIAMKH	DEVDILGLDG	HIYKGRMDTR	LPGILRKDSS	GTSKRKSQQP	RPHVHRPPSL
**570**	**580**	**590**	**600**	**610**	**620**	**630**	**640**
SSNGQLPSRP	QSAQISAGNT	SVSSRQQKDR	PSSEGRLSQP	NTAHPPSSAA	VANRGLERHV	DMPPGEGLEE	PTRGPRSSTA
**650**							
Q							

Cmpd.	No. of Cmpds.	m/z meas.	Δ m/z [ppm]	z	Rt [min]	Score	P	Range	Sequence	Modification
2513	1	410.0863	-280.49	2	61.4	19.3	1	68-74	R.ESQDRGK.A	
592	1	1105.2058	124.92	2	35.8	10.7	1	151-169	R.YEQLQDMVNSLAVSRPSKK.A	Oxidation: 7
16	1	537.2110	-148.15	2	29.8	16.9	0	429-438	K.VQIHFGGSTK.A	

Detailed Protein Report

Protein 191: sodium-driven chloride bicarbonate exchanger isoform 1 [Homo sapiens]

Accession: gi|295821221
Database: refseq_human(refseq_human_20140103.fasta)
Database Date: 2015-11-30
Modification(s): Oxidation

Score: 61.2
MW [kDa]: 125.9
pI: 6.0
Sequence Coverage [%]: 5.3
No. of unique Peptides: 3

Quantitation

Q:W	**Median:**	1.96	**CV:**	1.55 %	**No. of Peptides:**	2
M:Q	**Median:**	0.57	**CV:**	18.83 %	**No. of Peptides:**	2

10	20	30	40	50	60	70	80
MEIKDQGAQM	EPLLPTRNDE	EAVVDRGGTR	SILKTHFEKE	DLEGHRTLFI	GVHVPLGGRK	SHRRHRHRGH	KHRKRDRERD
90	100	110	120	130	140	150	160
SGLEDGRESP	SFDTPSQRVQ	FILGTEDDDE	EHIPHDLFTE	LDEICWREGE	DAEWRETARW	LKFEEDVEDG	GERWSKPYVA
170	180	190	200	210	220	230	240
TLSLHSLFEL	RSCILNGTVL	LDMHANTLEE	IADMVLDQQV	SSGQLNEDVR	HRVHEALMKQ	HHHQNQKKLT	NRIPIVRSFA
250	260	270	280	290	300	310	320
DIGKKQSEPN	SMDKNAGQVV	SPQSAPACVE	NKNDVSRENS	TVDFSKGLGG	QQKGHTSPCG	MKQRHEKGPP	HQQEREVDLH
330	340	350	360	370	380	390	400
FMKKIPPGAE	ASNILVGELE	FLDRTVVAFV	RLSPAVLLQG	LAEVPIPTRF	LFILLGPLGK	GQQYHEIGRS	IATLMTDEVF
410	420	430	440	450	460	470	480
HDVAYKAKDR	NDLVSGIDEF	LDQVTVLPPG	EWDPSIRIEP	PKNVPSQEKR	KIPAVPNGTA	AHGEAEPHGG	HSGPELQRTG
490	500	510	520	530	540	550	560
RIFGGLILDI	KRKAPYFWSD	FRDAFSLQCL	ASFLFLYCAC	MSPVITFGGL	LGEATEGRIS	AIESLFGASM	TGIAYSLFGG
570	580	590	600	610	620	630	640
QPLTILGSTG	PVLVFEKILF	KFCKEYGLSY	LSLRASIGLW	TATLCIILVA	TDASSLVCYI	TRFTEEAFAS	LICIIFIYEA
650	660	670	680	690	700	710	720
LEKLFELSEA	YPINMHNDLE	LLTQYSCNCV	EPHNPSNGTL	KEWRESNISA	SDIIWENLTV	SECKSLHGEY	VGRACGHDHP
730	740	750	760	770	780	790	800
YVPDVLFWSV	ILFFSTVTLS	ATLKQFKTSR	YFPTKVRSIV	SDFAVFLTIL	CMVLIDYAIG	IPSPKLQVPS	VFKPTRDDRG
810	820	830	840	850	860	870	880
WFVTPLGPNP	WWTVIAAIIP	ALLCTILIFM	DQQITAVIIN	RKEHKLKKGC	GYHLDLLMVA	VMLGVCSIMG	LPWFVAATVL
890	900	910	920	930	940	950	960
SITHVNSLKL	ESECSAPGEQ	PKFLGIREQR	VTGLMIFILM	GSSVFMTSIL	KFIPMPVLYG	VFLYMGASSL	KGIQFFDRIK
970	980	990	1000	1010	1020	1030	1040
LFWMPAKHQP	DFIYLRHVPL	RKVHLFTIIQ	MSCLGLLWII	KVSRAAIVFP	MMVLALVFVR	KLMDLLFTKR	ELSWLDDLMP
1050	1060	1070	1080	1090	1100	1110	1120
ESKKKKLEDA	EKEEEQSMLA	MEDEGTVQLP	LEGHYRDDPS	VINISDEMSK	TALWRNLLIT	ADNSKDKESS	FPSKSSPS

Cmpd.	No. of Cmpds.	m/z meas.	Δ m/z [ppm]	z	Rt [min]	Score	P	Range	Sequence	Modification	Ratios
63	1	724.6832	-193.81	2	29.7	11.3	1	128-139	R.EGEDAEWRETAR.W		M:Q 0.48<br>Q:W 1.99
1817	1	847.8355	-100.68	2	52.5	10.3	1	278-293	R.ENSTVDFSKGLGGQQK.G		
1399	1	783.2967	-72.60	2	47.2	11.8	0	1077-1090	R.DDPSVINISDEMSK.T	Oxidation: 12	Q:W 1.93<br>M:Q 0.69

Detailed Protein Report

Protein 192: **PREDICTED: UDP-glucose:glycoprotein glucosyltransferase 1 isoform X1 [Homo sapiens]**

**Accession:**	gi|578804601	**Score:**	60.9
**Database:**	refseq_human(refseq_human_20140103.fasta)	**MW [kDa]:**	176.5
**Database Date:**	2015-11-30	**pI:**	5.3
**Modification(s):**	Carbamidomethyl, Oxidation	**Sequence Coverage [%]:**	5.9
		**No. of unique Peptides:**	1

10	20	30	40	50	60	70	80
MGCKGDASGA	CAAGVCYKMG	VLVVLTVLWL	FSSVKADSKA	ITTSLTTKWF	STPLLLEASE	FLAEDSQEKF	WNFVEASQNI
**90**	**100**	**110**	**120**	**130**	**140**	**150**	**160**
GSSDHDGTDY	SYYHAILEAA	FQFLSPLQQN	LFKFCLSLRS	YSATIQAFQQ	IAADEPPPEG	CNSFFSVHGK	KTCESDTLEA
**170**	**180**	**190**	**200**	**210**	**220**	**230**	**240**
LLLTASERPK	PLLFKGDHRY	PSSNPESPVV	IFYSEIGSEE	FSNFHRQLIS	KSNAGKINYV	FRHYIFNPRK	EPVYLSGYGV
**250**	**260**	**270**	**280**	**290**	**300**	**310**	**320**
ELAIKSTEYK	AKDDTQVKGT	EVNTTVIGEN	DPIDEVQGFL	FGKLRDLHPD	LEGQLKELRK	HLVESTNEMA	PLKVWQLQDL
**330**	**340**	**350**	**360**	**370**	**380**	**390**	**400**
SFQTAARILA	SPVELALVVM	KDLSQNFPTK	ARAITKTAVS	SELRTEVEEN	QKYFKGTLGL	QPGDSALFIN	GLHMDLDTQD
**410**	**420**	**430**	**440**	**450**	**460**	**470**	**480**
IFSLFDVLRN	EARVMEGLHR	LGIEGLSLHN	VLKLNIQPSE	ADYAVDIRSP	AISWVNNLEV	DSRYNSWPSS	LQELLRPTFP
**490**	**500**	**510**	**520**	**530**	**540**	**550**	**560**
GVIRQIRKNL	HNMVFIVDPA	HETTAELMNT	AEMFLSNHIP	LRIGFIFVVN	DSEDVDGMQD	AGVAVLRAYN	YVAQEVDDYH
**570**	**580**	**590**	**600**	**610**	**620**	**630**	**640**
AFQTLTHIYN	KVRTGEKVKV	EHVVSVLEKK	YPYVEVNSIL	GIDSAYDRNR	KEARGYYEQT	GVGPLPVVLF	NGMPFEREQL
**650**	**660**	**670**	**680**	**690**	**700**	**710**	**720**
DPDELETITM	HKILETTTFF	QRAVYLGELP	HDQDVVEYIM	NQPNVVPRIN	SRILTAERDY	LDLTASNNFF	VDDYARFTIL
**730**	**740**	**750**	**760**	**770**	**780**	**790**	**800**
DSQGKTAAVA	NSMNYLTKKG	MSSKEIYDDS	FIRPVTFWIV	GDFDSPSGRQ	LLYDAIKHQK	SSNNVRISMI	NNPAKEISYE
**810**	**820**	**830**	**840**	**850**	**860**	**870**	**880**
NTQISRAIWA	ALQTQTSNAA	KNFITKMAKE	GAAEALAAGA	DIAEFSVGGM	DFSLFKEVFE	SSKMDFILSH	AVYCRDVLKL
**890**	**900**	**910**	**920**	**930**	**940**	**950**	**960**
KKGQRAVISN	GRIIGPLEDS	ELFNQDDFHL	LENIILKTSG	QKIKSHIQQL	RVEEDVASDL	VMKVDALLSA	QPKGDPRIEY
**970**	**980**	**990**	**1000**	**1010**	**1020**	**1030**	**1040**
QFFEDRHSAI	KLRPKEGETY	FDVVAVVDPV	TREAQRLAPL	LLVLAQLINM	NLRVFMNCQS	KLSDMPLKSF	YRYVLEPEIS
**1050**	**1060**	**1070**	**1080**	**1090**	**1100**	**1110**	**1120**
FTSDNSFAKG	PIAKFLDMPQ	SPLFTLNLNT	PESWMVESVR	TPYDLDNIYL	EEVDSVVAAE	YELEYLLLEG	HCYDITTGQP
**1130**	**1140**	**1150**	**1160**	**1170**	**1180**	**1190**	**1200**
PRGLQFTLGT	SANPVIVDTI	VMANLGYFQL	KANPGAWILR	LRKGRSEDIY	RIYSHDGTDS	PPDADEVVIV	LNNFKSKIIK
**1210**	**1220**	**1230**	**1240**	**1250**	**1260**	**1270**	**1280**
VKVQKKADMV	NEDLLSDGTS	ENESGFWDSF	KWGFTGQKTE	EVKQDKDDII	NIFSVASGHL	YERFLRIMML	SVLKNTKTPV
**1290**	**1300**	**1310**	**1320**	**1330**	**1340**	**1350**	**1360**
KFWFLKNYLS	PTFKEFIPYM	ANEYNFQYEL	VQYKWPRWLH	QQTEKQRIIW	GYKILFLDVL	FPLVVDKFLF	VDADQIVRTD
**1370**	**1380**	**1390**	**1400**	**1410**	**1420**	**1430**	**1440**
LKELRDFNLD	GAPYGYTPFC	DSRREMDGYR	FWKSGYWASH	LAGRKYHISA	LYVVDLKKFR	KIAAGDRLRG	QYQGLSQDPN
**1450**	**1460**	**1470**	**1480**	**1490**	**1500**	**1510**	**1520**
SLSNLDQDLP	NNMIHQVPIK	SLPQEWLWCE	TWCDDASKKR	AKTIDLCNNP	MTKEPKLEAA	VRIVPEWQDY	DQEIKQLQIR
**1530**	**1540**	**1550**					
FQKEKETGAL	YKEKTKEPSR	EGPQKREEL					

Cmpd.	No. of Cmpds.	m/z meas.	Δ m/z [ppm]	z	Rt [min]	Score	P	Range	Sequence	Modification
1283	1	882.7587	-117.26	2	45.6	10.6	1	1-18	-.MGCKGDASGACAAGVCYK.M	Carbamidomethyl: 11; Oxidation: 1

Detailed Protein Report

Protein 193: PREDICTED: RING finger protein 17 isoform X10 [Homo sapiens]

**Accession:**	gi|578824967	**Score:**	60.8
**Database:**	refseq_human(refseq_human_20140103.fasta)	**MW [kDa]:**	168.9
**Database Date:**	2015-11-30	**pI:**	5.0
		**Sequence Coverage [%]:**	3.6
		**No. of unique Peptides:**	2

10	20	30	40	50	60	70	80
MLDTNTAEEI	DEALNTAHHS	FEQLSIAGKA	LEHMQKQTIE	ERERVIEVVE	KQFDQLLAFF	DSRKKNLCEE	FARTTDDYLS
**90**	**100**	**110**	**120**	**130**	**140**	**150**	**160**
NLIKAKSYIE	EKKNNLNAAM	NIARALQLSP	SLRTYCDLNQ	IIRTLQLTSD	SELAQVSSPQ	LRNPPRLSVN	CSEIICMFNN
**170**	**180**	**190**	**200**	**210**	**220**	**230**	**240**
MGKIEFRDST	KCYPQENEIR	QNVQKKYNNK	KELSCYDTYP	PLEKKKVDMS	VLTSEAPPPP	LQPETNDVHL	EAKNFQPQKD
**250**	**260**	**270**	**280**	**290**	**300**	**310**	**320**
VATASPKTIA	VLPQMGSSPD	VIIEEIIEDN	VESSAELVFV	SHVIDPCHFY	IRKYSQIKDA	KVLEKKVNEF	CNRSSHLDPS
**330**	**340**	**350**	**360**	**370**	**380**	**390**	**400**
DILELGARIF	VSSIKNGMWC	RGTITELIPI	EGRNTRKPCS	PTRLFVHEVA	LIQIFMVDFG	NSEVLIVTGV	VDTHVRPEHS
**410**	**420**	**430**	**440**	**450**	**460**	**470**	**480**
AKQHIALNDL	CLVLRKSEPY	TEGLLKDIQP	LAQPCSLKDI	VPQNSNEGWE	EEAKVEFLKM	VNNKAVSMKV	FREEDGVLIV
**490**	**500**	**510**	**520**	**530**	**540**	**550**	**560**
DLQKPPPNKI	SSDMPVSLRD	ALVFMELAKF	KSQSLRSHFE	KNTTLHYHPP	ILPKEMTDVS	VTVCHINSPG	DFYLQLIEGL
**570**	**580**	**590**	**600**	**610**	**620**	**630**	**640**
DILFLLKTIE	EFYKSEDGEN	LEILCPVQDQ	ACVAKFEDGI	WYRAKVIGLP	GHQEVEVKYV	DFGNTAKITI	KDVRKIKDEF
**650**	**660**	**670**	**680**	**690**	**700**	**710**	**720**
LNAPEKAIKC	KLAYIEPYKR	TMQWSKEAKE	KFEEKAQDKF	MTCSVIKILE	DNVLLVELFD	SLGAPEMTTT	SINDQLVKEG
**730**	**740**	**750**	**760**	**770**	**780**	**790**	**800**
LASYEIGYIL	KDNSQKHIEV	WDPSPEEIIS	NEVHNLNPVS	AKSLPNENFQ	SLYNKELPVH	ICNVISPEKI	YVQWLLTENL
**810**	**820**	**830**	**840**	**850**	**860**	**870**	**880**
LNSLEEKMIA	AYENSKWEPV	KWENDMHCAV	KIQDKNQWRR	GQIIRMVTDT	LVEVLLYDVG	VELVVNVDCL	RKLEENLKTM
**890**	**900**	**910**	**920**	**930**	**940**	**950**	**960**
GRLSLECSLV	DIRPAGGSDK	WTATACDCLS	LYLTGAVATI	ILQVDSEENN	TTWPLPVKIF	CRDEKGERVD	VSKYLIKKGL
**970**	**980**	**990**	**1000**	**1010**	**1020**	**1030**	**1040**
ALRERRINNL	DNSHSLSEKS	LEVPLEQEDS	VVTNCIKTNF	DPDKKTADII	SEQKVSEFQE	KILEPRTTRG	YKPPAIPNMN
**1050**	**1060**	**1070**	**1080**	**1090**	**1100**	**1110**	**1120**
VFEATVSCVG	DDGTIFVVPK	LSEFELIKMT	NEIQSNLKCL	GLLEPYFWKK	GEACAVRGSD	TLWYRGKVME	VVGGAVRVQY
**1130**	**1140**	**1150**	**1160**	**1170**	**1180**	**1190**	**1200**
LDHGFTEKIP	QCHLYPILLY	PDIPQFCIPC	QLHNTTPVGN	VWQPDAIEVL	QQLLSKRQVD	IHIMELPKNP	WEKLSIHLYF
**1210**	**1220**	**1230**	**1240**	**1250**	**1260**	**1270**	**1280**
DGMSLSYFMA	YYKYCTSEHT	EEMLKEKPRS	DHDKKYEEEQ	WEIRFEELLS	AETDTPLLPP	YLSSSLPSPG	ELYAVQVKHV
**1290**	**1300**	**1310**	**1320**	**1330**	**1340**	**1350**	**1360**
VSPNEVYICL	DSIETSNQSN	QHSDTDDSGV	SGESESESLD	EALQRVNKKV	EALPPLTDFR	TEMPCLAEYD	DGLWYRAKIV
**1370**	**1380**	**1390**	**1400**	**1410**	**1420**	**1430**	**1440**
AIKEFNPLSI	LVQFVDYGST	AKLTLNRLCQ	IPSHLMRYPA	RAIKVLLAGF	KPPLRDLGET	RIPYCPKWSM	EALWAMIDCL
**1450**	**1460**	**1470**	**1480**	**1490**			
QGKQLYAVSM	APAPEQIVTL	YDDEQHPVHM	PLVEMGLADK	DE			

Cmpd.	No. of Cmpds.	m/z meas.	Δ m/z [ppm]	z	Rt [min]	Score	P	Range	Sequence	Modification
200	1	642.8816	92.45	2	32.2	16.1	1	596-605	K.FEDGIWYRAK.V	
2279	4	863.8727	-142.91	2	57.0	17.3	2	1326-1340	R.VNKKVEALPPLTDFR.T	

Detailed Protein Report

Protein 194: dedicator of cytokinesis protein 11 [Homo sapiens]

**Accession:**	gi|145699123	**Score:**	60.8
**Database:**	refseq_human(refseq_human_20140103.fasta)	**MW [kDa]:**	237.5
**Database Date:**	2015-11-30	**pI:**	8.6
**Modification(s):**	Oxidation	**Sequence Coverage [%]:**	1.6
		**No. of unique Peptides:**	4

Detailed Protein Report

10	20	30	40	50	60	70	80
MAEVRKFTKR	LSKPGTAAEL	RQSVSEAVRG	SVVLEKAKVV	EPLDYENVIA	QRKTQIYSDP	LRDLLMFPME	DISISVIGRQ
90	100	110	120	130	140	150	160
RRTVQSTVPE	DAEKRAQSLF	VKECIKTYST	DWHVVNYKYE	DFSGDFRMLP	CKSLRPEKIP	NHVFEIDEDC	EKDEDSSSLC
170	180	190	200	210	220	230	240
SQKGGVIKQG	WLHKANVNST	ITVTMKVFKR	RYFYLTQLPD	GSYILNSYKD	EKNSKESKGC	IYLDACIDVV	QCPKMRRHAF
250	260	270	280	290	300	310	320
ELKMLDKYSH	YLAAETEQEM	EEWLITLKKI	IQINTDSLVQ	EKKETVETAQ	DDETSSQGKA	ENIMASLERS	MHPELMKYGR
330	340	350	360	370	380	390	400
ETEQLNKLSR	GDGRQNLFSF	DSEVQRLDFS	GIEPDIKPFE	EKCNKRFLVN	CHDLTFNILG	QIGDNAKGPP	TNVEPFFINL
410	420	430	440	450	460	470	480
ALFDVKNNCK	ISADFHVDLN	PPSVREMLWG	SSTQLASDGS	PKGSSPESYI	HGIAESQLRY	IQQGIFSVTN	PHPEIFLVAR
490	500	510	520	530	540	550	560
IEKVLQGNIT	HCAEPYIKNS	DPVKTAQKVH	RTAKQVCSRL	GQYRMPFAWA	ARPIFKDTQG	SLDLDGRFSP	LYKQDSSKLS
570	580	590	600	610	620	630	640
SEDILKLLSE	YKKPEKTKLQ	IIPGQLNITV	ECVPVDLSNC	ITSSYVPLKP	FEKNCQNITV	EVEEFVPEMT	KYCYPFTIYK
650	660	670	680	690	700	710	720
NHLYVYPLQL	KYDSQKTFAK	ARNIAVCVEF	RDSDESDASA	LKCIYGKPAG	SVFTTNAYAV	VSHHNQNPEF	YDEIKIELPI
730	740	750	760	770	780	790	800
HLHQKHHLLF	TFYHVSCEIN	TKGTTKKQDT	VETPVGFAWV	PLLKDGRIIT	FEQQLPVSAN	LPPGYLNLND	AESRRQCNVD
810	820	830	840	850	860	870	880
IKWVDGAKPL	LKIKSHLEST	IYTQDLHVHK	FFHHCQLIQS	GSKEVPGELI	KYLKCLHAME	IQVMIQFLPV	ILMQLFRVLT
890	900	910	920	930	940	950	960
NMTHEDDVPI	NCTMVLLHIV	SKCHEEGLDS	YLRSFIKYSF	RPEKPSAPQA	QLIHETLATT	MIAILKQSAD	FLSINKLLKY
970	980	990	1000	1010	1020	1030	1040
SWFFFEIIAK	SMATYLLEEN	KIKLPRGQRF	PETYHHVLHS	LLLAIIPHVT	IRYAEIPDES	RNVNYSLASF	LKRCLTLMDR
1050	1060	1070	1080	1090	1100	1110	1120
GFIFNLINDY	ISGFSPKDPK	VLAEYKFEFL	QTICNHEHYI	PLNLPMAFAK	PKLQRVQDSN	LEYSLSDEYC	KHHFLVGLLL
1130	1140	1150	1160	1170	1180	1190	1200
RETSIALQDN	YEIRYTAISV	IKNLLIKHAF	DTRYQHKNQQ	AKIAQLYLPF	VGLLLENIQR	LAGRDTLYSC	AAMPNSASRD
1210	1220	1230	1240	1250	1260	1270	1280
EFPCGFTSPA	NRGSLSTDKD	TAYGSFQNGH	GIKREDSRGS	LIPEGATGFP	DQGNTGENTR	QSSTRSSVSQ	YNRLDQYEIR
1290	1300	1310	1320	1330	1340	1350	1360
SLLMCYLYIV	KMISEDTLLT	YWNKVSPQEL	INILILLEVC	LFHFRYMGKR	NIARVHDAWL	SKHFGIDRKS	QTMPALRNRS
1370	1380	1390	1400	1410	1420	1430	1440
GVMQARLQHL	SSLESSFTLN	HSSTTTEADI	FHQALLEGNT	ATEVSLTVLD	TISFFTQCFK	TQLLNNDGHN	PLMKKVFDIH
1450	1460	1470	1480	1490	1500	1510	1520
LAFLKNGQSE	VSLKHVFASL	RAFISKFPSA	FFKGRVNMCA	AFCYEVLKCC	TSKISSTRNE	ASALLYLLMR	NNFEYTKRKT
1530	1540	1550	1560	1570	1580	1590	1600
FLRTHLQIII	AVSQLIADVA	LSGGSRFQES	LFIINNFANS	DRPMKATAFP	AEVKDLTKRI	RTVLMATAQM	KEHEKDPEML
1610	1620	1630	1640	1650	1660	1670	1680
IDLQYSLAKS	YASTPELRKT	WLDSMAKIHV	KNGDFSEAAM	CYVHVAALVA	EFLHRKKLFP	NGCSAFKKIT	PNIDEEGAMK
1690	1700	1710	1720	1730	1740	1750	1760
EDAGMMDVHY	SEEVLLELLE	QCVDGLWKAE	RYEIISEISK	LIVPIYEKRR	EFEKLTQVYR	TLHGAYTKIL	EVMHTKKRLL
1770	1780	1790	1800	1810	1820	1830	1840
GTFFRVAFYG	QSFFEEEDGK	EYIYKEPKLT	GLSEISLRLV	KLYGEKFGTE	NVKIIQDSDK	VNAKELDPKY	AHIQVTYVKP
1850	1860	1870	1880	1890	1900	1910	1920
YFDDKELTER	KTEFERNHNI	SRFVFEAPYT	LSGKKQGCIE	EQCKRRTILT	TSNSFPYVKK	RIPINCEQQI	NLKPIDVATD
1930	1940	1950	1960	1970	1980	1990	2000
EIKDKTAELQ	KLCSSTDVDM	IQLQLKLQGC	VSVQVNAGPL	AYARAFLNDS	QASKYPPKKV	SELKDMFRKF	IQACSIALEL
2010	2020	2030	2040	2050	2060	2070	2080
NERLIKEDQV	EYHEGLKSNF	RDMVKELSDI	IHEQILQEDT	MHSPWMSNTL	HVFCAISGTS	SDRGYGSPRY	AEV

Cmpd.	No. of	m/z meas.	Δ m/z	z	Rt	Score	P	Range	Sequence	Modification

Detailed Protein Report

	Cmpds.		[ppm]		[min]					
180	1	1043.9250	-79.30	2	32.2	13.4	1	300-317	K.AENIMASLERSMHPELMK.Y	
2146	7	682.9130	127.21	2	57.2	18.0	1	310-320	R.SMHPELMKYGR.E	Oxidation: 7
1995	1	682.7325	-137.16	2	53.4	18.8	1	310-320	R.SMHPELMKYGR.E	Oxidation: 2
2045	1	731.3221	-60.47	2	56.0	10.6	1	1668-1680	K.KITPNIDEEGAMK.E	Oxidation: 12

Detailed Protein Report

Protein 195: **PREDICTED: N-alpha-acetyltransferase 15, NatA auxiliary subunit isoform X1 [Homo sapiens]**

**Accession:**	gi|530378196	**Score:**	60.7
**Database:**	refseq_human(refseq_human_20140103.fasta)	**MW [kDa]:**	101.1
**Database Date:**	2015-11-30	**pI:**	7.8
**Modification(s):**	Oxidation	**Sequence Coverage [%]:**	6.9
		**No. of unique Peptides:**	1

10	20	30	40	50	60	70	80
MPAVSLPPKE	NALFKRILRC	YEHKQYRNGL	KFCKQILSNP	KFAEHGETLA	MKGLTLNCLG	KKEEAYELVR	RGLRNDLKSH
**90**	**100**	**110**	**120**	**130**	**140**	**150**	**160**
VCWHVYGLLQ	RSDKKYDEAI	KCYRNALKWD	KDNLQILRDL	SLLQIQMRDL	EGYRETRYQL	LQLRPAQRAS	WIGYAIAYHL
**170**	**180**	**190**	**200**	**210**	**220**	**230**	**240**
LEDYEMAAKI	LEEFRKTQQT	SPDKVDYEYS	ELLLYQNQVL	REAGLYREAL	EHLCTYEKQI	CDKLAVEETK	GELLLQLCRL
**250**	**260**	**270**	**280**	**290**	**300**	**310**	**320**
EDAADVYRGL	QERNPENWAY	YKGLEKALKP	ANMLERLKIY	EEAWTKYPRG	LVPRRLPLNF	LSGEKFKECL	DKFLRMNFSK
**330**	**340**	**350**	**360**	**370**	**380**	**390**	**400**
GCPPVFNTLR	SLYKDKEKVA	IIEELVVGYE	TSLKSCRLFN	PNDDGKEEPP	TTLLWVQYYL	AQHYDKIGQP	SIALEYINTA
**410**	**420**	**430**	**440**	**450**	**460**	**470**	**480**
IESTPTLIEL	FLVKAKIYKH	AGNIKEAARW	MDEAQALDTA	DRFINSKCAK	YMLKANLIKE	AEEMCSKFTR	EGTSAVENLN
**490**	**500**	**510**	**520**	**530**	**540**	**550**	**560**
EMQCMWFQTE	CAQAYKAMNK	FGEALKKCHE	IERHFIEITD	DQFDFHTYCM	RKITLRSYVD	LLKLEDVLRQ	HPFYFKAARI
**570**	**580**	**590**	**600**	**610**	**620**	**630**	**640**
AIEIYLKLHD	NPLTDENKEH	EADTANMSDK	ELKKLRNKQR	RAQKKAQIEE	EKKNAEKEKQ	QRNQKKKKDD	DDEEIGGPKE
**650**	**660**	**670**	**680**	**690**	**700**	**710**	**720**
ELIPEKLAKV	ETPLEEAIKF	LTPLKNLVKN	KIETHLFAFE	IYFRKEKFLL	MLQSVKRAFA	IDSSHPWLHE	CMIRLFNTVC
**730**	**740**	**750**	**760**	**770**	**780**	**790**	**800**
ESKDLSDTVR	TVLKQEMNRL	FGATNPKNFN	ETFLKRNSDS	LPHRLSAAKM	VYYLDPSSQK	RAIELATTLD	ESLTNRNLQT
**810**	**820**	**830**	**840**	**850**	**860**	**870**	
CMEVLEALYD	GSLGDCKEAA	EIYRANCHKL	FPYALAFMPP	GYEEDMKITV	NGDSSAEAEE	LANEI	

Cmpd.	No. of Cmpds.	m/z meas.	Δ m/z [ppm]	z	Rt [min]	Score	P	Range	Sequence	Modification
2297	1	863.8705	-61.74	2	57.2	20.5	1	316-330	R.MNFSKGCPPVFNTLR.S	Oxidation: 1

Detailed Protein Report

Protein 196: spermatogenesis-associated protein 31E1 [Homo sapiens]

**Accession:**	gi|155029550	**Score:**	60.1
**Database:**	refseq_human(refseq_human_20140103.fasta)	**MW [kDa]:**	157.0
**Database Date:**	2015-11-30	**pI:**	10.3
**Modification(s):**	Oxidation	**Sequence Coverage [%]:**	3.5
		**No. of unique Peptides:**	4

```
        10         20         30         40         50         60         70         80
MGNLVIPLGK GRAGRVESGQ RIPPPAPRPS VECTGDDIAL QMEKMLFPLK SPSATWLSPS STPWMMDFIL TSVCGLVLLF
        90        100        110        120        130        140        150        160
LLLLYVHSDP PSPPPGRKRS SREPQRERSG RSRSRKISAL KACRILLREL EETRDLNYLL ESHLRKLAGE GSSHLPLGGD
       170        180        190        200        210        220        230        240
PLGDVCKPVP AKAHQPHGKC MQDPSPASLS PPAPPAPLAS TLSPGPMTFS EPFGPHSTLS ASGPPEPLLP LKCPATQPHV
       250        260        270        280        290        300        310        320
VFPPSPQPHG PLASSPPPPD SSLAGLQCGS TTCPVPQSSP LHNQVLPPPT RVISGLGCSS DPIWDLYCWR EAATTWGLST
       330        340        350        360        370        380        390        400
YSHGKSQPRH LPDHTSEASF WGDPTPKHME VGGCTFIHPD VQKLLETLIA KRALMKMWQE KERKRADHPH MTSLGKEWDI
       410        420        430        440        450        460        470        480
TTLNPFWNVS TQPQQLPRPQ QVSDATTVGN HLQQKRSQLF WDLPSLNSES LATTVWVSRN PSSQNAHSVP LDKASTSLPG
       490        500        510        520        530        540        550        560
EPEVEASSQL SQAPPQPHHM AQPQHFTPAW PQSQPPPLAE IQTQAHLSPP VPSLGCSSPP QIRGCGASYP TSQERTQSVI
       570        580        590        600        610        620        630        640
PTGKEYLEWP LKKRPKWKRV LPSLLKKSQA VLSQPTAHLP QERPASWSPK SAPILPGVVT SPELPEHWWQ GRNAIHQEQS
       650        660        670        680        690        700        710        720
CGPPSRLQAS GDLLQPDGEF PGRPQSQAED TQQALLPSQP SDFAGKGRKD VQKTGFRSSG RFSDKGCLGS KLGPDPSRDQ
       730        740        750        760        770        780        790        800
GSGRTSVKAL DEDKEAEGDL RRSWKYQSVS STPRDPDKEH LENKLQIHLA RKVGEIKEGW IPMPVRRSWL MAKCAVPKSD
       810        820        830        840        850        860        870        880
THRKPGKLAS WRGGKAHVNT SQELSFLHPC TQQILEVHLV RFCVRHSWGT DLQSLEPINV WSGEAQAPPF PQSTFTPWAS
       890        900        910        920        930        940        950        960
WVSRVESVPK VPIFLGKRPQ NGPGDNRTTS KSVPTVSGPL AAPPPEQEGV QRPPRGSQSA DTHGRSEAFP TGHKGRGCSQ
       970        980        990       1000       1010       1020       1030       1040
PPTCSLVGRT WQSRTVLESG KPKPRLEGSM GSEMAGNEAW LESESMSPGD PCSSRALQVL SIGSQWARAE DALQALKVGE
      1050       1060       1070       1080       1090       1100       1110       1120
KPPTWEVTLG ASVRASSGSV QEDLRSTGAL GTTGNPSASS VCVAQDPEQL HLKAQVVSEI ALIVQVDSEE QLPGRAPGIL
      1130       1140       1150       1160       1170       1180       1190       1200
LQDGATGLCL PGRHMDMLTA ADRLPTQAPL STSQSVSGKN MTASQGPCAL LWKGGDSPGQ QEPGSPKAKA PQKSQKTLGC
      1210       1220       1230       1240       1250       1260       1270       1280
ADKGEAHRRP RTGEQGHRSK GPRTSEASGR SHPAQAREIG DKQERKYNQL QLEKGQTPPE SHFQRKISHH PQGLHPRKGG
      1290       1300       1310       1320       1330       1340       1350       1360
TRWEDVLQKG KPGADAFQSW GSGPPRQFMD CMADKAWTIS RVVGQILVDK LGLQWGRGPS EVNRHKGDFR AQENVPSCCH
      1370       1380       1390       1400       1410       1420       1430       1440
RGHCHQERSR EMRALACSPK ATPKGHHCPV KNRGIRDRDS SWAPPPREPV SPAGPHHHRP RMASTSGGPH PQLQELMSAQ
      1450
RCLAS
```

Cmpd.	No. of Cmpds.	m/z meas.	Δ m/z [ppm]	z	Rt [min]	Score	P	Range	Sequence	Modification
2055	1	643.7814	-75.01	2	54.1	10.5	1	706-718	K.GCLGSKLGPDPSR.D	
1939	1	703.2728	-146.36	2	52.6	10.4	2	787-798	R.RSWLMAKCAVPK.S	Oxidation: 5
2526	1	574.6432	-239.26	2	60.3	12.8	0	1055-1065	R.ASSGSVQEDLR.S	
1848	1	805.3101	-128.16	2	53.1	14.3	2	1394-1407	R.GIRDRDSSWAPPPR.E	

Detailed Protein Report

Protein 197: **PREDICTED: leucine-rich PPR motif-containing protein, mitochondrial isoform X2 [Homo sapiens]**

**Accession:**	gi|578802653	**Score:**	60.0
**Database:**	refseq_human(refseq_human_20140103.fasta)	**MW [kDa]:**	119.1
**Database Date:**	2015-11-30	**pI:**	5.9
		**Sequence Coverage [%]:**	5.0
		**No. of unique Peptides:**	2

Quantitation

M:Q **Median:** 0.69 **CV:** 0.00 % **No. of Peptides:** 1

10	20	30	40	50	60	70	80
MAALLRSARW	LLRAGAAPRL	PLSLRLLPGG	PGRLHAASYL	PAARAGPVAG	GLLSPARLYA	IAAKEKDIQE	ESTFSSRKIS
90	100	110	120	130	140	150	160
NQFDWALMRL	DLSVRRTGRI	PKKLLQKVFN	DTCRSGGLGG	SHALLLLRSC	GSLLPELKLE	ERTEFAHRIW	DTLQKLGAVY
170	180	190	200	210	220	230	240
DVSHYNALLK	VYLQNEYKFS	PTDFLAKMEE	ANIQPNRVTY	QRLIASYCNV	GDIEGASKIL	GFMKTKDLPV	TEAVFSALVT
250	260	270	280	290	300	310	320
GHARAGDMEN	AENILTVMRD	AGIEPGPDTY	LALLNAYAEK	GDIDHVKQTL	EKVEKSELHL	MDRDLLQIIF	SFSKAGYPQY
330	340	350	360	370	380	390	400
VSEILEKVTC	ERRYIPDAMN	LILLLVTEKL	EDVALQILLA	CPVSKEDGPS	VFGSFFLQHC	VTMNTPVEKL	TDYCKKLKEV
410	420	430	440	450	460	470	480
QMHSFPLQFT	LHCALLANKT	DLAKALMKAV	KEEGFPIRPH	YFWPLLVGRR	KEKNVQGIIE	ILKGMQELGV	HPDQETYTDY
490	500	510	520	530	540	550	560
VIPCFDSVNS	ARAILQENGC	LSDSDMFSQA	GLRSEAANGN	LDFVLSFLKS	NTLPISLQSI	RSSLLLGFRR	SMNINLWSEI
570	580	590	600	610	620	630	640
TELLYKDGRY	CQEPRGPTEA	VGYFLYNLID	SMSDSEVQAK	EEHLRQYFHQ	LEKMNVKIPE	NIYRGIRNLL	ESYHVPELIK
650	660	670	680	690	700	710	720
DAHLLVESKN	LDFQKTVQLT	SSELESTLET	LKAENQPIRD	VLKQLILVLC	SEENMQKALE	LKAKYESDMV	TGGYAALINL
730	740	750	760	770	780	790	800
CCRHDKVEDA	LNLKEEFDRL	DSSAVLDTGK	YVGLVRVLAK	HGKLQDAINI	LKEMKEKDVL	IKDTTALSFF	HMLNGAALRG
810	820	830	840	850	860	870	880
EIETVKQLHE	AIVTLGLAEP	STNISFPLVT	VHLEKGDLST	ALEVAIDCYE	KYKVLPRIHD	VLCKLVEKGE	TDLIQKAMDF
890	900	910	920	930	940	950	960
VSQEQGEMVM	LYDLFFAFLQ	TGNYKEAKKI	IETPGIRARS	ARLQWFCDRC	VANNQVETLE	KLVELTQKLF	ECDRDQMYYN
970	980	990	1000	1010	1020	1030	1040
LLKLYKINGD	WQRADAVWNK	IQEENVIPRE	KTLRLLAEIL	REGNQEVPFD	VPELWYEDEK	HSLNSSSAST	TEPDFQKDIL
1050	1060						
IACRLNQKKR	GI						

Cmpd.	No. of Cmpds.	m/z meas.	Δ m/z [ppm]	z	Rt [min]	Score	P	Range	Sequence	Modification	Ratios	
1368	1	428.2118	-70.40	3	47.2	12.4	1	673-683	K.AENQPIRDVLK.Q			
386	5	627.3328	-88.01	2	34.8	18.2	2	909-919	K.KIIETPGIRAR.S		M:Q	0.69

Detailed Protein Report

Protein 198: G-protein coupled receptor 98 precursor [Homo sapiens]

**Accession:**	gi|113722120	**Score:**	60.0
**Database:**	refseq_human(refseq_human_20140103.fasta)	**MW [kDa]:**	692.6
**Database Date:**	2015-11-30	**pI:**	4.4
**Modification(s):**	Oxidation	**Sequence Coverage [%]:**	0.9
		**No. of unique Peptides:**	3

Detailed Protein Report

10	20	30	40	50	60	70	80
MSVFLGPGMP	SASLLVNLLS	ALLILFVFGE	TEIRFTGQTE	FVVNETSTTV	IRLIIERIGE	PANVTAIVSL	YGEDAGDFFD
90	100	110	120	130	140	150	160
TYAAAFIPAG	ETNRTVYIAV	CDDDLPEPDE	TFIFHLTLQK	PSANVKLGWP	RTVTVTILSN	DNAFGIISFN	MLPSIAVSEP
170	180	190	200	210	220	230	240
KGRNESMPLT	LIREKGTYGM	VMVTFEVEGG	PNPPDEDLSP	VKGNITFPPG	RATVIYNLTV	LDDEVPENDE	IFLIQLKSVE
250	260	270	280	290	300	310	320
GGAEINTSRN	SIEIIIKKND	SPVRFLQSIY	LVPEEDHILI	IPVVRGKDNN	GNLIGSDEYE	VSISYAVTTG	NSTAHAQQNL
330	340	350	360	370	380	390	400
DFIDLQPNTT	VVFPPFIHES	HLKFQIVDDT	IPEIAESFHI	MLLKDTLQGD	AVLISPSVVQ	VTIKPNDKPY	GVLSFNSVLF
410	420	430	440	450	460	470	480
ERTVIIDEDR	ISRYEEITVV	RNGGTHGNVS	ANWVLTRNST	DPSPVTADIR	PSSGVLHFAQ	GQMLATIPLT	VVDDDLPEEA
490	500	510	520	530	540	550	560
EAYLLQILPH	TIRGGAEVSE	PAELLFYIQD	SDDVYGLITF	FPMENQKIES	SPGERYLSLS	FTRLGGTKGD	VRLLYSVLYI
570	580	590	600	610	620	630	640
PAGAVDPLQA	KEGILNISRR	NDLIFPEQKT	QVTTKLPIRN	DAFLQNGAHF	LVQLETVELL	NIIPLIPPIS	PRFGEICNIS
650	660	670	680	690	700	710	720
LLVTPAIANG	EIGFLSNLPI	ILHEPEDFAA	EVVYIPLHRD	GTDGQATVYW	SLKPSGFNSK	AVTPDDIGPF	NGSVLFLSGQ
730	740	750	760	770	780	790	800
SDTTINITIK	GDDIPEMNET	VTLSLDRVNV	ENQVLKSGYT	SRDLIILEND	DPGGVFEFSP	ASRGPYVIKE	GESVELHIIR
810	820	830	840	850	860	870	880
SRGSLVKQFL	HYRVEPRDSN	EFYGNTGVLE	FKPGEREIVI	TLLARLDGIP	ELDEHYWVVL	SSHGERESKL	GSATIVNITI
890	900	910	920	930	940	950	960
LKNDDPHGII	EFVSDGLIVM	INESKGDAIY	SAVYDVVRNR	GNFGDVSVSW	VVSPDFTQDV	FPVQGTVVFG	DQEFSKNITI
970	980	990	1000	1010	1020	1030	1040
YSLPDEIPEE	MEEFTVILLN	GTGGAKVGNR	TTATLRIRRN	DDPIYFAEPR	VVRVQEGETA	NFTVLRNGSV	DVTCMVQYAT
1050	1060	1070	1080	1090	1100	1110	1120
KDGKATARER	DFIPVEKGET	LIFEVGSRQQ	SISIFVNEDG	IPETDEPFYI	ILLNSTGDTV	VYQYGVATVI	IEANDDPNGI
1130	1140	1150	1160	1170	1180	1190	1200
FSLEPIDKAV	EEGKTNAFWI	LRHRGYFGSV	SVSWQLFQND	SALQPGQEFY	ETSGTVNFMD	GEEAKPIILH	AFPDKIPEFN
1210	1220	1230	1240	1250	1260	1270	1280
EFYFLKLVNI	SGGSPGPGGQ	LAETNLQVTV	MVPFNDDPFG	VFILDPECLE	REVAEDVLSE	DDMSYITNFT	ILRQQGVFGD
1290	1300	1310	1320	1330	1340	1350	1360
VQLGWEILSS	EFPAGLPPMI	DFLLVGIFPT	TVHLQQHMRR	HHSGTDALYF	TGLEGAFGTV	NPKYHPSRNN	TIANFTFSAW
1370	1380	1390	1400	1410	1420	1430	1440
VMPNANTNGF	IIAKDDGNGS	IYYGVKIQTN	ESHVTLSLHY	KTLGSNATYI	AKTTVMKYLE	ESVWLHLLII	LEDGIIEFYL
1450	1460	1470	1480	1490	1500	1510	1520
DGNAMPRGIK	SLKGEAITDG	PGILRIGAGI	NGNDRFTGLM	QDVRSYERKL	TLEEIYELHA	MPAKSDLHPI	SGYLEFRQGE
1530	1540	1550	1560	1570	1580	1590	1600
TNKSFIISAR	DDNDEEGEEL	FILKLVSVYG	GARISEENTT	ARLTIQKSDN	ANGLFGFTGA	CIPEIAEEGS	TISCVVERTR
1610	1620	1630	1640	1650	1660	1670	1680
GALDYVHVFY	TISQIETDGI	NYLVDDFANA	SGTITFLPWQ	RSEVLNIYVL	DDDIPELNEY	FRVTLVSAIP	GDGKLGSTPT
1690	1700	1710	1720	1730	1740	1750	1760
SGASIDPEKE	TTDITIKASD	HPYGLLQFST	GLPPQPKDAM	TLPASSVPHI	TVEEEDGEIR	LLVIRAQGLL	GRVTAEFRTV
1770	1780	1790	1800	1810	1820	1830	1840
SLTAFSPEDY	QNVAGTLEFQ	PGERYKYIFI	NITDNSIPEL	EKSFKVELLN	LEGGVAELFR	VDGSGSGDGD	MEFFLPTIHK
1850	1860	1870	1880	1890	1900	1910	1920
RASLGVASQI	LVTIAASDHA	HGVFEFSPES	LFVSGTEPED	GYSTVTLNVI	RHHGTLSPVT	LHWNIDSDPD	GDLAFTSGNI
1930	1940	1950	1960	1970	1980	1990	2000
TFEIGQTSAN	ITVEILPDED	PELDKAFSVS	VLSVSSGSLG	AHINATLTVL	ASDDPYGIFI	FSEKNRPVKV	EEATQNITLS
2010	2020	2030	2040	2050	2060	2070	2080
IIRLKGLMGK	VLVSYATLDD	MEKPPYFPPN	LARATQGRDY	IPASGFALFG	ANQSEATIAI	SILDDDEPER	SESVFIELLN
2090	2100	2110	2120	2130	2140	2150	2160
STLVAKVQSR	SIPNSPRLGP	KVETIAQLII	IANDDAFGTL	QLSAPIVRVA	ENHVGPIINV	TRTGGAFADV	SVKFKAVPIT
2170	2180	2190	2200	2210	2220	2230	2240

Detailed Protein Report

Cmpd.	No. of Cmpds.	m/z meas.	Δ m/z [ppm]	z	Rt [min]	Score	P	Range	Sequence	Modification
112	4	793.8850	-70.56	2	30.7	12.9	0	1990-2003	K.VEEATQNITLSIIR.L	
1940	2	643.2750	-74.26	2	54.6	11.3	0	4809-4819	K.EQPIVTENAER.Q	
780	2	696.6836	-200.93	2	39.1	12.4	0	5266-5277	R.CAQMEPNALPFR.G	Oxidation: 4

Detailed Protein Report

Protein 199: protein PRR14L [Homo sapiens]

**Accession:**	gi|209969819	**Score:**	59.7
**Database:**	refseq_human(refseq_human_20140103.fasta)	**MW [kDa]:**	237.2
**Database Date:**	2015-11-30	**pI:**	5.9
**Modification(s):**	Carbamidomethyl	**Sequence Coverage [%]:**	2.6
		**No. of unique Peptides:**	2

Quantitation

Q:W	**Median:**	2.85	**CV:**	5.50 %	**No. of Peptides:**	2
M:Q	**Median:**	0.78	**CV:**	38.90 %	**No. of Peptides:**	2

Alias proteins:

Accession	Name	Description
gi|578837140	refseq_human (refseq_human_20140103.fasta)	PREDICTED: protein PRR14L isoform X3 [Homo sapiens]
gi|578837138	refseq_human (refseq_human_20140103.fasta)	PREDICTED: protein PRR14L isoform X2 [Homo sapiens]
gi|530419825	refseq_human (refseq_human_20140103.fasta)	PREDICTED: protein PRR14L isoform X1 [Homo sapiens]

Detailed Protein Report

10	20	30	40	50	60	70	80
MLSSGVETQP	VPLDSSMSAV	VQELYSELPV	SVSRELHADP	EPSVIPDVKP	GASSSLLSQN	RALPLELQRT	HVESCCEETY
90	100	110	120	130	140	150	160
ETLDHGSEPG	RCGLVDSTAG	GSVASGILDR	AKRSESMEPK	VFRDPGGQAG	IIREPSEGAK	EDPHQHSTAA	EEKTSPSQED
170	180	190	200	210	220	230	240
LLMQSSKELS	HVDLPEDFLR	SKEGNVQITA	ETLLKSAEVQ	GMKVNGTKTD	NNEGHKNGNV	SKDLSAGCGE	FQEVDKIMTS
250	260	270	280	290	300	310	320
DEVSETSTLV	TPEPLTFVDP	VLTEATPKEK	ECEELKSCPW	LSLPGNSAIS	NVDNGKEELC	KPNLVCEADD	NHQQLHGHHN
330	340	350	360	370	380	390	400
EQPSSTHDSP	TATSPLKENS	EVSCFTSDLS	GPESRTISLE	NCGFEGGGLL	KRSAEKTDSS	YFYRGDDQGK	NLASREENEE
410	420	430	440	450	460	470	480
RLLIPRSERG	GPFLFNAREP	EKEISGRCSG	EKEPVVSPKE	NIHNNCIQDS	LHTGNSSSLM	PNSFTEATEV	MLNKNDLKIT
490	500	510	520	530	540	550	560
VHVQGNLTNP	EDHKETFTNM	SHPGGHSEES	SFSSLMQIEE	AGQTTPVEPN	ILSKSFYTKD	CNSLVSIQRN	LEGNTQLNEA
570	580	590	600	610	620	630	640
SCNDFLFERK	SIVSLMPEDQ	ISPVSEVLKP	KQGTALLLPS	PEFDYRPESE	KVIQTSHDDI	PLLDEQSIAC	EMNELSCTNE
650	660	670	680	690	700	710	720
LVVNKVESEC	VLNQQVSLNS	QEHANLPTDS	LLHLNKEMPL	ATGRDAHQSH	HPPLEGRADV	IADIQTIPIQ	TKIKDISPPG
730	740	750	760	770	780	790	800
NQTCGASSNC	PTLNIKPVSL	ERKKEMADSG	TKALHSRLRS	NKREAAGFPQ	VVSVIECHSV	QSQDISSCHR	VRKNVSQENM
810	820	830	840	850	860	870	880
CSASAAFKSS	KISLQVDNSL	ITKYENAFQH	RDHCCQGTGH	SVEKSSCKVS	YTSQERELDG	KETNGSLPGD	KIRNKMVAGL
890	900	910	920	930	940	950	960
LNSGISNKTI	HTSSSIKLSE	EGLEGKEQDV	SKETVFCKYN	ISDHAIQELN	QTVNIPGPEK	VLDQSPTVMF	SSFKNVKSVE
970	980	990	1000	1010	1020	1030	1040
TLDQKADEVL	DCQSNQNRPD	ECKSEGQSAK	EMLSSDQRET	VTEPHGEVNH	NQKDLLVSSG	SNNSLPCGSP	KKCNLKGAFV
1050	1060	1070	1080	1090	1100	1110	1120
KMSGCDESTE	GMVDIVYTDC	SNKLAEGVLD	VKASNLLDCG	ARQEKLAFQE	DSRSTLSRRE	LDAAHTGTTG	QDSDFPVTAA
1130	1140	1150	1160	1170	1180	1190	1200
STVDFLKIKK	SCEENVCRSL	KDCEMEKCPD	SCAHEMESVA	DHEPNKRILG	RVNLSLNDSH	YGQQDKGTSL	RETQEMTEGS
1210	1220	1230	1240	1250	1260	1270	1280
RLEPNSEFGK	ESTFGISSKE	SMSCHDESSV	SLRSLKSIEI	MPSQENSETN	VNSEETDLKN	LCKPKDGEML	CENVKDCTVL
1290	1300	1310	1320	1330	1340	1350	1360
PEMKEIVSRD	WSNSSDRDSV	CTCVEKNACK	ACHPHENSSD	RHLPLTVKTD	IKVKGEETEE	HQRGRLGYLT	VGEQSEELVT
1370	1380	1390	1400	1410	1420	1430	1440
RETGDGDPVS	NISQTHFKCR	GILNHAEKQQ	SPEVLDYMLQ	KEEKYIRQQK	AHTISQQCIS	SSLLLDDAQN	QNQPKADKDE
1450	1460	1470	1480	1490	1500	1510	1520
STMINEITLA	KLAKDSIVAQ	TQKLEDQKEE	RLHHPLRKDT	ESCTSPCLLG	APRKAQDPSS	AGCDQIHGAF	AKKGVLPLKK
1530	1540	1550	1560	1570	1580	1590	1600
QPHRTCKKVS	YQEQIIVGRK	IGKIRSSAFL	KSSSNPIPTK	AHRLLSLCTL	SAPTRLEPET	APTKSLVSHI	PKQMSTPCHP
1610	1620	1630	1640	1650	1660	1670	1680
LRSLNFRKTT	KESALLNKLS	ILASKLAPAM	KTQKLRYRRC	SSELLPMAKS	YKRLRYKRLL	DGFSSSTEQL	NPYLAASGWD
1690	1700	1710	1720	1730	1740	1750	1760
KRPNSKPMAL	YSLESIKMTF	IDLSNKMPSL	LFGSEIFPVS	FHVKSSSSDC	TTESSRTFPE	HCAPARLALG	EALQCPSQPP
1770	1780	1790	1800	1810	1820	1830	1840
KWTFSFFLSH	GCPGMATFRE	DTGVHSQTHT	QAPPQPPAPL	QDYGGTAIVQ	TRADCSVLGL	HTLLALCSPG	CYRIWTKKRS
1850	1860	1870	1880	1890	1900	1910	1920
FSSHMPTMQR	LFMTQFTQGL	KGLRSPASIA	DKVFCSLPYS	VGRVLSIWSQ	HGPSVCSFEI	SSLHSPHCKR	QPSLGTTSSH
1930	1940	1950	1960	1970	1980	1990	2000
TMLPYVPLPG	MEATYNTSGS	QTRLEPPFPA	LVPKSCLVAE	SAVSKLLLSA	SEFQVRGLDE	LDGVKAACPC	PQSSPPEQKE
2010	2020	2030	2040	2050	2060	2070	2080
AEPEKRPKKV	SQIRIRKTIP	RPDPNLTPMG	LPRPKRLKKK	EFSLEEIYTN	KNYKSPPANR	CLETIFEEPK	ERNGTLISIS
2090	2100	2110	2120	2130	2140	2150	2160
QQKRKRVLEF	QDFTVPRKRR	ARGKVKVAGS	FTRAQKAAVQ	SRELDALLIQ	KLMELETFFA	KEEEQEQSSG	C

Detailed Protein Report

Cmpd.	No. of Cmpds.	m/z meas.	Δ m/z [ppm]	z	Rt [min]	Score	P	Range	Sequence	Modification	Ratios	
1682	1	917.4150	-55.34	2	49.6	13.4	2	407-422	R.SERGGPFLFNAREPEK.E		M:Q<br>Q:W	1.13<br>3.01
1702	2	867.8891	-25.32	2	49.8	17.0	1	1478-1493	R.KDTESCTSPCLLGAPR.K	Carbamidomethyl: 10	Q:W<br>M:Q	2.69<br>0.53

Detailed Protein Report

Protein 200: PREDICTED: E3 ubiquitin-protein ligase HERC2 isoform X4 [Homo sapiens]

**Accession:**	gi|578827505	**Score:**	59.6
**Database:**	refseq_human(refseq_human_20140103.fasta)	**MW [kDa]:**	526.3
**Database Date:**	2015-11-30	**pI:**	5.9
		**Sequence Coverage [%]:**	1.0
		**No. of unique Peptides:**	2

Detailed Protein Report

10	20	30	40	50	60	70	80
MPSESFCLAA	QARLDSKWLK	TDIQLAFTRD	GLCGLWNEMV	KDGEIVYTGT	ESTQNGELPP	RKDDSVEPSG	TKKEDLNDKE
90	100	110	120	130	140	150	160
KKDEEETPAP	IYRAKSILDS	WVWGKQPDVN	ELKECLSVLV	KEQQALAVQS	ATTTLSALRL	KQRLVILERY	FIALNRTVFQ
170	180	190	200	210	220	230	240
ENVKVKWKSS	GISLPPVDKK	SSRPAGKGVE	GLARVGSRAA	LSFAFAFLRR	AWRSGEDADL	CSELLQESLD	ALRALPEASL
250	260	270	280	290	300	310	320
FDESTVSSVW	LEVVERATRF	LRSVVTGDVH	GTPATKGPGS	IPLQDQHLAL	AILLELAVQR	GTLSQMLSAI	LLLLQLWDSG
330	340	350	360	370	380	390	400
AQETDNERSA	QGTSAPLLPL	LQRFQSIICR	KDAPHSEGDM	HLLSGPLSPN	ESFLRYLTLP	QDNELAIDLR	QTAVVVMAHL
410	420	430	440	450	460	470	480
DRLATPCMPP	LCSSPTSHKG	SLQEVIGWGL	IGWKYYANVI	GPIQCEGLAN	LGVTQIACAE	KRFLILSRNG	RVYTQAYNSD
490	500	510	520	530	540	550	560
TLAPQLVQGL	ASRNIVKIAA	HSDGHHYLAL	AATGEVYSWG	CGDGGRLGHG	DTVPLEEPKV	ISAFSGKQAG	KHVVHIACGS
570	580	590	600	610	620	630	640
TYSAAITAEG	ELYTWGRGNY	GRLGHGSSED	EAIPMLVAGL	KGLKVIDVAC	GSGDAQTLAV	TENGQVWSWG	DGDYGKLGRG
650	660	670	680	690	700	710	720
GSDGCKTPKL	IEKLQDLDVV	KVRCGSQFSI	ALTKDGQVYS	WGKGDNQRLG	HGTEEHVRYP	KLLEGLQGKK	VIDVAAGSTH
730	740	750	760	770	780	790	800
CLALTEDSEV	HSWGSNDQCQ	HFDTLRVTKP	EPAALPGLDT	KHIVGIACGP	AQSFAWSSCS	EWSIGLRVPF	VVDICSMTFE
810	820	830	840	850	860	870	880
QLDLLLRQVS	EGMDGSADWP	PPQEKECVAV	ATLNLLRLQL	HAAISHQVDP	EFLGLGLGSI	LLNSLKQTVV	TLASSAGVLS
890	900	910	920	930	940	950	960
TVQSAAQAVL	QSGWSVLLPT	AEERARALSA	LLPCAVSGNE	VNISPGRRFM	IDLLVGSLMA	DGGLESALHA	AITAEIQDIE
970	980	990	1000	1010	1020	1030	1040
AKKEAQKEKE	IDEQEANAST	FHRSRTPLDK	DLINTGICES	SGKQCLPLVQ	LIQQLLRNIA	SQTVARLKDV	ARRISSCLDF
1050	1060	1070	1080	1090	1100	1110	1120
EQHSRERSAS	LDLLLRFQRL	LISKLYPGES	IGQTSDISSP	ELMGVGSLLK	KYTALLCTHI	GDILPVAASI	ASTSWRHFAE
1130	1140	1150	1160	1170	1180	1190	1200
VAYIVEGDFT	GVLLPELVVS	IVLLLSKNAG	LMQEAGAVPL	LGGLLEHLDR	FNHLAPGKER	DDHEELAWPG	IMGQNCRNNE
1210	1220	1230	1240	1250	1260	1270	1280
EVTLIRKADL	ENHNKDGGFW	TVIDGKVYDI	KDFQTQSLTG	NSILAQFAGE	DPVVALEAAL	QFEDTRESMH	AFCVGQYLEP
1290	1300	1310	1320	1330	1340	1350	1360
DQEIVTIPDL	GSLSSPLIDT	ERNLGLLLGL	HASYLAMSTP	LSPVEIECAK	WLQSSIFSGG	LQTSQIHYSY	NEEKDEDHCS
1370	1380	1390	1400	1410	1420	1430	1440
SPGGTPASKS	RLCSHRRALG	DHSQAFLQAI	ADNNIQDHNV	KDFLCQIERY	CRQCHLTTPI	MFPPEHPVEE	VGRLLLCCLL
1450	1460	1470	1480	1490	1500	1510	1520
KHEDLGHVAL	SLVHAGALGI	EQVKHRTLPK	SVVDVCRVVY	QAKCSLIKTH	QEQGRSYKEV	CAPVIERLRF	LFNELRPAVC
1530	1540	1550	1560	1570	1580	1590	1600
NDLSIMSKFK	LLSSLPRWRR	IAQKIIRERR	KKRVPKKPES	TDDEEKIGNE	ESDLEEACIL	PHSPINVDKR	PIAIKSPKDK
1610	1620	1630	1640	1650	1660	1670	1680
WQPLLSTVTG	VHKYKWLKQN	VQGLYPQSPL	LSTIAEFALK	EEPVDVEKMR	KCLLKQLERA	EVRLEGIDTI	LKLASKNFLL
1690	1700	1710	1720	1730	1740	1750	1760
PSVQYAMFCG	WQRLIPEGID	IGEPLTDCLK	DVDLIPPFNR	MLLEVTFGKL	YAWAVQNIRN	VLMDASAKFK	ELGIQPVPLQ
1770	1780	1790	1800	1810	1820	1830	1840
TITNENPSGP	SLGTIPQARF	LLVMLSMLTL	QHGANNLDLL	LNSGMLALTQ	TALRLIGPSC	DNVEEDMNAS	AQGASATVLE
1850	1860	1870	1880	1890	1900	1910	1920
ETRKETAPVQ	LPVSGPELAA	MMKIGTRVMR	GVDWKWGDQD	GPPPGLGRVI	GELGEDGWIR	VQWDTGSTNS	YRMGKEGKYD
1930	1940	1950	1960	1970	1980	1990	2000
LKLAELPAAA	QPSAEDSDTE	DDSEAEQTER	NIHPTAMMFT	STINLLQTLC	LSAGVHAEIM	QSEATKTLCG	LLRMLVESGT
2010	2020	2030	2040	2050	2060	2070	2080
TDKTSSPNRL	VYREQHRSWC	TLGFVRSIAL	TPQVCGALSS	PQWITLLMKV	VEGHAPFTAT	SLQRQILAVH	LLQAVLPSWD
2090	2100	2110	2120	2130	2140	2150	2160
KTERARDMKC	LVEKLFDFLG	SLLTTCSSDV	PLLRESTLRR	RRVRPQASLT	ATHSSTLAEE	VVALLRTLHS	LTQWNGLINK
2170	2180	2190	2200	2210	2220	2230	2240

Detailed Protein Report

Cmpd.	No. of Cmpds.	m/z meas.	Δ m/z [ppm]	z	Rt [min]	Score	P	Range	Sequence	Modification
445	2	592.8266	-45.39	2	34.9	13.6	1	654-663	K.LQDLDVVKVR.C	
360	1	543.1311	-259.95	2	33.8	14.2	0	4001-4011	K.LYATGYGAGGR.L	

Detailed Protein Report

Protein 201: aldehyde oxidase [Homo sapiens]

Accession: gi|71773480
Database: refseq_human(refseq_human_20140103.fasta)
Database Date: 2015-11-30
Modification(s): Oxidation

Score: 59.5
MW [kDa]: 147.8
pI: 6.9
Sequence Coverage [%]: 4.9
No. of unique Peptides: 1

10	20	30	40	50	60	70	80
MDRASELLFY	VNGRKVIEKN	VDPETMLLPY	LRKKLRLTGT	KYGCGGGGCG	ACTVMISRYN	PITKRIRHHP	ANACLIPICS
90	100	110	120	130	140	150	160
LYGAAVTTVE	GIGSTHTRIH	PVQERIAKCH	GTQCGFCTPG	MVMSIYTLLR	NHPEPTLDQL	TDALGGNLCR	CTGYRPIIDA
170	180	190	200	210	220	230	240
CKTFCKTSGC	CQSKENGVCC	LDQGINGLPE	FEEGSKTSPK	LFAEEEFLPL	DPTQELIFPP	ELMIMAEKQS	QRTRVFGSER
250	260	270	280	290	300	310	320
MMWFSPVTLK	ELLEFKFKYP	QAPVIMGNTS	VGPEVKFKGV	FHPVIISPDR	IEELSVVNHA	YNGLTLGAGL	SLAQVKDILA
330	340	350	360	370	380	390	400
DVVQKLPEEK	TQMYHALLKH	LGTLAGSQIR	NMASLGGHII	SRHPDSDLNP	ILAVGNCTLN	LLSKEGKRQI	PLNEQFLSKC
410	420	430	440	450	460	470	480
PNADLKPQEI	LVSVNIPYSR	KWEFVSAFRQ	AQRQENALAI	VNSGMRVFFG	EGDGIIRELC	ISYGGVGPAT	ICAKNSCQKL
490	500	510	520	530	540	550	560
IGRHWNEQML	DIACRLILNE	VSLLGSAPGG	KVEFKRTLII	SFLFKFYLEV	SQILKKMDPV	HYPSLADKYE	SALEDLHSKH
570	580	590	600	610	620	630	640
HCSTLKYQNI	GPKQHPEDPI	GHPIMHLSGV	KHATGEAIYC	DDMPLVDQEL	FLTFVTSSRA	HAKIVSIDLS	EALSMPGVVD
650	660	670	680	690	700	710	720
IMTAEHLSDV	NSFCFFTEAE	KFLATDKVFC	VGQLVCAVLA	DSEVQAKRAA	KRVKIVYQDL	EPLILTIEES	IQHNSSFKPE
730	740	750	760	770	780	790	800
RKLEYGNVDE	AFKVVDQILE	GEIHMGGQEH	FYMETQSMLV	VPKGEDQEMD	VYVSTQFPKY	IQDIVASTLK	LPANKVMCHV
810	820	830	840	850	860	870	880
RRVGGAFGGK	VLKTGIIAAV	TAFAANKHGR	AVRCVLERGE	DMLITGGRHP	YLGKYKAGFM	NDGRILALDM	EHYSNAGASL
890	900	910	920	930	940	950	960
DESLFVIEMG	LLKMDNAYKF	PNLRCRGWAC	RTNLPSNTAF	RGFGFPQAAL	ITESCITEVA	AKCGLSPEKV	RIINMYKEID
970	980	990	1000	1010	1020	1030	1040
QTPYKQEINA	KNLIQCWREC	MAMSSYSLRK	VAVEKFNAEN	YWKKKGLAMV	PLKFPVGLGS	RAAGQAAALV	HIYLDGSVLV
1050	1060	1070	1080	1090	1100	1110	1120
THGGIEMGQG	VHTKMIQVVS	RELRMPMSNV	HLRGTSTETV	PNANISGGSV	VADLNGLAVK	DACQTLLKRL	EPIISKNPKG
1130	1140	1150	1160	1170	1180	1190	1200
TWKDWAQTAF	DESINLSAVG	YFRGYESDMN	WEKGEGQPFE	YFVYGAACSE	VEIDCLTGDH	KNIRTDIVMD	VGCSINPAID
1210	1220	1230	1240	1250	1260	1270	1280
IGQIEGAFIQ	GMGLYTIEEL	NYSPQGILHT	RGPDQYKIPA	ICDMPTELHI	ALLPPSQNSN	TLYSSKGLGE	SGVFLGCSVF
1290	1300	1310	1320	1330	1340		
FAIHDAVSAA	RQERGLHGPL	TLNSPLTPEK	IRMACEDKFT	KMIPRDEPGS	YVPWNVPI		

Cmpd.	No. of Cmpds.	m/z meas.	Δ m/z [ppm]	z	Rt [min]	Score	P	Range	Sequence	Modification
2323	1	965.9652	-16.68	2	57.3	10.5	1	235-250	R.VFGSERMMWFSPVTLK.E	Oxidation: 7

BRUKER

Detailed Protein Report

Protein 202: kinesin light chain 4 isoform a [Homo sapiens]

**Accession:**	gi|41871946	**Score:**	59.5
**Database:**	refseq_human(refseq_human_20140103.fasta)	**MW [kDa]:**	68.6
**Database Date:**	2015-11-30	**pI:**	5.8
		**Sequence Coverage [%]:**	11.6
		**No. of unique Peptides:**	2

Alias proteins:

Accession	Name	Description
gi|573459710	refseq_human (refseq_human_20140103.fasta)	kinesin light chain 4 isoform a [Homo sapiens]
gi|41871955	refseq_human (refseq_human_20140103.fasta)	kinesin light chain 4 isoform a [Homo sapiens]

10	20	30	40	50	60	70	80
MSGLVLGQRD	EPAGHRLSQE	EILGSTRLVS	QGLEALRSEH	QAVLQSLSQT	IECLQQGGHE	EGLVHEKARQ	LRRSMENIEL
**90**	**100**	**110**	**120**	**130**	**140**	**150**	**160**
GLSEAQVMLA	LASHLSTVES	EKQKLRAQVR	RLCQENQWLR	DELAGTQQRL	QRSEQAVAQL	EEEKKHLEFL	GQLRQYDEDG
**170**	**180**	**190**	**200**	**210**	**220**	**230**	**240**
HTSEEKEGDA	TKDSLDDLFP	NEEEEDPSNG	LSRGQGATAA	QQGGYEIPAR	LRTLHNLVIQ	YAAQGRYEVA	VPLCKQALED
**250**	**260**	**270**	**280**	**290**	**300**	**310**	**320**
LERTSGRGHP	DVATMLNILA	LVYRDQNKYK	EAAHLLNDAL	SIRESTLGPD	HPAVAATLNN	LAVLYGKRGK	YKEAEPLCQR
**330**	**340**	**350**	**360**	**370**	**380**	**390**	**400**
ALEIREKVLG	TNHPDVAKQL	NNLALLCQNQ	GKYEAVERYY	QRALAIYEGQ	LGPDNPNVAR	TKNNLASCYL	KQGKYAEAET
**410**	**420**	**430**	**440**	**450**	**460**	**470**	**480**
LYKEILTRAH	VQEFGSVDDD	HKPIWMHAEE	REEMSKSRHH	EGGTPYAEYG	GWYKACKVSS	PTVNTTLRNL	GALYRRQGKL
**490**	**500**	**510**	**520**	**530**	**540**	**550**	**560**
EAAETLEECA	LRSRRQGTDP	ISQTKVAELL	GESDGRRTSQ	EGPGDSVKFE	GGEDASVAVE	WSGDGSGTLQ	RSGSLGKIRD
**570**	**580**	**590**	**600**	**610**	**620**		
VLRRSSELLV	RKLQGTEPRP	SSSNMKRAAS	LNYLNQPSAA	PLQVSRGLSA	STMDLSSSS		

Cmpd.	No. of Cmpds.	m/z meas.	Δ m/z [ppm]	z	Rt [min]	Score	P	Range	Sequence	Modification
1691	1	1293.5862	-85.42	2	49.8	17.1	1	213-235	R.TLHNLVIQYAAQGRYEVAVPLCK.Q	
2084	1	900.3300	-81.09	3	54.4	16.9	2	432-454	R.EEMSKSRHHEGGTPYAEYGGWYK.A	

Detailed Protein Report

**Protein 203:**	**PREDICTED: leucine-rich repeat serine/threonine-protein kinase 2 isoform X1 [Homo sapiens]**		
**Accession:**	gi|530399725	**Score:**	59.2
**Database:**	refseq_human(refseq_human_20140103.fasta)	**MW [kDa]:**	285.2
**Database Date:**	2015-11-30	**pI:**	6.3
**Modification(s):**	Oxidation	**Sequence Coverage [%]:**	2.7
		**No. of unique Peptides:**	2

Quantitation

Q:W	**Median:**	1.19	**CV:**	0.00 %	**No. of Peptides:**	1
M:Q	**Median:**	1.52	**CV:**	0.00 %	**No. of Peptides:**	1

Detailed Protein Report

10	20	30	40	50	60	70	80
MASGSCQGCE	EDEETLKKLI	VRLNNVQEGK	QIETLVQILE	DLLVFTYSER	ASKLFQGKNI	HVPLLIVLDS	YMRVASVQQV
90	100	110	120	130	140	150	160
GWSLLCKLIE	VCPGTMQSLM	GPQDVGNDWE	VLGVHQLILK	MLTVHNASVN	LSVIGLKTLD	LLLTSGKITL	LILDEESDIF
170	180	190	200	210	220	230	240
MLIFDAMHSF	PANDEVQKLG	CKALHVLFER	VSEEQLTEFV	ENKDYMILLS	ALTNFKDEEE	IVLHVLHCLH	SLAIPCNNVE
250	260	270	280	290	300	310	320
VLMSGNVRCY	NIVVEAMKAF	PMSERIQEVS	CCLLHRLTLG	NFFNILVLNE	VHEFVVKAVQ	QYPENAALQI	SALSCLALLT
330	340	350	360	370	380	390	400
ETIFLNQDLE	EKNENQENDD	EGEEDKLFWL	EACYKALTWH	RKNKHVQEAA	CWALNNLLMY	QNSLHEKIGD	EDGHFPAHRE
410	420	430	440	450	460	470	480
VMLSMLMHSS	SKEVFQASAN	ALSTLLEQNV	NFRKILLSKG	IHLNVLELMQ	KHIHSPEVAE	SGCKMLNHLF	EGSNTSLDIM
490	500	510	520	530	540	550	560
AAVVPKILTV	MKRHETSLPV	QLEALRAILH	FIVPGMPEES	REDTEFHHKL	NMVKKQCFKN	DIHKLVLAAL	NRFIGNPGIQ
570	580	590	600	610	620	630	640
KCGLKVISSI	VHFPDALEML	SLEGAMDSVL	HTLQMYPDDQ	EIQCLGLSLI	GYLITKKNVF	IGTGHLLAKI	LVSSLYRFKD
650	660	670	680	690	700	710	720
VAEIQTKGFQ	TILAILKLSA	SFSKLLVHHS	FDLVIFHQMS	SNIMEQKDQQ	FLNLCCKCFA	KVAMDDYLKN	VMLERACDQN
730	740	750	760	770	780	790	800
NSIMVECLLL	LGADANQAKE	GSSLICQVCE	KESSPKLVEL	LLNSGSREQD	VRKALTISIG	KGDSQIISLL	LRRLALDVAN
810	820	830	840	850	860	870	880
NSICLGGFCI	GKVEPSWLGP	LFPDKTSNLR	KQTNIASTLA	RMVIRYQMKS	AVEEGTASGS	DGNFSEDVLS	KFDEWTFIPD
890	900	910	920	930	940	950	960
SSMDSVFAQS	DDLDSEGSEG	SFLVKKKSNS	ISVGEFYRDA	VLQRCSPNLQ	RHSNSLGPIF	DHEDLLKRKR	KILSSDDSLR
970	980	990	1000	1010	1020	1030	1040
SSKLQSHMRH	SDSISSLASE	REYITSLDLS	ANELRDIDAL	SQKCCISVHL	EHLEKLELHQ	NALTSFPQQL	CETLKSLTHL
1050	1060	1070	1080	1090	1100	1110	1120
DLHSNKFTSF	PSYLLKMSCI	ANLDVSRNDI	GPSVVLDPTV	KCPTLKQFNL	SYNQLSFVPE	NLTDVVEKLE	QLILEGNKIS
1130	1140	1150	1160	1170	1180	1190	1200
GICSPLRLKE	LKILNLSKNH	ISSLSENFLE	ACPKVESFSA	RMNFLAAMPF	LPPSMTILKL	SQNKFSCIPE	AILNLPHLRS
1210	1220	1230	1240	1250	1260	1270	1280
LDMSSNDIQY	LPGPAHWKSL	NLRELLFSHN	QISILDLSEK	AYLWSRVEKL	HLSHNKLKEI	PPEIGCLENL	TSLDVSYNLE
1290	1300	1310	1320	1330	1340	1350	1360
LRSFPNEMGK	LSKIWDLPLD	ELHLNFDFKH	IGCKAKDIIR	FLQQRLKKAV	PYNRMKLMIV	GNTGSGKTTL	LQQLMKTKKS
1370	1380	1390	1400	1410	1420	1430	1440
DLGMQSATVG	IDVKDWPIQI	RDKRKRDLVL	NVWDFAGREE	FYSTHPHFMT	QRALYLAVYD	LSKGQAEVDA	MKPWLFNIKA
1450	1460	1470	1480	1490	1500	1510	1520
RASSSPVILV	GTHLDVSDEK	QRKACMSKIT	KELLNKRGFP	AIRDYHFVNA	TEESDALAKL	RKTIINESLN	FKIRDQLVVG
1530	1540	1550	1560	1570	1580	1590	1600
QLIPDCYVEL	EKIILSERKN	VPIEFPVIDR	KRLLQLVREN	QLQLDENELP	HAVHFLNESG	VLLHFQDPAL	QLSDLYFVEP
1610	1620	1630	1640	1650	1660	1670	1680
KWLCKIMAQI	LTVKVEGCPK	HPKGIISRRD	VEKFLSKKRK	FPKNYMSQYF	KLLEKFQIAL	PIGEEYLLVP	SSLSDHRPVI
1690	1700	1710	1720	1730	1740	1750	1760
ELPHCENSEI	IIRLYEMPYF	PMGFWSRLIN	RLLEISPYML	SGRERALRPN	RMYWRQGIYL	NWSPEAYCLV	GSEVLDNHPE
1770	1780	1790	1800	1810	1820	1830	1840
SFLKITVPSC	RKGCILLGQV	VDHIDSLMEE	WFPGLLEIDI	CGEGETLLKK	WALYSFNDGE	EHQKILLDDL	MKKAEEGDLL
1850	1860	1870	1880	1890	1900	1910	1920
VNPDQPRLTI	PISQIAPDLI	LADLPRNIML	NNDELEFEQA	PEFLLGDGSF	GSVYRAAYEG	EEVAVKIFNK	HTSLRLLRQE
1930	1940	1950	1960	1970	1980	1990	2000
LVVLCHLHHP	SLISLLAAGI	RPRMLVMELA	SKGSLDRLLQ	QDKASLTRTL	QHRIALHVAD	GLRYLHSAMI	IYRDLKPHNV
2010	2020	2030	2040	2050	2060	2070	2080
LLFTLYPNAA	IIAKIADYGI	AQYCCRMGIK	TSEGTPGFRA	PEVARGNVIY	NQQADVYSFG	LLLYDILTTG	GRIVEGLKFP
2090	2100	2110	2120	2130	2140	2150	2160
NEFDELEIQG	KLPDPVKEYG	CAPWPMVEKL	IKQCLKENPQ	ERPTSAQVFD	ILNSAELVCL	TRRILLPKNV	IVECMVATHH
2170	2180	2190	2200	2210	2220	2230	2240

Detailed Protein Report

Cmpd.	No. of Cmpds.	m/z meas.	Δ m/z [ppm]	z	Rt [min]	Score	P	Range	Sequence	Modification	Ratios
394	1	591.8037	-59.20	2	34.9	10.0	1	1964-1973	K.ASLTRTLQHR.I		Q:W 1.19<br>M:Q 1.52
1730	1	796.0646	-20.88	3	51.4	11.0	1	2457-2477	R.VMMTAQLGSLKNVMLVLGYNR.K	Oxidation: 2, 3, 14	

Detailed Protein Report

Protein 204: **14-3-3 protein eta [Homo sapiens]**

**Accession:**	gi|4507951	**Score:**	59.1
**Database:**	refseq_human(refseq_human_20140103.fasta)	**MW [kDa]:**	28.2
**Database Date:**	2015-11-30	**pI:**	4.6
**Modification(s):**	Oxidation	**Sequence Coverage [%]:**	11.4
		**No. of unique Peptides:**	1

Quantitation

Q:W **Median:** 0.09 **CV:** 0.00 % **No. of Peptides:** 1

10	20	30	40	50	60	70	80
MGDREQLLQR	ARLAEQAERY	DDMASAMKAV	TELNEPLSNE	DRNLLSVAYK	NVVGARRSSW	RVISSIEQKT	MADGNEKKLE
**90**	**100**	**110**	**120**	**130**	**140**	**150**	**160**
KVKAYREKIE	KELETVCNDV	LSLLDKFLIK	NCNDFQYESK	VFYLKMKGDY	YRYLAEVASG	EKKNSVVEAS	EAAYKEAFEI
**170**	**180**	**190**	**200**	**210**	**220**	**230**	**240**
SKEQMQPTHP	IRLGLALNFS	VFYYEIQNAP	EQACLLAKQA	FDDAIAELDT	LNEDSYKDST	LIMQLLRDNL	TLWTSDQQDE
**250**							
EAGEGN							

Cmpd.	No. of Cmpds.	m/z meas.	Δ m/z [ppm]	z	Rt [min]	Score	P	Range	Sequence	Modification	Ratios	
2722	1	1044.5326	52.39	2	65.2	11.7	2	11-28	R.ARLAEQAERYDDMASAMK.A	Oxidation: 13, 17	Q:W	0.09

Detailed Protein Report

Protein 205: PDZ domain-containing protein 4 [Homo sapiens]

**Accession:**	gi|46559761	**Score:**	59.0
**Database:**	refseq_human(refseq_human_20140103.fasta)	**MW [kDa]:**	86.1
**Database Date:**	2015-11-30	**pI:**	5.7
**Modification(s):**	Carbamidomethyl, Oxidation	**Sequence Coverage [%]:**	10.5
		**No. of unique Peptides:**	2

10	20	30	40	50	60	70	80
MGCNMCVVQK	PEEQYKVMLQ	VNGKELSKLS	QEQTLQALRS	SKEPLVIQVL	RRSPRLRGDS	SCHDLQLVDS	GTQTDITFEH
**90**	**100**	**110**	**120**	**130**	**140**	**150**	**160**
IMALGKLRPP	TPPMVILEPP	PISHEYYDPA	EFMEGGPQEA	DRLDELEYEE	VELYKSSHRD	KLGLMVCYRT	DDEEDLGIYV
**170**	**180**	**190**	**200**	**210**	**220**	**230**	**240**
GEVNPNSIAA	KDGRIREGDR	IIQINGVDVQ	NREEAVAILS	QEENTNISLL	VARPESQLAK	RWKDSDRDDF	LDDFGSENEG
**250**	**260**	**270**	**280**	**290**	**300**	**310**	**320**
ELRARKLKSP	PAQQPGNEEE	KGAPDAGPGL	SNSQELDSGV	GRTDESTRNE	ESSEHDLLGD	EPPSSTNTPG	SLRKFGLQGD
**330**	**340**	**350**	**360**	**370**	**380**	**390**	**400**
ALQSRDFHFS	MDSLLAEGAG	LGGGDVPGLT	DEEYERYREL	LEIKCHLENG	NQLGLLFPRA	SGGNSALDVN	RNESLGHEMA
**410**	**420**	**430**	**440**	**450**	**460**	**470**	**480**
MLEEELRHLE	FKCRNILRAQ	KMQQLRERCM	KAWLLEEESL	YDLAASEPKK	HELSDISELP	EKSDKDSTSA	YNTGESCRST
**490**	**500**	**510**	**520**	**530**	**540**	**550**	**560**
PLLVEPLPES	PLRRAMAGNS	NLNRTPPGPA	VATPAKAAPP	PGSPAKFRSL	SRDPEAGRRQ	HAEERGRRNP	KTGLTLERVG
**570**	**580**	**590**	**600**	**610**	**620**	**630**	**640**
PESSPYLSRR	HRGQGQEGEH	YHSCVQLAPT	RGLEELGHGP	LSLAGGPRVG	GVAAAATEAP	RMEWKVKVRS	DGTRYVAKRP
**650**	**660**	**670**	**680**	**690**	**700**	**710**	**720**
VRDRLLKARA	LKIREERSGM	TTDDDAVSEM	KMGRYWSKEE	RKQHLIRARE	QRKRREFMMQ	SRLECLREQQ	NGDSKPELNI
**730**	**740**	**750**	**760**	**770**			
IALSHRKTMK	KRNKKILDNW	ITIQEMLAHG	ARSADGKRVY	NPLLSVTTV			

Cmpd.	No. of Cmpds.	m/z meas.	Δ m/z [ppm]	z	Rt [min]	Score	P	Range	Sequence	Modification
2759	1	905.4805	55.19	3	65.8	12.2	1	2-24	M.GCNMCVVQKPEEQYKVMLQVNGK.E	Carbamidomethyl: 2; Oxidation: 4, 17
450	1	1100.7316	-85.85	3	34.6	14.6	0	326-356	R.DFHFSMDSLLAEGAGLGGGDVPGLTDEEYER.Y	Oxidation: 6

Detailed Protein Report

Protein 206: fibronectin isoform 6 preproprotein [Homo sapiens]

**Accession:**	gi|47132549	**Score:**	59.0
**Database:**	refseq_human(refseq_human_20140103.fasta)	**MW [kDa]:**	239.5
**Database Date:**	2015-11-30	**pI:**	5.5
**Modification(s):**	Carbamidomethyl	**Sequence Coverage [%]:**	2.6
		**No. of unique Peptides:**	3

Detailed Protein Report

10	20	30	40	50	60	70	80
MLRGPGPGLL	LLAVQCLGTA	VPSTGASKSK	RQAQQMVQPQ	SPVAVSQSKP	GCYDNGKHYQ	INQQWERTYL	GNALVCTCYG
90	100	110	120	130	140	150	160
GSRGFNCESK	PEAEETCFDK	YTGNTYRVGD	TYERPKDSMI	WDCTCIGAGR	GRISCTIANR	CHEGGQSYKI	GDTWRRPHET
170	180	190	200	210	220	230	240
GGYMLECVCL	GNGKGEWTCK	PIAEKCFDHA	AGTSYVVGET	WEKPYQGWMM	VDCTCLGEGS	GRITCTSRNR	CNDQDTRTSY
250	260	270	280	290	300	310	320
RIGDTWSKKD	NRGNLLQCIC	TGNGRGEWKC	ERHTSVQTTS	SGSGPFTDVR	AAVYQPQPHP	QPPPYGHCVT	DSGVVYSVGM
330	340	350	360	370	380	390	400
QWLKTQGNKQ	MLCTCLGNGV	SCQETAVTQT	YGGNSNGEPC	VLPFTYNGRT	FYSCTTEGRQ	DGHLWCSTTS	NYEQDQKYSF
410	420	430	440	450	460	470	480
CTDHTVLVQT	RGGNSNGALC	HFPFLYNNHN	YTDCTSEGRR	DNMKWCGTTQ	NYDADQKFGF	CPMAAHEEIC	TTNEGVMYRI
490	500	510	520	530	540	550	560
GDQWDKQHDM	GHMMRCTCVG	NGRGEWTCIA	YSQLRDQCIV	DDITYNVNDT	FHKRHEEGHM	LNCTCFGQGR	GRWKCDPVDQ
570	580	590	600	610	620	630	640
CQDSETGTFY	QIGDSWEKYV	HGVRYQCYCY	GRGIGEWHCQ	PLQTYPSSSG	PVEVFITETP	SQPNSHPIQW	NAPQPSHISK
650	660	670	680	690	700	710	720
YILRWRPKNS	VGRWKEATIP	GHLNSYTIKG	LKPGVVYEGQ	LISIQQYGHQ	EVTRFDFTTT	STSTPVTSNT	VTGETTPFSP
730	740	750	760	770	780	790	800
LVATSESVTE	ITASSFVVSW	VSASDTVSGF	RVEYELSEEG	DEPQYLDLPS	TATSVNIPDL	LPGRKYIVNV	YQISEDGEQS
810	820	830	840	850	860	870	880
LILSTSQTTA	PDAPPDPTVD	QVDDTSIVVR	WSRPQAPITG	YRIVYSPSVE	GSSTELNLPE	TANSVTLSDL	QPGVQYNITI
890	900	910	920	930	940	950	960
YAVEENQEST	PVVIQQETTG	TPRSDTVPSP	RDLQFVEVTD	VKVTIMWTPP	ESAVTGYRVD	VIPVNLPGEH	GQRLPISRNT
970	980	990	1000	1010	1020	1030	1040
FAEVTGLSPG	VTYYFKVFAV	SHGRESKPLT	AQQTTKLDAP	TNLQFVNETD	STVLVRWTPP	RAQITGYRLT	VGLTRRGQPR
1050	1060	1070	1080	1090	1100	1110	1120
QYNVGPSVSK	YPLRNLQPAS	EYTVSLVAIK	GNQESPKATG	VFTTLQPGSS	IPPYNTEVTE	TTIVITWTPA	PRIGFKLGVR
1130	1140	1150	1160	1170	1180	1190	1200
PSQGGEAPRE	VTSDSGSIVV	SGLTPGVEYV	YTIQVLRDGQ	ERDAPIVNKV	VTPLSPPTNL	HLEANPDTGV	LTVSWERSTT
1210	1220	1230	1240	1250	1260	1270	1280
PDITGYRITT	TPTNGQQGNS	LEEVVHADQS	SCTFDNLSPG	LEYNVSVYTV	KDDKESVPIS	DTIIPAVPPP	TDLRFTNIGP
1290	1300	1310	1320	1330	1340	1350	1360
DTMRVTWAPP	PSIDLTNFLV	RYSPVKNEED	VAELSISPSD	NAVVLTNLLP	GTEYVVSVSS	VYEQHESTPL	RGRQKTGLDS
1370	1380	1390	1400	1410	1420	1430	1440
PTGIDFSDIT	ANSFTVHWIA	PRATITGYRI	RHHPEHFSGR	PREDRVPHSR	NSITLTNLTP	GTEYVVSIVA	LNGREESPLL
1450	1460	1470	1480	1490	1500	1510	1520
IGQQSTVSDV	PRDLEVVAAT	PTSLLISWDA	PAVTVRYYRI	TYGETGGNSP	VQEFTVPGSK	STATISGLKP	GVDYTITVYA
1530	1540	1550	1560	1570	1580	1590	1600
VTGRGDSPAS	SKPISINYRT	EIDKPSQMQV	TDVQDNSISV	KWLPSSSPVT	GYRVTTTPKN	GPGPTKTKTA	GPDQTEMTIE
1610	1620	1630	1640	1650	1660	1670	1680
GLQPTVEYVV	SVYAQNPSGE	SQPLVQTAVT	TIPAPTDLKF	TQVTPTSLSA	QWTPPNVQLT	GYRVRVTPKE	KTGPMKEINL
1690	1700	1710	1720	1730	1740	1750	1760
APDSSSVVVS	GLMVATKYEV	SVYALKDTLT	SRPAQGVVTT	LENVSPPRRA	RVTDATETTI	TISWRTKTET	ITGFQVDAVP
1770	1780	1790	1800	1810	1820	1830	1840
ANGQTPIQRT	IKPDVRSYTI	TGLQPGTDYK	IYLYTLNDNA	RSSPVVIDAS	TAIDAPSNLR	FLATTPNSLL	VSWQPPRARI
1850	1860	1870	1880	1890	1900	1910	1920
TGYIIKYEKP	GSPPREVVPR	PRPGVTEATI	TGLEPGTEYT	IYVIALKNNQ	KSEPLIGRKK	TGQEALSQTT	ISWAPFQDTS
1930	1940	1950	1960	1970	1980	1990	2000
EYIISCHPVG	TDEEPLQFRV	PGTSTSATLT	GLTRGATYNI	IVEALKDQQR	HKVREEVVTV	GNSVNEGLNQ	PTDDSCFDPY
2010	2020	2030	2040	2050	2060	2070	2080
TVSHYAVGDE	WERMSESGFK	LLCQCLGFGS	GHFRCDSSRW	CHDNGVNYKI	GEKWDRQGEN	GQMMSCTCLG	NGKGEFKCDP
2090	2100	2110	2120	2130	2140	2150	2160
HEATCYDDGK	TYHVGEQWQK	EYLGAICSCT	CFGGQRGWRC	DNCRRPGGEP	SPEGTTGQSY	NQYSQRYHQR	TNTNVNCPIE
2170	2180						

Detailed Protein Report

Cmpd.	No. of Cmpds.	m/z meas.	Δ m/z [ppm]	z	Rt [min]	Score	P	Range	Sequence	Modification
1763	1	1044.9953	44.20	2	51.9	16.6	1	534-550	K.RHEEGHMLNCTCFGQGR.G	Carbamidomethyl: 10, 12
2765	1	904.4689	14.28	2	65.7	16.9	0	923-938	K.VTIMWTPPESAVTGYR.V	
1706	1	796.3662	-51.72	2	51.1	15.3	0	1525-1539	R.GDSPASSKPISINYR.T	

Detailed Protein Report

Protein 207: androglobin [Homo sapiens]

**Accession:**	gi|168823435	**Score:**	58.9
**Database:**	refseq_human(refseq_human_20140103.fasta)	**MW [kDa]:**	189.6
**Database Date:**	2015-11-30	**pI:**	9.1
		**Sequence Coverage [%]:**	3.2
		**No. of unique Peptides:**	3

Quantitation

Q:W	**Median:**	0.57	**CV:**	0.00 %	**No. of Peptides:**	1
M:Q	**Median:**	1.92	**CV:**	0.00 %	**No. of Peptides:**	1

10	20	30	40	50	60	70	80
MASKQTKKKE	VHRINSAHGS	DKSKDFYPFG	SNVQSGSTEQ	KKGKFPLWPE	WSEADINSEK	WDAGKGAKEK	DKTGKSPVFH
90	100	110	120	130	140	150	160
FFEDPEGKIE	LPPSLKIYSW	KRPQDILFSQ	TPVVVKNEIT	FDLFSANEHL	LCSELMRWII	SEIYAVWKIF	NGGILSNYFK
170	180	190	200	210	220	230	240
GTSGEPPLLP	WKPWEHIYSL	CKAVKGHMPL	FNSYGKYVVK	LYWMGCWRKI	TIDDFLPFDE	DNNLLLPATT	YEFELWPMLL
250	260	270	280	290	300	310	320
SKAIIKLANI	DIHVADRREL	GEFTVIHALT	GWLPEVISLH	PGYMDKVWEL	LKEILPEFKL	SDEASSESKI	AVLDSKLKEP
330	340	350	360	370	380	390	400
GKEGKEGKEI	KDGKEVKDVK	EFKPESSLTT	LKAPEKSDKV	PKEKADARDI	GKKRSKDGEK	EKFKFSLHGS	RPSSEVQYSV
410	420	430	440	450	460	470	480
QSLSDCSSAI	QTSHMVVYAT	FTPLYLFENK	IFSLEKMADS	AEKLREYGLS	HICSHPVLVT	RSRSCPLVAP	PKPPPLPPWK
490	500	510	520	530	540	550	560
LIRQKKETVI	TDEAQELIVK	KPERFLEISS	PFLNYRMTPF	TIPTEMHFVR	SLIKKGIPPG	SDLPSVSETD	ETATHSQTDL
570	580	590	600	610	620	630	640
SQITKATSQG	NTASQVILGK	GTDEQTDFGL	GDAHQSDGLN	LEREIVSQTT	ATQEKSQEEL	PTTNNSVSKE	IWLDFEDFCV
650	660	670	680	690	700	710	720
CFQNIYIFHK	PSSYCLNFQK	SEFKFSEERV	SYYLFVDSLK	PIELLVCFSA	LVRWGEYGAL	TKDSPPIEPG	LLTAETFSWK
730	740	750	760	770	780	790	800
SLKPGSLVLK	IHTYATKATV	VRLPVGRHML	LFNAYSPVGH	SIHICSMVSF	VIGDEHVVLP	NFEPESCRFT	EQSLLIMKAI
810	820	830	840	850	860	870	880
GNVIANFKDK	GKLSAALKDL	QTAHYPVPFH	DKELTAQHFR	VFHLSLWRLM	KKVQITKPPP	NFKFAFRAMV	LDLELLNSSL
890	900	910	920	930	940	950	960
EEVSLVEWLD	VKYCMPTSDK	EYSAEEVAAA	IKIQAMWRGT	YVRLLMKARI	PDTKENISVA	DTLQKVWAVL	EMNLEQYAVS
970	980	990	1000	1010	1020	1030	1040
LLRLMFKSKC	KSLESYPCYQ	DEETKIAFAD	YTVTYQEQPP	NSWFIVFRET	FLVHQDMILV	PKVYTTLPIC	ILHIVNNDTM
1050	1060	1070	1080	1090	1100	1110	1120
EQVPKVFQKV	VPYLYTKNKK	GYTFVAEAFT	GDTYVAASRW	KLRLIGSSAP	LPCLSRDSPC	NSFAIKEIRD	YYIPNDKKIL
1130	1140	1150	1160	1170	1180	1190	1200
FRYSVKVLTP	QPATIQVRTS	KPDAFIKLQV	LENEETMVSS	TGKGQAIIPA	FHFLKSEKGL	SSQSSKHILS	FHSASKKEQE
1210	1220	1230	1240	1250	1260	1270	1280
VYVKKKAAQG	IQKSPKGRAV	SAIQDIGLPL	VEEETTSTPT	REDSSSTPLQ	NYKYIIQCSV	LYNSWPLTES	QLTFVQALKD
1290	1300	1310	1320	1330	1340	1350	1360
LKKSNTKAYG	ERHEELINLG	SPDSHTISEG	QKSSVTSKTT	RKGKEKSSEK	EKTAKEKQAP	RFEPQISTVH	PQQEDPNKPY
1370	1380	1390	1400	1410	1420	1430	1440
WILRLVTEHN	ESELFEVKKD	TERADEIRAM	KQAWETTEPG	RAIKASQARL	HYLSGFIKKT	SDAESPPISE	SQTKPKEEVE
1450	1460	1470	1480	1490	1500	1510	1520
TAARGVKEPN	SKNSAGSESK	EMTQTGSGSA	VWKKWQLTKG	LRDVAKSTSS	ESGGVSSPGK	EEREQSTRKE	NIQTGPRTRS
1530	1540	1550	1560	1570	1580	1590	1600
PTILETSPRL	IRKALEFMDL	SQYVRKTDTD	PLLQTDELNQ	QQAMQKAEEI	HQFRQHRTRV	LSIRNIDQEE	RLKLKDEVLD
1610	1620	1630	1640	1650	1660	1670	
MYKEMQDSLD	EARQKIFDIR	EEYRNKLLEA	EHLKLETLAA	QEAAMKLETE	KMTPAPDTQK	KKKGKKK	

Cmpd.	No. of Cmpds.	m/z meas.	Δ m/z [ppm]	z	Rt [min]	Score	P	Range	Sequence	Modification	Ratios

Detailed Protein Report

Cmpd.	No. of Cmpds.	m/z meas.	Δ m/z [ppm]	z	Rt [min]	Score	P	Range	Sequence	Modification	Ratios
2723	1	921.1582	-5.50	3	65.3	17.8	1	138-160	R.WIISEIYAVWKIFNGGILSNYFK.G		
2431	1	590.4218	113.13	3	60.3	14.9	2	849-863	R.LMKKVQITKPPPNFK.F		M:Q 1.92<br>Q:W 0.57
2707	2	933.4993	-17.55	2	65.1	26.2	2	913-927	K.IQAMWRGTYVRLLMK.A		

Detailed Protein Report

Protein 208: PREDICTED: collagen alpha-2(I) chain-like [Homo sapiens]

Accession: gi|530375734
Database: refseq_human(refseq_human_20140103.fasta)
Database Date: 2015-11-30

Score: 58.7
MW [kDa]: 28.8
pI: 12.9
Sequence Coverage [%]: 12.6
No. of unique Peptides: 2

10	20	30	40	50	60	70	80
MAPFHLSHAP	AGARKVKLRH	PRKWSPSGSP	QPPAQLQPLE	APRLPGEVRT	SGRLATWFCR	LLARRSAAQR	KSKSRKDLNR
**90**	**100**	**110**	**120**	**130**	**140**	**150**	**160**
RVQSPLTPHS	FHYFSSQGIG	GGGGGRDKFA	GRLAWSNPRG	WDRPRGSGGA	LPRPEGDCGG	ARPPAGGAGR	WASPGAAGRW
**170**	**180**	**190**	**200**	**210**	**220**	**230**	**240**
SLPGRVCGAF	AQPAPCPHLT	SSPPRPLGGP	LRCAAQARSV	AARAVRPRSL	GQGPGAQGVG	RAGRERGRPS	TASGSARAGA
**250**	**260**	**270**	**280**				
ALPAAVRPRE	GPRGPSACAL	GRRRLCVGRP	GGPRGTD				

Cmpd.	No. of Cmpds.	m/z meas.	Δ m/z [ppm]	z	Rt [min]	Score	P	Range	Sequence	Modification
2843	4	731.8776	7.32	2	66.0	30.6	0	1-14	-.MAPFHLSHAPAGAR.K	
1759	1	881.8978	-107.69	2	50.4	14.1	1	204-221	R.AVRPRSLGQGPGAQGVGR.A	

Detailed Protein Report

Protein 209: cation-independent mannose-6-phosphate receptor precursor [Homo sapiens]

**Accession:**	gi|119964726	**Score:**	58.7
**Database:**	refseq_human(refseq_human_20140103.fasta)	**MW [kDa]:**	274.1
**Database Date:**	2015-11-30	**pI:**	5.5
**Modification(s):**	Carbamidomethyl	**Sequence Coverage [%]:**	2.6
		**No. of unique Peptides:**	2

Detailed Protein Report

10	20	30	40	50	60	70	80
MGAAAGRSPH	LGPAPARRPQ	RSLLLLQLLL	LVAAPGSTQA	QAAPFPELCS	YTWEAVDTKN	NVLYKINICG	SVDIVQCGPS
90	100	110	120	130	140	150	160
SAVCMHDLKT	RTYHSVGDSV	LRSATRSLLE	FNTTVSCDQQ	GTNHRVQSSI	AFLCGKTLGT	PEFVTATECV	HYFEWRTTAA
170	180	190	200	210	220	230	240
CKKDIFKANK	EVPCYVFDEE	LRKHDLNPLI	KLSGAYLVDD	SDPDTSLFIN	VCRDIDTLRD	PGSQLRACPP	GTAACLVRGH
250	260	270	280	290	300	310	320
QAFDVGQPRD	GLKLVRKDRL	VLSYVREEAG	KLDFCDGHSP	AVTITFVCPS	ERREGTIPKL	TAKSNCRYEI	EWITEYACHR
330	340	350	360	370	380	390	400
DYLESKTCSL	SGEQQDVSID	LTPLAQSGGS	SYISDGKEYL	FYLNVCGETE	IQFCNKKQAA	VCQVKKSDTS	QVKAAGRYHN
410	420	430	440	450	460	470	480
QTLRYSDGDL	TLIYFGGDEC	SSGFQRMSVI	NFECNKTAGN	DGKGTPVFTG	EVDCTYFFTW	DTEYACVKEK	EDLLCGATDG
490	500	510	520	530	540	550	560
KKRYDLSALV	RHAEPEQNWE	AVDGSQTETE	KKHFFINICH	RVLQEGKARG	CPEDAAVCAV	DKNGSKNLGK	FISSPMKEKG
570	580	590	600	610	620	630	640
NIQLSYSDGD	DCGHGKKIKT	NITLVCKPGD	LESAPVLRTS	GEGGCFYEFE	WHTAAACVLS	KTEGENCTVF	DSQAGFSFDL
650	660	670	680	690	700	710	720
SPLTKKNGAY	KVETKKYDFY	INVCGPVSVS	PCQPDSGACQ	VAKSDEKTWN	LGLSNAKLSY	YDGMIQLNYR	GGTPYNNERH
730	740	750	760	770	780	790	800
TPRATLITFL	CDRDAGVGFP	EYQEEDNSTY	NFRWYTSYAC	PEEPLECVVT	DPSTLEQYDL	SSLAKSEGGL	GGNWYAMDNS
810	820	830	840	850	860	870	880
GEHVTWRKYY	INVCRPLNPV	PGCNRYASAC	QMKYEKDQGS	FTEVVSISNL	GMAKTGPVVE	DSGSLLLEYV	NGSACTTSDG
890	900	910	920	930	940	950	960
RQTTYTTRIH	LVCSRGRLNS	HPIFSLNWEC	VVSFLWNTEA	ACPIQTTTDT	DQACSIRDPN	SGFVFNLNPL	NSSQGYNVSG
970	980	990	1000	1010	1020	1030	1040
IGKIFMFNVC	GTMPVCGTIL	GKPASGCEAE	TQTEELKNWK	PARPVGIEKS	LQLSTEGFIT	LTYKGPLSAK	GTADAFIVRF
1050	1060	1070	1080	1090	1100	1110	1120
VCNDDVYSGP	LKFLHQDIDS	GQGIRNTYFE	FETALACVPS	PVDCQVTDLA	GNEYDLTGLS	TVRKPWTAVD	TSVDGRKRTF
1130	1140	1150	1160	1170	1180	1190	1200
YLSVCNPLPY	IPGCQGSAVG	SCLVSEGNSW	NLGVVQMSPQ	AAANGSLSIM	YVNGDKCGNQ	RFSTRITFEC	AQISGSPAFQ
1210	1220	1230	1240	1250	1260	1270	1280
LQDGCEYVFI	WRTVEACPVV	RVEGDNCEVK	DPRHGNLYDL	KPLGLNDTIV	SAGEYTYYFR	VCGKLSSDVC	PTSDKSKVVS
1290	1300	1310	1320	1330	1340	1350	1360
SCQEKREPQG	FHKVAGLLTQ	KLTYENGLLK	MNFTGGDTCH	KVYQRSTAIF	FYCDRGTQRP	VFLKETSDCS	YLFEWRTQYA
1370	1380	1390	1400	1410	1420	1430	1440
CPPFDLTECS	FKDGAGNSFD	LSSLSRYSDN	WEAITGTGDP	EHYLINVCKS	LAPQAGTEPC	PPEAAACLLG	GSKPVNLGRV
1450	1460	1470	1480	1490	1500	1510	1520
RDGPQWRDGI	IVLKYVDGDL	CPDGIRKKST	TIRFTCSESQ	VNSRPMFISA	VEDCEYTFAW	PTATACPMKS	NEHDDCQVTN
1530	1540	1550	1560	1570	1580	1590	1600
PSTGHLFDLS	SLSGRAGFTA	AYSEKGLVYM	SICGENENCP	PGVGACFGQT	RISVGKANKR	LRYVDQVLQL	VYKDGSPCPS
1610	1620	1630	1640	1650	1660	1670	1680
KSGLSYKSVI	SFVCRPEAGP	TNRPMLISLD	KQTCTLFFSW	HTPLACEQAT	ECSVRNGSSI	VDLSPLIHRT	GGYEAYDESE
1690	1700	1710	1720	1730	1740	1750	1760
DDASDTNPDF	YINICQPLNP	MHGVPCPAGA	AVCKVPIDGP	PIDIGRVAGP	PILNPIANEI	YLNFESSTPC	LADKHFNYTS
1770	1780	1790	1800	1810	1820	1830	1840
LIAFHCKRGV	SMGTPKLLRT	SECDFVFEWE	TPVVCPDEVR	MDGCTLTDEQ	LLYSFNLSSL	STSTFKVTRD	SRTYSVGVCT
1850	1860	1870	1880	1890	1900	1910	1920
FAVGPEQGGC	KDGGVCLLSG	TKGASFGRLQ	SMKLDYRHQD	EAVVLSYVNG	DRCPPETDDG	VPCVFPFIFN	GKSYEECIIE
1930	1940	1950	1960	1970	1980	1990	2000
SRAKLWCSTT	ADYDRDHEWG	FCRHSNSYRT	SSIIFKCDED	EDIGRPQVFS	EVRGCDVTFE	WKTKVVCPPK	KLECKFVQKH
2010	2020	2030	2040	2050	2060	2070	2080
KTYDLRLLSS	LTGSWSLVHN	GVSYYINLCQ	KIYKGPLGCS	ERASICRRTT	TGDVQVLGLV	HTQKLGVIGD	KVVVTYSKGY
2090	2100	2110	2120	2130	2140	2150	2160
PCGGNKTASS	VIELTCTKTV	GRPAFKRFDI	DSCTYYFSWD	SRAACAVKPQ	EVQMVNGTIT	NPINGKSFSL	GDIYFKLFRA
2170	2180	2190	2200	2210	2220	2230	2240

Detailed Protein Report

Cmpd.	No. of Cmpds.	m/z meas.	Δ m/z [ppm]	z	Rt [min]	Score	P	Range	Sequence	Modification
1181	1	955.7839	6.62	3	44.9	12.0	1	267-292	R.EEAGKLDFCDGHSPAVTITFVCPSER.R	Carbamidomethyl: 22
3000	1	703.3599	1.49	2	66.4	11.2	2	471-483	K.EDLLCGATDGKKR.Y	

Detailed Protein Report

Protein 210: collagen alpha-2(XI) chain isoform 2 preproprotein [Homo sapiens]

**Accession:**	gi|111118972	**Score:**	58.7
**Database:**	refseq_human(refseq_human_20140103.fasta)	**MW [kDa]:**	162.1
**Database Date:**	2015-11-30	**pI:**	8.8
		**Sequence Coverage [%]:**	3.5
		**No. of unique Peptides:**	2

Quantitation

Q:W	**Median:**	1.13	**CV:**	0.00 %	**No. of Peptides:**	1
M:Q	**Median:**	0.69	**CV:**	277.92 %	**No. of Peptides:**	2

10	20	30	40	50	60	70	80
MERCSRCHRL	LLLLPLVLGL	SAAPGWAGAP	PVDVLRALRF	PSLPDGVRRA	KGICPADVAY	RVARPAQLSA	PTRQLFPGGF
90	100	110	120	130	140	150	160
PKDFSLLTVV	RTRPGLQAPL	LTLYSAQGVR	QLGLELGRPV	RFLYEDQTGR	PQPPSQPVFR	GLSLADGKWH	RVAVAVKGQS
170	180	190	200	210	220	230	240
VTLIVDCKKR	VTRPLPRSAR	PVLDTHGVII	FGARILDEEV	FEGDVQELAI	VPGVQAAYES	CEQKELECEG	GQRERPQNQQ
250	260	270	280	290	300	310	320
PHRAQRSPQQ	QPSRLHRPQN	QEPQSQDPTP	GEEEEILESS	LLPPLEEAAH	GPRGLKGEKG	EPAVLEPGML	VEGPPGPEGP
330	340	350	360	370	380	390	400
AGLIGPPGIQ	GNPGPVGDPG	ERGPPGRAGL	PGSDGAPGPP	GTSLMLPFRF	GSGGGDKGPV	VAAQEAQAQA	ILQQARLALR
410	420	430	440	450	460	470	480
GPPGPMGYTG	RPGPLGQPGS	PGLKGESGDL	GPQGPRGPQG	LTGPPGKAGR	RGRAGADGAR	GMPGDPGVKG	DRGFDGLPGL
490	500	510	520	530	540	550	560
PGEKGHRGDT	GAQGLPGPPG	EDGERGDDGE	IGPRGLPGES	GPRGLLGPKG	PPGIPGPPGV	RGMDGPQGPK	GSLGPQGEPG
570	580	590	600	610	620	630	640
PPGQQGTPGT	QGLPGPQGAI	GPHGEKGPQG	KPGLPGMPGS	DGPPGHPGKE	GPPGTKGNQG	PSGPQGPLGY	PGPRGVKGVD
650	660	670	680	690	700	710	720
GIRGLKGHKG	EKGEDGFPGF	KGDIGVKGDR	GEVGVPGSRG	EDGPEGPKGR	TGPTGDPGPP	GLMGEKGKLG	VPGLPGYPGR
730	740	750	760	770	780	790	800
QGPKGSLGFP	GFPGASGEKG	ARGLSGKSGP	RGERGPTGPR	GQRGPRGATG	KSGAKGTSGG	DGPHGPPGER	GLPGPQGPNG
810	820	830	840	850	860	870	880
FPGPKGPPGP	PGKDGLPGHP	GQRGEVGFQG	KTGPPGPPGV	VGPQGAAGET	GPMGERGHPG	PPGPPGEQGL	PGTAGKEGTK
890	900	910	920	930	940	950	960
GDPGPPGAPG	KDGPAGLRGF	PGERGLPGTA	GGPGLKGNEG	PSGPPGPAGS	PGERGAAGSG	GPIGPPGRPG	PQGPPGAAGE
970	980	990	1000	1010	1020	1030	1040
KGVPGEKGPI	GPTGRDGVQG	PVGLPGPAGP	PGVAGEDGDK	GEVGDPGQKG	TKGNKGEHGP	PGPPGPIGPV	GQPGAAGADG
1050	1060	1070	1080	1090	1100	1110	1120
EPGARGPQGH	FGAKGDEGTR	GFNGPPGPIG	LQGLPGPSGE	KGETGDVGPM	GPPGPPGPRG	PAGPNGADGP	QGPPGGVGNL
1130	1140	1150	1160	1170	1180	1190	1200
GPPGEKGEPG	ESGSPGIQGE	PGVKGPRGER	GEKGESGQPG	EPGPPGPKGP	TGDDGPKGNP	GPVGFPGDPG	PPGEGGPRGQ
1210	1220	1230	1240	1250	1260	1270	1280
DGAKGDRGED	GEPGQPGSPG	PTGENGPPGP	LGKRGPAGSP	GSEGRQGGKG	AKGDPGAIGA	PGKTGPVGPA	GPAGKPGPDG
1290	1300	1310	1320	1330	1340	1350	1360
LRGLPGSVGQ	QGRPGATGQA	GPPGPVGPPG	LPGLRGDAGA	KGEKGHPGLI	GLIGPPGEQG	EKGDRGLPGP	QGSPGQKGEM
1370	1380	1390	1400	1410	1420	1430	1440
GIPGASGPIG	PGGPPGLPGP	AGPKGAKGAT	GPGGPKGEKG	VQGPPGHPGP	PGEVIQPLPI	QMPKKTRRSV	DGSRLMQEDE
1450	1460	1470	1480	1490	1500	1510	1520
AIPTGGAPGS	PGGLEEIFGS	LDSLREEIEQ	MRRPTGTQDS	PARTCQDLKL	CHPELPDGEY	WVDPNQGCAR	DAFRVFCNFT
1530	1540	1550	1560	1570	1580	1590	1600
AGGETCVTPR	DDVTQFSYVD	SEGSPVGVVQ	LTFLRLLSVS	AHQDVSYPCS	GAARDGPLRL	RGANEDELSP	ETSPYVKEFR
1610	1620	1630	1640	1650	1660		
DGCQTQQGRT	VLEVRTPVLE	QLPVLDASFS	DLGAPPRRGG	VLLGPVCFMG			

Cmpd.	No. of Cmpds.	m/z meas.	Δ m/z [ppm]	z	Rt [min]	Score	P	Range	Sequence	Modification	Ratios

Detailed Protein Report

Cmpd.	No. of Cmpds.	m/z meas.	Δ m/z [ppm]	z	Rt [min]	Score	P	Range	Sequence	Modification	Ratios	
2503	1	862.3584	-77.51	2	61.7	12.3	1	689-706	K.GRTGPTGDPGPPGLMGEK.G		M:Q	0.16
230	2	575.1791	-198.73	2	32.6	11.4	0	1639-1650	R.GGVLLGPVCFMG.-		M:Q<br>Q:W	3.00<br>1.13

Detailed Protein Report

Protein 211: **ATP-binding cassette sub-family A member 3 [Homo sapiens]**

**Accession:**	gi|116734710	**Score:**	58.7
**Database:**	refseq_human(refseq_human_20140103.fasta)	**MW [kDa]:**	191.2
**Database Date:**	2015-11-30	**pI:**	8.2
**Modification(s):**	Carbamidomethyl	**Sequence Coverage [%]:**	2.3
		**No. of unique Peptides:**	3

Quantitation

Q:W	**Median:**	0.48	**CV:**	62.97 %	**No. of Peptides:**	2
M:Q	**Median:**	1.00	**CV:**	4.58 %	**No. of Peptides:**	2

10	20	30	40	50	60	70	80
MAVLRQLALL	LWKNYTLQKR	KVLVTVLELF	LPLLFSGILI	WLRLKIQSEN	VPNATIYPGQ	SIQELPLFFT	FPPPGDTWEL
90	100	110	120	130	140	150	160
AYIPSHSDAA	KTVTETVRRA	LVINMRVRGF	PSEKDFEDYI	RYDNCSSSVL	AAVVFEHPFN	HSKEPLPLAV	KYHLRFSYTR
170	180	190	200	210	220	230	240
RNYMWTQTGS	FFLKETEGWH	TTSLFPLFPN	PGPREPTSPD	GGEPGYIREG	FLAVQHAVDR	AIMEYHADAA	TRQLFQRLTV
250	260	270	280	290	300	310	320
TIKRFPYPPF	IADPFLVAIQ	YQLPLLLLLS	FTYTALTIAR	AVVQEKERRL	KEYMRMMGLS	SWLHWSAWFL	LFFLFLLIAA
330	340	350	360	370	380	390	400
SFMTLLFCVK	VKPNVAVLSR	SDPSLVLAFL	LCFAISTISF	SFMVSTFFSK	ANMAAAFGGF	LYFFTYIPYF	FVAPRYNWMT
410	420	430	440	450	460	470	480
LSQKLCSCLL	SNVAMAMGAQ	LIGKFEAKGM	GIQWRDLLSP	VNVDDDFCFG	QVLGMLLLDS	VLYGLVTWYM	EAVFPGQFGV
490	500	510	520	530	540	550	560
PQPWYFFIMP	SYWCGKPRAV	AGKEEEDSDP	EKALRNEYFE	AEPEDLVAGI	KIKHLSKVFR	VGNKDRAAVR	DLNLNLYEGQ
570	580	590	600	610	620	630	640
ITVLLGHNGA	GKTTTLSMLT	GLFPPTSGRA	YISGYEISQD	MVQIRKSLGL	CPQHDILFDN	LTVAEHLYFY	AQLKGLSRQK
650	660	670	680	690	700	710	720
CPEEVKQMLH	IIGLEDKWNS	RSRFLSGGMR	RKLSIGIALI	AGSKVLILDE	PTSGMDAISR	RAIWDLLQRQ	KSDRTIVLTT
730	740	750	760	770	780	790	800
HFMDEADLLG	DRIAIMAKGE	LQCCGSSLFL	KQKYGAGYHM	TLVKEPHCNP	EDISQLVHHH	VPNATLESSA	GAELSFILPR
810	820	830	840	850	860	870	880
ESTHRFEGLF	AKLEKKQKEL	GIASFGASIT	TMEEVFLRVG	KLVDSSMDIQ	AIQLPALQYQ	HERRASDWAV	DSNLCGAMDP
890	900	910	920	930	940	950	960
SDGIGALIEE	ERTAVKLNTG	LALHCQQFWA	MFLKKAAYSW	REWKMVAAQV	LVPLTCVTLA	LLAINYSSEL	FDDPMLRLTL
970	980	990	1000	1010	1020	1030	1040
GEYGRTVVPF	SVPGTSQLGQ	QLSEHLKDAL	QAEGQEPREV	LGDLEEFLIF	RASVEGGGFN	ERCLVAASFR	DVGERTVVNA
1050	1060	1070	1080	1090	1100	1110	1120
LFNNQAYHSP	ATALAVVDNL	LFKLLCGPHA	SIVVSNFPQP	RSALQAAKDQ	FNEGRKGFDI	ALNLLFAMAF	LASTFSILAV
1130	1140	1150	1160	1170	1180	1190	1200
SERAVQAKHV	QFVSGVHVAS	FWLSALLWDL	ISFLIPSLLL	LVVFKAFDVR	AFTRDGHMAD	TLLLLLLYGW	AIIPLMYLMN
1210	1220	1230	1240	1250	1260	1270	1280
FFFLGAATAY	TRLTIFNILS	GIATFLMVTI	MRIPAVKLEE	LSKTLDHVFL	VLPNHCLGMA	VSSFYENYET	RRYCTSSEVA
1290	1300	1310	1320	1330	1340	1350	1360
AHYCKKYNIQ	YQENFYAWSA	PGVGRFVASM	AASGCAYLIL	LFLIETNLLQ	RLRGILCALR	RRRTLTELYT	RMPVLPEDQD
1370	1380	1390	1400	1410	1420	1430	1440
VADERTRILA	PSPDSLLHTP	LIIKELSKVY	EQRVPLLAVD	RLSLAVQKGE	CFGLLGFNGA	GKTTTFKMLT	GEESLTSGDA
1450	1460	1470	1480	1490	1500	1510	1520
FVGGHRISSD	VGKVRQRIGY	CPQFDALLDH	MTGREMLVMY	ARLRGIPERH	IGACVENTLR	GLLLEPHANK	LVRTYSGGNK
1530	1540	1550	1560	1570	1580	1590	1600
RKLSTGIALI	GEPAVIFLDE	PSTGMDPVAR	RLLWDTVARA	RESGKAIIIT	SHSMEECEAL	CTRLAIMVQG	QFKCLGSPQH
1610	1620	1630	1640	1650	1660	1670	1680
LKSKFGSGYS	LRAKVQSEGQ	QEALEEFKAF	VDLTFPGSVL	EDEHQGMVHY	HLPGRDLSWA	KVFGILEKAK	EKYGVDDYSV
1690	1700	1710					
SQISLEQVFL	SFAHLQPPTA	EEGR					

Detailed Protein Report

Cmpd.	No. of Cmpds.	m/z meas.	Δ m/z [ppm]	z	Rt [min]	Score	P	Range	Sequence	Modification	Ratios
198	1	424.2139	-143.01	3	32.2	15.7	1	672-684	R.KLSIGIALIAGSK.V		M:Q 0.95<br>Q:W 0.27
32	1	877.8644	-76.86	2	29.3	10.4	1	739-753	K.GELQCCGSSLFLKQK.Y	Carbamidomethyl: 5, 6	
535	8	607.3148	34.29	2	36.7	32.6	0	988-998	K.DALQAEGQEPR.E		M:Q 1.04<br>Q:W 0.86

Detailed Protein Report

Protein 212: semaphorin-5B isoform 3 precursor [Homo sapiens]

**Accession:**	gi|373432662	**Score:**	58.6
**Database:**	refseq_human(refseq_human_20140103.fasta)	**MW [kDa]:**	116.0
**Database Date:**	2015-11-30	**pI:**	9.0
**Modification(s):**	Carbamidomethyl	**Sequence Coverage [%]:**	5.1
		**No. of unique Peptides:**	1

10	20	30	40	50	60	70	80
MVLAGPLAVS	LLLPSLTLLV	SHLSSSQDVS	SEPSSEQQLC	ALSKHPTVAF	EDLQPWVSNF	TYPGARDFSQ	LALDPSGNQL
90	100	110	120	130	140	150	160
IVGARNYLFR	LSLANVSLLQ	ATEWASSEDT	RRSCQSKGKT	EEECQNYVRV	LIVAGRKVFM	CGTNAFSPMC	TSRQVGNLSR
170	180	190	200	210	220	230	240
TIEKINGVAR	CPYDPRHNST	AVISSQGELY	AATVIDFSGR	DPAIYRSLGS	GPPLRTAQYN	SKWLNEPNFV	AAYDIGLFAY
250	260	270	280	290	300	310	320
FFLRENAVEH	DCGRTVYSRV	ARVCKNDVGG	RFLLEDTWTT	FMKARLNCSR	PGEVPFYYNE	LQSAFHLPEQ	DLIYGVFTTN
330	340	350	360	370	380	390	400
VNSIAASAVC	AFNLSAISQA	FNGPFRYQEN	PRAAWLPIAN	PIPNFQCGTL	PETGPNENLT	ERSLQDAQRL	FLMSEAVQPV
410	420	430	440	450	460	470	480
TPEPCVTQDS	VRFSHLVVDL	VQAKDTLYHV	LYIGTESGTI	LKALSTASRS	LHGCYLEELH	VLPPGRREPL	RSLRILHSAR
490	500	510	520	530	540	550	560
ALFVGLRDGV	LRVPLERCAA	YRSQGACLGA	RDPYCGWDGK	QQRCSTLEDS	SNMSLWTQNI	TACPVRNVTR	DGGFGPWSPW
570	580	590	600	610	620	630	640
QPCEHLDGDN	SGSCLCRARS	CDSPRPRCGG	LDCLGPAIHI	ANCSRNGAWT	PWSSWALCST	SCGIGFQVRQ	RSCSNPAPRH
650	660	670	680	690	700	710	720
GGRICVGKSR	EERFCNENTP	CPVPIFWASW	GSWSKCSSNC	GGGMQSRRRA	CENGNSCLGC	GVEFKTCNPE	GCPEVRRNTP
730	740	750	760	770	780	790	800
WTPWLPVNVT	QGGARQEQRF	RFTCRAPLAD	PHGLQFGRRR	TETRTCPADG	SGSCDTDALV	EVLLRSGSTS	PHTVSGGWAA
810	820	830	840	850	860	870	880
WGPWSSCSRD	CELGFRVRKR	TCTNPEPRNG	GLPCVGDAAE	YQDCNPQACP	GEDICLGLHT	EEALCATQAC	PEGWSPWSEW
890	900	910	920	930	940	950	960
SKCTDDGAQS	RSRHCEELLP	GSSACAGNSS	QSRPCPYSEI	PVILPASSME	EATDCAGFNL	IHLVATGISC	FLGSGLLTLA
970	980	990	1000	1010	1020	1030	1040
VYLSCQHCQR	QSQESTLVHP	ATPNHLHYKG	GGTPKNEKYT	PMEFKTLNKN	NLIPDDRANF	YPLQQTNVYT	TTYYPSPLNK
1050	1060						
HSFRPEASPG	QRCFPNS						

Cmpd.	No. of Cmpds.	m/z meas.	Δ m/z [ppm]	z	Rt [min]	Score	P	Range	Sequence	Modification
2242	9	682.6902	75.49	3	58.4	22.5	2	113-129	R.SCQSKGKTEEECQNYVR.V	Carbamidomethyl: 2

Detailed Protein Report

Protein 213: **PREDICTED: pyridine nucleotide-disulfide oxidoreductase domain-containing protein 2 isoform X1 [Homo sapiens]**

**Accession:**	gi|578819959	**Score:**	58.6
**Database:**	refseq_human(refseq_human_20140103.fasta)	**MW [kDa]:**	61.0
**Database Date:**	2015-11-30	**pI:**	6.3
**Modification(s):**	Oxidation	**Sequence Coverage [%]:**	12.0
		**No. of unique Peptides:**	3

Quantitation

Q:W **Median:** 0.57 **CV:** 0.00 % **No. of Peptides:** 1

```
        10         20         30         40         50         60         70         80
MAASGRGLCK AVAASPFPAW RRDNTEARGG LKPEYDAVVI GAGHNGLVAA AYLQRLGVNT AVFERRHVIG GAAVTEEIIP
        90        100        110        120        130        140        150        160
GFKFSRASYL LSLLRPQIYT DLELKKHGLR LHLRNPYSFT PMLEEGAGSK VPRCLLLGTD MAENQKQIAQ FSQKDAQVFP
       170        180        190        200        210        220        230        240
KYEEFMHRLA LAIDPLLDAA PVDMAAFQHG SLLQRMRSLS TLKPLLKAGR ILGAQLPRYY EVLTAPITKV LDQWFESEPL
       250        260        270        280        290        300        310        320
KATLATDAVI GAMTSPHTPG SGYVLLHHVM GGLEGMQGAW GYVQGGMGAL SDAIASSATT HGASIFTEKT VAKVQVNSEG
       330        340        350        360        370        380        390        400
CVQGVVLEDG TEVRSKMVLS NTSPQITFLK LTPQEWLPEE FLERISQLDT RSPVTKINVA VDRLPSFLAA PNAPRGQPLP
       410        420        430        440        450        460        470        480
HHQCSIHLNC EDTLLLHQAF EDAMDGLPSH RPVIELCIPS SLDPTLAPPG CHVVSLFTQY MPYTLAGGKA WDEQERDAYA
       490        500        510        520        530        540        550        560
DRVFDCIEVY APGFKDSVVG RDILTPPDLE RIFGLPGGNI FHCAMSLDQL YFARPVPLHS GYRCPLQGLY LCGSGAHPGF
       570
```

Cmpd.	No. of Cmpds.	m/z meas.	Δ m/z [ppm]	z	Rt [min]	Score	P	Range	Sequence	Modification	Ratios	
2627	1	869.6010	138.74	2	61.1	14.0	0	67-83	R.HVIGGAAVTEEIIPGFK.F		Q:W	0.57
2482	1	836.1552	175.38	2	59.3	13.8	2	196-210	R.MRSLSTLKPLLKAGR.I			
2198	1	905.3670	-138.71	2	56.0	17.3	1	335-350	R.SKMVLSNTSPQITFLK.L	Oxidation: 3		

Detailed Protein Report

Protein 214: **protein kinase C-binding protein NELL2 isoform c precursor [Homo sapiens]**

Accession: gi|223029474
Database: refseq_human(refseq_human_20140103.fasta)
Database Date: 2015-11-30
Modification(s): Carbamidomethyl

Score: 58.6
MW [kDa]: 91.1
pI: 5.3
Sequence Coverage [%]: 8.0
No. of unique Peptides: 3

10	20	30	40	50	60	70	80
METGLGAPLF	KAWLLISVWG	LGVDPSLQID	VLTELELGES	TTGVRQVPGL	HNGTKAFLFQ	DTPRSIKAST	ATAEQFFQKL
90	100	110	120	130	140	150	160
RNKHEFTILV	TLKQTHLNSG	VILSIHHLDH	RYLELESSGH	RNEVRLHYRS	GSHRPHTEVF	PYILADDKWH	KLSLAISASH
170	180	190	200	210	220	230	240
LILHIDCNKI	YERVVEKPST	DLPLGTTFWL	GQRNNAHGYF	KGIMQDVQLL	VMPQGFIAQC	PDLNRTCPTC	NDFHGLVQKI
250	260	270	280	290	300	310	320
MELQDILAKT	SAKLSRAEQR	MNRLDQCYCE	RTCTMKGTTY	REFESWIDGC	KNCTCLNGTI	QCETLICPNP	DCPLKSALAY
330	340	350	360	370	380	390	400
VDGKCCKECK	SICQFQGRTY	FEGERNTVYS	SSGVCVLYEC	KDQTMKLVES	SGCPALDCPE	SHQITLSHSC	CKVCKGYDFC
410	420	430	440	450	460	470	480
SERHNCMENS	ICRNLNDRAV	CSCRDGFRAL	REDNAYCEDI	DECAEGRHYC	RENTMCVNTP	GSFMCICKTG	YIRIDDYSCT
490	500	510	520	530	540	550	560
EHDECITNQH	NCDENALCFN	TVGGHNCVCK	PGYTGNGTTC	KAFCKDGCRN	GGACIAANVC	ACPQGFTGPS	CETDIDECSD
570	580	590	600	610	620	630	640
GFVQCDSRAN	CINLPGWYHC	ECRDGYHDNG	MFSPSGESCE	DIDECGTGRH	SCANDTICFN	LDGGYDCRCP	HGKNCTGDCI
650	660	670	680	690	700	710	720
HDGKVKHNGQ	IWVLENDRCS	VCSCQNGFVM	CRRMVCDCEN	PTVDLFCCPE	CDPRLSSQCL	HQNGETLYNS	GDTWVQNCQQ
730	740	750	760	770	780	790	800
CRCLQGEVDC	WPLPCPDVEC	EFSILPENEC	CPRCVTDPCQ	ADTIRNDITK	TCLDEMNVVR	FTGSSWIKHG	TECTLCQCKN
810	820						
GHICCSVDPQ	CLQEL						

Cmpd.	No. of Cmpds.	m/z meas.	Δ m/z [ppm]	z	Rt [min]	Score	P	Range	Sequence	Modification
1786	1	916.3119	-31.66	2	52.7	10.3	0	432-447	R.EDNAYCEDIDECAEGR.H	
1492	1	450.0889	-239.47	2	48.4	12.4	1	522-529	K.AFCKDGCR.N	
1555	1	932.3749	-6.08	2	49.1	11.3	1	659-673	R.CSVCSCQNGFVMCRR.M	Carbamidomethyl: 1, 4, 6

Detailed Protein Report

Protein 215: insulin receptor-related protein precursor [Homo sapiens]

**Accession:**	gi|31657140	**Score:**	58.5
**Database:**	refseq_human(refseq_human_20140103.fasta)	**MW [kDa]:**	143.6
**Database Date:**	2015-11-30	**pI:**	6.0
**Modification(s):**	Carbamidomethyl	**Sequence Coverage [%]:**	4.2
		**No. of unique Peptides:**	2

10	20	30	40	50	60	70	80
MAVPSLWPWG	ACLPVIFLSL	GFGLDTVEVC	PSLDIRSEVA	ELRQLENCSV	VEGHLQILLM	FTATGEDFRG	LSFPRLTQVT
**90**	**100**	**110**	**120**	**130**	**140**	**150**	**160**
DYLLLFRVYG	LESLRDLFPN	LAVIRGTRLF	LGYALVIFEM	PHLRDVALPA	LGAVLRGAVR	VEKNQELCHL	STIDWGLLQP
**170**	**180**	**190**	**200**	**210**	**220**	**230**	**240**
APGANHIVGN	KLGEECADVC	PGVLGAAGEP	CAKTTFSGHT	DYRCWTSSHC	QRVCPCPHGM	ACTARGECCH	TECLGGCSQP
**250**	**260**	**270**	**280**	**290**	**300**	**310**	**320**
EDPRACVACR	HLYFQGACLW	ACPPGTYQYE	SWRCVTAERC	ASLHSVPGRA	STFGIHQGSC	LAQCPSGFTR	NSSSIFCHKC
**330**	**340**	**350**	**360**	**370**	**380**	**390**	**400**
EGLCPKECKV	GTKTIDSIQA	AQDLVGCTHV	EGSLILNLRQ	GYNLEPQLQH	SLGLVETITG	FLKIKHSFAL	VSLGFFKNLK
**410**	**420**	**430**	**440**	**450**	**460**	**470**	**480**
LIRGDAMVDG	NYTLYVLDNQ	NLQQLGSWVA	AGLTIPVGKI	YFAFNPRLCL	EHIYRLEEVT	GTRGRQNKAE	INPRTNGDRA
**490**	**500**	**510**	**520**	**530**	**540**	**550**	**560**
ACQTRTLRFV	SNVTEADRIL	LRWERYEPLE	ARDLLSFIVY	YKESPFQNAT	EHVGPDACGT	QSWNLLDVEL	PLSRTQEPGV
**570**	**580**	**590**	**600**	**610**	**620**	**630**	**640**
TLASLKPWTQ	YAVFVRAITL	TTEEDSPHQG	AQSPIVYLRT	LPAAPTVPQD	VISTSNSSSH	LLVRWKPPTQ	RNGNLTYYLV
**650**	**660**	**670**	**680**	**690**	**700**	**710**	**720**
LWQRLAEDGD	LYLNDYCHRG	LRLPTSNNDP	RFDGEDGDPE	AEMESDCCPC	QHPPPGQVLP	PLEAQEASFQ	KKFENFLHNA
**730**	**740**	**750**	**760**	**770**	**780**	**790**	**800**
ITIPISPWKV	TSINKSPQRD	SGRHRRAAGP	LRLGGNSSDF	EIQEDKVPRE	RAVLSGLRHF	TEYRIDIHAC	NHAAHTVGCS
**810**	**820**	**830**	**840**	**850**	**860**	**870**	**880**
AATFVFARTM	PHREADGIPG	KVAWEASSKN	SVLLRWLEPP	DPNGLILKYE	IKYRRLGEEA	TVLCVSRLRY	AKFGGVHLAL
**890**	**900**	**910**	**920**	**930**	**940**	**950**	**960**
LPPGNYSARV	RATSLAGNGS	WTDSVAFYIL	GPEEEDAGGL	HVLLTATPVG	LTLLIVLAAL	GFFYGKKRNR	TLYASVNPEY
**970**	**980**	**990**	**1000**	**1010**	**1020**	**1030**	**1040**
FSASDMYVPD	EWEVPREQIS	IIRELGQGSF	GMVYEGLARG	LEAGEESTPV	ALKTVNELAS	PRECIEFLKE	ASVMKAFKCH
**1050**	**1060**	**1070**	**1080**	**1090**	**1100**	**1110**	**1120**
HVVRLLGVVS	QGQPTLVIME	LMTRGDLKSH	LRSLRPEAEN	NPGLPQPALG	EMIQMAGEIA	DGMAYLAANK	FVHRDLAARN
**1130**	**1140**	**1150**	**1160**	**1170**	**1180**	**1190**	**1200**
CMVSQDFTVK	IGDFGMTRDV	YETDYYRKGG	KGLLPVRWMA	PESLKDGIFT	THSDVWSFGV	VLWEIVTLAE	QPYQGLSNEQ
**1210**	**1220**	**1230**	**1240**	**1250**	**1260**	**1270**	**1280**
VLKFVMDGGV	LEELEGCPLQ	LQELMSRCWQ	PNPRLRPSFT	HILDSIQEEL	RPSFRLLSFY	YSPECRGARG	SLPTTDAEPD
**1290**	**1300**						
SSPTPRDCSP	QNGGPGH						

Cmpd.	No. of Cmpds.	m/z meas.	Δ m/z [ppm]	z	Rt [min]	Score	P	Range	Sequence	Modification
561	3	730.2206	-117.87	2	36.3	16.7	0	213-225	R.VCPCPHGMACTAR.G	Carbamidomethyl: 2, 4
2751	1	922.4677	-48.57	2	65.7	17.3	2	499-512	R.ILLRWERYEPLEAR.D	

Detailed Protein Report

Protein 216: **PREDICTED: tetratricopeptide repeat protein 13 isoform X7 [Homo sapiens]**

Accession: gi|578802328
Database: refseq_human(refseq_human_20140103.fasta)
Database Date: 2015-11-30

Score: 58.5
MW [kDa]: 57.4
pI: 9.3
Sequence Coverage [%]: 10.8
No. of unique Peptides: 2

Quantitation

Q:W	**Median:**	0.30	**CV:**	0.00 %	**No. of Peptides:**	1
M:Q	**Median:**	1.60	**CV:**	0.00 %	**No. of Peptides:**	1

10	20	30	40	50	60	70	80
MMLYHHGSLQ	EALKNFKRCL	QLEPYNEVCQ	YMKGLSHVAM	GQFYEGIKAQ	TKVMLNDPLP	GQKASPEYLK	VKYLREYSRY
**90**	**100**	**110**	**120**	**130**	**140**	**150**	**160**
LHAHLDTPLT	EYNIDVDLPG	SFKDHWAKNL	PFLIEDYEEQ	PGLQPHIKDV	LHQNFESYKP	EVQELICVAD	RLGSLMQYET
**170**	**180**	**190**	**200**	**210**	**220**	**230**	**240**
PGFLPNKRIH	RAMGLAALEV	MQAVQRTWTN	SKVRMNGKTR	LMQWRDMFDI	AVKWRRIADP	DQPVLWLDQM	PARSLSRGFN
**250**	**260**	**270**	**280**	**290**	**300**	**310**	**320**
NHINLIRGQV	INMRYLEYFE	KILHFIKDRI	LVYHGANNPK	GLLEVREALE	KVHKVEDLLP	IMKQFNTKTK	DGFTVNTKVP
**330**	**340**	**350**	**360**	**370**	**380**	**390**	**400**
SLKDQGKEYD	GFTITITGDK	VGNILFSVET	QTTEERTQLY	HAEIDALYKD	LTAKGKVLIL	SSEFGEADAV	CNLILSLVYY
**410**	**420**	**430**	**440**	**450**	**460**	**470**	**480**
FYNLMPLSRG	SSVIAYSVIV	GALMASGKEV	AGKIPKGKLV	DFEAMTAPGS	EAFSKVAKSW	MNLKSISPSY	KTLPSVSETF
**490**	**500**	**510**					
PTLRSMIEVL	NTDSSPRCLK	KL					

Cmpd.	No. of Cmpds.	m/z meas.	Δ m/z [ppm]	z	Rt [min]	Score	P	Range	Sequence	Modification	Ratios
1682	5	820.8728	-97.18	2	49.4	15.9	1	234-247	R.SLSRGFNNHINLIR.G		
549	2	730.3654	19.41	2	36.2	15.9	0	328-340	K.EYDGFTITITGDK.V		M:Q 1.60<br>Q:W 0.30

Detailed Protein Report

Protein 217: PREDICTED: probable E3 ubiquitin-protein ligase HERC1 isoform X6 [Homo sapiens]

**Accession:**	gi|530406610	**Score:**	58.3
**Database:**	refseq_human(refseq_human_20140103.fasta)	**MW [kDa]:**	524.9
**Database Date:**	2015-11-30	**pI:**	5.7
**Modification(s):**	Carbamidomethyl, Oxidation	**Sequence Coverage [%]:**	2.1
		**No. of unique Peptides:**	4

Quantitation

M:Q	**Median:** 1.51	**CV:** 0.00 %	**No. of Peptides:** 1

10	20	30	40	50	60	70	80
MATMIPPVKL	KWLEHLNSSW	ITEDSESIAT	REGVAVLYSK	LVSNKEVVPL	PQQVLCLKGP	QLPDFERESL	SSDEQDHYLD
90	100	110	120	130	140	150	160
ALLSSQLALA	KMVCSDSPFA	GALRKRLLVL	QRVFYALSNK	YHDKGKVKQQ	QHSPESSSGS	ADVHSVSERP	RSSTDALIEM
170	180	190	200	210	220	230	240
GVRTGLSLLF	ALLRQSWMMP	VSGPGLSLCN	DVIHTAIEVV	SSLPPLSLAN	ESKIPPMGLD	CLSQVTTFLK	GVTIPNSGAD
250	260	270	280	290	300	310	320
TLGRRLASEL	LLGLAAQRGS	LRYLLEWIEM	ALGASAVVHT	MEKGKLLSSQ	EGMISFDCFM	TILMQMRRSL	GSSADRSQWR
330	340	350	360	370	380	390	400
EPTRTSDGLC	SLYEAALCLF	EEIEAGQYCT	FVISTDGSVR	ACGKGSYGRL	GLGDSNNQST	LKKLTFEPHR	SIKKVSSSKG
410	420	430	440	450	460	470	480
SDGHTLAFTT	EGEVFSWGDG	DYGKLGHGNS	STQKYPKLIQ	GPLQGKVVVC	VSAGYRHSAA	VTEDGELYTW	GEGDFGRLGH
490	500	510	520	530	540	550	560
GDSNSRNIPT	LVKDISNVGE	VSCGSSHTIA	LSKDGRTVWS	FGGGDNGKLG	HGDTNRVYKP	KVIEALQGMF	IRKVCAGSQS
570	580	590	600	610	620	630	640
SLALTSTGQV	YAWGCGACLG	CGSSEATALR	PKLIEELAAT	RIVDVSIGDS	HCLALSHDNE	VYAWGNNSMG	QCGQGNSTGP
650	660	670	680	690	700	710	720
ITKPKKVSGL	DGIAIQQISA	GTSHSLAWTA	LPRDRQVVAW	HRPYCVDLEE	STFSHLRSFL	ERYCDKINSE	IPPLPFPSSR
730	740	750	760	770	780	790	800
EHHSFLKLCL	KLLSNHLALA	LAGGVATSIL	GRQAGPLRNL	LFRLMDSTVP	DEIQEVVIET	LSVGATMLLP	PLRERMELLH
810	820	830	840	850	860	870	880
SLLPQGPDRW	ESLSKGQRMQ	LDIILTSLQD	HTHVASLLGY	SSPSDAADLS	SVCTGYGNLS	DQPYGTQSCH	PDTHLAEILM
890	900	910	920	930	940	950	960
KTLLRNLGFY	TDQAFGELEK	NSDKFLLGTS	SSENSQPAHL	HELLCSLQKQ	LLAFCHINNI	SENSSSVALL	HKHLQLLLPH
970	980	990	1000	1010	1020	1030	1040
ATDIYSRSAN	LLKESPWNGS	VGEKLRDVIY	VSAAGSMLCQ	IVNSLLLLPV	SVARPLLSYL	LDLLPPLDCL	NRLLPAADLL
1050	1060	1070	1080	1090	1100	1110	1120
EDQELQWPLH	GGPELIDPAG	LPLPQPAQSW	VWLVDLERTI	ALLIGRCLGG	MLQGSPVSPE	EQDTAYWMKT	PLFSDGVEMD
1130	1140	1150	1160	1170	1180	1190	1200
TPQLDKCMSC	LLEVALSGNE	EQKPFDYKLR	PEIAVYVDLA	LGCSKEPARS	LWISMQDYAV	SKDWDSATLS	NESLLDTVSR
1210	1220	1230	1240	1250	1260	1270	1280
FVLAALLKHT	NLLSQACGES	RYQPGKHLSE	VYRCVYKVRS	RLLACKNLEL	IQTRSSSRDR	WISENQDSAD	VDPQEHSFTR
1290	1300	1310	1320	1330	1340	1350	1360
TIDEEAEMEE	QAERDREEGH	PEPEDEEEER	EHEVMTAGKI	FQCFLSAREV	ARSRDRDRMN	SGAGSGARAD	DPPPQSQQER
1370	1380	1390	1400	1410	1420	1430	1440
RVSTDLPEGQ	DVYTAACNSV	IHRCALLILG	VSPVIDELQK	RREEGQLQQP	STSASEGGGL	MTRSESLTAE	SRLVHTSPNY
1450	1460	1470	1480	1490	1500	1510	1520
RLIKSRSESD	LSQPESDEEG	YALSGRRNVD	LDLAASHRKR	GPMHSQLESL	SDSWARLKHS	RDWLCNSSYS	FESDFDLTKS
1530	1540	1550	1560	1570	1580	1590	1600
LGVHTLIENV	VSFVSGDVGN	APGFKEPEES	MSTSPQASII	AMEQQQLRAE	LRLEALHQIL	VLLSGMEEKG	SISLAGSRLS
1610	1620	1630	1640	1650	1660	1670	1680
SGFQSSTLLT	SVRLQFLAGC	FGLGTVGHTG	GKGESGRLHH	YQDGIRAAKR	NIQIEIQVAV	HKIYQQLSAT	LERALQANKH
1690	1700	1710	1720	1730	1740	1750	1760
HIEAQQRLLL	VTVFALSVHY	QPVDVSLAIS	TGLLNVLSQL	CGTDTMLGQP	LQLLPKTGVS	QLSTALKVAS	TRLLQILAIT
1770	1780	1790	1800	1810	1820	1830	1840
TGTYADKLSP	KVVQSLLDLL	CSQLKNLLSQ	TGVLHMASFG	EGEQEDGEEE	EKKVDSSGET	EKKDFRAALR	KQHAAELHLG
1850	1860	1870	1880	1890	1900	1910	1920
DFLVFLRRVV	SSKAIQSKMA	SPKWTEVLLN	IASQKCSSGI	PLVGNLRTRL	LALHVLEAVL	PACESGVEDD	QMAQIVERLF
1930	1940	1950	1960	1970	1980	1990	2000
SLLSDCMWET	PIAQAKHAIQ	IKEKEQEIKL	QKQGELEEED	ENLPIQEVSF	DPEKAQCCLV	ENGQILTHGS	GGKGYGLAST
2010	2020	2030	2040	2050	2060	2070	2080
GVTSGCYQWK	FYIVKENRGN	EGTCVGVSRW	PVHDFNHRTT	SDMWLYRAYS	GNLYHNGEQT	LTLSSFTQGD	FITCVLDMEA
2090	2100	2110	2120	2130	2140	2150	2160
RTISFGKNGE	EPKLAFEDVD	AAELYPCVMF	YSSNPGEKVK	ICDMQMRGTP	RDLLPGDPIC	SPVAAVLAEA	TIQLIRILHR
2170	2180	2190	2200	2210	2220	2230	2240

Detailed Protein Report

Cmpd.	No. of Cmpds.	m/z meas.	Δ m/z [ppm]	z	Rt [min]	Score	P	Range	Sequence	Modification	Ratios
2832	1	923.8157	-4.48	3	64.6	12.0	1	487-513	R. NIPTLVKDISNVGEVSCGSSHTIAL D		
1613	1	954.4635	-82.64	2	49.9	11.5	1	537-552	R.VYKPKVIEALQGMFIR.K	Oxidation: 13	
1890	1	1023.4178	-75.16	2	53.5	10.4	1	1209-1226	K.HTNLLSQACGESRYQPGK.H	Carbamidomethyl: 9	
2149	1	701.2767	-121.55	3	57.0	11.4	1	4282-4301	R.QISAGRCHSAAWTAPPVPPR. A		M:Q 1.51

Detailed Protein Report

Protein 218: partitioning defective 3 homolog isoform 8 [Homo sapiens]

Accession: gi|296278200
Database: refseq_human(refseq_human_20140103.fasta)
Database Date: 2015-11-30
Modification(s): Oxidation

Score: 58.3
MW [kDa]: 138.2
pI: 8.4
Sequence Coverage [%]: 5.1
No. of unique Peptides: 3

Quantitation

Q:W	**Median:**	1.16	**CV:**	0.00 %	**No. of Peptides:**	1
M:Q	**Median:**	1.17	**CV:**	0.00 %	**No. of Peptides:**	1

10	20	30	40	50	60	70	80
MKVTVCFGRT	RVVVPCGDGH	MKVFSLIQQA	VTRYRKAIAK	DPNYWIQVHR	LEHGDGGILD	LDDILCDVAD	DKDRLVAVFD
90	100	110	120	130	140	150	160
EQDPHHGGDG	TSASSTGTQS	PEIFGSELGT	NNVSAFQPYQ	ATSEIEVTPS	VLRANMPLHV	RRSSDPALIG	LSTSVSDSNF
170	180	190	200	210	220	230	240
SSEEPSRKNP	TRWSTTAGFL	KQNTAGSPKT	CDRKDEDGTE	EDNSRVEPVG	HADTGLEHIP	NFSLDDMVKL	VEVPNDGGPL
250	260	270	280	290	300	310	320
GIHVVPFSAR	GGRTLGLLVK	RLEKGGKAEH	ENLFRENDCI	VRINDGDLRN	RRFEQAQHMF	RQAMRTPIIW	FHVVPAANKE
330	340	350	360	370	380	390	400
QYEQLSQSEK	NNYYSSRFSP	DSQYIDNRSV	NSAGLHTVQR	APRLNHPPEQ	IDSHSRLPHS	AHPSGKPPSA	PASAPQNVFS
410	420	430	440	450	460	470	480
TTVSSGYNTK	KIGKRLNIQL	KKGTEGLGFS	ITSRDVTIGG	SAPIYVKNIL	PRGAAIQDGR	LKAGDRLIEV	NGVDLVGKSQ
490	500	510	520	530	540	550	560
EEVVSLLRST	KMEGTVSLLV	FRQEDAFHPR	ELKAEDEDIV	LTPDGTREFL	TFEVPLNDSG	SAGLGVSVKG	NRSKENHADL
570	580	590	600	610	620	630	640
GIFVKSIING	GAASKDGRLR	VNDQLIAVNG	ESLLGKTNQD	AMETLRRSMS	TEGNKRGMIQ	LIVARRISKC	NELKSPGSPP
650	660	670	680	690	700	710	720
GPELPIETAL	DDRERRISHS	LYSGIEGLDE	SPSRNAALSR	IMGKYQLSPT	VNMPQDDTVI	IEDDRLPVLP	PHLSDQSSSS
730	740	750	760	770	780	790	800
SHDDVGFVTA	DAGTWAKAAI	SDSADCSLSP	DVDPVLAFQR	EGFGRQSMSE	KRTKQFSDAS	QLDFVKTRKS	KSMDLGSSPS
810	820	830	840	850	860	870	880
RDVGPSLGLK	KSSSLESLQT	AVAEVTLNGD	IPFHRPRPRI	IRGRGCNESF	RAAIDKSYDK	PAVDDDDEGM	ETLEEDTEES
890	900	910	920	930	940	950	960
SRSGRESVST	ASDQPSHSLE	RQMNGNQEKG	DKTDRKKDKT	GKEKKKDRDK	EKDKMKAKKG	MLKGLGDMFR	IQAKTREFRE
970	980	990	1000	1010	1020	1030	1040
RQARERDYAE	IQDFHRTFGC	DDELMYGGVS	SYEGSMALNA	RPQSPREGHM	MDALYAQVKK	PRNSKPSPVD	SNRSTPSNHD
1050	1060	1070	1080	1090	1100	1110	1120
RIQRLRQEFQ	QAKQDEDVED	RRRTYSFEQP	WPNARPATQS	GRHSVSVEVQ	MQRQRQEERE	SSQQAQRQYS	SLPRQSRKNA
1130	1140	1150	1160	1170	1180	1190	1200
SSVSQDSWEQ	NYSPGEGFQS	AKENPRYSSY	QGSRNGYLGG	HGFNARVMLE	TQELLRQEQR	RKEQQMKKQP	PSEGPSNYDS
1210	1220	1230	1240	1250			
YKKVQDPSYA	PPKGPFRQDV	PPSPSQVARL	NRLQTPEKGR	PFYS			

Cmpd.	No. of Cmpds.	m/z meas.	Δ m/z [ppm]	z	Rt [min]	Score	P	Range	Sequence	Modification	Ratios
2795	1	729.3498	-56.43	2	66.1	14.8	1	553-565	R.SKENHADLGIFVK.S		
2354	1	711.9476	-118.31	3	59.3	10.7	2	790-810	K.SKSMDLGSSPSRDVGPSLGLK.K	Oxidation: 4	
2929	4	937.4766	2.60	2	65.8	16.0	1	1007-1022	R.EGHMMDALYAQVKKPR.N		M:Q 1.17<br>Q:W 1.16

Detailed Protein Report

Protein 219: **cGMP-specific 3',5'-cyclic phosphodiesterase isoform 1 [Homo sapiens]**

**Accession:**	gi|61744435	**Score:**	57.9
**Database:**	refseq_human(refseq_human_20140103.fasta)	**MW [kDa]:**	99.9
**Database Date:**	2015-11-30	**pI:**	5.7
**Modification(s):**	Carbamidomethyl, Oxidation	**Sequence Coverage [%]:**	6.3
		**No. of unique Peptides:**	3

```
        10         20         30         40         50         60         70         80
MERAGPSFGQ QRQQQQPQQQ KQQQRDQDSV EAWLDDHWDF TFSYFVRKAT REMVNAWFAE RVHTIPVCKE GIRGHTESCS
        90        100        110        120        130        140        150        160
CPLQQSPRAD NSAPGTPTRK ISASEFDRPL RPIVVKDSEG TVSFLSDSEK KEQMPLTPPR FDHDEGDQCS RLLELVKDIS
       170        180        190        200        210        220        230        240
SHLDVTALCH KIFLHIHGLI SADRYSLFLV CEDSSNDKFL ISRLFDVAEG STLEEVSNNC IRLEWNKGIV GHVAALGEPL
       250        260        270        280        290        300        310        320
NIKDAYEDPR FNAEVDQITG YKTQSILCMP IKNHREEVVG VAQAINKKSG NGGTFTEKDE KDFAAYLAFC GIVLHNAQLY
       330        340        350        360        370        380        390        400
ETSLLENKRN QVLLDLASLI FEEQQSLEVI LKKIAATIIS FMQVQKCTIF IVDEDCSDSF SSVFHMECEE LEKSSDTLTR
       410        420        430        440        450        460        470        480
EHDANKINYM YAQYVKNTME PLNIPDVSKD KRFPWTTENT GNVNQQCIRS LLCTPIKNGK KNKVIGVCQL VNKMEENTGK
       490        500        510        520        530        540        550        560
VKPFNRNDEQ FLEAFVIFCG LGIQNTQMYE AVERAMAKQM VTLEVLSYHA SAAEEETREL QSLAAVVPS AQTLKITDFS
       570        580        590        600        610        620        630        640
FSDFELSDLE TALCTIRMFT DLNLVQNFQM KHEVLCRWIL SVKKNYRKNV AYHNWRHAFN TAQCMFAALK AGKIQNKLTD
       650        660        670        680        690        700        710        720
LEILALLIAA LSHDLDHRGV NNSYIQRSEH PLAQLYCHSI MEHHHFDQCL MILNSPGNQI LSGLSIEEYK TTLKIIKQAI
       730        740        750        760        770        780        790        800
LATDLALYIK RRGEFFELIR KNQFNLEDPH QKELFLAMLM TACDLSAITK PWPIQQRIAE LVATEFFDQG DRERKELNIE
       810        820        830        840        850        860        870        880
PTDLMNREKK NKIPSMQVGF IDAICLQLYE ALTHVSEDCF PLLDGCRKNR QKWQALAEQQ EKMLINGESG QAKRN
```

Cmpd.	No. of Cmpds.	m/z meas.	Δ m/z [ppm]	z	Rt [min]	Score	P	Range	Sequence	Modification
1989	1	616.7715	-62.66	2	54.8	10.4	1	2-12	M.ERAGPSFGQQR.Q	
2072	1	1071.4033	-85.02	2	56.3	13.5	1	70-88	K.EGIRGHTESCSCPLQQSPR.A	Carbamidomethyl: 10
2797	4	937.4532	-23.79	2	64.1	16.1	2	793-807	R.ERKELNIEPTDLMNR.E	Oxidation: 13

Detailed Protein Report

Protein 220: **PREDICTED: caspase-10 isoform X1 [Homo sapiens]**

Accession: gi|530371069
Database: refseq_human(refseq_human_20140103.fasta)
Database Date: 2015-11-30
Modification(s): Carbamidomethyl

Score: 57.8
MW [kDa]: 58.8
pI: 6.2
Sequence Coverage [%]: 9.8
No. of unique Peptides: 2

10	20	30	40	50	60	70	80
MKSQGQHWYS	SSDKNCKVSF	REKLLIIDSN	LGVQDVENLK	FLCIGLVPNK	KLEKSSSASD	VFEHLLAEDL	LSEEDPFFLA
**90**	**100**	**110**	**120**	**130**	**140**	**150**	**160**
ELLYIIRQKK	LLQHLNCTKE	EVERLLPTRQ	RVSLFRNLLY	ELSEGIDSEN	LKDMIFLLKD	SLPKTEMTSL	SFLAFLEKQG
**170**	**180**	**190**	**200**	**210**	**220**	**230**	**240**
KIDEDNLTCL	EDLCKTVVPK	LLRNIEKYKR	EKAIQIVTPP	VDKEAESYQG	EEELVSQTDV	KTFLEALPES	WQNKHAGSNG
**250**	**260**	**270**	**280**	**290**	**300**	**310**	**320**
NRATNGAPSL	VSRGMQGASA	NTLNSETSTK	RAAVYRMNRN	HRGLCVIVNN	HSFTSLKDRQ	GTHKDAEILS	HVFQWLGFTV
**330**	**340**	**350**	**360**	**370**	**380**	**390**	**400**
HIHNNVTKVE	MEMVLQKQKC	NPAHADGDCF	VFCILTHGRF	GAVYSSDEAL	IPIREIMSHF	TALQCPRLAE	KPKLFFIQAC
**410**	**420**	**430**	**440**	**450**	**460**	**470**	**480**
QGEEIQPSVS	IEADALNPEQ	APTSLQDSIP	AEADFLLGLA	TVPGYVSFRH	VEEGSWYIQS	LCNHLKKLVP	RHEDILSILT
**490**	**500**	**510**	**520**	**530**			
AVNDDVSRRV	DKQGTKKQMP	QPAFTLRKKL	VFPVPLDALS	L			

Cmpd.	No. of Cmpds.	m/z meas.	Δ m/z [ppm]	z	Rt [min]	Score	P	Range	Sequence	Modification
1677	1	933.3261	-154.07	2	50.9	26.9	1	235-253	K.HAGSNGNRATNGAPSLVSR.G	
2646	1	1145.5422	35.87	2	61.8	11.6	0	340-359	K.CNPAHADGDCFVFCILTHGR.F	Carbamidomethyl: 10, 14

Detailed Protein Report

Protein 221: PREDICTED: zinc finger protein 665 isoform X5 [Homo sapiens]

Accession: gi|578834915
Database: refseq_human(refseq_human_20140103.fasta)
Database Date: 2015-11-30
Modification(s): Carbamidomethyl, Oxidation

Score: 57.8
MW [kDa]: 76.3
pI: 10.3
Sequence Coverage [%]: 8.8
No. of unique Peptides: 4

Quantitation

Q:W	**Median:** 1.16	**CV:** 0.00 %	**No. of Peptides:** 2
M:Q	**Median:** 1.17	**CV:** 0.00 %	**No. of Peptides:** 2

```
        10         20         30         40         50         60         70         80
MVCPCSGLLL AVGGMPPRCQ GRTLEEEEEE GKGVRDGSSS DISCKCVNTD LPPKGKNNMG EAFYTVKLER LESCDTVGLS
        90        100        110        120        130        140        150        160
FQEVQKNTYD FECQWKDDEG NYKTVLMLQK ENLPGRRAQR DRRAAGNRHI ENQLGVSFQS HLPELQQFQH EGKIYEYNQV
       170        180        190        200        210        220        230        240
EKSPNNRGKH YKCDECGKVF SQNSRLTSHK RIHTGEKPYQ CNKCGKAFTV RSNLTIHQVI HTGEKPYKCN ECGKVFSQPS
       250        260        270        280        290        300        310        320
NLAGHQRIHT GEKPYKCNEC GKAFRAHSKL TTHQVIHTGE KPYKCKECGK CFTQNSHLAS HRRIHTGEKP YKCNECGKAF
       330        340        350        360        370        380        390        400
SVRSSLTTHQ TIHTGEKPYK CNECGKVFRH NSYLAKHRRI HTGEKPYKCN ECGKAFSMHS NLTKHQIIHT GEKPFKCNEC
       410        420        430        440        450        460        470        480
VKVFTQYSHL ANHRRIHTGE KPYRCDECGK AFSVRSSLTT HQAIHTGEKP YKCNDCGKVF TQNSHLASHR GIHSGEKPYK
       490        500        510        520        530        540        550        560
CDECGKAFSQ TSQLARHWRV HTGEKPYKCN ECGKAFSVHS SLTIHQTIHT GQKPYKCNDC GKVFRHNSYL AIHQRIHTGE
       570        580        590        600        610        620        630        640
KPYKCNECGK AFSVHSNLAT HQVIHTGEKP YKCNECGKVF TQNSHLANHR RIHTGEKPYR CNECGKAFSV RSTLTTHMAV
       650        660        670        680
HTGDKPYKCN QCGKVFTQNS NLAKHRRIHS G
```

Cmpd.	No. of Cmpds.	m/z meas.	Δ m/z [ppm]	z	Rt [min]	Score	P	Range	Sequence	Modification	Ratios
2105	7	937.3619	-106.09	2	56.5	15.4	0	1-18	-.MVCPCSGLLLAVGGMPPR.C	Carbamidomethyl: 5; Oxidation: 15	M:Q 1.17<br>Q:W 1.16
2293	5	937.4457	-16.78	2	57.0	19.3	0	1-18	-.MVCPCSGLLLAVGGMPPR.C	Carbamidomethyl: 3; Oxidation: 15	M:Q 1.17<br>Q:W 1.16
1354	1	684.3815	97.70	2	46.5	12.8	1	33-45	K.GVRDGSSSDISCK.C	Carbamidomethyl: 12	
1877	1	1057.5274	21.10	3	52.1	10.3	2	453-480	K.CNDCGKVFTQNSHLASHRGIHSGC	Carbamidomethyl: 4	

Detailed Protein Report

Protein 222: PREDICTED: lipoxygenase homology domain-containing protein 1 isoform X1 [Homo sapiens]

**Accession:**	gi|578832310	**Score:**	57.7
**Database:**	refseq_human(refseq_human_20140103.fasta)	**MW [kDa]:**	220.7
**Database Date:**	2015-11-30	**pI:**	5.2
**Modification(s):**	Carbamidomethyl	**Sequence Coverage [%]:**	2.7
		**No. of unique Peptides:**	4

Quantitation

M:Q	**Median:** 0.89	**CV:** 0.00 %	**No. of Peptides:** 1

Detailed Protein Report

10	20	30	40	50	60	70	80
MMQLTLNTLF	PVVSTPAITY	IVTVFTGDVR	GAGTKSKIYL	VMYGARGNKN	SGKIFLEGGV	FDRGRTDIFH	IELAVLLSPL
90	100	110	120	130	140	150	160
SRVSVGHGNV	GVNRGWFCEK	VVILCPFTGI	QQTFPCSNWL	DEKKADGLIE	RQLYEMVSLR	KKRLKKFPWS	LWVWTTDLKK
170	180	190	200	210	220	230	240
AGTNSPIFIQ	IYGQKGRTDE	ILLNPNNKWF	KPGIIEKFRI	ELPDLGRFYK	IRVWHDKRSS	GSGWHLERMT	LMNTLNKDKY
250	260	270	280	290	300	310	320
NFNCNRWLDA	NEDDNEIVRE	MTAEGPTVRR	IMGMARYHVT	VCTGELEGAG	TDANVYLCLF	GDVGDTGERL	LYNCRNNTDL
330	340	350	360	370	380	390	400
FEKGNADEFT	IESVTMRNVR	RVRIRHDGKG	SGSGWYLDRV	LVREEGQPES	DNVEFPCLRW	LDKDKDDGQL	VRELLPSDSS
410	420	430	440	450	460	470	480
ATLKNFRYHI	SLKTGDVSGA	STDSRVYIKL	YGDKSDTIKQ	VLLVSDNNLK	DYFERGRVDE	FTLETLNIGN	INRLVIGHDS
490	500	510	520	530	540	550	560
TGMHASWFLG	SVQIRVPRQG	KQYTFPANRW	LDKNQADGRL	EVELYPSEVV	EIQKLVHYEV	EIWTGDVGGA	GTSARVYMQI
570	580	590	600	610	620	630	640
YGEKGKTEVL	FLSSRSKVFE	RASKDTFQLE	AADVGEVYKL	RLGHTGEGFG	PSWFVDTVWL	RHLVVREVDL	TPEEEARKKK
650	660	670	680	690	700	710	720
EKDKLRQLLK	KERLKAKLQR	KKKKRKGSDE	EDEGEEEESS	SSEESSSEEE	EMEEEEEEEE	FGPGMQEVIE	QHKFEAHRWL
730	740	750	760	770	780	790	800
ARGKEDNELV	VELVPAGKPG	PERNTYEVQV	VTGNVPKAGT	DANVYLTIYG	EEYGDTGERP	LKKSDKSNKF	EQGQTDTFTI
810	820	830	840	850	860	870	880
YAIDLGALTK	IRIRHDNTGN	RAGWFLDRID	ITDMNNEITY	YFPCQRWLAV	EEDDGQLSRE	LLPVDESYVL	PQSEEGRGGG
890	900	910	920	930	940	950	960
DNNPLDNLAL	EQKDKSTTFS	VTIKTGVKKN	AGTDANVFIT	LFGTQDDTGM	TLLKSSKTNS	DKFERDSIEI	FTVETLDLGD
970	980	990	1000	1010	1020	1030	1040
LWKVRLGHDN	TGKAPGWFVD	WVEVDAPSLG	KCMTFPCGRW	LAKNEDDGSI	IRDLFHAELQ	TRLYTPFVPY	EITLYTSDVF
1050	1060	1070	1080	1090	1100	1110	1120
AAGTDANIFI	IIYGCDAVCT	QQKYLCTNKR	EQKQFFERKS	ASRFIVELED	VGEIIEKIRI	GHNNTGMNPG	WHCSHVDIRR
1130	1140	1150	1160	1170	1180	1190	1200
LLPDKDGAET	LTFPCDRWLA	TSEDDKKTIR	ELVPYDIFTE	KYMKDGSLRQ	VYKEVEEPLD	IVLYSVQIFT	GNIPGAGTDA
1210	1220	1230	1240	1250	1260	1270	1280
KVYITIYGDL	GDTGERYLGK	SENRTNKFER	GTADTFIIEA	ADLGVIYKIK	LRHDNSKWCA	DWYVEKVEIW	NDTNEDEFLF
1290	1300	1310	1320	1330	1340	1350	1360
LCGRWLSLKK	EDGRLERLFY	EKEYTGDRSS	NCSSPADFWE	IALSSKMADV	DISTVTGPMA	DYVQEGPIIP	YYVSVTTGKH
1370	1380	1390	1400	1410	1420	1430	1440
KDAATDSRAF	IFLIGEDDER	SKRIWLDYPR	GKRGFSRGSV	EEFYVAGLDV	GIIKKIEVLY	EMTVWTGDVV	GGGTDSNIFM
1450	1460	1470	1480	1490	1500	1510	1520
TLYGINGSTE	EMQLDKKKAR	FEREQNDTFI	MEILDIAPFT	KMRIRIDGLG	SRPEWFLERI	LLKNMNTGDL	TMFYYGDWLS
1530	1540	1550	1560	1570	1580	1590	1600
QRKGKKTLVC	EMCAVIDEEE	MMEWTSYTVA	VKTSDILGAG	TDANVFIIIF	GENGDSGTLA	LKQSANWNKF	ERNNTDTFNF
1610	1620	1630	1640	1650	1660	1670	1680
PDMLSLGHLC	KLRVWHDNKG	IFPGWHLSYV	DVKDNSRDET	FHFQCDCWLS	KSEGDGQTVR	DFACANNKIC	DELEETTYEI
1690	1700	1710	1720	1730	1740	1750	1760
VIETGNGGET	RENVWLILEG	RKNRSKEFLM	ENSSRQRAFR	KGTTDTFEFD	SIYLGDIASL	CVGHLAREDR	FIPKRELAWH
1770	1780	1790	1800	1810	1820	1830	1840
VKTITITEME	YGNVYFFNCD	CLIPLKRKRK	YFKVFEVTKT	TESFASKVQS	LVPVKYEVIV	TTGYEPGAGT	DANVFVTIFG
1850	1860	1870	1880	1890	1900	1910	1920
ANGDTGKREL	KQKMRNLFER	GSTDRFFLET	LELGELRKVR	LEHDSSGYCS	GWLVEKVEVT	NTSTGVATIF	NCGRWLDKKR
1930	1940						
GDGLTWRDLF	PSV						

Cmpd.	No. of Cmpds.	m/z meas.	Δ m/z [ppm]	z	Rt [min]	Score	P	Range	Sequence	Modification	Ratios
2434	1	1045.2535	-261.87	1	60.9	17.6	1	384-392	K.DKDDGQLVR.E		
88	1	516.5686	-74.24	3	30.5	13.2	0	744-757	R.NTYEVQVVTGNVPK.A		
2071	1	1052.4367	-86.97	2	56.3	14.8	2	1120-	R.RLLPDKDGAETLTFPCDR.W	Carbamidomethyl: 16	

Detailed Protein Report

Cmpd.	No. of Cmpds.	m/z meas.	Δ m/z [ppm]	z	Rt [min]	Score	P	Range	Sequence	Modification	Ratios
								1137			
1581	1	698.7063	-184.90	2	50.0	12.1	1	1707-1717	K.EFLMENSSRQR.A		M:Q 0.89

Detailed Protein Report

Protein 223: 14-3-3 protein zeta/delta [Homo sapiens]

**Accession:**	gi|4507953	**Score:**	57.6
**Database:**	refseq_human(refseq_human_20140103.fasta)	**MW [kDa]:**	27.7
**Database Date:**	2015-11-30	**pI:**	4.6
**Modification(s):**	Carbamidomethyl	**Sequence Coverage [%]:**	9.0
		**No. of unique Peptides:**	1

Quantitation

M:Q	**Median:** 0.88	**CV:** 0.00 %	**No. of Peptides:** 1

Alias proteins:

Accession	Name	Description
gi|530389321	refseq_human (refseq_human_20140103.fasta)	PREDICTED: 14-3-3 protein zeta/delta isoform X4 [Homo sapiens]
gi|530389319	refseq_human (refseq_human_20140103.fasta)	PREDICTED: 14-3-3 protein zeta/delta isoform X3 [Homo sapiens]
gi|530389317	refseq_human (refseq_human_20140103.fasta)	PREDICTED: 14-3-3 protein zeta/delta isoform X2 [Homo sapiens]
gi|208973244	refseq_human (refseq_human_20140103.fasta)	14-3-3 protein zeta/delta [Homo sapiens]
gi|208973242	refseq_human (refseq_human_20140103.fasta)	14-3-3 protein zeta/delta [Homo sapiens]
gi|208973240	refseq_human (refseq_human_20140103.fasta)	14-3-3 protein zeta/delta [Homo sapiens]
gi|208973238	refseq_human (refseq_human_20140103.fasta)	14-3-3 protein zeta/delta [Homo sapiens]
gi|21735625	refseq_human (refseq_human_20140103.fasta)	14-3-3 protein zeta/delta [Homo sapiens]

```
        10         20         30         40         50         60         70         80
MDKNELVQKA KLAEQAERYD DMAACMKSVT EQGAELSNEE RNLLSVAYKN VVGARRSSWR VVSSIEQKTE GAEKKQQMAR
        90        100        110        120        130        140        150        160
EYREKIETEL RDICNDVLSL LEKFLIPNAS QAESKVFYLK MKGDYYRYLA EVAAGDDKKG IVDQSQQAYQ EAFEISKKEM
       170        180        190        200        210        220        230        240
QPTHPIRLGL ALNFSVFYYE ILNSPEKACS LAKTAFDEAI AELDTLSEES YKDSTLIMQL LRDNLTLWTS DTQGDEAEAG
       250
EGGEN
```

Cmpd.	No. of Cmpds.	m/z meas.	Δ m/z [ppm]	z	Rt [min]	Score	P	Range	Sequence	Modification	Ratios	
2523	1	709.8534	-14.08	2	62.0	10.3	0	92-103	R.DICNDVLSLLEK.F	Carbamidomethyl: 3	M:Q	0.88

BRUKER

Detailed Protein Report

Protein 224: PREDICTED: zinc finger protein 646 isoform X3 [Homo sapiens]

**Accession:**	gi|530409439	**Score:**	57.3
**Database:**	refseq_human(refseq_human_20140103.fasta)	**MW [kDa]:**	198.5
**Database Date:**	2015-11-30	**pI:**	6.8
**Modification(s):**	Carbamidomethyl, Oxidation	**Sequence Coverage [%]:**	2.9
		**No. of unique Peptides:**	4

10	20	30	40	50	60	70	80
MEDTPPSLSC	SDCQRHFPSL	PELSRHRELL	HPSPNQDSEE	ADSIPRPYRC	QQCGRGYRHP	GSLVNHRRTH	ETGLFPCTTC
90	100	110	120	130	140	150	160
GKDFSNPMAL	KSHMRTHAPE	GRRRHRPPRP	KEATPHLQGE	TVSTDSWGQR	LGSSEGWENQ	TKHTEETPDC	ESVPDPRAAS
170	180	190	200	210	220	230	240
GTWEDLPTRQ	REGLASHPGP	EDGADGWGPS	TNSARAPPLP	IPASSLLSNL	EQYLAESVVN	FTGGQEPTQS	PPAEEERRYK
250	260	270	280	290	300	310	320
CSQCGKTYKH	AGSLTNHRQS	HTLGIYPCAI	CFKEFSNLMA	LKNHSRLHAQ	YRPYHCPHCP	RVFRLPRELL	EHQQSHEGER
330	340	350	360	370	380	390	400
QEPRWEEKGM	PTTNGHTDES	SQDQLPSAQM	LNGSAELSTS	GELEDSGLEE	YRPFRCGDCG	RTYRHAGSLI	NHRKSHQTGV
410	420	430	440	450	460	470	480
YPCSLCSKQL	FNAAALKNHV	RAHHRPRQGV	GENGQPSVPP	APLLLAETTH	KEEEDPTTTL	DHRPYKCSEC	GRAYRHRGSL
490	500	510	520	530	540	550	560
VNHRHSHRTG	EYQCSLCPRK	YPNLMALRNH	VRVHCKAARR	SADIGAEGAP	SHLKVELPPD	PVEAEAAPHT	DQDHVCKHEE
570	580	590	600	610	620	630	640
EATDITPAAD	KTAAHICSIC	GLLFEDAESL	ERHGLTHGAG	EKENSRTETT	MSPPRAFACR	DCGKSYRHSG	SLINHRQTHQ
650	660	670	680	690	700	710	720
TGDFSCGACA	KHFHTMAAMK	NHLRRHSRRR	SRRHRKRAGG	ASGGREAKLL	AAESWTRELE	DNEGLESPQD	PSGESPHGAE
730	740	750	760	770	780	790	800
GNLESDGDCL	QAESEGDKCG	LERDETHFQG	DKESGGTGEG	LERKDASLLD	NLDIPGEEGG	GTHFCDSLTG	VDEDQKPATG
810	820	830	840	850	860	870	880
QPNSSSHSAN	AVTGWQAGAA	HTCSDCGHSF	PHATGLLSHR	PCHPPGIYQC	SLCPKEFDSL	PALRSHFQNH	RPGEATSAQP
890	900	910	920	930	940	950	960
FLCCLCGMIF	PGRAGYRLHR	RQAHSSSGMT	EGSEEEGEEE	GVAEAAPARS	PPLQLSEAEL	LNQLQREVEA	LDSAGYGHIC
970	980	990	1000	1010	1020	1030	1040
GCCGQTYDDL	GSLERHHQSQ	SSGTTADKAP	SPLGVAGDAM	EMVVDSVLED	IVNSVSGEGG	DAKSQEGAGT	PLGDSLCIQG
1050	1060	1070	1080	1090	1100	1110	1120
GESLLEAQPR	PFRCNQCGKT	YRHGGSLVNH	RKIHQTGDFL	CPVCSRCYPN	LAAYRNHLRN	HPRCKGSEPQ	VGPIPEAAGS
1130	1140	1150	1160	1170	1180	1190	1200
SELQVGPIPE	GGSNKPQHMA	EEGPGQAEVE	KLQEELKVEP	LEEVARVKEE	VWEETTVKGE	EIEPRLETAE	KGCQTEASSE
1210	1220	1230	1240	1250	1260	1270	1280
RPFSCEVCGR	SYKHAGSLIN	HRQSHQTGHF	GCQACSKGFS	NLMSLKNHRR	IHADPRRFRC	SECGKAFRLR	KQLASHQRVH
1290	1300	1310	1320	1330	1340	1350	1360
MERRGGGGTR	KATREDRPFR	CGQCGRTYRH	AGSLLNHRRS	HETGQYSCPT	CPKTYSNRMA	LKDHQRLHSE	NRRRRAGRSR
1370	1380	1390	1400	1410	1420	1430	1440
RTAVRCALCG	RSFPGRGSLE	RHLREHEETE	REPANGQGGL	DGTAASEANL	TGSQGLETQL	GGAEPVPHLE	DGVPRPGERS
1450	1460	1470	1480	1490	1500	1510	1520
QSPIRAASSE	APEPLSWGAG	KAGGWPVGGG	LGNHSGGWVP	QFLTRSEEPE	DSVHRSPCHA	GDCQLNGPTL	SHMDSWDNRD
1530	1540	1550	1560	1570	1580	1590	1600
NSSQLQPGSH	SSCSQCGKTY	CQSGSLLNHN	TNKTDRHYCL	LCSKEFLNPV	ATKSHSHNHI	DAQTFACPDC	GKAFESHQEL
1610	1620	1630	1640	1650	1660	1670	1680
ASHLQAHARG	HSQVPAQMEE	ARDPKAGTGE	DQVVLPGQGK	AQEAPSETPR	GPGESVERAR	GGQAVTSMAA	EDKERPFRCT
1690	1700	1710	1720	1730	1740	1750	1760
QCGRSYRHAG	SLLNHQKAHT	TGLYPCSLCP	KLLPNLLSLK	NHSRTHTDPK	RHCCSICGKA	FRTAARLEGH	GRVHAPREGP
1770	1780	1790	1800	1810			
FTCPHCPRHF	RRRISFVQHQ	QQHQEEWTVA	GSGRGHEGSQ	EEEPQWHQ			

Cmpd.	No. of	m/z meas.	Δ m/z	z	Rt	Score	P	Range	Sequence	Modification

Detailed Protein Report

	Cmpds.		[ppm]		[min]					
2876	1	899.9560	111.44	2	64.7	16.6	0	1-15	-.MEDTPPSLSCSDCQR.H	Carbamidomethyl: 10, 13; Oxidation: 1
2169	1	701.2467	-130.95	3	57.3	10.5	1	308-324	R.ELLEHQQSHEGERQEPR.W	
1307	3	1023.7335	252.51	1	46.0	17.9	0	1557-1564	R.HYCLLCSK.E	Carbamidomethyl: 3
1325	1	640.6479	-226.44	2	46.2	12.3	0	1661-1673	R.GGQAVTSMAAEDK.E	Oxidation: 8

Detailed Protein Report

Protein 225: PREDICTED: LOW QUALITY PROTEIN: hemicentin-2 [Homo sapiens]

**Accession:**	gi|578818143	**Score:**	57.3
**Database:**	refseq_human(refseq_human_20140103.fasta)	**MW [kDa]:**	546.0
**Database Date:**	2015-11-30	**pI:**	5.5
**Modification(s):**	Carbamidomethyl	**Sequence Coverage [%]:**	1.5
		**No. of unique Peptides:**	2

Quantitation

Q:W	**Median:**	6.63	**CV:**	0.00 %	**No. of Peptides:**	1
M:Q	**Median:**	0.22	**CV:**	0.00 %	**No. of Peptides:**	1

Detailed Protein Report

10	20	30	40	50	60	70	80
MMPGAPLLRL	LTAVSAAVAV	AVAGAPGTVM	PPTTGDATLA	FVFDVTGSMW	DELMQVIDGA	SRILERSLSR	RSQAIANYAL
90	100	110	120	130	140	150	160
VPFHDPDIGP	VTLTADPTVF	QRELRELYVQ	GGGDCPEMSV	GAIKAAVEVA	NPGSFIYVFS	DARAKDYHKK	EELLRLLQLK
170	180	190	200	210	220	230	240
QSQVVFVLTG	DCGDRTHPGY	LAYEEIAATS	SGQVFHLDKQ	QVTEVLKWVE	SAIQASKVHL	LSTDHEEEGE	HTWRLPFDPS
250	260	270	280	290	300	310	320
LKEVTISLSG	PGPEIEVQDP	LGRILQEDEG	LNVLLNIPDS	AKVVAFKPEH	PGLWSIKVYS	SGRHSVRITG	VSNIDFRAGF
330	340	350	360	370	380	390	400
STQPLLDLNH	TLEWPLQGVP	ISLVINSTGL	KAPGRLDSVE	LAQSSGKPLL	TLPTKPLSNG	STHQLWGGPP	FHTPKERFYL
410	420	430	440	450	460	470	480
KVKGKDHEGN	PLLRVSGVSY	SGVAPGAPLV	SMAPRIHGYL	HQPLLVSCSV	HSALPFRLQL	RRGEARLGEE	RHFQESGNSS
490	500	510	520	530	540	550	560
WEILRASKAE	EGTYECTAVS	RAGTGRAKAQ	IVVTDPPPQL	VPAPNVTVSP	GETAVLSCRV	LGEAPYNLTW	VRDWRVLPAS
570	580	590	600	610	620	630	640
TGRVAQLADL	SLEISGIIPT	DGGRYQCVAS	NANGVTRASV	WLLVREAPQV	SIHTSSQHFS	QGVEVKVSCS	ASGYPTPHIS
650	660	670	680	690	700	710	720
WSRESQALQE	DSRIHVDAQG	TLIIQGVAPE	DAGNYSCQAT	NEVGTDQETV	TLYYTDPPSV	SAVNAVVLVA	VGEEAVLVCE
730	740	750	760	770	780	790	800
ASGVPPPRVI	WYRGGLEMIL	APEGSSSGKL	RIPAAQERDA	GTYTCRAVNE	LGDASAEIQL	AVGHAPQLTE	LPRDVTVELG
810	820	830	840	850	860	870	880
RSALLACRAT	GRPPPTVTWR	RGDGQPLGLR	LGAGRGSRSR	QPDSGVLFFE	SVAPEDQAPY	VCEARNVFGK	VQAEARLIVT
890	900	910	920	930	940	950	960
GHAPPQIASS	APTVRVLEGQ	PVSLPCIVLA	GRPLPERHWL	KDGRPLPPGS	RHSIRADGSL	HLDRALQEHA	GRYSCVATNT
970	980	990	1000	1010	1020	1030	1040
AGSQHRDVEL	VVQVPPRIHP	TATHHITNEG	VAASLPCVAS	GVPAPTITWT	KETNALTSRG	PHYNVSKEGT	LLIAQPSAQD
1050	1060	1070	1080	1090	1100	1110	1120
AGAYVCTATN	TVGFSSQEMR	LSVNTKPRIH	MNGSRNADVP	LQVTAKAGEE	VTLDCEAKGS	PPPLVTWTKD	SRPVPPITNR
1130	1140	1150	1160	1170	1180	1190	1200
YGLLPSGSLR	LAQVQVGDSG	HYECTASNPA	GSASHRYVLG	VQVPPQVQPG	PRVLKVLVGE	ALDLNCVAEG	NPEPQLSWSK
1210	1220	1230	1240	1250	1260	1270	1280
DGVVLQGRGP	QGSVHFAAIR	TSDAGRYRCE	ASNSAGVDAW	EVELRVLEPP	HWGADETSGL	LERVAGENAS	LPCPARGTPK
1290	1300	1310	1320	1330	1340	1350	1360
PQVTWRKGPS	SEPLHGQPGV	AVLEEGSLFL	ASVSPADSGD	YECQATNEVG	STSRRAKLVV	YVPPSIREDG	RKANVSGMAG
1370	1380	1390	1400	1410	1420	1430	1440
QSLTLECDAN	GFPVPEIVWL	KDAQLIPKVG	GHRLLDEGQS	LHFPRIQEGD	SGLYSCRAEN	QAGTAQRDFH	LLVLTPPSVL
1450	1460	1470	1480	1490	1500	1510	1520
GAGAAQEVLG	LAGADVELQC	WTSGVPTPQV	EWTKDRQPVL	PGGPHLQVQE	DGQVLRITGS	HVGDEGRYQC	VAFSPAGQQA
1530	1540	1550	1560	1570	1580	1590	1600
RDFQLRVHAP	PTIWGSNETG	EVAVMEDHLV	QLLCEARGVP	TPNITWFKDG	ALLPTSTKVV	YTRGGRQLQL	GRAQSSDAGV
1610	1620	1630	1640	1650	1660	1670	1680
YTCKASNAVG	AAEKATRLDV	YVPPTIEGAG	GRPYVVKAVA	GRPVALECVA	RGHPSPTLSW	HHEGLPVAES	NESRLETDGS
1690	1700	1710	1720	1730	1740	1750	1760
VLRLESPGEA	SSGLYSCVAS	SPAGEAVLQY	SVEVQVPPQL	LVAEGLGQVT	TIVGQPLELP	CQASGSPVPT	IQWLQNGRPA
1770	1780	1790	1800	1810	1820	1830	1840
EELAGVQVAS	QGTTLHIDHV	ELDHSGLFAC	QATNEAGTAG	AEVEVSVHGE	WAPGVLEVPI	GQYHWGXEHH	SSFPAACDPP
1850	1860	1870	1880	1890	1900	1910	1920
VHRGWGAHPK	PPLVEGWCSP	GSLWGEPTGI	PVAYSLPPPL	QIEKVDLRDE	GIYTCAATNL	AGESKREVAL	KVLVPPNIEP
1930	1940	1950	1960	1970	1980	1990	2000
GPVNKAVLEN	ASVTLECLAS	GVPPPDVSWF	KGHQPVSSWM	GVTVSVDGRV	LRIEQAQLSD	AGSYRCVASN	VAGSTELRYG
2010	2020	2030	2040	2050	2060	2070	2080
LRVNVPPRIT	LPPSLPGPVL	VNTPVRLTCN	ATGAPSPTLM	WLKDGNPVSP	AGTPGLQVFP	GGRVLTLASA	RASDSGRYSC
2090	2100	2110	2120	2130	2140	2150	2160
VAVSAVGEDR	QDVVLQVHMP	PSILGEELNV	SVVANESVAL	ECQSHAMPPP	VLSWWKDGRP	LEPRPGVHLS	ADKALLQVDR
2170	2180	2190	2200	2210	2220	2230	2240

Detailed Protein Report

Cmpd.	No. of Cmpds.	m/z meas.	Δ m/z [ppm]	z	Rt [min]	Score	P	Range	Sequence	Modification	Ratios	
2115	1	840.8591	-53.51	2	56.6	14.6	0	161-175	K.QSQVVFVLTGDCGDR.T	Carbamidomethyl: 12	M:Q<br>Q:W	0.22<br>6.63
61	1	623.8088	95.46	5	29.5	12.6	2	866-895	R.<br>NVFGKVQAEARLIVTGHAPPQIAS<br>V			

Detailed Protein Report

Protein 226: GH3 domain-containing protein isoform 2 precursor [Homo sapiens]

Accession: gi|217330592
Database: refseq_human(refseq_human_20140103.fasta)
Database Date: 2015-11-30

Score: 57.2
MW [kDa]: 53.5
pI: 10.0
Sequence Coverage [%]: 7.9
No. of unique Peptides: 3

Quantitation

Q:W	**Median:** 3.02	**CV:** 0.00 %	**No. of Peptides:**	1
M:Q	**Median:** 0.89	**CV:** 0.00 %	**No. of Peptides:**	1

10	20	30	40	50	60	70	80
MLLWPLLLLL	LLLPTLALLR	QQRSQDARLS	WLAGLQHRVA	WGALVWAATW	QRRRLEQSTL	HVHQSQQQAL	RWCLQGAQRP
90	100	110	120	130	140	150	160
HCSLRRSTDI	STFRNHLPLT	KAYPEVLAQG	RTARVTLTSP	WPRPLPWPGN	TLGQVGTPGT	KDPRALLLDA	LRSPGLRALE
170	180	190	200	210	220	230	240
AGTAVELLDV	FLGLETDGEE	LAGAIAAGNP	GAPLRERAAE	LREALEQGPR	GLALRLWPKL	QVVVTLDAGG	QAEAVAALGA
250	260	270	280	290	300	310	320
LWCQGLAFFS	PAYAASGGVL	GLNLQPEQPH	GLYLLPPGAP	FIELLPVKEG	TQEEAASTLL	LAEAQQGKEY	ELVLTDRASL
330	340	350	360	370	380	390	400
TRCRLGDVVR	VVGAYNQCPV	VRFICRLDQT	LSVRGEDIGE	DLFSEALGRA	VGQWAGAKLL	DHGCVESSIL	DSSAGSAPHY
410	420	430	440	450	460	470	480
EVFVALRGLR	NLSEENRDKL	DHCLQEASPR	YKSLRFWGSV	GPARVHLVGQ	GAFRALRAAL	AACPSSPFPP	AMPRVLRHRH
490	500						
LAQCLQERVV	S						

Cmpd.	No. of Cmpds.	m/z meas.	Δ m/z [ppm]	z	Rt [min]	Score	P	Range	Sequence	Modification	Ratios
273	1	442.6382	-324.42	2	32.1	14.2	0	145-152	R.ALLLDALR.S		Q:W 3.02<br>M:Q 0.89
2063	1	634.7944	-18.66	2	54.2	10.9	0	420-430	K.LDHCLQEASPR.Y		
734	1	712.3429	-104.91	2	39.2	10.3	1	445-457	R.VHLVGQGAFRALR.A		

Detailed Protein Report

Protein 227: PREDICTED: kinesin-like protein KIF23 isoform X8 [Homo sapiens]

**Accession:**	gi|578827640	**Score:**	57.2
**Database:**	refseq_human(refseq_human_20140103.fasta)	**MW [kDa]:**	56.1
**Database Date:**	2015-11-30	**pI:**	9.9
**Modification(s):**	Carbamidomethyl	**Sequence Coverage [%]:**	14.0
		**No. of unique Peptides:**	3

```
        10         20         30         40         50         60         70         80
MSFPPLPSCE ILDINDEQTL PRLIEALEKR HNLRQMMIDE FNKQSNAFKA LLQEFDNAVL SKENHMQGKL NEKEKMISGQ
        90        100        110        120        130        140        150        160
KLEIERLEKK NKTLEYKIEI LEKTTTIYEE DKRNLQQELE TQNQKLQRQF SDKRRLEARL QGMVTETTMK WEKECERRVA
       170        180        190        200        210        220        230        240
AKQLEMQNKL WVKDEKLKQL KAIVTEPKTE KPERPSRERD REKVTQRSVS PSPVPLSSNY IAQISNGQQL MSQPQLHRRS
       250        260        270        280        290        300        310        320
NSCSSISVAS CISEWEQKIP TYNTPLKVTS IARRRQQEPG QSKTCIVSDR RRGMYWTEGR EVVPTFRNEI EIEEDHCGRL
       330        340        350        360        370        380        390        400
LFQPDQNAPP IRLRHRRSRS AGDRWVDHKP ASNMQTETVM QPHVPHAITV SVANEKALAK CEKYMLTHQE LASDGEIETK
       410        420        430        440        450        460        470        480
LIKGDIYKTR GGGQSVQFTD IETLKQESPN GSRKRRSSTV APAQPDGAES EWTDVETRCS VAVEMRAGSQ LGPGYQHHAQ
       490
PKRKKP
```

Cmpd.	No. of Cmpds.	m/z meas.	Δ m/z [ppm]	z	Rt [min]	Score	P	Range	Sequence	Modification
2419	1	1051.5349	69.41	2	58.6	10.1	0	240-258	R.SNSCSSISVASCISEWEQK.I	Carbamidomethyl: 12
1489	2	1023.0105	58.00	2	48.8	13.4	0	240-258	R.SNSCSSISVASCISEWEQK.I	
243	1	838.3874	-37.26	2	31.8	11.3	0	467-482	R.AGSQLGPGYQHHAQPK.R	

Detailed Protein Report

Protein 228: fatty acid-binding protein, epidermal [Homo sapiens]

Accession: gi|4557581
Database: refseq_human(refseq_human_20140103.fasta)
Database Date: 2015-11-30
Modification(s): Carbamidomethyl

Score: 57.2
MW [kDa]: 15.2
pI: 7.5
Sequence Coverage [%]: 10.4
No. of unique Peptides: 1

Quantitation

M:Q **Median:** 1.10 **CV:** 0.00 % **No. of Peptides:** 1

10	20	30	40	50	60	70	80
MATVQQLEGR	WRLVDSKGFD	EYMKELGVGI	ALRKMGAMAK	PDCIITCDGK	NLTIKTESTL	KTTQFSCTLG	EKFEETTADG
**90**	**100**	**110**	**120**	**130**	**140**		
RKTQTVCNFT	DGALVQHQEW	DGKESTITRK	LKDGKLVVEC	VMNNVTCTRI	YEKVE		

Cmpd.	No. of Cmpds.	m/z meas.	Δ m/z [ppm]	z	Rt [min]	Score	P	Range	Sequence	Modification	Ratios	
1139	2	847.8676	-43.89	2	44.4	57.2	0	116-129	K.LVVECVMNNVTCTR.I	Carbamidomethyl: 5, 12	M:Q	1.10

Detailed Protein Report

Protein 229: **voltage-dependent P/Q-type calcium channel subunit alpha-1A isoform 3 [Homo sapiens]**

**Accession:**	gi|187828880	**Score:**	57.1
**Database:**	refseq_human(refseq_human_20140103.fasta)	**MW [kDa]:**	256.6
**Database Date:**	2015-11-30	**pI:**	9.3
		**Sequence Coverage [%]:**	2.6
		**No. of unique Peptides:**	1

Detailed Protein Report

10	20	30	40	50	60	70	80
MARFGDEMPA	RYGGGGSGAA	AGVVVGSGGG	RGAGGSRQGG	QPGAQRMYKQ	SMAQRARTMA	LYNPIPVRQN	CLTVNRSLFL
90	100	110	120	130	140	150	160
FSEDNVVRKY	AKKITEWPPF	EYMILATIIA	NCIVLALEQH	LPDDDKTPMS	ERLDDTEPYF	IGIFCFEAGI	KIIALGFAFH
170	180	190	200	210	220	230	240
KGSYLRNGWN	VMDFVVVLTG	ILATVGTEFD	LRTLRAVRVL	RPLKLVSGIP	SLQVVLKSIM	KAMIPLLQIG	LLLFFAILIF
250	260	270	280	290	300	310	320
AIIGLEFYMG	KFHTTCFEEG	TDDIQGESPA	PCGTEEPART	CPNGTKCQPY	WEGPNNGITQ	FDNILFAVLT	VFQCITMEGW
330	340	350	360	370	380	390	400
TDLLYNSNDA	SGNTWNWLYF	IPLIIIGSFF	MLNLVLGVLS	GEFAKERERV	ENRRAFLKLR	RQQQIERELN	GYMEWISKAE
410	420	430	440	450	460	470	480
EVILAEDETD	GEQRHPFDGA	LRRTTIKKSK	TDLLNPEEAE	DQLADIASVG	SPFARASIKS	AKLENSTFFH	KKERRMRFYI
490	500	510	520	530	540	550	560
RRMVKTQAFY	WTVLSLVALN	TLCVAIVHYN	QPEWLSDFLY	YAEFIFLGLF	MSEMFIKMYG	LGTRPYFHSS	FNCFDCGVII
570	580	590	600	610	620	630	640
GSIFEVIWAV	IKPGTSFGIS	VLRALRLLRI	FKVTKYWASL	RNLVVSLLNS	MKSIISLLFL	LFLFIVVFAL	LGMQLFGGQF
650	660	670	680	690	700	710	720
NFDEGTPPTN	FDTFPAAIMT	VFQILTGEDW	NEVMYDGIKS	QGGVQGGMVF	SIYFIVLTLF	GNYTLLNVFL	AIAVDNLANA
730	740	750	760	770	780	790	800
QELTKDEQEE	EEAANQKLAL	QKAKEVAEVS	PLSAANMSIA	VKEQQKNQKP	AKSVWEQRTS	EMRKQNLLAS	REALYNEMDP
810	820	830	840	850	860	870	880
DERWKAAYTR	HLRPDMKTHL	DRPLVVDPQE	NRNNNTNKSR	AAEPTVDQRL	GQQRAEDFLR	KQARYHDRAR	DPSGSAGLDA
890	900	910	920	930	940	950	960
RRPWAGSQEA	ELSREGPYGR	ESDHHAREGS	LEQPGFWEGE	AERGKAGDPH	RRHVHRQGGS	RESRSGSPRT	GADGEHRRHR
970	980	990	1000	1010	1020	1030	1040
AHRRPGEEGP	EDKAERRARH	REGSRPARGG	EGEGEGPDGG	ERRRRHRHGA	PATYEGDARR	EDKERRHRRR	KENQGSGVPV
1050	1060	1070	1080	1090	1100	1110	1120
SGPNLSTTRP	IQQDLGRQDP	PLAEDIDNMK	NNKLATAESA	APHGSLGHAG	LPQSPAKMGN	STDPGPMLAI	PAMATNPQNA
1130	1140	1150	1160	1170	1180	1190	1200
ASRRTPNNPG	NPSNPGPPKT	PENSLIVTNP	SGTQTNSAKT	ARKPDHTTVD	IPPACPPPLN	HTVVQVNKNA	NPDPLPKKEE
1210	1220	1230	1240	1250	1260	1270	1280
EKKEEEEDDR	GEDGPKPMPP	YSSMFILSTT	NPLRRLCHYI	LNLRYFEMCI	LMVIAMSSIA	LAAEDPVQPN	APRNNVLRYF
1290	1300	1310	1320	1330	1340	1350	1360
DYVFTGVFTF	EMVIKMIDLG	LVLHQGAYFR	DLWNILDFIV	VSGALVAFAF	TGNSKGKDIN	TIKSLRVLRV	LRPLKTIKRL
1370	1380	1390	1400	1410	1420	1430	1440
PKLKAVFDCV	VNSLKNVFNI	LIVYMLFMFI	FAVVAVQLFK	GKFFHCTDES	KEFEKDCRGK	YLLYEKNEVK	ARDREWKKYE
1450	1460	1470	1480	1490	1500	1510	1520
FHYDNVLWAL	LTLFTVSTGE	GWPQVLKHSV	DATFENQGPS	PGYRMEMSIF	YVVYFVVFPF	FFVNIFVALI	IITFQEQGDK
1530	1540	1550	1560	1570	1580	1590	1600
MMEEYSLEKN	ERACIDFAIS	AKPLTRHMPQ	NKQSFQYRMW	QFVVSPPFEY	TIMAMIALNT	IVLMMKFYGA	SVAYENALRV
1610	1620	1630	1640	1650	1660	1670	1680
FNIVFTSLFS	LECVLKVMAF	GILNYFRDAW	NIFDFVTVLG	SITDILVTEF	GNNFINLSFL	RLFRAARLIK	LLRQGYTIRI
1690	1700	1710	1720	1730	1740	1750	1760
LLWTFVQSFK	ALPYVCLLIA	MLFFIYAIIG	MQVFGNIGID	VEDEDSDEDE	FQITEHNNFR	TFFQALMLLF	RSATGEAWHN
1770	1780	1790	1800	1810	1820	1830	1840
IMLSCLSGKP	CDKNSGILTR	ECGNEFAYFY	FVSFIFLCSF	LMLNLFVAVI	MDNFEYLTRD	SSILGPHHLD	EYVRVWAEYD
1850	1860	1870	1880	1890	1900	1910	1920
PAACGRIHYK	DMYSLLRVIS	PPLGLGKKCP	HRVACKRLLR	MDLPVADDNT	VHFNSTLMAL	IRTALDIKIA	KGGADKQQMD
1930	1940	1950	1960	1970	1980	1990	2000
AELRKEMMAI	WPNLSQKTLD	LLVTPHKSTD	LTVGKIYAAM	MIMEYYRQSK	AKKLQAMREE	QDRTPLMFQR	MEPPSPTQEG
2010	2020	2030	2040	2050	2060	2070	2080
GPGQNALPST	QLDPGGALMA	HESGLKESPS	WVTQRAQEMF	QKTGTWSPEQ	GPPTDMPNSQ	PNSQSVEMRE	MGRDGYSDSE
2090	2100	2110	2120	2130	2140	2150	2160
HYLPMEGQGR	AASMPRLPAE	NQRRRGRPRG	NNLSTISDTS	PMKRSASVLG	PKARRLDDYS	LERVPPEENQ	RHHQRRRDRS
2170	2180	2190	2200	2210	2220	2230	2240

Detailed Protein Report

Cmpd.	No. of Cmpds.	m/z meas.	Δ m/z [ppm]	z	Rt [min]	Score	P	Range	Sequence	Modification
2837	1	913.4693	21.67	1	64.1	15.5	1	1869-1876	K.CPHRVACK.R	

Detailed Protein Report

Protein 230: **PREDICTED: lipopolysaccharide-responsive and beige-like anchor protein isoform X3 [Homo sapiens]**

**Accession:**	gi|530378475	**Score:**	56.9
**Database:**	refseq_human(refseq_human_20140103.fasta)	**MW [kDa]:**	318.2
**Database Date:**	2015-11-30	**pI:**	5.3
		**Sequence Coverage [%]:**	2.5
		**No. of unique Peptides:**	4

Quantitation

Q:W	**Median:**	2.36	**CV:**	0.00 %	**No. of Peptides:**	1
M:Q	**Median:**	1.38	**CV:**	0.00 %	**No. of Peptides:**	1

Detailed Protein Report

10	20	30	40	50	60	70	80
MASEDNRVPS	PPPTGDDGGG	GGREETPTEG	GALSLKPGLP	IRGIRMKFAV	LTGLVEVGEV	SNRDIVETVF	NLLVGGQFDL
90	100	110	120	130	140	150	160
EMNFIIQEGE	SINCMVDLLE	KCDITCQAEV	WSMFTAILKK	SIRNLQVCTE	VGLVEKVLGK	IEKVDNMIAD	LLVDMLGVLA
170	180	190	200	210	220	230	240
SYNLTVRELK	LFFSKLQGDK	GRWPPHAGKL	LSVLKHMPQK	YGPDAFFNFP	GKSAAAIALP	PIAKWPYQNG	FTFHTWLRMD
250	260	270	280	290	300	310	320
PVNNINVDKD	KPYLYCFRTS	KGLGYSAHFV	GGCLIVTSIK	SKGKGFQHCV	KFDFKPQKWY	MVTIVHIYNR	WKNSELRCYV
330	340	350	360	370	380	390	400
NGELASYGEI	TWFVNTSDTF	DKCFLGSSET	ADANRVFCGQ	MTAVYLFSEA	LNAAQIFAIY	QLGLGYKGTF	KFKAESDLFL
410	420	430	440	450	460	470	480
AEHHKLLLYD	GKLSSAIAFT	YNPRATDAQL	CLESSPKDNP	SIFVHSPHAL	MLQDVKAVLT	HSIQSAMHSI	GGVQVLFPLF
490	500	510	520	530	540	550	560
AQLDYRQYLS	DEIDLTICST	LLAFIMELLK	NSIAMQEQML	ACKGFLVIGY	SLEKSSKSHV	SRAVLELCLA	FSKYLSNLQN
570	580	590	600	610	620	630	640
GMPLLKQLCD	HVLLNPAIWI	HTPAKVQLML	YTYLSTEFIG	TVNIYNTIRR	VGTVLLIMHT	LKYYYWAVNP	QDRSGITPKG
650	660	670	680	690	700	710	720
LDGPRPNQKE	MLSLRAFLLM	FIKQLVMKDS	GVKEDELQAI	LNYLLTMHED	DNLMDVLQLL	VALMSEHPNS	MIPAFDQRNG
730	740	750	760	770	780	790	800
LRVIYKLLAS	KSEGIRVQAL	KAMGYFLKHL	APKRKAEVML	GHGLFSLLAE	RLMLQTNLIT	MTTYNVLFEI	LIEQIGTQVI
810	820	830	840	850	860	870	880
HKQHPDPDSS	VKIQNPQILK	VIATLLRNSP	QCPESMEVRR	AFLSDMIKLF	NNSRENRRSL	LQCSVWQEWM	LSLCYFNPKN
890	900	910	920	930	940	950	960
SDEQKITEMV	YAIFRILLYH	AVKYEWGGWR	VWVDTLSITH	SKVTFEIHKE	NLANIFREQQ	GKVDEEIGLC	SSTSVQAASG
970	980	990	1000	1010	1020	1030	1040
IRRDINVSVG	SQQPDTKDSP	VCPHFTTNGN	ENSSIEKTSS	LESASNIELQ	TTNTSYEEMK	AEQENQELPD	EGTLEETLTN
1050	1060	1070	1080	1090	1100	1110	1120
ETRNADDLEV	SSDIIEAVAI	SSNSFITTGK	DSMTVSEVTA	SISSPSEEDA	SEMPEFLDKS	IVEEEEDDDY	VELKVEGSPT
1130	1140	1150	1160	1170	1180	1190	1200
EEANLPTELQ	DNSLSPAASE	AGEKLDMFGN	DDKLIFQEGK	PVTEKQTDTE	TQDSKDSGIQ	TMTASGSSAM	SPETTVSQIA
1210	1220	1230	1240	1250	1260	1270	1280
VESDLGQMLE	EGKKATNLTR	ETKLINDCHG	SVSEASSEQK	IAKLDVSNVA	TDTERLELKA	SPNVEAPQPH	RHVLEISRQH
1290	1300	1310	1320	1330	1340	1350	1360
EQPGQGIAPD	AVNGQRRDSR	STVFRIPEFN	WSQMHQRLLT	DLLFSIETDI	QMWRSHSTKT	VMDFVNSSDN	VIFVHNTIHL
1370	1380	1390	1400	1410	1420	1430	1440
ISQVMDNMVM	ACGGILPLLS	AATSATHELE	NIEPTQGLSI	EASVTFLQRL	ISLVDVLIFA	SSLGFTEIEA	EKSMSSGGIL
1450	1460	1470	1480	1490	1500	1510	1520
RQCLRLVCAV	AVRNCLECQQ	HSQLKTRGDK	ALKPMHSLIP	LGKSAAKSPV	DIVTGGISPV	RDLDRLLQDM	DINRLRAVVF
1530	1540	1550	1560	1570	1580	1590	1600
RDIEDSKQAQ	FLALAVVYFI	SVLMVSKYRD	ILEPQNERHS	QSCTETGSEN	ENVSLSEITP	AAFSTLTTAS	VEESESTSSA
1610	1620	1630	1640	1650	1660	1670	1680
RRRDSGIGEE	TATGLGSHVE	VTPHTAPPGV	SAGPDAISEV	LSTLSLEVNK	SPETKNDRGN	DLDTKATPSV	SVSKNVNVKD
1690	1700	1710	1720	1730	1740	1750	1760
ILRSLVNIPA	DGVTVDPALL	PPACLGALGD	LSVEQPVQFR	SFDRSVIVAA	KKSAVSPSTF	NTSIPTNAVS	VVSSVDSAQA
1770	1780	1790	1800	1810	1820	1830	1840
SDMGGESPGS	RSSNAKLPSV	PTVDSVSQDP	VSNMSITERL	EHALEKAAPL	LREIFVDFAP	FLSRTLLGSH	GQELLIEGTS
1850	1860	1870	1880	1890	1900	1910	1920
LVCMKSSSSV	VELVMLLCSQ	EWQNSIQKNA	GLAFIELVNE	GRLLSQTMKD	HLVRVANEAE	FILSRQRAED	IHRHAEFESL
1930	1940	1950	1960	1970	1980	1990	2000
CAQYSADKRE	DEKMCDHLIR	AAKYRDHVTA	TQLIQKIINI	LTDKHGAWGN	SAVSRPLEFW	RLDYWEDDLR	RRRRFVRNPL
2010	2020	2030	2040	2050	2060	2070	2080
GSTHPEATLK	TAVEHATDED	ILAKGKQSIR	SQALGNQNSE	NEILLEGDDD	TLSSVDEKDL	ENLAGPVSLS	TPAQLVAPSV
2090	2100	2110	2120	2130	2140	2150	2160
VVKGTLSVTS	SELYFEVDEE	DPNFKKIDPK	ILAYTEGLHG	KWLFTEIRSI	FSRRYLLQNT	ALEIFMANRV	AVMFNFPDPA
2170	2180	2190	2200	2210	2220	2230	2240

Detailed Protein Report

Cmpd.	No. of Cmpds.	m/z meas.	Δ m/z [ppm]	z	Rt [min]	Score	P	Range	Sequence	Modification	Ratios
1142	1	555.2290	-64.32	2	44.4	10.2	0	803-812	K.QHPDPDSSVK.I		
1230	1	715.1053	93.99	3	43.7	12.4	1	821-839	K.VIATLLRNSPQCPESMEVR.R		
1642	1	698.8555	-54.14	2	50.8	10.1	0	1488-1501	K.SPVDIVTGGISPVR.D		
2042	1	766.2799	-83.49	2	55.4	11.9	0	2669-2684	K.CSGIGDNPGSETAAPR.A		M:Q 1.38<br>Q:W 2.36

Detailed Protein Report

Protein 231: filamin-A isoform 1 [Homo sapiens]

**Accession:**	gi|116063573	**Score:**	56.6
**Database:**	refseq_human(refseq_human_20140103.fasta)	**MW [kDa]:**	279.8
**Database Date:**	2015-11-30	**pI:**	5.6
		**Sequence Coverage [%]:**	2.4
		**No. of unique Peptides:**	2

Detailed Protein Report

10	20	30	40	50	60	70	80
MSSSHSRAGQ	SAAGAAPGGG	VDTRDAEMPA	TEKDLAEDAP	WKKIQQNTFT	RWCNEHLKCV	SKRIANLQTD	LSDGLRLIAL
90	100	110	120	130	140	150	160
LEVLSQKKMH	RKHNQRPTFR	QMQLENVSVA	LEFLDRESIK	LVSIDSKAIV	DGNLKLILGL	IWTLILHYSI	SMPMWDEEED
170	180	190	200	210	220	230	240
EEAKKQTPKQ	RLLGWIQNKL	PQLPITNFSR	DWQSGRALGA	LVDSCAPGLC	PDWDSWDASK	PVTNAREAMQ	QADDWLGIPQ
250	260	270	280	290	300	310	320
VITPEEIVDP	NVDEHSVMTY	LSQFPKAKLK	PGAPLRPKLN	PKKARAYGPG	IEPTGNMVKK	RAEFTVETRS	AGQGEVLVYV
330	340	350	360	370	380	390	400
EDPAGHQEEA	KVTANNDKNR	TFSVWYVPEV	TGTHKVTVLF	AGQHIAKSPF	EVYVDKSQGD	ASKVTAQGPG	LEPSGNIANK
410	420	430	440	450	460	470	480
TTYFEIFTAG	AGTGEVEVVI	QDPMGQKGTV	EPQLEARGDS	TYRCSYQPTM	EGVHTVHVTF	AGVPIPRSPY	TVTVGQACNP
490	500	510	520	530	540	550	560
SACRAVGRGL	QPKGVRVKET	ADFKVYTKGA	GSGELKVTVK	GPKGEERVKQ	KDLGDGVYGF	EYYPMVPGTY	IVTITWGGQN
570	580	590	600	610	620	630	640
IGRSPFEVKV	GTECGNQKVR	AWGPGLEGGV	VGKSADFVVE	AIGDDVGTLG	FSVEGPSQAK	IECDDKGDGS	CDVRYWPQEA
650	660	670	680	690	700	710	720
GEYAVHVLCN	SEDIRLSPFM	ADIRDAPQDF	HPDRVKARGP	GLEKTGVAVN	KPAEFTVDAK	HGGKAPLRVQ	VQDNEGCPVE
730	740	750	760	770	780	790	800
ALVKDNGNGT	YSCSYVPRKP	VKHTAMVSWG	GVSIPNSPFR	VNVGAGSHPN	KVKVYGPGVA	KTGLKAHEPT	YFTVDCAEAG
810	820	830	840	850	860	870	880
QGDVSIGIKC	APGVVGPAEA	DIDFDIIRND	NDTFTVKYTP	RGAGSYTIMV	LFADQATPTS	PIRVKVEPSH	DASKVKAEGP
890	900	910	920	930	940	950	960
GLSRTGVELG	KPTHFTVNAK	AAGKGKLDVQ	FSGLTKGDAV	RDVDIIDHHD	NTYTVKYTPV	QQGPVGVNVT	YGGDPIPKSP
970	980	990	1000	1010	1020	1030	1040
FSVAVSPSLD	LSKIKVSGLG	EKVDVGKDQE	FTVKSKGAGG	QGKVASKIVG	PSGAAVPCKV	EPGLGADNSV	VRFLPREEGP
1050	1060	1070	1080	1090	1100	1110	1120
YEVEVTYDGV	PVPGSPFPLE	AVAPTKPSKV	KAFGPGLQGG	SAGSPARFTI	DTKGAGTGGL	GLTVEGPCEA	QLECLDNGDG
1130	1140	1150	1160	1170	1180	1190	1200
TCSVSYVPTE	PGDYNINILF	ADTHIPGSPF	KAHVVPCFDA	SKVKCSGPGL	ERATAGEVGQ	FQVDCSSAGS	AELTIEICSE
1210	1220	1230	1240	1250	1260	1270	1280
AGLPAEVYIQ	DHGDGTHTIT	YIPLCPGAYT	VTIKYGGQPV	PNFPSKLQVE	PAVDTSGVQC	YGPGIEGQGV	FREATTEFSV
1290	1300	1310	1320	1330	1340	1350	1360
DARALTQTGG	PHVKARVANP	SGNLTETYVQ	DRGDGMYKVE	YTPYEEGLHS	VDVTYDGSPV	PSSPFQVPVT	EGCDPSRVRV
1370	1380	1390	1400	1410	1420	1430	1440
HGPGIQSGTT	NKPNKFTVET	RGAGTGGLGL	AVEGPSEAKM	SCMDNKDGSC	SVEYIPYEAG	TYSLNVTYGG	HQVPGSPFKV
1450	1460	1470	1480	1490	1500	1510	1520
PVHDVTDASK	VKCSGPGLSP	GMVRANLPQS	FQVDTSKAGV	APLQVKVQGP	KGLVEPVDVV	DNADGTQTVN	YVPSREGPYS
1530	1540	1550	1560	1570	1580	1590	1600
ISVLYGDEEV	PRSPFKVKVL	PTHDASKVKA	SGPGLNTTGV	PASLPVEFTI	DAKDAGEGLL	AVQITDPEGK	PKKTHIQDNH
1610	1620	1630	1640	1650	1660	1670	1680
DGTYTVAYVP	DVTGRYTILI	KYGGDEIPFS	PYRVRAVPTG	DASKCTVTGA	GIGPTIQIGE	ETVITVDTKA	AGKGKVTCTV
1690	1700	1710	1720	1730	1740	1750	1760
CTPDGSEVDV	DVVENEDGTF	DIFYTAPQPG	KYVICVRFGG	EHVPNSPFQV	TALAGDQPSV	QPPLRSQQLA	PQYTYAQGGQ
1770	1780	1790	1800	1810	1820	1830	1840
QTWAPERPLV	GVNGLDVTSL	RPFDLVIPFT	IKKGEITGEV	RMPSGKVAQP	TITDNKDGTV	TVRYAPSEAG	LHEMDIRYDN
1850	1860	1870	1880	1890	1900	1910	1920
MHIPGSPLQF	YVDYVNCGHV	TAYGPGLTHG	VVNKPATFTV	NTKDAGEGGL	SLAIEGPSKA	EISCTDNQDG	TCSVSYLPVL
1930	1940	1950	1960	1970	1980	1990	2000
PGDYSILVKY	NEQHVPGSPF	TARVTGDDSM	RMSHLKVGSA	ADIPINISET	DLSLLTATVV	PPSGREEPCL	LKRLRNGHVG
2010	2020	2030	2040	2050	2060	2070	2080
ISFVPKETGE	HLVHVKKNGQ	HVASSPIPVV	ISQSEIGDAS	RVRVSGQGLH	EGHTFEPAEF	IIDTRDAGYG	GLSLSIEGPS
2090	2100	2110	2120	2130	2140	2150	2160
KVDINTEDLE	DGTCRVTYCP	TEPGNYIINI	KFADQHVPGS	PFSVKVTGEG	RVKESITRRR	RAPSVANVGS	HCDLSLKIPE
2170	2180	2190	2200	2210	2220	2230	2240

Detailed Protein Report

Cmpd.	No. of Cmpds.	m/z meas.	Δ m/z [ppm]	z	Rt [min]	Score	P	Range	Sequence	Modification
1673	1	694.3984	51.21	2	51.2	15.2	1	1451-1464	K.VKCSGPGLSPGMVR.A	
418	1	686.6239	-116.77	3	33.8	10.2	1	2339-2359	K.VNQPASFAVSLNGAKGAIDAK.V	

Detailed Protein Report

Protein 232: **PREDICTED: uncharacterized aarF domain-containing protein kinase 1 isoform X2 [Homo sapiens]**

**Accession:**	gi|530404248	**Score:**	56.5
**Database:**	refseq_human(refseq_human_20140103.fasta)	**MW [kDa]:**	52.0
**Database Date:**	2015-11-30	**pI:**	9.7
**Modification(s):**	Carbamidomethyl	**Sequence Coverage [%]:**	10.1
		**No. of unique Peptides:**	2

Quantitation

Q:W	**Median:**	1.73	**CV:**	65.42 %	**No. of Peptides:**	2
M:Q	**Median:**	0.77	**CV:**	0.00 %	**No. of Peptides:**	1

10	20	30	40	50	60	70	80
MARKALKLAS	WTSMALAASG	IYFYSNKYLD	PNDFGAVRVG	RAVATTAVIS	YDYLTSLKSV	PYGSEEYLQL	RSKVHLRSAR
**90**	**100**	**110**	**120**	**130**	**140**	**150**	**160**
RLCELCCANR	GTFIKVGQHL	GALDYLLPEE	YTSTLKVLHS	QAPQSSMQEI	RQVIREDLGK	EIHDLFQSFD	DTPLGTASLA
**170**	**180**	**190**	**200**	**210**	**220**	**230**	**240**
QVHKAVLHDG	RTVAVKVQHP	KVRAQSSKDI	LLMEVLVLAV	KQLFPEFEFM	WLVDEAKKNL	PLELDFLNEG	RNAEKVSQML
**250**	**260**	**270**	**280**	**290**	**300**	**310**	**320**
RHFDFLKVPR	IHWDLSTERV	LLMEFVDGGQ	VNDRDYMERN	KIDVNEISRH	LGKMYSEMIF	VNGFVHCDPH	PGNVLVRKHP
**330**	**340**	**350**	**360**	**370**	**380**	**390**	**400**
GTGKAEIVLL	DHGLYQDLEI	RNNAANYLPQ	ISHLLNHVPR	QMLLILKTND	LLRGIEAALG	TRASASSFLN	MSRCCIRALA
**410**	**420**	**430**	**440**	**450**	**460**		
EHKKKNTCSF	FRRTQISFSE	AFNLWQINLH	ELILRVKGLK	LADRVLALIC	WLFPAPL		

Cmpd.	No. of Cmpds.	m/z meas.	Δ m/z [ppm]	z	Rt [min]	Score	P	Range	Sequence	Modification	Ratios	
2197	1	863.8529	-90.52	2	56.0	13.6	2	81-95	R.RLCELCCANRGTFIK.V		Q:W	3.14
5	1	1205.0356	-39.18	2	29.0	11.7	2	383-403	R.ASASSFLNMSRCCIRALAEHK.K	Carbamidomethyl: 12, 13	Q:W<br>M:Q	0.95<br>0.77

Detailed Protein Report

Protein 233: PREDICTED: zinc finger protein 675 isoform X4 [Homo sapiens]

Accession: gi|530414624
Database: refseq_human(refseq_human_20140103.fasta)
Database Date: 2015-11-30
Modification(s): Carbamidomethyl

Score: 56.5
MW [kDa]: 57.4
pI: 10.2
Sequence Coverage [%]: 5.7
No. of unique Peptides: 3

Alias proteins:

Accession	Name	Description
gi|578833063	refseq_human (refseq_human_20140103.fasta)	PREDICTED: zinc finger protein 675 isoform X6 [Homo sapiens]

10	20	30	40	50	60	70	80
MCSHFAQEFW	PEQNIKDSFE	KVTLRRYEKC	GNDNFQLKGC	KSVDECKLHK	GGYNGLNQCL	PTMQSKMFQC	DKYVKVFNKF
90	100	110	120	130	140	150	160
SHSDRHKIKH	MENKPFKCKE	CGRSFCMLSH	LTRHERNYTK	VNFCKCEECE	KAVNQSSKLT	KHKRIYTCEK	LYKCQECDRT
170	180	190	200	210	220	230	240
FNQFSNLTEY	KKDYAREKPY	KCEECGKAFN	QSSHLTTHKI	IHTGEKPYKC	EECGKAFNQF	SNLTTHKKIH	TGEQPYICEE
250	260	270	280	290	300	310	320
CGKAFTQSST	LTTHKRIHTG	EKPYKCEECG	KAFNRSSKLT	EHKNIHTGEQ	PYKCEECGKA	FNRSSNLTEH	RKIHTEEKPY
330	340	350	360	370	380	390	400
KCKECGKAFK	HSSALTTHKR	IHTGEKPYKC	EECGKAFNRS	SKLTEHKKLH	TGKKPYKCEE	CGKAFIQSSK	LTEHKKIHSG
410	420	430	440	450	460	470	480
EIPYKCEECG	KAFKHSSSLT	THKRIHTGEK	PYKCEECGKA	FSRSSKLTEH	KIIHTGEKPY	KCERCDKAFN	QSANLTKHKK
490	500						
IHTGEKLQNW	NV						

Cmpd.	No. of Cmpds.	m/z meas.	Δ m/z [ppm]	z	Rt [min]	Score	P	Range	Sequence	Modification
1613	2	682.3429	61.34	3	50.4	11.7	2	30-47	K.CGNDNFQLKGCKSVDECK.L	Carbamidomethyl: 17
2097	2	701.2282	-113.98	3	56.4	14.4	2	30-47	K.CGNDNFQLKGCKSVDECK.L	Carbamidomethyl: 1, 17
2266	1	682.3051	5.92	3	58.8	17.1	2	30-47	K.CGNDNFQLKGCKSVDECK.L	Carbamidomethyl: 1

Detailed Protein Report

Protein 234: matrix metalloproteinase-27 precursor [Homo sapiens]

Accession: gi|73808268
Database: refseq_human(refseq_human_20140103.fasta)
Database Date: 2015-11-30
Modification(s): Carbamidomethyl, Oxidation

Score: 56.3
MW [kDa]: 59.0
pI: 9.4
Sequence Coverage [%]: 9.7
No. of unique Peptides: 4

Quantitation

M:Q **Median:** 0.62 **CV:** 0.00 % **No. of Peptides:** 1

10	20	30	40	50	60	70	80
MKRLLLLFLF	FITFSSAFPL	VRMTENEENM	QLAQAYLNQF	YSLEIEGNHL	VQSKNRSLID	DKIREMQAFF	GLTVTGKLDS
**90**	**100**	**110**	**120**	**130**	**140**	**150**	**160**
NTLEIMKTPR	CGVPDVGQYG	YTLPGWRKYN	LTYRIINYTP	DMARAAVDEA	IQEGLEVWSK	VTPLKFTKIS	KGIADIMIAF
**170**	**180**	**190**	**200**	**210**	**220**	**230**	**240**
RTRVHGRCPR	YFDGPLGVLG	HAFPPGPGLG	GDTHFDEDEN	WTKDGAGFNL	FLVAAHEFGH	ALGLSHSNDQ	TALMFPNYVS
**250**	**260**	**270**	**280**	**290**	**300**	**310**	**320**
LDPRKYPLSQ	DDINGIQSIY	GGLPKEPAKP	KEPTIPHACD	PDLTFDAITT	FRREVMFFKG	RHLWRIYYDI	TDVEFELIAS
**330**	**340**	**350**	**360**	**370**	**380**	**390**	**400**
FWPSLPADLQ	AAYENPRDKI	LVFKDENFWM	IRGYAVLPDY	PKSIHTLGFP	GRVKKIDAAV	CDKTTRKTYF	FVGIWCWRFD
**410**	**420**	**430**	**440**	**450**	**460**	**470**	**480**
EMTQTMDKGF	PQRVVKHFPG	ISIRVDAAFQ	YKGFFFFSRG	SKQFEYDIKT	KNITRIMRTN	TWFQCKEPKN	SSFGFDINKE
**490**	**500**	**510**	**520**				
KAHSGGIKIL	YHKSLSLFIF	GIVHLLKNTS	IYQ				

Cmpd.	No. of Cmpds.	m/z meas.	Δ m/z [ppm]	z	Rt [min]	Score	P	Range	Sequence	Modification	Ratios	
2475	1	590.1610	-235.29	2	59.2	12.6	0	78-87	K.LDSNTLEIMK.T	Oxidation: 9	M:Q	0.62
2670	1	625.3045	-14.98	2	61.7	12.8	1	376-386	K.IDAAVCDKTTR.K	Carbamidomethyl: 6		
2792	4	626.8670	-20.54	2	63.5	14.4	1	414-424	R.VVKHFPGISIR.V			
2773	1	1051.5696	39.22	2	66.0	16.6	2	425-442	R.VDAAFQYKGFFFFSRGSK.Q			

Detailed Protein Report

Protein 235: PREDICTED: zinc finger protein 554 isoform X2 [Homo sapiens]

**Accession:**	gi|578832959	**Score:**	56.3
**Database:**	refseq_human(refseq_human_20140103.fasta)	**MW [kDa]:**	43.7
**Database Date:**	2015-11-30	**pI:**	9.8
**Modification(s):**	Carbamidomethyl	**Sequence Coverage [%]:**	13.4
		**No. of unique Peptides:**	3

10	20	30	40	50	60	70	80
MTVLRNQDST	YKKVALQEEP	ASGINMIKLI	REDGGWKQLE	DSHEDPQGLL	SQKASLHVVA	VPQEKATAWH	GFGENGNLSP
**90**	**100**	**110**	**120**	**130**	**140**	**150**	**160**
ALVLSQGSSK	GNHLCGSELD	ITSLASDSVL	NHHQLGYADR	RPCESNECGN	AIRQNSHFIQ	HGGKMFVYLE	NGQSLNHGMA
**170**	**180**	**190**	**200**	**210**	**220**	**230**	**240**
LTIHNKINTA	EKPFECHQCG	KVFNRRHSLS	EHQRIHTGEK	PYECQECGRA	FTHSSTLTRH	LRTHTGEKPY	GCGECGKAFN
**250**	**260**	**270**	**280**	**290**	**300**	**310**	**320**
RISSLTQHQR	IHTGEKPYKC	EDCGKSFCQS	SYLILHKRTH	TGEKPYECSE	CGKAFSDRSS	LNQHERTHTG	ENPYECKQCG
**330**	**340**	**350**	**360**	**370**	**380**	**390**	
RAFSQRSSLV	RHERTHTGEK	PYRCQECGKA	FSQSSSLVTH	QKTHSSQKTY	KIIDCGKAFY	QNRHLIGY	

Cmpd.	No. of Cmpds.	m/z meas.	Δ m/z [ppm]	z	Rt [min]	Score	P	Range	Sequence	Modification
1743	1	560.7234	-120.44	2	52.1	14.2	0	210-219	R.AFTHSSTLTR.H	
2623	1	812.4211	89.86	2	63.6	11.3	0	223-237	R.THTGEKPYGCGECGK.A	Carbamidomethyl: 13
2421	1	863.4315	78.18	2	58.6	10.2	0	279-293	R.THTGEKPYECSECGK.A	Carbamidomethyl: 13

Detailed Protein Report

Protein 236: collagen alpha-1(XXVIII) chain precursor [Homo sapiens]

Accession: gi|154759255
Database: refseq_human(refseq_human_20140103.fasta)
Database Date: 2015-11-30
Modification(s): Oxidation

Score: 56.2
MW [kDa]: 116.6
pI: 6.1
Sequence Coverage [%]: 6.6
No. of unique Peptides: 2

Quantitation

Q:W **Median:** 0.18 **CV:** 0.00 % **No. of Peptides:** 1

10	20	30	40	50	60	70	80
MWNRYFVFYL	LLLSAFTSQT	VSGQRKKGPK	SNLLARKSDV	QGSICFIDIV	FIVDSSESSK	IALFDKQKDF	VDSLSDKIFQ
90	100	110	120	130	140	150	160
LTPGRSLEYD	IKLAALQFSS	SVQIDPPFSS	WKDLQTFKQK	VKSMNLIGQG	TFSYYAISNA	TRLLKREGRK	DGVKVVLLMT
170	180	190	200	210	220	230	240
DGIDHPKNPD	VQSISEDARI	SGISFITIAL	STVVNEAKLR	LISGDSSSEP	TLLLSDPTLV	DKIQDRLDIL	FEKKCERKIC
250	260	270	280	290	300	310	320
ECEKGDPGDP	GPPGTHGNPG	IKGERGPKGN	PGNAQKGEAG	ERGPGGIPGY	KGDKGERGEC	GKPGIKGDKG	SPGPYGPKGP
330	340	350	360	370	380	390	400
RGIQGITGPP	GDPGPKGFQG	NKGEPGPPGP	YGSPGAPGIG	QQGIKGERGQ	EGRPGAPGPI	GVGEPGQPGP	RGPEGVPGER
410	420	430	440	450	460	470	480
GLPGEGFPGP	KGEKGSEGPT	GPQGLQGLSI	KGEKGDIGPV	GPQGPMGIPG	IGSQGEQGIQ	GPIGPPGPQG	PAGQGLPGSK
490	500	510	520	530	540	550	560
GEVGQMGPTG	PRGPVGIGVQ	GPKGEPGSIG	LPGQPGVPGE	DGAAGKKGEA	GLPGARGPEG	PPGKGQPGPK	GDEGKKGSKG
570	580	590	600	610	620	630	640
NQGQRGLPGP	EGPKGEPGIM	GPFGMPGTSI	PGPPGPKGDR	GGPGIPGFKG	EPGLSIRGPK	GVQGPRGPVG	APGLKGDGYP
650	660	670	680	690	700	710	720
GVPGPRGLPG	PPGPMGLRGV	GDTGAKGEPG	VRGPPGPSGP	RGVGTQGPKG	DTGQKGLPGP	PGPPGYGSQG	IKGEQGPQGF
730	740	750	760	770	780	790	800
PGPKGTMGHG	LPGQKGEHGE	RGDVGKKGDK	GEIGEPGSPG	KQGLQGPKGD	LGLTKEEIIK	LITEICGCGP	KCKETPLELV
810	820	830	840	850	860	870	880
FVIDSSESVG	PENFQIIKNF	VKTMADRVAL	DLATARIGII	NYSHKVEKVA	NLKQFSSKDD	FKLAVDNMQY	LGEGTYTATA
890	900	910	920	930	940	950	960
LQAANDMFED	ARPGVKKVAL	VITDGQTDSR	DKEKLTEVVK	NASDTNVEIF	VIGVVKKNDP	NFEIFHKEMN	LIATDPEHVY
970	980	990	1000	1010	1020	1030	1040
QFDDFFTLQD	TLKQKLFQKI	CEDFDSYLVQ	IFGSSSPQPG	FGMSGEELSE	STPEPQKEIS	ESLSVTRDQD	EDDKAPEPTW
1050	1060	1070	1080	1090	1100	1110	1120
ADDLPATTSS	EATTTPRPLL	STPVDGAEDP	RCLEALKPGN	CGEYVVRWYY	DKQVNSCARF	WFSGCNGSGN	RFNSEKECQE
1130							
TCIQG							

Cmpd.	No. of Cmpds.	m/z meas.	Δ m/z [ppm]	z	Rt [min]	Score	P	Range	Sequence	Modification	Ratios
1277	1	990.9708	-72.85	2	45.6	13.8	2	150-167	R.KDGVKVVLLMTDGIDHPK.N	Oxidation: 10	
201	1	714.3696	-12.54	3	32.5	18.4	2	667-689	K. GEPGVRGPPGPSGPRGVGTQGP G		Q:W 0.18

Detailed Protein Report

Protein 237: metabotropic glutamate receptor 4 isoform 6 [Homo sapiens]

Accession: gi|378548218
Database: refseq_human(refseq_human_20140103.fasta)
Database Date: 2015-11-30
Modification(s): Oxidation

Score: 56.1
MW [kDa]: 87.3
pI: 10.0
Sequence Coverage [%]: 7.8
No. of unique Peptides: 3

Quantitation

M:Q **Median:** 0.38 **CV:** 0.00 % **No. of Peptides:** 1

10	20	30	40	50	60	70	80
MAVPLGAPCW	ASALPAWAPP	GLPHRSLLTR	LLSQHVKPAK	IPQISYASTA	PDLSDNSRYD	FFSRVVPSDT	YQAQAMVDIV
**90**	**100**	**110**	**120**	**130**	**140**	**150**	**160**
RALKWNYVST	VASEGSYGES	GVEAFIQKSR	EDGGVCIAQS	VKIPREPKAG	EFDKIIRRLL	ETSNARAVII	FANEDDIRRV
**170**	**180**	**190**	**200**	**210**	**220**	**230**	**240**
LEAARRANQT	GHFFWMGSDS	WGSKIAPVLH	LEEVAEGAVT	ILPKRMSVRG	FDRYFSSRTL	DNNRRNIWFA	EFWEDNFHCK
**250**	**260**	**270**	**280**	**290**	**300**	**310**	**320**
LSRHALKKGS	HVKKCTNRER	IGQDSAYEQE	GKVQFVIDAV	YAMGHALHAM	HRDLCPGRVG	LCPRMDPVDG	TQLLKYIRNV
**330**	**340**	**350**	**360**	**370**	**380**	**390**	**400**
NFSGIAGNPV	TFNENGDAPG	RYDIYQYQLR	NDSAEYKVIG	SWTDHLHLRI	ERMHWPGSGQ	QLPRSICSLP	CQPGERKKTV
**410**	**420**	**430**	**440**	**450**	**460**	**470**	**480**
KGMPCCWHCE	PCTGYQYQVD	RYTCKTCPYD	MRPTENRTGC	RPIPIIKLEW	GSPWAVLPLF	LAVVGIAATL	FVVITFVRYN
**490**	**500**	**510**	**520**	**530**	**540**	**550**	**560**
DTPIVKASGR	ELSYVLLAGI	FLCYATTFLM	IAEPDLGTCS	LRRIFLGLGM	SISYAALLTK	TNRIYRIFEQ	GKRSVSAPRF
**570**	**580**	**590**	**600**	**610**	**620**	**630**	**640**
ISPASQLAIT	FSLISLQLLG	ICVWFVVDPS	HSVVDFQDQR	TLDPRFARGV	LKCDISDLSL	ICLLGYSMLL	MVTCTVYAIK
**650**	**660**	**670**	**680**	**690**	**700**	**710**	**720**
TRGVPETFNE	AKPIGFTMYT	TCIVWLAFIP	IFFGTSQSAD	KLYIQTTTLT	VSVSLSASVS	LGMLYMPKVY	IILFHPEQNV
**730**	**740**	**750**	**760**	**770**	**780**		
PKRKRSLKAV	VTAATMSNKF	TQKGNFRPNG	EAKSELCENL	EAPALATKQT	YVTYTNHAI		

Cmpd.	No. of Cmpds.	m/z meas.	Δ m/z [ppm]	z	Rt [min]	Score	P	Range	Sequence	Modification	Ratios
1677	1	619.9559	-55.34	3	51.2	11.2	1	299-315	R.VGLCPRMDPVDGTQLLK.Y	Oxidation: 7	M:Q 0.38
2594	1	741.8530	35.29	2	63.0	15.9	0	426-437	K.TCPYDMRPTENR.T		
1390	1	977.5200	-44.32	2	47.5	12.4	1	523-540	R.RIFLGLGMSISYAALLTK.T		

Detailed Protein Report

Protein 238: SH3 and multiple ankyrin repeat domains protein 3 [Homo sapiens]

Accession: gi|380748963
Database: refseq_human(refseq_human_20140103.fasta)
Database Date: 2015-11-30
Modification(s): Oxidation

Score: 56.0
MW [kDa]: 184.6
pI: 9.6
Sequence Coverage [%]: 3.3
No. of unique Peptides: 2

10	20	30	40	50	60	70	80
MDGPGASAVV	VRVGIPDLQQ	TKCLRLDPAA	PVWAAKQRVL	CALNHSLQDA	LNYGLFQPPS	RGRAGKFLDE	ERLLQEYPPN
90	100	110	120	130	140	150	160
LDTPLPYLEF	RYKRRVYAQN	LIDDKQFAKL	HTKANLKKFM	DYVQLHSTDK	VARLLDKGLD	PNFHDPDSGE	CPLSLAAQLD
170	180	190	200	210	220	230	240
NATDLLKVLK	NGGAHLDFRT	RDGLTAVHCA	TRQRNAAALT	TLLDLGASPD	YKDSRGLTPL	YHSALGGGDA	LCCELLLHDH
250	260	270	280	290	300	310	320
AQLGITDENG	WQEIHQACRF	GHVQHLEHLL	FYGADMGAQN	ASGNTALHIC	ALYNQESCAR	VLLFRGANRD	VRNYNSQTAF
330	340	350	360	370	380	390	400
QVAIIAGNFE	LAEVIKTHKD	SDVVPFRETP	SYAKRRRLAG	PSGLASPRPL	QRSASDINLK	GEAQPAASPG	PSLRSLPHQL
410	420	430	440	450	460	470	480
LLQRLQEEKD	RDRDADQESN	ISGPLAGRAG	QSKISPSGPG	GPGPAPGPGP	APPAPPAPPP	RGPKRKLYSA	VPGRKFIAVK
490	500	510	520	530	540	550	560
AHSPQGEGEI	PLHRGEAVKV	LSIGEGGFWE	GTVKGRTGWF	PADCVEEVQM	RQHDTRPETR	EDRTKRLFRH	YTVGSYDSLT
570	580	590	600	610	620	630	640
SHSDYVIDDK	VAVLQKRDHE	GFGFVLRGAK	AETPIEEFTP	TPAFPALQYL	ESVDVEGVAW	RAGLRTGDFL	IEVNGVNVVK
650	660	670	680	690	700	710	720
VGHKQVVALI	RQGGNRLVMK	VVSVTRKPEE	DGARRRAPPP	PKRAPSTTLT	LRSKSMTAEL	EELASIRRRK	GEKLDEMLAA
730	740	750	760	770	780	790	800
AAEPTLRPDI	ADADSRAATV	KQRPTSRRIT	PAEISSLFER	QGLPGPEKLP	GSLRKGIPRT	KSVGEDEKLA	SLLEGRFPRS
810	820	830	840	850	860	870	880
TSMQDPVREG	RGIPPPPQTA	PPPPPAPYYF	DSGPPPAFSP	PPPPGRAYDT	VRSSFKPGLE	ARLGAGAAGL	YEPGAALGPL
890	900	910	920	930	940	950	960
PYPERQKRAR	SMIILQDSAP	ESGDAPRPPP	AATPPERPKR	RPRPPGPDSP	YANLGAFSAS	LFAPSKPQRR	KSPLVKQLQV
970	980	990	1000	1010	1020	1030	1040
EDAQERAALA	VGSPGPGGGS	FAREPSPTHR	GPRPGGLDYG	AGDGPGLAFG	GPGPAKDRRL	EERRRSTVFL	SVGAIEGSAP
1050	1060	1070	1080	1090	1100	1110	1120
GADLPSLQPS	RSIDERLLGT	GPTAGRDLLL	PSPVSALKPL	VSGPSLGPSG	STFIHPLTGK	PLDPSSPLAL	ALAARERALA
1130	1140	1150	1160	1170	1180	1190	1200
SQAPSRSPTP	VHSPDADRPG	PLFVDVQARD	PERGSLASPA	FSPRSPAWIP	VPARREAEKV	PREERKSPED	KKSMILSVLD
1210	1220	1230	1240	1250	1260	1270	1280
TSLQRPAGLI	VVHATSNGQE	PSRLGGAEEE	RPGTPELAPA	PMQSAAVAEP	LPSPRAQPPG	GTPADAGPGQ	GSSEEEPELV
1290	1300	1310	1320	1330	1340	1350	1360
FAVNLPPAQL	SSSDEETREE	LARIGLVPPP	EEFANGVLLA	TPLAGPGPSP	TTVPSPASGK	PSSEPPPAPE	SAADSGVEEA
1370	1380	1390	1400	1410	1420	1430	1440
DTRSSSDPHL	ETTSTISTVS	SMSTLSSESG	ELTDTHTSFA	DGHTFLLEKP	PVPPKPKLKS	PLGKGPVTFR	DPLLKQSSDS
1450	1460	1470	1480	1490	1500	1510	1520
ELMAQQHHAA	SAGLASAAGP	ARPRYLFQRR	SKLWGDPVES	RGLPGPEDDK	PTVISELSSR	LQQLNKDTRS	LGEEPVGGLG
1530	1540	1550	1560	1570	1580	1590	1600
SLLDPAKKSP	IAAARLFSSL	GELSSISAQR	SPGGPGGGAS	YSVRPSGRYP	VARRAPSPVK	PASLERVEGL	GAGAGGAGRP
1610	1620	1630	1640	1650	1660	1670	1680
FGLTPPTILK	SSSLSIPHEP	KEVRFVVRSV	SARSRSPSPS	PLPSPASGPG	PGAPGPRRPF	QQKPLQLWSK	FDVGDWLESI
1690	1700	1710	1720	1730	1740		
HLGEHRDRFE	DHEIEGAHLP	ALTKDDFVEL	GVTRVGHRMN	IERALRQLDG	S		

Cmpd.	No. of Cmpds.	m/z meas.	Δ m/z [ppm]	z	Rt [min]	Score	P	Range	Sequence	Modification
1868	1	840.7873	-166.49	2	53.4	13.8	1	693-707	R.SKSMTAELEELASIR.R	Oxidation: 4
498	1	546.2697	-47.83	2	34.8	13.2	0	853-862	R.SSFKPGLEAR.L	

Detailed Protein Report

Protein 239: MAP kinase-activating death domain protein isoform j [Homo sapiens]

Accession: gi|209863002
Database: refseq_human(refseq_human_20140103.fasta)
Database Date: 2015-11-30
Modification(s): Carbamidomethyl

Score: 55.9
MW [kDa]: 170.9
pI: 5.8
Sequence Coverage [%]: 2.5
No. of unique Peptides: 2

Alias proteins:

Accession	Name	Description
gi|530395946	refseq_human (refseq_human_20140103.fasta)	PREDICTED: MAP kinase-activating death domain protein isoform X18 [Homo sapiens]

10	20	30	40	50	60	70	80
MVQKKKFCPR	LLDYLVIVGA	RHPSSDSVAQ	TPELLRRYPL	EDHTEFPLPP	DVVFFCQPEG	CLSVRQRRMS	LRDDTSFVFT
90	100	110	120	130	140	150	160
LTDKDTGVTR	YGICVNFYRS	FQKRISKEKG	EGGAGSRGKE	GTHATCASEE	GGTESSESGS	SLQPLSADST	PDVNQSPRGK
170	180	190	200	210	220	230	240
RRAKAGSRSR	NSTLTSLCVL	SHYPFFSTFR	ECLYTLKRLV	DCCSERLLGK	KLGIPRGVQR	DTMWRIFTGS	LLVEEKSSAL
250	260	270	280	290	300	310	320
LHDLREIEAW	IYRLLRSPVP	VSGQKRVDIE	VLPQELQPAL	TFALPDPSRF	TLVDFPLHLP	LELLGVDACL	QVLTCILLEH
330	340	350	360	370	380	390	400
KVVLQSRDYN	ALSMSVMAFV	AMIYPLEYMF	PVIPLLPTCM	ASAEQLLLAP	TPYIIGVPAS	FFLYKLDFKM	PDDVWLVDLD
410	420	430	440	450	460	470	480
SNRVIAPTNA	EVLPILPEPE	SLELKKHLKQ	ALASMSLNTQ	PILNLEKFHE	GQEIPLLLGR	PSNDLQSTPS	TEFNPLIYGN
490	500	510	520	530	540	550	560
DVDSVDVATR	VAMVRFFNSA	NVLQGFQMHT	RTLRLFPRPV	VAFQAGSFLA	SRPRQTPFAE	KLARTQAVEY	FGEWILNPTN
570	580	590	600	610	620	630	640
YAFQRIHNNM	FDPALIGDKP	KWYAHQLQPI	HYRVYDSNSQ	LAEALSVPPE	RDSDSEPTDD	SGSDSMDYDD	SSSSYSSLGD
650	660	670	680	690	700	710	720
FVSEMMKCDI	NGDTPNVDPL	THAALGDASE	VEIDELQNQK	EAEEPGPDSE	NSQENPPLRS	SSSTTASSSP	STVIHGANSE
730	740	750	760	770	780	790	800
PADSTEMDDK	AAVGVSKPLP	SVPPSIGKSN	VDRRQAEIGE	GAQKLLRPNS	LRLASDSDAE	SDSRASSPNS	TVSNTSTEGF
810	820	830	840	850	860	870	880
GGIMSFASSL	YRNHSTSFSL	SNLTLPTKGA	REKATPFPSL	KGNRRALVDQ	KSSVIKHSPT	VKREPPSPQG	RSSNSSENQQ
890	900	910	920	930	940	950	960
FLKEVVHSVL	DGQGVGWLNM	KKVRRLLESE	QLRVFVLSKL	NRMVQSEDDA	RQDIIPDVEI	SRKVYKGMLD	LLKCTVLSLE
970	980	990	1000	1010	1020	1030	1040
QSYAHAGLGG	MASIFGLLEI	AQTHYYSKEP	DKRKRSPTES	VNTPVGKDPG	LAGRGDPKAM	AQLRVPQLGP	RAPSATGKGP
1050	1060	1070	1080	1090	1100	1110	1120
KELDTRSLKE	ENFIASIGPE	VIKPVFDLGE	TEEKKSQISA	DSGVSLTSSS	QRTDQDSVIG	VSPAVMIRSS	SQDSEVSNSS
1130	1140	1150	1160	1170	1180	1190	1200
GETLGADSDL	SSNAGDGPGG	EGSVHLASSR	GTLSDSEIET	NSATSTIFGK	AHSLKPSIKE	KLAGSPIRTS	EDVSQRVYLY
1210	1220	1230	1240	1250	1260	1270	1280
EGLLGKERST	LWDQMQFWED	AFLDAVMLER	EGMGMDQGPQ	EMIDRYLSLG	EHDRKRLEDD	EDRLLATLLH	NLISYMLLMK
1290	1300	1310	1320	1330	1340	1350	1360
VNKNDIRKKV	RRLMGKSHIG	LVYSQQINEV	LDQLANLNGR	DLSIWSSGSR	HMKKQTFVVH	AGTDTNGDIF	FMEVCDDCVV
1370	1380	1390	1400	1410	1420	1430	1440
LRSNIGTVYE	RWWYEKLINM	TYCPKTKVLC	LWRRNGSETQ	LNKFYTKKCR	ELYYCVKDSM	ERAAARQQSI	KPGPELGGEF
1450	1460	1470	1480	1490	1500	1510	1520
PVQDLKTGEG	GLLQVTLEGI	NLKFMHNQVF	IELNHIKKCN	TVRGVFVLEE	FVPEIKEVVS	HKYKTPMAHE	ICYSVLCLFS
1530	1540	1550					
YVAAVHSSEE	DLRTPPRPVS	S					

Cmpd.	No. of Cmpds.	m/z meas.	Δ m/z [ppm]	z	Rt [min]	Score	P	Range	Sequence	Modification
1913	1	682.2568	-100.43	3	54.0	15.5	2	191-206	R.ECLYTLKRLVDCCSER.L	Carbamidomethyl: 12, 13
974	1	547.7654	-198.26	2	40.5	10.2	2	207-216	R.LLGKKLGIPR.G	

Detailed Protein Report

Protein 240: PREDICTED: serine/threonine-protein kinase WNK2 isoform X12 [Homo sapiens]

**Accession:**	gi|578817784	**Score:**	55.9
**Database:**	refseq_human(refseq_human_20140103.fasta)	**MW [kDa]:**	227.1
**Database Date:**	2015-11-30	**pI:**	5.4
		**Sequence Coverage [%]:**	3.0
		**No. of unique Peptides:**	2

Quantitation

Q:W	**Median:**	0.71	**CV:**	110.97 %	**No. of Peptides:**	2
M:Q	**Median:**	2.02	**CV:**	0.00 %	**No. of Peptides:**	1

Detailed Protein Report

10	20	30	40	50	60	70	80
MDGDGGRRDV	PGTLMEPGRG	AGPAGMAEPR	AKAARPGPQR	FLRRSVVESD	QEEPPGLEAA	EAPGPQPPQP	LQRRVLLLCK
90	100	110	120	130	140	150	160
TRRLIAERAR	GRPAAPAPAA	LVAQPGAPGA	PADAGPEPVG	TQEPGPDPIA	AAVETAPAPD	GGPREEAAAT	VRKEDEGAAE
170	180	190	200	210	220	230	240
AKPEPGRTRR	DEPEEEEDDE	DDLKAVATSL	DGRFLKFDIE	LGRGSFKTVY	KGLDTETWVE	VAWCELQDRK	LTKLERQRFK
250	260	270	280	290	300	310	320
EEAEMLKGLQ	HPNIVRFYDF	WESSAKGKRC	IVLVTELMTS	GTLKTYLKRF	KVMKPKVLRS	WCRQILKGLL	FLHTRTPPII
330	340	350	360	370	380	390	400
HRDLKCDNIF	ITGPTGSVKI	GDLGLATLKR	ASFAKSVIGT	PEFMAPEMYE	EHYDESVDVY	AFGMCMLEMA	TSEYPYSECQ
410	420	430	440	450	460	470	480
NAAQIYRKVT	CGIKPASFEK	VHDPEIKEII	GECICKNKEE	RYEIKDLLSH	AFFAEDTGVR	VELAEEDHGR	KSTIALRLWV
490	500	510	520	530	540	550	560
EDPKKLKGKP	KDNGAIEFTF	DLEKETPDEV	AQEMIESGFF	HESDVKIVAK	SIRDRVALIQ	WRRERIWPAL	QPKEQQDVGS
570	580	590	600	610	620	630	640
PDKARGPPVP	LQVQVTYHAQ	AGQPGPPEPE	EPEADQHLLP	PTLPTSATSL	ASDSTFDSGQ	GSTVYSDSQS	SQQSVMLGSL
650	660	670	680	690	700	710	720
ADAAPSPAQC	VCSPPVSEGP	VLPQSLPSLG	AYQQPTAAPG	LPVGSVPAPA	CPPSLQQHFP	DPAMSFAPVL	PPPSTPMPTG
730	740	750	760	770	780	790	800
PGQPAPPGQQ	PPPLAQPTPL	PQVLAPQPVV	PLQPVPPHLP	PYLAPASQVG	APAQLKPLQM	PQAPLQPLAQ	VPPQMPPIPV
810	820	830	840	850	860	870	880
VPPITPLAGI	DGLPPALPDL	PTATVPPVPP	PQYFSPAVIL	PSLAAPLPPA	SPALPLQAVK	LPHPPGAPLA	MPCRTIVPNA
890	900	910	920	930	940	950	960
PATIPLLAVA	PPGVAALSIH	SAVAQLPGQP	VYPAAFPQMA	PTDVPPSPHH	TVQNMRATPP	QPALPPQPTL	PPQPVLPPQP
970	980	990	1000	1010	1020	1030	1040
TLPPQPVLPP	QPTRPPQPVL	PPQPMLPPQP	VLPPQPALPV	RPEPLQPHLP	EQAAPAATPG	SQILLGHPAP	YAVDVAAQVP
1050	1060	1070	1080	1090	1100	1110	1120
TVPVPPAAVL	SPPLPEVLLP	AAPELLPQFP	SSLATVSASV	QSVPTQTATL	LPPANPPLPG	GPGIASPCPT	VQLTVEPVQE
1130	1140	1150	1160	1170	1180	1190	1200
EQASQDKPPG	LPQSCESYGG	SDVTSGKELS	DSCEGAFGGG	RLEGRAARKH	HRRSTRARSR	QERASRPRLT	ILNVCNTGDK
1210	1220	1230	1240	1250	1260	1270	1280
MVECQLETHN	HKMVTFKFDL	DGDAPDEIAT	YMVEHDFILQ	AERETFIEQM	KDVMDKAEDM	LSEDTDADRG	SDPGTSPPHL
1290	1300	1310	1320	1330	1340	1350	1360
STCGLGTGEE	SRQSQANAPV	YQQNVLHTGK	RWFIICPVAE	HPAPEAPESS	PPLPLSSLPP	EASQDSAPYK	DQLSSKEQPS
1370	1380	1390	1400	1410	1420	1430	1440
FLASQQLLSQ	AGPSNPPGAP	PAPLAPSSPP	VTALPQDGAA	PATSTMPEPA	SGTASQAGGP	GTPQGLTSEL	ETSQPLAETH
1450	1460	1470	1480	1490	1500	1510	1520
EAPLAVQPLV	VGLAPCTPAP	EAASTRDASA	PREPLPPPAP	EPSPHSGTPQ	PALGQPAPLL	PAAVGAVSLA	TSQLPSPPLG
1530	1540	1550	1560	1570	1580	1590	1600
PTVPPQPPSA	LESDGEGPPP	RVGFVDSTIK	SLDEKLRTLL	YQEHVPTSSA	SAGTPVEVGD	RDFTLEPLRG	DQPRSEVCGG
1610	1620	1630	1640	1650	1660	1670	1680
DLALPPVPKE	AVSGRVQLPQ	PLVEKSELAP	TRGAVMEQGT	SSSMTAESSP	RSMLGYDRDG	RQVASDSHVV	PSVPQDVPAF
1690	1700	1710	1720	1730	1740	1750	1760
VRPARVEPTD	RDGGEAGESS	AEPPPSDMGT	VGGQASHPQT	LGARALGSPR	KRPEQQDVSS	PAKTVGRFSV	VSTQDEWTLA
1770	1780	1790	1800	1810	1820	1830	1840
SPHSLRYSAP	PDVYLDEAPS	SPDVKLAVRR	AQTASSIEVG	VGEPVSSDSG	DEGPRARPPV	QKQASLPVSG	SVAGDFVKKA
1850	1860	1870	1880	1890	1900	1910	1920
TAFLQRPSRA	GSLGPETPSR	VGMKVPTISV	TSFHSQSSYI	SSDNDSELED	ADIKKELQSL	REKHLKEISE	LQSQQKQEIE
1930	1940	1950	1960	1970	1980	1990	2000
ALYRRLGKPL	PPNVGFFHTA	PPTGRRRKTS	KSKLKAGKLL	NPLVRQLKVV	ASSTGSSTSS	LAPGPEPGPQ	PALHVQAQVN
2010	2020	2030	2040	2050	2060	2070	2080
NSNNKKGTFT	DDLHKLVDEW	TSKTVGAAQL	KPTLNQLKQT	QKLQDMEAQA	GWAAPGEARA	MTAPRAGVGM	PRLPPAPGPL
2090	2100	2110	2120	2130	2140	2150	
STTVIPGAAP	TLSVPTPDGA	LGTARRNQVW	FGLRVPPTAC	CGHSTQPRGG	QRVGSKTASF	AASDPVRS	

Detailed Protein Report

Cmpd.	No. of Cmpds.	m/z meas.	Δ m/z [ppm]	z	Rt [min]	Score	P	Range	Sequence	Modification	Ratios
2405	1	904.4370	-32.64	2	60.5	15.1	1	323-339	R.DLKCDNIFITGPTGSVK.I		Q:W 0.29
1286	1	945.9319	-64.24	2	46.2	11.9	2	2129-2147	R.GGQRVGSKTASFAASDPVR.S		M:Q 2.02<br>Q:W 1.74

Detailed Protein Report

Protein 241: **PREDICTED: LOW QUALITY PROTEIN: ubiquitin carboxyl-terminal hydrolase 17-like protein 20 [Homo sapiens]**

Accession: gi|578843530
Database: refseq_human(refseq_human_20140103.fasta)
Database Date: 2015-11-30
Modification(s): Carbamidomethyl

Score: 55.8
MW [kDa]: 38.1
pI: 9.8
Sequence Coverage [%]: 6.0
No. of unique Peptides: 3

10	20	30	40	50	60	70	80
MEDDSLYLGA	SAKVLILVLK	RFSDVTGNKI	AKNVQYPECL	DMQPYMSQPN	TGPLVYVLYA	VLVHAGWSCH	NGHYFSYVKA
90	100	110	120	130	140	150	160
QEGQWYKMDD	AEVTASSITS	VLSQQAYVLF	YIQKSEWERH	SESVSRGREP	RALGAEDTDR	RATQGELKRD	HPCLQAPELD
170	180	190	200	210	220	230	240
EHLVERATQE	STLDHWKFLQ	EQNKTKPEFN	VRKVEGTLPP	DVLVIHQSXY	KCGMKNHHPE	QQSSLLNLSS	TTPTHQESMN
250	260	270	280	290	300	310	320
TGTLASLRGR	ARRSKGKNKH	SKRALLVCQX	SQWKYRPTRT	VIKVDQPQRK	AAQGTTQGSV	EPQNLGRNPA	QAPKCAYEQG
330	340						
LRVTTLYIRH	RKPSDG						

Cmpd.	No. of Cmpds.	m/z meas.	Δ m/z [ppm]	z	Rt [min]	Score	P	Range	Sequence	Modification
1896	1	599.9585	-51.71	3	52.4	13.5	2	260-274	K.HSKRALLVCQNSQWK.Y	
2567	1	709.8660	-0.12	2	62.5	14.7	0	264-274	R.ALLVCQWSQWK.Y	Carbamidomethyl: 5
1491	1	981.6005	60.92	2	47.4	15.8	1	264-279	R.ALLVCQISQWKYRPTR.T	

Detailed Protein Report

Protein 242: **PREDICTED: cleavage and polyadenylation specificity factor subunit 1 isoform X2 [Homo sapiens]**

**Accession:**	gi|578815945	**Score:**	55.7
**Database:**	refseq_human(refseq_human_20140103.fasta)	**MW [kDa]:**	151.9
**Database Date:**	2015-11-30	**pI:**	5.9
**Modification(s):**	Carbamidomethyl, Oxidation	**Sequence Coverage [%]:**	3.7
		**No. of unique Peptides:**	2

10	20	30	40	50	60	70	80
MSMASVQLAG	AKRDALLLSF	KDAKLSVVEY	DPGTHDLKTL	SLHYFEEPEL	RDGFVQNVHT	PRVRVDPDGR	CAAMLVYGTR
90	100	110	120	130	140	150	160
LVVLPFRRES	LAEEHEGLVG	EGQRSSFLPS	YIIDVRALDE	KLLNIIDLQF	LHGYYEPTLL	ILFEPNQTWP	GRVAVRQDTC
170	180	190	200	210	220	230	240
SIVAISLNIT	QKVHPVIWSL	TSLPFDCTQA	LAVPKPIGGV	VVFAVNSLLY	LNQSVPPYGV	ALNSLTTGTT	AFPLRTQEGV
250	260	270	280	290	300	310	320
RITLDCAQAT	FISYDKMVIS	LKGGEIYVLT	LITDGMRSVR	AFHFDKAAAS	VLTTSMVTME	PGYLFLGSRL	GNSLLLKYTE
330	340	350	360	370	380	390	400
KLQEPPASAV	REAADKEEPP	SKKKRVDATA	GWSAAGKSVP	QDEVDEIEVY	GSEAQSGTQL	ATYSFEVCDS	ILNIGPCANA
410	420	430	440	450	460	470	480
AVGEPAFLSE	EFQNSPEPDL	EIVVCSGHGK	NGALSVLQKS	IRPQVVTTFE	LPGCYDMWTV	IAPVRKEEED	NPKGEGTEQE
490	500	510	520	530	540	550	560
PSTTPEADDD	GRRHGFLILS	REDSTMILQT	GQEIMELDTS	GFATQGPTVF	AGNIGDNRYI	VQVSPLGIRL	LEGVNQLHFI
570	580	590	600	610	620	630	640
PVDLGAPIVQ	CAVADPYVVI	MSAEGHVTMF	LLKSDSYGGR	HHRLALHKPP	LHHQSKVITL	CLYRDLSGMF	TTESRLGGAR
650	660	670	680	690	700	710	720
DELGGRSGPE	AEGLGSETSP	TVDDEEEMLY	GDSGSLFSPS	KEEARRSSQP	PADRDPAPFR	AEPTHWCLLV	RENGTMEIYQ
730	740	750	760	770	780	790	800
LPDWRLVFLV	KNFPVGQRVL	VDSSFGQPTT	QGEARREEAT	RQGELPLVKE	VLLVALGSRQ	SRPYLLVHVD	QELLIYEAFP
810	820	830	840	850	860	870	880
HDSQLGQGNL	KVRFKKVPHN	INFREKKPKP	SKKKAEGGGA	EEGAGARGRV	ARFRYFEDIY	GYSGVFICGP	SPHWLLVTGR
890	900	910	920	930	940	950	960
GALRLHPMAI	DGPVDSFAPF	HNVNCPRGFL	YFNRQGELRI	SVLPAYLSYD	APWPVRKIPL	RCTAHYVAYH	VESKVYAVAT
970	980	990	1000	1010	1020	1030	1040
STNTPCARIP	RMTGEEKEFE	TIERDERYIH	PQQEAFSIQL	ISPVSWEAIP	NARIELQEWE	HVTCMKTVSL	RSEETVSGLK
1050	1060	1070	1080	1090	1100	1110	1120
GYVAAGTCLM	QGEEVTCRGR	ILIMDVIEVV	PEPGQPLTKN	KFKVLYEKEQ	KGPVTALCHC	NGHLVSAIGQ	KIFLWSLRAS
1130	1140	1150	1160	1170	1180	1190	1200
ELTGMAFIDT	QLYIHQMISV	KNFILAADVM	KSISLLRYQE	ESKTLSLVSR	DAKPLEVYSV	DFMVDNAQLG	FLVSDRDRNL
1210	1220	1230	1240	1250	1260	1270	1280
MVYMYLPEAK	ESFGGMRLLR	RADFHVGAHV	NTFWRTPCRG	ATEGLSKKSV	VWENKHITWF	ATLDGGIGLL	LPMQEKTYRR
1290	1300	1310	1320	1330	1340	1350	1360
LLMLQNALTT	MLPHHAGLNP	RAFRMLHVDR	RTLQNAVRNV	LDGELLNRYL	YLSTMERSEL	AKKIGTTPDI	ILDDLLETDR
1370							
VTAHF							

Cmpd.	No. of Cmpds.	m/z meas.	Δ m/z [ppm]	z	Rt [min]	Score	P	Range	Sequence	Modification
297	2	626.2973	-49.17	3	33.4	14.0	1	955-971	K.VYAVATSTNTPCARIPR.M	Carbamidomethyl: 12
2457	1	596.2158	-123.37	2	61.1	10.4	0	1329-1337	R.YLYLSTMER.S	Oxidation: 7

Detailed Protein Report

Protein 243:	PREDICTED: immunoglobulin-like and fibronectin type III domain-containing protein 1 isoform X2 [Homo sapiens]		
**Accession:**	gi|578801691	**Score:**	55.7
**Database:**	refseq_human(refseq_human_20140103.fasta)	**MW [kDa]:**	373.5
**Database Date:**	2015-11-30	**pI:**	5.6
**Modification(s):**	Oxidation	**Sequence Coverage [%]:**	1.1
		**No. of unique Peptides:**	3

Quantitation

M:Q	**Median:**	0.81	**CV:**	0.00 %	**No. of Peptides:**	1

Detailed Protein Report

10	20	30	40	50	60	70	80
MAGKLRKSHI	PGVSIWQLVE	EIPEGCSTPD	FEQKPVTSAL	PEGKNAVFRA	VVCGEPRPEV	RWQNSKGDLS	DSSKYKISSS
90	100	110	120	130	140	150	160
PGSKEHVLQI	NKLTGEDTDL	YRCTAVNAYG	EAACSVRLTV	IEVGFRKNRK	RHREPQEDLR	KELMDFRKLL	KKRAPPAPKK
170	180	190	200	210	220	230	240
KMDLEQIWQL	LMTADRKDYE	KICLKYGIVD	YRGMLRRLQE	MKKEQEDKMA	QYINTISSLR	HIRVTKDGNA	KFDLELDLKD
250	260	270	280	290	300	310	320
SQSKIYLYKD	GEMIPYGFNN	QTKHCLRRLG	KRYEFQIQDL	RPEDSGIYQV	KVEDAVVFST	ELEASAGKDK	DLQSTSADHK
330	340	350	360	370	380	390	400
LQRQGAQASG	AEESGSIESQ	GEKSREQGPR	GGSLEGAGPA	SGLQHIASPD	RDGLGRHGYS	LMGDKGTADS	AWGPGQEGEG
410	420	430	440	450	460	470	480
FPVAEGSRAT	LPRENQSHRE	GGWARSLAER	PHLQGESSES	GLGLPEKQQQ	DRGRDSNSDE	CWRKAGGWEA	GSSRLQAGGL
490	500	510	520	530	540	550	560
GSSREGKEHR	GDSGRQLDRH	APEQLWDARL	GPGRGKSDLQ	GCQSDPVGSW	PRGKQIEISQ	DDSLAEMDRG	DAPSRERGRG
570	580	590	600	610	620	630	640
IVVWGGGTGL	GEAGDSNGAG	GPGTLELTGG	RGSGSKVGMA	PESWGSQGGR	DADYGEARGY	WGSGELLEQI	PGGKDFQEPS
650	660	670	680	690	700	710	720
ISGGRKFLLG	DGSPEIKAED	SLQEADGICR	GESVVTGSAY	KTGPGGPGDP	RGCEGVLQEL	RGRDGQETAW	ASGEVEYDPR
730	740	750	760	770	780	790	800
SFQSSQGWTA	GHRAAGGIGR	IESKGTSPWD	DTPSSLRKTG	AHHGPGVLGP	SGGQEGMGGI	WVAGLTESGQ	GVDARSHWLS
810	820	830	840	850	860	870	880
RAPGLGAQGS	GGTLGDKKGL	RGPGSIGSEP	DFWNGSGSSR	VKGPRGETGY	KDGLEGPGRM	ESRYEGGLGY	SREISSKSGA
890	900	910	920	930	940	950	960
GYSYGSGVPG	EMGSGHGAGC	RVSPRAPAGV	ESEEGGGYRH	GSGAPGGVWS	GNEDSGPAGG	GSGRVASLKN	GSGGPDGAPM
970	980	990	1000	1010	1020	1030	1040
NDTRNWASAC	QAGMDPRGGH	HSDGGLGSPG	VTGSAGRGGL	KAPGVVETVG	MGCVEAEPES	SGRIRPWGQT	GNYGGFRASE
1050	1060	1070	1080	1090	1100	1110	1120
ALGAFGEGGY	EDGSGGPGAM	GPGSLRAGSK	VGEGDGTRCP	GAKASGAGAG	YRDDTRHPES	LAPHNGAASG	SQWAYGAGNV
1130	1140	1150	1160	1170	1180	1190	1200
LGYEDGSELP	GPQGTGVRTA	YGERSRGLGP	RSTGPGGEAG	FRDGSGGLQG	MGSADGPGCR	KGIGSSGEMG	SVDKEGYKKD
1210	1220	1230	1240	1250	1260	1270	1280
LGAPENMGSG	SKADYRDGVG	GSGAMGSMDE	AGYRKDLGAP	EGISSGSKAD	YRGGLQDSRE	AGSGSKADYS	GGLKGSREIG
1290	1300	1310	1320	1330	1340	1350	1360
SMDETDNRKD	LGVPEGMGAG	YRAGLRGPGE	MGSLDESGHR	NGIGGYGEMG	SGYREDLGAP	EGMGTGSKAG	YRDGLRGSGE
1370	1380	1390	1400	1410	1420	1430	1440
MRSMDEAGYR	KNLGAPERMD	SGSKAGYRGG	LRGSGEMGLI	EAGYRKDLGV	SEGGGSGSKA	GYRGGLGSGE	MGSVDKAGYR
1450	1460	1470	1480	1490	1500	1510	1520
KDLGASEAIG	SGSKAGFTDG	LGGSEEMGSV	NKAGYRKDLG	APKGMGSGSK	ASFRDGLGGS	GEMGSVNEAG	YRKDLGVPEG
1530	1540	1550	1560	1570	1580	1590	1600
IGSGSKAGFR	DGLGGSEEMG	SVNKAGYRKD	LGAPKGIGSG	SKAGFRDGLG	SSGEMGSMDE	AGYRKNLGAP	EGIGSGSKAG
1610	1620	1630	1640	1650	1660	1670	1680
FRDGLGSSVE	MGSVNEAGYR	KDLGAPEGMG	SGSKAGFRDG	LGGSGEMGSV	NEAGYRKDLG	APKGIGSGSK	ADFRDALGSS
1690	1700	1710	1720	1730	1740	1750	1760
GEMGSMDEAG	YRKDLWAPEG	IGSGSKAGFR	DGLGSSVEMG	SVNEAGYRKD	LGAPEGMGSG	SKAGFRDGLG	GSGEMGSVNE
1770	1780	1790	1800	1810	1820	1830	1840
AGYRKDLGAP	KGIGSGSKAD	FRDALGSSGE	MGSMDEAGYR	KDLWAPEGIG	SGSKAGFRDG	LGSSVEMGSV	NEAGYRKDLG
1850	1860	1870	1880	1890	1900	1910	1920
APEGMGSGSK	EGFRDGLGGS	EEMGSVNKAG	YRKDLGAPKG	MGSGSKEGFR	DGLGGSEEMG	SMDEAGYRKD	LGAPEGIGSG
1930	1940	1950	1960	1970	1980	1990	2000
SKAGFRDGLG	GSEEMRSMDE	AGYRKDLGAP	ERIGSGSKAG	FRDGLGSSVE	MGSVNEAGYR	KDLGAPKGMG	SGSKTGFRDG
2010	2020	2030	2040	2050	2060	2070	2080
LGGSEEMESM	DEAGYRKDLG	APEGIGSGSK	AGFRDGLGSS	TEMGSVNEAG	YRKDLGAPKG	MGSESKAGFR	DGLGSSGEMG
2090	2100	2110	2120	2130	2140	2150	2160
SMDEAGYRKD	LGAPEGMGSG	SKAGFRDGLG	GSEEMGSVNK	AGYRKDLGAP	KGMGSGSKAG	FRDGLGSSGE	MGSMDEADYR
2170	2180	2190	2200	2210	2220	2230	2240

Detailed Protein Report

Cmpd.	No. of Cmpds.	m/z meas.	Δ m/z [ppm]	z	Rt [min]	Score	P	Range	Sequence	Modification	Ratios
548	1	512.1851	-91.29	2	35.5	10.1	0	377-385	R.HGYSLMGDK.G	Oxidation: 6	M:Q 0.81
2697	1	596.2791	-81.45	2	62.6	20.8	2	841-851	R.VKGPRGETGYK.D		
2733	1	639.3478	43.14	2	62.6	14.3	1	1406-1419	R.KDLGVSEGGGSGSK.A		

Detailed Protein Report

Protein 244: **ubiquitin carboxyl-terminal hydrolase 6 [Homo sapiens]**

**Accession:**	gi|109638751	**Score:**	55.6
**Database:**	refseq_human(refseq_human_20140103.fasta)	**MW [kDa]:**	158.6
**Database Date:**	2015-11-30	**pI:**	8.8
**Modification(s):**	Oxidation	**Sequence Coverage [%]:**	5.3
		**No. of unique Peptides:**	3

Quantitation

Q:W	**Median:**	0.23	**CV:**	0.00 %	**No. of Peptides:**	1
M:Q	**Median:**	1.85	**CV:**	0.00 %	**No. of Peptides:**	1

Alias proteins:

Accession	Name	Description
gi|530410741	refseq_human (refseq_human_20140103.fasta)	PREDICTED: ubiquitin carboxyl-terminal hydrolase 6 isoform X1 [Homo sapiens]

10	20	30	40	50	60	70	80
MDMVENADSL	QAQERKDILM	KYDKGHRAGL	PEDKGPEPVG	INSSIDRFGI	LHETELPPVT	AREAKKIRRE	MTRTSKWMEM
**90**	**100**	**110**	**120**	**130**	**140**	**150**	**160**
LGEWETYKHS	SKLIDRVYKG	IPMNIRGPVW	SVLLNIQEIK	LKNPGRYQIM	KERGKRSSEH	IHHIDLDVRT	TLRNHVFFRD
**170**	**180**	**190**	**200**	**210**	**220**	**230**	**240**
RYGAKQRELF	YILLAYSEYN	PEVGYCRDLS	HITALFLLYL	PEEDAFWALV	QLLASERHSL	PGFHSPNGGT	VQGLQDQQEH
**250**	**260**	**270**	**280**	**290**	**300**	**310**	**320**
VVPKSQPKTM	WHQDKEGLCG	QCASLGCLLR	NLIDGISLGL	TLRLWDVYLV	EGEQVLMPIT	SIALKVQQKR	LMKTSRCGLW
**330**	**340**	**350**	**360**	**370**	**380**	**390**	**400**
ARLRNQFFDT	WAMNDDTVLK	HLRASTKKLT	RKQGDLPPPA	KREQGSLAPR	PVPASRGGKT	LCKGYRQAPP	GPPAQFQRPI
**410**	**420**	**430**	**440**	**450**	**460**	**470**	**480**
CSASPPWASR	FSTPCPGGAV	REDTYPVGTQ	GVPSLALAQG	GPQGSWRFLE	WKSMPRLPTD	LDIGGPWFPH	YDFEWSCWVR
**490**	**500**	**510**	**520**	**530**	**540**	**550**	**560**
AISQEDQLAT	CWQAEHCGEV	HNKDMSWPEE	MSFTANSSKI	DRQKVPTEKG	ATGLSNLGNT	CFMNSSIQCV	SNTQPLTQYF
**570**	**580**	**590**	**600**	**610**	**620**	**630**	**640**
ISGRHLYELN	RTNPIGMKGH	MAKCYGDLVQ	ELWSGTQKSV	APLKLRRTIA	KYAPKFDGFQ	QQDSQELLAF	LLDGLHEDLN
**650**	**660**	**670**	**680**	**690**	**700**	**710**	**720**
RVHEKPYVEL	KDSDGRPDWE	VAAEAWDNHL	RRNRSIIVDL	FHGQLRSQVK	CKTCGHISVR	FDPFNFLSLP	LPMDSYMDLE
**730**	**740**	**750**	**760**	**770**	**780**	**790**	**800**
ITVIKLDGTT	PVRYGLRLNM	DEKYTGLKKQ	LRDLCGLNSE	QILLAEVHDS	NIKNFPQDNQ	KVQLSVSGFL	CAFEIPVPSS
**810**	**820**	**830**	**840**	**850**	**860**	**870**	**880**
PISASSPTQI	DFSSSPSTNG	MFTLTTNGDL	PKPIFIPNGM	PNTVVPCGTE	KNFTNGMVNG	HMPSLPDSPF	TGYIIAVHRK
**890**	**900**	**910**	**920**	**930**	**940**	**950**	**960**
MMRTELYFLS	PQENRPSLFG	MPLIVPCTVH	TRKKDLYDAV	WIQVSWLARP	LPPQEASIHA	QDRDNCMGYQ	YPFTLRVVQK
**970**	**980**	**990**	**1000**	**1010**	**1020**	**1030**	**1040**
DGNSCAWCPQ	YRFCRGCKID	CGEDRAFIGN	AYIAVDWHPT	ALHLRYQTSQ	ERVVDKHESV	EQSRRAQAEP	INLDSCLRAF
**1050**	**1060**	**1070**	**1080**	**1090**	**1100**	**1110**	**1120**
TSEEELGESE	MYYCSKCKTH	CLATKKLDLW	RLPPFLIIHL	KRFQFVNDQW	IKSQKIVRFL	RESFDPSAFL	VPRDPALCQH
**1130**	**1140**	**1150**	**1160**	**1170**	**1180**	**1190**	**1200**
KPLTPQGDEL	SKPRILAREV	KKVDAQSSAG	KEDMLLSKSP	SSLSANISSS	PKGSPSSSRK	SGTSCPSSKN	SSPNSSPRTL
**1210**	**1220**	**1230**	**1240**	**1250**	**1260**	**1270**	**1280**
GRSKGRLRLP	QIGSKNKPSS	SKKNLDASKE	NGAGQICELA	DALSRGHMRG	GSQPELVTPQ	DHEVALANGF	LYEHEACGNG
**1290**	**1300**	**1310**	**1320**	**1330**	**1340**	**1350**	**1360**
CGDGYSNGQL	GNHSEEDSTD	DQREDTHIKP	IYNLYAISCH	SGILSGGHYI	TYAKNPNCKW	YCYNDSSCEE	LHPDEIDTDS
**1370**	**1380**	**1390**	**1400**	**1410**			
AYILFYEQQG	IDYAQFLPKI	DGKKMADTSS	TDEDSESDYE	KYSMLQ			

Cmpd.	No. of Cmpds.	m/z meas.	Δ m/z [ppm]	z	Rt [min]	Score	P	Range	Sequence	Modification	Ratios
2932	1	965.1226	-27.88	3	65.8	10.3	0	218-244	R. HSLPGFHSPNGGTVQGLQDQQEI S		

Detailed Protein Report

Cmpd.	No. of Cmpds.	m/z meas.	Δ m/z [ppm]	z	Rt [min]	Score	P	Range	Sequence	Modification	Ratios
2697	1	1175.5508	58.82	2	62.1	11.6	1	1039-1058	R.AFTSEEELGESEMYYCSKCK.T	Oxidation: 13	
598	1	823.8884	-6.53	2	37.5	15.8	0	1230-1245	K.ENGAGQICELADALSR.G		M:Q 1.85<br>Q:W 0.23

Detailed Protein Report

Protein 245: PREDICTED: bromodomain adjacent to zinc finger domain protein 2A isoform X11 [Homo sapiens]

Accession: gi|578823227
Database: refseq_human(refseq_human_20140103.fasta)
Database Date: 2015-11-30

Score: 55.6
MW [kDa]: 170.8
pI: 6.2
Sequence Coverage [%]: 3.0
No. of unique Peptides: 3

Quantitation

Q:W	**Median:** 0.94	**CV:** 0.00 %	**No. of Peptides:**	1
M:Q	**Median:** 0.86	**CV:** 0.00 %	**No. of Peptides:**	1

10	20	30	40	50	60	70	80
MEVACILHTR	GGVQKTGERR	EMEANDHFNF	TGLPPAPAAS	GLKPSPSSGE	GLYTNGSPMN	FPQQGKSLNG	DVNVNGLSTV
90	100	110	120	130	140	150	160
SHTTTSGILN	SAPHSSSTSH	LHHPSVAYDC	LWNYSQYPSA	NPGSNLKDPP	LLSQFSGGQY	PLNGILGGSR	QPSSPSHNTN
170	180	190	200	210	220	230	240
LRAGSQEFWA	NGTQSPMGLN	FDSQELYDSF	PDQNFEVMPN	GPPSFFTSPQ	TSPMLGSSIQ	TFAPSQEVGS	GIHPDEAAEK
250	260	270	280	290	300	310	320
EMTSVVAENG	TGLVGSLELE	EEQPELKMCG	YNGSVPSVES	LHQEVSVLVP	DPTVSCLDDP	SHLPDQLEDT	PILSEDSLEP
330	340	350	360	370	380	390	400
FNSLAPEPVS	GGLYGIDDTE	LMGAEDKLPL	EDSPVISALD	CPSLNNATAF	SLLADDSQTS	TSIFASPTSP	PVLGESVLQD
410	420	430	440	450	460	470	480
NSFDLNNGSD	AEQEEMETQS	SDFPPSLTQP	APDQSSTIQL	HPATSPAVSP	TTSPAVSLVV	SPAASPEISP	EVCPAASTVV
490	500	510	520	530	540	550	560
SPAVFSVVSP	ASSAVLPAVS	LEVPLTASVT	SPKASPVTSP	AAAFPTASPA	NKDVSSFLET	TADVEEITGE	GLTASGSGDV
570	580	590	600	610	620	630	640
MRRRIATPEE	VRLPLQHGWR	REVRIKKGSH	RWQGETWYYG	PCGKRMKQFP	EVIKYLSRNV	VHSVRREHFS	FSPRMPVGDF
650	660	670	680	690	700	710	720
FEERDTPEGL	QWVQLSAEEI	PSRIQAITGK	RGRPRNTEKA	KTKEVPKVKR	GRGRPPKVKI	TELLNKTDNR	PLKKLEAQET
730	740	750	760	770	780	790	800
LNEEDKAKIA	KSKKKMRQKV	QRGECQTTIQ	GQARNKRKQE	TKSLKQKEAK	KKSKAEKEKG	KTKQEKLKEK	VKREKKEKVK
810	820	830	840	850	860	870	880
MKEKEEVTKA	KPACKADKTL	ATQRRLEERQ	RQQMILEEMK	KPTEDMCLTD	HQPLPDFSRV	PGLTLPSGAF	SDCLTIVEFL
890	900	910	920	930	940	950	960
HSFGKVLGFD	PAKDVPSLGV	LQEGLLCQGD	SLGEVQDLLV	RLLKAALHDP	GFPSYCQSLK	ILGEKVSEIP	LTRDNVSEIL
970	980	990	1000	1010	1020	1030	1040
RCFLMAYGVE	PALCDRLRTQ	PFQAQPPQQK	AAVLAFLVHE	LNGSTLIINE	IDKTLESMSS	YRKNKWIVEG	RLRRLKTVLA
1050	1060	1070	1080	1090	1100	1110	1120
KRTGRSEVEM	EGPEECLGRR	RSSRIMEETS	GMEEEEEEES	IAAVPGRRGR	RDGEVDATAS	SIPELERQIE	KLSKRQLFFR
1130	1140	1150	1160	1170	1180	1190	1200
KKLLHSSQML	RAVSLGQDRY	RRRYWVLPYL	AGIFVEGTEG	NLVPEEVIKK	ETDSLKVAAH	ASLNPALFSM	KMELAGSNTT
1210	1220	1230	1240	1250	1260	1270	1280
ASSPARARGR	PRKTKPGSMQ	PRHLKSPVRG	QDSEQPQAQL	QPEAQLHAPA	QPQPQLQLQL	QSHKGFLEQE	GSPLSLGQSQ
1290	1300	1310	1320	1330	1340	1350	1360
HDLSQSAFLS	WLSQTQSHSS	LLSSSVLTPD	SSPGKLDPAP	SQPPEEPEPD	EAESSPDPQA	LWFNISAQMP	CNAAPTPPPA
1370	1380	1390	1400	1410	1420	1430	1440
VSEDQPTPSP	QQLASSKPMN	RPSAANPCSP	VQFSSTPLAG	LAPKRRAGDP	GEMPQSPTGL	GQPKRRGRPP	SKFFKQMEQR
1450	1460	1470	1480	1490	1500	1510	1520
YLTQLTAQPV	PPEMCSGWWW	IRDPEMLDAM	LKALHPRGIR	EKALHKHLNK	HRDFLQEVCL	RPSADPIFEP	RQLPAFQEGI
1530	1540	1550	1560				
MSWSPKEKTY	ETDLAVLQWV	EELEQRVIMS	DLQIR				

Cmpd.	No. of Cmpds.	m/z meas.	Δ m/z [ppm]	z	Rt [min]	Score	P	Range	Sequence	Modification	Ratios
											Q:W 0.94

Detailed Protein Report

Cmpd.	No. of Cmpds.	m/z meas.	Δ m/z [ppm]	z	Rt [min]	Score	P	Range	Sequence	Modification	Ratios
710	1	676.9374	32.63	2	38.2	15.0	2	664-675	R.IQAITGKRGRPR.N		M:Q 0.86
2577	1	941.5794	21.02	2	62.2	13.8	2	698-713	K.VKITELLNKTDNRPLK.K		
778	1	830.6256	153.36	1	39.7	11.3	0	700-706	K.ITELLNK.T		

Detailed Protein Report

Protein 246: PREDICTED: muskelin isoform X4 [Homo sapiens]

**Accession:**	gi|578814266	**Score:**	55.6
**Database:**	refseq_human(refseq_human_20140103.fasta)	**MW [kDa]:**	80.3
**Database Date:**	2015-11-30	**pI:**	6.1
		**Sequence Coverage [%]:**	6.3
		**No. of unique Peptides:**	1

Quantitation

Q:W	**Median:**	2.04	**CV:**	0.00 %	**No. of Peptides:**	1
M:Q	**Median:**	1.48	**CV:**	0.00 %	**No. of Peptides:**	1

10	20	30	40	50	60	70	80
MAAGGAVAAA	PECRLLPYAL	HKWSSFSSTY	LPENILVDKP	NDQSSRWSSE	SNYPPQYLIL	KLERPAIVQN	ITFGKYEKTH
**90**	**100**	**110**	**120**	**130**	**140**	**150**	**160**
VCNLKKFKVF	GGMNEENMTE	LLSSGLKNDY	NKETFTLKHK	IDEQMFPCRF	IKIVPLLSWG	PSFNFSIWYV	ELSGIDDPDI
**170**	**180**	**190**	**200**	**210**	**220**	**230**	**240**
VQPCLNWYSK	YREQEAIRLC	LKHFRQHNYT	EAFESLQKKT	KIALEHPMLT	DIHDKLVLKG	DFDACEELIE	KAVNDGLFNQ
**250**	**260**	**270**	**280**	**290**	**300**	**310**	**320**
YISQQEYKPR	WSQIIPKSTK	GDGEDNRPGM	RGGHQMVIDV	QTETVYLFGG	WDGTQDLADF	WAYSVKENQW	TCISRDTEKE
**330**	**340**	**350**	**360**	**370**	**380**	**390**	**400**
NGPSARSCHK	MCIDIQRRQI	YTLGRYLDSS	VRNSKSLKSD	FYRYDIDTNT	WMLLSEDTAA	DGGPKLVFDH	QMCMDSEKHM
**410**	**420**	**430**	**440**	**450**	**460**	**470**	**480**
IYTFGGRILT	CNGSVDDSRA	SEPQFSGLFA	FNCQCQTWKL	LREDSCNAGP	EDIQSRIGHC	MLFHSKNRCL	YVFGGQRSKT
**490**	**500**	**510**	**520**	**530**	**540**	**550**	**560**
YLNDFFSYDV	DSDHVDIISD	GTKKDSGMVP	MTGFTQRATI	DPELNEIHVL	SGLSKDKEKR	EENVRNSFWI	YDIVRNSWSC
**570**	**580**	**590**	**600**	**610**	**620**	**630**	**640**
VYKNDQAAKD	NPTKSLQEEE	PCPRFAHQLV	YDELHKVHYL	FGGNPGKSCS	PKMRLDDFWS	LKLCRPSKDY	LLRHCKYLIR
**650**	**660**	**670**	**680**	**690**	**700**		
KHRFEEKAQV	DPLSALKYLQ	NDLYITVDHS	DPEETKEFQL	LASALFKSGS	DFTALA		

Cmpd.	No. of Cmpds.	m/z meas.	Δ m/z [ppm]	z	Rt [min]	Score	P	Range	Sequence	Modification	Ratios
1158	1	477.6545	-288.11	2	44.1	17.4	0	15-22	R.LLPYALHK.W		Q:W 2.04<br>M:Q 1.48

Detailed Protein Report

Protein 247: sarcoplasmic/endoplasmic reticulum calcium ATPase 2 isoform a [Homo sapiens]

Accession: gi|4502285
Database: refseq_human(refseq_human_20140103.fasta)
Database Date: 2015-11-30
Modification(s): Carbamidomethyl, Oxidation

Score: 55.5
MW [kDa]: 109.6
pI: 5.1
Sequence Coverage [%]: 3.4
No. of unique Peptides: 3

Quantitation

Q:W **Median:** 0.74 **CV:** 131.09 % **No. of Peptides:** 2

10	20	30	40	50	60	70	80
MENAHTKTVE	EVLGHFGVNE	STGLSLEQVK	KLKERWGSNE	LPAEEGKTLL	ELVIEQFEDL	LVRILLLAAC	ISFVLAWFEE
**90**	**100**	**110**	**120**	**130**	**140**	**150**	**160**
GEETITAFVE	PFVILLILVA	NAIVGVWQER	NAENAIEALK	EYEPEMGKVY	RQDRKSVQRI	KAKDIVPGDI	VEIAVGDKVP
**170**	**180**	**190**	**200**	**210**	**220**	**230**	**240**
ADIRLTSIKS	TTLRVDQSIL	TGESVSVIKH	TDPVPDPRAV	NQDKKNMLFS	GTNIAAGKAM	GVVVATGVNT	EIGKIRDEMV
**250**	**260**	**270**	**280**	**290**	**300**	**310**	**320**
ATEQERTPLQ	QKLDEFGEQL	SKVISLICIA	VWIINIGHFN	DPVHGGSWIR	GAIYYFKIAV	ALAVAAIPEG	LPAVITTCLA
**330**	**340**	**350**	**360**	**370**	**380**	**390**	**400**
LGTRRMAKKN	AIVRSLPSVE	TLGCTSVICS	DKTGTLTTNQ	MSVCRMFILD	RVEGDTCSLN	EFTITGSTYA	PIGEVHKDDK
**410**	**420**	**430**	**440**	**450**	**460**	**470**	**480**
PVNCHQYDGL	VELATICALC	NDSALDYNEA	KGVYEKVGEA	TETALTCLVE	KMNVFDTELK	GLSKIERANA	CNSVIKQLMK
**490**	**500**	**510**	**520**	**530**	**540**	**550**	**560**
KEFTLEFSRD	RKSMSVYCTP	NKPSRTSMSK	MFVKGAPEGV	IDRCTHIRVG	STKVPMTSGV	KQKIMSVIRE	WGSGSDTLRC
**570**	**580**	**590**	**600**	**610**	**620**	**630**	**640**
LALATHDNPL	RREEMHLEDS	ANFIKYETNL	TFVGCVGMLD	PPRIEVASSV	KLCRQAGIRV	IMITGDNKGT	AVAICRRIGI
**650**	**660**	**670**	**680**	**690**	**700**	**710**	**720**
FGQDEDVTSK	AFTGREFDEL	NPSAQRDACL	NARCFARVEP	SHKSKIVEFL	QSFDEITAMT	GDGVNDAPAL	KKAEIGIAMG
**730**	**740**	**750**	**760**	**770**	**780**	**790**	**800**
SGTAVAKTAS	EMVLADDNFS	TIVAAVEEGR	AIYNNMKQFI	RYLISSNVGE	VVCIFLTAAL	GFPEALIPVQ	LLWVNLVTDG
**810**	**820**	**830**	**840**	**850**	**860**	**870**	**880**
LPATALGFNP	PDLDIMNKPP	RNPKEPLISG	WLFFRYLAIG	CYVGAATVGA	AAWWFIAADG	GPRVSFYQLS	HFLQCKEDNP
**890**	**900**	**910**	**920**	**930**	**940**	**950**	**960**
DFEGVDCAIF	ESPYPMTMAL	SVLVTIEMCN	ALNSLSENQS	LLRMPPWENI	WLVGSICLSM	SLHFLILYVE	PLPLIFQITP
**970**	**980**	**990**	**1000**				
LNVTQWLMVL	KISLPVILMD	ETLKFVARNY	LEPAILE				

Cmpd.	No. of Cmpds.	m/z meas.	Δ m/z [ppm]	z	Rt [min]	Score	P	Range	Sequence	Modification	Ratios	
1921	1	781.2702	-187.08	2	52.7	14.2	0	219-234	K.AMGVVVATGVNTEIGK.I	Oxidation: 2	Q:W	0.27
2182	2	682.2713	-118.44	3	57.7	13.8	2	511-528	K.MFVKGAPEGVIDRCTHIR.V	Oxidation: 1	Q:W	2.00
2121	1	701.2646	-134.99	3	56.7	14.0	2	511-528	K.MFVKGAPEGVIDRCTHIR.V	Carbamidomethyl: 14; Oxidation: 1		

Detailed Protein Report

Protein 248: PREDICTED: ubiquitin-protein ligase E3C isoform X3 [Homo sapiens]

**Accession:**	gi|530387311	**Score:**	55.3
**Database:**	refseq_human(refseq_human_20140103.fasta)	**MW [kDa]:**	116.7
**Database Date:**	2015-11-30	**pI:**	6.2
**Modification(s):**	Carbamidomethyl	**Sequence Coverage [%]:**	4.3
		**No. of unique Peptides:**	4

```
        10         20         30         40         50         60         70         80
MFSFEGDFKT RPKVSLGGAS RKEEKASLLH RTQEERRKRE EERRRLKNAI IIQSFIRGYR DRKQQYSIQR SAFDRCATLS
        90        100        110        120        130        140        150        160
QSGGAFPIAN GPNLTLLVRQ LLFFYKQNED SKRLIWLYQN LIKHSSLFVK QLDGSERLTC LFQIKRLMSL CCRLLQNCND
       170        180        190        200        210        220        230        240
DSLNVALPMR MLEVFSSENT YLPVLQDASY VVSVIEQILH YMIHNGYYRS LYLLINSKLP SSIEYSDLSR VPIAKILLEN
       250        260        270        280        290        300        310        320
VLKPLHFTYN SCPEGARQQV FTAFTEEFLA APFTDQIFHF IIPALADAQT VFPYEPFLNA LLLIESRCSR KSGGAPWLFY
       330        340        350        360        370        380        390        400
FVLTVGENYL GALSEEGLLV YLRVLQTFLS QLPVSPASAS CHDSASDSEE ESEEADKPSS PEDGRLSVSY ITEECLKKLD
       410        420        430        440        450        460        470        480
TKQQTNTLLN LVWRDSASEE VFTTMASVCH TLMVQHRMMV PKVRLLYSLA FNARFLRHLW FLISSMSTRM ITGSMVPLLQ
       490        500        510        520        530        540        550        560
VISRGSPMSF EDSSRIIPLF YLFSSLFSHS LISIHDNEFF GDPIEVVGQR QSSMMPFTLE ELIMLSRCLR DACLGIIKLA
       570        580        590        600        610        620        630        640
YPETKPEVRE EYITAFQSIG VTTSSEMQQC IQMEQKRWIQ LFKVITNLVK MLKSRDTRRN FCPPNHWLSE QEDIKADKIF
       650        660        670        680        690        700        710        720
QRLIYADKQE VQGDGPFLDG INVTIRRNYI YEDAYDKLSP ENEPDLKKRI RVHLLNAHGL DEAGIDGGGI FREFLNELLK
       730        740        750        760        770        780        790        800
SGFNPNQGFF KTTNEGLLYP NPAAQMLVGD SFARHYYFLG RMLGKALYEN MLVELPFAGF FLSKLLGTSA DVDIHHLASL
       810        820        830        840        850        860        870        880
DPEVYKNLLF LKSYEDDVEE LGLNFTVVNN DLGEAQVVEL KFGGKDIPVT SANRIAYIHL VADYRLNRQI RQHCLAFRQG
       890        900        910        920        930        940        950        960
LANVVSLEWL RMFDQQEIQV LISGAQVPIS LEDLKSFTNY SGGYSADHPV IKVFWRVVEG FTDEEKRKLL KFVTSCSRPP
       970        980        990       1000       1010       1020       1030
LLGFKELYPA FCIHNGGSDL ERLPTASTCM NLLKLPEFYD ETLLRSKLLY AIECAAGFEL S
```

Cmpd.	No. of Cmpds.	m/z meas.	Δ m/z [ppm]	z	Rt [min]	Score	P	Range	Sequence	Modification
2489	9	817.4835	32.82	1	61.7	16.0	0	124-130	K.HSSLFVK.Q	
259	1	862.8548	57.47	3	32.6	16.7	2	689-712	K.RIRVHLLNAHGLDEAGIDGGGIFR.E	
2651	1	856.4088	-69.02	2	64.2	10.9	2	866-878	R.LNRQIRQHCLAFR.Q	Carbamidomethyl: 9
2795	1	874.5325	111.23	1	64.0	11.6	0	872-878	R.QHCLAFR.Q	

Detailed Protein Report

Protein 249: DNA-directed RNA polymerase I subunit RPA2 isoform 4 [Homo sapiens]

**Accession:**	gi|544346215	**Score:**	55.3
**Database:**	refseq_human(refseq_human_20140103.fasta)	**MW [kDa]:**	107.8
**Database Date:**	2015-11-30	**pI:**	8.3
**Modification(s):**	Oxidation	**Sequence Coverage [%]:**	3.8
		**No. of unique Peptides:**	1

Quantitation

Q:W	**Median:**	0.15	**CV:**	0.00 %	**No. of Peptides:**	1
M:Q	**Median:**	1.43	**CV:**	0.00 %	**No. of Peptides:**	1

10	20	30	40	50	60	70	80
MDPGSRWRNL	PSGPSLKHLT	DPSYGIPREQ	QKAALQELTR	AHVESFNYAV	HEGLGLAVQA	IPPFEFAFKD	ERISFTILDA
90	100	110	120	130	140	150	160
VISPPTVPKG	TICKEANVYP	AECRGRRSTY	RGKLTADINW	AVNGISKGII	KQFLGYVPIM	VKSKLCNLRN	LPPQALIEHH
170	180	190	200	210	220	230	240
EEAEEMGGYF	IINGIEKVIR	MLIMPRRNFP	IAMIRPKWKT	RGPGYTQYGV	SMHCVREEHS	AVNMNLHYLE	NGTVMLNFIY
250	260	270	280	290	300	310	320
RKELFFLPLG	FALKALVSFS	DYQIFQELIK	GKEDDSFLRN	SVSQMLRIVM	EEGCSTQKQV	LNYLGECFRV	KLNVPDWYPN
330	340	350	360	370	380	390	400
EQAAEFLFNQ	CICIHLKSNT	EKFYMLCLMT	RKLFALAKGE	CMEDNPDSLV	NQEVLTPGQL	FLMFLKGSLP	LMELPTDHTV
410	420	430	440	450	460	470	480
LREKRIPPWM	EVVLIPMTGK	PSLYPGLFLF	TTPCRLVRPV	QNLALGKEEL	IGTMEQIFMN	VAIFEDEVFA	GVTTHQELFP
490	500	510	520	530	540	550	560
HSLLSVIANF	IPFSDHNQSP	RNMYQCQMGK	QTMGFPLLTY	QDRSDNKLYR	LQTPQSPLVR	PSMYDYYDMD	NYPIGTNAIV
570	580	590	600	610	620	630	640
AVISYTGYDM	EDAMIVNKAS	WERGFAHGSV	YKSEFIDLSE	KIKQGDSSLV	FGIKPGDPRV	LQKLDDDGLP	FIGAKLQYGD
650	660	670	680	690	700	710	720
PYYSYLNLNT	GESFVMYYKS	KENCVVDNIK	VCSNDTGSGK	FKCVCITMRV	PRNPTIGDKF	ASRHGQKGIL	SRLWPAEDMP
730	740	750	760	770	780	790	800
FTESGMVPDI	LFNPHGFPSR	MTIGMLIESM	AGKSAALHGL	CHDATPFIFS	EENSALEYFG	EMLKAAGYNF	YGTERLYSGI
810	820	830	840	850	860	870	880
SGLELEADIF	IGVVYYQRLR	HMVSDKFQVR	TTGARDRVTN	QPIGGRNVQG	GIRFGEMERD	ALLAHGTSFL	LHDRLFNCSD
890	900	910	920	930	940	950	960
RSVAHVCVKC	GSLLSPLLEK	PPPSWSAMRN	RKYNCTLCSR	SDTIDTVSVP	YVFRYFVAEL	AAMNIKVKLD	VV

Cmpd.	No. of Cmpds.	m/z meas.	Δ m/z [ppm]	z	Rt [min]	Score	P	Range	Sequence	Modification	Ratios	
354	1	1026.5219	-20.89	2	34.4	35.4	1	387-404	K.GSLPLMELPTDHTVLREK.R	Oxidation: 6	Q:W<br>M:Q	0.15<br>1.43

Detailed Protein Report

Protein 250: metallothionein-1G [Homo sapiens]

**Accession:**	gi|10835230	**Score:**	55.0
**Database:**	refseq_human(refseq_human_20140103.fasta)	**MW [kDa]:**	6.1
**Database Date:**	2015-11-30	**pI:**	10.5
**Modification(s):**	Carbamidomethyl	**Sequence Coverage [%]:**	100.0
		**No. of unique Peptides:**	3

```
        10         20         30         40         50         60         70
MDPNCSCAAG VSCTCASSCK CKECKCTSCK KSCCSCCPVG CAKCAQGCIC KGASEKCSCC A
```

Cmpd.	No. of Cmpds.	m/z meas.	Δ m/z [ppm]	z	Rt [min]	Score	P	Range	Sequence	Modification
834	1	703.6768	119.69	3	39.8	13.8	0	1-20	-.MDPNCSCAAGVSCTCASSCK.C	Carbamidomethyl: 5, 15, 19
1987	2	862.6346	-31.70	3	53.2	13.2	2	1-25	-.MDPNCSCAAGVSCTCASSCKCKECK.C	Carbamidomethyl: 7
2136	1	1019.7667	-122.67	2	56.9	14.5	2	26-43	K.CTSCKKSCCSCCPVGCAK.C	Carbamidomethyl: 1, 11, 12, 16

Detailed Protein Report

Protein 251: PREDICTED: ADAMTS-like protein 3 isoform X2 [Homo sapiens]

**Accession:**	gi|530406912	**Score:**	54.9
**Database:**	refseq_human(refseq_human_20140103.fasta)	**MW [kDa]:**	186.4
**Database Date:**	2015-11-30	**pI:**	9.5
		**Sequence Coverage [%]:**	4.5
		**No. of unique Peptides:**	1

10	20	30	40	50	60	70	80
MASWTSPWWV	LIGMVFMHSP	LPQTTAEKSP	GAYFLPEFAL	SPQGSFLEDT	TGEQFLTYRY	DDQTSRNTRS	DEDKDGNWDA
**90**	**100**	**110**	**120**	**130**	**140**	**150**	**160**
WGDWSDCSRT	CGGGASYSLR	RCLTGRNCEG	QNIRYKTCSN	HDCPPDAEDF	RAQQCSAYND	VQYQGHYYEW	LPRYNDPAAP
**170**	**180**	**190**	**200**	**210**	**220**	**230**	**240**
CALKCHAQGQ	NLVVELAPKV	LDGTRCNTDS	LDMCISGICQ	AVGCDRQLGS	NAKEDNCGVC	AGDGSTCRLV	RGQSKSHVSP
**250**	**260**	**270**	**280**	**290**	**300**	**310**	**320**
EKREENVIAV	PLGSRSVRIT	VKGPAHLFIE	SKTLQGSKGE	HSFNSPGVFL	VENTTVEFQR	GSERQTFKIP	GPLMADFIFK
**330**	**340**	**350**	**360**	**370**	**380**	**390**	**400**
TRYTAAKDSV	VQFFFYQPIS	HQWRQTDFFP	CTVTCGGGYQ	LNSAECVDIR	LKRVVPDHYC	HYYPENVKPK	PKLKECSMDP
**410**	**420**	**430**	**440**	**450**	**460**	**470**	**480**
CPSSDGFKEI	MPYDHFQPLP	RWEHNPWTAC	SVSCGGGIQR	RSFVCVEESM	HGEILQVEEW	KCMYAPKPKV	MQTCNLFDCP
**490**	**500**	**510**	**520**	**530**	**540**	**550**	**560**
KWIAMEWSQC	TVTCGRGLRY	RVVLCINHRG	EHVGGCNPQL	KLHIKEECVI	PIPCYKPKEK	SPVEAKLPWL	KQAQELEETR
**570**	**580**	**590**	**600**	**610**	**620**	**630**	**640**
IATEEPTFIP	EPWSACSTTC	GPGVQVREVK	CRVLLTFTQT	ETELPEEECE	GPKLPTERPC	LLEACDESPA	SRELDIPLPE
**650**	**660**	**670**	**680**	**690**	**700**	**710**	**720**
DSETTYDWEY	AGFTPCTATC	VGGHQEAIAV	CLHIQTQQTV	NDSLCDMVHR	PPAMSQACNT	EPCPPRWHVG	SWGPCSATCG
**730**	**740**	**750**	**760**	**770**	**780**	**790**	**800**
VGIQTRDVYC	LHPGETPAPP	EECRDEKPHA	LQACNQFDCP	PGWHIEEWQQ	CSRTCGGGTQ	NRRVTCRQLL	TDGSFLNLSD
**810**	**820**	**830**	**840**	**850**	**860**	**870**	**880**
ELCQGPKASS	HKSCARTDCP	PHLAVGDWSK	CSVSCGVGIQ	RRKQVCQRLA	AKGRRIPLSE	MMCRDLPGLP	LVRSCQMPEC
**890**	**900**	**910**	**920**	**930**	**940**	**950**	**960**
SKIKSEMKTK	LGEQGPQILS	VQRVYIQTRE	EKRINLTIGS	RAYLLPNTSV	IIKCPVRRFQ	KSLIQWEKDG	RCLQNSKRLG
**970**	**980**	**990**	**1000**	**1010**	**1020**	**1030**	**1040**
ITKSGSLKIH	GLAAPDIGVY	RCIAGSAQET	VVLKLIGTDN	RLIARPALRE	PMREYPGMDH	SEANSLGVTW	HKMRQMWNNK
**1050**	**1060**	**1070**	**1080**	**1090**	**1100**	**1110**	**1120**
NDLYLDDDHI	SNQPFLRALL	GHCSNSAGST	NSWELKNKQF	EAAVKQGAYS	MDTAQFDELI	RNMSQLMETG	EVSDDLASQL
**1130**	**1140**	**1150**	**1160**	**1170**	**1180**	**1190**	**1200**
IYQLVAELAK	AQPTHMQWRG	IQEETPPAAQ	LRGETGSVSQ	SSHAKNSGKL	TFKPKGPVLM	RQSQPPSISF	NKTINSRIGN
**1210**	**1220**	**1230**	**1240**	**1250**	**1260**	**1270**	**1280**
TVYITKRTEV	INILCDLITP	SEATYTWTKD	GTLLQPSVKI	ILDGTGKIQI	QNPTRKEQGI	YECSVANHLG	SDVESSSVLY
**1290**	**1300**	**1310**	**1320**	**1330**	**1340**	**1350**	**1360**
AEAPVILSVE	RNITKPEHNH	LSVVVGGIVE	AALGANVTIR	CPVKGVPQPN	ITWLKRGGSL	SGNVSLLFNG	SLLLQNVSLE
**1370**	**1380**	**1390**	**1400**	**1410**	**1420**	**1430**	**1440**
NEGTYVCIAT	NALGKAVATS	VLHLLERRWP	ESRIVFLQGH	KKYILQATNT	RTNSNDPTGE	PPPQEPFWEP	GNWSHCSATC
**1450**	**1460**	**1470**	**1480**	**1490**	**1500**	**1510**	**1520**
GHLGARIQRP	QCVMANGQEV	SEALCDHLQK	PLAGFEPCNI	RDCPARWFTS	VWSQCSVSCG	EGYHSRQVTC	KRTKANGTVQ
**1530**	**1540**	**1550**	**1560**	**1570**	**1580**	**1590**	**1600**
VVSPRACAPK	DRPLGRKPCF	GHPCVQWEPG	NRCPGRCMGR	AVRMQQRHTA	CQHNSSDSNC	DDRKRPTLRR	NCTSGACDVC
**1610**	**1620**	**1630**	**1640**	**1650**	**1660**	**1670**	**1680**
WHTGPWKPCT	AACGRGFQSR	KVDCIHTRSC	KPVAKRHCVQ	KKKPISWRHC	LGPSCDSTYT	SQETAQTQLT	TVCL

Cmpd.	No. of Cmpds.	m/z meas.	Δ m/z [ppm]	z	Rt [min]	Score	P	Range	Sequence	Modification
2080	1	1044.4112	-48.98	2	55.9	14.5	0	422-440	R.WEHNPWTACSVSCGGGIQR.R	

Detailed Protein Report

Protein 252: protein FAM194B [Homo sapiens]

**Accession:**	gi|210147567	**Score:**	54.8
**Database:**	refseq_human(refseq_human_20140103.fasta)	**MW [kDa]:**	81.6
**Database Date:**	2015-11-30	**pI:**	4.6
		**Sequence Coverage [%]:**	7.5
		**No. of unique Peptides:**	2

10	20	30	40	50	60	70	80
MSAENNQLSG	ASPPHPPTTP	QYSTQNLPSE	KEDTEVELDE	ESLQDESPFS	PEGESLEDKE	YLEEEEDLEE	EEYLGKEEYL
**90**	**100**	**110**	**120**	**130**	**140**	**150**	**160**
KEEEYLGKEE	HLEEEEYLEK	AGYLEEEEYI	EEEEYLGKEG	YLEEEEYLGK	EEHLEEEEYL	GKEGYLEKED	YIEEVDYLGK
**170**	**180**	**190**	**200**	**210**	**220**	**230**	**240**
KAYLEEEEYL	GKKSYLEEEK	ALEKEENLEE	EEALEKEENL	DGKENLYKKY	LKEPKASYSS	QTMLLRDARS	PDAGPSQVTT
**250**	**260**	**270**	**280**	**290**	**300**	**310**	**320**
FLTVPLTFAT	PSPVSESATE	SSELLLTLYR	RSQASQTDWC	YDRTAVKSLK	SKSETEQETT	TKLAPEEHVN	TKVQQKKEEN
**330**	**340**	**350**	**360**	**370**	**380**	**390**	**400**
VLEFASKENF	WDGITDESID	KLEVEDLDEN	FLNSSYQTVF	KTIIKEMAAH	NELEEDFDIP	LTKLLESENR	WKLVIMLKKN
**410**	**420**	**430**	**440**	**450**	**460**	**470**	**480**
YEKFKETILR	IKRRREAQKL	TEMTSFTFHL	MSKPTPEKPE	TEEIQKPQRV	VHHRKKLERD	KEWIQKKTVV	HQGDGKLILY
**490**	**500**	**510**	**520**	**530**	**540**	**550**	**560**
PNKNVYQILF	PDGTGQIHYP	SGNLAMLILY	AKMKKFTYII	LEDSLEGRIR	ALINNSGNAT	FYDENSDIWL	NLSSNLGYYF
**570**	**580**	**590**	**600**	**610**	**620**	**630**	**640**
PKDKRQKAWN	WWNLNIHVHA	PPVQPISLKI	NEYIQVQIRS	QDKIIFCFTY	EQKQICLNLG	TRYKFVIPEV	LSEMKKKTIL
**650**	**660**	**670**	**680**	**690**	**700**		
EAEPGPTAQK	IRVLLGKMNR	LLNYATTPDL	ENFIEAVSIS	LMDNKYLKKM	LSKLWF		

Cmpd.	No. of Cmpds.	m/z meas.	Δ m/z [ppm]	z	Rt [min]	Score	P	Range	Sequence	Modification
2059	1	1051.4459	-42.83	2	56.1	10.5	0	366-383	K.EMAAHNELEEDFDIPLTK.L	
2001	1	876.8626	-126.17	2	53.7	10.2	2	462-476	K.EWIQKKTVVHQGDGK.L	

Detailed Protein Report

Protein 253: origin recognition complex subunit 6 [Homo sapiens]

**Accession:**	gi|7657427	**Score:**	54.8
**Database:**	refseq_human(refseq_human_20140103.fasta)	**MW [kDa]:**	28.1
**Database Date:**	2015-11-30	**pI:**	9.8
**Modification(s):**	Carbamidomethyl	**Sequence Coverage [%]:**	20.6
		**No. of unique Peptides:**	3

Quantitation

Q:W	**Median:**	1.42	**CV:**	0.00 %	**No. of Peptides:**	1
M:Q	**Median:**	0.71	**CV:**	0.00 %	**No. of Peptides:**	1

```
        10         20         30         40         50         60         70         80
MGSELIGRLA PRLGLAEPDM LRKAEEYLRL SRVKCVGLSA RTTETSSAVM CLDLAASWMK CPLDRAYLIK LSGLNKETYQ
        90        100        110        120        130        140        150        160
SCLKSFECLL GLNSNIGIRD LAVQFSCIEA VNMASKILKS YESSLPQTQQ VDLDLSRPLF TSAALLSACK ILKLKVDKNK
       170        180        190        200        210        220        230        240
MVATSGVKKA IFDRLCKQLE KIGQQVDREP GDVATPPRKR KKIVVEAPAK EMEKVEEMPH KPQKDEDLTQ DYEEWKRKIL
       250        260
ENAASAQKAT AE
```

Cmpd.	No. of Cmpds.	m/z meas.	Δ m/z [ppm]	z	Rt [min]	Score	P	Range	Sequence	Modification	Ratios
2133	6	701.2686	-80.31	3	56.8	17.2	0	42-60	R.TTETSSAVMCLDLAASWMK.C	Carbamidomethyl: 10	
55	1	930.9009	-129.85	2	30.4	14.2	2	61-76	K.CPLDRAYLIKLSGLNK.E	Carbamidomethyl: 1	
2540	1	941.9343	-22.57	2	61.7	12.6	0	100-116	R.DLAVQFSCIEAVNMASK.I	Carbamidomethyl: 8	Q:W 1.42<br>M:Q 0.71

Detailed Protein Report

Protein 254: eosinophil peroxidase preproprotein [Homo sapiens]

Accession: gi|4503595
Database: refseq_human(refseq_human_20140103.fasta)
Database Date: 2015-11-30
Modification(s): Carbamidomethyl

Score: 54.7
MW [kDa]: 81.0
pI: 11.5
Sequence Coverage [%]: 6.2
No. of unique Peptides: 2

Alias proteins:

Accession	Name	Description
gi|578831504	refseq_human (refseq_human_20140103.fasta)	PREDICTED: eosinophil peroxidase isoform X1 [Homo sapiens]

```
        10         20         30         40         50         60         70         80
MHLLPALAGV LATLVLAQPC EGTDPASPGA VETSVLRDCI AEAKLLVDAA YNWTQKSIKQ RLRSGSASPM DLLSYFKQPV
        90        100        110        120        130        140        150        160
AATRTVVRAA DYMHVALGLL EEKLQPQRSG PFNVTDVLTE PQLRLLSQAS GCALRDQAER CSDKYRTITG RCNNKRRPLL
       170        180        190        200        210        220        230        240
GASNQALARW LPAEYEDGLS LPFGWTPSRR RNGFLLPLVR AVSNQIVRFP NERLTSDRGR ALMFMQWGQF IDHDLDFSPE
       250        260        270        280        290        300        310        320
SPARVAFTAG VDCERTCAQL PPCFPIKIPP NDPRIKNQRD CIPFFRSAPS CPQNKNRVRN QINALTSFVD ASMVYGSEVS
       330        340        350        360        370        380        390        400
LSLRLRNRTN YLGLLAINQR FQDNGRALLP FDNLHDDPCL LTNRSARIPC FLAGDTRSTE TPKLAAMHTL FMREHNRLAT
       410        420        430        440        450        460        470        480
ELRRLNPRWN GDKLYNEARK IMGAMVQIIT YRDFLPLVLG KARARRTLGH YRGYCSNVDP RVANVFTLAF RFGHTMLQPF
       490        500        510        520        530        540        550        560
MFRLDSQYRA SAPNSHVPLS SAFFASWRIV YEGGIDPILR GLMATPAKLN RQDAMLVDEL RDRLFRQVRR IGLDLAALNM
       570        580        590        600        610        620        630        640
QRSRDHGLPG YNAWRRFCGL SQPRNLAQLS RVLKNQDLAR KFLNLYGTPD NIDIWIGAIA EPLLPGARVG PLLACLFENQ
       650        660        670        680        690        700        710        720
FRRARDGDRF WWQKRGVFTK RQRKALSRIS LSRIICDNTG ITTVSRDIFR ANIYPRGFVN CSRIPRLNLS AWRGT
```

Cmpd.	No. of Cmpds.	m/z meas.	Δ m/z [ppm]	z	Rt [min]	Score	P	Range	Sequence	Modification
873	1	575.3008	19.33	2	40.5	19.5	0	368-377	R.IPCFLAGDTR.S	Carbamidomethyl: 3
2217	1	927.3936	-145.10	2	56.1	18.0	2	547-562	R.QVRRIGLDLAALNMQR.S	

Detailed Protein Report

Protein 255: WD repeat- and FYVE domain-containing protein 4 [Homo sapiens]

**Accession:**	gi|191252801	**Score:**	54.6
**Database:**	refseq_human(refseq_human_20140103.fasta)	**MW [kDa]:**	353.4
**Database Date:**	2015-11-30	**pI:**	5.9
**Modification(s):**	Oxidation	**Sequence Coverage [%]:**	1.6
		**No. of unique Peptides:**	1

Alias proteins:

Accession	Name	Description
gi|530394051	refseq_human (refseq_human_20140103.fasta)	PREDICTED: WD repeat- and FYVE domain-containing protein 4 isoform X2 [Homo sapiens]
gi|530394049	refseq_human (refseq_human_20140103.fasta)	PREDICTED: WD repeat- and FYVE domain-containing protein 4 isoform X1 [Homo sapiens]

Detailed Protein Report

10	20	30	40	50	60	70	80
MEAEDLSKAE	DRNEDPGSKN	EGQLAAVQPD	VPHGGQSSSP	TALWDMLERK	FLEYQQLTHK	SPIERQKSLL	SLLPLFLKAW
90	100	110	120	130	140	150	160
EHSVGIICFP	SLQRLAEDVS	DQLAQQLQKA	LVGKPAEQAR	LAAGQLLWWK	GDVDQDGYLL	LKSVYVLTGT	DSETLGRVAE
170	180	190	200	210	220	230	240
SGLPALLLQC	LYLFFVFPLD	KDELLESDLQ	VQKMFVQMLL	NICSDSQGLE	GLLSGSELQS	LLIATTCLRE	HSCCFWKEPT
250	260	270	280	290	300	310	320
FCVLRAISKA	QNLSIIQYLQ	ATDCVRLSLQ	NLSRLTDTLP	APEVSEAVSL	ILGFVKDSYP	VSSALFLEFE	NSEGYPLLLK
330	340	350	360	370	380	390	400
VLLRYDGLTQ	SEVDPHLEEL	LGLVVWLTTC	GRSELKVFDS	ITYPQLEGFK	FHHEASGVTV	KNLQAFQVLQ	NVFHKASDSV
410	420	430	440	450	460	470	480
LCIQVLSVIR	TMWAWNARNF	FLLEWTLQPI	SQFVEIMPLK	PAPVQEHFFQ	LLEALVFELH	YVPHEILRKV	QHLIKESPGP
490	500	510	520	530	540	550	560
SCTLMALQSI	LSIAGGDPLF	TDIFRDSGLL	GLLLAQLRKQ	AKIMRKSGNK	VSTPGVQDPE	RELTCVMLRI	VVTLLKGSVR
570	580	590	600	610	620	630	640
NAVVLKDHGM	VPFIKIFLDD	ECYREASLSI	LEQLSAINAE	EYMSIIVGAL	CSSTQGELQL	KLDLLKSLLR	ILVTPKGRAA
650	660	670	680	690	700	710	720
FRVSSGFNGL	LSLLSDLEGS	LQEPPLQAWG	AVSPRQTLEL	VLYTLCAVSA	ALHWDPVNGY	FFRRNGLFEK	LAEDLCLLGC
730	740	750	760	770	780	790	800
FGALEEEGNL	LRSWVDTKAR	PFADLLGTAF	SSSGSLPPRI	QSCLQILGFL	DSMASGTLHL	RGDLKESLRT	KQGPVVDVQK
810	820	830	840	850	860	870	880
GETGSDPQRN	FKQWPDLEER	MDEGDAAIMH	PGVVCIMVRL	LPRLYHEDHP	QLSEEIQCSL	ASHIQSLVKS	EKNRQVMCEA
890	900	910	920	930	940	950	960
GLLGTLMASC	HRALVTSGSP	LHSRLIRIFE	KLASQAIEPD	VLRQFLGLGI	PSSLSATTKI	LDSSHTHRGN	PGCSGSQTAQ
970	980	990	1000	1010	1020	1030	1040
GLAEGPWPAA	PDAGLHPGVT	QAPQPLGESQ	DSTTALQTAL	SLISMTSPRN	LQPQRAALAP	SFVEFDMSVE	GYGCLFIPTL
1050	1060	1070	1080	1090	1100	1110	1120
STVMGTSTEY	SVSGGIGTGA	TRPFPPPGGL	TFSCWFLISR	HGAATEGHPL	RFLTLVRHLA	RTEQPFVCFS	VSLCPDDLSL
1130	1140	1150	1160	1170	1180	1190	1200
VVSTEEKEFQ	PLDVMEPEDD	SEPSAGCQLQ	VRCGQLLACG	QWHHLAVVVT	KEMKRHCTVS	TCLDGQVIGS	AKMLYIQALP
1210	1220	1230	1240	1250	1260	1270	1280
GPFLSMDPSA	FVDVYGYIAT	PRVWKQKSSL	IWRLGPTYLF	EEAISMETLE	VINKLGPRYC	GNFQAVHVQG	EDLDSEATPF
1290	1300	1310	1320	1330	1340	1350	1360
VAEERVSFGL	HIASSSITSV	ADIRNAYNEV	DSRLIAKEMN	ISSRDNAMPV	FLLRNCAGHL	SGSLRTIGAV	AVGQLGVRVF
1370	1380	1390	1400	1410	1420	1430	1440
HSSPAASSLD	FIGGPAILLG	LISLATDDHT	MYAAVKVLHS	VLTSNAMCDF	LMQHICGYQI	MAFLLRKKAS	LLNHRIFQLI
1450	1460	1470	1480	1490	1500	1510	1520
LSVAGTVELG	FRSSAITNTG	VFQHILCNFE	LWMNTADNLE	LSLFSHLLEI	LQSPREGPRN	AEAAHQAQLI	PKLIFLFNEP
1530	1540	1550	1560	1570	1580	1590	1600
SLIPSKISTI	IGILACQLRG	HFSTQDLLRI	GLFVVYTLKP	SSVNERQICM	DGALDPSLPA	GSQTSGKTIW	LRNQLLEMLL
1610	1620	1630	1640	1650	1660	1670	1680
SVISSPQLHL	SSESKEEMFL	KLGPDWFLLL	LQGHLHASTT	VLALKLLLYF	LASPSLRTRF	RDGLCAGSWV	ERSTEGVDIV
1690	1700	1710	1720	1730	1740	1750	1760
MDNLKSQSPL	PEQSPCLLPG	FRVLNDFLAH	HVHIPEVYLI	VSTFFLQTPL	TELMDGPKDS	LDAMLQWLLQ	RHHQEEVLQA
1770	1780	1790	1800	1810	1820	1830	1840
GLCTEGALLL	LEMLKATMSQ	PLAGSEDGAW	AQTFPASVLQ	FLSLVHRTYP	QDPAWRAPEF	LQTLAIAAFP	LGAQKGVGAE
1850	1860	1870	1880	1890	1900	1910	1920
STRNTSSPEA	AAEGDSTVEG	LQAPTKAHPA	RRKLREFTQL	LLRELLLGAS	SPKQWLPLEV	LLEASPDHAT	SQQKRDFQSE
1930	1940	1950	1960	1970	1980	1990	2000
VLLSAMELFH	MTSGGDAAMF	RDGKEPQPSA	EAAAAPSLAN	ISCFTQKLVE	KLYSGMFSAD	PRHILLFILE	HIMVVIETAS
2010	2020	2030	2040	2050	2060	2070	2080
SQRDTVLSTL	YSSLNKVILY	CLSKPQQSLS	ECLGLLSILG	FLQEHWDVVF	ATYNSNISFL	LCLMHCLLLL	NERSYPEGFG
2090	2100	2110	2120	2130	2140	2150	2160
LEPKPRMSTY	HQVFLSPNED	VKEKREDLPS	LSDVQHNIQK	TVQTLWQQLV	AQRQQTLEDA	FKIDLSVKPG	EREVKIEEVT
2170	2180	2190	2200	2210	2220	2230	2240
PLWEETMLKA	WQHYLASEKK	SLASRSNVAH	HSKVTLWSGS	LSSAMKLMPG	RQAKDPECKT	EDFVSCIENY	RRRGQELYAS
2250	2260	2270	2280	2290	2300	2310	2320
LYKDHVQRRK	CGNIKAANAW	ARIQEQLFGE	LGLWSQGEET	KPCSPWELDW	REGPARMRKR	IKRLSPLEAL	SSGRHKESQD
2330	2340	2350	2360	2370	2380	2390	2400

Detailed Protein Report

Cmpd.	No. of Cmpds.	m/z meas.	Δ m/z [ppm]	z	Rt [min]	Score	P	Range	Sequence	Modification
1632	1	1073.5401	4.58	2	48.7	12.2	1	531-549	K.VSTPGVQDPERELTCVMLR.I	Oxidation: 17

Detailed Protein Report

Protein 256: **PREDICTED: histone-lysine N-methyltransferase 2B isoform X2 [Homo sapiens]**

**Accession:**	gi|578835223	**Score:**	54.6
**Database:**	refseq_human(refseq_human_20140103.fasta)	**MW [kDa]:**	212.5
**Database Date:**	2015-11-30	**pI:**	6.8
**Modification(s):**	Carbamidomethyl, Oxidation	**Sequence Coverage [%]:**	2.8
		**No. of unique Peptides:**	2

Detailed Protein Report

10	20	30	40	50	60	70	80
MPPLEKARIA	GVGSLPLSGV	EEKMFSLLKR	AKVQLFKIDQ	QQQQKVAASM	PLSPGGQMEE	VAGAVKQISD	RGPVRSEDES
90	100	110	120	130	140	150	160
VEAKRERPSG	PESPVQGPRI	KHVCRHAAVA	LGQARAMVPE	DVPRLSALPL	RDRQDLATED	TSSASETESV	PSRSRRGKVE
170	180	190	200	210	220	230	240
AAGPGGESEP	TGSGGTLAHT	PRRSLPSHHG	KKMRMARCGH	CRGCLRVQDC	GSCVNCLDKP	KFGGPNTKKQ	CCVYRKCDKI
250	260	270	280	290	300	310	320
EARKMERLAK	KGRTIVKTLL	PWDSDESPEA	SPGPPGPRRG	AGAGGPREEV	VAHPGPEEQD	SLLQRKSARR	CVKQRPSYDI
330	340	350	360	370	380	390	400
FEDSDDSEPG	GPPAPRRRTP	RENELPLPEP	EEQSRPRKPT	LQPVLQLKAR	RRLDKDALAP	GPFASFPNGW	TGKQKSPDGV
410	420	430	440	450	460	470	480
HRVRVDFKED	CDLENVWLMG	GLSVLTSVPG	GPPMVCLLCA	SKGLHELVFC	QVCCDPFHPF	CLEEAERPLP	QHHDTWCCRR
490	500	510	520	530	540	550	560
CKFCHVCGRK	GRGSKHLLEC	ERCRHAYHPA	CLGPSYPTRA	TRKRRHWVRD	EICSACVRCK	SCGATPGKNW	DVEWSGDYSL
570	580	590	600	610	620	630	640
CPRCTQLYEK	GNYCPICTRC	YEDNDYESKM	MQCAQCDHWV	HAKCEGLSDE	DYEILSGLPD	SVLYTCGPCA	GAAQPRWREA
650	660	670	680	690	700	710	720
LSGALQGGLR	QVLQGLLSSK	VVGPLLLCTQ	CGPDGKQLHP	GPCGLQAVSQ	RFEDGHYKSV	HSFMEDMVGI	LMRHSEEGET
730	740	750	760	770	780	790	800
PDRRAGGQMK	GLLLKLLESA	FGWFDAHDPK	YWRRSTRLPN	GVLPNAVLPP	SLDHVYAQWR	QQEPETPESG	QPPGDPSAAF
810	820	830	840	850	860	870	880
QGKDPAAFSH	LEDPRQCALC	LKYGDADSKE	AGRLLYIGQN	EWTHVNCAIW	SAEVFEENDG	SLKNVHAAVA	RGRQMRCELC
890	900	910	920	930	940	950	960
LKPGATVGCC	LSSCLSNFHF	MCARASYCIF	QDDKKVFCQK	HTDLLDGKEI	VNPDGFDVLR	RVYVDFEGIN	FKRKFLTGLE
970	980	990	1000	1010	1020	1030	1040
PDAINVLIGS	IRIDSLGTLS	DLSDCEGRLF	PIGYQCSRLY	WSTVDARRRC	WYRCRILEYR	PWGPREEPAH	LEAAEENQTI
1050	1060	1070	1080	1090	1100	1110	1120
VHSPAPSSEP	PGGEDPPLDT	DVLVPGAPER	HSPIQNLDPP	LRPDSGSAPP	PAPRSFSGAR	IKVPNYSPSR	RPLGGVSFGP
1130	1140	1150	1160	1170	1180	1190	1200
LPSPGSPSSL	THHIPTVGDP	DFPAPPRRSR	RPSPLAPRPP	PSRWASPPLK	TSPQLRVPPP	TSVVTALTPT	SGELAPPGPA
1210	1220	1230	1240	1250	1260	1270	1280
PSPPPPEDLG	PDFEDMEVVS	GLSAADLDFA	ASLLGTEPFQ	EEIVAAGAMG	SSHGGPGDSS	EEESSPTSRY	IHFPVTVVSA
1290	1300	1310	1320	1330	1340	1350	1360
PGLAPSATPG	APRIEQLDGV	DDGTDSEAEA	VQQPRGQGTP	PSGPGVVRAG	VLGAAGDRAR	PPEDLPSEIV	DFVLKNLGGP
1370	1380	1390	1400	1410	1420	1430	1440
GDGGAGPREE	SLPPAPPLAN	GSQPSQGLTA	SPADPTRTFA	WLPGAPGVRV	LSLGPAPEPP	KPATSKIILV	NKLGQVFVKM
1450	1460	1470	1480	1490	1500	1510	1520
AGEGEPVPPP	VKQPPLPPTI	SPTAPTSWTL	PPGPLLGVLP	VVGVVRPAPP	PPPPPLTLVL	SSGPASPPRQ	AIRVKRVSTF
1530	1540	1550	1560	1570	1580	1590	1600
SGRSPPAPPP	YKAPRLDEDG	EASEDTPQVP	GLGSGGFSRV	RMKTPTVRGV	LDLDRPGEPA	GEESPGPLQE	RSPLLPLPED
1610	1620	1630	1640	1650	1660	1670	1680
GPPQVPDGPP	DLLLESQWHH	YSGEASSSEE	EPPSPDDKEN	QAPKRTGPHL	RFEISSEDGF	SVEAESLEGA	WRTLIEKVQE
1690	1700	1710	1720	1730	1740	1750	1760
ARGHARLRHL	SFSGMSGARL	LGIHHDAVIF	LAEQLPGAQR	CQHYKFRYHQ	QGEGQEEPPL	NPHGAARAEV	YLRKCTFDMF
1770	1780	1790	1800	1810	1820	1830	1840
NFLASQHRVL	PEGATCDEEE	DEVQLRSTRR	ATSLELPMAM	RFRHLKKTSK	EAVGVYRSAI	HGRGLFCKRN	IDAGEMVIEY
1850	1860	1870	1880	1890	1900	1910	1920
SGIVIRSVLT	DKREKFYDGK	GIGCYMFRMD	DFDVVDATMH	GNAARFINHS	CEPNCFSRVI	HVEGQKHIVI	FALRRILRGE
1930	1940	1950	1960				
ELTYDYKFPI	EDASNKLPCN	CGAKRCRRFL	N				

Cmpd.	No. of Cmpds.	m/z meas.	Δ m/z [ppm]	z	Rt [min]	Score	P	Range	Sequence	Modification
2760	1	841.9053	-83.22	2	63.0	12.2	1	7-23	K.ARIAGVGSLPLSGVEEK.M	
2463	1	647.1849	-148.23	2	61.2	18.9	2	193-202	K.MRMARCGHCR.G	Carbamidomethyl: 6; Oxidation: 1

Detailed Protein Report

Protein 257: **unconventional myosin-IXb isoform 2 [Homo sapiens]**

**Accession:**	gi|194272142	**Score:**	54.5
**Database:**	refseq_human(refseq_human_20140103.fasta)	**MW [kDa]:**	229.0
**Database Date:**	2015-11-30	**pI:**	9.4
**Modification(s):**	Carbamidomethyl, Oxidation	**Sequence Coverage [%]:**	2.5
		**No. of unique Peptides:**	2

10	20	30	40	50	60	70	80
MSVKEAGSSG	RREQAAYHLH	IYPQLSTTES	QASCRVTATK	DSTTSDVIKD	AIASLRLDGT	KCYVLVEVKE	SGGEEWVLDA
90	100	110	120	130	140	150	160
NDSPVHRVLL	WPRRAQDEHP	QEDGYYFLLQ	ERNADGTIKY	VHMQLVAQAT	ATRRLVERGL	LPRQQADFDD	LCNLPELTEG
170	180	190	200	210	220	230	240
NLLKNLKHRF	LQQKIYTYAG	SILVAINPFK	FLPIYNPKYV	KMYENQQLGK	LEPHVFALAD	VAYYTMLRKR	VNQCIVISGE
250	260	270	280	290	300	310	320
SGSGKTQSTN	FLIHCLTALS	QKGYASGVER	TILGAGPVLE	AFGNAKTAHN	NNSSRFGKFI	QVSYLESGIV	RGAVVEKYLL
330	340	350	360	370	380	390	400
EKSRLVSQEK	DERNYHVFYY	LLLGVSEEER	QEFQLKQPED	YFYLNQHNLK	IEDGEDLKHD	FERLKQAMEM	VGFLPATKKQ
410	420	430	440	450	460	470	480
IFAVLSAILY	LGNVTYKKRA	TGREEGLEVG	PPEVLDTLSQ	LLKVKREILV	EVLTKRKTVT	VNDKLILPYS	LSEAITARDS
490	500	510	520	530	540	550	560
MAKSLYSALF	DWIVLRINHA	LLNKKDVEEA	VSCLSIGVLD	IFGFEDFERN	SFEQFCINYA	NEQLQYYFNQ	HIFKLEQEEY
570	580	590	600	610	620	630	640
QGEGITWHNI	GYTDNVGCIH	LISKKPTGLF	YLLDEESNFP	HATSQTLLAK	FKQQHEDNKY	FLGTPVMEPA	FIIQHFAGKV
650	660	670	680	690	700	710	720
KYQIKDFREK	NMDYMRPDIV	ALLRGSDSSY	VRELIGMDPV	AVFRWAVLRA	AIRAMAVLRE	AGRLRAERAE	KAAGMSSPGA
730	740	750	760	770	780	790	800
QSHPEELPRG	ASTPSEKLYR	DLHNQMIKSI	KGLPWQGEDP	RSLLQSLSRL	QKPRAFILKS	KGIKQKQIIP	KNLLDSKSLK
810	820	830	840	850	860	870	880
LIISMTLHDR	TTKSLLHLHK	KKKPPSISAQ	FQTSLNKLLE	ALGKAEPFFI	RCIRSNAEKK	ELCFDDELVL	QQLRYTGMLE
890	900	910	920	930	940	950	960
TVRIRRSGYS	AKYTFQDFTE	QFQVLLPKDA	QPCREVISTL	LEKMKIDKRN	YQIGKTKVFL	KETERQALQE	TLHREVVRKI
970	980	990	1000	1010	1020	1030	1040
LLLQSWFRMV	LERRHFLQMK	RAAVTIQACW	RSYRVRRALE	RTQAAVYLQA	SWRGYWQRKL	YRHQKQSIIR	LQSLCRGHLQ
1050	1060	1070	1080	1090	1100	1110	1120
RKSFSQMISE	KQKAEEKERE	ALEAARAGAE	EGGQGQAAGG	QQVAEQGPEP	AEDGGHLASE	PEVQPSDRSP	LEHSSPEKEA
1130	1140	1150	1160	1170	1180	1190	1200
PSPEKTLPPQ	KTVAAESHEK	VPSSREKRES	RRQRGLEHVK	FQNKHIQSCK	EESALREPSR	RVTQEQGVSL	LEDKKESRED
1210	1220	1230	1240	1250	1260	1270	1280
ETLLVVETEA	ENTSQKQPTE	QPQAMAVGKV	SEETEKTLPS	GSPRPGQLER	PTSLALDSRV	SPPAPGSAPE	TPEDKSKPCG
1290	1300	1310	1320	1330	1340	1350	1360
SPRVQEKPDS	PGGSTQIQRY	LDAERLASAV	ELWRGKKLVA	AASPSAMLSQ	SLDLSDRHRA	TGAALTPTEE	RRTSFSTSDV
1370	1380	1390	1400	1410	1420	1430	1440
SKLLPSLAKA	QPAAETTDGE	RSAKKPAVQK	KKPGDASSLP	DAGLSPGSQV	DSKSTFKRLF	LHKTKDKKYS	LEGAEELENA
1450	1460	1470	1480	1490	1500	1510	1520
VSGHVVLEAT	TMKKGLEAPS	GQQHRHAAGE	KRTKEPGGKG	KKNRNVKIGK	ITVSEKWRES	VFRQITNANE	LKYLDEFLLN
1530	1540	1550	1560	1570	1580	1590	1600
KINDLRSQKT	PIESLFIEAT	EKFRSNIKTM	YSVPNGKIHV	GYKDLMENYQ	IVVSNLATER	GQKDTNLVLN	LFQSLLDEFT
1610	1620	1630	1640	1650	1660	1670	1680
RGYTKNDFEP	VKQSKAQKKK	RKQERAVQEH	NGHVFASYQV	SIPQSCEQCL	SYIWLMDKAL	LCSVCKMTCH	KKCVHKIQSH
1690	1700	1710	1720	1730	1740	1750	1760
CSYTYGRKGE	PGVEPGHFGV	CVDSLTSDKA	SVPIVLEKLL	EHVEMHGLYT	EGLYRKSGAA	NRTRELRQAL	QTDPAAVKLE
1770	1780	1790	1800	1810	1820	1830	1840
NFPIHAITGV	LKQWLRELPE	PLMTFAQYGD	FLRAVELPEK	QEQLAAIYAV	LEHLPEANHN	SLERLIFHLV	KVALLEDVNR
1850	1860	1870	1880	1890	1900	1910	1920
MSPGALAIIF	APCLLRCPDN	SDPLTSMKDV	LKITTCVEML	IKEQMRKYKV	KMEEISQLEA	AESIAFRRLS	LLRQNAPWPL
1930	1940	1950	1960	1970	1980	1990	2000
KLGFSSPYEG	VLNKSPKTRD	IQEEELEVLL	EEEAAGGDED	REKEILIERI	QSIKEEKEDI	TYRLPELDPR	GSDEENLDSE
2010	2020	2030					
TSASTESLLE	ERAGRGASEG	QY					

Cmpd.	No. of Cmpds.	m/z meas.	Δ m/z [ppm]	z	Rt [min]	Score	P	Range	Sequence	Modification

Detailed Protein Report

Cmpd.	No. of Cmpds.	m/z meas.	Δ m/z [ppm]	z	Rt [min]	Score	P	Range	Sequence	Modification
1951	1	918.3471	-89.50	2	53.1	10.7	1	1857-1872	R.CPDNSDPLTSMKDVLK.I	Carbamidomethyl: 1; Oxidation: 11
1465	1	671.4099	1.89	2	47.1	16.6	1	1964-1974	K.EILIERIQSIK.E	

Detailed Protein Report

Protein 258: TRIO and F-actin-binding protein isoform 1 [Homo sapiens]

**Accession:**	gi|88501740	**Score:**	54.4
**Database:**	refseq_human(refseq_human_20140103.fasta)	**MW [kDa]:**	74.0
**Database Date:**	2015-11-30	**pI:**	6.5
		**Sequence Coverage [%]:**	8.9
		**No. of unique Peptides:**	2

Quantitation

M:Q **Median:** 0.38 **CV:** 0.00 % **No. of Peptides:** 1

10	20	30	40	50	60	70	80
MGGWKGPGQR	RGKEGPEARR	RAAERGGGGG	GGGVPAPRSP	AREPRPRSCL	LLPPPWGAAM	TPDLLNFKKG	WMSILDEPGE
**90**	**100**	**110**	**120**	**130**	**140**	**150**	**160**
PPSPSLTTTS	TSQWKKHWFV	LTDSSLKYYR	DSTAEEADEL	DGEIDLRSCT	DVTEYAVQRN	YGFQIHTKDA	VYTLSAMTSG
**170**	**180**	**190**	**200**	**210**	**220**	**230**	**240**
IRRNWIEALR	KTVRPTSAPD	VTKLSDSNKE	NALHSYSTQK	GPLKAGEQRA	GSEVISRGGP	RKADGQRQAL	DYVELSPLTQ
**250**	**260**	**270**	**280**	**290**	**300**	**310**	**320**
ASPQRARTPA	RTPDRLAKQE	ELERDLAQRS	EERRKWFEAT	DSRTPEVPAG	EGPRRGLGAP	LTEDQQNRLS	EEIEKKWQEL
**330**	**340**	**350**	**360**	**370**	**380**	**390**	**400**
EKLPLRENKR	VPLTALLNQS	RGERRGPPSD	GHEALEKEVQ	ALRAQLEAWR	LQGEAPQSAL	RSQEDGHIPP	GYISQEACER
**410**	**420**	**430**	**440**	**450**	**460**	**470**	**480**
SLAEMESSHQ	QVMEELQRHH	ERELQRLQQE	KEWLLAEETA	ATASAIEAMK	KAYQEELSRE	LSKTRSLQQG	PDGLRKQHQS
**490**	**500**	**510**	**520**	**530**	**540**	**550**	**560**
DVEALKRELQ	VLSEQYSQKC	LEIGALMRQA	EEREHTLRRC	QQEGQELLRH	NQELHGRLSE	EIDQLRGFIA	SQGMGNGCGR
**570**	**580**	**590**	**600**	**610**	**620**	**630**	**640**
SNERSSCELE	VLLRVKENEL	QYLKKEVQCL	RDELQMMQKD	KRFTSGKYQD	VYVELSHIKT	RSEREIEQLK	EHLRLAMAAL
**650**	**660**						
QEKESMRNSL	AE						

Cmpd.	No. of Cmpds.	m/z meas.	Δ m/z [ppm]	z	Rt [min]	Score	P	Range	Sequence	Modification	Ratios
1361	1	711.8629	-0.75	2	45.3	11.9	1	22-38	R.AAERGGGGGGGGVPAPR.S		M:Q 0.38
2821	3	1051.5459	43.13	2	65.7	13.0	1	276-294	K.WFEATDSRTPEVPAGEGPR.R		

Detailed Protein Report

Protein 259: PREDICTED: PHD and RING finger domain-containing protein 1 isoform X2 [Homo sapiens]

**Accession:**	gi|578839865	**Score:**	54.2
**Database:**	refseq_human(refseq_human_20140103.fasta)	**MW [kDa]:**	178.2
**Database Date:**	2015-11-30	**pI:**	10.1
**Modification(s):**	Carbamidomethyl, Oxidation	**Sequence Coverage [%]:**	2.8
		**No. of unique Peptides:**	3

Quantitation

Q:W	**Median:**	0.44	**CV:**	0.00 %	**No. of Peptides:**	1
M:Q	**Median:**	0.61	**CV:**	0.00 %	**No. of Peptides:**	1

10	20	30	40	50	60	70	80
MDDDSLDELV	ARSPGPDGHP	QVGPADPAEE	SSVGSSGDSG	DDSDSEHGDG	TDGEDEGASE	EEDLEDRSGS	EDSEDDGETL
90	100	110	120	130	140	150	160
LEVAGTQGKL	EAAGSFNSDD	DAESCPICLN	AFRDQAVGTP	ENCAHYFCLD	CIVEWSKNAN	SCPVDRTLFK	CICIRAQFGG
170	180	190	200	210	220	230	240
KILRKIPVEN	TKASEEEEDP	TFCEVCGRSD	REDRLLLCDG	CDAGYHMECL	DPPLQEVPVD	EWFCPECAAP	GVVLAADAGP
250	260	270	280	290	300	310	320
VSEEEVSLLL	ADVVPTTSRL	RPRAGRTRAI	ARTRQSERVR	ATVNRNRIST	ARRVQHTPGR	LGSSLLDEAI	EAVATGLSTA
330	340	350	360	370	380	390	400
VYQRPLTPRT	PARRKRKTRR	RKKVPGRKKT	PSGPSAKSKS	SATRSKKRQH	RVKKRRGKKV	KSEATTRSRI	ARTLGLRRPV
410	420	430	440	450	460	470	480
HSSCIPSVLK	PVEPSLGLLR	ADIGAASLSL	FGDPYELDPF	DSSEELSANP	LSPLSAKRRA	LSRSALQSHQ	PVARPVSVGL
490	500	510	520	530	540	550	560
SRRRLPAAVP	EPDLEEEPVP	DLLGSILSGQ	SLLMLGSSDV	IIHRDGSLSA	KRAAPVSFQR	NSGSLSRGEE	GFKGCLQPRA
570	580	590	600	610	620	630	640
LPSGSPAQGP	SGNRPQSTGL	SCQGRSRTPA	RTAGAPVRLD	LPAAPGAVQA	RNLSNGSVPG	FRQSHSPWFN	GTNKHTLPLA
650	660	670	680	690	700	710	720
SAASKISSRD	SKPPCRSVVP	GPPLKPAPRR	TDISELPRIP	KIRRDDGGGR	RDAAPAHGQS	IEIPSACISR	LTGREGTGQP
730	740	750	760	770	780	790	800
GRGTRAESEA	SSRVPREPGV	HTGSSRPPAP	SSHGSLAPLG	PSRGKGVGST	FESFRINIPG	NMAHSSQLSS	PGFCNTFRPV
810	820	830	840	850	860	870	880
DDKEQRKENP	SPLFSIKKTK	QLRSEVYDPS	DPTGSDSSAP	GSSPERSGPG	LLPSEITRTI	SINSPKAQTV	QAVRCVTSYT
890	900	910	920	930	940	950	960
VESIFGTEPE	PPLGPSSAMS	KLRGAVAAEG	ASDTEREEPT	ESQGLAARLR	RPSPPEPWDE	EDGASCSTFF	GSEERTVTCV
970	980	990	1000	1010	1020	1030	1040
TVVEPEAPPS	PDVLQAATHR	VVELRPPSRS	RSTSSSRSRK	KAKRKRVSRE	HGRTRSGTRS	ESRDRSSRSA	SPSVGEERPR
1050	1060	1070	1080	1090	1100	1110	1120
RQRSKAKSRR	SSSDRSSSRE	RAKRKKAKDK	SREHRRGPWG	HSRRTSRSRS	GSPGSSSYEH	YESRKKKKRR	SASRPRGREC
1130	1140	1150	1160	1170	1180	1190	1200
SPTSSLERLC	RHKHQRERSH	ERPDRKESVA	WPRDRRKRRS	RSPSSEHRAR	EHRRPRSREK	WPQTRSHSPE	RKGAVREASP
1210	1220	1230	1240	1250	1260	1270	1280
APLAQGEPGR	EDLPTRLPAL	GEAHVSPEVA	TADKAPLQAP	PVLEVAAECE	PDDLDLDYGD	SVEAGHVFDD	FSSDAVFIQL
1290	1300	1310	1320	1330	1340	1350	1360
DDMSSPPSPE	STDSSPERDF	PLKPALPPAS	LAVAAIQREV	SLMHDEDPSQ	PPPLPEGTQE	PHLLRPDAAE	KAEAPSSPDV
1370	1380	1390	1400	1410	1420	1430	1440
APAGKEDSPS	ASGRVQEAAR	PEEVVSQTPL	LRSRALVKRV	TWNLQESESS	APAEDRAPRA	PLHRPQKPRE	GAWDMEDVAP
1450	1460	1470	1480	1490	1500	1510	1520
TGVRQAFSEL	PFPSHVLPEP	GFPDTDPSQV	YSPGLPPAPA	QPSSIPPCAL	VSQPTVQFIL	QGSLPLVGCG	AAQTLAPVPA
1530	1540	1550	1560	1570	1580	1590	1600
ALTPASEPAS	QATAASNSEE	KTPAPRLAAE	KTKKEEYMKK	LHMQERAVEE	VKLAIKPFYQ	KREVTKEEYK	DILRKAVQKI
1610	1620	1630	1640	1650			
CHSKSGEINP	VKVANLVKAY	VDKYRHMRRH	KKPEAGEEPP	TQGAEG			

Detailed Protein Report

Cmpd.	No. of Cmpds.	m/z meas.	Δ m/z [ppm]	z	Rt [min]	Score	P	Range	Sequence	Modification	Ratios
1686	1	697.6744	-192.66	2	51.0	20.4	0	1-12	-.MDDDSLDELVAR.S	Oxidation: 1	
1064	2	500.6481	-289.29	2	42.9	11.8	2	348-357	R.KKTPSGPSAK.S		Q:W 0.44<br>M:Q 0.61
2635	1	644.4948	273.66	1	63.7	10.4	0	1600-1604	K.ICHSK.S	Carbamidomethyl: 2	

Detailed Protein Report

Protein 260: PREDICTED: integrin beta-7 isoform X3 [Homo sapiens]

Accession: gi|578823673
Database: refseq_human(refseq_human_20140103.fasta)
Database Date: 2015-11-30
Modification(s): Carbamidomethyl

Score: 54.2
MW [kDa]: 85.0
pI: 5.7
Sequence Coverage [%]: 6.6
No. of unique Peptides: 4

10	20	30	40	50	60	70	80
MVALPMVLVL	LLVLSRGESE	LDAKIPSTGD	ATEWRNPHLS	MLGSCQPAPS	CQKCILSHPS	CAWCKQLNFT	ASGEAEARRC
**90**	**100**	**110**	**120**	**130**	**140**	**150**	**160**
ARREELLARG	CPLEELEEPR	GQQEVLQDQP	LSQGARGEGA	TQLAPQRVRV	TLRPGEPQQL	QVRFLRAEGY	PVDLYYLMDL
**170**	**180**	**190**	**200**	**210**	**220**	**230**	**240**
SYSMKDDLER	VRQLGHALLV	RLQEVTHSVR	IGFGSFVDKT	VLPFVSTVPS	KLRHPCPTRL	ERCQSPFSFH	HVLSLTGDAQ
**250**	**260**	**270**	**280**	**290**	**300**	**310**	**320**
AFEREVGRQS	VSGNLDSPEG	GFDAILQAAL	CQEQIGWRNV	SRLLVFTSDD	TFHTAGDGKL	GGIFMPSDGH	CHLDSNGLYS
**330**	**340**	**350**	**360**	**370**	**380**	**390**	**400**
RSTEFDYPSV	GQVAQALSAA	NIQPIFAVTS	AALPVYQELS	KLIPKSAVGE	LSEDSSNVVQ	LIMDAYNSLS	STVTLEHSSL
**410**	**420**	**430**	**440**	**450**	**460**	**470**	**480**
PPGVHISYES	QCEGPEKREG	KAEDRGQCNH	VRINQTVTFW	VSLQATHCLP	EPHLLRLRAL	GFSEELIVEL	HTLCDCNCSD
**490**	**500**	**510**	**520**	**530**	**540**	**550**	**560**
TQPQAPHCSD	GQGHLQCGVC	SCAPGRLGRL	CECSVAELSS	PDLESGCRAP	NGTGPLCSGK	GHCQCGRCSC	SGQSSGHLCE
**570**	**580**	**590**	**600**	**610**	**620**	**630**	**640**
CDDASCERHE	GILCGGFGRC	QCGVCHCHAN	RTGRACECSG	DMDSCISPEG	GLCSGHGRCK	CNRCQCLDGY	YGALCDQCPG
**650**	**660**	**670**	**680**	**690**	**700**	**710**	**720**
CKTPCERHRD	CAECGAFRTG	PLATNCSTAC	AHTNVTLALA	PILDDGWCKE	RTLDNQLFFF	LVEDDARGTV	VLRVRPQEKG
**730**	**740**	**750**	**760**	**770**	**780**	**790**	
ADHTQAIVLG	CVGGIVAVGL	GLVLAYRLSV	EIYDRREYSR	FEKEQQQLNW	KQLLGFGSRL	AS	

Cmpd.	No. of Cmpds.	m/z meas.	Δ m/z [ppm]	z	Rt [min]	Score	P	Range	Sequence	Modification
2663	2	731.3721	63.85	2	64.5	13.4	0	54-65	K.CILSHPSCAWCK.Q	Carbamidomethyl: 1, 11
2734	1	772.4219	-54.41	2	65.4	16.0	1	200-213	K.TVLPFVSTVPSKLR.H	
2949	1	1019.4212	-21.13	3	66.0	11.9	2	569-594	R.HEGILCGGFGRCQCGVCHCHANRTGR.A	Carbamidomethyl: 6, 12, 14, 17, 19
143	1	722.7732	-4.06	2	30.5	13.0	0	580-591	R.CQCGVCHCHANR.T	Carbamidomethyl: 1, 3

Detailed Protein Report

Protein 261: **PREDICTED: keratin, type I cytoskeletal 26 isoform X2 [Homo sapiens]**

**Accession:**	gi|578830847	**Score:**	54.2
**Database:**	refseq_human(refseq_human_20140103.fasta)	**MW [kDa]:**	51.6
**Database Date:**	2015-11-30	**pI:**	4.7
**Modification(s):**	Oxidation	**Sequence Coverage [%]:**	10.5
		**No. of unique Peptides:**	4

Quantitation

M:Q	**Median:** 1.64	**CV:** 0.00 %	**No. of Peptides:** 1

10	20	30	40	50	60	70	80
MSFRLSGGSR	RICSRTGSGR	LSGGGTGFVA	GNVCVGSGAR	SSFSCTLEGI	SSGGSFCNSG	GGLGSGACAG	FLGNEHSLLS
**90**	**100**	**110**	**120**	**130**	**140**	**150**	**160**
GNEKVTMQNL	NDRLASYLDH	VHALEEANAD	LEQKIKGWYE	KCEPGSSREH	DHDYSRYFSV	IEDLKRQIIS	ATICNASIVL
**170**	**180**	**190**	**200**	**210**	**220**	**230**	**240**
QNDNARLTAD	DFRLKYENEL	ALHHSVEADT	SGLRRVLDEL	TLCTTDLEIQ	CETLSEELTY	LKKSHEEEME	VLQYTAGGNV
**250**	**260**	**270**	**280**	**290**	**300**	**310**	**320**
NVEMNATPGV	DLTVLLNNMR	AEYEDLAEQN	RKDAEAWFNE	RSATLQQQIS	DHEGAATAAR	NELTELKRNL	QTLEIELQSL
**330**	**340**	**350**	**360**	**370**	**380**	**390**	**400**
MAVKHSYECS	LAETEGNYCN	QLQQIQDQIG	VMEEQLQQIR	TETEGQKLEY	EQLLDVKIFL	EKEIDIYCNL	LDGEERKSKS
**410**	**420**	**430**	**440**	**450**	**460**	**470**	
TCYKSKGYRP	VNSGNQAKEE	TIVKTVVEEL	DQIGNLLSLR	VHSVEEKSSK	ISNITVEQRV	PSKAP	

Cmpd.	No. of Cmpds.	m/z meas.	Δ m/z [ppm]	z	Rt [min]	Score	P	Range	Sequence	Modification	Ratios
108	1	557.2530	-43.29	2	30.6	12.5	1	1-10	-.MSFRLSGGSR.R	Oxidation: 1	
383	3	625.4008	101.40	2	34.4	11.7	0	368-377	K.LEYEQLLDVK.I		
1697	1	905.3389	-109.42	2	49.8	10.8	1	383-397	K.EIDIYCNLLDGEERK.S		M:Q 1.64
2812	1	753.3845	-13.35	2	64.3	19.2	1	405-418	K.SKGYRPVNSGNQAK.E		

Detailed Protein Report

Protein 262: ATP-binding cassette sub-family A member 12 isoform b [Homo sapiens]

**Accession:**	gi|27881501	**Score:**	54.2
**Database:**	refseq_human(refseq_human_20140103.fasta)	**MW [kDa]:**	256.8
**Database Date:**	2015-11-30	**pI:**	7.6
**Modification(s):**	Oxidation	**Sequence Coverage [%]:**	2.2
		**No. of unique Peptides:**	3

Quantitation

M:Q	**Median:** 0.86	**CV:** 0.00 %	**No. of Peptides:** 1

Detailed Protein Report

10	20	30	40	50	60	70	80
MFTYIKIITS	GDSDNITHVW	NEDDGQTLSP	SSLAAQLLIL	ENFEDALLNI	SANSPYIPYL	ACVRNVTDSL	ARGSPENLRL
90	100	110	120	130	140	150	160
LQSTIRFKKS	FLRNGSYEDY	FPPVPEVLKS	KLSQLRNLTE	LLCESETFSL	IEKSCQLSDM	SFGSLCEESE	FDLQLLEAAE
170	180	190	200	210	220	230	240
LGTEIAASLL	YHDNVISKKV	RDLLTGDPSK	INLNMDQFLE	QALQMNYLEN	ITQLIPIIEA	MLHVNNSADA	SEKPGQLLEM
250	260	270	280	290	300	310	320
FKNVEELKED	LRRTTGMSNR	TIDKLLAIPI	PDNRAEIISQ	VFWLHSCDTN	ITTPKLEDAM	KEFCNLSLSE	RSRQSYLIGL
330	340	350	360	370	380	390	400
TLLHYLNIYN	FTYKVFFPRK	DQKPVEKMME	LFIRLKEILN	QMASGTHPLL	DKMRSLKQMH	LPRSVPLTQA	MYRSNRMNTP
410	420	430	440	450	460	470	480
QGSFSTISQA	LCSQGITTEY	LTAMLPSSQR	PKGNHTKDFL	TYKLTKEQIA	SKYGIPINST	PFCFSLYKDI	INMPAGPVIW
490	500	510	520	530	540	550	560
AFLKPMLLGR	ILYAPYNPVT	KAIMEKSNVT	LRQLAELREK	SQEWMDKSPL	FMNSFHLLNQ	AIPMLQNTLR	NPFVQVFVKF
570	580	590	600	610	620	630	640
SVGLDAVELL	KQIDELDILR	LKLENNIDII	DQLNTLSSLT	VNISSCVLYD	RIQAAKTIDE	MEREAKRLYK	SNELFGSVIF
650	660	670	680	690	700	710	720
KLPSNRSWHR	GYDSGNVFLP	PVIKYTIRMS	LKTAQTTRSL	RTKIWAPGPH	NSPSHNQIYG	RAFIYLQDSI	ERAIIELQTG
730	740	750	760	770	780	790	800
RNSQEIAVQV	QAIPYPCFMK	DNFLTSVSYS	LPIVLMVAWV	VFIAAFVKKL	VYEKDLRLHE	YMKMMGVNSC	SHFFAWLIES
810	820	830	840	850	860	870	880
VGFLLVTIVI	LIIILKFGNI	LPKTNGFILF	LYFSDYSFSV	IAMSYLISVF	FNNTNIAALI	GSLIYIIAFF	PFIVLVTVEN
890	900	910	920	930	940	950	960
ELSYVLKVFM	SLLSPTAFSY	ASQYIARYEE	QGIGLQWENM	YTSPVQDDTT	SFGWLCCLIL	ADSFIYFLIA	WYVRNVFPGT
970	980	990	1000	1010	1020	1030	1040
YGMAAPWYFP	ILPSYWKERF	GCAEVKPEKS	NGLMFTNIMM	QNTNPSASPE	YMFSSNIEPE	PKDLTVGVAL	HGVTKIYGSK
1050	1060	1070	1080	1090	1100	1110	1120
VAVDNLNLNF	YEGHITSLLG	PNGAGKTTTI	SMLTGLFGAS	AGTIFVYGKD	IKTDLHTVRK	NMGVCMQHDV	LFSYLTTKEH
1130	1140	1150	1160	1170	1180	1190	1200
LLLYGSIKVP	HWTKKQLHEE	VKRTLKDTGL	YSHRHKRVGT	LSGGMKRKLS	ISIALIGGSR	VVILDEPSTG	VDPCSRRSIW
1210	1220	1230	1240	1250	1260	1270	1280
DVISKNKTAR	TIILSTHHLD	EAEVLSDRIA	FLEQGGLRCC	GSPFYLKEAF	GDGYHLTLTK	KKSPNLNANA	VCDTMAVTAM
1290	1300	1310	1320	1330	1340	1350	1360
IQSHLPEAYL	KEDIGGELVY	VLPPFSTKVS	GAYLSLLRAL	DNGMGDLNIG	CYGISDTTVE	EVFLNLTKES	QKNSAMSLEH
1370	1380	1390	1400	1410	1420	1430	1440
LTQKKIGNSN	ANGISTPDDL	SVSSSNFTDR	DDKILTRGER	LDGFGLLLKK	IMAILIKRFH	HTRRNWKGLI	AQVILPIVFV
1450	1460	1470	1480	1490	1500	1510	1520
TTAMGLGTLR	NSSNSYPEIQ	ISPSLYGTSE	QTAFYANYHP	STEALVSAMW	DFPGIDNMCL	NTSDLQCLNK	DSLEKWNTSG
1530	1540	1550	1560	1570	1580	1590	1600
EPITNFGVCS	CSENVQECPK	FNYSPPHRRT	YSSQVIYNLT	GQRVENYLIS	TANEFVQKRY	GGWSFGLPLT	KDLRFDITGV
1610	1620	1630	1640	1650	1660	1670	1680
PANRTLAKVW	YDPEGYHSLP	AYLNSLNNFL	LRVNMSKYDA	ARHGIIMYSH	PYPGVQDQEQ	ATISSLIDIL	VALSILMGYS
1690	1700	1710	1720	1730	1740	1750	1760
VTTASFVTYV	VREHQTKAKQ	LQHISGIGVT	CYWVTNFIYD	MVFYLVPVAF	SIGIIAIFKL	PAFYSENNLG	AVSLLLLLFG
1770	1780	1790	1800	1810	1820	1830	1840
YATFSWMYLL	AGLFHETGMA	FITYVCVNLF	FGINSIVSLS	VVYFLSKEKP	NDPTLELISE	TLKRIFLIFP	QFCFGYGLIE
1850	1860	1870	1880	1890	1900	1910	1920
LSQQQSVLDF	LKAYGVEYPN	ETFEMNKLGA	MFVALVSQGT	MFFSLRLLIN	ESLIKKLRLF	FRKFNSSHVR	ETIDEDEDVR
1930	1940	1950	1960	1970	1980	1990	2000
AERLRVESGA	AEFDLVQLYC	LTKTYQLIHK	KIIAVNNISI	GIPAGECFGL	LGVNGAGKTT	IFKMLTGDII	PSSGNILIRN
2010	2020	2030	2040	2050	2060	2070	2080
KTGSLGHVDS	HSSLVGYCPQ	EDALDDLVTV	EEHLYFYARV	HGIPEKDIKE	TVHKLLRRLH	LMPFKDRATS	MCSYGTKRKL
2090	2100	2110	2120	2130	2140	2150	2160
STALALIGKP	SILLLDEPSS	GMDPKSKRHL	WKIISEEVQN	KCSVILTSHS	MEECEALCTR	LAIMVNGKFQ	CIGSLQHIKS
2170	2180	2190	2200	2210	2220	2230	2240

Detailed Protein Report

Cmpd.	No. of Cmpds.	m/z meas.	Δ m/z [ppm]	z	Rt [min]	Score	P	Range	Sequence	Modification	Ratios
1898	1	890.3676	-104.02	2	53.6	10.7	1	341-354	K.DQKPVEKMMELFIR.L	Oxidation: 8	
1953	1	590.7822	22.74	2	52.8	13.4	1	519-527	R.EKSQEWMDK.S		
2087	1	753.3822	-28.39	2	56.5	10.9	0	651-664	R.GYDSGNVFLPPVIK.Y		M:Q 0.86

Detailed Protein Report

Protein 263: peroxisome biogenesis factor 1 isoform 3 [Homo sapiens]

**Accession:**	gi|544186104	**Score:**	54.2
**Database:**	refseq_human(refseq_human_20140103.fasta)	**MW [kDa]:**	119.5
**Database Date:**	2015-11-30	**pI:**	5.7
		**Sequence Coverage [%]:**	3.4
		**No. of unique Peptides:**	1

10	20	30	40	50	60	70	80
MMKELQTKQL	QSNTVGITES	NENESEIPVD	SSSVASLWTM	IGSIFSFQSE	KKQETSWGLT	EINAFKNMQS	KVVPLDNIFR
**90**	**100**	**110**	**120**	**130**	**140**	**150**	**160**
VCKSQPPSIY	NASATSVFHK	HCAIHVFPWD	QEYFDVEPSF	TVTYGKLVKL	LSPKQQQSKT	KQNVLSPEKE	KQMSEPLDQK
**170**	**180**	**190**	**200**	**210**	**220**	**230**	**240**
KIRSDHNEED	EKACVLQVVW	NGLEELNNAI	KYTKNVEVLH	LGKVWIPDDL	RKRLNIEMHA	VVRITPVEVT	PKIPRSLKLQ
**250**	**260**	**270**	**280**	**290**	**300**	**310**	**320**
PRENLPKDIS	EEDIKTVFYS	WLQQSTTTML	PLVISEEEFI	KLETKDGLKE	FSLSIVHSWE	KEKDKNIFLL	SPNLLQKTTI
**330**	**340**	**350**	**360**	**370**	**380**	**390**	**400**
QVLLDPMVKE	ENSEEIDFIL	PFLKLSSLGG	VNSLGVSSLE	HITHSLLGRP	LSRQLMSLVA	GLRNGALLLT	GGKGSGKSTL
**410**	**420**	**430**	**440**	**450**	**460**	**470**	**480**
AKAICKEAFD	KLDAHVERVD	CKALRGKRLE	NIQKTLEVAF	SEAVWMQPSV	VLLDDLDLIA	GLPAVPEHEH	SPDAVQSQRL
**490**	**500**	**510**	**520**	**530**	**540**	**550**	**560**
AHALNDMIKE	FISMGSLVAL	IATSQSQQSL	HPLLVSAQGV	HIFQCVQHIQ	PPNQEQRCEI	LCNVIKNKLD	CDINKFTDLD
**570**	**580**	**590**	**600**	**610**	**620**	**630**	**640**
LQHVAKETGG	FVARDFTVLV	DRAIHSRLSR	QSISTREKLV	LTTLDFQKAL	RGFLPASLRS	VNLHKPRDLG	WDKIGGLHEV
**650**	**660**	**670**	**680**	**690**	**700**	**710**	**720**
RQILMDTIQL	PAKYPELFAN	LPIRQRTGIL	LYGPPGTGKT	LLAGVIARES	RMNFISVKGP	ELLSKYIGAS	EQAVRDIFIR
**730**	**740**	**750**	**760**	**770**	**780**	**790**	**800**
AQAAKPCILF	FDEFESIAPR	RGHDNTGVTD	RVVNQLLTQL	DGVEGLQGVY	VLAATSRPDL	IDPALLRPGR	LDKCVYCPPP
**810**	**820**	**830**	**840**	**850**	**860**	**870**	**880**
DQVSRLEILN	VLSDSLPLAD	DVDLQHVASV	TDSFTGADLK	ALLYNAQLEA	LHGMLLSSGL	QDGSSSSDSD	LSLSSMVFLN
**890**	**900**	**910**	**920**	**930**	**940**	**950**	**960**
HSSGSDDSAG	DGECGLDQSL	VSLEMSEILP	DESKFNMYRL	YFGSSYESEL	GNGTSSDLSS	QCLSAPSSMT	QDLPGVPGKD
**970**	**980**	**990**	**1000**	**1010**	**1020**	**1030**	**1040**
QLFSQPPVLR	TASQEGCQEL	TQEQRDQLRA	DISIIKGRYR	SQSGEDESMN	QPGPIKTRLA	ISQSHLMTAL	GHTRPSISED
**1050**	**1060**	**1070**	**1080**				
DWKNFAELYE	SFQNPKRRKN	QSGTMFRPGQ	KVTLA				

Cmpd.	No. of Cmpds.	m/z meas.	Δ m/z [ppm]	z	Rt [min]	Score	P	Range	Sequence	Modification
1073	10	1207.9210	130.55	1	43.0	31.9	2	233-242	K.IPRSLKLQPR.E	

Detailed Protein Report

Protein 264: utrophin [Homo sapiens]

**Accession:**	gi|110611228	**Score:**	54.0
**Database:**	refseq_human(refseq_human_20140103.fasta)	**MW [kDa]:**	394.2
**Database Date:**	2015-11-30	**pI:**	5.1
**Modification(s):**	Oxidation	**Sequence Coverage [%]:**	1.0
		**No. of unique Peptides:**	1

Alias proteins:

Accession	Name	Description
gi|530384033	refseq_human (refseq_human_20140103.fasta)	PREDICTED: utrophin isoform X4 [Homo sapiens]

Detailed Protein Report

10	20	30	40	50	60	70	80
MAKYGEHEAS	PDNGQNEFSD	IIKSRSDEHN	DVQKKTFTKW	INARFSKSGK	PPINDMFTDL	KDGRKLLDLL	EGLTGTSLPK
90	100	110	120	130	140	150	160
ERGSTRVHAL	NNVNRVLQVL	HQNNVELVNI	GGTDIVDGNH	KLTLGLLWSI	ILHWQVKDVM	KDVMSDLQQT	NSEKILLSWV
170	180	190	200	210	220	230	240
RQTTRPYSQV	NVLNFTTSWT	DGLAFNAVLH	RHKPDLFSWD	KVVKMSPIER	LEHAFSKAQT	YLGIEKLLDP	EDVAVQLPDK
250	260	270	280	290	300	310	320
KSIIMYLTSL	FEVLPQQVTI	DAIREVETLP	RKYKKECEEE	AINIQSTAPE	EEHESPRAET	PSTVTEVDMD	LDSYQIALEE
330	340	350	360	370	380	390	400
VLTWLLSAED	TFQEQDDISD	DVEEVKDQFA	THEAFMMELT	AHQSSVGSVL	QAGNQLITQG	TLSDEEEFEI	QEQMTLLNAR
410	420	430	440	450	460	470	480
WEALRVESMD	RQSRLHDVLM	ELQKKQLQQL	SAWLTLTEER	IQKMETCPLD	DDVKSLQKLL	EEHKSLQSDL	EAEQVKVNSL
490	500	510	520	530	540	550	560
THMVVIVDEN	SGESATAILE	DQLQKLGERW	TAVCRWTEER	WNRLQEINIL	WQELLEEQCL	LKAWLTEKEE	ALNKVQTSNF
570	580	590	600	610	620	630	640
KDQKELSVSV	RRLAILKEDM	EMKRQTLDQL	SEIGQDVGQL	LDNSKASKKI	NSDSEELTQR	WDSLVQRLED	SSNQVTQAVA
650	660	670	680	690	700	710	720
KLGMSQIPQK	DLLETVRVRE	QAITKKSKQE	LPPPPPPKKR	QIHVDIEAKK	KFDAISAELL	NWILKWKTAI	QTTEIKEYMK
730	740	750	760	770	780	790	800
MQDTSEMKKK	LKALEKEQRE	RIPRADELNQ	TGQILVEQMG	KEGLPTEEIK	NVLEKVSSEW	KNVSQHLEDL	ERKIQLQEDI
810	820	830	840	850	860	870	880
NAYFKQLDEL	EKVIKTKEEW	VKHTSISESS	RQSLPSLKDS	CQRELTNLLG	LHPKIEMARA	SCSALMSQPS	APDFVQRGFD
890	900	910	920	930	940	950	960
SFLGRYQAVQ	EAVEDRQQHL	ENELKGQPGH	AYLETLKTLK	DVLNDSENKA	QVSLNVLNDL	AKVEKALQEK	KTLDEILENQ
970	980	990	1000	1010	1020	1030	1040
KPALHKLAEE	TKALEKNVHP	DVEKLYKQEF	DDVQGKWNKL	KVLVSKDLHL	LEEIALTLRA	FEADSTVIEK	WMDGVKDFLM
1050	1060	1070	1080	1090	1100	1110	1120
KQQAAQGDDA	GLQRQLDQCS	AFVNEIETIE	SSLKNMKEIE	TNLRSGPVAG	IKTWVQTRLG	DYQTQLEKLS	KEIATQKSRL
1130	1140	1150	1160	1170	1180	1190	1200
SESQEKAANL	KKDLAEMQEW	MTQAEEEYLE	RDFEYKSPEE	LESAVEEMKR	AKEDVLQKEV	RVKILKDNIK	LLAAKVPSGG
1210	1220	1230	1240	1250	1260	1270	1280
QELTSELNVV	LENYQLLCNR	IRGKCHTLEE	VWSCWIELLH	YLDLETTWLN	TLEERMKSTE	VLPEKTDAVN	EALESLESVL
1290	1300	1310	1320	1330	1340	1350	1360
RHPADNRTQI	RELGQTLIDG	GILDDIISEK	LEAFNSRYED	LSHLAESKQI	SLEKQLQVLR	ETDQMLQVLQ	ESLGELDKQL
1370	1380	1390	1400	1410	1420	1430	1440
TTYLTDRIDA	FQVPQEAQKI	QAEISAHELT	LEELRRNMRS	QPLTSPESRT	ARGGSQMDVL	QRKLREVSTK	FQLFQKPANF
1450	1460	1470	1480	1490	1500	1510	1520
EQRMLDCKRV	LDGVKAELHV	LDVKDVDPDV	IQTHLDKCMK	LYKTLSEVKL	EVETVIKTGR	HIVQKQQTDN	PKGMDEQLTS
1530	1540	1550	1560	1570	1580	1590	1600
LKVLYNDLGA	QVTEGKQDLE	RASQLARKMK	KEAASLSEWL	SATETELVQK	STSEGLLGDL	DTEISWAKNV	LKDLEKRKAD
1610	1620	1630	1640	1650	1660	1670	1680
LNTITESSAA	LQNLIEGSEP	ILEERLCVLN	AGWSRVRTWT	EDWCNTLMNH	QNQLEIFDGN	VAHISTWLYQ	AEALLDEIEK
1690	1700	1710	1720	1730	1740	1750	1760
KPTSKQEEIV	KRLVSELDDA	NLQVENVRDQ	ALILMNARGS	SSRELVEPKL	AELNRNFEKV	SQHIKSAKLL	IAQEPLYQCL
1770	1780	1790	1800	1810	1820	1830	1840
VTTETFETGV	PFSDLEKLEN	DIENMLKFVE	KHLESSDEDE	KMDEESAQIE	EVLQRGEEML	HQPMEDNKKE	KIRLQLLLLH
1850	1860	1870	1880	1890	1900	1910	1920
TRYNKIKAIP	IQQRKMGQLA	SGIRSSLLPT	DYLVEINKIL	LCMDDVELSL	NVPELNTAIY	EDFSFQEDSL	KNIKDQLDKL
1930	1940	1950	1960	1970	1980	1990	2000
GEQIAVIHEK	QPDVILEASG	PEAIQIRDTL	TQLNAKWDRI	NRMYSDRKGC	FDRAMEEWRQ	FHCDLNDLTQ	WITEAEELLV
2010	2020	2030	2040	2050	2060	2070	2080
DTCAPGGSLD	LEKARIHQQE	LEVGISSHQP	SFAALNRTGD	GIVQKLSQAD	GSFLKEKLAG	LNQRWDAIVA	EVKDRQPRLK
2090	2100	2110	2120	2130	2140	2150	2160
GESKQVMKYR	HQLDEIICWL	TKAEHAMQKR	STTELGENLQ	ELRDLTQEME	VHAEKLKWLN	RTELEMLSDK	SLSLPERDKI
2170	2180	2190	2200	2210	2220	2230	2240
SESLRTVNMT	WNKICREVPT	TLKECIQEPS	SVSQTRIAAH	PNVQKVVLVS	SASDIPVQSH	RTSEISIPAD	LDKTITELAD
2250	2260	2270	2280	2290	2300	2310	2320
WLVLIDQMLK	SNIVTVGDVE	EINKTVSRMK	ITKADLEQRH	PQLDYVFTLA	QNLKNKASSS	DMRTAITEKL	ERVKNQWDGT
2330	2340	2350	2360	2370	2380	2390	2400

Detailed Protein Report

Cmpd.	No. of Cmpds.	m/z meas.	Δ m/z [ppm]	z	Rt [min]	Score	P	Range	Sequence	Modification
1249	1	764.3778	23.73	2	45.3	11.3	2	1957-1967	K.WDRINRMYSDR.K	Oxidation: 7

Detailed Protein Report

Protein 265: PREDICTED: pericentrin isoform X6 [Homo sapiens]

**Accession:**	gi|530419254	**Score:**	54.0
**Database:**	refseq_human(refseq_human_20140103.fasta)	**MW [kDa]:**	355.7
**Database Date:**	2015-11-30	**pI:**	5.2
		**Sequence Coverage [%]:**	1.2
		**No. of unique Peptides:**	2

Quantitation

Q:W	**Median:**	4.62	**CV:**	0.00 %	**No. of Peptides:**	1
M:Q	**Median:**	0.52	**CV:**	0.00 %	**No. of Peptides:**	1

Detailed Protein Report

10	20	30	40	50	60	70	80
MFTVSDHPPE	QHGMFTVGDH	PPEQRGMFTV	SDHPPEQHGM	FTVSDHPPEQ	RGMFTISDHQ	PEQRGMFTVS	DHTPEQRGIF
90	100	110	120	130	140	150	160
TISDHPAEQR	GMFTKECEQE	CELAITDLES	GREDEAGLHQ	SQAVHGLELE	ALRLSLSNMH	TAQLELTQAN	LQKEKETALT
170	180	190	200	210	220	230	240
ELREMLNSRR	AQELALLQSR	QQHELELLRE	QHAREKEEVV	LRCGQEAAEL	KEKLQSEMEK	NAQIVKTLKE	DWESEKDLCL
250	260	270	280	290	300	310	320
ENLRKELSAK	HQSEMEDLQN	QFQKELAEQR	AELEKIFQDK	NQAERALRNL	ESHHQAAIEK	LREDLQSEHG	RCLEDLEFKF
330	340	350	360	370	380	390	400
KESEKEKQLE	LENLQASYED	LKAQSQEEIR	RLWSQLDSAR	TSRQELSELH	EQLLARTSRV	EDLEQLKQRE	KTQHESELEQ
410	420	430	440	450	460	470	480
LRIYFEKKLR	DAEKTYQEDL	TLLQQRLQGA	REDALLDSVE	VGLSCVGLEE	KPEKGRKDHV	DELEPERHKE	SLPRFQAELE
490	500	510	520	530	540	550	560
ESHRHQLEAL	ESPLCIQHEG	HVSDRCCVET	SALGHEWRLE	PSEGHSQELP	WVHLQGVQDG	DLEADTERAA	RVLGLETEHK
570	580	590	600	610	620	630	640
VQLSLLQTEL	KEEIELLKIE	NRNLYGKLQH	ETRLKDDLEK	VKHNLIEDHQ	KELNNAKQKT	ELMKQEFQRK	ETDWKVMKEE
650	660	670	680	690	700	710	720
LQREAEEKLT	LMLLELREKA	ESEKQTIINK	FELREAEMRQ	LQDQQAAQIL	DLERSLTEQQ	GRLQQLEQDL	TSDDALHCSQ
730	740	750	760	770	780	790	800
CGREPPTAQD	GELAALHVKE	DCALQLMLAR	SRFLEERKEI	TEKFSAEQDA	FLQEAQEQHA	RELQLLQERH	QQQLLSVTAE
810	820	830	840	850	860	870	880
LEARHQAALG	ELTASLESKQ	GALLAARVAE	LQTKHAADLG	ALETRHLSSL	DSLESCYLSE	FQTIREEHRQ	ALELLRADFE
890	900	910	920	930	940	950	960
EQLWKKDSLH	QTILTQELEK	LKRKHEGELQ	SVRDHLRTEV	STELAGTVAH	ELQGVHQGEF	GSEKKTALHE	KEETLRLQSA
970	980	990	1000	1010	1020	1030	1040
QAQPFHQEEK	ESLSLQLQKK	NHQVQQLKDQ	VLSLSHEIEE	CRSELEVLQQ	RRERENREGA	NLLSMLKADV	NLSHSERGAL
1050	1060	1070	1080	1090	1100	1110	1120
QDALRRLLGL	FGETLRAAVT	LRSRIGERVG	LCLDDAGAGL	ALSTAPALEE	TWSDVALPEL	DRTLSECAEM	SSVAEISSHM
1130	1140	1150	1160	1170	1180	1190	1200
RESFLMSPES	VRECEQPIRR	VFQSLSLAVD	GLMEMALDSS	RQLEEARQIH	SRFEKEFSFK	NEETAQVVRK	HQELLECLKE
1210	1220	1230	1240	1250	1260	1270	1280
ESAAKAELAL	ELHKTQGTLE	GFKVETADLK	EVLAGKEDSE	HRLVLELESL	RRQLQQAAQE	QAALREECTR	LWSRGEATAT
1290	1300	1310	1320	1330	1340	1350	1360
DAEAREAALR	KEVEDLTKEQ	SETRKQAEKD	RSALLSQMKI	LESELEEQLS	QHRGCAKQAE	AVTALEQQVA	SLDKHLRNQR
1370	1380	1390	1400	1410	1420	1430	1440
QFMDEQAAER	EHEREEFQQE	IQRLEGQLRQ	AAKPQPWGPR	DSQQAPLDGE	VELLQQKLRE	KLDEFNELAI	QKESADRQVL
1450	1460	1470	1480	1490	1500	1510	1520
MQEEEIKRLE	EMNINIRKKV	AQLQEEVEKQ	KNIVKGLEQD	KEVLKKQQMS	SLLLASTLQS	TLDAGRCPEP	PSGSPPEGPE
1530	1540	1550	1560	1570	1580	1590	1600
IQLEVTQRAL	LRRESEVLDL	KEQLEKMKGD	LESKNEEILH	LNLKLDMQNS	QTAVSLRELE	EENTSLKVIY	TRSSEIEELK
1610	1620	1630	1640	1650	1660	1670	1680
ATIENLQENQ	KRLQKEKAEE	IEQLHEVIEK	LQHELSLMGP	VVHEVSDSQA	GSLQSELLCS	QAGGPRGQAL	QGELEAALEA
1690	1700	1710	1720	1730	1740	1750	1760
KEALSRLLAD	QERRHSQALE	ALQQRLQGAE	EAAELQLAEL	ERNVALREAE	VEDMASRIQE	FEAALKAKEA	TIAERNLEID
1770	1780	1790	1800	1810	1820	1830	1840
ALNQRKAAHS	AELEAVLLAL	ARIRRALEQQ	PLAAGAAPPE	LQWLRAQCAR	LSRQLQVLHQ	RFLRCQVELD	RRQARRATAH
1850	1860	1870	1880	1890	1900	1910	1920
TRVPGAHPQP	RMDGGAKAQV	TGDVEASHDA	ALEPVVPDPQ	GDLQPVLVTL	KDAPLCKQEG	VMSVLTVCQR	QLQSELLLVK
1930	1940	1950	1960	1970	1980	1990	2000
NEMRLSLEDG	GKGKEKVLED	CQLPKVDLVA	QVKQLQEKLN	RLLYSMTFQN	VDAADTKSLW	PMASAHLLES	SWSDDSCDGE
2010	2020	2030	2040	2050	2060	2070	2080
EPDISPHIDT	CDANTATGGV	TDVIKNQAID	ACDANTTPGG	VTDVIKNWDS	LIPDEMPDSP	IQEKSECQDM	SLSSPTSVLG
2090	2100	2110	2120	2130	2140	2150	2160
GSRHQSHTAE	AGPRKSPVGM	LDLSSWSSPE	VLRKDWTLEP	WPSLPVTPHS	GALSLCSADT	SLGDRADTSL	PQTQGPGLLC
2170	2180	2190	2200	2210	2220	2230	2240

Detailed Protein Report

Cmpd.	No. of Cmpds.	m/z meas.	Δ m/z [ppm]	z	Rt [min]	Score	P	Range	Sequence	Modification	Ratios
2710	4	1044.5270	3.44	2	65.0	25.0	2	1173-1189	R.FEKEFSFKNEETAQVVR.K		
490	3	472.1760	-159.67	2	34.6	13.3	1	3049-3056	R.TRESPPTR.D		Q:W 4.62<br>M:Q 0.52

Detailed Protein Report

Protein 266: tau-tubulin kinase 1 [Homo sapiens]

**Accession:**	gi|58761548	**Score:**	54.0
**Database:**	refseq_human(refseq_human_20140103.fasta)	**MW [kDa]:**	142.6
**Database Date:**	2015-11-30	**pI:**	5.4
		**Sequence Coverage [%]:**	4.6
		**No. of unique Peptides:**	3

Quantitation

M:Q **Median:** 1.51 **CV:** 0.00 % **No. of Peptides:** 1

10	20	30	40	50	60	70	80
MQCLAAALKD	ETNMSGGGEQ	ADILPANYVV	KDRWKVLKKI	GGGGFGEIYE	AMDLLTRENV	ALKVESAQQP	KQVLKMEVAV
**90**	**100**	**110**	**120**	**130**	**140**	**150**	**160**
LKKLQGKDHV	CRFIGCGRNE	KFNYVVMQLQ	GRNLADLRRS	QPRGTFTLST	TLRLGKQILE	SIEAIHSVGF	LHRDIKPSNF
**170**	**180**	**190**	**200**	**210**	**220**	**230**	**240**
AMGRLPSTYR	KCYMLDFGLA	RQYTNTTGDV	RPPRNVAGFR	GTVRYASVNA	HKNREMGRHD	DLWSLFYMLV	EFAVGQLPWR
**250**	**260**	**270**	**280**	**290**	**300**	**310**	**320**
KIKDKEQVGM	IKEKYEHRML	LKHMPSEFHL	FLDHIASLDY	FTKPDYQLIM	SVFENSMKER	GIAENEAFDW	EKAGTDALLS
**330**	**340**	**350**	**360**	**370**	**380**	**390**	**400**
TSTSTPPQQN	TRQTAAMFGV	VNVTPVPGDL	LRENTEDVLQ	GEHLSDQENA	PPILPGRPSE	GLGPSPHLVP	HPGGPEAEVW
**410**	**420**	**430**	**440**	**450**	**460**	**470**	**480**
EETDVNRNKL	RINIGKSPCV	EEEQSRGMGV	PSSPVRAPPD	SPTTPVRSLR	YRRVNSPESE	RLSTADGRVE	LPERRSRMDL
**490**	**500**	**510**	**520**	**530**	**540**	**550**	**560**
PGSPSRQACS	SQPAQMLSVD	TGHADRQASG	RMDVSASVEQ	EALSNAFRSV	PLAEEEDFDS	KEWVIIDKET	ELKDFPPGAE
**570**	**580**	**590**	**600**	**610**	**620**	**630**	**640**
PSTSGTTDEE	PEELRPLPEE	GEERRRLGAE	PTVRPRGRSM	QALAEEDLQH	LPPQPLPPQL	SQGDGRSETS	QPPTPGSPSH
**650**	**660**	**670**	**680**	**690**	**700**	**710**	**720**
SPLHSGPRPR	RRESDPTGPQ	RQVFSVAPPF	EVNGLPRAVP	LSLPYQDFKR	DLSDYRERAR	LLNRVRRVGF	SHMLLTTPQV
**730**	**740**	**750**	**760**	**770**	**780**	**790**	**800**
PLAPVQPQAN	GKEEEEEEEE	DEEEEEEDEE	EEEEEEEEEE	EEEEEEEEEE	EAAAAVALGE	VLGPRSGSSS	EGSERSTDRS
**810**	**820**	**830**	**840**	**850**	**860**	**870**	**880**
QEGAPSTLLA	DDQKESRGRA	SMADGDLEPE	EGSKTLVLVS	PGDMKKSPVT	AELAPDPDLG	TLAALTPQHE	RPQPTGSQLD
**890**	**900**	**910**	**920**	**930**	**940**	**950**	**960**
VSEPGTLSSV	LKSEPKPPGP	GAGLGAGTVT	TGVGGVAVTS	SPFTKVERTF	VHIAEKTHLN	VMSSGGQALR	SEEFSAGGEL
**970**	**980**	**990**	**1000**	**1010**	**1020**	**1030**	**1040**
GLELASDGGA	VEEGARAPLE	NGLALSGLNG	AEIEGSALSG	APRETPSEMA	TNSLPNGPAL	ADGPAPVSPL	EPSPEKVATI
**1050**	**1060**	**1070**	**1080**	**1090**	**1100**	**1110**	**1120**
SPRRHAMPGS	RPRSRIPVLL	SEEDTGSEPS	GSLSAKERWS	KRARPQQDLA	RLVMEKRQGR	LLLRLASGAS	SSSSEEQRRA
**1130**	**1140**	**1150**	**1160**	**1170**	**1180**	**1190**	**1200**
SETLSGTGSE	EDTPASEPAA	ALPRKSGRAA	ATRSRIPRPI	GLRMPMPVAA	QQPASRSHGA	APALDTAITS	RLQLQTPPGS
**1210**	**1220**	**1230**	**1240**	**1250**	**1260**	**1270**	**1280**
ATAADLRPKQ	PPGRGLGPGR	AQAGARPPAP	RSPRLPASTS	AARNASASPR	SQSLSRRESP	SPSHQARPGV	PPPRGVPPAR
**1290**	**1300**	**1310**	**1320**	**1330**			
AQPDGTPSPG	GSKKGPRGKL	QAQRATTKGR	AGGAEGRAGA	R			

Cmpd.	No. of Cmpds.	m/z meas.	Δ m/z [ppm]	z	Rt [min]	Score	P	Range	Sequence	Modification	Ratios
1658	1	697.8038	-143.18	2	50.5	11.5	1	124-136	R.GTFTLSTTLRLGK.Q		
2323	1	1175.1820	88.14	2	59.3	10.6	1	529-548	R.SVPLAEEEDFDSKEWVIIDK.E		
1595	1	932.5310	13.94	2	50.2	20.1	0	1192-1209	R.LQLQTPPGSATAADLRPK.Q		M:Q 1.51

Detailed Protein Report

Protein 267: conserved oligomeric Golgi complex subunit 1 [Homo sapiens]

Accession: gi|21237783
Database: refseq_human(refseq_human_20140103.fasta)
Database Date: 2015-11-30
Modification(s): Oxidation

Score: 54.0
MW [kDa]: 108.9
pI: 7.1
Sequence Coverage [%]: 6.3
No. of unique Peptides: 3

Quantitation

Q:W	**Median:**	2.26	**CV:**	49.32 %	**No. of Peptides:**	2
M:Q	**Median:**	0.71	**CV:**	0.00 %	**No. of Peptides:**	1

```
        10         20         30         40         50         60         70         80
MATAATSPAL KRLDLRDPAA LFETHGAEEI RGLERQVRAE IEHKKEELRQ MVGERYRDLI EAADTIGQMR RCAVGLVDAV
        90        100        110        120        130        140        150        160
KATDQYCARL RQAGSAAPRP PRAQQPQQPS QEKFYSMAAQ IKLLLEIPEK IWSSMEASQC LHATQLYLLC CHLHSLLQLD
       170        180        190        200        210        220        230        240
SSSSRYSPVL SRFPILIRQV AAASHFRSTI LHESKMLLKC QGVSDQAVAE ALCSIMLLEE SSPRQALTDF LLARKATIQK
       250        260        270        280        290        300        310        320
LLNQPHHGAG IKAQICSLVE LLATTLKQAH ALFYTLPEGL LPDPALPCGL LFSTLETITG QHPAGKGTGV LQEEMKLCSW
       330        340        350        360        370        380        390        400
FKHLPASIVE FQPTLRTLAH PISQEYLKDT LQKWIHMCNE DIKNGITNLL MYVKSMKGLA GIRDAMWELL TNESTNHSWD
       410        420        430        440        450        460        470        480
VLCRRLLEKP LLFWEDMMQQ LFLDRLQTLT KEGFDSISSS SKELLVSALQ ELESSTSNSP SNKHIHFEYN MSLFLWSESP
       490        500        510        520        530        540        550        560
NDLPSDAAWV SVANRGQFAS SGLSMKAQAI SPCVQNFCSA LDSKLKVKLD DLLAYLPSDD SSLPKDVSPT QAKSSAFDRY
       570        580        590        600        610        620        630        640
ADAGTVQEML RTQSVACIKH IVDCIRAELQ SIEEGVQGQQ DALNSAKLHS VLFMARLCQS LGELCPHLKQ CILGKSESSE
       650        660        670        680        690        700        710        720
KPAREFRALR KQGKVKTQEI IPTQAKWQEV KEVLLQQSVM GYQVWSSAVV KVLIHGFTQS LLLDDAGSVL ATATSWDELE
       730        740        750        760        770        780        790        800
IQEEAESGSS VTSKIRLPAQ PSWYVQSFLF SLCQEINRVG GHALPKVTLQ EMLKSCMVQV VAAYEKLSEE KQIKKEGAFP
       810        820        830        840        850        860        870        880
VTQNRALQLL YDLRYLNIVL TAKGDEVKSG RSKPDSRIEK VTDHLEALID PFDLDVFTPH LNSNLHRLVQ RTSVLFGLVT
       890        900        910        920        930        940        950        960
GTENQLAPRS STFNSQEPHN ILPLASSQIR FGLLPLSMTS TRKAKSTRNI ETKAQVVPPA RSTAGDPTVP GSLFRQLVSE
       970        980        990
EDNTSAPSLF KLGWLSSMTK
```

Cmpd.	No. of Cmpds.	m/z meas.	Δ m/z [ppm]	z	Rt [min]	Score	P	Range	Sequence	Modification	Ratios	
2917	1	724.8590	4.01	2	65.2	12.8	0	58-70	R.DLIEAADTIGQMR.R	Oxidation: 12	Q:W	3.60
2511	1	941.9690	8.39	2	62.0	16.9	1	72-89	R.CAVGLVDAVKATDQYCAR.L		Q:W<br>M:Q	1.42<br>0.71
1712	1	1045.0032	-51.34	2	49.7	13.3	1	527-545	K.VKLDDLLAYLPSDDSSLPK.D			

Detailed Protein Report

Protein 268: **PREDICTED: polyamine-modulated factor 1-binding protein 1 isoform X3 [Homo sapiens]**

**Accession:**	gi|578829261	**Score:**	53.9
**Database:**	refseq_human(refseq_human_20140103.fasta)	**MW [kDa]:**	118.6
**Database Date:**	2015-11-30	**pI:**	5.9
**Modification(s):**	Carbamidomethyl	**Sequence Coverage [%]:**	4.7
		**No. of unique Peptides:**	2

Quantitation

Q:W	**Median:**	0.38	**CV:**	1.63 %	**No. of Peptides:**	2
M:Q	**Median:**	1.29	**CV:**	22.99 %	**No. of Peptides:**	2

Alias proteins:

Accession	Name	Description
gi|578829263	r e f s e q _ h u m a n (refseq_human_20140103.fasta)	PREDICTED: polyamine-modulated factor 1-binding protein 1 isoform X4 [Homo sapiens]

10	20	30	40	50	60	70	80
MKDEAGERDR	EVSSLNSKLL	SLQLDIKNLH	DVCKRQRKTL	QDNQLCMEEA	MNSSHDKKQA	QALAFEESEV	EFGSSKQCHL
**90**	**100**	**110**	**120**	**130**	**140**	**150**	**160**
RQLQQLKKKL	LVLQQELEFH	TEELQTSYYS	LRQYQSILEK	QTSDLVLLHH	HCKLKEDEVI	LYEEEMGNHN	ENTGEKLHLA
**170**	**180**	**190**	**200**	**210**	**220**	**230**	**240**
QEQLALAGDK	IASLERSLNL	YRDKYQSSLS	NIELLECQVK	MLQGELGGIM	GQEPENKGDH	SKVRIYTSPC	MIQEHQETQK
**250**	**260**	**270**	**280**	**290**	**300**	**310**	**320**
RLSEVWQKVS	QQDDLIQELR	NKLACSNALV	LEREKALIKL	QADFASCTAT	HRYPPSSSEE	CEDIKKILKH	LQEQKDSQCL
**330**	**340**	**350**	**360**	**370**	**380**	**390**	**400**
HVEEYQNLVK	DLRVELEAVS	EQKRNIMKDM	MKLELDLHGL	REETSAHIER	KDKDITILQC	RLQELQLEFT	ETQKLTLKKD
**410**	**420**	**430**	**440**	**450**	**460**	**470**	**480**
KFLQEKDEML	QELEKKLTQV	QNSLLKKEKE	LEKQQCMATE	LEMTVKEAKQ	DKSKEAECKA	LQAEVQKLKN	SLEEAKQQER
**490**	**500**	**510**	**520**	**530**	**540**	**550**	**560**
LAAQQAAQCK	EEAALAGCHL	EDTQRKLQKG	LLLDKQKADT	IQELQRELQM	LQKESSMAEK	EQTSNRKRVE	ELSLELSEAL
**570**	**580**	**590**	**600**	**610**	**620**	**630**	**640**
RKLENSDKEK	RQLQKTVAEQ	DMKMNDMLDR	IKHQHREQGS	IKCKLEEDLQ	EATKLLEDKR	EQLKKSKEHE	KLMEGELEAL
**650**	**660**	**670**	**680**	**690**	**700**	**710**	**720**
RQEFKKKDKT	LKENSRKLEE	ENENLRAELQ	CCSTQLESSL	NKYNTSQQVI	QDLNKEIALQ	KESLMSLQAQ	LDKALQKEKH
**730**	**740**	**750**	**760**	**770**	**780**	**790**	**800**
YLQTTITKEA	YDALSRKSAA	CQDDLTQALE	KLNHVTSETK	SLQQSLTQTQ	EKKAQLEEEI	IAYEERMKKL	NTELRKLRGF
**810**	**820**	**830**	**840**	**850**	**860**	**870**	**880**
HQESELEVHA	FDKKLEEMSC	QVLQWQKQHQ	NDLKMLAAKE	EQLREFQEEM	AALKENLLED	DKEPCCLPQW	SVPKDTCRLY
**890**	**900**	**910**	**920**	**930**	**940**	**950**	**960**
RGNDQIMTNL	EQWAKQQKVA	NEKLGNQLRE	QVKYIAKLSG	EKDHLHSVMV	HLQQENKKLK	KEIEEKKMKA	ENTRLCTKAL
**970**	**980**	**990**	**1000**	**1010**	**1020**		
GPSRTESTQR	EKVCGTLGWK	GLPQDMGQRM	DLTKYIGMPH	CPGTSAICQK	NKCDFFL		

Cmpd.	No. of Cmpds.	m/z meas.	Δ m/z [ppm]	z	Rt [min]	Score	P	Range	Sequence	Modification	Ratios	
293	1	477.5768	-43.77	3	32.4	14.6	1	261-273	R.NKLACSNALVLER.E		Q:W<br>M:Q	0.37<br>1.03
1491	1	472.2634	90.28	2	48.8	14.3	1	1011-1017	K.NKCDFFL.-	Carbamidomethyl: 3	M:Q<br>Q:W	1.61<br>0.38

Detailed Protein Report

Protein 269: **complement C4-A isoform 2 preproprotein [Homo sapiens]**

**Accession:**	gi|356582273	**Score:**	53.8
**Database:**	refseq_human(refseq_human_20140103.fasta)	**MW [kDa]:**	187.6
**Database Date:**	2015-11-30	**pI:**	6.7
**Modification(s):**	Carbamidomethyl	**Sequence Coverage [%]:**	3.7
		**No. of unique Peptides:**	4

Quantitation

Q:W	**Median:** 1.99	**CV:** 0.00 %	**No. of Peptides:** 1

10	20	30	40	50	60	70	80
MRLLWGLIWA	SSFFTLSLQK	PRLLLFSPSV	VHLGVPLSVG	VQLQDVPRGQ	VVKGSVFLRN	PSRNNVPCSP	KVDFTLSSER
90	100	110	120	130	140	150	160
DFALLSLQVP	LKDAKSCGLH	QLLRGPEVQL	VAHSPWLKDS	LSRTTNIQGI	NLLFSSRRGH	LFLQTDQPIY	NPGQRVRYRV
170	180	190	200	210	220	230	240
FALDQKMRPS	TDTITVMVEN	SHGLRVRKKE	VYMPSSIFQD	DFVIPDISEP	GTWKISARFS	DGLESNSSTQ	FEVKKYVLPN
250	260	270	280	290	300	310	320
FEVKITPGKP	YILTVPGHLD	EMQLDIQARY	IYGKPVQGVA	YVRFGLLDED	GKKTFFRGLE	SQTKLVNGQS	HISLSKAEFQ
330	340	350	360	370	380	390	400
DALEKLNMGI	TDLQGLRLYV	AAAIIESPGG	EMEEAELTSW	YFVSSPFSLD	LSKTKRHLVP	GAPFLLQALV	REMSGSPASG
410	420	430	440	450	460	470	480
IPVKVSATVS	SPGSVPEVQD	IQQNTDGSGQ	VSIPIIIPQT	ISELQLSVSA	GSPHPAIARL	TVAAPPSGGP	GFLSIERPDS
490	500	510	520	530	540	550	560
RPPRVGDTLN	LNLRAVGSGA	TFSHYYYMIL	SRGQIVFMNR	EPKRTLTSVS	VFVDHHLAPS	FYFVAFYYHG	DHPVANSLRV
570	580	590	600	610	620	630	640
DVQAGACEGK	LELSVDGAKQ	YRNGESVKLH	LETDSLALVA	LGALDTALYA	AGSKSHKPLN	MGKVFEAMNS	YDLGCGPGGG
650	660	670	680	690	700	710	720
DSALQVFQAA	GLAFSDGDQW	TLSRKRLSCP	KEKTTRKKRN	VNFQKAINEK	LGQYASPTAK	RCCQDGVTRL	PMMRSCEQRA
730	740	750	760	770	780	790	800
ARVQQPDCRE	PFLSCCQFAE	SLRKKSRDKG	QAGLQRALEI	LQEEDLIDED	DIPVRSFFPE	NWLWRVETVD	RFQILTLWLP
810	820	830	840	850	860	870	880
DSLTTWEIHG	LSLSKTKGLC	VATPVQLRVF	REFHLHLRLP	MSVRRFEQLE	LRPVLYNYLD	KNLTVSVHVS	PVEGLCLAGG
890	900	910	920	930	940	950	960
GGLAQQVLVP	AGSARPVAFS	VVPTAAAAVS	LKVVARGSFE	FPVGDAVSKV	LQIEKEGAIH	REELVYELNP	LDHRGRTLEI
970	980	990	1000	1010	1020	1030	1040
PGNSDPNMIP	DGDFNSYVRV	TASDPLDTLG	SEGALSPGGV	ASLLRLPRGC	GEQTMIYLAP	TLAASRYLDK	TEQWSTLPPE
1050	1060	1070	1080	1090	1100	1110	1120
TKDHAVDLIQ	KGYMRIQQFR	KADGSYAAWL	SRDSSTWLTA	FVLKVLSLAQ	EQVGGSPEKL	QETSNWLLSQ	QQADGSFQDP
1130	1140	1150	1160	1170	1180	1190	1200
CPVLDRSMQG	GLVGNDETVA	LTAFVTIALH	HGLAVFQDEG	AEPLKQRVEA	SISKANSFLG	EKASAGLLGA	HAAAITAYAL
1210	1220	1230	1240	1250	1260	1270	1280
TLTKAPVDLL	GVAHNNLMAM	AQETGDNLYW	GSVTGSQSNA	VSPTPAPRNP	SDPMPQAPAL	WIETTAYALL	HLLLHEGKAE
1290	1300	1310	1320	1330	1340	1350	1360
MADQASAWLT	RQGSFQGGFR	STQDTVIALD	ALSAYWIASH	TTEERGLNVT	LSSTGRNGFK	SHALQLNNRQ	IRGLEEELQF
1370	1380	1390	1400	1410	1420	1430	1440
SLGSKINVKV	GGNSKGTLKV	LRTYNVLDMK	NTTCQDLQIE	VTVKGHVEYT	MEANEDYEDY	EYDELPAKDD	PDAPLQPVTP
1450	1460	1470	1480	1490	1500	1510	1520
LQLFEGRRNR	RRREAPKLTS	LSDRYVSHFE	TEGPHVLLYF	DSVPTSRECV	GFEAVQEVPV	GLVQPASATL	YDYYNPERRC
1530	1540	1550	1560	1570	1580	1590	1600
SVFYGAPSKS	RLLATLCSAE	VCQCAEGKCP	RQRRALERGL	QDEDGYRMKF	ACYYPRVEYG	FQVKVLREDS	RAAFRLFETK
1610	1620	1630	1640	1650	1660	1670	1680
ITQVLHFTKD	VKAAANQMRN	FLVRASCRLR	LEPGKEYLIM	GLDGATYDLE	GHPQYLLDSN	SWIEEMPSER	LCRSTRQRAA
1690	1700						
CAQLNDFLQE	YGTQGCQV						

Detailed Protein Report

Cmpd.	No. of Cmpds.	m/z meas.	Δ m/z [ppm]	z	Rt [min]	Score	P	Range	Sequence	Modification	Ratios
1978	2	656.8089	-24.39	2	54.8	11.3	1	60-71	R.NPSRNNVPCSPK.V		Q:W 1.99
1442	1	1061.4940	-67.98	2	47.8	19.8	1	119-137	K.DSLSRTTNIQGINLLFSSR.R		
1263	1	714.0054	-48.05	3	45.9	10.2	1	138-155	R.RGHLFLQTDQPIYNPGQR.V		
946	2	872.2583	-156.84	2	40.6	12.5	0	730-743	R.EPFLSCCQFAESLR.K	Carbamidomethyl: 6, 7	

BRUKER

Detailed Protein Report

Protein 270: PREDICTED: cortactin-binding protein 2 isoform X1 [Homo sapiens]

**Accession:**	gi|530386508	**Score:**	53.7
**Database:**	refseq_human(refseq_human_20140103.fasta)	**MW [kDa]:**	179.5
**Database Date:**	2015-11-30	**pI:**	9.5
**Modification(s):**	Carbamidomethyl	**Sequence Coverage [%]:**	2.8
		**No. of unique Peptides:**	2

Quantitation

Q:W	**Median:**	0.78	**CV:**	52.17 %	**No. of Peptides:**	2
M:Q	**Median:**	3.09	**CV:**	0.00 %	**No. of Peptides:**	1

```
        10         20         30         40         50         60         70         80
MIWCLHPQKK KEFDVDTLSK SELRMLLSVM EGELEARDLV IEALRARRKE VFIQERYGRF NLNDPFLALQ RDYEAGAGDK
        90        100        110        120        130        140        150        160
EKKPVCTNPL SILEAVMAHC KKMQERMSAQ LAAAESRQKK LEMEKLQLQA LEQEHKKLAA RLEEERGKNK QVVLMLVKEC
       170        180        190        200        210        220        230        240
KQLSGKVIEE AQKLEDVMAK LEEEKKKTNE LEEELSAEKR RSTEMEAQME KQLSEFDTER EQLRAKLNRE EAHTTDLKEE
       250        260        270        280        290        300        310        320
IDKMRKMIEQ LKRGSDSKPS LSLPRKTKDR RLVSISVGTE GTVTRSVACQ TDLVTENADH MKKLPLTMPV KPSTGSPLVS
       330        340        350        360        370        380        390        400
ANAKGSVCTS ATMARPGIDR QASYGDLIGA SVPAFPPPSA NKIEENGPST GSTPDPTSST PPLPSNAAPP TAQTPGIAPQ
       410        420        430        440        450        460        470        480
NSQAPPMHSL HSPCANTSLH PGLNPRIQAA RFRFQGNAND PDQNGNTTQS PPSRDVSPTS RDNLVAKQLA RNTVTQALSR
       490        500        510        520        530        540        550        560
FTSPQAGAPS RPGVPPTGDV GTHPPVGRTS LKTHGVARVD RGNPPPIPPK KPGLSQTPSP PHPQLKVIID SSRASNTGAK
       570        580        590        600        610        620        630        640
VDNKTVASTP SSLPQGNRVI NEENLPKSSS PQLPPKPSID LTVAPAGCAV SALATSQVGA WPAATPGLNQ PACSDSSLVI
       650        660        670        680        690        700        710        720
PTTIAFCSSI NPVSASSCRP GASDSLLVTA SGWSPSLTPL LMSGGPAPLA GRPTLLQQAA AQGNVTLLSM LLNEEGLDIN
       730        740        750        760        770        780        790        800
YSCEDGHSAL YSAAKNGHTD CVRLLLSAEA QVNAADKNGF TPLCAAAAQG HFECVELLIS YDANINHAAD GGQTPLYLAC
       810        820        830        840        850        860        870        880
KNGNKECIKL LLEAGTNRSV KTTDGWTPVH AAVDTGNVDS LKLLMYHRIP AHGNSFNEEE SESSVFDLDG GEESPEGISK
       890        900        910        920        930        940        950        960
PVVPADLINH ANREGWTAAH IAASKGFKNC LEILCRHGGL EPERRDKCNR TVHDVATDDC KHLLENLNAL KIPLRISVGE
       970        980        990       1000       1010       1020       1030       1040
IEPSNYGSDD LECENTICAL NIRKQTSWDD FSKAVSQALT NHFQAISSDG WWSLEDVTCN NTTDSNIGLS ARSIRSITLG
      1050       1060       1070       1080       1090       1100       1110       1120
NVPWSVGQSF AQSPWDFMRK NKAEHITVLL SGPQEGCLSS VTYASMIPLQ MMQNYLRLVE QYHNVIFHGP EGSLQDYIVH
      1130       1140       1150       1160       1170       1180       1190       1200
QLALCLKHRQ MAAGFSCEIV RAEVDAGFSK EQLLDLFISS ACLIPVKQSP SKKKIIIILE NLEKSSLSEL LRDFLAPLEN
      1210       1220       1230       1240       1250       1260       1270       1280
RSTESPCTFQ KGNGLSECYY FHENCFLMGT IAKACLQGSD LLVQQHFRWV QLRWDGEPMQ GLLQRFLRRK VVNKFKGQAP
      1290       1300       1310       1320       1330       1340       1350       1360
SPCDPVCKIV DWALSVWRQL NSCLARLGTP EALLGPKYFL SCPVVPGHAQ VTVKWMSKLW NGVIAPRVQE AILSRASVKR
      1370       1380       1390       1400       1410       1420       1430       1440
QPGFGQTTAK RHPSQGQQAV VKAALSILLN KAVLHGCPLP RAELDQHTAD FKGGSFPLSI VSSYNTCNKK KGESGAWRKV
      1450       1460       1470       1480       1490       1500       1510       1520
NTSPRRKSGR FSLPTWNKPD LSTEGMKNKT ISQLNCNRNA SLSKQKSLEN DLSLTLNLDQ RLSLGSDDEA DLVKELQSMC
      1530       1540       1550       1560       1570       1580       1590       1600
SSKSESDISK IADSRDDLRM FDSSGNNPVL SATINNLRMP VSQKEVSPLS SHQTTECSNS KSKTELGVSR VKSFLPVPRS
      1610       1620       1630       1640       1650
KVTQCSQNTK RSSSSSNTRQ IEINNNSKEV NWNLHKNEHL EKPNK
```

Cmpd.	No. of Cmpds.	m/z meas.	Δ m/z [ppm]	z	Rt [min]	Score	P	Range	Sequence	Modification	Ratios

Detailed Protein Report

Cmpd.	No. of Cmpds.	m/z meas.	Δ m/z [ppm]	z	Rt [min]	Score	P	Range	Sequence	Modification	Ratios	
493	1	464.6529	-266.41	2	34.8	10.9	0	38-45	R.DLVIEALR.A		M:Q<br>Q:W	3.09<br>0.48
2703	3	973.4604	-78.66	2	65.0	13.9	0	1151-1167	K.EQLLDLFISSACLIPVK.Q	Carbamidomethyl: 12	Q:W	1.27

Detailed Protein Report

Protein 271: ATP-dependent DNA helicase Q1 [Homo sapiens]

**Accession:**	gi|14591902	**Score:**	53.6
**Database:**	refseq_human(refseq_human_20140103.fasta)	**MW [kDa]:**	73.4
**Database Date:**	2015-11-30	**pI:**	9.0
		**Sequence Coverage [%]:**	7.7
		**No. of unique Peptides:**	2

Alias proteins:

Accession	Name	Description
gi|578823000	refseq_human (refseq_human_20140103.fasta)	PREDICTED: ATP-dependent DNA helicase Q1 isoform X5 [Homo sapiens]
gi|530399347	refseq_human (refseq_human_20140103.fasta)	PREDICTED: ATP-dependent DNA helicase Q1 isoform X4 [Homo sapiens]
gi|530399345	refseq_human (refseq_human_20140103.fasta)	PREDICTED: ATP-dependent DNA helicase Q1 isoform X3 [Homo sapiens]
gi|530399343	refseq_human (refseq_human_20140103.fasta)	PREDICTED: ATP-dependent DNA helicase Q1 isoform X2 [Homo sapiens]
gi|530399341	refseq_human (refseq_human_20140103.fasta)	PREDICTED: ATP-dependent DNA helicase Q1 isoform X1 [Homo sapiens]
gi|14591904	refseq_human (refseq_human_20140103.fasta)	ATP-dependent DNA helicase Q1 [Homo sapiens]

10	20	30	40	50	60	70	80
MASVSALTEE	LDSITSELHA	VEIQIQELTE	RQQELIQKKK	VLTKKIKQCL	EDSDAGASNE	YDSSPAAWNK	EDFPWSGKVK
**90**	**100**	**110**	**120**	**130**	**140**	**150**	**160**
DILQNVFKLE	KFRPLQLETI	NVTMAGKEVF	LVMPTGGGKS	LCYQLPALCS	DGFTLVICPL	ISLMEDQLMV	LKQLGISATM
**170**	**180**	**190**	**200**	**210**	**220**	**230**	**240**
LNASSSKEHV	KWVHAEMVNK	NSELKLIYVT	PEKIAKSKMF	MSRLEKAYEA	RRFTRIAVDE	VHCCSQWGHD	FRPDYKALGI
**250**	**260**	**270**	**280**	**290**	**300**	**310**	**320**
LKRQFPNASL	IGLTATATNH	VLTDAQKILC	IEKCFTFTAS	FNRPNLYYEV	RQKPSNTEDF	IEDIVKLING	RYKGQSGIIY
**330**	**340**	**350**	**360**	**370**	**380**	**390**	**400**
CFSQKDSEQV	TVSLQNLGIH	AGAYHANLEP	EDKTTVHRKW	SANEIQVVVA	TVAFGMGIDK	PDVRFVIHHS	MSKSMENYYQ
**410**	**420**	**430**	**440**	**450**	**460**	**470**	**480**
ESGRAGRDDM	KADCILYYGF	GDIFRISSMV	VMENVGQQKL	YEMVSYCQNI	SKCRRVLMAQ	HFDEVWNSEA	CNKMCDNCCK
**490**	**500**	**510**	**520**	**530**	**540**	**550**	**560**
DSAFERKNIT	EYCRDLIKIL	KQAEELNEKL	TPLKLIDSWM	GKGAAKLRVA	GVVAPTLPRE	DLEKIIAHFL	IQQYLKEDYS
**570**	**580**	**590**	**600**	**610**	**620**	**630**	**640**
FTAYATISYL	KIGPKANLLN	NEAHAITMQV	TKSTQNSFRA	ESSQTCHSEQ	GDKKMEEKNS	GNFQKKAANM	LQQSGSKNTG
**650**							
AKKRKIDDA							

Cmpd.	No. of Cmpds.	m/z meas.	Δ m/z [ppm]	z	Rt [min]	Score	P	Range	Sequence	Modification
2269	1	701.1276	94.64	3	58.2	13.1	1	540-556	R.EDLEKIIAHFLIQQYLK.E	
2931	1	753.8886	104.41	2	65.8	11.5	0	600-613	R.AESSQTCHSEQGDK.K	

Detailed Protein Report

Protein 272: PREDICTED: uncharacterized protein LOC285556 isoform X1 [Homo sapiens]

**Accession:**	gi|578809699	**Score:**	53.6
**Database:**	refseq_human(refseq_human_20140103.fasta)	**MW [kDa]:**	190.0
**Database Date:**	2015-11-30	**pI:**	9.8
**Modification(s):**	Carbamidomethyl	**Sequence Coverage [%]:**	2.6
		**No. of unique Peptides:**	3

Alias proteins:

Accession	Name	Description
gi|578809701	refseq_human (refseq_human_20140103.fasta)	PREDICTED: uncharacterized protein LOC285556 isoform X2 [Homo sapiens]

Detailed Protein Report

10	20	30	40	50	60	70	80
MLSFHFWKSR	GQPTDAASSV	ADGIQTPRCC	RRCQANNWTG	QLSYRTLATV	SAGAAAPQPQ	TTSTASSRSL	PTSLRLAAAP
**90**	**100**	**110**	**120**	**130**	**140**	**150**	**160**
PQGLKNWEVV	AAVAAVPTAL	GPVQIRGTLL	RATLQPLRGQ	RRTQDFPSDH	HCLFLSLKPG	QGLIMEAAPP	ELNSKARQAE
**170**	**180**	**190**	**200**	**210**	**220**	**230**	**240**
VGDGVSSAQD	SQELKQQLWP	LPKPSASSQR	EAKYVDMCAS	AEVQRESPQT	MKLTLGHCPG	GQRASRSPKE	KAQDEPSSKT
**250**	**260**	**270**	**280**	**290**	**300**	**310**	**320**
PSPQNNPASS	QLSRSQHSAS	EEGGNFSSSS	SSSPMNKAEE	DGLSKMEDST	TSTGALATSS	SSLGFESESG	ESEGCQAVGG
**330**	**340**	**350**	**360**	**370**	**380**	**390**	**400**
EGEKISGGGG	GGKGGGGGGA	GDGTECRDII	AKSQGSRDPP	KVEEAHYITT	HEIQLSEVEQ	DMDFDVGLAS	RWDFEDNNVI
**410**	**420**	**430**	**440**	**450**	**460**	**470**	**480**
YSFVDYASFG	GSDETPGDIT	SLTEEDDDNS	CYLSTTPSTN	TTRTPSPTSS	DLARPNAGRS	GRDTSSTEVG	SGPSDSGPTP
**490**	**500**	**510**	**520**	**530**	**540**	**550**	**560**
PPTGPGTAPL	TEPLPETPEA	ASGAAAAAAS	SCGSAASQIL	LSIKPASRAI	NEPSNVRAKQ	NIIYAAKHEG	DMSLRVSTAA
**570**	**580**	**590**	**600**	**610**	**620**	**630**	**640**
EHNSSSLKQN	PAAAVAQDHA	KKFIAVPARL	QTRCGAIRAK	ELVDYSSGAS	SAVSELDDAD	KEVRNLTSRA	FRSLAYPYFE
**650**	**660**	**670**	**680**	**690**	**700**	**710**	**720**
ALNISSRESS	TTLSEVGFGR	WSTFLDLKCG	GVGARVEQSL	LRSSAASVAA	GLRKGSGARA	TADQLYIQSK	KSQTKALEFV
**730**	**740**	**750**	**760**	**770**	**780**	**790**	**800**
VSKVEGEIKH	VETPLCFQKQ	VQTGSRVVTL	LEPLNVRSES	KASSAPGPGR	ATKGPGKGPG	SAYTDDGSET	SEGSKPTSRA
**810**	**820**	**830**	**840**	**850**	**860**	**870**	**880**
DGPQKSKFAS	SLLKNVISKK	MQREHEFKME	RGEVMDTSHH	LSGTSKETEG	ARGSERQRER	GLQRQSSRHS	EAGSEYTVVS
**890**	**900**	**910**	**920**	**930**	**940**	**950**	**960**
MSDAGGEGSV	AGSKSPVFKA	STPRERNAGP	GRNFTDGHTE	VCEIKKSASE	TVKGIFLRSQ	NSAFRSWKEK	EAEKREEQAP
**970**	**980**	**990**	**1000**	**1010**	**1020**	**1030**	**1040**
IGKLKLPKGG	DWRADLGEIS	ASKNTIMSRL	FVPNIQQTPK	DKQPRKQATK	YPAAQATSTA	VIRPKAPEIK	IRLGSVQQPS
**1050**	**1060**	**1070**	**1080**	**1090**	**1100**	**1110**	**1120**
SDFNIAKLLT	PKLAGGSASN	LFKTIEDNSR	AQQKLFRGDN	LEKVPHFQVR	DIRDKSKAQG	PLHQVRDVRK	LIKGSGDSSD
**1130**	**1140**	**1150**	**1160**	**1170**	**1180**	**1190**	**1200**
KGSVTPEQGL	TGPKPRQLSA	AAGGSGSLSP	MVITCQAVVN	QREDSMDREP	RESMGKGGGS	RVLNSSSPEG	TVLVHRASGR
**1210**	**1220**	**1230**	**1240**	**1250**	**1260**	**1270**	**1280**
LPVATIAPNK	PEQGSYLPVL	KIVSKASTQK	TPEKLKEEEV	KEEGKATKPA	RNALEKLTAA	VRSMEELYSF	NRNEWKRKSD
**1290**	**1300**	**1310**	**1320**	**1330**	**1340**	**1350**	**1360**
PLPMMMDSHV	LSLIASEERE	GVVVADGDHD	KLSKRLGEVE	ERGTGNKAGV	VLRGAPIERL	QRRNSNPSAE	SVSARAAAFE
**1370**	**1380**	**1390**	**1400**	**1410**	**1420**	**1430**	**1440**
NLARERPRSL	YIPPVHKDVE	RTQPLQPLPP	LPSNRNVFTV	SASSIQKTGG	VAGKFPQGPS	PESPSAAKGI	KSQGLRSLKI
**1450**	**1460**	**1470**	**1480**	**1490**	**1500**	**1510**	**1520**
SPATRAPPDE	VTNRKSGSNL	EKSNSDCENY	LTIPLKGSSA	AGELLSRPGA	SREGPPNSSA	ATLCSLPPLS	ARSQVPSSSK
**1530**	**1540**	**1550**	**1560**	**1570**	**1580**	**1590**	**1600**
GSQVSGTSRP	AWRTKPDNPR	ETVAAPPGPQ	SPEHPPTTIY	HQPPLPFTLQ	GAQPQVLCFS	PPSMPAPAPA	ASAPVPTDPF
**1610**	**1620**	**1630**	**1640**	**1650**	**1660**	**1670**	**1680**
QQAQPQQTQR	KMLLDVTTGQ	YYLVDTPVQP	MTRRLFDPET	GQYVDVPMTS	QQQAVAPMSI	SVPPLALSPG	AYGPTYMIYP
**1690**	**1700**	**1710**	**1720**	**1730**	**1740**	**1750**	**1760**
GFLPTVLPTN	ALQPTPIARA	PRGSELSPMV	AEPSSKEAAA	TFTEAPYFMA	SGQSPASSTS	SAPAATSQLL	GAKAFAQLHG
**1770**	**1780**	**1790**	**1800**				
KPVISITSQP	LGPRIIAPPS	FDGTTMSFVV	EHR				

Cmpd.	No. of Cmpds.	m/z meas.	Δ m/z [ppm]	z	Rt [min]	Score	P	Range	Sequence	Modification
886	1	575.7576	46.75	2	40.6	12.5	0	334-347	K.GGGGGGAGDGTECR.D	
1275	1	874.2865	-135.51	2	45.6	10.1	1	334-352	K.GGGGGGAGDGTECRDIIAK.S	Carbamidomethyl: 13
1984	1	634.7974	-113.89	2	54.7	11.7	2	955-965	K.REEQAPIGKLK.L	

Detailed Protein Report

Protein 273: PREDICTED: AP-3 complex subunit beta-2 isoform X2 [Homo sapiens]

**Accession:**	gi|578827454	**Score:**	53.6
**Database:**	refseq_human(refseq_human_20140103.fasta)	**MW [kDa]:**	114.4
**Database Date:**	2015-11-30	**pI:**	5.4
**Modification(s):**	Oxidation	**Sequence Coverage [%]:**	4.0
		**No. of unique Peptides:**	3

Quantitation

M:Q **Median:** 0.93 **CV:** 0.00 % **No. of Peptides:** 1

Alias proteins:

Accession	Name	Description
gi|578827456	refseq_human (refseq_human_20140103.fasta)	PREDICTED: AP-3 complex subunit beta-2 isoform X3 [Homo sapiens]

```
        10         20         30         40         50         60         70         80
MLDTNKDSLK LEAMKRIVAM IARGKNASDL FPAVVKNVAC KNIEVKKLVY VYLVRYAEEQ QDLALLSIST FQRGLKDPNQ
        90        100        110        120        130        140        150        160
LIRASALRVL SSIRVPIIVP IMMLAIKEAA SDMSPYVRKT AAHAIPKLYS LDSDQKDQLI EVIEKLLADK TTLVAGSVVM
       170        180        190        200        210        220        230        240
AFEEVCPERI DLIHKNYRKL CNLLIDVEEW GQVVIISMLT RYARTQFLSP TQNESLLEEN AEKAFYGSEE DEAKGAGSEE
       250        260        270        280        290        300        310        320
TAAAAAPSRK PYVMDPDHRL LLRNTKPLLQ SRSAAVVMAV AQLYFHLAPK AEVGVIAKAL VRLLRSHSEV QYVVLQNVAT
       330        340        350        360        370        380        390        400
MSIKRRGMFE PYLKSFYIRS TDPTQIKILK LEVLTNLANE TNIPTVLREF QTYIRSMDKD FVAATIQAIG RCATNIGRVR
       410        420        430        440        450        460        470        480
DTCLNGLVQL LSNRDELVVA ESVVVIKKLL QMQPAQHGEI IKHLAKLTDN IQVPMARASI LWLIGEYCEH VPRIAPDVLR
       490        500        510        520        530        540        550        560
KMAKSFTAEE DIVKLQVINL AAKLYLTNSK QTKLLTQYVL SLAKYDQNYD IRDRARFTRQ LIVPSEQGGA LSRHAKKLFL
       570        580        590        600        610        620        630        640
APKPAPVLES SFKDRDHFQL GSLSHLLNAK ATGYQELPDW PEEAPDPSVR NVEVPEWTKC SNREKRKEKE KPFYSDSEGE
       650        660        670        680        690        700        710        720
SGPTESADSD PESESESDSK SSSESGSGES SSESDNEDQD EDEEKGRGSE SEQSEEDGKR KTKKKVPERK GEASSSDEGS
       730        740        750        760        770        780        790        800
DSSSSSSESE MTSESEEEQL EPASWSRKTP PSSKSAPATK EISLLDLEDF TPPSVQPVSP PAIVSTSLAA DLEGLTLTDS
       810        820        830        840        850        860        870        880
TLVPSLLSPV SGVGRQELLH RVAGEGLAVD YTFSRQPFSG DPHMVSVHIH FSNSSDTPIK GLHVGTPKLP AGISIQEFPE
       890        900        910        920        930        940        950        960
IESLAPGESA TAVMGINFCD STQAANFQLC TQTRQFYVSI QPPVGELMAP VFMSENEFKK EQGKLMGMNE ITEKLMLPDT
       970        980        990       1000       1010       1020       1030       1040
CRSDHIVVQK VTATANLGRV PCGTSDEYRF AGRTLTGGSL VLLTLDARPA GAAQLTVNSE KMVIGTMLVK DVIQALTQ
```

Cmpd.	No. of Cmpds.	m/z meas.	Δ m/z [ppm]	z	Rt [min]	Score	P	Range	Sequence	Modification	Ratios
2503	2	974.2875	-301.77	1	59.6	11.1	1	17-25	R.IVAMIARGK.N	Oxidation: 4	
619	1	677.3962	18.75	2	36.6	11.7	1	77-88	K.DPNQLIRASALR.V		M:Q 0.93
1596	1	677.3556	42.21	2	50.2	10.6	1	108-119	K.EAASDMSPYVRK.T		

Detailed Protein Report

Protein 274: PREDICTED: zinc finger protein 729 isoform X1 [Homo sapiens]

**Accession:**	gi|578832877	**Score:**	53.6
**Database:**	refseq_human(refseq_human_20140103.fasta)	**MW [kDa]:**	144.0
**Database Date:**	2015-11-30	**pI:**	10.5
		**Sequence Coverage [%]:**	3.3
		**No. of unique Peptides:**	1

10	20	30	40	50	60	70	80
MAASLRDCAG	TTGGASGENP	DSGCGFMNGK	SFGPGPLTFR	DVTIEFSLEE	WQCLDTVQQN	LYRDVMLENY	RNLVFLGMAV
**90**	**100**	**110**	**120**	**130**	**140**	**150**	**160**
FKPDLITCLK	QGKEPWNMKR	HEMVTKPPVM	RSHFTQDLWP	DQSTKDSFQE	VILRTYARCG	HKNLRLRKDC	KSANEGKMHK
**170**	**180**	**190**	**200**	**210**	**220**	**230**	**240**
EGYNKLNQCR	TATQRKIFQC	NKHMKVFHKY	SNRNKVRHTK	KKTFKCIKCS	KSFFMLSCLI	RHKRIHIRQN	IYKCEERGKA
**250**	**260**	**270**	**280**	**290**	**300**	**310**	**320**
FKSFSTLTKH	KIIHTEDKPY	KYKKCGNAFK	FSSTFTKHKR	IHTGETPFRC	EECGKAFNQS	SNLTDHKRIH	TGEKTYKCEE
**330**	**340**	**350**	**360**	**370**	**380**	**390**	**400**
CGKAFKGSSN	FNAHKVIHTA	EKPYKCEDCG	KTFNHFSALR	KHKIIHTGKK	PYKREECGKA	FSQSSTLRKH	EIIHTGEKPY
**410**	**420**	**430**	**440**	**450**	**460**	**470**	**480**
KCEECGKAFK	WSSKLTVHKV	VHTGEKPYKC	EECGKAFSQF	STLKKHKIIH	TGKKPYKCEE	CGKAFNSSST	LMKHKIIHTG
**490**	**500**	**510**	**520**	**530**	**540**	**550**	**560**
EKPYKCEECG	KAFRQSSHLT	RHKAIHTGEK	PYKCEECGKA	FNHFSDLRRH	KIIHTGKKPY	KCEECGKAFS	QSSTLRNHQI
**570**	**580**	**590**	**600**	**610**	**620**	**630**	**640**
IHTGEKPYKC	EECGKAFKWS	SKLTVHKVIH	TGEKPCKCEE	CGKAFKHFSA	LRKHKVIHTR	EKLYKCEECG	KAFNNSSILA
**650**	**660**	**670**	**680**	**690**	**700**	**710**	**720**
KHKIIHTGKK	PYKCEECGKA	FRQSSHLTRH	KAIHTGEKPY	KCEECGKAFS	HFSALRRHKI	IHTGKKPYKC	EECGKAFSHF
**730**	**740**	**750**	**760**	**770**	**780**	**790**	**800**
SALRRHKIIH	TGEKPYKCEE	CGKAFKWSSK	LTVHKVIHTR	EKLYKCEECV	KAFNSFSALM	KHKVIHTGEK	PYKCEECGKA
**810**	**820**	**830**	**840**	**850**	**860**	**870**	**880**
FKWSSKLTVH	KVIHTGEKPC	KCEECGKAFK	HFSALRKHKV	IHTGKKPYKC	EECGKAFSQS	SSLRKHEIIH	SGEKPYKCEE
**890**	**900**	**910**	**920**	**930**	**940**	**950**	**960**
CGKAFKWLSK	LTVHKVIHTA	EKPCKCEECG	KAFKHFSALR	KHKIIHTGKK	PYKCEECGKA	FNDSSTLMKH	KIIHTGKKPY
**970**	**980**	**990**	**1000**	**1010**	**1020**	**1030**	**1040**
KCAECGKAFK	QSSHLTRHKA	IHTGEKPYKC	EECGKDFNNS	STLKKHKLIH	TREKLYKCEE	CVKAFNNFSA	LMKHKIIHTG
**1050**	**1060**	**1070**	**1080**	**1090**	**1100**	**1110**	**1120**
EKPYKCEECG	KAFKWSSKLT	EHKVIHTGEK	PCKCEECDKA	FKHFSALRKH	KVIHTGKKPY	QCDECGKAFN	NSSTLTKHKI
**1130**	**1140**	**1150**	**1160**	**1170**	**1180**	**1190**	**1200**
IHTGEKPYKC	EECGKAFSQS	SILTKHKIIH	SVEKPYKCEE	CGKAFNQSSH	LTRHKTIHTG	EKPYKCEECG	KAFIQCSYLI
**1210**	**1220**	**1230**	**1240**	**1250**			
RHKTIHTREK	PTNVKKVPKL	LSNPHTLLDK	TIHTGEKPYK	CEECAKAF			

Cmpd.	No. of Cmpds.	m/z meas.	Δ m/z [ppm]	z	Rt [min]	Score	P	Range	Sequence	Modification
2677	1	665.3406	42.21	2	61.8	13.9	2	621-631	R.EKLYKCEECGK.A	

Detailed Protein Report

Protein 275: coiled-coil domain-containing protein 38 [Homo sapiens]

Accession: gi|226491199
Database: refseq_human(refseq_human_20140103.fasta)
Database Date: 2015-11-30

Score: 53.5
MW [kDa]: 65.3
pI: 9.4
Sequence Coverage [%]: 10.8
No. of unique Peptides: 3

Quantitation

Q:W	**Median:**	1.51	**CV:**	0.00 %	**No. of Peptides:**	1
M:Q	**Median:**	0.58	**CV:**	0.00 %	**No. of Peptides:**	1

10	20	30	40	50	60	70	80
MSSNLLPTLN	SGGKVKDGST	KEDRPYKIFF	RDLFLVKENE	MAAKETEKFM	NRNMKVYQKT	TFSSRMKSHS	YLSQLAFYPK
**90**	**100**	**110**	**120**	**130**	**140**	**150**	**160**
RSGRSFEKFG	PGPAPIPRLI	EGSDTKRTVH	EFINDQRDRF	LLEYALSTKR	NTIKKFEKDI	AMRERQLKKA	EKKLQDDALA
**170**	**180**	**190**	**200**	**210**	**220**	**230**	**240**
FEEFLRENDQ	RSVDALKMAA	QETINKLQMT	AELKKASMEV	QAVKSEIAKT	EFLLREYMKY	GFFLLQMSPK	HWQIQQALKR
**250**	**260**	**270**	**280**	**290**	**300**	**310**	**320**
AQASKSKANI	ILPKILAKLS	LHSSNKEGIL	EESGRTAVLS	EDASQGRDSQ	GKPSRSLTRT	PEKKKSNLAE	SFGSEDSLEF
**330**	**340**	**350**	**360**	**370**	**380**	**390**	**400**
LLDDEMDVDL	EPALYFKEPE	ELLQVLRELE	EQNLTLFQYS	QDVDENLEEV	NKREKVIQDK	TNSNIEFLLE	QEKMLKANCV
**410**	**420**	**430**	**440**	**450**	**460**	**470**	**480**
REEEKAAELQ	LKSKLFSFGE	FNSDAQEILI	DSLSKKITQV	YKVCIGDAED	DGLNPIQKLV	KVESRLVELC	DLIESIPKEN
**490**	**500**	**510**	**520**	**530**	**540**	**550**	**560**
VEAIERMKQK	EWRQKFRDEK	MKEKQRHQQE	RLKAALEKAV	AQPKKKLGRR	LVFHSKPPSG	NKQQLPLVNE	TKTKSQEEEY
**570**							
FFT							

Cmpd.	No. of Cmpds.	m/z meas.	Δ m/z [ppm]	z	Rt [min]	Score	P	Range	Sequence	Modification	Ratios
29	1	1001.9624	-79.51	2	30.0	12.9	2	2-21	M.SSNLLPTLNSGGKVKDGSTK.E		M:Q 0.58
39	1	750.3346	-92.35	2	29.8	15.7	1	85-98	R.SFEKFGPGPAPIPR.L		
353	1	676.4443	133.31	3	33.1	11.6	1	443-461	K.VCIGDAEDDGLNPIQKLVK.V		

Detailed Protein Report

Protein 276: glutathione S-transferase P [Homo sapiens]

Accession: gi|4504183
Database: refseq_human(refseq_human_20140103.fasta)
Database Date: 2015-11-30

Score: 53.5
MW [kDa]: 23.3
pI: 5.3
Sequence Coverage [%]: 7.6
No. of unique Peptides: 1

Quantitation

Q:W	**Median:**	0.60	**CV:**	0.00 %	**No. of Peptides:**	1
M:Q	**Median:**	1.45	**CV:**	0.00 %	**No. of Peptides:**	1

```
        10         20         30         40         50         60         70         80
MPPYTVVYFP VRGRCAALRM LLADQGQSWK EEVVTVETWQ EGSLKASCLY GQLPKFQDGD LTLYQSNTIL RHLGRTLGLY
        90        100        110        120        130        140        150        160
GKDQQEAALV DMVNDGVEDL RCKYISLIYT NYEAGKDDYV KALPGQLKPF ETLLSQNQGG KTFIVGDQIS FADYNLLDLL
       170        180        190        200        210        220
LIHEVLAPGC LDAFPLLSAY VGRLSARPKL KAFLASPEYV NLPINGNGKQ
```

Cmpd.	No. of Cmpds.	m/z meas.	Δ m/z [ppm]	z	Rt [min]	Score	P	Range	Sequence	Modification	Ratios
1649	5	942.4739	-4.87	2	50.9	53.5	0	56-71	K.FQDGDLTLYQSNTILR.H		Q:W 0.60<br>M:Q 1.45

Detailed Protein Report

Protein 277: PREDICTED: latrophilin-1 isoform X1 [Homo sapiens]

**Accession:**	gi|530414661	**Score:**	53.5
**Database:**	refseq_human(refseq_human_20140103.fasta)	**MW [kDa]:**	164.7
**Database Date:**	2015-11-30	**pI:**	6.1
**Modification(s):**	Carbamidomethyl	**Sequence Coverage [%]:**	4.2
		**No. of unique Peptides:**	2

Quantitation

M:Q **Median:** 1.50 **CV:** 0.00 % **No. of Peptides:** 1

10	20	30	40	50	60	70	80
MARLAAVLWN	LCVTAVLVTS	ATQGLSRAGL	PFGLMRRELA	CEGYPIELRC	PGSDVIMVEN	ANYGRTDDKI	CDADPFQMEN
90	100	110	120	130	140	150	160
VQCYLPDAFK	IMSQRCNNRT	QCVVVAGSDA	FPDPCPGTYK	YLEVQYDCVP	YIFVCPGTLQ	KVLEPTSTHE	SEHQSGAWCK
170	180	190	200	210	220	230	240
DPLQAGDRIY	VMPWIPYRTD	TLTEYASWED	YVAARHTTTY	RLPNRVDGTG	FVVYDGAVFY	NKERTRNIVK	YDLRTRIKSG
250	260	270	280	290	300	310	320
ETVINTANYH	DTSPYRWGGK	TDIDLAVDEN	GLWVIYATEG	NNGRLVVSQL	NPYTLRFEGT	WETGYDKRSA	SNAFMVCGVL
330	340	350	360	370	380	390	400
YVLRSVYVDD	DSEAAGNRVD	YAFNTNANRE	EPVSLTFPNP	YQFISSVDYN	PRDNQLYVWN	NYFVVRYSLE	FGPPDPSAGP
410	420	430	440	450	460	470	480
ATSPPLSTTT	TARPTPLTST	ASPAATTPLR	RAPLTTHPVG	AINQLGPDLP	PATAPVPSTR	RPPAPNLHVS	PELFCEPREV
490	500	510	520	530	540	550	560
RRVQWPATQQ	GMLVERPCPK	GTRGIASFQC	LPALGLWNPR	GPDLSNCTSP	WVNQVAQKIK	SGENAANIAS	ELARHTRGSI
570	580	590	600	610	620	630	640
YAGDVSSSVK	LMEQLLDILD	AQLQALRPIE	RESAGKNYNK	MHKRERTCKD	YIKAVVETVD	NLLRPEALES	WKDMNATEQV
650	660	670	680	690	700	710	720
HTATMLLDVL	EEGAFLLADN	VREPARFLAA	KENVVLEVTV	LNTEGQVQEL	VFPQEEYPRK	NSIQLSAKTI	KQNSRNGVVK
730	740	750	760	770	780	790	800
VVFILYNNLG	LFLSTENATV	KLAGEAGPGG	PGGASLVVNS	QVIAASINKE	SSRVFLMDPV	IFTVAHLEDK	NHFNANCSFW
810	820	830	840	850	860	870	880
NYSERSMLGY	WSTQGCRLVE	SNKTHTTCAC	SHLTNFAVLM	AHREIYQGRI	NELLLSVITW	VGIVISLVCL	AICISTFCFL
890	900	910	920	930	940	950	960
RGLQTDRNTI	HKNLCINLFL	AELLFLVGID	KTQYEIACPI	FAGLLHYFFL	AAFSWLCLEG	VHLYLLLVEV	FESEYSRTKY
970	980	990	1000	1010	1020	1030	1040
YYLGGYCFPA	LVVGIAAAID	YRSYGTEKAC	WLRVDNYFIW	SFIGPVSFVI	VVNLVFLMVT	LHKMIRSSSV	LKPDSSRLDN
1050	1060	1070	1080	1090	1100	1110	1120
IKSWALGAIA	LLFLLGLTWA	FGLLFINKES	VVMAYLFTTF	NAFQGVFIFV	FHCALQKKVH	KEYSKCLRHS	YCCIRSPPGG
1130	1140	1150	1160	1170	1180	1190	1200
THGSLKTSAM	RSNTRYYTGT	QSRIRRMWND	TVRKQTESSF	MAGDINSTPT	LNRGTMGNHL	LTNPVLQPRG	GTSPYNTLIA
1210	1220	1230	1240	1250	1260	1270	1280
ESVGFNPSSP	PVFNSPELEP	GLAFRAHPCL	FSSLFWLPPA	GSYREPKHPL	GGREACGMDT	LPLNGNFNNS	YSLRSGDFPP
1290	1300	1310	1320	1330	1340	1350	1360
GDGGPEPPRG	RNLADAAAFE	KMIISELVHN	NLRGSSSAAK	GPPPPEPPVP	PVPGGGGEEE	AGGPGGADRA	EIELLYKALE
1370	1380	1390	1400	1410	1420	1430	1440
EPLLLPRAQS	VLYQSDLDES	ESCTAEDGAT	SRPLSSPPGR	DSLYASGANL	RDSPSYPDSS	PEGPSEALPP	PPPAPPGPPE
1450	1460	1470	1480	1490	1500		
IYYTSRPPAL	VARNPLQGYY	QVRRPSHEGY	LAAPGLEGPG	PDGDGQMQLV	TSL		

Cmpd.	No. of Cmpds.	m/z meas.	Δ m/z [ppm]	z	Rt [min]	Score	P	Range	Sequence	Modification	Ratios
2050	1	900.3480	-63.50	3	55.8	14.3	1	96-120	R. CNNRTQCVVVAGSDAFPDPCPGT Y	Carbamidomethyl: 7	
943	1	660.3451	-4.50	2	41.8	11.7	2	1099-1108	K.VHKEYSKCLR.H	Carbamidomethyl: 8	M:Q 1.50

Detailed Protein Report

Protein 278: PREDICTED: complement receptor type 1 isoform X4 [Homo sapiens]

**Accession:**	gi|578800553	**Score:**	53.4
**Database:**	refseq_human(refseq_human_20140103.fasta)	**MW [kDa]:**	224.1
**Database Date:**	2015-11-30	**pI:**	6.7
**Modification(s):**	Carbamidomethyl	**Sequence Coverage [%]:**	2.4
		**No. of unique Peptides:**	1

Quantitation

Q:W	**Median:**	0.38	**CV:**	0.00 %	**No. of Peptides:**	1
M:Q	**Median:**	0.77	**CV:**	0.00 %	**No. of Peptides:**	1

Detailed Protein Report

10	20	30	40	50	60	70	80
MCLGRMGASS	PRSPEPVGPP	APGLPFCCGG	SLLAVVVLLA	LPVAWGQCNA	PEWLPFARPT	NLTDEFEFPI	GTYLNYECRP
90	100	110	120	130	140	150	160
GYSGRPFSII	CLKNSVWTGA	KDRCRRKSCR	NPPDPVNGMV	HVIKGIQFGS	QIKYSCTKGY	RLIGSSSATC	IISGDTVIWD
170	180	190	200	210	220	230	240
NETPICDRIP	CGLPPTITNG	DFISTNRENF	HYGSVVTYRC	NPGSGGRKVF	ELVGEPSIYC	TSNDDQVGIW	SGPAPQCIIP
250	260	270	280	290	300	310	320
NKCTPPNVEN	GILVSDNRSL	FSLNEVVEFR	CQPGFVMKGP	RRVKCQALNK	WEPELPSCSR	VCQPPPDVLH	AERTQRDKDN
330	340	350	360	370	380	390	400
FSPGQEVFYS	CEPGYDLRGA	ASMRCTPQGD	WSPAAPTCEV	KSCDDFMGQL	LNGRVLFPVN	LQLGAKVDFV	CDEGFQLKGS
410	420	430	440	450	460	470	480
SASYCVLAGM	ESLWNSSVPV	CEQIFCPSPP	VIPNGRHTGK	PLEVFPFGKT	VNYTCDPHPD	RGTSFDLIGE	STIRCTSDPQ
490	500	510	520	530	540	550	560
GNGVWSSPAP	RCGILGHCQA	PDHFLFAKLK	TQTNASDFPI	GTSLKYECRP	EYYGRPFSIT	CLDNLVWSSP	KDVCKRKSCK
570	580	590	600	610	620	630	640
TPPDPVNGMV	HVITDIQVGS	RINYSCTTGH	RLIGHSSAEC	ILSGNAAHWS	TKPPICQRIP	CGLPPTIANG	DFISTNRENF
650	660	670	680	690	700	710	720
HYGSVVTYRC	NPGSGGRKVF	ELVGEPSIYC	TSNDDQVGIW	SGPAPQCIIP	NKCTPPNVEN	GILVSDNRSL	FSLNEVVEFR
730	740	750	760	770	780	790	800
CQPGFVMKGP	RRVKCQALNK	WEPELPSCSR	VCQPPPDVLH	AERTQRDKDN	FSPGQEVFYS	CEPGYDLRGA	ASMRCTPQGD
810	820	830	840	850	860	870	880
WSPAAPTCEV	KSCDDFMGQL	LNGRVLFPVN	LQLGAKVDFV	CDEGFQLKGS	SASYCVLAGM	ESLWNSSVPV	CEQIFCPSPP
890	900	910	920	930	940	950	960
VIPNGRHTGK	PLEVFPFGKT	VNYTCDPHPD	RGTSFDLIGE	STIRCTSDPQ	GNGVWSSPAP	RCGILGHCQA	PDHFLFAKLK
970	980	990	1000	1010	1020	1030	1040
TQTNASDFPI	GTSLKYECRP	EYYGRPFSIT	CLDNLVWSSP	KDVCKRKSCK	TPPDPVNGMV	HVITDIQVGS	RINYSCTTGH
1050	1060	1070	1080	1090	1100	1110	1120
RLIGHSSAEC	ILSGNAAHWS	TKPPICQRIP	CGLPPTIANG	DFISTNRENF	HYGSVVTYRC	NPGSGGRKVF	ELVGEPSIYC
1130	1140	1150	1160	1170	1180	1190	1200
TSNDDQVGIW	SGPAPQCIIP	NKCTPPNVEN	GILVSDNRSL	FSLNEVVEFR	CQPGFVMKGP	RRVKCQALNK	WEPELPSCSR
1210	1220	1230	1240	1250	1260	1270	1280
VCQPPPDVLH	AERTQRDKDN	FSPGQEVFYS	CEPGYDLRGA	ASLHCTPQGD	WSPEAPRCAV	KSCDDFLGQL	PHGRVLFPLN
1290	1300	1310	1320	1330	1340	1350	1360
LQLGAKVSFV	CDEGFRLKGS	SVSHCVLVGM	RSLWNNSVPV	CEHIFCPNPP	AILNGRHTGT	PSGDIPYGKE	ISYTCDPHPD
1370	1380	1390	1400	1410	1420	1430	1440
RGMTFNLIGE	STIRCTSDPH	GNGVWSSPAP	RCELSVRAGH	CKTPEQFPFA	SPTIPINDFE	FPVGTSLNYE	CRPGYFGKMF
1450	1460	1470	1480	1490	1500	1510	1520
SISCLENLVW	SSVEDNCRRK	SCGPPPEPFN	GMVHINTDTQ	FGSTVNYSCN	EGFRLIGSPS	TTCLVSGNNV	TWDKKAPICE
1530	1540	1550	1560	1570	1580	1590	1600
IISCEPPPTI	SNGDFYSNNR	TSFHNGTVVT	YQCHTGPDGE	QLFELVGERS	IYCTSKDDQV	GVWSSPPPRC	ISTNKCTAPE
1610	1620	1630	1640	1650	1660	1670	1680
VENAIRVPGN	RSFFTLTEII	RFRCQPGFVM	VGSHTVQCQT	NGRWGPKLPH	CSRVCQPPPE	ILHGEHTLSH	QDNFSPGQEV
1690	1700	1710	1720	1730	1740	1750	1760
FYSCEPSYDL	RGAASLHCTP	QGDWSPEAPR	CTVKSCDDFL	GQLPHGRVLL	PLNLQLGAKV	SFVCDEGFRL	KGRSASHCVL
1770	1780	1790	1800	1810	1820	1830	1840
AGMKALWNSS	VPVCEQIFCP	NPPAILNGRH	TGTPFGDIPY	GKEISYACDT	HPDRGMTFNL	IGESSIRCTS	DPQGNGVWSS
1850	1860	1870	1880	1890	1900	1910	1920
PAPRCELSVP	AACPHPPKIQ	NGHYIGGHVS	LYLPGMTISY	ICDPGYLLVG	KGFIFCTDQG	IWSQLDHYCK	EVNCSFPLFM
1930	1940	1950	1960	1970	1980	1990	2000
NGISKELEMK	KVYHYGDYVT	LKCEDGYTLE	GSPWSQCQAD	DRWDPPLAKC	TSRTHDALIV	GTLSGTIFFI	LLIIFLSWII
2010	2020	2030	2040	2050			
LKHRKGNNAH	ENPKEVAIHL	HSQGGSSVHP	RTLQTNEENS	RVLP			

Cmpd.	No. of Cmpds.	m/z meas.	Δ m/z [ppm]	z	Rt [min]	Score	P	Range	Sequence	Modification	Ratios	
73	4	826.3528	-22.94	3	30.6	30.9	1	339-361	R.GAASMRCTPQGDWSPAAPTCEV.S	Carbamidomethyl: 7, 20	Q:W<br>M:Q	0.38<br>0.77

Detailed Protein Report

Protein 279: **PREDICTED: sterol regulatory element-binding protein 1 isoform X2 [Homo sapiens]**

**Accession:**	gi|578830023	**Score:**	53.4
**Database:**	refseq_human(refseq_human_20140103.fasta)	**MW [kDa]:**	119.1
**Database Date:**	2015-11-30	**pI:**	9.9
**Modification(s):**	Oxidation	**Sequence Coverage [%]:**	4.4
		**No. of unique Peptides:**	2

Quantitation

Q:W	**Median:** 0.94	**CV:** 0.00 %	**No. of Peptides:**	1
M:Q	**Median:** 0.86	**CV:** 0.00 %	**No. of Peptides:**	1

```
        10         20         30         40         50         60         70         80
MDCTFEDMLQ LINNQDSDFP GLFDPPYAGS GAGGTDPASP DTSSPGSLSP PPATLSSSLE AFLSGPQAAP SPLSPPQPAP
        90        100        110        120        130        140        150        160
TPLKMYPSMP AFSPGPGIKE ESVPLSILQT PTPQPLPGAL LPQSFPAPAP PQFSSTPVLG YPSPPGGFST GSPPGNTQQP
       170        180        190        200        210        220        230        240
LPGLPLASPP GVPPVSLHTQ VQSVVPQQLL TVTAAPTAAP VTTTVTSQIQ QVPVLLQPHF IKADSLLLTA MKTDGATVKA
       250        260        270        280        290        300        310        320
AGLSPLVSGT TVQTGPLPTL VSGGTILATV PLVVDAEKLP INRLAAGSKA PASAQSRGEK RTAHNAIEKR YRSSINDKII
       330        340        350        360        370        380        390        400
ELKDLVVGTE AKLNKSAVLR KAIDYIRFLQ HSNQKLKQEN LSLRTAVHKS KSLKDLVSAC GSGGNTDVLM EGVKTEVEDT
       410        420        430        440        450        460        470        480
LTPPPSDAGS PFQSSPLSLG SRGSGSGGSG SDSEPDSPVF EDSKAKPEQR PSLHSRGMLD RSRLALCTLV FLCLSCNPLA
       490        500        510        520        530        540        550        560
SLLGARGLPS PSDTTSVYHS PGRNVLGTES RDGPGWAQWL LPPVVWLLNG LLVLVSLVLL FVYGEPVTRP HSGPAVYFWR
       570        580        590        600        610        620        630        640
HRKQADLDLA RGDFAQAAQQ LWLALRALGR PLPTSHLDLA CSLLWNLIRH LLQRLWVGRW LAGRAGGLQQ DCALRVDASA
       650        660        670        680        690        700        710        720
SARDAALVYH KLHQLHTMGK HTGGHLTATN LALSALNLAE CAGDAVSVAT LAEIYVAAAL RVKTSLPRAL HFLTRFFLSS
       730        740        750        760        770        780        790        800
ARQACLAQSG SVPPAMQWLC HPVGHRFFVD GDWSVLSTPW ESLYSLAGNP VDPLAQVTQL FREHLLERAL NCVTQPNPSP
       810        820        830        840        850        860        870        880
GSADGDKEFS DALGYLQLLN SCSDAAGAPA YSFSISSSMA TTTGVDPVAK WWASLTAVVI HWLRRDEEAA ERLCPLVEHL
       890        900        910        920        930        940        950        960
PRVLQESERP LPRAALHSFK AARALLGCAK AESGPASLTI CEKASGYLQD SLATTPASSS IDKAVQLFLC DLLLVVRTSL
       970        980        990       1000       1010       1020       1030       1040
WRQQQPPAPA PAAQGTSSRP QASALELRGF QRDLSSLRRL AQSFRPAMRR VFLHEATARL MAGASPTRTH QLLDRSLRRR
      1050       1060       1070       1080       1090       1100       1110       1120
AGPGGKGGAV AELEPRPTRR EHAEALLLAS CYLPPGFLSA PGQRVGMLAE AARTLEKLGD RRLLHDCQQM LMRLGGGTTV
      1130
TSS
```

Cmpd.	No. of Cmpds.	m/z meas.	Δ m/z [ppm]	z	Rt [min]	Score	P	Range	Sequence	Modification	Ratios
1578	1	798.3570	-33.20	2	49.4	11.7	0	85-99	K.MYPSMPAFSPGPGIK.E	Oxidation: 5	
783	1	676.8811	19.88	2	39.8	12.5	0	1047-1059	K.GGAVAELEPRPTR.R		Q:W 0.94<br>M:Q 0.86

Detailed Protein Report

Protein 280: **GRIP and coiled-coil domain-containing protein 1 [Homo sapiens]**

**Accession:**	gi|22208850	**Score:**	53.4
**Database:**	refseq_human(refseq_human_20140103.fasta)	**MW [kDa]:**	87.8
**Database Date:**	2015-11-30	**pI:**	5.2
		**Sequence Coverage [%]:**	6.5
		**No. of unique Peptides:**	3

Quantitation

Q:W	**Median:**	0.95	**CV:**	74.07 %	**No. of Peptides:**	3
M:Q	**Median:**	0.85	**CV:**	11.90 %	**No. of Peptides:**	2

10	20	30	40	50	60	70	80
MEKFGMNFGG	GPSKKDLLET	IETQKKQLLQ	YQARLKDVVR	AYKSLLKEKE	ALEASIKVLS	VSHEADVGLA	GVQLPGLTFP
**90**	**100**	**110**	**120**	**130**	**140**	**150**	**160**
DSVDDRCSTH	SEDSTGTATS	LDTAASLTST	KGEFGVEDDR	PARGPPPPKS	EEASWSESGV	SSSSGDGPFA	GGEVDKRLHQ
**170**	**180**	**190**	**200**	**210**	**220**	**230**	**240**
LKTQLATLTS	SLATVTQEKS	RMEASYLADK	KKMKQDLEDA	SNKAEEERAR	LEGELKGLQE	QIAETKARLI	TQQHDRAQEQ
**250**	**260**	**270**	**280**	**290**	**300**	**310**	**320**
SDHALMLREL	QKLLQEERTQ	RQDLELRLEE	TREALAGRAY	AAEQMEGFEL	QTKQLTREVE	ELKSELQAIR	DEKNQPDPRL
**330**	**340**	**350**	**360**	**370**	**380**	**390**	**400**
QELQEEAARL	KSHFQAQLQQ	EMRKTALAED	QLRQQSQVEE	QRVAALENQI	SEVSELLGTY	EKAKQKDQLA	IQKLKERILQ
**410**	**420**	**430**	**440**	**450**	**460**	**470**	**480**
LDLENKTLAL	AASSRSPLDS	HGEESSLDVN	VLKDKMEKLK	RLLQVAARKS	QVTLDVEKLC	DLEIMPSSEA	ADGEKATALY
**490**	**500**	**510**	**520**	**530**	**540**	**550**	**560**
YQQELKQLKE	EFERYKMRAQ	VVLKSKNTKD	GNLGKELEAA	QEQLAELKEK	YISLRLSCEE	LEHQHQQEAD	DWKQELARLQ
**570**	**580**	**590**	**600**	**610**	**620**	**630**	**640**
QLHRQELERC	QLDFRDRTLK	LEEELHKQRD	RALAVLTEKD	LELEQLRSVA	LASGLPGRRS	PVGGGGPGDP	ADTSSSDSLT
**650**	**660**	**670**	**680**	**690**	**700**	**710**	**720**
QALQLAAANE	PTFFLYAEQL	ARKEVEITSL	RKQKHRLEVE	VHQLQDRLLE	EGERHREEVA	ALQSHIEKNI	RDQSREGANL
**730**	**740**	**750**	**760**	**770**	**780**		
EYLKNIIYRF	LTLPDSLGRQ	QTLTAILTIL	HFSPEEKQVI	MRLPTSASWW	PSGKR		

Cmpd.	No. of Cmpds.	m/z meas.	Δ m/z [ppm]	z	Rt [min]	Score	P	Range	Sequence	Modification	Ratios
165	1	635.8027	-9.05	2	31.3	11.3	1	180-190	K.SRMEASYLADK.K		M:Q 0.95<br>Q:W 1.24
1918	4	572.7243	-166.46	2	54.0	17.8	1	344-353	R.KTALAEDQLR.Q		M:Q 0.75<br>Q:W 0.38
55	1	1096.9659	-93.00	2	30.1	11.7	0	363-382	R.VAALENQISEVSELLGTYEK.A		Q:W 1.80

Detailed Protein Report

Protein 281: chromosome-associated kinesin KIF4A [Homo sapiens]

**Accession:**	gi|116686122	**Score:**	53.3
**Database:**	refseq_human(refseq_human_20140103.fasta)	**MW [kDa]:**	139.8
**Database Date:**	2015-11-30	**pI:**	5.9
**Modification(s):**	Carbamidomethyl	**Sequence Coverage [%]:**	2.9
		**No. of unique Peptides:**	3

10	20	30	40	50	60	70	80
MKEEVKGIPV	RVALRCRPLV	PKEISEGCQM	CLSFVPGEPQ	VVVGTDKSFT	YDFVFDPSTE	QEEVFNTAVA	PLIKGVFKGY
90	100	110	120	130	140	150	160
NATVLAYGQT	GSGKTYSMGG	AYTAEQENEP	TVGVIPRVIQ	LLFKEIDKKS	DFEFTLKVSY	LEIYNEEILD	LLCPSREKAQ
170	180	190	200	210	220	230	240
INIREDPKEG	IKIVGLTEKT	VLVALDTVSC	LEQGNNSRTV	ASTAMNSQSS	RSHAIFTISL	EQRKKSDKNS	SFRSKLHLVD
250	260	270	280	290	300	310	320
LAGSERQKKT	KAEGDRLKEG	ININRGLLCL	GNVISALGDD	KKGGFVPYRD	SKLTRLLQDS	LGGNSHTLMI	ACVSPADSNL
330	340	350	360	370	380	390	400
EETLNTLRYA	DRARKIKNKP	IVNIDPQTAE	LNHLKQQVQQ	LQVLLLQAHG	GTLPGSITVE	PSENLQSLME	KNQSLVEENE
410	420	430	440	450	460	470	480
KLSRGLSEAA	GQTAQMLERI	ILTEQANEKM	NAKLEELRQH	AACKLDLQKL	VETLEDQELK	ENVEIICNLQ	QLITQLSDET
490	500	510	520	530	540	550	560
VACMAAAIDT	AVEQEAQVET	SPETSRSSDA	FTTQHALRQA	QMSKELVELN	KALALKEALA	RKMTQNDSQL	QPIQYQYQDN
570	580	590	600	610	620	630	640
IKELELEVIN	LQKEKEELVL	ELQTAKKDAN	QAKLSERRRK	RLQELEGQIA	DLKKKLNEQS	KLLKLKESTE	RTVSKLNQEI
650	660	670	680	690	700	710	720
RMMKNQRVQL	MRQMKEDAEK	FRQWKQKKDK	EVIQLKERDR	KRQYELLKLE	RNFQKQSNVL	RRKTEEAAAA	NKRLKDALQK
730	740	750	760	770	780	790	800
QREVADKRKE	TQSRGMEGTA	ARVKNWLGNE	IEVMVSTEEA	KRHLNDLLED	RKILAQDVAQ	LKEKKESGEN	PPPKLRRRTF
810	820	830	840	850	860	870	880
SLTEVRGQVS	ESEDSITKQI	ESLETEMEFR	SAQIADLQQK	LLDAESEDRP	KQRWENIATI	LEAKCALKYL	IGELVSSKIQ
890	900	910	920	930	940	950	960
VSKLESSLKQ	SKTSCADMQK	MLFEERNHFA	EIETELQAEL	VRMEQQHQEK	VLYLLSQLQQ	SQMAEKQLEE	SVSEKEQQLL
970	980	990	1000	1010	1020	1030	1040
STLKCQDEEL	EKMREVCEQN	QQLLRENEII	KQKLTLLQVA	SRQKHLPKDT	LLSPDSSFEY	VPPKPKPSRV	KEKFLEQSMD
1050	1060	1070	1080	1090	1100	1110	1120
IEDLKYCSEH	SVNEHEDGDG	DDDEGDDEEW	KPTKLVKVSR	KNIQGCSCKG	WCGNKQCGCR	KQKSDCGVDC	CCDPTKCRNR
1130	1140	1150	1160	1170	1180	1190	1200
QQGKDSLGTV	ERTQDSEGSF	KLEDPTEVTP	GLSFFNPVCA	TPNSKILKEM	CDVEQVLSKK	TPPAPSPFDL	PELKHVATEY
1210	1220	1230	1240				
QENKAPGKKK	KRALASNTSF	FSGCSPIEEE	AH				

Cmpd.	No. of Cmpds.	m/z meas.	Δ m/z [ppm]	z	Rt [min]	Score	P	Range	Sequence	Modification
2387	1	1051.5184	80.34	2	58.2	16.1	2	1082-1100	K.NIQGCSCKGWCGNKQCGCR.K	Carbamidomethyl: 11
2074	1	701.2275	-91.47	3	56.1	10.6	2	1082-1100	K.NIQGCSCKGWCGNKQCGCR.K	Carbamidomethyl: 18
1950	3	1023.4041	14.44	2	54.3	14.9	2	1104-1120	K.SDCGVDCCCDPTKCRNR.Q	Carbamidomethyl: 3, 7, 8

Detailed Protein Report

Protein 282: pregnancy zone protein precursor [Homo sapiens]

**Accession:**	gi|162809334	**Score:**	53.2
**Database:**	refseq_human(refseq_human_20140103.fasta)	**MW [kDa]:**	163.8
**Database Date:**	2015-11-30	**pI:**	5.9
		**Sequence Coverage [%]:**	1.6
		**No. of unique Peptides:**	1

10	20	30	40	50	60	70	80
MRKDRLLHLC	LVLLLILLSA	SDSNSTEPQY	MVLVPSLLHT	EAPKKGCVLL	SHLNETVTVS	ASLESGRENR	SLFTDLVAEK
90	100	110	120	130	140	150	160
DLFHCVSFTL	PRISASSEVA	FLSIQIKGPT	QDFRKRNTVL	VLNTQSLVFV	QTDKPMYKPG	QTVRFRVVSV	DENFRPRNEL
170	180	190	200	210	220	230	240
IPLIYLENPR	RNRIAQWQSL	KLEAGINQLS	FPLSSEPIQG	SYRVVVQTES	GGRIQHPFTV	EEFVLPKFEV	KVQVPKIISI
250	260	270	280	290	300	310	320
MDEKVNITVC	GEYTYGKPVP	GLATVSLCRK	LSRVLNCDKQ	EVCEEFSQQL	NSNGCITQQV	HTKMLQITNT	GFEMKLRVEA
330	340	350	360	370	380	390	400
RIREEGTDLE	VTANRISEIT	NIVSKLKFVK	VDSHFRQGIP	FFAQVLLVDG	KGVPIPNKLF	FISVNDANYY	SNATTNEQGL
410	420	430	440	450	460	470	480
AQFSINTTSI	SVNKLFVRVF	TVHPNLCFHY	SWVAEDHQGA	QHTANRVFSL	SGSYIHLEPV	AGTLPCGHTE	TITAHYTLNR
490	500	510	520	530	540	550	560
QAMGELSELS	FHYLIMAKGV	IVRSGTHTLP	VESGDMKGSF	ALSFPVESDV	APIARMFIFA	ILPDGEVVGD	SEKFEIENCL
570	580	590	600	610	620	630	640
ANKVDLSFSP	AQSPPASHAH	LQVAAAPQSL	CALRAVDQSV	LLMKPEAELS	VSSVYNLLTV	KDLTNFPDNV	DQQEEEQGHC
650	660	670	680	690	700	710	720
PRPFFIHNGA	IYVPLSSNEA	DIYSFLKGMG	LKVFTNSKIR	KPKSCSVIPS	VSAGAVGQGY	YGAGLGVVER	PYVPQLGTYN
730	740	750	760	770	780	790	800
VIPLNNEQSS	GPVPETVRSY	FPETWIWELV	AVNSSGVAEV	GVTVPDTITE	WKAGAFCLSE	DAGLGISSTA	SLRAFQPFFV
810	820	830	840	850	860	870	880
ELTMPYSVIR	GEVFTLKATV	LNYLPKCIRV	SVQLKASPAF	LASQNTKGEE	SYCICGNERQ	TLSWTVTPKT	LGNVNFSVSA
890	900	910	920	930	940	950	960
EAMQSLELCG	NEVVEVPEIK	RKDTVIKTLL	VEAEGIEQEK	TFSSMTCASG	ANVSEQLSLK	LPSNVVKESA	RASFSVLGDI
970	980	990	1000	1010	1020	1030	1040
LGSAMQNIQN	LLQMPYGCGE	QNMVLFAPNI	YVLNYLNETQ	QLTQEIKAKA	VGYLITGYQR	QLNYKHQDGS	YSTFGERYGR
1050	1060	1070	1080	1090	1100	1110	1120
NQGNTWLTAF	VLKTFAQARS	YIFIDEAHIT	QSLTWLSQMQ	KDNGCFRSSG	SLLNNAIKGG	VEDEATLSAY	VTIALLEIPL
1130	1140	1150	1160	1170	1180	1190	1200
PVTNPIVRNA	LFCLESAWNV	AKEGTHGSHV	YTKALLAYAF	SLLGKQNQNR	EILNSLDKEA	VKEDNLVHWE	RPQRPKAPVG
1210	1220	1230	1240	1250	1260	1270	1280
HLYQTQAPSA	EVEMTSYVLL	AYLTAQPAPT	SGDLTSATNI	VKWIMKQQNA	QGGFSSTQDT	VVALHALSRY	GAATFTRTEK
1290	1300	1310	1320	1330	1340	1350	1360
TAQVTVQDSQ	TFSTNFQVDN	NNLLLLQQIS	LPELPGEYVI	TVTGERCVYL	QTSMKYNILP	EKEDSPFALK	VQTVPQTCDG
1370	1380	1390	1400	1410	1420	1430	1440
HKAHTSFQIS	LTISYTGNRP	ASNMVIVDVK	MVSGFIPLKP	TVKMLERSSS	VSRTEVSNNH	VLIYVEQVTN	QTLSFSFMVL
1450	1460	1470	1480	1490			
QDIPVGDLKP	AIVKVYDYYE	TDESVVAEYI	APCSTDTEHG	NV			

Cmpd.	No. of Cmpds.	m/z meas.	Δ m/z [ppm]	z	Rt [min]	Score	P	Range	Sequence	Modification
705	1	802.0356	159.48	2	38.8	16.0	1	322-335	R.IREEGTDLEVTANR.I	

Detailed Protein Report

Protein 283: DNA polymerase zeta catalytic subunit isoform b [Homo sapiens]

**Accession:**	gi|556695361	**Score:**	53.2
**Database:**	refseq_human(refseq_human_20140103.fasta)	**MW [kDa]:**	343.9
**Database Date:**	2015-11-30	**pI:**	9.5
**Modification(s):**	Carbamidomethyl, Oxidation	**Sequence Coverage [%]:**	1.8
		**No. of unique Peptides:**	4

Alias proteins:

Accession	Name	Description
gi|578812913	refseq_human (refseq_human_20140103.fasta)	PREDICTED: DNA polymerase zeta catalytic subunit isoform X6 [Homo sapiens]
gi|556695404	refseq_human (refseq_human_20140103.fasta)	DNA polymerase zeta catalytic subunit isoform b [Homo sapiens]

Detailed Protein Report

10	20	30	40	50	60	70	80
MAFSIDRALN	VALGNPSSTA	QHVFKVSLVS	GMPFYGYHEK	ERHFMKIYLY	NPTMVKRICE	LLQSGAIMNK	FYQPHEAHIP
90	100	110	120	130	140	150	160
YLLQLFIDYN	LYGMNLINLA	AVKFRKARRK	SNTLHATGSC	KNHLSGNSLA	DTLFRWEQDE	IPSSLILEGV	EPQSTCELEV
170	180	190	200	210	220	230	240
DAVAADILNR	LDIEAQIGGN	PGLQAIWEDE	KQRRRNRNET	SQMSQPESQD	HRFVPATESE	KKFQKRLQEI	LKQNDFSVTL
250	260	270	280	290	300	310	320
SGSVDYSDGS	QEFSAELTLH	SEVLSPEMLQ	CTPANMVEVH	KDKESSKGHT	RHKVEEALIN	EEAILNLMEN	SQTFQPLTQR
330	340	350	360	370	380	390	400
LSESPVFMDS	SPDEALVHLL	AGLESDGYRG	ERNRMPSPCR	SFGNNKYPQN	SDDEENEPQI	EKEEMELSLV	MSQRWDSNIE
410	420	430	440	450	460	470	480
EHCAKKRSLC	RNTHRSSTED	DDSSSGEEME	WSDNSLLLAS	LSIPQLDGTA	DENSDNPLNN	ENSRTHSSVI	ATSKLSVKPS
490	500	510	520	530	540	550	560
IFHKDAATLE	PSSSAKITFQ	CKHTSALSSH	VLNKEDLIED	LSQTNKNTEK	GLDNSVTSFT	NESTYSMKYP	GSLSSTVHSE
570	580	590	600	610	620	630	640
NSHKENSKKE	ILPVSSCESS	IFDYEEDIPS	VTRQVPSRKY	TNIRKIEKDS	PFIHMHRHPN	ENTLGKNSFN	FSDLNHSKNK
650	660	670	680	690	700	710	720
VSSEGNEKGN	STALSSLFPS	SFTENCELLS	CSGENRTMVH	SLNSTADESG	LNKLKIRYEE	FQEHKTEKPS	LSQQAAHYMF
730	740	750	760	770	780	790	800
FPSVVLSNCL	TRPQKLSPVT	YKLQPGNKPS	RLKLNKRKLA	GHQETSTKSS	ETGSTKDNFI	QNNPCNSNPE	KDNALASDLT
810	820	830	840	850	860	870	880
KTTRGAFENK	TPTDGFIDCH	FGDGTLETEQ	SFGLYGNKYT	LRAKRKVNYE	TEDSESSFVT	HNSKISLPHP	MEIGESLDGT
890	900	910	920	930	940	950	960
LKSRKRRKMS	KKLPPVIIKY	IIINRFRGRK	NMLVKLGKID	SKEKQVILTE	EKMELYKKLA	PLKDFWPKVP	DSPATKYPIY
970	980	990	1000	1010	1020	1030	1040
PLTPKKSHRR	KSKHKSAKKK	TGKQQRTNNE	NIKRTLSFRK	KRSHAILSPP	SPSYNAETED	CDLNYSDVMS	KLGFLSERST
1050	1060	1070	1080	1090	1100	1110	1120
SPINSSPPRC	WSPTDPRAEE	IMAAAEKEAM	LFKGPNVYKK	TVNSRIGKTS	RARAQIKKSK	AKLANPSIVT	KKRNKRNQTN
1130	1140	1150	1160	1170	1180	1190	1200
KLVDDGKKKP	RAKQKTNEKG	TSRKHTTLKD	EKIKSQSGAE	VKFVLKHQNV	SEFASSSGGS	QLLFKQKDMP	LMGSAVDHPL
1210	1220	1230	1240	1250	1260	1270	1280
SASLPTGINA	QQKLSGCFSS	FLESKKSVDL	QTFPSSRDDL	HPSVVCNSIG	PGVSKINVQR	PHNQSAMFTL	KESTLIQKNI
1290	1300	1310	1320	1330	1340	1350	1360
FDLSNHLSQV	AQNTQISSGM	SSKIEDNANN	IQRNYLSSIG	KLSEYRNSLE	SKLDQAYTPN	FLHCKDSQQQ	IVCIAEQSKH
1370	1380	1390	1400	1410	1420	1430	1440
SETCSPGNTA	SEESQMPNNC	FVTSLRSPIK	QIAWEQKQRG	FILDMSNFKP	ERVKPRSLSE	AISQTKALSQ	CKNRNVSTPS
1450	1460	1470	1480	1490	1500	1510	1520
AFGEGQSGLA	VLKELLQKRQ	QKAQNANTTQ	DPLSNKHQPN	KNISGSLEHN	KANKRTRSVT	SPRKPRTPRS	TKQKEKIPKL
1530	1540	1550	1560	1570	1580	1590	1600
LKVDSLNLQN	SSQLDNSVSD	DSPIFFSDPG	FESCYSLEDS	LSPEHNYNFD	INTIGQTGFC	SFYSGSQFVP	ADQNLPQKFL
1610	1620	1630	1640	1650	1660	1670	1680
SDAVQDLFPG	QAIEKNEFLS	HDNQKCDEDK	HHTTDSASWI	RSGTLSPEIF	EKSTIDSNEN	RRHNQWKNSF	HPLTTRSNSI
1690	1700	1710	1720	1730	1740	1750	1760
MDSFCVQQAE	DCLSEKSRLN	RSSVSKEVFL	SLPQPNNSDW	IQGHTRKEMG	QSLDSANTSF	TAILSSPDGE	LVDVACEDLE
1770	1780	1790	1800	1810	1820	1830	1840
LYVSRNNDML	TPTPDSSPRS	TSSPSQSKNG	SFTPRTANIL	KPLMSPPSRE	EIMATLLDHD	LSETIYQEPF	CSNPSDVPEK
1850	1860	1870	1880	1890	1900	1910	1920
PREIGGRLLM	VETRLANDLA	EFEGDFSLEG	LRLWKTAFSA	MTQNPRPGSP	LRSGQGVVNK	GSSNSPKMVE	DKKIVIMPCK
1930	1940	1950	1960	1970	1980	1990	2000
CAPSRQLVQV	WLQAKEEYER	SKKLPKTKPT	GVVKSAENFS	SSVNPDDKPV	VPPKMDVSPC	ILPTTAHTKE	DVDNSQIALQ
2010	2020	2030	2040	2050	2060	2070	2080
APTTGCSQTA	SESQMLPPVA	SASDPEKDED	DDDNYYISYS	SPDSPVIPPW	QQPISPDSKA	LNGDDRPSSP	VEELPSLAFE
2090	2100	2110	2120	2130	2140	2150	2160
NFLKPIKDGI	QKSPCSEPQE	PLVISPINTR	ARTGKCESLC	FHSTPIIQRK	LLERLPEAPG	LSPLSTEPKT	QKLSNKKGSN
2170	2180	2190	2200	2210	2220	2230	2240

Detailed Protein Report

Cmpd.	No. of Cmpds.	m/z meas.	Δ m/z [ppm]	z	Rt [min]	Score	P	Range	Sequence	Modification
2757	1	710.3801	13.75	2	63.5	13.2	0	58-70	R.ICELLQSGAIMNK.F	
727	2	517.1500	-168.10	2	37.5	14.5	1	353-360	R.NRMPSPCR.S	Carbamidomethyl: 7; Oxidation: 3
671	1	648.3571	-103.32	2	36.8	12.0	2	1943-1954	K.KLPKTKPTGVVK.S	
1743	1	786.4023	-29.21	3	50.5	13.6	1	2643-2664	R.QLGLKLIANVTFGYTSANFSGR.M	

Detailed Protein Report

Protein 284: PREDICTED: 1-phosphatidylinositol 4,5-bisphosphate phosphodiesterase epsilon-1 isoform X6 [Homo sapiens]

**Accession:**	gi|578819549	**Score:**	53.2
**Database:**	refseq_human(refseq_human_20140103.fasta)	**MW [kDa]:**	225.1
**Database Date:**	2015-11-30	**pI:**	6.1
**Modification(s):**	Carbamidomethyl, Oxidation	**Sequence Coverage [%]:**	2.2
		**No. of unique Peptides:**	3

Quantitation

Q:W	**Median:**	17.81	**CV:**	0.00 %	**No. of Peptides:**	1
M:Q	**Median:**	0.51	**CV:**	14.44 %	**No. of Peptides:**	2

Detailed Protein Report

10	20	30	40	50	60	70	80
MVSEGSAAGR	DFAGMEEVRQ	LHVRFCKGIK	IWHQAWFLCS	LLGREPQERE	AGCQLWLCTL	SAVLKVGWLF	PLSEVPNFTL
90	100	110	120	130	140	150	160
LKDGCGCWRL	KEDQIYNAVR	REETENTVGS	LLHFLTKLPA	SETAHGRISV	GPCLKQCVRD	TVCEYRATLQ	RTSISQYITG
170	180	190	200	210	220	230	240
SLLEATTSLG	ARSGLLSTFG	GSTGRMMLKE	RQPGPSVANS	NALPSSSAGI	SKELIDLQPL	IQFPEEVASI	LMEQEQTIYR
250	260	270	280	290	300	310	320
RVLPVDYLCF	LTRDLGTPEC	QSSLPCLKAS	ISASILTTQN	GEHNALEDLV	MRFNEVSSWV	TWLILTAGSM	EEKREVFSYL
330	340	350	360	370	380	390	400
VHVAKCCWNM	GNYNAVMEFL	AGLRSRKVLK	MWQFMDQSDI	ETMRSLKDAM	AQHESSCEYR	KVVTRALHIP	GCKVVPFCGV
410	420	430	440	450	460	470	480
FLKELCEVLD	GASGLMKLCP	RYNSQEETLE	FVADYSGQDN	FLQRVGQNGL	KNSEKESTVN	SIFQVIRSCN	RSLETDEEDS
490	500	510	520	530	540	550	560
PSEGNSSRKS	SLKDKSRWQF	IIGDLLDSDN	DIFEQSKEYD	SHGSEDSQKA	FDHGTELIPW	YVLSIQADVH	QFLLQGATVI
570	580	590	600	610	620	630	640
HYDQDTHLSA	RCFLQLQPDN	STLTWVKPTT	ASPASSKAKL	GVLNNTAEPG	KFPLLGNAGL	SSLTEGVLDL	FAVKAVYMGH
650	660	670	680	690	700	710	720
PGIDIHTVCV	QNKLGSMFLS	ETGVTLLYGL	QTTDNRLLHF	VAPKHTAKML	FSGLLELTRA	VRKMRKFPDQ	RQQWLRKQYV
730	740	750	760	770	780	790	800
SLYQEDGRYE	GPTLAHAVEL	FGGRRWSARN	PSPGTSAKNA	EKPNMQRNNT	LGISTTKKKK	KILMRGESGE	VTDDEMATRK
810	820	830	840	850	860	870	880
AKMHKECRSR	SGSDPQDINE	QEESEVNAIA	NPPNPLPSRR	AHSLTTAGSP	NLAAGTSSPI	RPVSSPVLSS	SNKSPSSAWS
890	900	910	920	930	940	950	960
SSSWHGRIKG	GMKGFQSFMV	SDSNMSFVEF	VELFKSFSVR	SRKDLKDLFD	VYAVPCNRSG	SESAPLYTNL	TIDENTSDLQ
970	980	990	1000	1010	1020	1030	1040
PDLDLLTRNV	SDLGLFIKSK	QQLSDNQRQI	SDAIAAASIV	TNGTGIESTS	LGIFGVGILQ	LNDFLVNCQG	EHCTYDEILS
1050	1060	1070	1080	1090	1100	1110	1120
IIQKFEPSIS	MCHQGLMSFE	GFARFLMDKE	NFASKNDESQ	ENIKELQLPL	SYYYIESSHN	TYLTGHQLKG	ESSVELYSQV
1130	1140	1150	1160	1170	1180	1190	1200
LLQGCRSVEL	DCWDGDDGMP	IIYHGHTLTT	KIPFKEVVEA	IDRSAFINSD	LPIIISIENH	CSLPQQRKMA	EIFKTVFGEK
1210	1220	1230	1240	1250	1260	1270	1280
LVTKFLFETD	FSDDPMLPSP	DQLRKKVLLK	NKKLKAHQTP	VDILKQKAHQ	LASMQVQAYN	GGNANPRPAN	NEEEEDEEDE
1290	1300	1310	1320	1330	1340	1350	1360
YDYDYESLSD	ADVLTASPAP	NGQEDNILED	RPENKSCNDK	LQFEYNEEIP	KRIKKADNSA	CNKGKVYDME	LGEEFYLDQN
1370	1380	1390	1400	1410	1420	1430	1440
KKESRQIAPE	LSDLVIYCQA	VKFPGLSTLN	ASGSSRGKER	KSRKSIFGNN	PGRMSPGETA	SFNKTSGKSS	CEGIRQTWEE
1450	1460	1470	1480	1490	1500	1510	1520
SSSPLNPTTS	LSAIIRTPKC	YHISSLNENA	AKRLCRRYSQ	KLTQHTACQL	LRTYPAATRI	DSSNPNPLMF	WLHGIQLVAL
1530	1540	1550	1560	1570	1580	1590	1600
NYQTDDLPLH	LNAAMFEANG	GCGYVLKPPV	LWDKNCPMYQ	KFSPLERDLD	SMDPAVYSLT	IVSGQNVCPS	NSMGSPCIEV
1610	1620	1630	1640	1650	1660	1670	1680
DVLGMPLDSC	HFRTKPIHRN	TLNPMWNEQF	LFHVHFEDLV	FLRFAVVENN	SSAVTAQRII	PLKALKRGYR	HLQLRNLHNE
1690	1700	1710	1720	1730	1740	1750	1760
VLEISSLFIN	SRRMEENSSG	NTMSASSMFN	TEERKCLQTH	RVTVHGVPGP	EPFTVFTING	GTKAKQLLQQ	ILTNEQDIKP
1770	1780	1790	1800	1810	1820	1830	1840
VTTDYFLMEE	KYFISKEKNE	CRKQPFQRAI	GPEEEIMQIL	SSWFPEEGYM	GRIVLKTQQE	NLEEKNIVQD	DKEVILSSEE
1850	1860	1870	1880	1890	1900	1910	1920
ESFFVQVHDV	SPEQPRTVIK	APRVSTAQDV	IQQTLCKAKY	SYSILSNPNP	SDYVLLEEVV	KDTTNKKTTT	PKSSQRVLLD
1930	1940	1950	1960	1970	1980	1990	2000
QECVFQAQSK	WKGAGKFILK	LKEQVQASRE	DKKKGISFAS	ELKKLTKSTK	QPRGLTSPSQ	LLTSESIQTK	EEKPVGGLSS
2010							
SDTMDYRQ							

Cmpd.	No. of Cmpds.	m/z meas.	Δ m/z [ppm]	z	Rt [min]	Score	P	Range	Sequence	Modification	Ratios

Detailed Protein Report

Cmpd.	No. of Cmpds.	m/z meas.	Δ m/z [ppm]	z	Rt [min]	Score	P	Range	Sequence	Modification	Ratios	
2593	1	648.3609	8.98	2	62.4	10.3	0	689-699	K.MLFSGLLELTR.A	Oxidation: 1	M:Q<br>Q:W	0.59<br>17.81
2703	1	673.3636	55.13	2	64.1	13.1	1	1065-1075	R.FLMDKENFASK.N	Oxidation: 3		
547	1	624.6856	-218.06	2	35.5	11.3	2	1333-1343	R.IKKADNSACNK.G	Carbamidomethyl: 9	M:Q	0.44

Detailed Protein Report

Protein 285: **voltage-dependent P/Q-type calcium channel subunit alpha-1A isoform 4 [Homo sapiens]**

**Accession:**	gi|187828892	**Score:**	53.1
**Database:**	refseq_human(refseq_human_20140103.fasta)	**MW [kDa]:**	282.4
**Database Date:**	2015-11-30	**pI:**	9.6
		**Sequence Coverage [%]:**	2.6
		**No. of unique Peptides:**	1

Detailed Protein Report

10	20	30	40	50	60	70	80
MARFGDEMPA	RYGGGGSGAA	AGVVVGSGGG	RGAGGSRQGG	QPGAQRMYKQ	SMAQRARTMA	LYNPIPVRQN	CLTVNRSLFL
90	100	110	120	130	140	150	160
FSEDNVVRKY	AKKITEWPPF	EYMILATIIA	NCIVLALEQH	LPDDDKTPMS	ERLDDTEPYF	IGIFCFEAGI	KIIALGFAFH
170	180	190	200	210	220	230	240
KGSYLRNGWN	VMDFVVVLTG	ILATVGTEFD	LRTLRAVRVL	RPLKLVSGIP	SLQVVLKSIM	KAMIPLLQIG	LLLFFAILIF
250	260	270	280	290	300	310	320
AIIGLEFYMG	KFHTTCFEEG	TDDIQGESPA	PCGTEEPART	CPNGTKCQPY	WEGPNNGITQ	FDNILFAVLT	VFQCITMEGW
330	340	350	360	370	380	390	400
TDLLYNSNDA	SGNTWNWLYF	IPLIIIGSFF	MLNLVLGVLS	GEFAKERERV	ENRRAFLKLR	RQQQIERELN	GYMEWISKAE
410	420	430	440	450	460	470	480
EVILAEDETD	GEQRHPFDAL	RRTTIKKSKT	DLLNPEEAED	QLADIASVGS	PFARASIKSA	KLENSTFFHK	KERRMRFYIR
490	500	510	520	530	540	550	560
RMVKTQAFYW	TVLSLVALNT	LCVAIVHYNQ	PEWLSDFLYY	AEFIFLGLFM	SEMFIKMYGL	GTRPYFHSSF	NCFDCGVIIG
570	580	590	600	610	620	630	640
SIFEVIWAVI	KPGTSFGISV	LRALRLLRIF	KVTKYWASLR	NLVVSLLNSM	KSIISLLFLL	FLFIVVFALL	GMQLFGGQFN
650	660	670	680	690	700	710	720
FDEGTPPTNF	DTFPAAIMTV	FQILTGEDWN	EVMYDGIKSQ	GGVQGGMVFS	IYFIVLTLFG	NYTLLNVFLA	IAVDNLANAQ
730	740	750	760	770	780	790	800
ELTKDEQEEE	EAANQKLALQ	KAKEVAEVSP	LSAANMSIAV	KEQQKNQKPA	KSVWEQRTSE	MRKQNLLASR	EALYNEMDPD
810	820	830	840	850	860	870	880
ERWKAAYTRH	LRPDMKTHLD	RPLVVDPQEN	RNNNTNKSRA	AEPTVDQRLG	QQRAEDFLRK	QARYHDRARD	PSGSAGLDAR
890	900	910	920	930	940	950	960
RPWAGSQEAE	LSREGPYGRE	SDHHAREGSL	EQPGFWEGEA	ERGKAGDPHR	RHVHRQGGSR	ESRSGSPRTG	ADGEHRRHRA
970	980	990	1000	1010	1020	1030	1040
HRRPGEEGPE	DKAERRARHR	EGSRPARGGE	GEGEGPDGGE	RRRRHRHGAP	ATYEGDARRE	DKERRHRRRK	ENQGSGVPVS
1050	1060	1070	1080	1090	1100	1110	1120
GPNLSTTRPI	QQDLGRQDPP	LAEDIDNMKN	NKLATAESAA	PHGSLGHAGL	PQSPAKMGNS	TDPGPMLAIP	AMATNPQNAA
1130	1140	1150	1160	1170	1180	1190	1200
SRRTPNNPGN	PSNPGPPKTP	ENSLIVTNPS	GTQTNSAKTA	RKPDHTTVDI	PPACPPPLNH	TVVQVNKNAN	PDPLPKKEEE
1210	1220	1230	1240	1250	1260	1270	1280
KKEEEEDDRG	EDGPKPMPPY	SSMFILSTTN	PLRRLCHYIL	NLRYFEMCIL	MVIAMSSIAL	AAEDPVQPNA	PRNNVLRYFD
1290	1300	1310	1320	1330	1340	1350	1360
YVFTGVFTFE	MVIKMIDLGL	VLHQGAYFRD	LWNILDFIVV	SGALVAFAFT	GNSKGKDINT	IKSLRVLRVL	RPLKTIKRLP
1370	1380	1390	1400	1410	1420	1430	1440
KLKAVFDCVV	NSLKNVFNIL	IVYMLFMFIF	AVVAVQLFKG	KFFHCTDESK	EFEKDCRGKY	LLYEKNEVKA	RDREWKKYEF
1450	1460	1470	1480	1490	1500	1510	1520
HYDNVLWALL	TLFTVSTGEG	WPQVLKHSVD	ATFENQGPSP	GYRMEMSIFY	VVYFVVFPFF	FVNIFVALII	ITFQEQGDKM
1530	1540	1550	1560	1570	1580	1590	1600
MEEYSLEKNE	RACIDFAISA	KPLTRHMPQN	KQSFQYRMWQ	FVVSPPFEYT	IMAMIALNTI	VLMMKFYGAS	VAYENALRVF
1610	1620	1630	1640	1650	1660	1670	1680
NIVFTSLFSL	ECVLKVMAFG	ILNYFRDAWN	IFDFVTVLGS	ITDILVTEFG	NNFINLSFLR	LFRAARLIKL	LRQGYTIRIL
1690	1700	1710	1720	1730	1740	1750	1760
LWTFVQSFKA	LPYVCLLIAM	LFFIYAIIGM	QVFGNIGIDV	EDEDSDEDEF	QITEHNNFRT	FFQALMLLFR	SATGEAWHNI
1770	1780	1790	1800	1810	1820	1830	1840
MLSCLSGKPC	DKNSGILTRE	CGNEFAYFYF	VSFIFLCSFL	MLNLFVAVIM	DNFEYLTRDS	SILGPHHLDE	YVRVWAEYDP
1850	1860	1870	1880	1890	1900	1910	1920
AAWGRMPYLD	MYQMLRHMSP	PLGLGKKCPA	RVAYKRLLRM	DLPVADDNTV	HFNSTLMALI	RTALDIKIAK	GGADKQQMDA
1930	1940	1950	1960	1970	1980	1990	2000
ELRKEMMAIW	PNLSQKTLDL	LVTPHKSTDL	TVGKIYAAMM	IMEYYRQSKA	KKLQAMREEQ	DRTPLMFQRM	EPPSPTQEGG
2010	2020	2030	2040	2050	2060	2070	2080
PGQNALPSTQ	LDPGGALMAH	ESGLKESPSW	VTQRAQEMFQ	KTGTWSPEQG	PPTDMPNSQP	NSQSVEMREM	GRDGYSDSEH
2090	2100	2110	2120	2130	2140	2150	2160
YLPMEGQGRA	ASMPRLPAEN	QRRRGRPRGN	NLSTISDTSP	MKRSASVLGP	KARRLDDYSL	ERVPPEENQR	HHQRRRDRSH
2170	2180	2190	2200	2210	2220	2230	2240
RASERSLGRY	TDVDTGLGTD	LSMTTQSGDL	PSKERDQERG	RPKDRKHRQH	HHHHHHHHHP	PPPDKDRYAQ	ERPDHGRARA
2250	2260	2270	2280	2290	2300	2310	2320
RDQRWSRSPS	EGREHMAHRQ	GSSSVSGSPA	PSTSGTSTPR	RGRRQLPQTP	STPRPHVSYS	PVIRKAGGSG	PPQQQQQQQQ
2330	2340	2350	2360	2370	2380	2390	2400

Detailed Protein Report

Cmpd.	No. of Cmpds.	m/z meas.	Δ m/z [ppm]	z	Rt [min]	Score	P	Range	Sequence	Modification
2706	1	741.8621	-28.64	2	64.1	11.6	2	2371-2384	R.VPGPARSESPRACR.H	

Detailed Protein Report

Protein 286: PREDICTED: zinc finger protein 175 isoform X3 [Homo sapiens]

**Accession:**	gi|530417172	**Score:**	53.1
**Database:**	refseq_human(refseq_human_20140103.fasta)	**MW [kDa]:**	69.5
**Database Date:**	2015-11-30	**pI:**	10.1
**Modification(s):**	Carbamidomethyl	**Sequence Coverage [%]:**	3.5
		**No. of unique Peptides:**	3

Quantitation

Q:W	**Median:** 0.95	**CV:** 0.00 %	**No. of Peptides:**	1
M:Q	**Median:** 0.77	**CV:** 0.00 %	**No. of Peptides:**	1

10	20	30	40	50	60	70	80
MPADVNLSQK	PQVLGPEKQD	GSCEASVSFE	DVTVDFSREE	WQQLDPAQRC	LYRDVMLELY	SHLFAVGYHI	PNPEVIFRML
**90**	**100**	**110**	**120**	**130**	**140**	**150**	**160**
KEKEPRVEEA	EVSHQRCQES	LKLNLEVNGQ	NESNDTEQLD	DVVGSGQLFS	HSSSDACSKN	IHTGETFCKG	NQCRKVCGHK
**170**	**180**	**190**	**200**	**210**	**220**	**230**	**240**
QSLKQHQIHT	QKKPDGCSEC	GGSFTQKSHL	FAQQRIHSVG	NLHECGKCGK	AFMPQLKLSV	YLTDHTGDIP	CICKECGKVF
**250**	**260**	**270**	**280**	**290**	**300**	**310**	**320**
IQRSELLTHQ	KTHTRKKPYK	CHDCGKAFFQ	MLSLFRHQRT	HSREKLYECS	ECGKGFSQNS	TLIIHQKIHT	GERQYACSEC
**330**	**340**	**350**	**360**	**370**	**380**	**390**	**400**
GKAFTQKSTL	SLHQRIHSGQ	KSYVCIECGQ	AFIQKAHLIV	HQRSHTGEKP	YQCHNCGKSF	ISKSQLDIHH	RIHTGEKPYE
**410**	**420**	**430**	**440**	**450**	**460**	**470**	**480**
CSDCGKTFTQ	KSHLNIHQKI	HTGERHHVCS	ECGKAFNQKS	ILSMHQRIHT	GEKPYKCSEC	GKAFTSKSQF	KEHQRIHTGE
**490**	**500**	**510**	**520**	**530**	**540**	**550**	**560**
KPYVCTECGK	AFNGRSNFHK	HQITHTRERP	FVCYKCGKAF	VQKSELITHQ	RTHMGEKPYE	CLDCGKSFSK	KPQLKVHQRI
**570**	**580**	**590**	**600**	**610**			
HTGERPYVCS	ECGKAFNNRS	NFNKHQTTHT	RDKSYKCSYS	VKGFTKQ			

Cmpd.	No. of Cmpds.	m/z meas.	Δ m/z [ppm]	z	Rt [min]	Score	P	Range	Sequence	Modification	Ratios
30	3	1205.0059	-49.90	2	29.7	10.4	1	218-238	K.LSVYLTDHTGDIPCICKECGK.V	Carbamidomethyl: 16, 19	
22	1	1205.0462	-16.45	2	30.0	19.7	1	218-238	K.LSVYLTDHTGDIPCICKECGK.V	Carbamidomethyl: 14, 16	Q:W 0.95<br>M:Q 0.77
6	2	1205.0350	-25.71	2	29.5	22.9	1	218-238	K.LSVYLTDHTGDIPCICKECGK.V	Carbamidomethyl: 14, 19	

Detailed Protein Report

Protein 287: ubiquitin carboxyl-terminal hydrolase 32 [Homo sapiens]

**Accession:**	gi|22550104	**Score:**	52.9
**Database:**	refseq_human(refseq_human_20140103.fasta)	**MW [kDa]:**	181.5
**Database Date:**	2015-11-30	**pI:**	6.0
		**Sequence Coverage [%]:**	3.4
		**No. of unique Peptides:**	1

Quantitation

M:Q **Median:** 0.50 **CV:** 0.00 % **No. of Peptides:** 1

10	20	30	40	50	60	70	80
MGAKESRIGF	LSYEEALRRV	TDVELKRLKD	AFKRTCGLSY	YMGQHCFIRE	VLGDGVPPKV	AEVIYCSFGG	TSKGLHFNNL
90	100	110	120	130	140	150	160
IVGLVLLTRG	KDEEKAKYIF	SLFSSESGNY	VIREEMERML	HVVDGKVPDT	LRKCFSEGEK	VNYEKFRNWL	FLNKDAFTFS
170	180	190	200	210	220	230	240
RWLLSGGVYV	TLTDDSDTPT	FYQTLAGVTH	LEESDIIDLE	KRYWLLKAQS	RTGRFDLETF	GPLVSPPIRP	SLSEGLFNAF
250	260	270	280	290	300	310	320
DENRDNHIDF	KEISCGLSAC	CRGPLAERQK	FCFKVFDVDR	DGVLSRVELR	DMVVALLEVW	KDNRTDDIPE	LHMDLSDIVE
330	340	350	360	370	380	390	400
GILNAHDTTK	MGHLTLEDYQ	IWSVKNVLAN	EFLNLLFQVC	HIVLGLRPAT	PEEEGQIIRG	WLERESRYGL	QAGHNWFIIS
410	420	430	440	450	460	470	480
MQWWQQWKEY	VKYDANPVVI	EPSSVLNGGK	YSFGTAAHPM	EQVEDRIGSS	LSYVNTTEEK	FSDNISTASE	ASETAGSGFL
490	500	510	520	530	540	550	560
YSATPGADVC	FARQHNTSDN	NNQCLLGANG	NILLHLNPQK	PGAIDNQPLV	TQEPVKATSL	TLEGGRLKRT	PQLIHGRDYE
570	580	590	600	610	620	630	640
MVPEPVWRAL	YHWYGANLAL	PRPVIKNSKT	DIPELELFPR	YLLFLRQQPA	TRTQQSNIWV	NMGNVPSPNA	PLKRVLAYTG
650	660	670	680	690	700	710	720
CFSRMQTIKE	IHEYLSQRLR	IKEEDMRLWL	YNSENYLTLL	DDEDHKLEYL	KIQDEQHLVI	EVRNKDMSWP	EEMSFIANSS
730	740	750	760	770	780	790	800
KIDRHKVPTE	KGATGLSNLG	NTCFMNSSIQ	CVSNTQPLTQ	YFISGRHLYE	LNRTNPIGMK	GHMAKCYGDL	VQELWSGTQK
810	820	830	840	850	860	870	880
NVAPLKLRWT	IAKYAPRFNG	FQQQDSQELL	AFLLDGLHED	LNRVHEKPYV	ELKDSDGRPD	WEVAAEAWDN	HLRRNRSIVV
890	900	910	920	930	940	950	960
DLFHGQLRSQ	VKCKTCGHIS	VRFDPFNFLS	LPLPMDSYMH	LEITVIKLDG	TTPVRYGLRL	NMDEKYTGLK	KQLSDLCGLN
970	980	990	1000	1010	1020	1030	1040
SEQILLAEVH	GSNIKNFPQD	NQKVRLSVSG	FLCAFEIPVP	VSPISASSPT	QTDFSSSPST	NEMFTLTTNG	DLPRPIFIPN
1050	1060	1070	1080	1090	1100	1110	1120
GMPNTVVPCG	TEKNFTNGMV	NGHMPSLPDS	PFTGYIIAVH	RKMMRTELYF	LSSQKNRPSL	FGMPLIVPCT	VHTRKKDLYD
1130	1140	1150	1160	1170	1180	1190	1200
AVWIQVSRLA	SPLPPQEASN	HAQDCDDSMG	YQYPFTLRVV	QKDGNSCAWC	PWYRFCRGCK	IDCGEDRAFI	GNAYIAVDWD
1210	1220	1230	1240	1250	1260	1270	1280
PTALHLRYQT	SQERVVDEHE	SVEQSRRAQA	EPINLDSCLR	AFTSEEELGE	NEMYYCSKCK	THCLATKKLD	LWRLPPILII
1290	1300	1310	1320	1330	1340	1350	1360
HLKRFQFVNG	RWIKSQKIVK	FPRESFDPSA	FLVPRDPALC	QHKPLTPQGD	ELSEPRILAR	EVKKVDAQSS	AGEEDVLLSK
1370	1380	1390	1400	1410	1420	1430	1440
SPSSLSANII	SSPKGSPSSS	RKSGTSCPSS	KNSSPNSSPR	TLGRSKGRLR	LPQIGSKNKL	SSSKENLDAS	KENGAGQICE
1450	1460	1470	1480	1490	1500	1510	1520
LADALSRGHV	LGGSQPELVT	PQDHEVALAN	GFLYEHEACG	NGYSNGQLGN	HSEEDSTDDQ	REDTRIKPIY	NLYAISCHSG
1530	1540	1550	1560	1570	1580	1590	1600
ILGGGHYVTY	AKNPNCKWYC	YNDSSCKELH	PDEIDTDSAY	ILFYEQQGID	YAQFLPKTDG	KKMADTSSMD	EDFESDYKKY
1610							
CVLQ							

Cmpd.	No. of Cmpds.	m/z meas.	Δ m/z [ppm]	z	Rt [min]	Score	P	Range	Sequence	Modification	Ratios	
2778	1	1023.5416	14.56	2	63.2	11.2	1	1361-1381	K.SPSSLSANIISSPKGSPSSSR.K		M:Q	0.50

Detailed Protein Report

Protein 288: **putative pre-mRNA-splicing factor ATP-dependent RNA helicase DHX16 isoform 1 [Homo sapiens]**

**Accession:**	gi|255982614	**Score:**	52.9
**Database:**	refseq_human(refseq_human_20140103.fasta)	**MW [kDa]:**	119.2
**Database Date:**	2015-11-30	**pI:**	6.4
**Modification(s):**	Carbamidomethyl, Oxidation	**Sequence Coverage [%]:**	4.7
		**No. of unique Peptides:**	2

10	20	30	40	50	60	70	80
MATPAGLERW	VQDELHSVLG	LSERHVAQFL	IGTAQRCTSA	EEFVQRLRDT	DTLDLSGPAR	DFALRLWNKV	PRKAVVEKPA
**90**	**100**	**110**	**120**	**130**	**140**	**150**	**160**
RAAEREARAL	LEKNRSYRLL	EDSEESSEET	VSRAGSSLQK	KRKKRKHLRK	KREEEEEEEA	SEKGKKKTGG	SKQQTEKPES
**170**	**180**	**190**	**200**	**210**	**220**	**230**	**240**
EDEWERTERE	RLQDLEERDA	FAERVRQRDK	DRTRNVLERS	DKKAYEEAQK	RLKMAEEDRK	AMVPELRKKS	RREYLAKRER
**250**	**260**	**270**	**280**	**290**	**300**	**310**	**320**
EKLEDLEAEL	ADEEFLFGDV	ELSRHERQEL	KYKRRVRDLA	REYRAAGEQE	KLEATNRYHM	PKETRGQPAR	AVDLVEEESG
**330**	**340**	**350**	**360**	**370**	**380**	**390**	**400**
APGEEQRRWE	EARLGAASLK	FGARDAASQE	PKYQLVLEEE	ETIEFVRATQ	LQGDEEPSAP	PTSTQAQQKE	SIQAVRRSLP
**410**	**420**	**430**	**440**	**450**	**460**	**470**	**480**
VFPFREELLA	AIANHQVLII	EGETGSGKTT	QIPQYLFEEG	YTNKGMKIAC	TQPRRVAAMS	VAARVAREMG	VKLGNEVGYS
**490**	**500**	**510**	**520**	**530**	**540**	**550**	**560**
IRFEDCTSER	TVLRYMTDGM	LLREFLSEPD	LASYSVVMVD	EAHERTLHTD	ILFGLIKDVA	RFRPELKVLV	ASATMDTARF
**570**	**580**	**590**	**600**	**610**	**620**	**630**	**640**
STFFDDAPVF	RIPGRRFPVD	IFYTKAPEAD	YLEACVVSVL	QIHVTQPPGD	ILVFLTGQEE	IEAACEMLQD	RCRRLGSKIR
**650**	**660**	**670**	**680**	**690**	**700**	**710**	**720**
ELLVLPIYAN	LPSDMQARIF	QPTPPGARKV	VVATNIAETS	LTIEGIIYVL	DPGFCKQKSY	NPRTGMESLT	VTPCSKASAN
**730**	**740**	**750**	**760**	**770**	**780**	**790**	**800**
QRAGRAGRVA	AGKCFRLYTA	WAYQHELEET	TVPEIQRTSL	GNVVLLLKSL	GIHDLMHFDF	LDPPPYETLL	LALEQLYALG
**810**	**820**	**830**	**840**	**850**	**860**	**870**	**880**
ALNHLGELTT	SGRKMAELPV	DPMLSKMILA	SEKYSCSEEI	LTVAAMLSVN	NSIFYRPKDK	VVHADNARVN	FFLPGGDHLV
**890**	**900**	**910**	**920**	**930**	**940**	**950**	**960**
LLNVYTQWAE	SGYSSQWCYE	NFVQFRSMRR	ARDVREQLEG	LLERVEVGLS	SCQGDYIRVR	KAITAGYFYH	TARLTRSGYR
**970**	**980**	**990**	**1000**	**1010**	**1020**	**1030**	**1040**
TVKQQQTVFI	HPNSSLFEQQ	PRWLLYHELV	LTTKEFMRQV	LEIESSWLLE	VAPHYYKAKE	LEDPHAKKMP	KKIGKTREEL
**1050**							
G							

Cmpd.	No. of Cmpds.	m/z meas.	Δ m/z [ppm]	z	Rt [min]	Score	P	Range	Sequence	Modification
1748	1	1044.9652	-43.04	2	51.7	25.1	2	285-302	R.AAGEQEKLEATNRYHMPK.E	Oxidation: 16
2134	1	937.3539	-151.57	2	55.3	12.3	2	448-464	K.IACTQPRRVAAMSVAAR.V	Carbamidomethyl: 3; Oxidation: 12

Detailed Protein Report

Protein 289: PREDICTED: guanine nucleotide exchange factor VAV3 isoform X4 [Homo sapiens]

Accession: gi|530361574
Database: refseq_human(refseq_human_20140103.fasta)
Database Date: 2015-11-30
Modification(s): Carbamidomethyl, Oxidation

Score: 52.8
MW [kDa]: 72.1
pI: 6.9
Sequence Coverage [%]: 6.8
No. of unique Peptides: 3

Quantitation

Q:W	**Median:** 2.28	**CV:** 0.00 %	**No. of Peptides:**	1
M:Q	**Median:** 0.68	**CV:** 0.00 %	**No. of Peptides:**	1

10	20	30	40	50	60	70	80
MEPWKQCAQW	LIHCKVLPTN	HRVTWDSAQV	FDLAQTLRDG	VLLCQLLNNL	RAHSINLKEI	NLRPQMSQFL	CLKNIRTFLT
**90**	**100**	**110**	**120**	**130**	**140**	**150**	**160**
ACCETFGMRK	SELFEAFDLF	DVRDFGKVIE	TLSRLSRTPI	ALATGIRPFP	TEESINDEDI	YKGLPDLIDE	TLVEDEEDLY
**170**	**180**	**190**	**200**	**210**	**220**	**230**	**240**
DCVYGEDEGG	EVYEDLMKAE	EAHQPKCPEN	DIRSCCLAEI	KQTEEKYTET	LESIEKYFMA	PLKRFLTAAE	FDSVFINIPE
**250**	**260**	**270**	**280**	**290**	**300**	**310**	**320**
LVKLHRNLMQ	EIHDSIVNKN	DQNLYQVFIN	YKERLVIYGQ	YCSGVESAIS	SLDYISKTKE	DVKLKLEECS	KRANNGKFTL
**330**	**340**	**350**	**360**	**370**	**380**	**390**	**400**
RDLLVVPMQR	VLKYHLLLQE	LVKHTTDPTE	KANLKLALDA	MKDLAQYVNE	VKRDNETLRE	IKQFQLSIEN	LNQPVLLFGR
**410**	**420**	**430**	**440**	**450**	**460**	**470**	**480**
PQGDGEIRIT	TLDKHTKQER	HIFLFDLAVI	VCKRKGDNYE	MKEIIDLQQY	KIANNPTTDK	ENKKWSYGFY	LIHTQGQNGL
**490**	**500**	**510**	**520**	**530**	**540**	**550**	**560**
EFYCKTKDLK	KKWLEQFEMA	LSNIRPDYAD	SNFHDFKMHT	FTRVTSCKVC	QMLLRGTFYQ	GYLCFKCGAR	AHKECLGRVD
**570**	**580**	**590**	**600**	**610**	**620**	**630**	
NCGRVNSGEQ	GTLKLPEKRT	NGLRRTPKQV	DPDVPCLLHF	FISMAPATRS	IVKSQKKNKK	F	

Cmpd.	No. of Cmpds.	m/z meas.	Δ m/z [ppm]	z	Rt [min]	Score	P	Range	Sequence	Modification	Ratios
154	1	968.4666	19.86	2	31.6	10.4	1	74-89	K.NIRTFLTACCETFGMR.K	Carbamidomethyl: 10; Oxidation: 15	M:Q 0.68<br>Q:W 2.28
877	1	705.9834	77.48	2	41.0	16.1	1	322-333	R.DLLVVPMQRVLK.Y		
103	1	807.7358	-182.29	2	31.0	26.3	2	551-564	R.AHKECLGRVDNCGR.V	Carbamidomethyl: 12	

Detailed Protein Report

Protein 290: **PREDICTED: extracellular sulfatase Sulf-1 isoform X4 [Homo sapiens]**

**Accession:**	gi|578815634	**Score:**	52.7
**Database:**	refseq_human(refseq_human_20140103.fasta)	**MW [kDa]:**	101.9
**Database Date:**	2015-11-30	**pI:**	9.3
**Modification(s):**	Oxidation	**Sequence Coverage [%]:**	7.3
		**No. of unique Peptides:**	3

Quantitation

M:Q **Median:** 1.17 **CV:** 0.00 % **No. of Peptides:** 1

```
        10         20         30         40         50         60         70         80
MCYGTPSYNY APNMDKHWIM QYTGPMLPIH MEFTNILQRK RLQTLMSVDD SVERLYNMLV ETGELENTYI IYTADHGYHI
        90        100        110        120        130        140        150        160
GQFGLVKGKS MPYDFDIRVP FFIRGPSVEP GSIVPQIVLN IDLAPTILDI AGLDTPPDVD GKSVLKLLDP EKPGNRFRTN
       170        180        190        200        210        220        230        240
KKAKIWRDTF LVERGKFLRK KEESSKNIQQ SNHLPKYERV KELCQQARYQ TACEQPGQKW QCIEDTSGKL RIHKCKGPSD
       250        260        270        280        290        300        310        320
LLTVRQSTRN LYARGFHDKD KECSCRESGY RASRSQRKSQ RQFLRNQGTP KYKPRFVHTR QTRSLSVEFE GEIYDINLEE
       330        340        350        360        370        380        390        400
EEELQVLQPR NIAKRHDEGH KGPRDLQASS GGNRGRMLAD SSNAVGPPTT VRVTHKCFIL PNDSIHCERE LYQSARAWKD
       410        420        430        440        450        460        470        480
HKAYIDKEIE ALQDKIKNLR EVRGHLKRRK PEECSCSKQS YYNKEKGVKK QEKLKSHLHP FKEAAQEVDS KLQLFKENNR
       490        500        510        520        530        540        550        560
RRKKERKEKR RQRKGEECSL PGLTCFTHDN NHWQTAPFWN LGSFCACTSS NNNTYWCLRT VNETHNFLFC EFATGFLEYF
       570        580        590        600        610        620        630        640
DMNTDPYQLT NTVHTVERGI LNQLHVQLME LRSCQGYKQC NPRPKNLDVE DSYGMDGKVN QPRLTADINW QGLEELHSVN
       650        660        670        680        690        700        710        720
ENIYEYRQNY RLSLVDWTNY LKDLDRVFAL LKSHYEQNKT NKTQTAQSDG FLVVSAEHAV SMEMASADSD EDPRHKVGKT
       730        740        750        760        770        780        790        800
PHLTLPADLQ TLHLNRPTLS PESKLEWNND IPEVNHLNSE HWRKTEKWTG HEETNHLETD FSGDGMTELE LGPSPRLQPI
       810        820        830        840        850        860        870        880
RRHPKELPQY GGPGKDIFED QLYLPVHSDG ISVHQMFTMA TAEHRSNSSI AGKMLTKVEK NHEKEKSQHL EGSASSSLSS
       890
D
```

Cmpd.	No. of Cmpds.	m/z meas.	Δ m/z [ppm]	z	Rt [min]	Score	P	Range	Sequence	Modification	Ratios
2819	3	701.9509	-109.35	3	65.7	16.7	2	88-104	K.GKSMPYDFDIRVPFFIR.G	Oxidation: 4	
2524	1	813.8725	-59.59	2	61.5	11.6	1	187-199	K.NIQQSNHLPKYER.V		M:Q 1.17
2488	1	673.3201	-4.49	3	61.7	13.8	2	336-354	R.HDEGHKGPRDLQASSGGNR.G		

Detailed Protein Report

Protein 291: kinesin-like protein KIF1C [Homo sapiens]

**Accession:**	gi|40254834	**Score:**	52.5
**Database:**	refseq_human(refseq_human_20140103.fasta)	**MW [kDa]:**	122.9
**Database Date:**	2015-11-30	**pI:**	6.5
**Modification(s):**	Oxidation	**Sequence Coverage [%]:**	4.6
		**No. of unique Peptides:**	3

Alias proteins:

Accession	Name	Description
gi|530409731	refseq_human (refseq_human_20140103.fasta)	PREDICTED: kinesin-like protein KIF1C isoform X1 [Homo sapiens]

10	20	30	40	50	60	70	80
MAGASVKVAV	RVRPFNARET	SQDAKCVVSM	QGNTTSIINP	KQSKDAPKSF	TFDYSYWSHT	STEDPQFASQ	QQVYRDIGEE
**90**	**100**	**110**	**120**	**130**	**140**	**150**	**160**
MLLHAFEGYN	VCIFAYGQTG	AGKSYTMMGR	QEPGQQGIVP	QLCEDLFSRV	SENQSAQLSY	SVEVSYMEIY	CERVRDLLNP
**170**	**180**	**190**	**200**	**210**	**220**	**230**	**240**
KSRGSLRVRE	HPILGPYVQD	LSKLAVTSYA	DIADLMDCGN	KARTVAATNM	NETSSRSHAV	FTIVFTQRCH	DQLTGLDSEK
**250**	**260**	**270**	**280**	**290**	**300**	**310**	**320**
VSKISLVDLA	GSERADSSGA	RGMRLKEGAN	INKSLTTLGK	VISALADMQS	KKRKSDFIPY	RDSVLTWLLK	ENLGGNSRTA
**330**	**340**	**350**	**360**	**370**	**380**	**390**	**400**
MIAALSPADI	NYEETLSTLR	YADRTKQIRC	NAIINEDPNA	RLIRELQEEV	ARLRELLMAQ	GLSASALEGL	KTEEGSVRGA
**410**	**420**	**430**	**440**	**450**	**460**	**470**	**480**
LPAVSSPPAP	VSPSSPTTHN	GELEPSFSPN	TESQIGPEEA	MERLQETEKI	IAELNETWEE	KLRKTEALRM	EREALLAEMG
**490**	**500**	**510**	**520**	**530**	**540**	**550**	**560**
VAVREDGGTV	GVFSPKKTPH	LVNLNEDPLM	SECLLYHIKD	GVTRVGQVDM	DIKLTGQFIR	EQHCLFRSIP	QPDGEVVVTL
**570**	**580**	**590**	**600**	**610**	**620**	**630**	**640**
EPCEGAETYV	NGKLVTEPLV	LKSGNRIVMG	KNHVFRFNHP	EQARLERERG	VPPPPGPPSE	PVDWNFAQKE	LLEQQGIDIK
**650**	**660**	**670**	**680**	**690**	**700**	**710**	**720**
LEMEKRLQDL	ENQYRKEKEE	ADLLLEQQRL	YADSDSGDDS	DKRSCEESWR	LISSLREQLP	PTTVQTIVKR	CGLPSSGKRR
**730**	**740**	**750**	**760**	**770**	**780**	**790**	**800**
APRRVYQIPQ	RRRLQGKDPR	WATMADLKMQ	AVKEICYEVA	LADFRHGRAE	IEALAALKMR	ELCRTYGKPD	GPGDAWRAVA
**810**	**820**	**830**	**840**	**850**	**860**	**870**	**880**
RDVWDTVGEE	EGGGAGSGGG	SEEGARGAEV	EDLRAHIDKL	TGILQEVKLQ	NSSKDRELQA	LRDRMLRMER	VIPLAQDHED
**890**	**900**	**910**	**920**	**930**	**940**	**950**	**960**
ENEEGGEVPW	APPEGSEAAE	EAAPSDRMPS	ARPPSPPLSS	WERVSRLMEE	DPAFRRGRLR	WLKQEQLRLQ	GLQGSGGRGG
**970**	**980**	**990**	**1000**	**1010**	**1020**	**1030**	**1040**
GLRRPPARFV	PPHDCKLRFP	FKSNPQHRES	WPGMGSGEAP	TPLQPPEEVT	PHPATPARRP	PSPRRSHHPR	RNSLDGGGRS
**1050**	**1060**	**1070**	**1080**	**1090**	**1100**	**1110**	
RGAGSAQPEP	QHFQPKKHNS	YPQPPQPYPA	QRPPGPRYPP	YTTPPRMRRQ	RSAPDLKESG	AAV	

Cmpd.	No. of Cmpds.	m/z meas.	Δ m/z [ppm]	z	Rt [min]	Score	P	Range	Sequence	Modification
1891	1	902.3321	-145.45	2	53.7	11.9	1	244-261	K.ISLVDLAGSERADSSGAR.G	
911	1	512.1169	-231.37	2	41.5	10.9	1	255-264	R.ADSSGARGMR.L	Oxidation: 9
1261	1	1044.6916	131.82	2	44.1	13.7	2	630-646	K.ELLEQQGIDIKLEMEKR.L	Oxidation: 14

Detailed Protein Report

Protein 292: **testis-specific serine/threonine-protein kinase 2 [Homo sapiens]**

**Accession:**	gi|194294513	**Score:**	52.5
**Database:**	refseq_human(refseq_human_20140103.fasta)	**MW [kDa]:**	40.9
**Database Date:**	2015-11-30	**pI:**	9.8
**Modification(s):**	Carbamidomethyl	**Sequence Coverage [%]:**	5.3
		**No. of unique Peptides:**	2

10	20	30	40	50	60	70	80
MDDATVLRKK	GYIVGINLGK	GSYAKVKSAY	SERLKFNVAV	KIIDRKKTPT	DFVERFLPRE	MDILATVNHG	SIIKTYEIFE
**90**	**100**	**110**	**120**	**130**	**140**	**150**	**160**
TSDGRIYIIM	ELGVQGDLLE	FIKCQGALHE	DVARKMFRQL	SSAVKYCHDL	DIVHRDLKCE	NLLLDKDFNI	KLSDFGFSKR
**170**	**180**	**190**	**200**	**210**	**220**	**230**	**240**
CLRDSNGRII	LSKTFCGSAA	YAAPEVLQSI	PYQPKVYDIW	SLGVILYIMV	CGSMPYDDSD	IRKMLRIQKE	HRVDFPRSKN
**250**	**260**	**270**	**280**	**290**	**300**	**310**	**320**
LTCECKDLIY	RMLQPDVSQR	LHIDEILSHS	WLQPPKPKAT	SSASFKREGE	GKYRAECKLD	TKTGLRPDHR	PDHKLGAKTQ
**330**	**340**	**350**	**360**				
HRLLVVPENE	NRMEDRLAET	SRAKDHHISG	AEVGKAST				

Cmpd.	No. of Cmpds.	m/z meas.	Δ m/z [ppm]	z	Rt [min]	Score	P	Range	Sequence	Modification
1388	1	570.1598	-180.58	2	45.7	10.7	1	238-246	R.SKNLTCECK.D	Carbamidomethyl: 6, 8
174	4	642.2924	-49.65	2	31.4	20.1	2	293-302	K.YRAECKLDTK.T	Carbamidomethyl: 5

Detailed Protein Report

Protein 293: prolactin-inducible protein precursor [Homo sapiens]

Accession: gi|4505821
Database: refseq_human(refseq_human_20140103.fasta)
Database Date: 2015-11-30
Modification(s): Carbamidomethyl

Score: 52.5
MW [kDa]: 16.6
pI: 9.3
Sequence Coverage [%]: 18.5
No. of unique Peptides: 2

Quantitation

Q:W	**Median:** 0.58	**CV:** 15.99 %	**No. of Peptides:** 2
M:Q	**Median:** 0.94	**CV:** 9.42 %	**No. of Peptides:** 2

```
         10         20         30         40         50         60         70         80
MRLLQLLFRA SPATLLLVLC LQLGANKAQD NTRKIIIKNF DIPKSVRPND EVTAVLAVQT ELKECMVVKT YLISSIPLQG
         90        100        110        120        130        140        150
AFNYKYTACL CDDNPKTFYW DFYTNRTVQI AAVVDVIREL GICPDDAAVI PIKNNRFYTI EILKVE
```

Cmpd.	No. of Cmpds.	m/z meas.	Δ m/z [ppm]	z	Rt [min]	Score	P	Range	Sequence	Modification	Ratios
2176	2	642.3028	-132.15	2	57.4	28.1	0	107-118	R.TVQIAAVVDVIR.E		M:Q 0.86<br>Q:W 0.68
2317	1	805.9036	-28.46	2	57.6	24.3	0	119-133	R.ELGICPDDAAVIPIK.N	Carbamidomethyl: 5	Q:W 0.50<br>M:Q 1.04

Detailed Protein Report

Protein 294: PREDICTED: WD repeat-containing protein 88 isoform X1 [Homo sapiens]

Accession: gi|578833944
Database: refseq_human(refseq_human_20140103.fasta)
Database Date: 2015-11-30
Modification(s): Carbamidomethyl

Score: 52.4
MW [kDa]: 31.0
pI: 6.8
Sequence Coverage [%]: 14.7
No. of unique Peptides: 2

Quantitation

Q:W	**Median:**	1.04	**CV:**	0.00 %	**No. of Peptides:**	1
M:Q	**Median:**	1.65	**CV:**	0.00 %	**No. of Peptides:**	1

```
        10         20         30         40         50         60         70         80
MASPPRCSPT AHDRECKLPP PSAPASEYCP GKLSWGTMAR ALGRFKLSIP HTHLLATLDP LALDREPPPH LLPEKHQVPE
        90        100        110        120        130        140        150        160
KLIWGDQDPL SKIPFKILSG HEHAVSTCHF CVDDTKLLSG SYDCTVKLWD PVDGSVVRDF EHRPKAPVVE CSITGDSSRV
       170        180        190        200        210        220        230        240
IAASYDKTVR AWDLETGKLL WKVRYDTFIV SCKFSPDGKY VVSGFDVDHG ICIMDAENIT TVSVIKDHHT RSITSCCFDP
       250        260        270        280
DSQRVASVSL DRCIKIWDVT SQATLLTITK LFSHFWRV
```

Cmpd.	No. of Cmpds.	m/z meas.	Δ m/z [ppm]	z	Rt [min]	Score	P	Range	Sequence	Modification	Ratios	
2091	1	757.2566	-151.54	2	54.7	16.0	0	18-32	K.LPPPSAPASEYCPGK.L		Q:W	1.04
1628	1	882.4156	-3.95	2	48.7	14.8	1	185-199	R.YDTFIVSCKFSPDGK.Y	Carbamidomethyl: 8	M:Q	1.65

Detailed Protein Report

Protein 295: sushi domain-containing protein 1 isoform 2 precursor [Homo sapiens]

**Accession:**	gi|42476320	**Score:**	52.3
**Database:**	refseq_human(refseq_human_20140103.fasta)	**MW [kDa]:**	82.7
**Database Date:**	2015-11-30	**pI:**	6.0
**Modification(s):**	Carbamidomethyl	**Sequence Coverage [%]:**	7.8
		**No. of unique Peptides:**	2

Quantitation

M:Q **Median:** 0.57 **CV:** 0.00 % **No. of Peptides:** 1

10	20	30	40	50	60	70	80
MGRGPWDAGP	SRRLLPLLLL	LGLARGAAGA	PGPDGLDVCA	TCHEHATCQQ	REGKKICICN	YGFVGNGRTQ	CVDKNECQFG
**90**	**100**	**110**	**120**	**130**	**140**	**150**	**160**
ATLVCGNHTS	CHNTPGGFYC	ICLEGYRATN	NNKTFIPNDG	TFCTDIDECE	VSGLCRHGGR	CVNTHGSFEC	YCMDGYLPRN
**170**	**180**	**190**	**200**	**210**	**220**	**230**	**240**
GPEPFHPTTD	ATSCTEIDCG	TPPEVPDGYI	IGNYTSSLGS	QVRYACREGF	FSVPEDTVSS	CTGLGTWESP	KLHCQEINCG
**250**	**260**	**270**	**280**	**290**	**300**	**310**	**320**
NPPEMRHAIL	VGNHSSRLGG	VARYVCQEGF	ESPGGKITSV	CTEKGTWRES	TLTCTEILTK	INDVSLFNDT	CVRWQINSRR
**330**	**340**	**350**	**360**	**370**	**380**	**390**	**400**
INPKISYVIS	IKGQRLDPME	SVREETVNLT	TDSRTPEVCL	ALYPGTNYTV	NISTAPPRRS	MPAVIGFQTA	EVDLLEDDGS
**410**	**420**	**430**	**440**	**450**	**460**	**470**	**480**
FNISIFNETC	LKLNRRSRKV	GSEHMYQFTV	LGQRWYLANF	SHATSFNFTT	REQVPVVCLD	LYPTTDYTVN	VTLLRSPKRH
**490**	**500**	**510**	**520**	**530**	**540**	**550**	**560**
SVQITIATPP	AVKQTISNIS	GFNETCLRWR	SIKTADMEEM	YLFHIWGQRW	YQKEFAQEMT	FNISSSSRDP	EVCLDLRPGT
**570**	**580**	**590**	**600**	**610**	**620**	**630**	**640**
NYNVSLRALS	SELPVVISLT	TQITEPPLPE	VEFFTVHRGP	LPRLRLRKAK	EKNGPISSYQ	VLVLPLALQS	TFSCDSEGAS
**650**	**660**	**670**	**680**	**690**	**700**	**710**	**720**
SFFSNASDAD	GYVAAELLAK	DVPDDAMEIP	IGDRLYYGEY	YNAPLKRGSD	YCIILRITSE	WNKVRRHSCA	VWAQVKDSSL
**730**	**740**	**750**					
MLLQMAGVGL	GSLAVVIILT	FLSFSAV					

Cmpd.	No. of Cmpds.	m/z meas.	Δ m/z [ppm]	z	Rt [min]	Score	P	Range	Sequence	Modification	Ratios	
893	1	736.8280	-19.37	2	41.2	11.4	0	56-68	K.ICICNYGFVGNGR.T	Carbamidomethyl: 4	M:Q	0.57
844	1	776.9364	79.23	2	39.3	10.6	0	301-313	K.INDVSLFNDTCVR.W	Carbamidomethyl: 11		

Detailed Protein Report

Protein 296: **PREDICTED: neuroblastoma breakpoint family member 4 isoform X1 [Homo sapiens]**

**Accession:**	gi|530361858	**Score:**	52.3
**Database:**	refseq_human(refseq_human_20140103.fasta)	**MW [kDa]:**	80.2
**Database Date:**	2015-11-30	**pI:**	4.7
		**Sequence Coverage [%]:**	7.3
		**No. of unique Peptides:**	3

```
        10         20         30         40         50         60         70         80
MTPPPSNSVI ATADRYLVRW LLPPSLLSES ELQAVPSALS SGIPVPVFAV KDSKVLCRVH QLFLNFPGST SSATNVSMVV
        90        100        110        120        130        140        150        160
SADPLSSERA EMNILEINQE LRSQLAESNQ QFRDLKEKFL ITQATAYSLA NQLKKYKCEE YKDIIDSVLR DELQSMEKLA
       170        180        190        200        210        220        230        240
EKLRQAEELR QYKALVHSQA KELTQLREKL REGRDASRWL NKHLKTLLTP DDPDKSQGQD LREQLAEGHR LAEHLVHKLS
       250        260        270        280        290        300        310        320
PENDEDEDED EDDKDEEVEK VQESPAPREV QKTEEKEVPQ DSLEECAVTC SNSHNPSNSN QPHRSTKITF KEHEVDSALV
       330        340        350        360        370        380        390        400
VESEHPHDEE EEALNIPPEN QNDHEEEEGK APVPPRHHDK SNSYRHREVS FLALDEQKVC SAQDVARDYS NPKWDETSLG
       410        420        430        440        450        460        470        480
FLEKQSDLEE VKGQETVAPR LSRGPLRVDK HEIPQESLDG CCLTPSILPD LTPSYHPYWS TLYSFEDKQV SLALVDKIKK
       490        500        510        520        530        540        550        560
DQEEIEDQSP PCPRLSQELP EVKEQEVPED SVNEVYLTPS VHHDVSDCHQ PYSSTLSSLE DQLACSALDV ASPTEAACPQ
       570        580        590        600        610        620        630        640
GTWSGDLSHH QSEVQVSQAQ LEPSTLVPSC LRLQLDQGFH CGNGLAQRGL SSTTCSFSAN ADSGNQWPFQ ELVLEPSLGM
       650        660        670        680        690        700        710        720
KNPPQLEDDA LEGSASNTQG RQVTGRIRAS LVLILKTIRR RLPFSKWRLA FRFAGPHAES AEIPNTAGRT QRMAG
```

Cmpd.	No. of Cmpds.	m/z meas.	Δ m/z [ppm]	z	Rt [min]	Score	P	Range	Sequence	Modification
2547	4	1044.5458	-3.00	2	62.5	15.8	1	1-19	-.MTPPPSNSVIATADRYLVR.W	
573	2	666.3169	-41.31	2	37.1	12.9	2	192-202	R.EGRDASRWLNK.H	
601	1	460.1164	-243.14	2	35.9	12.8	1	361-367	K.SNSYRHR.E	

Detailed Protein Report

Protein 297: transcription regulator protein BACH1 [Homo sapiens]

Accession: gi|4502353
Database: refseq_human(refseq_human_20140103.fasta)
Database Date: 2015-11-30
Modification(s): Carbamidomethyl, Oxidation

Score: 52.3
MW [kDa]: 81.9
pI: 4.8
Sequence Coverage [%]: 5.8
No. of unique Peptides: 3

Quantitation

Q:W **Median:** 0.62 **CV:** 0.00 % **No. of Peptides:** 1

Alias proteins:

Accession	Name	Description
gi|530418993	refseq_human (refseq_human_20140103.fasta)	PREDICTED: transcription regulator protein BACH1 isoform X3 [Homo sapiens]
gi|530418991	refseq_human (refseq_human_20140103.fasta)	PREDICTED: transcription regulator protein BACH1 isoform X2 [Homo sapiens]
gi|530418989	refseq_human (refseq_human_20140103.fasta)	PREDICTED: transcription regulator protein BACH1 isoform X1 [Homo sapiens]
gi|45827690	refseq_human (refseq_human_20140103.fasta)	transcription regulator protein BACH1 [Homo sapiens]

```
        10         20         30         40         50         60         70         80
MSLSENSVFA YESSVHSTNV LLSLNDQRKK DVLCDVTIFV EGQRFRAHRS VLAACSSYFH SRIVGQADGE LNITLPEEVT
        90        100        110        120        130        140        150        160
VKGFEPLIQF AYTAKLILSK ENVDEVCKCV EFLSVHNIEE SCFQFLKFKF LDSTADQQEC PRKKCFSSHC QKTDLKLSLL
       170        180        190        200        210        220        230        240
DQRDLETDEV EEFLENKNVQ TPQCKLRRYQ GNAKASPPLQ DSASQTYESM CLEKDAALAL PSLCPKYRKF QKAFGTDRVR
       250        260        270        280        290        300        310        320
TGESSVKDIH ASVQPNERSE NECLGGVPEC RDLQVMLKCD ESKLAMEPEE TKKDPASQCP TEKSEVTPFP HNSSIDPHGL
       330        340        350        360        370        380        390        400
YSLSLLHTYD QYGDLNFAGM QNTTVLTEKP LSGTDVQEKT FGESQDLPLK SDLGTREDSS VASSDRSSVE REVAEHLAKG
       410        420        430        440        450        460        470        480
FWSDICSTDT PCQMQLSPAV AKDGSEQISQ KRSECPWLGI RISESPEPGQ RTFTTLSSVN CPFISTLSTE GCSSNLEIGN
       490        500        510        520        530        540        550        560
DDYVSEPQQE PCPYACVISL GDDSETDTEG DSESCSAREQ ECEVKLPFNA QRIISLSRND FQSLLKMHKL TPEQLDCIHD
       570        580        590        600        610        620        630        640
IRRRSKNRIA AQRCRKRKLD CIQNLESEIE KLQSEKESLL KERDHILSTL GETKQNLTGL CQKVCKEAAL SQEQIQILAK
       650        660        670        680        690        700        710        720
YSAADCPLSF LISEKDKSTP DGELALPSIF SLSDRPPAVL PPCARGNSEP GYARGQESQQ MSTATSEQAG PAEQCRQSGG
       730        740
ISDFCQQMTD KCTTDE
```

Cmpd.	No. of Cmpds.	m/z meas.	Δ m/z [ppm]	z	Rt [min]	Score	P	Range	Sequence	Modification	Ratios	
1691	1	1011.6416	141.51	2	51.4	17.7	1	547-562	K.MHKLTPEQLDCIHDIR.R	Carbamidomethyl: 11; Oxidation: 1		
504	1	667.7055	-215.81	2	35.6	22.1	1	615-626	K.QNLTGLCQKVCK.E		Q:W	0.62
1770	1	851.2192	-170.05	2	52.1	12.5	0	717-731	R.QSGGISDFCQQMTDK.C	Carbamidomethyl: 9		

Detailed Protein Report

Protein 298: **PREDICTED: insulin-like growth factor 1 receptor isoform X1 [Homo sapiens]**

**Accession:**	gi|530407100	**Score:**	52.1
**Database:**	refseq_human(refseq_human_20140103.fasta)	**MW [kDa]:**	154.7
**Database Date:**	2015-11-30	**pI:**	5.5
**Modification(s):**	Carbamidomethyl, Oxidation	**Sequence Coverage [%]:**	3.4
		**No. of unique Peptides:**	4

Quantitation

Q:W	**Median:** 0.76	**CV:** 0.00 %	**No. of Peptides:**	1
M:Q	**Median:** 4.18	**CV:** 0.00 %	**No. of Peptides:**	1

```
        10         20         30         40         50         60         70         80
MKSGSGGGSP TSLWGLLFLS AALSLWPTSG EICGPGIDIR NDYQQLKRLE NCTVIEGYLH ILLISKAEDY RSYRFPKLTV
        90        100        110        120        130        140        150        160
ITEYLLLFRV AGLESLGDLF PNLTVIRGWK LFYNYALVIF EMTNLKDIGL YNLRNITRGA IRIEKNADLC YLSTVDWSLI
       170        180        190        200        210        220        230        240
LDAVSNNYIV GNKPPKECGD LCPGTMEEKP MCEKTTINNE YNYRCWTTNR CQKMCPSTCG KRACTENNEC CHPECLGSCS
       250        260        270        280        290        300        310        320
APDNDTACVA CRHYYYAGVC VPACPPNTYR FEGWRCVDRD FCANILSAES SDSEGFVIHD GECMQECPSG FIRNGSQSMY
       330        340        350        360        370        380        390        400
CIPCEGPCPK VCEEEKKTKT IDSVTSAQML QGCTIFKGNL LINIRRGNNI ASELENFMGL IEVVTGYVKI RHSHALVSLS
       410        420        430        440        450        460        470        480
FLKNLRLILG EEQLEGNYSF YVLDNQNLQQ LWDWDHRNLT IKAGKMYFAF NPKLCVSEIY RMEEVTGTKG RQSKGDINTR
       490        500        510        520        530        540        550        560
NNGERASCES DVLHFTSTTT SKNRIIITWH RYRPPDYRDL ISFTVYYKEA PFKNVTEYDG QDACGSNSWN MVDVDLPPNK
       570        580        590        600        610        620        630        640
DVEPGILLHG LKPWTQYAVY VKAVTLTMVE NDHIRGAKSE ILYIRTNASV PSIPLDVLSA SNSSSQLIVK WNPPSLPNGN
       650        660        670        680        690        700        710        720
LSYYIVRWQR QPQDGYLYRH NYCSKDKIPI RKYADGTIDI EEVTENPKTE VCGGEKGPCC ACPKTEAEKQ AEKEEAEYRK
       730        740        750        760        770        780        790        800
VFENFLHNSI FVPRPERKRR DVMQVANTTM SSRSRNTTAA DTYNITDPEE LETEYPFFES RVDNKERTVI SNLRPFTLYR
       810        820        830        840        850        860        870        880
IDIHSCNHEA EKLGCSASNF VFARTMPAEG ADDIPGPVTW EPRPENSIFL KWPEPENPNG LILMYEIKYG SQVEDQRECV
       890        900        910        920        930        940        950        960
SRQEYRKYGG AKLNRLNPGN YTARIQATSL SGNGSWTDPV FFYVQAKRYE NFIHLIIALP VAVLLIVGGL VIMLYVFHRK
       970        980        990       1000       1010       1020       1030       1040
RNNSRLGNGV LYASVNPEYF SAADVYVPDE WEVAREKITM SRELGQGSFG MVYEGVAKGV VKDEPETRVA IKTVNEAASM
      1050       1060       1070       1080       1090       1100       1110       1120
RERIEFLNEA SVMKEFNCHH VVRLLGVVSQ GQPTLVIMEL MTRGDLKSYL RSLRPEMENN PVLAPPSLSK MIQMAGEIAD
      1130       1140       1150       1160       1170       1180       1190       1200
GMAYLNANKF VHRDLAARNC MVAEDFTVKI GDFGMTRDIY ETDYYRKGGK GLLPVRWMSP ESLKDGVFTT YSDVWSFGVV
      1210       1220       1230       1240       1250       1260       1270       1280
LWEIATLAEQ PYQGLSNEQV LRFVMEGGLL DKPDNCPDML FELMRMCWQY NPKMRPSFLE IISSIKEEME PGFREVSFYY
      1290       1300       1310       1320       1330       1340       1350       1360
SEENKLPEPE ELDLEPENME SVPLDPSASS SSLPLPDRHS GHKAENGPGP GVLVLRASFD ERQPYAHMNG GRKNERALPL
      1370
PQSSTC
```

Cmpd.	No. of Cmpds.	m/z meas.	Δ m/z [ppm]	z	Rt [min]	Score	P	Range	Sequence	Modification	Ratios
1849	3	1045.0301	118.60	2	51.5	16.2	0	177-194	K.ECGDLCPGTMEEKPMCEK.T	Carbamidomethyl: 16; Oxidation: 10, 15	
1628	1	716.0279	112.96	3	50.1	12.0	0	177-194	K.ECGDLCPGTMEEKPMCEK.T	Carbamidomethyl: 2, 16; Oxidation: 10, 15	
1529	2	669.8325	-70.94	2	49.3	13.1	0	392-403	R.HSHALVSLSFLK.N		M:Q 4.18<br>Q:W 0.76
2019	1	660.9088	-130.71	3	53.8	10.8	1	446-461	K.MYFAFNPKLCVSEIYR.M		

Detailed Protein Report

Protein 299: G protein-regulated inducer of neurite outgrowth 1 [Homo sapiens]

**Accession:**	gi|112821681	**Score:**	52.0
**Database:**	refseq_human(refseq_human_20140103.fasta)	**MW [kDa]:**	102.3
**Database Date:**	2015-11-30	**pI:**	9.3
**Modification(s):**	Oxidation	**Sequence Coverage [%]:**	6.6
		**No. of unique Peptides:**	3

Quantitation

Q:W	**Median:**	0.63	**CV:**	0.00 %	**No. of Peptides:**	1
M:Q	**Median:**	0.76	**CV:**	0.00 %	**No. of Peptides:**	1

Alias proteins:

Accession	Name	Description
gi|530380833	refseq_human (refseq_human_20140103.fasta)	PREDICTED: G protein-regulated inducer of neurite outgrowth 1 isoform X1 [Homo sapiens]

```
        10         20         30         40         50         60         70         80
MDTAEDPAWL QLLQKDSSPP GPRPTAFFCP QDGSLGAGSS AMRDYCPSQQ KASPAPPRHT PDQSPGMESR HRSPSGAGEG
        90        100        110        120        130        140        150        160
ASCSDGPRGS LACPSPTCFS PQESPSKETL EAHGASISGT PEATTSGKPE PVSSVKTEPK SSDDRNPMFL EKMDFKSSKQ
       170        180        190        200        210        220        230        240
ADSTSIGKED PGSSRKADPM FTGKAEPEIL GKGDPVAPGR MDPMTVRKED LGSLGKVDPL CSSKTYTVSP RKEDPGSLRK
       250        260        270        280        290        300        310        320
VDPVSSDKVD PVFPRKEEPR YSGKEHPVSS EKVAPTSAEK VDLVLSGKRD PGPSGKADPM PLESMDSAST GKTEPGLLGK
       330        340        350        360        370        380        390        400
LIPGSSGKNG PVSSGTGAPG SLGRLDPTCL GMADPASVGN VETVPATKED SRFLGKMDPA SSGEGRPVSG HTDTTASAKT
       410        420        430        440        450        460        470        480
DLTSLKNVDP MSSGKVDPVS LGKMDPMCSG KPELLSPGQA ERVSVGKAGT VSPGKEDPVS SRREDPISAG SRKTSSEKVN
       490        500        510        520        530        540        550        560
PESSGKTNPV SSGPGDPRSL GTAGPPSAVK AEPATGGKGD PLSSEKAGLV ASGKAAPTAS GKAEPLAVGK EDPVSKGKAD
       570        580        590        600        610        620        630        640
AGPSGQGDSV SIGKVVSTPG KTVPVPSGKV DPVSLGKAEA IPEGKVGSLP LEKGSPVTTT KADPRASGKA QPQSGGKAET
       650        660        670        680        690        700        710        720
KLPGQEGAAA PGEAGAVCLK KETPQASEKV DPGSCRKAEP LASGKGEPVS LGKADSAPSR KTESPSLGKV VPLSLEKTKP
       730        740        750        760        770        780        790        800
SSSSRQLDRK ALGSARSPEG ARGSEGRVEP KAEPVSSTEA SSLGQKDLEA AGAERSPCPE AAAPPGPRT  RDNFTKAPSW
       810        820        830        840        850        860        870        880
EASAPPPPRE DAGTQAGAQA CVSVAVSPMS PQDGAGGSAF SFQAAPRAPS PPSRRDAGLQ VSLGAAETRS VATGPMTPQA
       890        900        910        920        930        940        950        960
AAPPAFPEVR VRPGSALAAA VAPPEPAEPV RDVSWDEKGM TWEVYGAAME VEVLGMAIQK HLERQIEEHG RQGAPAPPPA
       970        980        990       1000       1010
ARAGPGRSGS VRTAPPDGAA KRPPGLFRAL LQSVRRPRCC SRAGPTAE
```

Cmpd.	No. of Cmpds.	m/z meas.	Δ m/z [ppm]	z	Rt [min]	Score	P	Range	Sequence	Modification	Ratios
2305	1	853.3930	-63.26	2	57.5	15.6	2	225-239	K.TYTVSPRKEDPGSLR.K		
2196	1	873.3355	-120.26	2	57.3	13.2	1	407-423	K.NVDPMSSGKVDPVSLGK.M	Oxidation: 5	M:Q 0.76<br>Q:W 0.63
2330	1	682.5941	-86.77	3	57.6	11.6	0	424-442	K.MDPMCSGKPELLSPGQAER.V		

Detailed Protein Report

Protein 300: thrombospondin type-1 domain-containing protein 7B [Homo sapiens]

**Accession:**	gi|122937257	**Score:**	52.0
**Database:**	refseq_human(refseq_human_20140103.fasta)	**MW [kDa]:**	175.5
**Database Date:**	2015-11-30	**pI:**	9.1
**Modification(s):**	Carbamidomethyl, Oxidation	**Sequence Coverage [%]:**	4.1
		**No. of unique Peptides:**	4

Quantitation

Q:W	**Median:**	1.50	**CV:**	0.00 %	**No. of Peptides:**	1
M:Q	**Median:**	0.54	**CV:**	0.00 %	**No. of Peptides:**	1

10	20	30	40	50	60	70	80
MHNTADEVVL	YHKLAGPWGR	CTGDCGPGGV	QSRAVWCFHV	DGWTSHLSNC	GESNRPPKER	SCFRVCDWHS	DLFQWEVSDW
**90**	**100**	**110**	**120**	**130**	**140**	**150**	**160**
HHCVLVPYAR	GEVKPRTAEC	VTAQHGLQHR	MVRCIQKLNR	TVVANEICEH	FALQPPTEQA	CLIPCPRDCV	VSEFLPWSNC
**170**	**180**	**190**	**200**	**210**	**220**	**230**	**240**
SKGCGKKLQH	RTRAVIAPPL	FGGLQCPNLT	ESRACDAPIS	CPLGEEEYTF	SLKVGPWSKC	RLPHLKEINP	SGRTVLDFNS
**250**	**260**	**270**	**280**	**290**	**300**	**310**	**320**
DSNERVTFKH	QSYKAHHHSK	SWAIEIGYQT	RQVSCTRSDG	QNAMLSLCLQ	DSFPLTVQSC	IMPKDCETSQ	WSSWSPCSKT
**330**	**340**	**350**	**360**	**370**	**380**	**390**	**400**
CRSGSLLPGF	RSRSRNVKHM	AIGGGKECPE	LLEKEACIVE	GELLQQCPRY	SWRTSEWKEC	QVSLLLEQQD	PHWHVTGPVC
**410**	**420**	**430**	**440**	**450**	**460**	**470**	**480**
GGGIQTREVY	CAQSVPAAAA	LRAKEVSRPV	EKALCVGPAP	LPSQLCNIPC	STDCIVSSWS	AWGLCIHENC	HDPQGKKGFR
**490**	**500**	**510**	**520**	**530**	**540**	**550**	**560**
TRQRHVLMES	TGPAGHCPHL	VESVPCEDPM	CYRWLASEGI	CFPDHGKCGL	GHRILKAVCQ	NDRGEDVSGS	LCPVPPPPER
**570**	**580**	**590**	**600**	**610**	**620**	**630**	**640**
KSCEIPCRMD	CVLSEWTEWS	SCSQSCSNKN	SDGKQTRSRT	ILALAGEGGK	PCPPSQALQE	HRLCNDHSCM	QLHWETSPWG
**650**	**660**	**670**	**680**	**690**	**700**	**710**	**720**
PCSEDTLVTA	LNATIGWNGE	ATCGVGIQTR	RVFCVKSHVG	QVMTKRCPDS	TRPETVRPCF	LPCKKDCIVT	AFSEWTPCPR
**730**	**740**	**750**	**760**	**770**	**780**	**790**	**800**
MCQAGNATVK	QSRYRIIIQE	AANGGQECPD	TLYEERECED	VSLCPVYRWK	PQKWSPCILV	PESVWQGITG	SSEACGKGLQ
**810**	**820**	**830**	**840**	**850**	**860**	**870**	**880**
TRAVSCISDD	NRSAEMMECL	KQTNGMPLLV	QECTVPCRED	CTFTAWSKFT	PCSTNCEATK	SRRRQLTGKS	RKKEKCQDSD
**890**	**900**	**910**	**920**	**930**	**940**	**950**	**960**
LYPLVETELC	PCDEFISQPY	GNWSDCILPE	GRREPHRGLR	VQADSKECGE	GLRFRAVACS	DKNGRPVDPS	FCSSSGYIQE
**970**	**980**	**990**	**1000**	**1010**	**1020**	**1030**	**1040**
KCVIPCPFDC	KLSDWSSWGS	CSSSCGIGVR	IRSKWLKEKP	YNGGRPCPKL	DLKNQAQVHE	AVPCYSECNQ	YSWVVEHWSS
**1050**	**1060**	**1070**	**1080**	**1090**	**1100**	**1110**	**1120**
CKINNELRSL	RCGGGTQSRK	IRCVNTADGE	GGAVDSNLCN	QDEIPPETQS	CSLMCPNECV	MSEWGLWSKC	PQSCDPHTMQ
**1130**	**1140**	**1150**	**1160**	**1170**	**1180**	**1190**	**1200**
RRTRHLLRPS	LNSRTCAEDS	QVQPCLLNEN	CFQFQYNLTE	WSTCQLSENA	PCGQGVRTRL	LSCVCSDGKP	VSMDQCEQHN
**1210**	**1220**	**1230**	**1240**	**1250**	**1260**	**1270**	**1280**
LEKPQRMSIP	CLVECVVNCQ	LSGWTAWTEC	SQTCGHGGRM	SRTRFIIMPT	QGEGRPCPTE	LTQEKTCPVT	PCYSWVLGNW
**1290**	**1300**	**1310**	**1320**	**1330**	**1340**	**1350**	**1360**
SACKLEGGDC	GEGVQIRSLS	CMVHSGSISH	AAGRVEDALC	GEMPFQDSIL	KQLCSVPCPG	DCHLTEWSEW	STCELTCIDG
**1370**	**1380**	**1390**	**1400**	**1410**	**1420**	**1430**	**1440**
RSFETVGRQS	RSRTFIIQSF	ENQDSCPQQV	LETRPCTGGK	CYHYTWKASL	WNNNERTVWC	QRSDGVNVTG	GCSPQARPAA
**1450**	**1460**	**1470**	**1480**	**1490**	**1500**	**1510**	**1520**
IRQCIPACRK	PFSYCTQGGV	CGCEKGYTEI	MKSNGFLDYC	MKVPGSEDKK	ADVKNLSGKN	RPVNSKIHDI	FKGWSLQPLD
**1530**	**1540**	**1550**	**1560**	**1570**	**1580**		
PDGRVKIWVY	GVSGGAFLIM	IFLIFTSYLV	CKKPKPHQST	PPQQKPLTLA	YDGDLDM		

Cmpd.	No. of Cmpds.	m/z meas.	Δ m/z [ppm]	z	Rt [min]	Score	P	Range	Sequence	Modification	Ratios
2544	1	885.4222	-9.12	2	61.8	10.9	2	721-735	R.MCQAGNATVKQSRYR.I	Carbamidomethyl: 2	
122	1	981.4639	-1.83	2	30.8	11.7	0	822-838	K.QTNGMPLLVQECTVPCR.E	Carbamidomethyl: 16; Oxidation: 5	M:Q 0.54<br>Q:W 1.50

Detailed Protein Report

Cmpd.	No. of Cmpds.	m/z meas.	Δ m/z [ppm]	z	Rt [min]	Score	P	Range	Sequence	Modification	Ratios
417	1	475.6019	-263.42	2	33.8	10.3	1	1052-1060	R.CGGGTQSRK.I	Carbamidomethyl: 1	
1180	1	883.7188	-3.03	3	44.4	19.1	1	1443-1465	R.QCIPACRKPFSYCTQGGVCGCEKG	Carbamidomethyl: 2, 6, 13	

Detailed Protein Report

Protein 301: dedicator of cytokinesis protein 6 [Homo sapiens]

**Accession:**	gi|157426887	**Score:**	51.9
**Database:**	refseq_human(refseq_human_20140103.fasta)	**MW [kDa]:**	229.4
**Database Date:**	2015-11-30	**pI:**	6.3
		**Sequence Coverage [%]:**	2.1
		**No. of unique Peptides:**	3

Quantitation

M:Q	**Median:**	0.95	**CV:**	0.00 %	**No. of Peptides:**	1	

Detailed Protein Report

10	20	30	40	50	60	70	80
MAASERRAFA	HKINRTVAAE	VRKQVSRERS	GSPHSSRRCS	SSLGVPLTEV	VEPLDFEDVL	LSRPPDAEPG	PLRDLVEFPA
90	100	110	120	130	140	150	160
DDLELLLQPR	ECRTTEPGIP	KDEKLDAQVR	AAVEMYIEDW	VIVHRRYQYL	SAAYSPVTTD	TQRERQKGLP	RQVFEQDASG
170	180	190	200	210	220	230	240
DERSGPEDSN	DSRRGSGSPE	DTPRSSGASS	IFDLRNLAAD	SLLPSLLERA	APEDVDRRNE	TLRRQHRPPA	LLTLYPAPDE
250	260	270	280	290	300	310	320
DEAVERCSRP	EPPREHFGQR	ILVKCLSLKF	EIEIEPIFGI	LALYDVREKK	KISENFYFDL	NSDSMKGLLR	AHGTHPAIST
330	340	350	360	370	380	390	400
LARSAIFSVT	YPSPDIFLVI	KLEKVLQQGD	ISECCEPYMV	LKEVDTAKNK	EKLEKLRLAA	EQFCTRLGRY	RMPFAWTAVH
410	420	430	440	450	460	470	480
LANIVSSAGQ	LDRDSDSEGE	RRPAWTDRRR	RGPQDRASSG	DDACSFSGFR	PATLTVTNFF	KQEAERLSDE	DLFKFLADMR
490	500	510	520	530	540	550	560
RPSSLLRRLR	PVTAQLKIDI	SPAPENPHFC	LSPELLHIKP	YPDPRGRPTK	EILEFPAREV	YAPHTSYRNL	LYVYPHSLNF
570	580	590	600	610	620	630	640
SSRQGSVRNL	AVRVQYMTGE	DPSQALPVIF	GKSSCSEFTR	EAFTPVVYHN	KSPEFYEEFK	LHLPACVTEN	HHLLFTFYHV
650	660	670	680	690	700	710	720
SCQPRPGTAL	ETPVGFTWIP	LLQHGRLRTG	PFCLPVSVDQ	PPPSYSVLTP	DVALPGMRWV	DGHKGVFSVE	LTAVSSVHPQ
730	740	750	760	770	780	790	800
DPYLDKFFTL	VHVLEEGAFP	FRLKDTVLSE	GNVEQELRAS	LAALRLASPE	PLVAFSHHVL	DKLVRLVIRP	PIISGQIVNL
810	820	830	840	850	860	870	880
GRGAFEAMAH	VVSLVHRSLE	AAQDARGHCP	QLAAYVHYAF	RLPGTEPSLP	DGAPPVTVQA	ATLARGSGRP	ASLYLARSKS
890	900	910	920	930	940	950	960
ISSSNPDLAV	APGSVDDEVS	RILASKLLHE	ELALQWVVSS	SAVREAILQH	AWFFFQLMVK	SMALHLLLGQ	RLDTPRKLRF
970	980	990	1000	1010	1020	1030	1040
PGRFLDDITA	LVGSVGLEVI	TRVHKDVELA	EHLNASLAFF	LSDLLSLVDR	GFVFSLVRAH	YKQVATRLQS	SPNPAALLTL
1050	1060	1070	1080	1090	1100	1110	1120
RMEFTRILCS	HEHYVTLNLP	CCPLSPPASP	SPSVSSTTSQ	SSTFSSQAPD	PKVTSMFELS	GPFRQQHFLA	GLLLTELALA
1130	1140	1150	1160	1170	1180	1190	1200
LEPEAEGAFL	LHKKAISAVH	SLLCGHDTDP	RYAEATVKAR	VAELYLPLLS	IARDTLPRLH	DFAEGPGQRS	RLASMLDSDT
1210	1220	1230	1240	1250	1260	1270	1280
EGEGDIAGTI	NPSVAMAIAG	GPLAPGSRAS	ISQGPPTASR	AGCALSAESS	RTLLACVLWV	LKNTEPALLQ	RWATDLTLPQ
1290	1300	1310	1320	1330	1340	1350	1360
LGRLLDLLYL	CLAAFEYKGK	KAFERINSLT	FKKSLDMKAR	LEEAILGTIG	ARQEMVRRSR	ERSPFGNPEN	VRWRKSVTHW
1370	1380	1390	1400	1410	1420	1430	1440
KQTSDRVDKT	KDEMEHEALV	EGNLATEASL	VVLDTLEIIV	QTVMLSEARE	SVLGAVLKVV	LYSLGSAQSA	LFLQHGLATQ
1450	1460	1470	1480	1490	1500	1510	1520
RALVSKFPEL	LFEEDTELCA	DLCLRLLRHC	GSRISTIRTH	ASASLYLLMR	QNFEIGHNFA	RVKMQVTMSL	SSLVGTTQNF
1530	1540	1550	1560	1570	1580	1590	1600
SEEHLRRSLK	TILTYAEEDM	GLRDSTFAEQ	VQDLMFNLHM	ILTDTVKMKE	HQEDPEMLID	LMYRIARGYQ	GSPDLRLTWL
1610	1620	1630	1640	1650	1660	1670	1680
QNMAGKHAEL	GNHAEAAQCM	VHAAALVAEY	LALLEDHRHL	PVGCVSFQNI	SSNVLEESAI	SDDILSPDEE	GFCSGKHFTE
1690	1700	1710	1720	1730	1740	1750	1760
LGLVGLLEQA	AGYFTMGGLY	EAVNEVYKNL	IPILEAHRDY	KKLAAVHGKL	QEAFTKIMHQ	SSGWERVFGT	YFRVGFYGAH
1770	1780	1790	1800	1810	1820	1830	1840
FGDLDEQEFV	YKEPSITKLA	EISHRLEEFY	TERFGDDVVE	IIKDSNPVDK	SKLDSQKAYI	QITYVEPYFD	TYELKDRVTY
1850	1860	1870	1880	1890	1900	1910	1920
FDRNYGLRTF	LFCTPFTPDG	RAHGELPEQH	KRKTLLSTDH	AFPYIKTRIR	VCHREETVLT	PVEVAIEDMQ	KKTRELAFAT
1930	1940	1950	1960	1970	1980	1990	2000
EQDPPDAKML	QMVLQGSVGP	TVNQGPLEVA	QVFLAEIPED	PKLFRHHNKL	RLCFKDFCKK	CEDALRKNKA	LIGPDQKEYH
2010	2020	2030	2040	2050			
RELERNYCRL	REALQPLLTQ	RLPQLMAPTP	PGLRNSLNRA	SFRKADL			

Cmpd.	No. of Cmpds.	m/z meas.	Δ m/z [ppm]	z	Rt [min]	Score	P	Range	Sequence	Modification	Ratios

Detailed Protein Report

Cmpd.	No. of Cmpds.	m/z meas.	Δ m/z [ppm]	z	Rt [min]	Score	P	Range	Sequence	Modification	Ratios
1750	1	628.2499	-105.50	2	51.8	11.8	2	28-38	R.ERSGSPHSSRR.C		
2956	1	753.3925	-14.65	2	66.1	10.4	0	1874-1886	K.TLLSTDHAFPYIK.T		
1355	1	695.8559	-59.65	2	46.5	14.1	0	2022-2034	R.LPQLMAPTPPGLR.N		M:Q 0.95

Detailed Protein Report

Protein 302: **protein FAM45A [Homo sapiens]**

**Accession:**	gi|46275835	**Score:**	51.9
**Database:**	refseq_human(refseq_human_20140103.fasta)	**MW [kDa]:**	40.5
**Database Date:**	2015-11-30	**pI:**	6.2
**Modification(s):**	Carbamidomethyl	**Sequence Coverage [%]:**	10.4
		**No. of unique Peptides:**	4

10	20	30	40	50	60	70	80
MAAAEVADTQ	LMLGVGLIEK	DTNGEVLWVW	CYPSTTATLR	NLLLRKCCLT	DENKLLHPFV	FGQYRRTWFY	ITTIEVPDSS
**90**	**100**	**110**	**120**	**130**	**140**	**150**	**160**
ILKKVTHFSI	VLTAKDFNPE	KYAAFTRILC	RMYLKHGSPV	KMMESYIAVL	TKGICQSEEN	GSFLSKDFDA	RKAYLAGSIK
**170**	**180**	**190**	**200**	**210**	**220**	**230**	**240**
DIVSQFGMET	VILHTALMLK	KRIVVYHPKI	EAVQEFTRTL	PALVWHRQDW	TILHSYVHLN	ADELEALQMC	TGYVAGFVDL
**250**	**260**	**270**	**280**	**290**	**300**	**310**	**320**
EVSNRPDLYD	VFVNLAESEI	TIAPLAKEAM	AMGKLHKEMG	QLIVQSAEDP	EKSESHVIQD	IALKTREIFT	NLAPFSEVSA
**330**	**340**	**350**	**360**				
DGEKRVLNLE	ALKQKRFPPA	TENFLYHLAA	AEQMLKI				

Cmpd.	No. of Cmpds.	m/z meas.	Δ m/z [ppm]	z	Rt [min]	Score	P	Range	Sequence	Modification
2766	1	804.4142	11.70	3	63.0	10.7	2	46-65	R.KCCLTDENKLLHPFVFGQYR.R	
837	1	491.6643	-76.81	2	38.9	11.9	0	47-54	K.CCLTDENK.L	Carbamidomethyl: 1
917	2	491.6624	-80.76	2	40.3	12.6	0	47-54	K.CCLTDENK.L	Carbamidomethyl: 2
2666	3	973.5093	-13.80	2	63.4	16.7	1	85-101	K.VTHFSIVLTAKDFNPEK.Y	

Detailed Protein Report

Protein 303: **PREDICTED: zinc finger protein 510 isoform X3 [Homo sapiens]**

**Accession:**	gi|530390743	**Score:**	51.9
**Database:**	refseq_human(refseq_human_20140103.fasta)	**MW [kDa]:**	72.1
**Database Date:**	2015-11-30	**pI:**	10.2
**Modification(s):**	Carbamidomethyl	**Sequence Coverage [%]:**	8.5
		**No. of unique Peptides:**	1

10	20	30	40	50	60	70	80
MAPVQKNLYR	DVMLENYSNL	VSVGYCCFKP	EVIFKLEQGE	EPWFSEEEFS	NQSHPKDYRG	DDLIKQNKKI	KDKHLEQAIC
**90**	**100**	**110**	**120**	**130**	**140**	**150**	**160**
INNKTLTTEE	EKVLGKPFTL	HVAAVASTKM	SCKCNSWEVN	LQSISEFIIN	NRNYSTKKIG	CGNVCENSPF	KINFEKTQTG
**170**	**180**	**190**	**200**	**210**	**220**	**230**	**240**
EKFYEHNKNM	KALNYNENLP	KHPKFQTLEQ	AFECNKIGKA	FNDKANCVKH	NSSHTGETSS	KDDEFRKNCD	KKTLFDHRRT
**250**	**260**	**270**	**280**	**290**	**300**	**310**	**320**
GTGKKHLHLN	QCGKSFEKST	VEEYNKLNMG	IKHYELNPSG	NNFNRKAHLT	DPQTAVIEEN	PLVSNDRTQT	WVKSSEYHEN
**330**	**340**	**350**	**360**	**370**	**380**	**390**	**400**
KKSYQTSVHR	VRRRSHSMMK	PYKCNECGKS	FCQKGHLIQH	QRTHTGEKPF	ECSECGKTFS	QKSHLSTHQR	IHTAEKPYKC
**410**	**420**	**430**	**440**	**450**	**460**	**470**	**480**
NECGKTFVQK	STLRGHQRIH	TGEKPYECSE	CGKTFVQKST	LRDHHRIHTG	EKSFQCNQCG	KTFGQKSNLR	IHQRTHTGEK
**490**	**500**	**510**	**520**	**530**	**540**	**550**	**560**
TYQCNECEKS	FWRKDHLIQH	QKTHTGEKPF	KCNECGKTFA	RTSTLRVHQR	IHTGEKPFKC	NECGKKFVRK	AILSDHQRIH
**570**	**580**	**590**	**600**	**610**	**620**	**630**	
TGEKPFQCNK	CGKTFGQKSN	LRIHQRTHSG	EKSYECNEYG	KLCKKSTLSL	YQKIQGEGNP	Y	

Cmpd.	No. of Cmpds.	m/z meas.	Δ m/z [ppm]	z	Rt [min]	Score	P	Range	Sequence	Modification
282	1	627.0085	98.28	3	33.2	10.2	2	481-494	K.TYQCNECEKSFWRK.D	Carbamidomethyl: 7

Detailed Protein Report

Protein 304: E3 ubiquitin-protein ligase TRIP12 isoform c [Homo sapiens]

**Accession:**	gi|10863903	**Score:**	51.8
**Database:**	refseq_human(refseq_human_20140103.fasta)	**MW [kDa]:**	220.3
**Database Date:**	2015-11-30	**pI:**	9.6
		**Sequence Coverage [%]:**	3.1
		**No. of unique Peptides:**	2

Quantitation

M:Q	**Median:** 0.38	**CV:** 0.00 %	**No. of Peptides:** 1

Detailed Protein Report

10	20	30	40	50	60	70	80
MSNRPNNNPG	GSLRRSQRNT	AGAQPQDDSI	GGRSCSSSSA	VIVPQPEDPD	RANTSERQKT	GQVPKKDNSR	GVKRSASPDY
90	100	110	120	130	140	150	160
NRTNSPSSAK	KPKALQHTES	PSETNKPHSK	SKKRHLDQEQ	QLKSAQSPST	SKAHTRKSGA	TGGSRSQKRK	RTESSCVKSG
170	180	190	200	210	220	230	240
SGSESTGAEE	RSAKPTKLAS	KSATSAKAGC	STITDSSSAA	STSSSSSAVA	SASSTVPPGA	RVKQGKDQNK	ARRSRSASSP
250	260	270	280	290	300	310	320
SPRRSSREKE	QSKTGGSSKF	DWAARFSPKV	SLPKTKLSLP	GSSKSETSKP	GPSGLQAKLA	SLRKSTKKRS	ESPPAELPSL
330	340	350	360	370	380	390	400
RRSTRQKTTG	SCASTSRRGS	GLGKRGAAEA	RRQEKMADPE	SNQEAVNSSA	ARTDEAPQGA	AGAVGMTTSG	ESESDDSEMG
410	420	430	440	450	460	470	480
RLQALLEARG	LPPHLFGPLG	PRMSQLFHRT	IGSGASSKAQ	QLLQGLQASD	ESQQLQAVIE	MCQLLVMGNE	ETLGGFPVKS
490	500	510	520	530	540	550	560
VVPALITLLQ	MEHNFDIMNH	ACRALTYMME	ALPRSSAVVV	DAIPVFLEKL	QVIQCIDVAE	QALTALEMLS	RRHSKAILQA
570	580	590	600	610	620	630	640
GGLADCLLYL	EFFSINAQRN	ALAIAANCCQ	SITPDEFHFV	ADSLPLLTQR	LTHQDKKSVE	STCLCFARLV	DNFQHEENLL
650	660	670	680	690	700	710	720
QQVASKDLLT	NVQQLLVVTP	PILSSGMFIM	VVRMFSLMCS	NCPTLAVQLM	KQNIAETLHF	LLCGASNGSC	QEQIDLVPRS
730	740	750	760	770	780	790	800
PQELYELTSL	ICELMPCLPK	EGIFAVDTML	KKGNAQNTDG	AIWQWRDDRG	LWHPYNRIDS	RIIEQINEDT	GTARAIQRKP
810	820	830	840	850	860	870	880
NPLANSNTSG	YSESKKDDAR	AQLMKEDPEL	AKSFIKTLFG	VLYEVYSSSA	GPAVRHKCLR	AILRIIYFAD	AELLKDVLKN
890	900	910	920	930	940	950	960
HAVSSHIASM	LSSQDLKIVV	GALQMAEILM	QKLPDIFSVY	FRREGVMHQV	KHLAESESLL	TSPPKACTNG	SGSMGSTTSV
970	980	990	1000	1010	1020	1030	1040
SSGTATAATH	AAADLGSPSL	QHSRDDSLDL	SPQGRLSDVL	KRKRLPKRGP	RRPKYSPPRD	DDKVDNQAKS	PTTTQSPKSS
1050	1060	1070	1080	1090	1100	1110	1120
FLASLNPKTW	GRLSTQSNSN	NIEPARTAGG	SGLARAASKD	TISNNREKIK	GWIKEQAHKF	VERYFSSENM	DGSNPALNVL
1130	1140	1150	1160	1170	1180	1190	1200
QRLCAATEQL	NLQVDGGAEC	LVEIRSIVSE	SDVSSFEIQH	SGFVKQLLLY	LTSKSEKDAV	SREIRLKRFL	HVFFSSPLPG
1210	1220	1230	1240	1250	1260	1270	1280
EEPIGRVEPV	GNAPLLALVH	KMNNCLSQME	QFPVKVHDFP	SGNGTGGSFS	LNRGSQALKF	FNTHQLKCQL	QRHPDCANVK
1290	1300	1310	1320	1330	1340	1350	1360
QWKGGPVKID	PLALVQAIER	YLVVRGYGRV	REDDEDSDDD	GSDEEIDESL	AAQFLNSGNV	RHRLQFYIGE	HLLPYNMTVY
1370	1380	1390	1400	1410	1420	1430	1440
QAVRQFSIQA	EDERESTDDE	SNPLGRAGIW	TKTHTIWYKP	VREDEESNKD	CVGGKRGRAQ	TAPTKTSPRN	AKKHDELWHD
1450	1460	1470	1480	1490	1500	1510	1520
GVCPSVSNPL	EVYLIPTPPE	NITFEDPSLD	VILLLRVLHA	ISRYWYYLYD	NAMCKEIIPT	SEFINSKLTA	KANRQLQDPL
1530	1540	1550	1560	1570	1580	1590	1600
VIMTGNIPTW	LTELGKTCPF	FFPFDTRQML	FYVTAFDRDR	AMQRLLDTNP	EINQSDSQDS	RVAPRLDRKK	RTVNREELLK
1610	1620	1630	1640	1650	1660	1670	1680
QAESVMQDLG	SSRAMLEIQY	ENEVGTGLGP	TLEFYALVSQ	ELQRADLGLW	RGEEVTLSNP	KGSQEGTKYI	QNLQGLFALP
1690	1700	1710	1720	1730	1740	1750	1760
FGRTAKPAHI	AKVKMKFRFL	GKLMAKAIMD	FRLVDLPLGL	PFYKWMLRQE	TSLTSHDLFD	IDPVVARSVY	HLEDIVRQKK
1770	1780	1790	1800	1810	1820	1830	1840
RLEQDKSQTK	ESLQYALETL	TMNGCSVEDL	GLDFTLPGFP	NIELKKGGKD	IPVTIHNLEE	YLRLVIFWAL	NEGVSRQFDS
1850	1860	1870	1880	1890	1900	1910	1920
FRDGFESVFP	LSHLQYFYPE	ELDQLLCGSK	ADTWDAKTLM	ECCRPDHGYT	HDSRAVKFLF	EILSSFDNEQ	QRLFLQFVTG
1930	1940	1950	1960	1970	1980	1990	2000
SPRLPVGGFR	SLNPPLTIVR	KTFESTENPD	DFLPSVMTCV	NYLKLPDYSS	IEIMREKLLI	AAREGQQSFH	LS

Cmpd.	No. of Cmpds.	m/z meas.	Δ m/z [ppm]	z	Rt [min]	Score	P	Range	Sequence	Modification	Ratios
1453	1	929.4464	3.04	2	46.5	10.5	1	16-33	R.SQRNTAGAQPQDDSIGGR.S		M:Q 0.38
1915	1	643.3072	-50.38	2	54.3	12.5	2	133-145	K.AHTRKSGATGGSR.S		

Detailed Protein Report

Protein 305: regulator of G-protein signaling 22 isoform 2 [Homo sapiens]

Accession: gi|557878614
Database: refseq_human(refseq_human_20140103.fasta)
Database Date: 2015-11-30
Modification(s): Carbamidomethyl

Score: 51.7
MW [kDa]: 145.7
pI: 8.7
Sequence Coverage [%]: 4.4
No. of unique Peptides: 4

10	20	30	40	50	60	70	80
MPEKRLTAEP	PTITEEEFED	SLATDDFLVD	YFNEFLSLPT	FSEAIRFNAD	YGVFEVANDA	PQFLEKQLKK	ILQNQQPRNP
90	100	110	120	130	140	150	160
IYDVVRKGKN	EVKPVQMNAP	DEDETINVNY	NIMCLSREEG	IKWIKKERLP	AFLESDCYFE	YRLAKLVSQV	RWSKSGMNFT
170	180	190	200	210	220	230	240
VGSNFSPWIV	KKPPSLPPPA	TEEDNLASYT	QTKDWFALAK	QSQQTVSTFS	LPCCVPYNKL	KSPAISSVSE	NFIFDDGVHP
250	260	270	280	290	300	310	320
RTKKDPSKTN	KLISEFEEEE	GEEEEVSVSL	QDTPSQALLR	VYLEKKQDVD	ESLTMHFSTC	EEFLSSYIYF	ILRGAIQQIV
330	340	350	360	370	380	390	400
GKPVGETPDY	INFNNITKVS	FDDCFESIHG	KNFLSELVQT	TKERSEEIEQ	TSLSSKNESA	GPESRADWCI	SHRTYDIGNR
410	420	430	440	450	460	470	480
KEFERFKKFI	KGTLGERYWW	LWMDIERLKV	LKDPGRHQRH	LEKMKKCYLV	SNGDYYLSAE	ILSKFKLLDG	SQWNEEHLRN
490	500	510	520	530	540	550	560
IQSEVLKPLL	LYWAPRFCVT	HSASTKYASA	ELKFWHLRQA	KPRKDIDPFP	QMATLLPLRP	KSCIPQIPEI	QKEEFSLSQP
570	580	590	600	610	620	630	640
PKSPNKSPEV	KTATQKPWKR	ELLYPGSSKD	DVIEKGSKYM	SESSKVIHLT	SFTDISECLK	PQLDRRYAYT	EEPRVKTVSD
650	660	670	680	690	700	710	720
VGALGGSDME	NLLQSLYVEN	RAGFFFTKFC	EHSGNKLWKN	SVYFWFDLQA	YHQLFYQETL	QPFKVCKQAQ	YLFATYVAPS
730	740	750	760	770	780	790	800
ATLDIGLQQE	KKKEIYMKIQ	PPFEDLFDTA	EEYILLLLLE	PWTKMVKSDQ	IAYKKVELVE	ETRQLDSTYF	RKLQALHKET
810	820	830	840	850	860	870	880
FSKKAEDTTC	EIGTGILSLS	NVSKRTEYWD	NVPAEYKHFK	FSDLLNNKLE	FEHFRQFLET	HSSSMDLMCW	TDIEQFRRIT
890	900	910	920	930	940	950	960
YRDRNQRKAK	SIYIKNKYLN	KKYFFGPNSP	ASLYQQNQVM	HLSGGWGKIL	HEQLDAPVLV	EIQKHVQNRL	ENVWLPLFLA
970	980	990	1000	1010	1020	1030	1040
SEQFAARQKI	KVQMKDIAEE	LLLQKAEKKI	GVWKPVESKW	ISSSCKIIAF	RKALLNPVTS	RQFQRFVALK	GDLLENGLLF
1050	1060	1070	1080	1090	1100	1110	1120
WQEVQKYKDL	CHSHCDESVI	QKKITTIINC	FINSSIPPAL	QIDIPVEQAQ	KIIEHRKELG	PYVFREAQMT	IFGVLFKFWP
1130	1140	1150	1160	1170	1180	1190	1200
QFCEFRKNLT	DENIMSVLER	RQEYNKQKKK	LAVLEDEKSG	KDGIKQYANT	SVPAIKTALL	SDSFLGLQPY	GRQPTWCYSK
1210	1220	1230	1240	1250	1260		
YIEALEQERI	LLKIQEELEK	KLFAGLQPLT	NFKASSSTMS	LKKNMSAHSS	QK		

Cmpd.	No. of Cmpds.	m/z meas.	Δ m/z [ppm]	z	Rt [min]	Score	P	Range	Sequence	Modification
2902	1	1192.7378	113.29	2	65.0	10.5	0	172-193	K.KPPSLPPPATEEDNLASYTQTK.D	
2013	1	703.2744	-90.02	2	53.5	13.4	1	669-679	K.FCEHSGNKLWK.N	Carbamidomethyl: 2
2278	1	557.5521	-50.47	3	58.7	12.0	0	1049-1062	K.DLCHSHCDESVIQK.K	Carbamidomethyl: 7
1789	1	916.3008	-215.79	1	52.4	15.8	0	1151-1158	K.LAVLEDEK.S	

Detailed Protein Report

Protein 306: neuron navigator 2 isoform 3 [Homo sapiens]

**Accession:**	gi|161169015	**Score:**	51.7
**Database:**	refseq_human(refseq_human_20140103.fasta)	**MW [kDa]:**	254.8
**Database Date:**	2015-11-30	**pI:**	9.7
**Modification(s):**	Carbamidomethyl	**Sequence Coverage [%]:**	2.0
		**No. of unique Peptides:**	1

Detailed Protein Report

10	20	30	40	50	60	70	80
MESVSESSQQ	QKRKPVIHGL	EDQKRIYTDW	ANHYLAKSGH	KRLIRDLQQD	VTDGVLLAQI	IQVVANEKIE	DINGCPKNRS
90	100	110	120	130	140	150	160
QMIENIDACL	NFLAAKGINI	QGLSAEEIRN	GNLKAILGLF	FSLSRYKQQQ	QQPQKQHLSS	PLPPAVSQVA	GAPSQCQAGT
170	180	190	200	210	220	230	240
PQQQVPVTPQ	APCQPHQPAP	HQQSKAQAEM	QSRLPGPTAR	VSAAGSEAKT	RGGSTTANNR	RSQSFNNYDK	SKPVTSPPPP
250	260	270	280	290	300	310	320
PSSHEKEPLA	SSASSHPGMS	DNAPASLESG	SSSTPTNCST	SSAIPQPGAA	TKPWRSKSLS	VKHSATVSML	SVKPPGPEAP
330	340	350	360	370	380	390	400
RPTPEAMKPA	PNNQKSMLEK	LKLFNSKGGS	KAGEGPGSRD	TSCERLETLP	SFEESEELEA	ASRMLTTVGP	ASSSPKIALK
410	420	430	440	450	460	470	480
GIAQRTFSRA	LTNKKSSLKG	NEKEKEKQQR	EKDKEKSKDL	AKRASVTERL	DLKEEPKEDP	SGAAVPEMPK	KSSKIASFIP
490	500	510	520	530	540	550	560
KGGKLNSAKK	EPMAPSHSGI	PKPGMKSMPG	KSPSAPAPSK	EGERSRSGKL	SSGLPQQKPQ	LDGRHSSSSS	SLASSEGKGP
570	580	590	600	610	620	630	640
GGTTLNHSIS	SQTVSGSVGT	TQTTGSNTVS	VQLPQPQQQY	NHPNTATVAP	FLYRSQTDTE	GNVTAESSST	GVSVEPSHFT
650	660	670	680	690	700	710	720
KTGQPALEEL	TGEDPEARRL	RTVKNIADLR	QNLEETMSSL	RGTQVTHSTL	ETTFDTNVTT	EMSGRSILSL	TGRPTPLSWR
730	740	750	760	770	780	790	800
LGQSSPRLQA	GDAPSMGNGY	PPRANASRFI	NTESGRYVYS	APLRRQLASR	GSSVCHVDVS	DKAGDEMDLE	GISMDAPGYM
810	820	830	840	850	860	870	880
SDGDVLSKNI	RTDDITSGYM	TDGGLGLYTR	RLNRLPDGMA	VVRETLQRNT	SLGLGDADSW	DDSSSVSSGI	SDTIDNLSTD
890	900	910	920	930	940	950	960
DINTSSSISS	YANTPASSRK	NLDVQTDAEK	HSQVERNSLW	SGDDVKKSDG	GSDSGIKMEP	GSKWRRNPSD	VSDESDKSTS
970	980	990	1000	1010	1020	1030	1040
GKKNPVISQT	GSWRRGMTAQ	VGITMPRTKP	SAPAGALKTP	GTGKTDDAKV	SEKGRLSPKA	SQVKRSPSDA	GRSSGDESKK
1050	1060	1070	1080	1090	1100	1110	1120
PLPSSSRTPT	ANANSFGFKK	QSGSAAGLAM	ITASGVTVTS	RSATLGKIPK	SSALVSRSAG	RKSSMDGAQN	QDDGYLALSS
1130	1140	1150	1160	1170	1180	1190	1200
RTNLQYRSLP	RPSKSNSRNG	AGNRSSTSSI	DSNISSKSAG	LPVPKLREPS	KTALGSSLPG	LVNQTDKEKG	ISSDNESVAS
1210	1220	1230	1240	1250	1260	1270	1280
CNSVKVNPAA	QPVSSPAQTS	LQPGAKYPDV	ASPTLRRLFG	GKPTKQVPIA	TAENMKNSVV	ISNPHATMTQ	QGNLDSPSGS
1290	1300	1310	1320	1330	1340	1350	1360
GVLSSGSSSP	LYSKNVDLNQ	SPLASSPSSA	HSAPSNSLTW	GTNASSSSAV	SKDGLGFQSV	SSLHTSCESI	DISLSSGGVP
1370	1380	1390	1400	1410	1420	1430	1440
SHNSSTGLIA	SSKDDSLTPF	VRTNSVKTTL	SESPLSSPAA	SPKFCRSTLP	RKQDSDPHLD	RNTLPKKGLR	YTPTSQLRTQ
1450	1460	1470	1480	1490	1500	1510	1520
EDAKEWLRSH	SAGGLQDTAA	NSPFSSGSSV	TSPSGTRFNF	SQLASPTTVT	QMSLSNPTML	RTHSLSNADG	QYDPYTDSRF
1530	1540	1550	1560	1570	1580	1590	1600
RNSSMSLDEK	SRTMSRSGSF	RDGFEEVHGS	SLSLVSSTSS	VYSTPEEKCQ	SEIRKLRREL	DASQEKVSAL	TTQLTANAHL
1610	1620	1630	1640	1650	1660	1670	1680
VAAFEQSLGN	MTIRLQSLTM	TAEQKDSELN	ELRKTIELLK	KQNAAAQAAI	NGVINTPELN	CKGNGTAQSA	DLRIRRQHSS
1690	1700	1710	1720	1730	1740	1750	1760
DSVSSINSAT	SHSSVGSNIE	SDSKKKKRKN	WLRSSFKQAF	GKKKSPKSAS	SHSDIEEMTD	SSLPSSPKLP	HNGSTGSTPL
1770	1780	1790	1800	1810	1820	1830	1840
LRNSHSNSLI	SECMDSEAET	VMQLRNELRD	KEMKLTDIRL	EALSSAHQLD	QLREAMNRMQ	SEIEKLKAEN	DRLKSESQGS
1850	1860	1870	1880	1890	1900	1910	1920
GCSRAPSQVS	ISASPRQSMG	LSQHSLNLTE	STSLDMLLDD	TGECSARKEG	GRHVKIVVSF	QEEMKWKEDS	RPHLFLIGCI
1930	1940	1950	1960	1970	1980	1990	2000
GVSGKTKWDV	LDGVVRRLFK	EYIIHVDPVS	QLGLNSDSVL	GYSIGEIKRS	NTSETPELLP	CGYLVGENTT	ISVTVKGLAE
2010	2020	2030	2040	2050	2060	2070	2080
NSLDSLVFES	LIPKPILQRY	VSLLIEHRRI	ILSGPSGTGK	TYLANRLSEY	IVLREGRELT	DGVIATFNVD	HKSSKELRQY
2090	2100	2110	2120	2130	2140	2150	2160
LSNLADQCNS	ENNAVDMPLV	IILDNLHHVS	SLGEIFNGLL	NCKYHKCPYI	IGTMNQATSS	TPNLQLHHNF	RWVLCANHTE
2170	2180	2190	2200	2210	2220	2230	2240

Detailed Protein Report

Cmpd.	No. of Cmpds.	m/z meas.	Δ m/z [ppm]	z	Rt [min]	Score	P	Range	Sequence	Modification
183	1	904.3719	-34.40	2	31.7	10.6	2	348-365	K.GGSKAGEGPGSRDTSCER.L	Carbamidomethyl: 16

Detailed Protein Report

Protein 307: PREDICTED: supervillin isoform X13 [Homo sapiens]

**Accession:**	gi|578818553	**Score:**	51.5
**Database:**	refseq_human(refseq_human_20140103.fasta)	**MW [kDa]:**	217.6
**Database Date:**	2015-11-30	**pI:**	6.6
**Modification(s):**	Oxidation	**Sequence Coverage [%]:**	3.4
		**No. of unique Peptides:**	3

Quantitation

Q:W	**Median:** 1.81	**CV:** 0.00 %	**No. of Peptides:** 1

Detailed Protein Report

```
        10         20         30         40         50         60         70         80
MKPNVFGREK SDFNKKNLSF LGFPRQRKVS SFQKEFSLED KEQLANHERG IDAQLLVALP KVAELRQIFE PKKKEFLEMK
        90        100        110        120        130        140        150        160
RKERIARRLE GIENDTQPIL LQSCTGLVTH RLLEEDTPRY MRASDPASPH IGRSNEEEET SDSSLEKQTR SKYCTETSGV
       170        180        190        200        210        220        230        240
HGDSPYGSGT MDTHSLESKA ERIARYKAER RRQLAEKYGL TLDPEADSEY LSRYTKSRKE PDAVEKRGGK SDKQEESSRD
       250        260        270        280        290        300        310        320
ASSLYPGTET MGLRTCAGES KDYALHVGDG SSDPEVLLNI ENQRRGQELS ATRQAHDLSP AAESSSTFSF SGRDSSFTEV
       330        340        350        360        370        380        390        400
PRSPKHAHSS SLQQAASRSP SFGDPQLSPE ARPRCTSHSE TPTVDDEEKV DERAKLSVAA KRLLFREMEK SFDEQNVPKR
       410        420        430        440        450        460        470        480
RSRNTAVEQR LRRLQDRSLT QPITTEEVVI AATEPIPASC SGGTHPVMAR LPSPTVARSA VQPARLQASA HQKALAKDQT
       490        500        510        520        530        540        550        560
NEGKELAEQG EPDSSTLSLA EKLALFNKLS QPVSKAISTR NRIDTRQRRM NARYQTQPVT LGEVEQVQSG KLIPFSPAVN
       570        580        590        600        610        620        630        640
TSVSTVASTV APMYAGDLRT KPPLDHNASA TDYKFSSSIE NSDSPVRSIL KSQAWQPLVE GSENKGMLRE YGETESKRAL
       650        660        670        680        690        700        710        720
TGRDSGMEKY GSFEEAEASY PILNRAREGD SHKESKYAVP RRGSLERANP PITHLGDEPK EFSMAKMNAQ GNLDLRDRLP
       730        740        750        760        770        780        790        800
FEEKVEVENV MKRKFSLRAA EFGEPTSEQT GTAAGKTIAQ TTAPVSWKPQ DSSEQPQEKL CKNPCAMFAA GEIKTPTGEG
       810        820        830        840        850        860        870        880
LLDSPSKTMS IKERLALLKK SGEEDWRNRL SRRQEGGKAP ASSLHTQEAG RSLIKKEEGG VADDSAISNL LWEPVYASTY
       890        900        910        920        930        940        950        960
SPAIPAAHKY LSFVSINQRV TESRESQMTI EERKQLITVR EEAWKTRGRG AANDSTQFTV AGRMVKKGLA SPTAITPVAS
       970        980        990       1000       1010       1020       1030       1040
PICGKTRGTT PVSKPLEDIE ARPDMQLESD LKLDRLETFL RRLNNKVGGM HETVLTVTGK SVKEVMKPDD DETFAKFYRS
      1050       1060       1070       1080       1090       1100       1110       1120
VDYNMPRSPV EMDEDFDVIF DPYAPKLTSS VAEHKRAVRP KRRVQASKNP LKMLAAREDL LQEYTEQRLN VAFMESKRMK
      1130       1140       1150       1160       1170       1180       1190       1200
VEKMSSNSNF SEVTLAGLAS KENFSNVSLR SVNLTEQNSN NSAVPYKRLM LLQIKGRRHV QTRLVEPRAS ALNSGDCFLL
      1210       1220       1230       1240       1250       1260       1270       1280
LSPHCCFLWV GEFANVIEKA KASELATLIQ TKRELGCRAT YIQTIEEGIN THTHAAKDFW KLLGGQTSYQ SAGDPKEDEL
      1290       1300       1310       1320       1330       1340       1350       1360
YEAAIIETNC IYRLMDDKLV PDDDYWGKIP KCSLLQPKEV LVFDFGSEVY VWHGKEVTLA QRKIAFQLAK HLWNGTFDYE
      1370       1380       1390       1400       1410       1420       1430       1440
NCDINPLDPG ECNPLIPRKG QGRPDWAIFG RLTEHNETIL FKEKFLDWTE LKRSNEKNPG ELAQHKEDPR TDVKAYDVTR
      1450       1460       1470       1480       1490       1500       1510       1520
MVSMPQTTAG TILDGVNVGR GYGLVEGHDR RQFEITSVSV DVWHILEFDY SRLPKQSIGQ FHEGDAYVVK WKFMVSTAVG
      1530       1540       1550       1560       1570       1580       1590       1600
SRQKGEHSVR AAGKEKCVYF FWQGRHSTVS EKGTSALMTV ELDEERGAQV QVLQGKEPPC FLQCFQGGMV VHSGRREEEE
      1610       1620       1630       1640       1650       1660       1670       1680
ENVQSEWRLY CVRGEVPVEG NLLEVACHCS SLRSRTSMVV LNVNKALIYL WHGCKAQAHT KEVGRTAANK IKEQCPLEAG
      1690       1700       1710       1720       1730       1740       1750       1760
LHSSSKVTIH ECDEGSEPLG FWDALGRRDR KAYDCMLQDP GSFNFAPRLF ILSSSSGDFA ATEFVYPARA PSVVSSMPFL
      1770       1780       1790       1800       1810       1820       1830       1840
QEDLYSAPQP ALFLVDNHHE VYLWQGWWPI ENKITGSARI RWASDRKSAM ETVLQYCKGK NLKKPAPKSY LIHAGLEPLT
      1850       1860       1870       1880       1890       1900       1910       1920
FTNMFPSWEH REDIAEITEM DTEVSNQITL VEDVLAKLCK TIYPLADLLA RPLPEGVDPL KLEIYLTDED FEFALDMTRD
      1930       1940       1950
EYNALPAWKQ VNLKKAKGLF
```

Cmpd.	No. of Cmpds.	m/z meas.	Δ m/z [ppm]	z	Rt [min]	Score	P	Range	Sequence	Modification	Ratios
874	1	903.4160	-143.87	2	40.5	11.0	1	50-66	R.GIDAQLLVALPKVAELR.Q		
2793	1	846.4331	0.98	2	66.1	19.3	1	323-338	R.SPKHAHSSSLQQAASR.S		Q:W 1.81
2904	1	974.3155	-109.14	2	65.1	10.9	0	1712-	K.AYDCMLQDPGSFNFAPR.L	Oxidation: 5	

Detailed Protein Report

Cmpd.	No. of Cmpds.	m/z meas.	Δ m/z [ppm]	z	Rt [min]	Score	P	Range	Sequence	Modification	Ratios
								1728			

Detailed Protein Report

Protein 308: elongation factor 1-alpha 2 [Homo sapiens]

Accession: gi|4503475
Database: refseq_human(refseq_human_20140103.fasta)
Database Date: 2015-11-30
Modification(s): Oxidation

Score: 51.4
MW [kDa]: 50.4
pI: 9.7
Sequence Coverage [%]: 4.3
No. of unique Peptides: 2

Quantitation

Q:W **Median:** 0.76 **CV:** 0.00 % **No. of Peptides:** 1

10	20	30	40	50	60	70	80
MGKEKTHINI	VVIGHVDSGK	STTTGHLIYK	CGGIDKRTIE	KFEKEAAEMG	KGSFKYAWVL	DKLKAERERG	ITIDISLWKF
90	100	110	120	130	140	150	160
ETTKYYITII	DAPGHRDFIK	NMITGTSQAD	CAVLIVAAGV	GEFEAGISKN	GQTREHALLA	YTLGVKQLIV	GVNKMDSTEP
170	180	190	200	210	220	230	240
AYSEKRYDEI	VKEVSAYIKK	IGYNPATVPF	VPISGWHGDN	MLEPSPNMPW	FKGWKVERKE	GNASGVSLLE	ALDTILPPTR
250	260	270	280	290	300	310	320
PTDKPLRLPL	QDVYKIGGIG	TVPVGRVETG	ILRPGMVVTF	APVNITTEVK	SVEMHHEALS	EALPGDNVGF	NVKNVSVKDI
330	340	350	360	370	380	390	400
RRGNVCGDSK	SDPPQEAAQF	TSQVIILNHP	GQISAGYSPV	IDCHTAHIAC	KFAELKEKID	RRSGKKLEDN	PKSLKSGDAA
410	420	430	440	450	460	470	
IVEMVPGKPM	CVESFSQYPP	LGRFAVRDMR	QTVAVGVIKN	VEKKSGGAGK	VTKSAQKAQK	AGK	

Cmpd.	No. of Cmpds.	m/z meas.	Δ m/z [ppm]	z	Rt [min]	Score	P	Range	Sequence	Modification	Ratios
2741	3	715.3112	-7.90	2	62.7	23.2	1	155-166	K.MDSTEPAYSEKR.Y	Oxidation: 1	
668	1	488.2507	-58.30	2	36.9	28.2	0	248-255	R.LPLQDVYK.I		Q:W 0.76

Detailed Protein Report

Protein 309: protein FAM115C isoform B [Homo sapiens]

Accession: gi|293651621
Database: refseq_human(refseq_human_20140103.fasta)
Database Date: 2015-11-30
Modification(s): Oxidation

Score: 51.4
MW [kDa]: 90.6
pI: 8.9
Sequence Coverage [%]: 3.8
No. of unique Peptides: 3

Quantitation

M:Q **Median:** 1.24 **CV:** 0.00 % **No. of Peptides:** 1

10	20	30	40	50	60	70	80
MKIFQKEFKF	ILKKCTPIQK	YREENGNSSS	SPHHQLQSHA	SGPRYGEDVR	QDQQQLLEGI	SELDIRTGGV	PSQLLVHGAL
**90**	**100**	**110**	**120**	**130**	**140**	**150**	**160**
AFPLGLDASL	NCFLAAAHYG	RGRVVLAAHE	CLLCAPKMGP	FLLNAVRWLA	RGQTGKVGVN	TNLKDLCPLL	SEHGLQCSLE
**170**	**180**	**190**	**200**	**210**	**220**	**230**	**240**
PHLNSDLCVY	CCKAYSDKEA	KQLQEFVAEG	GGLLIGGQAW	WWASQNPGHC	PLAGFPGNII	LNCFGLSILP	QTLKAGCFPV
**250**	**260**	**270**	**280**	**290**	**300**	**310**	**320**
PTPEMRSYHF	RKALSQFQAI	LNHENGNLEK	SCLAKLRVDG	AAFLQIPAEG	VPAYISLHRL	LRKMLRGSGL	PAVSRENPVA
**330**	**340**	**350**	**360**	**370**	**380**	**390**	**400**
SDSYEAAVLS	LATGLAHSGT	DCSQLAQGLG	TWTCSSSLYP	SKHPITVEIN	GINPESHSVI	QVGMQWRDLS	SCNLHLLGLS
**410**	**420**	**430**	**440**	**450**	**460**	**470**	**480**
NSSLSASCVA	GTTGTRHHAW	LIFVFLVERE	FHRKGNNDCW	VSTGLYLLEG	QNAEVSLSEA	AASAGLRVQI	GCHTDDLTKA
**490**	**500**	**510**	**520**	**530**	**540**	**550**	**560**
RKLSRAPVVT	HQCWMDRTER	SVSCLWGGLL	YVIVPKGSQL	GPVPVTIRGA	VPAPYYKLGK	TSLEEWKRQM	QENLAPWGEL
**570**	**580**	**590**	**600**	**610**	**620**	**630**	**640**
ATDNIILTVP	TTNLQALKDP	EPVLRLWDEM	MQAVARLAAE	PFPFRRPERI	VADVQISAGW	MHSGYPIMCH	LESVKEIINE
**650**	**660**	**670**	**680**	**690**	**700**	**710**	**720**
MDMRSRGVWG	PIHELGHNQQ	RHGWEFPPHT	TEATCNLWSV	YVHETVLGIP	RAQAHEALSP	PERERRIKAH	LGKGAPLCDW
**730**	**740**	**750**	**760**	**770**	**780**	**790**	**800**
NVWTALETYL	QLQEAFGWEP	FTQLFAEYQT	LSHLPKDNTG	RMNLWVKKFS	EKVKKNLVPF	FEAWGWPIQK	EVADSLASLP
**810**	**820**						
EWQENPMQVY	LRARK						

Cmpd.	No. of Cmpds.	m/z meas.	Δ m/z [ppm]	z	Rt [min]	Score	P	Range	Sequence	Modification	Ratios
126	1	822.3680	-116.44	2	30.8	17.1	1	118-131	K.MGPFLLNAVRWLAR.G		
495	2	705.1921	-194.39	2	35.1	10.8	1	636-646	K.EIINEMDMRSR.G	Oxidation: 6	M:Q 1.24
738	2	705.1981	-185.90	2	37.8	13.4	1	636-646	K.EIINEMDMRSR.G	Oxidation: 8	

Detailed Protein Report

Protein 310: **PREDICTED: unhealthy ribosome biogenesis protein 2 homolog isoform X1 [Homo sapiens]**

**Accession:**	gi|530366678	**Score:**	51.3
**Database:**	refseq_human(refseq_human_20140103.fasta)	**MW [kDa]:**	166.5
**Database Date:**	2015-11-30	**pI:**	6.9
		**Sequence Coverage [%]:**	2.6
		**No. of unique Peptides:**	1

Quantitation

M:Q **Median:** 1.55 **CV:** 0.00 % **No. of Peptides:** 1

10	20	30	40	50	60	70	80
MAAVYSGISL	KLKSKTTSWE	DKLKLAHFAW	ISHQCFLPNK	EQVLLDWARQ	SLVAFYKKKL	ELKEDIVERL	WIYIDNILHS
90	100	110	120	130	140	150	160
RKLQNLLKNG	KTINLQISLV	KIINERVAEF	SLSGSQRNIC	AVLRCCQGIL	STPALAVIYT	AKQELMVALL	SQLCWSACRQ
170	180	190	200	210	220	230	240
PEGAVVAQLF	EVIHLALGHY	LLILQQQVNP	RRAFGDVTAH	LLQPCLVLRH	LLSGGTWTQA	GQGQLRQVLS	RDIRSQIEAM
250	260	270	280	290	300	310	320
FRGGIFQPEL	LSSYKEGLLD	QQQGDVKTGA	MKNLLAPMDT	VLNRLVDAGY	CAASLHTSVV	ANSVALLYKL	FLDSYFKEGN
330	340	350	360	370	380	390	400
QLLCFQVLPR	LFGCLKISHL	QEEQSKALST	SDWTTELLVV	EQLLNSVANN	NIYNIAADRI	RHEEAQFRFY	RHVAELLINH
410	420	430	440	450	460	470	480
AQAPIPAWFR	CLKTLISLNH	LILEPDLDDL	LASAWIDAEV	TEFRTKKAQE	ALIRTVFQTY	AKLRQVPRLF	EEVLGVICRP
490	500	510	520	530	540	550	560
AAEALRQPVL	ASGPSTVLSA	CLLELPPSQI	LDTWSLVLEK	FQSLVLPYLQ	SDADMALKSL	SLSLLLHCIM	FNMRSLDSST
570	580	590	600	610	620	630	640
PLPIVRRTQC	MMERMMRELV	QPLLALLPDT	PGPEPELWLQ	KVSDSVLLLS	YTWAQVDAMF	SLNCSQYHSM	SGPLIGVALE
650	660	670	680	690	700	710	720
ISNLPSLLPG	VKTQHWKKIE	KFTAQFSSLG	TYCLEQLYLQ	KMKRTLMQTS	FRSEGAIQSL	RCDAAFIIGS	GRKSLNQRTT
730	740	750	760	770	780	790	800
ASWDGQVGMV	SGLTYPVAHW	HLIVSNLTIL	ISYLCPDDVG	YLASVLLRTL	PMGKAQEVSI	DEEAYITLEK	ISKAFLHSPL
810	820	830	840	850	860	870	880
FPEMQSLHSA	FLTCVTTSCS	SILCSGAQRD	SGLVSQQLPW	LFEKDHMVVG	HWENRFAKAG	PEGIEPRGEI	AQNLLSLVKS
890	900	910	920	930	940	950	960
DFPIQLEGEQ	LESILGLLEV	ISALQLDSLL	PPYHVHYFLV	LLSMAVTKLG	CSCSSSLALK	FLTTCYQLLG	YLQKGKSARS
970	980	990	1000	1010	1020	1030	1040
VFKIMYGSDI	FEVVLTSLFR	ASSRFLIEMD	DPAWLEFLQV	IGTFLEELMQ	MLIQMKLSLV	LNFRKITAFL	SSSKPYTEAA
1050	1060	1070	1080	1090	1100	1110	1120
SSKQLENQNP	QGRQLLLVSL	TRLCHVLGPF	LKEQKLGQEA	PAALSELLQQ	VVLQTGAVLQ	LCSVPGARGW	RLPSVLISSV
1130	1140	1150	1160	1170	1180	1190	1200
STLLEADLGQ	HCRDGGADIS	QGSDRTLLSH	VALYQGVYSQ	ILLELPALAG	HDQSFQAALQ	FLTLFFLAPE	LHPKKDSVFT
1210	1220	1230	1240	1250	1260	1270	1280
SMFHSVRRVL	ADPEIPVQVT	QDIEPHLGAL	FTQMLEVGTT	EDLRLVMQCI	LQGLDVSNMW	KADVQLLNRE	ASQEQPVSLT
1290	1300	1310	1320	1330	1340	1350	1360
VVGPVLDVLA	ALLRQGEEAI	GNPHHVSLAF	SILLTVPLDH	LKPLEYGSVF	PRLHNVLFSI	LQCHPKVMLK	AIPSFLNSFN
1370	1380	1390	1400	1410	1420	1430	1440
RLVFSVMREG	RQKDKGSIDD	LPTVLKCARL	VERMYSHIAA	RAEEFAVFSP	FMVAQYVLEV	QKVTLYPAVK	SLLQEGIYLI
1450	1460	1470	1480	1490			
LDLCIEPDVQ	FLRASLQPGM	RDIFKELYND	YLKYHKAKHE	GEKRYTA			

Cmpd.	No. of Cmpds.	m/z meas.	Δ m/z [ppm]	z	Rt [min]	Score	P	Range	Sequence	Modification	Ratios
598	1	661.8686	26.39	2	36.8	13.9	2	12-22	K.LKSKTTSWEDK.L		M:Q 1.55

Detailed Protein Report

Protein 311: **PREDICTED: macrophage mannose receptor 1 [Homo sapiens]**

**Accession:**	gi|578844889	**Score:**	51.3
**Database:**	refseq_human(refseq_human_20140103.fasta)	**MW [kDa]:**	88.9
**Database Date:**	2015-11-30	**pI:**	5.9
		**Sequence Coverage [%]:**	6.4
		**No. of unique Peptides:**	1

10	20	30	40	50	60	70	80
MGLELISEYT	RQFLIYNEDH	KRCVDAVSPS	AVQTAACNQD	AESQKFRWVS	ESQIMSVAFK	LCLGVPSKTD	WVAIPLYACD
**90**	**100**	**110**	**120**	**130**	**140**	**150**	**160**
SKSEFQKWEC	KNDTLLGIKG	EDLFFNYGNR	QEKNIMLYKG	SGLWSRWKIY	GTTDNLCSRG	YEAMYTLLGN	ANGATCAFPF
**170**	**180**	**190**	**200**	**210**	**220**	**230**	**240**
KFENKWYADC	TSAGRSDGWL	WCGTTTDYDT	DKLFGYCPLK	FEGSESLWNK	DPLTSVSYQI	NSKSALTWHQ	ARKSCQQQNA
**250**	**260**	**270**	**280**	**290**	**300**	**310**	**320**
ELLSITEIHE	QTYLTGKDMK	SRNVLGHENN	PAAAYEQFLG	YSVSTYPGKF	TVEDNNGIRT	EPNDELWIGL	NDIKIQMYFE
**330**	**340**	**350**	**360**	**370**	**380**	**390**	**400**
WSDGTPVTFT	KWLRGEPSHE	NNRQEDCVVM	KGKDGYWADR	GCEWPLGYIC	KMKSRSQGPE	IVEVEKGCRK	DKHQNLSQHQ
**410**	**420**	**430**	**440**	**450**	**460**	**470**	**480**
LLKTWKYVNR	NDAQSAYFIG	LLISLDKKFA	WMDGSKVDYV	SWATGEPNFA	NEDENCVTMY	SNSGFWNDIN	CGYPNAFICQ
**490**	**500**	**510**	**520**	**530**	**540**	**550**	**560**
RHNSSINATT	VMPTMPSVPS	GCKEGWNFYS	NKADCVVIIG	GASNEAGKWM	DDTCDSKRGY	ICQTRSEIPA	TEPPQLPGRC
**570**	**580**	**590**	**600**	**610**	**620**	**630**	**640**
PESDHTAWIP	FHGHCYYIES	SYTRNWGQAS	LECLRMGSSL	VSIESAAESS	FLSYRVEPLK	SKTNFWIGLF	RNVEGTWLWI
**650**	**660**	**670**	**680**	**690**	**700**	**710**	**720**
NNSPVSFVNW	NTGDPSGERN	DCVALHASSG	FWSNIHCSSY	KGYICKRPKI	IDAKPTHELL	TTKADTRKMD	PSKPSSNVAG
**730**	**740**	**750**	**760**	**770**	**780**	**790**	
VVIIVILLIL	TGAGLAAYFF	YKKRRVHLPQ	EGAFENTLYF	NSQSSPGTSD	MKDLVGNIEQ	NEHSVI	

Cmpd.	No. of Cmpds.	m/z meas.	Δ m/z [ppm]	z	Rt [min]	Score	P	Range	Sequence	Modification
2567	1	673.3649	-8.13	2	62.8	10.6	0	393-403	K.HQNLSQHQLLK.T	

Detailed Protein Report

Protein 312: ankyrin repeat domain-containing protein 12 isoform 2 [Homo sapiens]

**Accession:**	gi|134948605	**Score:**	51.3
**Database:**	refseq_human(refseq_human_20140103.fasta)	**MW [kDa]:**	232.9
**Database Date:**	2015-11-30	**pI:**	6.6
**Modification(s):**	Oxidation	**Sequence Coverage [%]:**	3.0
		**No. of unique Peptides:**	3

Quantitation

Q:W	**Median:**	1.54	**CV:**	0.00 %	**No. of Peptides:**	1
M:Q	**Median:**	1.54	**CV:**	0.00 %	**No. of Peptides:**	1

Alias proteins:

Accession	Name	Description
gi|323362985	refseq_human (refseq_human_20140103.fasta)	ankyrin repeat domain-containing protein 12 isoform 2 [Homo sapiens]

Detailed Protein Report

10	20	30	40	50	60	70	80
MPKSGFTKPI	QSENSDSDSN	MVEKPYGRKS	KDKIASYSKT	PKIERSDVSK	EMKEKSSMKR	KLPFTISPSR	NEERDSDTEK
90	100	110	120	130	140	150	160
EGPEKKKTKK	EAGNKKSTPV	SILFGYPLSE	RKQMALLMQM	TARDNSPDST	PNHPSQTTPA	QKKTPSSSSR	QKDKVNKRNE
170	180	190	200	210	220	230	240
RGETPLHMAA	IRGDVKQVKE	LISLGANVNV	KDFAGWTPLH	EACNVGYYDV	AKILIAAGAD	VNTQGLDDDT	PLHDSASSGH
250	260	270	280	290	300	310	320
RDIVKLLLRH	GGNPFQANKH	GERPVDVAET	EELELLLKRE	VPLSDDDESY	TDSEEAQSVN	PSSVDENIDS	ETEKDSLICE
330	340	350	360	370	380	390	400
SKQILPSKTP	LPSALDEYEF	KDDDDEEINK	MIDDRHILRK	EQRKENEPEA	EKTHLFAKQE	KAFYPKSFKS	KKQKPSRVLY
410	420	430	440	450	460	470	480
SSTESSDEEA	LQNKKISTSC	SVIPETSNSD	MQTKKEYVVS	GEHKQKGKVK	RKLKNQNKNK	ENQELKQEKE	GKENTRITNL
490	500	510	520	530	540	550	560
TVNTGLDCSE	KTREEGNFRK	SFSPKDDTSL	HLFHISTGKS	PKHSCGLSEK	QSTPLKQEHT	KTCLSPGSSE	MSLQPDLVRY
570	580	590	600	610	620	630	640
DNTESEFLPE	SSSVKSCKHK	EKSKHQKDFH	LEFGEKSNAK	IKDEDHSPTF	ENSDCTLKKM	DKEGKTLKKH	KLKHKEREKE
650	660	670	680	690	700	710	720
KHKKEIEGEK	EKYKTKDSAK	ELQRSVEFDR	EFWKENFFKS	DETEDLFLNM	EHESLTLEKK	SKLEKNIKDD	KSTKEKHVSK
730	740	750	760	770	780	790	800
ERNFKEERDK	IKKESEKSFR	EEKIKDLKEE	RENIPTDKDS	EFTSLGMSAI	EESIGLHLVE	KEIDIEKQEK	HIKESKEKPE
810	820	830	840	850	860	870	880
KRSQIKEKDI	EKMERKTFEK	EKKIKHEHKS	EKDKLDLSEC	VDKIKEKDKL	YSHHTEKCHK	EGEKSKNTAA	IKKTDDREKS
890	900	910	920	930	940	950	960
REKMDRKHDK	EKPEKERHLA	ESKEKHLMEK	KNKQSDNSEY	SKSEKGKNKE	KDRELDKKEK	SRDKESINIT	NSKHIQEEKK
970	980	990	1000	1010	1020	1030	1040
SSIVDGNKAQ	HEKPLSLKEK	TKDEPLKTPD	GKEKDKKDKD	IDRYKERDKH	KDKIQINSLL	KLKSEADKPK	PKSSPASKDT
1050	1060	1070	1080	1090	1100	1110	1120
RPKEKRLVND	DLMQTSFERM	LSLKDLEIEQ	WHKKHKEKIK	QKEKERLRNR	NCLELKIKDK	EKTKHTPTES	KNKELTRSKS
1130	1140	1150	1160	1170	1180	1190	1200
SEVTDAYTKE	KQPKDAVSNR	SQSVDTKNVM	TLGKSSFVSD	NSLNRSPRSE	NEKPGLSSRS	VSMISVASSE	DSCHTTVTTP
1210	1220	1230	1240	1250	1260	1270	1280
RPPVEYDSDF	MLESSESQMS	FSQSPFLSIA	KSPALHEREL	DSLADLPERI	KPPYANRLST	SHLRSSSVED	VKLIISEGRP
1290	1300	1310	1320	1330	1340	1350	1360
TIEVRRCSMP	SVICEHTKQF	QTISEESNQG	SLLTVPGDTS	PSPKPEVFSN	VPERDLSNVS	NIHSSFATSP	TGASNSKYVS
1370	1380	1390	1400	1410	1420	1430	1440
ADRNLIKNTA	PVNTVMDSPV	HLEPSSQVGV	IQNKSWEMPV	DRLETLSTRD	FICPNSNIPD	QESSLQSFCN	SENKVLKENA
1450	1460	1470	1480	1490	1500	1510	1520
DFLSLRQTEL	PGNSCAQDPA	SFMPPQQPCS	FPSQSLSDAE	SISKHMSLSY	VANQEPGILQ	QKNAVQIISS	ALDTDNESTK
1530	1540	1550	1560	1570	1580	1590	1600
DTENTFVLGD	VQKTDAFVPV	YSDSTIQEAS	PNFEKAYTLP	VLPSEKDFNG	SDASTQLNTH	YAFSKLTYKS	SSGHEVENST
1610	1620	1630	1640	1650	1660	1670	1680
TDTQVISHEK	ENKLESLVLT	HLSRCDSDLC	EMNAGMPKGN	LNEQDPKHCP	ESEKCLLSIE	DEESQQSILS	SLENHSQQST
1690	1700	1710	1720	1730	1740	1750	1760
QPEMHKYGQL	VKVELEENAE	DDKTENQIPQ	RMTRNKANTM	ANQSKQILAS	CTLLSEKDSE	SSSPRGRIRL	TEDDDPQIHH
1770	1780	1790	1800	1810	1820	1830	1840
PRKRKVSRVP	QPVQVSPSLL	QAKEKTQQSL	AAIVDSLKLD	EIQPYSSERA	NPYFEYLHIR	KKIEEKRKLL	CSVIPQAPQY
1850	1860	1870	1880	1890	1900	1910	1920
YDEYVTFNGS	YLLDGNPLSK	ICIPTITPPP	SLSDPLKELF	RQQEVVRMKL	RLQHSIEREK	LIVSNEQEVL	RVHYRAARTL
1930	1940	1950	1960	1970	1980	1990	2000
ANQTLPFSAC	TVLLDAEVYN	VPLDSQSDDS	KTSVRDRFNA	RQFMSWLQDV	DDKFDKLKTC	LLMRQQHEAA	ALNAVQRLEW
2010	2020	2030	2040				
QLKLQELDPA	TYKSISIYEI	QEFYVPLVDV	NDDFELTPI				

Cmpd.	No. of Cmpds.	m/z meas.	Δ m/z [ppm]	z	Rt [min]	Score	P	Range	Sequence	Modification	Ratios

Detailed Protein Report

Cmpd.	No. of Cmpds.	m/z meas.	Δ m/z [ppm]	z	Rt [min]	Score	P	Range	Sequence	Modification	Ratios	
468	1	572.6830	-230.04	2	34.3	15.5	2	717-725	K.HVSKERNFK.E		M:Q	1.54
110	1	1083.4576	-80.32	2	31.3	13.6	2	1135-1154	K.DAVSNRSQSVDTKNVMTLGK.S	Oxidation: 16		
623	1	824.1088	-20.46	3	37.4	10.9	2	1265-1286	R.SSSVEDVKLIISEGRPTIEVRR.C			

Detailed Protein Report

Protein 313: uncharacterized protein C9orf172 [Homo sapiens]

Accession: gi|148762978
Database: refseq_human(refseq_human_20140103.fasta)
Database Date: 2015-11-30

Score: 51.2
MW [kDa]: 106.6
pI: 10.2
Sequence Coverage [%]: 5.0
No. of unique Peptides: 2

10	20	30	40	50	60	70	80
MTRTDPPDLL	VSTVYQDIKV	ATPGPASKCS	PCERSVARPA	EPAPFNKRHC	RSFDFLEALD	GPAMETLPEP	PPPESAVPRA
90	100	110	120	130	140	150	160
RTREAEPRRR	ARSKSAPRAP	PGLTPAPASP	PVLPRRGREA	QRAARAEASP	RREPAYPALR	ALANELHPIK	LQPQRGGPGR
170	180	190	200	210	220	230	240
VAPLCAAAGR	CAPPEPPAGP	APHVRCRLDI	KPDDAVLQHA	TRGSRSCGPT	EAAHWARPAP	QFHGLTVPGP	RHMALSRTPT
250	260	270	280	290	300	310	320
PSDSYCADPR	AFYCDGPLPG	PRDYAERRSL	PFTTPPGPTQ	FFYTEEPQGF	RGSFAASPGP	TFDAYYPRPY	PSEELSGPSP
330	340	350	360	370	380	390	400
RRMGGYYAGE	VRTFPIQEPP	SRSYYGEAPR	AYGLPYGPRY	VPEEPRAHST	ARPFYTEDFG	RYRERDVLAR	TYPHPRSSPA
410	420	430	440	450	460	470	480
WADWGPRPYR	TLQVVPPSDP	DPLLASWHGG	TGTSPPRLAT	DSRHYSRSWD	NILAPGPRRE	DPLGRGRSYE	NLLGREVREP
490	500	510	520	530	540	550	560
RGVSPEGRRP	PVVVNLSTSP	RRYAALSLSE	TSLTEKGRAG	EGLGRNWYVT	PEITITDNDL	RATERPSARA	WELPGGRTRP
570	580	590	600	610	620	630	640
PPHAAPDGPT	SGRQRSLEQL	DELITDLVID	SRPTAGQASE	PAADCLGPQL	RRLLDSRPAG	SGAPALAPPR	SPPASAGSAE
650	660	670	680	690	700	710	720
EPAAPGEAAD	ASPEPSADED	DLMTCSNARC	RRTETMFNAC	LYFKSCHSCY	TYYCSRLCRR	EDWDAHKARC	VYGRVGSVCR
730	740	750	760	770	780	790	800
HVLQFCRDSG	PVHRAFSRIA	RVGFLSRGRG	VLFLGFPSPG	SADNFLRFGL	EGLLLSPTYL	SLRELATHAA	PLGSYARELA
810	820	830	840	850	860	870	880
AAGRLYEPAE	CFLLSVSVAV	GPGTAPPGTP	ALPAPAPRSH	GPTVRKFAKV	ALAAGSPARP	PPARSREPDM	ETLILTPPPG
890	900	910	920	930	940	950	960
TAGLDQDGEA	GRRAREVAFI	HIQRELRLRG	VFLRHEFPRV	YEQLCEFVEA	NRRFTPTTIY	PTDRRTGRPF	MCMIMAASEP
970	980						
RALDWVASAN	LLDDIM						

Cmpd.	No. of Cmpds.	m/z meas.	Δ m/z [ppm]	z	Rt [min]	Score	P	Range	Sequence	Modification
2104	1	977.4132	-55.55	2	54.9	10.8	1	945-961	R.RTGRPFMCMIMAASEPR.A	
1808	4	599.9802	55.20	3	51.3	13.7	0	946-961	R.TGRPFMCMIMAASEPR.A	

Detailed Protein Report

Protein 314: **pogo transposable element with ZNF domain isoform 3 [Homo sapiens]**

**Accession:**	gi|302699211	**Score:**	51.1
**Database:**	refseq_human(refseq_human_20140103.fasta)	**MW [kDa]:**	145.2
**Database Date:**	2015-11-30	**pI:**	6.8
**Modification(s):**	Carbamidomethyl, Oxidation	**Sequence Coverage [%]:**	2.8
		**No. of unique Peptides:**	4

Quantitation

M:Q **Median:** 0.55 **CV:** 0.00 % **No. of Peptides:** 1

10	20	30	40	50	60	70	80
MADTDLFMEC	EEEELEPWQK	ISDVIEDSVV	EDYNSVDKTT	TVSVSQQPVS	APVPIAAHAS	VAGHLSTSTT	VSSSGAQNSD
**90**	**100**	**110**	**120**	**130**	**140**	**150**	**160**
STKKTLVTLI	ANNNAPGTQF	VKPTVGVPQV	FSQMTPVRPG	STMPVRPTTN	TFTTVIPATL	TIRSTVPQSQ	SQQTKSTPST
**170**	**180**	**190**	**200**	**210**	**220**	**230**	**240**
STTPTATQPT	SLGQLAVQSP	GQSNQTTNPK	LAPSFPSPPA	VSIASFVTVK	RPGVTGENSN	EVAKLVNTLN	TIPSLGQSPG
**250**	**260**	**270**	**280**	**290**	**300**	**310**	**320**
PVVVSNNSSA	HGSQRTSGPE	SSMKVTSSIP	VFDLQDGGRK	ICPRCNAQFR	VTEALRGHMC	YCCPEMVEYQ	KKGKSLDSEP
**330**	**340**	**350**	**360**	**370**	**380**	**390**	**400**
SVPSAAKPPS	PEKTAPVAST	PSSTPIPALS	PPTKVPEPNE	NVGDAVQTKL	IMLVDDFYYG	RDGGKVAQLT	NFPKVATSFR
**410**	**420**	**430**	**440**	**450**	**460**	**470**	**480**
CPHCTKRLKN	NIRFMNHMKH	HVELDQQNGE	VDGHTICQHC	YRQFSTPFQL	QCHLENVHSP	YESTTKCKIC	EWAFESEPLF
**490**	**500**	**510**	**520**	**530**	**540**	**550**	**560**
LQHMKDTHKP	GEMPYVCQVC	QYRSSLYSEV	DVHFRMIHED	TRHLLCPYCL	KVFKNGNAFQ	QHYMRHQKRN	VYHCNKCRLQ
**570**	**580**	**590**	**600**	**610**	**620**	**630**	**640**
FLFAKDKIEH	KLQHHKTFRK	PKQLEGLKPG	TKVTIRASRG	QPRTVPVSSN	DTPPSALQEA	APLTSSMDPL	PVFLYPPVQR
**650**	**660**	**670**	**680**	**690**	**700**	**710**	**720**
SIQKRAVRKM	SVMGRQTCLE	CSFEIPDFPN	HFPTYVHCSL	CRYSTCCSRA	YANHMINNHV	PRKSPKYLAL	FKNSVSGIKL
**730**	**740**	**750**	**760**	**770**	**780**	**790**	**800**
ACTSCTFVTS	VGDAMAKHLV	FNPSHRSSSI	LPRGLTWIAH	SRHGQTRDRV	HDRNVKNMYP	PPSFPTNKAA	TVKSAGATPA
**810**	**820**	**830**	**840**	**850**	**860**	**870**	**880**
EPEELLTPLA	PALPSPASTA	TPPPTPTHPQ	ALALPPLATE	GAECLNVDDQ	DEGSPVTQEP	ELASGGGGSG	GVGKKEQLSV
**890**	**900**	**910**	**920**	**930**	**940**	**950**	**960**
KKLRVVLFAL	CCNTEQAAEH	FRNPQRRIRR	WLRRFQASQG	ENLEGKYLSF	EAEEKLAEWV	LTQREQQLPV	NEETLFQKAT
**970**	**980**	**990**	**1000**	**1010**	**1020**	**1030**	**1040**
KIGRSLEGGF	KISYEWAVRF	MLRHHLTPHA	RRAVAHTLPK	DVAENAGLFI	DFVQRQIHNQ	DLPLSMIVAI	DEISLFLDTE
**1050**	**1060**	**1070**	**1080**	**1090**	**1100**	**1110**	**1120**
VLSSDDRKEN	ALQTVGTGEP	WCDVVLAILA	DGTVLPTLVF	YRGQMDQPAN	MPDSILLEAK	ESGYSDDEIM	ELWSTRVWQK
**1130**	**1140**	**1150**	**1160**	**1170**	**1180**	**1190**	**1200**
HTACQRSKGM	LVMDCHRTHL	SEEVLAMLSA	SSTLPAVVPA	GCSSKIQPLD	VCIKRTVKNF	LHKKWKEQAR	EMADTACDSD
**1210**	**1220**	**1230**	**1240**	**1250**	**1260**	**1270**	**1280**
VLLQLVLVWL	GEVLGVIGDC	PELVQRSFLV	ASVLPGPDGN	INSPTRNADM	QEELIASLEE	QLKLSGEHSE	SSTPRPRSSP
**1290**	**1300**	**1310**	**1320**				
EETIEPESLH	QLFEGESETE	SFYGFEEADL	DLMEI				

Cmpd.	No. of Cmpds.	m/z meas.	Δ m/z [ppm]	z	Rt [min]	Score	P	Range	Sequence	Modification	Ratios
284	2	939.2416	-177.99	1	33.2	15.6	0	256-264	R.TSGPESSMK.V	Oxidation: 8	
240	1	732.8262	-63.03	2	31.7	14.2	1	285-296	R.CNAQFRVTEALR.G	Carbamidomethyl: 1	M:Q 0.55
2117	1	990.8950	2.39	2	55.0	10.3	1	297-312	R.GHMCYCCPEMVEYQKK.G	Oxidation: 3, 10	
2186	1	1031.8206	-95.59	2	55.9	11.0	1	297-312	R.GHMCYCCPEMVEYQKK.G	Carbamidomethyl: 4, 6	

Detailed Protein Report

Protein 315: PREDICTED: DNA (cytosine-5)-methyltransferase 1 isoform X1 [Homo sapiens]

**Accession:**	gi|578833065	**Score:**	51.1
**Database:**	refseq_human(refseq_human_20140103.fasta)	**MW [kDa]:**	169.3
**Database Date:**	2015-11-30	**pI:**	8.8
**Modification(s):**	Carbamidomethyl	**Sequence Coverage [%]:**	3.9
		**No. of unique Peptides:**	2

Quantitation

M:Q **Median:** 1.52 **CV:** 0.00 % **No. of Peptides:** 1

10	20	30	40	50	60	70	80
MADANSPPKP	LSKPRTPRRS	KSDGEAKPEP	SPSPRITRKS	TRQTTITSHF	AKGPAKRKPQ	EESERAKSDE	SIKEEDKDQD
90	100	110	120	130	140	150	160
EKRRRVTSRE	RVARPLPAEE	PERAKSGTRT	EKEEERDEKE	EKRLRSQTKE	PTPKQKLKEE	PDREARAGVQ	ADEDEDGDEK
170	180	190	200	210	220	230	240
DEKKHRSQPK	DLAAKRRPEE	KEPEKVNPQI	SDEKDEDEKE	EKRRKTTPKE	PTEKKMARAK	TVMNSKTHPP	KCIQCGQYLD
250	260	270	280	290	300	310	320
DPDLKYGQHP	PDAVDEPQML	TNEKLSIFDA	NESGFESYEA	LPQHKLTCFS	VYCKHGHLCP	IDTGLIEKNI	ELFFSGSAKP
330	340	350	360	370	380	390	400
IYDDDPSLEG	GVNGKNLGPI	NEWWITGFDG	GEKALIGFST	SFAEYILMDP	SPEYAPIFGL	MQEKIYISKI	VVEFLQSNSD
410	420	430	440	450	460	470	480
STYEDLINKI	ETTVPPSGLN	LNRFTEDSLL	RHAQFVVEQV	ESYDEAGDSD	EQPIFLTPCM	RDLIKLAGVT	LGQRRAQARR
490	500	510	520	530	540	550	560
QTIRHSTREK	DRGPTKATTT	KLVYQIFDTF	FAEQIEKDDR	EDKENAFKRR	RCGVCEVCQQ	PECGKCKACK	DMVKFGGSGR
570	580	590	600	610	620	630	640
SKQACQERRC	PNMAMKEADD	DEEVDDNIPE	MPSPKKMHQG	KKKKQNKNRI	SWVGEAVKTD	GKKSYYKKVC	IDAETLEVGD
650	660	670	680	690	700	710	720
CVSVIPDDSS	KPLYLARVTA	LWEDSSNGQM	FHAHWFCAGT	DTVLGATSDP	LELFLVDECE	DMQLSYIHSK	VKVIYKAPSE
730	740	750	760	770	780	790	800
NWAMEGGMDP	ESLLEGDDGK	TYFYQLWYDQ	DYARFESPPK	TQPTEDNKFK	FCVSCARLAE	MRQKEIPRVL	EQLEDLDSRV
810	820	830	840	850	860	870	880
LYYSATKNGI	LYRVGDGVYL	PPEAFTFNIK	LSSPVKRPRK	EPVDEDLYPE	HYRKYSDYIK	GSNLDAPEPY	RIGRIKEIFC
890	900	910	920	930	940	950	960
PKKSNGRPNE	TDIKIRVNKF	YRPENTHKST	PASYHADINL	LYWSDEEAVV	DFKAVQGRCT	VEYGEDLPEC	VQVYSMGGPN
970	980	990	1000	1010	1020	1030	1040
RFYFLEAYNA	KSKSFEDPPN	HARSPGNKGK	GKGKGKGKPK	SQACEPSEPE	IEIKLPKLRT	LDVFSGCGGL	SEGFHQAGIS
1050	1060	1070	1080	1090	1100	1110	1120
DTLWAIEMWD	PAAQAFRLNN	PGSTVFTEDC	NILLKLVMAG	ETTNSRGQRL	PQKGDVEMLC	GGPPCQGFSG	MNRFNSRTYS
1130	1140	1150	1160	1170	1180	1190	1200
KFKNSLVVSF	LSYCDYYRPR	FFLLENVRNF	VSFKRSMVLK	LTLRCLVRMG	YQCTFGVLQA	GQYGVAQTRR	RAIILAAAPG
1210	1220	1230	1240	1250	1260	1270	1280
EKLPLFPEPL	HVFAPRACQL	SVVVDDKKFV	SNITRLSSGP	FRTITVRDTM	SDLPEVRNGA	SALEISYNGE	PQSWFQRQLR
1290	1300	1310	1320	1330	1340	1350	1360
GAQYQPILRD	HICKDMSALV	AARMRHIPLA	PGSDWRDLPN	IEVRLSDGTM	ARKLRYTHHD	RKNGRSSSGA	LRGVCSCVEA
1370	1380	1390	1400	1410	1420	1430	1440
GKACDPAARQ	FNTLIPWCLP	HTGNRHNHWA	GLYGRLEWDG	FFSTTVTNPE	PMGKQGRVLH	PEQHRVVSVR	ECARSQGFPD
1450	1460	1470	1480	1490	1500		
TYRLFGNILD	KHRQVGNAVP	PPLAKAIGLE	IKLCMLAKAR	ESASAKIKEE	EAAKD		

Cmpd.	No. of Cmpds.	m/z meas.	Δ m/z [ppm]	z	Rt [min]	Score	P	Range	Sequence	Modification	Ratios	
273	1	685.7423	-204.14	2	32.8	12.1	2	778-788	R.LAEMRQKEIPR.V		M:Q	1.52
14	1	847.2854	-107.34	2	29.8	15.4	1	1353-1369	R.GVCSCVEAGKACDPAAR.Q	Carbamidomethyl: 5		

Detailed Protein Report

Protein 316: Fanconi anemia group I protein isoform 2 [Homo sapiens]

Accession: gi|82830440
Database: refseq_human(refseq_human_20140103.fasta)
Database Date: 2015-11-30

Score: 51.0
MW [kDa]: 142.5
pI: 6.3
Sequence Coverage [%]: 3.3
No. of unique Peptides: 3

Quantitation

Q:W	**Median:** 0.72	**CV:** 30.79 %	**No. of Peptides:**	2
M:Q	**Median:** 1.85	**CV:** 0.00 %	**No. of Peptides:**	1

10	20	30	40	50	60	70	80
MDQKILSLAA	EKTADKLQEF	LQTLREGDLT	NLLQNQAVKG	KVAGALLRAI	FKGSPCSEEA	GTLRRRKIYT	CCIQLVESGD
90	100	110	120	130	140	150	160
LQKEIASEII	GLLMLEAHHF	PGPLLVELAN	EFISAVREGS	LVNGKSLELL	PIILTALATK	KENLAYGKGV	LSGEECKKQL
170	180	190	200	210	220	230	240
INTLCSGRWD	QQYVIQLTSM	FKDVPLTAEE	VEFVVEKALS	MFSKMNLQEI	PPLVYQLLVL	SSKGSRKSVL	EGIIAFFSAL
250	260	270	280	290	300	310	320
DKQHNEEQSG	DELLDVVTVP	SGELRHVEGT	IILHIVFAIK	LDYELGRELV	KHLKVGQQGD	SNNNLSPFSI	ALLLSVTRIQ
330	340	350	360	370	380	390	400
RFQDQVLDLL	KTSVVKSFKD	LQLLQGSKFL	QNLVPHRSYV	STMILEVVKN	SVHSWDHVTQ	GLVELGFILM	DSYGPKKVLD
410	420	430	440	450	460	470	480
GKTIETSPSL	SRMPNQHACK	LGANILLETF	KIHEMIRQEI	LEQVLNRVVT	RASSPISHFL	DLLSNIVMYA	PLVLQSCSSK
490	500	510	520	530	540	550	560
VTEAFDYLSF	LPLQTVQRLL	KAVQPLLKVS	MSMRDCLILV	LRKAMFANQL	DARKSAVAGF	LLLLKNFKVL	GSLSSSQCSQ
570	580	590	600	610	620	630	640
SLSVSQVHVD	VHSHYNSVAN	ETFCLEIMDS	LRRCLSQQAD	VRLMLYEGFY	DVLRRNSQLA	NSVMQTLLSQ	LKQFYEPKPD
650	660	670	680	690	700	710	720
LLPPLKLEAC	ILTQGDKISL	QEPLDYLLCC	IQHCLAWYKN	TVIPLQQGEE	EEEEEEAFYE	DLDDILESIT	NRMIKSELED
730	740	750	760	770	780	790	800
FELDKSADFS	QSTSIGIKNN	ICAFLVMGVC	EVLIEYNFSI	SSFSKNRFED	ILSLFMCYKK	LSDILNEKAG	KAKTKMANKT
810	820	830	840	850	860	870	880
SDSLLSMKFV	SSLLTALFRV	LLWRYTSIPT	SVEESGKKEK	GKSISLLCLE	GLQKIFSAVQ	QFYQPKIQQF	LRALDVTDKE
890	900	910	920	930	940	950	960
GEEREDADVS	VTQRTAFQIR	QFQRSLLNLL	SSQEEDFNSK	EALLLVTVLT	SLSKLLEPSS	PQFVQMLSWT	SKICKENSRE
970	980	990	1000	1010	1020	1030	1040
DALFCKSLMN	LLFSLHVSYK	SPVILLRDLS	QDIHGHLGDI	DQDVEVEKTN	HFAIVNLRTA	APTVCLLVLS	QAEKVLEEVD
1050	1060	1070	1080	1090	1100	1110	1120
WLITKLKGQV	SQETLSEEAS	SQATLPNQPV	EKAIIMQLGT	LLTFFHELVQ	TALPSGSCVD	TLLKDLCKMY	TTLTALVRYY
1130	1140	1150	1160	1170	1180	1190	1200
LQVCQSSGGI	PKNMEKLVKL	SGSHLTPLCY	SFISYVQNKS	KSLNYTGEKK	EKPAAVATAM	ARVLRETKPI	PNLIFAIEQY
1210	1220	1230	1240	1250	1260	1270	
EKFLIHLSKK	SKVNLMQHMK	LSTSRDFKIK	GNILDMVLRE	DGEDENEEGT	ASEHGGQNKE	PAKKKRKK	

Cmpd.	No. of Cmpds.	m/z meas.	Δ m/z [ppm]	z	Rt [min]	Score	P	Range	Sequence	Modification	Ratios
426	4	658.3901	11.29	2	34.4	22.4	2	780-791	K.KLSDILNEKAGK.A		
1909	2	918.3239	-154.66	2	52.5	13.3	1	885-900	R.EDADVSVTQRTAFQIR.Q		Q:W 0.98
13	1	814.3623	-84.24	2	29.4	15.2	1	1105-1118	K.DLCKMYTTLTALVR.Y		Q:W 0.54<br>M:Q 1.85

Detailed Protein Report

Protein 317: **PREDICTED: cytosolic carboxypeptidase 2 isoform X10 [Homo sapiens]**

**Accession:**	gi|578820810	**Score:**	50.9
**Database:**	refseq_human(refseq_human_20140103.fasta)	**MW [kDa]:**	93.8
**Database Date:**	2015-11-30	**pI:**	9.8
**Modification(s):**	Oxidation	**Sequence Coverage [%]:**	7.0
		**No. of unique Peptides:**	2

10	20	30	40	50	60	70	80
MFPALETHLK	QTIPDPYEDF	MYRHLQYYGY	FKAQRGSLPN	SATHQHVRKN	NPQCLLNGSL	GEKDDLIPDT	LQKEKLLYSH
90	100	110	120	130	140	150	160
MLSLPHLRSR	QLLYDELDEV	NPRLREPQEL	FSILSTKRPL	QAPRWPIECE	VIKENIHHIE	WAPPQPEYFY	QPKGNEKVPE
170	180	190	200	210	220	230	240
IVGEKKGTVV	YQLDSVPIEG	SYFTSSRVGG	KRGIVKELAV	TLQGPEDNTL	LFESRFESGN	LQKAVRVDTY	EYELTLRTDL
250	260	270	280	290	300	310	320
YTNKHTQWFY	FRVQNTRKDA	TYRFTIVNLL	KPKSLYTVGM	KPLLYSQLDA	NTRNIGWRRE	GNEIKYYKNN	TDDGQQPFYC
330	340	350	360	370	380	390	400
LTWTIQFPYD	QDTCFFAHFY	PYTYTDLQCY	LLSVANNPIQ	SQFCKLQTLC	RSLAGNTVYL	LTITNPSQTP	QEAAAKKAVV
410	420	430	440	450	460	470	480
LSARVHPGES	NGSWVMKGFL	DFILSNSPDA	QLLRDIFVFK	VLPMLNPDGV	IVGNYRCSLA	GRDLNRHYKT	ILKESFPCIW
490	500	510	520	530	540	550	560
YTRNMIKRLL	EEREVLLYCD	FHGHSRKNNI	FLYGCNNNNR	KYWLHERVFP	LMLCKNAPDK	FSFHSCNFKV	QKCKEGTGRV
570	580	590	600	610	620	630	640
VMWRMGILNS	YTMESTFGGS	TLGNKRDTHF	TIEDLKSLGY	HVCDTLLDFC	DPDQMKFTQC	LAELKELLRQ	EIHKKFHELG
650	660	670	680	690	700	710	720
QDVDLEGSWS	DISLSDIESS	TSGSDSSLSD	GLPVHLANIA	DELTQKKKMF	KKKKKKSLQT	RKQRNEQYQK	KNLMQKLKLT
730	740	750	760	770	780	790	800
EDTSTNLNRR	DKDTPLDPSM	ATLILPKNKG	RMQNKKPGFT	VSCSPKRTIN	SSQEPAPGMK	PNWPRSRYPA	TKRGCAAMAA
810	820						
YPSLHIYTYP							

Cmpd.	No. of Cmpds.	m/z meas.	Δ m/z [ppm]	z	Rt [min]	Score	P	Range	Sequence	Modification
179	1	879.8295	-147.14	2	31.6	13.6	1	33-48	K.AQRGSLPNSATHQHVR.K	
1977	1	746.3572	-41.97	2	54.8	11.3	1	528-540	R.VFPLMLCKNAPDK.F	Oxidation: 5

Detailed Protein Report

Protein 318: mucin-19 precursor [Homo sapiens]

**Accession:**	gi|533112494	**Score:**	50.9
**Database:**	refseq_human(refseq_human_20140103.fasta)	**MW [kDa]:**	804.8
**Database Date:**	2015-11-30	**pI:**	4.8
**Modification(s):**	Carbamidomethyl	**Sequence Coverage [%]:**	0.9
		**No. of unique Peptides:**	1

BRUKER

Detailed Protein Report

10	20	30	40	50	60	70	80
MKLILWYLVV	ALWCFFKDVE	ALLYRQKSDG	KIAASRSGGF	SYGSSSSGDL	DRKKPLFSLE	FGSPGETEDK	SRQRQDAGSP
90	100	110	120	130	140	150	160
KSEDTPAGGF	FNSSSSSGDS	DRTKPFFSLG	LGAPGKAEDK	SGDSQDAGGS	KSEDTPPGGF	FYGSSSSGDS	DKKKPLFSFE
170	180	190	200	210	220	230	240
FGATGEDEDK	SRERWDAGNS	RSEDSPADST	NTRYGAGFSS	SGASLDVGFG	WGISDEKGLE	VSKADGRETR	GSGSAGGETI
250	260	270	280	290	300	310	320
VFGPDAGSSV	GTGSSGLKLG	AGKGDAAFGF	EVSDSNSFGD	TGISSKTVEG	NQTSSSGGSV	SIDLGDTSFR	SENQFVGGGS
330	340	350	360	370	380	390	400
LNSISNLWDS	GQEGFGINEI	GGNGMSGSVS	AEAGFKGFGS	DSSSSGDSSA	RNGFENSSGI	SEDSGVILGS	SDQHEVELSR
410	420	430	440	450	460	470	480
TGGNRKRSSD	PDEAGNLSPG	SDVSDSGGNT	WSSDSGSGGG	GVTSSSEYST	SGPLNTPEKG	SHIPEATPKY	SETNAIIGEI
490	500	510	520	530	540	550	560
STWSKGAYKS	FNGRIFFFES	SCPYTFCRHC	IESGGDFNIE	IKRNNDSEIE	KITVLIDNND	VSIFGDTILV	NGESVQIPYN
570	580	590	600	610	620	630	640
NKLIHIKKYG	EHNVLNSRRG	ILTLMWDKNN	KLSLTLHKQY	PTCGLCGNFN	STPGQDINEH	IANSKIPGDC	PNAVGKSYEV
650	660	670	680	690	700	710	720
CEDGIQHCNK	IIGTYFEKCG	KVAALSNDYK	MICIDEYCQT	RDKTSTCDTY	SELSRLCASD	GPGTFESWRS	DSDVVCGTQR
730	740	750	760	770	780	790	800
CPEQHIYKEC	GPSNPATCSN	VAPFQDSECV	SGCTCPEGYL	LDDIGEKGKC	VLKAECPCES	SGTVYQPGEV	REGPCGSQCT
810	820	830	840	850	860	870	880
CQDAKWSCTE	ALCPGRCKVE	GSSLTTFDGV	KYNFPGNCHF	LAVHNEDWSI	SVELRPCPSG	QTGTCLNSVT	LLLNSSVPVD
890	900	910	920	930	940	950	960
KYVFNSDGTV	TNDKIRNQGY	YYSDKIQIFN	ASSSYLQVET	YFHVKLQIQI	VPVMQLYVSM	PPNQFTDTVG	LCGSYNNKAE
970	980	990	1000	1010	1020	1030	1040
DDFMSSQNIL	EKTSQAFANS	WEMMSCPKGN	PSSCISIEKE	KFAERHCGIL	LDSSGPLASC	HPIVNPKPYH	EECKKYTCTC
1050	1060	1070	1080	1090	1100	1110	1120
ENSQDCLCTI	LGNYVKACAE	KETYIVGWRT	GLCEHSCPSG	LVFKYNVKAC	NSSCRSLSER	DRSCDVEDVP	VDGCTCPDAM
1130	1140	1150	1160	1170	1180	1190	1200
YQNNEGNCVL	KSQCDCYIND	EVMQPGKLIH	IDDNKCVCRD	GILLCQIPID	LTLQNCSGGA	EYVDCSDPKA	QRRTNRTCST
1210	1220	1230	1240	1250	1260	1270	1280
RNIPVFDENL	PCKRGCFCPE	GMVRNSKGIC	VFPNDCPCSF	GGREYDEGSV	TSVGCNECTC	IKGSWSCTQN	ECQTICHIYG
1290	1300	1310	1320	1330	1340	1350	1360
EGHVRTFDGK	SYSFDGLCQY	SFLEDYCGHE	NGTFRILTES	VPCCEDGLTC	SRKIIVAFQD	QNIVLQDGKV	TAVKSTESKK
1370	1380	1390	1400	1410	1420	1430	1440
CELNANAYSI	HTVGLYLILK	FQNGIIVIWD	KNTRLSVILD	PNWNGKVCGL	CGNNNGDLKD	DFTTRYSSVA	SGALEFGNSW
1450	1460	1470	1480	1490	1500	1510	1520
KTSQECSDTV	AQTFPCDSNP	YCKAWAVRKC	EILRDSTFRD	CHNKVDPSAY	HDACIEEACA	CDMEGKYLGF	CTAVAMYAEA
1530	1540	1550	1560	1570	1580	1590	1600
CSAVGVCVSW	RKPNLCPVYC	DYYNAPGECR	WHYEPCGTVT	AKTCKDQLVG	QKFSSLLEGC	YAKCPDSAPY	LDENTMKCVS
1610	1620	1630	1640	1650	1660	1670	1680
LSECSCFYND	VIPAGGVIED	NCGRTCYCIA	GQLECSETAP	TNSTFAVSTT	TATTILSTGA	AITLVTGGPS	TAASIPAITT
1690	1700	1710	1720	1730	1740	1750	1760
SSSETTGTTL	GPLTEPFTTG	ITETSVPIIS	TSGNAGMTGV	VSPTVTGASG	MAGTTGGVDA	ATTGAASENT	SERAGTPRVS
1770	1780	1790	1800	1810	1820	1830	1840
GETPAVGGGS	TPGEAGPGAT	VSGSTGVSAG	SITASPGASA	TSSESSKSGT	TGPSVGGKTG	ATSSEATSSE	GMSGVTGQSL
1850	1860	1870	1880	1890	1900	1910	1920
GSTAGSDSEI	TAKTSFTGSS	PPGKLTRPSP	GSPGHFSGGT	TEWGNVATTG	AAGENTSGAL	GSTEGSVEAT	TSAGSGNTAG
1930	1940	1950	1960	1970	1980	1990	2000
TSGTGDTGPG	NTAVSGTPVV	SPGATPGAPG	SSTPGEADIG	NTSFGKSGTP	TVSAASTTSS	PVSKHTDAAS	ATAVTISGSK
2010	2020	2030	2040	2050	2060	2070	2080
PGTPGTPGGA	TSGGKITSGW	SSSGTSTGAS	NTPGATGSST	GQTDTSGPSA	KVTGNYGQSS	EIPGTIKSSS	DVSGTMGQSD
2090	2100	2110	2120	2130	2140	2150	2160
TTSGPSVAVT	RTSEQSSGVT	VASEPSVGVS	GTTGPLAEIS	GTTRPLVSGL	RTTGSSAEGS	GTTGPSSRES	VTTRPLAEGS
2170	2180	2190	2200	2210	2220	2230	2240
GTSGQSVTGS	RATGLSATEL	GTTVSFTGGL	GTSRSSARET	RTTGPSADGS	GTTGPSVVRS	GTTRLSVGVT	RATESSPGVT
2250	2260	2270	2280	2290	2300	2310	2320
GTTTPSAEES	RTTGPSVLVT	GTTGQSGQGS	GTTGKSFIES	GPSVVGSGTT	GPTSAGLGTT	APSTRRSSTT	KPSVGRTGTT
2330	2340	2350	2360	2370	2380	2390	2400

Detailed Protein Report

Cmpd.	No. of Cmpds.	m/z meas.	Δ m/z [ppm]	z	Rt [min]	Score	P	Range	Sequence	Modification
2090	1	1131.3695	-118.05	2	56.3	11.2	1	8209-8227	K.LVKFQSNDTCCEIGYCEPR.T	Carbamidomethyl: 10

Detailed Protein Report

Protein 319: sorting nexin-8 [Homo sapiens]

**Accession:**	gi|23943858	**Score:**	50.8
**Database:**	refseq_human(refseq_human_20140103.fasta)	**MW [kDa]:**	52.5
**Database Date:**	2015-11-30	**pI:**	7.2
**Modification(s):**	Oxidation	**Sequence Coverage [%]:**	9.2
		**No. of unique Peptides:**	1

10	20	30	40	50	60	70	80
MTGRAMDPLP	AAAVGAAAEA	EADEEADPPA	SDLPTPQAIE	PQAIVQQVPA	PSRMQMPQGN	PLLLSHTLQE	LLARDTVQVE
**90**	**100**	**110**	**120**	**130**	**140**	**150**	**160**
LIPEKKGLFL	KHVEYEVSSQ	RFKSSVYRRY	NDFVVFQEML	LHKFPYRMVP	ALPPKRMLGA	DREFIEARRR	ALKRFVNLVA
**170**	**180**	**190**	**200**	**210**	**220**	**230**	**240**
RHPLFSEDVV	LKLFLSFSGS	DVQNKLKESA	QCVGDEFLNC	KLATRAKDFL	PADIQAQFAI	SRELIRNIYN	SFHKLRDRAE
**250**	**260**	**270**	**280**	**290**	**300**	**310**	**320**
RIASRAIDNA	ADLLIFGKEL	SAIGSDTTPL	PSWAALNSST	WGSLKQALKG	LSVEFALLAD	KAAQQGKQEE	NDVVEKLNLF
**330**	**340**	**350**	**360**	**370**	**380**	**390**	**400**
LDLLQSYKDL	CERHEKGVLH	KHQRALHKYS	LMKRQMMSAT	AQNREPESVE	QLESRIVEQE	NAIQTMELRN	YFSLYCLHQE
**410**	**420**	**430**	**440**	**450**	**460**	**470**	
TQLIHVYLPL	TSHILRAFVN	SQIQGHKEMS	KVWNDLRPKL	SCLFAGPHST	LTPPCSPPED	GLCPH	

Cmpd.	No. of Cmpds.	m/z meas.	Δ m/z [ppm]	z	Rt [min]	Score	P	Range	Sequence	Modification
1502	1	978.1127	119.49	2	48.6	18.2	1	109-123	R.RYNDFVVFQEMLLHK.F	Oxidation: 11

Detailed Protein Report

Protein 320: PREDICTED: ral GTPase-activating protein subunit alpha-2 isoform X2 [Homo sapiens]

**Accession:**	gi|578835487	**Score:**	50.8
**Database:**	refseq_human(refseq_human_20140103.fasta)	**MW [kDa]:**	221.0
**Database Date:**	2015-11-30	**pI:**	5.9
**Modification(s):**	Oxidation	**Sequence Coverage [%]:**	3.4
		**No. of unique Peptides:**	2

BRUKER

Detailed Protein Report

10	20	30	40	50	60	70	80
MFSRRSHGDV	KKSTQKVLDP	KKDVLTRLKH	LRALLDNVDA	NDLKQFFETN	YSQIYFIFYE	NFIALENSLK	LKGNNKSQRE
90	100	110	120	130	140	150	160
ELDSILFLFE	KILQFLPERI	FFRWHYQSIG	STLKKLLHTG	NSIKIRCEGI	RLFLLWLQAL	QTNCAEEQVL	IFACLVPGFP
170	180	190	200	210	220	230	240
AVMSSRGPCT	LETLINPSPS	VADVKIYPEE	ITPLLPAISG	EKIAEDQTCF	FLQILLKYMV	IQAASLEWKN	KENQDTGFKF
250	260	270	280	290	300	310	320
LFTLFRKYYL	PHLFPSFTKL	TNIYKPVLDI	PHLRPKPVYI	TTTRDNENIY	STKIPYMAAR	VVFIKWIVTF	FLEKKYLTAT
330	340	350	360	370	380	390	400
QNTKNGVDVL	PKIIQTVGGG	AVQERAPELD	GGGPTEQDKS	HSNSSTLSDR	RLSNSSLCSI	EEEHRMVYEM	VQRILLSTRG
410	420	430	440	450	460	470	480
YVNFVNEVFH	QAFLLPSCEI	AVTRKVVQVY	RKWILQDKPV	FMEEPDRKDV	AQEDAEKLGF	SETDSKEASS	ESSGHKRSSS
490	500	510	520	530	540	550	560
WGRTYSFTSA	MSRGCVTEEE	NTNVKAGVQA	LLQVFLTNSA	NIFLLEPCAE	VPVLLKEQVD	ACKAVLIIFR	RMIMELTMNK
570	580	590	600	610	620	630	640
KTWEQMLQIL	LRITEAVMQK	PKDKQIKDLF	AQSLAGLLFR	TLMVAWIRAN	LCVYISRELW	DDFLGVLSSL	TEWEELINEW
650	660	670	680	690	700	710	720
ANIMDSLTAV	LARTVYGVEM	TNLPLDKLSE	QKEKKQRGKG	CVLDPQKGTT	VGRSFSLSWR	SHPDVTEPMR	FRSATTSGAP
730	740	750	760	770	780	790	800
GVEKARNIVR	QKATAKRSQS	ISNCVHLSEA	LPATKSVPLL	LHTVSALLPG	LSYSSCSHRL	SEVEECQQSE	NAPAAGSGHL
810	820	830	840	850	860	870	880
TVGQQQQVLR	SSSTSDIPEP	LCSDSSQGQK	AENTQNSSSS	EPQPIQENKG	HVKREHEGIT	ILVRRSSSPA	ELDLKDDLQQ
890	900	910	920	930	940	950	960
TQGKCRERQK	SESTNSDTTL	GCTNEAELSM	GPWQTCEEDP	ELNTPTDVVA	DADARHWLQL	SPTDASNLTD	SSECLTDDCS
970	980	990	1000	1010	1020	1030	1040
IIAGGSLTGW	HPDSAAVLWR	RVLGILGDVN	NIQSPKIHAR	VFCYLYELWY	KLAKIRDNLA	ISLDNQSSPS	PPVLIPPLRM
1050	1060	1070	1080	1090	1100	1110	1120
FASWLFKAAT	LPNEYKEGKL	QAYRLICAMM	TRRQDVLPNS	DFLVHFYLVM	HLGLTSEDQD	ILNTIIRHCP	PRFFSLGFPG
1130	1140	1150	1160	1170	1180	1190	1200
FSMLVGDFIT	AAARVLSTDI	LTAPRSEAVT	VLGSLVCFPN	TYQEIPLLQS	VPEVNEAITG	TEDVKHYLIN	ILLKNATEEP
1210	1220	1230	1240	1250	1260	1270	1280
NEYARCIAVC	SLGVWICEEL	AQCTSHPQVK	EAINVIGVTL	KFPNKIVAQV	ACDVLQLLVS	YWEKLQMFET	SLPRKMAEIL
1290	1300	1310	1320	1330	1340	1350	1360
VATVAFLLPS	AEYSSVETDK	KFIVSLLLCL	LDWCMALPVS	VLLHPVSTAV	LEEQHSARAP	LLDYIYRVLH	CCVCGSSTYT
1370	1380	1390	1400	1410	1420	1430	1440
QQSHYILTLA	DLSSTDYDPF	LPLANVKSSE	PVQYHSSAEL	GNLLTVEEEK	KRRSLELIPL	TARMVMAHLV	NHLGHYPLSG
1450	1460	1470	1480	1490	1500	1510	1520
GPAILHSLVS	ENHDNAHVEG	SELSFEVFRS	PNLQLFVFND	STLISYLQTP	TEGPVGGSPV	GSLSDVRVIV	RDISGKYSWD
1530	1540	1550	1560	1570	1580	1590	1600
GKVLYGPLEG	CLAPNGRNPS	FLISSWHRDT	FGPQKDSSQV	EEGDDVLDKL	LENIGHTSPE	CLLPSQLNLN	EPSLTPCGMN
1610	1620	1630	1640	1650	1660	1670	1680
YDQEKEIIEV	ILRQNAQEDE	YIQSHNFDSA	MKVTSQGQPS	PVEPRGPFYF	CRLLLDDLGM	NSWDRRKNFH	LLKKNSKLLR
1690	1700	1710	1720	1730	1740	1750	1760
ELKNLDSRQC	RETHKIAVFY	IAEGQEDKCS	ILSNERGSQA	YEDFVAGLGW	EVDLSTHCGF	MGGLQRNGST	GQTAPYYATS
1770	1780	1790	1800	1810	1820	1830	1840
TVEVIFHVST	RMPSDSDDSL	TKKLRHLGND	EVHIVWSEHS	RDYRRGIIPT	AFGDVSIIIY	PMKNHMFFIA	ITKKPEVPFF
1850	1860	1870	1880	1890	1900	1910	1920
GPLFDGAIVS	GKLLPSLVCA	TCINASRAVK	CLIPLYQSFY	EERALYLEAI	IQNHREVMTF	EDFAAQVFSP	SPSYSLSGTG
1930	1940	1950	1960	1970	1980		
ALASSLSADL	EEPLSEEETE	HPLLPLPRAT	KPRISRKFRC	CTSALSRSSH			

Cmpd.	No. of Cmpds.	m/z meas.	Δ m/z [ppm]	z	Rt [min]	Score	P	Range	Sequence	Modification
1746	3	1023.9594	-58.70	2	51.6	16.7	1	433-448	K.WILQDKPVFMEEPDRK.D	Oxidation: 10
1736	1	1273.5367	-62.63	2	51.5	13.3	0	1388-1410	K.SSEPVQYHSSAELGNLLTVEEEK.K	

Detailed Protein Report

Protein 321: PREDICTED: midasin isoform X3 [Homo sapiens]

**Accession:**	gi|578812559	**Score:**	50.8
**Database:**	refseq_human(refseq_human_20140103.fasta)	**MW [kDa]:**	627.0
**Database Date:**	2015-11-30	**pI:**	5.4
		**Sequence Coverage [%]:**	0.9
		**No. of unique Peptides:**	2

Detailed Protein Report

10	20	30	40	50	60	70	80
MEHFLLEVAA	APLRLIAAKN	EKSRSELGRF	LAKQVWTPQD	RQCVLSTLAQ	LLLDKDCTVL	VGRQLRPLLL	DLLERNAEAI
90	100	110	120	130	140	150	160
KAGGQINHDL	HERLCVSMSK	LIGNHPDVLP	FALRYFKDTS	PVFQRLFLES	SDANPVRYGR	RRMKLRDLME	AAFKFLQQEQ
170	180	190	200	210	220	230	240
SVFRELWDWS	VCVPLLRSHD	TLVRWYTANC	LALVTCMNEE	HKLSFLKKIF	NSDELIHFRL	RLLEEAQLQD	LEKALVLANP
250	260	270	280	290	300	310	320
EVSLWRKQKE	LQYLQGHLVS	SDLSPRVTAV	CGVVLPGQLP	APGELGGNRS	SSREQELALR	SYVLVESVCK	SLQTLAMAVA
330	340	350	360	370	380	390	400
SQNAVLLEGP	IGCGKTSLVE	YLAAVTGRTK	PPQLLKVQLG	DQTDSKMLLG	MYRCTDVPGE	FVWQPGTLTQ	AATMGHWILL
410	420	430	440	450	460	470	480
EDIDYAPLDV	VSVLIPLLEN	GELLIPGRGD	CLKVAPGFQF	FATRRLLSCG	GNWYRPLNSH	ATLLDKYWTK	IHLDNLDKRE
490	500	510	520	530	540	550	560
LNEVLQSRYP	SLLAVVDHLL	DIYIQLTGEK	HHSWSDSSVG	CEQAPEEVSE	ARRENKRPTL	EGRELSLRDL	LNWCNRIAHS
570	580	590	600	610	620	630	640
FDSSSLSASL	NIFQEALDCF	TAMLSEHTSK	LKMAEVIGSK	LNISRKKAEF	FCQLYKPEIV	INELDLQVGR	VRLLRKQSEA
650	660	670	680	690	700	710	720
VHLQREKFTF	AATRPSSVLI	EQLAVCVSKG	EPVLLVGETG	TGKTSTIQYL	AHITGHRLRV	VNMNQQSDTA	DLLGGYKPVD
730	740	750	760	770	780	790	800
HKLIWLPLRE	AFEELFAQTF	SKKQNFTFLG	HIQTCYRQKR	WHDLLRLMQH	VHKSAVNKDG	KDSETGLLIK	EKWEAFGLRL
810	820	830	840	850	860	870	880
NHAQQQMKMT	ENTLLFAFVE	GTLAQAVKKG	EWILLDEINL	AAPEILECLS	GLLEGSSGSL	VLLDRGDTEP	LVRHPDFRLF
890	900	910	920	930	940	950	960
ACMNPATDVG	KRNLPPGIRN	RFTELYVEEL	ESKEDLQVLI	VDYLKGLSVN	KNTVQGIINF	YTALRKESGT	KLVDGTGHRP
970	980	990	1000	1010	1020	1030	1040
HYSLRTLCRA	LRFAASNPCG	NIQRSLYEGF	CLGFLTQLDR	ASHPIVQKLI	CQHIVPGNVK	SLLKQPIPEP	KGGRLIQVEG
1050	1060	1070	1080	1090	1100	1110	1120
YWIAVGDKEP	TIDETYILTS	SVKLNLRDIV	RVVSAGTYPV	LIQGETSVGK	TSLIQWLAAA	TGNHCVRINN	HEHTDIQEYI
1130	1140	1150	1160	1170	1180	1190	1200
GCYTSDSSGK	LVFKEGVLID	AMRKGYWIIL	DELNLAPTDV	LEALNRLLDD	NRELLVTETQ	EVVKAHPRFM	LFATQNPPGL
1210	1220	1230	1240	1250	1260	1270	1280
YGGRKVLSRA	FRNRFVELHF	DELPSSELET	ILHKRCSLPP	SYCSKLVKVM	LDLQSYRRSS	SVFAGKQGFI	TLRDLFRWAE
1290	1300	1310	1320	1330	1340	1350	1360
RYRLAEPTEK	EYDWLQHLAN	DGYMLLAGRV	RKQEEIDVIQ	EVLEKHFKKK	LCPQSLFSKE	NVLKLLGKLS	TQISTLECNF
1370	1380	1390	1400	1410	1420	1430	1440
GHIVWTEGMR	RLAMLVGRAL	EFGEPVLLVG	DTGCGKTTIC	QVFAALANQK	LYSVSCHLHM	ETSDFLGGLR	PVRQKPNDKE
1450	1460	1470	1480	1490	1500	1510	1520
EIDTSRLFEW	HDGPLVQAMK	EDGFFLLDEI	SLADDSVLER	LNSVLEVEKS	LVLAEKGSPE	DKDSEIELLT	AGKKFRILAT
1530	1540	1550	1560	1570	1580	1590	1600
MNPGGDFGKK	ELSPALRNRF	TEIWCPQSTS	REDLIQIISH	NLRPGLCLGR	IDPKGSDIPE	VMLDFIDWLT	HQEFGRKCVV
1610	1620	1630	1640	1650	1660	1670	1680
SIRDILSWVN	FMNKMGEEAA	LKRPEIISTV	TSFVHAACLV	YIDGIGSGVT	SSGFGTALLA	RKECLKFLIK	RLAKIVRLTE
1690	1700	1710	1720	1730	1740	1750	1760
YQKNELKIYD	RMKAKEFTGI	DNLWGIHPFF	IPRGPVLHRN	NIADYALSAG	TTAMNAQRLL	RATKLKKPIL	LEGSPGVGKT
1770	1780	1790	1800	1810	1820	1830	1840
SLVGALAKAS	GNTLVRINLS	EQTDITDLFG	ADLPVEGGKG	GEFAWRDGPL	LAALKAGHWV	VLDELNLASQ	SVLEGLNACF
1850	1860	1870	1880	1890	1900	1910	1920
DHRGEIYVPE	LGMSFQVQHE	KTKIFGCQNP	FRQGGGRKGL	PRSFLNRFTQ	VFVDPLTVID	MEFIASTLFP	AIEKNIVKKM
1930	1940	1950	1960	1970	1980	1990	2000
VAFNNQIDHE	VTVEKKWGQK	GGPWEFNLRD	LFRWCQLMLV	DQSPGCYDPG	QHVFLVYGER	MRTEEDKKKV	IAVFKDVFGS
2010	2020	2030	2040	2050	2060	2070	2080
NSNPYMGTRL	FRITPYDVQL	GYSVLSRGSC	VPHPSRHPLL	LLHQSFQPLE	SIMKCVQMSW	MVILVGPASV	GKTSLVQLLA
2090	2100	2110	2120	2130	2140	2150	2160
HLTGHTLKIM	AMNSAMDTTE	LLGGFEQVDL	IRPWRRLLEK	VEGTVRALLR	DSLLISADDA	EVVLRAWSHF	LLTYKPKCLG
2170	2180	2190	2200	2210	2220	2230	2240

Detailed Protein Report

Cmpd.	No. of Cmpds.	m/z meas.	Δ m/z [ppm]	z	Rt [min]	Score	P	Range	Sequence	Modification
558	1	512.6910	-181.41	2	35.4	13.3	2	1688-1695	K.IYDRMKAK.E	
1915	1	918.3456	-31.31	2	53.8	12.2	1	4832-4848	K.EEKEEAEADDGGQGEDK.I	

Detailed Protein Report

Protein 322: triosephosphate isomerase isoform 3 [Homo sapiens]

Accession: gi|384475517
Database: refseq_human(refseq_human_20140103.fasta)
Database Date: 2015-11-30
Modification(s): Carbamidomethyl

Score: 50.7
MW [kDa]: 17.9
pI: 5.3
Sequence Coverage [%]: 18.0
No. of unique Peptides: 1

10	20	30	40	50	60	70	80
MIKDCGATWV	VLGHSERRHV	FGESDELIGQ	KVAHALAEGL	GVIACIGEKL	DEREAGITEK	VVFEQTKVIA	DNVKDWSKVV
**90**	**100**	**110**	**120**	**130**	**140**	**150**	**160**
LAYEPVWAIG	TGKTATPQQA	QEVHEKLRGW	LKSNVSDAVA	QSTRIIYGGS	VTGATCKELA	SQPDVDGFLV	GGASLKPEFV
**170**							
DIINAKQ							

Cmpd.	No. of Cmpds.	m/z meas.	Δ m/z [ppm]	z	Rt [min]	Score	P	Range	Sequence	Modification
219	1	663.8152	-36.93	2	32.7	37.4	0	125-137	R.IIYGGSVTGATCK.E	Carbamidomethyl: 12

Detailed Protein Report

Protein 323: PREDICTED: nuclear receptor corepressor 1 isoform X13 [Homo sapiens]

**Accession:**	gi|578830118	**Score:**	50.6
**Database:**	refseq_human(refseq_human_20140103.fasta)	**MW [kDa]:**	277.7
**Database Date:**	2015-11-30	**pI:**	6.8
**Modification(s):**	Oxidation	**Sequence Coverage [%]:**	2.9
		**No. of unique Peptides:**	3

Quantitation

Q:W	**Median:**	1.09	**CV:**	0.00 %	**No. of Peptides:**	1
M:Q	**Median:**	0.83	**CV:**	0.00 %	**No. of Peptides:**	1

BRUKER

Detailed Protein Report

10	20	30	40	50	60	70	80
MSSSGYPPNQ	GAFSTEQSRY	PPHSVQYTFP	NTRHQQEFAV	PDYRSSHLEV	SQASQLLQQQ	QQQQLRRRPS	LLSEFHPGSD
90	100	110	120	130	140	150	160
RPQERRTSYE	PFHPGPSPVD	HDSLESKRPR	LEQVSDSHFQ	RVSAAVLPLV	HPLPEGLRAS	ADAKKDPAFG	GKHEAPSSPI
170	180	190	200	210	220	230	240
SGQPCGDDQN	ASPSKLSKEE	LIQSMDRVDR	EIAKVEQQIL	KLKKKQQQLE	EEAAKPPEPE	KPVSPPPVEQ	KHRSIVQIIY
250	260	270	280	290	300	310	320
DENRKKAEEA	HKIFEGLGPK	VELPLYNQPS	DTKVYHENIK	TGVPARRMMK	NQVMRKKLIL	FFKRRNHARK	QREQKICQRY
330	340	350	360	370	380	390	400
DQLMEAWEKK	VDRIENNPRR	KAKESKTREY	YEKQFPEIRK	QREQQERFQR	VGQRGAGLSA	TIARSEHEIS	EIIDGLSEQE
410	420	430	440	450	460	470	480
NNEKQMRQLS	VIPPMMFDAE	QRRVKFINMN	GLMEDPMKVY	KDRQFMNVWT	DHEKEIFKDK	FIQHPKNFGL	IASYLERKSV
490	500	510	520	530	540	550	560
PDCVLYYYLT	KKNENYKALV	RRNYGKRRGR	NQQQIARPSQ	EEKVEEKEED	KAEKTEKKEE	EKKDEEEKDE	KEDSKENTKE
570	580	590	600	610	620	630	640
KDKIDGTAEE	TEEREQATPR	GRKTANSQGR	RKGRITRSMT	NEAAAASAAA	AAATEEPPPP	LPPPPEPIST	EPVETSRWTE
650	660	670	680	690	700	710	720
EEMEVAKKGL	VEHGRNWAAI	AKMVGTKSEA	QCKNFYFNYK	RRHNLDNLLQ	QHKQKTSRKP	REERDVSQCE	SVASTVSAQE
730	740	750	760	770	780	790	800
DEDIEASNEE	ENPEDSEGAE	NSSDTESAPS	PSPVEAVKPS	EDSPENATSR	GNTEPAVELE	PTTETAPSTS	PSLAVPSTKP
810	820	830	840	850	860	870	880
AEDESVETQV	NDSISAETAE	QMDVDQQEHS	AEEGSVCDPP	PATKADSVDV	EVRVPENHAS	KVEGDNTKER	DLDRASEKVE
890	900	910	920	930	940	950	960
PRDEDLVVAQ	QINAQRPEPQ	SDNDSSATCS	ADEDVDGEPE	RQRMFPMDSK	PSLLNPTGSI	LVSSPLKPNP	LDLPQLQHRA
970	980	990	1000	1010	1020	1030	1040
AVIPPMVSCT	PCNIPIGTPV	SGYALYQRHI	KAMHESALLE	EQRQRQEQID	LECRSSTSPC	GTSKSPNREW	EGKSVAYMPY
1050	1060	1070	1080	1090	1100	1110	1120
AEVKRALEQE	AQMHNTAARS	ASPCRLSPRE	VSKAAPQPDM	SAARYSVPPV	LQPAPHQVIT	NLPEGVRLPT	TRPTRPPPPL
1130	1140	1150	1160	1170	1180	1190	1200
IPSSKTTVAS	EKPSFIMGGS	ISQGTPGTYL	TSHNQASYTQ	ETPKPSVGSI	SLGLPRQQES	AKSATLPYIK	QEEFSPRSQN
1210	1220	1230	1240	1250	1260	1270	1280
SQPEGLLVRA	QHEGVVRGTA	GAIQEGSITR	GTPTSKISVE	SIPSLRGSIT	QGTPALPQTG	IPTEALVKGS	ISRMPIEDSS
1290	1300	1310	1320	1330	1340	1350	1360
PEKGREEAAS	KGHVIYEGKS	GHILSYDNIK	NAREGTRSPR	TAHEISLKRS	YESVEGNIKQ	GMSMRESPVS	APLEGLICRA
1370	1380	1390	1400	1410	1420	1430	1440
LPRGSPHSDL	KERTVLSGSI	MQGTPRATTE	SFEDGLKYPK	QIKRESPPIR	AFEGAITKGK	PYDGITTIKE	MGRSIHEIPR
1450	1460	1470	1480	1490	1500	1510	1520
QDILTQESRK	TPEVVQSTRP	IIEGSISQGT	PIKFDNNSGQ	SAIKHNVKSL	ITGPSKLSRG	MPPLEIVPEN	IKVVERGKYE
1530	1540	1550	1560	1570	1580	1590	1600
DVKAGETVRS	RHTSVVSSGP	SVLRSTLHEA	PKAQLSPGIY	DDTSARRTPV	SYQNTMSRGS	PMMNRTSDVT	ISSNKSTNHE
1610	1620	1630	1640	1650	1660	1670	1680
RKSTLTPTQR	ESIPAKSPVP	GVDPVVSHSP	FDPHHRGSTA	GEVYRSHLPT	HLDPAMPFHR	ALDPAAAAYL	FQRQLSPTPG
1690	1700	1710	1720	1730	1740	1750	1760
YPSQYQLYAM	ENTRQTILND	YITSQQMQVN	LRPDVARGLS	PREQPLGLPY	PATRGIIDLT	NMPPTILVPH	PGGTSTPPMD
1770	1780	1790	1800	1810	1820	1830	1840
RITYIPGTQI	TFPPRPYNSA	SMSPGHPTHL	AAAASAERER	ERERERKERER	ERIAAASSDL	YLRPGSEQPG	RPGSHGYVRS
1850	1860	1870	1880	1890	1900	1910	1920
PSPSVRTQET	MLQQRPSVFQ	GTNGTSVITP	LDPTAQLRIM	PLPAGGPSIS	QGLPASRYNT	AADALAALVD	AAASAPQMDV
1930	1940	1950	1960	1970	1980	1990	2000
SKTKESKHEA	ARLEENLRSR	SAAVSEQQQL	EQKTLEVEKR	SVQCLYTSSA	FPSGKPQPHS	SVVYSEAGKD	KGPPPKSRYE
2010	2020	2030	2040	2050	2060	2070	2080
EELRTRGKTT	ITAANFIDVI	ITRQIASDKD	ARERGSQSSD	SSSSLSSHRY	ETPSDAIEVI	SPASSPAPPQ	EKLQTYQPEV
2090	2100	2110	2120	2130	2140	2150	2160
VKANQAENDP	TRQYEGPLHH	YRPQQESPSP	QQQLPPSSQA	EGMGQVPRTH	RLITLADHIC	QIITQDFARN	QVSSQTPQQP
2170	2180	2190	2200	2210	2220	2230	2240

Detailed Protein Report

Cmpd.	No. of Cmpds.	m/z meas.	Δ m/z [ppm]	z	Rt [min]	Score	P	Range	Sequence	Modification	Ratios
2870	1	973.5098	46.41	2	64.6	11.5	1	426-441	K.FINMNGLMEDPMKVYK.D	Oxidation: 8	
41	6	1068.4648	-20.43	2	29.7	15.6	2	2030-2049	K.DARERGSQSSDSSSSLSSHR.Y		M:Q 0.83<br>Q:W 1.09
911	1	492.2027	-52.98	2	40.9	10.7	0	2341-2349	K.ALMGSFDDK.V		

Detailed Protein Report

Protein 324: PREDICTED: zinc finger protein castor homolog 1 isoform X1 [Homo sapiens]

**Accession:**	gi|530360605	**Score:**	50.6
**Database:**	refseq_human(refseq_human_20140103.fasta)	**MW [kDa]:**	192.9
**Database Date:**	2015-11-30	**pI:**	6.6
**Modification(s):**	Carbamidomethyl	**Sequence Coverage [%]:**	2.6
		**No. of unique Peptides:**	2

10	20	30	40	50	60	70	80
MKVDYLSEFG	FRLSDYEFGT	KEKRMDLGTA	EGTRCTDPPA	GKPAMAPKRK	GGLKLNAICA	KLSRQVVVEK	RADAGSHTEG
90	100	110	120	130	140	150	160
SPSQPRDQER	SGPESGAARA	PRSEEDKRRA	VIEKWVNGEY	SEEPAPTPVL	GRIAREGLEL	PPEGVYMVQP	QGCSDEEDHA
170	180	190	200	210	220	230	240
EEPSKDGGAL	EEKDSDGAAS	KEDSGPSTRQ	ASGEASSLRD	YAASTMTEFL	GMFGYDDQNT	RDELARKISF	EKLHAGSTPE
250	260	270	280	290	300	310	320
AATSSMLPTS	EDTLSKRARF	SKYEEYIRKL	KAGEQLSWPA	PSTKTEERVG	KEVVGTLPGL	RLPSSTAHLE	TKATILPLPS
330	340	350	360	370	380	390	400
HSSVQMQNLV	ARASKYDFFI	QKLKTGENLR	PQNGSTYKKP	SKYDLENVKY	LHLFKPGEGS	PDMGGAIAFK	TGKVGRPSKY
410	420	430	440	450	460	470	480
DVRGIQKPGP	AKVPPTPSLA	PAPLASVPSA	PSAPGPGPEP	PASLSFNTPE	YLKSTFSKTD	SITTGTVSTV	KNGLPTDKPA
490	500	510	520	530	540	550	560
VTEDVNIYQK	YIARFSGSQH	CGHIHCAYQY	REHYHCLDPE	CNYQRFTSKQ	DVIRHYNMHK	KRDNSLQHGF	MRFSPLDDCS
570	580	590	600	610	620	630	640
VYYHGCHLNG	KSTHYHCMQV	GCNKVYTSTS	DVMTHENFHK	KNTQLINDGF	QRFRATEDCG	TADCQFYGQK	TTHFHCRRPG
650	660	670	680	690	700	710	720
CTFTFKNKCD	IEKHKSYHIK	DDAYAKDGFK	KFYKYEECKY	EGCVYSKATN	HFHCIRAGCG	FTFTSTSQMT	SHKRKHERRH
730	740	750	760	770	780	790	800
IRSSGALGLP	PSLLGAKDTE	HEESSNDDLV	DFSALSSKNS	SLSASPTSQQ	SSASLAAATA	ATEAGPSATK	PPNSKISGLL
810	820	830	840	850	860	870	880
PQGLPGSIPL	ALALSNSGLP	TPTPYFPILA	GRGSTSLPVG	TPSLLGAVSS	GSAASATPDT	PTLVASGAGD	SAPVAAASVP
890	900	910	920	930	940	950	960
APPASIMERI	SASKGLISPM	MARLAAAALK	PSATFDPGSG	QQVTPARFPP	AQVKPEPGES	TGAPGPHEAS	QDRSLDLTVK
970	980	990	1000	1010	1020	1030	1040
EPSNESNGHA	VPANSSLLSS	LMNKMSQGNP	GLGSLLNIKA	EAEGSPAAEP	SPFLGKAVKA	LVQEKLAEPW	KVYLRRFGTK
1050	1060	1070	1080	1090	1100	1110	1120
DFCDGQCDFL	HKAHFHCVVE	ECGALFSTLD	GAIKHANFHF	RTEGGAAKGN	TEAAFPASAA	ETKPPMAPSS	PPVPPVTTAT
1130	1140	1150	1160	1170	1180	1190	1200
VSSLEGPAPS	PASVPSTPTL	LAWKQLASTI	PQMPQIPASV	PHLPASPLAT	TSLENAKPQV	KPGFLQFQEN	DPCLATDCKY
1210	1220	1230	1240	1250	1260	1270	1280
ANKFHFHCLF	GNCKYVCKTS	GKAESHCLDH	INPNNNLVNV	RDQFAYYSLQ	CLCPNQHCEF	RMRGHYHCLR	TGCYFVTNIT
1290	1300	1310	1320	1330	1340	1350	1360
TKLPWHIKKH	EKAERRAANG	FKYFTKREEC	GRLGCKYNQV	NSHFHCIREG	CQFSFLLKHQ	MTSHARKHMR	RMLGKNFDRV
1370	1380	1390	1400	1410	1420	1430	1440
PPSQGPPGLM	DAETDECMDY	TGCSPGAMSS	ESSTMDRSCS	STPVGNESTA	AGNTISMPTA	SGAKKRFWII	EDMSPFGKRR
1450	1460	1470	1480	1490	1500	1510	1520
KTASSRKMLD	EGMMLEGFRR	FDLYEDCKDA	ACQFSLKVTH	YHCTRENCGY	KFCGRTHMYK	HAQHHDRVDN	LVLDDFKRFK
1530	1540	1550	1560	1570	1580	1590	1600
ASLSCHFADC	PFSGTSTHFH	CLRCRFRCTD	STKVTAHRKH	HGKQDVISAA	GFCQFSSSAD	CAVPDCKYKL	KCSHFHCTFP
1610	1620	1630	1640	1650	1660	1670	1680
GCRHTVVGMS	QMDSHKRKHE	KQERGEPAAE	GPAPGPPISL	DGSLSLGAEP	GSLLFLQSAA	AGLGLALGDA	GDPGPPDAAA
1690	1700	1710	1720	1730	1740	1750	1760
PGPREGAAAA	AAAAGESSQE	DEEEELELPE	EEAEDDEDED	DDEDDDDEDD	DEDDDDEDLR	TDSEESLPEA	AAEAAGAGAR
1770	1780	1790					
TPALAALAAL	GAPGPAPTAA	SSP					

Cmpd.	No. of	m/z meas.		z	Rt	Score	P	Range	Sequence	Modification

Detailed Protein Report

	Cmpds.		Δ m/z [ppm]		[min]					
2672	4	673.3651	67.68	2	63.5	13.0	2	1303-1312	K.YFTKREECGR.L	Carbamidomethyl: 8
1857	1	604.2655	-35.90	2	53.6	12.3	2	1307-1316	K.REECGRLGCK.Y	Carbamidomethyl: 9

Detailed Protein Report

Protein 325: PREDICTED: ankyrin repeat domain-containing protein 26 isoform X6 [Homo sapiens]

**Accession:**	gi|578818366	**Score:**	50.6
**Database:**	refseq_human(refseq_human_20140103.fasta)	**MW [kDa]:**	192.6
**Database Date:**	2015-11-30	**pI:**	5.4
		**Sequence Coverage [%]:**	1.7
		**No. of unique Peptides:**	1

Quantitation

M:Q **Median:** 2.22 **CV:** 0.00 % **No. of Peptides:** 1

10	20	30	40	50	60	70	80
MKKIFSKKGE	SPLGSFARRQ	RSSAGGGGEP	GEGAYSQPGY	HVRDRDLGKI	HKAASAGNVA	KVQQILLLRK	NGLNDRDKMN
**90**	**100**	**110**	**120**	**130**	**140**	**150**	**160**
RTALHLACAN	GHPEVVTLLV	DRKCQLNVCD	NENRTALMKA	VQCQEEKCAT	ILLEHGADPN	LADVHGNTAL	HYAVYNEDIS
**170**	**180**	**190**	**200**	**210**	**220**	**230**	**240**
VATKLLLYDA	NIEAKNKDDL	TPLLLAVSGK	KQQMVEFLIK	KKANVNAVDK	LESSHQLISE	YKEERIPKHS	SQNSNSVDES
**250**	**260**	**270**	**280**	**290**	**300**	**310**	**320**
SEDSLSRLSG	KPGVDDSWPT	SDDEDLNFDT	KNVPKPSLAK	LMTASQQSRK	NLEATYGTVR	TGNRTLFEDR	DSDSQDEVVV
**330**	**340**	**350**	**360**	**370**	**380**	**390**	**400**
ESLPTTSIKV	QCFSHPTYQS	PDLLPKPSHK	SLANPGLMKE	EPTKPGIAKK	ENGIDIIESA	PLEQTNNDNL	TYVDEVHKNN
**410**	**420**	**430**	**440**	**450**	**460**	**470**	**480**
RSDMMSALGL	GQEEDIESPW	DSESISENFP	QKYVDPLAGA	ADGKEKNIGN	EQAEESPERY	LHLKPTIEMK	DSVPNKAGGM
**490**	**500**	**510**	**520**	**530**	**540**	**550**	**560**
KDVQTSKAAE	HDLEVASEEE	QEREGSENNQ	PQVEEERKKH	RNNEMEVSAN	IHDGATDDAE	DDDDDDGLIQ	KRKSGETDHQ
**570**	**580**	**590**	**600**	**610**	**620**	**630**	**640**
QFPRKENKEY	ASSGPALQMK	EVKSTEKEKR	TSKESVNSPV	FGKASLLTGG	LLQVDDDSSL	SEIDEDEGRP	TKKTSNEKNK
**650**	**660**	**670**	**680**	**690**	**700**	**710**	**720**
VKNQIQSMDD	VDDLTQSSET	ASEDCELPHS	SYKNFMLLIE	QLGMECKDSV	SLLKIQDAAL	SCERLLELKK	NHCELLTVKI
**730**	**740**	**750**	**760**	**770**	**780**	**790**	**800**
KKMEDKVNVL	QRELSETKEI	KSQLEHQKVE	WERELCSLRF	SLNQEEEKRR	NADTLYEKIR	EQLRRKEEQY	RKEVEVKQQL
**810**	**820**	**830**	**840**	**850**	**860**	**870**	**880**
ELSLQTLEME	LRTVKSNLNQ	VVQERNDAQR	QLSREQNARM	LQDGILTNHL	SKQKEIEMAQ	KKMNSENSHS	HEEEKDLSHK
**890**	**900**	**910**	**920**	**930**	**940**	**950**	**960**
NSMLQEEIAM	LRLEIDTIKN	QNQEKEKKCF	EDLKIVKEKN	EDLQKTIKQN	EETLTQTISQ	YNGRLSVLTA	ENAMLNSKLE
**970**	**980**	**990**	**1000**	**1010**	**1020**	**1030**	**1040**
NEKQSKERLE	AEVESYHSRL	AAAIHDRDQS	ETSKRELELA	FQRARDECSR	LQDKMNFDVS	NLKDNNEILS	QQLFKTESKL
**1050**	**1060**	**1070**	**1080**	**1090**	**1100**	**1110**	**1120**
NSLEIEFHHT	RDALREKTLG	LERVQKDLSQ	TQCQMKEMEQ	KYQNEQVKVN	KYIGKQESVE	ERLSQLQSEN	MLLRQQLDDA
**1130**	**1140**	**1150**	**1160**	**1170**	**1180**	**1190**	**1200**
HNKADNKEKT	VINIQDQFHA	IVQKLQAESE	KQSLLLEERN	KELISECNHL	KERQYQYENE	KAEREVVVRQ	LQQELADTLK
**1210**	**1220**	**1230**	**1240**	**1250**	**1260**	**1270**	**1280**
KQSMSEASLE	VTSRYRINLE	DETQDLKKKL	GQIRNQLQEA	QDRHTEAVRC	AEKMQDHKQK	LEKDNAKLKV	TVKKQMDKIE
**1290**	**1300**	**1310**	**1320**	**1330**	**1340**	**1350**	**1360**
ELQKNLLNAN	LSEDEKEQLK	KLMELKQSLE	CNLDQEMKKN	VELEREITGF	KNLLKMTRKK	LNEYENGEFS	FHGDLKTSQF
**1370**	**1380**	**1390**	**1400**	**1410**	**1420**	**1430**	**1440**
EMDIQINKLK	HKIDDLTAEL	ETAGSKCLHL	DTKNQILQEE	LLSMKTVQKK	CEKLQKNKKK	LEQEVINLRS	HIERNMVELG
**1450**	**1460**	**1470**	**1480**	**1490**	**1500**	**1510**	**1520**
QVKQYKQEIE	ERARQEIAEK	LKEVNLFLQA	QAASQENLEQ	FRENNFASMK	SQMELRIKDL	ESELSKIKTS	QEDFNKTELE
**1530**	**1540**	**1550**	**1560**	**1570**	**1580**	**1590**	**1600**
KYKQLYLEEL	KVRKSLSSKL	TKTNERLAEV	NTKLLVEKQQ	SRSLFTTLTT	RPVMEPPCVG	NLNNSLDLNR	KLIPRENLVI
**1610**	**1620**	**1630**	**1640**	**1650**	**1660**	**1670**	**1680**
STSNPRASNN	SMENYLSKMQ	QELEKNITRE	LKEAAAELES	GSIASPLGST	DESNLNQDLV	WKASREYVQV	LKKNYMI

Cmpd.	No. of Cmpds.	m/z meas.	Δ m/z [ppm]	z	Rt [min]	Score	P	Range	Sequence	Modification	Ratios	
1740	1	555.2376	-81.69	2	51.7	17.4	1	770-778	R.RNADTLYEK.I		M:Q	2.22

Detailed Protein Report

Protein 326: nischarin isoform 1 [Homo sapiens]

**Accession:**	gi|66472382	**Score:**	50.5
**Database:**	refseq_human(refseq_human_20140103.fasta)	**MW [kDa]:**	166.5
**Database Date:**	2015-11-30	**pI:**	4.9
**Modification(s):**	Carbamidomethyl	**Sequence Coverage [%]:**	4.5
		**No. of unique Peptides:**	4

Quantitation

Q:W	**Median:**	0.89	**CV:**	0.00 %	**No. of Peptides:**	1
M:Q	**Median:**	1.40	**CV:**	0.00 %	**No. of Peptides:**	1

10	20	30	40	50	60	70	80
MATARTFGPE	REAEPAKEAR	VVGSELVDTY	TVYIIQVTDG	SHEWTVKHRY	SDFHDLHEKL	VAERKIDKNL	LPPKKIIGKN
90	100	110	120	130	140	150	160
SRSLVEKREK	DLEVYLQKLL	AAFPGVTPRV	LAHFLHFHFY	EINGITAALA	EELFEKGEQL	LGAGEVFAIG	PLQLYAVTEQ
170	180	190	200	210	220	230	240
LQQGKPTCAS	GDAKTDLGHI	LDFTCRLKYL	KVSGTEGPFG	TSNIQEQLLP	FDLSIFKSLH	QVEISHCDAK	HIRGLVASKP
250	260	270	280	290	300	310	320
TLATLSVRFS	ATSMKEVLVP	EASEFDEWEP	EGTTLEGPVT	AVIPTWQALT	TLDLSHNSIS	EIDESVKLIP	KIEFLDLSHN
330	340	350	360	370	380	390	400
GLLVVDNLQH	LYNLVHLDLS	YNKLSSLEGL	HTKLGNIKTL	NLAGNLLESL	SGLHKLYSLV	NLDLRDNRIE	QMEEVRSIGS
410	420	430	440	450	460	470	480
LPCLEHVSLL	NNPLSIIPDY	RTKVLAQFGE	RASEVCLDDT	VTTEKELDTV	EVLKAIQKAK	EVKSKLSNPE	KKGGEDSRLS
490	500	510	520	530	540	550	560
AAPCIRPSSS	PPTVAPASAS	LPQPILSNQG	IMFVQEEALA	SSLSSTDSLT	PEHQPIAQGC	SDSLESIPAG	QAASDDLRDV
570	580	590	600	610	620	630	640
PGAVGGASPE	HAEPEVQVVP	GSGQIIFLPF	TCIGYTATNQ	DFIQRLSTLI	RQAIERQLPA	WIEAANQREE	GQGEQGEEED
650	660	670	680	690	700	710	720
EEEEEEEDVA	ENRYFEMGPP	DVEEEEGGGQ	GEEEEEEEED	EEAEEEERLAL	EWALGADEDF	LLEHIRILKV	LWCFLIHVQG
730	740	750	760	770	780	790	800
SIRQFAACLV	LTDFGIAVFE	IPHQESRGSS	QHILSSLRFV	FCFPHGDLTE	FGFLMPELCL	VLKVRHSENT	LFIISDAANL
810	820	830	840	850	860	870	880
HEFHADLRSC	FAPQHMAMLC	SPILYGSHTS	LQEFLRQLLT	FYKVAGGCQE	RSQGCFPVYL	VYSDKRMVQT	AAGDYSGNIE
890	900	910	920	930	940	950	960
WASCTLCSAV	RRSCCAPSEA	VKSAAIPYWL	LLTPQHLNVI	KADFNPMPNR	GTHNCRNRNS	FKLSRVPLST	VLLDPTRSCT
970	980	990	1000	1010	1020	1030	1040
QPRGAFADGH	VLELLVGYRF	VTAIFVLPHE	KFHFLRVYNQ	LRASLQDLKT	VVIAKTPGTG	GSPQGSFADG	QPAERRASND
1050	1060	1070	1080	1090	1100	1110	1120
QRPQEVPAEA	LAPAPAEVPA	PAPAAASASG	PAKTPAPAEA	STSALVPEET	PVEAPAPPPA	EAPAQYPSEH	LIQATSEENQ
1130	1140	1150	1160	1170	1180	1190	1200
IPSHLPACPS	LRHVASLRGS	AIIELFHSSI	AEVENEELRH	LMWSSVVFYQ	TPGLEVTACV	LLSTKAVYFV	LHDGLRRYFS
1210	1220	1230	1240	1250	1260	1270	1280
EPLQDFWHQK	NTDYNNSPFH	ISQCFVLKLS	DLQSVNVGLF	DQHFRLTGST	PMQVVTCLTR	DSYLTHCFLQ	HLMVVLSSLE
1290	1300	1310	1320	1330	1340	1350	1360
RTPSPEPVDK	DFYSEFGNKT	TGKMENYELI	HSSRVKFTYP	SEEEIGDLTF	TVAQKMAEPE	KAPALSILLY	VQAFQVGMPP
1370	1380	1390	1400	1410	1420	1430	1440
PGCCRGPLRP	KTLLLTSSEI	FLLDEDCVHY	PLPEFAKEPP	QRDRYRLDDG	RRVRDLDRVL	MGYQTYPQAL	TLVFDDVQGH
1450	1460	1470	1480	1490	1500	1510	
DLMGSVTLDH	FGEVPGGPAR	ASQGREVQWQ	VFVPSAESRE	KLISLLARQW	EALCGRELPV	ELTG	

Cmpd.	No. of Cmpds.	m/z meas.	Δ m/z [ppm]	z	Rt [min]	Score	P	Range	Sequence	Modification	Ratios
2021	1	890.3901	-125.48	2	55.7	10.8	0	359-375	K.TLNLAGNLLESLSGLHK.L		M:Q 1.40<br>Q:W 0.89
2625	1	885.4475	-7.11	2	62.9	13.6	1	837-851	R.QLLTFYKVAGGCQER.S	Carbamidomethyl: 12	
2202	1	701.1391	108.48	3	57.9	10.8	1	980-996	R.FVTAIFVLPHEKFHFLR.V		
1738	1	1087.9436	-77.77	2	50.4	15.3	0	1317-1335	K.FTYPSEEEIGDLTFTVAQK.M		

Detailed Protein Report

Protein 327: zinc finger protein 573 isoform 4 [Homo sapiens]

**Accession:**	gi|289577063	**Score:**	50.5
**Database:**	refseq_human(refseq_human_20140103.fasta)	**MW [kDa]:**	77.9
**Database Date:**	2015-11-30	**pI:**	10.2
**Modification(s):**	Oxidation	**Sequence Coverage [%]:**	7.5
		**No. of unique Peptides:**	1

10	20	30	40	50	60	70	80
MFPVLEPHQV	GLIRSYNSKT	MTCELVTFRD	VAIDFSRQEW	EYLDPNQRDL	YRDVMLENYR	NLVSLGGHSI	SKPVVVDLLE
90	100	110	120	130	140	150	160
RGKEPWMILR	EETQFTDLDL	QCEIISYIEV	PTYETDISST	QLQSIYKREK	LYECKKCQKK	FSSGYQLILH	HRFHVIERPY
170	180	190	200	210	220	230	240
ECKECGKNFR	SGYQLTLHQR	FHTGEKPYEC	TECGKNFRSG	YQLTVHQRFH	TGEKTYECRQ	CGKAFIYASH	IVQHERIHTG
250	260	270	280	290	300	310	320
GKPYECQECG	RAFSQGGHLR	IHQRVHTGEK	PYKCKECGKT	FSRRSNLVEH	GQFHTDEKPY	ICEKCGKAFR	RGHQLTVHQR
330	340	350	360	370	380	390	400
VHTGKKPYEC	KECGKGYTTA	SYFLLHQRIH	KGGKPYECKE	CKKTFTLYRN	LTRHQNIHTG	EKLFECKQCG	KTYTTGSKLF
410	420	430	440	450	460	470	480
QHQKTHTGEK	PYECKECGKA	FSLYGYLKQH	QKIHTGMKHF	ECKECKKTFT	LYRNLTRHQN	IHTGKKLFEC	QECGKAYSTG
490	500	510	520	530	540	550	560
SNLIQHRKTH	TGEKPYKCKE	CGKTFSLHGY	LNQHQKIHTG	MKPYECKVCR	KTFTFYRNLT	LHQSIHTDEK	PFECKECGKT
570	580	590	600	610	620	630	640
FRRSSHLTAH	QSIHADKKPY	ECKECGKAFK	MYGYLTQHQK	IHTGGKPYEC	KECGKAFSRA	SNLVQHERIH	TGEKPYVCKQ
650	660	670					
CGKTFRYGSA	LKAHQRIHRS	IKV					

Cmpd.	No. of Cmpds.	m/z meas.	Δ m/z [ppm]	z	Rt [min]	Score	P	Range	Sequence	Modification
2167	1	900.3438	-97.52	3	55.5	11.8	2	15-37	R.SYNSKTMTCELVTFRDVAIDFSR.Q	Oxidation: 7

Detailed Protein Report

Protein 328: von Willebrand factor preproprotein [Homo sapiens]

**Accession:**	gi|89191868	**Score:**	50.4
**Database:**	refseq_human(refseq_human_20140103.fasta)	**MW [kDa]:**	309.1
**Database Date:**	2015-11-30	**pI:**	5.2
**Modification(s):**	Carbamidomethyl	**Sequence Coverage [%]:**	1.8
		**No. of unique Peptides:**	3

Quantitation

Q:W	**Median:**	1.01	**CV:**	22.46 %	**No. of Peptides:**	2
M:Q	**Median:**	0.51	**CV:**	158.88 %	**No. of Peptides:**	2

Detailed Protein Report

10	20	30	40	50	60	70	80
MIPARFAGVL	LALALILPGT	LCAEGTRGRS	STARCSLFGS	DFVNTFDGSM	YSFAGYCSYL	LAGGCQKRSF	SIIGDFQNGK
90	100	110	120	130	140	150	160
RVSLSVYLGE	FFDIHLFVNG	TVTQGDQRVS	MPYASKGLYL	ETEAGYYKLS	GEAYGFVARI	DGSGNFQVLL	SDRYFNKTCG
170	180	190	200	210	220	230	240
LCGNFNIFAE	DDFMTQEGTL	TSDPYDFANS	WALSSGEQWC	ERASPPSSSC	NISSGEMQKG	LWEQCQLLKS	TSVFARCHPL
250	260	270	280	290	300	310	320
VDPEPFVALC	EKTLCECAGG	LECACPALLE	YARTCAQEGM	VLYGWTDHSA	CSPVCPAGME	YRQCVSPCAR	TCQSLHINEM
330	340	350	360	370	380	390	400
CQERCVDGCS	CPEGQLLDEG	LCVESTECPC	VHSGKRYPPG	TSLSRDCNTC	ICRNSQWICS	NEECPGECLV	TGQSHFKSFD
410	420	430	440	450	460	470	480
NRYFTFSGIC	QYLLARDCQD	HSFSIVIETV	QCADDRDAVC	TRSVTVRLPG	LHNSLVKLKH	GAGVAMDGQD	VQLPLLKGDL
490	500	510	520	530	540	550	560
RIQHTVTASV	RLSYGEDLQM	DWDGRGRLLV	KLSPVYAGKT	CGLCGNYNGN	QGDDFLTPSG	LAEPRVEDFG	NAWKLHGDCQ
570	580	590	600	610	620	630	640
DLQKQHSDPC	ALNPRMTRFS	EEACAVLTSP	TFEACHRAVS	PLPYLRNCRY	DVCSCSDGRE	CLCGALASYA	AACAGRGVRV
650	660	670	680	690	700	710	720
AWREPGRCEL	NCPKGQVYLQ	CGTPCNLTCR	SLSYPDEECN	EACLEGCFCP	PGLYMDERGD	CVPKAQCPCY	YDGEIFQPED
730	740	750	760	770	780	790	800
IFSDHHTMCY	CEDGFMHCTM	SGVPGSLLPD	AVLSSPLSHR	SKRSLSCRPP	MVKLVCPADN	LRAEGLECTK	TCQNYDLECM
810	820	830	840	850	860	870	880
SMGCVSGCLC	PPGMVRHENR	CVALERCPCF	HQGKEYAPGE	TVKIGCNTCV	CRDRKWNCTD	HVCDATCSTI	GMAHYLTFDG
890	900	910	920	930	940	950	960
LKYLFPGECQ	YVLVQDYCGS	NPGTFRILVG	NKGCSHPSVK	CKKRVTILVE	GGEIELFDGE	VNVKRPMKDE	THFEVVESGR
970	980	990	1000	1010	1020	1030	1040
YIILLLGKAL	SVVWDRHLSI	SVVLKQTYQE	KVCGLCGNFD	GIQNNDLTSS	NLQVEEDPVD	FGNSWKVSSQ	CADTRKVPLD
1050	1060	1070	1080	1090	1100	1110	1120
SSPATCHNNI	MKQTMVDSSC	RILTSDVFQD	CNKLVDPEPY	LDVCIYDTCS	CESIGDCACF	CDTIAAYAHV	CAQHGKVVTW
1130	1140	1150	1160	1170	1180	1190	1200
RTATLCPQSC	EERNLRENGY	ECEWRYNSCA	PACQVTCQHP	EPLACPVQCV	EGCHAHCPPG	KILDELLQTC	VDPEDCPVCE
1210	1220	1230	1240	1250	1260	1270	1280
VAGRRFASGK	KVTLNPSDPE	HCQICHCDVV	NLTCEACQEP	GGLVVPPTDA	PVSPTTLYVE	DISEPPLHDF	YCSRLLDLVF
1290	1300	1310	1320	1330	1340	1350	1360
LLDGSSRLSE	AEFEVLKAFV	VDMMERLRIS	QKWVRVAVVE	YHDGSHAYIG	LKDRKRPSEL	RRIASQVKYA	GSQVASTSEV
1370	1380	1390	1400	1410	1420	1430	1440
LKYTLFQIFS	KIDRPEASRI	TLLLMASQEP	QRMSRNFVRY	VQGLKKKKVI	VIPVGIGPHA	NLKQIRLIEK	QAPENKAFVL
1450	1460	1470	1480	1490	1500	1510	1520
SSVDELEQQR	DEIVSYLCDL	APEAPPPTLP	PDMAQVTVGP	GLLGVSTLGP	KRNSMVLDVA	FVLEGSDKIG	EADFNRSKEF
1530	1540	1550	1560	1570	1580	1590	1600
MEEVIQRMDV	GQDSIHVTVL	QYSYMVTVEY	PFSEAQSKGD	ILQRVREIRY	QGGNRTNTGL	ALRYLSDHSF	LVSQGDREQA
1610	1620	1630	1640	1650	1660	1670	1680
PNLVYMVTGN	PASDEIKRLP	GDIQVVPIGV	GPNANVQELE	RIGWPNAPIL	IQDFETLPRE	APDLVLQRCC	SGEGLQIPTL
1690	1700	1710	1720	1730	1740	1750	1760
SPAPDCSQPL	DVILLLDGSS	SFPASYFDEM	KSFAKAFISK	ANIGPRLTQV	SVLQYGSITT	IDVPWNVVPE	KAHLLSLVDV
1770	1780	1790	1800	1810	1820	1830	1840
MQREGGPSQI	GDALGFAVRY	LTSEMHGARP	GASKAVVILV	TDVSVDSVDA	AADAARSNRV	TVFPIGIGDR	YDAAQLRILA
1850	1860	1870	1880	1890	1900	1910	1920
GPAGDSNVVK	LQRIEDLPTM	VTLGNSFLHK	LCSGFVRICM	DEDGNEKRPG	DVWTLPDQCH	TVTCQPDGQT	LLKSHRVNCD
1930	1940	1950	1960	1970	1980	1990	2000
RGLRPSCPNS	QSPVKVEETC	GCRWTCPCVC	TGSSTRHIVT	FDGQNFKLTG	SCSYVLFQNK	EQDLEVILHN	GACSPGARQG
2010	2020	2030	2040	2050	2060	2070	2080
CMKSIEVKHS	ALSVELHSDM	EVTVNGRLVS	VPYVGGNMEV	NVYGAIMHEV	RFNHLGHIFT	FTPQNNEFQL	QLSPKTFASK
2090	2100	2110	2120	2130	2140	2150	2160
TYGLCGICDE	NGANDFMLRD	GTVTTDWKTL	VQEWTVQRPG	QTCQPILEEQ	CLVPDSSHCQ	VLLLPLFAEC	HKVLAPATFY
2170	2180	2190	2200	2210	2220	2230	2240

Detailed Protein Report

Cmpd.	No. of Cmpds.	m/z meas.	Δ m/z [ppm]	z	Rt [min]	Score	P	Range	Sequence	Modification	Ratios	
1145	5	729.1568	-199.23	2	42.6	14.5	0	1944-1956	R.WTCPCVCTGSSTR.H	Carbamidomethyl: 5	M:Q<br>Q:W	1.55<br>1.27
1133	2	729.1918	-151.33	2	42.4	15.1	0	1944-1956	R.WTCPCVCTGSSTR.H	Carbamidomethyl: 3		
99	1	845.3517	-47.80	3	31.0	10.4	1	2289-2311	K.VNCTTQPCPTAKAPTCGLCEVAR L	Carbamidomethyl: 8, 16, 19	M:Q<br>Q:W	0.16<br>0.81

Detailed Protein Report

Protein 329: hemicentin-1 precursor [Homo sapiens]

**Accession:**	gi|118572606	**Score:**	50.4
**Database:**	refseq_human(refseq_human_20140103.fasta)	**MW [kDa]:**	613.0
**Database Date:**	2015-11-30	**pI:**	6.1
**Modification(s):**	Carbamidomethyl	**Sequence Coverage [%]:**	1.2
		**No. of unique Peptides:**	1

Detailed Protein Report

10	20	30	40	50	60	70	80
MISWEVVHTV	FLFALLYSSL	AQDASPQSEI	RAEEIPEGAS	TLAFVFDVTG	SMYDDLVQVI	EGASKILETS	LKRPKRPLFN
90	100	110	120	130	140	150	160
FALVPFHDPE	IGPVTITTDP	KKFQYELREL	YVQGGGDCPE	MSIGAIKIAL	EISLPGSFIY	VFTDARSKDY	RLTHEVLQLI
170	180	190	200	210	220	230	240
QQKQSQVVFV	LTGDCDDRTH	IGYKVYEEIA	STSSGQVFHL	DKKQVNEVLK	WVEEAVQASK	VHLLSTDHLE	QAVNTWRIPF
250	260	270	280	290	300	310	320
DPSLKEVTVS	LSGPSPMIEI	RNPLGKLIKK	GFGLHELLNI	HNSAKVVNVK	EPEAGMWTVK	TSSSGRHSVR	ITGLSTIDFR
330	340	350	360	370	380	390	400
AGFSRKPTLD	FKKTVSRPVQ	GIPTYVLLNT	SGISTPARID	LLELLSISGS	SLKTIPVKYY	PHRKPYGIWN	ISDFVPPNEA
410	420	430	440	450	460	470	480
FFLKVTGYDK	DDYLFQRVSS	VSFSSIVPDA	PKVTMPEKTP	GYYLQPGQIP	CSVDSLLPFT	LSFVRNGVTL	GVDQYLKESA
490	500	510	520	530	540	550	560
SVNLDIAKVT	LSDEGFYECI	AVSSAGTGRA	QTFFDVSEPP	PVIQVPNNVT	VTPGERAVLT	CLIISAVDYN	LTWQRNDRDV
570	580	590	600	610	620	630	640
RLAEPARIRT	LANLSLELKS	VKFNDAGEYH	CMVSSEGGSS	AASVFLTVQE	PPKVTVMPKN	QSFTGGSEVS	IMCSATGYPK
650	660	670	680	690	700	710	720
PKIAWTVNDM	FIVGSHRYRM	TSDGTLFIKN	AAPKDAGIYG	CLASNSAGTD	KQNSTLRYIE	APKLMVVQSE	LLVALGDITV
730	740	750	760	770	780	790	800
MECKTSGIPP	PQVKWFKGDL	ELRPSTFLII	DPLLGLLKIQ	ETQDLDAGDY	TCVAINEAGR	ATGKITLDVG	SPPVFIQEPA
810	820	830	840	850	860	870	880
DVSMEIGSNV	TLPCYVQGYP	EPTIKWRRLD	NMPIFSRPFS	VSSISQLRTG	ALFILNLWAS	DKGTYICEAE	NQFGKIQSET
890	900	910	920	930	940	950	960
TVTVTGLVAP	LIGISPSVAN	VIEGQQLTLP	CTLLAGNPIP	ERRWIKNSAM	LLQNPYITVR	SDGSLHIERV	QLQDGGEYTC
970	980	990	1000	1010	1020	1030	1040
VASNVAGTNN	KTTSVVVHVL	PTIQHGQQIL	STIEGIPVTL	PCKASGNPKP	SVIWSKKGEL	ISTSSAKFSA	GADGSLYVVS
1050	1060	1070	1080	1090	1100	1110	1120
PGGEESGEYV	CTATNTAGYA	KRKVQLTVYV	RPRVFGDQRG	LSQDKPVEIS	VLAGEEVTLP	CEVKSLPPPI	ITWAKETQLI
1130	1140	1150	1160	1170	1180	1190	1200
SPFSPRHTFL	PSGSMKITET	RTSDSGMYLC	VATNIAGNVT	QAVKLNVHVP	PKIQRGPKHL	KVQVGQRVDI	PCNAQGTPLP
1210	1220	1230	1240	1250	1260	1270	1280
VITWSKGGST	MLVDGEHHVS	NPDGTLSIDQ	ATPSDAGIYT	CVATNIAGTD	ETEITLHVQE	PPTVEDLEPP	YNTTFQERVA
1290	1300	1310	1320	1330	1340	1350	1360
NQRIEFPCPA	KGTPKPTIKW	LHNGRELTGR	EPGISILEDG	TLLVIASVTP	YDNGEYICVA	VNEAGTTERK	YNLKVHVPPV
1370	1380	1390	1400	1410	1420	1430	1440
IKDKEQVTNV	SVLLNQLTNL	FCEVEGTPSP	IIMWYKDNVQ	VTESSTIQTV	NNGKILKLFR	ATPEDAGRYS	CKAINIAGTS
1450	1460	1470	1480	1490	1500	1510	1520
QKYFNIDVLV	PPTIIGTNFP	NEVSVVLNRD	VALECQVKGT	PFPDIHWFKD	GKPLFLGDPN	VELLDRGQVL	HLKNARRNDK
1530	1540	1550	1560	1570	1580	1590	1600
GRYQCTVSNA	AGKQAKDIKL	TIYIPPSIKG	GNVTTDISVL	INSLIKLECE	TRGLPMPAIT	WYKDGQPIMS	SSQALYIDKG
1610	1620	1630	1640	1650	1660	1670	1680
QYLHIPRAQV	SDSATYTCHV	ANVAGTAEKS	FHVDVYVPPM	IEGNLATPLN	KQVVIAHSLT	LECKAAGNPS	PILTWLKDGV
1690	1700	1710	1720	1730	1740	1750	1760
PVKANDNIRI	EAGGKKLEIM	SAQEIDRGQY	ICVATSVAGE	KEIKYEVDVL	VPPAIEGGDE	TSYFIVMVNN	LLELDCHVTG
1770	1780	1790	1800	1810	1820	1830	1840
SPPPTIMWLK	DGQLIDERDG	FKILLNGRKL	VIAQAQVSNT	GLYRCMAANT	AGDHKKEFEV	TVHVPPTIKS	SGLSERVVVK
1850	1860	1870	1880	1890	1900	1910	1920
YKPVALQCIA	NGIPNPSITW	LKDDQPVNTA	QGNLKIQSSG	RVLQIAKTLL	EDAGRYTCVA	TNAAGETQQH	IQLHVHEPPS
1930	1940	1950	1960	1970	1980	1990	2000
LEDAGKMLNE	TVLVSNPVQL	ECKAAGNPVP	VITWYKDNRL	LSGSTSMTFL	NRGQIIDIES	AQISDAGIYK	CVAINSAGAT
2010	2020	2030	2040	2050	2060	2070	2080
ELFYSLQVHV	APSISGSNNM	VAVVVNNPVR	LECEARGIPA	PSLTWLKDGS	PVSSFSNGLQ	VLSGGRILAL	TSAQISDTGR
2090	2100	2110	2120	2130	2140	2150	2160
YTCVAVNAAG	EKQRDIDLRV	YVPPNIMGEE	QNVSVLISQA	VELLCQSDAI	PPPTLTWLKD	GHPLLKKPGL	SISENRSVLK
2170	2180	2190	2200	2210	2220	2230	2240

Detailed Protein Report

Cmpd.	No. of Cmpds.	m/z meas.	Δ m/z [ppm]	z	Rt [min]	Score	P	Range	Sequence	Modification
1984	1	883.9757	-72.13	3	53.5	15.0	1	5183-5205	R.RTSDGLSCQDINECQESSPCHQR.C	Carbamidomethyl: 8

Detailed Protein Report

Protein 330: **neuroblastoma breakpoint family member 3 isoform 2 [Homo sapiens]**

**Accession:**	gi|374088174	**Score:**	50.3
**Database:**	refseq_human(refseq_human_20140103.fasta)	**MW [kDa]:**	71.6
**Database Date:**	2015-11-30	**pI:**	4.3
**Modification(s):**	Oxidation	**Sequence Coverage [%]:**	7.6
		**No. of unique Peptides:**	2

Quantitation

M:Q **Median:** 1.95 **CV:** 0.00 % **No. of Peptides:** 1

10	20	30	40	50	60	70	80
MPLTPTVQGF	QWTLRGPDVE	TSPFGAPRAA	SHGVGRHQEL	RDPTVPGPTS	SATNVSMVVS	AGPWSGEKAE	MNILEINKKS
**90**	**100**	**110**	**120**	**130**	**140**	**150**	**160**
RPQLAENKQQ	FRNLKQKCLV	TQVAYFLANR	QNNYDYEDCK	DLIKSMLRDE	RLLTEEKLAE	ELGQAEELRQ	YKVLVHSQER
**170**	**180**	**190**	**200**	**210**	**220**	**230**	**240**
ELTQLREKLQ	EGRDASRSLN	QHLQALLTPD	EPDNSQGRDL	REQLAEGCRL	AQHLVQKLSP	ENDDDEDEDV	KVEEAEKVQE
**250**	**260**	**270**	**280**	**290**	**300**	**310**	**320**
LYAPREVQKA	EEKEVPEDSL	EECAITCSNS	HHPCESNQPY	GNTRITFEED	QVDSTLIDSS	SHDEWLDAVC	IIPENESDHE
**330**	**340**	**350**	**360**	**370**	**380**	**390**	**400**
QEEEKGPVSP	RSSGRFCCLI	SVGYIFCHPC	PAWLIRNLQE	SEEEEAPQES	WDEGHWCDQV	KKEDQEATSP	RLSRELLDEK
**410**	**420**	**430**	**440**	**450**	**460**	**470**	**480**
EPEVLQDSLD	RFYSTPFEYL	ELPDLCQPYR	SDFYSLQEQH	LGLALDLDRM	KKDQEEEEDQ	GPPCPRLSRE	LPEVVEPEDL
**490**	**500**	**510**	**520**	**530**	**540**	**550**	**560**
QDSLDRWYST	PFSYPELPDS	CQPYGSCFYS	LEEEHVGFSL	DVDEIEKYQE	GEEDQKPPCP	RLNEVLMEAE	EPEVLQDSLD
**570**	**580**	**590**	**600**	**610**	**620**	**630**	
RCYSTTSTYF	QLHASFQQYR	SAFYSFEEQD	VSLALDVDNR	FFTLTVIRHH	LAFQMGVIFP	H	

Cmpd.	No. of Cmpds.	m/z meas.	Δ m/z [ppm]	z	Rt [min]	Score	P	Range	Sequence	Modification	Ratios
2286	1	665.3174	-11.91	2	59.0	11.0	0	16-28	R.GPDVETSPFGAPR.A		M:Q 1.95
1974	2	696.2762	-133.49	2	54.8	13.9	2	121-131	K.DLIKSMLRDER.L	Oxidation: 6	

Detailed Protein Report

Protein 331: leukocyte receptor cluster member 1 [Homo sapiens]

**Accession:**	gi|24308289	**Score:**	50.2
**Database:**	refseq_human(refseq_human_20140103.fasta)	**MW [kDa]:**	30.5
**Database Date:**	2015-11-30	**pI:**	10.1
		**Sequence Coverage [%]:**	8.3
		**No. of unique Peptides:**	1

10	20	30	40	50	60	70	80
MNILPKKSWH	VRNKDNVARV	RRDEAQAREE	EKERERRVLL	AQQEARTEFL	RKKARHQNSL	PELEAAEAGA	PGSGPVDLFR
**90**	**100**	**110**	**120**	**130**	**140**	**150**	**160**
ELLEEGKGVI	RGNKEYEEEK	RQEKERQEKA	LGILTYLGQS	AAEAQTQPPW	YQLPPGRGGP	PPGPAPDEKI	KSRLDPLREM
**170**	**180**	**190**	**200**	**210**	**220**	**230**	**240**
QKHLGKKRQH	GGDEGSRSRK	EKEGSEKQRP	KEPPSLDQLR	AERLRREAAE	RSRAEALLAR	VQGRALQEGQ	PEEDETDDRR
**250**	**260**	**270**					
RRYNSQFNPQ	LARRPRQQDP	HLTH					

Cmpd.	No. of Cmpds.	m/z meas.	Δ m/z [ppm]	z	Rt [min]	Score	P	Range	Sequence	Modification
575	1	565.3015	-6.83	2	36.5	11.1	1	154-162	R.LDPLREMQK.H	

Detailed Protein Report

Protein 332: ubiquitin carboxyl-terminal hydrolase 43 isoform 2 [Homo sapiens]

Accession: gi|389616163
Database: refseq_human(refseq_human_20140103.fasta)
Database Date: 2015-11-30

Score: 50.1
MW [kDa]: 122.2
pI: 10.2
Sequence Coverage [%]: 3.5
No. of unique Peptides: 2

10	20	30	40	50	60	70	80
MDLGPGDAAG	GGPLAPRPRR	RRSLRRLFSR	FLLALGSRSR	PGDSPPRPQP	GHCDGDGEGG	FACAPGPVPA	APGSPGEERP
**90**	**100**	**110**	**120**	**130**	**140**	**150**	**160**
PGPQPQLQLP	AGDGARPPGA	QGLKNHGNTC	FMNAVVQCLS	NTDLLAEFLA	LGRYRAAPGR	AEVTEQLAAL	VRALWTREYT
**170**	**180**	**190**	**200**	**210**	**220**	**230**	**240**
PQLSAEFKNA	VSKYGSQFQG	NSQHDALEFL	LWLLDRVHED	LEGSSRGPVS	EKLPPEATKT	SENCLSPSAQ	LPLGQSFVQS
**250**	**260**	**270**	**280**	**290**	**300**	**310**	**320**
HFQAQYRSSL	TCPHCLKQSN	TFDPFLCVSL	PIPLRQTRFL	SVTLVFPSKS	QRFLRVGLAV	PILSTVAALR	KMVAEEGGVP
**330**	**340**	**350**	**360**	**370**	**380**	**390**	**400**
ADEVILVELY	PSGFQRSFFD	EEDLNTIAEG	DNVYAFQVPP	SPSQGTLSAH	PLGLSASPRL	AAREGQRFSL	SLHSESKVLI
**410**	**420**	**430**	**440**	**450**	**460**	**470**	**480**
LFCNLVGSGQ	QASRFGPPFL	IREDRAVSWA	QLQQSILSKV	RHLMKSEAPV	QNLGSLFSIR	VVGLSVACSY	LSPKDSRPLC
**490**	**500**	**510**	**520**	**530**	**540**	**550**	**560**
HWAVDRVLHL	RRPGGPPHVK	LAVEWDSSVK	ERLFGSLQEE	RAQDADSVWQ	QQQAHQQHSC	TLDECFQFYT	KEEQLAQDDA
**570**	**580**	**590**	**600**	**610**	**620**	**630**	**640**
WKCPHCQVLQ	QGMVKLSLWT	LPDILIIHLK	RFCQVGERRN	KLSTLVKFPL	SGLNMAPHVA	QRSTSPEAGL	GPWPSWKQPD
**650**	**660**	**670**	**680**	**690**	**700**	**710**	**720**
CLPTSYPLDF	LYDLYAVCNH	HGNLQAYCRN	SLDGQWYSYD	DSTVEPLRED	EVNTRGAYIL	FYQKRNSIPP	WSASSSMRGS
**730**	**740**	**750**	**760**	**770**	**780**	**790**	**800**
TSSSLSDHWL	LRLGSHAGST	RGSLLSWSSA	PCPSLPQVPD	SPIFTNSLCN	QEKGGLEPRR	LVRGVKGRSI	SMKAPTTSRA
**810**	**820**	**830**	**840**	**850**	**860**	**870**	**880**
KQGPFKTMPL	RWSFGSKEKP	PGASVELVEY	LESRRRPRST	SQSIVSLLTG	TAGEDEKSAS	PRSNVALPAN	SEDGGRAIER
**890**	**900**	**910**	**920**	**930**	**940**	**950**	**960**
GPAGVPCPSA	QPNHCLAPGN	SDGPNTARKL	KENAGQDIKL	PRKFDLPLTV	MPSVEHEKPA	RPEGQKAMNW	KESFQMGSKS
**970**	**980**	**990**	**1000**	**1010**	**1020**	**1030**	**1040**
SPPSPYMGFS	GNSKDSRRGT	SELDRPLQGT	LTLLRSVFRK	KENRRNERAE	VSPQVPPVSL	VSGGLSPAMD	GQAPGSPPAL
**1050**	**1060**	**1070**	**1080**	**1090**	**1100**	**1110**	**1120**
RIPEGLARGL	GSRLERDVWS	APSSLRLPRK	ASRAPRGSAL	GMSQRTVPGE	QASYGTFQRV	KYHTLSLGRK	KTLPESSF

Cmpd.	No. of Cmpds.	m/z meas.	Δ m/z [ppm]	z	Rt [min]	Score	P	Range	Sequence	Modification
2741	1	656.8579	50.64	2	64.6	11.9	0	158-168	R.EYTPQLSAEFK.N	
2554	5	812.4077	-74.32	2	62.4	18.3	2	1042-1056	R.IPEGLARGLGSRLER.D	

Detailed Protein Report

Protein 333: peroxiredoxin-2 [Homo sapiens]

**Accession:**	gi|32189392	**Score:**	50.1
**Database:**	refseq_human(refseq_human_20140103.fasta)	**MW [kDa]:**	21.9
**Database Date:**	2015-11-30	**pI:**	5.6
		**Sequence Coverage [%]:**	5.6
		**No. of unique Peptides:**	1

Quantitation

Q:W **Median:** 0.59 **CV:** 0.00 % **No. of Peptides:** 1

10	20	30	40	50	60	70	80
MASGNARIGK	PAPDFKATAV	VDGAFKEVKL	SDYKGKYVVL	FFYPLDFTFV	CPTEIIAFSN	RAEDFRKLGC	EVLGVSVDSQ
**90**	**100**	**110**	**120**	**130**	**140**	**150**	**160**
FTHLAWINTP	RKEGGLGPLN	IPLLADVTRR	LSEDYGVLKT	DEGIAYRGLF	IIDGKGVLRQ	ITVNDLPVGR	SVDEALRLVQ
**170**	**180**	**190**	**200**				
AFQYTDEHGE	VCPAGWKPGS	DTIKPNVDDS	KEYFSKHN				

Cmpd.	No. of Cmpds.	m/z meas.	Δ m/z [ppm]	z	Rt [min]	Score	P	Range	Sequence	Modification	Ratios	
766	1	606.3268	-23.04	2	38.4	50.1	0	140-150	R.QITVNDLPVGR.S		Q:W	0.59

Detailed Protein Report

Protein 334: PREDICTED: centrosomal protein of 135 kDa isoform X1 [Homo sapiens]

Accession: gi|530376879
Database: refseq_human(refseq_human_20140103.fasta)
Database Date: 2015-11-30
Modification(s): Oxidation

Score: 50.0
MW [kDa]: 91.8
pI: 5.7
Sequence Coverage [%]: 5.2
No. of unique Peptides: 1

Quantitation

M:Q **Median:** 0.71 **CV:** 0.00 % **No. of Peptides:** 1

10	20	30	40	50	60	70	80
MQDLEETMAK	LQLELNLCQK	EKERLSDELL	VKSDLETVVH	QLEQEKQRLS	KKVESFAVTE	RQLTLEVERM	RLEHGIKRRD
90	100	110	120	130	140	150	160
RSPSRLDTFL	KGIEEERDYY	KKELERLQHI	IQRRSCSTSY	SAREKSSIFR	TPEKGDYNSE	IHQITRERDE	LQRMLERFEK
170	180	190	200	210	220	230	240
YMEDIQSNVK	LLTAERDKLS	VLYNEAQEEL	SALRKESTQT	TAPHNIVSLM	EKEKELALSD	LRRIMAEKEA	LREKLEHIEE
250	260	270	280	290	300	310	320
VSLFGKSELE	KTIEHLTCVN	HQLESEKYEL	KSKVLIMKET	IESLENKLKV	QAQKFSHVAG	DSSHQKTEVN	SLRIVNEQLQ
330	340	350	360	370	380	390	400
RSVDDYQHRL	SIKRGELESA	QAQIKILEEK	IDELNLKMTS	QDEEAHVMKK	TIGVIDKEKD	FLQETVDEKT	EKIANLQENL
410	420	430	440	450	460	470	480
ANKEKAVAQM	KIMISECESS	VNQLKETLVN	RDREINSLRR	QLDAAHKELD	EVGRSREIAF	KENRRLQDDL	ATMARENQEI
490	500	510	520	530	540	550	560
SLELEAAVQE	KEEMKSRVHK	YITEVSRWES	LMAAKEKENQ	DLLDRFQMLH	NRAEDWEVKA	HQAEGESSSV	RLELLSIDTE
570	580	590	600	610	620	630	640
RRHLRERVEL	LEKEIQEHIN	AHHAYESQIS	SMAKAMSRLE	EELRHQEDEK	ATVLNDLSSL	RELCIKLDSG	KDIMTQQLNS
650	660	670	680	690	700	710	720
KNLEFERVVV	ELENVKSESD	LLKKQLSNER	HTVKNLESLL	ATNRDKEFHS	HLTSHEKDTE	IQLLKEKLTL	SESKLTSQSR
730	740	750	760	770	780	790	
ENTMLRAKVA	QLQTDYDALK	RQISTERYER	ERAIQEMRRH	GLATPPLSST	LRSPSHSPEH	RNV	

Cmpd.	No. of Cmpds.	m/z meas.	Δ m/z [ppm]	z	Rt [min]	Score	P	Range	Sequence	Modification	Ratios	
1060	1	540.7960	89.65	2	41.5	16.3	0	2-10	M.QDLEETMAK.L	Oxidation: 7	M:Q	0.71

Detailed Protein Report

Protein 335: protein piccolo isoform 2 [Homo sapiens]

**Accession:**	gi|150170670	**Score:**	50.0
**Database:**	refseq_human(refseq_human_20140103.fasta)	**MW [kDa]:**	537.6
**Database Date:**	2015-11-30	**pI:**	5.9
		**Sequence Coverage [%]:**	1.3
		**No. of unique Peptides:**	1

Quantitation

Q:W	**Median:**	0.84	**CV:**	0.00 %	**No. of Peptides:**	1
M:Q	**Median:**	0.99	**CV:**	0.00 %	**No. of Peptides:**	1

Detailed Protein Report

10	20	30	40	50	60	70	80
MGNEASLEGE	GLPEGLAAAA	AAGGGASGAG	SPSHTAIPAG	MEADLSQLSE	EERRQIAAVM	SRAQGLPKGS	VPPAAAESPS
90	100	110	120	130	140	150	160
MHRKQELDSS	HPPKQSGRPP	DPGRPAQPGL	SKSRTTDTFR	SEQKLPGRSP	STISLKESKS	RTDLKEEHKS	SMMPGFLSEV
170	180	190	200	210	220	230	240
NALSAVSSVV	NKFNPFDLIS	DSEASQEETT	KKQKVVQKEQ	GKPEGIIKPP	LQQQPPKPIP	KQQGPGRDPL	QQDGTPKSIS
250	260	270	280	290	300	310	320
SQQPEKIKSQ	PPGTGKPIQG	PTQTPQTDHA	KLPLQRDASR	PQTKQADIVR	GESVKPSLPS	PSKPPIQQPT	PGKPPAQQPG
330	340	350	360	370	380	390	400
HEKSQPGPAK	PPAQPSGLTK	PLAQQPGTVK	PPVQPPGTTK	PPAQPLGPAK	PPAQQTGSEK	PSSEQPGPKA	LAQPPGVGKT
410	420	430	440	450	460	470	480
PAQQPGPAKP	PTQQVGTPKP	LAQQPGLQSP	AKAPGPTKTP	VQQPGPGKIP	AQQAGPGKTS	AQQTGPTKPP	SQLPGPAKPP
490	500	510	520	530	540	550	560
PQQPGPAKPP	PQQPGSAKPP	SQQPGSTKPP	PQQPGPAKPS	PQQPGSTKPP	SQQPGSAKPS	AQQPSPAKPS	AQQSTKPVSQ
570	580	590	600	610	620	630	640
TGSGKPLQPP	TVSPSAKQPP	SQGLPKTICP	LCNTTELLLH	VPEKANFNTC	TECQTTVCSL	CGFNPNPHLT	EVKEWLCLNC
650	660	670	680	690	700	710	720
QMKRALGGDL	APVPSSPQPK	LKTAPVTTTS	AVSKSSPQPQ	QTSPKKDAAP	KQDLSKAPEP	KKPPPLVKQP	TLHGSPSAKA
730	740	750	760	770	780	790	800
KQPPEADSLS	KPAPPKEPSV	PSEQDKAPVA	DDKPKQPKMV	KPTTDLVSSS	SATTKPDIPS	SKVQSQAEEK	TTPPLKTDSA
810	820	830	840	850	860	870	880
KPSQSFPPTG	EKVSPFDSKA	IPRPASDSKI	ISHPGPSSES	KGQKQVDPVQ	KKEEPKKAQT	KMSPKPDAKP	MPKGSPTPPG
890	900	910	920	930	940	950	960
PRPTAGQTVP	TPQQSPKPQE	QSRRFSLNLG	SITDAPKSQP	TTPQETVTGK	LFGFGASIFS	QASNLISTAG	QPGPHSQSGP
970	980	990	1000	1010	1020	1030	1040
GAPMKQAPAP	SQPPTSQGPP	KSTGQAPPAP	AKSIPVKKET	KAPAAEKLEP	KAEQAPTVKR	TETEKKPPPI	KDSKSLTAEP
1050	1060	1070	1080	1090	1100	1110	1120
QKAVLPTKLE	KSPKPESTCP	LCKTELNIGS	KDPPNFNTCT	ECKNQVCNLC	GFNPTPHLTE	IQEWLCLNCQ	TQRAISGQLG
1130	1140	1150	1160	1170	1180	1190	1200
DIRKMPPAPS	GPKASPMPVP	TESSSQKTAV	PPQVKLVKKQ	EQEVKTEAEK	VILEKVKETL	SMEKIPPMVT	TDQKQEESKL
1210	1220	1230	1240	1250	1260	1270	1280
EKDKASALQE	KKPLPEEKKL	IPEEEKIRSE	EKKPLLEEKK	PTPEDKKLLP	EAKTSAPEEQ	KHDLLKSQVQ	IAEEKLEGRV
1290	1300	1310	1320	1330	1340	1350	1360
APKTVQEGKQ	PQTKMEGLPS	GTPQSLPKED	DKTTKTIKEQ	PQPPCTAKPD	QVEPGKEKTE	KEDDKSDTSS	SQQPKSPQGL
1370	1380	1390	1400	1410	1420	1430	1440
SDTGYSSDGI	SSSLGEIPSL	IPTDEKDILK	GLKKDSFSQE	SSPSSPSDLA	KLESTVLSIL	EAQASTLADE	KSEKKTQPHE
1450	1460	1470	1480	1490	1500	1510	1520
VSPEQPKDQE	KTQSLSETLE	ITISEEEIKE	SQEERKDTFK	KDSQQDIPSS	KDHKEKSEFV	DDITTRREPY	DSVEESSESE
1530	1540	1550	1560	1570	1580	1590	1600
NSPVPQRKRR	TSVGSSSSDE	YKQEDSQGSG	EEEDFIRKQI	IEMSADEDAS	GSEDDEFIRN	QLKEISSSTE	SQKKEETKGK
1610	1620	1630	1640	1650	1660	1670	1680
GKITAGKHRR	LTRKSSTSID	EDAGRRHSWH	DEDDEAFDES	PELKYRETKS	QESEELVVTG	GGGLRRFKTI	ELNSTIADKY
1690	1700	1710	1720	1730	1740	1750	1760
SAESSQKKTS	LYFDEEPELE	MESLTDSPED	RSRGEGSSSL	HASSFTPGTS	PTSVSSLDED	SDSSPSHKKG	ESKQQRKARH
1770	1780	1790	1800	1810	1820	1830	1840
RPHGPLLPTI	EDSSEEEELR	EEEELLKEQE	KQREIEQQQR	KSSSKKSKKD	KDELRAQRRR	ERPKTPPSNL	SPIEDASPTE
1850	1860	1870	1880	1890	1900	1910	1920
ELRQAAEMEE	LHRSSCSEYS	PSIESDPEGF	EISPEKIIEV	QKVYKLPTAV	SLYSPTDEQS	IMQKEGSQKA	LKSAEEMYEE
1930	1940	1950	1960	1970	1980	1990	2000
MMHKTHKYKA	FPAANERDEV	FEKEPLYGGM	LIEDYIYESL	VEDTYNGSVD	GSLLTRQEEE	NGFMQQKGRE	QKIRLSEQIY
2010	2020	2030	2040	2050	2060	2070	2080
EDPMQKITDL	QKEFYELESL	HSVVPQEDIV	SSSFIIPESH	EIVDLGTMVT	STEEERKLLD	ADAAYEELMK	RQQMQLTPGS
2090	2100	2110	2120	2130	2140	2150	2160
SPTQAPIGED	MTESTMDFDR	MPDASLTSSV	LSGASLTDST	SSATLSIPDV	KITQHFSTEE	IEDEYVTDYT	REIQEIIAHE
2170	2180	2190	2200	2210	2220	2230	2240
SLILTYSEPS	ESATSVPPSD	TPSLTSSVSS	VCTTDSSSPI	TTLDSITTVY	TEPVDMITKF	EDSEEISSST	YFPGSIIDYP
2250	2260	2270	2280	2290	2300	2310	2320
EEISVSLDRT	APPDGRASAD	HIVISLSDMA	SSIIESVVPK	PEGPVADTVS	TDLLISEKDP	VKKAKKETGN	GIILEVLEAY
2330	2340	2350	2360	2370	2380	2390	2400

Detailed Protein Report

Cmpd.	No. of Cmpds.	m/z meas.	Δ m/z [ppm]	z	Rt [min]	Score	P	Range	Sequence	Modification	Ratios
1917	1	902.8454	-85.60	2	54.0	12.9	0	3678-3694	R.SMSDPKPLSPTADESSR.A		M:Q 0.99<br>Q:W 0.84

Detailed Protein Report

Protein 336: death-inducer obliterator 1 isoform c [Homo sapiens]

**Accession:**	gi|301129165	**Score:**	50.0
**Database:**	refseq_human(refseq_human_20140103.fasta)	**MW [kDa]:**	243.7
**Database Date:**	2015-11-30	**pI:**	8.9
		**Sequence Coverage [%]:**	1.8
		**No. of unique Peptides:**	1

Alias proteins:

Accession	Name	Description
gi|301129170	refseq_human (refseq_human_20140103.fasta)	death-inducer obliterator 1 isoform c [Homo sapiens]

10	20	30	40	50	60	70	80
MDDKGDPSNE	EAPKAIKPTS	KEFRKTWGFR	RTTIAKREGA	GDAEADPLEP	PPPQQQLGLS	LRRSGRQPKR	TERVEQFLTI
90	100	110	120	130	140	150	160
ARRRGRRSMP	VSLEDSGEPT	SCPATDAETA	SEGSVESASE	TRSGPQSAST	AVKERPASSE	KVKGGDDHDD	TSDSDSDGLT
170	180	190	200	210	220	230	240
LKELQNRLRR	KREQEPTERP	LKGIQSRLRK	KRREEGPAET	VGSEASDTVE	GVLPSKQEPE	NDQGVVSQAG	KDDRESKLEG
250	260	270	280	290	300	310	320
KAAQDIKDEE	PGDLGRPKPE	CEGYDPNALY	CICRQPHNNR	FMICCDRCEE	WFHGDCVGIS	EARGRLLERN	GEDYICPNCT
330	340	350	360	370	380	390	400
ILQVQDETHS	ETADQQEAKW	RPGDADGTDC	TSIGTIEQKS	SEDQGIKGRI	EKAANPSGKK	KLKIFQPVIE	APGASKCIGP
410	420	430	440	450	460	470	480
GCCHVAQPDS	VYCSNDCILK	HAAATMKFLS	SGKEQKPKPK	EKMKMKPEKP	SLPKCGAQAG	IKISSVHKRP	APEKKETTVK
490	500	510	520	530	540	550	560
KAVVVPARSE	ALGKEAACES	STPSWASDHN	YNAVKPEKTA	APSPSLLYKS	TKEDRRSEEK	AAAMAASKKT	APPGSAVGKQ
570	580	590	600	610	620	630	640
PAPRNLVPKK	SSFANVAAAT	PAIKKPPSGF	KGTIPKRPWL	SATPSSGASA	ARQAGPAPAA	ATAASKKFPG	SAALVGAVRK
650	660	670	680	690	700	710	720
PVVPSVPMAS	PAPGRLGAMS	AAPSQPNSQI	RQNIRRSLKE	ILWKRVNDSD	DLIMTENEVG	KIALHIEKEM	FNLFQVTDNR
730	740	750	760	770	780	790	800
YKSKYRSIMF	NLKDPKNQGL	FHRVLREEIS	LAKLVRLKPE	ELVSKELSTW	KERPARSVME	SRTKLHNESK	KTAPRQEAIP
810	820	830	840	850	860	870	880
DLEDSPPVSD	SEEQQESARA	VPEKSTAPLL	DVFSSMLKDT	TSQHRAHLFD	LNCKICTGQV	PSAEDEPAPK	KQKLSASVKK
890	900	910	920	930	940	950	960
EDLKSKHDSS	APDPAPDSAD	EVMPEAVPEV	ASEPGLESAS	HPNVDRTYFP	GPPGDGHPEP	SPLEDLSPCP	ASCGSGVVTT
970	980	990	1000	1010	1020	1030	1040
VTVSGRDPRT	APSSSCTAVA	SAASRPDSTH	MVEARQDVPK	PVLTSVMVPK	SILAKPSSSP	DPRYLSVPPS	PNISTSESRS
1050	1060	1070	1080	1090	1100	1110	1120
PPEGDTTLFL	SRLSTIWKGF	INMQSVAKFV	TKAYPVSGCF	DYLSEDLPDT	IHIGGRIAPK	TVWDYVGKLK	SSVSKELCLI
1130	1140	1150	1160	1170	1180	1190	1200
RFHPATEEEE	VAYISLYSYF	SSRGRFGVVA	NNNRHVKDLY	LIPLSAQDPV	PSKLLPFEGP	GLESPRPNII	LGLVICQKIK
1210	1220	1230	1240	1250	1260	1270	1280
RPANSGELDK	MDEKRTRLQP	EEADVPAYPK	VATVPQSEKK	PSKYPLCSAD	AAVSTTPPGS	PPPPPPLPEP	PVLKVLSSLK
1290	1300	1310	1320	1330	1340	1350	1360
PAAPSPATAA	TTAAAASTAA	SSTASSASKT	ASPLEHILQT	LFGKKKSFDP	SAREPPGSTA	GLPQEPKTTA	EDGVPAPPLL
1370	1380	1390	1400	1410	1420	1430	1440
DPIVQQFGQF	SKDKALEEEE	DDRPYDPEEE	YDPERAFDTQ	LVERGRRHEV	ERAPEAAAAE	REEVAYDPED	ETILEEAKVT
1450	1460	1470	1480	1490	1500	1510	1520
VDDLPNRMCA	DVRRNSVERP	AEPVAGAATP	SLVEQQKMLE	ELNKQIEEQK	RQLEEQEEAL	RQQRAAVGVS	MAHFSVSDAL
1530	1540	1550	1560	1570	1580	1590	1600
MSPPPKSSLP	KAELFQQEQQ	SADKPASLPP	ASQASNHRDP	RQARRLATET	GEGEGEPLSR	LSARGAQGAL	PERDASRGGL
1610	1620	1630	1640	1650	1660	1670	1680
VGQAPMPVPE	EKEPASSPWA	SGEKPPAGSE	QDGWKAEPGE	GTRPATVGDS	SARPARRVLL	PTPPCGALQP	GFPLQHDGER
1690	1700	1710	1720	1730	1740	1750	1760
DPFTCPGFAS	QDKALGSAQY	EDPRNLHSAG	RSSSPAGETE	GDREPQARPG	EGTAPLPPPG	QKVGGSQPPF	QGQREPGPHA
1770	1780	1790	1800	1810	1820	1830	1840
LGMSGLHGPN	FPGPRGPAPP	FPEENIASND	GPRGPPPARF	GAQKGPIPSL	FSGQHGPPPY	GDSRGPSPSY	LGGPRGVAPS
1850	1860	1870	1880	1890	1900	1910	1920
QFEERKDPHG	EKREFQDAPY	NEVTGAPAQF	EGTEQAPFLG	SRGGAPFQFG	GQRRPLLSQL	KGPRGGPPPS	QFGGQRGPPP
1930	1940	1950	1960	1970	1980	1990	2000
GHFVGPRGPH	PSQFETARGP	HPNQFEGPRG	QAPNFMPGPR	GIQPQQFEDQ	RVHSPPRFTN	QRAPAPLQFG	GLRGSAPFSE
2010	2020	2030	2040	2050	2060	2070	2080
KNEQTPSRFH	FQGQAPQVMK	PGPRPLLELP	SHPPQHRKDR	WEEAGPPSAL	SSSAPGQGPE	ADGQWASADF	REGKGHEYRN
2090	2100	2110	2120	2130	2140	2150	2160
QTFEGRQRER	FDVGPKEKPL	EEPDAQGRAS	EDRRRERERG	RNWSRERDWD	RPREWDRHRD	KDSSRDWDRN	RERSANRDRE
2170	2180	2190	2200	2210	2220	2230	2240

Detailed Protein Report

Cmpd.	No. of Cmpds.	m/z meas.	Δ m/z [ppm]	z	Rt [min]	Score	P	Range	Sequence	Modification
411	1	701.3611	72.76	2	35.1	11.7	1	2-14	M.DDKGDPSNEEAPK.A	

Detailed Protein Report

Protein 337: PREDICTED: sperm flagellar protein 2 isoform X4 [Homo sapiens]

**Accession:**	gi|578809999	**Score:**	49.9
**Database:**	refseq_human(refseq_human_20140103.fasta)	**MW [kDa]:**	202.4
**Database Date:**	2015-11-30	**pI:**	5.2
**Modification(s):**	Oxidation	**Sequence Coverage [%]:**	3.4
		**No. of unique Peptides:**	3

Quantitation

Q:W	**Median:** 0.70	**CV:** 0.00 %	**No. of Peptides:** 1

Detailed Protein Report

10	20	30	40	50	60	70	80
MSEILCQWLN	KELKVSRTVS	PKSFAKAFSS	GYLLGEVLHK	FELQDDFSEF	LDSRVSSAKL	NNFSRLEPTL	NLLGVQFDQN
**90**	**100**	**110**	**120**	**130**	**140**	**150**	**160**
VAHGIITEKP	GVATKLLYQL	YIALQKKKKS	GLTGVEMQTM	QRLTNLRLQN	MKSDTFQEQY	LNRRRQNEIM	AKIQAAIIQI
**170**	**180**	**190**	**200**	**210**	**220**	**230**	**240**
PKPASNRTLK	ALEAQKMMKK	KKEAEDVADE	IKKFEALIKK	DLQAKESASK	TSLDTAGQTT	TDLLNTYSDD	EYIKKIQKRL
**250**	**260**	**270**	**280**	**290**	**300**	**310**	**320**
EEDAFAREQR	EKRRRKLLMD	QLIAHEAQEE	AYREEQLINR	LMRQSQQERR	IAVQLMHVRH	EKEVLWQNRI	FREKQHEERR
**330**	**340**	**350**	**360**	**370**	**380**	**390**	**400**
LKDFQDALDR	EAALAKQAKI	DFEEQFLKEK	RFHDQIAVER	AQARYEKHYS	VCAEILDQIV	DLSTKVADYR	MLTNNLIPYK
**410**	**420**	**430**	**440**	**450**	**460**	**470**	**480**
LMHDWKELFF	NAKPIYEQAS	VKTLPANPSR	EQLTELEKRD	LLDTNDYEEY	KNMVGEWALP	EEMVDNLPPS	NNCILGHILH
**490**	**500**	**510**	**520**	**530**	**540**	**550**	**560**
RLAEKSLPPR	AESTTPELPS	FAVKGCLLGK	TLSGKTTILR	SLQKDFPIQI	LSIDTLVQEA	IQAFHDNEKV	SEVLPIQKND
**570**	**580**	**590**	**600**	**610**	**620**	**630**	**640**
EEDALPVLQE	EIKESQDPQH	VFSAGPVSDE	VLPETEGETM	LSANADKTPK	AEEVKSSDSF	LKLTTRAQLG	AKSEQLLKKG
**650**	**660**	**670**	**680**	**690**	**700**	**710**	**720**
KSIPDVLLVD	IIVNAINEIP	VNQDCILDGF	PMTLNQAQLL	EEALTGCNRN	LTEVERKKAQ	KSTLAIDPAT	SKEIPLPSPA
**730**	**740**	**750**	**760**	**770**	**780**	**790**	**800**
FDFVILLDVS	DTSSMSRMND	IIAEELSYKT	AHEDISQRVA	AENQDKDGDQ	NLRDQIQHRI	IGFLDNWPLL	EQWFSEPENI
**810**	**820**	**830**	**840**	**850**	**860**	**870**	**880**
LIKINAEIDK	ESLCEKVKEI	LTTEIAKKKN	KVEKKLEEKE	AEKKAAASLA	ELPLPTPPPA	PPPEPEKEKE	IHQSHVASKT
**890**	**900**	**910**	**920**	**930**	**940**	**950**	**960**
PTAKGKPQSE	APHGKQESLQ	EGKGKKGETA	LKRKGSPKGK	SSGGKVPVKK	SPADSTDTSP	VAIVPQPPKP	GSEEWVYVNE
**970**	**980**	**990**	**1000**	**1010**	**1020**	**1030**	**1040**
PVPEEMPLFL	VPYWELIENS	YINTIKTVLR	HLREDQHTVL	AYLYEIRTSF	QEFLKRPDHK	QDFVAQWQAD	FNSLPDDLWD
**1050**	**1060**	**1070**	**1080**	**1090**	**1100**	**1110**	**1120**
DEETKAELHQ	RVNDLRDRLW	DICDARKEEA	EQERLDIINE	SWLQDTLGMT	MNHFFSLMQA	ELNRFQDTKR	LLQDYYWGME
**1130**	**1140**	**1150**	**1160**	**1170**	**1180**	**1190**	**1200**
SKIPVEDNKR	FTRIPLVQLD	SKDNSESQLR	IPLVPRISIS	LETVTPKPKT	KSVLKGKMDN	SLENVESNFE	ADEKLVMDTW
**1210**	**1220**	**1230**	**1240**	**1250**	**1260**	**1270**	**1280**
QQASLAVSHM	VAAEIHQRLM	EEEKENQPAD	PKEKSPQMGA	NKKVKKEPPK	KKQEDKKPKG	KSPPMAEATP	VIVTTEEIAE
**1290**	**1300**	**1310**	**1320**	**1330**	**1340**	**1350**	**1360**
IKRKNELRVK	IKEEHLAALQ	FEEIATQFRL	ELIKTKALAL	LEDLVTKVVD	VYKLMEKWLG	ERYLNEMAST	EKLTDVARYH
**1370**	**1380**	**1390**	**1400**	**1410**	**1420**	**1430**	**1440**
IETSTKIQNE	LYLSQEDFFI	NGNIKVFPDP	PPSIRPPPVE	KEEDGTLTIE	QLDSLRDQFL	DMAPKGIIGN	KAFTDILIDL
**1450**	**1460**	**1470**	**1480**	**1490**	**1500**	**1510**	**1520**
VTLNLGTNNF	PSNWMHLTQP	ELQELTSLLT	VNSEFVDWRK	FLLVTSMPWP	IPLEEELLET	LQKFKAVDKE	QLGTITFEQY
**1530**	**1540**	**1550**	**1560**	**1570**	**1580**	**1590**	**1600**
MQAGLWFTGD	EDIKIPENPL	EPLPFNRQEH	LIEFFFRLFA	DYEKDPPQLD	YTQMLLYFAC	HPDTVEGVYR	ALSVAVGTHV
**1610**	**1620**	**1630**	**1640**	**1650**	**1660**	**1670**	**1680**
FQQVKASIPS	AEKTSSTDAG	PAEEFPEPEE	NAAREERKLK	DDTEKREQKD	EEIPENANNE	KMSMETLLKV	FKGGSEAQDS
**1690**	**1700**	**1710**	**1720**	**1730**	**1740**	**1750**	**1760**
NRFASHLKIE	NIYAEGFIKT	FQDLGAKNLE	PIEVAVLLKH	PFIQDLISNY	SDYKFPVHII	YVVEMIVCAK	GWAPLPSGKQ
**1770**							
CARWRN							

Cmpd.	No. of Cmpds.	m/z meas.	Δ m/z [ppm]	z	Rt [min]	Score	P	Range	Sequence	Modification	Ratios
1496	1	1006.5889	17.49	3	47.4	12.9	2	1143-1169	K.DNSESQLRIPLVPRISISLETVTPKP T		
1569	1	480.6396	-230.14	2	48.1	14.6	1	1235-1243	K.SPQMGANKK.V		
2688	1	484.7720	59.46	2	62.0	10.3	0	1662-1669	K.MSMETLLK.V	Oxidation: 1	Q:W 0.70

Detailed Protein Report

Protein 338: **PREDICTED: STE20-like serine/threonine-protein kinase isoform X1 [Homo sapiens]**

**Accession:**	gi|530394656	**Score:**	49.9
**Database:**	refseq_human(refseq_human_20140103.fasta)	**MW [kDa]:**	138.9
**Database Date:**	2015-11-30	**pI:**	4.9
		**Sequence Coverage [%]:**	4.2
		**No. of unique Peptides:**	1

10	20	30	40	50	60	70	80
MSFFNFRKIF	KLGSEKKKKQ	YEHVKRDLNP	EDFWEIIGEL	GDGAFGKVYK	AQNKETSVLA	AAKVIDTKSE	EELEDYMVEI
**90**	**100**	**110**	**120**	**130**	**140**	**150**	**160**
DILASCDHPN	IVKLLDAFYY	ENNLWILIEF	CAGGAVDAVM	LELERPLTES	QIQVVCKQTL	DALNYLHDNK	IIHRDLKAGN
**170**	**180**	**190**	**200**	**210**	**220**	**230**	**240**
ILFTLDGDIK	LADFGVSAKN	TRTIQRRDSF	IGTPYWMAPE	VVMCETSKDR	PYDYKADVWS	LGITLIEMAE	IEPPHHELNP
**250**	**260**	**270**	**280**	**290**	**300**	**310**	**320**
MRVLLKIAKS	EPPTLAQPSR	WSSNFKDFLK	KCLEKNVDAR	WTTSQLLQHP	FVTVDSNKPI	RELIAEAKAE	VTEEVEDGKE
**330**	**340**	**350**	**360**	**370**	**380**	**390**	**400**
EDEEEETENS	LPIPASKRAS	SDLSIASSEE	DKLSQNACIL	ESVSEKTERS	NSEDKLNSKI	LNEKPTTDEP	EKAVEDINEH
**410**	**420**	**430**	**440**	**450**	**460**	**470**	**480**
ITDAQLEAMT	ELHDRTAVIK	ENEREKRPKL	ENLPDTEDQE	TVDINSVSEG	KENNIMITLE	TNIEHNLKSE	EEKDQEKQQM
**490**	**500**	**510**	**520**	**530**	**540**	**550**	**560**
FENKLIKSEE	IKDTILQTVD	LVSQETGEKE	ANIQAVDSEV	GLTKEDTQEK	LGEDDKTQKD	VISNTSDVIG	TCEAADVAQK
**570**	**580**	**590**	**600**	**610**	**620**	**630**	**640**
VDEDSAEDTQ	SNDGKEVVEV	GQKLINKPMV	GPEAGGTKEV	PIKEIVEMNE	IEEGKNKEQA	INSSENIMDI	NEEPGTTEGE
**650**	**660**	**670**	**680**	**690**	**700**	**710**	**720**
EITESSSTEE	MEVRSVVADT	DQKALGSEVQ	DASKVTTQID	KEKKEIPVSI	KKEPEVTVVS	QPTEPQPVLI	PSININSDSG
**730**	**740**	**750**	**760**	**770**	**780**	**790**	**800**
ENKEEIGSLS	KTETILPPES	ENPKENDNDS	GTGSTADTSS	IDLNLSISSF	LSKTKDSGSI	SLQETRRQKK	TLKKTRKFIV
**810**	**820**	**830**	**840**	**850**	**860**	**870**	**880**
DGVEVSVTTS	KIVTDSDSKT	EELRFLRRQE	LRELRFLQKE	EQRAQQQLNS	KLQQQREQIF	RRFEQEMMSK	KRQYDQEIEN
**890**	**900**	**910**	**920**	**930**	**940**	**950**	**960**
LEKQQKQTIE	RLEQEHTNRL	RDEAKRIKGE	QEKELSKFQN	MLKNRKKEEQ	EFVQKQQQEL	DGSLKKIIQQ	QKAELANIER
**970**	**980**	**990**	**1000**	**1010**	**1020**	**1030**	**1040**
ECLNNKQQLM	RAREAAIWEL	EERHLQEKHQ	LLKQQLKDQY	FMQRHQLLKR	HEKETEQMQR	YNQRLIEELK	NRQTQERARL
**1050**	**1060**	**1070**	**1080**	**1090**	**1100**	**1110**	**1120**
PKIQRSEAKT	RMAMFKKSLR	INSTATPDQD	RDKIKQFAAQ	EEKRQKNERM	AQHQKHENQM	RDLQLQCEAN	VRELHQLQNE
**1130**	**1140**	**1150**	**1160**	**1170**	**1180**	**1190**	**1200**
KCHLLVEHET	QKLKELDEEH	SQELKEWREK	LRPRKKTLEE	EFARKLQEQE	VFFKMTGESE	CLNPSTQSRI	SKFYPIPSLH
**1210**							
STGS							

Cmpd.	No. of Cmpds.	m/z meas.	Δ m/z [ppm]	z	Rt [min]	Score	P	Range	Sequence	Modification
1728	1	636.2813	-68.03	2	51.9	15.9	1	261-270	R.WSSNFKDFLK.K	

Detailed Protein Report

Protein 339: apolipoprotein B-100 precursor [Homo sapiens]

**Accession:**	gi|105990532	**Score:**	49.8
**Database:**	refseq_human(refseq_human_20140103.fasta)	**MW [kDa]:**	515.2
**Database Date:**	2015-11-30	**pI:**	6.6
**Modification(s):**	Oxidation	**Sequence Coverage [%]:**	0.9
		**No. of unique Peptides:**	1

Quantitation

Q:W	**Median:**	0.16	**CV:**	0.00 %	**No. of Peptides:**	1
M:Q	**Median:**	0.97	**CV:**	0.00 %	**No. of Peptides:**	1

Detailed Protein Report

10	20	30	40	50	60	70	80
MDPPRPALLA	LLALPALLLL	LLAGARAEEE	MLENVSLVCP	KDATRFKHLR	KYTYNYEAES	SSGVPGTADS	RSATRINCKV
90	100	110	120	130	140	150	160
ELEVPQLCSF	ILKTSQCTLK	EVYGFNPEGK	ALLKKTKNSE	EFAAAMSRYE	LKLAIPEGKQ	VFLYPEKDEP	TYILNIKRGI
170	180	190	200	210	220	230	240
ISALLVPPET	EEAKQVLFLD	TVYGNCSTHF	TVKTRKGNVA	TEISTERDLG	QCDRFKPIRT	GISPLALIKG	MTRPLSTLIS
250	260	270	280	290	300	310	320
SSQSCQYTLD	AKRKHVAEAI	CKEQHLFLPF	SYKNKYGMVA	QVTQTLKLED	TPKINSRFFG	EGTKKMGLAF	ESTKSTSPPK
330	340	350	360	370	380	390	400
QAEAVLKTLQ	ELKKLTISEQ	NIQRANLFNK	LVTELRGLSD	EAVTSLLPQL	IEVSSPITLQ	ALVQCGQPQC	STHILQWLKR
410	420	430	440	450	460	470	480
VHANPLLIDV	VTYLVALIPE	PSAQQLREIF	NMARDQRSRA	TLYALSHAVN	NYHKTNPTGT	QELLDIANYL	MEQIQDDCTG
490	500	510	520	530	540	550	560
DEDYTYLILR	VIGNMGQTME	QLTPELKSSI	LKCVQSTKPS	LMIQKAAIQA	LRKMEPKDKD	QEVLLQTFLD	DASPGDKRLA
570	580	590	600	610	620	630	640
AYLMLMRSPS	QADINKIVQI	LPWEQNEQVK	NFVASHIANI	LNSEELDIQD	LKKLVKEALK	ESQLPTVMDF	RKFSRNYQLY
650	660	670	680	690	700	710	720
KSVSLPSLDP	ASAKIEGNLI	FDPNNYLPKE	SMLKTTLTAF	GFASADLIEI	GLEGKGFEPT	LEALFGKQGF	FPDSVNKALY
730	740	750	760	770	780	790	800
WVNGQVPDGV	SKVLVDHFGY	TKDDKHEQDM	VNGIMLSVEK	LIKDLKSKEV	PEARAYLRIL	GEELGFASLH	DLQLLGKLLL
810	820	830	840	850	860	870	880
MGARTLQGIP	QMIGEVIRKG	SKNDFFLHYI	FMENAFELPT	GAGLQLQISS	SGVIAPGAKA	GVKLEVANMQ	AELVAKPSVS
890	900	910	920	930	940	950	960
VEFVTNMGII	IPDFARSGVQ	MNTNFFHESG	LEAHVALKAG	KLKFIIPSPK	RPVKLLSGGN	TLHLVSTTKT	EVIPPLIENR
970	980	990	1000	1010	1020	1030	1040
QSWSVCKQVF	PGLNYCTSGA	YSNASSTDSA	SYYPLTGDTR	LELELRPTGE	IEQYSVSATY	ELQREDRALV	DTLKFVTQAE
1050	1060	1070	1080	1090	1100	1110	1120
GAKQTEATMT	FKYNRQSMTL	SSEVQIPDFD	VDLGTILRVN	DESTEGKTSY	RLTLDIQNKK	ITEVALMGHL	SCDTKEERKI
1130	1140	1150	1160	1170	1180	1190	1200
KGVISIPRLQ	AEARSEILAH	WSPAKLLLQM	DSSATAYGST	VSKRVAWHYD	EEKIEFEWNT	GTNVDTKKMT	SNFPVDLSDY
1210	1220	1230	1240	1250	1260	1270	1280
PKSLHMYANR	LLDHRVPQTD	MTFRHVGSKL	IVAMSSWLQK	ASGSLPYTQT	LQDHLNSLKE	FNLQNMGLPD	FHIPENLFLK
1290	1300	1310	1320	1330	1340	1350	1360
SDGRVKYTLN	KNSLKIEIPL	PFGGKSSRDL	KMLETVRTPA	LHFKSVGFHL	PSREFQVPTF	TIPKLYQLQV	PLLGVLDLST
1370	1380	1390	1400	1410	1420	1430	1440
NVYSNLYNWS	ASYSGGNTST	DHFSLRARYH	MKADSVVDLL	SYNVQGSGET	TYDHKNTFTL	SCDGSLRHKF	LDSNIKFSHV
1450	1460	1470	1480	1490	1500	1510	1520
EKLGNNPVSK	GLLIFDASSS	WGPQMSASVH	LDSKKKQHLF	VKEVKIDGQF	RVSSFYAKGT	YGLSCQRDPN	TGRLNGESNL
1530	1540	1550	1560	1570	1580	1590	1600
RFNSSYLQGT	NQITGRYEDG	TLSLTSTSDL	QSGIIKNTAS	LKYENYELTL	KSDTNGKYKN	FATSNKMDMT	FSKQNALLRS
1610	1620	1630	1640	1650	1660	1670	1680
EYQADYESLR	FFSLLSGSLN	SHGLELNADI	LGTDKINSGA	HKATLRIGQD	GISTSATTNL	KCSLLVLENE	LNAELGLSGA
1690	1700	1710	1720	1730	1740	1750	1760
SMKLTTNGRF	REHNAKFSLD	GKAALTELSL	GSAYQAMILG	VDSKNIFNFK	VSQEGLKLSN	DMMGSYAEMK	FDHTNSLNIA
1770	1780	1790	1800	1810	1820	1830	1840
GLSLDFSSKL	DNIYSSDKFY	KQTVNLQLQP	YSLVTTLNSD	LKYNALDLTN	NGKLRLEPLK	LHVAGNLKGA	YQNNEIKHIY
1850	1860	1870	1880	1890	1900	1910	1920
AISSAALSAS	YKADTVAKVQ	GVEFSHRLNT	DIAGLASAID	MSTNYNSDSL	HFSNVFRSVM	APFTMTIDAH	TNGNGKLALW
1930	1940	1950	1960	1970	1980	1990	2000
GEHTGQLYSK	FLLKAEPLAF	TFSHDYKGST	SHHLVSRKSI	SAALEHKVSA	LLTPAEQTGT	WKLKTQFNNN	EYSQDLDAYN
2010	2020	2030	2040	2050	2060	2070	2080
TKDKIGVELT	GRTLADLTLL	DSPIKVPLLL	SEPINIIDAL	EMRDAVEKPQ	EFTIVAFVKY	DKNQDVHSIN	LPFFETLQEY
2090	2100	2110	2120	2130	2140	2150	2160
FERNRQTIIV	VLENVQRNLK	HINIDQFVRK	YRAALGKLPQ	QANDYLNSFN	WERQVSHAKE	KLTALTKKYR	ITENDIQIAL
2170	2180	2190	2200	2210	2220	2230	2240

Detailed Protein Report

Cmpd.	No. of Cmpds.	m/z meas.	Δ m/z [ppm]	z	Rt [min]	Score	P	Range	Sequence	Modification	Ratios	
222	1	493.5794	-31.61	3	32.8	10.5	0	513-525	K.CVQSTKPSLMIQK.A	Oxidation: 10	Q:W<br>M:Q	0.16<br>0.97

Detailed Protein Report

Protein 340: PREDICTED: PDZ domain-containing protein 2 isoform X8 [Homo sapiens]

**Accession:**	gi|578809880	**Score:**	49.7
**Database:**	refseq_human(refseq_human_20140103.fasta)	**MW [kDa]:**	289.4
**Database Date:**	2015-11-30	**pI:**	7.4
		**Sequence Coverage [%]:**	2.7
		**No. of unique Peptides:**	3

Detailed Protein Report

10	20	30	40	50	60	70	80
MPITQDNAVL	HLPLLYQWLQ	NSLQEGGDGP	EQRLCQAAIQ	KLQEYIQLNF	AVDESTVPPD	HSPPEMEICT	VYLTKELGDT
90	100	110	120	130	140	150	160
ETVGLSFGNI	PVFGDYGEKR	RGGKKRKTHQ	GPVLDVGCIW	VTELRKNSPA	GKSGKVRLRD	EILSLNGQLM	VGVDVSGASY
170	180	190	200	210	220	230	240
LAEQCWNGGF	IYLIMLRRFK	HKAHSTYNGN	SSNSSEPGET	PTLELGDRTA	KKGKRTRKFG	VISRPPANKA	PEESKGSAGC
250	260	270	280	290	300	310	320
EVSSDPSTEL	ENGPDPELGN	GHVFQLENGP	DSLKEVAGPH	LERSEVDRGT	EHRIPKTDAP	LTTSNDKRRF	SKGGKTDFQS
330	340	350	360	370	380	390	400
SDCLAREEVG	RIWKMELLKE	SDGLGIQVSG	GRGSKRSPHA	IVVTQVKEGG	AAHRDGRLSL	GDELLVINGH	LLVGLSHEEA
410	420	430	440	450	460	470	480
VAILRSATGM	VQLVVASKEN	SAEDLLRLTS	KSLPDLTSSV	EDVSSWTDNE	DQEADGEEDE	GTSSSVQRAM	PGTDEPQDVC
490	500	510	520	530	540	550	560
GAEESKGNLE	SPKQGSNKIK	LKSRLSGGVH	RLESVEEYNE	LMVRNGDPRI	RMLEVSRDGR	KHSLPQLLDS	SSASQEYHIV
570	580	590	600	610	620	630	640
KKSTRSLSTT	QVESPWRLIR	PSVISIIGLY	KEKGKGLGFS	IAGGRDCIRG	QMGIFVKTIF	PNGSAAEDGR	LKEGDEILDV
650	660	670	680	690	700	710	720
NGIPIKGLTF	QEAIHTFKQI	RSGLFVLTVR	TKLVSPSLTP	CSTPTHMSRS	ASPNFNTSGG	ASAGGSDEGS	SSSLGRKTPG
730	740	750	760	770	780	790	800
PKDRIVMEVT	LNKEPRVGLG	IGACCLALEN	SPPGIYIHSL	APGSVAKMES	NLSRGDQILE	VNSVNVRHAA	LSKVHAILSK
810	820	830	840	850	860	870	880
CPPGPVRLVI	GRHPNPKVSE	QEMDEVIARS	TYQESKEANS	SPGLGTPLKS	PSLAKKDSLI	SESELSQYFA	HDVPGPLSDF
890	900	910	920	930	940	950	960
MVAGSEDEDH	PGSGCSTSEE	GSLPPSTSTH	KEPGKPRANS	LVTLGSHRAS	GLFHKQVTVA	RQASLPGSPQ	ALRNPLLRQR
970	980	990	1000	1010	1020	1030	1040
KVGCYDANDA	SDEEEFDREG	DCISLPGALP	GPIRPLSEDD	PRRVSISSSK	GMDVHNQEER	PRKTLESLGK	LTTGDACVST
1050	1060	1070	1080	1090	1100	1110	1120
SCELASALSH	LDASHLTENL	PKAASELGQQ	PMTELDSSSD	LISSPGKKGA	AHPDPSKTSV	DTGQVSRPEN	PSQPASPRVT
1130	1140	1150	1160	1170	1180	1190	1200
KCKARSPVRL	PHEGSPSPGE	KAAAPPDYSK	TRSASETSTP	HNTRRVAALR	GAGPGAEGMT	PAGAVLPGDP	LTSQEQRQGA
1210	1220	1230	1240	1250	1260	1270	1280
PGNHSKALEM	TGIHAPESSQ	EPSLLEGADS	VSSRAPQASL	SMLPSTDNTK	EACGHVSGHC	CPGGSRESPV	TDIDSFIKEL
1290	1300	1310	1320	1330	1340	1350	1360
DASAARSPSS	QTGDSGSQEG	SAQGHPPAGA	GGGSSCRAEP	VPGGQTSSPR	RAWAAGAPAY	PQWASQPSVL	DSINPDKHFT
1370	1380	1390	1400	1410	1420	1430	1440
VNKNFLSNYS	RNFSSFHEDS	TSLSGLGDST	EPSLSSMYGD	AEDSSSDPES	LTEAPRASAR	DGWSPPRSRV	SLHKEDPSES
1450	1460	1470	1480	1490	1500	1510	1520
EEEQIEICST	RGCPNPPSSP	AHLPTQAAIC	PASAKVLSLK	YSTPRESVAS	PREKAACLPG	SYTSGPDSSQ	PSSLLEMSSQ
1530	1540	1550	1560	1570	1580	1590	1600
EHETHADIST	SQNHRPSCAE	ETTEVTSASS	AMENSPLSKV	ARHFHSPPII	LSSPNMVNGL	EHDLLDDETL	NQYETSINAA
1610	1620	1630	1640	1650	1660	1670	1680
ASLSSFSVDV	PKNGESVLEN	LHISESQDLD	DLLQKPKMIA	RRPIMAWFKE	INKHNQGTHL	RSKTEKEQPL	MPARSPDSKI
1690	1700	1710	1720	1730	1740	1750	1760
QMVSSSQKKG	VTVPHSPPQP	KTNLENKDLS	KKSPAEMLLT	NGQKAKCGPK	LKRLSLKGKA	KVNSEAPAAN	AVKAGGTDHR
1770	1780	1790	1800	1810	1820	1830	1840
KPLISPQTSH	KTLSKAVSQR	LHVADHEDPD	RNTTAAPRSP	QCVLESKPPL	ATSGPLKPSV	SDTSIRTFVS	PLTSPKPVPE
1850	1860	1870	1880	1890	1900	1910	1920
QGMWSRFHMA	VLSEPDRGCP	TTPKSPKCRA	EGRAPRADSG	PVSPAASRNG	MSVAGNRQSE	PRLASHVAAD	TAQPRPTGEK
1930	1940	1950	1960	1970	1980	1990	2000
GGNIMASDRL	ERTNQLKIVE	ISAEAVSETV	CGNKPAESDR	RGGCLAQGNC	QEKSEIRLYR	QVAESSTSHP	SSLPSHASQA
2010	2020	2030	2040	2050	2060	2070	2080
EQEMSRSFSM	AKLASSSSSL	QTAIRKAEYS	QGKSSLMSDS	RGVPRNSIPG	GPSGEDHLYF	TPRPATRTYS	MPAQFSSHFG
2090	2100	2110	2120	2130	2140	2150	2160
REGHPPHSLG	RSRDSQVPVT	SSVVPEAKAS	RGGLPSLANG	QGIYSVKPLL	DTSRNLPATD	EGDIISVQET	SCLVTDKIKV
2170	2180	2190	2200	2210	2220	2230	2240

Detailed Protein Report

Cmpd.	No. of Cmpds.	m/z meas.	Δ m/z [ppm]	z	Rt [min]	Score	P	Range	Sequence	Modification
892	1	675.8467	18.44	4	41.2	12.0	1	76-100	K.ELGDTETVGLSFGNIPVFGDYGEKR.R	
1875	2	917.8351	-138.97	2	53.5	15.2	1	2474-2492	R.KEGSGLGFSVAGGTDVEPK.S	
444	1	848.1390	136.74	2	35.2	10.0	1	2606-2621	R.IGRPTVLLGSSAVIRR.S	

Detailed Protein Report

Protein 341: keratin, type II cuticular Hb4 [Homo sapiens]

**Accession:**	gi|15431316	**Score:**	49.6
**Database:**	refseq_human(refseq_human_20140103.fasta)	**MW [kDa]:**	64.8
**Database Date:**	2015-11-30	**pI:**	9.0
**Modification(s):**	Carbamidomethyl, Oxidation	**Sequence Coverage [%]:**	5.8
		**No. of unique Peptides:**	1

Quantitation

Q:W **Median:** 0.99 **CV:** 0.00 % **No. of Peptides:** 1

10	20	30	40	50	60	70	80
MSCRSYRVSS	GHRVGNFSSC	SAMTPQNLNR	FRANSVSCWS	GPGFRGLGSF	GSRSVITFGS	YSPRIAAVGS	RPIHCGVRFG
**90**	**100**	**110**	**120**	**130**	**140**	**150**	**160**
AGCGMGFGDG	RGVGLGPRAD	SCVGLGFGAG	SGIGYGFGGP	GFGYRVGGVG	VPAAPSITAV	TVNKSLLTPL	NLEIDPNAQR
**170**	**180**	**190**	**200**	**210**	**220**	**230**	**240**
VKKDEKEQIK	TLNNKFASFI	DKVRFLEQQN	KLLETKWSFL	QEQKCIRSNL	EPLFESYITN	LRRQLEVLVS	DQARLQAERN
**250**	**260**	**270**	**280**	**290**	**300**	**310**	**320**
HLQDVLEGFK	KKYEEEVVCR	ANAENEFVAL	KKDVDAAFMN	KSDLEANVDT	LTQEIDFLKT	LYMEEIQLLQ	SHISETSVIV
**330**	**340**	**350**	**360**	**370**	**380**	**390**	**400**
KMDNSRDLNL	DGIIAEVKAQ	YEEVARRSRA	DAEAWYQTKY	EEMQVTAGQH	CDNLRNIRNE	INELTRLIQR	LKAEIEHAKA
**410**	**420**	**430**	**440**	**450**	**460**	**470**	**480**
QRAKLEAAVA	EAEQQGEATL	SDAKCKLADL	ECALQQAKQD	MARQLCEYQE	LMNAKLGLDI	EIATYRRLLE	GEESRLCEGV
**490**	**500**	**510**	**520**	**530**	**540**	**550**	**560**
GPVNISVSSS	RGGLVCGPEP	LVAGSTLSRG	GVTFSGSSSV	CATSGVLASC	GPSLGGARVA	PATGDLLSTG	TRSGSMLISE
**570**	**580**	**590**	**600**	**610**			
ACVPSVPCPL	PTQGGFSSCS	GGRSSSVRFV	STTTSCRTKY				

Cmpd.	No. of Cmpds.	m/z meas.	Δ m/z [ppm]	z	Rt [min]	Score	P	Range	Sequence	Modification	Ratios	
603	2	652.6252	-207.98	2	36.9	13.1	0	79-91	R.FGAGCGMGFGDGR.G	Carbamidomethyl: 5; Oxidation: 7	Q:W	0.99

Detailed Protein Report

Protein 342: TPR and ankyrin repeat-containing protein 1 [Homo sapiens]

**Accession:**	gi|257467636	**Score:**	49.5
**Database:**	refseq_human(refseq_human_20140103.fasta)	**MW [kDa]:**	336.0
**Database Date:**	2015-11-30	**pI:**	6.3
**Modification(s):**	Carbamidomethyl	**Sequence Coverage [%]:**	1.9
		**No. of unique Peptides:**	3

Quantitation

Q:W	**Median:**	1.18	**CV:**	0.00 %	**No. of Peptides:**	1
M:Q	**Median:**	0.70	**CV:**	0.00 %	**No. of Peptides:**	1

Detailed Protein Report

10	20	30	40	50	60	70	80
MWDPRAARVP	PRDLAVLLCN	KSNAFFSLGK	WNEAFVAAKE	CLQWDPTYVK	GYYRAGYSLL	RLHQPYEAAR	MFFEGLRLVQ
90	100	110	120	130	140	150	160
RSQDQAPVAD	FLVGVFTTMS	SDSIVLQSFL	PCFDHIFTTG	FPTEVWQSVI	EKLAKKGLWH	SFLLLSAKKD	RLPRNIHVPE
170	180	190	200	210	220	230	240
LSLKSLFEKY	VFIGLYEKME	QVPKLVQWLI	SIGASVETIG	PYPLHALMRL	CIQARENHLF	RWLMDHKPEW	KGRINQKDGD
250	260	270	280	290	300	310	320
GCTVLHVVAA	HSPGYLVKRQ	TEDVQMLLRF	GADPTLLDRQ	SRSVVDVLKR	NKNFKAIEKI	NSHLEKLATC	SKDLSGFSNG
330	340	350	360	370	380	390	400
DGPTSENDIF	RKVLEQLVKY	MNSGNRLLHK	NFLKQEVVQR	FLRLLSTLQE	IPPDLVCDIN	QDCATTVFKF	LLEKQRWPEV
410	420	430	440	450	460	470	480
LLLLTRKVSG	EPPLGDCLIK	DCNFSDLDIC	TIIPHLSTWD	QRKKQLLGCL	IDSGALPDGL	QESQERPVVT	CLKHEDFELA
490	500	510	520	530	540	550	560
FLLLTKGADP	RAISLTEGDT	PLHAALHIFL	EIKADIGFSF	LSHLLDLFWS	NPTEFDYLNP	NVQDSNGNTL	MHILFQKGML
570	580	590	600	610	620	630	640
KRVKKLLDLL	VKFDINFNLK	NKEGKDARHR	IKKNDSLLLA	WNKALMENRR	RSRQDSAAHL	GKLSKSTAPG	HTSQLKSQGS
650	660	670	680	690	700	710	720
FKSVPCGATA	RTLPEGSAVP	DSWETLPGTQ	VTRKEPGALR	PCSLRDCLMQ	DITVLIQQVE	VDPSFPEDCL	QSSEPLEAGA
730	740	750	760	770	780	790	800
GKEGKKDDKP	TLGAGAPDCS	EVGEGHAQVG	LGALQLVPDD	NRGKEGNDDQ	DDWSTQEIEA	CLQDFDNMTW	EIECTSEMLK
810	820	830	840	850	860	870	880
KLSSKVMTKV	IKKKIILAIQ	QLGNGEWTQG	LQKRLKHLKG	SIQLFEAKLD	KGARMLWELA	IDFSPRCSEN	PEKIIATEQN
890	900	910	920	930	940	950	960
TCAMEKSGRI	YTEIIRIWDI	VLDHCKLADS	IKAICNAYNR	GLSCVLRKKL	KGINKGQVSA	NMKIQKRIPR	CYVEDTEAEK
970	980	990	1000	1010	1020	1030	1040
GREHVNPEYF	PPASAVETEY	NIMKFHSFST	NMAFNILNDT	TATVEYPFRV	GELEYAVIDL	NPRPLEPIIL	IGRSGTGKTT
1050	1060	1070	1080	1090	1100	1110	1120
CCLYRLWKKF	HVYWEKAEQA	GSPLLAKQVW	LKRRLEVEPG	KESPGGEEEE	EEEDEEEEDS	IEVETVESID	EQEYEACAGG
1130	1140	1150	1160	1170	1180	1190	1200
AGVEPAGDGQ	AAEVCAPEHP	HQLEHLHQIF	VTKNHVLCQE	VQRNFIELSK	STKATSHYKP	LDPNIHKLQD	LRDENFPLFV
1210	1220	1230	1240	1250	1260	1270	1280
TSKQLLLLLD	ASLPKPFFLR	NEDGSLKRTI	IGWSAQEEST	IPSWQEDEEE	AEVDGDYSEE	DKAVEMRTGD	SDPRVYVTFE
1290	1300	1310	1320	1330	1340	1350	1360
VFKNEIWPKM	TKGRTAYNPA	LIWKEIKSFL	KGSFEALSCP	HGRLTEEVYK	KLGRKRCPNF	KEDRSEIYSL	FSLYQQIRSQ
1370	1380	1390	1400	1410	1420	1430	1440
KGYFDEEDVL	YNISRRLSKL	RVLPWSIHEL	YGDEIQDFTQ	AELALLMKCI	NDPNSMFLTG	DTAQSIMKGV	AFRFSDLRSL
1450	1460	1470	1480	1490	1500	1510	1520
FHYASRNTID	KQCAVRKPKK	IHQLYQNYRS	HSGILNLASG	VVDLLQFYFP	ESFDRLPRDS	GLFDGPKPTV	LESCSVSDLA
1530	1540	1550	1560	1570	1580	1590	1600
ILLRGNKRKT	QPIEFGAHQV	ILVANETAKE	KIPEELGLAL	VLTIYEAKGL	EFDDVLLYNF	FTDSEAYKEW	KIISSFTPTS
1610	1620	1630	1640	1650	1660	1670	1680
TDSREENRPL	VEVPLDKPGS	SQGRSLMVNP	EMYKLLNGEL	KQLYTAITRA	RVNLWIFDEN	REKRAPAFKY	FIRRDFVQVV
1690	1700	1710	1720	1730	1740	1750	1760
KTDENKDFDD	SMFVKTSTPA	EWIAQGDYYA	KHQCWKVAAK	CYQKGGAFEK	EKLALAHDTA	LSMKSKKVSP	KEKQLEYLEL
1770	1780	1790	1800	1810	1820	1830	1840
AKTYLECKEP	TLSLKCLSYA	KEFQLSAQLC	ERLGKIRDAA	YFYKRSQCYK	DAFRCFEQIQ	EFDLALKMYC	QEELFEEAAI
1850	1860	1870	1880	1890	1900	1910	1920
AVEKYEEMLK	TKTLPISKLS	YSASQFYLEA	AAKYLSANKM	KEMMAVLSKL	DIEDQLVFLK	SRKRLAEAAD	LLNREGRREE
1930	1940	1950	1960	1970	1980	1990	2000
AALLMKQHGC	LLEAARLTAD	KDFQASCLLG	AARLNVARDS	DIEHTKDILR	EALDICYQTG	QLSGIAEAHF	LQGVILRDFQ
2010	2020	2030	2040	2050	2060	2070	2080
KLRDAFFKFD	TLNHSAGVVE	ALYEAASQCE	AEPEKILGLA	PGGLEILLSL	VRALKRVTNN	AEKEMVKSCF	EFFGISQVDA
2090	2100	2110	2120	2130	2140	2150	2160
KYCQIAQNDP	GPILRIIFDL	DLNLREKKTK	DHFLIMTDQV	KLALNKHLLG	RLCQITRSLL	GKTYRGVCMR	FIVGLKCEDE
2170	2180	2190	2200	2210	2220	2230	2240

Detailed Protein Report

Cmpd.	No. of Cmpds.	m/z meas.	Δ m/z [ppm]	z	Rt [min]	Score	P	Range	Sequence	Modification	Ratios
825	1	575.2745	-105.17	2	40.3	12.1	0	155-164	R.NIHVPELSLK.S		M:Q 0.70<br>Q:W 1.18
1592	1	919.4423	-40.82	2	48.6	13.0	2	913-928	K.AICNAYNRGLSCVLRK.K	Carbamidomethyl: 3	
770	1	573.6008	-82.93	3	39.0	12.7	2	2682-2695	R.ACLVVSLCISWRRR.V	Carbamidomethyl: 8	

Detailed Protein Report

Protein 343: probable ATP-dependent RNA helicase DDX10 [Homo sapiens]

Accession: gi|13514831
Database: refseq_human(refseq_human_20140103.fasta)
Database Date: 2015-11-30
Score: 49.5
MW [kDa]: 100.8
pI: 9.3
Sequence Coverage [%]: 6.4
No. of unique Peptides: 2

10	20	30	40	50	60	70	80
MGKTANSPGS	GARPDPVRSF	NRWKKKHSHR	QNKKKQLRKQ	LKKPEWQVER	ESISRLMQNY	EKINVNEITR	FSDFPLSKKT
**90**	**100**	**110**	**120**	**130**	**140**	**150**	**160**
LKGLQEAQYR	LVTEIQKQTI	GLALQGKDVL	GAAKTGSGKT	LAFLVPVLEA	LYRLQWTSTD	GLGVLIISPT	RELAYQTFEV
**170**	**180**	**190**	**200**	**210**	**220**	**230**	**240**
LRKVGKNHDF	SAGLIIGGKD	LKHEAERINN	INILVCTPGR	LLQHMDETVS	FHATDLQMLV	LDEADRILDM	GFADTMNAVI
**250**	**260**	**270**	**280**	**290**	**300**	**310**	**320**
ENLPKKRQTL	LFSATQTKSV	KDLARLSLKN	PEYVWVHEKA	KYSTPATLEQ	NYIVCELQQK	ISVLYSFLRS	HLKKKSIVFF
**330**	**340**	**350**	**360**	**370**	**380**	**390**	**400**
SSCKEVQYLY	RVFCRLRPGV	SILALHGRQQ	QMRRMEVYNE	FVRKRAAVLF	ATDIAARGLD	FPAVNWVLQF	DCPEDANTYI
**410**	**420**	**430**	**440**	**450**	**460**	**470**	**480**
HRAGRTARYK	EDGEALLILL	PSEKAMVQQL	LQKKVPVKEI	KINPEKLIDV	QKKLESILAQ	DQDLKERAQR	CFVSYVRSVY
**490**	**500**	**510**	**520**	**530**	**540**	**550**	**560**
LMKDKEVFDV	SKLPIPEYAL	SLGLAVAPRV	RFLQKMQKQP	TKELVRSQAD	KVIEPRAPSL	TNDEVEEFRA	YFNEKMSILQ
**570**	**580**	**590**	**600**	**610**	**620**	**630**	**640**
KGGKRLEGTE	HRQDNDTGNE	EQEEEEDDEE	EMEEKLAKAK	GSQAPSLPNT	SEAQKIKEVP	TQFLDRDEEE	EDADFLKVKR
**650**	**660**	**670**	**680**	**690**	**700**	**710**	**720**
HNVFGLDLKD	EKTLQKKEPS	KSSIKKKMTK	VAEAKKVMKR	NFKVNKKITF	TDEGELVQQW	PQMQKSAIKD	AEEDDDTGGI
**730**	**740**	**750**	**760**	**770**	**780**	**790**	**800**
NLHKAKERLQ	EEDKFDKEEY	RKKIKAKHRE	KRLKEREARR	EANKRQAKAK	DEEEAFLDWS	DDDDDDDDGF	DPSTLPDPDK
**810**	**820**	**830**	**840**	**850**	**860**	**870**	**880**
YRSSEDSDSE	DMENKISDTK	KKQGMKKRSN	SEVEDVGPTS	HNRKKARWDT	LEPLDTGLSL	AEDEELVLHL	LRSQS

Cmpd.	No. of Cmpds.	m/z meas.	Δ m/z [ppm]	z	Rt [min]	Score	P	Range	Sequence	Modification
1771	5	599.9726	0.40	3	50.8	16.0	1	1-18	-.MGKTANSPGSGARPDPVR.S	
2761	2	713.3401	-111.86	2	65.7	11.2	2	638-649	K.VKRHNVFGLDLK.D	

Detailed Protein Report

Protein 344: PREDICTED: testis-expressed sequence 2 protein isoform X4 [Homo sapiens]

**Accession:**	gi|530412523	**Score:**	49.4
**Database:**	refseq_human(refseq_human_20140103.fasta)	**MW [kDa]:**	125.0
**Database Date:**	2015-11-30	**pI:**	5.6
**Modification(s):**	Carbamidomethyl, Oxidation	**Sequence Coverage [%]:**	3.9
		**No. of unique Peptides:**	3

Quantitation

Q:W	**Median:** 0.51	**CV:** 32.23 %	**No. of Peptides:**	2
M:Q	**Median:** 1.28	**CV:** 19.58 %	**No. of Peptides:**	2

```
        10         20         30         40         50         60         70         80
MTSLYGRHAE KTTDMPKPSA PKVHVQRSVS RDTIAIHFSA SGEEEEEEEE EFREYFEEGL DDQSIVTGLE AKEDLYLEPQ
        90        100        110        120        130        140        150        160
VGHDPAGPAA SPVLADGLSV SQAPAILPVS KNTVKLLESP VPAAQVLSTV PLAVSPGSSS SGPLASSPSV SSLSEQKTSS
       170        180        190        200        210        220        230        240
SSPLSSPSKS PILSSSASTS TLSSAKPFMS LVKSLSTEVE PKESPHPARH RHLMKTLVKS LSTDTSRQES DTVSYKPPDS
       250        260        270        280        290        300        310        320
KLNLHLFKQF TQPRNTGGDS KTAPSSPLTS PSDTRSFFKV PEMEAKIEDT KRRLSEVIYE PFQLLSKIIG EESGSHRPKA
       330        340        350        360        370        380        390        400
LSSSASELSN LSSLNGHLES NNNYSIKEEE CDSEGDGYGS DSNIPRSDHP KSTGEPTREI ELKSSQGSSL KDLGLKTSSL
       410        420        430        440        450        460        470        480
VLEKCSLSAL VSKEDEEFCE LYTEDFDLET EGESKVDKLS DIPLKPEVLA EDGVVLDSED EVDSAVQHPE LPVKTLGFFI
       490        500        510        520        530        540        550        560
MCVYVYLILP LPHYVSGLFL GIGLGFMTAV CVIWFFTPPS AHKYHKLHKN LRHWNTRSLD IKEPEILKGW MNEIYNYDPE
       570        580        590        600        610        620        630        640
TYHATLTHSV FVRLEGGTLR LSKPNKNISR RASYNEPKPE VTYISQKIYD LSDSKIYLVP KTLARKRIWN KKYPICIELG
       650        660        670        680        690        700        710        720
QQDDFMSKAQ TDKETSEEKP PAEGSEDPKK PPRPQEGTRS SQRDQILYLF GRTGREKEEW FRRFILASKL KSEIKKSSGV
       730        740        750        760        770        780        790        800
SGGKPGLLPA HSRHNSPSGH LTHSRSSSKG SVEEIMSQPK QKELAGSVRQ KMLLDYSVYM GRCVPQESRS PQRSPLQSAE
       810        820        830        840        850        860        870        880
SSPTAGKKLP EVPPSEEEEQ EAWVNALLGR IFWDFLGEKY WSDLVSKKIQ MKLSKIKLPY FMNELTLTEL DMGVAVPKIL
       890        900        910        920        930        940        950        960
QAFKPYVDHQ GLWIDLEMSY NGSFLMTLET KMNLTKLGKE PLVEALKVGE IGKEGPRAFC LADSDEESSS AGSSEEDDAP
       970        980        990       1000       1010       1020       1030       1040
EPSGGDKQLL PGAEGYVGGH RTSKIMRFVD KITKSKYFQK ATETEFIKKK IEEVSNTPLL LTVEVQECRG TLAVNIPPPP
      1050       1060       1070       1080       1090       1100       1110       1120
TDRVWYGFRK PPHVELKARP KLGEREVTLV HVTDWIEKKL EQEFQKVFVM PNMDDVYITI MHSAMDPRST SCLLKDPPVE
      1130
AADQP
```

Cmpd.	No. of Cmpds.	m/z meas.	Δ m/z [ppm]	z	Rt [min]	Score	P	Range	Sequence	Modification	Ratios	
1076	1	581.1694	-231.84	2	41.9	12.9	1	2-11	M.TSLYGRHAEK.T		M:Q	1.05
2499	1	1044.5086	15.43	2	61.7	15.0	1	632-648	K.KYPICIELGQQDDFMSK.A	Carbamidomethyl: 5; Oxidation: 15	Q:W	0.70
2481	1	449.6389	-282.20	2	61.0	10.9	0	920-927	K.EPLVEALK.V		M:Q<br>Q:W	1.55<br>0.37

Detailed Protein Report

Protein 345: contactin-associated protein-like 4 isoform 2 [Homo sapiens]

Accession: gi|148664242
Database: refseq_human(refseq_human_20140103.fasta)
Database Date: 2015-11-30
Modification(s): Oxidation

Score: 49.4
MW [kDa]: 137.4
pI: 6.1
Sequence Coverage [%]: 4.5
No. of unique Peptides: 2

Quantitation

M:Q **Median:** 1.51 **CV:** 0.00 % **No. of Peptides:** 1

10	20	30	40	50	60	70	80
MWNYDCDDPL	VSALPQASFS	SSSELSSSHG	PGFARLNRRD	GAGGWSPLVS	NKYQWLQIDL	GERMEVTAVA	TQGGYGSSNW
90	100	110	120	130	140	150	160
VTSYLLMFSD	SGWNWKQYRQ	EDSIWGFSGN	ANADSVVYYR	LQPSIKARFL	RFIPLEWNPK	GRIGMRIEVF	GCAYRSEVVD
170	180	190	200	210	220	230	240
LDGKSSLLYR	FDQKSLSPIK	DIISLKFKTM	QSDGILLHRE	GPNGDHITLQ	LRRARLFLLI	NSGEAKLPST	STLVNLTLGS
250	260	270	280	290	300	310	320
LLDDQHWHSV	LIQRLGKQVN	FTVDEHRHHF	HARGEFNLMN	LDYEGNVSFS	CSQPQSMPVT	FLSSRSYLAL	PDFSGEEEVS
330	340	350	360	370	380	390	400
ATFQFRTWNK	AGLLLFSELQ	LISGGILLFL	SDGKLKSNLY	QPGKLPSDIT	AGVELNDGQW	HSVSLSAKKN	HLSVAVDGQM
410	420	430	440	450	460	470	480
ASAAPLLGPE	QIYSGGTYYF	GGCPDKSFGS	KCKSPLGGFQ	GCMRLISISG	KVVDLISVQQ	GSLGNFSDLQ	IDSCGISDRC
490	500	510	520	530	540	550	560
LPNYCEHGGE	CSQSWSTFHC	NCTNTGYRGA	TCHNSIYEQS	CEAYKHRGNT	SGFYYIDSDG	SGPLEPFLLY	CNMTETAWTI
570	580	590	600	610	620	630	640
IQHNGSDLTR	VRNTNPENPY	AGFFEYVASM	EQLQATINRA	EHCEQEFTYY	CKKSRLVNKQ	DGTPLSWWVG	RTNETQTYWG
650	660	670	680	690	700	710	720
GSSPDLQKCT	CGLEGNCIDS	QYYCNCDADR	NEWTNDTGLL	AYKEHLPVTK	IVITDTGRLH	SEAAYKLGPL	LCQGDRSFWN
730	740	750	760	770	780	790	800
SASFDTEASY	LHFPTFHGEL	SADVSFFFKT	TASSGVFLEN	LGIADFIRIE	LRSPTVVTFS	FDVGNGPFEI	SVQSPTHFND
810	820	830	840	850	860	870	880
NQWHHVRVER	NMKEASLQVD	QLTPKTQPAP	ADGHVLLQLN	SQLFVGGTAT	RQRGFLGCIR	SLQLNGMTLD	LEERAQVTPE
890	900	910	920	930	940	950	960
VQPGCRGHCS	SYGKLCRNGG	KCRERPIGFF	CDCTFSAYTG	PFCSNEISAY	FGSGSSVIYN	FQENYLLSKN	SSSHAASFHG
970	980	990	1000	1010	1020	1030	1040
DMKLSREMIK	FSFRTTRTPS	LLLFVSSFYK	EYLSVIIAKN	GSLQIRYKLN	KYQEPDVVNF	DFKNMADGQL	HHIMINREEG
1050	1060	1070	1080	1090	1100	1110	1120
VVFIEIDDNR	RRQVHLSSGT	EFSAVKSLVL	GRILEHSDVD	QDTALAGAQG	FTGCLSAVQL	SHVAPLKAAL	HPSHPDPVTV
1130	1140	1150	1160	1170	1180	1190	1200
TGHVTESSCM	AQPGTDATSR	ERTHSFADHS	GTIDDREPLA	NAIKSDSAVI	GGLIAVVIFI	LLCITAIAVR	IYQQKRLYKR
1210	1220	1230	1240				
SEAKRSENVD	SAEAVLKSEL	NIQNAVNENQ	KEYFF				

Cmpd.	No. of Cmpds.	m/z meas.	Δ m/z [ppm]	z	Rt [min]	Score	P	Range	Sequence	Modification	Ratios	
2177	1	1051.4295	-113.84	2	55.7	21.4	2	808-825	R.VERNMKEASLQVDQLTPK.T	Oxidation: 5	M:Q	1.51
1571	1	1061.1650	-10.98	3	49.3	12.9	1	1024-1050	K.NMADGQLHHIMINREEGVVFIEIDD R	Oxidation: 2		

Detailed Protein Report

Protein 346: protein unc-13 homolog A [Homo sapiens]

Accession: gi|283837842
Database: refseq_human(refseq_human_20140103.fasta)
Database Date: 2015-11-30
Modification(s): Carbamidomethyl, Oxidation

Score: 49.4
MW [kDa]: 192.9
pI: 5.1
Sequence Coverage [%]: 3.8
No. of unique Peptides: 3

10	20	30	40	50	60	70	80
MSLLCVGVKK	AKFDGAQEKF	NTYVTLKVQN	VKSTTIAVRG	SQPSWEQDFM	FEINRLDLGL	TVEVWNKGLI	WDTMVGTVWI
90	100	110	120	130	140	150	160
PLRTIRQSNE	EGPGEWLTLD	SQVIMADSEI	CGTKDPTFHR	ILLDTRFELP	LDIPEEEARY	WAKKLEQLNA	MRDQDEYSFQ
170	180	190	200	210	220	230	240
DEQDKPLPVP	SNQCCNWNYF	GWGEQHNDDP	DSAVDDRDSD	YRSETSNSIP	PPYYTTSQPN	ASVHQYSVRP	PPLGSRESYS
250	260	270	280	290	300	310	320
DSMHSYEEFS	EPQALSPTGS	SRYASSGELS	QGSSQLSEDF	DPDEHSLQGS	DMEDERDRDS	YHSCHSSVSY	HKDSPRWDQD
330	340	350	360	370	380	390	400
EEELEEDLED	FLEEEELPED	EEELEEEEEE	VPDDLGSYAQ	REDVAVAEPK	DFKRISLPPA	APGKEDKAPV	APTEAPDMAK
410	420	430	440	450	460	470	480
VAPKPATPDK	VPAAEQIPEA	EPPKDEESFR	PREDEEGQEG	QDSMSRAKAN	WLRAFNKVRM	QLQEARGEGE	MSKSLWFKGG
490	500	510	520	530	540	550	560
PGGGLIIIDS	MPDIRKRKPI	PLVSDLAMSL	VQSRKAGITS	ALASSTLNNE	ELKNHVYKKT	LQALIYPISC	TTPHNFEVWT
570	580	590	600	610	620	630	640
ATTPTYCYEC	EGLLWGIARQ	GMRCTECGVK	CHEKCQDLLN	ADCLQRAAEK	SSKHGAEDRT	QNIIMVLKDR	MKIRERNKPE
650	660	670	680	690	700	710	720
IFELIQEIFA	VTKTAHTQQM	KAVKQSVLDG	TSKWSAKISI	TVVCAQGLQA	KDKTGSSDPY	VTVQVGKTKK	RTKTIYGNLN
730	740	750	760	770	780	790	800
PVWEENFHFE	CHNSSDRIKV	RVWDEDDDIK	SRVKQRFKRE	SDDFLGQTII	EVRTLSGEMD	VWYNLDKRTD	KSAVSGAIRL
810	820	830	840	850	860	870	880
HISVEIKGEE	KVAPYHVQYT	CLHENLFHFV	TDVQNNGVVK	IPDAKGDDAW	KVYYDETAQE	IVDEFAMRYG	VESIYQAMTH
890	900	910	920	930	940	950	960
FACLSSKYMC	PGVPAVMSTL	LANINAYYAH	TTASTNVSAS	DRFAASNFGK	ERFVKLLDQL	HNSLRIDLSM	YRNNFPASSP
970	980	990	1000	1010	1020	1030	1040
ERLQDLKSTV	DLLTSITFFR	MKVQELQSPP	RASQVVKDCV	KACLNSTYEY	IFNNCHELYS	REYQTDPAKK	GEVLPEEQGP
1050	1060	1070	1080	1090	1100	1110	1120
SIKNLDFWSK	LITLIVSIIE	EDKNSYTPCL	NQFPQELNVG	KISAEVMWNL	FAQDMKYAME	EHDKHRLCKS	ADYMNLHFKV
1130	1140	1150	1160	1170	1180	1190	1200
KWLYNEYVTE	LPAFKDRVPE	YPAWFEPFVI	QWLDENEEVS	RDFLHGALER	DKKDGFQQTS	EHALFSCSVV	DVFSQLNQSF
1210	1220	1230	1240	1250	1260	1270	1280
EIIKKLECPD	PQIVGHYMRR	FAKTISNVLL	QYADIISKDF	ASYCSKEKEK	VPCILMNNTQ	QLRVQLEKMF	EAMGGKELDA
1290	1300	1310	1320	1330	1340	1350	1360
EASDILKELQ	VKLNNVLDEL	SRVFATSFQP	HIEECVKQMG	DILSQVKGTG	NVPASACSSV	AQDADNVLQP	IMDLLDSNLT
1370	1380	1390	1400	1410	1420	1430	1440
LFAKICEKTV	LKRVLKELWK	LVMNTMEKTI	VLPPLTDQTM	IGNLLRKHGK	GLEKGRVKLP	SHSDGTQMIF	NAAKELGQLS
1450	1460	1470	1480	1490	1500	1510	1520
KLKDHMVREE	AKSLTPKQCA	VVELALDTIK	QYFHAGGVGL	KKTFLEKSPD	LQSLRYALSL	YTQATDLLIK	TFVQTQSAQG
1530	1540	1550	1560	1570	1580	1590	1600
LGVEDPVGEV	SVHVELFTHP	GTGEHKVTVK	VVAANDLKWQ	TSGIFRPFIE	VNIIGPQLSD	KKRKFATKSK	NNSWAPKYNE
1610	1620	1630	1640	1650	1660	1670	1680
SFQFTLSADA	GPECYELQVC	VKDYCFARED	RTVGLAVLQL	RELAQRGSAA	CWLPLGRRIH	MDDTGLTVLR	ILSQRSNDEV
1690	1700	1710					
AKEFVKLKSD	TRSAEEGGAA	PAP					

Cmpd.	No. of Cmpds.	m/z meas.	Δ m/z [ppm]	z	Rt [min]	Score	P	Range	Sequence	Modification
2705	1	805.4372	84.57	2	65.1	14.8	1	460-473	R.MQLQEARGEGEMSK.S	Oxidation: 1
2064	1	753.2991	-61.50	2	55.9	14.2	0	595-606	K.CQDLLNADCLQR.A	Carbamidomethyl: 1, 9

Detailed Protein Report

Cmpd.	No. of Cmpds.	m/z meas.	Δ m/z [ppm]	z	Rt [min]	Score	P	Range	Sequence	Modification
2215	1	682.1038	136.60	3	57.9	10.1	1	1122-1137	K.WLYNEYVTELPAFKDR.V	

Detailed Protein Report

Protein 347: PREDICTED: zinc finger protein 493 isoform X2 [Homo sapiens]

Accession: gi|530414758
Database: refseq_human(refseq_human_20140103.fasta)
Database Date: 2015-11-30
Modification(s): Carbamidomethyl, Oxidation

Score: 49.3
MW [kDa]: 81.4
pI: 10.4
Sequence Coverage [%]: 9.6
No. of unique Peptides: 3

10	20	30	40	50	60	70	80
MKGHSTVVKP	PVICSHFAED	FCPGPGIKDS	FQKVILREYV	KCGHKDLQLR	KGCKSMNECN	VHKEGYNELN	QYLTTTQSKI
**90**	**100**	**110**	**120**	**130**	**140**	**150**	**160**
FQCDKYVKVF	HKLLNSNRHN	TKHTGKKPFK	CKKCGKSFCM	LLHLCQHKRI	HIRENSYRCE	ECGKAFIWFS	TLTRHRRVHT
**170**	**180**	**190**	**200**	**210**	**220**	**230**	**240**
GEKSYKYECG	KSFNQDSNLT	THKRIHTGQK	PYKCEECGTS	FYQFSYLTRH	KLIHTREKPY	KCEQYGKTFN	QSSTLTGHKI
**250**	**260**	**270**	**280**	**290**	**300**	**310**	**320**
IHNGEKPYKC	EECGKAFSIF	STPTKHKIIH	TEEKSHRCEE	YCKAYKESSH	LTTHKRIHTG	EKPYKCEECG	KAFSIFSTLT
**330**	**340**	**350**	**360**	**370**	**380**	**390**	**400**
KHKIIHTEEK	SHRCEECGKA	YKESSHLTTH	KRIHTGEKPY	KCEECGKTFS	VFSILTKHKI	IHTEEKPYKC	EECGKAFKRS
**410**	**420**	**430**	**440**	**450**	**460**	**470**	**480**
STLTKHRIIH	TEEKPYKCEE	CGKAFNQSST	LSIHKIIHTG	EKPYKCEECG	KAFKRSSTLT	IHKMIHTGEK	PYKCEECGKA
**490**	**500**	**510**	**520**	**530**	**540**	**550**	**560**
FNRSSHLTTH	KRIHTGHKPY	KCKECGKSFS	VFSTLTKHKI	IHTDKKPYKC	EECGKAFNRS	SILSIHKKIH	TGEKPYKCEE
**570**	**580**	**590**	**600**	**610**	**620**	**630**	**640**
CGKAFKRSSH	LAGHKQIHSV	QKPYKCEECG	KAFSIFSTLT	KHKIIHTEEK	PYKCEKCGKT	FYRFSNLNTH	KIIHTGEKPC
**650**	**660**	**670**	**680**	**690**	**700**	**710**	
KCEECGKAFN	HSSNLIKHKL	IHTGDKPYKC	EACGKAFRRS	SHLSRHKIIH	IGIHTEETVQ	K	

Cmpd.	No. of Cmpds.	m/z meas.	Δ m/z [ppm]	z	Rt [min]	Score	P	Range	Sequence	Modification
1622	1	927.1215	-7.05	3	49.0	11.9	0	3-28	K.GHSTVVKPPVICSHFAEDFCPGPGIK.D	Carbamidomethyl: 12
2085	1	939.2954	-149.37	2	56.4	12.7	1	114-128	K.CGKSFCMLLHLCQHK.R	Carbamidomethyl: 1, 6; Oxidation: 7
1854	1	938.8329	-130.94	2	53.5	11.5	1	576-591	K.QIHSVQKPYKCEECGK.A	

Detailed Protein Report

Protein 348: MAM and LDL-receptor class A domain-containing protein C10orf112 precursor [Homo sapiens]

**Accession:**	gi|565671710	**Score:**	49.3
**Database:**	refseq_human(refseq_human_20140103.fasta)	**MW [kDa]:**	240.9
**Database Date:**	2015-11-30	**pI:**	4.9
**Modification(s):**	Carbamidomethyl	**Sequence Coverage [%]:**	2.6
		**No. of unique Peptides:**	2

Quantitation

Q:W	**Median:**	1.13	**CV:**	0.00 %	**No. of Peptides:**	1
M:Q	**Median:**	1.17	**CV:**	0.00 %	**No. of Peptides:**	1

Detailed Protein Report

10	20	30	40	50	60	70	80
MLFFLDRMLA	FPMNETFCCL	WIACVFNSTL	AQQGTESFQC	DNGVSLPPDS	ICDFTDQCGD	SSDERHCLNY	ERCDFEDGLC
90	100	110	120	130	140	150	160
HMTQDQSLQP	SWTKRSGMIG	LSPPFYDHNG	DVSAHFLSLV	SRVDSISSSL	RSRVFLPTND	QHDCQITFYY	FSCQVSGKLM
170	180	190	200	210	220	230	240
VGLQTACGGP	IQHLWQNTAA	LPNQWERNVI	KIQSSQRFQV	VFEGQMASTY	EQDEVIAIDD	ISFSSGCLPA	NDGILLCQEA
250	260	270	280	290	300	310	320
LNAERELCHP	DTDLCRFDAT	DEELRLCQAC	GFEFDMCEWT	SEASAGQISW	MRTKAREIPA	FESTPQQDQG	GDDEGYYVWV
330	340	350	360	370	380	390	400
GAKHGFTLNH	LDSRAYLNSS	VCHCLGKSCH	LQFYYAMESS	VLRVRLYNNK	EEEIFWTYNI	STHSQWVKAD	VLIPEDLKTF
410	420	430	440	450	460	470	480
KIIFEGTLLS	QRSFIALDHL	WVYACGQTQS	RKLCSADEFP	CTSGQCIAKE	SVCDSRQDCS	DESDEDPATC	SKHLTCDFES
490	500	510	520	530	540	550	560
GFCGWEPFLT	EDSHWKLMKG	LNNGEHHFPA	ADHTANINHG	SFIYLEAQRS	PGVAKLGSPV	LTKLLTASTP	CQVQFWYHLS
570	580	590	600	610	620	630	640
QHSNLSVFTR	TSLDGNLQKQ	GKIIRFSESQ	WSHAKIDLIA	EAGESTLPFQ	LILEATVLSS	NATVALDDIS	VSQECEISYK
650	660	670	680	690	700	710	720
SLPRTSTQSK	FSKCDFEANS	CDWFEAISGD	HFDWIRSSQS	ELSADFEHQA	PPRDHSLNAS	QGHFMFILKK	SSSLWQVAKL
730	740	750	760	770	780	790	800
QSPTFSQTGP	GCILSFWFYN	YGLSVGAAEL	QLHMENSHDS	TVIWRVLYNQ	GKQWLEATIQ	LGRLSQPFHL	SLDKVSLGIY
810	820	830	840	850	860	870	880
DGVSAIDDIR	FENCTLPLPA	ESCEGLDHFW	CRHTRACIEK	LRLCDLVDDC	GDRTDEVNCA	PELQCNFETG	ICNWEQDAKD
890	900	910	920	930	940	950	960
DFDWTRSQGP	TPTLNTGPMK	DNTLGTAKGH	YLYIESSEPQ	AFQDSAALLS	PILNATDTKG	CTFRFYYHMF	GKRIYRLAIY
970	980	990	1000	1010	1020	1030	1040
QRIWSDSRGQ	LLWQIFGNQG	NRWIRKHLNI	SSRQPFQILV	EASVGDGFTG	DIAIDDLSFM	DCTLYPGNLP	ADLPTPPETS
1050	1060	1070	1080	1090	1100	1110	1120
VPVTLPPHNC	TDNEFICRSD	GHCIEKMQKC	DFKYDCPDKS	DEASCVMEVC	SFEKRSLCKW	YQPIPVHLLQ	DSNTFRWGLG
1130	1140	1150	1160	1170	1180	1190	1200
NGISIHHGEE	NHRPSVDHTQ	NTTDGWYLYA	DSSNGKFGDT	ADILTPIISL	TGPKCTLVFW	THMNGATVGS	LQVLIKKDNV
1210	1220	1230	1240	1250	1260	1270	1280
TSKLWAQTGQ	QGAQWKRAEV	FLGIRSHTQI	VFRAKRGISY	IGDVAVDDIS	FQDCSPLLSP	ERKCTDHEFM	CANKHCIAKD
1290	1300	1310	1320	1330	1340	1350	1360
KLCDFVNDCA	DNSDETTFIC	RTSSGRCDFE	FDLCSWKQEK	DEDFDWNLKA	SSIPAAGTEP	AADHTLGNSS	GHYIFIKSLF
1370	1380	1390	1400	1410	1420	1430	1440
PQQPMRAARI	SSPVISKRSK	NCKIIFHYHM	YGNGIGALTL	MQVSVTNQTK	VLLNLTVEQG	NFWRREELSL	FGDEDFQLKF
1450	1460	1470	1480	1490	1500	1510	1520
EGRVGKGQRG	DIALDDIVLT	ENCLSLHDSV	QEELAVPLPT	GFCPLGYREC	HNGKCYRLEQ	SCNFVDNCGD	NTDENECGSS
1530	1540	1550	1560	1570	1580	1590	1600
CTFEKGWCGW	QNSQADNFDW	VLGVGSHQSL	RPPKDHTLGN	ENGHFMYLEA	TAVGLRGDKA	HFRSTMWRES	SAACTMSFWY
1610	1620	1630	1640	1650	1660	1670	1680
FVSAKATGSI	QILIKTEKGL	SKVWQESKQN	PGNHWQKADI	LLGKLRNFEV	IFQGIRTRDL	GGGAAIDDIE	FKNCTTVGEI
1690	1700	1710	1720	1730	1740	1750	1760
SELCPEITDF	LCRDKKCIAS	HLLCDYKPDC	SDRSDEAHCA	HYTSTTGSCN	FETSSGNWTT	ACSLTQDSED	DLDWAIGSRI
1770	1780	1790	1800	1810	1820	1830	1840
PAKALIPDSD	HTPGSGQHFL	YVNSSGSKEG	SVARITTSKS	FPASLGMCTV	RFWFYMIDPR	SMGILKVYTI	EESGLNILVW
1850	1860	1870	1880	1890	1900	1910	1920
SVIGNKRTGW	TYGSVPLSSN	SPFKVAFEAD	LDGNEDIFIA	LDDISFTPEC	VTGGPVPVQP	SPCEADQFSC	IYTLQCVPLS
1930	1940	1950	1960	1970	1980	1990	2000
GKCDGHEDCI	DGSDEMDCPL	SPTPPLCSNM	EFPCSTDECI	PSLLLCDGVP	DCHFNEDELI	CSNKSCSNGA	LVCASSNSCI
2010	2020	2030	2040	2050	2060	2070	2080
PAHQRCDGFA	DCMDFQLDES	SCSECPLNYC	RNGGTCVVEK	NGPMCRCRQG	WKGNRCHIKF	NPPATDFTYA	QNNTWTLLGI
2090	2100	2110	2120	2130	2140	2150	2160
GLAFLMTHIT	VAVLCFLANR	KVPIRKTEGS	GNCAFVNPVY	GNWSNPEKTE	SSVYSFSNPL	YGTTSGSLET	LSHHLK

Detailed Protein Report

Cmpd.	No. of Cmpds.	m/z meas.	Δ m/z [ppm]	z	Rt [min]	Score	P	Range	Sequence	Modification	Ratios
2517	1	913.4615	10.45	2	61.9	10.8	1	1425-1439	R.REELSLFGDEDFQLK.F		Q:W 1.13<br>M:Q 1.17
777	1	541.1764	-129.12	3	38.5	12.0	1	2032-2046	R.NGGTCVVEKNGPMCR.C	Carbamidomethyl: 5	

Detailed Protein Report

Protein 349: kinesin-like protein KIF20B isoform 2 [Homo sapiens]

**Accession:**	gi|46049114	**Score:**	49.1
**Database:**	refseq_human(refseq_human_20140103.fasta)	**MW [kDa]:**	206.0
**Database Date:**	2015-11-30	**pI:**	5.5
**Modification(s):**	Carbamidomethyl, Oxidation	**Sequence Coverage [%]:**	3.8
		**No. of unique Peptides:**	2

10	20	30	40	50	60	70	80
MESNFNQEGV	PRPSYVFSAD	PIARPSEINF	DGIKLDLSHE	FSLVAPNTEA	NSFESKDYLQ	VCLRIRPFTQ	SEKELESEGC
**90**	**100**	**110**	**120**	**130**	**140**	**150**	**160**
VHILDSQTVV	LKEPQCILGR	LSEKSSGQMA	QKFSFSKVFG	PATTQKEFFQ	GCIMQPVKDL	LKGQSRLIFT	YGLTNSGKTY
**170**	**180**	**190**	**200**	**210**	**220**	**230**	**240**
TFQGTEENIG	ILPRTLNVLF	DSLQERLYTK	MNLKPHRSRE	YLRLSSEQEK	EEIASKSALL	RQIKEVTVHN	DSDDTLYGSL
**250**	**260**	**270**	**280**	**290**	**300**	**310**	**320**
TNSLNISEFE	ESIKDYEQAN	LNMANSIKFS	VWVSFFEIYN	EYIYDLFVPV	SSKFQKRKML	RLSQDVKGYS	FIKDLQWIQV
**330**	**340**	**350**	**360**	**370**	**380**	**390**	**400**
SDSKEAYRLL	KLGIKHQSVA	FTKLNNASSR	SHSIFTVKIL	QIEDSEMSRV	IRVSELSLCD	LAGSERTMKT	QNEGERLRET
**410**	**420**	**430**	**440**	**450**	**460**	**470**	**480**
GNINTSLLTL	GKCINVLKNS	EKSKFQQHVP	FRESKLTHYF	QSFFNGKGKI	CMIVNISQCY	LAYDETLNVL	KFSAIAQKVC
**490**	**500**	**510**	**520**	**530**	**540**	**550**	**560**
VPDTLNSSQE	KLFGPVKSSQ	DVSLDSNSNS	KILNVKRATI	SWENSLEDLM	EDEDLVEELE	NAEETQNVET	KLLDEDLDKT
**570**	**580**	**590**	**600**	**610**	**620**	**630**	**640**
LEENKAFISH	EEKRKLLDLI	EDLKKKLINE	KKEKLTLEFK	IREEVTQEFT	QYWAQREADF	KETLLQEREI	LEENAERRLA
**650**	**660**	**670**	**680**	**690**	**700**	**710**	**720**
IFKDLVGKCD	TREEAAKDIC	ATKVETEEAT	ACLELKFNQI	KAELAKTKGE	LIKTKEELKK	RENESDSLIQ	ELETSNKKII
**730**	**740**	**750**	**760**	**770**	**780**	**790**	**800**
TQNQRIKELI	NIIDQKEDTI	NEFQNLKSHM	ENTFKCNDKA	DTSSLIINNK	LICNETVEVP	KDSKSKICSE	RKRVNENELQ
**810**	**820**	**830**	**840**	**850**	**860**	**870**	**880**
QDEPPAKKGS	IHVSSAITED	QKKSEEVRPN	IAEIEDIRVL	QENNEGLRAF	LLTIENELKN	EKEEKAELNK	QIVHFQQELS
**890**	**900**	**910**	**920**	**930**	**940**	**950**	**960**
LSEKKNLTLS	KEVQQIQSNY	DIAIAELHVQ	KSKNQEQEEK	IMKLSNEIET	ATRSITNNVS	QIKLMHTKID	ELRTLDSVSQ
**970**	**980**	**990**	**1000**	**1010**	**1020**	**1030**	**1040**
ISNIDLLNLR	DLSNGSEEDN	LPNTQLDLLG	NDYLVSKQVK	EYRIQEPNRE	NSFHSSIEAI	WEECKEIVKA	SSKKSHQIEE
**1050**	**1060**	**1070**	**1080**	**1090**	**1100**	**1110**	**1120**
LEQQIEKLQA	EVKGYKDENN	RLKEKEHKNQ	DDLLKEKETL	IQQLKEELQE	KNVTLDVQIQ	HVVEGKRALS	ELTQGVTCYK
**1130**	**1140**	**1150**	**1160**	**1170**	**1180**	**1190**	**1200**
AKIKELETIL	ETQKVECSHS	AKLEQDILEK	ESIILKLERN	LKEFQEHLQD	SVKNTKDLNV	KELKLKEEIT	QLTNNLQDMK
**1210**	**1220**	**1230**	**1240**	**1250**	**1260**	**1270**	**1280**
HLLQLKEEEE	ETNRQETEKL	KEELSASSAR	TQNLKADLQR	KEEDYADLKE	KLTDAKKQIK	QVQKEVSVMR	DEDKLLRIKI
**1290**	**1300**	**1310**	**1320**	**1330**	**1340**	**1350**	**1360**
NELEKKKNQC	SQELDMKQRT	IQQLKEQLNN	QKVEEAIQQY	ERACKDLNVK	EKIIEDMRMT	LEEQEQTQVE	QDQVLEAKLE
**1370**	**1380**	**1390**	**1400**	**1410**	**1420**	**1430**	**1440**
EVERLATELE	KWKEKCNDLE	TKNNQRSNKE	HENNTDVLGK	LTNLQDELQE	SEQKYNADRK	KWLEEKMMLI	TQAKEAENIR
**1450**	**1460**	**1470**	**1480**	**1490**	**1500**	**1510**	**1520**
NKEMKKYAED	RERFFKQQNE	MEILTAQLTE	KDSDLQKWRE	ERDQLVAALE	IQLKALISSN	VQKDNEIEQL	KRIISETSKI
**1530**	**1540**	**1550**	**1560**	**1570**	**1580**	**1590**	**1600**
ETQIMDIKPK	RISSADPDKL	QTEPLSTSFE	ISRNKIEDGS	VVLDSCEVST	ENDQSTRFPK	PELEIQFTPL	QPNKMAVKHP
**1610**	**1620**	**1630**	**1640**	**1650**	**1660**	**1670**	**1680**
GCTTPVTVKI	PKARKRKSNE	MEEDLVKCEN	KKNATPRTNL	KFPISDDRNS	SVKKEQKVAI	RPSSKKTYSL	RSQASIIGVN
**1690**	**1700**	**1710**	**1720**	**1730**	**1740**	**1750**	**1760**
LATKKKEGTL	QKFGDFLQHS	PSILQSKAKK	IIETMSSSKL	SNVEASKENV	SQPKRAKRKL	YTSEISSPID	ISGQVILMDQ
**1770**	**1780**	**1790**					
KMKESDHQII	KRRLRTKTAK						

Cmpd.	No. of	m/z meas.	Δ m/z	z	Rt	Score	P	Range	Sequence	Modification

Detailed Protein Report

	Cmpds.		[ppm]		[min]					
20	1	1103.5042	-42.73	2	29.9	14.1	2	93-112	K.EPQCILGRLSEKSSGQMAQK.F	Oxidation: 17
1683	1	954.5011	22.43	3	51.3	10.8	1	448-471	K.GKICMIVNISQCYLAYDETLNVLK.F	Carbamidomethyl: 4, 12; Oxidation: 5

Detailed Protein Report

Protein 350: neuroblastoma breakpoint family member 6 isoform 1 [Homo sapiens]

**Accession:**	gi|221136844	**Score:**	49.0
**Database:**	refseq_human(refseq_human_20140103.fasta)	**MW [kDa]:**	75.3
**Database Date:**	2015-11-30	**pI:**	4.7
**Modification(s):**	Oxidation	**Sequence Coverage [%]:**	5.8
		**No. of unique Peptides:**	1

Quantitation

M:Q **Median:** 1.58 **CV:** 0.00 % **No. of Peptides:** 1

10	20	30	40	50	60	70	80
MVVSADPLSS	ERAEMNILEI	NQELRSQLAE	SNQQFRDLKE	KFLITQATAY	SLANQLKKYK	CEEYKDIIDS	VLRDELQSME
90	100	110	120	130	140	150	160
KLAEKLRQAE	ELRQYKALVH	SQAKELTQLR	EKLREGRDAS	RWLNKHLKTL	LTPDDPDKSQ	GQDLREQLAE	GHRLAEHLVH
170	180	190	200	210	220	230	240
KLSPENDEDE	DEDEDDKDEE	VEKVQESPAP	REVQKTEEKE	VPQDSLEECA	VTCSNSHNPS	NSNQPHRSTK	ITFKEHEVDS
250	260	270	280	290	300	310	320
ALVVESEHPH	DEEEEALNIP	PENQNDHEEE	EGKAPVPPRN	RQSLEPGELT	NLRDFLISPV	VSHVANPGHH	DKSNSYRHRE
330	340	350	360	370	380	390	400
VSFLALDEQK	VCSAQDVARD	YSNPKWDETS	LGFLEKQSDL	EEVKGQETVA	PRLSRGPLRV	DKHEIPQESL	DGCCLTPSIL
410	420	430	440	450	460	470	480
PDLTPSYHPY	WSTLYSFEDK	QVSLALVDKI	KKDQEEIEDQ	SPPCPRLSQE	LPEVKEQEVP	EDSVNEVYLT	PSVHHDVSDC
490	500	510	520	530	540	550	560
HQPYSSTLSS	LEDQLACSAL	DVASPTEAAC	PQGTWSGDLS	HHRSEVQISQ	AQLEPSTLVP	SCLRLQLDQG	FHCGNGLAQR
570	580	590	600	610	620	630	640
GLSSTTCSFS	ANADSGNQWP	FQELVLEPSL	GMKNPPQLED	DALEGSASNT	QGRQVTGRIR	ASLVLILKTI	RRRLPFSKWR
650	660	670					
LAFRFAGPHA	ESAEIPNTAE	RMQRMIG					

Cmpd.	No. of Cmpds.	m/z meas.	Δ m/z [ppm]	z	Rt [min]	Score	P	Range	Sequence	Modification	Ratios	
1180	1	384.1589	-56.37	2	44.9	12.5	1	662-667	R.MQRMIG.-	Oxidation: 1, 4	M:Q	1.58

Detailed Protein Report

Protein 351: **cytospin-B isoform 2 [Homo sapiens]**

**Accession:**	gi|75750474	**Score:**	49.0
**Database:**	refseq_human(refseq_human_20140103.fasta)	**MW [kDa]:**	110.2
**Database Date:**	2015-11-30	**pI:**	5.6
		**Sequence Coverage [%]:**	6.0
		**No. of unique Peptides:**	1

10	20	30	40	50	60	70	80
MGNHSGRPED	PEPGAFTTTK	RTGIPAPREF	SVTVSRERSV	PRGPSNPRKS	VSSPTSSNTP	TPTKHLRTPS	TKPKQENEGG
**90**	**100**	**110**	**120**	**130**	**140**	**150**	**160**
EKAALESQVR	ELLAEAKAKD	SEINRLRSEL	KKYKEKRTLN	AEGTDALGPN	VDGTSVSPGD	TEPMIRALEE	KNKNFQKELS
**170**	**180**	**190**	**200**	**210**	**220**	**230**	**240**
DLEEENRVLK	EKLIYLEHSP	NSEGAASHTG	DSSCPTSITQ	ESSFGSPTGN	QMSSDIDEYK	KNIHGNALRT	SGSSSSDVTK
**250**	**260**	**270**	**280**	**290**	**300**	**310**	**320**
ASLSPDASDF	EHITAETPSR	PLSSTSNPFK	SSKCSTAGSS	PNSVSELSLA	SLTEKIQKME	ENHHSTAEEL	QATLQELSDQ
**330**	**340**	**350**	**360**	**370**	**380**	**390**	**400**
QQMVQELTAE	NEKLVDEKTI	LETSFHQHRE	RAEQLSQENE	KLMNLLQERV	KNEEPTTQEG	KIIELEQKCT	GILEQGRFER
**410**	**420**	**430**	**440**	**450**	**460**	**470**	**480**
EKLLNIQQQL	TCSLRKVEEE	NQGALEMIKR	LKEENEKLNE	FLELERHNNN	MMAKTLEECR	VTLEGLKMEN	GSLKSHLQGE
**490**	**500**	**510**	**520**	**530**	**540**	**550**	**560**
KQKATEASAV	EQTAESCEVQ	EMLKVARAEK	DLLELSCNEL	RQELLKANGE	IKHVSSLLAK	VEKDYSYLKE	ICDHQAEQLS
**570**	**580**	**590**	**600**	**610**	**620**	**630**	**640**
RTSLKLQEKA	SESDAEIKDM	KETIFELEDQ	VEQHRAVKLH	NNQLISELES	SVIKLEEQKS	DLERQLKTLT	KQMKEETEEW
**650**	**660**	**670**	**680**	**690**	**700**	**710**	**720**
RRFQADLQTA	VVVANDIKCE	AQQELRTVKR	KLLEEEEKNA	RLQKELGDVQ	GHGRVVTSRA	APPPVDEEPE	SSEVDAAGRW
**730**	**740**	**750**	**760**	**770**	**780**	**790**	**800**
PGVCVSRTSP	TPPESATTVK	SLIKSFDLGR	PGGAGQNISV	HKTPRSPLSG	IPVRTAPAAA	VSPMQRHSTY	SSVRPASRGV
**810**	**820**	**830**	**840**	**850**	**860**	**870**	**880**
TQRLDLPDLP	LSDILKGRTE	TLKPDPHLRK	SPSLESLSRP	PSLGFGDTRL	LSASTRAWKP	QSKLSVERKD	PLAALAREYG
**890**	**900**	**910**	**920**	**930**	**940**	**950**	**960**
GSKRNALLKW	CQKKTQGYAN	IDITNFSSSW	SDGLAFCALL	HTYLPAHIPY	QELNSQEKKR	NLLLAFEAAE	SVGIKPSLEL
**970**	**980**	**990**					
SEMLYTDRPD	WQSVMQYVAQ	IYKYFET					

Cmpd.	No. of Cmpds.	m/z meas.	Δ m/z [ppm]	z	Rt [min]	Score	P	Range	Sequence	Modification
14	1	1103.5194	-101.41	2	29.8	16.5	2	799-818	R.GVTQRLDLPDLPLSDILKGR.T	

Detailed Protein Report

Protein 352: SWI/SNF-related matrix-associated actin-dependent regulator of chromatin subfamily A member 5 [Homo sapiens]

**Accession:**	gi|325651836	**Score:**	49.0
**Database:**	refseq_human(refseq_human_20140103.fasta)	**MW [kDa]:**	121.8
**Database Date:**	2015-11-30	**pI:**	9.0
**Modification(s):**	Oxidation	**Sequence Coverage [%]:**	3.3
		**No. of unique Peptides:**	1

10	20	30	40	50	60	70	80
MSSAAEPPPP	PPPESAPSKP	AASIASGGSN	SSNKGGPEGV	AAQAVASAAS	AGPADAEMEE	IFDDASPGKQ	KEIQEPDPTY
**90**	**100**	**110**	**120**	**130**	**140**	**150**	**160**
EEKMQTDRAN	RFEYLLKQTE	LFAHFIQPAA	QKTPTSPLKM	KPGRPRIKKD	EKQNLLSVGD	YRHRRTEQEE	DEELLTESSK
**170**	**180**	**190**	**200**	**210**	**220**	**230**	**240**
ATNVCTRFED	SPSYVKWGKL	RDYQVRGLNW	LISLYENGIN	GILADEMGLG	KTLQTISLLG	YMKHYRNIPG	PHMVLVPKST
**250**	**260**	**270**	**280**	**290**	**300**	**310**	**320**
LHNWMSEFKR	WVPTLRSVCL	IGDKEQRAAF	VRDVLLPGEW	DVCVTSYEML	IKEKSVFKKF	NWRYLVIDEA	HRIKNEKSKL
**330**	**340**	**350**	**360**	**370**	**380**	**390**	**400**
SEIVREFKTT	NRLLLTGTPL	QNNLHELWSL	LNFLLPDVFN	SADDFDSWFD	TNNCLGDQKL	VERLHMVLRP	FLLRRIKADV
**410**	**420**	**430**	**440**	**450**	**460**	**470**	**480**
EKSLPPKKEV	KIYVGLSKMQ	REWYTRILMK	DIDILNSAGK	MDKMRLLNIL	MQLRKCCNHP	YLFDGAEPGP	PYTTDMHLVT
**490**	**500**	**510**	**520**	**530**	**540**	**550**	**560**
NSGKMVVLDK	LLPKLKEQGS	RVLIFSQMTR	VLDILEDYCM	WRNYEYCRLD	GQTPHDERQD	SINAYNEPNS	TKFVFMLSTR
**570**	**580**	**590**	**600**	**610**	**620**	**630**	**640**
AGGLGINLAT	ADVVILYDSD	WNPQVDLQAM	DRAHRIGQTK	TVRVFRFITD	NTVEERIVER	AEMKLRLDSI	VIQQGRLVDQ
**650**	**660**	**670**	**680**	**690**	**700**	**710**	**720**
NLNKIGKDEM	LQMIRHGATH	VFASKESEIT	DEDIDGILER	GAKKTAEMNE	KLSKMGESSL	RNFTMDTESS	VYNFEGEDYR
**730**	**740**	**750**	**760**	**770**	**780**	**790**	**800**
EKQKIAFTEW	IEPPKRERKA	NYAVDAYFRE	ALRVSEPKAP	KAPRPPKQPN	VQDFQFFPPR	LFELLEKEIL	FYRKTIGYKV
**810**	**820**	**830**	**840**	**850**	**860**	**870**	**880**
PRNPELPNAA	QAQKEEQLKI	DEAESLNDEE	LEEKEKLLTQ	GFTNWNKRDF	NQFIKANEKW	GRDDIENIAR	EVEGKTPEEV
**890**	**900**	**910**	**920**	**930**	**940**	**950**	**960**
IEYSAVFWER	CNELQDIEKI	MAQIERGEAR	IQRRISIKKA	LDTKIGRYKA	PFHQLRISYG	TNKGKNYTEE	EDRFLICMLH
**970**	**980**	**990**	**1000**	**1010**	**1020**	**1030**	**1040**
KLGFDKENVY	DELRQCIRNS	PQFRFDWFLK	SRTAMELQRR	CNTLITLIER	ENMELEEKEK	AEKKKRGPKP	STQKRKMDGA
**1050**	**1060**						
PDGRGRKKKL	KL						

Cmpd.	No. of Cmpds.	m/z meas.	Δ m/z [ppm]	z	Rt [min]	Score	P	Range	Sequence	Modification
971	1	706.9594	14.52	2	42.2	20.7	2	485-496	K.MVVLDKLLPKLK.E	Oxidation: 1

Detailed Protein Report

Protein 353: histone-lysine N-methyltransferase 2C [Homo sapiens]

**Accession:**	gi|91718902	**Score:**	48.7
**Database:**	refseq_human(refseq_human_20140103.fasta)	**MW [kDa]:**	541.0
**Database Date:**	2015-11-30	**pI:**	6.1
**Modification(s):**	Oxidation	**Sequence Coverage [%]:**	1.3
		**No. of unique Peptides:**	2

Quantitation

Q:W	**Median:** 0.59	**CV:** 0.00 %	**No. of Peptides:** 1

10	20	30	40	50	60	70	80
MSSEEDKSVE	QPQPPPPPPE	EPGAPAPSPA	AADKRPRGRP	RKDGASPFQR	ARKKPRSRGK	TAVEDEDSMD	GLETTETETI
90	100	110	120	130	140	150	160
VETEIKEQSA	EEDAEAEVDN	SKQLIPTLQR	SVSEESANSL	VSVGVEAKIS	EQLCAFCYCG	EKSSLGQGDL	KQFRITPGFI
170	180	190	200	210	220	230	240
LPWRNQPSNK	KDIDDNSNGT	YEKMQNSAPR	KQRGQRKERS	PQQNIVSCVS	VSTQTASDDQ	AGKLWDELSL	VGLPDAIDIQ
250	260	270	280	290	300	310	320
ALFDSTGTCW	AHHRCVEWSL	GVCQMEEPLL	VNVDKAVVSG	STERCAFCKH	LGATIKCCEE	KCTQMYHYPC	AAGAGTFQDF
330	340	350	360	370	380	390	400
SHIFLLCPEH	IDQAPERSKE	DANCAVCDSP	GDLLDQFFCT	TCGQHYHGMC	LDIAVTPLKR	AGWQCPECKV	CQNCKQSGED
410	420	430	440	450	460	470	480
SKMLVCDTCD	KGYHTFCLQP	VMKSVPTNGW	KCKNCRICIE	CGTRSSSQWH	HNCLICDNCY	QQQDNLCPFC	GKCYHPELQK
490	500	510	520	530	540	550	560
DMLHCNMCKR	WVHLECDKPT	DHELDTQLKE	EYICMYCKHL	GAEMDRLQPG	EEVEIAELTT	DYNNEMEVEG	PEDQMVFSEQ
570	580	590	600	610	620	630	640
AANKDVNGQE	STPGIVPDAV	QVHTEEQQKS	HPSESLDTDS	LLIAVSSQHT	VNTELEKQIS	NEVDSEDLKM	SSEVKHICGE
650	660	670	680	690	700	710	720
DQIEDKMEVT	ENIEVVTHQI	TVQQEQLQLL	EEPETVVSRE	ESRPPKLVME	SVTLPLETLV	SPHEESISLC	PEEQLVIERL
730	740	750	760	770	780	790	800
QGEKEQKENS	ELSTGLMDSE	MTPTIEGCVK	DVSYQGGKSI	KLSSETESSF	SSSADISKAD	VSSSPTPSSD	LPSHDMLHNY
810	820	830	840	850	860	870	880
PSALSSSAGN	IMPTTYISVT	PKIGMGKPAI	TKRKFSPGRP	RSKQGAWSTH	NTVSPPSWSP	DISEGREIFK	PRQLPGSAIW
890	900	910	920	930	940	950	960
SIKVGRGSGF	PGKRRPRGAG	LSGRGGRGRS	KLKSGIGAVV	LPGVSTADIS	SNKDDEENSM	HNTVVLFSSS	DKFTLNQDMC
970	980	990	1000	1010	1020	1030	1040
VVCGSFGQGA	EGRLLACSQC	GQCYHPYCVS	IKITKVVLSK	GWRCLECTVC	EACGKATDPG	RLLLCDDCDI	SYHTYCLDPP
1050	1060	1070	1080	1090	1100	1110	1120
LQTVPKGGWK	CKWCVWCRHC	GATSAGLRCE	WQNNYTQCAP	CASLSSCPVC	YRNYREEDLI	LQCRQCDRWM	HAVCQNLNTE
1130	1140	1150	1160	1170	1180	1190	1200
EEVENVADIG	FDCSMCRPYM	PASNVPSSDC	CESSLVAQIV	TKVKELDPPK	TYTQDGVCLT	ESGMTQLQSL	TVTVPRRKRS
1210	1220	1230	1240	1250	1260	1270	1280
KPKLKLKIIN	QNSVAVLQTP	PDIQSEHSRD	GEMDDSREGE	LMDCDGKSES	SPEREAVDDE	TKGVEGTDGV	KKRKRKPYRP
1290	1300	1310	1320	1330	1340	1350	1360
GIGGFMVRQR	SRTGQGKTKR	SVIRKDSSGS	ISEQLPCRDD	GWSEQLPDTL	VDESVSVTES	TEKIKKRYRK	RKNKLEETFP
1370	1380	1390	1400	1410	1420	1430	1440
AYLQEAFFGK	DLLDTSRQSK	ISLDNLSEDG	AQLLYKTNMN	TGFLDPSLDP	LLSSSSAPTK	SGTHGPADDP	LADISEVLNT
1450	1460	1470	1480	1490	1500	1510	1520
DDDILGIISD	DLAKSVDHSD	IGPVTDDPSS	LPQPNVNQSS	RPLSEEQLDG	ILSPELDKMV	TDGAILGKLY	KIPELGGKDV
1530	1540	1550	1560	1570	1580	1590	1600
EDLFTAVLSP	ANTQPTPLPQ	PPPPTQLLPI	HNQDAFSRMP	LMNGLIGSSP	HLPHNSLPPG	SGLGTFSAIA	QSSYPDARDK
1610	1620	1630	1640	1650	1660	1670	1680
NSAFNPMASD	PNNSWTSSAP	TVEGENDTMS	NAQRSTLKWE	KEEALGEMAT	VAPVLYTNIN	FPNLKEEFPD	WTTRVKQIAK
1690	1700	1710	1720	1730	1740	1750	1760
LWRKASSQER	APYVQKARDN	RAALRINKVQ	MSNDSMKRQQ	QQDSIDPSSR	IDSELFKDPL	KQRESEHEQE	WKFRQQMRQK
1770	1780	1790	1800	1810	1820	1830	1840
SKQQAKIEAT	QKLEQVKNEQ	QQQQQQQFGS	QHLLVQSGSD	TPSSGIQSPL	TPQPGNGNMS	PAQSFHKELF	TKQPPSTPTS
1850	1860	1870	1880	1890	1900	1910	1920
TSSDDVFVKP	QAPPPPPAPS	RIPIQDSLSQ	AQTSQPPSPQ	VFSPGSSNSR	PPSPMDPYAK	MVGTPRPPPV	GHSFSRRNSA
1930	1940	1950	1960	1970	1980	1990	2000
APVENCTPLS	SVSRPLQMNE	TTANRPSPVR	DLCSSSTTNN	DPYAKPPDTP	RPVMTDQFPK	SLGLSRSPVV	SEQTAKGPIA
2010	2020	2030	2040	2050	2060	2070	2080
AGTSDHFTKP	SPRADVFQRQ	RIPDSYARPL	LTPAPLDSGP	GPFKTPMQPP	PSSQDPYGSV	SQASRRLSVD	PYERPALTPR
2090	2100	2110	2120	2130	2140	2150	2160
PIDNFSHNQS	NDPYSQPPLT	PHPAVNESFA	HPSRAFSQPG	TISRPTSQDP	YSQPPGTPRP	VVDSYSQSSG	TARSNTDPYS
2170	2180	2190	2200	2210	2220	2230	2240

Detailed Protein Report

Cmpd.	No. of Cmpds.	m/z meas.	Δ m/z [ppm]	z	Rt [min]	Score	P	Range	Sequence	Modification	Ratios	
1824	1	495.2141	-161.73	2	51.3	13.4	1	3101-3109	K.MVALKGINK.V	Oxidation: 1	Q:W	0.59
132	1	846.0298	-52.12	3	31.6	13.7	1	4823-4845	R. GVYMFRMDNDHVIDATLTGGPAR Y			

Detailed Protein Report

Protein 354: zinc finger CCCH domain-containing protein 13 [Homo sapiens]

**Accession:**	gi|116008442	**Score:**	48.7
**Database:**	refseq_human(refseq_human_20140103.fasta)	**MW [kDa]:**	184.8
**Database Date:**	2015-11-30	**pI:**	10.0
**Modification(s):**	Oxidation	**Sequence Coverage [%]:**	2.4
		**No. of unique Peptides:**	3

Alias proteins:

Accession	Name	Description
gi|530402208	refseq_human (refseq_human_20140103.fasta)	PREDICTED: zinc finger CCCH domain-containing protein 13 isoform X12 [Homo sapiens]

10	20	30	40	50	60	70	80
MSKIRRKVTV	ENTKTISDST	SRRPSVFERL	GPSTGSTAET	QCRNWLKTGN	CLYGNTCRFV	HGPSPRGKGY	SSNYRRSPER
90	100	110	120	130	140	150	160
PTGDLRERMK	NKRQDVDTEP	QKRNTEESSS	PVRKESSRGR	HREKEDIKIT	KERTPESEEE	NVEWETNRDD	SDNGDINYDY
170	180	190	200	210	220	230	240
VHELSLEMKR	QKIQRELMKL	EQENMEKREE	IIIKKEVSPE	VVRSKLSPSP	SLRKSSKSPK	RKSSPKSSSA	SKKDRKTSAV
250	260	270	280	290	300	310	320
SSPLLDQQRN	SKTNQSKKKG	PRTPSPPPPI	PEDIALGKKY	KEKYKVKDRI	EEKTRDGKDR	GRDFERQREK	RDKPRSTSPA
330	340	350	360	370	380	390	400
GQHHSPISSR	HHSSSSQSGS	SIQRHSPSPR	RKRTPSPSYQ	RTLTPPLRRS	ASPYPSHSLS	SPQRKQSPPR	HRSPMREKGR
410	420	430	440	450	460	470	480
HDHERTSQSH	DRRHERREDT	RGKRDREKDS	REEREYEQDQ	SSSRDHRDDR	EPRDGRDRRD	ARDTRDREL	RDSRDMRDSR
490	500	510	520	530	540	550	560
EMRDYSRDTK	ESRDPRDSRS	TRDAHDYRDR	EGRDTHRKED	TYPEESRSYG	RNHLREESSR	TEIRNESRNE	SRSEIRNDRM
570	580	590	600	610	620	630	640
GRSRGRVPEL	PEKGSRGSRG	SQIDSHSSNS	NYHDSWETRS	SYPERDRYPE	RDNRDQARDS	SFERRHGERD	RRDNRERDQR
650	660	670	680	690	700	710	720
PSSPIRHQGR	NDELERDERR	EERRVDRVDD	RRDERARERD	RERERDRERE	RERERERDRE	REKERELERE	RARERERERE
730	740	750	760	770	780	790	800
KERDRERDRD	RDHDRERERE	RERDREKERE	REREERERER	ERERERERER	ERERERARER	DKERERQRDW	EDKDKGRDDR
810	820	830	840	850	860	870	880
REKREEIRED	RNPRDGHDER	KSKKRYRNEG	SPSPRQSPKR	RREHSPDSDA	YNSGDDKNEK	HRLLSQVVRP	QESRSLSPSH
890	900	910	920	930	940	950	960
LTEDRQGRWK	EEDRKPERKE	SSRRYEEQEL	KEKVSSVDKQ	REQTEILESS	RMRAQDIIGH	HQSEDRETSD	RAHDENKKKA
970	980	990	1000	1010	1020	1030	1040
KIQKKPIKKK	KEDDVGIERG	NIETTSEDGQ	VFSPKKGQKK	KSIEKKRKKS	KGDSDISDEE	AAQQSKKKRG	PRTPPITTKE
1050	1060	1070	1080	1090	1100	1110	1120
ELVEMCNGKN	GILEDSQKKE	DTAFSDWSDE	DVPDRTEVTE	AEHTATATTP	GSTPSPLSSL	LPPPPPVATA	TATTVPATLA
1130	1140	1150	1160	1170	1180	1190	1200
ATTAAAATSF	STSAITISTS	ATPTNTTNNT	FANEDSHRKC	HRTRVEKVET	PHVTIEDAQH	RKPMDQKRSS	SLGSNRSNRS
1210	1220	1230	1240	1250	1260	1270	1280
HTSGRLRSPS	NDSAHRSGDD	QSGRKRVLHS	GSRDREKTKS	LEITGERKSR	IDQLKRGEPS	RSTSSDRQDS	RSHSSRRSSP
1290	1300	1310	1320	1330	1340	1350	1360
ESDRQVHSRS	GSFDSRDRLQ	ERDRYEHDRE	RERERRDTRQ	REWDRDADKD	WPRNRDRDRL	RERERERERD	KRRDLDRERE
1370	1380	1390	1400	1410	1420	1430	1440
RLISDSVERD	RDRDRDRTFE	SSQIESVKRC	EAKLEGEHER	DLESTSRDSL	ALDKERMDKD	LGSVQGFEET	NKSERTESLE
1450	1460	1470	1480	1490	1500	1510	1520
AGDDESKLDD	AHSLGSGAGE	GYEPISDDEL	DEILAGDAEK	REDQQDEEKM	PDPLDVIDVD	WSGLMPKHPK	EPREPGAALL
1530	1540	1550	1560	1570			
KFTPGAVMLR	VGISKKLAGS	ELFAKVKETC	QRLLEKPKGS	FILL			

Cmpd.	No. of Cmpds.	m/z meas.	Δ m/z [ppm]	z	Rt [min]	Score	P	Range	Sequence	Modification
1252	1	918.2353	-315.87	1	45.8	10.9	1	7-14	R.KVTVENTK.T	
1864	1	606.2622	-6.93	2	53.3	12.3	2	475-483	R.DMRDSREMR.D	Oxidation: 8

Detailed Protein Report

Cmpd.	No. of Cmpds.	m/z meas.	Δ m/z [ppm]	z	Rt [min]	Score	P	Range	Sequence	Modification
1287	1	795.9839	117.71	2	45.8	13.8	2	528-540	R.SYGRNHLREESSR.T	

Detailed Protein Report

Protein 355: alpha-catulin isoform b [Homo sapiens]

Accession: gi|558695396
Database: refseq_human(refseq_human_20140103.fasta)
Database Date: 2015-11-30

Score: 48.6
MW [kDa]: 80.1
pI: 6.3
Sequence Coverage [%]: 4.7
No. of unique Peptides: 1

10	20	30	40	50	60	70	80
MAASPGPAGV	GGAGAVYGSG	SSGFALDSGL	EIKTRSVEQT	LLPLVSQITT	LINHKDNTKK	SDKTLQAIQR	VGQAVNLAVG
90	100	110	120	130	140	150	160
RFVKVGEAIA	NENWDLKEEI	NIACIEAKQA	GETIAALTDI	TNLNHLESDG	QITIFTDKTG	VIKAARLLLS	SVTKVLLLAD
170	180	190	200	210	220	230	240
RVVIKQIITS	RNKVLATMER	LEKVNSFQEF	VQIFSQFGNE	MVEFAHLSGD	RQNDLKDEKK	KAKMAAARAV	LEKCTMMLLT
250	260	270	280	290	300	310	320
ASKTCLRHPN	CESAHKNKEG	VFDRMKVALD	KVIEIVTDCK	PNGETDISSI	SIFTGIKEFK	MNIEALRENL	YFQSKENLSV
330	340	350	360	370	380	390	400
TLEVILERME	DFTDSAYTSH	EHRERILELS	TQARMELQQL	ISVWIQAQSK	KTKSIAEELE	LSILKISHSL	NELKKELHST
410	420	430	440	450	460	470	480
ATQLAADLLK	YHADHVVLKA	LKLTGVEGNL	EALAEYACKL	SEQKEQLVET	CRLLRHISGT	EPLEITCIHA	EETFQVTGQQ
490	500	510	520	530	540	550	560
IISAAETLTL	HPSSKIAKEN	LDVFCEAWES	QISDMSTLLR	EINDVFEGRR	GEKYGYLSLP	KPMKNNANLK	SLKPDKPDSE
570	580	590	600	610	620	630	640
EQAKIAKLGL	KLGLLTSDAD	CEIEKWEDQE	NEIVQYGRNM	SSMAYSLYLF	TRGEGPLKTS	QDLIHQLEVF	AAEGLKLTSS
650	660	670	680	690	700	710	720
VQAFSKQLKD	DDKLMLLLEI	NKLIPLCHQL	QTVTKTSLQN	KVFLKVDKCI	TKTRSMMALL	VQLLSLCYKL	LKKSETRY

Cmpd.	No. of Cmpds.	m/z meas.	Δ m/z [ppm]	z	Rt [min]	Score	P	Range	Sequence	Modification
2751	1	964.4855	0.82	1	63.4	16.8	1	257-264	K.NKEGVFDR.M	

Detailed Protein Report

Protein 356: storkhead-box protein 1 isoform a [Homo sapiens]

**Accession:**	gi|63025200	**Score:**	48.6
**Database:**	refseq_human(refseq_human_20140103.fasta)	**MW [kDa]:**	110.9
**Database Date:**	2015-11-30	**pI:**	8.7
**Modification(s):**	Carbamidomethyl	**Sequence Coverage [%]:**	4.4
		**No. of unique Peptides:**	2

Alias proteins:

Accession	Name	Description
gi|194328691	refseq_human (refseq_human_20140103.fasta)	storkhead-box protein 1 isoform a [Homo sapiens]

10	20	30	40	50	60	70	80
MARPVQLAPG	SLALVLCRLE	AQKAAGAAEE	PGGRAVFRAF	RRANARCFWN	ARLARAASRL	AFQGWLRRGV	LLVRAPPACL
**90**	**100**	**110**	**120**	**130**	**140**	**150**	**160**
QVLRDAWRRR	ALRPPRGFRI	RAVGDVFPVQ	MNPITQSQFV	PLGEVLCCAI	SDMNTAQIVV	TQESLLERLM	KHYPGIAIPS
**170**	**180**	**190**	**200**	**210**	**220**	**230**	**240**
EDILYTTLGT	LIKERKIYHT	GEGYFIVTPQ	TYFITNTTTQ	ENKRMLPSDE	SRLMPASMTY	LVSMESCAES	AQENAAPISH
**250**	**260**	**270**	**280**	**290**	**300**	**310**	**320**
CQSCQCFRDM	HTQDVQEAPV	AAEVTRKSHR	GLGESVSWVQ	NGAVSVSAEH	HICESTKPLP	YTRDKEKGKK	FGFSLLWRSL
**330**	**340**	**350**	**360**	**370**	**380**	**390**	**400**
SRKEKPKTEH	SSFSAQFPPE	EWPVRDEDDL	DNIPRDVEHE	IIKRINPILT	VDNLIKHTVL	MQKYEEQKKY	NSQGTSTDML
**410**	**420**	**430**	**440**	**450**	**460**	**470**	**480**
TIGHKYPSKE	GVKKRQGLSA	KPQGQGHSRR	DRHKARNQGS	EFQPGSIRLE	KHPKLPATQP	IPRIKSPNEM	VGQKPLGEIT
**490**	**500**	**510**	**520**	**530**	**540**	**550**	**560**
TVLGSHLIYK	KRISNPFQGL	SHRGSTISKG	HKIQKTSDLK	PSQTGPKEKP	FQKPRSLDSS	RIFDGKAKEP	YAEQPNDKME
**570**	**580**	**590**	**600**	**610**	**620**	**630**	**640**
AESIYINDPT	VKPINDDFRG	HLFSHPQQSM	LQNDGKCCPF	MESMLRYEVY	GGENEVIPEV	LRKSHSHFDK	LGETKQTPHS
**650**	**660**	**670**	**680**	**690**	**700**	**710**	**720**
LPSRGASFSD	RTPSACRLVD	NTIHQFQNLG	LLDYPVGVNP	LRQAARQDKD	SEELLRKGFV	QDAETTSLEN	EQLSNDDQAL
**730**	**740**	**750**	**760**	**770**	**780**	**790**	**800**
YQNEVEDDDG	ACSSLYLEED	DISENDDLRQ	MLPGHSQYSF	TGGSQGNHLG	KQKVIERSLT	EYNSTMERVE	SQVLKRNECY
**810**	**820**	**830**	**840**	**850**	**860**	**870**	**880**
KPTGLHATPG	ESQEPNLSAE	SCGLNSGAQF	GFNYEEEPSV	AKCVQASAPA	DERIFDYYSA	RKASFEAEVI	QDTIGDTGKK
**890**	**900**	**910**	**920**	**930**	**940**	**950**	**960**
PASWSQSPQN	QEMRKHFPQK	FQLFNTSHMP	VLAQDVQYEH	SHLEGTENHS	MAGDSGIDSP	RTQSLGSNNS	VILDGLKRRQ
**970**	**980**	**990**					
NFLQNVEGTK	SSQPLTSNSL	LPLTPVINV					

Cmpd.	No. of Cmpds.	m/z meas.	Δ m/z [ppm]	z	Rt [min]	Score	P	Range	Sequence	Modification
1506	1	881.6342	118.58	2	48.6	18.4	1	69-84	R.GVLLVRAPPACLQVLR.D	Carbamidomethyl: 11
1771	2	823.3088	-161.08	2	52.5	16.3	0	942-957	R.TQSLGSNNSVILDGLK.R	

BRUKER

Detailed Protein Report

Protein 357: PREDICTED: tudor domain-containing protein 7 isoform X1 [Homo sapiens]

Accession: gi|578817238
Database: refseq_human(refseq_human_20140103.fasta)
Database Date: 2015-11-30
Modification(s): Oxidation

Score: 48.6
MW [kDa]: 115.3
pI: 6.7
Sequence Coverage [%]: 5.9
No. of unique Peptides: 2

Quantitation

Q:W	**Median:**	0.58	**CV:**	0.00 %	**No. of Peptides:**	1
M:Q	**Median:**	2.47	**CV:**	0.00 %	**No. of Peptides:**	1

10	20	30	40	50	60	70	80
MACTETARIA	QLVARQRSSK	RKTGRQVNCQ	MRVKKTMPFF	LEGKPKATLR	QPGFASNFSV	GKKPNPAPLR	DKGNSVGVKP
**90**	**100**	**110**	**120**	**130**	**140**	**150**	**160**
DAEMSPYMLH	TTLGNEAFKD	IPVQRHVTMS	TNNRFSPKAS	LQPPLQMHLS	RTSTKEMSDN	LNQTVEKPNV	KPPASYTYKM
**170**	**180**	**190**	**200**	**210**	**220**	**230**	**240**
DEVQNRIKEI	LNKHNNGIWI	SKLPHFYKEL	YKEDLNQGIL	QQFEHWPHIC	TVEKPCSGGQ	DLLLYPAKRK	QLLRSELDTE
**250**	**260**	**270**	**280**	**290**	**300**	**310**	**320**
KVPLSPLPGP	KQTPPLKGCP	TVMAGDFKEK	VADLLVKYTS	GLWASALPKA	FEEMYKVKFP	EDALKNLASL	SDVCSIDYIS
**330**	**340**	**350**	**360**	**370**	**380**	**390**	**400**
GNPQKAILYA	KLPLPTDKIQ	KDAGQAHGDN	DIKAMVEQEY	LQVEESIAES	ANTFMEDITV	PPLMIPTEAS	PSVLVVELSN
**410**	**420**	**430**	**440**	**450**	**460**	**470**	**480**
TNEVVIRYVG	KDYSAAQELM	EDEMKEYYSK	NPKITPVQAV	NVGQLLAVNA	EEDAWLRAQV	ISTEENKIKV	CYVDYGFSEN
**490**	**500**	**510**	**520**	**530**	**540**	**550**	**560**
VEKSKAYKLN	PKFCSLSFQA	TKCKLAGLEV	LSDDPDLVKV	VESLTCGKIF	AVEILDKADI	PLVVLYDTSG	EDDININATC
**570**	**580**	**590**	**600**	**610**	**620**	**630**	**640**
LKAICDKSLE	VHLQVDAMYT	NVKVTNICSD	GTLYCQVPCK	GLNKLSDLLR	KIEDYFHCKH	MTSECFVSLP	FCGKICLFHC
**650**	**660**	**670**	**680**	**690**	**700**	**710**	**720**
KGKWLRVEIT	NVHSSRALDV	QFLDSGTVTS	VKVSELREIP	PRFLQEMIAI	PPQAIKCCLA	DLPQSIGMWT	PDAVLWLRDS
**730**	**740**	**750**	**760**	**770**	**780**	**790**	**800**
VLNCSDCSIK	VTKVDETRGI	AHVYLFTPKN	FPDPHRSINR	QITNADLWKH	QKDVFLSAIS	SGADSPNSKN	GNMPMSGNTG
**810**	**820**	**830**	**840**	**850**	**860**	**870**	**880**
ENFRKNLTDV	IKKSMVDHTS	AFSTEELPPP	VHLSKPGEHM	DVYVPVACHP	GYFVIQPWQE	IHKLEVLMEE	MILYYSVSEE
**890**	**900**	**910**	**920**	**930**	**940**	**950**	**960**
RHIAVEKDQV	YAAKVENKWH	RVLLKGILTN	GLVSVYELDY	GKHELVNIRK	VQPLVDMFRK	LPFQAVTAQL	AGVKCNQWSE
**970**	**980**	**990**	**1000**	**1010**	**1020**	**1030**	
EASMVFRNHV	EKKPLVALVQ	TVIENANPWD	RKVVVYLVDT	SLPDTDTWIH	DFMSEYLIEL	SKVN	

Cmpd.	No. of Cmpds.	m/z meas.	Δ m/z [ppm]	z	Rt [min]	Score	P	Range	Sequence	Modification	Ratios
2184	1	955.9047	-109.56	2	55.9	11.2	1	119-135	K.ASLQPPLQMHLSRTSTK.E	Oxidation: 9	
1797	1	851.2615	-133.71	2	52.3	11.4	0	620-634	K.HMTSECFVSLPFCGK.I	Oxidation: 2	Q:W 0.58<br>M:Q 2.47

Detailed Protein Report

Protein 358: PREDICTED: coiled-coil domain-containing protein 146 isoform X3 [Homo sapiens]

**Accession:**	gi|530386260	**Score:**	48.6
**Database:**	refseq_human(refseq_human_20140103.fasta)	**MW [kDa]:**	97.2
**Database Date:**	2015-11-30	**pI:**	9.8
**Modification(s):**	Oxidation	**Sequence Coverage [%]:**	4.6
		**No. of unique Peptides:**	4

Quantitation

Q:W	**Median:**	0.54	**CV:**	59.91 %	**No. of Peptides:**	2
M:Q	**Median:**	1.34	**CV:**	104.27 %	**No. of Peptides:**	2

```
        10         20         30         40         50         60         70         80
MQTSSPTTRL FLRIKEKSLK EEKIIIVKEF EKITKPGEME KKMKILREST EELRKEIMQK KLEIKNLRED LASKQKQLLK
        90        100        110        120        130        140        150        160
EQKELEELLG HQVVLKDEVA HHQTIPVQIG KEIEKITRKK VEMEKKKIVL EQEVKTLNDS LKKVENKVSA IVDEKENVIK
       170        180        190        200        210        220        230        240
EVEGKRALLE IKEREHNQLV KLLELARENE ATSLTERGIL DLNLRNSLID KQNYHDELSR KQREKERDFR NLRKMELLLK
       250        260        270        280        290        300        310        320
VSWDALRQTQ ALHQRLLLEM EAIPKDDSTL SERRELHKE  VEVAKRNLAQ QKIISEMESK LVEQQLAEEN KLLKEQENMK
       330        340        350        360        370        380        390        400
ELVVNLLRMT QIKIDEKEQK SKDFLKAQQK YTNIVKEMKA KDLEIRIHKK KKCEIYRRLR EFAKLYDTIR NERNKFVNLL
       410        420        430        440        450        460        470        480
HKAHQKVNEI KERHKMSLNE LEILRNSAVS QERKLQNSML KHANNVTIRE SMQNDVRKIV SKLQEMKEKK EAQLNNIDRL
       490        500        510        520        530        540        550        560
ANTITMIEEE MVQLRKRYEK AVQHRNESGV QLIEREEEIC IFYEKINIQE KMKLNGEIEI HLLEEKIQFL KMKIAEKQRQ
       570        580        590        600        610        620        630        640
ICVTQKLLPA KRSLDADLAV LQIQFSQCTD RIKDLEKQFV KPDGENRARF LPGKDLTEKE MIQKLDKLEL QLAKKEEKLL
       650        660        670        680        690        700        710        720
EKDFIYEQVS RLTDRLCSKT QGCKQDTLLL AKKMNGYQRR IKNATEKMMA LVAELSMKQA LTIELQKEVR EKEDFIFTCN
       730        740        750        760        770        780        790        800
SRIEKGLPLN KEIEKEWLKV LRDEEMHALA IAEKSQEFLE ADNRQLPNGV YTTAEQRPNA YIPEADATLP LPKPYGALAP
       810        820        830
FKPSEPGANM RHIRKPVIKP VEI
```

Cmpd.	No. of Cmpds.	m/z meas.	Δ m/z [ppm]	z	Rt [min]	Score	P	Range	Sequence	Modification	Ratios	
1670	1	524.8066	68.22	2	50.8	14.1	0	33-41	K.ITKPGEMEK.K	Oxidation: 7	Q:W<br>M:Q	0.31<br>3.15
2135	1	754.0400	192.03	2	57.1	12.8	2	329-340	R.MTQIKIDEKEQK.S	Oxidation: 1		
1783	1	870.2900	-263.69	1	52.3	10.4	0	396-402	K.FVNLLHK.A			
595	1	572.7739	-48.16	2	36.8	11.3	0	506-515	R.NESGVQLIER.E		M:Q<br>Q:W	0.57<br>0.94

Detailed Protein Report

Protein 359: sortilin-related receptor preproprotein [Homo sapiens]

**Accession:**	gi|4507157	**Score:**	48.4
**Database:**	refseq_human(refseq_human_20140103.fasta)	**MW [kDa]:**	248.3
**Database Date:**	2015-11-30	**pI:**	5.2
**Modification(s):**	Carbamidomethyl	**Sequence Coverage [%]:**	0.7
		**No. of unique Peptides:**	2

Quantitation

Q:W	**Median:**	1.52	**CV:**	0.00 %	**No. of Peptides:**	1
M:Q	**Median:**	1.49	**CV:**	0.00 %	**No. of Peptides:**	1

Detailed Protein Report

10	20	30	40	50	60	70	80
MATRSSRRES	RLPFLFTLVA	LLPPGALCEV	WTQRLHGGSA	PLPQDRGFLV	VQGDPRELRL	WARGDARGAS	RADEKPLRRK
90	100	110	120	130	140	150	160
RSAALQPEPI	KVYGQVSLND	SHNQMVVHWA	GEKSNVIVAL	ARDSLALARP	KSSDVYVSYD	YGKSFKKISD	KLNFGLGNRS
170	180	190	200	210	220	230	240
EAVIAQFYHS	PADNKRYIFA	DAYAQYLWIT	FDFCNTLQGF	SIPFRAADLL	LHSKASNLLL	GFDRSHPNKQ	LWKSDDFGQT
250	260	270	280	290	300	310	320
WIMIQEHVKS	FSWGIDPYDK	PNTIYIERHE	PSGYSTVFRS	TDFFQSRENQ	EVILEEVRDF	QLRDKYMFAT	KVVHLLGSEQ
330	340	350	360	370	380	390	400
QSSVQLWVSF	GRKPMRAAQF	VTRHPINEYY	IADASEDQVF	VCVSHSNNRT	NLYISEAEGL	KFSLSLENVL	YYSPGGAGSD
410	420	430	440	450	460	470	480
TLVRYFANEP	FADFHRVEGL	QGVYIATLIN	GSMNEENMRS	VITFDKGGTW	EFLQAPAFTG	YGEKINCELS	QGCSLHLAQR
490	500	510	520	530	540	550	560
LSQLLNLQLR	RMPILSKESA	PGLIIATGSV	GKNLASKTNV	YISSSAGARW	REALPGPHYY	TWGDHGGIIT	AIAQGMETNE
570	580	590	600	610	620	630	640
LKYSTNEGET	WKTFIFSEKP	VFVYGLLTEP	GEKSTVFTIF	GSNKENVHSW	LILQVNATDA	LGVPCTENDY	KLWSPSDERG
650	660	670	680	690	700	710	720
NECLLGHKTV	FKRRTPHATC	FNGEDFDRPV	VVSNCSCTRE	DYECDFGFKM	SEDLSLEVCV	PDPEFSGKSY	SPPVPCPVGS
730	740	750	760	770	780	790	800
TYRRTRGYRK	ISGDTCSGGD	VEARLEGELV	PCPLAEENEF	ILYAVRKSIY	RYDLASGATE	QLPLTGLRAA	VALDFDYEHN
810	820	830	840	850	860	870	880
CLYWSDLALD	VIQRLCLNGS	TGQEVIINSG	LETVEALAFE	PLSQLLYWVD	AGFKKIEVAN	PDGDFRLTIV	NSSVLDRPRA
890	900	910	920	930	940	950	960
LVLVPQEGVM	FWTDWGDLKP	GIYRSNMDGS	AAYHLVSEDV	KWPNGISVDD	QWIYWTDAYL	ECIERITFSG	QQRSVILDNL
970	980	990	1000	1010	1020	1030	1040
PHPYAIAVFK	NEIYWDDWSQ	LSIFRASKYS	GSQMEILANQ	LTGLMDMKIF	YKGKNTGSNA	CVPRPCSLLC	LPKANNSRSC
1050	1060	1070	1080	1090	1100	1110	1120
RCPEDVSSSV	LPSGDLMCDC	PQGYQLKNNT	CVKEENTCLR	NQYRCSNGNC	INSIWWCDFD	NDCGDMSDER	NCPTTICDLD
1130	1140	1150	1160	1170	1180	1190	1200
TQFRCQESGT	CIPLSYKCDL	EDDCGDNSDE	SHCEMHQCRS	DEYNCSSGMC	IRSSWVCDGD	NDCRDWSDEA	NCTAIYHTCE
1210	1220	1230	1240	1250	1260	1270	1280
ASNFQCRNGH	CIPQRWACDG	DTDCQDGSDE	DPVNCEKKCN	GFRCPNGTCI	PSSKHCDGLR	DCSDGSDEQH	CEPLCTHFMD
1290	1300	1310	1320	1330	1340	1350	1360
FVCKNRQQCL	FHSMVCDGII	QCRDGSDEDA	AFAGCSQDPE	FHKVCDEFGF	QCQNGVCISL	IWKCDGMDDC	GDYSDEANCE
1370	1380	1390	1400	1410	1420	1430	1440
NPTEAPNCSR	YFQFRCENGH	CIPNRWKCDR	ENDCGDWSDE	KDCGDSHILP	FSTPGPSTCL	PNYYRCSSGT	CVMDTWVCDG
1450	1460	1470	1480	1490	1500	1510	1520
YRDCADGSDE	EACPLLANVT	AASTPTQLGR	CDRFEFECHQ	PKTCIPNWKR	CDGHQDCQDG	RDEANCPTHS	TLTCMSREFQ
1530	1540	1550	1560	1570	1580	1590	1600
CEDGEACIVL	SERCDGFLDC	SDESDEKACS	DELTVYKVQN	LQWTADFSGD	VTLTWMRPKK	MPSASCVYNV	YYRVVGESIW
1610	1620	1630	1640	1650	1660	1670	1680
KTLETHSNKT	NTVLKVLKPD	TTYQVKVQVQ	CLSKAHNTND	FVTLRTPEGL	PDAPRNLQLS	LPREAEGVIV	GHWAPPIHTH
1690	1700	1710	1720	1730	1740	1750	1760
GLIREYIVEY	SRSGSKMWAS	QRAASNFTEI	KNLLVNTLYT	VRVAAVTSRG	IGNWSDSKSI	TTIKGKVIPP	PDIHIDSYGE
1770	1780	1790	1800	1810	1820	1830	1840
NYLSFTLTME	SDIKVNGYVV	NLFWAFDTHK	QERRTLNFRG	SILSHKVGNL	TAHTSYEISA	WAKTDLGDSP	LAFEHVMTRG
1850	1860	1870	1880	1890	1900	1910	1920
VRPPAPSLKA	KAINQTAVEC	TWTGPRNVVY	GIFYATSFLD	LYRNPKSLTT	SLHNKTVIVS	KDEQYLFLVR	VVVPYQGPSS
1930	1940	1950	1960	1970	1980	1990	2000
DYVVVKMIPD	SRLPPRHLHV	VHTGKTSVVI	KWESPYDSPD	QDLLYAIAVK	DLIRKTDRSY	KVKSRNSTVE	YTLNKLEPGG
2010	2020	2030	2040	2050	2060	2070	2080
KYHIIVQLGN	MSKDSSIKIT	TVSLSAPDAL	KIITENDHVL	LFWKSLALKE	KHFNESRGYE	IHMFDSAMNI	TAYLGNTTDN
2090	2100	2110	2120	2130	2140	2150	2160
FFKISNLKMG	HNYTFTVQAR	CLFGNQICGE	PAILLYDELG	SGADASATQA	ARSTDVAAVV	VPILFLILLS	LGVGFAILYT
2170	2180	2190	2200	2210	2220		

Detailed Protein Report

Cmpd.	No. of Cmpds.	m/z meas.	Δ m/z [ppm]	z	Rt [min]	Score	P	Range	Sequence	Modification	Ratios
2231	1	599.9323	-0.25	3	57.8	14.4	1	1239-1254	K.CNGFRCPNGTCIPSSK.H	Carbamidomethyl: 1, 11	
1162	2	610.6492	-203.57	2	44.2	14.0	0	1244-1254	R.CPNGTCIPSSK.H	Carbamidomethyl: 1, 6	Q:W 1.52<br>M:Q 1.49

Detailed Protein Report

Protein 360: neuropeptides B/W receptor type 1 [Homo sapiens]

**Accession:**	gi|53828924	**Score:**	48.3
**Database:**	refseq_human(refseq_human_20140103.fasta)	**MW [kDa]:**	36.1
**Database Date:**	2015-11-30	**pI:**	10.1
**Modification(s):**	Carbamidomethyl	**Sequence Coverage [%]:**	11.9
		**No. of unique Peptides:**	2

10	20	30	40	50	60	70	80
MDNASFSEPW	PANASGPDPA	LSCSNASTLA	PLPAPLAVAV	PVVYAVICAV	GLAGNSAVLY	VLLRAPRMKT	VTNLFILNLA
**90**	**100**	**110**	**120**	**130**	**140**	**150**	**160**
IADELFTLVL	PINIADFLLR	QWPFGELMCK	LIVAIDQYNT	FSSLYFLTVM	SADRYLVVLA	TAESRRVAGR	TYSAARAVSL
**170**	**180**	**190**	**200**	**210**	**220**	**230**	**240**
AVWGIVTLVV	LPFAVFARLD	DEQGRRQCVL	VFPQPEAFWW	RASRLYTLVL	GFAIPVSTIC	VLYTTLLCRL	HAMRLDSHAK
**250**	**260**	**270**	**280**	**290**	**300**	**310**	**320**
ALERAKKRVT	FLVVAILAVC	LLCWTPYHLS	TVVALTTDLP	QTPLVIAISY	FITSLSYANS	CLNPFLYAFL	DASFRRNLRQ
**330**							
LITCRAAA							

Cmpd.	No. of Cmpds.	m/z meas.	Δ m/z [ppm]	z	Rt [min]	Score	P	Range	Sequence	Modification
408	2	604.3472	17.95	2	34.1	23.5	2	146-156	R.RVAGRTYSAAR.A	
42	1	706.9621	-93.24	3	30.4	10.4	1	186-201	R.RQCVLVFPQPEAFWWR.A	Carbamidomethyl: 3

Detailed Protein Report

Protein 361: regulator of G-protein signaling 9 isoform 2 [Homo sapiens]

**Accession:**	gi|126366080	**Score:**	48.3
**Database:**	refseq_human(refseq_human_20140103.fasta)	**MW [kDa]:**	76.6
**Database Date:**	2015-11-30	**pI:**	10.0
		**Sequence Coverage [%]:**	6.3
		**No. of unique Peptides:**	2

Quantitation

Q:W	**Median:**	4.61	**CV:**	0.00 %	**No. of Peptides:**	1
M:Q	**Median:**	0.89	**CV:**	0.00 %	**No. of Peptides:**	1

10	20	30	40	50	60	70	80
MTIRHQGQQY	RPRMAFLQKI	EALVKDMQNP	ETGVRMQNQR	VLVTSVPHAM	TGSDVLQWIV	QRLWISSLEA	QNLGNFIVRY
**90**	**100**	**110**	**120**	**130**	**140**	**150**	**160**
GYIYPLQDPK	NLILKPDGSL	YRFQTPYFWP	TQQWPAEDTD	YAIYLAKRNI	KKKGILEEYE	KENYNFLNQK	MNYKWDFVIM
**170**	**180**	**190**	**200**	**210**	**220**	**230**	**240**
QAKEQYRAGK	ERNKADRYAL	DCQEKAYWLV	HRCPPGMDNV	LDYGLDRVTN	PNEVKKQTVV	AVKKEIMYYQ	QALMRSTVKS
**250**	**260**	**270**	**280**	**290**	**300**	**310**	**320**
SVSLGGIVKY	SEQFSSNDAI	MSGCLPSNPW	ITDDTQFWDL	NAKLVEIPTK	MRVERWAFNF	SELIRDPKGR	QSFQYFLKKE
**330**	**340**	**350**	**360**	**370**	**380**	**390**	**400**
FSGENLGFWE	ACEDLKYGDQ	SKVKEKAEEI	YKLFLAPGAR	RWINIDGKTM	DITVKGLKHP	HRYVLDAAQT	HIYMLMKKDS
**410**	**420**	**430**	**440**	**450**	**460**	**470**	**480**
YARYLKSPIY	KDMLAKAIEP	QETTKKSSTL	PFMRRHLRSS	PSPVILRQLE	EEAKAREAAN	TVDITQPGQH	MAPSPHLTVY
**490**	**500**	**510**	**520**	**530**	**540**	**550**	**560**
TGTCMPPSPS	SPFSSSCRSP	RKPFASPSRF	IRRPSTTICP	SPIRVALESS	SGLEQKGECS	GSMAPRGPSV	TESSEASLDT
**570**	**580**	**590**	**600**	**610**	**620**	**630**	**640**
SWPRSRPRAP	PKARMALSFS	RFLRRGCLAS	PVFARLSPKC	PAVSHGRVQP	LGDVGQQLPR	LKSKRVANFF	QIKMDVPTGS
**650**	**660**	**670**	**680**				
GTCLMDSEDA	GTGESGDRAT	EKEVICPWES	L				

Cmpd.	No. of Cmpds.	m/z meas.	Δ m/z [ppm]	z	Rt [min]	Score	P	Range	Sequence	Modification	Ratios
2782	1	681.4961	134.76	2	63.3	19.3	1	436-447	R.HLRSSPSPVILR.Q		
461	1	626.3269	-12.81	2	35.8	11.6	1	596-607	R.LSPKCPAVSHGR.V		Q:W 4.61<br>M:Q 0.89

Detailed Protein Report

Protein 362: cadherin EGF LAG seven-pass G-type receptor 1 precursor [Homo sapiens]

**Accession:**	gi|7656967	**Score:**	48.3
**Database:**	refseq_human(refseq_human_20140103.fasta)	**MW [kDa]:**	329.3
**Database Date:**	2015-11-30	**pI:**	5.5
		**Sequence Coverage [%]:**	1.6
		**No. of unique Peptides:**	1

Detailed Protein Report

10	20	30	40	50	60	70	80
MAPPPPPVLP	VLLLLAAAAA	LPAMGLRAAA	WEPRVPGGTR	AFALRPGCTY	AVGAACTPRA	PRELLDVGRD	GRLAGRRRVS
90	100	110	120	130	140	150	160
GAGRPLPLQV	RLVARSAPTA	LSRRLRARTH	LPGCGARARL	CGTGARLCGA	LCFPVPGGCA	AAQHSALAAP	TTLPACRCPP
170	180	190	200	210	220	230	240
RPRPRCPGRP	ICLPPGGSVR	LRLLCALRRA	AGAVRVGLAL	EAATAGTPSA	SPSPSPPLPP	NLPEARAGPA	RRARRGTSGR
250	260	270	280	290	300	310	320
GSLKFPMPNY	QVALFENEPA	GTLILQLHAH	YTIEGEEERV	SYYMEGLFDE	RSRGYFRIDS	ATGAVSTDSV	LDRETKETHV
330	340	350	360	370	380	390	400
LRVKAVDYST	PPRSATTYIT	VLVKDTNDHS	PVFEQSEYRE	RVRENLEVGY	EVLTIRASDR	DSPINANLRY	RVLGGAWDVF
410	420	430	440	450	460	470	480
QLNESSGVVS	TRAVLDREEA	AEYQLLVEAN	DQGRNPGPLS	ATATVYIEVE	DENDNYPQFS	EQNYVVQVPE	DVGLNTAVLR
490	500	510	520	530	540	550	560
VQATDRDQGQ	NAAIHYSILS	GNVAGQFYLH	SLSGILDVIN	PLDFEDVQKY	SLSIKAQDGG	RPPLINSSGV	VSVQVLDVND
570	580	590	600	610	620	630	640
NEPIFVSSPF	QATVLENVPL	GYPVVHIQAV	DADSGENARL	HYRLVDTAST	FLGGGSAGPK	NPAPTPDFPF	QIHNSSGWIT
650	660	670	680	690	700	710	720
VCAELDREEV	EHYSFGVEAV	DHGSPPMSSS	TSVSITVLDV	NDNDPVFTQP	TYELRLNEDA	AVGSSVLTLQ	ARDRDANSVI
730	740	750	760	770	780	790	800
TYQLTGGNTR	NRFALSSQRG	GGLITLALPL	DYKQEQQYVL	AVTASDGTRS	HTAHVLINVT	DANTHRPVFQ	SSHYTVSVSE
810	820	830	840	850	860	870	880
DRPVGTSIAT	LSANDEDTGE	NARITYVIQD	PVPQFRIDPD	SGTMYTMMEL	DYENQVAYTL	TIMAQDNGIP	QKSDTTTLEI
890	900	910	920	930	940	950	960
LILDANDNAP	QFLWDFYQGS	IFEDAPPSTS	ILQVSATDRD	SGPNGRLLYT	FQGGDDGDGD	FYIEPTSGVI	RTQRRLDREN
970	980	990	1000	1010	1020	1030	1040
VAVYNLWALA	VDRGSPTPLS	ASVEIQVTIL	DINDNAPMFE	KDELELFVEE	NNPVGSVVAK	IRANDPDEGP	NAQIMYQIVE
1050	1060	1070	1080	1090	1100	1110	1120
GDMRHFFQLD	LLNGDLRAMV	ELDFEVRREY	VLVVQATSAP	LVSRATVHIL	LVDQNDNPPV	LPDFQILFNN	YVTNKSNSFP
1130	1140	1150	1160	1170	1180	1190	1200
TGVIGCIPAH	DPDVSDSLNY	TFVQGNELRL	LLLDPATGEL	QLSRDLDNNR	PLEALMEVSV	SDGIHSVTAF	CTLRVTIITD
1210	1220	1230	1240	1250	1260	1270	1280
DMLTNSITVR	LENMSQEKFL	SPLLALFVEG	VAAVLSTTKD	DVFVFNVQND	TDVSSNILNV	TFSALLPGGV	RGQFFPSEDL
1290	1300	1310	1320	1330	1340	1350	1360
QEQIYLNRTL	LTTISTQRVL	PFDDNICLRE	PCENYMKCVS	VLRFDSSAPF	LSSTTVLFRP	IHPINGLRCR	CPPGFTGDYC
1370	1380	1390	1400	1410	1420	1430	1440
ETEIDLCYSD	PCGANGRCRS	REGGYTCECF	EDFTGEHCEV	DARSGRCANG	VCKNGGTCVN	LLIGGFHCVC	PPGEYERPYC
1450	1460	1470	1480	1490	1500	1510	1520
EVTTRSFPPQ	SFVTFRGLRQ	RFHFTISLTF	ATQERNGLLL	YNGRFNEKHD	FIALEIVDEQ	VQLTFSAGET	TTTVAPKVPS
1530	1540	1550	1560	1570	1580	1590	1600
GVSDGRWHSV	QVQYYNKPNI	GHLGLPHGPS	GEKMAVVTVD	DCDTTMAVRF	GKDIGNYSCA	AQGTQTGSKK	SLDLTGPLLL
1610	1620	1630	1640	1650	1660	1670	1680
GGVPNLPEDF	PVHNRQFVGC	MRNLSVDGKN	VDMAGFIANN	GTREGCAARR	NFCDGRRCQN	GGTCVNRWNM	YLCECPLRFG
1690	1700	1710	1720	1730	1740	1750	1760
GKNCEQAMPH	PQLFSGESVV	SWSDLNIIIS	VPWYLGLMFR	TRKEDSVLME	ATSGGPTSFR	LQILNNYLQF	EVSHGPSDVE
1770	1780	1790	1800	1810	1820	1830	1840
SVMLSGLRVT	DGEWHHLLIE	LKNVKEDSEM	KHLVTMTLDY	GMDQNKADIG	GMLPGLTVRS	VVVGGASEDK	VSVRRGFRGC
1850	1860	1870	1880	1890	1900	1910	1920
MQGVRMGGTP	TNVATLNMNN	ALKVRVKDGC	DVDDPCTSSP	CPPNSRCHDA	WEDYSCVCDK	GYLGINCVDA	CHLNPCENMG
1930	1940	1950	1960	1970	1980	1990	2000
ACVRSPGSPQ	GYVCECGPSH	YGPYCENKLD	LPCPRGWWGN	PVCGPCHCAV	SKGFDPDCNK	TNGQCQCKEN	YYKLLAQDTC
2010	2020	2030	2040	2050	2060	2070	2080
LPCDCFPHGS	HSRTCDMATG	QCACKPGVIG	RQCNRCDNPF	AEVTTLGCEV	IYNGCPKAFE	AGIWWPQTKF	GQPAAVPCPK
2090	2100	2110	2120	2130	2140	2150	2160
GSVGNAVRHC	SGEKGWLPPE	LFNCTTISFV	DLRAMNEKLS	RNETQVDGAR	ALQLVRALRS	ATQHTGTLFG	NDVRTAYQLL
2170	2180	2190	2200	2210	2220	2230	2240

Detailed Protein Report

Cmpd.	No. of Cmpds.	m/z meas.	Δ m/z [ppm]	z	Rt [min]	Score	P	Range	Sequence	Modification
155	1	855.3273	-115.70	2	31.7	12.6	0	715-730	R.DANSVITYQLTGGNTR.N	

Detailed Protein Report

Protein 363: phospholipase B1, membrane-associated isoform 2 precursor [Homo sapiens]

**Accession:**	gi|283436114	**Score:**	48.1
**Database:**	refseq_human(refseq_human_20140103.fasta)	**MW [kDa]:**	161.6
**Database Date:**	2015-11-30	**pI:**	5.5
**Modification(s):**	Carbamidomethyl	**Sequence Coverage [%]:**	3.0
		**No. of unique Peptides:**	3

```
        10         20         30         40         50         60         70         80
MGLRPGIFLL ELLLLLGQGT PQIHTSPRKS TLEGQLWPET LKNSPFPCNP NKLGVNMPSK SVHSLKPSDI KFVAAIGNLE
        90        100        110        120        130        140        150        160
IPPDPGTGDL EKQDWTERPQ QVCMGVMTVL SDIIRYFSPS VPMPVCHTGK RVIPHDGAED LWIQAQELVR NMKENLQLDF
       170        180        190        200        210        220        230        240
QFDWKLINVF FSNASQCYLC PSAQQAPSLS TVLLSQNGLA AGGVDELMGV LDYLQQEVPR AFVNLVDLSE VAEVSRQYHG
       250        260        270        280        290        300        310        320
TWLSPAPEPC NCSEETTRLA KVVMQWSYQE AWNSLLASSR YSEQESFTVV FQPFFYETTP SLHSEDPRLQ DSTTLAWHLW
       330        340        350        360        370        380        390        400
NRMMEPAGEK DEPLSVKHGR PMKCPSQESP YLFSYRNSNY LTRLQKPQDK LEVREGAEIR CPDKDPSDTV PTSVHRLKPA
       410        420        430        440        450        460        470        480
DINVIGALGD SLTAGNGAGS TPGNVLDVLT QYRGLSWSVG GDENIGTVTT LANILREFNP SLKGFSVGTG KETSPNAFLN
       490        500        510        520        530        540        550        560
QAVAGGRAED LPVQARRLVD LMKNDTRIHF QEDWKIITLF IGGNDLCDFC NDLVPRAFVN LVTVLEIVNL RELYQEKKVY
       570        580        590        600        610        620        630        640
CPRMILRSLC PCVLKFDDNS TELATLIEFN KKFQEKTHQL IESGRYDTRE DFTVVVQPFF ENVDMPKTSE GLPDNSFFAP
       650        660        670        680        690        700        710        720
DCFHFSSKSH SRAASALWNN MLEPVGQKTT RHKFENKINI TCPNQVQPFL RTYKNSMQGH GTWLPCRDRA PSALHPTSVH
       730        740        750        760        770        780        790        800
ALRPADIQVV AALGDSLTAG NGIGSKPDDL PDVTTQYRGL SYSAGGDGSL ENVTTLPNIL REFNRNLTGY AVGTGDANDT
       810        820        830        840        850        860        870        880
NAFLNQAVPG AKAEDLMSQV QTLMQKMKDD HRVNFHEDWK VITVLIGGSD LCDYCTDSNL YSAANFVHHL RNALDVLHRE
       890        900        910        920        930        940        950        960
VPRVLVNLVD FLNPTIMRQV FLGNPDKCPV QQASVLCNCV LTLRENSQEL ARLEAFSRAY RSSMRELVGS GRYDTQEDFS
       970        980        990       1000       1010       1020       1030       1040
VVLQPFFQNI QLPVLADGLP DTSFFAPDCI HPNQKFHSQL ARALWTNMLE PLGSKTETLD LRAEMPITCP TQNEPFLRTP
      1050       1060       1070       1080       1090       1100       1110       1120
RNSNYTYPIK PAIENWGSDF LCTEWKASNS VPTSVHQLRP ADIKVVAALG DSLTTAVGAR PNNSSDLPTS WRGLSWSIGG
      1130       1140       1150       1160       1170       1180       1190       1200
DGNLETHTTL PNILKKFNPY LLGFSTSTWE GTAGLNVAAE GARARDMPAQ AWDLVERMKN SPDINLEKDW KLVTLFIGVN
      1210       1220       1230       1240       1250       1260       1270       1280
DLCHYCENPE AHLATEYVQH IQQALDILSE ELPRAFVNVV EVMELASLYQ GQGGKCAMLA AQNNCTCLRH SQSSLEKQEL
      1290       1300       1310       1320       1330       1340       1350       1360
KKVNWNLQHG ISSFSYWHQY TQREDFAVVV QPFFQNTLTP LNERGDTDLT FFSEDCFHFS DRGHAEMAIA LWNNMLEPVG
      1370       1380       1390       1400       1410       1420       1430       1440
RKTTSNNFTH SRAKLKCPSP ESPYLYTLRN SRLLPDQAEE APEVLYWAVP VAAGVGLVVG IIGTVVWRCR RGGRREDPPM
      1450
SLRTVAL
```

Cmpd.	No. of Cmpds.	m/z meas.	Δ m/z [ppm]	z	Rt [min]	Score	P	Range	Sequence	Modification
428	2	587.8118	72.68	2	34.7	11.5	0	43-52	K.NSPFPCNPNK.L	Carbamidomethyl: 6
413	1	587.7939	24.16	2	34.5	11.6	1	375-384	R.EGAEIRCPDK.D	Carbamidomethyl: 7
2292	1	816.2731	-168.26	2	58.9	11.1	0	472-487	K.ETSPNAFLNQAVAGGR.A	

Detailed Protein Report

Protein 364: unconventional myosin-Id [Homo sapiens]

Accession: gi|51100974
Database: refseq_human(refseq_human_20140103.fasta)
Database Date: 2015-11-30

Score: 48.0
MW [kDa]: 116.1
pI: 10.0
Sequence Coverage [%]: 2.6
No. of unique Peptides: 1

Quantitation

Q:W **Median:** 2.02 **CV:** 0.00 % **No. of Peptides:** 1

10	20	30	40	50	60	70	80
MAEQESLEFG	KADFVLMDTV	SMPEFMANLR	LRFEKGRIYT	FIGEVVVSVN	PYKLLNIYGR	DTIEQYKGRE	LYERPPHLFA
90	100	110	120	130	140	150	160
IADAAYKAMK	RRSKDTCIVI	SGESGAGKTE	ASKYIMQYIA	AITNPSQRAE	VERVKNMLLK	SNCVLEAFGN	AKTNRNDNSS
170	180	190	200	210	220	230	240
RFGKYMDINF	DFKGDPIGGH	INNYLLEKSR	VIVQQPGERS	FHSFYQLLQG	GSEQMLRSLH	LQKSLSSYNY	IHVGAQLKSS
250	260	270	280	290	300	310	320
INDAAEFRVV	ADAMKVIGFK	PEEIQTVYKI	LAAILHLGNL	KFVVDGDTPL	IENGKVVSII	AELLSTKTDM	VEKALLYRTV
330	340	350	360	370	380	390	400
ATGRDIIDKQ	HTEQEASYGR	DAFAKAIYER	LFCWIVTRIN	DIIEVKNYDT	TIHGKNTVIG	VLDIYGFEIF	DNNSFEQFCI
410	420	430	440	450	460	470	480
NYCNEKLQQL	FIQLVLKQEQ	EEYQREGIPW	KHIDYFNNQI	IVDLVEQQHK	GIIAILDDAC	MNVGKVTDEM	FLEALNSKLG
490	500	510	520	530	540	550	560
KHAHFSSRKL	CASDKILEFD	RDFRIRHYAG	DVVYSVIGFI	DKNKDTLFQD	FKRLMYNSSN	PVLKNMWPEG	KLSITEVTKR
570	580	590	600	610	620	630	640
PLTAATLFKN	SMIALVDNLA	SKEPYYVRCI	KPNDKKSPQI	FDDERCRHQV	EYLGLLENVR	VRRAGFAFRQ	TYEKFLHRYK
650	660	670	680	690	700	710	720
MISEFTWPNH	DLPSDKEAVK	KLIERCGFQD	DVAYGKTKIF	IRTPRTLFTL	EELRAQMLIR	IVLFLQKVWR	GTLARMRYKR
730	740	750	760	770	780	790	800
TKAALTIIRY	YRRYKVKSYI	HEVARRFHGV	KTMRDYGKHV	KWPSPPKVLR	RFEEALQTIF	NRWRASQLIK	SIPASDLPQV
810	820	830	840	850	860	870	880
RAKVAAVEML	KGQRADLGLQ	RAWEGNYLAS	KPDTPQTSGT	FVPVANELKR	KDKYMNVLFS	CHVRKVNRFS	KVEDRAIFVT
890	900	910	920	930	940	950	960
DRHLYKMDPT	KQYKVMKTIP	LYNLTGLSVS	NGKDQLVVFH	TKDNKDLIVC	LFSKQPTHES	RIGELVGVLV	NHFKSEKRHL
970	980	990	1000	1010			
QVNVTNPVQC	SLHGKKCTVS	VETRLNQPQP	DFTKNRSGFI	LSVPGN			

Cmpd.	No. of Cmpds.	m/z meas.	Δ m/z [ppm]	z	Rt [min]	Score	P	Range	Sequence	Modification	Ratios	
2982	3	729.3741	-5.85	2	66.0	18.2	1	985-996	R.LNQPQPDFTKNR.S		Q:W	2.02

Detailed Protein Report

Protein 365: **PREDICTED: probable global transcription activator SNF2L1 isoform X5 [Homo sapiens]**

**Accession:**	gi|578838748	**Score:**	48.0
**Database:**	refseq_human(refseq_human_20140103.fasta)	**MW [kDa]:**	121.1
**Database Date:**	2015-11-30	**pI:**	9.0
**Modification(s):**	Oxidation	**Sequence Coverage [%]:**	3.5
		**No. of unique Peptides:**	3

Quantitation

Q:W	**Median:**	0.58	**CV:**	0.00 %	**No. of Peptides:**	1
M:Q	**Median:**	0.80	**CV:**	18.37 %	**No. of Peptides:**	2

10	20	30	40	50	60	70	80
MEQDTAAVAA	TVAAADATAT	IVVIEDEQPG	PSTSQEEGAA	AAATEATAAT	EKGEKKKEKN	VSSFQLKLAA	KAPKSEKEMD
**90**	**100**	**110**	**120**	**130**	**140**	**150**	**160**
PEYEEKMKAD	RAKRFEFLLK	QTELFAHFIQ	PSAQKSPTSP	LNMKLGRPRI	KKDEKQSLIS	AGDYRHRRTE	QEEDEELLSE
**170**	**180**	**190**	**200**	**210**	**220**	**230**	**240**
SRKTSNVCIR	FEVSPSYVKG	GPLRDYQIRG	LNWLISLYEN	GVNGILADEM	GLGKTLQTIA	LLGYLKHYRN	IPGPHMVLVP
**250**	**260**	**270**	**280**	**290**	**300**	**310**	**320**
KSTLHNWMNE	FKRWVPSLRV	ICFVGDKDAR	AAFIRDEMMP	GEWDVCVTSY	EMVIKEKSVF	KKFHWRYLVI	DEAHRIKNEK
**330**	**340**	**350**	**360**	**370**	**380**	**390**	**400**
SKLSEIVREF	KSTNRLLLTG	TPLQNNLHEL	WALLNFLLPD	VFNSADDFDS	WFDTKNCLGD	QKLVERLHAV	LKPFLLRRIK
**410**	**420**	**430**	**440**	**450**	**460**	**470**	**480**
TDVEKSLPPK	KEIKIYLGLS	KMQREWYTKI	LMKDIDVLNS	SGKMDKMRLL	NILMQLRKCC	NHPYLFDGAE	PGPPYTTDEH
**490**	**500**	**510**	**520**	**530**	**540**	**550**	**560**
IVSNSGKMVV	LDKLLAKLKE	QGSRVLIFSQ	MTRLLDILED	YCMWRGYEYC	RLDGQTPHEE	REEAIEAFNA	PNSSKFIFML
**570**	**580**	**590**	**600**	**610**	**620**	**630**	**640**
STRAGGLGIN	LASADVVILY	DSDWNPQVDL	QAMDRAHRIG	QKKPVRVFRL	ITDNTVEERI	VERAEIKLRL	DSIVIQQGRL
**650**	**660**	**670**	**680**	**690**	**700**	**710**	**720**
IDQQSNKLAK	EEMLQMIRHG	ATHVFASKES	ELTDEDITTI	LERGEKKTAE	MNERLQKMGE	SSLRNFRMDI	EQSLYKFEGE
**730**	**740**	**750**	**760**	**770**	**780**	**790**	**800**
DYREKQKLGM	VEWIEPPKRE	RKANYAVDAY	FREALRVSEP	KIPKAPRPPK	QPNVQDFQFF	PPRLFELLEK	EILYYRKTIG
**810**	**820**	**830**	**840**	**850**	**860**	**870**	**880**
YKVPRNPDIP	NPALAQREEQ	KKIDGAEPLT	PEETEEKEKL	LTQGFTNWTK	RDFNQFIKAN	EKYGRDDIDN	IAREVEGKSP
**890**	**900**	**910**	**920**	**930**	**940**	**950**	**960**
EEVMEYSAVF	WERCNELQDI	EKIMAQIERG	EARIQRRISI	KKALDAKIAR	YKAPFHQLRI	QYGTSKGKNY	TEEEDRFLIC
**970**	**980**	**990**	**1000**	**1010**	**1020**	**1030**	**1040**
MLHKMGFDRE	NVYEELRQCV	RNAPQFRFDW	FIKSRTAMEF	QRRCNTLISL	IEKENMEIEE	RERAEKKKRA	TKTPMVKFSA
**1050**							
FS							

Cmpd.	No. of Cmpds.	m/z meas.	Δ m/z [ppm]	z	Rt [min]	Score	P	Range	Sequence	Modification	Ratios
1545	1	765.2820	-48.68	2	49.2	16.1	1	75-86	K.SEKEMDPEYEEK.M	Oxidation: 5	
966	1	711.3525	2.57	2	40.4	21.3	1	434-446	K.DIDVLNSSGKMDK.M		M:Q 0.67
2348	1	646.6880	-199.21	2	58.0	10.6	2	684-694	R.GEKKTAEMNER.L		Q:W 0.58<br>M:Q 0.96

Detailed Protein Report

Protein 366: PREDICTED: T-complex protein 11 homolog isoform X4 [Homo sapiens]

**Accession:**	gi|578811876	**Score:**	48.0
**Database:**	refseq_human(refseq_human_20140103.fasta)	**MW [kDa]:**	61.2
**Database Date:**	2015-11-30	**pI:**	6.2
**Modification(s):**	Oxidation	**Sequence Coverage [%]:**	7.2
		**No. of unique Peptides:**	1

10	20	30	40	50	60	70	80
MHALASSQQF	QAILLEGREL	TGMNRPQRAA	RGLVGFELRR	DARACRFRLW	RGGHVPRPVA	PATAAVCSQA	PAFLTVRRPN
**90**	**100**	**110**	**120**	**130**	**140**	**150**	**160**
PPLRRGGTLQ	AARPRGPPSG	RQWMTRGGGG	GDTISKMPDV	KESVPPKYPG	DSEGRSCKPE	TSGPPQEDKS	GSEDPPPFLS
**170**	**180**	**190**	**200**	**210**	**220**	**230**	**240**
VTGLTETVNE	VSKLSNKIGM	NCDYYMEEKV	LPPSSLEGKV	KETVHNAFWD	HLKEQLSATP	PDFSCALELL	KEIKEILLSL
**250**	**260**	**270**	**280**	**290**	**300**	**310**	**320**
LLPRQNRLRI	EIEEALDMDL	LKQEAEHGAL	KVLYLSKYVL	NMMALLCAPV	RDEAVQKLEN	ITDPVWLLRG	IFQVLGRMKM
**330**	**340**	**350**	**360**	**370**	**380**	**390**	**400**
DMVNYTIQSL	QPHLQEHSIQ	YERAKFQELL	NKQPSLLNHT	TKWLTQAAGD	LTMSPPTCPD	TSDSSSVAGP	SPNEAANNPE
**410**	**420**	**430**	**440**	**450**	**460**	**470**	**480**
PLSPTMVLCQ	GFLNLLLWDL	ENEEFPETLL	MDRTRLQELK	SQLHQLTVMA	SVLLVASSFS	GSVLFGSPQF	VDKLKRITKS
**490**	**500**	**510**	**520**	**530**	**540**	**550**	**560**
LLEDFHSRPE	EAILTVSEQV	SQEIHQSLKN	MGLVALSSDN	TASLMGQLQN	IAKKENCVCS	VIAQALETKV	ESV

Cmpd.	No. of Cmpds.	m/z meas.	Δ m/z [ppm]	z	Rt [min]	Score	P	Range	Sequence	Modification
2154	1	1052.4435	-57.89	2	57.1	16.6	2	117-135	K.MPDVKESVPPKYPGDSEGR.S	Oxidation: 1

Detailed Protein Report

Protein 367: eukaryotic translation initiation factor 4 gamma 3 isoform 1 [Homo sapiens]

**Accession:**	gi|311771714	**Score:**	48.0
**Database:**	refseq_human(refseq_human_20140103.fasta)	**MW [kDa]:**	180.4
**Database Date:**	2015-11-30	**pI:**	5.2
**Modification(s):**	Carbamidomethyl, Oxidation	**Sequence Coverage [%]:**	2.8
		**No. of unique Peptides:**	1

10	20	30	40	50	60	70	80
MNSQPQTRSP	FFQRPQIQPP	RATIPNSSPS	IRPGAQTPTA	VYQANQHIMM	VNHLPMPYPV	PQGPQYCIPQ	YRHSGPPYVG
90	100	110	120	130	140	150	160
PPQQYPVQPP	GPGPFYPGPG	PGDFPNAYGT	PFYPSQPVYQ	SAPIIVPTQQ	QPPPAKREKK	TIRIRDPNQG	GKDITEEIMS
170	180	190	200	210	220	230	240
GGGSRNPTPP	IGRPTSTPTP	PQLPSQVPEH	SPVVYGTVES	AHLAASTPVT	AASDQKQEEK	PKPDPVLKSP	SPVLRLVLSG
250	260	270	280	290	300	310	320
EKKEQEGQTS	ETTAIVSIAE	LPLPPSPTTV	SSVARSTIAA	PTSSALSSQP	IFTTAIDDRC	ELSSPREDTI	PIPSLTSCTE
330	340	350	360	370	380	390	400
TSDPLPTNEN	DDDICKKPCS	VAPNDIPLVS	STNLINEING	VSEKLSATES	IVEIVKQEVL	PLTLELEILE	NPPEEMKLEC
410	420	430	440	450	460	470	480
IPAPITPSTV	PSFPPTPPTP	PASPPHTPVI	VPAAATTVSS	PSAAITVQRV	LEEDESIRTC	LSEDAKEIQN	KIEVEADGQT
490	500	510	520	530	540	550	560
EEILDSQNLN	SRRSPVPAQI	AITVPKTWKK	PKDRTRTTEE	MLEAELELKA	EEELSIDKVL	ESEQDKMSQG	FHPERDPSDL
570	580	590	600	610	620	630	640
KKVKAVEENG	EEAEPVRNGA	ESVSEGEGID	ANSGSTDSSG	DGVTFPFKPE	SWKPTDTEGK	KQYDREFLLD	FQFMPACIQK
650	660	670	680	690	700	710	720
PEGLPPISDV	VLDKINQPKL	PMRTLDPRIL	PRGPDFTPAF	ADFGRQTPGG	RGVPICKVQS	RHGLPILEQS	KAPTCPPLVM
730	740	750	760	770	780	790	800
SHPPMKSLPL	GLLNVGSRRS	QPGQRREPRK	IITVSVKEDV	HLKKAENAWK	PSQKRDSQAD	DPENIKTQEL	FRKVRSILNK
810	820	830	840	850	860	870	880
LTPQMFNQLM	KQVSGLTVDT	EERLKGVIDL	VFEKAIDEPS	FSVAYANMCR	CLVTLKVPMA	DKPGNTVNFR	KLLLNRCQKE
890	900	910	920	930	940	950	960
FEKDKADDDV	FEKKQKELEA	ASAPEERTRL	HDELEEAKDK	ARRRSIGNIK	FIGELFKLKM	LTEAIMHDCV	VKLLKNHDEE
970	980	990	1000	1010	1020	1030	1040
SLECLCRLLT	TIGKDLDFEK	AKPRMDQYFN	QMEKIVKERK	TSSRIRFMLQ	DVIDLRLCNW	VSRRADQGPK	TIEQIHKEAK
1050	1060	1070	1080	1090	1100	1110	1120
IEEQEEQRKV	QQLMTKEKRR	PGVQRVDEGG	WNTVQGAKNS	RVLDPSKFLK	ITKPTIDEKI	QLVPKAQLGS	WGKGSSGGAK
1130	1140	1150	1160	1170	1180	1190	1200
ASETDALRSS	ASSLNRFSAL	QPPAPSGSTP	STPVEFDSRR	TLTSRGSMGR	EKNDKPLPSA	TARPNTFMRG	GSSKDLLDNQ
1210	1220	1230	1240	1250	1260	1270	1280
SQEEQRREML	ETVKQLTGGV	DVERNSTEAE	RNKTRESAKP	EISAMSAHDK	AALSEEELER	KSKSIIDEFL	HINDFKEAMQ
1290	1300	1310	1320	1330	1340	1350	1360
CVEELNAQGL	LHVFVRVGVE	STLERSQITR	DHMGQLLYQL	VQSEKLSKQD	FFKGFSETLE	LADDMAIDIP	HIWLYLAELV
1370	1380	1390	1400	1410	1420	1430	1440
TPMLKEGGIS	MRELTIEFSK	PLLPVGRAGV	LLSEILHLLC	KQMSHKKVGA	LWREADLSWK	DFLPEGEDVH	NFLLEQKLDF
1450	1460	1470	1480	1490	1500	1510	1520
IESDSPCSSE	ALSKKELSAE	ELYKRLEKLI	IEDKANDEQI	FDWVEANLDE	IQMSSPTFLR	ALMTAVCKAA	IIADSSTFRV
1530	1540	1550	1560	1570	1580	1590	1600
DTAVIKQRVP	ILLKYLDSDT	EKELQALYAL	QASIVKLDQP	ANLLRMFFDC	LYDEEVISED	AFYKWESSKD	PAEQNGKGVA
1610	1620	1630					
LKSVTAFFTW	LREAEEESED	N					

Cmpd.	No. of Cmpds.	m/z meas.	Δ m/z [ppm]	z	Rt [min]	Score	P	Range	Sequence	Modification
2704	1	847.8687	-44.85	2	65.0	19.4	0	712-726	K.APTCPPLVMSHPPMK.S	Carbamidomethyl: 4; Oxidation: 9, 14

Detailed Protein Report

Protein 368: zinc finger protein 708 [Homo sapiens]

**Accession:**	gi|116642871	**Score:**	48.0
**Database:**	refseq_human(refseq_human_20140103.fasta)	**MW [kDa]:**	64.7
**Database Date:**	2015-11-30	**pI:**	10.3
**Modification(s):**	Carbamidomethyl	**Sequence Coverage [%]:**	5.3
		**No. of unique Peptides:**	2

Quantitation

Q:W	**Median:**	1.09	**CV:**	0.00 %	**No. of Peptides:**	1
M:Q	**Median:**	2.90	**CV:**	155.94 %	**No. of Peptides:**	2

```
        10         20         30         40         50         60         70         80
MGPLTFMDVA IEFSLEEWQC LDTAQQNLYR NVMLENYRNL VFLGIAVSNL DLITCLEQGK EPWNMKRHEM AAKPPAMCSH
        90        100        110        120        130        140        150        160
FAKDLRPEQY IKNSFQQVIL RRYGKCGYQK GCKSVDEHKL HKGGHKGLNR CVTTTQSKIV QCDKYVKVFH KYSNAKRHKI
       170        180        190        200        210        220        230        240
RHTGKNPFKC KECGKSFCML SQLTQHEIIH TGEKPYKCEE CGKAFKKSSN LTNHKIIHTG EKPYKCEECG KAFNQSSTLT
       250        260        270        280        290        300        310        320
RHKIIHTGEK LYKCEECGKA FNRSSNLTKH KIVHTGEKPY KCEECGKAFK QSSNLTNHKK IHTGEKPYKC GECGKAFTLS
       330        340        350        360        370        380        390        400
SHLTTHKRIH TGEKPYKCEE CGKAFSVFST LTKHKIIHTE EKPYKCEECG KAFNRSSHLT NHKVIHTGEK PYKCEECGKA
       410        420        430        440        450        460        470        480
FTKSSTLTYH KVIHTGKKPY KCEECGKAFS IFSILTKHKV IHTEDKPYKC EECGKTFNYS SNFTNHKKIH TGEKPYKCEE
       490        500        510        520        530        540        550        560
CGKSFILSSH LTTHKIIHTG EKPYKCKECG KAFNQSSTLM KHKIIHTGEK PYKCEECGKA FNQSPNLTKH KRIHTKEKPY
       570
KCK
```

Cmpd.	No. of Cmpds.	m/z meas.	Δ m/z [ppm]	z	Rt [min]	Score	P	Range	Sequence	Modification	Ratios	
328	5	605.6684	-199.06	2	32.8	13.8	2	166-175	K.NPFKCKECGK.S	Carbamidomethyl: 8	M:Q<br>Q:W	0.96<br>1.09
1258	8	586.7739	28.23	2	45.9	20.9	1	394-403	K.CEECGKAFTK.S	Carbamidomethyl: 4	M:Q	8.81

Detailed Protein Report

Protein 369: PREDICTED: NEDD8 ultimate buster 1 isoform X2 [Homo sapiens]

**Accession:**	gi|530387032	**Score:**	47.9
**Database:**	refseq_human(refseq_human_20140103.fasta)	**MW [kDa]:**	70.5
**Database Date:**	2015-11-30	**pI:**	5.6
**Modification(s):**	Carbamidomethyl	**Sequence Coverage [%]:**	3.4
		**No. of unique Peptides:**	2

Quantitation

Q:W	**Median:**	2.76	**CV:**	0.00 %	**No. of Peptides:**	1
M:Q	**Median:**	1.68	**CV:**	0.00 %	**No. of Peptides:**	1

```
        10         20         30         40         50         60         70         80
MAQKKYLQAK LTQFLREDRI QLWKPPYTDE NKKVGLALKD LAKQYSDRLE CCENEVEKVI EEIRCKAIER GTGNDNYRTT
        90        100        110        120        130        140        150        160
GIATIEVFLP PRLKKDRKNL LETRLHITGR ELRSKIAETF GLQENYIKIV INKKQLQLGK TLEEQGVAHN VKAMVLELKQ
       170        180        190        200        210        220        230        240
SEEDARKNFQ LEEEEQNEAK LKEKQIQRTK RGLEILAKRA AETVVDPEMT PYLDIANQTG RSIRIPPSER KALMLAMGYH
       250        260        270        280        290        300        310        320
EKGRAFLKRK EYGIALPCLL DADKYFCECC RELLDTVDNY AVLQLDIVWC YFRLEQLECL DDAEKKLNLA QKCFKNCYGE
       330        340        350        360        370        380        390        400
NHQRLVHIKG NCGKEKVLFL RLYLLQGIRN YHSGNDVEAY EYLNKARQLF KELYIDPSKV DNLLQLGFTA QEARLGLRAC
       410        420        430        440        450        460        470        480
DGNVDHAATH ITNRREELAQ IRKEEKEKKR RRLENIRFLK GMGYSTHAAQ QVLHAASGNL DEALKILLSN PQMWWLNDSN
       490        500        510        520        530        540        550        560
PETDNRQESP SQENIDRLVY MGFDALVAEA ALRVFRGNVQ LAAQTLAHNG GSLPPELPLS PEDSLSPPAT SPSDSAGTSS
       570        580        590        600        610        620
ASTDEDMETE AVNEILEDIP EHEEDYLDST LEDEEIIIAE YLSYVENRKS ATKKN
```

Cmpd.	No. of Cmpds.	m/z meas.	Δ m/z [ppm]	z	Rt [min]	Score	P	Range	Sequence	Modification	Ratios
2062	2	625.2581	-159.60	2	54.4	16.1	2	306-315	K.KLNLAQKCFK.N	Carbamidomethyl: 8	
2328	19	596.8262	-35.19	2	57.4	21.1	2	306-315	K.KLNLAQKCFK.N		M:Q 1.68<br>Q:W 2.76

Detailed Protein Report

Protein 370: zinc finger protein 540 isoform a [Homo sapiens]

Accession: gi|22749241
Database: refseq_human(refseq_human_20140103.fasta)
Database Date: 2015-11-30

Score: 47.9
MW [kDa]: 77.0
pI: 10.5
Sequence Coverage [%]: 7.4
No. of unique Peptides: 3

Alias proteins:

Accession	Name	Description
gi|288557351	refseq_human (refseq_human_20140103.fasta)	zinc finger protein 540 isoform a [Homo sapiens]

10	20	30	40	50	60	70	80
MAHALVTFRD	VAIDFSQKEW	ECLDTTQRKL	YRDVMLENYN	NLVSLGYSGS	KPDVITLLEQ	GKEPCVVARD	VTGRQCPGLL
**90**	**100**	**110**	**120**	**130**	**140**	**150**	**160**
SRHKTKKLSS	EKDIHEISLS	KESIIEKSKT	LRLKGSIFRN	EWQNKSEFEG	QQGLKERSIS	QKKIVSKKMS	TDRKRPSFTL
**170**	**180**	**190**	**200**	**210**	**220**	**230**	**240**
NQRIHNSEKS	CDSHLVQHGK	IDSDVKHDCK	ECGSTFNNVY	QLTLHQKIHT	GEKSCKCEKC	GKVFSHSYQL	TLHQRFHTGE
**250**	**260**	**270**	**280**	**290**	**300**	**310**	**320**
KPYECQECGK	TFTLYPQLNR	HQKIHTGKKP	YMCKKCDKGF	FSRLELTQHK	RIHTGKKSYE	CKECGKVFQL	IFYFKEHERI
**330**	**340**	**350**	**360**	**370**	**380**	**390**	**400**
HTGKKPYECK	ECGKAFSVCG	QLTRHQKIHT	GVKPYECKEC	GKTFRLSFYL	TEHRRTHAGK	KPYECKECGK	SFNVRGQLNR
**410**	**420**	**430**	**440**	**450**	**460**	**470**	**480**
HKTIHTGIKP	FACKVCEKAF	SYSGDLRVHS	RIHTGEKPYE	CKECGKAFML	RSVLTEHQRL	HTGVKPYECK	ECGKTFRVRS
**490**	**500**	**510**	**520**	**530**	**540**	**550**	**560**
QISLHKKIHT	DVKPYKCVRC	GKTFRFGFYL	TEHQRIHTGE	KPYKCKECGK	AFIRRGNLKE	HLKIHSGLKP	YDCKECGKSF
**570**	**580**	**590**	**600**	**610**	**620**	**630**	**640**
SRRGQFTEHQ	KIHTGVKPYK	CKECGKAFSR	SVDLRIHQRI	HTGEKPYECK	QCGKAFRLNS	HLTEHQRIHT	GEKPYECKVC
**650**	**660**	**670**					
RKAFRQYSHL	YQHQKTHNVI						

Cmpd.	No. of Cmpds.	m/z meas.	Δ m/z [ppm]	z	Rt [min]	Score	P	Range	Sequence	Modification
2641	1	701.3872	28.83	2	61.3	12.6	1	70-82	R.DVTGRQCPGLLSR.H	
765	1	541.3095	42.61	3	38.4	11.9	1	303-315	K.ECGKVFQLIFYFK.E	
818	1	946.4484	-19.61	2	39.1	10.2	2	331-347	K.ECGKAFSVCGQLTRHQK.I	

Detailed Protein Report

Protein 371: abnormal spindle-like microcephaly-associated protein isoform 1 [Homo sapiens]

**Accession:**	gi|126116596	**Score:**	47.8
**Database:**	refseq_human(refseq_human_20140103.fasta)	**MW [kDa]:**	409.5
**Database Date:**	2015-11-30	**pI:**	11.2
**Modification(s):**	Oxidation	**Sequence Coverage [%]:**	1.4
		**No. of unique Peptides:**	4

Quantitation

Q:W	**Median:** 1.99	**CV:** 0.00 %	**No. of Peptides:** 1

Detailed Protein Report

10	20	30	40	50	60	70	80
MANRRVGRGC	WEVSPTERRP	PAGLRGPAAE	EEASSPPVLS	LSHFCRSPFL	CFGDVLLGAS	RTLSLALDNP	NEEVAEVKIS
90	100	110	120	130	140	150	160
HFPAADLGFS	VSQRCFVLQP	KEKIVISVNW	TPLKEGRVRE	IMTFLVNDVL	KHQAILLGNA	EEQKKKKRSL	WDTIKKKKIS
170	180	190	200	210	220	230	240
ASTSHNRRVS	NIQNVNKTFS	VSQKVDRVRS	PLQACENLAM	NEGGPPTENN	SLILEENKIP	ISPISPAFNE	CHGATCLPLS
250	260	270	280	290	300	310	320
VRRSTTYSSL	HASENRELLN	VHSANVSKVS	FNEKAVTETS	FNSVNVNGQR	GENSKLSLTP	NCSSTLNITQ	SQIHFLSPDS
330	340	350	360	370	380	390	400
FVNNSHGANN	ELELVTCLSS	DMFMKDNSQP	VHLESTIAHE	IYQKILSPDS	FIKDNYGLNQ	DLESESVNPI	LSPNQFLKDN
410	420	430	440	450	460	470	480
MAYMCTSQQT	CKVPLSNENS	QVPQSPEDWR	KSEVSPRIPE	CQGSKSPKAI	FEELVEMKSN	YYSFIKQNNP	KFSAVQDISS
490	500	510	520	530	540	550	560
HSHNKQPKRR	PILSATVTKR	KATCTRENQT	EINKPKAKRC	LNSAVGEHEK	VINNQKEKED	FHSYLPIIDP	ILSKSKSYKN
570	580	590	600	610	620	630	640
EVTPSSTTAS	VARKRKSDGS	MEDANVRVAI	TEHTEVREIK	RIHFSPSEPK	TSAVKKTKNV	TTPISKRISN	REKLNLKKKT
650	660	670	680	690	700	710	720
DLSIFRTPIS	KTNKRTKPII	AVAQSSLTFI	KPLKTDIPRH	PMPFAAKNMF	YDERWKEKQE	QGFTWWLNFI	LTPDDFTVKT
730	740	750	760	770	780	790	800
NISEVNAATL	LLGIENQHKI	SVPRAPTKEE	MSLRAYTARC	RLNRLRRAAC	RLFTSEKMVK	AIKKLEIEIE	ARRLIVRKDR
810	820	830	840	850	860	870	880
HLWKDVGERQ	KVLNWLLSYN	PLWLRIGLET	TYGELISLED	NSDVTGLAMF	ILNRLLWNPD	IAAEYRHPTV	PHLYRDGHEE
890	900	910	920	930	940	950	960
ALSKFTLKKL	LLLVCFLDYA	KISRLIDHDP	CLFCKDAEFK	ASKEILLAFS	RDFLSGEGDL	SRHLGLLGLP	VNHVQTPFDE
970	980	990	1000	1010	1020	1030	1040
FDFAVTNLAV	DLQCGVRLVR	TMELLTQNWD	LSKKLRIPAI	SRLQKMHNVD	IVLQVLKSRG	IELSDEHGNT	ILSKDIVDRH
1050	1060	1070	1080	1090	1100	1110	1120
REKTLRLLWK	IAFAFQVDIS	LNLDQLKEEI	AFLKHTKSIK	KTISLLSCHS	DDLINKKKGK	RDSGSFEQYS	ENIKLLMDWV
1130	1140	1150	1160	1170	1180	1190	1200
NAVCAFYNKK	VENFTVSFSD	GRVLCYLIHH	YHPCYVPFDA	ICQRTTQTVE	CTQTGSVVLN	SSSESDDSSL	DMSLKAFDHE
1210	1220	1230	1240	1250	1260	1270	1280
NTSELYKELL	ENEKKNFHLV	RSAVRDLGGI	PAMINHSDMS	NTIPDEKVVI	TYLSFLCARL	LDLRKEIRAA	RLIQTTWRKY
1290	1300	1310	1320	1330	1340	1350	1360
KLKTDLKRHQ	EREKAARIIQ	LAVINFLAKQ	RLRKRVNAAL	VIQKYWRRVL	AQRKLLMLKK	EKLEKVQNKA	ASLIQGYWRR
1370	1380	1390	1400	1410	1420	1430	1440
YSTRQRFLKL	KYYSIILQSR	IRMIIAVTSY	KRYLWATVTI	QRHWRAYLRR	KQDQQRYEML	KSSTLIIQSM	FRKWKQRKMQ
1450	1460	1470	1480	1490	1500	1510	1520
SQVKATVILQ	RAFREWHLRK	QAKEENSAII	IQSWYRMHKE	LRKYIYIRSC	VVIIQKRFRC	FQAQKLYKRR	KESILTIQKY
1530	1540	1550	1560	1570	1580	1590	1600
YKAYLKGKIE	RTNYLQKRAA	AIQLQAAFRR	LKAHNLCRQI	RAACVIQSYW	RMRQDRVRFL	NLKKTIIKFQ	AHVRKHQQRQ
1610	1620	1630	1640	1650	1660	1670	1680
KYKKMKKAAV	IIQTHFRAYI	FAMKVLASYQ	KTRSAVIVLQ	SAYRGMQARK	MYIHILTSVI	KIQSYYRAYV	SKKEFLSLKN
1690	1700	1710	1720	1730	1740	1750	1760
ATIKLQSTVK	MKQTRKQYLH	LRAAALFIQQ	CYRSKKIAAQ	KREEYMQMRE	SCIKLQAFVR	GYLVRKQMRL	QRKAVISLQS
1770	1780	1790	1800	1810	1820	1830	1840
YFRMRKARQY	YLKMYKAIIV	IQNYYHAYKA	QVNQRKNFLQ	VKKAATCLQA	AYRGYKVRQL	IKQQSIAALK	IQSAFRGYNK
1850	1860	1870	1880	1890	1900	1910	1920
RVKYQSVLQS	IIKIQRWYRA	YKTLHDTRTH	FLKTKAAVIS	LQSAYRGWKV	RKQIRREHQA	ALKIQSAFRM	AKAQKQFRLF
1930	1940	1950	1960	1970	1980	1990	2000
KTAALVIQQN	FRAWTAGRKQ	CMEYIELRHA	VLVLQSMWKG	KTLRRQLQRQ	HKCAIIIQSY	YRMHVQQKKW	KIMKKAALLI
2010	2020	2030	2040	2050	2060	2070	2080
QKYYRAYSIG	REQNHLYLKT	KAAVVTLQSA	YRGMKVRKRI	KDCNKAAVTI	QSKYRAYKTK	KKYATYRASA	IIIQRWYRGI
2090	2100	2110	2120	2130	2140	2150	2160
KITNHQHKEY	LNLKKTAIKI	QSVYRGIRVR	RHIQHMHRAA	TFIKAMFKMH	QSRISYHTMR	KAAIVIQVRC	RAYYQGKMQR
2170	2180	2190	2200	2210	2220	2230	2240

Detailed Protein Report

Cmpd.	No. of Cmpds.	m/z meas.	Δ m/z [ppm]	z	Rt [min]	Score	P	Range	Sequence	Modification	Ratios
402	1	711.3339	-88.13	2	34.3	14.6	2	178-189	K.TFSVSQKVDRVR.S		
1960	1	731.2880	-192.12	2	54.6	11.0	1	1650-1661	R.KMYIHILTSVIK.I	Oxidation: 2	
1490	1	719.8392	-53.72	2	48.8	11.2	1	3030-3041	R.HRAACLIQAHYR.G		
1961	1	656.7950	-150.69	2	54.9	11.0	2	3182-3193	R.NRAASVIQKAVR.H		Q:W 1.99

Detailed Protein Report

Protein 372: PREDICTED: inositol 1,4,5-trisphosphate receptor type 2 isoform X1 [Homo sapiens]

**Accession:**	gi|578822836	**Score:**	47.6
**Database:**	refseq_human(refseq_human_20140103.fasta)	**MW [kDa]:**	212.9
**Database Date:**	2015-11-30	**pI:**	6.3
**Modification(s):**	Oxidation	**Sequence Coverage [%]:**	2.6
		**No. of unique Peptides:**	3

Quantitation

Q:W	**Median:**	2.69	**CV:**	0.00 %	**No. of Peptides:**	1
M:Q	**Median:**	0.53	**CV:**	0.00 %	**No. of Peptides:**	1

Detailed Protein Report

10	20	30	40	50	60	70	80
MTEKMSSFLY	IGDIVSLYAE	GSVNGFISTL	GLVDDRCVVH	PEAGDLANPP	KKFRDCLFKV	CPMNRYSAQK	QYWKAKQAKQ
90	100	110	120	130	140	150	160
GNHTEAALLK	KLQHAAELEQ	KQNESENKKL	LGEIVKYSNV	IQLLHIKSNK	YLTVNKRLPA	LLEKNAMRVS	LDAAGNEGSW
170	180	190	200	210	220	230	240
FYIHPFWKLR	SEGDNIVVGD	KVVLMPVNAG	QPLHASNIEL	LDNPGCKEVN	AVNCNTSWKI	TLFMKYSSYR	EDVLKGGDVV
250	260	270	280	290	300	310	320
RLFHAEQEKF	LTCDEYEKKQ	HIFLRTTLRQ	SATSATSSKA	LWEIEVVHHD	PCRGGAGQWN	SLFRFKHLAT	GNYLAAELNP
330	340	350	360	370	380	390	400
DYRDAQNEGK	NVRDGVPPTS	KKKRQAGEKI	MYTLVSVPHG	NDIASLFELD	ATTLQRADCL	VPRNSYVRLR	HLCTNTWVTS
410	420	430	440	450	460	470	480
TSIPIDTDEE	RPVMLKIGTC	QTKEDKEAFA	IVSVPLSEVR	DLDFANDANK	VLATTVKKLE	NGTITQNERR	FVTKLLEDLI
490	500	510	520	530	540	550	560
FFVADVPNNG	QEVLDVVITK	PNRERQKLMR	EQNILAQVFG	ILKAPFKEKA	GEGSMLRLED	LGDQRYAPYK	YMLRLCYRVL
570	580	590	600	610	620	630	640
RHSQQDYRKN	QEYIAKNFCV	MQSQIGYDIL	AEDTITALLH	NNRKLLEKHI	TAKEIETFVS	LLRRNREPRF	LDYLSDLCVS
650	660	670	680	690	700	710	720
NTTAIPVTQE	LICKFMLSPG	NADILIQTKV	VSMQADNPME	SSILSDDIDD	EEVWLYWIDS	NKEPHGKAIR	HLAQEAKEGT
730	740	750	760	770	780	790	800
KADLEVLTYY	RYQLNLFARM	CLDRQYLAIN	QISTQLSVDL	ILRCVSDESL	PFDLRASFCR	LMLHMHVDRD	PQESVVPVRY
810	820	830	840	850	860	870	880
ARLWTEIPTK	ITIHEYDSIT	DSSRNDMKRK	FALTMEFVEE	YLKEVVNQPF	PFGDKEKNKL	TFEVVHLARN	LIYFGFYSFS
890	900	910	920	930	940	950	960
ELLRLTRTLL	AILDIVQAPM	SSYFERLSKF	QDGGNNVMRT	IHGVGEMMTQ	MVLSRGSIFP	MSVPDVPPSI	HPSKQGSPTE
970	980	990	1000	1010	1020	1030	1040
HEDVTVMDTK	LKIIEILQFI	LSVRLDYRIS	YMLSIYKKEF	GEDNDNAETS	ASGSPDTLLP	SAIVPDIDEI	AAQAETMFAG
1050	1060	1070	1080	1090	1100	1110	1120
RKEKNPVQLD	DEGGRTFLRV	LIHLIMHDYP	PLLSGALQLL	FKHFSQRAEV	LQAFKQVQLL	VSNQDVDNYK	QIKADLDQLR
1130	1140	1150	1160	1170	1180	1190	1200
LTVEKSELWV	EKSSNYENGE	IGESQVKGGE	EPIEESNILS	PVQDGTKKPQ	IDSNKSNNYR	IVKEILIRLS	KLCVQNKKCR
1210	1220	1230	1240	1250	1260	1270	1280
NQHQRLLKNM	GAHSVVLDLL	QIPYEKNDEK	MNEVMNLAHT	FLQNFCRGNP	QNQVLLHKHL	NLFLTPGLLE	AETMRHIFMN
1290	1300	1310	1320	1330	1340	1350	1360
NYHLCNEISE	RVVQHFVHCI	ETHGRHVEYL	RFLQTIVKAD	GKYVKKCQDM	VMTELINGGE	DVLIFYNDRA	SFPILLHMMC
1370	1380	1390	1400	1410	1420	1430	1440
SERDRGDESG	PLAYHITLVE	LLAACTEGKN	VYTEIKCNSL	LPLDDIVRVV	THDDCIPEVK	IAYVNFVNHC	YVDTEVEMKE
1450	1460	1470	1480	1490	1500	1510	1520
IYTSNHIWKL	FENFLVDMAR	VCNTTTDRKH	ADIFLEKCVT	ESIMNIVSGF	FNSPFSDNST	SLQTHQPVFI	QLLQSAFRIY
1530	1540	1550	1560	1570	1580	1590	1600
NCTWPNPAQK	ASVESCIRTL	AEVAKNRGIA	IPVDLDSQVN	TLFMKSHSNM	VQRAAMGWRL	SARSGPRFKE	ALGGPAWDYR
1610	1620	1630	1640	1650	1660	1670	1680
NIIEKLQDVV	ASLEHQFSPM	MQAEFSVLVD	VLYSPELLFP	EGSDARIRCG	AFMSKLINHT	KKLMEKEEKL	CIKILQTLRE
1690	1700	1710	1720	1730	1740	1750	1760
MLEKKDSFVE	EGNTLRKILL	NRYFKGDYSI	GVNGHLSGAY	SKTAQVGGSF	SGQDSDKMGI	SMSDIQCLLD	KEGASELVID
1770	1780	1790	1800	1810	1820	1830	1840
VIVNTKNDRI	FSEGIFLGIA	LLEGGNTQTQ	YSFYQQLHEQ	KKSEKFFKVL	YDRMKAAQKE	IRSTVTVNTI	DLGNKKRDDD
1850	1860	1870					
NELMTSGPRM	REFWSWGDPK	TALQD					

Cmpd.	No. of Cmpds.	m/z meas.	Δ m/z [ppm]	z	Rt [min]	Score	P	Range	Sequence	Modification	Ratios
725	1	771.2336	-342.03	1	39.0	11.8	0	110-116	K.LLGEIVK.Y		
1690	7	648.2368	-188.91	2	51.4	14.8	2	1199-1208	K.CRNQHQRLLK.N		
1156	1	867.9132	-54.78	2	43.1	10.9	2	1647-1661	R.IRCGAFMSKLINHTK.K	Oxidation: 7	Q:W 2.69<br>M:Q 0.53

Detailed Protein Report

Protein 373: CCR4-NOT transcription complex subunit 1 isoform c [Homo sapiens]

**Accession:**	gi|388454220	**Score:**	47.6
**Database:**	refseq_human(refseq_human_20140103.fasta)	**MW [kDa]:**	266.2
**Database Date:**	2015-11-30	**pI:**	6.6
**Modification(s):**	Oxidation	**Sequence Coverage [%]:**	2.1
		**No. of unique Peptides:**	2

Quantitation

M:Q	**Median:** 1.08	**CV:** 0.00 %	**No. of Peptides:** 1

Detailed Protein Report

10	20	30	40	50	60	70	80
MNLDSLSLAL	SQISYLVDNL	TKKNYRASQQ	EIQHIVNRHG	PEADRHLLRC	LFSHVDFSGD	GKSSGKDFHQ	TQFLIQECAL
90	100	110	120	130	140	150	160
LITKPNFIST	LSYAIDNPLH	YQKSLKPAPH	LFAQLSKVLK	LSKVQEVIFG	LALLNSSSSD	LRGFAAQFIK	QKLPDLLRSY
170	180	190	200	210	220	230	240
IDADVSGNQE	GGFQDIAIEV	LHLLLSHLLF	GQKGAFGVGQ	EQIDAFLKTL	RRDFPQERCP	VVLAPLLYPE	KRDILMDRIL
250	260	270	280	290	300	310	320
PDSGGVAKTM	MESSLADFMQ	EVGYGFCASI	EECRNIIVQF	GVREVTAAQV	ARVLGMMART	HSGLTDGIPL	QSISAPGSGI
330	340	350	360	370	380	390	400
WSDGKDKSDG	AQAHTWNVEV	LIDVLKELNP	SLNFKEVTYE	LDHPGFQIRD	SKGLHNVVYG	IQRGLGMEVF	PVDLIYRPWK
410	420	430	440	450	460	470	480
HAEGQLSFIQ	HSLINPEIFC	FADYPCHTVA	TDILKAPPED	DNREIATWKS	LDLIESLLRL	AEVGQYEQVK	QLFSFPIKHC
490	500	510	520	530	540	550	560
PDMLVLALLQ	INTSWHTLRH	ELISTLMPIF	LGNHPNSAII	LHYAWHGQGQ	SPSIRQLIMH	AMAEWYMRGE	QYDQAKLSRI
570	580	590	600	610	620	630	640
LDVAQDLKAL	SMLLNGTPFA	FVIDLAALAS	RREYLKLDKW	LTDKIREHGE	PFIQACMTFL	KRRCPSILGG	LAPEKDQPKS
650	660	670	680	690	700	710	720
AQLPPETLAT	MLACLQACAG	SVSQELSETI	LTMVANCSNV	MNKARQPPPG	VMPKGRPPSA	SSLDAISPVQ	IDPLAGMTSL
730	740	750	760	770	780	790	800
SIGGSAAPHT	QSMQGFPPNL	GSAFSTPQSP	AKAFPPLSTP	NQTTAFSGIG	GLSSQLPVGG	LGTGSLTGIG	TGALGLPAVN
810	820	830	840	850	860	870	880
NDPFVQRKLG	TSGLNQPTFQ	QTDLSQVWPE	ANQHFSKEID	DEANSYFQRI	YNHPPHPTMS	VDEVLEMLQR	FKDSTIKRER
890	900	910	920	930	940	950	960
EVFNCMLRNL	FEEYRFFPQY	PDKELHITAC	LFGGIIEKGL	VTYMALGLAL	RYVLEALRKP	FGSKMYYFGI	AALDRFKNRL
970	980	990	1000	1010	1020	1030	1040
KDYPQYCQHL	ASISHFMQFP	HHLQEYIEYG	QQSRDPPVKM	QGSITTPGSI	ALAQAQAQAQ	VPAKAPLAGQ	VSTMVTTSTT
1050	1060	1070	1080	1090	1100	1110	1120
TTVAKTVTVT	RPTGVSFKKD	VPPSINTTNI	DTLLVATDQT	ERIVEPPENI	QEKIAFIFNN	LSQSNMTQKV	EELKETVKEE
1130	1140	1150	1160	1170	1180	1190	1200
FMPWVSQYLV	MKRVSIEPNF	HSLYSNFLDT	LKNPEFNKMV	LNETYRNIKV	LLTSDKAAAN	FSDRSLLKNL	GHWLGMITLA
1210	1220	1230	1240	1250	1260	1270	1280
KNKPILHTDL	DVKSLLLEAY	VKGQQELLYV	VPFVAKVLES	SIRSVVFRPP	NPWTMAIMNV	LAELHQEHDL	KLNLKFEIEV
1290	1300	1310	1320	1330	1340	1350	1360
LCKNLALDIN	ELKPGNLLKD	KDRLKNLDEQ	LSAPKKDVKQ	PEELPPITTT	TTSTTPATNT	TCTATVPPQP	QYSYHDINVY
1370	1380	1390	1400	1410	1420	1430	1440
SLAGLAPHIT	LNPTIPLFQA	HPQLKQCVRQ	AIERAVQELV	HPVVDRSIKI	AMTTCEQIVR	KDFALDSEES	RMRIAAHHMM
1450	1460	1470	1480	1490	1500	1510	1520
RNLTAGMAMI	TCREPLLMSI	STNLKNSFAS	ALRTASPQQR	EMMDQAAAQL	AQDNCELACC	FIQKTAVEKA	GPEMDKRLAT
1530	1540	1550	1560	1570	1580	1590	1600
EFELRKHARQ	EGRRYCDPVV	LTYQAERMPE	QIRLKVGGVD	PKQLAVYEEF	ARNVPGFLPT	NDLSQPTGFL	AQPMKQAWAT
1610	1620	1630	1640	1650	1660	1670	1680
DDVAQIYDKC	ITELEQHLHA	IPPTLAMNPQ	AQALRSLLEV	VVLSRNSRDA	IAALGLLQKA	VEGLLDATSG	ADADLLLRYR
1690	1700	1710	1720	1730	1740	1750	1760
ECHLLVLKAL	QDGRAYGSPW	CNKQITRCLI	ECRDEYKYNV	EAVELLIRNH	LVNMQQYDLH	LAQSMENGLN	YMAVAFAMQL
1770	1780	1790	1800	1810	1820	1830	1840
VKILLVDERS	VAHVTEADLF	HTIETLMRIN	AHSRGNAPEG	LPQLMEVVRS	NYEAMIDRAH	GGPNFMMHSG	ISQASEYDDP
1850	1860	1870	1880	1890	1900	1910	1920
PGLREKAEYL	LREWVNLYHS	AAAGRDSTKA	FSAFVGQMHQ	QGILKTDDLI	TRFFRLCTEM	CVEISYRAQA	EQQHNPAANP
1930	1940	1950	1960	1970	1980	1990	2000
TMIRAKCYHN	LDAFVRLIAL	LVKHSGEATN	TVTKINLLNK	VLGIVVGVLL	QDHDVRQSEF	QQLPYHRIFI	MLLLELNAPE
2010	2020	2030	2040	2050	2060	2070	2080
HVLETINFQT	LTAFCNTFHI	LRPTKAPGFV	YAWLELISHR	IFIARMLAHT	PQQKGWPMYA	QLLIDLFKYL	APFLRNVELT
2090	2100	2110	2120	2130	2140	2150	2160
KPMQILYKGT	LRVLLVLLHD	FPEFLCDYHY	GFCDVIPPNC	IQLRNLILSA	FPRNMRLPDP	FTPNLKVDML	SEINIAPRIL
2170	2180	2190	2200	2210	2220	2230	2240

Detailed Protein Report

Cmpd.	No. of Cmpds.	m/z meas.	Δ m/z [ppm]	z	Rt [min]	Score	P	Range	Sequence	Modification	Ratios	
1883	1	946.8565	-104.22	2	53.4	12.0	0	1908-1924	R.AQAEQQHNPAANPTMIR.A	Oxidation: 15		
585	1	603.8101	58.84	2	36.2	10.4	0	2360-2371	K.QAQQVMEGTGAS.-		M:Q	1.08

Detailed Protein Report

Protein 374: **PREDICTED: uncharacterized protein LOC101929845 [Homo sapiens]**

**Accession:**	gi|578845877	**Score:**	47.6
**Database:**	refseq_human(refseq_human_20140103.fasta)	**MW [kDa]:**	90.2
**Database Date:**	2015-11-30	**pI:**	12.2
**Modification(s):**	Carbamidomethyl	**Sequence Coverage [%]:**	9.3
		**No. of unique Peptides:**	2

10	20	30	40	50	60	70	80
MEESAEQAPR	CDETRVGRRR	HSWRNTCGGV	GRAGTAVRRD	AASQSCCPTC	TGVATRRHSW	RRRQSRQTKE	FIFSELLSNL
**90**	**100**	**110**	**120**	**130**	**140**	**150**	**160**
YSRGNEQTLV	EALAEQADQG	VHLLRAAVQP	ALAWEREDTR	GGVGRAGRPR	SSSSQSCCPN	CTRVGTRRHS	WRSRQSRHRG
**170**	**180**	**190**	**200**	**210**	**220**	**230**	**240**
VTRLAWEEED	TPGGVGRAGR	PRSSSSRSCC	PTCTRVGTCR	HSWRRRQSRQ	TKEFIFSELL	SNLYSRGNEK	TLVEASAEQA
**250**	**260**	**270**	**280**	**290**	**300**	**310**	**320**
DQGVHLLRAA	VQPVLAWERE	DTRGGVGRAG	RPRSSSSQSC	CPTCTRVGTR	RHSWRSRPSR	HSGVTRLAWE	EEDTPGGVGR
**330**	**340**	**350**	**360**	**370**	**380**	**390**	**400**
AGRPRSSSSR	SCCPTCTRVG	TCRHLWRSRQ	SRHSGATRCC	FSELLSNLHW	RGNEKTLVEA	LAEQADQGVH	LLRAAVQPVL
**410**	**420**	**430**	**440**	**450**	**460**	**470**	**480**
AWERADTRGG	VGRAGRPRSS	SSQSCCPTCT	RVGTRRHSWR	RRQSRQTKEF	IFSELLSKLH	SRGNEKTLME	ESAEQAQRCD
**490**	**500**	**510**	**520**	**530**	**540**	**550**	**560**
ETRVGRRRHS	WRSRQSRQTK	KFIFSELLSN	LYSRRNLQTL	VEASAEQADQ	GVHLLRAAVQ	PVLAWEREDT	RGGVGRAGRP
**570**	**580**	**590**	**600**	**610**	**620**	**630**	**640**
RSSSSQSCCP	TCTRVGTRRH	SWRRRQSRQT	KEFIFSELLS	NLHSRGNEKT	LMEESAEQAQ	RRDETRVGRR	RHSWRSRQSR
**650**	**660**	**670**	**680**	**690**	**700**	**710**	**720**
QTKEFIFSEL	LSNLYSRRNL	QTLVEESAEQ	AQRCDKMLLL	RAAVQPALAW	EREDTRGGVG	RAGRPRSSSS	QSCCPTCPRV
**730**	**740**	**750**	**760**	**770**	**780**	**790**	**800**
GTRRHSWRRR	QSRQTKEFIF	SELLSNLHSR	GNEKTLVEAS	AEQADQGVHL	LRAAVQTALA	WEREDTHGGV	GRAGTEV

Cmpd.	No. of Cmpds.	m/z meas.	Δ m/z [ppm]	z	Rt [min]	Score	P	Range	Sequence	Modification
2221	1	1032.3950	-68.06	2	56.4	11.4	2	1-18	-.MEESAEQAPRCDETRVGR.R	
48	1	1220.0219	-37.76	2	30.0	11.4	2	33-56	R.AGTAVRRDAASQSCCPTCTGVATR.R	Carbamidomethyl: 14

Detailed Protein Report

Protein 375: mis18-binding protein 1 [Homo sapiens]

**Accession:**	gi|42415492	**Score:**	47.5
**Database:**	refseq_human(refseq_human_20140103.fasta)	**MW [kDa]:**	129.0
**Database Date:**	2015-11-30	**pI:**	9.9
**Modification(s):**	Oxidation	**Sequence Coverage [%]:**	5.6
		**No. of unique Peptides:**	3

Alias proteins:

Accession	Name	Description
gi|530404134	refseq_human (refseq_human_20140103.fasta)	PREDICTED: mis18-binding protein 1 isoform X1 [Homo sapiens]

10	20	30	40	50	60	70	80
MIATPLKHSR	IYLPPEASSQ	RRNLPMDAIF	FDSIPSGTLT	PVKDLVKYQN	SSLKLNDHKK	NQFLKMTTFN	NKNIFQSTML
**90**	**100**	**110**	**120**	**130**	**140**	**150**	**160**
TEATTSNSSL	DISAIKPNKD	GLKNKANYES	PGKIFLRMKE	KVLRDKQEQP	SRNSSLLEPQ	KSGNNETFTP	NRVEKKKLQH
**170**	**180**	**190**	**200**	**210**	**220**	**230**	**240**
TYLCEEKENN	KSFQSDDSSL	RASVQGVPLE	SSNNDIFLPV	KQKIQCQQEK	KAPLHNLTYE	LPTLNQEQEN	FLAVEARNKT
**250**	**260**	**270**	**280**	**290**	**300**	**310**	**320**
LTRAQLAKQI	FHSKESIVAT	TKSKKDTFVL	ESVDSADEQF	QNTNAETLST	NCIPIKNGSL	LMVSDSERTT	EGTSQQKVKE
**330**	**340**	**350**	**360**	**370**	**380**	**390**	**400**
GNGKTVPGET	GLPGSMKDTC	KIVLATPRLH	ITIPRRSKRN	ISKLSPPRIF	QTVTNGLKKN	QVVQLQEWMI	KSINNNTAIC
**410**	**420**	**430**	**440**	**450**	**460**	**470**	**480**
VEGKLIDVTN	IYWHSNVIIE	RIEHNKLRTI	SGNVYILKGM	IDQISMKEAG	YPNYLIRKFM	FGFPENWKEH	IDNFLEQLRA
**490**	**500**	**510**	**520**	**530**	**540**	**550**	**560**
GEKNREKTKQ	KQKTGRSVRD	IRKSMKNDAR	ENQTDTAQRA	TTTYDFDCDN	LELKSNKHSE	SPGATELNMC	HSNCQNKPTL
**570**	**580**	**590**	**600**	**610**	**620**	**630**	**640**
RFPDDQVNNT	IQNGGGDDLS	NQELIGKKEY	KMSSKKLKIG	ERTNERIIKS	QKQETTEELD	VSIDILTSRE	QFFSDEERKY
**650**	**660**	**670**	**680**	**690**	**700**	**710**	**720**
MAINQKKAYI	LVTPLKSRKV	IEQRCMRYNL	SAGTIKAVTD	FVIPECQKKS	PISKSMGTLE	NTFEGHKSKN	KEDCDERDLL
**730**	**740**	**750**	**760**	**770**	**780**	**790**	**800**
TVNRKIKISN	LEKEQMLTSD	FKKNTRLLPK	LKKIENQVAM	SFYKHQSSPD	LSSEESETEK	EIKRKAEVKK	TKAGNTKEAV
**810**	**820**	**830**	**840**	**850**	**860**	**870**	**880**
VHLRKSTRNT	SNIPVILEPE	TEESENEFYI	KQKKARPSVK	ETLQKSGVRK	EFPITEAVGS	DKTNRHPLEC	LPGLIQDKEW
**890**	**900**	**910**	**920**	**930**	**940**	**950**	**960**
NEKELQKLHC	AFASLPKHKP	GFWSEVAAAV	GSRSPEECQR	KYMENPRGKG	SQKHVTKKKP	ANSKGQNGKR	GDADQKQTIK
**970**	**980**	**990**	**1000**	**1010**	**1020**	**1030**	**1040**
ITAKVGTLKR	KQQMREFLEQ	LPKDDHDDFF	STTPLQHQRI	LLPSFQDSED	DDDILPNMDK	NPTTPSSVIF	PLVKTPQCQH
**1050**	**1060**	**1070**	**1080**	**1090**	**1100**	**1110**	**1120**
VSPGMLGSIN	RNDCDKYVFR	MQKYHKSNGG	IVWGNIKKKL	VETDFSTPTP	RRKTPFNTDL	GENSGIGKLF	TNAVESLDEE
**1130**	**1140**						
EKDYYFSNSD	SA						

Cmpd.	No. of Cmpds.	m/z meas.	Δ m/z [ppm]	z	Rt [min]	Score	P	Range	Sequence	Modification
1865	1	1057.5367	-22.09	2	52.0	12.7	0	182-201	R.ASVQGVPLESSNNDIFLPVK.Q	
2837	1	868.9237	11.97	2	64.0	11.5	1	325-341	K.TVPGETGLPGSMKDTCK.I	Oxidation: 12
2772	1	733.8764	59.69	2	63.7	13.0	0	695-707	K.SMGTLENTFEGHK.S	Oxidation: 2

Detailed Protein Report

Protein 376: staphylococcal nuclease domain-containing protein 1 [Homo sapiens]

**Accession:**	gi|77404397	**Score:**	47.5
**Database:**	refseq_human(refseq_human_20140103.fasta)	**MW [kDa]:**	101.9
**Database Date:**	2015-11-30	**pI:**	6.8
**Modification(s):**	Oxidation	**Sequence Coverage [%]:**	5.9
		**No. of unique Peptides:**	1

Quantitation

M:Q **Median:** 0.50 **CV:** 0.00 % **No. of Peptides:** 1

10	20	30	40	50	60	70	80
MASSAQSGGS	SGGPAVPTVQ	RGIIKMVLSG	CAIIVRGQPR	GGPPPERQIN	LSNIRAGNLA	RRAAATQPDA	KDTPDEPWAF
90	100	110	120	130	140	150	160
PAREFLRKKL	IGKEVCFTIE	NKTPQGREYG	MIYLGKDTNG	ENIAESLVAE	GLATRREGMR	ANNPEQNRLS	ECEEQAKAAK
170	180	190	200	210	220	230	240
KGMWSEGNGS	HTIRDLKYTI	ENPRHFVDSH	HQKPVNAIIE	HVRDGSVVRA	LLLPDYYLVT	VMLSGIKCPT	FRREADGSET
250	260	270	280	290	300	310	320
PEPFAAEAKF	FTESRLLQRD	VQIILESCHN	QNILGTILHP	NGNITELLLK	EGFARCVDWS	IAVYTRGAEK	LRAAERFAKE
330	340	350	360	370	380	390	400
RRLRIWRDYV	APTANLDQKD	KQFVAKVMQV	LNADAIVVKL	NSGDYKTIHL	SSIRPPRLEG	ENTQDKNKKL	RPLYDIPYMF
410	420	430	440	450	460	470	480
EAREFLRKKL	IGKKVNVTVD	YIRPASPATE	TVPAFSERTC	ATVTIGGINI	AEALVSKGLA	TVIRYRQDDD	QRSSHYDELL
490	500	510	520	530	540	550	560
AAEARAIKNG	KGLHSKKEVP	IHRVADISGD	TQKAKQFLPF	LQRAGRSEAV	VEYVFSGSRL	KLYLPKETCL	ITFLLAGIEC
570	580	590	600	610	620	630	640
PRGARNLPGL	VQEGEPFSEE	ATLFTKELVL	QREVEVEVES	MDKAGNFIGW	LHIDGANLSV	LLVEHALSKV	HFTAERSSYY
650	660	670	680	690	700	710	720
KSLLSAEEAA	KQKKEKVWAH	YEEQPVEEVM	PVLEEKERSA	SYKPVFVTEI	TDDLHFYVQD	VETGTQLEKL	MENMRNDIAS
730	740	750	760	770	780	790	800
HPPVEGSYAP	RRGEFCIAKF	VDGEWYRARV	EKVESPAKIH	VFYIDYGNRE	VLPSTRLGTL	SPAFSTRVLP	AQATEYAFAF
810	820	830	840	850	860	870	880
IQVPQDDDAR	TDAVDSVVRD	IQNTQCLLNV	EHLSAGCPHV	TLQFADSKGD	VGLGLVKEGL	VMVEVRKEKQ	FQKVITEYLN
890	900	910	920				
AQESAKSARL	NLWRYGDFRA	DDADEFGYSR					

Cmpd.	No. of Cmpds.	m/z meas.	Δ m/z [ppm]	z	Rt [min]	Score	P	Range	Sequence	Modification	Ratios	
2148	1	966.3470	-130.61	2	56.7	12.8	2	161-177	K.KGMWSEGNGSHTIRDLK.Y	Oxidation: 3	M:Q	0.50

Detailed Protein Report

Protein 377: **PREDICTED: serine/threonine-protein kinase MRCK alpha isoform X7 [Homo sapiens]**

**Accession:**	gi|530366603	**Score:**	47.5
**Database:**	refseq_human(refseq_human_20140103.fasta)	**MW [kDa]:**	198.5
**Database Date:**	2015-11-30	**pI:**	5.8
		**Sequence Coverage [%]:**	3.4
		**No. of unique Peptides:**	2

10	20	30	40	50	60	70	80
MSGEVRLRQL	EQFILDGPAQ	TNGQCFSVET	LLDILICLYD	ECNNSPLRRE	KNILEYLEWA	KPFTSKVKQM	RLHREDFEIL
90	100	110	120	130	140	150	160
KVIGRGAFGE	VAVVKLKNAD	KVFAMKILNK	WEMLKRAETA	CFREERDVLV	NGDNKWITTL	HYAFQDDNNL	YLVMDYYVGG
170	180	190	200	210	220	230	240
DLLTLLSKFE	DRLPEDMARF	YLAEMVIAID	SVHQLHYVHR	DIKPDNILMD	MNGHIRLADF	GSCLKLMEDG	TVQSSVAVGT
250	260	270	280	290	300	310	320
PDYISPEILQ	AMEDGKGRYG	PECDWWSLGV	CMYEMLYGET	PFYAESLVET	YGKIMNHKER	FQFPAQVTDV	SENAKDLIRR
330	340	350	360	370	380	390	400
LICSREHRLG	QNGIEDFKKH	PFFSGIDWDN	IRNCEAPYIP	EVSSPTDTSN	FDVDDDCLKN	SETMPPPTHT	AFSGHHLPFV
410	420	430	440	450	460	470	480
GFTYTSSCVL	SDRSCLRVTA	GPTSLDLDVN	VQRTLDNNLA	TEAYERRIKR	LEQEKLELSR	KLQESTQTVQ	ALQYSTVDGP
490	500	510	520	530	540	550	560
LTASKDLEIK	NLKEEIEKLR	KQVTESSHLE	QQLEEANAVR	QELDDAFRQI	KAYEKQIKTL	QQEREDLNKL	EVHTEALAAE
570	580	590	600	610	620	630	640
ASKDRKLREQ	SEHYSKQLEN	ELEGLKQKQI	SYSPGVCSIE	HQQEITKLKT	DLEKKSIFYE	EELSKREGIH	ANEIKNLKKE
650	660	670	680	690	700	710	720
LHDSEGQQLA	LNKEIMILKD	KLEKTRRESQ	SEREEFESEF	KQQYEREKVL	LTEENKKLTS	ELDKLTTLYE	NLSIHNQQLE
730	740	750	760	770	780	790	800
EEVKDLADKK	ESVAHWEAQI	TEIIQWVSDE	KDARGYLQAL	ASKMTEELEA	LRNSSLGTRA	TDMPWKMRRF	AKLDMSARLE
810	820	830	840	850	860	870	880
LQSALDAEIR	AKQAIQEELN	KVKASNIITE	CKLKDSEKKN	LELLSEIEQL	IKDTEELRSE	KGIEHQDSQH	SFLAFLNTPT
890	900	910	920	930	940	950	960
DALDQFEDSF	SSSSSSLIDF	LDDTDPVENT	YVWNPSVKFH	IQSRSTSPST	SSEAEPVKTV	DSTPLSVHTP	TLRKKGCPGS
970	980	990	1000	1010	1020	1030	1040
TGFPPKRKTH	QFFVKSFTTP	TKCHQCTSLM	VGLIRQGCSC	EVCGFSCHIT	CVNKAPTTCP	VPPEQTKGPL	GIDPQKGIGT
1050	1060	1070	1080	1090	1100	1110	1120
AYEGHVRIPK	PAGVKKGWQR	ALAIVCDFKL	FLYDIAEGKA	SQPSVVISQV	IDMRDEEFSV	SSVLASDVIH	ASRKDIPCIF
1130	1140	1150	1160	1170	1180	1190	1200
RVTASQLSAS	NNKCSILMLA	DTENEKNKWV	GVLSELHKIL	KKNKFRDRSV	YVPKEAYDST	LPLIKTTQAA	AIIDHERIAL
1210	1220	1230	1240	1250	1260	1270	1280
GNEEGLFVVH	VTKDEIIRVG	DNKKIHQIEL	IPNDQLVAVI	SGRNRHVRLF	PMSALDGRET	DFYKLSETKG	CQTVTSGKVR
1290	1300	1310	1320	1330	1340	1350	1360
HGALTCLCVA	MKRQVLCYEL	FQSKTRHRKF	KEIQVPYNVQ	WMAIFSEQLC	VGFQSGFLRY	PLNGEGNPYS	MLHSNDHTLS
1370	1380	1390	1400	1410	1420	1430	1440
FIAHQPMDAI	CAVEISSKEY	LLCFNSIGIY	TDCQGRRSRQ	QELMWPANPS	SCCYNAPYLS	VYSENAVDIF	DVNSMEWIQT
1450	1460	1470	1480	1490	1500	1510	1520
LPLKKVRPLN	NEGSLNLLGL	ETIRLIYFKN	KMAEGDELVV	PETSDNSRKQ	MVRNINNKRR	YSFRVPEEER	MQQRREMLRD
1530	1540	1550	1560	1570	1580	1590	1600
PEMRNKLISN	PTNFNHIAHM	GPGDGIQILK	DLPMPGFPYP	SPHHHSGLIS	SPINFEHIYH	MTVNSAEKFL	SPDSINPEYS
1610	1620	1630	1640	1650	1660	1670	1680
PSLRSVPGTP	SFMTLRNPRP	QESRTVFSGS	VSIPSITKSR	PEPGRSMSAS	SGLSARSSAQ	NGSALKREFS	GGSYSAKRQP
1690	1700	1710	1720	1730	1740	1750	1760
MPSPSEGSLS	SGGMDQGSDA	PARDFDGEDS	DSPRHSTASN	SSNLSSPPSP	ASPRKTKSLS	LESTDRGSWD	P

Cmpd.	No. of Cmpds.	m/z meas.	Δ m/z [ppm]	z	Rt [min]	Score	P	Range	Sequence	Modification
249	2	679.3367	-48.57	2	32.5	11.4	0	799-810	R.LELQSALDAEIR.A	
2023	3	703.7681	-96.27	2	53.7	13.5	0	925-938	R.STSPSTSSEAEPVK.T	

Detailed Protein Report

Protein 378: OTU domain-containing protein 7B [Homo sapiens]

Accession: gi|118026942
Database: refseq_human(refseq_human_20140103.fasta)
Database Date: 2015-11-30
Modification(s): Oxidation

Score: 47.5
MW [kDa]: 92.5
pI: 6.3
Sequence Coverage [%]: 6.2
No. of unique Peptides: 2

Alias proteins:

Accession	Name	Description
gi|578801304	refseq_human (refseq_human_20140103.fasta)	PREDICTED: OTU domain-containing protein 7B isoform X5 [Homo sapiens]
gi|578801302	refseq_human (refseq_human_20140103.fasta)	PREDICTED: OTU domain-containing protein 7B isoform X4 [Homo sapiens]
gi|578801300	refseq_human (refseq_human_20140103.fasta)	PREDICTED: OTU domain-containing protein 7B isoform X3 [Homo sapiens]

10	20	30	40	50	60	70	80
MTLDMDAVLS	DFVRSTGAEP	GLARDLLEGK	NWDVNAALSD	FEQLRQVHAG	NLPPSFSEGS	GGSRTPEKGF	SDREPTRPPR
90	100	110	120	130	140	150	160
PILQRQDDIV	QEKRLSRGIS	HASSSIVSLA	RSHVSSNGGG	GGSNEHPLEM	PICAFQLPDL	TVYNEDFRSF	IERDLIEQSM
170	180	190	200	210	220	230	240
LVALEQAGRL	NWWVSVDPTS	QRLLPLATTG	DGNCLLHAAS	LGMWGFHDRD	LMLRKALYAL	MEKGVEKEAL	KRRWRWQQTQ
250	260	270	280	290	300	310	320
QNKESGLVYT	EDEWQKEWNE	LIKLASSEPR	MHLGTNGANC	GGVESSEEPV	YESLEEFHVF	VLAHVLRRPI	VVVADTMLRD
330	340	350	360	370	380	390	400
SGGEAFAPIP	FGGIYLPLEV	PASQCHRSPL	VLAYDQAHFS	ALVSMEQKEN	TKEQAVIPLT	DSEYKLLPLH	FAVDPGKGWE
410	420	430	440	450	460	470	480
WGKDDSDNVR	LASVILSLEV	KLHLLHSYMN	VKWIPLSSDA	QAPLAQPESP	TASAGDEPRS	TPESGDSDKE	SVGSSSTSNE
490	500	510	520	530	540	550	560
GGRRKEKSKR	DREKDKKRAD	SVANKLGSFG	KTLGSKLKKN	MGGLMHSKGS	KPGGVGTGLG	GSSGTETLEK	KKKNSLKSWK
570	580	590	600	610	620	630	640
GGKEEAAGDG	PVSEKPPAES	VGNGGSKYSQ	EVMQSLSILR	TAMQGEGKFI	FVGTLKMGHR	HQYQEEMIQR	YLSDAEERFL
650	660	670	680	690	700	710	720
AEQKQKEAER	KIMNGGIGGG	PPPAKKPEPD	AREEQPTGPP	AESRAMAFST	GYPGDFTIPR	PSGGGVHCQE	PRRQLAGGPC
730	740	750	760	770	780	790	800
VGGLPPYATF	PRQCPPGRPY	PHQDSIPSLE	PGSHSKDGLH	RGALLPPPYR	VADSYSNGYR	EPPEPDGWAG	GLRGLPPTQT
810	820	830	840	850			
KCKQPNCSFY	GHPETNNFCS	CCYREELRRR	EREPDGELLV	HRF			

Cmpd.	No. of Cmpds.	m/z meas.	Δ m/z [ppm]	z	Rt [min]	Score	P	Range	Sequence	Modification
2718	1	1175.5515	-33.60	2	62.4	11.6	0	348-368	R.SPLVLAYDQAHFSALVSMEQK.E	Oxidation: 18
2130	1	1052.4295	-112.31	2	56.8	17.0	1	529-551	K.GSKPGGVGTGLGGSSGTETLEKK.K	

Detailed Protein Report

Protein 379: MAP/microtubule affinity-regulating kinase 4 isoform 2 [Homo sapiens]

Accession: gi|33636756
Database: refseq_human(refseq_human_20140103.fasta)
Database Date: 2015-11-30

Score: 47.4
MW [kDa]: 75.3
pI: 10.5
Sequence Coverage [%]: 3.8
No. of unique Peptides: 1

Quantitation

M:Q **Median:** 0.40 **CV:** 0.00 % **No. of Peptides:** 1

10	20	30	40	50	60	70	80
MSSRTVLAPG	NDRNSDTHGT	LGSGRSSDKG	PSWSSRSLGA	RCRNSIASCP	EEQPHVGNYR	LLRTIGKGNF	AKVKLARHIL
90	100	110	120	130	140	150	160
TGREVAIKII	DKTQLNPSSL	QKLFREVRIM	KGLNHPNIVK	LFEVIETEKT	LYLVMEYASA	GEVFDYLVSH	GRMKEKEARA
170	180	190	200	210	220	230	240
KFRQIVSAVH	YCHQKNIVHR	DLKAENLLLD	AEANIKIADF	GFSNEFTLGS	KLDTFCGSPP	YAAPELFQGK	KYDGPEVDIW
250	260	270	280	290	300	310	320
SLGVILYTLV	SGSLPFDGHN	LKELRERVLR	GKYRVPFYMS	TDCESILRRF	LVLNPAKRCT	LEQIMKDKWI	NIGYEGEELK
330	340	350	360	370	380	390	400
PYTEPEEDFG	DTKRIEVMVG	MGYTREEIKE	SLTSQKYNEV	TATYLLLGRK	TEEGGDRGAP	GLALARVRAP	SDTTNGTSSS
410	420	430	440	450	460	470	480
KGTSHSKGQR	SSSSTYHRQR	RHSDFCGPSP	APLHPKRSPT	STGEAELKEE	RLPGRKASCS	TAGSGSRGLP	PSSPMVSSAH
490	500	510	520	530	540	550	560
NPNKAEIPER	RKDSTSTPNN	LPPSMMTRRN	TYVCTERPGA	ERPSLLPNGK	ENSSGTPRVP	PASPSSHSLA	PPSGERSRLA
570	580	590	600	610	620	630	640
RGSTIRSTFH	GGQVRDRRAG	GGGGGGVQNG	PPASPTLAHE	AAPLPAGRPR	PTTNLFTKLT	SKLTRRVTLD	PSKRQNSNRC
650	660	670	680	690			
VSGASLPQGS	KIRSQTNLRE	SGDLRSQVAI	YLGIKRKPPP	GCSDSPGV			

Cmpd.	No. of Cmpds.	m/z meas.	Δ m/z [ppm]	z	Rt [min]	Score	P	Range	Sequence	Modification	Ratios	
1367	1	388.0811	-379.40	2	45.6	16.7	1	106-111	R.EVRIMK.G		M:Q	0.40

Detailed Protein Report

Protein 380: AT-hook DNA-binding motif-containing protein 1 [Homo sapiens]

**Accession:**	gi|71274144	**Score:**	47.4
**Database:**	refseq_human(refseq_human_20140103.fasta)	**MW [kDa]:**	168.2
**Database Date:**	2015-11-30	**pI:**	10.0
**Modification(s):**	Oxidation	**Sequence Coverage [%]:**	4.1
		**No. of unique Peptides:**	2

Quantitation

Q:W	**Median:**	1.74	**CV:**	0.00 %	**No. of Peptides:**	1
M:Q	**Median:**	2.02	**CV:**	0.00 %	**No. of Peptides:**	1

Alias proteins:

Accession	Name	Description
gi|530361060	refseq_human (refseq_human_20140103.fasta)	PREDICTED: AT-hook DNA-binding motif-containing protein 1 isoform X6 [Homo sapiens]
gi|530361058	refseq_human (refseq_human_20140103.fasta)	PREDICTED: AT-hook DNA-binding motif-containing protein 1 isoform X5 [Homo sapiens]
gi|530361056	refseq_human (refseq_human_20140103.fasta)	PREDICTED: AT-hook DNA-binding motif-containing protein 1 isoform X4 [Homo sapiens]
gi|530361054	refseq_human (refseq_human_20140103.fasta)	PREDICTED: AT-hook DNA-binding motif-containing protein 1 isoform X3 [Homo sapiens]
gi|530361052	refseq_human (refseq_human_20140103.fasta)	PREDICTED: AT-hook DNA-binding motif-containing protein 1 isoform X2 [Homo sapiens]
gi|530361050	refseq_human (refseq_human_20140103.fasta)	PREDICTED: AT-hook DNA-binding motif-containing protein 1 isoform X1 [Homo sapiens]

Detailed Protein Report

10	20	30	40	50	60	70	80
MRVKPQGLVV	TSSAVCSSPD	YLREPKYYPG	GPPTPRPLLP	TRPPASPPDK	AFSTHAFSEN	PRPPPRRDPS	TRRPPVLAKG
90	100	110	120	130	140	150	160
DDPLPPRAAR	PVSQARCPTP	VGDGSSSRRC	WDNGRVNLRP	VVQLIDIMKD	LTRLSQDLQH	SGVHLDCGGL	RLSRPPAPPP
170	180	190	200	210	220	230	240
GDLQYSFFSS	PSLANSIRSP	EERATPHAKS	ERPSHPLYEP	EPEPRDSPQP	GQGHSPGATA	AATGLPPEPE	PDSTDYSELA
250	260	270	280	290	300	310	320
DADILSELAS	LTCPEAQLLE	AQALEPPSPE	PEPQLLDPQP	RFLDPQALEP	LGEALELPPL	QPLADPLGLP	GLALQALDTL
330	340	350	360	370	380	390	400
PDSLESQLLD	PQALDPLPKL	LDVPGRRLEP	QQPLGHCPLA	EPLRLDLCSP	HGPPGPEGHP	KYALRRTDRP	KILCRRRKAG
410	420	430	440	450	460	470	480
RGRKADAGPE	GRLLPLPMPT	GLVAALAEPP	PPPPPPPPAL	PGPGPVSVPE	LKPESSQTPV	VSTRKGKCRG	VRRMVVKMAK
490	500	510	520	530	540	550	560
IPVSLGRRNK	TTYKVSSLSS	SLSVEGKELG	LRVSAEPTPL	LKMKNNGRNV	VVVFPPGEMP	IILKRKRGRP	PKNLLLGPGK
570	580	590	600	610	620	630	640
PKEPAVVAAE	AATVAAATMA	MPEVKKRRRR	KQKLASPQPS	YAADANDSKA	EYSDVLAKLA	FLNRQSQCAG	RCSPPRCWTP
650	660	670	680	690	700	710	720
SEPESVHQAP	DTQSISHFLH	RVQGFRRRGG	KAGGFGGRGG	GHAAKSARCS	FSDFFEGIGK	KKKVVAVAAA	GVGGPGLTEL
730	740	750	760	770	780	790	800
GHPRKRGRGE	VDAVTGKPKR	KRRSRKNGTL	FPEQVPSGPG	FGEAGAEWAG	DKGGGWAPHH	GHPGGQAGRN	CGFQGTEARA
810	820	830	840	850	860	870	880
FASTGLESGA	SGRGSYYSTG	APSGQTELSQ	ERQNLFTGYF	RSLLDSDDSS	DLLDFALSAS	RPESRKASGT	YAGPPTSALP
890	900	910	920	930	940	950	960
AQRGLATFPS	RGAKASPVAV	GSSGAGADPS	FQPVLSARQT	FPPGRAASYG	LTPAASDCRA	AETFPKLVPP	PSAMARSPTT
970	980	990	1000	1010	1020	1030	1040
HPPANTYLPQ	YGGYGAGQSV	FAPTKPFTGQ	DCANSKDCSF	AYGSGNSLPA	SPSSAHSAGY	APPPTGGPCL	PPSKASFFSS
1050	1060	1070	1080	1090	1100	1110	1120
SEGAPFSGSA	PTPLRCDSRA	STVSPGGYMV	PKGTTASATS	AASAASSSSS	SFQPSPENCR	QFAGASQWPF	RQGYGGLDWA
1130	1140	1150	1160	1170	1180	1190	1200
SEAFSQLYNP	SFDCHVSEPN	VILDISNYTP	QKVKQQTAVS	ETFSESSSDS	TQFNQPVGGG	GFRRANSEAS	SSEGQSSLSS
1210	1220	1230	1240	1250	1260	1270	1280
LEKLMMDWNE	ASSAPGYNWN	QSVLFQSSSK	PGRGRRKKVD	LFEASHLGFP	TSASAAASGY	PSKRSTGPRQ	PRGGRGGGAC
1290	1300	1310	1320	1330	1340	1350	1360
SAKKERGGAA	AKAKFIPKPQ	PVNPLFQDSP	DLGLDYYSGD	SSMSPLPSQS	RAFGVGERDP	CDFIGPYSMN	PSTPSDGTFG
1370	1380	1390	1400	1410	1420	1430	1440
QGFHCDSPSL	GAPELDGKHF	PPLAHPPTVF	DAGLQKAYSP	TCSPTLGFKE	ELRPPPTKLA	ACEPLKHGLQ	GASLGHAAAA
1450	1460	1470	1480	1490	1500	1510	1520
QAHLSCRDLP	LGQPHYDSPS	CKGTAYWYPP	GSAARSPPYE	GKVGTGLLAD	FLGRTEAACL	SAPHLASPPA	TPKADKEPLE
1530	1540	1550	1560	1570	1580	1590	1600
MARPPGPPRG	PAAAAAGYGC	PLLSDLTLSP	VPRDSLLPLQ	DTAYRYPGFM	PQAHPGLGGG	PKSGFLGPMA	EPHPEDTFTV
1610							
TSL							

Cmpd.	No. of Cmpds.	m/z meas.	Δ m/z [ppm]	z	Rt [min]	Score	P	Range	Sequence	Modification	Ratios	
2595	1	665.2784	-146.69	2	60.7	10.1	1	513-524	R.VSAEPTPLLKMK.N	Oxidation: 11		
1735	1	945.9132	-52.92	2	50.2	11.2	1	592-609	K.QKLASPQPSYAADANDSK.A		M:Q<br>Q:W	2.02<br>1.74

Detailed Protein Report

Protein 381: SCO-spondin precursor [Homo sapiens]

**Accession:**	gi|134031945	**Score:**	47.3
**Database:**	refseq_human(refseq_human_20140103.fasta)	**MW [kDa]:**	547.1
**Database Date:**	2015-11-30	**pI:**	5.6
**Modification(s):**	Carbamidomethyl	**Sequence Coverage [%]:**	1.2
		**No. of unique Peptides:**	3

Quantitation

Q:W	**Median:**	2.74	**CV:**	0.00 %	**No. of Peptides:**	1
M:Q	**Median:**	0.55	**CV:**	21.30 %	**No. of Peptides:**	2

Detailed Protein Report

10	20	30	40	50	60	70	80
MLLPALLFGM	AWALADGRWC	EWTETIRVEE	EVAPRQEDLV	PCASLDHYSR	LGWRLDLPWS	GRSGLTRSPA	PGLCPIYKPP
90	100	110	120	130	140	150	160
ETRPAKWNRT	VRTCCPGWGG	AHCTEALAKA	SPEGHCFAMW	QCQLQAGSAN	ASAGSLEECC	ARPWGQSWWD	GSSQACRSCS
170	180	190	200	210	220	230	240
SRHLPGSASS	PALLQPLAGA	VGQLWSQHQR	PSATCASWSG	FHYRTFDGRH	YHFLGRCTYL	LAGAADSTWA	VHLTPGDRCP
250	260	270	280	290	300	310	320
QPGHCQRVTM	GPEEVLIQAG	NVSVKGQLVP	EGQSWLLHGL	SLQWLGDWLV	LSGGLGVVVR	LDRTGSISIS	VDHELWGQTQ
330	340	350	360	370	380	390	400
GLCGLYNGWP	EDDFMEPGGG	LAMLAATFGN	SWRLPGSESG	CLDAVEVAQG	CDPLGLIDAD	VEPGHLRAEA	QDVCHQLLEG
410	420	430	440	450	460	470	480
PFGQCHAQVS	PAEYHEACLF	AYCAGAMAGS	GQEGRQQAVC	ATFASYVQAC	ARRHIHIRWR	KPGFCERLCP	GGQLYSDCVS
490	500	510	520	530	540	550	560
LCPPSCEAVG	QGEEESCREE	CVSGCECPRG	LFWNGTLCVP	AAHCPCYYCR	QRYVPGDTVR	QLCNPCVCRD	GRWHCAQALC
570	580	590	600	610	620	630	640
PAECAVGGDG	HYLTFDGRSY	SFWGGQGCRY	SLVQDYVKGQ	LLILLEHGAC	DAGSCLHAIS	VSLEDTHIQL	RDSGAVLVNG
650	660	670	680	690	700	710	720
QDVGLPWIGA	EGLSVRRASS	AFLLLRWPGA	QVLWGLSDPV	AYITLDPRHA	HQVQGLCGTF	TQNQQDDFLT	PAGDVETSIA
730	740	750	760	770	780	790	800
AFASKFQVAG	KGRCPSEDSA	LLSPCTTHSQ	RHAFAEAACA	ILHSSVFQEC	HRLVDKEPFY	LRCLAAVCGC	DPGSDCLCPV
810	820	830	840	850	860	870	880
LSAYARRCAQ	EGASPPWRNQ	TLCPVMCPGG	QEYRECAPAC	GQHCGKPEDC	GELGSCVAGC	NCPLGLLWDP	EGQCVPPSLC
890	900	910	920	930	940	950	960
PCQLGARRYA	PGSATMKECN	RCICQERGLW	NCTARHCPSQ	AFCPRELVYA	PGACLLTCDS	PSANHSCPAG	STDGCVCPPG
970	980	990	1000	1010	1020	1030	1040
TVLLDERCVP	PDLCPCRHSG	QWYLPNATIQ	EDCNVCVCRG	RQWHCTGQRR	SGRCQASGAP	HYVTFDGLAF	TYPGACEYLL
1050	1060	1070	1080	1090	1100	1110	1120
VREASGLFTV	SAQNLPCGAS	GLTCTKALAV	RLEGTVVHML	RGRAVTVNGV	SVTPPKVYTG	PGLSLRRAGL	FLLLSTHLGL
1130	1140	1150	1160	1170	1180	1190	1200
TLLWDGGTRV	LVQLSPQFRG	RVAGLCGDFD	GDASNDLRSR	QGVLEPTAEL	AAHSWRLSPL	CPEPGDLPHP	CTMNTHRAGW
1210	1220	1230	1240	1250	1260	1270	1280
ARARCGALLQ	PLFTLCHAEV	PPQQHYEWCL	YDACGCDSGG	DCECLCSAIA	TYADECARHG	HHVRWRSQEL	CSLQCEGGQV
1290	1300	1310	1320	1330	1340	1350	1360
YEACGPTCPP	TCHEQHPEPG	WHCQVVACVE	GCFCPEGTLL	HGGACLEPAS	CPCEWGRNSF	PPGSVLQKDC	GNCTCQEGQW
1370	1380	1390	1400	1410	1420	1430	1440
HCGGDGGHCE	ELVPACAEGE	ALCQENGHCV	PHGWLCDNQD	DCGDGSDEEG	CAAPGCGEGQ	MTCSSGHCLP	LALLCDRQDD
1450	1460	1470	1480	1490	1500	1510	1520
CGDGTDEPSY	PCPQGLLACA	DGRCLPPALL	CDGHPDCLDA	ADEESCLGQV	TCVPGEVSCV	DGTCLGAIQL	CDGVWDCPDG
1530	1540	1550	1560	1570	1580	1590	1600
ADEGPGHCPL	PSLPTPPAST	LPGPSPGSLD	TASSPLASAS	PAPPCGPFEF	RCGSGECTPR	GWRCDQEEDC	ADGSDERGCG
1610	1620	1630	1640	1650	1660	1670	1680
GPCAPHHAPC	ARGPHCVSPE	QLCDGVRQCP	DGSDEGPDAC	GGLPALGGPN	RTGLPCPEYT	CPNGTCIGFQ	LVCDGQPDCG
1690	1700	1710	1720	1730	1740	1750	1760
RPGQVGPSPE	EQGCGAWGPW	SPWGPCSRTC	GPWGQGRSRR	CSPLGLLVLQ	NCPGPEHQSQ	ACFTAACPVD	GEWSTWSPWS
1770	1780	1790	1800	1810	1820	1830	1840
VCSEPCRGTM	TRQRQCHSPQ	NGGRTCAALP	GGLHSTRQTK	PCPQDGCPNA	TCSGELMFQP	CAPCPLTCDD	ISGQVTCPPD
1850	1860	1870	1880	1890	1900	1910	1920
WPCGSPGCWC	PEGQVLGSEG	WCVWPRQCPC	LVDGARYWPG	QRIKADCQLC	ICQDGRPRRC	RLNPDCAVDC	GWSSWSPWAK
1930	1940	1950	1960	1970	1980	1990	2000
CLGPCGSQSI	QWSFRSSNNP	RPSGRGRQCR	GIHRKARRCQ	TEPCEGCEHQ	GQVHRVGERW	HGGPCRVCQC	LHNLTAHCSP
2010	2020	2030	2040	2050	2060	2070	2080
YCPLGSCPQG	WVLVEGTGES	CCHCALPGEN	QTVQPMATPA	AAPAPSPQIR	FPLATYILPP	SGDPCYSPLG	LAGLAEGSLH
2090	2100	2110	2120	2130	2140	2150	2160
ASSQQLEHPT	QAALLGAPTQ	GPSPQGWHAG	GDAYAKWHTR	PHYLQLDLLQ	PRNLTGILVP	ETGSSNAYAS	SFSLQFSSNG
2170	2180	2190	2200	2210	2220	2230	2240

Detailed Protein Report

Cmpd.	No. of Cmpds.	m/z meas.	Δ m/z [ppm]	z	Rt [min]	Score	P	Range	Sequence	Modification	Ratios	
1387	1	927.1161	66.38	3	47.5	19.2	0	2250-2277	R. GGPCDGVLDCEDGSDEEGCVLLF F			
1675	2	1136.5319	16.36	2	49.5	14.8	1	2854-2875	R. GRSCSSLAPGDTTCPGPHSQTR. D	Carbamidomethyl: 4	M:Q	0.68
1364	1	669.3350	-3.76	2	45.8	13.3	1	3671-3682	R.RCARPPCPGPAR.Q	Carbamidomethyl: 2	M:Q<br>Q:W	0.44<br>2.74

Detailed Protein Report

Protein 382: **PREDICTED: conserved oligomeric Golgi complex subunit 4 isoform X1 [Homo sapiens]**

**Accession:**	gi|578828932	**Score:**	47.1
**Database:**	refseq_human(refseq_human_20140103.fasta)	**MW [kDa]:**	73.7
**Database Date:**	2015-11-30	**pI:**	5.1
		**Sequence Coverage [%]:**	11.0
		**No. of unique Peptides:**	1

Quantitation

Q:W	**Median:** 0.67	**CV:** 0.00 %	**No. of Peptides:**	1
M:Q	**Median:** 1.47	**CV:** 0.00 %	**No. of Peptides:**	1

```
        10         20         30         40         50         60         70         80
MDGVQTALRS EDYEQAAAHT HRYLCLDKSV IELSRQGKEG SMIDANLKLL QEAEQRLKAI VAEKFAIATK EGDLPQVERF
        90        100        110        120        130        140        150        160
FKIFPLLGLH EEGLRKFSEY LCKQVASKAE ENLLMVLGTD MSDRRAAVIF ADTLTLLFEG IARIVETHQP IVETYYGPGR
       170        180        190        200        210        220        230        240
LYTLIKYLQV ECDRQVEKVV DKFIKQRDYH QQFRHVQNNL MRNSTTEKIE PRELDPILTE VTLMNARSEL YLRFLKKRIS
       250        260        270        280        290        300        310        320
SDFEVGDSMA SEEVKQEHQK CLDKLLNNCL LSCTMQELIG LYVTMEEYFM RETVNKAVAL DTYEKGQLTS SMVDDVFYIV
       330        340        350        360        370        380        390        400
KKCIGRALSS SSIDCLCAMI NLATTELESD FRDVLCNKLR MGFPATTFQD IQRGVTSAVN IMHSSLQQGK FDTKGIESTD
       410        420        430        440        450        460        470        480
EAKMSFLVTL NNVEVCSENI STLKKTLESD CTKLFSQGIG GEQAQAKFDS CLSDLAAVSN KFRDLLQEGL TELNSTAIKP
       490        500        510        520        530        540        550        560
QVQPWINSFF SVSHNIEEEE FNDYEANDPW VQQFILNLEQ QMAEFKASLS PVIYDSLTGL MTSLVAVELE KVVLKSTFNR
       570        580        590        600        610        620        630        640
LGGLQFDKEL RSLIAYLTTV TTWTIRDKFA RLSQMATILN LERVTEILDY WGPNSGPLTW RLTPAEVRQV LALRIDFRSE
       650
DIKRLRL
```

Cmpd.	No. of Cmpds.	m/z meas.	Δ m/z [ppm]	z	Rt [min]	Score	P	Range	Sequence	Modification	Ratios
1637	1	993.4172	-42.79	2	50.7	12.5	1	238-255	K.RISSDFEVGDSMASEEVK.Q		M:Q 1.47<br>Q:W 0.67

Detailed Protein Report

Protein 383: tRNA wybutosine-synthesizing protein 1 homolog B isoform 1 [Homo sapiens]

**Accession:**	gi|224282123	**Score:**	47.1
**Database:**	refseq_human(refseq_human_20140103.fasta)	**MW [kDa]:**	76.9
**Database Date:**	2015-11-30	**pI:**	5.8
**Modification(s):**	Carbamidomethyl, Oxidation	**Sequence Coverage [%]:**	7.3
		**No. of unique Peptides:**	2

10	20	30	40	50	60	70	80
MDPSADTWDL	SSPLISLWIN	RFYIYLGFAV	SISLWICVQI	VIEMQGFATV	LAEAVTSLDL	PVAIINLKEY	DPDDHLIEEV
90	100	110	120	130	140	150	160
TSKNVCVFLV	ATYTDGLPTE	SAEWFCKWLE	EASIDFRFGK	TYLKGMRDAV	FGLGNSAYAS	HFNKVGKNVD	KWLWMLGVHR
170	180	190	200	210	220	230	240
VMSRGEGDCD	VVKSKHGSIE	ANFRAWKTKF	ISQLQALQKG	ERKKSCGGHC	KKGKCESHQH	GSEEREEGSQ	EQDELHHRDT
250	260	270	280	290	300	310	320
KEEEPFESSS	EEEFGGEDHQ	SLNSIVDVED	LGKIMDHVKK	EKREKEQQEE	KSGLFRNMGR	NEDGERRAMI	TPALREALTK
330	340	350	360	370	380	390	400
QVDAPRERSL	LQTHILWNES	HRCMETTPSL	ACANKCVFCW	WHHNNPVGTE	WLWKMDQPEM	ILKEAIENHQ	NMIKQFKGVP
410	420	430	440	450	460	470	480
GVKAERFEEG	MTVKHCALSL	VGEPIMYPEI	NRFLKLLHQC	KISSFLVTNA	QFPAEIRNLE	PVTQLYVSVD	ASTKDSLKKI
490	500	510	520	530	540	550	560
DRPLFKDFWQ	QFLDSLKALA	VKQQRTVYRL	TLVKAWNVDE	LQAYAQLVSL	GNPDFIEVKG	VTYCRESSAS	SLTMAHVPWH
570	580	590	600	610	620	630	640
EEVVQFVREL	VDLIPEYEIA	CEHEHSNCLL	IAHRKFKIGG	EWWTWIDYNR	FQELIQEYED	SGGSKTFSAK	DYMARTPHWA
650	660	670					
LFGANERSFD	PKDTRHQRKN	KSKAISGC					

Cmpd.	No. of Cmpds.	m/z meas.	Δ m/z [ppm]	z	Rt [min]	Score	P	Range	Sequence	Modification
161	1	793.8859	-88.32	2	31.7	10.4	2	307-320	R.RAMITPALREALTK.Q	Oxidation: 3
2915	3	713.3417	41.37	2	65.2	16.0	0	343-355	R.CMETTPSLACANK.C	Carbamidomethyl: 10

Detailed Protein Report

Protein 384: **PERQ amino acid-rich with GYF domain-containing protein 2 isoform c [Homo sapiens]**

**Accession:**	gi|156766047	**Score:**	47.1
**Database:**	refseq_human(refseq_human_20140103.fasta)	**MW [kDa]:**	149.4
**Database Date:**	2015-11-30	**pI:**	5.3
**Modification(s):**	Oxidation	**Sequence Coverage [%]:**	2.8
		**No. of unique Peptides:**	3

Quantitation

Q:W	**Median:**	0.41	**CV:**	0.00 %	**No. of Peptides:**	1
M:Q	**Median:**	2.86	**CV:**	152.73 %	**No. of Peptides:**	2

10	20	30	40	50	60	70	80
MAAETQTLNF	GPEWLRALSS	GGSITSPPLS	PALPKYKLAD	YRYGREEMLA	LFLKDNKIPS	DLLDKEFLPI	LQEEPLPPLA
**90**	**100**	**110**	**120**	**130**	**140**	**150**	**160**
LVPFTEEEQR	NFSMSVNSAA	VLRLTGRGGG	GTVVGAPRGR	SSSRGRGRGR	GECGFYQRSF	DEVEGVFGRG	GGREMHRSQS
**170**	**180**	**190**	**200**	**210**	**220**	**230**	**240**
WEERGDRRFE	KPGRKDVGRP	NFEEGGPTSV	GRKHEFIRSE	SENWRIFREE	QNGEDEDGGW	RLAGSRRDGE	RWRPHSPGWR
**250**	**260**	**270**	**280**	**290**	**300**	**310**	**320**
EHMERRRRFE	FDFRDRDDER	GYRRVRSGSG	SIDDDRDSLP	EWCLEDAEEE	MGTFDSSGAF	LSLKKVQKEP	IPEEQEMDFR
**330**	**340**	**350**	**360**	**370**	**380**	**390**	**400**
PVDEGEECSD	SEGSHNEEAK	EPDKTNKKEG	EKTDRVGVEA	SEETPQTSSS	SARPGTPSDH	QSQEASQFER	KDEPKTEQTE
**410**	**420**	**430**	**440**	**450**	**460**	**470**	**480**
KAEEETRMEN	SLPAKVPSRG	DEMVADVQQP	LSQIPSDTAS	PLLILPPPVP	NPSPTLRPVE	TPVVGAPGMG	SVSTEPDDEE
**490**	**500**	**510**	**520**	**530**	**540**	**550**	**560**
GLKHLEQQAE	KMVAYLQDSA	LDDERLASKL	QEHRAKGVSI	PLMHEAMQKW	YYKDPQGEIQ	GPFNNQEMAE	WFQAGYFTMS
**570**	**580**	**590**	**600**	**610**	**620**	**630**	**640**
LLVKRACDES	FQPLGDIMKM	WGRVPFSPGP	APPPHMGELD	QERLTRQQEL	TALYQMQHLQ	YQQFLIQQQY	AQVLAQQQKA
**650**	**660**	**670**	**680**	**690**	**700**	**710**	**720**
ALSSQQQQQL	ALLLQQFQTL	KMRISDQNII	PSVTRSVSVP	DTGSIWELQP	TASQPTVWEG	GSVWDLPLDT	TTPGPALEQL
**730**	**740**	**750**	**760**	**770**	**780**	**790**	**800**
QQLEKAKAAK	LEQERREAEM	RAKREEEERK	RQEELRRQQE	EILRRQQEEE	RKRREEEELA	RRKQEEALRR	QREQEIALRR
**810**	**820**	**830**	**840**	**850**	**860**	**870**	**880**
QREEEERQQQ	EEALRRLEER	RREEEERRKQ	EELLRKQEEE	AAKWAREEEE	AQRRLEENRL	RMEEEAARLR	HEEEERKRKE
**890**	**900**	**910**	**920**	**930**	**940**	**950**	**960**
LEVQRQKELM	RQRQQQQEAL	RRLQQQQQQQ	QLAQMKLPSS	STWGQQSNTT	ACQSQATLSL	AEIQKLEEER	ERQLREEQRR
**970**	**980**	**990**	**1000**	**1010**	**1020**	**1030**	**1040**
QQRELMKALQ	QQQQQQQQKL	SGWGNVSKPS	GTTKSLLEIQ	QEEARQMQKQ	QQQQQQHQQP	NRARNNTHSN	LHTSIGNSVW
**1050**	**1060**	**1070**	**1080**	**1090**	**1100**	**1110**	**1120**
GSINTGPPNQ	WASDLVSSIW	SNADTKNSNM	GFWDDAVKEV	GPRNSTNKNK	NNASLSKSVG	VSNRQNKKVE	EEEKLLKLFQ
**1130**	**1140**	**1150**	**1160**	**1170**	**1180**	**1190**	**1200**
GVNKAQDGFT	QWCEQMLHAL	NTANNLDVPT	FVSFLKEVES	PYEVHDYIRA	YLGDTSEAKE	FAKQFLERRA	KQKANQQRQQ
**1210**	**1220**	**1230**	**1240**	**1250**	**1260**	**1270**	**1280**
QQLPQQQQQQ	PPQQPPQQPQ	QQDSVWGMNH	STLHSVFQTN	QSNNQQSNFE	AVQSGKKKKK	QKMVRADPSL	LGFSVNASSE
**1290**	**1300**						
RLNMGEIETL	DDY						

Cmpd.	No. of Cmpds.	m/z meas.	Δ m/z [ppm]	z	Rt [min]	Score	P	Range	Sequence	Modification	Ratios	
1750	1	863.3936	-7.87	2	51.7	13.5	1	565-579	K.RACDESFQPLGDIMK.M	Oxidation: 14		
1628	1	1071.6266	33.70	1	50.2	10.0	1	758-765	R.QQEEILRR.Q		M:Q<br>Q:W	8.57<br>0.41
196	3	838.3951	-10.67	2	32.2	23.6	0	1010-1022	K.QQQQQQQHQQPNR.A		M:Q	0.95

Detailed Protein Report

Protein 385: PREDICTED: PR domain zinc finger protein 16 isoform X3 [Homo sapiens]

Accession: gi|530360370
Database: refseq_human(refseq_human_20140103.fasta)
Database Date: 2015-11-30
Modification(s): Carbamidomethyl

Score: 47.0
MW [kDa]: 129.7
pI: 5.9
Sequence Coverage [%]: 3.5
No. of unique Peptides: 2

Quantitation

Q:W **Median:** 8.65 **CV:** 0.00 % **No. of Peptides:** 1

10	20	30	40	50	60	70	80
MRSKARARKL	AKSDGDVVNN	MYEPNRDLLA	SHSAEDEAED	SAMSPIPVGP	PSPFPTSEDF	TPKEGSPYEA	PVYIPEDIPI
90	100	110	120	130	140	150	160
PADFELRESS	IPGAGLGVWA	KRKMEAGERL	GPCVVVPRAA	AKETDFGWEQ	ILTDVEVSPQ	EGCITKQISE	DLGSEKFCVD
170	180	190	200	210	220	230	240
ANQAGAGSWL	KYIRVACSCD	DQNLTMCQIS	EQIYYKVIKD	IEPGEELLVH	VKEGVYPLGT	VPPGLDEEPT	FRCDECDELF
250	260	270	280	290	300	310	320
QSKLDLRRHK	KYTCGSVGAA	LYEGLAEELK	PEGLGGGSGQ	AHECKDCERM	FPNKYSLEQH	MVIHTEEREY	KCDQCPKAFN
330	340	350	360	370	380	390	400
WKSNLIRHQM	SHDSGKRFEC	ENCVKVFTDP	SNLQRHIRSQ	HVGARAHACP	DCGKTFATSS	GLKQHKHIHS	TVKPFICEVC
410	420	430	440	450	460	470	480
HKSYTQFSNL	CRHKRMHADC	RTQIKCKDCG	QMFSTTSSLN	KHRRFCEGKN	HYTPGGIFAP	GLPLTPSPMM	DKAKPSPSLN
490	500	510	520	530	540	550	560
HASLGFNEYF	PSRPHPGSLP	FSTAPPTFPA	LTPGFPGIFP	PSLYPRPPLL	PPTSLLKSPL	NHTQDAKLPS	PLGNPALPLV
570	580	590	600	610	620	630	640
SAVSNSSQGT	TAAAGPEEKF	ESRLEDSCVE	KLKTRSSDMS	DGSDFEDVNT	TTGTDLDTTT	GTGSDLDSDV	DSDPDKDKGK
650	660	670	680	690	700	710	720
GKSAEGQPKF	GGGLAPPGAP	NSVAEVPVFY	SQHSFFPPPD	EQLLTATGAA	GDSIKAIASI	AEKYFGPGFM	GMQEKKLGSL
730	740	750	760	770	780	790	800
PYHSAFPFQF	LPNFPHSLYP	FTDRALAHNL	LVKAEPKSPR	DALKVGGPSA	ECPFDLTTKP	KDVKPILPMP	KGPSAPASGE
810	820	830	840	850	860	870	880
EQPLDLSIGS	RARASQNGGG	REPRKNHVYG	ERKLGAGEGL	PQVCPARMPQ	QPPLHYAKPS	PFFMDPIYSR	VEKRKVTDPV
890	900	910	920	930	940	950	960
GALKEKYLRP	SPLLFHPQMS	AIETMTEKLE	SFAAMKADSG	SSLQPLPHHP	FNFRSPPPTL	SDPILRKGKE	RYTCRYCGKI
970	980	990	1000	1010	1020	1030	1040
FPRSANLTRH	LRTHTGEQPY	RCKYCDRSFS	ISSNLQRHVR	NIHNKEKPFK	CHLCNRCFGQ	QTNLDRHLKK	HEHENAPVSQ
1050	1060	1070	1080	1090	1100	1110	1120
HPGVLTNHLG	TSASSPTSES	DNHALLDEKE	DSYFSEIRNF	IANSEMNQAS	TRTEKRADMQ	IVDGSAQCPG	LASEKQEDVE
1130	1140	1150	1160	1170	1180		
EEDDDDLEED	DEDSLAGKSQ	DDTVSPAPEP	QAAYEDEEDE	EPAASLAVGF	DHTRRHMQ		

Cmpd.	No. of Cmpds.	m/z meas.	Δ m/z [ppm]	z	Rt [min]	Score	P	Range	Sequence	Modification	Ratios
1511	1	1005.9710	-61.06	2	47.6	21.5	2	104-122	K.MEAGERLGPCVVVPRAAAK.E	Carbamidomethyl: 10	Q:W 8.65
2972	1	646.8226	-64.07	2	66.4	11.8	0	935-946	R.SPPPTLSDPILR.K		

Detailed Protein Report

Protein 386: centrosomal protein of 63 kDa isoform c [Homo sapiens]

**Accession:**	gi|109255226	**Score:**	47.0
**Database:**	refseq_human(refseq_human_20140103.fasta)	**MW [kDa]:**	57.9
**Database Date:**	2015-11-30	**pI:**	5.9
		**Sequence Coverage [%]:**	10.3
		**No. of unique Peptides:**	2

10	20	30	40	50	60	70	80
MEALLEGIQN	RGHGGGFLTS	CEAELQELMK	QIDIMVAHKK	SEWEGRTHAL	ETCLKIREQE	LKSLRSQLDV	THKEVGMLHQ
**90**	**100**	**110**	**120**	**130**	**140**	**150**	**160**
QVEEHEKIKQ	EMTMEYKQEL	KKLHEELCIL	KRSYEKLQKK	QMREFRGNTK	NHREDRSEIE	RLTAKIEEFR	QKSLDWEKQR
**170**	**180**	**190**	**200**	**210**	**220**	**230**	**240**
LIYQQQVSSL	EAQRKALAEQ	SEIIQAQLVN	RKQKLESVEL	SSQSEIQHLS	SKLERANDTI	CANELEIERL	TMRVNDLVGT
**250**	**260**	**270**	**280**	**290**	**300**	**310**	**320**
SMTVLQEQQQ	KEEKLRESEK	LLEALQEEKR	ELKAALQSQE	NLIHEARIQK	EKLQEKVKAT	NTQHAVEAIS	LESVSATCKQ
**330**	**340**	**350**	**360**	**370**	**380**	**390**	**400**
LSQELMEKYE	ELKRMEAHNN	EYKAEIKKLK	EQILQGEQSY	SSALEGMKME	ISHLTQELHQ	RDITIASTKG	SSSDMEKRLR
**410**	**420**	**430**	**440**	**450**	**460**	**470**	**480**
AEMQKAEDKA	VEHKEILDQL	ESLKLENRHL	SEMVMKLELG	LHECSLPVSP	LGSIATRFLE	EEELRSHHIL	ERLDAHIEEL
**490**	**500**						
KRESEKTVRQ	FTALK						

Cmpd.	No. of Cmpds.	m/z meas.	Δ m/z [ppm]	z	Rt [min]	Score	P	Range	Sequence	Modification
538	1	501.6964	-171.64	2	36.7	22.2	1	58-65	R.EQELKSLR.S	
2671	1	1079.8373	-57.12	3	64.6	12.7	1	437-465	K.LELGLHECSLPVSPLGSIATRFLEEEELR.S	

Detailed Protein Report

Protein 387: wee1-like protein kinase 2 [Homo sapiens]

**Accession:**	gi|157738687	**Score:**	47.0
**Database:**	refseq_human(refseq_human_20140103.fasta)	**MW [kDa]:**	62.9
**Database Date:**	2015-11-30	**pI:**	6.1
**Modification(s):**	Carbamidomethyl	**Sequence Coverage [%]:**	7.9
		**No. of unique Peptides:**	3

Quantitation

Q:W	**Median:**	1.20	**CV:**	0.00 %	**No. of Peptides:**	1
M:Q	**Median:**	2.10	**CV:**	0.00 %	**No. of Peptides:**	1

```
        10         20         30         40         50         60         70         80
MDDKDIDKEL RQKLNFSYCE ETEIEGQKKV EESREASSQT PEKGEVQDSE AKGTPPWTPL SNVHELDTSS EKDKESPDQI
        90        100        110        120        130        140        150        160
LRTPVSHPLK CPETPAQPDS RSKLLPSDSP STPKTMLSRL VISPTGKLPS RGPKHLKLTP APLKDEMTSL ALVNINPFTP
       170        180        190        200        210        220        230        240
ESYKKLFLQS GGKRKIRGDL EEAGPEEGKG GLPAKRCVLR ETNMASRYEK EFLEVEKIGV GEFGTVYKCI KRLDGCVYAI
       250        260        270        280        290        300        310        320
KRSMKTFTEL SNENSALHEV YAHAVLGHHP HVVRYYSSWA EDDHMIIQNE YCNGGSLQAA ISENTKSGNH FEEPKLKDIL
       330        340        350        360        370        380        390        400
LQISLGLNYI HNSSMVHLDI KPSNIFICHK MQSESSGVIE EVENEADWFL SANVMYKIGD LGHATSINKP KVEEGDSRFL
       410        420        430        440        450        460        470        480
ANEILQEDYR HLPKADIFAL GLTIAVAAGA ESLPTNGAAW HHIRKGNFPD VPQELSESFS SLLKNMIQPD AEQRPSAAAL
       490        500        510        520        530        540        550        560
ARNTVLRPSL GKTEELQQQL NLEKFKTATL ERELREAQQA QSPQGYTHHG DTGVSGTHTG SRSTKRLVGG KSARSSSFTS
       570
GEREPLH
```

Cmpd.	No. of Cmpds.	m/z meas.	Δ m/z [ppm]	z	Rt [min]	Score	P	Range	Sequence	Modification	Ratios	
1295	2	1023.3898	-86.03	2	45.9	14.1	1	12-28	R.QKLNFSYCEETEIEGQK.K			
1865	1	785.9040	-18.42	2	51.7	15.7	1	218-231	K.IGVGEFGTVYKCIK.R	Carbamidomethyl: 12	Q:W	1.20
135	1	725.9700	90.61	2	30.9	17.2	0	378-391	K.IGDLGHATSINKPK.V			

Detailed Protein Report

Protein 388: protein unc-13 homolog C [Homo sapiens]

**Accession:**	gi|122937514	**Score:**	47.0
**Database:**	refseq_human(refseq_human_20140103.fasta)	**MW [kDa]:**	250.8
**Database Date:**	2015-11-30	**pI:**	5.6
**Modification(s):**	Carbamidomethyl, Oxidation	**Sequence Coverage [%]:**	2.0
		**No. of unique Peptides:**	2

Quantitation

Q:W	**Median:**	0.62	**CV:**	0.00 %	**No. of Peptides:**	1
M:Q	**Median:**	0.62	**CV:**	0.00 %	**No. of Peptides:**	1

Alias proteins:

Accession	Name	Description
gi|530405876	refseq_human (refseq_human_20140103.fasta)	PREDICTED: protein unc-13 homolog C isoform X1 [Homo sapiens]

Detailed Protein Report

10	20	30	40	50	60	70	80
MVANFFKSLI	LPYIHKLCKG	MFTKKLGNTN	KNKEYRQQKK	DQDFPTAGQT	KSPKFSYTFK	STVKKIAKCS	STHNLSTEED
90	100	110	120	130	140	150	160
EASKEFSLSP	TFSYRVAIAN	GLQKNAKVTN	SDNEDLLQEL	SSIESSYSES	LNELRSSTEN	QAQSTHTMPV	RRNRKSSSSL
170	180	190	200	210	220	230	240
APSEGSSDGE	RTLHGLKLGA	LRKLRKWKKS	QECVSSDSEL	STMKKSWGIR	SKSLDRTVRN	PKTNALEPGF	SSSGCISQTH
250	260	270	280	290	300	310	320
DVMEMIFKEL	QGISQIETEL	SELRGHVNAL	KHSIDEISSS	VEVVQSEIEQ	LRTGFVQSRR	ETRDIHDYIK	HLGHMGSKAS
330	340	350	360	370	380	390	400
LRFLNVTEER	FEYVESVVYQ	ILIDKMGFSD	APNAIKIEFA	QRIGHQRDCP	NAKPRPILVY	FETPQQRDSV	LKKSYKLKGT
410	420	430	440	450	460	470	480
GIGISTDILT	HDIRERKEKG	IPSSQTYESM	AIKLSTPEPK	IKKNNWQSPD	DSDEDLESDL	NRNSYAVLSK	SELLTKGSTS
490	500	510	520	530	540	550	560
KPSSKSHSAR	SKNKTANSSR	ISNKSDYDKI	SSQLPESDIL	EKQTTTHYAD	ATPLWHSQSD	FFTAKLSRSE	SDFSKLCQSY
570	580	590	600	610	620	630	640
SEDFSENQFF	TRTNGSSLLS	SSDRELWQRK	QEGTATLYDS	PKDQHLNGGV	QGIQGQTETE	NTETVDSGMS	NGMVCASGDR
650	660	670	680	690	700	710	720
SHYSDSQLSL	HEDLSPWKEW	NQGADLGLDS	STQEGFDYET	NSLFDQQLDV	YNKDLEYLGK	CHSDLQDDSE	SYDLTQDDNS
730	740	750	760	770	780	790	800
SPCPGLDNEP	QGQWVGQYDS	YQGANSNELY	QNQNQLSMMY	RSQSELQSDD	SEDAPPKSWH	SRLSIDLSDK	TFSFPKFGST
810	820	830	840	850	860	870	880
LQRAKSALEV	VWNKSTQSLS	GYEDSGSSLM	GRFRTLSQST	ANESSTTLDS	DVYTEPYYYK	AEDEEDYTEP	VADNETDYVE
890	900	910	920	930	940	950	960
VMEQVLAKLE	NRTSITETDE	QMQAYDHLSY	ETPYETPQDE	GYDGPADDMV	SEEGLEPLNE	TSAEMEIRED	ENQNIPEQPV
970	980	990	1000	1010	1020	1030	1040
EITKPKRIRP	SFKEAALRAY	KKQMAELEEK	ILAGDSSSVD	EKARIVSGND	LDASKFSALQ	VCGGAGGGLY	GIDSMPDLRR
1050	1060	1070	1080	1090	1100	1110	1120
KKTLPIVRDV	AMTLAARKSG	LSLAMVIRTS	LNNEELKMHV	FKKTLQALIY	PMSSTIPHNF	EVWTATTPTY	CYECEGLLWG
1130	1140	1150	1160	1170	1180	1190	1200
IARQGMKCLE	CGVKCHEKCQ	DLLNADCLQR	AAEKSSKHGA	EDKTQTIITA	MKERMKIREK	NRPEVFEVIQ	EMFQISKEDF
1210	1220	1230	1240	1250	1260	1270	1280
VQFTKAAKQS	VLDGTSKWSA	KITITVVSAQ	GLQAKDKTGS	SDPYVTVQVG	KNKRRTKTIF	GNLNPVWDEK	FYFECHNSTD
1290	1300	1310	1320	1330	1340	1350	1360
RIKVRVWDED	DDIKSRVKQH	FKKESDDFLG	QTIVEVRTLS	GEMDVWYNLE	KRTDKSAVSG	AIRLKINVEI	KGEEKVAPYH
1370	1380	1390	1400	1410	1420	1430	1440
IQYTCLHENL	FHYLTEVKSN	GGVKIPEVKG	DEAWKVFFDD	ASQEIVDEFA	MRYGIESIYQ	AMTHFSCLSS	KYMCPGVPAV
1450	1460	1470	1480	1490	1500	1510	1520
MSTLLANINA	FYAHTTVSTN	IQVSASDRFA	ATNFGREKFI	KLLDQLHNSL	RIDLSKYREN	FPASNTERLQ	DLKSTVDLLT
1530	1540	1550	1560	1570	1580	1590	1600
SITFFRMKVL	ELQSPPKASM	VVKDCVRACL	DSTYKYIFDN	CHELYSQLTD	PSKKQDIPRE	DQGPTTKNLD	FWPQLITLMV
1610	1620	1630	1640	1650	1660	1670	1680
TIIDEDKTAY	TPVLNQFPQE	LNMGKISAEI	MWTLFALDMK	YALEEHENQR	LCKSTDYMNL	HFKVKWFYNE	YVRELPAFKD
1690	1700	1710	1720	1730	1740	1750	1760
AVPEYSLWFE	PFVMQWLDEN	EDVSMEFLHG	ALGRDKKDGF	QQTSEHALFS	CSVVDVFAQL	NQSFEIIKKL	ECPNPEALSH
1770	1780	1790	1800	1810	1820	1830	1840
LMRRFAKTIN	KVLLQYAAIV	SSDFSSHCDK	ENVPCILMNN	IQQLRVQLEK	MFESMGGKEL	DSEASTILKE	LQVKLSGVLD
1850	1860	1870	1880	1890	1900	1910	1920
ELSVTYGESF	QVIIEECIKQ	MSFELNQMRA	NGNTTSNKNS	AAMDAEIVLR	SLMDFLDKTL	SLSAKICEKT	VLKRVLKELW
1930	1940	1950	1960	1970	1980	1990	2000
KLVLNKIEKQ	IVLPPLTDQT	GPQMIFIAAK	DLGQLSKLKE	HMIREDARGL	TPRQCAIMEV	VLATIKQYFH	AGGNGLKKNF
2010	2020	2030	2040	2050	2060	2070	2080
LEKSPDLQSL	RYALSLYTQT	TDALIKKFID	TQTSQSRSSK	DAVGQISVHV	DITATPGTGD	HKVTVKVIAI	NDLNWQTTAM
2090	2100	2110	2120	2130	2140	2150	2160
FRPFVEVCIL	GPNLGDKKRK	QGTKTKSNTW	SPKYNETFQF	ILGKENRPGA	YELHLSVKDY	CFAREDRIIG	MTVIQLQNIA
2170	2180	2190	2200	2210	2220		

Detailed Protein Report

Cmpd.	No. of Cmpds.	m/z meas.	Δ m/z [ppm]	z	Rt [min]	Score	P	Range	Sequence	Modification	Ratios
1046	1	742.4006	68.40	2	41.8	12.0	1	1497-1508	K.YRENFPASNTER.L		M:Q 0.62<br>Q:W 0.62
1482	1	619.9673	-23.43	3	46.9	10.7	0	1791-1805	K.ENVPCILMNNIQQLR.V	Carbamidomethyl: 5; Oxidation: 8	

Detailed Protein Report

Protein 389: H/ACA ribonucleoprotein complex subunit 4 isoform 3 [Homo sapiens]

**Accession:**	gi|570700851	**Score:**	46.9
**Database:**	refseq_human(refseq_human_20140103.fasta)	**MW [kDa]:**	47.6
**Database Date:**	2015-11-30	**pI:**	10.0
**Modification(s):**	Oxidation	**Sequence Coverage [%]:**	10.5
		**No. of unique Peptides:**	3

```
        10         20         30         40         50         60         70         80
MADAEVIILP KKHKKKKERK SLPEEDVAEI QHAEEFLIKP ESKVAKLDTS QWPLLLKNFD KLNVRTTHYT PLACGSNPLK
        90        100        110        120        130        140        150        160
REIGDYIRTG FINLDKPSNP SSHEVVAWIR RILRVEKTGH SGTLDPKVTG CLIVCIERAT RLVKSQQSAG KEYVGIVRLH
       170        180        190        200        210        220        230        240
NAIEGGTQLS RALETLTGAL FQRPPLIAAV KRQLRVRTIY ESKMIEYDPE RRLGIFWVSC EAGTYIRTLC VHLGLLLGVG
       250        260        270        280        290        300        310        320
GQMQELRRVR SGVMSEKDHM VTMHDVLDAQ WLYDNHKDES YLRRVVYPLE KLLTSHKRLV MKDSAVNAIC YGAKIMLPGV
       330        340        350        360        370        380        390        400
LRYEDGIEVN QEIVVITTKG EAICMAIALM TTAVISTCDH GIVAKIKRVI MERDTYPRKW GLGPKASQKK LMIKQGLLDK
       410        420        430
HGKPTDSTPA TWKQEYVDYR
```

Cmpd.	No. of Cmpds.	m/z meas.	Δ m/z [ppm]	z	Rt [min]	Score	P	Range	Sequence	Modification
2374	1	1022.9281	-74.19	2	59.6	14.2	2	196-211	R.VRTIYESKMIEYDPER.R	Oxidation: 9
1944	1	618.9252	-105.31	3	53.0	18.6	2	298-314	K.RLVMKDSAVNAICYGAK.I	Oxidation: 4
2864	1	651.8581	-55.52	2	64.5	14.1	2	390-400	K.KLMIKQGLLDK.H	Oxidation: 3

Detailed Protein Report

Protein 390: **PREDICTED: MORC family CW-type zinc finger protein 4 isoform X2 [Homo sapiens]**

Accession: gi|578838478
Database: refseq_human(refseq_human_20140103.fasta)
Database Date: 2015-11-30

Score: 46.8
MW [kDa]: 92.9
pI: 6.4
Sequence Coverage [%]: 6.3
No. of unique Peptides: 2

10	20	30	40	50	60	70	80
MLLYRGAPAG	PGAPGCGLAR	PGGGPQAFGI	RLSTMSPRYL	QSNSSSHTRP	FSAIAELLDN	AVDPDVSART	VFIDVEEVKN
**90**	**100**	**110**	**120**	**130**	**140**	**150**	**160**
KSCLTFTDDG	CGMTPHKLHR	MLRNKNGKSE	LDFDTDQYDI	LVSDFDTEEK	MTGGVTSELP	ETEYSLRAFC	GILYMKPRMK
**170**	**180**	**190**	**200**	**210**	**220**	**230**	**240**
IFLRQKKVTT	QMIAKSLANV	EYDTYKPTFT	NKQVRITFGF	SCKNSNQFGI	MMYHNNRLIK	SFEKVGCQVK	PTRGEGVGVI
**250**	**260**	**270**	**280**	**290**	**300**	**310**	**320**
GVIECNFLKP	AYNKQDFEYT	KEYRLTINAL	AQKLNAYWKE	KTSQDNFETS	TVARPIPKVP	DQTWVQCDEC	LKWRKLPGKI
**330**	**340**	**350**	**360**	**370**	**380**	**390**	**400**
DPSMLPARWF	CYYNSHPKYR	RCSVPEEQEL	TDEDLCLSKA	KKQEQTVEEK	KKMPMENENH	QVFSNPPKIL	TVQEMAGLNN
**410**	**420**	**430**	**440**	**450**	**460**	**470**	**480**
KTIGYEGIHS	PSVLPSGGEE	SRSPSLQLKP	LDSSVLQFSS	KYKWILGEEP	VEKRRRLQNE	MTTPSLDYSM	PAPYRRVEAP
**490**	**500**	**510**	**520**	**530**	**540**	**550**	**560**
VAYPEGENSH	DKSSSERSTP	PYLFPEYPEA	SKNTGQNREV	SILYPGAKDQ	RQGSLLPEEL	EDQMPRLVAE	ESNRGSTTIN
**570**	**580**	**590**	**600**	**610**	**620**	**630**	**640**
KEEVNKGPFV	AVVGVAKGVR	DSGAPIQLIP	FNREELAERR	KAVESWNPVP	YSVASAAIPA	AAIGEKARGY	EESEGHNTPK
**650**	**660**	**670**	**680**	**690**	**700**	**710**	**720**
LKNQRELEEL	KRTTEKLERV	LAERNLFQQK	VEELEQERNH	WQSEFKKVQH	ELVIYSTQEA	EGLYWSKKHM	GYRQAEFQIL
**730**	**740**	**750**	**760**	**770**	**780**	**790**	**800**
KAELERTKEE	KQELKEKLKE	TETHLEMLQK	AQVSYRTPEG	DDLERALAKL	TRLRIHVSYL	LTSVLPHLEL	REIGYDSEQV
**810**	**820**						
DGILYTVLEA	NHILD						

Cmpd.	No. of Cmpds.	m/z meas.	Δ m/z [ppm]	z	Rt [min]	Score	P	Range	Sequence	Modification
2715	1	856.8870	34.41	2	65.2	15.1	0	82-97	K.SCLTFTDDGCGMTPHK.L	
256	1	715.8400	-65.97	2	33.2	10.1	0	389-401	K.ILTVQEMAGLNNK.T	

Detailed Protein Report

Protein 391: FCH domain only protein 1 isoform a [Homo sapiens]

**Accession:**	gi|239049440	**Score:**	46.8
**Database:**	refseq_human(refseq_human_20140103.fasta)	**MW [kDa]:**	97.0
**Database Date:**	2015-11-30	**pI:**	6.5
**Modification(s):**	Carbamidomethyl	**Sequence Coverage [%]:**	4.0
		**No. of unique Peptides:**	3

```
        10         20         30         40         50         60         70         80
MSYFGEHFWG EKNHGFEVLY HSVKQGPIST KELADFIRER ATIEETYSKA MAKLSKLASN GTPMGTFAPL WEVFRVSSDK
        90        100        110        120        130        140        150        160
LALCHLELTR KLQDLIKDVL RYGEEQLKTH KKCKEEVVST LDAVQVLSGV SQLLPKSREN YLNRCMDQER LRRESTSQKE
       170        180        190        200        210        220        230        240
MDKAETKTKK AAESLRRSVE KYNSARADFE QKMLDSALRF QAMEETHLRH MKALLGSYAH SVEDTHVQIG QVHEEFKQNI
       250        260        270        280        290        300        310        320
ENVSVEMLLR KFAESKGTGR EKPGPLDFEA YSAAALQEAM KRLRGAKAFR LPGLSRRERE PEPPAAVDFL EPDSGTCPEV
       330        340        350        360        370        380        390        400
DEEGFTVRPD VTQNSTAEPS RFSSSDSDFD DEEPRKFYVH IKPAPARAPA CSPEAAAAQL RATAGSLILP PGPGGTMKRH
       410        420        430        440        450        460        470        480
SSRDAAGKPQ RPRSAPRTSS CAERLQSEEQ VSKNLFGPPL ESAFDHEDFT GSSSLGFTSS PSPFSSSSPE NVEDSGLDSP
       490        500        510        520        530        540        550        560
SHAAPGPSPD SWVPRPGTPQ SPPSCRAPPP EARGIRAPPL PDSPQPLASS PGPWGLEALA GGDLMPAPAD PTAREGLAAP
       570        580        590        600        610        620        630        640
PRRLRSRKVS CPLTRSNGDL SRSLSPSPLG SSAASTALER PSFLSQTGHG VSRGPSPVVL GSQDALPIAT AFTEYVHAYF
       650        660        670        680        690        700        710        720
RGHSPSCLAR VTGELTMTFP AGIVRVFSGT PPPPVLSFRL VHTTAIEHFQ PNADLLFSDP SQSDPETKDF WLNMAALTEA
       730        740        750        760        770        780        790        800
LQRQAEQNPT ASYYNVVLLR YQFSRPGPQS VPLQLSAHWQ CGATLTQVSV EYGYRPGATA VPTPLTNVQI LLPVGEPVTN
       810        820        830        840        850        860        870        880
VRLQPAATWN LEEKRLTWRL PDVSEAGGSG RLSASWEPLS GPSTPSPVAA QFTSEGTTLS GVDLELVGSG YRMSLVKRRF
       890        900
ATAAPPQGCT W
```

Cmpd.	No. of Cmpds.	m/z meas.	Δ m/z [ppm]	z	Rt [min]	Score	P	Range	Sequence	Modification
1900	1	693.7929	-23.70	2	53.8	12.5	0	2-12	M.SYFGEHFWGEK.N	
195	1	572.8054	38.56	2	32.4	16.8	0	820-831	R.LPDVSEAGGSGR.L	
2590	1	731.8881	54.25	2	62.9	17.4	1	879-891	R.RFATAAPPQGCTW.-	Carbamidomethyl: 11

Detailed Protein Report

Protein 392: **PREDICTED: uncharacterized protein LOC101928409 [Homo sapiens]**

**Accession:**	gi|530358441	**Score:**	46.7
**Database:**	refseq_human(refseq_human_20140103.fasta)	**MW [kDa]:**	60.4
**Database Date:**	2015-11-30	**pI:**	11.3
**Modification(s):**	Carbamidomethyl	**Sequence Coverage [%]:**	7.0
		**No. of unique Peptides:**	1

Quantitation

Q:W	**Median:** 0.84	**CV:** 0.00 %	**No. of Peptides:**	1
M:Q	**Median:** 0.99	**CV:** 0.00 %	**No. of Peptides:**	1

10	20	30	40	50	60	70	80
MLGTVASPRP	GSLFSPPLAV	IKPGYEQGFK	SSLPHPQVYR	AAPELSDMLR	CGGQRRGGGI	ALCSLGVKSN	MNVKYCGKTE
**90**	**100**	**110**	**120**	**130**	**140**	**150**	**160**
SFQSRQRGPS	AGGPGRPESL	LPRVRLPEAL	LLAEDCRGNV	GEGEMDGGSR	AYKSDKYAHT	LTVTASHHAP	PPPTHMEGFE
**170**	**180**	**190**	**200**	**210**	**220**	**230**	**240**
LFHLPELCSP	SQDAQTTGRT	QMKPDHSPRP	SHRGRWVPKR	LLRLHPSSRV	VVRLVAEGAD	GARFGNTPHC	ILFHFTSTKE
**250**	**260**	**270**	**280**	**290**	**300**	**310**	**320**
ESRIGGRPPS	CFLMQITQHS	NRKSKLQGLL	LRGALEKEER	VVRARAGASE	AKSHSRGGWD	AWGLKANARG	RDGVGSGGRS
**330**	**340**	**350**	**360**	**370**	**380**	**390**	**400**
ALQSRPLPPA	SLEGGSGTCA	RSLSPGLCAS	AWRLPGKNLI	YFLPSRVSSL	PPRAAPFRRP	EPAPGPRGCS	GERGLGMSGG
**410**	**420**	**430**	**440**	**450**	**460**	**470**	**480**
VRAPPHPRAL	LGGDIQELKE	IPQEEVETSE	RRGNRLFCKK	TERRLEGLRK	AADGSQDNSV	RPVLQGNFNP	TRTQAKTIYI
**490**	**500**	**510**	**520**	**530**	**540**	**550**	**560**
YIYELTQNRA	TLVPTPPQVG	RHIQVALSED	GTWRLAGVAL	WRSRSPVSSY	EVSLLLQSSL	QRGDLATSLP	LGFPVNPL

Cmpd.	No. of Cmpds.	m/z meas.	Δ m/z [ppm]	z	Rt [min]	Score	P	Range	Sequence	Modification	Ratios	
1973	1	902.8715	-113.53	2	55.1	11.5	1	57-74	R.GGGIALCSLGVKSNMNVK.Y	Carbamidomethyl: 7	M:Q<br>Q:W	0.99<br>0.84

Detailed Protein Report

Protein 393: glutamine--tRNA ligase isoform b [Homo sapiens]

**Accession:**	gi|441478305	**Score:**	46.7
**Database:**	refseq_human(refseq_human_20140103.fasta)	**MW [kDa]:**	86.5
**Database Date:**	2015-11-30	**pI:**	6.6
**Modification(s):**	Oxidation	**Sequence Coverage [%]:**	7.7
		**No. of unique Peptides:**	1

10	20	30	40	50	60	70	80
MAALDSLSLF	TSLGLSEQKA	RETLKNSALS	AQLREAATQA	QQTLGSTIDK	ATGILLYGLA	SRLRDTRRLS	FLVSYIATAL
**90**	**100**	**110**	**120**	**130**	**140**	**150**	**160**
EYVRSHPLDP	IDTVDFEREC	GVGVIVTPEQ	IEEAVEAAIN	RHRPQLLVER	YHFNMGLLMG	EARAVLKWAD	GKMIKNEVDM
**170**	**180**	**190**	**200**	**210**	**220**	**230**	**240**
QVLHLLGPKL	EADLEKKFKV	AKARLEETDR	RTAKDVVENG	ETADQTLSLM	EQLRGEALKF	HKPGENYKTP	GYVVTPHTMN
**250**	**260**	**270**	**280**	**290**	**300**	**310**	**320**
LLKQHLEITG	GQVRTRFPPE	PNGILHIGHA	KAINFNFGYA	KANNGICFLR	FDDTNPEKEE	AKFFTAICDM	VAWLGYTPYK
**330**	**340**	**350**	**360**	**370**	**380**	**390**	**400**
VTYASDYFDQ	LYAWAVELIR	RGLAYVCHQR	GEELKGHNTL	PSPWRDRPME	ESLLLFEAMR	KGKFSEGEAT	LRMKLVMEDG
**410**	**420**	**430**	**440**	**450**	**460**	**470**	**480**
KMDPVAYRVK	YTPHHRTGDK	WCIYPTYDYT	HCLCDSIEHI	THSLCTKEFQ	ARRSSYFWLC	NALDVYCPVQ	WEYGRLNLHY
**490**	**500**	**510**	**520**	**530**	**540**	**550**	**560**
AVVSKRKILQ	LVATGAVRDW	DDPRLFTLTA	LRRRGFPPEA	INNFCARVGV	TVAQTTMEPH	LLEACVRDVL	NDTAPRAMAV
**570**	**580**	**590**	**600**	**610**	**620**	**630**	**640**
LESLRVIITN	FPAAKSLDIQ	VPNFPADETK	GFHQVPFAPI	VFIERTDFKE	EPEPGFKRLA	WGQPVGLRHT	GYVIELQHVV
**650**	**660**	**670**	**680**	**690**	**700**	**710**	**720**
KGPSGCVESL	EVTCRRADAG	EKPKAFIHWV	SQPLMCEVRL	YERLFQHKNP	EDPTEVPGGF	LSDLNLASLH	VVDAALVDCS
**730**	**740**	**750**	**760**	**770**			
VALAKPFDKF	QFERLGYFSV	DPDSHQGKLV	FNRTVTLKED	PGKV			

Cmpd.	No. of Cmpds.	m/z meas.	Δ m/z [ppm]	z	Rt [min]	Score	P	Range	Sequence	Modification
2780	1	841.6618	-115.85	3	65.2	10.5	2	131-152	R.YHFNMGLLMGEARAVLKWADGK.M	Oxidation: 9

Detailed Protein Report

Protein 394: stromal interaction molecule 2 isoform 2 precursor [Homo sapiens]

**Accession:**	gi|281182822	**Score:**	46.7
**Database:**	refseq_human(refseq_human_20140103.fasta)	**MW [kDa]:**	83.9
**Database Date:**	2015-11-30	**pI:**	6.3
**Modification(s):**	Carbamidomethyl	**Sequence Coverage [%]:**	6.0
		**No. of unique Peptides:**	2

Quantitation

M:Q **Median:** 1.50 **CV:** 0.00 % **No. of Peptides:** 1

10	20	30	40	50	60	70	80
MLVLGLLVAG	AADGCELVPR	HLRGRRATGS	AATAASSPAA	AAGDSPALMT	DPCMSLSPPC	FTEEDRFSLE	ALQTIHKQMD
**90**	**100**	**110**	**120**	**130**	**140**	**150**	**160**
DDKDGGIEVE	ESDEFIREDM	KYKDATNKHS	HLHREDKHIT	IEDLWKRWKT	SEVHNWTLED	TLQWLIEFVE	LPQYEKNFRD
**170**	**180**	**190**	**200**	**210**	**220**	**230**	**240**
NNVKGTTLPR	IAVHEPSFMI	SQLKISDRSH	RQKLQLKALD	VVLFGPLTRP	PHNWMKDFIL	TVSIVIGVGG	CWFAYTQNKT
**250**	**260**	**270**	**280**	**290**	**300**	**310**	**320**
SKEHVAKMMK	DLESLQTAEQ	SLMDLQERLE	KAQEENRNVA	VEKQNLERKM	MDEINYAKEE	ACRLRELREG	AECELSRRQY
**330**	**340**	**350**	**360**	**370**	**380**	**390**	**400**
AEQELEQVRM	ALKKAEKEFE	LRSSWSVPDA	LQKWLQLTHE	VEVQYYNIKR	QNAEMQLAIA	KDEAEKIKKK	RSTVFGTLHV
**410**	**420**	**430**	**440**	**450**	**460**	**470**	**480**
AHSSSLDEVD	HKILEAKKAL	SELTTCLRER	LFRWQQIEKI	CGFQIAHNSG	LPSLTSSLYS	DHSWVVMPRV	SIPPYPIAGG
**490**	**500**	**510**	**520**	**530**	**540**	**550**	**560**
VDDLDEDTPP	IVSQFPGTMA	KPPGSLARSS	SLCRSRRSIV	PSSPQPQRAQ	LAPHAPHPSH	PRHPHHPQHT	PHSLPSPDPD
**570**	**580**	**590**	**600**	**610**	**620**	**630**	**640**
ILSVSSCPAL	YRNEEEEEAI	YFSAEKQWEV	PDTASECDSL	NSSIGRKQSP	PLSLEIYQTL	SPRKISRDEV	SLEDSSRGDS
**650**	**660**	**670**	**680**	**690**	**700**	**710**	**720**
PVTVDVSWGS	PDCVGLTETK	SMIFSPASKV	YNGILEKSCS	MNQLSSGIPV	PKPRHTSCSS	AGNDSKPVQE	APSVARISSI
**730**	**740**	**750**					
PHDLCHNGEK	SKKPSKIKSL	FKKKSK					

Cmpd.	No. of Cmpds.	m/z meas.	Δ m/z [ppm]	z	Rt [min]	Score	P	Range	Sequence	Modification	Ratios
1585	1	942.4058	-100.34	2	49.7	13.0	1	661-677	K.SMIFSPASKVYNGILEK.S		M:Q 1.50
1467	1	619.9415	-65.11	3	46.7	10.7	0	678-694	K.SCSMNQLSSGIPVPKPR.H	Carbamidomethyl: 2	

Detailed Protein Report

Protein 395: serine/threonine-protein kinase WNK3 isoform 2 [Homo sapiens]

**Accession:**	gi|50845416	**Score:**	46.7
**Database:**	refseq_human(refseq_human_20140103.fasta)	**MW [kDa]:**	191.7
**Database Date:**	2015-11-30	**pI:**	5.4
		**Sequence Coverage [%]:**	3.8
		**No. of unique Peptides:**	2

Alias proteins:

**Accession**	**Name**	**Description**
gi|578838231	refseq_human (refseq_human_20140103.fasta)	PREDICTED: serine/threonine-protein kinase WNK3 isoform X2 [Homo sapiens]
gi|578838229	refseq_human (refseq_human_20140103.fasta)	PREDICTED: serine/threonine-protein kinase WNK3 isoform X1 [Homo sapiens]

BRUKER

Detailed Protein Report

10	20	30	40	50	60	70	80
MATDSGDPAS	TEDSEKPDGI	SFENRVPQVA	ATLTVEARLK	EKNSTFSASG	ETVERKRFFR	KSVEMTEDDK	VAESSPKDER
90	100	110	120	130	140	150	160
IKAAMNIPRV	DKLPSNVLRG	GQEVKYEQCS	KSTSEISKDC	FKEKNEKEME	EEAEMKAVAT	SPSGRFLKFD	IELGRGAFKT
170	180	190	200	210	220	230	240
VYKGLDTETW	VEVAWCELQD	RKLTKAEQQR	FKEEAEMLKG	LQHPNIVRFY	DSWESILKGK	KCIVLVTELM	TSGTLKTYLK
250	260	270	280	290	300	310	320
RFKVMKPKVL	RSWCRQILKG	LQFLHTRTPP	IIHRDLKCDN	IFITGPTGSV	KIGDLGLATL	MRTSFAKSVI	GTPEFMAPEM
330	340	350	360	370	380	390	400
YEEHYDESVD	VYAFGMCMLE	MATSEYPYSE	CQNAAQIYRK	VTSGIKPASF	NKVTDPEVKE	IIEGCIRQNK	SERLSIRDLL
410	420	430	440	450	460	470	480
NHAFFAEDTG	LRVELAEEDD	CSNSSLALRL	WVEDPKKLKG	KHKDNEAIEF	SFNLETDTPE	EVAYEMVKSG	FFHESDSKAV
490	500	510	520	530	540	550	560
AKSIRDRVTP	IKKTREKKPA	GCLEERRDSQ	CKSMGNVFPQ	PQNTTLPLAP	AQQTGAECEE	TEVDQHVRQQ	LLQRKPQQHC
570	580	590	600	610	620	630	640
SSVTGDNLSE	AGAASVIHSD	TSSQPSVAYS	SNQTMGSQMV	SNIPQAEVNV	PGQIYSSQQL	VGHYQQVSGL	QKHSKLTQPQ
650	660	670	680	690	700	710	720
ILPLVQGQST	VLPVHVLGPT	VVSQPQVSPL	TVQKVPQIKP	VSQPVGAEQQ	AALLKPDLVR	SLNQDVATTK	ENVSSPDNPS
730	740	750	760	770	780	790	800
GNGKQDRIKQ	RRASCPRPEK	GTKFQLTVLQ	VSTSGDNMVE	CQLETHNNKM	VTFKFDVDGD	APEDIADYMV	EDNFVLESEK
810	820	830	840	850	860	870	880
EKFVEELRAI	VGQAQEILHV	HFATERATGV	DSITVDSNSS	QTGSSEQVQI	NSTSTQTSNE	SAPQSSPVGR	WRFCINQTIR
890	900	910	920	930	940	950	960
NRETQSPPSL	QHSMSAVPGR	HPLPSPKNTS	NKEISRDTLL	TIENNPCHRA	LFTSKSEHKD	VVDGKISECA	SVETKQPAIL
970	980	990	1000	1010	1020	1030	1040
YQVEDNRQIM	APVTNSSSYS	TTSVRAVPAE	CEGLTKQASI	FIPVYPCHQT	ASQADALMSH	PGESTQTSGN	SLTTLAFDQK
1050	1060	1070	1080	1090	1100	1110	1120
PQTLSVQQPA	MDAEFISQEG	ETTVNTEASS	PKTVIPTQTP	GLEPTTLQPT	TVLESDGERP	PKLEFADNRI	KTLDEKLRNL
1130	1140	1150	1160	1170	1180	1190	1200
LYQEHSISSI	YPESQKDTQS	IDSPFSSSAE	DTLSCPVTEV	IAISHCGIKD	SPVQSPNFQQ	TGSKLLSNVA	ASQPANISVF
1210	1220	1230	1240	1250	1260	1270	1280
KRDLNVITSV	PSELCLHEMS	SDASLPGDPE	AYPAAVSSGG	AIHLQTGVET	EEMRSAIAPD	PIPLTRESTA	DTRALNRCKA
1290	1300	1310	1320	1330	1340	1350	1360
MSGSFQRGRF	QVITIPQQQS	AKMTSFGIEH	ISVFSETNHS	SEEAFIKTAK	SQLVEIEPAT	QNPKTSFSYE	KLQALQETCK
1370	1380	1390	1400	1410	1420	1430	1440
ENKGVPKQGD	NFLSFSAACE	TDVSSVTPEK	EFEETSATGS	SMQSGSELLL	KEREILTAGK	QPSSDSEFSA	SLAGSGKSVA
1450	1460	1470	1480	1490	1500	1510	1520
KTGPESNQCL	PHHEEQAYAQ	TQSSLFYSPS	SPMSSDDESE	IEDEDLKVEL	QRLREKHIQE	VVNLQTQQNK	ELQELYERLR
1530	1540	1550	1560	1570	1580	1590	1600
SIKDSKTQST	EIPLPPASPR	RPRSFKSKLR	SRPQSLTHVD	NGIVATDPLC	VESNAASCQQ	SPASKKGMFT	DDLHKLVDDW
1610	1620	1630	1640	1650	1660	1670	1680
TKEAVGNSLI	KPSLNQLKQS	QHKLETENWN	KVSENTPSTM	GYTSTWISSL	SQIRGAVPTS	LPQGLSLPSF	PGPLSSYGMP
1690	1700	1710	1720	1730	1740	1750	
HVCQYNAVAG	AGYPVQWGVI	SGTTQQSVVI	PAQSGGPFQP	GMNMQAFPTS	SVQNPATIPP	GPK	

Cmpd.	No. of Cmpds.	m/z meas.	Δ m/z [ppm]	z	Rt [min]	Score	P	Range	Sequence	Modification
1147	1	956.4746	-119.29	1	43.0	10.6	2	486-493	R.DRVTPIKK.T	
1472	1	918.1282	-10.85	3	46.9	10.8	2	1414-1441	R.EILTAGKQPSSDSEFSASLAGSGKSVAK.T	

Detailed Protein Report

Protein 396: RNA polymerase II subunit A C-terminal domain phosphatase isoform 3 [Homo sapiens]

**Accession:**	gi|321267522	**Score:**	46.6
**Database:**	refseq_human(refseq_human_20140103.fasta)	**MW [kDa]:**	92.3
**Database Date:**	2015-11-30	**pI:**	4.9
		**Sequence Coverage [%]:**	7.1
		**No. of unique Peptides:**	2

Quantitation

Q:W	**Median:**	1.23	**CV:**	0.00 %	**No. of Peptides:**	1
M:Q	**Median:**	0.72	**CV:**	0.00 %	**No. of Peptides:**	1

10	20	30	40	50	60	70	80
MKGLCAECGQ	DLTQLQSKNG	KQQVPLSTAT	VSMVHSVPEL	MVSSEQAEQL	GREDQQRLHR	NRKLVLMVDL	DQTLIHTTEQ
90	100	110	120	130	140	150	160
HCQQMSNKGI	FHFQLGRGEP	MLHTRLRPHC	KDFLEKIAKL	YELHVFTFGS	RLYAHTIAGF	LDPEKKLFSH	RILSRDECID
170	180	190	200	210	220	230	240
PFSKTGNLRN	LFPCGDSMVC	IIDDREDVWK	FAPNLITVKK	YVYFQGTGDM	NAPPGSRESQ	TRKKVNHSRG	TEVSEPSPPV
250	260	270	280	290	300	310	320
RDPEGVTQAP	GVEPSNGLEK	PARELNGSEA	ATPRDSPRPG	KPDERDIWPP	AQAPTSSQEL	AGAPEPQGSC	AQGGRVAPGQ
330	340	350	360	370	380	390	400
RPAQGATGTD	LDFDLSSDSE	SSSESEGTKS	SSSASDGESE	GKRGRQKPKA	APEGAGALAQ	GSSLEPGRPA	APSLPGEAEP
410	420	430	440	450	460	470	480
GAHAPDKEPE	LGGQEEGERD	GLCGLGNGCA	DRKEAETESQ	NSELSGVTAG	ESLDQSMEEE	EEEDTDEDDH	LIYLEEILVR
490	500	510	520	530	540	550	560
VHTDYYAKYD	RYLNKEIEEA	PDIRKIVPEL	KSKVLADVAI	IFSGLHPTNF	PIEKTREHYH	ATALGAKILT	RLVLSPDAPD
570	580	590	600	610	620	630	640
RATHLIAARA	GTEKVLQAQE	CGHLHVVNPD	WLWSCLERWD	KVEEQLFPLR	DDHTKAQREN	SPAAFPDREG	VPPTALFHPM
650	660	670	680	690	700	710	720
PVLPKAQPGP	EVRIYDSNTG	KLIRTGARGP	PAPSSSLPIR	QEPSSFRAVP	PPQPQMFGEE	LPDAQDGEQP	GPSRRKRQPS
730	740	750	760	770	780	790	800
MSETMPLYTL	CKEDLESMDK	EVDDILGEGS	DDSDSEKRRP	EEQEEEPQPR	KPGTRRERTL	GAPASSERSA	AGGRGPRGHK
810	820	830	840	850			
RKLNEEDAAS	ESSRESSNED	EGSSSEADEM	AKALEAELND	LM			

Cmpd.	No. of Cmpds.	m/z meas.	Δ m/z [ppm]	z	Rt [min]	Score	P	Range	Sequence	Modification	Ratios	
2677	1	973.4768	-94.08	2	63.6	15.5	2	662-680	K.LIRTGARGPPAPSSSLPIR.Q		Q:W	1.23
1965	3	899.3643	-116.81	2	52.9	16.3	2	779-797	R.TLGAPASSERSAAGGRGPR.G		M:Q	0.72

Detailed Protein Report

Protein 397: PREDICTED: protein TANC1 isoform X8 [Homo sapiens]

**Accession:**	gi|578805065	**Score:**	46.6
**Database:**	refseq_human(refseq_human_20140103.fasta)	**MW [kDa]:**	183.3
**Database Date:**	2015-11-30	**pI:**	9.8
**Modification(s):**	Oxidation	**Sequence Coverage [%]:**	2.9
		**No. of unique Peptides:**	2

10	20	30	40	50	60	70	80
MTVSYKQVLK	MLKAVLKKSR	EGGKGGKKEA	GSDFGPETSP	VLHLDHSADS	PVSSLPTAED	TYRVSLAKGV	SMSLPSSPLL
**90**	**100**	**110**	**120**	**130**	**140**	**150**	**160**
PRQSHLVQSR	VNKKSPGPVR	KPKYVESPRV	PGDAVIMPFR	EVAKPTEPDE	HATSSTKLED	LSYLDGQRNA	PLRTSIRLPW
**170**	**180**	**190**	**200**	**210**	**220**	**230**	**240**
HNTAGGRAQE	VKARFAPYKP	QDILLKPLLF	EVPSITTDSV	FVGRDWLFHQ	IEENLRNTEL	AENRGAVVVG	NVGFGKTAII
**250**	**260**	**270**	**280**	**290**	**300**	**310**	**320**
SKLVALSCHG	SRMRQIASNS	PGSSPKTSDP	TQDLHFTPLL	SPSSSTSASS	TAKTPLGSIS	AENQRPREDA	VKYLASKVVA
**330**	**340**	**350**	**360**	**370**	**380**	**390**	**400**
YHYCQADNTY	TCLVPEFVHS	IAALLCRSHQ	LAAYRDLLIK	EPQLQSMLSL	RSCVQDPVAA	FKRGVLEPLT	NLRNEQKIPE
**410**	**420**	**430**	**440**	**450**	**460**	**470**	**480**
EEYIILIDGL	NEAEFHKPDY	GDTLSSFITK	IISKFPAWLK	LIVTVRANFQ	EIISALPFVK	LSLDDFPDNK	DIHSDLHAYV
**490**	**500**	**510**	**520**	**530**	**540**	**550**	**560**
QHRVHSSQDI	LSNISLNGKA	DATLIGKVSS	HLVLRSLGSY	LYLKLTLDLF	QRGHLVIKSA	SYKVVPVSLS	ELYLLQCNMK
**570**	**580**	**590**	**600**	**610**	**620**	**630**	**640**
FMTQSAFERA	LPILNVALAS	LHPMTDEQIF	QAINAGHIQG	EQGWEDFQQR	MDALSCFLIK	RRDKTRMFCH	PSFREWLVWR
**650**	**660**	**670**	**680**	**690**	**700**	**710**	**720**
ADGENTAFLC	EPRNGHALLA	FMFSRQEGKL	NRQQTMELGH	HILKAHIFKG	LSKKTGISSS	HLQALWIGYS	TEGLSAALAS
**730**	**740**	**750**	**760**	**770**	**780**	**790**	**800**
LRNLYTPNVK	VSRLLILGGA	NVNYRTEVLN	NAPILCVQSH	LGHEEVVTLL	LEFGACLDGT	SENGMTALCY	AAAAGHMKLV
**810**	**820**	**830**	**840**	**850**	**860**	**870**	**880**
CLLTKKGVRV	DHLDKKGQCA	LVHSALRGHG	DILQYLLTCE	WSPGPPQPGT	LRKSHALQQA	LTAAASMGHS	SVVQCLLGME
**890**	**900**	**910**	**920**	**930**	**940**	**950**	**960**
KEHEVEVNGT	DTLWGETALT	AAAGRGKLEV	CELLLGHGAA	VSRTNRRGVP	PLFCAARQGH	WQIVRLLLER	GCDVNLSDKQ
**970**	**980**	**990**	**1000**	**1010**	**1020**	**1030**	**1040**
GRTPLMVAAC	EGHLSTVEFL	LSKGAALSSL	DKEGLSALSW	ACLKGHRAVV	QYLVEEGAAI	DQTDKNGRTP	LDLAAFYGDA
**1050**	**1060**	**1070**	**1080**	**1090**	**1100**	**1110**	**1120**
ETVLYLVEKG	AVIEHVDHSG	MRPLDRAIGC	RNTSVVVALL	RKGAKLGNAA	WAMATSKPDI	LIILLQKLME	EGNVMYKKGK
**1130**	**1140**	**1150**	**1160**	**1170**	**1180**	**1190**	**1200**
MKEAAQRYQY	ALRKFPREGF	GEDMRPFNEL	RVSLYLNLSR	CRRKTNDFGM	AEEFASKALE	LKPKSYEAFY	ARARAKRNSR
**1210**	**1220**	**1230**	**1240**	**1250**	**1260**	**1270**	**1280**
QFVAALADLQ	EAVKLCPTNQ	EVKRLLARVE	EECKQLQRSQ	QQKQQGPLPA	PLNDSENEED	TPTPGLSDHF	HSEETEEEET
**1290**	**1300**	**1310**	**1320**	**1330**	**1340**	**1350**	**1360**
SPQEESVSPT	PRSQPSSSVP	SSYIRNLQEG	LQSKGRPVSP	QSRAGIGKSL	REPVAQPGLL	LQPSKQAQIV	KTSQHLGSGQ
**1370**	**1380**	**1390**	**1400**	**1410**	**1420**	**1430**	**1440**
SAVRNGSMKV	QISSQNPPPS	PMPGRIAATP	AGSRTQHLEG	TGTFTTRAGC	GHFGDRLGPS	QNVRLQCGEN	GPAHPLPSKT
**1450**	**1460**	**1470**	**1480**	**1490**	**1500**	**1510**	**1520**
KTTERLLSHS	SVAVDAAPPN	QGGLATCSDV	RHPASLTSSG	SSGSPSSSIK	MSSSTSSLTS	SSSFSDGFKV	QGPDTRIKDK
**1530**	**1540**	**1550**	**1560**	**1570**	**1580**	**1590**	**1600**
VVTHVQSGTA	EHRPRNTPFM	GIMDKTARFQ	QQSNPPSRSW	HCPAPEGLLT	NTSSAAGLQS	ANTEKPSLMQ	VGGYNNQAKT
**1610**	**1620**	**1630**	**1640**	**1650**	**1660**	**1670**	**1680**
CSVSTLSASV	HNGAQVKELE	ESKCQIPVHS	QENRITKTVS	HLYQESISKQ	QPHISNEAHR	SHLTAAKPKR	SFIESNV

Cmpd.	No. of Cmpds.	m/z meas.	Δ m/z [ppm]	z	Rt [min]	Score	P	Range	Sequence	Modification
2221	1	953.0764	108.03	2	58.2	19.2	0	1050-1066	K.GAVIEHVDHSGMRPLDR.A	Oxidation: 12
2803	2	973.5138	96.14	2	64.1	10.3	0	1491-1509	K.MSSSTSSLTSSSSFSDGFK.V	Oxidation: 1

Detailed Protein Report

Protein 398: PREDICTED: rho guanine nucleotide exchange factor 5-like, partial [Homo sapiens]

**Accession:**	gi|578844357	**Score:**	46.6
**Database:**	refseq_human(refseq_human_20140103.fasta)	**MW [kDa]:**	165.0
**Database Date:**	2015-11-30	**pI:**	5.4
		**Sequence Coverage [%]:**	2.9
		**No. of unique Peptides:**	2

10	20	30	40	50	60	70	80
FSIIPEAPMR	SSQVSALGLE	AQEDEDPSYK	WREEHRLSAT	QQSELRDVCD	YAIETMPSFP	KEGSADVEPN	QESLVAEACD
**90**	**100**	**110**	**120**	**130**	**140**	**150**	**160**
TPEHWEAVPQ	SLAGRQARTL	APPELWACPI	QSEHLDMAPF	SSDLGSEEEE	VEFWPGLTSL	TLGSGQAEEE	EETSSDNSGQ
**170**	**180**	**190**	**200**	**210**	**220**	**230**	**240**
TRYYSPCEEH	PAETNQNEGS	ESGTIRQGEE	LPPEELQESQ	GLLHPQEVQV	LEEQGQQEAG	FRGEGTLRED	VCADGLLGEE
**250**	**260**	**270**	**280**	**290**	**300**	**310**	**320**
QMIEQVNDEK	GEQKQKQEQV	QDVMLGRQGE	RMGLTGEPEG	LNDGEWEQED	MERKAQGQGG	PEQGEERKRE	LQVPEENRAD
**330**	**340**	**350**	**360**	**370**	**380**	**390**	**400**
SQDEKSQIFL	GKSEEVTGKQ	EDHGIKEKGV	PVSGQEAKEP	ESWDGGRLGA	VGRARSREEE	NEHHGPSMPA	LIAPEDSPHC
**410**	**420**	**430**	**440**	**450**	**460**	**470**	**480**
DLFPGASYLM	TQIPGTQTES	RAEELSPAAL	SPSLEPIRCS	HQPISLLGSF	LTEESPDKEI	DQNSQQEGSR	LRKGTVSSQG
**490**	**500**	**510**	**520**	**530**	**540**	**550**	**560**
TEVVFASASV	TPPRTPDSAP	PSPAEAYPIT	PASVSARPPV	AFPRRETSCA	ARAPETASAP	LSMDDPSPCG	TSEMCPAALY
**570**	**580**	**590**	**600**	**610**	**620**	**630**	**640**
GFPSTGTSPP	RPPANSTGTV	QHLRSDSFPG	SHRTEQTPDL	VGMLLSYSHS	ELPQRPPKPA	IYSSVTPRRD	RRSGRDYSTV
**650**	**660**	**670**	**680**	**690**	**700**	**710**	**720**
SASPTALSTL	KQDSQESISN	LERPSSPPSI	QPWVSPHNPA	FATESPAYGS	SPSFVSMEDV	RIHEPLPPPP	PQRRDTHPSV
**730**	**740**	**750**	**760**	**770**	**780**	**790**	**800**
VETDGHARVV	VPTLKQHSHP	PPLALGSGLH	APHKGPLPQA	SDPAVARQHR	PLPSTPDSSH	HAQATPRWRY	NKPLPPTPDL
**810**	**820**	**830**	**840**	**850**	**860**	**870**	**880**
PQPHLPPISA	PGSSRIYRPL	PPLPIIDPPT	EPPPLPPKSR	GRSRSTRGGH	MNSGGHAKTR	PACQDWTVPL	PASAGRTSWP
**890**	**900**	**910**	**920**	**930**	**940**	**950**	**960**
PATARSTESF	TSTSRSKSEV	SPGMAFSNMT	NFLCPSSPTT	PWTPELQGPT	SKDEAGVSEH	PEAPAREPLR	RTTPQQGASG
**970**	**980**	**990**	**1000**	**1010**	**1020**	**1030**	**1040**
PGRSPVGQAR	QPEKPSHLHL	EKASSWPHRR	DSGRPPGDSS	GQAVAPSEGA	NKHKGWSRQG	LRRPSILPEG	SSDSRGPAVE
**1050**	**1060**	**1070**	**1080**	**1090**	**1100**	**1110**	**1120**
KHPGPSDTVV	FREKKPKEVM	GGFSRRCSKL	INSSQLLYQE	YSDVVLNKEI	QSQQRLESLS	ETPGPSSPRQ	PRKALVSSES
**1130**	**1140**	**1150**	**1160**	**1170**	**1180**	**1190**	**1200**
YLQRLSMASS	GSLWQEIPVV	RNSTVLLSMT	HEDQKLQEVK	FELIVSEASY	LRSLNIAVDH	FQLSTSLRAT	LSNQEHQWLF
**1210**	**1220**	**1230**	**1240**	**1250**	**1260**	**1270**	**1280**
SRLQDVRDVS	ATFLSDLEEN	FENNIFSFQV	CDVVLNHAPD	FRRVYLPYVT	NQTYQERTFQ	SLMNSNSNFR	EVLEKLESDP
**1290**	**1300**	**1310**	**1320**	**1330**	**1340**	**1350**	**1360**
VCQRLSLKSF	LILPFQRITR	LKLLLQNILK	RTQPGSSEEA	EATKAHHALE	QLIRDCNNNV	QSMRRTEELI	YLSQKIEFEC
**1370**	**1380**	**1390**	**1400**	**1410**	**1420**	**1430**	**1440**
KIFPLISQSR	WLVKSGELTA	LEFSASPGLR	RKLNTRPVHL	HLFNDCLLLS	RPREGSRFLV	FDHAPFSSIR	GEKCEMKLHG
**1450**	**1460**	**1470**	**1480**	**1490**	**1500**		
PHKNLFRLFL	RQNTQGAQAE	FLFRTETQSE	KLRWISALAM	PREELDLLEC	YTM		

Cmpd.	No. of Cmpds.	m/z meas.	Δ m/z [ppm]	z	Rt [min]	Score	P	Range	Sequence	Modification
2416	1	447.0974	-266.42	2	60.5	12.0	1	525-532	R.RETSCAAR.A	
1626	1	1058.4994	-29.00	2	49.0	12.0	2	933-951	K.DEAGVSEHPEAPAREPLRR.T	

Detailed Protein Report

Protein 399: cytoplasmic dynein 1 heavy chain 1 [Homo sapiens]

**Accession:**	gi|33350932	**Score:**	46.6
**Database:**	refseq_human(refseq_human_20140103.fasta)	**MW [kDa]:**	532.1
**Database Date:**	2015-11-30	**pI:**	6.0
**Modification(s):**	Oxidation	**Sequence Coverage [%]:**	0.8
		**No. of unique Peptides:**	1

Detailed Protein Report

10	20	30	40	50	60	70	80
MSEPGGGGGE	DGSAGLEVSA	VQNVADVSVL	QKHLRKLVPL	LLEDGGEAPA	ALEAALEEKS	ALEQMRKFLS	DPQVHTVLVE
90	100	110	120	130	140	150	160
RSTLKEDVGD	EGEEEKEFIS	YNINIDIHYG	VKSNSLAFIK	RTPVIDADKP	VSSQLRVLTL	SEDSPYETLH	SFISNAVAPF
170	180	190	200	210	220	230	240
FKSYIRESGK	ADRDGDKMAP	SVEKKIAELE	MGLLHLQQNI	EIPEISLPIH	PMITNVAKQC	YERGEKPKVT	DFGDKVEDPT
250	260	270	280	290	300	310	320
FLNQLQSGVN	RWIREIQKVT	KLDRDPASGT	ALQEISFWLN	LERALYRIQE	KRESPEVLLT	LDILKHGKRF	HATVSFDTDT
330	340	350	360	370	380	390	400
GLKQALETVN	DYNPLMKDFP	LNDLLSATEL	DKIRQALVAI	FTHLRKIRNT	KYPIQRALRL	VEAISRDLSS	QLLKVLGTRK
410	420	430	440	450	460	470	480
LMHVAYEEFE	KVMVACFEVF	QTWDDEYEKL	QVLLRDIVKR	KREENLKMVW	RINPAHRKLQ	ARLDQMRKFR	RQHEQLRAVI
490	500	510	520	530	540	550	560
VRVLRPQVTA	VAQQNQGEVP	EPQDMKVAEV	LFDAADANAI	EEVNLAYENV	KEVDGLDVSK	EGTEAWEAAM	KRYDERIDRV
570	580	590	600	610	620	630	640
ETRITARLRD	QLGTAKNANE	MFRIFSRFNA	LFVRPHIRGA	IREYQTQLIQ	RVKDDIESLH	DKFKVQYPQS	QACKMSHVRD
650	660	670	680	690	700	710	720
LPPVSGSIIW	AKQIDRQLTA	YMKRVEDVLG	KGWENHVEGQ	KLKQDGDSFR	MKLNTQEIFD	DWARKVQQRN	LGVSGRIFTI
730	740	750	760	770	780	790	800
ESTRVRGRTG	NVLKLKVNFL	PEIITLSKEV	RNLKWLGFRV	PLAIVNKAHQ	ANQLYPFAIS	LIESVRTYER	TCEKVEERNT
810	820	830	840	850	860	870	880
ISLLVAGLKK	EVQALIAEGI	ALVWESYKLD	PYVQRLAETV	FNFQEKVDDL	LIIEEKIDLE	VRSLETCMYD	HKTFSEILNR
890	900	910	920	930	940	950	960
VQKAVDDLNL	HSYSNLPIWV	NKLDMEIERI	LGVRLQAGLR	AWTQVLLGQA	EDKAEVDMDT	DAPQVSHKPG	GEPKIKNVVH
970	980	990	1000	1010	1020	1030	1040
ELRITNQVIY	LNPPIEECRY	KLYQEMFAWK	MVVLSLPRIQ	SQRYQVGVHY	ELTEEEKFYR	NALTRMPDGP	VALEESYSAV
1050	1060	1070	1080	1090	1100	1110	1120
MGIVSEVEQY	VKVWLQYQCL	WDMQAENIYN	RLGEDLNKWQ	ALLVQIRKAR	GTFDNAETKK	EFGPVVIDYG	KVQSKVNLKY
1130	1140	1150	1160	1170	1180	1190	1200
DSWHKEVLSK	FGQMLGSNMT	EFHSQISKSR	QELEQHSVDT	ASTSDAVTFI	TYVQSLKRKI	KQFEKQVELY	RNGQRLLEKQ
1210	1220	1230	1240	1250	1260	1270	1280
RFQFPPSWLY	IDNIEGEWGA	FNDIMRRKDS	AIQQQVANLQ	MKIVQEDRAV	ESRTTDLLTD	WEKTKPVTGN	LRPEEALQAL
1290	1300	1310	1320	1330	1340	1350	1360
TIYEGKFGRL	KDDREKCAKA	KEALELTDTG	LLSGSEERVQ	VALEELQDLK	GVWSELSKVW	EQIDQMKEQP	WVSVQPRKLR
1370	1380	1390	1400	1410	1420	1430	1440
QNLDALLNQL	KSFPARLRQY	ASYEFVQRLL	KGYMKINMLV	IELKSEALKD	RHWKQLMKRL	HVNWVVSELT	LGQIWDVDLQ
1450	1460	1470	1480	1490	1500	1510	1520
KNEAIVKDVL	LVAQGEMALE	EFLKQIREVW	NTYELDLVNY	QNKCRLIRGW	DDLFNKVKEH	INSVSAMKLS	PYYKVFEEDA
1530	1540	1550	1560	1570	1580	1590	1600
LSWEDKLNRI	MALFDVWIDV	QRRWVYLEGI	FTGSADIKHL	LPVETQRFQS	ISTEFLALMK	KVSKSPLVMD	VLNIQGVQRS
1610	1620	1630	1640	1650	1660	1670	1680
LERLADLLGK	IQKALGEYLE	RERSSFPRFY	FVGDEDLLEI	IGNSKNVAKL	QKHFKKMFAG	VSSIILNEDN	SVVLGISSRE
1690	1700	1710	1720	1730	1740	1750	1760
GEEVMFKTPV	SITEHPKINE	WLTLVEKEMR	VTLAKLLAES	VTEVEIFGKA	TSIDPNTYIT	WIDKYQAQLV	VLSAQIAWSE
1770	1780	1790	1800	1810	1820	1830	1840
NVETALSSMG	GGGDAAPLHS	VLSNVEVTLN	VLADSVLMEQ	PPLRRRKLEH	LITELVHQRD	VTRSLIKSKI	DNAKSFEWLS
1850	1860	1870	1880	1890	1900	1910	1920
QMRFYFDPKQ	TDVLQQLSIQ	MANAKFNYGF	EYLGVQDKLV	QTPLTDRCYL	TMTQALEARL	GGSPFGPAGT	GKTESVKALG
1930	1940	1950	1960	1970	1980	1990	2000
HQLGRFVLVF	NCDETFDFQA	MGRIFVGLCQ	VGAWGCFDEF	NRLEERMLSA	VSQQVQCIQE	ALREHSNPNY	DKTSAPITCE
2010	2020	2030	2040	2050	2060	2070	2080
LLNKQVKVSP	DMAIFITMNP	GYAGRSNLPD	NLKKLFRSLA	MTKPDRQLIA	QVMLYSQGFR	TAEVLANKIV	PFFKLCDEQL
2090	2100	2110	2120	2130	2140	2150	2160
SSQSHYDFGL	RALKSVLVSA	GNVKRERIQK	IKREKEERGE	AVDEGEIAEN	LPEQEILIQS	VCETMVPKLV	AEDIPLLFSL
2170	2180	2190	2200	2210	2220	2230	2240

Detailed Protein Report

Cmpd.	No. of Cmpds.	m/z meas.	Δ m/z [ppm]	z	Rt [min]	Score	P	Range	Sequence	Modification
1836	1	805.7966	-107.51	2	53.0	18.5	1	570-583	R.DQLGTAKNANEMFR.I	Oxidation: 12

Detailed Protein Report

Protein 400: PREDICTED: tau-tubulin kinase 2 isoform X5 [Homo sapiens]

**Accession:**	gi|578826647	**Score:**	46.6
**Database:**	refseq_human(refseq_human_20140103.fasta)	**MW [kDa]:**	129.8
**Database Date:**	2015-11-30	**pI:**	6.5
**Modification(s):**	Oxidation	**Sequence Coverage [%]:**	3.7
		**No. of unique Peptides:**	2

Quantitation

M:Q **Median:** 1.76 **CV:** 0.00 % **No. of Peptides:** 1

10	20	30	40	50	60	70	80
MLPGKDHVCR	FIGCGRNDRF	NYVVMQLQGR	NLADLRRSQS	RGTFTISTTL	RLGRQILESI	ESIHSVGFLH	RDIKPSNFAM
**90**	**100**	**110**	**120**	**130**	**140**	**150**	**160**
GRFPSTCRKC	YMLDFGLARQ	FTNSCGDVRP	PRAVAGFRGT	VRYASINAHR	NREMGRHDDL	WSLFYMLVEF	VVGQLPWRKI
**170**	**180**	**190**	**200**	**210**	**220**	**230**	**240**
KDKEQVGSIK	ERYDHRLMLK	HLPPEFSIFL	DHISSLDYFT	KPDYQLLTSV	FDNSIKTFGV	IESDPFDWEK	TGNDGSLTTT
**250**	**260**	**270**	**280**	**290**	**300**	**310**	**320**
TTSTTPQLHT	RLTPAAIGIA	NATPIPGDLL	RENTDEVFPD	EQLSDGENGI	PVGVSPDKLP	GSLGHPRPQE	KDVWEEMDAN
**330**	**340**	**350**	**360**	**370**	**380**	**390**	**400**
KNKIKLGICK	AATEEENSHG	QANGLLNAPS	LGSPIRVRSE	ITQPDRDIPL	VRKLRSIHSF	ELEKRLTLEP	KPDTDKFLET
**410**	**420**	**430**	**440**	**450**	**460**	**470**	**480**
CLEKMQKDTS	AGKESILPAL	LHKPCVPAVS	RTDHIWHYDE	EYLPDASKPA	SANTPEQADG	GGSNGFIAVN	LSSCKQEIDS
**490**	**500**	**510**	**520**	**530**	**540**	**550**	**560**
KEWVIVDKEQ	DLQDFRTNEA	VGHKTTGSPS	DEEPEVLQVL	EASPQDEKLQ	LGPWAENDHL	KKETSGVVLA	LSAEGPPTAA
**570**	**580**	**590**	**600**	**610**	**620**	**630**	**640**
SEQYTDRLEL	QPGAASQFIA	ATPTSLMEAQ	AEGPLTAITI	PRPSVASTQS	TSGSFHCGQQ	PEKKDLQPME	PTVELYSPRE
**650**	**660**	**670**	**680**	**690**	**700**	**710**	**720**
NFSGLVVTEG	EPPSGGSRTD	LGLQIDHIGH	DMLPNIRESN	KSQDLGPKEL	PDHNRLVVRE	FENLPGETEE	KSILLESDNE
**730**	**740**	**750**	**760**	**770**	**780**	**790**	**800**
DEKLSRGQHC	IEISSLPGDL	VIVEKDHSAT	TEPLDVTKTQ	TFSVVPNQDK	NNEIMKLLTV	GTSEISSRDI	DPHVEGQIGQ
**810**	**820**	**830**	**840**	**850**	**860**	**870**	**880**
VAEMQKNKIS	KDDDIMSEDL	PGHQGDLSTF	LHQEGKREKI	TPRNGELFHC	VSENEHGAPT	RKDMVRSSFV	TRHSRIPVLA
**890**	**900**	**910**	**920**	**930**	**940**	**950**	**960**
QEIDSTLESS	SPVSAKEKLL	QKKAYQPDLV	KLLVEKRQFK	SFLGDLSSAS	DKLLEEKLAT	VPAPFCEEEV	LTPFSRLTVD
**970**	**980**	**990**	**1000**	**1010**	**1020**	**1030**	**1040**
SHLSRSAEDS	FLSPIISQSR	KSKIPRPVSW	VNTDQVNSST	SSQFFPRPPP	GKPPTRPGVE	ARLRRYKVLG	SSNSDSDLFS
**1050**	**1060**	**1070**	**1080**	**1090**	**1100**	**1110**	**1120**
RLAQILQNGS	QKPRSTTQCK	SPGSPHNPKT	PPKSPVVPRR	SPSASPRSSS	LPRTSSSSPS	RAGRPHHDQR	SSSPHLGRSK
**1130**	**1140**	**1150**	**1160**	**1170**	**1180**		
SPPSHSGSSS	SRRSCQQEHC	KPSKNGLKGS	GSLHHHSAST	KTPQGKSKPA	SKLSR		

Cmpd.	No. of Cmpds.	m/z meas.	Δ m/z [ppm]	z	Rt [min]	Score	P	Range	Sequence	Modification	Ratios
1491	1	626.6231	-220.31	2	48.3	12.6	0	312-321	K.DVWEEMDANK.N	Oxidation: 6	
2024	1	694.3465	21.44	2	55.7	10.8	1	1119-1132	R.SKSPPSHSGSSSSR.R		M:Q 1.76

Detailed Protein Report

Protein 401: PREDICTED: G-protein coupled receptor 124 isoform X3 [Homo sapiens]

Accession: gi|578815277
Database: refseq_human(refseq_human_20140103.fasta)
Database Date: 2015-11-30
Modification(s): Carbamidomethyl

Score: 46.5
MW [kDa]: 140.0
pI: 9.9
Sequence Coverage [%]: 2.6
No. of unique Peptides: 2

10	20	30	40	50	60	70	80
MGAGGRRMRG	APARLLLPLL	PWLLLLLAPE	ARGAPGCPLS	IRSCKCSGER	PKGLSGGVPG	PARRRVVCSG	GDLPEPPEPG
90	100	110	120	130	140	150	160
LLPNGTVTLD	LRNNIISTVQ	PGAFLGLGEL	KRLDLSNNRI	GCLTSETFQG	LPRLLRLNIS	GNIFSSLQPG	VFDELPALKV
170	180	190	200	210	220	230	240
VDLGTEFLTC	DCHLRWLLPW	AQNRSLQLSE	HTLCAYPSAL	HAQALGSLQE	AQLCCEGALE	LHTHHLIPSL	RQVVFQGDRL
250	260	270	280	290	300	310	320
PFQCSASYLG	NDTRIRWYHN	RAPVEGDEQA	GILLAESLIH	DCTFITSELT	LSHIGVWASG	EWECTVSMAQ	GNASKKVEIV
330	340	350	360	370	380	390	400
VLETSASYCP	AERVANNRGD	FRWPRTLAGI	TAYQSCLQYP	FTSVPLGGGA	PGTRASRRCD	RAGRWEPGDY	SHCLYTNDIT
410	420	430	440	450	460	470	480
RVLYTFVLMP	INASNALTLA	HQLRVYTAEA	ASFSDMMDVV	YVAQMIQKFL	GYVDQIKELV	EVMVDMASNL	MLVDEHLLWL
490	500	510	520	530	540	550	560
AQREDKACSR	IVGALERIGG	AALSPHAQHI	SVNARNVALE	AYLIKPHSYV	GLTCTAFQRR	EGGVPGTRPG	SPGQNPPPEP
570	580	590	600	610	620	630	640
EPPADQQLRF	RCTTGRPNVS	LSSFHIKNSV	ALASIQLPPS	LFSSLPAALA	PPVPPDCTLQ	LLVFRNGRLF	HSHSNTSRPG
650	660	670	680	690	700	710	720
AAGPGKRRGV	ATPVIFAGTS	GCGVGNLTEP	VAVSLRHWAE	GAEPVAAWWS	QEGPGEAGGW	TSEGCQLRSS	QPNVSALHCQ
730	740	750	760	770	780	790	800
HLGNVAVLME	LSAFPREVGG	AGAGLHPVVY	PCTALLLLCL	FATIITYILN	HSSIRVSRKG	WHMLLNLCFH	IAMTSAVFAG
810	820	830	840	850	860	870	880
GITLTNYQMV	CQAVGITLHY	SSLSTLLWMG	VKARVLHKEL	TWRAPPPQEG	DPALPTPSPM	LRFYLIAGGI	PLIICGITAA
890	900	910	920	930	940	950	960
VNIHNYRDHS	PYCWLVWRPS	LGAFYIPVAL	ILLITWIYFL	CAGLRLRGPL	AQNPKAGNSR	ASLEAGEELR	GSTRLRGSGP
970	980	990	1000	1010	1020	1030	1040
LLSDSGSLLA	TGSARVGTPG	PPEDGDSLYS	PGVQLGALVT	THFLYLAMWA	CGALAVSQRW	LPRVVCSCLY	GVAASALGLF
1050	1060	1070	1080	1090	1100	1110	1120
VFTHHCARRR	DVRASWRACC	PPASPAAPHA	PPRALPAAAE	DGSPVFGEGP	PSLKSSPSGS	SGHPLALGPC	KLTNLQLAQS
1130	1140	1150	1160	1170	1180	1190	1200
QVCEAGAAAG	GEGEPEPAGT	RGNLAHRHPN	NVHHGRRAHK	SRAKGHRAGE	ACGKNRLKAL	RGGAAGALEL	LSSESGSLHN
1210	1220	1230	1240	1250	1260	1270	1280
SPTDSYLGSS	RNSPGAGLQL	EGEPMLTPSE	GSDTSAAPLS	EAGRAGQRRS	ASRDSLKGGG	ALEKESHRRS	YPLNAASLNG
1290	1300	1310	1320				
APKGGKYDDV	TLMGAEVASG	GCMKTGLWKS	ETTV				

Cmpd.	No. of Cmpds.	m/z meas.	Δ m/z [ppm]	z	Rt [min]	Score	P	Range	Sequence	Modification
2732	2	673.3595	29.93	2	64.5	15.8	1	33-45	R.GAPGCPLSIRSCK.C	Carbamidomethyl: 5
1660	1	716.0007	12.17	3	50.5	14.1	1	1284-1304	K.GGKYDDVTLMGAEVASGGCMK.T	Carbamidomethyl: 19

Detailed Protein Report

Protein 402: **PREDICTED: Golgin subfamily A member 8A isoform X1 [Homo sapiens]**

Accession: gi|578826741
Database: refseq_human(refseq_human_20140103.fasta)
Database Date: 2015-11-30

Score: 46.5
MW [kDa]: 67.1
pI: 6.4
Sequence Coverage [%]: 4.8
No. of unique Peptides: 1

10	20	30	40	50	60	70	80
MAEETGQSKL	AAAKKKFKEY	WQRNRPGVPA	AAKRNTKANG	SSPETAASGG	CHSSEASSSA	SSSLHARQSP	CQEQAAVLNS
90	100	110	120	130	140	150	160
RSIKISRLND	TIKSLKQQKK	QVEHQLEEEK	KANNEKQKAE	RELEGQIQRL	NTEKKKLNTD	LYHMKHSLRY	FEEESKDLAG
170	180	190	200	210	220	230	240
RLQRSSQRIG	ELEWSLCAVA	ATQKKKPDGF	SSRSKALLKR	QLEQSIREQI	LLKGHVTQLK	ESLKEVQLER	DQYAEQIKGE
250	260	270	280	290	300	310	320
RAQWQQRMRK	MSQEVCTLKE	EKKHDTHRVE	ELERSLSRLK	NQMAEPLPPD	APAVSSEVEL	QDLRKELERV	AGELQAQVEN
330	340	350	360	370	380	390	400
NQCISLLNRG	QKERLREQEE	RLQEQQERLR	EREKRLQQLA	EPQSDLEELH	ENKSALQLEQ	QVKELQEKLG	QVMETLTSAE
410	420	430	440	450	460	470	480
KEPEAAVPAS	GTGGESSGLM	DLLEEKADLR	EHVEKLELGF	IQYRRERCHQ	KVHRLLTEPG	DSAKDASPGG	GHHQAGPGQG
490	500	510	520	530	540	550	560
GEEGEAAGAA	GDGVAACGSY	SEGHGKFLAA	ARNPAAEPSP	GAPAPQELGA	ADKHGDLCEA	SLTNSVEPAQ	GEAREGSSQD
570	580	590	600	610			
NPTAQPVVQL	LGEMQDHQEH	PGLGSNCCVP	CFCWAWLPRR	RR			

Cmpd.	No. of Cmpds.	m/z meas.	Δ m/z [ppm]	z	Rt [min]	Score	P	Range	Sequence	Modification
865	1	647.7984	-105.89	2	40.3	15.4	1	436-445	K.LELGFIQYRR.E	

Detailed Protein Report

Protein 403: PREDICTED: proteasome-associated protein ECM29 homolog isoform X5 [Homo sapiens]

**Accession:**	gi|530390837	**Score:**	46.5
**Database:**	refseq_human(refseq_human_20140103.fasta)	**MW [kDa]:**	203.7
**Database Date:**	2015-11-30	**pI:**	6.7
**Modification(s):**	Oxidation	**Sequence Coverage [%]:**	3.0
		**No. of unique Peptides:**	1

Quantitation

Q:W	**Median:**	0.20	**CV:**	0.00 %	**No. of Peptides:**	1
M:Q	**Median:**	4.39	**CV:**	0.00 %	**No. of Peptides:**	1

Detailed Protein Report

10	20	30	40	50	60	70	80
METGSDSDQL	ERVFLRLGHA	ETDEQLQNII	SKFLPPVLLK	LSSTQEGVRK	KVMELLVHLN	KRIKSRPKIQ	LPVETLLVQY
90	100	110	120	130	140	150	160
QDPAAVSFVT	NFTIIYVKMG	YPRLPVEKQC	ELAPTLLTAM	EGKPQPQQDS	LMHLLIPTLF	HMKYPVESSK	SASPFNLAEK
170	180	190	200	210	220	230	240
PKTVQLLLDF	MLDVLLMPYG	YVLNESQSRQ	NSSSAQGSSS	NSGGGSGIPQ	PPPGMSFYAA	KRVIGDNPWT	PEQLEQCKLG
250	260	270	280	290	300	310	320
IVKFIEAEQV	PELEAVLHLV	IASSDTRHSV	ATAADLELKS	KQSLIDWNNP	AIINKMYKVY	LGDIPLKTKE	GAVLKPELKR
330	340	350	360	370	380	390	400
DPVSTRVKLK	IVPHLLRSRQ	AAETFPANIQ	VVYDGLFGTN	TNSKLRTLSL	QFVHHICITC	PEIKIKPLGP	MLLNGLTKLI
410	420	430	440	450	460	470	480
NEYKEDPKLL	SMAYSAVGKL	SSRMPHLFTK	DIALVQQLFE	ALCKEEPETR	LAIQEALSMM	VGAYSTLEGA	QRTLMEALVA
490	500	510	520	530	540	550	560
SYLIKPEVQV	RQVAVKFAST	VFPSDHIPSR	YLLLLAAGDP	REEVHGEAQR	VLRCLPGRNR	KESTSEQMPS	FPEMVYYIQE
570	580	590	600	610	620	630	640
KASHRMKTPV	KYMTGTTVLP	FNPAAFGEIV	LYLRMCLAHS	AGVVPTSQSL	ADMQDHAPAI	GRYIRTLMSS	GQMAPSSSNK
650	660	670	680	690	700	710	720
SGETNPVQIY	IGLLQQLLAG	VGGLPVMYCL	LEAVSVYPEK	LATKFVDKTE	WIKSLMNNSK	EEMRELAALF	YSVVVSTVSG
730	740	750	760	770	780	790	800
NELKSMIEQL	IKTTKDNHSP	EIQHGSLLAL	GFTVGRYLAK	KKMRMSEQQD	LERNADTLPD	QEELIQSATE	TIGSFLDSTS
810	820	830	840	850	860	870	880
PLLAIAACTA	LGEIGRNGPL	PIPSEGSGFT	KLHLVESLLS	RIPSSKETNK	MKERAIQTLG	YFPVGDGDFP	HQKLLLQGLM
890	900	910	920	930	940	950	960
DSVEAKQIEL	QFTIGEAITS	AAIGTSSVAA	RDAWQMTEEE	YTPPAGAKVN	DVVPWVLDVI	LNKHIISPNP	HVRQAACIWL
970	980	990	1000	1010	1020	1030	1040
LSLVRKLSTH	KEVKSHLKEI	QSAFVSVLSE	NDELSQDVAS	KGLGLVYELG	NEQDQQELVS	TLVETLMTGK	RVKHEVSGET
1050	1060	1070	1080	1090	1100	1110	1120
VVFQGGALGK	TPDGQGLSTY	KELCSLASDL	SQPDLVYKFM	NLANHHAMWN	SRKGAAFGFN	VIATRAGEQL	APFLPQLVPR
1130	1140	1150	1160	1170	1180	1190	1200
LYRYQFDPNL	GIRQAMTSIW	NALVTDKSMV	DKYLKEILQD	LVKNLTSNMW	RVRESSCLAL	NDLLRGRPLD	DIIDKLPEIW
1210	1220	1230	1240	1250	1260	1270	1280
ETLFRVQDDI	KESVRKAAEL	ALKTLSKVCV	KMCDPAKGAA	GQRTIAALLP	CLLDKGMMST	VTEVRALSIN	TLVKISKSAG
1290	1300	1310	1320	1330	1340	1350	1360
AMLKPHAPKL	IPALLESLSV	LEPQVLNYLS	LRATEQEKAA	MDSARLSAAK	SSPMMETINM	CLQYLDVSVL	GELVPRLCEL
1370	1380	1390	1400	1410	1420	1430	1440
IRSGVGLGTK	GGCASVIVSL	TTQCPQDLTP	YSGKLMSALL	SGLTDRNSVI	QKSCAFAMGH	LVRTSRDSST	EKLLQKLNGW
1450	1460	1470	1480	1490	1500	1510	1520
YMEKEEPIYK	TSCALTIHAI	GRYSPDVLKN	HAKEVLPLAF	LGMHEIADEE	KSEKEECNLW	TEVWQENVPG	SFGGIRLYLQ
1530	1540	1550	1560	1570	1580	1590	1600
ELITITQKAL	QSQSWKMKAQ	GAIAMASIAK	QTSSLVPPYL	GMILTALLQG	LAGRTWAGKE	ELLKAIACVV	TACSAELEKS
1610	1620	1630	1640	1650	1660	1670	1680
VPNQPSTNEI	LQAVLKECSK	ENVKYKIVAI	SCAADILKAT	KEDRFQEFSN	IVIPLIKKNS	LESSGVRTTK	NEEENEKEKE
1690	1700	1710	1720	1730	1740	1750	1760
LQLEYLLGAF	ESLGKAWPRN	AETQRCYRQE	LCKLMCERLK	LSTWKVQLGV	LQSMNAFFQG	LMLLEEEHAD	PEALAEILLE
1770	1780	1790	1800	1810	1820	1830	1840
TCKSITYSLE	NKTYSSVRTE	ALSVIELLLK	KLEESKQWEC	LTSECRVLLI	ESLATMEPDS	RPELQEKAAL	LKKTLENLE

Cmpd.	No. of Cmpds.	m/z meas.	Δ m/z [ppm]	z	Rt [min]	Score	P	Range	Sequence	Modification	Ratios	
681	1	625.0022	62.66	3	38.5	10.4	1	1079-1093	K.FMNLANHHAMWNSRK.G	Oxidation: 2	Q:W<br>M:Q	0.20<br>4.39

Detailed Protein Report

Protein 404: PREDICTED: centrosomal protein KIAA1731 isoform X7 [Homo sapiens]

**Accession:**	gi|578822447	**Score:**	46.5
**Database:**	refseq_human(refseq_human_20140103.fasta)	**MW [kDa]:**	268.9
**Database Date:**	2015-11-30	**pI:**	5.4
		**Sequence Coverage [%]:**	1.0
		**No. of unique Peptides:**	3

Quantitation

Q:W	**Median:**	2.54	**CV:**	0.00 %	**No. of Peptides:**	1
M:Q	**Median:**	0.83	**CV:**	0.00 %	**No. of Peptides:**	1

Detailed Protein Report

10	20	30	40	50	60	70	80
MERFEKAHVR	GFQAMKKIHL	AQNQEKLMKE	LKQLQQEDLA	RRRQTVAQMP	PQLVELPYKR	SEMKEDWQRE	LEFAFEDMYN
90	100	110	120	130	140	150	160
ADRKVKGNLI	LHLEPEPLPT	VTNQIQDEEL	DLSMEQENLG	AAEDLPVTEA	EICSSETDVP	LVMKTQQIPS	KVLFKKLLNK
170	180	190	200	210	220	230	240
IRSQKSLWTI	KSMSEDESEM	ITTVSEIESK	APTVESGTIA	SKERTLSSGQ	EQVVESDTLT	IESGPLASED	KPLSCGTNSG
250	260	270	280	290	300	310	320
KEQEINETLP	ITTVAQSSVL	LHPQEAAARI	RMSARQKQIM	EIEEQKQKQL	ELLEQIEQQK	LRLETDCFRA	QLEEEKRKKT
330	340	350	360	370	380	390	400
QPTGVGCRKS	HHYINHLVGI	APASCPVISD	EDSHRQMIRN	YQHQLLQQNR	LHRQSVETAR	KQLLEYQTML	KGRCPSVSAP
410	420	430	440	450	460	470	480
SLITDSVISV	PSWKSERPTA	ISEHWDQGQR	LKLSPNKYQP	IQPIQTSKLE	QDHFQVARQN	HFPQRQVETT	ETLRASDILT
490	500	510	520	530	540	550	560
NQALESQEHL	RQFSQTETQQ	RDYKLVPKDS	ETLSRALSHD	RQLISQDARK	ISETFGATTF	QSLESQQLFS	ENSENISYHL
570	580	590	600	610	620	630	640
TEPSSFVPLV	PQHSFSSLPV	KVESGKIQEP	FSAMSKSTVS	TSHSIISQMH	DRPLLPSENI	TAQQGNMKAL	QEQLDLQKKV
650	660	670	680	690	700	710	720
LQATQEAQEQ	LLLCKQKEVE	QQTGLSVFLP	LVTPDSSALL	PSAKADLGRI	QESSPTKNNI	AVSSDHHVIS	QLQDKRLSLS
730	740	750	760	770	780	790	800
QPILSQQNNF	KFLQEQLNIQ	KDSLQARREA	QEVLYVHKQS	ELDRRVCSEQ	AEPSFPFQVA	QHTFTSLPSA	DTKSGKIQEQ
810	820	830	840	850	860	870	880
HSSKSEKGLV	SCQSDIPISQ	DGSLSFLQQF	LPLHDSLKLL	QEQLTKQRDT	LQARHEAQVE	LLLHRQRDLG	DSKSGLVSSS
890	900	910	920	930	940	950	960
SSPVVVQHSV	ASQASAKAEP	RRIQELYLSE	KENVGPSCHL	IIPTFQDKSL	SFPQHSLAQQ	ENLTILQEQS	QIQRVILGAK
970	980	990	1000	1010	1020	1030	1040
EGTQEFVHTE	SELEKRISSE	QTGTSSSLSQ	VDESERFQEC	ISIKSDSTIP	LSHPKIPRCQ	ERLLRVSQHM	LPLQDNLEEH
1050	1060	1070	1080	1090	1100	1110	1120
QAWLDTEKEA	FHFSQKTQEN	TSSEQTGSSS	FIPQLVQLSF	TSLASAESGT	ILEPLFTESE	SKIFSSHLQI	PQLQDRLLRI
1130	1140	1150	1160	1170	1180	1190	1200
SQLIQPQQDN	LKALQEQLAT	QREAIILARQ	EAREELLLHQ	SEWEGRISPE	QVDTSSLPLV	PQHSFASLPL	NESERNQEPC
1210	1220	1230	1240	1250	1260	1270	1280
SINSDNIVSS	GHSEIPTLPD	GLLGLSHLVL	PQQDNLIALE	EHLHAQTDFL	PSIEKTQKEL	VLSKPCKFEE	KVSSEHFIQS
1290	1300	1310	1320	1330	1340	1350	1360
HHGDLQALQQ	QLDTQKKAIR	SIQEVQEELL	LQRLSELEKR	VSSEQVCSSS	FVSQVPVADS	ERTQKSFPTK	SNDTLPSSHR
1370	1380	1390	1400	1410	1420	1430	1440
EIPRLQDRLL	SLSKPILPQQ	DNMTAQLDAQ	REVMYSYEKP	QEELSLNKQR	KLNKSESAEH	TIPSLFLPKE	TEHSFIPLPF
1450	1460	1470	1480	1490	1500	1510	1520
AEAKPKSTCE	LYSSQNEHAA	PPSNPVIPGF	QDRLLSFSQS	VLTQQDNLGL	QKQLDLQREV	LHYSQKAQEK	LLVQRQTALQ
1530	1540	1550	1560	1570	1580	1590	1600
QQIQKHEETL	KDFFKDSQIS	KPTVENDLKT	QKMGQLRDWF	PNTQDLAGND	QENIRHADRN	NSDDNHLASE	DTSAKQSGEH
1610	1620	1630	1640	1650	1660	1670	1680
LEKDLGRRSS	KPPVAKVKCG	LDLNQHELSA	IQEVESPAIG	RTSILGKPGI	YEDRDPLRVS	ISREQSFFGS	PLAHDPFSCL
1690	1700	1710	1720	1730	1740	1750	1760
QLVGQENVCG	DDYDEAVKLK	ESVVENHAVL	SYAVEEEHAY	LGPTVKPDDK	AKTLSYEPLS	SATVSTGSLL	SYENTDLSLT
1770	1780	1790	1800	1810	1820	1830	1840
DPESFSEHMD	DSKQESTTSK	EEETNIISSI	VPSTQDIYQR	QNSSDVHKSL	LPAVDETTCG	HTHFQQMIDK	YINEANLIPE
1850	1860	1870	1880	1890	1900	1910	1920
KTDLQELEHI	FPNLHHQLFK	PLEPHPDFDL	SSSSSGISPD	NRDFYQRSDS	SSESHCATGL	SKSTVYFTAL	RRTSMHSSLN
1930	1940	1950	1960	1970	1980	1990	2000
TSPNQQPDTN	LAHVGAHSFA	TENIIGGSEQ	CFEQLQPEYS	SQEESQHADL	PSIFSIEARD	SSQGMKNQNY	PSEEHTEILQ
2010	2020	2030	2040	2050	2060	2070	2080
NKKKIVHFQL	SIGNLSSVYS	SSDEANVFDQ	LNVQHSTPCG	SNSSECSTKH	QLESRKESMG	FEELSKRGVV	TMLQSQGLIE
2090	2100	2110	2120	2130	2140	2150	2160
DNKNETCRVL	DINPQVEETD	SRLCVRTVEM	GTSIQAPYSL	TTQNEKYFEN	SAETDIPKIT	KKLSQLGESE	LFASSGSFSL
2170	2180	2190	2200	2210	2220	2230	2240

Detailed Protein Report

Cmpd.	No. of Cmpds.	m/z meas.	Δ m/z [ppm]	z	Rt [min]	Score	P	Range	Sequence	Modification	Ratios
488	1	686.3221	-73.56	2	36.1	13.0	1	748-758	R.REAQEVLYVHK.Q		M:Q 0.83<br>Q:W 2.54
131	2	608.2888	-54.72	2	30.9	17.7	0	749-758	R.EAQEVLYVHK.Q		
2043	1	709.3053	-139.25	2	55.7	15.7	1	2299-2311	K.EKPSISSSVSRLK.G		

Detailed Protein Report

Protein 405: PREDICTED: pecanex-like protein 3 isoform X4 [Homo sapiens]

**Accession:**	gi|530396772	**Score:**	46.5
**Database:**	refseq_human(refseq_human_20140103.fasta)	**MW [kDa]:**	115.4
**Database Date:**	2015-11-30	**pI:**	6.0
		**Sequence Coverage [%]:**	5.8
		**No. of unique Peptides:**	2

```
        10         20         30         40         50         60         70         80
MGSQVLQILR QGVWASLTGG WFFDPHQSTF SNCFHLYVWI FLLIFPFLLY MVLPPSLMVA GVYCLVVAVI FATIKTVNYR
        90        100        110        120        130        140        150        160
LHAMFDQGEI VEKRSSTMGE LEEEPAQGDS NPPRDPGVEM TVFRKVSSTP PVRCSSQHSV FGFNQVSELL PRMEDSGPLR
       170        180        190        200        210        220        230        240
DIKELVREQG SNNVIVTSAD REMLKLSSQE KLIGDLPQTP PGAVPDPSLA STDSSEPSPL AGDGAPWSGS SMADTPMSPL
       250        260        270        280        290        300        310        320
LKGSLSQELS KSFLTLTQPD RALVRTSSRR EQRRGAGGYQ PLDRRGSGEP TPQKAGSSDS CFSGTDRETL SSFKSEKTNS
       330        340        350        360        370        380        390        400
THLDSPPGGP APEGSDTDPP SEAELPASPD AGVPSDDTLR SFDTVIGAGT PPGLAEPLLV VRPKDLALLR PSKRQPPLRR
       410        420        430        440        450        460        470        480
HSPPGRAPRR PLLEGGGFFE DEDTSEGSEL SPASSLRSQR RYSTDSSSST SCYSPESSRG AAGGPRKRRA PHGAEEGTAV
       490        500        510        520        530        540        550        560
PPKRPYGTQR TPSTASAKTH ARVLSMDGAG GDVLRPPLAG CKAELEAQVG VEQAASEPVV LPAEARRGPA ANQPGWRGEL
       570        580        590        600        610        620        630        640
QEEGAVGGAA EETGRRDRSS SVRRTQAIRR RHNAGSNPTP PASVMGSPPS SLQEAQRGRA ASHSRALTLP SALHFASSLL
       650        660        670        680        690        700        710        720
LTRAGANVHE ACTFDDTSEG AVHYFYDESG VRRSYTFGLA GGGYENPVGQ QGEQTANGAW DRHSHSSSFH SADVPEATGG
       730        740        750        760        770        780        790        800
LNLLQPRPVV LQGMQVRRVP LEIPEFDLLD QDSLHESQEQ TLMEEAPPRA QHSYKYWLLP GRWTSVRYER LALLALLDRT
       810        820        830        840        850        860        870        880
RGVLENIFGV GLSSLVAFLG YLLLLKGFFT DIWVFQFCLV IASCQYSLLK SVQPDAASPM HGHNWVIAYS RPVYFCICCL
       890        900        910        920        930        940        950        960
LIWLLDALGS AQPFPPVSLY GLTLFSASFF FCARDVATVF TLCFPFVFLL GLLPQVNTCL MYLLEQIDMH GFGGTAATSP
       970        980        990       1000       1010       1020       1030       1040
LTAVFSLSRS LLAAALLYGF CLGAIKTPWP EQHVPVLFSV FCGLLVALSY HLSRQSSDPT VLWSLIRSKL FPELEERSLE
      1050       1060       1070
TARAEPPDPL PDKMRQSVLN VSPGPAA
```

Cmpd.	No. of Cmpds.	m/z meas.	Δ m/z [ppm]	z	Rt [min]	Score	P	Range	Sequence	Modification
2682	1	1051.5508	-5.70	2	64.7	10.8	2	115-133	R.DPGVEMTVFRKVSSTPPVR.C	
2754	1	1086.0034	31.79	2	64.8	11.5	1	286-307	R.GSGEPTPQKAGSSDSCFSGTDR.E	

Detailed Protein Report

Protein 406: PREDICTED: multiple epidermal growth factor-like domains protein 6 isoform X4 [Homo sapiens]

**Accession:**	gi|578798522	**Score:**	46.4
**Database:**	refseq_human(refseq_human_20140103.fasta)	**MW [kDa]:**	135.9
**Database Date:**	2015-11-30	**pI:**	5.9
**Modification(s):**	Carbamidomethyl	**Sequence Coverage [%]:**	4.4
		**No. of unique Peptides:**	2

10	20	30	40	50	60	70	80
MGASRDRGLA	ALWCLGLLGG	LARVAGTHYR	YLWRGCYPCH	LGQAGYPVSA	GDQRPDVDEC	RTHNGGCQHR	CVNTPGSYLC
**90**	**100**	**110**	**120**	**130**	**140**	**150**	**160**
ECKPGFRLHT	DSRTCLAINS	CALGNGGCQH	HCVQLTITRH	RCQCRPGFQL	QEDGRHCVRR	SPCANRNGSC	MHRCQVVRGL
**170**	**180**	**190**	**200**	**210**	**220**	**230**	**240**
ARCECHVGYQ	LAADGKACED	VDECAAGLAQ	CAHGCLNTQG	SFKCVCHAGY	ELGADGRQCY	RIEMEIVNSC	EANNGGCSHG
**250**	**260**	**270**	**280**	**290**	**300**	**310**	**320**
CSHTSAGPLC	TCPRGYELDT	DQRTCIDVDD	CADSPCCQQV	CTNNPGGYEC	GCYAGYRLSA	DGCGCEDVDE	CASSRGGCEH
**330**	**340**	**350**	**360**	**370**	**380**	**390**	**400**
HCTNLAGSFQ	CSCEAGYRLH	EDRRGCSPLE	EPMVDLDGEL	PFVRPLPHIA	VLQDELPQLF	QDDDVGADEE	EAELRGEHTL
**410**	**420**	**430**	**440**	**450**	**460**	**470**	**480**
TEKFVCLDDS	FGHDCSLTCD	DCRNGGTCLL	GLDGCDCPEG	WTGLICNETC	PPDTFGKNCS	FSCSCQNGGT	CDSVTGACRC
**490**	**500**	**510**	**520**	**530**	**540**	**550**	**560**
PPGVSGTNCE	DGCPKGYYGK	HCRKKCNCAN	RGRCHRLYGA	CLCDPGLYGR	FCHLTCPPWA	FGPGCSEECQ	CVQPHTQSCD
**570**	**580**	**590**	**600**	**610**	**620**	**630**	**640**
KRDGSCSCKA	GFRGERCQAE	CELGYFGPGC	WQACTCPVGV	ACDSVSGECG	KRCPAGFQGE	DCGQECPVGT	FGVNCSSSCS
**650**	**660**	**670**	**680**	**690**	**700**	**710**	**720**
CGGAPCHGVT	GQCRCPPGRT	GEDCEAECPQ	GHFGPGCEQR	CQCQHGAACD	HVSGACTCPA	GWRGTFCEHA	CPAGFFGLDC
**730**	**740**	**750**	**760**	**770**	**780**	**790**	**800**
RSACNCTAGA	ACDAVNGSCL	CPAGRRGPRC	AETCPAHTYG	HNCSQACACF	NGASCDPVHG	QCHCAPGWMG	PSCLQACPAG
**810**	**820**	**830**	**840**	**850**	**860**	**870**	**880**
LYGDNCRHSC	LCQNGGTCDP	VSGHCACPEG	WAGLACEKEC	LPRDVRAGCR	HSGGCLNGGL	CDPHTGRCLC	PAGWTGDKCQ
**890**	**900**	**910**	**920**	**930**	**940**	**950**	**960**
SPCLRGWFGE	ACAQRCSCPP	GAACHHVTGA	CRCPPGFTGS	GCEQACPPGS	FGEDCAQMCQ	CPGENPACHP	ATGTCSCAAG
**970**	**980**	**990**	**1000**	**1010**	**1020**	**1030**	**1040**
YHGPSCQQRC	PPGRYGPGCE	QLCGCLNGGS	CDAATGACRC	PTGFLGTDCN	LTCPQGRFGP	NCTHVCGCGQ	GAACDPVTGT
**1050**	**1060**	**1070**	**1080**	**1090**	**1100**	**1110**	**1120**
CLCPPGRAGV	RCERGCPQNR	FGVGCEHTCS	CRNGGLCHAS	NGSCSCGLGW	TGRHCELACP	PGRYGAACHL	ECSCHNNSTC
**1130**	**1140**	**1150**	**1160**	**1170**	**1180**	**1190**	**1200**
EPATGTCRCG	PGFYGQACEH	PCPPGFHGAG	CQGLCWCQHG	APCDPISGRC	LCPAGFHGHF	CERGCEPGSF	GEGCHQRCDC
**1210**	**1220**	**1230**	**1240**	**1250**	**1260**	**1270**	**1280**
DGGAPCDPVT	GLCLCPPGRS	GATCNLDCRR	GQFGPSCTLH	CDCGGGADCD	PVSGQCHCVD	GYMGPTCREG	GPLRLPENPS
**1290**	**1300**	**1310**					
LAQGSAGTLP	ASSRPTSRSG	GPARH					

Cmpd.	No. of Cmpds.	m/z meas.	Δ m/z [ppm]	z	Rt [min]	Score	P	Range	Sequence	Modification
12	2	1103.5102	57.86	2	29.4	10.6	0	1198-1219	R.CDCDGGAPCDPVTGLCLCPPGR.S	Carbamidomethyl: 16
17	1	1103.4962	45.23	2	29.9	11.4	0	1198-1219	R.CDCDGGAPCDPVTGLCLCPPGR.S	Carbamidomethyl: 18

Detailed Protein Report

Protein 407: zinc finger protein 90 [Homo sapiens]

Accession: gi|209862791
Database: refseq_human(refseq_human_20140103.fasta)
Database Date: 2015-11-30
Modification(s): Carbamidomethyl

Score: 46.3
MW [kDa]: 69.0
pI: 10.3
Sequence Coverage [%]: 6.0
No. of unique Peptides: 1

Quantitation

Q:W	**Median:** 0.50	**CV:** 0.00 %	**No. of Peptides:** 1
M:Q	**Median:** 1.01	**CV:** 0.00 %	**No. of Peptides:** 1

10	20	30	40	50	60	70	80
MGPLEFRDVA	IEFSLEEWHC	LDTAQQNLYR	DVMLENYRHL	VFLGIVVTKP	DLITCLEQGK	KPFTVKRHEM	IAKSPVMCFH
**90**	**100**	**110**	**120**	**130**	**140**	**150**	**160**
FAQDLCPEQS	LKDSFQKVIV	TRYEKREYGN	LELKKGCESV	DEGKVHKRGY	NGLNQCLTAT	QSKVFQCDTY	VKVSHIFSNS
**170**	**180**	**190**	**200**	**210**	**220**	**230**	**240**
NRHKIRDTGK	KPFKCIECGK	AFNQSSTLAT	HKKIHTGEIT	CKCEECGKAF	NRSSHLTSHK	RIHTGEKRYK	CEDCGKELKY
**250**	**260**	**270**	**280**	**290**	**300**	**310**	**320**
SSTLTAHKRI	HTGEKRYKCE	DCGKELKYSS	TLTAHKRIHT	GEKPYKCDKC	GRAFISSSIL	YVHKISHTEE	KPYKCEECGK
**330**	**340**	**350**	**360**	**370**	**380**	**390**	**400**
AFKLSSILST	HKRIHTGEKP	YKCEECGKAF	RRSLVLRTHK	RIHTGEKPYK	CDKCGKAFIS	SSLLYKHKIS	HSEKKPYKCE
**410**	**420**	**430**	**440**	**450**	**460**	**470**	**480**
ECGKAFKRSS	TLTIHKISHT	EEKPYKCQEC	DKVFKRSSAL	STHKIIHSGE	KPYKCEECGK	AFKRSSNLTT	HKISHTEEKL
**490**	**500**	**510**	**520**	**530**	**540**	**550**	**560**
YKCQECDKAF	KYSSALSTHK	IIHSGENPYK	CEECGKAFKR	SSVLSKHKII	HTGAKPYKCE	ECGKAFKRSS	QLTSHKISHT
**570**	**580**	**590**	**600**	**610**			
GEKPYKCEEC	GKAFNLSSDL	NTHKRIHIGQ	KAYIVKNMAN	L			

Cmpd.	No. of Cmpds.	m/z meas.	Δ m/z [ppm]	z	Rt [min]	Score	P	Range	Sequence	Modification	Ratios
1099	1	917.4452	-8.48	2	43.8	11.6	1	529-544	K.IIHTGAKPYKCEECGK.A	Carbamidomethyl: 11	M:Q 1.01<br>Q:W 0.50

Detailed Protein Report

Protein 408: transformation/transcription domain-associated protein isoform 2 [Homo sapiens]

**Accession:**	gi|4507691	**Score:**	46.3
**Database:**	refseq_human(refseq_human_20140103.fasta)	**MW [kDa]:**	434.1
**Database Date:**	2015-11-30	**pI:**	9.3
**Modification(s):**	Oxidation	**Sequence Coverage [%]:**	1.5
		**No. of unique Peptides:**	2

Detailed Protein Report

10	20	30	40	50	60	70	80
MAFVATQGAT	VVDQTTLMKK	YLQFVAALTD	VNTPDETKLK	MMQEVSENFE	NVTSSPQYST	FLEHIIPRFL	TFLQDGEVQF
90	100	110	120	130	140	150	160
LQEKPAQQLR	KLVLEIIHRI	PTNEHLRPHT	KNVLSVMFRF	LETENEENVL	ICLRIIIELH	KQFRPPITQE	IHHFLDFVKQ
170	180	190	200	210	220	230	240
IYKELPKVVN	RYFENPQVIP	ENTVPPPEMV	GMITTIAVKV	NPEREDSETR	THSIIPRGSL	SLKVLAELPI	IVVLMYQLYK
250	260	270	280	290	300	310	320
LNIHNVVAEF	VPLIMNTIAI	QVSAQARQHK	LYNKELYADF	IAAQIKTLSF	LAYIIRIYQE	LVTKYSQQMV	KGMLQLLSNC
330	340	350	360	370	380	390	400
PAETAHLRKE	LLIAAKHILT	TELRNQFIPC	MDKLFDESIL	IGSGYTARET	LRPLAYSTLA	DLVHHVRQHL	PLSDLSLAVQ
410	420	430	440	450	460	470	480
LFAKNIDDES	LPSSIQTMSC	KLLLNLVDCI	RSKSEQESGN	GRDVLMRMLE	VFVLKFHTIA	RYQLSAIFKK	CKPQSELGAV
490	500	510	520	530	540	550	560
EAALPGVPTA	PAAPGPAPSP	APVPAPPPPP	PPPPPATPVT	PAPVPPFEKQ	GEKDKEDKQT	FQVTDCRSLV	KTLVCGVKTI
570	580	590	600	610	620	630	640
TWGITSCKAP	GEAQFIPNKQ	LQPKETQIYI	KLVKYAMQAL	DIYQVQIAGN	GQTYIRVANC	QTVRMKEEKE	VLEHFAGVFT
650	660	670	680	690	700	710	720
MMNPLTFKEI	FQTTVPYMVE	RISKNYALQI	VANSFLANPT	TSALFATILV	EYLLDRLPEM	GSNVELSNLY	LKLFKLVFGS
730	740	750	760	770	780	790	800
VSLFAAENEQ	MLKPHLHKIV	NSSMELAQTA	KEPYNYFLLL	RALFRSIGGG	SHDLLYQEFL	PLLPNLLQGL	NMLQSGLHKQ
810	820	830	840	850	860	870	880
HMKDLFVELC	LTVPVRLSSL	LPYLPMLMDP	LVSALNGSQT	LVSQGLRTLE	LCVDNLQPDF	LYDHIQPVRA	ELMQALWRTL
890	900	910	920	930	940	950	960
RNPADSISHV	AYRVLGKFGG	SNRKMLKESQ	KLHYVVTEVQ	GPSITVEFSD	CKASLQLPME	KAIETALDCL	KSANTEPYYR
970	980	990	1000	1010	1020	1030	1040
RQAWEVIKCF	LVAMMSLEDN	KHALYQLLAH	PNFTEKTIPN	VIISHRYKAQ	DTPARKTFEQ	ALTGAFMSAV	IKDLRPSALP
1050	1060	1070	1080	1090	1100	1110	1120
FVASLIRHYT	MVAVAQQCGP	FLLPCYQVGS	QPSTAMFHSE	ENGSKGMDPL	VLIDAIAICM	AYEEKELCKI	GEVALAVIFD
1130	1140	1150	1160	1170	1180	1190	1200
VASIILGSKE	RACQLPLFSY	IVERLCACCY	EQAWYAKLGG	VVSIKFLMER	LPLTWVLQNQ	QTFLKALLFV	MMDLTGEVSN
1210	1220	1230	1240	1250	1260	1270	1280
GAVAMAKTTL	EQLLMRCATP	LKDEERAEEI	VAAQEKSFHH	VTHDLVREVT	SPNSTVRKQA	MHSLQVLAQV	TGKSVTVIME
1290	1300	1310	1320	1330	1340	1350	1360
PHKEVLQDMV	PPKKHLLRHQ	PANAQIGLME	GNTFCTTLQP	RLFTMDLNVV	EHKVFYTELL	NLCEAEDSAL	TKLPCYKSLP
1370	1380	1390	1400	1410	1420	1430	1440
SLVPLRIAAL	NALAACNYLP	QSREKIIAAL	FKALNSTNSE	LQEAGEACMR	KFLEGATIEV	DQIHTHMRPL	LMMLGDYRSL
1450	1460	1470	1480	1490	1500	1510	1520
TLNVVNRLTS	VTRLFPNSFN	DKFCDQMMQH	LRKWMEVVVI	THKGGQRSDG	NEMKICSAII	NLFHLIPAAP	QTLVKPLLEV
1530	1540	1550	1560	1570	1580	1590	1600
VMKTERAMLI	EAGSPFREPL	IKFLTRHPSQ	TVELFMMEAT	LNDPQWSRMF	MSFLKHKDAR	PLRDVLAANP	NRFITLLLPG
1610	1620	1630	1640	1650	1660	1670	1680
GAQTAVRPGS	PSTSTMRLDL	QFQAIKIISI	IVKNDDSWLA	SQHSLVSQLR	RVWVSENFQE	RHRKENMAAT	NWKEPKLLAY
1690	1700	1710	1720	1730	1740	1750	1760
CLLNYCKRNY	GDIELLFQLL	RAFTGRFLCN	MTFLKEYMEE	EIPKNYSIAQ	KRALFFRFVD	FNDPNFGDEL	KAKVLQHILN
1770	1780	1790	1800	1810	1820	1830	1840
PAFLYSFEKG	EGEQLLGPPN	PEGDNPESIT	SVFITKVLDP	EKQADMLDSL	RIYLLQYATL	LVEHAPHHIH	DNNKNRNSKL
1850	1860	1870	1880	1890	1900	1910	1920
RRLMTFAWPC	LLSKACVDPA	CKYSGHLLLA	HIIAKFAIHK	KIVLQVFHSL	LKAHAMEARA	IVRQAMAILT	PAVPARMEDG
1930	1940	1950	1960	1970	1980	1990	2000
HQMLTHWTRK	IIVEEGHTVP	QLVHILHLIV	QHFKVYYPVR	HHLVQHMVSA	MQRLGFTPSV	TIEQRRLAVD	LSEVVIKWEL
2010	2020	2030	2040	2050	2060	2070	2080
QRIKDQQPDS	DMDPNSSGEG	VNSVSSSIKR	GLSVDSAQEV	KRFRTATGAI	SAVFGRSQSL	PGADSLLAKP	IDKQHTDTVV
2090	2100	2110	2120	2130	2140	2150	2160
NFLIRVACQV	NDNTNTAGSP	GEVLSRRCVN	LLKTALRPDM	WPKSELKLQW	FDKLLMTVEQ	PNQVNYGNIC	TGLEVLSFLL
2170	2180	2190	2200	2210	2220	2230	2240
TVLQSPAILS	SFKPLQRGIA	ACMTCGNTKV	LRAVHSLLSR	LMSIFPTEPS	TSSVASKYEE	LECLYAAVGK	VIYEGLTNYE
2250	2260	2270	2280	2290	2300	2310	2320
KATNANPSQL	FGTLMILKSA	CSNNPSYIDR	LISVFMRSLQ	KMVREHLNPQ	AASGSTEATS	GTSELVMLSL	ELVKTRLAVM
2330	2340	2350	2360	2370	2380	2390	2400

Detailed Protein Report

Cmpd.	No. of Cmpds.	m/z meas.	Δ m/z [ppm]	z	Rt [min]	Score	P	Range	Sequence	Modification
873	1	946.4119	-18.65	3	40.4	15.0	1	2003-2029	R.IKDQQPDSDMDPNSSGEGVNSVSSSIK.R	Oxidation: 10
2507	1	913.4337	-85.65	2	61.8	14.5	2	2373-2387	R.EKSILLVKMMTYIEK.R	

Detailed Protein Report

Protein 409: kinesin-like protein KIF26B [Homo sapiens]

**Accession:**	gi|124430752	**Score:**	46.2
**Database:**	refseq_human(refseq_human_20140103.fasta)	**MW [kDa]:**	223.7
**Database Date:**	2015-11-30	**pI:**	9.7
**Modification(s):**	Carbamidomethyl	**Sequence Coverage [%]:**	3.1
		**No. of unique Peptides:**	3

Quantitation

Q:W	**Median:**	1.09	**CV:**	0.00 %	**No. of Peptides:**	1
M:Q	**Median:**	0.57	**CV:**	0.00 %	**No. of Peptides:**	1

Detailed Protein Report

10	20	30	40	50	60	70	80
MNSVAGNKER	LAVSTRGKKY	GVNEVCSPTK	PAAPFSPESW	YRKAYEESRA	GSRPTPEGAG	SALGSSGTPS	PGSGTSSPSS
90	100	110	120	130	140	150	160
FTGSPGPASP	GIGTSSPGSL	GGSPGFGTGS	PGSGSGGGSS	PGSDRGVWCE	NCNARLVELK	RQALRLLLPG	PFPGKDPAFS
170	180	190	200	210	220	230	240
AVIHDKLQVP	NTIRKAWNDR	DNRCDICATH	LNQLKQEAIQ	MVLTLEQAAG	SEHYDASPCS	PPPLSNIPTL	VGSRHVGGLQ
250	260	270	280	290	300	310	320
QPRDWAFVPA	PCATSNYTGF	ANKHGSKPSS	LGVSNGAEKK	SGSPTHQAKV	SLQMATSPSN	GNILNSVAIQ	AHQYLDGTWS
330	340	350	360	370	380	390	400
LSRTNGVTLY	PYQISQLMTE	SSREGLTEAV	LNRYNADKPS	ACSVPASQGS	CVASETSTGT	SVAASFFARA	AQKLNLSSKK
410	420	430	440	450	460	470	480
KKHRPSTSSA	AEPPLFATSF	SGILQTSPPP	APPCLLRAVN	KVKDTPGLGK	VKVMLRICST	LARDTSESSS	FLKVDPRKKQ
490	500	510	520	530	540	550	560
ITLYDPLTCG	GQNAFQKRGN	QVPPKMFAFD	AVFPQDASQA	EVCAGTVAEV	IQSVVNGADG	CVFCFGHAKL	GKSYTMIGKD
570	580	590	600	610	620	630	640
DSMQNLGIIP	CAISWLFKLI	NERKEKTGAR	FSVRVSAVEV	WGKEENLRDL	LSEVATGSLQ	DGQSPGVYLC	EDPICGTQLQ
650	660	670	680	690	700	710	720
NQSELRAPTA	EKAAFFLDAA	IASRRSHQQD	CDEDDHRNSH	VFFTLHIYQY	RMEKSGKGGM	SGGRSRLHLI	DLGSCVKALS
730	740	750	760	770	780	790	800
KNREGGSGLC	LSLSALGNVI	LALVNGSKHI	PYKESKLAML	LRESLGNMNC	RTTMIAHISA	AVGSYAETLS	TIQIASRVLR
810	820	830	840	850	860	870	880
MKKKKTKYTS	SSSGGESSCE	EGRMRRPTQL	RPFHTRATVD	PDFPIAHLSS	DPDYSSSSEQ	SCDTVIYIGP	NGTALSDKEL
890	900	910	920	930	940	950	960
TDNEGPPDFV	PIVPALQKTR	GDSRPAEAGE	AAAGKSERDC	LKCNTFAELQ	ERLDCIDGSE	EPSSFPFEEL	PAQFGPEQAS
970	980	990	1000	1010	1020	1030	1040
RGPRLSQAAG	ASPLSESDKE	DNGSEGQLTN	REGPELPASK	MQRSHSPVPA	AAPAHSPSPA	SPRSVPGSSS	QHSASPLVQS
1050	1060	1070	1080	1090	1100	1110	1120
PSLQSSRESL	NSCGFVEGKP	RPMGSPRLGI	ASLSKTSEYK	PPSSPSQRCK	VYTQKGVLPS	PAPLPPSSKD	SGVASRESLL
1130	1140	1150	1160	1170	1180	1190	1200
QPEVRTPPVG	MSPQVLKKSM	SAGSEGFPET	PVDDEQQAAT	PSESKKEILS	TTMVTVQQPL	ELNGEDELVF	TLVEELTISG
1210	1220	1230	1240	1250	1260	1270	1280
VLDSGRPTSI	ISFNSDCSAR	ALASGSRPVS	IISSISEDLE	CYSSTAPVSE	VSITQFLPLP	KMSLDEKAQD	AGSRRSSISS
1290	1300	1310	1320	1330	1340	1350	1360
WLSEMSAGSE	GEQSCHSFIA	QTCFGHGEAM	AEPVASEFVS	SLQNTAVVCR	EKPKASPDNL	LILSEMGDDS	FNKAAPIKGC
1370	1380	1390	1400	1410	1420	1430	1440
KISTVSKAMV	TISNTANLSS	CEGYIPMKTN	ITVYPCIAMS	PRNIQEPEAP	TATPKAGPTL	AQSRESKENS	AKKEMKFEDP
1450	1460	1470	1480	1490	1500	1510	1520
WLKREEEVKK	ETAHPNEEGM	MRCETATGPS	NAETRAEQEQ	DGKPSPGDRL	SSSSGEVSAS	PVTDNFRRVV	DGCEMALPGL
1530	1540	1550	1560	1570	1580	1590	1600
ATQSPVHPNK	SVKSSSLPRA	FQKASRQEEP	DSLSYYCAAE	TNGVGAASGT	PPSKATLEGK	VASPKHCVLA	RPKGTPPLPP
1610	1620	1630	1640	1650	1660	1670	1680
VRKSSLDQKN	RASPQHSASG	SGTSSPLNQP	AAFPAGLPDE	PSGKTKDASS	SSKLFSAKLE	QLASRSNSLG	RATVSHYECL
1690	1700	1710	1720	1730	1740	1750	1760
SLERAESLSS	VSSRLHAGKD	GTMPRAGRSL	GRSAGTSPPS	SGASPKAGQS	KISAVSRLLL	ASPRARGPSA	STTKTLSFST
1770	1780	1790	1800	1810	1820	1830	1840
KSLPQAVGQG	SSSPPGGKHT	PWSTQSLSRN	RSSGLASKLP	LRAVSGRISE	LLQGGAGARG	LQLRAGPEAE	ARGGALAEDE
1850	1860	1870	1880	1890	1900	1910	1920
PAAAHLLPSP	YSKITPPRRP	HRCSSGHGSD	NSSVLSGELP	PAMGKTALFY	HSGGSSGYES	VMRDSEATGS	ASSAQDSTSE
1930	1940	1950	1960	1970	1980	1990	2000
NSSSVGGRCR	SLKTPKKRSN	PGSQRRRLIP	ALSLDTSSPV	RKPPNSTGVR	WVDGPLRSSP	RGLGEPFEIK	VYEIDDVERL
2010	2020	2030	2040	2050	2060	2070	2080
QRRRGGASKE	AMCFNAKLKI	LEHRQQRIAE	VRAKYEWLMK	ELEATKQYLM	LDPNKWLSEF	DLEQVWELDS	LEYLEALECV
2090	2100	2110					
TERLESRVNF	CKAHLMMITC	FDITSRRR					

Detailed Protein Report

Cmpd.	No. of Cmpds.	m/z meas.	Δ m/z [ppm]	z	Rt [min]	Score	P	Range	Sequence	Modification	Ratios
2229	1	597.6218	-9.28	3	56.3	11.5	1	126-140	R.GVWCENCNARLVELK.R	Carbamidomethyl: 7	
1659	1	1273.7346	71.12	2	50.7	10.5	1	1335-1358	K.ASPDNLLILSEMGDDSFNKAAPIK.G		
2047	6	698.2586	-144.28	2	55.7	12.4	0	1403-1415	R.NIQEPEAPTATPK.A		M:Q 0.57<br>Q:W 1.09

Detailed Protein Report

Protein 410: **putative RNA polymerase II subunit B1 CTD phosphatase RPAP2 [Homo sapiens]**

Accession: gi|226246608
Database: refseq_human(refseq_human_20140103.fasta)
Database Date: 2015-11-30

Score: 46.2
MW [kDa]: 69.5
pI: 8.8
Sequence Coverage [%]: 6.4
No. of unique Peptides: 2

Quantitation

Q:W	**Median:** 0.18	**CV:** 0.00 %	**No. of Peptides:** 1
M:Q	**Median:** 1.78	**CV:** 0.00 %	**No. of Peptides:** 1

10	20	30	40	50	60	70	80
MADFAGPSSA	GRKAGAPRCS	RKAAGTKQTS	TLKQEDASKR	KAELEAAVRK	KIEFERKALH	IVEQLLEENI	TEEFLMECGR
**90**	**100**	**110**	**120**	**130**	**140**	**150**	**160**
FITPAHYSDV	VDERSIVKLC	GYPLCQKKLG	IVPKQKYKIS	TKTNKVYDIT	ERKSFCSNFC	YQASKFFEAQ	IPKTPVWVRE
**170**	**180**	**190**	**200**	**210**	**220**	**230**	**240**
EERHPDFQLL	KEEQSGHSGE	EVQLCSKAIK	TSDIDNPSHF	EKQYESSSSS	THSDSSSDNE	QDFVSSILPG	NRPNSTNIRP
**250**	**260**	**270**	**280**	**290**	**300**	**310**	**320**
QLHQKSIMKK	KAGHKANSKH	KDKEQTVVDV	TEQLGDCKLD	SQEKDATCEL	PLQKVNTQSS	SNSTLPERLK	ASENSESEYS
**330**	**340**	**350**	**360**	**370**	**380**	**390**	**400**
RSEITLVGIS	KKSAEHFKRK	FAKSNQVSRS	VSSSVQVCPE	VGKRNLLKVL	KETLIEWKTE	ETLRFLYGQN	YASVCLKPEA
**410**	**420**	**430**	**440**	**450**	**460**	**470**	**480**
SLVKEELDED	DIISDPDSHF	PAWRESQNSL	DESLPFRGSG	TAIKPLPSYE	NLKKETEKLN	LRIREFYRGR	YVLGEETTKS
**490**	**500**	**510**	**520**	**530**	**540**	**550**	**560**
QDSEEHDSTF	PLIDSSSQNQ	IRKRIVLEKL	SKVLPGLLVP	LQITLGDIYT	QLKNLVRTFR	LTNRNIIHKP	AEWTLIAMVL
**570**	**580**	**590**	**600**	**610**	**620**		
LSLLTPILGI	QKHSQEGMVF	TRFLDTLLEE	LHLKNEDLES	LTIIFRTSCL	PE		

Cmpd.	No. of Cmpds.	m/z meas.	Δ m/z [ppm]	z	Rt [min]	Score	P	Range	Sequence	Modification	Ratios
1581	1	583.8284	105.83	2	48.3	11.8	0	1-12	-.MADFAGPSSAGR.K		M:Q 1.78<br>Q:W 0.18
1728	1	725.3276	-76.17	2	50.1	11.1	0	595-606	K.NEDLESLTIIFR.T		

Detailed Protein Report

Protein 411: microtubule-associated protein 6 isoform 1 [Homo sapiens]

**Accession:**	gi|48375173	**Score:**	46.2
**Database:**	refseq_human(refseq_human_20140103.fasta)	**MW [kDa]:**	86.5
**Database Date:**	2015-11-30	**pI:**	9.8
**Modification(s):**	Carbamidomethyl, Oxidation	**Sequence Coverage [%]:**	5.5
		**No. of unique Peptides:**	3

10	20	30	40	50	60	70	80
MAWPCITRAC	CIARFWNQLD	KADIAVPLVF	TKYSEATEHP	GAPPQPPPPQ	QQAQPALAPP	SARAVAIETQ	PAQGELDAVA
**90**	**100**	**110**	**120**	**130**	**140**	**150**	**160**
RATGPAPGPT	GEREPAAGPG	RSGPGPGLGS	GSTSGPADSV	MRQDYRAWKV	QRPEPSCRPR	SEYQPSDAPF	ERETQYQKDF
**170**	**180**	**190**	**200**	**210**	**220**	**230**	**240**
RAWPLPRRGD	HPWIPKPVQI	SAASQASAPI	LGAPKRRPQS	QERWPVQAAA	EAREQEAAPG	GAGGLAAGKA	SGADERDTRR
**250**	**260**	**270**	**280**	**290**	**300**	**310**	**320**
KAGPAWIVRR	AEGLGHEQTP	LPAAQAQVQA	TGPEAGRGRA	AADALNRQIR	EEVASAVSSS	YRNEFRAWTD	IKPVKPIKAK
**330**	**340**	**350**	**360**	**370**	**380**	**390**	**400**
PQYKPPDDKM	VHETSYSAQF	KGEASKPTTA	DNKVIDRRRI	RSLYSEPFKE	PPKVEKPSVQ	SSKPKKTSAS	HKPTRKAKDK
**410**	**420**	**430**	**440**	**450**	**460**	**470**	**480**
QAVSGQAAKK	KSAEGPSTTK	PDDKEQSKEM	NNKLAEAKES	LAQPVSDSSK	TQGPVATEPD	KDQGSVVPGL	LKGQGPMVQE
**490**	**500**	**510**	**520**	**530**	**540**	**550**	**560**
PLKKQGSVVP	GPPKDLGPMI	PLPVKDQDHT	VPEPLKNESP	VISAPVKDQG	PSVPVPPKNQ	SPMVPAKVKD	QGSVVPESLK
**570**	**580**	**590**	**600**	**610**	**620**	**630**	**640**
DQGPRIPEPV	KNQAPMVPAP	VKDEGPMVSA	SVKDQGPMVS	APVKDQGPIV	PAPVKGEGPI	VPAPVKDEGP	MVSAPIKDQD
**650**	**660**	**670**	**680**	**690**	**700**	**710**	**720**
PMVPEHPKDE	SAMATAPIKN	QGSMVSEPVK	NQGLVVSGPV	KDQDVVVPEH	AKVHDSAVVA	PVKNQGPVVP	ESVKNQDPIL
**730**	**740**	**750**	**760**	**770**	**780**	**790**	**800**
PVLVKDQGPT	VLQPPKNQGR	IVPEPLKNQV	PIVPVPLKDQ	DPLVPVPAKD	QGPAVPEPLK	TQGPRDPQLP	TVSPLPRVMI
**810**	**820**						
PTAPHTEYIE	SSP						

Cmpd.	No. of Cmpds.	m/z meas.	Δ m/z [ppm]	z	Rt [min]	Score	P	Range	Sequence	Modification
2789	1	834.4737	105.67	2	63.4	11.4	1	1-14	-.MAWPCITRACCIAR.F	Carbamidomethyl: 11; Oxidation: 1
1658	1	1045.0497	8.34	2	49.4	18.6	1	528-547	K.DQGPSVPVPPKNQSPMVPAK.V	Oxidation: 16
1905	7	572.7262	-106.04	2	53.9	16.3	0	594-604	K.DQGPMVSAPVK.D	Oxidation: 5

Detailed Protein Report

Protein 412: PREDICTED: multiple PDZ domain protein isoform X3 [Homo sapiens]

**Accession:**	gi|530390235	**Score:**	46.2
**Database:**	refseq_human(refseq_human_20140103.fasta)	**MW [kDa]:**	213.7
**Database Date:**	2015-11-30	**pI:**	4.8
**Modification(s):**	Carbamidomethyl	**Sequence Coverage [%]:**	2.3
		**No. of unique Peptides:**	3

Detailed Protein Report

10	20	30	40	50	60	70	80
MLEAIDKNRA	LHAAERLQTK	LRERGDVANE	DKLSLLKSVL	QSPLFSQILS	LQTSVQQLKD	QVNIATSATS	NIEYAHVPHL
90	100	110	120	130	140	150	160
SPAVIPTLQN	ESFLLSPNNG	NLEALTGPGI	PHINGKPACD	EFDQLIKNMA	QGRHVEVFEL	LKPPSGGLGF	SVVGLRSENR
170	180	190	200	210	220	230	240
GELGIFVQEI	QEGSVAHRDG	RLKETDQILA	INGQALDQTI	THQQAISILQ	KAKDTVQLVI	ARGSLPQLVS	PIVSRSPSAA
250	260	270	280	290	300	310	320
STISAHSNPV	HWQHMETIEL	VNDGSGLGFG	IIGGKATGVI	VKTILPGGVA	DQHGRLCSGD	HILKIGDTDL	AGMSSEQVAQ
330	340	350	360	370	380	390	400
VLRQCGNRVK	LMIARGAIEE	RTAPTALGIT	LSSSPTSTPE	LRVDASTQKG	EESETFDVEL	TKNVQGLGIT	IAGYIGDKKL
410	420	430	440	450	460	470	480
EPSGIFVKSI	TKSSAVEHDG	RIQIGDQIIA	VDGTNLQGFT	NQQAVEVLRH	TGQTVLLTLM	RRGMKQEAEL	MSREDVTKDA
490	500	510	520	530	540	550	560
DLSPVNASII	KENYEKDEDF	LSSTRNTNIL	PTEEEGYPLL	SAEIEEIEDA	QKQEAALLTK	WQRIMGINYE	IVVAHVSKFS
570	580	590	600	610	620	630	640
ENSGLGISLE	ATVGHHFIRS	VLPEGPVGHS	GKLFSGDELL	EVNGITLLGE	NHQDVVNILK	ELPIEVTMVC	CRRTVPPTTQ
650	660	670	680	690	700	710	720
SELDSLDLCD	IELTEKPHVD	LGEFIGSSET	EDPVLAMTDA	GQSTEEVQAP	LAMWEAGIQH	IELEKGSKGL	GFSILDYQDP
730	740	750	760	770	780	790	800
IDPASTVIII	RSLVPGGIAE	KDGRLLPGDR	LMFVNDVNLE	NSSLEEAVEA	LKGAPSGTVR	IGVAKPLPLS	PEEGYVSAKE
810	820	830	840	850	860	870	880
DSFLYPPHSC	EEAGLADKPL	FRADLALVGT	NDADLVDEST	FESPYSPEND	SIYSTQASIL	SLHGSSCGDG	LNYGSSLPSS
890	900	910	920	930	940	950	960
PPKDVIENSC	DPVLDLHMSL	EELYTQNLLQ	RQDENTPSVD	ISMGPASGFT	INDYTPANAI	EQQYECENTI	VWTESHLPSE
970	980	990	1000	1010	1020	1030	1040
VISSAELPSV	LPDSAGKGSE	YLLEQSSLAC	NAECVMLQNV	SKESFERTIN	IAKGNSSLGM	TVSANKDGLG	MIVRSIIHGG
1050	1060	1070	1080	1090	1100	1110	1120
AISRDGRIAI	GDCILSINEE	STISVTNAQA	RAMLRRHSLI	GPDIKITYVP	AEHLEEFKIS	LGQQSGRVMA	LDIFSSYTGR
1130	1140	1150	1160	1170	1180	1190	1200
DIPELPEREE	GEGEESELQN	TAYSNWNQPR	RVELWREPSK	SLGISIVGGR	GMGSRLSNGE	VMRGIFIKHV	LEDSPAGKNG
1210	1220	1230	1240	1250	1260	1270	1280
TLKPGDRIVE	APSQSESEPE	KAPLCSVPPP	PPSAFAEMGS	DHTQSSASKI	SQDVDKEDEF	GYSWKNIRER	YGTLTGELHM
1290	1300	1310	1320	1330	1340	1350	1360
IELEKGHSGL	GLSLAGNKDR	SRMSVFIVGI	DPNGAAGKDG	RLQIADELLE	INGQILYGRS	HQNASSIIKC	APSKVKIIFI
1370	1380	1390	1400	1410	1420	1430	1440
RNKDAVNQMA	VCPGNAVEPL	PSNSENLQNK	ETEPTVTTSD	AAVDLSSFKN	VQHLELPKDQ	GGLGIAISEE	DTLSGVIIKS
1450	1460	1470	1480	1490	1500	1510	1520
LTEHGVAATD	GRLKVGDQIL	AVDDEIVVGY	PIEKFISLLK	TAKMTVKLTI	HAENPDSQAV	PSAAGAASGE	KKNSSQSLMV
1530	1540	1550	1560	1570	1580	1590	1600
PQSGSPEPES	IRNTSRSSTP	AIFASDPATC	PIIPGCETTI	EISKGRTGLG	LSIVGGSDTL	LGAIIIHEVY	EEGAACKDGR
1610	1620	1630	1640	1650	1660	1670	1680
LWAGDQILEV	NGIDLRKATH	DEAINVLRQT	PQRVRLTLYR	DEAPYKEEEV	CDTLTIELQK	KPGKGLGLSI	VGKRNDTGVF
1690	1700	1710	1720	1730	1740	1750	1760
VSDIVKGGIA	DADGRLMQGD	QILMVNGEDV	RNATQEAVAA	LLKCSLGTVT	LEVGRIKAGP	FHSERRPSQS	SQVSEGSLSS
1770	1780	1790	1800	1810	1820	1830	1840
FTFPLSGSST	SESLESSSKK	NALASEIQGL	RTVEMKKGPT	DSLGISIAGG	VGSPLGDVPI	FIAMMHPTGV	AAQTQKLRVG
1850	1860	1870	1880	1890	1900	1910	1920
DRIVTICGTS	TEGMTHTQAV	NLLKNASGSI	EMQVVAGGDV	SVVTGHQQEP	ASSSLSFTGL	TSSSIFQDDL	GPPQCKSITL
1930	1940	1950	1960	1970	1980	1990	2000
ERGPDGLGFS	IVGGYGSPHG	DLPIYVKTVF	AKGAASEDGR	LKRGDQIIAV	NGQSLEGVTH	EEAVAILKRT	KGTVTLMVLS
2010							

Cmpd.	No. of Cmpds.	m/z meas.	Δ m/z [ppm]	z	Rt [min]	Score	P	Range	Sequence	Modification

Detailed Protein Report

Cmpd.	No. of Cmpds.	m/z meas.	Δ m/z [ppm]	z	Rt [min]	Score	P	Range	Sequence	Modification
2017	1	788.3019	-141.73	2	55.1	14.1	0	1303-1318	R.MSVFIVGIDPNGAAGK.D	
1954	1	618.9203	-128.74	3	53.1	10.4	2	1340-1356	R.SHQNASSIIKCAPSKVK.I	Carbamidomethyl: 11
1435	1	627.2993	-161.59	2	47.6	10.2	1	1661-1673	K.KPGKGLGLSIVGK.R	

Detailed Protein Report

Protein 413: PREDICTED: SH3 domain-containing protein 21 isoform X4 [Homo sapiens]

Accession: gi|578799774
Database: refseq_human(refseq_human_20140103.fasta)
Database Date: 2015-11-30

Score: 46.2
MW [kDa]: 84.7
pI: 5.6
Sequence Coverage [%]: 4.3
No. of unique Peptides: 2

Quantitation

Q:W **Median:** 0.56 **CV:** 0.00 % **No. of Peptides:** 1

10	20	30	40	50	60	70	80
MEVLVLAGYR	AQKEDELSLA	PGDVVRQVRW	VPARGWLRGE	FGGRYGLFPE	RLVQEIPETL	RGSGEARRPR	CARRRGHPAK
**90**	**100**	**110**	**120**	**130**	**140**	**150**	**160**
HPRPQRWCKV	NFSYSPEQAD	ELKLQAGEIV	EMIKEIEDGW	WLGKKNGQLG	AFPSNFVELL	DSGPPSLGNP	DMPSVSPGPQ
**170**	**180**	**190**	**200**	**210**	**220**	**230**	**240**
RPPKLSSLAY	DSPPDYLQTV	SHPEVYRVLF	DYQPEAPDEL	ALRRGDVVKV	LSKTTEDKGW	WEGECQGRRG	VFPDNFVLPP
**250**	**260**	**270**	**280**	**290**	**300**	**310**	**320**
PPIKKLVPRK	VVSRESAPIK	EPKKLMPKTS	LPTVKKLATA	TTGPSKAKTS	RTPSRDSQKL	TSRDSGPNGG	FQSGGSYHPG
**330**	**340**	**350**	**360**	**370**	**380**	**390**	**400**
RKRSKTQTPQ	QRSVSSQEEE	HSSPVKAPSV	KRTPMPDKTA	TPERPPAPEN	APSSKKIPAP	DKVPSPEKTL	TLGDKASIPG
**410**	**420**	**430**	**440**	**450**	**460**	**470**	**480**
NSTSGKIPAP	DKVPTPEKMV	TPEDKASIPE	NSIIPEETLT	VDKPSTPERV	FSVEESPALE	APPMDKVPNP	KMAPLGDEAP
**490**	**500**	**510**	**520**	**530**	**540**	**550**	**560**
TLEKVLTPEL	SEEEVSTRDD	IQFHHFSSEE	ALQKVKYFVA	KEDPSSQEEA	HTPEAPPPQP	PSSERCLGEM	KCTLVRGDSS
**570**	**580**	**590**	**600**	**610**	**620**	**630**	**640**
PRQAELKSGP	ASRPALEKPH	PHEEATTLPE	EAPSNDERTP	EEEAPPNEQR	PLREEVLPKE	GVASKEEVTL	KEELPPKEEV
**650**	**660**	**670**	**680**	**690**	**700**	**710**	**720**
APKEEVPPIE	RAFAQKTRPI	KPPPDSQETL	ALPSLVPQNY	TENKNEGVDV	TSLRGEVESL	RRALELMEEE	ADRHLGGAEE
**730**	**740**	**750**	**760**	**770**	**780**		
REGAAPAAGG	PVYEPRALWG	QGDVCPGVCR	GLPWPSQARW	EKRQPCPGGF			

Cmpd.	No. of Cmpds.	m/z meas.	Δ m/z [ppm]	z	Rt [min]	Score	P	Range	Sequence	Modification	Ratios
328	1	658.3980	85.56	2	32.8	13.8	2	62-73	R.GSGEARRPRCAR.R		
2858	1	805.9291	-2.54	2	66.2	12.9	2	751-763	R.GLPWPSQARWEKR.Q		Q:W 0.56

Detailed Protein Report

Protein 414: laminin subunit beta-2 precursor [Homo sapiens]

**Accession:**	gi|119703755	**Score:**	46.2
**Database:**	refseq_human(refseq_human_20140103.fasta)	**MW [kDa]:**	195.9
**Database Date:**	2015-11-30	**pI:**	6.1
		**Sequence Coverage [%]:**	3.6
		**No. of unique Peptides:**	2

Alias proteins:

Accession	Name	Description
gi|530372442	refseq_human (refseq_human_20140103.fasta)	PREDICTED: laminin subunit beta-2 isoform X1 [Homo sapiens]

Detailed Protein Report

10	20	30	40	50	60	70	80
MELTSRERGR	GQPLPWELRL	GLLLSVLAAT	LAQAPAPDVP	GCSRGSCYPA	TGDLLVGRAD	RLTASSTCGL	NGPQPYCIVS
90	100	110	120	130	140	150	160
HLQDEKKCFL	CDSRRPFSAR	DNPHSHRIQN	VVTSFAPQRR	AAWWQSENGI	PAVTIQLDLE	AEFHFTHLIM	TFKTFRPAAM
170	180	190	200	210	220	230	240
LVERSADFGR	TWHVYRYFSY	DCGADFPGVP	LAPPRHWDDV	VCESRYSEIE	PSTEGEVIYR	VLDPAIPIPD	PYSSRIQNLL
250	260	270	280	290	300	310	320
KITNLRVNLT	RLHTLGDNLL	DPRREIREKY	YYALYELVVR	GNCFCYGHAS	ECAPAPGAPA	HAEGMVHGAC	ICKHNTRGLN
330	340	350	360	370	380	390	400
CEQCQDFYRD	LPWRPAEDGH	SHACRKCECH	GHTHSCHFDM	AVYLASGNVS	GGVCDGCQHN	TAGRHCELCR	PFFYRDPTKD
410	420	430	440	450	460	470	480
LRDPAVCRSC	DCDPMGSQDG	GRCDSHDDPA	LGLVSGQCRC	KEHVVGTRCQ	QCRDGFFGLS	ISDRLGCRRC	QCNARGTVPG
490	500	510	520	530	540	550	560
STPCDPNSGS	CYCKRLVTGR	GCDRCLPGHW	GLSHDLLGCR	PCDCDVGGAL	DPQCDEGTGQ	CHCRQHMVGR	RCEQVQPGYF
570	580	590	600	610	620	630	640
RPFLDHLIWE	AEDTRGQVLD	VVERLVTPGE	TPSWTGSGFV	RLQEGQTLEF	LVASVPKAMD	YDLLLRLEPQ	VPEQWAELEL
650	660	670	680	690	700	710	720
IVQRPGPVPA	HSLCGHLVPK	DDRIQGTLQP	HARYLIFPNP	VCLEPGISYK	LHLKLVRTGG	SAQPETPYSG	PGLLIDSLVL
730	740	750	760	770	780	790	800
LPRVLVLEMF	SGGDAAALER	QATFERYQCH	EEGLVPSKTS	PSEACAPLLI	SLSTLIYNGA	LPCQCNPQGS	LSSECNPHGG
810	820	830	840	850	860	870	880
QCLCKPGVVG	RRCDLCAPGY	YGFGPTGCQA	CQCSHEGALS	SLCEKTSGQC	LCRTGAFGLR	CDRCQRGQWG	FPSCRPCVCN
890	900	910	920	930	940	950	960
GHADECNTHT	GACLGCRDHT	GGEHCERCIA	GFHGDPRLPY	GGQCRPCPCP	EGPGSQRHFA	TSCHQDEYSQ	QIVCHCRAGY
970	980	990	1000	1010	1020	1030	1040
TGLRCEACAP	GHFGDPSRPG	GRCQLCECSG	NIDPMDPDAC	DPHTGQCLRC	LHHTEGPHCA	HCKPGFHGQA	ARQSCHRCTC
1050	1060	1070	1080	1090	1100	1110	1120
NLLGTNPQQC	PSPDQCHCDP	SSGQCPCLPN	VQGPSCDRCA	PNFWNLTSGH	GCQPCACHPS	RARGPTCNEF	TGQCHCRAGF
1130	1140	1150	1160	1170	1180	1190	1200
GGRTCSECQE	LHWGDPGLQC	HACDCDSRGI	DTPQCHRFTG	HCSCRPGVSG	VRCDQCARGF	SGIFPACHPC	HACFGDWDRV
1210	1220	1230	1240	1250	1260	1270	1280
VQDLAARTQR	LEQRAQELQQ	TGVLGAFESS	FWHMQEKLGI	VQGIVGARNT	SAASTAQLVE	ATEELRREIG	EATEHLTQLE
1290	1300	1310	1320	1330	1340	1350	1360
ADLTDVQDEN	FNANHALSGL	ERDRLALNLT	LRQLDQHLDL	LKHSNFLGAY	DSIRHAHSQS	AEAERRANTS	ALAVPSPVSN
1370	1380	1390	1400	1410	1420	1430	1440
SASARHRTEA	LMDAQKEDFN	SKHMANQRAL	GKLSAHTHTL	SLTDINELVC	GAPGDAPCAT	SPCGGAGCRD	EDGQPRCGGL
1450	1460	1470	1480	1490	1500	1510	1520
SCNGAAATAD	LALGRARHTQ	AELQRALAEG	GSILSRVAET	RRQASEAQQR	AQAALDKANA	SRGQVEQANQ	ELQELIQSVK
1530	1540	1550	1560	1570	1580	1590	1600
DFLNQEGADP	DSIEMVATRV	LELSIPASAE	QIQHLAGAIA	ERVRSLADVD	AILARTVGDV	RRAEQLLQDA	RRARSWAEDE
1610	1620	1630	1640	1650	1660	1670	1680
KQKAETVQAA	LEEAQRAQGI	AQGAIRGAVA	DTRDTEQTLY	QVQERMAGAE	RALSSAGERA	RQLDALLEAL	KLKRAGNSLA
1690	1700	1710	1720	1730	1740	1750	1760
ASTAEETAGS	AQGRAQEAEQ	LLRGPLGDQY	QTVKALAERK	AQGVLAAQAR	AEQLRDEARD	LLQAAQDKLQ	RLQELEGTYE
1770	1780	1790	1800				
ENERALESKA	AQLDGLEARM	RSVLQAINLQ	VQIYNTCQ				

Cmpd.	No. of Cmpds.	m/z meas.	Δ m/z [ppm]	z	Rt [min]	Score	P	Range	Sequence	Modification
1490	1	977.1102	99.37	2	48.4	12.1	0	674-690	R.YLIFPNPVCLEPGISYK.L	
2931	1	1051.5566	80.57	2	65.4	10.0	0	918-937	R.LPYGGQCRPCPCPEGPGSQR.H	

Detailed Protein Report

Protein 415: alpha-1-acid glycoprotein 2 precursor [Homo sapiens]

**Accession:**	gi|4505529	**Score:**	46.2
**Database:**	refseq_human(refseq_human_20140103.fasta)	**MW [kDa]:**	23.6
**Database Date:**	2015-11-30	**pI:**	4.9
		**Sequence Coverage [%]:**	5.0
		**No. of unique Peptides:**	1

10	20	30	40	50	60	70	80
MALSWVLTVL	SLLPLLEAQI	PLCANLVPVP	ITNATLDRIT	GKWFYIASAF	RNEEYNKSVQ	EIQATFFYFT	PNKTEDTIFL
**90**	**100**	**110**	**120**	**130**	**140**	**150**	**160**
REYQTRQNQC	FYNSSYLNVQ	RENGTVSRYE	GGREHVAHLL	FLRDTKTLMF	GSYLDDEKNW	GLSFYADKPE	TTKEQLGEFY
**170**	**180**	**190**	**200**	**210**			
EALDCLCIPR	SDVMYTDWKK	DKCEPLEKQH	EKERKQEEGE	S			

Cmpd.	No. of Cmpds.	m/z meas.	Δ m/z [ppm]	z	Rt [min]	Score	P	Range	Sequence	Modification
1023	3	617.8379	-30.01	2	41.5	46.2	0	114-123	R.EHVAHLLFLR.D	

Detailed Protein Report

Protein 416: TATA element modulatory factor [Homo sapiens]

**Accession:**	gi|110347443	**Score:**	46.1
**Database:**	refseq_human(refseq_human_20140103.fasta)	**MW [kDa]:**	122.8
**Database Date:**	2015-11-30	**pI:**	4.7
**Modification(s):**	Oxidation	**Sequence Coverage [%]:**	4.8
		**No. of unique Peptides:**	2

Quantitation

M:Q **Median:** 0.51 **CV:** 0.00 % **No. of Peptides:** 1

10	20	30	40	50	60	70	80
MSWFNASQLS	SFAKQALSQA	QKSIDRVLDI	QEEEPSIWAE	TIPYGEPGIS	SPVSGGWDTS	TWGLKSNTEP	QSPPIASPKA
90	100	110	120	130	140	150	160
ITKPVRRTVV	DESENFFSAF	LSPTDVQTIQ	KSPVVSKPPA	KSQRPEEEVK	SSLHESLHIG	QSRTPETTES	QVKDSSLCVS
170	180	190	200	210	220	230	240
GETLAAGTSS	PKTEGKHEET	VNKESDMKVP	TVSLKVSESV	IDVKTTMESI	SNTSTQSLTA	ETKDIALEPK	EQKHEDRQSN
250	260	270	280	290	300	310	320
TPSPPVSTFS	SGTSTTSDIE	VLDHESVISE	SSASSRQETT	DSKSSLHLMQ	TSFQLLSASA	CPEYNRLDDF	QKLTESCCSS
330	340	350	360	370	380	390	400
DAFERIDSFS	VQSLDSRSVS	EINSDDELSG	KGYALVPIIV	NSSTPKSKTV	ESAEGKSEEV	NETLVIPTEE	AEMEESGRSA
410	420	430	440	450	460	470	480
TPVNCEQPDI	LVSSTPINEG	QTVLDKVAEQ	CEPAESQPEA	LSEKEDVCKT	VEFLNEKLEK	REAQLLSLSK	EKALLEEAFD
490	500	510	520	530	540	550	560
NLKDEMFRVK	EESSSISSLK	DEFTQRIAEA	EKKVQLACKE	RDAAKKEIKN	IKEELATRLN	SSETADLLKE	KDEQIRGLME
570	580	590	600	610	620	630	640
EGEKLSKQQL	HNSNIIKKLR	AKDKENENMV	AKLNKKVKEL	EEELQHLKQV	LDGKEEVEKQ	HRENIKKLNS	MVERQEKDLG
650	660	670	680	690	700	710	720
RLQVDMDELE	EKNRSIQAAL	DSAYKELTDL	HKANAAKDSE	AQEAALSREM	KAKEELSAAL	EKAQEEARQQ	QETLAIQVGD
730	740	750	760	770	780	790	800
LRLALQRTEQ	AAARKEDYLR	HEIGELQQRL	QEAENRNQEL	SQSVSSTTRP	LLRQIENLQA	TLGSQTSSWE	KLEKNLSDRL
810	820	830	840	850	860	870	880
GESQTLLAAA	VERERAATEE	LLANKIQMSS	MESQNSLLRQ	ENSRFQAQLE	SEKNRLCKLE	DENNRYQVEL	ENLKDEYVRT
890	900	910	920	930	940	950	960
LEETRKEKTL	LNSQLEMERM	KVEQERKKAI	FTQETIKEKE	RKPFSVSSTP	TMSRSSSISG	VDMAGLQTSF	LSQDESHDHS
970	980	990	1000	1010	1020	1030	1040
FGPMPISANG	SNLYDAVRMG	AGSSIIENLQ	SQLKLREGEI	THLQLEIGNL	EKTRSIMAEE	LVKLTNQNDE	LEEKVKEIPK
1050	1060	1070	1080	1090	1100		
LRTQLRDLDQ	RYNTILQMYG	EKAEEAEELR	LDLEDVKNMY	KTQIDELLRQ	SLS		

Cmpd.	No. of Cmpds.	m/z meas.	Δ m/z [ppm]	z	Rt [min]	Score	P	Range	Sequence	Modification	Ratios	
1573	1	1023.5251	43.32	2	49.9	12.3	0	205-223	K.TTMESISNTSTQSLTAETK.D	Oxidation: 3		
1116	1	555.1899	-177.69	2	42.4	11.2	0	741-749	R.HEIGELQQR.L		M:Q	0.51

Detailed Protein Report

Protein 417: **PREDICTED: nascent polypeptide-associated complex subunit alpha isoform X5 [Homo sapiens]**

**Accession:**	gi|578823770	**Score:**	46.1
**Database:**	refseq_human(refseq_human_20140103.fasta)	**MW [kDa]:**	203.6
**Database Date:**	2015-11-30	**pI:**	10.2
		**Sequence Coverage [%]:**	2.9
		**No. of unique Peptides:**	1

BRUKER

Detailed Protein Report

10	20	30	40	50	60	70	80
MPGEATETVP	ATEQELPQPQ	AETAVLPMSS	ALSVTAALGQ	PGPTLPPPCS	PAPQQCPLSA	ANQASPFPSP	STIASTPLEV
90	100	110	120	130	140	150	160
PFPQSSSGTA	LPLGTAPEAP	TFLPNLIGPP	ISPAALALAS	PMIAPTLKGT	PSSSAPLALV	ALAPHSVQKS	SAFPPNLLTS
170	180	190	200	210	220	230	240
PPSVAVAESG	SVITLSAPIA	PSEPKTNLNK	VPSEVVPNPK	GTPSPPCIVS	TVPYHCVTPM	ASIQSGVASL	PQTTPTTTLA
250	260	270	280	290	300	310	320
IASPQVKDTT	ISSVLISPQN	PGSLSLKGPV	SPPAALSLST	QSLPVVTSSQ	KTAGPNTPPD	FPISLGSHLA	PLHQSSFGSV
330	340	350	360	370	380	390	400
QLLGQTGPSA	LSDPTVKTIS	VDHSSTGASY	PSQRSVIPPL	PSRNEVVPAT	VAAFPVVAPS	VDKGPSTISS	ITCSPSGSLN
410	420	430	440	450	460	470	480
VATSFSLSPT	TSLILKSSPN	ATYHYPLVAQ	MPVSSVGTTP	LVVTNPCTIA	AAPTTTFEVA	TCVSPPMSSG	PISNIEPTSP
490	500	510	520	530	540	550	560
AALVMAPVAP	KEPSTQVATT	LRIPVSPPLP	DPEDLKNLPS	SVLVKFPTQK	DLQTVPASLE	GAPFSPAQAG	LTTKKDPTVL
570	580	590	600	610	620	630	640
PLVQAAPKNS	PSFQSTSSSP	EIPLSPEATL	AKKSLGEPLP	IGKPASSMTS	PLGVNSSASV	IKTDSYAGPD	SAGPLLKSSL
650	660	670	680	690	700	710	720
ITPTVAAFPL	ESADPAGVAP	TTAKGTSTYT	TTASPFLEGT	VSLAPKNHPV	KEGTLTTLPL	VPTASENCPV	APSPQNTCAP
730	740	750	760	770	780	790	800
LATLVLAPEI	PKSVPSPSLP	PAGTPPGTKK	VDGISHTSAL	APVASSPKEC	PTEDSGASAT	ASSKGTLTYL	ADSPSPLGVS
810	820	830	840	850	860	870	880
VSPQTKRPPT	KKGSAGPDTP	IGNLSSPVSP	VEASFLPENS	LSFQGSKDSP	ATTHSPTPPS	PKGAPTPSAV	TPLSPKGVTL
890	900	910	920	930	940	950	960
PPKETPTPSV	VNLPFPKEGP	ATPAPKQAPA	LSMTSSSPKK	ARATPAPKGI	PASPSPKGAP	TPPAATPPSP	KGGPATPSPK
970	980	990	1000	1010	1020	1030	1040
WAPTPPAATP	PSPKGGPATP	SPKGAPTPPA	ATPPSPKGSP	AATPFPKGAS	TPPAATPPSP	KGSPAATPLP	KGAPTTPAAT
1050	1060	1070	1080	1090	1100	1110	1120
LPSPKGGPAT	PSLKGAPTPP	AATPPSPKGG	PATPSPKGAP	MPPAATPPSP	KGGLATPPHK	GAPTTPAATP	PSPKGGLATP
1130	1140	1150	1160	1170	1180	1190	1200
PPKGAPTTPA	ATPPSPKGGL	ATPPPKGAPT	TPAATPPSPK	GGLATPSPKG	APTTPAATPP	SPKGGLATPS	PKGAPTTPAA
1210	1220	1230	1240	1250	1260	1270	1280
TPPSPKGGLA	TPSPKGAPTT	PAATPPSPKG	GPATPPPKGA	PTPPAATPPS	LKGGLATPPH	KGAPNPAVVT	PPSPKGGPAT
1290	1300	1310	1320	1330	1340	1350	1360
SPPKGAPTPP	AATPPSPKGS	PGTPPPKGAP	TPPAVTPPSP	KGTPTLPATT	PSSKGGPTTP	SSKEGPTPPA	ATPSHKGGPA
1370	1380	1390	1400	1410	1420	1430	1440
MTPPSPKRGP	AIPSPKGDPT	SPAVIPLSPK	KAPATPVTRE	GAATPSKGDL	TPPAVTPVSL	KKAPATSAPK	GGPATPSSKG
1450	1460	1470	1480	1490	1500	1510	1520
DPTLPAVTPP	SPKEPPAPKQ	VATSSSPKKA	PATPAPMGAP	TLPAVIPSSP	KEVPATPSSR	RDPIAPTATL	LSKKTPATLA
1530	1540	1550	1560	1570	1580	1590	1600
PKEALIPPAM	TVPSPKKTPA	IPTPKEAPAT	PSSKEASSPP	AVTPSTYKGA	PSPKELLIPP	AVTSPSPKEA	PTPPAVTPPS
1610	1620	1630	1640	1650	1660	1670	1680
PEKGPATPAP	KGTPTSPPVT	PSSLKDSPTS	PASVTCKMGA	TVPQASKGLP	AKKGPTALKE	VLVAPAPEST	PIITAPTRKG
1690	1700	1710	1720	1730	1740	1750	1760
PQTKKSSATS	PPICPDPSAK	NGSKGPLSTV	APAPLLPVQK	DSSKTAKGKD	ASHSPKGPLA	PPESKASTPL	TAAAFEKVLP
1770	1780	1790	1800	1810	1820	1830	1840
KPESASVSAA	PSPPVSLPLA	PSPVPTLPPK	QQFLPSSPGL	VLESPSKPLA	PADEDELLPL	IPPEPISGGV	PFQSVLVNMP
1850	1860	1870	1880	1890	1900	1910	1920
TPKSAGIPVP	TPSAKQPVTK	NNKGSGTESD	SDESVPELEE	QDSTQATTQQ	AQLAAAAEID	EEPVSKAKQS	RSEKKARKAM
1930	1940	1950	1960	1970	1980	1990	2000
SKLGLRQVTG	VTRVTIRKSK	NILFVITKPD	VYKSPASDTY	IVFGEAKIED	LSQQAQLAAA	EKFKVQGEAV	SNIQENTQTP
2010	2020	2030	2040	2050	2060		
TVQEESEEEE	VDETGVEVKD	IELVMSQANV	SRAKAVRALK	NNSNDIVNAI	MVSVQAFVP		

Cmpd.	No. of Cmpds.	m/z meas.	Δ m/z [ppm]	z	Rt [min]	Score	P	Range	Sequence	Modification
52	1	717.7555	-144.48	2	30.5	11.1	0	1555-1568	K.EASSPPAVTPSTYK.G	

Detailed Protein Report

Protein 418: CUB and sushi domain-containing protein 3 isoform 3 [Homo sapiens]

**Accession:**	gi|205277354	**Score:**	46.0
**Database:**	refseq_human(refseq_human_20140103.fasta)	**MW [kDa]:**	387.1
**Database Date:**	2015-11-30	**pI:**	5.5
**Modification(s):**	Carbamidomethyl	**Sequence Coverage [%]:**	1.3
		**No. of unique Peptides:**	2

10	20	30	40	50	60	70	80
MKGIRKGESR	AKESKPWEPG	KRRCAKCGRL	DFILMKKMGI	KSGFTFWNLV	FLLTVSCVKG	FIYTCGGTLK	GLNGTIESPG
90	100	110	120	130	140	150	160
FPYGYPNGAN	CTWVIIAEER	NRIQIVFQSF	ALEEEYDYLS	LYDGHPHPTN	FRTRLTGFHL	PPPVTSTKSV	FSLRLTSDFA
170	180	190	200	210	220	230	240
VSAHGFKVYY	EELQSSSCGN	PGVPPKGVLY	GTRFDVGDKI	RYSCVTGYIL	DGHPQLTCIA	NSVNTASWDF	PVPICRAEDA
250	260	270	280	290	300	310	320
CGGTMRGSSG	IISSPSFPNE	YHNNADCTWT	IVAEPGDTIS	LIFTDFQMEE	KYDYLEIEGS	EPPTIWLSGM	NIPPPIISNK
330	340	350	360	370	380	390	400
NWLRLHFVTD	SNHRYRGFSA	PYQVKKAIDF	KSRGFKLFPG	KDNSNKFSIL	NEGGIKTASN	LCPDPGEPEN	GKRIGSDFSL
410	420	430	440	450	460	470	480
GSTVQFSCDE	DYVLQGAKSI	TCQRIAEVFA	AWSDHRPVCK	VKTCGSNLQG	PSGTFTSPNF	PFQYDSNAQC	VWVITAVNTN
490	500	510	520	530	540	550	560
KVIQINFEEF	DLEIGYDTLT	IGDGGEVGDP	RTVLQVLTGS	FVPDLIVSMS	SQMWLHLQTD	ESVGSVGFKV	NYKEIEKESC
570	580	590	600	610	620	630	640
GDPGTPLYGI	REGDGFSNRD	VLRFECQFGF	ELIGEKSIVC	QENNQWSANI	PICIFPCLSN	FTAPMGTVLS	PDYPEGYGNN
650	660	670	680	690	700	710	720
LNCIWTIISD	PGSRIHLSFN	DFDLESQFDF	LAVKDGDSPE	SPILGTFTGA	EVPSHLTSNS	HILRLEFQAD	HSMSGRGFNI
730	740	750	760	770	780	790	800
TYNTFGHNEC	PDPGIPINAR	RFGDNFQLGS	SISVICEEGF	IKTQGTETIT	CILMDGKVMW	SGLIPKCGAP	CGGHFSAPSG
810	820	830	840	850	860	870	880
VILSPGWPGY	YKDSLNCEWV	IEAEPGHSIK	ITFERFQTEL	NYDVLEVHDG	PNLLSPLLGS	YNGTQVPQFL	FSSSNFIYLL
890	900	910	920	930	940	950	960
FTTDNSRSNN	GFKIHYESVT	VNTYSCLDPG	IPVHGRRYGH	DFSIGSTVSF	SCDSGYRLSH	EEPLLCEKNH	WWSHPLPTCD
970	980	990	1000	1010	1020	1030	1040
ALCGGDVRGP	SGTILSPGYP	EFYPNSLNCT	WTVDVTHGKG	VQFNFHTFHL	EDHHDYLLIT	ENGSFTQPLA	RLTGSDLPPT
1050	1060	1070	1080	1090	1100	1110	1120
INAGLYGNFR	AQLRFISDFS	ISYEGFNITF	SEYNLEPCED	PGIPQYGSRI	GFNFGIGDTL	TFSCSSGYRL	EGTSEIICLG
1130	1140	1150	1160	1170	1180	1190	1200
GGRRVWSAPL	PRCVAECGAS	ATNNEGILLS	PNYPLNYENN	HECIYSIQVQ	AGKGINISAR	TFHLAQGDVL	KIYDGKDKTT
1210	1220	1230	1240	1250	1260	1270	1280
HLLGAFTGAS	MRGLTLSSTS	NQLWLEFNSD	TEGTDEGFQL	VYTSFELSHC	EDPGIPQFGY	KISDQGHFAG	STIIYGCNPG
1290	1300	1310	1320	1330	1340	1350	1360
YTLHGSSLLK	CMTGERRAWD	YPLPSCIAEC	GGRFKGESSG	RILSPGYPFP	YDNNLRCMWM	IEVDPGNIVS	LQFLAFDTEA
1370	1380	1390	1400	1410	1420	1430	1440
SHDILRVWDG	PPENDMLLKE	ISGSLIPEGI	HSTLNIVTIQ	FDTDFYISKS	GFAIQFSSSV	ATACRDPGVP	MNGTRNGDGR
1450	1460	1470	1480	1490	1500	1510	1520
EPGDTVVFQC	DPGYELQGEE	RITCIQVENR	YFWQPSPPVC	IAPCGGNLTG	SSGFILSPNF	PHPYPHSRDC	DWTITVNADY
1530	1540	1550	1560	1570	1580	1590	1600
VISLAFISFS	IEPNYDFLYI	YDGPDSNSPL	IGSFQDSKLP	ERIESSSNTM	HLAFRSDGSV	SYTGFHLEYK	AKLRESCFDP
1610	1620	1630	1640	1650	1660	1670	1680
GNIMNGTRLG	MDYKLGSTVT	YYCDAGYVLQ	GYSTLTCIMG	DDGRPGWNRA	LPSCHAPCGS	RSTGSEGTVL	SPNYPKNYSV
1690	1700	1710	1720	1730	1740	1750	1760
GHNCVYSIAV	PKEFVVFGQF	VFFQTSLHDV	VEVYDGPTQQ	SSLLSSLSGS	HSGESLPLSS	GNQITIRFTS	VGPITAKGFH
1770	1780	1790	1800	1810	1820	1830	1840
FVYQAVPRTS	STQCSSVPEP	RFGRRIGNEF	AVGSSVLFDC	NPGYILHGSI	AIRCETVPNS	LAQWNDSLPT	CIVPCGGILT
1850	1860	1870	1880	1890	1900	1910	1920
KRKGTILSPG	YPEPYDNNLN	CVWKITVPEG	AGIQVQVVSF	ATEHNWDSLD	FYDGGDNNAP	RLGSYSGTTI	PHLLNSTSNN
1930	1940	1950	1960	1970	1980	1990	2000
LYLNFQSDIS	VSAAGFHLEY	TAIGLDSCPE	PQTPSSGIKI	GDRYMVGDVV	SFQCDQGYSL	QGHSHITCMP	GPVRRWNYPI
2010	2020	2030	2040	2050	2060	2070	2080
PICLAQCGGA	MSDFSGVILS	PGFPGNYPSS	LDCTWTINLP	IGFGVHLQFV	NFSTETIHDY	LEVRSGSSET	STVIGRLSGP
2090	2100	2110	2120	2130	2140	2150	2160
QIPSSLFSTT	HETSLYFHSD	YSQNKQGFHI	VYQAYQLQSC	PDPRPFRNGF	VIGNDFTVGQ	TISFECFPGY	TLIGNSALTC
2170	2180	2190	2200	2210	2220	2230	2240

Detailed Protein Report

Cmpd.	No. of Cmpds.	m/z meas.	Δ m/z [ppm]	z	Rt [min]	Score	P	Range	Sequence	Modification
1865	1	897.8571	-115.65	2	53.2	10.9	1	321-334	K.NWLRLHFVTDSNHR.Y	
1304	1	661.3930	144.57	1	46.4	11.2	0	2937-2941	R.QPECK.A	Carbamidomethyl: 4

Detailed Protein Report

Protein 419: **PREDICTED: transcriptional regulator ATRX isoform X8 [Homo sapiens]**

**Accession:**	gi|578838421	**Score:**	46.0
**Database:**	refseq_human(refseq_human_20140103.fasta)	**MW [kDa]:**	211.9
**Database Date:**	2015-11-30	**pI:**	6.7
		**Sequence Coverage [%]:**	2.0
		**No. of unique Peptides:**	2

10	20	30	40	50	60	70	80
MTAEPMSESK	LNTLVQKLHD	FLAHSSEESE	ETSSPPRLAM	NQNTDKISGS	GSNSDMMENS	KEEGTSSSEK	SKSSGSSRSK
**90**	**100**	**110**	**120**	**130**	**140**	**150**	**160**
RKPSIVTKYV	ESDDEKPLDD	ETVNEDASNE	NSENDITMQS	LPKGTVIVQP	EPVLNEDKDD	FKGPEFRSRS	KMKTENLKKR
**170**	**180**	**190**	**200**	**210**	**220**	**230**	**240**
GEDGLHGIVS	CTACGQQVNH	FQKDSIYRHP	SLQVLICKNC	FKYYMSDDIS	RDSDGMDEQC	RWCAEGGNLI	CCDFCHNAFC
**250**	**260**	**270**	**280**	**290**	**300**	**310**	**320**
KKCILRNLGR	KELSTIMDEN	NQWYCYICHP	EPLLDLVTAC	NSVFENLEQL	LQQNKKKIKV	DSEKSNKVYE	HTSRFSPKKT
**330**	**340**	**350**	**360**	**370**	**380**	**390**	**400**
SSNCNGEEKK	LDDSCSGSVT	YSYSALIVPK	EMIKKAKKLI	ETTANMNSSY	VKFLKQATDN	SEISSATKLR	QLKAFKSVLA
**410**	**420**	**430**	**440**	**450**	**460**	**470**	**480**
DIKKAHLALE	EDLNSEFRAM	DAVNKEKNTK	EHKVIDAKFE	TKARKGEKPC	ALEKKDISKS	EAKLSRKQVD	SEHMHQNVPT
**490**	**500**	**510**	**520**	**530**	**540**	**550**	**560**
EEQRTNKSTG	GEHKKSDRKE	EPQYEPANTS	EDLDMDIVSV	PSSVPEDIFE	NLETAMEVQS	SVDHQGDGSS	GTEQEVESSS
**570**	**580**	**590**	**600**	**610**	**620**	**630**	**640**
VKLNISSKDN	RGGIKSKTTA	KVTKELYVKL	TPVSLSNSPI	KGADCQEVPQ	DKDGYKSCGL	NPKLEKCGLG	QENSDNEHLV
**650**	**660**	**670**	**680**	**690**	**700**	**710**	**720**
ENEVSLLLEE	SDLRRSPRVK	TTPLRRPTET	NPVTSNSDEE	CNETVKEKQK	LSVPVRKKDK	RNSSDSAIDN	PKPNKLPKSK
**730**	**740**	**750**	**760**	**770**	**780**	**790**	**800**
QSETVDQNSD	SDEMLAILKE	VSRMSHSSSS	DTDINEIHTN	HKTLYDLKTQ	AGKDDKGKRK	RKSSTSGSDF	DTKKGKSAKS
**810**	**820**	**830**	**840**	**850**	**860**	**870**	**880**
SIISKKKRQT	QSESSNYDSE	LEKEIKSMSK	IGAARTTKKR	IPNTKDFDSS	EDEKHSKKGM	DNQGHKNLKT	SQEGSSDDAE
**890**	**900**	**910**	**920**	**930**	**940**	**950**	**960**
RKQERETFSS	AEGTVDKDTT	IMELRDRLPK	KQQASASTDG	VDKLSGKEES	FTSLEVRKVA	ETKEKSKHLK	TKTCKKVQDG
**970**	**980**	**990**	**1000**	**1010**	**1020**	**1030**	**1040**
LSDIAEKFLK	KDQSDETSED	DKKQSKKGTE	EKKKPSDFKK	KVIKMEQQYE	SSSDGTEKLP	EREEICHFPK	GIKQIKNGTT
**1050**	**1060**	**1070**	**1080**	**1090**	**1100**	**1110**	**1120**
DGEKKSKKIR	DKTSKKKDEL	SDYAEKSTGK	GDSCDSSEDK	KSKNGAYGRE	KKRCKLLGKS	SRKRQDCSSS	DTEKYSMKED
**1130**	**1140**	**1150**	**1160**	**1170**	**1180**	**1190**	**1200**
GCNSSDKRLK	RIELRERRNL	SSKRNTKEIQ	SGSSSSDAEE	SSEDNKKKKQ	RTSSKKKAVI	VKEKKRNSLR	TSTKRKQADI
**1210**	**1220**	**1230**	**1240**	**1250**	**1260**	**1270**	**1280**
TSSSSSDIED	DDQNSIGEGS	SDEQKIKPVT	ENLVLSSHTG	FCQSSGDEAL	SKSVPVTVDD	DDDDNDPENR	IAKKMLLEEI
**1290**	**1300**	**1310**	**1320**	**1330**	**1340**	**1350**	**1360**
KANLSSDEDG	SSDDEPEEGK	KRTGKQNEEN	PGDEEAKNQV	NSESDSDSEE	SKKPRYRHRL	LRHKLTVSDG	ESGEEKKTKP
**1370**	**1380**	**1390**	**1400**	**1410**	**1420**	**1430**	**1440**
KEHKEVKGRN	RRKVSSEDSE	DSDFQESGVS	EEVSESEDEQ	RPRTRSAKKA	ELEENQRSYK	QKKKRRRIKV	QEDSSSENKS
**1450**	**1460**	**1470**	**1480**	**1490**	**1500**	**1510**	**1520**
NSEEEEEEKE	EEEEEEEEEE	EEEEDENDDS	KSPGKGRKKI	RKILKDDKLR	TETQNALKEE	EERRKRIAER	EREREKLREV
**1530**	**1540**	**1550**	**1560**	**1570**	**1580**	**1590**	**1600**
IEIEDASPTK	CPITTKLVLD	EDEETKEPLV	QVHRNMVIKL	KPHQVDGVQF	MWDCCCESVK	KTKKSPGSGC	ILAHCMGLGK
**1610**	**1620**	**1630**	**1640**	**1650**	**1660**	**1670**	**1680**
TLQVVSFLHT	VLLCDKLDFS	TALVVCPLNT	ALNWMNEFEK	WQEGLKDDEK	LEVSELATVK	RPQERSYMLQ	RWQEDGGVMI
**1690**	**1700**	**1710**	**1720**	**1730**	**1740**	**1750**	**1760**
IGYEMYRNLA	QGRNVKSRKL	KEIFNKALVD	PGPDFVVCDE	GHILKNEASA	VSKAMNSIRS	RRRIILTGTP	LQNNLIEYHC
**1770**	**1780**	**1790**	**1800**	**1810**	**1820**	**1830**	**1840**
MVNFIKENLL	GSIKEFRNRF	INPIQNGQCA	DSTMVDVRVM	KKRAHILYEM	LAGCVQRKDY	TALTKFLPPK	HEYVLAVRMT
**1850**	**1860**	**1870**	**1880**				
SIQCKLYQYY	LDHLTVLHLN	KMGMLKSIKY	F				

Detailed Protein Report

Cmpd.	No. of Cmpds.	m/z meas.	Δ m/z [ppm]	z	Rt [min]	Score	P	Range	Sequence	Modification
1627	1	1010.4939	-28.29	1	50.6	13.2	2	71-80	K.SKSSGSSRSK.R	
2633	1	589.1691	-335.46	1	61.2	10.3	1	572-577	R.GGIKSK.T	

Detailed Protein Report

Protein 420: mucin-2 precursor [Homo sapiens]

**Accession:**	gi|557129045	**Score:**	46.0
**Database:**	refseq_human(refseq_human_20140103.fasta)	**MW [kDa]:**	539.8
**Database Date:**	2015-11-30	**pI:**	5.4
**Modification(s):**	Carbamidomethyl	**Sequence Coverage [%]:**	1.1
		**No. of unique Peptides:**	1

BRUKER

Detailed Protein Report

10	20	30	40	50	60	70	80
MGLPLARLAA	VCLALSLAGG	SELQTEGRTR	NHGHNVCSTW	GNFHYKTFDG	DVFRFPGPCD	YNFASDCRGS	YKEFAVHLKR
90	100	110	120	130	140	150	160
GPGQAEAPAG	VESILLTIKD	DTIYLTRHLA	VLNGAVVSTP	HYSPGLLIEK	SDAYTKVYSR	AGLTLMWNRE	DALMLELDTK
170	180	190	200	210	220	230	240
FRNHTCGLCG	DYNGLQSYSE	FLSDGVLFSP	LEFGNMQKIN	QPDVVCEDPE	EEVAPASCSE	HRAECERLLT	AEAFADCQDL
250	260	270	280	290	300	310	320
VPLEPYLRAC	QQDRCRCPGG	DTCVCSTVAE	FSRQCSHAGG	RPGNWRTATL	CPKTCPGNLV	YLESGSPCMD	TCSHLEVSSL
330	340	350	360	370	380	390	400
CEEHRMDGCF	CPEGTVYDDI	GDSGCVPVSQ	CHCRLHGHLY	TPGQEITNDC	EQCVCNAGRW	VCKDLPCPGT	CALEGGSHIT
410	420	430	440	450	460	470	480
TFDGKTYTFH	GDCYYVLAKG	DHNDSYALLG	ELAPCGSTDK	QTCLKTVVLL	ADKKKNVVVF	KSDGSVLLNE	LQVNLPHVTA
490	500	510	520	530	540	550	560
SFSVFRPSSY	HIMVSMAIGV	RLQVQLAPVM	QLFVTLDQAS	QGQVQGLCGN	FNGLEGDDFK	TASGLVEATG	AGFANTWKAQ
570	580	590	600	610	620	630	640
STCHDKLDWL	DDPCSLNIES	ANYAEHWCSL	LKKTETPFGR	CHSAVDPAEY	YKRCKYDTCN	CQNNEDCLCA	ALSSYARACT
650	660	670	680	690	700	710	720
AKGVMLWGWR	EHVCNKDVGS	CPNSQVFLYN	LTTCQQTCRS	LSEADSHCLE	GFAPVDGCGC	PDHTFLDEKG	RCVPLAKCSC
730	740	750	760	770	780	790	800
YHRGLYLEAG	DVVVRQEERC	VCRDGRLHCR	QIRLIGQSCT	APKIHMDCSN	LTALATSKPR	ALSCQTLAAG	YYHTECVSGC
810	820	830	840	850	860	870	880
VCPDGLMDDG	RGGCVVEKEC	PCVHNNDLYS	SGAKIKVDCN	TCTCKRGRWV	CTQAVCHGTC	SIYGSGHYIT	FDGKYYDFDG
890	900	910	920	930	940	950	960
HCSYVAVQDY	CGQNSSLGSF	SIITENVPCG	TTGVTCSKAI	KIFMGRTELK	LEDKHRVVIQ	RDEGHHVAYT	TREVGQYLVV
970	980	990	1000	1010	1020	1030	1040
ESSTGIIVIW	DKRTTVFIKL	APSYKGTVCG	LCGNFDHRSN	NDFTTRDHMV	VSSELDFGNS	WKEAPTCPDV	STNPEPCSLN
1050	1060	1070	1080	1090	1100	1110	1120
PHRRSWAEKQ	CSILKSSVFS	ICHSKVDPKP	FYEACVHDSC	SCDTGGDCEC	FCSAVASYAQ	ECTKEGACVF	WRTPDLCPIF
1130	1140	1150	1160	1170	1180	1190	1200
CDYYNPPHEC	EWHYEPCGNR	SFETCRTING	IHSNISVSYL	EGCYPRCPKD	RPIYEEDLKK	CVTADKCGCY	VEDTHYPPGA
1210	1220	1230	1240	1250	1260	1270	1280
SVPTEETCKS	CVCTNSSQVV	CRPEEGKILN	QTQDGAFCYW	EICGPNGTVE	KHFNICSITT	RPSTLTTFTT	ITLPTTPTTF
1290	1300	1310	1320	1330	1340	1350	1360
TTTTTTTTPT	SSTVLSTTPK	LCCLWSDWIN	EDHPSSGSDD	GDRETFDGVC	GAPEDIECRS	VKDPHLSLEQ	LGQKVQCDVS
1370	1380	1390	1400	1410	1420	1430	1440
VGFICKNEDQ	FGNGPFGLCY	DYKIRVNCCW	PMDKCITTPS	PPTTTPSPPP	TSTTTLPPTT	TPSPPTTTTT	TPPPTTTPSP
1450	1460	1470	1480	1490	1500	1510	1520
PITTTTTPPP	TTTPSPPIST	TTTPPPTTTP	SPPTTTPSPP	TTTPSPPTTT	TTTPPPTTTP	SPPTTTPITP	PASTTTLPPT
1530	1540	1550	1560	1570	1580	1590	1600
TTPSPPTTTT	TTPPPTTTPS	PPTTTPITPP	TSTTTLPPTT	TPSPPPTTTT	TPPPTTTPSP	PTTTTPSPPT	ITTTTPPPTT
1610	1620	1630	1640	1650	1660	1670	1680
TPSPPTTTTT	TPPPTTTPSP	PTTTPITPPT	STTTLPPTTT	PSPPPTTTTT	PPPTTTPSPP	TTTTPSPPIT	TTTTPPPTTT
1690	1700	1710	1720	1730	1740	1750	1760
PSSPITTTPS	PPTTTMTTPS	PTTTPSSPIT	TTTTPSSTTT	PSPPPTTMTT	PSPTTTPSPP	TTTMTTLPPT	TTSSPLTTTP
1770	1780	1790	1800	1810	1820	1830	1840
LPPSITPPTF	SPFSTTTPTT	PCVPLCNWTG	WLDSGKPNFH	KPGGDTELIG	DVCGPGWAAN	ISCRATMYPD	VPIGQLGQTV
1850	1860	1870	1880	1890	1900	1910	1920
VCDVSVGLIC	KNEDQKPGGV	IPMAFCLNYE	INVQCCECVT	QPTTMTTTTT	ENPTPPTTTP	ITTTTTVTPT	PTPTGTQTPT
1930	1940	1950	1960	1970	1980	1990	2000
TTPITTTTTV	TPTPTPTGTQ	TPTTTPITTT	TTVTPTPTPT	GTQTPTTTPI	TTTTTVTPTP	TPTGTQTPTT	TPITTTTTVT
2010	2020	2030	2040	2050	2060	2070	2080
PTPTPTGTQT	PTTTPITTTT	TVTPTPTPTG	TQTPTTTPIT	TTTTVTPTPT	PTGTQTPTTT	PITTTTTVTP	TPTPTGTQTP
2090	2100	2110	2120	2130	2140	2150	2160
TTTPITTTTT	VTPTPTPTGT	QTPTTTPITT	TTTVTPTPTP	TGTQTPTTTP	ITTTTTVTPT	PTPTGTQTPT	TTPITTTTTV
2170	2180	2190	2200	2210	2220	2230	2240
TPTPTPTGTQ	TPTTTPITTT	TTVTPTPTPT	GTQTPTTTPI	TTTTTVTPTP	TPTGTQTPTT	TPITTTTTVT	PTPTPTGTQT
2250	2260	2270	2280	2290	2300	2310	2320
PTTTPITTTT	TVTPTPTPTG	TQTPTTTPIT	TTTTVTPTPT	PTGTQTPTTT	PITTTTTVTP	TPTPTGTQTP	TTTPITTTTT
2330	2340	2350	2360	2370	2380	2390	2400

Detailed Protein Report

Cmpd.	No. of Cmpds.	m/z meas.	Δ m/z [ppm]	z	Rt [min]	Score	P	Range	Sequence	Modification
2678	1	857.6944	26.58	3	64.7	10.2	0	616-637	K.YDTCNCQNNEDCLCAALSSYAR.A	Carbamidomethyl: 4, 12

Detailed Protein Report

Protein 421: polycystic kidney disease protein 1-like 3 precursor [Homo sapiens]

**Accession:**	gi|31559825	**Score:**	45.9
**Database:**	refseq_human(refseq_human_20140103.fasta)	**MW [kDa]:**	195.8
**Database Date:**	2015-11-30	**pI:**	9.6
		**Sequence Coverage [%]:**	3.0
		**No. of unique Peptides:**	2

Quantitation

M:Q **Median:** 6.32 **CV:** 0.00 % **No. of Peptides:** 1

10	20	30	40	50	60	70	80
MFFKGGSWLW	LYIRTSIILG	SELNSPAPHG	QNNCYQLNRF	QCSFEEAQHY	CHVQRGFLAH	IWNKEVQDLI	RDYLEEGKKW
90	100	110	120	130	140	150	160
WIGQNVMPLK	KHQDNKYPAD	VAANGPPKPL	SCTYLSRNFI	RISSKGDKCL	LKYYFICQTG	DFLDGDAHYE	RNGNNSHLYQ
170	180	190	200	210	220	230	240
RHKKTKRGVA	IARDKMPPGP	GHLPTTCHYP	LPAHLSKTLC	HPISQFPSVL	SSITSQVTSA	ASEPSSQPLP	VITQLTMPVS
250	260	270	280	290	300	310	320
VTHAGQSLAE	TTSSPKEEGH	PNTFTSYLQV	SLQKASGQVI	DEIAGNFSRA	VHGLQALNKL	QEACEFLQKL	TALTPRFSKP
330	340	350	360	370	380	390	400
AQVNLINSLI	YLSEELLRIP	FQNNNSLGFK	VPPTVCPFHS	LNNVTKAGEG	SWLESKRHTE	PVEDILEMSL	VEFGNIGEAF
410	420	430	440	450	460	470	480
LEQNQSPESS	VTLTSANATL	LLSRQNISTL	PLSSYTLGHP	APVRLGFPSA	LALKELLNKH	PGVNVQITGL	AFNPFKDLDN
490	500	510	520	530	540	550	560
RNIVGSIGSV	LLSANRKLLQ	VHDLMEDIEI	MLWRNVSLET	HPTSLNMSTH	QLTITVNVTS	LEKSLIVSID	PDSPLLMTLY
570	580	590	600	610	620	630	640
LGFQYQPNCT	HFHLNITLPK	DKVWQKDEEY	TWVLNPEHLQ	HGIGTYYITA	VLSERQEGAQ	QTPSLVSVIT	AVTQCYYWEI
650	660	670	680	690	700	710	720
HNQTWSSAGC	QVGPQSTILR	TQCLCNHLTF	FASDFFVVPR	TVNVEDTIKL	FLRVTNNPVG	VSLLASLLGF	YVITVVWARK
730	740	750	760	770	780	790	800
KDQADMQKVK	VTVLADNDPS	AQFHYLIQVY	TGYRRSAATT	AKVVITLYGS	EGRSEPHHLC	DPQKTVFERG	GLDVFLLTTW
810	820	830	840	850	860	870	880
TSLGNLHSLR	LWHDNSGVSP	SWYVSQVIVC	DMAVKRKWHF	LCNCWLAVDL	GDCELDRVFI	PVSKRELFSF	RHLFSSMIVE
890	900	910	920	930	940	950	960
KFTQDYLWLS	IATRHPWNQF	TRVQRLSCCM	TLLLCNMVIN	VMFWKINSTT	AKRDEQMRPF	AVAWSELLVS	IHTAVILFPI
970	980	990	1000	1010	1020	1030	1040
NLVIGRLFPL	IEPQETLPLF	PPIQASCLSD	ASVEPLSATM	VVEELKETVR	FLLRRNTYLL	SKCEQPPWSS	WDITKLVKLL
1050	1060	1070	1080	1090	1100	1110	1120
SSLVSSHLEG	QGCHQQGERH	WARVVPENHH	HFCCYLHRVL	QRLKSHLGTL	GLTQGHQSCD	FLDAASQLQK	LQELLETHIL
1130	1140	1150	1160	1170	1180	1190	1200
PTEQEPSREV	TSFAILSSEE	GKKPISNGLS	KWLTSVCWLL	LGFTSLASAF	FTALYSLELS	KDQATSWMIS	IILSVLQNIF
1210	1220	1230	1240	1250	1260	1270	1280
ISQPVKVVFF	TFLYSLMMSR	MPRLNKENEQ	QTKRILALLA	KCSSSVPGSR	DKNNPVYVAP	AINSPTKHPE	RTLKKKKLFK
1290	1300	1310	1320	1330	1340	1350	1360
LTGDILVQIL	FLTLLMTAIY	SAKNSNRFYL	HQAIWKTFSH	QFSEIKLLQD	FYPWANHILL	PSLYGDYRGK	NAVLEPSHCK
1370	1380	1390	1400	1410	1420	1430	1440
CGVQLIFQIP	RTKTYEKVDE	GQLAFCDNGH	TCGRPKSLFP	GLHLRRFSYI	CSPRPMVLIP	TDELHERLTS	KNENGFSYIM
1450	1460	1470	1480	1490	1500	1510	1520
RGAFFTSLRL	ESFTSLQMSK	KGCVWSIISQ	VIYYLLVCYY	AFIQGCQLKQ	QKWRFFTGKR	NILDTSIILI	SFILLGLDMK
1530	1540	1550	1560	1570	1580	1590	1600
SISLHKKNMA	RYRDDQDRFI	SFYEAVKVNS	AATHLVGFPV	LLATVQLWNL	LRHSPRLRVI	SRTLSRAWDE	VVGFLLIILI
1610	1620	1630	1640	1650	1660	1670	1680
LLTGYAIAFN	LLFGCSISDY	RTFFSSAVTV	VGLLMGISHQ	EEVFALDPVL	GTFLILTSVI	LMVLVVINLF	VSAILMAFGK
1690	1700	1710	1720	1730	1740		
ERKSLKKEAA	LIDTLLQKLS	NLLGISWPQK	TSSEQAATTA	VGSDTEVLDE	LP		

Detailed Protein Report

Cmpd.	No. of Cmpds.	m/z meas.	Δ m/z [ppm]	z	Rt [min]	Score	P	Range	Sequence	Modification	Ratios
78	1	877.8658	-164.02	2	30.9	13.9	2	1227-1241	K.ENEQQTKRILALLAK.C		
146	1	896.8772	102.59	3	31.2	18.7	2	1242-1267	K. CSSSVPGSRDKNNPVYVAPAINS[illegible] H		M:Q 6.32

Detailed Protein Report

Protein 422: lysosomal-trafficking regulator [Homo sapiens]

**Accession:**	gi|54292123	**Score:**	45.9
**Database:**	refseq_human(refseq_human_20140103.fasta)	**MW [kDa]:**	428.9
**Database Date:**	2015-11-30	**pI:**	6.2
**Modification(s):**	Oxidation	**Sequence Coverage [%]:**	1.5
		**No. of unique Peptides:**	4

Alias proteins:

Accession	Name	Description
gi|530365986	refseq_human (refseq_human_20140103.fasta)	PREDICTED: lysosomal-trafficking regulator isoform X1 [Homo sapiens]

BRUKER

Detailed Protein Report

10	20	30	40	50	60	70	80
MSTDSNSLAR	EFLTDVNRLC	NAVVQRVEAR	EEEEEETHMA	TLGQYLVHGR	GFLLLTKLNS	IIDQALTCRE	ELLTLLLSLL
90	100	110	120	130	140	150	160
PLVWKIPVQE	EKATDFNLPL	SADIILTKEK	NSSSQRSTQE	KLHLEGSALS	SQVSAKVNVF	RKSRRQRKIT	HRYSVRDARK
170	180	190	200	210	220	230	240
TQLSTSDSEA	NSDEKGIAMN	KHRRPHLLHH	FLTSFPKQDH	PKAKLDRLAT	KEQTPPDAMA	LENSREIIPR	QGSNTDILSE
250	260	270	280	290	300	310	320
PAALSVISNM	NNSPFDLCHV	LLSLLEKVCK	FDVTLNHNSP	LAASVVPTLT	EFLAGFGDCC	SLSDNLESRV	VSAGWTEEPV
330	340	350	360	370	380	390	400
ALIQRMLFRT	VLHLLSVDVS	TAEMMPENLR	KNLTELLRAA	LKIRICLEKQ	PDPFAPRQKK	TLQEVQEDFV	FSKYRHRALL
410	420	430	440	450	460	470	480
LPELLEGVLQ	ILICCLQSAA	SNPFYFSQAM	DLVQEFIQHH	GFNLFETAVL	QMEWLVLRDG	VPPEASEHLK	ALINSVMKIM
490	500	510	520	530	540	550	560
STVKKVKSEQ	LHHSMCTRKR	HRRCEYSHFM	HHHRDLSGLL	VSAFKNQVSK	NPFEETADGD	VYYPERCCCI	AVCAHQCLRL
570	580	590	600	610	620	630	640
LQQASLSSTC	VQILSGVHNI	GICCCMDPKS	VIIPLLHAFK	LPALKNFQQH	ILNILNKLIL	DQLGGAEISP	KIKKAACNIC
650	660	670	680	690	700	710	720
TVDSDQLAQL	EETLQGNLCD	AELSSSLSSP	SYRFQGILPS	SGSEDLLWKW	DALKAYQNFV	FEEDRLHSIQ	IANHICNLIQ
730	740	750	760	770	780	790	800
KGNIVVQWKL	YNYIFNPVLQ	RGVELAHHCQ	HLSVTSAQSH	VCSHHNQCLP	QDVLQIYVKT	LPILLKSRVI	RDLFLSCNGV
810	820	830	840	850	860	870	880
SQIIELNCLN	GIRSHSLKAF	ETLIISLGEQ	QKDASVPDID	GIDIEQKELS	SVHVGTSFHH	QQAYSDSPQS	LSKFYAGLKE
890	900	910	920	930	940	950	960
AYPKRRKTVN	QDVHINTINL	FLCVAFLCVS	KEAESDRESA	NDSEDTSGYD	STASEPLSHM	LPCISLESLV	LPSPEHMHQA
970	980	990	1000	1010	1020	1030	1040
ADIWSMCRWI	YMLSSVFQKQ	FYRLGGFRVC	HKLIFMIIQK	LFRSHKEEQG	KKEGDTSVNE	NQDLNRISQP	KRTMKEDLLS
1050	1060	1070	1080	1090	1100	1110	1120
LAIKSDPIPS	ELGSLKKSAD	SLGKLELQHI	SSINVEEVSA	TEAAPEEAKL	FTSQESETSL	QSIRLLEALL	AICLHGARTS
1130	1140	1150	1160	1170	1180	1190	1200
QQKMELELPN	QNLSVESILF	EMRDHLSQSK	VIETQLAKPL	FDALLRVALG	NYSADFEHND	AMTEKSHQSA	EELSSQPGDF
1210	1220	1230	1240	1250	1260	1270	1280
SEEAEDSQCC	SFKLLVEEEG	YEADSESNPE	DGETQDDGVD	LKSETEGFSA	SSSPNDLLEN	LTQGEIIYPE	ICMLELNLLS
1290	1300	1310	1320	1330	1340	1350	1360
ASKAKLDVLA	HVFESFLKII	RQKEKNVFLL	MQQGTVKNLL	GGFLSILTQD	DSDFQACQRV	LVDLLVSLMS	SRTCSEELTL
1370	1380	1390	1400	1410	1420	1430	1440
LLRIFLEKSP	CTKILLLGIL	KIIESDTTMS	PSQYLTFPLL	HAPNLSNGVS	SQKYPGILNS	KAMGLLRRAR	VSRSKKEADR
1450	1460	1470	1480	1490	1500	1510	1520
ESFPHRLLSS	WHIAPVHLPL	LGQNCWPHLS	EGFSVSLWFN	VECIHEAEST	TEKGKKIKKR	NKSLILPDSS	FDGTESDRPE
1530	1540	1550	1560	1570	1580	1590	1600
GAEYINPGER	LIEEGCIHII	SLGSKALMIQ	VWADPHNATL	IFRVCMDSND	DMKAVLLAQV	ESQENIFLPS	KWQHLVLTYL
1610	1620	1630	1640	1650	1660	1670	1680
QQPQGKRRIH	GKISIWVSGQ	RKPDVTLDFM	LPRKTSLSSD	SNKTFCMIGH	CLSSQEEFLQ	LAGKWDLGNL	LLFNGAKVGS
1690	1700	1710	1720	1730	1740	1750	1760
QEAFYLYACG	PNHTSVMPCK	YGKPVNDYSK	YINKEILRCE	QIRELFMTKK	DVDIGLLIES	LSVVYTTYCP	AQYTIYEPVI
1770	1780	1790	1800	1810	1820	1830	1840
RLKGQMKTQL	SQRPFSSKEV	QSILLEPHHL	KNLQPTEYKT	IQGILHEIGG	TGIFVFLFAR	VVELSSCEET	QALALRVILS
1850	1860	1870	1880	1890	1900	1910	1920
LIKYNQQRVH	ELENCNGLSM	IHQVLIKQKC	IVGFYILKTL	LEGCCGEDII	YMNENGEFKL	DVDSNAIIQD	VKLLEELLLD
1930	1940	1950	1960	1970	1980	1990	2000
WKIWSKAEQG	VWETLLAALE	VLIRADHHQQ	MFNIKQLLKA	QVVHHFLLTC	QVLQEYKEGQ	LTPMPREVCR	SFVKIIAEVL
2010	2020	2030	2040	2050	2060	2070	2080
GSPPDLELLT	IIFNFLLAVH	PPTNTYVCHN	PTNFYFSLHI	DGKIFQEKVR	SIMYLRHSSS	GGRSLMSPGF	MVISPSGFTA
2090	2100	2110	2120	2130	2140	2150	2160
SPYEGENSSN	IIPQQMAAHM	LRSRSLPAFP	TSSLLTQSQK	LTGSLGCSID	RLQNIADTYV	ATQSKKQNSL	GSSDTLKKGK
2170	2180	2190	2200	2210	2220	2230	2240

Detailed Protein Report

Cmpd.	No. of Cmpds.	m/z meas.	Δ m/z [ppm]	z	Rt [min]	Score	P	Range	Sequence	Modification
1974	1	757.2741	-159.87	2	55.1	11.1	1	365-377	R.ICLEKQPDPFAPR.Q	
1683	1	853.4016	-89.30	2	49.4	11.6	1	515-530	R.DLSGLLVSAFKNQVSK.N	
2340	1	953.1627	116.28	2	57.7	13.1	2	989-1003	R.VCHKLIFMIIQKLFR.S	Oxidation: 8
2723	1	639.3585	31.42	2	62.4	10.1	1	2146-2157	K.KQNSLGSSDTLK.K	

Detailed Protein Report

Protein 423: tetratricopeptide repeat protein 37 [Homo sapiens]

**Accession:**	gi|7662078	**Score:**	45.9
**Database:**	refseq_human(refseq_human_20140103.fasta)	**MW [kDa]:**	175.4
**Database Date:**	2015-11-30	**pI:**	8.1
**Modification(s):**	Carbamidomethyl	**Sequence Coverage [%]:**	2.4
		**No. of unique Peptides:**	2

Quantitation

Q:W	**Median:** 1.00	**CV:** 0.00 %	**No. of Peptides:**	1
M:Q	**Median:** 1.10	**CV:** 0.00 %	**No. of Peptides:**	1

10	20	30	40	50	60	70	80
MSSKEVKTAL	KSARDAIRNK	EYKEALKHCK	TVLKQEKNNY	NAWVFIGVAA	AELEQPDQAQ	SAYKKAAELE	PDQLLAWQGL
90	100	110	120	130	140	150	160
ANLYEKYNHI	NAKDDLPGVY	QKLLDLYESV	DKQKWCDVCK	KLVDLYYQEK	KHLEVARTWH	KLIKTRQEQG	AENEELHQLW
170	180	190	200	210	220	230	240
RKLTQFLAES	TEDQNNETQQ	LLFTAFENAL	GLSDKIPSED	HQVLYRHFIQ	SLSKFPHESA	RLKKACEGMI	NIYPTVQYPL
250	260	270	280	290	300	310	320
EVLCLHLIES	GNLTDEGQQY	CCRLVEMDSK	SGPGLIGLGI	KALQDKKYED	AVRNLTEGLK	ESPVCTSGWY	HLAEAQVKMH
330	340	350	360	370	380	390	400
RPKEAVLSCS	QALKIVDNLG	ASGNSLYQRN	LCLHLKAEAL	IKLSDYDSSE	EAIRTLDQIS	DADNIPGLLV	LKSLAYRNKG
410	420	430	440	450	460	470	480
SFDEAAKIME	DLLSSYPDLA	EVHALEALIH	FTKKDYLQAE	KCFQRALEKD	TEVAEYHYQL	GLTYWFMGEE	TRKDKTKALT
490	500	510	520	530	540	550	560
HFLKAARLDT	YMGKVFCYLG	HYYRDVVGDK	NRARGCYRKA	FELDDTDAES	GAAAVDLSVE	LEDMEMALAI	LTTVTQKASA
570	580	590	600	610	620	630	640
GTAKWAWLRR	GLYYLKAGQH	SQAVADLQAA	LRADPKDFNC	WESLGEAYLS	RGGYTTALKS	FTKASELNPE	SIYSVFKVAA
650	660	670	680	690	700	710	720
IQQILGKYKE	AVAQYQMIIK	KKEDYVPALK	GLGECHLMMA	KAALVDYLDG	KAVDYIEKAL	EYFTCALQHR	ADVSCLWKLA
730	740	750	760	770	780	790	800
GDACTCLYAV	APSKVNVHVL	GVLLGQKEGK	QVLKKNELLH	LGGRCYGRAL	KLMSTSNTWC	DLGINYYRQA	QHLAETGSNM
810	820	830	840	850	860	870	880
NDLKELLEKS	LHCLKKAVRL	DSNNHLYWNA	LGVVACYSGI	GNYALAQHCF	IKSIQSEQIN	AVAWTNLGVL	YLTNENIEQA
890	900	910	920	930	940	950	960
HEAFKMAQSL	DPSYLMCWIG	QALIAEAVGS	YDTMDLFRHT	TELNMHTEGA	LGYAYWVCTT	LQDKSNRETE	LYQYNILQMN
970	980	990	1000	1010	1020	1030	1040
AIPAAQVILN	KYVERIQNYA	PAFTMLGYLN	EHLQLKKEAA	NAYQRAILLL	QTAEDQDTYN	VAIRNYGRLL	CSTGEYDKAI
1050	1060	1070	1080	1090	1100	1110	1120
QAFKSTPLEV	LEDIIGFALA	LFMKGLYKES	SKAYERALSI	VESEQDKAHI	LTALAITEYK	QGKTDVAKTL	LFKCSILKEP
1130	1140	1150	1160	1170	1180	1190	1200
TTESLQALCA	LGLAMQDATL	SKAALNELLK	HIKHKDSNYQ	RCLLTSAIYA	LQGRSVAVQK	QISKAVHSNP	GDPALWSLLS
1210	1220	1230	1240	1250	1260	1270	1280
RVVAQYAQRN	AKGGVVAGNV	AHILDSNHGK	KALLYTAVNQ	LAMGSSSAED	EKNTALKTIQ	KAALLSPGDP	AIWAGLMAAC
1290	1300	1310	1320	1330	1340	1350	1360
HADDKLALVN	NTQPKRIDLY	LALLSAVSAS	IKDEKFFENY	NQSLEKWSLS	QAVTGLIDTG	RISEAETLCT	KNLKSNPDQP
1370	1380	1390	1400	1410	1420	1430	1440
AVILLLRQVQ	CKPLLESQKP	LPDAVLEELQ	KTVMSNSTSV	PAWQWLAHVY	QSQGMMRAAE	MCYRKSLQLA	SQRGSWSGKL
1450	1460	1470	1480	1490	1500	1510	1520
SSLLRLALLA	LKVCMANISN	DHWPSLVQEA	TTEALKLCFC	PLAVLLQALL	QFKRKMGARE	TRRLLERVVY	QPGYPKSIAS
1530	1540	1550	1560	1570			
TARWYLLRHL	YAKDDYELID	VLVNNAKTHG	DTRALELNQR	LSSQ			

Cmpd.	No. of Cmpds.	m/z meas.	Δ m/z [ppm]	z	Rt [min]	Score	P	Range	Sequence	Modification	Ratios
1224	1	533.6546	-167.61	2	44.0	14.5	1	113-120	K.QKWCDVCK.K	Carbamidomethyl: 7	M:Q 1.10<br>Q:W 1.00
728	1	705.9560	-25.50	2	37.6	14.3	1	1440-	K.LSSLLRLALLALK.V		

Detailed Protein Report

Cmpd.	No. of Cmpds.	m/z meas.	Δ m/z [ppm]	z	Rt [min]	Score	P	Range	Sequence	Modification	Ratios
								1452			

Detailed Protein Report

Protein 424: **PREDICTED: GAS2-like protein 2 isoform X1 [Homo sapiens]**

Accession: gi|578830659
Database: refseq_human(refseq_human_20140103.fasta)
Database Date: 2015-11-30

Score: 45.9
MW [kDa]: 92.5
pI: 10.2
Sequence Coverage [%]: 5.7
No. of unique Peptides: 2

Quantitation

M:Q **Median:** 0.78 **CV:** 0.00 % **No. of Peptides:** 1

10	20	30	40	50	60	70	80
MSQPAGGRRK	PRTLGPPVCS	IRPFKSSEQY	LEAMKEDLAE	WLRDLYGLDI	DAANFLQVLE	TGLVLCQHAN	VVTDAALAFL
90	100	110	120	130	140	150	160
AEAPAQAQKI	PMPRVGVSCN	GAAQPGTFQA	RDNVSNFIQW	CRKEMGIQEV	LMFETEDLVL	RKNVKNVVLC	LLELGRRAWR
170	180	190	200	210	220	230	240
FGVAAPTLVQ	LEEEIEEEVR	RELALPPPDP	SPPAPPRRQP	CHFRNLDQMI	LRNHVMVRVG	GGWDTLGHYL	DKHDPCRCTS
250	260	270	280	290	300	310	320
LSHKPGSFLK	PPAPPVQHEV	RVQDGPSQTQ	PTMTISRSQS	PPPPVDWKTY	TSSDRRLRPP	TPSSPRPRRE	RGAGTGASRE
330	340	350	360	370	380	390	400
MAPFLRCQER	SLIPSWRQPT	AGDSPPSPQS	SSTQKGRDPQ	CTSSGKREER	YPPELPRGRI	PTSWVHEETD	SWGTDAGNPT
410	420	430	440	450	460	470	480
PQRLRAIEAT	TKGISARGPS	PLPRSFGPAE	CLGLRLPLRD	EAKGAFFQFR	EPESVRSPTP	VQGLTKIPIR	LPPARPPTPG
490	500	510	520	530	540	550	560
RSFPGATSGS	PRTELGRDPI	PLRAVTVDLA	GSTHGDCSVE	VRQEDQQLDI	QVMAEARESW	DLGLQEQEGR	YTPLPLGGNK
570	580	590	600	610	620	630	640
EQAIYCSLEE	EILGNMKLLE	VRSACPQGTR	SGVIPRSGVY	IPRLAGQWPE	PGGPYDKAIQ	ELAQGSPSLL	KVDLEAWKAA
650	660	670	680	690	700	710	720
PTGSPKPAVT	PGPGSLKGKL	GARQSGPRTK	ASLSAKGTHM	RKVPPQGGQD	CSASTVSASP	EAPTPSPLDP	NSDKAKACLS
730	740	750	760	770	780	790	800
KGRRTLRKPK	RVPSIYKLKL	RPRIRPRRDH	RPEKQPSRIP	RPLAYVFLGP	ARQPPKDRLL	RAVLGSKGGE	ASRVDGASVG
810	820	830	840	850			
EEEEEGKEEK	EPAAPLESSP	QPPEGLQPHW	LNQAPLPPEE	ESWV			

Cmpd.	No. of Cmpds.	m/z meas.	Δ m/z [ppm]	z	Rt [min]	Score	P	Range	Sequence	Modification	Ratios
599	1	690.3992	97.92	2	36.8	10.9	1	320-330	R.EMAPFLRCQER.S		M:Q 0.78
1978	1	643.7875	-102.56	2	54.6	14.5	2	671-682	K.ASLSAKGTHMRK.V		

Detailed Protein Report

Protein 425: leucine-, glutamate- and lysine-rich protein 1 isoform 1 [Homo sapiens]

**Accession:**	gi|262263420	**Score:**	45.9
**Database:**	refseq_human(refseq_human_20140103.fasta)	**MW [kDa]:**	81.2
**Database Date:**	2015-11-30	**pI:**	8.7
**Modification(s):**	Oxidation	**Sequence Coverage [%]:**	5.8
		**No. of unique Peptides:**	1

Alias proteins:

Accession	Name	Description
gi|530374591	refseq_human (refseq_human_20140103.fasta)	PREDICTED: leucine-, glutamate- and lysine-rich protein 1 isoform X1 [Homo sapiens]

10	20	30	40	50	60	70	80
MDHHIPMHAL	PEEIQKMLPE	EKVCKYCGVS	YLILHEFKAM	EEKVKAMEKE	MKFYQGSVDR	EKRLQEKLHS	LSQELEQYKI
**90**	**100**	**110**	**120**	**130**	**140**	**150**	**160**
DNKSKTERIY	DVGMQLKSQQ	NEFQKVKKQL	SHLQDELKIK	YRQSYIFSQR	LSEYKYFWNK	TLSLLTFTKR	ELTSIKNEVY
**170**	**180**	**190**	**200**	**210**	**220**	**230**	**240**
DNYQNWTSLK	GAVFLQIKSI	SETALTEIDI	LNKSLTVSQR	NKVCLEKEMK	NLKLLSDAAI	LRSQQIRTSR	QQEVNLQTRC
**250**	**260**	**270**	**280**	**290**	**300**	**310**	**320**
YDLQKEVLDL	QCLVEALGLK	LQKAVTEMDN	YKEMLMNKSN	EADDCQRELK	KLKFESIISE	SQHTMLLKEK	EDSLMTCQQI
**330**	**340**	**350**	**360**	**370**	**380**	**390**	**400**
YKALQEELTV	KEKQEEDIKR	RINLAENELE	ITKTLLNQTR	EEVLTLKNER	ELMLISHQKS	IEQLQETLRQ	KLLSDDNWKE
**410**	**420**	**430**	**440**	**450**	**460**	**470**	**480**
KIEAELAKER	AQHLVEFEEQ	ALLFKEETKL	QLDIEKEKHQ	DVIQKYKKEQ	EELQMKISDL	ITGATRDLRQ	EVTTLKEKLH
**490**	**500**	**510**	**520**	**530**	**540**	**550**	**560**
KSHIRYTEES	NSKEKEIENL	KNLVAEFESR	LKKEIDSNDS	VSENLRKEME	QKSDELKRVM	LAQTQLIEQF	NQSQEENTFL
**570**	**580**	**590**	**600**	**610**	**620**	**630**	**640**
QETVRRECEE	RFELTEALSQ	AREQLLELSK	LRGSLPFSPC	SLSKGSLTSP	AAAVSNHGER	SLARLNSEKG	IQIPNLRGVS
**650**	**660**	**670**	**680**	**690**	**700**		
KPTTFPTSDK	PKRVRSGVPI	LPQPHPPRGG	ASSANETRQR	LAAILRRRRS	QQ		

Cmpd.	No. of Cmpds.	m/z meas.	Δ m/z [ppm]	z	Rt [min]	Score	P	Range	Sequence	Modification
1884	1	734.7526	-169.10	2	53.9	10.2	1	86-97	K.TERIYDVGMQLK.S	Oxidation: 9

Detailed Protein Report

Protein 426: dynein heavy chain domain-containing protein 1 isoform 1 [Homo sapiens]

**Accession:**	gi|222144249	**Score:**	45.9
**Database:**	refseq_human(refseq_human_20140103.fasta)	**MW [kDa]:**	533.3
**Database Date:**	2015-11-30	**pI:**	6.3
**Modification(s):**	Oxidation	**Sequence Coverage [%]:**	1.1
		**No. of unique Peptides:**	1

Detailed Protein Report

10	20	30	40	50	60	70	80
MVPEERRVGL	SSDETSSDSL	KSWHSICVLD	SKEQPLACQQ	KQRQFVKPVT	ESEQPTVLEL	LLAELRTLFS	AVLQDSSPAA
90	100	110	120	130	140	150	160
WRYLHAVLGL	LPPYRELLVG	HLDLLPFLEQ	LYCWAPWVQT	HLHLDLLGAI	VQAFPPDSSL	LDSASHADCC	PQKRRLHHRP
170	180	190	200	210	220	230	240
PCPACPFVQA	QWSRQQVKEE	LATWLRPLTL	PELQRCLGIV	GAQVALEEAV	WLDGLSLLPL	ALAADIPVRY	ESSDTDNAEV
250	260	270	280	290	300	310	320
EPVGRKETRS	QLDYEVPREK	AFQKSSTGFS	PETSFLDSQV	MTALKMERYL	KKIHFLYLNV	APSRYFRPYS	LMVVPPDKVN
330	340	350	360	370	380	390	400
PEHYIFSPFG	ILHVHPVEGS	ETMTLGTWHH	HCVLWQQLQF	IPFFKYCLLR	KSFTCWKKNV	RLQGLHRLQK	FLENHLLLAV
410	420	430	440	450	460	470	480
PHFGAGLLHI	SRLLQELHSV	SWLPQELDRC	YELLDLQTAL	AEEKHKALRL	LHRCLNLCTS	ILRLVHEDTY	HMQQCLQERV
490	500	510	520	530	540	550	560
QNCDRIRTGQ	GSIYLQRVQH	KQLEQKLKQA	EAWWLQLGKF	ARLVDYMICQ	SLISVLEEQI	TSFVANILQA	PRQKPFLSSQ
570	580	590	600	610	620	630	640
LVFDDHGQLS	HVPCVENMIQ	TLTGGLQSVK	TSALQVVQSA	DLKTSSDSLY	SEEEDEEEDS	KDEFLMPKFQ	GQPSDAVSIF
650	660	670	680	690	700	710	720
CGPNVGLVWP	WKSHPIAGIL	EVRGCRLRGQ	YFPHNYKQLE	EDLDNNPKIQ	QALNIQQVLL	EGVLCKVQEF	CREHHWITGI
730	740	750	760	770	780	790	800
YEFLQSWGPQ	KLEDMRGGPI	KNYVTLVSRL	NVWQARVSSM	PIELLTKGGL	LLLSCHDVQA	EMESKLNSIR	KDILAHVQNE
810	820	830	840	850	860	870	880
CWNLSQQLMT	ELTDFMHIFR	TINSDIHAIA	QCTQKLNEAN	EQYVELEERM	EYVRALHELI	RNHFSLFSAE	NEALDISVRR
890	900	910	920	930	940	950	960
QFGESPIPPC	PPPPQPHLLH	CPLLAPQLLD	MWEAFQFEKS	QASEFLLSKR	HAIMPKLQQL	MAAALAELEG	LLAKALSGPF
970	980	990	1000	1010	1020	1030	1040
MDPTQDQRST	EHQLVSLERQ	FQNTVSDLSE	LHHAYAIFTE	DETPVPLPIC	GTRPIVQQQR	IWHLYRVISE	NISEWKCMAF
1050	1060	1070	1080	1090	1100	1110	1120
AKFSPAMAQE	KTEGWLTEAA	RMSTTLELHS	PVLQHCMRIL	GEFRSYLPLL	TKLGSLHPQS	LNCQCLLRAL	GLGSLQTIEL
1130	1140	1150	1160	1170	1180	1190	1200
LTLGQLLTYP	LLEFADRINQ	VWQNENERIH	AQETIRRLQR	YWEARQLRLL	NFILHVPYEP	PASERSKRQV	LRSPQWEVVD
1210	1220	1230	1240	1250	1260	1270	1280
KDSGTFILSD	YSNLQDSIQE	SLQVLSKILA	IEKSGDLNKI	ALEWVAIMHG	LGALLEVWLT	FQQKWIFLNK	VLHEMKIQFP
1290	1300	1310	1320	1330	1340	1350	1360
NADLNSRFKV	MDDQYRTLMR	ISVADPMVLS	LVVPSAERSP	YFQGQQLQQL	LQAGSVELEG	IIMSLESVLY	GVCAHFPRLF
1370	1380	1390	1400	1410	1420	1430	1440
FLSDSELVAL	LAARLESCEA	QLWVRRCFPH	VHAVSFRSCP	TGEKNTDDWE	SSPNTQTQVE	ALAVLGAGGE	EVKLQGPLPL
1450	1460	1470	1480	1490	1500	1510	1520
HPDLPKWLAS	LEKCLRLALV	HMLQGCVAAR	LARGPSLGEA	LKQLPKQNKL	YLQLYVQHWI	DLVQAFPWQC	VLVAEEVVWR
1530	1540	1550	1560	1570	1580	1590	1600
AEMEEALLEW	GTLAMVSMHM	RKLEVLVNFM	RAQRASQGGQ	SLPSVRQTSL	LSALLVMAVT	HRDIAQLLEQ	HQVSDLTDFH
1610	1620	1630	1640	1650	1660	1670	1680
WVRQLKYHLG	SPHIIPKSPL	QSLKTIASSE	PSLSPAACWI	DVLGRSFLYN	YEYLGPRLGP	LPSLLPERPA	LVLLLALEEV
1690	1700	1710	1720	1730	1740	1750	1760
ACGTVLGPNG	VGKRAIVNSL	AQALGRQLVM	LPCSPQIEAQ	CLSNYLNGAL	QGGAWLLLEK	VHQLPPGLLS	ALGQRLGELH
1770	1780	1790	1800	1810	1820	1830	1840
HLYAPLYQEA	SRNTSTIDPT	QPQLLGSSFF	EKHHVSVRLG	YGCLLVLRAL	SSAVPANLHL	LLRPVALALP	DLRQVAELTL
1850	1860	1870	1880	1890	1900	1910	1920
LGAGMRDAFQ	MATRLSKFFS	LERELVSGPL	PCRLPLLKQI	LEDTIRTLNV	TKEEPKCQKP	RSLAAIEEAA	LLRSPLFSIL
1930	1940	1950	1960	1970	1980	1990	2000
NGLHLHNLRG	LLCALFPSAS	QVLAEPMTYK	LMKPLVVEEL	QQVGLDPSPD	ILGSLEQLSQ	ALSRASGILL	LGPAGSGKTT
2010	2020	2030	2040	2050	2060	2070	2080
CWHSLFKIQN	RLAAMEDTST	QGCQPVEITH	LYPSGLSPQE	FLGWLEGSCW	HHGIFPKVLR	AAGQCNNMGQ	KRQTEESIGI
2090	2100	2110	2120	2130	2140	2150	2160
QHWIICDGAS	NGAWLDSITC	LLSELPQLSL	PSGQQIARPP	GTFLLMEVAD	TTGISPTVVG	CCALVWCGGE	QTWQCILSAL
2170	2180	2190	2200	2210	2220	2230	2240
MASLPYEYRL	QHRTVAELNH	MAEVLVPATL	RFLTCQGVSS	LLQVHGQQAV	CAGVAEVTSM	ARILHSLLDL	HLRLKEEKAP
2250	2260	2270	2280	2290	2300	2310	2320
GPEDLSYSDP	VAQSFRSSKS	SFLNRSQVDS	DDVPDKCREH	LLAVSSFLFA	LIWGFAHLP	SRFWPIFDTF	IRDSISRLSN
2330	2340	2350	2360	2370	2380	2390	2400

Detailed Protein Report

Cmpd.	No. of Cmpds.	m/z meas.	Δ m/z [ppm]	z	Rt [min]	Score	P	Range	Sequence	Modification
1775	1	1023.8652	29.43	3	52.2	11.8	2	265-291	K.SSTGFSPETSFLDSQVMTALKMERYLK.K	Oxidation: 17

Detailed Protein Report

Protein 427: protein-methionine sulfoxide oxidase MICAL3 isoform 1 [Homo sapiens]

**Accession:**	gi|209862789	**Score:**	45.7
**Database:**	refseq_human(refseq_human_20140103.fasta)	**MW [kDa]:**	224.2
**Database Date:**	2015-11-30	**pI:**	5.3
		**Sequence Coverage [%]:**	2.8
		**No. of unique Peptides:**	2

Quantitation

Q:W	**Median:**	2.07	**CV:**	0.00 %	**No. of Peptides:**	1
M:Q	**Median:**	2.56	**CV:**	0.00 %	**No. of Peptides:**	1

Alias proteins:

Accession	Name	Description
gi|530426255	refseq_human (refseq_human_20140103.fasta)	PREDICTED: protein-methionine sulfoxide oxidase MICAL3 isoform X6 [Homo sapiens]

Detailed Protein Report

10	20	30	40	50	60	70	80
MEERKHETMN	PAHVLFDRFV	QATTCKGTLK	AFQELCDHLE	LKPKDYRSFY	HKLKSKLNYW	KAKALWAKLD	KRGSHKDYKK
90	100	110	120	130	140	150	160
GKACTNTKCL	IIGAGPCGLR	TAIDLSLLGA	KVVVIEKRDA	FSRNNVLHLW	PFTIHDLRGL	GAKKFYGKFC	AGAIDHISIR
170	180	190	200	210	220	230	240
QLQLILLKVA	LILGIEIHVN	VEFQGLIQPP	EDQENERIGW	RALVHPKTHP	VSEYEFEVII	GGDGRRNTLE	GFRRKEFRGK
250	260	270	280	290	300	310	320
LAIAITANFI	NRNTTAEAKV	EEISGVAFIF	NQKFFQELRE	ATGIDLENIV	YYKDDTHYFV	MTAKKQSLLD	KGVILHDYAD
330	340	350	360	370	380	390	400
TELLLSRENV	DQEALLSYAR	EAADFSTQQQ	LPSLDFAINH	YGQPDVAMFD	FTCMYASENA	ALVREQNGHQ	LLVALVGDSL
410	420	430	440	450	460	470	480
LEPFWPMGTG	IARGFLAAMD	SAWMVRSWSL	GTSPLEVLAE	RESIYRLLPQ	TTPENVSKNF	SQYSIDPVTR	YPNINVNFLR
490	500	510	520	530	540	550	560
PSQVRHLYDT	GETKDIHLEM	ESLVNSRTTP	KLTRNESVAR	SSKLLGWCQR	QTDGYAGVNV	TDLTMSWKSG	LALCAIIHRY
570	580	590	600	610	620	630	640
RPDLIDFDSL	DEQNVEKNNQ	LAFDIAEKEL	GISPIMTGKE	MASVGEPDKL	SMVMYLTQFY	EMFKDSLPSS	DTLDLNAEEK
650	660	670	680	690	700	710	720
AVLIASTRSP	ISFLSKLGQT	ISRKRSPKDK	KEKDLDGAGK	RRKTSQSEEE	EAPRGHRGER	PTLVSTLTDR	RMDVAVGNQN
730	740	750	760	770	780	790	800
KVKYMATQLL	AKFEENAPAQ	SIGIRRQGSM	KKEFPQNLGG	SDTCYFCQKR	VYVMERLSAE	GKFFHRSCFK	CEYCATTLRL
810	820	830	840	850	860	870	880
SAYAYDIEDG	KFYCKPHYCY	RLSGYAQRKR	PAVAPLSGKE	AKGPLQDGAT	TDANGRANAV	ASSTERTPGS	GVNGLEEPSI
890	900	910	920	930	940	950	960
AKRLRGTPER	IELENYRLSL	RQAEALQEVP	EETQAEHNLS	SVLDTGAEED	VASSSSESEM	EEEGEEEEEE	PRLPPSDLGG
970	980	990	1000	1010	1020	1030	1040
VPWKEAVRIH	ALLKGKSEEE	LEASKSFGPG	NEEEEEEEEE	YEEEEEEDYD	EEEEESSEAG	NQRLQQVMHA	ADPLEIQADV
1050	1060	1070	1080	1090	1100	1110	1120
HWTHIREREE	EERMAPASES	SASGAPLDEN	DLEEDVDSEP	AEIEGEAAED	GDPGDTGAEL	DDDQHWSDSP	SDADRELRLP
1130	1140	1150	1160	1170	1180	1190	1200
CPAEGEAELE	LRVSEDEEKL	PASPKHQERG	PSQATSPIRS	PQESALLFIP	VHSPSTEGPQ	LPPVPAATQE	KSPEERLFPE
1210	1220	1230	1240	1250	1260	1270	1280
PLLPKEKPKA	DAPSDLKAVH	SPIRSQPVTL	PEARTPVSPG	SPQPQPPVAA	STPPPSPLPI	CSQPQPSTEA	TVPSPTQSPI
1290	1300	1310	1320	1330	1340	1350	1360
RFQPAPAKTS	TPLAPLPVQS	QSDTKDRLGS	PLAVDEALRR	SDLVEEFWMK	SAEIRRSLGL	TPVDRSKGPE	PSFPTPAFRP
1370	1380	1390	1400	1410	1420	1430	1440
VSLKSYSVEK	SPQDEGLHLL	KPLSIPKRLG	LPKPEGEPLS	LPTPRSPSDR	ELRSAQEERR	ELSSSSGLGL	HGSSSNMKTL
1450	1460	1470	1480	1490	1500	1510	1520
GSQSFNTSDS	AMLTPPSSPP	PPPPPGEEPA	TLRRKLREAE	PNASVVPPPL	PATWMRPPRE	PAQPPREEVR	KSFVESVEEI
1530	1540	1550	1560	1570	1580	1590	1600
PFADDVEDTY	DDKTEDSSLQ	EKFFTPPSCW	PRPEKPRHPP	LAKENGRLPA	LEGTLQPQKR	GLPLVSAEAK	ELAEERMRAR
1610	1620	1630	1640	1650	1660	1670	1680
EKSVKSQALR	DAMARQLSRM	QQMELASGAP	RPRKASSAPS	QGKERRPDSP	TRPTLRGSEE	PTLKHEATSE	EVLSPPSDSG
1690	1700	1710	1720	1730	1740	1750	1760
GPDGSFTSSE	GSSGKSKKRS	SLFSPRRNKK	EKKSKGEGRP	PEKPSSNLLE	EAAAKPKSLW	KSVFSGYKKD	KKKKADDKSC
1770	1780	1790	1800	1810	1820	1830	1840
PSTPSSGATV	DSGKHRVLPV	VRAELQLRRQ	LSFSEDSDLS	SDDVLEKSSQ	KSRREPRTYT	EEELNAKLTR	RVQKAARRQA
1850	1860	1870	1880	1890	1900	1910	1920
KQEELKRLHR	AQIIQRQLQQ	VEERQRRLEE	RGVAVEKALR	GEAGMGKKDD	PKLMQEWFKL	VQEKNAMVRY	ESELMIFARE
1930	1940	1950	1960	1970	1980	1990	2000
LELEDRQSRL	QQELRERMAV	EDHLKTEEEL	SEEKQILNEM	LEVVEQRDSL	VALLEEQRLR	EREEDKDLEA	AMLSKGFSLN
2010							
WS							

Cmpd.	No. of Cmpds.	m/z meas.	Δ m/z [ppm]	z	Rt [min]	Score	P	Range	Sequence	Modification	Ratios
1700	1	538.2445	-35.71	2	51.5	12.2	0	712-721	R.MDVAVGNQNK.V		M:Q 2.56<br>Q:W 2.07
2512	1	834.4289	-40.88	2	61.8	10.6	2	1306-1320	K.DRLGSPLAVDEALRR.S		

Detailed Protein Report

Protein 428: PREDICTED: ankyrin-3 isoform X13 [Homo sapiens]

**Accession:**	gi|578819310	**Score:**	45.7
**Database:**	refseq_human(refseq_human_20140103.fasta)	**MW [kDa]:**	478.5
**Database Date:**	2015-11-30	**pI:**	6.0
		**Sequence Coverage [%]:**	0.9
		**No. of unique Peptides:**	1

Quantitation

Q:W	**Median:**	0.94	**CV:**	0.00 %	**No. of Peptides:**	1
M:Q	**Median:**	0.86	**CV:**	0.00 %	**No. of Peptides:**	1

Detailed Protein Report

10	20	30	40	50	60	70	80
MSDANASYLR	AARAGHLEKA	LDYIKNGVDI	NICNQNGLNA	LHLASKEGHV	EVVSELLQRE	ANVDAATKKG	NTALHIASLA
90	100	110	120	130	140	150	160
GQAEVVKVLV	TNGANVNAQS	QNGFTPLYMA	AQENHLEVVK	FLLDNGASQS	LATEDGFTPL	AVALQQGHDQ	VVSLLLENDT
170	180	190	200	210	220	230	240
KGKVRLPALH	IAARKDDTKA	AALLLQNDNN	ADVESKSGFT	PLHIAAHYGN	INVATLLLNR	AAAVDFTARN	DITPLHVASK
250	260	270	280	290	300	310	320
RGNANMVKLL	LDRGAKIDAK	TRDGLTPLHC	GARSGHEQVV	EMLLDRAAPI	LSKTKNGLSP	LHMATQGDHL	NCVQLLLQHN
330	340	350	360	370	380	390	400
VPVDDVTNDY	LTALHVAAHC	GHYKVAKVLL	DKKANPNAKA	LNGFTPLHIA	CKKNRIKVME	LLLKHGASIQ	AVTESGLTPI
410	420	430	440	450	460	470	480
HVAAFMGHVN	IVSQLMHHGA	SPNTTNVRGE	TALHMAARSG	QAEVVRYLVQ	DGAQVEAKAK	DDQTPLHISA	RLGKADIVQQ
490	500	510	520	530	540	550	560
LLQQGASPNA	ATTSGYTPLH	LSAREGHEDV	AAFLLDHGAS	LSITTKKGFT	PLHVAAKYGK	LEVANLLLQK	SASPDAAGKS
570	580	590	600	610	620	630	640
GLTPLHVAAH	YDNQKVALLL	LDQGASPHAA	AKNGYTPLHI	AAKKNQMDIA	TTLLEYGADA	NAVTRQGIAS	VHLAAQEGHV
650	660	670	680	690	700	710	720
DMVSLLLGRN	ANVNLSNKSG	LTPLHLAAQE	DRVNVAEVLV	NQGAHVDAQT	KMGYTPLHVG	CHYGNIKIVN	FLLQHSAKVN
730	740	750	760	770	780	790	800
AKTKNGYTPL	HQAAQQGHTH	IINVLLQNNA	SPNELTVNGN	TALGIARRLG	YISVVDTLKI	VTEETMTTTT	VTEKHKMNVP
810	820	830	840	850	860	870	880
ETMNEVLDMS	DDEVRKANAP	EMLSDGEYIS	DVEEGNRCTW	YKIPKVQEFT	VKSEDAMTGD	TDKYLGPQDL	KELGDDSLPA
890	900	910	920	930	940	950	960
EGYMGFSLGA	RSASPKISLR	SFSSDRSYTL	NRSSYARDSM	MIEELLVPSK	EQHLTFTREF	DSDSLRHYSW	AADTLDNVNL
970	980	990	1000	1010	1020	1030	1040
VSSPIHSGFL	VSFMVDARGG	SMRGSRHHGM	RIIIPPRKCT	APTRITCRLV	KRHKLANPPP	MVEGEGLASR	LVEMGPAGAQ
1050	1060	1070	1080	1090	1100	1110	1120
FLGPVIVEIP	HFGSMRGKER	ELIVLRSENG	ETWKEHQFDS	KNEDLTELLN	GMDEELDSPE	ELGKKRICRI	ITKDFPQYFA
1130	1140	1150	1160	1170	1180	1190	1200
VVSRIKQESN	QIGPEGGILS	STTVPLVQAS	FPEGALTKRI	RVGLQAQPVP	DEIVKKILGN	KATFSPIVTV	EPRRRKFHKP
1210	1220	1230	1240	1250	1260	1270	1280
ITMTIPVPPP	SGEGVSNGYK	GDTTPNLRLL	CSITGGTSPA	QWEDITGTTP	LTFIKDCVSF	TTNVSARFWL	ADCHQVLETV
1290	1300	1310	1320	1330	1340	1350	1360
GLATQLYREL	ICVPYMAKFV	VFAKMNDPVE	SSLRCFCMTD	DKVDKTLEQQ	ENFEEVARSK	DIEVLEGKPI	YVDCYGNLAP
1370	1380	1390	1400	1410	1420	1430	1440
LTKGGQQLVF	NFYSFKENRL	PFSIKIRDTS	QEPCGRLSFL	KEPKTTKGLP	QTAVCNLNIT	LPAHKKETES	DQDDEIEKTD
1450	1460	1470	1480	1490	1500	1510	1520
RRQSFASLAL	RKRYSYLTEP	GMKTVERSTG	ATRSLPTTYS	YKPFFSTRPY	QSWTTAPITV	PGPAKSGFTS	LSSSSSNTPS
1530	1540	1550	1560	1570	1580	1590	1600
ASPLKSIWSV	STPSPIKSTL	GASTTSSVKS	ISDVASPIRS	FRTMSSPIKT	VVSQSPYNIQ	VSSGTLARAP	AVTEATPLKG
1610	1620	1630	1640	1650	1660	1670	1680
LASNSTFSSR	TSPVTTAGSL	LERSSITMTP	PASPKSNINM	YSSSLPFKSI	ITSAAPLISS	PLKSVVSPVK	SAVDVISSAK
1690	1700	1710	1720	1730	1740	1750	1760
ITMASSLSSP	VKQMPGHAEV	ALVNGSISPL	KYPSSSTLIN	GCKATATLQE	KISSATNSVS	SVVSAATDTV	EKVFSTTTAM
1770	1780	1790	1800	1810	1820	1830	1840
PFSPLRSYVS	AAPSAFQSLR	TPSASALYTS	LGSSISATTS	SVTSSIITVP	VYSVVNVLPE	PALKKLPDSN	SFTKSAAALL
1850	1860	1870	1880	1890	1900	1910	1920
SPIKTLTTET	HPQPHFSRTS	SPVKSSLFLA	PSALKLSTPS	SLSSSQEILK	DVAEMKEDLM	RMTAILQTDV	PEEKPFQPEL
1930	1940	1950	1960	1970	1980	1990	2000
PKEGRIDDEE	PFKIVEKVKE	DLVKVSEILK	KDVCVDNKGS	PKSPKSDKGH	SPEDDWIEFS	SEEIREARQQ	AAASQSPSLP
2010	2020	2030	2040	2050	2060	2070	2080
ERVQVKAKAA	SEKDYNLTKV	IDYLTNDIGS	SSLTNLKYKF	EDAKKDGEER	QKRVLKPAIA	LQEHKLKMPP	ASMRTSTSEK
2090	2100	2110	2120	2130	2140	2150	2160
ELCKMADSFF	GTDTILESPD	DFSQHDQDKS	PLSDSGFETR	SEKTPSAPQS	AESTGPKPLF	HEVPIPPVIT	ETRTEVVHVI
2170	2180	2190	2200	2210	2220	2230	2240

Detailed Protein Report

Cmpd.	No. of Cmpds.	m/z meas.	Δ m/z [ppm]	z	Rt [min]	Score	P	Range	Sequence	Modification	Ratios
534	1	676.8481	-14.81	2	35.1	20.1	1	3885-3897	K.TKALTTSSCVDVK.S		Q:W 0.94<br>M:Q 0.86

Detailed Protein Report

Protein 429: transmembrane protein 169 [Homo sapiens]

**Accession:**	gi|19923913	**Score:**	45.6
**Database:**	refseq_human(refseq_human_20140103.fasta)	**MW [kDa]:**	33.6
**Database Date:**	2015-11-30	**pI:**	4.6
**Modification(s):**	Oxidation	**Sequence Coverage [%]:**	13.8
		**No. of unique Peptides:**	2

Alias proteins:

Accession	Name	Description
gi|578805184	refseq_human (refseq_human_20140103.fasta)	PREDICTED: transmembrane protein 169 isoform X1 [Homo sapiens]
gi|214831834	refseq_human (refseq_human_20140103.fasta)	transmembrane protein 169 [Homo sapiens]
gi|214831799	refseq_human (refseq_human_20140103.fasta)	transmembrane protein 169 [Homo sapiens]
gi|214831780	refseq_human (refseq_human_20140103.fasta)	transmembrane protein 169 [Homo sapiens]

```
        10         20         30         40         50         60         70         80
MEEPTAVEGQ VQLPSPHQGS LRKAVAAALA LDGESTMGHR KKKRKESRPE SIIIYRSDNE KTDEEPGESE GGDQPKEEEG
        90        100        110        120        130        140        150        160
DDFLDYPVDD DMWNLPLDSR YVTLTGTITR GKKKGQMVDI HVTLTEKELQ ELTKPKESSR ETTPEGRMAC QMGADRGPHV
       170        180        190        200        210        220        230        240
VLWTLICLPV VFILSFVVSF YYGTITWYNI FLVYNEERTF WHKISYCPCL VLFYPVLIMA MASSLGLYAA VVQLSWSWEA
       250        260        270        280        290        300
WWQAARDMEK GFCGWLCSKL GLEDCSPYSI VELLESDNIS STLSNKDPIQ EVETSTV
```

Cmpd.	No. of Cmpds.	m/z meas.	Δ m/z [ppm]	z	Rt [min]	Score	P	Range	Sequence	Modification
77	1	845.3458	-96.16	3	30.7	10.3	1	1-23	-.MEEPTAVEGQVQLPSPHQGSLRK.A	Oxidation: 1
1888	1	899.4768	10.43	2	52.3	12.3	1	24-41	K.AVAAALALDGESTMGHRK.K	

Detailed Protein Report

Protein 430: **PREDICTED: ATP-dependent RNA helicase DDX55 isoform X1 [Homo sapiens]**

**Accession:**	gi|530401708	**Score:**	45.5
**Database:**	refseq_human(refseq_human_20140103.fasta)	**MW [kDa]:**	50.9
**Database Date:**	2015-11-30	**pI:**	9.9
		**Sequence Coverage [%]:**	7.3
		**No. of unique Peptides:**	1

Quantitation

Q:W	**Median:**	1.05	**CV:**	0.00 %	**No. of Peptides:**	1
M:Q	**Median:**	0.48	**CV:**	0.00 %	**No. of Peptides:**	1

10	20	30	40	50	60	70	80
MEHVTEGSWE	SLPVPLHPQV	LGALRELGFP	YMTPVQSATI	PLFMRNKDVA	AEAVTGSGKT	LAFVIPILEI	LLRREEKLKK
**90**	**100**	**110**	**120**	**130**	**140**	**150**	**160**
SQVGAIIITP	TRELAIQIDE	VLSHFTKHFP	EFSQILWIGG	RNPGEDVERF	KQQGGNIIVA	TPGRLEDMFR	RKAEGLDLAS
**170**	**180**	**190**	**200**	**210**	**220**	**230**	**240**
CVRSLDVLVL	DEADRLLDMG	FEASINTILE	FLPKQRRTGL	FSATQTQEVE	NLVRAGLRNP	VRVSVKEKGV	AASSAQKTPS
**250**	**260**	**270**	**280**	**290**	**300**	**310**	**320**
RLENYYMVCK	ADEKFNQLVH	FLRNHKQEKH	LVFFSTCACV	EYYGKALEVL	VKGVKIMCIH	GKMKYKRNKI	FMEFRKLQSG
**330**	**340**	**350**	**360**	**370**	**380**	**390**	**400**
ILVCTDVMAR	GIDIPEVNWV	LQYDPPSNAS	AFVHRCGRTA	RIGHGGSALV	FLLPMEESYI	NFLAINQKCP	LQEMKPQRNT
**410**	**420**	**430**	**440**	**450**	**460**		
ADLLPKLKSM	ALADRAVFEK	GMKAFVSYVQ	AYAKHECNLI	FRLKALLEVL	PC		

Cmpd.	No. of Cmpds.	m/z meas.	Δ m/z [ppm]	z	Rt [min]	Score	P	Range	Sequence	Modification	Ratios
408	1	655.8392	-35.43	2	35.1	10.7	0	132-144	K.QQGGNIIVATPGR.L		Q:W 1.05<br>M:Q 0.48

Detailed Protein Report

Protein 431: PREDICTED: protein HEXIM2 isoform X6 [Homo sapiens]

**Accession:**	gi|578830381	**Score:**	45.5
**Database:**	refseq_human(refseq_human_20140103.fasta)	**MW [kDa]:**	35.1
**Database Date:**	2015-11-30	**pI:**	7.9
		**Sequence Coverage [%]:**	8.4
		**No. of unique Peptides:**	1

10	20	30	40	50	60	70	80
MALWCKRAGV	TSSRRLLKDL	EQKMMATPNQ	TACNAESPVA	LEEAKTSGAP	GSPQTPPERH	DSGGSLPLTP	RMESHSEDED
**90**	**100**	**110**	**120**	**130**	**140**	**150**	**160**
LAGAVGGLGW	NSRSPRTQSP	GGCSAEAVLA	RKKHRRRPSK	RKRHWRPYLE	LSWAEKQQRD	ERQSQRASRV	REEMFAKGQP
**170**	**180**	**190**	**200**	**210**	**220**	**230**	**240**
VAPYNTTQFL	MNDRDPEEPN	LDVPHGISHP	GSSGESEAGD	SDGRGRAHGE	FQRKDFSETY	ERFHTESLQG	RSKQELVRDY
**250**	**260**	**270**	**280**	**290**	**300**	**310**	
LELEKRLSQA	EEETRRLQQL	QACTGQQSCR	QVEELAAEVQ	RLRTENQRLR	QENQMWNREG	CRCDEEPGT	

Cmpd.	No. of Cmpds.	m/z meas.	Δ m/z [ppm]	z	Rt [min]	Score	P	Range	Sequence	Modification
1676	1	916.3092	-203.70	1	50.9	12.0	1	284-290	R.TENQRLR.Q	

BRUKER

Detailed Protein Report

Protein 432: **myomesin-1 isoform b [Homo sapiens]**

**Accession:**	gi|140560917	**Score:**	45.5
**Database:**	refseq_human(refseq_human_20140103.fasta)	**MW [kDa]:**	177.6
**Database Date:**	2015-11-30	**pI:**	6.4
		**Sequence Coverage [%]:**	2.3
		**No. of unique Peptides:**	2

Quantitation

Q:W	**Median:**	1.92	**CV:**	0.00 %	**No. of Peptides:**	1
M:Q	**Median:**	0.76	**CV:**	0.00 %	**No. of Peptides:**	1

```
        10         20         30         40         50         60         70         80
MSLPFYQRCH QHYDLSYRNK DVRSTVSHYQ REKKRSAVYT QGSTAYSSRS SAAHRRESEA FRRASASSSQ QQASQHALSS
        90        100        110        120        130        140        150        160
EVSRKAASAY DYGSSHGLTD SSLLLDDYSS KLSPKPKRAK HSLLSGEEKE NLPSDYMVPI FSGRQKHVSG ITDTEEERIK
       170        180        190        200        210        220        230        240
EAAAYIAQRN LLASEEGITT SKQSTASKQT TASKQSTASK QSTASKQSTA SRQSTASRQS VVSKQATSAL QQEETSEKKS
       250        260        270        280        290        300        310        320
RKVVIREKAE RLSLRKTLEE TETYHAKLNE DHLLHAPEFI IKPRSHTVWE KENVKLHCSI AGWPEPRVTW YKNQVPINVH
       330        340        350        360        370        380        390        400
ANPGKYIIES RYGMHTLEIN GCDFEDTAQY RASAMNVKGE LSAYASVVVK RYKGEFDETR FHAGASTMPL SFGVTPYGYA
       410        420        430        440        450        460        470        480
SRFEIHFDDK FDVSFGREGE TMSLGCRVVI TPEIKHFQPE IQWYRNGVPL SPSKWVQTLW SGERATLTFS HLNKEDEGLY
       490        500        510        520        530        540        550        560
TIRVRMGEYY EQYSAYVFVR DADAEIEGAP AAPLDVKCLE ANKDYIIISW KQPAVDGGSP ILGYFIDKCE VGTDSWSQCN
       570        580        590        600        610        620        630        640
DTPVKFARFP VTGLIEGRSY IFRVRAVNKM GIGFPSRVSE PVAALDPAEK ARLKSRPSAP WTGQIIVTEE EPSEGIVPGP
       650        660        670        680        690        700        710        720
PTDLSVTEAT RSYVVLSWKP PGQRGHEGIM YFVEKCEAGT ENWQRVNTEL PVKSPRFALF DLAEGKSYCF RVRCSNSAGV
       730        740        750        760        770        780        790        800
GEPSEATEVT VVGDKLDIPK APGKIIPSRN TDTSVVVSWE ESKDAKELVG YYIEASVAGS GKWEPCNNNP VKGSRFTCHG
       810        820        830        840        850        860        870        880
LVTGQSYIFR VRAVNAAGLS EYSQDSEAIE VKAAIAPPSP PCDITCLESF RDSMVLGWKQ PDKIGGAEIT GYYVNYREVI
       890        900        910        920        930        940        950        960
DGVPGKWREA NVKAVSEEAY KISNLKENMV YQFQVAAMNM AGLGAPSAVS ECFKCEEWTI AVPGPPHSLK CSEVRKDSLV
       970        980        990       1000       1010       1020       1030       1040
LQWKPPVHSG RTPVTGYFVD LKEAKAKEDQ WRGLNEAAIK NVYLKVRGLK EGVSYVFRVR AINQAGVGKP SDLAGPVVAE
      1050       1060       1070       1080       1090       1100       1110       1120
TRPGTKEVVV NVDDDGVISL NFECDKMTPK SEFSWSKDYV STEDSPRLEV ESKGNKTKMT FKDLGMDDLG IYSCDVTDTD
      1130       1140       1150       1160       1170       1180       1190       1200
GIASSYLIDE EELKRLLALS HEHKFPTVPV KSELAVEILE KGQVRFWMQA EKLSGNAKVN YIFNEKEIFE GPKYKMHIDR
      1210       1220       1230       1240       1250       1260       1270       1280
NTGIIEMFME KLQDEDEGTY TFQLQDGKAT NHSTVVLVGD VFKKLQKEAE FQRQEWIRKQ GPHFVEYLSW EVTGECNVLL
      1290       1300       1310       1320       1330       1340       1350       1360
KCKVANIKKE THIVWYKDER EISVDEKHDF KDGICTLLIT EFSKKDAGIY EVILKDDRGK DKSRLKLVDE AFKELMMEVC
      1370       1380       1390       1400       1410       1420       1430       1440
KKIALSATDL KIQSTAEGIQ LYSFVTYYVE DLKVNWSHNG SAIRYSDRVK TGVTGEQIWL QINEPTPNDK GKYVMELFDG
      1450       1460       1470       1480       1490       1500       1510       1520
KTGHQKTVDL SGQAYDEAYA EFQRLKQAAI AEKNRARVLG GLPDVVTIQE GKALNLTCNV WGDPPPEVSW LKNEKALASD
      1530       1540       1550       1560       1570       1580       1590
DHCNLKFEAG RTAYFTINGV STADSGKYGL VVKNKYGSET SDFTVSVFIP EEEARMAALE SLKGGKKAK
```

Cmpd.	No. of Cmpds.	m/z meas.	Δ m/z [ppm]	z	Rt [min]	Score	P	Range	Sequence	Modification	Ratios
1772	1	807.4927	102.23	2	52.1	14.0	1	147-160	K.HVSGITDTEEERIK.E		
792	2	555.7879	-13.08	2	38.3	14.3	0	697-706	R.FALFDLAEGK.S		Q:W 1.92<br>M:Q 0.76

Detailed Protein Report

Protein 433: PREDICTED: fatty-acid amide hydrolase 2 isoform X4 [Homo sapiens]

Accession: gi|530426388
Database: refseq_human(refseq_human_20140103.fasta)
Database Date: 2015-11-30

Score: 45.5
MW [kDa]: 49.2
pI: 9.3
Sequence Coverage [%]: 8.5
No. of unique Peptides: 1

10	20	30	40	50	60	70	80
MINGIVKYRF	EEAMKEAHAV	DQKLAEKQED	EATLENKWPF	LGVPLTVKEA	FQLQGMPNSS	GLMNRRDAIA	KTDATVVALL
**90**	**100**	**110**	**120**	**130**	**140**	**150**	**160**
KGAGAIPLGI	TNCSELCMWY	ESSNKIYGRS	NNPYDLQHIV	GGSSGGEGCT	LAAACSVIGV	GSDIGGSIRM	PAFFNGIFGH
**170**	**180**	**190**	**200**	**210**	**220**	**230**	**240**
KPSPGVVPNK	GQFPLAVGAQ	ELFLCTGPMC	RYAEDLAPML	KVMAGPGIKR	LKLDTKVHLK	DLKFYWMEHD	GGSFLMSKVD
**250**	**260**	**270**	**280**	**290**	**300**	**310**	**320**
QDLIMTQKKV	VVHLETILGA	SVQHVKLKKM	KYSFQLWIAM	MSAKGHDGKE	PVKFVDLLGD	HGKHVSPLWE	LIKWCLGLSV
**330**	**340**	**350**	**360**	**370**	**380**	**390**	**400**
YTIPSIGLAL	LEEKLRYSNE	KYQKFKAVEE	SLRKELVDML	GDDGVFLYPS	HPTVAPKHHV	PLTRPFNFAY	TGVFSALGLP
**410**	**420**	**430**	**440**	**450**			
VTQCPLGLNA	KGLPLGIQVV	AGPFNDHLTL	AVAQYLEKTF	GGWVCPGKF			

Cmpd.	No. of Cmpds.	m/z meas.	Δ m/z [ppm]	z	Rt [min]	Score	P	Range	Sequence	Modification
701	1	1099.6923	17.73	2	38.7	22.7	2	249-268	K.KVVVHLETILGASVQHVKLK.K	

Detailed Protein Report

Protein 434: PREDICTED: nipped-B-like protein isoform X4 [Homo sapiens]

**Accession:**	gi|530378766	**Score:**	45.5
**Database:**	refseq_human(refseq_human_20140103.fasta)	**MW [kDa]:**	257.1
**Database Date:**	2015-11-30	**pI:**	9.1
**Modification(s):**	Oxidation	**Sequence Coverage [%]:**	2.0
		**No. of unique Peptides:**	2

10	20	30	40	50	60	70	80
MVSIDLHQAG	RVDSQASITQ	DSDSIKKPEE	IKQCNDAPVS	VLQEDIVGSL	KSTPENHPET	PKKKSDPELS	KSEMKQSESR
90	100	110	120	130	140	150	160
LAESKPNENR	LVETKSSENK	LETKVETQTE	ELKQNESRTT	ECKQNESTIV	EPKQNENRLS	DTKPNDNKQN	NGRSETTKSR
170	180	190	200	210	220	230	240
PETPKQKGES	RPETPKQKSD	GHPETPKQKG	DGRPETPKQK	GESRPETPKQ	KNEGRPETPK	HRHDNRRDSG	KPSTEKKPEV
250	260	270	280	290	300	310	320
SKHKQDTKSD	SPRLKSERAE	ALKQRPDGRS	VSESLRRDHD	NKQKSDDRGE	SERHRGDQSR	VRRPETLRSS	SRNEHGIKSD
330	340	350	360	370	380	390	400
SSKTDKLERK	HRHESGDSRE	RPSSGEQKSR	PDSPRVKQGD	SNKSRSDKLG	FKSPTSKDDK	RTEGNKSKVD	TNKAHPDNKA
410	420	430	440	450	460	470	480
EFPSYLLGGR	SGALKNFVIP	KIKRDKDGNV	TQETKKMEMK	GEPKDKVEKI	GLVEDLNKGA	KPVVVLQKLS	LDDVQKLIKD
490	500	510	520	530	540	550	560
REDKSRSSLK	PIKNKPSKSN	KGSIDQSVLK	ELPPELLAEI	ESTMPLCERV	KMNKRKRSTV	NEKPKYAEIS	SDEDNDSDEA
570	580	590	600	610	620	630	640
FESSRKRHKK	DDDKAWEYEE	RDRRSSGDHR	RSGHSHEGRR	SSGGGRYRNR	SPSDSDMEDY	SPPPSLSEVA	RKMKKKEKQK
650	660	670	680	690	700	710	720
KRKAYEPKLT	PEEMMDSSTF	KRFTASIENI	LDNLEDMDFT	AFGDDDEIPQ	ELLLGKHQLN	ELGSESAKIK	AMGIMDKLST
730	740	750	760	770	780	790	800
DKTVKVLNIL	EKNIQDGSKL	STLLNHNNDT	EEEERLWRDL	IMERVTKSAD	ACLTTINIMT	SPNMPKAVYI	EDVIERVIQY
810	820	830	840	850	860	870	880
TKFHLQNTLY	PQYDPVYRLD	PHGGGLLSSK	AKRAKCSTHK	QRVIVMLYNK	VCDIVSSLSE	LLEIQLLTDT	TILQVSSMGI
890	900	910	920	930	940	950	960
TPFFVENVSE	LQLCAIKLVT	AVFSRYEKHR	QLILEEIFTS	LARLPTSKRS	LRNFRLNSSD	MDGEPMYIQM	VTALVLQLIQ
970	980	990	1000	1010	1020	1030	1040
CVVHLPSSEK	DSNAEEDSNK	KIDQDVVITN	SYETAMRTAQ	NFLSIFLKKC	GSKQGEEDYR	PLFENFVQDL	LSTVNKPEWP
1050	1060	1070	1080	1090	1100	1110	1120
AAELLLSLLG	RLLVHQFSNK	STEMALRVAS	LDYLGTVAAR	LRKDAVTSKM	DQGSIERILK	QVSGGEDEIQ	QLQKALLDYL
1130	1140	1150	1160	1170	1180	1190	1200
DENTETDPSL	VFSRKFYIAQ	WFRDTTLETE	KAMKSQKDEE	SSEGTHHAKE	IETTGQIMHR	AENRKKFLRS	IIKTTPSQFS
1210	1220	1230	1240	1250	1260	1270	1280
TLKMNSDTVD	YDDACLIVRY	LASMRPFAQS	FDIYLTQILR	VLGENAIAVR	TKAMKCLSEV	VAVDPSILAR	LDMQRGVHGR
1290	1300	1310	1320	1330	1340	1350	1360
LMDNSTSVRE	AAVELLGRFV	LCRPQLAEQY	YDMLIERILD	TGISVRKRVI	KILRDICIEQ	PTFPKITEMC	VKMIRRVNDE
1370	1380	1390	1400	1410	1420	1430	1440
EGIKKLVNET	FQKLWFTPTP	HNDKEAMTRK	ILNITDVVAA	CRDTGYDWFE	QLLQNLLKSE	EDSSYKPVKK	ACTQLVDNLV
1450	1460	1470	1480	1490	1500	1510	1520
EHILKYEESL	ADSDNKGVNS	GRLVACITTL	FLFSKIRPQL	MVKHAMTMQP	YLTTKCSTQN	DFMVICNVAK	ILELVVPLME
1530	1540	1550	1560	1570	1580	1590	1600
HPSETFLATI	EEDLMKLIIK	YGMTVVQHCV	SCLGAVVNKV	TQNFKFVWAC	FNRYYGAISK	LKSQHQEDPN	NTSLLTNKPA
1610	1620	1630	1640	1650	1660	1670	1680
LLRSLFTVGA	LCRHFDFDLE	DFKGNSKVNI	KDKVLELLMY	FTKHSDEEVQ	TKAIIGLGFA	FIQHPSLMFE	QEVKNLYNNI
1690	1700	1710	1720	1730	1740	1750	1760
LSDKNSSVNL	KIQVLKNLQT	YLQEEDTRMQ	QADRDWKKVA	KQEDLKEMGD	VSSGMSSSIM	QLYLKQVLEA	FFHTQSSVRH
1770	1780	1790	1800	1810	1820	1830	1840
FALNVIALTL	NQGLIHPVQC	VPYLIAMGTD	PEPAMRNKAD	QQLVEIDKKY	AGFIHMKAVA	GMKMSYQVQQ	AINTCLKDPV
1850	1860	1870	1880	1890	1900	1910	1920
RGFRQDESSS	ALCSHLYSMI	RGNRQHRRAF	LISLLNLFDD	TAKTDVTMLL	YIADNLACFP	YQTQEEPLFI	MHHIDITLSV
1930	1940	1950	1960	1970	1980	1990	2000
SGSNLLQSFK	ESMVKDKRKE	RKSSPSKENE	SSDSEEEVSR	PRKSRKRVDS	DSDSDSEDDI	NSVMKCLPEN	SAPLIEFANV
2010	2020	2030	2040	2050	2060	2070	2080
SQGILLLLML	KQHLKNLCGF	SDSKIQKYSP	SESAKVYDKA	INRKTGVHFH	PKQTLDFLRS	DMANSKITEE	VKRSIVKQYL
2090	2100	2110	2120	2130	2140	2150	2160
DFKLLMEHLD	PDEEEEEGEV	SASTNARNKA	ITSLLGGGSP	KNNTAAETED	DESDGEDRGG	GTSGSLRRSK	RNSDSTELAA
2170	2180	2190	2200	2210	2220	2230	2240
QMNESVDVMD	VIAICCPKYK	DRPQIARVVQ	KTSSGFSVQW	MAGSYSGSWT	EAKRRDGRKL	VPWVDTIKES	DIIYKKIALT
2250	2260	2270					
SANKLTNKVV	QTLRSLYAAK	DGTSS					

Detailed Protein Report

Cmpd.	No. of Cmpds.	m/z meas.	Δ m/z [ppm]	z	Rt [min]	Score	P	Range	Sequence	Modification
2607	1	913.4633	-59.64	2	62.6	12.0	1	909-923	K.HRQLILEEIFTSLAR.L	
425	1	946.2023	58.66	3	35.0	14.6	2	1692-1714	K.IQVLKNLQTYLQEEDTRMQQADR.D	Oxidation: 18

Detailed Protein Report

Protein 435: rho GTPase-activating protein 36 isoform 1 precursor [Homo sapiens]

**Accession:**	gi|40255080	**Score:**	45.4
**Database:**	refseq_human(refseq_human_20140103.fasta)	**MW [kDa]:**	61.6
**Database Date:**	2015-11-30	**pI:**	10.2
		**Sequence Coverage [%]:**	5.9
		**No. of unique Peptides:**	2

10	20	30	40	50	60	70	80
MGGCIPFLKA	ARALCPRIMP	PLLLLSAFIF	LVSVLGGAPG	HNPDRRTKMV	SIHSLSELER	LKLQETAYHE	LVARHFLSEF
90	100	110	120	130	140	150	160
KPDRALPIDR	PNTLDKWFLI	LRGQQRAVSH	KTFGISLEEV	LVNEFTRRKH	LELTATMQVE	EATGQAAGRR	RGNVVRRVFG
170	180	190	200	210	220	230	240
RIRRFFSRRR	NEPTLPREFT	RRGRRGAVSV	DSLAELEDGA	LLLQTLQLSK	ISFPIGQRLL	GSKRKMSLNP	IAKQIPQVVE
250	260	270	280	290	300	310	320
ACCQFIEKHG	LSAVGIFTLE	YSVQRVRQLR	EEFDQGLDVV	LDDNQNVHDV	AALLKEFFRD	MKDSLLPDDL	YMSFLLTATL
330	340	350	360	370	380	390	400
KPQDQLSALQ	LLVYLMPPCH	SDTLERLLKA	LHKITENCED	SIGIDGQLVP	GNRMTSTNLA	LVFGSALLKK	GKFGKRESRK
410	420	430	440	450	460	470	480
TKLGIDHYVA	SVNVVRAMID	NWDVLFQVPP	HIQRQVAKRV	WKSSPEALDF	IRRRNLRKIQ	SARIKMEEDA	LLSDPVETSA
490	500	510	520	530	540	550	
EARAAVLAQS	KPSDEGSSEE	PAVPSGTARS	HDDEEGAGNP	PIPEQDRPLL	RVPREKEAKT	GVSYFFP	

Cmpd.	No. of Cmpds.	m/z meas.	Δ m/z [ppm]	z	Rt [min]	Score	P	Range	Sequence	Modification
1422	1	459.2842	44.39	2	47.9	16.9	0	211-218	K.ISFPIGQR.L	
2474	1	822.4025	-186.55	1	61.5	16.1	1	347-353	R.LLKALHK.I	

Detailed Protein Report

Protein 436: PREDICTED: testis-specific gene 10 protein isoform X9 [Homo sapiens]

Accession: gi|578804973
Database: refseq_human(refseq_human_20140103.fasta)
Database Date: 2015-11-30
Modification(s): Carbamidomethyl, Oxidation

Score: 45.4
MW [kDa]: 103.2
pI: 5.3
Sequence Coverage [%]: 3.9
No. of unique Peptides: 2

Quantitation

Q:W	**Median:** 0.95	**CV:** 0.00 %	**No. of Peptides:** 1
M:Q	**Median:** 0.88	**CV:** 57.09 %	**No. of Peptides:** 2

```
        10         20         30         40         50         60         70         80
MSSGQDGGPR SPGRDPELQV EAAEVTTKEY DFGLGAPVSE AENYQNTLQL EQEVRNQDRF ISTLKLQIED LKQTNHGLEE
        90        100        110        120        130        140        150        160
YVRKLLDSKE VVSSQVDDLT SHNEHLCKEL IKIDQLAEQL EKEKNFVVDS ANKELEEAKI DLICQQNNII VLEDTIKRLK
       170        180        190        200        210        220        230        240
SIILDTEKAQ NKSPSRLDSF VKTLEADKDH YKSEAQHLRK MMRSRSKSPR RPSPTARGAN CDVELLKTTT RDREELKCML
       250        260        270        280        290        300        310        320
EKYERHLAEI QGNVKVLKSE RDKIFLLYEQ AQEEITRLRR EMMKSCKSPK STTAHAILRR VETERDVAFT DLRRMTTERD
       330        340        350        360        370        380        390        400
SLRERLKIAQ ETAFNEKAHL EQRIEELECT VHNLDDERME QMSNMTLMKE TISTVEKEMK SLARKAMDTE SELGRQKAEN
       410        420        430        440        450        460        470        480
NSLRLLYENT EKDLSDTQRH LAKKKYELQL TQEKIMCLDE KIDNFTRQNI AQREEISILG GTLNDLAKEK ECLQACLDKK
       490        500        510        520        530        540        550        560
SENIASLGES LAMKEKTISG MKNIIAEMEQ ASRQCTEALI VCEQDVSRMR RQLDETNDEL AQIARERDIL AHDNDNLQEQ
       570        580        590        600        610        620        630        640
FAKAKQENQA LSKKLNDTHN ELNDIKQKVQ DTNLEVNKLK NILKSEESEN RQMMEQLRKA NEDAENWENK ARQSEADNNT
       650        660        670        680        690        700        710        720
LKLELITAEA EGNRLKEKVD SLNREVEQHL NAERSYKSQI STLHKSVVKM EEELQKVQFE KVSALADLSS TRELCIKLDS
       730        740        750        760        770        780        790        800
SKELLNRQLV AKDQEIEMRE NELDSAHSEI ELLRSQMANE RISMQNLEAL LVANRDKEYQ SQIALQEKES EIQLLKEHLC
       810        820        830        840        850        860        870        880
LAENKMAIQS RDVAQFRNVV TQLEADLDIT KRQLGTERFE RERAVQELRR QNYSSNAYHM SSTMKPNTKC HSPERAHHRS
       890        900
PDRGLDRSLE E
```

Cmpd.	No. of Cmpds.	m/z meas.	Δ m/z [ppm]	z	Rt [min]	Score	P	Range	Sequence	Modification	Ratios	
1178	1	760.8598	-40.38	2	43.4	12.7	1	218-231	R.GANCDVELLKTTTR.D		M:Q<br>Q:W	1.49<br>0.95
1031	3	598.1512	-229.57	2	43.0	21.1	1	234-242	R.EELKCMLEK.Y	Carbamidomethyl: 5; Oxidation: 6	M:Q	0.52

Detailed Protein Report

Protein 437: PREDICTED: integrin beta-8 isoform X2 [Homo sapiens]

**Accession:**	gi|530384769	**Score:**	45.4
**Database:**	refseq_human(refseq_human_20140103.fasta)	**MW [kDa]:**	71.2
**Database Date:**	2015-11-30	**pI:**	9.2
**Modification(s):**	Carbamidomethyl	**Sequence Coverage [%]:**	7.4
		**No. of unique Peptides:**	2

Quantitation

Q:W	**Median:**	4.04	**CV:**	0.00 %	**No. of Peptides:**	1
M:Q	**Median:**	15.38	**CV:**	0.00 %	**No. of Peptides:**	1

Alias proteins:

Accession	Name	Description
gi|530384771	refseq_human (refseq_human_20140103.fasta)	PREDICTED: integrin beta-8 isoform X3 [Homo sapiens]

10	20	30	40	50	60	70	80
MLKVHPLKKY	PVDLYYLVDV	SASMHNNIEK	LNSVGNDLSR	KMAFFSRDFR	LGFGSYVDKT	VSPYISIHPE	RIHNQCSDYN
**90**	**100**	**110**	**120**	**130**	**140**	**150**	**160**
LDCMPPHGYI	HVLSLTENIT	EFEKAVHRQK	ISGNIDTPEG	GFDAMLQAAV	CESHIGWRKE	AKRLLLVMTD	QTSHLALDSK
**170**	**180**	**190**	**200**	**210**	**220**	**230**	**240**
LAGIVVPNDG	NCHLKNNVYV	KSTTMEHPSL	GQLSEKLIDN	NINVIFAVQG	KQFHWYKDLL	PLLPGTIAGE	IESKAANLNN
**250**	**260**	**270**	**280**	**290**	**300**	**310**	**320**
LVVEAYQKLI	SEVKVQVENQ	VQGIYFNITA	ICPDGSRKPG	MEGCRNVTSN	DEVLFNVTVT	MKKCDVTGGK	NYAIIKPIGF
**330**	**340**	**350**	**360**	**370**	**380**	**390**	**400**
NETAKIHIHR	NCSCQCEDNR	GPKGKCVDET	FLDSKCFQCD	ENKCHFDEDQ	FSSESCKSHK	DQPVCSGRGV	CVCGKCSCHK
**410**	**420**	**430**	**440**	**450**	**460**	**470**	**480**
IKLGKVYGKY	CEKDDFSCPY	HHGNLCAGHG	ECEAGRCQCF	SGWEGDRCQC	PSAAAQHCVN	SKGQVCSGRG	TCVCGRCECT
**490**	**500**	**510**	**520**	**530**	**540**	**550**	**560**
DPRSIGRFCE	HCPTCYTACK	ENWNCMQCLH	PHNLSQAILD	QCKTSCALME	QQHYVDQTSE	CFSSPSYLRI	FFIIFIVTFL
**570**	**580**	**590**	**600**	**610**	**620**	**630**	**640**
IGLLKVLIIR	QVILQWNSNK	IKSSSDYRVS	ASKKDKLILQ	SVCTRAVTYR	REKPEEIKMD	ISKLNAHETF	RCNF

Cmpd.	No. of Cmpds.	m/z meas.	Δ m/z [ppm]	z	Rt [min]	Score	P	Range	Sequence	Modification	Ratios
1358	1	955.9298	-52.51	2	46.6	10.6	0	286-302	R.NVTSNDEVLFNVTVTMK.K		
2775	1	746.3843	84.07	3	66.0	16.7	2	381-400	K. DQPVCSGRGVCVCGKCSCHK.I	Carbamidomethyl: 5, 11, 18	

Detailed Protein Report

Protein 438: **PREDICTED: cyclin-dependent kinase 7 isoform X1 [Homo sapiens]**

**Accession:**	gi|530379082	**Score:**	45.3
**Database:**	refseq_human(refseq_human_20140103.fasta)	**MW [kDa]:**	32.7
**Database Date:**	2015-11-30	**pI:**	9.8
		**Sequence Coverage [%]:**	11.4
		**No. of unique Peptides:**	1

Alias proteins:

Accession	Name	Description
gi|530430719	refseq_human (refseq_human_20140103.fasta)	PREDICTED: cyclin-dependent kinase 7 isoform X1 [Homo sapiens]

10	20	30	40	50	60	70	80
MRSWTSLGRD	SLPPFTRPEI	RTPTKLSPLR	KSNLDIDQKL	KMVIIKDNSL	VLTPSHIKAY	MLMTLQGLEY	LHQHWILHRD
**90**	**100**	**110**	**120**	**130**	**140**	**150**	**160**
LKPNNLLLDE	NGVLKLADFG	LAKSFGSPNR	AYTHQVVTRW	YRAPELLFGA	RMYGVGVDMW	AVGCILAELL	LRVPFLPGDS
**170**	**180**	**190**	**200**	**210**	**220**	**230**	**240**
DLDQLTRIFE	TLGTPTEEQW	PDMCSLPDYV	TFKSFPGIPL	HHIFSAAGDD	LLDLIQGLFL	FNPCARITAT	QALKMKYFSN
**250**	**260**	**270**	**280**	**290**			
RPGPTPGCQL	PRPNCPVETL	KEQSNPALAI	KRKRTEALEQ	GGLPKKLIF			

Cmpd.	No. of Cmpds.	m/z meas.	Δ m/z [ppm]	z	Rt [min]	Score	P	Range	Sequence	Modification
50	1	662.2443	-185.28	2	29.3	10.8	0	47-58	K.DNSLVLTPSHIK.A	

Detailed Protein Report

Protein 439: **PREDICTED: chromodomain-helicase-DNA-binding protein 4 isoform X6 [Homo sapiens]**

**Accession:**	gi|578822562	**Score:**	45.3
**Database:**	refseq_human(refseq_human_20140103.fasta)	**MW [kDa]:**	216.7
**Database Date:**	2015-11-30	**pI:**	5.7
**Modification(s):**	Carbamidomethyl	**Sequence Coverage [%]:**	1.5
		**No. of unique Peptides:**	2

Detailed Protein Report

10	20	30	40	50	60	70	80
MASGLGSPSP	CSAGSEEEDM	DALLNNSLPP	PHPENEEDPE	EDLSETETPK	LKKKKKPKKP	RDPKIPKSKR	QKKERMLLCR
90	100	110	120	130	140	150	160
QLGDSSGEGP	EFVEEEEEVA	LRSDSEGSDY	TPGKKKKKKL	GPKKEKKSKS	KRKEEEEEED	DDDDSKEPKS	SAQLLEDWGM
170	180	190	200	210	220	230	240
EDIDHVFSEE	DYRTLTNYKA	FSQFVRPLIA	AKNPKIAVSK	MMMVLGAKWR	EFSTNNPFKG	SSGASVAAAA	AAAVAVVESM
250	260	270	280	290	300	310	320
VTATEVAPPP	PPVEVPIRKA	KTKEGKGPNA	RRKPKGSPRV	PDAKKPKPKK	VAPLKIKLGG	FGSKRKRSSS	EDDDLDVESD
330	340	350	360	370	380	390	400
FDDASINSYS	VSDGSTSRSS	RSRKKLRTTK	KKKKDHQDYC	EVCQQGGEII	LCDTCPRAYH	MVCLDPDMEK	APEGKWSCPH
410	420	430	440	450	460	470	480
CEKEGIQWEA	KEDNSEGEEI	LEEVGGDLEE	EDDHHMEFCR	VCKDGGELLC	CDTCPSSYHI	HCLNPPLPEI	PNGEWLCPRC
490	500	510	520	530	540	550	560
TCPALKGKVQ	KILIWKWGQP	PSPTPVPRPP	DADPNTPSPK	PLEGRPERQF	FVKWQGMSYW	HCSWVSELQL	ELHCQVMFRN
570	580	590	600	610	620	630	640
YQRKNDMDEP	PSGDFGGDEE	KSRKRKNKDP	KFAEMEERFY	RYGIKPEWMM	IHRILNHSVD	KKGHVHYLIK	WRDLPYDQAS
650	660	670	680	690	700	710	720
WESEDVEIQD	YDLFKQSYWN	HRELMRGEEG	RPGKKLKKVK	LRKLERPPET	PTVDPTVKYE	RQPEYLDATG	GTLHPYQMEG
730	740	750	760	770	780	790	800
LNWLRFSWAQ	GTDTILADEM	GLGKTVQTAV	FLYSLYKEGH	SKGPFLVSAP	LSTIINWERE	FEMWAPDMYV	VTYVGDKDSR
810	820	830	840	850	860	870	880
AIIRENEFSF	EDNAIRGGKK	ASRMKKEASV	KFHVLLTSYE	LITIDMAILG	SIDWACLIVD	EAHRLKNNQS	KFFRVLNGYS
890	900	910	920	930	940	950	960
LQHKLLLTGT	PLQNNLEELF	HLLNFLTPER	FHNLEGFLEE	FADIAKEDQI	KKLHDMLGPH	MLRRLKADVF	KNMPSKTELI
970	980	990	1000	1010	1020	1030	1040
VRVELSPMQK	KYYKYILTRN	FEALNARGGG	NQVSLLNVVM	DLKKCCNHPY	LFPVAAMEAP	KMPNGMYDGS	ALIRASGKLL
1050	1060	1070	1080	1090	1100	1110	1120
LLQKMLKNLK	EGGHRVLIFS	QMTKMLDLLE	DFLEHEGYKY	ERIDGGITGN	MRQEAIDRFN	APGAQQFCFL	LSTRAGGLGI
1130	1140	1150	1160	1170	1180	1190	1200
NLATADTVII	YDSDWNPHND	IQAFSRAHRI	GQNKKVMIYR	FVTRASVEER	ITQVAKKKMM	LTHLVVRPGL	GSKTGSMSKQ
1210	1220	1230	1240	1250	1260	1270	1280
ELDDILKFGT	EELFKDEATD	GGGDNKEGED	SSVIHYDDKA	IERLLDRNQD	ETEDTELQGM	NEYLSSFKVA	QYVVREEEMG
1290	1300	1310	1320	1330	1340	1350	1360
EEEEVEREII	KQEESVDPDY	WEKLLRHHYE	QQQEDLARNL	GKGKRIRKQV	NYNDGSQEDR	DWQDDQSDNQ	SDYSVASEEG
1370	1380	1390	1400	1410	1420	1430	1440
DEDFDERSEA	PRRPSRKGLR	NDKDKPLPPL	LARVGGNIEV	LGFNARQRKA	FLNAIMRYGM	PPQDAFTTQW	LVRDLRGKSE
1450	1460	1470	1480	1490	1500	1510	1520
KEFKAYVSLF	MRHLCEPGAD	GAETFADGVP	REGLSRQHVL	TRIGVMSLIR	KKVQEFEHVN	GRWSMPELAE	VEENKKMSQP
1530	1540	1550	1560	1570	1580	1590	1600
GSPSPKTPTP	STPGDTQPNT	PAPVPPAEDG	IKIEENSLKE	EESIEGEKEV	KSTAPETAIE	CTQAPAPASE	DEKVVVEPPE
1610	1620	1630	1640	1650	1660	1670	1680
GEEKVEKAEV	KERTEEPMET	EPKGKGAADV	EKVEEKSAID	LTPIVVEDKE	EKKEEEEKKE	VMLQNGETPK	DLNDEKQKKN
1690	1700	1710	1720	1730	1740	1750	1760
IKQRFMFNIA	DGGFTELHSL	WQNEERAATV	TKKTYEIWHR	RHDYWLLAGI	INHGYARWQD	IQNDPRYAIL	NEPFKGEMNR
1770	1780	1790	1800	1810	1820	1830	1840
GNFLEIKNKF	LARRFKLLEQ	ALVIEEQLRR	AAYLNMSEDP	SHPSMALNTR	FAEVECLAES	HQHLSKESMA	GNKPANAVLH
1850	1860	1870	1880	1890	1900	1910	
KGILKQLEEL	LSDMKADVTR	LPATIARIPP	VAVRLQMSER	NILSRLANRA	PEPTPQQVAQ	QQ	

Cmpd.	No. of Cmpds.	m/z meas.	Δ m/z [ppm]	z	Rt [min]	Score	P	Range	Sequence	Modification
2537	3	649.8872	-125.27	3	60.0	13.8	0	1005-1021	K.CCNHPYLFPVAAMEAPK.M	Carbamidomethyl: 2
2522	3	649.8743	-145.17	3	59.8	13.9	0	1005-1021	K.CCNHPYLFPVAAMEAPK.M	Carbamidomethyl: 1

Detailed Protein Report

Protein 440: PREDICTED: lysine-specific demethylase 2A isoform X3 [Homo sapiens]

**Accession:**	gi|578821239	**Score:**	45.2
**Database:**	refseq_human(refseq_human_20140103.fasta)	**MW [kDa]:**	120.2
**Database Date:**	2015-11-30	**pI:**	9.2
**Modification(s):**	Carbamidomethyl, Oxidation	**Sequence Coverage [%]:**	3.4
		**No. of unique Peptides:**	3

Quantitation

Q:W	**Median:**	0.33	**CV:**	0.00 %	**No. of Peptides:**	1
M:Q	**Median:**	0.80	**CV:**	0.00 %	**No. of Peptides:**	1

```
        10         20         30         40         50         60         70         80
MVDVMDVNTQ KGIEMTMAQW TRYYETPEEE REKLYNVISL EFSHTRLENM VQRPSTVDFI DWVDNMWPRH LKESQTESTN
        90        100        110        120        130        140        150        160
AILEMQYPKV QKYCLMSVRG CYTDFHVDFG GTSVWYHIHQ GGKVFWLIPP TAHNLELYEN WLLSGKQGDI FLGDRVSDCQ
       170        180        190        200        210        220        230        240
RIELKQGYTF VIPSGWIHAV YTPTDTLVFG GNFLHSFNIP MQLKIYNIED RTRVPNKFRY PFYYEMCWYV LERYVYCITN
       250        260        270        280        290        300        310        320
RSHLTKEFQK ESLSMDLELN GLESGNGDEE AVDREPRRLS SRRSVLTSPV ANGVNLDYDG LGKTCRSLPS LKKTLAGDSS
       330        340        350        360        370        380        390        400
SDCSRGSHNG QVWDPQCAPR KDRQVHLTHF ELEGLRCLVD KLESLPLHKK CVPTGIEDED ALIADVKILL EELANSDPKL
       410        420        430        440        450        460        470        480
ALTGVPIVQW PKRDKLKFPT RPKVRVPTIP ITKPHTMKPA PRLTPVRPAA ASPIVSGARR RRVRCRKCKA CVQGECGVCH
       490        500        510        520        530        540        550        560
YCRDMKKFGG PGRMKQSCVL RQCLAPRLPH SVTCSLCGEV DQNEETQDFE KKLMECCICN EIVHPGCLQM DGEGLLNEEL
       570        580        590        600        610        620        630        640
PNCWECPKCY QEDSSEKAQK RKMEESDEEA VQAKVLRPLR SCDEPLTPPP HSPTSMLQLI HDPVSPRGMV TRSSPGAGPS
       650        660        670        680        690        700        710        720
DHHSASRDER FKRRQLLRLQ ATERTMVREK ENNPSGKKEL SEVEKAKIRG SYLTVTLQRP TKELHGTSIV PKLQAITASS
       730        740        750        760        770        780        790        800
ANLRHSPRVL VQHCPARTPQ RGDEEGLGGE EEEEEEEEEE DDSAEEGGAA RLNGRGSWAQ DGDESWMQRE VWMSVFRYLS
       810        820        830        840        850        860        870        880
RRELCECMRV CKTWYKWCCD KRLWTKIDLS RCKAIVPQAL SGIIKRQPVS LDLSWTNISK KQLTWLVNRL PGLKDLLLAG
       890        900        910        920        930        940        950        960
CSWSAVSALS TSSCPLLRTL DLRWAVGIKD PQIRDLLTPP ADKPGQDNRS KLRNMTDFRL AGLDITDATL RLIIRHMPLL
       970        980        990       1000       1010       1020       1030       1040
SRLDLSHCSH LTDQSSNLLT AVGSSTRYSL TELNMAGCNK LTDQTLIYLR RIANVTLIDL RGCKQITRKA CEHFISDLSI
      1050       1060
NSLYCLSDEK LIQKIS
```

Cmpd.	No. of Cmpds.	m/z meas.	Δ m/z [ppm]	z	Rt [min]	Score	P	Range	Sequence	Modification	Ratios	
846	1	663.6790	-184.30	2	40.6	10.3	1	313-325	K.KTLAGDSSSDCSR.G		Q:W	0.33
762	1	599.6213	-221.03	2	39.5	10.5	0	314-325	K.TLAGDSSSDCSR.G			
1689	1	932.9135	-0.89	2	51.0	12.0	2	803-816	R.ELCECMRVCKTWYK.W	Carbamidomethyl: 9; Oxidation: 6	M:Q	0.80

Detailed Protein Report

Protein 441: PREDICTED: transmembrane channel-like protein 8 isoform X4 [Homo sapiens]

**Accession:**	gi|578830481	**Score:**	45.2
**Database:**	refseq_human(refseq_human_20140103.fasta)	**MW [kDa]:**	45.7
**Database Date:**	2015-11-30	**pI:**	10.7
		**Sequence Coverage [%]:**	4.2
		**No. of unique Peptides:**	1

Quantitation

Q:W	**Median:** 3.58	**CV:** 0.00 %	**No. of Peptides:** 1
M:Q	**Median:** 1.38	**CV:** 0.00 %	**No. of Peptides:** 1

```
        10         20         30         40         50         60         70         80
MLLPRSVSSE RAPGVPEPEE LWEAEMERLR GSGTPVRGLP YAMMDKRLIW QLREPAGVQT LRWQRWQRRR QTVERRLREA
        90        100        110        120        130        140        150        160
AQRLARGLGL WEGALYEIGG LFGTGIRSYF TFLRFLLLLN LLSLLLTASF VLLPLVWLRP PDPGPTLNLT LQCPGSRQSP
       170        180        190        200        210        220        230        240
PGVLRFHNQL WHVLTGRAFT NTYLFYGAYR VGPESSSVYS IRLAYLLSPL ACLLLCFCGT LRRMVKGLPQ KTLLGQGYQA
       250        260        270        280        290        300        310        320
PLSAKVFSSW DFCIRVQEAA TIKKHEISNE FKVELEEGRR FQLMQQQTRA QTACRLLSYL RVNVLNGLLV VGAISAIFWA
       330        340        350        360        370        380        390        400
TKYSQDNKEV SGVPVSAAPV PAPWGHRPGQ LPGSPAVHIS GPAGELPSQH GGQPHSDLVR GAEAGQLGDV LRLPGSDHTV
       410
HWQRQEQL
```

Cmpd.	No. of Cmpds.	m/z meas.	Δ m/z [ppm]	z	Rt [min]	Score	P	Range	Sequence	Modification	Ratios
704	1	457.7465	-33.68	2	37.6	18.2	1	79-86	R.EAAQRLAR.G		M:Q 1.38<br>Q:W 3.58

Detailed Protein Report

Protein 442: integrin beta-like protein 1 isoform 3 [Homo sapiens]

**Accession:**	gi|418203907	**Score:**	45.2
**Database:**	refseq_human(refseq_human_20140103.fasta)	**MW [kDa]:**	43.9
**Database Date:**	2015-11-30	**pI:**	5.0
**Modification(s):**	Carbamidomethyl	**Sequence Coverage [%]:**	10.0
		**No. of unique Peptides:**	2

Quantitation

Q:W	**Median:**	1.18	**CV:**	0.00 %	**No. of Peptides:**	1
M:Q	**Median:**	0.43	**CV:**	0.00 %	**No. of Peptides:**	1

10	20	30	40	50	60	70	80
MNLVAVMGAF	NKGHGKCDCG	KCKCDQGWYG	DACQYPTNCD	LTKKKSNQMC	KNSQDIICSN	AGTCHCGRCK	CDNSDGSGLV
**90**	**100**	**110**	**120**	**130**	**140**	**150**	**160**
YGKFCECDDR	ECIDDETEEI	CGGHGKCYCG	NCYCKAGWHG	DKCEFQCDIT	PWESKRRCTS	PDGKICSNRG	TCVCGECTCH
**170**	**180**	**190**	**200**	**210**	**220**	**230**	**240**
DVDPTGDWGD	IHGDTCECDE	RDCRAVYDRY	SDDFCSGHGQ	CNCGRCDCKA	GWYGKKCEHP	QSCTLSAEES	IRKCQGSSDL
**250**	**260**	**270**	**280**	**290**	**300**	**310**	**320**
PCSGRGKCEC	GKCTCYPPGD	RRVYGKTCEC	DDRRCEDLDG	VVCGGHGTCS	CGRCVCERGW	FGKLCQHPRK	CNMTEEQSKN
**330**	**340**	**350**	**360**	**370**	**380**	**390**	**400**
LCESADGILC	SGKGSCHCGK	CICSAEEWYI	SGEFCDCDDR	DCDKHDGLIC	TGNGICSCGN	CECWDGWNGN	ACEIWLGSEY
**410**							
P							

Cmpd.	No. of Cmpds.	m/z meas.	Δ m/z [ppm]	z	Rt [min]	Score	P	Range	Sequence	Modification	Ratios
864	1	647.7154	-186.04	2	40.9	19.5	0	1-12	-.MNLVAVMGAFNK.G		Q:W 1.18<br>M:Q 0.43
2134	1	1062.3814	-60.33	2	56.8	10.7	1	52-70	K.NSQDIICSNAGTCHCGRCK.C	Carbamidomethyl: 13, 15	

Detailed Protein Report

Protein 443: dedicator of cytokinesis protein 8 isoform 2 [Homo sapiens]

**Accession:**	gi|299473744	**Score:**	45.1
**Database:**	refseq_human(refseq_human_20140103.fasta)	**MW [kDa]:**	227.3
**Database Date:**	2015-11-30	**pI:**	6.3
**Modification(s):**	Oxidation	**Sequence Coverage [%]:**	1.3
		**No. of unique Peptides:**	1

Detailed Protein Report

10	20	30	40	50	60	70	80
MTHLNSLDVQ	LAQELGDFTD	DDLDVVFTPK	ECRTLQPSLP	EEGVELDPHV	RDCVQTYIRE	WLIVNRKNQG	SPEICGFKKT
90	100	110	120	130	140	150	160
GSRKDFHKTL	PKQTFESETL	ECSEPAAQAG	PRHLNVLCDV	SGKGPVTACD	FDLRSLQPDK	RLENLLQQVS	AEDFEKQNEE
170	180	190	200	210	220	230	240
ARRTNRQAEL	FALYPSVDEE	DAVEIRPVPE	CPKEHLGNRI	LVKLLTLKFE	IEIEPLFASI	ALYDVKERKK	ISENFHCDLN
250	260	270	280	290	300	310	320
SDQFKGFLRA	HTPSVAASSQ	ARSAVFSVTY	PSSDIYLVVK	IEKVLQQGEI	GDCAEPYTVI	KESDGGKSKE	KIEKLKLQAE
330	340	350	360	370	380	390	400
SFCQRLGKYR	MPFAWAPISL	SSFFNVSTLE	REVTDVDSVV	GRSSVGERRT	LAQSRRLSER	ALSLEENGVG	SNFKTSTLSV
410	420	430	440	450	460	470	480
SSFFKQEGDR	LSDEDLFKFL	ADYKRSSSLQ	RRVKSIPGLL	RLEISTAPEI	INCCLTPEML	PVKPFPENRT	RPHKEILEFP
490	500	510	520	530	540	550	560
TREVYVPHTV	YRNLLYVYPQ	RLNFVNKLAS	ARNITIKIQF	MCGEDASNAM	PVIFGKSSGP	EFLQEVYTAV	TYHNKSPDFY
570	580	590	600	610	620	630	640
EEVKIKLPAK	LTVNHHLLFT	FYHISCQQKQ	GASVETLLGY	SWLPILLNER	LQTGSYCLPV	ALEKLPPNYS	MHSAEKVPLQ
650	660	670	680	690	700	710	720
NPPIKWAEGH	KGVFNIEVQA	VSSVHTQDNH	LEKFFTLCHS	LESQVTFPIR	VLDQKISEMA	LEHELKLSII	CLNSSRLEPL
730	740	750	760	770	780	790	800
VLFLHLVLDK	LFQLSVQPMV	IAGQTANFSQ	FAFESVVAIA	NSLHNSKDLS	KDQHGRNCLL	ASYVHYVFRL	PEVQRDVPKS
810	820	830	840	850	860	870	880
GAPTALLDPR	SYHTYGRTSA	AAVSSKLLQA	RVMSSSNPDL	AGTHSAADEE	VKNIMSSKHF	HEELALQMVV	STGMVRETVF
890	900	910	920	930	940	950	960
KYAWFFFELL	VKSMAQHVHN	MDKRDSFRRT	RFSDRFMDDI	TTIVNVVTSE	IAALLVKPQK	ENEQAEKMNI	SLAFFLYDLL
970	980	990	1000	1010	1020	1030	1040
SLMDRGFVFN	LIRHYCSQLS	AKLSNLPTLI	SMRLEFLRIL	CSHEHYLNLN	LFFMNADTAP	TSPCPSISSQ	NSSSCSSFQD
1050	1060	1070	1080	1090	1100	1110	1120
QKIASMFDLT	SEYRQQHFLT	GLLFTELAAA	LDAEGEGISK	VQRKAVSAIH	SLLSSHDLDP	RCVKPEVKVK	IAALYLPLVG
1130	1140	1150	1160	1170	1180	1190	1200
IILDALPQLC	DFTVADTRRY	RTSGSDEEQE	GAGAINQNVA	LAIAGNNFNL	KTSGIVLSSL	PYKQYNMLNA	DTTRNLMICF
1210	1220	1230	1240	1250	1260	1270	1280
LWIMKNADQS	LIRKWIADLP	STQLNRILDL	LFICVLCFEY	KGKQSSDKVS	TQVLQKSRDV	KARLEEALLR	GEGARGEMMR
1290	1300	1310	1320	1330	1340	1350	1360
RRAPGNDRFP	GLNENLRWKK	EQTHWRQANE	KLDKTKAELD	QEALISGNLA	TEAHLIILDM	QENIIQASSA	LDCKDSLLGG
1370	1380	1390	1400	1410	1420	1430	1440
VLRVLVNSLN	CDQSTTYLTH	CFATLRALIA	KFGDLLFEEE	VEQCFDLCHQ	VLHHCSSSMD	VTRSQACATL	YLLMRFSFGA
1450	1460	1470	1480	1490	1500	1510	1520
TSNFARVKMQ	VTMSLASLVG	RAPDFNEEHL	RRSLRTILAY	SEEDTAMQMT	PFPTQVEELL	CNLNSILYDT	VKMREFQEDP
1530	1540	1550	1560	1570	1580	1590	1600
EMLMDLMYRI	AKSYQASPDL	RLTWLQNMAE	KHTKKKCYTE	AAMCLVHAAA	LVAEYLSMLE	DHSYLPVGSV	SFQNISSNVL
1610	1620	1630	1640	1650	1660	1670	1680
EESVVSEDTL	SPDEDGVCAG	QYFTESGLVG	LLEQAAELFS	TGGLYETVNE	VYKLVIPILE	AHREFRKLTL	THSKLQRAFD
1690	1700	1710	1720	1730	1740	1750	1760
SIVNKDHKRM	FGTYFRVGFF	GSKFGDLDEQ	EFVYKEPAIT	KLPEISHRLE	AFYGQCFGAE	FVEVIKDSTP	VDKTKLDPNK
1770	1780	1790	1800	1810	1820	1830	1840
AYIQITFVEP	YFDEYEMKDR	VTYFEKNFNL	RRFMYTTPFT	LEGRPRGELH	EQYRRNTVLT	TMHAFPYIKT	RISVIQKEEF
1850	1860	1870	1880	1890	1900	1910	1920
VLTPIEVAIE	DMKKKTLQLA	VAINQEPPDA	KMLQMVLQGS	VGATVNQGPL	EVAQVFLAEI	PADPKLYRHH	NKLRLCFKEF
1930	1940	1950	1960	1970	1980	1990	2000
IMRCGEAVEK	NKRLITADQR	EYQQELKKNY	NKLKENLRPM	IERKIPELYK	PIFRVESQKR	DSFHRSSFRK	CETQLSQGS

Cmpd.	No. of Cmpds.	m/z meas.	Δ m/z [ppm]	z	Rt [min]	Score	P	Range	Sequence	Modification
2772	1	826.4371	9.54	2	66.0	17.2	0	1816-1829	R.NTVLTTMHAFPYIK.T	Oxidation: 7

Detailed Protein Report

Protein 444: cyclic nucleotide-gated cation channel beta-1 isoform c [Homo sapiens]

**Accession:**	gi|554790418	**Score:**	45.1
**Database:**	refseq_human(refseq_human_20140103.fasta)	**MW [kDa]:**	139.1
**Database Date:**	2015-11-30	**pI:**	4.6
		**Sequence Coverage [%]:**	2.9
		**No. of unique Peptides:**	2

Quantitation

Q:W	**Median:**	0.42	**CV:**	0.00 %	**No. of Peptides:**	1
M:Q	**Median:**	1.43	**CV:**	0.00 %	**No. of Peptides:**	1

10	20	30	40	50	60	70	80
MLGWVQRVLP	QPPGTPRKTK	MQEEEEVEPE	PEMEAEVEPE	PNPEEAETES	ESMPPEESFK	EEEVAVADPS	PQETKEAALT
90	100	110	120	130	140	150	160
STISLRAQGA	EISEMNSPSR	RVLTWLMKGV	EKVIPQPVHS	ITEDPAQILG	HGSTGDTGCT	DEPNEALEAQ	DTRPGLRLLL
170	180	190	200	210	220	230	240
WLEQNLERVL	PQPPKSSEVW	RDEPAVATAP	PGRPQEMGPK	LQARETPSLP	TPIPLQPKEE	PKEAPAPEPQ	PGSQAQTSSL
250	260	270	280	290	300	310	320
PPTRDPARLV	AWVLHRLEMA	LPQPVLHGKI	GEQEPDSPGI	CDVQTISILP	GGQVEPDLVL	EEVEPPWEDA	HQDVSTSPQG
330	340	350	360	370	380	390	400
TEVVPAYEEE	NKAVEKMPRE	LSRIEEEKED	EEEEEEEEEE	EEEEEVTEVL	LDSCVVSQVG	VGQSEEDGTR	PQSTSDQKLW
410	420	430	440	450	460	470	480
EEVGEEAKKE	AEEKAKEEAE	EVAEEEAEKE	PQDWAETKEE	PEAEAEAASS	GVPATKQHPE	VQVEDTDADS	CPLMAEENPP
490	500	510	520	530	540	550	560
STVLPPPSPA	KSDTLIVPSS	ASGTHRKKLP	SEDDEAEELK	ALSPAESPVV	AWSDPTTPKD	TDGQDRAAST	ASTNSAIIND
570	580	590	600	610	620	630	640
RLQELVKLFK	ERTEKVKEKL	IDPDVTSDEE	SPKPSPAKKA	PEPAPDTKPA	EAEPVEEEHY	CDMLCCKFKH	RPWKKYQFPQ
650	660	670	680	690	700	710	720
SIDPLTNLMY	VLWLFFVVMA	WNWNCWLIPV	RWAFPYQTPD	NIHHWLLMDY	LCDLIYFLDI	TVFQTRLQFV	RGGDIITDKK
730	740	750	760	770	780	790	800
DMRNNYLKSR	RFKMDLLSLL	PLDFLYLKVG	VNPLLRLPRC	LKYMAFFEFN	SRLESILSKA	YVYRVIRTTA	YLLYSLHLNS
810	820	830	840	850	860	870	880
CLYYWASAYQ	GLGSTHWVYD	GVGNSYIRCY	YFAVKTLITI	GGLPDPKTLF	EIVFQLLNYF	TGVFAFSVMI	GQMRDVVGAA
890	900	910	920	930	940	950	960
TAGQTYYRSC	MDSTVKYMNF	YKIPKSVQNR	VKTWYEYTWH	SQGMLDESEL	MVQLPDKMRL	DLAIDVNYNI	VSKVALFQGC
970	980	990	1000	1010	1020	1030	1040
DRQMIFDMLK	RLRSVVYLPN	DYVCKKGEIG	REMYIIQAGQ	VQVLGGPDGK	SVLVTLKAGS	VFGEISLLAV	GGGNRRTANV
1050	1060	1070	1080	1090	1100	1110	1120
VAHGFTNLFI	LDKKDLNEIL	VHYPESQKLL	RKKARRMLRS	NNKPKEEKSV	LILPPRAGTP	KLFNAALAMT	GKMGGKGAKG
1130	1140	1150	1160	1170	1180	1190	1200
GKLAHLRARL	KELAALEAAA	KQQELVEQAK	SSQDVKGEEG	SAAPDQHTHP	KEAATDPPAP	RTPPEPPGSP	PSSPPPASLG
1210	1220	1230	1240	1250			
RPEGEEEGPA	EPEEHSVRIC	MSPGPEPGEQ	ILSVKMPEER	EEKAE			

Cmpd.	No. of Cmpds.	m/z meas.	Δ m/z [ppm]	z	Rt [min]	Score	P	Range	Sequence	Modification	Ratios
1888	3	612.7759	-142.99	2	54.0	15.4	0	836-847	K.TLITIGGLPDPK.T		M:Q 1.43<br>Q:W 0.42
1512	1	851.5801	69.44	1	48.6	11.1	1	1120-1127	K.GGKLAHLR.A		

Detailed Protein Report

Protein 445: PREDICTED: armadillo repeat-containing protein 3 isoform X3 [Homo sapiens]

**Accession:**	gi|530392014	**Score:**	45.0
**Database:**	refseq_human(refseq_human_20140103.fasta)	**MW [kDa]:**	86.5
**Database Date:**	2015-11-30	**pI:**	5.8
		**Sequence Coverage [%]:**	2.6
		**No. of unique Peptides:**	1

10	20	30	40	50	60	70	80
MIFGILASNN	DVKKLLRELD	VMNSVIAQLA	PEEEVVIHEF	ASLCLANMSA	EYTSKVQIFE	HGGLEPLIRL	LSSPDPDVKK
90	100	110	120	130	140	150	160
NSMECIYNLV	QDFQCRAKLQ	ELNAIPPILD	LLKSEYPVIQ	LLALKTLGVI	ANDKESRTML	RDNQGLDHLI	KILETKELND
170	180	190	200	210	220	230	240
LHIEALAVIA	NCLEDMDTMV	QIQQTGGLKK	LLSFAENSTI	PDIQKNAAKA	ITKAAYDPEN	RKLFHEQEVE	KCLVALLGSE
250	260	270	280	290	300	310	320
NDGTKIAASQ	AISAMCENSG	SKDFFNNQGI	PQLIQLLKSD	NEEVREAAAL	ALANLTTCNP	ANANAAAEAD	GIDPLINLLS
330	340	350	360	370	380	390	400
SKRDGAIANA	ATVLTNMAMQ	EPLRLNIQNH	DIMHAIISPL	RSANTVVQSK	AALAVTATAC	DVEARTELRN	SGGLEPLVEL
410	420	430	440	450	460	470	480
LRSKNDEVRK	HASWAVMVCA	GDELTANELC	RLGALDILEE	VNVSGTRKNK	FSEAAYNKLL	NNNLSLKYSQ	TGYLSSSNII
490	500	510	520	530	540	550	560
NDGFYDYGRI	NPGTKLLPLK	ELCLQEPSDL	RAVLLINSKS	YVSPPSSMED	KSDVGYGRSI	SSSSSLRRSS	KEKNKKNSYH
570	580	590	600	610	620	630	640
FSAGFGSPIE	DKSEPASGRN	TVLSKSATKE	KGWRKSKGKK	EEEKVKEEEE	VMVVPKFVGE	GSSDKEWCPP	SDPDFSMYVY
650	660	670	680	690	700	710	720
EVTKSILPIT	NIKEQIEDLA	KYVAEKMGGK	IPKEKLPDFS	WELHISELKF	QLKSNVIPIG	HVKKGIFYHR	ALLFKALADR
730	740	750	760	770	780	790	
IGIGCSLVRG	EYGRAWNEVM	LQNDSRKGVI	GGLPAPEMYV	IDLMFHPGGL	MKLRSREADL	YRFI	

Cmpd.	No. of Cmpds.	m/z meas.	Δ m/z [ppm]	z	Rt [min]	Score	P	Range	Sequence	Modification
2698	1	709.8343	-97.82	2	64.9	17.6	1	2-14	M.IFGILASNNDVKK.L	

Detailed Protein Report

Protein 446: mitochondrial intermediate peptidase [Homo sapiens]

Accession: gi|156105687
Database: refseq_human(refseq_human_20140103.fasta)
Database Date: 2015-11-30
Modification(s): Oxidation

Score: 45.0
MW [kDa]: 80.6
pI: 6.6
Sequence Coverage [%]: 5.2
No. of unique Peptides: 2

10	20	30	40	50	60	70	80
MLCVGRLGGL	GARAAALPPR	RAGRGSLEAG	IRARRVSTSW	SPVGAAFNVK	PQGSRLDLFG	ERRGLFGVPE	LSAPEGFHIA
**90**	**100**	**110**	**120**	**130**	**140**	**150**	**160**
QEKALRKTEL	LVDRACSTPP	GPQTVLIFDE	LSDSLCRVAD	LADFVKIAHP	EPAFREAAEE	ACRSIGTMVE	KLNTNVDLYQ
**170**	**180**	**190**	**200**	**210**	**220**	**230**	**240**
SLQKLLADKK	LVDSLDPETR	RVAELFMFDF	EISGIHLDKE	KRKRAVDLNV	KILDLSSTFL	MGTNFPNKIE	KHLLPEHIRR
**250**	**260**	**270**	**280**	**290**	**300**	**310**	**320**
NFTSAGDHII	IDGLHAESPD	DLVREAAYKI	FLYPNAGQLK	CLEELLSSRD	LLAKLVGYST	FSHRALQGTI	AKNPETVMQF
**330**	**340**	**350**	**360**	**370**	**380**	**390**	**400**
LEKLSDKLSE	RTLKDFEMIR	GMKMKLNPQN	SEVMPWDPPY	YSGVIRAERY	NIEPSLYCPF	FSLGACMEGL	NILLNRLLGI
**410**	**420**	**430**	**440**	**450**	**460**	**470**	**480**
SLYAEQPAKG	EVWSEDVRKL	AVVHESEGLL	GYIYCDFFQR	ADKPHQDCHF	TIRGGRLKED	GDYQLPVVVL	MLNLPRSSRS
**490**	**500**	**510**	**520**	**530**	**540**	**550**	**560**
SPTLLTPGMM	ENLFHEMGHA	MHSMLGRTRY	QHVTGTRCPT	DFAEVPSILM	EYFANDYRVV	NQFARHYQTG	QPLPKNMVSR
**570**	**580**	**590**	**600**	**610**	**620**	**630**	**640**
LCESKKVCAA	ADMQLQVFYA	TLDQIYHGKH	PLRNSTTDIL	KETQEKFYGL	PYVPNTAWQL	RFSHLVGYGA	RYYSYLMSRA
**650**	**660**	**670**	**680**	**690**	**700**	**710**	**720**
VASMVWKECF	LQDPFNRAAG	ERYRREMLAH	GGGREPMLMV	EGMLQKCPSV	DDFVSALVSD	LDLDFETFLM	DSE

Cmpd.	No. of Cmpds.	m/z meas.	Δ m/z [ppm]	z	Rt [min]	Score	P	Range	Sequence	Modification
2189	1	993.8848	-92.03	2	56.0	11.7	1	632-647	R.YYSYLMSRAVASMVWK.E	Oxidation: 6, 13
2877	5	703.3682	28.70	2	66.7	15.9	0	675-686	R.EPMLMVEGMLQK.C	

Detailed Protein Report

Protein 447: PREDICTED: rootletin-like [Homo sapiens]

**Accession:**	gi|578843302	**Score:**	44.8
**Database:**	refseq_human(refseq_human_20140103.fasta)	**MW [kDa]:**	147.5
**Database Date:**	2015-11-30	**pI:**	5.5
		**Sequence Coverage [%]:**	4.9
		**No. of unique Peptides:**	2

Quantitation

Q:W	**Median:** 1.23	**CV:** 0.00 %	**No. of Peptides:**	1
M:Q	**Median:** 1.93	**CV:** 0.00 %	**No. of Peptides:**	1

```
        10         20         30         40         50         60         70         80
MRAAIERRWR REQELCLQLK SSQALVASLQ EQLSESRREL WAAQKLQQER AREQAREREA LRGQLEAQRL EVQQCRASCK
        90        100        110        120        130        140        150        160
LLGREKAALE MVVEELKGKA DAADAEKQGL EAEAAELQRS LLLQAERREE LALRRERSCR ALETSQGRLQ QLEEKVSGLR
       170        180        190        200        210        220        230        240
EELASVREAL STAQLQRDVV ESEREGLRSA LARAECSNAD LELLVRRLKS EGVEQRDSLA AMAALMEGLA QDKSALNHLA
       250        260        270        280        290        300        310        320
LQLEQERDQL REQRKTLEQE RARAGEQLAQ AEQQLALERA ERRGLQQACG RLEQRQEQLE GQAALLGREK AQLQEQVGQV
       330        340        350        360        370        380        390        400
TCQKQALEEQ LAQSLQDQEA QMGTLQQALQ GKDALSEERA QLLAKQEALE RQGRLAAEEA ADLRVERDSL ESSLLEAQQL
       410        420        430        440        450        460        470        480
ATKLQEQLEE EARSAGLARQ ALQVEMEQLQ SDWEVQEMKL RQDTVRLQRQ VAQQEREAQR ALESQALAHR EALAQLQREK
       490        500        510        520        530        540        550        560
ETLSLTLAEE KEVARCQLEQ EKELVTKSAA EREALKGEIQ SLKQERDESL LQLEHKMQQA LSLKETERSL LSEELSRARR
       570        580        590        600        610        620        630        640
TLERVQQEAQ SQQEQAQATI SATTEELKAL QAQFEDAITA HQRETTALRE SLQDLAAERG DVEREVRGRA GGLLGMPEDR
       650        660        670        680        690        700        710        720
RKQGRRKVSF QQISKSGPVS DHTGKQNEIA KLGAAGGGGG GGVERAERLR AQLTVAQEGL AALRQELQGV EESREGLHRE
       730        740        750        760        770        780        790        800
AQEARRALSD EAREKDVLLL FNSELRATIC RAEQEKASFK RSKEEKEQKL LILEEAQAAL QQEASALRAH LWELEQAGGD
       810        820        830        840        850        860        870        880
ARQELRELHR QVLGLQRKLA EVEAAGEAHG QRLQEHLRES RGAEQTLRAE LHSVTRKLQE ASGVADALQA RLDQACHRIH
       890        900        910        920        930        940        950        960
SLEQELAQAE GARQDAEAQL GRLCSTLRRG LGLQRQSPWA SPEQPGSPTK GSDSSQALPG QQGTSPPARP HSPLRWPSPT
       970        980        990       1000       1010       1020       1030       1040
PGGRSSELMD VATVQDILRD FVQKLREAQR ERDDSRIQMA TLSSRLSEAE CRCARAQSRV GQLQKALAEA EEGQRRVEGA
      1050       1060       1070       1080       1090       1100       1110       1120
LSSARAARAL QKEALRRLEL EHLASVRAAG QEKRRLQEQL ETLRQALEES RRHSQGLAKQ GKLLEEQLTN LEHRCQKAEV
      1130       1140       1150       1160       1170       1180       1190       1200
SLEPLRQVLC RPQRLSGGQE AAEAQAERRV LQEQTAALRT ERARLQGELA ALRAQLAQME QETLKREEDV ARLGAEKEQL
      1210       1220       1230       1240       1250       1260       1270       1280
DQSLNSLHQE VDGALRQNQQ LQAQMTEMEQ AHTQRLQDLT AQHQRDLATE AERLHGARPQ ATQALESQEW THQQRVKVLE
      1290       1300       1310       1320
EQVASLKEQL DQEVQWRQQA HLGQAFQTGQ
```

Cmpd.	No. of Cmpds.	m/z meas.	Δ m/z [ppm]	z	Rt [min]	Score	P	Range	Sequence	Modification	Ratios
2904	5	973.5046	-21.62	2	65.1	13.6	2	479-495	R.EKETLSLTLAEEKEVAR.C		M:Q 1.93<br>Q:W 1.23
1510	1	899.4718	8.92	2	47.2	10.7	1	666-685	K.QNEIAKLGAAGGGGGGGVER.A		

Detailed Protein Report

Protein 448: PREDICTED: dolichyl-phosphate beta-glucosyltransferase isoform X1 [Homo sapiens]

Accession: gi|530402309
Database: refseq_human(refseq_human_20140103.fasta)
Database Date: 2015-11-30

Score: 44.8
MW [kDa]: 32.8
pI: 10.1
Sequence Coverage [%]: 16.8
No. of unique Peptides: 1

Quantitation

Q:W **Median:** 0.26 **CV:** 0.00 % **No. of Peptides:** 1

10	20	30	40	50	60	70	80
MAPLLLQLAV	LGAALAAAAL	VLISIVAFTT	ATKMPALHRH	EEEKFFLNAK	GQKETLPSIW	DSPTKQLSVV	VPSYNEEKRL
90	100	110	120	130	140	150	160
PVMMDEALSY	LEKRQKRDPA	FTYEVIVVDD	GSKDQTSKVA	FKYCQKYGSD	KVRVITLVKN	RGKGGAIRMN	QMAIACGSRA
170	180	190	200	210	220	230	240
HLEKESIAQR	SYFRTLLMYG	FHFLVWFLCV	KGIRDTQCGF	KLFTREAASR	TFSSLHVERW	AFDVELLYIA	QFFKIPIAEI
250	260	270	280	290			
AVNWTEIEGS	KLVPFWSWLQ	MGKDLLFIRL	RYLTGAWRLE	QTRKMN			

Cmpd.	No. of Cmpds.	m/z meas.	Δ m/z [ppm]	z	Rt [min]	Score	P	Range	Sequence	Modification	Ratios	
2024	1	687.3070	-64.03	2	55.4	10.8	0	54-65	K.ETLPSIWDSPTK.Q		Q:W	0.26

Detailed Protein Report

Protein 449: **PREDICTED: zinc finger protein 467 isoform X5 [Homo sapiens]**

**Accession:**	gi|578813954	**Score:**	44.8
**Database:**	refseq_human(refseq_human_20140103.fasta)	**MW [kDa]:**	66.0
**Database Date:**	2015-11-30	**pI:**	11.0
**Modification(s):**	Carbamidomethyl	**Sequence Coverage [%]:**	9.3
		**No. of unique Peptides:**	3

Quantitation

Q:W	**Median:** 0.46	**CV:** 0.00 %	**No. of Peptides:** 1

10	20	30	40	50	60	70	80
MKFPTPHPGL	PPSSLSPENR	ILPALSQVLW	PDPQEELLWV	TMRETLEALS	SLGFSVGQPE	MAPQSEPREG	SHNAQEQMSS
**90**	**100**	**110**	**120**	**130**	**140**	**150**	**160**
SREERALGVC	SGEEWMIRKV	KVEDEDQEAE	EEVEWPQHLS	LLPSPFPAPD	LGHLAAAYKL	EPGAPGALSG	LALSGWGPMP
**170**	**180**	**190**	**200**	**210**	**220**	**230**	**240**
EKPYGCGECE	RRFRDQLTLR	LHQRLHRGEG	PCACPDCGRS	FTQRAHMLLH	QRSHRGERPF	PCSECDKRFS	KKAHLTRHLR
**250**	**260**	**270**	**280**	**290**	**300**	**310**	**320**
THTGERPYPC	AECGKRFSQK	IHLGSHQKTH	TGERPFPCTE	CEKRFRKKTH	LIRHQRIHTG	ERPYQCAQCA	RSFTHKQHLV
**330**	**340**	**350**	**360**	**370**	**380**	**390**	**400**
RHQRVHQTAG	PARPSPDSSA	SPHSTAPSPT	PSFPGPKPFA	CSDCGLSFGW	KKNLATHQCL	HRSEGRPFGC	DECALGATVD
**410**	**420**	**430**	**440**	**450**	**460**	**470**	**480**
APAAKPLASA	PGGPGCGPGS	DPVVPQRAPS	GERSFFCPDC	GRGFSHGQHL	ARHPRVHTGE	RPFACTQCDR	RFGSRPNLVA
**490**	**500**	**510**	**520**	**530**	**540**	**550**	**560**
HSRAHSGARP	FACAQCGRRF	SRKSHLGRHQ	AVHTGSRPHA	CAVCARSFSS	KTNLVRHQAI	HTGSRPFSCP	QCGKSFSRKT
**570**	**580**	**590**	**600**				
HLVRHQLIHG	EAAHAAPDAA	LAAPAWSAPP	EVAPPPLFF				

Cmpd.	No. of Cmpds.	m/z meas.	Δ m/z [ppm]	z	Rt [min]	Score	P	Range	Sequence	Modification	Ratios	
1902	1	785.7933	-55.89	2	52.1	10.0	1	185-199	R.LHRGEGPCACPDCGR.S			
1119	3	555.1856	-176.56	2	44.1	13.3	0	443-452	R.GFSHGQHLAR.H		Q:W	0.46
2174	1	849.0086	-71.63	3	57.0	10.8	1	537-558	R.HQAIHTGSRPFSCPQCGKSFSR.K	Carbamidomethyl: 13, 16		

Detailed Protein Report

Protein 450: **PREDICTED: centrosomal protein of 95 kDa isoform X4 [Homo sapiens]**

**Accession:**	gi|530413079	**Score:**	44.6
**Database:**	refseq_human(refseq_human_20140103.fasta)	**MW [kDa]:**	76.2
**Database Date:**	2015-11-30	**pI:**	10.0
**Modification(s):**	Carbamidomethyl	**Sequence Coverage [%]:**	4.1
		**No. of unique Peptides:**	1

Quantitation

Q:W	**Median:**	0.09	**CV:**	0.00 %	**No. of Peptides:**	1
M:Q	**Median:**	0.18	**CV:**	0.00 %	**No. of Peptides:**	1

```
        10         20         30         40         50         60         70         80
MLGPSWDGDE AESTGEIIRL GDTAHTFSLR SNGAQCPNEM LSKKALASPS SKSHEDMLYP PSVLSKSRTS FVEDTETLSV
        90        100        110        120        130        140        150        160
SGIPNARKLG EPIRAAIPLH PPYHPSEPRA PCPIGKEYLH SSHCSPAVNS TGEHTEFSGD LDDGLFLISK LPKGSKWEVY
       170        180        190        200        210        220        230        240
PAQVQGPRTR KPPKGKRNEN RATASSCNSP FPQRPRKRLT EQELHDVSEK LSQRLSELDW MLKSALGDRI KEKTDHKEEN
       250        260        270        280        290        300        310        320
TGNEEVEDGT EETLSQHSDG IVEYGPKKSR PGLSMRRKPP YRSHSLSPSP VNKHKQFHLE RKRQRKPRET DVRQFQAQAF
       330        340        350        360        370        380        390        400
TEAFERELRR HKVQENIGPL RIHEKEEETE KIYRGEAVRK GTPECSQPWK IYSRKTTTQS LRGGLPKPNK AVPMKVSEHS
       410        420        430        440        450        460        470        480
LLPLMLEQFP FLYVSGPTLS KMWKQQIAQV EQLKKEACRE NRSKKKLQDE IEEALRRHDL LTTLVKKEYE HNKRLQDFKD
       490        500        510        520        530        540        550        560
CIRRQRLTQS KIKENRQQIV RARKYYDDYR VQLCAKMMRM RTREEMIFKK LFEEGLNIQK QRLRDLRNYA KEKRDEQRRR
       570        580        590        600        610        620        630        640
HQDELDSMEN YYKDQFSLLA EAISQEHQEL KAREKSQAQV IIKIEAKSCT AWQCFFQQGT SYIGGWSNRL YHIAPVCSGL
       650        660
YSLGLCKYTD ILHNNKII
```

Cmpd.	No. of Cmpds.	m/z meas.	Δ m/z [ppm]	z	Rt [min]	Score	P	Range	Sequence	Modification	Ratios
1748	1	568.7494	-72.31	2	52.2	14.3	1	511-519	R.VQLCAKMMR.M	Carbamidomethyl: 4	M:Q 0.18<br>Q:W 0.09

Detailed Protein Report

Protein 451: transmembrane protein 151A [Homo sapiens]

Accession: gi|23397566
Database: refseq_human(refseq_human_20140103.fasta)
Database Date: 2015-11-30
Modification(s): Carbamidomethyl, Oxidation

Score: 44.6
MW [kDa]: 51.2
pI: 9.3
Sequence Coverage [%]: 13.0
No. of unique Peptides: 2

10	20	30	40	50	60	70	80
MPEDGAGDGG	EVPALIPDGE	PLREEQRPLK	QSLGSSLCRE	SHWKCLLLTL	LIHACGAVVA	WCRLATVPRL	VLGPEAALAR
**90**	**100**	**110**	**120**	**130**	**140**	**150**	**160**
GAGGPPPTYP	ASPCSDGYLY	IPLAFVSLLY	LLYLAECWHC	HVRSCQAPRT	DAHTVLALIR	RLQQAPPCVW	WKATSYHYVR
**170**	**180**	**190**	**200**	**210**	**220**	**230**	**240**
RTRQITRYRN	GDAYTTTQVY	HERADSRTAR	GEFDYSAHGV	RDVSKELVGL	AEHAATRLRF	TKCFSFGSAE	AEASYLTQRA
**250**	**260**	**270**	**280**	**290**	**300**	**310**	**320**
RFFSANEGLD	DYLEAREGMH	LKDVDFRESL	MVFADPRSPP	WYARAWVFWL	VSAATLSWPL	RVVAAYGTAH	VHYQVEKLFG
**330**	**340**	**350**	**360**	**370**	**380**	**390**	**400**
ASSPPPGAVP	SGPPLSRVAT	VDFTELEWHI	CSNRQLVPSY	SEAVVMGAGS	GAYLRGCQRC	RRSVSSNSLP	PARPSGPRLP
**410**	**420**	**430**	**440**	**450**	**460**	**470**	
FSRSRLSLGA	GGRATPGVFR	SLSGGPLGRR	GEDTEPLESP	PCYEDALYFP	VLIVHGDSGC	QGDGQGAL	

Cmpd.	No. of Cmpds.	m/z meas.	Δ m/z [ppm]	z	Rt [min]	Score	P	Range	Sequence	Modification
136	1	977.7569	-49.80	3	31.1	10.2	2	355-381	R.QLVPSYSEAVVMGAGSGAYLRGCQRCR.R	Carbamidomethyl: 23; Oxidation: 12
1863	1	864.4736	-15.26	2	52.0	17.8	2	414-430	R.ATPGVFRSLSGGPLGRR.G	

Detailed Protein Report

Protein 452: **constitutive coactivator of peroxisome proliferator-activated receptor gamma isoform a [Homo sapiens]**

**Accession:**	gi|556503397	**Score:**	44.6
**Database:**	refseq_human(refseq_human_20140103.fasta)	**MW [kDa]:**	105.1
**Database Date:**	2015-11-30	**pI:**	5.4
**Modification(s):**	Carbamidomethyl, Oxidation	**Sequence Coverage [%]:**	6.8
		**No. of unique Peptides:**	2

10	20	30	40	50	60	70	80
MDRQMILSRS	SVMGVRGLQG	FVGSTCPHIC	TVVNFKELAE	HHRSKYPGCT	PTIVVDAMCC	LRYWYTPESW	ICGGQWREYF
**90**	**100**	**110**	**120**	**130**	**140**	**150**	**160**
SALRDFVKTF	TAAGIKLIFF	FDGMVEQDKR	DEWVKRRLKN	NREISRIFHY	IKSHKEQPGR	NMFFIPSGLA	VFTRFALKTL
**170**	**180**	**190**	**200**	**210**	**220**	**230**	**240**
GQETLCSLQE	ADYEVASYGL	QHNCLGILGE	DTDYLIYDTC	PYFSISELCL	ESLDTVMLCR	EKLCESLGLC	VADLPLLACL
**250**	**260**	**270**	**280**	**290**	**300**	**310**	**320**
LGNDIIPEGM	FESFRYKCLS	SYTSVKENFD	KKGNIILAVS	DHISKVLYLY	QGEKKLEEIL	PLGPNKALFY	KGMASYLLPG
**330**	**340**	**350**	**360**	**370**	**380**	**390**	**400**
QKSPWFFQKP	KGVITLDKQV	ISTSSDAESR	EEVPMCSDAE	SRQEVPMCTG	PESRREVPVY	TDSEPRQEVP	MCSDPEPRQE
**410**	**420**	**430**	**440**	**450**	**460**	**470**	**480**
VPTCTGPESR	REVPMCSDPE	PRQEVPMCTG	PEARQEVPMY	TDSEPRQEVP	MYTDSEPRQE	VPMYTGSEPR	QEVPMYTGPE
**490**	**500**	**510**	**520**	**530**	**540**	**550**	**560**
SRQEVPMYTG	PESRQEVLIR	TDPESRQEIM	CTGHESKQEV	PICTDPISKQ	EDSMCTHAEI	NQKLPVATDF	EFKLEALMCT
**570**	**580**	**590**	**600**	**610**	**620**	**630**	**640**
NPEIKQEDPT	NVGPEVKQQV	TMVSDTEILK	VARTHHVQAE	SYLVYNIMSS	GEIECSNTLE	DELDQALPSQ	AFIYRPIRQR
**650**	**660**	**670**	**680**	**690**	**700**	**710**	**720**
VYSLLLEDCQ	DVTSTCLAVK	EWFVYPGNPL	RHPDLVRPLQ	MTIPGGTPSL	KILWLNQEPE	IQVRRLDTLL	ACFNLSSSRE
**730**	**740**	**750**	**760**	**770**	**780**	**790**	**800**
ELQAVESPFQ	ALCCLLIYLF	VQVDTLCLED	LHAFIAQALC	LQGKSTSQLV	NLQPDYINPR	AVQLGSLLVR	GLTTLVLVNS
**810**	**820**	**830**	**840**	**850**	**860**	**870**	**880**
ACGFPWKTSD	FMPWNVFDGK	LFHQKYLQSE	KGYAVEVLLE	QNRSRLTKFH	NLKAVVCKAC	MKENRRITGR	AHWGSHHAGR
**890**	**900**	**910**	**920**	**930**			
WGRQGSSYHR	TGSGYSRSSQ	GQPWRDQGPG	SRQYEHDQWR	RY			

Cmpd.	No. of Cmpds.	m/z meas.	Δ m/z [ppm]	z	Rt [min]	Score	P	Range	Sequence	Modification
1639	4	839.3743	-22.36	2	49.0	11.4	1	258-271	K.CLSSYTSVKENFDK.K	Carbamidomethyl: 1
1242	1	666.6715	-165.15	2	45.6	11.1	0	412-422	R.EVPMCSDPEPR.Q	Carbamidomethyl: 5; Oxidation: 4

Detailed Protein Report

Protein 453: MAP7 domain-containing protein 2 isoform 4 [Homo sapiens]

**Accession:**	gi|270483747	**Score:**	44.6
**Database:**	refseq_human(refseq_human_20140103.fasta)	**MW [kDa]:**	76.6
**Database Date:**	2015-11-30	**pI:**	9.4
		**Sequence Coverage [%]:**	4.7
		**No. of unique Peptides:**	2

10	20	30	40	50	60	70	80
MEGFLKSDER	QRLAKERREE	REKCLAAREQ	QILEKQKRAR	LQYEKQMEER	WRKLEEQRQR	EDQKRAAVEE	KRKQKLREEE
**90**	**100**	**110**	**120**	**130**	**140**	**150**	**160**
ERLEAMMRRS	LERTQQLELK	KKYSWGAPLA	IGPGGHDACD	KLSTSTMSLP	KPTEPPMNKR	LSSSTVAISY	SPDRAPLGPL
**170**	**180**	**190**	**200**	**210**	**220**	**230**	**240**
NPSYKSSPTR	NIEKKKATST	STSGAGDVGK	EALSGGEASL	VEKVKRGQRT	ATSLPVVNFG	SPLRRCEFSG	GIPKRPSSPV
**250**	**260**	**270**	**280**	**290**	**300**	**310**	**320**
ISKTATKAYP	QSPKTTKPPY	PGSPVKYRLP	ALSGQDMPKR	KAEKEKSNKE	REGTLAQQAA	GPQGEEALEK	HVVDKHASEK
**330**	**340**	**350**	**360**	**370**	**380**	**390**	**400**
HAAAAGGKAE	NSAALGKPTA	GTTDAGEAAK	ILAEKRRQAR	LQKEQEEQER	LEKEEQDRLE	REELKRKAEE	ERLRLEEEAR
**410**	**420**	**430**	**440**	**450**	**460**	**470**	**480**
KQEEERKRQE	EEKKKQEGEE	KRKAGEEAKR	KAEEELLLKE	KQEQEKQEKA	MIEKQKEAAE	TKAREVAEQM	RLEREQIMLQ
**490**	**500**	**510**	**520**	**530**	**540**	**550**	**560**
IEQERLERKK	RIDEIMKRTR	KSDVSPQVKK	EDPKVGVQPA	VCVEKKTKLV	VPNKMEINGL	NTCQEVNGVD	HAAPETYPQD
**570**	**580**	**590**	**600**	**610**	**620**	**630**	**640**
IFSNGLKPAG	GLIHLDALDG	KSNSLDDSTE	EVQSMDVSPV	SKEELISIPE	FSPVSEMIPG	VSLDQNGTGN	ARALQDLLDF
**650**	**660**	**670**	**680**	**690**			
TGPPTFPKRS	SENLSLDDCN	KNLIEGFNSP	GQETPLNTFC				

Cmpd.	No. of Cmpds.	m/z meas.	Δ m/z [ppm]	z	Rt [min]	Score	P	Range	Sequence	Modification
1648	1	575.1737	-226.87	2	50.5	13.4	1	311-320	K.HVVDKHASEK.H	
547	1	472.6490	-252.92	2	35.3	10.2	0	432-439	K.AEEELLLK.E	

Detailed Protein Report

Protein 454: cytochrome b5 reductase 4 [Homo sapiens]

**Accession:**	gi|84875541	**Score:**	44.6
**Database:**	refseq_human(refseq_human_20140103.fasta)	**MW [kDa]:**	59.4
**Database Date:**	2015-11-30	**pI:**	8.5
		**Sequence Coverage [%]:**	8.6
		**No. of unique Peptides:**	1

10	20	30	40	50	60	70	80
MLNVPSQSFP	APRSQQRVAS	GGRSKVPLKQ	GRSLMDWIRL	TKSGKDLTGL	KGRLIEVTEE	ELKKHNKKDD	CWICIRGFVY
**90**	**100**	**110**	**120**	**130**	**140**	**150**	**160**
NVSPYMEYHP	GGEDELMRAA	GSDGTELFDQ	VHRWVNYESM	LKECLVGRMA	IKPAVLKDYR	EEEKKVLNGM	LPKSQVTDTL
**170**	**180**	**190**	**200**	**210**	**220**	**230**	**240**
AKEGPSYPSY	DWFQTDSLVT	IAIYTKQKDI	NLDSIIVDHQ	NDSFRAETII	KDCLYLIHIG	LSHEVQEDFS	VRVVESVGKI
**250**	**260**	**270**	**280**	**290**	**300**	**310**	**320**
EIVLQKKENT	SWDFLGHPLK	NHNSLIPRKD	TGLYYRKCQL	ISKEDVTHDT	RLFCLMLPPS	THLQVPIGQH	VYLKLPITGT
**330**	**340**	**350**	**360**	**370**	**380**	**390**	**400**
EIVKPYTPVS	GSLLSEFKEP	VLPNNKYIYF	LIKIYPTGLF	TPELDRLQIG	DFVSVSSPEG	NFKISKFQEL	EDLFLLAAGT
**410**	**420**	**430**	**440**	**450**	**460**	**470**	**480**
GFTPMVKILN	YALTDIPSLR	KVKLMFFNKT	EDDIIWRSQL	EKLAFKDKRL	DVEFVLSAPI	SEWNGKQGHI	SPALLSEFLK
**490**	**500**	**510**	**520**	**530**			
RNLDKSKVLV	CICGPVPFTE	QGVRLLHDLN	FSKNEIHSFT	A			

Cmpd.	No. of Cmpds.	m/z meas.	Δ m/z [ppm]	z	Rt [min]	Score	P	Range	Sequence	Modification
156	1	822.7809	-149.81	2	31.7	17.5	1	278-291	K.CQLISKEDVTHDTR.L	

Detailed Protein Report

Protein 455: tyrosine-protein kinase Fgr [Homo sapiens]

**Accession:**	gi|4885235	**Score:**	44.5
**Database:**	refseq_human(refseq_human_20140103.fasta)	**MW [kDa]:**	59.4
**Database Date:**	2015-11-30	**pI:**	5.3
**Modification(s):**	Carbamidomethyl, Oxidation	**Sequence Coverage [%]:**	8.5
		**No. of unique Peptides:**	3

Alias proteins:

Accession	Name	Description
gi|578798632	refseq_human (refseq_human_20140103.fasta)	PREDICTED: tyrosine-protein kinase Fgr isoform X1 [Homo sapiens]
gi|112382244	refseq_human (refseq_human_20140103.fasta)	tyrosine-protein kinase Fgr [Homo sapiens]
gi|112382241	refseq_human (refseq_human_20140103.fasta)	tyrosine-protein kinase Fgr [Homo sapiens]

10	20	30	40	50	60	70	80
MGCVFCKKLE	PVATAKEDAG	LEGDFRSYGA	ADHYGPDPTK	ARPASSFAHI	PNYSNFSSQA	INPGFLDSGT	IRGVSGIGVT
**90**	**100**	**110**	**120**	**130**	**140**	**150**	**160**
LFIALYDYEA	RTEDDLTFTK	GEKFHILNNT	EGDWWEARSL	SSGKTGCIPS	NYVAPVDSIQ	AEEWYFGKIG	RKDAERQLLS
**170**	**180**	**190**	**200**	**210**	**220**	**230**	**240**
PGNPQGAFLI	RESETTKGAY	SLSIRDWDQT	RGDHVKHYKI	RKLDMGGYYI	TTRVQFNSVQ	ELVQHYMEVN	DGLCNLLIAP
**250**	**260**	**270**	**280**	**290**	**300**	**310**	**320**
CTIMKPQTLG	LAKDAWEISR	SSITLERRLG	TGCFGDVWLG	TWNGSTKVAV	KTLKPGTMSP	KAFLEEAQVM	KLLRHDKLVQ
**330**	**340**	**350**	**360**	**370**	**380**	**390**	**400**
LYAVVSEEPI	YIVTEFMCHG	SLLDFLKNPE	GQDLRLPQLV	DMAAQVAEGM	AYMERMNYIH	RDLRAANILV	GERLACKIAD
**410**	**420**	**430**	**440**	**450**	**460**	**470**	**480**
FGLARLIKDD	EYNPCQGSKF	PIKWTAPEAA	LFGRFTIKSD	VWSFGILLTE	LITKGRIPYP	GMNKREVLEQ	VEQGYHMPCP
**490**	**500**	**510**	**520**	**530**			
PGCPASLYEA	MEQTWRLDPE	ERPTFEYLQS	FLEDYFTSAE	PQYQPGDQT			

Cmpd.	No. of Cmpds.	m/z meas.	Δ m/z [ppm]	z	Rt [min]	Score	P	Range	Sequence	Modification
2000	2	899.3791	-86.20	2	53.4	11.8	2	1-16	-.MGCVFCKKLEPVATAK.E	Carbamidomethyl: 6; Oxidation: 1
2074	1	682.6660	-32.64	3	56.3	10.3	2	8-26	K.KLEPVATAKEDAGLEGDFR.S	
1458	1	1022.6515	107.92	2	46.6	10.7	0	73-91	R.GVSGIGVTLFIALYDYEAR.T	

Detailed Protein Report

Protein 456: spectrin beta chain, non-erythrocytic 5 [Homo sapiens]

**Accession:**	gi|485837026	**Score:**	44.5
**Database:**	refseq_human(refseq_human_20140103.fasta)	**MW [kDa]:**	416.5
**Database Date:**	2015-11-30	**pI:**	6.2
		**Sequence Coverage [%]:**	1.2
		**No. of unique Peptides:**	3

Quantitation

Q:W	**Median:**	1.12	**CV:**	0.00 %	**No. of Peptides:**	1
M:Q	**Median:**	0.66	**CV:**	0.00 %	**No. of Peptides:**	1

Detailed Protein Report

10	20	30	40	50	60	70	80
MAGQPHSPRE	LLGAAGHRSR	RPSTELRVPP	SPSLTMDSQY	ETGHIRKLQA	RHMQMQEKTF	TKWINNVFQC	GQAGIKIRNL
90	100	110	120	130	140	150	160
YTELADGIHL	LRLLELISGE	ALPPPSRGRL	RVHFLENSSR	ALAFLRAKVP	VPLIGPENIV	DGDQTLILGL	IWVIILRFQI
170	180	190	200	210	220	230	240
SHISLDKEEF	GASAALLSTK	EALLVWCQRK	TASYTNVNIT	DFSRSWSDGL	GFNALIHAHR	PDLLDYGSLR	PDRPLHNLAF
250	260	270	280	290	300	310	320
AFLVAEQELG	IAQLLDPEDV	AAAQPDERSI	MTYVSLYYHY	CSRLHQGQTV	QRRLTKILLQ	LQETELLQTQ	YEQLVADLLR
330	340	350	360	370	380	390	400
WIAEKQMQLE	ARDFPDSLPA	MRQLLAAFTI	FRTQEKPPRL	QQRGAAEALL	FRLQTALQAQ	NRRPFLPHEG	LGLAELSQCW
410	420	430	440	450	460	470	480
AGLEWAEAAR	SQALQQRLLQ	LQRLETLARR	FQHKAALRES	FLKDAEQVLD	QARAPPASLA	TVEAAVQRLG	MLEAGILPQE
490	500	510	520	530	540	550	560
GRFQALAEIA	DILRQEQYHS	WADVARRQEE	VTVRWQRLLQ	HLQGQRKQVA	DMQAVLSLLQ	EVEAASHQLE	ELQEPARSTA
570	580	590	600	610	620	630	640
CGQQLAEVVE	LLQRHDLLEA	QVSAHGAHVS	HLAQQTAELD	SSLGTSVEVL	QAKARTLAQL	QQSLVALVRA	RRALLEQTLQ
650	660	670	680	690	700	710	720
RAEFLRNCEE	EEAWLKECGQ	RVGNAALGRD	LSQIAGALQK	HKALEAEVHR	HQAVCVDLVR	RGRDLSARRP	PTQPDPGERA
730	740	750	760	770	780	790	800
EAVQGGWQLL	QTRVVGRGAR	LQTALLVLQY	FADAAEAASW	LRERRSSLER	ASCGQDQAAA	ETLLRRHVRL	ERVLRAFAAE
810	820	830	840	850	860	870	880
LRRLEEQGRA	ASARASLFTV	NSALSPPGES	LRNPGPWSEA	SCHPGPGDAW	KMALPAEPDP	DFDPNTILQT	QDHLSQDYES
890	900	910	920	930	940	950	960
LRALAQLRRA	RLEEAMALFG	FCSSCGELQL	WLEKQTVLLQ	RVQPQADTLE	VMQLKYENFL	TALAVGKGLW	AEVSSSAEQL
970	980	990	1000	1010	1020	1030	1040
RQRYPGNSTQ	IQRQQEELSQ	RWGQLEALKR	EKAVQLAHSV	EVCSFLQECG	PTQVQLRDVL	LQLEALQPGS	SEDTCHALQL
1050	1060	1070	1080	1090	1100	1110	1120
AQKKTLVLER	RVHFLQSVVV	KVEEPGYAES	QPLQGQVETL	QGLLKQVQEQ	VAQRARRQAE	TQARQSFLQE	SQQLLLWAES
1130	1140	1150	1160	1170	1180	1190	1200
VQAQLRSKEV	SVDVASAQRL	LREHQDLLEE	IHLWQERLQQ	LDAQSQPMAA	LDCPDSQEVP	NTLRVLGQQG	QELKVLWEQR
1210	1220	1230	1240	1250	1260	1270	1280
QQWLQEGLEL	QKFGREVDGF	TATCANHQAW	LHLDNLGEDV	REALSLLQQH	REFGRLLSTL	GPRAEALRAH	GEKLVQSQHP
1290	1300	1310	1320	1330	1340	1350	1360
AAHTVREQLQ	SIQAQWTRLQ	GRSEQRRRQL	LASLQLQEWK	QDVAELMQWM	EEKGLMAAHE	PSGARRNILQ	TLKRHEAAES
1370	1380	1390	1400	1410	1420	1430	1440
ELLATRRHVE	ALQQVGRELL	SRRPCGQEDI	QTRLQGLRSK	WEALNRKMTE	RGDELQQAGQ	QEQLLRQLQD	AKEQLEQLEG
1450	1460	1470	1480	1490	1500	1510	1520
ALQSSETGQD	LRSSQRLQKR	HQQLESESRT	LAAKMAALAS	MAHGMAASPA	ILEETQKHLR	RLELLQGHLA	IRGLQLQASV
1530	1540	1550	1560	1570	1580	1590	1600
ELHQFCHLSN	MELSWVAEHM	PHGSPTSYTE	CLNGAQSLHR	KHKELQVEVK	AHQGQVQRVL	SSGRSLAASG	HPQAQHIVEQ
1610	1620	1630	1640	1650	1660	1670	1680
CQELEGHWAE	LERACEARAQ	CLQQAVTFQQ	YFLDVSELEG	WVEEKRPLVS	SRDYGRDEAA	TLRLINKHQA	LQEELAIYWS
1690	1700	1710	1720	1730	1740	1750	1760
SMEELDQTAQ	TLTGPEVPEQ	QRVVQERLRE	QLRALQELAA	TRDRELEGTL	RLHEFLREAE	DLQGWLASQK	QAAKGGESLG
1770	1780	1790	1800	1810	1820	1830	1840
EDPEHALHLC	TKFAKFQHQV	EMGSQRVAAC	RLLAESLLER	GHSAGPMVRQ	RQQDLQTAWS	ELWELTQARG	HALRDTETTL
1850	1860	1870	1880	1890	1900	1910	1920
RVHRDLLEVL	TQVQEKATSL	PNNVARDLCG	LEAQLRSHQG	LERELVGTER	QLQELLETAG	RVQKLCPGPQ	AHAVQQRQQA
1930	1940	1950	1960	1970	1980	1990	2000
VTQAWAVLQR	RMEQRRAQLE	RARLLARFRT	AVRDYASWAA	RVRQDLQVEE	SSQEPSSGPL	KLSAHQWLRA	ELEAREKLWQ
2010	2020	2030	2040	2050	2060	2070	2080
QATQLGQQAL	LAAGTPTKEV	QEELRALQDQ	RDQVYQTWAR	KQERLQAEQQ	EQLFLRECGR	LEEILAAQEV	SLKTSALGSS
2090	2100	2110	2120	2130	2140	2150	2160
VEEVEQLIRK	HEVFLKVLTA	QDKKEAALRE	RLKTLRRPRV	RDRLPILLQR	RMRVKELAES	RGHALHASLL	MASFTQAATQ
2170	2180	2190	2200	2210	2220	2230	2240

Detailed Protein Report

Cmpd.	No. of Cmpds.	m/z meas.	Δ m/z [ppm]	z	Rt [min]	Score	P	Range	Sequence	Modification	Ratios
1932	1	724.3207	-73.16	2	54.2	11.4	1	962-973	R.QRYPGNSTQIQR.Q		
1262	1	598.7915	-6.95	2	45.9	11.3	0	1334-1345	K.GLMAAHEPSGAR.R		Q:W 1.12<br>M:Q 0.66
2799	2	665.3651	-30.72	2	65.4	11.8	0	3278-3291	R.LGQLHPAAPGGLAK.V		

Detailed Protein Report

Protein 457: myotubularin-related protein 8 [Homo sapiens]

Accession: gi|21361759
Database: refseq_human(refseq_human_20140103.fasta)
Database Date: 2015-11-30
Modification(s): Oxidation

Score: 44.5
MW [kDa]: 78.9
pI: 6.2
Sequence Coverage [%]: 4.5
No. of unique Peptides: 2

10	20	30	40	50	60	70	80
MDHITVPKVE	NVKLVDRYVS	KKPANGILYL	TATHLIYVEA	SGAARKETWI	ALHHIATVEK	LPITSLGCPL	TLRCKNFRVA
**90**	**100**	**110**	**120**	**130**	**140**	**150**	**160**
HFVLDSDLVC	HEVYISLLKL	SQPALPEDLY	AFSYNPKSSK	EMRESGWKLI	DPISDFGRMG	IPNRNWTITD	ANRNYEICST
**170**	**180**	**190**	**200**	**210**	**220**	**230**	**240**
YPPEIVVPKS	VTLGTVVGSS	KFRSKERVPV	LSYLYKENNA	AICRCSQPLS	GFYTRCVDDE	LLLEAISQTN	PGSQFMYVVD
**250**	**260**	**270**	**280**	**290**	**300**	**310**	**320**
TRPKLNAMAN	RAAGKGYENE	DNYANIRFRF	MGIENIHVMR	SSLQKLLEVC	ELKTPTMSEF	LSGLESSGWL	RHIKAIMDAG
**330**	**340**	**350**	**360**	**370**	**380**	**390**	**400**
IFITKAVKVE	KASVLVHCSD	GWDRTAQVCS	VASILLDPFY	RTFKGLMILI	EKEWISMGHK	FSQRCGHLDG	DSKEVSPIFT
**410**	**420**	**430**	**440**	**450**	**460**	**470**	**480**
QFLDCIWQLM	EQFPCAFEFN	ENFLLEIHDH	VFSCQFGNFL	GNCQKDREDL	RVYEKTHSVW	PFLVQRKPDF	RNPLYKGFTM
**490**	**500**	**510**	**520**	**530**	**540**	**550**	**560**
YGVLNPSTVP	YNIQFWCGMY	NRFDKGLQPK	QSMLESLLEI	KKQRAMLETD	VHELEKKLKV	RDEPPEEICT	CSQLGNILSQ
**570**	**580**	**590**	**600**	**610**	**620**	**630**	**640**
HLGSPLTNPL	GFMGINGDLN	TLMENGTLSR	EGGLRAQMDQ	VKSQGADLHH	NCCEIVGSLR	AINISGDVGI	SEAMGISGDM
**650**	**660**	**670**	**680**	**690**	**700**	**710**	
CTFEATGFSK	DLGICGAMDI	SEATGISGNL	GISEARGFSG	DMGILGDTGI	SKASTKEADY	SKHQ	

Cmpd.	No. of Cmpds.	m/z meas.	Δ m/z [ppm]	z	Rt [min]	Score	P	Range	Sequence	Modification
1315	9	956.5835	100.93	1	46.6	16.9	0	1-8	-.MDHITVPK.V	Oxidation: 1
2921	1	729.3717	73.33	2	65.3	10.5	0	256-267	K.GYENEDNYANIR.F	

Detailed Protein Report

Protein 458: PREDICTED: serine/threonine-protein kinase B-raf isoform X2 [Homo sapiens]

**Accession:**	gi|530387108	**Score:**	44.5
**Database:**	refseq_human(refseq_human_20140103.fasta)	**MW [kDa]:**	83.9
**Database Date:**	2015-11-30	**pI:**	7.9
**Modification(s):**	Oxidation	**Sequence Coverage [%]:**	3.4
		**No. of unique Peptides:**	1

Quantitation

Q:W	**Median:**	1.60	**CV:**	0.00 %	**No. of Peptides:**	1
M:Q	**Median:**	0.83	**CV:**	0.00 %	**No. of Peptides:**	1

10	20	30	40	50	60	70	80
MAALSGGGGG	GAEPGQALFN	GDMEPEAGAG	AGAAASSAAD	PAIPEEVWNI	KQMIKLTQEH	IEALLDKFGG	EHNPPSIYLE
**90**	**100**	**110**	**120**	**130**	**140**	**150**	**160**
AYEEYTSKLD	ALQQREQQLL	ESLGNGTDFS	VSSSASMDTV	TSSSSSSLSV	LPSSLSVFQN	PTDVARSNPK	SPQKPIVRVF
**170**	**180**	**190**	**200**	**210**	**220**	**230**	**240**
LPNKQRTVVP	ARCGVTVRDS	LKKALMMRGL	IPECCAVYRI	QDGEKKPIGW	DTDISWLTGE	ELHVEVLENV	PLTTHNFVRK
**250**	**260**	**270**	**280**	**290**	**300**	**310**	**320**
TFFTLAFCDF	CRKLLFQGFR	CQTCGYKFHQ	RCSTEVPLMC	VNYDQLDLLF	VSKFFEHHPI	PQEEASLAET	ALTSGSSPSA
**330**	**340**	**350**	**360**	**370**	**380**	**390**	**400**
PASDSIGPQI	LTSPSPSKSI	PIPQPFRPAD	EDHRNQFGQR	DRSSSAPNVH	INTIEPVNID	DLIRDQGFRG	DGGSTTGLSA
**410**	**420**	**430**	**440**	**450**	**460**	**470**	**480**
TPPASLPGSL	TNVKALQKSP	GPQRERKSSS	SSEDRNRMKT	LGRRDSSDDW	EIPDGQITVG	QRIGSGSFGT	VYKGKWHGDV
**490**	**500**	**510**	**520**	**530**	**540**	**550**	**560**
AVKMLNVTAP	TPQQLQAFKN	EVGVLRKTRH	VNILLFMGYS	TKPQLAIVTQ	WCEGSSLYHH	LHIIETKFEM	IKLIDIARQT
**570**	**580**	**590**	**600**	**610**	**620**	**630**	**640**
AQGMDYLHAK	SIIHRDLKSN	NIFLHEDLTV	KIGDFGLATV	KSRWSGSHQF	EQLSGSILWM	APEVIRMQDK	NPYSFQSDVY
**650**	**660**	**670**	**680**	**690**	**700**	**710**	**720**
AFGIVLYELM	TGQLPYSNIN	NRDQIIFMVG	RGYLSPDLSK	VRSNCPKAMK	RLMAECLKKK	RDERPLFPQE	NLQPSSSHHH
**730**	**740**	**750**	**760**				
GSICSYFLSL	VFVQFVNIKT	QFCSSNLFLK	IQNFQCIS				

Cmpd.	No. of Cmpds.	m/z meas.	Δ m/z [ppm]	z	Rt [min]	Score	P	Range	Sequence	Modification	Ratios	
116	1	929.4622	17.68	2	30.9	16.0	1	184-199	K.ALMMRGLIPECCAVYR.I	Oxidation: 3, 4	Q:W<br>M:Q	1.60<br>0.83

Detailed Protein Report

Protein 459: PREDICTED: plexin-A1 isoform X1 [Homo sapiens]

**Accession:**	gi|578807485	**Score:**	44.5
**Database:**	refseq_human(refseq_human_20140103.fasta)	**MW [kDa]:**	212.5
**Database Date:**	2015-11-30	**pI:**	6.6
**Modification(s):**	Oxidation	**Sequence Coverage [%]:**	2.3
		**No. of unique Peptides:**	1

10	20	30	40	50	60	70	80
MMLTPAGPEH	RGPRPQPAMP	LPPRSLQVLL	LLLLLLLLLP	GMWAEAGLPR	AGGGSQPPFR	TFSASDWGLT	HLVVHEQTGE
90	100	110	120	130	140	150	160
VYVGAVNRIY	KLSGNLTLLR	AHVTGPVEDN	EKCYPPPSVQ	SCPHGLGSTD	NVNKLLLLDY	AANRLLACGS	ASQGICQFLR
170	180	190	200	210	220	230	240
LDDLFKLGEP	HHRKEHYLSS	VQEAGSMAGV	LIAGPPGQGQ	AKLFVGTPID	GKSEYFPTLS	SRRLMANEED	ADMFGFVYQD
250	260	270	280	290	300	310	320
EFVSSQLKIP	SDTLSKFPAF	DIYYVYSFRS	EQFVYYLTLQ	LDTQLTSPDA	AGEHFFTSKI	VRLCVDDPKF	YSYVEFPIGC
330	340	350	360	370	380	390	400
EQAGVEYRLV	QDAYLSRPGR	ALAHQLGLAE	DEDVLFTVFA	QGQKNRVKPP	KESALCLFTL	RAIKEKIKER	IQSCYRGEGK
410	420	430	440	450	460	470	480
LSLPWLLNKE	LGCINSPLQI	DDDFCGQDFN	QPLGGTVTIE	GTPLFVDKDD	GLTAVAAYDY	RGRTVVFAGT	RSGRIRKILV
490	500	510	520	530	540	550	560
DLSNPGGRPA	LAYESVVAQE	GSPILRDLVL	SPNHQYLYAM	TEKQVTRVPV	ESCVQYTSCE	LCLGSRDPHC	GWCVLHSICS
570	580	590	600	610	620	630	640
RRDACERADE	PQRFAADLLQ	CVQLTVQPRN	VSVTMSQVPL	VLQAWNVPDL	SAGVNCSFED	FTESESVLED	GRIHCRSPSA
650	660	670	680	690	700	710	720
REVAPITRGQ	GDQRVVKLYL	KSKETGKKFA	SVDFVFYNCS	VHQSCLSCVN	GSFPCHWCKY	RHVCTHNVAD	CAFLEGRVNV
730	740	750	760	770	780	790	800
SEDCPQILPS	TQIYVPVGVV	KPITLAARNL	PQPQSGQRGY	ECLFHIPGSP	ARVTALRFNS	SSLQCQNSSY	SYEGNDVSDL
810	820	830	840	850	860	870	880
PVNLSVVWNG	NFVIDNPQNI	QAHLYKCPAL	RESCGLCLKA	DPRFECGWCV	AERRCSLRHH	CAADTPASWM	HARHGSSRCT
890	900	910	920	930	940	950	960
DPKILKLSPE	TGPRQGGTRL	TITGENLGLR	FEDVRLGVRV	GKVLCSPVES	EYISAEQIVC	EIGDASSVRA	HDALVEVCVR
970	980	990	1000	1010	1020	1030	1040
DCSPHYRALS	PKRFTFVTPT	FYRVSPSRGP	LSGGTWIGIE	GSHLNAGSDV	AVSVGGRPCS	FSWRNSREIR	CLTPPGQSPG
1050	1060	1070	1080	1090	1100	1110	1120
SAPIIININR	AQLTNPEVKY	NYTEDPTILR	IDPEWSINSG	GTLLTVTGTN	LATVREPRIR	AKYGGIEREN	GCLVYNDTTM
1130	1140	1150	1160	1170	1180	1190	1200
VCRAPSVANP	VRSPPELGER	PDELGFVMDN	VRSLLVLNST	SFLYYPDPVL	EPLSPTGLLE	LKPSSPLILK	GRNLLPPAPG
1210	1220	1230	1240	1250	1260	1270	1280
NSRLNYTVLI	GSTPCTLTVS	ETQLLCEAPN	LTGQHKVTVR	AGGFEFSPGT	LQVYSDSLLT	LPAIVGIGGG	GGLLLLVIVA
1290	1300	1310	1320	1330	1340	1350	1360
VLIAYKRKSR	DADRTLKRLQ	LQMDNLESRV	ALECKEAFAE	LQTDIHELTN	DLDGAGIPFL	DYRTYAMRVL	FPGIEDHPVL
1370	1380	1390	1400	1410	1420	1430	1440
KEMEVQANVE	KSLTLFGQLL	TKKHFLLTFI	RTLEAQRSFS	MRDRGNVASL	IMTALQGEME	YATGVLKQLL	SDLIEKNLES
1450	1460	1470	1480	1490	1500	1510	1520
KNHPKLLLRR	TESVAEKMLT	NWFTFLLYKF	LKECAGEPLF	MLYCAIKQQM	EKGPIDAITG	EARYSLSEDK	LIRQQIDYKT
1530	1540	1550	1560	1570	1580	1590	1600
LTLNCVNPEN	ENAPEVPVKG	LDCDTVTQAK	EKLLDAAYKG	VPYSQRPKAA	DMDLEWRQGR	MARIILQDED	VTTKIDNDWK
1610	1620	1630	1640	1650	1660	1670	1680
RLNTLAHYQV	TDGSSVALVP	KQTSAYNISN	SSTFTKSLSR	YESMLRTASS	PDSLRSRTPM	ITPDLESGTK	LWHLVKNHDH
1690	1700	1710	1720	1730	1740	1750	1760
LDQREGDRGS	KMVSEIYLTR	LLATKKFVDD	LFETIFSTAH	RGSALPLAIK	YMFDFLDEQA	DKHQIHDADV	RHTWKSNCLP
1770	1780	1790	1800	1810	1820	1830	1840
LRFWVNVIKN	PQFVFDIHKN	SITDACLSVV	AQTFMDSCST	SEHKLGKDSP	SNKLLYAKDI	PNYKSWVERY	YADIAKMPAI
1850	1860	1870	1880	1890	1900	1910	1920
SDQDMSAYLA	EQSRLHLSQF	NSMSALHEIY	SYITKYKDEI	LAALEKDEQA	RRQRLRSKLE	QVVDTMALSS	

Cmpd.	No. of Cmpds.	m/z meas.	Δ m/z [ppm]	z	Rt [min]	Score	P	Range	Sequence	Modification

Detailed Protein Report

Cmpd.	No. of Cmpds.	m/z meas.	Δ m/z [ppm]	z	Rt [min]	Score	P	Range	Sequence	Modification
1881	1	628.2724	-43.47	2	53.9	15.1	0	1-11	-.MMLTPAGPEHR.G	Oxidation: 1

Detailed Protein Report

Protein 460: PREDICTED: mucin-6 isoform X1, partial [Homo sapiens]

**Accession:**	gi|578841512	**Score:**	44.4
**Database:**	refseq_human(refseq_human_20140103.fasta)	**MW [kDa]:**	194.5
**Database Date:**	2015-11-30	**pI:**	6.9
**Modification(s):**	Carbamidomethyl	**Sequence Coverage [%]:**	1.5
		**No. of unique Peptides:**	3

10	20	30	40	50	60	70	80
MVQRWLLLSC	CGALLSAGLA	NTSYTSPGLQ	RLKDSPQTAP	DKGQCSTWGA	GHFSTFDHHV	YDFSGTCNYI	FAATCKDAFP
90	100	110	120	130	140	150	160
TFSVQLRRGP	DGSISRIIVE	LGASVVTVSE	AIISVKDIGV	ISLPYTSNGL	QITPFGQSVR	LVAKQLELEL	EVVWGPDSHL
170	180	190	200	210	220	230	240
MVLVERKYMG	QMCGLCGNFD	GKVTNEFVSE	EGKFLEPHKF	AALQKLDDPG	EICTFQDIPS	THVRQAQHAR	ICTQLLTLVA
250	260	270	280	290	300	310	320
PECSVSKEPF	VLSCQADVAA	APQPGPQNSS	CATLSEYSRQ	CSMVGQPVRR	WRSPGLCSVG	QCPANQVYQE	CGSACVKTCS
330	340	350	360	370	380	390	400
NPQHSCSSSC	TFGCFCPEGT	VLNDLSNNHT	CVPVTQCPCV	LHGAMYAPGE	VTIAACQTCR	CTLGRWVCTE	RPCPGHCSLE
410	420	430	440	450	460	470	480
GGSFVTTFDA	RPYRFHGTCT	YILLQSPQLP	EDGALMAVYD	KSGVSHSETS	LVAVVYLSRQ	DKIVISQDEV	VTNNGEAKWL
490	500	510	520	530	540	550	560
PYKTRNITVF	RQTSTHLQMA	TSFGLELVVQ	LRPIFQAYVT	VGPQFRGQTR	GLCGNFNGDT	TDDFTTSMGI	AEGTASLFVD
570	580	590	600	610	620	630	640
SWRAGNCPAA	LERETDPCSM	SQLNKVCAET	HCSMLLRTGT	VFERCHATVN	PAPFYKRCVY	QACNYEETFP	HICAALGDYV
650	660	670	680	690	700	710	720
HACSLRGVLL	WGWRSSVDNC	TIPCTGNTTF	SYNSQACERT	CLSLSDRATE	CHHSAVPVDG	CNCPDGTYLN	QKGECVRKAQ
730	740	750	760	770	780	790	800
CPCILEGYKF	ILAEQSTVIN	GITCHCINGR	LSCPQRPQMF	LASCQAPKTF	KSCSQSSENK	FGAACAPTCQ	MLATGVACVP
810	820	830	840	850	860	870	880
TKCEPGCVCA	EGLYENADGQ	CVPPEECPCE	FSGVSYPGGA	ELHTDCRTCS	CSRGRWACQQ	GTHCPSTCTL	YGEGHVITFD
890	900	910	920	930	940	950	960
GQRFVFDGNC	EYILATDVCG	VNDSQPTFKI	LTENVICGNS	GVTCSRAIKI	FLGGLSVVLA	DRNYTVTGEE	PHVQLGVTPG
970	980	990	1000	1010	1020	1030	1040
ALSLVVDISI	PGRYNLTLIW	NRHMTILIRI	ARASQDPLCG	LCGNFNGNMK	DDFETRSRYV	ASSELELVNS	WKESPLCGDV
1050	1060	1070	1080	1090	1100	1110	1120
SFVTDPCSLN	AFRRSWAERK	CSVINSQTFA	TCHSKVYHLP	YYEACVRDAC	GCDSGGDCEC	LCDAVAAYAQ	ACLDKGVCVD
1130	1140	1150	1160	1170	1180	1190	1200
WRTPAFCPIY	CGFYNTHTQD	GHGEYQYTQE	ANCTWHYQPC	LCPSQPQSVP	GSNIEGCYNC	SQDEYFDHEE	GVCVPCMPPT
1210	1220	1230	1240	1250	1260	1270	1280
TPQPPTTPQL	PTTGSRPTQV	WPMTGTSTTI	GLLSSTGPSP	SSNHTPASPT	QTPLLPATLT	SSKPTASSGE	PPRPTTAVTP
1290	1300	1310	1320	1330	1340	1350	1360
QATSGLPPTA	TLRSTATKPT	VTQATTRATA	STASPATTST	AQSTTRTTMT	LPTPATSGTS	PTLPKSTNQE	LPGTTATQTT
1370	1380	1390	1400	1410	1420	1430	1440
GPRPTPASTT	GPTTPQPGQP	TRPTATETTQ	TRTTTEYTTP	QTPHTTHSPP	TAGSPVPSTG	PVTATSFHAT	TTYPTPSHPE
1450	1460	1470	1480	1490	1500	1510	1520
TTLPTHVPPF	STSLVTPSTH	TVITPTHAQM	ATSASNHSAP	TGTIPPPTTL	KATGSTHTAP	PITPTTSGTS	QAHSSFSTNK
1530	1540	1550	1560	1570	1580	1590	1600
TPTSLHSHTS	STHHPEVTPT	STTTITPNPT	STRTRTPVAH	TNSATSSRPP	PPFTTHSPPT	GSSPFSSTGP	MTATSFKTTT
1610	1620	1630	1640	1650	1660	1670	1680
TYPTPSLPQT	TLLTHVPPFS	TSLVTPITHT	VITPTHPQMS	TSAYIHSTPT	GTIAPPTTVK	ATRSTYTAPL	MTATTSRISQ
1690	1700	1710	1720	1730	1740	1750	1760
AHSSISTAKT	STSLHSHASS	THHPEVTPTS	TTNVTPKSTS	RGTSTPVTHT	TSATSSRPPT	PITTHSSPTR	SSPLSSTGRM
1770	1780	1790	1800	1810	1820	1830	
TATSLKTTTT	YPTPSQAHIT	LPIHVPPFST	SSVTPSTHTV	ITPTHPQMST	SASNHSTSTG	TIPPL	

Detailed Protein Report

Cmpd.	No. of Cmpds.	m/z meas.	Δ m/z [ppm]	z	Rt [min]	Score	P	Range	Sequence	Modification
748	1	1010.1872	71.34	3	39.3	11.6	2	167-193	R.KYMGQMCGLCGNFDGKVTNEFVSEEGK.F	Carbamidomethyl: 7
2899	4	840.9173	97.31	2	65.0	13.7	0	168-182	K.YMGQMCGLCGNFDGK.V	Carbamidomethyl: 6
1896	4	840.7895	-54.74	2	53.8	19.2	0	168-182	K.YMGQMCGLCGNFDGK.V	Carbamidomethyl: 9

Detailed Protein Report

Protein 461: ATP-binding cassette sub-family A member 13 [Homo sapiens]

**Accession:**	gi|31657092	**Score:**	44.3
**Database:**	refseq_human(refseq_human_20140103.fasta)	**MW [kDa]:**	575.8
**Database Date:**	2015-11-30	**pI:**	6.0
		**Sequence Coverage [%]:**	0.8
		**No. of unique Peptides:**	1

Detailed Protein Report

10	20	30	40	50	60	70	80
MGHAGCQFKA	LLWKNWLCRL	RNPVLFLAEF	FWPCILFVIL	TVLRFQEPPR	YRDICYLQPR	DLPSCGVIPF	VQSLLCNTGS
90	100	110	120	130	140	150	160
RCRNFSYEGS	MEHHFRLSRF	QTAADPKKVN	NLAFLKEIQD	LAEEIHGMMD	KAKNLKRLWV	ERSNTPDSSY	GSSFFTMDLN
170	180	190	200	210	220	230	240
KTEEVILKLE	SLHQQPHIWD	FLLLLPRLHT	SHDHVEDGMD	VAVNLLQTIL	NSLISLEDLD	WLPLNQTFSQ	VSELVLNVTI
250	260	270	280	290	300	310	320
STLTFLQQHG	VAVTEPVYHL	SMQNIVWDPQ	KVQYDLKSQF	GFDDLHTEQI	LNSSAELKEI	PTDTSLEKMV	CSVLSSTSED
330	340	350	360	370	380	390	400
EAEKWGHVGG	CHPKWSEAKN	YLVHAVSWLR	VYQQVFVQWQ	QGSLLQKTLT	GMGHSLEALR	NQFEEESKPW	KVVEALHTAL
410	420	430	440	450	460	470	480
LLLNDSLSAD	GPKDNHTFPK	ILQHLWKLQS	LLQNLPQWPA	LKRFLQLDGA	LRNAIAQNLH	FVQEVLICLE	TSANDFKWFE
490	500	510	520	530	540	550	560
LNQLKLEKDV	FFWELKQMLA	KNAVCPNGRF	SEKEVFLPPG	NSSIWGGLQG	LLCYCNSSET	SVLNKLLGSV	EDADRILQEV
570	580	590	600	610	620	630	640
ITWHKNMSVL	IPEEYLDWQE	LEMQLSEASL	SCTRLFLLLG	ADPSPENDVF	SSDCKHQLVS	TVIFHTLEKT	QFFLEQAYYW
650	660	670	680	690	700	710	720
KAFKKFIRKT	CEVAQYVNMQ	ESFQNRLLAF	PEESPCFEEN	MDWKMISDNY	FQFLNNLLKS	PTASISRALN	FTKHLLMMEK
730	740	750	760	770	780	790	800
KLHTLEDEQM	NFLLSFVEFF	EKLLLPNLFD	SSIVPSFHSL	PSLTEDILNI	SSLWTNHLKS	LKRDPSATDA	QKLLEFGNEV
810	820	830	840	850	860	870	880
IWKMQTLGSH	WIRKEPKNLL	RFIELILFEI	NPKLLELWAY	GISKGKRAKL	ENFFTLLNFS	VPENEILSTS	FNFSQLFHSD
890	900	910	920	930	940	950	960
WPKSPAMNID	FVRLSEAIIT	SLHEFGFLEQ	EQISEALNTV	YAIRNASDLF	SALSEPQKQE	VDKILTHIHL	NVFQDKDSAL
970	980	990	1000	1010	1020	1030	1040
LLQIYSSFYR	YIYELLNIQS	RGSSLTFLTQ	ISKHILDIIK	QFNFQNISKA	FAFLFKTAEV	LGGISNVSYC	QQLLSIFNFL
1050	1060	1070	1080	1090	1100	1110	1120
ELQAQSFMST	EGQELEVIHT	TLTGLKQLLI	IDEDFRISLF	QYMSQFFNSS	VEDLLDNKCL	ISDNKHISSV	NYSTSEESSF
1130	1140	1150	1160	1170	1180	1190	1200
VFPLAQIFSN	LSANVSVFNK	FMSIHCTVSW	LQMWTEIWET	ISQLFKFDMN	VFTSLHHGFT	QLLDELEDDV	KVSKSCQGIL
1210	1220	1230	1240	1250	1260	1270	1280
PTHNVARLIL	NLFKNVTQAN	DFHNWEDFLD	LRDFLVALGN	ALVSVKKLNL	EQVEKSLFTM	EAALHQLKTF	PFNESTSREF
1290	1300	1310	1320	1330	1340	1350	1360
LNSLLEVFIE	FSSTSEYIVR	NLDSINDFLS	NNLTNYGEKF	ENIITELREA	IVFLRNVSHD	RDLFSCADIF	QNVTECILED
1370	1380	1390	1400	1410	1420	1430	1440
GFLYVNTSQR	MLRILDTLNS	TFSSENTISS	LKGCIVWLDV	INHLYLLSNS	SFSQGHLQNI	LGNFRDIENK	MNSILKIVTW
1450	1460	1470	1480	1490	1500	1510	1520
VLNIKKPLCS	SNGSHINCVN	IYLKDVTDFL	NIVLTTVFEK	EKKPKFEILL	ALLNDSTKQV	RMSINNLTTD	FDFASQSNWR
1530	1540	1550	1560	1570	1580	1590	1600
YFTELILRPI	EMSDEIPNQF	QNIWLHLITL	GKEFQKLVKG	IYFNILENNS	SSKTENLLNI	FATSPKEKDV	NSVGNSIYHL
1610	1620	1630	1640	1650	1660	1670	1680
ASYLAFSLSH	DLQNSPKIII	SPEIMKATGL	GIQLIRDVFN	SLMPVVHHTS	PQNAGYMQAL	KKVTSVMRTL	KKADIDLLVD
1690	1700	1710	1720	1730	1740	1750	1760
QLEQVSVNLM	DFFKNISSVG	TGNLVVNLLV	GLMEKFADSS	HSWNVNHLLQ	LSRLFPKDVV	DAVIDVYYVL	PHAVRLLQGV
1770	1780	1790	1800	1810	1820	1830	1840
PGKNITEGLK	DVYSFTLLHG	ITISNITKED	FAIVIKILLD	TIELVSDKPD	IISEALACFP	VVWCWNHTNS	GFRQNSKIDP
1850	1860	1870	1880	1890	1900	1910	1920
CNVHGLMSSS	FYGKVASILD	HFHLSPQGED	SPCSNESSRM	EITRKVVCII	HELVDWNSIL	LELSEVFHVN	ISLVKTVQKF
1930	1940	1950	1960	1970	1980	1990	2000
WHKILPFVPP	SINQTRDSIS	ELCPSGSIKQ	VALQIIEKLK	NVNFTKVTSG	ENILDKLSSL	NKILNINEDT	ETSVQNIISS
2010	2020	2030	2040	2050	2060	2070	2080
NLERTVQLIS	EDWSLEKSTH	NLLSLFMMLQ	NANVTGSSLE	ALSSFIEKSE	TPYNFEELWP	KFQQIMKDLT	QDFRIRHLLS
2090	2100	2110	2120	2130	2140	2150	2160
EMNKGIKSIN	SMALQKITLQ	FAHFLEILDS	PSLKTLEIIE	DFLLVTKNWL	QEYANEDYSR	MIETLFIPVT	NESSTEDIAL
2170	2180	2190	2200	2210	2220	2230	2240
LAKAIATFWG	SLKNISRAGN	FDVAFLTHLL	NQEQLTNFSV	VQLLFENILI	NLINNLAGNS	QEAAWNLNDT	DLQIMNFINL
2250	2260	2270	2280	2290	2300	2310	2320
ILNHMQSETS	RKTVLSLRSI	VDFTEQFLKT	FFSLFLKEDS	ENKISLLLKY	FHKDVIAEMS	FVPKDKILEI	LKLDQFLTLM
2330	2340	2350	2360	2370	2380	2390	2400

Detailed Protein Report

Cmpd.	No. of Cmpds.	m/z meas.	Δ m/z [ppm]	z	Rt [min]	Score	P	Range	Sequence	Modification
2092	1	1030.3822	-211.70	1	56.3	15.0	1	3972-3981	K.QTRALSGGLK.R	

Detailed Protein Report

Protein 462: intraflagellar transport protein 122 homolog isoform 7 [Homo sapiens]

**Accession:**	gi|526253060	**Score:**	44.3
**Database:**	refseq_human(refseq_human_20140103.fasta)	**MW [kDa]:**	118.4
**Database Date:**	2015-11-30	**pI:**	6.8
		**Sequence Coverage [%]:**	3.0
		**No. of unique Peptides:**	2

Quantitation

Q:W	**Median:** 0.29	**CV:** 0.00 %	**No. of Peptides:**	1
M:Q	**Median:** 2.22	**CV:** 12.87 %	**No. of Peptides:**	2

10	20	30	40	50	60	70	80
MFNGIISIRN	KNGEEKVKIE	RPGGSLSPIW	SICWNPSREE	RNDILAVADW	GQKVSFYQLS	GKQIGKDRAL	NFDPCCISYF
**90**	**100**	**110**	**120**	**130**	**140**	**150**	**160**
TKGEYILLGG	SDKQVSLFTK	DGVRLGTVGE	QNSWVWTCQA	KPDSNYVVVG	CQDGTISFYQ	LIFSTVHGLY	KDRYAYRDSM
**170**	**180**	**190**	**200**	**210**	**220**	**230**	**240**
TDVIVQHLIT	EQKVRIKCKE	LVKKIAIYRN	RLAIQLPEKI	LIYELYSEDL	SDMHYRVKEK	IIKKFECNLL	VVCANHIILC
**250**	**260**	**270**	**280**	**290**	**300**	**310**	**320**
QEKRLQCLSF	SGVKEREWQM	ESLIRYIKVI	GGPPGREGLL	VGLKNGQILK	IFVDNLFAIV	LLKQATAVRC	LDMSASRKKL
**330**	**340**	**350**	**360**	**370**	**380**	**390**	**400**
AVVDENDTCL	VYDIDTKELL	FQEPNANSVA	WNTQCEDMLC	FSGGGYLNIK	ASTFPVHRQK	LQGFVVGYNG	SKIFCLHVFS
**410**	**420**	**430**	**440**	**450**	**460**	**470**	**480**
ISAVEVPQSA	PMYQYLDRKL	FKEAYQIACL	GVTDTDWREL	AMEALEGLDF	ETAKKAFIRV	QDLRYLELIS	SIEERKKRGE
**490**	**500**	**510**	**520**	**530**	**540**	**550**	**560**
TNNDLFLADV	FSYQGKFHEA	AKLYKRSGHE	NLALEMYTDL	CMFEYAKDFL	GSGDPKETKM	LITKQADWAR	NIKEPKAAVE
**570**	**580**	**590**	**600**	**610**	**620**	**630**	**640**
MYISAGEHVK	AIEICGDHGW	VDMLIDIARK	LDKAEREPLL	LCATYLKKLD	SPGYAAETYL	KMGDLKSLVQ	LHVETQRWDE
**650**	**660**	**670**	**680**	**690**	**700**	**710**	**720**
AFALGEKHPE	FKDDIYMPYA	QWLAENDRFE	EAQKAFHKAG	RQREAVQVLE	QLTNNAVAES	RFNDAAYYYW	MLSMQCLDIA
**730**	**740**	**750**	**760**	**770**	**780**	**790**	**800**
QDPAQKDTML	GKFYHFQRLA	ELYHGYHAIH	RHTEDPFSVH	RPETLFNISR	FLLHSLPKDT	PSGISKVKIL	FTLAKQSKAL
**810**	**820**	**830**	**840**	**850**	**860**	**870**	**880**
GAYRLARHAY	DKLRGLYIPA	RFQKSIELGT	LTIRAKPFHD	SEELVPLCYR	CSTNNPLLNN	LGNVCINCRQ	PFIFSASSYD
**890**	**900**	**910**	**920**	**930**	**940**	**950**	**960**
VLHLVEFYLE	EGITDEEAIS	LIDLEVLRPK	RDDRQLEIAN	NSSQILRLVE	TKDSIGDEDP	FTAKLSFEQG	GSEFVPVVVS
**970**	**980**	**990**	**1000**	**1010**	**1020**	**1030**	**1040**
RLVLRSMSRR	DVLIKRWPPP	LRWQYFRSLL	PDASITMCPS	CFQMFHSEDY	ELLVLQHGCC	PYCRRCKDDP	GP

Cmpd.	No. of Cmpds.	m/z meas.	Δ m/z [ppm]	z	Rt [min]	Score	P	Range	Sequence	Modification	Ratios	
2627	3	665.3628	29.07	2	61.5	19.4	0	42-53	R.NDILAVADWGQK.V		M:Q	1.95
2085	1	731.3255	-153.98	2	56.2	10.9	2	813-824	K.LRGLYIPARFQK.S		Q:W<br>M:Q	0.29<br>2.52

Detailed Protein Report

Protein 463: R-spondin-1 isoform 3 precursor [Homo sapiens]

Accession: gi|339276103
Database: refseq_human(refseq_human_20140103.fasta)
Database Date: 2015-11-30
Modification(s): Carbamidomethyl, Oxidation

Score: 44.1
MW [kDa]: 21.8
pI: 11.3
Sequence Coverage [%]: 32.0
No. of unique Peptides: 2

10	20	30	40	50	60	70	80
MRLGLCVVAL	VLSWTHLTIS	SRGIKGKRQR	RISAEGSQAC	AKGCELCSEV	NGCLKCSPKL	FILLERNDIR	QVGVCLPSCP
**90**	**100**	**110**	**120**	**130**	**140**	**150**	**160**
PGYFDARNPD	MNKCIKCKIE	HCEACFSHNF	CTKCKEGLYL	HKGRCYPACP	EGSSAANGTM	ECSSPGQKRR	KGGQGRRENA
**170**	**180**	**190**	**200**	**210**			
NRNLARKESK	EAGAGSRRRK	GQQQQQQGT	VGPLTSAGPA				

Cmpd.	No. of Cmpds.	m/z meas.	Δ m/z [ppm]	z	Rt [min]	Score	P	Range	Sequence	Modification
2673	1	913.9847	93.98	2	63.5	12.9	1	43-59	K.GCELCSEVNGCLKCSPK.L	Carbamidomethyl: 2
1684	1	1273.9976	-19.18	2	50.8	10.8	1	125-149	R.CYPACPEGSSAANGTMECSSPGQKR.R	Oxidation: 16

Detailed Protein Report

Protein 464: centrosome-associated protein 350 [Homo sapiens]

**Accession:**	gi|171184451	**Score:**	44.0
**Database:**	refseq_human(refseq_human_20140103.fasta)	**MW [kDa]:**	350.7
**Database Date:**	2015-11-30	**pI:**	5.9
		**Sequence Coverage [%]:**	1.5
		**No. of unique Peptides:**	2

Quantitation

Q:W	**Median:**	1.24	**CV:**	0.00 %	**No. of Peptides:**	1
M:Q	**Median:**	0.80	**CV:**	0.00 %	**No. of Peptides:**	1

Detailed Protein Report

10	20	30	40	50	60	70	80
MRSSKSKEVP	LPNPRNSQSK	DTVQADITTS	WDALSQTKAA	LRHIENKLEV	APTSTAVCDS	VMDTKKSSTS	ATRKISRKDG
90	100	110	120	130	140	150	160
RYLDDSWVNA	PISKSTKSRK	EKSRSPLRAT	TLESNVKKNN	RVEFREPLVS	YREIHGAPSN	FSSSHLESKH	VYCVDVNEEK
170	180	190	200	210	220	230	240
TESGNWMIGS	REERNIRSCD	FESSQSSVIN	DTVVRFLNDR	PAIDALQNSE	CLIRMGASMR	TEEEMPNRTK	GSENNLKLSV
250	260	270	280	290	300	310	320
NNMAHDTDPK	ALRLTDSSPS	STSTSNSQRL	DILKRRQHDV	KLEKLKERIR	KQWEHSEETN	GRGQKLGHID	HPVMVVNVDN
330	340	350	360	370	380	390	400
SVTAKVRKVA	TAPPAPAYKG	FNPSETKIRT	PDGKVWQEAE	FQNMSRELYR	DLALHFADDI	SIKEKPAEKS	KEKKVVKPVR
410	420	430	440	450	460	470	480
KVQKVAQLSS	TECRTGSSHL	ISTSSWRDGQ	KLVKKILGPA	PRMEPKEQRT	ASSDRGGRER	TAKSGGHIGR	AESDPRLDVL
490	500	510	520	530	540	550	560
HRHLQRNSER	SRSKSRSENN	IKKLASSLPD	NKQEENTALN	KDFLPIEIRG	ILDDLQLDST	AHTAKQDTVE	LQNQKSSAPV
570	580	590	600	610	620	630	640
HAPRSHSPVK	RKPDKITANE	DPPVISKRRH	YDTDEVRQYI	VRQQEERKRK	QNEEKKAQKE	ATEQKNKRLQ	ELYRKQKEAF
650	660	670	680	690	700	710	720
TKVKNVPPSE	PSATRRLQET	YSKLLLEKTL	LEEPSHQHVT	QETQAKPGYQ	PSGESDKENK	VQERPPSASS	SSDMSLSEPP
730	740	750	760	770	780	790	800
QPLARKDLME	STWMQPERLS	PQVHHSQPQP	FAGTAGSLLS	HLLSLEHVGI	LHKDFESILP	TRKNHNMASR	PLTFTPQPYV
810	820	830	840	850	860	870	880
TSPAAYTDAL	LKPSASQYKS	KLDRIEALKA	TAASLSSRIE	SEAKKLAGAS	INYGSAWNTE	YDVQQAPQED	GPWTKAVTPP
890	900	910	920	930	940	950	960
VKDDNEDVFS	ARIQKMLGSC	VSHATFDDDL	PGVGNLSEFK	KLPEMIRPQS	AISSFRVRSP	GPKPEGLLAQ	LCKRQTDSSS
970	980	990	1000	1010	1020	1030	1040
SDMQACSQDK	AKISLGSSID	SVSEGPLLSE	GSLSEEEGDQ	DGQPLLKVAE	ILKEKEFCPG	ERNSYEPIKE	FQKEAEKFLP
1050	1060	1070	1080	1090	1100	1110	1120
LFGHIGGTQS	KGPWEELAKG	SPHSVINIFT	KSYQLYGKGF	EDKLDRGTST	SRPLNATATP	LSGVSYEDDF	VSSPGTGTST
1130	1140	1150	1160	1170	1180	1190	1200
EKKSTLEPHS	TLSPQEDHSN	RKSAYDPSSV	DVTSQHSSGA	QSAASSRSST	SSKGKKGKKE	KTEWLDSFTG	NVQNSLLDEE
1210	1220	1230	1240	1250	1260	1270	1280
KAERGSHQGK	KSGTSSKLSV	KDFEQTLDTD	STLEDLSGHS	VSVSSDKGRS	QKTPTSPLSP	SSQKSLQFDV	AGTSSERSKS
1290	1300	1310	1320	1330	1340	1350	1360
SVMPPTITGF	KPNAPLTDLN	PAASRTTTEN	MAPIPGSKRF	SPAGLHHRMA	AELSYLNAIE	ESVRQLSDVE	RVRGISLAQQ
1370	1380	1390	1400	1410	1420	1430	1440
ESVSLAQIIK	AQQQRHERDL	ALLKLKAEQE	ALESQRQLEE	TRNKAAQVHA	ESLQQVVQSQ	REVTEVLQEA	TCKIAAQQSE
1450	1460	1470	1480	1490	1500	1510	1520
TARLTTDAAR	QICEMAELTR	THISDAVVAS	GAPLAILYDH	QRQHLPDFVK	QLRTRTETDR	KSPSVSLSQS	KEGTLDSKHQ
1530	1540	1550	1560	1570	1580	1590	1600
KYSASYDSYS	ESSGYKNHDR	RSSSGSSRQE	SPSVPSCKEN	EKKLNGEKIE	SSIDEQVQTA	ADDSLRSDSV	PSLPDEKDST
1610	1620	1630	1640	1650	1660	1670	1680
SIATEYSLKF	DESMTEDEIE	EQSFRSLLPS	ESHRRFNMEK	RRGHHDDSDE	EASPEKTTLS	TAKELNMPFS	GGQDSFSKFT
1690	1700	1710	1720	1730	1740	1750	1760
MEMVRQYMKE	EEMRAAHQSS	LLRLREKALK	EKTKAELAWL	EHQKKHLRDK	GEDDKMPPLR	KKQRGLLLRL	QQEKAEIKRL
1770	1780	1790	1800	1810	1820	1830	1840
QEANKAARKE	RQLILKQQEE	IEKIRQTTIK	LQEKLKSAGE	SKLDSHSDDD	TKDNKATSPG	PTDLETRSPS	PISISSSETS
1850	1860	1870	1880	1890	1900	1910	1920
SIMQKLKKMR	SRMDEKFLTK	REQKLMQRRQ	HAEELLEWKR	RLDAEEAEIR	QMEKQALAAW	DKELIKPKTP	KKELEDQRTE
1930	1940	1950	1960	1970	1980	1990	2000
QKEIASEEES	PVPLYSHLNS	ESSIPEELGS	PAVEYVPSES	IGQEQPGSPD	HSILTEEMIC	SQELESSTSP	SKHSLPKSCT
2010	2020	2030	2040	2050	2060	2070	2080
SVSKQESSKG	SHRTGGQCHL	PIKSHQHCYS	WSDESLSMTQ	SETTSDQSDI	EGRIRALKDE	LRKRKSVVNQ	LKKEQKKRQK
2090	2100	2110	2120	2130	2140	2150	2160
ERLKAQEASL	IKQLESYDEF	IKKTEAELSQ	DLETSPTAKP	QIKTLSSASE	KPKIKPLTPL	HRSETAKNWK	SLTESERSRG
2170	2180	2190	2200	2210	2220	2230	2240

Detailed Protein Report

Cmpd.	No. of Cmpds.	m/z meas.	Δ m/z [ppm]	z	Rt [min]	Score	P	Range	Sequence	Modification	Ratios
2849	14	973.4915	-13.36	2	64.3	20.6	1	329-347	K.VATAPPAPAYKGFNPSETK.I		
316	1	966.9340	-27.85	2	33.3	11.1	2	1795-1812	K.LKSAGESKLDSHSDDDTK.D		M:Q 0.80<br>Q:W 1.24

Detailed Protein Report

Protein 465: **PREDICTED: autoimmune regulator isoform X1 [Homo sapiens]**

**Accession:**	gi|530419216	**Score:**	44.0
**Database:**	refseq_human(refseq_human_20140103.fasta)	**MW [kDa]:**	36.5
**Database Date:**	2015-11-30	**pI:**	6.4
**Modification(s):**	Carbamidomethyl	**Sequence Coverage [%]:**	14.9
		**No. of unique Peptides:**	2

10	20	30	40	50	60	70	80
MWLVYSSGAP	GTQQPARNRV	FFPIGMAPGG	VCWRPDGWGT	GGQGRISGPG	SMGAGQRLGS	SGTQRCCWGS	CFGKEVALRR
**90**	**100**	**110**	**120**	**130**	**140**	**150**	**160**
VLHPSPVCMG	VSCLCQKNED	ECAVCRDGGE	LICCDGCPRA	FHLACLSPPL	REIPSGTWRC	SSCLQATVQE	VQPRAEEPRP
**170**	**180**	**190**	**200**	**210**	**220**	**230**	**240**
QEPPVETPLP	PGLRSAGEEV	RGPPGEPLAG	MDTTLVYKHL	PAPPSAAPLP	GLDSSALHPL	LCVGPEGQQN	LAPGARCGVC
**250**	**260**	**270**	**280**	**290**	**300**	**310**	**320**
GDGTDVLRCT	HCAAAFHWRC	HFPAGTSRPG	TGLRCRSCSG	DVTPAPVEGV	LAPSPARLAP	GPAKDDTASH	EPALHRDDLE
**330**	**340**	**350**					
SLLSEHTFDG	ILQWAIQSMA	RPAAPFPS					

Cmpd.	No. of Cmpds.	m/z meas.	Δ m/z [ppm]	z	Rt [min]	Score	P	Range	Sequence	Modification
1699	1	1011.4214	-66.62	3	51.2	12.9	1	18-45	R.NRVFFPIGMAPGGVCWRPDGWGTGGQGR.I	Carbamidomethyl: 15
1351	1	669.2815	20.48	2	45.7	14.6	0	107-119	R.DGGELICCDGCPR.A	

Detailed Protein Report

Protein 466: **PREDICTED: exosome complex exonuclease RRP44 isoform X1 [Homo sapiens]**

Accession: gi|530402172
Database: refseq_human(refseq_human_20140103.fasta)
Database Date: 2015-11-30

Score: 44.0
MW [kDa]: 90.5
pI: 6.5
Sequence Coverage [%]: 3.5
No. of unique Peptides: 1

10	20	30	40	50	60	70	80
MSADNQLQVI	FITNDRRNKE	KAIEEGIPAF	TCEEYVKSLT	ANPELIDRLA	CLSEEGNEIE	SGKIIFSEHL	PLSKLQQGIK
**90**	**100**	**110**	**120**	**130**	**140**	**150**	**160**
SGTYLQGTFR	ASRENYLEAT	VWIHGDNEEN	KEIILQGLKH	LNRAVHEDIV	AVELLPKSQW	VAPSSVVLHD	EGQNEEDVEK
**170**	**180**	**190**	**200**	**210**	**220**	**230**	**240**
EEETERMLKT	AVSEKMLKPT	GRVVGIIKRN	WRPYCGMLSK	SDIKESRRHL	FTPADKRIPR	IRIETRQAST	LEGRRIIVAI
**250**	**260**	**270**	**280**	**290**	**300**	**310**	**320**
DGWPRNSRYP	NGHFVRNLGD	VGEKETETEV	LLLEHDVPHQ	PFSQAVLSFL	PKMPWSITEK	DMKNREDLRH	LCICSVDPPG
**330**	**340**	**350**	**360**	**370**	**380**	**390**	**400**
CTDIDDALHC	RELENGNLEV	GVHIADVSHF	IRPGNALDQE	SARRGTTVYL	CEKRIDMVPE	LLSSNLCSLK	CDVDRLAFSC
**410**	**420**	**430**	**440**	**450**	**460**	**470**	**480**
IWEMNHNAEI	LKTKFTKSVI	NSKASLTYAE	AQLRIDSANM	NDDITTSLRG	LNKLAKILKK	RRIEKGALTL	SSPEVRFHMD
**490**	**500**	**510**	**520**	**530**	**540**	**550**	**560**
SETHDPIDLQ	TKELRETNSM	VEEFMLLANI	SVAKKIHEEF	SEHALLRKHP	APPPSNYEIL	VKAARSRNLE	IKTDTAKSLA
**570**	**580**	**590**	**600**	**610**	**620**	**630**	**640**
ESLDQAESPT	FPYLNTLLRI	LATRCMMQAV	YFCSGMDNDF	HHYGLASPIY	THFTSPIRRY	ADVIVHRLLA	VAIGADCTYP
**650**	**660**	**670**	**680**	**690**	**700**	**710**	**720**
ELTDKHKLAD	ICKNLNFRHK	MAQYAQRASV	AFHTQLFFKS	KGIVSEEAYI	LFVRKNAIVV	LIPKYGLEGT	VFFEEKDKPN
**730**	**740**	**750**	**760**	**770**	**780**	**790**	**800**
PQLIYDDEIP	SLKIEDTVFH	VFDKVKVKIM	LDSSNLQHQK	IRMSLVEPQI	PGISIPTDTS	NMDLNGPKKK	KMKLGK

Cmpd.	No. of Cmpds.	m/z meas.	Δ m/z [ppm]	z	Rt [min]	Score	P	Range	Sequence	Modification
1167	1	977.5217	29.45	2	44.2	19.3	2	189-204	K.RNWRPYCGMLSKSDIK.E	

Detailed Protein Report

Protein 467: kinesin-like protein KIF6 isoform 3 [Homo sapiens]

**Accession:**	gi|573459724	**Score:**	44.0
**Database:**	refseq_human(refseq_human_20140103.fasta)	**MW [kDa]:**	86.2
**Database Date:**	2015-11-30	**pI:**	6.0
**Modification(s):**	Oxidation	**Sequence Coverage [%]:**	5.7
		**No. of unique Peptides:**	2

Quantitation

Q:W	**Median:**	1.54	**CV:**	0.00 %	**No. of Peptides:**	1
M:Q	**Median:**	0.73	**CV:**	0.00 %	**No. of Peptides:**	1

10	20	30	40	50	60	70	80
MVKQTIQIFA	RVKPPVRKHQ	QGIYSIDEDE	KLIPSLEIIL	PRDLADGFVN	NKRESYKFKF	QRIFDQDANQ	ETVFENIAKP
**90**	**100**	**110**	**120**	**130**	**140**	**150**	**160**
VAGSVLAGYN	GTIFAYGQTG	SGKTFTITGG	AERYSDRGII	PRTLSYIFEQ	LQKDSSKIYT	THISYLEIYN	ECGYDLLDPR
**170**	**180**	**190**	**200**	**210**	**220**	**230**	**240**
HEASSLEDLP	KVTILEDPDQ	NIHLKNLTLH	QATTEEEALN	LLFLGDTNRM	IAETPMNQAS	TRSHCIFTIH	LSSKEPGSAT
**250**	**260**	**270**	**280**	**290**	**300**	**310**	**320**
VRHAKLHLVD	LAGSERVAKT	GVGGHLLTEA	KYINLSLHYL	EQVIIALSEK	HRSHIPYRNS	MMTSVLRDSL	GGNCMTTMIA
**330**	**340**	**350**	**360**	**370**	**380**	**390**	**400**
TLSLEKRNLD	ESISTCRFAQ	RVALIKNEAV	LNEEINPRLV	IKRLQKEIQE	LKDELAMVTG	EQRTEALTEA	ELLQLEKLIT
**410**	**420**	**430**	**440**	**450**	**460**	**470**	**480**
SFLEDQDSDS	RLEVGADMRK	VHHCFHHLKK	LLNDKKILEN	NTVSSESKDQ	DCQEPLKEEE	YRKLRDILKQ	RDNEISILSE
**490**	**500**	**510**	**520**	**530**	**540**	**550**	**560**
VMKKLPLSWF	ERGMREEMSL	GCQEAFEIFK	RDHADSVTID	DNKQILKQRF	SEAKALGESI	NEARSKIGHL	KEEITQRHIQ
**570**	**580**	**590**	**600**	**610**	**620**	**630**	**640**
QVALGISENM	AVPLMPDQQE	EKLRSQLEEE	KRRYKTMFTR	LKALKVEIEH	LQLLMDKAKV	KLQKEFEVWW	AEEATNLQVN
**650**	**660**	**670**	**680**	**690**	**700**	**710**	**720**
SPAVNSLDHT	KPFLQTSDSQ	HEWSQLLSNK	SSGGWEVQDQ	GTGRFDVCDV	NARKILPSPC	PSPHSQKQSS	TSTPLEDSIP
**730**	**740**	**750**	**760**				
KRPVSSIPLT	GDSQTDSDII	AFIKARQSIL	QKQCLGSN				

Cmpd.	No. of Cmpds.	m/z meas.	Δ m/z [ppm]	z	Rt [min]	Score	P	Range	Sequence	Modification	Ratios
2648	1	994.9726	-29.06	2	63.2	15.8	1	367-383	K.EIQELKDELAMVTGEQR.T		
1583	1	839.3761	-61.20	2	48.3	11.5	1	470-483	K.QRDNEISILSEVMK.K	Oxidation: 13	M:Q 0.73<br>Q:W 1.54

BRUKER

Detailed Protein Report

Protein 468: PREDICTED: coiled-coil domain-containing protein 88B isoform X1 [Homo sapiens]

**Accession:**	gi|578821335	**Score:**	44.0
**Database:**	refseq_human(refseq_human_20140103.fasta)	**MW [kDa]:**	173.8
**Database Date:**	2015-11-30	**pI:**	5.0
		**Sequence Coverage [%]:**	2.8
		**No. of unique Peptides:**	2

Quantitation

Q:W	**Median:**	0.37	**CV:**	0.00 %	**No. of Peptides:**	1
M:Q	**Median:**	1.03	**CV:**	0.00 %	**No. of Peptides:**	1

10	20	30	40	50	60	70	80
MEGGKGPRLR	DFLSGSLATW	ALGLAGLVGE	AEDSEGEEEE	EEEEPPLWLE	KRFLRLSDGA	LLLRVLGIIA	PSSRGGPRML
90	100	110	120	130	140	150	160
RGLDGPAAWR	VWNLNHLWGR	LRDFYQEELQ	LLILSPPPDL	QTLGFDPLSE	EAVEQLEGVL	RLLLGASVQC	EHRELFIRHI
170	180	190	200	210	220	230	240
QGLSLEVQSE	LAAAIQEVTQ	PGAGVVLALS	GPDPGELAPA	ELEMLSRSLM	GTLSKLARER	DLGAQRLAEL	LLEREPLCLR
250	260	270	280	290	300	310	320
PEAPSRAPAE	GPSHHLALQL	ANAKAQLRRL	RQELEEKAEL	LLDSQAEVQG	LEAEIRRLRQ	EAQALSGQAK	RAELYREEAE
330	340	350	360	370	380	390	400
ALRERAGRLP	RLQEELRRCR	ERLQAAEAYK	SQLEEERVLS	GVLEASKALL	EEQLEAARER	CARLHETQRE	NLLLRTRLGE
410	420	430	440	450	460	470	480
AHAELDSLRH	QVDQLAEENV	ELELELQRSL	EPPPGSPGEA	PLAGAAPSLQ	DEVREAEAGR	LRTLERENRE	LRGLLQVLQG
490	500	510	520	530	540	550	560
QPGGQHPLLE	APREDPVLPV	LEEAPQTPVA	FDHSPQGLVQ	KARDGGPQAL	DLAPPALDSV	LEASAECPQA	PDSDPQEAES
570	580	590	600	610	620	630	640
PLQAAAMDPQ	ASDWSPQESG	SPVETQESPE	KAGRRSSLQS	PASVAPPQGP	GTKIQAPQLL	GGETEGREAP	QGELVPEAWG
650	660	670	680	690	700	710	720
LRQEGPEHKP	GPSEPSSVQL	EEQEGPNQGL	DLATGQAEAR	EHDQRLEGTV	RDPAWQKPQQ	KSEGALEVQV	WEGPIPGESL
730	740	750	760	770	780	790	800
ASGVAEQEAL	REEVAQLRRK	AEALGDELEA	QARKLEAQNT	EAARLSKELA	QARRAEAEAH	REAEAQAWEQ	ARLREAVEAA
810	820	830	840	850	860	870	880
GQELESASQE	REALVEALAA	AGRERRQWER	EGSRLRAQSE	AAEERMQVLE	SEGRQHLEEA	ERERREKEAL	QAELEKAVVR
890	900	910	920	930	940	950	960
GKELGDRLEH	LQRELEQAAL	ERQEFLREKE	SQHQRYQGLE	QRLEAELQAA	ATSKEEALME	LKTRALQLEE	ELFQLRQGPA
970	980	990	1000	1010	1020	1030	1040
GLGPKKRAEP	QLVETQNVRL	IEVERSNAML	VAEKAALQGQ	LQHLEGQLGS	LQGRAQELLL	QSQRAQEHSS	RLQAEKSVLE
1050	1060	1070	1080	1090	1100	1110	1120
IQGQELHRKL	EVLEEEVRAA	RQSQEETRGQ	QQALLRDHKA	LAQLQRRQEA	ELEGLLVRHR	DLKANMRALE	LAHRELQGRH
1130	1140	1150	1160	1170	1180	1190	1200
EQLQAQRASV	EAQEVALLAE	RERLMQDGHR	QRGLEEELRR	LQSEHDRAQM	LLAELSRERG	ELQGERGELR	GRLARLELER
1210	1220	1230	1240	1250	1260	1270	1280
AQLEMQSQQL	RESNQQLDLS	ACRLTTQCEL	LTQLRSAQEE	ENRQLLAEVQ	ALSRENRELL	ERSLESRDHL	HREQREYLDQ
1290	1300	1310	1320	1330	1340	1350	1360
LNALRREKQK	LVEKIMDQYR	VLEPVPLPRT	KKGSWLADKG	PLPRHPCAGP	RAPSACGMRP	WQAGSGGNSA	QGSRWGEALS
1370	1380	1390	1400	1410	1420	1430	1440
HSALGTPLGN	DSDSAIQAPW	GRPSPTAKDL	VWDGRTPLRP	CRNTKQMPTE	RALRYRNRRN	VPSPHPSASD	TVGTAGLGVQ
1450	1460	1470	1480	1490	1500	1510	1520
PSRHWSVSGG	PRQPKSSGSQ	GPQGESLDKE	AWALRSSTVS	AGARRWSWDE	CVDRGDGWPP	RAAPGWSSGS	SRWLPLRQRS
1530	1540	1550	1560	1570			
LGDPPAEGGW	QELAREPPAL	SRWEAESQCW	GTVAWADLEP				

Cmpd.	No. of Cmpds.	m/z meas.	Δ m/z [ppm]	z	Rt [min]	Score	P	Range	Sequence	Modification	Ratios
245	1	715.7361	-205.90	2	33.1	13.1	1	755-767	K.LEAQNTEAARLSK.E		Q:W 0.37<br>M:Q 1.03
2188	1	945.6234	129.84	2	57.8	19.6	2	1258-	R.ELLERSLESRDHLHR.E		

Detailed Protein Report

Cmpd.	No. of Cmpds.	m/z meas.	Δ m/z [ppm]	z	Rt [min]	Score	P	Range	Sequence	Modification	Ratios
								1272			

Detailed Protein Report

Protein 469: selenium-binding protein 1 isoform 1 [Homo sapiens]

Accession: gi|16306550
Database: refseq_human(refseq_human_20140103.fasta)
Database Date: 2015-11-30
Modification(s): Oxidation

Score: 43.9
MW [kDa]: 52.4
pI: 5.9
Sequence Coverage [%]: 9.3
No. of unique Peptides: 2

10	20	30	40	50	60	70	80
MATKCGNCGP	GYSTPLEAMK	GPREEIVYLP	CIYRNTGTEA	PDYLATVDVD	PKSPQYCQVI	HRLPMPNLKD	ELHHSGWNTC
90	100	110	120	130	140	150	160
SSCFGDSTKS	RTKLVLPSLI	SSRIYVVDVG	SEPRAPKLHK	VIEPKDIHAK	CELAFLHTSH	CLASGEVMIS	SLGDVKGNGK
170	180	190	200	210	220	230	240
GGFVLLDGET	FEVKGTWERP	GGAAPLGYDF	WYQPRHNVMI	STEWAAPNVL	RDGFNPADVE	AGLYGSHLYV	WDWQRHEIVQ
250	260	270	280	290	300	310	320
TLSLKDGLIP	LEIRFLHNPD	AAQGFVGCAL	SSTIQRFYKN	EGGTWSVEKV	IQVPPKKVKG	WLLPEMPGLI	TDILLSLDDR
330	340	350	360	370	380	390	400
FLYFSNWLHG	DLRQYDISDP	QRPRLTGQLF	LGGSIVKGGP	VQVLEDEELK	SQPEPLVVKG	KRVAGGPQMI	QLSLDGKRLY
410	420	430	440	450	460	470	480
ITTSLYSAWD	KQFYPDLIRE	GSVMLQVDVD	TVKGGLKLNP	NFLVDFGKEP	LGPALAHELR	YPGGDCSSDI	WI

Cmpd.	No. of Cmpds.	m/z meas.	Δ m/z [ppm]	z	Rt [min]	Score	P	Range	Sequence	Modification
1766	1	1045.8477	-101.94	2	50.4	14.3	1	1-20	-.MATKCGNCGPGYSTPLEAMK.G	Oxidation: 1, 19
827	1	1106.2441	46.85	3	38.8	13.1	2	5-34	K.CGNCGPGYSTPLEAMKGPREEIVYLPCIYR.N	

Detailed Protein Report

Protein 470: putative ribosomal RNA methyltransferase NOP2 isoform 4 [Homo sapiens]

Accession: gi|385198063
Database: refseq_human(refseq_human_20140103.fasta)
Database Date: 2015-11-30

Score: 43.8
MW [kDa]: 69.6
pI: 6.2
Sequence Coverage [%]: 6.2
No. of unique Peptides: 2

Quantitation

Q:W **Median:** 0.58 **CV:** 0.00 % **No. of Peptides:** 1

10	20	30	40	50	60	70	80
MGRKLDPTKE	KRGPGRKARK	QKGAETELVR	FLPAVSDENS	KRLSSRARKR	AAKRRLGSVE	APKTNKSPEA	KPLPGKLPKG
90	100	110	120	130	140	150	160
AVQTAGKKGP	QSLFNAPRGK	KRPAPGSDEE	EEEEDSEEDG	MVNHGDLWGS	EDDADTVDDY	GADSNSEDEE	EGEALLPIER
170	180	190	200	210	220	230	240
AARKQKAREA	AAGIQWSEEE	TEDEEEEKEV	TPESGPPKVE	EADGGLQINV	DEEPFVLPPA	GEMEQDAQAP	DLQRVHKRIQ
250	260	270	280	290	300	310	320
DIVGILRDFG	AQREEGRSRS	EYLNRLKKDL	AIYYSYGDFL	LGKLMDLFPL	SELVEFLEAN	EVPRPVTLRT	NTLKTRRRDL
330	340	350	360	370	380	390	400
AQALINRGVN	LDPLGKWSKT	GLVVYDSSVP	IGATPEYLAG	HYMLQGASSM	LPVMALAPQE	HERILDMCCA	PGGKTSYMAQ
410	420	430	440	450	460	470	480
LMKNTGVILA	NDANAERLKS	VVGNLHRLGV	TNTIISHYDG	RQFPKVVGGF	DRVLLDAPCS	GTGVISKDPA	VKTNKDEKDI
490	500	510	520	530	540	550	560
LRCAHLQKEL	LLSAIDSVNA	TSKTGGYLVY	CTCSITVEEN	EWVVDYALKK	RNVRLVPTGL	DFGQEGFTRF	RERRFHPSLR
570	580	590	600	610	620	630	
STRRFYPHTH	NMDGFFIAKF	KKFSNSIPQS	QTDGVLLCRS	GWTAVVQSQL	IATSTFQVQA	ILVPQTPK	

Cmpd.	No. of Cmpds.	m/z meas.	Δ m/z [ppm]	z	Rt [min]	Score	P	Range	Sequence	Modification	Ratios
2658	1	625.2870	-81.65	2	61.5	11.2	1	442-452	R.QFPKVVGGFDR.V		Q:W 0.58
2674	1	933.1060	151.43	2	64.6	13.8	0	583-599	K.FSNSIPQSQTDGVLLCR.S		

Detailed Protein Report

Protein 471: keratin, type II cuticular Hb5 [Homo sapiens]

**Accession:**	gi|4504935	**Score:**	43.8
**Database:**	refseq_human(refseq_human_20140103.fasta)	**MW [kDa]:**	55.8
**Database Date:**	2015-11-30	**pI:**	6.3
**Modification(s):**	Carbamidomethyl	**Sequence Coverage [%]:**	6.5
		**No. of unique Peptides:**	1

Quantitation

Q:W **Median:** 0.50 **CV:** 0.00 % **No. of Peptides:** 1

10	20	30	40	50	60	70	80
MSCRSYRISS	GCGVTRNFSS	CSAVAPKTGN	RCCISAAPYR	GVSCYRGLTG	FGSRSLCNLG	SCGPRIAVGG	FRAGSCGRSF
**90**	**100**	**110**	**120**	**130**	**140**	**150**	**160**
GYRSGGVCGP	SPPCITTVSV	NESLLTPLNL	EIDPNAQCVK	QEEKEQIKSL	NSRFAAFIDK	VRFLEQQNKL	LETKWQFYQN
**170**	**180**	**190**	**200**	**210**	**220**	**230**	**240**
QRCCESNLEP	LFSGYIETLR	REAECVEADS	GRLASELNHV	QEVLEGYKKK	YEEEVALRAT	AENEFVVLKK	DVDCAYLRKS
**250**	**260**	**270**	**280**	**290**	**300**	**310**	**320**
DLEANVEALV	EESSFLRRLY	EEEIRVLQAH	ISDTSVIVKM	DNSRDLNMDC	IIAEIKAQYD	DVASRSRAEA	ESWYRSKCEE
**330**	**340**	**350**	**360**	**370**	**380**	**390**	**400**
MKATVIRHGE	TLRRTKEEIN	ELNRMIQRLT	AEIENAKCQR	AKLEAAVAEA	EQQGEAALSD	ARCKLAELEG	ALQKAKQDMA
**410**	**420**	**430**	**440**	**450**	**460**	**470**	**480**
CLLKEYQEVM	NSKLGLDIEI	ATYRRLLEGE	EHRLCEGVGS	VNVCVSSSRG	GVSCGGLSYS	TTPGRQITSG	PSAIGGSITV
**490**	**500**	**510**					
VAPDSCAPCQ	PRSSSFSCGS	SRSVRFA					

Cmpd.	No. of Cmpds.	m/z meas.	Δ m/z [ppm]	z	Rt [min]	Score	P	Range	Sequence	Modification	Ratios	
953	1	531.2210	2.88	2	40.3	11.1	0	493-502	R.SSSFSCGSSR.S	Carbamidomethyl: 6	Q:W	0.50

Detailed Protein Report

Protein 472: **PREDICTED: polyribonucleotide nucleotidyltransferase 1, mitochondrial isoform X1 [Homo sapiens]**

**Accession:**	gi|530368250	**Score:**	43.8
**Database:**	refseq_human(refseq_human_20140103.fasta)	**MW [kDa]:**	77.3
**Database Date:**	2015-11-30	**pI:**	6.2
		**Sequence Coverage [%]:**	3.8
		**No. of unique Peptides:**	1

10	20	30	40	50	60	70	80
MVTAVSKTKP	SPSQFMPLVV	DYRQKAAAAG	RIPTNYLRRE	IGTSDKEILT	SRIIDRSIRP	LFPAGYFYDT	QVLCNLLAVD
**90**	**100**	**110**	**120**	**130**	**140**	**150**	**160**
GVNEPDVLAI	NGASVALSLS	DIPWNGPVGA	VRIGIIDGEY	VVNPTRKEMS	SSTLNLVVAG	APKSQIVMLE	ASAENILQQD
**170**	**180**	**190**	**200**	**210**	**220**	**230**	**240**
FCHAIKVGVK	YTQQIIQGIQ	QLVKETGVTK	RTPQKLFTPS	PEIVKYTHKL	AMERLYAVFT	DYEHDKVSRD	EAVNKIRLDT
**250**	**260**	**270**	**280**	**290**	**300**	**310**	**320**
EEQLKEKFPE	ADPYEIIESF	NVVAKEVFRS	IVLNEYKRCD	GRDLTSLRNV	SCEVDMFKTL	HGSALFQRGQ	TQVLCTVTFD
**330**	**340**	**350**	**360**	**370**	**380**	**390**	**400**
SLESGIKSDQ	VITAINGIKD	KNFMLHYEFP	PYATNEIGKV	TGLNRRELGH	GALAEKALYP	VIPRDFPFTI	RVTSEVLESN
**410**	**420**	**430**	**440**	**450**	**460**	**470**	**480**
GSSSMASACG	GSLALMDSGV	PISSAVAGVA	IGLVTKTDPE	KGEIEDYRLL	TDILGIEDYN	GDMDFKIAGT	NKGITALQAD
**490**	**500**	**510**	**520**	**530**	**540**	**550**	**560**
IKLPGIPIKI	VMEAIQQASV	AKKEILQIMN	KTISKPRASR	KENGPVVETV	QVPLSKRAKF	VGPGGYNLKK	LQAETGVTIS
**570**	**580**	**590**	**600**	**610**	**620**	**630**	**640**
QVDEETFSVF	APTPSAMHEA	RDFITEICKD	DQEQQLEFGA	VYTATITEIR	DTGVMVKLYP	NMTAVLLHNT	QLDQRKIKHP
**650**	**660**	**670**	**680**	**690**	**700**	**710**	
TALGLEVGQE	IQVKYFGRDP	ADGRMRLSRK	VLQSPATTVV	RTLNDRSSIV	MGEPISQSSS	NSQ	

Cmpd.	No. of Cmpds.	m/z meas.	Δ m/z [ppm]	z	Rt [min]	Score	P	Range	Sequence	Modification
80	1	586.3280	116.43	2	30.9	28.4	0	289-298	R.NVSCEVDMFK.T	

Detailed Protein Report

Protein 473: zinc finger protein 518A isoform a [Homo sapiens]

**Accession:**	gi|57242807	**Score:**	43.8
**Database:**	refseq_human(refseq_human_20140103.fasta)	**MW [kDa]:**	166.7
**Database Date:**	2015-11-30	**pI:**	10.2
**Modification(s):**	Carbamidomethyl, Oxidation	**Sequence Coverage [%]:**	3.4
		**No. of unique Peptides:**	2

Alias proteins:

Accession	Name	Description
gi|512388675	refseq_human (refseq_human_20140103.fasta)	zinc finger protein 518A isoform a [Homo sapiens]
gi|512388673	refseq_human (refseq_human_20140103.fasta)	zinc finger protein 518A isoform a [Homo sapiens]

```
        10         20         30         40         50         60         70         80
MPSEQKQLFC DEKQTTLKKD YDVKNEIVDR SAPKPKISGS IHYALKNVKI DLPKINIPNE VLLKHEVDKY RKLFQSKQQT
        90        100        110        120        130        140        150        160
ARKSISIKTV SCVEECTLLH KSERAEEEGV KMSAKILNFS CLKCRDNTRY SPNDLQKHFQ MWHHGELPSY PCEMCNFSAN
       170        180        190        200        210        220        230        240
DFQVFKQHRR THRSTLVKCD ICNNESVYTL LNLTKHFTST HCVNGNFQCE KCKFSTQDVG TFVQHIHRHN EIHYKCGKCH
       250        260        270        280        290        300        310        320
HVCFTKGELQ KHLHIHSGTF PFTCQYCSYG ATRREHLVRH VITLHKEHLY AKEKLEKDKY EKRMAKTSAG LKLILKRYKI
       330        340        350        360        370        380        390        400
GASRKTFWKR KKINSGSDRS IEKNTQVLKK MNKTQTKSED QSHVVQEHLS EEKDERLHCE NNDKAPESES EKPTPLSTGQ
       410        420        430        440        450        460        470        480
GNRAEEGPNA SSGFMKTAVL GPTLKNVMMK NNKLAVSPNY NATFMGFKMM DGKQHIVLKL VPIKQNVCSP GSQSGAAKDG
       490        500        510        520        530        540        550        560
TANLQPQTLD TNGFLTGVTT ELNDTVYMKA ATPFSCSSSI LSGKASSEKE MTLISQRNNM LQTMDYEKSV SSLSATSELV
       570        580        590        600        610        620        630        640
TASVNLTTKF ETRDNVDFWG NHLTQSHPEV LGTTIKSPDK VNCVAKPNAY NSGDMHNYCI NYGNCELPVE SSNQGSLPFH
       650        660        670        680        690        700        710        720
NYSKVNNSNK RRRFSGTAVY ENPQRESSSS KTVVQQPISE SFLSLVRQES SKPDSLLASI SLLNDKDGTL KAKSEIEEQY
       730        740        750        760        770        780        790        800
VLEKGQNIDG QNLYSNENQN LECATEKSKW EDFSNVDSPM MPRITSVFSL QSQQASEFLP PEVNQLLQDV LKIKPDVKQD
       810        820        830        840        850        860        870        880
SSNTPNKGLP LHCDQSFQKH EREGKIVESS KDFKVQGIFP VPPGSVGINV PTNDLNLKFG KEKQVSSIPQ DVRDSEKMPR
       890        900        910        920        930        940        950        960
ISGFGTLLKT QSDAIITQQL VKDKLRATTQ NLGSFYMQSP LLNSEQKKTI IVQTSKGFLI PLNITNKPGL PVIPGNALPL
       970        980        990       1000       1010       1020       1030       1040
VNSQGIPASL FVNKKPGMVL TLNNGKLEGV SAVKTEGAPA RGTVTKEPCK TPILKVEPNN NCLTPGLCSS IGSCLSMKSS
      1050       1060       1070       1080       1090       1100       1110       1120
SENTLPLKGP YILKPTSSVK AVLIPNMLSE QQSTKLNISD SVKQQNEIFP KPPLYTFLPD GKQAVFLKCV MPNKTELLKP
      1130       1140       1150       1160       1170       1180       1190       1200
KLVQNSTYQN IQPKKPEGTP QRILLKIFNP VLNVTAANNL SVSNSASSLQ KDNVPSNQII GGEQKEPESR DALPFLLDDL
      1210       1220       1230       1240       1250       1260       1270       1280
MPANEIVITS TATCPESSEE PICVSDCSES RVLRCKTNCR IERNFNRKKT SKKIFSKTKT HGSKDSETAF VSRNRNCKRK
      1290       1300       1310       1320       1330       1340       1350       1360
CRDSYQEPPR RKATLHRKCK EKAKPEDVRE TFGFSRPRLS KDSIRTLRLF PFSSKQLVKC PRRNQPVVVL NHPDADAPEV
      1370       1380       1390       1400       1410       1420       1430       1440
VSVMKTIAKF NGHVLKVSLS KRTINALLKP VCYNPPKTTY DDFSKRHKTF KPVSSVKERF VLKLTLKKTS KNNYQIVKTT
      1450       1460       1470       1480       1490
SENILKAKFN CWFCGRVFDN QDTWAGHGQR HLMEATRDWN MLE
```

Cmpd.	No. of Cmpds.	m/z meas.	Δ m/z [ppm]	z	Rt [min]	Score	P	Range	Sequence	Modification
1117	1	1019.4619	-129.58	2	43.5	17.7	2	31-49	R.SAPKPKISGSIHYALKNVK.I	
63	1	814.0267	-13.11	3	30.1	14.3	0	1016-	K.VEPNNNCLTPGLCSSIGSCLSMK.S	Carbamidomethyl: 7;

Detailed Protein Report

Cmpd.	No. of Cmpds.	m/z meas.	Δ m/z [ppm]	z	Rt [min]	Score	P	Range	Sequence	Modification
								1038		Oxidation: 22

Detailed Protein Report

Protein 474: PREDICTED: filamin-B isoform X6 [Homo sapiens]

**Accession:**	gi|530372129	**Score:**	43.7
**Database:**	refseq_human(refseq_human_20140103.fasta)	**MW [kDa]:**	233.6
**Database Date:**	2015-11-30	**pI:**	5.2
		**Sequence Coverage [%]:**	1.7
		**No. of unique Peptides:**	2

Quantitation

M:Q	**Median:** 0.48	**CV:** 0.00 %	**No. of Peptides:** 1

Detailed Protein Report

10	20	30	40	50	60	70	80
MPVTEKDLAE	DAPWKKIQQN	TFTRWCNEHL	KCVNKRIGNL	QTDLSDGLRL	IALLEVLSQK	RMYRKYHQRP	TFRQMQLENV
90	100	110	120	130	140	150	160
SVALEFLDRE	SIKLVSIDSK	AIVDGNLKLI	LGLVWTLILH	YSISMPVWED	EGDDDAKKQT	PKQRLLGWIQ	NKIPYLPITN
170	180	190	200	210	220	230	240
FNQNWQDGKA	LGALVDSCAP	GLCPDWESWD	PQKPVDNARE	AMQQADDWLG	VPQVITPEEI	IHPDVDEHSV	MTYLSQFPKA
250	260	270	280	290	300	310	320
KLKPGAPLKP	KLNPKKARAY	GRGIEPTGNM	VKQPAKFTVD	TISAGQGDVM	VFVEDPEGNK	EEAQVTPDSD	KNKTYSVEYL
330	340	350	360	370	380	390	400
PKVTGLHKVT	VLFAGQHISK	SPFEVSVDKA	QGDASKVTAK	GPGLEAVGNI	ANKPTYFDIY	TAGAGVGDIG	VEVEDPQGKN
410	420	430	440	450	460	470	480
TVELLVEDKG	NQVYRCVYKP	MQPGPHVVKI	FFAGDTIPKS	PFVVQVGEAC	NPNACRASGR	GLQPKGVRIR	ETTDFKVDTK
490	500	510	520	530	540	550	560
AAGSGELGVT	MKGPKGLEEL	VKQKDFLDGV	YAFEYYPSTP	GRYSIAITWG	GHHIPKSPFE	VQVGPEAGMQ	KVRAWGPGLH
570	580	590	600	610	620	630	640
GGIVGRSADF	VVESIGSEVG	SLGFAIEGPS	QAKIEYNDQN	DGSCDVKYWP	KEPGEYAVHI	MCDDEDIKDS	PYMAFIHPAT
650	660	670	680	690	700	710	720
GGYNPDLVRA	YGPGLEKSGC	IVNNLAEFTV	DPKDAGKAPL	KIFAQDGEGQ	RIDIQMKNRM	DGTYACSYTP	VKAIKHTIAV
730	740	750	760	770	780	790	800
VWGGVNIPHS	PYRVNIGQGS	HPQKVKVFGP	GVERSGLKAN	EPTHFTVDCT	EAGEGDVSVG	IKCDARVLSE	DEEDVDFDII
810	820	830	840	850	860	870	880
HNANDTFTVK	YVPPAAGRYT	IKVLFASQEI	PASPFRVKVD	PSHDASKVKA	EGPGLSKAGV	ENGKPTHFTV	YTKGAGKAPL
890	900	910	920	930	940	950	960
NVQFNSPLPG	DAVKDLDIID	NYDYSHTVKY	TPTQQGNMQV	LVTYGGDPIP	KSPFTVGVAA	PLDLSKIKLN	GLENRVEVGK
970	980	990	1000	1010	1020	1030	1040
DQEFTVDTRG	AGGQGKLDVT	ILSPSRKVVP	CLVTPVTGRE	NSTAKFIPRE	EGLYAVDVTY	DGHPVPGSPY	TVEASLPPDP
1050	1060	1070	1080	1090	1100	1110	1120
SKVKAHGPGL	EGGLVGKPAE	FTIDTKGAGT	GGLGLTVEGP	CEAKIECSDN	GDGTCSVSYL	PTKPGEYFVN	ILFEEVHIPG
1130	1140	1150	1160	1170	1180	1190	1200
SPFKADIEMP	FDPSKVVASG	PGLEHGKVGE	AGLLSVDCSE	AGPGALGLEA	VSDSGTKAEV	SIQNNKDGTY	AVTYVPLTAG
1210	1220	1230	1240	1250	1260	1270	1280
MYTLTMKYGG	ELVPHFPARV	KVEPAVDTSR	IKVFGPGIEG	KDVFREATTD	FTVDSRPLTQ	VGGDHIKAHI	ANPSGASTEC
1290	1300	1310	1320	1330	1340	1350	1360
FVTDNADGTY	QVEYTPFEKG	LHVVEVTYDD	VPIPNSPFKV	AVTEGCQPSR	VQAQGPGLKE	AFTNKPNVFT	VVTRGAGIGG
1370	1380	1390	1400	1410	1420	1430	1440
LGITVEGPSE	SKINCRDNKD	GSCSAEYIPF	APGDYDVNIT	YGGAHIPGSP	FRVPVKDVVD	PSKVKIAGPG	LGSGVRARVL
1450	1460	1470	1480	1490	1500	1510	1520
QSFTVDSSKA	GLAPLEVRVL	GPRADDTDSQ	SWRSPLKALS	EFFKGDPKGD	FNKTGLVEPV	NVVDNGDGTH	TVTYTPSQEG
1530	1540	1550	1560	1570	1580	1590	1600
PYMVSVKYAD	EEIPRSPFKV	KVLPTYDASK	VTASGPGLSS	YGVPASLPVD	FAIDARDAGE	GLLAVQITDQ	EGKPKRAIVH
1610	1620	1630	1640	1650	1660	1670	1680
DNKDGTYAVT	YIPDKTGRYM	IGVTYGGDDI	PLSPYRIRAT	QTGDASKCLA	TGPGIASTVK	TGEEVGFVVD	AKTAGKGKVT
1690	1700	1710	1720	1730	1740	1750	1760
CTVLTPDGTE	AEADVIENED	GTYDIFYTAA	KPGTYVIYVR	FGGVDIPNSP	FTVMATDGEV	TAVEEAPVNA	CPPGFRPWVT
1770	1780	1790	1800	1810	1820	1830	1840
EEAYVPVSDM	NGLGFKPFDL	VIPFAVRKGE	ITGEVHMPSG	KTATPEIVDN	KDGTVTVRYA	PTEVGLHEMH	IKYMGSHIPE
1850	1860	1870	1880	1890	1900	1910	1920
SPLQFYVNYP	NSGSVSAYGP	GLVYGVANKT	ATFTIVTEDA	GEGGLDLAIE	GPSKAEISCI	DNKDGTCTVT	YLPTLPGDYS
1930	1940	1950	1960	1970	1980	1990	2000
ILVKYNDKHI	PGSPFTAKIT	DDSRRCSQVK	LGSAADFLLD	ISETDLSSLT	ASIKAPSGRD	EPCLLKRLPN	NHIGISFIPR
2010	2020	2030	2040	2050	2060	2070	2080
EVGEHLVSIK	KNGNHVANSP	VSIMVVQSEI	GDARRAKVYG	RGLSEGRTFE	MSDFIVDTRD	AGYGGISLAV	EGPSKVDIQT
2090	2100	2110	2120	2130	2140	2150	2160
EDLEDGTCKV	SYFPTVPGVY	IVSTKFADEH	VPGSPFTVKI	SGEGRVKESI	TRTSRAPSVA	TVGSICDLNL	KIPGVRVMNC
2170	2180						

Detailed Protein Report

Cmpd.	No. of Cmpds.	m/z meas.	Δ m/z [ppm]	z	Rt [min]	Score	P	Range	Sequence	Modification	Ratios
171	1	614.7837	-134.87	2	30.9	13.4	1	977-987	K.LDVTILSPSRK.V		
224	1	575.6716	-248.03	2	31.6	10.4	0	1136-1147	K.VVASGPGLEHGK.V		M:Q 0.48

Detailed Protein Report

Protein 475: FERM, RhoGEF and pleckstrin domain-containing protein 2 isoform b [Homo sapiens]

Accession: gi|545478372
Database: refseq_human(refseq_human_20140103.fasta)
Database Date: 2015-11-30

Score: 43.7
MW [kDa]: 73.2
pI: 9.6
Sequence Coverage [%]: 7.7
No. of unique Peptides: 1

10	20	30	40	50	60	70	80
MGEIEGTYRV	LQTAGMRLGA	QTPVGVSTLE	PGQTLLPRMQ	EKHLHLRVKL	LDNTMEIFDI	EPKCDGQVLL	TQVWKRLNLV
**90**	**100**	**110**	**120**	**130**	**140**	**150**	**160**
ECDYFGMEFQ	NTQSYWIWLE	PMKPIIRQIR	RPKNVVLRLA	VKFFPPDPGQ	LQEEYTRYLF	ALQLKRDLLE	ERLTCADTTA
**170**	**180**	**190**	**200**	**210**	**220**	**230**	**240**
ALLTSHLLQS	EIGDYDETLD	REHLKVNEYL	PGQQHCLEKI	LEFHQKHVGQ	TPAESDFQVL	EIARKLEMYG	IRFHMASDRE
**250**	**260**	**270**	**280**	**290**	**300**	**310**	**320**
GTKIQLAVSH	MGVLVFQGTT	KINTFNWSKV	RKLSFKRKRF	LIKLHPEVHG	PYQDTLEFLL	GSRDECKNFW	KICVEYHTFF
**330**	**340**	**350**	**360**	**370**	**380**	**390**	**400**
RLLDQPKPKA	KAVFFSRGSS	FRYSGRTQKQ	LVDYFKDSGM	KRIPYERRHS	KTHTSVRALT	ADLPKQSISF	PEGLRTPASP
**410**	**420**	**430**	**440**	**450**	**460**	**470**	**480**
SSANAFYSLS	PSTLVPSGLP	EFKDSSSSLT	DPQVSYVKSP	AAERRSGAVA	GGPDTPSAQP	LGPPALQPGP	GLSTKSPQPS
**490**	**500**	**510**	**520**	**530**	**540**	**550**	**560**
PSSRKSPLSL	SPAFQVPLGP	AEQGSSPLLS	PVLSDAGGAG	MDCEEPRHKR	VPADEAYFIV	KEILATERTY	LKDLEVITVW
**570**	**580**	**590**	**600**	**610**	**620**	**630**	**640**
FRSAVVKEDA	MPATLMTLLF	SNIDPIYEFH	RGFLREVEQR	LALWEGPSKA	HTKGSHQRIG	DILLRNMRQL	KVFQLHEGHV
**650**							
AGVTKME							

Cmpd.	No. of Cmpds.	m/z meas.	Δ m/z [ppm]	z	Rt [min]	Score	P	Range	Sequence	Modification
22	1	1001.9616	-70.42	2	29.6	16.6	2	601-618	R.LALWEGPSKAHTKGSHQR.I	

Detailed Protein Report

Protein 476: zinc finger protein 177 isoform b [Homo sapiens]

Accession: gi|289547607
Database: refseq_human(refseq_human_20140103.fasta)
Database Date: 2015-11-30
Modification(s): Carbamidomethyl

Score: 43.7
MW [kDa]: 36.4
pI: 9.8
Sequence Coverage [%]: 10.6
No. of unique Peptides: 3

Quantitation

Q:W	**Median:** 1.27	**CV:** 0.00 %	**No. of Peptides:** 1
M:Q	**Median:** 0.81	**CV:** 0.00 %	**No. of Peptides:** 1

Alias proteins:

Accession	Name	Description
gi|289547609	refseq_huma (refseq_human_20140103.fasta)	zinc finger protein 177 isoform b [Homo sapiens]

```
        10         20         30         40         50         60         70         80
MAAGWLTTWS QNSVTFQEVA VDFSQEEWAL LDPAQKNLYK DVMLENFRNL ASVGYQLCRH SLISKVDQEQ LKTDERGILQ
        90        100        110        120        130        140        150        160
GDCADWETQL KPKDTIAMQN IPGGKTSNGI NTNCVRTHSG EMPYECSDCG KAFIFQSSLK KHMRSHTGEK PYECDHCGKS
       170        180        190        200        210        220        230        240
FSQSSHLNVH KRTHTGEKPY DCKECGKAFT VPSSLQKHVR THTGEKPYEC SDCGKAFIDQ SSLKKHTRSH TGEKPYECNQ
       250        260        270        280        290        300        310        320
CGKSFSTGSY LIVHKRTHTG EKTYECKECG KAFRNSSCLR VHVRTHTGEK PYKCIQCEKA FSTSTNLIMH KRIHNGQKLH
       330
E
```

Cmpd.	No. of Cmpds.	m/z meas.	Δ m/z [ppm]	z	Rt [min]	Score	P	Range	Sequence	Modification	Ratios
977	1	512.1767	-143.10	2	41.0	10.1	0	41-48	K.DVMLENFR.N		M:Q 0.81
2497	4	973.4783	-40.26	2	61.8	16.7	1	49-65	R.NLASVGYQLCRHSLISK.V	Carbamidomethyl: 10	Q:W 1.27
1517	1	1008.6274	90.62	1	48.8	16.8	0	216-224	K.AFIDQSSLK.K		

Detailed Protein Report

Protein 477: FERM and PDZ domain-containing protein 1 [Homo sapiens]

Accession: gi|239582741
Database: refseq_human(refseq_human_20140103.fasta)
Database Date: 2015-11-30
Modification(s): Carbamidomethyl, Oxidation

Score: 43.6
MW [kDa]: 173.3
pI: 5.0
Sequence Coverage [%]: 2.7
No. of unique Peptides: 3

Quantitation

Q:W **Median:** 1.18 **CV:** 0.00 % **No. of Peptides:** 1

10	20	30	40	50	60	70	80
MEELETSLFQ	TRKAHRIEQM	VARWLRRSRD	SSARAKVAAA	DGPARNPTQT	LIPVRHTVKI	DKDTLLQDYG	FHISESLPLT
90	100	110	120	130	140	150	160
VVAVTAGGSA	HGKLFPGDQI	LQMNNEPAED	LSWERAVDIL	REAEDSLSIT	VVRCTSGVPK	SSFLTEEKRA	RLKTNPVKVH
170	180	190	200	210	220	230	240
FAEEVLISGH	SQGNSLLCMP	NVLKLYLENG	QTKAFKFEAN	TTVKDIILTV	KEKLSIRSIE	YFALALEEQY	SISRLHLLHE
250	260	270	280	290	300	310	320
EELIQQVVER	EESHDYRCLF	RVCFVPKDPL	DLLKEDPVAF	EYLYLQSCSD	VLQERFAVEM	KCSSALRLAA	LHIQERIYAC
330	340	350	360	370	380	390	400
AQPQKISLKY	IEKDWGIENF	ISPTLLRNMK	GKDIKKAISF	HMKRNQNLLE	PRQKQLISAA	QLRLNYLQIL	GELKTYGGRI
410	420	430	440	450	460	470	480
FNATLMLQDR	ESYIALLVGA	KYGISQVINS	KLNIMSTLAE	FANISRVELT	EESEKVSVVK	VYLQDVKVLT	LLLESNSAKD
490	500	510	520	530	540	550	560
LACLIAGYYR	LLVDPVTSIF	LWPGNKQQAH	RVSAEEGYES	RACSDSEESS	EVDCVLEPLS	DRRLVKLAPC	RSLIKEEQPP
570	580	590	600	610	620	630	640
GNSPTPEVAR	RGPSTCGASS	TTDSAESEAS	DSANTESRGY	RTSGSSESMD	ALEEDDLDTC	SSSRSTFFHF	GSPGLAESID
650	660	670	680	690	700	710	720
SDSQEERSGI	ETSGFLCLLD	LAQRANPQCQ	KTEFSESAAL	ETFGWAPELS	TVRLDPRLYE	GSHADYYSLC	SSVSPASYLS
730	740	750	760	770	780	790	800
DSSESTASRQ	GGAPPAWGQQ	GWTEAQPSSM	LEPLALHPPL	AFEDGSSDEE	YYDAADKLTP	PGPPSGPRDV	STAEPSATSL
810	820	830	840	850	860	870	880
QNKASTSSPE	NSLPCGPDGR	QPSRRGGVKK	YAKTLRKRRS	FLQTDYTSQV	SFPLVPSASL	ESVDDVCYYD	REPYLALGAP
890	900	910	920	930	940	950	960
SPTVSSLQDM	QGEPGLLETK	ALGLLAPLRE	TKSTNPASRV	MEMEPETMET	KSVIDSRVSS	ISAIRFRIDP	NNKENSGVVP
970	980	990	1000	1010	1020	1030	1040
AASSSASTPH	CSNPGSSGPD	TAQARPSQIL	PLSQDLDGIA	PKEPTIEHGD	SSFSLSSGDP	NPDRACLASN	PGLNNVSQGD
1050	1060	1070	1080	1090	1100	1110	1120
TLELQLEPHV	QLEMGLESFC	TNHIQETAPK	YTEPLLSPRD	EPRSDECGIN	PGEKIASIPT	KEEPQGQLSL	ERDREVTNKN
1130	1140	1150	1160	1170	1180	1190	1200
GTNVFQEESR	KDSGDSPGDV	SNNVSQTLDI	SSPAGKIVTS	LSLDAPVTGT	EQIPPHPPRD	PQGQSREPPG	QGCQAQEQKL
1210	1220	1230	1240	1250	1260	1270	1280
FVELDLDPDF	FLGKQTVSPA	VPPEGIKAEA	PNHVTGQDIA	PRDSPEWVCF	NPEPSLPEPL	PCPQEDPHLE	TSNHCLLSEG
1290	1300	1310	1320	1330	1340	1350	1360
KSDSSSICLS	AEKSFLCFAP	ESHPEVSASL	RVATSLGFAG	MNEMVAPRIG	MDQCSCQFSY	ATCFRGPQPE	TEEEDRDLEA
1370	1380	1390	1400	1410	1420	1430	1440
HPMAPLTSPP	SAGSPVVLPW	RPARAHSCTT	APLSRKSHIW	PEYCSRALRQ	LKATPASTPE	GFIQLMESLL	ELQDILETSW
1450	1460	1470	1480	1490	1500	1510	1520
GVGNKHPPEK	CTWHFTESRS	RLCMGSQKLL	SSCRHVIRMD	QSPEEMQGAV	RDTFQHLVQL	AGLCFQFTDC	SRCSARHREA
1530	1540	1550	1560	1570	1580		
AGNLRDVVYT	YHQFIEAAKS	TCERGYHDLS	VKLLARQCTA	LTAAVFCLTQ	KFRASTAL		

Cmpd.	No. of Cmpds.	m/z meas.	Δ m/z [ppm]	z	Rt [min]	Score	P	Range	Sequence	Modification	Ratios
2745	1	849.4011	-32.55	1	65.4	14.4	0	357-363	K.AISFHMK.R	Oxidation: 6	
2634	1	994.5127	-8.52	2	63.7	13.9	2	1462-1478	R.LCMGSQKLLSSCRHVIR.M	Carbamidomethyl: 2	
2077	1	1158.9066	-123.27	2	55.9	15.3	1	1526-	R.DVVYTYHQFIEAAKSTCER.G	Carbamidomethyl: 17	Q:W 1.18

Detailed Protein Report

Cmpd.	No. of Cmpds.	m/z meas.	Δ m/z [ppm]	z	Rt [min]	Score	P	Range	Sequence	Modification	Ratios
								1544			

Detailed Protein Report

Protein 478: PREDICTED: coiled-coil domain-containing protein 91 isoform X6 [Homo sapiens]

**Accession:**	gi|578822930	**Score:**	43.6
**Database:**	refseq_human(refseq_human_20140103.fasta)	**MW [kDa]:**	51.6
**Database Date:**	2015-11-30	**pI:**	5.4
**Modification(s):**	Oxidation	**Sequence Coverage [%]:**	5.8
		**No. of unique Peptides:**	2

10	20	30	40	50	60	70	80
MWVQQHRPEN	TFGDQRETCN	FHCELPSRVY	GRTSLKLLMP	MWNKVSGVHL	SPSSPEIVLD	RDHSSSIGCL	SSDAIISSPE
**90**	**100**	**110**	**120**	**130**	**140**	**150**	**160**
NTHAANSIVS	QTIPKAQIQQ	STHTHLDISL	FPLGLTDEKS	NGTIALVDDS	EDPGANVSNI	QLQQKISSLE	IKLKVSEEEK
**170**	**180**	**190**	**200**	**210**	**220**	**230**	**240**
QRIKQDVESL	MEKHNVLEKG	FLKEKEQEAI	SFQDRYKELQ	EKHKQELEDM	RKAGHEALSI	IVDEYKALLQ	SSVKQQVEAI
**250**	**260**	**270**	**280**	**290**	**300**	**310**	**320**
EKQYISAIEK	QAHKCEELLN	AQHQRLLEML	DTEKELLKEK	IKEALIQQSQ	EQKEILEKCL	EEERQRNKEA	LVSAAKLEKE
**330**	**340**	**350**	**360**	**370**	**380**	**390**	**400**
AVKDAVLKVV	EEERKNLEKA	HAEERELWKT	EHAKDQEKVS	QEIQKAIQEQ	RKISQETVKA	AIIEEQKRSE	KAVEEAVKRT
**410**	**420**	**430**	**440**	**450**			
RDELIEYIKE	QKRLDQVIRQ	RSLSSLELFL	SCAQKQLSAL	IATEPVDIE			

Cmpd.	No. of Cmpds.	m/z meas.	Δ m/z [ppm]	z	Rt [min]	Score	P	Range	Sequence	Modification
1931	7	1022.8987	-73.55	2	54.2	16.6	0	1-16	-.MWVQQHRPENTFGDQR.E	Oxidation: 1
1996	1	665.3129	-29.71	2	55.1	11.2	2	203-212	K.HKQELEDMRK.A	Oxidation: 8

Detailed Protein Report

Protein 479: coiled-coil domain-containing protein 88B precursor [Homo sapiens]

**Accession:**	gi|262118216	**Score:**	43.5
**Database:**	refseq_human(refseq_human_20140103.fasta)	**MW [kDa]:**	164.7
**Database Date:**	2015-11-30	**pI:**	4.9
		**Sequence Coverage [%]:**	2.8
		**No. of unique Peptides:**	1

Quantitation

Q:W **Median:** 1.49 **CV:** 0.00 % **No. of Peptides:** 1

10	20	30	40	50	60	70	80
MEGGKGPRLR	DFLSGSLATW	ALGLAGLVGE	AEDSEGEEEE	EEEEPPLWLE	KRFLRLSDGA	LLLRVLGIIA	PSSRGGPRML
90	100	110	120	130	140	150	160
RGLDGPAAWR	VWNLNHLWGR	LRDFYQEELQ	LLILSPPPDL	QTLGFDPLSE	EAVEQLEGVL	RLLLGASVQC	EHRELFIRHI
170	180	190	200	210	220	230	240
QGLSLEVQSE	LAAAIQEVTQ	PGAGVVLALS	GPDPGELAPA	ELEMLSRSLM	GTLSKLARER	DLGAQRLAEL	LLEREPLCLR
250	260	270	280	290	300	310	320
PEAPSRAPAE	GPSHHLALQL	ANAKAQLRRL	RQELEEKAEL	LLDSQAEVQG	LEAEIRRLRQ	EAQALSGQAK	RAELYREEAE
330	340	350	360	370	380	390	400
ALRERAGRLP	RLQEELRRCR	ERLQAAEAYK	SQLEEERVLS	GVLEASKALL	EEQLEAARER	CARLHETQRE	NLLLRTRLGE
410	420	430	440	450	460	470	480
AHAELDSLRH	QVDQLAEENV	ELELELQRSL	EPPPGSPGEA	PLAGAAPSLQ	DEVREAEAGR	LRTLERENRE	LRGLLQVLQG
490	500	510	520	530	540	550	560
QPGGQHPLLE	APREDPVLPV	LEEAPQTPVA	FDHSPQGLVQ	KARDGGPQAL	DLAPPALDSV	LEASAECPQA	PDSDPQEAES
570	580	590	600	610	620	630	640
PLQAAAMDPQ	ASDWSPQESG	SPVETQESPE	KAGRRSSLQS	PASVAPPQGP	GTKIQAPQLL	GGETEGREAP	QGELVPEAWG
650	660	670	680	690	700	710	720
LRQEGPEHKP	GPSEPSSVQL	EEQEGPNQGL	DLATGQAEAR	EHDQRLEGTV	RDPAWQKPQQ	KSEGALEVQV	WEGPIPGESL
730	740	750	760	770	780	790	800
ASGVAEQEAL	REEVAQLRRK	AEALGDELEA	QARKLEAQNT	EAARLSKELA	QARRAEAEAH	REAEAQAWEQ	ARLREAVEAA
810	820	830	840	850	860	870	880
GQELESASQE	REALVEALAA	AGRERRQWER	EGSRLRAQSE	AAEERMQVLE	SEGRQHLEEA	ERERREKEAL	QAELEKAVVR
890	900	910	920	930	940	950	960
GKELGDRLEH	LQRELEQAAL	ERQEFLREKE	SQHQRYQGLE	QRLEAELQAA	ATSKEEALME	LKTRALQLEE	ELFQLRQGPA
970	980	990	1000	1010	1020	1030	1040
GLGPKKRAEP	QLVETQNVRL	IEVERSNAML	VAEKAALQGQ	LQHLEGQLGS	LQGRAQELLL	QSQRAQEHSS	RLQAEKSVLE
1050	1060	1070	1080	1090	1100	1110	1120
IQGQELHRKL	EVLEEEVRAA	RQSQEETRGQ	QQALLRDHKA	LAQLQRRQEA	ELEGLLVRHR	DLKANMRALE	LAHRELQGRH
1130	1140	1150	1160	1170	1180	1190	1200
EQLQAQRASV	EAQEVALLAE	RERLMQDGHR	QRGLEEELRR	LQSEHDRAQM	LLAELSRERG	ELQGERGELR	GRLARLELER
1210	1220	1230	1240	1250	1260	1270	1280
AQLEMQSQQL	RESNQQLDLS	ACRLTTQCEL	LTQLRSAQEE	ENRQLLAEVQ	ALSRENRELL	ERSLESRDHL	HREQREYLDQ
1290	1300	1310	1320	1330	1340	1350	1360
LNALRREKQK	LVEKIMDQYR	VLEPVPLPRT	KKGSWLADKV	KRLMRPRREG	GPPGGLRLGA	DGAGSTESLG	GPPETELPEG
1370	1380	1390	1400	1410	1420	1430	1440
READGTGSPS	PAPMRRAQSS	LCLRDETLAG	GQRRKLSSRF	PVGRSSESFS	PGDTPRQRFR	QRHPGPLGAP	VSHSKGPGVG
1450	1460	1470	1480				
WENSAETLQE	HETDANREGP	EVQEPEKRPL	TPSLSQ				

Cmpd.	No. of Cmpds.	m/z meas.	Δ m/z [ppm]	z	Rt [min]	Score	P	Range	Sequence	Modification	Ratios
177	1	642.3095	-57.49	2	31.6	10.8	0	1423-1435	R.HPGPLGAPVSHSK.G		Q:W 1.49

Detailed Protein Report

Protein 480: protein furry homolog-like [Homo sapiens]

**Accession:**	gi|119874201	**Score:**	43.5
**Database:**	refseq_human(refseq_human_20140103.fasta)	**MW [kDa]:**	339.4
**Database Date:**	2015-11-30	**pI:**	5.3
		**Sequence Coverage [%]:**	1.4
		**No. of unique Peptides:**	1

Detailed Protein Report

10	20	30	40	50	60	70	80
MSNITIDPDV	KPGEYVIKSL	FAEFAVQAEK	KIEVVMAEPL	EKLLSRSLQR	GEDLQFDQLI	SSMSSVAEHC	LPSLLRTLFD
90	100	110	120	130	140	150	160
WYRRQNGTED	ESYEYRPRSS	TKSKGDEQQR	ERDYLLERRD	LAVDFIFCLV	LVEVLKQIPV	HPVPDPLVHE	VLNLAFKHFK
170	180	190	200	210	220	230	240
HKEGYSGTNT	GNVHIIADLY	AEVIGVLAQS	KFQAVRKKFV	TELKELRQKE	QSPHVVQSVI	SLIMGMKFFR	VKMYPVEDFE
250	260	270	280	290	300	310	320
ASFQFMQECA	QYFLEVKDKD	IKHALAGLFV	EILIPVAAAV	KNEVNVPCLK	NFVEMLYQTT	FELSSRKKHS	LALYPLITCL
330	340	350	360	370	380	390	400
LCVSQKQFFL	NNWHIFLQNC	LSHLKNKDPK	MSRVALESLY	RLLWVYVIRI	KCESNTVTQS	RLMSIVSALF	PKGSRSVVPR
410	420	430	440	450	460	470	480
DTPLNIFVKI	IQFIAQERLD	FAMKEIIFDL	LSVGKSTKTF	TINPERMNIG	LRVFLVIADS	LQQKDGEPPM	PTTGVILPSG
490	500	510	520	530	540	550	560
NTLRVKKIFL	NKTLTDEEAK	VIGMSVYYPQ	VRKALDSILR	HLDKEVGRPM	CMTSVQMSNK	EPEDMITGER	KPKIDLFRTC
570	580	590	600	610	620	630	640
IAAIPRLIPD	GMSRTDLIEL	LARLTIHMDE	ELRALAFNTL	QALMLDFPDW	REDVLSGFVY	FIVREVTDVH	PTLLDNAVKM
650	660	670	680	690	700	710	720
LVQLINQWKQ	AAQMHNKNQD	TQHGVANGAS	HPPPLERSPY	SNVFHVVEGF	ALVILCSSRP	ATRRLAVSVL	REIRALFALL
730	740	750	760	770	780	790	800
EIPKGDDELA	IDVMDRLSPS	ILESFIHLTG	ADQTTLLYCP	SSIDLQTLAE	WNSSPISHQF	DVISPSHIWI	FAHVTQGQDP
810	820	830	840	850	860	870	880
WIISLSSFLK	QENLPKHCST	AVSYAWMFAY	TRLQLLSPQV	DINSPINAKK	VNTTTSSDSY	IGLWRNYLIL	CCSAATSSSS
890	900	910	920	930	940	950	960
TSAGSVRCSP	PETLASTPDS	GYSIDSKIIG	IPSPSSLFKH	IVPMMRSESM	EITESLVLGL	GRTNPGAFRE	LIEELHPIIK
970	980	990	1000	1010	1020	1030	1040
EALERRPENM	KRRRRRDILR	VQLVRIFELL	ADAGVISHSA	SGGLDNETHF	LNNTLLEYVD	LTRQLLEAEN	EKDSDTLKDI
1050	1060	1070	1080	1090	1100	1110	1120
RCHFSALVAN	IIQNVPVHQR	RSIFPQQSLR	HSLFMLFSHW	AGPFSIMFTP	LDRYSDRNMQ	INRHQYCALK	AMSAVLCCGP
1130	1140	1150	1160	1170	1180	1190	1200
VADNVGLSSD	GYLYKWLDNI	LDSLDKKVHQ	LGCEAVTLLL	ELNPDQSNLM	YWAVDRCYTG	SGRVAAGCFK	AIANVFQNRD
1210	1220	1230	1240	1250	1260	1270	1280
YQCDTVMLLN	LILFKAADSS	RSIYEVAMQL	LQILEPKMFR	YAHKLEVQRT	DGVLSQLSPL	PHLYSVSYYQ	LSEELARAYP
1290	1300	1310	1320	1330	1340	1350	1360
ELTLAIFSEI	SQRIQTAHPA	GRQVMLHYLL	PWMNNIELVD	LKPLPTARRH	DEDEDDSLKD	RELMVTSRRW	LRGEGWGSPQ
1370	1380	1390	1400	1410	1420	1430	1440
ATAMVLNNLM	YMTAKYGDEL	AWSEVENVWT	TLADGWPKNL	KIILHFLISI	CGVNSEPSLL	PYVKKVIVYL	GRDKTMQLLE
1450	1460	1470	1480	1490	1500	1510	1520
ELVSELQLTD	PVSSGVTHMD	NPPYYRITSS	YKIPSVTSGT	TSSSNTMVAP	TDGNPDNKPI	KENIEESYVH	LDIYSGLNSH
1530	1540	1550	1560	1570	1580	1590	1600
LNRQHHRLES	RYSSSSGGSY	EEEKSDSMPL	YSNWRLKVME	HNQGEPLPFP	PAGGCWSPLV	DYVPETSSPG	LPLHRCNIAV
1610	1620	1630	1640	1650	1660	1670	1680
ILLTDLIIDH	SVKVEWGSYL	HLLLHAIFIG	FDHCHPEVYE	HCKRLLLHLL	IVMGPNSNIR	TVASVLLRNK	EFNEPRVLTV
1690	1700	1710	1720	1730	1740	1750	1760
KQVAHLDYNF	TAGINDFIPD	YQPSPMTDSG	LSSSSTSSSI	SLGNNSAAIS	HLHTTILNEV	DISVEQDGKV	KTLMEFITSR
1770	1780	1790	1800	1810	1820	1830	1840
KRGPLWNHED	VSAKNPSIKS	AEQLTTFLKH	VVSVFKQSSS	EGIHLEHHLS	EVALQTALSC	SSRHYAGRSF	QIFRALKQPL
1850	1860	1870	1880	1890	1900	1910	1920
TATTLSDVLS	RLVETVGDPG	EDAQGFVIEL	LLTLESAIDT	LAETMKHYDL	LSALSQTSYH	DPIMGNKYAA	NRKSTGQLNL
1930	1940	1950	1960	1970	1980	1990	2000
STSPINSSSY	LGYNSNARSN	SLRLSLIGDR	RGDRRRSNTL	DIMDGRINHS	SSLARTRSLS	SLREKGMYDV	QSTTEPTNLM
2010	2020	2030	2040	2050	2060	2070	2080
ATIFWIAASL	LESDYEYEYL	LALRLLNKLL	IHLPLDKSES	REKIENVQSK	LKWTNFPGLQ	QLFLKGFTSA	STQEMTVHLL
2090	2100	2110	2120	2130	2140	2150	2160
SKLISVSKHT	LVDPSQLSGF	PLNILCLLPH	LIQHFDSPTQ	FCKETASRIA	KVCAEEKCPT	LVNLAHMMSL	YSTHTYSRDC
2170	2180	2190	2200	2210	2220	2230	2240

Detailed Protein Report

Cmpd.	No. of Cmpds.	m/z meas.	Δ m/z [ppm]	z	Rt [min]	Score	P	Range	Sequence	Modification
2730	1	725.3873	9.20	3	65.2	13.7	2	541-558	K.EPEDMITGERKPKIDLFR.T	

Detailed Protein Report

Protein 481: PREDICTED: diacylglycerol kinase alpha isoform X2 [Homo sapiens]

**Accession:**	gi|530399854	**Score:**	43.4
**Database:**	refseq_human(refseq_human_20140103.fasta)	**MW [kDa]:**	69.9
**Database Date:**	2015-11-30	**pI:**	9.3
**Modification(s):**	Carbamidomethyl	**Sequence Coverage [%]:**	3.9
		**No. of unique Peptides:**	1

Alias proteins:

Accession	Name	Description
gi|530399856	refseq_human (refseq_human_20140103.fasta)	PREDICTED: diacylglycerol kinase alpha isoform X3 [Homo sapiens]

10	20	30	40	50	60	70	80
MVLRNRRGWG	ERLSPLGLNL	KEVDKIILQM	MRVAEYLDWD	VSELRPILQE	MMKEIDYDGS	GSVSQAEWVR	AGATTVPLLV
**90**	**100**	**110**	**120**	**130**	**140**	**150**	**160**
LLGLEMTLKD	DGQHMWRPKR	FPRPVYCNLC	ESSIGLGKQG	LSCNLCKYTV	HDQCAMKALP	CEVSTYAKSR	KDIGVQSHVW
**170**	**180**	**190**	**200**	**210**	**220**	**230**	**240**
VRGGCESGRC	DRCQKKIRIY	HSLTGLHCVW	CHLEIHDDCL	QAVGHECDCG	LLRDHILPPS	SIYPSVLASG	PDRKNSKTSQ
**250**	**260**	**270**	**280**	**290**	**300**	**310**	**320**
KTMDDLNLST	SEALRIDPVP	NTHPLLVFVN	PKSGGKQGQR	VLWKFQYILN	PRQVFNLLKD	GPEIGLRLFK	DVPDSRILVC
**330**	**340**	**350**	**360**	**370**	**380**	**390**	**400**
GGDGTVGWIL	ETIDKANLPV	LPPVAVLPLG	TGNDLARCLR	WGGGYEGQNL	AKILKDLEMS	KVVHMDRWSV	EVIPQQTEEK
**410**	**420**	**430**	**440**	**450**	**460**	**470**	**480**
SDPVPFQIIN	NYFSIGVDAS	IAHRFHIMRE	KYPEKFNSRM	KNKLWYFEFA	TSESIFSTCK	KLEESLTVEI	CGKPLDLSNL
**490**	**500**	**510**	**520**	**530**	**540**	**550**	**560**
SLEGIAVLNI	PSMHGGSNLW	GDTRRPHGDI	YGINQALGAT	AKVITDPDIL	KTCVPDLSDK	RLEVVGLEGA	IEMGQIYTKL
**570**	**580**	**590**	**600**	**610**	**620**	**630**	
KNAGRRLAKC	SEITFHTTKT	LPMQIDGEPW	MQTPCTIKIT	HKNQMPMLMG	PPPRSTNFFG	FLS	

Cmpd.	No. of Cmpds.	m/z meas.	Δ m/z [ppm]	z	Rt [min]	Score	P	Range	Sequence	Modification
1847	1	577.2815	93.89	2	52.9	16.5	1	163-172	R.GGCESGRCDR.C	Carbamidomethyl: 3, 8

Detailed Protein Report

Protein 482: protein Hook homolog 1 [Homo sapiens]

**Accession:**	gi|7705415	**Score:**	43.3
**Database:**	refseq_human(refseq_human_20140103.fasta)	**MW [kDa]:**	84.6
**Database Date:**	2015-11-30	**pI:**	5.0
**Modification(s):**	Oxidation	**Sequence Coverage [%]:**	4.8
		**No. of unique Peptides:**	2

Quantitation

M:Q	**Median:**	2.02	**CV:**	0.00 %	**No. of Peptides:**	1	

Alias proteins:

Accession	Name	Description
gi|530362743	refseq_human (refseq_human_20140103.fasta)	PREDICTED: protein Hook homolog 1 isoform X1 [Homo sapiens]

10	20	30	40	50	60	70	80
MEETQPPPQP	KLPLCDSLMI	WLQTFNTASP	CQDVKQLTSG	VAMAQVLHQI	DAAWFNESWL	SRIKEDVGDN	WRIKASNVKK
**90**	**100**	**110**	**120**	**130**	**140**	**150**	**160**
VLQGIMSYYH	EFLGQQISEA	LIPDLNQITE	CSDPVELGRL	LQLILGCAIN	CEKKQEHIQN	IMTLEESVQH	VVMTAIQELM
**170**	**180**	**190**	**200**	**210**	**220**	**230**	**240**
SKEILSSPPN	DAVGELEQQL	KRALEELQEA	LAEKEELRQR	CEELDMQVTT	LQDEKNSLVS	ENEMMNEKLD	QLDGSFDDPN
**250**	**260**	**270**	**280**	**290**	**300**	**310**	**320**
TVVAKKYFHA	QLQLEQLQEE	NFRLEAAKDD	YRVHCEELEK	QLIEFQHRND	ELTSLAEETR	ALKDEIDVLR	ATSDKANKLE
**330**	**340**	**350**	**360**	**370**	**380**	**390**	**400**
STVEIYRQKL	QDLNDLRKQV	KTLQETNMMY	MHNTVSLEEE	LKKANAARTQ	LETYKRQVQD	LHVKLSSESK	RADTLAFEMK
**410**	**420**	**430**	**440**	**450**	**460**	**470**	**480**
RLEEKHEALL	KEKERLIEQR	DTLKETNEEL	RCSQVQQDHL	NQTDASATKS	YENLAAEIMP	VEYREVFIRL	QHENKMLRLQ
**490**	**500**	**510**	**520**	**530**	**540**	**550**	**560**
QEGSENERIE	ELQEQLEQKH	RKMNELETEQ	RLSKERIREL	QQQIEDLQKS	LQEQGSKSEG	ESSSKLKQKL	EAHMEKLTEV
**570**	**580**	**590**	**600**	**610**	**620**	**630**	**640**
HEELQKKQEL	IEDLQPDINQ	NVQKINELEA	ALQKKDEDMK	AMEERYKMYL	EKARNVIKTL	DPKLNPASAE	IMLLRKQLAE
**650**	**660**	**670**	**680**	**690**	**700**	**710**	**720**
KERRIEILES	ECKVAKFRDY	EEKLIVSAWY	NKSLAFQKLG	MESRLVSGGG	ACSDTGACTP	ARSFLAQQRH	ITNTRRNLSV
**730**							
KVPATTSD							

Cmpd.	No. of Cmpds.	m/z meas.	Δ m/z [ppm]	z	Rt [min]	Score	P	Range	Sequence	Modification	Ratios
1597	1	685.8641	-18.30	2	48.3	10.6	2	546-556	K.LKQKLEAHMEK.L	Oxidation: 9	
1143	1	630.9795	-12.57	3	43.0	11.9	2	585-600	K.INELEAALQKKDEDMK.A	Oxidation: 15	M:Q 2.02

Detailed Protein Report

Protein 483: zinc finger protein 674 isoform 3 [Homo sapiens]

**Accession:**	gi|298676524	**Score:**	43.3
**Database:**	refseq_human(refseq_human_20140103.fasta)	**MW [kDa]:**	66.6
**Database Date:**	2015-11-30	**pI:**	10.2
**Modification(s):**	Carbamidomethyl	**Sequence Coverage [%]:**	12.0
		**No. of unique Peptides:**	3

Quantitation

Q:W	**Median:**	4.42	**CV:**	40.14 %	**No. of Peptides:**	2
M:Q	**Median:**	1.51	**CV:**	0.00 %	**No. of Peptides:**	1

```
        10         20         30         40         50         60         70         80
MAMSQESLTF KDVFVDFTLE EWQQLDSAQK NLYRDVMLEN YSHLVSVGHL VGKPDVIFRL GPGDESWMAD GGTPVRTCAE
        90        100        110        120        130        140        150        160
VWEVDEQIDH YKESQDKFLW QAAFIGKETL KDESGQECKI CRKIIYLNTD FVSVKQRLPK YYSWERCSKH HLNFLGQNRS
       170        180        190        200        210        220        230        240
YVRKKDDGCK AYWKVCLHYN LHKAQPAERF FDPNQRGKAL HQKQALRKSQ RSQTGEKLYK CTECGKVFIQ KANLVVHQRT
       250        260        270        280        290        300        310        320
HTGEKPYECC ECAKAFSQKS TLIAHQRTHT GEKPYECSEC GKTFIQKSTL IKHQRTHTGE KPFVCDKCPK AFKSSYHLIR
       330        340        350        360        370        380        390        400
HEKTHIRQAF YKGIKCTTSS LIYQRIHTSE KPQCSEHGKA SDEKPSPTKH WRTHTKENIY ECSKCGKSFR GKSHLSVHQR
       410        420        430        440        450        460        470        480
IHTGEKPYEC SICGKTFSGK SHLSVHHRTH TGEKPYECRR CGKAFGEKST LIVHQRMHTG EKPYKCNECG KAFSEKSPLI
       490        500        510        520        530        540        550        560
KHQRIHTGER PYECTDCKKA FSRKSTLIKH QRIHTGEKPY KCSECGKAFS VKSTLIVHHR THTGEKPYEC RDCGKAFSGK
       570        580
STLIKHQRSH TGDKNL
```

Cmpd.	No. of Cmpds.	m/z meas.	Δ m/z [ppm]	z	Rt [min]	Score	P	Range	Sequence	Modification	Ratios	
2101	1	599.9147	-53.58	3	56.4	11.0	2	373-387	R.THTKENIYECSKCGK.S	Carbamidomethyl: 10	Q:W	3.00
2175	1	701.2622	-138.53	3	57.6	10.2	2	522-540	K.CSECGKAFSVKSTLIVHHR.T		M:Q<br>Q:W	1.51<br>6.50
1639	1	1136.1099	81.18	2	50.4	11.8	2	541-560	R.THTGEKPYECRDCGKAFSGK.S	Carbamidomethyl: 13		

Detailed Protein Report

Protein 484: E3 SUMO-protein ligase CBX4 [Homo sapiens]

**Accession:**	gi|55770830	**Score:**	43.3
**Database:**	refseq_human(refseq_human_20140103.fasta)	**MW [kDa]:**	61.3
**Database Date:**	2015-11-30	**pI:**	10.0
**Modification(s):**	Oxidation	**Sequence Coverage [%]:**	6.8
		**No. of unique Peptides:**	1

10	20	30	40	50	60	70	80
MELPAVGEHV	FAVESIEKKR	IRKGRVEYLV	KWRGWSPKYN	TWEPEENILD	PRLLIAFQNR	ERQEQLMGYR	KRGPKPKPLV
**90**	**100**	**110**	**120**	**130**	**140**	**150**	**160**
VQVPTFARRS	NVLTGLQDSS	TDNRAKLDLG	AQGKGQGHQY	ELNSKKHHQY	QPHSKERAGK	PPPPGKSGKY	YYQLNSKKHH
**170**	**180**	**190**	**200**	**210**	**220**	**230**	**240**
PYQPDPKMYD	LQYQGGHKEA	PSPTCPDLGA	KSHPPDKWAQ	GAGAKGYLGA	VKPLAGAAGA	PGKGSEKGPP	NGMMPAPKEA
**250**	**260**	**270**	**280**	**290**	**300**	**310**	**320**
VTGNGIGGKM	KIVKNKNKNG	RIVIVMSKYM	ENGMQAVKIK	SGEVAEGEAR	SPSHKKRAAD	ERHPPADRTF	KKAAGAEEKK
**330**	**340**	**350**	**360**	**370**	**380**	**390**	**400**
VEAPPKRREE	EVSGVSDPQP	QDAGSRKLSP	TKEAFGEQPL	QLTTKPDLLA	WDPARNTHPP	SHHPHPHPHH	HHHHHHHHHH
**410**	**420**	**430**	**440**	**450**	**460**	**470**	**480**
AVGLNLSHVR	KRCLSETHGE	REPCKKRLTA	RSISTPTCLG	GSPAAERPAD	LPPAAALPQP	EVILLDSDLD	EPIDLRCVKT
**490**	**500**	**510**	**520**	**530**	**540**	**550**	**560**
RSEAGEPPSS	LQVKPETPAS	AAVAVAAAAA	PTTTAEKPPA	EAQDEPAESL	SEFKPFFGNI	IITDVTANCL	TVTFKEYVTV
**570**							

Cmpd.	No. of Cmpds.	m/z meas.	Δ m/z [ppm]	z	Rt [min]	Score	P	Range	Sequence	Modification
443	1	699.2695	-110.76	3	34.5	10.6	1	228-249	K.GPPNGMMPAPKEAVTGNGIGGK.M	Oxidation: 6

Detailed Protein Report

Protein 485: probable G-protein coupled receptor 179 precursor [Homo sapiens]

**Accession:**	gi|93352554	**Score:**	43.3
**Database:**	refseq_human(refseq_human_20140103.fasta)	**MW [kDa]:**	257.2
**Database Date:**	2015-11-30	**pI:**	5.4
**Modification(s):**	Carbamidomethyl, Oxidation	**Sequence Coverage [%]:**	1.2
		**No. of unique Peptides:**	2

Quantitation

Q:W	**Median:**	1.61	**CV:**	0.00 %	**No. of Peptides:**	1
M:Q	**Median:**	0.68	**CV:**	9.61 %	**No. of Peptides:**	2

Detailed Protein Report

10	20	30	40	50	60	70	80
MGTRGAVMPP	PMWGLLGCCF	VCAWALGGPR	PIRSLPPLSS	QVKPGSVPMQ	VPLEGAEAAL	AYLYSGDAQQ	LSQVNCSERY
90	100	110	120	130	140	150	160
EARGAGAMPG	LPPSLQGAAG	TLAQAANFLN	MLLQANDIRE	SSVEEDVEWY	QALVRSVAEG	DPRVYRALLT	FNPPPGASHL
170	180	190	200	210	220	230	240
QLALQATRTG	EETILQDLSG	NWVQEENPPG	DLDTPALKKR	VLTNDLGSLG	SPKWPQADGY	VGDTQQVRLS	PPFLECQEGR
250	260	270	280	290	300	310	320
LRPGWLITLS	ATFYGLKPDL	SPEVRGQVQM	DVDLQSVDIN	QCASGPGWYS	NTHLCDLNST	QCVPLESQGF	VLGRYLCRCR
330	340	350	360	370	380	390	400
PGFYGASPSG	GLEESDFQTT	GQFGFPEGRS	GRLLQCLPCP	EGCTSCMDAT	PCLVEEAAVL	RAAVLACQAC	CMLAIFLSML
410	420	430	440	450	460	470	480
VSYRCRRNKR	IWASGVVLLE	TVLFGFLLLY	FPVFILYFKP	SVFRCIALRW	VRLLGFAIVY	GTIILKLYRV	LQLFLSRTAQ
490	500	510	520	530	540	550	560
RSALLSSGRL	LRRLGLLLLP	VLGFLAVWTV	GALERGIQHA	PLVIRGHTPS	GRHFYLCHHD	RWDYIMVVAE	LLLLCWGSFL
570	580	590	600	610	620	630	640
CYATRAVLSA	FHEPRYMGIA	LHNELLLSAA	FHTARFVLVP	SLHPDWTLLL	FFFHTHSTVT	TTLALIFIPK	FWKLGAPPRE
650	660	670	680	690	700	710	720
EMVDEVCEDE	LDLQHSGSYL	GSSIASAWSE	HSLDPGDIRD	ELKKLYAQLE	VHKTKEMAAN	NPHLPKKRGS	SCQGLGRSFM
730	740	750	760	770	780	790	800
RYLAEFPEAL	ARQHSRDSGS	PGHGSLPGSS	RRRLLSSSLQ	EPEGTPALHK	SRSTYDQRRE	QDPPLLDSLL	RRKLAKKASR
810	820	830	840	850	860	870	880
TESRESVEGP	PALGFRSASA	HNLTVGERLP	RARPASLQKS	LSVASSREKA	LLMASQAYLE	ETYRQAKERE	ERKKAKAAMA
890	900	910	920	930	940	950	960
SLVRRPSARR	LERPRGAPLS	APPSPAKSSS	VDSSHTSGRL	HEEARRRLPH	PPIRHQVSTP	ILALSGGLGE	PRMLSPTSTL
970	980	990	1000	1010	1020	1030	1040
APALLPALAP	TPAPALAPVP	VSPQSPNLLT	YICPWENAEL	PAKQENVPQE	GPSGPERGHH	SPAPARARLW	RALSVAVEKS
1050	1060	1070	1080	1090	1100	1110	1120
RAGENEMDAE	DAHHQREAND	VDEDRPKIFP	KSHSLKAPVQ	QGSMRSLGLA	IKALTRSRST	YREKESVEES	PEGQNSGTAG
1130	1140	1150	1160	1170	1180	1190	1200
ESMGAPSRSP	RLGRPKAVSK	QAALIPSDDK	ESLQNQQNAH	TSRMLQVCQR	EGSREQEDRG	RRMTQGLGER	KAERAGKTGL
1210	1220	1230	1240	1250	1260	1270	1280
AMLRQVSRDK	NIKQSKETPV	GWQELPKAGL	QSLGSADHRV	AEVCPWEVTE	SETRQPDSGN	KAEICPWETS	EGAPESRALR
1290	1300	1310	1320	1330	1340	1350	1360
QDPGDSQKKR	GEARGKSEPI	DVVPMMRKKP	ERLVREQEAV	CPWESADRGG	LSPGSAPQDP	GRIRDKSEAG	DSVEARKVEK
1370	1380	1390	1400	1410	1420	1430	1440
PGWEAAGPEA	HTPDITKAEP	CPWEASEGGE	DGKPAQEAVK	DLPQEKQKTR	KATFWKEQKP	GGDLESLCPW	ESTDFRGPSA
1450	1460	1470	1480	1490	1500	1510	1520
VSIQAPGSSE	CSGSLGSGIA	EVCLWEAGDA	PAIQKAEICP	WELDDNVMGQ	EMLSLGTGRE	SLQEKEKASR	KGSFGEMGEQ
1530	1540	1550	1560	1570	1580	1590	1600
TVKAVQKLSQ	QQESVCPRES	TVPGHSSPCL	DNSSSKAGSQ	FLCNGGSRAT	QVCPQEDLRP	EAQEATPAKT	EICPWEVNER
1610	1620	1630	1640	1650	1660	1670	1680
TREEWTSAQV	PRGGESQKDK	EKMPGKSEIE	DVTAWEKPEG	QIQKQEAVGP	WESVDPGSFS	PQPRPQDTER	PQTLLQMSGS
1690	1700	1710	1720	1730	1740	1750	1760
VGSKAADICP	LDVEENLTAG	KAEICPWEVG	AGAGEERALG	AEAIRKSPND	TGKVSADLGP	RERAVTAPEK	PQKPTPEWEV
1770	1780	1790	1800	1810	1820	1830	1840
ACPWGSVGPG	ACSQHPGTLD	ADGPKAGFQE	LDHMGCRPGE	VCPWEAQEAA	TSEKAKICPW	EVSEGTTGKG	LDQKAGSESA
1850	1860	1870	1880	1890	1900	1910	1920
EQREKALEKG	RLTSLGEDVS	KGMAKLCQQQ	ETICIWENKD	LRESPAQAPK	ISDLPSSMSS	EVAEGHSLEA	TEKGDLRQDP
1930	1940	1950	1960	1970	1980	1990	2000
KTGSFPEHIT	QEKAPAADTE	EFTTEDGEKT	SHELQSVCPW	ETTAPADSVS	HLDRQRPDQP	KASSQRLVST	GGRAADVCPW
2010	2020	2030	2040	2050	2060	2070	2080
DVPDAGVYKS	DSSAKAETCP	WEVTERIPVK	GVSRQDGKGD	SQEEKGRAPE	KSEPKGVPVQ	KKPEMADFRQ	QEAVCPWESQ
2090	2100	2110	2120	2130	2140	2150	2160
DGKGLSPQPA	PDASDRSRGS	SEAAGSVETR	VAEVCLWEVV	EAPSAKKAEI	CPWEAGGGAA	EEGEQERESQ	GQGEMFLQKA
2170	2180	2190	2200	2210	2220	2230	2240

Detailed Protein Report

Cmpd.	No. of Cmpds.	m/z meas.	Δ m/z [ppm]	z	Rt [min]	Score	P	Range	Sequence	Modification	Ratios	
479	1	475.6002	-274.91	2	35.6	13.9	0	1164-1170	R.MLQVCQR.E	Carbamidomethyl: 5; Oxidation: 1	M:Q	0.75
813	11	509.1525	-284.31	2	38.6	17.2	1	1195-1204	R.AGKTGLAMLR.Q		Q:W<br>M:Q	1.61<br>0.62

Detailed Protein Report

Protein 486: prosaposin receptor GPR37 precursor [Homo sapiens]

Accession: gi|4885323
Database: refseq_human(refseq_human_20140103.fasta)
Database Date: 2015-11-30
Modification(s): Oxidation

Score: 43.2
MW [kDa]: 67.1
pI: 9.9
Sequence Coverage [%]: 4.2
No. of unique Peptides: 1

Quantitation

Q:W	**Median:**	0.31	**CV:**	0.00 %	**No. of Peptides:**	1
M:Q	**Median:**	1.22	**CV:**	0.00 %	**No. of Peptides:**	1

10	20	30	40	50	60	70	80
MRAPGALLAR	MSRLLLLLLL	KVSASSALGV	APASRNETCL	GESCAPTVIQ	RRGRDAWGPG	NSARDVLRAR	APREEQGAAF
90	100	110	120	130	140	150	160
LAGPSWDLPA	APGRDPAAGR	GAEASAAGPP	GPPTRPPGPW	RWKGARGQEP	SETLGRGNPT	ALQLFLQISE	EEEKGPRGAG
170	180	190	200	210	220	230	240
ISGRSQEQSV	KTVPGASDLF	YWPRRAGKLQ	GSHHKPLSKT	ANGLAGHEGW	TIALPGRALA	QNGSLGEGIH	EPGGPRRGNS
250	260	270	280	290	300	310	320
TNRRVRLKNP	FYPLTQESYG	AYAVMCLSVV	IFGTGIIGNL	AVMCIVCHNY	YMRSISNSLL	ANLAFWDFLI	IFFCLPLVIF
330	340	350	360	370	380	390	400
HELTKKWLLE	DFSCKIVPYI	EVASLGVTTF	TLCALCIDRF	RAATNVQMYY	EMIENCSSTT	AKLAVIWVGA	LLLALPEVVL
410	420	430	440	450	460	470	480
RQLSKEDLGF	SGRAPAERCI	IKISPDLPDT	IYVLALTYDS	ARLWWYFGCY	FCLPTLFTIT	CSLVTARKIR	KAEKACTRGN
490	500	510	520	530	540	550	560
KRQIQLESQM	NCTVVALTIL	YGFCIIPENI	CNIVTAYMAT	GVSQQTMDLL	NIISQFLLFF	KSCVTPVLLF	CLCKPFSRAF
570	580	590	600	610	620		
MECCCCCCEE	CIQKSSTVTS	DDNDNEYTTE	LELSPFSTIR	REMSTFASVG	THC		

Cmpd.	No. of Cmpds.	m/z meas.	Δ m/z [ppm]	z	Rt [min]	Score	P	Range	Sequence	Modification	Ratios
2128	2	721.2347	-109.30	2	56.8	13.5	1	601-613	R.REMSTFASVGTHC.-	Oxidation: 3	M:Q 1.22<br>Q:W 0.31

Detailed Protein Report

Protein 487: ATP-dependent RNA helicase DDX42 [Homo sapiens]

**Accession:**	gi|45446743	**Score:**	43.2
**Database:**	refseq_human(refseq_human_20140103.fasta)	**MW [kDa]:**	102.9
**Database Date:**	2015-11-30	**pI:**	6.6
		**Sequence Coverage [%]:**	5.1
		**No. of unique Peptides:**	3

Alias proteins:

Accession	Name	Description
gi|578830314	refseq_human (refseq_human_20140103.fasta)	PREDICTED: ATP-dependent RNA helicase DDX42 isoform X1 [Homo sapiens]
gi|45446747	refseq_human (refseq_human_20140103.fasta)	ATP-dependent RNA helicase DDX42 [Homo sapiens]

```
        10         20         30         40         50         60         70         80
MNWNKGGPGT KRGFGFGGFA ISAGKKEEPK LPQQSHSAFG ATSSSSGFGK SAPPQLPSFY KIGSKRANFD EENAYFEDEE
        90        100        110        120        130        140        150        160
EDSSNVDLPY IPAENSPTRQ QFHSKPVDSD SDDDPLEAFM AEVEDQAARD MKRLEEKDKE RKNVKGIRDD IEEEDDQEAY
       170        180        190        200        210        220        230        240
FRYMAENPTA GVVQEEEEDN LEYDSDGNPI APTKKIIDPL PPIDHSEIDY PPFEKNFYNE HEEITNLTPQ QLIDLRHKLN
       250        260        270        280        290        300        310        320
LRVSGAAPPR PGSSFAHFGF DEQLMHQIRK SEYTQPTPIQ CQGVPVALSG RDMIGIAKTG SGKTAAFIWP MLIHIMDQKE
       330        340        350        360        370        380        390        400
LEPGDGPIAV IVCPTRELCQ QIHAECKRFG KAYNLRSVAV YGGGSMWEQA KALQEGAEIV VCTPGRLIDH VKKKATNLQR
       410        420        430        440        450        460        470        480
VSYLVFDEAD RMFDMGFEYQ VRSIASHVRP DRQTLLFSAT FRKKIEKLAR DILIDPIRVV QGDIGEANED VTQIVEILHS
       490        500        510        520        530        540        550        560
GPSKWNWLTR RLVEFTSSGS VLLFVTKKAN AEELANNLKQ EGHNLGLLHG DMDQSERNKV ISDFKKKDIP VLVATDVAAR
       570        580        590        600        610        620        630        640
GLDIPSIKTV INYDVARDID THTHRIGRTG RAGEKGVAYT LLTPKDSNFA GDLVRNLEGA NQHVSKELLD LAMQNAWFRK
       650        660        670        680        690        700        710        720
SRFKGGKGKK LNIGGGGLGY RERPGLGSEN MDRGNNNVMS NYEAYKPSTG AMGDRLTAMK AAFQSQYKSH FVAASLSNQK
       730        740        750        760        770        780        790        800
AGSSAAGASG WTSAGSLNSV PTNSAQQGHN SPDSPVTSAA KGIPGFGNTG NISGAPVTYP SAGAQGVNNT ASGNNSREGT
       810        820        830        840        850        860        870        880
GGSNGKRERY TENRGSSRHS HGETGNRHSD SPRHGDGGRH GDGYRHPESS SRHTDGHRHG ENRHGGSAGR HGENRGANDG
       890        900        910        920        930        940
RNGESRKEAF NRESKMEPKM EPKVDSSKMD KVDSKTDKTA DGFAVPEPPK RKKSRWDS
```

Cmpd.	No. of Cmpds.	m/z meas.	Δ m/z [ppm]	z	Rt [min]	Score	P	Range	Sequence	Modification
1875	1	990.8378	-137.62	2	51.9	12.0	0	31-50	K.LPQQSHSAFGATSSSSGFGK.S	
2705	1	558.6699	147.14	3	64.9	15.6	1	819-833	R.HSHGETGNRHSDSPR.H	
2100	1	753.3769	8.39	2	56.6	15.7	2	896-908	K.MEPKMEPKVDSSK.M	

Detailed Protein Report

Protein 488: PREDICTED: serine/arginine repetitive matrix protein 2 isoform X3 [Homo sapiens]

**Accession:**	gi|530407861	**Score:**	43.2
**Database:**	refseq_human(refseq_human_20140103.fasta)	**MW [kDa]:**	281.3
**Database Date:**	2015-11-30	**pI:**	12.6
		**Sequence Coverage [%]:**	1.5
		**No. of unique Peptides:**	3

Quantitation

Q:W	**Median:**	0.23	**CV:**	0.00 %	**No. of Peptides:**	1	

Detailed Protein Report

10	20	30	40	50	60	70	80
MYNGIGLPTP	RGSGTNGYVQ	RNLSLVRGRR	GERPDYKGEE	ELRRLEAALV	KRPNPDILDH	ERKRRVELRC	LELEEMMEEQ
90	100	110	120	130	140	150	160
GYEEQQIQEK	VATFRLMLLE	KDVNPGGKEE	TPGQRPAVTE	THQLAELNEK	KNERLRAAFG	ISDSYVDGSS	FDPQRRAREA
170	180	190	200	210	220	230	240
KQPAPEPPKP	YSLVRESSSS	RSPTPKQKKK	KKKKDRGRRS	ESSSPRRERK	KSSKKKKHRS	ESESKKRKHR	SPTPKSKRKS
250	260	270	280	290	300	310	320
KDKKRKRSRS	TTPAPKSRRA	HRSTSADSAS	SSDTSRSRSR	SAAAKTHTTA	LAGRSPSPAS	GRRGEGDAPF	SEPGTTSTQR
330	340	350	360	370	380	390	400
PSSPETATKQ	PSSPYEDKDK	DKKEKSATRP	SPSPERSSTG	PEPPAPTPLL	AERHGGSPQP	LATTPLSQEP	VNPPSEASPT
410	420	430	440	450	460	470	480
RDRSPPKSPE	KLPQSSSSES	SPPSPQPTKV	SRHASSSPES	PKPAPAPGSH	REISSSPTSK	NRSHGRAKRD	KSHSHTPSRR
490	500	510	520	530	540	550	560
MGRSRSPATA	KRGRSRSRTP	TKRGHSRSRS	PQWRRSRSAQ	RWGRSRSPQR	RGRSRSPQRP	GWSRSRNTQR	RGRSRSARRG
570	580	590	600	610	620	630	640
RSHSRSPATR	GRSRSRTPAR	RGRSRSRTPA	RRRSRSRTPT	RRRSRSRTPA	RRGRSRSRTP	ARRRSRTRSP	VRRRSRSRSP
650	660	670	680	690	700	710	720
ARRSGRSRSR	TPARRGRSRS	RTPARRGRSR	SRTPARRSGR	SRSRTPARRG	RSRSRTPRRG	RSRSRSLVRR	GRSHSRTPQR
730	740	750	760	770	780	790	800
RGRSGSSSER	KNKSRTSQRR	SRSNSSPEMK	KSRISSRRSR	SLSSPRSKAK	SRLSLRRSLS	GSSPCPKQKS	QTPPRRSRSG
810	820	830	840	850	860	870	880
SSQPKAKSRT	PPRRSRSSSS	PPPKQKSKTP	SRQSHSSSSP	HPKVKSGTPP	RQGSITSPQA	NEQSVTPQRR	SCFESSPDPE
890	900	910	920	930	940	950	960
LKSRTPSRHS	CSGSSPPRVK	SSTPPRQSPS	RSSSPQPKVK	AIISPRQRSH	SGSSSPSPSR	VTSRTTPRRS	RSVSPCSNVE
970	980	990	1000	1010	1020	1030	1040
SRLLPRYSHS	GSSSPDTKVK	PETPPRQSHS	GSISPYPKVK	AQTPPGPSLS	GSKSPCPQEK	SKDSLVQSCP	GSLSLCAGVK
1050	1060	1070	1080	1090	1100	1110	1120
SSTPPGESYF	GVSSLQLKGQ	SQTSPDHRSD	TSSPEVRQSH	SESPSLQSKS	QTSPKGGRSR	SSSPVTELAS	RSPIRQDRGE
1130	1140	1150	1160	1170	1180	1190	1200
FSASPMLKSG	MSPEQSRFQS	DSSSYPTVDS	NSLLGQSRLE	TAESKEKMAL	PPQEDATASP	PRQKDKFSPF	PVQDRPESSL
1210	1220	1230	1240	1250	1260	1270	1280
VFKDTLRTPP	RERSGAGSSP	ETKEQNSALP	TSSQDEELME	VVEKSEEPAG	QILSHLSSEL	KEMSTSNFES	SPEVEERPAV
1290	1300	1310	1320	1330	1340	1350	1360
SLTLDQSQSQ	ASLEAVEVPS	MASSWGGPHF	SPEHKELSNS	PLRENSFGSP	LEFRNSGPLG	TEMNTGFSSE	VKEDLNGPFL
1370	1380	1390	1400	1410	1420	1430	1440
NQLETDPSLD	MKEQSTRSSG	HSSSELSPDA	VEKAGMSSNQ	SISSPVLDAV	PRTPSRERSS	SASSPEMKDG	LPRTPSRRSR
1450	1460	1470	1480	1490	1500	1510	1520
SGSSPGLRDG	SGTPSRHSLS	GSSPGMKDIP	RTPSRGRSEC	DSSPEPKALP	QTPRPRSRSP	SSPELNNKCL	TPQRERSGSE
1530	1540	1550	1560	1570	1580	1590	1600
SSVDQKTVAR	TPLGQRSRSG	SSQELDVKPS	ASPQERSESD	SSPDSKAKTR	TPLRQRSRSG	SSPEVDSKSR	LSPRRSRSGS
1610	1620	1630	1640	1650	1660	1670	1680
SPEVKDKPRA	APRAQSGSDS	SPEPKAPAPR	ALPRRSRSGS	SSKGRGPSPE	GSSSTESSPE	HPPKSRTARR	GSRSSPEPKT
1690	1700	1710	1720	1730	1740	1750	1760
KSRTPPRRRS	SRSSPELTRK	ARLSRRSRSA	SSSPETRSRT	PPRHRRSPSV	SSPEPAEKSR	SSRRRRSASS	PRTKTTSRRG
1770	1780	1790	1800	1810	1820	1830	1840
RSPSPKPRGL	QRSRSRSRRE	KTRTTRRRDR	SGSSQSTSRR	RQRSRSRSRV	TRRRRGGSGY	HSRSPARQES	SRTSSRRRRG
1850	1860	1870	1880	1890	1900	1910	1920
RSRTPPTSRK	RSRSRTSPAP	WKRSRSRASP	ATHRRSRSRT	PLISRRRSRS	RTSPVSRRRS	RSRTSVTRRR	SRSRASPVSR
1930	1940	1950	1960	1970	1980	1990	2000
RRSRSRTPPV	TRRRSRSRTP	TTRRRSRSRT	PPVTRRRSRS	RTPPVTRRRS	RSRTSPITRR	RSRSRTSPVT	RRRSRSRTSP
2010	2020	2030	2040	2050	2060	2070	2080
VTRRRSRSRT	SPVTRRRSRS	RTPPAIRRRS	RSRTPLLPRK	RSRSRSPLAI	RRRSRSRTPR	TARGKRSLTR	SPPAIRRRSA
2090	2100	2110	2120	2130	2140	2150	2160
SGSSSDRSRS	ATPPATRNHS	GSRTPPVALN	SSRMSCFSRP	SMSPTPLDRC	RSPGMLEPLG	SSRTPMSVLQ	QAGGSMMDGP
2170	2180	2190	2200	2210	2220	2230	2240

Detailed Protein Report

Cmpd.	No. of Cmpds.	m/z meas.	Δ m/z [ppm]	z	Rt [min]	Score	P	Range	Sequence	Modification	Ratios
2951	11	703.3519	-15.42	2	65.6	16.8	1	833-845	R.QSHSSSSPHPKVK.S		
2324	1	841.5088	116.64	2	57.3	10.3	1	1614-1630	R.AQSGSDSSPEPKAPAPR.A		Q:W 0.23
873	1	491.7878	51.39	2	39.7	16.0	1	1866-1874	R.SRASPATHR.R		

Detailed Protein Report

Protein 489: PREDICTED: tripartite motif-containing protein 2 isoform X2 [Homo sapiens]

Accession: gi|578808962
Database: refseq_human(refseq_human_20140103.fasta)
Database Date: 2015-11-30

Score: 43.2
MW [kDa]: 84.7
pI: 7.3
Sequence Coverage [%]: 4.5
No. of unique Peptides: 1

Quantitation

M:Q **Median:** 2.24 **CV:** 0.00 % **No. of Peptides:** 1

10	20	30	40	50	60	70	80
MHRSGRYGTQ	QRAGSKTAGP	PCQWSRMASE	GTNIPSPVVR	QIDKQFLICS	ICLERYKNPK	VLPCLHTFCE	RCLQNYIPAH
90	100	110	120	130	140	150	160
SLTLSCPVCR	QTSILPEKGV	AALQNNFFIT	NLMDVLQRTP	GSNAEESSIL	ETVTAVAAGK	PLSCPNHDGN	VMEFYCQSCE
170	180	190	200	210	220	230	240
TAMCRECTEG	EHAEHPTVPL	KDVVEQHKAS	LQVQLDAVNK	RLPEIDSALQ	FISEIIHQLT	NQKASIVDDI	HSTFDELQKT
250	260	270	280	290	300	310	320
LNVRKSVLLM	ELEVNYGLKH	KVLQSQLDTL	LQGQESIKSC	SNFTAQALNH	GTETEVLLVK	KQMSEKLNEL	ADQDFPLHPR
330	340	350	360	370	380	390	400
ENDQLDFIVE	TEGLKKSIHN	LGTILTTNAV	ASETVATGEG	LRQTIIGQPM	SVTITTKDKD	GELCKTGNAY	LTAELSTPDG
410	420	430	440	450	460	470	480
SVADGEILDN	KNGTYEFLYT	VQKEGDFTLS	LRLYDQHIRG	SPFKLKVIRS	ADVSPTTEGV	KRRVKSPGSG	HVKQKAVKRP
490	500	510	520	530	540	550	560
ASMYSTGKRK	ENPIEDDLIF	RVGTKGRNKG	EFTNLQGVAA	STNGKILIAD	SNNQCVQIFS	NDGQFKSRFG	IRGRSPGQLQ
570	580	590	600	610	620	630	640
RPTGVAVHPS	GDIIIADYDN	KWVSIFSSDG	KFKTKIGSGK	LMGPKGVSVD	RNGHIIVVDN	KACCVFIFQP	NGKIVTRFGS
650	660	670	680	690	700	710	720
RGNGDRQFAG	TLDGPHFAAV	NSNNEIIITD	FHNHSVKVFN	QEGEFMLKFG	SNGEGNGQFN	APTGVAVDSN	GNIIVADWGN
730	740	750	760	770	780		
SRIQVFDGSG	SFLSYINTSA	DPLYGPQGLA	LTSDGHVVVA	DSGNHCFKVY	RYLQ		

Cmpd.	No. of Cmpds.	m/z meas.	Δ m/z [ppm]	z	Rt [min]	Score	P	Range	Sequence	Modification	Ratios
2668	1	673.3612	23.64	2	63.5	12.0	2	2-12	M.HRSGRYGTQQR.A		M:Q 2.24

Detailed Protein Report

Protein 490: zinc finger protein 831 [Homo sapiens]

**Accession:**	gi|123701326	**Score:**	43.2
**Database:**	refseq_human(refseq_human_20140103.fasta)	**MW [kDa]:**	177.8
**Database Date:**	2015-11-30	**pI:**	9.7
**Modification(s):**	Carbamidomethyl	**Sequence Coverage [%]:**	2.7
		**No. of unique Peptides:**	2

Alias proteins:

Accession	Name	Description
gi|578835748	refseq_human (refseq_human_20140103.fasta)	PREDICTED: zinc finger protein 831 isoform X5 [Homo sapiens]
gi|530417822	refseq_human (refseq_human_20140103.fasta)	PREDICTED: zinc finger protein 831 isoform X3 [Homo sapiens]
gi|530417820	refseq_human (refseq_human_20140103.fasta)	PREDICTED: zinc finger protein 831 isoform X2 [Homo sapiens]
gi|530417818	refseq_human (refseq_human_20140103.fasta)	PREDICTED: zinc finger protein 831 isoform X1 [Homo sapiens]

Detailed Protein Report

10	20	30	40	50	60	70	80
MEVPEPTCPA	PPARDQPAPT	PGPPGAPGGQ	ASPHLTLGPV	LLPPEQGLAP	PTVFLKALPI	PLYHTVPPGG	LQPRAPLVTG
90	100	110	120	130	140	150	160
SLDGGNVPFI	LSPVLQPEGP	GPTQVGKPAA	PTLTVNIVGT	LPVLSPGLGP	TLGSPGKVRN	AGKYLCPHCG	RDCLKPSVLE
170	180	190	200	210	220	230	240
KHIRSHTGER	PFPCATCGIA	FKTQSNLYKH	RRTQTHLNNS	RLSSESEGAG	GGLLEEGDKA	GEPPRPEGRG	ESRCQGMHEG
250	260	270	280	290	300	310	320
ASERPLSPGA	HVPLLAKNLD	VRTEAAPCPG	SAFADREAPW	DSAPMASPGL	PAASTQPWRK	LPEQKSPTAG	KPCALQRQQA
330	340	350	360	370	380	390	400
TAAEKPWDAK	APEGRLRKCE	STDSGYLSRS	DSAEQPHAPC	SPLHSLSEHS	AESEGEGGPG	PGPGVAGAEP	GAREAGLELE
410	420	430	440	450	460	470	480
KKRLEERIAQ	LISHNQAVVD	DAQLDNVRPR	KTGLSKQGSI	DLPTPYTYKD	SFHFDIRALE	PGRRRAPGPV	RSTWTPPDKS
490	500	510	520	530	540	550	560
RPLFFHSVPT	QLSTTVECVP	VTRSNSLPFV	EGSRTWLEPR	EPRDPWSRTQ	KPLSPRPGPA	RLGCRSGLSS	TDVPSGHPRA
570	580	590	600	610	620	630	640
LVRQAAVEDL	PGTPIGDALV	PAEDTDAKRT	AAREAMAGKG	RAGGRKCGQR	RLKMFSQEKW	QVYGDETFKR	IYQKMKASPH
650	660	670	680	690	700	710	720
GGKKAREVGM	GSGAELGFPL	QKEAAGSSGT	VPTQDRRTPV	HEDISAGATP	EPWGNPPALE	ASLVTEPTKH	GETVARRGDS
730	740	750	760	770	780	790	800
DRPRVEEAVS	SPALGGRDSP	CSGSRSPLVS	PNGRLELGWQ	MPPAPGPLKG	GDVEAPRPVW	PDPKLEGGAR	GVGDVQETCL
810	820	830	840	850	860	870	880
WAQTVLRWPS	RGSGEDKLPS	ERKKLKVEDL	HSWKQPEPVS	AETPGGPTQP	ASLSSQKQDA	DPGEVPGGSK	ESARQVGEPL
890	900	910	920	930	940	950	960
ESSGASLAAA	SVALKRVGPR	DKATPLHPAA	PAPAEHPSLA	TPPQAPRVLS	ALADNAFSPK	YLLRLPQAET	PLPLPIPWGP
970	980	990	1000	1010	1020	1030	1040
RHSQDSLCSS	GWPEERASFV	GSGLGTPLSP	SPASGPSPGE	ADSILEDPSC	SRPQDGRKGA	QLGGDKGDRM	ATSRPAAREL
1050	1060	1070	1080	1090	1100	1110	1120
PISAPGAPRE	ATSSPPTPTC	EAHLVQDMEG	DSHRIHRLCM	GSTLARARLS	GDVLNPWVPN	WELGEPPGNA	PEDPSSGPLV
1130	1140	1150	1160	1170	1180	1190	1200
GPDPCSPLQP	GSFLTALTRP	QGVPPGWPEL	ALSSHSGTSR	SHSTRSPHST	QNPFPSLKAE	PRLTWCCLSR	SVPLPAEQKA
1210	1220	1230	1240	1250	1260	1270	1280
KAASVYLAVH	FPGSSLRDEG	PNGPPGSNGG	WTWTSPGEGG	PAQMSKFSYP	TVPGVMPQHQ	VSEPEWKKGL	PWRAKMSRGN
1290	1300	1310	1320	1330	1340	1350	1360
SKQRKLKINP	KRYKGNFLQS	CVQLRASRLR	TPTWVRRRSR	HPPALEGLKP	CRTPGQTSSE	IAGLNLQEEP	SCATSESPPC
1370	1380	1390	1400	1410	1420	1430	1440
CGKEEKKEGD	CRQTLGTLSL	GTSSRIVREM	DKRTVKDISP	SAGEHGDCTT	HSTAATSGLS	LQSDTCLAVV	NDVPLPPGKG
1450	1460	1470	1480	1490	1500	1510	1520
LDLGLLETQL	LASQDSVSTD	PKPYIFSDAQ	RPSSFGSKGT	FPHHDIATSV	AAVCISLPVR	TDHIAQEIHS	AESRDHSQTA
1530	1540	1550	1560	1570	1580	1590	1600
GRTLTSSSPD	SKVTEEGRAQ	TLLPGRPSSG	QRISDSVPLE	STEKTHLEIP	ASGPSSASSH	HKEGRHKTFF	PSRGQYGCGE
1610	1620	1630	1640	1650	1660	1670	1680
MTVPCPSLGS	DGRKRQVSGL	ITRKDSVVPS	KPEQPIEIPE	APSKSLKKRS	LEGMRKQTRV	EFSDTSSDDE	DRLVIEI

Cmpd.	No. of Cmpds.	m/z meas.	Δ m/z [ppm]	z	Rt [min]	Score	P	Range	Sequence	Modification
2187	11	1023.8998	-84.84	2	57.2	18.3	1	258-276	K.NLDVRTEAAPCPGSAFADR.E	Carbamidomethyl: 11
1503	1	626.6200	-108.01	3	48.5	13.1	1	928-944	R.VLSALADNAFSPKYLLR.L	

Detailed Protein Report

Protein 491: RB1-inducible coiled-coil protein 1 isoform 2 [Homo sapiens]

**Accession:**	gi|134304846	**Score:**	43.2
**Database:**	refseq_human(refseq_human_20140103.fasta)	**MW [kDa]:**	182.7
**Database Date:**	2015-11-30	**pI:**	5.2
		**Sequence Coverage [%]:**	1.9
		**No. of unique Peptides:**	2

Quantitation

M:Q	**Median:**	0.21	**CV:**	0.00 %	**No. of Peptides:**	1	

Alias proteins:

Accession	Name	Description
gi|578815756	refseq_human (refseq_human_20140103.fasta)	PREDICTED: RB1-inducible coiled-coil protein 1 isoform X1 [Homo sapiens]

10	20	30	40	50	60	70	80
MKLYVFLVNT	GTTLTFDTEL	TVQTVADLKH	AIQSKYKIAI	QHQVLVVNGG	ECMAADRRVC	TYSAGTDTNP	IFLFNKEMIL
90	100	110	120	130	140	150	160
CDRPPAIPKT	TFSTENDMEI	KVEESLMMPA	VFHTVASRTQ	LALEMYEVAK	KLCSFCEGLV	HDEHLQHQGW	AAIMANLEDC
170	180	190	200	210	220	230	240
SNSYQKLLFK	FESIYSNYLQ	SIEDIKLKLT	HLGTAVSVMA	KIPLLECLTR	HSYRECLGRL	DSLPEHEDSE	KAEMKRSTEL
250	260	270	280	290	300	310	320
VLSPDMPRTT	NESLLTSFPK	SVEHVSPDTA	DAESGKEIRE	SCQSTVHQQD	ETTIDTKDGD	LPFFNVSLLD	WINVQDRPND
330	340	350	360	370	380	390	400
VESLVRKCFD	SMSRLDPRII	RPFIAECRQT	IAKLDNQNMK	AIKGLEDRLY	ALDQMIASCG	RLVNEQKELA	QGFLANQKRA
410	420	430	440	450	460	470	480
ENLKDASVLP	DLCLSHANQL	MIMLQNHRKL	LDIKQKCTTA	KQELANNLHV	RLKWCCFVML	HADQDGEKLQ	ALLRLVIELL
490	500	510	520	530	540	550	560
ERVKIVEALS	TVPQMYCLAV	VEVVRRKMFI	KHYREWAGAL	VKDGKRLYEA	EKSKRESFGK	LFRKSFLRNR	LFRGLDSWPP
570	580	590	600	610	620	630	640
SFCTQKPRKF	DCELPDISLK	DLQFLQSFCP	SEVQPFLRVP	LLCDFEPLHQ	HVLALHNLVK	AAQSLDEMSQ	TITDLLSEQK
650	660	670	680	690	700	710	720
ASVSQTSPQS	ASSPRMESTA	GITTTTSPRT	PPPLTVQDPL	CPAVCPLEEL	SPDSIDAHTF	DFETIPHPNI	EQTIHQVSLD
730	740	750	760	770	780	790	800
LDSLAESPES	DFMSAVNEFV	IEENLSSPNP	ISDPQSPEMM	VESLYSSVIN	AIDSRRMQDT	NVCGKEDFGD	HTSLNVQLER
810	820	830	840	850	860	870	880
CRVVAQDSHF	SIQTIKEDLC	HFRTFVQKEQ	CDFSNSLKCT	AVEIRNIIEK	VKCSLEITLK	EKHQKELLSL	KNEYEGKLDG
890	900	910	920	930	940	950	960
LIKETEENEN	KIKKLKGELV	CLEEVLQNKD	NEFALVKHEK	EAVICLQNEK	DQKLLEMENI	MHSQNCEIKE	LKQSREIVLE
970	980	990	1000	1010	1020	1030	1040
DLKKLHVEND	EKLQLLRAEL	QSLEQSHLKE	LEDTLQVRHI	QEFEKVMTDH	RVSLEELKKE	NQQIINQIQE	SHAEIIQEKE
1050	1060	1070	1080	1090	1100	1110	1120
KQLQELKLKV	SDLSDTRCKL	EVELALKEAE	TDEIKILLEE	SRAQQKETLK	SLLEQETENL	RTEISKLNQK	IQDNNENYQV
1130	1140	1150	1160	1170	1180	1190	1200
GLAELRTLMT	IEKDQCISEL	ISRHEEESNI	LKAELNKVTS	LHNQAFEIEK	NLKEQIIELQ	SKLDSELSAL	ERQKDEKITQ
1210	1220	1230	1240	1250	1260	1270	1280
QEEKYEAIIQ	NLEKDRQKLV	SSQEQDREQL	IQKLNCEKDE	AIQTALKEFK	LEREVVEKEL	LEKVKHLENQ	IAKSPAIDST
1290	1300	1310	1320	1330	1340	1350	1360
RGDSSSLVAE	LQEKLQEEKA	KFLEQLEEQE	KRKNEEMQNV	RTSLIAEQQT	NFNTVLTREK	MRKENIINDL	SDKLKSTMQQ
1370	1380	1390	1400	1410	1420	1430	1440
QERDKDLIES	LSEDRARLLE	EKKKLEEEVS	KLRSSSFVPS	PYVATAPELY	GACAPELPGE	SDRSAVETAD	EGRVDSAMET
1450	1460	1470	1480	1490	1500	1510	1520
SMMSVQENIH	MLSEEKQRIM	LLERTLQLKE	EENKRLNQRL	MSQSMSSVSS	RHSEKIAIRD	FQVGDLVLII	LDERHDNYVL
1530	1540	1550	1560	1570	1580	1590	1600
FTVSPTLYFL	HSESLPALDL	KPASGASRRP	WVLGKVMEKE	YCQAKKAQNR	FKVPLGTKFY	RVKAVSWNKK	V

Detailed Protein Report

Cmpd.	No. of Cmpds.	m/z meas.	Δ m/z [ppm]	z	Rt [min]	Score	P	Range	Sequence	Modification	Ratios
2032	1	687.8227	-80.45	2	55.5	10.2	1	388-399	K.ELAQGFLANQKR.A		
1335	1	497.7323	-72.04	2	46.4	14.6	1	508-514	K.MFIKHYR.E		M:Q 0.21

Detailed Protein Report

Protein 492: **PREDICTED: leucine-rich repeat-containing protein 16C isoform X1 [Homo sapiens]**

**Accession:**	gi|530423607	**Score:**	43.1
**Database:**	refseq_human(refseq_human_20140103.fasta)	**MW [kDa]:**	148.1
**Database Date:**	2015-11-30	**pI:**	6.1
**Modification(s):**	Oxidation	**Sequence Coverage [%]:**	2.8
		**No. of unique Peptides:**	2

10	20	30	40	50	60	70	80
MAQTPDGISC	ELRGEITRFL	WPKEVELLLK	TWLPGEGAVQ	NHVLALLRWR	AYLLHTTCLP	LRVDCTFSYL	EVQAMALQET
**90**	**100**	**110**	**120**	**130**	**140**	**150**	**160**
PPQVTFELES	LRELVLEFPG	VAALEQLAQH	VAAAIKKVFP	RSTLGKLFRR	PTPASMLARL	ERSSPSESTD	PCSPCGGFLE
**170**	**180**	**190**	**200**	**210**	**220**	**230**	**240**
TYEALCDYNG	FPFREEIQWD	VDTIYHRQGC	RHFSLGDFSH	LGSRDLALSV	AALSYNLWFR	CLSCVDMKLS	LEVSEQILHM
**250**	**260**	**270**	**280**	**290**	**300**	**310**	**320**
MSQSSHLEEL	VLETCSLRGD	FVRRLAQALA	GHSSSGLREL	SLAGNLLDDR	GMTALSRHLE	RCPGALRRLS	LAQTGLTPRG
**330**	**340**	**350**	**360**	**370**	**380**	**390**	**400**
MRALGRALAT	NAAFDSTLTH	LDLSGNPGAL	GASEDSGGLY	SFLSRPNVLS	FLNLAGTDTA	LDTLFAAVSR	GCCTSLTHLD
**410**	**420**	**430**	**440**	**450**	**460**	**470**	**480**
ASRNVFSRTK	SRAAPAALQL	FLSRARTLRH	LGLAGCKLPP	DALRALLDGL	ALNTHLRDLH	LDLSACELRS	AGAQVIQDLV
**490**	**500**	**510**	**520**	**530**	**540**	**550**	**560**
CDAGAVSSLD	LADNGFGSDM	VTLVLAIGRS	RSLRHVALGR	NFNVRCKETL	DDVLHRIVQL	MQDDDCPLQS	LSVAESRLKL
**570**	**580**	**590**	**600**	**610**	**620**	**630**	**640**
GASVLLRALA	TNPNLTALDI	SGNAMGDAGA	KLLAKALRVN	SRLRSVVWDR	NHTSALGLLD	VAQALEQNHS	LKAMPLPLND
**650**	**660**	**670**	**680**	**690**	**700**	**710**	**720**
VAQAQRSRPE	LTARAVHQIQ	ACLLRNNRAD	PASSDHTTRL	QPLGLVSDPS	EQEVNELCQS	VQEHVELLGC	GAGPQGEAAV
**730**	**740**	**750**	**760**	**770**	**780**	**790**	**800**
RQAEDAIQNA	NFSLSILPIL	YEAGSSPSHH	WQLGQKLEGL	LRQVGEVCRQ	DIQDFTQATL	DTARSLCPQM	LQGSSWREQL
**810**	**820**	**830**	**840**	**850**	**860**	**870**	**880**
EGVLAGSRGL	PELLPEQLLQ	DAFTRLRDMR	LSITGTLAES	IVAQALAGLS	AARDQLVESL	AQQATVTMPP	ALPAPDGGEP
**890**	**900**	**910**	**920**	**930**	**940**	**950**	**960**
SLLEPGELEG	LFFPEEKEEE	KEKDDSPPQK	WPELSHGLHL	VPFIHSAAEE	AEPEPELAAP	GEDAEPQAGP	SARGSPSPAA
**970**	**980**	**990**	**1000**	**1010**	**1020**	**1030**	**1040**
PGPPAGPLPR	MDLPLAGQPL	RHPTRARPRP	RRQHHHRPPP	GGPQVPPALP	QEGNGLSARV	DEGVEEFFSK	RLIQQDRLWA
**1050**	**1060**	**1070**	**1080**	**1090**	**1100**	**1110**	**1120**
PEEDPATEGG	ATPVPRTLRK	KLGTLFAFKK	PRSTRGPRTD	LETSPGAAPR	TRKTTFGDLL	RPPTRPSRGE	ELGGAEGDTS
**1130**	**1140**	**1150**	**1160**	**1170**	**1180**	**1190**	**1200**
SPDPAGRSRP	RYTRDSKAYS	MILLPAEEEA	TLGARPDKRR	PLERGETELA	PSFEQRVQVM	LQRIGVSRGS	GGAEGKRKQS
**1210**	**1220**	**1230**	**1240**	**1250**	**1260**	**1270**	**1280**
KDGEIKKAGS	DGDIMDSSTE	APPISIKSRT	HSVSADPSCR	PGPGSQGPES	ATWKTLGQQL	NAELRSRGWG	QQDGPGPPSP
**1290**	**1300**	**1310**	**1320**	**1330**	**1340**	**1350**	**1360**
GQSPSPCRTS	PSPDSLGLPE	DPCLGPRNEE	RPLRLQRSPV	LKRRPKLEAP	PSPSLGSGLG	TEPLPPQPTE	PSSPERSPPS
**1370**	**1380**						
PATDQRGGGP	NP						

Cmpd.	No. of Cmpds.	m/z meas.	Δ m/z [ppm]	z	Rt [min]	Score	P	Range	Sequence	Modification
2608	1	698.3486	-78.93	2	61.3	15.4	2	425-437	R.ARTLRHLGLAGCK.L	
2909	1	682.3620	-10.00	3	65.1	17.7	2	1177-1196	R.VQVMLQRIGVSRGSGGAEGK.R	Oxidation: 4

Detailed Protein Report

Protein 493: Golgi apparatus protein 1 isoform 3 precursor [Homo sapiens]

Accession: gi|224586817
Database: refseq_human(refseq_human_20140103.fasta)
Database Date: 2015-11-30
Modification(s): Carbamidomethyl, Oxidation

Score: 43.1
MW [kDa]: 134.5
pI: 6.5
Sequence Coverage [%]: 2.5
No. of unique Peptides: 1

10	20	30	40	50	60	70	80
MAACGRVRRM	FRLSAALHLL	LLFAAGAEKL	PGQGVHSQGQ	GPGANFVSFV	GQAGGGGPAG	QQLPQLPQSS	QLQQQQQQQQ
90	100	110	120	130	140	150	160
QQQQPQPPQP	PFPAGGPPAR	RGGAGAGGGW	KLAEEESCRE	DVTRVCPKHT	WSNNLAVLEC	LQDVREPENE	ISSDCNHLLW
170	180	190	200	210	220	230	240
NYKLNLTTDP	KFESVAREVC	KSTITEIKEC	ADEPVGKGYM	VSCLVDHRGN	ITEYQCHQYI	TKMTAIIFSD	YRLICGFMDD
250	260	270	280	290	300	310	320
CKNDINILKC	GSIRLGEKDA	HSQGEVVSCL	EKGLVKEAEE	REPKIQVSEL	CKKAILRVAE	LSSDDFHLDR	HLYFACRDDR
330	340	350	360	370	380	390	400
ERFCENTQAG	EGRVYKCLFN	HKFEESMSEK	CREALTTRQK	LIAQDYKVSY	SLAKSCKSDL	KKYRCNVENL	PRSREARLSY
410	420	430	440	450	460	470	480
LLMCLESAVH	RGRQVSSECQ	GEMLDYRRML	MEDFSLSPEI	ILSCRGEIEH	HCSGLHRKGR	TLHCLMKVVR	GEKGNLGMNC
490	500	510	520	530	540	550	560
QQALQTLIQE	TDPGADYRID	RALNEACESV	IQTACKHIRS	GDPMILSCLM	EHLYTEKMVE	DCEHRLLELQ	YFISRDWKLD
570	580	590	600	610	620	630	640
PVLYRKCQGD	ASRLCHTHGW	NETSEFMPQG	AVFSCLYRHA	YRTEEQGRRL	SRECRAEVQR	ILHQRAMDVK	LDPALQDKCL
650	660	670	680	690	700	710	720
IDLGKWCSEK	TETGQELECL	QDHLDDLVVE	CRDIVGNLTE	LESEDIQIEA	LLMRACEPII	QNFCHDVADN	QIDSGDLMEC
730	740	750	760	770	780	790	800
LIQNKHQKDM	NEKCAIGVTH	FQLVQMKDFR	FSYKFKMACK	EDVLKLCPNI	KKKVDVVICL	STTVRNDTLQ	EAKEHRVSLK
810	820	830	840	850	860	870	880
CRRQLRVEEL	EMTEDIRLEP	DLYEACKSDI	KNFCSAVQYG	NAQIIECLKE	NKKQLSTRCH	QKVFKLQETE	MMDPELDYTL
890	900	910	920	930	940	950	960
MRVCKQMIKR	FCPEADSKTM	LQCLKQNKNS	ELMDPKCKQM	ITKRQITQNT	DYRLNPMLRK	ACKADIPKFC	HGILTKAKDD
970	980	990	1000	1010	1020	1030	1040
SELEGQVISC	LKLRYADQRL	SSDCEDQIRI	IIQESALDYR	LDPQLQLHCS	DEISSLCAEE	AAAQEQTGQV	EECLKVNLLK
1050	1060	1070	1080	1090	1100	1110	1120
IKTELCKKEV	LNMLKESKAD	IFVDPVLHTA	CALDIKHHCA	AITPGRGRQM	SCLMEALEDK	RVRLQPECKK	RLNDRIEMWS
1130	1140	1150	1160	1170	1180		
YAAKVAPADG	FSDLAMQVMT	SPSKNYILSV	ISGSICILFL	IGLMCGRITK	RVTRELKDR		

Cmpd.	No. of Cmpds.	m/z meas.	Δ m/z [ppm]	z	Rt [min]	Score	P	Range	Sequence	Modification
484	1	546.2461	55.39	2	34.7	15.7	0	538-545	K.MVEDCEHR.L	Carbamidomethyl: 5; Oxidation: 1

Detailed Protein Report

Protein 494: PREDICTED: amphiphysin isoform X1 [Homo sapiens]

**Accession:**	gi|578813410	**Score:**	43.1
**Database:**	refseq_human(refseq_human_20140103.fasta)	**MW [kDa]:**	134.8
**Database Date:**	2015-11-30	**pI:**	4.3
**Modification(s):**	Carbamidomethyl, Oxidation	**Sequence Coverage [%]:**	3.7
		**No. of unique Peptides:**	2

10	20	30	40	50	60	70	80
MADIKTGIFA	KNVQKRLNRA	QEKVLQKLGK	ADETKDEQFE	EYVQNFKRQE	AEGTRLQREL	RGYLAAIKGM	QEASMKLTES
**90**	**100**	**110**	**120**	**130**	**140**	**150**	**160**
LHEVYEPDWY	GREDVKMVGE	KCDVLWEDFH	QKLVDGSLLT	LDTYLGQFPD	IKNRIAKRSR	KLVDYDSARH	HLEALQSSKR
**170**	**180**	**190**	**200**	**210**	**220**	**230**	**240**
KDESRISKAE	EEFQKAQKVF	EEFNVDLQEE	LPSLWSRRVG	FYVNTFKNVS	SLEAKFHKEI	AVLCHKLYEV	MTKLGDQHAD
**250**	**260**	**270**	**280**	**290**	**300**	**310**	**320**
KAFTIQGAPS	DSGPLRIAKT	PSPPEEPSPL	PSPTASPNHT	LAPASPAPAR	PRSPSQTRKG	PPVPPLPKVT	PTKELQQENI
**330**	**340**	**350**	**360**	**370**	**380**	**390**	**400**
ISFFEDNFVP	EISVTTPSQN	EVPEVKKEET	LLDLDFDPFK	PEVTPAGSAG	VTHSPMSQTL	PWDLWTTDSS	ESLSLCNLIM
**410**	**420**	**430**	**440**	**450**	**460**	**470**	**480**
EETPDSGLAE	EIQRSQNDIG	AFTWGPDAST	DRVSQEVTSG	GFGEDSACPS	ETEQDIRLST	SLLSSADWPT	VAEESEHAPG
**490**	**500**	**510**	**520**	**530**	**540**	**550**	**560**
PAFPGGNEQL	PPKPAPEAGV	AIAACVEMEQ	LYDPLDSDMP	AMDTAGLFKE	SHEDMKKSDE	EEEKQKMEDS	LWAGVEACQK
**570**	**580**	**590**	**600**	**610**	**620**	**630**	**640**
VDTGAIDIKT	LEGTEEFEEK	ACERTGNRVV	ELLNTIGKED	HKTLKINEVE	TRKDLRAGYK	ESTNANENYF	EKTDLLEKDM
**650**	**660**	**670**	**680**	**690**	**700**	**710**	**720**
EGGDMAKELS	DRDQGLHDGI	QKVINNCGMI	EGFGAKPANH	PDILNPSLQT	YSVSNIEDGS	PEGWDLTRKD	IKKNAIDTPN
**730**	**740**	**750**	**760**	**770**	**780**	**790**	**800**
HDMIFYPAQD	STDAEEIQCE	TVLNALVGDR	DKNQAVLSIK	LEVEAQSRTD	LNYSLGRDAN	RRDLCSCKAE	SSLIEGSERT
**810**	**820**	**830**	**840**	**850**	**860**	**870**	**880**
VTENRSHTVN	LPRIAEIDSW	GTDPGQIWQP	SLDSAMNNLD	ITGFSGIPDG	QASGFASCLD	SINHWPIRDT	EGLDNSWTHS
**890**	**900**	**910**	**920**	**930**	**940**	**950**	**960**
DVVEHKEELK	VDLLAGDCRE	QAIASCWEQS	QEIARPLILG	TSWNASTESS	ASSRDQDINN	SDLSEDEIAN	QRYGLLYQEI
**970**	**980**	**990**	**1000**	**1010**	**1020**	**1030**	**1040**
EADKDEASGG	SFNGFTQPQD	TSLFTMQTDQ	SMICNLIIPG	ADADAAVGTL	VSAAEGAPGE	EAEAEKATVP	AGEGVSLEEA
**1050**	**1060**	**1070**	**1080**	**1090**	**1100**	**1110**	**1120**
KIGTETTEGA	ESAQPEAEEL	EATVPQEKVI	PSVVIEPASN	HEEEGENEIT	IGAEPKETTE	DAAPPGPTSE	TPELATEQKP
**1130**	**1140**	**1150**	**1160**	**1170**	**1180**	**1190**	**1200**
IQDPQPTPSA	PAMGAADQLA	SAREASQELP	PGFLYKVETL	HDFEAANSDE	LTLQRGDVVL	VVPSDSEADQ	DAGWLVGVKE
**1210**	**1220**	**1230**					
SDWLQYRDLA	TYKGLFPENF	TRRLD					

Cmpd.	No. of Cmpds.	m/z meas.	Δ m/z [ppm]	z	Rt [min]	Score	P	Range	Sequence	Modification
2902	1	836.7384	18.81	3	65.4	13.6	2	93-112	R.EDVKMVGEKCDVLWEDFHQK.L	Carbamidomethyl: 10; Oxidation: 5
1311	1	1115.7434	90.99	1	46.1	17.0	1	751-760	R.DKNQAVLSIK.L	

Detailed Protein Report

Protein 495: PREDICTED: protein unc-13 homolog B isoform X1 [Homo sapiens]

**Accession:**	gi|578816361	**Score:**	43.0
**Database:**	refseq_human(refseq_human_20140103.fasta)	**MW [kDa]:**	268.9
**Database Date:**	2015-11-30	**pI:**	5.4
		**Sequence Coverage [%]:**	1.7
		**No. of unique Peptides:**	1

Quantitation

Q:W	**Median:** 0.83	**CV:** 0.00 %	**No. of Peptides:** 1

Detailed Protein Report

10	20	30	40	50	60	70	80
MSLLCVRVKR	AKFQGSPDKF	NTYVTLKVQN	VKSTTVAVRG	DQPSWEQDFM	FEISRLDLGL	SVEVWNKGLI	WDTMVGTVWI
90	100	110	120	130	140	150	160
ALKTIRQSDE	EGPGEWSTLE	AETLMKDDEI	CGTRNPTPHK	ILLDTRFELP	FDIPEEEARY	WTYKWEQINA	LGADNEYSSQ
170	180	190	200	210	220	230	240
EESQRKPLPT	AAAQCCHWTY	LGWGEQQTFE	DPDSAVDDRD	SDYRSETSNS	FPPPYHTASQ	PNASVHQFPV	PVRSPQQLLL
250	260	270	280	290	300	310	320
QGSSRDSCND	SMQSYDLDYP	ERRAISPTSS	SRYGSSCNVS	QGSSQLSELD	QYHEQDDDHR	ETDSIHSCHS	SHSLSRDGQA
330	340	350	360	370	380	390	400
GFGEQEKPLE	VTGQAEKEAA	CEPKEMKEDA	TTHPPPDLVL	QKDHFLGPQE	SFPEENASSP	FTQARAHWIR	AVTKVRLQLQ
410	420	430	440	450	460	470	480
EVGNLFYILT	RNKRKKQRTP	QTLREKMKRL	LQESEEEIMV	TLGPSSRLSP	DKAKAETMCG	TKSKSSGPAG	SLPEDNSLPP
490	500	510	520	530	540	550	560
CCGSAALAIG	GDRDGDLAQL	CSFGQQNNSQ	LPLGSTAYVS	GSGSQDPSSV	SITTSCQEPS	ERNQTKTLLS	RGHGQGCSRE
570	580	590	600	610	620	630	640
QREPLGDVVE	YIIRELQGIS	RLQTEIAELQ	QHLSQVQGSV	DEVSSCVDSV	LSEIEGLQVS	TCSLAKVCEG	EKAQEPHVDR
650	660	670	680	690	700	710	720
PSEEAILYLY	GLPEQDGENT	MELVESFLAK	HLCVNGMQCN	RYIKKAYRAG	TSPSPRPTVV	KLAHLEHRDF	ILQKSILLQN
730	740	750	760	770	780	790	800
VGVRIATREE	PSCPQGNKNP	QKESISFFQQ	QHQDYSQTSL	NQDEPVLQME	TGDRGPITGT	YQMKAQDQQR	EHQAPEQQGP
810	820	830	840	850	860	870	880
CFLLKNNLSK	ESDVPKLGDE	IKGASRTSQV	ISGSCDELSE	KKASLSTPHQ	FEEPALVLIS	KEEASGKSQF	FKQYSQKHEA
890	900	910	920	930	940	950	960
CKVGKPENDC	HDKSEASSCL	SLSGLLKTER	INAEDKLLGC	EAGLDILSSK	ELEDLLADKS	RRLAALSCDS	MMEEIIIGPE
970	980	990	1000	1010	1020	1030	1040
TFSDMVHIDL	NEEEECAAHV	LKDVFDKSSC	VLGGSQEDED	VEIKFYTSKL	GRAIHHFRSA	LQGVFQKLEN	SGSISPEDLE
1050	1060	1070	1080	1090	1100	1110	1120
SNESGSQSEN	SDRLLGTVSS	GGAQDCSLES	PGSQGSESLL	SVVSGGVGVS	TQGEQTPQDP	STFSLASNNS	LPSVALAPCL
1130	1140	1150	1160	1170	1180	1190	1200
GSETCSRPGS	PKQGRLSLEQ	VCTETVYLNK	CINNFKNVLR	EKRLRQKKLL	HELVQKANRL	SVEDIHSEEK	RGALQIPDDG
1210	1220	1230	1240	1250	1260	1270	1280
DPSLPQWLPE	GPAGGLYGID	SMPDLRRKKP	LPLVSDLAMS	LVQSRKAGIT	SAMATRTSLK	DEELKSHVYK	KTLQALIYPI
1290	1300	1310	1320	1330	1340	1350	1360
SCTTPHNFEV	WTATTPTYCY	ECEGLLWGIA	RQGMRCSECG	VKCHEKCQDL	LNADCLQRAA	EKSCKHGAED	RTQNIIMAMK
1370	1380	1390	1400	1410	1420	1430	1440
DRMKIRERNK	PEIFEVIRDV	FTVNKAAHVQ	QMKTVKQSVL	DGTSKWSAKI	TITVVCAQGL	QAKDKTGSSD	PYVTVQVSKT
1450	1460	1470	1480	1490	1500	1510	1520
KKRTKTIFGN	LNPVWEEKFH	FECHNSSDRI	KVRVWDEDDD	IKSRVKQRLK	RESDDFLGQT	IIEVRTLSGE	MDVWYNLEKR
1530	1540	1550	1560	1570	1580	1590	1600
TDKSAVSGAI	RLQISVEIKG	EEKVAPYHVQ	YTCLHENLFH	YLTDIQGSGG	VRIPEARGDD	AWKVYFDETA	QEIVDEFAMR
1610	1620	1630	1640	1650	1660	1670	1680
YGIESIYQAM	THFACLSSKY	MCPGVPAVMS	TLLANINAYY	AHTTASTNVS	ASDRFAASNF	GKERFVKLLD	QLHNSLRIDL
1690	1700	1710	1720	1730	1740	1750	1760
STYRNNFPAG	SPERLQDLKS	TVDLLTSITF	FRMKVQELQS	PPRASQVVKD	CVKACLNSTY	EYIFNNCHDL	YSRQYQLKQE
1770	1780	1790	1800	1810	1820	1830	1840
LPPEEQGPSI	RNLDFWPKLI	TLIVSIIEED	KNSYTPVLNQ	FPQELNVGKV	SAEVMWHLFA	QDMKYALEEH	EKDHLCKSAD
1850	1860	1870	1880	1890	1900	1910	1920
YMNLHFKVKW	LHNEYVRDLP	VLQGQVPEYP	AWFEQFVLQW	LDENEDVSLE	FLRGALERDK	KDGFQQTSEH	ALFSCSVVDV
1930	1940	1950	1960	1970	1980	1990	2000
FTQLNQSFEI	IRKLECPDPS	ILAHYMRRFA	KTIGKVLMQY	ADILSKDFPA	YCTKEKLPCI	LMNNVQQLRV	QLEKMFEAMG
2010	2020	2030	2040	2050	2060	2070	2080
GKELDLEAAD	SLKELQVKLN	TVLDELSMVF	GNSFQVRIDE	CVRQMADILG	QVRGTGNASP	DARASAAQDA	DSVLRPLMDF
2090	2100	2110	2120	2130	2140	2150	2160
LDGNLTLFAT	VCEKTVLKRV	LKELWRVVMN	TMERMIVLPP	LTDQTGTQLI	FTAAKELSHL	SKLKDHMVRE	ETRNLTPKQC
2170	2180	2190	2200	2210	2220	2230	2240
AVLDLALDTI	KQYFHAGGNG	LKKTFLEKSP	DLQSLRYALS	LYTQTTDTLI	KTFVRSQTTQ	GSGVDDPVGE	VSIQVDLFTH
2250	2260	2270	2280	2290	2300	2310	2320
PGTGEHKVTV	KVVAANDLKW	QTAGMFRPFV	EVTMVGPHQS	DKKRKFTTKS	KSNNWAPKYN	ETFHFLLGNE	EGPESYELQI
2330	2340	2350	2360	2370	2380	2390	2400

Detailed Protein Report

Cmpd.	No. of Cmpds.	m/z meas.	Δ m/z [ppm]	z	Rt [min]	Score	P	Range	Sequence	Modification	Ratios	
2611	1	709.8596	-31.02	2	63.3	11.5	0	917-930	K.LLGCEAGLDILSSK.E		Q:W	0.83

Detailed Protein Report

Protein 496: PREDICTED: protein DBF4 homolog B isoform X7 [Homo sapiens]

Accession: gi|578831452
Database: refseq_human(refseq_human_20140103.fasta)
Database Date: 2015-11-30
Modification(s): Carbamidomethyl

Score: 43.0
MW [kDa]: 48.4
pI: 10.4
Sequence Coverage [%]: 7.4
No. of unique Peptides: 1

10	20	30	40	50	60	70	80
MSEPGKGDDC	LELESSMAES	RLRAPDLGVS	RCLGKCQKNS	PGARKHPFSG	KSFYLDLPAG	KNLQFLTGAI	QQLGGVIEGF
90	100	110	120	130	140	150	160
LSKEVSYIVS	SRREVKAESS	GKSHRGCPSP	SPSEVRVETS	AMVDPKGSHP	RPSRKPVDSV	PLSRGKELLQ	KAIRNQGSIS
170	180	190	200	210	220	230	240
GGGSGGSSSL	LTNARSWGVR	ILHVDEMMMH	VQQLSLASLC	VKKQQPKKPE	GTCPAAESRT	RKVARLKAPF	LKIEDESRKF
250	260	270	280	290	300	310	320
RPFHHQFKSF	PEISFLGPKD	ASPFEAPTTL	GSMHHTRESK	DGEPSPRSAA	HTMPRRKKGY	CECCQEAFEE	LHVHLQSAQH
330	340	350	360	370	380	390	400
RSFALEAHLY	AEVDRIIAQL	SHSFADIPFQ	AGLPSDPRQG	CGPAAMDRIT	RWCDGTSCKS	HMCECHNPPA	GLAQGLQGAG
410	420	430	440	450			
LPLSLPSLLY	PVSSGHFLGS	AAWGVVACRG	HAPPSLPRKL	LCPGGHSC			

Cmpd.	No. of Cmpds.	m/z meas.	Δ m/z [ppm]	z	Rt [min]	Score	P	Range	Sequence	Modification
1527	2	472.2297	42.07	2	49.3	18.1	0	440-448	K.LLCPGGHSC.-	Carbamidomethyl: 9

Detailed Protein Report

Protein 497: zinc finger protein 605 isoform 1 [Homo sapiens]

**Accession:**	gi|34222391	**Score:**	43.0
**Database:**	refseq_human(refseq_human_20140103.fasta)	**MW [kDa]:**	74.3
**Database Date:**	2015-11-30	**pI:**	10.4
**Modification(s):**	Carbamidomethyl	**Sequence Coverage [%]:**	9.0
		**No. of unique Peptides:**	2

10	20	30	40	50	60	70	80
MIQSQISFED	VAVDFTLEEW	QLLNPTQKNL	YRDVMLENYS	NLVFLEVWLD	NPKMWLRDNQ	DNLKSMERGH	KYDVFGKIFN
**90**	**100**	**110**	**120**	**130**	**140**	**150**	**160**
SSINIVHVGL	RSHKCGTGEK	SLKCPFDLLI	PKNNCERKKI	DELNKKLLFC	IKPGRTHGGI	KYCDCSTCRK	SSNEEPWLTA
**170**	**180**	**190**	**200**	**210**	**220**	**230**	**240**
NHITHTGVYL	CMECGRFFNK	KSQLVIHQRT	HTGEKPYQCS	ECGKAFSQKS	LLTVHQRTHS	GEKPHGCSEC	QKAFSRKSLL
**250**	**260**	**270**	**280**	**290**	**300**	**310**	**320**
ILHQRIHTGE	KPYGCSECGK	AFSRKSQLKR	HQITHTIEKP	YSCSECGKAF	SQKLKLITHQ	RAHTGEKPYP	CSHCGKAFFW
**330**	**340**	**350**	**360**	**370**	**380**	**390**	**400**
KSQLITHQRT	HTGKKPYGCG	ECQKAFSRNS	LLIRHQRIHT	GEKPYECNEC	GEAFIRKPQL	IKHQITHTGE	KNYRCSDCEE
**410**	**420**	**430**	**440**	**450**	**460**	**470**	**480**
AFFKKSELIR	HQKIHLGEKP	YGCIQCGKTF	FGKSQLLTHH	RTHTGEKPYE	CSECGKAFTQ	KSSLISHQRT	HTGEKPYECS
**490**	**500**	**510**	**520**	**530**	**540**	**550**	**560**
ECRKTFSEKS	SLIHHQRTHT	GEKPFECSEC	RKAFAWKPQL	LRHQRIHTGE	KPYECSECGK	AFVQKVQLIK	HQRNHTGEKT
**570**	**580**	**590**	**600**	**610**	**620**	**630**	**640**
YGCSDCAKAF	FEKAQLIIHQ	RIHTGERPYK	CGECGKSFTR	KSHLMRHQRI	HTGDKYYGCN	ECGTTFNRKS	QLMIHQRNHI
**650**							
I							

Cmpd.	No. of Cmpds.	m/z meas.	Δ m/z [ppm]	z	Rt [min]	Score	P	Range	Sequence	Modification
1971	1	1079.8531	-128.17	2	53.3	10.5	1	523-540	R.HQRIHTGEKPYECSECGK.A	Carbamidomethyl: 16
1767	1	856.7921	-80.88	2	52.1	10.3	1	616-629	K.YYGCNECGTTFNRK.S	Carbamidomethyl: 4

Detailed Protein Report

Protein 498: leucine-rich repeat-containing protein 16A isoform 2 [Homo sapiens]

**Accession:**	gi|291190783	**Score:**	42.9
**Database:**	refseq_human(refseq_human_20140103.fasta)	**MW [kDa]:**	150.8
**Database Date:**	2015-11-30	**pI:**	8.8
**Modification(s):**	Oxidation	**Sequence Coverage [%]:**	3.2
		**No. of unique Peptides:**	1

10	20	30	40	50	60	70	80
MTEESSDVPR	ELIESIKDVI	GRKIKISVKK	KVKLEVKGDK	VENKVLVLTS	CRAFLVTARI	PTKLELTFSY	LEIHGVVCSK
90	100	110	120	130	140	150	160
SAQMIVETEK	CSISMKMASP	EDVSEVLAHI	GTCLRKIFPG	LSPVRIMKKV	SMEPSERLAS	LQALWDSQTV	AEQGPCGGFS
170	180	190	200	210	220	230	240
QMYACVCDWL	GFSYREEVQW	DVDTIYLTQD	TRELNLQDFS	HLDHRDLIPI	IAALEYNQWF	TKLSSKDLKL	STDVCEQILR
250	260	270	280	290	300	310	320
VVSRSNRLEE	LVLENAGLRT	DFAQKLASAL	AHNPNSGLHT	INLAGNPLED	RGVSSLSIQF	AKLPKGLKHL	NLSKTSLSPK
330	340	350	360	370	380	390	400
GVNSLSQSLS	ANPLTASTLV	HLDLSGNVLR	GDDLSHMYNF	LAQPNAIVHL	DLSNTECSLD	MVCGALLRGC	LQYLAVLNLS
410	420	430	440	450	460	470	480
RTVFSHRKGK	EVPPSFKQFF	SSSLALMHIN	LSGTKLSPEP	LKALLLGLAC	NHNLKGVSLD	LSNCELRSGG	AQVLEGCIAE
490	500	510	520	530	540	550	560
IHNITSLDIS	DNGLESDLST	LIVWLSKNRS	IQHLALGKNF	NNMKSKNLTP	VLDNLVQMIQ	DEESPLQSLS	LADSKLKTEV
570	580	590	600	610	620	630	640
TIIINALGSN	TSLTKVDISG	NGMGDMGAKM	LAKALQINTK	LRTVIWDKNN	ITAQGFQDIA	VAMEKNYTLR	FMPIPMYDAS
650	660	670	680	690	700	710	720
QALKTNPEKT	EDALQKIENY	LLRNHETRKY	LQEQAYRLQQ	GIVTSTTQQM	IDRICVKVQD	HLNSLRNCGG	DAIQEDLKSA
730	740	750	760	770	780	790	800
ERLMRDAKNS	KTLLPNLYHV	GGASWAGASG	LLSSPIQETL	ESMAGEVTRV	VDEQLKALLE	SMVDAAENLC	PNVMKKAHIR
810	820	830	840	850	860	870	880
QDLIHASTEK	ISIPRTFVKN	VLLEQSGIDI	LNKISEVKLT	VASFLSDRIV	DEILDALSHC	HHKLADHFSR	RGKTLPQQES
890	900	910	920	930	940	950	960
LEIELAEEKP	VKRSIITVEE	LTEIERLEDL	DTCMMTPKSK	RKSIHSRMLR	PVSRAFEMEF	DLDKALEEVP	IHIEDPPFPS
970	980	990	1000	1010	1020	1030	1040
LRQEKRSSGF	ISELPSEEGK	KLEHFTKLRP	KRNKKQQPTQ	AAVCAANIVS	QDGEQNGLMG	RVDEGVDEFF	TKKVTKMDSK
1050	1060	1070	1080	1090	1100	1110	1120
KWSTRGSESH	ELNEGGDEKK	KRDSRKSSGF	LNLIKSRSKS	ERPPTILMTE	EPSSPKGAVR	SPPVDCPRKD	TKAAEHNGNS
1130	1140	1150	1160	1170	1180	1190	1200
ERIEEIKTPD	SFEESQGEEI	GKVERSDSKS	SPQAGRRYGV	QVMGSGLLAE	MKAKQEKRAA	CAQKKLGNDA	VSQDSSSPAL
1210	1220	1230	1240	1250	1260	1270	1280
SGVERSDGGG	AGLPENRFGL	GTPEKNTKAE	PKAEAGSRSR	SSSSTPTSPK	PLLQSPKPSL	AARPVIPQKP	RTASRPDDIP
1290	1300	1310	1320	1330	1340	1350	1360
DSPSSPKVAL	LPPVLKKVPS	DKERDGQSSP	QPSPRTFSQE	VSRRSWGQQA	QEYQEQKQRS	SSKDGHQGSK	SNDSGEEAEK
1370							
EFIFV							

Cmpd.	No. of Cmpds.	m/z meas.	Δ m/z [ppm]	z	Rt [min]	Score	P	Range	Sequence	Modification
843	1	946.5224	65.61	3	40.1	18.7	2	91-116	K.CSISMKMASPEDVSEVLAHIGTCLRK.I	Oxidation: 5, 7

Detailed Protein Report

Protein 499: DNA-directed RNA polymerase III subunit RPC5 isoform 4 [Homo sapiens]

**Accession:**	gi|384475537	**Score:**	42.9
**Database:**	refseq_human(refseq_human_20140103.fasta)	**MW [kDa]:**	75.7
**Database Date:**	2015-11-30	**pI:**	6.0
**Modification(s):**	Oxidation	**Sequence Coverage [%]:**	5.4
		**No. of unique Peptides:**	2

Quantitation

Q:W	**Median:** 0.52	**CV:** 0.00 %	**No. of Peptides:**	1
M:Q	**Median:** 0.86	**CV:** 0.00 %	**No. of Peptides:**	1

```
        10         20         30         40         50         60         70         80
MANEEDDPVV QEIKPKQQKV ELEMAIDTLN PNYCRSKGEQ IALNVDGACA DETSTYSSKL MDKQTFCSSQ TTSNTSRYAA
        90        100        110        120        130        140        150        160
ALYRQGELHL TPLHGILQLR PSFSYLDKAD AKHREREAAN EAGDSSQDEA EDDVKQITVR FSRPESEQAR QRRVQSYEFL
       170        180        190        200        210        220        230        240
QKKHAEEPWV HLHYYGLRDS RSEHERQYLL CPGSSGVENT ELVKSPSEYL MMLMPPSQEE EKDKPVAPSN VLSMAQLRTL
       250        260        270        280        290        300        310        320
PLADQIKILM KNVKVMPFAN LMSLLGPSID SVAVLRGIQK VAMLVQGNWV VKSDILYPKD SSSPHSGVPA EVLCRGRDFV
       330        340        350        360        370        380        390        400
MWKFTQSRWV VRKEVATVTK LCAEDVKDFL EHMAVVRINK GWEFILPYDG EFIKKHPDVV QRQHMLWTGI QAKLEKVYNL
       410        420        430        440        450        460        470        480
VKETMPKKPD AQSGPAGLVC GDQRIQVAKT KAQQNHALLE RELQRRKEQL RVPAVPPGVR IKEEPVSEEG EEDEEQEAEE
       490        500        510        520        530        540        550        560
EPMDTSPSGL HSKLANGLPL GRAAGTDSFN GHPPQGCAST PVARELKAFV EATFQRQFVL TLSELKRLFN LHLASLPPGH
       570        580        590        600        610        620        630        640
TLFSGISDRM LQDTVLAAGC KQILVPFPPQ TAASPDEQKV FALWESGDMS DQHRQVLLEI FSKNYRVRRN MIQSRLTQEC
       650        660        670        680
GEDLSKQEVD KVLKDCCVSY GGMWYLKGTV QS
```

Cmpd.	No. of Cmpds.	m/z meas.	Δ m/z [ppm]	z	Rt [min]	Score	P	Range	Sequence	Modification	Ratios
2534	2	742.5453	129.06	2	62.1	15.7	1	239-251	R.TLPLADQIKILMK.N		
828	1	897.4861	96.86	2	40.4	13.7	0	600-614	K.VFALWESGDMSDQHR.Q	Oxidation: 10	M:Q 0.86<br>Q:W 0.52

Detailed Protein Report

Protein 500: PREDICTED: uncharacterized protein LOC102725205, partial [Homo sapiens]

Accession: gi|578845222
Database: refseq_human(refseq_human_20140103.fasta)
Database Date: 2015-11-30
Modification(s): Oxidation

Score: 42.9
MW [kDa]: 95.5
pI: 6.0
Sequence Coverage [%]: 5.8
No. of unique Peptides: 4

Quantitation

Q:W	**Median:** 2.48	**CV:** 0.00 %	**No. of Peptides:** 1
M:Q	**Median:** 0.43	**CV:** 0.00 %	**No. of Peptides:** 1

10	20	30	40	50	60	70	80
TLSHTLHPPT	HTPNLTHSTH	THTTPPTHTP	HPTHHSTPLT	HSTPHTSHTH	STPLTHSTPH	SWAAKSLERL	LWVVASSPPR
**90**	**100**	**110**	**120**	**130**	**140**	**150**	**160**
RTAVVGRIPG	AGMPAGAGGP	ACRCPCLSRV	GWRDLDAEPS	SQEFRQLSLD	SHQVAAFQTE	MECWARGSGM	DATEPLCCDC
**170**	**180**	**190**	**200**	**210**	**220**	**230**	**240**
QTSDLVCWSQ	QGHQGPERGA	LGSLEEPGLL	SAREAWPVSC	SSIFLEVSGR	RLESRMPPGG	AAINISPGDK	RTNQSPAGYR
**250**	**260**	**270**	**280**	**290**	**300**	**310**	**320**
GEDKSGKRLM	SELWLQVAAA	RDGLQDFPGL	LVAGRGSSSG	DWSERAYGWE	LEELMEKRGR	VGWSSREALW	SGEGEEEPNK
**330**	**340**	**350**	**360**	**370**	**380**	**390**	**400**
EKQQSTWNIS	IPPSKAVLEE	QPQSPIVPKS	GKSVKTFIRN	RYEPAKRCEE	LICAELIRMN	KVTTDSAMGI	ADKDLSEELQ
**410**	**420**	**430**	**440**	**450**	**460**	**470**	**480**
HRLQHALASL	QVAQGEDLTA	DIMYSPSASE	PLPFMQCDGE	QETGTASPIW	LWNLWAGESH	PHGVHALATL	GWEGPMVNAG
**490**	**500**	**510**	**520**	**530**	**540**	**550**	**560**
EKVQEEEGAP	ATGPSCGQHR	EGCCGGEDVG	VEGCGGGHLL	ALGCPSKTLT	ARGILPVGGA	ASTTSLQEAM	FVFRTTLIRA
**570**	**580**	**590**	**600**	**610**	**620**	**630**	**640**
VGCKEQRPLK	GEGGAVKDVE	SRRCAGLWFR	EPCGRQVKME	LHKSAGETPG	KLKVEYADGG	MPEARLWEGT	HIFETKVKLS
**650**	**660**	**670**	**680**	**690**	**700**	**710**	**720**
TVIHEAAVQW	PSGDIEKTHL	SSGLFSELIR	GLCSFDMFLP	DTVVCWPLPW	SAFHPVLLQT	MSAPGLSPAL	DPLCPQLLAD
**730**	**740**	**750**	**760**	**770**	**780**	**790**	**800**
LGSEKQSCPS	REFGWGLAEV	PSVSNLCKGG	LALQAADGGS	GGGVRTPSYY	LNECMGITAA	LYEHLKTTLS	NPSFSKEKES
**810**	**820**	**830**	**840**	**850**	**860**	**870**	**880**
VNFPFSMHEV	TGVCHSGGPV	FKKNREETVR	LSAGGAARQK	ELMLYPVVSF	PKGLHQLPSF	LALIMTISVV	TSLLSKESIF
**890**							
SSCQE							

Cmpd.	No. of Cmpds.	m/z meas.	Δ m/z [ppm]	z	Rt [min]	Score	P	Range	Sequence	Modification	Ratios	
1138	1	983.4246	-85.91	2	44.4	11.1	1	82-103	R. TAVVGRIPGAGMPAGAGGPACR. C		M:Q	0.43
2662	1	933.9882	34.09	2	64.5	10.3	0	194-210	R.EAWPVSCSSIFLEVSGR.R			
992	2	647.6724	-181.96	2	40.9	10.3	0	614-625	K.VEYADGGMPEAR.L		Q:W	2.48
2897	1	655.6487	-212.02	2	65.0	11.2	0	614-625	K.VEYADGGMPEAR.L	Oxidation: 8		

Detailed Protein Report

Protein 501: undifferentiated embryonic cell transcription factor 1 [Homo sapiens]

Accession: gi|71043876
Database: refseq_human(refseq_human_20140103.fasta)
Database Date: 2015-11-30

Score: 42.9
MW [kDa]: 36.4
pI: 11.8
Sequence Coverage [%]: 13.2
No. of unique Peptides: 2

Quantitation

M:Q **Median:** 0.20 **CV:** 0.00 % **No. of Peptides:** 1

10	20	30	40	50	60	70	80
MLLRPRRPPP	LAPPAPPSPA	SPDPEPRTPG	DAPGTPPRRP	ASPSALGELG	LPVSPGSAQR	TPWSARETEL	LLGTLLQPAV
**90**	**100**	**110**	**120**	**130**	**140**	**150**	**160**
WRALLLDRRQ	ALPTYRRVSA	ALAQQQVRRT	PAQCRRRYKF	LKDKFREAHG	QPPGPFDEQI	RKLMGLLGDN	GRKRPRRRSP
**170**	**180**	**190**	**200**	**210**	**220**	**230**	**240**
GSGRPQRARR	PVPNAHAPAP	SEPDATPLPT	ARDRDADPTW	TLRFSPSPPK	SADASPAPGS	PPAPAPTALA	TCIPEDRAPV
**250**	**260**	**270**	**280**	**290**	**300**	**310**	**320**
RGPGSPPPPP	AREDPDSPPG	RPEDCAPPPA	APPSLNTALL	QTLGHLGDIA	NILGPLRDQL	LTLNQHVEQL	RGAFDQTVSL
**330**	**340**	**350**					
AVGFILGSAA	AERGVLRDPC	Q					

Cmpd.	No. of Cmpds.	m/z meas.	Δ m/z [ppm]	z	Rt [min]	Score	P	Range	Sequence	Modification	Ratios
953	1	663.8439	-59.89	2	40.4	10.1	1	97-108	R.RVSAALAQQQVR.R		M:Q 0.20
2319	1	788.1227	53.82	3	59.3	15.2	0	170-192	R. RPVPNAHAPAPSEPDATPLPTAR. D		

Detailed Protein Report

Protein 502: **PREDICTED: suppression of tumorigenicity 18 protein isoform X4 [Homo sapiens]**

**Accession:**	gi|578815754	**Score:**	42.8
**Database:**	refseq_human(refseq_human_20140103.fasta)	**MW [kDa]:**	106.1
**Database Date:**	2015-11-30	**pI:**	5.8
		**Sequence Coverage [%]:**	3.6
		**No. of unique Peptides:**	1

Quantitation

Q:W **Median:** 0.97 **CV:** 0.00 % **No. of Peptides:** 1

10	20	30	40	50	60	70	80
MDAEAEDKTL	RTRSKGTEVP	MDSLIQELSV	AYDCSMAKKR	TAEDQALGVP	VNKRKSLLMK	PRHYSPKADC	QEDRSDRTED
90	100	110	120	130	140	150	160
DGPLETHGHS	TAEEIMIKPM	DESLLSTAQE	NSSRKEDRYS	CYQELMVKSL	MHLGKFEKNV	SVQTVSENLN	DSGIQSLKAE
170	180	190	200	210	220	230	240
SDEADECFLI	HSDDGRDKID	DSQPPFCSSD	DNESNSESAE	NGWDSGSNFS	EETKPPRVPK	YVLTDHKKDL	LEVPEIKTEG
250	260	270	280	290	300	310	320
DKFIPCENRC	DSETERKDPQ	NALAEPLDGN	AQPSFPDVEE	EDSESLAVMT	EEGSDLEKAK	GNLSLLEQAI	ALQAERGCVF
330	340	350	360	370	380	390	400
HNTYKELDRF	LLEHLAGERR	QTKVIDMGGR	QIFNNKHSPR	PEKRETKCPI	PGCDGTGHVT	GLYPHHRSLS	GCPHKVRVPL
410	420	430	440	450	460	470	480
EILAMHENVL	KCPTPGCTGR	GHVNSNRNTH	RSLSGCPIAA	AEKLAMSQDK	NQLDSPQTGQ	CPDQAHRTSL	VKQIEFNFPS
490	500	510	520	530	540	550	560
QAITSPRATV	SKEQEKFGKV	PFDYASFDAQ	VFGKRPLIQT	VQGRKTPPFP	ESKHFPNPVK	FPNRLPSAGA	HTQSPGRASS
570	580	590	600	610	620	630	640
YSYGQCSEDT	HIAAAAAILN	LSTRCREATD	ILSNKPQSLH	AKKDPVSSLE	NLEEKKFPGE	ASIPSPKPKL	HARDLKKELI
650	660	670	680	690	700	710	720
TCPTPGCDGS	GHVTGNYASH	RSVSGCPLAD	KTLKSLMAAN	SQELKCPTPG	CDGSGHVTGN	YASHRSLSGC	PRARKGGVKM
730	740	750	760	770	780	790	800
TPTKEEKEDP	ELKCPVIGCD	GQGHISGKYT	SHRTASGCPL	AAKRQKENPL	NGASLSWKLN	KQELPHCPLP	GCNGLGHVNN
810	820	830	840	850	860	870	880
VFVTHRSLSG	CPLNAQVIKK	GKVSEELMTI	KLKATGGIES	DEEIRHLDEE	IKELNESNLK	IEADMMKLQT	QITSMESNLK
890	900	910	920	930	940	950	960
TIEEENKLIE	QNNESLLKEL	AGLSQALISS	LADIQLPQMG	PISEQNFEAY	VNTLTDMYSN	LERDYSPECK	ALLESIKQAV
970							
KGIHV							

Cmpd.	No. of Cmpds.	m/z meas.	Δ m/z [ppm]	z	Rt [min]	Score	P	Range	Sequence	Modification	Ratios	
1772	1	793.3983	-26.82	2	50.8	13.3	1	229-242	K.DLLEVPEIKTEGDK.F		Q:W	0.97

Detailed Protein Report

Protein 503: estrogen sulfotransferase [Homo sapiens]

**Accession:**	gi|4885617	**Score:**	42.8
**Database:**	refseq_human(refseq_human_20140103.fasta)	**MW [kDa]:**	35.1
**Database Date:**	2015-11-30	**pI:**	6.2
		**Sequence Coverage [%]:**	13.3
		**No. of unique Peptides:**	1

10	20	30	40	50	60	70	80
MNSELDYYEK	FEEVHGILMY	KDFVKYWDNV	EAFQARPDDL	VIATYPKSGT	TWVSEIVYMI	YKEGDVEKCK	EDVIFNRIPF
**90**	**100**	**110**	**120**	**130**	**140**	**150**	**160**
LECRKENLMN	GVKQLDEMNS	PRIVKTHLPP	ELLPASFWEK	DCKIIYLCRN	AKDVAVSFYY	FFLMVAGHPN	PGSFPEFVEK
**170**	**180**	**190**	**200**	**210**	**220**	**230**	**240**
FMQGQVPYGS	WYKHVKSWWE	KGKSPRVLFL	FYEDLKEDIR	KEVIKLIHFL	ERKPSEELVD	RIIHHTSFQE	MKNNPSTNYT
**250**	**260**	**270**	**280**	**290**	**300**		
TLPDEIMNQK	LSPFMRKGIT	GDWKNHFTVA	LNEKFDKHYE	QQMKESTLKF	RTEI		

Cmpd.	No. of Cmpds.	m/z meas.	Δ m/z [ppm]	z	Rt [min]	Score	P	Range	Sequence	Modification
2243	1	945.9550	-47.59	2	58.5	10.9	2	78-93	R.IPFLECRKENLMNGVK.Q	

Detailed Protein Report

Protein 504: **interferon-induced guanylate-binding protein 2 [Homo sapiens]**

**Accession:**	gi|38327558	**Score:**	42.8
**Database:**	refseq_human(refseq_human_20140103.fasta)	**MW [kDa]:**	67.2
**Database Date:**	2015-11-30	**pI:**	5.4
**Modification(s):**	Oxidation	**Sequence Coverage [%]:**	7.1
		**No. of unique Peptides:**	1

10	20	30	40	50	60	70	80
MAPEINLPGP	MSLIDNTKGQ	LVVNPEALKI	LSAITQPVVV	VAIVGLYRTG	KSYLMNKLAG	KKNGFSLGST	VKSHTKGIWM
**90**	**100**	**110**	**120**	**130**	**140**	**150**	**160**
WCVPHPKKPE	HTLVLLDTEG	LGDIEKGDNE	NDSWIFALAI	LLSSTFVYNS	MGTINQQAMD	QLHYVTELTD	RIKANSSPGN
**170**	**180**	**190**	**200**	**210**	**220**	**230**	**240**
NSVDDSADFV	SFFPAFVWTL	RDFTLELEVD	GEPITADDYL	ELSLKLRKGT	DKKSKSFNDP	RLCIRKFFPK	RKCFVFDWPA
**250**	**260**	**270**	**280**	**290**	**300**	**310**	**320**
PKKYLAHLEQ	LKEEELNPDF	IEQVAEFCSY	ILSHSNVKTL	SGGIPVNGPR	LESLVLTYVN	AISSGDLPCM	ENAVLALAQI
**330**	**340**	**350**	**360**	**370**	**380**	**390**	**400**
ENSAAVEKAI	AHYEQQMGQK	VQLPTETLQE	LLDLHRDSER	EAIEVFMKNS	FKDVDQMFQR	KLGAQLEARR	DDFCKQNSKA
**410**	**420**	**430**	**440**	**450**	**460**	**470**	**480**
SSDCCMALLQ	DIFGPLEEDV	KQGTFSKPGG	YRLFTQKLQE	LKNKYYQVPR	KGIQAKEVLK	KYLESKEDVA	DALLQTDQSL
**490**	**500**	**510**	**520**	**530**	**540**	**550**	**560**
SEKEKAIEVE	RIKAESAEAA	KKMLEEIQKK	NEEMMEQKEK	SYQEHVKQLT	EKMERDRAQL	MAEQEKTLAL	KLQEQERLLK
**570**	**580**	**590**	**600**				
EGFENESKRL	QKDIWDIQMR	SKSLEPICNI	L				

Cmpd.	No. of Cmpds.	m/z meas.	Δ m/z [ppm]	z	Rt [min]	Score	P	Range	Sequence	Modification
2870	1	1027.5482	62.66	2	65.0	11.1	2	503-518	K.MLEEIQKKNEEMMEQK.E	Oxidation: 12

Detailed Protein Report

Protein 505: synaptojanin-1 isoform d [Homo sapiens]

**Accession:**	gi|237757326	**Score:**	42.8
**Database:**	refseq_human(refseq_human_20140103.fasta)	**MW [kDa]:**	168.1
**Database Date:**	2015-11-30	**pI:**	6.9
		**Sequence Coverage [%]:**	3.4
		**No. of unique Peptides:**	2

10	20	30	40	50	60	70	80
MAFSKGFRIY	HKLDPPPFSL	IVETRHKEEC	LMFESGAVAV	LSSAEKEAIK	GTYSKVLDAY	GLLGVLRLNL	GDTMLHYLVL
**90**	**100**	**110**	**120**	**130**	**140**	**150**	**160**
VTGCMSVGKI	QESEVFRVTS	TEFISLRIDS	SDEDRISEVR	KVLNSGNFYF	AWSASGISLD	LSLNAHRSMQ	EQTTDNRFFW
**170**	**180**	**190**	**200**	**210**	**220**	**230**	**240**
NQSLHLHLKH	YGVNCDDWLL	RLMCGGVEIR	TIYAAHKQAK	ACLISRLSCE	RAGTRFNVRG	TNDDGHVANF	VETEQVVYLD
**250**	**260**	**270**	**280**	**290**	**300**	**310**	**320**
DSVSSFIQIR	GSVPLFWEQP	GLQVGSHRVR	MSRGFEANAP	AFDRHFRTLK	NLYGKQIIVN	LLGSKEGEHM	LSKAFQSHLK
**330**	**340**	**350**	**360**	**370**	**380**	**390**	**400**
ASEHAADIQM	VNFDYHQMVK	GGKAEKLHSV	LKPQVQKFLD	YGFFYFNGSE	VQRCQSGTVR	TNCLDCLDRT	NSVQAFLGLE
**410**	**420**	**430**	**440**	**450**	**460**	**470**	**480**
MLAKQLEALG	LAEKPQLVTR	FQEVFRSMWS	VNGDSISKIY	AGTGALEGKA	KAGKLKDGAR	SVTRTIQNNF	FDSSKQEAID
**490**	**500**	**510**	**520**	**530**	**540**	**550**	**560**
VLLLGNTLNS	DLADKARALL	TTGSLRASSK	VLKSMCENFY	KYSKPKKIRV	CVGTWNVNGG	KQFRSIAFKN	QTLTDWLLDA
**570**	**580**	**590**	**600**	**610**	**620**	**630**	**640**
PKLAGIQEFQ	DKRSKPTDIF	AIGFEEMVEL	NAGNIVSAST	TNQKLWAVEL	QKTISRDNKY	VLLASEQLVG	VCLFVFIRPQ
**650**	**660**	**670**	**680**	**690**	**700**	**710**	**720**
HAPFIRDVAV	DTVKTGMGGA	TGNKGAVAIR	MLFHTTSLCF	VCSHFAAGQS	QVKERNEDFI	EIARKLSFPM	GRMLFSHDYV
**730**	**740**	**750**	**760**	**770**	**780**	**790**	**800**
FWCGDFNYRI	DLPNEEVKEL	IRQQNWDSLI	AGDQLINQKN	AGQVFRGFLE	GKVTFAPTYK	YDLFSDDYDT	SEKCRTPAWT
**810**	**820**	**830**	**840**	**850**	**860**	**870**	**880**
DRVLWRRRKW	PFDRSAEDLD	LLNASFQDES	KILYTWTPGT	LLHYGRAELK	TSDHRPVVAL	IDIDIFEVEA	EERQNIYKEV
**890**	**900**	**910**	**920**	**930**	**940**	**950**	**960**
IAVQGPPDGT	VLVSIKSSLP	ENNFFDDALI	DELLQQFASF	GEVILIRFVE	DKMWVTFLEG	SSALNVLSLN	GKELLNRTIT
**970**	**980**	**990**	**1000**	**1010**	**1020**	**1030**	**1040**
IALKSPDWIK	NLEEEMSLEK	ISIALPSSTS	STLLGEDAEV	AADFDMEGDV	DDYSAEVEEL	LPQHLQPSSS	SGLGTSPSSS
**1050**	**1060**	**1070**	**1080**	**1090**	**1100**	**1110**	**1120**
PRTSPCQSPT	ISEGPVPSLP	IRPSRAPSRT	PGPPSAQSSP	IDAQPATPLP	QKDPAQPLEP	KRPPPPRPVA	PPTRPAPPQR
**1130**	**1140**	**1150**	**1160**	**1170**	**1180**	**1190**	**1200**
PPPPSGRSQP	SPQAGLAGPG	PAGYSTARPT	IPPRAGVISA	PQSHARASAG	RLTPESQSKT	SETSKGSTFL	PEPLKPQAAF
**1210**	**1220**	**1230**	**1240**	**1250**	**1260**	**1270**	**1280**
PPQSSLPPPA	QRLQEPLVPV	AAPMPQSGPQ	PNLETPPQPP	PRSRSSHSLP	SEASSQPQVK	TNGISDGKRE	SPLKIDPFED
**1290**	**1300**	**1310**	**1320**	**1330**	**1340**	**1350**	**1360**
LSFNLLAVSK	AQLSVQTSPV	PTPDPKRLIQ	LPSATQSNVL	SSVSCMPTMP	PIPARSQSQE	NMRSSPNPFI	TGLTRTNPFS
**1370**	**1380**	**1390**	**1400**	**1410**	**1420**	**1430**	**1440**
DRTAAPGNPF	RAKSEESEAT	SWFSKEEPVT	ISPFPSLQPL	GHNKSRASSS	LDGFKDSFDL	QGQSTLKISN	PKGWVTFEEE
**1450**	**1460**	**1470**	**1480**	**1490**	**1500**	**1510**	**1520**
EDFGVKGKSK	SACSDLLGNQ	PSSFSGSNLT	LNDDWNKGTN	VSFCVLPSRR	PPPPPVPLLP	PGTSPPVDPF	TTLASKASPT
**1530**							
LDFTER							

Cmpd.	No. of Cmpds.	m/z meas.	Δ m/z [ppm]	z	Rt [min]	Score	P	Range	Sequence	Modification
1994	1	1044.9084	-141.16	2	55.3	14.4	2	182-200	R.LMCGGVEIRTIYAAHKQAK.A	
1652	1	626.6317	-42.50	3	50.4	12.3	1	1416-1432	K.DSFDLQGQSTLKISNPK.G	

Detailed Protein Report

Protein 506: probable ATP-dependent RNA helicase DDX58 [Homo sapiens]

Accession: gi|27881482
Database: refseq_human(refseq_human_20140103.fasta)
Database Date: 2015-11-30
Modification(s): Oxidation

Score: 42.7
MW [kDa]: 106.5
pI: 6.0
Sequence Coverage [%]: 3.8
No. of unique Peptides: 1

10	20	30	40	50	60	70	80
MTTEQRRSLQ	AFQDYIRKTL	DPTYILSYMA	PWFREEEVQY	IQAEKNNKGP	MEAATLFLKF	LLELQEEGWF	RGFLDALDHA
90	100	110	120	130	140	150	160
GYSGLYEAIE	SWDFKKIEKL	EEYRLLLKRL	QPEFKTRIIP	TDIISDLSEC	LINQECEEIL	QICSTKGMMA	GAEKLVECLL
170	180	190	200	210	220	230	240
RSDKENWPKT	LKLALEKERN	KFSELWIVEK	GIKDVETEDL	EDKMETSDIQ	IFYQEDPECQ	NLSENSCPPS	EVSDTNLYSP
250	260	270	280	290	300	310	320
FKPRNYQLEL	ALPAMKGKNT	IICAPTGCGK	TFVSLLICEH	HLKKFPQGQK	GKVVFFANQI	PVYEQQKSVF	SKYFERHGYR
330	340	350	360	370	380	390	400
VTGISGATAE	NVPVEQIVEN	NDIIILTPQI	LVNNLKKGTI	PSLSIFTLMI	FDECHNTSKQ	HPYNMIMFNY	LDQKLGGSSG
410	420	430	440	450	460	470	480
PLPQVIGLTA	SVGVGDAKNT	DEALDYICKL	CASLDASVIA	TVKHNLEELE	QVVYKPQKFF	RKVESRISDK	FKYIIAQLMR
490	500	510	520	530	540	550	560
DTESLAKRIC	KDLENLSQIQ	NREFGTQKYE	QWIVTVQKAC	MVFQMPDKDE	ESRICKALFL	YTSHLRKYND	ALIISEHARM
570	580	590	600	610	620	630	640
KDALDYLKDF	FSNVRAAGFD	EIEQDLTQRF	EEKLQELESV	SRDPSNENPK	LEDLCFILQE	EYHLNPETIT	ILFVKTRALV
650	660	670	680	690	700	710	720
DALKNWIEGN	PKLSFLKPGI	LTGRGKTNQN	TGMTLPAQKC	ILDAFKASGD	HNILIATSVA	DEGIDIAQCN	LVILYEYVGN
730	740	750	760	770	780	790	800
VIKMIQTRGR	GRARGSKCFL	LTSNAGVIEK	EQINMYKEKM	MNDSILRLQT	WDEAVFREKI	LHIQTHEKFI	RDSQEKPKPV
810	820	830	840	850	860	870	880
PDKENKKLLC	RKCKALACYT	ADVRVIEECH	YTVLGDAFKE	CFVSRPHPKP	KQFSSFEKRA	KIFCARQNCS	HDWGIHVKYK
890	900	910	920	930			
TFEIPVIKIE	SFVVEDIATG	VQTLYSKWKD	FHFEKIPFDP	AEMSK			

Cmpd.	No. of Cmpds.	m/z meas.	Δ m/z [ppm]	z	Rt [min]	Score	P	Range	Sequence	Modification
1833	1	599.7809	-18.91	2	52.9	13.8	1	751-759	K.EQINMYKEK.M	Oxidation: 5

Detailed Protein Report

Protein 507: mothers against decapentaplegic homolog 3 isoform 1 [Homo sapiens]

Accession: gi|5174513
Database: refseq_human(refseq_human_20140103.fasta)
Database Date: 2015-11-30
Modification(s): Carbamidomethyl, Oxidation

Score: 42.7
MW [kDa]: 48.0
pI: 6.8
Sequence Coverage [%]: 10.8
No. of unique Peptides: 3

Quantitation

Q:W	**Median:**	0.76	**CV:**	0.00 %	**No. of Peptides:**	1
M:Q	**Median:**	1.04	**CV:**	0.00 %	**No. of Peptides:**	1

```
        10         20         30         40         50         60         70         80
MSSILPFTPP IVKRLLGWKK GEQNGQEEKW CEKAVKSLVK KLKKTGQLDE LEKAITTQNV NTKCITIPRS LDGRLQVSHR
        90        100        110        120        130        140        150        160
KGLPHVIYCR LWRWPDLHSH HELRAMELCE FAFNMKKDEV CVNPYHYQRV ETPVLPPVLV PRHTEIPAEF PPLDDYSHSI
       170        180        190        200        210        220        230        240
PENTNFPAGI EPQSNIPETP PPGYLSEDGE TSDHQMNHSM DAGSPNLSPN PMSPAHNNLD LQPVTYCEPA FWCSISYYEL
       250        260        270        280        290        300        310        320
NQRVGETFHA SQPSMTVDGF TDPSNSERFC LGLLSNVNRN AAVELTRRHI GRGVRLYYIG GEVFAECLSD SAIFVQSPNC
       330        340        350        360        370        380        390        400
NQRYGWHPAT VCKIPPGCNL KIFNNQEFAA LLAQSVNQGF EAVYQLTRMC TIRMSFVKGW GAEYRRQTVT STPCWIELHL
       410        420        430
NGPLQWLDKV LTQMGSPSIR CSSVS
```

Cmpd.	No. of Cmpds.	m/z meas.	Δ m/z [ppm]	z	Rt [min]	Score	P	Range	Sequence	Modification	Ratios	
1610	1	1100.1365	-2.93	2	50.0	20.3	2	1-19	-.MSSILPFTPPIVKRLLGWK.K	Oxidation: 1		
1152	4	644.3310	-22.52	2	43.1	11.4	1	64-74	K.CITIPRSLDGR.L	Carbamidomethyl: 1	Q:W<br>M:Q	0.76<br>1.04
2171	1	862.9449	30.12	2	57.5	11.0	1	410-425	K.VLTQMGSPSIRCSSVS.-	Carbamidomethyl: 12;<br>Oxidation: 5		

Detailed Protein Report

Protein 508: PREDICTED: MAGUK p55 subfamily member 6 isoform X2 [Homo sapiens]

**Accession:**	gi|530384825	**Score:**	42.7
**Database:**	refseq_human(refseq_human_20140103.fasta)	**MW [kDa]:**	48.7
**Database Date:**	2015-11-30	**pI:**	9.5
**Modification(s):**	Oxidation	**Sequence Coverage [%]:**	12.1
		**No. of unique Peptides:**	2

10	20	30	40	50	60	70	80
MNNSSINNQL	LPVDAIRILG	IHKRAGEPLG	VTFRVENNDL	VIARILHGGM	IDRQGLLHVG	DIIKEVNGHE	VGNNPKELQE
**90**	**100**	**110**	**120**	**130**	**140**	**150**	**160**
LLKNISGSVT	LKILPSYRDT	ITPQQVFVKC	HFDYNPYNDN	LIPCKEAGLK	FSKGEILQIV	NREDPNWWQA	SHVKEGGSAG
**170**	**180**	**190**	**200**	**210**	**220**	**230**	**240**
LIPSQFLEEK	RKAFVRRDWD	NSGPFCGTIS	SKKKKKMMYL	TTRNAEFDRH	EIQIYEEVAK	MPPFQRKTLV	LIGAQGVGRR
**250**	**260**	**270**	**280**	**290**	**300**	**310**	**320**
SLKNRFIVLN	PTRFGTTVPF	TSRKPREDEK	DGQAYKFVSR	SEMEADIKAG	KYLEHGEYEG	NLYGTKIDSI	LEVVQTGRTC
**330**	**340**	**350**	**360**	**370**	**380**	**390**	**400**
ILDVNPQALK	VLRTSEFMPY	VVFIAAPELE	TLRAMHKAVV	DAGITTKLLT	DSDLKKTVDE	SARIQRAYNH	YFDLIIINDN
**410**	**420**	**430**					
LDKAFEKLQT	AIEKLRMEPQ	WVPISWVY					

Cmpd.	No. of Cmpds.	m/z meas.	Δ m/z [ppm]	z	Rt [min]	Score	P	Range	Sequence	Modification
2335	1	938.2188	84.20	3	57.5	10.4	1	334-357	R.TSEFMPYVVFIAAPELETLRAMHK.A	Oxidation: 5, 22
2850	1	994.4934	-74.35	2	64.3	22.2	2	358-376	K.AVVDAGITTKLLTDSDLKK.T	

Detailed Protein Report

Protein 509: PREDICTED: kinesin-like protein KIF13B isoform X2 [Homo sapiens]

**Accession:**	gi|530387685	**Score:**	42.6
**Database:**	refseq_human(refseq_human_20140103.fasta)	**MW [kDa]:**	195.4
**Database Date:**	2015-11-30	**pI:**	5.4
**Modification(s):**	Oxidation	**Sequence Coverage [%]:**	1.4
		**No. of unique Peptides:**	1

Alias proteins:

Accession	Name	Description
gi|578815259	refseq_human (refseq_human_20140103.fasta)	PREDICTED: kinesin-like protein KIF13B isoform X4 [Homo sapiens]
gi|578815257	refseq_human (refseq_human_20140103.fasta)	PREDICTED: kinesin-like protein KIF13B isoform X3 [Homo sapiens]

Detailed Protein Report

10	20	30	40	50	60	70	80
MDESVKEKYA	GQDIVFKCLG	ENILQNAFDG	YNACIFAYGQ	TGSGKSYTMM	GTADQPGLIP	RLCSGLFERT	QKEENEEQSF
90	100	110	120	130	140	150	160
KVEVSYMEIY	NEKVRDLLDP	KGSRQTLKVR	EHSVLGPYVD	GLSKLAVTSY	KDIESLMSEG	NKSRTVAATN	MNEESSRSHA
170	180	190	200	210	220	230	240
VFKITLTHTL	YDVKSGTSGE	KVGKLSLVDL	AGSERATKTG	AAGDRLKEGS	NINKSLTTLG	LVISALADQS	AGKNKNKFVP
250	260	270	280	290	300	310	320
YRDSVLTWLL	KDSLGGNSKT	AMVATVSPAA	DNYDETLSTL	RYADRAKHIV	NHAVVNEDPN	ARIIRDLREE	VEKLREQLTK
330	340	350	360	370	380	390	400
AEAMKSPELK	DRLEESEKLI	QEMTVTWEEK	LRKTEEIAQE	RQKQLESLGI	SLQSSGIKVG	DDKCFLVNLN	ADPALNELLV
410	420	430	440	450	460	470	480
YYLKEHTLIG	SANSQDIQLC	GMGILPEHCI	IDITSEGQVM	LTPQKNTRTF	VNGSSVSSPI	QLHHGDRILW	GNNHFFRLNL
490	500	510	520	530	540	550	560
PKKKKKAERE	DEDQDPSMKN	ENSSEQLDVD	GDSSSEVSSE	VNFNYEYAQM	EVTMKALGSN	DPMQSILNSL	EQQHEEEKRS
570	580	590	600	610	620	630	640
ALERQRLMYE	HELEQLRRRL	SPEKQNCRSM	DRFSFHSPSA	QQRLRQWAEE	REATLNNSLM	RLREQIVKAN	LLVREANYIA
650	660	670	680	690	700	710	720
EELDKRTEYK	VTLQIPASSL	DANRKRGSLL	SEPAIQVRRK	GKGKQIWSLE	KLDNRLLDMR	DLYQEWKECE	EDNPVIRSYF
730	740	750	760	770	780	790	800
KRADPFYDEQ	ENHSLIGVAN	VFLESLFYDV	KLQYAVPIIN	QKGEVAGRLH	VEVMRLSGDV	GERIAGGDEV	AEVSFEKETQ
810	820	830	840	850	860	870	880
ENKLVCMVKI	LQATGLPQHL	SHFVFCKYSF	WDQQEPVIVA	PEVDTSSSSV	SKEPHCMVVF	DHCNEFSVNI	TEDFIEHLSE
890	900	910	920	930	940	950	960
GALAIEVYGH	KINDPRKNPA	LWDLGIIQAK	TRSLRDRWSE	VTRKLEFWVQ	ILEQNENGEY	CPVEVISAKD	VPTGGIFQLR
970	980	990	1000	1010	1020	1030	1040
QGQSRRVQVE	VKSVQESGTL	PLMEECILSV	GIGCVKVRPL	RAPRTHETFH	EEEEDMDSYQ	DRDLERLRRK	WLNALTKRQE
1050	1060	1070	1080	1090	1100	1110	1120
YLDQQLQKLV	SKRDKTEDDA	DREAQLLEMR	LTLTEERNAV	MVPSAGSGIP	GAPAEWTPVP	GMETHIPVIF	LDLNADDFSS
1130	1140	1150	1160	1170	1180	1190	1200
QDNLDDPEAG	GWDATLTGEE	EEEFFELQIV	KQHDGEVKAE	ASWDSAVHGC	PQLSRGTPVD	ERLFLIVRVT	VQLSHPADMQ
1210	1220	1230	1240	1250	1260	1270	1280
LVLRKRICVN	VHGRQGFAQS	LLKKMSHRSS	IPGCGVTFEI	VSNIPEDAQG	VEEREALARM	AANVENPASA	DSEAYIEKYL
1290	1300	1310	1320	1330	1340	1350	1360
RSVLAVENLL	TLDRLRQEVA	VKEQLTGKGK	LSRRSISSPN	VNRLSGSRQD	LIPSYSLGSN	KGRWESQQDV	SQTTVSRGIA
1370	1380	1390	1400	1410	1420	1430	1440
PAPALSVSPQ	NNHSPDPGLS	NLAASYLNPV	KSFVPQMPKL	LKSLFPVRDE	KRGKRPSPLA	HQPVPRIMVQ	SASPDIRVTR
1450	1460	1470	1480	1490	1500	1510	1520
MEEAQPEMGP	DVLVQTMGAP	ALKICDKPAK	VPSPPPVIAV	TAVTPAPEAQ	DGPPSPLSEA	SSGYFSHSVS	TATLSDALGP
1530	1540	1550	1560	1570	1580	1590	1600
GLDAAAPPGS	MPTAPEAEPE	APISHPPPPT	AVPAEEPPGP	QQLVSPGRER	PDLEAPAPGS	PFRVRRVRAS	ELRSFSRMLA
1610	1620	1630	1640	1650	1660	1670	1680
GDPGCSPGAE	GNAPAPGAGG	QALASDSEEA	DEVPEWLREG	EFVTVGAHKT	GVVRYVGPAD	FQEGTWVGVE	LDLPSGKNDG
1690	1700	1710	1720	1730	1740	1750	1760
SIGGKQYFRC	NPGYGLLVRP	SRVRRATGPV	RRRSTGLRLG	APEARRSATL	SGSATNLASL	TAALAKADRS	HKNPENRKSW
1770							
AS							

Cmpd.	No. of Cmpds.	m/z meas.	Δ m/z [ppm]	z	Rt [min]	Score	P	Range	Sequence	Modification
1541	1	513.1056	-215.54	2	48.0	12.4	1	585-592	K.QNCRSMDR.F	Oxidation: 6

Detailed Protein Report

Protein 510: **PREDICTED: protein ELYS isoform X2 [Homo sapiens]**

**Accession:**	gi|578802192	**Score:**	42.6
**Database:**	refseq_human(refseq_human_20140103.fasta)	**MW [kDa]:**	252.3
**Database Date:**	2015-11-30	**pI:**	6.2
		**Sequence Coverage [%]:**	1.9
		**No. of unique Peptides:**	1

Detailed Protein Report

10	20	30	40	50	60	70	80
MRDLRAQVTS	GLLPFPEVTL	QALGEDEITL	ESVLRGKFAA	GKNGLACLAC	GPQLEVVNSI	TGERLSAYRF	SGVNEQPPVV
90	100	110	120	130	140	150	160
LAVKEFSWQK	RTGLLIGLEE	TEGSVLCLYD	LGISKVVKAV	VLPGRVTAIE	PIINHGGASA	STQHLHPSLR	WLFGVAAVVT
170	180	190	200	210	220	230	240
DVGQILLVDL	CLDDLSCNQN	EVEASDLEVL	TGIPAEVPHI	RESVMRQGRH	LCFQLVSPTG	TAVSTLSYIS	RTNQLAVGFS
250	260	270	280	290	300	310	320
DGYLALWNMK	SMKREYYIQL	ESGQVPVYAV	TFQEPENDPR	NCCYLWAVQS	TQDSEGDVLS	LHLLQLAFGN	RKCLASGQIL
330	340	350	360	370	380	390	400
YEGLEYCEER	YTLDLTGGMF	PLRGQTSNTK	LLGCQSIEKF	RSHGDREEGV	NEALSPDTSV	SVFTWQVNIY	GQGKPSVYLG
410	420	430	440	450	460	470	480
LFDINRWYHA	QMPDSLRSGE	YLHNCSYFAL	WSLESVVSRT	SPHGILDILV	HERSLNRGVP	PSYPPPEQFF	NPSTYNFDAT
490	500	510	520	530	540	550	560
CLLNSGVVHL	TCTGFQKETL	TFLKKSGPSL	NELIPDGYNR	CLVAGLLSPR	FVDVQPSSLS	QEEQLEAILS	AAIQTSSLGL
570	580	590	600	610	620	630	640
LTGYIRRWIT	EEQPNSATNL	RFVLEWTWNK	VVLTKEEFDR	LCVPLFDGSC	HFMDPQTIQS	IQQCYLLLSN	LNIVLSCFAS
650	660	670	680	690	700	710	720
EAREITERGL	IDLSNKFVVS	HLICQYAQVV	LWFSHSGLLP	EGIDDSVQLS	RLCYNYPVIQ	NYYTSRRQKF	ERLSRGKWNP
730	740	750	760	770	780	790	800
DCLMIDGLVS	QLGERIEKLW	KRDEGGTGKY	PPASLHAVLD	MYLLDGVTEA	AKHSITIYLL	LDIMYSFPNK	TDTPIESFPT
810	820	830	840	850	860	870	880
VFAISWGQVK	LIQGFWLIDH	NDYESGLDLL	FHPATAKPLS	WQHSKIIQAF	MSQGEHRQAL	RYIQTMKPTV	SSGNDVILHL
890	900	910	920	930	940	950	960
TVLLFNRCMV	EAWNFLRQHC	NRLNIEELLK	HMYEVCQEMG	LMEDLLKLPF	TDTEQECLVK	FLQSSASVQN	HEFLLVHHLQ
970	980	990	1000	1010	1020	1030	1040
RANYVPALKL	NQTLKINVMN	DRDPRLRERS	LARNSILDQY	GKILPRVHRK	LAIERAKPYH	LSTSSVFRLV	SRPKPLSAVP
1050	1060	1070	1080	1090	1100	1110	1120
KQVVTGTVLT	RSVFINNVLS	KIGEVWASKE	PINSTTPFNS	SKIEEPSPIV	YSLPAPELPE	AFFGTPISKA	SQKISRLLDL
1130	1140	1150	1160	1170	1180	1190	1200
VVQPVPRPSQ	CSEFIQQSSM	KSPLYLVSRS	LPSSSQLKGS	PQAISRASEL	HLLETPLVVK	KAKSLAMSVT	TSGFSEFTPQ
1210	1220	1230	1240	1250	1260	1270	1280
SILRSTLRST	PLASPSPSPG	RSPQRLKETR	ISFVEEDVHP	KWIPGAADDS	KLEVFTTPKK	CAVPVETEWL	KSKDRTTSFF
1290	1300	1310	1320	1330	1340	1350	1360
LNSPEKEHQE	MDEGSQSLEK	LDVSKGNSSV	SITSDETTLE	YQDAPSPEDL	EETVFTASKP	KSSSTALTTN	VTEQTEKDGD
1370	1380	1390	1400	1410	1420	1430	1440
KDVFASEVTP	SDLQKQMGNL	EDAETKDLLV	AAEAFSELNH	LSPVQGTEAS	LCAPSVYEGK	IFTQKSKVPV	LDEGLTSVET
1450	1460	1470	1480	1490	1500	1510	1520
YTPAIRANDN	KSMADVLGDG	GNSSLTISEG	PIVSERRLNQ	EVALNLKEDH	EVEVGVLKES	VDLPEEKLPI	SDSPPDTQEI
1530	1540	1550	1560	1570	1580	1590	1600
HVIEQEKLEA	QDSGEEARNL	SFNELYPSGT	LKLQYNFDTI	DQQFCDLADN	KDTAECDIAE	VDGELFVAQS	NFTLILEGEE
1610	1620	1630	1640	1650	1660	1670	1680
GEVEPGDFAS	SDVLPKAANT	ATEEKLVCSG	ENDNHGQIAN	LPSAVTSDQK	SQKVDTLPYV	PEPIKVAIAE	NLLDVIKDTR
1690	1700	1710	1720	1730	1740	1750	1760
SKEITSDTME	QSIHETIPLV	SQNIMCPTKL	VKSAFKTAQE	TSTMTMNVSQ	VDDVVSSKTR	TRGQRIQNVN	VKSAQQEASA
1770	1780	1790	1800	1810	1820	1830	1840
DVATPKMPGQ	SVRKKTRKAK	EISEASENIY	SDVRGLSQNQ	QIPQNSVTPR	RGRRKKEVNQ	DILENTSSVE	QELQITTGRE
1850	1860	1870	1880	1890	1900	1910	1920
SKRLKSSQLL	EPAVEETTKK	EVKVSSVTKR	TPRRIKRSVE	NQESVEIIND	LKVSTVTSPS	RMIRKLRSTN	LDASENTGNK
1930	1940	1950	1960	1970	1980	1990	2000
QDDKSSDKQL	RIKHVRRVRG	REVSPSDVRE	DSNLESSQLT	VQAEFDMSAI	PRKRGRPRKI	NPSEDVGSKA	VKEERSPKKK
2010	2020	2030	2040	2050	2060	2070	2080
EAPSIRRRST	RNTPAKSENV	DVGKPALGKS	ILVPNEELSM	VMSSKKKLTK	KTESQSQKRS	LHSVSEERTD	EMTHKETNEQ
2090	2100	2110	2120	2130	2140	2150	2160
EERLLATASF	TKSSRSSRTR	SSKAILLPDL	SEPNNEPLFS	PASEVPRKAK	AKKIEVPAQL	KELVSDLSSQ	FVISPPALRS
2170	2180	2190	2200	2210	2220	2230	2240
RQKNTSNKNK	LEDELKDDAQ	SVETLGKPKA	KRIRTSKTKQ	ASKNTEKESA	WSPPPIEIRL	ISPLASPADG	VKSKPRKTTE
2250	2260	2270					
VTGTGLGRNR	KKLSSYPKQI	LRRKML					

Detailed Protein Report

Cmpd.	No. of Cmpds.	m/z meas.	Δ m/z [ppm]	z	Rt [min]	Score	P	Range	Sequence	Modification
2789	1	735.0657	-23.60	3	66.4	12.6	2	1010-1028	R.KLAIERAKPYHLSTSSVFR.L	

Detailed Protein Report

Protein 511: PREDICTED: uncharacterized protein C7orf63 isoform X1 [Homo sapiens]

**Accession:**	gi|530386429	**Score:**	42.5
**Database:**	refseq_human(refseq_human_20140103.fasta)	**MW [kDa]:**	101.8
**Database Date:**	2015-11-30	**pI:**	6.2
		**Sequence Coverage [%]:**	5.1
		**No. of unique Peptides:**	1

10	20	30	40	50	60	70	80
MWTEEAGATA	EAQESGIRNK	SSSSSQIPVV	GVVTEDDEAQ	DVFKPMDLNR	VIKLLEETDK	DGLEEKQLKF	VKKLVQCYQN
90	100	110	120	130	140	150	160
GLPLRDLAQI	FKILNLCSGK	IKNQPRFIES	AYDIIKLCGL	PFLKKKVSDE	ITYAEDTANS	IALLGDLMKI	PSSELRIQIC
170	180	190	200	210	220	230	240
KCIVDFYHAE	PPKKHIPGYQ	QASSSYKIQM	AEVGGLAKTM	VQSMTLLENQ	LVEKLWVLKV	LQHLSTSEVN	CTIMMKAQAA
250	260	270	280	290	300	310	320
SGICTHLNDP	DPSGQLLFRS	SEILWNLLEK	SSKEEVIQQL	SNLECLLALK	EVFKNLFMRG	FSHYDRQLRN	DILVITTIIA
330	340	350	360	370	380	390	400
QNPEAPMIEC	GFTKDLILFA	TFNEVKSQNL	LVKGLKLSNS	YEDFELKKLL	FNVIVILCKD	LPTVQLLIDG	KVILALFTYV
410	420	430	440	450	460	470	480
KKPEKQKIID	WSAAQHEELQ	LHAIATLSSV	APLLIEEYMS	CQGNARVLAF	LEWCESEDPF	FSHGNSFHGT	GGRGNKFAQM
490	500	510	520	530	540	550	560
RYSLRLLRAV	VYLEDETVNK	DLCEKGTIQQ	MIGIFKNIIS	KPNEKEEAIV	LEIQSDILLI	LSGLCENHIQ	RKEIFGTEGV
570	580	590	600	610	620	630	640
DIVLHVMKTD	PRKLQSGLGY	NVLLFSTLDS	IWCCILGCYP	SEDYFLEKEG	IFLLLDLLAL	NQKKFCNLIL	GIMVEFCDNP
650	660	670	680	690	700	710	720
KTAAHVNAWQ	GKKDQTAASL	LIKLWRKEEK	ELGVKRDKNG	KIIDTKKPLF	TSFQEEQKII	PLPANCPSIA	VMDVSENIRA
730	740	750	760	770	780	790	800
KIYAILGKLD	FENLPGLSAE	DFVTLCIIHR	YLDFKIGEIW	NEIYEEIKLE	KLRPVTTDKK	ALEAITTASE	NIGKMVASLQ
810	820	830	840	850	860	870	880
SDIIESQACQ	DMQNEQKVYA	KIQATHKQRE	LANKSWEDFL	ARTSNAKTLK	VPSGGVVTVE	STPARLVGGP	LVDTDIALKK
890	900	910					
LPIRGGALQR	VKAVKIVDAP	KKSIPT					

Cmpd.	No. of Cmpds.	m/z meas.	Δ m/z [ppm]	z	Rt [min]	Score	P	Range	Sequence	Modification
2785	1	711.3716	-1.34	3	66.0	10.9	2	157-174	R.IQICKCIVDFYHAEPPKK.H	

Detailed Protein Report

Protein 512: ectodysplasin-A isoform 2 [Homo sapiens]

**Accession:**	gi|54112101	**Score:**	42.5
**Database:**	refseq_human(refseq_human_20140103.fasta)	**MW [kDa]:**	41.0
**Database Date:**	2015-11-30	**pI:**	9.6
		**Sequence Coverage [%]:**	8.7
		**No. of unique Peptides:**	2

10	20	30	40	50	60	70	80
MGYPEVERRE	LLPAAAPRER	GSQGCGCGGA	PARAGEGNSC	LLFLGFFGLS	LALHLLTLCC	YLELRSELRR	ERGAESRLGG
**90**	**100**	**110**	**120**	**130**	**140**	**150**	**160**
SGTPGTSGTL	SSLGGLDPDS	PITSHLGQPS	PKQQPLEPGE	AALHSDSQDG	HQMALLNFFF	PDEKPYSEEE	SRRVRRNKRS
**170**	**180**	**190**	**200**	**210**	**220**	**230**	**240**
KSNEGADGPV	KNKKKGKKAG	PPGPNGPPGP	PGPPGPQGPP	GIPGIPGIPG	TTVMGPPGPP	GPPGPQGPPG	LQGPSGAADK
**250**	**260**	**270**	**280**	**290**	**300**	**310**	**320**
AGTRENQPAV	VHLQGQGSAI	QVKNDLSGGV	LNDWSRITMN	PKVFKLHPRS	GELEVLVDGT	YFIYSQVYYI	NFTDFASYEV
**330**	**340**	**350**	**360**	**370**	**380**	**390**	
VVDEKPFLQC	TRSIETGKTN	YNTCYTAGVC	LLKARQKIAV	KMVHADISIN	MSKHTTFFGA	IRLGEAPAS	

Cmpd.	No. of Cmpds.	m/z meas.	Δ m/z [ppm]	z	Rt [min]	Score	P	Range	Sequence	Modification
1964	4	703.2668	-56.59	2	52.9	13.6	1	19-33	R.ERGSQGCGCGGAPAR.A	
1518	3	1059.0796	50.76	2	48.7	16.6	1	264-282	K.NDLSGGVLNDWSRITMNPK.V	

Detailed Protein Report

Protein 513: PREDICTED: talin-2 isoform X15 [Homo sapiens]

**Accession:**	gi|578827491	**Score:**	42.5
**Database:**	refseq_human(refseq_human_20140103.fasta)	**MW [kDa]:**	267.3
**Database Date:**	2015-11-30	**pI:**	5.4
		**Sequence Coverage [%]:**	1.9
		**No. of unique Peptides:**	1

Quantitation

M:Q	**Median:** 0.47	**CV:** 0.00 %	**No. of Peptides:** 1

Detailed Protein Report

10	20	30	40	50	60	70	80
MVALSLKICV	RHCNVVKTMQ	FEPSTAVYDA	CRVIRERVPE	AQTGQASDYG	LFLSDEDPRK	GIWLEAGRTL	DYYMLRNGDI
90	100	110	120	130	140	150	160
LEYKKKQRPQ	KIRMLDGSVK	TVMVDDSKTV	GELLVTICSR	IGITNYEEYS	LIQETIEEKK	EEGTGTLKKD	RTLLRDERKM
170	180	190	200	210	220	230	240
EKLKAKLHTD	DDLNWLDHSR	TFREQGVDEN	ETLLLRRKFF	YSDQNVDSRD	PVQLNLLYVQ	ARDDILNGSH	PVSFEKACEF
250	260	270	280	290	300	310	320
GGFQAQIQFG	PHVEHKHKPG	FLDLKEFLPK	EYIKQRGAEK	RIFQEHKNCG	EMSEIEAKVK	YVKLARSLRT	YGVSFFLVKE
330	340	350	360	370	380	390	400
KMKGKNKLVP	RLLGITKDSV	MRVDEKTKEV	LQEWPLTTVK	RWAASPKSFT	LDFGEYQESY	YSVQTTEGEQ	ISQLIAGYID
410	420	430	440	450	460	470	480
IILKKKQSKD	RFGLEGDEES	TMLEESVSPK	KSTILQQQFN	RTGKAEHGSV	ALPAVMRSGS	SGPETFNVGS	MPSPQQQVMV
490	500	510	520	530	540	550	560
GQMHRGHMPP	LTSAQQALMG	TINTSMHAVQ	QAQDDLSELD	SLPPLGQDMA	SRVWVQNKVD	ESKHEIHSQV	DAITAGTASV
570	580	590	600	610	620	630	640
VNLTAGDPAD	TDYTAVGCAI	TTISSNLTEM	SKGVKLLAAL	MDDEVGSGED	LLRAARTLAG	AVSDLLKAVQ	PTSGEPRQTV
650	660	670	680	690	700	710	720
LTAAGSIGQA	SGDLLRQIGE	NETDERFQDV	LMSLAKAVAN	AAAMLVLKAK	NVAQVAEDTV	LQNRVIAAAT	QCALSTSQLV
730	740	750	760	770	780	790	800
ACAKVVSPTI	SSPVCQEQLI	EAGKLVDRSV	ENCVRACQAA	TTDSELLKQV	SAAASVVSQA	LHDLLQHVRQ	FASRGEPIGR
810	820	830	840	850	860	870	880
YDQATDTIMC	VTESIFSSMG	DAGEMVRQAR	VLAQATSDLV	NAMRSDAEAE	IDMENSKKLL	AAAKLLADST	ARMVEAAKGA
890	900	910	920	930	940	950	960
AANPENEDQQ	QRLREAAEGL	RVATNAAAQN	AIKKKIVNRL	EVAAKQAAAA	ATQTIAASQN	AAVSNKNPAA	QQQLVQSCKA
970	980	990	1000	1010	1020	1030	1040
VADHIPQLVQ	GVRGSQAQAE	DLSAQLALII	SSQNFLQPGS	KMVSSAKAAV	PTVSDQAAAM	QLSQCAKNLA	TSLAELRTAS
1050	1060	1070	1080	1090	1100	1110	1120
QKAHEACGPM	EIDSALNTVQ	TLKNELQDAK	MAAVESQLKP	LPGETLEKCA	QDLGSTSKAV	GSSMAQLLTC	AAQGNEHYTG
1130	1140	1150	1160	1170	1180	1190	1200
VAARETAQAL	KTLAQAARGV	AASTTDPAAA	HAMLDSARDV	MEGSAMLIQE	AKQALIAPGD	AERQQRLAQV	AKAVSHSLNN
1210	1220	1230	1240	1250	1260	1270	1280
CVNCLPGQKD	VDVALKSIGE	SSKKLLVDSL	PPSTKPFQEA	QSELNQAAAD	LNQSAGEVVH	ATRGQSGELA	AASGKFSDDF
1290	1300	1310	1320	1330	1340	1350	1360
DEFLDAGIEM	AGQAQTKEDQ	IQVIGNLKNI	SMASSKLLLA	AKSLSVDPGA	PNAKNLLAAA	ARAVTESINQ	LITLCTQQAP
1370	1380	1390	1400	1410	1420	1430	1440
GQKECDNALR	ELETVKGMLD	NPNEPVSDLS	YFDCIESVME	NSKVLGESMA	GISQNAKTGD	LPAFGECVGI	ASKALCGLTE
1450	1460	1470	1480	1490	1500	1510	1520
AAAQAAYLVG	ISDPNSQAGH	QGLVDPIQFA	RANQAIQMAC	QNLVDPGSSP	SQVLSAATIV	AKHTSALCNA	CRIASSKTAN
1530	1540	1550	1560	1570	1580	1590	1600
PVAKRHFVQS	AKEVANSTAN	LVKTIKALDG	DFSEDNRNKC	RIATAPLIEA	VENLTAFASN	PEFVSIPAQI	SSEGSQAQEP
1610	1620	1630	1640	1650	1660	1670	1680
ILVSAKTMLE	SSSYLIRTAR	SLAINPKDPP	TWSVLAGHSH	TVSDSIKSLI	TSIRDKAPGQ	RECDYSIDGI	NRCIRDIEQA
1690	1700	1710	1720	1730	1740	1750	1760
SLAAVSQSLA	TRDDISVEAL	QEQLTSVVQE	IGHLIDPIAT	AARGEAAQLG	HKVTQLASYF	EPLILAAVGV	ASKILDHQQQ
1770	1780	1790	1800	1810	1820	1830	1840
MTVLDQTKTL	AESALQMLYA	AKEGGGNPKA	QHTHDAITEA	AQLMKEAVDD	IMVTLNEAAS	EVGLVGGMVD	AIAEAMSKLD
1850	1860	1870	1880	1890	1900	1910	1920
EGTPPEPKGT	FVDYQTTVVK	YSKAIAVTAQ	EMIGFQIRTR	VQDLGHGCIF	LVQKAGALQV	CPTDSYTKRE	LIECARAVTE
1930	1940	1950	1960	1970	1980	1990	2000
KVSLVLSALQ	AGNKGTQACI	TAATAVSGII	ADLDTTIMFA	TAGTLNAENS	ETFADHRENI	LKTAKALVED	TKLLVSGAAS
2010	2020	2030	2040	2050	2060	2070	2080
TPDKLAQAAQ	SSAATITQLA	EVVKLGAASL	GSDDPETQVV	LINAIKDVAK	ALSDLISATK	GAASKPVDDP	SMYQLKGAAK
2090	2100	2110	2120	2130	2140	2150	2160
VMVTNVTSLL	KTVKAVEDEA	TRGTRALEAT	IECIKQELTV	FQSKDVPEKT	SSPEESIRMT	KGITMATAKA	VAAGNSCRQE
2170	2180	2190	2200	2210	2220	2230	2240
DVIATANLSR	KAVSDMLTAC	KQASFHPDVS	DEVRTRALRF	GTECTLGYLD	LLEHVLVILQ	KPTPEFKQQL	AAFSKRVAGA
2250	2260	2270	2280	2290	2300	2310	2320
VTELIQAAEA	MKGTEWVDPE	DPTVIAETEL	LGAAASIEAA	AKKLEQLKPR	AKPKQADETL	DFEEQILEAA	KSIAAATSAL
2330	2340	2350	2360	2370	2380	2390	2400

Detailed Protein Report

Cmpd.	No. of Cmpds.	m/z meas.	Δ m/z [ppm]	z	Rt [min]	Score	P	Range	Sequence	Modification	Ratios	
2049	1	812.3468	-109.11	2	56.0	14.1	1	442-457	R.TGKAEHGSVALPAVMR.S		M:Q	0.47

Detailed Protein Report

Protein 514: cytoplasmic phosphatidylinositol transfer protein 1 isoform a [Homo sapiens]

Accession: gi|32307140
Database: refseq_human(refseq_human_20140103.fasta)
Database Date: 2015-11-30
Modification(s): Carbamidomethyl

Score: 42.5
MW [kDa]: 38.4
pI: 6.0
Sequence Coverage [%]: 15.4
No. of unique Peptides: 2

10	20	30	40	50	60	70	80
MLLKEYRICM	PLTVDEYKIG	QLYMISKHSH	EQSDRGEGVE	VVQNEPFEDP	HHGNGQFTEK	RVYLNSKLPS	WARAVVPKIF
**90**	**100**	**110**	**120**	**130**	**140**	**150**	**160**
YVTEKAWNYY	PYTITEYTCS	FLPKFSIHIE	TKYEDNKGSN	DTIFDNEAKD	VEREVCFIDI	ACDEIPERYY	KESEDPKHFK
**170**	**180**	**190**	**200**	**210**	**220**	**230**	**240**
SEKTGRGQLR	EGWRDSHQPI	MCSYKLVTVK	FEVWGLQTRV	EQFVHKVVRD	ILLIGHRQAF	AWVDEWYDMT	MDEVREFERA
**250**	**260**	**270**	**280**	**290**	**300**	**310**	**320**
TQEATNKKIG	IFPPAISISS	IPLLPSSVRS	APSSAPSTPL	STDAPEFLSV	PKDRPRKKSA	PETLTLPDPE	KKATLNLPGM
**330**	**340**						
HSSDKPCRPK	SE						

Cmpd.	No. of Cmpds.	m/z meas.	Δ m/z [ppm]	z	Rt [min]	Score	P	Range	Sequence	Modification
38	1	918.9134	2.10	2	29.2	16.1	1	171-185	R.EGWRDSHQPIMCSYK.L	
26	2	712.9803	-81.39	3	29.3	11.2	1	312-330	K.KATLNLPGMHSSDKPCRPK.S	Carbamidomethyl: 16

Detailed Protein Report

Protein 515: **PREDICTED: protein Shroom4 isoform X2 [Homo sapiens]**

Accession: gi|578838225
Database: refseq_human(refseq_human_20140103.fasta)
Database Date: 2015-11-30
Modification(s): Carbamidomethyl, Oxidation

Score: 42.5
MW [kDa]: 147.5
pI: 6.0
Sequence Coverage [%]: 2.8
No. of unique Peptides: 2

Quantitation

Q:W	**Median:** 2.40	**CV:** 0.00 %	**No. of Peptides:** 1
M:Q	**Median:** 0.81	**CV:** 0.00 %	**No. of Peptides:** 1

```
        10         20         30         40         50         60         70         80
MESLEQPGQA TYESHLLPID QNMYPNQRDS AYSSFSASSN ASDCALSLRP EEPASTDCIM QGPGPTKAPS GRPNVAETSG
        90        100        110        120        130        140        150        160
GSRRTNGGHL TPSSQMSSRP QEGYQSGPAK AVRGPPQPPV RRDSLQASRA QLLNGEQRRA SEPVVPLPQK EKLSLEPVLP
       170        180        190        200        210        220        230        240
ARNPNRFCCL SGHDQVTSEG HQNCEFSQPP ESSQQGSEHL LMQASTKAVG SPKACDRASS VDSNPLNEAS AELAKASFGR
       250        260        270        280        290        300        310        320
PPHLIGPTGH RHSAPEQLLA SHLQHVHLDT RGSKGMELPP VQDGHQWTLS PLHSSHKGKK SPCPPTGGTH DQSSKERKTR
       330        340        350        360        370        380        390        400
QVDDRSLVLG HQSQSSPPHG EADGHPSEKG FLDPNRTSRA ASELANQQPS ASGSLVQQAT DCSSTTKAAS GTEAGEEGDS
       410        420        430        440        450        460        470        480
EPKECSRMGG RRSGGTRGRS IQNRRKSERF ATNLRNEIQR RKAQLQKSKG PLSQLCDTKE PVEETQEPPE SPPLTASNTS
       490        500        510        520        530        540        550        560
LLSSCKKPPS PRDKLFNKSM MLRARSSECL SQAPESHESR TGLEGRISPG QRPGQSSLGL NTWWKAPDPS SSDPEKAHAH
       570        580        590        600        610        620        630        640
CGVRGGHWRW SPEHNSQPLV AAAMEGPSNP GDNKELKAST AQAGEDAILL PFADRRKFFE ESSKSLSTSH LPGLTTHSNK
       650        660        670        680        690        700        710        720
TFTQRPKPID QNFQPMSSSC RELRRHPMDQ SYHSADQPYH ATDQSYHSMS PLQSETPTYS ECFASKGLEN SMCCKPLHCG
       730        740        750        760        770        780        790        800
DFDYHRTCSY SCSVQGALVH DPCIYCSGEI CPALLKRNMM PNCYNCRCHH HQCIRCSVCY HNPQHSALED SSLAPGNTWK
       810        820        830        840        850        860        870        880
PRKLTVQEFP GDKWNPITGN RKTSQSGREM AHSKTSFSWA TPFHPCLENP ALDLSSYRAI SSLDLLGDFK HALKKSEETS
       890        900        910        920        930        940        950        960
VYEEGSSLAS MPHPLRSRAF SESHISLAPQ STRAWGQHRR ELFSKGDETQ SDLLGARKKA FPPPRPPPPN WEKYRLFRAA
       970        980        990       1000       1010       1020       1030       1040
QQQKQQQQQQ KQQEEEEEEE EEEEEEEEEE EEEAEEEEEE LPPQYFSSET SGSCALNPEE VLEQPQPLSF GHLEGSRQGS
      1050       1060       1070       1080       1090       1100       1110       1120
QSVPAEQESF ALHSSDFLPP IRGHLGSQPE QAQPPCYYGI GGLWRTSGQE ATESAKQEFQ HFSPPSGAPG IPTSYSAYYN
      1130       1140       1150       1160       1170       1180       1190       1200
ISVAKAELLN KLKDQPEMAE IGLGEEEVDH ELAQKKIQLI ESISRKLSVL REAQRGLLED INANSALGEE VEANLKAVCK
      1210       1220       1230       1240       1250       1260       1270       1280
SNEFEKYHLF VGDLDKVVNL LLSLSGRLAR VENALNSIDS EANQEKLVLI EKKQQLTGQL ADAKELKEHV DRREKLVFGM
      1290       1300       1310       1320       1330       1340
VSRYLPQDQL QDYQHFVKMK SALIIEQREL EEKIKLGEEQ LKCLRESLLL GPSNF
```

Cmpd.	No. of Cmpds.	m/z meas.	Δ m/z [ppm]	z	Rt [min]	Score	P	Range	Sequence	Modification	Ratios
43	1	750.8112	-65.69	2	30.4	14.4	1	557-569	K.AHAHCGVRGGHWR.W	Carbamidomethyl: 5	Q:W 2.40<br>M:Q 0.81
2564	1	731.7179	-190.01	2	60.7	10.2	2	822-834	R.KTSQSGREMAHSK.T	Oxidation: 9	

Detailed Protein Report

Protein 516: dynein heavy chain 7, axonemal [Homo sapiens]

**Accession:**	gi|151301127	**Score:**	42.5
**Database:**	refseq_human(refseq_human_20140103.fasta)	**MW [kDa]:**	460.9
**Database Date:**	2015-11-30	**pI:**	5.6
		**Sequence Coverage [%]:**	1.0
		**No. of unique Peptides:**	1

Detailed Protein Report

10	20	30	40	50	60	70	80
MSSEQDKSAS	KEKSKKPVRF	LPQLSMEKLA	SKEKFKAPAR	ALPQLSMVST	KPHWQQAAPS	FHLSVKQDDE	SPEPFSVKNE
90	100	110	120	130	140	150	160
QSHAEYMERF	GKKGKLPHQV	DDSYVGPSTS	KSKGKSPHKE	RENFRSTLVN	VIMQQDADLD	SAVPDGSTIP	KPTASAIEKD
170	180	190	200	210	220	230	240
ILRYYYYIHH	GIDTDHVAPM	EDSWLEHVLD	LVPQHLKVFT	DSIVTLSDEM	REDYLLSVRK	SIVDFVLKDP	REKGDDKKTD
250	260	270	280	290	300	310	320
ELPAHRAEME	ILPKPWRKSF	LAASSYIRDH	LNAMNPTMLA	VLDLWHTNFK	KLRLVDIKEF	HNCQDALELS	SFQNIIMRHM
330	340	350	360	370	380	390	400
DSAKETLLKM	WFPEVQNIYY	QGNKKKQLPT	GDSSAKLESF	FNCAAALMTL	QLQDLTLVSM	QDFTDLIAQP	PDSVRAFEHP
410	420	430	440	450	460	470	480
GFIMRLILDN	DTIKFEPELS	DYIDIFLNVY	DVMIKAVSFV	PRVETKLYSK	WESKSKPTTL	KPIILNEIVD	AHKEKIKEVI
490	500	510	520	530	540	550	560
MKESVAPTEH	LRLYDKYDFL	ITRKAERDVD	NFLAENHSYE	KIIDEICKYQ	KLIEEIQYTS	IKTIRLGMFE	MHCEELIRAL
570	580	590	600	610	620	630	640
VKRADIICGK	LLAKMFRDHQ	EVNTRLCDEF	ERIAEKALST	PPNTAELMEM	KAYIQKVEVT	DMIELEQRLV	DSKNCLAFLI
650	660	670	680	690	700	710	720
EYVNFSPADM	RLNNSVFQWY	GRMGEIFEEH	RKIIKEKIEQ	YQEGLKLRCE	RFVEELESYA	KQSEEFYSFG	DLQDVQRYLK
730	740	750	760	770	780	790	800
KAQILNGKLD	LAADKIEQFN	AEEEAFGWLP	SVYPQRKKIQ	DGLNPYLRLY	ETAVEFSSNY	RAWTEGPYHK	VNPDQVEADI
810	820	830	840	850	860	870	880
GNYWRGLYKL	EKTFHDSPYA	LAMTKKVRSK	VEDFKQHIPL	IQVICNPGLR	PRHWEAMSAI	VGYPLQPSDD	STVSSFLDMN
890	900	910	920	930	940	950	960
LEPYIDRFEG	ISEAASKEYS	LEKAMEKMIT	EWDAVEFVIH	SYRETGTFIL	ASVDEIQMLL	DDHIIKTQTM	RGSPFIKPYE
970	980	990	1000	1010	1020	1030	1040
KQMREWEGKL	LLLQEILDEW	LKVQATWLYL	EPIFSSPDIM	SQMPEEGRRF	TAVDKTWRDI	MRSVMQDKHV	LTVVTIDRML
1050	1060	1070	1080	1090	1100	1110	1120
ERLKKSNELL	ELILKGLNEY	LEKKRLFFPR	FFFLSNDELL	EILSETKDPT	RVQPHLKKCF	EGIAKVEFTE	TLDITHMKSS
1130	1140	1150	1160	1170	1180	1190	1200
EGEVVELIEI	ISTAKARGQV	EKWLVELERV	MINSIHKVTG	DATFAYTKYE	RINWVRDWPG	QTVLCVSQIF	WTKEVQTAIP
1210	1220	1230	1240	1250	1260	1270	1280
MGIKALEQYL	KTCNRQIDDI	VTLVRGKLSM	QNRVTLGALV	VLDVHARDVL	SSLVKKNISD	DSDFEWLSQL	RYYWQENHLE
1290	1300	1310	1320	1330	1340	1350	1360
TKMINAGLRY	GYEYLGNSPR	LVITPLTDRC	YRTLFGALHL	HLGGAPEGPA	GTGKTETTKD	LAKAVAKQCV	VFNCSDGLDY
1370	1380	1390	1400	1410	1420	1430	1440
LALGKFFKGL	LSCGAWACFD	EFNRIDLEVL	SVVAQQILTI	QRGINAGADI	LMFEGTELKL	DPTCAVFITM	NPGYAGRSEL
1450	1460	1470	1480	1490	1500	1510	1520
PDNLKALFRT	VAMMVPDYAM	IAEIVLYSCG	FVTARPLSVK	IVATYRLCSE	QLSSQHHYDY	GMRAVKSVLT	AAGNLKLKYP
1530	1540	1550	1560	1570	1580	1590	1600
NENEEILLLR	SIIDVNLPKF	LSHDLPLFEG	ITSDLFPGVK	LPKPDYNDLL	AAIKDNCASM	NLQMTAFFSE	KILQVYEMMI
1610	1620	1630	1640	1650	1660	1670	1680
VRHGFMIVGE	PFGGKTSAYR	VLAGALNDIC	EKGLMEENKV	QITVLNPKSV	TMGQLYGQFD	SVSHEWSDGV	LAVSFRAFAS
1690	1700	1710	1720	1730	1740	1750	1760
SVTPDRKWLI	FDGPVDAVWI	ENMNTVLDDN	KKLCLMSGEI	IQMSPQMNLI	FEPMDLEVAS	PATVSRCGMI	YMEPHMLGWR
1770	1780	1790	1800	1810	1820	1830	1840
PLMLSWVNLL	PASVSVIQKE	FIMGLFDRMV	PVSVEFIRKH	TKELSPTSDT	NLVRSLMNLI	DCFMDDFADE	VKLKERNDRE
1850	1860	1870	1880	1890	1900	1910	1920
TYSLLEGIFL	FSLIWSVGAS	CTDDDRLKFN	KILRELMESP	ISDRTRNTFK	LQSGTEQTSS	KALTVPFPEK	GTIYDYQFVT
1930	1940	1950	1960	1970	1980	1990	2000
EGIGKWEPWI	KKLKEAPPIP	KDVMFNEIIV	PTLDTIRYSA	LMELLTTHQK	PSIFVGPTGT	GKSVYITNFL	LNQLNKEIYK
2010	2020	2030	2040	2050	2060	2070	2080
PLLINFSAQT	TAAQTQNIVM	SKLDKRRKGV	FGPPLGKRMV	VFVDDVNMPA	REVYGAQPPI	ELLRQWLDHW	NWYDLKDCSM
2090	2100	2110	2120	2130	2140	2150	2160
IKLVDIQIMC	AMGPPGGGRN	PVTPRYMRHF	NIITINEFSD	KSMYTIFSRI	LTWHLEICYK	FPDEFLDLTT	QIVNGTMTLY
2170	2180	2190	2200	2210	2220	2230	2240

Detailed Protein Report

Cmpd.	No. of Cmpds.	m/z meas.	Δ m/z [ppm]	z	Rt [min]	Score	P	Range	Sequence	Modification
2726	1	862.4253	-75.80	2	65.2	11.4	2	436-450	K.AVSFVPRVETKLYSK.W	

Detailed Protein Report

Protein 517: **short transient receptor potential channel 7 isoform 3 [Homo sapiens]**

**Accession:**	gi|262399375	**Score:**	42.4
**Database:**	refseq_human(refseq_human_20140103.fasta)	**MW [kDa]:**	86.2
**Database Date:**	2015-11-30	**pI:**	8.4
		**Sequence Coverage [%]:**	5.8
		**No. of unique Peptides:**	2

10	20	30	40	50	60	70	80
MLRNSTFKNM	QRRHTTLREK	GRRQAIRGPA	YMFNEKGTSL	TPEEERFLDS	AEYGNIPVVR	KMLEESKTLN	FNCVDYMGQN
**90**	**100**	**110**	**120**	**130**	**140**	**150**	**160**
ALQLAVGNEH	LEVTELLLKK	ENLARVGDAL	LLAISKGYVR	IVEAILNHPA	FAQGQRLTLS	PLEQELRDDD	FYAYDEDGTR
**170**	**180**	**190**	**200**	**210**	**220**	**230**	**240**
FSHDITPIIL	AAHCQEYEIV	HILLLKGARI	ERPHDYFCKC	NECTEKQRKD	SFSHSRSRMN	AYKGLASAAY	LSLSSEDPVL
**250**	**260**	**270**	**280**	**290**	**300**	**310**	**320**
TALELSNELA	RLANIETEFK	LGRTLRSPFM	KFVAHAVSFT	IFLGLLVVNA	SDRFEGVKTL	PNETFTDYPK	QIFRVKTTQF
**330**	**340**	**350**	**360**	**370**	**380**	**390**	**400**
SWTEMLIMKW	VLGMIWSECK	EIWEEGPREY	VLHLWNLLDF	GMLSIFVASF	TARFMAFLKA	TEAQLYVDQH	VQDDTLHNVS
**410**	**420**	**430**	**440**	**450**	**460**	**470**	**480**
LPPEVAYFTY	ARDKWWPSDP	QIISEGLYAI	AVVLSFSRIA	YILPANESFG	PLQISLGRTV	KDIFKFMVIF	IMVFVAFMIG
**490**	**500**	**510**	**520**	**530**	**540**	**550**	**560**
MFNLYSYYRG	AKYNPAFTTV	EESFKTLFWS	IFGLSEVISV	VLKYDHKFIE	NIGYVLYGVY	NVTMVVVLLN	MLIAMINNSY
**570**	**580**	**590**	**600**	**610**	**620**	**630**	**640**
QEIEEDADVE	WKFARAKLWL	SYFDEGRTLP	APFNLVPSPK	SFYYLIMRIK	MCLIKLCKSK	AKSCENDLEM	GMLNSKFKKT
**650**	**660**	**670**	**680**	**690**	**700**	**710**	**720**
RYQAGMRNSE	NLTANNTLSK	PTRYQKIMKR	LIKRYVLKAQ	VDRENDEVNE	GELKEIKQDI	SSLRYELLEE	KSQATGELAD
**730**	**740**	**750**					
LIQQLSEKFG	KNLNKDHLRV	NKGKDI					

Cmpd.	No. of Cmpds.	m/z meas.	Δ m/z [ppm]	z	Rt [min]	Score	P	Range	Sequence	Modification
81	1	1120.1328	21.81	2	30.9	11.6	1	117-136	K.GYVRIVEAILNHPAFAQGQR.L	
965	1	607.1283	-333.12	1	41.6	14.2	0	611-615	K.MCLIK.L	

Detailed Protein Report

Protein 518: phosphatidylinositol phosphatase PTPRQ precursor [Homo sapiens]

**Accession:**	gi|222537743	**Score:**	42.4
**Database:**	refseq_human(refseq_human_20140103.fasta)	**MW [kDa]:**	257.1
**Database Date:**	2015-11-30	**pI:**	5.4
**Modification(s):**	Oxidation	**Sequence Coverage [%]:**	1.7
		**No. of unique Peptides:**	2

Quantitation

Q:W	**Median:** 1.20	**CV:** 0.00 %	**No. of Peptides:** 1

10	20	30	40	50	60	70	80
MDFLIIFLLL	FIGTSETQVD	VSNVVPGTRY	DITISSISTT	YTSPVTRIVT	TNVTKPGPPV	FLAGERVGSA	GILLSWNTPP
90	100	110	120	130	140	150	160
NPNGRIISYI	VKYKEVCPWM	QTVYTQVRSK	PDSLEVLLTN	LNPGTTYEIK	VAAENSAGIG	VFSDPFLFQT	AESAPGKVVN
170	180	190	200	210	220	230	240
LTVEAYNASA	VKLIWYLPRQ	PNGKITSFKI	SVKHARSGIV	VKDVSIRVED	ILTGKLPECN	ENSESFLWST	ASPSPTLGRV
250	260	270	280	290	300	310	320
TPPSRTTHSS	STLTQNEISS	VWKEPISFVV	THLRPYTTYL	FEVSAATTEA	GYIDSTIVRT	PESVPEGPPQ	NCVTGNITGK
330	340	350	360	370	380	390	400
SFSILWDPPT	IVTGKFSYRV	ELYGPSGRIL	DNSTKDLKFA	FTNLTPFTMY	DVYIAAETSA	GTGPKSNISV	FTPPDVPGAV
410	420	430	440	450	460	470	480
FDLQLAEVES	TQVRITWKKP	RQPNGIINQY	RVKVLVPETG	IILENTLLTG	NNEYINDPMA	PEIVNIVEPM	VGLYEGSAEM
490	500	510	520	530	540	550	560
SSDLHSLATF	IYNSHPDKNF	PARNRAEDQT	SPVVTTRNQY	ITDIAAEQLS	YVIRRLVPFT	EHMISVSAFT	IMGEGPPTVL
570	580	590	600	610	620	630	640
SVRTRQQVPS	SIKIINYKNI	SSSSILLYWD	PPEYPNGKIT	HYTIYAMELD	TNRAFQITTI	DNSFLITGLK	KYTKYKMRVA
650	660	670	680	690	700	710	720
ASTHVGESSL	SEENDIFVRT	SEDEPESSPQ	DVEVIDVTAD	EIRLKWSPPE	KPNGIIIAYE	VLYKNIDTLY	MKNTSTTDII
730	740	750	760	770	780	790	800
LRNLRPHTLY	NISVRSYTRF	GHGNQVSSLL	SVRTSETVPD	SAPENITYKN	ISSGEIELSF	LPPSSPNGII	QKYTIYLKRS
810	820	830	840	850	860	870	880
NGNEERTINT	TSLTQNIKVL	KKYTQYIIEV	SASTLKGEGV	RSAPISILTE	EDAPDSPPQD	FSVKQLSGVT	VKLSWQPPLE
890	900	910	920	930	940	950	960
PNGIILYYTV	YVWNRSSLKT	INVTETSLEL	SDLDYNVEYS	AYVTASTRFG	DGKTRSNIIS	FQTPEGAPSD	PPKDVYYANL
970	980	990	1000	1010	1020	1030	1040
SSSSIILFWT	PPSKPNGIIQ	YYSVYYRNTS	GTFMQNFTLH	EVTNDFDNMT	VSTIIDKLTI	FSYYTFWLTA	STSVGNGNKS
1050	1060	1070	1080	1090	1100	1110	1120
SDIIEVYTDQ	DIPEGFVGNL	TYESISSTAI	NVSWVPPAQP	NGLVFYYVSL	ILQQTPRHVR	PPLVTYERSI	YFDNLEKYTD
1130	1140	1150	1160	1170	1180	1190	1200
YILKITPSTE	KGFSDTYTAQ	LYIKTEEDVP	ETSPIINTFK	NLSSTSVLLS	WDPPVKPNGA	IISYDLTLQG	PNENYSFITS
1210	1220	1230	1240	1250	1260	1270	1280
DNYIILEELS	PFTLYSFFAA	ARTRKGLGPS	SILFFYTDES	VPLAPPQNLT	LINCTSDFVW	LKWSPSPLPG	GIVKVYSFKI
1290	1300	1310	1320	1330	1340	1350	1360
HEHETDTIYY	KNISGFKTEA	KLVGLEPVST	YSIRVSAFTK	VGNGNQFSNV	VKFTTQESVP	DVVQNMQCMA	TSWQSVLVKW
1370	1380	1390	1400	1410	1420	1430	1440
DPPKKANGII	TQYMVTVERN	STKVSPQDHM	YTFIKLLANT	SYVFKVRAST	SAGEGDESTC	HVSTLPETVP	SVPTNIAFSD
1450	1460	1470	1480	1490	1500	1510	1520
VQSTSATLTW	IRPDTILGYF	QNYKITTQLR	AQKCKEWESE	ECVEYQKIQY	LYEAHLTEET	VYGLKKFRWY	RFQVAASTNA
1530	1540	1550	1560	1570	1580	1590	1600
GYGNASNWIS	TKTLPGPPDG	PPENVHVVAT	SPFSISISWS	EPAVITGPTC	YLIDVKSVDN	DEFNISFIKS	NEENKTIEIK
1610	1620	1630	1640	1650	1660	1670	1680
DLEIFTRYSV	VITAFTGNIS	AAYVEGKSSA	EMIVTTLESA	PKDPPNNMTF	QKIPDEVTKF	QLTFLPPSQP	NGNIQVYQAL
1690	1700	1710	1720	1730	1740	1750	1760
VYREDDPTAV	QIHNLSIIQK	TNTFVIAMLE	GLKGGHTYNI	SVYAVNSAGA	GPKVPMRITM	DIKAPARPKT	KPTPIYDATG
1770	1780	1790	1800	1810	1820	1830	1840
KLLVTSTTIT	IRMPICYYSD	DHGPIKNVQV	LVTETGAQHD	GNVTKWYDAY	FNKARPYFTN	EGFPNPPCTE	GKTKFSGNEE
1850	1860	1870	1880	1890	1900	1910	1920
IYIIGADNAC	MIPGNEDKIC	NGPLKPKKQY	LFKFRATNIM	GQFTDSDYSD	PVKTLGEGLS	ERTVEIILSV	TLCILSIILL
1930	1940	1950	1960	1970	1980	1990	2000
GTAIFAFARI	RQKQKEGGTY	SPQDAEIIDT	KLKLDQLITV	ADLELKDERL	TRLLSYRKSI	KPISKKSFLQ	HVEELCTNNN
2010	2020	2030	2040	2050	2060	2070	2080
LKFQEEFSEL	PKFLQDLSST	DADLPWNRAK	NRFPNIKPYN	NNRVKLIADA	SVPGSDYINA	SYISGYLCPN	EFIATQGPLP
2090	2100	2110	2120	2130	2140	2150	2160
GTVGDFWRMV	WETRAKTLVM	LTQCFEKGRI	RCHQYWPEDN	KPVTVFGDIV	ITKLMEDVQI	DWTIRDLKIE	RHGDCMTVRQ
2170	2180	2190	2200	2210	2220	2230	2240

Detailed Protein Report

Cmpd.	No. of Cmpds.	m/z meas.	Δ m/z [ppm]	z	Rt [min]	Score	P	Range	Sequence	Modification	Ratios
13	1	1002.9641	-45.61	2	29.1	11.9	0	30-47	R.YDITISSISTTYTSPVTR.I		
627	1	604.2948	34.08	2	36.7	13.9	0	1643-1652	K.DPPNNMTFQK.I	Oxidation: 6	Q:W 1.20

Detailed Protein Report

Protein 519: **PREDICTED: probable JmjC domain-containing histone demethylation protein 2C isoform X6 [Homo sapiens]**

**Accession:**	gi|530393271	**Score:**	42.3
**Database:**	refseq_human(refseq_human_20140103.fasta)	**MW [kDa]:**	250.9
**Database Date:**	2015-11-30	**pI:**	9.0
**Modification(s):**	Oxidation	**Sequence Coverage [%]:**	1.3
		**No. of unique Peptides:**	2

Detailed Protein Report

10	20	30	40	50	60	70	80
MNSQAAVPKQ	NTHQQQQQRS	IRPNKRKGSD	SSIPDEEKMK	EEKYDYISRG	ENPKGKNKHL	MNKRRKPEED	EKKLNMKRLR
90	100	110	120	130	140	150	160
TDNVSDFSES	SDSENSNKRI	IDNSSEQKPE	NELKNKNTSK	INGEEGKPHN	NEKAGEETLK	NSQPPWDQIQ	EDKKHEEAEK
170	180	190	200	210	220	230	240
RKSVDTQLQE	DMIIHSSEQS	TVSDHNSNDL	LPQECNMDKT	HTMELLPKEK	FVSRPPTPKC	VIDITNDTNL	EKVAQENSST
250	260	270	280	290	300	310	320
FGLQTLQKMD	PNVSDSKHSI	ANAKFLETAK	KDSDQSWVSD	VVKVDLTQSS	VTNASSGNDH	LNMEKEKYVS	YISPLSAVSV
330	340	350	360	370	380	390	400
MEDKLHKRSP	PPETIKSKLN	TSVDTHKIKS	SPSPEVVKPK	ITHSPDSVKS	KATYVNSQAT	GERRLANKIE	HELSRCSFHP
410	420	430	440	450	460	470	480
IPTRSSTLET	TKSPLIIDKN	EHFTVYRDPA	LIGSETGANH	ISPFLSQHPF	PLHSSSHRTC	LNPGTHHPAL	TPAPHLLAGS
490	500	510	520	530	540	550	560
SSQTPLPTIN	THPLTSGPHH	AVHHPHLLPT	VLPGVPTASL	LGGHPRLESA	HASSLSHLAL	AHQQQQQLLQ	HQSPHLLGQA
570	580	590	600	610	620	630	640
HPSASYNQLG	LYPIIWQYPN	GTHAYSGLGL	PSSKWVHPEN	AVNAEASLRR	NSPSPWLHQP	TPVTSADGIG	LLSHIPVRPS
650	660	670	680	690	700	710	720
SAEPHRPLKI	TAHSSPPLTK	TLVDHHKEEL	ERKAFMEPLR	SVASTSAKND	LDLNRSQTGK	DCHLHRHFVD	PVLNQLQRPP
730	740	750	760	770	780	790	800
QETGERLNKY	KEEHRRILQE	SIDVAPFTTK	IKGLEGEREN	YSRVASSSSS	PKSHIIKQDM	DVERSVSDLY	KMKHSVPQSL
810	820	830	840	850	860	870	880
PQSNYFTTLS	NSVVNEPPRS	YPSKEVSNIY	GDKQSNALAA	AAANPQTLTS	FITSLSKPPP	LIKHQPESEG	LVGKIPEHLP
890	900	910	920	930	940	950	960
HQIASHSVTT	FRNDCRSPTH	LTVSSTNTLR	SMPALHRAPV	FHPPIHHSLE	RKEGSYSSLS	PPTLTPVMPV	NAGGKVQESQ
970	980	990	1000	1010	1020	1030	1040
KPPTLIPEPK	DSQANFKSSS	EQSLTEMWRP	NNNLSKEKTE	WHVEKSSGKL	QAAMASVIVR	PSSSTKTDSM	PAMQLASKDR
1050	1060	1070	1080	1090	1100	1110	1120
VSERSSAGAH	KTDCLKLAEA	GETGRIILPN	VNSDSVHTKS	EKNFQAVSQG	SVPSSVMSAV	NTMCNTKTDV	ITSAADTTSV
1130	1140	1150	1160	1170	1180	1190	1200
SSWGGSEVIS	SLSNTILAST	SSECVSSKSV	SQPVAQKQEC	KVSTTAPVTL	ASSKTGSVVQ	PSSGFSGTTD	FIHLKKHKAA
1210	1220	1230	1240	1250	1260	1270	1280
LAAAQYKSSN	ASETEPNAIK	NQTLSASLPL	DSTVICSTIN	KANSVGNGQA	SQTSQPNYHT	KLKKAWLTRH	SEEDKNTNKM
1290	1300	1310	1320	1330	1340	1350	1360
ENSGNSVSEI	IKPCSVNLIA	STSSDIQNSV	DSKIIVDKYV	KDDKVNRRKA	KRTYESGSES	GDSDESESKS	EQRTKRQPKP
1370	1380	1390	1400	1410	1420	1430	1440
TYKKKQNDLQ	KRKGEIEEDL	KPNGVLSRSA	KERSKLKLQS	NSNTGIPRSV	LKDWRKVKKL	KQTGESFLQD	DSCCEIGPNL
1450	1460	1470	1480	1490	1500	1510	1520
QKCRECRLIR	SKKGEEPAHS	PVFCRFYYFR	RLSFSKNGVV	RIDGFSSPDQ	YDDEAMSLWT	HENFEDDELD	IETSKYILDI
1530	1540	1550	1560	1570	1580	1590	1600
IGDKFCQLVT	SEKTALSWVK	KDAKIAWKRA	VRGVREMCDA	CEATLFNIHW	VCQKCGFVVC	LDCYKAKERK	SSRDKELYAW
1610	1620	1630	1640	1650	1660	1670	1680
MKCVKGQPHD	HKHLMPTQII	PGSVLTDLLD	AMHTLREKYG	IKSHCHCTNK	QNLQVGNFPT	MNGVSQVLQN	VLNHSNKISL
1690	1700	1710	1720	1730	1740	1750	1760
CMPESQQQNT	PPKSEKNGGS	SPESDVGTDN	KLTPPESQSP	LHWLADLAEQ	KAREEKKENK	ELTLENQIKE	EREQDNSESP
1770	1780	1790	1800	1810	1820	1830	1840
NGRTSPLVSQ	NNEQGSTLRD	LLTTTAGKLR	VGSTDAGIAF	APVYSMGAPS	SKSGRTMPNI	LDDIIASVVE	NKIPPSKTSK
1850	1860	1870	1880	1890	1900	1910	1920
INVKPELKEE	PEESIISAVD	ENNKLYSDIP	HSWICEKHIL	WLKDYKNSSN	WKLFKECWKQ	GQPAVVSGVH	KKMNISLWKA
1930	1940	1950	1960	1970	1980	1990	2000
ESISLDFGDH	QADLLNCKDS	IISNANVKEF	WDGFEEVSKR	QKNKSGETVV	LKLKDWPSGE	DFKTMMPARY	EDLLKSLPLP
2010	2020	2030	2040	2050	2060	2070	2080
EYCNPEGKFN	LASHLPGFFV	RPDLGPRLCS	AYGVVAAKDH	DIGTTNLHIE	VSDVVNILVY	VGIAKGNGIL	SKAGILKKFE
2090	2100	2110	2120	2130	2140	2150	2160
EEDLDDILRK	RLKDSSEIPG	ALWHIYAGKD	VDKIREFLQK	ISKEQGLEVL	PEHDPIRDQS	WYVNKKLRQR	LLEEYGVRTC
2170	2180	2190	2200	2210	2220	2230	2240

Detailed Protein Report

Cmpd.	No. of Cmpds.	m/z meas.	Δ m/z [ppm]	z	Rt [min]	Score	P	Range	Sequence	Modification
343	1	528.7540	-68.44	2	33.9	11.7	2	57-64	K.NKHLMNKR.R	Oxidation: 5
1263	1	1019.5782	7.40	1	45.5	17.4	1	1912-1919	K.KMNISLWK.A	

Detailed Protein Report

Protein 520: **PREDICTED: nuclear receptor subfamily 1 group D member 2 isoform X2 [Homo sapiens]**

**Accession:**	gi|578806859	**Score:**	42.3
**Database:**	refseq_human(refseq_human_20140103.fasta)	**MW [kDa]:**	49.9
**Database Date:**	2015-11-30	**pI:**	9.5
**Modification(s):**	Oxidation	**Sequence Coverage [%]:**	12.1
		**No. of unique Peptides:**	2

10	20	30	40	50	60	70	80
MEVNAGGVIA	YISSSSSASS	PASCHSEGSE	NSFQSSSSSV	PSSPNSSNSD	TNGNPKNGDL	ANIEGILKND	RIDCSMKTSK
**90**	**100**	**110**	**120**	**130**	**140**	**150**	**160**
SSAPGMTKSH	SGVTKFSGMV	LLCKVCGDVA	SGFHYGVHAC	EGCKGFFRRS	IQQNIQYKKC	LKNENCSIMR	MNRNRCQQCR
**170**	**180**	**190**	**200**	**210**	**220**	**230**	**240**
FKKCLSVGMS	RDAVRFGRIP	KREKQRMLIE	MQSAMKTMMN	SQFSGHLQND	TLVEHHEQTA	LPAQEQLRPK	PQLEQENIKS
**250**	**260**	**270**	**280**	**290**	**300**	**310**	**320**
SSPPSSDFAK	EEVIGMVTRA	HKDTFMYNQE	QQENSAESMQ	PQRGERIPKN	MEQYNLNHDH	CGNGLSSHFP	CSESQQHLNG
**330**	**340**	**350**	**360**	**370**	**380**	**390**	**400**
QFKGRNIMHY	PNGHAICIAN	GHCMNFSNAY	TQRVCDRVPI	DGFSQNENKN	SYLCNTGGRM	HLVCPMSKSP	YVDPHKSGHE
**410**	**420**	**430**	**440**	**450**			
IWEEFSMSFT	PAVKEVVEFA	KRIPGFRDLS	QHDQVNLLKA	GTFEIDLE			

Cmpd.	No. of Cmpds.	m/z meas.	Δ m/z [ppm]	z	Rt [min]	Score	P	Range	Sequence	Modification
1576	1	1011.7169	-72.08	3	49.6	10.7	1	96-124	K.FSGMVLLCKVCGDVASGFHYGVHACEGCK.G	Oxidation: 4
80	1	877.8666	-76.26	2	30.7	12.1	2	183-196	R.EKQRMLIEMQSAMK.T	Oxidation: 9, 13

Detailed Protein Report

Protein 521: PREDICTED: zinc finger protein 114 isoform X2 [Homo sapiens]

**Accession:**	gi|530415846	**Score:**	42.3
**Database:**	refseq_human(refseq_human_20140103.fasta)	**MW [kDa]:**	43.8
**Database Date:**	2015-11-30	**pI:**	10.1
**Modification(s):**	Carbamidomethyl	**Sequence Coverage [%]:**	12.5
		**No. of unique Peptides:**	1

10	20	30	40	50	60	70	80
MLENSRNLAF	IDWATPCKTK	DATPQPDILP	KRTFPEANRV	CLTSISSQHS	TLREDWRCPK	TEEPHRQGVN	NVKPPAVAPE
**90**	**100**	**110**	**120**	**130**	**140**	**150**	**160**
KDESPVSICE	DHEMRNHSKP	TCRLVPSQGD	SIRQCILTRD	SSIFKYNPVL	NDSQKTHENN	EDDGVLGWNI	QWVPCGRKTE
**170**	**180**	**190**	**200**	**210**	**220**	**230**	**240**
LKSSTWTGSQ	NTVHHIRDEI	DTGANRHQRN	PFGKAFREDG	SLRAHNTHGR	EKMYDFTQCE	NTSRNNSIHA	MQMQLYTAET
**250**	**260**	**270**	**280**	**290**	**300**	**310**	**320**
NKKDCQTGAT	SANAPNSGSH	KSHCTGEKTH	KCPECGRAFF	YQSFLMRHMK	IHTGEKPYEC	GKCGKAFRYS	LHLNKHLRKH
**330**	**340**	**350**	**360**	**370**	**380**	**390**	
VVQKKPYECE	ECGKVIRESS	KYTHIRSHTG	EKPYKCKTCG	KDFAKSSGLK	KHLKTHKDEK	PCE	

Cmpd.	No. of Cmpds.	m/z meas.	Δ m/z [ppm]	z	Rt [min]	Score	P	Range	Sequence	Modification
1695	1	671.2184	-127.57	3	51.5	15.8	1	272-287	K.CPECGRAFFYQSFLMR.H	Carbamidomethyl: 1

Detailed Protein Report

Protein 522: rho GTPase-activating protein 42 [Homo sapiens]

**Accession:**	gi|221307575	**Score:**	42.2
**Database:**	refseq_human(refseq_human_20140103.fasta)	**MW [kDa]:**	98.5
**Database Date:**	2015-11-30	**pI:**	8.9
**Modification(s):**	Oxidation	**Sequence Coverage [%]:**	3.4
		**No. of unique Peptides:**	1

Quantitation

Q:W	**Median:**	0.99	**CV:**	0.00 %	**No. of Peptides:**	1
M:Q	**Median:**	0.87	**CV:**	0.00 %	**No. of Peptides:**	1

10	20	30	40	50	60	70	80
MGLPTLEFSD	SYLDSPDFRE	RLQCHEIELE	RTNKFIKELI	KDGSLLIGAL	RNLSMAVQKF	SQSLQDFQFE	CIGDAETDDE
**90**	**100**	**110**	**120**	**130**	**140**	**150**	**160**
ISIAQSLKEF	ARLLIAVEEE	RRRLIQNAND	VLIAPLEKFR	KEQIGAAKDG	KKKFDKESEK	YYSILEKHLN	LSAKKKESHL
**170**	**180**	**190**	**200**	**210**	**220**	**230**	**240**
QEADTQIDRE	HQNFYEASLE	YVFKIQEVQE	KKKFEFVEPL	LSFLQGLFTF	YHEGYELAQE	FAPYKQQLQF	NLQNTRNNFE
**250**	**260**	**270**	**280**	**290**	**300**	**310**	**320**
STRQEVERLM	QRMKSANQDY	RPPSQWTMEG	YLYVQEKRPL	GFTWIKHYCT	YDKGSKTFTM	SVSEMKSSGK	MNGLVTSSPE
**330**	**340**	**350**	**360**	**370**	**380**	**390**	**400**
MFKLKSCIRR	KTDSIDKRFC	FDIEVVERHG	IITLQAFSEA	NRKLWLEAMD	GKEPIYTLPA	IISKKEEMYL	NEAGFNFVRK
**410**	**420**	**430**	**440**	**450**	**460**	**470**	**480**
CIQAVETRGI	TILGLYRIGG	VNSKVQKLMN	TTFSPKSPPD	IDIDIELWDN	KTITSGLKNY	LRCLAEPLMT	YKLHKDFIIA
**490**	**500**	**510**	**520**	**530**	**540**	**550**	**560**
VKSDDQNYRV	EAVHALVHKL	PEKNREMLDI	LIKHLVKVSL	HSQQNLMTVS	NLGVIFGPTL	MRAQEETVAA	MMNIKFQNIV
**570**	**580**	**590**	**600**	**610**	**620**	**630**	**640**
VEILIEHYEK	IFHTAPDPSI	PLPQPQSRSG	SRRTRAICLS	TGSRKPRGRY	TPCLAEPDSD	SYSSSPDSTP	MGSIESLSSH
**650**	**660**	**670**	**680**	**690**	**700**	**710**	**720**
SSEQNSTTKS	ASCQPREKSG	GIPWIATPSS	SNGQKSLGLW	TTSPESSSRE	DATKTDAESD	CQSVASVTSP	GDVSPPIDLV
**730**	**740**	**750**	**760**	**770**	**780**	**790**	**800**
KKEPYGLSGL	KRASASSLRS	ISAAEGNKSY	SGSIQSLTSV	GSKETPKASP	NPDLPPKMCR	RLRLDTASSN	GYQRPGSVVA
**810**	**820**	**830**	**840**	**850**	**860**	**870**	**880**
AKAQLFENVG	SPKPVSSGRQ	AKAMYSCKAE	HSHELSFPQG	AIFSNVYPSV	EPGWLKATYE	GKTGLVPENY	VVFL

Cmpd.	No. of Cmpds.	m/z meas.	Δ m/z [ppm]	z	Rt [min]	Score	P	Range	Sequence	Modification	Ratios
150	1	849.3532	-94.77	2	31.6	11.8	1	311-325	K.MNGLVTSSPEMFKLK.S	Oxidation: 11	M:Q 0.87<br>Q:W 0.99

Detailed Protein Report

Protein 523: frizzled-10 precursor [Homo sapiens]

Accession: gi|6005762
Database: refseq_human(refseq_human_20140103.fasta)
Database Date: 2015-11-30
Modification(s): Carbamidomethyl, Oxidation

Score: 42.2
MW [kDa]: 65.3
pI: 9.9
Sequence Coverage [%]: 6.4
No. of unique Peptides: 2

10	20	30	40	50	60	70	80
MQRPGPRLWL	VLQVMGSCAA	ISSMDMERPG	DGKCQPIEIP	MCKDIGYNMT	RMPNLMGHEN	QREAAIQLHE	FAPLVEYGCH
**90**	**100**	**110**	**120**	**130**	**140**	**150**	**160**
GHLRFFLCSL	YAPMCTEQVS	TPIPACRVMC	EQARLKCSPI	MEQFNFKWPD	SLDCRKLPNK	NDPNYLCMEA	PNNGSDEPTR
**170**	**180**	**190**	**200**	**210**	**220**	**230**	**240**
GSGLFPPLFR	PQRPHSAQEH	PLKDGGPGRG	GCDNPGKFHH	VEKSASCAPL	CTPGVDVYWS	REDKRFAVVW	LAIWAVLCFF
**250**	**260**	**270**	**280**	**290**	**300**	**310**	**320**
SSAFTVLTFL	IDPARFRYPE	RPIIFLSMCY	CVYSVGYLIR	LFAGAESIAC	DRDSGQLYVI	QEGLESTGCT	LVFLVLYYFG
**330**	**340**	**350**	**360**	**370**	**380**	**390**	**400**
MASSLWWVVL	TLTWFLAAGK	KWGHEAIEAN	SSYFHLAAWA	IPAVKTILIL	VMRRVAGDEL	TGVCYVGSMD	VNALTGFVLI
**410**	**420**	**430**	**440**	**450**	**460**	**470**	**480**
PLACYLVIGT	SFILSGFVAL	FHIRRVMKTG	GENTDKLEKL	MVRIGLFSVL	YTVPATCVIA	CYFYERLNMD	YWKILAAQHK
**490**	**500**	**510**	**520**	**530**	**540**	**550**	**560**
CKMNNQTKTL	DCLMAASIPA	VEIFMVKIFM	LLVVGITSGM	WIWTSKTLQS	WQQVCSRRLK	KKSRRKPASV	ITSGGIYKKA
**570**	**580**	**590**					
QHPQKTHHGK	YEIPAQSPTC	V					

Cmpd.	No. of Cmpds.	m/z meas.	Δ m/z [ppm]	z	Rt [min]	Score	P	Range	Sequence	Modification
1958	2	655.2730	-62.48	2	52.8	10.4	0	281-292	R.LFAGAESIACDR.D	Carbamidomethyl: 10
2186	1	928.7350	-68.15	3	55.7	17.1	1	483-507	K.MNNQTKTLDCLMAASIPAVEIFMVK.I	Oxidation: 23

Detailed Protein Report

Protein 524: protein-glutamine gamma-glutamyltransferase 5 isoform 2 [Homo sapiens]

Accession: gi|4759230
Database: refseq_human(refseq_human_20140103.fasta)
Database Date: 2015-11-30
Modification(s): Carbamidomethyl

Score: 42.2
MW [kDa]: 71.9
pI: 5.9
Sequence Coverage [%]: 8.5
No. of unique Peptides: 2

10	20	30	40	50	60	70	80
MAQGLEVALT	DLQSSRNNVR	HHTEEITVDH	LLVRRGQAFN	LTLYFRNRSF	QPGLDNIIFV	VETEDAVYLD	SEPQRQEYVM
90	100	110	120	130	140	150	160
NDYGFIYQGS	KNWIRPCPWN	YGQFEDKIID	ICLKLLDKSL	HFQTDPATDC	ALRGSPVYVS	RVVCAMINSN	DDNGVLNGNW
170	180	190	200	210	220	230	240
SENYTDGANP	AEWTGSVAIL	KQWNATGCQP	VRYGQCWVFA	AVMCTVMRCL	GIPTRVITNF	DSGHDTDGNL	IIDEYYDNTG
250	260	270	280	290	300	310	320
RILGNKKKDT	IWNFHVWNEC	WMARKDLPPA	YGGWQVLDAT	PQEMSNGVYC	CGPASVRAIK	EGEVDLNYDT	PFVFSMVNAD
330	340	350	360	370	380	390	400
CMSWLVQGGK	EQKLHQDTSS	VGNFISTKSI	QSDERDDITE	NYKYEEGSLQ	ERQVFLKALQ	KLKARSFHGS	QRGAELQPSR
410	420	430	440	450	460	470	480
PTSLSQDSPR	SLHTPSLRPS	DVVQVSLKFK	LLDPPNMGQD	ICFVLLALNM	SSQFKDLKVN	LSAQSLLHDG	SPLSPFWQDT
490	500	510	520	530	540	550	560
AFITLSPKEA	KTYPCKISYS	QYSQYLSTDK	LIRISALGEE	KSSPEKILVN	KIITLSYPSI	TINVLGAAVV	NQPLSIQVIF
570	580	590	600	610	620	630	640
SNPLSEQVED	CVLTVEGSGL	FKKQQKVFLG	VLKPQHQASI	ILETVPFKSG	QRQIQANMRS	NKFKDIKGYR	NVYVDFAL

Cmpd.	No. of Cmpds.	m/z meas.	Δ m/z [ppm]	z	Rt [min]	Score	P	Range	Sequence	Modification
2196	1	961.3531	-82.76	2	56.1	16.4	0	193-208	R.YGQCWVFAAVMCTVMR.C	Carbamidomethyl: 4
1787	1	1023.8570	-1.46	3	52.3	10.0	1	429-455	K.FKLLDPPNMGQDICFVLLALNMSSQFK.D	

Detailed Protein Report

Protein 525: **immunoglobulin superfamily member 22 [Homo sapiens]**

**Accession:**	gi|253970427	**Score:**	42.1
**Database:**	refseq_human(refseq_human_20140103.fasta)	**MW [kDa]:**	147.9
**Database Date:**	2015-11-30	**pI:**	9.0
		**Sequence Coverage [%]:**	3.4
		**No. of unique Peptides:**	1

10	20	30	40	50	60	70	80
MTTIHSRQML	QEHVSMEFSS	STTHVQTFSQ	TTKIVGEEVV	RRKSSSIVEF	FSLVTRSSNI	PAGDSVPEFV	EKPQPVTAPE
90	100	110	120	130	140	150	160
GDKAVFRARV	QGNAKPHISW	KRESGIPIKE	SAKIFYDSIN	KEHVLKLEPL	TSDDSDNYKC	IASNDHADAI	YTVSLLVTEG
170	180	190	200	210	220	230	240
QEKMDFKKML	KKRAPPAPKK	KQKKVANEKE	MLEILSKVPK	KDFEKVCMEY	GFTDFRGLLR	KLKEMKKKVE	VEAIRILKPL
250	260	270	280	290	300	310	320
EDKETKVDTT	VVFDCIMELK	DPNVKMIWIK	GTEPLRIQYS	LGKYDVKQMG	TKYMLVISNV	NMNDAGIYSL	SVGDKRMSAE
330	340	350	360	370	380	390	400
LTVLDEPLKF	LGEMKPVKVT	ERQTAVFEIR	LSKKEPNFVW	KFNGKELKRD	DKYEITVSED	GLTHTLKIKD	ARLSDSGEFS
410	420	430	440	450	460	470	480
AEAGNLVQKA	QLTIDRIPIK	FVSNLKNVRV	KERSRACLEC	ELTSKDVTLR	WKKDGQLLMH	GTKYSMNHEG	KRAELIIEDA
490	500	510	520	530	540	550	560
QLSDGGEYTV	VAMQDGDPTE	YYSTAIVTVE	ERLATVKSGM	SDVHAATGSP	AELCVVLNDE	KVEGVWLKDG	KEITDLPGMQ
570	580	590	600	610	620	630	640
IVKQGAVHKL	IFPSMGPEHE	GKYTFRAKGT	ESEASVFIAD	PPTIDPSVLE	ALAAHAITVK	VGHTAHIKVP	FRGKPLPKVT
650	660	670	680	690	700	710	720
WYKDGMEVTE	EERVSMERGE	DQALLTISNC	VREDSGLILL	KLKNDHGSAT	ATLHLSVLDR	PKPPQGRVEF	LELSGSCVHM
730	740	750	760	770	780	790	800
KWKAPKDNGG	RPVTQFIVER	RAVGKKSWIK	IGEVDGKVTN	FSTNKVEEGK	AYQFRILAVN	SEGVSDPLET	EEVFAGNPIE
810	820	830	840	850	860	870	880
PPGFASQPQV	TDVTKEAVTI	TWNAPTQDGG	APVLGYIVER	RKKGSNLWVP	VNKDPIQGTK	CTVDGLLEDT	EYEFRVIAVN
890	900	910	920	930	940	950	960
KAGPGQPSVP	SSSVVAKDPV	KPPGLVQDLH	VSDSSNSSIS	LAWREPAEGD	PPSGYILEMR	AEDTKEWSKC	TKIPISGTCY
970	980	990	1000	1010	1020	1030	1040
TVGGLIERQK	YFFRIRAVNE	AGVGEPVELD	KGVRAMPPPA	APKFDLSARL	KSHMVVRAGT	ALCIHAAFSG	SPPPDVIWQK
1050	1060	1070	1080	1090	1100	1110	1120
DGVPTKGRET	ITKSKNHSQF	LINSTKRSDS	GVYRILLQNE	FGEARYDIHV	RVADFPRPPT	NLRLFEEVPN	TVTLTWNHSP
1130	1140	1150	1160	1170	1180	1190	1200
DVQEDGEAHY	IIMKRDASTA	TWYTAAERVF	SNKYTVTGLL	PGRKYYFRVV	ARNEIGDSEP	LDSRDTWLIN	KDQIQDLSAK
1210	1220	1230	1240	1250	1260	1270	1280
LKPYEKKDWR	HAPRFVTPLK	PHTVLRGQDC	TMTCAFLGNP	RPTVTLYKGD	VNITANSKFW	YNSTSGVCTL	VIPTCTLKDS
1290	1300	1310	1320	1330			
GDYSVLVENE	LGKDRSSCTL	TVYDKDDKSV	VASITESLQK	KSKHLM			

Cmpd.	No. of Cmpds.	m/z meas.	Δ m/z [ppm]	z	Rt [min]	Score	P	Range	Sequence	Modification
2097	1	900.3887	-98.78	3	56.6	12.0	2	977-1003	R.AVNEAGVGEPVELDKGVRAMPPPAAPK.F	

Detailed Protein Report

Protein 526: **PREDICTED: ubiquitin carboxyl-terminal hydrolase 40 isoform X4 [Homo sapiens]**

**Accession:**	gi|530371419	**Score:**	42.1
**Database:**	refseq_human(refseq_human_20140103.fasta)	**MW [kDa]:**	125.8
**Database Date:**	2015-11-30	**pI:**	5.5
		**Sequence Coverage [%]:**	2.7
		**No. of unique Peptides:**	1

10	20	30	40	50	60	70	80
MRQHDVQELN	RILFSALETS	LVGTSGHDLI	YRLYHGTIVN	QIVCKECKNV	SERQEDFLDL	TVAVKNVSGL	EDALWNMYVE
90	100	110	120	130	140	150	160
EEVFDCDNLY	HCGTCDRLVK	AAKSAKLRKL	PPFLTVSLLR	FNFDFVKCER	YKETSCYTFP	LRINLKPFCE	QSELDDLEYI
170	180	190	200	210	220	230	240
YDLFSVIIHK	GGCYGGHYHV	YIKDVDHLGN	WQFQEEKSKP	DVNLKDLQSE	EEIDHPLMIL	KAILLEEENN	LIPVDQLGQK
250	260	270	280	290	300	310	320
LLKKIGISWN	KKYRKQHGPL	RKFLQLHSQI	FLLSSDESTV	RLLKNSSLQA	ESDFQRNDQQ	IFKMLPPESP	GLNNSISCPH
330	340	350	360	370	380	390	400
WFDINDSKVQ	PIREKDIEQQ	FQGKESAYML	FYRKSQLQRP	PEARANPRYG	VPCHLLNEMD	AANIELQTKR	AECDSANNTF
410	420	430	440	450	460	470	480
ELHLHLGPQY	HFFNGALHPV	VSQTESVWDL	TFDKRKTLGD	LRQSIFQLLE	FWEGDMVLSV	AKLVPAGLHI	YQSLGGDELT
490	500	510	520	530	540	550	560
LCETEIADGE	DIFVWNGVEV	GGVHIQTGID	CEPLLLNVLH	LDTSSDGEKC	CQVIESPHVF	PANAEVGTVL	TALAIPAGVI
570	580	590	600	610	620	630	640
FINSAGCPGG	EGWTAIPKED	MRKTFREQGL	RNGSSILIQD	SHDDNSLLTK	EEKWVTSMNE	IDWLHVKNLC	QLESEEKQVK
650	660	670	680	690	700	710	720
ISATVNTMVF	DIRIKAIKEL	KLMKELADNS	CLRPIDRNGK	LLCPVPDSYT	LKEAELKMGS	SLGLCLGKAP	SSSQLFLFFA
730	740	750	760	770	780	790	800
MGSDVQPGTE	MEIVVEETIS	VRDCLKLMLK	KSGLQGDAWH	LRKMDWCYEA	GEPLCEEDAT	LKELLICSGD	TLLLIEGQLP
810	820	830	840	850	860	870	880
PLGFLKVPIW	WYQLQGPSGH	WESHQDQTNC	TSSWGRVWRA	TSSQGASGNE	PAQVSLLYLG	DIEISEDATL	AELKSQAMTL
890	900	910	920	930	940	950	960
PPFLEFGVPS	PAHLRAWTVE	RKRPGRLLRT	DRQPLREYKL	GRRIEICLEP	LQKGENLGPQ	DVLLRTQVRI	PGERTYAPAL
970	980	990	1000	1010	1020	1030	1040
DLVWNAAQGG	TAGSLRQRVA	DFYRLPVEKI	EIAKYFPEKF	EWLPISSWNQ	QITKRKKKKK	QDYLQGAPYY	LKDGDTIGVK
1050	1060	1070	1080	1090	1100	1110	
NLLIDDDDDF	STIRDDTGKE	KQKQRALGRR	KSQEALHEQS	SYILSSAETP	ARPRAPETSL	SIHVGSFR	

Cmpd.	No. of Cmpds.	m/z meas.	Δ m/z [ppm]	z	Rt [min]	Score	P	Range	Sequence	Modification
2823	1	826.4280	41.02	2	65.7	30.1	0	1041-1054	K.NLLIDDDDDFSTIR.D	

Detailed Protein Report

Protein 527: 5'-nucleotidase isoform 1 preproprotein [Homo sapiens]

Accession: gi|4505467
Database: refseq_human(refseq_human_20140103.fasta)
Database Date: 2015-11-30
Modification(s): Carbamidomethyl, Oxidation

Score: 42.1
MW [kDa]: 63.3
pI: 6.6
Sequence Coverage [%]: 4.4
No. of unique Peptides: 1

10	20	30	40	50	60	70	80
MCPRAARAPA	TLLLALGAVL	WPAAGAWELT	ILHTNDVHSR	LEQTSEDSSK	CVNASRCMGG	VARLFTKVQQ	IRRAEPNVLL
**90**	**100**	**110**	**120**	**130**	**140**	**150**	**160**
LDAGDQYQGT	IWFTVYKGAE	VAHFMNALRY	DAMALGNHEF	DNGVEGLIEP	LLKEAKFPIL	SANIKAKGPL	ASQISGLYLP
**170**	**180**	**190**	**200**	**210**	**220**	**230**	**240**
YKVLPVGDEV	VGIVGYTSKE	TPFLSNPGTN	LVFEDEITAL	QPEVDKLKTL	NVNKIIALGH	SGFEMDKLIA	QKVRGVDVVV
**250**	**260**	**270**	**280**	**290**	**300**	**310**	**320**
GGHSNTFLYT	GNPPSKEVPA	GKYPFIVTSD	DGRKVPVVQA	YAFGKYLGYL	KIEFDERGNV	ISSHGNPILL	NSSIPEDPSI
**330**	**340**	**350**	**360**	**370**	**380**	**390**	**400**
KADINKWRIK	LDNYSTQELG	KTIVYLDGSS	QSCRFRECNM	GNLICDAMIN	NNLRHTDEMF	WNHVSMCILN	GGGIRSPIDE
**410**	**420**	**430**	**440**	**450**	**460**	**470**	**480**
RNNGTITWEN	LAAVLPFGGT	FDLVQLKGST	LKKAFEHSVH	RYGQSTGEFL	QVGGIHVVYD	LSRKPGDRVV	KLDVLCTKCR
**490**	**500**	**510**	**520**	**530**	**540**	**550**	**560**
VPSYDPLKMD	EVYKVILPNF	LANGGDGFQM	IKDELLRHDS	GDQDINVVST	YISKMKVIYP	AVEGRIKFST	GSHCHGSFSL
**570**	**580**						
IFLSLWAVIF	VLYQ						

Cmpd.	No. of Cmpds.	m/z meas.	Δ m/z [ppm]	z	Rt [min]	Score	P	Range	Sequence	Modification
1850	1	727.3153	-15.31	2	51.6	13.1	1	51-63	K.CVNASRCMGGVAR.L	Carbamidomethyl: 1, 7; Oxidation: 8

Detailed Protein Report

Protein 528: **A disintegrin and metalloproteinase with thrombospondin motifs 16 preproprotein [Homo sapiens]**

Accession: gi|110735441
Database: refseq_human(refseq_human_20140103.fasta)
Database Date: 2015-11-30
Modification(s): Carbamidomethyl, Oxidation

Score: 42.1
MW [kDa]: 136.1
pI: 10.2
Sequence Coverage [%]: 3.8
No. of unique Peptides: 2

10	20	30	40	50	60	70	80
MKPRARGWRG	LAALWMLLAQ	VAEQAPACAM	GPAAAAPGSP	SVPRPPPPAE	RPGWMEKGEY	DLVSAYEVDH	RGDYVSHEIM
90	100	110	120	130	140	150	160
HHQRRRRAVP	VSEVESLHLR	LKGSRHDFHM	DLRTSSSLVA	PGFIVQTLGK	TGTKSVQTLP	PEDFCFYQGS	LRSHRNSSVA
170	180	190	200	210	220	230	240
LSTCQGLSGM	IRTEEADYFL	RPLPSHLSWK	LGRAAQGSSP	SHVLYKRSTE	PHAPGASEVL	VTSRTWELAH	QPLHSSDLRL
250	260	270	280	290	300	310	320
GLPQKQHFCG	RRKKYMPQPP	KEDLFILPDE	YKSCLRHKRS	LLRSHRNEEL	NVETLVVVDK	KMMQNHGHEN	ITTYVLTILN
330	340	350	360	370	380	390	400
MVSALFKDGT	IGGNINIAIV	GLILLEDEQP	GLVISHHADH	TLSSFCQWQS	GLMGKDGTRH	DHAILLTGLD	ICSWKNEPCD
410	420	430	440	450	460	470	480
TLGFAPISGM	CSKYRSCTIN	EDTGLGLAFT	IAHESGHNFG	MIHDGEGNMC	KKSEGNIMSP	TLAGRNGVFS	WSPCSRQYLH
490	500	510	520	530	540	550	560
KFLSTAQAIC	LADQPKPVKE	YKYPEKLPGE	LYDANTQCKW	QFGEKAKLCM	LDFKKDICKA	LWCHRIGRKC	ETKFMPAAEG
570	580	590	600	610	620	630	640
TICGHDMWCR	GGQCVKYGDE	GPKPTHGHWS	DWSSWSPCSR	TCGGGVSHRS	RLCTNPKPSH	GGKFCEGSTR	TLKLCNSQKC
650	660	670	680	690	700	710	720
PRDSVDFRAA	QCAEHNSRRF	RGRHYKWKPY	TQVEDQDLCK	LYCIAEGFDF	FFSLSNKVKD	GTPCSEDSRN	VCIDGICERV
730	740	750	760	770	780	790	800
GCDNVLGSDA	VEDVCGVCNG	NNSACTIHRG	LYTKHHHTNQ	YYHMVTIPSG	ARSIRIYEMN	VSTSYISVRN	ALRRYYLNGH
810	820	830	840	850	860	870	880
WTVDWPGRYK	FSGTTFDYRR	SYNEPENLIA	TGPTNETLIV	ELLFQGRNPG	VAWEYSMPRL	GTEKQPPAQP	SYTWAIVRSE
890	900	910	920	930	940	950	960
CSVSCGGGQM	TVREGCYRDL	KFQVNMSFCN	PKTRPVTGLV	PCKVSACPPS	WSVGNWSACS	RTCGGGAQSR	PVQCTRRVHY
970	980	990	1000	1010	1020	1030	1040
DSEPVPASLC	PQPAPSSRQA	CNSQSCPPAW	SAGPWAECSH	TCGKGWRKRA	VACKSTNPSA	RAQLLPDAVC	TSEPKPRMHE
1050	1060	1070	1080	1090	1100	1110	1120
ACLLQRCHKP	KKLQWLVSAW	SQCSVTCERG	TQKRFLKCAE	KYVSGKYREL	ASKKCSHLPK	PSLELERACA	PLPCPRHPPF
1130	1140	1150	1160	1170	1180	1190	1200
AAAGPSRGSW	FASPWSQCTA	SCGGGVQTRS	VQCLAGGRPA	SGCLLHQKPS	ASLACNTHFC	PIAEKKDAFC	KDYFHWCYLV
1210	1220	1230					
PQHGMCSHKF	YGKQCCKTCS	KSNL					

Cmpd.	No. of Cmpds.	m/z meas.	Δ m/z [ppm]	z	Rt [min]	Score	P	Range	Sequence	Modification
1904	1	724.3383	-56.32	2	54.2	12.7	2	631-642	R.TLKLCNSQKCPR.D	Carbamidomethyl: 10
2449	1	1025.1077	-9.04	3	60.5	13.5	2	1186-1209	K.KDAFCKDYFHWCYLVPQHGMCSHK.F	Carbamidomethyl: 5, 12; Oxidation: 20

Detailed Protein Report

Protein 529: **PREDICTED: LOW QUALITY PROTEIN: UPF0627 protein ENSP00000364708 [Homo sapiens]**

**Accession:**	gi|410171120	**Score:**	42.1
**Database:**	refseq_human(refseq_human_20140103.fasta)	**MW [kDa]:**	18.2
**Database Date:**	2015-11-30	**pI:**	9.4
**Modification(s):**	Carbamidomethyl	**Sequence Coverage [%]:**	8.9
		**No. of unique Peptides:**	1

10	20	30	40	50	60	70	80
MGCSKGLWKE	RPSAHTSECF	STTACPVAFI	LLVWNSQSPA	GLQSFCTGRH	PSLSVRAQRA	GPGASREEGT	XWTECVGQER
**90**	**100**	**110**	**120**	**130**	**140**	**150**	**160**
RLIHSGSSEN	ESQEDEGADL	IPYTGLKADN	RRKSSTWANE	VEDRRPQSTP	ALNLTPSHPH	PPRPLITFLR	SVIGIQILPG
**170**							
LVAAGGTVA							

Cmpd.	No. of Cmpds.	m/z meas.	Δ m/z [ppm]	z	Rt [min]	Score	P	Range	Sequence	Modification
1988	2	918.3938	-29.29	2	55.2	12.1	1	67-81	R.EEGTVWTECVGQERR.L	Carbamidomethyl: 9

BRUKER

Detailed Protein Report

Protein 530: PREDICTED: zinc finger protein 728 isoform X1 [Homo sapiens]

**Accession:**	gi|578833255	**Score:**	41.9
**Database:**	refseq_human(refseq_human_20140103.fasta)	**MW [kDa]:**	68.5
**Database Date:**	2015-11-30	**pI:**	10.3
**Modification(s):**	Carbamidomethyl	**Sequence Coverage [%]:**	5.9
		**No. of unique Peptides:**	1

10	20	30	40	50	60	70	80
MGSLTFRDVA	IQFSLEEWQC	LDTAQQNLYR	NVMLENYRNL	VFLGIAAPKP	DLIIFLEQGK	EPWNMKRHEL	VKEPPVICSH
**90**	**100**	**110**	**120**	**130**	**140**	**150**	**160**
FAQDLWPEQG	REDSFQKVIL	RRYEKCGHEN	LQLKIGCTNV	DECKVHKKGY	NKLNQSLTTT	QSKVFQCGKY	ANIFHKCSNS
**170**	**180**	**190**	**200**	**210**	**220**	**230**	**240**
KRHKIRHTGK	KLLKCKEYVR	SFCMLSHLSQ	HKRIYTRENS	YKSEEHGKAF	NWSSALTYKR	IHTGEKPCKC	EECGKAFSKF
**250**	**260**	**270**	**280**	**290**	**300**	**310**	**320**
SILTKHKVIH	TGEKHYKCEE	CGKAFTRSSS	LIEHKRSHAG	EKPYKCEECG	KAFSKASTLT	AHKTIHAGEK	PYKCEECGKA
**330**	**340**	**350**	**360**	**370**	**380**	**390**	**400**
FNRSSNLMEH	KRIHTGEKPC	KCEECGKAFG	NFSTLTKHKV	IHTGEKPYKC	EECGKAFSWP	SSLTEHKRIH	AGDKPYKCEE
**410**	**420**	**430**	**440**	**450**	**460**	**470**	**480**
CGKTFKWSST	LTKHKIIHTG	EKPYKCEECG	KAFTTFSSLT	KHKVIHTGEK	HYKCEECGKV	FSWSSSLTTH	KRIHTGEKPY
**490**	**500**	**510**	**520**	**530**	**540**	**550**	**560**
KCEECGKAFS	KVANLTKHKV	IHTGEKQYKC	EECGKAFIWS	SRLSEHKRIH	TGEKPYKCEE	CGKAFSWVSV	LNKHKKIHAG
**570**	**580**	**590**	**600**				
KKFYKCEECG	KDFNQSSHLT	THKRIHTGGK	TLQM				

Cmpd.	No. of Cmpds.	m/z meas.	Δ m/z [ppm]	z	Rt [min]	Score	P	Range	Sequence	Modification
2120	1	924.3321	-133.13	2	56.4	11.9	2	230-245	K.CEECGKAFSKFSILTK.H	Carbamidomethyl: 1

Detailed Protein Report

Protein 531: putative hexokinase HKDC1 [Homo sapiens]

**Accession:**	gi|156151420	**Score:**	41.9
**Database:**	refseq_human(refseq_human_20140103.fasta)	**MW [kDa]:**	102.4
**Database Date:**	2015-11-30	**pI:**	7.2
[truncated: 435,405 more chars]
